# *Hif1a* inactivation rescues photoreceptor degeneration induced by a chronic hypoxia-like stress

**DOI:** 10.1038/s41418-018-0094-7

**Published:** 2018-04-17

**Authors:** Maya Barben, Divya Ail, Federica Storti, Katrin Klee, Christian Schori, Marijana Samardzija, Stylianos Michalakis, Martin Biel, Isabelle Meneau, Frank Blaser, Daniel Barthelmes, Christian Grimm

**Affiliations:** 1 Lab for Retinal Cell Biology, Department of Ophthalmology, University of Zürich, Zürich, Switzerland; 2 Neuroscience Center Zurich (ZNZ), University of Zürich, Zürich, Switzerland; 3 Center for Integrative Human Physiology (ZIHP), University of Zürich, Zürich, Switzerland; 4 Munich Center for Integrated Protein Science at the Department of Pharmacy, Center for Drug Research, Ludwig-Maximilians-Universität München, Munich, Germany; 5 Dept. Ophthalmology, University Hospital Zürich, Zürich, Switzerland; 6 Save Sight Institute, The University of Sydney, Sydney, Australia; 7 Present Address: Paris-Saclay Institute of Neuroscience, CNRS, Univ. Paris-Sud, Université Paris-Saclay, Orsay, France

## Abstract

Reduced choroidal blood flow and tissue changes in the ageing human eye impair oxygen delivery to photoreceptors and the retinal pigment epithelium. As a consequence, mild but chronic hypoxia may develop and disturb cell metabolism, function and ultimately survival, potentially contributing to retinal pathologies such as age-related macular degeneration (AMD). Here, we show that several hypoxia-inducible genes were expressed at higher levels in the aged human retina suggesting increased activity of hypoxia-inducible transcription factors (HIFs) during the physiological ageing process. To model chronically elevated HIF activity and investigate ensuing consequences for photoreceptors, we generated mice lacking von Hippel Lindau (VHL) protein in rods. This activated HIF transcription factors and led to a slowly progressing retinal degeneration in the ageing mouse retina. Importantly, this process depended mainly on HIF1 with only a minor contribution of HIF2. A gene therapy approach using AAV-mediated RNA interference through an anti-*Hif1a* shRNA significantly mitigated the degeneration suggesting a potential intervention strategy that may be applicable to human patients.

## Introduction

Several blinding diseases of the retina are characterized by the progressive loss of photoreceptors and retinal pigment epithelium (RPE) cells. Underlying causes are manifold and include gene mutations, age-related tissue changes, systemic alterations and environmental factors. Another relevant condition that can lead to retinal pathology is hypoxia. Reduced tissue oxygenation is causative for the production of vascular endothelial growth factor (VEGF), a main factor involved in the development of diabetic macular oedema and neovascularization in age-related macular degeneration (AMD) [1–3]. However, tissue hypoxia may also be of significance for retinal pathologies not associated with abnormal vessel growth such as the highly prevalent non-exudative form of AMD [4–6]. Reduced choroidal blood flow in the ageing eye [7, 8] and in the foveolar region of AMD patients [9], choroidal ischemia in dry AMD [9–11] and the correlation between drusen accumulation and decreased choroidal blood volume in AMD [12] has led to the hypothesis that reduced oxygen availability to retinal cells might be a significant factor that contributes—likely together with other factors—to disease development and progression [4, 13]. Since both rods and cones have an extraordinarily high demand for energy [14], their function and survival might be especially sensitive to reduced tissue oxygenation.

Hypoxia-inducible transcription factors (HIFs) are the major regulators of the cellular response to reduced oxygen levels [15]. They are composed of a constitutively expressed β-subunit (HIFB) and an oxygen-labile α-subunit (HIFA). In the presence of O_2_, prolyl hydroxylases hydroxylate the α-subunit that is then recognized by the von Hippel Lindau (VHL) protein complex. An E3 ligase in this complex ubiquitinates the hydroxylated HIFAs targeting them for rapid proteasomal degradation. In hypoxia, HIFAs are less hydroxylated, escape recognition by VHL, ubiquitination and degradation, and can thus function as transcription factors [16, 17]. Prominent HIF target genes are *VEGF* and erythropoietin (*EPO*). Both play eminent roles in the response to hypoxia and are key factors for neovascularization and the increase in haematocrit, respectively. Although HIF1 and HIF2 share several common targets, they also have their own set of genes for specific regulation [18].

Inactivation of VHL prevents degradation of HIF-alpha subunits and leads to increased HIF1 and HIF2 activity. This allows to model a major part of the molecular response to hypoxia in normoxic conditions. Inactivation of the *Vhl* gene in retinal cells already during development causes a severe vessel phenotype and retinal degeneration [19, 20]. Deletion of *Vhl* in rods after postnatal development, however, leads to a late onset and age-dependent loss of photoreceptors and retinal function [21]. Inactivation of *Vhl* in the RPE alters RPE morphology and metabolism leading to cell death in a HIF2-dependent manner [22].

Here, we show that the ageing human retina may indeed experience increased hypoxic stress, identify HIF1 as the factor being mainly responsible for photoreceptor degeneration in a model of chronic hypoxia-like conditions and demonstrate that AAV-mediated RNA interference targeting *Hif1a* mitigates the degenerative phenotype.

## Results

### Increased expression of hypoxia-related genes in the aged human retina

**Fig. 1:**
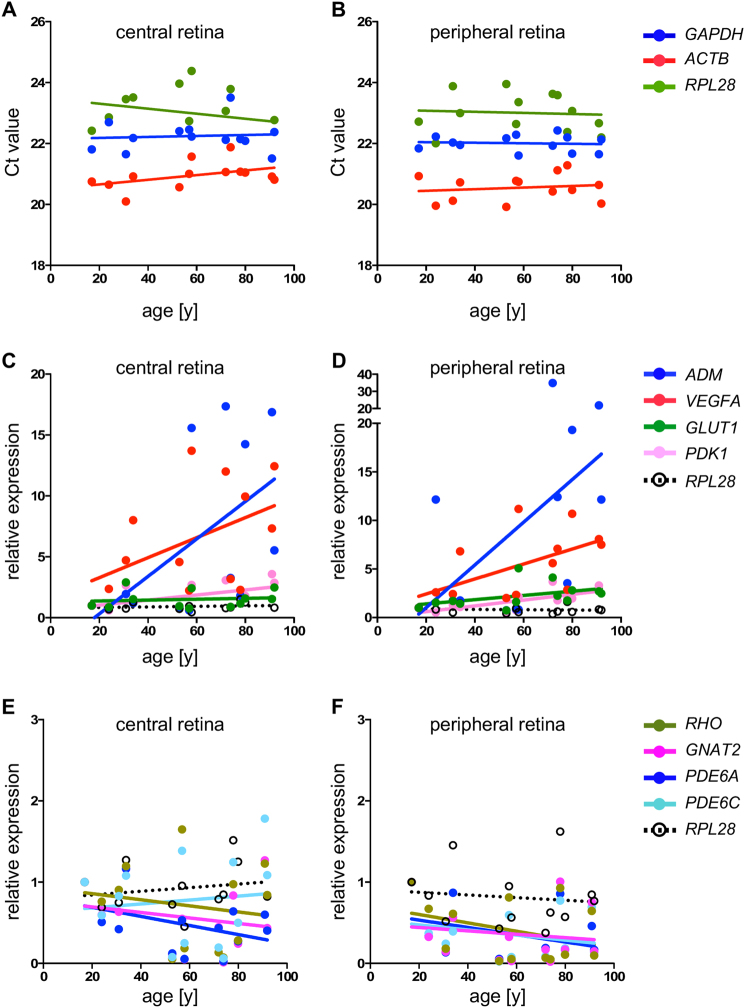
Expression of HIF controlled genes in the human retina. **a**, **b** Ct-values of housekeeping genes *GAPDH, RPL28*, and *ACTB* in the central and peripheral retina of 13 donor eyes. The central retina included the macular region whereas peripheral tissue was isolated from the mid periphery of the nasal retina. Ten nanogram cDNA were used as template. **c**, **d** Expression of HIF-regulated genes in the central and peripheral retina of 13 donor eyes relative to the expression level in a 17-year-old donor. **e**, **f** Expression of rod-specific and cone-specific genes in the central and peripheral retina of 13 donor eyes relative to the expression level in a 17-year-old donor. Expression of genes was normalized to *ACTB* and the housekeeping gene *RPL28* served as control (**c**–**f**). Dots: individual values. Lines: linear regression through all values. Note the tendency of hypoxia-regulated genes to be expressed at higher levels and photoreceptor-specific genes to be expressed at lower levels in aged human retinas

Reduced choroidal blood flow and tissue changes may reduce oxygen availability for photoreceptors in the aged retina. To test this hypothesis, we analysed expression of hypoxia-related genes in the central and peripheral retina from 13 human donors between the age of 17 and 92 years without diagnosed retinal pathologies (Table S1). Although post-mortem times differed considerably, Ct-values of the housekeeping genes glyceraldehyde 3-phosphate dehydrogenase (*GAPDH*), ribosomal protein L28 (*RPL28*) and beta-actin (*ACTB*) were relatively similar in all samples (Fig. 1a,b). Expression levels of genes of interest, however, varied strongly between donor eyes. Although *R*^2^ values were low (not shown), linear regression indicated a tendency for an age-dependent increased retinal expression of the hypoxia-regulated HIF-target genes adrenomedullin (*ADM*), *VEGFA* and to a lower extent also of pyruvate dehydrogenase kinase 1 (*PDK1*) and glucose transporter 1 (*GLUT1*) (Fig. 1c,d). In contrast, rod and cone-specific genes involved in phototransduction including rhodopsin (*RHO*), G protein subunit alpha transducin 2 (cone transducin, *GNAT2*) and rod phosphodiesterase 6A (*PDE6A*) showed the opposite trend and seemed to be expressed at reduced levels in the aged retina. The only exception was cone phosphodiesterase 6C (*PDE6C*) in the central retina that followed expression of the reference gene *RPL28* (Fig. 1e,f). Thus, the aged human retina may upregulate expression of hypoxia-related factors and reduce expression of photoreceptor-specific genes. Given that photoreceptors receive their oxygen largely from the choroidal blood with its reduced flow in older eyes, it is plausible that photoreceptors contribute to the upregulation of hypoxia-induced HIF target genes in response to a mild but chronic hypoxia that may develop during ageing.

### HIF1-dependent rod photoreceptor degeneration

Since rods are among the first cells to die in AMD [23] we inactivated *Vhl* specifically in rods to model a state of chronically activated HIF transcription factors in photoreceptors as it may be found in the aged human retina. Excision of floxed sequences starts at around PND7 and affects about 50% of rods at around 6 weeks [21, 24]. Although increased HIF1A levels were detected already at 3 weeks of age (Fig. S1A), expression of the HIF1 target genes *Adm* and *Vegf* was significantly increased only by 6 (*Adm;* Fig. S1B) and 11 weeks (*Vegf*; Fig. 5), respectively. At 3 and 6 weeks of age, ONL thickness, retinal morphology and expression of photoreceptor-specific as well as of survival (*Lif*, *Fgf2*), and stress (glial fibrillary acidic protein; *Gfap*) related genes were not affected in *rod*^*ΔVhl*^ mice (Fig. S1B-F), suggesting that development of retinal cells in *rod*^*ΔVhl*^ mice was not disturbed.

**Fig. 2:**
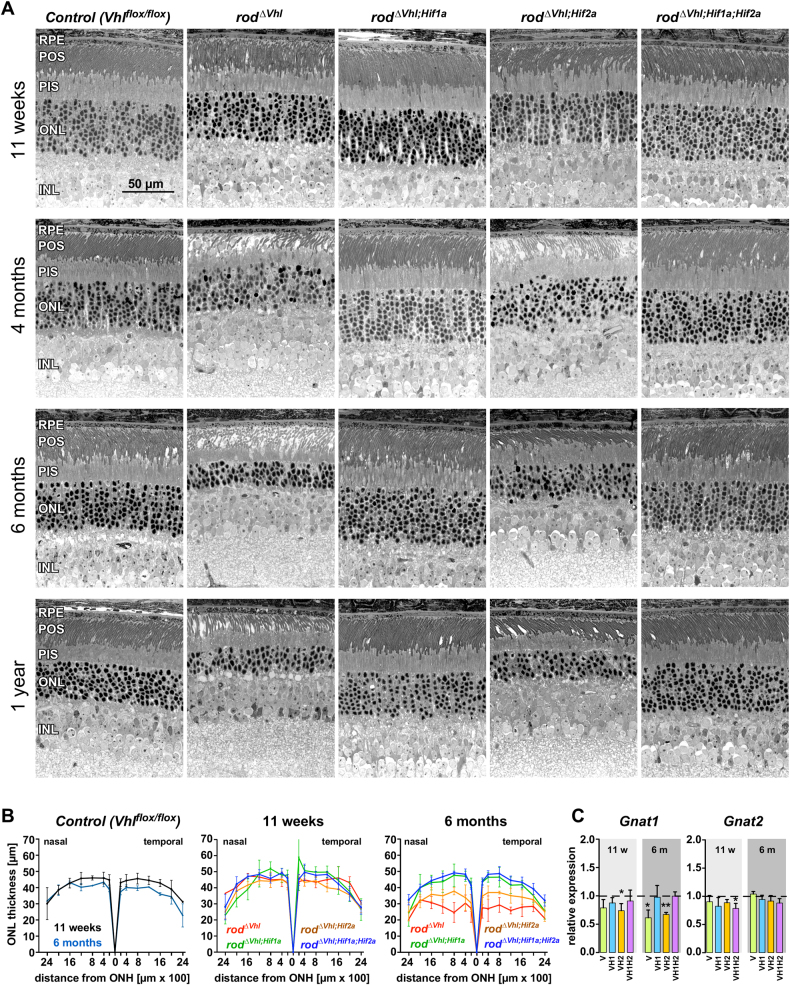
Consequences of chronically activated HIF transcription factors in rods. **a** Retinal morphology was tested at 11 weeks, 4 months, 6 months, and 1 year of age as indicated. Cre-negative *Vhl*^*flox/flox*^ mice served as controls. RPE retinal pigment epithelium, POS photoreceptor outer segments, PIS photoreceptor inner segments, ONL outer nuclear layer, INL inner nuclear layer. Scale bar: 50 μm. *N* ≥ 3. **b** ONL thicknesses in indicated strains were determined at 11 weeks and 6 months of age and are presented as spidergrams. Shown are means ± SD of *N* ≥ 3, except for *Vhl*^*flox/flox*^ mice at 6 months (*N* = 2). **c** Expression of *Gnat1* and *Gnat2* in *rod*^*ΔVhl*^ (V), *rod*^*ΔVhl;Hif1a*^ (VH1), *rod*^*ΔVhl;Hif2a*^ (VH2), and *rod*^*ΔVhl;Hif1a;Hif2a*^ (VH1H2) mice at 11 weeks and 6 months of age. Expression levels were calculated relative to their respective Cre-negative controls (set to 1; dotted line). Shown are means ± SD of *N* = 3-4. *P < 0.05; **P < 0.01. Individual comparisons between Cre-positive and Cre-negative mice of each genotype and time point were done using Student’s *t*-test

Genomic excision of floxed sequences was verified by PCR (Fig. S2A) and normoxic stabilization of HIF1A and HIF2A confirmed by Western blotting in retinas of 11-weeks-old *rod*^*ΔVhl*^ mice (Fig. S2B) [21]. Additional inactivation of *Hif1a* and/or *Hif2a* resulted in increased levels of HIF2A in *rod*^*ΔVhl;Hif1a*^, of HIF1A in *rod*^*ΔVhl;Hif2a*^, or in unchanged levels of both HIFA transcription factors in *rod*^*ΔVhl;Hif1a;Hif2a*^ mice (Fig. S2B). Rod-specific inactivation of *Vhl* caused a late onset and slowly progressing retinal degeneration reaching its maximal extent around 6 months of age (Fig. 2a,b). Since only about 50% of rods express Cre [21, 24], it is likely that surviving photoreceptors were Cre-negative and may thus not have activated HIF transcription factors. In contrast to other models of retina-specific *Vhl* inactivation [19, 20], *rod*^*ΔVhl*^ mice lacked a strong vessel phenotype. However, since some retinal sections suggested the presence of very few displaced vessels in the ONL (not shown), we cannot completely rule out that the retinal vasculature of *rod*^*ΔVhl*^ mice was mildly affected as well.

**Fig. 3:**
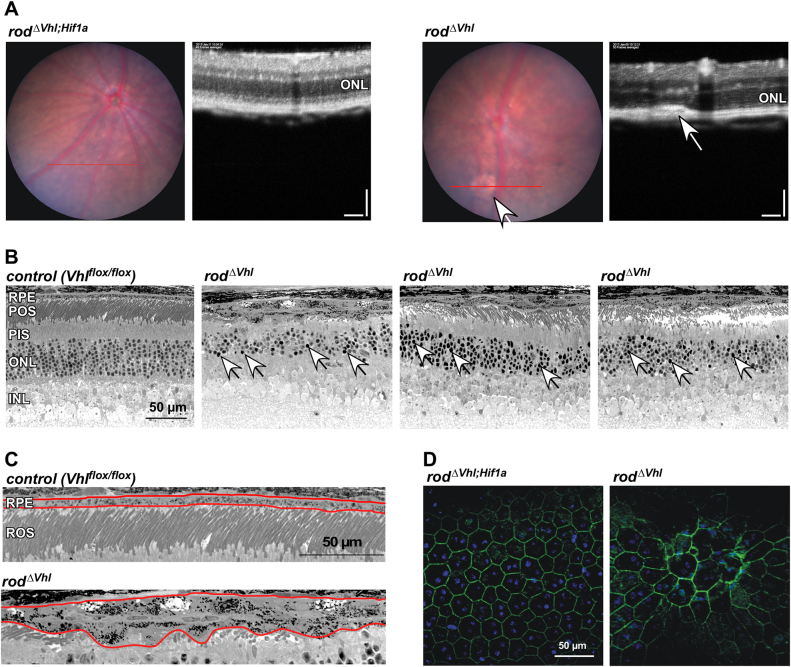
RPE phenotype in *rod*^*ΔVhl*^ mice at 4 months of age. **a** Fundus imaging and OCT scans of *rod*^*ΔVhl;Hif1a*^ mice (left, served as controls) and *rod*^*ΔVhl*^ mice (right) at 4 months of age. Red lines indicate the positions of the OCT scans. White arrows point to RPE irregularities in *rod*^*ΔVhl*^ mice. **b** Morphology of a control and three different *rod*^*ΔVhl*^ mice at 4 months of age. Shown are the focused regions where RPE irregularities were detected. Arrows: examples of pyknotic nuclei. **c** Higher magnifications of the RPE of a control and a *rod*^*ΔVhl*^ mouse. Red lines indicate borders of the RPE. **d** RPE flatmounts of *rod*^*ΔVhl;Hif1a*^ and *rod*^*ΔVhl*^ mice as indicated. Green: F-actin stained with phalloidin. Blue: nuclei stained with DAPI. RPE retinal pigment epithelium, POS photoreceptor outer segments, PIS photoreceptor inner segments, ONL outer nuclear layer, INL inner nuclear layer, Scale bars: 100 μm (**a**) and 50 µm (**b**–**d**)

Although an earlier cohort of *rod*^*ΔVhl*^ mice showed no functional loss or degeneration at 17 weeks of age [21], we detected photoreceptor degeneration already at 4 months in the cohort presented here (Fig. 2a). This slightly accelerated degeneration may be based on the prolonged light period (14 h instead of 12 h) and slightly increased light levels in our new animal facility. Interestingly, aged control mice that were housed under the same conditions showed a slight thinning of the ONL with time (Fig. 2a,b). Importantly, however, the slowly progressing, age-dependent photoreceptor degeneration in *rod*^*ΔVhl*^ mice was completely rescued by the additional inactivation of *Hif1a* or of *Hif1a* and *Hif2a* together, whereas inactivation of *Hif2a* alone had only a minor protective effect (Fig. 2a,b).

We tested expression of *Gnat1* and *Gnat2* as correlates for the presence of rods and cones, respectively. Both genes were expressed at control levels in *rod*^*ΔVhl*^ mice up to 6 weeks of age (Fig. S1B). At 11 weeks only *rod*^*ΔVhl;Hif2a*^ mice showed a slight reduction of *Gnat1* expression (Fig. 2c). At 6 months, however, *Gnat1* was significantly reduced in both *rod*^*ΔVhl*^ and *rod*^*ΔVhl;Hif2a*^ mice. *Gnat2* was not affected up to 6 months of age. These data support the conclusion that the degenerative phenotype had a late onset, primarily affected rods and depended on HIF1. HIF1-dependency was also reflected by the retinal stress marker GFAP that was elevated in *rod*^*ΔVhl*^ and *rod*^*ΔVhl;Hif2a*^ retinas but remained at basal levels when *Hif1a* was inactivated (Fig. S4).

Surprisingly, *rod*^*ΔVhl*^ mice also showed an RPE phenotype, albeit with variable severity and only in isolated areas. Fundus imaging detected few pale flecks that appeared in the OCT scans as hyperreflective regions in or close to the RPE at 4 months of age (Fig. 3a). Similar flecks and hyperreflective OCT signals were also described for human retinal degenerative diseases such as Stargardt dystrophy [25]. Thus, even in the presence of causative gene mutations for instance in *ABCA4*, degeneration of retinal cells and fundus appearance may not always be uniform across the retina. Cross sections of *rod*^*ΔVhl*^ retinas showed that the RPE was thicker and multi-layered in focused regions. Here, RPE cells appeared more heavily pigmented, partially vesiculated and enlarged (Fig. 3b,c). Some RPE cells had a less regular shape and made contact to an unusually high number of neighboring cells (Fig. 3d). Photoreceptors below affected RPE regions seemed less viable as many pyknotic nuclei were detectable in the ONL (Fig. 3b, arrows). Since this RPE phenotype was never observed in *rod*^*ΔVhl;Hif1a*^ mice we conclude that chronic activation of HIF1 in rods not only caused cell death *in cis* but also affected neighboring RPE cells in some parts of the retina.

**Fig. 4:**
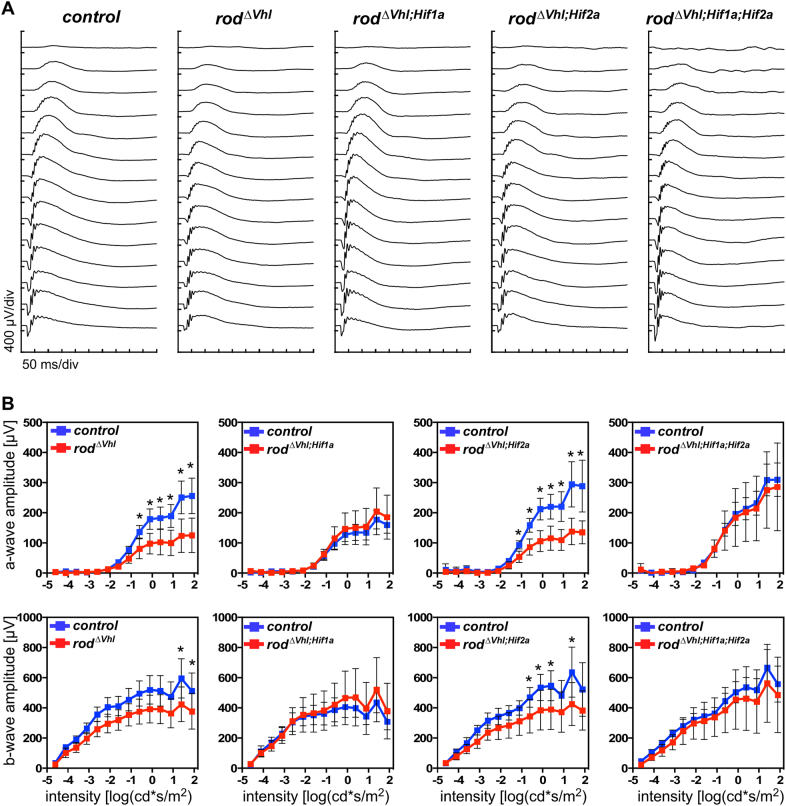
Scotopic retinal function of *rod*^*ΔVhl*^, *rod*^*ΔVhl;Hif1a*^, *rod*^*ΔVhl;Hif2a*^, and *rod*^*ΔVhl;Hif1a;Hif2a*^ mice at 6 months of age. **a** Scotopic ERG traces were recorded after light stimuli of increasing light intensities. Shown are representative traces. Cre-negative *Vhl*^*flox/flox*^ mice served as controls. **b** Scotopic a-wave and b-wave amplitudes plotted as a function of stimulus intensity. Control mice were Cre-negative littermates of the respective strains. Shown are averages ± SD. *N* = 6 eyes (3 mice), except for controls of *rod*^*ΔVhl;Hif2a*^ (*N* = 5) and of *rod*^*ΔVhl;Hif2a*^ (*N* = 7), and for *rod*^*ΔVhl;Hif1a;Hif2a*^ (*N* = 4). *P < 0.05. Two-way ANOVA with Sidak’s multiple comparison test

The tissue phenotype was mirrored by retinal function. At 6 months of age, *rod*^*ΔVhl*^ and *rod*^*ΔVhl;Hif2a*^ mice had significantly reduced scotopic a- and b-wave amplitudes at higher flash intensities. Mice lacking *Hif1a* in addition to *Vhl* (*rod*^*ΔVhl;Hif1a*^ and *rod*^*ΔVhl;Hif1a;Hif2a*^ mice), however, retained normal function (Fig. 4). This shows that adult photoreceptors do not require HIF1 for function or survival. Although *Vhl* inactivation was rod-specific, cone-driven photopic b-wave amplitudes at higher light intensities were also reduced in *rod*^*ΔVhl*^ mice suggesting that loss of VHL in rods affected cone function or survival. Interestingly, the reduction in cone-driven ERG responses was prevented by the additional inactivation of *Hif1a* and/or *Hif2a* (Fig. S3). The reason for this is unclear but may point to mechanisms in rods that can affect cone function in a HIF1- and HIF2-dependent manner. However, this needs further testing.

**Fig. 5:**
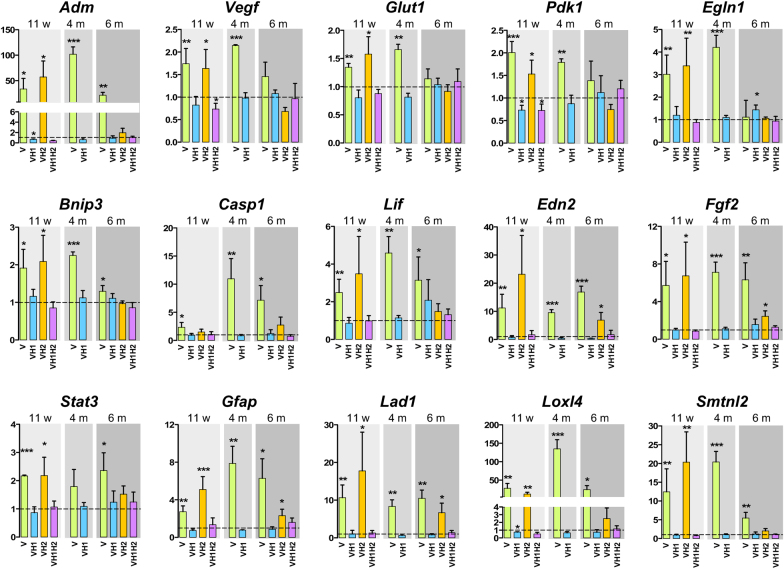
Retinal gene expression. Retinal expression of indicated genes was tested in retinas of *rod*^*ΔVhl*^ (V), *rod*^*ΔVhl;Hif1a*^ (VH1), *rod*^*ΔVhl;Hif2a*^ (VH2), and *rod*^*ΔVhl;Hif1a;Hif2a*^ (VH1H2) mice at 11 weeks, 4 months and 6 months of age. Expression levels were calculated relative to their respective Cre-negative controls (set to 1; dotted line). Shown are means ± SD of *N* = 3-4. *P < 0.05; **P < 0.01; ***P < 0.001. Individual comparisons between Cre-positive and Cre-negative mice of each genotype were done using Student’s t-test

### The transcriptomic response

**Fig. 6:**
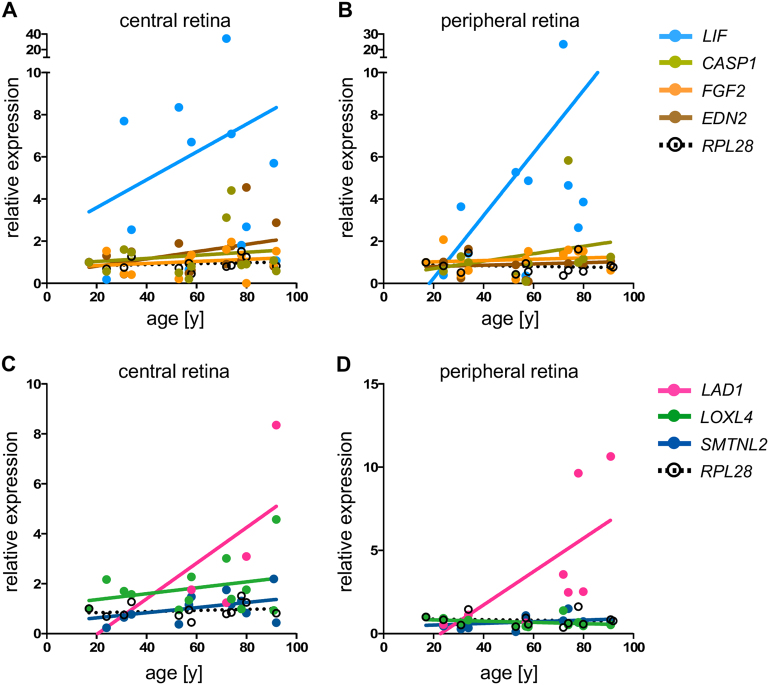
Age-dependent gene expression in the human retina. **a**, **b** Expression of *LIF*, *CASP1*, *FGF2,* and *EDN2* in the central and peripheral human retina of 13 donor eyes. **c**, **d** Expression of *LOXL4*, *LAD1,* and *SMTNL2* in the central and peripheral human retina of 13 donor eyes. Expression levels were normalized to *ACTB* and are shown relative to the levels in the retina of a 17-year-old donor. The housekeeping gene RPL28 served as control. Dots: individual values. Lines: linear regression through all values

Increased expression of the HIF1 targets *Adm*, *Vegf*, *Glut1*, *Pdk1*, and Egl-9 family hypoxia-inducible factor 1 (*Egln1*) in *rod*^*ΔVhl*^ and *rod*^*ΔVhl;Hif2a*^ mice verified that HIF1 was transcriptionally active at 11 weeks of age (Fig. 5). Normal expression levels of these genes in *rod*^*ΔVhl;Hif1a*^ and *rod*^*ΔVhl;Hif1a;Hif2a*^ mice confirmed their HIF1-dependency. Expression of these genes was less increased at 6 months, most likely because most rods lacking *Vhl* have already degenerated at this time point (Fig. 2a). BCL2 interacting protein 3 (*Bnip3*), caspase-1 (*Casp1*), leukemia inhibitory factor (*Lif*), endothelin-2 (*Edn2*), fibroblast growth factor-2 (*Fgf2*), signal transducer and activator of transcription-3 (*Stat3*) and *Gfap* are upregulated in degenerating retinas and connected to cell death or cell survival [26–29]. These genes were activated exclusively in the degenerating retinas of *rod*^*ΔVhl*^ and *rod*^*ΔVhl;Hif2a*^ mice. It is noteworthy that *LIF*, and to a lesser extent also *EDN2* and *CASP1*, showed a trend of increased expression in the aged human retina (Fig. 6) pointing to a stress response that is more likely to be activated in the senescent retina.

In addition to HIF1A and HIF2A, levels of pSTAT3 were also increased in *rod*^*ΔVhl*^ and *rod*^*ΔVhl;Hif2a*^ mice (Fig. S4). HIF1 may cooperate with STAT3 to regulate HIF1-specific gene expression [30], may increase STAT3 activity through decreasing suppressor of cytokine signalling 3 (SOCS3) [31] and may directly interact with constitutively active STAT3 [32]. Thus, regulation of genes in *rod*^*ΔVhl*^ and *rod*^*ΔVhl;Hif2a*^ mice may be attributed to HIF1, STAT3 or to both transcription factors.

To detect novel genes that were regulated by chronically active HIFs in rods, we determined the retinal transcriptomes of *rod*^*ΔVhl*^, *rod*^*ΔVhl;Hif1a*^ and of *Vhl*^*flox/flox*^*;Hif1a*^*flox/flox*^ controls at 11 weeks of age (Fig. S5). Tables S2-S4 show the top up-regulated and down-regulated genes, and list genes that may be regulated by HIF1, HIF2 or STAT3, or a combination of those (Table S2); by HIF1, STAT3 or both of them (Table S3); and by mainly HIF2 (Table S4). For a detailed description of the transcriptomic data, see the supplemental information including Tables S2-S7 and Files S1-S3. Among the top upregulated genes in *rod*^*ΔVhl*^ mice we identified and verified *Adm* and *Edn2* by real-time PCR (Fig. 5). Among the genes with less well known functions, we verified lysyl oxidase like 4 (*Loxl4*), ladinin 1 (*Lad1*) and smoothelin like 2 (*Smtnl2*) establishing them as HIF-responsive genes in mouse rods (Fig. 5).

### Gene expression in the human retina

The gene expression pattern in mice may be relevant to understand ageing processes in human retinas. *LIF* levels were not only increased in the stressed (degenerating) mouse retina (Fig. 5) but also in the aged human retina. *EDN2*, another gene of the LIF-signalling pathway [27], and *CASP1*, a proinflammatory protein [33] implicated in inflammasome-triggered pyroptosis [34], also revealed trends of increased expression in aged human retinas providing evidence of potential inflammatory and stress-related processes in the old eye (Fig. 6a,b).

Although *LAD1* was detected in only five of the central and nine of the peripheral retinal samples, it showed a clear tendency of increased expression with age. *LAD1* encodes an anchoring filament [35] and might thus be involved in the structural adaptation to reduced oxygen levels in rods. Expression of *LOXL4* and *SMTNL2* followed a similar trend as observed for *RPL28* and were thus without apparent regulation during ageing (Fig. 6c,d). *LOXL4* encodes a lysyl oxidase-like protein implicated in collagen remodelling and metastasis formation in cancer [36]. Its expression in the retina has not yet been described but it might be involved in extracellular matrix remodelling during hypoxic periods. Even less is known about *SMTNL2*, except that it may be a target for c-Jun N-terminal kinase [37]. It will be of interest to localize these proteins in the normal and hypoxic retina and to elucidate their functions.

### Anti-*Hif1a* gene therapy

**Fig. 7:**
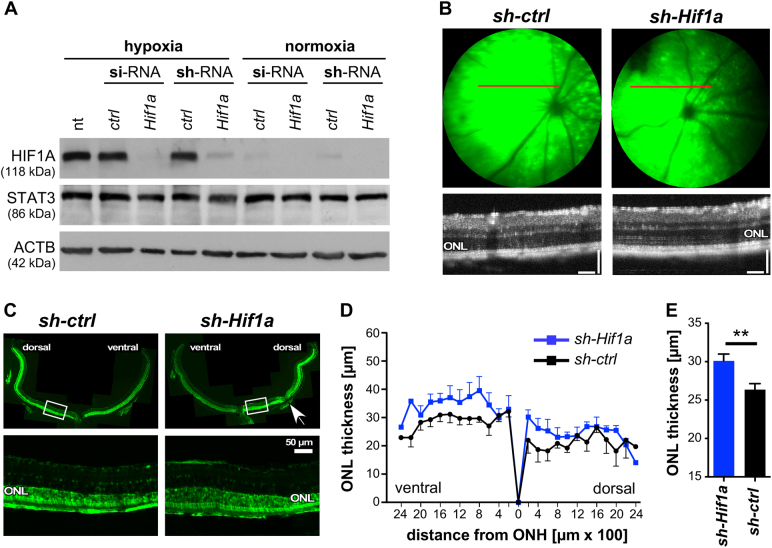
Efficacy of RNA interference and anti-*Hif1a* gene therapy. **a** NIH3T3 cells were transiently transfected with *si-Hif1a* RNA or stably transfected with *sh-Hif1a*, or treated with the respective scrambled controls as indicated, followed by exposure to 0.2% oxygen (hypoxia) or normoxia. Not transfected cells (nt) exposed to hypoxia served as controls. Levels of HIF1A, STAT3, and ACTB were detected by Western blotting. **b** Fluorescent fundus imaging (upper panels) and OCT scans (lower panels) of eyes that received a subretinal injection of AAV2/8(Y733F) particles expressing either the control sh-RNA sequence (left, *sh-ctrl*) or the *sh-Hif1a* sequence (*sh-Hif1a*, right) together with EGFP. The red line in the fundus image indicates the position of the OCT scan. **c** Retinal cross sections of mice injected with the control (left, *sh-ctrl*) or the *sh-Hif1a* virus (right, *sh-Hif1a*). Lower panels are higher magnifications of retinal areas marked with a white square in the upper panels. White arrow: damage due to injection. Scale bar: 50 µm. **d** Spidergram of the ONL thickness 5 months after injection of the control (black line, *sh-ctrl*) or the *sh-Hif1a* virus (blue line, *sh-Hif1a*). All mice were injected at 5 weeks and analysed at 6 months of age. Areas of obvious injection-inflicted damage (see white arrow in (**c**) as an example) were excluded from quantification. Shown are means ± SEM. *N* = 6. **e** Quantification of the ONL thickness of *sh-Hif1a* (blue bar) and *sh-ctrl* (black bar) virus injected mice at 6 months of age. All data points (115 for *sh-Hif1a*; 99 for *sh-ctrl*) shown in (**d**) were included. Shown are means ± SEM. **P < 0.01. ONL outer nuclear layer

To reduce toxic levels of HIF1 and protect photoreceptors in *rod*^*ΔVhl*^ mice by a gene therapy approach, we used AAV-mediated expression of an shRNA against *Hif1a*. Test of the shRNA and corresponding siRNA in NIH3T3 cells showed a highly efficient downregulation of HIF1A, whereas STAT3 was not affected (Fig. 7a). AAV2/8(Y733F) viral particles carrying the *sh-Hif1a* or a scrambled sequence (*sh-ctrl*) as well as an *Egfp* expression cassette (Fig. S6) were injected into the subretinal space of *rod*^*ΔVhl*^ mice at 5 weeks of age. Fundus fluorescence imaging at 6 months showed surprisingly widespread EGFP expression (Fig. 7b) that was largely confined to ONL and RPE (Fig. 7c). OCT scans indicated a more regular ONL layering and measurements showed significantly increased ONL thickness in retinas of *sh-Hif1a* injected eyes. The strongest effect was observed in the ventral retina and dorsally close to the optic nerve head (Fig. 7d,e). These data strongly support the hypothesis that an anti-*Hif1a* therapy may protect photoreceptors in situations of chronic tissue hypoxia.

## Discussion

Tissue hypoxia is relevant for many pathologies affecting the retinal and choroidal vasculature in diseases such as diabetic retinopathy and neovascular AMD. Targeting the HIF-regulated growth factor VEGF shows great benefit for patients suffering from wet AMD. However, chronic hypoxia may also develop in the normal retina during ageing as indicated by the increased expression of HIF target genes in retinas of older donors (Fig. 1). Since chronic HIF activity led to age-dependent photoreceptor degeneration in mice, we and others hypothesize that chronically increased HIF activity in aged human retinas may be involved in AMD pathogenesis in at least some patients [4, 13]. This hypothesis, however, does not imply that elevated HIF activity is toxic *per se* but that it may be one of several factors contributing to multifactorial pathologies found in diseases such as dry AMD. Thus, reducing HIF levels may be a potential strategy to eliminate one of the disease-contributing factors. This may lessen the burden for cells and potentially result in delaying or even preventing disease onset and/or progression.

Earlier we showed that a short period of systemic hypoxic preconditioning induces a response that protects photoreceptors [38]. Protection is either HIF-independent or requires HIF activity in cells other than photoreceptors [39, 40]. Also, after mice are removed from acute hypoxia, retinal HIF1A returns to basal levels in less than one hour allowing cells to quickly re-establish a normoxic gene expression profile [38, 41]. In contrast, the degeneration-inducing chronic activation of HIF1 in rods over weeks or months may induce lasting changes in the cellular metabolism, which may lead to deficits such as reduced energy production and finally to cell death. Indeed, increased expression of *Pdk1* and *Glut1* in *rod*^*ΔVhl*^ mice indicated a metabolic shift that may have resulted in reduced oxidative phosphorylation and thus reduced production of ATP in rods. If long-lasting, this may curtail metabolic support and weaken the cells’ ability to survive periods of stress.

Gene expression profiling revealed that HIF1-induced degeneration in *rod*^*ΔVhl*^ mice followed similar signalling mechanisms as detected in other models of retinal degeneration. This included activation of the *Lif*/*Edn2*/*Fgf2* pathway [27, 28, 42] with a late increase in *Casp1* expression [29]. In addition, we detected a variety of differentially expressed HIF1 target genes in retinas of *rod*^*ΔVhl*^ mice, even before the onset of extensive degeneration. Among those, *LAD1, LOXL4*, and *SMTNL2* were also detected in the human retina, with *LAD1* showing a tendency of increased expression with age.

Photoreceptor degeneration in *rod*^*ΔVhl*^ mice depended on intrinsic HIF1, with only a minor contribution of HIF2. This is of relevance and in marked contrast to RPE cells where chronically active HIF2, but not HIF1, leads to RPE loss [22]. Intriguingly, the changes in cellular metabolism resulting from chronically active HIF1 in rods or HIF2 in RPE not only caused cell death *in cis* but also affected neighboring cells (Fig. 3 and [22]) either through secreted factors, accumulation of toxic cellular debris or reduced metabolic support. The differential toxicity of chronically active HIF1 and HIF2 for rods and RPE may reflect the highly divergent function of these two cell types and indicates that different aspects of the hypoxic response can be toxic. These might be altered lipid handling for RPE cells [22] and energy metabolism (see above) or other factors for rods.

Our rescue experiments showed that inactivation of HIF1A led to a thicker ONL in *rod*^*∆Vhl*^ mice. This could result either from prevention of cell death or from cell proliferation leading to tissue regeneration. However, the second explanation is unlikely since the ONL in mice lacking HIF1A in rods *(rod*^*∆Vhl;Hif1a*^ (Fig. 2), *rod*^*∆Hif1a*^ and *rod*^*∆Hif1a;Hif2a*^ mice [39, 40]) retained a comparable thickness to the ONL in wild type mice. Even the inactivation of *Hif1a* in most retinal cells during development does not increase ONL thickness [43]. Thus, lack of *Hif1a* may not affect cell proliferation but rescues rods by preventing cell death.

Although it will be important to define the processes that lead to HIF1-dependent rod cell death and to HIF2-dependent degeneration of RPE cells, their detailed knowledge might not be essential to establish therapeutic approaches. Our genetic experiments showed that inactivation of *Hif1a* at the beginning of the hypoxic response rescued photoreceptor cells. Patients, however, may seek medical advice only once pathological processes have commenced. Thus, it is important to establish an interventional therapy. We showed that an RNA interference strategy through the AAV-based delivery of an sh-RNA against *Hif1a* may be an applicable strategy to protect photoreceptors in conditions of chronic HIF activity. However, from our data and data published by others [22] it seems clear that a therapy targeting solely *Hif1a* in photoreceptors will not be sufficient for patients. Since reduced choroidal blood flow in the ageing eye affects oxygenation of both photoreceptors and RPE, a combination therapy that targets both cell types and both *HIF1* and *HIF2* transcription factors may be needed. For a therapy to be successful it is therefore mandatory that inactivation of HIF1 and HIF2 in adult photoreceptors and RPE does not lead to toxic effects. We recently showed that *Hif1a* and *Hif2a* can be safely inactivated in adult rods [39]. Similarly, inactivation of *Hif1* alone in RPE cells had no obvious consequences [44] and we have collected preliminary evidence that simultaneous inactivation of both *Hif1a* and *Hif2a* did not adversely affect RPE (not shown). This is further supported by the normal appearance of the retina and RPE in mice lacking *Vhl*, *Hif1a* and *Hif2a* in the RPE [22]. However, since a beneficial effect of *Hif1a* after retinal detachment has been suggested [45], it may be advisable not to inhibit HIF1 completely but to merely reduce its expression or activity.

In conclusion, our data show that a chronic activation of HIF transcription factors in photoreceptors induces retinal degeneration in a HIF1-dependent manner. Since several hypoxia-related genes may be expressed at higher levels in the retina of older donors, hypoxia-related mechanisms may be relevant in the ageing human retina and contribute to retinal diseases such as AMD. As HIF transcription factors do not seem essential for adult photoreceptors and RPE, anti-HIF therapies may prove beneficial for patients.

## Materials and methods

### Mice, genotyping and excision of floxed sequences

All mice were maintained as breeding colonies at the Laboratory Animal Services Center (LASC) of the University of Zurich in a 14 h: 10 h light-dark cycle with lights on at 6 am and lights off at 8 pm. Mice had access to food and water *ad libitum*. Average light intensity at cage levels was 60–150 lux, depending on the position in the rack.

*Vhl*^*flox/flox*^ [46], *Hif1a*^*flox/flox*^ [47], *Hif2a*^*flox/flox*^ [48], and OpsinCre (LMOPC1; [24]) mice were intercrossed to obtain *rod*^*ΔVhl*^ (*Vhl*^*flox/flox*^*;OpsinCre*), *rod*^*ΔVhl;Hif1a*^ (*Vhl*^*flox/flox*^*;Hif1a*^*flox/flox*^*;OpsinCre*), *rod*^*ΔVhl;Hif2a*^ (*Vhl*^*flox/flox*^*;Hif2a*^*flox/flox*^*;OpsinCre*), and *rod*^*ΔVhl;Hif1a;Hif2a*^ (*Vhl*^*flox/flox*^*;Hif1a*^*flox/flox*^*;Hif2a*^*flox/flox*^*;OpsinCre*) mice. All breeding pairs were heterozygous for *OpsinCre* and pups without *OpsinCre* served as littermate controls. Rod-specific *Cre* expression in *OpsinCre* mice starts around postnatal day 7 and increases up to 6 weeks of age [24]. All mice were homozygous for the *Rpe65*_*450Leu*_ variant [49]. Genotyping was performed by conventional PCR using pairs as specified in Table S8. To detect alleles carrying CRE-mediated deletions of the floxed sequences, genomic DNA was isolated from retinal tissue and amplified by PCR using the primer pairs shown in Table S9. All PCR products were run on agarose gels and visualized using ethidium bromide.

### Human retina samples

Peripheral nasal retina and central retina including the macula were isolated and frozen separately. RNA was isolated using the RNeasy kit (Qiagen, Hilden, Germany). cDNA synthesis and real-time PCR were performed as described for the mouse samples (see below) using human-specific primer pairs (Table S10).

### Western blotting

Isolated retinas were sonicated in 200 µl of 100 mM Tris/HCl (pH 8,0). After centrifugation (1000 × *g*; 3 min) protein concentrations were determined in the supernatants using Bradford reagent (BioRad, Hercules, CA, USA). Standard SDS-PAGE and Western blotting were performed using the following primary antibodies: rabbit anti-HIF1A (1:2000–1:4000, NB100-479, Novus Biologicals, Cambridge, UK); rabbit anti-HIF2A (1:1000, PAB12124, Abnova, Aachen, Germany); rabbit anti-pSTAT3_Tyr705_ (1:500, #913L, Cell Signaling Technology, Danvers, MA, USA); rabbit anti-STAT3 (1:1000, D3Z2G, Cell Signaling Technology); mouse anti-GFAP (1:1000, G3893-Clone G-A-5, Sigma, Buchs, Switzerland); mouse anti-ACTB (1:10,000, A5441, Sigma). Primary antibodies were diluted in 5% non-fat blocking milk (BioRad, Cressier, Switzerland) in TBST, added to the membrane and incubated over night at 4 °C with gentle agitation. Appropriate HRP-conjugated secondary antibodies were added and signals detected using the Western lightning chemiluminescence reagent (PerkinElmer, Waltham, MA, USA). Signals were analysed using X-ray films.

### Morphology, RPE flatmounts, and immunofluorescence

Eyes were marked at the dorsal limbus, enucleated, fixed in glutaraldehyde (2.5% in cacodylate buffer) for 12–24 h at 4 °C, trimmed, post-fixed in 1% osmium tetroxide and embedded in Epon 812 as described [28]. Tempero-nasal cross-sections of 0.5 µm were cut through the optic nerve head, stained with toluidine blue and analysed by light microscopy (Zeiss, Axioplan, Jena, Germany). The thickness of the outer nuclear layer was measured at indicated distances from the optic nerve head using the Adobe Photoshop CS6 ruler tool (Adobe Systems, Inc., San Jose, CA, USA). RPE flatmounts were prepared and stained as described [50]. Briefly, eyes were enucleated and incubated in 2% paraformaldehyde for 5 min. After removal of cornea and lens and incubation in phosphate buffer containing 140 mM NaCl and 2.7 mM KCl for 20 min, the detached retina was gently removed and the eyecup prepared for flat mounting by making four incisions. The resulting clover-leafed eyecup was post-fixed in 4% PFA for 1 h. Alexa Fluor 488-phalloidin (1:100, A12379, Thermo Fischer Scientific, Waltham, MA, USA) was applied for 2 h and nuclei stained with DAPI for 30 min. Flatmounts were analysed using a fluorescence microscope (Axioplan 2, Zeiss, Switzerland).

### Electroretinography, fundus imaging, and OCT

Pupils of dark-adapted mice were dilated with Cyclogyl 1% (Alcon Pharmaceuticals, Fribourg, Switzerland) and Neosynephrine 5% (Ursapharm Schweiz GmbH, Roggwil, Switzerland). Mice were anesthetized by a subcutaneous injection of ketamine (85 mg/kg, Parke-Davis, Berlin, Germany) and xylazine (4 mg/kg, Bayer AG, Leverkusen, Germany). A drop of atropin 0.5% (Thea Pharma, Schaffhausen, Switzerland) was applied to each cornea just prior to placing gold ring electrodes onto each cornea. Recordings were done with an LKC UTAS Bigshot unit (LKC Technologies, Inc. Gaithersburg, MD, USA) using flash intensities from −50 db (0.000025 cd*s/m^2^) to 15 db (79 cd*s/m^2^) for scotopic and from −10 db (25 cd*s/m^2^) to 25 db (790 cd*s/m^2^) for photopic responses. Before photopic responses were recorded, mice were light-adapted for 5 min. Ten recordings were averaged per light intensity.

For fundus imaging and OCT scans, pupils were dilated and mice anesthetized as described above. A drop of 2% methocel (OmniVision AG, Neuhausen, Switzerland) was applied to keep eyes moist. Fundus images and OCT scans were acquired using the Micron IV system (Phoenix Research Labs, Pleasanton, CA, USA) as described [51].

### RNA isolation, gene chip analysis, and semi-quantitative real-time PCR

Total RNA was purified from retinas using RNA isolation kits (RNeasy, Qiagen, Hilden, Germany; Macherey-Nagel, Düren, Germany) with an on-column DNAse treatment. RNA concentrations were measured using a Nanodrop spectrophotometer (Thermo Fisher Scientific). The retinal transcriptomes of 11-weeks-old *rod*^*ΔVhl*^*, rod*^*ΔVhl;Hif1a*^, and *Vhl*^*flox/flox*^*;Hif1a*^*flox/flox*^ (controls, ctrl) mice were determined at the Functional Genomics Center of the University of Zurich using 'Agilent Mouse 4 × 44k V2' gene chips. RNA isolates from four individual mice per genotype were analysed.

For real-time PCR, cDNA was prepared from total RNA using oligo(dT) and M-MLV reverse transcriptase (Promega, Dübendorf, Switzerland). Ten nanogram cDNA was amplified in a LightCycler480 with SYBR Green I master mix (Roche Diagnostics). Primer pairs (Table S10) avoided known SNPs and were designed to span large intronic regions. Levels were normalized to *Actb* as reference gene and relative expression was calculated using the comparative threshold cycle method (ΔΔC_T_). At least three mice per strain were used for each time point and strain. Deletion strains were compared to their respective control strain, which expression was set to 1 for each time point.

### siRNA and shRNA-mediated gene silencing in NIH3T3 cells

NIH3T3 cells (ATCC® CRL-1658™) were plated on 6-well plates and grown in DMEM + 10% heat-inactivated fetal bovine serum (FBS, Gibco, Thermo Fisher Scientific) and 1% penicillin-streptomycin (Gibco) at 37 °C and 5% CO_2_ for 24 h. Cells were transfected with 80 pmol anti-*Hif1a* siRNA (5′-GUGGAUAGCGAUAUGGUCAUU-3′) using lipofectamine RNAiMAX (Invitrogen, Thermo Fisher Scientific) and Opti-MEM (Gibco). A scrambled sequence (AllStars negative control siRNA; Qiagen) served as control. Twenty four hours after transfection, cells were or were not exposed to hypoxia (0.2% O_2_, 5% CO_2_) at 37 °C for 6 h. After washing with pre-warmed PBS, cells were collected with sample buffer and Western blotting was performed as described above.

To test the efficiency of the corresponding anti-*Hif1a* shRNA, we used lentivirus-pseudotyped particles that were produced using HEK293T cells (ATCC® CRL-3216). Briefly, cells were plated in 75 cm^2^ culture flasks and co-transfected with anti-*Hif1a* shRNA or non-target shRNA (Sigma) using the ViraPower lentiviral expression vector system and lipofectamine 3000 (Invitrogen, Thermo Fisher Scientific). The following day, the medium was replaced with fresh medium containing 10% FBS and 1% penicillin-streptomycin. The supernatant was collected 72 h post transfection, centrifuged to pellet large particles and debris, and filtered through a 0.45 μm filter (Merck&Cie, Schaffhausen, Switzerland). The filtrate was used to transduce NIH3T3 cells with lentiviral particles containing *sh-Hif1a* or *sh-ctrl* and 6 μg/mL polybrene, followed by selection with 2 μg/mL puromycin. To test *sh-Hif1a*-mediated downregulation of HIF1A in hypoxia, cells were exposed to 0.2% O_2_ for 6 h and harvested immediately thereafter. Protein homogenates were used for Western blotting as described above.

### AAV–mediated shRNA expression and analysis

pAAV2.1-U6-*shHif1a*-CMV-EGFP (3 × 10^11^ vector genomes (vg)/μL) and pAAV2.1-U6-*shcontrol*-CMV-EGFP (3 × 10^10^ vg/μL) (Fig. S6) were packaged as AAV2/8Y733F and produced as described recently [52]. For subretinal injections, the pupils were dilated and mice were anesthetized as described above. Viscotears (Bausch & Lomb Swiss AG, Zug, Switzerland) were applied to keep the eyes moist. 1.5 × 10^10^ total vg were injected into the subretinal space using the NanoFil Intraocular Injection Kit (WPI, Berlin, Germany). To visualize and control the injection, we added a small amount of fluorescein (0.1 mg/mL, Akorn Inc., IL, USA) to the AAV solution. Mice were injected at 5 weeks and analysed at 6 months of age. After euthanasia, eyes were marked nasally, enucleated and fixed in 4% paraformaldehyde for 1 h at 4 °C as described [53]. Dorsoventral cryosections (12 μm) were cut, counterstained with DAPI (4′,6-Diamidine-2′-phenylindole dihydrochloride, Roche, Basel, Switzerland) and analysed by fluorescence microscopy (Axioplan; Zeiss, Jena, Germany). The thickness of the outer nuclear layer was measured as described above. Tissue areas that were damaged due to injections were excluded from measurements. The average ONL thicknesses after treatment with *sh-Hif1a (*115 measurements) or *sh-ctrl* (99 measurements) viruses, excluding the optic nerve head, were calculated [54] and compared.

### Statistical analysis

a-wave and b-wave amplitudes of ERG recordings were tested using 2-way ANOVA with Sidak’s multiple comparison test. Gene expression in deletion strains was compared to their respective control strains at each time point individually and evaluated by Student’s *t*-test (GraphPad Prism, San Diego, CA, USA). Student’s *t*-test was also used to compare the overall ONL thickness of *sh-Hif1a* and *sh-ctrl* treated mice. *P*-values < 0.05 were considered to show significant differences. Linear regression of gene expression in human samples was calculated using Prism software (GraphPad, La Jolla, USA).

### Study approval

Mouse experiments were performed in accordance with the regulations of the Veterinary Authority of Zurich (ZH109/2013; ZH219/2012; ZH216/2015; ZH141/2016) and with the statement of ‘The Association for Research in Vision and Ophthalmology’ for the use of animals in research. Human retinas were collected from donor eyes that were enucleated post-mortem at the University Hospital Zurich, Switzerland. Collection of retinas was approved by the ethics committee of Zurich, Switzerland (BASEC-Nr: PB_2017-00550) and adhered to the tenets of the Declaration of Helsinki.

## Electronic supplementary material

Supplemental Material

Supplemental File S1

Supplemental File S2

Supplemental File S3
