# Supplementary material for: Hif1a inactivation rescues photoreceptor degeneration induced by a chronic hypoxia-like stress
Source: Cell Death Differ. 2018 Apr 17;25(12):2071–85. doi: 10.1038/s41418-018-0094-7 (PMC6261999; doi:10.1038/s41418-018-0094-7)
Supplement: Supplementary file 1 — Supplemental Material [file 41418_2018_94_MOESM1_ESM.docx]

**Supplemental Information**

***Impact of rod-specific Vhl deletion in 3 and 6 weeks old mice***

To verify that rod-specific deletion of *Vhl* resulted in a late onset degeneration in the adult retina, we investigated 3 weeks and 6 weeks old *rod^ΔVhl^* and control (*Vhl^flox/flox^*) mice. Although Opsin-Cre-mediated excision of floxed sequences starts at around PND7, it reaches its maximal extent around 6 weeks of age ^1^. Nevertheless, levels of HIF1A were elevated already at 3 weeks of age (Fig, S1A). The increased presence of HIF1A, however, did not result in increased expression of HIF1 target genes at this time point. *Adm* and *Vegf* were not significantly different from controls and only showed increased expression levels at 6 weeks (*Adm*, Fig S1B) or 11 weeks (*Vegf*, Fig. 5) of age, respectively. The reason for the delayed activation of transcription by elevated HIF1A levels is currently unknown and will be elucidated in future experiments. At 3 and 6 weeks of age, ONL thickness and retinal morphology were not affected in *rod^ΔVhl^* mice (Fig. S1C-F), and expression levels of rod and cone specific genes such as *Gnat1* and *Gnat2* were not different from control levels (Fig. S1B). This suggests that rod-specific deletion of Vhl did not affect survival of photoreceptors up to 6 weeks of age. This is further supported by normal expression levels of survival (*Lif*, *Fgf2*) and stress (glial fibrillary acidic protein; *Gfap*) related genes at 3 and 6 weeks of age (Fig. S1B). We conclude that retinal degeneration in *rod^ΔVhl^* mice started in the adult retina and was not caused by developmental deficits.

***Verification of mouse strains***

To verify successful deletion of floxed sequences, genomic DNA was isolated from the retina and PCR-amplified using the three primers shown in Table S9. Since genomic DNA was isolated from all retinal cells and Opsin-Cre is specifically expressed in rods ^1, 2^, the *2-loxP* fragment indicating the presence of non-excised DNA was always detected. Nevertheless, the *1-loxP* fragment indicating Cre-mediated deletion of floxed sequences was present in all Cre-positive mice (Figure S2A). Deletion of *Vhl* caused increased levels of HIF1A and HIF2A proteins already at 11 weeks of age (Figure S2B). Additional deletion of *Hif1a* reduced HIF1A but not HIF2A to basal levels. In contrast, additional deletion of *Hif2a* reduced levels of HIF2A but not of HIF1A to controls. Additional inactivation of both, *Hif1a* and *Hif2a* prevented accumulation of both proteins. Retinal samples of hypoxic and normoxic wild type mice served as controls. These data show that Cre-mediated deletion of floxed sequences was successful and resulted in the anticipated levels of HIF transcription factors.

***Deletion of Vhl in rods may affect function in cones***

Photopic ERG recordings of *rod^ΔVhl^* mice at 6 months of age showed reduced b-wave amplitudes suggesting that cones were affected by the hypoxic response in rods. After the additional rod-specific deletion of *Hif1a*, of *Hif2a* or of both *Hif1a* and *Hif2a* photopic b-wave amplitudes of deletion strains were not distinguishable from their respective controls (Fig. S3). Importantly, photopic ERGs were recorded in the same mice and during the same experimental session used to determine the scotopic ERGs shown in Fig. 4. The reduced photopic response in *rod^ΔVhl^* mice may not have been caused by a significant loss of cones since expression of cone specific transducin (*Gnat2*) was not affected at 6 months of age (Fig. 2C), the timepoint of the functional tests. Thus, the hypoxic response of rods between 10 weeks and 6 months of age may have induced a lasting effect on cone function by unknown mechanisms. These putative mechanisms may depend on both HIF1 and HIF2 transcription factors as additional deletion of either factor in *rod^ΔVhl^* mice prevented the effect.

***STAT3 is activated in absence of Vhl***

Inactivation of *Vhl* in rod photoreceptors stabilized (Figs. S1A,S2B) and activated (Figure 5) HIF1A and HIF2A transcription factors. In addition, inactivation of *Vhl* resulted in the phosphorylation of STAT3 in an HIF1A dependent manner as the additional deletion of *Hif1a* resulted in basal pSTAT3 levels (Fig. S4). It has been reported that HIF1 can cooperate with STAT3 to regulate gene expression of target genes ^3^. Furthermore, it has been shown that HIF1 increases STAT3 activity through decreasing the levels of suppressor of cytokine signalling 3 (SOCS3) ^4^ and that constitutively active STAT3 directly interacts with the C-terminal domain of HIF1A ^5^. Thus, HIF1 and STAT3 may cooperate to regulate expression of specific target genes in rod photoreceptors.

***Transcriptomic analysis***

To detect genes that may be under transcriptional control of HIF1 and/or HIF2 in rods and that may contribute to the degenerative phenotype in *rod^ΔVhl^* mice, we determined the retinal transcriptome of *rod^ΔVhl^*, *rod^ΔVhl;Hif1a^* and *Vhl^flox/flox^*;*Hif1a^flox/flox^* control mice at 11 weeks of age. Tables S2 – S4 show the top 20 up- and down regulated genes of each individual comparison. The full lists of genes analysed can be found in Files S1 – S3. Inactivation of *Vhl* in rods had a strong impact on the retinal transcriptome with 657 and 245 genes that were more than 2-fold (P < 0.05) up- or down-regulated, respectively. In contrast, only 30 genes were more than 2-fold (P < 0.05) up- and 29 genes down-regulated in *rod^ΔVhl;Hif1a^* mice when compared to controls. Whereas the differentially regulated genes in *rod^ΔVhl^* mice may be mostly controlled by HIF1, HIF2 and/or STAT3, the genes found in *rod^ΔVhl;Hif1a^* mice may be primarily regulated by HIF2. When we compared differentially regulated genes in *rod^ΔVhl^* to those in *rod^ΔVhl;Hif1a^* mice we identified 493 genes that were expressed at least 2-fold higher than in *rod^ΔVhl^* mice and 246 genes that were down regulated. These genes may be primarily regulated by HIF1 and/or STAT3, but not by HIF2. Venn diagrams of up- and down-regulated genes showed that genes differentially regulated in *rod^ΔVhl^* mice were largely overlapping with those regulated in *rod^ΔVhl^* when compared to *rod^ΔVhl;Hif1a^* mice. But both had only little overlap to the differentially regulated genes *rod^ΔVhl;Hif1a^* mice (Fig. S5A). *Rod^ΔVhl;Hif1a^* mice had overlaps only with *rod^ΔVhl^* mice since both mice had HIF2 activated. This was not the case in the list of genes that resulted from the comparison of *rod^ΔVhl^* mice with *rod^ΔVhl;Hif1a^* mice as already stated above. The gene that was commonly downregulated in all three gene lists was *Igh-VJ558*, a gene that has been implicated in the immune response, leukocyte migration and apoptosis ^6, 7^. The strongly different retinal transcriptome of *rod^ΔVhl^* mice and the similarity of *rod^ΔVhl;Hif1a^* to controls is also obvious from the cluster heat map (Fig. S5B). Thus, inactivating *Hif1a* in mice lacking *Vhl* results in only few transcriptomic changes that may be attributable to HIF2.

To evaluate the regulation of the genes in more detail, we cross-compared the top regulated genes of all three lists. Table S5 uses the top 20 up- and down regulated genes in *rod^ΔVhl^* mice (see Table S2) as reference list. Fold-changes of these genes as detected in the other lists are shown for direct comparison. The same was done using the 20 top regulated genes in *rod^ΔVhl^* when compared to *rod^ΔVhl;Hif1a^* (see Table S3) as reference list (Table S6); and using the 20 top regulated in genes in *rod^ΔVhl;Hif1a^* (see Table S4) as reference list (Table S7). It is apparent that the transcriptome of *rod^ΔVhl;Hif1a^* mice differs strongly from *rod^ΔVhl^* mice. Genes that were differentially regulated in *rod^ΔVhl;Hif1a^ and rod^ΔVhl^* mice compared to their respective controls, but not in *rod^ΔVhl^* mice when compared to *rod^ΔVhl;Hif1a^* are good candidates to be regulated by HIF2 in rod photoreceptors. These genes included five (*Prim2*, *Gadl1*, *Xlrb4*, *Slurp1*, *Mybph)* up- and eight (*Mns1*, *Lmod1*, *Lce1i*, *Lce1d*, *Lce1f*, *Cd27*, *Ak7*, *Calm4*) down-regulated genes that were not defined as HIF2 targets so far. Clearly, however, HIF2-regulated expression of these genes needs to be verified by additional tests in future experiments.

***References***

1. Le YZ *et al.* Mouse opsin promoter-directed Cre recombinase expression in transgenic mice. *Mol Vis* 2006; **12**: 389-398.

2. Lange C *et al.* Normoxic activation of hypoxia-inducible factors in photoreceptors provides transient protection against light-induced retinal degeneration. *Invest Ophthalmol Vis Sci* 2011; **52**: 5872-5880.

3. Pawlus MR, Wang L, Murakami A, Dai G, Hu CJ. STAT3 or USF2 contributes to HIF target gene specificity. *PLoS One* 2013; **8**: e72358.

4. Yokogami K, Yamashita S, Takeshima H. Hypoxia-induced decreases in SOCS3 increase STAT3 activation and upregulate VEGF gene expression. *Brain Tumor Pathol* 2013; **30**: 135-143.

5. Jung JE *et al.* STAT3 inhibits the degradation of HIF-1alpha by pVHL-mediated ubiquitination. *Exp Mol Med* 2008; **40**: 479-485.

6. Bong JJ, Kang YM, Shin SC, Choi SJ, Lee KM, Kim HS. Identification of radiation-sensitive expressed genes in the ICR and AKR/J mouse thymus. *Cell Biol Int* 2013; **37**: 485-494.

7. Lin M *et al.* Impacts of hypoxia-inducible factor-1 knockout in the retinal pigment epithelium on choroidal neovascularization. *Invest Ophthalmol Vis Sci* 2012; **53**: 6197-6206.

**Figure S1**


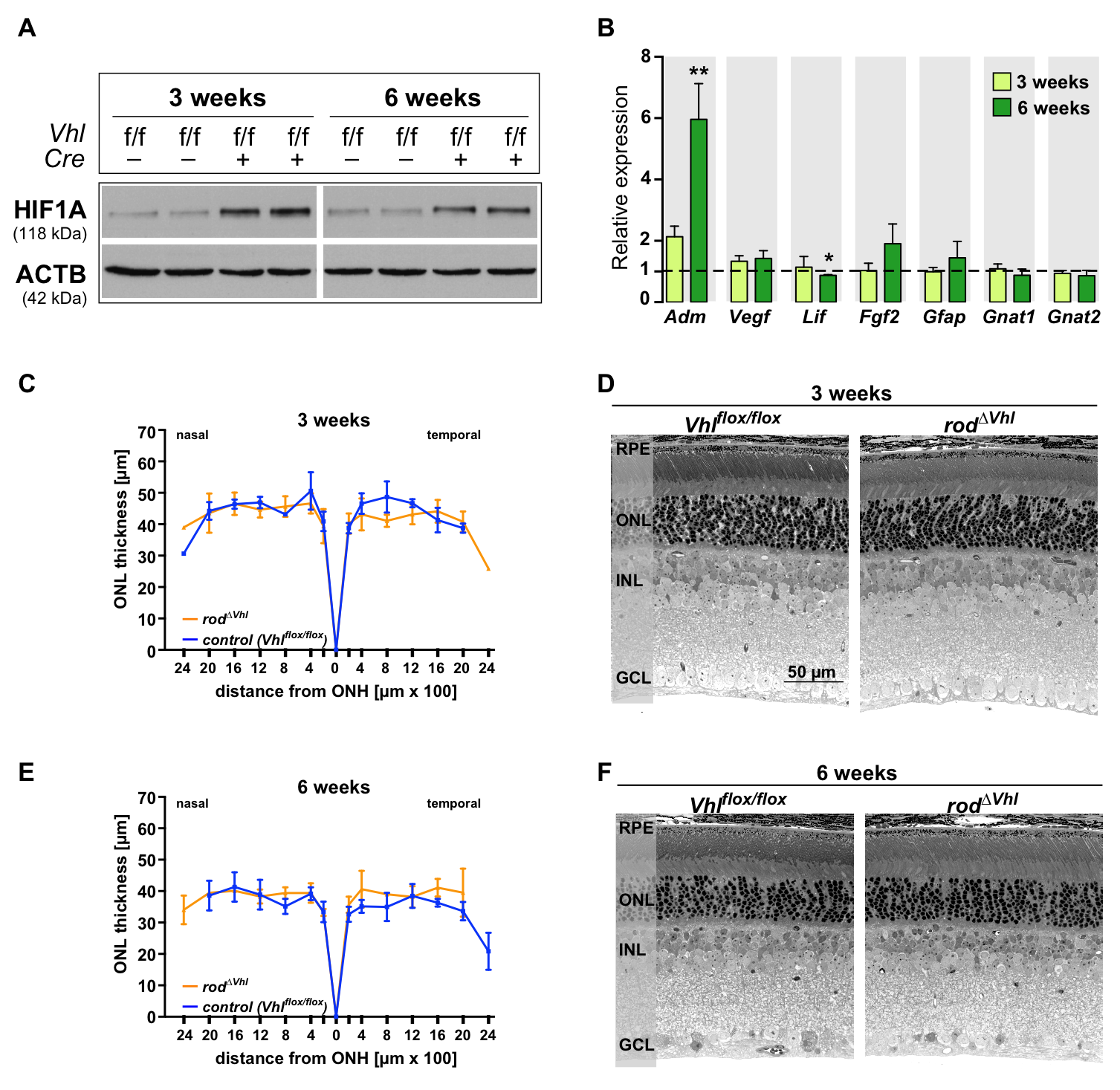


**Fig S1.** ***Impact of rod-specific Vhl deletion in 3 and 6 weeks old mice***.

**A)** Western blots for HIF1A in retinal homogenates of 3 and 6 weeks old *rod^ΔVhl^* and control mice. ACTB was used as loading control. **B)** Retinal expression of indicated genes was tested in retinas of *rod^ΔVhl^* mice at 3 and 6 weeks of age. Expression levels were calculated relative to their respective Cre-negative controls (set to 1; dotted line). Shown are means ± SD of *N* = 3-5. *: P < 0.05; **: P < 0.01. Individual comparisons between Cre-positive and Cre-negative mice of each genotype were done using Student’s t-test. **C, E)** ONL thicknesses of *rod^ΔVhl^* and control mice were determined at 3 and 6 weeks of age and are presented as spidergrams. Shown are means ± SD of *N* = 3-4, except for *rod^ΔVhl^* mice at 6 months (*N* = 2). **D, F)** Retinal morphology of *rod^ΔVhl^* and control mice at 3 and 6 weeks of age. Cre-negative *Vhl^flox/flox^* mice served as controls. RPE: retinal pigment epithelium; ONL: outer nuclear layer; INL: inner nuclear layer. GCL: ganglion cell layer. Scale bar: 50 µm. *N* = 3-4, except for *rod^ΔVhl^* mice at 6 months (*N* = 2).

**Figure S2**

**Fig S2.** Characterization of mouse strains.

**A)** Cre-positive (+) and Cre-negative (-) strains carrying floxed (f/f) alleles of *Vhl*, *Hif1a* and *Hif2a* were tested for excision of floxed sequences from genomic DNA of the retina by conventional PCR. *2-loxP*: not excised; *1-loxP*: excised; NA: not applicable. Note that genomic DNA was isolated from total retina that included cells without CRE expression. Thus, the 2-*loxP* band was expected in all samples. **B)** Western blots for HIF1A and HIF2A in retinal homogenates of strains with genotypes as indicated. ACTB was used as control. Mice were 10-11 weeks of age. Retinal homogenates of wild type mice exposed to 6 h of hypoxia (7% O_2_) were used as positive controls. Homogenates of normoxic wild type mice served as negative controls. *N* = 3.

**Figure S3**

**
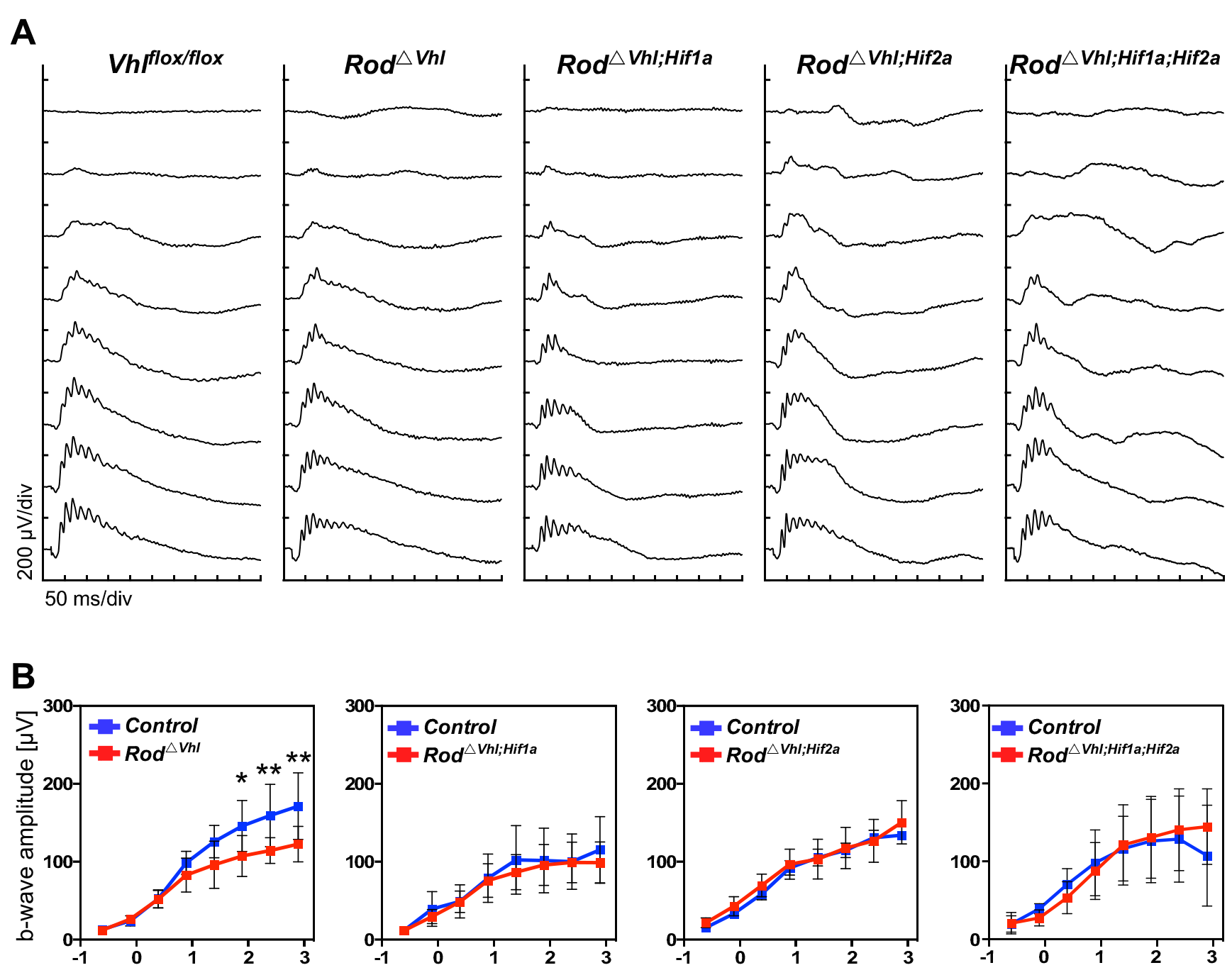
**

**Fig S3.** Retinal function. Photopic retinal function was tested in 6 months old *rod^ΔVhl^*, *rod^ΔVhl;Hif1a^*, *rod^ΔVhl;Hif2a^*, and *rod^ΔVhl;Hif1a;Hif2a^* mice. Cre-negative *Vhl^flos/flox^* mice served as controls. **A)** Representative photopic ERG traces recorded after light stimuli of increasing light intensities. **B)** Photopic b-wave amplitudes plotted as a function of stimulus intensity. Control mice were Cre-negative littermates of the respective strains. Shown are averages ± SD. *N* = 6 eyes (3 mice), except for controls of *rod^ΔVhl;Hif2a^* (N = 5), *rod^ΔVhl;Hif2a^* (N = 7), controls of *rod^ΔVhl;Hif1a;Hif2a^* (N = 5) and *rod^ΔVhl;Hif1a;Hif2a^* (N = 4). *: P < 0.05; **: P < 0.01. 2-way ANOVA with Sidak’s multiple comparison test.

**Figure S4**

**Fig S4.** Protein levels in retinal homogenates.

Western blot for indicated proteins in mice at 10-11 weeks of age. Retinal homogenates of wild type mice exposed to 6 h of hypoxia (7% O_2_) were used as positive controls. Homogenates of normoxic wild type mice served as negative controls. Note that homogenates were the same as those used for Fig. 2B and the same ACTB control is shown. *N* = 3.

**Figure S5**


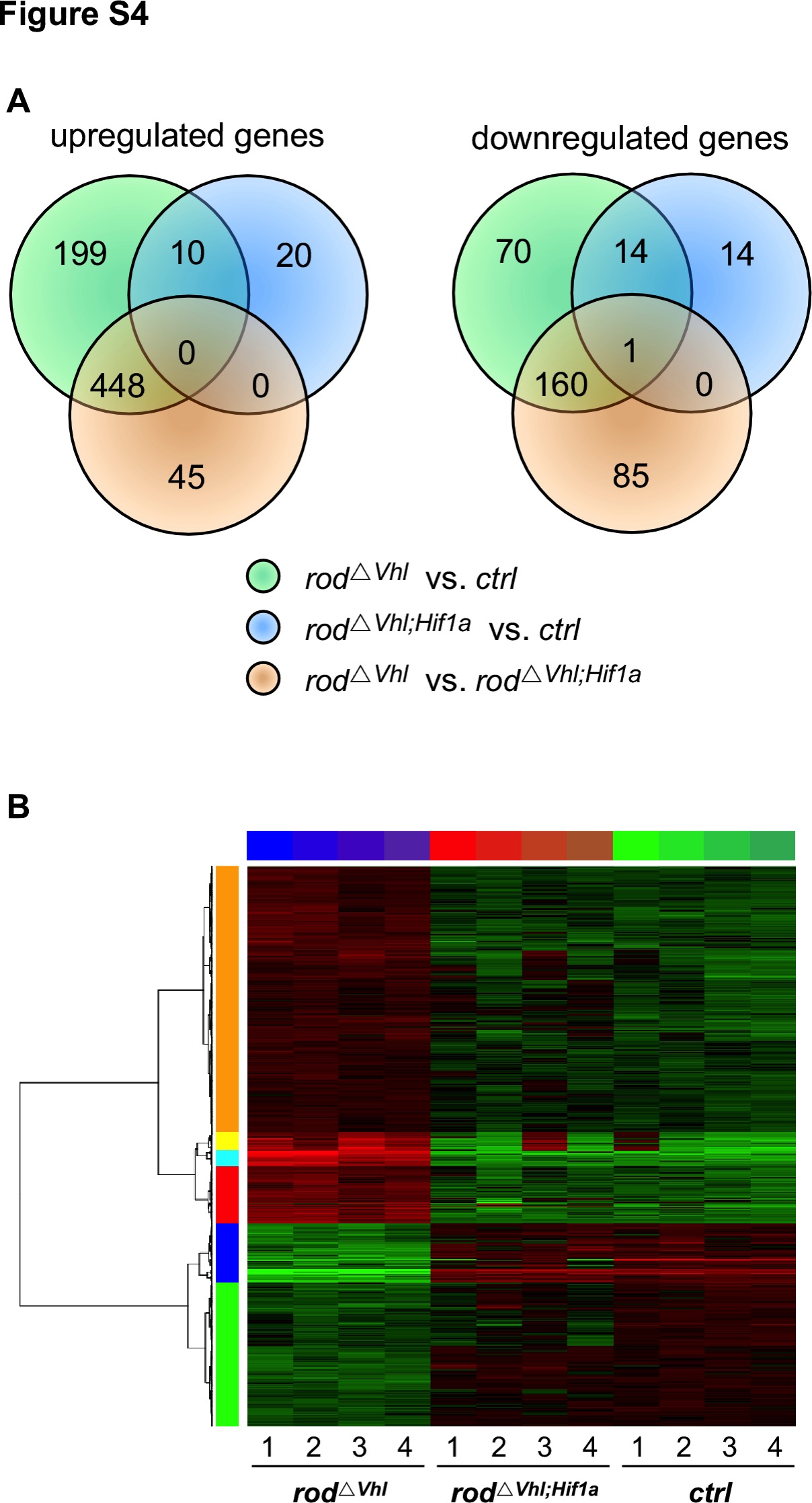


**Fig S5.** Differentially regulated genes. **A)** Venn diagrams of genes differentially regulated in *rod^ΔVhl^* (compared to controls; green), *rod^ΔVhl;Hif1a^* (compared to controls; blue) and *rod^ΔVhl^* (compared to *rod^ΔVhl;Hif1a^*; orange). Controls were *Vhl^flox/flox^*;*Hif1a^flox/flox^* mice. Genes with a fold change of ≥ 2 and a P-value < 0.05 were included in the analysis. **B)** Cluster heat map of the transcriptomic data. Note the close relationship between *rod^ΔVhl;Hif1a^* and control mice.

**Figure S6**

**Fig S6.** Schematic representation of viral construct. Expression of *sh-Hif1a* is controlled by the U6 promoter. Expression of EGFP is driven by the CMV enhancer and CMV promoter. A woodchuck hepatitis post-transcriptional regulatory element (WPRE) is added before the bovine growth hormone (bGH) poly(A) signal. ITR: inverted terminal repeat. The entire cassette is 3001 bp in length.

**Table S1. Human retina samples used in the study**

| **Nr** | **Age** | **Gender** | **Post mortem-time** | **Cause of death** |
| --- | --- | --- | --- | --- |
| HE19 | 17 | male | 30 h | Accident |
| HE4 | 24 | male | 21 h | Heart failure |
| HE25 | 31 | male | 29 h | Accident |
| HE27 | 34 | female | 24 h | Accident |
| HE26 | 53 | male | 20 h | Cerebrellar stroke |
| HE6 | 57 | male | 13 h | Multiorgan failure |
| HE11 | 58 | male | 31 h | Accident / brain trauma |
| HE1 | 72 | male | 13 h | Cardiogenic shock |
| HE16 | 74 | male | 35 h | Prostata adenocarcinoma |
| HE17 | 78 | male | 27 h | Heart failure |
| HE13 | 80 | female | 15 h | Cerebral ischemia |
| HE15 | 91 | female | 23 h | Aortic stenosis |
| HE3 | 92 | male | 36 h | Gastrointestinal bleeding |

**Table S2**

**Top regulated genes in retinas of *rod^ΔVhl^* mice, relative to *Vhl^flox/flox^;Hif1a^flox/flox^* ctrl mice**

| **Top 20 genes upregulated in retinas of *rod^ΔVhl^* mice relative to ctrl** | | | | |
| --- | --- | --- | --- | --- |
| **Entrez**  **Gene ID** | **Gene Symbol** | **ratio** | **pValue** | **Description** |
| 71957 | *Ints11* | 162.9 | 4.09E-07 | integrator complex subunit 11 |
| 76487 | *Ppp1r3g* | 79.7 | 8.93E-10 | protein phosphatase 1, regulatory (inhibitor) subunit 3G |
| 223780 | *Adm2* | 71.9 | 9.09E-08 | adrenomedullin 2 |
| 13615 | *Edn2* | 62.7 | 4.30E-08 | endothelin 2 |
| 67573 | *Loxl4* | 33.9 | 1.83E-05 | lysyl oxidase-like 4 |
| 240913 | *Adamts4* | 31.9 | 1.61E-04 | a disintegrin-like and metallopeptidase (reprolysin type) with thrombospondin type 1 motif, 4 |
| 16763 | *Lad1* | 30.3 | 6.58E-07 | ladinin |
| 14663 | *Glycam1* | 25.2 | 3.39E-05 | glycosylation dependent cell adhesion molecule 1 |
| 11535 | *Adm* | 23.7 | 1.12E-07 | adrenomedullin |
| 17339 | *Mip* | 22.9 | 8.08E-03 | major intrinsic protein of eye lens fiber |
| 214301 | *Crygn* | 22.3 | 6.26E-03 | crystallin, gamma N |
| 192199 | *Rspo1* | 21.7 | 7.82E-03 | R-spondin homolog (Xenopus laevis) |
| 233187 | *Lim2* | 21.6 | 4.19E-03 | lens intrinsic membrane protein 2 |
| 12954 | *Cryaa* | 20.9 | 7.34E-03 | crystallin, alpha A |
| 20856 | *Stc2* | 20.7 | 1.13E-06 | stanniocalcin 2 |
| 77998 | *Grifin* | 19.8 | 3.58E-03 | galectin-related inter-fiber protein |
| 12051 | *Bcl3* | 19.5 | 1.62E-06 | B-cell leukemia/lymphoma 3 |
| 276829 | *Smtnl2* | 17.0 | 9.83E-08 | smoothelin-like 2 |
| 381359 | *Prdm12* | 16.2 | 1.72E-05 | PR domain containing 12 |
| 100039660 | *Ect2l* | 15.5 | 1.72E-05 | epithelial cell transforming sequence 2 oncogene-like |
|  |  |  |  |  |
| **Top 20 genes downregulated in retinas of *rod^ΔVhl^* mice relative to ctrl** | | | | |
| **Entrez**  **Gene ID** | **Gene Symbol** | **ratio** | **pValue** | **Description** |
| 258198 | *Olfr224* | 0.015 | 2.21E-06 | olfactory receptor 224 |
| 80509 | *Med8* | 0.015 | 3.21E-07 | mediator of RNA polymerase II transcription, subunit 8 homolog (yeast) |
| 76681 | *Trim12a* | 0.024 | 2.23E-08 | tripartite motif-containing 12A |
| 30939 | *Pttg1* | 0.026 | 1.03E-09 | pituitary tumor-transforming gene 1 |
| 54631 | *Nphs1* | 0.032 | 1.02E-07 | nephrosis 1 homolog, nephrin (human) |
| 14537 | *Gcnt1* | 0.039 | 1.14E-09 | glucosaminyl (N-acetyl) transferase 1, core 2 |
| 75764 | *Slx1b* | 0.051 | 2.71E-09 | SLX1 structure-specific endonuclease subunit homolog B (S. cerevisiae) |
| 72154 | *Zfp157* | 0.058 | 4.90E-07 | zinc finger protein 157 |
| 269209 | *Stk36* | 0.091 | 4.27E-06 | serine/threonine kinase 36 (fused homolog, Drosophila) |
| 214523 | *Tmprss4* | 0.095 | 2.39E-08 | transmembrane protease, serine 4 |
| 17427 | *Mns1* | 0.100 | 1.01E-06 | meiosis-specific nuclear structural protein 1 |
| 72461 | *Prcp* | 0.115 | 1.35E-08 | prolylcarboxypeptidase (angiotensinase C) |
| 16061 | *Igh-VJ558* | 0.117 | 4.18E-04 | immunoglobulin heavy chain (J558 family) |
| 140709 | *Emid2* | 0.120 | 1.01E-06 | EMI domain containing 2 |
| 17921 | *Myo7a* | 0.124 | 1.77E-07 | myosin VIIA |
| 94216 | *Col4a6* | 0.129 | 3.29E-04 | collagen, type IV, alpha 6 |
| 69611 | *Lce1d* | 0.148 | 1.60E-02 | late cornified envelope 1D |
| 71687 | *Tmem25* | 0.154 | 6.43E-08 | transmembrane protein 25 |
| 12769 | *Ccr9* | 0.163 | 5.52E-03 | chemokine (C-C motif) receptor 9 |
| 66442 | *Spc25* | 0.168 | 7.49E-05 | SPC25, NDC80 kinetochore complex component, homolog (S. cerevisiae) |

Genes with P > 0.05 were excluded from the analysis, as were ‘Rik’ sequences, ‘expressed sequences’, ‘predicted genes’ and ‘hypothetical proteins’. Also excluded was ‘*Xist*’ as this gene is X-linked and the number of females and males in the gene chip analyses were not balanced.

**Table S3**

**Top regulated genes in retinas of *rod^ΔVhl^* mice, relative to *rod^ΔVhl;Hif1a^* mice**

| **Top 20 genes upregulated in retinas of *rod^ΔVhl^* mice relative to *rod^ΔVhl;Hif1a^* mice** | | | | |
| --- | --- | --- | --- | --- |
| **Entrez**  **Gene ID** | **Gene Symbol** | **ratio** | **pValue** | **Description** |
| 71957 | *Ints11* | 282.8 | 5.20E-09 | integrator complex subunit 11 |
| 223780 | *Adm2* | 70.9 | 1.19E-06 | adrenomedullin 2 |
| 13615 | *Edn2* | 65.8 | 3.12E-08 | endothelin 2 |
| 76487 | *Ppp1r3g* | 59.2 | 5.15E-06 | protein phosphatase 1, regulatory (inhibitor) subunit 3G |
| 240913 | *Adamts4* | 24.7 | 2.89E-04 | a disintegrin-like and metallopeptidase (reprolysin type) with thrombospondin type 1 motif, 4 |
| 11535 | *Adm* | 23.5 | 6.34E-08 | adrenomedullin |
| 16763 | *Lad1* | 23.3 | 3.38E-05 | ladinin |
| 67573 | *Loxl4* | 22.4 | 2.51E-04 | lysyl oxidase-like 4 |
| 381359 | *Prdm12* | 19.5 | 1.37E-05 | PR domain containing 12 |
| 12051 | *Bcl3* | 18.7 | 4.52E-06 | B-cell leukemia/lymphoma 3 |
| 233187 | *Lim2* | 17.0 | 2.37E-02 | lens intrinsic membrane protein 2 |
| 17339 | *Mip* | 16.3 | 2.49E-02 | major intrinsic protein of eye lens fiber |
| 21818 | *Tgm3* | 15.6 | 4.76E-04 | transglutaminase 3, E polypeptide |
| 20856 | *Stc2* | 13.8 | 5.69E-07 | stanniocalcin 2 |
| 276829 | *Smtnl2* | 13.2 | 2.79E-07 | smoothelin-like 2 |
| 14663 | *Glycam1* | 13.0 | 1.27E-03 | glycosylation dependent cell adhesion molecule 1 |
| 15006 | *H2-Q1* | 12.2 | 9.49E-06 | histocompatibility 2, Q region locus 1 |
| 14173 | *Fgf2* | 11.6 | 7.27E-06 | fibroblast growth factor 2 |
| 192199 | *Rspo1* | 11.3 | 2.04E-02 | R-spondin homolog (Xenopus laevis) |
| 12954 | *Cryaa* | 10.9 | 1.64E-02 | crystallin, alpha A |
|  |  |  |  |  |
| **Top 20 genes downregulated in retinas of *rod^ΔVhl^* mice relative to *rod^ΔVhl;Hif1a^* mice** | | | | |
| **Entrez**  **Gene ID** | **Gene Symbol** | **ratio** | **pValue** | **Description** |
| 76681 | *Trim12a* | 0.014 | 8.54E-09 | tripartite motif-containing 12A |
| 80509 | *Med8* | 0.025 | 1.54E-06 | mediator of RNA polymerase II transcription, subunit 8 homolog (yeast) |
| 30939 | *Pttg1* | 0.026 | 1.81E-10 | pituitary tumor-transforming gene 1 |
| 258198 | *Olfr224* | 0.027 | 2.80E-05 | olfactory receptor 224 |
| 54631 | *Nphs1* | 0.028 | 4.80E-07 | nephrosis 1 homolog, nephrin (human) |
| 75764 | *Slx1b* | 0.034 | 4.22E-09 | SLX1 structure-specific endonuclease subunit homolog B (S. cerevisiae) |
| 14537 | *Gcnt1* | 0.035 | 3.59E-11 | glucosaminyl (N-acetyl) transferase 1, core 2 |
| 109685 | *Hyal3* | 0.052 | 3.57E-06 | hyaluronoglucosaminidase 3 |
| 72154 | *Zfp157* | 0.053 | 2.11E-07 | zinc finger protein 157 |
| 17921 | *Myo7a* | 0.074 | 3.64E-08 | myosin VIIA |
| 214523 | *Tmprss4* | 0.084 | 2.07E-08 | transmembrane protease, serine 4 |
| 72461 | *Prcp* | 0.101 | 9.91E-09 | prolylcarboxypeptidase (angiotensinase C) |
| 269209 | *Stk36* | 0.103 | 4.68E-06 | serine/threonine kinase 36 (fused homolog, Drosophila) |
| 94216 | *Col4a6* | 0.112 | 3.70E-04 | collagen, type IV, alpha 6 |
| 140709 | *Emid2* | 0.112 | 9.73E-07 | EMI domain containing 2 |
| 209760 | *Tmc7* | 0.129 | 1.24E-05 | transmembrane channel-like gene family 7 |
| 68668 | *Klk5* | 0.134 | 1.22E-02 | kallikrein related-peptidase 5 |
| 71687 | *Tmem25* | 0.156 | 7.66E-08 | transmembrane protein 25 |
| 19223 | *Ptgis* | 0.167 | 2.71E-06 | prostaglandin I2 (prostacyclin) synthase |
| 18261 | *Ocm* | 0.175 | 1.41E-05 | oncomodulin |

Genes with P > 0.05 were excluded from the analysis, as were ‘Rik’ sequences, ‘expressed sequences’, ‘predicted genes’ and ‘hypothetical proteins’. Also excluded was ‘*Xist*’ as this gene is X-linked and the number of females and males in the gene chip analyses were not balanced.

**Table S4**

**To Top regulated genes in retinas of *rod^ΔVhl;Hif1a^* mice, relative to *Vhl^flox/flox^;Hif1a^flox/flox^* control mice**

| **Top 20 genes upregulated in retinas of *rod^ΔVhl;Hif1a^* mice relative to ctrl** | | | | |
| --- | --- | --- | --- | --- |
| **Entrez**  **Gene ID** | **Gene Symbol** | **ratio** | **pValue** | **Description** |
| 109685 | *Hyal3* | 7.0 | 1.26E-02 | hyaluronoglucosaminidase 3 |
| 19076 | *Prim2* | 5.3 | 7.60E-09 | DNA primase, p58 subunit |
| 77044 | *Arid2* | 4.8 | 3.88E-02 | AT rich interactive domain 2 (ARID, RFX-like) |
| 434794 | *Xlr4a* | 4.5 | 8.59E-03 | X-linked lymphocyte-regulated 4A |
| 628171 | *Olfr936* | 3.4 | 5.31E-03 | olfactory receptor 936 |
| 73748 | *Gadl1* | 3.3 | 1.48E-02 | glutamate decarboxylase-like 1 |
| 545548 | *Lce3a* | 2.9 | 3.65E-03 | late cornified envelope 3A |
| 12534 | *Cdk1* | 2.9 | 8.44E-03 | cyclin-dependent kinase 1 |
| 27083 | *Xlr4b* | 2.9 | 3.77E-02 | X-linked lymphocyte-regulated 4B |
| 57277 | *Slurp1* | 2.8 | 2.82E-02 | secreted Ly6/Plaur domain containing 1 |
| 17748 | *Mt1* | 2.7 | 6.82E-08 | metallothionein 1 |
| 53311 | *Mybph* | 2.7 | 3.58E-02 | myosin binding protein H |
| 67652 | *Spaca1* | 2.7 | 8.29E-05 | sperm acrosome associated 1 |
| 194597 | *Tmprss11a* | 2.5 | 4.75E-03 | transmembrane protease, serine 11a |
| 230678 | *Tmem125* | 2.4 | 1.02E-03 | transmembrane protein 125 |
| 244332 | *Defb14* | 2.3 | 5.00E-03 | defensin beta 14 |
| 14428 | *Galr2* | 2.3 | 1.65E-02 | galanin receptor 2 |
| 238393 | *Serpina3f* | 2.3 | 2.42E-05 | serine (or cysteine) peptidase inhibitor, clade A, member 3F |
| 22326 | *Vax1* | 2.2 | 3.28E-02 | ventral anterior homeobox containing gene 1 |
| 69930 | *Zfp715* | 2.2 | 2.04E-02 | zinc finger protein 715 |
|  |  |  |  |  |
| **Top 20 genes downregulated in retinas of *rod^ΔVhl;Hif1a^* mice relative to ctrl** | | | | |
| **Entrez**  **Gene ID** | **Gene Symbol** | **ratio** | **pValue** | **Description** |
| 353371 | *Oxct2b* | 0.183 | 1.24E-02 | 3-oxoacid CoA transferase 2B |
| 17427 | *Mns1* | 0.254 | 1.58E-02 | meiosis-specific nuclear structural protein 1 |
| 51925 | *D2Ertd640e* | 0.260 | 2.78E-04 | DNA segment, Chr 2, ERATO Doi 640, expressed |
| 93689 | *Lmod1* | 0.324 | 3.44E-02 | leiomodin 1 (smooth muscle) |
| 76585 | *Lce1i* | 0.335 | 5.87E-03 | late cornified envelope 1I |
| 69611 | *Lce1d* | 0.338 | 2.88E-02 | late cornified envelope 1D |
| 56057 | *Btg4* | 0.356 | 4.73E-02 | B-cell translocation gene 4 |
| 16061 | *Igh-VJ558* | 0.357 | 7.34E-03 | immunoglobulin heavy chain (J558 family) |
| 236904 | *Klhl15* | 0.375 | 1.83E-02 | kelch-like 15 (Drosophila) |
| 67828 | *Lce1f* | 0.376 | 1.43E-02 | late cornified envelope 1F |
| 20521 | *Slc22a12* | 0.377 | 3.41E-04 | solute carrier family 22 (organic anion/cation transporter), member 12 |
| 21940 | *Cd27* | 0.380 | 1.84E-02 | CD27 antigen |
| 78801 | *Ak7* | 0.385 | 4.67E-04 | adenylate kinase 7 |
| 68694 | *Lce1e* | 0.394 | 2.09E-02 | late cornified envelope 1E |
| 258692 | *Olfr1442* | 0.395 | 1.93E-02 | olfactory receptor 1442 |
| 76117 | *Arhgap15* | 0.397 | 2.29E-03 | Rho GTPase activating protein 15 |
| 246746 | *Cd300lf* | 0.398 | 4.89E-02 | CD300 antigen like family member F |
| 96875 | *Prg4* | 0.400 | 1.36E-04 | proteoglycan 4 (megakaryocyte stimulating factor, articular superficial zone protein) |
| 15130 | *Hbb-b2* | 0.402 | 2.54E-02 | hemoglobin, beta adult minor chain |
| 80796 | *Calm4* | 0.402 | 2.60E-02 | calmodulin 4 |

Genes with P > 0.05 were excluded from the analysis, as were ‘Rik’ sequences, ‘expressed sequences’, ‘predicted genes’ and ‘hypothetical proteins’. Also excluded was ‘*Xist*’ as this gene is X-linked and the number of females and males in the gene chip analyses were not balanced.

**Table S5**

**Comparison to top 20 genes that were differentially regulated in *rod^ΔVhl^* mice relative to *Vhl^flox/flox^;Hif1a^flox/flox^* control mice**

| **Reference list: Table S2: *rod^ΔVhl^ vs ctrl***  **Upregulated genes**  **Potential targets of: HIF1; HIF2; pSTAT3** | | | | | | | | ***rod^ΔVhl^ vs rod^ΔVhl;Hif1a^*** | | ***rod^ΔVhl;Hif1a^ vs ctrl*** | |
| --- | --- | --- | --- | --- | --- | --- | --- | --- | --- | --- | --- |
|  |  |  |  |  |  |  |  | **potential targets of:**  **HIF1; pSTAT3** | | **potential targets of:**  **HIF2** | |
| **Entrez**  **Gene ID** | | **Gene Symbol** | | **ratio** | | **pValue** | | **ratio** | **pValue** | **ratio** | **pValue** |
| 71957 | | ***Ints11*** | | 162.9 | | 4.09E-07 | | 282.8 | 5.20E-09 | – | – |
| 76487 | | ***Ppp1r3g*** | | 79.7 | | 8.93E-10 | | 59.2 | 5.15E-06 | – | – |
| 223780 | | ***Adm2*** | | 71.9 | | 9.09E-08 | | 70.9 | 1.19E-06 | – | – |
| 13615 | | ***Edn2*** | | 62.7 | | 4.30E-08 | | 65.8 | 3.12E-08 | – | – |
| 67573 | | ***Loxl4*** | | 33.9 | | 1.83E-05 | | 22.4 | 2.51E-04 | – | – |
| 240913 | | ***Adamts4*** | | 31.9 | | 1.61E-04 | | 24.7 | 2.89E-04 | – | – |
| 16763 | | ***Lad1*** | | 30.3 | | 6.58E-07 | | 23.3 | 3.38E-05 | – | – |
| 14663 | | ***Glycam1*** | | 25.2 | | 3.39E-05 | | 13.0 | 1.27E-03 | – | – |
| 11535 | | ***Adm*** | | 23.7 | | 1.12E-07 | | 23.5 | 6.34E-08 | – | – |
| 17339 | | ***Mip*** | | 22.9 | | 8.08E-03 | | 16.3 | 2.49E-02 | – | – |
| 214301 | | ***Crygn*** | | 22.3 | | 6.26E-03 | | † 10.3 | † 1.19E-02 | – | – |
| 192199 | | ***Rspo1*** | | 21.7 | | 7.82E-03 | | 11.3 | 2.04E-02 | – | – |
| 233187 | | ***Lim2*** | | 21.6 | | 4.19E-03 | | 17.0 | 2.37E-02 | – | – |
| 12954 | | ***Cryaa*** | | 20.9 | | 7.34E-03 | | 10.9 | 1.64E-02 | – | – |
| 20856 | | ***Stc2*** | | 20.7 | | 1.13E-06 | | 13.8 | 5.69E-07 | – | – |
| 77998 | | ***Grifin*** | | 19.8 | | 3.58E-03 | | † 10.7 | † 1.75E-02 | – | – |
| 12051 | | ***Bcl3*** | | 19.5 | | 1.62E-06 | | 18.7 | 4.52E-06 | – | – |
| 276829 | | ***Smtnl2*** | | 17.0 | | 9.83E-08 | | 13.2 | 2.79E-07 | † 1.3 | † 1.34E-02 |
| 381359 | | ***Prdm12*** | | 16.2 | | 1.72E-05 | | 19.5 | 1.37E-05 | – | – |
| 100039660 | | ***Ect2l*** | | 15.5 | | 1.72E-05 | | † 10.8 | † 3.46E-05 | – | – |
|  |  | |  | |  | |  | | | | |
| **Reference list: Table S2: *rod^ΔVhl^ vs ctrl***  **Downregulated genes**  **Potential targets of: HIF1; HIF2; pSTAT3** | | | | | | | | ***rod^ΔVhl^ vs rod^ΔVhl;Hif1a^*** | | ***rod^ΔVhl;Hif1a^ vs ctrl*** | |
|  |  |  |  |  |  |  |  | **potential targets of:**  **HIF1; pSTAT3** | | **potential targets of:**  **HIF2** | |
| **Entrez**  **Gene ID** | | **Gene Symbol** | | **ratio** | | **pValue** | | **ratio** | **pValue** | **ratio** | **pValue** |
| 258198 | | ***Olfr224*** | | 0.015 | | 2.21E-06 | | 0.027 | 2.80E-05 | – | – |
| 80509 | | ***Med8*** | | 0.015 | | 3.21E-07 | | 0.025 | 1.54E-06 | – | – |
| 76681 | | ***Trim12a*** | | 0.024 | | 2.23E-08 | | 0.014 | 8.54E-09 | † 1.68 | † 3.77E-03 |
| 30939 | | ***Pttg1*** | | 0.026 | | 1.03E-09 | | 0.026 | 1.81E-10 | – | – |
| 54631 | | ***Nphs1*** | | 0.032 | | 1.02E-07 | | 0.028 | 4.80E-07 | – | – |
| 14537 | | ***Gcnt1*** | | 0.039 | | 1.14E-09 | | 0.035 | 3.59E-11 | – | – |
| 75764 | | ***Slx1b*** | | 0.051 | | 2.71E-09 | | 0.034 | 4.22E-09 | † 1.49 | † 9.70E-03 |
| 72154 | | ***Zfp157*** | | 0.058 | | 4.90E-07 | | 0.053 | 2.11E-07 | – | – |
| 269209 | | ***Stk36*** | | 0.091 | | 4.27E-06 | | 0.103 | 4.68E-06 | – | – |
| 214523 | | ***Tmprss4*** | | 0.095 | | 2.39E-08 | | 0.084 | 2.07E-08 | – | – |
| 17427 | | ***Mns1*** | | 0.100 | | 1.01E-06 | | – | – | † 0.25 | † 1.58E-02 |
| 72461 | | ***Prcp*** | | 0.115 | | 1.35E-08 | | 0.101 | 9.91E-09 | † 1.14 | † 2.83E-02 |
| 16061 | | ***Igh-VJ558*** | | 0.117 | | 4.18E-04 | | † 0.313 | † 2.28E-02 | † 0.37 | † 1.28E-02 |
| 140709 | | ***Emid2*** | | 0.120 | | 1.01E-06 | | 0.112 | 9.73E-07 | – | – |
| 17921 | | ***Myo7a*** | | 0.124 | | 1.77E-07 | | 0.074 | 3.64E-08 | † 1.75 | † 1.24E-04 |
| 94216 | | ***Col4a6*** | | 0.129 | | 3.29E-04 | | 0.112 | 3.70E-04 | – | – |
| 69611 | | ***Lce1d*** | | 0.148 | | 1.60E-02 | | – | – | † 0.34 | † 2.88E-02 |
| 71687 | | ***Tmem25*** | | 0.154 | | 6.43E-08 | | 0.156 | 7.66E-08 | – | – |
| 12769 | | ***Ccr9*** | | 0.163 | | 5.52E-03 | | – | – | – | – |
| 66442 | | ***Spc25*** | | 0.168 | | 7.49E-05 | | † 0.183 | † 1.35E-04 | – | – |

Genes with P > 0.05 were excluded from the analysis, as were ‘Rik’ sequences, ‘expressed sequences’, ‘predicted genes’ and ‘hypothetical proteins’. Also excluded was ‘*Xist*’ as this gene is X-linked and the number of females and males in the gene chip analyses were not balanced.

† : not present in the top 20 regulated genes of the respective list

– : not present in the respective list of genes, or P > 0.05

**Table S6**

**Comparison to top 20 genes that were differentially regulated in *rod^ΔVhl^* relative to *rod^Δrod;Hif1a^* mice**

| **Reference list: Table S3: *rod^ΔVhl^ vs rod^ΔVhl;Hif1a^***  **Upregulated genes**  **Potential targets of: HIF1; pSTAT3** | | | | | | | | ***rod^ΔVhl;Hif1a^ vs ctrl*** | | ***rod^ΔVhl^ vs ctrl*** | |
| --- | --- | --- | --- | --- | --- | --- | --- | --- | --- | --- | --- |
|  |  |  |  |  |  |  |  | **potential targets of:**  **HIF2** | | **potential targets of:**  **HIF1; HIF2; pSTAT3** | |
| **Entrez**  **Gene ID** | | **Gene Symbol** | | **ratio** | | **pValue** | | **ratio** | **pValue** | **ratio** | **pValue** |
| 71957 | | *Ints11* | | 282.8 | | 5.20E-09 | | – | – | 162.9 | 4.09E-07 |
| 223780 | | *Adm2* | | 70.9 | | 1.19E-06 | | – | – | 71.9 | 9.09E-08 |
| 13615 | | *Edn2* | | 65.8 | | 3.12E-08 | | – | – | 62.7 | 4.30E-08 |
| 76487 | | *Ppp1r3g* | | 59.2 | | 5.15E-06 | | – | – | 79.7 | 8.93E-10 |
| 240913 | | *Adamts4* | | 24.7 | | 2.89E-04 | | – | – | 31.9 | 1.61E-04 |
| 11535 | | *Adm* | | 23.5 | | 6.34E-08 | | – | – | 23.7 | 1.12E-07 |
| 16763 | | *Lad1* | | 23.3 | | 3.38E-05 | | – | – | 30.3 | 6.58E-07 |
| 67573 | | *Loxl4* | | 22.4 | | 2.51E-04 | | – | – | 33.9 | 1.83E-05 |
| 381359 | | *Prdm12* | | 19.5 | | 1.37E-05 | | – | – | 16.2 | 1.72E-05 |
| 12051 | | *Bcl3* | | 18.7 | | 4.52E-06 | | – | – | 19.5 | 1.62E-06 |
| 233187 | | *Lim2* | | 17.0 | | 2.37E-02 | | – | – | 21.6 | 4.19E-03 |
| 17339 | | *Mip* | | 16.3 | | 2.49E-02 | | – | – | 22.9 | 8.08E-03 |
| 21818 | | *Tgm3* | | 15.6 | | 4.76E-04 | | – | – | † 14.5 | † 2.99E-05 |
| 20856 | | *Stc2* | | 13.8 | | 5.69E-07 | | – | – | 20.7 | 1.13E-06 |
| 276829 | | *Smtnl2* | | 13.2 | | 2.79E-07 | | † 1.3 | † 1.34E-02 | 17.0 | 9.83E-08 |
| 14663 | | *Glycam1* | | 13.0 | | 1.27E-03 | | – | – | 25.2 | 3.39E-05 |
| 15006 | | *H2-Q1* | | 12.2 | | 9.49E-06 | | – | – | † 10.3 | † 2.26E-06 |
| 14173 | | *Fgf2* | | 11.6 | | 7.27E-06 | | – | – | † 9.86 | † 1.01E-05 |
| 192199 | | *Rspo1* | | 11.3 | | 2.04E-02 | | – | – | 21.7 | 7.82E-03 |
| 12954 | | *Cryaa* | | 10.9 | | 1.64E-02 | | – | – | 20.9 | 7.34E-03 |
|  |  | |  | |  | |  | | | | |
| **Reference list: Table S3: *rod^ΔVhl^ vs rod^ΔVhl;Hif1a^***  **Downregulated genes**  **Potential targets of: HIF1; pSTAT3** | | | | | | | | ***rod^ΔVhl;Hif1a^ vs ctrl*** | | ***rod^ΔVhl^ vs ctrl*** | |
|  |  |  |  |  |  |  |  | **potential targets of:**  **HIF2** | | **potential targets of:**  **HIF1; HIF2; pSTAT3** | |
| **Entrez**  **Gene ID** | | **Gene Symbol** | | **ratio** | | **pValue** | | **ratio** | **pValue** | **ratio** | **pValue** |
| 76681 | | *Trim12a* | | 0.014 | | 8.54E-09 | | † 1.68 | † 3.77E-03 | 0.024 | 2.23E-08 |
| 80509 | | *Med8* | | 0.025 | | 1.54E-06 | | – | – | 0.015 | 3.21E-07 |
| 30939 | | *Pttg1* | | 0.026 | | 1.81E-10 | | – | – | 0.026 | 1.03E-09 |
| 258198 | | *Olfr224* | | 0.027 | | 2.80E-05 | | – | – | 0.015 | 2.21E-06 |
| 54631 | | *Nphs1* | | 0.028 | | 4.80E-07 | | – | – | 0.032 | 1.02E-07 |
| 75764 | | *Slx1b* | | 0.034 | | 4.22E-09 | | † 1.49 | † 9.70E-03 | 0.051 | 2.71E-09 |
| 14537 | | *Gcnt1* | | 0.035 | | 3.59E-11 | | – | – | 0.039 | 1.14E-09 |
| 109685 | | *Hyal3* | | 0.052 | | 3.57E-06 | | 6.96 | 1.26E-02 | – | – |
| 72154 | | *Zfp157* | | 0.053 | | 2.11E-07 | | – | – | 0.058 | 4.90E-07 |
| 17921 | | *Myo7a* | | 0.074 | | 3.64E-08 | | † 1.75 | † 1.24E-04 | 0.124 | 1.77E-07 |
| 214523 | | *Tmprss4* | | 0.084 | | 2.07E-08 | | – | – | 0.095 | 2.39E-08 |
| 72461 | | *Prcp* | | 0.101 | | 9.91E-09 | | † 1.14 | † 2.83E-02 | 0.115 | 1.35E-08 |
| 269209 | | *Stk36* | | 0.103 | | 4.68E-06 | | – | – | 0.091 | 4.27E-06 |
| 94216 | | *Col4a6* | | 0.112 | | 3.70E-04 | | – | – | 0.129 | 3.29E-04 |
| 140709 | | *Emid2* | | 0.112 | | 9.73E-07 | | – | – | 0.120 | 1.01E-06 |
| 209760 | | *Tmc7* | | 0.129 | | 1.24E-05 | | † 1.45 | † 2.99E-03 | † 0.187 | † 4.51E-05 |
| 68668 | | *Klk5* | | 0.134 | | 1.22E-02 | | – | – | – | – |
| 71687 | | *Tmem25* | | 0.156 | | 7.66E-08 | | – | – | 0.154 | 6.43E-08 |
| 19223 | | *Ptgis* | | 0.167 | | 2.71E-06 | | † 1.49 | † 9.72E-03 | † 0.249 | † 1.39E-06 |
| 18261 | | *Ocm* | | 0.175 | | 1.41E-05 | | † 1.42 | † 3.63E-02 | † 0.403 | † 4.41E-06 |

Genes with P > 0.05 were excluded from the analysis, as were ‘Rik’ sequences, ‘expressed sequences’, ‘predicted genes’ and ‘hypothetical proteins’. Also excluded was ‘*Xist*’ as this gene is X-linked and the number of females and males in the gene chip analyses were not balanced.

† : not present in the top 20 regulated genes of the respective list

– : not present in the respective list of genes, or P > 0.05

**Table S7**

**Comparison to top 20 genes that were differentially regulated in *rod^ΔVhl;Hif1a^* relative to *Vhl^flox/flox^;Hif1a^flox/flox^* control mice**

| **Reference list: Table S4: *rod^ΔVhl;Hif1a^ vs ctrl***  **Upregulated genes**  **Potential targets of: HIF2** | | | | | | | | ***rod^ΔVhl^ vs rod^ΔVhl;Hif1a^*** | | ***rod^ΔVhl^ vs ctrl*** | |
| --- | --- | --- | --- | --- | --- | --- | --- | --- | --- | --- | --- |
|  |  |  |  |  |  |  |  | **potential targets of: HIF1; pSTAT3** | | **potential targets of:**  **HIF1; HIF2; pSTAT3** | |
| **Entrez**  **Gene ID** | | **Gene Symbol** | | **ratio** | | **pValue** | | **ratio** | **pValue** | **ratio** | **pValue** |
| 109685 | | *Hyal3* | | 7.0 | | 1.26E-02 | | 0.05 | 3.57E-06 | – | – |
| 19076 | | *Prim2* | | 5.3 | | 7.60E-09 | | – | – | † 4.7 | † 3.02E-05 |
| 77044 | | *Arid2* | | 4.8 | | 3.88E-02 | | – | – | – | – |
| 434794 | | *Xlr4a* | | 4.5 | | 8.59E-03 | | – | – | – | – |
| 628171 | | *Olfr936* | | 3.4 | | 5.31E-03 | | – | – | – | – |
| 73748 | | *Gadl1* | | 3.3 | | 1.48E-02 | | – | – | † 5.0 | † 9.08E-04 |
| 545548 | | *Lce3a* | | 2.9 | | 3.65E-03 | | † 0.2 | † 2.17E-03 | – | – |
| 12534 | | *Cdk1* | | 2.9 | | 8.44E-03 | | – | – | – | – |
| 27083 | | *Xlr4b* | | 2.9 | | 3.77E-02 | | – | – | † 2.6 | † 5.27E-03 |
| 57277 | | *Slurp1* | | 2.8 | | 2.82E-02 | | – | – | † 2.5 | † 8.05E-03 |
| 17748 | | *Mt1* | | 2.7 | | 6.82E-08 | | † 0.8 | † 9.22E-03 | † 2.0 | † 4.22E-05 |
| 53311 | | *Mybph* | | 2.7 | | 3.58E-02 | | – | – | † 1.9 | † 3.72E-02 |
| 67652 | | *Spaca1* | | 2.7 | | 8.29E-05 | | – | – | – | – |
| 194597 | | *Tmprss11a* | | 2.5 | | 4.75E-03 | | – | – | – | – |
| 230678 | | *Tmem125* | | 2.4 | | 1.02E-03 | | – | – | – | – |
| 244332 | | *Defb14* | | 2.3 | | 5.00E-03 | | – | – | – | – |
| 14428 | | *Galr2* | | 2.3 | | 1.65E-02 | | † 0.3 | † 1.33E-02 | – | – |
| 238393 | | *Serpina3f* | | 2.3 | | 2.42E-05 | | † 1.44 | † 2.38E-03 | † 3.3 | † 2.46E-07 |
| 22326 | | *Vax1* | | 2.2 | | 3.28E-02 | | – | – | – | – |
| 69930 | | *Zfp715* | | 2.2 | | 2.04E-02 | | † 0.4 | † 1.87E-02 | † 0.8 | † 9.99E-03 |
|  |  | |  | |  | |  | | | | |
| **Reference list: Table S4: *rod^ΔVhl;Hif1a^ vs ctrl***  **Downregulated genes**  **Potential targets of: HIF2** | | | | | | | | ***rod^ΔVhl^ vs rod^ΔVhl;Hif1a^*** | | ***rod^ΔVhl^ vs ctrl*** | |
|  |  |  |  |  |  |  |  | **potential targets of:**  **HIF1; pSTAT3** | | **potential targets of:**  **HIF1; HIF2; pSTAT3** | |
| **Entrez**  **Gene ID** | | **Gene Symbol** | | **ratio** | | **pValue** | | **ratio** | **pValue** | **ratio** | **pValue** |
| 353371 | | *Oxct2b* | | 0.183 | | 1.24E-02 | | – | – | – | – |
| 17427 | | *Mns1* | | 0.254 | | 1.58E-02 | | – | – | 0.100 | 1.01E-06 |
| 51925 | | *D2Ertd640e* | | 0.260 | | 2.78E-04 | | – | – | – | – |
| 93689 | | *Lmod1* | | 0.324 | | 3.44E-02 | | – | – | † 0.322 | † 3.25E-02 |
| 76585 | | *Lce1i* | | 0.335 | | 5.87E-03 | | – | – | † 0.207 | † 1.09E-02 |
| 69611 | | *Lce1d* | | 0.338 | | 2.88E-02 | | – | – | 0.148 | 1.60E-02 |
| 56057 | | *Btg4* | | 0.356 | | 4.73E-02 | | – | – | – | – |
| 16061 | | *Igh-VJ558* | | 0.357 | | 7.34E-03 | | † 0.31 | † 2.28E-02 | 0.117 | 4.18E-04 |
| 236904 | | *Klhl15* | | 0.375 | | 1.83E-02 | | – | – | – | – |
| 67828 | | *Lce1f* | | 0.376 | | 1.43E-02 | | – | – | † 0.175 | † 4.43E-03 |
| 20521 | | *Slc22a12* | | 0.377 | | 3.41E-04 | | – | – | – | – |
| 21940 | | *Cd27* | | 0.380 | | 1.84E-02 | | – | – | † 0.329 | † 3.02E-02 |
| 78801 | | *Ak7* | | 0.385 | | 4.67E-04 | | – | – | – | – |
| 68694 | | *Lce1e* | | 0.394 | | 2.09E-02 | | – | – | † 0.180 | † 3.60E-03 |
| 258692 | | *Olfr1442* | | 0.395 | | 1.93E-02 | | † 4.50 | † 4.86E-02 | – | – |
| 76117 | | *Arhgap15* | | 0.397 | | 2.29E-03 | | † 0.68 | † 8.86E-04 | – | – |
| 246746 | | *Cd300lf* | | 0.398 | | 4.89E-02 | | † 0.74 | † 4.34E-03 | – | – |
| 96875 | | *Prg4* | | 0.400 | | 1.36E-04 | | – | – | – | – |
| 15130 | | *Hbb-b2* | | 0.402 | | 2.54E-02 | | † 2.29 | † 1.86E-02 | – | – |
| 80796 | | *Calm4* | | 0.402 | | 2.60E-02 | | – | – | † 0.239 | † 1.79E-02 |

Genes with P > 0.05 were excluded from the analysis, as were ‘Rik’ sequences, ‘expressed sequences’, ‘predicted genes’ and ‘hypothetical proteins’. Also excluded was ‘*Xist*’ as this gene is X-linked and the number of females and males in the gene chip analyses were not balanced.

† : not present in the top 20 regulated genes of the respective list

– : not present in the respective list of genes, or P > 0.05

**Table S8**

**Primers used for genotyping**

| **Gene** | **Primer sequence (5’ – 3’)** | **Products [bp]** | |
| --- | --- | --- | --- |
| *Vhl* | forward: TGAGTATGGGATAACGGGTTGAAC | wt: 125 | floxed: 317 |
|  | reverse: AGAACTGACTGACTTCCACTGATGC |  |  |
| *Hif1a* | forward: GGAGCTATCTCTCTAGACC | wt: 215 | floxed: 260 |
|  | reverse: GCAGTTAAGAGCACTAGTTG |  |  |
| *Hif2a* | forward: TGTAGGCAAGGAAACCAAGG | wt: 182 | floxed: 220 |
|  | reverse: GAGAGCAGCTTCTCCTGGAA |  |  |
| *OpsinCre* | forward: AGGTGTAGAGAAGGCACTTAGC | wt: – | cre: 441 |
|  | reverse: CTAATCGCCATCTTCCAGCAGG |  |  |

**Table S9**

**Primers used to test for genomic excision**

| **Gene** | **Primer sequence (5’ – 3’)** | **Product [bp]** | | |
| --- | --- | --- | --- | --- |
|  |  | **wt** | **1-loxP** | **2-loxP** |
| *Vhl* | forward-1 ctggtacccacgaaactgtc | 286 | ~260 | ~460 |
|  | forward-2 ctaggcaccgagcttagaggtttgcg |  |  |  |
|  | reverse-1+2: ctgacttccactgatgcttgtcacag |  |  |  |
| *Hif1a* | forward-1: TTGGGGATGAAAACATCTGC | 215 | ~270 | ~260 |
|  | forward-2 GGAGCTATCTCTCTAGACC |  |  |  |
|  | reverse-1+2: GCAGTTAAGAGCACTAGTTG |  |  |  |
| *Hif2a* | forward-1: GCTAACACTGTACTGTCTGAAAGAGTAGC | 457 | ~300 | ~550 |
|  | forward-2 CTTCTTCCATCATCTGGGATCTGGGACT |  |  |  |
|  | reverse-1+2: CAGGCAGTATGCCTGGCTAATTCCAGTT |  |  |  |

**Table S10**

**Primers used for real-time PCR**

| **Gene** | **Forward 5’ – 3’** | **Reverse 5’ – 3’** | **Product [bp]** |
| --- | --- | --- | --- |
| **Human-specific primers** | | | |
| *ACTB* | CCTGGCACCCAGCACAAT | GGGCCGGACTCGTCATAC | 144 |
| *GAPDH* | CTTCGCTCTCTGCTCCTCCT | ATCCGTTGACTCCGACCTTC | 111 |
| *RPL28* | gcaattccttccgctacaac | TGTTCTTGCGGATCATGTGT | 198 |
| *ADM* | ATCACTCTCTTAGCAGGGTCT | CCACTTATTCCACTTCTTTCG | 148 |
| *VEGFA* | GTGGACATCTTCCAGGAGTACC | TGTTGTGCTGTAGGAAGCTCAT | 205 |
| *GLUT1* | ACTGTCGTGTCGCTGTTTG | CCAGGACCCACTTCAAAGAA | 191 |
| *PDK1* | CACGCTGGGTAATGAGGATT | GGAGGTCTCAACACGAGGT | 127 |
| *RHO* | ATCATGGTCATCGCTTTCCT | CTTGGACACGGTAGCAGAG | 252 |
| *GNAT2* | CTGCTACTGCTGGGTGCT | TGGTGAATAGCCATCCTGGT | 84 |
| *PDE6A* | CTACCAGATGAAATCCCAGAAC | CTTGAAATACAGGGCGAGGT | 202 |
| *PDE6C* | AACTGTTTCAAACTGCCGCT | TATTTTGGAGAGGCACCACC | 100 |
| *LOXL4* | AACTGCCTCTCCAAGTCTGC | AGGAGGTCGTAGTGGGTGAA | 191 |
| *LAD1* | CCTTTCGGATGAAACCCAAGA | CCCACAGGAGCCACGAATA | 187 |
| *SMTNL2* | AACTTCGAGCTGGCTTTCA | CATCACCATCATGTCCTCCA | 81 |
| *LIF* | TGCCAATGCCCTCTTTATTC | AGGTGCCAAGGTACACGACT | 170 |
| *EDN2* | TGTGCCACCTTCTGCCTTC | TGGAAATGTCCCTCAGCCTT | 139 |
| *FGF2* | AGAAGAGCGACCCTCACATCAAG | TCATCCGTAACACATTTAGAAGCCAG | 139 |
| *CASP1* | CCACAATGGGCTCTGTTTTT | GCTCTACCATCTGGCTGCTC | 124 |
|  |  |  |  |
| **Mouse-specific primers** | | | |
| *Actb* | CAACGGCTCCGGCATGTGC | CTCTTGCTCTGGGCCTCG | 153 |
| *Gnat1* | GAGGATGCTGAGAAGGATGC | TGAATGTTGAGCGTGGTCAT | 209 |
| *Gnat2* | GCATCAGTGCTGAGGACAAA | CTAGGCACTCTTCGGGTGAG | 192 |
| *Adm* | TCCTGGTTTCTCGGCTTCTC | ATTCTGTGGCGATGCTCTGA | 133 |
| *Vegf* | ACTTGTGTTGGGAGGAGGATGTC | AATGGGTTTGTCGTGTTTCTGG | 171 |
| *Bnip3* | cctgtcgcagttgggttc | gaagtgcagttctacccaggag | 93 |
| *Egln1* | cattgttggcagaaggtgtg | caaaggactacagggtctcca | 70 |
| *Lif* | AATGCCACCTGTGCCATACG | CAACTTGGTCTTCTCTGTCCCG | 216 |
| *Edn2* | agacctcctccgaaagctg | Ctggctgtagctggcaaag | 64 |
| *Fgf2* | tgtgtctatcaagggagtgtgtgc | accaactggagtatttccgtgaccg | 158 |
| *Casp1* | ggcaggaattctggagcttcaa | gtcagtcctggaaatgtgcc | 138 |
| *Pdk1* | GTTGAAACGTCCCGTGCT | AGTCTCTCGACGGATTCTGT | 170 |
| *Glut1* | CAGTGTATCCTGTTGCCCTTCTG | GCCGACCCTCTTCTTTCATCTC | 151 |
| *Stat3* | CAAAACCCTCAAGAGCCAAGG | TCACTCACAATGCTTCTCCGC | 132 |
| *Gfap* | ccaccaaactggctgatgtctac | ttctctccaaatccacacgagc | 240 |
| *Smtnl2* | GCATCTTGGAGAACGGACA | CAGAGAATTTCTCGGAGACTCG | 168 |
| *Lad1* | CACCCAGAATGGAGCTCAG | CACCTGCCGCCTCTGTCTCCG | 154 |
| *Loxl4* | GTTGCACAACTGCCACACA | GGAGTGCAGTAATGGCTTC | 108 |
