# Supplementary material for: Hif1a inactivation rescues photoreceptor degeneration induced by a chronic hypoxia-like stress
Source: Cell Death Differ. 2018 Apr 17;25(12):2071–85. doi: 10.1038/s41418-018-0094-7 (PMC6261999; doi:10.1038/s41418-018-0094-7)
Supplement: Supplementary file 2 — Supplemental File S1 [file 41418_2018_94_MOESM2_ESM.pdf]

Suppl. Table S1: Comparison: Average expression in *rod*$^{\Delta Vhl}$ divided by average expression in *Vhl*$^{f/f}$;*Hif1a*$^{f/f}$ (control)

Entrez Gene ID [Agilent]	Gene Symbol [Agilent]	Description [Agilent]	ratio	pValue	fdr
213742	Xist	inactive X specific transcripts	472.813239	6.51E-09	1.44E-05
71957	Cpsf3l	cleavage and polyadenylation specific factor 3-like	162.919518	4.09E-07	0.0001309
619297	C430049E01Rik	RIKEN cDNA C430049E01 gene	151.080224	4.52E-09	1.18E-05
76487	Ppp1r3g	protein phosphatase 1, regulatory (inhibitor) subunit 3G	79.6812749	8.93E-10	5.20E-06
213742	Xist	inactive X specific transcripts	78.9889415	2.37E-07	9.42E-05
223780	Adm2	adrenomedullin 2	71.942446	9.09E-08	5.73E-05
13615	Edn2	endothelin 2	62.6566416	4.30E-08	4.13E-05
67573	Loxl4	lysyl oxidase-like 4	33.9558574	1.83E-05	0.001389
240913	Adamts4	a disintegrin-like and metallopeptidase (reprolysin type) with thrombospondin type 1 motif, 4	31.9081047	0.0001611	0.004382
70045	2610528A11Rik	RIKEN cDNA 2610528A11 gene	30.816641	2.05E-08	3.05E-05
16763	Lad1	ladinin	30.2938503	6.58E-07	0.000182
236220	LOC236220	hypothetical protein LOC236220	26.3504611	9.73E-06	0.0009814
14663	Glycam1	glycosylation dependent cell adhesion molecule 1	25.1825737	3.39E-05	0.001912
11535	Adm	adrenomedullin	23.7022991	1.12E-07	6.00E-05
17339	Mip	major intrinsic protein of eye lens fiber	22.9095074	0.008083	0.04646
214301	Crygn	crystallin, gamma N	22.3313979	0.006261	0.03944
192199	Rspo1	R-spondin homolog (Xenopus laevis)	21.6825672	0.007823	0.04555
233187	Lim2	lens intrinsic membrane protein 2	21.6076059	0.004186	0.03061
12954	Cryaa	crystallin, alpha A	20.9205021	0.007343	0.04379
20856	Stc2	stanniocalcin 2	20.7425845	1.13E-06	0.0002853
77998	Grifin	galectin-related inter-fiber protein	19.8059022	0.003584	0.02785
12051	Bcl3	B-cell leukemia/lymphoma 3	19.4552529	1.62E-06	0.0003488
276829	Smtnl2	smoothelin-like 2	17.0299728	9.83E-08	5.80E-05
381359	Prdm12	PR domain containing 12	16.1576991	1.72E-05	0.001327
75290	4930557B15Rik	RIKEN cDNA 4930557B15 gene	16.025641	0.008547	0.04815
100039660	Ect2l	epithelial cell transforming sequence 2 oncogene-like	15.4511743	1.72E-05	0.001327
67573	Loxl4	lysyl oxidase-like 4	15.3092468	9.15E-06	0.000955
12962	Crybb3	crystallin, beta B3	14.5391102	0.003442	0.02711
21818	Tgm3	transglutaminase 3, E polypeptide	14.5327714	2.99E-05	0.001781
667977	Gm8909	predicted gene 8909	14.1743444	0.007455	0.04417
74492	Kbtbd13	kelch repeat and BTB (POZ) domain containing 13	13.3280021	1.03E-05	0.0009954
22300	Vmn2r123	vomeronasal 2, receptor 123	12.9651238	4.27E-09	1.18E-05
68662	Scgb3a1	secretoglobin, family 3A, member 1	11.8764846	4.83E-05	0.00234
20296	Ccl2	chemokine (C-C motif) ligand 2	11.8652112	0.0003137	0.006358
210321	BC048679	cDNA sequence BC048679	11.8553646	0.01211	0.06044
12268	C4b	complement component 4B (Childo blood group)	11.7938436	3.69E-06	0.0005564
15019	H2-Q8	histocompatibility 2, Q region locus 8	11.4784206	6.27E-08	4.89E-05
68380	0610042G04Rik	RIKEN cDNA 0610042G04 gene	11.4246544	1.66E-07	7.98E-05
18073	Nid1	nidogen 1	11.1869337	0.007656	0.04497
12310	Calca	calcitonin/calcitonin-related polypeptide, alpha	11.055832	1.77E-07	8.07E-05
18542	Pcolce	procollagen C-endopeptidase enhancer protein	10.9902187	0.0001002	0.003427

12957	Cryba1	crystallin, beta A1	10.9613066	0.009598	0.05187
12958	Cryba2	crystallin, beta A2	10.9194147	0.009546	0.05171
14611	Gja3	gap junction protein, alpha 3	10.8401084	0.003981	0.02967
238692	Zfp874a	zinc finger protein 874a	10.8307159	5.79E-06	0.0007388
12960	Crybb1	crystallin, beta B1	10.8201688	0.01173	0.05935
12609	Cebpd	CCAAT/enhancer binding protein (C/EBP), delta	10.7307651	1.59E-06	0.0003464
100043899	R3hdml	R3H domain containing-like	10.7032003	0.001889	0.01898
12960	Crybb1	crystallin, beta B1	10.6746371	0.01213	0.06051
19153	Prx	periaxin	10.5764146	0.003291	0.02638
19013	Ppara	peroxisome proliferator activated receptor alpha	10.5529759	3.56E-06	0.0005503
30923	Foxe3	forkhead box E3	10.4569696	0.005314	0.03552
12961	Crybb2	crystallin, beta B2	10.3896104	0.009309	0.05078
54382	Tcstv1	2-cell-stage, variable group, member 1	10.3691414	0.0002298	0.005414
235435	Lctl	lactase-like	10.3541106	0.004704	0.03296
15006	H2-Q1	histocompatibility 2, Q region locus 1	10.2986612	2.26E-06	0.0004246
14184	Fgfr3	fibroblast growth factor receptor 3	10.2061645	0.007933	0.04591
70989	4931429I11Rik	RIKEN cDNA 4931429I11 gene	10.2009589	1.78E-06	0.0003698
12630	Cfi	complement component factor i	10.0512614	3.49E-06	0.0005482
19013	Ppara	peroxisome proliferator activated receptor alpha	9.94035785	2.37E-05	0.001568
14173	Fgf2	fibroblast growth factor 2	9.86193294	1.01E-05	0.0009931
94224	Srd5a2	steroid 5 alpha-reductase 2	9.45179584	0.006427	0.0401
94346	Tmem40	transmembrane protein 40	9.37207123	0.006733	0.04137
14863	Gstm2	glutathione S-transferase, mu 2	9.36329588	0.002156	0.02029
13646	Klk1b22	kallikrein 1-related peptidase b22	9.33706816	9.99E-06	0.0009857
12955	Cryab	crystallin, alpha B	9.27643785	0.01013	0.05374
12961	Crybb2	crystallin, beta B2	9.10746812	0.01003	0.05343
12964	Cryga	crystallin, gamma A	8.91265597	0.002072	0.01983
94346	Tmem40	transmembrane protein 40	8.89679715	0.006332	0.03975
319506	7530428D23Rik	RIKEN cDNA 7530428D23 gene	8.89679715	0.01068	0.05545
79554	Gltpd1	glycolipid transfer protein domain containing 1	8.79507476	6.39E-05	0.002753
56312	Nupr1	nuclear protein 1	8.77963126	0.001623	0.01733
12959	Cryba4	crystallin, beta A4	8.77192982	0.0139	0.06607
12955	Cryab	crystallin, alpha B	8.59106529	0.007605	0.04477
14616	Gja8	gap junction protein, alpha 8	8.5106383	0.006404	0.03998
12182	Bst1	bone marrow stromal cell antigen 1	8.4317032	6.05E-06	0.0007456
14580	Gfap	glial fibrillary acidic protein	8.42459983	3.60E-07	0.0001173
22422	Wnt7b	wingless-related MMTV integration site 7B	7.98084597	0.0044	0.03163
18742	Pitx3	paired-like homeodomain transcription factor 3	7.82472613	0.006582	0.0407
434280	Gm5607	predicted gene 5607	7.80640125	0.0135	0.06474
21956	Tnnt2	troponin T2, cardiac	7.58725341	2.30E-07	9.34E-05
266744	Lgsn	lengsin, lens protein with glutamine synthetase domain	7.56429652	0.001688	0.01769
117167	Steap4	STEAP family member 4	7.51314801	0.0003453	0.006759
16453	Jak3	Janus kinase 3	7.50187547	7.83E-06	0.0008661
18050	Klk1b3	kallikrein 1-related peptidase b3	7.42942051	5.11E-06	0.0006957
11807	Apoa2	apolipoprotein A-II	7.31528895	0.02262	0.09122
12959	Cryba4	crystallin, beta A4	7.27802038	0.01249	0.06156
14462	Gata3	GATA binding protein 3	7.21500722	0.002053	0.01973
330004	Gm833	predicted gene 833	7.20980534	2.50E-05	0.001622
14863	Gstm2	glutathione S-transferase, mu 2	7.20980534	0.007895	0.04579
100041143	Gm3161	predicted gene 3161	7.18390805	0.008456	0.04775

69291	1700001L05Rik	RIKEN cDNA 1700001L05 gene	7.06713781	1.30E-06	0.0003081
12372	Casq1	calsequestrin 1	7.06713781	0.007068	0.04284
20564	Slit3	slit homolog 3 (Drosophila)	7.04721635	0.001355	0.01555
83961	Nrg4	neuregulin 4	6.95410292	5.64E-05	0.002567
15945	Cxcl10	chemokine (C-X-C motif) ligand 10	6.90131125	7.87E-05	0.003048
15160	Serpind1	serine (or cysteine) peptidase inhibitor, clade D, member 1	6.77506775	0.002793	0.02375
18295	Ogn	osteoglycin	6.65778961	0.0164	0.07339
112407	Egln3	EGL nine homolog 3 (C. elegans)	6.62251656	2.70E-06	0.0004772
12965	Crygb	crystallin, gamma B	6.57894737	0.003804	0.02892
12966	Crygc	crystallin, gamma C	6.57030223	0.006187	0.03908
327766	Tmem26	transmembrane protein 26	6.55737705	0.0001263	0.003875
20377	Sfrp1	secreted frizzled-related protein 1	6.54022237	0.006347	0.03983
12266	C3	complement component 3	6.41025641	5.74E-05	0.00259
112407	Egln3	EGL nine homolog 3 (C. elegans)	6.39795266	1.70E-05	0.001321
228796	Bpil3	bactericidal/permeability-increasing protein-like 3	6.39386189	1.78E-06	0.0003698
108105	B3gnt5	UDP-GlcNAc:betaGal beta-1,3-N-acetylglucosaminyltransferase 5	6.29326621	0.006051	0.03851
69291	1700001L05Rik	RIKEN cDNA 1700001L05 gene	6.24219725	1.47E-06	0.0003262
12966	Crygc	crystallin, gamma C	6.23441397	0.004186	0.03061
108105	B3gnt5	UDP-GlcNAc:betaGal beta-1,3-N-acetylglucosaminyltransferase 5	6.22665006	0.002786	0.02371
103988	Gck	glucokinase	6.18046972	2.74E-06	0.0004772
14580	Gfap	glial fibrillary acidic protein	6.10873549	1.30E-05	0.001118
18073	Nid1	nidogen 1	6.10873549	0.0003946	0.00733
320893	6430562O15Rik	RIKEN cDNA 6430562O15 gene	6.00961538	1.71E-06	0.0003637
68355	2010204K13Rik	RIKEN cDNA 2010204K13 gene	6.00600601	6.41E-06	0.0007597
14580	Gfap	glial fibrillary acidic protein	5.99161174	5.59E-05	0.002567
11670	Aldh3a1	aldehyde dehydrogenase family 3, subfamily A1	5.97728631	0.007963	0.046
379043	Raet1e	retinoic acid early transcript 1E	5.92768228	0.01281	0.06264
69354	Slc38a4	solute carrier family 38, member 4	5.88581519	0.00391	0.02936
14863	Gstm2	glutathione S-transferase, mu 2	5.83771162	0.007089	0.04292
12075	Bfsp1	beaded filament structural protein 1, in lens-CP94	5.76036866	0.006033	0.03843
667034	Pnp2	purine-nucleoside phosphorylase 2	5.74052813	0.0001062	0.003565
329977	Fhad1	forkhead-associated (FHA) phosphopeptide binding domain 1	5.73394495	0.000445	0.007902
232966	Zfp114	zinc finger protein 114	5.70450656	1.32E-07	6.89E-05
21857	Timp1	tissue inhibitor of metalloproteinase 1	5.6980057	3.18E-05	0.001855
26910	Figla	folliculogenesis specific basic helix-loop-helix	5.69151964	0.01367	0.0653
654812	Angptl7	angiopoietin-like 7	5.67214974	0.003531	0.02754
12970	Crygs	crystallin, gamma S	5.66893424	0.01565	0.07134
57814	Kcne4	potassium voltage-gated channel, Isk-related subfamily, gene 4	5.65930956	1.92E-05	0.001424
75600	Calml4	calmodulin-like 4	5.64015792	8.23E-07	0.0002243
12484	Cd24a	CD24a antigen	5.52181115	0.007127	0.04302
12774	Ccr5	chemokine (C-C motif) receptor 5	5.50055006	5.47E-05	0.002524
14526	Gcg	glucagon	5.48245614	0.00111	0.0139
12182	Bst1	bone marrow stromal cell antigen 1	5.43478261	4.72E-05	0.002319
100040018	Gm9835	predicted pseudogene 9835	5.41418517	5.01E-06	0.000688
18073	Nid1	nidogen 1	5.3361793	0.007503	0.04439
12967	Crygd	crystallin, gamma D	5.27983105	0.005186	0.03495
12832	Col5a2	collagen, type V, alpha 2	5.18403318	4.25E-07	0.0001317
319189	Hist2h2bb	histone cluster 2, H2bb	5.14668039	3.51E-06	0.0005482
58866	Treh	trehalase (brush-border membrane glycoprotein)	5.13347023	1.25E-05	0.001101
116812	Zfp264	zinc finger protein 264	5.07614213	0.002751	0.02359

104027	Synpo	synaptopodin	5.07099391	0.0002659	0.005839
20198	S100a4	S100 calcium binding protein A4	5.06842372	0.003883	0.02923
109901	Cela1	chymotrypsin-like elastase family, member 1	5.06072874	6.20E-05	0.002691
67951	Tubb6	tubulin, beta 6	5.05561173	0.001966	0.01937
100041089	Gm3134	predicted gene 3134	5.03778338	0.00337	0.02678
73748	Gadl1	glutamate decarboxylase-like 1	5.02008032	0.0009084	0.01224
12966	Crygc	crystallin, gamma C	5	0.006059	0.03851
56727	Miox	myo-inositol oxygenase	4.99001996	1.22E-06	0.0003014
69368	Wdfy1	WD repeat and FYVE domain containing 1	4.97017893	1.38E-06	0.0003151
20198	S100a4	S100 calcium binding protein A4	4.9382716	0.004066	0.03003
83961	Nrg4	neuregulin 4	4.85908649	0.0001293	0.003934
12738	Cldn2	claudin 2	4.85436893	0.03056	0.1116
15007	H2-Q10	histocompatibility 2, Q region locus 10	4.77783086	8.33E-06	0.000893
224008	2310008H04Rik	RIKEN cDNA 2310008H04 gene	4.76644423	5.97E-06	0.0007433
19076	Prim2	DNA primase, p58 subunit	4.6728972	3.02E-05	0.001781
18933	Prrx1	paired related homeobox 1	4.66200466	0.003236	0.02621
69253	Hspb2	heat shock protein 2	4.6253469	0.004739	0.03313
192136	5033411D12Rik	RIKEN cDNA 5033411D12 gene	4.62107209	0.007733	0.04528
214105	Sox30	SRY-box containing gene 30	4.61254613	9.46E-06	0.0009655
12954	Cryaa	crystallin, alpha A	4.60617227	0.02434	0.09552
67712	Slc25a37	solute carrier family 25, member 37	4.58926113	1.69E-05	0.001318
71004	4931440P22Rik	RIKEN cDNA 4931440P22 gene	4.53103761	1.13E-05	0.001054
12968	Cryge	crystallin, gamma E	4.50653447	7.96E-05	0.003065
71176	Fbxo24	F-box protein 24	4.48430493	0.0001534	0.004303
353211	Prune2	prune homolog 2 (Drosophila)	4.43262411	4.12E-06	0.0005976
242620	Dmrta2	doublesex and mab-3 related transcription factor like family A2	4.39947206	0.008159	0.04678
621304	Gm6209	predicted gene 6209	4.38404209	0.01011	0.05371
100041143	Gm3161	predicted gene 3161	4.38404209	0.02855	0.1065
12394	Runx1	runt related transcription factor 1	4.36681223	0.001428	0.01602
15360	Hmgcs2	3-hydroxy-3-methylglutaryl-Coenzyme A synthase 2	4.3554007	0.002349	0.02135
12550	Cdh1	cadherin 1	4.34027778	0.006482	0.04027
75998	5033425G24Rik	RIKEN cDNA 5033425G24 gene	4.33275563	2.11E-05	0.001458
100043229	Gm10046	predicted gene 10046	4.32338954	0.001206	0.01452
14283	Fosl1	fos-like antigen 1	4.3047783	1.99E-05	0.001424
226041	Pgm5	phosphoglucomutase 5	4.29922614	8.55E-05	0.00317
12258	Serping1	serine (or cysteine) peptidase inhibitor, clade G, member 1	4.28816467	0.0001001	0.003427
71862	Gpr160	G protein-coupled receptor 160	4.28082192	0.0053	0.03547
22421	Wnt7a	wingless-related MMTV integration site 7A	4.27899016	0.005364	0.03573
12258	Serping1	serine (or cysteine) peptidase inhibitor, clade G, member 1	4.2625746	0.0001883	0.004749
80885	Niacr1	niacin receptor 1	4.20521447	0.0004597	0.008073
271424	Ip6k3	inositol hexaphosphate kinase 3	4.20344683	3.55E-07	0.0001173
64706	Scube1	signal peptide, CUB domain, EGF-like 1	4.15973378	3.52E-05	0.001958
112405	Egln1	EGL nine homolog 1 (C. elegans)	4.14078675	1.86E-06	0.0003729
12642	Ch25h	cholesterol 25-hydroxylase	4.1322314	0.009222	0.05051
66812	Ppcdc	phosphopantothenoylcysteine decarboxylase	4.12371134	0.003734	0.02856
12702	Socs3	suppressor of cytokine signaling 3	4.12031314	1.02E-05	0.0009941
620382	Gm6146	predicted gene 6146	4.11692054	5.42E-06	0.0007224
18400	Slc22a18	solute carrier family 22 (organic cation transporter), member 18	4.11184211	0.0004306	0.007763
105355	Slc17a3	solute carrier family 17 (sodium phosphate), member 3	4.11015208	3.75E-05	0.002047
80879	Slc16a3	solute carrier family 16 (monocarboxylic acid transporters), member 3	4.08496732	9.21E-06	0.000956

232345	A2m	alpha-2-macroglobulin	4.08163265	0.008129	0.04664
320323	A930038B10Rik	RIKEN cDNA A930038B10 gene	4.04367165	7.90E-08	5.26E-05
17380	Mme	membrane metallo endopeptidase	4.03877221	0.008301	0.04723
74199	Vit	vitrin	4.01767778	0.002718	0.02338
100041358	Gm3289	predicted gene 3289	3.99520575	0.003097	0.02545
100038632	F830016D02Rik	RIKEN cDNA F830016D02 gene	3.97140588	0.005682	0.03705
11670	Aldh3a1	aldehyde dehydrogenase family 3, subfamily A1	3.96825397	0.009879	0.0529
214425	Cilp	cartilage intermediate layer protein, nucleotide pyrophosphohydrolase	3.94944708	0.01403	0.06652
66643	Lix1	limb expression 1 homolog (chicken)	3.93855849	0.004769	0.03323
74249	Lrrc2	leucine rich repeat containing 2	3.92772977	7.59E-06	0.0008451
80879	Slc16a3	solute carrier family 16 (monocarboxylic acid transporters), member 3	3.88048118	7.17E-06	0.0008127
272636	Esyt3	extended synaptotagmin-like protein 3	3.85653683	0.01013	0.05373
77619	Prelid2	PRELI domain containing 2	3.85356455	2.45E-05	0.001607
258689	Olfr1466	olfactory receptor 1466	3.85356455	0.001427	0.01602
14183	Fgfr2	fibroblast growth factor receptor 2	3.82262997	0.001382	0.01572
100382	AW011738	expressed sequence AW011738	3.77786173	0.0129	0.06293
100040022	LOC100040022	mCG1037230	3.73412995	0.0287	0.1069
76722	Ckmt2	creatine kinase, mitochondrial 2	3.72856078	0.006147	0.03889
12444	Ccnd2	cyclin D2	3.72439479	0.002326	0.02128
230099	Car9	carbonic anhydrase 9	3.71747212	0.02318	0.09259
666422	Gm8096	3-phosphoglycerate dehydrogenase pseudogene	3.71471025	0.001961	0.01936
72267	Lrrc8e	leucine rich repeat containing 8 family, member E	3.71057514	0.008087	0.04647
170706	Tmem37	transmembrane protein 37	3.69959304	0.000756	0.01098
24088	Tlr2	toll-like receptor 2	3.69276219	4.06E-05	0.002124
76905	Lrg1	leucine-rich alpha-2-glycoprotein 1	3.67376929	0.0001682	0.004465
545798	Tmem233	transmembrane protein 233	3.67376929	0.008849	0.04914
110253	Triobp	TRIO and F-actin binding protein	3.66972477	0.0002277	0.00538
14862	Gstm1	glutathione S-transferase, mu 1	3.66972477	0.002275	0.02098
11604	Agrp	agouti related protein	3.66837858	0.0003354	0.006637
72361	Ces2g	carboxylesterase 2G	3.62581581	0.01958	0.08261
74645	Fam46c	family with sequence similarity 46, member C	3.61925443	0.00531	0.03551
329581	Birc7	baculoviral IAP repeat-containing 7 (livin)	3.61532899	0.002702	0.02329
17345	Mki67	antigen identified by monoclonal antibody Ki 67	3.60880549	0.003237	0.02621
100689	Spon2	spondin 2, extracellular matrix protein	3.6036036	0.002893	0.02426
76718	Catsperg2	cation channel, sperm-associated, gamma 2	3.60100828	0.001248	0.01485
67315	Ceacam12	carcinoembryonic antigen-related cell adhesion molecule 12	3.59066427	0.03699	0.1263
11812	Apoc1	apolipoprotein C-I	3.58808755	2.02E-07	8.79E-05
100040235	LOC100040235	y-linked testis-specific protein 1-like	3.57398142	0.1711	0.3539
20200	S100a6	S100 calcium binding protein A6 (calcyclin)	3.57270454	0.0003967	0.007339
319555	Nwd1	NACHT and WD repeat domain containing 1	3.56760614	0.04645	0.1469
56847	Aldh1a3	aldehyde dehydrogenase family 1, subfamily A3	3.56252227	0.001063	0.01357
243755	Slc13a4	solute carrier family 13 (sodium/sulfate symporters), member 4	3.56125356	0.01723	0.07571
20969	Sdc1	syndecan 1	3.55871886	0.003761	0.0287
56012	Pgam2	phosphoglycerate mutase 2	3.55492357	0.0001911	0.004808
236539	Phgdh	3-phosphoglycerate dehydrogenase	3.54609929	0.003386	0.02687
280635	Emilin3	elastin microfibril interfacer 3	3.54358611	0.01962	0.08268
12353	Car6	carbonic anhydrase 6	3.52858151	6.88E-06	0.0008003
12266	C3	complement component 3	3.51864884	0.0002311	0.005432
14114	Fbln1	fibulin 1	3.51123596	0.06634	0.1879
236539	Phgdh	3-phosphoglycerate dehydrogenase	3.49528137	0.004359	0.03144

67410	4930449I24Rik	RIKEN cDNA 4930449I24 gene	3.4904014	0.0001261	0.003875
246177	Myo1g	myosin IG	3.47342827	6.93E-06	0.0008007
14962	Cfb	complement factor B	3.46860909	0.007448	0.04417
14864	Gstm3	glutathione S-transferase, mu 3	3.46260388	0.006396	0.03996
11639	Ak4	adenylate kinase 4	3.45901072	6.32E-07	0.0001795
12338	Capn6	calpain 6	3.45542502	0.009924	0.05301
72850	2900024J01Rik	RIKEN cDNA 2900024J01 gene	3.44827586	1.56E-05	0.001256
72088	Ush1c	Usher syndrome 1C homolog (human)	3.44352617	8.14E-05	0.003104
99543	Olfml3	olfactomedin-like 3	3.44352617	0.002332	0.02131
50909	C1ra	complement component 1, r subcomponent A	3.43170899	8.21E-05	0.003113
226527	BC026585	cDNA sequence BC026585	3.40831629	4.58E-05	0.002264
20293	Ccl12	chemokine (C-C motif) ligand 12	3.38753388	0.003302	0.02641
666048	Gm12824	predicted gene 12824	3.38180588	0.006012	0.03834
22041	Trf	transferrin	3.38066261	0.0001638	0.004406
329502	Pla2g4e	phospholipase A2, group IVE	3.36360579	0.0006366	0.00991
66898	Baiap2l1	BAI1-associated protein 2-like 1	3.36134454	0.000197	0.004911
17380	Mme	membrane metallo endopeptidase	3.35908633	0.005755	0.03732
432494	Gm5427	predicted gene 5427	3.354579	0.00167	0.01758
11639	Ak4	adenylate kinase 4	3.33889816	7.63E-05	0.003006
545648	Gm13272	predicted gene 13272	3.32005312	5.35E-05	0.002492
71846	Syce2	synaptonemal complex central element protein 2	3.3101622	1.09E-05	0.001036
238393	Serpina3f	serine (or cysteine) peptidase inhibitor, clade A, member 3F	3.30360093	2.46E-07	9.54E-05
241624	Exd1	exonuclease 3'-5' domain containing 1	3.2905561	1.15E-05	0.001065
268482	Krt12	keratin 12	3.2808399	0.0124	0.06128
18414	Osmr	oncostatin M receptor	3.27225131	6.43E-05	0.002762
74249	Lrrc2	leucine rich repeat containing 2	3.25839036	0.0001383	0.004072
338417	Scgb1c1	secretoglobin, family 1C, member 1	3.25626832	0.03081	0.1123
22041	Trf	transferrin	3.24991875	4.20E-05	0.002168
67434	Ankrd33b	ankyrin repeat domain 33B	3.21233537	0.02225	0.09044
73040	2900052N01Rik	RIKEN cDNA 2900052N01 gene	3.20821303	1.93E-06	0.0003823
231832	Tmem184a	transmembrane protein 184a	3.20102433	3.03E-07	0.0001086
21950	Tnfsf9	tumor necrosis factor (ligand) superfamily, member 9	3.2	1.20E-05	0.001077
14537	Gcnt1	glucosaminyl (N-acetyl) transferase 1, core 2	3.2	0.001178	0.01434
11830	Aqp5	aquaporin 5	3.19897633	0.0001162	0.003741
12654	Chi3l1	chitinase 3-like 1	3.19795331	3.29E-05	0.001877
54139	Irf6	interferon regulatory factor 6	3.18268619	2.89E-06	0.000488
69368	Wdfy1	WD repeat and FYVE domain containing 1	3.17359568	1.34E-06	0.0003132
252830	Obox6	oocyte specific homeobox 6	3.17258883	5.66E-08	4.88E-05
60531	Npvf	neuropeptide VF precursor	3.16656111	0.08603	0.225
224079	Atp13a4	ATPase type 13A4	3.16355584	0.003251	0.02623
12124	Bik	BCL2-interacting killer	3.15955766	0.001596	0.01716
71950	Nanog	Nanog homeobox	3.15756236	0.004901	0.03372
11830	Aqp5	aquaporin 5	3.15656566	0.0001372	0.00406
50909	C1ra	complement component 1, r subcomponent A	3.15556958	0.0001125	0.00369
22774	Zic4	zinc finger protein of the cerebellum 4	3.14070352	0.02046	0.08526
14114	Fbln1	fibulin 1	3.13381385	0.04673	0.1475
76293	Mfap4	microfibrillar-associated protein 4	3.12597687	0.06417	0.1839
110958	D6Mm5e	DNA segment, Chr 6, Miriam Meisler 5, expressed	3.12109863	0.003419	0.02702
12614	Celsr1	cadherin, EGF LAG seven-pass G-type receptor 1 (flamingo homolog, Drosophila)	3.11429461	0.008006	0.04618
74121	Acoxl	acyl-Coenzyme A oxidase-like	3.11138768	1.31E-05	0.001122

14972	H2-K1	histocompatibility 2, K1, K region	3.11041991	2.88E-06	0.000488
16774	Lama3	laminin, alpha 3	3.10462589	0.002932	0.02444
23882	Gadd45g	growth arrest and DNA-damage-inducible 45 gamma	3.09789343	3.66E-06	0.0005564
26968	Islr	immunoglobulin superfamily containing leucine-rich repeat	3.09693404	0.01107	0.05676
628900	Serpina3i	serine (or cysteine) peptidase inhibitor, clade A, member 3I	3.09501702	0.0004295	0.00775
12505	Cd44	CD44 antigen	3.09310238	0.0005382	0.008967
21785	Tff2	trefoil factor 2 (spasmolytic protein 1)	3.08546745	0.0002084	0.005075
66101	Ppih	peptidyl prolyl isomerase H	3.0835646	0.02084	0.0864
75860	4930588N13Rik	RIKEN cDNA 4930588N13 gene	3.08071473	0.000863	0.01191
14012	Mpzl2	myelin protein zero-like 2	3.07881773	0.002837	0.024
75853	4930592I03Rik	RIKEN cDNA 4930592I03 gene	3.07503075	0.001302	0.01518
246084	Defb35	defensin beta 35	3.06278714	0.14	0.3095
14865	Gstm4	glutathione S-transferase, mu 4	3.05810398	0.0007244	0.01077
76459	Car12	carbonic anyhydrase 12	3.05250305	7.03E-05	0.00286
14862	Gstm1	glutathione S-transferase, mu 1	3.04971028	0.004411	0.03164
15403	Hoxa6	homeobox A6	3.04043782	0.000272	0.005893
230145	Galnt12	UDP-N-acetyl-alpha-D-galactosamine:polypeptide N-acetylgalactosaminyltransferase 12	3.02297461	0.0089	0.04925
17873	Gadd45b	growth arrest and DNA-damage-inducible 45 beta	3.01932367	2.48E-05	0.001619
270685	Mthfd1l	methylenetetrahydrofolate dehydrogenase (NADP+ dependent) 1-like	3.01659125	3.00E-06	0.0005021
209195	Clic6	chloride intracellular channel 6	3.0102348	3.36E-05	0.001912
73424	1700064H15Rik	RIKEN cDNA 1700064H15 gene	3.00661455	0.0003109	0.006337
14102	Fas	Fas (TNF receptor superfamily member 6)	3.003003	1.94E-05	0.001424
12969	Crygf	crystallin, gamma F	3.00030003	0.005117	0.03465
11876	Artn	artemin	2.99760192	3.83E-05	0.002066
16453	Jak3	Janus kinase 3	2.99670363	5.64E-05	0.002567
270685	Mthfd1l	methylenetetrahydrofolate dehydrogenase (NADP+ dependent) 1-like	2.98953662	1.29E-05	0.001113
241490	Rbm45	RNA binding motif protein 45	2.97619048	4.67E-05	0.002303
14264	Fmod	fibromodulin	2.97265161	0.02183	0.08926
71583	9130008F23Rik	RIKEN cDNA 9130008F23 gene	2.97000297	2.44E-05	0.001607
408063	BC062258	cDNA sequence BC062258	2.93944738	2.67E-07	9.96E-05
14609	Gja1	gap junction protein, alpha 1	2.9385836	0.02309	0.09245
57746	Piwil2	piwi-like homolog 2 (Drosophila)	2.93772033	2.03E-05	0.001424
16997	Ltbp2	latent transforming growth factor beta binding protein 2	2.92312189	0.1053	0.2567
105355	Slc17a3	solute carrier family 17 (sodium phosphate), member 3	2.92226768	0.001162	0.01422
57435	Plin4	perilipin 4	2.90782204	0.0003187	0.006424
258165	Olfr965	olfactory receptor 965	2.89939113	0.02012	0.08412
14961	H2-Ab1	histocompatibility 2, class II antigen A, beta 1	2.89519398	0.002184	0.02039
19725	Rfx2	regulatory factor X, 2 (influences HLA class II expression)	2.89100896	0.001297	0.01514
66141	Ifitm3	interferon induced transmembrane protein 3	2.89017341	0.0001319	0.003982
50905	Il17rb	interleukin 17 receptor B	2.88850376	0.001646	0.0174
98267	Stk17b	serine/threonine kinase 17b (apoptosis-inducing)	2.87935502	2.11E-07	8.90E-05
67551	4933409L14Rik	RIKEN cDNA 4933409L14 gene	2.87191269	0.1449	0.3171
14961	H2-Ab1	histocompatibility 2, class II antigen A, beta 1	2.86532951	0.002138	0.02017
102265	AV026068	expressed sequence AV026068	2.86204923	0.08865	0.2293
78458	1700064N11Rik	RIKEN cDNA 1700064N11 gene	2.85714286	0.01175	0.05939
71355	Col24a1	collagen, type XXIV, alpha 1	2.85469597	0.008771	0.04889
68616	Gdpd3	glycerophosphodiester phosphodiesterase domain containing 3	2.84414107	0.0002251	0.005338
83457	Fthl17	ferritin, heavy polypeptide-like 17	2.83848992	6.00E-05	0.002628
12345	Capzb	capping protein (actin filament) muscle Z-line, beta	2.83205891	0.0009037	0.01221
13685	Eif4ebp1	eukaryotic translation initiation factor 4E binding protein 1	2.82725474	6.40E-06	0.0007597

74121	Acoxl	acyl-Coenzyme A oxidase-like	2.81769513	5.02E-05	0.002398
71738	Mamdc2	MAM domain containing 2	2.81373101	3.48E-05	0.001946
227753	Gsn	gelsolin	2.80662363	0.005993	0.03828
102436	Lars2	leucyl-tRNA synthetase, mitochondrial	2.80504909	5.88E-08	4.88E-05
791282	Gm10030	predicted gene 10030	2.80347631	2.34E-06	0.0004324
269120	Optc	opticin	2.79876854	0.1044	0.2553
21938	Tnfrsf1b	tumor necrosis factor receptor superfamily, member 1b	2.79642058	0.05351	0.1627
768271	BE949265	cDNA sequence BE949265	2.79173646	7.78E-05	0.00303
17084	Ly86	lymphocyte antigen 86	2.78318954	2.02E-05	0.001424
72088	Ush1c	Usher syndrome 1C homolog (human)	2.77623542	0.0004162	0.007604
620419	Zfp963	zinc finger protein 963	2.77623542	0.002103	0.02001
242608	Podn	podocan	2.76472215	0.007433	0.04414
22774	Zic4	zinc finger protein of the cerebellum 4	2.75862069	0.01655	0.07369
14362	Fzd1	frizzled homolog 1 (Drosophila)	2.74348422	5.98E-06	0.0007433
628900	Serpina3i	serine (or cysteine) peptidase inhibitor, clade A, member 3I	2.74122807	0.0002521	0.005712
67410	4930449I24Rik	RIKEN cDNA 4930449I24 gene	2.72182907	7.59E-05	0.003005
63959	Slc29a1	solute carrier family 29 (nucleoside transporters), member 1	2.72182907	9.91E-05	0.003415
240913	Adamts4	a disintegrin-like and metallopeptidase (reprolysin type) with thrombospondin type 1 motif, 4	2.7203482	0.005216	0.03511
20848	Stat3	signal transducer and activator of transcription 3	2.71960838	1.98E-05	0.001424
70258	1500035N22Rik	RIKEN cDNA 1500035N22 gene	2.71886895	0.002103	0.02001
77090	Ocel1	occludin/ELL domain containing 1	2.71591526	0.0002475	0.005635
71162	4933421I07Rik	RIKEN cDNA 4933421I07 gene	2.70197244	0.0001442	0.004169
218630	Ccno	cyclin O	2.70124257	0.0001529	0.004299
434729	Gm5635	predicted gene 5635	2.7005131	2.48E-05	0.001619
12223	Btc	betacellulin, epidermal growth factor family member	2.69541779	0.0005794	0.009333
14728	Lilrb4	leukocyte immunoglobulin-like receptor, subfamily B, member 4	2.69396552	0.02765	0.1042
16592	Fabp5	fatty acid binding protein 5, epidermal	2.68889486	0.008734	0.04878
100042782	Gm14458	predicted gene 14458	2.68384326	3.27E-05	0.001876
387345	Tas2r113	taste receptor, type 2, member 113	2.68312316	0.006613	0.04084
80876	Ifitm2	interferon induced transmembrane protein 2	2.68024658	0.001978	0.0194
11534	Adk	adenosine kinase	2.67594327	1.58E-05	0.00126
14555	Gpd1	glycerol-3-phosphate dehydrogenase 1 (soluble)	2.67522739	0.002078	0.01984
17970	Ncf2	neutrophil cytosolic factor 2	2.66880171	0.0004225	0.007669
622384	Fabp5l2	fatty acid binding protein 5-like 2	2.6652452	0.005842	0.03767
14587	Gfra3	glial cell line derived neurotrophic factor family receptor alpha 3	2.66098989	1.20E-05	0.001077
245884	Fam71f2	family with sequence similarity 71, member F2	2.65322367	0.0001041	0.003516
20491	Sla	src-like adaptor	2.65322367	0.002459	0.02189
434341	Nlrc5	NLR family, CARD domain containing 5	2.64410365	0.001143	0.01408
71760	Agxt2l1	alanine-glyoxylate aminotransferase 2-like 1	2.64270613	3.23E-05	0.001866
14999	H2-DMb1	histocompatibility 2, class II, locus Mb1	2.64061262	0.002774	0.02366
100043600	Gm4544	predicted gene 4544	2.63991552	0.0001135	0.003703
20657	Sod3	superoxide dismutase 3, extracellular	2.63782643	0.004635	0.03265
27083	Xlr4b	X-linked lymphocyte-regulated 4B	2.63782643	0.005274	0.03534
245572	Tbx22	T-box 22	2.63296472	0.05549	0.1668
73649	Cybrd1	cytochrome b reductase 1	2.62812089	3.77E-05	0.002047
107993	Bfsp2	beaded filament structural protein 2, phakinin	2.61917234	0.1739	0.3575
225187	Ankrd29	ankyrin repeat domain 29	2.61711594	5.70E-05	0.002584
21930	Tnfaip6	tumor necrosis factor alpha induced protein 6	2.61711594	0.0002548	0.005743
666317	Prl2c1	Prolactin family 2, subfamily c, member 1	2.61028452	1.26E-06	0.0003029
497106	Rnase12	ribonuclease, RNase A family, 12 (non-active)	2.60892252	0.0001577	0.004382

21937	Tnfrsf1a	tumor necrosis factor receptor superfamily, member 1a	2.60281104	4.57E-06	0.0006321
94352	Loxl2	lysyl oxidase-like 2	2.59807742	5.18E-05	0.002449
68813	Dock5	dedicator of cytokinesis 5	2.59000259	0.01016	0.05383
319207	Pgbd1	piggyBac transposable element derived 1	2.58531541	0.0006767	0.01026
20664	Sox1	SRY-box containing gene 1	2.58331181	0.01719	0.07557
22608	Ybx1	Y box protein 1	2.57997936	0.08257	0.219
15512	Hspa2	heat shock protein 2	2.5793139	2.26E-05	0.001511
78004	Prr15	proline rich 15	2.57599176	4.46E-05	0.002232
242481	Palm2	paralemmin 2	2.57201646	0.003213	0.02608
234130	Dkk4	dickkopf homolog 4 (Xenopus laevis)	2.5713551	0.09159	0.2344
434729	Gm5635	predicted gene 5635	2.57069409	0.002399	0.02155
14726	Pdpn	podoplanin	2.57003341	2.90E-05	0.001747
12505	Cd44	CD44 antigen	2.57003341	0.0001739	0.004549
70904	4921515G04Rik	RIKEN cDNA 4921515G04 gene	2.56213169	0.1158	0.2733
69382	1700024P04Rik	RIKEN cDNA 1700024P04 gene	2.55754476	0.0002235	0.005328
94226	S1pr5	sphingosine-1-phosphate receptor 5	2.55297421	5.85E-05	0.002604
218215	Rnf144b	ring finger protein 144B	2.55297421	0.0001759	0.004568
246079	Defb9	defensin beta 9	2.55297421	0.08022	0.2148
77558	9330179D12Rik	RIKEN cDNA 9330179D12 gene	2.54841998	0.1137	0.27
625360	BC147527	cDNA sequence BC147527	2.54517689	0.01643	0.07346
72415	Sgol1	shugoshin-like 1 (S. pombe)	2.53485425	4.46E-06	0.0006223
20538	Slc6a2	solute carrier family 6 (neurotransmitter transporter, noradrenalin), member 2	2.53164557	0.01753	0.07657
252967	Ropn1l	ropporin 1-like	2.52397779	5.87E-06	0.0007433
245450	Slitrk2	SLIT and NTRK-like family, member 2	2.5220681	1.97E-05	0.001424
67896	Ccdc80	coiled-coil domain containing 80	2.5220681	0.008267	0.04716
23880	Fyb	FYN binding protein	2.52079657	0.03644	0.1251
108832	5430405G05Rik	RIKEN cDNA 5430405G05 gene	2.52016129	7.01E-05	0.002856
18431	Oca2	oculocutaneous albinism II	2.51889169	8.16E-05	0.003105
21937	Tnfrsf1a	tumor necrosis factor receptor superfamily, member 1a	2.51445813	3.26E-05	0.001876
15007	H2-Q10	histocompatibility 2, Q region locus 10	2.51445813	0.0005887	0.009389
67606	Fibin	fin bud initiation factor homolog (zebrafish)	2.51319427	2.72E-05	0.001668
69382	1700024P04Rik	RIKEN cDNA 1700024P04 gene	2.51130085	0.001117	0.01392
26436	Psg16	pregnancy specific glycoprotein 16	2.5025025	4.28E-05	0.002195
170484	Nphs2	nephrosis 2 homolog, podocin (human)	2.5025025	0.01024	0.05414
66058	Tmem176a	transmembrane protein 176A	2.50062516	8.35E-05	0.003125
57277	Slurp1	secreted Ly6/Plaur domain containing 1	2.50062516	0.008048	0.04631
17133	Maff	v-maf musculoaponeurotic fibrosarcoma oncogene family, protein F (avian)	2.49812641	2.56E-05	0.001648
26570	Slc7a11	solute carrier family 7 (cationic amino acid transporter, y+ system), member 11	2.4888004	0.009849	0.05285
26415	Mapk13	mitogen-activated protein kinase 13	2.48632521	0.0006764	0.01026
27371	Sh2d2a	SH2 domain protein 2A	2.48200546	0.001273	0.01501
14735	Gpc4	glypican 4	2.48138958	0.01787	0.07768
56868	Psg23	pregnancy-specific glycoprotein 23	2.4789291	0.0001676	0.004465
12983	Csf2rb	colony stimulating factor 2 receptor, beta, low-affinity (granulocyte-macrophage)	2.47524752	0.01768	0.07716
58521	Eid1	EP300 interacting inhibitor of differentiation 1	2.47096615	3.33E-06	0.0005329
12803	Cntf	ciliary neurotrophic factor	2.47096615	0.0001567	0.004363
72778	Dnajc22	DnaJ (Hsp40) homolog, subfamily C, member 22	2.47096615	0.02396	0.09474
12870	Cp	ceruloplasmin	2.46974562	9.88E-05	0.003415
13003	Vcan	versican	2.45881485	0.08264	0.2191
71914	Antxr2	anthrax toxin receptor 2	2.45639892	8.23E-05	0.003113
57741	Noc2l	nucleolar complex associated 2 homolog (S. cerevisiae)	2.45098039	3.18E-06	0.0005236

76747	Dapl1	death associated protein-like 1	2.44857982	0.01395	0.06624
16403	Itga6	integrin alpha 6	2.44798042	0.002495	0.02209
633640	Gm7120	predicted gene 7120	2.44618395	0.009347	0.05088
70186	Fam162a	family with sequence similarity 162, member A	2.4431957	1.33E-05	0.001135
633640	Gm7120	predicted gene 7120	2.4431957	0.004102	0.03025
67410	4930449I24Rik	RIKEN cDNA 4930449I24 gene	2.43013366	0.03663	0.1255
50908	C1s	complement component 1, s subcomponent	2.42424242	6.55E-05	0.002788
19128	Pros1	protein S (alpha)	2.42248062	0.00096	0.01267
327959	Xaf1	XIAP associated factor 1	2.42189392	0.0001149	0.003728
381868	Gm1082	predicted gene 1082	2.42189392	0.003551	0.02765
17831	Muc2	mucin 2	2.41954996	0.0004427	0.007895
66338	Cdrt4	CMT1A duplicated region transcript 4	2.41954996	0.02697	0.1025
68151	Wls	wntless homolog (Drosophila)	2.41312741	0.005128	0.03469
78896	1500015O10Rik	RIKEN cDNA 1500015O10 gene	2.41312741	0.02652	0.1014
667742	Fam38b	family with sequence similarity 38, member B	2.41312741	0.03426	0.12
14412	Slc6a13	solute carrier family 6 (neurotransmitter transporter, GABA), member 13	2.40905806	0.04022	0.1333
20848	Stat3	signal transducer and activator of transcription 3	2.40673887	3.87E-05	0.002066
21817	Tgm2	transglutaminase 2, C polypeptide	2.40384615	9.74E-05	0.003408
52502	Carhsp1	calcium regulated heat stable protein 1	2.40269101	0.0008824	0.01208
15162	Hck	hemopoietic cell kinase	2.39520958	0.001074	0.01361
666048	Gm12824	predicted gene 12824	2.39520958	0.003865	0.02918
70415	2610018G03Rik	RIKEN cDNA 2610018G03 gene	2.38777459	0.0001794	0.004623
73914	Irak3	interleukin-1 receptor-associated kinase 3	2.38720458	0.001692	0.0177
14963	H2-Bl	histocompatibility 2, blastocyst	2.38038562	0.0001622	0.004382
75773	Adad2	adenosine deaminase domain containing 2	2.37812128	0.1552	0.3314
17181	Matn2	matrilin 2	2.37755587	0.009005	0.04966
14972	H2-K1	histocompatibility 2, K1, K region	2.37699073	9.45E-06	0.0009655
15530	Hspg2	perlecan (heparan sulfate proteoglycan 2)	2.37529691	0.009818	0.05273
108832	5430405G05Rik	RIKEN cDNA 5430405G05 gene	2.37473284	1.55E-05	0.001256
56485	Slc2a5	solute carrier family 2 (facilitated glucose transporter), member 5	2.37473284	0.001551	0.01696
20341	Selenbp1	selenium binding protein 1	2.37360551	3.17E-05	0.001853
218215	Rnf144b	ring finger protein 144B	2.36686391	0.000266	0.005839
242481	Palm2	paralemmin 2	2.36630383	0.00396	0.02955
212989	Best2	bestrophin 2	2.36239074	0.1475	0.3206
76486	Ly6k	lymphocyte antigen 6 complex, locus K	2.36016049	0.001348	0.01551
12159	Bmp4	bone morphogenetic protein 4	2.35626767	0.04297	0.1392
18205	Ntf3	neurotrophin 3	2.3551578	0.01648	0.07357
236285	Lancl3	LanC lantibiotic synthetase component C-like 3 (bacterial)	2.35460325	0.0002406	0.005548
14469	Gbp2	guanylate binding protein 2	2.35294118	0.0001267	0.003881
70370	Fbln7	fibulin 7	2.34852043	0.003299	0.0264
14867	Gstm6	glutathione S-transferase, mu 6	2.34576589	5.00E-05	0.002397
57275	Lenep	lens epithelial protein	2.34356691	0.0126	0.06201
14275	Folr1	folate receptor 1 (adult)	2.34192037	0.001267	0.01497
67269	Agtpbp1	ATP/GTP binding protein 1	2.34137204	2.09E-05	0.001458
22626	Slc23a3	solute carrier family 23 (nucleobase transporters), member 3	2.33154581	2.14E-06	0.000415
14107	Fat1	FAT tumor suppressor homolog 1 (Drosophila)	2.33100233	0.007907	0.04584
56185	Hao2	hydroxyacid oxidase 2	2.32991612	3.96E-05	0.002101
245269	E130304F04Rik	RIKEN cDNA E130304F04 gene	2.32991612	0.007617	0.04482
619289	Rfx8	regulatory factor X 8	2.32883093	0.002817	0.02388
18011	Neurl1a	neuralized homolog 1A (Drosophila)	2.32666356	2.65E-05	0.001661

11910	Atf3	activating transcription factor 3	2.32288037	1.77E-05	0.001349
21788	Tfpi	tissue factor pathway inhibitor	2.32126277	0.001097	0.01379
100038577	Gm10790	predicted gene 10790	2.31535078	0.02185	0.08932
100043272	5430417L22Rik	RIKEN cDNA 5430417L22 gene	2.31107003	0.00131	0.01523
626175	Gm6654	predicted pseudogene 6654	2.31107003	0.006869	0.04197
21817	Tgm2	transglutaminase 2, C polypeptide	2.30840259	4.88E-05	0.002356
317677	Gm5077	predicted gene 5077	2.3057413	0.0001446	0.004169
320159	Fam179a	family with sequence similarity 179, member A	2.30414747	0.0006182	0.009706
18566	Pdcd1	programmed cell death 1	2.30361668	0.01456	0.06815
242705	E2f2	E2F transcription factor 2	2.30308614	0.0002427	0.005581
19041	Ppl	periplakin	2.30255584	0.01671	0.0741
631145	4932442L08Rik	RIKEN cDNA 4932442L08 gene	2.30255584	0.01715	0.07547
15486	Hsd17b2	hydroxysteroid (17-beta) dehydrogenase 2	2.30096641	0.0007452	0.01095
12176	Bnip3	BCL2/adenovirus E1B interacting protein 3	2.29726625	9.79E-05	0.003415
263803	Pkn3	protein kinase N3	2.29726625	0.0395	0.1317
380878	AF067063	cDNA sequence AF067063	2.29673863	8.02E-05	0.003077
228026	Pdk1	pyruvate dehydrogenase kinase, isoenzyme 1	2.29621125	5.54E-06	0.000723
545056	Gm5801	ubiquitin-conjugating enzyme E2, J2 homolog pseudogene	2.29410415	0.01244	0.06144
207839	Galnt6	UDP-N-acetyl-alpha-D-galactosamine:polypeptide N-acetylgalactosaminyltransferase 6	2.29357798	0.005552	0.03651
74589	Kbtbd12	kelch repeat and BTB (POZ) domain containing 12	2.29042602	0.003813	0.02893
100038504	Gm10369	predicted gene 10369	2.28885328	0.05345	0.1626
56615	Mgst1	microsomal glutathione S-transferase 1	2.28310502	0.001113	0.0139
20702	Serpina1c	serine (or cysteine) peptidase inhibitor, clade A, member 1C	2.27998176	0.002609	0.02279
12176	Bnip3	BCL2/adenovirus E1B interacting protein 3	2.27946205	4.41E-05	0.002225
102242	AU024180	expressed sequence AU024180	2.27583068	0.0007826	0.01123
210029	Metrnl	meteorin, glial cell differentiation regulator-like	2.27479527	0.002217	0.02057
22673	Zfp185	zinc finger protein 185	2.27221086	0.01347	0.06465
236285	Lancl3	LanC lantibiotic synthetase component C-like 3 (bacterial)	2.2675737	2.67E-05	0.001661
12575	Cdkn1a	cyclin-dependent kinase inhibitor 1A (P21)	2.26449275	0.000239	0.005541
56615	Mgst1	microsomal glutathione S-transferase 1	2.25835592	0.002371	0.02141
219134	Shisa2	shisa homolog 2 (Xenopus laevis)	2.25479143	8.31E-05	0.003125
78353	2500002B13Rik	RIKEN cDNA 2500002B13 gene	2.25377507	0.003853	0.02913
76166	6330545A04Rik	RIKEN cDNA 6330545A04 gene	2.25123818	1.56E-05	0.001256
226041	Pgm5	phosphoglucomutase 5	2.25073149	0.02126	0.08763
241989	Pabpc4l	poly(A) binding protein, cytoplasmic 4-like	2.25022502	6.30E-06	0.0007573
52502	Carhsp1	calcium regulated heat stable protein 1	2.24971879	0.002161	0.02031
14936	Gys1	glycogen synthase 1, muscle	2.24466891	0.0002558	0.005746
58198	Sall1	sal-like 1 (Drosophila)	2.24466891	0.001601	0.0172
210876	Vmn2r111	vomeronasal 2, receptor 111	2.24466891	0.01283	0.06272
18440	P2rx6	purinergic receptor P2X, ligand-gated ion channel, 6	2.2426553	0.01493	0.06916
69706	Ppil5	peptidylprolyl isomerase (cyclophilin) like 5	2.23813787	0.00597	0.03817
56857	Slc37a2	solute carrier family 37 (glycerol-3-phosphate transporter), member 2	2.23713647	0.0001027	0.003493
226421	5430435G22Rik	RIKEN cDNA 5430435G22 gene	2.2366361	6.65E-05	0.00279
212108	Rln3	relaxin 3	2.23613596	6.44E-05	0.002762
18011	Neurl1a	neuralized homolog 1A (Drosophila)	2.23463687	6.18E-06	0.0007474
12971	Crym	crystallin, mu	2.23264121	4.45E-05	0.002231
21858	Timp2	tissue inhibitor of metalloproteinase 2	2.23015165	1.78E-05	0.001352
20204	Prrx2	paired related homeobox 2	2.22866057	0.0004646	0.008135
244864	Layn	layilin	2.22766763	0.00127	0.01499
14695	Gnb3	guanine nucleotide binding protein (G protein), beta 3	2.22518914	0.0001596	0.004382

56306	Fam60a	family with sequence similarity 60, member A	2.22321032	0.0003255	0.006504
19215	Ptgds	prostaglandin D2 synthase (brain)	2.22321032	0.003147	0.0257
216725	Adamts2	a disintegrin-like and metallopeptidase (reprolysin type) with thrombospondin type 1 motif, 2	2.22271616	0.01535	0.07056
19329	Rab17	RAB17, member RAS oncogene family	2.22024867	0.01488	0.06901
14867	Gstm6	glutathione S-transferase, mu 6	2.21680337	0.0001188	0.003767
71145	Scara5	scavenger receptor class A, member 5 (putative)	2.21680337	0.0002022	0.005006
12176	Bnip3	BCL2/adenovirus E1B interacting protein 3	2.21533008	3.29E-06	0.0005329
84004	Mcam	melanoma cell adhesion molecule	2.21385876	0.001878	0.01889
240444	Kcng2	potassium voltage-gated channel, subfamily G, member 2	2.21385876	0.009603	0.05188
23833	Cd52	CD52 antigen	2.21141088	0.001388	0.01574
66857	Plbd1	phospholipase B domain containing 1	2.20945647	0.02057	0.08555
12306	Anxa2	annexin A2	2.20848057	7.55E-05	0.002998
329727	Dennd2c	DENN/MADD domain containing 2C	2.20604456	0.001588	0.01712
97458	C80012	expressed sequence C80012	2.20215811	6.61E-06	0.0007742
19013	Ppara	peroxisome proliferator activated receptor alpha	2.20022002	0.0005872	0.009381
74071	Ifltd1	intermediate filament tail domain containing 1	2.19828534	0.3438	0.5547
14936	Gys1	glycogen synthase 1, muscle	2.19731927	5.76E-05	0.00259
54357	Epb4.1l4b	erythrocyte protein band 4.1-like 4b	2.19731927	0.01995	0.08371
64058	Perp	PERP, TP53 apoptosis effector	2.19683656	0.006674	0.04113
227545	5430407P10Rik	RIKEN cDNA 5430407P10 gene	2.19587176	0.00026	0.0058
20852	Stat6	signal transducer and activator of transcription 6	2.19538968	0.04239	0.1381
14468	Gbp1	guanylate binding protein 1	2.19394471	0.0006593	0.01007
545260	Arsi	arylsulfatase i	2.19394471	0.1131	0.2691
17909	Myo10	myosin X	2.19202104	0.001531	0.01682
73671	Sult6b1	sulfotransferase family, cytosolic, 6B, member 1	2.1872266	0.0002876	0.006071
414801	Itprip	inositol 1,4,5-triphosphate receptor interacting protein	2.18531469	3.23E-05	0.001866
242939	Cpz	carboxypeptidase Z	2.17864924	0.001419	0.01597
12803	Cntf	ciliary neurotrophic factor	2.17817469	0.0004773	0.008292
80290	Gpr146	G protein-coupled receptor 146	2.17770035	1.86E-05	0.001397
69706	Ppil5	peptidylprolyl isomerase (cyclophilin) like 5	2.17155266	5.38E-06	0.0007224
11745	Anxa3	annexin A3	2.1691974	0.007149	0.04314
69376	Zpbp2	zona pellucida binding protein 2	2.16872696	0.002338	0.02132
14130	Fcgr2b	Fc receptor, IgG, low affinity IIb	2.16778669	0.003586	0.02785
18452	P4ha2	procollagen-proline, 2-oxoglutarate 4-dioxygenase (proline 4-hydroxylase), alpha II polypeptide	2.16684724	1.98E-05	0.001424
547349	LOC547349	similar to MHC class I antigen precursor	2.16684724	0.001414	0.01593
102589	AI835735	expressed sequence AI835735	2.16684724	0.1364	0.3039
21788	Tfpi	tissue factor pathway inhibitor	2.16309756	0.0002913	0.006115
52670	Cpsf4l	cleavage and polyadenylation specific factor 4-like	2.16262976	0.01674	0.07416
104816	Aspg	asparaginase homolog (S. cerevisiae)	2.16076059	0.0002301	0.005416
14407	Gabrg3	gamma-aminobutyric acid (GABA) A receptor, subunit gamma 3	2.15889465	5.85E-05	0.002604
100038740	Gm10325	predicted gene 10325	2.1574973	0.0001188	0.003767
330004	Gm833	predicted gene 833	2.15100022	0.0002203	0.005273
71660	Rarres2	retinoic acid receptor responder (tazarotene induced) 2	2.1486893	0.007067	0.04284
100169874	Gm11110	predicted gene 11110	2.14730513	7.14E-05	0.002897
20730	Spink3	serine peptidase inhibitor, Kazal type 3	2.14730513	0.0003552	0.006863
21884	Fabp9	fatty acid binding protein 9, testis	2.14638334	0.0001685	0.004466
17750	Mt2	metallothionein 2	2.14362272	0.0001107	0.003663
20971	Sdc4	syndecan 4	2.14041096	3.44E-06	0.0005461
50874	Tmod4	tropomodulin 4	2.13949508	0.001219	0.0146
74341	G630025P09Rik	RIKEN cDNA G630025P09 gene	2.13675214	2.25E-05	0.001507

211896	Depdc7	DEP domain containing 7	2.13629566	0.002515	0.0222
103743	Tmem98	transmembrane protein 98	2.13401622	0.0001557	0.004342
230145	Galnt12	UDP-N-acetyl-alpha-D-galactosamine:polypeptide N-acetylgalactosaminyltransferase 12	2.13401622	0.0148	0.06879
70358	Steap1	six transmembrane epithelial antigen of the prostate 1	2.13265089	0.0005382	0.008967
208439	Klhl29	kelch-like 29 (Drosophila)	2.13219616	3.94E-06	0.0005852
15002	H2-Ob	histocompatibility 2, O region beta locus	2.13219616	0.09683	0.243
394435	Ugt1a6b	UDP glucuronosyltransferase 1 family, polypeptide A6B	2.1312873	0.0003395	0.006688
30794	Pdlim4	PDZ and LIM domain 4	2.12992545	0.0003638	0.006971
22352	Vim	vimentin	2.12947189	0.0008868	0.01209
67712	Slc25a37	solute carrier family 25, member 37	2.12856535	1.04E-05	0.0009972
13836	Epha2	Eph receptor A2	2.12720698	0.0001529	0.004299
14635	Galk1	galactokinase 1	2.12675457	8.45E-05	0.00315
68794	Flnc	filamin C, gamma	2.12359312	8.68E-05	0.003189
243084	Tmprss11e	transmembrane protease, serine 11e	2.12359312	0.07356	0.2024
13590	Lefty1	left right determination factor 1	2.12179079	0.03617	0.1246
74580	Pyroxd2	pyridine nucleotide-disulphide oxidoreductase domain 2	2.12089077	0.0005392	0.008976
106648	Cyp4f15	cytochrome P450, family 4, subfamily f, polypeptide 15	2.12089077	0.03836	0.1294
22419	Wnt5b	wingless-related MMTV integration site 5B	2.11954218	0.001156	0.01418
320528	Vps13c	vacuolar protein sorting 13C (yeast)	2.11909303	0.0002148	0.00519
56722	Litaf	LPS-induced TN factor	2.11864407	6.57E-05	0.00279
22772	Zic2	zinc finger protein of the cerebellum 2	2.11729833	0.002173	0.02033
67269	Agtpbp1	ATP/GTP binding protein 1	2.11505922	0.000294	0.006161
17919	Myo5b	myosin VB	2.11148649	0.01053	0.05502
217353	Tmc6	transmembrane channel-like gene family 6	2.11059519	0.0009257	0.01241
243914	Lgi4	leucine-rich repeat LGI family, member 4	2.10216523	2.21E-05	0.001504
93694	Clec2d	C-type lectin domain family 2, member d	2.10128178	0.0002238	0.005328
24064	Spry2	sprouty homolog 2 (Drosophila)	2.10128178	0.001052	0.01349
195531	Gm13152	predicted gene 13152	2.10084034	0.03386	0.1192
233744	Spon1	spondin 1, (f-spondin) extracellular matrix protein	2.09951711	0.0002677	0.005855
639774	Skint8	selection and upkeep of intraepithelial T cells 8	2.09819555	0.2026	0.3956
240638	Slc16a12	solute carrier family 16 (monocarboxylic acid transporters), member 12	2.09424084	0.07266	0.2006
69634	Clybl	citrate lyase beta like	2.09205021	6.73E-05	0.002797
12259	C1qa	complement component 1, q subcomponent, alpha polypeptide	2.08724692	0.0001608	0.004382
17240	Mdfi	MyoD family inhibitor	2.08420175	0.0006571	0.01006
12919	Crhbp	corticotropin releasing hormone binding protein	2.08246564	0.02174	0.08898
320004	A930002H24Rik	RIKEN cDNA A930002H24 gene	2.08116545	0.001161	0.01422
13649	Egfr	epidermal growth factor receptor	2.07856994	0.01265	0.06214
14675	Gna14	guanine nucleotide binding protein, alpha 14	2.07296849	0.01416	0.06696
227545	5430407P10Rik	RIKEN cDNA 5430407P10 gene	2.07082212	8.58E-05	0.00317
17476	Mpeg1	macrophage expressed gene 1	2.06953642	3.28E-05	0.001877
66066	Gng11	guanine nucleotide binding protein (G protein), gamma 11	2.06953642	0.0001853	0.004709
19039	Lgals3bp	lectin, galactoside-binding, soluble, 3 binding protein	2.06654267	6.68E-05	0.00279
16949	Loxl1	lysyl oxidase-like 1	2.0661157	0.001579	0.01708
64929	Scel	sciellin	2.06526229	0.03179	0.1145
69376	Zpbp2	zona pellucida binding protein 2	2.06355757	0.09574	0.2416
108052	Slc14a1	solute carrier family 14 (urea transporter), member 1	2.06100577	0.0001959	0.004901
100041143	Gm3161	predicted gene 3161	2.05296654	0.04088	0.1347
16669	Krt19	keratin 19	2.05128205	0.007856	0.04564
239368	BC030476	cDNA sequence BC030476	2.0500205	0.000932	0.01245
18726	Lilra6	leukocyte immunoglobulin-like receptor, subfamily A (with TM domain), member 6	2.04918033	0.002847	0.02405

385380	Tex28	testis expressed 28	2.0487605	0.05963	0.1747
16161	Il12rb1	interleukin 12 receptor, beta 1	2.04792136	0.0004274	0.007719
69640	Fam83g	family with sequence similarity 83, member G	2.04582651	0.0005056	0.008613
245050	Fam198a	family with sequence similarity 198, member A	2.04290092	0.003062	0.02521
252967	Ropn1l	ropporin 1-like	2.04206657	3.32E-06	0.0005329
100043335	Gm4371	eukaryotic translation initiation factor 3, subunit I pseudogene	2.04206657	0.04226	0.1379
15016	H2-Q5	histocompatibility 2, Q region locus 5	2.04164965	0.0003734	0.007103
78403	2900041M22Rik	RIKEN cDNA 2900041M22 gene	2.04081633	0.002912	0.02433
14468	Gbp1	guanylate binding protein 1	2.03998368	0.0006117	0.009639
78523	Mrpl9	mitochondrial ribosomal protein L9	2.03790503	2.24E-05	0.001507
17748	Mt1	metallothionein 1	2.03583062	4.22E-05	0.00217
18824	Plp2	proteolipid protein 2	2.03252033	0.0004056	0.007462
229534	Pbxip1	pre-B-cell leukemia transcription factor interacting protein 1	2.03128174	9.46E-05	0.003368
208439	Klhl29	kelch-like 29 (Drosophila)	2.03086921	9.27E-05	0.003316
17314	Mgmt	O-6-methylguanine-DNA methyltransferase	2.03086921	0.001906	0.01908
328035	Fads6	fatty acid desaturase domain family, member 6	2.03004466	0.1352	0.3024
94352	Loxl2	lysyl oxidase-like 2	2.02922078	0.004517	0.03212
16773	Lama2	laminin, alpha 2	2.02798621	0.01345	0.0646
18654	Pgf	placental growth factor	2.02757502	0.001067	0.01359
56295	Higd1a	HIG1 domain family, member 1A	2.02552157	2.84E-05	0.001724
22146	Tuba1c	tubulin, alpha 1C	2.02511138	0.0001123	0.003687
27368	Tbl2	transducin (beta)-like 2	2.0242915	9.84E-06	0.0009816
16423	Cd47	CD47 antigen (Rh-related antigen, integrin-associated signal transducer)	2.0242915	0.0001236	0.003839
217830	9030617O03Rik	RIKEN cDNA 9030617O03 gene	2.0242915	0.0001271	0.003887
69634	Clybl	citrate lyase beta like	2.02388181	0.0001086	0.003626
69820	1810059H22Rik	RIKEN cDNA 1810059H22 gene	2.02061022	5.91E-05	0.002619
16165	Il13ra2	interleukin 13 receptor, alpha 2	2.01979398	0.006827	0.04178
320085	B830012L14Rik	RIKEN cDNA B830012L14 gene	2.0189784	0.005934	0.03801
171504	Apob48r	apolipoprotein B48 receptor	2.01857085	0.004403	0.03164
13835	Epha1	Eph receptor A1	2.01612903	0.002135	0.02017
668147	Gm13284	predicted gene 13284	2.01572264	0.008288	0.04721
16949	Loxl1	lysyl oxidase-like 1	2.01450443	0.002426	0.02168
76408	Abcc3	ATP-binding cassette, sub-family C (CFTR/MRP), member 3	2.01288245	0.0008575	0.01185
22146	Tuba1c	tubulin, alpha 1C	2.01126307	0.000145	0.004169
67304	3110070M22Rik	RIKEN cDNA 3110070M22 gene	2.01045436	0.002298	0.02112
98733	Obsl1	obscurin-like 1	2.01045436	0.003778	0.0288
219033	Ang4	angiogenin, ribonuclease A family, member 4	2.0096463	0.0005408	0.008995
230971	Megf6	multiple EGF-like-domains 6	2.0096463	0.001076	0.01361
223646	Naprt1	nicotinate phosphoribosyltransferase domain containing 1	2.00722601	3.02E-05	0.001781
14867	Gstm6	glutathione S-transferase, mu 6	2.00722601	0.0007091	0.01063
18712	Pim1	proviral integration site 1	2.0068232	0.0002908	0.006115
18817	Plk1	polo-like kinase 1 (Drosophila)	2.00642055	2.23E-06	0.0004235
53318	Pdlim3	PDZ and LIM domain 3	2.00601805	0.0003661	0.006994
76238	Grhpr	glyoxylate reductase/hydroxypyruvate reductase	2.00280393	3.01E-05	0.001781
15360	Hmgcs2	3-hydroxy-3-methylglutaryl-Coenzyme A synthase 2	2.00240288	0.3381	0.5483
30955	Pik3cg	phosphoinositide-3-kinase, catalytic, gamma polypeptide	2.002002	0.00466	0.03278
100041734	4930522L14Rik	RIKEN cDNA 4930522L14 gene	2.00080032	0.0001261	0.003875
319269	A130040M12Rik	RIKEN cDNA A130040M12 gene	2	0.0008871	0.01209
20700	Serpina1a	serine (or cysteine) peptidase inhibitor, clade A, member 1A	2	0.001409	0.0159
69571	2310034O05Rik	RIKEN cDNA 2310034O05 gene	2	0.002314	0.02123

625098	Slc38a6	solute carrier family 38, member 6	1.998002	0.0002437	0.005582
78771	Mctp1	multiple C2 domains, transmembrane 1	1.99720391	0.01447	0.06785
22117	Tst	thiosulfate sulfurtransferase, mitochondrial	1.99600798	0.004652	0.03274
211151	Churc1	churchill domain containing 1	1.99362041	1.57E-05	0.001256
545652	Gm13275	predicted gene 13275	1.99203187	0.0006945	0.01048
107373	Fam111a	family with sequence similarity 111, member A	1.99163513	0.01703	0.07504
229534	Pbxip1	pre-B-cell leukemia transcription factor interacting protein 1	1.98925801	3.91E-05	0.002082
380930	9330188P03Rik	RIKEN cDNA 9330188P03 gene	1.98886237	0.0003249	0.006503
231125	Zfyve28	zinc finger, FYVE domain containing 28	1.98491465	4.29E-05	0.002195
434782	Gm5637	predicted pseudogene 5637	1.98176774	9.68E-05	0.003408
100503257	LOC100503257	hypothetical protein LOC100503257	1.97902236	0.1497	0.324
329316	A930038G18	hypothetical protein A930038G18	1.97823937	8.89E-06	0.0009331
18755	Prkch	protein kinase C, eta	1.9778481	0.002921	0.02438
110095	Pygl	liver glycogen phosphorylase	1.97745699	0.006492	0.04032
448987	Fbxl7	F-box and leucine-rich repeat protein 7	1.97706603	0.004467	0.03195
107585	Dio3	deiodinase, iodothyronine type III	1.97706603	0.02326	0.09275
18018	Nfatc1	nuclear factor of activated T-cells, cytoplasmic, calcineurin-dependent 1	1.97628458	0.003212	0.02608
434402	Gm5617	predicted gene 5617	1.97550375	0.0001311	0.003976
330108	4732457N14	hypothetical protein 4732457N14	1.97550375	0.03631	0.1249
18034	Nfkb2	nuclear factor of kappa light polypeptide gene enhancer in B-cells 2, p49/p100	1.97316496	0.001304	0.01519
432855	Zfhx2as	zinc finger homeobox 2, antisense	1.97238659	0.0001418	0.004142
100042484	4732419C18Rik	RIKEN cDNA 4732419C18 gene	1.97238659	0.00299	0.02477
67860	S100a16	S100 calcium binding protein A16	1.97199763	0.0003005	0.006228
233571	P2ry6	pyrimidinergic receptor P2Y, G-protein coupled, 6	1.97160883	0.001267	0.01497
380712	Tlcd2	TLC domain containing 2	1.97160883	0.003144	0.0257
27368	Tbl2	transducin (beta)-like 2	1.96966713	1.68E-05	0.001313
227631	Sohlh1	spermatogenesis and oogenesis specific basic helix-loop-helix 1	1.96772924	0.01201	0.06015
242125	BC037703	cDNA sequence BC037703	1.96463654	0.002351	0.02135
84035	Kremen1	kringle containing transmembrane protein 1	1.96463654	0.02747	0.1038
66892	Eif4e3	eukaryotic translation initiation factor 4E member 3	1.96232339	1.20E-05	0.001077
208936	Adamts18	a disintegrin-like and metallopeptidase (reprolysin type) with thrombospondin type 1 motif, 18	1.96232339	0.005439	0.03603
407790	Ndufa4l2	NADH dehydrogenase (ubiquinone) 1 alpha subcomplex, 4-like 2	1.9619384	0.0001371	0.00406
20716	Serpina3n	serine (or cysteine) peptidase inhibitor, clade A, member 3N	1.9619384	0.001122	0.01394
229228	Nudt6	nudix (nucleoside diphosphate linked moiety X)-type motif 6	1.96155355	0.0001044	0.003516
22339	Vegfa	vascular endothelial growth factor A	1.96001568	0.003893	0.02928
67784	Plxnd1	plexin D1	1.95963159	3.02E-05	0.001781
15482	Hspa1l	heat shock protein 1-like	1.95963159	0.001199	0.01447
17476	Mpeg1	macrophage expressed gene 1	1.95809673	0.02051	0.08534
11607	Agtr1a	angiotensin II receptor, type 1a	1.95656427	0.000562	0.009188
14184	Fgfr3	fibroblast growth factor receptor 3	1.95618153	0.005301	0.03547
73542	Tssk5	testis-specific serine kinase 5	1.95618153	0.02578	0.0995
68588	Cthrc1	collagen triple helix repeat containing 1	1.95579894	0.03283	0.1169
270160	Rab39	RAB39, member RAS oncogene family	1.9554165	8.43E-05	0.003149
235631	Prss50	protease, serine, 50	1.9554165	0.1832	0.3703
18858	Pmp22	peripheral myelin protein 22	1.9527436	0.001575	0.01707
71839	Osgin1	oxidative stress induced growth inhibitor 1	1.95160031	0.002528	0.02226
58998	Pvrl3	poliovirus receptor-related 3	1.95121951	0.0002179	0.005232
22117	Tst	thiosulfate sulfurtransferase, mitochondrial	1.95121951	0.00285	0.02405
226041	Pgm5	phosphoglucomutase 5	1.95045836	0.02398	0.09478
67776	Vwa5a	von Willebrand factor A domain containing 5A	1.950078	2.62E-05	0.001661

228778	6820408C15Rik	RIKEN cDNA 6820408C15 gene	1.94893783	0.0043	0.03116
12053	Bcl6	B-cell leukemia/lymphoma 6	1.94779899	1.41E-05	0.001182
13732	Emp3	epithelial membrane protein 3	1.9470405	0.002614	0.02281
170768	Pfkfb3	6-phosphofructo-2-kinase/fructose-2,6-biphosphatase 3	1.94552529	0.004678	0.03283
66395	Ahnak	AHNAK nucleoprotein (desmoyokin)	1.94552529	0.00979	0.05264
14067	F5	coagulation factor V	1.94439043	0.1021	0.2521
66599	Rdm1	RAD52 motif 1	1.9432569	0.000346	0.006759
12580	Cdkn2c	cyclin-dependent kinase inhibitor 2C (p18, inhibits CDK4)	1.94287935	4.07E-06	0.0005946
98660	Atp1a2	ATPase, Na+/K+ transporting, alpha 2 polypeptide	1.94287935	0.01321	0.06403
320736	E130203B14Rik	RIKEN cDNA E130203B14 gene	1.94250194	0.008585	0.04829
72136	Chst14	carbohydrate (N-acetylgalactosamine 4-0) sulfotransferase 14	1.93836015	0.0001776	0.004607
20675	Sox3	SRY-box containing gene 3	1.9379845	0.00114	0.01406
20305	Ccl6	chemokine (C-C motif) ligand 6	1.9379845	0.02957	0.1091
229534	Pbxip1	pre-B-cell leukemia transcription factor interacting protein 1	1.93610842	5.65E-05	0.002567
80837	Rhoj	ras homolog gene family, member J	1.93236715	0.01087	0.05611
217353	Tmc6	transmembrane channel-like gene family 6	1.93199382	0.002239	0.0207
110835	Chrna5	cholinergic receptor, nicotinic, alpha polypeptide 5	1.93087469	0.002538	0.0223
12827	Col4a2	collagen, type IV, alpha 2	1.93050193	0.01169	0.05918
20708	Serpinb6b	serine (or cysteine) peptidase inhibitor, clade B, member 6b	1.92975685	0.0005799	0.009333
63873	Trpv4	transient receptor potential cation channel, subfamily V, member 4	1.92938453	0.001065	0.01359
16952	Anxa1	annexin A1	1.92901235	0.02775	0.1044
107817	Jmjd6	jumonji domain containing 6	1.92678227	8.24E-06	0.0008901
21391	Tbxas1	thromboxane A synthase 1, platelet	1.92492782	0.005791	0.03746
14969	H2-Eb1	histocompatibility 2, class II antigen E beta	1.92381685	0.008598	0.04832
21877	Tk1	thymidine kinase 1	1.92307692	0.03864	0.1298
12826	Col4a1	collagen, type IV, alpha 1	1.91864927	0.009032	0.04976
18619	Penk	preproenkephalin	1.91864927	0.05461	0.165
20390	Sftpd	surfactant associated protein D	1.91681043	0.3117	0.5214
100529082	Gm11127	predicted gene 11127	1.91644308	0.002463	0.02189
100038552	4732463B04Rik	RIKEN cDNA 4732463B04 gene	1.91534189	0.02955	0.1091
13665	Eif2s1	eukaryotic translation initiation factor 2, subunit 1 alpha	1.91350938	6.63E-05	0.00279
278795	Lrrc10b	leucine rich repeat containing 10B	1.91350938	0.03476	0.1213
22139	Ttr	transthyretin	1.91094974	0.007689	0.0451
18824	Plp2	proteolipid protein 2	1.90985485	0.0005078	0.008641
16852	Lgals1	lectin, galactose binding, soluble 1	1.90949017	0.002934	0.02444
20692	Sparc	secreted acidic cysteine rich glycoprotein	1.90876121	0.0002695	0.005858
17919	Myo5b	myosin VB	1.90839695	0.0005858	0.009376
630499	H2-K2	histocompatibility 2, K region locus 2	1.90621426	0.001176	0.01433
252903	Ap1s3	adaptor-related protein complex AP-1, sigma 3	1.9054878	9.91E-06	0.0009832
20130	Rras	Harvey rat sarcoma oncogene, subgroup R	1.90439916	5.22E-05	0.002449
12606	Cebpa	CCAAT/enhancer binding protein (C/EBP), alpha	1.90439916	0.00672	0.04133
613117	4930571B16Rik	RIKEN cDNA 4930571B16 gene	1.90150219	0.005074	0.03446
12549	Arhgap31	Rho GTPase activating protein 31	1.89861401	0.000205	0.005031
77570	3930401B19Rik	RIKEN cDNA 3930401B19 gene	1.89825361	0.002077	0.01983
71994	Cnn3	calponin 3, acidic	1.89753321	6.49E-05	0.002774
229228	Nudt6	nudix (nucleoside diphosphate linked moiety X)-type motif 6	1.89681335	0.001114	0.0139
20135	Rrm2	ribonucleotide reductase M2	1.89609405	0.001893	0.019
80285	Parp9	poly (ADP-ribose) polymerase family, member 9	1.8957346	2.54E-05	0.00164
14778	Gpx3	glutathione peroxidase 3	1.89465707	0.1291	0.2945
100042265	Gm9790	predicted gene 9790	1.89322226	8.57E-05	0.00317

353504	Dio3os	deiodinase, iodothyronine type III, opposite strand	1.89250568	0.03476	0.1213
70355	Gprc5c	G protein-coupled receptor, family C, group 5, member C	1.89214759	0.00279	0.02373
14633	Gli2	GLI-Kruppel family member GLI2	1.89214759	0.008782	0.04894
53311	Mybph	myosin binding protein H	1.89214759	0.03719	0.1268
56016	Hebp2	heme binding protein 2	1.89143181	0.002346	0.02135
17702	Msx2	homeobox, msh-like 2	1.89107413	0.03837	0.1294
66905	Plin3	perilipin 3	1.89071658	0.0002368	0.005524
16008	Igfbp2	insulin-like growth factor binding protein 2	1.89035917	0.0007577	0.01098
320295	C920006O11Rik	RIKEN cDNA C920006O11 gene	1.89000189	0.003753	0.02867
170768	Pfkfb3	6-phosphofructo-2-kinase/fructose-2,6-biphosphatase 3	1.88893087	1.39E-05	0.00117
23985	Slc26a4	solute carrier family 26, member 4	1.88857413	0.2333	0.4348
16323	Inhba	inhibin beta-A	1.88786105	0.00113	0.01399
11622	Ahr	aryl-hydrocarbon receptor	1.88786105	0.001371	0.01565
16423	Cd47	CD47 antigen (Rh-related antigen, integrin-associated signal transducer)	1.88750472	0.0007439	0.01095
17150	Mfap2	microfibrillar-associated protein 2	1.88643652	0.002019	0.01961
67269	Agtpbp1	ATP/GTP binding protein 1	1.88465888	0.0002806	0.005973
56213	Htra1	HtrA serine peptidase 1	1.88288458	4.08E-05	0.002124
66715	4921515J06Rik	RIKEN cDNA 4921515J06 gene	1.8821758	0.008352	0.0474
21898	Tlr4	toll-like receptor 4	1.88146754	0.007303	0.0437
18241	Gpr143	G protein-coupled receptor 143	1.88146754	0.0294	0.1086
16477	Junb	Jun-B oncogene	1.88075983	0.01059	0.05519
73649	Cybrd1	cytochrome b reductase 1	1.88040617	0.001459	0.01622
77037	Mrap	melanocortin 2 receptor accessory protein	1.87934599	0.01133	0.05788
14275	Folr1	folate receptor 1 (adult)	1.87899286	0.00199	0.01947
12759	Clu	clusterin	1.87863986	0.0001971	0.004911
20613	Snai1	snail homolog 1 (Drosophila)	1.87863986	0.0198	0.08322
228785	Mylk2	myosin, light polypeptide kinase 2, skeletal muscle	1.87863986	0.06044	0.1763
30963	Ptpla	protein tyrosine phosphatase-like (proline instead of catalytic arginine), member a	1.878287	7.43E-05	0.002977
70370	Fbln7	fibulin 7	1.87441425	0.003075	0.0253
94352	Loxl2	lysyl oxidase-like 2	1.87441425	0.005104	0.03461
207474	Kctd12b	potassium channel tetramerisation domain containing 12b	1.87265918	9.95E-05	0.003415
105785	Kdelr3	KDEL (Lys-Asp-Glu-Leu) endoplasmic reticulum protein retention receptor 3	1.87265918	0.005952	0.03808
320528	Vps13c	vacuolar protein sorting 13C (yeast)	1.87265918	0.01295	0.06305
12560	Cdh3	cadherin 3	1.87160771	0.000646	0.009989
15040	H2-T23	histocompatibility 2, T region locus 23	1.87055743	0.002018	0.01961
235472	Prtg	protogenin homolog (Gallus gallus)	1.86880957	0.004065	0.03003
16773	Lama2	laminin, alpha 2	1.86776242	0.04328	0.1397
17916	Myo1f	myosin IF	1.86671645	0.01633	0.07319
234155	Mboat4	membrane bound O-acyltransferase domain containing 4	1.86671645	0.06777	0.1907
68487	Tmem140	transmembrane protein 140	1.86636805	0.0004988	0.008528
171382	Trpm8	transient receptor potential cation channel, subfamily M, member 8	1.86601978	0.02744	0.1037
12830	Col4a5	collagen, type IV, alpha 5	1.86462801	0.004375	0.0315
353504	Dio3os	deiodinase, iodothyronine type III, opposite strand	1.86428039	0.03259	0.1165
14726	Pdpn	podoplanin	1.86289121	0.0001031	0.003493
72789	Veph1	ventricular zone expressed PH domain homolog 1 (zebrafish)	1.86289121	0.02833	0.106
70560	Wars2	tryptophanyl tRNA synthetase 2 (mitochondrial)	1.86254424	4.04E-06	0.0005943
66214	1190002H23Rik	RIKEN cDNA 1190002H23 gene	1.86219739	1.20E-06	0.0002997
223650	Eppk1	epiplakin 1	1.86185068	0.005984	0.03824
225187	Ankrd29	ankyrin repeat domain 29	1.86115764	0.000934	0.01246
100040294	Gm2694	predicted gene 2694	1.86046512	0.000361	0.006931

13731	Emp2	epithelial membrane protein 2	1.85873606	0.01405	0.06654
74589	Kbtbd12	kelch repeat and BTB (POZ) domain containing 12	1.85839063	0.003637	0.02808
436022	6030429G01Rik	RIKEN cDNA 6030429G01 gene	1.85839063	0.005419	0.03596
64297	Gprc5b	G protein-coupled receptor, family C, group 5, member B	1.85735513	0.0006085	0.009604
11475	Acta2	actin, alpha 2, smooth muscle, aorta	1.85597624	0.06913	0.1936
69573	2310016C08Rik	RIKEN cDNA 2310016C08 gene	1.85563184	1.96E-05	0.001424
78465	1700084C01Rik	RIKEN cDNA 1700084C01 gene	1.85563184	0.00372	0.02847
17916	Myo1f	myosin IF	1.85563184	0.01957	0.0826
69573	2310016C08Rik	RIKEN cDNA 2310016C08 gene	1.85494342	2.01E-05	0.001424
217430	Pqlc3	PQ loop repeat containing	1.84979652	0.002352	0.02135
16194	Il6ra	interleukin 6 receptor, alpha	1.84979652	0.002524	0.02225
545030	Wdfy4	WD repeat and FYVE domain containing 4	1.84945441	0.0004192	0.007633
109267	Srcrb4d	scavenger receptor cysteine rich domain containing, group B (4 domains)	1.84911243	0.001049	0.01346
18641	Pfkl	phosphofructokinase, liver, B-type	1.84911243	0.004809	0.03339
17178	Fxyd3	FXYD domain-containing ion transport regulator 3	1.84877057	2.18E-05	0.001496
223650	Eppk1	epiplakin 1	1.84877057	0.003275	0.02633
100342	Fam46b	family with sequence similarity 46, member B	1.84808723	0.003442	0.02711
110959	Nudt19	nudix (nucleoside diphosphate linked moiety X)-type motif 19	1.8474044	0.0001802	0.004634
108097	Prkab2	protein kinase, AMP-activated, beta 2 non-catalytic subunit	1.8474044	0.0001817	0.00466
26886	Cenph	centromere protein H	1.84672207	0.06277	0.1812
231125	Zfyve28	zinc finger, FYVE domain containing 28	1.84638109	5.95E-05	0.002623
16007	Cyr61	cysteine rich protein 61	1.84604024	1.21E-05	0.001077
630499	H2-K2	histocompatibility 2, K region locus 2	1.84535892	0.002355	0.02135
17000	Ltbr	lymphotoxin B receptor	1.8446781	0.0001826	0.004665
72287	Plekhf1	pleckstrin homology domain containing, family F (with FYVE domain) member 1	1.8446781	0.001921	0.01916
12822	Col18a1	collagen, type XVIII, alpha 1	1.84433788	0.04055	0.134
18439	P2rx7	purinergic receptor P2X, ligand-gated ion channel, 7	1.84399779	0.104	0.2548
15507	Hspb1	heat shock protein 1	1.84297825	0.0009024	0.01221
13051	Cx3cr1	chemokine (C-X3-C) receptor 1	1.84229919	0.004208	0.03071
15013	H2-Q2	histocompatibility 2, Q region locus 2	1.84162063	0.001786	0.0183
243362	Stard13	StAR-related lipid transfer (START) domain containing 13	1.84094256	0.01036	0.05448
99929	Tiparp	TCDD-inducible poly(ADP-ribose) polymerase	1.84060372	0.0001186	0.003767
238393	Serpina3f	serine (or cysteine) peptidase inhibitor, clade A, member 3F	1.840265	0.007325	0.04377
12527	Cd9	CD9 antigen	1.83958793	1.16E-05	0.001065
15013	H2-Q2	histocompatibility 2, Q region locus 2	1.83958793	0.003628	0.02805
17110	Lyz1	lysozyme 1	1.83891136	0.0004488	0.007941
74174	Gtsf1	gametocyte specific factor 1	1.83891136	0.03415	0.1198
20347	Sema3b	sema domain, immunoglobulin domain (Ig), short basic domain, secreted, (semaphorin) 3B	1.83857327	1.43E-05	0.001195
242700	Il28ra	interleukin 28 receptor alpha	1.83755972	0.005266	0.03533
319651	Usp37	ubiquitin specific peptidase 37	1.83688464	2.64E-05	0.001661
16573	Kif5b	kinesin family member 5B	1.83654729	0.0129	0.06293
11853	Rhoc	ras homolog gene family, member C	1.83621006	3.99E-05	0.002105
69073	1810019J16Rik	RIKEN cDNA 1810019J16 gene	1.83519912	2.01E-05	0.001424
12262	C1qc	complement component 1, q subcomponent, C chain	1.83385292	0.001946	0.01926
66902	Mtap	methylthioadenosine phosphorylase	1.83284457	0.004298	0.03116
320910	Itgb8	integrin beta 8	1.83284457	0.09384	0.2381
54353	Skap2	src family associated phosphoprotein 2	1.8294914	7.73E-05	0.003015
100040591	Kcnj13	potassium inwardly-rectifying channel, subfamily J, member 13	1.82882224	0.04511	0.144
15442	Hpse	heparanase	1.82315406	0.0002261	0.005349
69665	2310043J07Rik	RIKEN cDNA 2310043J07 gene	1.82215743	0.002573	0.02254

53381	Prdx4	peroxiredoxin 4	1.82149362	2.92E-05	0.001749
20852	Stat6	signal transducer and activator of transcription 6	1.8211619	1.60E-05	0.001266
72318	Cyth4	cytohesin 4	1.81950509	0.0003872	0.007251
230766	Fam167b	family with sequence similarity 167, member B	1.81950509	0.02304	0.09231
67099	Fam119a	family with sequence similarity 119, member A	1.81884322	0.0008428	0.01175
98365	Slamf9	SLAM family member 9	1.81818182	0.0005913	0.009422
65963	Tmem176b	transmembrane protein 176B	1.8175209	0.0003784	0.007158
23880	Fyb	FYN binding protein	1.8175209	0.01788	0.0777
16819	Lcn2	lipocalin 2	1.81686047	0.007753	0.04532
666794	Rbm24	RNA binding motif protein 24	1.81620051	0.01433	0.06749
216343	Tph2	tryptophan hydroxylase 2	1.81488203	0.05569	0.1672
58187	Cldn10	claudin 10	1.8115942	0.02721	0.1031
77890	6720407P12Rik	RIKEN cDNA 6720407P12 gene	1.8089725	0.001125	0.01395
68797	Pdgfrl	platelet-derived growth factor receptor-like	1.80864532	0.0002425	0.005581
18733	Lilrb3	leukocyte immunoglobulin-like receptor, subfamily B (with TM and ITIM domains), member 3	1.80831826	0.07504	0.2049
232560	Caprin2	caprin family member 2	1.80799132	0.001074	0.01361
18826	Lcp1	lymphocyte cytosolic protein 1	1.80635838	0.000161	0.004382
66922	Rras2	related RAS viral (r-ras) oncogene homolog 2	1.80440274	0.0002659	0.005839
84035	Kremen1	kringle containing transmembrane protein 1	1.80277628	3.99E-05	0.002105
217154	Stac2	SH3 and cysteine rich domain 2	1.80277628	0.001278	0.01502
69305	Dcps	decapping enzyme, scavenger	1.80212651	8.14E-06	0.0008851
72669	2810032G03Rik	RIKEN cDNA 2810032G03 gene	1.80212651	0.002073	0.01983
13713	Elk3	ELK3, member of ETS oncogene family	1.8018018	0.003451	0.02713
12977	Csf1	colony stimulating factor 1 (macrophage)	1.80018002	0.1254	0.2887
13175	Dclk1	doublecortin-like kinase 1	1.79985601	0.0008556	0.01183
11815	Apod	apolipoprotein D	1.79920835	1.50E-05	0.001231
70788	Klhl30	kelch-like 30 (Drosophila)	1.79920835	0.000388	0.00726
18828	Plscr2	phospholipid scramblase 2	1.79920835	0.002171	0.02032
216974	Proca1	protein interacting with cyclin A1	1.79823773	0.00133	0.01541
69550	Bst2	bone marrow stromal cell antigen 2	1.79694519	0.0004455	0.007902
13040	Ctss	cathepsin S	1.79436569	0.0003787	0.007158
333452	Gm5132	predicted gene 5132	1.79307872	0.002463	0.02189
13175	Dclk1	doublecortin-like kinase 1	1.79115171	0.0002373	0.005526
69665	2310043J07Rik	RIKEN cDNA 2310043J07 gene	1.79083095	0.003601	0.02787
54324	Arhgef5	Rho guanine nucleotide exchange factor (GEF) 5	1.79018976	0.001582	0.01708
100040377	LOC100040377	hypothetical protein LOC100040377	1.7885888	8.92E-05	0.003257
16952	Anxa1	annexin A1	1.78826896	0.0621	0.1798
71893	Noxo1	NADPH oxidase organizer 1	1.7876296	0.0003665	0.006994
13506	Dsc2	desmocollin 2	1.7876296	0.0006168	0.009693
16854	Lgals3	lectin, galactose binding, soluble 3	1.78635227	0.009961	0.05315
664862	Gpr137b-ps	G protein-coupled receptor 137B, pseudogene	1.78603322	0.003106	0.02549
68458	Ppp1r14a	protein phosphatase 1, regulatory (inhibitor) subunit 14A	1.78443969	2.50E-05	0.001622
22324	Vav1	vav 1 oncogene	1.78348493	0.00512	0.03465
50934	Slc7a8	solute carrier family 7 (cationic amino acid transporter, y+ system), member 8	1.7831669	6.70E-05	0.002795
50722	Dkkl1	dickkopf-like 1	1.7831669	0.000342	0.006709
140497	AF251705	cDNA sequence AF251705	1.78284899	5.42E-05	0.002503
16008	Igfbp2	insulin-like growth factor binding protein 2	1.78253119	9.07E-05	0.003288
378460	Pram1	PML-RAR alpha-regulated adaptor molecule 1	1.78062678	0.0001059	0.003561
21816	Tgm1	transglutaminase 1, K polypeptide	1.78062678	0.05929	0.174
71943	Tom1l1	target of myb1-like 1 (chicken)	1.7796761	0.004049	0.03

69987	1700026L06Rik	RIKEN cDNA 1700026L06 gene	1.7796761	0.007853	0.04564
13846	Ephb4	Eph receptor B4	1.77904287	0.0005784	0.009331
574437	Xlr3b	X-linked lymphocyte-regulated 3B	1.77904287	0.04889	0.1525
14166	Fgf11	fibroblast growth factor 11	1.77872643	0.00812	0.04661
68396	Nat8	N-acetyltransferase 8 (GCN5-related, putative)	1.77777778	0.001009	0.01312
258692	Olfr1442	olfactory receptor 1442	1.77746178	0.4367	0.6401
106878	2010002N04Rik	RIKEN cDNA 2010002N04 gene	1.7758835	0.001581	0.01708
71145	Scara5	scavenger receptor class A, member 5 (putative)	1.7758835	0.02898	0.1076
78405	Ntf5	neurotrophin 5	1.77462289	0.00535	0.0357
94242	Tinagl1	tubulointerstitial nephritis antigen-like 1	1.77336407	0.01707	0.07522
56392	Shoc2	soc-2 (suppressor of clear) homolog (C. elegans)	1.77242113	0.0007135	0.01066
12862	Cox6a2	cytochrome c oxidase, subunit VI a, polypeptide 2	1.77147919	0.006739	0.04139
58861	Cysltr1	cysteinyl leukotriene receptor 1	1.77085178	0.04745	0.1492
20692	Sparc	secreted acidic cysteine rich glycoprotein	1.77053824	0.1513	0.3261
242747	Zfp933	zinc finger protein 933	1.77022482	0.01681	0.07442
242484	D630039A03Rik	RIKEN cDNA D630039A03 gene	1.77022482	0.05747	0.1706
72293	Nkd2	naked cuticle 2 homolog (Drosophila)	1.7683466	0.004563	0.03233
19011	Endou	endonuclease, polyU-specific	1.76740898	0.004789	0.0333
20193	S100a1	S100 calcium binding protein A1	1.76709666	0.0002956	0.006162
27276	Plekhb1	pleckstrin homology domain containing, family B (evectins) member 1	1.76397954	2.62E-05	0.001661
21859	Timp3	tissue inhibitor of metalloproteinase 3	1.76304654	0.004197	0.03065
63873	Trpv4	transient receptor potential cation channel, subfamily V, member 4	1.76180409	0.002164	0.02031
380712	Tlcd2	TLC domain containing 2	1.76025348	0.0001198	0.003786
53896	Slc7a10	solute carrier family 7 (cationic amino acid transporter, y+ system), member 10	1.759634	0.1231	0.2854
67392	4833420G17Rik	RIKEN cDNA 4833420G17 gene	1.75901495	0.002635	0.02295
14701	Gng12	guanine nucleotide binding protein (G protein), gamma 12	1.7580872	0.0001098	0.003646
71683	Gypc	glycophorin C	1.7580872	0.01637	0.07331
19662	Rbp4	retinol binding protein 4, plasma	1.75777817	8.33E-05	0.003125
16816	Lcat	lecithin cholesterol acyltransferase	1.75777817	0.002924	0.02439
18214	Ddr2	discoidin domain receptor family, member 2	1.75746924	0.003334	0.02658
83554	Fstl3	follistatin-like 3	1.75716043	0.0007752	0.01115
100044874	LOC100044874	h-2 class I histocompatibility antigen, K-W28 alpha chain-like	1.75716043	0.03625	0.1247
56226	Espn	espin	1.75685172	0.01892	0.08067
17313	Mgp	matrix Gla protein	1.75592625	1.24E-05	0.001101
12739	Cldn3	claudin 3	1.75500176	0.08214	0.2183
57370	B4galt3	UDP-Gal:betaGlcNAc beta 1,4-galactosyltransferase, polypeptide 3	1.75346309	0.0391	0.1308
54526	Syt10	synaptotagmin X	1.75315568	0.152	0.3268
18787	Serpine1	serine (or cysteine) peptidase inhibitor, clade E, member 1	1.75254118	0.1451	0.3173
57265	Fzd2	frizzled homolog 2 (Drosophila)	1.7522341	0.003595	0.02787
100042198	Gm3716	predicted gene 3716	1.75192712	0.002046	0.0197
11853	Rhoc	ras homolog gene family, member C	1.75162025	0.0005119	0.008665
12036	Bcat2	branched chain aminotransferase 2, mitochondrial	1.75100683	0.01535	0.07056
237360	Adamts14	a disintegrin-like and metallopeptidase (reprolysin type) with thrombospondin type 1 motif, 14	1.75070028	0.003258	0.02625
68603	Pmvk	phosphomevalonate kinase	1.74978128	9.82E-05	0.003415
18858	Pmp22	peripheral myelin protein 22	1.74886324	0.001453	0.0162
50766	Crim1	cysteine rich transmembrane BMP regulator 1 (chordin like)	1.74886324	0.02465	0.09647
12257	Tspo	translocator protein	1.74733531	0.0001179	0.003764
94242	Tinagl1	tubulointerstitial nephritis antigen-like 1	1.74703005	0.005762	0.03734
268935	Scube3	signal peptide, CUB domain, EGF-like 3	1.74672489	0.004548	0.03225
216019	Hkdc1	hexokinase domain containing 1	1.74641984	0.0004998	0.008535

116914	Slc19a2	solute carrier family 19 (thiamine transporter), member 2	1.74611489	0.000233	0.005462
78376	Ng23	Ng23 protein	1.74611489	0.05051	0.1561
212933	Pm20d1	peptidase M20 domain containing 1	1.74581006	0.002138	0.02017
13167	Dbi	diazepam binding inhibitor	1.74550532	0.0004218	0.007669
58521	Eid1	EP300 interacting inhibitor of differentiation 1	1.7452007	0.0002638	0.005839
70355	Gprc5c	G protein-coupled receptor, family C, group 5, member C	1.74185682	0.002059	0.01978
17888	Myh6	myosin, heavy polypeptide 6, cardiac muscle, alpha	1.74155347	0.005618	0.03676
216445	Arhgap9	Rho GTPase activating protein 9	1.74003828	0.0004222	0.007669
78558	Htra3	HtrA serine peptidase 3	1.74003828	0.01393	0.06615
11542	Adora3	adenosine A3 receptor	1.73913043	0.01013	0.05373
23962	Oasl2	2'-5' oligoadenylate synthetase-like 2	1.73882803	0.001624	0.01733
70355	Gprc5c	G protein-coupled receptor, family C, group 5, member C	1.73852573	0.003075	0.0253
75627	Snapc1	small nuclear RNA activating complex, polypeptide 1	1.73822354	9.78E-06	0.0009814
14734	Gpc3	glypican 3	1.73792145	0.0002577	0.005776
78388	Mvp	major vault protein	1.73490632	0.002027	0.01963
14955	H19	H19 fetal liver mRNA	1.73400381	0.002018	0.01961
68190	5330426P16Rik	RIKEN cDNA 5330426P16 gene	1.73370319	0.004154	0.03048
240725	Sulf1	sulfatase 1	1.73310225	0.002562	0.02248
26432	Plod2	procollagen lysine, 2-oxoglutarate 5-dioxygenase 2	1.73280194	0.009081	0.04993
109218	Tmem139	transmembrane protein 139	1.73070267	0.0002343	0.005471
219151	Scara3	scavenger receptor class A, member 3	1.73070267	0.001778	0.01828
66968	Plin5	perilipin 5	1.72980453	0.003043	0.02507
329977	Fhad1	forkhead-associated (FHA) phosphopeptide binding domain 1	1.72950536	0.002011	0.01959
16543	Mdfic	MyoD family inhibitor domain containing	1.72651934	0.008796	0.04897
20704	Serpina1e	serine (or cysteine) peptidase inhibitor, clade A, member 1E	1.72651934	0.008938	0.04941
18074	Nid2	nidogen 2	1.72592337	0.0374	0.1273
67392	4833420G17Rik	RIKEN cDNA 4833420G17 gene	1.72265289	0.01022	0.05407
207175	Cetn4	centrin 4	1.72146669	0.0005852	0.009376
17150	Mfap2	microfibrillar-associated protein 2	1.7211704	0.0001695	0.004472
16770	Lalba	lactalbumin, alpha	1.72057811	0.004646	0.03272
246747	Adig	adipogenin	1.71939477	0.000323	0.006475
71930	2310043M15Rik	RIKEN cDNA 2310043M15 gene	1.71880371	0.2781	0.4863
18140	Uhrf1	ubiquitin-like, containing PHD and RING finger domains, 1	1.71703297	0.003831	0.02899
12521	Cd82	CD82 antigen	1.7158545	0.002181	0.02037
434280	Gm5607	predicted gene 5607	1.7158545	0.1322	0.2987
13636	Efna1	ephrin A1	1.71526587	0.000125	0.003855
171286	Slc12a8	solute carrier family 12 (potassium/chloride transporters), member 8	1.7149717	0.03961	0.1319
666704	Samd1	sterile alpha motif domain containing 1	1.71467764	6.01E-05	0.002628
13167	Dbi	diazepam binding inhibitor	1.71438368	0.0002049	0.005031
213208	Il20rb	interleukin 20 receptor beta	1.71438368	0.03891	0.1305
240725	Sulf1	sulfatase 1	1.71379606	0.0007573	0.01098
107747	Aldh1l1	aldehyde dehydrogenase 1 family, member L1	1.7135024	0.0001482	0.004216
67073	Pi4k2b	phosphatidylinositol 4-kinase type 2 beta	1.7135024	0.005967	0.03817
12047	Bcl2a1d	B-cell leukemia/lymphoma 2 related protein A1d	1.71291538	0.003584	0.02785
14182	Fgfr1	fibroblast growth factor receptor 1	1.71203561	0.0006132	0.009653
12521	Cd82	CD82 antigen	1.7114496	2.89E-05	0.001747
67217	2810055F11Rik	RIKEN cDNA 2810055F11 gene	1.71086399	2.63E-05	0.001661
239405	Rspo2	R-spondin 2 homolog (Xenopus laevis)	1.71057133	0.02727	0.1032
622282	Gm6306	predicted gene 6306	1.70910955	2.12E-05	0.001458
50765	Trfr2	transferrin receptor 2	1.7088175	0.0007626	0.01103

19132	Prph	peripherin	1.70794193	0.02168	0.08884
15900	Irf8	interferon regulatory factor 8	1.70735872	0.0004962	0.008499
100040591	Kcnj13	potassium inwardly-rectifying channel, subfamily J, member 13	1.70561146	0.05739	0.1704
17117	Amacr	alpha-methylacyl-CoA racemase	1.70415815	0.0004885	0.00842
71918	Zcchc24	zinc finger, CCHC domain containing 24	1.70386778	0.0001156	0.003741
319239	Npsr1	neuropeptide S receptor 1	1.70386778	0.02298	0.09216
380912	Zfp395	zinc finger protein 395	1.70328734	0.006956	0.0424
15018	H2-Q7	histocompatibility 2, Q region locus 7	1.7027073	0.001373	0.01566
22771	Zic1	zinc finger protein of the cerebellum 1	1.7027073	0.02478	0.09673
17972	Ncf4	neutrophil cytosolic factor 4	1.70241743	0.007094	0.04294
100041230	Hist1h4m	histone cluster 1, H4m	1.70125893	0.067	0.1892
56226	Espn	espin	1.70068027	0.009954	0.05315
20525	Slc2a1	solute carrier family 2 (facilitated glucose transporter), member 1	1.69981302	5.22E-05	0.002449
72386	2610035D17Rik	RIKEN cDNA 2610035D17 gene	1.69894665	0.04407	0.1415
22177	Tyrobp	TYRO protein tyrosine kinase binding protein	1.69865806	0.0007314	0.01084
22271	Upp1	uridine phosphorylase 1	1.69865806	0.001039	0.01337
12156	Bmp2	bone morphogenetic protein 2	1.69836957	0.06073	0.177
12608	Cebpb	CCAAT/enhancer binding protein (C/EBP), beta	1.69808117	0.0003474	0.006763
24117	Wif1	Wnt inhibitory factor 1	1.69692856	0.1302	0.2958
18008	Nes	nestin	1.69664065	0.0006359	0.009907
72433	Rab38	RAB38, member of RAS oncogene family	1.69664065	0.009484	0.05147
319251	9630001P10Rik	RIKEN cDNA 9630001P10 gene	1.69635284	0.001061	0.01355
74482	Ifitm7	interferon induced transmembrane protein 7	1.69635284	0.001916	0.01913
56524	Mpp6	membrane protein, palmitoylated 6 (MAGUK p55 subfamily member 6)	1.69577751	0.0002779	0.00595
12841	Col9a3	collagen, type IX, alpha 3	1.6943409	0.02033	0.08487
14735	Gpc4	glypican 4	1.69405387	0.002811	0.02387
16543	Mdfic	MyoD family inhibitor domain containing	1.69405387	0.02248	0.09081
171506	H1foo	H1 histone family, member O, oocyte-specific	1.69376694	0.0001044	0.003516
192166	Sardh	sarcosine dehydrogenase	1.6934801	0.001418	0.01597
68178	Cgnl1	cingulin-like 1	1.69262018	0.003765	0.02871
70240	Ufsp1	UFM1-specific peptidase 1	1.69204738	0.0005012	0.008545
17132	Maf	avian musculoaponeurotic fibrosarcoma (v-maf) AS42 oncogene homolog	1.69176112	0.01186	0.05971
20324	Sdpr	serum deprivation response	1.69147497	0.009462	0.05138
381925	Ppapdc1a	phosphatidic acid phosphatase type 2 domain containing 1A	1.69061708	0.1314	0.2976
12799	Cnp	2',3'-cyclic nucleotide 3' phosphodiesterase	1.69004563	0.004062	0.03003
14991	H2-M3	histocompatibility 2, M region locus 3	1.68976005	0.002204	0.02051
20720	Serpine2	serine (or cysteine) peptidase inhibitor, clade E, member 2	1.68976005	0.002895	0.02426
14127	Fcer1g	Fc receptor, IgE, high affinity I, gamma polypeptide	1.68947457	0.003249	0.02623
68603	Pmvk	phosphomevalonate kinase	1.68861871	3.16E-05	0.001853
13032	Ctsc	cathepsin C	1.68861871	6.55E-05	0.002788
26410	Map3k8	mitogen-activated protein kinase kinase kinase 8	1.68861871	0.2253	0.4251
98396	Slc41a1	solute carrier family 41, member 1	1.68634064	0.0001665	0.004452
17698	Msn	moesin	1.68605631	0.0001093	0.003637
108114	Slc22a7	solute carrier family 22 (organic anion transporter), member 7	1.68577208	0.005787	0.03745
654455	Defb48-ps	defensin beta 48 pseudogene	1.68577208	0.06014	0.1756
12628	Cfh	complement component factor h	1.68520391	0.0008202	0.01153
18805	Pld1	phospholipase D1	1.68435237	0.008063	0.04637
208795	Tmem63a	transmembrane protein 63a	1.68321831	0.0009589	0.01267
338320	Mia2	melanoma inhibitory activity 2	1.68321831	0.005353	0.0357
30935	Tor3a	torsin family 3, member A	1.68236878	2.35E-05	0.001563

110606	Fntb	farnesyltransferase, CAAX box, beta	1.68236878	0.09176	0.2348
101477	AA960618	expressed sequence AA960618	1.68236878	0.1196	0.2797
70843	Krt28	keratin 28	1.68123739	0.002334	0.02131
14119	Fbn2	fibrillin 2	1.68067227	0.04202	0.1373
70274	Ly6g6e	lymphocyte antigen 6 complex, locus G6E	1.68010753	0.0001688	0.004467
67795	Rnls	renalase, FAD-dependent amine oxidase	1.68010753	0.0007674	0.01106
245945	Rbm47	RNA binding motif protein 47	1.67954316	0.001362	0.01559
13731	Emp2	epithelial membrane protein 2	1.67954316	0.01169	0.05918
117109	Pop5	processing of precursor 5, ribonuclease P/MRP family (S. cerevisiae)	1.67785235	0.01073	0.05557
67621	Bend5	BEN domain containing 5	1.6772895	7.19E-05	0.00291
269823	Pon3	paraoxonase 3	1.67672703	0.01146	0.05834
71918	Zcchc24	zinc finger, CCHC domain containing 24	1.67560322	7.68E-05	0.003014
12837	Col8a1	collagen, type VIII, alpha 1	1.67560322	0.07531	0.2054
19039	Lgals3bp	lectin, galactoside-binding, soluble, 3 binding protein	1.67504188	7.60E-05	0.003005
12984	Csf2rb2	colony stimulating factor 2 receptor, beta 2, low-affinity (granulocyte-macrophage)	1.67504188	0.001007	0.01312
21815	Tgif1	TGFB-induced factor homeobox 1	1.67392032	0.0005456	0.009033
230674	Kdm4a	lysine (K)-specific demethylase 4A	1.67392032	0.0006333	0.00989
574428	Zmynd15	zinc finger, MYND-type containing 15	1.67392032	0.01441	0.0677
22350	Ezr	ezrin	1.67336011	0.01224	0.06077
75571	Spata9	spermatogenesis associated 9	1.67196121	0.007119	0.04301
16909	Lmo2	LIM domain only 2	1.67112299	0.0004558	0.008026
18442	P2ry2	purinergic receptor P2Y, G-protein coupled 2	1.67000668	0.007625	0.04486
66775	Ptplad2	protein tyrosine phosphatase-like A domain containing 2	1.66889186	0.01088	0.05612
70355	Gprc5c	G protein-coupled receptor, family C, group 5, member C	1.66861338	0.003958	0.02955
56188	Fxyd1	FXYD domain-containing ion transport regulator 1	1.668335	0.001009	0.01312
217344	Rhbdf2	rhomboid 5 homolog 2 (Drosophila)	1.66805671	7.46E-05	0.002979
12978	Csf1r	colony stimulating factor 1 receptor	1.66805671	0.0001709	0.004497
74202	Fblim1	filamin binding LIM protein 1	1.66805671	0.004111	0.03031
668880	Stard9	START domain containing 9	1.66777852	0.0005471	0.009042
408065	Zfp456	zinc finger protein 456	1.66777852	0.01631	0.07315
94175	Hrg	histidine-rich glycoprotein	1.66750042	0.09614	0.242
54381	Pgcp	plasma glutamate carboxypeptidase	1.6655563	0.001075	0.01361
243362	Stard13	StAR-related lipid transfer (START) domain containing 13	1.66500167	0.0002746	0.005921
15024	H2-T10	histocompatibility 2, T region locus 10	1.66417041	0.000626	0.009796
19285	Ptrf	polymerase I and transcript release factor	1.66389351	0.0002164	0.005208
11761	Aox1	aldehyde oxidase 1	1.66333999	0.002019	0.01961
66952	2310030G06Rik	RIKEN cDNA 2310030G06 gene	1.66140555	0.006923	0.04222
65963	Tmem176b	transmembrane protein 176B	1.66112957	7.71E-05	0.003014
19049	Ppp1r1b	protein phosphatase 1, regulatory (inhibitor) subunit 1B	1.66030217	0.002075	0.01983
338368	Fam109b	family with sequence similarity 109, member B	1.66002656	0.1472	0.3203
11727	Ang	angiogenin, ribonuclease, RNase A family, 5	1.65947561	0.001134	0.01401
171095	Il17rc	interleukin 17 receptor C	1.65920027	0.01696	0.0748
67454	Ikbip	IKBKB interacting protein	1.65782493	0.0002776	0.00595
790956	LOC790956	5.8S ribosomal RNA	1.65700083	0.3234	0.5331
114584	Clic1	chloride intracellular channel 1	1.6559033	5.58E-06	0.0007232
67784	Plxnd1	plexin D1	1.6559033	0.0002015	0.005006
68753	Mybphl	myosin binding protein H-like	1.65562914	0.01248	0.06156
16792	Laptm5	lysosomal-associated protein transmembrane 5	1.6550811	0.005033	0.03428
16693	Krtap11-1	keratin associated protein 11-1	1.65480721	0.005419	0.03596
21991	Tpi1	triosephosphate isomerase 1	1.65398611	9.15E-05	0.003297

21922	Clec3b	C-type lectin domain family 3, member b	1.65398611	0.01035	0.05446
14368	Fzd6	frizzled homolog 6 (Drosophila)	1.65289256	0.03434	0.1202
14066	F3	coagulation factor III	1.6526194	0.00147	0.01631
97064	Wwtr1	WW domain containing transcription regulator 1	1.65180046	0.0003129	0.006357
74048	4632428N05Rik	RIKEN cDNA 4632428N05 gene	1.65180046	0.001444	0.01615
574437	Xlr3b	X-linked lymphocyte-regulated 3B	1.65125495	0.1178	0.2765
73690	Glipr1	GLI pathogenesis-related 1 (glioma)	1.65070981	0.0007997	0.01139
67099	Fam119a	family with sequence similarity 119, member A	1.64962059	9.66E-05	0.003408
224224	Impg2	interphotoreceptor matrix proteoglycan 2	1.64934851	0.001113	0.0139
11622	Ahr	aryl-hydrocarbon receptor	1.64934851	0.01613	0.0725
17345	Mki67	antigen identified by monoclonal antibody Ki 67	1.64934851	0.07537	0.2055
14263	Fmo5	flavin containing monooxygenase 5	1.64907652	0.0001583	0.004382
171095	Il17rc	interleukin 17 receptor C	1.64853281	0.002188	0.02041
107581	Col16a1	collagen, type XVI, alpha 1	1.64798945	0.0001213	0.003807
100043074	Gm4211	predicted gene 4211	1.64798945	0.001024	0.01322
66889	Rnf128	ring finger protein 128	1.64690382	0.00182	0.01852
70673	Prdm16	PR domain containing 16	1.64690382	0.0104	0.05458
13610	S1pr3	sphingosine-1-phosphate receptor 3	1.64636154	0.000133	0.004001
67451	Pkp2	plakophilin 2	1.64609053	0.000152	0.004291
72512	Tmem173	transmembrane protein 173	1.64609053	0.002864	0.02413
216616	Efemp1	epidermal growth factor-containing fibulin-like extracellular matrix protein 1	1.64581962	0.000303	0.00626
12977	Csf1	colony stimulating factor 1 (macrophage)	1.64581962	0.0004805	0.008332
67608	Narf	nuclear prelamin A recognition factor	1.64554879	5.60E-05	0.002567
208922	Cpeb3	cytoplasmic polyadenylation element binding protein 3	1.64446637	0.0007669	0.01106
215456	Gpat2	glycerol-3-phosphate acyltransferase 2, mitochondrial	1.64446637	0.006157	0.03894
11668	Aldh1a1	aldehyde dehydrogenase family 1, subfamily A1	1.64446637	0.01129	0.0577
11867	Arpc1b	actin related protein 2/3 complex, subunit 1B	1.64419599	0.00455	0.03225
381232	5830416P10Rik	RIKEN cDNA 5830416P10 gene	1.64365549	0.001648	0.01741
66253	Aig1	androgen-induced 1	1.64284541	0.001402	0.01585
384009	Glipr2	GLI pathogenesis-related 2	1.64203612	0.0004667	0.008155
100041489	Gm3367	predicted gene 3367	1.64203612	0.003785	0.02882
30053	Reg3d	regenerating islet-derived 3 delta	1.64203612	0.13	0.2957
633640	Gm7120	predicted gene 7120	1.64068909	0.002465	0.02189
52535	Mett11d1	methyltransferase 11 domain containing 1	1.64015089	0.000258	0.005776
215819	Nhsl1	NHS-like 1	1.63988193	0.004169	0.03053
66395	Ahnak	AHNAK nucleoprotein (desmoyokin)	1.63961305	0.1162	0.2738
17289	Mertk	c-mer proto-oncogene tyrosine kinase	1.63880695	0.0001503	0.004261
17919	Myo5b	myosin VB	1.63880695	0.0003716	0.007084
13846	Ephb4	Eph receptor B4	1.63880695	0.002766	0.02362
22351	Vill	villin-like	1.63853842	0.01694	0.07478
67512	Agpat2	1-acylglycerol-3-phosphate O-acyltransferase 2 (lysophosphatidic acid acyltransferase, beta)	1.63826999	0.00332	0.0265
14230	Fkbp10	FK506 binding protein 10	1.63800164	0.0008898	0.0121
233328	Lrrk1	leucine-rich repeat kinase 1	1.63773338	0.0002249	0.005338
252973	Grhl2	grainyhead-like 2 (Drosophila)	1.6374652	0.02474	0.09665
69660	Tmbim1	transmembrane BAX inhibitor motif containing 1	1.63692912	3.63E-05	0.002004
76568	Ift46	intraflagellar transport 46 homolog (Chlamydomonas)	1.63666121	8.03E-06	0.0008786
64177	Trpv6	transient receptor potential cation channel, subfamily V, member 6	1.63612565	0.02868	0.1068
66491	Polr2l	polymerase (RNA) II (DNA directed) polypeptide L	1.63478829	0.008203	0.04692
60527	Fads3	fatty acid desaturase 3	1.63452109	3.62E-05	0.002004
320004	A930002H24Rik	RIKEN cDNA A930002H24 gene	1.63452109	9.72E-05	0.003408

12229	Btk	Bruton agammaglobulinemia tyrosine kinase	1.63425396	0.006964	0.04243
15368	Hmox1	heme oxygenase (decycling) 1	1.63398693	0.0657	0.1868
57916	Tnfrsf13b	tumor necrosis factor receptor superfamily, member 13b	1.63371998	0.008018	0.0462
21401	Tcea3	transcription elongation factor A (SII), 3	1.63371998	0.07072	0.1965
67454	Ikbip	IKBKB interacting protein	1.63291966	0.0004238	0.007685
110606	Fntb	farnesyltransferase, CAAX box, beta	1.63291966	0.00291	0.02433
22339	Vegfa	vascular endothelial growth factor A	1.63132137	0.0009404	0.01251
71742	Ulk3	unc-51-like kinase 3 (C. elegans)	1.63105529	0.0006055	0.009582
19329	Rab17	RAB17, member RAS oncogene family	1.62972621	0.007398	0.04399
72215	1700001P01Rik	RIKEN cDNA 1700001P01 gene	1.62892979	0.01748	0.07647
100198	H6pd	hexose-6-phosphate dehydrogenase (glucose 1-dehydrogenase)	1.62654522	0.02172	0.08898
72807	Zfp429	zinc finger protein 429	1.62522347	0.001596	0.01716
214424	Parp16	poly (ADP-ribose) polymerase family, member 16	1.62522347	0.01261	0.06201
55987	Cpxm2	carboxypeptidase X 2 (M14 family)	1.62495938	0.009916	0.05298
56175	Bace2	beta-site APP-cleaving enzyme 2	1.62390386	0.01069	0.05547
207819	4930539E08Rik	RIKEN cDNA 4930539E08 gene	1.62390386	0.1339	0.301
71962	Gatsl3	GATS protein-like 3	1.62337662	0.008191	0.04686
20284	Scrg1	scrapie responsive gene 1	1.6225864	0.06244	0.1805
213211	Rnf26	ring finger protein 26	1.62153397	0.002481	0.02201
66350	Pla2g12a	phospholipase A2, group XIIA	1.62127108	0.0003834	0.007234
14314	Fstl1	follistatin-like 1	1.62127108	0.001946	0.01926
18115	Nnt	nicotinamide nucleotide transhydrogenase	1.62127108	0.1477	0.3211
12260	C1qb	complement component 1, q subcomponent, beta polypeptide	1.62100827	0.004803	0.03336
434246	Trim72	tripartite motif-containing 72	1.6196955	0.008359	0.0474
19054	Ppp2r3d	protein phosphatase 2 (formerly 2A), regulatory subunit B'', delta	1.6196955	0.01166	0.05909
403346	A730062M13Rik	RIKEN cDNA A730062M13 gene	1.6196955	0.01852	0.07964
18979	Pon1	paraoxonase 1	1.61812298	0.08177	0.2175
53622	Krt85	keratin 85	1.61733786	0.1024	0.2524
60322	Chst7	carbohydrate (N-acetylglucosamino) sulfotransferase 7	1.61707633	0.08267	0.2191
18783	Pla2g4a	phospholipase A2, group IVA (cytosolic, calcium-dependent)	1.61681487	0.0002439	0.005582
14182	Fgfr1	fibroblast growth factor receptor 1	1.61629223	0.0002091	0.005084
56524	Mpp6	membrane protein, palmitoylated 6 (MAGUK p55 subfamily member 6)	1.61629223	0.0008311	0.01162
338367	Myo1d	myosin ID	1.61524794	6.68E-05	0.00279
110058	Syt17	synaptotagmin XVII	1.61498708	0.004374	0.0315
14089	Fap	fibroblast activation protein	1.61498708	0.0242	0.09525
76629	Wbscr28	Williams-Beuren syndrome chromosome region 28 (human)	1.61498708	0.08564	0.2244
12335	Capn3	calpain 3	1.61394448	0.0007525	0.01098
16792	Laptm5	lysosomal-associated protein transmembrane 5	1.61394448	0.003994	0.02969
15275	Hk1	hexokinase 1	1.61316341	0.003273	0.02633
112422	2610305D13Rik	RIKEN cDNA 2610305D13 gene	1.61316341	0.02862	0.1067
15277	Hk2	hexokinase 2	1.61108426	2.37E-06	0.0004324
20852	Stat6	signal transducer and activator of transcription 6	1.60875161	0.002908	0.02433
14854	Gss	glutathione synthetase	1.60849284	0.01316	0.06385
20498	Slc12a4	solute carrier family 12, member 4	1.60823416	0.0001839	0.004683
72565	Uaca	uveal autoantigen with coiled-coil domains and ankyrin repeats	1.60797556	0.0008065	0.01144
100669	9930105H17Rik	RIKEN cDNA 9930105H17 gene	1.60771704	0.001124	0.01395
20439	Siah2	seven in absentia 2	1.60694199	1.25E-05	0.001101
268977	Ltbp1	latent transforming growth factor beta binding protein 1	1.60590975	0.03821	0.129
11854	Rhod	ras homolog gene family, member D	1.60539412	7.33E-06	0.000821
67621	Bend5	BEN domain containing 5	1.60539412	0.001572	0.01706

20193	S100a1	S100 calcium binding protein A1	1.60513644	0.000572	0.009267
12508	Cd53	CD53 antigen	1.60513644	0.01977	0.08318
385343	Rhox1	reproductive homeobox 1	1.60436387	0.07053	0.1961
17969	Ncf1	neutrophil cytosolic factor 1	1.60307791	0.04615	0.1463
67216	Mboat2	membrane bound O-acyltransferase domain containing 2	1.60205062	0.000817	0.0115
71138	Tmem217	transmembrane protein 217	1.60153748	0.003209	0.02608
12661	Chl1	cell adhesion molecule with homology to L1CAM	1.60128102	0.004538	0.03224
72112	Ppp1r14d	protein phosphatase 1, regulatory (inhibitor) subunit 14D	1.60076837	0.006922	0.04222
12406	Serpinh1	serine (or cysteine) peptidase inhibitor, clade H, member 1	1.59948816	0.001071	0.01361
210027	Slc35f3	solute carrier family 35, member F3	1.59846547	0.0036	0.02787
12177	Bnip3l	BCL2/adenovirus E1B interacting protein 3-like	1.59821	0.000169	0.004467
435791	Gm13271	predicted gene 13271	1.59693389	0.003214	0.02608
319899	Dock6	dedicator of cytokinesis 6	1.59616919	0.0009903	0.01295
195046	Nlrp1a	NLR family, pyrin domain containing 1A	1.59565981	0.0004132	0.007561
59010	Sqrdl	sulfide quinone reductase-like (yeast)	1.59540523	0.01343	0.06455
214359	Tmem51	transmembrane protein 51	1.59515074	0.0003665	0.006994
14598	Ggt1	gamma-glutamyltransferase 1	1.59489633	0.005339	0.03565
22035	Tnfsf10	tumor necrosis factor (ligand) superfamily, member 10	1.594642	0.04074	0.1344
56175	Bace2	beta-site APP-cleaving enzyme 2	1.594642	0.1435	0.3149
98845	Eps8l2	EPS8-like 2	1.59413359	0.005904	0.03786
258830	Olfr103	olfactory receptor 103	1.5938795	0.015	0.0694
654818	C030030A07Rik	RIKEN cDNA C030030A07 gene	1.5936255	0.1015	0.2511
227612	A830007P12Rik	RIKEN cDNA A830007P12 gene	1.59337157	0.008747	0.04882
70652	Tmem144	transmembrane protein 144	1.59235669	0.0001624	0.004382
107272	Psat1	phosphoserine aminotransferase 1	1.59083678	0.0001745	0.004559
22601	Yap1	yes-associated protein 1	1.59083678	0.0001879	0.004749
12633	Cflar	CASP8 and FADD-like apoptosis regulator	1.59058374	0.0006725	0.01022
114654	Ly6g6d	lymphocyte antigen 6 complex, locus G6D	1.59033079	0.0002027	0.005009
75805	Nln	neurolysin (metallopeptidase M3 family)	1.58931977	0.0001507	0.004267
19659	Rbp1	retinol binding protein 1, cellular	1.58906722	0.0001731	0.004541
104445	Cdc42ep1	CDC42 effector protein (Rho GTPase binding) 1	1.58906722	0.0004103	0.00753
58222	Rab37	RAB37, member of RAS oncogene family	1.58856235	0.001558	0.01698
107503	Atf5	activating transcription factor 5	1.58805781	0.0001161	0.003741
382083	Snx22	sorting nexin 22	1.58730159	0.008684	0.04863
320685	Dctd	dCMP deaminase	1.58679784	0.1099	0.2639
231805	Pilra	paired immunoglobin-like type 2 receptor alpha	1.58654609	0.0003505	0.006801
319508	Syt15	synaptotagmin XV	1.58629442	0.002136	0.02017
15170	Ptpn6	protein tyrosine phosphatase, non-receptor type 6	1.58579131	0.06934	0.194
67532	Mfap1a	microfibrillar-associated protein 1A	1.58528852	0.0001452	0.004169
53404	Atoh7	atonal homolog 7 (Drosophila)	1.58528852	0.02262	0.09122
329384	Ptrh1	peptidyl-tRNA hydrolase 1 homolog (S. cerevisiae)	1.58503725	0.0001014	0.00346
100503215	LOC100503215	hypothetical protein LOC100503215	1.58478605	0.02622	0.1007
26401	Map3k1	mitogen-activated protein kinase kinase kinase 1	1.58403295	0.004414	0.03164
329828	AI464131	expressed sequence AI464131	1.58403295	0.01244	0.06144
16391	Irf9	interferon regulatory factor 9	1.58353127	0.0001084	0.003626
67622	Mxra7	matrix-remodelling associated 7	1.58328056	0.01902	0.08105
66775	Ptplad2	protein tyrosine phosphatase-like A domain containing 2	1.58302992	0.01027	0.05422
319259	9930021D14Rik	RIKEN cDNA 9930021D14 gene	1.58252888	0.0318	0.1145
54630	Prickle3	prickle homolog 3 (Drosophila)	1.58152776	0.004479	0.03198
14807	Grik3	glutamate receptor, ionotropic, kainate 3	1.58077774	0.002245	0.02074

16624	Klk1b8	kallikrein 1-related peptidase b8	1.5805279	0.01594	0.07211
231633	Tmem119	transmembrane protein 119	1.57977883	0.00188	0.0189
75472	1700009P17Rik	RIKEN cDNA 1700009P17 gene	1.5795293	0.002024	0.01962
11504	Adamts1	a disintegrin-like and metallopeptidase (reprolysin type) with thrombospondin type 1 motif, 1	1.5795293	0.008483	0.04786
17259	Mef2b	myocyte enhancer factor 2B	1.5795293	0.01093	0.05624
268739	Arhgef40	Rho guanine nucleotide exchange factor (GEF) 40	1.57927985	0.001277	0.01502
18552	Pcsk5	proprotein convertase subtilisin/kexin type 5	1.57828283	0.002491	0.02208
12521	Cd82	CD82 antigen	1.57828283	0.003586	0.02785
17123	Madcam1	mucosal vascular addressin cell adhesion molecule 1	1.57828283	0.0508	0.1567
268595	D430019H16Rik	RIKEN cDNA D430019H16 gene	1.57703832	0.002164	0.02031
56722	Litaf	LPS-induced TN factor	1.57678966	0.01686	0.07453
16206	Lrig1	leucine-rich repeats and immunoglobulin-like domains 1	1.57654107	0.004927	0.03383
14132	Fcgrt	Fc receptor, IgG, alpha chain transporter	1.57629256	0.006678	0.04113
544717	1190007I07Rik	RIKEN cDNA 1190007I07 gene	1.57629256	0.03442	0.1204
70784	Rasl12	RAS-like, family 12	1.57505119	0.003751	0.02866
67009	Ttc23	tetratricopeptide repeat domain 23	1.5743073	0.0005428	0.009012
16427	Itih4	inter alpha-trypsin inhibitor, heavy chain 4	1.57381177	0.01566	0.07138
14707	Gng5	guanine nucleotide binding protein (G protein), gamma 5	1.57306906	0.0001722	0.004525
319236	9230105E10Rik	RIKEN cDNA 9230105E10 gene	1.57183276	0.004875	0.03361
18030	Nfil3	nuclear factor, interleukin 3, regulated	1.57133878	1.10E-05	0.001039
108978	4930555G01Rik	RIKEN cDNA 4930555G01 gene	1.57133878	0.1301	0.2958
26401	Map3k1	mitogen-activated protein kinase kinase kinase 1	1.57084511	0.005298	0.03547
12452	Ccng2	cyclin G2	1.57084511	0.00984	0.05282
381175	Ccdc68	coiled-coil domain containing 68	1.57084511	0.02849	0.1064
12978	Csf1r	colony stimulating factor 1 receptor	1.5701052	0.0003345	0.006637
16188	Il3ra	interleukin 3 receptor, alpha chain	1.56985871	0.02852	0.1064
100042092	Gm3662	predicted gene 3662	1.56887355	0.002934	0.02444
17060	Blnk	B-cell linker	1.56887355	0.01291	0.06293
320608	D930023I05Rik	RIKEN cDNA D930023I05 gene	1.56862745	0.02114	0.08723
78829	Tsc22d4	TSC22 domain family, member 4	1.56862745	0.03883	0.1304
320405	Cadps2	Ca2+-dependent activator protein for secretion 2	1.56838143	0.0004449	0.007902
16576	Kif7	kinesin family member 7	1.56838143	0.01268	0.06221
231287	Atp10d	ATPase, class V, type 10D	1.56838143	0.04261	0.1385
170750	Xpnpep1	X-prolyl aminopeptidase (aminopeptidase P) 1, soluble	1.56813549	3.66E-05	0.002012
56484	Foxo3	forkhead box O3	1.56813549	0.003208	0.02608
13733	Emr1	EGF-like module containing, mucin-like, hormone receptor-like sequence 1	1.56764383	0.0679	0.1908
19221	Ptgfrn	prostaglandin F2 receptor negative regulator	1.56739812	0.0001404	0.004114
218121	Mboat1	membrane bound O-acyltransferase domain containing 1	1.56592546	0.02281	0.09167
71670	Acy3	aspartoacylase (aminoacylase) 3	1.56519017	0.01278	0.06257
15064	Mr1	major histocompatibility complex, class I-related	1.56494523	0.01073	0.05557
12661	Chl1	cell adhesion molecule with homology to L1CAM	1.56396622	0.001682	0.01766
226422	Rab7l1	RAB7, member RAS oncogene family-like 1	1.56323277	0.001214	0.01457
213056	Fam126b	family with sequence similarity 126, member B	1.56274418	0.0001937	0.004858
12983	Csf2rb	colony stimulating factor 2 receptor, beta, low-affinity (granulocyte-macrophage)	1.56274418	0.003686	0.02833
269132	Glt25d2	glycosyltransferase 25 domain containing 2	1.5622559	2.63E-05	0.001661
12362	Casp1	caspase 1	1.56176792	0.06701	0.1892
245855	BC026513	cDNA sequence BC026513	1.56152405	0.002042	0.01969
97908	Hist1h3g	histone cluster 1, H3g	1.56128025	0.02295	0.09211
224805	Aars2	alanyl-tRNA synthetase 2, mitochondrial (putative)	1.56054931	0.01869	0.08012
319508	Syt15	synaptotagmin XV	1.5600624	0.0002955	0.006162

403347	F830014O18Rik	RIKEN cDNA F830014O18 gene	1.55933261	0.03156	0.1139
16651	Sspn	sarcospan	1.55860349	0.006558	0.04061
66929	Asf1b	ASF1 anti-silencing function 1 homolog B (S. cerevisiae)	1.55666252	0.0008759	0.01202
228550	Itpka	inositol 1,4,5-trisphosphate 3-kinase A	1.55666252	0.005512	0.03634
69675	Pxdn	peroxidasin homolog (Drosophila)	1.55666252	0.02885	0.1073
546546	Serpina3h	serine (or cysteine) peptidase inhibitor, clade A, member 3H	1.55666252	0.2085	0.4034
68119	Cmtm3	CKLF-like MARVEL transmembrane domain containing 3	1.55545186	0.0002438	0.005582
76088	Dock8	dedicator of cytokinesis 8	1.55545186	0.02091	0.08661
12822	Col18a1	collagen, type XVIII, alpha 1	1.55520995	0.03713	0.1267
71956	Rnf135	ring finger protein 135	1.55496812	0.01103	0.05663
68239	Krt42	keratin 42	1.55424308	0.04346	0.1401
14693	Gnb2	guanine nucleotide binding protein (G protein), beta 2	1.55327742	0.00964	0.05202
66101	Ppih	peptidyl prolyl isomerase H	1.55303619	0.008349	0.0474
626058	E330020D12Rik	Riken cDNA E330020D12 gene	1.55303619	0.06177	0.1793
625237	Gm6566	predicted gene 6566	1.55231295	0.05509	0.166
20446	St6galnac2	ST6 (alpha-N-acetyl-neuraminyl-2,3-beta-galactosyl-1,3)-N-acetylgalactosaminide alpha-2,6-sialyltransferase 2	1.55159038	0.0001881	0.004749
72141	Adpgk	ADP-dependent glucokinase	1.550628	0.04603	0.146
99543	Olfml3	olfactomedin-like 3	1.550628	0.0569	0.1696
12177	Bnip3l	BCL2/adenovirus E1B interacting protein 3-like	1.54990701	0.0001824	0.004665
433594	Gm5537	phosphoglycerate kinase 1-like	1.54966682	0.0005758	0.009313
11746	Anxa4	annexin A4	1.54942671	0.0005156	0.008702
13713	Elk3	ELK3, member of ETS oncogene family	1.54942671	0.0005704	0.009258
16498	Kcnab2	potassium voltage-gated channel, shaker-related subfamily, beta member 2	1.54918668	0.1054	0.2568
77634	Snapc3	small nuclear RNA activating complex, polypeptide 3	1.54894672	0.003714	0.02845
70005	1700029I01Rik	RIKEN cDNA 1700029I01 gene	1.54846702	0.01719	0.07557
14067	F5	coagulation factor V	1.54846702	0.1745	0.3583
67991	Nacc2	nucleus accumbens associated 2, BEN and BTB (POZ) domain containing	1.5470297	0.2441	0.4478
20345	Selplg	selectin, platelet (p-selectin) ligand	1.54655119	0.005694	0.03708
27276	Plekhb1	pleckstrin homology domain containing, family B (evectins) member 1	1.54631205	0.0002986	0.00621
13168	Dbil5	diazepam binding inhibitor-like 5	1.54607297	0.0002775	0.00595
116701	Fgfrl1	fibroblast growth factor receptor-like 1	1.54607297	0.001613	0.01726
209200	Dtx3l	deltex 3-like (Drosophila)	1.54583398	0.01047	0.05485
226250	Afap1l2	actin filament associated protein 1-like 2	1.5453562	0.003777	0.0288
21632	Tcrg-V1	T-cell receptor gamma, variable 1	1.54511743	0.02686	0.1022
234577	Cpne2	copine II	1.5446401	0.001104	0.01386
19124	Procr	protein C receptor, endothelial	1.54440154	0.005817	0.03756
20391	Sgca	sarcoglycan, alpha (dystrophin-associated glycoprotein)	1.54440154	0.03105	0.1128
12153	Bmp1	bone morphogenetic protein 1	1.54368632	0.0003592	0.006916
213326	Scyl2	SCY1-like 2 (S. cerevisiae)	1.54249576	0.0005443	0.009025
382018	Unc13a	unc-13 homolog A (C. elegans)	1.54249576	0.01078	0.05576
20868	Stk10	serine/threonine kinase 10	1.54225787	0.00133	0.01541
228839	Tgif2	TGFB-induced factor homeobox 2	1.54225787	0.01077	0.05575
319188	Hist1h2bp	histone cluster 1, H2bp	1.54225787	0.0227	0.09144
67306	Fam164a	family with sequence similarity 164, member A	1.54202005	0.0004243	0.007686
73182	Pear1	platelet endothelial aggregation receptor 1	1.54202005	0.03598	0.1242
110557	H2-Q6	histocompatibility 2, Q region locus 6	1.54130703	0.003283	0.02634
13848	Ephb6	Eph receptor B6	1.5410695	0.0008857	0.01209
66835	Snord123	small nucleolar RNA, C/D box 123	1.53964588	0.001569	0.01704
57765	Tbx21	T-box 21	1.53893506	0.004063	0.03003
387131	Ssxb9	synovial sarcoma, X member B, breakpoint 9	1.53893506	0.009021	0.04974

54519	Apbb1ip	amyloid beta (A4) precursor protein-binding, family B, member 1 interacting protein	1.53822489	0.03688	0.1261
12177	Bnip3l	BCL2/adenovirus E1B interacting protein 3-like	1.53775181	0.0008313	0.01162
213019	Pdlim2	PDZ and LIM domain 2	1.53751538	0.001856	0.01877
238252	Gpr135	G protein-coupled receptor 135	1.53727902	0.001592	0.01714
110596	Rgnef	Rho-guanine nucleotide exchange factor	1.53727902	0.002673	0.02314
106762	AW047481	expressed sequence AW047481	1.53727902	0.002753	0.02359
628705	Gm6907	predicted gene 6907	1.53727902	0.01867	0.08009
234839	Fam38a	family with sequence similarity 38, member A	1.53704273	0.006407	0.03998
19224	Ptgs1	prostaglandin-endoperoxide synthase 1	1.53704273	0.04056	0.134
244864	Layn	layilin	1.53680652	0.004573	0.03235
100040736	9130206I24Rik	RIKEN cDNA 9130206I24 gene	1.53680652	0.03253	0.1164
72284	Oraov1	oral cancer overexpressed 1	1.53562654	0.03088	0.1124
68328	Rab13	RAB13, member RAS oncogene family	1.53515505	0.002107	0.02004
69675	Pxdn	peroxidasin homolog (Drosophila)	1.53491942	0.02682	0.1022
69922	Vrk2	vaccinia related kinase 2	1.53468386	0.02393	0.09468
319153	Hist1h3i	histone cluster 1, H3i	1.53421295	0.06691	0.189
277468	Slc39a12	solute carrier family 39 (zinc transporter), member 12	1.53421295	0.07143	0.1979
111975	Igf2as	insulin-like growth factor 2, antisense	1.5339776	0.0002789	0.005956
72014	Btbd17	BTB (POZ) domain containing 17	1.5339776	0.002111	0.02006
227099	Pms1	postmeiotic segregation increased 1 (S. cerevisiae)	1.53350713	0.0002516	0.005707
19746	Rhd	Rh blood group, D antigen	1.53350713	0.005531	0.0364
12177	Bnip3l	BCL2/adenovirus E1B interacting protein 3-like	1.533272	0.001293	0.01513
216974	Proca1	protein interacting with cyclin A1	1.53303695	0.01438	0.06761
83454	Nxf2	nuclear RNA export factor 2	1.53256705	0.01026	0.05417
207175	Cetn4	centrin 4	1.53233221	0.001476	0.01635
233328	Lrrk1	leucine-rich repeat kinase 1	1.53209744	0.002722	0.02341
100039432	Gm2233	predicted gene 2233	1.53139357	0.1223	0.2841
23828	Bves	blood vessel epicardial substance	1.53092468	0.0002738	0.005915
104099	Itga9	integrin alpha 9	1.53092468	0.001128	0.01399
65969	Cubn	cubilin (intrinsic factor-cobalamin receptor)	1.53045608	0.01693	0.07476
17768	Mthfd2	methylenetetrahydrofolate dehydrogenase (NAD+ dependent), methenyltetrahydrofolate cyclohydrolase	1.52975371	0.001399	0.01583
259300	Ehd2	EH-domain containing 2	1.52951973	0.00257	0.02253
73230	Bmper	BMP-binding endothelial regulator	1.52951973	0.00697	0.04244
21748	Terc	telomerase RNA component	1.52928582	0.0008534	0.01181
74627	4930413E15Rik	RIKEN cDNA 4930413E15 gene	1.52928582	0.2048	0.3984
56735	Krt71	keratin 71	1.52905199	0.01588	0.07195
104174	Gldc	glycine decarboxylase	1.52858453	0.0004385	0.007852
17025	Alad	aminolevulinate, delta-, dehydratase	1.52858453	0.001545	0.01693
21677	Tead2	TEA domain family member 2	1.52741714	0.001736	0.01804
69080	Gmppa	GDP-mannose pyrophosphorylase A	1.52741714	0.002871	0.02417
20527	Slc2a3	solute carrier family 2 (facilitated glucose transporter), member 3	1.52718387	0.0009632	0.01267
67092	Gatm	glycine amidinotransferase (L-arginine:glycine amidinotransferase)	1.52671756	0.001353	0.01555
231125	Zfyve28	zinc finger, FYVE domain containing 28	1.52648451	0.0009688	0.01272
16617	Klk1b24	kallikrein 1-related peptidase b24	1.52648451	0.05703	0.1699
140703	Emid1	EMI domain containing 1	1.52625153	0.02048	0.0853
21828	Thbs4	thrombospondin 4	1.52625153	0.02737	0.1035
170756	Slc24a6	solute carrier family 24 (sodium/potassium/calcium exchanger), member 6	1.52625153	0.1444	0.3162
215627	Zbtb8b	zinc finger and BTB domain containing 8b	1.52601862	0.009893	0.05291
72947	Agxt2l2	alanine-glyoxylate aminotransferase 2-like 2	1.52508769	0.002946	0.0245
17701	Msx1	homeobox, msh-like 1	1.52508769	0.05771	0.171

75563	Dnali1	dynein, axonemal, light intermediate polypeptide 1	1.52462266	0.001996	0.0195
13866	Erbb2	v-erb-b2 erythroblastic leukemia viral oncogene homolog 2, neuro/glioblastoma derived oncogene homolog (avian)	1.5241579	0.006678	0.04113
16774	Lama3	laminin, alpha 3	1.52392563	0.0151	0.06975
78892	Crispld2	cysteine-rich secretory protein LCCL domain containing 2	1.5234613	0.000168	0.004465
239122	Setdb2	SET domain, bifurcated 2	1.52322925	0.001718	0.01791
21677	Tead2	TEA domain family member 2	1.52276534	0.001149	0.01412
16776	Lama5	laminin, alpha 5	1.52276534	0.001629	0.01733
13036	Ctsh	cathepsin H	1.5225335	0.003647	0.02812
18552	Pcsk5	proprotein convertase subtilisin/kexin type 5	1.5225335	0.01027	0.05422
74365	Lonrf3	LON peptidase N-terminal domain and ring finger 3	1.52230172	0.004146	0.03047
226781	Slc30a10	solute carrier family 30, member 10	1.52207002	0.1048	0.2559
65973	Asph	aspartate-beta-hydroxylase	1.52160682	0.005755	0.03732
57342	Parva	parvin, alpha	1.51998784	0.0001606	0.004382
235406	Snx33	sorting nexin 33	1.51906426	0.004315	0.03125
19332	Rab20	RAB20, member RAS oncogene family	1.51883354	0.006228	0.0393
100042092	Gm3662	predicted gene 3662	1.5183723	0.004293	0.03116
83436	Plekha2	pleckstrin homology domain-containing, family A (phosphoinositide binding specific) member 2	1.51768098	9.51E-05	0.00338
56050	Cyp39a1	cytochrome P450, family 39, subfamily a, polypeptide 1	1.51722045	0.001747	0.01811
83701	Srrt	serrate RNA effector molecule homolog (Arabidopsis)	1.51699029	2.64E-05	0.001661
27055	Fkbp9	FK506 binding protein 9	1.51699029	0.005472	0.03614
67473	Slc47a1	solute carrier family 47, member 1	1.5167602	0.01284	0.06273
230824	Grhl3	grainyhead-like 3 (Drosophila)	1.51653018	0.0046	0.03247
54325	Elovl1	elongation of very long chain fatty acids (FEN1/Elo2, SUR4/Elo3, yeast)-like 1	1.51584053	0.06348	0.1825
71994	Cnn3	calponin 3, acidic	1.51538112	0.0004386	0.007852
21824	Thbd	thrombomodulin	1.51538112	0.0005884	0.009389
78286	Nav2	neuron navigator 2	1.51538112	0.1221	0.2839
545253	Gm5820	predicted gene 5820	1.51469252	0.1294	0.2949
21928	Tnfaip2	tumor necrosis factor, alpha-induced protein 2	1.51400454	0.009672	0.05216
68303	Fam114a1	family with sequence similarity 114, member A1	1.51263046	0.01073	0.05558
100040880	Gm3020	predicted gene 3020	1.51263046	0.1751	0.3592
272551	Gins2	GINS complex subunit 2 (Psf2 homolog)	1.51240169	0.0001317	0.003982
21407	Tcf15	transcription factor 15	1.51240169	0.005284	0.03539
78829	Tsc22d4	TSC22 domain family, member 4	1.51194436	0.00866	0.04855
622640	Gm6337	predicted gene 6337	1.5117158	0.2248	0.4245
12633	Cflar	CASP8 and FADD-like apoptosis regulator	1.5114873	0.002582	0.02258
71586	Ifih1	interferon induced with helicase C domain 1	1.51080224	0.00324	0.02622
212974	Athl1	ATH1, acid trehalase-like 1 (yeast)	1.51080224	0.004359	0.03144
17480	Mpl	myeloproliferative leukemia virus oncogene	1.50943396	0.0005644	0.009194
228608	Smox	spermine oxidase	1.50897842	0.00177	0.01823
50496	E2f6	E2F transcription factor 6	1.50897842	0.007273	0.0436
14919	Gucy2e	guanylate cyclase 2e	1.50875075	0.0007342	0.01087
63913	Fam129a	family with sequence similarity 129, member A	1.50852316	0.0909	0.2333
74744	5830408C22Rik	RIKEN cDNA 5830408C22 gene	1.50715901	0.03865	0.1298
170721	Papln	papilin, proteoglycan-like sulfated glycoprotein	1.50670484	0.08506	0.2232
14187	Akr1b8	aldo-keto reductase family 1, member B8	1.5060241	0.004632	0.03265
75736	Bcl2l12	BCL2-like 12 (proline rich)	1.50579732	0.02085	0.0864
16149	Cd74	CD74 antigen (invariant polypeptide of major histocompatibility complex, class II antigen-associated)	1.50557061	0.1339	0.3009
17349	Mlf1	myeloid leukemia factor 1	1.5051174	0.000891	0.0121
26934	Racgap1	Rac GTPase-activating protein 1	1.50466446	0.001553	0.01696
109019	Obfc2a	oligonucleotide/oligosaccharide-binding fold containing 2A	1.50443809	0.006819	0.04177

107272	Psat1	phosphoserine aminotransferase 1	1.50398556	0.0002494	0.005672
12368	Casp6	caspase 6	1.50398556	0.001019	0.01321
234582	Ccdc102a	coiled-coil domain containing 102A	1.50217816	0.0008273	0.01159
16362	Irf1	interferon regulatory factor 1	1.50195254	8.60E-05	0.003173
75495	Morn5	MORN repeat containing 5	1.50195254	0.001455	0.0162
240479	Fam69c	family with sequence similarity 69, member C	1.50060024	0.002849	0.02405
13713	Elk3	ELK3, member of ETS oncogene family	1.50060024	0.1075	0.2598
14725	Lrp2	low density lipoprotein receptor-related protein 2	1.50037509	0.0002072	0.005066
381716	1700015F17Rik	RIKEN cDNA 1700015F17 gene	1.49970006	0.00962	0.05194
20024	Sub1	SUB1 homolog (S. cerevisiae)	1.49925037	0.0003638	0.006971
13039	Ctsl	cathepsin L	1.49925037	0.001759	0.0182
100306953	4122401K19Rik	RIKEN cDNA 4122401K19 gene	1.49902563	0.001067	0.01359
547253	Parp14	poly (ADP-ribose) polymerase family, member 14	1.49902563	0.001546	0.01694
19331	Rab19	RAB19, member RAS oncogene family	1.49857635	5.21E-05	0.002449
384059	Tlr12	toll-like receptor 12	1.49857635	0.01346	0.0646
229841	Cenpe	centromere protein E	1.49835181	0.002159	0.0203
226422	Rab7l1	RAB7, member RAS oncogene family-like 1	1.49790294	0.002027	0.01963
258505	Olfr97	olfactory receptor 97	1.49790294	0.09835	0.2458
192657	Ell2	elongation factor RNA polymerase II 2	1.4976786	0.00203	0.01964
27392	Pign	phosphatidylinositol glycan anchor biosynthesis, class N	1.4976786	0.04164	0.1364
12705	Cited1	Cbp/p300-interacting transactivator with Glu/Asp-rich carboxy-terminal domain 1	1.49745433	0.0002804	0.005973
109594	Lmo1	LIM domain only 1	1.49745433	0.0004324	0.007787
20315	Cxcl12	chemokine (C-X-C motif) ligand 12	1.49745433	0.01502	0.06945
20196	S100a13	S100 calcium binding protein A13	1.49633398	0.0006435	0.009975
217830	9030617O03Rik	RIKEN cDNA 9030617O03 gene	1.49611011	0.004517	0.03212
100040259	Gm16379	predicted pseudogene 16379	1.49588631	0.001776	0.01828
21818	Tgm3	transglutaminase 3, E polypeptide	1.49566258	0.2969	0.506
12331	Cap1	CAP, adenylate cyclase-associated protein 1 (yeast)	1.49543891	0.0002641	0.005839
12412	Cbx1	chromobox homolog 1 (Drosophila HP1 beta)	1.49543891	0.002837	0.024
100039027	Gm2011	predicted gene 2011	1.49476831	0.01467	0.06841
240667	Sec31b	Sec31 homolog B (S. cerevisiae)	1.49432158	0.0003528	0.006833
319625	Galm	galactose mutarotase	1.49432158	0.01234	0.0611
252864	Dusp15	dual specificity phosphatase-like 15	1.49409831	0.0149	0.06903
14066	F3	coagulation factor III	1.49387511	0.0001158	0.003741
18158	Nppb	natriuretic peptide type B	1.49365198	0.04204	0.1373
52588	Tspan14	tetraspanin 14	1.49320591	0.000238	0.005529
226432	Ipo9	importin 9	1.49276011	0.0001425	0.004152
11593	Aga	aspartylglucosaminidase	1.49276011	0.005319	0.03554
66873	Tril	TLR4 interactor with leucine-rich repeats	1.49186931	0.02413	0.09508
319765	Igf2bp2	insulin-like growth factor 2 mRNA binding protein 2	1.49142431	0.0008709	0.01198
20409	Ostf1	osteoclast stimulating factor 1	1.4905351	0.006504	0.04036
225341	Lims2	LIM and senescent cell antigen like domains 2	1.4905351	0.04183	0.1368
74856	4930418C01Rik	RIKEN cDNA 4930418C01 gene	1.49031297	0.5874	0.7577
231440	Parm1	prostate androgen-regulated mucin-like protein 1	1.4900909	0.0001607	0.004382
100503359	LOC100503359	hypothetical LOC100503359	1.4900909	0.1841	0.371
230837	Asap3	ArfGAP with SH3 domain, ankyrin repeat and PH domain 3	1.48964695	0.02936	0.1085
81910	Rrbp1	ribosome binding protein 1	1.48920328	0.0009259	0.01241
67454	Ikbip	IKBKB interacting protein	1.48898154	0.001174	0.01433
170721	Papln	papilin, proteoglycan-like sulfated glycoprotein	1.48898154	0.07979	0.2141
58214	Cst10	cystatin 10 (chondrocytes)	1.48898154	0.4432	0.6463

57274	Slc16a8	solute carrier family 16 (monocarboxylic acid transporters), member 8	1.48853826	0.0411	0.1352
235461	Fam63b	family with sequence similarity 63, member B	1.48765248	0.0005596	0.009165
19207	Ptch2	patched homolog 2	1.48765248	0.3002	0.5095
56421	Pfkp	phosphofructokinase, platelet	1.48654675	0.000117	0.003744
239530	Gpr20	G protein-coupled receptor 20	1.4863258	0.005585	0.03663
71004	4931440P22Rik	RIKEN cDNA 4931440P22 gene	1.4863258	0.1029	0.2531
13162	Slc6a3	solute carrier family 6 (neurotransmitter transporter, dopamine), member 3	1.4858841	0.1666	0.3473
70572	Ipo5	importin 5	1.48522204	0.000391	0.007292
69668	Ccdc115	coiled-coil domain containing 115	1.48456057	0.000512	0.008665
52187	Rragd	Ras-related GTP binding D	1.48345943	0.05216	0.1597
21390	Tbxa2r	thromboxane A2 receptor	1.48323939	0.0004856	0.0084
83433	Trem2	triggering receptor expressed on myeloid cells 2	1.48301943	0.006322	0.03972
64899	Lpin3	lipin 3	1.48257969	0.001855	0.01877
216795	Wnt9a	wingless-type MMTV integration site 9A	1.48235992	4.07E-05	0.002124
107581	Col16a1	collagen, type XVI, alpha 1	1.48170099	0.001027	0.01325
74145	F13a1	coagulation factor XIII, A1 subunit	1.48060409	0.2254	0.4252
15937	Ier3	immediate early response 3	1.4803849	7.97E-05	0.003065
319158	Hist1h4i	histone cluster 1, H4i	1.4803849	0.009916	0.05298
81015	Vmn1r56	vomeronasal 1 receptor 56	1.4803849	0.3345	0.5452
11629	Aif1	allograft inflammatory factor 1	1.48016578	0.003145	0.0257
231103	Gckr	glucokinase regulatory protein	1.48016578	0.006231	0.0393
67512	Agpat2	1-acylglycerol-3-phosphate O-acyltransferase 2 (lysophosphatidic acid acyltransferase, beta)	1.47994672	0.001787	0.0183
99929	Tiparp	TCDD-inducible poly(ADP-ribose) polymerase	1.47994672	0.00472	0.03302
81910	Rrbp1	ribosome binding protein 1	1.47994672	0.03945	0.1316
73822	F630110N24Rik	RIKEN cDNA F630110N24 gene	1.47972773	0.03495	0.1217
12931	Crlf1	cytokine receptor-like factor 1	1.47928994	0.001973	0.01938
330260	Pon2	paraoxonase 2	1.47841514	0.001057	0.01352
71729	Rgs12	regulator of G-protein signaling 12	1.47754137	0.01243	0.06143
69386	Hist1h4h	histone cluster 1, H4h	1.47754137	0.01448	0.06787
403175	Tigd4	tigger transposable element derived 4	1.47732309	0.07427	0.2037
72947	Agxt2l2	alanine-glyoxylate aminotransferase 2-like 2	1.47666864	0.002636	0.02295
72057	Phf10	PHD finger protein 10	1.47623265	0.0001748	0.004559
23876	Fbln5	fibulin 5	1.47623265	0.04128	0.1357
76974	1190003J15Rik	RIKEN cDNA 1190003J15 gene	1.47557916	0.09314	0.237
244723	Olfm2	olfactomedin 2	1.47536146	0.08514	0.2234
76448	2310014H01Rik	RIKEN cDNA 2310014H01 gene	1.47492625	0.03167	0.1142
432825	Gm5458	predicted gene 5458	1.47492625	0.2564	0.4615
74318	Hopx	HOP homeobox	1.47427392	0.004515	0.03212
17319	Mif	macrophage migration inhibitory factor	1.47340504	0.001574	0.01706
69974	2810405F15Rik	RIKEN cDNA 2810405F15 gene	1.47297098	0.001245	0.01483
58222	Rab37	RAB37, member of RAS oncogene family	1.47297098	0.003663	0.02819
20723	Serpinb9	serine (or cysteine) peptidase inhibitor, clade B, member 9	1.47275405	0.007424	0.0441
52118	Pvr	poliovirus receptor	1.47210364	0.005687	0.03708
16779	Lamb2	laminin, beta 2	1.47188696	0.0009338	0.01246
13215	Defb2	defensin beta 2	1.4714538	0.4246	0.6301
78771	Mctp1	multiple C2 domains, transmembrane 1	1.47102089	0.1511	0.3258
12263	C2	complement component 2 (within H-2S)	1.470372	0.0002826	0.005997
230073	Ddx58	DEAD (Asp-Glu-Ala-Asp) box polypeptide 58	1.470372	0.002546	0.02237
233274	Siglech	sialic acid binding Ig-like lectin H	1.46972369	0.02013	0.08412
271047	Serpina3b	serine (or cysteine) peptidase inhibitor, clade A, member 3B	1.46972369	0.02504	0.09752

207683	Igsf11	immunoglobulin superfamily, member 11	1.46950771	0.001682	0.01766
380912	Zfp395	zinc finger protein 395	1.46799765	0.01344	0.06459
102614	Rpp25	ribonuclease P 25 subunit (human)	1.46799765	0.03663	0.1255
51798	Ech1	enoyl coenzyme A hydratase 1, peroxisomal	1.46756677	0.0003659	0.006994
17686	Msh3	mutS homolog 3 (E. coli)	1.46756677	0.001582	0.01708
12633	Cflar	CASP8 and FADD-like apoptosis regulator	1.46756677	0.004568	0.03233
20818	Srprb	signal recognition particle receptor, B subunit	1.46756677	0.007736	0.04528
93840	Vangl2	vang-like 2 (van gogh, Drosophila)	1.46670578	0.03291	0.1171
225659	Cep76	centrosomal protein 76	1.46649069	0.01095	0.05631
100042149	Gm3696	predicted gene 3696	1.46649069	0.192	0.3816
667572	Gm8709	glyceraldehyde-3-phosphate dehydrogenase pseudogene	1.46627566	0.00137	0.01565
18080	Nin	ninein	1.46606069	0.0001595	0.004382
66949	Trim59	tripartite motif-containing 59	1.46606069	0.004353	0.03142
22329	Vcam1	vascular cell adhesion molecule 1	1.46498682	0.005009	0.03418
108897	Aif1l	allograft inflammatory factor 1-like	1.46477223	0.0002005	0.004988
17087	Ly96	lymphocyte antigen 96	1.46477223	0.008961	0.04948
15288	Hmbs	hydroxymethylbilane synthase	1.4645577	0.0001855	0.004709
54204	Sep-01	septin 1	1.46434324	0.04142	0.1361
232748	Fam115c	family with sequence similarity 115, member C	1.46391451	0.00826	0.04714
75472	1700009P17Rik	RIKEN cDNA 1700009P17 gene	1.46262981	0.008437	0.04769
16206	Lrig1	leucine-rich repeats and immunoglobulin-like domains 1	1.46241591	0.0004563	0.008028
12969	Crygf	crystallin, gamma F	1.46177459	0.1494	0.3233
12946	Cr1l	complement component (3b/4b) receptor 1-like	1.46113384	0.000962	0.01267
11799	Birc5	baculoviral IAP repeat-containing 5	1.46092038	0.001656	0.01747
106869	Tnfaip8	tumor necrosis factor, alpha-induced protein 8	1.46092038	0.002689	0.02321
326623	Tnfsf15	tumor necrosis factor (ligand) superfamily, member 15	1.46092038	0.03578	0.1237
63872	Zfp296	zinc finger protein 296	1.46070698	0.002208	0.02054
79554	Gltpd1	glycolipid transfer protein domain containing 1	1.46049365	0.002449	0.02183
232334	Vgll4	vestigial like 4 (Drosophila)	1.45964093	0.0006069	0.009595
76633	1700112E06Rik	RIKEN cDNA 1700112E06 gene	1.45964093	0.00111	0.0139
72772	Rint1	RAD50 interactor 1	1.45964093	0.01582	0.07185
66707	Nkapl	NFKB activating protein-like	1.45921494	0.004189	0.03062
11877	Arvcf	armadillo repeat gene deleted in velo-cardio-facial syndrome	1.45900204	0.02361	0.09379
18103	Nme2	non-metastatic cells 2, protein (NM23B) expressed in	1.4587892	0.000537	0.008964
16562	Kif1c	kinesin family member 1C	1.45836372	0.01008	0.05364
241303	Fam78a	family with sequence similarity 78, member A	1.45836372	0.1023	0.2522
52639	Wipi1	WD repeat domain, phosphoinositide interacting 1	1.45815106	0.001744	0.0181
71790	Anxa9	annexin A9	1.45815106	0.003405	0.02694
67880	Dcxr	dicarbonyl L-xylulose reductase	1.45815106	0.02311	0.09246
545007	ENSMUSG00000068790	predicted gene, ENSMUSG00000068790	1.45772595	0.275	0.4829
54219	Cd320	CD320 antigen	1.45751348	0.001255	0.0149
76281	Tax1bp3	Tax1 (human T-cell leukemia virus type I) binding protein 3	1.45708874	0.0008879	0.01209
74410	Ttll11	tubulin tyrosine ligase-like family, member 11	1.45708874	0.003244	0.02623
245638	Tbc1d8b	TBC1 domain family, member 8B	1.45708874	0.01856	0.07976
118449	Synpo2	synaptopodin 2	1.45708874	0.02337	0.0931
93897	Fzd10	frizzled homolog 10 (Drosophila)	1.45687646	0.001011	0.01313
116847	Prelp	proline arginine-rich end leucine-rich repeat	1.45539223	0.2955	0.5044
72982	Tmem138	transmembrane protein 138	1.45496872	0.03129	0.1133
102545	Cmtm7	CKLF-like MARVEL transmembrane domain containing 7	1.45454545	0.00262	0.02285
12587	Mia1	melanoma inhibitory activity 1	1.45433392	0.02102	0.08698

52231	Ankzf1	ankyrin repeat and zinc finger domain containing 1	1.45412244	0.0008126	0.01148
30049	Scd3	stearoyl-coenzyme A desaturase 3	1.45285486	0.001893	0.019
28010	Miip	migration and invasion inhibitory protein	1.45264381	0.04745	0.1492
13008	Csrp2	cysteine and glycine-rich protein 2	1.45201104	0.01338	0.06448
234878	BC021891	cDNA sequence BC021891	1.45201104	0.07756	0.2099
30060	Mfi2	antigen p97 (melanoma associated) identified by monoclonal antibodies 133.2 and 96.5	1.45201104	0.07993	0.2144
71368	5430431A17Rik	RIKEN cDNA 5430431A17 gene	1.45201104	0.2341	0.4356
110596	Rgnef	Rho-guanine nucleotide exchange factor	1.45180023	0.004284	0.03112
58998	Pvrl3	poliovirus receptor-related 3	1.45180023	0.004489	0.03202
229214	Qrfpr	pyroglutamylated RFamide peptide receptor	1.45137881	0.0007476	0.01095
83436	Plekha2	pleckstrin homology domain-containing, family A (phosphoinositide binding specific) member 2	1.45137881	0.00158	0.01708
319269	A130040M12Rik	RIKEN cDNA A130040M12 gene	1.45116819	0.04378	0.1409
76571	Styxl1	serine/threonine/tyrosine interacting-like 1	1.45095763	0.001494	0.01649
234757	BC024137	cDNA sequence BC024137	1.45074713	0.03632	0.1249
676894	Gm9694	predicted gene 9694	1.44969556	0.004294	0.03116
13733	Emr1	EGF-like module containing, mucin-like, hormone receptor-like sequence 1	1.44969556	0.02571	0.09933
11898	Ass1	argininosuccinate synthetase 1	1.44948543	0.009041	0.04979
12843	Col1a2	collagen, type I, alpha 2	1.44927536	0.1317	0.298
12566	Cdk2	cyclin-dependent kinase 2	1.44906535	0.06511	0.1857
69590	Gpx8	glutathione peroxidase 8 (putative)	1.4488554	0.0006049	0.009581
13051	Cx3cr1	chemokine (C-X3-C) receptor 1	1.44843569	0.03032	0.111
14388	Gab1	growth factor receptor bound protein 2-associated protein 1	1.44801622	0.004171	0.03053
75580	Zbtb4	zinc finger and BTB domain containing 4	1.44780657	0.005097	0.03459
17130	Smad6	MAD homolog 6 (Drosophila)	1.44759699	0.007253	0.04355
63986	Gmfg	glia maturation factor, gamma	1.447178	0.04143	0.1361
69034	4930579G22Rik	RIKEN cDNA 4930579G22 gene	1.44654998	0.0001788	0.004623
213522	Plekhg6	pleckstrin homology domain containing, family G (with RhoGef domain) member 6	1.44654998	0.03761	0.1278
50527	Ero1l	ERO1-like (S. cerevisiae)	1.44634076	0.001687	0.01769
18797	Plcb3	phospholipase C, beta 3	1.44634076	0.004365	0.03147
66838	0610009L18Rik	RIKEN cDNA 0610009L18 gene	1.44571346	0.1679	0.3486
104444	Rexo2	REX2, RNA exonuclease 2 homolog (S. cerevisiae)	1.44550448	0.0001192	0.003772
11933	Atp1b3	ATPase, Na+/K+ transporting, beta 3 polypeptide	1.44529556	0.008858	0.04914
432825	Gm5458	predicted gene 5458	1.44529556	0.2331	0.4346
627872	Dnahc7a	dynein, axonemal, heavy chain 7A	1.44466917	0.0009849	0.01289
320405	Cadps2	Ca2+-dependent activator protein for secretion 2	1.44425188	0.0005667	0.009215
19255	Ptpn2	protein tyrosine phosphatase, non-receptor type 2	1.44404332	0.0009345	0.01246
18103	Nme2	non-metastatic cells 2, protein (NM23B) expressed in	1.44362639	0.001811	0.01845
170756	Slc24a6	solute carrier family 24 (sodium/potassium/calcium exchanger), member 6	1.44341801	0.02509	0.09761
100039258	Gm10290	glyceraldehyde-3-phosphate dehydrogenase pseudogene	1.4432097	0.02366	0.0939
19354	Rac2	RAS-related C3 botulinum substrate 2	1.44300144	0.01055	0.05509
381549	Zfp69	zinc finger protein 69	1.44300144	0.1164	0.2742
328424	Kcnrg	potassium channel regulator	1.44300144	0.1518	0.3265
16195	Il6st	interleukin 6 signal transducer	1.44237704	0.000437	0.007846
56448	Cyp2d22	cytochrome P450, family 2, subfamily d, polypeptide 22	1.44237704	0.001766	0.01823
74075	Syce1	synaptonemal complex central element protein 1	1.44237704	0.07163	0.1984
381489	Rxfp1	relaxin/insulin-like family peptide receptor 1	1.44196107	0.02442	0.0958
71544	Arhgap42	Rho GTPase activating protein 42	1.44175317	0.005572	0.03661
26362	Axl	AXL receptor tyrosine kinase	1.44133756	0.002778	0.02369
67317	1700022I11Rik	RIKEN cDNA 1700022I11 gene	1.44133756	0.01391	0.0661
73847	Fam110a	family with sequence similarity 110, member A	1.44112985	0.0006935	0.01047

216792	A230051G13Rik	RIKEN cDNA A230051G13 gene	1.44112985	0.001532	0.01682
77707	9130604C24Rik	RIKEN cDNA 9130604C24 gene	1.44112985	0.009579	0.05183
114644	Slc13a3	solute carrier family 13 (sodium-dependent dicarboxylate transporter), member 3	1.44112985	0.07516	0.2051
64817	Svep1	sushi, von Willebrand factor type A, EGF and pentraxin domain containing 1	1.44092219	0.02154	0.08843
83453	Chrdl1	chordin-like 1	1.44029958	0.00221	0.02055
332579	Card9	caspase recruitment domain family, member 9	1.44029958	0.06825	0.1916
270192	Rab6b	RAB6B, member RAS oncogene family	1.43967751	2.77E-05	0.001692
229445	Ctso	cathepsin O	1.43947027	0.001075	0.01361
102626	Mapkapk3	mitogen-activated protein kinase-activated protein kinase 3	1.4392631	0.002132	0.02017
70810	Krt25	keratin 25	1.4392631	0.007793	0.04545
223696	Tomm22	translocase of outer mitochondrial membrane 22 homolog (yeast)	1.4392631	0.008229	0.04702
67860	S100a16	S100 calcium binding protein A16	1.43884892	0.005129	0.03469
11686	Alox12b	arachidonate 12-lipoxygenase, 12R type	1.43864192	0.04177	0.1367
26941	Slc9a3r1	solute carrier family 9 (sodium/hydrogen exchanger), member 3 regulator 1	1.43843498	0.02082	0.08633
69430	1700048O20Rik	RIKEN cDNA 1700048O20 gene	1.4382281	0.001696	0.01774
107995	Cdc20	cell division cycle 20 homolog (S. cerevisiae)	1.4382281	0.01088	0.05612
66953	Cdca7	cell division cycle associated 7	1.43802128	0.003215	0.02608
246728	Oas2	2'-5' oligoadenylate synthetase 2	1.43802128	0.004145	0.03047
170799	Rtkn2	rhotekin 2	1.43698807	0.01411	0.06675
69068	1810011O10Rik	RIKEN cDNA 1810011O10 gene	1.43678161	0.0009136	0.01229
72040	Cdhr5	cadherin-related family member 5	1.4365752	0.04335	0.1398
20345	Selplg	selectin, platelet (p-selectin) ligand	1.43636886	0.01012	0.05371
67689	Aldh3b1	aldehyde dehydrogenase 3 family, member B1	1.43616257	0.007045	0.04274
21788	Tfpi	tissue factor pathway inhibitor	1.43595635	0.1346	0.3015
67464	Entpd4	ectonucleoside triphosphate diphosphohydrolase 4	1.43554407	0.001765	0.01823
100038941	Vmn2r121	vomeronasal 2, receptor 121	1.43513203	0.1407	0.3105
65972	Ifi30	interferon gamma inducible protein 30	1.4349261	0.01185	0.05969
207965	Gm71	predicted gene 71	1.43472023	0.0003461	0.006759
26559	Hunk	hormonally upregulated Neu-associated kinase	1.43410297	0.01435	0.06754
60344	Fign	fidgetin	1.43389733	0.00968	0.05216
100038433	9130213A22Rik	RIKEN cDNA 9130213A22 gene	1.43348624	0.005729	0.03722
78892	Crispld2	cysteine-rich secretory protein LCCL domain containing 2	1.43307538	0.003663	0.02819
12829	Col4a4	collagen, type IV, alpha 4	1.43287004	0.01537	0.07062
328035	Fads6	fatty acid desaturase domain family, member 6	1.43245953	7.64E-05	0.003006
76281	Tax1bp3	Tax1 (human T-cell leukemia virus type I) binding protein 3	1.43163923	0.002419	0.02168
75690	Vsig10l	ZV-set and immunoglobulin domain containing 10 like	1.43163923	0.02424	0.09531
107029	Me2	malic enzyme 2, NAD(+)-dependent, mitochondrial	1.4314343	0.0002626	0.005835
654824	Ankrd37	ankyrin repeat domain 37	1.4314343	0.0004515	0.007966
100039258	Gm10290	glyceraldehyde-3-phosphate dehydrogenase pseudogene	1.43122943	0.01868	0.08012
18217	Ntsr2	neurotensin receptor 2	1.43122943	0.0291	0.1078
74692	4930442P07Rik	RIKEN cDNA 4930442P07 gene	1.43122943	0.03123	0.1132
320383	B230317F23Rik	RIKEN cDNA B230317F23 gene	1.43122943	0.03803	0.1286
53608	Map3k6	mitogen-activated protein kinase kinase kinase 6	1.43102461	0.01361	0.06511
11829	Aqp4	aquaporin 4	1.43061516	0.0005429	0.009012
52685	Cd300lg	CD300 antigen like family member G	1.43061516	0.04174	0.1366
66729	Ankrd61	ankyrin repeat domain 61	1.43041053	0.009447	0.05131
18576	Pde3b	phosphodiesterase 3B, cGMP-inhibited	1.42979697	0.004632	0.03265
56190	Rbm38	RNA binding motif protein 38	1.42959257	0.04871	0.1521
74485	4933430H15Rik	RIKEN cDNA 4933430H15 gene	1.42938822	0.1005	0.2494
544678	2010015L04Rik	RIKEN cDNA 2010015L04 gene	1.42918394	0.003589	0.02786

57811	Rgr	retinal G protein coupled receptor	1.42918394	0.06644	0.1881
246081	Defb11	defensin beta 11	1.42918394	0.133	0.3
238333	Samd15	sterile alpha motif domain containing 15	1.42918394	0.1451	0.3173
216864	Mgl2	macrophage galactose N-acetyl-galactosamine specific lectin 2	1.42836738	0.0001673	0.004465
26934	Racgap1	Rac GTPase-activating protein 1	1.42795945	0.006112	0.03873
68861	1190002N15Rik	RIKEN cDNA 1190002N15 gene	1.42755175	0.01818	0.07861
13057	Cyba	cytochrome b-245, alpha polypeptide	1.42755175	0.02012	0.08412
22771	Zic1	zinc finger protein of the cerebellum 1	1.42734799	0.01232	0.06106
74191	P2ry13	purinergic receptor P2Y, G-protein coupled 13	1.42734799	0.08434	0.2221
233115	Dpy19l3	dpy-19-like 3 (C. elegans)	1.42714428	0.0002403	0.005548
100038846	Gm1973	predicted gene 1973	1.42714428	0.3227	0.5325
266620	Defb36	defensin beta 36	1.42714428	0.4636	0.6639
14369	Fzd7	frizzled homolog 7 (Drosophila)	1.42694064	0.0009192	0.01234
66181	Nop10	NOP10 ribonucleoprotein homolog (yeast)	1.42694064	0.001141	0.01407
18212	Ntrk2	neurotrophic tyrosine kinase, receptor, type 2	1.42673705	0.0006221	0.009748
11832	Aqp7	aquaporin 7	1.42673705	0.03322	0.1178
626575	Gm6687	predicted gene 6687	1.42653352	0.02598	0.09995
14198	Fhit	fragile histidine triad gene	1.42653352	0.1037	0.2544
20197	S100a3	S100 calcium binding protein A3	1.42633005	0.001894	0.019
15214	Hey2	hairy/enhancer-of-split related with YRPW motif 2	1.42612664	0.05968	0.1747
74319	1110005A03Rik	RIKEN cDNA 1110005A03 gene	1.42592329	0.00164	0.01738
12494	Cd38	CD38 antigen	1.42592329	0.04894	0.1526
76432	2310001H17Rik	RIKEN cDNA 2310001H17 gene	1.42571999	0.2131	0.4091
320701	Fam19a4	family with sequence similarity 19, member A4	1.42551675	0.004967	0.03398
12869	Cox8b	cytochrome c oxidase, subunit VIIIb	1.42511045	0.1129	0.2688
79201	Tnfrsf23	tumor necrosis factor receptor superfamily, member 23	1.42490738	0.157	0.334
58998	Pvrl3	poliovirus receptor-related 3	1.42470437	0.001704	0.01782
19682	Rdh5	retinol dehydrogenase 5	1.42470437	0.01834	0.07911
56228	Ube2j1	ubiquitin-conjugating enzyme E2, J1	1.42429853	0.0002116	0.005139
18822	Plod1	procollagen-lysine, 2-oxoglutarate 5-dioxygenase 1	1.42429853	0.002384	0.02147
74747	Ddit4	DNA-damage-inducible transcript 4	1.4240957	0.0005089	0.008648
13631	Eef2k	eukaryotic elongation factor-2 kinase	1.42389292	0.03384	0.1191
29810	Bag3	BCL2-associated athanogene 3	1.42348754	0.0001142	0.003716
338369	Tmem220	transmembrane protein 220	1.42348754	0.02003	0.08385
54485	Dll4	delta-like 4 (Drosophila)	1.42328494	0.3136	0.5232
71729	Rgs12	regulator of G-protein signaling 12	1.4230824	0.006793	0.04164
64099	Parvg	parvin, gamma	1.4230824	0.2086	0.4034
106869	Tnfaip8	tumor necrosis factor, alpha-induced protein 8	1.42287991	0.0009543	0.01264
107607	Nod1	nucleotide-binding oligomerization domain containing 1	1.42207053	0.0003059	0.00629
21813	Tgfbr2	transforming growth factor, beta receptor II	1.42186833	0.005809	0.03754
50529	Mrps7	mitchondrial ribosomal protein S7	1.42166619	0.001196	0.01445
624855	Gm6531	predicted gene 6531	1.42166619	0.05662	0.169
546519	Gm12581	predicted gene 12581	1.42146411	0.01216	0.06055
71994	Cnn3	calponin 3, acidic	1.42126208	0.0004411	0.007873
69513	1700030C10Rik	RIKEN cDNA 1700030C10 gene	1.42045455	0.02404	0.09495
18655	Pgk1	phosphoglycerate kinase 1	1.4202528	0.002068	0.01983
385138	BC061237	cDNA sequence BC061237	1.42005112	0.1899	0.3787
229214	Qrfpr	pyroglutamylated RFamide peptide receptor	1.4198495	0.002315	0.02123
67432	Hoga1	4-hydroxy-2-oxoglutarate aldolase 1	1.4198495	0.05683	0.1695
18230	Nxn	nucleoredoxin	1.41964793	0.00123	0.01469

71226	4933433G19Rik	RIKEN cDNA 4933433G19 gene	1.41904356	0.01094	0.05628
16912	Psmb9	proteasome (prosome, macropain) subunit, beta type 9 (large multifunctional peptidase 2)	1.41884222	0.02345	0.09332
268935	Scube3	signal peptide, CUB domain, EGF-like 3	1.41864094	0.0348	0.1213
67724	Pop1	processing of precursor 1, ribonuclease P/MRP family, (S. cerevisiae)	1.41843972	0.009876	0.0529
22255	Uncx	UNC homeobox	1.41843972	0.03348	0.1184
75777	Ttc23l	tetratricopeptide repeat domain 23-like	1.41843972	0.251	0.4558
20431	Pmel	premelanosome protein	1.41803744	0.03084	0.1123
11920	Atm	ataxia telangiectasia mutated homolog (human)	1.41783638	0.03213	0.1153
12215	Bsg	basigin	1.41763538	0.006873	0.04197
20356	Sema5a	sema domain, seven thrombospondin repeats (type 1 and type 1-like), transmembrane domain (TM) and short cytoplasmic domain, (semaphorin) 5A	1.41723356	0.0006503	0.01001
72240	1600014C23Rik	RIKEN cDNA 1600014C23 gene	1.41723356	0.005186	0.03495
70530	Lrfn2	leucine rich repeat and fibronectin type III domain containing 2	1.41723356	0.01373	0.06555
77041	Arsk	arylsulfatase K	1.41703273	0.00292	0.02438
18746	Pkm2	pyruvate kinase, muscle	1.41703273	0.007881	0.04572
66395	Ahnak	AHNAK nucleoprotein (desmoyokin)	1.41703273	0.04629	0.1465
103080	Sep-10	septin 10	1.41683196	0.03822	0.129
78928	Pigt	phosphatidylinositol glycan anchor biosynthesis, class T	1.41623	0.007527	0.04442
100041019	Gm3099	predicted gene 3099	1.41602945	0.2069	0.4015
68428	Steap3	STEAP family member 3	1.41582897	0.00581	0.03754
78829	Tsc22d4	TSC22 domain family, member 4	1.41582897	0.007518	0.04441
19245	Ptp4a3	protein tyrosine phosphatase 4a3	1.41582897	0.02241	0.09076
319765	Igf2bp2	insulin-like growth factor 2 mRNA binding protein 2	1.41542817	0.002351	0.02135
234362	Zfp868	zinc finger protein 868	1.41502759	0.001129	0.01399
11443	Chrnb1	cholinergic receptor, nicotinic, beta polypeptide 1 (muscle)	1.41502759	0.004133	0.03041
70294	Rnf126	ring finger protein 126	1.41502759	0.01475	0.0687
73016	Kremen2	kringle containing transmembrane protein 2	1.41482739	0.0382	0.129
114601	Ehbp1l1	EH domain binding protein 1-like 1	1.41422712	0.004821	0.03344
232156	Slc4a5	solute carrier family 4, sodium bicarbonate cotransporter, member 5	1.41382723	0.01517	0.07003
50530	Mfap5	microfibrillar associated protein 5	1.41382723	0.1528	0.3281
14739	S1pr2	sphingosine-1-phosphate receptor 2	1.41362737	0.02187	0.08933
70021	Nt5dc2	5'-nucleotidase domain containing 2	1.41342756	0.02861	0.1067
109857	Cbr3	carbonyl reductase 3	1.41322781	0.001119	0.01392
18230	Nxn	nucleoredoxin	1.41302812	0.004862	0.03358
11818	Apoh	apolipoprotein H	1.41282848	0.06571	0.1868
73916	Ift57	intraflagellar transport 57 homolog (Chlamydomonas)	1.41242938	0.001471	0.01631
27280	Phlda3	pleckstrin homology-like domain, family A, member 3	1.41242938	0.001768	0.01823
66913	Kdelr2	KDEL (Lys-Asp-Glu-Leu) endoplasmic reticulum protein retention receptor 2	1.41222991	0.001374	0.01567
105501	Abhd4	abhydrolase domain containing 4	1.41183114	0.0001129	0.003694
52348	Vps37a	vacuolar protein sorting 37A (yeast)	1.41183114	0.0001239	0.00384
20917	Suclg2	succinate-Coenzyme A ligase, GDP-forming, beta subunit	1.4114326	0.01747	0.07645
53871	Pkd2l2	polycystic kidney disease 2-like 2	1.41103429	0.1026	0.2526
83493	Sacm1l	SAC1 (suppressor of actin mutations 1, homolog)-like (S. cerevisiae)	1.41083521	0.002101	0.02001
100042342	Gm10375	predicted gene 10375	1.41023833	0.2211	0.4201
27381	Tcl1b2	T-cell leukemia/lymphoma 1B, 2	1.41023833	0.5585	0.7378
109552	Sri	sorcin	1.40984069	0.0007612	0.01102
74091	Npl	N-acetylneuraminate pyruvate lyase	1.40984069	0.01613	0.0725
11936	Fxyd2	FXYD domain-containing ion transport regulator 2	1.40904608	0.03188	0.1147
19731	Rgl1	ral guanine nucleotide dissociation stimulator,-like 1	1.40884756	0.001427	0.01602
319158	Hist1h4i	histone cluster 1, H4i	1.4084507	0.001844	0.01868
13829	Epb4.9	erythrocyte protein band 4.9	1.4084507	0.009281	0.05069

227737	Fam129b	family with sequence similarity 129, member B	1.40805407	0.0002377	0.005528
64297	Gprc5b	G protein-coupled receptor, family C, group 5, member B	1.40785584	0.004714	0.033
14701	Gng12	guanine nucleotide binding protein (G protein), gamma 12	1.40765766	0.002341	0.02133
13650	Rhbdf1	rhomboid family 1 (Drosophila)	1.40765766	0.002443	0.02178
16913	Psmb8	proteasome (prosome, macropain) subunit, beta type 8 (large multifunctional peptidase 7)	1.40745954	0.01196	0.06006
13649	Egfr	epidermal growth factor receptor	1.40706346	0.0008101	0.01147
54132	Pdlim1	PDZ and LIM domain 1 (elfin)	1.40706346	0.1191	0.279
71956	Rnf135	ring finger protein 135	1.40646976	0.01064	0.05534
67075	Magt1	magnesium transporter 1	1.40607424	0.02693	0.1024
56078	Car5b	carbonic anhydrase 5b, mitochondrial	1.40528387	0.02187	0.08933
15461	Hras1	Harvey rat sarcoma virus oncogene 1	1.40508641	0.02575	0.09945
68632	Myct1	myc target 1	1.40488901	0.09658	0.2427
12476	Cd151	CD151 antigen	1.40429715	0.001346	0.0155
103220	BC030307	cDNA sequence BC030307	1.40429715	0.02191	0.08941
19672	Rcn1	reticulocalbin 1	1.40409997	0.0006617	0.01009
11520	Plin2	perilipin 2	1.40409997	0.003122	0.02558
554327	2610042L04Rik	RIKEN cDNA 2610042L04 gene	1.40370578	0.3204	0.5303
76281	Tax1bp3	Tax1 (human T-cell leukemia virus type I) binding protein 3	1.40331182	0.0003386	0.006681
239096	Cdh24	cadherin-like 24	1.40311492	0.0086	0.04832
23849	Klf6	Kruppel-like factor 6	1.40291807	0.002175	0.02033
113845	Vmn1r48	vomeronasal 1 receptor 48	1.40272128	0.0061	0.03869
225182	Rbbp8	retinoblastoma binding protein 8	1.40193467	0.00703	0.04266
12577	Cdkn1c	cyclin-dependent kinase inhibitor 1C (P57)	1.4015417	0.02642	0.1011
268291	Rnf217	ring finger protein 217	1.40134529	0.01113	0.05704
70572	Ipo5	importin 5	1.40114894	0.0004164	0.007604
11758	Prdx6	peroxiredoxin 6	1.40075641	0.0003127	0.006357
67683	2610029G23Rik	RIKEN cDNA 2610029G23 gene	1.40056022	0.001558	0.01698
320662	Casc1	cancer susceptibility candidate 1	1.40036409	0.01402	0.06647
109624	Cald1	caldesmon 1	1.40036409	0.0453	0.1445
228608	Smox	spermine oxidase	1.399972	0.00233	0.02131
269633	Wdr86	WD repeat domain 86	1.399972	0.1127	0.2684
17390	Mmp2	matrix metallopeptidase 2	1.39918847	0.07259	0.2004
14119	Fbn2	fibrillin 2	1.3986014	0.01143	0.05824
13448	Dok1	docking protein 1	1.39840582	0.0007856	0.01126
66961	Neat1	nuclear paraspeckle assembly transcript 1 (non-protein coding)	1.39821029	0.05022	0.1555
12977	Csf1	colony stimulating factor 1 (macrophage)	1.39821029	0.3568	0.5667
27756	Lsm2	LSM2 homolog, U6 small nuclear RNA associated (S. cerevisiae)	1.3978194	0.05924	0.1739
70001	1700028B04Rik	RIKEN cDNA 1700028B04 gene	1.39762404	0.003674	0.02826
19255	Ptpn2	protein tyrosine phosphatase, non-receptor type 2	1.39762404	0.01071	0.05557
22329	Vcam1	vascular cell adhesion molecule 1	1.39762404	0.0559	0.1677
214944	Mobkl2b	MOB1, Mps One Binder kinase activator-like 2B (yeast)	1.39723348	0.003291	0.02638
22342	Lin7b	lin-7 homolog B (C. elegans)	1.39723348	0.01302	0.06333
22004	Tpm2	tropomyosin 2, beta	1.39703828	0.001671	0.01758
11898	Ass1	argininosuccinate synthetase 1	1.39703828	0.02274	0.09152
18242	Oat	ornithine aminotransferase	1.39664804	0.0007133	0.01066
104444	Rexo2	REX2, RNA exonuclease 2 homolog (S. cerevisiae)	1.39645301	0.005251	0.03528
13190	Dct	dopachrome tautomerase	1.39645301	0.2089	0.4039
227737	Fam129b	family with sequence similarity 129, member B	1.3960631	0.0001117	0.003683
629557	Gm6981	glyceraldehyde-3-phosphate dehydrogenase pseudogene	1.39586823	0.0007461	0.01095
17961	Nat2	N-acetyltransferase 2 (arylamine N-acetyltransferase)	1.39547865	0.005274	0.03534

19091	Prkg1	protein kinase, cGMP-dependent, type I	1.39528394	0.006503	0.04036
30791	Slc39a1	solute carrier family 39 (zinc transporter), member 1	1.39508929	0.0001244	0.003851
70511	Fam86	family with sequence similarity 86	1.39508929	0.001008	0.01312
17387	Mmp14	matrix metallopeptidase 14 (membrane-inserted)	1.39508929	0.01606	0.07239
381544	Gm1661	predicted gene 1661	1.39508929	0.07581	0.2065
72123	2010109K11Rik	RIKEN cDNA 2010109K11 gene	1.39489469	0.001228	0.01468
20363	Sepp1	selenoprotein P, plasma, 1	1.39489469	0.007767	0.04537
18129	Notch2	Notch gene homolog 2 (Drosophila)	1.39411683	0.0004552	0.008023
140703	Emid1	EMI domain containing 1	1.3939225	0.01321	0.06403
791273	B630006N21Rik	RIKEN cDNA B630006N21 gene	1.393534	0.03811	0.1288
68738	Acss1	acyl-CoA synthetase short-chain family member 1	1.39314572	0.03037	0.1111
109624	Cald1	caldesmon 1	1.39314572	0.03285	0.1169
100038637	Gm10134	predicted gene 10134	1.39256371	0.02417	0.09514
16619	Klk1b27	kallikrein 1-related peptidase b27	1.39082058	0.1318	0.2982
68153	Gtf2e2	general transcription factor II E, polypeptide 2 (beta subunit)	1.39062717	0.001189	0.0144
74048	4632428N05Rik	RIKEN cDNA 4632428N05 gene	1.39024051	0.02641	0.1011
232201	Arhgap25	Rho GTPase activating protein 25	1.39004726	0.01065	0.05538
106759	Ticam1	toll-like receptor adaptor molecule 1	1.38985407	0.01718	0.07557
11936	Fxyd2	FXYD domain-containing ion transport regulator 2	1.3892748	0.01354	0.06483
70550	5730416F02Rik	RIKEN cDNA 5730416F02 gene	1.38908182	0.1531	0.3283
320183	Msrb3	methionine sulfoxide reductase B3	1.38888889	0.004671	0.03282
382253	Cdkl5	cyclin-dependent kinase-like 5	1.38850319	0.01482	0.06884
12829	Col4a4	collagen, type IV, alpha 4	1.38792505	0.1397	0.3092
76142	Ppp1r14c	protein phosphatase 1, regulatory (inhibitor) subunit 14c	1.38792505	0.159	0.3367
76995	1700095A13Rik	RIKEN cDNA 1700095A13 gene	1.38753989	0.01096	0.05638
74055	Plce1	phospholipase C, epsilon 1	1.38715495	0.01569	0.07144
114664	Hsd17b11	hydroxysteroid (17-beta) dehydrogenase 11	1.38638569	0.01387	0.06596
72446	Prr5l	proline rich 5 like	1.38600139	0.001535	0.01684
66494	Prelid1	PRELI domain containing 1	1.38542533	0.0001428	0.004152
14865	Gstm4	glutathione S-transferase, mu 4	1.38484974	0.01662	0.07384
13190	Dct	dopachrome tautomerase	1.38465799	0.18	0.3656
21955	Tnnt1	troponin T1, skeletal, slow	1.38446629	0.001334	0.01542
18080	Nin	ninein	1.38446629	0.001426	0.01602
100042545	Gm3896	predicted gene 3896	1.38427464	0.03286	0.117
68922	Dnaic1	dynein, axonemal, intermediate chain 1	1.38408304	0.005863	0.03772
320701	Fam19a4	family with sequence similarity 19, member A4	1.38408304	0.01097	0.0564
320655	Pgap3	post-GPI attachment to proteins 3	1.38370001	0.008991	0.04962
67739	Slc48a1	solute carrier family 48 (heme transporter), member 1	1.38350858	0.0003134	0.006358
56338	Txnip	thioredoxin interacting protein	1.38350858	0.001959	0.01935
12010	B2m	beta-2 microglobulin	1.38350858	0.003055	0.02516
545007	ENSMUSG00000068790	predicted gene, ENSMUSG00000068790	1.38331719	0.2415	0.4442
207683	Igsf11	immunoglobulin superfamily, member 11	1.38293459	0.03327	0.1179
15203	Heph	hephaestin	1.38217001	0.00521	0.03509
103142	Rdh9	retinol dehydrogenase 9	1.38197899	0.0004697	0.008192
74465	4933421H12Rik	RIKEN cDNA 4933421H12 gene	1.38178803	0.1866	0.3744
11758	Prdx6	peroxiredoxin 6	1.38159713	0.008295	0.04723
320398	Lrig3	leucine-rich repeats and immunoglobulin-like domains 3	1.38140627	0.0006505	0.01001
171580	Mical1	microtubule associated monoxygenase, calponin and LIM domain containing 1	1.38140627	0.0009432	0.01254
435333	LOC435333	similar to monoclonal antibody heavy chain	1.38140627	0.01379	0.06572
57394	Tmem27	transmembrane protein 27	1.38121547	6.62E-05	0.00279

229320	Clrn1	clarin 1	1.38121547	0.001568	0.01704
11536	Gpr182	G protein-coupled receptor 182	1.38121547	0.003813	0.02893
24001	Tiam2	T-cell lymphoma invasion and metastasis 2	1.38102472	0.02296	0.09211
69772	Bdh2	3-hydroxybutyrate dehydrogenase, type 2	1.38083402	0.002753	0.02359
21925	Tnnc2	troponin C2, fast	1.38083402	0.01058	0.05516
243373	AI854703	expressed sequence AI854703	1.38045279	0.01184	0.05967
14858	Gsta2	glutathione S-transferase, alpha 2 (Yc2)	1.38045279	0.234	0.4356
56534	Hspb3	heat shock protein 3	1.38026225	0.006622	0.04087
100038941	Vmn2r121	vomeronasal 2, receptor 121	1.38026225	0.0764	0.2078
97122	Hist2h4	histone cluster 2, H4	1.38026225	0.1467	0.3195
216161	Sbno2	strawberry notch homolog 2 (Drosophila)	1.37969095	0.02633	0.1009
320712	Abi3bp	ABI gene family, member 3 (NESH) binding protein	1.37969095	0.07932	0.2132
26377	Dapp1	dual adaptor for phosphotyrosine and 3-phosphoinositides 1	1.37950062	0.03477	0.1213
69034	4930579G22Rik	RIKEN cDNA 4930579G22 gene	1.37892995	0.00198	0.0194
545366	BC026782	cDNA sequence BC026782	1.37892995	0.0177	0.07721
208263	Tor1aip1	torsin A interacting protein 1	1.37873983	0.1797	0.3654
14431	Gamt	guanidinoacetate methyltransferase	1.37854977	0.02911	0.1079
12840	Col9a2	collagen, type IX, alpha 2	1.37835975	0.01138	0.05806
381045	Ccdc58	coiled-coil domain containing 58	1.37797988	0.003015	0.0249
16322	Inha	inhibin alpha	1.37797988	0.005507	0.03633
26559	Hunk	hormonally upregulated Neu-associated kinase	1.37779002	0.002143	0.02019
99586	Dpyd	dihydropyrimidine dehydrogenase	1.37760022	0.01321	0.06403
69563	2310015B20Rik	RIKEN cDNA 2310015B20 gene	1.37741047	0.001824	0.01852
74153	Uba7	ubiquitin-like modifier activating enzyme 7	1.37703112	0.001092	0.01374
380705	Tmem102	transmembrane protein 102	1.37703112	0.1346	0.3015
66859	Slc16a9	solute carrier family 16 (monocarboxylic acid transporters), member 9	1.37684153	0.009171	0.05028
67916	Ppap2b	phosphatidic acid phosphatase type 2B	1.37646249	0.003875	0.02922
22417	Wnt4	wingless-related MMTV integration site 4	1.37608367	0.03387	0.1192
217294	BC006965	cDNA sequence BC006965	1.37589433	0.03263	0.1165
230899	Nppa	natriuretic peptide type A	1.37570505	0.4894	0.6838
55942	Sertad1	SERTA domain containing 1	1.37513751	0.0009288	0.01244
16796	Lasp1	LIM and SH3 protein 1	1.37513751	0.04491	0.1436
16855	Lgals4	lectin, galactose binding, soluble 4	1.37494844	0.01672	0.07412
14088	Fancc	Fanconi anemia, complementation group C	1.37475942	0.01237	0.06119
76566	Fam101b	family with sequence similarity 101, member B	1.37457045	0.02818	0.1056
408190	Wfdc13	WAP four-disulfide core domain 13	1.37457045	0.09847	0.246
669149	Vmn2r88	vomeronasal 2, receptor 88	1.37438153	0.03478	0.1213
66578	2610039C10Rik	RIKEN cDNA 2610039C10 gene	1.37400385	0.0009305	0.01245
19012	Ppap2a	phosphatidic acid phosphatase type 2A	1.37400385	0.02755	0.104
22420	Wnt6	wingless-related MMTV integration site 6	1.37381508	0.1202	0.2806
65973	Asph	aspartate-beta-hydroxylase	1.37362637	0.0002515	0.005707
22229	Ucp3	uncoupling protein 3 (mitochondrial, proton carrier)	1.37343771	0.0367	0.1257
19317	Qk	quaking	1.37324911	0.001362	0.01559
329934	Foxo6	forkhead box O6	1.37324911	0.1231	0.2854
76566	Fam101b	family with sequence similarity 101, member B	1.37306055	0.04327	0.1397
319996	Casc4	cancer susceptibility candidate 4	1.37230685	0.001258	0.01493
12123	Hrk	harakiri, BCL2 interacting protein (contains only BH3 domain)	1.37230685	0.06788	0.1908
100049162	BC001981	cDNA sequence BC001981	1.37193031	0.5655	0.7422
13639	Efna4	ephrin A4	1.37174211	0.01185	0.05967
216964	Trp53i13	transformation related protein 53 inducible protein 13	1.37155397	0.001193	0.01443

231668	Vsig10	V-set and immunoglobulin domain containing 10	1.37155397	0.007981	0.04607
75224	4930528J11Rik	RIKEN cDNA 4930528J11 gene	1.37117784	0.000825	0.01157
18115	Nnt	nicotinamide nucleotide transhydrogenase	1.37080192	0.0007028	0.01055
246691	Prok1	prokineticin 1	1.37080192	0.004246	0.03092
110611	Hdlbp	high density lipoprotein (HDL) binding protein	1.37080192	0.01008	0.05365
14118	Fbn1	fibrillin 1	1.37061404	0.1624	0.3412
22178	Tyrp1	tyrosinase-related protein 1	1.37061404	0.2608	0.4668
52639	Wipi1	WD repeat domain, phosphoinositide interacting 1	1.37023842	0.0001439	0.004169
14871	Gstt1	glutathione S-transferase, theta 1	1.37023842	0.003238	0.02621
76441	Daam2	dishevelled associated activator of morphogenesis 2	1.37023842	0.01558	0.07122
104080	Nxph4	neurexophilin 4	1.37005069	0.001279	0.01502
28114	Nsun2	NOL1/NOP2/Sun domain family member 2	1.36986301	0.0001624	0.004382
319757	Smo	smoothened homolog (Drosophila)	1.36967539	0.001118	0.01392
15205	Hes1	hairy and enhancer of split 1 (Drosophila)	1.36948781	0.1054	0.2568
433182	Gm5506	predicted gene 5506	1.36930029	0.01478	0.06875
665225	Gm7544	predicted gene 7544	1.36911281	0.1746	0.3586
171463	Il17rd	interleukin 17 receptor D	1.3685507	0.01165	0.05906
218693	Paip1	polyadenylate binding protein-interacting protein 1	1.3685507	0.02597	0.09993
18938	Ppp1r14b	protein phosphatase 1, regulatory (inhibitor) subunit 14B	1.36836344	0.0002758	0.005932
232966	Zfp114	zinc finger protein 114	1.36817622	0.0001466	0.004189
12483	Cd22	CD22 antigen	1.36817622	0.04461	0.1427
545013	Gm5797	predicted gene 5797	1.36817622	0.2986	0.5081
234094	Arhgef10	Rho guanine nucleotide exchange factor (GEF) 10	1.36780194	0.06514	0.1858
170829	Tram2	translocating chain-associating membrane protein 2	1.36742787	0.005376	0.03579
382035	Pabpn1l	poly(A)binding protein nuclear 1-like	1.36686714	0.002682	0.02319
14794	Spsb2	splA/ryanodine receptor domain and SOCS box containing 2	1.36668033	0.003277	0.02633
16880	Lifr	leukemia inhibitory factor receptor	1.36668033	0.0061	0.03869
100039781	Hrct1	histidine rich carboxyl terminus 1	1.36649358	0.0008735	0.01201
192976	BC046404	cDNA sequence BC046404	1.36612022	0.001384	0.01573
224705	Vps52	vacuolar protein sorting 52 (yeast)	1.36612022	0.03187	0.1147
20591	Kdm5c	lysine (K)-specific demethylase 5C	1.36612022	0.08066	0.2155
68991	Ssu72	Ssu72 RNA polymerase II CTD phosphatase homolog (yeast)	1.36593362	0.002383	0.02146
13631	Eef2k	eukaryotic elongation factor-2 kinase	1.36556056	0.006532	0.0405
17841	Mup2	major urinary protein 2	1.36537411	0.1478	0.3212
268741	Tox4	TOX high mobility group box family member 4	1.36500137	0.08711	0.2269
13363	Dhh	desert hedgehog	1.36481507	0.0004109	0.007534
66170	Chchd5	coiled-coil-helix-coiled-coil-helix domain containing 5	1.36444263	0.00143	0.01603
22439	Xk	Kell blood group precursor (McLeod phenotype) homolog	1.36444263	0.03613	0.1245
16848	Lfng	LFNG O-fucosylpeptide 3-beta-N-acetylglucosaminyltransferase	1.36444263	0.1302	0.2959
260296	Trim61	tripartite motif-containing 61	1.36407039	0.2827	0.4912
57394	Tmem27	transmembrane protein 27	1.36388434	0.0004449	0.007902
17136	Mag	myelin-associated glycoprotein	1.36388434	0.02645	0.1012
52377	Rcn3	reticulocalbin 3, EF-hand calcium binding domain	1.36369835	0.0105	0.05492
11568	Aebp1	AE binding protein 1	1.36369835	0.01572	0.07153
638833	Gm7251	glyceraldehyde-3-phosphate dehydrogenase pseudogene	1.36369835	0.1151	0.2723
240479	Fam69c	family with sequence similarity 69, member C	1.36351241	0.01552	0.07109
12007	Azgp1	alpha-2-glycoprotein 1, zinc	1.36351241	0.09967	0.2478
14345	Fut4	fucosyltransferase 4	1.36351241	0.1395	0.3089
69547	Nkpd1	NTPase, KAP family P-loop domain containing 1	1.36332652	0.0218	0.0892
171171	Ntng2	netrin G2	1.36332652	0.0637	0.1829

319504	Nrcam	neuron-glia-CAM-related cell adhesion molecule	1.36239782	0.000333	0.006628
545700	Vmn2r-ps14	vomeronasal 2, receptor, pseudogene 14	1.36221223	0.0894	0.2304
384198	Fam47e	family with sequence similarity 47, member E	1.3620267	0.4764	0.6736
210622	Pamr1	peptidase domain containing associated with muscle regeneration 1	1.36184121	0.001984	0.01942
15223	Foxj1	forkhead box J1	1.36128505	0.003918	0.02938
227682	Trub2	TruB pseudouridine (psi) synthase homolog 2 (E. coli)	1.36091453	0.0003854	0.007246
94279	Sfxn2	sideroflexin 2	1.36091453	0.009877	0.0529
75465	Dynlrb2	dynein light chain roadblock-type 2	1.36091453	0.01489	0.06901
436188	Gm5751	predicted gene 5751	1.36072935	0.06995	0.1951
69551	2310022B05Rik	RIKEN cDNA 2310022B05 gene	1.36054422	0.002965	0.0246
30935	Tor3a	torsin family 3, member A	1.36054422	0.007861	0.04566
19766	Ripk1	receptor (TNFRSF)-interacting serine-threonine kinase 1	1.3601741	0.006309	0.03966
12831	Col5a1	collagen, type V, alpha 1	1.3601741	0.02057	0.08555
219132	D14Ertd668e	DNA segment, Chr 14, ERATO Doi 668, expressed	1.3601741	0.02592	0.09976
56289	Rassf1	Ras association (RalGDS/AF-6) domain family member 1	1.35980419	0.0005995	0.009529
13052	Cxadr	coxsackie virus and adenovirus receptor	1.35980419	0.01649	0.07358
56795	Arl10	ADP-ribosylation factor-like 10	1.35961931	0.03068	0.1119
14431	Gamt	guanidinoacetate methyltransferase	1.35943448	0.02228	0.09047
16439	Itpr2	inositol 1,4,5-triphosphate receptor 2	1.35924969	0.001518	0.01672
11922	Neurod6	neurogenic differentiation 6	1.35924969	0.5373	0.7215
64009	Syne1	synaptic nuclear envelope 1	1.35888028	0.001428	0.01602
66180	Leprel4	leprecan-like 4	1.35869565	0.001837	0.01863
27386	Npas3	neuronal PAS domain protein 3	1.35869565	0.00288	0.02421
12038	Bche	butyrylcholinesterase	1.35869565	0.009325	0.0508
14282	Fosb	FBJ osteosarcoma oncogene B	1.35869565	0.06274	0.1811
15212	Hexb	hexosaminidase B	1.35832654	0.0003926	0.007301
269338	Vps39	vacuolar protein sorting 39 (yeast)	1.35832654	0.007633	0.04489
380912	Zfp395	zinc finger protein 395	1.35795763	0.02567	0.09924
108017	Fxyd4	FXYD domain-containing ion transport regulator 4	1.35795763	0.06636	0.1879
12829	Col4a4	collagen, type IV, alpha 4	1.35795763	0.1531	0.3283
12517	Cd72	CD72 antigen	1.35777325	0.02184	0.08929
23801	Aloxe3	arachidonate lipoxygenase 3	1.35758892	0.008415	0.04762
545611	Gm13298	predicted gene 13298	1.35758892	0.3488	0.5592
26459	Slc27a5	solute carrier family 27 (fatty acid transporter), member 5	1.35722041	0.004575	0.03236
242406	Rgp1	RGP1 retrograde golgi transport homolog (S. cerevisiae)	1.35722041	0.1079	0.2605
16596	Klf1	Kruppel-like factor 1 (erythroid)	1.35666802	0.09535	0.2409
20681	Sox8	SRY-box containing gene 8	1.35630001	0.007182	0.04328
217837	Itpk1	inositol 1,3,4-triphosphate 5/6 kinase	1.35630001	0.01872	0.08017
791308	Gm9933	predicted gene 9933	1.35630001	0.02759	0.104
75033	Mei4	meiosis-specific, MEI4 homolog (S. cerevisiae)	1.35630001	0.08042	0.2151
380997	Cyp2d12	cytochrome P450, family 2, subfamily d, polypeptide 12	1.35574837	0.002644	0.02299
13649	Egfr	epidermal growth factor receptor	1.35574837	0.02158	0.08856
68440	Dusp23	dual specificity phosphatase 23	1.35574837	0.02875	0.107
12512	Cd63	CD63 antigen	1.35538086	0.008261	0.04714
100041677	Gm13157	predicted gene 13157	1.35538086	0.05875	0.1731
16855	Lgals4	lectin, galactose binding, soluble 4	1.35519718	0.02475	0.09665
56421	Pfkp	phosphofructokinase, platelet	1.35464644	0.004744	0.03315
71742	Ulk3	unc-51-like kinase 3 (C. elegans)	1.35427952	0.003649	0.02813
18595	Pdgfra	platelet derived growth factor receptor, alpha polypeptide	1.35427952	0.02763	0.1042
11747	Anxa5	annexin A5	1.35409614	0.002378	0.02144

192976	BC046404	cDNA sequence BC046404	1.35409614	0.002859	0.02409
74185	Gbe1	glucan (1,4-alpha-), branching enzyme 1	1.35409614	0.008736	0.04878
65973	Asph	aspartate-beta-hydroxylase	1.35409614	0.09929	0.2472
76670	Ttc18	tetratricopeptide repeat domain 18	1.35391281	0.0317	0.1143
67622	Mxra7	matrix-remodelling associated 7	1.35354629	0.001716	0.01789
59289	Ccbp2	chemokine binding protein 2	1.35354629	0.02932	0.1084
381822	1190002F15Rik	RIKEN cDNA 1190002F15 gene	1.35317997	0.02631	0.1008
22359	Vldlr	very low density lipoprotein receptor	1.35299689	0.01549	0.071
668158	Ccdc85c	coiled-coil domain containing 85C	1.35299689	0.08299	0.2196
623172	Gm6403	predicted gene 6403	1.35299689	0.1094	0.2632
243219	2900026A02Rik	RIKEN cDNA 2900026A02 gene	1.35281385	0.02908	0.1078
338368	Fam109b	family with sequence similarity 109, member B	1.35281385	0.0457	0.1454
259277	Klk8	kallikrein related-peptidase 8	1.35281385	0.1198	0.2799
268291	Rnf217	ring finger protein 217	1.35263087	0.0005226	0.008804
56277	Tmem45a	transmembrane protein 45a	1.35263087	0.2742	0.4822
58859	Efemp2	epidermal growth factor-containing fibulin-like extracellular matrix protein 2	1.35244793	0.001638	0.01738
259300	Ehd2	EH-domain containing 2	1.35244793	0.01639	0.07337
14433	Gapdh	glyceraldehyde-3-phosphate dehydrogenase	1.35226504	0.002122	0.02011
74318	Hopx	HOP homeobox	1.35226504	0.003488	0.02734
66102	Cxcl16	chemokine (C-X-C motif) ligand 16	1.35189942	0.0258	0.0995
102448	Xylb	xylulokinase homolog (H. influenzae)	1.35171668	0.001295	0.01514
67724	Pop1	processing of precursor 1, ribonuclease P/MRP family, (S. cerevisiae)	1.35171668	0.003896	0.02929
19348	Kif20a	kinesin family member 20A	1.35135135	0.005273	0.03534
76742	Snx27	sorting nexin family member 27	1.35080373	0.0007368	0.01089
66940	Shisa5	shisa homolog 5 (Xenopus laevis)	1.35080373	0.01	0.05332
83679	Pde4dip	phosphodiesterase 4D interacting protein (myomegalin)	1.35080373	0.01428	0.06735
12228	Btg3	B-cell translocation gene 3	1.35043889	0.009894	0.05291
16402	Itga5	integrin alpha 5 (fibronectin receptor alpha)	1.35043889	0.3112	0.5212
54391	Rfk	riboflavin kinase	1.35025655	0.001971	0.01938
233561	A430054B03	hypothetical protein A430054B03	1.34989201	0.01294	0.06305
320825	Samd5	sterile alpha motif domain containing 5	1.34970981	0.0009141	0.01229
18515	Pbx2	pre B-cell leukemia transcription factor 2	1.34898152	0.01854	0.07971
77836	Mlana	melan-A	1.34898152	0.2549	0.4599
16331	Inpp5d	inositol polyphosphate-5-phosphatase D	1.34879957	0.003923	0.02939
12125	Bcl2l11	BCL2-like 11 (apoptosis facilitator)	1.34879957	0.07066	0.1965
20963	Sykb	spleen tyrosine kinase	1.34879957	0.1461	0.3188
78550	E130119H09Rik	RIKEN cDNA E130119H09 gene	1.34843581	0.002378	0.02144
68576	Hbxip	hepatitis B virus x interacting protein	1.34825401	0.001905	0.01908
212124	E030019B06Rik	RIKEN cDNA E030019B06 gene	1.34825401	0.01532	0.07055
72388	Ripk4	receptor-interacting serine-threonine kinase 4	1.34752729	0.007939	0.04591
171469	Gpr37l1	G protein-coupled receptor 37-like 1	1.34752729	0.144	0.3155
20619	Snap23	synaptosomal-associated protein 23	1.34734573	0.001208	0.01454
100040608	Fancf	Fanconi anemia, complementation group F	1.34734573	0.02304	0.09231
70435	Inf2	inverted formin, FH2 and WH2 domain containing	1.34716422	0.001993	0.01949
58207	Slc43a3	solute carrier family 43, member 3	1.34716422	0.02109	0.08718
14433	Gapdh	glyceraldehyde-3-phosphate dehydrogenase	1.34698276	0.05962	0.1747
52570	Ccdc69	coiled-coil domain containing 69	1.34680135	0.09464	0.2395
231872	Aimp2	aminoacyl tRNA synthetase complex-interacting multifunctional protein 2	1.34661998	0.0001925	0.004836
66494	Prelid1	PRELI domain containing 1	1.34643867	0.0002974	0.006193
11928	Atp1a1	ATPase, Na+/K+ transporting, alpha 1 polypeptide	1.3462574	0.003372	0.02678

328353	D030051J21Rik	RIKEN cDNA D030051J21 gene	1.3462574	0.07286	0.201
225266	Klhl14	kelch-like 14 (Drosophila)	1.3462574	0.07436	0.2039
140792	Colec12	collectin sub-family member 12	1.34607619	0.06423	0.1839
72691	Calhm2	calcium homeostasis modulator 2	1.34553283	0.01981	0.08324
70839	P2ry12	purinergic receptor P2Y, G-protein coupled 12	1.34498991	0.03946	0.1316
13019	Ctf1	cardiotrophin 1	1.34480904	0.008844	0.04914
71853	Pdia6	protein disulfide isomerase associated 6	1.34462821	5.28E-05	0.002466
230917	Tmem201	transmembrane protein 201	1.34462821	0.0001963	0.004904
18938	Ppp1r14b	protein phosphatase 1, regulatory (inhibitor) subunit 14B	1.34462821	0.003602	0.02787
73009	2900057B20Rik	RIKEN cDNA 2900057B20 gene	1.34462821	0.6961	0.8321
77956	A930026B05Rik	RIKEN cDNA A930026B05 gene	1.34444743	0.01072	0.05557
14176	Fgf5	fibroblast growth factor 5	1.34444743	0.01562	0.07134
230809	Pdik1l	PDLIM1 interacting kinase 1 like	1.34444743	0.05969	0.1747
18791	Plat	plasminogen activator, tissue	1.3442667	0.0001121	0.003687
277360	Prex1	phosphatidylinositol-3,4,5-trisphosphate-dependent Rac exchange factor 1	1.3442667	0.001516	0.0167
13078	Cyp1b1	cytochrome P450, family 1, subfamily b, polypeptide 1	1.34408602	0.04718	0.1485
16162	Il12rb2	interleukin 12 receptor, beta 2	1.34372481	0.1689	0.35
246228	Vwa1	von Willebrand factor A domain containing 1	1.34354427	0.06421	0.1839
104759	Pld4	phospholipase D family, member 4	1.34354427	0.1086	0.2617
18822	Plod1	procollagen-lysine, 2-oxoglutarate 5-dioxygenase 1	1.34336378	0.0417	0.1365
268564	Zbtb1	zinc finger and BTB domain containing 1	1.34210173	0.01181	0.05962
268756	Gulo	gulonolactone (L-) oxidase	1.34192163	0.009497	0.05149
545005	LOC545005	hypothetical protein LOC545005	1.34156158	0.2909	0.4993
12476	Cd151	CD151 antigen	1.34120172	0.002459	0.02189
252838	Tox	thymocyte selection-associated high mobility group box	1.34084205	0.01268	0.06221
207607	Ccdc40	coiled-coil domain containing 40	1.34084205	0.1677	0.3485
68191	5330430P22Rik	RIKEN cDNA 5330430P22 gene	1.34048257	0.1032	0.2536
100039316	Gm9843	predicted gene 9843	1.34030291	0.006063	0.03851
79362	Bhlhe41	basic helix-loop-helix family, member e41	1.33994372	0.002214	0.02056
242602	BC055111	cDNA sequence BC055111	1.3397642	0.4345	0.6383
18451	P4ha1	procollagen-proline, 2-oxoglutarate 4-dioxygenase (proline 4-hydroxylase), alpha 1 polypeptide	1.33958473	0.000346	0.006759
330790	Hapln4	hyaluronan and proteoglycan link protein 4	1.33958473	0.1249	0.288
12927	Bcar1	breast cancer anti-estrogen resistance 1	1.3390466	0.0001753	0.004568
640530	Gm7298	predicted gene 7298	1.3390466	0.4217	0.6278
170947	Myoz3	myozenin 3	1.33886732	0.2394	0.4419
18476	Pafah1b3	platelet-activating factor acetylhydrolase, isoform 1b, subunit 3	1.3385089	0.03332	0.118
18301	Fxyd5	FXYD domain-containing ion transport regulator 5	1.33832976	0.0321	0.1153
546336	Prrg1	proline rich Gla (G-carboxyglutamic acid) 1	1.33797164	0.006865	0.04197
57028	Pdxp	pyridoxal (pyridoxine, vitamin B6) phosphatase	1.33797164	0.06184	0.1794
100041241	Gm3227	predicted gene 3227	1.3374348	0.0609	0.1773
77634	Snapc3	small nuclear RNA activating complex, polypeptide 3	1.33725595	0.004821	0.03344
101437	Dhx32	DEAH (Asp-Glu-Ala-His) box polypeptide 32	1.33725595	0.01836	0.07916
67946	Spata6	spermatogenesis associated 6	1.33725595	0.1134	0.2696
16456	F11r	F11 receptor	1.33707715	0.02692	0.1024
74525	8430419L09Rik	RIKEN cDNA 8430419L09 gene	1.33707715	0.2904	0.4987
545013	Gm5797	predicted gene 5797	1.33654103	0.306	0.5154
20229	Sat1	spermidine/spermine N1-acetyl transferase 1	1.33600534	0.01588	0.07195
116891	Derl2	Der1-like domain family, member 2	1.33582688	0.0002798	0.005969
17295	Met	met proto-oncogene	1.33547009	0.03959	0.1319
71207	Nudt4	nudix (nucleoside diphosphate linked moiety X)-type motif 4	1.33511348	0.000748	0.01095

667572	Gm8709	glyceraldehyde-3-phosphate dehydrogenase pseudogene	1.33493526	0.004404	0.03164
233335	Synm	synemin, intermediate filament protein	1.33493526	0.02069	0.08595
216850	Kdm6b	KDM1 lysine (K)-specific demethylase 6B	1.33457894	0.005998	0.0383
14560	Gdf10	growth differentiation factor 10	1.33457894	0.02912	0.1079
238377	Gpr68	G protein-coupled receptor 68	1.33440085	0.01206	0.06029
16476	Jun	Jun oncogene	1.33422282	0.01437	0.06758
17319	Mif	macrophage migration inhibitory factor	1.33404482	0.00241	0.02161
231583	Slc26a1	solute carrier family 26 (sulfate transporter), member 1	1.33404482	0.04627	0.1465
503692	Aym1	activator of yeast meiotic promoters 1	1.33386688	0.01607	0.07239
109232	Sccpdh	saccharopine dehydrogenase (putative)	1.33368898	0.004494	0.03202
77056	Tmco4	transmembrane and coiled-coil domains 4	1.33368898	0.05512	0.166
75805	Nln	neurolysin (metallopeptidase M3 family)	1.33368898	0.06542	0.1863
108655	Foxp1	forkhead box P1	1.33351113	0.05961	0.1747
218506	Mrps27	mitochondrial ribosomal protein S27	1.33333333	0.06019	0.1757
630836	2010315B03Rik	RIKEN cDNA 2010315B03 gene	1.33315558	0.03773	0.128
11813	Apoc2	apolipoprotein C-II	1.33297787	0.3438	0.5547
328779	Hs3st6	heparan sulfate (glucosamine) 3-O-sulfotransferase 6	1.3326226	0.01711	0.07532
21356	Tapbp	TAP binding protein	1.33244504	0.01022	0.05407
12350	Car3	carbonic anhydrase 3	1.33244504	0.03565	0.1235
100039528	1110002E22Rik	RIKEN cDNA 1110002E22 gene	1.33244504	0.05619	0.1682
66864	Clec14a	C-type lectin domain family 14, member a	1.33244504	0.07882	0.2124
80749	Lrfn1	leucine rich repeat and fibronectin type III domain containing 1	1.33209005	0.0009357	0.01246
100037283	Rnaset2a	ribonuclease T2A	1.33209005	0.01213	0.06053
228775	Trib3	tribbles homolog 3 (Drosophila)	1.33209005	0.01559	0.07128
100217450	Snora47	small nucleolar RNA, H/ACA box 47	1.33191263	0.104	0.2548
67448	Plxdc2	plexin domain containing 2	1.33173525	0.002178	0.02035
231986	Jazf1	JAZF zinc finger 1	1.33173525	0.01034	0.05446
213389	Prdm9	PR domain containing 9	1.33155792	0.003427	0.02703
24059	Slco2a1	solute carrier organic anion transporter family, member 2a1	1.33155792	0.01611	0.07247
72536	Tagap	T-cell activation Rho GTPase-activating protein	1.33138064	0.009436	0.05127
12155	Bmp15	bone morphogenetic protein 15	1.33138064	0.06613	0.1876
217306	Cd300e	CD300e antigen	1.33138064	0.1969	0.3881
19106	Eif2ak2	eukaryotic translation initiation factor 2-alpha kinase 2	1.33084908	0.005694	0.03708
78977	Popdc3	popeye domain containing 3	1.33084908	0.01055	0.05509
109305	Orai1	ORAI calcium release-activated calcium modulator 1	1.33031795	0.02239	0.09076
84682	Cox4i2	cytochrome c oxidase subunit IV isoform 2	1.33014099	0.1021	0.2521
18451	P4ha1	procollagen-proline, 2-oxoglutarate 4-dioxygenase (proline 4-hydroxylase), alpha 1 polypeptide	1.32996409	0.003158	0.02575
338350	9330129D05Rik	RIKEN cDNA 9330129D05 gene	1.32996409	0.01049	0.05492
269423	3110057O12Rik	RIKEN cDNA 3110057O12 gene	1.32978723	0.0446	0.1427
16190	Il4ra	interleukin 4 receptor, alpha	1.32925695	0.04542	0.1446
57028	Pdxp	pyridoxal (pyridoxine, vitamin B6) phosphatase	1.32908028	0.007125	0.04302
100042659	Gm3952	predicted gene 3952	1.32908028	0.5569	0.7366
192656	Ripk2	receptor (TNFRSF)-interacting serine-threonine kinase 2	1.32872708	0.005327	0.03558
11624	Ahrr	aryl-hydrocarbon receptor repressor	1.32837407	0.007917	0.04585
68312	Gstm7	glutathione S-transferase, mu 7	1.32819764	0.07775	0.2102
66855	Tcf25	transcription factor 25 (basic helix-loop-helix)	1.32784491	0.02962	0.1092
224024	Scarf2	scavenger receptor class F, member 2	1.32766861	0.01292	0.06297
209039	Tenc1	tensin like C1 domain-containing phosphatase	1.32766861	0.02929	0.1083
15228	Foxg1	forkhead box G1	1.32749237	0.002962	0.02459
17215	Mcm3	minichromosome maintenance deficient 3 (S. cerevisiae)	1.32714001	0.0006803	0.01031

319469	A230056J06Rik	RIKEN cDNA A230056J06 gene	1.32661183	0.003491	0.02735
71726	Smug1	single-strand selective monofunctional uracil DNA glycosylase	1.32643587	0.0006533	0.01003
72042	Cotl1	coactosin-like 1 (Dictyostelium)	1.32625995	0.001075	0.01361
28193	Reep3	receptor accessory protein 3	1.32590825	0.0002224	0.005316
21350	Tal2	T-cell acute lymphocytic leukemia 2	1.32590825	0.3589	0.5687
208777	Sned1	sushi, nidogen and EGF-like domains 1	1.32573247	0.2418	0.4446
56228	Ube2j1	ubiquitin-conjugating enzyme E2, J1	1.32555673	0.0008128	0.01148
68312	Gstm7	glutathione S-transferase, mu 7	1.32555673	0.134	0.3011
17319	Mif	macrophage migration inhibitory factor	1.32538105	0.04993	0.1549
378954	3000002C10Rik	glyceraldehyde-3-phosphate dehydrogenase pseudogene	1.32502981	0.06956	0.1945
23802	Amfr	autocrine motility factor receptor	1.32485427	0.00435	0.03141
18648	Pgam1	phosphoglycerate mutase 1	1.32467877	0.007362	0.04386
105511	Fam170b	family with sequence similarity 170, member B	1.32467877	0.157	0.334
20583	Snai2	snail homolog 2 (Drosophila)	1.32450331	0.1308	0.2967
20364	Sepw1	selenoprotein W, muscle 1	1.32415254	0.01036	0.05448
20186	Nr1h4	nuclear receptor subfamily 1, group H, member 4	1.32415254	0.05111	0.1574
75016	4930480K23Rik	RIKEN cDNA 4930480K23 gene	1.32380196	0.0247	0.09658
20516	Slc20a2	solute carrier family 20, member 2	1.32362674	0.002909	0.02433
22333	Vdac1	voltage-dependent anion channel 1	1.32345156	0.01273	0.06239
544707	Gm5779	predicted gene 5779	1.32310135	0.01064	0.05534
244417	Gm501	predicted gene 501	1.32310135	0.01747	0.07645
225608	Sh3tc2	SH3 domain and tetratricopeptide repeats 2	1.32310135	0.0343	0.1201
108655	Foxp1	forkhead box P1	1.32310135	0.04409	0.1416
13853	Epm2a	epilepsy, progressive myoclonic epilepsy, type 2 gene alpha	1.32310135	0.1039	0.2546
67397	Erp29	endoplasmic reticulum protein 29	1.32292631	0.0007926	0.01133
67704	1810037I17Rik	RIKEN cDNA 1810037I17 gene	1.32292631	0.007278	0.0436
22004	Tpm2	tropomyosin 2, beta	1.32275132	0.0008696	0.01198
15242	Hhex	hematopoietically expressed homeobox	1.32275132	0.02232	0.09056
20660	Sorl1	sortilin-related receptor, LDLR class A repeats-containing	1.32257638	0.01344	0.06459
83984	Tssk6	testis-specific serine kinase 6	1.32257638	0.09681	0.243
100037278	Fam129c	family with sequence similarity 129, member C	1.32222663	0.002074	0.01983
24082	Gm16516	predicted gene, Gm16516	1.32187707	0.006462	0.04019
104080	Nxph4	neurexophilin 4	1.32187707	0.03455	0.1207
414116	D630024D03Rik	RIKEN cDNA D630024D03 gene	1.32152769	0.04247	0.1382
109731	Maob	monoamine oxidase B	1.32152769	0.08769	0.2279
328699	Gabrr3	gamma-aminobutyric acid (GABA) receptor, rho 3	1.32152769	0.115	0.2722
434325	Tmem221	transmembrane protein 221	1.32152769	0.4264	0.6315
14433	Gapdh	glyceraldehyde-3-phosphate dehydrogenase	1.32135307	0.01063	0.05531
66129	1110018J18Rik	RIKEN cDNA 1110018J18 gene	1.32082948	0.00252	0.02223
11829	Aqp4	aquaporin 4	1.32082948	0.004995	0.03411
13033	Ctsd	cathepsin D	1.32065504	0.002279	0.02098
11409	Acads	acyl-Coenzyme A dehydrogenase, short chain	1.32065504	0.08785	0.2281
72388	Ripk4	receptor-interacting serine-threonine kinase 4	1.32048065	0.01104	0.05663
224530	Acat3	acetyl-Coenzyme A acetyltransferase 3	1.32030631	0.001899	0.01904
385109	Gm1499	predicted gene 1499	1.32030631	0.03763	0.1278
57764	Ntn4	netrin 4	1.32030631	0.2247	0.4245
433966	5730422E09Rik	RIKEN cDNA 5730422E09 gene	1.32013201	0.02736	0.1035
320204	4833442J19Rik	RIKEN cDNA 4833442J19 gene	1.31995776	0.1054	0.2568
625342	Gm13315	lactate dehydrogenase A pseudogene	1.3196094	0.007682	0.04509
100503238	LOC100503238	hypothetical LOC100503238	1.31943528	0.01914	0.0813

67331	Atp8b3	ATPase, class I, type 8B, member 3	1.31926121	0.08206	0.2181
77113	Klhl2	kelch-like 2, Mayven (Drosophila)	1.31908719	0.001029	0.01327
320502	Lmod3	leiomodin 3 (fetal)	1.31908719	0.02214	0.09011
16904	Gzmm	granzyme M (lymphocyte met-ase 1)	1.31839156	0.01091	0.05621
277089	Gm5068	predicted gene 5068	1.31804402	0.1874	0.3756
231832	Tmem184a	transmembrane protein 184a	1.31769667	0.1339	0.301
73847	Fam110a	family with sequence similarity 110, member A	1.31717597	0.005228	0.03515
213649	Arhgef19	Rho guanine nucleotide exchange factor (GEF) 19	1.31717597	0.0583	0.1722
70693	Gpr125	G protein-coupled receptor 125	1.3170025	0.1344	0.3015
11596	Ager	advanced glycosylation end product-specific receptor	1.31682908	0.008715	0.04874
68701	Dysfip1	dysferlin interacting protein 1	1.31682908	0.08032	0.215
107526	Gimap4	GTPase, IMAP family member 4	1.31682908	0.109	0.2624
13400	Dmpk	dystrophia myotonica-protein kinase	1.31682908	0.2399	0.4422
66251	Arfgap3	ADP-ribosylation factor GTPase activating protein 3	1.31665569	0.00217	0.02032
257635	Sdsl	serine dehydratase-like	1.31665569	0.004601	0.03247
21939	Cd40	CD40 antigen	1.31648236	0.057	0.1698
213311	Fbxl21	F-box and leucine-rich repeat protein 21	1.31648236	0.09803	0.2452
11606	Agt	angiotensinogen (serpin peptidase inhibitor, clade A, member 8)	1.31648236	0.1625	0.3413
100505122	LOC100505122	hypothetical LOC100505122	1.31630907	0.003716	0.02845
74039	Nfam1	Nfat activating molecule with ITAM motif 1	1.31630907	0.07944	0.2135
55932	Gbp3	guanylate binding protein 3	1.31596263	0.05449	0.1649
18983	Cnot7	CCR4-NOT transcription complex, subunit 7	1.31578947	0.002558	0.02246
66494	Prelid1	PRELI domain containing 1	1.31561637	0.01458	0.06819
14451	Gas1	growth arrest specific 1	1.3154433	0.1087	0.262
329941	Col8a2	collagen, type VIII, alpha 2	1.3154433	0.1263	0.2899
14183	Fgfr2	fibroblast growth factor receptor 2	1.31527029	0.07182	0.1988
269423	3110057O12Rik	RIKEN cDNA 3110057O12 gene	1.31527029	0.07659	0.2081
231162	Cytl1	cytokine-like 1	1.31527029	0.08599	0.2249
14634	Gli3	GLI-Kruppel family member GLI3	1.31509732	0.01426	0.0673
53378	Sdcbp	syndecan binding protein	1.31509732	0.01511	0.0698
11624	Ahrr	aryl-hydrocarbon receptor repressor	1.31509732	0.1506	0.3255
100041487	Gm3366	predicted gene 3366	1.31509732	0.1774	0.3624
67075	Magt1	magnesium transporter 1	1.31475151	0.04275	0.1388
27273	Pdk4	pyruvate dehydrogenase kinase, isoenzyme 4	1.31475151	0.1951	0.3856
101488	Slco2b1	solute carrier organic anion transporter family, member 2b1	1.31423314	0.09187	0.235
76742	Snx27	sorting nexin family member 27	1.31354262	0.01049	0.05492
381175	Ccdc68	coiled-coil domain containing 68	1.31354262	0.3584	0.5683
20163	Rsu1	Ras suppressor protein 1	1.31337011	0.005528	0.03639
69953	2810025M15Rik	RIKEN cDNA 2810025M15 gene	1.31319764	0.001605	0.01722
217069	Trim25	tripartite motif-containing 25	1.31302521	0.007868	0.04567
66720	Klhl10	kelch-like 10 (Drosophila)	1.31302521	0.06615	0.1876
57742	Abhd1	abhydrolase domain containing 1	1.31302521	0.1079	0.2605
16002	Igf2	insulin-like growth factor 2	1.31285283	0.07462	0.2042
234395	Ushbp1	Usher syndrome 1C binding protein 1	1.3125082	0.01381	0.0658
77857	9430065F17Rik	RIKEN cDNA 9430065F17 gene	1.3125082	0.115	0.2722
16784	Lamp2	lysosomal-associated membrane protein 2	1.31233596	0.00783	0.04557
66129	1110018J18Rik	RIKEN cDNA 1110018J18 gene	1.31233596	0.01856	0.07976
54396	Irgm2	immunity-related GTPase family M member 2	1.31233596	0.025	0.09745
66251	Arfgap3	ADP-ribosylation factor GTPase activating protein 3	1.31181949	0.0125	0.06159
326618	Tpm4	tropomyosin 4	1.31130344	0.00194	0.01924

214239	A430105I19Rik	RIKEN cDNA A430105I19 gene	1.31113151	0.0004597	0.008073
329470	Accs	1-aminocyclopropane-1-carboxylate synthase homolog (Arabidopsis)(non-functional)	1.31078778	0.02158	0.08856
70829	Ccdc93	coiled-coil domain containing 93	1.31044424	0.009318	0.05078
19698	Relb	avian reticuloendotheliosis viral (v-rel) oncogene related B	1.31044424	0.03085	0.1123
277468	Slc39a12	solute carrier family 39 (zinc transporter), member 12	1.31027254	0.03777	0.1281
13641	Efnb1	ephrin B1	1.31010088	0.003153	0.02573
107522	Ece2	endothelin converting enzyme 2	1.30975769	0.01509	0.06972
20591	Kdm5c	lysine (K)-specific demethylase 5C	1.30941469	0.02544	0.09862
18669	Abcb1b	ATP-binding cassette, sub-family B (MDR/TAP), member 1B	1.30924326	0.0001371	0.00406
16828	Ldha	lactate dehydrogenase A	1.30924326	0.007099	0.04295
94090	Trim9	tripartite motif-containing 9	1.30907187	0.00266	0.02307
100503637	LOC100503637	envelope glycoprotein-like	1.30907187	0.01433	0.06749
15213	Hey1	hairy/enhancer-of-split related with YRPW motif 1	1.30890052	0.004065	0.03003
19024	Ppfibp2	PTPRF interacting protein, binding protein 2 (liprin beta 2)	1.30872922	0.01555	0.07119
22371	Vwf	Von Willebrand factor homolog	1.30872922	0.01563	0.07134
68957	Paqr6	progestin and adipoQ receptor family member VI	1.30872922	0.024	0.09482
381306	BC055324	cDNA sequence BC055324	1.30855797	0.0228	0.09167
240427	Setbp1	SET binding protein 1	1.30855797	0.0872	0.227
14709	Gng8	guanine nucleotide binding protein (G protein), gamma 8	1.30855797	0.1118	0.2669
75355	4930553P18Rik	RIKEN cDNA 4930553P18 gene	1.30838676	0.03281	0.1169
68957	Paqr6	progestin and adipoQ receptor family member VI	1.30821559	0.1092	0.2628
27218	Slamf1	signaling lymphocytic activation molecule family member 1	1.30821559	0.6213	0.7826
64138	Ctsz	cathepsin Z	1.30804447	0.008405	0.04759
70296	Tbc1d13	TBC1 domain family, member 13	1.30804447	0.09903	0.2468
67509	1810063B07Rik	RIKEN cDNA 1810063B07 gene	1.3078734	0.01634	0.07323
71755	Dhdh	dihydrodiol dehydrogenase (dimeric)	1.30770237	0.007381	0.04394
67389	Fam132a	family with sequence similarity 132, member A	1.30753138	0.0002449	0.005596
268709	Fam107a	family with sequence similarity 107, member A	1.30718954	0.1909	0.3802
21948	Cd70	CD70 antigen	1.30718954	0.2092	0.4044
80752	Fam20c	family with sequence similarity 20, member C	1.30684788	0.03839	0.1294
545136	Fam186b	family with sequence similarity 186, member B	1.30684788	0.0616	0.1789
69332	Lelp1	late cornified envelope-like proline-rich 1	1.30684788	0.1889	0.3771
15586	Hyal1	hyaluronoglucosaminidase 1	1.30667712	0.05314	0.1618
12365	Casp14	caspase 14	1.3065064	0.2372	0.4392
385138	BC061237	cDNA sequence BC061237	1.30633573	0.1416	0.312
213311	Fbxl21	F-box and leucine-rich repeat protein 21	1.30633573	0.2068	0.4015
269774	Aak1	AP2 associated kinase 1	1.30599451	0.01936	0.082
104086	Cyp27a1	cytochrome P450, family 27, subfamily a, polypeptide 1	1.30599451	0.02195	0.08955
83675	Bicc1	bicaudal C homolog 1 (Drosophila)	1.30582397	0.004792	0.03331
219022	Ttc5	tetratricopeptide repeat domain 5	1.30565348	0.006276	0.03951
21454	Tcp1	t-complex protein 1	1.30548303	0.002502	0.02213
15903	Id3	inhibitor of DNA binding 3	1.30548303	0.01218	0.06059
16172	Il17ra	interleukin 17 receptor A	1.30548303	0.08712	0.2269
20544	Slc9a1	solute carrier family 9 (sodium/hydrogen exchanger), member 1	1.30548303	0.1598	0.338
386612	Thoc6	THO complex 6 homolog (Drosophila)	1.30531262	0.004997	0.03411
12495	Entpd1	ectonucleoside triphosphate diphosphohydrolase 1	1.30531262	0.05938	0.1742
16412	Itgb1	integrin beta 1 (fibronectin receptor beta)	1.30514226	0.001347	0.0155
353172	Gars	glycyl-tRNA synthetase	1.30480167	0.01432	0.06749
170753	Zfp704	zinc finger protein 704	1.30480167	0.05183	0.1591
76974	1190003J15Rik	RIKEN cDNA 1190003J15 gene	1.30480167	0.1932	0.3832

20318	Sdf4	stromal cell derived factor 4	1.30463144	0.004675	0.03283
319757	Smo	smoothened homolog (Drosophila)	1.30463144	0.1893	0.3778
16156	Il11	interleukin 11	1.30429112	0.01172	0.05929
223752	Gramd4	GRAM domain containing 4	1.30395097	0.01279	0.06259
12475	Cd14	CD14 antigen	1.303611	0.004457	0.03189
16977	Lrrc23	leucine rich repeat containing 23	1.303611	0.01739	0.07617
71772	Plbd2	phospholipase B domain containing 2	1.30344108	0.0028	0.02379
77805	Esco1	establishment of cohesion 1 homolog 1 (S. cerevisiae)	1.30344108	0.0111	0.05689
213539	Bag2	BCL2-associated athanogene 2	1.30344108	0.01599	0.07222
15976	Ifnar2	interferon (alpha and beta) receptor 2	1.30327121	0.01914	0.08132
16978	Lrrfip1	leucine rich repeat (in FLII) interacting protein 1	1.30327121	0.05783	0.1713
59044	Rnf130	ring finger protein 130	1.30310138	0.001981	0.0194
21682	Tec	tec protein tyrosine kinase	1.30310138	0.008174	0.04683
59027	Nampt	nicotinamide phosphoribosyltransferase	1.3029316	0.004843	0.03351
69479	1700029J07Rik	RIKEN cDNA 1700029J07 gene	1.3029316	0.04954	0.154
22354	Vipr1	vasoactive intestinal peptide receptor 1	1.30259216	0.008758	0.04883
68431	Fbxl15	F-box and leucine-rich repeat protein 15	1.30259216	0.01788	0.07769
260296	Trim61	tripartite motif-containing 61	1.30259216	0.07329	0.202
15458	Hpx	hemopexin	1.30174434	0.0844	0.2222
18048	Klk1b4	kallikrein 1-related pepidase b4	1.30174434	0.192	0.3816
217700	Acot6	acyl-CoA thioesterase 6	1.30157491	0.003546	0.02764
74347	4632415K11Rik	RIKEN cDNA 4632415K11 gene	1.30157491	0.008951	0.04944
71726	Smug1	single-strand selective monofunctional uracil DNA glycosylase	1.30157491	0.2315	0.4323
70110	Ifi35	interferon-induced protein 35	1.30140552	0.01185	0.05967
13849	Ephx1	epoxide hydrolase 1, microsomal	1.30106687	0.008179	0.04683
52013	D19Ertd386e	DNA segment, Chr 19, ERATO Doi 386, expressed	1.30106687	0.02458	0.09625
78781	Zc3hav1	zinc finger CCCH type, antiviral 1	1.30106687	0.02784	0.1047
66197	Cks2	CDC28 protein kinase regulatory subunit 2	1.30072841	0.028	0.1051
66170	Chchd5	coiled-coil-helix-coiled-coil-helix domain containing 5	1.30039012	0.005509	0.03633
69942	Rnf113a1	ring finger protein 113A1	1.30022104	0.0007468	0.01095
16889	Lipa	lysosomal acid lipase A	1.300052	0.01759	0.07681
72123	2010109K11Rik	RIKEN cDNA 2010109K11 gene	1.300052	0.01911	0.08126
18596	Pdgfrb	platelet derived growth factor receptor, beta polypeptide	1.300052	0.02485	0.09695
16414	Itgb2	integrin beta 2	1.300052	0.05029	0.1556
109280	9330176C04Rik	solute carrier family 22 (organic cation transporter), member 13 gene:pseudogene	1.29971406	0.001412	0.01593
269608	Plekhg5	pleckstrin homology domain containing, family G (with RhoGef domain) member 5	1.29971406	0.05093	0.157
100038847	Gm10406	predicted gene 10406	1.29971406	0.4058	0.6134
242109	Zfp697	zinc finger protein 697	1.29954516	0.03305	0.1175
18755	Prkch	protein kinase C, eta	1.29954516	0.1048	0.2559
71445	5530601H04Rik	RIKEN cDNA 5530601H04 gene	1.29920748	0.03157	0.1139
330004	Gm833	predicted gene 833	1.29920748	0.1274	0.2918
217721	Mfsd7c	major facilitator superfamily domain containing 7C	1.29886998	0.04327	0.1397
22289	Kdm6a	4lysine (K)-specific demethylase 6A	1.2987013	0.01569	0.07144
76483	Lmf1	lipase maturation factor 1	1.2987013	0.01608	0.07239
68861	1190002N15Rik	RIKEN cDNA 1190002N15 gene	1.29819551	0.001919	0.01915
80859	Nfkbiz	nuclear factor of kappa light polypeptide gene enhancer in B-cells inhibitor, zeta	1.298027	0.006245	0.03936
22228	Ucp2	uncoupling protein 2 (mitochondrial, proton carrier)	1.298027	0.1652	0.3452
212516	BC060267	cDNA sequence BC060267	1.298027	0.1919	0.3816
100041724	Gm15217	predicted gene 15217	1.29785853	0.1265	0.2902
208634	Tspan10	tetraspanin 10	1.29785853	0.1468	0.3195

330323	Fam188b	family with sequence similarity 188, member B	1.29752173	0.1875	0.3758
93688	Klhl1	kelch-like 1 (Drosophila)	1.2973534	0.002351	0.02135
18032	Nfix	nuclear factor I/X	1.29718511	0.0127	0.06226
72465	Zfp131	zinc finger protein 131	1.29718511	0.01954	0.0825
14017	Evi2a	ecotropic viral integration site 2a	1.29718511	0.07431	0.2038
98660	Atp1a2	ATPase, Na+/K+ transporting, alpha 2 polypeptide	1.29718511	0.2236	0.4232
208777	Sned1	sushi, nidogen and EGF-like domains 1	1.29718511	0.3829	0.5919
69745	Pold4	polymerase (DNA-directed), delta 4	1.29701686	0.0146	0.06822
16412	Itgb1	integrin beta 1 (fibronectin receptor beta)	1.29701686	0.1088	0.2621
67238	2810453I06Rik	RIKEN cDNA 2810453I06 gene	1.29684866	0.02338	0.0931
223433	Fam105a	family with sequence similarity 105, member A	1.29634431	0.1109	0.2656
11481	Acvr2b	activin receptor IIB	1.29617628	0.002202	0.0205
224109	Lrrc33	leucine rich repeat containing 33	1.29617628	0.01136	0.05796
19317	Qk	quaking	1.29617628	0.02724	0.1031
231842	Amz1	archaelysin family metallopeptidase 1	1.29600829	0.04792	0.1502
632764	5730471H19Rik	RIKEN cDNA 5730471H19 gene	1.29600829	0.1319	0.2983
269604	Gpr157	G protein-coupled receptor 157	1.29600829	0.1809	0.367
26408	Map3k5	mitogen-activated protein kinase kinase kinase 5	1.29584035	0.01017	0.05388
63828	Fn3k	fructosamine 3 kinase	1.29567245	0.002627	0.02289
100009600	Zglp1	zinc finger, GATA-like protein 1	1.2955046	0.007816	0.04554
78412	3110062M04Rik	RIKEN cDNA 3110062M04 gene	1.29533679	0.004375	0.0315
624367	Gm6498	glyceraldehyde-3-phosphate dehydrogenase pseudogene	1.29516902	0.1217	0.2831
18802	Plcd4	phospholipase C, delta 4	1.29483361	0.05515	0.1661
107650	Pi4kb	phosphatidylinositol 4-kinase, catalytic, beta polypeptide	1.29466598	0.03114	0.1129
319537	B230334C09Rik	RIKEN cDNA B230334C09 gene	1.29466598	0.1718	0.3547
100038847	Gm10406	predicted gene 10406	1.29466598	0.3233	0.5329
18081	Ninj1	ninjurin 1	1.29433083	0.001627	0.01733
68328	Rab13	RAB13, member RAS oncogene family	1.29433083	0.04868	0.152
246694	Hps5	Hermansky-Pudlak syndrome 5 homolog (human)	1.29433083	0.1993	0.3911
56358	Copz2	coatomer protein complex, subunit zeta 2	1.29416332	0.01415	0.06694
21991	Tpi1	triosephosphate isomerase 1	1.29416332	0.02149	0.08836
108037	Shmt2	serine hydroxymethyltransferase 2 (mitochondrial)	1.29416332	0.02321	0.09265
22763	Zfr	zinc finger RNA binding protein	1.29416332	0.09479	0.2398
19073	Srgn	serglycin	1.29416332	0.1119	0.267
116891	Derl2	Der1-like domain family, member 2	1.29399586	0.002039	0.01967
192970	Dhrs11	dehydrogenase/reductase (SDR family) member 11	1.29399586	0.2574	0.4626
320923	Mtap7d3	MAP7 domain containing 3	1.29382844	0.1003	0.249
27356	Insl6	insulin-like 6	1.29366106	0.02846	0.1063
319513	Fam113a	family with sequence similarity 113, member A	1.29349373	0.00333	0.02655
78878	B230206F22Rik	RIKEN cDNA B230206F22 gene	1.29315919	0.1603	0.3383
110075	Bmp3	bone morphogenetic protein 3	1.29299198	0.01805	0.07822
81877	Tnxb	tenascin XB	1.29249063	0.03837	0.1294
55927	Hes6	hairy and enhancer of split 6 (Drosophila)	1.2923236	0.001376	0.01568
84094	Plvap	plasmalemma vesicle associated protein	1.2923236	0.01319	0.06395
14370	Fzd8	frizzled homolog 8 (Drosophila)	1.29198966	0.0191	0.08125
67141	Fbxo5	F-box protein 5	1.2916559	0.004326	0.03131
17532	Mras	muscle and microspikes RAS	1.2916559	0.02106	0.08709
16828	Ldha	lactate dehydrogenase A	1.29148909	0.008713	0.04874
16145	Igtp	interferon gamma induced GTPase	1.29148909	0.0469	0.1478
20732	Spint1	serine protease inhibitor, Kunitz type 1	1.29132231	0.04052	0.134

66205	Cd302	CD302 antigen	1.29132231	0.06525	0.186
66991	2410004A20Rik	RIKEN cDNA 2410004A20 gene	1.29115558	0.01267	0.06219
209837	Slc38a5	solute carrier family 38, member 5	1.29115558	0.07363	0.2025
225020	Fez2	fasciculation and elongation protein zeta 2 (zygin II)	1.2909889	0.001947	0.01926
629557	Gm6981	glyceraldehyde-3-phosphate dehydrogenase pseudogene	1.2909889	0.04577	0.1455
140859	Nek8	NIMA (never in mitosis gene a)-related expressed kinase 8	1.29065565	0.001779	0.01828
12445	Ccnd3	cyclin D3	1.29065565	0.007787	0.04543
14027	Evpl	envoplakin	1.29015611	0.02272	0.09146
545366	BC026782	cDNA sequence BC026782	1.29015611	0.09831	0.2457
12506	Cd48	CD48 antigen	1.28949065	0.02426	0.09533
70419	2810408A11Rik	RIKEN cDNA 2810408A11 gene	1.28932439	0.001907	0.01908
16668	Krt18	keratin 18	1.28932439	0.002878	0.02421
66756	4933411K20Rik	RIKEN cDNA 4933411K20 gene	1.28915818	0.1345	0.3015
208924	A730045E13Rik	RIKEN cDNA A730045E13 gene	1.28915818	0.2322	0.4333
170472	Recql5	RecQ protein-like 5	1.28915818	0.2612	0.4672
21961	Tns1	tensin 1	1.28899201	0.007023	0.04263
212670	Catsper2	cation channel, sperm associated 2	1.28899201	0.01583	0.07186
171210	Acot2	acyl-CoA thioesterase 2	1.28882588	0.09836	0.2458
14683	Gnas	GNAS (guanine nucleotide binding protein, alpha stimulating) complex locus	1.28882588	0.535	0.7196
74519	Cyp2j9	cytochrome P450, family 2, subfamily j, polypeptide 9	1.28865979	0.001605	0.01722
236900	Pdk3	pyruvate dehydrogenase kinase, isoenzyme 3	1.28865979	0.1087	0.2619
102644	Oaf	OAF homolog (Drosophila)	1.28865979	0.1818	0.3683
71617	9130011E15Rik	RIKEN cDNA 9130011E15 gene	1.28849375	0.01007	0.05362
12684	Cideb	cell death-inducing DNA fragmentation factor, alpha subunit-like effector B	1.28849375	0.2	0.3918
77074	4930426I24Rik	RIKEN cDNA 4930426I24 gene	1.28832775	0.02086	0.08642
56358	Copz2	coatomer protein complex, subunit zeta 2	1.28832775	0.1361	0.3036
320020	6330415G19Rik	RIKEN cDNA 6330415G19 gene	1.28816179	0.3177	0.5272
59042	Cope	coatomer protein complex, subunit epsilon	1.28766418	0.003715	0.02845
70419	2810408A11Rik	RIKEN cDNA 2810408A11 gene	1.28749839	0.06445	0.1844
13206	Ddx4	DEAD (Asp-Glu-Ala-Asp) box polypeptide 4	1.28749839	0.1083	0.2613
21803	Tgfb1	transforming growth factor, beta 1	1.28749839	0.1984	0.3898
11650	Alppl2	alkaline phosphatase, placental-like 2	1.28749839	0.5205	0.7089
108682	Gpt2	glutamic pyruvate transaminase (alanine aminotransferase) 2	1.28733265	0.002018	0.01961
22232	Slc35a2	solute carrier family 35 (UDP-galactose transporter), member A2	1.28716695	0.01662	0.07384
73005	2900072G11Rik	RIKEN cDNA 2900072G11 gene	1.28700129	0.2372	0.4391
14793	Cdca3	cell division cycle associated 3	1.28683567	0.01657	0.07372
327959	Xaf1	XIAP associated factor 1	1.28683567	0.1511	0.3258
319197	Gpr4	G protein-coupled receptor 4	1.28683567	0.1959	0.3865
621080	AI429214	expressed sequence AI429214	1.2866701	0.009291	0.05073
13200	Ddost	dolichyl-di-phosphooligosaccharide-protein glycotransferase	1.28650457	0.005907	0.03786
503610	Zdhhc18	zinc finger, DHHC domain containing 18	1.28650457	0.006062	0.03851
67341	Ascl4	achaete-scute complex homolog 4 (Drosophila)	1.28650457	0.09196	0.2352
226025	Trpm3	transient receptor potential cation channel, subfamily M, member 3	1.28633908	0.04714	0.1485
224090	Tmem44	transmembrane protein 44	1.28617363	0.00926	0.05063
19892	Rpe65	retinal pigment epithelium 65	1.28617363	0.204	0.3975
241303	Fam78a	family with sequence similarity 78, member A	1.28600823	0.004473	0.03197
20732	Spint1	serine protease inhibitor, Kunitz type 1	1.28600823	0.1005	0.2494
76453	Prss23	protease, serine, 23	1.28567755	0.01823	0.07871
216188	Aldh1l2	aldehyde dehydrogenase 1 family, member L2	1.28567755	0.02861	0.1067
213391	Rassf4	Ras association (RalGDS/AF-6) domain family member 4	1.28567755	0.06503	0.1856

170654	Krtap16-4	keratin associated protein 16-4	1.28567755	0.2019	0.3946
11758	Prdx6	peroxiredoxin 6	1.28551228	0.008668	0.04859
16419	Itgb5	integrin beta 5	1.28551228	0.01817	0.07857
108960	Irak2	interleukin-1 receptor-associated kinase 2	1.28518185	0.007346	0.0438
17536	Meis2	Meis homeobox 2	1.28501671	0.002617	0.02283
269999	Orai3	ORAI calcium release-activated calcium modulator 3	1.28501671	0.03987	0.1326
67701	Wfdc2	WAP four-disulfide core domain 2	1.28501671	0.05054	0.1561
19369	Raet1b	retinoic acid early transcript beta	1.28501671	0.1789	0.3643
223752	Gramd4	GRAM domain containing 4	1.28468654	0.0001548	0.00433
107375	Slc25a45	solute carrier family 25, member 45	1.28452152	0.04837	0.1512
74032	Sdr42e1	short chain dehydrogenase/reductase family 42E, member 1	1.28435654	0.002642	0.02299
386612	Thoc6	THO complex 6 homolog (Drosophila)	1.2841916	0.005859	0.03771
235302	D630033O11Rik	RIKEN cDNA D630033O11 gene	1.2841916	0.01622	0.07286
18080	Nin	ninein	1.2841916	0.02971	0.1094
12153	Bmp1	bone morphogenetic protein 1	1.28402671	0.0505	0.1561
13555	E2f1	E2F transcription factor 1	1.28386186	0.0384	0.1294
56863	Cldn9	claudin 9	1.28369705	0.1609	0.3392
72080	2010317E24Rik	RIKEN cDNA 2010317E24 gene	1.28369705	0.2426	0.4456
76267	Fads1	fatty acid desaturase 1	1.28353228	0.006057	0.03851
14177	Fgf6	fibroblast growth factor 6	1.28353228	0.04071	0.1344
319159	Hist1h4j	histone cluster 1, H4j	1.28320287	0.2365	0.4383
332397	Nanos1	nanos homolog 1 (Drosophila)	1.28303823	0.002751	0.02359
330695	Ctxn1	cortexin 1	1.28287364	0.3485	0.559
545007	ENSMUSG00000068790	predicted gene, ENSMUSG00000068790	1.28287364	0.4334	0.637
353287	Clec18a	C-type lectin domain family 18, member A	1.2823801	0.1614	0.3398
68612	Ube2c	ubiquitin-conjugating enzyme E2C	1.28205128	0.2939	0.5029
218100	Zfp322a	zinc finger protein 322A	1.28188694	0.0331	0.1176
246221	Mpst	mercaptopyruvate sulfurtransferase	1.28188694	0.03566	0.1235
239528	Eif2c2	eukaryotic translation initiation factor 2C, 2	1.28188694	0.2495	0.4539
213211	Rnf26	ring finger protein 26	1.28172264	0.0004058	0.007462
268420	Alkbh5	alkB, alkylation repair homolog 5 (E. coli)	1.28172264	0.0005687	0.00924
109929	Zbtb25	zinc finger and BTB domain containing 25	1.28172264	0.01342	0.06455
215387	Ncaph	non-SMC condensin I complex, subunit H	1.28172264	0.05614	0.1681
22320	Vamp8	vesicle-associated membrane protein 8	1.28155837	0.003647	0.02812
14919	Gucy2e	guanylate cyclase 2e	1.28155837	0.01198	0.06007
68214	Gsto2	glutathione S-transferase omega 2	1.28122998	0.04538	0.1446
17988	Ndrg1	N-myc downstream regulated gene 1	1.28090175	0.0004914	0.008442
52150	Kcnk6	potassium inwardly-rectifying channel, subfamily K, member 6	1.28090175	0.06581	0.187
22689	Zfp27	zinc finger protein 27	1.2807377	0.01011	0.05371
20818	Srprb	signal recognition particle receptor, B subunit	1.2807377	0.03841	0.1294
266632	Irak4	interleukin-1 receptor-associated kinase 4	1.28008193	0.381	0.5903
14581	Gfi1	growth factor independent 1	1.27926314	0.1254	0.2887
71207	Nudt4	nudix (nucleoside diphosphate linked moiety X)-type motif 4	1.27909951	0.006765	0.04153
11816	Apoe	apolipoprotein E	1.27893593	0.01306	0.06345
218314	Zfp595	zinc finger protein 595	1.27877238	0.007237	0.04352
15944	Irgm1	immunity-related GTPase family M member 1	1.27860887	0.006049	0.03851
244418	D8Ertd82e	DNA segment, Chr 8, ERATO Doi 82, expressed	1.27860887	0.006167	0.039
12496	Entpd2	ectonucleoside triphosphate diphosphohydrolase 2	1.27860887	0.0279	0.1049
67884	1810043G02Rik	RIKEN cDNA 1810043G02 gene	1.27828199	0.01727	0.07581
19729	Slc50a1	solute carrier family 50 (sugar transporter), member 1	1.27828199	0.05354	0.1627

15229	Foxd1	forkhead box D1	1.27795527	0.2552	0.4602
106572	Rab31	RAB31, member RAS oncogene family	1.27779198	0.04398	0.1414
320332	Hist4h4	histone cluster 4, H4	1.27779198	0.1869	0.3748
22241	Ulk1	Unc-51 like kinase 1 (C. elegans)	1.27746551	0.002111	0.02006
84094	Plvap	plasmalemma vesicle associated protein	1.27746551	0.04075	0.1345
170748	BC017612	cDNA sequence BC017612	1.27730234	0.003597	0.02787
54366	Ctnnal1	catenin (cadherin associated protein), alpha-like 1	1.27713921	0.0353	0.1227
404330	Olfr1198	olfactory receptor 1198	1.27713921	0.459	0.6601
329554	Gm826	predicted gene 826	1.27681307	0.04998	0.1551
20475	Six5	sine oculis-related homeobox 5 homolog (Drosophila)	1.27665007	0.07	0.1951
18053	Ngfr	nerve growth factor receptor (TNFR superfamily, member 16)	1.27648711	0.07653	0.208
574428	Zmynd15	zinc finger, MYND-type containing 15	1.27632419	0.002047	0.0197
64685	Nmi	N-myc (and STAT) interactor	1.27632419	0.006103	0.0387
20502	Slc16a2	solute carrier family 16 (monocarboxylic acid transporters), member 2	1.27632419	0.00814	0.04668
78284	Creb3l4	cAMP responsive element binding protein 3-like 4	1.27632419	0.03023	0.1109
20018	Polr1d	polymerase (RNA) I polypeptide D	1.27599847	0.003127	0.02561
99382	Abtb2	ankyrin repeat and BTB (POZ) domain containing 2	1.27599847	0.01339	0.0645
17775	Laptm4a	lysosomal-associated protein transmembrane 4A	1.27599847	0.0252	0.0979
211612	Ptchd1	patched domain containing 1	1.27599847	0.05595	0.1678
230857	Ece1	endothelin converting enzyme 1	1.27599847	0.09873	0.2464
620986	Gm6195	predicted pseudogene 6195	1.27567292	0.01133	0.05788
381280	Hjurp	Holliday junction recognition protein	1.27567292	0.4031	0.6106
15108	Hsd17b10	hydroxysteroid (17-beta) dehydrogenase 10	1.2755102	0.004512	0.03212
277333	Gm5069	glyceraldehyde-3-phosphate dehydrogenase pseudogene	1.2755102	0.05612	0.1681
64654	Fgf23	fibroblast growth factor 23	1.2755102	0.1301	0.2958
69540	Klk10	kallikrein related-peptidase 10	1.27534753	0.05545	0.1667
68038	Chid1	chitinase domain containing 1	1.27534753	0.0988	0.2464
20878	Aurka	aurora kinase A	1.2751849	0.04673	0.1475
14613	Gja5	gap junction protein, alpha 5	1.27502231	0.1063	0.258
19024	Ppfibp2	PTPRF interacting protein, binding protein 2 (liprin beta 2)	1.27485977	0.0563	0.1683
16818	Lck	lymphocyte protein tyrosine kinase	1.27469726	0.01228	0.06091
67103	Ptgr1	prostaglandin reductase 1	1.27453479	0.07733	0.2094
78323	2310046O06Rik	RIKEN cDNA 2310046O06 gene	1.27404765	0.007566	0.04461
73167	Arhgap8	Rho GTPase activating protein 8	1.27388535	0.0418	0.1367
73191	Fezf1	Fez family zinc finger 1	1.27388535	0.4223	0.628
19125	Prodh	proline dehydrogenase	1.2733987	0.05855	0.1726
17754	Mtap1a	microtubule-associated protein 1 A	1.27307447	0.03007	0.1105
20104	Rps6	ribosomal protein S6	1.27258844	0.008121	0.04661
66578	2610039C10Rik	RIKEN cDNA 2610039C10 gene	1.27258844	0.03497	0.1218
56274	Stk3	serine/threonine kinase 3 (Ste20, yeast homolog)	1.27242652	0.4139	0.6204
16418	Eif6	eukaryotic translation initiation factor 6	1.27226463	0.0939	0.2382
213389	Prdm9	PR domain containing 9	1.27210279	0.02318	0.09259
16780	Lamb3	laminin, beta 3	1.27210279	0.169	0.3503
171286	Slc12a8	solute carrier family 12 (potassium/chloride transporters), member 8	1.27194098	0.003486	0.02733
55938	Apom	apolipoprotein M	1.27177922	0.1347	0.3015
626870	Gm11992	predicted gene 11992	1.27177922	0.164	0.3436
52668	Ifi27l1	interferon, alpha-inducible protein 27 like 1	1.2716175	0.01466	0.06841
11491	Adam17	a disintegrin and metallopeptidase domain 17	1.2716175	0.05909	0.1737
67732	Iah1	isoamyl acetate-hydrolyzing esterase 1 homolog (S. cerevisiae)	1.27145582	0.01445	0.0678
619329	F420015M19Rik	RIKEN cDNA F420015M19 gene	1.27145582	0.03292	0.1171

18212	Ntrk2	neurotrophic tyrosine kinase, receptor, type 2	1.27145582	0.05589	0.1677
60532	Wtap	Wilms' tumour 1-associating protein	1.27129418	0.003635	0.02808
98733	Obsl1	obscurin-like 1	1.27129418	0.004055	0.03001
56293	Amac1	acyl-malonyl condensing enzyme 1	1.27129418	0.111	0.2658
22409	Wnt10a	wingless related MMTV integration site 10a	1.27129418	0.1263	0.2899
545989	Gm5901	predicted gene 5901	1.27129418	0.2784	0.4866
74487	5430405H02Rik	RIKEN cDNA 5430405H02 gene	1.27113258	0.01024	0.05414
381810	Lpar5	lysophosphatidic acid receptor 5	1.27097102	0.3485	0.559
20449	St8sia1	ST8 alpha-N-acetyl-neuraminide alpha-2,8-sialyltransferase 1	1.27080951	0.05818	0.1719
277333	Gm5069	glyceraldehyde-3-phosphate dehydrogenase pseudogene	1.27064803	0.02746	0.1038
73824	Snhg6	small nucleolar RNA host gene (non-protein coding) 6	1.27064803	0.05709	0.17
20357	Sema5b	sema domain, seven thrombospondin repeats (type 1 and type 1-like), transmembrane domain (TM) and short cytoplasmic domain, (semaphorin) 5B	1.27064803	0.0766	0.2081
230577	Pars2	prolyl-tRNA synthetase (mitochondrial)(putative)	1.27064803	0.1228	0.2849
66985	Rassf7	Ras association (RalGDS/AF-6) domain family (N-terminal) member 7	1.2704866	0.003657	0.02817
17215	Mcm3	minichromosome maintenance deficient 3 (S. cerevisiae)	1.2704866	0.08874	0.2294
68481	Mpzl1	myelin protein zero-like 1	1.27016385	0.001366	0.01562
66079	Tmem42	transmembrane protein 42	1.26935771	0.008852	0.04914
102502	Pls1	plastin 1 (I-isoform)	1.26935771	0.2525	0.4574
67513	2610002J02Rik	RIKEN cDNA 2610002J02 gene	1.2691966	0.01644	0.07346
69724	Rnaseh2a	ribonuclease H2, large subunit	1.2691966	0.03159	0.114
241770	Rims4	regulating synaptic membrane exocytosis 4	1.2691966	0.03383	0.1191
242785	Klhl21	kelch-like 21 (Drosophila)	1.26903553	0.04319	0.1396
107476	Acaca	acetyl-Coenzyme A carboxylase alpha	1.26887451	0.4152	0.6213
15245	Hhip	Hedgehog-interacting protein	1.26871352	0.09333	0.2372
26905	Eif2s3x	eukaryotic translation initiation factor 2, subunit 3, structural gene X-linked	1.26855258	0.02559	0.0991
69008	Cab39l	calcium binding protein 39-like	1.26807	0.007742	0.04529
230979	Tnfrsf14	tumor necrosis factor receptor superfamily, member 14 (herpesvirus entry mediator)	1.26807	0.5198	0.7087
58172	Sertad2	SERTA domain containing 2	1.26790922	0.01072	0.05557
329002	Zfp236	zinc finger protein 236	1.26790922	0.1088	0.2621
70847	4733401D01Rik	RIKEN cDNA 4733401D01 gene	1.26790922	0.1159	0.2735
56349	Net1	neuroepithelial cell transforming gene 1	1.26742712	0.02825	0.1058
16515	Kcnj12	potassium inwardly-rectifying channel, subfamily J, member 12	1.26726651	0.02611	0.1004
332397	Nanos1	nanos homolog 1 (Drosophila)	1.26726651	0.02678	0.1021
68957	Paqr6	progestin and adipoQ receptor family member VI	1.26726651	0.1422	0.3128
67041	Oxct1	3-oxoacid CoA transferase 1	1.26710593	0.001938	0.01923
268709	Fam107a	family with sequence similarity 107, member A	1.26710593	0.2623	0.4683
381286	Serpinb3c	serine (or cysteine) peptidase inhibitor, clade B, member 3C	1.26710593	0.3398	0.5501
666752	Gm8273	predicted gene 8273	1.2667849	0.01083	0.05595
18221	Nudc	nuclear distribution gene C homolog (Aspergillus)	1.26662445	0.008857	0.04914
212442	Lactb2	lactamase, beta 2	1.26662445	0.02348	0.09342
26408	Map3k5	mitogen-activated protein kinase kinase kinase 5	1.26646403	0.04916	0.1532
20195	S100a11	S100 calcium binding protein A11 (calgizzarin)	1.26646403	0.2166	0.4139
13169	Dbnl	drebrin-like	1.26630366	0.01902	0.08105
18207	Nthl1	nth (endonuclease III)-like 1 (E.coli)	1.26630366	0.02013	0.08412
226359	C1ql2	complement component 1, q subcomponent-like 2	1.26630366	0.06675	0.1888
21346	Tagln2	transgelin 2	1.26614333	0.1034	0.254
69065	Chac1	ChaC, cation transport regulator-like 1 (E. coli)	1.26614333	0.1588	0.3364
78330	Ndufv3	NADH dehydrogenase (ubiquinone) flavoprotein 3	1.26598304	0.01526	0.07038
14411	Slc6a12	solute carrier family 6 (neurotransmitter transporter, betaine/GABA), member 12	1.26598304	0.07885	0.2124
16678	Krt1	keratin 1	1.26598304	0.2842	0.4927

666464	Gm8120	predicted gene 8120	1.26582278	0.1779	0.363
216527	Ccm2	cerebral cavernous malformation 2 homolog (human)	1.26566257	0.0008184	0.01151
67464	Entpd4	ectonucleoside triphosphate diphosphohydrolase 4	1.2655024	0.002279	0.02098
67528	Nudt7	nudix (nucleoside diphosphate linked moiety X)-type motif 7	1.2655024	0.004197	0.03065
102209	Snapc2	small nuclear RNA activating complex, polypeptide 2	1.2655024	0.06413	0.1838
21415	Tcf7l1	transcription factor 7-like 1 (T-cell specific, HMG box)	1.2655024	0.09689	0.243
18111	Nnat	neuronatin	1.2655024	0.1561	0.3331
20682	Sox9	SRY-box containing gene 9	1.26534228	0.05467	0.1652
245638	Tbc1d8b	TBC1 domain family, member 8B	1.26518219	0.0801	0.2147
21949	Tnfsf8	tumor necrosis factor (ligand) superfamily, member 8	1.26502214	0.1124	0.268
67204	Eif2s2	eukaryotic translation initiation factor 2, subunit 2 (beta)	1.26486213	0.01573	0.07153
18753	Prkcd	protein kinase C, delta	1.26470216	0.01036	0.05448
66548	Adamtsl5	ADAMTS-like 5	1.26454224	0.05633	0.1684
629557	Gm6981	glyceraldehyde-3-phosphate dehydrogenase pseudogene	1.26438235	0.08304	0.2196
11652	Akt2	thymoma viral proto-oncogene 2	1.2642225	0.005428	0.03599
15202	Hemt1	hematopoietic cell transcript 1	1.2642225	0.5551	0.735
56516	Rbms2	RNA binding motif, single stranded interacting protein 2	1.26374321	0.007152	0.04314
100038712	Gm10516	predicted gene 10516	1.26358352	0.01661	0.07383
27419	Naglu	alpha-N-acetylglucosaminidase (Sanfilippo disease IIIB)	1.26342388	0.01143	0.05824
68234	2400009B08Rik	RIKEN cDNA 2400009B08 gene	1.26342388	0.1074	0.2597
50927	Nasp	nuclear autoantigenic sperm protein (histone-binding)	1.26342388	0.2156	0.4127
497210	Tpt1p	tumor protein, translationally-controlled 1 pseudogene	1.26326427	0.01822	0.07869
669389	Gm9456	predicted gene 9456	1.26326427	0.1166	0.2745
71877	Efhc1	EF-hand domain (C-terminal) containing 1	1.26326427	0.1204	0.2809
27015	Polk	polymerase (DNA directed), kappa	1.26326427	0.1624	0.3412
75570	Nhej1	nonhomologous end-joining factor 1	1.26310471	0.02972	0.1095
74743	5830403F22Rik	RIKEN cDNA 5830403F22 gene	1.26294519	0.2237	0.4233
227683	Coq4	coenzyme Q4 homolog (yeast)	1.26262626	0.0184	0.07929
15275	Hk1	hexokinase 1	1.26262626	0.02898	0.1076
16782	Lamc2	laminin, gamma 2	1.26262626	0.03479	0.1213
321022	Cdv3	carnitine deficiency-associated gene expressed in ventricle 3	1.2623075	0.01557	0.07122
83560	Tex14	testis expressed gene 14	1.2623075	0.05909	0.1737
15464	Hrc	histidine rich calcium binding protein	1.26214818	0.005266	0.03533
57441	Gmnn	geminin	1.26214818	0.02658	0.1015
73121	Fam101a	family with sequence similarity 101, member A	1.26198889	0.06273	0.1811
71721	Fam13c	family with sequence similarity 13, member C	1.26198889	0.5413	0.7243
12305	Ddr1	discoidin domain receptor family, member 1	1.26167045	0.007831	0.04557
21858	Timp2	tissue inhibitor of metalloproteinase 2	1.26151129	0.007703	0.04514
78134	Lpar4	lysophosphatidic acid receptor 4	1.26151129	0.2409	0.4436
230073	Ddx58	DEAD (Asp-Glu-Ala-Asp) box polypeptide 58	1.26151129	0.2752	0.483
71929	Tmem123	transmembrane protein 123	1.26135217	0.002375	0.02143
72690	Grrp1	glycine/arginine rich protein 1	1.26135217	0.01576	0.07165
16440	Itpr3	inositol 1,4,5-triphosphate receptor 3	1.26135217	0.01659	0.07377
19823	Rnf7	ring finger protein 7	1.26119309	0.002649	0.02301
14109	Fau	Finkel-Biskis-Reilly murine sarcoma virus (FBR-MuSV) ubiquitously expressed (fox derived)	1.26103405	0.01009	0.05365
68507	Ppfia4	protein tyrosine phosphatase, receptor type, f polypeptide (PTPRF), interacting protein (liprin), alpha 4	1.26103405	0.02895	0.1076
21346	Tagln2	transgelin 2	1.26071609	0.04985	0.1548
666385	Gm8075	predicted gene 8075	1.26071609	0.296	0.5049
26377	Dapp1	dual adaptor for phosphotyrosine and 3-phosphoinositides 1	1.26008065	0.1561	0.333
99681	Tchh	trichohyalin	1.25992188	0.2524	0.4574

232933	Ccdc61	coiled-coil domain containing 61	1.25976316	0.005052	0.03437
231147	Sh3tc1	SH3 domain and tetratricopeptide repeats 1	1.25976316	0.01545	0.07092
23836	Cdh20	cadherin 20	1.25944584	0.005879	0.03779
73608	Marveld3	MARVEL (membrane-associating) domain containing 3	1.25944584	0.009425	0.05124
18176	Nras	neuroblastoma ras oncogene	1.25928724	0.007922	0.04587
67247	Mosc2	MOCO sulphurase C-terminal domain containing 2	1.25912868	0.001968	0.01937
66795	Atg10	autophagy-related 10 (yeast)	1.25897016	0.01208	0.06035
268934	Grm4	glutamate receptor, metabotropic 4	1.25897016	0.1212	0.2825
238317	C130039O16Rik	RIKEN cDNA C130039O16 gene	1.25897016	0.1993	0.3911
241950	Bbs12	Bardet-Biedl syndrome 12 (human)	1.25881168	0.003305	0.02641
70804	Pgrmc2	progesterone receptor membrane component 2	1.25881168	0.0545	0.1649
381113	Cdkl4	cyclin-dependent kinase-like 4	1.25865324	0.1259	0.2892
271849	Shc4	SHC (Src homology 2 domain containing) family, member 4	1.25865324	0.1574	0.3345
230810	Slc30a2	solute carrier family 30 (zinc transporter), member 2	1.25849484	0.02367	0.0939
109305	Orai1	ORAI calcium release-activated calcium modulator 1	1.25849484	0.05495	0.1658
106581	Itfg3	integrin alpha FG-GAP repeat containing 3	1.25833648	0.01771	0.07722
11981	Atp9a	ATPase, class II, type 9A	1.25817816	0.009563	0.05177
67397	Erp29	endoplasmic reticulum protein 29	1.25801988	0.0006533	0.01003
20871	Aurkc	aurora kinase C	1.25801988	0.01267	0.06219
20649	Sntb1	syntrophin, basic 1	1.25801988	0.1688	0.35
69953	2810025M15Rik	RIKEN cDNA 2810025M15 gene	1.25786164	0.02051	0.08534
110749	Chaf1b	chromatin assembly factor 1, subunit B (p60)	1.25786164	0.137	0.3046
403178	Plcxd1	phosphatidylinositol-specific phospholipase C, X domain containing 1	1.25770343	0.009814	0.05273
74614	4833422F24Rik	RIKEN cDNA 4833422F24 gene	1.25754527	0.4797	0.6759
108655	Foxp1	forkhead box P1	1.25738715	0.02141	0.08807
19712	Rest	RE1-silencing transcription factor	1.25738715	0.03395	0.1193
170790	Mlc1	megalencephalic leukoencephalopathy with subcortical cysts 1 homolog (human)	1.25722907	0.0009441	0.01254
72614	Pih1d2	PIH1 domain containing 2	1.25722907	0.01553	0.07111
101437	Dhx32	DEAH (Asp-Glu-Ala-His) box polypeptide 32	1.25722907	0.1478	0.3211
319865	E130114P18Rik	RIKEN cDNA E130114P18 gene	1.25707102	0.01564	0.07134
66788	5430414B12Rik	RIKEN cDNA 5430414B12 gene	1.25707102	0.06876	0.1927
17001	Ltc4s	leukotriene C4 synthase	1.25691302	0.04077	0.1345
432879	Gm5465	predicted gene 5465	1.25675506	0.1106	0.2649
232533	Stk38l	serine/threonine kinase 38 like	1.25659713	0.01211	0.06044
11491	Adam17	a disintegrin and metallopeptidase domain 17	1.25659713	0.01351	0.06474
16332	Inppl1	inositol polyphosphate phosphatase-like 1	1.25643925	0.007526	0.04442
381229	Ccdc147	coiled-coil domain containing 147	1.25643925	0.08442	0.2222
67866	Wfdc1	WAP four-disulfide core domain 1	1.25643925	0.2312	0.4321
216456	Gls2	glutaminase 2 (liver, mitochondrial)	1.2561236	0.001731	0.018
75234	Rnf19b	ring finger protein 19B	1.25596584	0.0415	0.1363
11676	Aldoc	aldolase C, fructose-bisphosphate	1.25580811	0.06981	0.1949
66824	Pycard	PYD and CARD domain containing	1.25565043	0.003993	0.02969
54123	Irf7	interferon regulatory factor 7	1.25565043	0.1762	0.3609
277744	Gm694	predicted gene 694	1.25549278	0.0733	0.202
19703	Renbp	renin binding protein	1.25549278	0.1077	0.2602
434446	Ccdc13	coiled-coil domain containing 13	1.25533517	0.1713	0.3541
330010	Ttll10	tubulin tyrosine ligase-like family, member 10	1.25517761	0.2147	0.4116
20509	Slc19a1	solute carrier family 19 (sodium/hydrogen exchanger), member 1	1.25502008	0.004481	0.03198
27386	Npas3	neuronal PAS domain protein 3	1.25502008	0.008306	0.04724
236573	Gbp9	guanylate-binding protein 9	1.25454774	0.05205	0.1595

78713	D530017H19Rik	RIKEN cDNA D530017H19 gene	1.25439037	0.08688	0.2264
547431	Btnl2	butyrophilin-like 2	1.25439037	0.4369	0.6403
67268	2900073G15Rik	RIKEN cDNA 2900073G15 gene	1.25423304	0.01034	0.05446
107435	Hat1	histone aminotransferase 1	1.25407575	0.05048	0.156
24004	Rai2	retinoic acid induced 2	1.2539185	0.00791	0.04584
57756	Fhl5	four and a half LIM domains 5	1.2539185	0.3371	0.5474
64934	Pes1	pescadillo homolog 1, containing BRCT domain (zebrafish)	1.25376128	0.0202	0.08434
68527	Ucma	upper zone of growth plate and cartilage matrix associated	1.25344698	0.1426	0.3134
72611	Zfp655	zinc finger protein 655	1.25313283	0.0196	0.08263
17259	Mef2b	myocyte enhancer factor 2B	1.25297582	0.1839	0.3709
98363	Efhd1	EF hand domain containing 1	1.25281884	0.03212	0.1153
100039220	Gm12751	predicted gene 12751	1.25266191	0.007879	0.04572
111173	Erc1	ELKS/RAB6-interacting/CAST family member 1	1.25266191	0.2243	0.424
100037283	Rnaset2a	ribonuclease T2A	1.25250501	0.04617	0.1463
68667	Trpm4	transient receptor potential cation channel, subfamily M, member 4	1.25250501	0.07491	0.2047
105559	Mbnl2	muscleblind-like 2	1.25219133	0.05208	0.1595
67122	Nrarp	Notch-regulated ankyrin repeat protein	1.25219133	0.1171	0.2753
78689	Naa35	N(alpha)-acetyltransferase 35, NatC auxiliary subunit	1.25203456	0.009211	0.05048
27279	Tnfrsf12a	tumor necrosis factor receptor superfamily, member 12a	1.25187782	0.0126	0.06201
277360	Prex1	phosphatidylinositol-3,4,5-trisphosphate-dependent Rac exchange factor 1	1.25187782	0.01377	0.06567
338362	Ust	uronyl-2-sulfotransferase	1.25187782	0.02789	0.1049
12908	Crat	carnitine acetyltransferase	1.25187782	0.04427	0.1421
69009	Thap7	THAP domain containing 7	1.25172112	0.1525	0.3277
74286	Tbc1d21	TBC1 domain family, member 21	1.25172112	0.4769	0.674
20111	Rps6ka1	ribosomal protein S6 kinase polypeptide 1	1.25140783	0.004826	0.03346
21766	Tex261	testis expressed gene 261	1.2509382	0.001791	0.01832
230903	Fbxo44	F-box protein 44	1.2509382	0.07073	0.1965
28200	Dhrs4	dehydrogenase/reductase (SDR family) member 4	1.25078174	0.004475	0.03197
101502	Hsd3b7	hydroxy-delta-5-steroid dehydrogenase, 3 beta- and steroid delta-isomerase 7	1.25078174	0.0351	0.1222
546071	Mast3	microtubule associated serine/threonine kinase 3	1.25062531	0.08709	0.2269
21460	Tcp10a	t-complex protein 10a	1.25062531	0.2226	0.4221
545123	Cyp2d11	cytochrome P450, family 2, subfamily d, polypeptide 11	1.25046893	0.0267	0.1019
107242	AI837181	expressed sequence AI837181	1.25046893	0.1125	0.2682
70445	Cd248	CD248 antigen, endosialin	1.25046893	0.1602	0.3383
68337	Crip2	cysteine rich protein 2	1.25031258	0.001331	0.01541
382139	Gm1715	predicted gene 1715	1.25031258	0.2651	0.4715
209086	Samd9l	sterile alpha motif domain containing 9-like	1.25015627	0.03565	0.1235
100017	Ldlrap1	low density lipoprotein receptor adaptor protein 1	1.25015627	0.05544	0.1667
353287	Clec18a	C-type lectin domain family 18, member A	1.25015627	0.1574	0.3346
15208	Hes5	hairy and enhancer of split 5 (Drosophila)	1.24984377	0.02393	0.09468
78244	Dnajc21	DnaJ (Hsp40) homolog, subfamily C, member 21	1.24984377	0.04206	0.1374
54325	Elovl1	elongation of very long chain fatty acids (FEN1/Elo2, SUR4/Elo3, yeast)-like 1	1.24984377	0.08057	0.2154
68385	Tlcd1	TLC domain containing 1	1.24953143	0.02004	0.08386
207521	Dtx4	deltex 4 homolog (Drosophila)	1.24937531	0.001274	0.01501
73713	Rbm20	RNA binding motif protein 20	1.24937531	0.07224	0.1997
212898	Dse	dermatan sulfate epimerase	1.24921924	0.001632	0.01734
12479	Cd1d1	CD1d1 antigen	1.24921924	0.01869	0.08012
16418	Eif6	eukaryotic translation initiation factor 6	1.2490632	0.07746	0.2097
67282	Ccdc53	coiled-coil domain containing 53	1.24875125	0.001923	0.01917
14711	Gnmt	glycine N-methyltransferase	1.24859533	0.04275	0.1388

14600	Ghr	growth hormone receptor	1.24843945	0.001209	0.01454
20516	Slc20a2	solute carrier family 20, member 2	1.24828361	0.0116	0.05892
27392	Pign	phosphatidylinositol glycan anchor biosynthesis, class N	1.24812781	0.2578	0.4632
72523	2700005E23Rik	RIKEN cDNA 2700005E23 gene	1.24797205	0.02241	0.09076
214579	Aldh5a1	aldhehyde dehydrogenase family 5, subfamily A1	1.24781632	0.002758	0.0236
93742	Pard3	par-3 (partitioning defective 3) homolog (C. elegans)	1.24781632	0.01912	0.08126
13848	Ephb6	Eph receptor B6	1.24781632	0.1983	0.3898
13096	Cyp2c37	cytochrome P450, family 2. subfamily c, polypeptide 37	1.24766064	0.231	0.432
100040671	Gm2897	predicted gene 2897	1.24766064	0.4388	0.6417
97122	Hist2h4	histone cluster 2, H4	1.24750499	0.2218	0.421
228913	Zfp217	zinc finger protein 217	1.24734938	0.03839	0.1294
59020	Pdzk1	PDZ domain containing 1	1.24734938	0.05375	0.1632
13478	Dpagt1	dolichyl-phosphate (UDP-N-acetylglucosamine) acetylglucosaminephosphotransferase 1 (GlcNAc-1-P transferase)	1.24719381	0.002647	0.02301
109272	Mybpc1	myosin binding protein C, slow-type	1.24719381	0.2797	0.4879
77134	Hnrnpa0	heterogeneous nuclear ribonucleoprotein A0	1.24688279	0.06412	0.1838
16855	Lgals4	lectin, galactose binding, soluble 4	1.24672734	0.04484	0.1434
13544	Dvl3	dishevelled 3, dsh homolog (Drosophila)	1.24672734	0.0987	0.2463
50794	Klf13	Kruppel-like factor 13	1.24626122	0.06119	0.1778
71562	Afmid	arylformamidase	1.24626122	0.101	0.2503
72454	Ccdc71	coiled-coil domain containing 71	1.24610592	0.01932	0.08185
93840	Vangl2	vang-like 2 (van gogh, Drosophila)	1.24610592	0.05794	0.1716
74096	Hvcn1	hydrogen voltage-gated channel 1	1.24610592	0.08782	0.2281
77630	Prdm8	PR domain containing 8	1.24564026	0.0007412	0.01092
105278	Cdk20	cyclin-dependent kinase 20	1.24564026	0.001223	0.01463
68066	Slc25a39	solute carrier family 25, member 39	1.24564026	0.05697	0.1698
17153	Mal	myelin and lymphocyte protein, T-cell differentiation protein	1.24564026	0.1084	0.2614
21452	Tcn2	transcobalamin 2	1.24548512	0.004735	0.03312
93880	Pcdhb9	protocadherin beta 9	1.24548512	0.2289	0.4292
110954	Rpl10	ribosomal protein 10	1.24533001	0.005527	0.03639
17826	Mtvr2	mammary tumor virus receptor 2	1.24517495	0.003277	0.02633
212442	Lactb2	lactamase, beta 2	1.24517495	0.02646	0.1012
22418	Wnt5a	wingless-related MMTV integration site 5A	1.24470998	0.02258	0.09109
66042	Sostdc1	sclerostin domain containing 1	1.24470998	0.0959	0.2419
666257	Gm8008	predicted gene 8008	1.24470998	0.1605	0.3387
12445	Ccnd3	cyclin D3	1.24455507	0.0005561	0.009124
74720	Tmem114	transmembrane protein 114	1.24455507	0.08637	0.2256
13003	Vcan	versican	1.2444002	0.2224	0.4219
50782	Rgs11	regulator of G-protein signaling 11	1.24424537	0.03841	0.1294
52838	Dnlz	DNL-type zinc finger	1.24409057	0.03394	0.1193
20287	Sct	secretin	1.24393581	0.04461	0.1427
235461	Fam63b	family with sequence similarity 63, member B	1.24378109	0.1008	0.25
21767	Tex264	testis expressed gene 264	1.24362641	0.005392	0.03584
13491	Drd4	dopamine receptor D4	1.24347177	0.0104	0.05458
57913	Lrdd	leucine-rich and death domain containing	1.24347177	0.2339	0.4356
75695	Rilpl1	Rab interacting lysosomal protein-like 1	1.24316261	0.01876	0.08028
71709	Syde1	synapse defective 1, Rho GTPase, homolog 1 (C. elegans)	1.24316261	0.05803	0.1716
67513	2610002J02Rik	RIKEN cDNA 2610002J02 gene	1.24285359	0.02579	0.0995
16995	Ltb4r1	leukotriene B4 receptor 1	1.24285359	0.03332	0.118
14910	Gt(ROSA)26Sor	gene trap ROSA 26, Philippe Soriano	1.24254473	0.03963	0.1319
72701	Zfp618	zinc fingerprotein 618	1.24254473	0.3331	0.5436

14919	Gucy2e	guanylate cyclase 2e	1.24239036	0.02578	0.0995
71949	Lass5	LAG1 homolog, ceramide synthase 5	1.24223602	0.002038	0.01967
209497	Tmem164	transmembrane protein 164	1.24223602	0.00351	0.02745
18111	Nnat	neuronatin	1.24208173	0.1153	0.2725
20862	Stfa2	stefin A2	1.24208173	0.2953	0.5042
319160	Hist1h4k	histone cluster 1, H4k	1.24192747	0.2805	0.4887
328971	Spink10	serine peptidase inhibitor, Kazal type 10	1.24177325	0.2597	0.4654
17294	Mest	mesoderm specific transcript	1.24131082	0.003529	0.02754
67460	Decr1	2,4-dienoyl CoA reductase 1, mitochondrial	1.24115676	0.008625	0.04843
67690	Prss37	protease, serine, 37	1.24115676	0.04494	0.1436
52276	Cdca8	cell division cycle associated 8	1.24115676	0.06692	0.189
67706	Tmem179b	transmembrane protein 179B	1.24115676	0.07868	0.212
22773	Zic3	zinc finger protein of the cerebellum 3	1.24084874	0.034	0.1194
229900	Gbp6	guanylate binding protein 6	1.24054088	0.08096	0.216
12520	Cd81	CD81 antigen	1.240387	0.002675	0.02315
66756	4933411K20Rik	RIKEN cDNA 4933411K20 gene	1.240387	0.007334	0.04378
72828	Ubash3b	ubiquitin associated and SH3 domain containing, B	1.24023316	0.009269	0.05066
16470	Ush1g	Usher syndrome 1G homolog (human)	1.24023316	0.06586	0.187
100216343	LOC100216343	plasma membrane Ca2+ pump interacting protein	1.24007937	0.05325	0.1621
20908	Stx3	syntaxin 3	1.23977188	0.01589	0.07197
17293	Mesp2	mesoderm posterior 2	1.2396182	0.2831	0.4916
56275	Rbm14	RNA binding motif protein 14	1.23946455	0.1173	0.2757
238405	Adam6b	a disintegrin and metallopeptidase domain 6B	1.23946455	0.6196	0.7816
54445	Unc93b1	unc-93 homolog B1 (C. elegans)	1.23931094	0.1348	0.3017
232969	Zfp428	zinc finger protein 428	1.23915737	0.01205	0.06025
30956	Aass	aminoadipate-semialdehyde synthase	1.23915737	0.02217	0.09018
97998	Deptor	DEP domain containing MTOR-interacting protein	1.23900384	0.03281	0.1169
69216	Ccdc23	coiled-coil domain containing 23	1.23869689	0.009057	0.04981
68240	Rpa3	replication protein A3	1.23854347	0.02891	0.1075
12540	Cdc42	cell division cycle 42 homolog (S. cerevisiae)	1.23839009	0.003916	0.02938
20871	Aurkc	aurora kinase C	1.23839009	0.2999	0.5093
101612	Grwd1	glutamate-rich WD repeat containing 1	1.23823675	0.1325	0.2992
269951	Idh2	isocitrate dehydrogenase 2 (NADP+), mitochondrial	1.23808345	0.02113	0.08721
15481	Hspa8	heat shock protein 8	1.23793018	0.04319	0.1396
229488	Fam160a1	family with sequence similarity 160, member A1	1.23777695	0.06965	0.1946
193286	BC049762	cDNA sequence BC049762	1.23762376	0.1795	0.3651
75202	Ncrna00085	non-protein coding RNA 85	1.23747061	0.08325	0.22
26921	Map4k4	mitogen-activated protein kinase kinase kinase kinase 4	1.23747061	0.1071	0.2592
109108	Slc30a9	solute carrier family 30 (zinc transporter), member 9	1.23716442	0.08569	0.2244
235312	C1qtnf5	C1q and tumor necrosis factor related protein 5	1.23716442	0.08892	0.2298
19700	Rem1	rad and gem related GTP binding protein 1	1.23716442	0.2047	0.3984
231125	Zfyve28	zinc finger, FYVE domain containing 28	1.23716442	0.4418	0.6447
14457	Gas7	growth arrest specific 7	1.23701138	0.02244	0.09076
74090	Paqr5	progestin and adipoQ receptor family member V	1.23701138	0.03898	0.1306
18249	Obp1a	odorant binding protein Ia	1.23701138	0.09977	0.248
106628	Trip10	thyroid hormone receptor interactor 10	1.23685838	0.00449	0.03202
240058	Cpne5	copine V	1.23685838	0.06917	0.1937
14247	Fli1	Friend leukemia integration 1	1.23685838	0.09414	0.2386
104383	Rcor2	REST corepressor 2	1.23670542	0.001182	0.01435
70394	Kptn	kaptin	1.23655249	0.0215	0.08838

229541	Dennd4b	DENN/MADD domain containing 4B	1.2363996	0.05609	0.168
24109	Ubl3	ubiquitin-like 3	1.23624675	0.00571	0.03713
229279	Hnrnpa3	heterogeneous nuclear ribonucleoprotein A3	1.23624675	0.04734	0.1489
75064	Zcchc13	zinc finger, CCHC domain containing 13	1.23594117	0.1172	0.2754
240025	Dact2	dapper homolog 2, antagonist of beta-catenin (xenopus)	1.23578843	0.181	0.367
11652	Akt2	thymoma viral proto-oncogene 2	1.23533045	0.003003	0.02484
72658	2700097O09Rik	RIKEN cDNA 2700097O09 gene	1.23533045	0.004867	0.03358
667572	Gm8709	glyceraldehyde-3-phosphate dehydrogenase pseudogene	1.23533045	0.02582	0.09955
218214	Kdm1b	lysine (K)-specific demethylase 1B	1.23533045	0.04011	0.133
70686	Dusp16	dual specificity phosphatase 16	1.23517787	0.002221	0.0206
20467	Sin3b	transcriptional regulator, SIN3B (yeast)	1.23502532	0.001594	0.01716
319601	Zfp653	zinc finger protein 653	1.23502532	0.003823	0.02895
109042	Prkcdbp	protein kinase C, delta binding protein	1.23502532	0.0736	0.2024
14433	Gapdh	glyceraldehyde-3-phosphate dehydrogenase	1.23502532	0.1359	0.3033
94089	Trim7	tripartite motif-containing 7	1.23502532	0.1417	0.3122
71279	Slc29a3	solute carrier family 29 (nucleoside transporters), member 3	1.23502532	0.2819	0.49
319565	Syne2	synaptic nuclear envelope 2	1.23472034	0.01975	0.0831
19727	Rfxank	regulatory factor X-associated ankyrin-containing protein	1.2344155	0.07475	0.2044
78816	Gmip	Gem-interacting protein	1.2344155	0.07596	0.2069
664968	2210411K11Rik	RIKEN cDNA 2210411K11 gene	1.2344155	0.1199	0.28
192120	Bspry	B-box and SPRY domain containing	1.2344155	0.1414	0.3118
319156	Hist1h4d	histone cluster 1, H4d	1.2344155	0.3055	0.5147
19823	Rnf7	ring finger protein 7	1.23426314	0.008568	0.04821
192188	Stab2	stabilin 2	1.23411082	0.03259	0.1165
13661	Ehf	ets homologous factor	1.23411082	0.09261	0.2362
17063	Muc13	mucin 13, epithelial transmembrane	1.23395854	0.06846	0.1921
11982	Atp10a	ATPase, class V, type 10A	1.23380629	0.04918	0.1532
12398	Cbfa2t3	core-binding factor, runt domain, alpha subunit 2, translocated to, 3 (human)	1.23380629	0.2557	0.4607
100042371	Gm3807	predicted gene 3807	1.23365408	0.2586	0.4641
76311	1110019D14Rik	RIKEN cDNA 1110019D14 gene	1.23334978	0.02756	0.104
76100	5830454E08Rik	RIKEN cDNA 5830454E08 gene	1.23319768	0.01527	0.07041
56398	1500003O03Rik	RIKEN cDNA 1500003O03 gene	1.23319768	0.03138	0.1135
54698	Crtam	cytotoxic and regulatory T cell molecule	1.23319768	0.1362	0.3036
16155	Il10rb	interleukin 10 receptor, beta	1.23304562	0.01273	0.06239
20352	Sema4b	sema domain, immunoglobulin domain (Ig), transmembrane domain (TM) and short cytoplasmic domain, (semaphorin) 4B	1.23304562	0.03176	0.1144
234309	Cbr4	carbonyl reductase 4	1.2328936	0.04159	0.1364
22433	Xbp1	X-box binding protein 1	1.23274162	0.01204	0.06022
71729	Rgs12	regulator of G-protein signaling 12	1.23274162	0.2548	0.4599
545047	Gm5800	predicted gene 5800	1.23274162	0.4367	0.6401
68352	Aspdh	aspartate dehydrogenase domain containing	1.23258967	0.1193	0.2792
223732	Ldoc1l	leucine zipper, down-regulated in cancer 1-like	1.23258967	0.514	0.7042
207958	Alg11	asparagine-linked glycosylation 11 homolog (yeast, alpha-1,2-mannosyltransferase)	1.23243776	0.09126	0.2338
100169878	Gm10941	predicted gene 10941	1.23228589	0.09677	0.243
208618	Etl4	enhancer trap locus 4	1.23228589	0.172	0.355
54188	Cpsf4	cleavage and polyadenylation specific factor 4	1.23213406	0.07538	0.2055
16190	Il4ra	interleukin 4 receptor, alpha	1.23213406	0.1786	0.3637
100037282	Rsph3b	radial spoke 3B homolog (Chlamydomonas)	1.23198226	0.0009592	0.01267
78372	Snrnp25	small nuclear ribonucleoprotein 25 (U11/U12)	1.23198226	0.004602	0.03247
75475	Oplah	5-oxoprolinase (ATP-hydrolysing)	1.23198226	0.06788	0.1908
68939	Rasl11b	RAS-like, family 11, member B	1.23167878	0.0913	0.2339

69178	Snx5	sorting nexin 5	1.23167878	0.09467	0.2396
103220	BC030307	cDNA sequence BC030307	1.23152709	0.1308	0.2967
102680	Slc6a20a	solute carrier family 6 (neurotransmitter transporter), member 20A	1.23152709	0.2074	0.4021
214812	Zfp609	zinc finger protein 609	1.23137545	0.3213	0.5311
11695	Alx4	aristaless-like homeobox 4	1.23137545	0.4713	0.67
12332	Capg	capping protein (actin filament), gelsolin-like	1.23122384	0.1643	0.3441
235320	Zbtb16	zinc finger and BTB domain containing 16	1.23107226	0.1112	0.2659
319160	Hist1h4k	histone cluster 1, H4k	1.23092073	0.2447	0.4484
237504	Rassf9	Ras association (RalGDS/AF-6) domain family (N-terminal) member 9	1.23076923	0.2341	0.4356
13002	Dnajc5	DnaJ (Hsp40) homolog, subfamily C, member 5	1.23061777	0.03065	0.1118
100201	Tmem64	transmembrane protein 64	1.22986103	0.004759	0.03321
320869	4732415M23Rik	RIKEN cDNA 4732415M23 gene	1.22955859	0.03136	0.1134
67753	4930579C15Rik	RIKEN cDNA 4930579C15 gene	1.22940743	0.1026	0.2526
77569	Limch1	LIM and calponin homology domains 1	1.22910521	0.03898	0.1306
69903	Rasip1	Ras interacting protein 1	1.22895416	0.03968	0.132
19229	Ptk2b	PTK2 protein tyrosine kinase 2 beta	1.22895416	0.2213	0.4204
67337	Cstf1	cleavage stimulation factor, 3' pre-RNA, subunit 1	1.22880315	0.007187	0.0433
102141	Snx25	sorting nexin 25	1.22880315	0.3095	0.5193
14584	Gfpt2	glutamine fructose-6-phosphate transaminase 2	1.22865217	0.0815	0.217
20846	Stat1	signal transducer and activator of transcription 1	1.22850123	0.01328	0.06418
83962	Btbd1	BTB (POZ) domain containing 1	1.22850123	0.02876	0.1071
15199	Hebp1	heme binding protein 1	1.22835033	0.01426	0.0673
105450	Mmrn2	multimerin 2	1.22835033	0.08	0.2145
67338	Rffl	ring finger and FYVE like domain containing protein	1.22819946	0.1418	0.3124
54199	Ccrl2	chemokine (C-C motif) receptor-like 2	1.22804863	0.2045	0.3982
218518	Marveld2	MARVEL (membrane-associating) domain containing 2	1.22774708	0.06243	0.1805
70693	Gpr125	G protein-coupled receptor 125	1.22774708	0.08383	0.2211
15361	Hmga1	high mobility group AT-hook 1	1.22759637	0.05353	0.1627
101488	Slco2b1	solute carrier organic anion transporter family, member 2b1	1.22759637	0.09364	0.2378
51801	Ramp1	receptor (calcitonin) activity modifying protein 1	1.22744569	0.02073	0.08604
56173	Cldn14	claudin 14	1.22744569	0.5418	0.7246
100126228	Gm9856	predicted gene 9856	1.22729504	0.01804	0.07819
18793	Plaur	plasminogen activator, urokinase receptor	1.22729504	0.2022	0.3949
20465	Sim2	single-minded homolog 2 (Drosophila)	1.22729504	0.3735	0.5822
73910	Arhgap18	Rho GTPase activating protein 18	1.22714443	0.02198	0.08959
71607	Snx20	sorting nexin 20	1.22714443	0.0338	0.1191
57279	Slc25a20	solute carrier family 25 (mitochondrial carnitine/acylcarnitine translocase), member 20	1.22699387	0.03675	0.1258
68743	Anln	anillin, actin binding protein	1.22699387	0.1778	0.3629
140499	Ube2j2	ubiquitin-conjugating enzyme E2, J2 homolog (yeast)	1.22684333	0.007361	0.04386
170728	Rtn4ip1	reticulon 4 interacting protein 1	1.22639195	0.04503	0.1438
228094	Cerkl	ceramide kinase-like	1.22639195	0.2211	0.4201
17301	Foxd2	forkhead box D2	1.22579063	0.09308	0.237
16578	Kif9	kinesin family member 9	1.22579063	0.1464	0.3191
109342	Slc5a10	solute carrier family 5 (sodium/glucose cotransporter), member 10	1.22579063	0.3995	0.6072
51793	Ddah2	dimethylarginine dimethylaminohydrolase 2	1.2256404	0.01221	0.06066
67884	1810043G02Rik	RIKEN cDNA 1810043G02 gene	1.2256404	0.03783	0.1282
277743	Fam131c	family with sequence similarity 131, member C	1.2256404	0.1847	0.3719
434438	Ccdc36	coiled-coil domain containing 36	1.2256404	0.3688	0.5779
101602	AI467606	expressed sequence AI467606	1.2254902	0.05126	0.1577
117149	Tirap	toll-interleukin 1 receptor (TIR) domain-containing adaptor protein	1.22534003	0.007749	0.04531

74756	5830408B19Rik	RIKEN cDNA 5830408B19 gene	1.22534003	0.03273	0.1168
218865	Chdh	choline dehydrogenase	1.2251899	0.0526	0.1606
19118	Prm1	protamine 1	1.2251899	0.6689	0.8139
226861	Hhat	hedgehog acyltransferase	1.22503981	0.07018	0.1954
321022	Cdv3	carnitine deficiency-associated gene expressed in ventricle 3	1.22503981	0.2389	0.4413
100040382	Gm2745	predicted gene 2745	1.22488976	0.3353	0.5457
58238	Fam181b	family with sequence similarity 181, member B	1.22458976	0.06004	0.1754
74761	Mxra8	matrix-remodelling associated 8	1.22458976	0.308	0.5177
171281	Acot3	acyl-CoA thioesterase 3	1.22443982	0.05502	0.1659
223453	Dap	death-associated protein	1.22428991	0.1418	0.3124
214424	Parp16	poly (ADP-ribose) polymerase family, member 16	1.22414004	0.02953	0.109
381678	Zcwpw1	zinc finger, CW type with PWWP domain 1	1.22414004	0.03784	0.1282
12334	Capn2	calpain 2	1.22384041	0.006448	0.04015
11423	Ache	acetylcholinesterase	1.22384041	0.1098	0.2637
268420	Alkbh5	alkB, alkylation repair homolog 5 (E. coli)	1.22384041	0.1432	0.3143
58182	Prokr1	prokineticin receptor 1	1.22384041	0.5902	0.7601
192212	Prom2	prominin 2	1.22369065	0.8194	0.9066
71137	Rfx4	regulatory factor X, 4 (influences HLA class II expression)	1.22354093	0.4066	0.6137
66606	Lrrc57	leucine rich repeat containing 57	1.22324159	0.02356	0.09363
50779	Rgs6	regulator of G-protein signaling 6	1.22309198	0.005576	0.03661
93695	Gpnmb	glycoprotein (transmembrane) nmb	1.22309198	0.2791	0.4872
12499	Entpd5	ectonucleoside triphosphate diphosphohydrolase 5	1.2229424	0.004858	0.03358
240660	Tmem20	transmembrane protein 20	1.2229424	0.01222	0.06069
101148	B630005N14Rik	RIKEN cDNA B630005N14 gene	1.2229424	0.0424	0.1381
75784	1700007G11Rik	RIKEN cDNA 1700007G11 gene	1.2229424	0.08131	0.2167
11945	Atp4b	ATPase, H+/K+ exchanging, beta polypeptide	1.22279286	0.2404	0.4429
228545	Vps18	vacuolar protein sorting 18 (yeast)	1.22264335	0.08677	0.2262
241769	Kcnk15	potassium channel, subfamily K, member 15	1.22264335	0.08865	0.2293
26921	Map4k4	mitogen-activated protein kinase kinase kinase kinase 4	1.22249389	0.01937	0.082
54135	Lsr	lipolysis stimulated lipoprotein receptor	1.22249389	0.02431	0.09545
320135	BC049715	cDNA sequence BC049715	1.22249389	0.1511	0.3258
13865	Nr2f1	nuclear receptor subfamily 2, group F, member 1	1.22234446	0.006118	0.03875
99503	AA517023	expressed sequence AA517023	1.22234446	0.2307	0.4316
64658	Mrps25	mitochondrial ribosomal protein S25	1.2220457	0.002164	0.02031
26459	Slc27a5	solute carrier family 27 (fatty acid transporter), member 5	1.2220457	0.03735	0.1272
11979	Atp7b	ATPase, Cu++ transporting, beta polypeptide	1.2220457	0.0885	0.2291
20401	Sh3bp1	SH3-domain binding protein 1	1.2220457	0.1516	0.3264
75695	Rilpl1	Rab interacting lysosomal protein-like 1	1.22189638	0.01215	0.06055
66473	Ctrb1	chymotrypsinogen B1	1.22189638	0.1379	0.3062
11837	Rplp0	ribosomal protein, large, P0	1.2217471	0.007106	0.04298
75212	Rnf121	ring finger protein 121	1.22159785	0.003958	0.02955
100702	Mpa2l	macrophage activation 2 like	1.22159785	0.2264	0.4262
233186	Siglec5	sialic acid binding Ig-like lectin 5	1.22144864	0.1577	0.335
52430	Echdc2	enoyl Coenzyme A hydratase domain containing 2	1.22129946	0.05446	0.1648
63955	Cables1	CDK5 and Abl enzyme substrate 1	1.22129946	0.08098	0.216
19701	Ren1	renin 1 structural	1.22115032	0.13	0.2957
74012	Rap2b	RAP2B, member of RAS oncogene family	1.22100122	0.1584	0.3359
74268	Aven	apoptosis, caspase activation inhibitor	1.22085215	0.01354	0.06483
14198	Fhit	fragile histidine triad gene	1.22085215	0.1713	0.354
320135	BC049715	cDNA sequence BC049715	1.22085215	0.1899	0.3788

68180	Hyi	hydroxypyruvate isomerase homolog (E. coli)	1.22070313	0.006313	0.03967
70333	Cd3eap	CD3E antigen, epsilon polypeptide associated protein	1.22070313	0.006784	0.0416
16562	Kif1c	kinesin family member 1C	1.22055413	0.005068	0.03443
14872	Gstt2	glutathione S-transferase, theta 2	1.22055413	0.04019	0.1332
20729	Spin1	spindlin 1	1.22040517	0.01836	0.07916
52466	Slc46a1	solute carrier family 46, member 1	1.22040517	0.07208	0.1993
21808	Tgfb2	transforming growth factor, beta 2	1.22025625	0.003322	0.0265
73710	Tubb2b	tubulin, beta 2B	1.22010737	0.01037	0.05448
77115	6030451C04Rik	RIKEN cDNA 6030451C04 gene	1.22010737	0.2081	0.4028
29811	Ndrg2	N-myc downstream regulated gene 2	1.21995852	0.007486	0.0443
245532	Awat2	acyl-CoA wax alcohol acyltransferase 2	1.21980971	0.2064	0.4009
76646	Wdr38	WD repeat domain 38	1.21980971	0.3411	0.5516
109754	Cyb5r3	cytochrome b5 reductase 3	1.2195122	0.01332	0.06429
19317	Qk	quaking	1.2195122	0.137	0.3046
12363	Casp4	caspase 4, apoptosis-related cysteine peptidase	1.2195122	0.5398	0.7232
17294	Mest	mesoderm specific transcript	1.21936349	0.01223	0.06074
102103	Mtus1	mitochondrial tumor suppressor 1	1.21936349	0.03553	0.1232
67711	Nsmce1	non-SMC element 1 homolog (S. cerevisiae)	1.2190662	0.01404	0.06652
102141	Snx25	sorting nexin 25	1.2190662	0.01529	0.07045
78294	Rps27a	ribosomal protein S27A	1.2190662	0.04257	0.1385
74140	Tm9sf1	transmembrane 9 superfamily member 1	1.2190662	0.09082	0.2331
226252	Fam160b1	family with sequence similarity 160, member B1	1.2189176	0.02279	0.09167
233733	Galntl4	UDP-N-acetyl-alpha-D-galactosamine:polypeptide N-acetylgalactosaminyltransferase-like 4	1.2189176	0.05554	0.1669
77209	8030453O22Rik	RIKEN cDNA 8030453O22 gene	1.21876904	0.2739	0.4818
381694	B3galtl	beta 1,3-galactosyltransferase-like	1.21862052	0.03259	0.1165
381560	Xkr8	X Kell blood group precursor related family member 8 homolog	1.21862052	0.05145	0.1582
56760	Clec1b	C-type lectin domain family 1, member b	1.21832359	0.2675	0.4744
109108	Slc30a9	solute carrier family 30 (zinc transporter), member 9	1.21817517	0.004384	0.03154
407816	BC023202	cDNA sequence BC023202	1.2180268	0.05451	0.1649
59050	Nsa2	NSA2 ribosome biogenesis homolog (S. cerevisiae)	1.21787846	0.002704	0.02329
14566	Gdf9	growth differentiation factor 9	1.21787846	0.1056	0.257
14678	Gnai2	guanine nucleotide binding protein (G protein), alpha inhibiting 2	1.21758188	0.002852	0.02406
218311	Zfp455	zinc finger protein 455	1.21758188	0.05796	0.1716
26885	Casp8ap2	caspase 8 associated protein 2	1.21743365	0.06307	0.1816
233335	Synm	synemin, intermediate filament protein	1.21728545	0.008284	0.04721
109889	Mzf1	myeloid zinc finger 1	1.21728545	0.03652	0.1252
101497	Plekhg2	pleckstrin homology domain containing, family G (with RhoGef domain) member 2	1.21713729	0.03356	0.1185
77533	C030034I22Rik	RIKEN cDNA C030034I22 gene	1.21713729	0.03695	0.1262
258285	Olfr122	olfactory receptor 122	1.21713729	0.6098	0.7744
14579	Gem	GTP binding protein (gene overexpressed in skeletal muscle)	1.21669303	0.05315	0.1618
26357	Abcg2	ATP-binding cassette, sub-family G (WHITE), member 2	1.21639703	0.01326	0.06411
21678	Tead3	TEA domain family member 3	1.21639703	0.02417	0.09514
15107	Hadh	hydroxyacyl-Coenzyme A dehydrogenase	1.21639703	0.03425	0.12
14751	Gpi1	glucose phosphate isomerase 1	1.21639703	0.05895	0.1734
24063	Spry1	sprouty homolog 1 (Drosophila)	1.21639703	0.07143	0.1979
14011	Etv6	ets variant gene 6 (TEL oncogene)	1.21639703	0.07447	0.2041
67554	Slc25a30	solute carrier family 25, member 30	1.21639703	0.07677	0.2083
56787	Ascl3	achaete-scute complex homolog 3 (Drosophila)	1.21639703	0.226	0.4257
235330	Ttc12	tetratricopeptide repeat domain 12	1.21624909	0.1345	0.3015
319236	9230105E10Rik	RIKEN cDNA 9230105E10 gene	1.21610118	0.3116	0.5214

633387	LOC633387	nucleophosmin-like	1.21580547	0.01459	0.06819
21665	Tdg	thymine DNA glycosylase	1.21580547	0.03238	0.116
23986	Peci	peroxisomal delta3, delta2-enoyl-Coenzyme A isomerase	1.21580547	0.2365	0.4383
19152	Prtn3	proteinase 3	1.21565767	0.2806	0.4888
54402	Stk19	serine/threonine kinase 19	1.21550991	0.004142	0.03046
381933	6430531B16Rik	RIKEN cDNA 6430531B16 gene	1.21550991	0.04618	0.1463
21825	Thbs1	thrombospondin 1	1.21550991	0.2326	0.4338
17869	Myc	myelocytomatosis oncogene	1.21536218	0.1395	0.3089
21917	Tmpo	thymopoietin	1.21521449	0.02169	0.08886
18176	Nras	neuroblastoma ras oncogene	1.21521449	0.02841	0.1062
67861	Akr1b10	aldo-keto reductase family 1, member B10 (aldose reductase)	1.21506683	0.02322	0.09265
69269	Scnm1	sodium channel modifier 1	1.21491921	0.1134	0.2696
21665	Tdg	thymine DNA glycosylase	1.21491921	0.2787	0.4868
252903	Ap1s3	adaptor-related protein complex AP-1, sigma 3	1.21477162	0.03585	0.1238
378937	Lrrc24	leucine rich repeat containing 24	1.21462407	0.05198	0.1594
69071	Tmem97	transmembrane protein 97	1.21447656	0.003819	0.02895
76263	Gstk1	glutathione S-transferase kappa 1	1.21447656	0.03812	0.1288
70083	Metrn	meteorin, glial cell differentiation regulator	1.21447656	0.07691	0.2086
15365	Hmga2-ps1	high mobility group AT-hook 2, pseudogene 1	1.21447656	0.08938	0.2304
12663	Chml	choroideremia-like	1.21447656	0.3591	0.5689
244141	Nars2	asparaginyl-tRNA synthetase 2 (mitochondrial)(putative)	1.21403424	0.1183	0.2773
11423	Ache	acetylcholinesterase	1.21403424	0.1742	0.358
98711	Rdh10	retinol dehydrogenase 10 (all-trans)	1.21388687	0.00471	0.03299
240084	Cchcr1	coiled-coil alpha-helical rod protein 1	1.21373953	0.07143	0.1979
19271	Ptprj	protein tyrosine phosphatase, receptor type, J	1.21359223	0.3768	0.5858
106407	Osta	organic solute transporter alpha	1.21329774	0.1045	0.2555
81489	Dnajb1	DnaJ (Hsp40) homolog, subfamily B, member 1	1.21315055	0.00177	NA
211798	Mfsd9	major facilitator superfamily domain containing 9	1.21315055	0.1169	0.275
622657	Gm6340	predicted pseudogene 6340	1.21315055	0.2121	0.408
632793	Vmn1r204	vomeronasal 1 receptor 204	1.2130034	0.275	0.4829
19155	Npepps	aminopeptidase puromycin sensitive	1.21285628	0.01058	0.05516
64540	Tspan4	tetraspanin 4	1.21285628	0.01406	0.06657
100039042	Gm2016	predicted gene 2016	1.21285628	0.08641	0.2257
72893	2900040C04Rik	RIKEN cDNA 2900040C04 gene	1.21270919	0.1315	0.2978
20649	Sntb1	syntrophin, basic 1	1.21256214	0.02244	0.09076
14864	Gstm3	glutathione S-transferase, mu 3	1.21256214	0.8184	0.9059
74155	Errfi1	ERBB receptor feedback inhibitor 1	1.21241513	0.01544	0.07088
11669	Aldh2	aldehyde dehydrogenase 2, mitochondrial	1.21226815	0.008039	0.04629
217864	Rcor1	REST corepressor 1	1.21226815	0.1391	0.3082
13664	Eif1a	eukaryotic translation initiation factor 1A	1.21212121	0.02247	0.09081
209086	Samd9l	sterile alpha motif domain containing 9-like	1.21212121	0.052	0.1594
17250	Abcc1	ATP-binding cassette, sub-family C (CFTR/MRP), member 1	1.21197431	0.001946	NA
12579	Cdkn2b	cyclin-dependent kinase inhibitor 2B (p15, inhibits CDK4)	1.21197431	0.2126	0.4085
100039181	LOC100039181	uncharacterized protein C4orf3 homolog	1.21182744	0.006193	0.03909
100040766	Heatr7b1	HEAT repeat containing 7B1	1.21182744	0.6292	0.7891
233335	Synm	synemin, intermediate filament protein	1.2116806	0.01918	0.08143
78586	Srbd1	S1 RNA binding domain 1	1.2116806	0.02573	0.09938
12097	Bglap2	bone gamma-carboxyglutamate protein 2	1.2116806	0.1104	0.2647
18173	Slc11a1	solute carrier family 11 (proton-coupled divalent metal ion transporters), member 1	1.2116806	0.2311	0.432
213211	Rnf26	ring finger protein 26	1.2115338	0.1544	0.3302

74245	Ctbs	chitobiase, di-N-acetyl-	1.21138704	0.02271	0.09144
228019	Mettl8	methyltransferase like 8	1.21138704	0.04792	0.1502
60611	Foxj2	forkhead box J2	1.21124031	0.09412	0.2386
320343	Lypd6	LY6/PLAUR domain containing 6	1.21065375	0.02882	0.1072
13714	Elk4	ELK4, member of ETS oncogene family	1.2105072	0.07903	0.2128
227289	Gpbar1	G protein-coupled bile acid receptor 1	1.21036069	0.0213	0.08773
72324	Plxdc1	plexin domain containing 1	1.21021421	0.02697	0.1025
230459	Cyp2j13	cytochrome P450, family 2, subfamily j, polypeptide 13	1.21021421	0.05459	0.165
106389	Eaf2	ELL associated factor 2	1.20992136	0.05833	0.1722
27222	Atp1a4	ATPase, Na+/K+ transporting, alpha 4 polypeptide	1.20992136	0.2943	0.5032
50779	Rgs6	regulator of G-protein signaling 6	1.20977498	0.0152	0.07017
67733	Itgb3bp	integrin beta 3 binding protein (beta3-endonexin)	1.20977498	0.02709	0.1028
16513	Kcnj10	potassium inwardly-rectifying channel, subfamily J, member 10	1.20977498	0.05425	0.1643
227720	Nup214	nucleoporin 214	1.20962864	0.1556	0.3322
74158	Josd1	Josephin domain containing 1	1.20948234	0.003805	0.02892
19353	Rac1	RAS-related C3 botulinum substrate 1	1.20948234	0.007479	0.04427
106952	Arap3	ArfGAP with RhoGAP domain, ankyrin repeat and PH domain 3	1.20948234	0.1342	0.3013
72938	Hspb11	heat shock protein family B (small), member 11	1.20918984	0.006537	0.04051
22121	Rpl13a	ribosomal protein L13A	1.20918984	0.02365	0.0939
57373	D930014E17Rik	RIKEN cDNA D930014E17 gene	1.20904365	0.02413	0.09508
26450	Rbbp9	retinoblastoma binding protein 9	1.20904365	0.05084	0.1568
78323	2310046O06Rik	RIKEN cDNA 2310046O06 gene	1.20889749	0.09113	0.2337
19823	Rnf7	ring finger protein 7	1.20875136	0.006823	0.04177
18643	Pfn1	profilin 1	1.20875136	0.2774	0.4857
170736	Parvb	parvin, beta	1.20860527	0.01928	0.08176
12554	Cdh13	cadherin 13	1.20860527	0.1081	0.2609
17254	Slc3a2	solute carrier family 3 (activators of dibasic and neutral amino acid transport), member 2	1.20845921	0.06617	0.1876
17201	Mc3r	melanocortin 3 receptor	1.20845921	0.0823	0.2184
105268	AU016916	expressed sequence AU016916	1.20845921	0.08878	0.2295
225579	Slc27a6	solute carrier family 27 (fatty acid transporter), member 6	1.20845921	0.09674	0.2429
140494	Atp6v0a4	ATPase, H+ transporting, lysosomal V0 subunit A4	1.20845921	0.254	0.4591
74142	Lonp1	lon peptidase 1, mitochondrial	1.20831319	0.005344	0.03567
100502982	LOC100502982	hypothetical LOC100502982	1.20816721	0.01909	0.08124
69538	Antxr1	anthrax toxin receptor 1	1.20802126	0.03555	0.1233
210135	Zfp180	zinc finger protein 180	1.20802126	0.03555	0.1233
230903	Fbxo44	F-box protein 44	1.20802126	0.1406	0.3104
319991	Kif6	kinesin family member 6	1.20802126	0.1631	0.3422
243025	Tmem156	transmembrane protein 156	1.20802126	0.2051	0.3989
12411	Cbs	cystathionine beta-synthase	1.20802126	0.2204	0.4196
14115	Fbln2	fibulin 2	1.20787535	0.01732	0.07591
12315	Calm3	calmodulin 3	1.20787535	0.02515	0.09777
108151	Sema3d	sema domain, immunoglobulin domain (Ig), short basic domain, secreted, (semaphorin) 3D	1.20787535	0.158	0.3352
16400	Itga3	integrin alpha 3	1.20787535	0.2286	0.4288
231093	Agbl5	ATP/GTP binding protein-like 5	1.20772947	0.04988	0.1548
327956	Vmo1	vitelline membrane outer layer 1 homolog (chicken)	1.20772947	0.0709	0.1969
652925	4930420K17Rik	RIKEN cDNA 4930420K17 gene	1.20758363	0.02343	0.09326
104479	Ccdc117	coiled-coil domain containing 117	1.20758363	0.05628	0.1683
67733	Itgb3bp	integrin beta 3 binding protein (beta3-endonexin)	1.20743782	0.01815	0.07852
211612	Ptchd1	patched domain containing 1	1.20743782	0.09678	0.243
18019	Nfatc2	nuclear factor of activated T-cells, cytoplasmic, calcineurin-dependent 2	1.20743782	0.1587	0.3362

76477	Pcolce2	procollagen C-endopeptidase enhancer 2	1.20729204	0.06304	0.1816
140577	Ankrd6	ankyrin repeat domain 6	1.20714631	0.2395	0.4419
11565	Adssl1	adenylosuccinate synthetase like 1	1.20685494	0.03046	0.1114
67844	Rab32	RAB32, member RAS oncogene family	1.20685494	0.03433	0.1202
18861	Pms2	postmeiotic segregation increased 2 (S. cerevisiae)	1.2067093	0.00295	NA
28135	Cep63	centrosomal protein 63	1.2067093	0.02313	0.09249
16367	Irs1	insulin receptor substrate 1	1.2067093	0.2624	0.4683
69219	Ddah1	dimethylarginine dimethylaminohydrolase 1	1.20656371	0.01731	0.0759
16768	Lag3	lymphocyte-activation gene 3	1.20656371	0.1033	0.2538
19848	Rnu2	U2 small nuclear RNA	1.20656371	0.4073	0.6142
116811	Zim3	zinc finger, imprinted 3	1.20641814	0.6165	0.7793
408070	B930036G03Rik	RIKEN cDNA B930036G03 gene	1.20627262	0.001009	NA
74843	Zmynd17	zinc finger, MYND domain containing 17	1.20627262	0.1487	0.3226
67819	Derl1	Der1-like domain family, member 1	1.20612713	0.01236	0.06117
14708	Gng7	guanine nucleotide binding protein (G protein), gamma 7	1.20612713	0.09874	0.2464
12845	Comp	cartilage oligomeric matrix protein	1.20612713	0.19	0.3788
170743	Tlr7	toll-like receptor 7	1.20612713	0.4457	0.6484
229707	Fam40a	family with sequence similarity 40, member A	1.20598167	0.002188	NA
218454	Lhfpl2	lipoma HMGIC fusion partner-like 2	1.20598167	0.004682	0.03285
70579	Zc3h11a	zinc finger CCCH type containing 11A	1.20583625	0.005873	0.03778
67013	Oma1	OMA1 homolog, zinc metallopeptidase (S. cerevisiae)	1.20583625	0.006691	0.04118
100039707	Gm2382	predicted gene 2382	1.20583625	0.0182	0.07868
16413	Itgb1bp1	integrin beta 1 binding protein 1	1.20583625	0.2311	0.432
194655	Klf11	Kruppel-like factor 11	1.20583625	0.406	0.6135
13607	Eda	ectodysplasin-A	1.20583625	0.4134	0.6198
215493	A3galt2	alpha 1,3-galactosyltransferase 2 (isoglobotriaosylceramide synthase)	1.20569086	0.1377	0.3058
230279	6330416G13Rik	RIKEN cDNA 6330416G13 gene	1.20569086	0.212	0.4079
73863	4930415O20Rik	RIKEN cDNA 4930415O20 gene	1.20569086	0.2617	0.4675
67282	Ccdc53	coiled-coil domain containing 53	1.20554551	0.01553	0.07111
105348	Golm1	golgi membrane protein 1	1.20554551	0.03055	0.1115
381306	BC055324	cDNA sequence BC055324	1.20554551	0.03438	0.1203
108655	Foxp1	forkhead box P1	1.20554551	0.04791	0.1502
97640	C78653	expressed sequence C78653	1.20554551	0.3635	0.5725
16416	Itgb3	integrin beta 3	1.20554551	0.386	0.5947
70024	Mcm10	minichromosome maintenance deficient 10 (S. cerevisiae)	1.20540019	0.02115	0.08725
66365	Ccdc90b	coiled-coil domain containing 90B	1.20540019	0.05098	0.157
213673	9530068E07Rik	RIKEN cDNA 9530068E07 gene	1.20525491	0.001075	NA
71720	Osbpl3	oxysterol binding protein-like 3	1.20525491	0.0315	0.1138
60613	Kcnq4	potassium voltage-gated channel, subfamily Q, member 4	1.20496445	0.02069	0.08595
433375	Creg1	cellular repressor of E1A-stimulated genes 1	1.20496445	0.03542	0.123
673094	Cd99	CD99 antigen	1.20481928	0.03743	0.1273
104027	Synpo	synaptopodin	1.20481928	0.1748	0.3588
66222	Serpinb1a	serine (or cysteine) peptidase inhibitor, clade B, member 1a	1.20481928	0.2276	0.4278
57811	Rgr	retinal G protein coupled receptor	1.20481928	0.4438	0.6467
319195	Rpl17	ribosomal protein L17	1.20452903	0.1438	0.3154
11730	Ang3	angiogenin, ribonuclease A family, member 3	1.20452903	0.3065	0.5159
18221	Nudc	nuclear distribution gene C homolog (Aspergillus)	1.20438396	0.03975	0.1322
56356	Gltp	glycolipid transfer protein	1.20423892	0.08631	0.2255
69765	1500004F05Rik	RIKEN cDNA 1500004F05 gene	1.20409392	0.01483	0.06887
97848	Serpinb6c	serine (or cysteine) peptidase inhibitor, clade B, member 6c	1.20409392	0.03609	0.1244

319370	Fam100b	family with sequence similarity 100, member B	1.20394895	0.005267	0.03533
27973	Vkorc1	vitamin K epoxide reductase complex, subunit 1	1.20380402	0.004951	NA
78309	Cul9	cullin 9	1.20380402	0.09096	0.2334
22240	Dpysl3	dihydropyrimidinase-like 3	1.20380402	0.092	0.2352
20974	Syngr3	synaptogyrin 3	1.20365912	0.0823	0.2184
68859	1190007F08Rik	RIKEN cDNA 1190007F08 gene	1.20365912	0.1529	0.3283
74347	4632415K11Rik	RIKEN cDNA 4632415K11 gene	1.20351426	0.03131	0.1134
14579	Gem	GTP binding protein (gene overexpressed in skeletal muscle)	1.20351426	0.06482	0.1853
268490	Lsm12	LSM12 homolog (S. cerevisiae)	1.20351426	0.07823	0.2111
20357	Sema5b	sema domain, seven thrombospondin repeats (type 1 and type 1-like), transmembrane domain (TM) and short cytoplasmic domain, (semaphorin) 5B	1.20351426	0.09298	0.2368
23827	Bpnt1	bisphosphate 3'-nucleotidase 1	1.20351426	0.1345	0.3015
329421	Myo3b	myosin IIIB	1.20336943	0.1068	0.2588
193796	Kdm4b	lysine (K)-specific demethylase 4B	1.20322464	0.1304	0.2961
16510	Kcnh1	potassium voltage-gated channel, subfamily H (eag-related), member 1	1.20322464	0.2811	0.4892
100042834	Gm4055	predicted gene 4055	1.20307988	0.07922	0.2131
99377	Sall4	sal-like 4 (Drosophila)	1.20307988	0.09737	0.2438
26568	Slc27a3	solute carrier family 27 (fatty acid transporter), member 3	1.20293516	0.002152	NA
66077	Aurkaip1	aurora kinase A interacting protein 1	1.20293516	0.01565	0.07134
11553	Adra2c	adrenergic receptor, alpha 2c	1.20293516	0.05071	0.1565
246710	Rhobtb2	Rho-related BTB domain containing 2	1.20279047	0.05378	0.1633
105083	Pelo	pelota homolog (Drosophila)	1.20264582	0.01823	0.07871
77889	Lbh	limb-bud and heart	1.20264582	0.02366	0.0939
243780	E330009J07Rik	RIKEN cDNA E330009J07 gene	1.20264582	0.04303	0.1393
231672	Fbxw8	F-box and WD-40 domain protein 8	1.20264582	0.1235	0.2859
64295	Tmub1	transmembrane and ubiquitin-like domain containing 1	1.2025012	0.003465	NA
73296	Rhobtb3	Rho-related BTB domain containing 3	1.2025012	0.0165	0.07359
52585	Dhrs1	dehydrogenase/reductase (SDR family) member 1	1.2025012	0.03114	0.1129
16985	Lsp1	lymphocyte specific 1	1.20221207	0.01995	0.08371
76573	1700027D21Rik	RIKEN cDNA 1700027D21 gene	1.20221207	0.064	0.1836
20932	Surf4	surfeit gene 4	1.20206756	0.01187	0.05975
27060	Tcirg1	T-cell, immune regulator 1, ATPase, H+ transporting, lysosomal V0 protein A3	1.20206756	0.1188	0.2783
320244	Ttll5	tubulin tyrosine ligase-like family, member 5	1.20206756	0.1886	0.3768
18753	Prkcd	protein kinase C, delta	1.20192308	0.03349	0.1184
432754	LOC432754	similar to glyceraldehyde-3-phosphate dehydrogenase	1.20192308	0.235	0.4366
15374	Hn1	hematological and neurological expressed sequence 1	1.20163422	0.05261	0.1606
207818	Smagp	small cell adhesion glycoprotein	1.20163422	0.1206	0.2813
320226	4930473A06Rik	RIKEN cDNA 4930473A06 gene	1.20148985	0.3659	0.575
171279	Vmn1r216	vomeronasal 1 receptor 216	1.20148985	0.4909	0.6851
11641	Akap2	A kinase (PRKA) anchor protein 2	1.20134551	0.1	0.2485
56693	Crtap	cartilage associated protein	1.20091269	0.1172	0.2754
11433	Acp5	acid phosphatase 5, tartrate resistant	1.20091269	0.2343	0.436
13527	Dtna	dystrobrevin alpha	1.20048019	0.01239	0.06125
69071	Tmem97	transmembrane protein 97	1.20048019	0.02449	0.09601
11837	Rplp0	ribosomal protein, large, P0	1.20048019	0.02527	0.09807
71585	9130403I23Rik	RIKEN cDNA 9130403I23 gene	1.20048019	0.1032	0.2536
72083	Mzt2	mitotic spindle organizing protein 2	1.20033609	0.01043	0.05471
14158	Fert2	fer (fms/fps related) protein kinase, testis specific 2	1.20033609	0.06484	0.1853
20598	Smpd2	sphingomyelin phosphodiesterase 2, neutral	1.20019203	0.05477	0.1653
258662	Olfr738	olfactory receptor 738	1.20019203	0.4693	0.6687
73363	1700056E22Rik	RIKEN cDNA 1700056E22 gene	1.200048	0.0183	0.07896

77312	C030010L15Rik	RIKEN cDNA C030010L15 gene	1.200048	0.2303	0.4313
102414	Clk3	CDC-like kinase 3	1.19990401	0.01686	0.07453
11541	Adora2b	adenosine A2b receptor	1.19990401	0.04779	0.15
209378	Itih5	inter-alpha (globulin) inhibitor H5	1.19990401	0.2453	0.4489
13589	Mapre1	microtubule-associated protein, RP/EB family, member 1	1.19947223	0.01529	0.07045
114663	Impa2	inositol (myo)-1(or 4)-monophosphatase 2	1.19947223	0.02424	0.09531
100503619	LOC100503619	hypothetical LOC100503619	1.19932838	0.1218	0.2834
66191	Ier3ip1	immediate early response 3 interacting protein 1	1.19918455	0.01915	0.08132
74753	5830415F09Rik	RIKEN cDNA 5830415F09 gene	1.19918455	0.08688	0.2264
53417	Hif3a	hypoxia inducible factor 3, alpha subunit	1.19918455	0.3075	0.5172
78244	Dnajc21	DnaJ (Hsp40) homolog, subfamily C, member 21	1.19904077	0.1174	0.2758
12517	Cd72	CD72 antigen	1.19889701	0.04278	0.1388
244650	Phlpp2	PH domain and leucine rich repeat protein phosphatase 2	1.19889701	0.1289	0.2942
231130	Tnip2	TNFAIP3 interacting protein 2	1.1987533	0.004094	NA
238037	BC068281	cDNA sequence BC068281	1.1987533	0.1232	0.2855
244238	Mrgpre	MAS-related GPR, member E	1.19846596	0.1054	0.2568
68591	Mocos	molybdenum cofactor sulfurase	1.19846596	0.1317	0.298
235415	Cplx3	complexin 3	1.19846596	0.2311	0.432
71213	Cage1	cancer antigen 1	1.19832235	0.1507	0.3255
214189	Scgn	secretagogin, EF-hand calcium binding protein	1.19832235	0.1916	0.3812
319887	E030030I06Rik	RIKEN cDNA E030030I06 gene	1.19817877	0.01165	0.05906
21646	Tcte2	t-complex-associated testis expressed 2	1.19817877	0.2005	0.3927
230972	Arhgef16	Rho guanine nucleotide exchange factor (GEF) 16	1.19803522	0.1156	0.273
93686	Rbfox2	RNA binding protein, fox-1 homolog (C. elegans) 2	1.19803522	0.1325	0.299
23890	Gpr34	G protein-coupled receptor 34	1.19803522	0.1857	0.3731
67457	Frmd8	FERM domain containing 8	1.19789171	0.02803	0.1052
53378	Sdcbp	syndecan binding protein	1.19789171	0.1256	0.2889
75284	Bcdin3d	BCDIN3 domain containing	1.19774823	0.01647	0.07356
117599	Helb	helicase (DNA) B	1.19774823	0.02846	0.1063
100043823	Gm4673	predicted gene 4673	1.19774823	0.1641	0.3438
234378	Klhl26	kelch-like 26 (Drosophila)	1.19760479	0.05751	0.1707
24055	Sh3bp2	SH3-domain binding protein 2	1.19746138	0.01445	0.0678
114643	Oas1c	2'-5' oligoadenylate synthetase 1C	1.19731801	0.04886	0.1525
66431	1810049H13Rik	RIKEN cDNA 1810049H13 gene	1.19717467	0.03802	0.1286
213233	Tapbpl	TAP binding protein-like	1.19717467	0.07675	0.2083
224170	Dzip3	DAZ interacting protein 3, zinc finger	1.19717467	0.105	0.2562
21990	Tph1	tryptophan hydroxylase 1	1.19717467	0.1919	0.3815
226351	Tmem185b	transmembrane protein 185B	1.19703136	0.02775	0.1044
12348	Car11	carbonic anhydrase 11	1.19703136	0.0641	0.1838
15561	Htr3a	5-hydroxytryptamine (serotonin) receptor 3A	1.19703136	0.272	0.4794
94185	Tnfrsf21	tumor necrosis factor receptor superfamily, member 21	1.19688809	0.08021	0.2148
69146	Gsdmd	gasdermin D	1.19688809	0.1067	0.2585
17960	Nat1	N-acetyl transferase 1	1.19688809	0.1959	0.3865
245038	Dclk3	doublecortin-like kinase 3	1.19688809	0.2249	0.4246
18260	Ocln	occludin	1.19688809	0.3744	0.5832
14950	H13	histocompatibility 13	1.19674485	0.03646	0.1251
235256	Olfr149	olfactory receptor 149	1.19674485	0.6412	0.797
242819	Rundc3b	RUN domain containing 3B	1.19631535	0.05506	0.166
66101	Ppih	peptidyl prolyl isomerase H	1.19631535	0.08401	0.2214
215615	Rnpep	arginyl aminopeptidase (aminopeptidase B)	1.19617225	0.1227	0.2847

20132	Rrh	retinal pigment epithelium derived rhodopsin homolog	1.19602918	0.07117	0.1974
140570	Plxnb2	plexin B2	1.19602918	0.1468	0.3195
67106	Zbtb8os	zinc finger and BTB domain containing 8 opposite strand	1.19588615	0.03462	0.1209
12490	Cd34	CD34 antigen	1.19588615	0.06632	0.1879
57896	Krcc1	lysine-rich coiled-coil 1	1.19560019	0.06905	0.1934
17229	Tpsb2	tryptase beta 2	1.19560019	0.4536	0.6553
235330	Ttc12	tetratricopeptide repeat domain 12	1.19545726	0.02816	0.1056
15896	Icam2	intercellular adhesion molecule 2	1.19531437	0.04326	0.1397
100049569	A730015C16Rik	RIKEN cDNA A730015C16 gene	1.19531437	0.3096	0.5193
100041121	Gm10144	predicted pseudogene 10144	1.19531437	0.3629	0.5721
83453	Chrdl1	chordin-like 1	1.19502868	0.01383	0.06583
66240	Kcne1l	potassium voltage-gated channel, Isk-related family, member 1-like, pseudogene	1.19502868	0.1058	0.2573
235587	Parp3	poly (ADP-ribose) polymerase family, member 3	1.19488589	0.1472	0.3202
77609	Ccdc151	coiled-coil domain containing 151	1.19474313	0.01455	0.06815
230868	Igsf21	immunoglobin superfamily, member 21	1.19460041	0.005456	NA
29873	Cspg5	chondroitin sulfate proteoglycan 5	1.19460041	0.05727	0.1702
19698	Relb	avian reticuloendotheliosis viral (v-rel) oncogene related B	1.19460041	0.1406	0.3104
68545	Ecscr	endothelial cell-specific chemotaxis regulator	1.19445772	0.109	0.2624
19065	Ppyr1	pancreatic polypeptide receptor 1	1.19431506	0.4177	0.6239
56838	Ccl28	chemokine (C-C motif) ligand 28	1.19431506	0.4775	0.6746
218952	Fermt2	fermitin family homolog 2 (Drosophila)	1.19417244	0.03453	0.1207
77053	Sun1	Sad1 and UNC84 domain containing 1	1.19417244	0.06303	0.1816
58175	Rgs20	regulator of G-protein signaling 20	1.19417244	0.1266	0.2904
18624	Pepd	peptidase D	1.19402985	0.01144	0.05824
16840	Lect1	leukocyte cell derived chemotaxin 1	1.19402985	0.06495	0.1854
434204	Whamm	WAS protein homolog associated with actin, golgi membranes and microtubules	1.19402985	0.3487	0.5591
12798	Cnn2	calponin 2	1.1938873	0.09392	0.2382
207911	Mchr1	melanin-concentrating hormone receptor 1	1.1938873	0.3607	0.5703
59052	Mettl9	methyltransferase like 9	1.19374478	0.01953	0.08247
56486	Gabarap	gamma-aminobutyric acid receptor associated protein	1.19374478	0.09711	0.2434
320923	Mtap7d3	MAP7 domain containing 3	1.19374478	0.1565	0.3334
13537	Dusp2	dual specificity phosphatase 2	1.19360229	0.2346	0.4363
67885	1500011K16Rik	RIKEN cDNA 1500011K16 gene	1.19345984	0.05505	0.166
13025	Ctla2b	cytotoxic T lymphocyte-associated protein 2 beta	1.19345984	0.06267	0.181
227095	Hibch	3-hydroxyisobutyryl-Coenzyme A hydrolase	1.19331742	0.01246	0.06146
100900	Hscb	HscB iron-sulfur cluster co-chaperone homolog (E. coli)	1.19331742	0.02711	0.1028
108837	Ibtk	inhibitor of Bruton agammaglobulinemia tyrosine kinase	1.19331742	0.03455	0.1207
23972	Papss2	3'-phosphoadenosine 5'-phosphosulfate synthase 2	1.19331742	0.05604	0.168
15191	Hdgf	hepatoma-derived growth factor	1.19317504	0.006621	NA
217340	Rnf157	ring finger protein 157	1.19303269	0.08417	0.2217
20608	Sstr4	somatostatin receptor 4	1.19303269	0.301	0.5104
11783	Apaf1	apoptotic peptidase activating factor 1	1.19289037	0.07795	0.2106
68845	Pih1d1	PIH1 domain containing 1	1.19274809	0.02154	0.08843
27027	Tspan32	tetraspanin 32	1.19274809	0.09952	0.2476
20378	Frzb	frizzled-related protein	1.19232145	0.02019	0.08431
12558	Cdh2	cadherin 2	1.19203719	0.08869	0.2294
107271	Yars	tyrosyl-tRNA synthetase	1.19175307	0.1407	0.3105
66075	Chchd3	coiled-coil-helix-coiled-coil-helix domain containing 3	1.19161106	0.009522	NA
20335	Sec61g	SEC61, gamma subunit	1.19161106	0.01325	0.06411
232089	Elmod3	ELMO/CED-12 domain containing 3	1.19161106	0.01608	0.07239

75686	Nudt16	nudix (nucleoside diphosphate linked moiety X)-type motif 16	1.19161106	0.03829	0.1292
18802	Plcd4	phospholipase C, delta 4	1.19161106	0.05035	0.1558
100503637	LOC100503637	envelope glycoprotein-like	1.19161106	0.06209	0.1798
22259	Nr1h3	nuclear receptor subfamily 1, group H, member 3	1.19161106	0.1144	0.2713
13166	Dbh	dopamine beta hydroxylase	1.19161106	0.3109	0.5208
19340	Rab3d	RAB3D, member RAS oncogene family	1.19146908	0.1148	0.272
218734	3830406C13Rik	RIKEN cDNA 3830406C13 gene	1.19146908	0.1443	0.3161
239273	Abcc4	ATP-binding cassette, sub-family C (CFTR/MRP), member 4	1.19118523	0.06648	0.1882
73721	1110017D15Rik	RIKEN cDNA 1110017D15 gene	1.19118523	0.3214	0.5312
69066	1810010H24Rik	RIKEN cDNA 1810010H24 gene	1.19104335	0.07799	0.2107
17164	Mapkapk2	MAP kinase-activated protein kinase 2	1.19104335	0.09547	0.2411
66487	2010107H07Rik	RIKEN cDNA 2010107H07 gene	1.19090151	0.006145	NA
320039	A030010E16Rik	RIKEN cDNA A030010E16 gene	1.19090151	0.07603	0.207
319480	Itga11	integrin alpha 11	1.19090151	0.1514	0.3261
76407	Sun5	Sad1 and UNC84 domain containing 5	1.1907597	0.1058	0.2573
100043060	Gm4200	predicted gene 4200	1.19061793	0.1068	0.2588
320790	Chd7	chromodomain helicase DNA binding protein 7	1.19019281	0.02266	0.09134
207728	Pde2a	phosphodiesterase 2A, cGMP-stimulated	1.19019281	0.0459	0.1457
14751	Gpi1	glucose phosphate isomerase 1	1.19019281	0.06177	0.1793
237221	Gemin8	gem (nuclear organelle) associated protein 8	1.189768	0.01263	0.06211
19255	Ptpn2	protein tyrosine phosphatase, non-receptor type 2	1.189768	0.1994	0.3912
66659	Acp6	acid phosphatase 6, lysophosphatidic	1.18962646	0.003561	NA
219189	1300010F03Rik	RIKEN cDNA 1300010F03 gene	1.18962646	0.0363	0.1249
20476	Six6	sine oculis-related homeobox 6 homolog (Drosophila)	1.18962646	0.05407	0.1639
233016	Blvrb	biliverdin reductase B (flavin reductase (NADPH))	1.18948495	0.08743	0.2274
328788	Gm749	predicted gene 749	1.18948495	0.5301	0.7158
269954	Ttll13	tubulin tyrosine ligase-like family, member 13	1.18934348	0.1069	0.2588
56812	Dnajb2	DnaJ (Hsp40) homolog, subfamily B, member 2	1.18906064	0.04029	0.1335
16001	Igf1r	insulin-like growth factor I receptor	1.18906064	0.04455	0.1427
67399	Pdlim7	PDZ and LIM domain 7	1.18891927	0.05957	0.1746
22153	Tubb4	tubulin, beta 4	1.18891927	0.1406	0.3104
13388	Dll1	delta-like 1 (Drosophila)	1.18877794	0.04572	0.1454
76850	Eif2c4	eukaryotic translation initiation factor 2C, 4	1.18863663	0.04347	0.1401
269233	Fam171a1	family with sequence similarity 171, member A1	1.18863663	0.09125	0.2338
242037	Gm410	predicted gene 410	1.18863663	0.4884	0.6832
14467	Gbas	glioblastoma amplified sequence	1.18849536	0.03442	0.1204
435376	Atp6ap1l	ATPase, H+ transporting, lysosomal accessory protein 1-like	1.18849536	0.08811	0.2285
73049	2900054C01Rik	RIKEN cDNA 2900054C01 gene	1.18821293	0.04828	0.1511
22186	Uba52	ubiquitin A-52 residue ribosomal protein fusion product 1	1.18821293	0.07905	0.2128
76936	Hnrnpm	heterogeneous nuclear ribonucleoprotein M	1.18807176	0.07651	0.208
192662	Arhgdia	Rho GDP dissociation inhibitor (GDI) alpha	1.18778952	0.03995	0.1327
171207	Arhgap4	Rho GTPase activating protein 4	1.18778952	0.0608	0.1771
18028	Nfib	nuclear factor I/B	1.18764846	0.02898	0.1076
16410	Itgav	integrin alpha V	1.18764846	0.09427	0.2389
12169	Bmx	BMX non-receptor tyrosine kinase	1.18722545	0.145	0.3172
329934	Foxo6	forkhead box O6	1.18694362	0.03324	0.1179
665669	Gm7742	predicted gene 7742	1.18694362	0.04985	0.1548
67605	Akt1s1	AKT1 substrate 1 (proline-rich)	1.18694362	0.1303	0.296
19340	Rab3d	RAB3D, member RAS oncogene family	1.18694362	0.2883	0.4967
100040353	2810416G20Rik	RIKEN cDNA 2810416G20 gene	1.18680275	0.07397	0.2031

319370	Fam100b	family with sequence similarity 100, member B	1.18666192	0.01248	NA
13138	Dag1	dystroglycan 1	1.18666192	0.04756	0.1494
72572	Spats2	spermatogenesis associated, serine-rich 2	1.18666192	0.09976	0.248
242960	Fbxl5	F-box and leucine-rich repeat protein 5	1.18652112	0.01666	0.07397
22754	Zfp92	zinc finger protein 92	1.18652112	0.1277	0.2922
229898	Gbp5	guanylate binding protein 5	1.18652112	0.1834	0.3706
216820	Dhrs7b	dehydrogenase/reductase (SDR family) member 7B	1.18623962	0.01964	0.08273
319164	Hist1h2ac	histone cluster 1, H2ac	1.18623962	0.2101	0.4057
74127	Krt80	keratin 80	1.18623962	0.3596	0.5692
65107	Lrp10	low-density lipoprotein receptor-related protein 10	1.18595825	0.03062	0.1117
14082	Fadd	Fas (TNFRSF6)-associated via death domain	1.18595825	0.1192	0.2792
100043736	Gm10762	predicted gene 10762	1.18595825	0.3508	0.561
216443	Mars	methionine-tRNA synthetase	1.18581762	0.007116	NA
11652	Akt2	thymoma viral proto-oncogene 2	1.18567702	0.02872	0.107
330189	Tmem120b	transmembrane protein 120B	1.18567702	0.02901	0.1077
11461	Actb	actin, beta	1.18567702	0.1745	0.3584
21336	Tacr1	tachykinin receptor 1	1.18567702	0.1843	0.3712
71275	4933437F05Rik	RIKEN cDNA 4933437F05 gene	1.18539592	0.02882	0.1072
68758	Abhd11	abhydrolase domain containing 11	1.18525542	0.03279	0.1169
20905	Sts	steroid sulfatase	1.18511496	0.2289	0.4292
243371	Lrrc61	leucine rich repeat containing 61	1.18497452	0.08608	0.2251
14062	F2r	coagulation factor II (thrombin) receptor	1.18497452	0.1593	0.3373
78177	Ninl	ninein-like	1.18483412	0.08707	0.2268
14824	Grn	granulin	1.18483412	0.0927	0.2364
21416	Tcf7l2	transcription factor 7-like 2, T-cell specific, HMG-box	1.18483412	0.1506	0.3255
338350	9330129D05Rik	RIKEN cDNA 9330129D05 gene	1.18469376	0.00332	NA
72748	Hdhd3	haloacid dehalogenase-like hydrolase domain containing 3	1.18469376	0.02708	0.1028
20719	Serpinb6a	serine (or cysteine) peptidase inhibitor, clade B, member 6a	1.18469376	0.04176	0.1367
77271	9430024F10Rik	RIKEN cDNA 9430024F10 gene	1.18469376	0.08171	0.2174
19719	Rfng	RFNG O-fucosylpeptide 3-beta-N-acetylglucosaminyltransferase	1.18455342	0.2	0.3918
636901	LOC636901	40S ribosomal protein SA-like	1.18441312	0.02407	0.09502
236069	Gm13238	predicted gene 13238	1.18441312	0.06843	0.192
442812	B930049P21Rik	RIKEN cDNA B930049P21 gene	1.18441312	0.09262	0.2362
69047	Atp2c2	ATPase, Ca++ transporting, type 2C, member 2	1.18441312	0.3078	0.5176
104725	1110002B05Rik	RIKEN cDNA 1110002B05 gene	1.18427286	0.01583	NA
14782	Gsr	glutathione reductase	1.18427286	0.05644	0.1686
64292	Ptges	prostaglandin E synthase	1.18427286	0.2292	0.4296
51944	D2Ertd750e	DNA segment, Chr 2, ERATO Doi 750, expressed	1.18427286	0.4132	0.6195
19823	Rnf7	ring finger protein 7	1.18385226	0.01618	NA
320926	C730029A08Rik	RIKEN cDNA C730029A08 gene	1.18385226	0.0696	0.1946
72050	Kdelc1	KDEL (Lys-Asp-Glu-Leu) containing 1	1.18385226	0.4624	0.6629
320348	C130051F05Rik	RIKEN cDNA C130051F05 gene	1.18371212	0.3166	0.526
75614	2610019E17Rik	RIKEN cDNA 2610019E17 gene	1.18371212	0.3482	0.5587
404331	Olfr1252	olfactory receptor 1252	1.18371212	0.5039	0.6954
414085	9330151L19Rik	RIKEN cDNA 9330151L19 gene	1.18357202	0.01554	NA
234796	Klhl36	kelch-like 36 (Drosophila)	1.18357202	0.1259	0.2892
71803	Slc25a18	solute carrier family 25 (mitochondrial carrier), member 18	1.18315192	0.2717	0.4791
12492	Scarb2	scavenger receptor class B, member 2	1.18301195	0.02456	0.09625
17974	Nck2	non-catalytic region of tyrosine kinase adaptor protein 2	1.18301195	0.0437	0.1408
66624	Spcs2	signal peptidase complex subunit 2 homolog (S. cerevisiae)	1.18301195	0.2468	0.4509

67177	Cdt1	chromatin licensing and DNA replication factor 1	1.18287201	0.1139	0.2704
78372	Snrnp25	small nuclear ribonucleoprotein 25 (U11/U12)	1.18273211	0.03405	0.1195
70225	Ppil3	peptidylprolyl isomerase (cyclophilin)-like 3	1.18273211	0.04331	0.1397
18453	P4hb	prolyl 4-hydroxylase, beta polypeptide	1.18259224	0.03095	0.1126
629557	Gm6981	glyceraldehyde-3-phosphate dehydrogenase pseudogene	1.18259224	0.08934	0.2304
26897	Acot1	acyl-CoA thioesterase 1	1.18245241	0.1277	0.2922
27384	Akr1c13	aldo-keto reductase family 1, member C13	1.18245241	0.1428	0.3136
72590	Ppme1	protein phosphatase methylesterase 1	1.18245241	0.1439	0.3155
214779	Zfp879	zinc figer protein 879	1.1823126	0.01223	NA
78560	Gpr124	G protein-coupled receptor 124	1.1823126	0.0173	NA
30046	Zfp292	zinc finger protein 292	1.1823126	0.1455	0.3181
270627	Taf1	TAF1 RNA polymerase II, TATA box binding protein (TBP)-associated factor	1.1823126	0.5695	0.7449
66510	Rnf181	ring finger protein 181	1.18217283	0.0173	NA
67169	Nradd	neurotrophin receptor associated death domain	1.18217283	0.1174	0.2758
231713	Naa25	N(alpha)-acetyltransferase 25, NatB auxiliary subunit	1.1820331	0.03225	0.1156
66175	Mustn1	musculoskeletal, embryonic nuclear protein 1	1.1820331	0.4123	0.6185
319186	Hist1h2bm	histone cluster 1, H2bm	1.18175372	0.05512	0.166
14453	Gas2	growth arrest specific 2	1.18147448	0.06936	0.194
72230	Zfp558	zinc finger protein 558	1.18147448	0.2996	0.509
19729	Slc50a1	solute carrier family 50 (sugar transporter), member 1	1.18133491	0.01319	NA
12972	Cryz	crystallin, zeta	1.18133491	0.02391	0.09465
233064	Wdr62	WD repeat domain 62	1.18119537	0.123	0.2852
17095	Lyl1	lymphoblastomic leukemia 1	1.18119537	0.1236	0.2861
118452	Baalc	brain and acute leukemia, cytoplasmic	1.18119537	0.1528	0.3281
20677	Sox4	SRY-box containing gene 4	1.18105586	0.02587	0.09966
59030	Mkks	McKusick-Kaufman syndrome protein	1.18091639	0.01067	NA
73379	Dcbld2	discoidin, CUB and LCCL domain containing 2	1.18091639	0.08928	0.2303
78781	Zc3hav1	zinc finger CCCH type, antiviral 1	1.18091639	0.3201	0.5301
110637	Grik4	glutamate receptor, ionotropic, kainate 4	1.18077695	0.01361	NA
16978	Lrrfip1	leucine rich repeat (in FLII) interacting protein 1	1.18063754	0.0303	0.111
16784	Lamp2	lysosomal-associated membrane protein 2	1.18063754	0.1156	0.273
70745	6330418B08Rik	RIKEN cDNA 6330418B08 gene	1.18049817	0.39	0.5983
99480	Dnttip2	deoxynucleotidyltransferase, terminal, interacting protein 2	1.18021952	0.04233	0.138
16006	Igfbp1	insulin-like growth factor binding protein 1	1.18021952	0.2425	0.4454
20091	Rps3a	ribosomal protein S3A	1.18021952	0.4069	0.6139
242022	Frem2	Fras1 related extracellular matrix protein 2	1.17980179	0.04581	0.1455
320946	A930035D04Rik	RIKEN cDNA A930035D04 gene	1.17980179	0.1644	0.3442
66176	Nat9	N-acetyltransferase 9 (GCN5-related, putative)	1.17966262	0.02977	0.1096
170770	Bbc3	BCL2 binding component 3	1.17966262	0.03283	0.1169
22003	Tpm1	tropomyosin 1, alpha	1.17966262	0.06208	0.1798
75299	4930547M16Rik	RIKEN cDNA 4930547M16 gene	1.17966262	0.1614	0.3398
74737	Pcf11	cleavage and polyadenylation factor subunit homolog (S. cerevisiae)	1.17966262	0.2931	0.5019
11569	Aebp2	AE binding protein 2	1.17952347	0.167	0.3479
100041184	Gm3187	predicted gene 3187	1.17952347	0.2932	0.502
238205	Lrfn5	leucine rich repeat and fibronectin type III domain containing 5	1.17952347	0.3281	0.5381
19416	Rasd1	RAS, dexamethasone-induced 1	1.17938436	0.1308	0.2968
70961	4921532D01Rik	RIKEN cDNA 4921532D01 gene	1.17938436	0.2109	0.4065
13038	Ctsk	cathepsin K	1.17924528	0.0338	0.1191
74666	4930432K21Rik	RIKEN cDNA 4930432K21 gene	1.17924528	0.08204	0.2181
214048	Larp1b	La ribonucleoprotein domain family, member 1B	1.17910624	0.04931	0.1535

67305	Gpx7	glutathione peroxidase 7	1.17910624	0.2108	0.4063
21354	Tap1	transporter 1, ATP-binding cassette, sub-family B (MDR/TAP)	1.17910624	0.376	0.585
231503	Tmem150c	transmembrane protein 150C	1.17896722	0.04452	0.1427
213603	Slc44a3	solute carrier family 44, member 3	1.17896722	0.5892	0.7593
21917	Tmpo	thymopoietin	1.17882824	0.02256	0.09103
236690	Nyx	nyctalopin	1.1786893	0.0344	0.1203
12684	Cideb	cell death-inducing DNA fragmentation factor, alpha subunit-like effector B	1.1786893	0.1972	0.3885
72901	2900011F02Rik	RIKEN cDNA 2900011F02 gene	1.17855038	0.04163	0.1364
105855	Nckap1l	NCK associated protein 1 like	1.17855038	0.06428	0.184
13838	Epha4	Eph receptor A4	1.17855038	0.06597	0.1872
14083	Ptk2	PTK2 protein tyrosine kinase 2	1.17855038	0.3536	0.5634
321022	Cdv3	carnitine deficiency-associated gene expressed in ventricle 3	1.1784115	0.009859	NA
13222	Defa-rs2	defensin, alpha, related sequence 2	1.1784115	0.1118	0.2669
70040	2610037D02Rik	RIKEN cDNA 2610037D02 gene	1.1784115	0.6315	0.7905
244421	Lonrf1	LON peptidase N-terminal domain and ring finger 1	1.17827265	0.06457	0.1847
13163	Daxx	Fas death domain-associated protein	1.17827265	0.1159	0.2734
68348	Serpina1f	serine (or cysteine) peptidase inhibitor, clade A, member 1F	1.17813384	0.4722	0.6705
12763	Cmah	cytidine monophospho-N-acetylneuraminic acid hydroxylase	1.17813384	0.7573	0.8701
57357	Srd5a3	steroid 5 alpha-reductase 3	1.17799505	0.08227	0.2184
218952	Fermt2	fermitin family homolog 2 (Drosophila)	1.1778563	0.01944	NA
64136	Sdf2l1	stromal cell-derived factor 2-like 1	1.1778563	0.1614	0.3398
60510	Syt9	synaptotagmin IX	1.17771758	0.01008	NA
381101	BC048355	cDNA sequence BC048355	1.17771758	0.04313	0.1395
58867	Syngr4	synaptogyrin 4	1.17771758	0.04706	0.1482
18736	Pou1f1	POU domain, class 1, transcription factor 1	1.17771758	0.1388	0.3077
53605	Nap1l1	nucleosome assembly protein 1-like 1	1.1775789	0.005581	NA
404311	Olfr209	olfactory receptor 209	1.1775789	0.4174	0.6236
67486	Polr3g	polymerase (RNA) III (DNA directed) polypeptide G	1.17744024	0.005484	NA
17955	Nap1l4	nucleosome assembly protein 1-like 4	1.17744024	0.02484	0.09694
66346	1700029P11Rik	RIKEN cDNA 1700029P11 gene	1.17744024	0.3162	0.5256
69287	Odf3	outer dense fiber of sperm tails 3	1.17744024	0.4156	0.6219
66536	Nipsnap3b	nipsnap homolog 3B (C. elegans)	1.17716304	0.04813	0.1506
77533	C030034I22Rik	RIKEN cDNA C030034I22 gene	1.17716304	0.07793	0.2106
56376	Pdlim5	PDZ and LIM domain 5	1.17702448	0.02172	NA
80752	Fam20c	family with sequence similarity 20, member C	1.17702448	0.1921	0.3818
93722	Pcdhga10	protocadherin gamma subfamily A, 10	1.17702448	0.2314	0.4322
23794	Adamts5	a disintegrin-like and metallopeptidase (reprolysin type) with thrombospondin type 1 motif, 5 (aggrecanase-2)	1.17688596	0.02802	0.1051
16408	Itgal	integrin alpha L	1.17688596	0.4448	0.6476
14588	Gfra4	glial cell line derived neurotrophic factor family receptor alpha 4	1.17688596	0.5123	0.7029
18571	Pdcd6ip	programmed cell death 6 interacting protein	1.17674747	0.03388	0.1192
236920	Stard8	START domain containing 8	1.17674747	0.03847	0.1294
60411	Cenpk	centromere protein K	1.17674747	0.1995	0.3913
57745	Zfp112	zinc finger protein 112	1.17660901	0.363	0.5721
67623	Tm7sf3	transmembrane 7 superfamily member 3	1.1763322	0.03195	0.1149
74777	Sepn1	selenoprotein N, 1	1.1763322	0.07308	0.2016
52466	Slc46a1	solute carrier family 46, member 1	1.1763322	0.1758	0.3603
230796	Wdtc1	WD and tetratricopeptide repeats 1	1.1763322	0.2579	0.4632
384482	Gm5316	predicted gene 5316	1.17619384	0.1055	0.257
71093	Atoh8	atonal homolog 8 (Drosophila)	1.17605551	0.2402	0.4427
224912	Crb3	crumbs homolog 3 (Drosophila)	1.17591722	0.2463	0.4502

384179	Gm5292	predicted gene 5292	1.17577895	0.1151	0.2723
108015	Chrnb4	cholinergic receptor, nicotinic, beta polypeptide 4	1.17577895	0.1197	0.2797
100043902	Six3os1	Six3 opposite strand transcript 1	1.17577895	0.6508	0.8034
18213	Ntrk3	neurotrophic tyrosine kinase, receptor, type 3	1.17550253	0.03572	0.1236
239217	Kctd12	potassium channel tetramerisation domain containing 12	1.17550253	0.06518	0.1859
19205	Ptbp1	polypyrimidine tract binding protein 1	1.17550253	0.1176	0.2761
269881	Map3k10	mitogen-activated protein kinase kinase kinase 10	1.17550253	0.2431	0.4464
16367	Irs1	insulin receptor substrate 1	1.17536436	0.01601	NA
69638	Enho	energy homeostasis associated	1.17536436	0.01611	NA
233806	Tmem159	transmembrane protein 159	1.17536436	0.08301	0.2196
74471	4933440N22Rik	RIKEN cDNA 4933440N22 gene	1.17536436	0.1357	0.3033
21682	Tec	tec protein tyrosine kinase	1.17522623	0.1254	0.2887
56087	Dnahc10	dynein, axonemal, heavy chain 10	1.17522623	0.2779	0.4861
83396	Glis2	GLIS family zinc finger 2	1.17508813	0.0223	NA
211389	Suox	sulfite oxidase	1.17495006	0.01413	NA
30945	Rnf19a	ring finger protein 19A	1.17495006	0.02413	NA
66365	Ccdc90b	coiled-coil domain containing 90B	1.17495006	0.09535	0.2409
71990	Ddx54	DEAD (Asp-Glu-Ala-Asp) box polypeptide 54	1.17481203	0.05778	0.1712
93960	Nkd1	naked cuticle 1 homolog (Drosophila)	1.17467403	0.0339	0.1192
20623	Snrk	SNF related kinase	1.17453606	0.02196	NA
66510	Rnf181	ring finger protein 181	1.17453606	0.02638	0.101
18003	Nedd9	neural precursor cell expressed, developmentally down-regulated gene 9	1.17453606	0.04712	0.1484
360220	Speer4d	spermatogenesis associated glutamate (E)-rich protein 4d	1.17453606	0.5433	0.7259
11974	Atp6v0e	ATPase, H+ transporting, lysosomal V0 subunit E	1.17439812	0.005124	NA
223254	Farp1	FERM, RhoGEF (Arhgef) and pleckstrin domain protein 1 (chondrocyte-derived)	1.17439812	0.03623	0.1247
11464	Actc1	actin, alpha, cardiac muscle 1	1.17439812	0.1617	0.3402
20318	Sdf4	stromal cell derived factor 4	1.17426022	0.0951	0.2404
74721	4930519P11Rik	RIKEN cDNA 4930519P11 gene	1.17426022	0.3884	0.5969
319888	5330437I02Rik	RIKEN cDNA 5330437I02 gene	1.17426022	0.4887	0.6834
433375	Creg1	cellular repressor of E1A-stimulated genes 1	1.17412234	0.018	NA
75712	Tmem14a	transmembrane protein 14A	1.17412234	0.3492	0.5596
69307	Pxt1	peroxisomal, testis specific 1	1.17370892	0.03905	0.1307
16578	Kif9	kinesin family member 9	1.17370892	0.1527	0.3279
232449	Dera	2-deoxyribose-5-phosphate aldolase homolog (C. elegans)	1.17357118	0.06759	0.1903
16796	Lasp1	LIM and SH3 protein 1	1.17343347	0.01586	NA
67330	1700047M11Rik	RIKEN cDNA 1700047M11 gene	1.17343347	0.02771	0.1044
56193	Plek	pleckstrin	1.17343347	0.09807	0.2452
791308	Gm9933	predicted gene 9933	1.17343347	0.4277	0.6322
67674	Trmt112	tRNA methyltransferase 11-2 homolog (S. cerevisiae)	1.17329579	0.07799	0.2107
414120	E330018M18Rik	RIKEN cDNA E330018M18 gene	1.17315814	0.5586	0.7379
22632	Yy1	YY1 transcription factor	1.17302053	0.01798	NA
68137	Kdelr1	KDEL (Lys-Asp-Glu-Leu) endoplasmic reticulum protein retention receptor 1	1.17302053	0.01908	NA
50788	Fbxl8	F-box and leucine-rich repeat protein 8	1.17302053	0.1608	0.3391
72750	Fam117b	family with sequence similarity 117, member B	1.1727454	0.06424	0.1839
67941	Rps27l	ribosomal protein S27-like	1.17260788	0.006721	NA
269109	Dpp10	dipeptidylpeptidase 10	1.17260788	0.1072	0.2594
192662	Arhgdia	Rho GDP dissociation inhibitor (GDI) alpha	1.17260788	0.1693	0.3505
244418	D8Ertd82e	DNA segment, Chr 8, ERATO Doi 82, expressed	1.1724704	0.2305	0.4314
21425	Tcfeb	transcription factor EB	1.1724704	0.2617	0.4676
13110	Cyp2j6	cytochrome P450, family 2, subfamily j, polypeptide 6	1.17233294	0.03411	0.1197

627110	Tubb2a-ps2	tubulin, beta 2a, pseudogene 2	1.17233294	0.03907	0.1307
22757	Zkscan5	zinc finger with KRAB and SCAN domains 5	1.17233294	0.06702	0.1892
15451	Hpn	hepsin	1.17219552	0.02131	NA
13024	Ctla2a	cytotoxic T lymphocyte-associated protein 2 alpha	1.17205813	0.08481	0.2228
51800	Bok	BCL2-related ovarian killer protein	1.17205813	0.08551	0.2242
12499	Entpd5	ectonucleoside triphosphate diphosphohydrolase 5	1.17205813	0.1058	0.2573
114606	Tle6	transducin-like enhancer of split 6, homolog of Drosophila E(spl)	1.17192078	0.01398	NA
70885	Ints10	integrator complex subunit 10	1.17192078	0.01409	NA
70396	Asnsd1	asparagine synthetase domain containing 1	1.17192078	0.02687	NA
69666	Psmg4	proteasome (prosome, macropain) assembly chaperone 4	1.17192078	0.1535	0.3289
66294	Fam3a	family with sequence similarity 3, member A	1.17178345	0.03769	0.1279
79362	Bhlhe41	basic helix-loop-helix family, member e41	1.17178345	0.4221	0.6279
52428	Rhpn2	rhophilin, Rho GTPase binding protein 2	1.17164616	0.04085	0.1347
269955	Rccd1	RCC1 domain containing 1	1.17164616	0.1061	0.2577
74568	Mlkl	mixed lineage kinase domain-like	1.17164616	0.4858	0.6811
229644	Trim45	tripartite motif-containing 45	1.1715089	0.214	0.4105
100047183	LOC100047183	protein AHNAK2-like	1.1715089	0.3144	0.5239
319176	Hist2h2ac	histone cluster 2, H2ac	1.17137168	0.03652	0.1252
11632	Aip	aryl-hydrocarbon receptor-interacting protein	1.17137168	0.08362	0.2208
13709	Elf1	E74-like factor 1	1.17137168	0.167	0.3479
382769	Gm5196	predicted gene 5196	1.17123448	0.1099	0.2639
19249	Ptpn13	protein tyrosine phosphatase, non-receptor type 13	1.17109732	0.02244	NA
22388	Wdr1	WD repeat domain 1	1.17096019	0.07341	0.2023
170742	Sertad3	SERTA domain containing 3	1.17096019	0.3027	0.5117
71609	Tradd	TNFRSF1A-associated via death domain	1.17082309	0.02068	NA
12042	Bcl10	B-cell leukemia/lymphoma 10	1.17082309	0.1074	0.2596
16998	Ltbp3	latent transforming growth factor beta binding protein 3	1.17068602	0.143	0.314
14700	Gng10	guanine nucleotide binding protein (G protein), gamma 10	1.17041199	0.01254	NA
108735	Sft2d2	SFT2 domain containing 2	1.17041199	0.032	0.115
71853	Pdia6	protein disulfide isomerase associated 6	1.17041199	0.03415	0.1198
57320	Park7	Parkinson disease (autosomal recessive, early onset) 7	1.17041199	0.0411	0.1352
110326	Tas1r1	taste receptor, type 1, member 1	1.17027501	0.2808	0.489
12479	Cd1d1	CD1d1 antigen	1.17013808	0.03151	0.1138
14874	Gstz1	glutathione transferase zeta 1 (maleylacetoacetate isomerase)	1.17013808	0.1081	0.2609
77053	Sun1	Sad1 and UNC84 domain containing 1	1.17013808	0.2768	0.4849
14674	Gna13	guanine nucleotide binding protein, alpha 13	1.17000117	0.05821	0.1719
13595	Ebp	phenylalkylamine Ca2+ antagonist (emopamil) binding protein	1.17000117	0.07153	0.1981
66617	Mettl11a	methyltransferase like 11A	1.17000117	0.08691	0.2265
228993	Slc17a9	solute carrier family 17, member 9	1.17000117	0.09053	0.2326
19981	Rpl37a	ribosomal protein L37a	1.17000117	0.1075	0.2598
320795	Pkn1	protein kinase N1	1.1698643	0.0627	0.181
63954	Rbp7	retinol binding protein 7, cellular	1.1698643	0.06721	0.1895
70873	4921517L17Rik	RIKEN cDNA 4921517L17 gene	1.16972745	0.04454	0.1427
68050	Akirin1	akirin 1	1.16972745	0.1838	0.3709
56460	Pkp3	plakophilin 3	1.16972745	0.2012	0.3936
74004	Jakmip3	janus kinase and microtubule interacting protein 3	1.16959064	0.2095	0.4049
269954	Ttll13	tubulin tyrosine ligase-like family, member 13	1.16959064	0.5932	0.7626
69537	Dnase1l1	deoxyribonuclease 1-like 1	1.16945387	0.03348	0.1184
12192	Zfp36l1	zinc finger protein 36, C3H type-like 1	1.16945387	0.08533	0.2238
101148	B630005N14Rik	RIKEN cDNA B630005N14 gene	1.16945387	0.1975	0.3886

16410	Itgav	integrin alpha V	1.16945387	0.3099	0.5196
71733	Susd2	sushi domain containing 2	1.16931712	0.1402	0.31
15191	Hdgf	hepatoma-derived growth factor	1.16931712	0.1678	0.3485
435684	Shf	Src homology 2 domain containing F	1.16931712	0.3026	0.5117
22003	Tpm1	tropomyosin 1, alpha	1.16931712	0.422	0.6278
72591	2700022O18Rik	RIKEN cDNA 2700022O18 gene	1.1691804	0.1798	0.3654
77569	Limch1	LIM and calponin homology domains 1	1.16904372	0.0128	NA
13875	Erf	Ets2 repressor factor	1.16904372	0.01662	NA
20474	Six4	sine oculis-related homeobox 4 homolog (Drosophila)	1.16877045	0.0956	0.2413
30940	Usp25	ubiquitin specific peptidase 25	1.16877045	0.0961	0.242
320178	4921529L05Rik	RIKEN cDNA 4921529L05 gene	1.16877045	0.1971	0.3884
224055	Rtp2	receptor transporter protein 2	1.16877045	0.6377	0.7948
19358	Rad23a	RAD23a homolog (S. cerevisiae)	1.16863387	0.2375	0.4396
56488	Nxt1	NTF2-related export protein 1	1.16836079	0.008436	NA
71685	Galnt14	UDP-N-acetyl-alpha-D-galactosamine:polypeptide N-acetylgalactosaminyltransferase 14	1.16836079	0.04314	0.1395
100042539	Gm3893	predicted gene 3893	1.16836079	0.3338	0.5442
20512	Slc1a3	solute carrier family 1 (glial high affinity glutamate transporter), member 3	1.1682243	0.06488	0.1853
110595	Timp4	tissue inhibitor of metalloproteinase 4	1.16808784	0.01737	NA
101604	E430018J23Rik	RIKEN cDNA E430018J23 gene	1.16795141	0.05233	0.1601
13002	Dnajc5	DnaJ (Hsp40) homolog, subfamily C, member 5	1.16781502	0.01454	NA
97484	Cog8	component of oligomeric golgi complex 8	1.16781502	0.1041	0.2548
246154	Vasn	vasorin	1.16767865	0.07794	0.2106
18028	Nfib	nuclear factor I/B	1.16754232	0.01575	NA
215160	Rhbdd2	rhomboid domain containing 2	1.16740602	0.04255	0.1384
76866	Morn1	MORN repeat containing 1	1.16740602	0.2877	0.4961
100561	Slc15a4	solute carrier family 15, member 4	1.16726976	0.009749	NA
56496	Tspan6	tetraspanin 6	1.16713352	0.02853	NA
74365	Lonrf3	LON peptidase N-terminal domain and ring finger 3	1.16699732	0.03585	0.1238
225642	Grp	gastrin releasing peptide	1.16686114	0.2515	0.4563
56338	Txnip	thioredoxin interacting protein	1.166725	0.04659	0.1472
229731	Slc25a24	solute carrier family 25 (mitochondrial carrier, phosphate carrier), member 24	1.166725	0.1026	0.2526
208213	Tmem132c	transmembrane protein 132C	1.16658889	0.01275	NA
64659	Mrps14	mitochondrial ribosomal protein S14	1.16658889	0.01674	NA
16601	Klf9	Kruppel-like factor 9	1.16658889	0.05844	0.1724
231805	Pilra	paired immunoglobin-like type 2 receptor alpha	1.16658889	0.6569	0.8063
80509	Med8	mediator of RNA polymerase II transcription, subunit 8 homolog (yeast)	1.16618076	0.03125	NA
14674	Gna13	guanine nucleotide binding protein, alpha 13	1.16618076	0.08441	0.2222
22354	Vipr1	vasoactive intestinal peptide receptor 1	1.16618076	0.1993	0.3911
75530	Lyrm7	LYR motif containing 7	1.16618076	0.2146	0.4114
12367	Casp3	caspase 3	1.16604478	0.1805	0.3665
98314	D2hgdh	D-2-hydroxyglutarate dehydrogenase	1.16604478	0.2552	0.4602
382118	Zfp167	zinc finger protein 167	1.16604478	0.2966	0.5056
233335	Synm	synemin, intermediate filament protein	1.16590883	0.2743	0.4823
12346	Car1	carbonic anhydrase 1	1.16590883	0.445	0.6478
14395	Gabra2	gamma-aminobutyric acid (GABA) A receptor, subunit alpha 2	1.16577291	0.2425	0.4454
72085	Osgepl1	O-sialoglycoprotein endopeptidase-like 1	1.16563702	0.03487	NA
66460	Sys1	SYS1 Golgi-localized integral membrane protein homolog (S. cerevisiae)	1.16563702	0.05268	0.1608
76138	Ccdc138	coiled-coil domain containing 138	1.16563702	0.079	0.2127
12144	Blm	Bloom syndrome, RecQ helicase-like	1.16563702	0.08588	0.2246
103140	Gstt3	glutathione S-transferase, theta 3	1.16563702	0.1383	0.3069

241113	Prkag3	protein kinase, AMP-activated, gamma 3 non-catatlytic subunit	1.16563702	0.151	0.3258
664808	Gm7349	predicted gene 7349	1.16563702	0.2242	0.424
70478	Mipep	mitochondrial intermediate peptidase	1.16550117	0.06679	0.1888
70361	Lman1	lectin, mannose-binding, 1	1.16536534	0.02582	NA
67760	Slc38a2	solute carrier family 38, member 2	1.16522955	0.003247	NA
240752	Pik3c2b	phosphoinositide-3-kinase, class 2, beta polypeptide	1.16522955	0.4056	0.6131
18088	Nkx2-2	NK2 transcription factor related, locus 2 (Drosophila)	1.16522955	0.4146	0.6209
271005	Klhdc1	kelch domain containing 1	1.16509379	0.0295	NA
103784	Wdr92	WD repeat domain 92	1.16509379	0.2056	0.3996
12442	Ccnb2	cyclin B2	1.16495806	0.03707	NA
74564	9.13E+15	hypothetical 9130022E09	1.16495806	0.06678	0.1888
215015	Fam20b	family with sequence similarity 20, member B	1.16495806	0.1644	0.3442
54188	Cpsf4	cleavage and polyadenylation specific factor 4	1.16482236	0.1389	0.3079
668592	Gm9258	predicted gene 9258	1.16482236	0.3149	0.5243
110253	Triobp	TRIO and F-actin binding protein	1.16482236	0.3292	0.5395
19119	Prm2	protamine 2	1.16482236	0.638	0.7951
12912	Creb1	cAMP responsive element binding protein 1	1.1646867	0.03506	NA
223722	Mcat	malonyl CoA:ACP acyltransferase (mitochondrial)	1.1646867	0.06878	0.1928
70377	Derl3	Der1-like domain family, member 3	1.1646867	0.1918	0.3815
12443	Ccnd1	cyclin D1	1.1646867	0.2106	0.4061
71743	Coasy	Coenzyme A synthase	1.16455107	0.007053	NA
27267	Cars	cysteinyl-tRNA synthetase	1.16455107	0.05952	0.1745
22070	Tpt1	tumor protein, translationally-controlled 1	1.16455107	0.05966	0.1747
77945	Rpgrip1	retinitis pigmentosa GTPase regulator interacting protein 1	1.16455107	0.2069	0.4015
20402	Zfp106	zinc finger protein 106	1.16441546	0.03137	NA
227298	Fam134a	family with sequence similarity 134, member A	1.16441546	0.05415	0.1641
108900	Fam72a	family with sequence similarity 72, member A	1.16441546	0.1048	0.2559
83997	Slmap	sarcolemma associated protein	1.16427989	0.1922	0.3819
20480	Clpb	ClpB caseinolytic peptidase B homolog (E. coli)	1.16427989	0.221	0.4201
56724	Cript	cysteine-rich PDZ-binding protein	1.16414435	0.0298	NA
380993	Zfat	zinc finger and AT hook domain containing	1.16414435	0.0823	0.2184
11856	Arhgap6	Rho GTPase activating protein 6	1.16414435	0.106	0.2576
60411	Cenpk	centromere protein K	1.16414435	0.3311	0.5415
78425	9530053H05Rik	RIKEN cDNA 9530053H05 gene	1.16400885	0.14	0.3095
232089	Elmod3	ELMO/CED-12 domain containing 3	1.16387337	0.0147	NA
14451	Gas1	growth arrest specific 1	1.16387337	0.0167	NA
67861	Akr1b10	aldo-keto reductase family 1, member B10 (aldose reductase)	1.16387337	0.03142	NA
12995	Csnk2a1	casein kinase 2, alpha 1 polypeptide	1.16360251	0.1833	0.3704
14998	H2-DMa	histocompatibility 2, class II, locus DMa	1.16346713	0.2305	0.4314
18969	Pola2	polymerase (DNA directed), alpha 2	1.16333178	0.3229	0.5327
544817	Arhgap27	Rho GTPase activating protein 27	1.16333178	0.5377	0.7216
22032	Traf4	TNF receptor associated factor 4	1.16306118	0.009498	NA
105352	Dusp22	dual specificity phosphatase 22	1.16292592	0.023	NA
27973	Vkorc1	vitamin K epoxide reductase complex, subunit 1	1.16292592	0.07525	0.2053
433182	Gm5506	predicted gene 5506	1.16292592	0.145	0.3172
108096	Slco1a5	solute carrier organic anion transporter family, member 1a5	1.16292592	0.3512	0.5612
71990	Ddx54	DEAD (Asp-Glu-Ala-Asp) box polypeptide 54	1.1627907	0.05347	0.1626
102580	Alg9	asparagine-linked glycosylation 9 homolog (yeast, alpha 1,2 mannosyltransferase)	1.16265551	0.1035	0.2541
192187	Stab1	stabilin 1	1.16265551	0.2445	0.4483
16528	Kcnk4	potassium channel, subfamily K, member 4	1.16252034	0.1923	0.3821

20840	Stac	src homology three (SH3) and cysteine rich domain	1.16238521	0.2278	0.428
17534	Mrc2	mannose receptor, C type 2	1.16225012	0.5683	0.744
98488	Gtf3c3	general transcription factor IIIC, polypeptide 3	1.16211505	0.007721	NA
74143	Opa1	optic atrophy 1 homolog (human)	1.16211505	0.01886	NA
399510	Map4k5	mitogen-activated protein kinase kinase kinase kinase 5	1.16211505	0.04532	0.1445
70887	Dmrtc1a	DMRT-like family C1a	1.16211505	0.2114	0.4071
24110	Usp18	ubiquitin specific peptidase 18	1.16211505	0.2865	0.4949
74315	Rnf145	ring finger protein 145	1.16198001	0.02258	NA
20643	Snrpe	small nuclear ribonucleoprotein E	1.16198001	0.05249	0.1604
216505	Pik3ip1	phosphoinositide-3-kinase interacting protein 1	1.16198001	0.1314	0.2976
20729	Spin1	spindlin 1	1.16184501	0.01731	NA
14158	Fert2	fer (fms/fps related) protein kinase, testis specific 2	1.16184501	0.01873	NA
13543	Dvl2	dishevelled 2, dsh homolog (Drosophila)	1.16184501	0.04939	0.1536
78921	9130019O22Rik	RIKEN cDNA 9130019O22 gene	1.16184501	0.3742	0.5829
672125	Gm14496	predicted gene 14496	1.16184501	0.4736	0.6715
19276	Ptprn2	protein tyrosine phosphatase, receptor type, N polypeptide 2	1.16171004	0.2688	0.4759
23936	Lynx1	Ly6/neurotoxin 1	1.16171004	0.3019	0.5111
619331	Zfp551	zinc fingr protein 551	1.16171004	0.3459	0.5567
229658	Vangl1	vang-like 1 (van gogh, Drosophila)	1.16144019	0.03285	NA
321022	Cdv3	carnitine deficiency-associated gene expressed in ventricle 3	1.16144019	0.1841	0.371
76846	Rps9	ribosomal protein S9	1.16117046	0.02208	NA
17454	Mov10	Moloney leukemia virus 10	1.16117046	0.1883	0.3765
21810	Tgfbi	transforming growth factor, beta induced	1.16117046	0.3775	0.5865
77125	Il33	interleukin 33	1.16090086	0.02133	NA
269261	Rpl12	ribosomal protein L12	1.16090086	0.04651	0.147
320244	Ttll5	tubulin tyrosine ligase-like family, member 5	1.16090086	0.04798	0.1503
11827	Aqp2	aquaporin 2	1.16090086	0.3412	0.5517
56739	Rec8	REC8 homolog (yeast)	1.16090086	0.3613	0.5709
14629	Gclc	glutamate-cysteine ligase, catalytic subunit	1.16076611	0.0594	0.1742
16847	Lepr	leptin receptor	1.16076611	0.7061	0.838
12238	Commd3	COMM domain containing 3	1.16049669	0.0185	NA
232798	Leng8	leukocyte receptor cluster (LRC) member 8	1.16049669	0.323	0.5327
269994	Gsg1l	GSG1-like	1.16049669	0.4514	0.6536
435684	Shf	Src homology 2 domain containing F	1.16036203	0.1008	0.2499
67106	Zbtb8os	zinc finger and BTB domain containing 8 opposite strand	1.16036203	0.146	0.3186
19038	Ppic	peptidylprolyl isomerase C	1.16036203	0.1761	0.3606
54125	Polm	polymerase (DNA directed), mu	1.16036203	0.3844	0.5936
14055	Ezh1	enhancer of zeste homolog 1 (Drosophila)	1.1602274	0.2027	0.3957
54397	Ppt2	palmitoyl-protein thioesterase 2	1.1602274	0.239	0.4415
64436	Inpp5e	inositol polyphosphate-5-phosphatase E	1.1602274	0.2633	0.4694
20170	Hps6	Hermansky-Pudlak syndrome 6	1.16009281	0.02021	NA
74025	Nphp3	nephronophthisis 3 (adolescent)	1.16009281	0.1118	0.2669
257926	Olfr544	olfactory receptor 544	1.15995824	0.4165	0.6227
13661	Ehf	ets homologous factor	1.15995824	0.501	0.693
67993	Nudt12	nudix (nucleoside diphosphate linked moiety X)-type motif 12	1.15982371	0.2476	0.4518
57319	Smpdl3a	sphingomyelin phosphodiesterase, acid-like 3A	1.1596892	0.1514	0.3261
26417	Mapk3	mitogen-activated protein kinase 3	1.15955473	0.1129	0.2688
75995	5033417F24Rik	RIKEN cDNA 5033417F24 gene	1.15955473	0.1552	0.3314
79196	Osbpl5	oxysterol binding protein-like 5	1.15942029	0.01516	NA
104184	Blmh	bleomycin hydrolase	1.15942029	0.04338	NA

224022	Slc7a4	solute carrier family 7 (cationic amino acid transporter, y+ system), member 4	1.15942029	0.08127	0.2166
230279	6330416G13Rik	RIKEN cDNA 6330416G13 gene	1.15942029	0.09602	0.2419
640979	LOC640979	ig heavy chain V region B1-8/186-2-like	1.15928588	0.1158	0.2733
68087	Dcakd	dephospho-CoA kinase domain containing	1.15928588	0.2264	0.4261
12729	Clns1a	chloride channel, nucleotide-sensitive, 1A	1.15901715	0.08901	0.23
56349	Net1	neuroepithelial cell transforming gene 1	1.15901715	0.1067	0.2586
109077	Ints5	integrator complex subunit 5	1.15901715	0.2107	0.4061
70686	Dusp16	dual specificity phosphatase 16	1.15901715	0.23	0.4308
319169	Hist1h2ak	histone cluster 1, H2ak	1.15888284	0.01492	NA
19170	Psmb1	proteasome (prosome, macropain) subunit, beta type 1	1.15888284	0.0266	NA
15163	Hcls1	hematopoietic cell specific Lyn substrate 1	1.15874855	0.03757	NA
380732	Gm885	predicted gene 885	1.15874855	0.5449	0.7269
227613	Tubb2c	tubulin, beta 2C	1.1586143	0.01024	NA
70984	4931406C07Rik	RIKEN cDNA 4931406C07 gene	1.1586143	0.06079	0.1771
12453	Ccni	cyclin I	1.15848007	0.01573	NA
19043	Ppm1b	protein phosphatase 1B, magnesium dependent, beta isoform	1.15848007	0.0275	NA
69270	Gins1	GINS complex subunit 1 (Psf1 homolog)	1.15834588	0.1428	0.3137
114570	Crip3	cysteine-rich protein 3	1.15834588	0.1904	0.3793
77219	Ptgr2	prostaglandin reductase 2	1.15821172	0.03094	NA
104215	Rhoq	ras homolog gene family, member Q	1.15821172	0.06711	0.1893
66170	Chchd5	coiled-coil-helix-coiled-coil-helix domain containing 5	1.15807759	0.03598	NA
23980	Pebp1	phosphatidylethanolamine binding protein 1	1.15807759	0.2199	0.4188
67091	Trappc6a	trafficking protein particle complex 6A	1.15794349	0.03313	NA
69638	Enho	energy homeostasis associated	1.15794349	0.04686	NA
328633	4930515I15	hypothetical protein 4930515I15	1.15794349	0.1691	0.3503
56484	Foxo3	forkhead box O3	1.15794349	0.3613	0.5709
69870	Polr3gl	polymerase (RNA) III (DNA directed) polypeptide G like	1.15767539	0.02971	NA
71904	Paqr7	progestin and adipoQ receptor family member VII	1.15767539	0.05222	0.1598
402751	C430019N01Rik	RIKEN cDNA C430019N01 gene	1.15767539	0.2272	0.4271
54601	Foxo4	forkhead box O4	1.15767539	0.4731	0.6712
12333	Capn1	calpain 1	1.15754138	0.1358	0.3033
228911	Tshz2	teashirt zinc finger family member 2	1.15754138	0.1924	0.3821
20448	St6galnac4	ST6 (alpha-N-acetyl-neuraminyl-2,3-beta-galactosyl-1,3)-N-acetylgalactosaminide alpha-2,6-sialyltransferase 4	1.15754138	0.2422	0.4452
56401	Lepre1	leprecan 1	1.15754138	0.251	0.4558
56707	Zfp111	zinc finger protein 111	1.15727346	0.1775	0.3624
71983	Tmco6	transmembrane and coiled-coil domains 6	1.15713955	0.1564	0.3334
13885	Esd	esterase D/formylglutathione hydrolase	1.15700567	0.01339	NA
57258	Xpo4	exportin 4	1.15700567	0.04674	NA
12404	Cbln1	cerebellin 1 precursor protein	1.15700567	0.2896	0.498
73486	1700084J12Rik	RIKEN cDNA 1700084J12 gene	1.15700567	0.4337	0.6374
320343	Lypd6	LY6/PLAUR domain containing 6	1.15687182	0.01949	NA
81500	Sil1	endoplasmic reticulum chaperone SIL1 homolog (S. cerevisiae)	1.156738	0.01311	NA
319939	Tns3	tensin 3	1.156738	0.02188	NA
55949	Eef1b2	eukaryotic translation elongation factor 1 beta 2	1.15660421	0.008879	NA
72614	Pih1d2	PIH1 domain containing 2	1.15647045	0.0466	NA
73363	1700056E22Rik	RIKEN cDNA 1700056E22 gene	1.15647045	0.1029	0.2531
229658	Vangl1	vang-like 1 (van gogh, Drosophila)	1.15647045	0.3506	0.5609
381062	2210404J11Rik	RIKEN cDNA 2210404J11 gene	1.15633673	0.09944	0.2475
268933	Wdr24	WD repeat domain 24	1.15620303	0.2158	0.413
52040	Ppp1r10	protein phosphatase 1, regulatory subunit 10	1.15606936	0.1353	0.3025

668339	Gm9112	predicted gene 9112	1.15606936	0.3237	0.5334
67729	Mansc1	MANSC domain containing 1	1.15606936	0.3974	0.6054
76938	Rbm17	RNA binding motif protein 17	1.15593573	0.01918	NA
192678	Rassf3	Ras association (RalGDS/AF-6) domain family member 3	1.15593573	0.1673	0.3481
666938	Bend4	BEN domain containing 4	1.15580213	0.1726	0.3559
215472	Gm4792	predicted gene 4792	1.15580213	0.5585	0.7378
76718	Catsperg2	cation channel, sperm-associated, gamma 2	1.15580213	0.5599	0.7386
266690	Cyb5r4	cytochrome b5 reductase 4	1.15566855	0.04433	NA
227733	Pip5kl1	phosphatidylinositol-4-phosphate 5-kinase-like 1	1.15566855	0.2106	0.4061
57748	Jmy	junction-mediating and regulatory protein	1.15566855	0.3461	0.5567
17826	Mtvr2	mammary tumor virus receptor 2	1.15553501	0.09253	0.2361
26395	Map2k1	mitogen-activated protein kinase kinase 1	1.1554015	0.04248	NA
654472	Gm12070	glyceraldehyde-3-phosphate dehydrogenase pseudogene	1.1554015	0.06581	0.187
73419	1700052N19Rik	RIKEN cDNA 1700052N19 gene	1.15526802	0.06091	0.1773
12017	Bag1	BCL2-associated athanogene 1	1.15526802	0.09118	0.2337
12388	Ctnnd1	catenin (cadherin associated protein), delta 1	1.15526802	0.09858	0.2462
232430	Crebl2	cAMP responsive element binding protein-like 2	1.15526802	0.1294	0.2949
239796	1600021P15Rik	RIKEN cDNA 1600021P15 gene	1.15526802	0.3753	0.5841
103710	Slc35e4	solute carrier family 35, member E4	1.15500116	0.1406	0.3104
381280	Hjurp	Holliday junction recognition protein	1.15486777	0.04021	NA
74196	Ttc27	tetratricopeptide repeat domain 27	1.15473441	0.2034	0.3968
20661	Sort1	sortilin 1	1.15473441	0.4845	0.6798
56088	Psmg1	proteasome (prosome, macropain) assembly chaperone 1	1.15460109	0.009231	NA
70807	Arrdc2	arrestin domain containing 2	1.15460109	0.2951	0.5041
331046	Tgm4	transglutaminase 4 (prostate)	1.15446779	0.1385	0.3072
328121	Abhd12b	abhydrolase domain containing 12B	1.15433453	0.2936	0.5025
71365	Pdss2	prenyl (solanesyl) diphosphate synthase, subunit 2	1.15420129	0.105	0.2562
78902	4833447P13Rik	RIKEN cDNA 4833447P13 gene	1.15420129	0.2407	0.4433
20604	Sst	somatostatin	1.15420129	0.3949	0.6029
70375	Ica1l	islet cell autoantigen 1-like	1.15406809	0.1152	0.2725
72640	Mex3a	mex3 homolog A (C. elegans)	1.15406809	0.1699	0.3518
70281	2310068J16Rik	RIKEN cDNA 2310068J16 gene	1.15406809	0.2078	0.4025
13007	Csrp1	cysteine and glycine-rich protein 1	1.15393492	0.004044	NA
22781	Ikzf4	IKAROS family zinc finger 4	1.15393492	0.08012	0.2147
66191	Ier3ip1	immediate early response 3 interacting protein 1	1.15380178	0.06659	0.1885
100038635	Gm10621	predicted gene 10621	1.15380178	0.07573	0.2063
20055	Rps16	ribosomal protein S16	1.15380178	0.1146	0.2716
19047	Ppp1cc	protein phosphatase 1, catalytic subunit, gamma isoform	1.15380178	0.1284	0.2935
19301	Pxmp2	peroxisomal membrane protein 2	1.15380178	0.3443	0.5549
71743	Coasy	Coenzyme A synthase	1.15366867	0.01153	NA
21376	Tbrg1	transforming growth factor beta regulated gene 1	1.15353559	0.03414	NA
17067	Ly6c1	lymphocyte antigen 6 complex, locus C1	1.15353559	0.07819	0.2111
11641	Akap2	A kinase (PRKA) anchor protein 2	1.15353559	0.1335	0.3004
106522	Pkdcc	protein kinase domain containing, cytoplasmic	1.15353559	0.1351	0.3021
58865	Tdh	L-threonine dehydrogenase	1.15353559	0.5658	0.7423
72043	Sulf2	sulfatase 2	1.15340254	0.1395	0.3089
19943	Rpl28	ribosomal protein L28	1.15326952	0.03567	NA
27965	Spg21	spastic paraplegia 21 homolog (human)	1.15326952	0.03998	NA
218734	3830406C13Rik	RIKEN cDNA 3830406C13 gene	1.15313653	0.09372	0.2379
12480	Cd1d2	CD1d2 antigen	1.15313653	0.1573	0.3344

194908	Pld6	phospholipase D family, member 6	1.15313653	0.188	0.3762
98970	Fibcd1	fibrinogen C domain containing 1	1.15313653	0.2944	0.5033
108907	Nusap1	nucleolar and spindle associated protein 1	1.15313653	0.3975	0.6055
93960	Nkd1	naked cuticle 1 homolog (Drosophila)	1.15313653	0.4566	0.658
675985	Rps6-ps2	ribosomal protein S6, pseudogene 2	1.15287065	0.05562	NA
27205	Podxl	podocalyxin-like	1.15287065	0.06568	0.1867
110198	Akr7a5	aldo-keto reductase family 7, member A5 (aflatoxin aldehyde reductase)	1.15287065	0.07052	0.1961
67902	Sumf2	sulfatase modifying factor 2	1.15287065	0.1043	0.2552
223665	C030006K11Rik	RIKEN cDNA C030006K11 gene	1.15273775	0.217	0.4146
319748	Zfp865	zinc finger protein 865	1.15260489	0.2545	0.4594
12390	Cav2	caveolin 2	1.15260489	0.278	0.4862
12804	Cntfr	ciliary neurotrophic factor receptor	1.15247205	0.1675	0.3483
106582	Nrm	nurim (nuclear envelope membrane protein)	1.15247205	0.18	0.3657
69274	Ctdspl	CTD (carboxy-terminal domain, RNA polymerase II, polypeptide A) small phosphatase-like	1.15220648	0.1022	0.2521
66628	Thg1l	tRNA-histidine guanylyltransferase 1-like (S. cerevisiae)	1.15220648	0.1182	0.2772
19046	Ppp1cb	protein phosphatase 1, catalytic subunit, beta isoform	1.15220648	0.1649	0.345
56336	B4galt5	UDP-Gal:betaGlcNAc beta 1,4-galactosyltransferase, polypeptide 5	1.15207373	0.05201	NA
245631	Mum1l1	melanoma associated antigen (mutated) 1-like 1	1.15207373	0.1113	0.2661
244958	Mrap2	melanocortin 2 receptor accessory protein 2	1.15207373	0.2099	0.4054
104457	0610010K14Rik	RIKEN cDNA 0610010K14 gene	1.15194102	0.04353	NA
67628	Anp32b	acidic (leucine-rich) nuclear phosphoprotein 32 family, member B	1.15194102	0.04913	NA
66795	Atg10	autophagy-related 10 (yeast)	1.15194102	0.07867	0.212
231830	Micall2	MICAL-like 2	1.15180834	0.3775	0.5865
70003	1700028I16Rik	RIKEN cDNA 1700028I16 gene	1.15180834	0.4464	0.6489
242570	Raver2	ribonucleoprotein, PTB-binding 2	1.15167569	0.05657	NA
93883	Pcdhb12	protocadherin beta 12	1.15167569	0.07723	0.2092
69008	Cab39l	calcium binding protein 39-like	1.15154307	0.06509	0.1857
20813	Srp14	signal recognition particle 14	1.15141048	0.02166	NA
52331	Stbd1	starch binding domain 1	1.15141048	0.04611	NA
27643	Ubl4	ubiquitin-like 4	1.15141048	0.04648	NA
213499	Fbxo42	F-box protein 42	1.15127792	0.07127	0.1976
214855	Arid5a	AT rich interactive domain 5A (MRF1-like)	1.15127792	0.08815	0.2285
16478	Jund	Jun proto-oncogene related gene d	1.15127792	0.3503	0.5607
67763	Prpsap1	phosphoribosyl pyrophosphate synthetase-associated protein 1	1.15114539	0.03678	NA
100877	AV074028	expressed sequence AV074028	1.15101289	0.207	0.4016
105722	Ano6	anoctamin 6	1.15088042	0.2838	0.4924
239570	Ttc38	tetratricopeptide repeat domain 38	1.15088042	0.392	0.6003
69008	Cab39l	calcium binding protein 39-like	1.15074799	0.02206	NA
67469	Abhd5	abhydrolase domain containing 5	1.15074799	0.03777	NA
218639	Arl15	ADP-ribosylation factor-like 15	1.15074799	0.1183	0.2773
207667	Skor1	SKI family transcriptional corepressor 1	1.15074799	0.1857	0.3731
66230	Mrps28	mitochondrial ribosomal protein S28	1.1504832	0.02323	NA
546164	Gm5921	predicted gene 5921	1.1504832	0.1353	0.3025
58809	Rnase4	ribonuclease, RNase A family 4	1.1504832	0.2394	0.4419
18606	Enpp2	ectonucleotide pyrophosphatase/phosphodiesterase 2	1.15035086	0.0346	NA
21917	Tmpo	thymopoietin	1.15035086	0.06926	0.1938
16917	Lmx1b	LIM homeobox transcription factor 1 beta	1.15035086	0.4875	0.6824
56369	Apip	APAF1 interacting protein	1.15021854	0.03319	NA
11674	Aldoa	aldolase A, fructose-bisphosphate	1.15021854	0.08286	0.2194
72169	Trim29	tripartite motif-containing 29	1.15008626	0.4258	0.6309

19739	Rgs9	regulator of G-protein signaling 9	1.149954	0.05847	NA	
14630	Gclm	glutamate-cysteine ligase, modifier subunit	1.14982178	0.03625	NA	
69802	Cox11	COX11 homolog, cytochrome c oxidase assembly protein (yeast)	1.14982178	0.05984	NA	
66111	Tmed3	transmembrane emp24 domain containing 3	1.14982178	0.06222	NA	
76096	5830468K08Rik	RIKEN cDNA 5830468K08 gene	1.14982178	0.1248		0.2878
105670	Rcbtb2	regulator of chromosome condensation (RCC1) and BTB (POZ) domain containing protein 2	1.14968958	0.0149	NA	
22158	Tulp3	tubby-like protein 3	1.14968958	0.1286		0.2937
67078	Pgp	phosphoglycolate phosphatase	1.14955742	0.03821	NA	
233335	Synm	synemin, intermediate filament protein	1.14955742	0.1488		0.3227
12426	Cckbr	cholecystokinin B receptor	1.14942529	0.2112		0.4068
101543	Wtip	WT1-interacting protein	1.14929318	0.009323	NA	
71720	Osbpl3	oxysterol binding protein-like 3	1.14929318	0.01743	NA	
211666	Mgst2	microsomal glutathione S-transferase 2	1.14929318	0.158		0.3353
263876	Spata2	spermatogenesis associated 2	1.14916111	0.08024		0.2148
75692	Nr2c2ap	nuclear receptor 2C2-associated protein	1.14902907	0.2475		0.4517
20472	Six2	sine oculis-related homeobox 2 homolog (Drosophila)	1.14902907	0.3018		0.511
84652	Fam126a	family with sequence similarity 126, member A	1.14902907	0.3756		0.5845
69397	1700019A02Rik	RIKEN cDNA 1700019A02 gene	1.14902907	0.3993		0.607
195208	Dcdc2a	doublecortin domain containing 2a	1.14889706	0.105		0.2562
226548	Aph1a	anterior pharynx defective 1a homolog (C. elegans)	1.14889706	0.146		0.3185
59038	Pxmp4	peroxisomal membrane protein 4	1.14889706	0.1643		0.3441
228961	Npepl1	aminopeptidase-like 1	1.14889706	0.1845		0.3715
353282	Sfmbt2	Scm-like with four mbt domains 2	1.14889706	0.3179		0.5274
30838	Fbxw4	F-box and WD-40 domain protein 4	1.14876508	0.0699		0.195
74670	Zfp943	zinc finger prtoein 943	1.14876508	0.09891		0.2466
233826	Palb2	partner and localizer of BRCA2	1.14876508	0.1371		0.3049
20652	Soat1	sterol O-acyltransferase 1	1.14876508	0.2012		0.3936
73137	Prrc1	proline-rich coiled-coil 1	1.14863313	0.05631	NA	
54411	Atp6ap1	ATPase, H+ transporting, lysosomal accessory protein 1	1.14850121	0.1199		0.28
22154	Tubb5	tubulin, beta 5	1.14836932	0.1826		0.3696
17261	Mef2d	myocyte enhancer factor 2D	1.14836932	0.2069		0.4015
226548	Aph1a	anterior pharynx defective 1a homolog (C. elegans)	1.14823746	0.04583	NA	
14086	Fscn1	fascin homolog 1, actin bundling protein (Strongylocentrotus purpuratus)	1.14823746	0.2715		0.479
407802	BC028789	cDNA sequence BC028789	1.14823746	0.641		0.7969
16890	Lipe	lipase, hormone sensitive	1.14810563	0.06694	NA	
100042173	Gm11968	Rps15a pseudogene	1.14797383	0.1099		0.2639
30058	Timm8a1	translocase of inner mitochondrial membrane 8 homolog a1 (yeast)	1.14797383	0.1387		0.3075
238123	Cog5	component of oligomeric golgi complex 5	1.14797383	0.1732		0.3568
72184	Klhl35	kelch-like 35 (Drosophila)	1.14797383	0.2775		0.4857
223775	Pim3	proviral integration site 3	1.14784206	0.2022		0.3949
65971	Tbata	thymus, brain and testes associated	1.14784206	0.2784		0.4866
407243	Tmem189	transmembrane protein 189	1.14784206	0.4268		0.6318
66642	Ctnnbl1	catenin, beta like 1	1.14771032	0.01059	NA	
212986	Scfd2	Sec1 family domain containing 2	1.14771032	0.05773	NA	
73736	Fcf1	FCF1 small subunit (SSU) processome component homolog (S. cerevisiae)	1.14771032	0.06844	NA	
22755	Zfp93	zinc finger protein 93	1.14757861	0.007363	NA	
13844	Ephb2	Eph receptor B2	1.14744693	0.2751		0.4829
21750	Terf2	telomeric repeat binding factor 2	1.14731528	0.07363		0.2025
93742	Pard3	par-3 (partitioning defective 3) homolog (C. elegans)	1.14731528	0.07803		0.2108
69129	Pex11c	peroxisomal biogenesis factor 11 gamma	1.14731528	0.1367		0.3042

22030	Traf2	TNF receptor-associated factor 2	1.14718366	0.06264	NA
65973	Asph	aspartate-beta-hydroxylase	1.14718366	0.114	0.2707
21429	Ubtf	upstream binding transcription factor, RNA polymerase I	1.14718366	0.1288	0.294
64385	Cyp4f14	cytochrome P450, family 4, subfamily f, polypeptide 14	1.14718366	0.2446	0.4483
76820	Fam49a	family with sequence similarity 49, member A	1.14692052	0.03336	NA
235416	Lman1l	lectin, mannose-binding 1 like	1.14692052	0.1325	0.2991
78586	Srbd1	S1 RNA binding domain 1	1.14692052	0.2999	0.5093
74062	Speer8-ps1	spermatogenesis associated glutamate (E)-rich protein 8, pseudogene 1	1.14692052	0.3494	0.5599
239408	Tmem74	transmembrane protein 74	1.14692052	0.3549	0.5647
234076	Tmco3	transmembrane and coiled-coil domains 3	1.14678899	0.3268	0.537
114230	Aipl1	aryl hydrocarbon receptor-interacting protein-like 1	1.14665749	0.0767	0.2082
121022	Mrps6	mitochondrial ribosomal protein S6	1.14652603	0.01976	NA
16828	Ldha	lactate dehydrogenase A	1.14652603	0.1059	0.2573
20605	Sstr1	somatostatin receptor 1	1.14652603	0.2711	0.4788
240832	Tor1aip2	torsin A interacting protein 2	1.14639459	0.02281	NA
67042	Ift27	intraflagellar transport 27 homolog (Chlamydomonas)	1.14639459	0.08419	0.2217
223604	Kcnk9	potassium channel, subfamily K, member 9	1.14626318	0.4092	0.6156
399619	A930027P06Rik	RIKEN cDNA A930027P06 gene	1.14626318	0.4662	0.6661
19012	Ppap2a	phosphatidic acid phosphatase type 2A	1.14613181	0.05917	NA
100042916	Gm4107	predicted gene 4107	1.14613181	0.3603	0.5701
319168	Hist1h2ah	histone cluster 1, H2ah	1.14600046	0.0356	NA
230767	Iqcc	IQ motif containing C	1.14586914	0.03729	NA
109115	Supt3h	suppressor of Ty 3 homolog (S. cerevisiae)	1.14586914	0.09364	0.2378
22003	Tpm1	tropomyosin 1, alpha	1.14586914	0.1341	0.3011
22362	Vpreb1	pre-B lymphocyte gene 1	1.14586914	0.1776	0.3626
15465	Hrh1	histamine receptor H1	1.14573786	0.3441	0.5548
68736	1110034B05Rik	RIKEN cDNA 1110034B05 gene	1.1456066	0.1292	0.2947
70772	Ggnbp1	gametogenetin binding protein 1	1.1456066	0.2895	0.4979
246293	Klhl8	kelch-like 8 (Drosophila)	1.14547537	0.0195	NA
74244	Atg7	autophagy-related 7 (yeast)	1.14547537	0.2597	0.4654
72285	1810073O08Rik	RIKEN cDNA 1810073O08 gene	1.14534418	0.5208	0.7091
18685	Phtf1	putative homeodomain transcription factor 1	1.14521301	0.02131	NA
239827	Pigz	phosphatidylinositol glycan anchor biosynthesis, class Z	1.14521301	0.1242	0.2869
75840	4930558N01Rik	RIKEN cDNA 4930558N01 gene	1.14521301	0.1469	0.3198
18429	Oxt	oxytocin	1.14521301	0.2676	0.4745
69216	Ccdc23	coiled-coil domain containing 23	1.14521301	0.4394	0.6422
71711	Mus81	MUS81 endonuclease homolog (yeast)	1.14508187	0.2012	0.3936
100040016	Rhox2e	reproductive homeobox 2E	1.14508187	0.4393	0.6422
20972	Syngr1	synaptogyrin 1	1.14495077	0.06938	NA
214779	Zfp879	zinc figer protein 879	1.14495077	0.1506	0.3255
66733	Kcng4	potassium voltage-gated channel, subfamily G, member 4	1.14495077	0.1584	0.3359
209225	Zfp710	zinc finger protein 710	1.14481969	0.03662	NA
66092	Ghitm	growth hormone inducible transmembrane protein	1.14481969	0.06516	NA
57329	Otor	otoraplin	1.14481969	0.1196	0.2797
13808	Eno3	enolase 3, beta muscle	1.14468864	0.1371	0.3048
15504	Dnajb3	DnaJ (Hsp40) homolog, subfamily B, member 3	1.14468864	0.2559	0.4609
100503659	Dos	downstream of Stk11	1.14455763	0.1955	0.386
26417	Mapk3	mitogen-activated protein kinase 3	1.14455763	0.2194	0.4182
18096	Nkx6-1	NK6 homeobox 1	1.14442664	0.2008	0.3931
54366	Ctnnal1	catenin (cadherin associated protein), alpha-like 1	1.14442664	0.5601	0.7386

14828	Hspa5	heat shock protein 5	1.14429569	0.01481	NA
434179	Gm5595	predicted gene 5595	1.14429569	0.02645	NA
67503	1700001G17Rik	RIKEN cDNA 1700001G17 gene	1.14429569	0.09601	0.2419
18220	Nucb1	nucleobindin 1	1.14429569	0.273	0.4805
57915	Tbc1d1	TBC1 domain family, member 1	1.14416476	0.04902	NA
12162	Bmp7	bone morphogenetic protein 7	1.14416476	0.3151	0.5245
110960	Tars	threonyl-tRNA synthetase	1.143903	0.04211	NA
74013	Rftn2	raftlin family member 2	1.143903	0.07419	NA
231296	Lrrc66	leucine rich repeat containing 66	1.143903	0.1548	0.3309
67889	Rbm18	RNA binding motif protein 18	1.14377216	0.02987	NA
67774	Loh12cr1	loss of heterozygosity, 12, chromosomal region 1 homolog (human)	1.14377216	0.09176	0.2348
13531	Dub1	deubiquitinating enzyme 1	1.14377216	0.3035	0.5127
56440	Snx1	sorting nexin 1	1.14364135	0.07352	NA
212167	Pion	pigeon homolog (Drosophila)	1.14364135	0.2102	0.4058
100216474	Ttll2	tubulin tyrosine ligase-like family, member 2	1.14351058	0.1511	0.3258
270151	Nlrx1	NLR family member X1	1.14351058	0.362	0.5715
12400	Cbfb	core binding factor beta	1.14337983	0.1168	0.2748
101533	Klk9	kallikrein related-peptidase 9	1.14337983	0.1562	0.3331
68846	Rnf208	ring finger protein 208	1.14337983	0.2102	0.4058
13033	Ctsd	cathepsin D	1.14324911	0.04365	NA
76892	Rnft1	ring finger protein, transmembrane 1	1.14324911	0.1052	0.2566
11987	Slc7a1	solute carrier family 7 (cationic amino acid transporter, y+ system), member 1	1.14324911	0.1688	0.3499
328092	6530401N04Rik	RIKEN cDNA 6530401N04 gene	1.14311843	0.09272	0.2364
50723	Icosl	icos ligand	1.14311843	0.2269	0.4267
73453	1700067K01Rik	RIKEN cDNA 1700067K01 gene	1.14311843	0.6528	0.8041
100678	Psph	phosphoserine phosphatase	1.14298777	0.06902	NA
245424	Gpr101	G protein-coupled receptor 101	1.14298777	0.1281	0.2929
67458	Ergic1	endoplasmic reticulum-golgi intermediate compartment (ERGIC) 1	1.14298777	0.2218	0.4211
231050	Galnt11	UDP-N-acetyl-alpha-D-galactosamine:polypeptide N-acetylgalactosaminyltransferase 11	1.14298777	0.2858	0.4943
270152	Amica1	adhesion molecule, interacts with CXADR antigen 1	1.14298777	0.4968	0.6899
50917	Galns	galactosamine (N-acetyl)-6-sulfate sulfatase	1.14285714	0.3115	0.5214
70726	Angptl6	angiopoietin-like 6	1.14272655	0.06447	NA
219140	Spata13	spermatogenesis associated 13	1.14272655	0.1751	0.3592
14670	Gnl1	guanine nucleotide binding protein-like 1	1.14259598	0.04111	NA
671535	Parp10	poly (ADP-ribose) polymerase family, member 10	1.14259598	0.04191	NA
77018	Col25a1	collagen, type XXV, alpha 1	1.14259598	0.4854	0.6807
399558	Flrt2	fibronectin leucine rich transmembrane protein 2	1.14246544	0.03789	NA
14687	Gnaz	guanine nucleotide binding protein, alpha z subunit	1.14246544	0.1285	0.2937
252875	Mios	missing oocyte, meiosis regulator, homolog (Drosophila)	1.14246544	0.1332	0.3001
70361	Lman1	lectin, mannose-binding, 1	1.14246544	0.4486	0.6507
68836	Mrpl52	mitochondrial ribosomal protein L52	1.14233493	0.02652	NA
66682	Trappc5	trafficking protein particle complex 5	1.14233493	0.2542	0.4592
319276	A230038B01Rik	RIKEN cDNA A230038B01 gene	1.14233493	0.4673	0.6672
228911	Tshz2	teashirt zinc finger family member 2	1.14220445	0.03855	NA
71966	Nkiras2	NFKB inhibitor interacting Ras-like protein 2	1.14220445	0.151	0.3258
227682	Trub2	TruB pseudouridine (psi) synthase homolog 2 (E. coli)	1.14220445	0.1592	0.3372
29815	Bcar3	breast cancer anti-estrogen resistance 3	1.14207401	0.04659	NA
74777	Sepn1	selenoprotein N, 1	1.14194359	0.1254	0.2887
243529	H1fx	H1 histone family, member X	1.14194359	0.3615	0.5711
667433	Gm8630	predicted gene 8630	1.14194359	0.4645	0.6647

15473	Hrsp12	heat-responsive protein 12	1.1418132	0.1652	0.3452
73239	3110054G05Rik	RIKEN cDNA 3110054G05 gene	1.14168284	0.2005	0.3927
108101	Fermt3	fermitin family homolog 3 (Drosophila)	1.14168284	0.2677	0.4746
12035	Bcat1	branched chain aminotransferase 1, cytosolic	1.14155251	0.08327	NA
76950	2900001G08Rik	RIKEN cDNA 2900001G08 gene	1.14155251	0.1676	0.3485
77446	Heg1	HEG homolog 1 (zebrafish)	1.14155251	0.19	0.3788
19328	Rab12	RAB12, member RAS oncogene family	1.14142221	0.08522	NA
320907	B430105G09Rik	RIKEN cDNA B430105G09 gene	1.14142221	0.1489	0.3227
70808	4632415L05Rik	RRS1 ribosome biogenesis regulator homolog pseudogene	1.14142221	0.2714	0.4789
14827	Pdia3	protein disulfide isomerase associated 3	1.14142221	0.3013	0.5108
209773	Dennd2a	DENN/MADD domain containing 2A	1.14129194	0.04349	NA
100502683	LOC100502683	40S ribosomal protein S29-like	1.14129194	0.1679	0.3486
230514	Leprot	leptin receptor overlapping transcript	1.1411617	0.005736	NA
223732	Ldoc1l	leucine zipper, down-regulated in cancer 1-like	1.1411617	0.18	0.3656
229543	Ints3	integrator complex subunit 3	1.1411617	0.234	0.4356
70617	5730508B09Rik	RIKEN cDNA 5730508B09 gene	1.14103149	0.08862	0.2293
19411	Rarg	retinoic acid receptor, gamma	1.14103149	0.1731	0.3565
19142	Prss12	protease, serine, 12 neurotrypsin (motopsin)	1.14090131	0.4384	0.6415
270624	Spin4	spindlin family, member 4	1.14077116	0.122	0.2836
234362	Zfp868	zinc finger protein 868	1.14077116	0.1664	0.3471
68050	Akirin1	akirin 1	1.14077116	0.2306	0.4314
100036569	Gm16525	predicted gene, 16525	1.14064104	0.3584	0.5683
100043484	Gm4470	predicted gene 4470	1.14064104	0.5688	0.7446
235582	Glyctk	glycerate kinase	1.14051095	0.4242	0.6297
16681	Krt2	keratin 2	1.14051095	0.5401	0.7233
232440	H2afj	H2A histone family, member J	1.14038089	0.04444	NA
20185	Ncor1	nuclear receptor co-repressor 1	1.14038089	0.09742	0.2439
74026	Msl1	male-specific lethal 1 homolog (Drosophila)	1.14038089	0.1196	0.2797
17129	Smad5	MAD homolog 5 (Drosophila)	1.14025086	0.1735	0.357
14373	G0s2	G0/G1 switch gene 2	1.14025086	0.184	0.3709
15251	Hif1a	hypoxia inducible factor 1, alpha subunit	1.14012085	0.03433	NA
230761	Zfp362	zinc finger protein 362	1.14012085	0.1377	0.3059
791088	C630016N16Rik	RIKEN cDNA C630016N16 gene	1.14012085	0.1763	0.3609
11534	Adk	adenosine kinase	1.13999088	0.2674	0.4744
64010	Sav1	salvador homolog 1 (Drosophila)	1.13986094	0.03866	NA
66343	Tmem177	transmembrane protein 177	1.13986094	0.2053	0.3991
628919	Gm6934	predicted gene 6934	1.13986094	0.4784	0.6752
22781	Ikzf4	IKAROS family zinc finger 4	1.13973102	0.05047	NA
73067	Tmem192	transmembrane protein 192	1.13960114	0.0392	NA
14387	Gaa	glucosidase, alpha, acid	1.13960114	0.1163	0.2739
72462	Rrp1b	ribosomal RNA processing 1 homolog B (S. cerevisiae)	1.13960114	0.3336	0.5441
446101	Xrra1	X-ray radiation resistance associated 1	1.13947129	0.4367	0.6401
214444	Cdk5rap2	CDK5 regulatory subunit associated protein 2	1.13934146	0.1836	0.3708
67106	Zbtb8os	zinc finger and BTB domain containing 8 opposite strand	1.13921167	0.1235	0.286
246278	Cd207	CD207 antigen	1.13921167	0.2887	0.4971
72018	Fundc1	FUN14 domain containing 1	1.1390819	0.03361	NA
229285	Spg20	spastic paraplegia 20, spartin (Troyer syndrome) homolog (human)	1.1390819	0.1256	0.2889
105833	Ccdc65	coiled-coil domain containing 65	1.1390819	0.185	0.3721
381319	Batf3	basic leucine zipper transcription factor, ATF-like 3	1.1390819	0.3495	0.5599
80708	Pacsin3	protein kinase C and casein kinase substrate in neurons 3	1.13895216	0.02739	NA

54201	Zfp316	zinc finger protein 316	1.13895216	0.09981	0.248
100041231	Gm3219	B-cell CLL/lymphoma 7C pseudogene	1.13895216	0.3298	0.5401
24061	Smc1a	structural maintenance of chromosomes 1A	1.13882246	0.09499	0.2402
71409	Fmnl2	formin-like 2	1.13882246	0.5193	0.7085
55983	Pdzrn3	PDZ domain containing RING finger 3	1.13882246	0.561	0.7393
68075	1520402A15Rik	RIKEN cDNA 1520402A15 gene	1.13869278	0.1197	0.2797
60406	Sap30	sin3 associated polypeptide	1.13869278	0.1838	0.3709
66815	Ccdc109b	coiled-coil domain containing 109B	1.13869278	0.2354	0.4371
626359	Wdr93	WD repeat domain 93	1.13869278	0.2928	0.5016
224079	Atp13a4	ATPase type 13A4	1.13869278	0.889	0.943
214804	Syde2	synapse defective 1, Rho GTPase, homolog 2 (C. elegans)	1.13856313	0.1489	0.3227
14433	Gapdh	glyceraldehyde-3-phosphate dehydrogenase	1.13856313	0.2722	0.4796
432870	Gm5464	predicted gene 5464	1.13856313	0.376	0.585
11459	Acta1	actin, alpha 1, skeletal muscle	1.13843352	0.1973	0.3885
107971	Frs3	fibroblast growth factor receptor substrate 3	1.13843352	0.7826	0.8858
319167	Hist1h2ag	histone cluster 1, H2ag	1.13830393	0.04945	NA
20509	Slc19a1	solute carrier family 19 (sodium/hydrogen exchanger), member 1	1.13830393	0.09777	0.2446
620592	Tmem28	transmembrane protein 28	1.13830393	0.1037	0.2543
68703	Rere	arginine glutamic acid dipeptide (RE) repeats	1.13817437	0.1857	0.3731
20442	St3gal1	ST3 beta-galactoside alpha-2,3-sialyltransferase 1	1.13804484	0.1788	0.3642
54125	Polm	polymerase (DNA directed), mu	1.13778587	0.07153	NA
78934	4930581F22Rik	RIKEN cDNA 4930581F22 gene	1.13778587	0.1313	0.2975
75114	4930516B21Rik	RIKEN cDNA 4930516B21 gene	1.13778587	0.497	0.6899
12540	Cdc42	cell division cycle 42 homolog (S. cerevisiae)	1.13765643	0.03071	NA
210530	Leprel1	leprecan-like 1	1.13765643	0.3028	0.5118
59290	Gpa33	glycoprotein A33 (transmembrane)	1.13765643	0.5085	0.6993
76438	Rftn1	raftlin lipid raft linker 1	1.13752702	0.08336	NA
14775	Gpx1	glutathione peroxidase 1	1.13752702	0.1586	0.3362
20116	Rps8	ribosomal protein S8	1.13739763	0.2047	0.3984
67168	Lpar6	lysophosphatidic acid receptor 6	1.13739763	0.2181	0.4163
54635	Pdgfc	platelet-derived growth factor, C polypeptide	1.13739763	0.3115	0.5214
100038402	BC025933	cDNA sequence BC025933	1.13739763	0.4484	0.6505
434858	Gm5643	heterogeneous nuclear ribonucleoprotein A1 pseudogene	1.13726828	0.08117	NA
67203	Nde1	nuclear distribution gene E homolog 1 (A nidulans)	1.13726828	0.1358	0.3033
14081	Acsl1	acyl-CoA synthetase long-chain family member 1	1.13726828	0.1488	0.3227
67752	4930579J09Rik	RIKEN cDNA 4930579J09 gene	1.13726828	0.3116	0.5214
381693	Wdr95	WD40 repeat domain 95	1.13726828	0.4003	0.6081
66047	Mrpl54	mitochondrial ribosomal protein L54	1.13713896	0.05784	NA
66379	2310016M24Rik	RIKEN cDNA 2310016M24 gene	1.13713896	0.08245	NA
15493	Hsd3b2	hydroxy-delta-5-steroid dehydrogenase, 3 beta- and steroid delta-isomerase 2	1.13713896	0.48	0.676
22022	Tpst2	protein-tyrosine sulfotransferase 2	1.13700966	0.05927	NA
19155	Npepps	aminopeptidase puromycin sensitive	1.13700966	0.1016	0.2512
14687	Gnaz	guanine nucleotide binding protein, alpha z subunit	1.13700966	0.1924	0.3822
12493	Cd37	CD37 antigen	1.13700966	0.5593	0.7383
320244	Ttll5	tubulin tyrosine ligase-like family, member 5	1.1368804	0.4479	0.6501
14254	Flt1	FMS-like tyrosine kinase 1	1.13662196	0.1269	0.2909
319173	Hist1h2af	histone cluster 1, H2af	1.13649278	0.04455	NA
19718	Rfc2	replication factor C (activator 1) 2	1.13636364	0.1457	0.3184
224705	Vps52	vacuolar protein sorting 52 (yeast)	1.13636364	0.1793	0.3649
100046302	LOC100046302	protein disulfide-isomerase A6-like	1.13636364	0.1854	0.3728

12741	Cldn5	claudin 5	1.13636364	0.2177	0.4156
433586	Maml3	mastermind like 3 (Drosophila)	1.13636364	0.4396	0.6424
17863	Myb	myeloblastosis oncogene	1.13623452	0.03686	NA
52892	Sco1	SCO cytochrome oxidase deficient homolog 1 (yeast)	1.13623452	0.09404	NA
53333	Tomm40	translocase of outer mitochondrial membrane 40 homolog (yeast)	1.13623452	0.1343	0.3014
100038443	9330195I24Rik	RIKEN cDNA 9330195I24 gene	1.13610543	0.09445	NA
76927	1700021C14Rik	RIKEN cDNA 1700021C14 gene	1.13610543	0.1085	0.2615
665189	Gm7536	predicted gene 7536	1.13610543	0.3587	0.5686
21645	Tcte1	t-complex-associated testis expressed 1	1.13610543	0.5843	0.7553
27029	Sgsh	N-sulfoglucosamine sulfohydrolase (sulfamidase)	1.13610543	0.5913	0.761
21808	Tgfb2	transforming growth factor, beta 2	1.13597637	0.04294	NA
26927	Foxl2	forkhead box L2	1.13597637	0.05737	NA
24068	Sra1	steroid receptor RNA activator 1	1.13597637	0.07522	NA
11848	Rhoa	ras homolog gene family, member A	1.13597637	0.09677	NA
57278	Bcam	basal cell adhesion molecule	1.13597637	0.5045	0.6957
19773	Rln1	relaxin 1	1.13597637	0.5378	0.7217
56486	Gabarap	gamma-aminobutyric acid receptor associated protein	1.13584734	0.03325	NA
546164	Gm5921	predicted gene 5921	1.13571834	0.1059	0.2574
67059	Ola1	Obg-like ATPase 1	1.13571834	0.2434	0.4468
625540	Gm6598	predicted gene 6598	1.13571834	0.2523	0.4573
100061	Lrrc19	leucine rich repeat containing 19	1.13571834	0.2903	0.4987
233905	Zfp646	zinc finger protein 646	1.13558937	0.5651	0.742
71891	Cdadc1	cytidine and dCMP deaminase domain containing 1	1.13546043	0.05184	NA
110826	Etfb	electron transferring flavoprotein, beta polypeptide	1.13546043	0.06031	NA
68758	Abhd11	abhydrolase domain containing 11	1.13546043	0.121	0.282
69890	Zfp219	zinc finger protein 219	1.13546043	0.1245	0.2873
17865	Mybl2	myeloblastosis oncogene-like 2	1.13546043	0.1446	0.3167
70605	Zdhhc24	zinc finger, DHHC domain containing 24	1.13546043	0.2403	0.4429
13445	Cdk2ap1	CDK2 (cyclin-dependent kinase 2)-associated protein 1	1.13533152	0.02463	NA
67528	Nudt7	nudix (nucleoside diphosphate linked moiety X)-type motif 7	1.13533152	0.09875	NA
17132	Maf	avian musculoaponeurotic fibrosarcoma (v-maf) AS42 oncogene homolog	1.13533152	0.493	0.687
65019	Rpl23	ribosomal protein L23	1.13520263	0.05203	NA
70396	Asnsd1	asparagine synthetase domain containing 1	1.13507378	0.04089	NA
629159	1700008J07Rik	RIKEN cDNA 1700008J07 gene	1.13494496	0.2755	0.4834
210027	Slc35f3	solute carrier family 35, member F3	1.13494496	0.5	0.6922
26378	Decr2	2-4-dienoyl-Coenzyme A reductase 2, peroxisomal	1.13481616	0.1225	0.2844
70153	2210016F16Rik	RIKEN cDNA 2210016F16 gene	1.13468739	0.06	NA
69306	Efcab9	EF-hand calcium binding domain 9	1.13468739	0.4028	0.6103
329360	Gm757	predicted gene 757	1.13468739	0.4514	0.6536
75423	Arl5a	ADP-ribosylation factor-like 5A	1.13455866	0.1521	0.327
11981	Atp9a	ATPase, class II, type 9A	1.13455866	0.2274	0.4275
57914	Crlf2	cytokine receptor-like factor 2	1.13442995	0.02657	NA
66278	1810013D10Rik	RIKEN cDNA 1810013D10 gene	1.13442995	0.1062	0.2579
100929	Tyw1	tRNA-yW synthesizing protein 1 homolog (S. cerevisiae)	1.13442995	0.1324	0.2989
56771	Med20	mediator complex subunit 20	1.13442995	0.1544	0.3302
54124	Cks1b	CDC28 protein kinase 1b	1.13442995	0.3707	0.5794
66700	Vps24	vacuolar protein sorting 24 (yeast)	1.13430127	0.03563	NA
320799	Zhx3	zinc fingers and homeoboxes 3	1.134044	0.01698	NA
18759	Prkci	protein kinase C, iota	1.13391541	0.02211	NA
72446	Prr5l	proline rich 5 like	1.13391541	0.4502	0.6525

13112	Cyp3a11	cytochrome P450, family 3, subfamily a, polypeptide 11	1.13391541	0.5426	0.7253
258851	Olfr1339	olfactory receptor 1339	1.13391541	0.5651	0.742
56494	Gosr2	golgi SNAP receptor complex member 2	1.13378685	0.00913	NA
12805	Cntn1	contactin 1	1.13365832	0.05635	NA
236576	Spry3	sprouty homolog 3 (Drosophila)	1.13365832	0.07343	NA
68201	Ccdc34	coiled-coil domain containing 34	1.13365832	0.1267	0.2905
19730	Ralgds	ral guanine nucleotide dissociation stimulator	1.13352981	0.1431	0.3141
232089	Elmod3	ELMO/CED-12 domain containing 3	1.13340134	0.06681	NA
53883	Celsr2	cadherin, EGF LAG seven-pass G-type receptor 2 (flamingo homolog, Drosophila)	1.13327289	0.3066	0.516
69444	Lyzl6	lysozyme-like 6	1.13327289	0.4255	0.6307
16409	Itgam	integrin alpha M	1.13327289	0.6358	0.7933
107702	Rnh1	ribonuclease/angiogenin inhibitor 1	1.13314448	0.08317	NA
59007	Ngly1	N-glycanase 1	1.13314448	0.316	0.5255
19344	Rab5b	RAB5B, member RAS oncogene family	1.13301609	0.1602	0.3383
71446	Wrb	tryptophan rich basic protein	1.13301609	0.2721	0.4794
71766	Raver1	ribonucleoprotein, PTB-binding 1	1.13301609	0.3826	0.5917
18176	Nras	neuroblastoma ras oncogene	1.13288773	0.05948	NA
332937	Tcfap2e	transcription factor AP-2, epsilon	1.13288773	0.2692	0.4763
270669	Mbtps2	membrane-bound transcription factor peptidase, site 2	1.13288773	0.4576	0.659
217370	BC017643	cDNA sequence BC017643	1.1327594	0.01613	NA
22195	Ube2l3	ubiquitin-conjugating enzyme E2L 3	1.1327594	0.1853	0.3726
241226	Itga8	integrin alpha 8	1.1327594	0.1932	0.3832
19296	Pvt1	plasmacytoma variant translocation 1	1.1327594	0.3218	0.5315
66832	Rsph3a	radial spoke 3A homolog (Chlamydomonas)	1.1326311	0.07238	NA
66848	Fuca2	fucosidase, alpha-L- 2, plasma	1.1326311	0.0898	NA
106512	Gpsm3	G-protein signalling modulator 3 (AGS3-like, C. elegans)	1.1326311	0.1883	0.3765
217674	Gphb5	glycoprotein hormone beta 5	1.1326311	0.5861	0.7566
73598	1700001O22Rik	RIKEN cDNA 1700001O22 gene	1.13250283	0.3274	0.5375
77766	Elp4	elongation protein 4 homolog (S. cerevisiae)	1.13237459	0.08079	NA
66220	Zdhhc12	zinc finger, DHHC domain containing 12	1.13224638	0.04474	NA
75665	Ccdc64	coiled-coil domain containing 64	1.13224638	0.2451	0.4486
545238	G430049J08Rik	RIKEN cDNA G430049J08 gene	1.13224638	0.7432	0.8613
242687	Wasf2	WAS protein family, member 2	1.13211819	0.1346	0.3015
15368	Hmox1	heme oxygenase (decycling) 1	1.13211819	0.3349	0.5455
67898	Pef1	penta-EF hand domain containing 1	1.13199004	0.07371	NA
56363	Tmeff2	transmembrane protein with EGF-like and two follistatin-like domains 2	1.13199004	0.133	0.3
17858	Mx2	myxovirus (influenza virus) resistance 2	1.13199004	0.245	0.4486
258028	Olfr901	olfactory receptor 901	1.13186191	0.8331	0.9139
57320	Park7	Parkinson disease (autosomal recessive, early onset) 7	1.13160575	0.05833	NA
56491	Vapb	vesicle-associated membrane protein, associated protein B and C	1.13160575	0.07938	NA
57738	Slc15a2	solute carrier family 15 (H+/peptide transporter), member 2	1.13160575	0.2593	0.465
117606	Boc	biregional cell adhesion molecule-related/down-regulated by oncogenes (Cdon) binding protein	1.13160575	0.2744	0.4823
103537	Mbtd1	mbt domain containing 1	1.13160575	0.4193	0.6255
66662	5730577I03Rik	zinc finger protein pseudogene	1.13160575	0.4733	0.6713
235533	Gk5	glycerol kinase 5 (putative)	1.13160575	0.4963	0.6895
23872	Ets2	E26 avian leukemia oncogene 2, 3' domain	1.13147771	0.04216	NA
668224	Gm9054	predicted gene 9054	1.13147771	0.2393	0.4419
319564	C230012O17Rik	RIKEN cDNA C230012O17 gene	1.13147771	0.2647	0.4711
381933	6430531B16Rik	RIKEN cDNA 6430531B16 gene	1.13147771	0.2664	0.4731
57230	Sap30bp	SAP30 binding protein	1.1313497	0.2051	0.3989

93761	Smarca1	SWI/SNF related, matrix associated, actin dependent regulator of chromatin, subfamily a, member 1	1.1313497	0.2934	0.5022
257913	Olfr141	olfactory receptor 141	1.1313497	0.4594	0.6604
171273	Vmn1r217	vomeronasal 1 receptor 217	1.1313497	0.4873	0.6824
12167	Bmpr1b	bone morphogenetic protein receptor, type 1B	1.13122172	0.2875	0.4959
654472	Gm12070	glyceraldehyde-3-phosphate dehydrogenase pseudogene	1.13122172	0.3762	0.5851
101490	Inpp5f	inositol polyphosphate-5-phosphatase F	1.13122172	0.5094	0.7001
67669	l7Rn6	lethal, Chr 7, Rinchik 6	1.13109377	0.01765	NA
20751	Spr	sepiapterin reductase	1.13109377	0.05738	NA
18247	Oaz2-ps	ornithine decarboxylase antizyme 2, pseudogene	1.13109377	0.07242	NA
19242	Ptn	pleiotrophin	1.13109377	0.3443	0.5549
17217	Mcm4	minichromosome maintenance deficient 4 homolog (S. cerevisiae)	1.13096584	0.03057	NA
70508	Bbx	bobby sox homolog (Drosophila)	1.13096584	0.03231	NA
223267	A2ld1	AIG2-like domain 1	1.13096584	0.1049	NA
66483	Rpl36al	ribosomal protein L36A-like	1.13083795	0.02042	NA
19015	Ppard	peroxisome proliferator activator receptor delta	1.13083795	0.2099	0.4054
67872	Nsmce4a	non-SMC element 4 homolog A (S. cerevisiae)	1.13071009	0.02067	NA
436062	Fam92b	family with sequence similarity 92, member B	1.13071009	0.2317	0.4326
14433	Gapdh	glyceraldehyde-3-phosphate dehydrogenase	1.13058225	0.06174	NA
60315	Myg1	melanocyte proliferating gene 1	1.13058225	0.1615	0.3399
67248	Rpl39	ribosomal protein L39	1.13058225	0.2524	0.4574
319934	Sbf2	SET binding factor 2	1.13058225	0.3119	0.5216
791379	C030009H01Rik	RIKEN cDNA C030009H01 gene	1.13058225	0.5155	0.7051
234371	Tmem161a	transmembrane protein 161A	1.13045444	0.1528	0.3281
26458	Slc27a2	solute carrier family 27 (fatty acid transporter), member 2	1.13045444	0.2604	0.4662
67763	Prpsap1	phosphoribosyl pyrophosphate synthetase-associated protein 1	1.13032666	0.05302	NA
76826	Nubpl	nucleotide binding protein-like	1.13032666	0.1735	0.357
66231	Thoc7	THO complex 7 homolog (Drosophila)	1.13032666	0.2566	0.4617
268739	Arhgef40	Rho guanine nucleotide exchange factor (GEF) 40	1.13007119	0.09574	NA
402767	A830052D11Rik	RIKEN cDNA A830052D11 gene	1.13007119	0.4469	0.6491
56520	Nme4	non-metastatic cells 4, protein expressed in	1.1299435	0.08454	NA
20088	Rps24	ribosomal protein S24	1.1299435	0.1317	0.298
12389	Cav1	caveolin 1, caveolae protein	1.1299435	0.164	0.3436
75216	4930534B04Rik	RIKEN cDNA 4930534B04 gene	1.1299435	0.4614	0.6619
59047	Pnkp	polynucleotide kinase 3'- phosphatase	1.12981584	0.01419	NA
14151	Fech	ferrochelatase	1.12981584	0.02176	NA
217216	BC030867	cDNA sequence BC030867	1.12981584	0.3616	0.5712
229003	BC006779	cDNA sequence BC006779	1.12968821	0.1034	NA
15481	Hspa8	heat shock protein 8	1.12968821	0.1596	0.3378
106740	LOC106740	hypothetical LOC106740	1.12968821	0.1736	0.3571
71890	Mad2l2	MAD2 mitotic arrest deficient-like 2 (yeast)	1.1295606	0.03812	NA
69241	Polr2d	polymerase (RNA) II (DNA directed) polypeptide D	1.1295606	0.09857	NA
69099	1810009N02Rik	RIKEN cDNA 1810009N02 gene	1.1295606	0.1184	NA
69581	Rhou	ras homolog gene family, member U	1.1295606	0.2264	0.4261
22294	Uxt	ubiquitously expressed transcript	1.1295606	0.2588	0.4644
67726	Fam114a2	family with sequence similarity 114, member A2	1.12930548	0.3144	0.5239
114249	Npnt	nephronectin	1.12930548	0.5434	0.726
230718	Nt5c1a	5'-nucleotidase, cytosolic IA	1.12930548	0.6834	0.8232
66294	Fam3a	family with sequence similarity 3, member A	1.12905047	0.0805	NA
225912	Cybasc3	cytochrome b, ascorbate dependent 3	1.12905047	0.1041	NA
56327	Arl2	ADP-ribosylation factor-like 2	1.12905047	0.1164	NA

73666	Thoc3	THO complex 3	1.12905047	0.1165	NA
12916	Crem	cAMP responsive element modulator	1.12905047	0.1254	0.2887
105446	Gmpr2	guanosine monophosphate reductase 2	1.12905047	0.2126	0.4085
11550	Adra1d	adrenergic receptor, alpha 1d	1.12905047	0.5582	0.7377
109264	Me3	malic enzyme 3, NADP(+)-dependent, mitochondrial	1.12892301	0.109	NA
268527	Greb1	gene regulated by estrogen in breast cancer protein	1.12892301	0.1289	0.2942
328274	Zfp459	zinc finger protein 459	1.12892301	0.3055	0.5147
19241	Tmsb4x	thymosin, beta 4, X chromosome	1.12879558	0.03909	NA
22661	Zfp148	zinc finger protein 148	1.12879558	0.1153	NA
70397	Tmem70	transmembrane protein 70	1.12866817	0.0386	NA
319170	Hist1h2an	histone cluster 1, H2an	1.12866817	0.04036	NA
50868	Keap1	kelch-like ECH-associated protein 1	1.12866817	0.1041	NA
224671	Btbd9	BTB (POZ) domain containing 9	1.1285408	0.03658	NA
100210	Gpn2	GPN-loop GTPase 2	1.1285408	0.08435	NA
100503302	LOC100503302	40S ribosomal protein S19-like	1.1285408	0.1493	0.3233
622459	Gm12216	predicted gene 12216	1.1285408	0.2671	0.4742
64339	Fndc4	fibronectin type III domain containing 4	1.12841345	0.02803	NA
22027	Hsp90b1	heat shock protein 90, beta (Grp94), member 1	1.12841345	0.04374	NA
68969	Eif1b	eukaryotic translation initiation factor 1B	1.12841345	0.06685	NA
70478	Mipep	mitochondrial intermediate peptidase	1.12841345	0.1322	0.2987
69574	Cmbl	carboxymethylenebutenolidase-like (Pseudomonas)	1.12828613	0.1875	0.3757
234734	Aars	alanyl-tRNA synthetase	1.12828613	0.2327	0.434
100505352	LOC100505352	hypothetical LOC100505352	1.12828613	0.2505	0.4553
442802	C330011M18Rik	RIKEN cDNA C330011M18 gene	1.12828613	0.355	0.5648
14768	Lancl1	LanC (bacterial lantibiotic synthetase component C)-like 1	1.12828613	0.409	0.6155
23986	Peci	peroxisomal delta3, delta2-enoyl-Coenzyme A isomerase	1.12815884	0.07883	NA
14229	Fkbp5	FK506 binding protein 5	1.12815884	0.1709	0.3534
380753	Atxn7l1	ataxin 7-like 1	1.12815884	0.2792	0.4873
70785	Dennd1c	DENN/MADD domain containing 1C	1.12815884	0.3982	0.6062
627035	Gm6729	predicted gene 6729	1.12815884	0.4517	0.6539
212127	2810046L04Rik	RIKEN cDNA 2810046L04 gene	1.12803158	0.2936	0.5025
215690	Nav1	neuron navigator 1	1.12803158	0.294	0.503
68736	1110034B05Rik	RIKEN cDNA 1110034B05 gene	1.12803158	0.3425	0.5534
382097	Gm1123	predicted gene 1123	1.12803158	0.4656	0.6657
319468	Ppm1h	protein phosphatase 1H (PP2C domain containing)	1.12790435	0.05453	NA
227154	Stradb	STE20-related kinase adaptor beta	1.12790435	0.0704	NA
243510	Ccdc142	coiled-coil domain containing 142	1.12790435	0.3644	0.5733
21907	Nr2e1	nuclear receptor subfamily 2, group E, member 1	1.12777715	0.02834	NA
20850	Stat5a	signal transducer and activator of transcription 5A	1.12777715	0.1151	NA
68188	Sympk	symplekin	1.12777715	0.1628	0.3418
72723	Zfp74	zinc finger protein 74	1.12764998	0.1059	NA
108664	Atp6v1h	ATPase, H+ transporting, lysosomal V1 subunit H	1.12752283	0.04929	NA
20933	Med22	mediator complex subunit 22	1.12752283	0.1489	0.3227
74778	Rrp7a	ribosomal RNA processing 7 homolog A (S. cerevisiae)	1.12752283	0.1606	0.3387
67178	Zmat5	zinc finger, matrin type 5	1.12752283	0.2659	0.4726
22169	Cmpk2	cytidine monophosphate (UMP-CMP) kinase 2, mitochondrial	1.12752283	0.2864	0.4949
12905	Cradd	CASP2 and RIPK1 domain containing adaptor with death domain	1.12739572	0.1933	0.3832
56458	Foxo1	forkhead box O1	1.12739572	0.2272	0.4271
15964	Ifna11	interferon alpha 11	1.12739572	0.7364	0.8575
233744	Spon1	spondin 1, (f-spondin) extracellular matrix protein	1.12726863	0.08615	NA

18186	Nrp1	neuropilin 1	1.12726863	0.09504	NA
170736	Parvb	parvin, beta	1.12726863	0.5165	0.706
52440	Tax1bp1	Tax1 (human T-cell leukemia virus type I) binding protein 1	1.12701454	0.01708	NA
107885	Mthfs	5, 10-methenyltetrahydrofolate synthetase	1.12701454	0.249	0.4531
83962	Btbd1	BTB (POZ) domain containing 1	1.12688754	0.01521	NA
72333	Palld	palladin, cytoskeletal associated protein	1.12688754	0.1327	0.2994
328156	C230037L09	hypothetical protein C230037L09	1.12688754	0.2259	0.4256
100038452	Gm13372	predicted gene 13372	1.12688754	0.4479	0.6501
76947	2310030N02Rik	RIKEN cDNA 2310030N02 gene	1.12676056	0.0553	NA
14055	Ezh1	enhancer of zeste homolog 1 (Drosophila)	1.12663362	0.02575	NA
330171	Kctd10	potassium channel tetramerisation domain containing 10	1.12663362	0.03502	NA
76809	Bri3bp	Bri3 binding protein	1.12663362	0.07142	NA
278507	Wfikkn2	WAP, follistatin/kazal, immunoglobulin, kunitz and netrin domain containing 2	1.12663362	0.2261	0.4257
333473	Zfp36l3	zinc finger protein 36, C3H type-like 3	1.12663362	0.6007	0.7684
381455	6430573P05Rik	RIKEN cDNA 6430573P05 gene	1.1265067	0.05247	NA
104318	Csnk1d	casein kinase 1, delta	1.1265067	0.06334	NA
67991	Nacc2	nucleus accumbens associated 2, BEN and BTB (POZ) domain containing	1.1265067	0.1002	NA
76763	Mospd2	motile sperm domain containing 2	1.1265067	0.2626	0.4687
320435	Rinl	Ras and Rab interactor-like	1.12637982	0.4237	0.6293
19782	Rmrp	RNA component of mitochondrial RNAase P	1.12637982	0.5708	0.746
230696	AU022252	expressed sequence AU022252	1.12625296	0.0739	NA
100037260	9430060I03Rik	RIKEN cDNA 9430060I03 gene	1.12599932	0.1816	0.368
54418	Fmn2	formin 2	1.12599932	0.301	0.5104
97761	Sgsm2	small G protein signaling modulator 2	1.12587255	0.09197	NA
382019	Zfp882	zinc finger protein 882	1.12587255	0.1552	0.3314
12317	Calr	calreticulin	1.12587255	0.2477	0.4519
239691	AU021092	expressed sequence AU021092	1.12587255	0.3146	0.524
632883	LOC632883	protein transport protein Sec61 subunit gamma-like	1.12587255	0.4124	0.6186
70134	2210011C24Rik	RIKEN cDNA 2210011C24 gene	1.12587255	0.559	0.7382
69577	Fastkd3	FAST kinase domains 3	1.12574581	0.0659	NA
380701	Slc47a2	solute carrier family 47, member 2	1.12574581	0.1944	0.3847
227541	Camk1d	calcium/calmodulin-dependent protein kinase ID	1.12574581	0.4726	0.6708
19210	Ptdss1	phosphatidylserine synthase 1	1.12561909	0.0255	NA
50927	Nasp	nuclear autoantigenic sperm protein (histone-binding)	1.12561909	0.09015	NA
18000	Sep-02	septin 2	1.12561909	0.105	NA
74840	Manf	mesencephalic astrocyte-derived neurotrophic factor	1.1254924	0.1252	NA
13400	Dmpk	dystrophia myotonica-protein kinase	1.1254924	0.3548	0.5647
230866	C230096C10Rik	RIKEN cDNA C230096C10 gene	1.12536574	0.1933	0.3832
100039795	Ildr2	immunoglobulin-like domain containing receptor 2	1.12536574	0.2415	0.4442
22724	Zbtb7b	zinc finger and BTB domain containing 7B	1.12536574	0.3024	0.5115
233912	Armc5	armadillo repeat containing 5	1.12523911	0.02862	NA
67213	Cmtm6	CKLF-like MARVEL transmembrane domain containing 6	1.12523911	0.0494	NA
67865	Rgs10	regulator of G-protein signalling 10	1.12523911	0.06559	NA
14221	Fjx1	four jointed box 1 (Drosophila)	1.12523911	0.09331	NA
236904	Klhl15	kelch-like 15 (Drosophila)	1.12523911	0.3162	0.5256
71856	Wfdc3	WAP four-disulfide core domain 3	1.12523911	0.6044	0.7712
75642	1700020C07Rik	RIKEN cDNA 1700020C07 gene	1.12523911	0.7723	0.8788
66487	2010107H07Rik	RIKEN cDNA 2010107H07 gene	1.12511251	0.04779	NA
18631	Pex11a	peroxisomal biogenesis factor 11 alpha	1.12511251	0.07305	NA
104069	Sncb	synuclein, beta	1.12511251	0.5051	0.6962

28084	Vps25	vacuolar protein sorting 25 (yeast)	1.12498594	0.2822	0.4904
98496	Pid1	phosphotyrosine interaction domain containing 1	1.12485939	0.1424	0.3131
73711	Fam125a	family with sequence similarity 125, member A	1.12485939	0.172	0.355
66132	1110008L16Rik	RIKEN cDNA 1110008L16 gene	1.12485939	0.3439	0.5547
69736	Nup37	nucleoporin 37	1.12473288	0.1257	NA
23831	Car14	carbonic anhydrase 14	1.12473288	0.2678	0.4747
230584	Yipf1	Yip1 domain family, member 1	1.12460639	0.03487	NA
18038	Nfkbil1	nuclear factor of kappa light polypeptide gene enhancer in B-cells inhibitor-like 1	1.12460639	0.1028	NA
16578	Kif9	kinesin family member 9	1.12460639	0.3619	0.5714
76737	Creld2	cysteine-rich with EGF-like domains 2	1.12447993	0.01993	NA
27275	Nufip1	nuclear fragile X mental retardation protein interacting protein 1	1.12447993	0.103	NA
76721	1700085B13Rik	RIKEN cDNA 1700085B13 gene	1.12447993	0.3213	0.5311
70676	Gulp1	GULP, engulfment adaptor PTB domain containing 1	1.1243535	0.2612	0.4672
19210	Ptdss1	phosphatidylserine synthase 1	1.1243535	0.3259	0.5359
71699	Slc41a3	solute carrier family 41, member 3	1.1243535	0.3806	0.5897
665095	Cyp2j8-ps	cytochrome P450, family 2, subfamily j, polypeptide 8, pseudogene	1.12422709	0.04777	NA
11489	Adam12	a disintegrin and metallopeptidase domain 12 (meltrin alpha)	1.12422709	0.1481	0.3218
230582	Cyb5rl	cytochrome b5 reductase-like	1.12422709	0.2217	0.421
72106	Jmjd8	jumonji domain containing 8	1.12422709	0.3205	0.5305
231999	Plekha8	pleckstrin homology domain containing, family A (phosphoinositide binding specific) member 8	1.12397437	0.02449	NA
216829	Mmgt2	membrane magnesium transporter 2	1.12384806	0.07407	NA
16562	Kif1c	kinesin family member 1C	1.12384806	0.1415	0.312
19186	Psme1	proteasome (prosome, macropain) 28 subunit, alpha	1.12384806	0.2747	0.4827
268816	Gm628	predicted gene 628	1.12384806	0.408	0.6145
116748	Lsm10	U7 snRNP-specific Sm-like protein LSM10	1.12372177	0.1429	0.3138
53614	Reck	reversion-inducing-cysteine-rich protein with kazal motifs	1.12372177	0.1495	0.3235
22259	Nr1h3	nuclear receptor subfamily 1, group H, member 3	1.12372177	0.1672	0.3481
18028	Nfib	nuclear factor I/B	1.12372177	0.1807	0.3666
20090	Rps29	ribosomal protein S29	1.12372177	0.1816	0.3681
276952	Rasl10b	RAS-like, family 10, member B	1.12372177	0.2924	0.5012
16351	Ipp	IAP promoted placental gene	1.12359551	0.1005	NA
57912	Cdc42se1	CDC42 small effector 1	1.12346927	0.2409	0.4436
74570	Zkscan1	zinc finger with KRAB and SCAN domains 1	1.12346927	0.4536	0.6553
12867	Cox7c	cytochrome c oxidase, subunit VIIc	1.12346927	0.6481	0.8016
638008	Gm7227	predicted gene 7227	1.12334307	0.08439	NA
320302	Glt28d2	glycosyltransferase 28 domain containing 2	1.12321689	0.26	0.4657
77800	4933406J10Rik	RIKEN cDNA 4933406J10 gene	1.12321689	0.5071	0.698
20908	Stx3	syntaxin 3	1.12321689	0.665	0.8113
19188	Psme2	proteasome (prosome, macropain) 28 subunit, beta	1.12309075	0.03145	NA
546143	Gm5918	predicted gene 5918	1.12309075	0.09437	NA
75604	Tm4sf5	transmembrane 4 superfamily member 5	1.12309075	0.226	0.4256
223527	Eny2	enhancer of yellow 2 homolog (Drosophila)	1.12296463	0.04855	NA
76916	4930455C21Rik	RIKEN cDNA 4930455C21 gene	1.12283854	0.2467	0.4508
73068	Fut11	fucosyltransferase 11	1.12283854	0.3266	0.5368
15206	Hes2	hairy and enhancer of split 2 (Drosophila)	1.12283854	0.5034	0.695
71413	5430410E06Rik	RIKEN cDNA 5430410E06 gene	1.12283854	0.6519	0.8037
19858	Rnu3b1	U3B small nuclear RNA 1	1.12271247	0.4714	0.67
50764	Fbxo15	F-box protein 15	1.12258644	0.3379	0.5481
72055	Slc38a10	solute carrier family 38, member 10	1.12258644	0.3617	0.5713
22321	Vars	valyl-tRNA synthetase	1.12258644	0.494	0.6879

621998	Gm6277	predicted gene 6277	1.12258644	0.5	0.6922
57342	Parva	parvin, alpha	1.12233446	0.2799	0.4881
16475	Jub	ajuba	1.12233446	0.4119	0.6182
67997	Ddx59	DEAD (Asp-Glu-Ala-Asp) box polypeptide 59	1.12220851	0.1341	NA
18231	Nxph1	neurexophilin 1	1.12220851	0.2813	0.4894
21769	Zfand3	zinc finger, AN1-type domain 3	1.12208259	0.03494	NA
14950	H13	histocompatibility 13	1.12208259	0.1109	NA
235459	Gtf2a2	general transcription factor II A, 2	1.12208259	0.1482	0.3218
76916	4930455C21Rik	RIKEN cDNA 4930455C21 gene	1.12208259	0.1902	0.3791
402735	B230114P17Rik	RIKEN cDNA B230114P17 gene	1.12208259	0.2433	0.4466
229707	Fam40a	family with sequence similarity 40, member A	1.12208259	0.3365	0.5469
75930	4930567H12Rik	RIKEN cDNA 4930567H12 gene	1.12208259	0.7718	0.8787
18674	Slc25a3	solute carrier family 25 (mitochondrial carrier, phosphate carrier), member 3	1.12195669	0.1189	NA
69263	Rfc3	replication factor C (activator 1) 3	1.12195669	0.2138	0.4102
17920	Myo6	myosin VI	1.12195669	0.2485	0.4527
12572	Cdk7	cyclin-dependent kinase 7	1.12195669	0.3271	0.5373
13849	Ephx1	epoxide hydrolase 1, microsomal	1.12183083	0.3213	0.5311
102657	Cd276	CD276 antigen	1.12183083	0.3636	0.5726
76826	Nubpl	nucleotide binding protein-like	1.12170499	0.1341	NA
15452	Hprt	hypoxanthine guanine phosphoribosyl transferase	1.12170499	0.1737	0.3572
112422	2610305D13Rik	RIKEN cDNA 2610305D13 gene	1.12170499	0.5231	0.7103
69358	Lrrc51	leucine rich repeat containing 51	1.12157918	0.01989	NA
13205	Ddx3x	DEAD/H (Asp-Glu-Ala-Asp/His) box polypeptide 3, X-linked	1.12157918	0.02533	NA
216551	1110067D22Rik	RIKEN cDNA 1110067D22 gene	1.12157918	0.08779	NA
109264	Me3	malic enzyme 3, NADP(+)-dependent, mitochondrial	1.1214534	0.05254	NA
99470	Magi3	membrane associated guanylate kinase, WW and PDZ domain containing 3	1.1214534	0.2549	0.4599
233893	Zfp764	zinc finger protein 764	1.12132765	0.05508	NA
53872	Caprin1	cell cycle associated protein 1	1.12132765	0.08341	NA
22319	Vamp3	vesicle-associated membrane protein 3	1.12132765	0.1546	0.3306
72773	2810449G22Rik	RIKEN cDNA 2810449G22 gene	1.12132765	0.3851	0.5942
76815	Calcoco2	calcium binding and coiled-coil domain 2	1.12132765	0.4892	0.6837
383243	Olfr128	olfactory receptor 128	1.12132765	0.8015	0.8959
407803	BC051226	cDNA sequence BC051226	1.12120193	0.1766	0.3613
17300	Foxc1	forkhead box C1	1.12120193	0.2062	0.4005
432582	E130309D14Rik	RIKEN cDNA E130309D14 gene	1.12120193	0.4116	0.6179
214685	Chadl	chondroadherin-like	1.12107623	0.1244	NA
52276	Cdca8	cell division cycle associated 8	1.12107623	0.4576	0.659
68311	Lypd2	Ly6/Plaur domain containing 2	1.12107623	0.6072	0.7728
13205	Ddx3x	DEAD/H (Asp-Glu-Ala-Asp/His) box polypeptide 3, X-linked	1.12095057	0.0586	NA
56351	Ptges3	prostaglandin E synthase 3 (cytosolic)	1.12095057	0.0737	NA
69480	Ttc9	tetratricopeptide repeat domain 9	1.12095057	0.07383	NA
66391	2310061J03Rik	RIKEN cDNA 2310061J03 gene	1.12095057	0.1932	0.3832
70426	Tekt5	tektin 5	1.12095057	0.5763	0.7495
19337	Rab33a	RAB33A, member of RAS oncogene family	1.12082493	0.09441	NA
74577	Glb1l	galactosidase, beta 1-like	1.12082493	0.2594	0.465
320022	Ccdc79	coiled-coil domain containing 79	1.12082493	0.3728	0.5816
101118	Tmem168	transmembrane protein 168	1.12082493	0.5173	0.7067
211922	Fam116a	family with sequence similarity 116, member A	1.12069932	0.01788	NA
230936	Phf13	PHD finger protein 13	1.12069932	0.05505	NA
14385	Slc37a4	solute carrier family 37 (glucose-6-phosphate transporter), member 4	1.12069932	0.1272	NA

19943	Rpl28	ribosomal protein L28	1.12069932	0.1608	0.3391
13489	Drd2	dopamine receptor D2	1.12069932	0.3183	0.5279
319192	Hist2h2aa2	histone cluster 2, H2aa2	1.12057373	0.05879	NA
319493	A430078G23Rik	RIKEN cDNA A430078G23 gene	1.12057373	0.1813	0.3676
20091	Rps3a	ribosomal protein S3A	1.12044818	0.04341	NA
21968	Tom1	target of myb1 homolog (chicken)	1.12044818	0.05563	NA
83964	Jam3	junction adhesion molecule 3	1.12019715	0.02228	NA
75593	2410003K15Rik	RIKEN cDNA 2410003K15 gene	1.12019715	0.1214	NA
65970	Lima1	LIM domain and actin binding 1	1.12019715	0.2332	0.4347
68394	Ccdc163	coiled-coil domain containing 163	1.12019715	0.3288	0.5389
100088	Rcc1	regulator of chromosome condensation 1	1.12007168	0.02595	NA
100505098	LOC100505098	60S ribosomal protein L19-like	1.12007168	0.04297	NA
66313	Smurf2	SMAD specific E3 ubiquitin protein ligase 2	1.12007168	0.308	0.5177
232533	Stk38l	serine/threonine kinase 38 like	1.11982083	0.1033	NA
19352	Rabggtb	RAB geranylgeranyl transferase, b subunit	1.11982083	0.1531	NA
19847	Rnu1b6	U1b6 small nuclear RNA	1.11982083	0.4926	0.6867
18193	Nsd1	nuclear receptor-binding SET-domain protein 1	1.11969544	0.03434	NA
28077	Med10	mediator of RNA polymerase II transcription, subunit 10 homolog (NUT2, S. cerevisiae)	1.11969544	0.09105	NA
319177	Hist1h2ba	histone cluster 1, H2ba	1.11969544	0.2021	0.3949
666105	Gm7932	predicted gene 7932	1.11969544	0.6024	0.7693
28193	Reep3	receptor accessory protein 3	1.11957009	0.08647	NA
433050	Rpl26-ps4	ribosomal protein L26, pseudogene 4	1.11944476	0.01818	NA
52250	Reep1	receptor accessory protein 1	1.11944476	0.01975	NA
13205	Ddx3x	DEAD/H (Asp-Glu-Ala-Asp/His) box polypeptide 3, X-linked	1.11944476	0.2259	0.4256
69537	Dnase1l1	deoxyribonuclease 1-like 1	1.11944476	0.4092	0.6156
67898	Pef1	penta-EF hand domain containing 1	1.11931945	0.04509	NA
67903	Gipc1	GIPC PDZ domain containing family, member 1	1.11931945	0.3588	0.5687
18618	Pemt	phosphatidylethanolamine N-methyltransferase	1.11931945	0.4464	0.6489
66910	Tmem107	transmembrane protein 107	1.11919418	0.01768	NA
66939	Aagab	alpha- and gamma-adaptin binding protein	1.11919418	0.05134	NA
20220	Sap18	Sin3-associated polypeptide 18	1.11919418	0.0842	NA
100039478	Gm11810	predicted gene 11810	1.11919418	0.136	NA
225049	Ttc7	tetratricopeptide repeat domain 7	1.11919418	0.1486	NA
234396	Ankle1	ankyrin repeat and LEM domain containing 1	1.11919418	0.3319	0.5423
331623	Bend3	BEN domain containing 3	1.11906893	0.087	NA
18700	Piga	phosphatidylinositol glycan anchor biosynthesis, class A	1.11906893	0.1275	NA
14731	Gpaa1	GPI anchor attachment protein 1	1.11906893	0.3191	0.5289
78878	B230206F22Rik	RIKEN cDNA B230206F22 gene	1.11906893	0.592	0.7617
18441	P2ry1	purinergic receptor P2Y, G-protein coupled 1	1.11894372	0.2024	0.3952
380753	Atxn7l1	ataxin 7-like 1	1.11894372	0.206	0.4003
14786	Grb7	growth factor receptor bound protein 7	1.11894372	0.4284	0.6329
12015	Bad	BCL2-associated agonist of cell death	1.11881853	0.1388	NA
229517	Slc25a44	solute carrier family 25, member 44	1.11881853	0.279	0.4872
74915	Atp6v1e2	ATPase, H+ transporting, lysosomal V1 subunit E2	1.11881853	0.5881	0.7583
241289	Gm347	predicted gene 347	1.11869337	0.0892	NA
24100	Tpra1	transmembrane protein, adipocyte asscociated 1	1.11869337	0.149	NA
77422	C330018D20Rik	RIKEN cDNA C330018D20 gene	1.11869337	0.1643	0.3441
100502854	LOC100502854	hypothetical LOC100502854	1.11856823	0.1218	NA
75705	Eif4b	eukaryotic translation initiation factor 4B	1.11856823	0.139	NA
12015	Bad	BCL2-associated agonist of cell death	1.11856823	0.2321	0.4331

69697	2310057J16Rik	RIKEN cDNA 2310057J16 gene	1.11856823	0.3434	0.5545
239559	A4galt	alpha 1,4-galactosyltransferase	1.11856823	0.6456	0.7997
67628	Anp32b	acidic (leucine-rich) nuclear phosphoprotein 32 family, member B	1.11844313	0.0377	NA
72392	Tmem175	transmembrane protein 175	1.11844313	0.1015	NA
75430	3200002M19Rik	RIKEN cDNA 3200002M19 gene	1.11844313	0.2572	0.4623
12796	Camp	cathelicidin antimicrobial peptide	1.11844313	0.3254	0.5353
11421	Ace	angiotensin I converting enzyme (peptidyl-dipeptidase A) 1	1.11844313	0.6167	0.7795
67885	1500011K16Rik	RIKEN cDNA 1500011K16 gene	1.11831805	0.05517	NA
12295	Cacnb1	calcium channel, voltage-dependent, beta 1 subunit	1.11831805	0.2396	0.4421
69761	1600015I10Rik	RIKEN cDNA 1600015I10 gene	1.11831805	0.2469	0.4511
68612	Ube2c	ubiquitin-conjugating enzyme E2C	1.11831805	0.4044	0.6121
56398	1500003O03Rik	RIKEN cDNA 1500003O03 gene	1.118193	0.02968	NA
223697	Sun2	Sad1 and UNC84 domain containing 2	1.11806798	0.05169	NA
100039697	Gm2375	predicted gene 2375	1.11806798	0.259	0.4646
319236	9230105E10Rik	RIKEN cDNA 9230105E10 gene	1.11806798	0.7054	0.8377
67963	Npc2	Niemann Pick type C2	1.11794298	0.1058	NA
50873	Park2	Parkinson disease (autosomal recessive, juvenile) 2, parkin	1.11794298	0.1353	NA
217119	Xylt2	xylosyltransferase II	1.11794298	0.3107	0.5205
56709	Dnajb12	DnaJ (Hsp40) homolog, subfamily B, member 12	1.11781802	0.05086	NA
623131	Prr19	proline rich 19	1.11769308	0.5395	0.723
384701	Dub2a	deubiquitinating enzyme 2a	1.11769308	0.7693	0.8776
68212	Tmbim4	transmembrane BAX inhibitor motif containing 4	1.11756817	0.07142	NA
72171	Shq1	SHQ1 homolog (S. cerevisiae)	1.11756817	0.1569	NA
18036	Nfkbib	nuclear factor of kappa light polypeptide gene enhancer in B-cells inhibitor, beta	1.11756817	0.239	0.4415
17346	Mknk1	MAP kinase-interacting serine/threonine kinase 1	1.11756817	0.4141	0.6205
211577	Mrgprf	MAS-related GPR, member F	1.11756817	0.5814	0.7534
108816	4933409K07Rik	RIKEN cDNA 4933409K07 gene	1.11756817	0.7658	0.8756
27224	Tceb3	transcription elongation factor B (SIII), polypeptide 3	1.11744329	0.08931	NA
26363	Btd	biotinidase	1.11744329	0.1972	0.3885
100038969	Gm14958	predicted gene 14958	1.11731844	0.04211	NA
210126	Lpp	LIM domain containing preferred translocation partner in lipoma	1.11731844	0.05382	NA
231912	Katnal1	katanin p60 subunit A-like 1	1.11731844	0.13	NA
19035	Ppib	peptidylprolyl isomerase B	1.11719361	0.1578	NA
107435	Hat1	histone aminotransferase 1	1.11719361	0.2114	0.4071
272359	Irf2bp1	interferon regulatory factor 2 binding protein 1	1.11719361	0.2482	0.4525
18826	Lcp1	lymphocyte cytosolic protein 1	1.11719361	0.4415	0.6445
20362	Sep-08	septin 8	1.11719361	0.4726	0.6708
277343	Wfdc8	WAP four-disulfide core domain 8	1.11719361	0.4795	0.6759
16898	Rps2	ribosomal protein S2	1.11706881	0.1399	NA
74268	Aven	apoptosis, caspase activation inhibitor	1.11706881	0.1401	NA
381801	Tatdn2	TatD DNase domain containing 2	1.11706881	0.3416	0.5521
17364	Trpm1	transient receptor potential cation channel, subfamily M, member 1	1.11706881	0.5312	0.7165
385605	Rundc2a	RUN domain containing 2A	1.11706881	0.6327	0.7914
72205	Eml2	echinoderm microtubule associated protein like 2	1.1168193	0.07018	NA
20102	Rps4x	ribosomal protein S4, X-linked	1.11669458	0.04629	NA
234723	Txnl4b	thioredoxin-like 4B	1.11669458	0.1139	NA
80880	Kank3	KN motif and ankyrin repeat domains 3	1.11669458	0.1838	0.3709
69681	Cdk3-ps	cyclin-dependent kinase 3, pseudogene	1.11669458	0.3154	0.5248
28075	Pppde2	PPPDE peptidase domain containing 2	1.1165699	0.06062	NA
235442	Rab8b	RAB8B, member RAS oncogene family	1.1165699	0.09711	NA

76366	Mtif3	mitochondrial translational initiation factor 3	1.1165699	0.2318	0.4326
70598	Filip1	filamin A interacting protein 1	1.1165699	0.2459	0.4497
328265	A530001N23Rik	RIKEN cDNA A530001N23 gene	1.1165699	0.5287	0.7147
100503670	Rpl5	ribosomal protein L5	1.11644524	0.1781	0.3632
72992	2900076A07Rik	RIKEN cDNA 2900076A07 gene	1.11644524	0.2077	0.4025
16814	Lbx1	ladybird homeobox homolog 1 (Drosophila)	1.11644524	0.3321	0.5423
73687	2410087M07Rik	RIKEN cDNA 2410087M07 gene	1.11644524	0.5325	0.7176
27357	Gyg	glycogenin	1.11632061	0.1005	NA
93691	Klf7	Kruppel-like factor 7 (ubiquitous)	1.11632061	0.1377	NA
106757	Tmem146	transmembrane protein 146	1.11632061	0.2087	0.4035
75552	Paqr9	progestin and adipoQ receptor family member IX	1.11632061	0.4556	0.6571
234309	Cbr4	carbonyl reductase 4	1.116196	0.0693	NA
67115	Rpl14	ribosomal protein L14	1.116196	0.2295	0.4301
106046	AW413774	expressed sequence AW413774	1.116196	0.4893	0.6838
93896	Glp2r	glucagon-like peptide 2 receptor	1.116196	0.6482	0.8016
100045396	Gm9908	predicted gene 9908	1.116196	0.8404	0.9177
22245	Uck1	uridine-cytidine kinase 1	1.11607143	0.02648	NA
246256	Fcgr4	Fc receptor, IgG, low affinity IV	1.11594688	0.4415	0.6445
11852	Rhob	ras homolog gene family, member B	1.11582236	0.1578	NA
69976	Galk2	galactokinase 2	1.11582236	0.2069	0.4015
20340	Glg1	golgi apparatus protein 1	1.11582236	0.2487	0.4529
231148	Ablim2	actin-binding LIM protein 2	1.11582236	0.2941	0.503
54352	Irx5	Iroquois related homeobox 5 (Drosophila)	1.11582236	0.3734	0.5821
56351	Ptges3	prostaglandin E synthase 3 (cytosolic)	1.11569787	0.192	0.3817
66868	Mfsd1	major facilitator superfamily domain containing 1	1.11569787	0.2873	0.4958
232539	Klhdc5	kelch domain containing 5	1.1155734	0.04189	NA
70382	Kctd2	potassium channel tetramerisation domain containing 2	1.1155734	0.1303	NA
12467	Cct6b	chaperonin containing Tcp1, subunit 6b (zeta)	1.1155734	0.1819	0.3684
11603	Agrn	agrin	1.11544897	0.1155	NA
76820	Fam49a	family with sequence similarity 49, member A	1.11544897	0.1852	0.3724
73075	Ppil6	peptidylprolyl isomerase (cyclophilin)-like 6	1.11532456	0.17	NA
68982	1500015A07Rik	RIKEN cDNA 1500015A07 gene	1.11532456	0.305	0.5142
223754	Tbc1d22a	TBC1 domain family, member 22a	1.11520018	0.02906	NA
229725	Clcc1	chloride channel CLIC-like 1	1.11507583	0.05373	NA
68135	Eif3h	eukaryotic translation initiation factor 3, subunit H	1.11507583	0.1126	NA
70420	2610034B18Rik	RIKEN cDNA 2610034B18 gene	1.11507583	0.2619	0.4677
258818	Olfr629	olfactory receptor 629	1.11507583	0.4209	0.6271
100038580	6820445E23Rik	RIKEN cDNA 6820445E23 gene	1.1149515	0.3322	0.5423
110052	Dek	DEK oncogene (DNA binding)	1.1148272	0.05627	NA
19201	Pstpip2	proline-serine-threonine phosphatase-interacting protein 2	1.1148272	0.3678	0.5768
107227	Macrod1	MACRO domain containing 1	1.11470293	0.3039	0.513
208211	Alg1	asparagine-linked glycosylation 1 homolog (yeast, beta-1,4-mannosyltransferase)	1.11457869	0.06737	NA
666955	Gm8378	predicted gene 8378	1.11457869	0.3439	0.5547
170459	Stard4	StAR-related lipid transfer (START) domain containing 4	1.11457869	0.416	0.6222
216987	Utp6	UTP6, small subunit (SSU) processome component, homolog (yeast)	1.11457869	0.4509	0.653
66536	Nipsnap3b	nipsnap homolog 3B (C. elegans)	1.11445447	0.3437	0.5547
74895	4930455F23Rik	RIKEN cDNA 4930455F23 gene	1.11420613	0.03028	NA
225876	Kdm2a	lysine (K)-specific demethylase 2A	1.11420613	0.3265	0.5367
233046	Rasgrp4	RAS guanyl releasing protein 4	1.11420613	0.4734	0.6714
14083	Ptk2	PTK2 protein tyrosine kinase 2	1.114082	0.07449	NA

219022	Ttc5	tetratricopeptide repeat domain 5	1.114082	0.1881	0.3763
278507	Wfikkn2	WAP, follistatin/kazal, immunoglobulin, kunitz and netrin domain containing 2	1.114082	0.1888	0.3771
67526	Atg12	autophagy-related 12 (yeast)	1.114082	0.3965	0.6043
19981	Rpl37a	ribosomal protein L37a	1.11395789	0.2991	0.5084
320237	Ncrna00086	non-protein coding RNA 86	1.11395789	0.3674	0.5765
22325	Vav2	vav 2 oncogene	1.11383382	0.3714	0.5801
71520	Grap	GRB2-related adaptor protein	1.11383382	0.4922	0.6863
69123	1810022C23Rik	RIKEN cDNA 1810022C23 gene	1.11370977	0.1639	NA
193742	Abhd16a	abhydrolase domain containing 16A	1.11370977	0.1675	NA
67469	Abhd5	abhydrolase domain containing 5	1.11370977	0.379	0.588
268782	Agxt2	alanine-glyoxylate aminotransferase 2	1.11370977	0.7747	0.8801
58172	Sertad2	SERTA domain containing 2	1.11358575	0.09097	NA
269181	Mgat4a	mannoside acetylglucosaminyltransferase 4, isoenzyme A	1.11358575	0.1925	0.3823
544872	Gm5786	predicted pseudogene 5786	1.11358575	0.2942	0.5032
18707	Pik3cd	phosphatidylinositol 3-kinase catalytic delta polypeptide	1.11358575	0.3964	0.6043
67501	Ccdc50	coiled-coil domain containing 50	1.11346175	0.3493	0.5597
68488	1110002J07Rik	RIKEN cDNA 1110002J07 gene	1.11346175	0.3706	0.5794
50523	Lats2	large tumor suppressor 2	1.11333779	0.4051	0.6129
18483	Palm	paralemmin	1.11333779	0.4122	0.6185
170779	Cd209d	CD209d antigen	1.11333779	0.4273	0.6321
74934	Armc4	armadillo repeat containing 4	1.11333779	0.6004	0.7682
18174	Slc11a2	solute carrier family 11 (proton-coupled divalent metal ion transporters), member 2	1.11321385	0.0683	NA
226849	Ppp2r5a	protein phosphatase 2, regulatory subunit B (B56), alpha isoform	1.11321385	0.1366	NA
56455	Dynll1	dynein light chain LC8-type 1	1.11321385	0.1795	NA
101700	Trim68	tripartite motif-containing 68	1.11321385	0.2248	0.4245
319215	4932413F04Rik	RIKEN cDNA 4932413F04 gene	1.11321385	0.4783	0.6752
72103	Aplf	aprataxin and PNKP like factor	1.11308994	0.1015	NA
378702	Serf2	small EDRK-rich factor 2	1.11308994	0.2306	0.4314
399558	Flrt2	fibronectin leucine rich transmembrane protein 2	1.11308994	0.3739	0.5825
65971	Tbata	thymus, brain and testes associated	1.11308994	0.7279	0.8519
68634	Tm2d3	TM2 domain containing 3	1.11296605	0.06804	NA
16785	Rpsa	ribosomal protein SA	1.11296605	0.1516	NA
14761	Gpr27	G protein-coupled receptor 27	1.11296605	0.351	0.5611
14548	Mrps33	mitochondrial ribosomal protein S33	1.1128422	0.06678	NA
70057	2210008F06Rik	RIKEN cDNA 2210008F06 gene	1.1128422	0.3013	0.5108
100504876	LOC100504876	60S ribosomal protein L7a-like	1.11271837	0.03652	NA
216560	AV249152	expressed sequence AV249152	1.11271837	0.1402	NA
100042959	Gm4130	predicted gene 4130	1.11271837	0.2282	0.4282
626150	Gm12271	predicted gene 12271	1.11271837	0.2722	0.4795
228769	Psmf1	proteasome (prosome, macropain) inhibitor subunit 1	1.11271837	0.3134	0.5231
14852	Gspt1	G1 to S phase transition 1	1.11259457	0.08022	NA
71966	Nkiras2	NFKB inhibitor interacting Ras-like protein 2	1.11259457	0.08948	NA
192231	Hexim1	hexamethylene bis-acetamide inducible 1	1.11259457	0.1499	NA
79566	Sh3bp5l	SH3 binding domain protein 5 like	1.1124708	0.02279	NA
11465	Actg1	actin, gamma, cytoplasmic 1	1.1124708	0.1103	NA
69101	Ydjc	YdjC homolog (bacterial)	1.1124708	0.1297	NA
11541	Adora2b	adenosine A2b receptor	1.1124708	0.5905	0.7603
56348	Hsd17b12	hydroxysteroid (17-beta) dehydrogenase 12	1.11234705	0.2939	0.5029
76123	Gpsm2	G-protein signalling modulator 2 (AGS3-like, C. elegans)	1.11222333	0.03147	NA
68514	Efha1	EF hand domain family A1	1.11222333	0.03615	NA

381629	0610007C21Rik	RIKEN cDNA 0610007C21 gene	1.11222333	0.08696	NA
72355	2210021J22Rik	RIKEN cDNA 2210021J22 gene	1.11222333	0.1595	NA
66988	Lap3	leucine aminopeptidase 3	1.11209964	0.07143	NA
209683	Ttc28	tetratricopeptide repeat domain 28	1.11209964	0.0828	NA
77775	A430103D13Rik	RIKEN cDNA A430103D13 gene	1.11209964	0.1731	NA
69259	Kctd5	potassium channel tetramerisation domain containing 5	1.11209964	0.3653	0.5743
14055	Ezh1	enhancer of zeste homolog 1 (Drosophila)	1.11197598	0.1664	NA
74104	Abcb6	ATP-binding cassette, sub-family B (MDR/TAP), member 6	1.11197598	0.2168	0.4143
211488	Ado	2-aminoethanethiol (cysteamine) dioxygenase	1.11197598	0.3995	0.6073
75958	5033403F01Rik	RIKEN cDNA 5033403F01 gene	1.11197598	0.4926	0.6866
74026	Msl1	male-specific lethal 1 homolog (Drosophila)	1.11185235	0.05965	NA
229644	Trim45	tripartite motif-containing 45	1.11172874	0.08684	NA
27979	Eif3b	eukaryotic translation initiation factor 3, subunit B	1.11160516	0.04241	NA
66588	Cmpk1	cytidine monophosphate (UMP-CMP) kinase 1	1.11160516	0.2399	0.4423
16980	Lrrn2	leucine rich repeat protein 2, neuronal	1.1114816	0.2498	0.4542
22294	Uxt	ubiquitously expressed transcript	1.1114816	0.3024	0.5115
77065	Ints7	integrator complex subunit 7	1.11135808	0.05627	NA
56314	Zfp113	zinc finger protein 113	1.11135808	0.1774	NA
101095	Zfp282	zinc finger protein 282	1.11135808	0.2452	0.4488
72711	2810037O22Rik	RIKEN cDNA 2810037O22 gene	1.11135808	0.2501	0.4547
16785	Rpsa	ribosomal protein SA	1.11123458	0.1217	NA
67198	Spats2l	spermatogenesis associated, serine-rich 2-like	1.11123458	0.2925	0.5013
67121	Mastl	microtubule associated serine/threonine kinase-like	1.11123458	0.4963	0.6895
19401	Rara	retinoic acid receptor, alpha	1.11111111	0.1395	NA
17385	Mmp11	matrix metallopeptidase 11	1.11111111	0.3928	0.6011
76915	Mnd1	meiotic nuclear divisions 1 homolog (S. cerevisiae)	1.11111111	0.3967	0.6045
329659	E130311K13Rik	RIKEN cDNA E130311K13 gene	1.11111111	0.4022	0.6098
66357	Ostc	oligosaccharyltransferase complex subunit	1.11098767	0.04311	NA
19739	Rgs9	regulator of G-protein signaling 9	1.11098767	0.1071	NA
106795	Tcf19	transcription factor 19	1.11098767	0.2745	0.4825
320309	1520401A03Rik	RIKEN cDNA 1520401A03 gene	1.11098767	0.7436	0.8615
225896	Ubxn1	UBX domain protein 1	1.11086425	0.1472	NA
329416	Nostrin	nitric oxide synthase trafficker	1.11086425	0.2254	0.4252
14425	Galnt3	UDP-N-acetyl-alpha-D-galactosamine:polypeptide N-acetylgalactosaminyltransferase 3	1.11086425	0.2888	0.4972
231452	Sdad1	SDA1 domain containing 1	1.11074086	0.1644	NA
16706	Ksr1	kinase suppressor of ras 1	1.11074086	0.2606	0.4665
20588	Smarcc1	SWI/SNF related, matrix associated, actin dependent regulator of chromatin, subfamily c, member 1	1.11074086	0.389	0.5973
654796	9530036O11Rik	RIKEN cDNA 9530036O11Rik	1.1106175	0.07843	NA
13681	Eif4a1	eukaryotic translation initiation factor 4A1	1.1106175	0.1435	NA
258353	Olfr521	olfactory receptor 521	1.1106175	0.2116	0.4073
11550	Adra1d	adrenergic receptor, alpha 1d	1.1106175	0.6237	0.7845
22282	Usf2	upstream transcription factor 2	1.11049417	0.1478	NA
13449	Dok2	docking protein 2	1.11049417	0.221	0.4201
67255	Zfp422	zinc finger protein 422	1.11049417	0.526	0.7126
70356	St13	suppression of tumorigenicity 13	1.11037086	0.03048	NA
76108	Rap2a	RAS related protein 2a	1.11037086	0.04602	NA
19934	Rpl22	ribosomal protein L22	1.11037086	0.1456	NA
66307	Isoc1	isochorismatase domain containing 1	1.11037086	0.2357	0.4375
230500	Efcab7	EF-hand calcium binding domain 7	1.11037086	0.4518	0.654
329003	Zfp516	zinc finger protein 516	1.11024759	0.377	0.5861

72927	Hepacam	hepatocyte cell adhesion molecule	1.11024759	0.4753	0.6729
66894	Wwp2	WW domain containing E3 ubiquitin protein ligase 2	1.11012433	0.1079	NA
11479	Acvr1b	activin A receptor, type 1B	1.11012433	0.5091	0.6999
621852	Rhox3f	reproductive homeobox 3F	1.11012433	0.813	0.9031
56431	Dstn	destrin	1.11000111	0.11	NA
384071	Slc25a34	solute carrier family 25, member 34	1.11000111	0.4942	0.688
70757	Ptplb	protein tyrosine phosphatase-like (proline instead of catalytic arginine), member b	1.10987791	0.02773	NA
18549	Pcsk2	proprotein convertase subtilisin/kexin type 2	1.10987791	0.3667	0.5757
69743	Casz1	castor homolog 1, zinc finger (Drosophila)	1.10987791	0.4481	0.6503
100043380	Gm4402	predicted gene 4402	1.10987791	0.5725	0.7473
70044	Tut1	terminal uridylyl transferase 1, U6 snRNA-specific	1.10975474	0.07864	NA
114585	D17H6S53E	DNA segment, Chr 17, human D6S53E	1.10975474	0.2711	0.4788
17155	Man1a	mannosidase 1, alpha	1.10975474	0.4699	0.6691
17192	Mbd3	methyl-CpG binding domain protein 3	1.1096316	0.1294	NA
15381	Hnrnpc	heterogeneous nuclear ribonucleoprotein C	1.1096316	0.1613	NA
110954	Rpl10	ribosomal protein 10	1.1096316	0.2514	0.4561
17773	Mtnr1a	melatonin receptor 1A	1.10950849	0.3769	0.5858
241118	Accn4	amiloride-sensitive cation channel 4, pituitary	1.1093854	0.09709	NA
11671	Aldh3a2	aldehyde dehydrogenase family 3, subfamily A2	1.1093854	0.1218	NA
66056	Zfp524	zinc finger protein 524	1.1093854	0.1269	NA
67264	Ndufb8	NADH dehydrogenase (ubiquinone) 1 beta subcomplex 8	1.1093854	0.1355	NA
229791	D3Bwg0562e	DNA segment, Chr 3, Brigham & Women's Genetics 0562 expressed	1.1093854	0.1821	NA
68810	Nexn	nexilin	1.1093854	0.3556	0.5654
78655	Eif3j	eukaryotic translation initiation factor 3, subunit J	1.10926234	0.1289	NA
22143	Tuba1b	tubulin, alpha 1B	1.10926234	0.1828	NA
67997	Ddx59	DEAD (Asp-Glu-Ala-Asp) box polypeptide 59	1.10926234	0.1852	NA
20112	Rps6ka2	ribosomal protein S6 kinase, polypeptide 2	1.10926234	0.2221	0.4214
246277	Csad	cysteine sulfinic acid decarboxylase	1.10926234	0.2839	0.4924
20810	Srm	spermidine synthase	1.10926234	0.3223	0.5321
17127	Smad3	MAD homolog 3 (Drosophila)	1.10926234	0.4561	0.6576
67838	Dnajb11	DnaJ (Hsp40) homolog, subfamily B, member 11	1.10913931	0.05012	NA
27357	Gyg	glycogenin	1.10913931	0.1009	NA
71295	4933431K14Rik	RIKEN cDNA 4933431K14 gene	1.10913931	0.1137	NA
67726	Fam114a2	family with sequence similarity 114, member A2	1.10913931	0.1355	NA
20444	St3gal2	ST3 beta-galactoside alpha-2,3-sialyltransferase 2	1.10913931	0.2472	0.4515
22185	U2af2	U2 small nuclear ribonucleoprotein auxiliary factor (U2AF) 2	1.10913931	0.3096	0.5193
107970	Hist1h1t	histone cluster 1, H1t	1.10913931	0.4357	0.6394
50781	Dkk3	dickkopf homolog 3 (Xenopus laevis)	1.1090163	0.2944	0.5033
56296	Dmrtb1	DMRT-like family B with proline-rich C-terminal, 1	1.1090163	0.4727	0.6709
69010	Anapc13	anaphase promoting complex subunit 13	1.10889332	0.08845	NA
56375	B4galt4	UDP-Gal:betaGlcNAc beta 1,4-galactosyltransferase, polypeptide 4	1.10889332	0.1917	NA
93757	Immp2l	IMP2 inner mitochondrial membrane peptidase-like (S. cerevisiae)	1.10889332	0.3231	0.5329
17191	Mbd2	methyl-CpG binding domain protein 2	1.10877037	0.1081	NA
225888	Suv420h1	suppressor of variegation 4-20 homolog 1 (Drosophila)	1.10877037	0.4723	0.6705
71461	Ptk7	PTK7 protein tyrosine kinase 7	1.10864745	0.2328	0.4341
109246	Tspan9	tetraspanin 9	1.10852455	0.4078	0.6144
58200	Ppp1r1a	protein phosphatase 1, regulatory (inhibitor) subunit 1A	1.10840168	0.3337	0.5441
102093	Phkb	phosphorylase kinase beta	1.10840168	0.36	0.5696
68841	1110054M08Rik	RIKEN cDNA 1110054M08 gene	1.10840168	0.3923	0.6005
381059	Gm1604b	predicted gene 1604b	1.10840168	0.5088	0.6996

17151	Ccndbp1	cyclin D-type binding-protein 1	1.10827884	0.1447	NA
15382	Hnrnpa1	heterogeneous nuclear ribonucleoprotein A1	1.10827884	0.1817	NA
209018	Vps8	vacuolar protein sorting 8 homolog (S. cerevisiae)	1.10827884	0.1937	NA
216613	Ccdc85a	coiled-coil domain containing 85A	1.10827884	0.2684	0.4755
237387	Lrrc3	leucine rich repeat containing 3	1.10827884	0.3044	0.5135
16536	Kcnq2	potassium voltage-gated channel, subfamily Q, member 2	1.10827884	0.5849	0.7556
64934	Pes1	pescadillo homolog 1, containing BRCT domain (zebrafish)	1.10815603	0.06561	NA
93892	Pcdhb21	protocadherin beta 21	1.10815603	0.122	NA
110078	Pygb	brain glycogen phosphorylase	1.10815603	0.3524	0.5625
54371	Chst2	carbohydrate sulfotransferase 2	1.10815603	0.4298	0.634
100038410	Gm10536	predicted gene 10536	1.10815603	0.7929	0.8914
78792	4930432F04Rik	RIKEN cDNA 4930432F04 gene	1.10803324	0.1293	NA
58244	Stx6	syntaxin 6	1.10803324	0.1356	NA
21961	Tns1	tensin 1	1.10803324	0.1961	NA
69683	2310044H10Rik	RIKEN cDNA 2310044H10 gene	1.10791048	0.424	0.6295
320933	D230017M19Rik	RIKEN cDNA D230017M19 gene	1.10791048	0.7082	0.8395
67399	Pdlim7	PDZ and LIM domain 7	1.10778775	0.2761	0.4841
69408	Dnajc17	DnaJ (Hsp40) homolog, subfamily C, member 17	1.10778775	0.5459	0.7278
69511	Klk12	kallikrein related-peptidase 12	1.10778775	0.6126	0.7766
12035	Bcat1	branched chain aminotransferase 1, cytosolic	1.10766504	0.06865	NA
76889	Adck4	aarF domain containing kinase 4	1.10766504	0.192	NA
71769	Bbs10	Bardet-Biedl syndrome 10 (human)	1.10766504	0.2388	0.4413
18036	Nfkbib	nuclear factor of kappa light polypeptide gene enhancer in B-cells inhibitor, beta	1.10766504	0.4611	0.6616
319986	A130042O14Rik	RIKEN cDNA A130042O14 gene	1.10766504	0.5545	0.7345
114666	Krtap5-5	keratin associated protein 5-5	1.10754236	0.2685	0.4755
12181	Bop1	block of proliferation 1	1.10754236	0.3586	0.5685
20447	St6galnac3	ST6 (alpha-N-acetyl-neuraminyl-2,3-beta-galactosyl-1,3)-N-acetylgalactosaminide alpha-2,6-sialyltransferase 3	1.10754236	0.6382	0.7951
17722	ND6	NADH dehydrogenase subunit 6	1.10754236	0.6448	0.7994
94220	Cnnm4	cyclin M4	1.10741971	0.1013	NA
18412	Sqstm1	sequestosome 1	1.10741971	0.3961	0.604
12725	Clcn3	chloride channel 3	1.10729709	0.08974	NA
15939	Ier5	immediate early response 5	1.10729709	0.4272	0.632
320119	Rps6kc1	ribosomal protein S6 kinase polypeptide 1	1.10729709	0.5666	0.7428
17434	Mocs2	molybdenum cofactor synthesis 2	1.10705192	0.09861	NA
80733	Car15	carbonic anhydrase 15	1.10705192	0.1383	NA
58178	Sorcs1	VPS10 domain receptor protein SORCS 1	1.10705192	0.3172	0.5267
74096	Hvcn1	hydrogen voltage-gated channel 1	1.10705192	0.4199	0.626
232286	Tmf1	TATA element modulatory factor 1	1.10692938	0.2663	0.4731
13427	Dync1i2	dynein cytoplasmic 1 intermediate chain 2	1.10680686	0.1594	NA
21416	Tcf7l2	transcription factor 7-like 2, T-cell specific, HMG-box	1.10680686	0.288	0.4965
19364	Rad51l3	RAD51-like 3 (S. cerevisiae)	1.10680686	0.3305	0.5408
24099	Tnfsf13b	tumor necrosis factor (ligand) superfamily, member 13b	1.10680686	0.458	0.6592
69072	Ebna1bp2	EBNA1 binding protein 2	1.10668437	0.08496	NA
74025	Nphp3	nephronophthisis 3 (adolescent)	1.10656191	0.1162	NA
268395	Mpg	N-methylpurine-DNA glycosylase	1.10656191	0.1983	NA
73668	Ttc21b	tetratricopeptide repeat domain 21B	1.10656191	0.2865	0.4949
74243	2210009G21Rik	RIKEN cDNA 2210009G21 gene	1.10656191	0.4333	0.6369
74343	Crtc2	CREB regulated transcription coactivator 2	1.10656191	0.4738	0.6717
70101	Cyp4f16	cytochrome P450, family 4, subfamily f, polypeptide 16	1.10643948	0.4324	0.6364
17175	Masp2	mannan-binding lectin serine peptidase 2	1.10643948	0.5697	0.745

224671	Btbd9	BTB (POZ) domain containing 9	1.10631707	0.117	NA
54725	Cadm1	cell adhesion molecule 1	1.10631707	0.1426	NA
75430	3200002M19Rik	RIKEN cDNA 3200002M19 gene	1.10631707	0.1929	NA
21814	Tgfbr3	transforming growth factor, beta receptor III	1.10631707	0.1996	NA
109801	Glo1	glyoxalase 1	1.10631707	0.2096	NA
71910	Ppapdc1b	phosphatidic acid phosphatase type 2 domain containing 1B	1.10631707	0.2315	0.4323
380686	Cnrip1	cannabinoid receptor interacting protein 1	1.10619469	0.1936	NA
17172	Ascl1	achaete-scute complex homolog 1 (Drosophila)	1.10619469	0.2151	0.4122
67912	1600012H06Rik	RIKEN cDNA 1600012H06 gene	1.10619469	0.2227	0.4221
12808	Cobl	cordon-bleu	1.10619469	0.2346	0.4363
380836	Mrs2	MRS2 magnesium homeostasis factor homolog (S. cerevisiae)	1.10619469	0.3353	0.5457
67151	Psmd9	proteasome (prosome, macropain) 26S subunit, non-ATPase, 9	1.10607234	0.1377	NA
279618	Gm715	predicted pseudogene 715	1.10607234	0.2557	0.4607
98417	Cnih4	cornichon homolog 4 (Drosophila)	1.10595001	0.1532	NA
54709	Eif3i	eukaryotic translation initiation factor 3, subunit I	1.10595001	0.1892	NA
319880	Tmcc3	transmembrane and coiled coil domains 3	1.10595001	0.2078	NA
83924	Gpr137b	G protein-coupled receptor 137B	1.10595001	0.2697	0.477
12417	Cbx3	chromobox homolog 3 (Drosophila HP1 gamma)	1.10595001	0.3384	0.5486
73795	4930405D01Rik	RIKEN cDNA 4930405D01 gene	1.10595001	0.6968	0.8325
56486	Gabarap	gamma-aminobutyric acid receptor associated protein	1.10582771	0.05603	NA
20779	Src	Rous sarcoma oncogene	1.10582771	0.4192	0.6254
12511	Cd6	CD6 antigen	1.10582771	0.5361	0.7207
15211	Hexa	hexosaminidase A	1.10570544	0.07995	NA
20741	Spnb1	spectrin beta 1	1.10570544	0.2623	0.4683
52855	Lair1	leukocyte-associated Ig-like receptor 1	1.10570544	0.286	0.4945
71685	Galnt14	UDP-N-acetyl-alpha-D-galactosamine:polypeptide N-acetylgalactosaminyltransferase 14	1.10570544	0.3117	0.5214
231430	Cox18	COX18 cytochrome c oxidase assembly homolog (S. cerevisiae)	1.10570544	0.3279	0.5379
108802	Calr4	calreticulin 4	1.10570544	0.3727	0.5814
100038347	Fam174b	family with sequence similarity 174, member B	1.1055832	0.1572	NA
226610	Fam78b	family with sequence similarity 78, member B	1.10533879	0.1854	NA
58250	Chst11	carbohydrate sulfotransferase 11	1.10533879	0.3174	0.527
16765	Stmn1	stathmin 1	1.10521662	0.1351	NA
24075	Taf10	TAF10 RNA polymerase II, TATA box binding protein (TBP)-associated factor	1.10521662	0.2701	0.4776
74123	Foxp4	forkhead box P4	1.10521662	0.3454	0.5562
56317	Anapc7	anaphase promoting complex subunit 7	1.10521662	0.4345	0.6383
13601	Ecm1	extracellular matrix protein 1	1.10521662	0.6216	0.7828
68350	Mul1	mitochondrial ubiquitin ligase activator of NFKB 1	1.10509449	0.08202	NA
70510	Rnf167	ring finger protein 167	1.10509449	0.1721	NA
67236	Cinp	cyclin-dependent kinase 2 interacting protein	1.10509449	0.2921	0.5009
20102	Rps4x	ribosomal protein S4, X-linked	1.10497238	0.04513	NA
59043	Wsb2	WD repeat and SOCS box-containing 2	1.10497238	0.0874	NA
225745	Haus1	HAUS augmin-like complex, subunit 1	1.10497238	0.1136	NA
100040220	Gm9892	predicted gene 9892	1.10497238	0.1265	NA
66624	Spcs2	signal peptidase complex subunit 2 homolog (S. cerevisiae)	1.10497238	0.2052	NA
72119	Tpx2	TPX2, microtubule-associated protein homolog (Xenopus laevis)	1.10497238	0.4328	0.6366
107976	Bre	brain and reproductive organ-expressed protein	1.10485029	0.4059	0.6134
57815	Spata5	spermatogenesis associated 5	1.10472824	0.06876	NA
68095	Ociad1	OCIA domain containing 1	1.10472824	0.06942	NA
11482	Acvrl1	activin A receptor, type II-like 1	1.10472824	0.1528	NA
17896	Myl4	myosin, light polypeptide 4	1.10472824	0.2543	0.4593

16956	Lpl	lipoprotein lipase	1.10472824	0.3402	0.5506
74097	Pop7	processing of precursor 7, ribonuclease P family, (S. cerevisiae)	1.10460621	0.05731	NA
99167	Ssx2ip	synovial sarcoma, X breakpoint 2 interacting protein	1.10460621	0.1222	NA
15587	Hyal2	hyaluronoglucosaminidase 2	1.10460621	0.2157	NA
68174	4930534H18Rik	RIKEN cDNA 4930534H18 gene	1.10460621	0.4569	0.6583
27078	B9d1	B9 protein domain 1	1.10448421	0.03121	NA
101351	A130022J15Rik	RIKEN cDNA A130022J15 gene	1.10448421	0.09465	NA
75565	Ccdc101	coiled-coil domain containing 101	1.10448421	0.1521	NA
20588	Smarcc1	SWI/SNF related, matrix associated, actin dependent regulator of chromatin, subfamily c, member 1	1.10448421	0.1892	NA
100504871	LOC100504871	ATP synthase lipid-binding protein, mitochondrial-like	1.10448421	0.2532	0.458
59029	Psmd14	proteasome (prosome, macropain) 26S subunit, non-ATPase, 14	1.10448421	0.3559	0.5656
83771	Tas1r3	taste receptor, type 1, member 3	1.10448421	0.3719	0.5807
228801	U46068	cDNA sequence U46068	1.10448421	0.4536	0.6553
14451	Gas1	growth arrest specific 1	1.10436223	0.2258	0.4256
70423	Tspan15	tetraspanin 15	1.10436223	0.2307	0.4316
231134	Dok7	docking protein 7	1.10436223	0.4369	0.6403
258493	Olfr319	olfactory receptor 319	1.10436223	0.8018	0.8959
27176	Rpl7a	ribosomal protein L7A	1.10424028	0.02463	NA
21855	Timm17b	translocase of inner mitochondrial membrane 17b	1.10424028	0.05478	NA
223864	Rapgef3	Rap guanine nucleotide exchange factor (GEF) 3	1.10424028	0.07455	NA
230119	Zbtb5	zinc finger and BTB domain containing 5	1.10424028	0.1968	NA
272350	Gm5065	predicted gene 5065	1.10424028	0.6709	0.8155
268420	Alkbh5	alkB, alkylation repair homolog 5 (E. coli)	1.10411836	0.03412	NA
226646	Ndufs2	NADH dehydrogenase (ubiquinone) Fe-S protein 2	1.10411836	0.1221	NA
26369	Cetn1	centrin 1	1.10411836	0.7149	0.844
101568	Vrk3	vaccinia related kinase 3	1.10399647	0.08525	NA
66874	1200014J11Rik	RIKEN cDNA 1200014J11 gene	1.10399647	0.1523	NA
71921	2310058N22Rik	RIKEN cDNA 2310058N22 gene	1.1038746	0.2253	0.4251
56473	Fads2	fatty acid desaturase 2	1.1038746	0.2715	0.479
24058	Sigirr	single immunoglobulin and toll-interleukin 1 receptor (TIR) domain	1.1038746	0.2984	0.5079
52504	Cenpo	centromere protein O	1.1038746	0.3152	0.5246
328778	Rab26	RAB26, member RAS oncogene family	1.1038746	0.3377	0.5479
53608	Map3k6	mitogen-activated protein kinase kinase kinase 6	1.1038746	0.4706	0.6697
13445	Cdk2ap1	CDK2 (cyclin-dependent kinase 2)-associated protein 1	1.10375276	0.2426	0.4456
380787	A230065H16Rik	RIKEN cDNA A230065H16 gene	1.10375276	0.3749	0.5838
68846	Rnf208	ring finger protein 208	1.10375276	0.408	0.6145
22722	Zfp64	zinc finger protein 64	1.10363095	0.06947	NA
18477	Prdx1	peroxiredoxin 1	1.10363095	0.0745	NA
74883	4930461C15Rik	RIKEN cDNA 4930461C15 gene	1.10363095	0.3866	0.5952
230917	Tmem201	transmembrane protein 201	1.10363095	0.4709	0.6698
105193	Nhlrc1	NHL repeat containing 1	1.10350916	0.2178	NA
69358	Lrrc51	leucine rich repeat containing 51	1.10350916	0.3152	0.5246
72007	Fndc3b	fibronectin type III domain containing 3B	1.1033874	0.2063	NA
14085	Fah	fumarylacetoacetate hydrolase	1.1033874	0.2224	NA
109754	Cyb5r3	cytochrome b5 reductase 3	1.1033874	0.2382	0.4405
23871	Ets1	E26 avian leukemia oncogene 1, 5' domain	1.1033874	0.3927	0.601
19317	Qk	quaking	1.10326567	0.1321	NA
621580	Gm13308	predicted gene 13308	1.10326567	0.156	NA
14758	Gpm6b	glycoprotein m6b	1.10326567	0.2053	NA
18222	Numb	numb gene homolog (Drosophila)	1.10326567	0.2229	NA

20533	Slc4a1	solute carrier family 4 (anion exchanger), member 1	1.10326567	0.6598	0.8085
16950	Loxl3	lysyl oxidase-like 3	1.10314396	0.2474	0.4517
228071	Sestd1	SEC14 and spectrin domains 1	1.10314396	0.3224	0.5322
276952	Rasl10b	RAS-like, family 10, member B	1.10314396	0.3893	0.5975
22062	Trp73	transformation related protein 73	1.10314396	0.5554	0.7353
320554	Tcp11l1	t-complex 11 like 1	1.10302228	0.3529	0.5629
68942	Chmp2b	chromatin modifying protein 2B	1.10290063	0.2867	0.4951
68709	Cilp2	cartilage intermediate layer protein 2	1.10290063	0.3247	0.5345
13143	Dapk2	death-associated protein kinase 2	1.10290063	0.4274	0.6321
54644	Otud5	OTU domain containing 5	1.102779	0.06537	NA
210992	Lpcat1	lysophosphatidylcholine acyltransferase 1	1.102779	0.5083	0.6991
246085	Defb10	defensin beta 10	1.102779	0.5525	0.7331
214987	Chtf8	CTF8, chromosome transmission fidelity factor 8 homolog (S. cerevisiae)	1.1026574	0.06852	NA
14539	Opn1mw	opsin 1 (cone pigments), medium-wave-sensitive (color blindness, deutan)	1.1026574	0.2567	0.4619
74729	Setmar	SET domain and mariner transposase fusion gene	1.1026574	0.3753	0.5841
320022	Ccdc79	coiled-coil domain containing 79	1.1026574	0.4193	0.6254
667141	Gm8479	thiopurine S-methyltransferase pseudogene	1.1026574	0.4698	0.6691
242050	Igsf10	immunoglobulin superfamily, member 10	1.1026574	0.5321	0.7172
381572	9430007A20Rik	RIKEN cDNA 9430007A20 gene	1.1026574	0.5721	0.7469
70612	5730494N06Rik	RIKEN cDNA 5730494N06 gene	1.10253583	0.05476	NA
19921	Rpl19	ribosomal protein L19	1.10253583	0.1693	NA
14367	Fzd5	frizzled homolog 5 (Drosophila)	1.10253583	0.2578	0.4631
12033	Bcap29	B-cell receptor-associated protein 29	1.10253583	0.3465	0.5571
66972	Slc25a23	solute carrier family 25 (mitochondrial carrier; phosphate carrier), member 23	1.10253583	0.6418	0.7974
67087	Ctnnbip1	catenin beta interacting protein 1	1.10241429	0.1421	NA
12385	Ctnna1	catenin (cadherin associated protein), alpha 1	1.10229277	0.09998	NA
244667	Disc1	disrupted in schizophrenia 1	1.10229277	0.1197	NA
30926	Glrx3	glutaredoxin 3	1.10229277	0.1497	NA
668936	Gm14217	predicted gene 14217	1.10229277	0.1977	NA
56321	Aatf	apoptosis antagonizing transcription factor	1.10229277	0.209	NA
73293	Ccdc103	coiled-coil domain containing 103	1.10229277	0.3461	0.5567
83962	Btbd1	BTB (POZ) domain containing 1	1.10229277	0.4408	0.6436
20336	Exoc4	exocyst complex component 4	1.10217128	0.1918	NA
19249	Ptpn13	protein tyrosine phosphatase, non-receptor type 13	1.10217128	0.2317	0.4326
67168	Lpar6	lysophosphatidic acid receptor 6	1.10217128	0.3202	0.5302
71665	Fuca1	fucosidase, alpha-L- 1, tissue	1.10217128	0.3392	0.5494
77945	Rpgrip1	retinitis pigmentosa GTPase regulator interacting protein 1	1.10217128	0.3828	0.5918
81535	Sgpp1	sphingosine-1-phosphate phosphatase 1	1.10204981	0.1062	NA
29805	Znhit2-ps	zinc finger, HIT domain containing 2, pseudogene	1.10204981	0.1188	NA
74134	Cyp2s1	cytochrome P450, family 2, subfamily s, polypeptide 1	1.10192837	0.2469	0.451
408022	Ccdc111	coiled-coil domain containing 111	1.10192837	0.2954	0.5043
243912	Hspb6	heat shock protein, alpha-crystallin-related, B6	1.10192837	0.3814	0.5907
320802	6330512M04Rik	RIKEN cDNA 6330512M04 gene	1.10192837	0.4599	0.6607
20317	Serpinf1	serine (or cysteine) peptidase inhibitor, clade F, member 1	1.10192837	0.4604	0.6612
66830	Nacc1	nucleus accumbens associated 1, BEN and BTB (POZ) domain containing	1.10192837	0.4842	0.6796
68533	Mphosph6	M phase phosphoprotein 6	1.10180696	0.1751	NA
212862	Chpt1	choline phosphotransferase 1	1.10180696	0.2405	0.443
217333	Trim47	tripartite motif-containing 47	1.10180696	0.367	0.576
235041	Kank2	KN motif and ankyrin repeat domains 2	1.10168558	0.1157	NA
319832	Tmem229a	transmembrane protein 229A	1.10168558	0.2236	NA

13841	Epha7	Eph receptor A7	1.10168558	0.2326	NA
29816	Hip1r	huntingtin interacting protein 1 related	1.10168558	0.2359	0.4378
78908	Igsf3	immunoglobulin superfamily, member 3	1.10168558	0.2793	0.4874
103677	Smg6	Smg-6 homolog, nonsense mediated mRNA decay factor (C. elegans)	1.10144289	0.1085	NA
78329	2310010J17Rik	RIKEN cDNA 2310010J17 gene	1.10144289	0.2709	0.4786
26433	Plod3	procollagen-lysine, 2-oxoglutarate 5-dioxygenase 3	1.10144289	0.3471	0.5575
100125931	A130049A11Rik	RIKEN cDNA A130049A11 gene	1.10132159	0.2472	0.4515
70574	Cpm	carboxypeptidase M	1.10132159	0.2529	0.4579
545861	Gm5878	predicted gene 5878	1.10132159	0.2786	0.4868
232946	Bloc1s3	biogenesis of lysosome-related organelles complex-1, subunit 3	1.10120031	0.2863	0.4949
53945	Slc40a1	solute carrier family 40 (iron-regulated transporter), member 1	1.10120031	0.3255	0.5354
26961	Rpl8	ribosomal protein L8	1.10107906	0.1898	NA
14297	Fxn	frataxin	1.10107906	0.1926	NA
54371	Chst2	carbohydrate sulfotransferase 2	1.10107906	0.5444	0.7265
56275	Rbm14	RNA binding motif protein 14	1.10095783	0.08238	NA
20911	Stxbp2	syntaxin binding protein 2	1.10083664	0.5613	0.7395
80281	Cttnbp2nl	CTTNBP2 N-terminal like	1.10059432	0.1354	NA
59126	Nek6	NIMA (never in mitosis gene a)-related expressed kinase 6	1.10059432	0.1681	NA
66568	Rwdd3	RWD domain containing 3	1.10059432	0.3625	0.5716
19262	Ptpra	protein tyrosine phosphatase, receptor type, A	1.1004732	0.06385	NA
68463	Mrpl14	mitochondrial ribosomal protein L14	1.1004732	0.08661	NA
66109	Tspan13	tetraspanin 13	1.1004732	0.1355	NA
224823	Rrp36	ribosomal RNA processing 36 homolog (S. cerevisiae)	1.1004732	0.1946	NA
14009	Etv1	ets variant gene 1	1.1004732	0.3238	0.5335
27375	Tjp3	tight junction protein 3	1.10035211	0.2777	0.486
17886	Myh9	myosin, heavy polypeptide 9, non-muscle	1.10035211	0.3206	0.5305
319455	Pld5	phospholipase D family, member 5	1.10035211	0.4377	0.6409
19731	Rgl1	ral guanine nucleotide dissociation stimulator,-like 1	1.10023105	0.05212	NA
54683	Prdx5	peroxiredoxin 5	1.10023105	0.1715	NA
67685	Dyx1c1	dyslexia susceptibility 1 candidate 1 homolog (human)	1.10023105	0.2579	0.4632
17354	Mllt10	myeloid/lymphoid or mixed-lineage leukemia (trithorax homolog, Drosophila); translocated to, 10	1.10023105	0.5166	0.706
58231	Stk4	serine/threonine kinase 4	1.10011001	0.1779	NA
54411	Atp6ap1	ATPase, H+ transporting, lysosomal accessory protein 1	1.10011001	0.2031	NA
75732	Iqcd	IQ motif containing D	1.10011001	0.2543	0.4593
21827	Thbs3	thrombospondin 3	1.099989	0.0576	NA
19942	Rpl27	ribosomal protein L27	1.099989	0.06226	NA
18538	Pcna	proliferating cell nuclear antigen	1.099989	0.1692	NA
56335	Mettl3	methyltransferase like 3	1.09986802	0.0745	NA
14651	Hagh	hydroxyacyl glutathione hydrolase	1.09986802	0.2013	NA
14084	Faf1	Fas-associated factor 1	1.09986802	0.2685	0.4755
66541	Immp1l	IMP1 inner mitochondrial membrane peptidase-like (S. cerevisiae)	1.09986802	0.2789	0.487
69318	1700007K09Rik	RIKEN cDNA 1700007K09 gene	1.09986802	0.3887	0.5971
108014	Srsf9	serine/arginine-rich splicing factor 9	1.09974706	0.03589	NA
20055	Rps16	ribosomal protein S16	1.09974706	0.1403	NA
71567	Mcm9	minichromosome maintenance complex component 9	1.09974706	0.2817	0.4896
114889	Vsx1	visual system homeobox 1 homolog (zebrafish)	1.09962613	0.0748	NA
238690	Zfp458	zinc finger protein 458	1.09962613	0.1818	NA
14866	Gstm5	glutathione S-transferase, mu 5	1.09962613	0.2514	0.4562
108960	Irak2	interleukin-1 receptor-associated kinase 2	1.09962613	0.31	0.5198
72522	Atxn7l2	ataxin 7-like 2	1.09962613	0.494	0.6879

69723	Rpain	RPA interacting protein	1.09950522	0.07864	NA
27354	Nbn	nibrin	1.09950522	0.4104	0.6168
100038474	K230015D01Rik	RIKEN cDNA K230015D01 gene	1.09950522	0.4688	0.6685
66641	Sike1	suppressor of IKBKE 1	1.09926349	0.1405	NA
80906	Kcnip2	Kv channel-interacting protein 2	1.09926349	0.5602	0.7386
241621	Gm13981	predicted gene 13981	1.09914267	0.04576	NA
110006	Gusb	glucuronidase, beta	1.09914267	0.2121	NA
50918	Myadm	myeloid-associated differentiation marker	1.09914267	0.2369	NA
70178	Fam108c	family with sequence similarity 108, member C	1.09902187	0.1176	NA
68185	Chchd8	coiled-coil-helix-coiled-coil-helix domain containing 8	1.09902187	0.1852	NA
68975	Med27	mediator complex subunit 27	1.0989011	0.1976	NA
70544	5730437N04Rik	RIKEN cDNA 5730437N04 gene	1.09878035	0.06206	NA
68347	0610011F06Rik	RIKEN cDNA 0610011F06 gene	1.09878035	0.1454	NA
14284	Fosl2	fos-like antigen 2	1.09865964	0.3723	0.5811
71133	4933422A05Rik	RIKEN cDNA 4933422A05 gene	1.09865964	0.637	0.7943
14390	Gabpa	GA repeat binding protein, alpha	1.09853894	0.287	0.4956
238799	Tnpo1	transportin 1	1.09853894	0.6539	0.8045
67267	2900010M23Rik	RIKEN cDNA 2900010M23 gene	1.09841828	0.1132	NA
69195	Tmem121	transmembrane protein 121	1.09841828	0.2136	NA
269152	Kif26b	kinesin family member 26B	1.09841828	0.4144	0.6207
56690	Mlycd	malonyl-CoA decarboxylase	1.09829764	0.0983	NA
18120	Mrpl49	mitochondrial ribosomal protein L49	1.09829764	0.1081	NA
23881	G3bp2	GTPase activating protein (SH3 domain) binding protein 2	1.09829764	0.2979	0.5073
381280	Hjurp	Holliday junction recognition protein	1.09829764	0.3323	0.5424
52683	Ncaph2	non-SMC condensin II complex, subunit H2	1.09829764	0.3679	0.5768
216881	Wscd1	WSC domain containing 1	1.09829764	0.4468	0.6491
230904	Fbxo2	F-box protein 2	1.09817703	0.2951	0.504
11532	Adh5	alcohol dehydrogenase 5 (class III), chi polypeptide	1.09817703	0.3282	0.5382
72113	Adck1	aarF domain containing kinase 1	1.09805644	0.08426	NA
286942	Kif19a	kinesin family member 19A	1.09805644	0.3106	0.5204
22704	Zfp46	zinc finger protein 46	1.09793588	0.1	NA
109593	Lmo3	LIM domain only 3	1.09793588	0.3936	0.6017
216197	Ckap4	cytoskeleton-associated protein 4	1.09793588	0.757	0.8701
234683	Elmo3	engulfment and cell motility 3, ced-12 homolog (C. elegans)	1.09781535	0.4692	0.6686
218793	Ube2e2	ubiquitin-conjugating enzyme E2E 2 (UBC4/5 homolog, yeast)	1.09769484	0.1095	NA
668144	Gm9000	predicted gene 9000	1.09769484	0.2226	NA
20272	Scn7a	sodium channel, voltage-gated, type VII, alpha	1.09769484	0.454	0.6555
108012	Ap1s2	adaptor-related protein complex 1, sigma 2 subunit	1.09769484	0.4626	0.6632
67603	Dusp6	dual specificity phosphatase 6	1.09745391	0.08276	NA
66069	Snupn	snurportin 1	1.09745391	0.09509	NA
21807	Tsc22d1	TSC22 domain family, member 1	1.09745391	0.1135	NA
194309	Vps37d	vacuolar protein sorting 37D (yeast)	1.09745391	0.1299	NA
20042	Rps12	ribosomal protein S12	1.09745391	0.1325	NA
18226	Nup62	nucleoporin 62	1.09745391	0.157	NA
12616	Cenpb	centromere protein B	1.09733348	0.28	0.4882
11632	Aip	aryl-hydrocarbon receptor-interacting protein	1.09733348	0.4111	0.6174
75698	Fam35a	family with sequence similarity 35, member A	1.09721308	0.4433	0.6463
56378	Arpc3	actin related protein 2/3 complex, subunit 3	1.0970927	0.1287	NA
101646	B830008H07Rik	RIKEN cDNA B830008H07 gene	1.0970927	0.1347	NA
12896	Cpt2	carnitine palmitoyltransferase 2	1.0970927	0.2589	0.4645

18974	Pole2	polymerase (DNA directed), epsilon 2 (p59 subunit)	1.0970927	0.5327	0.7176
69833	Polr2f	polymerase (RNA) II (DNA directed) polypeptide F	1.09697236	0.1688	NA
103534	Mgat4b	mannoside acetylglucosaminyltransferase 4, isoenzyme B	1.09697236	0.2784	0.4866
67236	Cinp	cyclin-dependent kinase 2 interacting protein	1.09697236	0.2973	0.5065
637916	LOC637916	midline-1-like	1.09697236	0.4534	0.6552
22165	Txk	TXK tyrosine kinase	1.09697236	0.5754	0.7491
216156	Wdr18	WD repeat domain 18	1.09685203	0.3713	0.5801
19737	Rgs5	regulator of G-protein signaling 5	1.09685203	0.4213	0.6273
66482	Exoc2	exocyst complex component 2	1.09673174	0.1204	NA
13839	Epha5	Eph receptor A5	1.09673174	0.1379	NA
66087	Tmem111	transmembrane protein 111	1.09673174	0.3151	0.5245
74123	Foxp4	forkhead box P4	1.09673174	0.3249	0.5347
232156	Slc4a5	solute carrier family 4, sodium bicarbonate cotransporter, member 5	1.09673174	0.3604	0.5701
11785	Apbb1	amyloid beta (A4) precursor protein-binding, family B, member 1	1.09673174	0.4376	0.6409
74218	1700016H13Rik	RIKEN cDNA 1700016H13 gene	1.09673174	0.7264	0.8513
14733	Gpc1	glypican 1	1.09661147	0.08323	NA
78246	Phf23	PHD finger protein 23	1.09661147	0.2385	NA
14227	Fkbp2	FK506 binding protein 2	1.09649123	0.11	NA
66077	Aurkaip1	aurora kinase A interacting protein 1	1.09649123	0.1144	NA
27414	Sergef	secretion regulating guanine nucleotide exchange factor	1.09649123	0.3139	0.5235
66491	Polr2l	polymerase (RNA) II (DNA directed) polypeptide L	1.09637101	0.1313	NA
68024	Hist1h2bc	histone cluster 1, H2bc	1.09637101	0.3185	0.5281
104110	Adcy4	adenylate cyclase 4	1.09637101	0.4839	0.6794
628012	Gm16441	predicted pseudogene 16441	1.09637101	0.773	0.8791
30058	Timm8a1	translocase of inner mitochondrial membrane 8 homolog a1 (yeast)	1.09625082	0.1167	NA
12167	Bmpr1b	bone morphogenetic protein receptor, type 1B	1.09625082	0.3326	0.5428
74559	Elovl7	ELOVL family member 7, elongation of long chain fatty acids (yeast)	1.09625082	0.3664	0.5755
71529	9030409G11Rik	RIKEN cDNA 9030409G11 gene	1.09625082	0.3732	0.582
77359	9430063H18Rik	RIKEN cDNA 9430063H18 gene	1.09625082	0.4106	0.617
20280	Scp2	sterol carrier protein 2, liver	1.09613066	0.05672	NA
72124	Seh1l	SEH1-like (S. cerevisiae	1.09613066	0.1492	NA
26914	H2afy	H2A histone family, member Y	1.09613066	0.2666	0.4735
66060	0610010O12Rik	RIKEN cDNA 0610010O12 gene	1.09613066	0.298	0.5074
26927	Foxl2	forkhead box L2	1.09613066	0.4502	0.6525
13397	Dlx6as	distal-less homeobox 6, antisense	1.09613066	0.6563	0.806
21855	Timm17b	translocase of inner mitochondrial membrane 17b	1.09601052	0.126	NA
71545	9030625G05Rik	RIKEN cDNA 9030625G05 gene	1.09601052	0.2135	NA
70532	5730433K22Rik	RIKEN cDNA 5730433K22 gene	1.09601052	0.2246	NA
97086	Nhedc2	Na+/H+ exchanger domain containing 2	1.09601052	0.4233	0.629
17067	Ly6c1	lymphocyte antigen 6 complex, locus C1	1.09589041	0.1105	NA
14841	Gsg2	germ cell-specific gene 2	1.09589041	0.4089	0.6154
18399	Slc22a6	solute carrier family 22 (organic anion transporter), member 6	1.09589041	0.6947	0.831
241118	Accn4	amiloride-sensitive cation channel 4, pituitary	1.09577033	0.1344	NA
76260	Ttc8	tetratricopeptide repeat domain 8	1.09577033	0.2191	NA
109263	Rlf	rearranged L-myc fusion sequence	1.09577033	0.4097	0.6161
223922	Atf7	activating transcription factor 7	1.09577033	0.5129	0.7035
380855	Rsl1	regulator of sex limited protein 1	1.09565027	0.1871	NA
15239	Hgs	HGF-regulated tyrosine kinase substrate	1.09565027	0.1911	NA
77644	C330007P06Rik	RIKEN cDNA C330007P06 gene	1.09565027	0.2149	NA
67115	Rpl14	ribosomal protein L14	1.09565027	0.2187	NA

15371	Hmx1	H6 homeobox 1	1.09565027	0.3201	0.5301
24069	Sufu	suppressor of fused homolog (Drosophila)	1.09565027	0.3531	0.5631
20218	Khdrbs1	KH domain containing, RNA binding, signal transduction associated 1	1.09553024	0.1824	NA
105298	Epdr1	ependymin related protein 1 (zebrafish)	1.09553024	0.3311	0.5415
14137	Fdft1	farnesyl diphosphate farnesyl transferase 1	1.09553024	0.5667	0.7428
237320	Aldh8a1	aldehyde dehydrogenase 8 family, member A1	1.09553024	0.6607	0.8089
81630	Zbtb22	zinc finger and BTB domain containing 22	1.09541023	0.1996	NA
239027	Arhgap22	Rho GTPase activating protein 22	1.09541023	0.3068	0.5162
18976	Pomc	pro-opiomelanocortin-alpha	1.09541023	0.312	0.5216
12461	Cct2	chaperonin containing Tcp1, subunit 2 (beta)	1.09529025	0.08095	NA
432713	Gm5441	predicted gene 5441	1.09529025	0.367	0.576
654795	Sdr39u1	short chain dehydrogenase/reductase family 39U, member 1	1.0951703	0.5426	0.7253
545198	LOC545198	hypothetical LOC545198	1.09505037	0.05124	NA
110157	Raf1	v-raf-leukemia viral oncogene 1	1.09505037	0.1123	NA
21894	Tln1	talin 1	1.09505037	0.1377	NA
20005	Rpl9	ribosomal protein L9	1.09505037	0.1879	NA
66356	2310008H09Rik	RIKEN cDNA 2310008H09 gene	1.09505037	0.289	0.4975
58227	Fam184b	family with sequence similarity 184, member B	1.09505037	0.4069	0.6139
14676	Gna15	guanine nucleotide binding protein, alpha 15	1.09505037	0.4694	0.6687
12122	Bid	BH3 interacting domain death agonist	1.09505037	0.595	0.7638
70757	Ptplb	protein tyrosine phosphatase-like (proline instead of catalytic arginine), member b	1.09493047	0.1291	NA
68618	1110012L19Rik	RIKEN cDNA 1110012L19 gene	1.09493047	0.132	NA
22359	Vldlr	very low density lipoprotein receptor	1.09493047	0.1431	NA
93737	Pard6g	par-6 partitioning defective 6 homolog gamma (C. elegans)	1.09493047	0.1463	NA
50850	Spast	spastin	1.09493047	0.2001	NA
546100	Gm5914	predicted gene 5914	1.09493047	0.3321	0.5423
26386	Hsf4	heat shock transcription factor 4	1.09493047	0.6996	0.8337
22142	Tuba1a	tubulin, alpha 1A	1.0948106	0.3008	0.5103
13885	Esd	esterase D/formylglutathione hydrolase	1.0948106	0.3209	0.5309
242406	Rgp1	RGP1 retrograde golgi transport homolog (S. cerevisiae)	1.0948106	0.3909	0.5992
218772	Rarb	retinoic acid receptor, beta	1.09469075	0.05555	NA
66313	Smurf2	SMAD specific E3 ubiquitin protein ligase 2	1.09469075	0.1094	NA
225742	St8sia5	ST8 alpha-N-acetyl-neuraminide alpha-2,8-sialyltransferase 5	1.09469075	0.1516	NA
70448	2610204G22Rik	RIKEN cDNA 2610204G22 gene	1.09469075	0.2411	NA
66229	Rpl7l1	ribosomal protein L7-like 1	1.09469075	0.276	0.484
75044	4930506A18Rik	RIKEN cDNA 4930506A18 gene	1.09469075	0.4096	0.6159
17160	Man2b2	mannosidase 2, alpha B2	1.09469075	0.4989	0.6914
70020	Ino80b	INO80 complex subunit B	1.09469075	0.5043	0.6956
18775	Prl3d1	prolactin family 3, subfamily d, member 1	1.09457093	0.7091	0.84
20529	Slc31a1	solute carrier family 31, member 1	1.09445113	0.1025	NA
69089	Oxa1l	oxidase assembly 1-like	1.09445113	0.1649	NA
384185	Arl9	ADP-ribosylation factor-like 9	1.09445113	0.5623	0.7401
19982	Rpl36a	ribosomal protein L36A	1.09433136	0.1014	NA
94184	Pdxdc1	pyridoxal-dependent decarboxylase domain containing 1	1.09433136	0.227	NA
66320	Tmem208	transmembrane protein 208	1.09421162	0.08106	NA
16777	Lamb1	laminin B1	1.09421162	0.1677	NA
93893	Pcdhb22	protocadherin beta 22	1.09421162	0.1919	NA
27355	X99384	cDNA sequence X99384	1.09421162	0.2811	0.4892
20729	Spin1	spindlin 1	1.09421162	0.3105	0.5203
269952	D330012F22Rik	RIKEN cDNA D330012F22 gene	1.09421162	0.411	0.6174

99470	Magi3	membrane associated guanylate kinase, WW and PDZ domain containing 3	1.09421162	0.4839	0.6794
23950	Dnajb6	DnaJ (Hsp40) homolog, subfamily B, member 6	1.09421162	0.4874	0.6824
57810	Cdon	cell adhesion molecule-related/down-regulated by oncogenes	1.09421162	0.5228	0.7102
76954	St5	suppression of tumorigenicity 5	1.0940919	0.1152	NA
93886	Pcdhb15	protocadherin beta 15	1.0940919	0.2176	NA
235330	Ttc12	tetratricopeptide repeat domain 12	1.0940919	0.6139	0.7775
12424	Cck	cholecystokinin	1.09397221	0.0991	NA
14751	Gpi1	glucose phosphate isomerase 1	1.09397221	0.2283	NA
76763	Mospd2	motile sperm domain containing 2	1.09397221	0.4026	0.6102
18016	Nf2	neurofibromatosis 2	1.09397221	0.5439	0.7261
20610	Sumo3	SMT3 suppressor of mif two 3 homolog 3 (yeast)	1.09385255	0.1332	NA
72093	2010320M18Rik	RIKEN cDNA 2010320M18 gene	1.09385255	0.5164	0.706
66552	2010106G01Rik	RIKEN cDNA 2010106G01 gene	1.09373291	0.1866	NA
624086	A230045G11Rik	RIKEN cDNA A230045G11 gene	1.09373291	0.2583	NA
620155	Gm6133	60S ribosomal protein L17 pseudogene	1.09373291	0.3074	0.517
22038	Plscr1	phospholipid scramblase 1	1.0936133	0.3504	0.5607
27388	Ptdss2	phosphatidylserine synthase 2	1.09349371	0.3574	0.5672
18701	Pigf	phosphatidylinositol glycan anchor biosynthesis, class F	1.09337415	0.2796	0.4878
12021	Bard1	BRCA1 associated RING domain 1	1.09337415	0.4202	0.6263
59046	Arpp19	cAMP-regulated phosphoprotein 19	1.09337415	0.4967	0.6899
17772	Mtm1	X-linked myotubular myopathy gene 1	1.09337415	0.5106	0.7012
104156	Etv5	ets variant gene 5	1.09325462	0.2024	NA
75751	Ipo4	importin 4	1.09325462	0.2815	0.4895
71776	Tha1	threonine aldolase 1	1.09325462	0.4728	0.6709
19712	Rest	RE1-silencing transcription factor	1.09325462	0.5903	0.7601
21827	Thbs3	thrombospondin 3	1.09313511	0.08046	NA
14569	Gdi2	guanosine diphosphate (GDP) dissociation inhibitor 2	1.09313511	0.08918	NA
17305	Mfng	MFNG O-fucosylpeptide 3-beta-N-acetylglucosaminyltransferase	1.09313511	0.1837	NA
14964	H2-D1	histocompatibility 2, D region locus 1	1.09313511	0.2391	NA
66821	Bcs1l	BCS1-like (yeast)	1.09313511	0.2539	NA
53817	Bat1a	HLA-B-associated transcript 1A	1.09313511	0.2903	0.4987
100042757	Gm4013	predicted gene 4013	1.09313511	0.407	0.6139
97998	Deptor	DEP domain containing MTOR-interacting protein	1.09313511	0.4237	0.6293
13850	Ephx2	epoxide hydrolase 2, cytoplasmic	1.09313511	0.4291	0.6336
67332	Snrpd3	small nuclear ribonucleoprotein D3	1.09301563	0.0774	NA
17688	Msh6	mutS homolog 6 (E. coli)	1.09301563	0.1172	NA
270097	Vat1l	vesicle amine transport protein 1 homolog-like (T. californica)	1.09301563	0.1512	NA
66374	2310011J03Rik	RIKEN cDNA 2310011J03 gene	1.09301563	0.2133	NA
231912	Katnal1	katanin p60 subunit A-like 1	1.09301563	0.238	NA
68021	Bphl	biphenyl hydrolase-like (serine hydrolase, breast epithelial mucin-associated antigen)	1.09289617	0.2419	NA
68597	1110021J02Rik	RIKEN cDNA 1110021J02 gene	1.09289617	0.2512	NA
23863	Dand5	DAN domain family, member 5	1.09289617	0.3145	0.524
667250	Gm12657	predicted gene 12657	1.09277675	0.07547	NA
71667	0610007L01Rik	RIKEN cDNA 0610007L01 gene	1.09277675	0.09312	NA
17210	Mcl1	myeloid cell leukemia sequence 1	1.09265734	0.08754	NA
73680	Zbtb8a	zinc finger and BTB domain containing 8a	1.09265734	0.6173	0.7797
66396	Ccdc82	coiled-coil domain containing 82	1.09253797	0.1736	NA
67889	Rbm18	RNA binding motif protein 18	1.09253797	0.5789	0.7515
69871	2010007H12Rik	RIKEN cDNA 2010007H12 gene	1.09253797	0.6051	0.7717
107045	Lars	leucyl-tRNA synthetase	1.09241861	0.09916	NA

209018	Vps8	vacuolar protein sorting 8 homolog (S. cerevisiae)	1.09241861	0.1541	NA
66162	Bola2	bolA-like 2 (E. coli)	1.09229929	0.04636	NA
17984	Ndn	necdin	1.09229929	0.1741	NA
69384	Tmem89	transmembrane protein 89	1.09229929	0.5476	0.7292
21349	Tal1	T-cell acute lymphocytic leukemia 1	1.09229929	0.5977	0.7664
638532	Gm7241	predicted pseudogene 7241	1.09217999	0.09058	NA
66235	Eif1ax	eukaryotic translation initiation factor 1A, X-linked	1.09217999	0.2089	NA
27050	Rps3	ribosomal protein S3	1.09217999	0.2352	NA
227298	Fam134a	family with sequence similarity 134, member A	1.09217999	0.2647	NA
545156	Kalrn	kalirin, RhoGEF kinase	1.09217999	0.4275	0.6321
76429	Lhpp	phospholysine phosphohistidine inorganic pyrophosphate phosphatase	1.09217999	0.4682	0.6679
56378	Arpc3	actin related protein 2/3 complex, subunit 3	1.09206072	0.1744	NA
100043682	Gm10584	predicted gene 10584	1.09206072	0.801	0.8957
16976	Lrpap1	low density lipoprotein receptor-related protein associated protein 1	1.09194147	0.1519	NA
69171	1810031K17Rik	RIKEN cDNA 1810031K17 gene	1.09194147	0.1526	NA
22755	Zfp93	zinc finger protein 93	1.09194147	0.1874	NA
229782	Slc35a3	solute carrier family 35 (UDP-N-acetylglucosamine (UDP-GlcNAc) transporter), member 3	1.09194147	0.2007	NA
434204	Whamm	WAS protein homolog associated with actin, golgi membranes and microtubules	1.09194147	0.3952	0.6031
192191	Med9	mediator of RNA polymerase II transcription, subunit 9 homolog (yeast)	1.09182225	0.08717	NA
70320	2610008G14Rik	RIKEN cDNA 2610008G14 gene	1.09182225	0.1687	NA
244958	Mrap2	melanocortin 2 receptor accessory protein 2	1.09182225	0.2579	NA
22143	Tuba1b	tubulin, alpha 1B	1.09182225	0.3718	0.5806
12111	Bgn	biglycan	1.09182225	0.8527	0.9244
19173	Psmb5	proteasome (prosome, macropain) subunit, beta type 5	1.09170306	0.2902	0.4987
28084	Vps25	vacuolar protein sorting 25 (yeast)	1.09170306	0.4015	0.6091
241062	Pgap1	post-GPI attachment to proteins 1	1.09170306	0.4861	0.6813
52589	Ncald	neurocalcin delta	1.09170306	0.5262	0.7128
73103	3110009E18Rik	RIKEN cDNA 3110009E18 gene	1.09158389	0.3148	0.5241
239759	Liph	lipase, member H	1.09158389	0.4745	0.6722
665618	Gm7715	predicted gene 7715	1.09158389	0.4851	0.6805
278097	Armcx6	armadillo repeat containing, X-linked 6	1.09158389	0.6063	0.7725
74190	1200009I06Rik	RIKEN cDNA 1200009I06 gene	1.09158389	0.6126	0.7766
76113	Lpo	lactoperoxidase	1.09158389	0.629	0.789
21968	Tom1	target of myb1 homolog (chicken)	1.09146475	0.1141	NA
73212	3110082I17Rik	RIKEN cDNA 3110082I17 gene	1.09146475	0.1181	NA
378702	Serf2	small EDRK-rich factor 2	1.09146475	0.1268	NA
17252	Rdh11	retinol dehydrogenase 11	1.09146475	0.1402	NA
66884	Appbp2	amyloid beta precursor protein (cytoplasmic tail) binding protein 2	1.09146475	0.2153	NA
22634	Plagl1	pleiomorphic adenoma gene-like 1	1.09146475	0.3905	0.5988
14158	Fert2	fer (fms/fps related) protein kinase, testis specific 2	1.09146475	0.5225	0.7102
17188	Maz	MYC-associated zinc finger protein (purine-binding transcription factor)	1.09146475	0.5896	0.7596
14087	Fanca	Fanconi anemia, complementation group A	1.09146475	0.6718	0.8163
236794	Slc9a6	solute carrier family 9 (sodium/hydrogen exchanger), member 6	1.09134563	0.2247	NA
110893	Slc8a3	solute carrier family 8 (sodium/calcium exchanger), member 3	1.09134563	0.4741	0.6718
74629	4930426L09Rik	RIKEN cDNA 4930426L09 gene	1.09134563	0.5377	0.7216
56420	Ppp4c	protein phosphatase 4, catalytic subunit	1.09134563	0.5733	0.7476
67050	Nkap	NFKB activating protein	1.09122654	0.2758	NA
56347	Eif3c	eukaryotic translation initiation factor 3, subunit C	1.09122654	0.327	0.5371
16969	Zbtb7a	zinc finger and BTB domain containing 7a	1.09122654	0.4351	0.639
230890	Gm436	predicted gene 436	1.09122654	0.6052	0.7718

268527	Greb1	gene regulated by estrogen in breast cancer protein	1.09110747	0.1423	NA
73828	Dcaf4	DDB1 and CUL4 associated factor 4	1.09110747	0.3113	0.5213
101985	AA960436	expressed sequence AA960436	1.09110747	0.4948	0.6883
18049	Ngf	nerve growth factor	1.09110747	0.5244	0.7112
104263	Kdm3a	lysine (K)-specific demethylase 3A	1.09098844	0.1906	NA
16011	Igfbp5	insulin-like growth factor binding protein 5	1.09086942	0.1747	NA
69572	Mfsd3	major facilitator superfamily domain containing 3	1.09086942	0.1956	NA
22051	Trip6	thyroid hormone receptor interactor 6	1.09086942	0.4402	0.6431
225416	Gm4838	predicted gene 4838	1.09075044	0.1035	NA
231130	Tnip2	TNFAIP3 interacting protein 2	1.09075044	0.2237	NA
57810	Cdon	cell adhesion molecule-related/down-regulated by oncogenes	1.09063148	0.1561	NA
26426	Nubp2	nucleotide binding protein 2	1.09063148	0.2195	NA
19703	Renbp	renin binding protein	1.09063148	0.6336	0.7918
107605	Rdh1	retinol dehydrogenase 1 (all trans)	1.09063148	0.6407	0.7968
224045	Eif2b5	eukaryotic translation initiation factor 2B, subunit 5 epsilon	1.09051254	0.3087	0.5185
13885	Esd	esterase D/formylglutathione hydrolase	1.09051254	0.3544	0.5643
320021	C430042M11Rik	RIKEN cDNA C430042M11 gene	1.09051254	0.5054	0.6966
320698	C530042K13Rik	RIKEN cDNA C530042K13 gene	1.09051254	0.5355	0.72
18232	Nxph2	neurexophilin 2	1.09051254	0.5413	0.7243
50724	Sap30l	SAP30-like	1.09039363	0.07604	NA
70349	Copb1	coatomer protein complex, subunit beta 1	1.09039363	0.1508	NA
74585	Sppl3	signal peptide peptidase 3	1.09039363	0.2113	NA
13804	Endog	endonuclease G	1.09039363	0.2271	NA
52504	Cenpo	centromere protein O	1.09039363	0.2467	NA
209200	Dtx3l	deltex 3-like (Drosophila)	1.09039363	0.3831	0.592
66112	Mosc1	MOCO sulphurase C-terminal domain containing 1	1.09039363	0.4558	0.6573
17686	Msh3	mutS homolog 3 (E. coli)	1.09027475	0.162	NA
69009	Thap7	THAP domain containing 7	1.09027475	0.2565	NA
77254	Yif1b	Yip1 interacting factor homolog B (S. cerevisiae)	1.09027475	0.3706	0.5794
66880	Rsrc1	arginine/serine-rich coiled-coil 1	1.09027475	0.404	0.6114
338523	Jhdm1d	jumonji C domain-containing histone demethylase 1 homolog D (S. cerevisiae)	1.09027475	0.4601	0.6608
235086	Igsf9b	immunoglobulin superfamily, member 9B	1.09027475	0.6072	0.7728
59050	Nsa2	NSA2 ribosome biogenesis homolog (S. cerevisiae)	1.09015589	0.1193	NA
237353	Sh3rf3	SH3 domain containing ring finger 3	1.09015589	0.2158	NA
78294	Rps27a	ribosomal protein S27A	1.09015589	0.5531	0.7336
14079	Fabp2	fatty acid binding protein 2, intestinal	1.09015589	0.6311	0.7903
228413	Prrg4	proline rich Gla (G-carboxyglutamic acid) 4 (transmembrane)	1.09015589	0.6398	0.7962
385380	Tex28	testis expressed 28	1.09003706	0.6028	0.7696
19108	Prkx	protein kinase, X-linked	1.08991826	0.1922	NA
73139	Cenpv	centromere protein V	1.08991826	0.2571	NA
20851	Stat5b	signal transducer and activator of transcription 5B	1.08991826	0.269	NA
67582	Slc25a26	solute carrier family 25 (mitochondrial carrier, phosphate carrier), member 26	1.08991826	0.3162	0.5256
17318	Mid1	midline 1	1.08991826	0.4646	0.6648
93790	Nipa2	non imprinted in Prader-Willi/Angelman syndrome 2 homolog (human)	1.08979948	0.1094	NA
108098	Med21	mediator complex subunit 21	1.08979948	0.2784	NA
319586	Celf5	CUGBP, Elav-like family member 5	1.08979948	0.602	0.7692
229571	Gm4858	predicted gene 4858	1.08979948	0.6153	0.7784
353169	Slc2a12	solute carrier family 2 (facilitated glucose transporter), member 12	1.08968072	0.08601	NA
233552	Gdpd5	glycerophosphodiester phosphodiesterase domain containing 5	1.08968072	0.2991	0.5084
11548	Adra1b	adrenergic receptor, alpha 1b	1.08968072	0.3858	0.5946

665992	Krtap4-8	keratin associated protein 4-8	1.08968072	0.5232	0.7103
272790	Magee2	melanoma antigen, family E, 2	1.08968072	0.5648	0.7419
78151	4930404H24Rik	RIKEN cDNA 4930404H24 gene	1.08968072	0.7058	0.8379
13026	Pcyt1a	phosphate cytidylyltransferase 1, choline, alpha isoform	1.089562	0.2838	NA
319875	Tmprss11bnl	transmembrane protease, serine 11b N terminal like	1.089562	0.3163	0.5257
16998	Ltbp3	latent transforming growth factor beta binding protein 3	1.089562	0.4176	0.6238
67946	Spata6	spermatogenesis associated 6	1.089562	0.4709	0.6698
223664	Lrrc14	leucine rich repeat containing 14	1.089562	0.5223	0.7101
59002	Wdr8	WD repeat domain 8	1.08944329	0.3296	0.5399
237339	L3mbtl3	l(3)mbt-like 3 (Drosophila)	1.08932462	0.1975	NA
56791	Ube2l6	ubiquitin-conjugating enzyme E2L 6	1.08932462	0.2343	NA
75605	Kdm5b	lysine (K)-specific demethylase 5B	1.08932462	0.2954	NA
20438	Siah1b	seven in absentia 1B	1.08920597	0.1926	NA
28084	Vps25	vacuolar protein sorting 25 (yeast)	1.08920597	0.3458	0.5566
16898	Rps2	ribosomal protein S2	1.08920597	0.508	0.6988
67212	Mrpl55	mitochondrial ribosomal protein L55	1.08896875	0.1412	NA
67869	Paip2	polyadenylate-binding protein-interacting protein 2	1.08896875	0.2462	NA
13057	Cyba	cytochrome b-245, alpha polypeptide	1.08896875	0.2911	NA
30926	Glrx3	glutaredoxin 3	1.08885017	0.1069	NA
55991	Panx1	pannexin 1	1.08885017	0.117	NA
18810	Plec	plectin	1.08885017	0.346	0.5567
18028	Nfib	nuclear factor I/B	1.08885017	0.5783	0.751
52357	Wwc2	WW, C2 and coiled-coil domain containing 2	1.08873163	0.07964	NA
67005	Polr3k	polymerase (RNA) III (DNA directed) polypeptide K	1.08873163	0.2562	NA
94064	Mrpl27	mitochondrial ribosomal protein L27	1.08873163	0.3074	0.517
20425	Shmt1	serine hydroxymethyltransferase 1 (soluble)	1.08873163	0.7256	0.8507
67669	l7Rn6	lethal, Chr 7, Rinchik 6	1.08861311	0.2708	NA
12539	Cdc37	cell division cycle 37 homolog (S. cerevisiae)	1.08849461	0.2117	NA
12567	Cdk4	cyclin-dependent kinase 4	1.08849461	0.3338	0.5442
13436	Dnmt3b	DNA methyltransferase 3B	1.08849461	0.3729	0.5816
66523	2810004N23Rik	RIKEN cDNA 2810004N23 gene	1.08837614	0.1336	NA
22590	Xpa	xeroderma pigmentosum, complementation group A	1.08837614	0.1598	NA
67287	Parp6	poly (ADP-ribose) polymerase family, member 6	1.08837614	0.2077	NA
70568	Cpne3	copine III	1.08837614	0.2945	NA
209334	Gen1	Gen homolog 1, endonuclease (Drosophila)	1.08837614	0.5875	0.7578
624713	Gm6525	ribosomal protein L36a pseudogene	1.0882577	0.05231	NA
78406	2900041H08Rik	RIKEN cDNA 2900041H08 gene	1.0882577	0.4138	0.6203
26451	Rpl27a	ribosomal protein L27A	1.08813928	0.08854	NA
17434	Mocs2	molybdenum cofactor synthesis 2	1.08813928	0.2539	NA
67226	Tmem19	transmembrane protein 19	1.08813928	0.2724	NA
68292	Stt3b	STT3, subunit of the oligosaccharyltransferase complex, homolog B (S. cerevisiae)	1.08813928	0.3283	0.5383
100038850	A130082M07Rik	RIKEN cDNA A130082M07 gene	1.08813928	0.3384	0.5486
56305	Pitpnb	phosphatidylinositol transfer protein, beta	1.08802089	0.3846	0.5937
14667	Gm2a	GM2 ganglioside activator protein	1.08802089	0.4004	0.6083
16906	Lmnb1	lamin B1	1.08802089	0.4067	0.6139
14269	Fnbp1	formin binding protein 1	1.08802089	0.4263	0.6315
16168	Il15	interleukin 15	1.08802089	0.5375	0.7215
18247	Oaz2-ps	ornithine decarboxylase antizyme 2, pseudogene	1.08790252	0.1402	NA
101358	Fbxl14	F-box and leucine-rich repeat protein 14	1.08790252	0.1446	NA
109082	Fbxw17	F-box and WD-40 domain protein 17	1.08790252	0.2326	NA

258852	Olfr1341	olfactory receptor 1341	1.08790252	0.7834	0.8864
22785	Slc30a4	solute carrier family 30 (zinc transporter), member 4	1.08778418	0.08305	NA
17966	Nbr1	neighbor of Brca1 gene 1	1.08778418	0.1965	NA
216119	Ybey	ybeY metallopeptidase	1.08778418	0.3123	0.522
100047044	LOC100047044	hypothetical protein LOC100047044	1.08778418	0.6965	0.8324
103583	Fbxw11	F-box and WD-40 domain protein 11	1.08766587	0.1111	NA
74080	Nmnat3	nicotinamide nucleotide adenylyltransferase 3	1.08766587	0.3465	0.5571
11877	Arvcf	armadillo repeat gene deleted in velo-cardio-facial syndrome	1.08766587	0.468	0.6678
16504	Kcnc3	potassium voltage gated channel, Shaw-related subfamily, member 3	1.08766587	0.5446	0.7266
54216	Pcdh7	protocadherin 7	1.08766587	0.6599	0.8085
69748	Aldh16a1	aldehyde dehydrogenase 16 family, member A1	1.08754758	0.1307	NA
277010	Marveld1	MARVEL (membrane-associating) domain containing 1	1.08754758	0.3141	0.5236
21968	Tom1	target of myb1 homolog (chicken)	1.08754758	0.6562	0.8059
69195	Tmem121	transmembrane protein 121	1.08742932	0.1797	NA
384864	Gm1943	WD repeat domain 70 pseudogene	1.08742932	0.2194	NA
75212	Rnf121	ring finger protein 121	1.08742932	0.2803	NA
545551	BC021767	cingulin-like	1.08742932	0.2859	NA
67939	Prorsd1	prolyl-tRNA synthetase domain containing 1	1.08742932	0.2904	NA
654795	Sdr39u1	short chain dehydrogenase/reductase family 39U, member 1	1.08731108	0.09387	NA
223978	Cpped1	calcineurin-like phosphoesterase domain containing 1	1.08719287	0.1252	NA
80385	Tusc2	tumor suppressor candidate 2	1.08719287	0.1415	NA
23971	Papss1	3'-phosphoadenosine 5'-phosphosulfate synthase 1	1.08719287	0.2305	NA
76898	B3gat1	beta-1,3-glucuronyltransferase 1 (glucuronosyltransferase P)	1.08707468	0.4037	0.6112
18717	Pip5k1c	phosphatidylinositol-4-phosphate 5-kinase, type 1 gamma	1.08707468	0.5415	0.7244
72129	Pex13	peroxisomal biogenesis factor 13	1.08695652	0.1504	NA
66356	2310008H09Rik	RIKEN cDNA 2310008H09 gene	1.08695652	0.1728	NA
75482	Hspb9	heat shock protein, alpha-crystallin-related, B9	1.08695652	0.3056	NA
68304	Kdelc2	KDEL (Lys-Asp-Glu-Leu) containing 2	1.08695652	0.3731	0.5819
110074	Dut	deoxyuridine triphosphatase	1.08695652	0.4063	0.6135
100046950	LOC100046950	hypothetical LOC100046950	1.08695652	0.6097	0.7744
76411	1700019E19Rik	RIKEN cDNA 1700019E19 gene	1.08683839	0.09461	NA
70737	Cgn	cingulin	1.08683839	0.2838	NA
69367	Glrx2	glutaredoxin 2 (thioltransferase)	1.08683839	0.767	0.8763
72454	Ccdc71	coiled-coil domain containing 71	1.08672028	0.1195	NA
215493	A3galt2	alpha 1,3-galactosyltransferase 2 (isoglobotriaosylceramide synthase)	1.08672028	0.38	0.5891
72003	Synpr	synaptoporin	1.08672028	0.4703	0.6695
18121	Nog	noggin	1.08672028	0.7744	0.8801
18750	Prkca	protein kinase C, alpha	1.08660219	0.06946	NA
71919	Rpap3	RNA polymerase II associated protein 3	1.08660219	0.1304	NA
103694	Tmed4	transmembrane emp24 protein transport domain containing 4	1.08660219	0.1404	NA
20833	Ssrp1	structure specific recognition protein 1	1.08660219	0.1763	NA
93881	Pcdhb10	protocadherin beta 10	1.08660219	0.25	NA
66131	Tipin	timeless interacting protein	1.08660219	0.2947	NA
54615	Npff	neuropeptide FF-amide peptide precursor	1.08660219	0.3054	NA
76429	Lhpp	phospholysine phosphohistidine inorganic pyrophosphate phosphatase	1.08660219	0.3441	0.5548
19946	Rpl30	ribosomal protein L30	1.08648414	0.1973	NA
14679	Gnai3	guanine nucleotide binding protein (G protein), alpha inhibiting 3	1.08648414	0.2091	NA
15939	Ier5	immediate early response 5	1.08648414	0.3464	0.557
16369	Irs3	insulin receptor substrate 3	1.08648414	0.5681	0.7439
194388	Tet3	tet oncogene family member 3	1.08648414	0.6364	0.7938

545363	Gm5833		predicted gene 5833	1.08648414	0.6782	0.8202
667370	I830012O16Rik		RIKEN cDNA I830012O16 gene	1.0862481	0.42	0.6261
230709	Zmpste24		zinc metallopeptidase, STE24 homolog (S. cerevisiae)	1.08613012	0.09318	NA
66394	Nosip		nitric oxide synthase interacting protein	1.08613012	0.2867	NA
20362		Sep-08	septin 8	1.08613012	0.3013	NA
77613	Prss36		protease, serine, 36	1.08601216	0.1222	NA
13123	Cyp7b1		cytochrome P450, family 7, subfamily b, polypeptide 1	1.08601216	0.2073	NA
226594	Rcsd1		RCSD domain containing 1	1.08601216	0.491	0.6851
12558	Cdh2		cadherin 2	1.08601216	0.5369	0.7213
26901	Deb1		differentially expressed in B16F10 1	1.08589423	0.1738	NA
74682	Wdr35		WD repeat domain 35	1.08589423	0.2398	NA
77697	Mmab		methylmalonic aciduria (cobalamin deficiency) type B homolog (human)	1.08589423	0.2747	NA
100678	Psph		phosphoserine phosphatase	1.08589423	0.3081	NA
237436	Gas2l3		growth arrest-specific 2 like 3	1.08589423	0.4407	0.6436
66153	Fbxo36		F-box protein 36	1.08589423	0.4833	0.6789
170460	Stard5		StAR-related lipid transfer (START) domain containing 5	1.08589423	0.6601	0.8086
77870	E130116L18Rik		RIKEN cDNA E130116L18 gene	1.08589423	0.8892	0.9431
102162	Taf5l		TAF5-like RNA polymerase II, p300/CBP-associated factor (PCAF)-associated factor	1.08577633	0.1191	NA
319169	Hist1h2ak		histone cluster 1, H2ak	1.08577633	0.1822	NA
66706	Ndufaf3		NADH dehydrogenase (ubiquinone) 1 alpha subcomplex, assembly factor 3	1.08577633	0.3458	0.5566
70604	Dnajb14		DnaJ (Hsp40) homolog, subfamily B, member 14	1.08577633	0.3639	0.5729
504193	Npcd		neuronal pentraxin chromo domain	1.08577633	0.5071	0.698
17191	Mbd2		methyl-CpG binding domain protein 2	1.08565845	0.06344	NA
106344	Rfc4		replication factor C (activator 1) 4	1.08565845	0.2821	NA
171257	Vmn1r195		vomeronasal 1 receptor 195	1.08565845	0.7939	0.8917
19707	Reps1		RalBP1 associated Eps domain containing protein	1.0855406	0.212	NA
228812	Pigu		phosphatidylinositol glycan anchor biosynthesis, class U	1.0855406	0.3548	0.5646
106393	Srl		sarcalumenin	1.0855406	0.6552	0.8056
239555	Smcr7l		Smith-Magenis syndrome chromosome region, candidate 7-like (human)	1.08542277	0.1445	NA
69234	Zfp688		zinc finger protein 688	1.08542277	0.165	NA
233332	Adamts17		a disintegrin-like and metallopeptidase (reprolysin type) with thrombospondin type 1 motif, 17	1.08542277	0.225	NA
56458	Foxo1		forkhead box O1	1.08530497	0.09055	NA
72925		Mar-01	membrane-associated ring finger (C3HC4) 1	1.08530497	0.7155	0.8444
55934	rp9		retinitis pigmentosa 9 (human)	1.08518719	0.1842	NA
12166	Bmpr1a		bone morphogenetic protein receptor, type 1A	1.08518719	0.2033	NA
17772	Mtm1		X-linked myotubular myopathy gene 1	1.08518719	0.2184	NA
64656	Mrps23		mitochondrial ribosomal protein S23	1.08518719	0.2696	NA
21833	Thra		thyroid hormone receptor alpha	1.08518719	0.3138	NA
229588	Gm128		predicted gene 128	1.08518719	0.4347	0.6385
66323	1700001K19Rik		RIKEN cDNA 1700001K19 gene	1.08518719	0.5933	0.7627
22183	Zrsr1		zinc finger (CCCH type), RNA binding motif and serine/arginine rich 1	1.08506944	0.2569	NA
233280	Nipa1		non imprinted in Prader-Willi/Angelman syndrome 1 homolog (human)	1.08506944	0.3039	NA
66568	Rwdd3		RWD domain containing 3	1.08506944	0.4564	0.6579
78785	Clip4		CAP-GLY domain containing linker protein family, member 4	1.08506944	0.9249	0.9618
69860	Eif1ad		eukaryotic translation initiation factor 1A domain containing	1.08495172	0.2612	NA
19989	Rpl7		ribosomal protein L7	1.08495172	0.3214	NA
54124	Cks1b		CDC28 protein kinase 1b	1.08495172	0.3869	0.5956
65254	Dpysl5		dihydropyrimidinase-like 5	1.08495172	0.5634	0.7409
331046	Tgm4		transglutaminase 4 (prostate)	1.08495172	0.5907	0.7604
67371	Gtf3c6		general transcription factor IIIC, polypeptide 6, alpha	1.08483402	0.1746	NA

66340	Psenen	presenilin enhancer 2 homolog (C. elegans)	1.08483402	0.3523	0.5624
13074	Cyp17a1	cytochrome P450, family 17, subfamily a, polypeptide 1	1.08483402	0.7331	0.856
109154	Mlec	malectin	1.08471635	0.1576	NA
67856	Echdc3	enoyl Coenzyme A hydratase domain containing 3	1.08471635	0.4119	0.6182
239706	BC024814	cDNA sequence BC024814	1.08471635	0.4361	0.6396
104910	Slc25a47	solute carrier family 25, member 47	1.08471635	0.4496	0.6518
20482	Skil	SKI-like	1.08471635	0.6832	0.8231
233887	Zfp553	zinc finger protein 553	1.0845987	0.1639	NA
71960	Myh14	myosin, heavy polypeptide 14	1.0845987	0.3305	0.5408
16548	Khk	ketohexokinase	1.0845987	0.3908	0.599
67623	Tm7sf3	transmembrane 7 superfamily member 3	1.0845987	0.4127	0.6188
19401	Rara	retinoic acid receptor, alpha	1.0845987	0.4917	0.6857
67664	Rnf125	ring finger protein 125	1.0845987	0.5618	0.7398
54139	Irf6	interferon regulatory factor 6	1.0845987	0.6716	0.8161
14652	Glp1r	glucagon-like peptide 1 receptor	1.0845987	0.8176	0.9055
22324	Vav1	vav 1 oncogene	1.0845987	0.8339	0.9141
270906	Prr11	proline rich 11	1.08448108	0.1256	NA
68916	Cdkal1	CDK5 regulatory subunit associated protein 1-like 1	1.08448108	0.1425	NA
72999	Insig2	insulin induced gene 2	1.08448108	0.189	NA
98303	D630023F18Rik	RIKEN cDNA D630023F18 gene	1.08448108	0.2604	NA
20055	Rps16	ribosomal protein S16	1.08448108	0.297	NA
75452	Ascc2	activating signal cointegrator 1 complex subunit 2	1.08448108	0.3121	NA
75812	Tasp1	taspase, threonine aspartase 1	1.08448108	0.3547	0.5646
667766	Gm8801	protein phosphatase 1, regulatory subunit 10 pseudogene	1.08448108	0.4618	0.6624
66404	2410001C21Rik	RIKEN cDNA 2410001C21 gene	1.08448108	0.4619	0.6625
386463	Cdsn	corneodesmosin	1.08448108	0.4985	0.6912
68938	Aspscr1	alveolar soft part sarcoma chromosome region, candidate 1 (human)	1.08448108	0.6075	0.773
211550	Tifa	TRAF-interacting protein with forkhead-associated domain	1.08436348	0.1531	NA
18799	Plcd1	phospholipase C, delta 1	1.08436348	0.1676	NA
224454	Zdhhc14	zinc finger, DHHC domain containing 14	1.08436348	0.1932	NA
67826	Snap47	synaptosomal-associated protein, 47	1.08436348	0.3446	0.5553
12778	Cxcr7	chemokine (C-X-C motif) receptor 7	1.08436348	0.3781	0.5871
431706	Zfp457	zinc finger protein 457	1.08436348	0.6086	0.7735
98682	Mfsd6	major facilitator superfamily domain containing 6	1.08424591	0.07384	NA
66789	Alg14	asparagine-linked glycosylation 14 homolog (yeast)	1.08424591	0.1515	NA
237459	Cdk17	cyclin-dependent kinase 17	1.08412836	0.2729	NA
210719	Mkx	mohawk homeobox	1.08412836	0.2794	NA
14763	Gpr37	G protein-coupled receptor 37	1.08412836	0.356	0.5658
15968	Ifna5	interferon alpha 5	1.08412836	0.7825	0.8858
53310	Dlg3	discs, large homolog 3 (Drosophila)	1.08401084	0.05969	NA
240121	Fsd1	fibronectin type 3 and SPRY domain-containing protein	1.08401084	0.3688	0.5779
77994	2810055G20Rik	RIKEN cDNA 2810055G20 gene	1.08401084	0.4469	0.6491
66860	Tanc1	tetratricopeptide repeat, ankyrin repeat and coiled-coil containing 1	1.08401084	0.5073	0.6981
67693	2310003F16Rik	RIKEN cDNA 2310003F16 gene	1.08389334	0.2298	NA
396184	Flrt1	fibronectin leucine rich transmembrane protein 1	1.08389334	0.4532	0.6551
17527	Mpv17	MpV17 mitochondrial inner membrane protein	1.08377588	0.09065	NA
209212	Osgin2	oxidative stress induced growth inhibitor family member 2	1.08377588	0.1873	NA
77219	Ptgr2	prostaglandin reductase 2	1.08377588	0.2772	NA
103963	Rpn1	ribophorin I	1.08377588	0.3025	NA
106407	Osta	organic solute transporter alpha	1.08377588	0.5821	0.7538

231151	Tada2b	transcriptional adaptor 2B	1.08377588	0.6144	0.7778
56395	Tmem115	transmembrane protein 115	1.08365843	0.5423	0.725
319163	Hist1h2aa	histone cluster 1, H2aa	1.08365843	0.6625	0.8097
71911	Bdh1	3-hydroxybutyrate dehydrogenase, type 1	1.08354101	0.1943	NA
20132	Rrh	retinal pigment epithelium derived rhodopsin homolog	1.08354101	0.1984	NA
56361	Pus1	pseudouridine synthase 1	1.08354101	0.3647	0.5736
23829	C1ql1	complement component 1, q subcomponent-like 1	1.08354101	0.6236	0.7844
320411	A730089K16Rik	RIKEN cDNA A730089K16 gene	1.08354101	0.7278	0.8519
12226	Btg1	B-cell translocation gene 1, anti-proliferative	1.08342362	0.1118	NA
16157	Il11ra1	interleukin 11 receptor, alpha chain 1	1.08342362	0.1161	NA
66497	2610528E23Rik	RIKEN cDNA 2610528E23 gene	1.08342362	0.2269	NA
20893	Bhlhe40	basic helix-loop-helix family, member e40	1.08342362	0.2611	NA
21345	Tagln	transgelin	1.08342362	0.4033	0.6108
236293	D630002G06Rik	RIKEN cDNA D630002G06 gene	1.08342362	0.4849	0.6803
246782	Atpaf2	ATP synthase mitochondrial F1 complex assembly factor 2	1.08330625	0.2049	NA
68828	Sync	syncoilin	1.08330625	0.349	0.5593
319638	Nt5dc1	5'-nucleotidase domain containing 1	1.08330625	0.5012	0.6931
66398	Commd5	COMM domain containing 5	1.08318891	0.08963	NA
76282	Gpt	glutamic pyruvic transaminase, soluble	1.08318891	0.139	NA
13226	Defa-rs7	defensin, alpha, related sequence 7	1.08318891	0.3499	0.5602
21917	Tmpo	thymopoietin	1.08318891	0.3905	0.5988
54608	Abhd2	abhydrolase domain containing 2	1.08318891	0.5852	0.756
69288	Rhobtb1	Rho-related BTB domain containing 1	1.08318891	0.6007	0.7684
67287	Parp6	poly (ADP-ribose) polymerase family, member 6	1.08307159	0.3022	NA
75613	Med25	mediator of RNA polymerase II transcription, subunit 25 homolog (yeast)	1.08307159	0.4637	0.664
100034361	Mfap1b	microfibrillar-associated protein 1B	1.08307159	0.6395	0.7959
102866	Pls3	plastin 3 (T-isoform)	1.0829543	0.1272	NA
233890	Zfp768	zinc finger protein 768	1.0829543	0.1783	NA
23980	Pebp1	phosphatidylethanolamine binding protein 1	1.0829543	0.2547	NA
22068	Trpc6	transient receptor potential cation channel, subfamily C, member 6	1.0829543	0.3328	NA
67246	2810474O19Rik	RIKEN cDNA 2810474O19 gene	1.0829543	0.3879	0.5964
17196	Mbp	myelin basic protein	1.0829543	0.7263	0.8513
100169	Phactr4	phosphatase and actin regulator 4	1.08283703	0.1355	NA
68250	Fam96a	family with sequence similarity 96, member A	1.08283703	0.1843	NA
224794	Enpp4	ectonucleotide pyrophosphatase/phosphodiesterase 4	1.08283703	0.283	NA
93878	Pcdhb7	protocadherin beta 7	1.08283703	0.3024	NA
69020	Zfp707	zinc finger protein 707	1.08283703	0.3341	NA
66611	Ribc1	RIB43A domain with coiled-coils 1	1.08283703	0.3705	0.5794
100101457	A830035O19Rik	RIKEN cDNA A830035O19 gene	1.08283703	0.4105	0.6169
320279	C630007K24Rik	RIKEN cDNA C630007K24 gene	1.08283703	0.421	0.6271
67974	Ccny	cyclin Y	1.08283703	0.6688	0.8139
16452	Jak2	Janus kinase 2	1.08271979	0.1609	NA
72479	Hsdl2	hydroxysteroid dehydrogenase like 2	1.08271979	0.1636	NA
20250	Scd2	stearoyl-Coenzyme A desaturase 2	1.08271979	0.1786	NA
17865	Mybl2	myeloblastosis oncogene-like 2	1.08271979	0.2568	NA
81879	Tcfcp2l1	transcription factor CP2-like 1	1.08271979	0.7959	0.8927
246229	Bivm	basic, immunoglobulin-like variable motif containing	1.08260258	0.07902	NA
13436	Dnmt3b	DNA methyltransferase 3B	1.08260258	0.247	NA
18028	Nfib	nuclear factor I/B	1.08260258	0.2883	NA
69072	Ebna1bp2	EBNA1 binding protein 2	1.08260258	0.3446	0.5553

268445	Ankrd13b	ankyrin repeat domain 13b	1.08260258	0.5254	0.7122
268288	Samd3	sterile alpha motif domain containing 3	1.08260258	0.5902	0.7601
27364	Srr	serine racemase	1.08248539	0.1471	NA
69171	1810031K17Rik	RIKEN cDNA 1810031K17 gene	1.08248539	0.1635	NA
11848	Rhoa	ras homolog gene family, member A	1.08248539	0.1647	NA
74901	Kbtbd11	kelch repeat and BTB (POZ) domain containing 11	1.08248539	0.1932	NA
70546	Zdhhc2	zinc finger, DHHC domain containing 2	1.08248539	0.1974	NA
71795	Pitpnc1	phosphatidylinositol transfer protein, cytoplasmic 1	1.08248539	0.2888	NA
53860	Sep-09	septin 9	1.08248539	0.4874	0.6824
114641	Rpl31	ribosomal protein L31	1.08236822	0.08808	NA
434179	Gm5595	predicted gene 5595	1.08236822	0.3389	NA
74044	Ttf2	transcription termination factor, RNA polymerase II	1.08236822	0.3649	0.5739
21909	Tlx2	T-cell leukemia, homeobox 2	1.08236822	0.5571	0.7366
436440	Gpr31c	G protein-coupled receptor 31, D17Leh66c region	1.08236822	0.7435	0.8615
12017	Bag1	BCL2-associated athanogene 1	1.08225108	0.2695	NA
654472	Gm12070	glyceraldehyde-3-phosphate dehydrogenase pseudogene	1.08225108	0.3402	0.5506
74325	Cltb	clathrin, light polypeptide (Lcb)	1.08213397	0.316	NA
58178	Sorcs1	VPS10 domain receptor protein SORCS 1	1.08213397	0.429	0.6336
72434	Lypd3	Ly6/Plaur domain containing 3	1.08213397	0.7248	0.8502
212670	Catsper2	cation channel, sperm associated 2	1.08201688	0.1278	NA
103583	Fbxw11	F-box and WD-40 domain protein 11	1.08201688	0.2286	NA
69535	2310004N24Rik	RIKEN cDNA 2310004N24 gene	1.08201688	0.2916	NA
73368	Col20a1	collagen, type XX, alpha 1	1.08201688	0.478	0.6749
242653	Cldn19	claudin 19	1.08201688	0.8016	0.8959
60455	Tmem8	transmembrane protein 8 (five membrane-spanning domains)	1.08189982	0.2869	NA
140579	Elmo2	engulfment and cell motility 2, ced-12 homolog (C. elegans)	1.08189982	0.2965	NA
70807	Arrdc2	arrestin domain containing 2	1.08189982	0.5132	0.7038
791344	E330037I15Rik	RIKEN cDNA E330037I15 gene	1.08189982	0.6997	0.8337
26374	Rfwd2	ring finger and WD repeat domain 2	1.08178278	0.1476	NA
239157	Pnma2	paraneoplastic antigen MA2	1.08178278	0.1707	NA
209361	Taf3	TAF3 RNA polymerase II, TATA box binding protein (TBP)-associated factor	1.08178278	0.178	NA
14714	Gnrh1	gonadotropin releasing hormone 1	1.08166577	0.2146	NA
26961	Rpl8	ribosomal protein L8	1.08166577	0.2288	NA
54720	Rcan1	regulator of calcineurin 1	1.08166577	0.3858	0.5946
70544	5730437N04Rik	RIKEN cDNA 5730437N04 gene	1.08154878	0.2295	NA
81909	Zfpl1	zinc finger like protein 1	1.08154878	0.4501	0.6524
436493	H2-Gs10	MHC class I like protein GS10	1.08154878	0.6873	0.826
77333	C030007I01Rik	RIKEN cDNA C030007I01 gene	1.08154878	0.7682	0.877
56369	Apip	APAF1 interacting protein	1.08143182	0.1333	NA
68968	Cdan1	congenital dyserythropoietic anemia, type I (human)	1.08143182	0.2015	NA
239029	Antxrl	anthrax toxin receptor-like	1.08143182	0.6611	0.809
54409	Ramp2	receptor (calcitonin) activity modifying protein 2	1.08131488	0.1327	NA
73172	Dem1	defects in morphology 1 homolog (S. cerevisiae)	1.08131488	0.1778	NA
66938	1700029G01Rik	RIKEN cDNA 1700029G01 gene	1.08131488	0.1985	NA
22590	Xpa	xeroderma pigmentosum, complementation group A	1.08131488	0.2799	NA
225207	Zfp521	zinc finger protein 521	1.08131488	0.2897	NA
23999	Twf2	twinfilin, actin-binding protein, homolog 2 (Drosophila)	1.08119797	0.4296	0.6339
52463	Tet1	tet oncogene 1	1.08119797	0.4632	0.6635
629499	4922505G16Rik	RIKEN cDNA 4922505G16 gene	1.08119797	0.5536	0.7339
19230	Twf1	twinfilin, actin-binding protein, homolog 1 (Drosophila)	1.08108108	0.0783	NA

72103	Aplf	aprataxin and PNKP like factor	1.08108108	0.1379	NA
80748	BC004004	cDNA sequence BC004004	1.08108108	0.1741	NA
15239	Hgs	HGF-regulated tyrosine kinase substrate	1.08108108	0.3355	NA
639606	LOC639606	40S ribosomal protein S2-like	1.08108108	0.3624	0.5716
19730	Ralgds	ral guanine nucleotide dissociation stimulator	1.08108108	0.712	0.842
106052	Fbxo4	F-box protein 4	1.08096422	0.1255	NA
102791	Tcta	T-cell leukemia translocation altered gene	1.08096422	0.4798	0.6759
19303	Pxn	paxillin	1.08096422	0.5032	0.6949
70252	2010000I03Rik	RIKEN cDNA 2010000I03 gene	1.08096422	0.5208	0.7091
66682	Trappc5	trafficking protein particle complex 5	1.08084738	0.1375	NA
68327	0610007P22Rik	RIKEN cDNA 0610007P22 gene	1.08084738	0.2115	NA
19164	Psen1	presenilin 1	1.08084738	0.3466	NA
72310	Nkg7	natural killer cell group 7 sequence	1.08084738	0.602	0.7692
328309	Gm9776	predicted gene 9776	1.08073057	0.5013	0.6932
50768	Dlc1	deleted in liver cancer 1	1.08061379	0.4353	0.6391
55934	rp9	retinitis pigmentosa 9 (human)	1.08049703	0.3329	NA
50789	Fbxl3	F-box and leucine-rich repeat protein 3	1.08049703	0.3784	0.5873
66256	Ssr2	signal sequence receptor, beta	1.08049703	0.395	0.6029
69379	C8g	complement component 8, gamma polypeptide	1.08049703	0.4178	0.6239
68339	Ccdc88c	coiled-coil domain containing 88C	1.08049703	0.7016	0.8349
69723	Rpain	RPA interacting protein	1.08038029	0.2302	NA
237759	Col23a1	collagen, type XXIII, alpha 1	1.08038029	0.3841	0.5933
103978	Gpc5	glypican 5	1.08038029	0.601	0.7685
236598	LOC236598	28S ribosomal RNA	1.08026358	0.4551	0.6566
17064	Cd93	CD93 antigen	1.08026358	0.5059	0.697
26419	Mapk8	mitogen-activated protein kinase 8	1.08026358	0.5673	0.7432
12808	Cobl	cordon-bleu	1.08026358	0.6036	0.7705
223723	Ttll12	tubulin tyrosine ligase-like family, member 12	1.0801469	0.2659	NA
11539	Adora1	adenosine A1 receptor	1.08003024	0.1734	NA
17344	Pias2	protein inhibitor of activated STAT 2	1.08003024	0.2147	NA
68870	Ak8	adenylate kinase 8	1.08003024	0.5498	0.7312
72931	2900010J23Rik	RIKEN cDNA 2900010J23 gene	1.07991361	0.09464	NA
434215	Lrrc32	leucine rich repeat containing 32	1.07991361	0.1298	NA
75871	Zfp821	zinc finger protein 821	1.07991361	0.2702	NA
67878	Tmem33	transmembrane protein 33	1.07991361	0.3136	NA
23939	Mapk7	mitogen-activated protein kinase 7	1.07991361	0.3551	0.5648
74392	Specc1l	sperm antigen with calponin homology and coiled-coil domains 1-like	1.07991361	0.3778	0.5867
16764	Aff3	AF4/FMR2 family, member 3	1.07991361	0.4455	0.6481
76573	1700027D21Rik	RIKEN cDNA 1700027D21 gene	1.079797	0.3952	0.6031
67971	Tppp3	tubulin polymerization-promoting protein family member 3	1.079797	0.4997	0.6922
230801	Pigv	phosphatidylinositol glycan anchor biosynthesis, class V	1.07968041	0.2931	NA
231123	Haus3	HAUS augmin-like complex, subunit 3	1.07956386	0.1976	NA
230597	Zfyve9	zinc finger, FYVE domain containing 9	1.07956386	0.2308	NA
192185	Nadk	NAD kinase	1.07956386	0.2703	NA
229473	D930015E06Rik	RIKEN cDNA D930015E06 gene	1.07956386	0.276	NA
65103	Arl6ip6	ADP-ribosylation factor-like 6 interacting protein 6	1.07956386	0.6316	0.7905
74116	Pi16	peptidase inhibitor 16	1.07944732	0.3358	NA
233020	Hipk4	homeodomain interacting protein kinase 4	1.07944732	0.5799	0.7522
259105	Olfr549	olfactory receptor 549	1.07944732	0.8572	0.9263
102866	Pls3	plastin 3 (T-isoform)	1.07933081	0.08687	NA

666676	Gm8230	predicted gene 8230	1.07933081	0.2587	NA
208449	Sgms1	sphingomyelin synthase 1	1.07933081	0.3264	NA
231861	Tnrc18	trinucleotide repeat containing 18	1.07933081	0.3387	NA
217365	Nploc4	nuclear protein localization 4 homolog (S. cerevisiae)	1.07933081	0.3699	0.5789
74277	Chic2	cysteine-rich hydrophobic domain 2	1.07933081	0.4747	0.6723
72248	1700014B07Rik	RIKEN cDNA 1700014B07 gene	1.07933081	0.514	0.7042
76854	Gpr30	G protein-coupled receptor 30	1.07921433	0.3093	NA
192173	Fam195b	family with sequence similarity 195, member B	1.07921433	0.3257	NA
17872	Ppp1r15a	protein phosphatase 1, regulatory (inhibitor) subunit 15A	1.07921433	0.3518	NA
76025	Cant1	calcium activated nucleotidase 1	1.07921433	0.4281	0.6326
107173	Gpr137	G protein-coupled receptor 137	1.07921433	0.5623	0.74
17246	Mdm2	transformed mouse 3T3 cell double minute 2	1.07909787	0.05973	NA
63985	Gmfb	glia maturation factor, beta	1.07909787	0.2605	NA
73103	3110009E18Rik	RIKEN cDNA 3110009E18 gene	1.07909787	0.3116	NA
71584	Gdpd2	glycerophosphodiester phosphodiesterase domain containing 2	1.07909787	0.3122	NA
271786	Galnt13	UDP-N-acetyl-alpha-D-galactosamine:polypeptide N-acetylgalactosaminyltransferase 13	1.07909787	0.3293	NA
68611	Mrpl28	mitochondrial ribosomal protein L28	1.07909787	0.3756	0.5845
24075	Taf10	TAF10 RNA polymerase II, TATA box binding protein (TBP)-associated factor	1.07909787	0.3915	0.5997
58233	Dnaja4	DnaJ (Hsp40) homolog, subfamily A, member 4	1.07898144	0.1923	NA
67288	Srek1ip1	splicing regulatory glutamine/lysine-rich protein 1interacting protein 1	1.07898144	0.2463	NA
66340	Psenen	presenilin enhancer 2 homolog (C. elegans)	1.07898144	0.3883	0.5968
110784	Nr3c2	nuclear receptor subfamily 3, group C, member 2	1.07898144	0.4748	0.6724
107056	AW742475	expressed sequence AW742475	1.07898144	0.515	0.7049
100855	Tbc1d14	TBC1 domain family, member 14	1.07886503	0.08335	NA
633947	Gm6225	predicted gene 6225	1.07886503	0.2896	NA
228019	Mettl8	methyltransferase like 8	1.07886503	0.3565	NA
69250	2610029K11Rik	RIKEN cDNA 2610029K11 gene	1.07886503	0.4099	0.6162
50798	Gne	glucosamine	1.07886503	0.4126	0.6188
433586	Maml3	mastermind like 3 (Drosophila)	1.07886503	0.4471	0.6493
66174	Nudt14	nudix (nucleoside diphosphate linked moiety X)-type motif 14	1.07886503	0.4587	0.6597
83704	Slc12a9	solute carrier family 12 (potassium/chloride transporters), member 9	1.07886503	0.5116	0.7021
620695	Gm13889	predicted gene 13889	1.07874865	0.105	NA
12570	Cdk5r2	cyclin-dependent kinase 5, regulatory subunit 2 (p39)	1.07874865	0.2077	NA
73736	Fcf1	FCF1 small subunit (SSU) processome component homolog (S. cerevisiae)	1.07874865	0.2126	NA
14088	Fancc	Fanconi anemia, complementation group C	1.07874865	0.3051	NA
232533	Stk38l	serine/threonine kinase 38 like	1.07874865	0.5771	0.7502
624784	Gm9855	thymine DNA glycosylase pseudogene	1.07874865	0.7117	0.8417
19982	Rpl36a	ribosomal protein L36A	1.07863229	0.1078	NA
12450	Ccng1	cyclin G1	1.07863229	0.2964	NA
78611	Btbd19	BTB (POZ) domain containing 19	1.07863229	0.4186	0.6249
68114	Mum1	melanoma associated antigen (mutated) 1	1.07863229	0.4804	0.6765
11890	Asgr2	asialoglycoprotein receptor 2	1.07863229	0.6283	0.7884
70097	Sash1	SAM and SH3 domain containing 1	1.07851596	0.3498	NA
67630	Samd8	sterile alpha motif domain containing 8	1.07851596	0.5023	0.6941
101359	Prrt4	proline-rich transmembrane protein 4	1.07851596	0.5621	0.74
114875	Plcz1	phospholipase C, zeta 1	1.07851596	0.6269	0.7871
67504	Rnf151	ring finger protein 151	1.07851596	0.7905	0.8902
140559	Igsf8	immunoglobulin superfamily, member 8	1.07839965	0.4534	0.6552
75995	5033417F24Rik	RIKEN cDNA 5033417F24 gene	1.07839965	0.5065	0.6975
57776	Ttyh1	tweety homolog 1 (Drosophila)	1.07828337	0.1213	NA

74504	Fam53a	family with sequence similarity 53, member A	1.07828337	0.1404	NA
76547	Tmem101	transmembrane protein 101	1.07828337	0.206	NA
232370	Clstn3	calsyntenin 3	1.07828337	0.2292	NA
72828	Ubash3b	ubiquitin associated and SH3 domain containing, B	1.07828337	0.3167	NA
69399	1700025G04Rik	RIKEN cDNA 1700025G04 gene	1.07828337	0.3658	0.5749
320844	Amigo3	adhesion molecule with Ig like domain 3	1.07828337	0.4094	0.6157
208177	Phldb2	pleckstrin homology-like domain, family B, member 2	1.07828337	0.4832	0.6788
22682	Zfand5	zinc finger, AN1-type domain 5	1.07828337	0.5959	0.7646
22042	Tfrc	transferrin receptor	1.07828337	0.6804	0.8213
11666	Abcd1	ATP-binding cassette, sub-family D (ALD), member 1	1.07828337	0.7112	0.8414
16542	Kdr	kinase insert domain protein receptor	1.07816712	0.189	NA
13666	Eif2ak3	eukaryotic translation initiation factor 2 alpha kinase 3	1.07816712	0.2684	NA
267019	Rps15a	ribosomal protein S15A	1.07805088	0.08005	NA
210711	Mcmbp	MCM (minichromosome maintenance deficient) binding protein	1.07805088	0.1316	NA
12925	Crip1	cysteine-rich protein 1 (intestinal)	1.07805088	0.1643	NA
68436	Rpl34	ribosomal protein L34	1.07805088	0.2881	NA
13841	Epha7	Eph receptor A7	1.07805088	0.3962	0.6041
52538	Acaa2	acetyl-Coenzyme A acyltransferase 2 (mitochondrial 3-oxoacyl-Coenzyme A thiolase)	1.07805088	0.3998	0.6076
67437	Ssr3	signal sequence receptor, gamma	1.07805088	0.7011	0.8345
226654	Tstd1	thiosulfate sulfurtransferase (rhodanese)-like domain containing 1	1.07793468	0.21	NA
12757	Clta	clathrin, light polypeptide (Lca)	1.07793468	0.354	NA
626858	Gm6713	predicted gene 6713	1.07793468	0.8032	0.8971
73251	Setd7	SET domain containing (lysine methyltransferase) 7	1.0778185	0.2222	NA
110954	Rpl10	ribosomal protein 10	1.0778185	0.2382	NA
67131	Acbd4	acyl-Coenzyme A binding domain containing 4	1.0778185	0.2897	NA
217219	Fam171a2	family with sequence similarity 171, member A2	1.0778185	0.3775	0.5865
56473	Fads2	fatty acid desaturase 2	1.0778185	0.4026	0.6102
14020	Evi5	ecotropic viral integration site 5	1.07770234	0.1536	NA
13992	Khdrbs3	KH domain containing, RNA binding, signal transduction associated 3	1.07770234	0.1974	NA
67781	Ilf2	interleukin enhancer binding factor 2	1.07770234	0.2291	NA
68691	1110028C15Rik	RIKEN cDNA 1110028C15 gene	1.07770234	0.4188	0.625
270151	Nlrx1	NLR family member X1	1.07770234	0.5287	0.7146
78317	Ccdc88b	coiled-coil domain containing 88B	1.07758621	0.3717	0.5805
56470	Rgs19	regulator of G-protein signaling 19	1.07758621	0.3917	0.5999
75758	9130401M01Rik	RIKEN cDNA 9130401M01 gene	1.07758621	0.4526	0.6548
319939	Tns3	tensin 3	1.07758621	0.4728	0.6709
14561	Gdf11	growth differentiation factor 11	1.07758621	0.5497	0.7312
15957	Ifit1	interferon-induced protein with tetratricopeptide repeats 1	1.07758621	0.6332	0.7916
16170	Il16	interleukin 16	1.07758621	0.6595	0.8084
22070	Tpt1	tumor protein, translationally-controlled 1	1.0774701	0.1999	NA
237553	Trhde	TRH-degrading enzyme	1.0774701	0.2536	NA
77044	Arid2	AT rich interactive domain 2 (ARID, RFX-like)	1.0774701	0.267	NA
13000	Csnk2a2	casein kinase 2, alpha prime polypeptide	1.0774701	0.2724	NA
67422	Dhdds	dehydrodolichyl diphosphate synthase	1.0774701	0.3817	0.591
69710	Arap1	ArfGAP with RhoGAP domain, ankyrin repeat and PH domain 1	1.0774701	0.4061	0.6135
114229	Kiss1r	KISS1 receptor	1.0774701	0.4579	0.6591
57776	Ttyh1	tweety homolog 1 (Drosophila)	1.07735402	0.1933	NA
69757	Leng1	leukocyte receptor cluster (LRC) member 1	1.07735402	0.2751	NA
12289	Cacna1d	calcium channel, voltage-dependent, L type, alpha 1D subunit	1.07735402	0.375	0.5838
12321	Calu	calumenin	1.07735402	0.3822	0.5914

258418	Olfr469	olfactory receptor 469	1.07735402	0.797	0.8932
76773	Wdyhv1	WDYHV motif containing 1	1.07723796	0.1335	NA
71817	Tmem50a	transmembrane protein 50A	1.07723796	0.2647	NA
16871	Lhx3	LIM homeobox protein 3	1.07723796	0.2755	NA
67204	Eif2s2	eukaryotic translation initiation factor 2, subunit 2 (beta)	1.07723796	0.2761	NA
53605	Nap1l1	nucleosome assembly protein 1-like 1	1.07723796	0.2955	NA
22630	Ywhaq	tyrosine 3-monooxygenase/tryptophan 5-monooxygenase activation protein, theta polypeptide	1.07723796	0.5096	0.7003
73747	1110034G24Rik	RIKEN cDNA 1110034G24 gene	1.07712193	0.1962	NA
723988	A330062J17Rik	RIKEN cDNA A330062J17 gene	1.07712193	0.2862	NA
18104	Nqo1	NAD(P)H dehydrogenase, quinone 1	1.07712193	0.4798	0.6759
110880	Scn4a	sodium channel, voltage-gated, type IV, alpha	1.07712193	0.6425	0.7978
213402	Armc2	armadillo repeat containing 2	1.07700592	0.1841	NA
17330	Minpp1	multiple inositol polyphosphate histidine phosphatase 1	1.07700592	0.2034	NA
75605	Kdm5b	lysine (K)-specific demethylase 5B	1.07700592	0.396	0.6039
67484	Eepd1	endonuclease/exonuclease/phosphatase family domain containing 1	1.07688994	0.09955	NA
66193	1110049F12Rik	RIKEN cDNA 1110049F12 gene	1.07688994	0.2036	NA
14239	Foxs1	forkhead box S1	1.07688994	0.2483	NA
636808	Cntnap5a	contactin associated protein-like 5A	1.07688994	0.5566	0.7363
626231	Gm6658	predicted gene 6658	1.07688994	0.6498	0.8028
66488	Fam136a	family with sequence similarity 136, member A	1.07677399	0.1958	NA
71702	Cdc5l	cell division cycle 5-like (S. pombe)	1.07677399	0.2247	NA
224640	Lemd2	LEM domain containing 2	1.07677399	0.2367	NA
223435	Trio	triple functional domain (PTPRF interacting)	1.07677399	0.7738	0.8797
54342	Gnpnat1	glucosamine-phosphate N-acetyltransferase 1	1.07665805	0.2142	NA
226866	Gm106	predicted gene 106	1.07665805	0.3556	NA
170459	Stard4	StAR-related lipid transfer (START) domain containing 4	1.07665805	0.387	0.5957
67399	Pdlim7	PDZ and LIM domain 7	1.07665805	0.5644	0.7418
545474	Scrt2	scratch homolog 2, zinc finger protein (Drosophila)	1.07665805	0.5645	0.7418
333654	Ppp1r13l	protein phosphatase 1, regulatory (inhibitor) subunit 13 like	1.07654215	0.2313	NA
240613	9930021J03Rik	RIKEN cDNA 9930021J03 gene	1.07654215	0.326	NA
26446	Psmb3	proteasome (prosome, macropain) subunit, beta type 3	1.07654215	0.4689	0.6685
223186	Gm4822	predicted pseudogene 4822	1.07654215	0.5671	0.7431
105734	Tigd5	tigger transposable element derived 5	1.07654215	0.6765	0.8189
112406	Egln2	EGL nine homolog 2 (C. elegans)	1.07642626	0.1485	NA
66116	Cml1	camello-like 1	1.07642626	0.2609	NA
100041294	Gm3258	predicted gene 3258	1.07642626	0.2765	NA
67281	Rpl37	ribosomal protein L37	1.07642626	0.2789	NA
73608	Marveld3	MARVEL (membrane-associating) domain containing 3	1.07642626	0.3979	0.6058
72306	Zfp777	zinc finger protein 777	1.07631041	0.2497	NA
18194	Nsdhl	NAD(P) dependent steroid dehydrogenase-like	1.07631041	0.3189	NA
231382	Tmprss11d	transmembrane protease, serine 11d	1.07631041	0.6376	0.7948
26364	Cd97	CD97 antigen	1.07619458	0.2153	NA
15288	Hmbs	hydroxymethylbilane synthase	1.07619458	0.2628	NA
23837	Cfdp1	craniofacial development protein 1	1.07619458	0.2757	NA
66333	Aqp11	aquaporin 11	1.07619458	0.2926	NA
67678	Lsm3	LSM3 homolog, U6 small nuclear RNA associated (S. cerevisiae)	1.07619458	0.3815	0.5907
65116	Prrg2	proline-rich Gla (G-carboxyglutamic acid) polypeptide 2	1.07619458	0.4716	0.67
277333	Gm5069	glyceraldehyde-3-phosphate dehydrogenase pseudogene	1.07619458	0.5267	0.7132
13178	Dck	deoxycytidine kinase	1.07619458	0.6203	0.7819
17113	M6pr	mannose-6-phosphate receptor, cation dependent	1.07607877	0.1625	NA

66868	Mfsd1	major facilitator superfamily domain containing 1	1.07607877	0.4692	0.6686
76960	Bcas1	breast carcinoma amplified sequence 1	1.07607877	0.5011	0.693
15078	H3f3a	H3 histone, family 3A	1.07596299	0.214	NA
11750	Anxa7	annexin A7	1.07596299	0.3017	NA
16522	Kcnj6	potassium inwardly-rectifying channel, subfamily J, member 6	1.07596299	0.4923	0.6864
103220	BC030307	cDNA sequence BC030307	1.07596299	0.6114	0.7757
53859	Map3k14	mitogen-activated protein kinase kinase kinase 14	1.07596299	0.6667	0.8122
223732	Ldoc1l	leucine zipper, down-regulated in cancer 1-like	1.07584723	0.3498	NA
98238	Lrrc59	leucine rich repeat containing 59	1.07584723	0.3761	NA
73130	Tmed5	transmembrane emp24 protein transport domain containing 5	1.07584723	0.4142	0.6206
11548	Adra1b	adrenergic receptor, alpha 1b	1.07584723	0.5458	0.7278
67045	Riok2	RIO kinase 2 (yeast)	1.0757315	0.1789	NA
231841	AA881470	EST AA881470	1.0757315	0.4313	0.6353
78755	Fam122b	family with sequence similarity 122, member B	1.0757315	0.5704	0.7456
11350	Abl1	c-abl oncogene 1, non-receptor tyrosine kinase	1.0757315	0.6055	0.7719
109815	H47	histocompatibility 47	1.07561579	0.1381	NA
68520	Zfyve21	zinc finger, FYVE domain containing 21	1.07561579	0.3577	NA
231997	Fkbp14	FK506 binding protein 14	1.07561579	0.4451	0.6479
105847	Lmf2	lipase maturation factor 2	1.07561579	0.5676	0.7434
114896	Afg3l1	AFG3(ATPase family gene 3)-like 1 (yeast)	1.07550011	0.3216	NA
13638	Efna3	ephrin A3	1.07550011	0.651	0.8034
219249	Tdrd3	tudor domain containing 3	1.07538445	0.3314	NA
74030	Rin2	Ras and Rab interactor 2	1.07538445	0.545	0.727
100039252	Gm12693	predicted gene 12693	1.07526882	0.1726	NA
65116	Prrg2	proline-rich Gla (G-carboxyglutamic acid) polypeptide 2	1.07526882	0.2089	NA
103266	AI597468	expressed sequence AI597468	1.07526882	0.2745	NA
224997	Dlgap1	discs, large (Drosophila) homolog-associated protein 1	1.07526882	0.4048	0.6124
12890	Cplx2	complexin 2	1.07526882	0.4209	0.6271
72895	Setd5	SET domain containing 5	1.07515321	0.1349	NA
218914	Wapal	wings apart-like homolog (Drosophila)	1.07515321	0.2373	NA
319468	Ppm1h	protein phosphatase 1H (PP2C domain containing)	1.07515321	0.2525	NA
67398	Srpr	signal recognition particle receptor ('docking protein')	1.07515321	0.304	NA
14252	Flot2	flotillin 2	1.07515321	0.3205	NA
20194	S100a10	S100 calcium binding protein A10 (calpactin)	1.07515321	0.3934	0.6016
18477	Prdx1	peroxiredoxin 1	1.07515321	0.3946	0.6028
66589	Ube2v1	ubiquitin-conjugating enzyme E2 variant 1	1.07515321	0.4271	0.6319
67747	Ribc2	RIB43A domain with coiled-coils 2	1.07515321	0.4469	0.6491
56215	Acin1	apoptotic chromatin condensation inducer 1	1.07515321	0.566	0.7423
218490	Btf3	basic transcription factor 3	1.07503763	0.1355	NA
72083	Mzt2	mitotic spindle organizing protein 2	1.07503763	0.3909	0.5992
66848	Fuca2	fucosidase, alpha-L- 2, plasma	1.07503763	0.4782	0.675
433926	Lrrc8b	leucine rich repeat containing 8 family, member B	1.07503763	0.6462	0.8003
226162	Dpcd	deleted in primary ciliary dyskinesia	1.07492207	0.1883	NA
268470	Ube2z	ubiquitin-conjugating enzyme E2Z (putative)	1.07492207	0.2487	NA
243963	Zfp473	zinc finger protein 473	1.07492207	0.3438	NA
215445	Rab11fip3	RAB11 family interacting protein 3 (class II)	1.07492207	0.3861	0.5947
76942	Lypd5	Ly6/Plaur domain containing 5	1.07492207	0.4903	0.6846
75079	Zbtb49	zinc finger and BTB domain containing 49	1.07492207	0.5615	0.7396
18436	P2rx1	purinergic receptor P2X, ligand-gated ion channel, 1	1.07492207	0.8079	0.9004
74255	Smu1	smu-1 suppressor of mec-8 and unc-52 homolog (C. elegans)	1.07480653	0.2636	NA

116871	Mta3	metastasis associated 3	1.07480653	0.5666	0.7428
78365	1500016L03Rik	RIKEN cDNA 1500016L03 gene	1.07480653	0.6067	0.7727
72008	Zfyve19	zinc finger, FYVE domain containing 19	1.07469103	0.09872	NA
20091	Rps3a	ribosomal protein S3A	1.07469103	0.121	NA
226169	Pprc1	peroxisome proliferative activated receptor, gamma, coactivator-related 1	1.07469103	0.4156	0.6219
217700	Acot6	acyl-CoA thioesterase 6	1.07469103	0.5815	0.7535
78420	9530048J24Rik	RIKEN cDNA 9530048J24 gene	1.07469103	0.6555	0.8056
103737	Pex12	peroxisomal biogenesis factor 12	1.07457554	0.229	NA
56371	Fzr1	fizzy/cell division cycle 20 related 1 (Drosophila)	1.07457554	0.4571	0.6584
18131	Notch3	Notch gene homolog 3 (Drosophila)	1.07457554	0.4764	0.6736
23983	Pcbp1	poly(rC) binding protein 1	1.07446008	0.2069	NA
12040	Bckdhb	branched chain ketoacid dehydrogenase E1, beta polypeptide	1.07446008	0.2514	NA
56550	Ube2d2	ubiquitin-conjugating enzyme E2D 2	1.07446008	0.3142	NA
53893	Nudt5	nudix (nucleoside diphosphate linked moiety X)-type motif 5	1.07446008	0.3715	NA
208092	Chmp6	chromatin modifying protein 6	1.07446008	0.4564	0.6579
211586	Tfdp2	transcription factor Dp 2	1.07446008	0.4704	0.6696
217695	Zfyve1	zinc finger, FYVE domain containing 1	1.07434465	0.4463	0.6489
15936	Ier2	immediate early response 2	1.07434465	0.5383	0.7221
77870	E130116L18Rik	RIKEN cDNA E130116L18 gene	1.07434465	0.5393	0.7228
68106	Nt5c3l	5'-nucleotidase, cytosolic III-like	1.07422924	0.1566	NA
101604	E430018J23Rik	RIKEN cDNA E430018J23 gene	1.07422924	0.2183	NA
223978	Cpped1	calcineurin-like phosphoesterase domain containing 1	1.07422924	0.2438	NA
56368	Cyb561d2	cytochrome b-561 domain containing 2	1.07411386	0.1544	NA
26894	Cops7a	COP9 (constitutive photomorphogenic) homolog, subunit 7a (Arabidopsis thaliana)	1.07411386	0.5234	0.7104
268395	Mpg	N-methylpurine-DNA glycosylase	1.0739985	0.2101	NA
20615	Snapin	SNAP-associated protein	1.0739985	0.2704	NA
70369	Bag5	BCL2-associated athanogene 5	1.0739985	0.2912	NA
26893	Cops6	COP9 (constitutive photomorphogenic) homolog, subunit 6 (Arabidopsis thaliana)	1.0739985	0.2948	NA
319463	C230057M02Rik	RIKEN cDNA C230057M02 gene	1.0739985	0.5166	0.706
13660	Ehd1	EH-domain containing 1	1.07388316	0.1386	NA
66890	Lman2	lectin, mannose-binding 2	1.07388316	0.1752	NA
69752	Zfp511	zinc finger protein 511	1.07388316	0.2051	NA
242915	Fam59b	family with sequence similarity 59, member B	1.07388316	0.4246	0.6301
23950	Dnajb6	DnaJ (Hsp40) homolog, subfamily B, member 6	1.07376785	0.07317	NA
105014	Rdh14	retinol dehydrogenase 14 (all-trans and 9-cis)	1.07376785	0.2688	NA
242702	Myom3	myomesin family, member 3	1.07376785	0.3493	NA
75731	5133401N09Rik	RIKEN cDNA 5133401N09 gene	1.07376785	0.3666	NA
67149	Nkain1	Na+/K+ transporting ATPase interacting 1	1.07365257	0.1779	NA
21761	Morf4l1	mortality factor 4 like 1	1.07365257	0.2672	NA
229699	Slc16a4	solute carrier family 16 (monocarboxylic acid transporters), member 4	1.07365257	0.5315	0.7168
242083	Ppm1l	protein phosphatase 1 (formerly 2C)-like	1.07353731	0.2711	NA
230459	Cyp2j13	cytochrome P450, family 2, subfamily j, polypeptide 13	1.07353731	0.3532	NA
56212	Rhog	ras homolog gene family, member G	1.07353731	0.491	0.6851
78827	5830426C09Rik	RIKEN cDNA 5830426C09 gene	1.07353731	0.6012	0.7687
77057	Ston1	stonin 1	1.07353731	0.8071	0.8999
387565	Cd300c	CD300C antigen	1.07353731	0.8252	0.9099
19088	Prkar2b	protein kinase, cAMP dependent regulatory, type II beta	1.07342207	0.1244	NA
16430	Stt3a	STT3, subunit of the oligosaccharyltransferase complex, homolog A (S. cerevisiae)	1.07342207	0.5259	0.7126
214763	E330016A19Rik	RIKEN cDNA E330016A19 gene	1.07342207	0.6211	0.7825
52856	Gtpbp5	GTP binding protein 5	1.07330686	0.2445	NA

74513	Neto2	neuropilin (NRP) and tolloid (TLL)-like 2	1.07330686	0.3904	NA
67117	Dynlt3	dynein light chain Tctex-type 3	1.07330686	0.4943	0.688
276905	Armc7	armadillo repeat containing 7	1.07330686	0.5229	0.7102
67675	Cuta	cutA divalent cation tolerance homolog (E. coli)	1.07330686	0.6307	0.7902
19702	Ren2	renin 2 tandem duplication of Ren1	1.07330686	0.7245	0.85
66500	Slc30a7	solute carrier family 30 (zinc transporter), member 7	1.07319167	0.2839	NA
12982	Csf2ra	colony stimulating factor 2 receptor, alpha, low-affinity (granulocyte-macrophage)	1.07319167	0.3601	NA
403187	Opa3	optic atrophy 3 (human)	1.07319167	0.3848	NA
19941	Rpl26	ribosomal protein L26	1.07307651	0.1733	NA
100502680	LOC100502680	ubiquitin-conjugating enzyme E2 L3-like	1.07307651	0.3784	NA
224904	2410015M20Rik	RIKEN cDNA 2410015M20 gene	1.07296137	0.1805	NA
103836	Zfp692	zinc finger protein 692	1.07296137	0.531	0.7164
19171	Psmb10	proteasome (prosome, macropain) subunit, beta type 10	1.07284626	0.1509	NA
100415785	Gm11559	predicted gene 11559	1.07273117	0.3006	NA
71838	Phf7	PHD finger protein 7	1.07273117	0.365	NA
67393	Cxxc5	CXXC finger 5	1.07261611	0.1598	NA
235441	Usp3	ubiquitin specific peptidase 3	1.07261611	0.1774	NA
108673	Ccdc86	coiled-coil domain containing 86	1.07261611	0.2956	NA
67883	Uxs1	UDP-glucuronate decarboxylase 1	1.07261611	0.3783	NA
66940	Shisa5	shisa homolog 5 (Xenopus laevis)	1.07261611	0.5214	0.7096
16527	Kcnk3	potassium channel, subfamily K, member 3	1.07261611	0.5514	0.7323
68581	Tmed10	transmembrane emp24-like trafficking protein 10 (yeast)	1.07250107	0.3097	NA
229007	Zgpat	zinc finger, CCCH-type with G patch domain	1.07250107	0.5271	0.7135
232430	Crebl2	cAMP responsive element binding protein-like 2	1.07250107	0.5672	0.7431
67826	Snap47	synaptosomal-associated protein, 47	1.07238606	0.415	0.6213
68427	Slc39a13	solute carrier family 39 (metal ion transporter), member 13	1.07227107	0.362	NA
21420	Tcfap2c	transcription factor AP-2, gamma	1.07215611	0.167	NA
231642	Alkbh2	alkB, alkylation repair homolog 2 (E. coli)	1.07215611	0.1853	NA
16795	Large	like-glycosyltransferase	1.07215611	0.2362	NA
77591	Ddx10	DEAD (Asp-Glu-Ala-Asp) box polypeptide 10	1.07215611	0.3583	NA
791360	Gm9853	predicted gene 9853	1.07215611	0.484	0.6795
22720	Zfp62	zinc finger protein 62	1.07215611	0.6187	0.7808
71517	9030624J02Rik	RIKEN cDNA 9030624J02 gene	1.07204117	0.2764	NA
54399	Bet1l	blocked early in transport 1 homolog (S. cerevisiae)-like	1.07204117	0.3198	NA
73390	Msl3l2	male-specific lethal 3-like 2 (Drosophila)	1.07204117	0.3537	NA
381110	Fam82a1	family with sequence similarity 82, member A1	1.07204117	0.4018	NA
60530	Fignl1	fidgetin-like 1	1.07204117	0.5342	0.7189
67248	Rpl39	ribosomal protein L39	1.07192625	0.2452	NA
57342	Parva	parvin, alpha	1.07192625	0.2882	NA
16539	Kcns2	K+ voltage-gated channel, subfamily S, 2	1.07192625	0.5947	0.7637
100705	Acacb	acetyl-Coenzyme A carboxylase beta	1.07192625	0.6635	0.8105
67830	Rer1	RER1 retention in endoplasmic reticulum 1 homolog (S. cerevisiae)	1.07181136	0.1504	NA
52468	Ctdsp2	CTD (carboxy-terminal domain, RNA polymerase II, polypeptide A) small phosphatase 2	1.07181136	0.2106	NA
56248	Ak3	adenylate kinase 3	1.07181136	0.2725	NA
76894	Mett5d1	methyltransferase 5 domain containing 1	1.07181136	0.4364	0.6401
230459	Cyp2j13	cytochrome P450, family 2, subfamily j, polypeptide 13	1.0716965	0.1961	NA
16599	Klf3	Kruppel-like factor 3 (basic)	1.0716965	0.3433	NA
75104	Mmd2	monocyte to macrophage differentiation-associated 2	1.0716965	0.4265	0.6315
11350	Abl1	c-abl oncogene 1, non-receptor tyrosine kinase	1.0716965	0.4268	0.6318
15267	Hist2h2aa1	histone cluster 2, H2aa1	1.07158165	0.1406	NA

57815	Spata5	spermatogenesis associated 5	1.07158165	0.1421	NA
216818	Gm4802	predicted gene 4802	1.07158165	0.1883	NA
140499	Ube2j2	ubiquitin-conjugating enzyme E2, J2 homolog (yeast)	1.07158165	0.216	NA
104303	Arl1	ADP-ribosylation factor-like 1	1.07158165	0.3683	NA
22782	Slc30a1	solute carrier family 30 (zinc transporter), member 1	1.07158165	0.3832	NA
103236	Csnk1g2	casein kinase 1, gamma 2	1.07158165	0.4023	NA
75602	1810062O18Rik	RIKEN cDNA 1810062O18 gene	1.07158165	0.4941	0.688
78668	E130112N10Rik	RIKEN cDNA E130112N10 gene	1.07158165	0.6199	0.7816
18991	Pou3f1	POU domain, class 3, transcription factor 1	1.07146684	0.3668	NA
57869	Adck2	aarF domain containing kinase 2	1.07146684	0.4236	0.6293
76813	Armc6	armadillo repeat containing 6	1.07146684	0.4969	0.6899
30957	Mapk8ip3	mitogen-activated protein kinase 8 interacting protein 3	1.07146684	0.5111	0.7017
17294	Mest	mesoderm specific transcript	1.07146684	0.5285	0.7146
66585	Snrnp40	small nuclear ribonucleoprotein 40 (U5)	1.07135205	0.3054	NA
104884	Tdp1	tyrosyl-DNA phosphodiesterase 1	1.07135205	0.3562	NA
66973	Mrps18b	mitochondrial ribosomal protein S18B	1.07135205	0.3634	NA
381059	Gm1604b	predicted gene 1604b	1.07135205	0.4827	0.6784
14814	Grin2d	glutamate receptor, ionotropic, NMDA2D (epsilon 4)	1.07135205	0.7488	0.8649
17113	M6pr	mannose-6-phosphate receptor, cation dependent	1.07123728	0.1556	NA
13660	Ehd1	EH-domain containing 1	1.07123728	0.2909	NA
68961	Phkg2	phosphorylase kinase, gamma 2 (testis)	1.07123728	0.3929	NA
241053	Rpl12-ps1	ribosomal protein L12, pseudogene 1	1.07123728	0.4024	NA
14705	Bscl2	Bernardinelli-Seip congenital lipodystrophy 2 homolog (human)	1.07123728	0.4035	NA
67474	Snap29	synaptosomal-associated protein 29	1.07112254	0.2413	NA
27756	Lsm2	LSM2 homolog, U6 small nuclear RNA associated (S. cerevisiae)	1.07112254	0.2808	NA
246154	Vasn	vasorin	1.07112254	0.3452	NA
270110	Irf2bp2	interferon regulatory factor 2 binding protein 2	1.07112254	0.5246	0.7114
69217	Plekha4	pleckstrin homology domain containing, family A (phosphoinositide binding specific) member 4	1.07100782	0.1706	NA
19899	Rpl18	ribosomal protein L18	1.07100782	0.1783	NA
22247	Umps	uridine monophosphate synthetase	1.07100782	0.3224	NA
18607	Pdpk1	3-phosphoinositide dependent protein kinase 1	1.07100782	0.4254	0.6307
74770	Hhatl	hedgehog acyltransferase-like	1.07100782	0.6137	0.7774
14748	Gpr3	G-protein coupled receptor 3	1.07100782	0.6666	0.8121
28018	Ubfd1	ubiquitin family domain containing 1	1.07089312	0.2822	NA
22668	Sf1	splicing factor 1	1.07089312	0.4036	NA
67789	Dalrd3	DALR anticodon binding domain containing 3	1.07089312	0.444	0.6468
67017	2010011I20Rik	RIKEN cDNA 2010011I20 gene	1.07077846	0.2002	NA
27207	Rps11	ribosomal protein S11	1.07077846	0.2598	NA
545718	Gm13141	predicted gene 13141	1.07077846	0.2828	NA
67026	Thap4	THAP domain containing 4	1.07077846	0.2872	NA
52463	Tet1	tet oncogene 1	1.07077846	0.3188	NA
217011	Nle1	notchless homolog 1 (Drosophila)	1.07077846	0.4373	0.6407
353237	Pcdhac2	protocadherin alpha subfamily C, 2	1.07077846	0.4858	0.6811
22635	Zan	zonadhesin	1.07077846	0.4944	0.688
106639	Vmac	vimentin-type intermediate filament associated coiled-coil protein	1.07077846	0.5004	0.6924
20867	Stip1	stress-induced phosphoprotein 1	1.07077846	0.5144	0.7044
70503	Ddo	D-aspartate oxidase	1.07066381	0.2943	NA
246196	Zfp277	zinc finger protein 277	1.07066381	0.2995	NA
98193	Dcaf8	DDB1 and CUL4 associated factor 8	1.07066381	0.413	NA
27528	D0H4S114	DNA segment, human D4S114	1.07066381	0.5355	0.72

11911	Atf4	activating transcription factor 4	1.07054919	0.2312	NA
384864	Gm1943	WD repeat domain 70 pseudogene	1.07054919	0.3067	NA
15505	Hsph1	heat shock 105kDa/110kDa protein 1	1.07054919	0.3401	NA
107071	Wdr74	WD repeat domain 74	1.07054919	0.3982	NA
225164	Mib1	mindbomb homolog 1 (Drosophila)	1.07054919	0.4187	0.625
140482	Zfp358	zinc finger protein 358	1.0704346	0.2513	NA
74167	Nudt9	nudix (nucleoside diphosphate linked moiety X)-type motif 9	1.0704346	0.4181	0.6244
215999	Ccdc109a	coiled-coil domain containing 109A	1.0704346	0.641	0.7969
69577	Fastkd3	FAST kinase domains 3	1.07032003	0.3996	NA
11433	Acp5	acid phosphatase 5, tartrate resistant	1.07032003	0.5606	0.739
244650	Phlpp2	PH domain and leucine rich repeat protein phosphatase 2	1.07020548	0.1515	NA
69596	2310035K24Rik	RIKEN cDNA 2310035K24 gene	1.07020548	0.1574	NA
68048	Aen	apoptosis enhancing nuclease	1.07020548	0.2071	NA
68001	1110004E09Rik	RIKEN cDNA 1110004E09 gene	1.07020548	0.2693	NA
216152	BC005764	cDNA sequence BC005764	1.07020548	0.2776	NA
66333	Aqp11	aquaporin 11	1.07020548	0.3177	NA
66971	Cdk5rap1	CDK5 regulatory subunit associated protein 1	1.07020548	0.3359	NA
17766	Nudt1	nudix (nucleoside diphosphate linked moiety X)-type motif 1	1.07020548	0.4198	0.6259
74648	S100pbp	S100P binding protein	1.07020548	0.6797	0.821
68525	Evc2	Ellis van Creveld syndrome 2 homolog (human)	1.07020548	0.735	0.8568
20729	Spin1	spindlin 1	1.07009096	0.2442	NA
111175	Pecr	peroxisomal trans-2-enoyl-CoA reductase	1.07009096	0.3582	NA
68653	Samm50	sorting and assembly machinery component 50 homolog (S. cerevisiae)	1.07009096	0.4064	NA
208624	Alg3	asparagine-linked glycosylation 3 homolog (yeast, alpha-1,3-mannosyltransferase)	1.07009096	0.6577	0.8066
320492	A830018L16Rik	RIKEN cDNA A830018L16 gene	1.07009096	0.6726	0.8167
26451	Rpl27a	ribosomal protein L27A	1.06997646	0.09485	NA
102632	Acad11	acyl-Coenzyme A dehydrogenase family, member 11	1.06997646	0.1746	NA
14590	Ggh	gamma-glutamyl hydrolase	1.06997646	0.2635	NA
69228	Zfp746	zinc finger protein 746	1.06997646	0.4077	NA
77697	Mmab	methylmalonic aciduria (cobalamin deficiency) type B homolog (human)	1.06997646	0.4628	0.6633
319748	Zfp865	zinc finger protein 865	1.06997646	0.5873	0.7577
67778	Zfp639	zinc finger protein 639	1.06986199	0.2315	NA
24051	Sgcb	sarcoglycan, beta (dystrophin-associated glycoprotein)	1.06986199	0.3125	NA
74351	Ddx23	DEAD (Asp-Glu-Ala-Asp) box polypeptide 23	1.06986199	0.6707	0.8154
106585	Ankrd12	ankyrin repeat domain 12	1.06986199	0.6724	0.8166
238799	Tnpo1	transportin 1	1.06974754	0.199	NA
18769	Pkig	protein kinase inhibitor, gamma	1.06974754	0.377	NA
16880	Lifr	leukemia inhibitory factor receptor	1.06974754	0.4235	0.6292
233067	Lrfn3	leucine rich repeat and fibronectin type III domain containing 3	1.06974754	0.4356	0.6393
73692	2410089E03Rik	RIKEN cDNA 2410089E03 gene	1.06974754	0.5223	0.7101
27207	Rps11	ribosomal protein S11	1.06963312	0.1423	NA
52065	Mfhas1	malignant fibrous histiocytoma amplified sequence 1	1.06963312	0.2636	NA
11477	Acvr1	activin A receptor, type 1	1.06963312	0.277	NA
102294	Cyp4v3	cytochrome P450, family 4, subfamily v, polypeptide 3	1.06963312	0.3056	NA
108903	Tbcd	tubulin-specific chaperone d	1.06963312	0.4048	NA
67320	Iqcf4	IQ motif containing F4	1.06963312	0.8336	0.9141
30949	Lcmt1	leucine carboxyl methyltransferase 1	1.06951872	0.1609	NA
268417	Zkscan17	zinc finger with KRAB and SCAN domains 17	1.06951872	0.298	NA
12483	Cd22	CD22 antigen	1.06951872	0.8055	0.8989
21752	Tert	telomerase reverse transcriptase	1.06951872	0.842	0.9183

19942	Rpl27	ribosomal protein L27	1.06940434	0.4434	0.6464
140489	Bhlhe23	basic helix-loop-helix family, member e23	1.06940434	0.4633	0.6637
327954	Dnahc2	dynein, axonemal, heavy chain 2	1.06940434	0.466	0.6659
54601	Foxo4	forkhead box O4	1.06940434	0.7302	0.8537
217342	Ube2o	ubiquitin-conjugating enzyme E2O	1.06928999	0.4185	NA
225888	Suv420h1	suppressor of variegation 4-20 homolog 1 (Drosophila)	1.06928999	0.6631	0.8102
73844	Ankrd45	ankyrin repeat domain 45	1.06928999	0.6895	0.8276
19941	Rpl26	ribosomal protein L26	1.06917567	0.1806	NA
57376	Smarce1	SWI/SNF related, matrix associated, actin dependent regulator of chromatin, subfamily e, member 1	1.06917567	0.2151	NA
268741	Tox4	TOX high mobility group box family member 4	1.06917567	0.2151	NA
52700	Txndc17	thioredoxin domain containing 17	1.06917567	0.3017	NA
215494	C85492	expressed sequence C85492	1.06917567	0.3736	NA
233812	BC030336	cDNA sequence BC030336	1.06917567	0.4149	NA
20333	Sec22b	SEC22 vesicle trafficking protein homolog B (S. cerevisiae)	1.06906136	0.1052	NA
108099	Prkag2	protein kinase, AMP-activated, gamma 2 non-catalytic subunit	1.06906136	0.1276	NA
20085	Rps19	ribosomal protein S19	1.06906136	0.1393	NA
18590	Pdgfa	platelet derived growth factor, alpha	1.06906136	0.1999	NA
80914	Uck2	uridine-cytidine kinase 2	1.06906136	0.2372	NA
19989	Rpl7	ribosomal protein L7	1.06906136	0.2469	NA
69928	Apitd1	apoptosis-inducing, TAF9-like domain 1	1.06906136	0.5571	0.7366
231717	Fam109a	family with sequence similarity 109, member A	1.06894709	0.178	NA
72754	Arhgef10l	Rho guanine nucleotide exchange factor (GEF) 10-like	1.06894709	0.269	NA
67489	Ap4b1	adaptor-related protein complex AP-4, beta 1	1.06894709	0.3287	NA
22375	Wars	tryptophanyl-tRNA synthetase	1.06894709	0.3991	NA
72108	Ddhd2	DDHD domain containing 2	1.06894709	0.5178	0.7071
13144	Dapk3	death-associated protein kinase 3	1.06894709	0.63	0.7896
69191	Pdia2	protein disulfide isomerase associated 2	1.06894709	0.6946	0.831
26407	Map3k4	mitogen-activated protein kinase kinase kinase 4	1.06883283	0.1648	NA
432999	A930007A09Rik	RIKEN cDNA A930007A09 gene	1.06883283	0.2558	NA
56461	Kcnip3	Kv channel interacting protein 3, calsenilin	1.06883283	0.3254	NA
67160	Eef1g	eukaryotic translation elongation factor 1 gamma	1.06883283	0.4785	0.6753
100039864	Snhg12	small nucleolar RNA host gene 12	1.06883283	0.4919	0.686
66861	Dnajc10	DnaJ (Hsp40) homolog, subfamily C, member 10	1.06871861	0.2217	NA
98221	Eif3m	eukaryotic translation initiation factor 3, subunit M	1.06871861	0.2712	NA
22134	Tgoln1	trans-golgi network protein	1.06871861	0.3937	NA
330474	Zc3h4	zinc finger CCCH-type containing 4	1.06871861	0.4774	0.6744
104457	0610010K14Rik	RIKEN cDNA 0610010K14 gene	1.0686044	0.1635	NA
73668	Ttc21b	tetratricopeptide repeat domain 21B	1.0686044	0.2499	NA
320916	Wscd2	WSC domain containing 2	1.0686044	0.2772	NA
20166	Rtkn	rhotekin	1.0686044	0.3054	NA
102060	Gadd45gip1	growth arrest and DNA-damage-inducible, gamma interacting protein 1	1.0686044	0.4388	0.6417
23844	Clca3	chloride channel calcium activated 3	1.0686044	0.5507	0.7317
58202	Cobra1	cofactor of BRCA1	1.06849022	0.1273	NA
320595	Phf8	PHD finger protein 8	1.06849022	0.1434	NA
66480	Rpl15	ribosomal protein L15	1.06849022	0.1765	NA
67673	Tceb2	transcription elongation factor B (SIII), polypeptide 2	1.06849022	0.2358	NA
13665	Eif2s1	eukaryotic translation initiation factor 2, subunit 1 alpha	1.06849022	0.2638	NA
266690	Cyb5r4	cytochrome b5 reductase 4	1.06849022	0.5001	0.6922
231874	Ccz1	CCZ1 vacuolar protein trafficking and biogenesis associated homolog (S. cerevisiae)	1.06837607	0.184	NA
16532	Kcnu1	potassium channel, subfamily U, member 1	1.06837607	0.4268	NA

11465	Actg1	actin, gamma, cytoplasmic 1	1.06826194	0.1653	NA
208718	Dis3l2	DIS3 mitotic control homolog (S. cerevisiae)-like 2	1.06826194	0.1822	NA
228368	Slc35c1	solute carrier family 35, member C1	1.06826194	0.269	NA
67067	Romo1	reactive oxygen species modulator 1	1.06826194	0.2759	NA
80861	Dhx58	DEXH (Asp-Glu-X-His) box polypeptide 58	1.06826194	0.3211	NA
68051	Nutf2	nuclear transport factor 2	1.06826194	0.3475	NA
381792	2310040G24Rik	RIKEN cDNA 2310040G24 gene	1.06826194	0.5065	0.6975
239167	D930020E02Rik	RIKEN cDNA D930020E02 gene	1.06826194	0.706	0.8379
75036	4930488B01Rik	RIKEN cDNA 4930488B01 gene	1.06814783	0.2851	NA
80898	Erap1	endoplasmic reticulum aminopeptidase 1	1.06814783	0.5195	0.7085
103841	Cuedc1	CUE domain containing 1	1.06814783	0.5758	0.7492
21847	Klf10	Kruppel-like factor 10	1.06803375	0.2468	NA
26931	Ppp2r5c	protein phosphatase 2, regulatory subunit B (B56), gamma isoform	1.06803375	0.2685	NA
319880	Tmcc3	transmembrane and coiled coil domains 3	1.06803375	0.3802	NA
215114	Hip1	huntingtin interacting protein 1	1.06803375	0.4789	0.6755
213573	Efcab4a	EF-hand calcium binding domain 4A	1.06803375	0.5397	0.7231
15944	Irgm1	immunity-related GTPase family M member 1	1.06803375	0.6066	0.7727
666747	Trim43b	tripartite motif-containing 43B	1.06803375	0.8619	0.9287
329910	Acot11	acyl-CoA thioesterase 11	1.06791969	0.1527	NA
11682	Alk	anaplastic lymphoma kinase	1.06791969	0.2491	NA
56209	Gde1	glycerophosphodiester phosphodiesterase 1	1.06791969	0.2849	NA
21975	Top3a	topoisomerase (DNA) III alpha	1.06791969	0.3041	NA
76510	Trappc9	trafficking protein particle complex 9	1.06791969	0.4877	0.6825
70951	Spata1	spermatogenesis associated 1	1.06791969	0.5642	0.7416
50785	Hs6st1	heparan sulfate 6-O-sulfotransferase 1	1.06791969	0.5921	0.7617
19697	Rela	v-rel reticuloendotheliosis viral oncogene homolog A (avian)	1.06780566	0.1672	NA
67067	Romo1	reactive oxygen species modulator 1	1.06780566	0.2402	NA
22323	Vasp	vasodilator-stimulated phosphoprotein	1.06780566	0.3244	NA
68731	Rbfa	ribosome binding factor A	1.06780566	0.5552	0.7351
71268	Lrrfip2	leucine rich repeat (in FLII) interacting protein 2	1.06780566	0.591	0.7607
57740	Stk32c	serine/threonine kinase 32C	1.06769165	0.7102	0.8408
227835	Gtdc1	glycosyltransferase-like domain containing 1	1.06757767	0.2121	NA
217149	Cisd3	CDGSH iron sulfur domain 3	1.06757767	0.3131	NA
218138	Gmds	GDP-mannose 4, 6-dehydratase	1.06757767	0.355	NA
108150	Galnt7	UDP-N-acetyl-alpha-D-galactosamine: polypeptide N-acetylgalactosaminyltransferase 7	1.06757767	0.3732	NA
21750	Terf2	telomeric repeat binding factor 2	1.06757767	0.5019	0.6937
66253	Aig1	androgen-induced 1	1.06757767	0.5136	0.704
18986	Pou2f1	POU domain, class 2, transcription factor 1	1.06757767	0.5656	0.7423
331461	Il1rapl1	interleukin 1 receptor accessory protein-like 1	1.06757767	0.7719	0.8787
791383	Gm9962	predicted gene 9962	1.06757767	0.7832	0.8863
217779	Lysmd1	LysM, putative peptidoglycan-binding, domain containing 1	1.06746371	0.2208	NA
67673	Tceb2	transcription elongation factor B (SIII), polypeptide 2	1.06746371	0.2374	NA
75608	Chmp4b	chromatin modifying protein 4B	1.06746371	0.2385	NA
65106	Arl6ip5	ADP-ribosylation factor-like 6 interacting protein 5	1.06746371	0.2612	NA
78600	Pde6h	phosphodiesterase 6H, cGMP-specific, cone, gamma	1.06746371	0.3435	NA
56233	Hdac7	histone deacetylase 7	1.06746371	0.4269	NA
384179	Gm5292	predicted gene 5292	1.06734977	0.1757	NA
68250	Fam96a	family with sequence similarity 96, member A	1.06734977	0.2247	NA
233107	Kctd15	potassium channel tetramerisation domain containing 15	1.06734977	0.2716	NA
319162	Hist3h2a	histone cluster 3, H2a	1.06734977	0.3706	NA

71974	Prmt3	protein arginine N-methyltransferase 3	1.06734977	0.6132	0.7771
67263	Zswim6	zinc finger, SWIM domain containing 6	1.06734977	0.7076	0.8391
17129	Smad5	MAD homolog 5 (Drosophila)	1.06723586	0.1487	NA
66266	Eapp	E2F-associated phosphoprotein	1.06723586	0.2986	NA
240028	Lnpep	leucyl/cystinyl aminopeptidase	1.06723586	0.4826	0.6783
56700	0610031J06Rik	RIKEN cDNA 0610031J06 gene	1.06723586	0.5849	0.7556
70873	4921517L17Rik	RIKEN cDNA 4921517L17 gene	1.06723586	0.6172	0.7797
66990	Tmem134	transmembrane protein 134	1.06712197	0.2166	NA
22393	Wfs1	Wolfram syndrome 1 homolog (human)	1.06712197	0.2875	NA
67222	Srfbp1	serum response factor binding protein 1	1.06712197	0.2928	NA
677044	Gm10653	ribosomal protein S2 pseudogene	1.06712197	0.3193	NA
14466	Gba	glucosidase, beta, acid	1.06712197	0.5014	0.6932
56217	Mpp5	membrane protein, palmitoylated 5 (MAGUK p55 subfamily member 5)	1.06712197	0.5143	0.7044
17878	Myf6	myogenic factor 6	1.06712197	0.5704	0.7456
26358	Aldh1a7	aldehyde dehydrogenase family 1, subfamily A7	1.06712197	0.647	0.8009
19188	Psme2	proteasome (prosome, macropain) 28 subunit, beta	1.06700811	0.1753	NA
22724	Zbtb7b	zinc finger and BTB domain containing 7B	1.06700811	0.2074	NA
99152	Anapc2	anaphase promoting complex subunit 2	1.06689427	0.277	NA
68718	Rnf166	ring finger protein 166	1.06689427	0.2839	NA
232536	Mrps35	mitochondrial ribosomal protein S35	1.06689427	0.323	NA
67410	4930449I24Rik	RIKEN cDNA 4930449I24 gene	1.06689427	0.5715	0.7465
79044	Mrps34	mitochondrial ribosomal protein S34	1.06678046	0.1687	NA
98932	Myl9	myosin, light polypeptide 9, regulatory	1.06678046	0.177	NA
667338	Gm8580	ribosomal protein L29 pseudogene	1.06678046	0.3171	NA
58887	Repin1	replication initiator 1	1.06666667	0.1662	NA
13000	Csnk2a2	casein kinase 2, alpha prime polypeptide	1.06666667	0.3744	NA
72338	Wdr89	WD repeat domain 89	1.06666667	0.4737	0.6717
234779	Plcg2	phospholipase C, gamma 2	1.06666667	0.5126	0.7031
52563	Cdc23	CDC23 (cell division cycle 23, yeast, homolog)	1.06666667	0.5263	0.7128
15166	Hcn2	hyperpolarization-activated, cyclic nucleotide-gated K+ 2	1.06666667	0.6105	0.775
268301	Ankrd57	ankyrin repeat domain 57	1.0665529	0.2491	NA
19075	Prim1	DNA primase, p49 subunit	1.0665529	0.2679	NA
75712	Tmem14a	transmembrane protein 14A	1.0665529	0.2884	NA
23897	Hax1	HCLS1 associated X-1	1.0665529	0.4715	0.67
14696	Gnb4	guanine nucleotide binding protein (G protein), beta 4	1.0665529	0.4985	0.6912
22619	Siae	sialic acid acetylesterase	1.06643916	0.2667	NA
11690	Alox5ap	arachidonate 5-lipoxygenase activating protein	1.06643916	0.3769	NA
108899	2700081O15Rik	RIKEN cDNA 2700081O15 gene	1.06643916	0.6022	0.7693
208647	Creb3l2	cAMP responsive element binding protein 3-like 2	1.06643916	0.6442	0.7989
272589	Tbcel	tubulin folding cofactor E-like	1.06632544	0.2299	NA
12567	Cdk4	cyclin-dependent kinase 4	1.06632544	0.2329	NA
23938	Map2k5	mitogen-activated protein kinase kinase 5	1.06632544	0.3348	NA
66830	Nacc1	nucleus accumbens associated 1, BEN and BTB (POZ) domain containing	1.06632544	0.4757	0.673
14570	Arhgdig	Rho GDP dissociation inhibitor (GDI) gamma	1.06632544	0.5536	0.7339
14300	Frg1	FSHD region gene 1	1.06632544	0.6138	0.7775
18795	Plcb1	phospholipase C, beta 1	1.06632544	0.6915	0.8289
20442	St3gal1	ST3 beta-galactoside alpha-2,3-sialyltransferase 1	1.06621175	0.2005	NA
19294	Pvrl2	poliovirus receptor-related 2	1.06621175	0.3221	NA
12322	Camk2a	calcium/calmodulin-dependent protein kinase II alpha	1.06621175	0.3251	NA
547008	Gm6010	predicted gene 6010	1.06621175	0.3605	NA

436332	Gm5766	ribosomal protein L7a pseudogene	1.06609808	0.1192	NA
13353	Dgcr6	DiGeorge syndrome critical region gene 6	1.06609808	0.4128	NA
20901	Strap	serine/threonine kinase receptor associated protein	1.06598444	0.2605	NA
18789	Papola	poly (A) polymerase alpha	1.06598444	0.6289	0.7889
20224	Sar1a	SAR1 gene homolog A (S. cerevisiae)	1.06598444	0.652	0.8037
110265	Msra	methionine sulfoxide reductase A	1.06587082	0.2412	NA
67955	Sugt1	SGT1, suppressor of G2 allele of SKP1 (S. cerevisiae)	1.06587082	0.2778	NA
72075	Ogfr	opioid growth factor receptor	1.06587082	0.4994	0.692
16549	Khsrp	KH-type splicing regulatory protein	1.06587082	0.5217	0.7097
69890	Zfp219	zinc finger protein 219	1.06587082	0.5518	0.7326
21672	Prdx2	peroxiredoxin 2	1.06575722	0.135	NA
74167	Nudt9	nudix (nucleoside diphosphate linked moiety X)-type motif 9	1.06575722	0.3148	NA
208618	Etl4	enhancer trap locus 4	1.06575722	0.4111	NA
56702	Hist1h1b	histone cluster 1, H1b	1.06575722	0.4702	0.6694
19271	Ptprj	protein tyrosine phosphatase, receptor type, J	1.06575722	0.6068	0.7727
75659	Wdr54	WD repeat domain 54	1.06575722	0.6897	0.8277
231571	Rpap2	RNA polymerase II associated protein 2	1.06564365	0.3621	NA
244144	Usp35	ubiquitin specific peptidase 35	1.06564365	0.6262	0.7865
16859	Lgals9	lectin, galactose binding, soluble 9	1.06564365	0.6465	0.8004
26356	Ing1	inhibitor of growth family, member 1	1.0655301	0.3452	NA
19063	Ppt1	palmitoyl-protein thioesterase 1	1.0655301	0.3518	NA
20587	Smarcb1	SWI/SNF related, matrix associated, actin dependent regulator of chromatin, subfamily b, member 1	1.0655301	0.3565	NA
20750	Spp1	secreted phosphoprotein 1	1.0655301	0.4013	NA
22003	Tpm1	tropomyosin 1, alpha	1.0655301	0.4151	NA
246048	Chodl	chondrolectin	1.0655301	0.7198	0.8469
66360	Bbip1	BBSome interacting protein 1	1.06541658	0.3609	NA
58802	Kcnmb4	potassium large conductance calcium-activated channel, subfamily M, beta member 4	1.06541658	0.5817	0.7535
381418	Ctxn2	cortexin 2	1.06541658	0.5847	0.7556
19336	Rab24	RAB24, member RAS oncogene family	1.06530308	0.1771	NA
19172	Psmb4	proteasome (prosome, macropain) subunit, beta type 4	1.06530308	0.3094	NA
57875	Angptl4	angiopoietin-like 4	1.06530308	0.4584	0.6595
70829	Ccdc93	coiled-coil domain containing 93	1.06530308	0.6536	0.8045
654810	D630032N06Rik	RIKEN cDNA D630032N06 gene	1.06530308	0.6602	0.8086
68048	Aen	apoptosis enhancing nuclease	1.0651896	0.2406	NA
57376	Smarce1	SWI/SNF related, matrix associated, actin dependent regulator of chromatin, subfamily e, member 1	1.0651896	0.4985	0.6912
15078	H3f3a	H3 histone, family 3A	1.06507615	0.2301	NA
80880	Kank3	KN motif and ankyrin repeat domains 3	1.06507615	0.2465	NA
28075	Pppde2	PPPDE peptidase domain containing 2	1.06507615	0.2493	NA
218734	3830406C13Rik	RIKEN cDNA 3830406C13 gene	1.06507615	0.382	NA
102366	BB211804	expressed sequence BB211804	1.06507615	0.3984	NA
100604	Lrrc8c	leucine rich repeat containing 8 family, member C	1.06507615	0.4317	NA
235043	Tmem205	transmembrane protein 205	1.06507615	0.5323	0.7174
241528	Lrrc55	leucine rich repeat containing 55	1.06507615	0.5612	0.7395
19045	Ppp1ca	protein phosphatase 1, catalytic subunit, alpha isoform	1.06496273	0.4267	NA
627096	LOC627096	hypothetical LOC627096	1.06496273	0.7709	0.8784
70314	Rabep2	rabaptin, RAB GTPase binding effector protein 2	1.06484932	0.3053	NA
73738	Haus7	HAUS augmin-like complex, subunit 7	1.06484932	0.3133	NA
237211	Fancb	Fanconi anemia, complementation group B	1.06484932	0.4227	NA
230582	Cyb5rl	cytochrome b5 reductase-like	1.06484932	0.6464	0.8003
17125	Smad1	MAD homolog 1 (Drosophila)	1.06484932	0.6512	0.8034

22276	Uros	uroporphyrinogen III synthase	1.06484932	0.7913	0.8906
26370	Cetn2	centrin 2	1.06473595	0.1995	NA
72543	Fam125b	family with sequence similarity 125, member B	1.06473595	0.2249	NA
109129	Mmadhc	methylmalonic aciduria (cobalamin deficiency) cblD type, with homocystinuria	1.06473595	0.3122	NA
66899	Fip1l1	FIP1 like 1 (S. cerevisiae)	1.06473595	0.3427	NA
22286	Utf1	undifferentiated embryonic cell transcription factor 1	1.06473595	0.4248	NA
66193	1110049F12Rik	RIKEN cDNA 1110049F12 gene	1.06462259	0.2228	NA
219024	Tmem55b	transmembrane protein 55b	1.06462259	0.3967	NA
68039	Nmb	neuromedin B	1.06462259	0.5194	0.7085
19878	Rock2	Rho-associated coiled-coil containing protein kinase 2	1.06462259	0.55	0.7313
75452	Ascc2	activating signal cointegrator 1 complex subunit 2	1.06450926	0.2838	NA
19944	Rpl29	ribosomal protein L29	1.06450926	0.3032	NA
75304	4930563E22Rik	RIKEN cDNA 4930563E22 gene	1.06450926	0.3231	NA
26436	Psg16	pregnancy specific glycoprotein 16	1.06450926	0.3523	NA
20638	Snrpb	small nuclear ribonucleoprotein B	1.06450926	0.468	0.6678
83815	Cenpq	centromere protein Q	1.06450926	0.5652	0.742
11600	Angpt1	angiopoietin 1	1.06450926	0.6203	0.7819
66914	Vps28	vacuolar protein sorting 28 (yeast)	1.06439596	0.2228	NA
68581	Tmed10	transmembrane emp24-like trafficking protein 10 (yeast)	1.06439596	0.3709	NA
20182	Rxrb	retinoid X receptor beta	1.06439596	0.3933	NA
30938	Fgd3	FYVE, RhoGEF and PH domain containing 3	1.06439596	0.4209	NA
70231	Gorasp2	golgi reassembly stacking protein 2	1.06439596	0.4388	NA
207704	Gtpbp10	GTP-binding protein 10 (putative)	1.06439596	0.5031	0.6949
225724	Mapk4	mitogen-activated protein kinase 4	1.06428267	0.3775	NA
30805	Slc22a4	solute carrier family 22 (organic cation transporter), member 4	1.06428267	0.4865	0.6815
631906	Gm7075	predicted gene 7075	1.06428267	0.6557	0.8057
224912	Crb3	crumbs homolog 3 (Drosophila)	1.06428267	0.6932	0.83
58193	Extl2	exostoses (multiple)-like 2	1.06416942	0.2802	NA
69094	Tmem160	transmembrane protein 160	1.06416942	0.334	NA
56438	Rbx1	ring-box 1	1.06416942	0.3631	NA
71769	Bbs10	Bardet-Biedl syndrome 10 (human)	1.06416942	0.3786	NA
75544	1700016K05Rik	RIKEN cDNA 1700016K05 gene	1.06416942	0.5055	0.6966
70093	Ube2q1	ubiquitin-conjugating enzyme E2Q (putative) 1	1.06405618	0.1225	NA
671641	Gm10063	predicted gene 10063	1.06405618	0.162	NA
278097	Armcx6	armadillo repeat containing, X-linked 6	1.06405618	0.4495	NA
20535	Slc4a2	solute carrier family 4 (anion exchanger), member 2	1.06405618	0.6514	0.8034
268857	Nlrc3	NLR family, CARD domain containing 3	1.06405618	0.6925	0.8298
56336	B4galt5	UDP-Gal:betaGlcNAc beta 1,4-galactosyltransferase, polypeptide 5	1.06405618	0.7884	0.8891
240476	Zfp407	zinc finger protein 407	1.06394297	0.3231	NA
20174	Ruvbl2	RuvB-like protein 2	1.06394297	0.329	NA
319342	C230034O21Rik	RIKEN cDNA C230034O21 gene	1.06394297	0.3558	NA
270669	Mbtps2	membrane-bound transcription factor peptidase, site 2	1.06394297	0.5016	0.6934
105837	Mtbp	Mdm2, transformed 3T3 cell double minute p53 binding protein	1.06382979	0.3944	NA
73845	Ankrd42	ankyrin repeat domain 42	1.06382979	0.4367	NA
381823	Apold1	apolipoprotein L domain containing 1	1.06382979	0.5797	0.7521
74649	Cpa5	carboxypeptidase A5	1.06382979	0.6298	0.7895
192173	Fam195b	family with sequence similarity 195, member B	1.06371663	0.2372	NA
26462	Txnrd2	thioredoxin reductase 2	1.06371663	0.2456	NA
18769	Pkig	protein kinase inhibitor, gamma	1.06371663	0.5674	0.7433
56356	Gltp	glycolipid transfer protein	1.06371663	0.6204	0.7819

74711	Ttll9	tubulin tyrosine ligase-like family, member 9	1.06371663	0.6382	0.7951
66827	Ttc1	tetratricopeptide repeat domain 1	1.06360349	0.3187	NA
15525	Hspa4	heat shock protein 4	1.06360349	0.339	NA
64340	Dhx38	DEAH (Asp-Glu-Ala-His) box polypeptide 38	1.06360349	0.3983	NA
24070	Mpdu1	mannose-P-dolichol utilization defect 1	1.06360349	0.5096	0.7002
76355	Tgds	TDP-glucose 4,6-dehydratase	1.06360349	0.595	0.7638
212163	8030462N17Rik	RIKEN cDNA 8030462N17 gene	1.06360349	0.695	0.8312
382051	Pdp2	pyruvate dehyrogenase phosphatase catalytic subunit 2	1.06360349	0.7663	0.8759
67135	2310021H06Rik	RIKEN cDNA 2310021H06 gene	1.06360349	0.8376	0.9161
319195	Rpl17	ribosomal protein L17	1.06349038	0.1433	NA
68563	Dpm3	dolichyl-phosphate mannosyltransferase polypeptide 3	1.06349038	0.2595	NA
68763	1110038B12Rik	RIKEN cDNA 1110038B12 gene	1.06349038	0.4598	NA
23789	Coro1b	coronin, actin binding protein 1B	1.06349038	0.5371	0.7214
20603	Sms	spermine synthase	1.06326422	0.1739	NA
99349	Dnajc24	DnaJ (Hsp40) homolog, subfamily C, member 24	1.06326422	0.2526	NA
97287	Mtmr14	myotubularin related protein 14	1.06326422	0.505	0.6962
380608	Tagap1	T-cell activation GTPase activating protein 1	1.06326422	0.6135	0.7773
14674	Gna13	guanine nucleotide binding protein, alpha 13	1.06326422	0.7408	0.8598
68911	Pygo2	pygopus 2	1.06315118	0.3816	NA
56418	Ykt6	YKT6 homolog (S. Cerevisiae)	1.06315118	0.4675	0.6674
242687	Wasf2	WAS protein family, member 2	1.06315118	0.552	0.7328
110379	Sec13	SEC13 homolog (S. cerevisiae)	1.06303816	0.2864	NA
103806	Maml1	mastermind like 1 (Drosophila)	1.06303816	0.3005	NA
231571	Rpap2	RNA polymerase II associated protein 2	1.06303816	0.3731	NA
68118	9430023L20Rik	RIKEN cDNA 9430023L20 gene	1.06303816	0.434	NA
17385	Mmp11	matrix metallopeptidase 11	1.06303816	0.5021	0.6939
18803	Plcg1	phospholipase C, gamma 1	1.06303816	0.6362	0.7936
218975	Mapk1ip1l	mitogen-activated protein kinase 1 interacting protein 1-like	1.06303816	0.8464	0.9205
18119	Nodal	nodal	1.06292517	0.8269	0.9104
107734	Mrpl30	mitochondrial ribosomal protein L30	1.0628122	0.218	NA
545487	Gm14439	predicted gene 14439	1.0628122	0.2401	NA
14593	Ggps1	geranylgeranyl diphosphate synthase 1	1.0628122	0.2841	NA
216858	Kctd11	potassium channel tetramerisation domain containing 11	1.0628122	0.4007	NA
18109	Mycn	v-myc myelocytomatosis viral related oncogene, neuroblastoma derived (avian)	1.0628122	0.4172	NA
20787	Srebf1	sterol regulatory element binding transcription factor 1	1.0628122	0.687	0.8258
192285	Phf21a	PHD finger protein 21A	1.06269926	0.2358	NA
66148	Dnajc15	DnaJ (Hsp40) homolog, subfamily C, member 15	1.06269926	0.2509	NA
217869	Eif5	eukaryotic translation initiation factor 5	1.06258634	0.4146	NA
100465	Mobkl2c	MOB1, Mps One Binder kinase activator-like 2C (yeast)	1.06258634	0.5184	0.7078
231464	Cnot6l	CCR4-NOT transcription complex, subunit 6-like	1.06247344	0.1612	NA
14225	Fkbp1a	FK506 binding protein 1a	1.06247344	0.3415	NA
19889	Rp2h	retinitis pigmentosa 2 homolog (human)	1.06247344	0.6099	0.7744
207952	Klhl25	kelch-like 25 (Drosophila)	1.06236057	0.2695	NA
77254	Yif1b	Yip1 interacting factor homolog B (S. cerevisiae)	1.06236057	0.399	NA
69724	Rnaseh2a	ribonuclease H2, large subunit	1.06236057	0.5035	0.695
67867	Lrrc28	leucine rich repeat containing 28	1.06224772	0.2197	NA
72183	Snx6	sorting nexin 6	1.06224772	0.4961	0.6894
27370	Rps26	ribosomal protein S26	1.06213489	0.2671	NA
67685	Dyx1c1	dyslexia susceptibility 1 candidate 1 homolog (human)	1.06213489	0.2706	NA
170930	Sumo2	SMT3 suppressor of mif two 3 homolog 2 (yeast)	1.06213489	0.3047	NA

22184	Zrsr2		zinc finger (CCCH type), RNA binding motif and serine/arginine rich 2	1.06213489	0.3323	NA
69051	Pycr2		pyrroline-5-carboxylate reductase family, member 2	1.06202209	0.2406	NA
235028	Zfp426		zinc finger protein 426	1.06202209	0.2891	NA
216558	Ugp2		UDP-glucose pyrophosphorylase 2	1.06202209	0.2923	NA
15505	Hsph1		heat shock 105kDa/110kDa protein 1	1.06202209	0.2981	NA
57438		Mar-07	membrane-associated ring finger (C3HC4) 7	1.06202209	0.3188	NA
71131	Zfp689		zinc finger protein 689	1.06202209	0.5741	0.748
19230	Twf1		twinfilin, actin-binding protein, homolog 1 (Drosophila)	1.06190931	0.3049	NA
382864	Colq		collagen-like tail subunit (single strand of homotrimer) of asymmetric acetylcholinesterase	1.06190931	0.3802	NA
110173	Manba		mannosidase, beta A, lysosomal	1.06190931	0.4174	NA
15356	Hmgcl		3-hydroxy-3-methylglutaryl-Coenzyme A lyase	1.06179656	0.2063	NA
319195	Rpl17		ribosomal protein L17	1.06179656	0.2446	NA
233765	Plekha7		pleckstrin homology domain containing, family A member 7	1.06179656	0.3784	NA
23954	Nek3		NIMA (never in mitosis gene a)-related expressed kinase 3	1.06179656	0.3854	NA
74479	Snx11		sorting nexin 11	1.06179656	0.5437	0.7261
68975	Med27		mediator complex subunit 27	1.06179656	0.5488	0.7305
328133	Slc39a9		solute carrier family 39 (zinc transporter), member 9	1.06179656	0.5831	0.7543
18618	Pemt		phosphatidylethanolamine N-methyltransferase	1.06179656	0.652	0.8037
12501	Cd3e		CD3 antigen, epsilon polypeptide	1.06179656	0.7219	0.8483
69274	Ctdspl		CTD (carboxy-terminal domain, RNA polymerase II, polypeptide A) small phosphatase-like	1.06168383	0.563	0.7406
12789	Cnga2		cyclic nucleotide gated channel alpha 2	1.06168383	0.656	0.8058
110749	Chaf1b		chromatin assembly factor 1, subunit B (p60)	1.06157113	0.4122	NA
64659	Mrps14		mitochondrial ribosomal protein S14	1.06157113	0.5624	0.7402
320237	Ncrna00086		non-protein coding RNA 86	1.06157113	0.6152	0.7783
546663	Gm5963		predicted pseudogene 5963	1.06145844	0.2331	NA
56530	Cnpy2		canopy 2 homolog (zebrafish)	1.06145844	0.3678	NA
381201	Gm962		predicted gene 962	1.06145844	0.3719	NA
28071	Twistnb		TWIST neighbor	1.06145844	0.4813	0.6775
71941	Cars2		cysteinyl-tRNA synthetase 2 (mitochondrial)(putative)	1.06145844	0.4987	0.6913
320700	A930033H14Rik		RIKEN cDNA A930033H14 gene	1.06145844	0.6293	0.7892
17175	Masp2		mannan-binding lectin serine peptidase 2	1.06145844	0.6347	0.7924
77996	D730039F16Rik		RIKEN cDNA D730039F16 gene	1.06145844	0.6452	0.7995
20416	Shc1		src homology 2 domain-containing transforming protein C1	1.06134579	0.3504	NA
16870	Lhx2		LIM homeobox protein 2	1.06134579	0.3805	NA
230734	Yrdc		yrdC domain containing (E.coli)	1.06134579	0.3967	NA
667250	Gm12657		predicted gene 12657	1.06134579	0.4971	0.69
69104		Mar-05	membrane-associated ring finger (C3HC4) 5	1.06123315	0.5652	0.742
66525	Timm50		translocase of inner mitochondrial membrane 50 homolog (yeast)	1.06123315	0.5712	0.7461
67511	Tmed9		transmembrane emp24 protein transport domain containing 9	1.06112054	0.33	NA
72568	Lin9		lin-9 homolog (C. elegans)	1.06112054	0.3648	NA
11826	Aqp1		aquaporin 1	1.06112054	0.5628	0.7404
353170	Txlng		taxilin gamma	1.06112054	0.7057	0.8379
66070	Cwc15		CWC15 homolog (S. cerevisiae)	1.06100796	0.1772	NA
68185	Chchd8		coiled-coil-helix-coiled-coil-helix domain containing 8	1.06100796	0.3198	NA
226778	Mark1		MAP/microtubule affinity-regulating kinase 1	1.06100796	0.3656	NA
19179	Psmc1		protease (prosome, macropain) 26S subunit, ATPase 1	1.06100796	0.402	NA
238317	C130039O16Rik		RIKEN cDNA C130039O16 gene	1.06100796	0.6503	0.8031
57248	Ly6i		lymphocyte antigen 6 complex, locus I	1.06100796	0.7749	0.8802
100041294	Gm3258		predicted gene 3258	1.0608954	0.3732	NA
75221	Dpp3		dipeptidylpeptidase 3	1.0608954	0.5285	0.7146

213491	D4Ertd22e	DNA segment, Chr 4, ERATO Doi 22, expressed	1.0608954	0.6353	0.7929
381827	1700073E17Rik	ribosomal protein L7 pseudogene	1.06078286	0.1842	NA
16797	Lat	linker for activation of T cells	1.06078286	0.3386	NA
621312	Gm6211	predicted gene 6211	1.06067034	0.2675	NA
209018	Vps8	vacuolar protein sorting 8 homolog (S. cerevisiae)	1.06067034	0.4228	NA
14538	Gcnt2	glucosaminyl (N-acetyl) transferase 2, I-branching enzyme	1.06067034	0.4657	NA
269378	Ahcy	S-adenosylhomocysteine hydrolase	1.06067034	0.4906	0.6848
118445	Klf16	Kruppel-like factor 16	1.06067034	0.534	0.7188
12048	Bcl2l1	BCL2-like 1	1.06067034	0.6602	0.8086
17874	Myd88	myeloid differentiation primary response gene 88	1.06055785	0.4201	NA
232947	Lrrc68	leucine rich repeat containing 68	1.06055785	0.4209	NA
67119	Ccdc159	coiled-coil domain containing 159	1.06055785	0.4524	NA
16865	Eif2d	eukaryotic translation initiation factor 2D	1.06055785	0.506	0.697
677073	Gm16477	ribosomal protein L7a pseudogene	1.06044539	0.1292	NA
107686	Snrpd2	small nuclear ribonucleoprotein D2	1.06044539	0.3449	NA
665891	Krtap4-1	keratin associated protein 4-1	1.06044539	0.8539	0.9249
75062	Sf3a3	splicing factor 3a, subunit 3	1.06033294	0.2103	NA
53320	Folh1	folate hydrolase	1.06033294	0.2756	NA
75565	Ccdc101	coiled-coil domain containing 101	1.06033294	0.2885	NA
14760	Gpr19	G protein-coupled receptor 19	1.06033294	0.2897	NA
69190	Dym	dymeclin	1.06033294	0.309	NA
381356	5930434B04Rik	RIKEN cDNA 5930434B04 gene	1.06033294	0.3656	NA
110012	Gm16517	predicted gene, Gm16517	1.06033294	0.375	NA
11821	Aprt	adenine phosphoribosyl transferase	1.06033294	0.4156	NA
76561	Snx7	sorting nexin 7	1.06033294	0.6437	0.7985
432763	Prr7	proline rich 7 (synaptic)	1.06022053	0.2644	NA
66863	Lztr1	leucine-zipper-like transcriptional regulator, 1	1.06022053	0.3049	NA
26992	Brd7	bromodomain containing 7	1.06010813	0.1818	NA
224742	Abcf1	ATP-binding cassette, sub-family F (GCN20), member 1	1.06010813	0.2784	NA
22637	Zap70	zeta-chain (TCR) associated protein kinase	1.06010813	0.4396	NA
68691	1110028C15Rik	RIKEN cDNA 1110028C15 gene	1.06010813	0.4745	NA
53380	Psmd10	proteasome (prosome, macropain) 26S subunit, non-ATPase, 10	1.06010813	0.5633	0.7408
238317	C130039O16Rik	RIKEN cDNA C130039O16 gene	1.06010813	0.5707	0.7459
94218	Cnnm3	cyclin M3	1.06010813	0.6023	0.7693
18590	Pdgfa	platelet derived growth factor, alpha	1.05999576	0.1931	NA
78802	Ttc30a1	tetratricopeptide repeat domain 30A1	1.05999576	0.205	NA
27207	Rps11	ribosomal protein S11	1.05988341	0.195	NA
26451	Rpl27a	ribosomal protein L27A	1.05988341	0.3444	NA
69757	Leng1	leukocyte receptor cluster (LRC) member 1	1.05988341	0.362	NA
237336	Tbpl1	TATA box binding protein-like 1	1.05988341	0.3747	NA
22095	Tshr	thyroid stimulating hormone receptor	1.05988341	0.6912	0.8287
27176	Rpl7a	ribosomal protein L7A	1.05988341	0.8259	0.9102
56695	Pnkd	paroxysmal nonkinesiogenic dyskinesia	1.05977109	0.3538	NA
21676	Tead1	TEA domain family member 1	1.05977109	0.3621	NA
15516	Hsp90ab1	heat shock protein 90 alpha (cytosolic), class B member 1	1.05977109	0.4436	NA
67252	Cap2	CAP, adenylate cyclase-associated protein, 2 (yeast)	1.05977109	0.5276	0.7138
12902	Cr2	complement receptor 2	1.05977109	0.797	0.8932
15488	Hsd17b4	hydroxysteroid (17-beta) dehydrogenase 4	1.05965879	0.2177	NA
215201	Trmt2b	TRM2 tRNA methyltransferase 2 homolog B (S. cerevisiae)	1.05965879	0.2701	NA
14231	Fkbp7	FK506 binding protein 7	1.05965879	0.3074	NA

30934	Tor1b	torsin family 1, member B	1.05965879	0.3962	NA
17906	Myl2	myosin, light polypeptide 2, regulatory, cardiac, slow	1.05965879	0.5174	0.7067
319636	Fsd1l	fibronectin type III and SPRY domain containing 1-like	1.05965879	0.5963	0.7651
75040	Efcab10	EF-hand calcium binding domain 10	1.05965879	0.794	0.8917
15529	Sdc2	syndecan 2	1.05954651	0.2401	NA
30928	Zfp238	zinc finger protein 238	1.05954651	0.3785	NA
18181	Nrf1	nuclear respiratory factor 1	1.05954651	0.4586	NA
27407	Abcf2	ATP-binding cassette, sub-family F (GCN20), member 2	1.05954651	0.477	NA
102115	Dohh	deoxyhypusine hydroxylase/monooxygenase	1.05943426	0.3136	NA
83383	Tcfap4	transcription factor AP4	1.05943426	0.4652	NA
107372	C030016D13Rik	RIKEN cDNA C030016D13 gene	1.05943426	0.5341	0.7188
53332	Mtmr1	myotubularin related protein 1	1.05932203	0.2341	NA
67923	Tceb1	transcription elongation factor B (SIII), polypeptide 1	1.05932203	0.3692	NA
107686	Snrpd2	small nuclear ribonucleoprotein D2	1.05932203	0.3871	NA
50876	Tmod2	tropomodulin 2	1.05932203	0.4504	NA
19156	Psap	prosaposin	1.05932203	0.4558	NA
107022	Gramd3	GRAM domain containing 3	1.05932203	0.5417	0.7245
28295	D10Jhu81e	DNA segment, Chr 10, Johns Hopkins University 81 expressed	1.05920983	0.2397	NA
73738	Haus7	HAUS augmin-like complex, subunit 7	1.05920983	0.3032	NA
27404	Abca8b	ATP-binding cassette, sub-family A (ABC1), member 8b	1.05920983	0.3109	NA
208638	Slc25a38	solute carrier family 25, member 38	1.05920983	0.3161	NA
223691	Eif3l	eukaryotic translation initiation factor 3, subunit L	1.05920983	0.3568	NA
56088	Psmg1	proteasome (prosome, macropain) assembly chaperone 1	1.05920983	0.4448	NA
79565	Wbscr27	Williams Beuren syndrome chromosome region 27 (human)	1.05909765	0.5274	0.7138
27222	Atp1a4	ATPase, Na+/K+ transporting, alpha 4 polypeptide	1.05909765	0.5732	0.7476
333315	Frem3	Fras1 related extracellular matrix protein 3	1.05909765	0.6758	0.8184
20588	Smarcc1	SWI/SNF related, matrix associated, actin dependent regulator of chromatin, subfamily c, member 1	1.05909765	0.6936	0.8302
209478	Tbc1d12	TBC1D12: TBC1 domain family, member 12	1.05909765	0.7476	0.864
320858	L3mbtl4	l(3)mbt-like 4 (Drosophila)	1.05898549	0.3414	NA
19359	Rad23b	RAD23b homolog (S. cerevisiae)	1.05898549	0.381	NA
245828	Trappc1	trafficking protein particle complex 1	1.05898549	0.5406	0.7238
23989	Med24	mediator complex subunit 24	1.05898549	0.6062	0.7725
56856	Insm2	insulinoma-associated 2	1.05898549	0.6618	0.8095
236749	Gm4907	predicted gene 4907	1.05887336	0.4228	NA
17986	Ndp	Norrie disease (pseudoglioma) (human)	1.05887336	0.4588	NA
66072	Sdhaf2	succinate dehydrogenase complex assembly factor 2	1.05876125	0.4374	NA
67811	Poldip2	polymerase (DNA-directed), delta interacting protein 2	1.05876125	0.4992	0.6917
23921	Sh2b2	SH2B adaptor protein 2	1.05876125	0.5918	0.7614
103511	Fam26e	family with sequence similarity 26, member E	1.05876125	0.7583	0.871
235504	Slc17a5	solute carrier family 17 (anion/sugar transporter), member 5	1.05876125	0.835	0.9148
18148	Npm1	nucleophosmin 1	1.05864916	0.254	NA
19182	Psmc3	proteasome (prosome, macropain) 26S subunit, ATPase 3	1.05864916	0.4307	NA
69582	Plekhm2	pleckstrin homology domain containing, family M (with RUN domain) member 2	1.0585371	0.2113	NA
66169	Tomm7	translocase of outer mitochondrial membrane 7 homolog (yeast)	1.0585371	0.2409	NA
66073	Txndc12	thioredoxin domain containing 12 (endoplasmic reticulum)	1.0585371	0.2523	NA
76457	Ccdc134	coiled-coil domain containing 134	1.0585371	0.2982	NA
110208	Pgd	phosphogluconate dehydrogenase	1.0585371	0.314	NA
19877	Rock1	Rho-associated coiled-coil containing protein kinase 1	1.0585371	0.6182	0.7804
75420	Secisbp2	SECIS binding protein 2	1.0585371	0.6258	0.7862
241035	Pkhd1	polycystic kidney and hepatic disease 1	1.0585371	0.6662	0.8119

93735	Wnt16	wingless-related MMTV integration site 16	1.0585371	0.8339	0.9141
103819	AI663975	expressed sequence AI663975	1.05842506	0.2458	NA
65103	Arl6ip6	ADP-ribosylation factor-like 6 interacting protein 6	1.05842506	0.5065	0.6975
56068	Ammecr1	Alport syndrome, mental retardation, midface hypoplasia and elliptocytosis chromosomal region gene 1 homolog (human)	1.05842506	0.6144	0.7778
217826	Kcnk13	potassium channel, subfamily K, member 13	1.05842506	0.7505	0.8659
268996	Ss18	synovial sarcoma translocation, Chromosome 18	1.05831305	0.1627	NA
66844	Ormdl2	ORM1-like 2 (S. cerevisiae)	1.05831305	0.2975	NA
72147	Zbtb46	zinc finger and BTB domain containing 46	1.05831305	0.5226	0.7102
72823	Pard3b	par-3 partitioning defective 3 homolog B (C. elegans)	1.05831305	0.7728	0.879
628596	Gm6900	predicted gene 6900	1.05820106	0.3538	NA
67326	1700037H04Rik	RIKEN cDNA 1700037H04 gene	1.05820106	0.3813	NA
74919	4930471M23Rik	RIKEN cDNA 4930471M23 gene	1.05820106	0.4617	NA
207667	Skor1	SKI family transcriptional corepressor 1	1.05820106	0.5441	0.7262
27207	Rps11	ribosomal protein S11	1.05808909	0.1789	NA
68047	Mpnd	MPN domain containing	1.05808909	0.3841	NA
321006	Vprbp	Vpr (HIV-1) binding protein	1.05808909	0.419	NA
103844	Inca1	inhibitor of CDK, cyclin A1 interacting protein 1	1.05808909	0.6402	0.7964
236643	Sytl5	synaptotagmin-like 5	1.05808909	0.6767	0.819
233058	Zfp420	zinc finger protein 420	1.05808909	0.6974	0.8327
22194	Ube2e1	ubiquitin-conjugating enzyme E2E 1, UBC4/5 homolog (yeast)	1.05797715	0.368	NA
243937	Zfp536	zinc finger protein 536	1.05797715	0.4568	NA
70160	Vps36	vacuolar protein sorting 36 (yeast)	1.05797715	0.5098	0.7004
77862	Thyn1	thymocyte nuclear protein 1	1.05786523	0.2842	NA
23945	Mgll	monoglyceride lipase	1.05786523	0.3282	NA
14081	Acsl1	acyl-CoA synthetase long-chain family member 1	1.05786523	0.4797	NA
432779	Lrrc14b	leucine rich repeat containing 14B	1.05786523	0.5318	0.717
68090	Yif1a	Yip1 interacting factor homolog A (S. cerevisiae)	1.05786523	0.5343	0.719
21934	Tnfrsf11a	tumor necrosis factor receptor superfamily, member 11a	1.05786523	0.6072	0.7728
434758	Rhox3h	reproductive homeobox 3H	1.05786523	0.7514	0.8663
57377	Mogs	mannosyl-oligosaccharide glucosidase	1.05775333	0.294	NA
76366	Mtif3	mitochondrial translational initiation factor 3	1.05775333	0.5622	0.74
268481	Krt222	keratin 222	1.05775333	0.8357	0.9152
104776	Aldh6a1	aldehyde dehydrogenase family 6, subfamily A1	1.05764146	0.411	NA
20090	Rps29	ribosomal protein S29	1.05764146	0.4299	NA
212398	Frat2	frequently rearranged in advanced T-cell lymphomas 2	1.05764146	0.6155	0.7785
106200	Txndc11	thioredoxin domain containing 11	1.05752961	0.1709	NA
80981	Arl4d	ADP-ribosylation factor-like 4D	1.05752961	0.5759	0.7493
17532	Mras	muscle and microspikes RAS	1.05752961	0.7033	0.836
66455	Cnpy4	canopy 4 homolog (zebrafish)	1.05741779	0.3142	NA
98221	Eif3m	eukaryotic translation initiation factor 3, subunit M	1.05741779	0.4475	NA
80987	Nckipsd	NCK interacting protein with SH3 domain	1.05741779	0.6542	0.8047
66627	Ogfod2	2-oxoglutarate and iron-dependent oxygenase domain containing 2	1.05730598	0.2382	NA
223527	Eny2	enhancer of yellow 2 homolog (Drosophila)	1.05730598	0.2675	NA
58217	Trem1	triggering receptor expressed on myeloid cells 1	1.05730598	0.4118	NA
20610	Sumo3	SMT3 suppressor of mif two 3 homolog 3 (yeast)	1.05730598	0.5506	0.7317
223774	Alg12	asparagine-linked glycosylation 12 homolog (yeast, alpha-1,6-mannosyltransferase)	1.05730598	0.631	0.7903
75422	Mettl5	methyltransferase like 5	1.05719421	0.4104	NA
242894	Actr3b	ARP3 actin-related protein 3 homolog B (yeast)	1.05719421	0.4454	NA
319618	Dcp1b	DCP1 decapping enzyme homolog b (S. cerevisiae)	1.05708245	0.2446	NA
75291	Zbtb3	zinc finger and BTB domain containing 3	1.05708245	0.2778	NA

19108	Prkx	protein kinase, X-linked	1.05708245	0.5353	0.7199
192775	Kcnh6	potassium voltage-gated channel, subfamily H (eag-related), member 6	1.05708245	0.6085	0.7734
12695	Inadl	InaD-like (Drosophila)	1.05708245	0.7136	0.8431
100039895	Gm2479	predicted gene 2479	1.05708245	0.761	0.8728
407819	BC031181	cDNA sequence BC031181	1.05697072	0.2618	NA
83815	Cenpq	centromere protein Q	1.05697072	0.4243	NA
668940	Myh7b	myosin, heavy chain 7B, cardiac muscle, beta	1.05697072	0.7578	0.8706
245866	Ift52	intraflagellar transport 52 homolog (Chlamydomonas)	1.05685902	0.3378	NA
11881	Arsb	arylsulfatase B	1.05685902	0.4075	NA
71954	Suds3	suppressor of defective silencing 3 homolog (S. cerevisiae)	1.05685902	0.546	0.7279
20743	Spnb3	spectrin beta 3	1.05685902	0.5906	0.7604
239408	Tmem74	transmembrane protein 74	1.05685902	0.6036	0.7705
435145	AI848285	expressed sequence AI848285	1.05685902	0.6258	0.7862
13097	Cyp2c38	cytochrome P450, family 2, subfamily c, polypeptide 38	1.05685902	0.7142	0.8436
68278	Ddx39	DEAD (Asp-Glu-Ala-Asp) box polypeptide 39	1.05674733	0.2256	NA
68015	Trap1	TNF receptor-associated protein 1	1.05674733	0.3207	NA
232970	Phldb3	pleckstrin homology-like domain, family B, member 3	1.05674733	0.3621	NA
74498	Gorasp1	golgi reassembly stacking protein 1	1.05674733	0.3652	NA
67452	Pnpla8	patatin-like phospholipase domain containing 8	1.05674733	0.4285	NA
66293	1810032O08Rik	RIKEN cDNA 1810032O08 gene	1.05663567	0.1813	NA
226757	Wdr26	WD repeat domain 26	1.05663567	0.2048	NA
69786	Tprkb	Tp53rk binding protein	1.05663567	0.2299	NA
27057	Ncoa4	nuclear receptor coactivator 4	1.05663567	0.3221	NA
56187	Rabggta	Rab geranylgeranyl transferase, a subunit	1.05663567	0.3784	NA
56844	Tssc4	tumor-suppressing subchromosomal transferable fragment 4	1.05663567	0.4321	NA
68039	Nmb	neuromedin B	1.05663567	0.5514	0.7323
625603	Gm6607	40S ribosomal protein S20 pseudogene	1.05663567	0.5873	0.7577
76467	Msrb2	methionine sulfoxide reductase B2	1.05652404	0.3437	NA
732482	Gm9731	Fam58a pseudogene	1.05652404	0.4556	NA
59050	Nsa2	NSA2 ribosome biogenesis homolog (S. cerevisiae)	1.05652404	0.4663	NA
13865	Nr2f1	nuclear receptor subfamily 2, group F, member 1	1.05641242	0.4713	NA
74325	Cltb	clathrin, light polypeptide (Lcb)	1.05630083	0.2287	NA
12457	Ccrn4l	CCR4 carbon catabolite repression 4-like (S. cerevisiae)	1.05630083	0.4076	NA
100043404	Gm4416	predicted gene 4416	1.05630083	0.4342	NA
67487	Dhx40	DEAH (Asp-Glu-Ala-His) box polypeptide 40	1.05630083	0.4529	NA
50877	Neu3	neuraminidase 3	1.05630083	0.4921	NA
94282	Sfxn5	sideroflexin 5	1.05630083	0.62	0.7817
14260	Fmn1	formin 1	1.05630083	0.7488	0.8649
66260	Tmem54	transmembrane protein 54	1.05630083	0.8311	0.9126
242126	Slc22a15	solute carrier family 22 (organic anion/cation transporter), member 15	1.05618927	0.2883	NA
212514	Spice1	spindle and centriole associated protein 1	1.05618927	0.4338	NA
81897	Tlr9	toll-like receptor 9	1.05618927	0.4525	NA
26427	Creb3l1	cAMP responsive element binding protein 3-like 1	1.05618927	0.5227	0.7102
58875	Hibadh	3-hydroxyisobutyrate dehydrogenase	1.05618927	0.5709	0.746
213350	Pddc1	Parkinson disease 7 domain containing 1	1.05618927	0.6948	0.831
14276	Folr2	folate receptor 2 (fetal)	1.05618927	0.7725	0.8789
432940	Fam105b	family with sequence similarity 105, member B	1.05607773	0.4014	NA
15171	Hcrt	hypocretin	1.05607773	0.5653	0.742
71787	Trnau1ap	tRNA selenocysteine 1 associated protein 1	1.05596621	0.3599	NA
619547	Rpl34-ps1	ribosomal protein L34, pseudogene 1	1.05596621	0.4808	NA

70552	Lrrc56	leucine rich repeat containing 56	1.05596621	0.5255	0.7122
140781	Myh7	myosin, heavy polypeptide 7, cardiac muscle, beta	1.05596621	0.6758	0.8184
80891	Fcrls	Fc receptor-like S, scavenger receptor	1.05585471	0.3636	NA
68133	Gcsh	glycine cleavage system protein H (aminomethyl carrier)	1.05585471	0.3666	NA
67973	Mphosph10	M-phase phosphoprotein 10 (U3 small nucleolar ribonucleoprotein)	1.05585471	0.4296	NA
230917	Tmem201	transmembrane protein 201	1.05585471	0.455	NA
80886	Senp3	SUMO/sentrin specific peptidase 3	1.05585471	0.4647	NA
81018	Rnf114	ring finger protein 114	1.05585471	0.5496	0.7312
108116	Slco3a1	solute carrier organic anion transporter family, member 3a1	1.05574324	0.1904	NA
217057	Ptrh2	peptidyl-tRNA hydrolase 2	1.05574324	0.3124	NA
330662	Dock1	dedicator of cytokinesis 1	1.05574324	0.3642	NA
17347	Mknk2	MAP kinase-interacting serine/threonine kinase 2	1.05574324	0.4191	NA
67702	Rnf149	ring finger protein 149	1.05574324	0.5227	0.7102
74569	Ttc17	tetratricopeptide repeat domain 17	1.05574324	0.7232	0.8491
667847	Gm8842	predicted gene 8842	1.0556318	0.3823	NA
270166	Clpx	caseinolytic peptidase X (E.coli)	1.0556318	0.4453	NA
12777	Ccr10	chemokine (C-C motif) receptor 10	1.0556318	0.4475	NA
16177	Il1r1	interleukin 1 receptor, type I	1.0556318	0.4642	NA
231672	Fbxw8	F-box and WD-40 domain protein 8	1.0556318	0.6579	0.8067
73067	Tmem192	transmembrane protein 192	1.05552037	0.3417	NA
12916	Crem	cAMP responsive element modulator	1.05552037	0.4205	NA
666676	Gm8230	predicted gene 8230	1.05552037	0.4792	NA
171486	Cd99l2	CD99 antigen-like 2	1.05552037	0.4821	NA
68544	2310036O22Rik	RIKEN cDNA 2310036O22 gene	1.05552037	0.5472	0.7289
20664	Sox1	SRY-box containing gene 1	1.05552037	0.7509	0.8662
22781	Ikzf4	IKAROS family zinc finger 4	1.05552037	0.8309	0.9126
78512	3300005D01Rik	RIKEN cDNA 3300005D01 gene	1.05552037	0.8419	0.9183
14773	Grk5	G protein-coupled receptor kinase 5	1.05540897	0.3289	NA
11993	Aup1	ancient ubiquitous protein 1	1.05540897	0.4249	NA
494504	Apcdd1	adenomatosis polyposis coli down-regulated 1	1.05540897	0.5437	0.7261
67602	Necap1	NECAP endocytosis associated 1	1.05529759	0.2291	NA
228139	P2rx3	purinergic receptor P2X, ligand-gated ion channel, 3	1.05529759	0.3709	NA
14113	Fbl	fibrillarin	1.05529759	0.4258	NA
106073	Mfsd5	major facilitator superfamily domain containing 5	1.05529759	0.4886	NA
67938	Myl12b	myosin, light chain 12B, regulatory	1.05529759	0.5071	NA
381338	Lonrf2	LON peptidase N-terminal domain and ring finger 2	1.05529759	0.5595	0.7385
229715	Amigo1	adhesion molecule with Ig like domain 1	1.05529759	0.5816	0.7535
100042693	Gm3970	predicted gene 3970	1.05529759	0.6014	0.7688
22129	Ttc3	tetratricopeptide repeat domain 3	1.05529759	0.7342	0.8565
56745	C1qtnf1	C1q and tumor necrosis factor related protein 1	1.05529759	0.749	0.8649
68634	Tm2d3	TM2 domain containing 3	1.05518624	0.4096	NA
320919	A230107N01Rik	RIKEN cDNA A230107N01 gene	1.05518624	0.4346	NA
215653	Rassf2	Ras association (RalGDS/AF-6) domain family member 2	1.05518624	0.7053	0.8377
72301	1810041L15Rik	RIKEN cDNA 1810041L15 gene	1.05518624	0.7153	0.8442
232493	Gys2	glycogen synthase 2	1.05518624	0.7346	0.8566
232337	Zfp637	zinc finger protein 637	1.05507491	0.254	NA
14357	Dtx1	deltex 1 homolog (Drosophila)	1.05507491	0.5057	NA
16542	Kdr	kinase insert domain protein receptor	1.05507491	0.5495	0.7312
65962	Slc9a3r2	solute carrier family 9 (sodium/hydrogen exchanger), member 3 regulator 2	1.05507491	0.5576	0.7371
19210	Ptdss1	phosphatidylserine synthase 1	1.05507491	0.5726	0.7473

13358	Slc25a1	solute carrier family 25 (mitochondrial carrier, citrate transporter), member 1	1.05507491	0.5786	0.7512
20511	Slc1a2	solute carrier family 1 (glial high affinity glutamate transporter), member 2	1.05507491	0.6198	0.7816
66656	Eef1d	eukaryotic translation elongation factor 1 delta (guanine nucleotide exchange protein)	1.0549636	0.2125	NA
622301	Rhox2h	reproductive homeobox 2H	1.0549636	0.3634	NA
207704	Gtpbp10	GTP-binding protein 10 (putative)	1.0549636	0.3698	NA
70719	Hmha1	histocompatibility (minor) HA-1	1.0549636	0.4224	NA
21953	Tnni2	troponin I, skeletal, fast 2	1.0549636	0.796	0.8927
68023	Pdf	peptide deformylase (mitochondrial)	1.05485232	0.3166	NA
21681	Thoc4	THO complex 4	1.05485232	0.3222	NA
244579	Tox3	TOX high mobility group box family member 3	1.05485232	0.3661	NA
66179	1110031I02Rik	RIKEN cDNA 1110031I02 gene	1.05485232	0.3666	NA
242681	Rab42-ps	RAB42, member RAS oncogene family, pseudogene	1.05485232	0.5352	0.7198
67442	Retsat	retinol saturase (all trans retinol 13,14 reductase)	1.05485232	0.5689	0.7446
195576	Gm13229	predicted gene 13229	1.05485232	0.7233	0.8491
225131	Wac	WW domain containing adaptor with coiled-coil	1.05474106	0.2554	NA
104009	Qsox1	quiescin Q6 sulfhydryl oxidase 1	1.05474106	0.3218	NA
19387	Rangap1	RAN GTPase activating protein 1	1.05474106	0.5242	NA
109672	Cyb5	cytochrome b-5	1.05462982	0.297	NA
12677	Vsx2	visual system homeobox 2	1.05462982	0.4872	NA
18033	Nfkb1	nuclear factor of kappa light polypeptide gene enhancer in B-cells 1, p105	1.05462982	0.6013	0.7688
13830	Stom	stomatin	1.05462982	0.6477	0.8012
384309	Trim56	tripartite motif-containing 56	1.05462982	0.6527	0.804
76425	2310003C23Rik	RIKEN cDNA 2310003C23 gene	1.05451861	0.2774	NA
72542	Pgam5	phosphoglycerate mutase family member 5	1.05451861	0.3426	NA
231997	Fkbp14	FK506 binding protein 14	1.05451861	0.3922	NA
242687	Wasf2	WAS protein family, member 2	1.05451861	0.4138	NA
635470	Gm14407	predicted gene 14407	1.05451861	0.4318	NA
66855	Tcf25	transcription factor 25 (basic helix-loop-helix)	1.05451861	0.4843	NA
347722	Agap1	ArfGAP with GTPase domain, ankyrin repeat and PH domain 1	1.05451861	0.4942	NA
20226	Sars	seryl-aminoacyl-tRNA synthetase	1.05451861	0.625	0.7856
330814	Lphn1	latrophilin 1	1.05451861	0.6861	0.8251
13404	Dmc1	DMC1 dosage suppressor of mck1 homolog, meiosis-specific homologous recombination (yeast)	1.05451861	0.7494	0.8652
18007	Neo1	neogenin	1.05440742	0.2337	NA
14755	Pigq	phosphatidylinositol glycan anchor biosynthesis, class Q	1.05440742	0.3053	NA
66091	Ndufa3	NADH dehydrogenase (ubiquinone) 1 alpha subcomplex, 3	1.05440742	0.4183	NA
432555	Gm5431	predicted gene 5431	1.05440742	0.6017	0.769
70310	Plscr3	phospholipid scramblase 3	1.05440742	0.6115	0.7757
217864	Rcor1	REST corepressor 1	1.05429626	0.2588	NA
20444	St3gal2	ST3 beta-galactoside alpha-2,3-sialyltransferase 2	1.05429626	0.3085	NA
665533	Gm13004	60S ribosomal protein L31 pseudogene	1.05418511	0.2449	NA
13726	Emd	emerin	1.05418511	0.409	NA
67080	1700019D03Rik	RIKEN cDNA 1700019D03 gene	1.05418511	0.4295	NA
68735	Mrps18c	mitochondrial ribosomal protein S18C	1.05418511	0.4415	NA
70239	Gtf3c5	general transcription factor IIIC, polypeptide 5	1.05418511	0.4811	NA
433966	5730422E09Rik	RIKEN cDNA 5730422E09 gene	1.05418511	0.6349	0.7926
55990	Fmo2	flavin containing monooxygenase 2	1.05418511	0.7405	0.8596
13177	Dci	dodecenoyl-Coenzyme A delta isomerase (3,2 trans-enoyl-Coenyme A isomerase)	1.054074	0.4095	NA
68472	Tmem126b	transmembrane protein 126B	1.054074	0.4881	NA
12867	Cox7c	cytochrome c oxidase, subunit VIIc	1.0539629	0.267	NA
75847	Ispd	isoprenoid synthase domain containing	1.0539629	0.5657	0.7423

20729	Spin1	spindlin 1	1.0539629	0.632	0.7909
70354	Secisbp2l	SECIS binding protein 2-like	1.0539629	0.8082	0.9005
245688	Rbbp7	retinoblastoma binding protein 7	1.05385183	0.3029	NA
227937	Pkp4	plakophilin 4	1.05385183	0.3198	NA
67922	Fam32a	family with sequence similarity 32, member A	1.05385183	0.3771	NA
11844	Arf5	ADP-ribosylation factor 5	1.05385183	0.4216	NA
12922	Crhr2	corticotropin releasing hormone receptor 2	1.05385183	0.5939	0.7633
20821	Trim21	tripartite motif-containing 21	1.05374078	0.4982	NA
66462	2810428I15Rik	RIKEN cDNA 2810428I15 gene	1.05374078	0.5799	0.7522
18000	Sep-02	septin 2	1.05374078	0.7047	0.8372
329739	Fam102b	family with sequence similarity 102, member B	1.05362975	0.2686	NA
30954	Siva1	SIVA1, apoptosis-inducing factor	1.05362975	0.2991	NA
59045	Stard3	START domain containing 3	1.05362975	0.3374	NA
16443	Itsn1	intersectin 1 (SH3 domain protein 1A)	1.05362975	0.3656	NA
19143	St14	suppression of tumorigenicity 14 (colon carcinoma)	1.05362975	0.467	NA
116870	Mta1	metastasis associated 1	1.05362975	0.6076	0.7731
66156	Anapc11	anaphase promoting complex subunit 11	1.05351875	0.2666	NA
17128	Smad4	MAD homolog 4 (Drosophila)	1.05351875	0.4756	NA
67429	Nudcd1	NudC domain containing 1	1.05351875	0.4797	NA
53413	Exoc7	exocyst complex component 7	1.05351875	0.5048	NA
211187	Lrtm2	leucine-rich repeats and transmembrane domains 2	1.05351875	0.6185	0.7806
100038385	F830115B05Rik	RIKEN cDNA F830115B05 gene	1.05351875	0.7587	0.8713
66055	0610009D07Rik	RIKEN cDNA 0610009D07 gene	1.05340777	0.4533	NA
70350	Basp1	brain abundant, membrane attached signal protein 1	1.05340777	0.5747	0.7485
19286	Pts	6-pyruvoyl-tetrahydropterin synthase	1.05340777	0.5917	0.7614
16974	Lrp6	low density lipoprotein receptor-related protein 6	1.05340777	0.623	0.7838
56463	Snd1	staphylococcal nuclease and tudor domain containing 1	1.05340777	0.6282	0.7884
67707	Mrpl24	mitochondrial ribosomal protein L24	1.05329682	0.294	NA
17127	Smad3	MAD homolog 3 (Drosophila)	1.05329682	0.3008	NA
218461	Pde8b	phosphodiesterase 8B	1.05329682	0.3736	NA
20021	Polr2c	polymerase (RNA) II (DNA directed) polypeptide C	1.05329682	0.4378	NA
54401	Ywhab	tyrosine 3-monooxygenase/tryptophan 5-monooxygenase activation protein, beta polypeptide	1.05329682	0.4407	NA
110842	Etfa	electron transferring flavoprotein, alpha polypeptide	1.05329682	0.5253	NA
217708	Lin52	lin-52 homolog (C. elegans)	1.05329682	0.5267	NA
18717	Pip5k1c	phosphatidylinositol-4-phosphate 5-kinase, type 1 gamma	1.05329682	0.6034	0.7703
14457	Gas7	growth arrest specific 7	1.05329682	0.7713	0.8785
381126	Fam59a	family with sequence similarity 59, member A	1.05318589	0.2483	NA
100044951	LOC100044951	hypothetical LOC100044951	1.05318589	0.2733	NA
20088	Rps24	ribosomal protein S24	1.05318589	0.4603	NA
235559	Topbp1	topoisomerase (DNA) II binding protein 1	1.05318589	0.4794	NA
67308	Mrpl46	mitochondrial ribosomal protein L46	1.05318589	0.5164	NA
270106	Rpl13	ribosomal protein L13	1.05307498	0.3707	NA
109113	Uhrf2	ubiquitin-like, containing PHD and RING finger domains 2	1.05307498	0.3832	NA
22666	Zfp161	zinc finger protein 161	1.05307498	0.4862	NA
66933	1700025L06Rik	RIKEN cDNA 1700025L06 gene	1.05307498	0.6792	0.8207
14129	Fcgr1	Fc receptor, IgG, high affinity I	1.05307498	0.6975	0.8327
68968	Cdan1	congenital dyserythropoietic anemia, type I (human)	1.05307498	0.7141	0.8435
102462	Imp3	IMP3, U3 small nucleolar ribonucleoprotein, homolog (yeast)	1.05296409	0.4127	NA
67792	Rgs8	regulator of G-protein signaling 8	1.05296409	0.4604	NA
66192	Lage3	L antigen family, member 3	1.05296409	0.4805	NA

385454	Gm5396	predicted pseudogene 5396	1.05296409	0.6297	0.7895
27056	Irf5	interferon regulatory factor 5	1.05296409	0.6509	0.8034
12796	Camp	cathelicidin antimicrobial peptide	1.05296409	0.7276	0.8518
70853	Vwa3b	von Willebrand factor A domain containing 3B	1.05296409	0.7719	0.8787
67710	Polr2g	polymerase (RNA) II (DNA directed) polypeptide G	1.05285323	0.2969	NA
75646	Rai14	retinoic acid induced 14	1.05285323	0.3461	NA
94044	Bcl2l13	BCL2-like 13 (apoptosis facilitator)	1.05285323	0.3746	NA
625123	Gm6557	predicted gene 6557	1.05285323	0.5277	NA
407824	BC020402	cDNA sequence BC020402	1.05285323	0.6362	0.7936
12297	Cacnb3	calcium channel, voltage-dependent, beta 3 subunit	1.05285323	0.6425	0.7978
73571	1700096K18Rik	RIKEN cDNA 1700096K18 gene	1.05285323	0.6828	0.8229
57784	Bin3	bridging integrator 3	1.05274239	0.3737	NA
269037	Gm672	predicted gene 672	1.05274239	0.4567	NA
110213	Tmbim6	transmembrane BAX inhibitor motif containing 6	1.05274239	0.4991	NA
233271	Luzp2	leucine zipper protein 2	1.05274239	0.5609	0.7392
18174	Slc11a2	solute carrier family 11 (proton-coupled divalent metal ion transporters), member 2	1.05274239	0.6315	0.7905
65105	Arl6ip4	ADP-ribosylation factor-like 6 interacting protein 4	1.05263158	0.2485	NA
12522	Cd83	CD83 antigen	1.05263158	0.5726	0.7473
215085	Slc35f1	solute carrier family 35, member F1	1.05263158	0.6812	0.8221
70422	Ints2	integrator complex subunit 2	1.05263158	0.7524	0.8669
57783	Tnip1	TNFAIP3 interacting protein 1	1.05252079	0.2824	NA
26936	Mprip	myosin phosphatase Rho interacting protein	1.05252079	0.5048	NA
83602	Gtf2a1	general transcription factor II A, 1	1.05252079	0.7383	0.8582
26374	Rfwd2	ring finger and WD repeat domain 2	1.05241002	0.31	NA
71228	Dlg5	discs, large homolog 5 (Drosophila)	1.05241002	0.3295	NA
223978	Cpped1	calcineurin-like phosphoesterase domain containing 1	1.05241002	0.5113	NA
11924	Neurog2	neurogenin 2	1.05241002	0.514	NA
230259	E130308A19Rik	RIKEN cDNA E130308A19 gene	1.05241002	0.6503	0.8031
52163	Camk1	calcium/calmodulin-dependent protein kinase I	1.05241002	0.7247	0.8501
21337	Tacr2	tachykinin receptor 2	1.05241002	0.8387	0.9169
106672	AI413582	expressed sequence AI413582	1.05229927	0.2439	NA
11951	Atp5g1	ATP synthase, H+ transporting, mitochondrial F0 complex, subunit c1 (subunit 9)	1.05229927	0.2704	NA
245676	Gm4997	predicted gene 4997	1.05229927	0.3358	NA
30953	Schip1	schwannomin interacting protein 1	1.05229927	0.559	0.7382
101351	A130022J15Rik	RIKEN cDNA A130022J15 gene	1.05229927	0.5886	0.7588
68519	Eml1	echinoderm microtubule associated protein like 1	1.05229927	0.5988	0.7672
212862	Chpt1	choline phosphotransferase 1	1.05229927	0.6038	0.7706
13132	Dab2	disabled homolog 2 (Drosophila)	1.05229927	0.7194	0.8467
100041154	Gm3167	predicted gene 3167	1.05218855	0.1975	NA
108934	BC024659	cDNA sequence BC024659	1.05218855	0.2925	NA
53600	Timm23	translocase of inner mitochondrial membrane 23 homolog (yeast)	1.05218855	0.2972	NA
66585	Snrnp40	small nuclear ribonucleoprotein 40 (U5)	1.05218855	0.4753	NA
56264	Cpxm1	carboxypeptidase X 1 (M14 family)	1.05218855	0.5352	NA
74307	1700092M07Rik	RIKEN cDNA 1700092M07 gene	1.05207785	0.5049	NA
19141	Lgmn	legumain	1.05196718	0.3336	NA
108030	Lin7a	lin-7 homolog A (C. elegans)	1.05196718	0.3601	NA
66948	Acad8	acyl-Coenzyme A dehydrogenase family, member 8	1.05196718	0.4707	NA
329763	Gm5105	predicted gene 5105	1.05196718	0.6381	0.7951
243369	Sspo	SCO-spondin	1.05196718	0.6759	0.8184
21673	Dntt	deoxynucleotidyltransferase, terminal	1.05196718	0.7633	0.8744

52521	Zfp622	zinc finger protein 622	1.05185653	0.194	NA
69597	Afg3l2	AFG3(ATPase family gene 3)-like 2 (yeast)	1.05185653	0.3942	NA
69920	Polr2i	polymerase (RNA) II (DNA directed) polypeptide I	1.05185653	0.4126	NA
319149	Hist1h3d	histone cluster 1, H3d	1.05185653	0.4809	NA
22365	Vps45	vacuolar protein sorting 45 (yeast)	1.05185653	0.5938	0.7632
74154	Unkl	unkempt-like (Drosophila)	1.05185653	0.6054	0.7719
97213	C77137	expressed sequence C77137	1.05185653	0.6829	0.8229
66637	Tsen15	tRNA splicing endonuclease 15 homolog (S. cerevisiae)	1.0517459	0.2624	NA
677044	Gm10653	ribosomal protein S2 pseudogene	1.0517459	0.3736	NA
74243	2210009G21Rik	RIKEN cDNA 2210009G21 gene	1.0517459	0.4987	NA
67874	Rprm	reprimo, TP53 dependent G2 arrest mediator candidate	1.0517459	0.5288	NA
67429	Nudcd1	NudC domain containing 1	1.05163529	0.7404	0.8596
243872	Rpl7a-ps8	ribosomal protein L7A, pseudogene 8	1.05152471	0.2513	NA
53627	Porcn	porcupine homolog (Drosophila)	1.05152471	0.3869	NA
219158	2610301G19Rik	RIKEN cDNA 2610301G19 gene	1.05152471	0.4324	NA
18555	Cdk16	cyclin-dependent kinase 16	1.05152471	0.6131	0.7771
20511	Slc1a2	solute carrier family 1 (glial high affinity glutamate transporter), member 2	1.05152471	0.7343	0.8565
19084	Prkar1a	protein kinase, cAMP dependent regulatory, type I, alpha	1.05141415	0.285	NA
68801	Elovl5	ELOVL family member 5, elongation of long chain fatty acids (yeast)	1.05141415	0.3623	NA
22230	Ufd1l	ubiquitin fusion degradation 1 like	1.05141415	0.4182	NA
21780	Tfam	transcription factor A, mitochondrial	1.05141415	0.4885	NA
78381	2310047N11Rik	RIKEN cDNA 2310047N11 gene	1.05141415	0.8087	0.9006
11833	Aqp8	aquaporin 8	1.05141415	0.8637	0.9294
79566	Sh3bp5l	SH3 binding domain protein 5 like	1.05130362	0.2612	NA
68106	Nt5c3l	5'-nucleotidase, cytosolic III-like	1.05130362	0.4209	NA
13712	Elk1	ELK1, member of ETS oncogene family	1.05130362	0.4663	NA
77462	Tmem116	transmembrane protein 116	1.05130362	0.5616	0.7396
76574	Mfsd2a	major facilitator superfamily domain containing 2A	1.05130362	0.662	0.8096
54422	Barhl1	BarH-like 1 (Drosophila)	1.05130362	0.8286	0.9113
107869	Cth	cystathionase (cystathionine gamma-lyase)	1.0511931	0.2986	NA
231413	Grsf1	G-rich RNA sequence binding factor 1	1.0511931	0.3744	NA
72046	Urgcp	upregulator of cell proliferation	1.0511931	0.5704	0.7456
270802	BC048403	cDNA sequence BC048403	1.0511931	0.5818	0.7535
16480	Jup	junction plakoglobin	1.0511931	0.8352	0.9148
20641	Snrpd1	small nuclear ribonucleoprotein D1	1.05108262	0.2303	NA
19988	Rpl6	ribosomal protein L6	1.05108262	0.3294	NA
328370	Rft1	RFT1 homolog (S. cerevisiae)	1.05108262	0.4505	NA
94279	Sfxn2	sideroflexin 2	1.05108262	0.5861	0.7566
544736	Glipr1l3	GLI pathogenesis-related 1 like 3	1.05108262	0.6865	0.8254
107771	Bmyc	brain expressed myelocytomatosis oncogene	1.05108262	0.6896	0.8276
320595	Phf8	PHD finger protein 8	1.05108262	0.7288	0.8526
74205	Acsl3	acyl-CoA synthetase long-chain family member 3	1.05108262	0.7472	0.8638
223337	Ugt3a2	UDP glycosyltransferases 3 family, polypeptide A2	1.05108262	0.8366	0.9158
73796	4930402F11Rik	RIKEN cDNA 4930402F11 gene	1.05108262	0.9375	0.9682
23991	Cib1	calcium and integrin binding 1 (calmyrin)	1.05097215	0.3439	NA
68027	Tmem178	transmembrane protein 178	1.05097215	0.4135	NA
98314	D2hgdh	D-2-hydroxyglutarate dehydrogenase	1.05097215	0.4722	NA
20810	Srm	spermidine synthase	1.05097215	0.6408	0.7968
56347	Eif3c	eukaryotic translation initiation factor 3, subunit C	1.05097215	0.6712	0.8158
20588	Smarcc1	SWI/SNF related, matrix associated, actin dependent regulator of chromatin, subfamily c, member 1	1.05097215	0.7625	0.8738

192652	Wdr81	WD repeat domain 81	1.05086171	0.288	NA
66212	Sec61b	Sec61 beta subunit	1.05086171	0.3092	NA
118454	Gjc2	gap junction protein, gamma 2	1.05086171	0.4722	NA
140721	Caskin2	CASK-interacting protein 2	1.05086171	0.4752	NA
54380	Smarcal1	SWI/SNF related matrix associated, actin dependent regulator of chromatin, subfamily a-like 1	1.05086171	0.4889	NA
18041	Nfs1	nitrogen fixation gene 1 (S. cerevisiae)	1.05086171	0.4926	NA
319179	Hist1h2be	histone cluster 1, H2be	1.05086171	0.5052	NA
226409	Zranb3	zinc finger, RAN-binding domain containing 3	1.05086171	0.5706	0.7458
623174	Gm6404	predicted gene 6404	1.05086171	0.6163	0.7793
109095	Rbm15b	RNA binding motif protein 15B	1.05086171	0.679	0.8206
75292	Prkd3	protein kinase D3	1.05075129	0.3623	NA
216558	Ugp2	UDP-glucose pyrophosphorylase 2	1.05075129	0.383	NA
66427	Cyb5b	cytochrome b5 type B	1.05075129	0.443	NA
64657	Mrps10	mitochondrial ribosomal protein S10	1.05075129	0.4822	NA
20892	Stra13	stimulated by retinoic acid 13	1.05075129	0.5407	NA
434203	Slc28a1	solute carrier family 28 (sodium-coupled nucleoside transporter), member 1	1.05075129	0.8047	0.8983
13929	Amz2	archaelysin family metallopeptidase 2	1.05064089	0.3811	NA
74164	Nfx1	nuclear transcription factor, X-box binding 1	1.05064089	0.6451	0.7995
26430	Parg	poly (ADP-ribose) glycohydrolase	1.05053052	0.3252	NA
257633	Acsf3	acyl-CoA synthetase family member 3	1.05053052	0.5087	NA
29861	Dpf1	D4, zinc and double PHD fingers family 1	1.05053052	0.5856	0.7563
67477	Abhd15	abhydrolase domain containing 15	1.05053052	0.7588	0.8713
621818	Gm9785	BMI1-like	1.05053052	0.7681	0.877
12793	Cnih	cornichon homolog (Drosophila)	1.05042017	0.4343	NA
331026	Gmppb	GDP-mannose pyrophosphorylase B	1.05042017	0.4363	NA
59079	Erbb2ip	Erbb2 interacting protein	1.05042017	0.4819	NA
67026	Thap4	THAP domain containing 4	1.05042017	0.5757	0.7492
16443	Itsn1	intersectin 1 (SH3 domain protein 1A)	1.05042017	0.6427	0.7978
74764	Klc4	kinesin light chain 4	1.05042017	0.6456	0.7997
56462	Mtch1	mitochondrial carrier homolog 1 (C. elegans)	1.05042017	0.6523	0.8039
382019	Zfp882	zinc finger protein 882	1.05042017	0.6573	0.8065
97031	Tprn	taperin	1.05042017	0.7101	0.8408
76952	Nt5c2	5'-nucleotidase, cytosolic II	1.05030984	0.4867	NA
54375	Azin1	antizyme inhibitor 1	1.05030984	0.7139	0.8434
76687	Spcs3	signal peptidase complex subunit 3 homolog (S. cerevisiae)	1.05030984	0.8044	0.8981
69692	Hddc2	HD domain containing 2	1.05019954	0.2815	NA
77809	Lrrc42	leucine rich repeat containing 42	1.05019954	0.3253	NA
72137	Wdsub1	WD repeat, SAM and U-box domain containing 1	1.05019954	0.4677	NA
110821	Pcca	propionyl-Coenzyme A carboxylase, alpha polypeptide	1.05019954	0.552	NA
65247	Asb1	ankyrin repeat and SOCS box-containing 1	1.05019954	0.608	0.7732
235627	Nbeal2	neurobeachin-like 2	1.05019954	0.6477	0.8012
73161	3110035C09Rik	RIKEN cDNA 3110035C09 gene	1.05019954	0.8135	0.9033
57357	Srd5a3	steroid 5 alpha-reductase 3	1.05008926	0.488	NA
14815	Nr3c1	nuclear receptor subfamily 3, group C, member 1	1.05008926	0.5619	NA
66082	Abhd6	abhydrolase domain containing 6	1.05008926	0.6145	0.7779
76816	Sdccag8	serologically defined colon cancer antigen 8	1.05008926	0.7669	0.8763
228061	Agps	alkylglycerone phosphate synthase	1.04986877	0.38	NA
26572	Cops3	COP9 (constitutive photomorphogenic) homolog, subunit 3 (Arabidopsis thaliana)	1.04986877	0.4604	NA
24050	Sep-03	septin 3	1.04986877	0.4803	NA
16800	Arhgef2	rho/rac guanine nucleotide exchange factor (GEF) 2	1.04975856	0.3454	NA

50791	Magi2	membrane associated guanylate kinase, WW and PDZ domain containing 2	1.04975856	0.473	NA
20183	Rxrg	retinoid X receptor gamma	1.04975856	0.5134	NA
226971	Plekhb2	pleckstrin homology domain containing, family B (evectins) member 2	1.04975856	0.5586	NA
16549	Khsrp	KH-type splicing regulatory protein	1.04975856	0.6009	0.7684
66665	5730528L13Rik	RIKEN cDNA 5730528L13 gene	1.04964837	0.3053	NA
68292	Stt3b	STT3, subunit of the oligosaccharyltransferase complex, homolog B (S. cerevisiae)	1.04964837	0.3263	NA
320706	9830001H06Rik	RIKEN cDNA 9830001H06 gene	1.04964837	0.6062	0.7725
11465	Actg1	actin, gamma, cytoplasmic 1	1.0495382	0.4139	NA
13430	Dnm2	dynamin 2	1.0495382	0.5612	NA
11431	Acp1	acid phosphatase 1, soluble	1.0495382	0.5867	0.7572
382062	AB124611	cDNA sequence AB124611	1.0495382	0.6405	0.7967
74563	Rasgef1c	RasGEF domain family, member 1C	1.0495382	0.6699	0.8147
100049077	LOC100049077	hypothetical protein LOC100049077	1.0495382	0.681	0.8219
320683	Zfp629	zinc finger protein 629	1.04942806	0.2154	NA
100041953	Gm10094	predicted gene 10094	1.04942806	0.2348	NA
66975	2410002O22Rik	RIKEN cDNA 2410002O22 gene	1.04942806	0.4012	NA
104522	AU040972	expressed sequence AU040972	1.04942806	0.5423	NA
69161	Manbal	mannosidase, beta A, lysosomal-like	1.04942806	0.6742	0.8176
20181	Rxra	retinoid X receptor alpha	1.04942806	0.7047	0.8372
240283	Dmxl1	Dmx-like 1	1.04942806	0.7228	0.849
22195	Ube2l3	ubiquitin-conjugating enzyme E2L 3	1.04931794	0.2679	NA
76602	1700040D17Rik	RIKEN cDNA 1700040D17 gene	1.04931794	0.3875	NA
20351	Sema4a	sema domain, immunoglobulin domain (Ig), transmembrane domain (TM) and short cytoplasmic domain, (semaphorin) 4A	1.04931794	0.3921	NA
66074	Tmem167	transmembrane protein 167	1.04931794	0.4253	NA
71448	Tmem80	transmembrane protein 80	1.04931794	0.4344	NA
232853	Zfp954	zinc finger protein 954	1.04931794	0.5254	NA
67703	Kirrel3	kin of IRRE like 3 (Drosophila)	1.04931794	0.5976	0.7662
19946	Rpl30	ribosomal protein L30	1.04920785	0.2894	NA
320301	E530011L22Rik	RIKEN cDNA E530011L22 gene	1.04920785	0.3929	NA
12870	Cp	ceruloplasmin	1.04920785	0.4503	NA
53380	Psmd10	proteasome (prosome, macropain) 26S subunit, non-ATPase, 10	1.04920785	0.4925	NA
76787	Ppfia3	protein tyrosine phosphatase, receptor type, f polypeptide (PTPRF), interacting protein (liprin), alpha 3	1.04920785	0.6231	0.7839
30960	Vapa	vesicle-associated membrane protein, associated protein A	1.04909778	0.3943	NA
67738	Ppid	peptidylprolyl isomerase D (cyclophilin D)	1.04909778	0.4127	NA
66587	Fastk	Fas-activated serine/threonine kinase	1.04909778	0.5751	0.749
328451	Gm5088	poly(A)-binding protein, cytoplasmic pseudogene	1.04909778	0.7605	0.8724
73689	Bloc1s2	biogenesis of lysosome-related organelles complex-1, subunit 2	1.04898773	0.2753	NA
53421	Sec61a1	Sec61 alpha 1 subunit (S. cerevisiae)	1.04898773	0.3516	NA
11430	Acox1	acyl-Coenzyme A oxidase 1, palmitoyl	1.04898773	0.3866	NA
72096	Mettl10	methyltransferase like 10	1.04898773	0.3917	NA
66942	Ddx18	DEAD (Asp-Glu-Ala-Asp) box polypeptide 18	1.04898773	0.4923	NA
105428	Fam149b	family with sequence similarity 149, member B	1.04898773	0.6079	0.7732
70235	Poc1a	POC1 centriolar protein homolog A (Chlamydomonas)	1.0488777	0.3002	NA
268970	Arhgap28	Rho GTPase activating protein 28	1.0488777	0.6433	0.7982
11844	Arf5	ADP-ribosylation factor 5	1.0487677	0.5134	NA
70997	Spef1	sperm flagellar 1	1.04865772	0.3113	NA
107686	Snrpd2	small nuclear ribonucleoprotein D2	1.04865772	0.4392	NA
66684	Tceal8	transcription elongation factor A (SII)-like 8	1.04865772	0.5165	NA
56294	Ptpn9	protein tyrosine phosphatase, non-receptor type 9	1.04865772	0.5632	NA
67576	4930429B21Rik	RIKEN cDNA 4930429B21 gene	1.04865772	0.6082	0.7733

67501	Ccdc50	coiled-coil domain containing 50	1.04865772	0.7928	0.8914
67808	Tprgl	transformation related protein 63 regulated like	1.04854776	0.3127	NA
71923	2310047M10Rik	RIKEN cDNA 2310047M10 gene	1.04854776	0.3785	NA
68135	Eif3h	eukaryotic translation initiation factor 3, subunit H	1.04854776	0.4047	NA
242521	Klhl9	kelch-like 9 (Drosophila)	1.04854776	0.4413	NA
72124	Seh1l	SEH1-like (S. cerevisiae	1.04854776	0.5346	NA
19663	Rbpms	RNA binding protein gene with multiple splicing	1.04854776	0.54	NA
67445	C1qtnf4	C1q and tumor necrosis factor related protein 4	1.04854776	0.6487	0.802
51801	Ramp1	receptor (calcitonin) activity modifying protein 1	1.04854776	0.7952	0.8926
13030	Ctsb	cathepsin B	1.04843783	0.3839	NA
19283	Ptprz1	protein tyrosine phosphatase, receptor type Z, polypeptide 1	1.04843783	0.4865	NA
72075	Ogfr	opioid growth factor receptor	1.04843783	0.5102	NA
654801	Zfp784	zinc finger protein 784	1.04832792	0.2817	NA
18141	Nup50	nucleoporin 50	1.04832792	0.4898	NA
100044339	LOC100044339	zinc finger protein 764-like	1.04832792	0.5829	0.7543
278240	Spin2	spindlin family, member 2	1.04832792	0.596	0.7648
230908	Tardbp	TAR DNA binding protein	1.04832792	0.6279	0.7882
383766	Gm1332	predicted gene 1332	1.04832792	0.6968	0.8325
18016	Nf2	neurofibromatosis 2	1.04821803	0.531	NA
54393	Gabbr1	gamma-aminobutyric acid (GABA) B receptor, 1	1.04821803	0.6051	0.7717
26390	Mapkbp1	mitogen-activated protein kinase binding protein 1	1.04821803	0.6515	0.8034
319195	Rpl17	ribosomal protein L17	1.04810816	0.3525	NA
114249	Npnt	nephronectin	1.04810816	0.4783	NA
74919	4930471M23Rik	RIKEN cDNA 4930471M23 gene	1.04810816	0.6071	0.7728
272589	Tbcel	tubulin folding cofactor E-like	1.04810816	0.6251	0.7857
246707	Emilin2	elastin microfibril interfacer 2	1.04810816	0.7118	0.8418
67840	Mrp63	mitochondrial ribosomal protein 63	1.04799832	0.2684	NA
70451	Dhrs13	dehydrogenase/reductase (SDR family) member 13	1.04799832	0.3596	NA
52551	Sgta	small glutamine-rich tetratricopeptide repeat (TPR)-containing, alpha	1.04799832	0.3969	NA
66916	Ndufb7	NADH dehydrogenase (ubiquinone) 1 beta subcomplex, 7	1.04799832	0.4726	NA
101513	2700078K21Rik	RIKEN cDNA 2700078K21 gene	1.04799832	0.5272	NA
18132	Notch4	Notch gene homolog 4 (Drosophila)	1.04799832	0.615	0.7782
106344	Rfc4	replication factor C (activator 1) 4	1.04799832	0.6491	0.8021
320328	B130024M06Rik	RIKEN cDNA B130024M06 gene	1.04799832	0.7381	0.8581
68918	1190005I06Rik	RIKEN cDNA 1190005I06 gene	1.0478885	0.3407	NA
677044	Gm10653	ribsomal protein S2 pseudogene	1.0478885	0.4211	NA
66231	Thoc7	THO complex 7 homolog (Drosophila)	1.0478885	0.4324	NA
94064	Mrpl27	mitochondrial ribosomal protein L27	1.0478885	0.4617	NA
19826	Rnps1	ribonucleic acid binding protein S1	1.0478885	0.5563	NA
69710	Arap1	ArfGAP with RhoGAP domain, ankyrin repeat and PH domain 1	1.0478885	0.7453	0.8627
330260	Pon2	paraoxonase 2	1.0478885	0.7911	0.8905
227656	Rexo4	REX4, RNA exonuclease 4 homolog (S. cerevisiae)	1.04777871	0.3462	NA
24135	Zfp68	zinc finger protein 68	1.04777871	0.5193	NA
97550	C130081A10Rik	RIKEN cDNA C130081A10 gene	1.04777871	0.535	NA
226356	Gm101	predicted gene 101	1.04777871	0.8309	0.9126
257902	Olfr704	olfactory receptor 704	1.04777871	0.8654	0.9302
67581	Tbc1d23	TBC1 domain family, member 23	1.04766894	0.3066	NA
380601	Fastkd5	FAST kinase domains 5	1.04766894	0.3449	NA
74374	Clec16a	C-type lectin domain family 16, member A	1.04766894	0.4236	NA
56374	Tmem59	transmembrane protein 59	1.04766894	0.5577	NA

14570	Arhgdig	Rho GDP dissociation inhibitor (GDI) gamma	1.04766894	0.6837	0.8233
11774	Ap3b1	adaptor-related protein complex 3, beta 1 subunit	1.04755919	0.3334	NA
73836	Slc35b2	solute carrier family 35, member B2	1.04755919	0.4331	NA
20443	St3gal4	ST3 beta-galactoside alpha-2,3-sialyltransferase 4	1.04755919	0.506	NA
102060	Gadd45gip1	growth arrest and DNA-damage-inducible, gamma interacting protein 1	1.04755919	0.5682	NA
433904	Ociad2	OCIA domain containing 2	1.04755919	0.5893	0.7594
69539	Trnp1	TMF1-regulated nuclear protein 1	1.04755919	0.6095	0.7744
17318	Mid1	midline 1	1.04755919	0.698	0.8328
226178	D19Wsu162e	DNA segment, Chr 19, Wayne State University 162, expressed	1.04744946	0.3316	NA
57423	Atp5j2	ATP synthase, H+ transporting, mitochondrial F0 complex, subunit F2	1.04744946	0.3322	NA
69790	Med30	mediator complex subunit 30	1.04744946	0.3531	NA
66240	Kcne1l	potassium voltage-gated channel, Isk-related family, member 1-like, pseudogene	1.04744946	0.5503	NA
98386	Lbr	lamin B receptor	1.04744946	0.5687	NA
71750	R3hdm2	R3H domain containing 2	1.04744946	0.5979	0.7665
65967	Eefsec	eukaryotic elongation factor, selenocysteine-tRNA-specific	1.04744946	0.6768	0.8191
270035	Letm2	leucine zipper-EF-hand containing transmembrane protein 2	1.04744946	0.7777	0.8822
16512	Kcnh3	potassium voltage-gated channel, subfamily H (eag-related), member 3	1.04744946	0.8704	0.933
268656	Sptlc1	serine palmitoyltransferase, long chain base subunit 1	1.04733976	0.465	NA
55950	Bri3	brain protein I3	1.04733976	0.4653	NA
19428	Rasl2-9-ps	RAS-like, family 2, locus 9, pseudogene	1.04733976	0.5083	NA
217578	Baz1a	bromodomain adjacent to zinc finger domain 1A	1.04733976	0.6473	0.8012
246316	Lgi2	leucine-rich repeat LGI family, member 2	1.04733976	0.661	0.809
230126	Shb	src homology 2 domain-containing transforming protein B	1.04733976	0.6992	0.8336
234159	Gm4889	predicted gene 4889	1.04723008	0.3576	NA
108148	Galnt2	UDP-N-acetyl-alpha-D-galactosamine:polypeptide N-acetylgalactosaminyltransferase 2	1.04723008	0.4951	NA
72630	Hspa12b	heat shock protein 12B	1.04723008	0.5237	NA
71912	Jsrp1	junctional sarcoplasmic reticulum protein 1	1.04723008	0.5745	NA
620592	Tmem28	transmembrane protein 28	1.04723008	0.5858	0.7564
72440	5930416I19Rik	RIKEN cDNA 5930416I19 gene	1.04723008	0.6351	0.7928
69104	Mar-05	membrane-associated ring finger (C3HC4) 5	1.04712042	0.392	NA
272465	Fam70b	family with sequence similarity 70, member B	1.04701078	0.3289	NA
12340	Capza1	capping protein (actin filament) muscle Z-line, alpha 1	1.04701078	0.4214	NA
66242	Mrps16	mitochondrial ribosomal protein S16	1.04701078	0.4218	NA
14789	Leprel2	leprecan-like 2	1.04701078	0.4636	NA
27393	Mrpl39	mitochondrial ribosomal protein L39	1.04701078	0.4797	NA
234356	Csgalnact1	chondroitin sulfate N-acetylgalactosaminyltransferase 1	1.04701078	0.5429	NA
22142	Tuba1a	tubulin, alpha 1A	1.04701078	0.5582	NA
627798	Gm6790	tumor protein, translationally-controlled 1 pseudogene	1.04701078	0.6138	0.7775
71086	4933412E12Rik	RIKEN cDNA 4933412E12 gene	1.04701078	0.6731	0.817
271887	BC066135	cDNA sequence BC066135	1.04701078	0.8444	0.9198
432812	BC052688	cDNA sequence BC052688	1.04701078	0.849	0.9221
68554	1110001A16Rik	RIKEN cDNA 1110001A16 gene	1.04690117	0.3943	NA
78903	Wrnip1	Werner helicase interacting protein 1	1.04690117	0.408	NA
212679	Mars2	methionine-tRNA synthetase 2 (mitochondrial)	1.04690117	0.6014	0.7688
66840	Wdr45l	Wdr45 like	1.04690117	0.643	0.798
622935	Krtap20-2	keratin associated protein 20-2	1.04690117	0.7448	0.8624
224247	E330017A01Rik	RIKEN cDNA E330017A01 gene	1.04690117	0.8186	0.9061
21754	Tesk1	testis specific protein kinase 1	1.04679158	0.384	NA
21780	Tfam	transcription factor A, mitochondrial	1.04679158	0.4209	NA
225207	Zfp521	zinc finger protein 521	1.04679158	0.4701	NA

68572	Ict1	immature colon carcinoma transcript 1	1.04679158	0.5611	NA
69702	Ndufaf1	NADH dehydrogenase (ubiquinone) 1 alpha subcomplex, assembly factor 1	1.04679158	0.5806	NA
231807	BC037034	cDNA sequence BC037034	1.04679158	0.5988	0.7672
224897	Dpp9	dipeptidylpeptidase 9	1.04679158	0.6508	0.8034
70957	4921530L18Rik	RIKEN cDNA 4921530L18 gene	1.04679158	0.7702	0.8781
621893	Hist2h2ab	histone cluster 2, H2ab	1.04679158	0.7809	0.8845
74241	Chpf	chondroitin polymerizing factor	1.04668202	0.4718	NA
66263	1810014B01Rik	RIKEN cDNA 1810014B01 gene	1.04668202	0.509	NA
22631	Ywhaz	tyrosine 3-monooxygenase/tryptophan 5-monooxygenase activation protein, zeta polypeptide	1.04668202	0.5767	NA
23794	Adamts5	a disintegrin-like and metallopeptidase (reprolysin type) with thrombospondin type 1 motif, 5 (aggrecanase-2)	1.04668202	0.6888	0.827
69125	Cnot8	CCR4-NOT transcription complex, subunit 8	1.04657248	0.3198	NA
22694	Zfp35	zinc finger protein 35	1.04657248	0.399	NA
22195	Ube2l3	ubiquitin-conjugating enzyme E2L 3	1.04657248	0.4707	NA
98238	Lrrc59	leucine rich repeat containing 59	1.04657248	0.4924	NA
240058	Cpne5	copine V	1.04657248	0.6208	0.7823
11479	Acvr1b	activin A receptor, type 1B	1.04657248	0.6341	0.792
68070	Pdzd2	PDZ domain containing 2	1.04657248	0.7934	0.8915
382423	Atxn7l3b	ataxin 7-like 3B	1.04646296	0.3911	NA
230125	Mcart1	mitochondrial carrier triple repeat 1	1.04646296	0.4675	NA
320634	Ocrl	oculocerebrorenal syndrome of Lowe	1.04646296	0.5065	NA
69150	Snx4	sorting nexin 4	1.04646296	0.6197	0.7816
26400	Map2k7	mitogen-activated protein kinase kinase 7	1.04646296	0.6429	0.798
230673	Ipo13	importin 13	1.04646296	0.7545	0.8682
23807	Arih2	ariadne homolog 2 (Drosophila)	1.04635346	0.2525	NA
14661	Glud1	glutamate dehydrogenase 1	1.04635346	0.4364	NA
234374	Ddx49	DEAD (Asp-Glu-Ala-Asp) box polypeptide 49	1.04635346	0.4504	NA
83997	Slmap	sarcolemma associated protein	1.04635346	0.4839	NA
20980	Syt2	synaptotagmin II	1.04635346	0.6747	0.8178
232187	Smyd5	SET and MYND domain containing 5	1.04635346	0.7048	0.8372
20450	St8sia2	ST8 alpha-N-acetyl-neuraminide alpha-2,8-sialyltransferase 2	1.04635346	0.7727	0.879
100042314	Gm10639	predicted gene 10639	1.04635346	0.8101	0.9015
14356	Fxc1	fractured callus expressed transcript 1	1.04624398	0.2455	NA
59048	C1galt1c1	C1GALT1-specific chaperone 1	1.04624398	0.2804	NA
99311	Commd7	COMM domain containing 7	1.04624398	0.4889	NA
56422	Hbs1l	Hbs1-like (S. cerevisiae)	1.04624398	0.5014	NA
434179	Gm5595	predicted gene 5595	1.04624398	0.5341	NA
100206	Adprhl2	ADP-ribosylhydrolase like 2	1.04624398	0.5583	NA
94332	Cadm3	cell adhesion molecule 3	1.04624398	0.6724	0.8166
23936	Lynx1	Ly6/neurotoxin 1	1.04624398	0.7105	0.841
67863	Slc25a11	solute carrier family 25 (mitochondrial carrier oxoglutarate carrier), member 11	1.04613453	0.3116	NA
434460	Gm5623	predicted gene 5623	1.04613453	0.338	NA
66596	Gtf3a	general transcription factor III A	1.04613453	0.4163	NA
66416	Ndufa7	NADH dehydrogenase (ubiquinone) 1 alpha subcomplex, 7 (B14.5a)	1.04613453	0.4387	NA
12798	Cnn2	calponin 2	1.04613453	0.7345	0.8565
66124	Josd2	Josephin domain containing 2	1.04613453	0.7531	0.8673
100042784	Prdm11	PR domain containing 11	1.04613453	0.8263	0.9104
106258	AI790442	expressed sequence AI790442	1.04613453	0.8857	0.9412
19247	Ptpn11	protein tyrosine phosphatase, non-receptor type 11	1.0460251	0.3153	NA
57170	Dolpp1	dolichyl pyrophosphate phosphatase 1	1.0460251	0.4344	NA
72692	Hnrpll	heterogeneous nuclear ribonucleoprotein L-like	1.0460251	0.4568	NA

208618	Etl4	enhancer trap locus 4	1.0460251	0.502	NA
107476	Acaca	acetyl-Coenzyme A carboxylase alpha	1.0459157	0.4761	NA
72588	2700012I20Rik	RIKEN cDNA 2700012I20 gene	1.0459157	0.5593	NA
353156	Egfl7	EGF-like domain 7	1.04580632	0.2867	NA
67023	Use1	unconventional SNARE in the ER 1 homolog (S. cerevisiae)	1.04580632	0.3608	NA
93670	Tac4	tachykinin 4	1.04580632	0.6508	0.8034
319915	A830049F12Rik	RIKEN cDNA A830049F12 gene	1.04580632	0.6686	0.8138
108912	Cdca2	cell division cycle associated 2	1.04580632	0.7974	0.8934
56811	Dkk2	dickkopf homolog 2 (Xenopus laevis)	1.04580632	0.8188	0.9062
11603	Agrn	agrin	1.04580632	0.8541	0.925
72023	Cyb561d1	cytochrome b-561 domain containing 1	1.04569696	0.3508	NA
107305	Vps37c	vacuolar protein sorting 37C (yeast)	1.04569696	0.6144	0.7778
68479	Phf5a	PHD finger protein 5A	1.04569696	0.6193	0.7814
97848	Serpinb6c	serine (or cysteine) peptidase inhibitor, clade B, member 6c	1.04569696	0.7935	0.8915
73830	Eif3k	eukaryotic translation initiation factor 3, subunit K	1.04558762	0.344	NA
12905	Cradd	CASP2 and RIPK1 domain containing adaptor with death domain	1.04558762	0.4186	NA
11911	Atf4	activating transcription factor 4	1.04558762	0.4857	NA
28146	Serp1	stress-associated endoplasmic reticulum protein 1	1.04558762	0.521	NA
17118	Marcks	myristoylated alanine rich protein kinase C substrate	1.04558762	0.5801	NA
27058	Srp9	signal recognition particle 9	1.04558762	0.6426	0.7978
22784	Slc30a3	solute carrier family 30 (zinc transporter), member 3	1.04558762	0.6525	0.804
12616	Cenpb	centromere protein B	1.04558762	0.7474	0.8639
68816	Ppil1	peptidylprolyl isomerase (cyclophilin)-like 1	1.04547831	0.4361	NA
268759	9930012K11Rik	RIKEN cDNA 9930012K11 gene	1.04547831	0.7075	0.839
18802	Plcd4	phospholipase C, delta 4	1.04547831	0.7801	0.8837
22163	Tnfrsf4	tumor necrosis factor receptor superfamily, member 4	1.04536902	0.548	NA
69788	1600023N17Rik	RIKEN cDNA 1600023N17 gene	1.04536902	0.553	NA
217869	Eif5	eukaryotic translation initiation factor 5	1.04536902	0.6066	0.7727
233875	Ino80e	INO80 complex subunit E	1.04536902	0.6104	0.775
56215	Acin1	apoptotic chromatin condensation inducer 1	1.04536902	0.8283	0.9112
19317	Qk	quaking	1.04525975	0.6027	0.7696
18747	Prkaca	protein kinase, cAMP dependent, catalytic, alpha	1.04525975	0.6163	0.7793
54151	Cyhr1	cysteine and histidine rich 1	1.0451505	0.362	NA
57913	Lrdd	leucine-rich and death domain containing	1.0451505	0.5123	NA
22644	Rnf103	ring finger protein 103	1.0451505	0.5454	NA
67474	Snap29	synaptosomal-associated protein 29	1.0451505	0.5461	NA
67771	Arpc5	actin related protein 2/3 complex, subunit 5	1.0451505	0.628	0.7882
100038991	Gm2000	predicted gene 2000	1.0451505	0.6372	0.7945
329693	Fcrl5	Fc receptor-like 5	1.0451505	0.6981	0.8328
15950	Ifi203	interferon activated gene 203	1.0451505	0.8162	0.9047
19229	Ptk2b	PTK2 protein tyrosine kinase 2 beta	1.0451505	0.8328	0.9138
67437	Ssr3	signal sequence receptor, gamma	1.04504128	0.3533	NA
12725	Clcn3	chloride channel 3	1.04504128	0.4561	NA
21355	Tap2	transporter 2, ATP-binding cassette, sub-family B (MDR/TAP)	1.04504128	0.4786	NA
12616	Cenpb	centromere protein B	1.04504128	0.5041	NA
69104	Mar-05	membrane-associated ring finger (C3HC4) 5	1.04504128	0.5273	NA
67836	Wdr83	WD repeat domain containing 83	1.04504128	0.6626	0.8097
77590	Chst15	carbohydrate (N-acetylgalactosamine 4-sulfate 6-O) sulfotransferase 15	1.04504128	0.6735	0.8171
140740	Sec63	SEC63-like (S. cerevisiae)	1.04493208	0.5252	NA
319748	Zfp865	zinc finger protein 865	1.04493208	0.6191	0.7812

21881	Tkt	transketolase	1.04493208	0.6682	0.8135
102566	Ano10	anoctamin 10	1.04493208	0.7136	0.8431
69392	1700024P12Rik	RIKEN cDNA 1700024P12 gene	1.04493208	0.8151	0.9045
74043	Pex26	peroxisomal biogenesis factor 26	1.0448229	0.3606	NA
100042720	Gm3988	predicted gene 3988	1.0448229	0.4614	NA
66309	Tmem128	transmembrane protein 128	1.0448229	0.4647	NA
97423	R74862	expressed sequence R74862	1.0448229	0.537	NA
18181	Nrf1	nuclear respiratory factor 1	1.0448229	0.5553	NA
112403	Dom3z	DOM-3 homolog Z (C. elegans)	1.0448229	0.5901	NA
16697	LOC16697	keratin associated protein LOC16697	1.0448229	0.7698	0.8779
26429	Orc5	origin recognition complex, subunit 5	1.04471375	0.6921	0.8295
76524	Cln6	ceroid-lipofuscinosis, neuronal 6	1.04471375	0.6981	0.8328
51812	Mcrs1	microspherule protein 1	1.04471375	0.7647	0.8753
12387	Ctnnb1	catenin (cadherin associated protein), beta 1	1.04460462	0.3386	NA
234159	Gm4889	predicted gene 4889	1.04460462	0.454	NA
78339	Ttyh3	tweety homolog 3 (Drosophila)	1.04460462	0.4676	NA
14043	Ext2	exostoses (multiple) 2	1.04460462	0.5018	NA
74316	Isca2	iron-sulfur cluster assembly 2 homolog (S. cerevisiae)	1.04460462	0.5351	NA
319901	Dsel	dermatan sulfate epimerase-like	1.04460462	0.5691	NA
66355	Gmpr	guanosine monophosphate reductase	1.04460462	0.6176	0.7798
72584	Cul4b	cullin 4B	1.04460462	0.6263	0.7866
21856	Timm44	translocase of inner mitochondrial membrane 44	1.04460462	0.6998	0.8337
230936	Phf13	PHD finger protein 13	1.04460462	0.7785	0.8828
66420	Polr2e	polymerase (RNA) II (DNA directed) polypeptide E	1.04449551	0.4742	NA
78251	Zfp712	zinc finger protein 712	1.04449551	0.4921	NA
17156	Man1a2	mannosidase, alpha, class 1A, member 2	1.04449551	0.5241	NA
19047	Ppp1cc	protein phosphatase 1, catalytic subunit, gamma isoform	1.04449551	0.5243	NA
67125	Tspan31	tetraspanin 31	1.04449551	0.53	NA
268749	Rnf31	ring finger protein 31	1.04449551	0.5606	NA
17703	Msx3	homeobox, msh-like 3	1.04449551	0.6739	0.8173
16874	Lhx6	LIM homeobox protein 6	1.04449551	0.869	0.9325
103517	BB187676	expressed sequence BB187676	1.04438642	0.4928	NA
664894	Gm13215	predicted gene 13215	1.04438642	0.5269	NA
15975	Ifnar1	interferon (alpha and beta) receptor 1	1.04438642	0.7311	0.8544
69806	Slc39a11	solute carrier family 39 (metal ion transporter), member 11	1.04427736	0.4959	NA
226118	AI606181	expressed sequence AI606181	1.04427736	0.5857	NA
50994	Mtag2	metastasis associated gene 2	1.04427736	0.6236	0.7844
67326	1700037H04Rik	RIKEN cDNA 1700037H04 gene	1.04427736	0.7918	0.891
217201	Rundc1	RUN domain containing 1	1.04416832	0.5155	NA
230757	5730409E04Rik	RIKEN cDNA 5730409E04Rik gene	1.04416832	0.6847	0.8238
17388	Mmp15	matrix metallopeptidase 15	1.04416832	0.6934	0.8302
22687	Zfp259	zinc finger protein 259	1.0440593	0.4894	NA
623174	Gm6404	predicted gene 6404	1.0440593	0.5968	NA
73166	Tm7sf2	transmembrane 7 superfamily member 2	1.0440593	0.5973	NA
74320	Wdr33	WD repeat domain 33	1.0440593	0.6227	0.7837
224796	Clic5	chloride intracellular channel 5	1.0440593	0.6644	0.8111
208846	Daam1	dishevelled associated activator of morphogenesis 1	1.0440593	0.6969	0.8325
104923	Adi1	acireductone dioxygenase 1	1.04395031	0.4147	NA
108735	Sft2d2	SFT2 domain containing 2	1.04395031	0.4293	NA
243867	Fbxo46	F-box protein 46	1.04395031	0.5258	NA

53817	Bat1a	HLA-B-associated transcript 1A	1.04395031	0.5637	NA
56445	Dnaja2	DnaJ (Hsp40) homolog, subfamily A, member 2	1.04395031	0.5728	NA
21894	Tln1	talin 1	1.04395031	0.7229	0.849
18193	Nsd1	nuclear receptor-binding SET-domain protein 1	1.04395031	0.7842	0.8869
432767	Gm5450	predicted gene 5450	1.04384134	0.4442	NA
627908	LOC627908	hypothetical LOC627908	1.04384134	0.4556	NA
242291	Impad1	inositol monophosphatase domain containing 1	1.04384134	0.5652	NA
94245	Dtnbp1	dystrobrevin binding protein 1	1.04384134	0.6173	0.7797
73750	Whrn	whirlin	1.04384134	0.6729	0.8169
18951	Sep-05	septin 5	1.04384134	0.741	0.8599
28199	Dcaf11	DDB1 and CUL4 associated factor 11	1.04373239	0.3328	NA
69672	Txndc15	thioredoxin domain containing 15	1.04373239	0.3881	NA
67869	Paip2	polyadenylate-binding protein-interacting protein 2	1.04373239	0.4394	NA
50797	Copb2	coatomer protein complex, subunit beta 2 (beta prime)	1.04373239	0.5469	NA
14408	Gabrr1	gamma-aminobutyric acid (GABA) C receptor, subunit rho 1	1.04373239	0.5783	NA
233863	Gtf3c1	general transcription factor III C 1	1.04373239	0.6464	0.8003
67773	Myst1	MYST histone acetyltransferase 1	1.04362346	0.3753	NA
547267	Gm6030	predicted gene 6030	1.04362346	0.5204	NA
18798	Plcb4	phospholipase C, beta 4	1.04362346	0.5892	NA
109168	Atl3	atlastin GTPase 3	1.04362346	0.6652	0.8114
235416	Lman1l	lectin, mannose-binding 1 like	1.04362346	0.7101	0.8408
12928	Crk	v-crk sarcoma virus CT10 oncogene homolog (avian)	1.04362346	0.7179	0.8459
12390	Cav2	caveolin 2	1.04362346	0.7272	0.8516
19347	Dennd5a	DENN/MADD domain containing 5A	1.04351456	0.5104	NA
20354	Sema4d	sema domain, immunoglobulin domain (Ig), transmembrane domain (TM) and short cytoplasmic domain, (semaphorin) 4D	1.04351456	0.5683	NA
11431	Acp1	acid phosphatase 1, soluble	1.04351456	0.6427	0.7978
20499	Slc12a7	solute carrier family 12, member 7	1.04351456	0.7081	0.8395
57316	C1d	C1D nuclear receptor co-repressor	1.04351456	0.7858	0.8877
76709	Arpc2	actin related protein 2/3 complex, subunit 2	1.04340568	0.3604	NA
433813	Pusl1	pseudouridylate synthase-like 1	1.04340568	0.4322	NA
13389	Dll3	delta-like 3 (Drosophila)	1.04340568	0.6138	NA
195209	Gm22	predicted gene 22	1.04340568	0.7755	0.8806
69038	1810006K21Rik	RIKEN cDNA 1810006K21 gene	1.04329682	0.3898	NA
19989	Rpl7	ribosomal protein L7	1.04329682	0.4149	NA
94315	Prcc	papillary renal cell carcinoma (translocation-associated)	1.04329682	0.6152	NA
72999	Insig2	insulin induced gene 2	1.04329682	0.7684	0.8772
15510	Hspd1	heat shock protein 1 (chaperonin)	1.04318798	0.4153	NA
52840	Dbndd2	dysbindin (dystrobrevin binding protein 1) domain containing 2	1.04318798	0.4743	NA
14356	Fxc1	fractured callus expressed transcript 1	1.04318798	0.4936	NA
668253	Dleu2	deleted in lymphocytic leukemia, 2	1.04307917	0.3538	NA
212090	Tmem60	transmembrane protein 60	1.04307917	0.5231	NA
224105	Pak2	p21 protein (Cdc42/Rac)-activated kinase 2	1.04307917	0.5436	NA
66680	3230401D17Rik	RIKEN cDNA 3230401D17 gene	1.04297038	0.3615	NA
67203	Nde1	nuclear distribution gene E homolog 1 (A nidulans)	1.04297038	0.4594	NA
106489	Sft2d1	SFT2 domain containing 1	1.04297038	0.6214	0.7826
20510	Slc1a1	solute carrier family 1 (neuronal/epithelial high affinity glutamate transporter, system Xag), member 1	1.04297038	0.7681	0.877
623483	LOC623483	60S ribosomal protein L22-like	1.04286161	0.4668	NA
52123	Agpat5	1-acylglycerol-3-phosphate O-acyltransferase 5 (lysophosphatidic acid acyltransferase, epsilon)	1.04286161	0.5504	NA
210529	Mettl14	methyltransferase like 14	1.04286161	0.5766	NA
239833	Lmln	leishmanolysin-like (metallopeptidase M8 family)	1.04286161	0.5779	NA

11807	Apoa2	apolipoprotein A-II	1.04286161	0.7731	0.8791
234684	Lrrc29	leucine rich repeat containing 29	1.04286161	0.788	0.8889
226823	Kctd3	potassium channel tetramerisation domain containing 3	1.04275287	0.3765	NA
381582	Gm5151	predicted gene 5151	1.04275287	0.4702	NA
18845	Plxna2	plexin A2	1.04275287	0.4883	NA
52838	Dnlz	DNL-type zinc finger	1.04264415	0.5672	NA
18604	Pdk2	pyruvate dehydrogenase kinase, isoenzyme 2	1.04264415	0.7977	0.8936
66548	Adamtsl5	ADAMTS-like 5	1.04264415	0.8561	0.9256
16450	Jag2	jagged 2	1.04253545	0.5548	NA
321006	Vprbp	Vpr (HIV-1) binding protein	1.04253545	0.6284	0.7885
243963	Zfp473	zinc finger protein 473	1.04242677	0.5253	NA
66404	2410001C21Rik	RIKEN cDNA 2410001C21 gene	1.04242677	0.6122	NA
20203	S100b	S100 protein, beta polypeptide, neural	1.04242677	0.6146	NA
67030	Fancl	Fanconi anemia, complementation group L	1.04242677	0.6598	0.8085
12752	Cln3	ceroid lipofuscinosis, neuronal 3, juvenile (Batten, Spielmeyer-Vogt disease)	1.04242677	0.6644	0.8111
330171	Kctd10	potassium channel tetramerisation domain containing 10	1.04231812	0.3956	NA
72008	Zfyve19	zinc finger, FYVE domain containing 19	1.04231812	0.464	NA
233826	Palb2	partner and localizer of BRCA2	1.04231812	0.5916	NA
19353	Rac1	RAS-related C3 botulinum substrate 1	1.04231812	0.6333	0.7916
100504710	LOC100504710	camello-like 3-like	1.04231812	0.8212	0.9077
72931	2900010J23Rik	RIKEN cDNA 2900010J23 gene	1.04220948	0.3937	NA
77605	H2afv	H2A histone family, member V	1.04220948	0.4868	NA
66853	Pnpla2	patatin-like phospholipase domain containing 2	1.04220948	0.7025	0.8356
18518	Igbp1	immunoglobulin (CD79A) binding protein 1	1.04210088	0.4118	NA
17692	Msl3	male-specific lethal 3 homolog (Drosophila)	1.04199229	0.4624	NA
66489	Rpl35	ribosomal protein L35	1.04199229	0.5575	NA
320858	L3mbtl4	l(3)mbt-like 4 (Drosophila)	1.04199229	0.5978	NA
70552	Lrrc56	leucine rich repeat containing 56	1.04199229	0.6246	NA
67736	Ccdc130	coiled-coil domain containing 130	1.04199229	0.6888	0.827
237300	Gm4922	predicted gene 4922	1.04199229	0.7421	0.8607
67345	Herc4	hect domain and RLD 4	1.04188373	0.4377	NA
226551	AI848100	expressed sequence AI848100	1.04188373	0.5069	NA
116871	Mta3	metastasis associated 3	1.04188373	0.6703	0.8151
100039128	LOC100039128	nuclear pore complex protein Nup93-like	1.04188373	0.7626	0.8738
670211	Gm12508	predicted gene 12508	1.04177518	0.388	NA
229906	Gtf2b	general transcription factor IIB	1.04177518	0.414	NA
22428	Dctn6	dynactin 6	1.04177518	0.428	NA
53331	Stx7	syntaxin 7	1.04177518	0.4873	NA
69740	Dph5	DPH5 homolog (S. cerevisiae)	1.04177518	0.489	NA
13418	Dnajc1	DnaJ (Hsp40) homolog, subfamily C, member 1	1.04166667	0.5777	NA
217125	Samd14	sterile alpha motif domain containing 14	1.04166667	0.6521	0.8037
24083	Gm16515	predicted gene, Gm16515	1.04155817	0.3554	NA
20042	Rps12	ribosomal protein S12	1.04155817	0.591	NA
226122	Ubtd1	ubiquitin domain containing 1	1.04155817	0.6752	0.8183
66541	Immp1l	IMP1 inner mitochondrial membrane peptidase-like (S. cerevisiae)	1.04155817	0.8291	0.9116
54195	Gucy1b3	guanylate cyclase 1, soluble, beta 3	1.0414497	0.3824	NA
101113	Snx21	sorting nexin family member 21	1.0414497	0.4945	NA
218772	Rarb	retinoic acid receptor, beta	1.0414497	0.5759	NA
625054	Gm6548	eukaryotic translation elongation factor 1 alpha 1 pseudogene	1.0414497	0.6384	0.7952
54723	Tfip11	tuftelin interacting protein 11	1.0414497	0.6643	0.8111

71891	Cdadc1	cytidine and dCMP deaminase domain containing 1	1.04134125	0.3485	NA
69221	2410006H16Rik	RIKEN cDNA 2410006H16 gene	1.04134125	0.5034	NA
105352	Dusp22	dual specificity phosphatase 22	1.04134125	0.6043	NA
268780	Egflam	EGF-like, fibronectin type III and laminin G domains	1.04134125	0.6333	0.7916
56044	Rala	v-ral simian leukemia viral oncogene homolog A (ras related)	1.04134125	0.6416	0.7973
319618	Dcp1b	DCP1 decapping enzyme homolog b (S. cerevisiae)	1.04123282	0.3239	NA
432572	Specc1	sperm antigen with calponin homology and coiled-coil domains 1	1.04123282	0.5393	NA
11303	Abca1	ATP-binding cassette, sub-family A (ABC1), member 1	1.04123282	0.5839	NA
74098	0610037L13Rik	RIKEN cDNA 0610037L13 gene	1.04123282	0.6053	NA
102570	Slc22a13	solute carrier family 22 (organic cation transporter), member 13	1.04123282	0.641	0.7969
12054	Bcl7b	B-cell CLL/lymphoma 7B	1.04123282	0.7743	0.8801
66144	Atp6v1f	ATPase, H+ transporting, lysosomal V1 subunit F	1.04112441	0.4066	NA
232164	Paip2b	poly(A) binding protein interacting protein 2B	1.04112441	0.4456	NA
70382	Kctd2	potassium channel tetramerisation domain containing 2	1.04112441	0.4543	NA
243937	Zfp536	zinc finger protein 536	1.04112441	0.5057	NA
12398	Cbfa2t3	core-binding factor, runt domain, alpha subunit 2, translocated to, 3 (human)	1.04112441	0.5058	NA
53424	Tsnax	translin-associated factor X	1.04112441	0.5283	NA
66165	Bccip	BRCA2 and CDKN1A interacting protein	1.04101603	0.5355	NA
20322	Sord	sorbitol dehydrogenase	1.04101603	0.543	NA
50760	Fbxo17	F-box protein 17	1.04101603	0.7279	0.8519
16704	Krtap8-2	keratin associated protein 8-2	1.04101603	0.749	0.8649
58520	0610007P14Rik	RIKEN cDNA 0610007P14 gene	1.04090767	0.2991	NA
71989	Rpusd4	RNA pseudouridylate synthase domain containing 4	1.04090767	0.4377	NA
67210	Gatad1	GATA zinc finger domain containing 1	1.04090767	0.4598	NA
217371	Rab40b	Rab40b, member RAS oncogene family	1.04090767	0.4916	NA
16201	Ilf3	interleukin enhancer binding factor 3	1.04090767	0.542	NA
23945	Mgll	monoglyceride lipase	1.04090767	0.6089	NA
245578	Pcdh11x	protocadherin 11 X-linked	1.04090767	0.6168	NA
108767	Pnrc1	proline-rich nuclear receptor coactivator 1	1.04090767	0.6558	0.8058
212508	Mtg1	mitochondrial GTPase 1 homolog (S. cerevisiae)	1.04079933	0.3801	NA
69747	Zswim7	zinc finger, SWIM-type containing 7	1.04079933	0.4369	NA
26396	Map2k2	mitogen-activated protein kinase kinase 2	1.04079933	0.4806	NA
104570	Smek2	SMEK homolog 2, suppressor of mek1 (Dictyostelium)	1.04079933	0.4947	NA
56386	B4galt6	UDP-Gal:betaGlcNAc beta 1,4-galactosyltransferase, polypeptide 6	1.04079933	0.5421	NA
118454	Gjc2	gap junction protein, gamma 2	1.04079933	0.5457	NA
66847	Hint3	histidine triad nucleotide binding protein 3	1.04079933	0.674	0.8174
70031	Cmtm8	CKLF-like MARVEL transmembrane domain containing 8	1.04079933	0.676	0.8184
19062	Inpp5k	inositol polyphosphate 5-phosphatase K	1.04079933	0.7003	0.8338
103724	Tbc1d10a	TBC1 domain family, member 10a	1.04069102	0.4425	NA
69568	Vkorc1l1	vitamin K epoxide reductase complex, subunit 1-like 1	1.04069102	0.4889	NA
56517	Slc22a21	solute carrier family 22 (organic cation transporter), member 21	1.04069102	0.5508	NA
432770	Rslcan18	regulator of sex-limitation candidate 18	1.04069102	0.5831	NA
77038	Arfgap2	ADP-ribosylation factor GTPase activating protein 2	1.04069102	0.6164	NA
69202	Ptms	parathymosin	1.04069102	0.7365	0.8575
20249	Scd1	stearoyl-Coenzyme A desaturase 1	1.04069102	0.7454	0.8627
20815	Srpk1	serine/arginine-rich protein specific kinase 1	1.04058273	0.4649	NA
66844	Ormdl2	ORM1-like 2 (S. cerevisiae)	1.04058273	0.5639	NA
380732	Gm885	predicted gene 885	1.04058273	0.5933	NA
106529	Tecr	trans-2,3-enoyl-CoA reductase	1.04058273	0.7498	0.8654
11865	Arntl	aryl hydrocarbon receptor nuclear translocator-like	1.04047446	0.294	NA

66105	Ube2d3	ubiquitin-conjugating enzyme E2D 3 (UBC4/5 homolog, yeast)	1.04047446	0.3335	NA	
69136	Tusc1	tumor suppressor candidate 1	1.04047446	0.4202	NA	
57908	Zfp318	zinc finger protein 318	1.04047446	0.5745	NA	
227449	Zcchc2	zinc finger, CCHC domain containing 2	1.04047446	0.6339	NA	
210126	Lpp	LIM domain containing preferred translocation partner in lipoma	1.04047446	0.6879		0.8264
269019	Stk32a	serine/threonine kinase 32A	1.04047446	0.7012		0.8345
626578	Gbp10	guanylate-binding protein 10	1.04047446	0.792		0.8911
665306	3930402G23Rik	RIKEN cDNA 3930402G23 gene	1.04047446	0.9152		0.957
16443	Itsn1	intersectin 1 (SH3 domain protein 1A)	1.04036621	0.4977	NA	
234797	6430548M08Rik	RIKEN cDNA 6430548M08 gene	1.04036621	0.6045	NA	
72183	Snx6	sorting nexin 6	1.04036621	0.6464		0.8003
11837	Rplp0	ribosomal protein, large, P0	1.04036621	0.722		0.8483
18012	Neurod1	neurogenic differentiation 1	1.04036621	0.7277		0.8519
16660	Krt31	keratin 31	1.04036621	0.779		0.883
12165	Gdf2	growth differentiation factor 2	1.04036621	0.8169		0.9051
68695	Hddc3	HD domain containing 3	1.04025798	0.406	NA	
330902	4930565O14	hypothetical protein 4930565O14	1.04025798	0.441	NA	
77559	Agl	amylo-1,6-glucosidase, 4-alpha-glucanotransferase	1.04025798	0.497	NA	
244913	Gm4978	ribosomal protein L7A pseudogene	1.04025798	0.5893	NA	
93728	Pabpc5	poly(A) binding protein, cytoplasmic 5	1.04025798	0.6423		0.7978
73293	Ccdc103	coiled-coil domain containing 103	1.04025798	0.6731		0.817
237400	Mex3d	mex3 homolog D (C. elegans)	1.04014978	0.5247	NA	
16593	Klc1	kinesin light chain 1	1.04014978	0.6124	NA	
22070	Tpt1	tumor protein, translationally-controlled 1	1.04014978	0.6217	NA	
104303	Arl1	ADP-ribosylation factor-like 1	1.04014978	0.6366	NA	
218294	Cdc14b	CDC14 cell division cycle 14 homolog B (S. cerevisiae)	1.0400416	0.5298	NA	
13626	Eed	embryonic ectoderm development	1.0400416	0.5442	NA	
55943	Stx8	syntaxin 8	1.0400416	0.5542	NA	
77095	D330022H12Rik	RIKEN cDNA D330022H12 gene	1.0400416	0.6125	NA	
27373	Csnk1e	casein kinase 1, epsilon	1.0400416	0.6182	NA	
72341	2610002I17Rik	RIKEN cDNA 2610002I17	1.0400416	0.6257	NA	
320014	B930025P03Rik	RIKEN cDNA B930025P03 gene	1.0400416	0.6385	NA	
170758	Rac3	RAS-related C3 botulinum substrate 3	1.0400416	0.6503		0.8031
16815	Lbx2	ladybird homeobox homolog 2 (Drosophila)	1.0400416	0.7719		0.8787
69961	2810432D09Rik	RIKEN cDNA 2810432D09 gene	1.03993344	0.393	NA	
245688	Rbbp7	retinoblastoma binding protein 7	1.03993344	0.5038	NA	
217038	Mrm1	mitochondrial rRNA methyltransferase 1 homolog (S. cerevisiae)	1.03993344	0.5338	NA	
66412	Arrdc4	arrestin domain containing 4	1.03993344	0.5717	NA	
228019	Mettl8	methyltransferase like 8	1.03993344	0.6003	NA	
22213	Ube2g2	ubiquitin-conjugating enzyme E2G 2	1.03993344	0.7373		0.8578
242316	Gdf6	growth differentiation factor 6	1.03993344	0.7405		0.8596
109660	Ctrl	chymotrypsin-like	1.03993344	0.7662		0.8758
77975	Tmem50b	transmembrane protein 50B	1.03982531	0.384	NA	
14547	Gdap2	ganglioside-induced differentiation-associated-protein 2	1.03982531	0.6116	NA	
80884	Maged2	melanoma antigen, family D, 2	1.03982531	0.6815		0.8221
217218	Atxn7l3	ataxin 7-like 3	1.03982531	0.7176		0.8458
71897	Lypd6b	LY6/PLAUR domain containing 6B	1.03982531	0.7701		0.8781
77087	Ankrd11	ankyrin repeat domain 11	1.0397172	0.3273	NA	
20909	Stx4a	syntaxin 4A (placental)	1.0397172	0.4078	NA	
66506	Psmg3	proteasome (prosome, macropain) assembly chaperone 3	1.0397172	0.4535	NA	

68137	Kdelr1	KDEL (Lys-Asp-Glu-Leu) endoplasmic reticulum protein retention receptor 1	1.0397172	0.6039	NA
232853	Zfp954	zinc finger protein 954	1.0397172	0.618	NA
78521	B230219D22Rik	RIKEN cDNA B230219D22 gene	1.0397172	0.7091	0.84
78926	Gas2l1	growth arrest-specific 2 like 1	1.0397172	0.8603	0.9278
12161	Bmp6	bone morphogenetic protein 6	1.0397172	0.9001	0.9492
26425	Nubp1	nucleotide binding protein 1	1.03960911	0.3631	NA
101706	Numa1	nuclear mitotic apparatus protein 1	1.03960911	0.5302	NA
269831	Tspan12	tetraspanin 12	1.03960911	0.5721	NA
29869	Ulk2	Unc-51 like kinase 2 (C. elegans)	1.03960911	0.5776	NA
225743	Rnf165	ring finger protein 165	1.03960911	0.6303	NA
382073	Ccdc84	coiled-coil domain containing 84	1.03960911	0.707	0.8386
627967	Gm6816	predicted gene 6816	1.03960911	0.7369	0.8576
18844	Plxna1	plexin A1	1.03960911	0.8312	0.9126
19988	Rpl6	ribosomal protein L6	1.03950104	0.3895	NA
53859	Map3k14	mitogen-activated protein kinase kinase kinase 14	1.03950104	0.3945	NA
66839	0610009O20Rik	RIKEN cDNA 0610009O20 gene	1.03950104	0.4309	NA
73830	Eif3k	eukaryotic translation initiation factor 3, subunit K	1.03950104	0.4528	NA
19383	Raly	hnRNP-associated with lethal yellow	1.03950104	0.4996	NA
12282	Hyou1	hypoxia up-regulated 1	1.03950104	0.6967	0.8325
54218	B3galt4	UDP-Gal:betaGlcNAc beta 1,3-galactosyltransferase, polypeptide 4	1.03950104	0.7199	0.8469
627626	3110082D06Rik	RIKEN cDNA 3110082D06 gene	1.03950104	0.8723	0.9344
19025	Ctsa	cathepsin A	1.03939299	0.3121	NA
54405	Ndufa1	NADH dehydrogenase (ubiquinone) 1 alpha subcomplex, 1	1.03939299	0.4521	NA
67067	Romo1	reactive oxygen species modulator 1	1.03939299	0.5002	NA
12462	Cct3	chaperonin containing Tcp1, subunit 3 (gamma)	1.03939299	0.5509	NA
17216	Mcm2	minichromosome maintenance deficient 2 mitotin (S. cerevisiae)	1.03939299	0.6876	0.8262
214601	Slc10a3	solute carrier family 10 (sodium/bile acid cotransporter family), member 3	1.03928497	0.5121	NA
72727	B3gat3	beta-1,3-glucuronyltransferase 3 (glucuronosyltransferase I)	1.03928497	0.5462	NA
20872	Stk16	serine/threonine kinase 16	1.03928497	0.673	0.817
13819	Epas1	endothelial PAS domain protein 1	1.03928497	0.6975	0.8327
100040632	AA684185	expressed sequence AA684185	1.03928497	0.7228	0.849
12359	Cat	catalase	1.03928497	0.7618	0.8734
224405	Cyyr1	cysteine and tyrosine-rich protein 1	1.03928497	0.8577	0.9267
619883	Gm6109	predicted gene 6109	1.03917697	0.4477	NA
28081	D11Wsu99e	DNA segment, Chr 11, Wayne State University 99, expressed	1.03917697	0.4694	NA
19935	Mrpl23	mitochondrial ribosomal protein L23	1.03917697	0.5074	NA
71296	4933436C20Rik	RIKEN cDNA 4933436C20 gene	1.03917697	0.7358	0.8572
72096	Mettl10	methyltransferase like 10	1.03906899	0.4038	NA
57315	Wdr46	WD repeat domain 46	1.03906899	0.4375	NA
75758	9130401M01Rik	RIKEN cDNA 9130401M01 gene	1.03906899	0.4404	NA
19377	Rai1	retinoic acid induced 1	1.03906899	0.4783	NA
67096	Mmachc	methylmalonic aciduria cblC type, with homocystinuria	1.03906899	0.5101	NA
94178	Mcoln1	mucolipin 1	1.03906899	0.5182	NA
19183	Psmc3ip	proteasome (prosome, macropain) 26S subunit, ATPase 3, interacting protein	1.03906899	0.5868	NA
19364	Rad51l3	RAD51-like 3 (S. cerevisiae)	1.03906899	0.6459	NA
66999	Med28	mediator of RNA polymerase II transcription, subunit 28 homolog (yeast)	1.03896104	0.3059	NA
65099	Irak1bp1	interleukin-1 receptor-associated kinase 1 binding protein 1	1.03896104	0.3578	NA
68606	Ppm1f	protein phosphatase 1F (PP2C domain containing)	1.03896104	0.4957	NA
328243	E230012P03	hypothetical protein E230012P03	1.03896104	0.5992	NA
26611	Rcn2	reticulocalbin 2	1.03896104	0.6048	NA

11737	Anp32a	acidic (leucine-rich) nuclear phosphoprotein 32 family, member A	1.03896104	0.6521	NA
75985	Rab30	RAB30, member RAS oncogene family	1.03896104	0.7411	0.8599
18550	Furin	furin (paired basic amino acid cleaving enzyme)	1.03885311	0.568	NA
74237	Tubgcp2	tubulin, gamma complex associated protein 2	1.03885311	0.6464	NA
66480	Rpl15	ribosomal protein L15	1.03885311	0.6845	0.8237
69627	Fam89a	family with sequence similarity 89, member A	1.0387452	0.4929	NA
14885	Gtf2h4	general transcription factor II H, polypeptide 4	1.0387452	0.5767	NA
232910	Ap2s1	adaptor-related protein complex 2, sigma 1 subunit	1.0387452	0.6048	NA
83602	Gtf2a1	general transcription factor II A, 1	1.0387452	0.6605	0.8088
77034	2510039O18Rik	RIKEN cDNA 2510039O18 gene	1.03863731	0.4203	NA
114301	Palmd	palmdelphin	1.03863731	0.6035	NA
27418	Mkln1	muskelin 1, intracellular mediator containing kelch motifs	1.03863731	0.6785	0.8202
15566	Htr7	5-hydroxytryptamine (serotonin) receptor 7	1.03863731	0.7309	0.8543
622645	Tmem200c	transmembrane protein 200C	1.03863731	0.7461	0.8632
103149	Upb1	ureidopropionase, beta	1.03863731	0.787	0.8885
56433	Vps29	vacuolar protein sorting 29 (S. pombe)	1.0384216	0.3356	NA
66845	Mrpl33	mitochondrial ribosomal protein L33	1.0384216	0.4277	NA
54484	Mkrn1	makorin, ring finger protein, 1	1.0384216	0.4363	NA
268822	Adck5	aarF domain containing kinase 5	1.0384216	0.4846	NA
230917	Tmem201	transmembrane protein 201	1.0384216	0.604	NA
225058	Gm4832	predicted gene 4832	1.0384216	0.7157	0.8445
237221	Gemin8	gem (nuclear organelle) associated protein 8	1.0384216	0.78	0.8837
66525	Timm50	translocase of inner mitochondrial membrane 50 homolog (yeast)	1.03831378	0.5348	NA
26931	Ppp2r5c	protein phosphatase 2, regulatory subunit B (B56), gamma isoform	1.03831378	0.5996	NA
20955	Vamp7	vesicle-associated membrane protein 7	1.03831378	0.6008	NA
17444	Grap2	GRB2-related adaptor protein 2	1.03831378	0.6253	NA
100042583	Gm10268	predicted gene 10268	1.03831378	0.6383	NA
66832	Rsph3a	radial spoke 3A homolog (Chlamydomonas)	1.03831378	0.7553	0.8688
268396	Sh3pxd2b	SH3 and PX domains 2B	1.03831378	0.7655	0.8755
53869	Rab11a	RAB11a, member RAS oncogene family	1.03820598	0.482	NA
245000	Atr	ataxia telangiectasia and Rad3 related	1.03820598	0.5158	NA
67771	Arpc5	actin related protein 2/3 complex, subunit 5	1.03820598	0.5235	NA
54198	Snx3	sorting nexin 3	1.03820598	0.5424	NA
20336	Exoc4	exocyst complex component 4	1.03820598	0.5649	NA
19271	Ptprj	protein tyrosine phosphatase, receptor type, J	1.03820598	0.5957	NA
104130	Ndufb11	NADH dehydrogenase (ubiquinone) 1 beta subcomplex, 11	1.03820598	0.7088	0.8399
12879	Cys1	cystin 1	1.03820598	0.7524	0.8669
26893	Cops6	COP9 (constitutive photomorphogenic) homolog, subunit 6 (Arabidopsis thaliana)	1.0380982	0.5649	NA
80981	Arl4d	ADP-ribosylation factor-like 4D	1.0380982	0.6753	0.8183
19228	Pth1r	parathyroid hormone 1 receptor	1.0380982	0.6982	0.8328
22701	Zfp41	zinc finger protein 41	1.0380982	0.7335	0.8561
223658	Heatr7a	HEAT repeat containing 7A	1.0380982	0.7663	0.8759
230848	Zbtb40	zinc finger and BTB domain containing 40	1.03799045	0.4309	NA
56812	Dnajb2	DnaJ (Hsp40) homolog, subfamily B, member 2	1.03799045	0.4815	NA
66464	Taf12	TAF12 RNA polymerase II, TATA box binding protein (TBP)-associated factor	1.03799045	0.5634	NA
17192	Mbd3	methyl-CpG binding domain protein 3	1.03799045	0.5832	NA
230959	Ajap1	adherens junction associated protein 1	1.03799045	0.598	NA
23955	Nek4	NIMA (never in mitosis gene a)-related expressed kinase 4	1.03799045	0.6189	NA
101489	Ric8	resistance to inhibitors of cholinesterase 8 homolog (C. elegans)	1.03799045	0.6202	NA
85029	Rpph1	ribonuclease P RNA component H1	1.03799045	0.8548	0.9251

72736	Tmx1	thioredoxin-related transmembrane protein 1	1.03788272	0.4907	NA
229681	St7l	suppression of tumorigenicity 7-like	1.03788272	0.5104	NA
66270	Fam134b	family with sequence similarity 134, member B	1.03788272	0.6671	0.8125
237073	Rbm41	RNA binding motif protein 41	1.03788272	0.8063	0.8994
99730	Taf13	TAF13 RNA polymerase II, TATA box binding protein (TBP)-associated factor	1.03788272	0.8223	0.9085
69639	Exosc8	exosome component 8	1.03777501	0.5634	NA
24113	Vax2	ventral anterior homeobox containing gene 2	1.03777501	0.6694	0.8143
69034	4930579G22Rik	RIKEN cDNA 4930579G22 gene	1.03777501	0.7286	0.8524
50759	Fbxo16	F-box protein 16	1.03777501	0.8372	0.9158
18617	Rhox5	reproductive homeobox 5	1.03766732	0.6178	NA
791318	Gm10125	predicted gene 10125	1.03766732	0.7589	0.8713
100040276	Gm11595	predicted gene 11595	1.03766732	0.8644	0.9296
76987	Hdhd2	haloacid dehalogenase-like hydrolase domain containing 2	1.03766732	0.9095	0.9538
77106	Tmem181a	transmembrane protein 181A	1.03755966	0.471	NA
21843	Tial1	Tia1 cytotoxic granule-associated RNA binding protein-like 1	1.03755966	0.664	NA
53332	Mtmr1	myotubularin related protein 1	1.03755966	0.6793	0.8207
14159	Fes	feline sarcoma oncogene	1.03755966	0.6978	0.8327
23886	Gdf15	growth differentiation factor 15	1.03755966	0.713	0.8426
20609	Sstr5	somatostatin receptor 5	1.03755966	0.7515	0.8663
12915	Atf6b	activating transcription factor 6 beta	1.03755966	0.7693	0.8776
11692	Gfer	growth factor, erv1 (S. cerevisiae)-like (augmenter of liver regeneration)	1.03745202	0.4725	NA
68441	Rraga	Ras-related GTP binding A	1.03745202	0.5112	NA
70575	Gfod2	glucose-fructose oxidoreductase domain containing 2	1.03745202	0.6302	NA
50878	Stag3	stromal antigen 3	1.03745202	0.6538	NA
381922	D830044I16Rik	RIKEN cDNA D830044I16 gene	1.03745202	0.6853	0.8244
66622	Ubr7	ubiquitin protein ligase E3 component n-recognin 7 (putative)	1.0373444	0.5274	NA
269470	Wdr3	WD repeat domain 3	1.0373444	0.5578	NA
12576	Cdkn1b	cyclin-dependent kinase inhibitor 1B	1.0373444	0.5894	NA
320538	Ubn2	ubinuclein 2	1.0373444	0.6439	NA
80294	Pofut2	protein O-fucosyltransferase 2	1.0373444	0.807	0.8999
98193	Dcaf8	DDB1 and CUL4 associated factor 8	1.0372368	0.421	NA
232337	Zfp637	zinc finger protein 637	1.0372368	0.5626	NA
66053	Ppil2	peptidylprolyl isomerase (cyclophilin)-like 2	1.0372368	0.629	NA
12049	Bcl2l10	Bcl2-like 10	1.03712923	0.5707	NA
109075	Exosc4	exosome component 4	1.03712923	0.6302	NA
76850	Eif2c4	eukaryotic translation initiation factor 2C, 4	1.03712923	0.6865	0.8254
70059	Degs2	degenerative spermatocyte homolog 2 (Drosophila), lipid desaturase	1.03712923	0.6978	0.8327
68230	1700102H20Rik	RIKEN cDNA 1700102H20 gene	1.03712923	0.7358	0.8572
320496	C230076A16Rik	RIKEN cDNA C230076A16 gene	1.03712923	0.7567	0.8699
12023	Barx2	BarH-like homeobox 2	1.03712923	0.8237	0.9093
353190	Edc3	enhancer of mRNA decapping 3 homolog (S. cerevisiae)	1.03702167	0.6785	0.8202
66768	Pacrgl	PARK2 co-regulated-like	1.03691414	0.436	NA
622459	Gm12216	predicted gene 12216	1.03691414	0.6227	NA
209550	Rad51ap2	RAD51 associated protein 2	1.03691414	0.6929	0.83
105638	Dph3	DPH3 homolog (KTI11, S. cerevisiae)	1.03691414	0.7056	0.8378
27681	Snf8	SNF8, ESCRT-II complex subunit, homolog (S. cerevisiae)	1.03680664	0.512	NA
68149	Otub2	OTU domain, ubiquitin aldehyde binding 2	1.03680664	0.5391	NA
71393	Kctd6	potassium channel tetramerisation domain containing 6	1.03680664	0.5699	NA
12041	Bckdk	branched chain ketoacid dehydrogenase kinase	1.03680664	0.6835	0.8232
277854	Depdc5	DEP domain containing 5	1.03680664	0.7986	0.8943

434171	Gm5591	predicted gene 5591	1.03680664	0.8706	0.9332
319604	Fam168a	family with sequence similarity 168, member A	1.03669915	0.5779	NA
69697	2310057J16Rik	RIKEN cDNA 2310057J16 gene	1.03669915	0.6153	NA
19684	Rdx	radixin	1.03669915	0.648	NA
26441	Psma4	proteasome (prosome, macropain) subunit, alpha type 4	1.03659169	0.5844	NA
17342	Mitf	microphthalmia-associated transcription factor	1.03659169	0.7417	0.8604
20480	Clpb	ClpB caseinolytic peptidase B homolog (E. coli)	1.03659169	0.7536	0.8676
57752	Tacc2	transforming, acidic coiled-coil containing protein 2	1.03648425	0.3644	NA
68559	Pdrg1	p53 and DNA damage regulated 1	1.03648425	0.4583	NA
69109	Fam58b	family with sequence similarity 58, member B	1.03648425	0.4961	NA
17979	Ncoa3	nuclear receptor coactivator 3	1.03648425	0.6346	NA
57813	Tk2	thymidine kinase 2, mitochondrial	1.03648425	0.6396	NA
240186	Zfp438	zinc finger protein 438	1.03648425	0.7528	0.8672
215449	Rap1b	RAS related protein 1b	1.03637683	0.538	NA
70266	Ccbl1	cysteine conjugate-beta lyase 1	1.03637683	0.6841	0.8235
545156	Kalrn	kalirin, RhoGEF kinase	1.03637683	0.7779	0.8824
78250	Iqch	IQ motif containing H	1.03637683	0.8105	0.9016
89867	Sec16b	SEC16 homolog B (S. cerevisiae)	1.03626943	0.5463	NA
66167	Ccdc72	coiled-coil domain containing 72	1.03626943	0.5557	NA
77697	Mmab	methylmalonic aciduria (cobalamin deficiency) type B homolog (human)	1.03626943	0.6759	0.8184
14654	Glra1	glycine receptor, alpha 1 subunit	1.03626943	0.7237	0.8494
56788	Scube2	signal peptide, CUB domain, EGF-like 2	1.03626943	0.8402	0.9175
100233208	Gm10778	predicted gene 10778	1.03616206	0.5745	NA
73490	Mipol1	mirror-image polydactyly gene 1 homolog (human)	1.03616206	0.6458	NA
170788	Crb1	crumbs homolog 1 (Drosophila)	1.03616206	0.7459	0.8631
380967	Tmem106c	transmembrane protein 106C	1.03616206	0.7603	0.8723
71795	Pitpnc1	phosphatidylinositol transfer protein, cytoplasmic 1	1.03616206	0.8104	0.9016
12417	Cbx3	chromobox homolog 3 (Drosophila HP1 gamma)	1.0360547	0.3794	NA
22213	Ube2g2	ubiquitin-conjugating enzyme E2G 2	1.0360547	0.4918	NA
68262	Agpat4	1-acylglycerol-3-phosphate O-acyltransferase 4 (lysophosphatidic acid acyltransferase, delta)	1.0360547	0.4973	NA
66496	Ppdpf	pancreatic progenitor cell differentiation and proliferation factor homolog (zebrafish)RIKEN cDNA 2700038C09 gene	1.0360547	0.5935	NA
71785	Pdgfd	platelet-derived growth factor, D polypeptide	1.0360547	0.6356	NA
67905	Ppm1m	protein phosphatase 1M	1.0360547	0.6662	NA
64652	Nisch	nischarin	1.03594737	0.5458	NA
69257	Elf2	E74-like factor 2	1.03594737	0.5654	NA
69072	Ebna1bp2	EBNA1 binding protein 2	1.03594737	0.6684	NA
20068	Rps17	ribosomal protein S17	1.03584007	0.481	NA
70533	Btf3l4	basic transcription factor 3-like 4	1.03584007	0.5095	NA
17973	Nck1	non-catalytic region of tyrosine kinase adaptor protein 1	1.03584007	0.5265	NA
52468	Ctdsp2	CTD (carboxy-terminal domain, RNA polymerase II, polypeptide A) small phosphatase 2	1.03584007	0.6865	0.8254
53607	Snrpa	small nuclear ribonucleoprotein polypeptide A	1.03584007	0.753	0.8673
75430	3200002M19Rik	RIKEN cDNA 3200002M19 gene	1.03584007	0.7596	0.8718
65246	Xpo7	exportin 7	1.03573278	0.4396	NA
107652	Uap1	UDP-N-acetylglucosamine pyrophosphorylase 1	1.03573278	0.4508	NA
56438	Rbx1	ring-box 1	1.03573278	0.4533	NA
56299	Fkbpl	FK506 binding protein-like	1.03573278	0.513	NA
24045	Scamp3	secretory carrier membrane protein 3	1.03573278	0.5939	NA
269881	Map3k10	mitogen-activated protein kinase kinase kinase 10	1.03573278	0.6734	NA
235441	Usp3	ubiquitin specific peptidase 3	1.03573278	0.7181	0.8459
24131	Ldb3	LIM domain binding 3	1.03573278	0.7952	0.8926

269514	Fbxl4	F-box and leucine-rich repeat protein 4	1.03562552	0.5791	NA
73533	1700080G18Rik	RIKEN cDNA 1700080G18 gene	1.03562552	0.6395	NA
15289	Hmgb1	high mobility group box 1	1.03562552	0.6837	0.8233
66679	Rae1	RAE1 RNA export 1 homolog (S. pombe)	1.03562552	0.6882	0.8266
67333	Stk35	serine/threonine kinase 35	1.03562552	0.7041	0.8367
19347	Dennd5a	DENN/MADD domain containing 5A	1.03551828	0.4963	NA
77675	5033406O09Rik	RIKEN cDNA 5033406O09 gene	1.03551828	0.7024	0.8356
383815	Rps24-ps2	ribosomal protein S24, pseudogene 2	1.03551828	0.7067	0.8384
381379	Med19	mediator of RNA polymerase II transcription, subunit 19 homolog (yeast)	1.03541106	0.5831	NA
269997	Zfp747	zinc finger protein 747	1.03541106	0.5912	NA
380918	Siah3	seven in absentia homolog 3 (Drosophila)	1.03541106	0.6011	NA
107734	Mrpl30	mitochondrial ribosomal protein L30	1.03541106	0.6398	NA
171508	Creld1	cysteine-rich with EGF-like domains 1	1.03541106	0.6882	0.8266
225876	Kdm2a	lysine (K)-specific demethylase 2A	1.03541106	0.6978	0.8327
213350	Pddc1	Parkinson disease 7 domain containing 1	1.03541106	0.7979	0.8936
69192	Dhx16	DEAH (Asp-Glu-Ala-His) box polypeptide 16	1.03530386	0.4053	NA
67126	Atp5e	ATP synthase, H+ transporting, mitochondrial F1 complex, epsilon subunit	1.03530386	0.5544	NA
14661	Glud1	glutamate dehydrogenase 1	1.03530386	0.656	NA
76497	Ppp1r11	protein phosphatase 1, regulatory (inhibitor) subunit 11	1.03530386	0.7291	0.8529
12518	Cd79a	CD79A antigen (immunoglobulin-associated alpha)	1.03530386	0.7732	0.8791
54613	St3gal6	ST3 beta-galactoside alpha-2,3-sialyltransferase 6	1.03519669	0.7383	0.8582
66836	Tmem223	transmembrane protein 223	1.03508954	0.3777	NA
20733	Spint2	serine protease inhibitor, Kunitz type 2	1.03508954	0.6574	NA
17134	Mafg	v-maf musculoaponeurotic fibrosarcoma oncogene family, protein G (avian)	1.03508954	0.6935	0.8302
666445	Gm13035	predicted gene 13035	1.03498241	0.4991	NA
228012	Tlk1	tousled-like kinase 1	1.03498241	0.5322	NA
12057	Opn1sw	opsin 1 (cone pigments), short-wave-sensitive (color blindness, tritan)	1.03498241	0.6014	NA
69654	Dctn2	dynactin 2	1.03498241	0.6328	NA
19726	Rfx3	regulatory factor X, 3 (influences HLA class II expression)	1.03498241	0.715	0.844
57321	Terf2ip	telomeric repeat binding factor 2, interacting protein	1.03498241	0.8084	0.9006
50873	Park2	Parkinson disease (autosomal recessive, juvenile) 2, parkin	1.03498241	0.8121	0.9023
230908	Tardbp	TAR DNA binding protein	1.0348753	0.4425	NA
68401	G6pc3	glucose 6 phosphatase, catalytic, 3	1.0348753	0.6367	NA
68910	Zfp467	zinc finger protein 467	1.0348753	0.6955	0.8317
224143	Poglut1	protein O-glucosyltransferase 1	1.0348753	0.7306	0.8541
327749	Gm5079	predicted gene 5079	1.0348753	0.7896	0.8901
14950	H13	histocompatibility 13	1.03476821	0.567	NA
67980	Gnpda2	glucosamine-6-phosphate deaminase 2	1.03476821	0.5802	NA
27398	Mrpl2	mitochondrial ribosomal protein L2	1.03476821	0.5992	NA
68092	Ncbp2	nuclear cap binding protein subunit 2	1.03476821	0.7317	0.8549
19047	Ppp1cc	protein phosphatase 1, catalytic subunit, gamma isoform	1.03466115	0.565	NA
270096	Mon1b	MON1 homolog b (yeast)	1.03466115	0.568	NA
20187	Ryk	receptor-like tyrosine kinase	1.03466115	0.5787	NA
234700	Nrn1l	neuritin 1-like	1.03466115	0.6307	NA
71069	Stox2	storkhead box 2	1.03466115	0.6412	NA
319755	6330566A10Rik	RIKEN cDNA 6330566A10 gene	1.03466115	0.739	0.8588
241556	Tspan18	tetraspanin 18	1.03466115	0.747	0.8637
629557	Gm6981	glyceraldehyde-3-phosphate dehydrogenase pseudogene	1.03466115	0.8268	0.9104
67063	2810432L12Rik	RIKEN cDNA 2810432L12 gene	1.03455411	0.5593	NA
107951	Cdk9	cyclin-dependent kinase 9 (CDC2-related kinase)	1.03455411	0.7796	0.8834

245880	Wasf3	WAS protein family, member 3	1.03455411	0.8231	0.909
22764	Zfx	zinc finger protein X-linked	1.03444709	0.3744	NA
68067	3010026O09Rik	RIKEN cDNA 3010026O09 gene	1.03444709	0.637	NA
68867	Rnf122	ring finger protein 122	1.03444709	0.6872	NA
72181	Nsun4	NOL1/NOP2/Sun domain family, member 4	1.03444709	0.7107	0.8412
19325	Rab10	RAB10, member RAS oncogene family	1.03444709	0.7188	0.8464
209630	Frmd4a	FERM domain containing 4A	1.03444709	0.7758	0.8808
664805	Skor2	SKI family transcriptional corepressor 2	1.03444709	0.8008	0.8956
50773	Nt5c	5',3'-nucleotidase, cytosolic	1.03434009	0.4764	NA
19165	Psen2	presenilin 2	1.03434009	0.5086	NA
27632	Rdbp	RD RNA-binding protein	1.03434009	0.5981	NA
113865	Vmn1r25	vomeronasal 1 receptor 25	1.03434009	0.6791	NA
28126	Nop16	NOP16 nucleolar protein homolog (yeast)	1.03434009	0.7303	0.8537
18514	Pbx1	pre B-cell leukemia transcription factor 1	1.03434009	0.782	0.8853
67338	Rffl	ring finger and FYVE like domain containing protein	1.03434009	0.7978	0.8936
66266	Eapp	E2F-associated phosphoprotein	1.03423312	0.4398	NA
66406	Sac3d1	SAC3 domain containing 1	1.03423312	0.4978	NA
319195	Rpl17	ribosomal protein L17	1.03423312	0.5008	NA
56794	Hacl1	2-hydroxyacyl-CoA lyase 1	1.03423312	0.5242	NA
26936	Mprip	myosin phosphatase Rho interacting protein	1.03423312	0.6584	NA
70005	1700029I01Rik	RIKEN cDNA 1700029I01 gene	1.03423312	0.7285	0.8524
238076	Kcns3	potassium voltage-gated channel, delayed-rectifier, subfamily S, member 3	1.03423312	0.7714	0.8785
54722	Dfna5	deafness, autosomal dominant 5 (human)	1.03412616	0.6319	NA
17152	Mak	male germ cell-associated kinase	1.03412616	0.7281	0.852
319172	Hist1h2ab	histone cluster 1, H2ab	1.03412616	0.8529	0.9244
101966	D8Ertd738e	DNA segment, Chr 8, ERATO Doi 738, expressed	1.03401923	0.4305	NA
24051	Sgcb	sarcoglycan, beta (dystrophin-associated glycoprotein)	1.03401923	0.6674	NA
216169	Fam108a	family with sequence similarity 108, member A	1.03401923	0.6814	NA
382051	Pdp2	pyruvate dehyrogenase phosphatase catalytic subunit 2	1.03401923	0.6942	0.8308
111175	Pecr	peroxisomal trans-2-enoyl-CoA reductase	1.03401923	0.7293	0.853
94066	Mrpl36	mitochondrial ribosomal protein L36	1.03391232	0.4564	NA
68539	Tmem109	transmembrane protein 109	1.03391232	0.5464	NA
57750	Wdr12	WD repeat domain 12	1.03391232	0.5786	NA
14784	Grb2	growth factor receptor bound protein 2	1.03391232	0.816	0.9047
232944	Mark4	MAP/microtubule affinity-regulating kinase 4	1.03391232	0.824	0.9093
269941	Chsy1	chondroitin sulfate synthase 1	1.03380544	0.4691	NA
64660	Mrps24	mitochondrial ribosomal protein S24	1.03380544	0.4889	NA
68501	Nsmce2	non-SMC element 2 homolog (MMS21, S. cerevisiae)	1.03380544	0.6366	NA
232078	Thnsl2	threonine synthase-like 2 (bacterial)	1.03380544	0.6653	NA
69310	Pacrg	PARK2 co-regulated	1.03380544	0.6716	NA
387314	Tmtc1	transmembrane and tetratricopeptide repeat containing 1	1.03380544	0.7597	0.8719
59050	Nsa2	NSA2 ribosome biogenesis homolog (S. cerevisiae)	1.03380544	0.7657	0.8756
402749	B430316J06Rik	RIKEN cDNA B430316J06 gene	1.03380544	0.8657	0.9304
50529	Mrps7	mitochondrial ribosomal protein S7	1.03369857	0.5595	NA
72278	Ccpg1	cell cycle progression 1	1.03369857	0.5919	NA
18674	Slc25a3	solute carrier family 25 (mitochondrial carrier, phosphate carrier), member 3	1.03369857	0.6144	NA
330369	Fbxo41	F-box protein 41	1.03369857	0.7074	0.839
11499	Adam5	a disintegrin and metallopeptidase domain 5	1.03369857	0.7266	0.8514
68644	Abhd14a	abhydrolase domain containing 14A	1.03369857	0.8049	0.8983
217558	G2e3	G2/M-phase specific E3 ubiquitin ligase	1.03369857	0.8117	0.9022

67978	Tctn2	tectonic family member 2	1.03359173	0.5326	NA
20286	Zc3h7b	zinc finger CCCH type containing 7B	1.03359173	0.5866	NA
230959	Ajap1	adherens junction associated protein 1	1.03359173	0.6355	NA
108012	Ap1s2	adaptor-related protein complex 1, sigma 2 subunit	1.03359173	0.6575	NA
109674	Ampd2	adenosine monophosphate deaminase 2	1.03359173	0.715	0.844
19216	Ptger1	prostaglandin E receptor 1 (subtype EP1)	1.03359173	0.766	0.8757
66313	Smurf2	SMAD specific E3 ubiquitin protein ligase 2	1.03359173	0.8288	0.9115
75786	Ckap5	cytoskeleton associated protein 5	1.03348491	0.4173	NA
20289	Scx	scleraxis	1.03348491	0.6181	NA
53312	Nub1	negative regulator of ubiquitin-like proteins 1	1.03348491	0.7056	0.8379
84113	Ptov1	prostate tumor over expressed gene 1	1.03348491	0.7102	0.8408
78926	Gas2l1	growth arrest-specific 2 like 1	1.03337811	0.4389	NA
216853	Wrap53	WD repeat containing, antisense to TP53	1.03337811	0.4914	NA
69731	Gemin7	gem (nuclear organelle) associated protein 7	1.03337811	0.5091	NA
233490	Crebzf	CREB/ATF bZIP transcription factor	1.03337811	0.6551	NA
71146	Golga7b	golgi autoantigen, golgin subfamily a, 7B	1.03337811	0.6589	NA
78541	Asb8	ankyrin repeat and SOCS box-containing 8	1.03337811	0.6604	NA
102566	Ano10	anoctamin 10	1.03337811	0.7214	0.8479
53885	Nphp1	nephronophthisis 1 (juvenile) homolog (human)	1.03337811	0.8542	0.925
16876	Lhx9	LIM homeobox protein 9	1.03337811	0.8585	0.9271
330173	2610524H06Rik	RIKEN cDNA 2610524H06 gene	1.03327134	0.5063	NA
58911	Sumf1	sulfatase modifying factor 1	1.03327134	0.5774	NA
77644	C330007P06Rik	RIKEN cDNA C330007P06 gene	1.03327134	0.6185	NA
100038538	Gm10767	predicted gene 10767	1.03327134	0.6603	NA
12564	Cdh8	cadherin 8	1.03327134	0.7104	0.841
74354	Lrguk	leucine-rich repeats and guanylate kinase domain containing	1.03327134	0.8743	0.9351
330863	Trim67	tripartite motif-containing 67	1.03327134	0.8883	0.9427
268996	Ss18	synovial sarcoma translocation, Chromosome 18	1.03316458	0.4441	NA
67552	H2afy3	H2A histone family, member Y3	1.03316458	0.4543	NA
17190	Mbd1	methyl-CpG binding domain protein 1	1.03316458	0.4952	NA
70316	Ndufab1	NADH dehydrogenase (ubiquinone) 1, alpha/beta subcomplex, 1	1.03316458	0.5728	NA
216028	Lrrtm3	leucine rich repeat transmembrane neuronal 3	1.03316458	0.7379	0.858
23894	Gtf2h2	general transcription factor II H, polypeptide 2	1.03305785	0.5762	NA
19185	Psmd4	proteasome (prosome, macropain) 26S subunit, non-ATPase, 4	1.03305785	0.6097	NA
100504975	LOC100504975	hypothetical protein LOC100504975	1.03305785	0.7213	0.8478
17532	Mras	muscle and microspikes RAS	1.03305785	0.7708	0.8784
73710	Tubb2b	tubulin, beta 2B	1.03305785	0.9023	0.9504
67097	Rps10	ribosomal protein S10	1.03295114	0.5005	NA
11927	Atox1	ATX1 (antioxidant protein 1) homolog 1 (yeast)	1.03295114	0.5142	NA
223693	Tmem184b	transmembrane protein 184b	1.03295114	0.5468	NA
67832	Brix1	BRX1, biogenesis of ribosomes, homolog (S. cerevisiae)	1.03295114	0.5809	NA
99426	AW742560	expressed sequence AW742560	1.03295114	0.6215	NA
192169	Ufsp2	UFM1-specific peptidase 2	1.03295114	0.6285	NA
58802	Kcnmb4	potassium large conductance calcium-activated channel, subfamily M, beta member 4	1.03295114	0.6569	NA
67511	Tmed9	transmembrane emp24 protein transport domain containing 9	1.03295114	0.662	NA
107951	Cdk9	cyclin-dependent kinase 9 (CDC2-related kinase)	1.03295114	0.6856	NA
210925	Ints9	integrator complex subunit 9	1.03295114	0.711	0.8413
14068	F7	coagulation factor VII	1.03295114	0.8452	0.92
269682	Golga3	golgi autoantigen, golgin subfamily a, 3	1.03284445	0.3961	NA
27176	Rpl7a	ribosomal protein L7A	1.03284445	0.4924	NA

268469	Zfp652	zinc finger protein 652	1.03284445	0.525	NA
15258	Hipk2	homeodomain interacting protein kinase 2	1.03284445	0.646	NA
75984	5031415H12Rik	RIKEN cDNA 5031415H12 gene	1.03284445	0.7795	0.8834
217615	Ctage5	CTAGE family, member 5	1.03273779	0.3999	NA
20104	Rps6	ribosomal protein S6	1.03273779	0.4592	NA
277396	Klhl23	kelch-like 23 (Drosophila)	1.03273779	0.5757	NA
19384	Ran	RAN, member RAS oncogene family	1.03273779	0.6447	NA
67890	Ufm1	ubiquitin-fold modifier 1	1.03273779	0.6613	NA
106931	Kctd1	potassium channel tetramerisation domain containing 1	1.03273779	0.6767	NA
71924	Tube1	epsilon-tubulin 1	1.03263114	0.4067	NA
70369	Bag5	BCL2-associated athanogene 5	1.03263114	0.5782	NA
20280	Scp2	sterol carrier protein 2, liver	1.03263114	0.6844	NA
435518	Pdxk-ps	pyridoxal (pyridoxine, vitamin B6) kinase, pseudogene	1.03263114	0.8926	0.945
56428	Mtch2	mitochondrial carrier homolog 2 (C. elegans)	1.03252452	0.5313	NA
64209	Herpud1	homocysteine-inducible, endoplasmic reticulum stress-inducible, ubiquitin-like domain member 1	1.03252452	0.6519	NA
277978	Exoc3l	exocyst complex component 3-like	1.03252452	0.7856	0.8877
107358	Tm9sf3	transmembrane 9 superfamily member 3	1.03252452	0.812	0.9023
67228	Wdr85	WD repeat domain 85	1.03241792	0.5628	NA
98732	Rab3gap2	RAB3 GTPase activating protein subunit 2	1.03241792	0.6587	NA
69090	Ascc1	activating signal cointegrator 1 complex subunit 1	1.03241792	0.8009	0.8957
68995	Mcts1	malignant T cell amplified sequence 1	1.03231135	0.6528	NA
71986	Ddx28	DEAD (Asp-Glu-Ala-Asp) box polypeptide 28	1.03231135	0.7037	NA
100040320	Gm2710	predicted gene 2710	1.03231135	0.7209	0.8475
19290	Pura	purine rich element binding protein A	1.03231135	0.7401	0.8595
26889	Cln8	ceroid-lipofuscinosis, neuronal 8	1.03220479	0.5854	NA
66420	Polr2e	polymerase (RNA) II (DNA directed) polypeptide E	1.03220479	0.6287	NA
55960	Ebag9	estrogen receptor-binding fragment-associated gene 9	1.03220479	0.6351	NA
21411	Tcf20	transcription factor 20	1.03220479	0.775	0.8802
27225	Ddx24	DEAD (Asp-Glu-Ala-Asp) box polypeptide 24	1.03209826	0.5619	NA
100038554	Gm15348	predicted gene 15348	1.03209826	0.6787	NA
14377	G6pc	glucose-6-phosphatase, catalytic	1.03209826	0.8807	0.9389
112406	Egln2	EGL nine homolog 2 (C. elegans)	1.03199174	0.5646	NA
107250	Kazald1	Kazal-type serine peptidase inhibitor domain 1	1.03199174	0.6384	NA
53333	Tomm40	translocase of outer mitochondrial membrane 40 homolog (yeast)	1.03199174	0.6645	NA
207393	Elfn2	leucine rich repeat and fibronectin type III, extracellular 2	1.03199174	0.7523	0.8669
71844	Nupl1	nucleoporin like 1	1.03199174	0.8253	0.9099
17165	Mapkapk5	MAP kinase-activated protein kinase 5	1.03188525	0.4601	NA
665268	1600029O15Rik	ribosomal protein L17 pseudogene	1.03188525	0.4632	NA
385658	Fam55c	family with sequence similarity 55, member C	1.03188525	0.6422	NA
268934	Grm4	glutamate receptor, metabotropic 4	1.03188525	0.6506	NA
20979	Syt1	synaptotagmin I	1.03188525	0.7699	0.8779
110751	Adam33	a disintegrin and metallopeptidase domain 33	1.03188525	0.8731	0.9348
68021	Bphl	biphenyl hydrolase-like (serine hydrolase, breast epithelial mucin-associated antigen)	1.03177879	0.535	NA
217198	Plekhh3	pleckstrin homology domain containing, family H (with MyTH4 domain) member 3	1.03177879	0.5878	NA
211949	Spsb4	splA/ryanodine receptor domain and SOCS box containing 4	1.03177879	0.605	NA
93742	Pard3	par-3 (partitioning defective 3) homolog (C. elegans)	1.03177879	0.6892	NA
396184	Flrt1	fibronectin leucine rich transmembrane protein 1	1.03177879	0.7087	NA
80748	BC004004	cDNA sequence BC004004	1.03177879	0.7268	0.8514
69379	C8g	complement component 8, gamma polypeptide	1.03177879	0.7714	0.8785
22408	Wnt1	wingless-related MMTV integration site 1	1.03177879	0.8201	0.9069

21983	Tpbg	trophoblast glycoprotein	1.03167234	0.7164	0.8451
791286	Gm9886	predicted gene 9886	1.03167234	0.8141	0.9038
17203	Mc5r	melanocortin 5 receptor	1.03167234	0.8339	0.9141
14194	Fh1	fumarate hydratase 1	1.03156592	0.5281	NA
20005	Rpl9	ribosomal protein L9	1.03156592	0.6699	NA
71306	Mfap3l	microfibrillar-associated protein 3-like	1.03156592	0.6872	NA
243931	Tshz3	teashirt zinc finger family member 3	1.03156592	0.6931	NA
328699	Gabrr3	gamma-aminobutyric acid (GABA) receptor, rho 3	1.03156592	0.7511	0.8662
16418	Eif6	eukaryotic translation initiation factor 6	1.03156592	0.7801	0.8837
212153	2610015P09Rik	RIKEN cDNA 2610015P09 gene	1.03145952	0.4988	NA
66104	Tceal6	transcription elongation factor A (SII)-like 6	1.03145952	0.5739	NA
193116	Slu7	SLU7 splicing factor homolog (S. cerevisiae)	1.03135314	0.5164	NA
319475	Zfp672	zinc finger protein 672	1.03135314	0.5976	NA
228139	P2rx3	purinergic receptor P2X, ligand-gated ion channel, 3	1.03135314	0.6031	NA
107823	Whsc1	Wolf-Hirschhorn syndrome candidate 1 (human)	1.03135314	0.7903	0.8901
12325	Camk2g	calcium/calmodulin-dependent protein kinase II gamma	1.03124678	0.5208	NA
72500	Ier5l	immediate early response 5-like	1.03124678	0.5252	NA
18481	Pak3	p21 protein (Cdc42/Rac)-activated kinase 3	1.03124678	0.5418	NA
13495	Drg2	developmentally regulated GTP binding protein 2	1.03124678	0.7154	NA
18032	Nfix	nuclear factor I/X	1.03124678	0.8458	0.9203
100039317	Gm2155	predicted gene 2155	1.03124678	0.8623	0.9289
223745	Gm4825	predicted pseudogene 4825	1.03114044	0.4623	NA
54451	Cpsf3	cleavage and polyadenylation specificity factor 3	1.03114044	0.5167	NA
29864	Rnf11	ring finger protein 11	1.03114044	0.6349	NA
54473	Tollip	toll interacting protein	1.03114044	0.6782	NA
100505195	LOC100505195	hypothetical LOC100505195	1.03114044	0.8106	0.9016
67163	Ccdc47	coiled-coil domain containing 47	1.03114044	0.8281	0.9112
19014	Med1	mediator complex subunit 1	1.03103413	0.5434	NA
22646	Zfp105	zinc finger protein 105	1.03103413	0.5472	NA
266781	Snx17	sorting nexin 17	1.03103413	0.6212	NA
208869	Dock3	dedicator of cyto-kinesis 3	1.03103413	0.7715	0.8786
18704	Pik3c2a	phosphatidylinositol 3-kinase, C2 domain containing, alpha polypeptide	1.03103413	0.8451	0.92
14804	Grid2	glutamate receptor, ionotropic, delta 2	1.03103413	0.868	0.9321
71787	Trnau1ap	tRNA selenocysteine 1 associated protein 1	1.03092784	0.4805	NA
78651	Lsm6	LSM6 homolog, U6 small nuclear RNA associated (S. cerevisiae)	1.03092784	0.5217	NA
67306	Fam164a	family with sequence similarity 164, member A	1.03092784	0.6295	NA
66513	Tab1	TGF-beta activated kinase 1/MAP3K7 binding protein 1	1.03092784	0.6569	NA
74407	Ttc25	tetratricopeptide repeat domain 25	1.03092784	0.6798	NA
236082	Dhrsx	dehydrogenase/reductase (SDR family) X chromosome	1.03092784	0.7359	0.8572
19944	Rpl29	ribosomal protein L29	1.03092784	0.7604	0.8723
66112	Mosc1	MOCO sulphurase C-terminal domain containing 1	1.03092784	0.795	0.8924
75796	Cdyl2	chromodomain protein, Y chromosome-like 2	1.03092784	0.8334	0.9141
73827	1110012D08Rik	RIKEN cDNA 1110012D08 gene	1.03082156	0.5881	NA
226151	Fam178a	family with sequence similarity 178, member A	1.03082156	0.6026	NA
56278	Gkap1	G kinase anchoring protein 1	1.03082156	0.6085	NA
76900	Ssbp4	single stranded DNA binding protein 4	1.03082156	0.6503	NA
243510	Ccdc142	coiled-coil domain containing 142	1.03082156	0.6572	NA
75597	Ndufaf2	NADH dehydrogenase (ubiquinone) 1 alpha subcomplex, assembly factor 2	1.03082156	0.6931	NA
109979	Art3	ADP-ribosyltransferase 3	1.03082156	0.7462	0.8632
66158	Cxx1a	CAAX box 1 homolog A (human)	1.03082156	0.7712	0.8785

52874	D19Bwg1357e	DNA segment, Chr 19, Brigham & Women's Genetics 1357 expressed	1.03082156	0.7863	0.8881
12292	Cacna1s	calcium channel, voltage-dependent, L type, alpha 1S subunit	1.03082156	0.7901	0.8901
319269	A130040M12Rik	RIKEN cDNA A130040M12 gene	1.03082156	0.8525	0.9242
270328	Gsdmc3	gasdermin C3	1.03082156	0.8626	0.929
225283	Rprd1a	regulation of nuclear pre-mRNA domain containing 1A	1.03071532	0.4035	NA
20365	Serf1	small EDRK-rich factor 1	1.03071532	0.5258	NA
69920	Polr2i	polymerase (RNA) II (DNA directed) polypeptide I	1.03071532	0.6101	NA
16202	Ilk	integrin linked kinase	1.03071532	0.71	NA
100503185	LOC100503185	BTB/POZ domain-containing protein 8-like	1.03071532	0.7815	0.885
436332	Gm5766	ribosomal protein L7a pseudogene	1.03060909	0.4051	NA
74287	Kcmf1	potassium channel modulatory factor 1	1.03060909	0.6037	NA
30055	Timm13	translocase of inner mitochondrial membrane 13 homolog (yeast)	1.03060909	0.6046	NA
76646	Wdr38	WD repeat domain 38	1.03060909	0.6643	NA
67623	Tm7sf3	transmembrane 7 superfamily member 3	1.03060909	0.739	0.8588
103768	Tubg2	tubulin, gamma 2	1.03050289	0.7076	NA
238037	BC068281	cDNA sequence BC068281	1.03050289	0.7569	0.8701
57355	BC051019	cDNA sequence BC051019	1.03050289	0.7604	0.8724
21849	Trim28	tripartite motif-containing 28	1.0303967	0.7513	0.8663
18488	Cntn3	contactin 3	1.0303967	0.7622	0.8737
12091	Glb1	galactosidase, beta 1	1.0303967	0.7864	0.8882
233545	2210018M11Rik	RIKEN cDNA 2210018M11 gene	1.03029054	0.5683	NA
70661	Sik3	SIK family kinase 3	1.03029054	0.5724	NA
20524	Slc25a17	solute carrier family 25 (mitochondrial carrier, peroxisomal membrane protein), member 17	1.03029054	0.6342	NA
27054	Sec23b	SEC23B (S. cerevisiae)	1.03029054	0.6735	NA
20238	Atxn1	ataxin 1	1.03029054	0.7654	0.8755
13385	Dlg4	discs, large homolog 4 (Drosophila)	1.03029054	0.7755	0.8806
58805	Mlxipl	MLX interacting protein-like	1.03029054	0.8846	0.9407
12703	Socs1	suppressor of cytokine signaling 1	1.0301844	0.7238	NA
15289	Hmgb1	high mobility group box 1	1.03007829	0.542	NA
71968	Wdr73	WD repeat domain 73	1.03007829	0.6225	NA
56749	Dhodh	dihydroorotate dehydrogenase	1.03007829	0.6248	NA
12752	Cln3	ceroid lipofuscinosis, neuronal 3, juvenile (Batten, Spielmeyer-Vogt disease)	1.03007829	0.7728	0.879
67553	Gstcd	glutathione S-transferase, C-terminal domain containing	1.03007829	0.8017	0.8959
108160	Fam50a	family with sequence similarity 50, member A	1.02997219	0.4168	NA
216440	Os9	amplified in osteosarcoma	1.02997219	0.6371	NA
319513	Fam113a	family with sequence similarity 113, member A	1.02997219	0.8067	0.8997
217340	Rnf157	ring finger protein 157	1.02997219	0.8556	0.9255
52184	Odf2l	outer dense fiber of sperm tails 2-like	1.02997219	0.8598	0.9276
74552	Nipal3	NIPA-like domain containing 3	1.02986612	0.5594	NA
27367	Rpl3	ribosomal protein L3	1.02986612	0.624	NA
67031	Upf3a	UPF3 regulator of nonsense transcripts homolog A (yeast)	1.02986612	0.7103	NA
66201	Vta1	Vps20-associated 1 homolog (S. cerevisiae)	1.02986612	0.7517	0.8665
57890	Il17re	interleukin 17 receptor E	1.02986612	0.8145	0.904
70561	Txndc16	thioredoxin domain containing 16	1.02976007	0.5997	NA
102693	Phldb1	pleckstrin homology-like domain, family B, member 1	1.02976007	0.6236	NA
68915	Vars2	valyl-tRNA synthetase 2, mitochondrial (putative)	1.02976007	0.7753	0.8804
100434	Slc44a1	solute carrier family 44, member 1	1.02976007	0.8547	0.9251
98766	Ubac1	ubiquitin associated domain containing 1	1.02965404	0.5984	NA
76251	0610007P08Rik	RIKEN cDNA 0610007P08 gene	1.02965404	0.612	NA
216395	Tmem5	transmembrane protein 5	1.02965404	0.6671	NA

30957	Mapk8ip3	mitogen-activated protein kinase 8 interacting protein 3	1.02965404	0.7248	NA
66626	5730403B10Rik	RIKEN cDNA 5730403B10 gene	1.02965404	0.7372	0.8578
622320	Kctd21	potassium channel tetramerisation domain containing 21	1.02965404	0.742	0.8606
18570	Pdcd6	programmed cell death 6	1.02954803	0.54	NA
26894	Cops7a	COP9 (constitutive photomorphogenic) homolog, subunit 7a (Arabidopsis thaliana)	1.02954803	0.608	NA
67242	Gemin6	gem (nuclear organelle) associated protein 6	1.02954803	0.7843	0.887
208718	Dis3l2	DIS3 mitotic control homolog (S. cerevisiae)-like 2	1.02954803	0.7868	0.8884
320078	Olfml2b	olfactomedin-like 2B	1.02954803	0.7942	0.8919
640374	Gm7293	glyceraldehyde-3-phosphate dehydrogenase pseudogene	1.02954803	0.8263	0.9104
100756	Usp30	ubiquitin specific peptidase 30	1.02944204	0.529	NA
66590	Farsa	phenylalanyl-tRNA synthetase, alpha subunit	1.02944204	0.5346	NA
83767	Wasf1	WASP family 1	1.02944204	0.5387	NA
56470	Rgs19	regulator of G-protein signaling 19	1.02944204	0.6246	NA
14533	Bloc1s1	biogenesis of lysosome-related organelles complex-1, subunit 1	1.02944204	0.6367	NA
19338	Rab33b	RAB33B, member of RAS oncogene family	1.02944204	0.7118	NA
70537	5730419F03Rik	RIKEN cDNA 5730419F03 gene	1.02944204	0.9193	0.9591
237759	Col23a1	collagen, type XXIII, alpha 1	1.02933608	0.7106	NA
72555	Shisa9	shisa homolog 9 (Xenopus laevis)	1.02933608	0.7144	NA
100040288	Gm2692	predicted gene 2692	1.02933608	0.8571	0.9263
210544	Wdr67	WD repeat domain 67	1.02933608	0.8979	0.948
28295	D10Jhu81e	DNA segment, Chr 10, Johns Hopkins University 81 expressed	1.02923014	0.5331	NA
70266	Ccbl1	cysteine conjugate-beta lyase 1	1.02923014	0.5729	NA
66446	Exosc7	exosome component 7	1.02923014	0.6863	NA
13864	Nr2f6	nuclear receptor subfamily 2, group F, member 6	1.02923014	0.7271	NA
108737	Oxsr1	oxidative-stress responsive 1	1.02923014	0.8	0.8952
319455	Pld5	phospholipase D family, member 5	1.02923014	0.8448	0.9198
78921	9130019O22Rik	RIKEN cDNA 9130019O22 gene	1.02923014	0.8643	0.9296
53610	Nono	non-POU-domain-containing, octamer binding protein	1.02912422	0.6055	NA
21923	Tnc	tenascin C	1.02912422	0.8775	0.937
70508	Bbx	bobby sox homolog (Drosophila)	1.02912422	0.8837	0.9401
18035	Nfkbia	nuclear factor of kappa light polypeptide gene enhancer in B-cells inhibitor, alpha	1.02901832	0.6147	NA
19982	Rpl36a	ribosomal protein L36A	1.02901832	0.6219	NA
20103	Rps5	ribosomal protein S5	1.02901832	0.6554	NA
54130	Actr1a	ARP1 actin-related protein 1 homolog A, centractin alpha (yeast)	1.02901832	0.6979	NA
56699	Cdc42ep4	CDC42 effector protein (Rho GTPase binding) 4	1.02901832	0.7118	NA
78830	Slc25a12	solute carrier family 25 (mitochondrial carrier, Aralar), member 12	1.02891244	0.6316	NA
170460	Stard5	StAR-related lipid transfer (START) domain containing 5	1.02891244	0.6573	NA
106143	Cggbp1	CGG triplet repeat binding protein 1	1.02891244	0.6837	NA
170461	Stard6	StAR-related lipid transfer (START) domain containing 6	1.02891244	0.6987	NA
237403	Lingo3	leucine rich repeat and Ig domain containing 3	1.02891244	0.7772	0.8816
75580	Zbtb4	zinc finger and BTB domain containing 4	1.02880658	0.4934	NA
78303	Hist3h2ba	histone cluster 3, H2ba	1.02880658	0.5637	NA
231889	Bud31	BUD31 homolog (yeast)	1.02880658	0.6124	NA
228869	Ncoa5	nuclear receptor coactivator 5	1.02880658	0.6416	NA
230648	4732418C07Rik	RIKEN cDNA 4732418C07 gene	1.02880658	0.6856	NA
73261	1700037C18Rik	RIKEN cDNA 1700037C18 gene	1.02880658	0.7232	NA
319728	E030042N06Rik	RIKEN cDNA E030042N06 gene	1.02880658	0.8063	0.8994
100041951	Gm3587	predicted gene 3587	1.02880658	0.8904	0.9437
71591	Zfp251	zinc finger protein 251	1.02870075	0.563	NA
16404	Itga7	integrin alpha 7	1.02870075	0.685	NA

73634	1700125H20Rik	RIKEN cDNA 1700125H20 gene	1.02870075	0.8693	0.9326
74302	Mtmr3	myotubularin related protein 3	1.02859494	0.6153	NA
233870	Tufm	Tu translation elongation factor, mitochondrial	1.02859494	0.6455	NA
66367	2310022A10Rik	RIKEN cDNA 2310022A10 gene	1.02859494	0.6586	NA
67489	Ap4b1	adaptor-related protein complex AP-4, beta 1	1.02859494	0.6753	NA
214137	Arhgap29	Rho GTPase activating protein 29	1.02859494	0.7024	NA
54326	Elovl2	elongation of very long chain fatty acids (FEN1/Elo2, SUR4/Elo3, yeast)-like 2	1.02859494	0.7298	NA
83924	Gpr137b	G protein-coupled receptor 137B	1.02859494	0.7402	NA
66915	Myeov2	myeloma overexpressed 2	1.02859494	0.7455	0.8628
240476	Zfp407	zinc finger protein 407	1.02859494	0.8266	0.9104
214058	Megf11	multiple EGF-like-domains 11	1.02859494	0.8565	0.9259
70026	Tspo2	translocator protein 2	1.02859494	0.9015	0.9499
70821	4921507P07Rik	RIKEN cDNA 4921507P07 gene	1.02859494	0.905	0.9516
66448	Mrpl20	mitochondrial ribosomal protein L20	1.02848915	0.5907	NA
70233	Cd2bp2	CD2 antigen (cytoplasmic tail) binding protein 2	1.02848915	0.5916	NA
74164	Nfx1	nuclear transcription factor, X-box binding 1	1.02848915	0.6052	NA
74132	Rnf6	ring finger protein (C3H2C3 type) 6	1.02848915	0.6149	NA
74533	Gzf1	GDNF-inducible zinc finger protein 1	1.02848915	0.6161	NA
396184	Flrt1	fibronectin leucine rich transmembrane protein 1	1.02848915	0.6184	NA
50996	Pdcd7	programmed cell death 7	1.02848915	0.6387	NA
15081	H3f3b	H3 histone, family 3B	1.02848915	0.728	NA
327860	Gm11961	predicted gene 11961	1.02848915	0.8097	0.9013
613254	AA465934	expressed sequence AA465934	1.02848915	0.8309	0.9126
66245	Hspbp1	HSPA (heat shock 70kDa) binding protein, cytoplasmic cochaperone 1	1.02848915	0.8372	0.9158
216198	Tcp11l2	t-complex 11 (mouse) like 2	1.02838338	0.5712	NA
70315	Hdac8	histone deacetylase 8	1.02838338	0.6062	NA
64143	Ralb	v-ral simian leukemia viral oncogene homolog B (ras related)	1.02838338	0.6476	NA
14977	Slc39a7	solute carrier family 39 (zinc transporter), member 7	1.02838338	0.6622	NA
64833	Acot10	acyl-CoA thioesterase 10	1.02838338	0.7293	NA
13637	Efna2	ephrin A2	1.02838338	0.7416	NA
109333	Pkn2	protein kinase N2	1.02838338	0.8221	0.9085
14009	Etv1	ets variant gene 1	1.02838338	0.8642	0.9296
234852	Chmp1a	chromatin modifying protein 1A	1.02827763	0.5194	NA
20826	Nhp2l1	NHP2 non-histone chromosome protein 2-like 1 (S. cerevisiae)	1.02827763	0.5953	NA
317755	Zar1	zygote arrest 1	1.02827763	0.7975	0.8934
276919	Gemin4	gem (nuclear organelle) associated protein 4	1.02827763	0.8339	0.9141
18389	Oprl1	opioid receptor-like 1	1.02827763	0.8863	0.9413
80751	Rnf34	ring finger protein 34	1.02817191	0.6014	NA
68263	Pdhb	pyruvate dehydrogenase (lipoamide) beta	1.02817191	0.7276	NA
228913	Zfp217	zinc finger protein 217	1.02817191	0.7714	0.8785
57837	Eral1	Era (G-protein)-like 1 (E. coli)	1.02817191	0.792	0.8911
67680	Sdhb	succinate dehydrogenase complex, subunit B, iron sulfur (Ip)	1.02806621	0.6133	NA
78925	Srd5a1	steroid 5 alpha-reductase 1	1.02806621	0.7004	NA
108071	Grm5	glutamate receptor, metabotropic 5	1.02806621	0.7373	NA
20677	Sox4	SRY-box containing gene 4	1.02806621	0.7936	0.8915
19271	Ptprj	protein tyrosine phosphatase, receptor type, J	1.02806621	0.847	0.9208
260423	Hist1h3f	histone cluster 1, H3f	1.02806621	0.9058	0.952
68970	Dcaf12	DDB1 and CUL4 associated factor 12	1.02796053	0.6041	NA
108755	Lyrm2	LYR motif containing 2	1.02796053	0.6941	NA
16413	Itgb1bp1	integrin beta 1 binding protein 1	1.02796053	0.7277	NA

622512	Gm6328	predicted gene 6328	1.02796053	0.8698	0.9327
59047	Pnkp	polynucleotide kinase 3'- phosphatase	1.02785487	0.5413	NA
14409	Gabrr2	gamma-aminobutyric acid (GABA) C receptor, subunit rho 2	1.02785487	0.8423	0.9184
238021	Fscn2	fascin homolog 2, actin-bundling protein, retinal (Strongylocentrotus purpuratus)	1.02774923	0.6008	NA
71883	Coq2	coenzyme Q2 homolog, prenyltransferase (yeast)	1.02774923	0.6431	NA
107476	Acaca	acetyl-Coenzyme A carboxylase alpha	1.02774923	0.6502	NA
18715	Pim2	proviral integration site 2	1.02774923	0.7318	NA
19018	Scand1	SCAN domain-containing 1	1.02764361	0.6561	NA
18438	P2rx4	purinergic receptor P2X, ligand-gated ion channel 4	1.02753802	0.5386	NA
23922	Jtb	jumping translocation breakpoint	1.02753802	0.5612	NA
17165	Mapkapk5	MAP kinase-activated protein kinase 5	1.02753802	0.6143	NA
74617	Scpep1	serine carboxypeptidase 1	1.02753802	0.6294	NA
16905	Lmna	lamin A	1.02753802	0.7852	0.8875
71887	Ppm1j	protein phosphatase 1J	1.02753802	0.8243	0.9095
83922	Tsga14	testis specific gene A14	1.02753802	0.8285	0.9113
667284	Gm8556	predicted gene 8556	1.02753802	0.8453	0.92
68944	Tmco1	transmembrane and coiled-coil domains 1	1.02743245	0.556	NA
14790	Grcc10	gene rich cluster, C10 gene	1.02743245	0.6313	NA
99712	Cept1	choline/ethanolaminephosphotransferase 1	1.02743245	0.6832	NA
68115	9430016H08Rik	RIKEN cDNA 9430016H08 gene	1.02743245	0.6968	NA
100043534	Gm11686	predicted gene 11686	1.02743245	0.7298	NA
67381	Med4	mediator of RNA polymerase II transcription, subunit 4 homolog (yeast)	1.02743245	0.7745	0.8801
56480	Tbk1	TANK-binding kinase 1	1.0273269	0.6508	NA
15461	Hras1	Harvey rat sarcoma virus oncogene 1	1.0273269	0.6911	NA
226591	Tiprl	TIP41, TOR signalling pathway regulator-like (S. cerevisiae)	1.0273269	0.6999	NA
69962	2810422O20Rik	RIKEN cDNA 2810422O20 gene	1.0273269	0.7165	NA
26913	Gprin1	G protein-regulated inducer of neurite outgrowth 1	1.0273269	0.8015	0.8959
20918	Eif1	eukaryotic translation initiation factor 1	1.0273269	0.8071	0.8999
17364	Trpm1	transient receptor potential cation channel, subfamily M, member 1	1.0273269	0.8168	0.9051
56044	Rala	v-ral simian leukemia viral oncogene homolog A (ras related)	1.02722137	0.5092	NA
69544	Wdr5b	WD repeat domain 5B	1.02722137	0.6594	NA
28000	Prpf19	PRP19/PSO4 pre-mRNA processing factor 19 homolog (S. cerevisiae)	1.02722137	0.751	NA
74486	Osbpl10	oxysterol binding protein-like 10	1.02722137	0.7514	NA
101612	Grwd1	glutamate-rich WD repeat containing 1	1.02722137	0.764	0.8748
13857	Epor	erythropoietin receptor	1.02722137	0.7939	0.8917
21770	Ppp2r5d	protein phosphatase 2, regulatory subunit B (B56), delta isoform	1.02722137	0.8412	0.918
13043	Cttn	cortactin	1.02711586	0.6167	NA
170952	Prima1	proline rich membrane anchor 1	1.02711586	0.6449	NA
19941	Rpl26	ribosomal protein L26	1.02711586	0.668	NA
227154	Stradb	STE20-related kinase adaptor beta	1.02711586	0.7203	NA
58186	Rad18	RAD18 homolog (S. cerevisiae)	1.02711586	0.7445	NA
100043404	Gm4416	predicted gene 4416	1.02711586	0.7664	0.8759
30947	Adat1	adenosine deaminase, tRNA-specific 1	1.02711586	0.865	0.93
71885	2310003H01Rik	RIKEN cDNA 2310003H01 gene	1.02711586	0.8836	0.9401
53317	Plrg1	pleiotropic regulator 1, PRL1 homolog (Arabidopsis)	1.02701037	0.5837	NA
629732	Rps8-ps1	ribosomal protein S8, pseudogene 1	1.02701037	0.6822	NA
436583	Snora74a	small nucleolar RNA, H/ACA box 74A	1.02701037	0.8363	0.9155
57437	Golga7	golgi autoantigen, golgin subfamily a, 7	1.02690491	0.6126	NA
244216	Zfp771	zinc finger protein 771	1.02690491	0.7397	NA
77739	Adamtsl1	ADAMTS-like 1	1.02690491	0.7714	0.8785

16857	Lgals6	lectin, galactose binding, soluble 6	1.02690491	0.7742	0.88
333050	Ksr2	kinase suppressor of ras 2	1.02690491	0.7858	0.8877
230587	Glis1	GLIS family zinc finger 1	1.02690491	0.788	0.8889
11881	Arsb	arylsulfatase B	1.02690491	0.8297	0.912
52897	Rbfox3	RNA binding protein, fox-1 homolog (C. elegans) 3	1.02690491	0.8461	0.9204
98402	Sh3bp4	SH3-domain binding protein 4	1.02690491	0.8816	0.9394
67698	Fam174a	family with sequence similarity 174, member A	1.02679947	0.5794	NA
227743	Mapkap1	mitogen-activated protein kinase associated protein 1	1.02679947	0.667	NA
18188	Nrtn	neurturin	1.02679947	0.7281	NA
243872	Rpl7a-ps8	ribosomal protein L7A, pseudogene 8	1.02679947	0.7534	NA
73062	Ppp1r16a	protein phosphatase 1, regulatory (inhibitor) subunit 16A	1.02679947	0.767	0.8763
69752	Zfp511	zinc finger protein 511	1.02679947	0.7685	0.8772
70235	Poc1a	POC1 centriolar protein homolog A (Chlamydomonas)	1.02679947	0.783	0.8861
26987	Eif4e2	eukaryotic translation initiation factor 4E member 2	1.02679947	0.8173	0.9054
68157	6720475J19Rik	RIKEN cDNA 6720475J19 gene	1.02679947	0.9006	0.9494
106610	AI504002	expressed sequence AI504002	1.02679947	0.9208	0.9596
69234	Zfp688	zinc finger protein 688	1.02669405	0.5618	NA
53610	Nono	non-POU-domain-containing, octamer binding protein	1.02669405	0.591	NA
18567	Pdcd2	programmed cell death 2	1.02669405	0.648	NA
14366	Fzd4	frizzled homolog 4 (Drosophila)	1.02669405	0.6703	NA
74781	Wipi2	WD repeat domain, phosphoinositide interacting 2	1.02669405	0.7417	NA
17357	Marcksl1	MARCKS-like 1	1.02669405	0.7843	0.887
68992	Zfp580	zinc finger protein 580	1.02669405	0.8081	0.9005
12354	Car7	carbonic anhydrase 7	1.02669405	0.9104	0.9541
17128	Smad4	MAD homolog 4 (Drosophila)	1.02658865	0.561	NA
114641	Rpl31	ribosomal protein L31	1.02658865	0.5791	NA
22169	Cmpk2	cytidine monophosphate (UMP-CMP) kinase 2, mitochondrial	1.02658865	0.7106	NA
170711	Otud7a	OTU domain containing 7A	1.02658865	0.762	0.8735
15979	Ifngr1	interferon gamma receptor 1	1.02648327	0.4915	NA
68275	Rpa1	replication protein A1	1.02648327	0.6292	NA
75729	4933432B09Rik	RIKEN cDNA 4933432B09 gene	1.02648327	0.791	0.8905
73086	Rps6ka5	ribosomal protein S6 kinase, polypeptide 5	1.02648327	0.7921	0.8911
233056	Zfp790	zinc finger protein 790	1.02648327	0.8635	0.9294
11842	Arf3	ADP-ribosylation factor 3	1.02637791	0.6231	NA
330216	Mblac1	metallo-beta-lactamase domain containing 1	1.02637791	0.66	NA
170759	Atp13a1	ATPase type 13A1	1.02637791	0.6872	NA
68915	Vars2	valyl-tRNA synthetase 2, mitochondrial (putative)	1.02637791	0.7672	0.8764
12160	Bmp5	bone morphogenetic protein 5	1.02637791	0.7746	0.8801
30947	Adat1	adenosine deaminase, tRNA-specific 1	1.02637791	0.7899	0.8901
67867	Lrrc28	leucine rich repeat containing 28	1.02637791	0.8347	0.9146
18005	Nek2	NIMA (never in mitosis gene a)-related expressed kinase 2	1.02637791	0.8953	0.9464
52712	Zkscan6	zinc finger with KRAB and SCAN domains 6	1.02627258	0.5466	NA
78651	Lsm6	LSM6 homolog, U6 small nuclear RNA associated (S. cerevisiae)	1.02627258	0.6354	NA
21689	Tekt1	tektin 1	1.02627258	0.7128	NA
99696	Ankrd50	ankyrin repeat domain 50	1.02627258	0.876	0.936
54351	Rai12	retinoic acid induced 12	1.02616727	0.6467	NA
271305	Phf21b	PHD finger protein 21B	1.02616727	0.656	NA
73833	Fam98c	family with sequence similarity 98, member C	1.02616727	0.6937	NA
13195	Ddc	dopa decarboxylase	1.02616727	0.8757	0.9357
69737	Ttl	tubulin tyrosine ligase	1.02606197	0.6326	NA

11569	Aebp2	AE binding protein 2	1.02606197	0.722	NA
270210	Zfp651	zinc finger protein 651	1.02606197	0.7757	0.8807
259144	Olfr456	olfactory receptor 456	1.02606197	0.9322	0.9653
83921	Tmem2	transmembrane protein 2	1.0259567	0.6261	NA
74393	4933403G14Rik	RIKEN cDNA 4933403G14 gene	1.0259567	0.6913	NA
20788	Srebf2	sterol regulatory element binding factor 2	1.0259567	0.8191	0.9064
11889	Asgr1	asialoglycoprotein receptor 1	1.0259567	0.8255	0.91
69053	1810013L24Rik	RIKEN cDNA 1810013L24 gene	1.0259567	0.8583	0.927
19057	Ppp3cc	protein phosphatase 3, catalytic subunit, gamma isoform	1.02585146	0.6075	NA
20316	Sdf2	stromal cell derived factor 2	1.02585146	0.6579	NA
227715	Exosc2	exosome component 2	1.02585146	0.7553	NA
71834	Zbtb43	zinc finger and BTB domain containing 43	1.02585146	0.7632	NA
20349	Sema3e	sema domain, immunoglobulin domain (Ig), short basic domain, secreted, (semaphorin) 3E	1.02585146	0.8379	0.9163
76640	1700113H08Rik	RIKEN cDNA 1700113H08 gene	1.02585146	0.9196	0.9591
13877	Erh	enhancer of rudimentary homolog (Drosophila)	1.02574623	0.5525	NA
75292	Prkd3	protein kinase D3	1.02574623	0.5798	NA
66590	Farsa	phenylalanyl-tRNA synthetase, alpha subunit	1.02574623	0.6996	NA
13589	Mapre1	microtubule-associated protein, RP/EB family, member 1	1.02574623	0.7624	NA
214254	Nudt15	nudix (nucleoside diphosphate linked moiety X)-type motif 15	1.02574623	0.8791	0.9381
66496	Ppdpf	pancreatic progenitor cell differentiation and proliferation factor homolog (zebrafish)RIKEN cDNA 2700038C09 gene	1.02564103	0.6593	NA
17904	Myl6	myosin, light polypeptide 6, alkali, smooth muscle and non-muscle	1.02564103	0.6651	NA
73826	Poldip3	polymerase (DNA-directed), delta interacting protein 3	1.02564103	0.7218	NA
14359	Fxr1	fragile X mental retardation gene 1, autosomal homolog	1.02564103	0.7781	0.8825
68294	Mfsd10	major facilitator superfamily domain containing 10	1.02564103	0.787	0.8885
320674	4921534A09Rik	RIKEN cDNA 4921534A09 gene	1.02564103	0.8962	0.9471
227700	Sh3glb2	SH3-domain GRB2-like endophilin B2	1.02553584	0.602	NA
269261	Rpl12	ribosomal protein L12	1.02553584	0.6233	NA
233075	Gm4883	predicted gene 4883	1.02553584	0.6609	NA
110796	Tshz1	teashirt zinc finger family member 1	1.02553584	0.6999	NA
242584	Wdr78	WD repeat domain 78	1.02553584	0.8034	0.8972
68526	Gpr155	G protein-coupled receptor 155	1.02553584	0.8072	0.8999
208228	Mobkl2a	MOB1, Mps One Binder kinase activator-like 2A (yeast)	1.02553584	0.8507	0.9235
67057	Yaf2	YY1 associated factor 2	1.02543068	0.5935	NA
224703	Mar-02	membrane-associated ring finger (C3HC4) 2	1.02543068	0.6898	NA
18458	Pabpc1	poly(A) binding protein, cytoplasmic 1	1.02543068	0.721	NA
76871	4930422N03Rik	RIKEN cDNA 4930422N03 gene	1.02543068	0.7731	0.8791
23994	Dazap2	DAZ associated protein 2	1.02532554	0.6011	NA
19179	Psmc1	protease (prosome, macropain) 26S subunit, ATPase 1	1.02532554	0.6075	NA
50498	Ebi3	Epstein-Barr virus induced gene 3	1.02532554	0.6842	NA
70138	2210417A02Rik	RIKEN cDNA 2210417A02 gene	1.02532554	0.9041	0.9513
240411	Loxhd1	lipoxygenase homology domains 1	1.02532554	0.9231	0.961
76820	Fam49a	family with sequence similarity 49, member A	1.02522042	0.6071	NA
19345	Rab5c	RAB5C, member RAS oncogene family	1.02522042	0.702	NA
245857	Ssh3	slingshot homolog 3 (Drosophila)	1.02522042	0.7202	NA
67843	Slc35a4	solute carrier family 35, member A4	1.02522042	0.8006	0.8956
100042982	Gm4146	predicted gene 4146	1.02522042	0.818	0.9057
217356	Tmc8	transmembrane channel-like gene family 8	1.02522042	0.9399	0.9692
65019	Rpl23	ribosomal protein L23	1.02511533	0.5893	NA
114615	Elac1	elaC homolog 1 (E. coli)	1.02511533	0.6031	NA
75616	2810008M24Rik	RIKEN cDNA 2810008M24 gene	1.02511533	0.6614	NA

21379	Tbrg4	transforming growth factor beta regulated gene 4	1.02511533	0.7316	NA
69908	Rab3b	RAB3B, member RAS oncogene family	1.02511533	0.7605	NA
246229	Bivm	basic, immunoglobulin-like variable motif containing	1.02511533	0.766	NA
22637	Zap70	zeta-chain (TCR) associated protein kinase	1.02511533	0.7736	0.8795
78102	8430426J06Rik	RIKEN cDNA 8430426J06 gene	1.02511533	0.881	0.939
66292	Mrps21	mitochondrial ribosomal protein S21	1.02501025	0.5985	NA
242864	Napepld	N-acyl phosphatidylethanolamine phospholipase D	1.02501025	0.6444	NA
22693	Zfp30	zinc finger protein 30	1.02501025	0.6867	NA
66177	Ubl5	ubiquitin-like 5	1.02501025	0.6927	NA
69219	Ddah1	dimethylarginine dimethylaminohydrolase 1	1.02501025	0.7257	NA
18633	Pex16	peroxisomal biogenesis factor 16	1.02501025	0.8156	0.9045
73723	Sh3bgrl3	SH3 domain binding glutamic acid-rich protein-like 3	1.02501025	0.8263	0.9104
71365	Pdss2	prenyl (solanesyl) diphosphate synthase, subunit 2	1.0249052	0.5453	NA
67025	Rpl11	ribosomal protein L11	1.0249052	0.7506	NA
85305	Kars	lysyl-tRNA synthetase	1.0249052	0.7565	NA
11477	Acvr1	activin A receptor, type 1	1.0249052	0.7866	0.8883
57905	Isy1	ISY1 splicing factor homolog (S. cerevisiae)	1.02480016	0.6793	NA
50709	Hist1h1e	histone cluster 1, H1e	1.02480016	0.8945	0.946
21343	Taf6	TAF6 RNA polymerase II, TATA box binding protein (TBP)-associated factor	1.02469515	0.5822	NA
114301	Palmd	palmdelphin	1.02469515	0.6387	NA
18574	Pde1b	phosphodiesterase 1B, Ca2+-calmodulin dependent	1.02469515	0.7438	NA
22427	Wrn	Werner syndrome homolog (human)	1.02469515	0.8312	0.9126
320333	D830030K20Rik	RIKEN cDNA D830030K20 gene	1.02469515	0.8555	0.9255
13543	Dvl2	dishevelled 2, dsh homolog (Drosophila)	1.02469515	0.8914	0.9445
68465	Adipor2	adiponectin receptor 2	1.02459016	0.5896	NA
20851	Stat5b	signal transducer and activator of transcription 5B	1.02459016	0.662	NA
213573	Efcab4a	EF-hand calcium binding domain 4A	1.02459016	0.8175	0.9054
258642	Olfr1165-ps	olfactory receptor 1165, pseudogene	1.02459016	0.9411	0.9698
28081	D11Wsu99e	DNA segment, Chr 11, Wayne State University 99, expressed	1.0244852	0.6955	NA
72368	2310045N01Rik	RIKEN cDNA 2310045N01 gene	1.0244852	0.7107	NA
14782	Gsr	glutathione reductase	1.0244852	0.7367	NA
231510	Agpat9	1-acylglycerol-3-phosphate O-acyltransferase 9	1.0244852	0.7411	NA
192986	Cyb5d2	cytochrome b5 domain containing 2	1.0244852	0.758	NA
21380	Tbx1	T-box 1	1.0244852	0.79	0.8901
282619	Sbsn	suprabasin	1.0244852	0.7996	0.8949
20088	Rps24	ribosomal protein S24	1.0244852	0.8396	0.9173
70458	2610318N02Rik	RIKEN cDNA 2610318N02 gene	1.0244852	0.9433	0.9713
21402	Skp1a	S-phase kinase-associated protein 1A	1.02427533	0.6936	NA
330059	A430017K17	hypothetical protein A430017K17	1.02427533	0.7176	NA
72726	Tbcc	tubulin-specific chaperone C	1.02427533	0.7428	NA
77701	Lcn12	lipocalin 12	1.02427533	0.7559	NA
68375	Ndufa8	NADH dehydrogenase (ubiquinone) 1 alpha subcomplex, 8	1.02427533	0.8207	0.9073
69187	Erp27	endoplasmic reticulum protein 27	1.02427533	0.8564	0.9258
67942	Atp5g2	ATP synthase, H+ transporting, mitochondrial F0 complex, subunit C2 (subunit 9)	1.02417042	0.6116	NA
627049	Zfp800	zinc finger protein 800	1.02417042	0.7743	NA
20054	Rps15	ribosomal protein S15	1.02417042	0.8096	0.9013
27361	Sepx1	selenoprotein X 1	1.02406554	0.6033	NA
98417	Cnih4	cornichon homolog 4 (Drosophila)	1.02406554	0.7059	NA
74150	Slc35f5	solute carrier family 35, member F5	1.02406554	0.7466	NA
66694	Uqcrfs1	ubiquinol-cytochrome c reductase, Rieske iron-sulfur polypeptide 1	1.02406554	0.7477	NA

56325	Abcb9	ATP-binding cassette, sub-family B (MDR/TAP), member 9	1.02406554	0.748	NA
71339	5430400D12Rik	RIKEN cDNA 5430400D12 gene	1.02406554	0.7588	NA
27058	Srp9	signal recognition particle 9	1.02406554	0.7756	NA
67229	Prpf18	PRP18 pre-mRNA processing factor 18 homolog (yeast)	1.02396068	0.5827	NA
19205	Ptbp1	polypyrimidine tract binding protein 1	1.02396068	0.6415	NA
268390	Ahsa2	AHA1, activator of heat shock protein ATPase homolog 2 (yeast)	1.02396068	0.6853	NA
20869	Stk11	serine/threonine kinase 11	1.02396068	0.7252	NA
67369	Qpctl	glutaminyl-peptide cyclotransferase-like	1.02396068	0.8235	0.9091
435965	Lrp3	low density lipoprotein receptor-related protein 3	1.02396068	0.8481	0.9215
101685	Spty2d1	SPT2, Suppressor of Ty, domain containing 1 (S. cerevisiae)	1.02396068	0.851	0.9235
76294	Asb5	ankyrin repeat and SOCs box-containing 5	1.02396068	0.8614	0.9285
52690	Setd3	SET domain containing 3	1.02385584	0.6111	NA
192176	Flna	filamin, alpha	1.02385584	0.7004	NA
14539	Opn1mw	opsin 1 (cone pigments), medium-wave-sensitive (color blindness, deutan)	1.02385584	0.7391	NA
68275	Rpa1	replication protein A1	1.02385584	0.7503	NA
56407	Trpc4ap	transient receptor potential cation channel, subfamily C, member 4 associated protein	1.02375102	0.6244	NA
11637	Ak2	adenylate kinase 2	1.02375102	0.6287	NA
22629	Ywhah	tyrosine 3-monooxygenase/tryptophan 5-monooxygenase activation protein, eta polypeptide	1.02375102	0.6432	NA
19122	Prnp	prion protein	1.02375102	0.7178	NA
75953	Samd7	sterile alpha motif domain containing 7	1.02375102	0.7727	NA
27984	Efhd2	EF hand domain containing 2	1.02375102	0.7798	NA
20511	Slc1a2	solute carrier family 1 (glial high affinity glutamate transporter), member 2	1.02375102	0.7964	0.8928
17283	Men1	multiple endocrine neoplasia 1	1.02375102	0.8255	0.91
69917	Obfc2b	oligonucleotide/oligosaccharide-binding fold containing 2B	1.02375102	0.8372	0.9158
71778	Klhl5	kelch-like 5 (Drosophila)	1.02364623	0.6751	NA
436049	Gm5741	predicted gene 5741	1.02364623	0.7232	NA
242662	Rims3	regulating synaptic membrane exocytosis 3	1.02364623	0.8608	0.928
546052	Gm5908	predicted gene 5908	1.02354145	0.6508	NA
103199	Fig4	FIG4 homolog (S. cerevisiae)	1.02354145	0.7677	NA
244682	Cntn5	contactin 5	1.02354145	0.8163	0.9047
76483	Lmf1	lipase maturation factor 1	1.02354145	0.8592	0.9274
245405	Gm4987	predicted gene 4987	1.0234367	0.6065	NA
14272	Fnta	farnesyltransferase, CAAX box, alpha	1.0234367	0.6105	NA
11737	Anp32a	acidic (leucine-rich) nuclear phosphoprotein 32 family, member A	1.0234367	0.6199	NA
12493	Cd37	CD37 antigen	1.0234367	0.69	NA
56176	Pigp	phosphatidylinositol glycan anchor biosynthesis, class P	1.0234367	0.6951	NA
54218	B3galt4	UDP-Gal:betaGlcNAc beta 1,3-galactosyltransferase, polypeptide 4	1.0234367	0.8328	0.9138
233824	Cog7	component of oligomeric golgi complex 7	1.02333197	0.8078	0.9004
13436	Dnmt3b	DNA methyltransferase 3B	1.02333197	0.8331	0.9139
260298	Fev	FEV (ETS oncogene family)	1.02333197	0.8802	0.9387
213956	Fam83f	family with sequence similarity 83, member F	1.02333197	0.9062	0.9523
57808	Rpl35a	ribosomal protein L35A	1.02322726	0.5645	NA
267019	Rps15a	ribosomal protein S15A	1.02322726	0.5759	NA
19866	Rnu7	U7 small nuclear RNA	1.02322726	0.6583	NA
674321	LOC674321	glycine cleavage system H protein, mitochondrial-like	1.02322726	0.7212	NA
53611	Vti1a	vesicle transport through interaction with t-SNAREs homolog 1A (yeast)	1.02312257	0.6206	NA
68558	Ankra2	ankyrin repeat, family A (RFXANK-like), 2	1.02312257	0.7241	NA
66248	Alg5	asparagine-linked glycosylation 5 homolog (yeast, dolichyl-phosphate beta-glucosyltransferase)	1.02312257	0.7249	NA
70237	Bhlhb9	basic helix-loop-helix domain containing, class B9	1.02312257	0.7457	NA
11544	Adprh	ADP-ribosylarginine hydrolase	1.02312257	0.7484	NA

227624	B230208H17Rik	RIKEN cDNA B230208H17 gene	1.02312257	0.8222	0.9085
380768	Gm1568	predicted gene 1568	1.0230179	0.7383	NA
68567	Cgref1	cell growth regulator with EF hand domain 1	1.0230179	0.7517	NA
380629	Heca	headcase homolog (Drosophila)	1.0230179	0.7665	NA
66496	Ppdpf	pancreatic progenitor cell differentiation and proliferation factor homolog (zebrafish)RIKEN cDNA 2700038C09 gene	1.0230179	0.7856	NA
68051	Nutf2	nuclear transport factor 2	1.0230179	0.7966	0.893
226499	BC003331	cDNA sequence BC003331	1.0230179	0.8333	0.9141
27041	G3bp1	Ras-GTPase-activating protein SH3-domain binding protein 1	1.02291326	0.5919	NA
68366	Tmem129	transmembrane protein 129	1.02291326	0.707	NA
665610	Gm7710	predicted gene 7710	1.02291326	0.7244	NA
67956	Setd8	SET domain containing (lysine methyltransferase) 8	1.02291326	0.7265	NA
74302	Mtmr3	myotubularin related protein 3	1.02291326	0.7885	NA
69773	1810026J23Rik	RIKEN cDNA 1810026J23 gene	1.02291326	0.7959	0.8927
22038	Plscr1	phospholipid scramblase 1	1.02291326	0.8	0.8952
20975	Synj2	synaptojanin 2	1.02291326	0.8299	0.912
11686	Alox12b	arachidonate 12-lipoxygenase, 12R type	1.02291326	0.8417	0.9182
57246	Tbx20	T-box 20	1.02291326	0.8517	0.9237
108797	Mex3b	mex3 homolog B (C. elegans)	1.02291326	0.8626	0.929
17463	Psmd7	proteasome (prosome, macropain) 26S subunit, non-ATPase, 7	1.02280863	0.6528	NA
20663	Sos2	son of sevenless homolog 2 (Drosophila)	1.02280863	0.6593	NA
66108	Ndufa9	NADH dehydrogenase (ubiquinone) 1 alpha subcomplex, 9	1.02280863	0.7441	NA
67895	Ppa1	pyrophosphatase (inorganic) 1	1.02280863	0.7706	NA
71913	Tmem79	transmembrane protein 79	1.02280863	0.7957	0.8927
56726	Sh3bgrl	SH3-binding domain glutamic acid-rich protein like	1.02280863	0.8154	0.9045
71472	Usp19	ubiquitin specific peptidase 19	1.02280863	0.8648	0.9299
57267	Apba3	amyloid beta (A4) precursor protein-binding, family A, member 3	1.02280863	0.8716	0.9339
226499	BC003331	cDNA sequence BC003331	1.02270403	0.7472	NA
246782	Atpaf2	ATP synthase mitochondrial F1 complex assembly factor 2	1.02270403	0.7644	NA
269514	Fbxl4	F-box and leucine-rich repeat protein 4	1.02270403	0.7935	0.8915
16348	Invs	inversin	1.02259945	0.7305	NA
78929	Polr3h	polymerase (RNA) III (DNA directed) polypeptide H	1.02259945	0.7584	NA
20586	Smarca4	SWI/SNF related, matrix associated, actin dependent regulator of chromatin, subfamily a, member 4	1.02259945	0.7803	NA
333315	Frem3	Fras1 related extracellular matrix protein 3	1.02259945	0.8087	0.9006
17766	Nudt1	nudix (nucleoside diphosphate linked moiety X)-type motif 1	1.02259945	0.8149	0.9043
15289	Hmgb1	high mobility group box 1	1.02259945	0.8227	0.9088
71532	9030418K01Rik	RIKEN cDNA 9030418K01 gene	1.02259945	0.8229	0.9089
13110	Cyp2j6	cytochrome P450, family 2, subfamily j, polypeptide 6	1.02259945	0.8797	0.9384
381259	Als2cr4	amyotrophic lateral sclerosis 2 (juvenile) chromosome region, candidate 4	1.02259945	0.8989	0.9487
666113	Gm7935	predicted pseudogene 7935	1.02249489	0.8929	0.9451
66117	1110001J03Rik	RIKEN cDNA 1110001J03 gene	1.02239035	0.6442	NA
234366	Gatad2a	GATA zinc finger domain containing 2A	1.02239035	0.748	NA
100040632	AA684185	expressed sequence AA684185	1.02239035	0.7511	NA
69716	Trip13	thyroid hormone receptor interactor 13	1.02239035	0.8324	0.9137
114886	Cygb	cytoglobin	1.02228583	0.7518	NA
68877	Maf1	MAF1 homolog (S. cerevisiae)	1.02228583	0.7951	NA
77631	4930554H23Rik	RIKEN cDNA 4930554H23 gene	1.02228583	0.7993	0.8949
217353	Tmc6	transmembrane channel-like gene family 6	1.02228583	0.8825	0.9397
71836	1700012A16Rik	RIKEN cDNA 1700012A16 gene	1.02228583	0.9276	0.9629
64945	Cldn12	claudin 12	1.02218133	0.6796	NA
60530	Fignl1	fidgetin-like 1	1.02218133	0.8585	0.9271

381353	Gm996	predicted gene 996	1.02218133	0.9169	0.9577
14359	Fxr1	fragile X mental retardation gene 1, autosomal homolog	1.02207686	0.6562	NA
229675	Rsbn1	rosbin, round spermatid basic protein 1	1.02207686	0.7442	NA
104130	Ndufb11	NADH dehydrogenase (ubiquinone) 1 beta subcomplex, 11	1.02207686	0.8046	0.8982
667338	Gm8580	ribosomal protein L29 pseudogene	1.02207686	0.8119	0.9023
239570	Ttc38	tetratricopeptide repeat domain 38	1.02207686	0.8893	0.9431
224090	Tmem44	transmembrane protein 44	1.02207686	0.9194	0.9591
117109	Pop5	processing of precursor 5, ribonuclease P/MRP family (S. cerevisiae)	1.02197241	0.6918	NA
56207	Uchl5	ubiquitin carboxyl-terminal esterase L5	1.02197241	0.7166	NA
77717	6030408B16Rik	RIKEN cDNA 6030408B16 gene	1.02197241	0.8607	0.9279
12175	Bnip2	BCL2/adenovirus E1B interacting protein 2	1.02186797	0.6621	NA
14396	Gabra3	gamma-aminobutyric acid (GABA) A receptor, subunit alpha 3	1.02186797	0.6634	NA
108159	Ubxn8	UBX domain protein 8	1.02186797	0.7496	NA
70417	Megf10	multiple EGF-like-domains 10	1.02176356	0.6931	NA
381605	Tbc1d2	TBC1 domain family, member 2	1.02176356	0.7451	NA
329584	Gm14462	predicted gene 14462	1.02176356	0.7823	NA
209268	Igsf1	immunoglobulin superfamily, member 1	1.02176356	0.855	0.9251
18218	Dusp8	dual specificity phosphatase 8	1.02165917	0.7257	NA
66942	Ddx18	DEAD (Asp-Glu-Ala-Asp) box polypeptide 18	1.02165917	0.758	NA
73284	Ddit4l	DNA-damage-inducible transcript 4-like	1.02165917	0.8103	0.9016
27176	Rpl7a	ribosomal protein L7A	1.02165917	0.8701	0.9328
76302	Pcnp	PEST proteolytic signal containing nuclear protein	1.02155481	0.7438	NA
217410	Trib2	tribbles homolog 2 (Drosophila)	1.02155481	0.8252	0.9099
382034	Gse1	genetic suppressor element 1	1.02145046	0.7496	NA
14628	Ostm1	osteopetrosis associated transmembrane protein 1	1.02145046	0.7749	NA
225583	A730017C20Rik	RIKEN cDNA A730017C20 gene	1.02145046	0.8402	0.9175
69019	Spcs1	signal peptidase complex subunit 1 homolog (S. cerevisiae)	1.02134613	0.6297	NA
21853	Timeless	timeless homolog (Drosophila)	1.02134613	0.722	NA
69707	Iqcg	IQ motif containing G	1.02134613	0.7441	NA
66052	Sdhc	succinate dehydrogenase complex, subunit C, integral membrane protein	1.02134613	0.7703	NA
68276	Toe1	target of EGR1, member 1 (nuclear)	1.02134613	0.7747	NA
211253	Mtrf1	mitochondrial translational release factor 1	1.02134613	0.7762	NA
77087	Ankrd11	ankyrin repeat domain 11	1.02134613	0.7922	NA
68839	Ankrd46	ankyrin repeat domain 46	1.02134613	0.839	0.917
73723	Sh3bgrl3	SH3 domain binding glutamic acid-rich protein-like 3	1.02134613	0.8451	0.92
14423	Galnt1	UDP-N-acetyl-alpha-D-galactosamine:polypeptide N-acetylgalactosaminyltransferase 1	1.02134613	0.8653	0.9302
71474	Ppp6r2	protein phosphatase 6, regulatory subunit 2	1.02134613	0.8751	0.9354
12581	Cdkn2d	cyclin-dependent kinase inhibitor 2D (p19, inhibits CDK4)	1.02124183	0.6851	NA
74023	Rd3	retinal degeneration 3	1.02124183	0.7702	NA
19268	Ptprf	protein tyrosine phosphatase, receptor type, F	1.02124183	0.7718	NA
233189	Ctu1	cytosolic thiouridylase subunit 1 homolog (S. pombe)	1.02124183	0.8448	0.9198
218820	Zfp503	zinc finger protein 503	1.02124183	0.8528	0.9244
319314	A930001C03Rik	RIKEN cDNA A930001C03 gene	1.02124183	0.9461	0.973
69957	Cdc16	CDC16 cell division cycle 16 homolog (S. cerevisiae)	1.02113755	0.6057	NA
72482	Acbd6	acyl-Coenzyme A binding domain containing 6	1.02113755	0.6616	NA
67150	Rnf141	ring finger protein 141	1.02113755	0.6624	NA
224023	Klhl22	kelch-like 22 (Drosophila)	1.02113755	0.6765	NA
22194	Ube2e1	ubiquitin-conjugating enzyme E2E 1, UBC4/5 homolog (yeast)	1.02113755	0.7141	NA
66530	Ubxn6	UBX domain protein 6	1.02113755	0.722	NA
20301	Ccl27a	chemokine (C-C motif) ligand 27A	1.02113755	0.7558	NA

66609	Cryzl1	crystallin, zeta (quinone reductase)-like 1	1.02113755	0.7645	NA
56637	Gsk3b	glycogen synthase kinase 3 beta	1.02113755	0.7923	NA
73183	5430402O13Rik	RIKEN cDNA 5430402O13 gene	1.02103329	0.616	NA
223593	E430025E21Rik	RIKEN cDNA E430025E21 gene	1.02103329	0.6493	NA
217666	L2hgdh	L-2-hydroxyglutarate dehydrogenase	1.02103329	0.7178	NA
72205	Eml2	echinoderm microtubule associated protein like 2	1.02103329	0.7614	NA
353170	Txlng	taxilin gamma	1.02103329	0.7869	NA
56513	Pard6a	par-6 (partitioning defective 6,) homolog alpha (C. elegans)	1.02103329	0.8147	0.9041
224273	Crybg3	beta-gamma crystallin domain containing 3	1.02092905	0.7167	NA
72140	Ccdc123	coiled-coil domain containing 123	1.02092905	0.7184	NA
94221	Gopc	golgi associated PDZ and coiled-coil motif containing	1.02092905	0.7496	NA
75516	Ttc32	tetratricopeptide repeat domain 32	1.02092905	0.8123	0.9025
106840	Unc119b	unc-119 homolog B (C. elegans)	1.02092905	0.8531	0.9245
66999	Med28	mediator of RNA polymerase II transcription, subunit 28 homolog (yeast)	1.02082483	0.7107	NA
11769	Ap1s1	adaptor protein complex AP-1, sigma 1	1.02082483	0.7168	NA
320522	Bhlha9	basic helix-loop-helix family, member a9	1.02082483	0.7397	NA
640636	Gm7303	predicted gene 7303	1.02082483	0.8206	0.9073
14168	Fgf13	fibroblast growth factor 13	1.02082483	0.8924	0.9449
20271	Scn5a	sodium channel, voltage-gated, type V, alpha	1.02082483	0.9192	0.9591
64339	Fndc4	fibronectin type III domain containing 4	1.02072063	0.7415	NA
230603	Ttc39a	tetratricopeptide repeat domain 39A	1.02072063	0.7926	NA
11972	Atp6v0d1	ATPase, H+ transporting, lysosomal V0 subunit D1	1.02072063	0.8068	NA
667350	Gm8587	predicted pseudogene 8587	1.02072063	0.8688	0.9324
16337	Insr	insulin receptor	1.02072063	0.8884	0.9427
54376	Cacng3	calcium channel, voltage-dependent, gamma subunit 3	1.02072063	0.8946	0.946
16783	Lamp1	lysosomal-associated membrane protein 1	1.02061645	0.6789	NA
243983	Zdhhc13	zinc finger, DHHC domain containing 13	1.02061645	0.6861	NA
76425	2310003C23Rik	RIKEN cDNA 2310003C23 gene	1.02061645	0.7637	NA
67131	Acbd4	acyl-Coenzyme A binding domain containing 4	1.0205123	0.6529	NA
18100	Mrpl40	mitochondrial ribosomal protein L40	1.0205123	0.6776	NA
13219	Defa-rs10	defensin, alpha, related sequence 10	1.0205123	0.756	NA
227120	Plcl1	phospholipase C-like 1	1.0205123	0.8502	0.9231
26930	Ppnr	per-pentamer repeat gene	1.0205123	0.8511	0.9235
57267	Apba3	amyloid beta (A4) precursor protein-binding, family A, member 3	1.0205123	0.8784	0.9377
208092	Chmp6	chromatin modifying protein 6	1.02040816	0.7404	NA
74388	Dpp8	dipeptidylpeptidase 8	1.02040816	0.8035	NA
67463	Poc5	POC5 centriolar protein homolog (Chlamydomonas)	1.02030405	0.6886	NA
59050	Nsa2	NSA2 ribosome biogenesis homolog (S. cerevisiae)	1.02030405	0.7568	NA
212111	Inpp5a	inositol polyphosphate-5-phosphatase A	1.02030405	0.757	NA
667682	Gm8759	predicted gene 8759	1.02019996	0.7312	NA
57908	Zfp318	zinc finger protein 318	1.02019996	0.7556	NA
72544	Exosc6	exosome component 6	1.02019996	0.7592	NA
70325	Pigw	phosphatidylinositol glycan anchor biosynthesis, class W	1.02019996	0.9074	0.9528
237038	Nox1	NADPH oxidase 1	1.02019996	0.9078	0.953
56378	Arpc3	actin related protein 2/3 complex, subunit 3	1.02009589	0.6758	NA
67115	Rpl14	ribosomal protein L14	1.02009589	0.709	NA
75991	Slain2	SLAIN motif family, member 2	1.02009589	0.7358	NA
74349	Fam160a2	family with sequence similarity 160, member A2	1.02009589	0.7445	NA
20866	Stim1	stromal interaction molecule 1	1.02009589	0.7745	NA
330788	Zfp866	zinc finger protein 866	1.02009589	0.7867	NA

72831	Dhx30	DEAH (Asp-Glu-Ala-His) box polypeptide 30	1.02009589	0.8113	NA
66241	Tmem9	transmembrane protein 9	1.02009589	0.8307	0.9126
98682	Mfsd6	major facilitator superfamily domain containing 6	1.02009589	0.8423	0.9184
229600	BC028528	cDNA sequence BC028528	1.02009589	0.8873	0.942
59016	Thap11	THAP domain containing 11	1.01999184	0.6574	NA
70573	Tbccd1	TBCC domain containing 1	1.01999184	0.7145	NA
24045	Scamp3	secretory carrier membrane protein 3	1.01999184	0.721	NA
76943	Psapl1	prosaposin-like 1	1.01999184	0.7229	NA
66890	Lman2	lectin, mannose-binding 2	1.01999184	0.752	NA
20603	Sms	spermine synthase	1.01999184	0.8008	NA
209361	Taf3	TAF3 RNA polymerase II, TATA box binding protein (TBP)-associated factor	1.01999184	0.8625	0.929
331004	Slc9a9	solute carrier family 9 (sodium/hydrogen exchanger), member 9	1.01999184	0.8633	0.9293
100041686	Gm15427	predicted pseudogene 15427	1.01988781	0.7119	NA
109065	1110034A24Rik	RIKEN cDNA 1110034A24 gene	1.01988781	0.7813	NA
16525	Kcnk1	potassium channel, subfamily K, member 1	1.01988781	0.7879	NA
100039948	Rhox2c	reproductive homeobox 2C	1.01988781	0.8628	0.9291
232491	Pyroxd1	pyridine nucleotide-disulphide oxidoreductase domain 1	1.01978381	0.7072	NA
26434	Prnd	prion protein dublet	1.01978381	0.7118	NA
269999	Orai3	ORAI calcium release-activated calcium modulator 3	1.01978381	0.7341	NA
57294	Rps27	ribosomal protein S27	1.01978381	0.7459	NA
105377	Ankrd32	ankyrin repeat domain 32	1.01978381	0.7947	NA
68017	Ftsj2	FtsJ homolog 2 (E. coli)	1.01978381	0.8154	NA
11416	Slc33a1	solute carrier family 33 (acetyl-CoA transporter), member 1	1.01978381	0.8432	0.919
14797	Aes	amino-terminal enhancer of split	1.01978381	0.8605	0.9279
14269	Fnbp1	formin binding protein 1	1.01978381	0.8934	0.9453
223254	Farp1	FERM, RhoGEF (Arhgef) and pleckstrin domain protein 1 (chondrocyte-derived)	1.01978381	0.906	0.9521
73102	Slc22a23	solute carrier family 22, member 23	1.01967982	0.6504	NA
66200	Commd6	COMM domain containing 6	1.01967982	0.7578	NA
224697	Adamts10	a disintegrin-like and metallopeptidase (reprolysin type) with thrombospondin type 1 motif, 10	1.01967982	0.8652	0.9302
665434	Gm7634	predicted gene 7634	1.01967982	0.8968	0.9474
68170	B230118H07Rik	RIKEN cDNA B230118H07 gene	1.01957586	0.7062	NA
66911	Nudt16l1	nudix (nucleoside diphosphate linked moiety X)-type motif 16-like 1	1.01957586	0.7522	NA
68401	G6pc3	glucose 6 phosphatase, catalytic, 3	1.01957586	0.7876	NA
212427	A730008H23Rik	RIKEN cDNA A730008H23 gene	1.01957586	0.7935	NA
67158	Sft2d3	SFT2 domain containing 3	1.01957586	0.8407	0.918
320207	Pik3r5	phosphoinositide-3-kinase, regulatory subunit 5, p101	1.01957586	0.8656	0.9304
19346	Rab6	RAB6, member RAS oncogene family	1.01947191	0.7242	NA
117589	Asb7	ankyrin repeat and SOCS box-containing 7	1.01947191	0.7411	NA
11973	Atp6v1e1	ATPase, H+ transporting, lysosomal V1 subunit E1	1.01947191	0.7904	NA
27784	Commd8	COMM domain containing 8	1.01947191	0.8374	0.916
56417	Adar	adenosine deaminase, RNA-specific	1.01936799	0.6464	NA
19946	Rpl30	ribosomal protein L30	1.01936799	0.6837	NA
93684	Sep-15	selenoprotein	1.01936799	0.6939	NA
68837	Foxk2	forkhead box K2	1.01936799	0.7736	NA
669393	Gm9457	predicted gene 9457	1.01926409	0.6842	NA
15275	Hk1	hexokinase 1	1.01926409	0.7237	NA
232987	B9d2	B9 protein domain 2	1.01926409	0.7559	NA
13056	Cyb561	cytochrome b-561	1.01926409	0.8392	0.9171
21853	Timeless	timeless homolog (Drosophila)	1.01926409	0.8596	0.9274
100986	Akap9	A kinase (PRKA) anchor protein (yotiao) 9	1.01916021	0.7466	NA

77798	A930009A15Rik	RIKEN cDNA A930009A15 gene	1.01916021	0.8179	NA
66662	5730577I03Rik	zinc finger protein pseudogene	1.01916021	0.89	0.9435
26926	Aifm1	apoptosis-inducing factor, mitochondrion-associated 1	1.01905635	0.7023	NA
67245	Peli1	pellino 1	1.01905635	0.71	NA
18292	Sebox	SEBOX homeobox	1.01905635	0.7592	NA
75841	Rnf139	ring finger protein 139	1.01905635	0.7753	NA
13361	Dhfr	dihydrofolate reductase	1.01905635	0.8072	NA
28064	Yipf3	Yip1 domain family, member 3	1.01905635	0.8137	NA
56360	Acot9	acyl-CoA thioesterase 9	1.01905635	0.8438	0.9193
74107	Cep55	centrosomal protein 55	1.01905635	0.8858	0.9412
14840	Gsg1	germ cell-specific gene 1	1.01895252	0.763	NA
66314	Tpd52l2	tumor protein D52-like 2	1.01895252	0.7918	NA
217335	Fbf1	Fas (TNFRSF6) binding factor 1	1.01895252	0.8559	0.9256
80911	Acox3	acyl-Coenzyme A oxidase 3, pristanoyl	1.01895252	0.8797	0.9384
270106	Rpl13	ribosomal protein L13	1.01895252	0.9058	0.952
270106	Rpl13	ribosomal protein L13	1.0188487	0.7056	NA
22196	Ube2i	ubiquitin-conjugating enzyme E2I	1.0188487	0.7221	NA
76522	Naa38	N(alpha)-acetyltransferase 38, NatC auxiliary subunit	1.0188487	0.7594	NA
330657	Prss53	protease, serine, 53	1.0188487	0.9068	0.9524
225339	Ammecr1l	AMME chromosomal region gene 1-like	1.01874491	0.7452	NA
54673	Sh3glb1	SH3-domain GRB2-like B1 (endophilin)	1.01874491	0.7959	NA
75458	Cklf	chemokine-like factor	1.01874491	0.8239	NA
28077	Med10	mediator of RNA polymerase II transcription, subunit 10 homolog (NUT2, S. cerevisiae)	1.01874491	0.8574	0.9265
13043	Cttn	cortactin	1.01874491	0.897	0.9475
103537	Mbtd1	mbt domain containing 1	1.01864113	0.7693	NA
66725	Lrrk2	leucine-rich repeat kinase 2	1.01864113	0.7701	NA
19212	Pter	phosphotriesterase related	1.01864113	0.7882	NA
18216	Ntsr1	neurotensin receptor 1	1.01864113	0.8285	NA
109648	Npy	neuropeptide Y	1.01864113	0.8716	0.9339
19687	Rfc1	replication factor C (activator 1) 1	1.01853738	0.688	NA
108072	Grm6	glutamate receptor, metabotropic 6	1.01853738	0.7241	NA
192652	Wdr81	WD repeat domain 81	1.01853738	0.7787	NA
74122	Tmem43	transmembrane protein 43	1.01853738	0.8224	NA
20312	Cx3cl1	chemokine (C-X3-C motif) ligand 1	1.01853738	0.8261	NA
16180	Il1rap	interleukin 1 receptor accessory protein	1.01853738	0.874	0.9351
76688	Arfrp1	ADP-ribosylation factor related protein 1	1.01843365	0.6922	NA
69807	Trim32	tripartite motif-containing 32	1.01843365	0.7198	NA
76824	Fam54b	family with sequence similarity 54, member B	1.01843365	0.7378	NA
67899	Cmc1	COX assembly mitochondrial protein homolog (S. cerevisiae)	1.01843365	0.7749	NA
108654	4933403F05Rik	RIKEN cDNA 4933403F05 gene	1.01843365	0.8336	0.9141
474156	Zbtb9	zinc finger and BTB domain containing 9	1.01843365	0.847	0.9208
21453	Tcof1	Treacher Collins Franceschetti syndrome 1, homolog	1.01843365	0.8517	0.9237
212518	Sprn	shadow of prion protein	1.01843365	0.856	0.9256
17312	Clec10a	C-type lectin domain family 10, member A	1.01843365	0.8696	0.9327
50794	Klf13	Kruppel-like factor 13	1.01843365	0.8819	0.9395
22151	Tubb2a	tubulin, beta 2A	1.01832994	0.7396	NA
225888	Suv420h1	suppressor of variegation 4-20 homolog 1 (Drosophila)	1.01832994	0.7493	NA
26992	Brd7	bromodomain containing 7	1.01832994	0.7857	NA
81703	Jdp2	Jun dimerization protein 2	1.01832994	0.8514	0.9235
268880	AI480653	expressed sequence AI480653	1.01832994	0.8533	0.9246

140483	Hnmt	histamine N-methyltransferase	1.01832994	0.8674	0.9318
19044	Ppox	protoporphyrinogen oxidase	1.01832994	0.8997	0.949
20826	Nhp2l1	NHP2 non-histone chromosome protein 2-like 1 (S. cerevisiae)	1.01822625	0.7253	NA
434674	Gm5631	predicted gene 5631	1.01822625	0.8834	0.9401
74123	Foxp4	forkhead box P4	1.01822625	0.9338	0.9661
100040233	Gm10334	predicted gene 10334	1.01822625	0.9512	0.9756
96935	Susd4	sushi domain containing 4	1.01812258	0.6958	NA
59069	Tpm3	tropomyosin 3, gamma	1.01812258	0.7053	NA
17242	Mdk	midkine	1.01812258	0.808	NA
11819	Nr2f2	nuclear receptor subfamily 2, group F, member 2	1.01812258	0.8218	NA
67893	Tmem86a	transmembrane protein 86A	1.01812258	0.8233	NA
23825	Banf1	barrier to autointegration factor 1	1.01812258	0.8431	0.919
71481	Alpk1	alpha-kinase 1	1.01812258	0.8447	0.9198
81701	Egfl8	EGF-like domain 8	1.01791531	0.7147	NA
238276	Akap5	A kinase (PRKA) anchor protein 5	1.01791531	0.7837	NA
56708	Clcf1	cardiotrophin-like cytokine factor 1	1.01791531	0.8306	NA
24071	Synj2bp	synaptojanin 2 binding protein	1.01791531	0.841	0.918
170461	Stard6	StAR-related lipid transfer (START) domain containing 6	1.01791531	0.8423	0.9184
100039024	LOC100039024	arf-GAP with SH3 domain, ANK repeat and PH domain-containing protein 1-like	1.01791531	0.8593	0.9274
330188	Ccdc63	coiled-coil domain containing 63	1.01791531	0.91	0.954
70960	4921531P14Rik	RIKEN cDNA 4921531P14 gene	1.01791531	0.9155	0.9572
81013	Vmn1r65	vomeronasal 1 receptor 65	1.01791531	0.9301	0.9641
237775	Zfp867	zinc finger protein 867	1.0178117	0.7671	NA
56707	Zfp111	zinc finger protein 111	1.0178117	0.806	NA
13972	Gnb1l	guanine nucleotide binding protein (G protein), beta polypeptide 1-like	1.0178117	0.8241	NA
76829	Dok5	docking protein 5	1.0178117	0.9361	0.9673
107377	AW492981	expressed sequence AW492981	1.0178117	0.9431	0.9712
67052	Ndc80	NDC80 homolog, kinetochore complex component (S. cerevisiae)	1.0178117	0.9482	0.9743
71902	Cand1	cullin associated and neddylation disassociated 1	1.01770812	0.6437	NA
230075	Ndufb6	NADH dehydrogenase (ubiquinone) 1 beta subcomplex, 6	1.01770812	0.6744	NA
20019	Polr1a	polymerase (RNA) I polypeptide A	1.01770812	0.7	NA
595136	Ndufs5	NADH dehydrogenase (ubiquinone) Fe-S protein 5	1.01770812	0.7019	NA
628596	Gm6900	predicted gene 6900	1.01770812	0.7203	NA
57437	Golga7	golgi autoantigen, golgin subfamily a, 7	1.01770812	0.736	NA
17222	Anapc1	anaphase promoting complex subunit 1	1.01770812	0.7594	NA
228859	Fitm2	fat storage-inducing transmembrane protein 2	1.01770812	0.7918	NA
14407	Gabrg3	gamma-aminobutyric acid (GABA) A receptor, subunit gamma 3	1.01770812	0.8234	NA
75409	Slitrk5	SLIT and NTRK-like family, member 5	1.01770812	0.84	0.9175
235047	Zfp809	zinc finger protein 809	1.01770812	0.8602	0.9278
217337	Srp68	signal recognition particle 68	1.01760456	0.7942	NA
94214	Spock2	sparc/osteonectin, cwcv and kazal-like domains proteoglycan 2	1.01760456	0.8003	NA
74600	Mrpl47	mitochondrial ribosomal protein L47	1.01760456	0.8333	NA
67109	Zfp787	zinc finger protein 787	1.01760456	0.8963	0.9471
434174	Hmg1l1	high-mobility group (nonhistone chromosomal) protein 1-like 1	1.01760456	0.9052	0.9516
52662	D18Ertd653e	DNA segment, Chr 18, ERATO Doi 653, expressed	1.01750102	0.8422	0.9184
78473	Skap1	src family associated phosphoprotein 1	1.01750102	0.8935	0.9453
20641	Snrpd1	small nuclear ribonucleoprotein D1	1.0173975	0.6961	NA
66495	Ndufb3	NADH dehydrogenase (ubiquinone) 1 beta subcomplex 3	1.0173975	0.7026	NA
68523	Fam96b	family with sequence similarity 96, member B	1.0173975	0.7291	NA
328099	Prps1l3	phosphoribosyl pyrophosphate synthetase 1-like 3	1.0173975	0.8071	NA

20440	St6gal1	beta galactoside alpha 2,6 sialyltransferase 1	1.0173975	0.8255	NA
22793	Zyx	zyxin	1.0173975	0.8406	0.9179
20907	Stx1a	syntaxin 1A (brain)	1.0173975	0.9	0.9491
77805	Esco1	establishment of cohesion 1 homolog 1 (S. cerevisiae)	1.0173975	0.9035	0.951
12352	Car5a	carbonic anhydrase 5a, mitochondrial	1.0173975	0.9166	0.9577
78651	Lsm6	LSM6 homolog, U6 small nuclear RNA associated (S. cerevisiae)	1.017294	0.6655	NA
68468	Ly6g6c	lymphocyte antigen 6 complex, locus G6C	1.017294	0.7467	NA
69076	Triap1	TP53 regulated inhibitor of apoptosis 1	1.017294	0.7566	NA
68283	9530077C05Rik	RIKEN cDNA 9530077C05 gene	1.017294	0.7576	NA
19089	Prkcsh	protein kinase C substrate 80K-H	1.01719052	0.6991	NA
78808	Stxbp5	syntaxin binding protein 5 (tomosyn)	1.01719052	0.7704	NA
107358	Tm9sf3	transmembrane 9 superfamily member 3	1.01719052	0.847	0.9208
58186	Rad18	RAD18 homolog (S. cerevisiae)	1.01719052	0.8487	0.9219
18082	Nipsnap1	4-nitrophenylphosphatase domain and non-neuronal SNAP25-like protein homolog 1 (C. elegans)	1.01719052	0.8549	0.9251
78321	Ankrd23	ankyrin repeat domain 23	1.01719052	0.8614	0.9285
67871	Mrrf	mitochondrial ribosome recycling factor	1.01719052	0.8692	0.9325
66320	Tmem208	transmembrane protein 208	1.01708706	0.6619	NA
18187	Nrp2	neuropilin 2	1.01708706	0.7137	NA
258943	Olfr353	olfactory receptor 353	1.01708706	0.9765	0.9887
94181	Nans	N-acetylneuraminic acid synthase (sialic acid synthase)	1.01698363	0.7321	NA
68059	Tm9sf2	transmembrane 9 superfamily member 2	1.01698363	0.778	NA
232679	Zc3hc1	zinc finger, C3HC type 1	1.01698363	0.8638	0.9294
107324	AV313155	expressed sequence AV313155	1.01698363	0.9066	0.9524
552877	LOC552877	hypothetical LOC552877	1.01688021	0.7272	NA
238330	6430527G18Rik	RIKEN cDNA 6430527G18 gene	1.01688021	0.7529	NA
100039359	Gm10399	predicted gene 10399	1.01688021	0.8549	0.9251
76406	1700019B03Rik	RIKEN cDNA 1700019B03 gene	1.01688021	0.8677	0.9318
227738	Lrsam1	leucine rich repeat and sterile alpha motif containing 1	1.01688021	0.9077	0.953
72309	Tmem158	transmembrane protein 158	1.01677682	0.7433	NA
28075	Pppde2	PPPDE peptidase domain containing 2	1.01677682	0.7756	NA
268749	Rnf31	ring finger protein 31	1.01677682	0.7891	NA
22123	Psmd3	proteasome (prosome, macropain) 26S subunit, non-ATPase, 3	1.01677682	0.8152	NA
66700	Vps24	vacuolar protein sorting 24 (yeast)	1.01677682	0.8443	NA
319876	Cobll1	Cobl-like 1	1.01677682	0.8683	0.9322
20371	Foxp3	forkhead box P3	1.01677682	0.9709	0.9857
13014	Cstb	cystatin B	1.01667344	0.7516	NA
103742	1810046J19Rik	RIKEN cDNA 1810046J19 gene	1.01667344	0.8272	NA
68607	Serhl	serine hydrolase-like	1.01667344	0.8513	0.9235
54138	Atxn10	ataxin 10	1.01667344	0.8523	0.924
109658	Txlna	taxilin alpha	1.01667344	0.8619	0.9287
83672	Sytl3	synaptotagmin-like 3	1.01667344	0.9196	0.9591
384525	Gm5321	predicted gene 5321	1.01657009	0.6596	NA
68537	Mrpl13	mitochondrial ribosomal protein L13	1.01657009	0.7261	NA
100126824	Sco2	SCO cytochrome oxidase deficient homolog 2 (yeast)	1.01657009	0.8401	NA
212198	Wdr25	WD repeat domain 25	1.01657009	0.8674	0.9318
72155	Cenpn	centromere protein N	1.01657009	0.8849	0.9408
54633	Pqbp1	polyglutamine binding protein 1	1.01657009	0.8851	0.9408
66449	Pam16	presequence translocase-asssociated motor 16 homolog (S. cerevisiae)	1.01646676	0.6965	NA
76687	Spcs3	signal peptidase complex subunit 3 homolog (S. cerevisiae)	1.01646676	0.6982	NA
67211	Armc10	armadillo repeat containing 10	1.01646676	0.7469	NA

66834	Acot13	acyl-CoA thioesterase 13	1.01646676	0.7744	NA
100040851	Gm9767	predicted gene 9767	1.01646676	0.8112	NA
67563	Narfl	nuclear prelamin A recognition factor-like	1.01646676	0.8262	NA
58231	Stk4	serine/threonine kinase 4	1.01646676	0.8858	0.9412
192216	Tmem47	transmembrane protein 47	1.01646676	0.9208	0.9596
19207	Ptch2	patched homolog 2	1.01646676	0.9317	0.965
674419	Rpl7a-ps5	ribosomal protein L7A, pseudogene 5	1.01636345	0.7073	NA
110842	Etfa	electron transferring flavoprotein, alpha polypeptide	1.01636345	0.7233	NA
22030	Traf2	TNF receptor-associated factor 2	1.01636345	0.7292	NA
269997	Zfp747	zinc finger protein 747	1.01636345	0.7725	NA
71538	Fbxo9	f-box protein 9	1.01636345	0.7938	NA
18571	Pdcd6ip	programmed cell death 6 interacting protein	1.01636345	0.8236	NA
67006	Cisd2	CDGSH iron sulfur domain 2	1.01636345	0.8421	NA
12567	Cdk4	cyclin-dependent kinase 4	1.01636345	0.857	0.9263
68755	Cgrrf1	cell growth regulator with ring finger domain 1	1.01636345	0.8744	0.9351
76261	0610040J01Rik	RIKEN cDNA 0610040J01 gene	1.01636345	0.8778	0.9372
75578	Fggy	FGGY carbohydrate kinase domain containing	1.01636345	0.9039	0.9513
27397	Mrpl17	mitochondrial ribosomal protein L17	1.01626016	0.7694	NA
107951	Cdk9	cyclin-dependent kinase 9 (CDC2-related kinase)	1.01626016	0.8336	NA
223770	Brd1	bromodomain containing 1	1.01626016	0.8344	NA
12978	Csf1r	colony stimulating factor 1 receptor	1.01626016	0.8989	0.9487
14128	Fcer2a	Fc receptor, IgE, low affinity II, alpha polypeptide	1.01626016	0.924	0.9615
19072	Prep	prolyl endopeptidase	1.01615689	0.7503	NA
21927	Tnfaip1	tumor necrosis factor, alpha-induced protein 1 (endothelial)	1.01615689	0.7701	NA
103468	Nup107	nucleoporin 107	1.01615689	0.7809	NA
26919	Zfp346	zinc finger protein 346	1.01615689	0.8537	0.9249
406218	Panx2	pannexin 2	1.01615689	0.8892	0.9431
56031	Ppie	peptidylprolyl isomerase E (cyclophilin E)	1.01615689	0.8927	0.945
210973	Kbtbd2	kelch repeat and BTB (POZ) domain containing 2	1.01605365	0.7179	NA
66943	Pqlc1	PQ loop repeat containing 1	1.01605365	0.7861	NA
20788	Srebf2	sterol regulatory element binding factor 2	1.01605365	0.8458	NA
212503	Paox	polyamine oxidase (exo-N4-amino)	1.01605365	0.8699	0.9327
21924	Tnnc1	troponin C, cardiac/slow skeletal	1.01605365	0.873	0.9348
230584	Yipf1	Yip1 domain family, member 1	1.01605365	0.8762	0.936
20418	Shc3	src homology 2 domain-containing transforming protein C3	1.01605365	0.8874	0.942
225358	Fam13b	family with sequence similarity 13, member B	1.01605365	0.9024	0.9504
432768	Gm5451	predicted gene 5451	1.01605365	0.9165	0.9577
228366	Gyltl1b	glycosyltransferase-like 1B	1.01605365	0.9489	0.9746
193670	Rnf185	ring finger protein 185	1.01595042	0.7171	NA
68196	Hsbp1	heat shock factor binding protein 1	1.01595042	0.7462	NA
24018	Rngtt	RNA guanylyltransferase and 5'-phosphatase	1.01595042	0.7498	NA
75580	Zbtb4	zinc finger and BTB domain containing 4	1.01595042	0.8099	NA
320541	Slc35e2	solute carrier family 35, member E2	1.01595042	0.8191	NA
11651	Akt1	thymoma viral proto-oncogene 1	1.01595042	0.8406	NA
19173	Psmb5	proteasome (prosome, macropain) subunit, beta type 5	1.01595042	0.8791	0.9381
15381	Hnrnpc	heterogeneous nuclear ribonucleoprotein C	1.01595042	0.893	0.9452
229937	Znhit6	zinc finger, HIT type 6	1.01584722	0.7757	NA
110948	Hlcs	holocarboxylase synthetase (biotin- [propriony-Coenzyme A-carboxylase (ATP-hydrolysing)] ligase)	1.01584722	0.8123	NA
84652	Fam126a	family with sequence similarity 126, member A	1.01584722	0.836	NA
117005	Olfr74	olfactory receptor 74	1.01584722	0.953	0.9764

67819	Derl1	Der1-like domain family, member 1	1.01564087	0.7049	NA
19182	Psmc3	proteasome (prosome, macropain) 26S subunit, ATPase 3	1.01564087	0.7896	NA
263876	Spata2	spermatogenesis associated 2	1.01564087	0.8118	NA
223701	Mkl1	MKL (megakaryoblastic leukemia)/myocardin-like 1	1.01564087	0.8324	NA
66273	1810020D17Rik	RIKEN cDNA 1810020D17 gene	1.01564087	0.8367	NA
381236	Lipo1	lipase, member O1	1.01564087	0.8623	0.9289
211484	Tsga10	testis specific 10	1.01564087	0.8699	0.9327
12048	Bcl2l1	BCL2-like 1	1.01564087	0.8823	0.9397
20370	Sez6	seizure related gene 6	1.01564087	0.921	0.9597
12660	Chka	choline kinase alpha	1.01553773	0.7628	NA
360216	Zranb1	zinc finger, RAN-binding domain containing 1	1.01553773	0.7907	NA
13642	Efnb2	ephrin B2	1.01553773	0.7937	NA
67671	Rpl38	ribosomal protein L38	1.01553773	0.8562	NA
245190	Gm4980	predicted gene 4980	1.01553773	0.9014	0.9497
74343	Crtc2	CREB regulated transcription coactivator 2	1.01543461	0.7431	NA
21351	Taldo1	transaldolase 1	1.01543461	0.7449	NA
59054	Mrps30	mitochondrial ribosomal protein S30	1.01543461	0.7656	NA
215210	Tmem120a	transmembrane protein 120A	1.01543461	0.7846	NA
66701	Spryd4	SPRY domain containing 4	1.01543461	0.8261	NA
12611	Cebpg	CCAAT/enhancer binding protein (C/EBP), gamma	1.01543461	0.8309	NA
77578	Bcl9	B-cell CLL/lymphoma 9	1.01543461	0.8556	NA
13135	Dad1	defender against cell death 1	1.01543461	0.8594	0.9274
448850	Znhit3	zinc finger, HIT type 3	1.01543461	0.8595	0.9274
14464	Gata5	GATA binding protein 5	1.01543461	0.9021	0.9502
11431	Acp1	acid phosphatase 1, soluble	1.01543461	0.9317	0.965
171212	Galnt10	UDP-N-acetyl-alpha-D-galactosamine:polypeptide N-acetylgalactosaminyltransferase 10	1.01533151	0.7742	NA
217715	Eif2b2	eukaryotic translation initiation factor 2B, subunit 2 beta	1.01533151	0.7995	NA
380629	Heca	headcase homolog (Drosophila)	1.01533151	0.7996	NA
68796	Tmem214	transmembrane protein 214	1.01533151	0.8353	NA
108737	Oxsr1	oxidative-stress responsive 1	1.01533151	0.8558	NA
78938	Fbxo34	F-box protein 34	1.01533151	0.8683	0.9322
19288	Ptx3	pentraxin related gene	1.01533151	0.8875	0.942
76982	3110035E14Rik	RIKEN cDNA 3110035E14 gene	1.01533151	0.8899	0.9435
72244	1600014C10Rik	RIKEN cDNA 1600014C10 gene	1.01533151	0.8953	0.9464
74230	1700016K19Rik	RIKEN cDNA 1700016K19 gene	1.01522843	0.9089	0.9534
170472	Recql5	RecQ protein-like 5	1.01522843	0.9187	0.9587
20422	Shfm1	split hand/foot malformation (ectrodactyly) type 1	1.01512537	0.7533	NA
55950	Bri3	brain protein I3	1.01512537	0.7571	NA
17342	Mitf	microphthalmia-associated transcription factor	1.01512537	0.7732	NA
170718	Idh3b	isocitrate dehydrogenase 3 (NAD+) beta	1.01512537	0.8126	NA
69126	1810022K09Rik	RIKEN cDNA 1810022K09 gene	1.01512537	0.8231	NA
234683	Elmo3	engulfment and cell motility 3, ced-12 homolog (C. elegans)	1.01512537	0.8243	NA
12068	Bet1	blocked early in transport 1 homolog (S. cerevisiae)	1.01512537	0.8335	NA
66192	Lage3	L antigen family, member 3	1.01512537	0.855	NA
12704	Cit	citron	1.01512537	0.9098	0.9539
69731	Gemin7	gem (nuclear organelle) associated protein 7	1.01502233	0.7736	NA
67211	Armc10	armadillo repeat containing 10	1.01502233	0.7979	NA
56529	Sec11a	SEC11 homolog A (S. cerevisiae)	1.01502233	0.8101	NA
20775	Sqle	squalene epoxidase	1.01502233	0.8367	NA
217030	Synrg	synergin, gamma	1.01502233	0.9013	0.9497

117589	Asb7	ankyrin repeat and SOCS box-containing 7	1.01502233	0.9197	0.9592
67433	Ccdc127	coiled-coil domain containing 127	1.01491931	0.8749	0.9353
320664	Cass4	Cas scaffolding protein family member 4	1.01491931	0.9716	0.9861
211770	Trib1	tribbles homolog 1 (Drosophila)	1.01481632	0.7772	NA
218490	Btf3	basic transcription factor 3	1.01481632	0.8269	NA
81904	Cacng7	calcium channel, voltage-dependent, gamma subunit 7	1.01481632	0.8382	NA
72787	Tmem48	transmembrane protein 48	1.01481632	0.8533	NA
56228	Ube2j1	ubiquitin-conjugating enzyme E2, J1	1.01481632	0.9164	0.9577
329993	Gm438	predicted gene 438	1.01481632	0.9351	0.9669
68045	2700060E02Rik	RIKEN cDNA 2700060E02 gene	1.01471334	0.7523	NA
66849	Ppp1r2	protein phosphatase 1, regulatory (inhibitor) subunit 2	1.01471334	0.797	NA
407785	Ndufs6	NADH dehydrogenase (ubiquinone) Fe-S protein 6	1.01471334	0.8281	NA
52443	Mrpl48	mitochondrial ribosomal protein L48	1.01471334	0.852	NA
67289	3110021A11Rik	RIKEN cDNA 3110021A11 gene	1.01471334	0.9073	0.9528
68133	Gcsh	glycine cleavage system protein H (aminomethyl carrier)	1.01461039	0.7707	NA
12464	Cct4	chaperonin containing Tcp1, subunit 4 (delta)	1.01461039	0.7961	NA
17967	Ncam1	neural cell adhesion molecule 1	1.01461039	0.8602	NA
52713	Ccdc59	coiled-coil domain containing 59	1.01461039	0.9052	0.9516
66725	Lrrk2	leucine-rich repeat kinase 2	1.01461039	0.9066	0.9524
241489	Pde11a	phosphodiesterase 11A	1.01461039	0.9157	0.9574
76479	Smndc1	survival motor neuron domain containing 1	1.01461039	0.9487	0.9745
19414	Rasa3	RAS p21 protein activator 3	1.01450746	0.7619	NA
56453	Mbtps1	membrane-bound transcription factor peptidase, site 1	1.01450746	0.7689	NA
103425	Ncln	nicalin homolog (zebrafish)	1.01450746	0.8667	0.9313
225280	Ino80c	INO80 complex subunit C	1.01450746	0.8869	0.9416
266692	Cpne1	copine I	1.01450746	0.9296	0.964
16646	Kpna1	karyopherin (importin) alpha 1	1.01440454	0.7987	NA
211945	Plekhh1	pleckstrin homology domain containing, family H (with MyTH4 domain) member 1	1.01440454	0.814	NA
68971	1500001M20Rik	RIKEN cDNA 1500001M20 gene	1.01440454	0.8166	NA
69962	2810422O20Rik	RIKEN cDNA 2810422O20 gene	1.01440454	0.8443	NA
56351	Ptges3	prostaglandin E synthase 3 (cytosolic)	1.01440454	0.8612	NA
70004	1700028J19Rik	RIKEN cDNA 1700028J19 gene	1.01440454	0.8757	0.9357
319262	Fchsd1	FCH and double SH3 domains 1	1.01440454	0.8815	0.9394
22631	Ywhaz	tyrosine 3-monooxygenase/tryptophan 5-monooxygenase activation protein, zeta polypeptide	1.01440454	0.9377	0.9682
75767	Rab11fip1	RAB11 family interacting protein 1 (class I)	1.01440454	0.9481	0.9742
15353	Hmg20b	high mobility group 20 B	1.01430165	0.7234	NA
243373	AI854703	expressed sequence AI854703	1.01430165	0.8506	NA
320965	4831440E17Rik	RIKEN cDNA 4831440E17 gene	1.01430165	0.8951	0.9464
319659	A730091E23Rik	RIKEN cDNA A730091E23 gene	1.01430165	0.9037	0.9512
624696	Gm6522	predicted gene 6522	1.01430165	0.9533	0.9766
99683	Sec24b	Sec24 related gene family, member B (S. cerevisiae)	1.01419878	0.77	NA
106947	Slc39a3	solute carrier family 39 (zinc transporter), member 3	1.01419878	0.7864	NA
15468	Prmt2	protein arginine N-methyltransferase 2	1.01419878	0.8009	NA
245841	Polr2h	polymerase (RNA) II (DNA directed) polypeptide H	1.01419878	0.8134	NA
79565	Wbscr27	Williams Beuren syndrome chromosome region 27 (human)	1.01419878	0.8339	NA
30940	Usp25	ubiquitin specific peptidase 25	1.01409593	0.7685	NA
73192	Xpot	exportin, tRNA (nuclear export receptor for tRNAs)	1.01409593	0.8073	NA
14156	Fen1	flap structure specific endonuclease 1	1.01409593	0.8213	NA
12847	Copa	coatomer protein complex subunit alpha	1.01409593	0.8461	NA
14433	Gapdh	glyceraldehyde-3-phosphate dehydrogenase	1.01409593	0.8471	NA

66665	5730528L13Rik	RIKEN cDNA 5730528L13 gene	1.01409593	0.8531	NA
71131	Zfp689	zinc finger protein 689	1.01409593	0.8674	NA
14403	Gabrd	gamma-aminobutyric acid (GABA) A receptor, subunit delta	1.0139931	0.8142	NA
66151	Prr13	proline rich 13	1.0139931	0.8271	NA
93891	Pcdhb20	protocadherin beta 20	1.0139931	0.8271	NA
14923	Guk1	guanylate kinase 1	1.0139931	0.8399	NA
69804	Tmem147	transmembrane protein 147	1.0139931	0.8411	NA
93762	Smarca5	SWI/SNF related, matrix associated, actin dependent regulator of chromatin, subfamily a, member 5	1.0139931	0.8514	NA
628596	Gm6900	predicted gene 6900	1.0139931	0.8791	0.9381
18673	Phb	prohibitin	1.0139931	0.9215	0.96
75573	2310007L24Rik	RIKEN cDNA 2310007L24 gene	1.0139931	0.9222	0.9606
100043474	Gm4461	predicted gene 4461	1.0139931	0.9382	0.9684
241520	Fam171b	family with sequence similarity 171, member B	1.0138903	0.7602	NA
70312	2510012J08Rik	RIKEN cDNA 2510012J08 gene	1.0138903	0.7778	NA
94067	Mrpl43	mitochondrial ribosomal protein L43	1.0138903	0.787	NA
216001	Cbara1	calcium binding atopy-related autoantigen 1	1.0138903	0.8184	NA
59125	Nek7	NIMA (never in mitosis gene a)-related expressed kinase 7	1.0138903	0.8285	NA
81896	Ift122	intraflagellar transport 122 homolog (Chlamydomonas)	1.0138903	0.8752	0.9354
14706	Gng4	guanine nucleotide binding protein (G protein), gamma 4	1.0138903	0.9153	0.9571
51875	Tmem141	transmembrane protein 141	1.01378751	0.8174	NA
22217	Usp12	ubiquitin specific peptidase 12	1.01378751	0.8582	NA
217071	Gm525	predicted gene 525	1.01378751	0.8802	0.9387
170759	Atp13a1	ATPase type 13A1	1.01378751	0.8828	0.9398
84095	Pi4k2a	phosphatidylinositol 4-kinase type 2 alpha	1.01378751	0.8891	0.943
14756	Gpld1	glycosylphosphatidylinositol specific phospholipase D1	1.01378751	0.9095	0.9538
53324	Nptx2	neuronal pentraxin 2	1.01378751	0.9098	0.9539
56745	C1qtnf1	C1q and tumor necrosis factor related protein 1	1.01378751	0.9329	0.9656
270106	Rpl13	ribosomal protein L13	1.01368474	0.7781	NA
68927	Ptcd2	pentatricopeptide repeat domain 2	1.01368474	0.7947	NA
74114	Crot	carnitine O-octanoyltransferase	1.01368474	0.808	NA
72344	Usp36	ubiquitin specific peptidase 36	1.01368474	0.8517	NA
217262	Abca9	ATP-binding cassette, sub-family A (ABC1), member 9	1.01368474	0.8743	0.9351
111173	Erc1	ELKS/RAB6-interacting/CAST family member 1	1.01368474	0.8824	0.9397
66973	Mrps18b	mitochondrial ribosomal protein S18B	1.013582	0.797	NA
67943	Mesdc2	mesoderm development candidate 2	1.013582	0.8122	NA
99334	Zscan29	zinc finger SCAN domains 29	1.013582	0.8273	NA
69882	2010321M09Rik	RIKEN cDNA 2010321M09 gene	1.013582	0.8332	NA
50789	Fbxl3	F-box and leucine-rich repeat protein 3	1.013582	0.8658	NA
16785	Rpsa	ribosomal protein SA	1.01347927	0.8438	NA
117197	Cno	cappuccino	1.01347927	0.8453	NA
16772	Lama1	laminin, alpha 1	1.01347927	0.8509	NA
385668	Lca5l	Leber congenital amaurosis 5-like	1.01347927	0.9133	0.956
170574	Sp7	Sp7 transcription factor 7	1.01347927	0.9491	0.9746
66878	Riok3	RIO kinase 3 (yeast)	1.01337657	0.7714	NA
53317	Plrg1	pleiotropic regulator 1, PRL1 homolog (Arabidopsis)	1.01337657	0.8063	NA
20520	Slc22a5	solute carrier family 22 (organic cation transporter), member 5	1.01337657	0.8341	NA
244745	Dpy19l1	dpy-19-like 1 (C. elegans)	1.01337657	0.8387	NA
494448	Cbx6	chromobox homolog 6	1.01337657	0.8571	NA
52696	Zwint	ZW10 interactor	1.01337657	0.8596	NA
77044	Arid2	AT rich interactive domain 2 (ARID, RFX-like)	1.01337657	0.9245	0.9618

71254	Naif1	nuclear apoptosis inducing factor 1	1.01337657	0.9712	0.9859
209966	Pgbd5	piggyBac transposable element derived 5	1.01327389	0.7406	NA
68875	Tmcc2	transmembrane and coiled-coil domains 2	1.01327389	0.8043	NA
59040	Rhot1	ras homolog gene family, member T1	1.01327389	0.8116	NA
72611	Zfp655	zinc finger protein 655	1.01327389	0.8237	NA
71952	2410016O06Rik	RIKEN cDNA 2410016O06 gene	1.01327389	0.8449	NA
13869	Erbb4	v-erb-a erythroblastic leukemia viral oncogene homolog 4 (avian)	1.01327389	0.8473	NA
20973	Syngr2	synaptogyrin 2	1.01327389	0.8576	NA
68954	1500012K07Rik	RIKEN cDNA 1500012K07 gene	1.01327389	0.8824	0.9397
19166	Psma2	proteasome (prosome, macropain) subunit, alpha type 2	1.01317123	0.7802	NA
67542	Cog6	component of oligomeric golgi complex 6	1.01317123	0.7828	NA
22377	Wbp1	WW domain binding protein 1	1.01317123	0.7914	NA
59022	Edf1	endothelial differentiation-related factor 1	1.01317123	0.7952	NA
14683	Gnas	GNAS (guanine nucleotide binding protein, alpha stimulating) complex locus	1.01317123	0.8188	NA
223776	1300018J18Rik	RIKEN cDNA 1300018J18 gene	1.01317123	0.8388	NA
231670	Fbxo21	F-box protein 21	1.01317123	0.8421	NA
74383	Ubap2l	ubiquitin associated protein 2-like	1.01317123	0.8654	NA
73316	Calr3	calreticulin 3	1.01306858	0.817	NA
217449	Ttc15	tetratricopeptide repeat domain 15	1.01306858	0.8172	NA
12468	Cct7	chaperonin containing Tcp1, subunit 7 (eta)	1.01306858	0.8486	NA
22225	Usp5	ubiquitin specific peptidase 5 (isopeptidase T)	1.01306858	0.8514	NA
73261	1700037C18Rik	RIKEN cDNA 1700037C18 gene	1.01306858	0.8541	NA
71458	Bcor	BCL6 interacting corepressor	1.01306858	0.867	NA
101471	Phrf1	PHD and ring finger domains 1	1.01306858	0.8678	NA
57440	Ehd3	EH-domain containing 3	1.01296596	0.7477	NA
100038683	Gm10775	predicted gene 10775	1.01296596	0.7806	NA
13722	Aimp1	aminoacyl tRNA synthetase complex-interacting multifunctional protein 1	1.01296596	0.7892	NA
66492	Zmat2	zinc finger, matrin type 2	1.01296596	0.7937	NA
210035	Tmem194	transmembrane protein 194	1.01296596	0.8261	NA
67145	Tomm34	translocase of outer mitochondrial membrane 34	1.01296596	0.8394	NA
74414	Polr3c	polymerase (RNA) III (DNA directed) polypeptide C	1.01296596	0.8609	NA
232944	Mark4	MAP/microtubule affinity-regulating kinase 4	1.01296596	0.8972	0.9477
381582	Gm5151	predicted gene 5151	1.01296596	0.9293	0.9638
18114	Rrp1	ribosomal RNA processing 1 homolog (S. cerevisiae)	1.01286336	0.8599	NA
71147	Oxsm	3-oxoacyl-ACP synthase, mitochondrial	1.01286336	0.8691	NA
240261	Ccdc112	coiled-coil domain containing 112	1.01286336	0.8853	0.9409
192157	Socs7	suppressor of cytokine signaling 7	1.01276079	0.7986	NA
66881	Pcyox1	prenylcysteine oxidase 1	1.01276079	0.8005	NA
107392	Brms1	breast cancer metastasis-suppressor 1	1.01276079	0.8836	0.9401
20615	Snapin	SNAP-associated protein	1.01276079	0.8887	0.9428
66492	Zmat2	zinc finger, matrin type 2	1.01265823	0.7449	NA
74192	Arpc5l	actin related protein 2/3 complex, subunit 5-like	1.01265823	0.7988	NA
56612	Pfdn5	prefoldin 5	1.01265823	0.803	NA
170644	Ubn1	ubinuclein 1	1.01265823	0.8496	NA
98432	Phlpp1	PH domain and leucine rich repeat protein phosphatase 1	1.01265823	0.8585	NA
228033	Atp5g3	ATP synthase, H+ transporting, mitochondrial F0 complex, subunit C3 (subunit 9)	1.01265823	0.8702	NA
59001	Pole3	polymerase (DNA directed), epsilon 3 (p17 subunit)	1.01265823	0.9011	0.9497
380752	Tssc1	tumor suppressing subtransferable candidate 1	1.01265823	0.9049	0.9516
19400	Rapsn	receptor-associated protein of the synapse	1.01265823	0.9309	0.9647
11861	Arl4a	ADP-ribosylation factor-like 4A	1.01255569	0.8454	NA

269774	Aak1	AP2 associated kinase 1	1.01255569	0.8599	NA
27998	Exosc5	exosome component 5	1.01255569	0.8637	NA
72273	2210404O07Rik	RIKEN cDNA 2210404O07 gene	1.01255569	0.915	0.9569
20021	Polr2c	polymerase (RNA) II (DNA directed) polypeptide C	1.01255569	0.9252	0.9619
217356	Tmc8	transmembrane channel-like gene family 8	1.01255569	0.9442	0.9719
14208	Ppm1g	protein phosphatase 1G (formerly 2C), magnesium-dependent, gamma isoform	1.01245317	0.8384	NA
67703	Kirrel3	kin of IRRE like 3 (Drosophila)	1.01245317	0.8964	0.9471
67054	Paics	phosphoribosylaminoimidazole carboxylase, phosphoribosylaminoribosylaminoimidazole, succinocarboxamide synthetase	1.01235068	0.7931	NA
17420	Mnat1	menage a trois 1	1.01235068	0.8131	NA
386655	Eid2	EP300 interacting inhibitor of differentiation 2	1.01235068	0.8157	NA
18472	Pafah1b1	platelet-activating factor acetylhydrolase, isoform 1b, subunit 1	1.01235068	0.817	NA
106504	Stk38	serine/threonine kinase 38	1.01235068	0.8931	0.9452
269941	Chsy1	chondroitin sulfate synthase 1	1.0122482	0.8049	NA
20088	Rps24	ribosomal protein S24	1.0122482	0.8253	NA
74117	Actr3	ARP3 actin-related protein 3 homolog (yeast)	1.0122482	0.8314	NA
27387	Sh2d3c	SH2 domain containing 3C	1.0122482	0.847	NA
100042150	Nrg2	neuregulin 2	1.0122482	0.8752	NA
229905	Ccbl2	cysteine conjugate-beta lyase 2	1.0122482	0.8754	NA
55992	Trim3	tripartite motif-containing 3	1.0122482	0.9208	0.9596
71754	Cyp2d40	cytochrome P450, family 2, subfamily d, polypeptide 40	1.0122482	0.9662	0.9834
19043	Ppm1b	protein phosphatase 1B, magnesium dependent, beta isoform	1.01214575	0.8017	NA
270066	Slc35e1	solute carrier family 35, member E1	1.01214575	0.8496	NA
77220	Tmem200a	transmembrane protein 200A	1.01214575	0.8778	NA
100303732	Gm14431	predicted gene 14431	1.01214575	0.8885	0.9428
433667	Ankrd13c	ankyrin repeat domain 13c	1.01214575	0.9002	0.9492
72522	Atxn7l2	ataxin 7-like 2	1.01214575	0.9202	0.9595
19663	Rbpms	RNA binding protein gene with multiple splicing	1.01214575	0.9288	0.9635
68219	Nudt21	nudix (nucleoside diphosphate linked moiety X)-type motif 21	1.01204332	0.8035	NA
102595	Plekho2	pleckstrin homology domain containing, family O member 2	1.01204332	0.8369	NA
18992	Pou3f2	POU domain, class 3, transcription factor 2	1.01204332	0.9291	0.9637
94219	Cnnm2	cyclin M2	1.0119409	0.7517	NA
66691	Gapvd1	GTPase activating protein and VPS9 domains 1	1.0119409	0.8005	NA
21982	Tmem165	transmembrane protein 165	1.0119409	0.8216	NA
72117	Naa50	N(alpha)-acetyltransferase 50, NatE catalytic subunit	1.0119409	0.8616	NA
140741	Gpr6	G protein-coupled receptor 6	1.0119409	0.8744	NA
16974	Lrp6	low density lipoprotein receptor-related protein 6	1.0119409	0.901	0.9496
382562	Pfn4	profilin family, member 4	1.01183851	0.8656	NA
403187	Opa3	optic atrophy 3 (human)	1.01183851	0.8729	NA
66500	Slc30a7	solute carrier family 30 (zinc transporter), member 7	1.01183851	0.9067	0.9524
18211	Ntrk1	neurotrophic tyrosine kinase, receptor, type 1	1.01183851	0.9324	0.9654
228866	Pcif1	PDX1 C-terminal inhibiting factor 1	1.01173614	0.9187	0.9587
56456	Actl6a	actin-like 6A	1.01163379	0.8761	NA
245828	Trappc1	trafficking protein particle complex 1	1.01153146	0.8137	NA
225887	Ndufs8	NADH dehydrogenase (ubiquinone) Fe-S protein 8	1.01153146	0.86	NA
76178	6330578E17Rik	RIKEN cDNA 6330578E17 gene	1.01153146	0.8973	0.9477
73825	Klraq1	KLRAQ motif containing 1	1.01153146	0.9124	0.9555
23808	Ash2l	ash2 (absent, small, or homeotic)-like (Drosophila)	1.01142915	0.8135	NA
66407	Mrps15	mitochondrial ribosomal protein S15	1.01142915	0.8141	NA
233073	U2af1l4	U2 small nuclear RNA auxiliary factor 1-like 4	1.01142915	0.891	NA
59008	Anapc5	anaphase-promoting complex subunit 5	1.01142915	0.895	0.9463

28017	D7Wsu130e	DNA segment, Chr 7, Wayne State University 130, expressed	1.01142915	0.9396	0.969
76416	Znrd1as	ZNRD1 antisense RNA	1.01132686	0.7978	NA
67248	Rpl39	ribosomal protein L39	1.01132686	0.8134	NA
104112	Acly	ATP citrate lyase	1.01132686	0.8362	NA
77041	Arsk	arylsulfatase K	1.01132686	0.8798	NA
77462	Tmem116	transmembrane protein 116	1.01132686	0.9052	0.9516
12552	Cdh11	cadherin 11	1.01132686	0.9062	0.9523
22173	Tyr	tyrosinase	1.01132686	0.9582	0.9792
70661	Sik3	SIK family kinase 3	1.01122459	0.8264	NA
18806	Pld2	phospholipase D2	1.01122459	0.8401	NA
212772	2700007P21Rik	RIKEN cDNA 2700007P21 gene	1.01122459	0.8866	NA
217827	BC002230	cDNA sequence BC002230	1.01122459	0.9042	0.9513
97159	A430005L14Rik	RIKEN cDNA A430005L14 gene	1.01112235	0.82	NA
108671	Dnajc9	DnaJ (Hsp40) homolog, subfamily C, member 9	1.01112235	0.8472	NA
229877	Rap1gds1	RAP1, GTP-GDP dissociation stimulator 1	1.01112235	0.8601	NA
76580	Mib2	mindbomb homolog 2 (Drosophila)	1.01112235	0.8682	NA
66314	Tpd52l2	tumor protein D52-like 2	1.01112235	0.8868	NA
16523	Kcnj8	potassium inwardly-rectifying channel, subfamily J, member 8	1.01112235	0.921	0.9597
192174	Rwdd4a	RWD domain containing 4A	1.01112235	0.9281	0.9632
17713	Grpel1	GrpE-like 1, mitochondrial	1.01102012	0.8006	NA
17975	Ncl	nucleolin	1.01102012	0.8223	NA
18146	Npdc1	neural proliferation, differentiation and control gene 1	1.01102012	0.8601	NA
20054	Rps15	ribosomal protein S15	1.01102012	0.8793	NA
23961	Oas1b	2'-5' oligoadenylate synthetase 1B	1.01102012	0.8929	NA
22612	Yes1	Yamaguchi sarcoma viral (v-yes) oncogene homolog 1	1.01102012	0.9034	0.951
70510	Rnf167	ring finger protein 167	1.01102012	0.9172	0.9579
210998	D15Ertd621e	DNA segment, Chr 15, ERATO Doi 621, expressed	1.01091791	0.8343	NA
234825	Klhdc4	kelch domain containing 4	1.01091791	0.852	NA
104776	Aldh6a1	aldehyde dehydrogenase family 6, subfamily A1	1.01091791	0.8629	NA
216965	Taok1	TAO kinase 1	1.01091791	0.8662	NA
20532	Slc3a1	solute carrier family 3, member 1	1.01091791	0.8788	NA
381062	2210404J11Rik	RIKEN cDNA 2210404J11 gene	1.01091791	0.9031	0.951
20116	Rps8	ribosomal protein S8	1.01091791	0.904	0.9513
270362	LOC270362	60S acidic ribosomal protein P1-like	1.01091791	0.9063	0.9523
114666	Krtap5-5	keratin associated protein 5-5	1.01091791	0.923	0.9609
227867	Epc2	enhancer of polycomb homolog 2 (Drosophila)	1.01091791	0.9248	0.9618
67725	Nudt13	nudix (nucleoside diphosphate linked moiety X)-type motif 13	1.01081573	0.8325	NA
22209	Ube2a	ubiquitin-conjugating enzyme E2A, RAD6 homolog (S. cerevisiae)	1.01081573	0.8515	NA
68059	Tm9sf2	transmembrane 9 superfamily member 2	1.01081573	0.8613	NA
382089	Ripply2	ripply2 homolog (zebrafish)	1.01081573	0.8672	NA
56298	Atl2	atlastin GTPase 2	1.01081573	0.8829	NA
72265	Tram1	translocating chain-associating membrane protein 1	1.01081573	0.9004	0.9493
71367	Chst9	carbohydrate (N-acetylgalactosamine 4-0) sulfotransferase 9	1.01081573	0.9201	0.9595
19079	Prkab1	protein kinase, AMP-activated, beta 1 non-catalytic subunit	1.01081573	0.9274	0.9629
621542	Gm6238	predicted pseudogene 6238	1.01071356	0.8052	NA
15361	Hmga1	high mobility group AT-hook 1	1.01071356	0.8638	NA
328855	E330032C10Rik	RIKEN cDNA E330032C10 gene	1.01071356	0.8936	NA
12928	Crk	v-crk sarcoma virus CT10 oncogene homolog (avian)	1.01071356	0.8984	NA
108645	Mat2b	methionine adenosyltransferase II, beta	1.01061142	0.8678	NA
26965	Cul1	cullin 1	1.01061142	0.9021	0.9502

67871	Mrrf	mitochondrial ribosome recycling factor	1.01061142	0.9026	0.9506
93746	Gprc5d	G protein-coupled receptor, family C, group 5, member D	1.01061142	0.9352	0.9669
14148	Fdx1	ferredoxin 1	1.0105093	0.8279	NA
70823	Hmgxb4	HMG box domain containing 4	1.0105093	0.8304	NA
68011	Snrpg	small nuclear ribonucleoprotein polypeptide G	1.0105093	0.8353	NA
237775	Zfp867	zinc finger protein 867	1.0105093	0.8631	NA
66821	Bcs1l	BCS1-like (yeast)	1.0105093	0.8875	NA
240038	Gm4944	predicted gene 4944	1.0105093	0.8973	NA
233870	Tufm	Tu translation elongation factor, mitochondrial	1.0105093	0.9136	0.956
67701	Wfdc2	WAP four-disulfide core domain 2	1.0105093	0.9198	0.9593
52815	Ldhd	lactate dehydrogenase D	1.0105093	0.9204	0.9596
14559	Gdf1	growth differentiation factor 1	1.0105093	0.9491	0.9746
672214	Gm10136	predicted pseudogene 10136	1.01040719	0.7885	NA
72587	Pan3	PAN3 polyA specific ribonuclease subunit homolog (S. cerevisiae)	1.01040719	0.8458	NA
16324	Inhbb	inhibin beta-B	1.01040719	0.8975	NA
110279	Bcr	breakpoint cluster region	1.01040719	0.8991	NA
19273	Ptpru	protein tyrosine phosphatase, receptor type, U	1.01040719	0.9075	0.9529
67495	Tmem167b	transmembrane protein 167B	1.01040719	0.9129	0.9559
99349	Dnajc24	DnaJ (Hsp40) homolog, subfamily C, member 24	1.01030511	0.8534	NA
69556	Bod1	biorientation of chromosomes in cell division 1	1.01030511	0.8613	NA
71131	Zfp689	zinc finger protein 689	1.01030511	0.8668	NA
13560	E4f1	E4F transcription factor 1	1.01030511	0.8804	NA
104027	Synpo	synaptopodin	1.01030511	0.908	0.953
74375	Gcc1	golgi coiled coil 1	1.01030511	0.9137	0.9561
101187	Parp11	poly (ADP-ribose) polymerase family, member 11	1.01030511	0.9175	0.958
20544	Slc9a1	solute carrier family 9 (sodium/hydrogen exchanger), member 1	1.01020305	0.7869	NA
24070	Mpdu1	mannose-P-dolichol utilization defect 1	1.01020305	0.8263	NA
17159	Man2b1	mannosidase 2, alpha B1	1.01020305	0.8913	NA
12370	Casp8	caspase 8	1.01020305	0.9635	0.9823
66406	Sac3d1	SAC3 domain containing 1	1.01010101	0.8153	NA
67949	Mki67ip	Mki67 (FHA domain) interacting nucleolar phosphoprotein	1.01010101	0.8228	NA
21809	Tgfb3	transforming growth factor, beta 3	1.01010101	0.8664	NA
224807	Tmem63b	transmembrane protein 63b	1.01010101	0.8804	NA
100040999	Gm3086	RuvB-like protein 1 pseudogene	1.01010101	0.9002	NA
67246	2810474O19Rik	RIKEN cDNA 2810474O19 gene	1.01010101	0.927	0.9627
19053	Ppp2cb	protein phosphatase 2 (formerly 2A), catalytic subunit, beta isoform	1.00999899	0.8159	NA
234865	Nup133	nucleoporin 133	1.00999899	0.8397	NA
20224	Sar1a	SAR1 gene homolog A (S. cerevisiae)	1.00999899	0.864	NA
545459	Gm10766	predicted gene 10766	1.00999899	0.8794	NA
54128	Pmm2	phosphomannomutase 2	1.00999899	0.9511	0.9755
17955	Nap1l4	nucleosome assembly protein 1-like 4	1.00999899	0.9663	0.9834
404634	H2afy2	H2A histone family, member Y2	1.00989699	0.8176	NA
100038734	Gm10845	predicted gene 10845	1.00989699	0.8473	NA
268449	Rpl23a	ribosomal protein L23A	1.00989699	0.8744	NA
11431	Acp1	acid phosphatase 1, soluble	1.00989699	0.9045	NA
674810	LOC674810	60S ribosomal protein L3-like	1.00989699	0.9251	0.9618
76959	Chmp5	chromatin modifying protein 5	1.00979501	0.8213	NA
70123	2210013O21Rik	RIKEN cDNA 2210013O21 gene	1.00979501	0.8646	NA
17305	Mfng	MFNG O-fucosylpeptide 3-beta-N-acetylglucosaminyltransferase	1.00979501	0.8784	NA
20441	St3gal3	ST3 beta-galactoside alpha-2,3-sialyltransferase 3	1.00979501	0.9073	NA

58246	Slc35b4	solute carrier family 35, member B4	1.00979501	0.9134	0.956
210766	Brcc3	BRCA1/BRCA2-containing complex, subunit 3	1.00979501	0.9205	0.9596
74136	Sec14l1	SEC14-like 1 (S. cerevisiae)	1.00979501	0.9286	0.9634
67387	Unc50	unc-50 homolog (C. elegans)	1.00969305	0.84	NA
56386	B4galt6	UDP-Gal:betaGlcNAc beta 1,4-galactosyltransferase, polypeptide 6	1.00969305	0.8694	NA
67150	Rnf141	ring finger protein 141	1.00969305	0.8775	NA
13617	Ednra	endothelin receptor type A	1.00969305	0.9117	0.955
381375	Dfnb59	deafness, autosomal recessive 59 (human)	1.00969305	0.9283	0.9632
13714	Elk4	ELK4, member of ETS oncogene family	1.00969305	0.9407	0.9696
68051	Nutf2	nuclear transport factor 2	1.00959112	0.8048	NA
19719	Rfng	RFNG O-fucosylpeptide 3-beta-N-acetylglucosaminyltransferase	1.00959112	0.8176	NA
26416	Mapk14	mitogen-activated protein kinase 14	1.00959112	0.8469	NA
12531	Cdc25b	cell division cycle 25 homolog B (S. pombe)	1.00959112	0.8688	NA
67364	1700121C10Rik	RIKEN cDNA 1700121C10 gene	1.00959112	0.9166	0.9577
20619	Snap23	synaptosomal-associated protein 23	1.0094892	0.9697	0.9853
66059	Krtcap2	keratinocyte associated protein 2	1.0093873	0.8194	NA
338363	6030446N20Rik	RIKEN cDNA 6030446N20 gene	1.0093873	0.9196	0.9591
72097	2010300C02Rik	RIKEN cDNA 2010300C02 gene	1.0093873	0.9555	0.9778
13202	Ddt	D-dopachrome tautomerase	1.00928543	0.8403	NA
16432	Itm2b	integral membrane protein 2B	1.00928543	0.8485	NA
22335	Vdac3	voltage-dependent anion channel 3	1.00928543	0.8513	NA
73431	1700052K11Rik	RIKEN cDNA 1700052K11 gene	1.00928543	0.8774	NA
71436	Flrt3	fibronectin leucine rich transmembrane protein 3	1.00928543	0.8882	NA
75416	Nop14	NOP14 nucleolar protein homolog (yeast)	1.00928543	0.9207	0.9596
69944	2810021J22Rik	RIKEN cDNA 2810021J22 gene	1.00928543	0.9264	0.9626
15220	Foxq1	forkhead box Q1	1.00928543	0.9301	0.9641
56808	Cacna2d2	calcium channel, voltage-dependent, alpha 2/delta subunit 2	1.00918357	0.8742	NA
57294	Rps27	ribosomal protein S27	1.00918357	0.8822	NA
212516	BC060267	cDNA sequence BC060267	1.00918357	0.8833	NA
74111	Rbm19	RNA binding motif protein 19	1.00918357	0.8977	NA
19363	Rad51l1	RAD51-like 1 (S. cerevisiae)	1.00918357	0.9425	0.9706
16764	Aff3	AF4/FMR2 family, member 3	1.00918357	0.9593	0.98
28015	Grinl1a	glutamate receptor, ionotropic, N-methyl D-aspartate-like 1A	1.00908174	0.8444	NA
68977	Haghl	hydroxyacylglutathione hydrolase-like	1.00908174	0.8699	NA
104416	Bap1	Brca1 associated protein 1	1.00908174	0.9147	NA
11419	Accn2	amiloride-sensitive cation channel 2, neuronal	1.00908174	0.9218	0.9602
22092	Rsph1	radial spoke head 1 homolog (Chlamydomonas)	1.00908174	0.9325	0.9654
74842	4833419G08Rik	RIKEN cDNA 4833419G08 gene	1.00908174	0.9333	0.9658
76594	Dnajc18	DnaJ (Hsp40) homolog, subfamily C, member 18	1.00897992	0.849	NA
353310	Zfp703	zinc finger protein 703	1.00897992	0.8939	NA
218850	D14Abb1e	DNA segment, Chr 14, Abbott 1 expressed	1.00897992	0.9186	0.9587
235379	Gldn	gliomedin	1.00897992	0.9347	0.9668
56703	Pigo	phosphatidylinositol glycan anchor biosynthesis, class O	1.00887813	0.8626	NA
69126	1810022K09Rik	RIKEN cDNA 1810022K09 gene	1.00887813	0.8988	NA
66176	Nat9	N-acetyltransferase 9 (GCN5-related, putative)	1.00887813	0.9152	NA
382421	Gm5176	predicted gene 5176	1.00887813	0.9379	0.9682
78285	5330426L24Rik	RIKEN cDNA 5330426L24 gene	1.00887813	0.9399	0.9692
433931	Pigg	phosphatidylinositol glycan anchor biosynthesis, class G	1.00887813	0.9444	0.972
22640	Zfp1	zinc finger protein 1	1.00877635	0.863	NA
232785	Zfp783	zinc finger protein 783	1.00877635	0.8863	NA

66194	Pycrl	pyrroline-5-carboxylate reductase-like	1.00877635	0.9247	0.9618
60321	Wbp11	WW domain binding protein 11	1.0086746	0.8216	NA
67089	Psmc6	proteasome (prosome, macropain) 26S subunit, ATPase, 6	1.0086746	0.8792	NA
71679	Atp5h	ATP synthase, H+ transporting, mitochondrial F0 complex, subunit d	1.0086746	0.8811	NA
71147	Oxsm	3-oxoacyl-ACP synthase, mitochondrial	1.0086746	0.8992	NA
101471	Phrf1	PHD and ring finger domains 1	1.0086746	0.8994	NA
21762	Psmd2	proteasome (prosome, macropain) 26S subunit, non-ATPase, 2	1.0086746	0.8995	NA
11685	Alox12e	arachidonate lipoxygenase, epidermal	1.0086746	0.9115	NA
791365	6430601O08Rik	RIKEN cDNA 6430601O08 gene	1.0086746	0.9211	0.9597
66508	2400001E08Rik	RIKEN cDNA 2400001E08 gene	1.0086746	0.9271	0.9627
69431	1700022N22Rik	RIKEN cDNA 1700022N22 gene	1.0086746	0.9435	0.9714
319887	E030030I06Rik	RIKEN cDNA E030030I06 gene	1.0086746	0.9642	0.9826
664862	Gpr137b-ps	G protein-coupled receptor 137B, pseudogene	1.00857287	0.9059	NA
22063	Trpc1	transient receptor potential cation channel, subfamily C, member 1	1.00857287	0.9408	0.9696
11857	Arhgdib	Rho, GDP dissociation inhibitor (GDI) beta	1.00857287	0.941	0.9698
78255	Ralgps2	Ral GEF with PH domain and SH3 binding motif 2	1.00847116	0.8574	NA
20334	Sec23a	SEC23A (S. cerevisiae)	1.00847116	0.8946	NA
100504876	LOC100504876	60S ribosomal protein L7a-like	1.00847116	0.8993	NA
68229	AI846148	expressed sequence AI846148	1.00847116	0.9	NA
17756	Mtap2	microtubule-associated protein 2	1.00847116	0.9522	0.9761
56409	Nudt3	nudix (nucleotide diphosphate linked moiety X)-type motif 3	1.00836947	0.8463	NA
66498	Dda1	DET1 and DDB1 associated 1	1.00836947	0.8698	NA
13877	Erh	enhancer of rudimentary homolog (Drosophila)	1.00836947	0.88	NA
14470	Rabac1	Rab acceptor 1 (prenylated)	1.00836947	0.8846	NA
70533	Btf3l4	basic transcription factor 3-like 4	1.00836947	0.9033	NA
93719	Ear6	eosinophil-associated, ribonuclease A family, member 6	1.00836947	0.963	0.9821
234396	Ankle1	ankyrin repeat and LEM domain containing 1	1.00836947	0.9728	0.9867
56692	Mapksp1	MAPK scaffold protein 1	1.0082678	0.8475	NA
13823	Epb4.1l3	erythrocyte protein band 4.1-like 3	1.0082678	0.9012	NA
17686	Msh3	mutS homolog 3 (E. coli)	1.0082678	0.9735	0.9872
93888	Pcdhb17	protocadherin beta 17	1.00816615	0.886	NA
75234	Rnf19b	ring finger protein 19B	1.00816615	0.9026	NA
69684	Aarsd1	alanyl-tRNA synthetase domain containing 1	1.00816615	0.9084	NA
73845	Ankrd42	ankyrin repeat domain 42	1.00816615	0.9276	0.9629
100336	Ppp1r8	protein phosphatase 1, regulatory (inhibitor) subunit 8	1.00816615	0.9335	0.9659
66043	Atp5d	ATP synthase, H+ transporting, mitochondrial F1 complex, delta subunit	1.00816615	0.9346	0.9668
406218	Panx2	pannexin 2	1.00816615	0.9474	0.9738
70383	Cox10	COX10 homolog, cytochrome c oxidase assembly protein, heme A: farnesyltransferase (yeast)	1.00816615	0.9515	0.9757
101095	Zfp282	zinc finger protein 282	1.00816615	0.9515	0.9757
68079	Pdcd2l	programmed cell death 2-like	1.00806452	0.822	NA
241846	Lsm14b	LSM14 homolog B (SCD6, S. cerevisiae)	1.00806452	0.8935	NA
75740	Egfem1	EGF-like and EMI domain containing 1	1.00806452	0.9486	0.9744
381356	5930434B04Rik	RIKEN cDNA 5930434B04 gene	1.00796291	0.8585	NA
93761	Smarca1	SWI/SNF related, matrix associated, actin dependent regulator of chromatin, subfamily a, member 1	1.00796291	0.879	NA
71951	Gpc2	glypican 2 (cerebroglycan)	1.00796291	0.8807	NA
78653	Bola3	bolA-like 3 (E. coli)	1.00796291	0.8892	NA
232337	Zfp637	zinc finger protein 637	1.00796291	0.8974	NA
170935	Grid2ip	glutamate receptor, ionotropic, delta 2 (Grid2) interacting protein 1	1.00796291	0.9259	NA
227659	Slc2a6	solute carrier family 2 (facilitated glucose transporter), member 6	1.00796291	0.955	0.9774
68770	Phtf2	putative homeodomain transcription factor 2	1.00796291	0.9658	0.9831

13806	Eno1	enolase 1, alpha non-neuron	1.00786132	0.8937	NA
56297	Arl6	ADP-ribosylation factor-like 6	1.00786132	0.9005	NA
217935	Wdr60	WD repeat domain 60	1.00786132	0.9057	NA
68929	Mospd3	motile sperm domain containing 3	1.00786132	0.9301	0.9641
434174	Hmg1l1	high-mobility group (nonhistone chromosomal) protein 1-like 1	1.00786132	0.9432	0.9712
109113	Uhrf2	ubiquitin-like, containing PHD and RING finger domains 2	1.00786132	0.9572	0.9787
67532	Mfap1a	microfibrillar-associated protein 1A	1.00775975	0.8804	NA
18679	Phka1	phosphorylase kinase alpha 1	1.00775975	0.8905	NA
74255	Smu1	smu-1 suppressor of mec-8 and unc-52 homolog (C. elegans)	1.00775975	0.891	NA
56353	Rybp	RING1 and YY1 binding protein	1.00775975	0.8945	NA
54151	Cyhr1	cysteine and histidine rich 1	1.00775975	0.9015	NA
21781	Tfdp1	transcription factor Dp 1	1.00775975	0.932	0.9651
71890	Mad2l2	MAD2 mitotic arrest deficient-like 2 (yeast)	1.00775975	0.954	0.9768
14071	F9	coagulation factor IX	1.00775975	0.9778	0.9893
78317	Ccdc88b	coiled-coil domain containing 88B	1.0076582	0.9017	NA
234371	Tmem161a	transmembrane protein 161A	1.0076582	0.9132	NA
71820	Wdr34	WD repeat domain 34	1.0076582	0.9332	0.9658
72061	2010111I01Rik	RIKEN cDNA 2010111I01 gene	1.0076582	0.9352	0.9669
105377	Ankrd32	ankyrin repeat domain 32	1.0076582	0.9417	0.9702
228543	Rhov	ras homolog gene family, member V	1.0076582	0.9494	0.9747
93871	Brwd1	bromodomain and WD repeat domain containing 1	1.0076582	0.957	0.9787
12953	Cry2	cryptochrome 2 (photolyase-like)	1.00755668	0.8745	NA
213575	Dync2li1	dynein cytoplasmic 2 light intermediate chain 1	1.00755668	0.9273	NA
74610	Abcb8	ATP-binding cassette, sub-family B (MDR/TAP), member 8	1.00755668	0.9349	0.9668
225743	Rnf165	ring finger protein 165	1.00755668	0.9354	0.967
68283	9530077C05Rik	RIKEN cDNA 9530077C05 gene	1.00755668	0.9381	0.9684
245537	Nlgn3	neuroligin 3	1.00755668	0.9619	0.9815
21888	Tle4	transducin-like enhancer of split 4, homolog of Drosophila E(spl)	1.00745517	0.8755	NA
13799	En2	engrailed 2	1.00745517	0.9226	NA
268807	Klhl38	kelch-like 38 (Drosophila)	1.00745517	0.935	0.9669
226139	Cox15	COX15 homolog, cytochrome c oxidase assembly protein (yeast)	1.00745517	0.9372	0.9679
270066	Slc35e1	solute carrier family 35, member E1	1.00745517	0.9504	0.9753
99132	AI956758	expressed sequence AI956758	1.00745517	0.9572	0.9787
665434	Gm7634	predicted gene 7634	1.00735368	0.9061	NA
56199	Abcb10	ATP-binding cassette, sub-family B (MDR/TAP), member 10	1.00735368	0.9126	NA
76983	Scfd1	Sec1 family domain containing 1	1.00735368	0.9202	NA
66172	Med11	mediator of RNA polymerase II transcription, subunit 11 homolog (S. cerevisiae)	1.00735368	0.9302	NA
14489	Mtpn	myotrophin	1.00735368	0.9368	0.9676
67801	Pllp	plasma membrane proteolipid	1.00735368	0.9603	0.9806
66979	Pole4	polymerase (DNA-directed), epsilon 4 (p12 subunit)	1.00735368	0.9709	0.9857
26384	Gnpda1	glucosamine-6-phosphate deaminase 1	1.00725222	0.8687	NA
195333	Gsc2	goosecoid homebox 2	1.00725222	0.918	NA
20496	Slc12a2	solute carrier family 12, member 2	1.00725222	0.9365	0.9676
56380	Arid3b	AT rich interactive domain 3B (BRIGHT-like)	1.00725222	0.9527	0.9762
22634	Plagl1	pleiomorphic adenoma gene-like 1	1.00725222	0.9609	0.981
17083	Tmed1	transmembrane emp24 domain containing 1	1.00715077	0.8903	NA
12785	Cnbp	cellular nucleic acid binding protein	1.00715077	0.9016	NA
105513	Chmp7	CHMP family, member 7	1.00715077	0.9022	NA
72180	Zfp661	zinc finger protein 661	1.00715077	0.9033	NA
319481	Wdr59	WD repeat domain 59	1.00715077	0.9047	NA

26895	Cops7b	COP9 (constitutive photomorphogenic) homolog, subunit 7b (Arabidopsis thaliana)	1.00715077	0.9051	NA
66647	Ndnl2	necdin-like 2	1.00704935	0.895	NA
21685	Tef	thyrotroph embryonic factor	1.00704935	0.9184	NA
16337	Insr	insulin receptor	1.00704935	0.9257	NA
80860	Ghdc	GH3 domain containing	1.00704935	0.9564	0.9784
266690	Cyb5r4	cytochrome b5 reductase 4	1.00704935	0.9575	0.9788
13609	S1pr1	sphingosine-1-phosphate receptor 1	1.00704935	0.9666	0.9834
54380	Smarcal1	SWI/SNF related matrix associated, actin dependent regulator of chromatin, subfamily a-like 1	1.00694794	0.8828	NA
52432	Ppp2r2d	protein phosphatase 2, regulatory subunit B, delta isoform	1.00694794	0.8856	NA
69106	Stoml1	stomatin-like 1	1.00694794	0.9078	NA
71213	Cage1	cancer antigen 1	1.00694794	0.9144	NA
66966	Trit1	tRNA isopentenyltransferase 1	1.00694794	0.922	NA
52837	Tmx4	thioredoxin-related transmembrane protein 4	1.00694794	0.9223	NA
56471	Stmn4	stathmin-like 4	1.00694794	0.9228	NA
224727	Bat3	HLA-B-associated transcript 3	1.00694794	0.9568	0.9786
66155	Ufc1	ubiquitin-fold modifier conjugating enzyme 1	1.00684656	0.873	NA
100038999	Gm13552	predicted gene 13552	1.00684656	0.907	NA
22608	Ybx1	Y box protein 1	1.00684656	0.9168	NA
12183	Bpgm	2,3-bisphosphoglycerate mutase	1.00684656	0.9212	NA
230793	Ahdc1	AT hook, DNA binding motif, containing 1	1.00684656	0.9388	0.9685
67803	Limd2	LIM domain containing 2	1.00684656	0.9525	0.9761
55992	Trim3	tripartite motif-containing 3	1.00684656	0.9585	0.9794
218763	Lrrc3b	leucine rich repeat containing 3B	1.00684656	0.9812	0.9909
24010	Ik	IK cytokine	1.00674519	0.9037	NA
70750	Kdsr	3-ketodihydrosphingosine reductase	1.00674519	0.9116	NA
74296	1700093J21Rik	RIKEN cDNA 1700093J21 gene	1.00674519	0.9217	NA
114893	Dcun1d1	DCN1, defective in cullin neddylation 1, domain containing 1 (S. cerevisiae)	1.00674519	0.926	NA
217682	3830431G21Rik	RIKEN cDNA 3830431G21 gene	1.00674519	0.927	NA
72699	Lime1	Lck interacting transmembrane adaptor 1	1.00674519	0.9332	NA
73467	1700066M21Rik	RIKEN cDNA 1700066M21 gene	1.00674519	0.9408	0.9696
227399	Ppip5k2	diphosphoinositol pentakisphosphate kinase 2	1.00674519	0.9548	0.9773
252870	Usp7	ubiquitin specific peptidase 7	1.00664385	0.9038	NA
22156	Tuft1	tuftelin 1	1.00664385	0.9277	NA
75838	4930560O18Rik	RIKEN cDNA 4930560O18 gene	1.00664385	0.9597	0.9803
26754	Cops5	COP9 (constitutive photomorphogenic) homolog, subunit 5 (Arabidopsis thaliana)	1.00654253	0.8582	NA
16911	Lmo4	LIM domain only 4	1.00654253	0.8761	NA
69168	Bola1	bolA-like 1 (E. coli)	1.00654253	0.8847	NA
225995	D030056L22Rik	RIKEN cDNA D030056L22 gene	1.00654253	0.8892	NA
226098	Hectd2	HECT domain containing 2	1.00654253	0.9065	NA
108652	Slc35b3	solute carrier family 35, member B3	1.00654253	0.9164	NA
231571	Rpap2	RNA polymerase II associated protein 2	1.00654253	0.9237	NA
232989	Hnrnpul1	heterogeneous nuclear ribonucleoprotein U-like 1	1.00654253	0.9417	0.9702
66358	2310004I24Rik	RIKEN cDNA 2310004I24 gene	1.00654253	0.9516	0.9757
223921	Aaas	achalasia, adrenocortical insufficiency, alacrimia	1.00654253	0.9595	0.9802
319195	Rpl17	ribosomal protein L17	1.00654253	0.9665	0.9834
11810	Apobec1	apolipoprotein B mRNA editing enzyme, catalytic polypeptide 1	1.00654253	0.9719	0.9862
11883	Arsa	arylsulfatase A	1.00644122	0.9013	NA
99890	Prmt6	protein arginine N-methyltransferase 6	1.00644122	0.9363	NA
58867	Syngr4	synaptogyrin 4	1.00644122	0.9688	0.985
170458	Gpha2	glycoprotein hormone alpha 2	1.00644122	0.9813	0.9909

70533	Btf3l4	basic transcription factor 3-like 4	1.00633994	0.8902	NA
19671	Rce1	RCE1 homolog, prenyl protein peptidase (S. cerevisiae)	1.00633994	0.9265	NA
76308	Rab1b	RAB1B, member RAS oncogene family	1.00633994	0.9265	NA
72562	Pcbd2	pterin 4 alpha carbinolamine dehydratase/dimerization cofactor of hepatocyte nuclear factor 1 alpha (TCF1) 2	1.00633994	0.9424	0.9706
18604	Pdk2	pyruvate dehydrogenase kinase, isoenzyme 2	1.00633994	0.9474	0.9738
14694	Gnb2l1	guanine nucleotide binding protein (G protein), beta polypeptide 2 like 1	1.00633994	0.9499	0.975
66997	Psmd12	proteasome (prosome, macropain) 26S subunit, non-ATPase, 12	1.00623868	0.8735	NA
24051	Sgcb	sarcoglycan, beta (dystrophin-associated glycoprotein)	1.00623868	0.9026	NA
67211	Armc10	armadillo repeat containing 10	1.00623868	0.9231	NA
625929	Gm6636	predicted gene 6636	1.00623868	0.9321	NA
212123	Dcaf15	DDB1 and CUL4 associated factor 15	1.00623868	0.9456	0.9728
114675	4932431P20Rik	RIKEN cDNA 4932431P20 gene	1.00623868	0.9541	0.9768
26416	Mapk14	mitogen-activated protein kinase 14	1.00623868	0.9615	0.9813
20276	Scnn1a	sodium channel, nonvoltage-gated 1 alpha	1.00623868	0.9771	0.9891
73112	3110003A17Rik	RIKEN cDNA 3110003A17 gene	1.00613744	0.9136	NA
381605	Tbc1d2	TBC1 domain family, member 2	1.00613744	0.9352	NA
434179	Gm5595	predicted gene 5595	1.00613744	0.9537	0.9767
100043431	Gm4430	predicted gene 4430	1.00613744	0.9628	0.982
50793	Orc3	origin recognition complex, subunit 3	1.00603622	0.8756	NA
11302	Aatk	apoptosis-associated tyrosine kinase	1.00603622	0.9147	NA
93841	Uchl4	ubiquitin carboxyl-terminal esterase L4	1.00603622	0.9173	NA
14455	Gas5	growth arrest specific 5	1.00603622	0.9445	0.972
67186	Rplp2	ribosomal protein, large P2	1.00603622	0.9471	0.9737
15929	Idh3g	isocitrate dehydrogenase 3 (NAD+), gamma	1.00593502	0.9177	NA
21689	Tekt1	tektin 1	1.00593502	0.9399	NA
26384	Gnpda1	glucosamine-6-phosphate deaminase 1	1.00593502	0.9484	0.9744
56380	Arid3b	AT rich interactive domain 3B (BRIGHT-like)	1.00593502	0.9595	0.9802
212281	A530054K11Rik	RIKEN cDNA A530054K11 gene	1.00593502	0.9708	0.9857
19355	Rad1	RAD1 homolog (S. pombe)	1.00583384	0.9031	NA
69923	Agk	acylglycerol kinase	1.00583384	0.9127	NA
72185	Dbndd1	dysbindin (dystrobrevin binding protein 1) domain containing 1	1.00573268	0.8878	NA
56249	Actr8	ARP8 actin-related protein 8 homolog (S. cerevisiae)	1.00573268	0.9198	NA
67044	Higd2a	HIG1 domain family, member 2A	1.00573268	0.9217	NA
74335	Xrcc3	X-ray repair complementing defective repair in Chinese hamster cells 3	1.00573268	0.9388	NA
19085	Prkar1b	protein kinase, cAMP dependent regulatory, type I beta	1.00573268	0.946	NA
13079	Cyp21a1	cytochrome P450, family 21, subfamily a, polypeptide 1	1.00573268	0.9821	0.9913
18761	Prkcq	protein kinase C, theta	1.00563154	0.9456	NA
75747	Sesn3	sestrin 3	1.00563154	0.9654	0.983
226352	Epb4.1l5	erythrocyte protein band 4.1-like 5	1.00563154	0.9658	0.9831
319181	Hist1h2bg	histone cluster 1, H2bg	1.00563154	0.97	0.9855
227674	Ddx31	DEAD/H (Asp-Glu-Ala-Asp/His) box polypeptide 31	1.00553042	0.9072	NA
66268	Pigyl	phosphatidylinositol glycan anchor biosynthesis, class Y-like	1.00553042	0.911	NA
13244	Degs1	degenerative spermatocyte homolog 1 (Drosophila)	1.00553042	0.9278	NA
56494	Gosr2	golgi SNAP receptor complex member 2	1.00553042	0.93	NA
235459	Gtf2a2	general transcription factor II A, 2	1.00553042	0.9308	NA
66078	Tsen34	tRNA splicing endonuclease 34 homolog (S. cerevisiae)	1.00553042	0.9313	NA
68133	Gcsh	glycine cleavage system protein H (aminomethyl carrier)	1.00553042	0.9526	0.9762
18972	Pold2	polymerase (DNA directed), delta 2, regulatory subunit	1.00553042	0.96	0.9805
14788	Gpr162	G protein-coupled receptor 162	1.00553042	0.9669	0.9836
18105	Nqo2	NAD(P)H dehydrogenase, quinone 2	1.00542932	0.9132	NA

619605	Zcchc17	zinc finger, CCHC domain containing 17	1.00542932	0.92	NA
74440	4933407C03Rik	RIKEN cDNA 4933407C03 gene	1.00542932	0.9246	NA
381038	Parl	presenilin associated, rhomboid-like	1.00542932	0.9247	NA
100042786	Gm16381	predicted gene 16381	1.00542932	0.9255	NA
224129	Adcy5	adenylate cyclase 5	1.00542932	0.9361	NA
330812	Rnf150	ring finger protein 150	1.00542932	0.9509	0.9755
240672	Dusp5	dual specificity phosphatase 5	1.00542932	0.9572	0.9787
17762	Mapt	microtubule-associated protein tau	1.00532824	0.9428	NA
20540	Slc7a7	solute carrier family 7 (cationic amino acid transporter, y+ system), member 7	1.00532824	0.9546	0.9771
171567	Nme7	non-metastatic cells 7, protein expressed in (nucleoside-diphosphate kinase)	1.00532824	0.9568	0.9786
215051	Bud13	BUD13 homolog (yeast)	1.00532824	0.9621	0.9815
80748	BC004004	cDNA sequence BC004004	1.00522718	0.9126	NA
104721	Ddx1	DEAD (Asp-Glu-Ala-Asp) box polypeptide 1	1.00522718	0.927	NA
68073	Fam173b	family with sequence similarity 173, member B	1.00522718	0.9344	NA
56412	2610024G14Rik	RIKEN cDNA 2610024G14 gene	1.00522718	0.936	NA
218335	Clptm1l	CLPTM1-like	1.00522718	0.9497	NA
276919	Gemin4	gem (nuclear organelle) associated protein 4	1.00522718	0.9499	NA
69662	2310061I04Rik	RIKEN cDNA 2310061I04 gene	1.00522718	0.9505	NA
19177	Psmb7	proteasome (prosome, macropain) subunit, beta type 7	1.00522718	0.9584	0.9793
56043	Akr1e1	aldo-keto reductase family 1, member E1	1.00512614	0.9112	NA
66164	Nip7	nuclear import 7 homolog (S. cerevisiae)	1.00512614	0.9152	NA
224648	Uhrf1bp1	UHRF1 (ICBP90) binding protein 1	1.00512614	0.9274	NA
216169	Fam108a	family with sequence similarity 108, member A	1.00512614	0.9291	NA
68889	Ubac2	ubiquitin associated domain containing 2	1.00512614	0.9503	NA
19674	Rcvrn	recoverin	1.00512614	0.9689	0.985
56454	Aldh18a1	aldehyde dehydrogenase 18 family, member A1	1.00512614	0.9737	0.9873
56444	Actr10	ARP10 actin-related protein 10 homolog (S. cerevisiae)	1.00502513	0.9088	NA
94067	Mrpl43	mitochondrial ribosomal protein L43	1.00502513	0.9201	NA
51789	Tnk2	tyrosine kinase, non-receptor, 2	1.00502513	0.9274	NA
67148	Fam103a1	family with sequence similarity 103, member A1	1.00502513	0.9361	NA
320508	Cachd1	cache domain containing 1	1.00502513	0.9381	NA
68036	Zfp706	zinc finger protein 706	1.00492413	0.9033	NA
16881	Lig1	ligase I, DNA, ATP-dependent	1.00492413	0.909	NA
382090	4922501C03Rik	RIKEN cDNA 4922501C03 gene	1.00492413	0.937	NA
242773	Slc45a1	solute carrier family 45, member 1	1.00492413	0.9421	NA
14588	Gfra4	glial cell line derived neurotrophic factor family receptor alpha 4	1.00492413	0.9605	0.9807
109689	Arrb1	arrestin, beta 1	1.00482315	0.9416	NA
214791	Sertad4	SERTA domain containing 4	1.00482315	0.9471	NA
20539	Slc7a5	solute carrier family 7 (cationic amino acid transporter, y+ system), member 5	1.00482315	0.9544	NA
68047	Mpnd	MPN domain containing	1.00482315	0.9574	0.9788
66549	Aggf1	angiogenic factor with G patch and FHA domains 1	1.00472219	0.9273	NA
19988	Rpl6	ribosomal protein L6	1.00472219	0.9602	0.9805
380924	Olfm4	olfactomedin 4	1.00472219	0.9722	0.9864
53622	Krt85	keratin 85	1.00472219	0.9735	0.9872
66899	Fip1l1	FIP1 like 1 (S. cerevisiae)	1.00472219	0.9735	0.9872
22687	Zfp259	zinc finger protein 259	1.00462126	0.9192	NA
66989	Kctd20	potassium channel tetramerisation domain containing 20	1.00462126	0.9382	NA
76137	Ccdc90a	coiled-coil domain containing 90A	1.00462126	0.9558	NA
13644	Efs	embryonal Fyn-associated substrate	1.00462126	0.9606	0.9807
241128	Fam124b	family with sequence similarity 124, member B	1.00462126	0.9653	0.9829

19291	Purb	purine rich element binding protein B	1.00462126	0.9706	0.9857
57808	Rpl35a	ribosomal protein L35A	1.00452034	0.9545	NA
104130	Ndufb11	NADH dehydrogenase (ubiquinone) 1 beta subcomplex, 11	1.00452034	0.9593	0.9801
208151	Tmem132b	transmembrane protein 132B	1.00452034	0.9801	0.9906
93687	Csnk1a1	casein kinase 1, alpha 1	1.00452034	0.981	0.9909
68011	Snrpg	small nuclear ribonucleoprotein polypeptide G	1.00441945	0.9131	NA
66589	Ube2v1	ubiquitin-conjugating enzyme E2 variant 1	1.00441945	0.9359	NA
70527	Stambp	STAM binding protein	1.00441945	0.9398	NA
276852	D11Wsu47e	DNA segment, Chr 11, Wayne State University 47, expressed	1.00441945	0.9435	NA
193740	Hspa1a	heat shock protein 1A	1.00441945	0.9453	NA
68857	Dtwd2	DTW domain containing 2	1.00441945	0.9477	NA
72267	Lrrc8e	leucine rich repeat containing 8 family, member E	1.00441945	0.979	0.9899
13723	Emb	embigin	1.00431857	0.9216	NA
228361	Ambra1	autophagy/beclin 1 regulator 1	1.00431857	0.9285	NA
239985	Arid1b	AT rich interactive domain 1B (SWI-like)	1.00431857	0.9576	NA
243616	Slc6a11	solute carrier family 6 (neurotransmitter transporter, GABA), member 11	1.00431857	0.9596	NA
209462	Hace1	HECT domain and ankyrin repeat containing, E3 ubiquitin protein ligase 1	1.00431857	0.9621	0.9815
73998	Herc3	hect domain and RLD 3	1.00431857	0.9623	0.9816
24105	Rbck1	RanBP-type and C3HC4-type zinc finger containing 1	1.00431857	0.9633	0.9823
277010	Marveld1	MARVEL (membrane-associating) domain containing 1	1.00431857	0.9637	0.9824
12465	Cct5	chaperonin containing Tcp1, subunit 5 (epsilon)	1.00421771	0.9194	NA
53319	Nxf1	nuclear RNA export factor 1 homolog (S. cerevisiae)	1.00421771	0.9395	NA
18002	Nedd8	neural precursor cell expressed, developmentally down-regulated gene 8	1.00421771	0.9396	NA
56018	Stard10	START domain containing 10	1.00421771	0.9434	NA
192170	Eif4a3	eukaryotic translation initiation factor 4A3	1.00421771	0.9578	NA
22282	Usf2	upstream transcription factor 2	1.00421771	0.9643	0.9826
110893	Slc8a3	solute carrier family 8 (sodium/calcium exchanger), member 3	1.00421771	0.9712	0.9859
229697	Cym	chymosin	1.00421771	0.9716	0.9861
66273	1810020D17Rik	RIKEN cDNA 1810020D17 gene	1.00411688	0.9358	NA
17237	Mgrn1	mahogunin, ring finger 1	1.00411688	0.9425	NA
70239	Gtf3c5	general transcription factor IIIC, polypeptide 5	1.00411688	0.9427	NA
209462	Hace1	HECT domain and ankyrin repeat containing, E3 ubiquitin protein ligase 1	1.00411688	0.9492	NA
72667	Zfp444	zinc finger protein 444	1.00411688	0.9724	0.9864
30841	Kdm2b	lysine (K)-specific demethylase 2B	1.00401606	0.9309	NA
244219	Zfp668	zinc finger protein 668	1.00401606	0.9536	NA
97487	Cmtm4	CKLF-like MARVEL transmembrane domain containing 4	1.00401606	0.9555	NA
231821	Adap1	ArfGAP with dual PH domains 1	1.00401606	0.9588	NA
108100	Baiap2	brain-specific angiogenesis inhibitor 1-associated protein 2	1.00401606	0.96	NA
241275	Noxa1	NADPH oxidase activator 1	1.00401606	0.9691	0.9851
16205	Gimap1	GTPase, IMAP family member 1	1.00401606	0.9836	0.9921
68552	1110003E01Rik	RIKEN cDNA 1110003E01 gene	1.00391527	0.9394	NA
239606	Slc2a13	solute carrier family 2 (facilitated glucose transporter), member 13	1.00391527	0.9472	NA
78832	2700078E11Rik	RIKEN cDNA 2700078E11 gene	1.00391527	0.9575	NA
53881	Slc5a3	solute carrier family 5 (inositol transporters), member 3	1.00391527	0.9807	0.9909
259053	Olfr362	olfactory receptor 362	1.00391527	0.9855	0.9931
22166	Txn1	thioredoxin 1	1.0038145	0.9538	NA
70425	Csnk1g3	casein kinase 1, gamma 3	1.0038145	0.9556	NA
68043	N6amt2	N-6 adenine-specific DNA methyltransferase 2 (putative)	1.0038145	0.9575	NA
22630	Ywhaq	tyrosine 3-monooxygenase/tryptophan 5-monooxygenase activation protein, theta polypeptide	1.0038145	0.9599	NA
109241	Mbd5	methyl-CpG binding domain protein 5	1.0038145	0.9606	NA

381085	Tbc1d22b	TBC1 domain family, member 22B	1.0038145	0.9673	0.9838
67201	Glod4	glyoxalase domain containing 4	1.00371374	0.9311	NA
77407	Rab35	RAB35, member RAS oncogene family	1.00371374	0.9314	NA
13640	Efna5	ephrin A5	1.00371374	0.9448	NA
17828	Muted	muted	1.00371374	0.949	NA
22218	Sumo1	SMT3 suppressor of mif two 3 homolog 1 (yeast)	1.00371374	0.9509	NA
72012	1600020E01Rik	RIKEN cDNA 1600020E01 gene	1.00371374	0.956	NA
17283	Men1	multiple endocrine neoplasia 1	1.00371374	0.9577	NA
210992	Lpcat1	lysophosphatidylcholine acyltransferase 1	1.00371374	0.961	NA
232157	Mobkl1b	MOB1, Mps One Binder kinase activator-like 1B (yeast)	1.00371374	0.9789	0.9898
66511	2500003M10Rik	RIKEN cDNA 2500003M10 gene	1.00361301	0.9441	NA
18484	Pam	peptidylglycine alpha-amidating monooxygenase	1.00361301	0.965	NA
54612	Sfrp5	secreted frizzled-related sequence protein 5	1.00361301	0.9794	0.9902
26949	Vat1	vesicle amine transport protein 1 homolog (T californica)	1.00361301	0.9809	0.9909
66381	Rnf113a2	ring finger protein 113A2	1.00351229	0.9315	NA
216136	Ilvbl	ilvB (bacterial acetolactate synthase)-like	1.00351229	0.9613	NA
64931	Folr4	folate receptor 4 (delta)	1.00351229	0.985	0.9928
109791	Clps	colipase, pancreatic	1.00351229	0.9889	0.9946
69786	Tprkb	Tp53rk binding protein	1.0034116	0.9454	NA
53330	Vamp4	vesicle-associated membrane protein 4	1.0034116	0.9475	NA
100039826	Gm2444	predicted gene 2444	1.0034116	0.9578	NA
12466	Cct6a	chaperonin containing Tcp1, subunit 6a (zeta)	1.0034116	0.9607	NA
73469	Rnf38	ring finger protein 38	1.00331093	0.9343	NA
56505	Ruvbl1	RuvB-like protein 1	1.00331093	0.9479	NA
217716	Mlh3	mutL homolog 3 (E coli)	1.00331093	0.9544	NA
242585	Slc35d1	solute carrier family 35 (UDP-glucuronic acid/UDP-N-acetylgalactosamine dual transporter), member D1	1.00331093	0.96	NA
217119	Xylt2	xylosyltransferase II	1.00331093	0.9661	NA
14904	Gtpbp1	GTP binding protein 1	1.00331093	0.9778	0.9893
94190	Ophn1	oligophrenin 1	1.00321027	0.9429	NA
56506	Cib2	calcium and integrin binding family member 2	1.00321027	0.9562	NA
72831	Dhx30	DEAH (Asp-Glu-Ala-His) box polypeptide 30	1.00321027	0.9581	NA
100042958	Gm4129	predicted gene 4129	1.00321027	0.9617	NA
16179	Irak1	interleukin-1 receptor-associated kinase 1	1.00321027	0.9647	NA
12055	Bcl7c	B-cell CLL/lymphoma 7C	1.00321027	0.9706	0.9857
66404	2410001C21Rik	RIKEN cDNA 2410001C21 gene	1.00321027	0.9757	0.9884
11610	Agtrap	angiotensin II, type I receptor-associated protein	1.00321027	0.9767	0.9889
239114	Il17d	interleukin 17D	1.00321027	0.9779	0.9894
654822	D330041H03Rik	RIKEN cDNA D330041H03 gene	1.00321027	0.9868	0.9937
15260	Hira	histone cell cycle regulation defective homolog A (S. cerevisiae)	1.00310964	0.9601	NA
11496	Adam22	a disintegrin and metallopeptidase domain 22	1.00310964	0.9617	NA
19052	Ppp2ca	protein phosphatase 2 (formerly 2A), catalytic subunit, alpha isoform	1.00310964	0.9651	NA
12408	Cbr1	carbonyl reductase 1	1.00310964	0.9674	NA
432530	Adcy1	adenylate cyclase 1	1.00310964	0.9723	0.9864
54351	Rai12	retinoic acid induced 12	1.00310964	0.9744	0.9878
217666	L2hgdh	L-2-hydroxyglutarate dehydrogenase	1.00310964	0.9788	0.9898
66330	1700020L24Rik	RIKEN cDNA 1700020L24 gene	1.00310964	0.9797	0.9904
234729	Vac14	Vac14 homolog (S. cerevisiae)	1.00310964	0.9802	0.9906
215632	Psd4	pleckstrin and Sec7 domain containing 4	1.00310964	0.9833	0.9919
72198	Skiv2l2	superkiller viralicidic activity 2-like 2 (S. cerevisiae)	1.00300903	0.9381	NA
73830	Eif3k	eukaryotic translation initiation factor 3, subunit K	1.00300903	0.9443	NA

99167	Ssx2ip	synovial sarcoma, X breakpoint 2 interacting protein	1.00300903	0.9461	NA
234135	Whsc1l1	Wolf-Hirschhorn syndrome candidate 1-like 1 (human)	1.00300903	0.9608	NA
100715	Papd4	PAP associated domain containing 4	1.00300903	0.9632	NA
56438	Rbx1	ring-box 1	1.00300903	0.9635	NA
223918	Spryd3	SPRY domain containing 3	1.00300903	0.9668	NA
234740	Tmem231	transmembrane protein 231	1.00300903	0.9813	0.9909
52705	Krr1	KRR1, small subunit (SSU) processome component, homolog (yeast)	1.00290843	0.9478	NA
382492	Gm12034	predicted gene 12034	1.00290843	0.9623	NA
67128	Ube2g1	ubiquitin-conjugating enzyme E2G 1 (UBC7 homolog, C. elegans)	1.00290843	0.9702	NA
69227	2810407C02Rik	RIKEN cDNA 2810407C02 gene	1.00290843	0.9726	NA
19070	Mobkl3	MOB1, Mps One Binder kinase activator-like 3 (yeast)	1.00290843	0.9731	NA
80743	Vps16	vacuolar protein sorting 16 (yeast)	1.00280786	0.9437	NA
17470	Cd200	CD200 antigen	1.00280786	0.951	NA
64296	Abhd8	abhydrolase domain containing 8	1.00280786	0.9716	NA
330319	Wipf3	WAS/WASL interacting protein family, member 3	1.00280786	0.9719	NA
56613	Rps6ka4	ribosomal protein S6 kinase, polypeptide 4	1.00280786	0.9732	NA
14371	Fzd9	frizzled homolog 9 (Drosophila)	1.00280786	0.9751	0.9882
12667	Chrd	chordin	1.00280786	0.9856	0.9931
18430	Oxtr	oxytocin receptor	1.00270731	0.9562	NA
54160	Copg2	coatomer protein complex, subunit gamma 2	1.00270731	0.9608	NA
73205	3110043O21Rik	RIKEN cDNA 3110043O21 gene	1.00270731	0.9667	NA
19243	Ptp4a1	protein tyrosine phosphatase 4a1	1.00270731	0.967	NA
22273	Uqcrc1	ubiquinol-cytochrome c reductase core protein 1	1.00270731	0.9786	0.9898
209318	Gps1	G protein pathway suppressor 1	1.00260678	0.9537	NA
21807	Tsc22d1	TSC22 domain family, member 1	1.00260678	0.9725	NA
215951	Lace1	lactation elevated 1	1.00260678	0.9793	0.9902
27050	Rps3	ribosomal protein S3	1.00260678	0.9827	0.9916
67181	Ctdnep1	CTD nuclear envelope phosphatase 1	1.00260678	0.983	0.9917
17356	Mllt4	myeloid/lymphoid or mixed-lineage leukemia (trithorax homolog, Drosophila); translocated to, 4	1.00260678	0.9843	0.9924
622446	Gm6323	predicted gene 6323	1.00260678	0.987	0.9937
52206	Anapc4	anaphase promoting complex subunit 4	1.00250627	0.9573	NA
14148	Fdx1	ferredoxin 1	1.00250627	0.9583	NA
225724	Mapk4	mitogen-activated protein kinase 4	1.00250627	0.9736	NA
15980	Ifngr2	interferon gamma receptor 2	1.00250627	0.9768	0.9889
107260	Otub1	OTU domain, ubiquitin aldehyde binding 1	1.00250627	0.9797	0.9904
17175	Masp2	mannan-binding lectin serine peptidase 2	1.00250627	0.9823	0.9915
76987	Hdhd2	haloacid dehalogenase-like hydrolase domain containing 2	1.00250627	0.9894	0.9947
228730	Plk1s1	polo-like kinase 1 substrate 1	1.00240577	0.9588	NA
102339	Cog4	component of oligomeric golgi complex 4	1.00240577	0.9634	NA
224650	Anks1	ankyrin repeat and SAM domain containing 1	1.00240577	0.9647	NA
19330	Rab18	RAB18, member RAS oncogene family	1.00240577	0.9651	NA
74451	Pgs1	phosphatidylglycerophosphate synthase 1	1.00240577	0.9687	NA
245847	Amdhd2	amidohydrolase domain containing 2	1.00240577	0.9743	NA
433702	Ncbp1	nuclear cap binding protein subunit 1	1.00240577	0.9746	NA
72003	Synpr	synaptoporin	1.0023053	0.9607	NA
18012	Neurod1	neurogenic differentiation 1	1.0023053	0.9749	NA
621407	Gm9970	predicted gene 9970	1.0023053	0.982	0.9913
76611	1700071A11Rik	RIKEN cDNA 1700071A11 gene	1.0023053	0.9825	0.9916
67112	Fgf22	fibroblast growth factor 22	1.0023053	0.9843	0.9923
56698	Phax	phosphorylated adaptor for RNA export	1.0023053	0.9877	0.9942

71746	Rgl3	ral guanine nucleotide dissociation stimulator-like 3	1.00220485	0.97	NA
67288	Srek1ip1	splicing regulatory glutamine/lysine-rich protein 1interacting protein 1	1.00220485	0.9712	NA
192734	AI646023	expressed sequence AI646023	1.00220485	0.9769	NA
52137	D7Ertd595e	DNA segment, Chr 7, ERATO Doi 595, expressed	1.00220485	0.9806	0.9909
56535	Pex3	peroxisomal biogenesis factor 3	1.00210442	0.9662	NA
109778	Blvra	biliverdin reductase A	1.00210442	0.9729	NA
27360	Add3	adducin 3 (gamma)	1.00210442	0.9769	NA
276770	Eif5a	eukaryotic translation initiation factor 5A	1.00210442	0.9779	NA
69993	Chn2	chimerin (chimaerin) 2	1.00210442	0.98	NA
19826	Rnps1	ribonucleic acid binding protein S1	1.00210442	0.9813	0.9909
70611	Fbxo33	F-box protein 33	1.00210442	0.9842	0.9923
226757	Wdr26	WD repeat domain 26	1.00200401	0.9683	NA
80291	Rilpl2	Rab interacting lysosomal protein-like 2	1.00200401	0.9767	NA
329777	Pigk	phosphatidylinositol glycan anchor biosynthesis, class K	1.00200401	0.9782	NA
14732	Gpam	glycerol-3-phosphate acyltransferase, mitochondrial	1.00200401	0.9866	0.9937
11431	Acp1	acid phosphatase 1, soluble	1.00200401	0.989	0.9946
23997	Psmd13	proteasome (prosome, macropain) 26S subunit, non-ATPase, 13	1.00190362	0.97	NA
74386	Rmi1	RMI1, RecQ mediated genome instability 1, homolog (S. cerevisiae)	1.00190362	0.973	NA
97613	C85181	expressed sequence C85181	1.00190362	0.9793	NA
216438	Mar-09	membrane-associated ring finger (C3HC4) 9	1.00190362	0.98	NA
72649	Tmem209	transmembrane protein 209	1.00190362	0.9811	NA
14137	Fdft1	farnesyl diphosphate farnesyl transferase 1	1.00190362	0.9839	0.9923
68079	Pdcd2l	programmed cell death 2-like	1.00180325	0.9698	NA
224829	Trerf1	transcriptional regulating factor 1	1.00180325	0.9882	0.9944
74137	Nuak2	NUAK family, SNF1-like kinase, 2	1.00180325	0.9896	0.9948
338372	Map3k9	mitogen-activated protein kinase kinase kinase 9	1.00180325	0.9902	0.9949
66152	Uqcr10	ubiquinol-cytochrome c reductase, complex III subunit X	1.00170289	0.9706	NA
13194	Ddb1	damage specific DNA binding protein 1	1.00170289	0.9718	NA
68515	Myadml2	myeloid-associated differentiation marker-like 2	1.00170289	0.9797	NA
13116	Cyp46a1	cytochrome P450, family 46, subfamily a, polypeptide 1	1.00170289	0.9813	NA
68531	1110020A21Rik	RIKEN cDNA 1110020A21 gene	1.00170289	0.983	NA
229615	Pias3	protein inhibitor of activated STAT 3	1.00170289	0.9859	0.9934
67170	2610306M01Rik	RIKEN cDNA 2610306M01 gene	1.00170289	0.9866	0.9937
18738	Pitpna	phosphatidylinositol transfer protein, alpha	1.00160256	0.9707	NA
66302	Fam82b	family with sequence similarity 82, member B	1.00160256	0.9771	NA
225326	Pik3c3	phosphoinositide-3-kinase, class 3	1.00160256	0.9793	NA
68152	Fam133b	family with sequence similarity 133, member B	1.00160256	0.9823	NA
231148	Ablim2	actin-binding LIM protein 2	1.00160256	0.9827	NA
232333	Slc6a1	solute carrier family 6 (neurotransmitter transporter, GABA), member 1	1.00160256	0.9841	NA
100037258	Dnajc3	DnaJ (Hsp40) homolog, subfamily C, member 3	1.00150225	0.9848	NA
78921	9130019O22Rik	RIKEN cDNA 9130019O22 gene	1.00150225	0.9867	0.9937
170755	Sgk3	serum/glucocorticoid regulated kinase 3	1.00150225	0.9874	0.9939
269704	Zfp664	zinc finger protein 664	1.00150225	0.989	0.9946
20116	Rps8	ribosomal protein S8	1.00150225	0.9898	0.9949
114896	Afg3l1	AFG3(ATPase family gene 3)-like 1 (yeast)	1.00140196	0.9789	NA
78893	Cnot10	CCR4-NOT transcription complex, subunit 10	1.00140196	0.9807	NA
68988	Prpf31	PRP31 pre-mRNA processing factor 31 homolog (yeast)	1.00140196	0.9821	NA
114255	Dok4	docking protein 4	1.00140196	0.9822	NA
14470	Rabac1	Rab acceptor 1 (prenylated)	1.00140196	0.9826	NA
67862	2310033P09Rik	RIKEN cDNA 2310033P09 gene	1.00140196	0.9832	NA

57230	Sap30bp	SAP30 binding protein	1.00140196	0.9841	NA
18181	Nrf1	nuclear respiratory factor 1	1.00140196	0.9878	0.9942
233877	Kctd13	potassium channel tetramerisation domain containing 13	1.00140196	0.9902	0.9949
14588	Gfra4	glial cell line derived neurotrophic factor family receptor alpha 4	1.00140196	0.991	0.9954
20937	Suv39h1	suppressor of variegation 3-9 homolog 1 (Drosophila)	1.00140196	0.992	0.996
13819	Epas1	endothelial PAS domain protein 1	1.00130169	0.9808	NA
107815	Scml2	sex comb on midleg-like 2 (Drosophila)	1.00130169	0.9823	NA
17134	Mafg	v-maf musculoaponeurotic fibrosarcoma oncogene family, protein G (avian)	1.00130169	0.9877	NA
13046	Celf1	CUGBP, Elav-like family member 1	1.00130169	0.9905	0.995
12757	Clta	clathrin, light polypeptide (Lca)	1.00120144	0.9766	NA
67003	Uqcrc2	ubiquinol cytochrome c reductase core protein 2	1.00120144	0.9775	NA
66409	Rsl1d1	ribosomal L1 domain containing 1	1.00120144	0.9814	NA
13198	Ddit3	DNA-damage inducible transcript 3	1.00120144	0.982	NA
619331	Zfp551	zinc fingr protein 551	1.00120144	0.988	NA
246735	AY074887	cDNA sequence AY074887	1.00120144	0.9897	0.9949
72514	Fgfbp3	fibroblast growth factor binding protein 3	1.00120144	0.9902	0.9949
26903	Dysf	dysferlin	1.00120144	0.9913	0.9956
12304	Pdia4	protein disulfide isomerase associated 4	1.00120144	0.9936	0.9968
100505330	LOC100505330	NHP2-like protein 1-like	1.00110121	0.9851	NA
101240	Wdr91	WD repeat domain 91	1.00110121	0.9879	NA
240185	9430020K01Rik	RIKEN cDNA 9430020K01 gene	1.00110121	0.989	NA
12613	Cel	carboxyl ester lipase	1.00110121	0.9917	0.9959
226180	Ina	internexin neuronal intermediate filament protein, alpha	1.00110121	0.9926	0.9964
94192	C1galt1	core 1 synthase, glycoprotein-N-acetylgalactosamine 3-beta-galactosyltransferase, 1	1.00110121	0.9945	0.997
637004	Vmn2r3	vomeronasal 2, receptor 3	1.00110121	0.9964	0.9981
20454	St3gal5	ST3 beta-galactoside alpha-2,3-sialyltransferase 5	1.001001	0.9793	NA
217031	Tada2a	transcriptional adaptor 2A	1.001001	0.983	NA
211922	Fam116a	family with sequence similarity 116, member A	1.001001	0.984	NA
56372	1110004F10Rik	RIKEN cDNA 1110004F10 gene	1.001001	0.9878	NA
14979	H2-Ke6	H2-K region expressed gene 6	1.001001	0.9897	NA
69161	Manbal	mannosidase, beta A, lysosomal-like	1.001001	0.9918	0.9959
432971	A130088B03Rik	RIKEN cDNA A130088B03 gene	1.001001	0.9939	0.9968
18764	Pkd2	polycystic kidney disease 2	1.00090081	0.988	NA
208967	Thnsl1	threonine synthase-like 1 (bacterial)	1.00090081	0.9885	NA
26371	Ciao1	cytosolic iron-sulfur protein assembly 1 homolog (S. cerevisiae)	1.00090081	0.9909	NA
66521	Rwdd1	RWD domain containing 1	1.00090081	0.9929	0.9964
269473	Lrig2	leucine-rich repeats and immunoglobulin-like domains 2	1.00090081	0.9934	0.9967
268958	Capn11	calpain 11	1.00090081	0.9953	0.9976
68193	Rpl24	ribosomal protein L24	1.00080064	0.983	NA
76246	Rtf1	Rtf1, Paf1/RNA polymerase II complex component, homolog (S. cerevisiae)	1.00080064	0.9884	NA
433273	Gm5523	glyceraldehyde-3-phosphate dehydrogenase pseudogene	1.00080064	0.9919	NA
232232	Hdac11	histone deacetylase 11	1.00080064	0.9938	0.9968
100273	Osbpl9	oxysterol binding protein-like 9	1.00080064	0.9955	0.9976
23970	Pacsin2	protein kinase C and casein kinase substrate in neurons 2	1.00070049	0.9876	NA
68298	Ncapd2	non-SMC condensin I complex, subunit D2	1.00070049	0.9929	NA
67459	Nvl	nuclear VCP-like	1.00070049	0.9933	NA
68505	1110014N23Rik	RIKEN cDNA 1110014N23 gene	1.00070049	0.9934	0.9967
100910	Chpf2	chondroitin polymerizing factor 2	1.00070049	0.9941	0.997
22185	U2af2	U2 small nuclear ribonucleoprotein auxiliary factor (U2AF) 2	1.00070049	0.9952	0.9975
320267	Fubp3	far upstream element (FUSE) binding protein 3	1.00070049	0.9952	0.9975

381287	A530032D15Rik	RIKEN cDNA A530032D15Rik gene	1.00070049	0.9974	0.9986
72567	Bclaf1	BCL2-associated transcription factor 1	1.00060036	0.9914	NA
53895	Clpp	caseinolytic peptidase, ATP-dependent, proteolytic subunit homolog (E. coli)	1.00060036	0.9926	NA
76178	6330578E17Rik	RIKEN cDNA 6330578E17 gene	1.00060036	0.9927	NA
66121	Chchd1	coiled-coil-helix-coiled-coil-helix domain containing 1	1.00060036	0.9929	NA
67458	Ergic1	endoplasmic reticulum-golgi intermediate compartment (ERGIC) 1	1.00060036	0.9931	NA
71684	Rbm43	RNA binding motif protein 43	1.00060036	0.9938	NA
210293	Dock10	dedicator of cytokinesis 10	1.00060036	0.9939	NA
23966	Odz4	odd Oz/ten-m homolog 4 (Drosophila)	1.00050025	0.99	NA
233490	Crebzf	CREB/ATF bZIP transcription factor	1.00050025	0.9935	NA
17120	Mad1l1	mitotic arrest deficient 1-like 1	1.00050025	0.9955	0.9976
110902	Chrna2	cholinergic receptor, nicotinic, alpha polypeptide 2 (neuronal)	1.00050025	0.9966	0.9982
20842	Stag1	stromal antigen 1	1.00040016	0.9913	NA
70120	Yars2	tyrosyl-tRNA synthetase 2 (mitochondrial)	1.00040016	0.9925	NA
223666	Arhgap39	Rho GTPase activating protein 39	1.00040016	0.9935	NA
381038	Parl	presenilin associated, rhomboid-like	1.00040016	0.9935	NA
11769	Ap1s1	adaptor protein complex AP-1, sigma 1	1.00040016	0.9943	NA
81896	Ift122	intraflagellar transport 122 homolog (Chlamydomonas)	1.00040016	0.9956	NA
72886	Ccdc94	coiled-coil domain containing 94	1.00040016	0.9958	NA
56424	Stub1	STIP1 homology and U-Box containing protein 1	1.00040016	0.996	NA
330660	Btbd16	BTB (POZ) domain containing 16	1.00040016	0.9972	0.9986
382099	Gm5161	predicted pseudogene 5161	1.00040016	0.9973	0.9986
269389	Tox2	TOX high mobility group box family member 2	1.00040016	0.9974	0.9986
240444	Kcng2	potassium voltage-gated channel, subfamily G, member 2	1.00040016	0.9977	0.9989
230596	Prpf38a	PRP38 pre-mRNA processing factor 38 (yeast) domain containing A	1.00030009	0.9946	NA
12866	Cox7a2	cytochrome c oxidase, subunit VIIa 2	1.00030009	0.9952	NA
74617	Scpep1	serine carboxypeptidase 1	1.00030009	0.9955	NA
66887	Lonp2	lon peptidase 2, peroxisomal	1.00030009	0.9965	NA
406217	Bex4	brain expressed gene 4	1.00030009	0.9966	NA
76299	Erp44	endoplasmic reticulum protein 44	1.00030009	0.9968	NA
320707	Atp2b3	ATPase, Ca++ transporting, plasma membrane 3	1.00030009	0.997	0.9985
17167	Marco	macrophage receptor with collagenous structure	1.00030009	0.9988	0.9996
14933	Gyk	glycerol kinase	1.00020004	0.997	NA
20655	Sod1	superoxide dismutase 1, soluble	1.00020004	0.9973	NA
27660	1700088E04Rik	RIKEN cDNA 1700088E04 gene	1.00020004	0.9974	NA
71715	Dhx35	DEAH (Asp-Glu-Ala-His) box polypeptide 35	1.00020004	0.9975	NA
12018	Bak1	BCL2-antagonist/killer 1	1.00020004	0.9977	NA
18148	Npm1	nucleophosmin 1	1.00020004	0.9985	NA
544963	Iqgap2	IQ motif containing GTPase activating protein 2	1.00020004	0.9992	0.9998
27411	Slc14a2	solute carrier family 14 (urea transporter), member 2	1.00020004	0.9996	0.9999
18245	Oaz1	ornithine decarboxylase antizyme 1	1.00010001	0.9989	NA
212503	Paox	polyamine oxidase (exo-N4-amino)	1.00010001	0.9991	0.9998
56503	Ankrd49	ankyrin repeat domain 49	1.00010001	0.9991	NA
72049	Tnfrsf13c	tumor necrosis factor receptor superfamily, member 13c	1.00010001	0.9993	0.9998
97212	Hadha	hydroxyacyl-Coenzyme A dehydrogenase/3-ketoacyl-Coenzyme A thiolase/enoyl-Coenzyme A hydratase (trifunctional protein), alpha subunit	1.00010001	0.9993	0.9998
17937	Nab2	Ngfi-A binding protein 2	1.00010001	0.9998	1
11840	Arf1	ADP-ribosylation factor 1	1	0.9934	NA
19299	Abcd3	ATP-binding cassette, sub-family D (ALD), member 3	1	0.9937	NA
54636	Wdr45	WD repeat domain 45	1	0.9937	NA
211739	Vstm2a	V-set and transmembrane domain containing 2A	1	0.9941	NA

66771	4933439F18Rik	RIKEN cDNA 4933439F18 gene	1	0.9946	NA
432768	Gm5451	predicted gene 5451	1	0.995	NA
66343	Tmem177	transmembrane protein 177	1	0.9953	NA
71991	Ercc8	excision repaiross-complementing rodent repair deficiency, complementation group 8	1	0.9956	NA
76915	Mnd1	meiotic nuclear divisions 1 homolog (S. cerevisiae)	1	0.9956	NA
217874	BC048943	cDNA sequence BC048943	1	0.9957	NA
76612	Lrrc27	leucine rich repeat containing 27	1	0.9962	0.998
100503304	LOC100503304	STAT3-interacting protein as a repressor-like	1	0.9964	NA
66653	Brf2	BRF2, subunit of RNA polymerase III transcription initiation factor, BRF1-like	1	0.9965	0.9982
242506	Frmd3	FERM domain containing 3	1	0.9968	0.9984
16981	Lrrn3	leucine rich repeat protein 3, neuronal	1	0.9976	NA
107829	Thoc5	THO complex 5	1	0.998	0.9991
65079	Rtn4r	reticulon 4 receptor	1	0.9982	0.9991
216543	Cep68	centrosomal protein 68	1	0.9982	NA
320076	C630001G18Rik	RIKEN cDNA C630001G18 gene	1	0.9987	0.9996
22210	Ube2b	ubiquitin-conjugating enzyme E2B, RAD6 homology (S. cerevisiae)	1	0.9987	NA
67695	Ost4	oligosaccharyltransferase 4 homolog (S. cerevisiae)	1	0.9989	0.9997
384281	Gatc	glutamyl-tRNA(Gln) amidotransferase, subunit C homolog (bacterial)	1	0.9989	NA
23897	Hax1	HCLS1 associated X-1	1	0.9991	NA
242506	Frmd3	FERM domain containing 3	1	0.9991	NA
67456	Ergic2	ERGIC and golgi 2	1	0.9992	0.9998
65102	Nif3l1	Ngg1 interacting factor 3-like 1 (S. pombe)	1	0.9992	NA
73247	1600027N09Rik	RIKEN cDNA 1600027N09 gene	1	0.9992	NA
214063	Dnajc16	DnaJ (Hsp40) homolog, subfamily C, member 16	1	0.9992	NA
30954	Siva1	SIVA1, apoptosis-inducing factor	1	0.9993	NA
433273	Gm5523	glyceraldehyde-3-phosphate dehydrogenase pseudogene	1	0.9994	0.9998
80284	BC003266	cDNA sequence BC003266	1	0.9996	NA
320500	Tmem215	transmembrane protein 215	1	0.9997	NA
56373	Cpb2	carboxypeptidase B2 (plasma)	1	0.9999	1
619937	Gm6115	predicted gene 6115	1	0.9999	1
70571	Tcerg1l	transcription elongation regulator 1-like	1	1	1
13185	Dscr3	Down syndrome critical region gene 3	1	1	NA
12995	Csnk2a1	casein kinase 2, alpha 1 polypeptide	0.999001	0.9744	NA
14356	Fxc1	fractured callus expressed transcript 1	0.999001	0.9774	NA
66184	Rps4y2	ribosomal protein S4, Y-linked 2	0.999001	0.9775	NA
73754	Thap1	THAP domain containing, apoptosis associated protein 1	0.999001	0.9781	NA
67378	Bbs2	Bardet-Biedl syndrome 2 (human)	0.999001	0.9792	NA
102247	Agpat6	1-acylglycerol-3-phosphate O-acyltransferase 6 (lysophosphatidic acid acyltransferase, zeta)	0.999001	0.9807	NA
67019	Actr6	ARP6 actin-related protein 6 homolog (yeast)	0.999001	0.9814	NA
69955	Fars2	phenylalanine-tRNA synthetase 2 (mitochondrial)	0.999001	0.9816	NA
100504872	LOC100504872	60S ribosomal protein L32-like	0.999001	0.9822	NA
233204	Tbc1d17	TBC1 domain family, member 17	0.999001	0.983	NA
67767	Jagn1	jagunal homolog 1 (Drosophila)	0.999001	0.9832	NA
229593	Golph3l	golgi phosphoprotein 3-like	0.999001	0.984	NA
18024	Nfe2l2	nuclear factor, erythroid derived 2, like 2	0.999001	0.9843	NA
71163	Zfp626	zinc finger protein 626	0.999001	0.985	NA
100042332	2810410L24Rik	RIKEN cDNA 2810410L24 gene	0.999001	0.985	NA
69091	Vps26b	vacuolar protein sorting 26 homolog B (yeast)	0.999001	0.9853	NA
14802	Gria4	glutamate receptor, ionotropic, AMPA4 (alpha 4)	0.999001	0.9854	NA
66070	Cwc15	CWC15 homolog (S. cerevisiae)	0.999001	0.9862	NA

227695	D2Wsu81e	DNA segment, Chr 2, Wayne State University 81, expressed	0.999001	0.9863	NA
223753	Cerk	ceramide kinase	0.999001	0.9865	NA
22330	Vcl	vinculin	0.999001	0.9867	NA
17993	Ndufs4	NADH dehydrogenase (ubiquinone) Fe-S protein 4	0.999001	0.9868	NA
12034	Phb2	prohibitin 2	0.999001	0.9872	NA
666372	Gm8066	predicted gene 8066	0.999001	0.9872	NA
77579	Myh10	myosin, heavy polypeptide 10, non-muscle	0.999001	0.9875	NA
229524	Msto1	misato homolog 1 (Drosophila)	0.999001	0.9877	NA
224023	Klhl22	kelch-like 22 (Drosophila)	0.999001	0.9882	NA
29876	Clic4	chloride intracellular channel 4 (mitochondrial)	0.999001	0.9885	NA
53599	Cd164	CD164 antigen	0.999001	0.9885	NA
72569	Bbs5	Bardet-Biedl syndrome 5 (human)	0.999001	0.9887	NA
107767	Scamp1	secretory carrier membrane protein 1	0.999001	0.9889	NA
227801	Dennd1a	DENN/MADD domain containing 1A	0.999001	0.9891	0.9947
22348	Slc32a1	solute carrier family 32 (GABA vesicular transporter), member 1	0.999001	0.9893	0.9947
103850	Nt5m	5',3'-nucleotidase, mitochondrial	0.999001	0.9895	NA
103213	Traf3ip2	TRAF3 interacting protein 2	0.999001	0.9899	NA
54152	Dnalc4	dynein, axonemal, light chain 4	0.999001	0.9901	NA
56459	Sae1	SUMO1 activating enzyme subunit 1	0.999001	0.9901	NA
69724	Rnaseh2a	ribonuclease H2, large subunit	0.999001	0.9909	0.9954
14533	Bloc1s1	biogenesis of lysosome-related organelles complex-1, subunit 1	0.999001	0.9909	NA
15926	Idh1	isocitrate dehydrogenase 1 (NADP+), soluble	0.999001	0.9909	NA
225644	Cplx4	complexin 4	0.999001	0.9909	NA
14792	Lpcat3	lysophosphatidylcholine acyltransferase 3	0.999001	0.991	0.9954
30937	Lmcd1	LIM and cysteine-rich domains 1	0.999001	0.9917	0.9959
12928	Crk	v-crk sarcoma virus CT10 oncogene homolog (avian)	0.999001	0.9918	0.9959
67510	Fam18b	family with sequence similarity 18, member B	0.999001	0.992	0.996
243302	Gm4963	predicted gene 4963	0.999001	0.9922	NA
100042539	Gm3893	predicted gene 3893	0.999001	0.9924	0.9962
20405	Sh3gl1	SH3-domain GRB2-like 1	0.999001	0.9926	0.9963
12336	Capns1	calpain, small subunit 1	0.999001	0.9927	NA
17684	Cited2	Cbp/p300-interacting transactivator, with Glu/Asp-rich carboxy-terminal domain, 2	0.999001	0.9932	0.9967
242050	Igsf10	immunoglobulin superfamily, member 10	0.999001	0.9934	0.9967
68592	Syf2	SYF2 homolog, RNA splicing factor (S. cerevisiae)	0.999001	0.9936	0.9968
16506	Kcnd1	potassium voltage-gated channel, Shal-related family, member 1	0.999001	0.9941	0.997
66471	Anp32e	acidic (leucine-rich) nuclear phosphoprotein 32 family, member E	0.999001	0.9944	0.997
433961	Gm5565	predicted gene 5565	0.999001	0.9949	0.9973
78751	Zc3h6	zinc finger CCCH type containing 6	0.999001	0.9956	0.9976
100978	Nfxl1	nuclear transcription factor, X-box binding-like 1	0.999001	0.9958	0.9978
19246	Ptpn1	protein tyrosine phosphatase, non-receptor type 1	0.999001	0.9962	0.998
20678	Sox5	SRY-box containing gene 5	0.999001	0.9962	0.998
22761	Zfpm1	zinc finger protein, multitype 1	0.99800399	0.96	NA
68051	Nutf2	nuclear transport factor 2	0.99800399	0.9626	NA
59008	Anapc5	anaphase-promoting complex subunit 5	0.99800399	0.9635	NA
66126	Elof1	elongation factor 1 homolog (ELF1, S. cerevisiae)	0.99800399	0.9653	NA
69601	Dab2ip	disabled homolog 2 (Drosophila) interacting protein	0.99800399	0.9654	NA
18970	Polb	polymerase (DNA directed), beta	0.99800399	0.9666	NA
68342	Ndufb10	NADH dehydrogenase (ubiquinone) 1 beta subcomplex, 10	0.99800399	0.9666	NA
12995	Csnk2a1	casein kinase 2, alpha 1 polypeptide	0.99800399	0.9682	NA
12055	Bcl7c	B-cell CLL/lymphoma 7C	0.99800399	0.9687	NA

381813	Prmt8	protein arginine N-methyltransferase 8	0.99800399	0.9696	NA
12951	Crx	cone-rod homeobox containing gene	0.99800399	0.9703	NA
223701	Mkl1	MKL (megakaryoblastic leukemia)/myocardin-like 1	0.99800399	0.9705	NA
13426	Dync1i1	dynein cytoplasmic 1 intermediate chain 1	0.99800399	0.9707	NA
70123	2210013O21Rik	RIKEN cDNA 2210013O21 gene	0.99800399	0.9722	NA
381066	Zfp948	zinc finger protein 948	0.99800399	0.9722	NA
16825	Ldb1	LIM domain binding 1	0.99800399	0.9724	NA
70238	Rnf168	ring finger protein 168	0.99800399	0.9726	NA
228994	Ythdf1	YTH domain family 1	0.99800399	0.9728	NA
14360	Fyn	Fyn proto-oncogene	0.99800399	0.9735	NA
114641	Rpl31	ribosomal protein L31	0.99800399	0.9737	NA
53901	Rcan2	regulator of calcineurin 2	0.99800399	0.9741	NA
12848	Cops2	COP9 (constitutive photomorphogenic) homolog, subunit 2 (Arabidopsis thaliana)	0.99800399	0.9744	NA
15975	Ifnar1	interferon (alpha and beta) receptor 1	0.99800399	0.9745	NA
109145	Gins4	GINS complex subunit 4 (Sld5 homolog)	0.99800399	0.9748	NA
72151	Rfc5	replication factor C (activator 1) 5	0.99800399	0.9754	NA
67188	2700046G09Rik	RIKEN cDNA 2700046G09 gene	0.99800399	0.9755	NA
270076	Gcdh	glutaryl-Coenzyme A dehydrogenase	0.99800399	0.9755	NA
68525	Evc2	Ellis van Creveld syndrome 2 homolog (human)	0.99800399	0.976	NA
103172	Chchd10	coiled-coil-helix-coiled-coil-helix domain containing 10	0.99800399	0.9766	NA
12909	Crcp	calcitonin gene-related peptide-receptor component protein	0.99800399	0.9767	NA
100705	Acacb	acetyl-Coenzyme A carboxylase beta	0.99800399	0.9775	NA
54126	Arhgef7	Rho guanine nucleotide exchange factor (GEF7)	0.99800399	0.9776	NA
100039968	Gm12942	predicted gene 12942	0.99800399	0.9777	NA
77766	Elp4	elongation protein 4 homolog (S. cerevisiae)	0.99800399	0.978	NA
106014	Fam19a5	family with sequence similarity 19, member A5	0.99800399	0.9783	NA
71384	5430440L12Rik	RIKEN cDNA 5430440L12 gene	0.99800399	0.9789	NA
56382	Rab9	RAB9, member RAS oncogene family	0.99800399	0.9794	NA
58810	Akr1a4	aldo-keto reductase family 1, member A4 (aldehyde reductase)	0.99800399	0.9801	NA
66616	Snx9	sorting nexin 9	0.99800399	0.9811	0.9909
170638	Hpcal4	hippocalcin-like 4	0.99800399	0.9816	0.9911
211006	Sepsecs	Sep (O-phosphoserine) tRNA:Sec (selenocysteine) tRNA synthase	0.99800399	0.9817	0.9911
19988	Rpl6	ribosomal protein L6	0.99800399	0.9828	0.9917
74600	Mrpl47	mitochondrial ribosomal protein L47	0.99800399	0.9834	NA
68089	Arpc4	actin related protein 2/3 complex, subunit 4	0.99800399	0.9841	NA
72962	Tymp	thymidine phosphorylase	0.99800399	0.9845	NA
27261	Dok3	docking protein 3	0.99800399	0.9849	0.9928
628847	Gm6921	predicted pseudogene 6921	0.99800399	0.9853	0.993
233899	Gm166	predicted gene 166	0.99800399	0.986	0.9934
238328	Vash1	vasohibin 1	0.99800399	0.9863	0.9936
11298	Aanat	arylalkylamine N-acetyltransferase	0.99800399	0.9864	0.9936
382867	Zfp488	zinc finger protein 488	0.99800399	0.9866	0.9937
71599	Senp8	SUMO/sentrin specific peptidase 8	0.99800399	0.9868	0.9937
105892	9030619P08Rik	RIKEN cDNA 9030619P08 gene	0.99800399	0.987	0.9937
19342	Rab4b	RAB4B, member RAS oncogene family	0.99800399	0.9872	0.9939
74340	Ahcyl2	S-adenosylhomocysteine hydrolase-like 2	0.99800399	0.9873	0.9939
73442	Hspa12a	heat shock protein 12A	0.99800399	0.9886	0.9946
545700	Vmn2r-ps14	vomeronasal 2, receptor, pseudogene 14	0.99800399	0.9893	0.9947
224807	Tmem63b	transmembrane protein 63b	0.99800399	0.9896	0.9948
237433	Gm4925	predicted gene 4925	0.99800399	0.9903	0.995

68453	Gpihbp1	GPI-anchored HDL-binding protein 1	0.99800399	0.9927	0.9964
93690	Gpr45	G protein-coupled receptor 45	0.99800399	0.9937	0.9968
27425	Atp5l	ATP synthase, H+ transporting, mitochondrial F0 complex, subunit g	0.99800399	0.9945	0.997
100038415	F830034J09Rik	RIKEN cDNA F830034J09 gene	0.99800399	0.9945	0.997
19201	Pstpip2	proline-serine-threonine phosphatase-interacting protein 2	0.99800399	0.9952	0.9975
11739	Slc25a4	solute carrier family 25 (mitochondrial carrier, adenine nucleotide translocator), member 4	0.99700897	0.9304	NA
15525	Hspa4	heat shock protein 4	0.99700897	0.9307	NA
386649	Nsfl1c	NSFL1 (p97) cofactor (p47)	0.99700897	0.9394	NA
68183	Bcas2	breast carcinoma amplified sequence 2	0.99700897	0.9411	NA
67851	1700021F05Rik	RIKEN cDNA 1700021F05 gene	0.99700897	0.943	NA
71927	Itfg1	integrin alpha FG-GAP repeat containing 1	0.99700897	0.9434	NA
98053	Gtf2f1	general transcription factor IIF, polypeptide 1	0.99700897	0.9434	NA
13684	Eif4e	eukaryotic translation initiation factor 4E	0.99700897	0.9479	NA
19280	Ptprs	protein tyrosine phosphatase, receptor type, S	0.99700897	0.9493	NA
140499	Ube2j2	ubiquitin-conjugating enzyme E2, J2 homolog (yeast)	0.99700897	0.952	NA
67615	Ube2r2	ubiquitin-conjugating enzyme E2R 2	0.99700897	0.9535	NA
30932	Zfp330	zinc finger protein 330	0.99700897	0.9552	NA
68295	0610011L14Rik	RIKEN cDNA 0610011L14 gene	0.99700897	0.9556	NA
59022	Edf1	endothelial differentiation-related factor 1	0.99700897	0.956	NA
75425	Tti1	Tel2 interacting protein 1 homolog (S. pombe)	0.99700897	0.959	NA
192285	Phf21a	PHD finger protein 21A	0.99700897	0.9594	NA
12034	Phb2	prohibitin 2	0.99700897	0.9598	NA
13542	Dvl1	dishevelled, dsh homolog 1 (Drosophila)	0.99700897	0.9608	NA
66225	Llph	LLP homolog, long-term synaptic facilitation (Aplysia)	0.99700897	0.9608	NA
14911	Thumpd3	THUMP domain containing 3	0.99700897	0.9615	NA
106952	Arap3	ArfGAP with RhoGAP domain, ankyrin repeat and PH domain 3	0.99700897	0.9618	NA
208820	Gm11818	predicted gene 11818	0.99700897	0.9618	NA
67179	Ccdc25	coiled-coil domain containing 25	0.99700897	0.9633	NA
382030	Tmem188	transmembrane protein 188	0.99700897	0.9633	NA
11843	Arf4	ADP-ribosylation factor 4	0.99700897	0.9639	NA
72729	Cdc42se2	CDC42 small effector 2	0.99700897	0.9646	NA
67064	Chmp1b	chromatin modifying protein 1B	0.99700897	0.9648	NA
77605	H2afv	H2A histone family, member V	0.99700897	0.9648	NA
80888	Hspb8	heat shock protein 8	0.99700897	0.9651	NA
72290	Lsm11	U7 snRNP-specific Sm-like protein LSM11	0.99700897	0.9667	NA
52708	Zfp410	zinc finger protein 410	0.99700897	0.9669	NA
20595	Smn1	survival motor neuron 1	0.99700897	0.9674	NA
23938	Map2k5	mitogen-activated protein kinase kinase 5	0.99700897	0.9675	NA
217737	Ahsa1	AHA1, activator of heat shock protein ATPase homolog 1 (yeast)	0.99700897	0.9675	NA
72749	Tonsl	tonsoku-like, DNA repair protein	0.99700897	0.9677	NA
319229	Sctr	secretin receptor	0.99700897	0.9683	0.9846
16842	Lef1	lymphoid enhancer binding factor 1	0.99700897	0.9689	0.985
15312	Hmgn1	high mobility group nucleosomal binding domain 1	0.99700897	0.9699	NA
244329	Mcph1	microcephaly, primary autosomal recessive 1	0.99700897	0.9701	NA
16558	Kif16b	kinesin family member 16B	0.99700897	0.9706	NA
433938	Mn1	meningioma 1	0.99700897	0.9706	NA
214897	Csnk1g1	casein kinase 1, gamma 1	0.99700897	0.9725	NA
217140	Scrn2	secernin 2	0.99700897	0.9731	NA
72552	Hsdl1	hydroxysteroid dehydrogenase like 1	0.99700897	0.9737	NA
380912	Zfp395	zinc finger protein 395	0.99700897	0.9752	0.9882

57912	Cdc42se1	CDC42 small effector 1	0.99700897	0.976	0.9885
66311	Cenpw	centromere protein W	0.99700897	0.9762	0.9886
228852	Ppp1r16b	protein phosphatase 1, regulatory (inhibitor) subunit 16B	0.99700897	0.9765	0.9887
13714	Elk4	ELK4, member of ETS oncogene family	0.99700897	0.9775	0.9892
15502	Dnaja1	DnaJ (Hsp40) homolog, subfamily A, member 1	0.99700897	0.9788	0.9898
269862	Olfr1349	olfactory receptor 1349	0.99700897	0.9834	0.992
93742	Pard3	par-3 (partitioning defective 3) homolog (C. elegans)	0.99700897	0.9889	0.9946
57312	Mrps31	mitochondrial ribosomal protein S31	0.99601594	0.9129	NA
20397	Sgpl1	sphingosine phosphate lyase 1	0.99601594	0.9161	NA
330222	Sdk1	sidekick homolog 1 (chicken)	0.99601594	0.9165	NA
69743	Casz1	castor homolog 1, zinc finger (Drosophila)	0.99601594	0.9184	NA
109674	Ampd2	adenosine monophosphate deaminase 2	0.99601594	0.9254	NA
20909	Stx4a	syntaxin 4A (placental)	0.99601594	0.9261	NA
68642	Tmem216	transmembrane protein 216	0.99601594	0.9268	NA
68028	Rpl22l1	ribosomal protein L22 like 1	0.99601594	0.9278	NA
54678	Zfp108	zinc finger protein 108	0.99601594	0.9293	NA
69792	Med6	mediator of RNA polymerase II transcription, subunit 6 homolog (yeast)	0.99601594	0.9301	NA
66352	Blzf1	basic leucine zipper nuclear factor 1	0.99601594	0.9311	NA
594844	Tceal3	transcription elongation factor A (SII)-like 3	0.99601594	0.9312	NA
66212	Sec61b	Sec61 beta subunit	0.99601594	0.935	NA
69821	Mterfd2	MTERF domain containing 2	0.99601594	0.9362	NA
21769	Zfand3	zinc finger, AN1-type domain 3	0.99601594	0.9368	NA
26891	Cops4	COP9 (constitutive photomorphogenic) homolog, subunit 4 (Arabidopsis thaliana)	0.99601594	0.9372	NA
227290	Aamp	angio-associated migratory protein	0.99601594	0.9395	NA
19027	Sypl	synaptophysin-like protein	0.99601594	0.9419	NA
75608	Chmp4b	chromatin modifying protein 4B	0.99601594	0.9421	NA
54197	Rnf5	ring finger protein 5	0.99601594	0.9438	NA
76688	Arfrp1	ADP-ribosylation factor related protein 1	0.99601594	0.9452	NA
11793	Atg5	autophagy-related 5 (yeast)	0.99601594	0.9467	NA
338348	Ttc16	tetratricopeptide repeat domain 16	0.99601594	0.9469	NA
101869	Unc45a	unc-45 homolog A (C. elegans)	0.99601594	0.9492	NA
71667	0610007L01Rik	RIKEN cDNA 0610007L01 gene	0.99601594	0.9508	NA
27366	Txnl4a	thioredoxin-like 4A	0.99601594	0.9513	NA
14375	Xrcc6	X-ray repair complementing defective repair in Chinese hamster cells 6	0.99601594	0.9525	NA
68507	Ppfia4	protein tyrosine phosphatase, receptor type, f polypeptide (PTPRF), interacting protein (liprin), alpha 4	0.99601594	0.9534	NA
11538	Adnp	activity-dependent neuroprotective protein	0.99601594	0.9548	NA
23879	Fxr2	fragile X mental retardation, autosomal homolog 2	0.99601594	0.9552	NA
235574	Atp2c1	ATPase, Ca++-sequestering	0.99601594	0.9552	NA
16834	Cog1	component of oligomeric golgi complex 1	0.99601594	0.9556	NA
100163	Pafah2	platelet-activating factor acetylhydrolase 2	0.99601594	0.9557	NA
74008	Arsg	arylsulfatase G	0.99601594	0.9562	NA
327900	Ubtd2	ubiquitin domain containing 2	0.99601594	0.9571	NA
16865	Eif2d	eukaryotic translation initiation factor 2D	0.99601594	0.9572	NA
12572	Cdk7	cyclin-dependent kinase 7	0.99601594	0.9573	NA
100039043	Gm10731	predicted gene 10731	0.99601594	0.9582	NA
433313	Rpl17-ps1	ribosomal protein L17, pseudogene 1	0.99601594	0.9583	NA
64424	Polr1e	polymerase (RNA) I polypeptide E	0.99601594	0.9584	NA
56529	Sec11a	SEC11 homolog A (S. cerevisiae)	0.99601594	0.9595	NA
192196	Luc7l2	LUC7-like 2 (S. cerevisiae)	0.99601594	0.9609	NA
20167	Rtn2	reticulon 2 (Z-band associated protein)	0.99601594	0.9614	NA

434632	BC085271	cDNA sequence BC085271	0.99601594	0.9618	0.9814
329641	6030405A18Rik	RIKEN cDNA 6030405A18 gene	0.99601594	0.9621	NA
18127	Nos3	nitric oxide synthase 3, endothelial cell	0.99601594	0.9625	NA
76740	Efr3a	EFR3 homolog A (S. cerevisiae)	0.99601594	0.9631	NA
666173	Vps13b	vacuolar protein sorting 13B (yeast)	0.99601594	0.9642	NA
224598	Zfp758	zinc finger protein 758	0.99601594	0.9655	0.983
110750	Cse1l	chromosome segregation 1-like (S. cerevisiae)	0.99601594	0.9665	0.9834
13627	Eef1a1	eukaryotic translation elongation factor 1 alpha 1	0.99601594	0.9681	0.9844
225363	Etf1	eukaryotic translation termination factor 1	0.99601594	0.9686	0.9848
16911	Lmo4	LIM domain only 4	0.99601594	0.9694	0.9852
22715	Zfp57	zinc finger protein 57	0.99601594	0.9699	0.9854
20382	Srsf2	serine/arginine-rich splicing factor 2	0.99601594	0.9703	0.9856
17218	Mcm5	minichromosome maintenance deficient 5, cell division cycle 46 (S. cerevisiae)	0.99601594	0.9706	0.9857
72511	2610316D01Rik	RIKEN cDNA 2610316D01 gene	0.99601594	0.9736	0.9873
69080	Gmppa	GDP-mannose pyrophosphorylase A	0.99601594	0.9744	0.9878
622116	Gm6287	predicted gene 6287	0.99601594	0.9747	0.988
101476	Plekha1	pleckstrin homology domain containing, family A (phosphoinositide binding specific) member 1	0.99601594	0.9751	0.9882
18155	Pnoc	prepronociceptin	0.99601594	0.9755	0.9884
231798	Lrch4	leucine-rich repeats and calponin homology (CH) domain containing 4	0.99601594	0.9757	0.9884
236266	Alms1	Alstrom syndrome 1 homolog (human)	0.99601594	0.9775	0.9892
213827	Arcn1	archain 1	0.99601594	0.9786	0.9898
14260	Fmn1	formin 1	0.99601594	0.9801	0.9906
16705	Krtap9-1	keratin associated protein 9-1	0.99601594	0.9801	0.9906
59004	Pias4	protein inhibitor of activated STAT 4	0.99601594	0.9809	0.9909
282663	Serpinb1b	serine (or cysteine) peptidase inhibitor, clade B, member 1b	0.99601594	0.987	0.9937
628324	S100a2	S100 calcium binding protein A2	0.99601594	0.9879	0.9943
11474	Actn3	actinin alpha 3	0.99601594	0.9887	0.9946
547168	Rhox7	reproductive homeobox 7	0.99601594	0.9894	0.9947
76808	Rpl18a	ribosomal protein L18A	0.99502488	0.908	NA
80795	Selk	selenoprotein K	0.99502488	0.91	NA
140887	Lnx2	ligand of numb-protein X 2	0.99502488	0.914	NA
68349	Ndufs3	NADH dehydrogenase (ubiquinone) Fe-S protein 3	0.99502488	0.9142	NA
12366	Casp2	caspase 2	0.99502488	0.9165	NA
232146	Fam176a	family with sequence similarity 176, member A	0.99502488	0.92	NA
68365	Rab14	RAB14, member RAS oncogene family	0.99502488	0.922	NA
108707	1810008A18Rik	RIKEN cDNA 1810008A18 gene	0.99502488	0.9256	NA
668661	2410002F23Rik	RIKEN cDNA 2410002F23 gene	0.99502488	0.9262	NA
19283	Ptprz1	protein tyrosine phosphatase, receptor type Z, polypeptide 1	0.99502488	0.9284	NA
71348	5430439M09Rik	RIKEN cDNA 5430439M09 gene	0.99502488	0.934	NA
67443	Map1lc3b	microtubule-associated protein 1 light chain 3 beta	0.99502488	0.9369	NA
192651	Zfp286	zinc finger protein 286	0.99502488	0.9372	NA
66498	Dda1	DET1 and DDB1 associated 1	0.99502488	0.9381	NA
20698	Sphk1	sphingosine kinase 1	0.99502488	0.9391	NA
545389	Cep170	centrosomal protein 170	0.99502488	0.9394	NA
268354	Fam19a2	family with sequence similarity 19, member A2	0.99502488	0.9399	NA
18045	Nfyb	nuclear transcription factor-Y beta	0.99502488	0.9427	NA
12292	Cacna1s	calcium channel, voltage-dependent, L type, alpha 1S subunit	0.99502488	0.9451	NA
494448	Cbx6	chromobox homolog 6	0.99502488	0.9459	NA
67433	Ccdc127	coiled-coil domain containing 127	0.99502488	0.9465	NA
54342	Gnpnat1	glucosamine-phosphate N-acetyltransferase 1	0.99502488	0.9467	NA

29865	Cabp5	calcium binding protein 5	0.99502488	0.9479	NA
320292	Rasgef1b	RasGEF domain family, member 1B	0.99502488	0.948	NA
382117	D9Ertd402e	DNA segment, Chr 9, ERATO Doi 402, expressed	0.99502488	0.9501	NA
12552	Cdh11	cadherin 11	0.99502488	0.9537	0.9767
16973	Lrp5	low density lipoprotein receptor-related protein 5	0.99502488	0.9559	0.9779
229357	Gpr149	G protein-coupled receptor 149	0.99502488	0.9559	0.9779
227210	Ccnyl1	cyclin Y-like 1	0.99502488	0.9568	0.9786
17304	Mfge8	milk fat globule-EGF factor 8 protein	0.99502488	0.9579	0.979
224523	4732491K20Rik	RIKEN cDNA 4732491K20 gene	0.99502488	0.9579	0.979
107732	Mrpl10	mitochondrial ribosomal protein L10	0.99502488	0.9601	0.9805
672284	Nkx1-1	NK1 transcription factor related, locus 1 (Drosophila)	0.99502488	0.9616	0.9813
14395	Gabra2	gamma-aminobutyric acid (GABA) A receptor, subunit alpha 2	0.99502488	0.9634	0.9823
242466	Zfp462	zinc finger protein 462	0.99502488	0.9645	0.9827
17436	Me1	malic enzyme 1, NADP(+)-dependent, cytosolic	0.99502488	0.9647	0.9828
234069	Pcid2	PCI domain containing 2	0.99502488	0.9668	0.9835
66766	4933425O20Rik	RIKEN cDNA 4933425O20 gene	0.99502488	0.9672	0.9837
67291	Ccdc137	coiled-coil domain containing 137	0.99502488	0.9678	0.9842
236266	Alms1	Alstrom syndrome 1 homolog (human)	0.99502488	0.9695	0.9852
69709	2410017P09Rik	RIKEN cDNA 2410017P09 gene	0.99502488	0.9728	0.9867
74646	Spsb1	splA/ryanodine receptor domain and SOCS box containing 1	0.99502488	0.9773	0.9892
57916	Tnfrsf13b	tumor necrosis factor receptor superfamily, member 13b	0.99502488	0.9823	0.9915
381994	E030018B13Rik	RIKEN cDNA E030018B13 gene	0.99502488	0.9842	0.9923
70963	4931402H11Rik	RIKEN cDNA 4931402H11 gene	0.99502488	0.9885	0.9946
635756	Gm7166	predicted gene 7166	0.99502488	0.9886	0.9946
66511	2500003M10Rik	RIKEN cDNA 2500003M10 gene	0.99403579	0.8861	NA
68193	Rpl24	ribosomal protein L24	0.99403579	0.8916	NA
57783	Tnip1	TNFAIP3 interacting protein 1	0.99403579	0.8958	NA
106042	Prickle1	prickle homolog 1 (Drosophila)	0.99403579	0.8974	NA
74450	Pank2	pantothenate kinase 2	0.99403579	0.9016	NA
94213	Ddx50	DEAD (Asp-Glu-Ala-Asp) box polypeptide 50	0.99403579	0.905	NA
68675	Fam172a	family with sequence similarity 172, member A	0.99403579	0.9084	NA
71946	Endod1	endonuclease domain containing 1	0.99403579	0.9103	NA
19167	Psma3	proteasome (prosome, macropain) subunit, alpha type 3	0.99403579	0.9106	NA
216157	ORF61	open reading frame 61	0.99403579	0.9124	NA
66258	Mrps17	mitochondrial ribosomal protein S17	0.99403579	0.9127	NA
52829	D4Bwg0951e	DNA segment, Chr 4, Brigham & Women's Genetics 0951 expressed	0.99403579	0.9138	NA
68972	Tatdn3	TatD DNase domain containing 3	0.99403579	0.9141	NA
19139	Prps1	phosphoribosyl pyrophosphate synthetase 1	0.99403579	0.9155	NA
68083	Pak1ip1	PAK1 interacting protein 1	0.99403579	0.916	NA
381598	2610005L07Rik	cadherin 11 pseudogene	0.99403579	0.9166	NA
215748	Cnksr3	Cnksr family member 3	0.99403579	0.9189	NA
67025	Rpl11	ribosomal protein L11	0.99403579	0.9203	NA
18645	Pfn2	profilin 2	0.99403579	0.9207	NA
74427	Eaf1	ELL associated factor 1	0.99403579	0.921	NA
22099	Tsn	translin	0.99403579	0.9213	NA
227738	Lrsam1	leucine rich repeat and sterile alpha motif containing 1	0.99403579	0.9228	NA
252966	Cables2	CDK5 and Abl enzyme substrate 2	0.99403579	0.9253	NA
66885	Acadsb	acyl-Coenzyme A dehydrogenase, short/branched chain	0.99403579	0.9258	NA
57874	Ptplad1	protein tyrosine phosphatase-like A domain containing 1	0.99403579	0.9261	NA
18784	Pla2g5	phospholipase A2, group V	0.99403579	0.9286	NA

66164	Nip7	nuclear import 7 homolog (S. cerevisiae)	0.99403579	0.9295	NA
53624	Cldn7	claudin 7	0.99403579	0.9299	NA
67453	Slc25a46	solute carrier family 25, member 46	0.99403579	0.9323	NA
30050	Fbxw2	F-box and WD-40 domain protein 2	0.99403579	0.9345	NA
18231	Nxph1	neurexophilin 1	0.99403579	0.9352	NA
234076	Tmco3	transmembrane and coiled-coil domains 3	0.99403579	0.9371	NA
28035	Usp39	ubiquitin specific peptidase 39	0.99403579	0.9384	NA
73420	1700054N08Rik	RIKEN cDNA 1700054N08 gene	0.99403579	0.939	NA
78920	Dlst	dihydrolipoamide S-succinyltransferase (E2 component of 2-oxo-glutarate complex)	0.99403579	0.9394	NA
60425	Doc2g	double C2, gamma	0.99403579	0.9419	0.9702
21833	Thra	thyroid hormone receptor alpha	0.99403579	0.9419	NA
241732	Tspyl3	TSPY-like 3	0.99403579	0.9464	NA
11516	Adcyap1	adenylate cyclase activating polypeptide 1	0.99403579	0.9485	0.9744
14433	Gapdh	glyceraldehyde-3-phosphate dehydrogenase	0.99403579	0.9507	0.9754
94218	Cnnm3	cyclin M3	0.99403579	0.9507	0.9754
50907	Preb	prolactin regulatory element binding	0.99403579	0.9545	0.9771
380928	Lmo7	LIM domain only 7	0.99403579	0.9556	0.9778
22253	Unc5c	unc-5 homolog C (C. elegans)	0.99403579	0.9575	0.9788
330914	Arhgap32	Rho GTPase activating protein 32	0.99403579	0.9581	0.9792
242553	Kank4	KN motif and ankyrin repeat domains 4	0.99403579	0.9626	0.9818
545700	Vmn2r-ps14	vomeronasal 2, receptor, pseudogene 14	0.99403579	0.9644	0.9826
67203	Nde1	nuclear distribution gene E homolog 1 (A nidulans)	0.99403579	0.9651	0.9829
14380	G6pd2	glucose-6-phosphate dehydrogenase 2	0.99403579	0.9652	0.9829
15446	Hpgd	hydroxyprostaglandin dehydrogenase 15 (NAD)	0.99403579	0.967	0.9836
71186	4933417D19Rik	RIKEN cDNA 4933417D19 gene	0.99403579	0.972	0.9862
209318	Gps1	G protein pathway suppressor 1	0.99304866	0.8605	NA
227102	Ormdl1	ORM1-like 1 (S. cerevisiae)	0.99304866	0.874	NA
72068	Cnot2	CCR4-NOT transcription complex, subunit 2	0.99304866	0.8748	NA
210148	Slc30a6	solute carrier family 30 (zinc transporter), member 6	0.99304866	0.8764	NA
107035	Fbxo38	F-box protein 38	0.99304866	0.8786	NA
107733	Mrpl41	mitochondrial ribosomal protein L41	0.99304866	0.8822	NA
78610	Uvrag	UV radiation resistance associated gene	0.99304866	0.8855	NA
56330	Pdcd5	programmed cell death 5	0.99304866	0.8863	NA
66614	Gpatch4	G patch domain containing 4	0.99304866	0.8884	NA
72144	Slc37a3	solute carrier family 37 (glycerol-3-phosphate transporter), member 3	0.99304866	0.8888	NA
28036	Larp7	La ribonucleoprotein domain family, member 7	0.99304866	0.8951	NA
12904	Crabp2	cellular retinoic acid binding protein II	0.99304866	0.8966	NA
231889	Bud31	BUD31 homolog (yeast)	0.99304866	0.8972	NA
69534	Avpi1	arginine vasopressin-induced 1	0.99304866	0.9	NA
67381	Med4	mediator of RNA polymerase II transcription, subunit 4 homolog (yeast)	0.99304866	0.9001	NA
268860	Abat	4-aminobutyrate aminotransferase	0.99304866	0.9024	NA
66101	Ppih	peptidyl prolyl isomerase H	0.99304866	0.9034	NA
384061	Fndc5	fibronectin type III domain containing 5	0.99304866	0.9077	NA
67936	Wdr55	WD repeat domain 55	0.99304866	0.9088	NA
68818	Zfand2b	zinc finger, AN1 type domain 2B	0.99304866	0.913	NA
93687	Csnk1a1	casein kinase 1, alpha 1	0.99304866	0.9153	NA
98366	Smap1	stromal membrane-associated protein 1	0.99304866	0.9173	NA
53951	Ccdc75	coiled-coil domain containing 75	0.99304866	0.9185	NA
100042332	2810410L24Rik	RIKEN cDNA 2810410L24 gene	0.99304866	0.9186	NA
414098	C230096K16Rik	RIKEN cDNA C230096K16 gene	0.99304866	0.9196	NA

234797	6430548M08Rik	RIKEN cDNA 6430548M08 gene	0.99304866	0.9204	NA
71653	4930506M07Rik	RIKEN cDNA 4930506M07 gene	0.99304866	0.9212	NA
70291	2510049J12Rik	RIKEN cDNA 2510049J12 gene	0.99304866	0.9224	NA
70681	Fam175a	family with sequence similarity 175, member A	0.99304866	0.9233	NA
112418	1700102P08Rik	RIKEN cDNA 1700102P08 gene	0.99304866	0.9234	NA
19663	Rbpms	RNA binding protein gene with multiple splicing	0.99304866	0.9249	NA
320184	Lrrc58	leucine rich repeat containing 58	0.99304866	0.9255	NA
72050	Kdelc1	KDEL (Lys-Asp-Glu-Leu) containing 1	0.99304866	0.9266	NA
99375	Cul4a	cullin 4A	0.99304866	0.931	NA
17762	Mapt	microtubule-associated protein tau	0.99304866	0.9317	NA
21453	Tcof1	Treacher Collins Franceschetti syndrome 1, homolog	0.99304866	0.9318	NA
74254	Gpn1	GPN-loop GTPase 1	0.99304866	0.9321	NA
93672	Il24	interleukin 24	0.99304866	0.9335	0.9659
103733	Tubg1	tubulin, gamma 1	0.99304866	0.9365	0.9676
67727	Stx17	syntaxin 17	0.99304866	0.9373	NA
69185	Dtwd1	DTW domain containing 1	0.99304866	0.9384	0.9684
106200	Txndc11	thioredoxin domain containing 11	0.99304866	0.9387	0.9685
76303	Osbp	oxysterol binding protein	0.99304866	0.9401	0.9693
20318	Sdf4	stromal cell derived factor 4	0.99304866	0.9406	0.9696
16970	Lrmp	lymphoid-restricted membrane protein	0.99304866	0.9408	0.9696
20845	Star	steroidogenic acute regulatory protein	0.99304866	0.9413	0.97
77622	Apex2	apurinic/apyrimidinic endonuclease 2	0.99304866	0.9416	0.9702
76482	3110002H16Rik	RIKEN cDNA 3110002H16 gene	0.99304866	0.9463	0.9731
30932	Zfp330	zinc finger protein 330	0.99304866	0.9496	0.9748
22722	Zfp64	zinc finger protein 64	0.99304866	0.9511	0.9755
20637	Snrnp70	small nuclear ribonucleoprotein 70 (U1)	0.99304866	0.9538	0.9767
30941	Usp21	ubiquitin specific peptidase 21	0.99304866	0.9556	0.9778
75705	Eif4b	eukaryotic translation initiation factor 4B	0.99304866	0.9632	0.9822
243819	Ppp6r1	protein phosphatase 6, regulatory subunit 1	0.99304866	0.9648	0.9828
69714	Tfpt	TCF3 (E2A) fusion partner	0.99304866	0.9658	0.9831
234964	Ccdc67	coiled-coil domain containing 67	0.99304866	0.9664	0.9834
380780	Serpina11	serine (or cysteine) peptidase inhibitor, clade A (alpha-1 antiproteinase, antitrypsin), member 11	0.99304866	0.9703	0.9856
16000	Igf1	insulin-like growth factor 1	0.99304866	0.9718	0.9862
22376	Was	Wiskott-Aldrich syndrome homolog (human)	0.99304866	0.9749	0.9881
414084	Tnip3	TNFAIP3 interacting protein 3	0.99304866	0.983	0.9917
64095	Gpr35	G protein-coupled receptor 35	0.99304866	0.9901	0.9949
78308	Gpr108	G protein-coupled receptor 108	0.99206349	0.8326	NA
69870	Polr3gl	polymerase (RNA) III (DNA directed) polypeptide G like	0.99206349	0.8462	NA
68259	Ift80	intraflagellar transport 80 homolog (Chlamydomonas)	0.99206349	0.8532	NA
268449	Rpl23a	ribosomal protein L23A	0.99206349	0.8552	NA
69556	Bod1	biorientation of chromosomes in cell division 1	0.99206349	0.8642	NA
216767	Mrpl22	mitochondrial ribosomal protein L22	0.99206349	0.865	NA
69674	Mif4gd	MIF4G domain containing	0.99206349	0.8724	NA
22031	Traf3	TNF receptor-associated factor 3	0.99206349	0.875	NA
22428	Dctn6	dynactin 6	0.99206349	0.8754	NA
19324	Rab1	RAB1, member RAS oncogene family	0.99206349	0.8756	NA
22234	Ugcg	UDP-glucose ceramide glucosyltransferase	0.99206349	0.8758	NA
17196	Mbp	myelin basic protein	0.99206349	0.8809	NA
56430	Clip1	CAP-GLY domain containing linker protein 1	0.99206349	0.8903	NA
52717	Anapc16	anaphase promoting complex subunit 16	0.99206349	0.8923	NA

232157	Mobkl1b	MOB1, Mps One Binder kinase activator-like 1B (yeast)	0.99206349	0.8934	NA
55944	Eif3d	eukaryotic translation initiation factor 3, subunit D	0.99206349	0.8944	NA
94186	Strn3	striatin, calmodulin binding protein 3	0.99206349	0.8947	NA
383712	Gm13637	predicted gene 13637	0.99206349	0.8968	NA
66233	Dmap1	DNA methyltransferase 1-associated protein 1	0.99206349	0.8986	NA
57816	Tesc	tescalcin	0.99206349	0.9	NA
210711	Mcmbp	MCM (minichromosome maintenance deficient) binding protein	0.99206349	0.9009	NA
50927	Nasp	nuclear autoantigenic sperm protein (histone-binding)	0.99206349	0.9049	NA
27416	Abcc5	ATP-binding cassette, sub-family C (CFTR/MRP), member 5	0.99206349	0.9063	NA
101197	Zfp956	zinc finger protein 956	0.99206349	0.9064	NA
18673	Phb	prohibitin	0.99206349	0.9065	NA
54152	Dnalc4	dynein, axonemal, light chain 4	0.99206349	0.9096	NA
12812	Coil	coilin	0.99206349	0.9141	NA
239336	Rxfp3	relaxin family peptide receptor 3	0.99206349	0.9147	NA
12857	Cox4i1	cytochrome c oxidase subunit IV isoform 1	0.99206349	0.9165	NA
108679	Cops8	COP9 (constitutive photomorphogenic) homolog, subunit 8 (Arabidopsis thaliana)	0.99206349	0.9187	NA
399616	A130078K24Rik	RIKEN cDNA A130078K24 gene	0.99206349	0.9249	0.9618
76857	Spopl	speckle-type POZ protein-like	0.99206349	0.9267	NA
269513	Nkain3	Na+/K+ transporting ATPase interacting 3	0.99206349	0.9286	0.9634
76375	Det1	de-etiolated homolog 1 (Arabidopsis)	0.99206349	0.9297	0.964
69981	Tmem30a	transmembrane protein 30A	0.99206349	0.9299	0.9641
100043133	9130023H24Rik	RIKEN cDNA 9130023H24 gene	0.99206349	0.9365	0.9676
21888	Tle4	transducin-like enhancer of split 4, homolog of Drosophila E(spl)	0.99206349	0.9372	0.9679
28106	D17Wsu104e	DNA segment, Chr 17, Wayne State University 104, expressed	0.99206349	0.9377	0.9682
76964	2610028H24Rik	RIKEN cDNA 2610028H24 gene	0.99206349	0.9383	0.9684
243274	Tmem132d	transmembrane protein 132D	0.99206349	0.9402	0.9693
257971	Olfr1382	olfactory receptor 1382	0.99206349	0.9449	0.9724
229725	Clcc1	chloride channel CLIC-like 1	0.99206349	0.9461	0.973
233276	Tubgcp5	tubulin, gamma complex associated protein 5	0.99206349	0.9533	0.9766
20619	Snap23	synaptosomal-associated protein 23	0.99206349	0.9615	0.9813
230654	Lrrc41	leucine rich repeat containing 41	0.99206349	0.9642	0.9826
69655	Cd164l2	CD164 sialomucin-like 2	0.99206349	0.9655	0.983
214922	Slc39a2	solute carrier family 39 (zinc transporter), member 2	0.99206349	0.9755	0.9884
30925	Slamf6	SLAM family member 6	0.99206349	0.9759	0.9885
67313	5730559C18Rik	RIKEN cDNA 5730559C18 gene	0.99206349	0.977	0.9891
72320	2510003E04Rik	RIKEN cDNA 2510003E04 gene	0.99108028	0.8107	NA
53323	Ube2k	ubiquitin-conjugating enzyme E2K (UBC1 homolog, yeast)	0.99108028	0.8134	NA
19042	Ppm1a	protein phosphatase 1A, magnesium dependent, alpha isoform	0.99108028	0.8221	NA
50995	Uba2	ubiquitin-like modifier activating enzyme 2	0.99108028	0.8309	NA
214572	Prmt7	protein arginine N-methyltransferase 7	0.99108028	0.8359	NA
15526	Hspa9	heat shock protein 9	0.99108028	0.8437	NA
18100	Mrpl40	mitochondrial ribosomal protein L40	0.99108028	0.846	NA
214917	Fam173a	family with sequence similarity 173, member A	0.99108028	0.851	NA
12419	Cbx5	chromobox homolog 5 (Drosophila HP1a)	0.99108028	0.8515	NA
78287	Zfyve20	zinc finger, FYVE domain containing 20	0.99108028	0.8557	NA
66082	Abhd6	abhydrolase domain containing 6	0.99108028	0.858	NA
74198	Dtx2	deltex 2 homolog (Drosophila)	0.99108028	0.8651	NA
278279	Tmtc2	transmembrane and tetratricopeptide repeat containing 2	0.99108028	0.8675	NA
72053	Tmub2	transmembrane and ubiquitin-like domain containing 2	0.99108028	0.8696	NA
67674	Trmt112	tRNA methyltransferase 11-2 homolog (S. cerevisiae)	0.99108028	0.8707	NA

22258	Usp4	ubiquitin specific peptidase 4 (proto-oncogene)	0.99108028	0.8725	NA
14791	Emg1	EMG1 nucleolar protein homolog (S. cerevisiae)	0.99108028	0.8761	NA
66580	Esf1	ESF1, nucleolar pre-rRNA processing protein, homolog (S. cerevisiae)	0.99108028	0.8786	NA
75129	4930524J08Rik	RIKEN cDNA 4930524J08 gene	0.99108028	0.8815	NA
22184	Zrsr2	zinc finger (CCCH type), RNA binding motif and serine/arginine rich 2	0.99108028	0.8816	NA
17308	Mgat1	mannoside acetylglucosaminyltransferase 1	0.99108028	0.8834	NA
66511	2500003M10Rik	RIKEN cDNA 2500003M10 gene	0.99108028	0.8834	NA
14251	Flot1	flotillin 1	0.99108028	0.8842	NA
66234	Sc4mol	sterol-C4-methyl oxidase-like	0.99108028	0.8845	NA
208595	Gm9897	predicted gene 9897	0.99108028	0.8848	NA
68493	Ndufaf4	NADH dehydrogenase (ubiquinone) 1 alpha subcomplex, assembly factor 4	0.99108028	0.886	NA
209354	Eif2b1	eukaryotic translation initiation factor 2B, subunit 1 (alpha)	0.99108028	0.8863	NA
21333	Tac1	tachykinin 1	0.99108028	0.8866	NA
239099	Homez	homeodomain leucine zipper-encoding gene	0.99108028	0.8896	NA
67568	Mrfap1	Morf4 family associated protein 1	0.99108028	0.8902	NA
83945	Dnaja3	DnaJ (Hsp40) homolog, subfamily A, member 3	0.99108028	0.8902	NA
68550	1110002N22Rik	RIKEN cDNA 1110002N22 gene	0.99108028	0.8916	NA
78785	Clip4	CAP-GLY domain containing linker protein family, member 4	0.99108028	0.8933	NA
246703	Apoa1bp	apolipoprotein A-I binding protein	0.99108028	0.8964	NA
11964	Atp6v1a	ATPase, H+ transporting, lysosomal V1 subunit A	0.99108028	0.9021	NA
15525	Hspa4	heat shock protein 4	0.99108028	0.9048	NA
234624	A330008L17Rik	RIKEN cDNA A330008L17 gene	0.99108028	0.9055	NA
74144	Robo4	roundabout homolog 4 (Drosophila)	0.99108028	0.9058	NA
80280	Cdk5rap3	CDK5 regulatory subunit associated protein 3	0.99108028	0.9065	NA
78600	Pde6h	phosphodiesterase 6H, cGMP-specific, cone, gamma	0.99108028	0.9077	NA
69602	Otop3	otopetrin 3	0.99108028	0.9092	NA
54006	Deaf1	deformed epidermal autoregulatory factor 1 (Drosophila)	0.99108028	0.911	NA
67873	Mri1	methylthioribose-1-phosphate isomerase homolog (S. cerevisiae)	0.99108028	0.9136	NA
229603	Otud7b	OTU domain containing 7B	0.99108028	0.916	0.9575
224613	Flywch1	FLYWCH-type zinc finger 1	0.99108028	0.9206	0.9596
54375	Azin1	antizyme inhibitor 1	0.99108028	0.9218	0.9602
12903	Crabp1	cellular retinoic acid binding protein I	0.99108028	0.9245	0.9618
13602	Sparcl1	SPARC-like 1	0.99108028	0.9247	0.9618
23892	Grem1	gremlin 1	0.99108028	0.9269	0.9627
235534	Acpl2	acid phosphatase-like 2	0.99108028	0.9293	0.9638
19646	Rbbp4	retinoblastoma binding protein 4	0.99108028	0.931	0.9647
67561	Wdr48	WD repeat domain 48	0.99108028	0.9384	0.9684
17684	Cited2	Cbp/p300-interacting transactivator, with Glu/Asp-rich carboxy-terminal domain, 2	0.99108028	0.9388	0.9685
80913	Pum2	pumilio 2 (Drosophila)	0.99108028	0.9423	0.9705
74901	Kbtbd11	kelch repeat and BTB (POZ) domain containing 11	0.99108028	0.9441	0.9718
72865	Cxx1c	CAAX box 1 homolog C (human)	0.99108028	0.9478	0.9741
12856	Cox17	cytochrome c oxidase, subunit XVII assembly protein homolog (yeast)	0.99108028	0.9495	0.9748
58233	Dnaja4	DnaJ (Hsp40) homolog, subfamily A, member 4	0.99108028	0.9544	0.9771
224703	Mar-02	membrane-associated ring finger (C3HC4) 2	0.99108028	0.9578	0.979
20665	Sox10	SRY-box containing gene 10	0.99108028	0.965	0.9829
77018	Col25a1	collagen, type XXV, alpha 1	0.99108028	0.9652	0.9829
100504388	LOC100504388	histone acetyltransferase KAT2B-like	0.99108028	0.9665	0.9834
67130	Ndufa6	NADH dehydrogenase (ubiquinone) 1 alpha subcomplex, 6 (B14)	0.99009901	0.79	NA
72047	Ddx42	DEAD (Asp-Glu-Ala-Asp) box polypeptide 42	0.99009901	0.7973	NA
17187	Max	Max protein	0.99009901	0.8079	NA

66594	Uqcr11	ubiquinol-cytochrome c reductase, complex III subunit XI	0.99009901	0.8109	NA
20832	Ssr4	signal sequence receptor, delta	0.99009901	0.8156	NA
67673	Tceb2	transcription elongation factor B (SIII), polypeptide 2	0.99009901	0.8161	NA
66854	Trim35	tripartite motif-containing 35	0.99009901	0.8179	NA
28028	Mrpl50	mitochondrial ribosomal protein L50	0.99009901	0.8355	NA
69754	Fbxo7	F-box protein 7	0.99009901	0.8374	NA
24017	Rnf13	ring finger protein 13	0.99009901	0.8397	NA
216363	Rab3ip	RAB3A interacting protein	0.99009901	0.8459	NA
77134	Hnrnpa0	heterogeneous nuclear ribonucleoprotein A0	0.99009901	0.8522	NA
11778	Ap3s2	adaptor-related protein complex 3, sigma 2 subunit	0.99009901	0.8531	NA
271564	Vps13a	vacuolar protein sorting 13A (yeast)	0.99009901	0.8548	NA
27402	Pdhx	pyruvate dehydrogenase complex, component X	0.99009901	0.8611	NA
107976	Bre	brain and reproductive organ-expressed protein	0.99009901	0.8621	NA
26444	Psma7	proteasome (prosome, macropain) subunit, alpha type 7	0.99009901	0.8628	NA
68161	A930005H10Rik	RIKEN cDNA A930005H10 gene	0.99009901	0.8676	NA
66964	Golt1b	golgi transport 1 homolog B (S. cerevisiae)	0.99009901	0.8705	NA
66925	Sdhd	succinate dehydrogenase complex, subunit D, integral membrane protein	0.99009901	0.8728	NA
20133	Rrm1	ribonucleotide reductase M1	0.99009901	0.8736	NA
22761	Zfpm1	zinc finger protein, multitype 1	0.99009901	0.8737	NA
67123	Ubap1	ubiquitin-associated protein 1	0.99009901	0.8824	NA
108077	Skiv2l	superkiller viralicidic activity 2-like (S. cerevisiae)	0.99009901	0.8825	NA
72219	Fam75d3	family with sequence similarity 75, member D3	0.99009901	0.8873	NA
229473	D930015E06Rik	RIKEN cDNA D930015E06 gene	0.99009901	0.8885	NA
69487	2310003L22Rik	RIKEN cDNA 2310003L22 gene	0.99009901	0.889	NA
100040563	Dynlt1c	dynein light chain Tctex-type 1C	0.99009901	0.8911	NA
22682	Zfand5	zinc finger, AN1-type domain 5	0.99009901	0.8996	NA
17762	Mapt	microtubule-associated protein tau	0.99009901	0.901	NA
27050	Rps3	ribosomal protein S3	0.99009901	0.9033	NA
225348	Wdr36	WD repeat domain 36	0.99009901	0.9039	NA
381823	Apold1	apolipoprotein L domain containing 1	0.99009901	0.9058	NA
11773	Ap2m1	adaptor protein complex AP-2, mu1	0.99009901	0.9083	0.9531
627049	Zfp800	zinc finger protein 800	0.99009901	0.9105	0.9542
56224	Tspan5	tetraspanin 5	0.99009901	0.911	0.9544
654409	4932416H05Rik	RIKEN cDNA 4932416H05 gene	0.99009901	0.9122	0.9554
331474	Rgag4	retrotransposon gag domain containing 4	0.99009901	0.9175	0.958
53600	Timm23	translocase of inner mitochondrial membrane 23 homolog (yeast)	0.99009901	0.9183	0.9584
75299	4930547M16Rik	RIKEN cDNA 4930547M16 gene	0.99009901	0.9225	0.9608
99738	Kcnc4	potassium voltage gated channel, Shaw-related subfamily, member 4	0.99009901	0.9228	0.9609
68229	AI846148	expressed sequence AI846148	0.99009901	0.9235	0.9613
11937	Atp2a1	ATPase, Ca++ transporting, cardiac muscle, fast twitch 1	0.99009901	0.9264	0.9626
243374	Gimap8	GTPase, IMAP family member 8	0.99009901	0.9297	0.964
235442	Rab8b	RAB8B, member RAS oncogene family	0.99009901	0.9319	0.9651
399572	E430010N07Rik	RIKEN cDNA E430010N07 gene	0.99009901	0.9358	0.9671
52463	Tet1	tet oncogene 1	0.99009901	0.9384	0.9684
170638	Hpcal4	hippocalcin-like 4	0.99009901	0.9461	0.973
22268	Upk1b	uroplakin 1B	0.99009901	0.9489	0.9746
72171	Shq1	SHQ1 homolog (S. cerevisiae)	0.99009901	0.9501	0.9751
279572	Tlr13	toll-like receptor 13	0.99009901	0.9546	0.9771
231866	Zfp12	zinc finger protein 12	0.99009901	0.9549	0.9773
433294	A530098C11Rik	RIKEN cDNA A530098C11 gene	0.99009901	0.9599	0.9804

75403	1010001B22Rik	RIKEN cDNA 1010001B22 gene	0.99009901	0.9628	0.982
675749	Gm10693	predicted pseudogene 10693	0.99009901	0.9693	0.9851
67236	Cinp	cyclin-dependent kinase 2 interacting protein	0.99009901	0.9696	0.9852
14455	Gas5	growth arrest specific 5	0.99009901	0.9816	0.9911
17938	Naca	nascent polypeptide-associated complex alpha polypeptide	0.98911968	0.7604	NA
109229	Fam118b	family with sequence similarity 118, member B	0.98911968	0.7692	NA
216821	Tmem11	transmembrane protein 11	0.98911968	0.7982	NA
107328	Trpt1	tRNA phosphotransferase 1	0.98911968	0.7991	NA
70351	Ppp4r1	protein phosphatase 4, regulatory subunit 1	0.98911968	0.8087	NA
68597	1110021J02Rik	RIKEN cDNA 1110021J02 gene	0.98911968	0.8113	NA
66413	Psmd6	proteasome (prosome, macropain) 26S subunit, non-ATPase, 6	0.98911968	0.8131	NA
213550	Dis3l	DIS3 mitotic control homolog (S. cerevisiae)-like	0.98911968	0.8153	NA
12261	C1qbp	complement component 1, q subcomponent binding protein	0.98911968	0.8204	NA
17828	Muted	muted	0.98911968	0.8265	NA
14869	Gstp2	glutathione S-transferase, pi 2	0.98911968	0.8266	NA
232784	Zfp212	Zinc finger protein 212	0.98911968	0.8379	NA
69186	1810027O10Rik	RIKEN cDNA 1810027O10 gene	0.98911968	0.8387	NA
78248	Armcx1	armadillo repeat containing, X-linked 1	0.98911968	0.8396	NA
66397	Sar1b	SAR1 gene homolog B (S. cerevisiae)	0.98911968	0.8425	NA
66358	2310004I24Rik	RIKEN cDNA 2310004I24 gene	0.98911968	0.8456	NA
320806	Gfm2	G elongation factor, mitochondrial 2	0.98911968	0.8477	NA
13667	Eif2b4	eukaryotic translation initiation factor 2B, subunit 4 delta	0.98911968	0.8499	NA
387524	Znrf2	zinc and ring finger 2	0.98911968	0.8523	NA
22196	Ube2i	ubiquitin-conjugating enzyme E2I	0.98911968	0.8544	NA
66199	Commd4	COMM domain containing 4	0.98911968	0.8583	NA
17827	Mtx1	metaxin 1	0.98911968	0.8593	NA
93747	Echs1	enoyl Coenzyme A hydratase, short chain, 1, mitochondrial	0.98911968	0.8626	NA
432479	4930404N11Rik	RIKEN cDNA 4930404N11 gene	0.98911968	0.8637	NA
74868	Tmem65	transmembrane protein 65	0.98911968	0.865	NA
67370	Zfp606	zinc finger protein 606	0.98911968	0.8711	NA
14852	Gspt1	G1 to S phase transition 1	0.98911968	0.8751	NA
76332	Cog2	component of oligomeric golgi complex 2	0.98911968	0.8757	NA
116940	Tgs1	trimethylguanosine synthase homolog (S. cerevisiae)	0.98911968	0.878	NA
214572	Prmt7	protein arginine N-methyltransferase 7	0.98911968	0.8783	NA
217310	C630004H02Rik	RIKEN cDNA C630004H02 gene	0.98911968	0.879	NA
68169	A930038C07Rik	RIKEN cDNA A930038C07 gene	0.98911968	0.8791	NA
13990	Smarcad1	SWI/SNF-related, matrix-associated actin-dependent regulator of chromatin, subfamily a, containing DEAD/H box 1	0.98911968	0.8804	NA
102442	Dennd4a	DENN/MADD domain containing 4A	0.98911968	0.8842	NA
232566	Amn1	antagonist of mitotic exit network 1 homolog (S. cerevisiae)	0.98911968	0.8851	NA
108143	Taf9	TAF9 RNA polymerase II, TATA box binding protein (TBP)-associated factor	0.98911968	0.8856	NA
16468	Jarid2	jumonji, AT rich interactive domain 2	0.98911968	0.8862	NA
64242	Ngb	neuroglobin	0.98911968	0.8894	NA
14225	Fkbp1a	FK506 binding protein 1a	0.98911968	0.8895	NA
108067	Eif2b3	eukaryotic translation initiation factor 2B, subunit 3	0.98911968	0.8935	NA
72238	Tbc1d5	TBC1 domain family, member 5	0.98911968	0.8948	NA
11792	Apex1	apurinic/apyrimidinic endonuclease 1	0.98911968	0.8974	0.9477
384619	Ccdc155	coiled-coil domain containing 155	0.98911968	0.8979	NA
269800	Zfp384	zinc finger protein 384	0.98911968	0.8982	NA
19099	Mapk8ip1	mitogen-activated protein kinase 8 interacting protein 1	0.98911968	0.8997	0.949
71567	Mcm9	minichromosome maintenance complex component 9	0.98911968	0.9002	0.9492

13134	Dach1	dachshund 1 (Drosophila)	0.98911968	0.9032	0.951
68636	Fahd1	fumarylacetoacetate hydrolase domain containing 1	0.98911968	0.9034	0.951
74637	Shpk	sedoheptulokinase	0.98911968	0.9034	0.951
68215	Fam98b	family with sequence similarity 98, member B	0.98911968	0.9042	0.9513
214669	L3mbtl2	l(3)mbt-like 2 (Drosophila)	0.98911968	0.9088	0.9534
22746	Zfp85-rs1	zinc finger protein 85, related sequence 1	0.98911968	0.9101	0.954
71617	9130011E15Rik	RIKEN cDNA 9130011E15 gene	0.98911968	0.9101	0.954
21389	Tbx6	T-box 6	0.98911968	0.9104	0.9541
30049	Scd3	stearoyl-coenzyme A desaturase 3	0.98911968	0.9118	0.9551
235504	Slc17a5	solute carrier family 17 (anion/sugar transporter), member 5	0.98911968	0.9159	0.9575
100041488	Gm11937	predicted gene 11937	0.98911968	0.9164	0.9577
11488	Adam11	a disintegrin and metallopeptidase domain 11	0.98911968	0.9182	0.9584
212285	Arap2	ArfGAP with RhoGAP domain, ankyrin repeat and PH domain 2	0.98911968	0.9229	0.9609
110197	Dgkg	diacylglycerol kinase, gamma	0.98911968	0.9236	0.9614
244152	Tsku	tsukushin	0.98911968	0.9289	0.9636
75396	Spp2	secreted phosphoprotein 2	0.98911968	0.9309	0.9647
14815	Nr3c1	nuclear receptor subfamily 3, group C, member 1	0.98911968	0.9525	0.9761
100038491	A730068I03Rik	RIKEN cDNA A730068I03 gene	0.98911968	0.9614	0.9813
14803	Grid1	glutamate receptor, ionotropic, delta 1	0.98911968	0.962	0.9815
14061	F2	coagulation factor II	0.98911968	0.9623	0.9816
68176	6230427J02Rik	RIKEN cDNA 6230427J02 gene	0.98911968	0.9655	0.983
74435	Lrriq3	leucine-rich repeats and IQ motif containing 3	0.98911968	0.9693	0.9851
66371	Chmp4c	chromatin modifying protein 4C	0.98911968	0.9719	0.9862
15384	Hnrnpab	heterogeneous nuclear ribonucleoprotein A/B	0.98814229	0.7779	NA
216150	Cdc34	cell division cycle 34 homolog (S. cerevisiae)	0.98814229	0.787	NA
70699	Nup205	nucleoporin 205	0.98814229	0.788	NA
353258	Ltv1	LTV1 homolog (S. cerevisiae)	0.98814229	0.7884	NA
66959	Dusp26	dual specificity phosphatase 26 (putative)	0.98814229	0.7944	NA
68140	Tigd2	tigger transposable element derived 2	0.98814229	0.7953	NA
14718	Got1	glutamate oxaloacetate transaminase 1, soluble	0.98814229	0.8147	NA
66377	Ndufc1	NADH dehydrogenase (ubiquinone) 1, subcomplex unknown, 1	0.98814229	0.815	NA
68598	Dnajc8	DnaJ (Hsp40) homolog, subfamily C, member 8	0.98814229	0.8272	NA
20646	Snrpn	small nuclear ribonucleoprotein N	0.98814229	0.8296	NA
71116	Stx18	syntaxin 18	0.98814229	0.83	NA
71843	R3hcc1	R3H domain and coiled-coil containing 1	0.98814229	0.83	NA
66213	Med7	mediator complex subunit 7	0.98814229	0.8309	NA
78541	Asb8	ankyrin repeat and SOCS box-containing 8	0.98814229	0.8316	NA
66142	Cox7b	cytochrome c oxidase subunit VIIb	0.98814229	0.8379	NA
622534	Gm13611	predicted gene 13611	0.98814229	0.8383	NA
66354	Snw1	SNW domain containing 1	0.98814229	0.8429	NA
20843	Stag2	stromal antigen 2	0.98814229	0.846	NA
54473	Tollip	toll interacting protein	0.98814229	0.8486	NA
20773	Sptlc2	serine palmitoyltransferase, long chain base subunit 2	0.98814229	0.8609	NA
78321	Ankrd23	ankyrin repeat domain 23	0.98814229	0.8612	NA
230259	E130308A19Rik	RIKEN cDNA E130308A19 gene	0.98814229	0.8626	NA
217331	Unk	unkempt homolog (Drosophila)	0.98814229	0.8659	NA
72438	2510016G02Rik	RIKEN cDNA 2510016G02 gene	0.98814229	0.8672	NA
18861	Pms2	postmeiotic segregation increased 2 (S. cerevisiae)	0.98814229	0.8674	NA
233073	U2af1l4	U2 small nuclear RNA auxiliary factor 1-like 4	0.98814229	0.868	NA
51885	Tubgcp4	tubulin, gamma complex associated protein 4	0.98814229	0.8683	NA

110454	Ly6a	lymphocyte antigen 6 complex, locus A	0.98814229	0.8702	NA
78100	8430410K20Rik	RIKEN cDNA 8430410K20 gene	0.98814229	0.8726	NA
15312	Hmgn1	high mobility group nucleosomal binding domain 1	0.98814229	0.8752	NA
230025	Prdm13	PR domain containing 13	0.98814229	0.8793	NA
103406	Zfr2	zinc finger RNA binding protein 2	0.98814229	0.8805	NA
80750	N4bp1	NEDD4 binding protein 1	0.98814229	0.8903	0.9437
68281	4930430F08Rik	RIKEN cDNA 4930430F08 gene	0.98814229	0.8909	NA
108909	Aida	axin interactor, dorsalization associated	0.98814229	0.8946	0.946
66185	1110037F02Rik	RIKEN cDNA 1110037F02 gene	0.98814229	0.8993	0.9489
64291	Osbpl1a	oxysterol binding protein-like 1A	0.98814229	0.9008	0.9495
56310	Gps2	G protein pathway suppressor 2	0.98814229	0.903	0.9509
234577	Cpne2	copine II	0.98814229	0.9054	0.9518
381310	6330403A02Rik	RIKEN cDNA 6330403A02 gene	0.98814229	0.9119	0.9551
67160	Eef1g	eukaryotic translation elongation factor 1 gamma	0.98814229	0.9132	0.956
69524	Esam	endothelial cell-specific adhesion molecule	0.98814229	0.9143	0.9565
14860	Gsta4	glutathione S-transferase, alpha 4	0.98814229	0.9145	0.9566
53883	Celsr2	cadherin, EGF LAG seven-pass G-type receptor 2 (flamingo homolog, Drosophila)	0.98814229	0.9208	0.9596
217866	Cdc42bpb	CDC42 binding protein kinase beta	0.98814229	0.9255	0.9621
20410	Sorbs3	sorbin and SH3 domain containing 3	0.98814229	0.9257	0.9622
16872	Lhx4	LIM homeobox protein 4	0.98814229	0.9269	0.9627
18612	Etv4	ets variant gene 4 (E1A enhancer binding protein, E1AF)	0.98814229	0.9281	0.9632
217369	Uts2r	urotensin 2 receptor	0.98814229	0.9329	0.9656
625281	Gm6570	predicted gene 6570	0.98814229	0.9331	0.9658
434178	Zfp141	zinc finger protein 141	0.98814229	0.9379	0.9682
100047702	LOC100047702	battenin-like	0.98814229	0.9417	0.9702
328066	C920021A13	hypothetical protein C920021A13	0.98814229	0.9456	0.9728
74910	4930480E11Rik	RIKEN cDNA 4930480E11 gene	0.98814229	0.9527	0.9762
327942	Pigl	phosphatidylinositol glycan anchor biosynthesis, class L	0.98814229	0.9681	0.9844
15565	Htr6	5-hydroxytryptamine (serotonin) receptor 6	0.98814229	0.9692	0.9851
226957	Gm4850	THO complex 4 pseudogene	0.98814229	0.9701	0.9855
210146	Irgq	immunity-related GTPase family, Q	0.98716683	0.7375	NA
57370	B4galt3	UDP-Gal:betaGlcNAc beta 1,4-galactosyltransferase, polypeptide 3	0.98716683	0.767	NA
27096	Trappc3	trafficking protein particle complex 3	0.98716683	0.7714	NA
65247	Asb1	ankyrin repeat and SOCS box-containing 1	0.98716683	0.7745	NA
16531	Kcnma1	potassium large conductance calcium-activated channel, subfamily M, alpha member 1	0.98716683	0.781	NA
68295	0610011L14Rik	RIKEN cDNA 0610011L14 gene	0.98716683	0.786	NA
58230	Rnf8	ring finger protein 8	0.98716683	0.7877	NA
94245	Dtnbp1	dystrobrevin binding protein 1	0.98716683	0.7924	NA
381802	Tsen2	tRNA splicing endonuclease 2 homolog (S. cerevisiae)	0.98716683	0.7951	NA
69657	2310047D07Rik	RIKEN cDNA 2310047D07 gene	0.98716683	0.7958	NA
233902	Fbxl19	F-box and leucine-rich repeat protein 19	0.98716683	0.7989	NA
216549	Aftph	aftiphilin	0.98716683	0.7999	NA
257871	Olfr1372-ps1	olfactory receptor 1372, pseudogene 1	0.98716683	0.8031	NA
109136	Mmaa	methylmalonic aciduria (cobalamin deficiency) type A	0.98716683	0.8033	NA
54354	Rassf5	Ras association (RalGDS/AF-6) domain family member 5	0.98716683	0.806	NA
209318	Gps1	G protein pathway suppressor 1	0.98716683	0.8066	NA
227522	Rpp38	ribonuclease P/MRP 38 subunit (human)	0.98716683	0.8099	NA
54624	Paf1	Paf1, RNA polymerase II associated factor, homolog (S. cerevisiae)	0.98716683	0.8122	NA
66279	Tmem218	transmembrane protein 218	0.98716683	0.8136	NA
21385	Tbx2	T-box 2	0.98716683	0.8146	NA

215708	Fam73a	family with sequence similarity 73, member A	0.98716683	0.8147	NA
19822	Rnf4	ring finger protein 4	0.98716683	0.8152	NA
67249	Tbc1d19	TBC1 domain family, member 19	0.98716683	0.8186	NA
21418	Tcfap2a	transcription factor AP-2, alpha	0.98716683	0.8199	NA
59021	Rab2a	RAB2A, member RAS oncogene family	0.98716683	0.8241	NA
13709	Elf1	E74-like factor 1	0.98716683	0.8279	NA
17158	Man2a1	mannosidase 2, alpha 1	0.98716683	0.8299	NA
78938	Fbxo34	F-box protein 34	0.98716683	0.8302	NA
67048	Vma21	VMA21 vacuolar H+-ATPase homolog (S. cerevisiae)	0.98716683	0.8313	NA
243300	6430598A04Rik	RIKEN cDNA 6430598A04 gene	0.98716683	0.8314	NA
108067	Eif2b3	eukaryotic translation initiation factor 2B, subunit 3	0.98716683	0.8322	NA
18670	Abcb4	ATP-binding cassette, sub-family B (MDR/TAP), member 4	0.98716683	0.833	NA
67738	Ppid	peptidylprolyl isomerase D (cyclophilin D)	0.98716683	0.8421	NA
237400	Mex3d	mex3 homolog D (C. elegans)	0.98716683	0.8443	NA
56471	Stmn4	stathmin-like 4	0.98716683	0.8507	NA
243339	Tmem130	transmembrane protein 130	0.98716683	0.8524	NA
26394	Lypla2	lysophospholipase 2	0.98716683	0.8543	NA
227746	Rabepk	Rab9 effector protein with kelch motifs	0.98716683	0.8546	NA
105440	Kctd9	potassium channel tetramerisation domain containing 9	0.98716683	0.8565	NA
72323	Asb6	ankyrin repeat and SOCS box-containing 6	0.98716683	0.8569	NA
69993	Chn2	chimerin (chimaerin) 2	0.98716683	0.8596	NA
72898	Asphd2	aspartate beta-hydroxylase domain containing 2	0.98716683	0.8618	NA
14686	Gnat2	guanine nucleotide binding protein, alpha transducing 2	0.98716683	0.863	NA
237313	Il20ra	interleukin 20 receptor, alpha	0.98716683	0.8658	NA
22066	Trpc4	transient receptor potential cation channel, subfamily C, member 4	0.98716683	0.8694	NA
18749	Prkacb	protein kinase, cAMP dependent, catalytic, beta	0.98716683	0.8711	NA
20379	Sfrp4	secreted frizzled-related protein 4	0.98716683	0.8726	NA
74229	Paqr8	progestin and adipoQ receptor family member VIII	0.98716683	0.8754	NA
64436	Inpp5e	inositol polyphosphate-5-phosphatase E	0.98716683	0.8777	0.9371
228598	Ebf4	early B-cell factor 4	0.98716683	0.8796	0.9384
320534	Tmem104	transmembrane protein 104	0.98716683	0.8826	0.9397
67151	Psmd9	proteasome (prosome, macropain) 26S subunit, non-ATPase, 9	0.98716683	0.8847	0.9407
66993	Smarcd3	SWI/SNF related, matrix associated, actin dependent regulator of chromatin, subfamily d, member 3	0.98716683	0.8868	0.9416
106389	Eaf2	ELL associated factor 2	0.98716683	0.8895	0.9433
216705	Clint1	clathrin interactor 1	0.98716683	0.9018	0.95
77252	9430038I01Rik	RIKEN cDNA 9430038I01 gene	0.98716683	0.9048	0.9515
230815	Man1c1	mannosidase, alpha, class 1C, member 1	0.98716683	0.9067	0.9524
51789	Tnk2	tyrosine kinase, non-receptor, 2	0.98716683	0.9148	0.9568
23950	Dnajb6	DnaJ (Hsp40) homolog, subfamily B, member 6	0.98716683	0.9174	0.958
18709	Pik3r2	phosphatidylinositol 3-kinase, regulatory subunit, polypeptide 2 (p85 beta)	0.98716683	0.9204	0.9596
17242	Mdk	midkine	0.98716683	0.9227	0.9609
13680	Ddx19a	DEAD (Asp-Glu-Ala-Asp) box polypeptide 19a	0.98716683	0.9228	0.9609
231605	Galnt9	UDP-N-acetyl-alpha-D-galactosamine:polypeptide N-acetylgalactosaminyltransferase 9	0.98716683	0.9318	0.9651
19120	Prm3	protamine 3	0.98716683	0.9757	0.9884
72900	Ndufv2	NADH dehydrogenase (ubiquinone) flavoprotein 2	0.98619329	0.7372	NA
14886	Gtf2i	general transcription factor II I	0.98619329	0.7495	NA
218885	Oxnad1	oxidoreductase NAD-binding domain containing 1	0.98619329	0.7602	NA
60595	Actn4	actinin alpha 4	0.98619329	0.7691	NA
80795	Selk	selenoprotein K	0.98619329	0.77	NA
67841	Atg3	autophagy-related 3 (yeast)	0.98619329	0.7788	NA

67290	3110040N11Rik	RIKEN cDNA 3110040N11 gene	0.98619329	0.78	NA
19243	Ptp4a1	protein tyrosine phosphatase 4a1	0.98619329	0.7862	NA
15574	Hus1	Hus1 homolog (S. pombe)	0.98619329	0.7869	NA
67273	Ndufa10	NADH dehydrogenase (ubiquinone) 1 alpha subcomplex 10	0.98619329	0.7895	NA
70681	Fam175a	family with sequence similarity 175, member A	0.98619329	0.7941	NA
381760	Ssbp1	single-stranded DNA binding protein 1	0.98619329	0.7943	NA
13016	Ctbp1	C-terminal binding protein 1	0.98619329	0.7981	NA
59093	Pcbp3	poly(rC) binding protein 3	0.98619329	0.8046	NA
68170	B230118H07Rik	RIKEN cDNA B230118H07 gene	0.98619329	0.806	NA
19175	Psmb6	proteasome (prosome, macropain) subunit, beta type 6	0.98619329	0.8075	NA
18607	Pdpk1	3-phosphoinositide dependent protein kinase 1	0.98619329	0.8123	NA
64144	Mllt1	myeloid/lymphoid or mixed-lineage leukemia (trithorax homolog, Drosophila); translocated to, 1	0.98619329	0.8125	NA
56085	Ubqln1	ubiquilin 1	0.98619329	0.8207	NA
22627	Ywhae	tyrosine 3-monooxygenase/tryptophan 5-monooxygenase activation protein, epsilon polypeptide	0.98619329	0.8259	NA
66136	Znrd1	zinc ribbon domain containing, 1	0.98619329	0.8266	NA
14211	Smc2	structural maintenance of chromosomes 2	0.98619329	0.8293	NA
11966	Atp6v1b2	ATPase, H+ transporting, lysosomal V1 subunit B2	0.98619329	0.8335	NA
622474	Smok3b	sperm motility kinase 3B	0.98619329	0.8336	NA
26414	Mapk10	mitogen-activated protein kinase 10	0.98619329	0.8373	NA
212276	Zfp748	zinc finger protein 748	0.98619329	0.8416	NA
72201	Otud6b	OTU domain containing 6B	0.98619329	0.8428	NA
100039203	Gm2099	predicted gene 2099	0.98619329	0.8457	NA
66593	Diablo	diablo homolog (Drosophila)	0.98619329	0.8484	NA
230784	Sesn2	sestrin 2	0.98619329	0.8493	NA
12488	Cd2ap	CD2-associated protein	0.98619329	0.8535	NA
104570	Smek2	SMEK homolog 2, suppressor of mek1 (Dictyostelium)	0.98619329	0.8537	NA
51960	Kctd18	potassium channel tetramerisation domain containing 18	0.98619329	0.8553	NA
241727	Snph	syntaphilin	0.98619329	0.8561	NA
108853	Mtrf1l	mitochondrial translational release factor 1-like	0.98619329	0.8576	NA
279067	Gm13777	predicted gene 13777	0.98619329	0.8585	NA
224129	Adcy5	adenylate cyclase 5	0.98619329	0.86	NA
56376	Pdlim5	PDZ and LIM domain 5	0.98619329	0.8624	NA
69627	Fam89a	family with sequence similarity 89, member A	0.98619329	0.8629	NA
57776	Ttyh1	tweety homolog 1 (Drosophila)	0.98619329	0.8662	NA
226641	Atf6	activating transcription factor 6	0.98619329	0.8674	NA
74849	4930412F12Rik	RIKEN cDNA 4930412F12 gene	0.98619329	0.873	0.9348
622208	Gm6297	predicted gene 6297	0.98619329	0.8762	0.936
67154	Mtdh	metadherin	0.98619329	0.8786	0.9378
73043	2900064F13Rik	RIKEN cDNA 2900064F13 gene	0.98619329	0.8804	0.9387
76980	Ube2ql1	ubiquitin-conjugating enzyme E2Q family-like 1	0.98619329	0.8822	0.9397
14546	Gdap10	ganglioside-induced differentiation-associated-protein 10	0.98619329	0.8825	0.9397
229487	Pet112l	PET112-like (yeast)	0.98619329	0.8842	0.9404
11855	Arhgap5	Rho GTPase activating protein 5	0.98619329	0.8944	0.946
214951	Rhbdl1	rhomboid, veinlet-like 1 (Drosophila)	0.98619329	0.8956	0.9467
22026	Nr2c2	nuclear receptor subfamily 2, group C, member 2	0.98619329	0.8963	0.9471
22253	Unc5c	unc-5 homolog C (C. elegans)	0.98619329	0.8976	0.9479
27426	Nagpa	N-acetylglucosamine-1-phosphodiester alpha-N-acetylglucosaminidase	0.98619329	0.8992	0.9489
67775	Rtp4	receptor transporter protein 4	0.98619329	0.9045	0.9514
103767	A530047J11Rik	RIKEN cDNA A530047J11 gene	0.98619329	0.9065	0.9524
12660	Chka	choline kinase alpha	0.98619329	0.9074	0.9528

192156	Mvd	mevalonate (diphospho) decarboxylase	0.98619329	0.9083	0.9531
18194	Nsdhl	NAD(P) dependent steroid dehydrogenase-like	0.98619329	0.9094	0.9538
320534	Tmem104	transmembrane protein 104	0.98619329	0.9102	0.9541
56839	Lgi1	leucine-rich repeat LGI family, member 1	0.98619329	0.9109	0.9544
106627	AI646383	expressed sequence AI646383	0.98619329	0.9171	0.9579
72805	Zfp839	zinc finger protein 839	0.98619329	0.9207	0.9596
16704	Krtap8-2	keratin associated protein 8-2	0.98619329	0.9239	0.9615
22240	Dpysl3	dihydropyrimidinase-like 3	0.98619329	0.9268	0.9627
20501	Slc16a1	solute carrier family 16 (monocarboxylic acid transporters), member 1	0.98619329	0.9281	0.9632
17190	Mbd1	methyl-CpG binding domain protein 1	0.98619329	0.9366	0.9676
14260	Fmn1	formin 1	0.98619329	0.9367	0.9676
80982	9930013L23Rik	RIKEN cDNA 9930013L23 gene	0.98619329	0.9572	0.9787
11764	Ap1b1	adaptor protein complex AP-1, beta 1 subunit	0.98522167	0.6862	NA
81845	Gpank1	G patch domain and ankyrin repeats 1	0.98522167	0.6929	NA
76222	Magef1	melanoma antigen family F, 1	0.98522167	0.697	NA
66592	Stoml2	stomatin (Epb7.2)-like 2	0.98522167	0.7108	NA
26466	Zfp260	zinc finger protein 260	0.98522167	0.7221	NA
14376	Ganab	alpha glucosidase 2 alpha neutral subunit	0.98522167	0.724	NA
545600	Gm12372	predicted gene 12372	0.98522167	0.7282	NA
53598	Dctn3	dynactin 3	0.98522167	0.7467	NA
234730	Fuk	fucokinase	0.98522167	0.7517	NA
68449	Tbc1d10b	TBC1 domain family, member 10b	0.98522167	0.7616	NA
68499	Mrpl53	mitochondrial ribosomal protein L53	0.98522167	0.7703	NA
108689	Obfc1	oligonucleotide/oligosaccharide-binding fold containing 1	0.98522167	0.7725	NA
21887	Tle3	transducin-like enhancer of split 3, homolog of Drosophila E(spl)	0.98522167	0.7773	NA
244373	Erlin2	ER lipid raft associated 2	0.98522167	0.7829	NA
23802	Amfr	autocrine motility factor receptor	0.98522167	0.7832	NA
76832	Hyls1	hydrolethalus syndrome 1	0.98522167	0.7885	NA
235323	Usp28	ubiquitin specific peptidase 28	0.98522167	0.7885	NA
105732	Fam83h	family with sequence similarity 83, member H	0.98522167	0.7965	NA
192651	Zfp286	zinc finger protein 286	0.98522167	0.7966	NA
229279	Hnrnpa3	heterogeneous nuclear ribonucleoprotein A3	0.98522167	0.7981	NA
67187	Zmynd19	zinc finger, MYND domain containing 19	0.98522167	0.8064	NA
20104	Rps6	ribosomal protein S6	0.98522167	0.8086	NA
77782	Polq	polymerase (DNA directed), theta	0.98522167	0.8103	NA
22210	Ube2b	ubiquitin-conjugating enzyme E2B, RAD6 homology (S. cerevisiae)	0.98522167	0.8105	NA
19654	Rbm6	RNA binding motif protein 6	0.98522167	0.818	NA
20844	Stam	signal transducing adaptor molecule (SH3 domain and ITAM motif) 1	0.98522167	0.8188	NA
21428	Mlx	MAX-like protein X	0.98522167	0.8194	NA
237877	Atad5	ATPase family, AAA domain containing 5	0.98522167	0.8196	NA
24017	Rnf13	ring finger protein 13	0.98522167	0.8279	NA
19027	Sypl	synaptophysin-like protein	0.98522167	0.8329	NA
14042	Ext1	exostoses (multiple) 1	0.98522167	0.8361	NA
13929	Amz2	archaelysin family metallopeptidase 2	0.98522167	0.8364	NA
17126	Smad2	MAD homolog 2 (Drosophila)	0.98522167	0.8371	NA
330817	Dhps	deoxyhypusine synthase	0.98522167	0.8503	NA
18163	Ctnnd2	catenin (cadherin associated protein), delta 2	0.98522167	0.8579	NA
72022	Slc35f2	solute carrier family 35, member F2	0.98522167	0.8612	NA
74100	Arpp21	cyclic AMP-regulated phosphoprotein, 21	0.98522167	0.8668	0.9313
270156	AU019823	expressed sequence AU019823	0.98522167	0.8685	0.9324

22724	Zbtb7b	zinc finger and BTB domain containing 7B	0.98522167	0.8761	0.936
217480	Dgkb	diacylglycerol kinase, beta	0.98522167	0.8768	0.9364
382423	Atxn7l3b	ataxin 7-like 3B	0.98522167	0.8832	0.94
328789	Lhfpl5	lipoma HMGIC fusion partner-like 5	0.98522167	0.8903	0.9437
96957	Tmem62	transmembrane protein 62	0.98522167	0.8931	0.9452
107701	Sf3b4	splicing factor 3b, subunit 4	0.98522167	0.8934	0.9453
22026	Nr2c2	nuclear receptor subfamily 2, group C, member 2	0.98522167	0.8935	0.9453
71765	Klhdc3	kelch domain containing 3	0.98522167	0.8946	0.946
20346	Sema3a	sema domain, immunoglobulin domain (Ig), short basic domain, secreted, (semaphorin) 3A	0.98522167	0.905	0.9516
75691	Anks6	ankyrin repeat and sterile alpha motif domain containing 6	0.98522167	0.9166	0.9577
70713	Gpr137c	G protein-coupled receptor 137C	0.98522167	0.9323	0.9653
66183	1110032A04Rik	RIKEN cDNA 1110032A04 gene	0.98522167	0.9327	0.9655
73658	Spns1	spinster homolog 1 (Drosophila)	0.98425197	0.6775	NA
15194	Htt	huntingtin	0.98425197	0.6966	NA
22209	Ube2a	ubiquitin-conjugating enzyme E2A, RAD6 homolog (S. cerevisiae)	0.98425197	0.7148	NA
227334	Usp40	ubiquitin specific peptidase 40	0.98425197	0.7544	NA
108909	Aida	axin interactor, dorsalization associated	0.98425197	0.7612	NA
11440	Chrna6	cholinergic receptor, nicotinic, alpha polypeptide 6	0.98425197	0.7707	NA
105428	Fam149b	family with sequence similarity 149, member B	0.98425197	0.772	NA
26394	Lypla2	lysophospholipase 2	0.98425197	0.7723	NA
20916	Sucla2	succinate-Coenzyme A ligase, ADP-forming, beta subunit	0.98425197	0.7753	NA
70054	Ccdc89	coiled-coil domain containing 89	0.98425197	0.7762	NA
54217	Rpl36	ribosomal protein L36	0.98425197	0.7773	NA
101592	Eftud1	elongation factor Tu GTP binding domain containing 1	0.98425197	0.7773	NA
71832	Csl	citrate synthase like	0.98425197	0.7812	NA
66390	Slmo2	slowmo homolog 2 (Drosophila)	0.98425197	0.7858	NA
22210	Ube2b	ubiquitin-conjugating enzyme E2B, RAD6 homology (S. cerevisiae)	0.98425197	0.7894	NA
28036	Larp7	La ribonucleoprotein domain family, member 7	0.98425197	0.7901	NA
272396	Tarsl2	threonyl-tRNA synthetase-like 2	0.98425197	0.7902	NA
226830	Smyd2	SET and MYND domain containing 2	0.98425197	0.7938	NA
68598	Dnajc8	DnaJ (Hsp40) homolog, subfamily C, member 8	0.98425197	0.7985	NA
100042049	LOC100042049	ribosomal protein L22 like 1 pseudogene	0.98425197	0.7988	NA
330502	Zfp82	zinc finger protein 82	0.98425197	0.8064	NA
65113	Ndfip1	Nedd4 family interacting protein 1	0.98425197	0.8101	NA
30931	Tor1a	torsin family 1, member A (torsin A)	0.98425197	0.8102	NA
215194	Kri1	KRI1 homolog (S. cerevisiae)	0.98425197	0.8126	NA
103817	AI662501	expressed sequence AI662501	0.98425197	0.8132	NA
11564	Adsl	adenylosuccinate lyase	0.98425197	0.8173	NA
320234	Ccdc66	coiled-coil domain containing 66	0.98425197	0.8206	NA
66078	Tsen34	tRNA splicing endonuclease 34 homolog (S. cerevisiae)	0.98425197	0.8215	NA
227738	Lrsam1	leucine rich repeat and sterile alpha motif containing 1	0.98425197	0.8219	NA
67655	Ctdp1	CTD (carboxy-terminal domain, RNA polymerase II, polypeptide A) phosphatase, subunit 1	0.98425197	0.8258	NA
67945	Rpl41	ribosomal protein L41	0.98425197	0.826	NA
72157	Pgm2	phosphoglucomutase 2	0.98425197	0.8302	NA
277463	Gpr107	G protein-coupled receptor 107	0.98425197	0.8434	NA
19656	Rbmxrt	RNA binding motif protein, X chromosome retrogene	0.98425197	0.8469	NA
26943	Serinc3	serine incorporator 3	0.98425197	0.8489	NA
70122	Mllt3	myeloid/lymphoid or mixed-lineage leukemia (trithorax homolog, Drosophila); translocated to, 3	0.98425197	0.8497	NA
12330	Canx	calnexin	0.98425197	0.854	0.925
68036	Zfp706	zinc finger protein 706	0.98425197	0.8557	0.9255

83796	Smarcd2	SWI/SNF related, matrix associated, actin dependent regulator of chromatin, subfamily d, member 2	0.98425197	0.8581	0.9269
18391	Sigmar1	sigma non-opioid intracellular receptor 1	0.98425197	0.8621	0.9288
100503659	Dos	downstream of Stk11	0.98425197	0.8623	0.9289
13831	Epc1	enhancer of polycomb homolog 1 (Drosophila)	0.98425197	0.8639	0.9295
26411	Map4k1	mitogen-activated protein kinase kinase kinase kinase 1	0.98425197	0.8683	0.9322
93843	Pnck	pregnancy upregulated non-ubiquitously expressed CaM kinase	0.98425197	0.8708	0.9333
18175	Nrap	nebulin-related anchoring protein	0.98425197	0.8743	0.9351
67035	Dnajb4	DnaJ (Hsp40) homolog, subfamily B, member 4	0.98425197	0.8847	0.9407
105171	Arrdc3	arrestin domain containing 3	0.98425197	0.8853	0.9409
217708	Lin52	lin-52 homolog (C. elegans)	0.98425197	0.8918	0.9447
317750	Slc24a5	solute carrier family 24, member 5	0.98425197	0.8924	0.9449
18027	Nfia	nuclear factor I/A	0.98425197	0.8967	0.9473
11749	Anxa6	annexin A6	0.98425197	0.9097	0.9539
382010	BC088983	cDNA sequence BC088983	0.98425197	0.918	0.9583
224585	Zfp160	zinc finger protein 160	0.98425197	0.9183	0.9584
17965	Nbl1	neuroblastoma, suppression of tumorigenicity 1	0.98425197	0.9294	0.9639
330217	Gal3st4	galactose-3-O-sulfotransferase 4	0.98425197	0.9348	0.9668
56734	Tulp2	tubby-like protein 2	0.98425197	0.9349	0.9668
544710	Krtap10-10	keratin associated protein 10-10	0.98425197	0.945	0.9724
234788	Slc38a8	solute carrier family 38, member 8	0.98425197	0.9522	0.9761
319195	Rpl17	ribosomal protein L17	0.98425197	0.9523	0.9761
211286	Cln5	ceroid-lipofuscinosis, neuronal 5	0.98328417	0.6277	NA
56041	Uso1	USO1 homolog, vesicle docking protein (yeast)	0.98328417	0.6601	NA
66515	Cul7	cullin 7	0.98328417	0.6639	NA
259279	Tubgcp3	tubulin, gamma complex associated protein 3	0.98328417	0.6802	NA
227619	Man1b1	mannosidase, alpha, class 1B, member 1	0.98328417	0.704	NA
57773	Wdr4	WD repeat domain 4	0.98328417	0.7105	NA
56046	Uqcc	ubiquinol-cytochrome c reductase complex chaperone, CBP3 homolog (yeast)	0.98328417	0.7151	NA
66213	Med7	mediator complex subunit 7	0.98328417	0.7275	NA
81904	Cacng7	calcium channel, voltage-dependent, gamma subunit 7	0.98328417	0.7317	NA
74254	Gpn1	GPN-loop GTPase 1	0.98328417	0.7402	NA
234736	Rfwd3	ring finger and WD repeat domain 3	0.98328417	0.7459	NA
12653	Chgb	chromogranin B	0.98328417	0.749	NA
11502	Adam9	a disintegrin and metallopeptidase domain 9 (meltrin gamma)	0.98328417	0.751	NA
68770	Phtf2	putative homeodomain transcription factor 2	0.98328417	0.7533	NA
76000	5033430I15Rik	RIKEN cDNA 5033430I15 gene	0.98328417	0.7546	NA
50505	Ercc4	excision repair cross-complementing rodent repair deficiency, complementation group 4	0.98328417	0.7577	NA
20630	Snrpc	U1 small nuclear ribonucleoprotein C	0.98328417	0.7644	NA
241915	Phc3	polyhomeotic-like 3 (Drosophila)	0.98328417	0.7748	NA
56354	Dnajc7	DnaJ (Hsp40) homolog, subfamily C, member 7	0.98328417	0.7757	NA
246198	Mllt6	myeloid/lymphoid or mixed-lineage leukemia (trithorax homolog, Drosophila); translocated to, 6	0.98328417	0.7798	NA
240263	Fem1c	fem-1 homolog c (C.elegans)	0.98328417	0.7849	NA
223648	2410075B13Rik	RIKEN cDNA 2410075B13 gene	0.98328417	0.7864	NA
18120	Mrpl49	mitochondrial ribosomal protein L49	0.98328417	0.7873	NA
11820	App	amyloid beta (A4) precursor protein	0.98328417	0.7889	NA
73368	Col20a1	collagen, type XX, alpha 1	0.98328417	0.791	NA
229504	Isg20l2	interferon stimulated exonuclease gene 20-like 2	0.98328417	0.7925	NA
64704	Htra2	HtrA serine peptidase 2	0.98328417	0.7931	NA
232821	Ccdc106	coiled-coil domain containing 106	0.98328417	0.8024	NA
22680	Zfp207	zinc finger protein 207	0.98328417	0.8042	NA

66566	Ntpcr	nucleoside-triphosphatase, cancer-related	0.98328417	0.8074	NA
72569	Bbs5	Bardet-Biedl syndrome 5 (human)	0.98328417	0.808	NA
67980	Gnpda2	glucosamine-6-phosphate deaminase 2	0.98328417	0.8122	NA
18762	Prkcz	protein kinase C, zeta	0.98328417	0.8137	NA
94043	Tm2d1	TM2 domain containing 1	0.98328417	0.8144	NA
110651	Rps6ka3	ribosomal protein S6 kinase polypeptide 3	0.98328417	0.8329	NA
20044	Rps14	ribosomal protein S14	0.98328417	0.8346	NA
625730	Gm6616	predicted gene 6616	0.98328417	0.8378	NA
13367	Diap1	diaphanous homolog 1 (Drosophila)	0.98328417	0.8408	NA
17256	Mea1	male enhanced antigen 1	0.98328417	0.8437	0.9193
74754	Dhcr24	24-dehydrocholesterol reductase	0.98328417	0.8437	NA
170767	Rfxap	regulatory factor X-associated protein	0.98328417	0.8444	0.9198
382406	Poc1b	POC1 centriolar protein homolog B (Chlamydomonas)	0.98328417	0.8447	0.9198
66412	Arrdc4	arrestin domain containing 4	0.98328417	0.8476	0.9211
227059	Slc39a10	solute carrier family 39 (zinc transporter), member 10	0.98328417	0.8487	0.9219
53858	Rwdd2b	RWD domain containing 2B	0.98328417	0.8497	0.9227
235582	Glyctk	glycerate kinase	0.98328417	0.8514	0.9235
76133	6230400D17Rik	RIKEN cDNA 6230400D17 gene	0.98328417	0.8545	0.925
66071	Ethe1	ethylmalonic encephalopathy 1	0.98328417	0.8595	0.9274
245405	Gm4987	predicted gene 4987	0.98328417	0.8601	0.9278
20747	Spop	speckle-type POZ protein	0.98328417	0.8715	0.9338
17957	Napb	N-ethylmaleimide sensitive fusion protein attachment protein beta	0.98328417	0.8718	0.934
66054	Cndp2	CNDP dipeptidase 2 (metallopeptidase M20 family)	0.98328417	0.872	0.9341
18798	Plcb4	phospholipase C, beta 4	0.98328417	0.8733	0.9348
320368	A730063M14Rik	RIKEN cDNA A730063M14 gene	0.98328417	0.8736	0.9348
12912	Creb1	cAMP responsive element binding protein 1	0.98328417	0.8762	0.936
27984	Efhd2	EF hand domain containing 2	0.98328417	0.8797	0.9384
232816	Zfp628	zinc finger protein 628	0.98328417	0.8816	0.9394
67897	Rnmt	RNA (guanine-7-) methyltransferase	0.98328417	0.8836	0.9401
14724	Gp1bb	glycoprotein Ib, beta polypeptide	0.98328417	0.8858	0.9412
17299	Mettl1	methyltransferase like 1	0.98328417	0.89	0.9435
232879	Zbtb45	zinc finger and BTB domain containing 45	0.98328417	0.8927	0.945
11689	Alox5	arachidonate 5-lipoxygenase	0.98328417	0.9006	0.9494
15254	Hint1	histidine triad nucleotide binding protein 1	0.98328417	0.9041	0.9513
433102	Sfta2	surfactant associated 2	0.98328417	0.9097	0.9539
20595	Smn1	survival motor neuron 1	0.98328417	0.911	0.9545
240518	Peli3	pellino 3	0.98328417	0.9112	0.9546
30840	Fbxl6	F-box and leucine-rich repeat protein 6	0.98328417	0.9139	0.9562
19664	Rbpj	recombination signal binding protein for immunoglobulin kappa J region	0.98328417	0.9155	0.9572
74434	Sohlh2	spermatogenesis and oogenesis specific basic helix-loop-helix 2	0.98328417	0.9228	0.9609
73174	Tbkbp1	TBK1 binding protein 1	0.98328417	0.9254	0.962
75964	Trappc8	trafficking protein particle complex 8	0.98328417	0.9357	0.9671
330502	Zfp82	zinc finger protein 82	0.98328417	0.9376	0.9682
66691	Gapvd1	GTPase activating protein and VPS9 domains 1	0.98231827	0.6519	NA
65105	Arl6ip4	ADP-ribosylation factor-like 6 interacting protein 4	0.98231827	0.6584	NA
74356	4931428F04Rik	RIKEN cDNA 4931428F04 gene	0.98231827	0.6679	NA
22596	Xrcc5	X-ray repair complementing defective repair in Chinese hamster cells 5	0.98231827	0.6692	NA
67526	Atg12	autophagy-related 12 (yeast)	0.98231827	0.6779	NA
104303	Arl1	ADP-ribosylation factor-like 1	0.98231827	0.678	NA
100972	Rab28	RAB28, member RAS oncogene family	0.98231827	0.6965	NA

76799	2510006D16Rik	RIKEN cDNA 2510006D16 gene	0.98231827	0.6974	NA
69276	Sec62	SEC62 homolog (S. cerevisiae)	0.98231827	0.7091	NA
12349	Car2	carbonic anhydrase 2	0.98231827	0.7097	NA
80292	Zxdc	ZXD family zinc finger C	0.98231827	0.7135	NA
237898	Usp32	ubiquitin specific peptidase 32	0.98231827	0.7149	NA
69225	Carkd	carbohydrate kinase domain containing	0.98231827	0.7246	NA
17527	Mpv17	MpV17 mitochondrial inner membrane protein	0.98231827	0.7281	NA
237781	Smcr7	Smith-Magenis syndrome chromosome region, candidate 7 homolog (human)	0.98231827	0.7363	NA
66048	Tmem93	transmembrane protein 93	0.98231827	0.741	NA
56692	Mapksp1	MAPK scaffold protein 1	0.98231827	0.7452	NA
245841	Polr2h	polymerase (RNA) II (DNA directed) polypeptide H	0.98231827	0.7453	NA
98386	Lbr	lamin B receptor	0.98231827	0.7473	NA
170833	Hook2	hook homolog 2 (Drosophila)	0.98231827	0.7498	NA
215693	Zmat1	zinc finger, matrin type 1	0.98231827	0.7531	NA
12237	Bub3	budding uninhibited by benzimidazoles 3 homolog (S. cerevisiae)	0.98231827	0.7539	NA
11841	Arf2	ADP-ribosylation factor 2	0.98231827	0.7595	NA
11958	Atp5k	ATP synthase, H+ transporting, mitochondrial F1F0 complex, subunit e	0.98231827	0.7603	NA
27061	Bcap31	B-cell receptor-associated protein 31	0.98231827	0.7669	NA
218210	Nup153	nucleoporin 153	0.98231827	0.7728	NA
12846	Comt	catechol-O-methyltransferase	0.98231827	0.7781	NA
19736	Rgs4	regulator of G-protein signaling 4	0.98231827	0.7848	NA
72068	Cnot2	CCR4-NOT transcription complex, subunit 2	0.98231827	0.7879	NA
19656	Rbmxrt	RNA binding motif protein, X chromosome retrogene	0.98231827	0.7939	NA
74412	Gle1	GLE1 RNA export mediator (yeast)	0.98231827	0.7973	NA
12895	Cpt1b	carnitine palmitoyltransferase 1b, muscle	0.98231827	0.8008	NA
24056	Sh3bp5	SH3-domain binding protein 5 (BTK-associated)	0.98231827	0.8067	NA
69162	Sec31a	Sec31 homolog A (S. cerevisiae)	0.98231827	0.8108	NA
75406	Ndufs7	NADH dehydrogenase (ubiquinone) Fe-S protein 7	0.98231827	0.8143	NA
19193	Pipox	pipecolic acid oxidase	0.98231827	0.8193	NA
27399	Ip6k1	inositol hexaphosphate kinase 1	0.98231827	0.8201	NA
24060	Slc35a1	solute carrier family 35 (CMP-sialic acid transporter), member 1	0.98231827	0.8202	NA
50880	Scly	selenocysteine lyase	0.98231827	0.8213	NA
269105	Gm5048	predicted gene 5048	0.98231827	0.8303	NA
22156	Tuft1	tuftelin 1	0.98231827	0.8357	NA
108147	Atic	5-aminoimidazole-4-carboxamide ribonucleotide formyltransferase/IMP cyclohydrolase	0.98231827	0.8384	0.9167
22186	Uba52	ubiquitin A-52 residue ribosomal protein fusion product 1	0.98231827	0.8389	0.9169
238023	Hexdc	hexosaminidase (glycosyl hydrolase family 20, catalytic domain) containing	0.98231827	0.8391	0.9171
208188	Ghsr	growth hormone secretagogue receptor	0.98231827	0.8393	0.9171
26448	Stk30	serine/threonine kinase 30	0.98231827	0.8453	0.92
71928	2310047K21Rik	RIKEN cDNA 2310047K21 gene	0.98231827	0.846	0.9204
20817	Srpk2	serine/arginine-rich protein specific kinase 2	0.98231827	0.8486	0.9219
50776	Polg2	polymerase (DNA directed), gamma 2, accessory subunit	0.98231827	0.849	0.9221
67025	Rpl11	ribosomal protein L11	0.98231827	0.85	0.923
68480	1110007C09Rik	RIKEN cDNA 1110007C09 gene	0.98231827	0.8513	0.9235
20014	Rpn2	ribophorin II	0.98231827	0.8606	0.9279
108013	Celf4	CUGBP, Elav-like family member 4	0.98231827	0.8617	0.9287
78070	Cpt1c	carnitine palmitoyltransferase 1c	0.98231827	0.8633	0.9293
51812	Mcrs1	microspherule protein 1	0.98231827	0.87	0.9328
56371	Fzr1	fizzy/cell division cycle 20 related 1 (Drosophila)	0.98231827	0.8732	0.9348
67891	Rpl4	ribosomal protein L4	0.98231827	0.8763	0.9361

266614	Ly6g5b	lymphocyte antigen 6 complex, locus G5B	0.98231827	0.8804	0.9387
16370	Irs4	insulin receptor substrate 4	0.98231827	0.8822	0.9397
101831	C230052I12Rik	RIKEN cDNA C230052I12 gene	0.98231827	0.8829	0.9399
71837	1700003E16Rik	RIKEN cDNA 1700003E16 gene	0.98231827	0.8838	0.9402
52850	Sgsm1	small G protein signaling modulator 1	0.98231827	0.8918	0.9447
269116	Nfasc	neurofascin	0.98231827	0.8987	0.9487
14548	Mrps33	mitochondrial ribosomal protein S33	0.98231827	0.9014	0.9497
381314	Iars2	isoleucine-tRNA synthetase 2, mitochondrial	0.98231827	0.9069	0.9525
18569	Pdcd4	programmed cell death 4	0.98231827	0.9159	0.9575
71521	Pds5a	PDS5, regulator of cohesion maintenance, homolog A (S. cerevisiae)	0.98231827	0.9243	0.9618
546648	Klhdc7b	kelch domain containing 7B	0.98231827	0.9262	0.9626
12390	Cav2	caveolin 2	0.98231827	0.928	0.9632
219170	AU021034	expressed sequence AU021034	0.98231827	0.9405	0.9696
320106	Slc38a11	solute carrier family 38, member 11	0.98231827	0.9496	0.9748
226861	Hhat	hedgehog acyltransferase	0.98231827	0.9587	0.9795
67665	Dctn4	dynactin 4	0.98135427	0.6158	NA
67604	Get4	golgi to ER traffic protein 4 homolog (S. cerevisiae)	0.98135427	0.6573	NA
269401	Znf512b	zinc finger protein 512B	0.98135427	0.6659	NA
52392	D1Ertd622e	DNA segment, Chr 1, ERATO Doi 622, expressed	0.98135427	0.6718	NA
243833	Zfp128	zinc finger protein 128	0.98135427	0.6843	NA
75734	Mff	mitochondrial fission factor	0.98135427	0.6853	NA
235623	Scap	SREBF chaperone	0.98135427	0.6886	NA
85308	Fam158a	family with sequence similarity 158, member A	0.98135427	0.6963	NA
56332	Amotl2	angiomotin-like 2	0.98135427	0.6985	NA
73078	Pmpcb	peptidase (mitochondrial processing) beta	0.98135427	0.6986	NA
69724	Rnaseh2a	ribonuclease H2, large subunit	0.98135427	0.7101	NA
67870	Enoph1	enolase-phosphatase 1	0.98135427	0.7158	NA
102774	Bbs4	Bardet-Biedl syndrome 4 (human)	0.98135427	0.7186	NA
51788	H2afz	H2A histone family, member Z	0.98135427	0.7204	NA
73174	Tbkbp1	TBK1 binding protein 1	0.98135427	0.7241	NA
241593	Pin1-ps1	peptidylprolyl cis/trans isomerase, NIMA-interacting 1, pseudogene 1	0.98135427	0.7243	NA
268445	Ankrd13b	ankyrin repeat domain 13b	0.98135427	0.7378	NA
268783	Mtmr12	myotubularin related protein 12	0.98135427	0.7379	NA
380702	Shisa6	shisa homolog 6 (Xenopus laevis)	0.98135427	0.7421	NA
66314	Tpd52l2	tumor protein D52-like 2	0.98135427	0.7433	NA
66556	Drap1	Dr1 associated protein 1 (negative cofactor 2 alpha)	0.98135427	0.7437	NA
18127	Nos3	nitric oxide synthase 3, endothelial cell	0.98135427	0.749	NA
53605	Nap1l1	nucleosome assembly protein 1-like 1	0.98135427	0.7494	NA
102182	Prmt10	protein arginine methyltransferase 10 (putative)	0.98135427	0.7496	NA
269529	Fbxo10	F-box protein 10	0.98135427	0.76	NA
71722	Cic	capicua homolog (Drosophila)	0.98135427	0.7613	NA
106766	Stap2	signal transducing adaptor family member 2	0.98135427	0.7619	NA
22658	Pcgf2	polycomb group ring finger 2	0.98135427	0.7655	NA
209357	Gtf2h3	general transcription factor IIH, polypeptide 3	0.98135427	0.7661	NA
263876	Spata2	spermatogenesis associated 2	0.98135427	0.7673	NA
100043902	Six3os1	Six3 opposite strand transcript 1	0.98135427	0.7689	NA
67444	Ilkap	integrin-linked kinase-associated serine/threonine phosphatase 2C	0.98135427	0.7707	NA
382030	Tmem188	transmembrane protein 188	0.98135427	0.7711	NA
17904	Myl6	myosin, light polypeptide 6, alkali, smooth muscle and non-muscle	0.98135427	0.7756	NA
103284	Zc3h10	zinc finger CCCH type containing 10	0.98135427	0.7791	NA

264064	Cdk8	cyclin-dependent kinase 8	0.98135427	0.782	NA
18693	Pick1	protein interacting with C kinase 1	0.98135427	0.7845	NA
98258	Txndc9	thioredoxin domain containing 9	0.98135427	0.7936	NA
59005	Trappc2l	trafficking protein particle complex 2-like	0.98135427	0.7995	NA
14797	Aes	amino-terminal enhancer of split	0.98135427	0.8034	NA
66818	9130011J15Rik	RIKEN cDNA 9130011J15 gene	0.98135427	0.8037	NA
69878	Snrpf	small nuclear ribonucleoprotein polypeptide F	0.98135427	0.8063	NA
14432	Gap43	growth associated protein 43	0.98135427	0.8065	NA
67681	Mrpl18	mitochondrial ribosomal protein L18	0.98135427	0.8078	NA
211007	Trim41	tripartite motif-containing 41	0.98135427	0.8118	NA
100043580	Gm4532	predicted gene 4532	0.98135427	0.8165	NA
210297	Lrch2	leucine-rich repeats and calponin homology (CH) domain containing 2	0.98135427	0.8174	NA
16151	Ikbkg	inhibitor of kappaB kinase gamma	0.98135427	0.8248	0.9097
14057	Sfxn1	sideroflexin 1	0.98135427	0.8256	0.91
14816	Grm1	glutamate receptor, metabotropic 1	0.98135427	0.8269	0.9104
22116	Tsks	testis-specific serine kinase substrate	0.98135427	0.8277	0.911
213582	Mtap9	microtubule-associated protein 9	0.98135427	0.8296	0.912
18611	Pea15a	phosphoprotein enriched in astrocytes 15A	0.98135427	0.8318	0.9131
80707	Wwox	WW domain-containing oxidoreductase	0.98135427	0.8342	0.9143
76014	Zc3h18	zinc finger CCCH-type containing 18	0.98135427	0.8359	0.9153
14390	Gabpa	GA repeat binding protein, alpha	0.98135427	0.8412	0.918
216963	Git1	G protein-coupled receptor kinase-interactor 1	0.98135427	0.844	0.9194
93717	Pcdhga9	protocadherin gamma subfamily A, 9	0.98135427	0.8448	0.9198
50912	Exosc10	exosome component 10	0.98135427	0.8477	0.9212
242466	Zfp462	zinc finger protein 462	0.98135427	0.8505	0.9233
623483	LOC623483	60S ribosomal protein L22-like	0.98135427	0.8519	0.9238
272031	E130309F12Rik	RIKEN cDNA E130309F12 gene	0.98135427	0.8605	0.9279
13136	Cd55	CD55 antigen	0.98135427	0.8626	0.929
320387	D930030O05Rik	RIKEN cDNA D930030O05 gene	0.98135427	0.8688	0.9324
20317	Serpinf1	serine (or cysteine) peptidase inhibitor, clade F, member 1	0.98135427	0.8713	0.9338
622675	Zfp827	zinc finger protein 827	0.98135427	0.8742	0.9351
68598	Dnajc8	DnaJ (Hsp40) homolog, subfamily C, member 8	0.98135427	0.8867	0.9415
67382	Brd3	bromodomain containing 3	0.98135427	0.8992	0.9489
72554	Utp14a	UTP14, U3 small nucleolar ribonucleoprotein, homolog A (yeast)	0.98135427	0.8993	0.9489
238799	Tnpo1	transportin 1	0.98135427	0.913	0.956
382221	Gm1141	predicted gene 1141	0.98135427	0.9135	0.956
30938	Fgd3	FYVE, RhoGEF and PH domain containing 3	0.98135427	0.9168	0.9577
634650	Gbp11	guanylate binding protein 11	0.98135427	0.9317	0.965
241627	Wdr76	WD repeat domain 76	0.98135427	0.9456	0.9728
74969	4930466K18Rik	RIKEN cDNA 4930466K18 gene	0.98135427	0.9516	0.9757
20611	Ssty1	spermiogenesis specific transcript on the Y 1	0.98135427	0.966	0.9832
99650	4933434E20Rik	RIKEN cDNA 4933434E20 gene	0.98039216	0.6301	NA
226252	Fam160b1	family with sequence similarity 160, member B1	0.98039216	0.6449	NA
105148	Iars	isoleucine-tRNA synthetase	0.98039216	0.6461	NA
71911	Bdh1	3-hydroxybutyrate dehydrogenase, type 1	0.98039216	0.6527	NA
76233	Dnttip1	deoxynucleotidyltransferase, terminal, interacting protein 1	0.98039216	0.6662	NA
106821	AI314976	expressed sequence AI314976	0.98039216	0.6829	NA
56451	Suclg1	succinate-CoA ligase, GDP-forming, alpha subunit	0.98039216	0.6934	NA
50884	Nckap1	NCK-associated protein 1	0.98039216	0.6936	NA
338365	Slc41a2	solute carrier family 41, member 2	0.98039216	0.6938	NA

15247	Hiat1	hippocampus abundant gene transcript 1	0.98039216	0.6972	NA
30957	Mapk8ip3	mitogen-activated protein kinase 8 interacting protein 3	0.98039216	0.7006	NA
19878	Rock2	Rho-associated coiled-coil containing protein kinase 2	0.98039216	0.7054	NA
108699	Chn1	chimerin (chimaerin) 1	0.98039216	0.7063	NA
227697	Dolk	dolichol kinase	0.98039216	0.7141	NA
56307	Metap2	methionine aminopeptidase 2	0.98039216	0.7157	NA
68949	1500012F01Rik	RIKEN cDNA 1500012F01 gene	0.98039216	0.7157	NA
19291	Purb	purine rich element binding protein B	0.98039216	0.7162	NA
12070	Ngfrap1	nerve growth factor receptor (TNFRSF16) associated protein 1	0.98039216	0.7208	NA
66797	Cntnap2	contactin associated protein-like 2	0.98039216	0.7256	NA
241547	Harbi1	harbinger transposase derived 1	0.98039216	0.7316	NA
76577	Faf2	Fas associated factor family member 2	0.98039216	0.7338	NA
56443	Arpc1a	actin related protein 2/3 complex, subunit 1A	0.98039216	0.7341	NA
26443	Psma6	proteasome (prosome, macropain) subunit, alpha type 6	0.98039216	0.7343	NA
67205	Utp11l	UTP11-like, U3 small nucleolar ribonucleoprotein, (yeast)	0.98039216	0.739	NA
19376	Rab34	RAB34, member of RAS oncogene family	0.98039216	0.7439	NA
71524	8430432A02Rik	RIKEN cDNA 8430432A02 gene	0.98039216	0.7505	NA
75770	Brsk2	BR serine/threonine kinase 2	0.98039216	0.7522	NA
66441	Magohb	mago-nashi homolog B (Drosophila)	0.98039216	0.7649	NA
22064	Trpc2	transient receptor potential cation channel, subfamily C, member 2	0.98039216	0.7658	NA
16979	Lrrn1	leucine rich repeat protein 1, neuronal	0.98039216	0.7728	NA
66067	Gtpbp8	GTP-binding protein 8 (putative)	0.98039216	0.7748	NA
23934	Ly6h	lymphocyte antigen 6 complex, locus H	0.98039216	0.7796	NA
226751	Cdc42bpa	CDC42 binding protein kinase alpha	0.98039216	0.7817	NA
13643	Efnb3	ephrin B3	0.98039216	0.7892	NA
56710	Dbc1	deleted in bladder cancer 1 (human)	0.98039216	0.7914	NA
382913	Neil2	nei like 2 (E. coli)	0.98039216	0.7916	NA
52064	Coq5	coenzyme Q5 homolog, methyltransferase (yeast)	0.98039216	0.7937	NA
18968	Pola1	polymerase (DNA directed), alpha 1	0.98039216	0.796	NA
16330	Inpp5b	inositol polyphosphate-5-phosphatase B	0.98039216	0.8059	NA
66368	Rtcd1	RNA terminal phosphate cyclase domain 1	0.98039216	0.8173	NA
56716	Mlst8	MTOR associated protein, LST8 homolog (S. cerevisiae)	0.98039216	0.8233	0.9091
68268	Zdhhc21	zinc finger, DHHC domain containing 21	0.98039216	0.8285	0.9113
72844	Kctd17	potassium channel tetramerisation domain containing 17	0.98039216	0.8337	0.9141
19087	Prkar2a	protein kinase, cAMP dependent regulatory, type II alpha	0.98039216	0.8352	0.9148
76246	Rtf1	Rtf1, Paf1/RNA polymerase II complex component, homolog (S. cerevisiae)	0.98039216	0.8356	0.9151
19305	Pex5	peroxisomal biogenesis factor 5	0.98039216	0.8409	0.918
66926	Trmt6	tRNA methyltransferase 6 homolog (S. cerevisiae)	0.98039216	0.8457	0.9203
76787	Ppfia3	protein tyrosine phosphatase, receptor type, f polypeptide (PTPRF), interacting protein (liprin), alpha 3	0.98039216	0.85	0.923
107975	Pacs1	phosphofurin acidic cluster sorting protein 1	0.98039216	0.8509	0.9235
68339	Ccdc88c	coiled-coil domain containing 88C	0.98039216	0.8537	0.9249
228714	Csrp2bp	cysteine and glycine-rich protein 2 binding protein	0.98039216	0.8538	0.9249
75564	Rsph9	radial spoke head 9 homolog (Chlamydomonas)	0.98039216	0.8556	0.9255
232974	Gm4881	predicted gene 4881	0.98039216	0.8592	0.9274
97387	Strn4	striatin, calmodulin binding protein 4	0.98039216	0.8607	0.928
67357	1700092C02Rik	RIKEN cDNA 1700092C02 gene	0.98039216	0.8629	0.9291
13688	Eif4ebp2	eukaryotic translation initiation factor 4E binding protein 2	0.98039216	0.8653	0.9302
22690	Zfp28	zinc finger protein 28	0.98039216	0.8668	0.9313
224824	Pex6	peroxisomal biogenesis factor 6	0.98039216	0.8688	0.9324
73072	BC068157	cDNA sequence BC068157	0.98039216	0.8696	0.9327

77371	Sec24a	Sec24 related gene family, member A (S. cerevisiae)	0.98039216	0.8738	0.935
17391	Mmp24	matrix metallopeptidase 24	0.98039216	0.8741	0.9351
73316	Calr3	calreticulin 3	0.98039216	0.8816	0.9394
246779	Il27	interleukin 27	0.98039216	0.8837	0.9401
78369	Icam4	intercellular adhesion molecule 4, Landsteiner-Wiener blood group	0.98039216	0.8858	0.9412
56526	Sep-06	septin 6	0.98039216	0.8863	0.9413
67516	Kctd4	potassium channel tetramerisation domain containing 4	0.98039216	0.8877	0.9422
74466	4933427G17Rik	RIKEN cDNA 4933427G17 gene	0.98039216	0.8936	0.9453
257921	Olfr1229	olfactory receptor 1229	0.98039216	0.8991	0.9489
210172	Zfp526	zinc finger protein 526	0.98039216	0.9272	0.9627
66594	Uqcr11	ubiquinol-cytochrome c reductase, complex III subunit XI	0.97943193	0.5506	NA
56397	Morf4l2	mortality factor 4 like 2	0.97943193	0.6162	NA
13929	Amz2	archaelysin family metallopeptidase 2	0.97943193	0.6347	NA
21371	Tbca	tubulin cofactor A	0.97943193	0.6347	NA
17855	Mvk	mevalonate kinase	0.97943193	0.642	NA
75665	Ccdc64	coiled-coil domain containing 64	0.97943193	0.6437	NA
100504663	Atg14	VATG14 autophagy related 14 homolog (S. cerevisiae)	0.97943193	0.6607	NA
240396	Mex3c	mex3 homolog C (C. elegans)	0.97943193	0.6745	NA
67180	Yipf5	Yip1 domain family, member 5	0.97943193	0.6786	NA
70601	Ecd	ecdysoneless homolog (Drosophila)	0.97943193	0.6837	NA
78889	Wsb1	WD repeat and SOCS box-containing 1	0.97943193	0.6848	NA
17749	Polr2k	polymerase (RNA) II (DNA directed) polypeptide K	0.97943193	0.6867	NA
114713	Rasa2	RAS p21 protein activator 2	0.97943193	0.6872	NA
19653	Rbm4	RNA binding motif protein 4	0.97943193	0.6962	NA
235040	Atg4d	autophagy-related 4D (yeast)	0.97943193	0.6973	NA
50907	Preb	prolactin regulatory element binding	0.97943193	0.7074	NA
80883	Ntng1	netrin G1	0.97943193	0.7103	NA
67490	1810074P20Rik	RIKEN cDNA 1810074P20 gene	0.97943193	0.7109	NA
73823	4930401B11Rik	RIKEN cDNA 4930401B11 gene	0.97943193	0.7163	NA
209446	Tcfe3	transcription factor E3	0.97943193	0.7232	NA
53416	Stk39	serine/threonine kinase 39, STE20/SPS1 homolog (yeast)	0.97943193	0.7286	NA
20024	Sub1	SUB1 homolog (S. cerevisiae)	0.97943193	0.7319	NA
114893	Dcun1d1	DCN1, defective in cullin neddylation 1, domain containing 1 (S. cerevisiae)	0.97943193	0.7467	NA
59025	Usp14	ubiquitin specific peptidase 14	0.97943193	0.7526	NA
68145	Etaa1	Ewing's tumor-associated antigen 1	0.97943193	0.7529	NA
216877	Dhx33	DEAH (Asp-Glu-Ala-His) box polypeptide 33	0.97943193	0.7548	NA
225326	Pik3c3	phosphoinositide-3-kinase, class 3	0.97943193	0.758	NA
83397	Akap12	A kinase (PRKA) anchor protein (gravin) 12	0.97943193	0.7588	NA
73341	Arhgef6	Rac/Cdc42 guanine nucleotide exchange factor (GEF) 6	0.97943193	0.761	NA
69841	2010002M09Rik	RIKEN cDNA 2010002M09 gene	0.97943193	0.7659	NA
100039982	Gm2533	predicted gene 2533	0.97943193	0.7704	NA
269994	Gsg1l	GSG1-like	0.97943193	0.779	NA
16502	Kcnc1	potassium voltage gated channel, Shaw-related subfamily, member 1	0.97943193	0.7801	NA
72981	Prkrir	protein-kinase, interferon-inducible double stranded RNA dependent inhibitor, repressor of (P58 repressor)	0.97943193	0.7994	NA
319638	Nt5dc1	5'-nucleotidase domain containing 1	0.97943193	0.8079	NA
94186	Strn3	striatin, calmodulin binding protein 3	0.97943193	0.8128	0.9028
21976	Top3b	topoisomerase (DNA) III beta	0.97943193	0.8156	0.9045
93889	Pcdhb18	protocadherin beta 18	0.97943193	0.8161	0.9047
18618	Pemt	phosphatidylethanolamine N-methyltransferase	0.97943193	0.8209	0.9075
67654	4930558C23Rik	RIKEN cDNA 4930558C23 gene	0.97943193	0.8221	0.9085

17957	Napb	N-ethylmaleimide sensitive fusion protein attachment protein beta	0.97943193	0.8246	0.9096
243961	Shank1	SH3/ankyrin domain gene 1	0.97943193	0.8269	0.9104
56320	Dbn1	drebrin 1	0.97943193	0.8316	0.913
114774	Pawr	PRKC, apoptosis, WT1, regulator	0.97943193	0.8368	0.9158
18029	Nfic	nuclear factor I/C	0.97943193	0.8422	0.9184
20383	Srsf3	serine/arginine-rich splicing factor 3	0.97943193	0.8431	0.919
666945	Gm10638	predicted gene 10638	0.97943193	0.8453	0.92
65960	Twsg1	twisted gastrulation homolog 1 (Drosophila)	0.97943193	0.8574	0.9265
71991	Ercc8	excision repaiross-complementing rodent repair deficiency, complementation group 8	0.97943193	0.8588	0.9273
74102	Slc35a5	solute carrier family 35, member A5	0.97943193	0.8588	0.9273
211329	Ncoa7	nuclear receptor coactivator 7	0.97943193	0.8677	0.9318
72459	Htatsf1	HIV TAT specific factor 1	0.97943193	0.8691	0.9325
246179	Fktn	fukutin	0.97943193	0.8785	0.9378
78754	Galntl2	UDP-N-acetyl-alpha-D-galactosamine:polypeptide N-acetylgalactosaminyltransferase-like 2	0.97943193	0.8804	0.9387
16578	Kif9	kinesin family member 9	0.97943193	0.886	0.9412
216565	Ehbp1	EH domain binding protein 1	0.97943193	0.8942	0.9459
72076	Mospd4	motile sperm domain containing 4	0.97943193	0.8993	0.9489
19227	Pthlh	parathyroid hormone-like peptide	0.97943193	0.9041	0.9513
76987	Hdhd2	haloacid dehalogenase-like hydrolase domain containing 2	0.97943193	0.909	0.9534
74696	4930519A11Rik	RIKEN cDNA 4930519A11 gene	0.97943193	0.9159	0.9575
13017	Ctbp2	C-terminal binding protein 2	0.97847358	0.5729	NA
100046282	LOC100046282	selenoprotein K pseudogene	0.97847358	0.6037	NA
67742	Samsn1	SAM domain, SH3 domain and nuclear localization signals, 1	0.97847358	0.6197	NA
15547	Trmt2a	TRM2 tRNA methyltransferase 2 homolog A (S. cerevisiae)	0.97847358	0.6263	NA
104771	Jkamp	JNK1/MAPK8-associated membrane protein	0.97847358	0.6405	NA
27410	Abca3	ATP-binding cassette, sub-family A (ABC1), member 3	0.97847358	0.646	NA
226751	Cdc42bpa	CDC42 binding protein kinase alpha	0.97847358	0.6467	NA
12568	Cdk5	cyclin-dependent kinase 5	0.97847358	0.6471	NA
56374	Tmem59	transmembrane protein 59	0.97847358	0.6508	NA
21761	Morf4l1	mortality factor 4 like 1	0.97847358	0.6542	NA
228491	Zfp770	zinc finger protein 770	0.97847358	0.6648	NA
74479	Snx11	sorting nexin 11	0.97847358	0.6655	NA
631470	Gm7065	predicted gene 7065	0.97847358	0.6671	NA
71883	Coq2	coenzyme Q2 homolog, prenyltransferase (yeast)	0.97847358	0.6682	NA
70769	Nolc1	nucleolar and coiled-body phosphoprotein 1	0.97847358	0.674	NA
108116	Slco3a1	solute carrier organic anion transporter family, member 3a1	0.97847358	0.6752	NA
51788	H2afz	H2A histone family, member Z	0.97847358	0.6881	NA
230075	Ndufb6	NADH dehydrogenase (ubiquinone) 1 beta subcomplex, 6	0.97847358	0.6905	NA
110417	Pigh	phosphatidylinositol glycan anchor biosynthesis, class H	0.97847358	0.6967	NA
193813	Mcfd2	multiple coagulation factor deficiency 2	0.97847358	0.7018	NA
252870	Usp7	ubiquitin specific peptidase 7	0.97847358	0.7066	NA
77929	Yipf6	Yip1 domain family, member 6	0.97847358	0.7143	NA
66400	Alkbh7	alkB, alkylation repair homolog 7 (E. coli)	0.97847358	0.7193	NA
50780	Rgs3	regulator of G-protein signaling 3	0.97847358	0.7246	NA
74270	Usp20	ubiquitin specific peptidase 20	0.97847358	0.7252	NA
18975	Polg	polymerase (DNA directed), gamma	0.97847358	0.731	NA
76161	6330527O06Rik	RIKEN cDNA 6330527O06 gene	0.97847358	0.733	NA
70233	Cd2bp2	CD2 antigen (cytoplasmic tail) binding protein 2	0.97847358	0.735	NA
50753	Fbxo8	F-box protein 8	0.97847358	0.7412	NA
93836	Rnf111	ring finger 111	0.97847358	0.7417	NA

107371	Exoc6	exocyst complex component 6	0.97847358	0.7536	NA
665055	Gm7467	predicted gene 7467	0.97847358	0.7592	NA
11655	Alas1	aminolevulinic acid synthase 1	0.97847358	0.7619	NA
66098	Chchd6	coiled-coil-helix-coiled-coil-helix domain containing 6	0.97847358	0.7619	NA
56878	Rbms1	RNA binding motif, single stranded interacting protein 1	0.97847358	0.762	NA
268469	Zfp652	zinc finger protein 652	0.97847358	0.7665	NA
12709	Ckb	creatine kinase, brain	0.97847358	0.7693	NA
50523	Lats2	large tumor suppressor 2	0.97847358	0.7714	NA
94223	Dgcr8	DiGeorge syndrome critical region gene 8	0.97847358	0.7877	NA
67028	2610002M06Rik	RIKEN cDNA 2610002M06 gene	0.97847358	0.7923	NA
244579	Tox3	TOX high mobility group box family member 3	0.97847358	0.8006	NA
56351	Ptges3	prostaglandin E synthase 3 (cytosolic)	0.97847358	0.8017	0.8959
217779	Lysmd1	LysM, putative peptidoglycan-binding, domain containing 1	0.97847358	0.8049	0.8983
243274	Tmem132d	transmembrane protein 132D	0.97847358	0.8079	0.9004
234388	Ccdc124	coiled-coil domain containing 124	0.97847358	0.8155	0.9045
69399	1700025G04Rik	RIKEN cDNA 1700025G04 gene	0.97847358	0.8205	0.9071
12864	Cox6c	cytochrome c oxidase, subunit VIc	0.97847358	0.8235	0.9091
72836	Pot1b	protection of telomeres 1B	0.97847358	0.8325	0.9137
104307	Rnu12	RNA U12, small nuclear	0.97847358	0.8344	0.9145
56637	Gsk3b	glycogen synthase kinase 3 beta	0.97847358	0.8362	0.9155
52389	Gpr123	G protein-coupled receptor 123	0.97847358	0.8369	0.9158
21687	Tek	endothelial-specific receptor tyrosine kinase	0.97847358	0.8464	0.9205
56363	Tmeff2	transmembrane protein with EGF-like and two follistatin-like domains 2	0.97847358	0.8467	0.9207
15446	Hpgd	hydroxyprostaglandin dehydrogenase 15 (NAD)	0.97847358	0.8479	0.9213
434282	Gm5608	predicted gene 5608	0.97847358	0.8542	0.925
330050	Fam185a	family with sequence similarity 185, member A	0.97847358	0.8581	0.9269
320707	Atp2b3	ATPase, Ca++ transporting, plasma membrane 3	0.97847358	0.8632	0.9293
338355	Fkbp15	FK506 binding protein 15	0.97847358	0.8641	0.9296
12530	Cdc25a	cell division cycle 25 homolog A (S. pombe)	0.97847358	0.8728	0.9348
114600	Gm4736	predicted gene 4736	0.97847358	0.8743	0.9351
67420	Far1	fatty acyl CoA reductase 1	0.97847358	0.8787	0.9378
20698	Sphk1	sphingosine kinase 1	0.97847358	0.8847	0.9407
434057	Nat8b	N-acetyltransferase 8B	0.97847358	0.885	0.9408
77644	C330007P06Rik	RIKEN cDNA C330007P06 gene	0.97847358	0.8897	0.9434
224088	Atp13a3	ATPase type 13A3	0.97847358	0.8951	0.9464
17274	Rab8a	RAB8A, member RAS oncogene family	0.97847358	0.8957	0.9467
21353	Tank	TRAF family member-associated Nf-kappa B activator	0.97847358	0.9138	0.9562
102545	Cmtm7	CKLF-like MARVEL transmembrane domain containing 7	0.97847358	0.9171	0.9579
208884	Zdhhc9	zinc finger, DHHC domain containing 9	0.97751711	0.5538	NA
93765	Ube2n	ubiquitin-conjugating enzyme E2N	0.97751711	0.5955	NA
69113	Alkbh3	alkB, alkylation repair homolog 3 (E. coli)	0.97751711	0.6068	NA
27045	Nit1	nitrilase 1	0.97751711	0.6164	NA
140858	Wdr5	WD repeat domain 5	0.97751711	0.6246	NA
78581	Utp23	UTP23, small subunit (SSU) processome component, homolog (yeast)	0.97751711	0.6375	NA
20523	Slc25a14	solute carrier family 25 (mitochondrial carrier, brain), member 14	0.97751711	0.6455	NA
78653	Bola3	bolA-like 3 (E. coli)	0.97751711	0.66	NA
69470	Tmem127	transmembrane protein 127	0.97751711	0.6622	NA
67994	Mrps11	mitochondrial ribosomal protein S11	0.97751711	0.6758	NA
230700	Foxj3	forkhead box J3	0.97751711	0.6779	NA
216049	Zfp365	zinc finger protein 365	0.97751711	0.6826	NA

75210	Prr3	proline-rich polypeptide 3	0.97751711	0.6981	NA
76688	Arfrp1	ADP-ribosylation factor related protein 1	0.97751711	0.7027	NA
72026	Trmu	tRNA 5-methylaminomethyl-2-thiouridylate methyltransferase	0.97751711	0.7039	NA
23797	Akt3	thymoma viral proto-oncogene 3	0.97751711	0.7058	NA
100233175	AK010878	cDNA sequence AK010878	0.97751711	0.7062	NA
14312	Brd2	bromodomain containing 2	0.97751711	0.7074	NA
75732	Iqcd	IQ motif containing D	0.97751711	0.7075	NA
211446	Exoc3	exocyst complex component 3	0.97751711	0.7116	NA
69478	2300009A05Rik	RIKEN cDNA 2300009A05 gene	0.97751711	0.7183	NA
20807	Srf	serum response factor	0.97751711	0.7249	NA
67390	Rnmtl1	RNA methyltransferase like 1	0.97751711	0.7259	NA
78895	Pus7l	pseudouridylate synthase 7 homolog (S. cerevisiae)-like	0.97751711	0.7271	NA
11637	Ak2	adenylate kinase 2	0.97751711	0.7285	NA
12345	Capzb	capping protein (actin filament) muscle Z-line, beta	0.97751711	0.736	NA
216767	Mrpl22	mitochondrial ribosomal protein L22	0.97751711	0.7396	NA
68936	Fam165b	family with sequence similarity 165, member B	0.97751711	0.7411	NA
74005	6330412A17Rik	RIKEN cDNA 6330412A17 gene	0.97751711	0.7474	NA
224111	Ubxn7	UBX domain protein 7	0.97751711	0.75	NA
606496	Gsk3a	glycogen synthase kinase 3 alpha	0.97751711	0.7571	NA
19720	Trim27	tripartite motif-containing 27	0.97751711	0.7573	NA
67582	Slc25a26	solute carrier family 25 (mitochondrial carrier, phosphate carrier), member 26	0.97751711	0.7581	NA
77574	Fam115a	family with sequence similarity 115, member A	0.97751711	0.7648	NA
13476	Reep5	receptor accessory protein 5	0.97751711	0.7689	NA
94184	Pdxdc1	pyridoxal-dependent decarboxylase domain containing 1	0.97751711	0.7845	NA
114679	Selm	selenoprotein M	0.97751711	0.7848	NA
67588	Rnf41	ring finger protein 41	0.97751711	0.7888	NA
217030	Synrg	synergin, gamma	0.97751711	0.7889	0.8894
75769	4833424O15Rik	RIKEN cDNA 4833424O15 gene	0.97751711	0.7901	0.8901
58240	Hs1bp3	HCLS1 binding protein 3	0.97751711	0.7931	0.8914
98415	Nucks1	nuclear casein kinase and cyclin-dependent kinase substrate 1	0.97751711	0.797	0.8932
209224	Enox2	ecto-NOX disulfide-thiol exchanger 2	0.97751711	0.8018	0.8959
26399	Map2k6	mitogen-activated protein kinase kinase 6	0.97751711	0.8087	0.9006
12140	Fabp7	fatty acid binding protein 7, brain	0.97751711	0.8102	0.9015
109006	Ciapin1	cytokine induced apoptosis inhibitor 1	0.97751711	0.8113	0.902
97654	C81189	expressed sequence C81189	0.97751711	0.8135	0.9033
18293	Ogdh	oxoglutarate dehydrogenase (lipoamide)	0.97751711	0.8178	0.9055
99237	Tm9sf4	transmembrane 9 superfamily protein member 4	0.97751711	0.8198	0.9068
22379	Fmnl3	formin-like 3	0.97751711	0.8235	0.9091
21388	Tbx5	T-box 5	0.97751711	0.8282	0.9112
11845	Arf6	ADP-ribosylation factor 6	0.97751711	0.8292	0.9116
114143	Atp6v0b	ATPase, H+ transporting, lysosomal V0 subunit B	0.97751711	0.8312	0.9126
237107	Gnl3l	guanine nucleotide binding protein-like 3 (nucleolar)-like	0.97751711	0.833	0.9139
22343	Lin7c	lin-7 homolog C (C. elegans)	0.97751711	0.8331	0.9139
319477	6030419C18Rik	RIKEN cDNA 6030419C18 gene	0.97751711	0.8485	0.9218
110639	Prps2	phosphoribosyl pyrophosphate synthetase 2	0.97751711	0.8823	0.9397
319876	Cobll1	Cobl-like 1	0.97751711	0.8995	0.9489
433485	Tmem90b	transmembrane protein 90B	0.97751711	0.9037	0.9512
76971	2810007J24Rik	RIKEN cDNA 2810007J24 gene	0.97751711	0.9042	0.9513
14857	Gsta1	glutathione S-transferase, alpha 1 (Ya)	0.97751711	0.9166	0.9577
258248	Olfr576	olfactory receptor 576	0.97751711	0.9267	0.9627

23808	Ash2l	ash2 (absent, small, or homeotic)-like (Drosophila)	0.9765625	0.6041	NA
71514	Sfpq	splicing factor proline/glutamine rich (polypyrimidine tract binding protein associated)	0.9765625	0.6157	NA
20603	Sms	spermine synthase	0.9765625	0.6195	NA
14194	Fh1	fumarate hydratase 1	0.9765625	0.6384	NA
234733	Ddx19b	DEAD (Asp-Glu-Ala-Asp) box polypeptide 19b	0.9765625	0.639	NA
67877	Naa20	N(alpha)-acetyltransferase 20, NatB catalytic subunit	0.9765625	0.6448	NA
66125	Sf3b5	splicing factor 3b, subunit 5	0.9765625	0.6545	NA
27373	Csnk1e	casein kinase 1, epsilon	0.9765625	0.6636	NA
67857	Ppp6c	protein phosphatase 6, catalytic subunit	0.9765625	0.6719	NA
269023	Zfp608	zinc finger protein 608	0.9765625	0.6763	NA
213541	Ythdf2	YTH domain family 2	0.9765625	0.6843	NA
380664	Lemd3	LEM domain containing 3	0.9765625	0.686	NA
93790	Nipa2	non imprinted in Prader-Willi/Angelman syndrome 2 homolog (human)	0.9765625	0.6876	NA
11957	Atp5j	ATP synthase, H+ transporting, mitochondrial F0 complex, subunit F	0.9765625	0.6915	NA
66437	Fis1	fission 1 (mitochondrial outer membrane) homolog (yeast)	0.9765625	0.6938	NA
68617	1110012J17Rik	RIKEN cDNA 1110012J17 gene	0.9765625	0.6987	NA
68879	Prpf6	PRP6 pre-mRNA splicing factor 6 homolog (yeast)	0.9765625	0.7046	NA
68198	Ndufb2	NADH dehydrogenase (ubiquinone) 1 beta subcomplex, 2	0.9765625	0.7068	NA
223255	Stk24	serine/threonine kinase 24 (STE20 homolog, yeast)	0.9765625	0.7097	NA
18263	Odc1	ornithine decarboxylase, structural 1	0.9765625	0.714	NA
237082	Nxt2	nuclear transport factor 2-like export factor 2	0.9765625	0.7168	NA
245020	Tmem22	transmembrane protein 22	0.9765625	0.7216	NA
19349	Rab7	RAB7, member RAS oncogene family	0.9765625	0.7286	NA
73062	Ppp1r16a	protein phosphatase 1, regulatory (inhibitor) subunit 16A	0.9765625	0.7346	NA
100040322	3830408C21Rik	RIKEN cDNA 3830408C21 gene	0.9765625	0.7425	NA
20255	Scg3	secretogranin III	0.9765625	0.7547	NA
69975	2810405F17Rik	RIKEN cDNA 2810405F17 gene	0.9765625	0.7618	NA
230935	Dnajc11	DnaJ (Hsp40) homolog, subfamily C, member 11	0.9765625	0.7639	NA
74257	Tspan17	tetraspanin 17	0.9765625	0.7679	NA
66314	Tpd52l2	tumor protein D52-like 2	0.9765625	0.7701	NA
230103	Npr2	natriuretic peptide receptor 2	0.9765625	0.7749	NA
226025	Trpm3	transient receptor potential cation channel, subfamily M, member 3	0.9765625	0.7878	0.8887
29861	Dpf1	D4, zinc and double PHD fingers family 1	0.9765625	0.7885	0.8892
56470	Rgs19	regulator of G-protein signaling 19	0.9765625	0.7963	0.8928
72567	Bclaf1	BCL2-associated transcription factor 1	0.9765625	0.7995	0.8949
434768	Rhox8	reproductive homeobox 8	0.9765625	0.8001	0.8953
77767	Ermn	ermin, ERM-like protein	0.9765625	0.8115	0.9021
100336	Ppp1r8	protein phosphatase 1, regulatory (inhibitor) subunit 8	0.9765625	0.8238	0.9093
72486	Rnf219	ring finger protein 219	0.9765625	0.826	0.9103
71602	Myo1e	myosin IE	0.9765625	0.827	0.9105
12995	Csnk2a1	casein kinase 2, alpha 1 polypeptide	0.9765625	0.8272	0.9106
18126	Nos2	nitric oxide synthase 2, inducible	0.9765625	0.8299	0.912
21780	Tfam	transcription factor A, mitochondrial	0.9765625	0.8307	0.9126
27801	Zdhhc8	zinc finger, DHHC domain containing 8	0.9765625	0.8362	0.9155
105988	Espl1	extra spindle poles-like 1 (S. cerevisiae)	0.9765625	0.8366	0.9158
328087	D930001B02	hypothetical protein D930001B02	0.9765625	0.8399	0.9174
26562	Ncdn	neurochondrin	0.9765625	0.8448	0.9198
66832	Rsph3a	radial spoke 3A homolog (Chlamydomonas)	0.9765625	0.8512	0.9235
224630	Bnip1	BCL2/adenovirus E1B interacting protein 1	0.9765625	0.8619	0.9287
18798	Plcb4	phospholipase C, beta 4	0.9765625	0.8668	0.9313

70425	Csnk1g3	casein kinase 1, gamma 3	0.9765625	0.8693	0.9326
381337	Fam178b	family with sequence similarity 178, member B	0.9765625	0.8741	0.9351
75341	4930564C03Rik	RIKEN cDNA 4930564C03 gene	0.9765625	0.8864	0.9413
244059	Chd2	chromodomain helicase DNA binding protein 2	0.9765625	0.8902	0.9437
58234	Shank3	SH3/ankyrin domain gene 3	0.9765625	0.8904	0.9437
20460	Stil	Scl/Tal1 interrupting locus	0.9765625	0.8959	0.9469
84092	Usp8	ubiquitin specific peptidase 8	0.9765625	0.9247	0.9618
14058	F10	coagulation factor X	0.9765625	0.9513	0.9756
79043	Spsb3	splA/ryanodine receptor domain and SOCS box containing 3	0.97560976	0.5056	NA
66152	Uqcr10	ubiquinol-cytochrome c reductase, complex III subunit X	0.97560976	0.5594	NA
69938	Scrn1	secernin 1	0.97560976	0.5694	NA
73024	2900064A13Rik	RIKEN cDNA 2900064A13 gene	0.97560976	0.5701	NA
76179	Usp31	ubiquitin specific peptidase 31	0.97560976	0.577	NA
100045367	LOC100045367	60S ribosomal protein L19-like	0.97560976	0.5926	NA
98758	Hnrnpf	heterogeneous nuclear ribonucleoprotein F	0.97560976	0.5975	NA
66469	2810405K02Rik	RIKEN cDNA 2810405K02 gene	0.97560976	0.6144	NA
79464	Lias	lipoic acid synthetase	0.97560976	0.6148	NA
27878	Tada1	transcriptional adaptor 1	0.97560976	0.6221	NA
66236	1500011B03Rik	RIKEN cDNA 1500011B03 gene	0.97560976	0.6247	NA
67109	Zfp787	zinc finger protein 787	0.97560976	0.6328	NA
243302	Gm4963	predicted gene 4963	0.97560976	0.633	NA
16179	Irak1	interleukin-1 receptor-associated kinase 1	0.97560976	0.638	NA
26901	Deb1	differentially expressed in B16F10 1	0.97560976	0.644	NA
384214	Ephx4	epoxide hydrolase 4	0.97560976	0.6474	NA
20639	Snrpb2	U2 small nuclear ribonucleoprotein B	0.97560976	0.6523	NA
217517	Stxbp6	syntaxin binding protein 6 (amisyn)	0.97560976	0.6601	NA
239760	Gm4943	predicted pseudogene 4943	0.97560976	0.665	NA
30933	Tor2a	torsin family 2, member A	0.97560976	0.6686	NA
59003	Maea	macrophage erythroblast attacher	0.97560976	0.6689	NA
230752	Fam176b	family with sequence similarity 176, member B	0.97560976	0.6711	NA
20729	Spin1	spindlin 1	0.97560976	0.676	NA
56208	Becn1	beclin 1, autophagy related	0.97560976	0.6797	NA
52174	Tmem222	transmembrane protein 222	0.97560976	0.6804	NA
14682	Gnaq	guanine nucleotide binding protein, alpha q polypeptide	0.97560976	0.6901	NA
51869	Rif1	Rap1 interacting factor 1 homolog (yeast)	0.97560976	0.6954	NA
668166	Zxdb	zinc finger, X-linked, duplicated B	0.97560976	0.7061	NA
93887	Pcdhb16	protocadherin beta 16	0.97560976	0.7072	NA
434632	BC085271	cDNA sequence BC085271	0.97560976	0.7074	NA
21848	Trim24	tripartite motif-containing 24	0.97560976	0.7158	NA
245595	Zfp711	zinc finger protein 711	0.97560976	0.7184	NA
99311	Commd7	COMM domain containing 7	0.97560976	0.722	NA
72729	Cdc42se2	CDC42 small effector 2	0.97560976	0.7319	NA
15081	H3f3b	H3 histone, family 3B	0.97560976	0.7326	NA
19344	Rab5b	RAB5B, member RAS oncogene family	0.97560976	0.7393	NA
11908	Atf1	activating transcription factor 1	0.97560976	0.747	NA
56297	Arl6	ADP-ribosylation factor-like 6	0.97560976	0.7514	NA
22385	Baz1b	bromodomain adjacent to zinc finger domain, 1B	0.97560976	0.7524	NA
94232	Ubqln4	ubiquilin 4	0.97560976	0.7537	NA
72667	Zfp444	zinc finger protein 444	0.97560976	0.7558	NA
12469	Cct8	chaperonin containing Tcp1, subunit 8 (theta)	0.97560976	0.7572	NA

19246	Ptpn1	protein tyrosine phosphatase, non-receptor type 1	0.97560976	0.7587	NA
100038760	LOC100038760	hypothetical LOC100038760	0.97560976	0.7615	NA
100044193	LOC100044193	hypothetical protein LOC100044193	0.97560976	0.7629	NA
217207	Dhx8	DEAH (Asp-Glu-Ala-His) box polypeptide 8	0.97560976	0.7669	NA
21848	Trim24	tripartite motif-containing 24	0.97560976	0.7759	0.8808
56464	Ctsf	cathepsin F	0.97560976	0.7762	0.881
53323	Ube2k	ubiquitin-conjugating enzyme E2K (UBC1 homolog, yeast)	0.97560976	0.7763	0.8811
20866	Stim1	stromal interaction molecule 1	0.97560976	0.7772	0.8816
73130	Tmed5	transmembrane emp24 protein transport domain containing 5	0.97560976	0.7865	0.8882
399603	Fam84b	family with sequence similarity 84, member B	0.97560976	0.7924	0.8911
626391	Zfp951	zinc finger protein 951	0.97560976	0.7936	0.8915
108653	Rimklb	ribosomal modification protein rimK-like family member B	0.97560976	0.7998	0.8952
218820	Zfp503	zinc finger protein 503	0.97560976	0.801	0.8957
97244	C80140	expressed sequence C80140	0.97560976	0.8023	0.8962
14248	Flii	flightless I homolog (Drosophila)	0.97560976	0.8068	0.8999
20679	Sox6	SRY-box containing gene 6	0.97560976	0.8081	0.9005
71678	Brox	BRO1 domain and CAAX motif containing	0.97560976	0.8083	0.9005
110695	Aldh7a1	aldehyde dehydrogenase family 7, member A1	0.97560976	0.8084	0.9006
75422	Mettl5	methyltransferase like 5	0.97560976	0.8098	0.9013
272381	Lrrc4b	leucine rich repeat containing 4B	0.97560976	0.8162	0.9047
320110	B230369F24Rik	RIKEN cDNA B230369F24 gene	0.97560976	0.8174	0.9054
13877	Erh	enhancer of rudimentary homolog (Drosophila)	0.97560976	0.8265	0.9104
381833	Prb1	proline-rich protein BstNI subfamily 1	0.97560976	0.8291	0.9116
26356	Ing1	inhibitor of growth family, member 1	0.97560976	0.8306	0.9126
56174	Nagk	N-acetylglucosamine kinase	0.97560976	0.8344	0.9145
18029	Nfic	nuclear factor I/C	0.97560976	0.8517	0.9237
106369	Ypel1	yippee-like 1 (Drosophila)	0.97560976	0.8557	0.9255
23965	Odz3	odd Oz/ten-m homolog 3 (Drosophila)	0.97560976	0.8585	0.9271
211499	Tmem87a	transmembrane protein 87A	0.97560976	0.8735	0.9348
100042464	2610203C20Rik	RIKEN cDNA 2610203C20 gene	0.97560976	0.8792	0.9381
50772	Mapk6	mitogen-activated protein kinase 6	0.97560976	0.8861	0.9412
94047	Cecr6	cat eye syndrome chromosome region, candidate 6 homolog (human)	0.97560976	0.9017	0.95
71274	4933433G15Rik	RIKEN cDNA 4933433G15 gene	0.97560976	0.9146	0.9567
26442	Psma5	proteasome (prosome, macropain) subunit, alpha type 5	0.97465887	0.4748	NA
230751	Oscp1	organic solute carrier partner 1	0.97465887	0.5649	NA
80909	Gatsl2	GATS protein-like 2	0.97465887	0.5653	NA
74778	Rrp7a	ribosomal RNA processing 7 homolog A (S. cerevisiae)	0.97465887	0.5773	NA
69834	Rab43	RAB43, member RAS oncogene family	0.97465887	0.5783	NA
50708	Hist1h1c	histone cluster 1, H1c	0.97465887	0.5887	NA
23894	Gtf2h2	general transcription factor II H, polypeptide 2	0.97465887	0.5988	NA
55936	Ctps2	cytidine 5'-triphosphate synthase 2	0.97465887	0.6062	NA
108888	Atad3a	ATPase family, AAA domain containing 3A	0.97465887	0.6221	NA
70620	Ube2v2	ubiquitin-conjugating enzyme E2 variant 2	0.97465887	0.6435	NA
232196	C87436	expressed sequence C87436	0.97465887	0.6441	NA
15958	Ifit2	interferon-induced protein with tetratricopeptide repeats 2	0.97465887	0.6447	NA
66421	2410004B18Rik	RIKEN cDNA 2410004B18 gene	0.97465887	0.6498	NA
68273	Pomgnt1	protein O-linked mannose beta1,2-N-acetylglucosaminyltransferase	0.97465887	0.6517	NA
11983	Atpif1	ATPase inhibitory factor 1	0.97465887	0.6715	NA
76174	6330526H18Rik	RIKEN cDNA 6330526H18 gene	0.97465887	0.672	NA
100137727	9130230N09Rik	RIKEN cDNA 9130230N09 gene	0.97465887	0.679	NA

23945	Mgll	monoglyceride lipase	0.97465887	0.6802	NA
67111	Naaa	N-acylethanolamine acid amidase	0.97465887	0.6857	NA
56317	Anapc7	anaphase promoting complex subunit 7	0.97465887	0.6871	NA
71198	Otud1	OTU domain containing 1	0.97465887	0.6982	NA
71723	Dhx34	DEAH (Asp-Glu-Ala-His) box polypeptide 34	0.97465887	0.7	NA
69082	Zc3h15	zinc finger CCCH-type containing 15	0.97465887	0.7035	NA
106021	Topors	topoisomerase I binding, arginine/serine-rich	0.97465887	0.7112	NA
102032	AI316807	expressed sequence AI316807	0.97465887	0.7213	NA
20683	Sp1	trans-acting transcription factor 1	0.97465887	0.7236	NA
100213	Rusc2	RUN and SH3 domain containing 2	0.97465887	0.7293	NA
54713	Fezf2	Fez family zinc finger 2	0.97465887	0.7325	NA
268480	Rapgefl1	Rap guanine nucleotide exchange factor (GEF)-like 1	0.97465887	0.7335	NA
53374	Chst3	carbohydrate (chondroitin 6/keratan) sulfotransferase 3	0.97465887	0.7336	NA
70564	5730469M10Rik	RIKEN cDNA 5730469M10 gene	0.97465887	0.7381	NA
19734	Rgs16	regulator of G-protein signaling 16	0.97465887	0.7454	NA
246746	Cd300lf	CD300 antigen like family member F	0.97465887	0.7476	NA
21405	Hnf1a	HNF1 homeobox A	0.97465887	0.7483	NA
19434	Rax	retina and anterior neural fold homeobox	0.97465887	0.7583	NA
216148	Shc2	SHC (Src homology 2 domain containing) transforming protein 2	0.97465887	0.7647	0.8753
237730	Fbll1	fibrillarin-like 1	0.97465887	0.7691	0.8775
107732	Mrpl10	mitochondrial ribosomal protein L10	0.97465887	0.7726	0.8789
432516	Myo1a	myosin IA	0.97465887	0.7738	0.8797
75647	1700025E21Rik	RIKEN cDNA 1700025E21 gene	0.97465887	0.7796	0.8834
11886	Asah1	N-acylsphingosine amidohydrolase 1	0.97465887	0.7832	0.8863
13805	Eng	endoglin	0.97465887	0.7844	0.887
12764	Cmas	cytidine monophospho-N-acetylneuraminic acid synthetase	0.97465887	0.7856	0.8877
14164	Fgf1	fibroblast growth factor 1	0.97465887	0.7871	0.8885
75624	Metap1	methionyl aminopeptidase 1	0.97465887	0.7873	0.8885
14391	Gabpb1	GA repeat binding protein, beta 1	0.97465887	0.7877	0.8887
20181	Rxra	retinoid X receptor alpha	0.97465887	0.7987	0.8943
19206	Ptch1	patched homolog 1	0.97465887	0.8049	0.8983
338364	Trim65	tripartite motif-containing 65	0.97465887	0.8091	0.9008
68094	Smarcc2	SWI/SNF related, matrix associated, actin dependent regulator of chromatin, subfamily c, member 2	0.97465887	0.8092	0.9009
26419	Mapk8	mitogen-activated protein kinase 8	0.97465887	0.8132	0.9032
241274	Pnpla7	patatin-like phospholipase domain containing 7	0.97465887	0.8163	0.9047
16842	Lef1	lymphoid enhancer binding factor 1	0.97465887	0.8215	0.9079
19242	Ptn	pleiotrophin	0.97465887	0.8268	0.9104
74302	Mtmr3	myotubularin related protein 3	0.97465887	0.8283	0.9112
66997	Psmd12	proteasome (prosome, macropain) 26S subunit, non-ATPase, 12	0.97465887	0.829	0.9116
216991	Adap2	ArfGAP with dual PH domains 2	0.97465887	0.8394	0.9171
23966	Odz4	odd Oz/ten-m homolog 4 (Drosophila)	0.97465887	0.8412	0.918
14391	Gabpb1	GA repeat binding protein, beta 1	0.97465887	0.8544	0.925
226525	Rasal2	RAS protein activator like 2	0.97465887	0.8646	0.9298
12507	Cd5	CD5 antigen	0.97465887	0.8727	0.9347
14235	Foxm1	forkhead box M1	0.97465887	0.8802	0.9387
668489	Gm9199	glycine cleavage system protein H (aminomethyl carrier) pseudogene	0.97465887	0.8814	0.9394
229363	Gmps	guanine monophosphate synthetase	0.97465887	0.9087	0.9534
14131	Fcgr3	Fc receptor, IgG, low affinity III	0.97465887	0.9264	0.9626
67676	Rpp21	ribonuclease P 21 subunit (human)	0.97370983	0.4962	NA
52477	Angel2	angel homolog 2 (Drosophila)	0.97370983	0.503	NA

106894	Hmgxb3	HMG box domain containing 3	0.97370983	0.5329	NA
16650	Kpna6	karyopherin (importin) alpha 6	0.97370983	0.5474	NA
11655	Alas1	aminolevulinic acid synthase 1	0.97370983	0.5756	NA
108121	U2af1	U2 small nuclear ribonucleoprotein auxiliary factor (U2AF) 1	0.97370983	0.5831	NA
280408	Rilp	Rab interacting lysosomal protein	0.97370983	0.5894	NA
93877	Pcdhb6	protocadherin beta 6	0.97370983	0.639	NA
66308	2810021B07Rik	RIKEN cDNA 2810021B07 gene	0.97370983	0.6398	NA
22758	Zscan12	zinc finger and SCAN domain containing 12	0.97370983	0.6427	NA
237988	Cdr2l	cerebellar degeneration-related protein 2-like	0.97370983	0.6467	NA
17991	Ndufa2	NADH dehydrogenase (ubiquinone) 1 alpha subcomplex, 2	0.97370983	0.6524	NA
20084	Rps18	ribosomal protein S18	0.97370983	0.6662	NA
58523	Elp2	elongation protein 2 homolog (S. cerevisiae)	0.97370983	0.6762	NA
229007	Zgpat	zinc finger, CCCH-type with G patch domain	0.97370983	0.6798	NA
12695	Inadl	InaD-like (Drosophila)	0.97370983	0.6824	NA
69742	Tm2d2	TM2 domain containing 2	0.97370983	0.6947	NA
64933	Ap3m2	adaptor-related protein complex 3, mu 2 subunit	0.97370983	0.7004	NA
16697	LOC16697	keratin associated protein LOC16697	0.97370983	0.7088	NA
67914	Coq9	coenzyme Q9 homolog (yeast)	0.97370983	0.7098	NA
77891	Ube2s	ubiquitin-conjugating enzyme E2S	0.97370983	0.7111	NA
433693	Akirin2	akirin 2	0.97370983	0.7127	NA
433771	2310028O11Rik	RIKEN cDNA 2310028O11 gene	0.97370983	0.7138	NA
66092	Ghitm	growth hormone inducible transmembrane protein	0.97370983	0.7166	NA
56447	Copz1	coatomer protein complex, subunit zeta 1	0.97370983	0.7354	NA
268451	Rab11fip4	RAB11 family interacting protein 4 (class II)	0.97370983	0.7386	NA
231946	D330028D13Rik	RIKEN cDNA D330028D13 gene	0.97370983	0.7537	NA
13170	Dbp	D site albumin promoter binding protein	0.97370983	0.7619	0.8734
223690	Ankrd54	ankyrin repeat domain 54	0.97370983	0.7648	0.8753
56218	Patz1	POZ (BTB) and AT hook containing zinc finger 1	0.97370983	0.7664	0.8759
54637	Praf2	PRA1 domain family 2	0.97370983	0.7768	0.8814
77462	Tmem116	transmembrane protein 116	0.97370983	0.7789	0.883
14528	Gch1	GTP cyclohydrolase 1	0.97370983	0.7798	0.8835
52513	Ddx56	DEAD (Asp-Glu-Ala-Asp) box polypeptide 56	0.97370983	0.802	0.8961
399604	C530014P21Rik	RIKEN cDNA C530014P21 gene	0.97370983	0.8197	0.9068
319655	Podxl2	podocalyxin-like 2	0.97370983	0.8297	0.912
677368	LOC677368	hypothetical protein LOC677368	0.97370983	0.8367	0.9158
67839	Gpsm1	G-protein signalling modulator 1 (AGS3-like, C. elegans)	0.97370983	0.8398	0.9174
269472	LOC269472	hypothetical LOC269472	0.97370983	0.8433	0.919
66356	2310008H09Rik	RIKEN cDNA 2310008H09 gene	0.97370983	0.8436	0.9193
20684	Sp100	nuclear antigen Sp100	0.97370983	0.8467	0.9208
381290	Atp2b4	ATPase, Ca++ transporting, plasma membrane 4	0.97370983	0.8529	0.9244
75782	Lca5	Leber congenital amaurosis 5 (human)	0.97370983	0.8601	0.9278
117160	Ttyh2	tweety homolog 2 (Drosophila)	0.97370983	0.8601	0.9278
545428	Ccdc141	coiled-coil domain containing 141	0.97370983	0.8634	0.9294
384719	Gm5341	predicted pseudogene 5341	0.97370983	0.8654	0.9302
14407	Gabrg3	gamma-aminobutyric acid (GABA) A receptor, subunit gamma 3	0.97370983	0.866	0.9306
68936	Fam165b	family with sequence similarity 165, member B	0.97370983	0.8826	0.9397
30946	Abt1	activator of basal transcription 1	0.97370983	0.8909	0.9441
12175	Bnip2	BCL2/adenovirus E1B interacting protein 2	0.97370983	0.9419	0.9702
218811	Sec24c	Sec24 related gene family, member C (S. cerevisiae)	0.97276265	0.4602	NA
228790	Asxl1	additional sex combs like 1 (Drosophila)	0.97276265	0.4604	NA

27366	Txnl4a	thioredoxin-like 4A	0.97276265	0.5043	NA
11958	Atp5k	ATP synthase, H+ transporting, mitochondrial F1F0 complex, subunit e	0.97276265	0.5102	NA
218194	Phactr1	phosphatase and actin regulator 1	0.97276265	0.5162	NA
18432	Mybbp1a	MYB binding protein (P160) 1a	0.97276265	0.5575	NA
76983	Scfd1	Sec1 family domain containing 1	0.97276265	0.5622	NA
210544	Wdr67	WD repeat domain 67	0.97276265	0.5707	NA
19344	Rab5b	RAB5B, member RAS oncogene family	0.97276265	0.5941	NA
66290	Atp6v1g1	ATPase, H+ transporting, lysosomal V1 subunit G1	0.97276265	0.5983	NA
21915	Dtymk	deoxythymidylate kinase	0.97276265	0.6003	NA
11992	Auh	AU RNA binding protein/enoyl-coenzyme A hydratase	0.97276265	0.6107	NA
66520	2610001J05Rik	RIKEN cDNA 2610001J05 gene	0.97276265	0.6182	NA
16993	Lta4h	leukotriene A4 hydrolase	0.97276265	0.6297	NA
22193	Ube2e3	ubiquitin-conjugating enzyme E2E 3, UBC4/5 homolog (yeast)	0.97276265	0.6362	NA
101314	6720456B07Rik	RIKEN cDNA 6720456B07 gene	0.97276265	0.6381	NA
68203	Diras2	DIRAS family, GTP-binding RAS-like 2	0.97276265	0.6455	NA
69131	Cdk12	cyclin-dependent kinase 12	0.97276265	0.6561	NA
235459	Gtf2a2	general transcription factor II A, 2	0.97276265	0.6588	NA
15469	Prmt1	protein arginine N-methyltransferase 1	0.97276265	0.6622	NA
97541	Qars	glutaminyl-tRNA synthetase	0.97276265	0.6872	NA
68277	2310057M21Rik	RIKEN cDNA 2310057M21 gene	0.97276265	0.6892	NA
20624	Eftud2	elongation factor Tu GTP binding domain containing 2	0.97276265	0.6982	NA
18389	Oprl1	opioid receptor-like 1	0.97276265	0.7088	NA
606735	A330069E16Rik	RIKEN cDNA A330069E16 gene	0.97276265	0.7157	NA
230648	4732418C07Rik	RIKEN cDNA 4732418C07 gene	0.97276265	0.7289	NA
67223	Rrp15	ribosomal RNA processing 15 homolog (S. cerevisiae)	0.97276265	0.7324	NA
12361	Cask	calcium/calmodulin-dependent serine protein kinase (MAGUK family)	0.97276265	0.7333	NA
69674	Mif4gd	MIF4G domain containing	0.97276265	0.7402	NA
52014	Nus1	nuclear undecaprenyl pyrophosphate synthase 1 homolog (S. cerevisiae)	0.97276265	0.7579	0.8706
217379	Ubxn2a	UBX domain protein 2A	0.97276265	0.7871	0.8885
15460	Hr	hairless	0.97276265	0.7972	0.8932
101206	Tada3	transcriptional adaptor 3	0.97276265	0.798	0.8937
216613	Ccdc85a	coiled-coil domain containing 85A	0.97276265	0.8004	0.8954
76499	Clasp2	CLIP associating protein 2	0.97276265	0.8009	0.8957
229011	Samd10	sterile alpha motif domain containing 10	0.97276265	0.8134	0.9033
69318	1700007K09Rik	RIKEN cDNA 1700007K09 gene	0.97276265	0.8204	0.9071
76551	Ccdc6	coiled-coil domain containing 6	0.97276265	0.8471	0.9208
243376	Doxl2	diamine oxidase-like protein 2	0.97276265	0.8518	0.9237
72549	Reep4	receptor accessory protein 4	0.97276265	0.8547	0.9251
59287	Ncstn	nicastrin	0.97276265	0.8636	0.9294
18015	Nf1	neurofibromatosis 1	0.97276265	0.8779	0.9373
17755	Mtap1b	microtubule-associated protein 1B	0.97276265	0.881	0.939
211556	Ap1ar	adaptor-related protein complex 1 associated regulatory protein	0.97276265	0.8817	0.9394
626683	Gm11362	predicted gene 11362	0.97276265	0.8864	0.9413
270627	Taf1	TAF1 RNA polymerase II, TATA box binding protein (TBP)-associated factor	0.97276265	0.9179	0.9583
75909	Tmem49	transmembrane protein 49	0.9718173	0.5213	NA
67530	Uqcrb	ubiquinol-cytochrome c reductase binding protein	0.9718173	0.5386	NA
28088	D10Wsu52e	DNA segment, Chr 10, Wayne State University 52, expressed	0.9718173	0.5518	NA
545725	Mterf	mitochondrial transcription termination factor	0.9718173	0.5719	NA
232236	C130022K22Rik	RIKEN cDNA C130022K22 gene	0.9718173	0.5761	NA
64704	Htra2	HtrA serine peptidase 2	0.9718173	0.5861	NA

20301	Ccl27a	chemokine (C-C motif) ligand 27A	0.9718173	0.5931	NA
104885	Tmem179	transmembrane protein 179	0.9718173	0.6015	NA
69549	2310009B15Rik	RIKEN cDNA 2310009B15 gene	0.9718173	0.6043	NA
230125	Mcart1	mitochondrial carrier triple repeat 1	0.9718173	0.6061	NA
68394	Ccdc163	coiled-coil domain containing 163	0.9718173	0.6291	NA
170930	Sumo2	SMT3 suppressor of mif two 3 homolog 2 (yeast)	0.9718173	0.6379	NA
67557	Larp6	La ribonucleoprotein domain family, member 6	0.9718173	0.643	NA
140629	Ubox5	U box domain containing 5	0.9718173	0.646	NA
14784	Grb2	growth factor receptor bound protein 2	0.9718173	0.6468	NA
19346	Rab6	RAB6, member RAS oncogene family	0.9718173	0.6478	NA
74106	Dcaf6	DDB1 and CUL4 associated factor 6	0.9718173	0.6501	NA
74257	Tspan17	tetraspanin 17	0.9718173	0.6553	NA
213006	Mfsd4	major facilitator superfamily domain containing 4	0.9718173	0.6611	NA
381314	Iars2	isoleucine-tRNA synthetase 2, mitochondrial	0.9718173	0.6735	NA
12790	Cnga3	cyclic nucleotide gated channel alpha 3	0.9718173	0.6751	NA
68682	Slc44a2	solute carrier family 44, member 2	0.9718173	0.677	NA
217692	Sipa1l1	signal-induced proliferation-associated 1 like 1	0.9718173	0.6774	NA
319322	Sf3b2	splicing factor 3b, subunit 2	0.9718173	0.692	NA
26912	Gcat	glycine C-acetyltransferase (2-amino-3-ketobutyrate-coenzyme A ligase)	0.9718173	0.694	NA
208715	Hmgcs1	3-hydroxy-3-methylglutaryl-Coenzyme A synthase 1	0.9718173	0.7086	NA
108071	Grm5	glutamate receptor, metabotropic 5	0.9718173	0.711	NA
76795	Tbc1d9b	TBC1 domain family, member 9B	0.9718173	0.7157	NA
18163	Ctnnd2	catenin (cadherin associated protein), delta 2	0.9718173	0.7182	NA
64424	Polr1e	polymerase (RNA) I polypeptide E	0.9718173	0.7238	NA
22194	Ube2e1	ubiquitin-conjugating enzyme E2E 1, UBC4/5 homolog (yeast)	0.9718173	0.7382	NA
226971	Plekhb2	pleckstrin homology domain containing, family B (evectins) member 2	0.9718173	0.7452	0.8626
68920	1110065P20Rik	RIKEN cDNA 1110065P20 gene	0.9718173	0.7536	0.8676
234664	Nae1	NEDD8 activating enzyme E1 subunit 1	0.9718173	0.7548	0.8685
56224	Tspan5	tetraspanin 5	0.9718173	0.7734	0.8793
68371	Pbld1	phenazine biosynthesis-like protein domain containing 1	0.9718173	0.7816	0.885
83558	Tex11	testis expressed gene 11	0.9718173	0.7987	0.8943
13642	Efnb2	ephrin B2	0.9718173	0.8007	0.8956
74235	1700020G03Rik	RIKEN cDNA 1700020G03 gene	0.9718173	0.8053	0.8987
114142	Foxp2	forkhead box P2	0.9718173	0.8105	0.9016
108800	Ston2	stonin 2	0.9718173	0.8211	0.9077
18575	Pde1c	phosphodiesterase 1C	0.9718173	0.8347	0.9146
319155	Hist1h4c	histone cluster 1, H4c	0.9718173	0.8361	0.9154
66932	Rexo1	REX1, RNA exonuclease 1 homolog (S. cerevisiae)	0.9718173	0.837	0.9158
51813	Ccnc	cyclin C	0.9718173	0.8379	0.9163
241489	Pde11a	phosphodiesterase 11A	0.9718173	0.8513	0.9235
435784	Gm12471	predicted gene 12471	0.9718173	0.8549	0.9251
50799	Slc25a13	solute carrier family 25 (mitochondrial carrier, adenine nucleotide translocator), member 13	0.9718173	0.856	0.9256
11517	Adcyap1r1	adenylate cyclase activating polypeptide 1 receptor 1	0.9718173	0.858	0.9269
107766	Haao	3-hydroxyanthranilate 3,4-dioxygenase	0.9718173	0.8707	0.9332
100046692	LOC100046692	40S ribosomal protein S10-like	0.9718173	0.878	0.9373
194388	Tet3	tet oncogene family member 3	0.9718173	0.8809	0.939
347740	2900097C17Rik	RIKEN cDNA 2900097C17 gene	0.9718173	0.891	0.9441
59050	Nsa2	NSA2 ribosome biogenesis homolog (S. cerevisiae)	0.9718173	0.8919	0.9447
72902	Spock3	sparc/osteonectin, cwcv and kazal-like domains proteoglycan 3	0.9718173	0.9325	0.9654
69048	Slc30a5	solute carrier family 30 (zinc transporter), member 5	0.97087379	0.4778	NA

72399	Brap	BRCA1 associated protein	0.97087379	0.485	NA
76779	Cluap1	clusterin associated protein 1	0.97087379	0.4859	NA
319719	4732471D19Rik	RIKEN cDNA 4732471D19 gene	0.97087379	0.5027	NA
28042	Ept1	ethanolaminephosphotransferase 1 (CDP-ethanolamine-specific)	0.97087379	0.5109	NA
28019	Ing4	inhibitor of growth family, member 4	0.97087379	0.511	NA
170755	Sgk3	serum/glucocorticoid regulated kinase 3	0.97087379	0.5177	NA
170625	Snx18	sorting nexin 18	0.97087379	0.5303	NA
103711	Pnpo	pyridoxine 5'-phosphate oxidase	0.97087379	0.5304	NA
102323	Dcun1d2	DCN1, defective in cullin neddylation 1, domain containing 2 (S. cerevisiae)	0.97087379	0.5384	NA
22375	Wars	tryptophanyl-tRNA synthetase	0.97087379	0.5402	NA
20534	Slc4a1ap	solute carrier family 4 (anion exchanger), member 1, adaptor protein	0.97087379	0.5518	NA
76967	2700049A03Rik	RIKEN cDNA 2700049A03 gene	0.97087379	0.5646	NA
56434	Tspan3	tetraspanin 3	0.97087379	0.5704	NA
27967	Cherp	calcium homeostasis endoplasmic reticulum protein	0.97087379	0.5787	NA
22404	Wiz	widely-interspaced zinc finger motifs	0.97087379	0.5852	NA
74569	Ttc17	tetratricopeptide repeat domain 17	0.97087379	0.5887	NA
109359	Fam175b	family with sequence similarity 175, member B	0.97087379	0.6055	NA
66387	Nudt8	nudix (nucleoside diphosphate linked moiety X)-type motif 8	0.97087379	0.6083	NA
67921	Ube2f	ubiquitin-conjugating enzyme E2F (putative)	0.97087379	0.609	NA
67383	2410127L17Rik	RIKEN cDNA 2410127L17 gene	0.97087379	0.6094	NA
68521	Fam189b	family with sequence similarity 189, member B	0.97087379	0.6112	NA
66385	Ppp1r7	protein phosphatase 1, regulatory (inhibitor) subunit 7	0.97087379	0.6128	NA
74352	Zfp84	zinc finger protein 84	0.97087379	0.6269	NA
67468	Mmd	monocyte to macrophage differentiation-associated	0.97087379	0.6426	NA
18263	Odc1	ornithine decarboxylase, structural 1	0.97087379	0.6481	NA
110920	Hspa13	heat shock protein 70 family, member 13	0.97087379	0.6513	NA
83768	Dpp7	dipeptidylpeptidase 7	0.97087379	0.6628	NA
22762	Zfpm2	zinc finger protein, multitype 2	0.97087379	0.6639	NA
74326	Hnrnpr	heterogeneous nuclear ribonucleoprotein R	0.97087379	0.6677	NA
329154	Ankrd44	ankyrin repeat domain 44	0.97087379	0.6679	NA
22235	Ugdh	UDP-glucose dehydrogenase	0.97087379	0.6704	NA
71957	Cpsf3l	cleavage and polyadenylation specific factor 3-like	0.97087379	0.6839	NA
66597	Trim13	tripartite motif-containing 13	0.97087379	0.6851	NA
234373	Sugp2	SURP and G patch domain containing 2	0.97087379	0.7047	NA
109275	Actr5	ARP5 actin-related protein 5 homolog (yeast)	0.97087379	0.714	NA
192654	Pla2g15	phospholipase A2, group XV	0.97087379	0.7156	NA
104799	Vipar	VPS33B interacting protein, apical-basolateral polarity regulator	0.97087379	0.7253	NA
228136	Zdhhc5	zinc finger, DHHC domain containing 5	0.97087379	0.7361	0.8573
68112	Sdccag3	serologically defined colon cancer antigen 3	0.97087379	0.7391	0.8588
381738	Gm1060	predicted gene 1060	0.97087379	0.7469	0.8637
244144	Usp35	ubiquitin specific peptidase 35	0.97087379	0.7497	0.8654
58249	Fibp	fibroblast growth factor (acidic) intracellular binding protein	0.97087379	0.7503	0.8657
104112	Acly	ATP citrate lyase	0.97087379	0.752	0.8668
22200	Uba3	ubiquitin-like modifier activating enzyme 3	0.97087379	0.7543	0.8681
225362	Reep2	receptor accessory protein 2	0.97087379	0.7624	0.8738
18302	Oit3	oncoprotein induced transcript 3	0.97087379	0.7681	0.877
626055	Gm15645	predicted gene 15645	0.97087379	0.7716	0.8786
107823	Whsc1	Wolf-Hirschhorn syndrome candidate 1 (human)	0.97087379	0.7789	0.883
74430	4930452B06Rik	RIKEN cDNA 4930452B06 gene	0.97087379	0.7852	0.8875
72121	Dennd2d	DENN/MADD domain containing 2D	0.97087379	0.7971	0.8932

229722	5330417C22Rik	RIKEN cDNA 5330417C22 gene	0.97087379	0.8011	0.8957
52665	Echdc1	enoyl Coenzyme A hydratase domain containing 1	0.97087379	0.8124	0.9025
30924	Angptl3	angiopoietin-like 3	0.97087379	0.8235	0.9091
19733	Rgn	regucalcin	0.97087379	0.8239	0.9093
54201	Zfp316	zinc finger protein 316	0.97087379	0.8253	0.9099
232431	Gprc5a	G protein-coupled receptor, family C, group 5, member A	0.97087379	0.8305	0.9126
75019	Rnase10	ribonuclease, RNase A family, 10 (non-active)	0.97087379	0.8598	0.9276
237073	Rbm41	RNA binding motif protein 41	0.97087379	0.8648	0.9299
59032	Ppp2r3c	protein phosphatase 2, regulatory subunit B'', gamma	0.9699321	0.4038	NA
21422	Tcfcp2	transcription factor CP2	0.9699321	0.4068	NA
52666	Arhgef25	Rho guanine nucleotide exchange factor (GEF) 25	0.9699321	0.4235	NA
76167	Snrnp35	small nuclear ribonucleoprotein 35 (U11/U12)	0.9699321	0.4576	NA
66218	Ndufb9	NADH dehydrogenase (ubiquinone) 1 beta subcomplex, 9	0.9699321	0.4674	NA
68497	1110018G07Rik	RIKEN cDNA 1110018G07 gene	0.9699321	0.4747	NA
66753	Erlec1	endoplasmic reticulum lectin 1	0.9699321	0.4751	NA
217707	Coq6	coenzyme Q6 homolog (yeast)	0.9699321	0.4857	NA
20469	Sipa1	signal-induced proliferation associated gene 1	0.9699321	0.5165	NA
72615	Anks3	ankyrin repeat and sterile alpha motif domain containing 3	0.9699321	0.5205	NA
242418	Dcaf10	DDB1 and CUL4 associated factor 10	0.9699321	0.5303	NA
269704	Zfp664	zinc finger protein 664	0.9699321	0.5395	NA
108660	Rnf187	ring finger protein 187	0.9699321	0.5402	NA
20930	Surf1	surfeit gene 1	0.9699321	0.5481	NA
56278	Gkap1	G kinase anchoring protein 1	0.9699321	0.5556	NA
56748	Nfu1	NFU1 iron-sulfur cluster scaffold homolog (S. cerevisiae)	0.9699321	0.5668	NA
320469	9930014A18Rik	RIKEN cDNA 9930014A18 gene	0.9699321	0.5818	NA
73181	Nfatc4	nuclear factor of activated T-cells, cytoplasmic, calcineurin-dependent 4	0.9699321	0.5944	NA
449521	Zfp213	zinc finger protein 213	0.9699321	0.6045	NA
624866	Lekr1	leucine, glutamate and lysine rich 1	0.9699321	0.608	NA
71706	Slc46a3	solute carrier family 46, member 3	0.9699321	0.6134	NA
18585	Pde9a	phosphodiesterase 9A	0.9699321	0.6141	NA
53890	Sart3	squamous cell carcinoma antigen recognized by T-cells 3	0.9699321	0.6187	NA
231014	9330182L06Rik	RIKEN cDNA 9330182L06 gene	0.9699321	0.6223	NA
21981	Ppp1r13b	protein phosphatase 1, regulatory (inhibitor) subunit 13B	0.9699321	0.6247	NA
58239	Dexi	dexamethasone-induced transcript	0.9699321	0.6289	NA
66272	Cox16	COX16 cytochrome c oxidase assembly homolog (S. cerevisiae)	0.9699321	0.6292	NA
100609	Nsun5	NOL1/NOP2/Sun domain family, member 5	0.9699321	0.6301	NA
54632	Ftsj1	FtsJ homolog 1 (E. coli)	0.9699321	0.642	NA
107513	Ssr1	signal sequence receptor, alpha	0.9699321	0.6494	NA
117198	Ivns1abp	influenza virus NS1A binding protein	0.9699321	0.6525	NA
20585	Hltf	helicase-like transcription factor	0.9699321	0.6593	NA
72254	1700030K09Rik	RIKEN cDNA 1700030K09 gene	0.9699321	0.6662	NA
68031	Rnf146	ring finger protein 146	0.9699321	0.669	NA
17709	COX2	cytochrome c oxidase subunit II	0.9699321	0.6723	NA
107999	Gtpbp6	GTP binding protein 6 (putative)	0.9699321	0.6762	NA
14958	H1f0	H1 histone family, member 0	0.9699321	0.6771	NA
68193	Rpl24	ribosomal protein L24	0.9699321	0.6805	NA
72091	Snhg7	small nucleolar RNA host gene (non-protein coding) 7	0.9699321	0.6838	NA
19876	Robo1	roundabout homolog 1 (Drosophila)	0.9699321	0.6879	NA
260297	Prrt1	proline-rich transmembrane protein 1	0.9699321	0.6901	NA
67922	Fam32a	family with sequence similarity 32, member A	0.9699321	0.6911	NA

52036	Ppp6r3	protein phosphatase 6, regulatory subunit 3	0.9699321	0.6913	NA
11858	Rnd2	Rho family GTPase 2	0.9699321	0.6949	NA
228807	Zfp341	zinc finger protein 341	0.9699321	0.7015	NA
23792	Adam23	a disintegrin and metallopeptidase domain 23	0.9699321	0.7055	NA
114674	Gtf2ird2	GTF2I repeat domain containing 2	0.9699321	0.7076	NA
21664	Phlda1	pleckstrin homology-like domain, family A, member 1	0.9699321	0.7103	NA
666731	Trim43c	tripartite motif-containing 43C	0.9699321	0.7336	0.8561
213417	Klhdc8a	kelch domain containing 8A	0.9699321	0.7347	0.8566
93734	Mpv17l	Mpv17 transgene, kidney disease mutant-like	0.9699321	0.7411	0.8599
56809	Gmeb1	glucocorticoid modulatory element binding protein 1	0.9699321	0.7417	0.8604
22402	Wisp1	WNT1 inducible signaling pathway protein 1	0.9699321	0.7912	0.8905
26403	Map3k11	mitogen-activated protein kinase kinase kinase 11	0.9699321	0.8023	0.8962
72140	Ccdc123	coiled-coil domain containing 123	0.9699321	0.8108	0.9017
414872	Zyg11b	zyg-ll homolog B (C. elegans)	0.9699321	0.8136	0.9033
71889	Epn3	epsin 3	0.9699321	0.8171	0.9053
228777	Nrsn2	neurensin 2	0.9699321	0.8174	0.9054
108723	Card11	caspase recruitment domain family, member 11	0.9699321	0.8226	0.9088
80287	Apobec3	apolipoprotein B mRNA editing enzyme, catalytic polypeptide 3	0.9699321	0.831	0.9126
665536	Gm7676	interferon induced transmembrane protein 1 pseudogene	0.9699321	0.8619	0.9287
71059	Hexim2	hexamthylene bis-acetamide inducible 2	0.9699321	0.894	0.9457
258055	Olfr524	olfactory receptor 524	0.9699321	0.9228	0.9609
16866	Lhb	luteinizing hormone beta	0.9699321	0.9267	0.9627
230101	Gba2	glucosidase beta 2	0.96899225	0.4161	NA
56736	Rnf14	ring finger protein 14	0.96899225	0.4882	NA
17967	Ncam1	neural cell adhesion molecule 1	0.96899225	0.5006	NA
12934	Dpysl2	dihydropyrimidinase-like 2	0.96899225	0.5023	NA
69902	Mrto4	MRT4, mRNA turnover 4, homolog (S. cerevisiae)	0.96899225	0.5048	NA
67105	1700034H14Rik	RIKEN cDNA 1700034H14 gene	0.96899225	0.5172	NA
193043	Zfp3	zinc finger protein 3	0.96899225	0.5176	NA
66128	Mrps36	mitochondrial ribosomal protein S36	0.96899225	0.5288	NA
319277	A230046K03Rik	RIKEN cDNA A230046K03 gene	0.96899225	0.5394	NA
67979	Atad1	ATPase family, AAA domain containing 1	0.96899225	0.5443	NA
50497	Hspa14	heat shock protein 14	0.96899225	0.5587	NA
94112	Med15	mediator complex subunit 15	0.96899225	0.5603	NA
52132	Ccdc97	coiled-coil domain containing 97	0.96899225	0.5701	NA
17149	Magoh	mago-nashi homolog, proliferation-associated (Drosophila)	0.96899225	0.5733	NA
71767	Tysnd1	trypsin domain containing 1	0.96899225	0.5751	NA
231326	Aasdh	aminoadipate-semialdehyde dehydrogenase	0.96899225	0.5794	NA
99890	Prmt6	protein arginine N-methyltransferase 6	0.96899225	0.5804	NA
67270	Mrpl42	mitochondrial ribosomal protein L42	0.96899225	0.5817	NA
101197	Zfp956	zinc finger protein 956	0.96899225	0.5923	NA
22709	Zfp51	zinc finger protein 51	0.96899225	0.5938	NA
231834	Snx8	sorting nexin 8	0.96899225	0.5989	NA
244895	C230081A13Rik	RIKEN cDNA C230081A13 gene	0.96899225	0.6035	NA
52846	D1Bwg0212e	DNA segment, Chr 1, Brigham & Women's Genetics 0212 expressed	0.96899225	0.6102	NA
67241	Smc6	structural maintenance of chromosomes 6	0.96899225	0.6144	NA
56330	Pdcd5	programmed cell death 5	0.96899225	0.6228	NA
552902	LOC552902	hypothetical LOC552902	0.96899225	0.6237	NA
78891	Scyl1	SCY1-like 1 (S. cerevisiae)	0.96899225	0.6264	NA
67785	Zmym4	zinc finger, MYM-type 4	0.96899225	0.6331	NA

70439	Taf15	TAF15 RNA polymerase II, TATA box binding protein (TBP)-associated factor	0.96899225	0.6372	NA
67206	2810013P06Rik	RIKEN cDNA 2810013P06 gene	0.96899225	0.6375	NA
56430	Clip1	CAP-GLY domain containing linker protein 1	0.96899225	0.6383	NA
230577	Pars2	prolyl-tRNA synthetase (mitochondrial)(putative)	0.96899225	0.6408	NA
20842	Stag1	stromal antigen 1	0.96899225	0.6446	NA
18971	Pold1	polymerase (DNA directed), delta 1, catalytic subunit	0.96899225	0.6527	NA
108657	Rnpepl1	arginyl aminopeptidase (aminopeptidase B)-like 1	0.96899225	0.6536	NA
634012	LOC634012	ubiquitin-conjugating enzyme E2 Q2-like	0.96899225	0.6583	NA
54646	Ppp1r3f	protein phosphatase 1, regulatory (inhibitor) subunit 3F	0.96899225	0.663	NA
20674	Sox2	SRY-box containing gene 2	0.96899225	0.6674	NA
73635	1700113I22Rik	RIKEN cDNA 1700113I22 gene	0.96899225	0.6686	NA
22340	Vegfb	vascular endothelial growth factor B	0.96899225	0.6741	NA
68436	Rpl34	ribosomal protein L34	0.96899225	0.6807	NA
245404	Dcaf12l1	DDB1 and CUL4 associated factor 12-like 1	0.96899225	0.6863	NA
26398	Map2k4	mitogen-activated protein kinase kinase 4	0.96899225	0.6901	NA
107869	Cth	cystathionase (cystathionine gamma-lyase)	0.96899225	0.6929	NA
216846	Cntrob	centrobin, centrosomal BRCA2 interacting protein	0.96899225	0.7068	NA
629967	Gm11677	predicted gene 11677	0.96899225	0.7114	NA
11441	Chrna7	cholinergic receptor, nicotinic, alpha polypeptide 7	0.96899225	0.7128	NA
56626	Poll	polymerase (DNA directed), lambda	0.96899225	0.7131	0.8426
75430	3200002M19Rik	RIKEN cDNA 3200002M19 gene	0.96899225	0.7184	0.846
18213	Ntrk3	neurotrophic tyrosine kinase, receptor, type 3	0.96899225	0.7208	0.8475
72137	Wdsub1	WD repeat, SAM and U-box domain containing 1	0.96899225	0.7338	0.8563
26409	Map3k7	mitogen-activated protein kinase kinase kinase 7	0.96899225	0.7345	0.8565
21871	Atp6v0a2	ATPase, H+ transporting, lysosomal V0 subunit A2	0.96899225	0.7382	0.8581
70717	6330406I15Rik	RIKEN cDNA 6330406I15 gene	0.96899225	0.7405	0.8596
66714	4921524J17Rik	RIKEN cDNA 4921524J17 gene	0.96899225	0.7412	0.8599
56086	Set	SET nuclear oncogene	0.96899225	0.7677	0.8768
59043	Wsb2	WD repeat and SOCS box-containing 2	0.96899225	0.7722	0.8788
20112	Rps6ka2	ribosomal protein S6 kinase, polypeptide 2	0.96899225	0.7822	0.8855
226414	Dars	aspartyl-tRNA synthetase	0.96899225	0.788	0.8889
53867	Col5a3	collagen, type V, alpha 3	0.96899225	0.7962	0.8928
114304	Slc28a3	solute carrier family 28 (sodium-coupled nucleoside transporter), member 3	0.96899225	0.8008	0.8956
70911	Phyhipl	phytanoyl-CoA hydroxylase interacting protein-like	0.96899225	0.8015	0.8959
11907	Ate1	arginyltransferase 1	0.96899225	0.8106	0.9016
72535	Aldh1b1	aldehyde dehydrogenase 1 family, member B1	0.96899225	0.8109	0.9017
403185	4932443I19Rik	RIKEN cDNA 4932443I19 gene	0.96899225	0.8264	0.9104
217258	Abca8a	ATP-binding cassette, sub-family A (ABC1), member 8a	0.96899225	0.835	0.9148
245880	Wasf3	WAS protein family, member 3	0.96899225	0.8382	0.9166
20975	Synj2	synaptojanin 2	0.96899225	0.8686	0.9324
258954	Olfr522	olfactory receptor 522	0.96899225	0.8714	0.9338
622675	Zfp827	zinc finger protein 827	0.96899225	0.8934	0.9453
78887	Sfi1	Sfi1 homolog, spindle assembly associated (yeast)	0.96899225	0.9193	0.9591
68251	5430437P03Rik	RIKEN cDNA 5430437P03 gene	0.96805421	0.412	NA
215814	Ccdc28a	coiled-coil domain containing 28A	0.96805421	0.4617	NA
66414	Ndufa12	NADH dehydrogenase (ubiquinone) 1 alpha subcomplex, 12	0.96805421	0.4663	NA
68980	Wdr53	WD repeat domain 53	0.96805421	0.4781	NA
68730	Dus1l	dihydrouridine synthase 1-like (S. cerevisiae)	0.96805421	0.4882	NA
228715	Gm561	predicted gene 561	0.96805421	0.4995	NA
228960	Stx16	syntaxin 16	0.96805421	0.5033	NA

668501	Zfp507	zinc finger protein 507	0.96805421	0.5049	NA
218194	Phactr1	phosphatase and actin regulator 1	0.96805421	0.5203	NA
63856	Taf8	TAF8 RNA polymerase II, TATA box binding protein (TBP)-associated factorq	0.96805421	0.5219	NA
26413	Mapk1	mitogen-activated protein kinase 1	0.96805421	0.5221	NA
227615	Tmem203	transmembrane protein 203	0.96805421	0.5221	NA
72722	Fam98a	family with sequence similarity 98, member A	0.96805421	0.5328	NA
22380	Wbp4	WW domain binding protein 4	0.96805421	0.5413	NA
68519	Eml1	echinoderm microtubule associated protein like 1	0.96805421	0.5503	NA
69064	1810014F10Rik	RIKEN cDNA 1810014F10 gene	0.96805421	0.5533	NA
70428	Polr3b	polymerase (RNA) III (DNA directed) polypeptide B	0.96805421	0.5533	NA
67674	Trmt112	tRNA methyltransferase 11-2 homolog (S. cerevisiae)	0.96805421	0.5598	NA
66945	Sdha	succinate dehydrogenase complex, subunit A, flavoprotein (Fp)	0.96805421	0.5666	NA
213819	Casd1	CAS1 domain containing 1	0.96805421	0.5752	NA
22224	Usp10	ubiquitin specific peptidase 10	0.96805421	0.5802	NA
269224	Pask	PAS domain containing serine/threonine kinase	0.96805421	0.5833	NA
100039815	Gm2436	predicted gene 2436	0.96805421	0.5839	NA
14164	Fgf1	fibroblast growth factor 1	0.96805421	0.5853	NA
27103	Eif2ak4	eukaryotic translation initiation factor 2 alpha kinase 4	0.96805421	0.6059	NA
19650	Rbl1	retinoblastoma-like 1 (p107)	0.96805421	0.6111	NA
56086	Set	SET nuclear oncogene	0.96805421	0.6111	NA
170930	Sumo2	SMT3 suppressor of mif two 3 homolog 2 (yeast)	0.96805421	0.6188	NA
223455	Mar-06	membrane-associated ring finger (C3HC4) 6	0.96805421	0.6264	NA
24013	Grk1	G protein-coupled receptor kinase 1	0.96805421	0.6323	NA
72803	2810454L23Rik	RIKEN cDNA 2810454L23 gene	0.96805421	0.6358	NA
27494	Amot	angiomotin	0.96805421	0.6556	NA
170930	Sumo2	SMT3 suppressor of mif two 3 homolog 2 (yeast)	0.96805421	0.6563	NA
68750	Rreb1	ras responsive element binding protein 1	0.96805421	0.6599	NA
209011	Sirt7	sirtuin 7 (silent mating type information regulation 2, homolog) 7 (S. cerevisiae)	0.96805421	0.6769	NA
260299	Cadm4	cell adhesion molecule 4	0.96805421	0.6838	NA
74630	4930432N10Rik	RIKEN cDNA 4930432N10 gene	0.96805421	0.6881	NA
72650	2810006K23Rik	RIKEN cDNA 2810006K23 gene	0.96805421	0.6921	NA
67455	Klhl13	kelch-like 13 (Drosophila)	0.96805421	0.7037	NA
218613	Mier3	mesoderm induction early response 1, family member 3	0.96805421	0.7048	0.8372
15289	Hmgb1	high mobility group box 1	0.96805421	0.7057	0.8379
23992	Prkra	protein kinase, interferon inducible double stranded RNA dependent activator	0.96805421	0.706	NA
54393	Gabbr1	gamma-aminobutyric acid (GABA) B receptor, 1	0.96805421	0.7082	0.8395
70047	Trnt1	tRNA nucleotidyl transferase, CCA-adding, 1	0.96805421	0.7097	0.8406
66911	Nudt16l1	nudix (nucleoside diphosphate linked moiety X)-type motif 16-like 1	0.96805421	0.7102	0.8408
18189	Nrxn1	neurexin I	0.96805421	0.7172	0.8456
74513	Neto2	neuropilin (NRP) and tolloid (TLL)-like 2	0.96805421	0.7191	0.8466
83409	Robld3	roadblock domain containing 3	0.96805421	0.7276	0.8518
54004	Diap2	diaphanous homolog 2 (Drosophila)	0.96805421	0.7294	0.853
244666	Gm505	predicted gene 505	0.96805421	0.7441	0.8618
223499	Dcaf13	DDB1 and CUL4 associated factor 13	0.96805421	0.753	0.8673
68142	Ino80	INO80 homolog (S. cerevisiae)	0.96805421	0.7532	0.8673
66090	Ypel3	yippee-like 3 (Drosophila)	0.96805421	0.7577	0.8706
232314	Ppp4r2	protein phosphatase 4, regulatory subunit 2	0.96805421	0.7591	0.8714
666043	Gm7904	predicted gene 7904	0.96805421	0.7687	0.8772
105522	Ankrd28	ankyrin repeat domain 28	0.96805421	0.7704	0.8782
67542	Cog6	component of oligomeric golgi complex 6	0.96805421	0.7705	0.8783

16590	Kit	kit oncogene	0.96805421	0.7709	0.8784
108068	Grm2	glutamate receptor, metabotropic 2	0.96805421	0.7724	0.8789
70427	Mier2	mesoderm induction early response 1, family member 2	0.96805421	0.7856	0.8877
13682	Eif4a2	eukaryotic translation initiation factor 4A2	0.96805421	0.792	0.8911
100503757	LOC100503757	putative transposase element L1Md-A101/L1Md-A102/L1Md-A2-like	0.96805421	0.7923	0.8911
223726	Mpped1	metallophosphoesterase domain containing 1	0.96805421	0.7939	0.8917
66793	Efcab1	EF hand calcium binding domain 1	0.96805421	0.8164	0.9047
100504173	LOC100504173	hypothetical protein LOC100504173	0.96805421	0.8335	0.9141
546886	Ccdc42b	coiled-coil domain containing 42B	0.96805421	0.8757	0.9357
11518	Add1	adducin 1 (alpha)	0.96711799	0.3704	NA
66928	3110001D03Rik	RIKEN cDNA 3110001D03 gene	0.96711799	0.403	NA
102278	Cpne7	copine VII	0.96711799	0.4254	NA
16969	Zbtb7a	zinc finger and BTB domain containing 7a	0.96711799	0.4796	NA
67382	Brd3	bromodomain containing 3	0.96711799	0.5113	NA
74610	Abcb8	ATP-binding cassette, sub-family B (MDR/TAP), member 8	0.96711799	0.523	NA
66454	Nmnat1	nicotinamide nucleotide adenylyltransferase 1	0.96711799	0.5342	NA
72605	Car10	carbonic anhydrase 10	0.96711799	0.5556	NA
74841	Usp38	ubiquitin specific peptidase 38	0.96711799	0.5618	NA
109205	Sobp	sine oculis-binding protein homolog (Drosophila)	0.96711799	0.574	NA
66085	Eif3f	eukaryotic translation initiation factor 3, subunit F	0.96711799	0.5764	NA
18046	Nfyc	nuclear transcription factor-Y gamma	0.96711799	0.5807	NA
57354	Cramp1l	Crm, cramped-like (Drosophila)	0.96711799	0.586	NA
71679	Atp5h	ATP synthase, H+ transporting, mitochondrial F0 complex, subunit d	0.96711799	0.5867	NA
66713	Actr2	ARP2 actin-related protein 2 homolog (yeast)	0.96711799	0.5879	NA
67933	Hcfc2	host cell factor C2	0.96711799	0.5985	NA
668137	Gm8994	predicted gene 8994	0.96711799	0.6027	NA
71807	Tars2	threonyl-tRNA synthetase 2, mitochondrial (putative)	0.96711799	0.6235	NA
211548	Nomo1	nodal modulator 1	0.96711799	0.631	NA
242557	Atg4c	autophagy-related 4C (yeast)	0.96711799	0.6359	NA
402737	A130014A01Rik	RIKEN cDNA A130014A01 gene	0.96711799	0.6398	NA
239114	Il17d	interleukin 17D	0.96711799	0.6406	NA
28240	Trpm2	transient receptor potential cation channel, subfamily M, member 2	0.96711799	0.6424	NA
66676	Tmed7	transmembrane emp24 protein transport domain containing 7	0.96711799	0.6451	NA
19664	Rbpj	recombination signal binding protein for immunoglobulin kappa J region	0.96711799	0.6575	NA
101206	Tada3	transcriptional adaptor 3	0.96711799	0.6698	NA
71728	Stk11ip	serine/threonine kinase 11 interacting protein	0.96711799	0.6771	NA
12895	Cpt1b	carnitine palmitoyltransferase 1b, muscle	0.96711799	0.6774	NA
320878	Mical2	microtubule associated monoxygenase, calponin and LIM domain containing 2	0.96711799	0.6862	NA
192292	Nrbp1	nuclear receptor binding protein 1	0.96711799	0.7122	0.8421
100047133	LOC100047133	hypothetical LOC100047133	0.96711799	0.7125	0.8423
235431	Coro2b	coronin, actin binding protein, 2B	0.96711799	0.7126	0.8424
11757	Prdx3	peroxiredoxin 3	0.96711799	0.7235	0.8493
13548	Dyrk1a	dual-specificity tyrosine-(Y)-phosphorylation regulated kinase 1a	0.96711799	0.7241	0.8498
320404	Itpkb	inositol 1,4,5-trisphosphate 3-kinase B	0.96711799	0.7274	0.8518
194952	Jmjd4	jumonji domain containing 4	0.96711799	0.7351	0.8568
29871	Scmh1	sex comb on midleg homolog 1	0.96711799	0.747	0.8637
217124	Ppp1r9b	protein phosphatase 1, regulatory subunit 9B	0.96711799	0.747	0.8637
68092	Ncbp2	nuclear cap binding protein subunit 2	0.96711799	0.7558	0.8692
230674	Kdm4a	lysine (K)-specific demethylase 4A	0.96711799	0.7607	0.8725
213236	Dnd1	dead end homolog 1 (zebrafish)	0.96711799	0.7612	0.8729

12095	Bglap-rs1	bone gamma-carboxyglutamate protein, related sequence 1	0.96711799	0.7655	0.8755
68272	Rbm28	RNA binding motif protein 28	0.96711799	0.7703	0.8781
78688	Nol3	nucleolar protein 3 (apoptosis repressor with CARD domain)	0.96711799	0.7718	0.8787
78108	4930414L22Rik	RIKEN cDNA 4930414L22 gene	0.96711799	0.7745	0.8801
94332	Cadm3	cell adhesion molecule 3	0.96711799	0.7821	0.8853
75146	Tmem180	transmembrane protein 180	0.96711799	0.7876	0.8886
238330	6430527G18Rik	RIKEN cDNA 6430527G18 gene	0.96711799	0.8002	0.8953
12607	Cebpz	CCAAT/enhancer binding protein zeta	0.96711799	0.8301	0.9123
353025	Caps2	calcyphosphine 2	0.96711799	0.8741	0.9351
230737	Gnl2	guanine nucleotide binding protein-like 2 (nucleolar)	0.96618357	0.4094	NA
99412	Golga2	golgi autoantigen, golgin subfamily a, 2	0.96618357	0.4261	NA
56322	Timm22	translocase of inner mitochondrial membrane 22 homolog (yeast)	0.96618357	0.4549	NA
110147	Ehmt2	euchromatic histone lysine N-methyltransferase 2	0.96618357	0.4606	NA
72322	Xpo5	exportin 5	0.96618357	0.4721	NA
66128	Mrps36	mitochondrial ribosomal protein S36	0.96618357	0.4961	NA
170789	Acot8	acyl-CoA thioesterase 8	0.96618357	0.5042	NA
67468	Mmd	monocyte to macrophage differentiation-associated	0.96618357	0.509	NA
230157	Tmeff1	transmembrane protein with EGF-like and two follistatin-like domains 1	0.96618357	0.5098	NA
226976	4632411B12Rik	RIKEN cDNA 4632411B12 gene	0.96618357	0.5272	NA
69029	1500032L24Rik	RIKEN cDNA 1500032L24 gene	0.96618357	0.5408	NA
193452	Zfp184	zinc finger protein 184 (Kruppel-like)	0.96618357	0.552	NA
67568	Mrfap1	Morf4 family associated protein 1	0.96618357	0.5584	NA
28018	Ubfd1	ubiquitin family domain containing 1	0.96618357	0.5718	NA
19377	Rai1	retinoic acid induced 1	0.96618357	0.5784	NA
75669	Pik3r4	phosphatidylinositol 3 kinase, regulatory subunit, polypeptide 4, p150	0.96618357	0.5804	NA
238266	Syt16	synaptotagmin XVI	0.96618357	0.5998	NA
22196	Ube2i	ubiquitin-conjugating enzyme E2I	0.96618357	0.6014	NA
613262	BC029722	cDNA sequence BC029722	0.96618357	0.6052	NA
235469	Zfp280d	zinc finger protein 280D	0.96618357	0.6081	NA
227867	Epc2	enhancer of polycomb homolog 2 (Drosophila)	0.96618357	0.6252	NA
66597	Trim13	tripartite motif-containing 13	0.96618357	0.6499	NA
66860	Tanc1	tetratricopeptide repeat, ankyrin repeat and coiled-coil containing 1	0.96618357	0.6548	NA
30930	Vps26a	vacuolar protein sorting 26 homolog A (yeast)	0.96618357	0.6572	NA
106564	Ppcs	phosphopantothenoylcysteine synthetase	0.96618357	0.6634	NA
381668	Fbrsl1	fibrosin-like 1	0.96618357	0.6635	NA
213541	Ythdf2	YTH domain family 2	0.96618357	0.6664	NA
67008	1600012F09Rik	RIKEN cDNA 1600012F09 gene	0.96618357	0.6674	NA
66626	5730403B10Rik	RIKEN cDNA 5730403B10 gene	0.96618357	0.67	NA
76932	Arfip2	ADP-ribosylation factor interacting protein 2	0.96618357	0.6716	NA
19296	Pvt1	plasmacytoma variant translocation 1	0.96618357	0.6719	NA
11858	Rnd2	Rho family GTPase 2	0.96618357	0.6727	NA
227580	C1ql3	C1q-like 3	0.96618357	0.6762	NA
66616	Snx9	sorting nexin 9	0.96618357	0.6849	0.824
76642	1700113A16Rik	RIKEN cDNA 1700113A16 gene	0.96618357	0.6936	0.8302
245522	Zc4h2	zinc finger, C4H2 domain containing	0.96618357	0.7002	0.8338
226026	Smc5	structural maintenance of chromosomes 5	0.96618357	0.701	0.8344
69188	Mll5	myeloid/lymphoid or mixed-lineage leukemia 5	0.96618357	0.7173	0.8456
22779	Ikzf2	IKAROS family zinc finger 2	0.96618357	0.7196	0.8468
72978	Cnih3	cornichon homolog 3 (Drosophila)	0.96618357	0.72	0.8469
68792	Srpx2	sushi-repeat-containing protein, X-linked 2	0.96618357	0.7275	0.8518

73754	Thap1	THAP domain containing, apoptosis associated protein 1	0.96618357	0.732	0.8552
68045	2700060E02Rik	RIKEN cDNA 2700060E02 gene	0.96618357	0.74	0.8594
68235	2410066E13Rik	RIKEN cDNA 2410066E13 gene	0.96618357	0.7424	0.8607
14547	Gdap2	ganglioside-induced differentiation-associated-protein 2	0.96618357	0.7436	0.8615
211770	Trib1	tribbles homolog 1 (Drosophila)	0.96618357	0.7483	0.8644
27966	Rrp9	RRP9, small subunit (SSU) processome component, homolog (yeast)	0.96618357	0.7493	0.8651
50492	Thop1	thimet oligopeptidase 1	0.96618357	0.7501	0.8657
17913	Myo1c	myosin IC	0.96618357	0.7512	0.8663
100210	Gpn2	GPN-loop GTPase 2	0.96618357	0.7534	0.8674
67897	Rnmt	RNA (guanine-7-) methyltransferase	0.96618357	0.7542	0.8681
76668	Mdh1b	malate dehydrogenase 1B, NAD (soluble)	0.96618357	0.7562	0.8694
110854	Ppp2r4	protein phosphatase 2A, regulatory subunit B (PR 53)	0.96618357	0.7586	0.8713
67824	Nmral1	NmrA-like family domain containing 1	0.96618357	0.7593	0.8716
70470	Rprd1b	regulation of nuclear pre-mRNA domain containing 1B	0.96618357	0.7627	0.874
19334	Rab22a	RAB22A, member RAS oncogene family	0.96618357	0.7639	0.8748
56457	Clptm1	cleft lip and palate associated transmembrane protein 1	0.96618357	0.7706	0.8783
192285	Phf21a	PHD finger protein 21A	0.96618357	0.7865	0.8882
625638	Fam43b	family with sequence similarity 43, member B	0.96618357	0.787	0.8885
14068	F7	coagulation factor VII	0.96618357	0.8044	0.8981
320204	4833442J19Rik	RIKEN cDNA 4833442J19 gene	0.96618357	0.8117	0.9022
56273	Pex14	peroxisomal biogenesis factor 14	0.96618357	0.8201	0.9069
100039133	Gm2058	predicted gene 2058	0.96618357	0.8203	0.9071
229694	AI504432	expressed sequence AI504432	0.96618357	0.8256	0.91
71242	Spata24	spermatogenesis associated 24	0.96618357	0.8432	0.919
22644	Rnf103	ring finger protein 103	0.96618357	0.8543	0.925
16560	Kif1a	kinesin family member 1A	0.96618357	0.856	0.9256
212285	Arap2	ArfGAP with RhoGAP domain, ankyrin repeat and PH domain 2	0.96618357	0.8734	0.9348
68344	Tmem174	transmembrane protein 174	0.96618357	0.8832	0.94
94353	Hmgn3	high mobility group nucleosomal binding domain 3	0.96525097	0.3655	NA
22275	Urod	uroporphyrinogen decarboxylase	0.96525097	0.4043	NA
67680	Sdhb	succinate dehydrogenase complex, subunit B, iron sulfur (Ip)	0.96525097	0.4132	NA
72475	Ssbp3	single-stranded DNA binding protein 3	0.96525097	0.4223	NA
11958	Atp5k	ATP synthase, H+ transporting, mitochondrial F1F0 complex, subunit e	0.96525097	0.4243	NA
18744	Pja1	praja1, RING-H2 motif containing	0.96525097	0.4354	NA
229776	Cdc14a	CDC14 cell division cycle 14 homolog A (S. cerevisiae)	0.96525097	0.4615	NA
14712	Gnpat	glyceronephosphate O-acyltransferase	0.96525097	0.4808	NA
225215	Rsl24d1	ribosomal L24 domain containing 1	0.96525097	0.4869	NA
433297	Gm5526	predicted pseudogene 5526	0.96525097	0.494	NA
218194	Phactr1	phosphatase and actin regulator 1	0.96525097	0.4961	NA
99982	Kdm1a	lysine (K)-specific demethylase 1A	0.96525097	0.5034	NA
98952	Fam102a	family with sequence similarity 102, member A	0.96525097	0.5102	NA
382252	A830080D01Rik	RIKEN cDNA A830080D01 gene	0.96525097	0.5303	NA
142688	Asb13	ankyrin repeat and SOCS box-containing 13	0.96525097	0.5359	NA
52696	Zwint	ZW10 interactor	0.96525097	0.5406	NA
66046	Ndufb5	NADH dehydrogenase (ubiquinone) 1 beta subcomplex, 5	0.96525097	0.5407	NA
78783	Brpf1	bromodomain and PHD finger containing, 1	0.96525097	0.5516	NA
100043585	Gm4535	predicted gene 4535	0.96525097	0.552	NA
74238	Mterfd3	MTERF domain containing 3	0.96525097	0.5594	NA
66975	2410002O22Rik	RIKEN cDNA 2410002O22 gene	0.96525097	0.5601	NA
14605	Tsc22d3	TSC22 domain family, member 3	0.96525097	0.565	NA

69434	Snhg10	small nucleolar RNA host gene (non-protein coding) 10	0.96525097	0.5807	NA
74781	Wipi2	WD repeat domain, phosphoinositide interacting 2	0.96525097	0.5845	NA
68117	Apool	apolipoprotein O-like	0.96525097	0.5869	NA
69527	Mrps9	mitochondrial ribosomal protein S9	0.96525097	0.5872	NA
20493	Slc10a1	solute carrier family 10 (sodium/bile acid cotransporter family), member 1	0.96525097	0.5887	NA
14450	Gart	phosphoribosylglycinamide formyltransferase	0.96525097	0.5899	NA
229488	Fam160a1	family with sequence similarity 160, member A1	0.96525097	0.5902	NA
71777	Ing3	inhibitor of growth family, member 3	0.96525097	0.5982	NA
14025	Bcl11a	B-cell CLL/lymphoma 11A (zinc finger protein)	0.96525097	0.6031	NA
77629	Sphkap	SPHK1 interactor, AKAP domain containing	0.96525097	0.6062	NA
103203	AI413759	expressed sequence AI413759	0.96525097	0.6124	NA
69641	Wdr20a	WD repeat domain 20A	0.96525097	0.6176	NA
53872	Caprin1	cell cycle associated protein 1	0.96525097	0.6218	NA
234344	Naf1	nuclear assembly factor 1 homolog (S. cerevisiae)	0.96525097	0.6287	NA
67068	Dynlrb1	dynein light chain roadblock-type 1	0.96525097	0.6386	NA
217732	2310044G17Rik	RIKEN cDNA 2310044G17 gene	0.96525097	0.6401	NA
17449	Mdh1	malate dehydrogenase 1, NAD (soluble)	0.96525097	0.6626	NA
21835	Thrsp	thyroid hormone responsive SPOT14 homolog (Rattus)	0.96525097	0.6731	NA
97165	Hmgb2	high mobility group box 2	0.96525097	0.6754	0.8183
19243	Ptp4a1	protein tyrosine phosphatase 4a1	0.96525097	0.6945	0.831
94230	Cpsf1	cleavage and polyadenylation specific factor 1	0.96525097	0.705	0.8374
108121	U2af1	U2 small nuclear ribonucleoprotein auxiliary factor (U2AF) 1	0.96525097	0.7102	0.8408
18563	Pcx	pyruvate carboxylase	0.96525097	0.7181	0.8459
232227	Iqsec1	IQ motif and Sec7 domain 1	0.96525097	0.7183	0.846
353502	Hcfc1r1	host cell factor C1 regulator 1 (XPO1-dependent)	0.96525097	0.7366	0.8575
270627	Taf1	TAF1 RNA polymerase II, TATA box binding protein (TBP)-associated factor	0.96525097	0.7375	0.8579
268451	Rab11fip4	RAB11 family interacting protein 4 (class II)	0.96525097	0.7386	0.8585
74653	4930444A02Rik	RIKEN cDNA 4930444A02 gene	0.96525097	0.7438	0.8616
52892	Sco1	SCO cytochrome oxidase deficient homolog 1 (yeast)	0.96525097	0.7463	0.8633
224824	Pex6	peroxisomal biogenesis factor 6	0.96525097	0.7493	0.8651
74476	4933439C10Rik	RIKEN cDNA 4933439C10 gene	0.96525097	0.7532	0.8673
72000	1600016N20Rik	RIKEN cDNA 1600016N20 gene	0.96525097	0.7652	0.8755
20359	Sema6b	sema domain, transmembrane domain (TM), and cytoplasmic domain, (semaphorin) 6B	0.96525097	0.7841	0.8869
74051	Steap2	six transmembrane epithelial antigen of prostate 2	0.96525097	0.7845	0.8871
66935	Cir1	corepressor interacting with RBPJ, 1	0.96525097	0.8278	0.911
117198	Ivns1abp	influenza virus NS1A binding protein	0.96525097	0.8416	0.9182
21427	Vps72	vacuolar protein sorting 72 (yeast)	0.96525097	0.8555	0.9255
67737	Ttc39d	tetratricopeptide repeat domain 39D	0.96525097	0.8695	0.9326
12839	Col9a1	collagen, type IX, alpha 1	0.96525097	0.8976	0.9479
230234	BC026590	cDNA sequence BC026590	0.96432015	0.3582	NA
67279	Med31	mediator of RNA polymerase II transcription, subunit 31 homolog (yeast)	0.96432015	0.3681	NA
18139	Zfml	zinc finger, matrin-like	0.96432015	0.3714	NA
69713	Pin4	protein (peptidyl-prolyl cis/trans isomerase) NIMA-interacting, 4 (parvulin)	0.96432015	0.4361	NA
102093	Phkb	phosphorylase kinase beta	0.96432015	0.4561	NA
12848	Cops2	COP9 (constitutive photomorphogenic) homolog, subunit 2 (Arabidopsis thaliana)	0.96432015	0.4686	NA
67008	1600012F09Rik	RIKEN cDNA 1600012F09 gene	0.96432015	0.4897	NA
107513	Ssr1	signal sequence receptor, alpha	0.96432015	0.5009	NA
103236	Csnk1g2	casein kinase 1, gamma 2	0.96432015	0.5084	NA
381062	2210404J11Rik	RIKEN cDNA 2210404J11 gene	0.96432015	0.521	NA
56191	Tro	trophinin	0.96432015	0.525	NA

330817	Dhps	deoxyhypusine synthase	0.96432015	0.531	NA
433375	Creg1	cellular repressor of E1A-stimulated genes 1	0.96432015	0.5355	NA
56389	Stx5a	syntaxin 5A	0.96432015	0.5449	NA
18107	Nmt1	N-myristoyltransferase 1	0.96432015	0.5465	NA
170789	Acot8	acyl-CoA thioesterase 8	0.96432015	0.5516	NA
72084	Pigx	phosphatidylinositol glycan anchor biosynthesis, class X	0.96432015	0.5554	NA
668096	Gm13698	predicted gene 13698	0.96432015	0.5639	NA
67610	Rspry1	ring finger and SPRY domain containing 1	0.96432015	0.5644	NA
227197	Ndufs1	NADH dehydrogenase (ubiquinone) Fe-S protein 1	0.96432015	0.5695	NA
224624	Rab40c	Rab40c, member RAS oncogene family	0.96432015	0.57	NA
21912	Tspan7	tetraspanin 7	0.96432015	0.5836	NA
70155	Ogfrl1	opioid growth factor receptor-like 1	0.96432015	0.5848	NA
66286	Sec11c	SEC11 homolog C (S. cerevisiae)	0.96432015	0.5991	NA
77219	Ptgr2	prostaglandin reductase 2	0.96432015	0.6107	NA
11429	Aco2	aconitase 2, mitochondrial	0.96432015	0.6169	NA
99887	Tmem56	transmembrane protein 56	0.96432015	0.6172	NA
66958	Tmx2	thioredoxin-related transmembrane protein 2	0.96432015	0.6185	NA
20115	Rps7	ribosomal protein S7	0.96432015	0.6205	NA
20408	Sh3gl3	SH3-domain GRB2-like 3	0.96432015	0.6211	NA
26441	Psma4	proteasome (prosome, macropain) subunit, alpha type 4	0.96432015	0.6281	NA
212880	Ddx46	DEAD (Asp-Glu-Ala-Asp) box polypeptide 46	0.96432015	0.6415	NA
67636	Lyrm5	LYR motif containing 5	0.96432015	0.6509	NA
269233	Fam171a1	family with sequence similarity 171, member A1	0.96432015	0.6606	NA
224902	Safb2	scaffold attachment factor B2	0.96432015	0.6681	0.8135
107823	Whsc1	Wolf-Hirschhorn syndrome candidate 1 (human)	0.96432015	0.6706	0.8154
19989	Rpl7	ribosomal protein L7	0.96432015	0.6739	0.8173
380977	A330009N23Rik	RIKEN cDNA A330009N23 gene	0.96432015	0.6743	0.8176
100434	Slc44a1	solute carrier family 44, member 1	0.96432015	0.6935	0.8302
68035	Rbm42	RNA binding motif protein 42	0.96432015	0.6994	0.8337
14706	Gng4	guanine nucleotide binding protein (G protein), gamma 4	0.96432015	0.7169	0.8454
624866	Lekr1	leucine, glutamate and lysine rich 1	0.96432015	0.7178	0.8458
243764	Chrm2	cholinergic receptor, muscarinic 2, cardiac	0.96432015	0.7218	0.8482
19265	Ptprcap	protein tyrosine phosphatase, receptor type, C polypeptide-associated protein	0.96432015	0.7366	0.8575
11784	Apba2	amyloid beta (A4) precursor protein-binding, family A, member 2	0.96432015	0.7393	0.8589
207742	Rnf43	ring finger protein 43	0.96432015	0.7405	0.8596
70729	Nos1ap	nitric oxide synthase 1 (neuronal) adaptor protein	0.96432015	0.7713	0.8785
241589	D430041D05Rik	RIKEN cDNA D430041D05 gene	0.96432015	0.7839	0.8868
233210	Prr12	proline rich 12	0.96432015	0.7873	0.8885
70747	Tspan2	tetraspanin 2	0.96432015	0.7888	0.8894
104886	Rab15	RAB15, member RAS oncogene family	0.96432015	0.804	0.8977
228852	Ppp1r16b	protein phosphatase 1, regulatory (inhibitor) subunit 16B	0.96432015	0.8072	0.8999
27380	Tcl1b4	T-cell leukemia/lymphoma 1B, 4	0.96432015	0.8162	0.9047
245583	Tgif2lx1	TGFB-induced factor homeobox 2-like, X-linked 1	0.96432015	0.8278	0.911
67037	Pmf1	polyamine-modulated factor 1	0.96432015	0.8411	0.918
75827	4930542N06Rik	RIKEN cDNA 4930542N06 gene	0.96432015	0.8519	0.9238
26888	Clec4a2	C-type lectin domain family 4, member a2	0.96432015	0.9246	0.9618
195046	Nlrp1a	NLR family, pyrin domain containing 1A	0.96432015	0.9354	0.967
66152	Uqcr10	ubiquinol-cytochrome c reductase, complex III subunit X	0.96339114	0.3215	NA
66249	Pno1	partner of NOB1 homolog (S. cerevisiae)	0.96339114	0.3303	NA
66282	1810029B16Rik	RIKEN cDNA 1810029B16 gene	0.96339114	0.3557	NA

78304	Lsmd1	LSM domain containing 1	0.96339114	0.3778	NA
75617	Rps25	ribosomal protein S25	0.96339114	0.3806	NA
239719	Mkl2	MKL/myocardin-like 2	0.96339114	0.385	NA
230752	Fam176b	family with sequence similarity 176, member B	0.96339114	0.393	NA
434394	Gm5614	predicted gene 5614	0.96339114	0.4027	NA
80877	Lrba	LPS-responsive beige-like anchor	0.96339114	0.4035	NA
217995	Heatr1	HEAT repeat containing 1	0.96339114	0.4228	NA
217684	4933426M11Rik	RIKEN cDNA 4933426M11 gene	0.96339114	0.4536	NA
230085	N28178	expressed sequence N28178	0.96339114	0.4647	NA
15278	Tfb2m	transcription factor B2, mitochondrial	0.96339114	0.4689	NA
12386	Ctnna2	catenin (cadherin associated protein), alpha 2	0.96339114	0.4849	NA
70082	Lysmd2	LysM, putative peptidoglycan-binding, domain containing 2	0.96339114	0.5054	NA
13356	Dgcr2	DiGeorge syndrome critical region gene 2	0.96339114	0.5098	NA
76932	Arfip2	ADP-ribosylation factor interacting protein 2	0.96339114	0.5125	NA
13048	Cux2	cut-like homeobox 2	0.96339114	0.5136	NA
75698	Fam35a	family with sequence similarity 35, member A	0.96339114	0.515	NA
20621	Snn	stannin	0.96339114	0.5222	NA
59009	Sh3rf1	SH3 domain containing ring finger 1	0.96339114	0.529	NA
104010	Cdh22	cadherin 22	0.96339114	0.5421	NA
58799	Crbn	cereblon	0.96339114	0.544	NA
68852	Lrrn4cl	LRRN4 C-terminal like	0.96339114	0.5456	NA
70998	Phf6	PHD finger protein 6	0.96339114	0.5573	NA
56452	Orc6	origin recognition complex, subunit 6	0.96339114	0.5587	NA
67180	Yipf5	Yip1 domain family, member 5	0.96339114	0.5605	NA
13481	Dpm2	dolichol-phosphate (beta-D) mannosyltransferase 2	0.96339114	0.5612	NA
109934	Abr	active BCR-related gene	0.96339114	0.563	NA
70441	2610100L16Rik	RIKEN cDNA 2610100L16 gene	0.96339114	0.5679	NA
22017	Tpmt	thiopurine methyltransferase	0.96339114	0.5685	NA
210094	Iglon5	IgLON family member 5	0.96339114	0.573	NA
67028	2610002M06Rik	RIKEN cDNA 2610002M06 gene	0.96339114	0.5825	NA
109136	Mmaa	methylmalonic aciduria (cobalamin deficiency) type A	0.96339114	0.5828	NA
13496	Arid3a	AT rich interactive domain 3A (BRIGHT-like)	0.96339114	0.5833	NA
269254	Setx	senataxin	0.96339114	0.5944	NA
242506	Frmd3	FERM domain containing 3	0.96339114	0.5959	NA
76857	Spopl	speckle-type POZ protein-like	0.96339114	0.6086	NA
69215	Sat2	spermidine/spermine N1-acetyl transferase 2	0.96339114	0.6126	NA
27374	Prmt5	protein arginine N-methyltransferase 5	0.96339114	0.6258	NA
24044	Scamp2	secretory carrier membrane protein 2	0.96339114	0.6377	NA
75941	4930570B17Rik	RIKEN cDNA 4930570B17 gene	0.96339114	0.6481	NA
67487	Dhx40	DEAH (Asp-Glu-Ala-His) box polypeptide 40	0.96339114	0.659	0.8078
70591	5730455P16Rik	RIKEN cDNA 5730455P16 gene	0.96339114	0.6614	NA
18029	Nfic	nuclear factor I/C	0.96339114	0.6634	0.8105
15904	Id4	inhibitor of DNA binding 4	0.96339114	0.6652	0.8114
71963	Cdca4	cell division cycle associated 4	0.96339114	0.6676	0.813
11886	Asah1	N-acylsphingosine amidohydrolase 1	0.96339114	0.6744	0.8176
71361	Aifm2	apoptosis-inducing factor, mitochondrion-associated 2	0.96339114	0.6746	0.8177
245469	Pdzd4	PDZ domain containing 4	0.96339114	0.678	0.82
16971	Lrp1	low density lipoprotein receptor-related protein 1	0.96339114	0.6877	0.8263
20466	Sin3a	transcriptional regulator, SIN3A (yeast)	0.96339114	0.6885	0.8268
14367	Fzd5	frizzled homolog 5 (Drosophila)	0.96339114	0.6976	0.8327

98258	Txndc9		thioredoxin domain containing 9	0.96339114	0.7026	0.8356
73728	Psd		pleckstrin and Sec7 domain containing	0.96339114	0.7037	0.8365
16371	Irx1		Iroquois related homeobox 1 (Drosophila)	0.96339114	0.7178	0.8458
56531	Ylpm1		YLP motif containing 1	0.96339114	0.7198	0.8469
667510	Gm8675		predicted gene 8675	0.96339114	0.7208	0.8475
272381	Lrrc4b		leucine rich repeat containing 4B	0.96339114	0.728	0.8519
57438		Mar-07	membrane-associated ring finger (C3HC4) 7	0.96339114	0.7327	0.8557
103432	6430706H07Rik		RIKEN cDNA 6430706H07 gene	0.96339114	0.7349	0.8568
21414	Tcf7		transcription factor 7, T-cell specific	0.96339114	0.7358	0.8572
242409	Tmem8b		transmembrane protein 8B	0.96339114	0.7424	0.8607
15531	Ndst1		N-deacetylase/N-sulfotransferase (heparan glucosaminyl) 1	0.96339114	0.7449	0.8625
378462	Morn2		MORN repeat containing 2	0.96339114	0.7562	0.8694
57295	Icmt		isoprenylcysteine carboxyl methyltransferase	0.96339114	0.7588	0.8713
14724	Gp1bb		glycoprotein Ib, beta polypeptide	0.96339114	0.7652	0.8755
434008	Gm5567		predicted gene 5567	0.96339114	0.7652	0.8755
100515	Zfp518b		zinc finger protein 518B	0.96339114	0.7681	0.877
71900	Tmem106b		transmembrane protein 106B	0.96339114	0.7727	0.879
100040711	Gm2921		predicted gene 2921	0.96339114	0.7782	0.8825
67956	Setd8		SET domain containing (lysine methyltransferase) 8	0.96339114	0.7858	0.8877
106338	Nsun3		NOL1/NOP2/Sun domain family member 3	0.96339114	0.8088	0.9006
654805	F930015N05Rik		RIKEN cDNA F930015N05 gene	0.96339114	0.8245	0.9095
667277	C1rb		complement component 1, r subcomponent B	0.96339114	0.8348	0.9147
546837	Cyp2j7-ps		cytochrome P450, family 2, subfamily j, polypeptide 7, pseudogene	0.96339114	0.8388	0.9169
258383	Olfr1347		olfactory receptor 1347	0.96339114	0.8747	0.9352
225058	Gm4832		predicted gene 4832	0.96339114	0.8779	0.9373
77022	2700099C18Rik		NDC80 homolog, kinetochore complex component pseudogene	0.96339114	0.8996	0.949
104318	Csnk1d		casein kinase 1, delta	0.96246391	0.3111	NA
66855	Tcf25		transcription factor 25 (basic helix-loop-helix)	0.96246391	0.3311	NA
67665	Dctn4		dynactin 4	0.96246391	0.3378	NA
66589	Ube2v1		ubiquitin-conjugating enzyme E2 variant 1	0.96246391	0.3545	NA
56491	Vapb		vesicle-associated membrane protein, associated protein B and C	0.96246391	0.3786	NA
66258	Mrps17		mitochondrial ribosomal protein S17	0.96246391	0.3824	NA
243302	Gm4963		predicted gene 4963	0.96246391	0.3974	NA
56384	Letm1		leucine zipper-EF-hand containing transmembrane protein 1	0.96246391	0.3975	NA
16570	Kif3c		kinesin family member 3C	0.96246391	0.4203	NA
67467	1200011I18Rik		RIKEN cDNA 1200011I18 gene	0.96246391	0.4301	NA
210009	Mtrr		5-methyltetrahydrofolate-homocysteine methyltransferase reductase	0.96246391	0.436	NA
66461	Ptpmt1		protein tyrosine phosphatase, mitochondrial 1	0.96246391	0.4389	NA
66317	Wdr61		WD repeat domain 61	0.96246391	0.4427	NA
94062	Mrpl3		mitochondrial ribosomal protein L3	0.96246391	0.4695	NA
75725	Phf14		PHD finger protein 14	0.96246391	0.4862	NA
14155	Fem1b		feminization 1 homolog b (C. elegans)	0.96246391	0.5023	NA
99696	Ankrd50		ankyrin repeat domain 50	0.96246391	0.506	NA
66433	Chchd7		coiled-coil-helix-coiled-coil-helix domain containing 7	0.96246391	0.5086	NA
72349	Dusp3		dual specificity phosphatase 3 (vaccinia virus phosphatase VH1-related)	0.96246391	0.5168	NA
66308	2810021B07Rik		RIKEN cDNA 2810021B07 gene	0.96246391	0.521	NA
71701	Pnpt1		polyribonucleotide nucleotidyltransferase 1	0.96246391	0.5239	NA
69726	Smyd3		SET and MYND domain containing 3	0.96246391	0.5268	NA
13629	Eef2		eukaryotic translation elongation factor 2	0.96246391	0.5369	NA
546134	Gramd2		GRAM domain containing 2	0.96246391	0.546	NA

75302	Asxl2	additional sex combs like 2 (Drosophila)	0.96246391	0.5498	NA
328801	Zfp414	zinc finger protein 414	0.96246391	0.556	NA
19951	Rpl32	ribosomal protein L32	0.96246391	0.5581	NA
94061	Mrpl1	mitochondrial ribosomal protein L1	0.96246391	0.5635	NA
66493	Mrpl51	mitochondrial ribosomal protein L51	0.96246391	0.5642	NA
66257	Nicn1	nicolin 1	0.96246391	0.5709	NA
19092	Prkg2	protein kinase, cGMP-dependent, type II	0.96246391	0.5736	NA
98376	Gorab	golgin, RAB6-interacting	0.96246391	0.5748	NA
13528	Dtnb	dystrobrevin, beta	0.96246391	0.5782	NA
223881	Rnd1	Rho family GTPase 1	0.96246391	0.5842	NA
100910	Chpf2	chondroitin polymerizing factor 2	0.96246391	0.5912	NA
233724	Tmem41b	transmembrane protein 41B	0.96246391	0.5933	NA
68098	Rchy1	ring finger and CHY zinc finger domain containing 1	0.96246391	0.5973	NA
18148	Npm1	nucleophosmin 1	0.96246391	0.602	NA
73047	Camk2n2	calcium/calmodulin-dependent protein kinase II inhibitor 2	0.96246391	0.6123	NA
76192	Abhd12	abhydrolase domain containing 12	0.96246391	0.6169	NA
76183	Celf6	CUGBP, Elav-like family member 6	0.96246391	0.6174	NA
231326	Aasdh	aminoadipate-semialdehyde dehydrogenase	0.96246391	0.6174	NA
69792	Med6	mediator of RNA polymerase II transcription, subunit 6 homolog (yeast)	0.96246391	0.6229	NA
71452	Ankrd40	ankyrin repeat domain 40	0.96246391	0.6353	NA
75553	Zc3h14	zinc finger CCCH type containing 14	0.96246391	0.6466	NA
432768	Gm5451	predicted gene 5451	0.96246391	0.6534	NA
21945	Dedd	death effector domain-containing	0.96246391	0.6544	0.805
22045	Trhr	thyrotropin releasing hormone receptor	0.96246391	0.6589	0.8078
74147	Ehhadh	enoyl-Coenzyme A, hydratase/3-hydroxyacyl Coenzyme A dehydrogenase	0.96246391	0.6607	0.8089
52004	Cdk2ap2	CDK2-associated protein 2	0.96246391	0.6618	0.8095
93874	Pcdhb3	protocadherin beta 3	0.96246391	0.6618	0.8095
328162	Trmt61a	tRNA methyltransferase 61 homolog A (S. cerevisiae)	0.96246391	0.6733	0.817
104570	Smek2	SMEK homolog 2, suppressor of mek1 (Dictyostelium)	0.96246391	0.6778	0.8199
18293	Ogdh	oxoglutarate dehydrogenase (lipoamide)	0.96246391	0.6919	0.8293
71833	Dcaf7	DDB1 and CUL4 associated factor 7	0.96246391	0.7031	0.8359
385668	Lca5l	Leber congenital amaurosis 5-like	0.96246391	0.7054	0.8377
66399	Tsfm	Ts translation elongation factor, mitochondrial	0.96246391	0.7068	0.8385
19116	Prlr	prolactin receptor	0.96246391	0.7181	0.8459
17207	Mcf2l	mcf.2 transforming sequence-like	0.96246391	0.7237	0.8494
15273	Hivep2	human immunodeficiency virus type I enhancer binding protein 2	0.96246391	0.7315	0.8547
19791	Rn18s	18S ribosomal RNA	0.96246391	0.7447	0.8624
68795	Ubr3	ubiquitin protein ligase E3 component n-recognin 3	0.96246391	0.7473	0.8639
100502708	LOC100502708	hypothetical LOC100502708	0.96246391	0.7482	0.8644
100043597	Srcap	Snf2-related CREBBP activator protein	0.96246391	0.7848	0.8872
321003	Xpnpep3	X-prolyl aminopeptidase (aminopeptidase P) 3, putative	0.96246391	0.7896	0.8901
320103	A730080H06Rik	RIKEN cDNA A730080H06 gene	0.96246391	0.7904	0.8901
65945	Clstn1	calsyntenin 1	0.96246391	0.8039	0.8977
110304	Glra3	glycine receptor, alpha 3 subunit	0.96246391	0.8285	0.9113
268566	Gphn	gephyrin	0.96153846	0.3774	NA
15200	Hbegf	heparin-binding EGF-like growth factor	0.96153846	0.3896	NA
11487	Adam10	a disintegrin and metallopeptidase domain 10	0.96153846	0.4067	NA
80283	Abtb1	ankyrin repeat and BTB (POZ) domain containing 1	0.96153846	0.4138	NA
110323	Cox6b1	cytochrome c oxidase, subunit VIb polypeptide 1	0.96153846	0.4177	NA
75717	Cul5	cullin 5	0.96153846	0.4609	NA

641340	Nrbf2	nuclear receptor binding factor 2	0.96153846	0.4614	NA
100040631	Dynlt1e	dynein light chain Tctex-type 1E	0.96153846	0.4864	NA
66460	Sys1	SYS1 Golgi-localized integral membrane protein homolog (S. cerevisiae)	0.96153846	0.4906	NA
66537	Pomp	proteasome maturation protein	0.96153846	0.4965	NA
50524	Sall2	sal-like 2 (Drosophila)	0.96153846	0.498	NA
98170	Tmem132a	transmembrane protein 132A	0.96153846	0.4986	NA
107569	Nt5c3	5'-nucleotidase, cytosolic III	0.96153846	0.4991	NA
237222	Ofd1	oral-facial-digital syndrome 1 gene homolog (human)	0.96153846	0.4994	NA
66538	Rps19bp1	ribosomal protein S19 binding protein 1	0.96153846	0.5134	NA
320352	Lrrc31	leucine rich repeat containing 31	0.96153846	0.534	NA
110391	Qdpr	quinoid dihydropteridine reductase	0.96153846	0.537	NA
74772	Atp13a2	ATPase type 13A2	0.96153846	0.5391	NA
74778	Rrp7a	ribosomal RNA processing 7 homolog A (S. cerevisiae)	0.96153846	0.5584	NA
101835	AW146154	expressed sequence AW146154	0.96153846	0.5622	NA
383341	Gm5239	ubiquitin A-52 residue ribosomal protein fusion product 1 pseudogene	0.96153846	0.5673	NA
12700	Cish	cytokine inducible SH2-containing protein	0.96153846	0.5687	NA
66615	Atg4b	autophagy-related 4B (yeast)	0.96153846	0.5763	NA
52421	D4Ertd681e	DNA segment, Chr 4, ERATO Doi 681, expressed	0.96153846	0.5858	NA
75540	Fpgt	fucose-1-phosphate guanylyltransferase	0.96153846	0.5935	NA
11767	Ap1m1	adaptor-related protein complex AP-1, mu subunit 1	0.96153846	0.5948	NA
18771	Pknox1	Pbx/knotted 1 homeobox	0.96153846	0.601	NA
72056	1810055G02Rik	RIKEN cDNA 1810055G02 gene	0.96153846	0.6168	NA
97351	E230006M18Rik	RIKEN cDNA E230006M18 gene	0.96153846	0.6188	NA
56350	Arl3	ADP-ribosylation factor-like 3	0.96153846	0.6327	NA
56406	Ncoa6	nuclear receptor coactivator 6	0.96153846	0.64	NA
30934	Tor1b	torsin family 1, member B	0.96153846	0.644	NA
50758	Fbxl17	F-box and leucine-rich repeat protein 17	0.96153846	0.6503	0.8031
52897	Rbfox3	RNA binding protein, fox-1 homolog (C. elegans) 3	0.96153846	0.6508	0.8034
69726	Smyd3	SET and MYND domain containing 3	0.96153846	0.6539	0.8045
17274	Rab8a	RAB8A, member RAS oncogene family	0.96153846	0.677	0.8192
210035	Tmem194	transmembrane protein 194	0.96153846	0.6858	0.825
108086	Rnf216	ring finger protein 216	0.96153846	0.691	0.8286
69694	Tatdn1	TatD DNase domain containing 1	0.96153846	0.6998	0.8337
212139	Cc2d1a	coiled-coil and C2 domain containing 1A	0.96153846	0.7007	0.8342
56086	Set	SET nuclear oncogene	0.96153846	0.7242	0.8499
232854	Zfp418	zinc finger protein 418	0.96153846	0.7324	0.8555
71985	Acad10	acyl-Coenzyme A dehydrogenase family, member 10	0.96153846	0.7408	0.8598
223739	5031439G07Rik	RIKEN cDNA 5031439G07 gene	0.96153846	0.7479	0.8642
77614	C030044M21Rik	RIKEN cDNA C030044M21 gene	0.96153846	0.7508	0.8661
100041098	Gm15431	predicted gene 15431	0.96153846	0.7518	0.8666
11556	Adrb3	adrenergic receptor, beta 3	0.96153846	0.7642	0.875
20668	Sox13	SRY-box containing gene 13	0.96153846	0.7648	0.8753
106633	Ift140	intraflagellar transport 140 homolog (Chlamydomonas)	0.96153846	0.7782	0.8825
68833	Pdcl3	phosducin-like 3	0.96153846	0.7846	0.8871
20370	Sez6	seizure related gene 6	0.96153846	0.7904	0.8901
71860	Wdr16	WD repeat domain 16	0.96153846	0.7982	0.8939
67507	1700019N19Rik	RIKEN cDNA 1700019N19 gene	0.96153846	0.7995	0.8949
20481	Ski	ski sarcoma viral oncogene homolog (avian)	0.96153846	0.8152	0.9045
14886	Gtf2i	general transcription factor II I	0.96153846	0.8183	0.9059
319807	3110047P20Rik	RIKEN cDNA 3110047P20 gene	0.96153846	0.8236	0.9092

83766	Actl6b	actin-like 6B	0.96153846	0.8543	0.925
17145	Mageb1	melanoma antigen, family B, 1	0.96153846	0.858	0.9269
621967	Gm6272	predicted pseudogene 6272	0.96153846	0.8726	0.9347
320011	Uggt1	UDP-glucose glycoprotein glucosyltransferase 1	0.96061479	0.2897	NA
218038	Amph	amphiphysin	0.96061479	0.313	NA
12460	Ccs	copper chaperone for superoxide dismutase	0.96061479	0.3358	NA
229524	Msto1	misato homolog 1 (Drosophila)	0.96061479	0.3418	NA
234730	Fuk	fucokinase	0.96061479	0.3451	NA
52637	Cisd1	CDGSH iron sulfur domain 1	0.96061479	0.4003	NA
68705	Gtf2f2	general transcription factor IIF, polypeptide 2	0.96061479	0.4188	NA
66046	Ndufb5	NADH dehydrogenase (ubiquinone) 1 beta subcomplex, 5	0.96061479	0.4239	NA
668272	Gm9079	transmembrane emp24 domain trafficking protein 2 pseudogene	0.96061479	0.4264	NA
330401	Tmcc1	transmembrane and coiled coil domains 1	0.96061479	0.4313	NA
20643	Snrpe	small nuclear ribonucleoprotein E	0.96061479	0.4395	NA
19046	Ppp1cb	protein phosphatase 1, catalytic subunit, beta isoform	0.96061479	0.4517	NA
55979	Agpat1	1-acylglycerol-3-phosphate O-acyltransferase 1 (lysophosphatidic acid acyltransferase, alpha)	0.96061479	0.4579	NA
70638	Fam189a1	family with sequence similarity 189, member A1	0.96061479	0.4612	NA
227195	Ino80d	INO80 complex subunit D	0.96061479	0.466	NA
319673	9330159M07Rik	RIKEN cDNA 9330159M07 gene	0.96061479	0.4759	NA
67391	Fundc2	FUN14 domain containing 2	0.96061479	0.4808	NA
69260	Ing2	inhibitor of growth family, member 2	0.96061479	0.4897	NA
66841	Etfdh	electron transferring flavoprotein, dehydrogenase	0.96061479	0.4912	NA
74164	Nfx1	nuclear transcription factor, X-box binding 1	0.96061479	0.5061	NA
67118	Bfar	bifunctional apoptosis regulator	0.96061479	0.5065	NA
276852	D11Wsu47e	DNA segment, Chr 11, Wayne State University 47, expressed	0.96061479	0.5099	NA
66658	Ccdc51	coiled-coil domain containing 51	0.96061479	0.5132	NA
56280	Mrpl37	mitochondrial ribosomal protein L37	0.96061479	0.5233	NA
100226	Stx12	syntaxin 12	0.96061479	0.5407	NA
19732	Rgl2	ral guanine nucleotide dissociation stimulator-like 2	0.96061479	0.5427	NA
72061	2010111I01Rik	RIKEN cDNA 2010111I01 gene	0.96061479	0.5457	NA
80985	Trim44	tripartite motif-containing 44	0.96061479	0.5459	NA
73166	Tm7sf2	transmembrane 7 superfamily member 2	0.96061479	0.5518	NA
26757	Dpysl4	dihydropyrimidinase-like 4	0.96061479	0.6047	NA
232440	H2afj	H2A histone family, member J	0.96061479	0.6056	NA
218877	Sema3g	sema domain, immunoglobulin domain (Ig), short basic domain, secreted, (semaphorin) 3G	0.96061479	0.6114	NA
66439	2010012O05Rik	RIKEN cDNA 2010012O05 gene	0.96061479	0.6128	NA
223918	Spryd3	SPRY domain containing 3	0.96061479	0.6147	NA
230775	Bai2	brain-specific angiogenesis inhibitor 2	0.96061479	0.6173	NA
27226	Pla2g7	phospholipase A2, group VII (platelet-activating factor acetylhydrolase, plasma)	0.96061479	0.633	0.7916
56314	Zfp113	zinc finger protein 113	0.96061479	0.6341	NA
12626	Cetn3	centrin 3	0.96061479	0.6454	0.7996
227638	Qsox2	quiescin Q6 sulfhydryl oxidase 2	0.96061479	0.6612	0.809
320404	Itpkb	inositol 1,4,5-trisphosphate 3-kinase B	0.96061479	0.6622	0.8096
66163	Mrpl4	mitochondrial ribosomal protein L4	0.96061479	0.6694	0.8143
57294	Rps27	ribosomal protein S27	0.96061479	0.6778	0.8199
75552	Paqr9	progestin and adipoQ receptor family member IX	0.96061479	0.6821	0.8227
16154	Il10ra	interleukin 10 receptor, alpha	0.96061479	0.6915	0.8289
240817	5830403L16Rik	RIKEN cDNA 5830403L16 gene	0.96061479	0.7045	0.8371
791393	A230065N10Rik	RIKEN cDNA A230065N10 gene	0.96061479	0.7147	0.844
110891	Slc8a2	solute carrier family 8 (sodium/calcium exchanger), member 2	0.96061479	0.7344	0.8565

108687	Edem2	ER degradation enhancer, mannosidase alpha-like 2	0.96061479	0.7432	0.8613
217166	Nr1d1	nuclear receptor subfamily 1, group D, member 1	0.96061479	0.7444	0.8621
330324	6430584L05	hypothetical protein 6430584L05	0.96061479	0.7489	0.8649
70568	Cpne3	copine III	0.96061479	0.7702	0.8781
626903	Gm6718	predicted gene 6718	0.96061479	0.8035	0.8973
72459	Htatsf1	HIV TAT specific factor 1	0.96061479	0.8114	0.9021
72992	2900076A07Rik	RIKEN cDNA 2900076A07 gene	0.96061479	0.8165	0.9048
433416	Gm13547	predicted gene 13547	0.96061479	0.8267	0.9104
20939	Sva	seminal vesicle antigen	0.96061479	0.8345	0.9146
244653	Hydin	hydrocephalus inducing	0.96061479	0.8809	0.939
225870	Rin1	Ras and Rab interactor 1	0.96061479	0.8973	0.9477
100502698	1700021K19Rik	RIKEN cDNA 1700021K19 gene	0.9596929	0.309	NA
69902	Mrto4	MRT4, mRNA turnover 4, homolog (S. cerevisiae)	0.9596929	0.3565	NA
235132	Zbtb44	zinc finger and BTB domain containing 44	0.9596929	0.3742	NA
223773	Zbed4	zinc finger, BED domain containing 4	0.9596929	0.3907	NA
68776	Taf11	TAF11 RNA polymerase II, TATA box binding protein (TBP)-associated factor	0.9596929	0.4016	NA
214895	Lman2l	lectin, mannose-binding 2-like	0.9596929	0.4197	NA
214345	Lrrc1	leucine rich repeat containing 1	0.9596929	0.434	NA
81018	Rnf114	ring finger protein 114	0.9596929	0.4483	NA
27883	D16H22S680E	DNA segment, Chr 16, human D22S680E, expressed	0.9596929	0.4545	NA
21834	Thrb	thyroid hormone receptor beta	0.9596929	0.4564	NA
67556	Pigm	phosphatidylinositol glycan anchor biosynthesis, class M	0.9596929	0.4606	NA
210853	Zfp947	zinc finger protein 947	0.9596929	0.4735	NA
75458	Cklf	chemokine-like factor	0.9596929	0.4837	NA
104885	Tmem179	transmembrane protein 179	0.9596929	0.494	NA
56786	Tmem9b	TMEM9 domain family, member B	0.9596929	0.4977	NA
16975	Lrp8	low density lipoprotein receptor-related protein 8, apolipoprotein e receptor	0.9596929	0.5037	NA
66154	Tmem14c	transmembrane protein 14C	0.9596929	0.5052	NA
23934	Ly6h	lymphocyte antigen 6 complex, locus H	0.9596929	0.5067	NA
67101	2310039H08Rik	RIKEN cDNA 2310039H08 gene	0.9596929	0.5131	NA
15114	Hap1	huntingtin-associated protein 1	0.9596929	0.5204	NA
12013	Bach1	BTB and CNC homology 1	0.9596929	0.5218	NA
56541	Habp4	hyaluronic acid binding protein 4	0.9596929	0.5449	NA
14683	Gnas	GNAS (guanine nucleotide binding protein, alpha stimulating) complex locus	0.9596929	0.5561	NA
239250	Slitrk6	SLIT and NTRK-like family, member 6	0.9596929	0.5621	NA
76899	Golga1	golgi autoantigen, golgin subfamily a, 1	0.9596929	0.5772	NA
56404	Trip4	thyroid hormone receptor interactor 4	0.9596929	0.5827	NA
268859	Rbfox1	RNA binding protein, fox-1 homolog (C. elegans) 1	0.9596929	0.5874	NA
76375	Det1	de-etiolated homolog 1 (Arabidopsis)	0.9596929	0.5932	NA
13131	Dab1	disabled homolog 1 (Drosophila)	0.9596929	0.598	NA
246696	Slc25a28	solute carrier family 25, member 28	0.9596929	0.6077	NA
57138	Slc12a5	solute carrier family 12, member 5	0.9596929	0.6153	NA
211480	Kcnj14	potassium inwardly-rectifying channel, subfamily J, member 14	0.9596929	0.6227	NA
231070	Insig1	insulin induced gene 1	0.9596929	0.6237	NA
13684	Eif4e	eukaryotic translation initiation factor 4E	0.9596929	0.631	NA
16728	L1cam	L1 cell adhesion molecule	0.9596929	0.6326	0.7914
23989	Med24	mediator complex subunit 24	0.9596929	0.6452	0.7995
16528	Kcnk4	potassium channel, subfamily K, member 4	0.9596929	0.6516	0.8034
66336	Cenpp	centromere protein P	0.9596929	0.666	0.8118
22437	Xirp1	xin actin-binding repeat containing 1	0.9596929	0.6778	0.8199

245880	Wasf3	WAS protein family, member 3	0.9596929	0.6894	0.8276
57432	Zc3h8	zinc finger CCCH type containing 8	0.9596929	0.6965	0.8324
11465	Actg1	actin, gamma, cytoplasmic 1	0.9596929	0.7066	0.8383
13356	Dgcr2	DiGeorge syndrome critical region gene 2	0.9596929	0.7085	0.8397
56527	Mast1	microtubule associated serine/threonine kinase 1	0.9596929	0.711	0.8413
242408	4930412F15Rik	RIKEN cDNA 4930412F15 gene	0.9596929	0.7263	0.8513
21399	Tcea1	transcription elongation factor A (SII) 1	0.9596929	0.732	0.8552
69024	Snx15	sorting nexin 15	0.9596929	0.7373	0.8578
58220	Pard6b	par-6 (partitioning defective 6) homolog beta (C. elegans)	0.9596929	0.7587	0.8713
75079	Zbtb49	zinc finger and BTB domain containing 49	0.9596929	0.7697	0.8779
381921	Taok2	TAO kinase 2	0.9596929	0.7934	0.8915
71782	Ankle2	ankyrin repeat and LEM domain containing 2	0.9596929	0.8073	0.8999
69129	Pex11c	peroxisomal biogenesis factor 11 gamma	0.9596929	0.8098	0.9013
68089	Arpc4	actin related protein 2/3 complex, subunit 4	0.9596929	0.8146	0.904
213484	Nudt18	nudix (nucleoside diphosphate linked moiety X)-type motif 18	0.9596929	0.8251	0.9099
110862	Kcnq3	potassium voltage-gated channel, subfamily Q, member 3	0.9596929	0.8523	0.924
18117	Cox4nb	COX4 neighbor	0.95877277	0.3605	NA
22591	Xpc	xeroderma pigmentosum, complementation group C	0.95877277	0.3857	NA
72843	Prdm4	PR domain containing 4	0.95877277	0.3941	NA
212307	Mapre2	microtubule-associated protein, RP/EB family, member 2	0.95877277	0.3965	NA
17925	Myo9b	myosin IXb	0.95877277	0.4185	NA
66743	Rnf220	ring finger protein 220	0.95877277	0.419	NA
18634	Pex7	peroxisomal biogenesis factor 7	0.95877277	0.4361	NA
27395	Mrpl15	mitochondrial ribosomal protein L15	0.95877277	0.4393	NA
63958	Ube4b	ubiquitination factor E4B, UFD2 homolog (S. cerevisiae)	0.95877277	0.4455	NA
14976	H2-Ke2	H2-K region expressed gene 2	0.95877277	0.4459	NA
18950	Pnp	purine-nucleoside phosphorylase	0.95877277	0.452	NA
235315	Rnf214	ring finger protein 214	0.95877277	0.461	NA
234404	Nxnl1	nucleoredoxin-like 1	0.95877277	0.4699	NA
105638	Dph3	DPH3 homolog (KTI11, S. cerevisiae)	0.95877277	0.47	NA
73124	Golim4	golgi integral membrane protein 4	0.95877277	0.4742	NA
72084	Pigx	phosphatidylinositol glycan anchor biosynthesis, class X	0.95877277	0.4758	NA
70223	Nars	asparaginyl-tRNA synthetase	0.95877277	0.4938	NA
22195	Ube2l3	ubiquitin-conjugating enzyme E2L 3	0.95877277	0.5	NA
64655	Mrps22	mitochondrial ribosomal protein S22	0.95877277	0.502	NA
70300	Fuz	fuzzy homolog (Drosophila)	0.95877277	0.5165	NA
230162	Zfp189	zinc finger protein 189	0.95877277	0.5364	NA
70625	Med26	mediator complex subunit 26	0.95877277	0.5397	NA
68778	1110038D17Rik	RIKEN cDNA 1110038D17 gene	0.95877277	0.5415	NA
53881	Slc5a3	solute carrier family 5 (inositol transporters), member 3	0.95877277	0.5453	NA
71182	4933417G07Rik	RIKEN cDNA 4933417G07 gene	0.95877277	0.5569	NA
98766	Ubac1	ubiquitin associated domain containing 1	0.95877277	0.5574	NA
208266	Dot1l	DOT1-like, histone H3 methyltransferase (S. cerevisiae)	0.95877277	0.5757	NA
114641	Rpl31	ribosomal protein L31	0.95877277	0.5817	NA
14405	Gabrg1	gamma-aminobutyric acid (GABA) A receptor, subunit gamma 1	0.95877277	0.5886	NA
234959	Med17	mediator complex subunit 17	0.95877277	0.5894	NA
108954	Ppp1r15b	protein phosphatase 1, regulatory (inhibitor) subunit 15b	0.95877277	0.5899	NA
11750	Anxa7	annexin A7	0.95877277	0.5973	NA
58244	Stx6	syntaxin 6	0.95877277	0.5993	NA
67803	Limd2	LIM domain containing 2	0.95877277	0.6016	NA

66932	Rexo1	REX1, RNA exonuclease 1 homolog (S. cerevisiae)	0.95877277	0.6081	NA
71678	Brox	BRO1 domain and CAAX motif containing	0.95877277	0.6115	NA
20392	Sgce	sarcoglycan, epsilon	0.95877277	0.6116	NA
30839	Fbxw5	F-box and WD-40 domain protein 5	0.95877277	0.6131	NA
70620	Ube2v2	ubiquitin-conjugating enzyme E2 variant 2	0.95877277	0.6206	0.7822
22264	Prap1	proline-rich acidic protein 1	0.95877277	0.6258	0.7862
78825	Pppde1	PPPDE peptidase domain containing 1	0.95877277	0.6268	0.7871
64707	Suv39h2	suppressor of variegation 3-9 homolog 2 (Drosophila)	0.95877277	0.6338	0.792
15369	Hmox2	heme oxygenase (decycling) 2	0.95877277	0.6368	0.7941
212996	Wbscr17	Williams-Beuren syndrome chromosome region 17 homolog (human)	0.95877277	0.6455	0.7997
14339	Aktip	thymoma viral proto-oncogene 1 interacting protein	0.95877277	0.6463	0.8003
209416	Gpkow	G patch domain and KOW motifs	0.95877277	0.6511	0.8034
66958	Tmx2	thioredoxin-related transmembrane protein 2	0.95877277	0.6514	0.8034
107197	AI462493	expressed sequence AI462493	0.95877277	0.6534	0.8044
214952	Rhot2	ras homolog gene family, member T2	0.95877277	0.6612	0.809
12005	Axin1	axin 1	0.95877277	0.669	0.814
18023	Nfe2l1	nuclear factor, erythroid derived 2,-like 1	0.95877277	0.676	0.8184
14007	Celf2	CUGBP, Elav-like family member 2	0.95877277	0.6841	0.8235
21933	Tnfrsf10b	tumor necrosis factor receptor superfamily, member 10b	0.95877277	0.6965	0.8324
80749	Lrfn1	leucine rich repeat and fibronectin type III domain containing 1	0.95877277	0.7022	0.8355
60441	Mrpl38	mitochondrial ribosomal protein L38	0.95877277	0.7042	0.8368
15166	Hcn2	hyperpolarization-activated, cyclic nucleotide-gated K+ 2	0.95877277	0.7152	0.8441
231931	Gimap6	GTPase, IMAP family member 6	0.95877277	0.7336	0.8561
12847	Copa	coatomer protein complex subunit alpha	0.95877277	0.7417	0.8604
252907	Vmn1r192	vomeronasal 1 receptor 192	0.95877277	0.7471	0.8637
229599	Gm129	predicted gene 129	0.95877277	0.7572	0.8701
13859	Eps15l1	epidermal growth factor receptor pathway substrate 15-like 1	0.95877277	0.7584	0.8711
100317	AU040320	expressed sequence AU040320	0.95877277	0.772	0.8787
215789	Phactr2	phosphatase and actin regulator 2	0.95877277	0.7748	0.8802
269999	Orai3	ORAI calcium release-activated calcium modulator 3	0.95877277	0.7795	0.8834
225997	Trpm6	transient receptor potential cation channel, subfamily M, member 6	0.95877277	0.7923	0.8911
219150	Hmbox1	homeobox containing 1	0.95877277	0.7946	0.8921
217473	Ankmy2	ankyrin repeat and MYND domain containing 2	0.95877277	0.803	0.8969
71740	Pvrl4	poliovirus receptor-related 4	0.95877277	0.8038	0.8976
30937	Lmcd1	LIM and cysteine-rich domains 1	0.95877277	0.8122	0.9024
67143	Ikzf5	IKAROS family zinc finger 5	0.95785441	0.3045	NA
66163	Mrpl4	mitochondrial ribosomal protein L4	0.95785441	0.3162	NA
13726	Emd	emerin	0.95785441	0.338	NA
433759	Hdac1	histone deacetylase 1	0.95785441	0.3515	NA
211961	Asxl3	additional sex combs like 3 (Drosophila)	0.95785441	0.3563	NA
50755	Fbxo18	F-box protein 18	0.95785441	0.3606	NA
57423	Atp5j2	ATP synthase, H+ transporting, mitochondrial F0 complex, subunit F2	0.95785441	0.3612	NA
68646	1110020G09Rik	RIKEN cDNA 1110020G09 gene	0.95785441	0.3679	NA
69349	1700008O03Rik	RIKEN cDNA 1700008O03 gene	0.95785441	0.3692	NA
12859	Cox5b	cytochrome c oxidase, subunit Vb	0.95785441	0.3726	NA
20646	Snrpn	small nuclear ribonucleoprotein N	0.95785441	0.3811	NA
20847	Stat2	signal transducer and activator of transcription 2	0.95785441	0.3852	NA
71805	Nup93	nucleoporin 93	0.95785441	0.3889	NA
68046	2700062C07Rik	RIKEN cDNA 2700062C07 gene	0.95785441	0.4113	NA
27008	Micall1	microtubule associated monoxygenase, calponin and LIM domain containing -like 1	0.95785441	0.4143	NA

18145	Npc1	Niemann Pick type C1	0.95785441	0.4293	NA
22628	Ywhag	tyrosine 3-monooxygenase/tryptophan 5-monooxygenase activation protein, gamma polypeptide	0.95785441	0.4294	NA
239739	Lamp3	lysosomal-associated membrane protein 3	0.95785441	0.4327	NA
243272	Sbno1	sno, strawberry notch homolog 1 (Drosophila)	0.95785441	0.4401	NA
234776	Atmin	ATM interactor	0.95785441	0.4506	NA
71793	Ints12	integrator complex subunit 12	0.95785441	0.4577	NA
70767	Prpf3	PRP3 pre-mRNA processing factor 3 homolog (yeast)	0.95785441	0.4586	NA
218442	Serinc5	serine incorporator 5	0.95785441	0.4639	NA
73112	3110003A17Rik	RIKEN cDNA 3110003A17 gene	0.95785441	0.4689	NA
52683	Ncaph2	non-SMC condensin II complex, subunit H2	0.95785441	0.472	NA
215476	C330019G07Rik	RIKEN cDNA C330019G07 gene	0.95785441	0.4722	NA
57330	Gigyf1	GRB10 interacting GYF protein 1	0.95785441	0.475	NA
78593	Nrip3	nuclear receptor interacting protein 3	0.95785441	0.4922	NA
234515	Inpp4b	inositol polyphosphate-4-phosphatase, type II	0.95785441	0.4928	NA
66871	Cpne8	copine VIII	0.95785441	0.4979	NA
74340	Ahcyl2	S-adenosylhomocysteine hydrolase-like 2	0.95785441	0.4984	NA
12652	Chga	chromogranin A	0.95785441	0.5	NA
74585	Sppl3	signal peptide peptidase 3	0.95785441	0.5025	NA
114715	Spred1	sprouty protein with EVH-1 domain 1, related sequence	0.95785441	0.5077	NA
76917	Flywch2	FLYWCH family member 2	0.95785441	0.5307	NA
233489	Picalm	phosphatidylinositol binding clathrin assembly protein	0.95785441	0.5353	NA
66614	Gpatch4	G patch domain containing 4	0.95785441	0.5482	NA
72141	Adpgk	ADP-dependent glucokinase	0.95785441	0.5528	NA
15382	Hnrnpa1	heterogeneous nuclear ribonucleoprotein A1	0.95785441	0.5541	NA
15568	Elavl1	ELAV (embryonic lethal, abnormal vision, Drosophila)-like 1 (Hu antigen R)	0.95785441	0.5581	NA
224481	Tfb1m	transcription factor B1, mitochondrial	0.95785441	0.5671	NA
71169	Nbas	neuroblastoma amplified sequence	0.95785441	0.5697	NA
76800	Usp42	ubiquitin specific peptidase 42	0.95785441	0.5773	NA
114716	Spred2	sprouty-related, EVH1 domain containing 2	0.95785441	0.5778	NA
210376	Mtmr9	myotubularin related protein 9	0.95785441	0.581	NA
18813	Pa2g4	proliferation-associated 2G4	0.95785441	0.5984	NA
18479	Pak1	p21 protein (Cdc42/Rac)-activated kinase 1	0.95785441	0.5987	NA
56526	Sep-06	septin 6	0.95785441	0.5987	NA
67255	Zfp422	zinc finger protein 422	0.95785441	0.6021	NA
18813	Pa2g4	proliferation-associated 2G4	0.95785441	0.6083	NA
21372	Tbl1x	transducin (beta)-like 1 X-linked	0.95785441	0.6341	0.792
14724	Gp1bb	glycoprotein Ib, beta polypeptide	0.95785441	0.644	0.7987
553127	Cxx1b	CAAX box 1 homolog B (human)	0.95785441	0.6623	0.8097
66467	Gtf2h5	general transcription factor IIH, polypeptide 5	0.95785441	0.6642	0.811
21400	Tcea2	transcription elongation factor A (SII), 2	0.95785441	0.6657	0.8117
66299	2610019N06Rik	RIKEN cDNA 2610019N06 gene	0.95785441	0.6812	0.8221
19298	Pex19	peroxisomal biogenesis factor 19	0.95785441	0.6927	0.8299
12028	Bax	BCL2-associated X protein	0.95785441	0.6928	0.83
14704	Gng3	guanine nucleotide binding protein (G protein), gamma 3	0.95785441	0.6956	0.8317
18642	Pfkm	phosphofructokinase, muscle	0.95785441	0.6966	0.8324
104600	AW125324	expressed sequence AW125324	0.95785441	0.7025	0.8356
77980	Sbf1	SET binding factor 1	0.95785441	0.7068	0.8385
75273	Pelp1	proline, glutamic acid and leucine rich protein 1	0.95785441	0.7176	0.8458
66309	Tmem128	transmembrane protein 128	0.95785441	0.7227	0.849
231863	Fbxl18	F-box and leucine-rich repeat protein 18	0.95785441	0.7302	0.8537

320376	Bcorl1	BCL6 co-repressor-like 1	0.95785441	0.7364	0.8575
331474	Rgag4	retrotransposon gag domain containing 4	0.95785441	0.7408	0.8598
66212	Sec61b	Sec61 beta subunit	0.95785441	0.7521	0.8668
435285	Krtap4-16	keratin associated protein 4-16	0.95785441	0.7696	0.8779
102442	Dennd4a	DENN/MADD domain containing 4A	0.95785441	0.7926	0.8912
14238	Foxf2	forkhead box F2	0.95785441	0.796	0.8927
66438	Hamp2	hepcidin antimicrobial peptide 2	0.95785441	0.8177	0.9055
64654	Fgf23	fibroblast growth factor 23	0.95785441	0.8748	0.9353
218232	Ptpdc1	protein tyrosine phosphatase domain containing 1	0.9569378	0.3288	NA
57321	Terf2ip	telomeric repeat binding factor 2, interacting protein	0.9569378	0.3724	NA
433864	Nom1	nucleolar protein with MIF4G domain 1	0.9569378	0.413	NA
66591	Mad2l1bp	MAD2L1 binding protein	0.9569378	0.4199	NA
72148	2610019F03Rik	RIKEN cDNA 2610019F03 gene	0.9569378	0.4217	NA
30045	Dnajc12	DnaJ (Hsp40) homolog, subfamily C, member 12	0.9569378	0.4296	NA
66609	Cryzl1	crystallin, zeta (quinone reductase)-like 1	0.9569378	0.4315	NA
24128	Xrn2	5'-3' exoribonuclease 2	0.9569378	0.4506	NA
212627	Prpsap2	phosphoribosyl pyrophosphate synthetase-associated protein 2	0.9569378	0.4507	NA
13435	Dnmt3a	DNA methyltransferase 3A	0.9569378	0.4551	NA
544696	D630037F22Rik	RIKEN cDNA D630037F22 gene	0.9569378	0.4675	NA
52552	Parp8	poly (ADP-ribose) polymerase family, member 8	0.9569378	0.4697	NA
226090	Ermp1	endoplasmic reticulum metallopeptidase 1	0.9569378	0.4707	NA
407786	Taf9b	TAF9B RNA polymerase II, TATA box binding protein (TBP)-associated factor	0.9569378	0.4722	NA
57754	Cend1	cell cycle exit and neuronal differentiation 1	0.9569378	0.4735	NA
668459	Gm9182	predicted gene 9182	0.9569378	0.4878	NA
68475	Ssna1	Sjogren's syndrome nuclear autoantigen 1	0.9569378	0.4931	NA
226153	Peo1	progressive external ophthalmoplegia 1 (human)	0.9569378	0.495	NA
67666	Hapln3	hyaluronan and proteoglycan link protein 3	0.9569378	0.507	NA
57317	Srsf4	serine/arginine-rich splicing factor 4	0.9569378	0.5116	NA
66449	Pam16	presequence translocase-asssociated motor 16 homolog (S. cerevisiae)	0.9569378	0.5124	NA
83429	Ctns	cystinosis, nephropathic	0.9569378	0.5125	NA
215201	Trmt2b	TRM2 tRNA methyltransferase 2 homolog B (S. cerevisiae)	0.9569378	0.5147	NA
210044	Adcy2	adenylate cyclase 2	0.9569378	0.5242	NA
17220	Mcm7	minichromosome maintenance deficient 7 (S. cerevisiae)	0.9569378	0.5293	NA
74769	Pik3cb	phosphatidylinositol 3-kinase, catalytic, beta polypeptide	0.9569378	0.5345	NA
380773	1810035L17Rik	RIKEN cDNA 1810035L17 gene	0.9569378	0.5366	NA
269003	Sap130	Sin3A associated protein	0.9569378	0.537	NA
67788	6330577E15Rik	RIKEN cDNA 6330577E15 gene	0.9569378	0.5376	NA
56040	Rplp1	ribosomal protein, large, P1	0.9569378	0.5506	NA
101943	Sf3b3	splicing factor 3b, subunit 3	0.9569378	0.5553	NA
21393	Tcap	titin-cap	0.9569378	0.5656	NA
56422	Hbs1l	Hbs1-like (S. cerevisiae)	0.9569378	0.5823	NA
80890	Trim2	tripartite motif-containing 2	0.9569378	0.5829	NA
67139	Mis12	MIS12 homolog (yeast)	0.9569378	0.5839	NA
27369	Dguok	deoxyguanosine kinase	0.9569378	0.5897	NA
192950	Nacad	NAC alpha domain containing	0.9569378	0.5916	NA
66870	Serbp1	serpine1 mRNA binding protein 1	0.9569378	0.5935	NA
320299	Iqcb1	IQ calmodulin-binding motif containing 1	0.9569378	0.6081	0.7732
330192	Vps37b	vacuolar protein sorting 37B (yeast)	0.9569378	0.6143	0.7778
74355	Smchd1	SMC hinge domain containing 1	0.9569378	0.6286	0.7887
50916	Irx4	Iroquois related homeobox 4 (Drosophila)	0.9569378	0.6445	0.7992

625054	Gm6548	eukaryotic translation elongation factor 1 alpha 1 pseudogene	0.9569378	0.6468	0.8008
142682	Zcchc14	zinc finger, CCHC domain containing 14	0.9569378	0.6534	0.8044
19360	Rad50	RAD50 homolog (S. cerevisiae)	0.9569378	0.6554	0.8056
71777	Ing3	inhibitor of growth family, member 3	0.9569378	0.6555	0.8056
108943	Rg9mtd2	RNA (guanine-9-) methyltransferase domain containing 2	0.9569378	0.6599	0.8085
50772	Mapk6	mitogen-activated protein kinase 6	0.9569378	0.6664	0.8119
12043	Bcl2	B-cell leukemia/lymphoma 2	0.9569378	0.7079	0.8393
404337	Olfr1383	olfactory receptor 1383	0.9569378	0.7113	0.8414
239647	Fam113b	family with sequence similarity 113, member B	0.9569378	0.7166	0.8452
240055	Neurl1b	neuralized homolog 1b (Drosophila)	0.9569378	0.7347	0.8566
192185	Nadk	NAD kinase	0.9569378	0.7374	0.8578
78929	Polr3h	polymerase (RNA) III (DNA directed) polypeptide H	0.9569378	0.7377	0.858
227120	Plcl1	phospholipase C-like 1	0.9569378	0.7467	0.8636
639281	Gm10382	predicted gene 10382	0.9569378	0.7903	0.8901
258778	Olfr921	olfactory receptor 921	0.9569378	0.8108	0.9017
70951	Spata1	spermatogenesis associated 1	0.9569378	0.9085	0.9532
26413	Mapk1	mitogen-activated protein kinase 1	0.95602294	0.2665	NA
20084	Rps18	ribosomal protein S18	0.95602294	0.2787	NA
77048	Ccdc41	coiled-coil domain containing 41	0.95602294	0.2974	NA
225791	Zadh2	zinc binding alcohol dehydrogenase, domain containing 2	0.95602294	0.3527	NA
68979	Nol11	nucleolar protein 11	0.95602294	0.3553	NA
241296	Lrrc8a	leucine rich repeat containing 8A	0.95602294	0.3624	NA
268729	Gm626	predicted gene 626	0.95602294	0.3649	NA
67160	Eef1g	eukaryotic translation elongation factor 1 gamma	0.95602294	0.4031	NA
56334	Tmed2	transmembrane emp24 domain trafficking protein 2	0.95602294	0.4082	NA
56551	Txn2	thioredoxin 2	0.95602294	0.4238	NA
22668	Sf1	splicing factor 1	0.95602294	0.4295	NA
22344	Vezf1	vascular endothelial zinc finger 1	0.95602294	0.4416	NA
69554	Klhdc2	kelch domain containing 2	0.95602294	0.4438	NA
11949	Atp5c1	ATP synthase, H+ transporting, mitochondrial F1 complex, gamma polypeptide 1	0.95602294	0.4489	NA
68981	Snrpa1	small nuclear ribonucleoprotein polypeptide A'	0.95602294	0.4555	NA
66443	Tnfaip8l1	tumor necrosis factor, alpha-induced protein 8-like 1	0.95602294	0.4585	NA
635504	Gm7160	predicted gene 7160	0.95602294	0.4644	NA
73822	F630110N24Rik	RIKEN cDNA F630110N24 gene	0.95602294	0.4656	NA
70797	Ankib1	ankyrin repeat and IBR domain containing 1	0.95602294	0.473	NA
93709	Pcdhga1	protocadherin gamma subfamily A, 1	0.95602294	0.479	NA
208990	Npb	neuropeptide B	0.95602294	0.479	NA
71835	Lancl2	LanC (bacterial lantibiotic synthetase component C)-like 2	0.95602294	0.482	NA
14799	Gria1	glutamate receptor, ionotropic, AMPA1 (alpha 1)	0.95602294	0.5131	NA
66361	Zfand1	zinc finger, AN1-type domain 1	0.95602294	0.5154	NA
52064	Coq5	coenzyme Q5 homolog, methyltransferase (yeast)	0.95602294	0.5187	NA
22187	Ubb	ubiquitin B	0.95602294	0.5274	NA
16438	Itpr1	inositol 1,4,5-triphosphate receptor 1	0.95602294	0.5289	NA
19058	Ppp3r1	protein phosphatase 3, regulatory subunit B, alpha isoform (calcineurin B, type I)	0.95602294	0.529	NA
56279	Fam69b	family with sequence similarity 69, member B	0.95602294	0.5347	NA
15481	Hspa8	heat shock protein 8	0.95602294	0.5366	NA
414073	BC040756	cDNA sequence BC040756	0.95602294	0.5399	NA
228998	Arfgap1	ADP-ribosylation factor GTPase activating protein 1	0.95602294	0.5556	NA
27395	Mrpl15	mitochondrial ribosomal protein L15	0.95602294	0.5561	NA
233812	BC030336	cDNA sequence BC030336	0.95602294	0.5626	NA

226123	Morn4	MORN repeat containing 4	0.95602294	0.5682	NA
56747	Sez6l	seizure related 6 homolog like	0.95602294	0.5694	NA
16649	Kpna4	karyopherin (importin) alpha 4	0.95602294	0.5993	0.7675
72117	Naa50	N(alpha)-acetyltransferase 50, NatE catalytic subunit	0.95602294	0.6058	0.7722
20666	Sox11	SRY-box containing gene 11	0.95602294	0.6107	0.7751
22157	Tulp1	tubby like protein 1	0.95602294	0.6119	0.776
18162	Npr3	natriuretic peptide receptor 3	0.95602294	0.6459	0.8001
231474	Paqr3	progestin and adipoQ receptor family member III	0.95602294	0.6475	0.8012
54721	Tyk2	tyrosine kinase 2	0.95602294	0.6489	0.802
20256	Clec11a	C-type lectin domain family 11, member a	0.95602294	0.6555	0.8056
104896	AI852580	expressed sequence AI852580	0.95602294	0.6567	0.8062
217473	Ankmy2	ankyrin repeat and MYND domain containing 2	0.95602294	0.6575	0.8065
17751	Mt3	metallothionein 3	0.95602294	0.6733	0.817
433171	Gm10549	predicted gene 10549	0.95602294	0.6769	0.8191
66101	Ppih	peptidyl prolyl isomerase H	0.95602294	0.6816	0.8222
14073	Faah	fatty acid amide hydrolase	0.95602294	0.6825	0.8228
78305	1500032F14Rik	RIKEN cDNA 1500032F14 gene	0.95602294	0.7161	0.8449
228366	Gyltl1b	glycosyltransferase-like 1B	0.95602294	0.7243	0.8499
384569	Nova2	neuro-oncological ventral antigen 2	0.95602294	0.7268	0.8514
104570	Smek2	SMEK homolog 2, suppressor of mek1 (Dictyostelium)	0.95602294	0.7294	0.853
76484	Kndc1	kinase non-catalytic C-lobe domain (KIND) containing 1	0.95602294	0.735	0.8568
116972	Fam57a	family with sequence similarity 57, member A	0.95602294	0.7358	0.8572
229589	Prune	prune homolog (Drosophila)	0.95602294	0.7394	0.859
78670	Plekhj1	pleckstrin homology domain containing, family J member 1	0.95602294	0.7475	0.8639
76653	Cby3	chibby homolog 3 (Drosophila)	0.95602294	0.7541	0.8679
213054	Gabpb2	GA repeat binding protein, beta 2	0.95602294	0.7549	0.8685
26564	Ror2	receptor tyrosine kinase-like orphan receptor 2	0.95602294	0.7699	0.8779
78439	A930037H05Rik	RIKEN cDNA A930037H05 gene	0.95602294	0.7763	0.8811
338523	Jhdm1d	jumonji C domain-containing histone demethylase 1 homolog D (S. cerevisiae)	0.95602294	0.7819	0.8852
171262	Vmn1r70	vomeronasal 1 receptor 70	0.95602294	0.7846	0.8871
330938	Dixdc1	DIX domain containing 1	0.95602294	0.7869	0.8885
23871	Ets1	E26 avian leukemia oncogene 1, 5' domain	0.95602294	0.7932	0.8914
381350	BC061194	cDNA sequence BC061194	0.95602294	0.8119	0.9023
76965	Slitrk1	SLIT and NTRK-like family, member 1	0.95602294	0.8415	0.9181
622408	Gm6320	predicted gene 6320	0.95602294	0.8675	0.9318
624483	Gm6507	predicted gene 6507	0.95602294	0.8735	0.9348
244668	Sipa1l2	signal-induced proliferation-associated 1 like 2	0.95510984	0.2129	NA
98828	Cdc123	cell division cycle 123 homolog (S. cerevisiae)	0.95510984	0.2627	NA
68948	1500011H22Rik	RIKEN cDNA 1500011H22 gene	0.95510984	0.2702	NA
105782	Scrib	scribbled homolog (Drosophila)	0.95510984	0.2757	NA
66048	Tmem93	transmembrane protein 93	0.95510984	0.3243	NA
76582	Ipo11	importin 11	0.95510984	0.3406	NA
20463	Cox7a2l	cytochrome c oxidase subunit VIIa polypeptide 2-like	0.95510984	0.3474	NA
100042150	Nrg2	neuregulin 2	0.95510984	0.3613	NA
19821	Rnf2	ring finger protein 2	0.95510984	0.3698	NA
108911	Rcc2	regulator of chromosome condensation 2	0.95510984	0.3739	NA
74718	Snx16	sorting nexin 16	0.95510984	0.3782	NA
98404	AI597479	expressed sequence AI597479	0.95510984	0.3843	NA
233315	Mtmr10	myotubularin related protein 10	0.95510984	0.3899	NA
66054	Cndp2	CNDP dipeptidase 2 (metallopeptidase M20 family)	0.95510984	0.3919	NA

98999	Znfx1	zinc finger, NFX1-type containing 1	0.95510984	0.405	NA
216618	Ccdc104	coiled-coil domain containing 104	0.95510984	0.4218	NA
67493	Mett10d	methyltransferase 10 domain containing	0.95510984	0.4245	NA
17979	Ncoa3	nuclear receptor coactivator 3	0.95510984	0.4279	NA
19769	Rit1	Ras-like without CAAX 1	0.95510984	0.4292	NA
22654	Zfp13	zinc finger protein 13	0.95510984	0.4302	NA
21652	Phf1	PHD finger protein 1	0.95510984	0.4343	NA
224129	Adcy5	adenylate cyclase 5	0.95510984	0.441	NA
12421	Rb1cc1	RB1-inducible coiled-coil 1	0.95510984	0.4425	NA
72515	Wdr43	WD repeat domain 43	0.95510984	0.4551	NA
73674	Wdr75	WD repeat domain 75	0.95510984	0.4558	NA
20970	Sdc3	syndecan 3	0.95510984	0.463	NA
13494	Drg1	developmentally regulated GTP binding protein 1	0.95510984	0.4745	NA
19891	Rpa2	replication protein A2	0.95510984	0.477	NA
76788	Klhdc10	kelch domain containing 10	0.95510984	0.4826	NA
320720	Fastkd1	FAST kinase domains 1	0.95510984	0.4867	NA
19357	Rad21	RAD21 homolog (S. pombe)	0.95510984	0.4992	NA
67136	Kbtbd4	kelch repeat and BTB (POZ) domain containing 4	0.95510984	0.5133	NA
12709	Ckb	creatine kinase, brain	0.95510984	0.5228	NA
50935	St6galnac6	ST6 (alpha-N-acetyl-neuraminyl-2,3-beta-galactosyl-1,3)-N-acetylgalactosaminide alpha-2,6-sialyltransferase 6	0.95510984	0.5232	NA
665563	Mthfd2l	methylenetetrahydrofolate dehydrogenase (NADP+ dependent) 2-like	0.95510984	0.5252	NA
16511	Kcnh2	potassium voltage-gated channel, subfamily H (eag-related), member 2	0.95510984	0.5253	NA
108653	Rimklb	ribosomal modification protein rimK-like family member B	0.95510984	0.5493	NA
329872	Frem1	Fras1 related extracellular matrix protein 1	0.95510984	0.5594	NA
494448	Cbx6	chromobox homolog 6	0.95510984	0.5658	NA
21872	Tjp1	tight junction protein 1	0.95510984	0.5708	NA
69934	Rg9mtd3	RNA (guanine-9-) methyltransferase domain containing 3	0.95510984	0.5733	NA
12039	Bckdha	branched chain ketoacid dehydrogenase E1, alpha polypeptide	0.95510984	0.5919	0.7616
71881	2310001A20Rik	RIKEN cDNA 2310001A20 gene	0.95510984	0.6008	0.7684
75051	4930578N16Rik	RIKEN cDNA 4930578N16 gene	0.95510984	0.6019	0.7691
382492	Gm12034	predicted gene 12034	0.95510984	0.632	0.791
224250	Cldnd1	claudin domain containing 1	0.95510984	0.6343	0.7921
218440	Ankrd34b	ankyrin repeat domain 34B	0.95510984	0.6391	0.7956
270893	Tmem132e	transmembrane protein 132E	0.95510984	0.656	0.8058
208228	Mobkl2a	MOB1, Mps One Binder kinase activator-like 2A (yeast)	0.95510984	0.6574	0.8065
232976	Zfp574	zinc finger protein 574	0.95510984	0.6599	0.8085
21987	Tpd52l1	tumor protein D52-like 1	0.95510984	0.6637	0.8105
381760	Ssbp1	single-stranded DNA binding protein 1	0.95510984	0.6707	0.8154
230721	Pabpc4	poly(A) binding protein, cytoplasmic 4	0.95510984	0.6759	0.8184
654470	Tctn1	tectonic family member 1	0.95510984	0.6785	0.8202
77066	5930409G06Rik	RIKEN cDNA 5930409G06 gene	0.95510984	0.6823	0.8228
74251	Ankrd9	ankyrin repeat domain 9	0.95510984	0.6837	0.8233
12918	Crh	corticotropin releasing hormone	0.95510984	0.6931	0.83
208898	Unc13c	unc-13 homolog C (C. elegans)	0.95510984	0.7271	0.8516
93765	Ube2n	ubiquitin-conjugating enzyme E2N	0.95510984	0.7379	0.858
328108	Fam179b	family with sequence similarity 179, member B	0.95510984	0.7534	0.8674
76937	2810429I04Rik	RIKEN cDNA 2810429I04 gene	0.95510984	0.8157	0.9045
435392	Gm5670	predicted gene 5670	0.95510984	0.8544	0.925
79560	Ublcp1	ubiquitin-like domain containing CTD phosphatase 1	0.95419847	0.2748	NA
56550	Ube2d2	ubiquitin-conjugating enzyme E2D 2	0.95419847	0.2989	NA

170833	Hook2	hook homolog 2 (Drosophila)	0.95419847	0.3137	NA
625347	Gm6578	mitochondrial ribosomal protein L32 pseudogene	0.95419847	0.3188	NA
54636	Wdr45	WD repeat domain 45	0.95419847	0.3441	NA
101685	Spty2d1	SPT2, Suppressor of Ty, domain containing 1 (S. cerevisiae)	0.95419847	0.3564	NA
102122	Fam192a	family with sequence similarity 192, member A	0.95419847	0.382	NA
57436	Gabarapl1	gamma-aminobutyric acid (GABA) A receptor-associated protein-like 1	0.95419847	0.3852	NA
83485	Ngrn	neugrin, neurite outgrowth associated	0.95419847	0.4047	NA
70870	4921516I12Rik	RIKEN cDNA 4921516I12 gene	0.95419847	0.4192	NA
319740	Zfyve27	zinc finger, FYVE domain containing 27	0.95419847	0.4222	NA
214133	Tet2	tet oncogene family member 2	0.95419847	0.442	NA
102124	E130303B06Rik	RIKEN cDNA E130303B06 gene	0.95419847	0.4423	NA
50776	Polg2	polymerase (DNA directed), gamma 2, accessory subunit	0.95419847	0.4751	NA
11636	Ak1	adenylate kinase 1	0.95419847	0.4846	NA
52589	Ncald	neurocalcin delta	0.95419847	0.488	NA
72357	2210016L21Rik	RIKEN cDNA 2210016L21 gene	0.95419847	0.4921	NA
66052	Sdhc	succinate dehydrogenase complex, subunit C, integral membrane protein	0.95419847	0.4924	NA
13480	Dpm1	dolichol-phosphate (beta-D) mannosyltransferase 1	0.95419847	0.4941	NA
235028	Zfp426	zinc finger protein 426	0.95419847	0.4991	NA
242505	Rasef	RAS and EF hand domain containing	0.95419847	0.5079	NA
67171	Dram2	VDNA-damage regulated autophagy modulator 2	0.95419847	0.5157	NA
68323	Nudt22	nudix (nucleoside diphosphate linked moiety X)-type motif 22	0.95419847	0.5179	NA
269854	Nat14	N-acetyltransferase 14	0.95419847	0.5185	NA
67897	Rnmt	RNA (guanine-7-) methyltransferase	0.95419847	0.5257	NA
319945	Flad1	RFad1, flavin adenine dinucleotide synthetase, homolog (yeast)	0.95419847	0.5319	NA
237436	Gas2l3	growth arrest-specific 2 like 3	0.95419847	0.535	NA
75608	Chmp4b	chromatin modifying protein 4B	0.95419847	0.5371	NA
263764	Creg2	cellular repressor of E1A-stimulated genes 2	0.95419847	0.5403	NA
69632	Arhgef12	Rho guanine nucleotide exchange factor (GEF) 12	0.95419847	0.5498	NA
278679	Apol7b	apolipoprotein L 7b	0.95419847	0.5581	NA
214158	Trim38	tripartite motif-containing 38	0.95419847	0.6086	0.7735
97485	C88045	expressed sequence C88045	0.95419847	0.615	0.7782
17997	Nedd1	neural precursor cell expressed, developmentally down-regulated gene 1	0.95419847	0.6226	0.7836
21770	Ppp2r5d	protein phosphatase 2, regulatory subunit B (B56), delta isoform	0.95419847	0.6295	0.7894
27053	Asns	asparagine synthetase	0.95419847	0.6302	0.7897
66202	1110059G10Rik	RIKEN cDNA 1110059G10 gene	0.95419847	0.6366	0.7939
107770	Tm6sf2	transmembrane 6 superfamily member 2	0.95419847	0.6431	0.7981
252972	Tpcn1	two pore channel 1	0.95419847	0.6495	0.8026
13393	Dlx3	distal-less homeobox 3	0.95419847	0.6556	0.8056
72881	Zdhhc4	zinc finger, DHHC domain containing 4	0.95419847	0.6732	0.817
12631	Cfl1	cofilin 1, non-muscle	0.95419847	0.6791	0.8207
320265	Fam19a1	family with sequence similarity 19, member A1	0.95419847	0.6797	0.821
212980	Slc45a3	solute carrier family 45, member 3	0.95419847	0.6803	0.8213
230603	Ttc39a	tetratricopeptide repeat domain 39A	0.95419847	0.6804	0.8213
66882	Bzw1	basic leucine zipper and W2 domains 1	0.95419847	0.682	0.8226
99296	Hrh3	histamine receptor H3	0.95419847	0.6826	0.8228
70560	Wars2	tryptophanyl tRNA synthetase 2 (mitochondrial)	0.95419847	0.6871	0.8259
100038371	Zfp389	zinc finger protein 389	0.95419847	0.691	0.8286
269252	Gtf3c4	general transcription factor IIIC, polypeptide 4	0.95419847	0.7188	0.8464
65962	Slc9a3r2	solute carrier family 9 (sodium/hydrogen exchanger), member 3 regulator 2	0.95419847	0.7273	0.8518
226751	Cdc42bpa	CDC42 binding protein kinase alpha	0.95419847	0.736	0.8572

100045736	LOC100045736	hypothetical protein LOC100045736	0.95419847	0.7422	0.8607
56543	Kcnd3	potassium voltage-gated channel, Shal-related family, member 3	0.95419847	0.7503	0.8657
14528	Gch1	GTP cyclohydrolase 1	0.95419847	0.7522	0.8669
70713	Gpr137c	G protein-coupled receptor 137C	0.95419847	0.757	0.8701
22042	Tfrc	transferrin receptor	0.95419847	0.7613	0.8729
72117	Naa50	N(alpha)-acetyltransferase 50, NatE catalytic subunit	0.95419847	0.7769	0.8814
58208	Bcl11b	B-cell leukemia/lymphoma 11B	0.95419847	0.7772	0.8816
17764	Mtf1	metal response element binding transcription factor 1	0.95419847	0.7835	0.8864
13131	Dab1	disabled homolog 1 (Drosophila)	0.95419847	0.7883	0.8891
22691	Zscan2	zinc finger and SCAN domain containing 2	0.95419847	0.79	0.8901
210544	Wdr67	WD repeat domain 67	0.95419847	0.7912	0.8905
16975	Lrp8	low density lipoprotein receptor-related protein 8, apolipoprotein e receptor	0.95419847	0.7987	0.8943
71151	Eri2	exoribonuclease 2	0.95419847	0.8286	0.9113
14810	Grin1	glutamate receptor, ionotropic, NMDA1 (zeta 1)	0.95419847	0.846	0.9204
21763	Tex2	testis expressed gene 2	0.95328885	0.2509	NA
70790	Ubr5	ubiquitin protein ligase E3 component n-recognin 5	0.95328885	0.2699	NA
14376	Ganab	alpha glucosidase 2 alpha neutral subunit	0.95328885	0.2843	NA
16499	Kcnab3	potassium voltage-gated channel, shaker-related subfamily, beta member 3	0.95328885	0.2932	NA
214505	Gnptg	N-acetylglucosamine-1-phosphotransferase, gamma subunit	0.95328885	0.296	NA
14894	Gtl3	gene trap locus 3	0.95328885	0.3093	NA
68316	Apoo	apolipoprotein O	0.95328885	0.3144	NA
11950	Atp5f1	ATP synthase, H+ transporting, mitochondrial F0 complex, subunit B1	0.95328885	0.3214	NA
231014	9330182L06Rik	RIKEN cDNA 9330182L06 gene	0.95328885	0.333	NA
229211	Acad9	acyl-Coenzyme A dehydrogenase family, member 9	0.95328885	0.3609	NA
229363	Gmps	guanine monophosphate synthetase	0.95328885	0.3712	NA
77031	Slc9a8	solute carrier family 9 (sodium/hydrogen exchanger), member 8	0.95328885	0.3887	NA
445007	Nup85	nucleoporin 85	0.95328885	0.4008	NA
67109	Zfp787	zinc finger protein 787	0.95328885	0.4017	NA
23961	Oas1b	2'-5' oligoadenylate synthetase 1B	0.95328885	0.4042	NA
19418	Rasgrf2	RAS protein-specific guanine nucleotide-releasing factor 2	0.95328885	0.4196	NA
67059	Ola1	Obg-like ATPase 1	0.95328885	0.4257	NA
60363	Cldn15	claudin 15	0.95328885	0.4347	NA
67609	4930453N24Rik	RIKEN cDNA 4930453N24 gene	0.95328885	0.4501	NA
66390	Slmo2	slowmo homolog 2 (Drosophila)	0.95328885	0.476	NA
72612	2700029M09Rik	RIKEN cDNA 2700029M09 gene	0.95328885	0.4897	NA
69035	Zdhhc3	zinc finger, DHHC domain containing 3	0.95328885	0.4919	NA
53412	Ppp1r3c	protein phosphatase 1, regulatory (inhibitor) subunit 3C	0.95328885	0.4934	NA
52009	Hn1l	hematological and neurological expressed 1-like	0.95328885	0.4993	NA
18114	Rrp1	ribosomal RNA processing 1 homolog (S. cerevisiae)	0.95328885	0.506	NA
107733	Mrpl41	mitochondrial ribosomal protein L41	0.95328885	0.5069	NA
68205	Urm1	ubiquitin related modifier 1 homolog (S. cerevisiae)	0.95328885	0.5102	NA
14401	Gabrb2	gamma-aminobutyric acid (GABA) A receptor, subunit beta 2	0.95328885	0.5139	NA
73296	Rhobtb3	Rho-related BTB domain containing 3	0.95328885	0.5261	NA
69918	2610020C07Rik	RIKEN cDNA 2610020C07 gene	0.95328885	0.5363	NA
234086	Erich1	glutamate-rich 1	0.95328885	0.5368	NA
18105	Nqo2	NAD(P)H dehydrogenase, quinone 2	0.95328885	0.5486	NA
56323	Dnajb5	DnaJ (Hsp40) homolog, subfamily B, member 5	0.95328885	0.5491	NA
229521	Syt11	synaptotagmin XI	0.95328885	0.5582	NA
18424	Otx2	orthodenticle homolog 2 (Drosophila)	0.95328885	0.5641	NA
319945	Flad1	RFad1, flavin adenine dinucleotide synthetase, homolog (yeast)	0.95328885	0.5795	0.7519

74020	Cpne4	copine IV	0.95328885	0.5842	0.7552
78438	A930028N01Rik	RIKEN cDNA A930028N01 gene	0.95328885	0.6045	0.7712
269585	Zscan20	zinc finger and SCAN domains 20	0.95328885	0.607	0.7728
71276	Ccdc57	coiled-coil domain containing 57	0.95328885	0.6296	0.7895
72273	2210404O07Rik	RIKEN cDNA 2210404O07 gene	0.95328885	0.6526	0.804
17711	CYTB	cytochrome b	0.95328885	0.6569	0.8063
20295	Ccl17	chemokine (C-C motif) ligand 17	0.95328885	0.6704	0.8152
381832	Prpmp5	proline-rich protein MP5	0.95328885	0.6707	0.8154
56807	Scamp5	secretory carrier membrane protein 5	0.95328885	0.6735	0.8171
666938	Bend4	BEN domain containing 4	0.95328885	0.6753	0.8183
26428	Orc4	origin recognition complex, subunit 4	0.95328885	0.6829	0.8229
233651	Dchs1	dachsous 1 (Drosophila)	0.95328885	0.6832	0.8231
13047	Cux1	cut-like homeobox 1	0.95328885	0.7192	0.8466
74019	Traf3ip1	TRAF3 interacting protein 1	0.95328885	0.7192	0.8466
75697	C2cd4b	C2 calcium-dependent domain containing 4B	0.95328885	0.7218	0.8482
12631	Cfl1	cofilin 1, non-muscle	0.95328885	0.739	0.8588
21406	Tcf12	transcription factor 12	0.95328885	0.7521	0.8668
73242	Atat1	alpha tubulin acetyltransferase 1	0.95328885	0.7549	0.8685
24047	Ccl19	chemokine (C-C motif) ligand 19	0.95328885	0.7664	0.8759
109880	Braf	Braf transforming gene	0.95328885	0.7668	0.8763
114604	Prdm15	PR domain containing 15	0.95328885	0.7686	0.8772
97863	C78339	expressed sequence C78339	0.95328885	0.7792	0.8832
619937	Gm6115	predicted gene 6115	0.95328885	0.7874	0.8885
77583	Notum	notum pectinacetylesterase homolog (Drosophila)	0.95328885	0.7945	0.8921
68713	Ifitm1	interferon induced transmembrane protein 1	0.95328885	0.7962	0.8928
633057	Gm7102	predicted gene 7102	0.95328885	0.8244	0.9095
11852	Rhob	ras homolog gene family, member B	0.95328885	0.8277	0.911
100191037	Krtap10-4	keratin associated protein 10-4	0.95328885	0.8875	0.942
16553	Kif13a	kinesin family member 13A	0.95238095	0.2323	NA
30795	Fkbp3	FK506 binding protein 3	0.95238095	0.271	NA
219181	Akap11	A kinase (PRKA) anchor protein 11	0.95238095	0.2717	NA
78334	Cdk19	cyclin-dependent kinase 19	0.95238095	0.2906	NA
66400	Alkbh7	alkB, alkylation repair homolog 7 (E. coli)	0.95238095	0.3219	NA
228829	Phf20	PHD finger protein 20	0.95238095	0.3278	NA
269593	Luzp1	leucine zipper protein 1	0.95238095	0.3374	NA
17977	Ncoa1	nuclear receptor coactivator 1	0.95238095	0.3426	NA
65961	Utp3	UTP3, small subunit (SSU) processome component, homolog (S. cerevisiae)	0.95238095	0.3468	NA
20257	Stmn2	stathmin-like 2	0.95238095	0.3582	NA
19211	Pten	phosphatase and tensin homolog	0.95238095	0.369	NA
233802	Thumpd1	THUMP domain containing 1	0.95238095	0.3748	NA
217364	Engase	endo-beta-N-acetylglucosaminidase	0.95238095	0.3783	NA
434377	Zfp560	zinc finger protein 560	0.95238095	0.3971	NA
216760	Mfap3	microfibrillar-associated protein 3	0.95238095	0.3972	NA
64656	Mrps23	mitochondrial ribosomal protein S23	0.95238095	0.4089	NA
68126	Fahd2a	fumarylacetoacetate hydrolase domain containing 2A	0.95238095	0.4101	NA
232341	Wnk1	WNK lysine deficient protein kinase 1	0.95238095	0.4147	NA
72203	2610507I01Rik	RIKEN cDNA 2610507I01 gene	0.95238095	0.423	NA
74125	Armc8	armadillo repeat containing 8	0.95238095	0.437	NA
70380	Mospd1	motile sperm domain containing 1	0.95238095	0.4467	NA
54383	Phc2	polyhomeotic-like 2 (Drosophila)	0.95238095	0.4609	NA

56382	Rab9	RAB9, member RAS oncogene family	0.95238095	0.461	NA
69020	Zfp707	zinc finger protein 707	0.95238095	0.4784	NA
228788	BC020535	cDNA sequence BC020535	0.95238095	0.4889	NA
74477	4933427D14Rik	RIKEN cDNA 4933427D14 gene	0.95238095	0.4897	NA
668661	2410002F23Rik	RIKEN cDNA 2410002F23 gene	0.95238095	0.4899	NA
620631	Ttc30a2	tetratricopeptide repeat domain 30A2	0.95238095	0.4917	NA
66980	Zdhhc6	zinc finger, DHHC domain containing 6	0.95238095	0.4955	NA
19684	Rdx	radixin	0.95238095	0.4961	NA
229663	Csde1	cold shock domain containing E1, RNA binding	0.95238095	0.5047	NA
16835	Ldlr	low density lipoprotein receptor	0.95238095	0.51	NA
216810	Tom1l2	target of myb1-like 2 (chicken)	0.95238095	0.5123	NA
218341	Rfesd	Rieske (Fe-S) domain containing	0.95238095	0.5185	NA
76041	Ccdc125	coiled-coil domain containing 125	0.95238095	0.5247	NA
71728	Stk11ip	serine/threonine kinase 11 interacting protein	0.95238095	0.5279	NA
15490	Hsd17b7	hydroxysteroid (17-beta) dehydrogenase 7	0.95238095	0.5341	NA
14397	Gabra4	gamma-aminobutyric acid (GABA) A receptor, subunit alpha 4	0.95238095	0.5379	NA
29819	Stau2	staufen (RNA binding protein) homolog 2 (Drosophila)	0.95238095	0.5502	NA
58242	Nudt11	nudix (nucleoside diphosphate linked moiety X)-type motif 11	0.95238095	0.5609	NA
77582	Mboat7	membrane bound O-acyltransferase domain containing 7	0.95238095	0.5616	NA
211401	Mtss1	metastasis suppressor 1	0.95238095	0.5739	0.7479
226856	Lpgat1	lysophosphatidylglycerol acyltransferase 1	0.95238095	0.5758	0.7492
320615	Dopey1	dopey family member 1	0.95238095	0.5769	0.7502
12283	Cab39	calcium binding protein 39	0.95238095	0.579	0.7515
18807	Pld3	phospholipase D family, member 3	0.95238095	0.5827	0.7543
170787	Hdac10	histone deacetylase 10	0.95238095	0.6025	0.7694
320100	Relt	RELT tumor necrosis factor receptor	0.95238095	0.6073	0.7728
280645	B3gat2	beta-1,3-glucuronyltransferase 2 (glucuronosyltransferase S)	0.95238095	0.6096	0.7744
215446	Entpd3	ectonucleoside triphosphate diphosphohydrolase 3	0.95238095	0.6109	0.7753
68184	Denr	density-regulated protein	0.95238095	0.6115	0.7757
57340	Jph3	junctophilin 3	0.95238095	0.6165	0.7793
71640	Zfp949	zinc finger protein 949	0.95238095	0.6359	0.7933
384783	Irs2	insulin receptor substrate 2	0.95238095	0.6428	0.7978
19294	Pvrl2	poliovirus receptor-related 2	0.95238095	0.6503	0.8031
264064	Cdk8	cyclin-dependent kinase 8	0.95238095	0.6569	0.8063
68631	Cryl1	crystallin, lambda 1	0.95238095	0.6648	0.8113
70454	Cenpl	centromere protein L	0.95238095	0.672	0.8164
51792	Ppp2r1a	protein phosphatase 2 (formerly 2A), regulatory subunit A (PR 65), alpha isoform	0.95238095	0.6814	0.8221
12589	Ift81	intraflagellar transport 81 homolog (Chlamydomonas)	0.95238095	0.6859	0.825
26914	H2afy	H2A histone family, member Y	0.95238095	0.7007	0.8342
20500	Slc13a2	solute carrier family 13 (sodium-dependent dicarboxylate transporter), member 2	0.95238095	0.7086	0.8398
70796	Zdhhc1	zinc finger, DHHC domain containing 1	0.95238095	0.7275	0.8518
408068	Zfp738	zinc finger protein 738	0.95238095	0.7301	0.8537
381199	Tmem151a	transmembrane protein 151A	0.95238095	0.7328	0.8557
224344	Rbm11	RNA binding motif protein 11	0.95238095	0.7357	0.8572
100505291	LOC100505291	60S ribosomal protein L36-like	0.95238095	0.7427	0.8609
432442	Akap7	A kinase (PRKA) anchor protein 7	0.95238095	0.7749	0.8802
20404	Sh3gl2	SH3-domain GRB2-like 2	0.95238095	0.7849	0.8872
16495	Kcna7	potassium voltage-gated channel, shaker-related subfamily, member 7	0.95238095	0.7899	0.8901
20022	Polr2j	polymerase (RNA) II (DNA directed) polypeptide J	0.95147479	0.2303	NA
78914	Nadsyn1	NAD synthetase 1	0.95147479	0.2567	NA

54393	Gabbr1	gamma-aminobutyric acid (GABA) B receptor, 1	0.95147479	0.336	NA
57080	Gtf2ird1	general transcription factor II I repeat domain-containing 1	0.95147479	0.3372	NA
76952	Nt5c2	5'-nucleotidase, cytosolic II	0.95147479	0.37	NA
75497	Fabp12	fatty acid binding protein 12	0.95147479	0.3705	NA
231659	Gcn1l1	GCN1 general control of amino-acid synthesis 1-like 1 (yeast)	0.95147479	0.3786	NA
80886	Senp3	SUMO/sentrin specific peptidase 3	0.95147479	0.3864	NA
83925	Trps1	trichorhinophalangeal syndrome I (human)	0.95147479	0.3973	NA
28030	Gfm1	G elongation factor, mitochondrial 1	0.95147479	0.401	NA
54217	Rpl36	ribosomal protein L36	0.95147479	0.4228	NA
76233	Dnttip1	deoxynucleotidyltransferase, terminal, interacting protein 1	0.95147479	0.4264	NA
67239	Rpf2	ribosome production factor 2 homolog (S. cerevisiae)	0.95147479	0.4329	NA
68565	Mrps18a	mitochondrial ribosomal protein S18A	0.95147479	0.4356	NA
13496	Arid3a	AT rich interactive domain 3A (BRIGHT-like)	0.95147479	0.4372	NA
67883	Uxs1	UDP-glucuronate decarboxylase 1	0.95147479	0.4427	NA
13972	Gnb1l	guanine nucleotide binding protein (G protein), beta polypeptide 1-like	0.95147479	0.4528	NA
12226	Btg1	B-cell translocation gene 1, anti-proliferative	0.95147479	0.4595	NA
20513	Slc1a6	solute carrier family 1 (high affinity aspartate/glutamate transporter), member 6	0.95147479	0.4723	NA
104079	Nxph3	neurexophilin 3	0.95147479	0.4869	NA
26922	Mecr	mitochondrial trans-2-enoyl-CoA reductase	0.95147479	0.5027	NA
68052	Rps13	ribosomal protein S13	0.95147479	0.5407	NA
70257	2010107E04Rik	RIKEN cDNA 2010107E04 gene	0.95147479	0.5421	NA
382245	Tmem29	transmembrane protein 29	0.95147479	0.5503	NA
11908	Atf1	activating transcription factor 1	0.95147479	0.5511	NA
11984	Atp6v0c	ATPase, H+ transporting, lysosomal V0 subunit C	0.95147479	0.5697	0.7451
239099	Homez	homeodomain leucine zipper-encoding gene	0.95147479	0.5729	0.7474
213980	Fbxw10	F-box and WD-40 domain protein 10	0.95147479	0.5752	0.749
15476	Hs3st1	heparan sulfate (glucosamine) 3-O-sulfotransferase 1	0.95147479	0.5811	0.7532
56375	B4galt4	UDP-Gal:betaGlcNAc beta 1,4-galactosyltransferase, polypeptide 4	0.95147479	0.5818	0.7535
554160	BB283564	expressed sequence BB283564	0.95147479	0.5839	0.755
12095	Bglap-rs1	bone gamma-carboxyglutamate protein, related sequence 1	0.95147479	0.5859	0.7565
14200	Fhl2	four and a half LIM domains 2	0.95147479	0.6081	0.7732
240853	Gm4953	ATP synthase, H+ transporting, mitochondrial F0 complex, subunit d pseudogene	0.95147479	0.61	0.7745
17448	Mdh2	malate dehydrogenase 2, NAD (mitochondrial)	0.95147479	0.6133	0.7771
229474	Fhdc1	FH2 domain containing 1	0.95147479	0.6222	0.7832
208194	Exog	endo/exonuclease (5'-3'), endonuclease G-like	0.95147479	0.6362	0.7936
224897	Dpp9	dipeptidylpeptidase 9	0.95147479	0.6424	0.7978
231946	D330028D13Rik	RIKEN cDNA D330028D13 gene	0.95147479	0.6606	0.8088
14420	Galc	galactosylceramidase	0.95147479	0.6621	0.8096
236733	Usp11	ubiquitin specific peptidase 11	0.95147479	0.6785	0.8202
626231	Gm6658	predicted gene 6658	0.95147479	0.6792	0.8207
23877	Fiz1	Flt3 interacting zinc finger protein 1	0.95147479	0.6803	0.8213
76867	Rhbdd1	rhomboid domain containing 1	0.95147479	0.6817	0.8222
93875	Pcdhb4	protocadherin beta 4	0.95147479	0.6843	0.8236
66923	Pbrm1	polybromo 1	0.95147479	0.7365	0.8575
320360	Ric3	resistance to inhibitors of cholinesterase 3 homolog (C. elegans)	0.95147479	0.7377	0.858
619301	G630016D24Rik	RIKEN cDNA G630016D24 gene	0.95147479	0.7538	0.8677
77756	A230101C19Rik	RIKEN cDNA A230101C19 gene	0.95147479	0.7634	0.8745
11828	Aqp3	aquaporin 3	0.95147479	0.8392	0.9171
16579	Kifap3	kinesin-associated protein 3	0.95057034	0.2284	NA
67440	Mtpap	mitochondrial poly(A) polymerase	0.95057034	0.2644	NA

224742	Abcf1	ATP-binding cassette, sub-family F (GCN20), member 1	0.95057034	0.2784	NA
69894	2010107G23Rik	RIKEN cDNA 2010107G23 gene	0.95057034	0.3006	NA
15441	Hp1bp3	heterochromatin protein 1, binding protein 3	0.95057034	0.3186	NA
627214	Fam196a	family with sequence similarity 196, member A	0.95057034	0.3261	NA
71745	Cul2	cullin 2	0.95057034	0.3443	NA
26440	Psma1	proteasome (prosome, macropain) subunit, alpha type 1	0.95057034	0.3524	NA
72180	Zfp661	zinc finger protein 661	0.95057034	0.3721	NA
22630	Ywhaq	tyrosine 3-monooxygenase/tryptophan 5-monooxygenase activation protein, theta polypeptide	0.95057034	0.3772	NA
54160	Copg2	coatomer protein complex, subunit gamma 2	0.95057034	0.3805	NA
15185	Hdac6	histone deacetylase 6	0.95057034	0.3999	NA
68077	Gltscr2	glioma tumor suppressor candidate region gene 2	0.95057034	0.4022	NA
17524	Mpp1	membrane protein, palmitoylated	0.95057034	0.4144	NA
242083	Ppm1l	protein phosphatase 1 (formerly 2C)-like	0.95057034	0.4186	NA
226823	Kctd3	potassium channel tetramerisation domain containing 3	0.95057034	0.4316	NA
67057	Yaf2	YY1 associated factor 2	0.95057034	0.4491	NA
23881	G3bp2	GTPase activating protein (SH3 domain) binding protein 2	0.95057034	0.4498	NA
17714	Grpel2	GrpE-like 2, mitochondrial	0.95057034	0.4517	NA
66440	Cdc26	cell division cycle 26	0.95057034	0.4564	NA
244911	C2cd4a	C2 calcium-dependent domain containing 4A	0.95057034	0.4615	NA
109778	Blvra	biliverdin reductase A	0.95057034	0.4688	NA
433424	Gm13476	predicted gene 13476	0.95057034	0.4839	NA
27226	Pla2g7	phospholipase A2, group VII (platelet-activating factor acetylhydrolase, plasma)	0.95057034	0.4929	NA
16706	Ksr1	kinase suppressor of ras 1	0.95057034	0.4954	NA
60533	Cd274	CD274 antigen	0.95057034	0.4997	NA
77032	2610029I01Rik	RIKEN cDNA 2610029I01 gene	0.95057034	0.5009	NA
109785	Pgm3	phosphoglucomutase 3	0.95057034	0.5064	NA
433287	Gm15455	predicted gene 15455	0.95057034	0.5113	NA
68796	Tmem214	transmembrane protein 214	0.95057034	0.516	NA
214547	She	src homology 2 domain-containing transforming protein E	0.95057034	0.5206	NA
18491	Pappa	pregnancy-associated plasma protein A	0.95057034	0.5264	NA
380959	Alg10b	asparagine-linked glycosylation 10 homolog B (yeast, alpha-1,2-glucosyltransferase)	0.95057034	0.5555	0.7353
74498	Gorasp1	golgi reassembly stacking protein 1	0.95057034	0.5614	0.7396
22717	Zfp59	zinc finger protein 59	0.95057034	0.5665	0.7428
216459	Myl6b	myosin, light polypeptide 6B	0.95057034	0.5715	0.7465
170787	Hdac10	histone deacetylase 10	0.95057034	0.5717	0.7467
93896	Glp2r	glucagon-like peptide 2 receptor	0.95057034	0.5738	0.7479
381510	Dpy19l4	dpy-19-like 4 (C. elegans)	0.95057034	0.584	0.7551
66435	Uggt2	UDP-glucose glycoprotein glucosyltransferase 2	0.95057034	0.6063	0.7725
16907	Lmnb2	lamin B2	0.95057034	0.6174	0.7797
74741	5730419I09Rik	RIKEN cDNA 5730419I09 gene	0.95057034	0.6245	0.7852
18948	Pnmt	phenylethanolamine-N-methyltransferase	0.95057034	0.6277	0.7879
14226	Fkbp1b	FK506 binding protein 1b	0.95057034	0.6313	0.7905
22685	Zfp239	zinc finger protein 239	0.95057034	0.6378	0.7949
69944	2810021J22Rik	RIKEN cDNA 2810021J22 gene	0.95057034	0.6436	0.7984
67184	Ndufa13	NADH dehydrogenase (ubiquinone) 1 alpha subcomplex, 13	0.95057034	0.651	0.8034
105675	Ppif	peptidylprolyl isomerase F (cyclophilin F)	0.95057034	0.6537	0.8045
12854	Cort	cortistatin	0.95057034	0.6545	0.805
75599	Pcdh1	protocadherin 1	0.95057034	0.6625	0.8097
665180	Clec2l	C-type lectin domain family, member L	0.95057034	0.6755	0.8184
20897	Stra6	stimulated by retinoic acid gene 6	0.95057034	0.6771	0.8193

224647	D17Wsu92e	DNA segment, Chr 17, Wayne State University 92, expressed	0.95057034	0.7109	0.8412
246179	Fktn	fukutin	0.95057034	0.7141	0.8435
64082	Popdc2	popeye domain containing 2	0.95057034	0.7205	0.8473
108013	Celf4	CUGBP, Elav-like family member 4	0.95057034	0.7282	0.8521
21812	Tgfbr1	transforming growth factor, beta receptor I	0.95057034	0.7362	0.8574
56846	Necab3	N-terminal EF-hand calcium binding protein 3	0.95057034	0.7374	0.8578
23797	Akt3	thymoma viral proto-oncogene 3	0.95057034	0.7514	0.8663
666168	Cyp4a31	cytochrome P450, family 4, subfamily a, polypeptide 31	0.95057034	0.7922	0.8911
381334	Gal3st2	galactose-3-O-sulfotransferase 2	0.95057034	0.8086	0.9006
100038485	Gm10398	predicted gene 10398	0.95057034	0.9248	0.9618
76895	Bicd2	bicaudal D homolog 2 (Drosophila)	0.94966762	0.1987	NA
79264	Krit1	KRIT1, ankyrin repeat containing	0.94966762	0.2164	NA
231887	Pdap1	PDGFA associated protein 1	0.94966762	0.2515	NA
102857	Slc6a8	solute carrier family 6 (neurotransmitter transporter, creatine), member 8	0.94966762	0.2704	NA
72701	Zfp618	zinc fingerprotein 618	0.94966762	0.2744	NA
56876	Nelf	nasal embryonic LHRH factor	0.94966762	0.2761	NA
321008	6330408A02Rik	RIKEN cDNA 6330408A02 gene	0.94966762	0.3097	NA
68202	Ndufa5	NADH dehydrogenase (ubiquinone) 1 alpha subcomplex, 5	0.94966762	0.3116	NA
227054	Rpl23a-ps1	ribosomal protein 23A, pseudogene 1	0.94966762	0.3219	NA
236537	Zfp352	zinc finger protein 352	0.94966762	0.3271	NA
75454	Phpt1	phosphohistidine phosphatase 1	0.94966762	0.3402	NA
244962	Snx14	sorting nexin 14	0.94966762	0.3475	NA
210582	Coq10a	coenzyme Q10 homolog A (yeast)	0.94966762	0.3563	NA
78408	Fam131a	family with sequence similarity 131, member A	0.94966762	0.3591	NA
67515	Ttc33	tetratricopeptide repeat domain 33	0.94966762	0.4002	NA
15077	Hist2h3c1	histone cluster 2, H3c1	0.94966762	0.4055	NA
18854	Pml	promyelocytic leukemia	0.94966762	0.406	NA
12371	Casp9	caspase 9	0.94966762	0.4187	NA
66849	Ppp1r2	protein phosphatase 1, regulatory (inhibitor) subunit 2	0.94966762	0.4284	NA
320127	Dgki	diacylglycerol kinase, iota	0.94966762	0.4423	NA
26914	H2afy	H2A histone family, member Y	0.94966762	0.4483	NA
64658	Mrps25	mitochondrial ribosomal protein S25	0.94966762	0.4616	NA
56032	Nprl2	nitrogen permease regulator-like 2 (S. cerevisiae)	0.94966762	0.4674	NA
16661	Krt10	keratin 10	0.94966762	0.4925	NA
76893	Lass2	LAG1 homolog, ceramide synthase 2	0.94966762	0.4935	NA
56543	Kcnd3	potassium voltage-gated channel, Shal-related family, member 3	0.94966762	0.5046	NA
27041	G3bp1	Ras-GTPase-activating protein SH3-domain binding protein 1	0.94966762	0.5213	NA
100710	Pds5b	PDS5, regulator of cohesion maintenance, homolog B (S. cerevisiae)	0.94966762	0.5262	NA
66799	Ube2w	ubiquitin-conjugating enzyme E2W (putative)	0.94966762	0.5463	0.7282
21389	Tbx6	T-box 6	0.94966762	0.5494	0.7311
236848	BC023829	cDNA sequence BC023829	0.94966762	0.5543	0.7345
69900	Mfsd11	major facilitator superfamily domain containing 11	0.94966762	0.5597	0.7385
78890	2310079F23Rik	RIKEN cDNA 2310079F23 gene	0.94966762	0.5616	0.7396
15569	Elavl2	ELAV (embryonic lethal, abnormal vision, Drosophila)-like 2 (Hu antigen B)	0.94966762	0.5696	0.745
229782	Slc35a3	solute carrier family 35 (UDP-N-acetylglucosamine (UDP-GlcNAc) transporter), member 3	0.94966762	0.5967	0.7653
13803	Enc1	ectodermal-neural cortex 1	0.94966762	0.5972	0.7658
15369	Hmox2	heme oxygenase (decycling) 2	0.94966762	0.6152	0.7784
414077	BC056474	cDNA sequence BC056474	0.94966762	0.6153	0.7784
23948	Mmp17	matrix metallopeptidase 17	0.94966762	0.6484	0.8018
13627	Eef1a1	eukaryotic translation elongation factor 1 alpha 1	0.94966762	0.6604	0.8088

22029	Traf1	TNF receptor-associated factor 1	0.94966762	0.6983	0.8328
52808	Tspyl2	TSPY-like 2	0.94966762	0.7026	0.8356
258294	Olfr1115	olfactory receptor 1115	0.94966762	0.7397	0.8592
22412	Wnt9b	wingless-type MMTV integration site 9B	0.94966762	0.7725	0.8789
16333	Ins1	insulin I	0.94966762	0.7973	0.8934
632687	Mar-10	membrane-associated ring finger (C3HC4) 10	0.94966762	0.8851	0.9408
244646	Pkd1l3	polycystic kidney disease 1 like 3	0.94966762	0.8886	0.9428
72354	Ttc4	tetratricopeptide repeat domain 4	0.9487666	0.188	NA
68917	Hint2	histidine triad nucleotide binding protein 2	0.9487666	0.2374	NA
66593	Diablo	diablo homolog (Drosophila)	0.9487666	0.2525	NA
233902	Fbxl19	F-box and leucine-rich repeat protein 19	0.9487666	0.2572	NA
71999	Fbxo22	F-box protein 22	0.9487666	0.279	NA
108946	Zzz3	zinc finger, ZZ domain containing 3	0.9487666	0.3266	NA
235626	Setd2	SET domain containing 2	0.9487666	0.334	NA
66576	Uqcrh	ubiquinol-cytochrome c reductase hinge protein	0.9487666	0.3376	NA
78798	Eml4	echinoderm microtubule associated protein like 4	0.9487666	0.3403	NA
20947	Swap70	SWA-70 protein	0.9487666	0.3478	NA
100101807	1700047I17Rik2	RIKEN cDNA 1700047I17 gene 2	0.9487666	0.3695	NA
70434	2610201A13Rik	RIKEN cDNA 2610201A13 gene	0.9487666	0.3734	NA
23988	Pin1	protein (peptidyl-prolyl cis/trans isomerase) NIMA-interacting 1	0.9487666	0.3826	NA
225289	AW554918	expressed sequence AW554918	0.9487666	0.383	NA
414072	BC031361	cDNA sequence BC031361	0.9487666	0.3899	NA
78394	Ddx52	DEAD (Asp-Glu-Ala-Asp) box polypeptide 52	0.9487666	0.3966	NA
108705	Pttg1ip	pituitary tumor-transforming 1 interacting protein	0.9487666	0.4051	NA
52469	Ccdc56	coiled-coil domain containing 56	0.9487666	0.4089	NA
20844	Stam	signal transducing adaptor molecule (SH3 domain and ITAM motif) 1	0.9487666	0.4103	NA
100532	Rell1	RELT-like 1	0.9487666	0.4224	NA
22066	Trpc4	transient receptor potential cation channel, subfamily C, member 4	0.9487666	0.4395	NA
72826	Fam76b	family with sequence similarity 76, member B	0.9487666	0.4433	NA
66190	Acer3	alkaline ceramidase 3	0.9487666	0.4475	NA
21411	Tcf20	transcription factor 20	0.9487666	0.4572	NA
230484	Usp1	ubiquitin specific peptidase 1	0.9487666	0.4628	NA
77207	8030425K09Rik	RIKEN cDNA 8030425K09 gene	0.9487666	0.4644	NA
208677	Creb3l3	cAMP responsive element binding protein 3-like 3	0.9487666	0.4675	NA
11781	Ap4m1	adaptor-related protein complex AP-4, mu 1	0.9487666	0.468	NA
243043	Kctd8	potassium channel tetramerisation domain containing 8	0.9487666	0.4695	NA
231871	Daglb	diacylglycerol lipase, beta	0.9487666	0.4768	NA
13559	E2f5	E2F transcription factor 5	0.9487666	0.4777	NA
15387	Hnrnpk	heterogeneous nuclear ribonucleoprotein K	0.9487666	0.485	NA
16568	Kif3a	kinesin family member 3A	0.9487666	0.5047	NA
228807	Zfp341	zinc finger protein 341	0.9487666	0.5105	NA
246102	Rttn	rotatin	0.9487666	0.523	NA
54403	Slc4a4	solute carrier family 4 (anion exchanger), member 4	0.9487666	0.5245	NA
22678	Zfp2	zinc finger protein 2	0.9487666	0.5691	0.7447
171207	Arhgap4	Rho GTPase activating protein 4	0.9487666	0.5827	0.7543
100503330	LOC100503330	hypothetical LOC100503330	0.9487666	0.583	0.7543
58180	Hic2	hypermethylated in cancer 2	0.9487666	0.5992	0.7674
67803	Limd2	LIM domain containing 2	0.9487666	0.604	0.7708
228858	Gdap1l1	ganglioside-induced differentiation-associated protein 1-like 1	0.9487666	0.6087	0.7736
11677	Akr1b3	aldo-keto reductase family 1, member B3 (aldose reductase)	0.9487666	0.6148	0.7781

218973	Wdhd1	WD repeat and HMG-box DNA binding protein 1	0.9487666	0.6311	0.7903
215512	Fam117a	family with sequence similarity 117, memberA	0.9487666	0.6329	0.7915
74749	5830405M20Rik	RIKEN cDNA 5830405M20 gene	0.9487666	0.6523	0.8039
238328	Vash1	vasohibin 1	0.9487666	0.6801	0.8213
13426	Dync1i1	dynein cytoplasmic 1 intermediate chain 1	0.9487666	0.686	0.825
81907	Tmem108	transmembrane protein 108	0.9487666	0.686	0.825
665211	Gm14326	predicted gene 14326	0.9487666	0.7082	0.8395
258772	Olfr821	olfactory receptor 821	0.9487666	0.7149	0.844
67112	Fgf22	fibroblast growth factor 22	0.9487666	0.7238	0.8495
18998	Pou4f3	POU domain, class 4, transcription factor 3	0.9487666	0.726	0.8512
68675	Fam172a	family with sequence similarity 172, member A	0.9487666	0.7314	0.8547
217232	Cdc27	cell division cycle 27 homolog (S. cerevisiae)	0.9487666	0.7322	0.8553
109294	Prex2	phosphatidylinositol-3,4,5-trisphosphate-dependent Rac exchange factor 2	0.9487666	0.8084	0.9006
93746	Gprc5d	G protein-coupled receptor, family C, group 5, member D	0.9487666	0.8358	0.9152
213696	Duoxa1	dual oxidase maturation factor 1	0.9487666	0.8746	0.9352
19181	Psmc2	proteasome (prosome, macropain) 26S subunit, ATPase 2	0.9478673	0.2219	NA
66866	Nhlrc2	NHL repeat containing 2	0.9478673	0.2466	NA
268294	Zbtb24	zinc finger and BTB domain containing 24	0.9478673	0.2576	NA
24071	Synj2bp	synaptojanin 2 binding protein	0.9478673	0.2745	NA
67942	Atp5g2	ATP synthase, H+ transporting, mitochondrial F0 complex, subunit C2 (subunit 9)	0.9478673	0.2795	NA
320504	5930403N24Rik	RIKEN cDNA 5930403N24 gene	0.9478673	0.2799	NA
15387	Hnrnpk	heterogeneous nuclear ribonucleoprotein K	0.9478673	0.3085	NA
16588	Kin	antigenic determinant of rec-A protein	0.9478673	0.314	NA
66369	Dus2l	dihydrouridine synthase 2-like (SMM1, S. cerevisiae)	0.9478673	0.3251	NA
19896	Rpl10a	ribosomal protein L10A	0.9478673	0.3544	NA
20300	Ccl25	chemokine (C-C motif) ligand 25	0.9478673	0.3828	NA
231128	Fam193a	family with sequence similarity 193, member A	0.9478673	0.3852	NA
17763	Mtcp1	mature T-cell proliferation 1	0.9478673	0.3937	NA
19126	Prom1	prominin 1	0.9478673	0.4043	NA
338366	Mia3	melanoma inhibitory activity 3	0.9478673	0.4104	NA
23790	Coro1c	coronin, actin binding protein 1C	0.9478673	0.413	NA
74775	Lmbr1l	limb region 1 like	0.9478673	0.4407	NA
70088	Meaf6	MYST/Esa1-associated factor 6	0.9478673	0.4422	NA
13384	Mpp3	membrane protein, palmitoylated 3 (MAGUK p55 subfamily member 3)	0.9478673	0.4494	NA
73873	Fam161a	family with sequence similarity 161, member A	0.9478673	0.4604	NA
208990	Npb	neuropeptide B	0.9478673	0.4717	NA
320376	Bcorl1	BCL6 co-repressor-like 1	0.9478673	0.4877	NA
328417	Parp4	poly (ADP-ribose) polymerase family, member 4	0.9478673	0.5153	NA
55980	Impa1	inositol (myo)-1(or 4)-monophosphatase 1	0.9478673	0.5197	NA
170772	Glcci1	glucocorticoid induced transcript 1	0.9478673	0.5334	0.7181
57751	Rnf25	ring finger protein 25	0.9478673	0.5629	0.7406
108800	Ston2	stonin 2	0.9478673	0.5771	0.7502
233208	Scaf1	SR-related CTD-associated factor 1	0.9478673	0.5823	0.7539
75623	1700029F09Rik	RIKEN cDNA 1700029F09 gene	0.9478673	0.6114	0.7757
76612	Lrrc27	leucine rich repeat containing 27	0.9478673	0.6169	0.7795
18786	Plaa	phospholipase A2, activating protein	0.9478673	0.6308	0.7902
381884	Slc6a16	solute carrier family 6, member 16	0.9478673	0.634	0.792
71367	Chst9	carbohydrate (N-acetylgalactosamine 4-0) sulfotransferase 9	0.9478673	0.6496	0.8027
380785	Begain	brain-enriched guanylate kinase-associated	0.9478673	0.6605	0.8088
243308	A430033K04Rik	RIKEN cDNA A430033K04 gene	0.9478673	0.6682	0.8135

380614	Intu	inturned planar cell polarity effector homolog (Drosophila)	0.9478673	0.6698	0.8146
66705	Dnase1l2	deoxyribonuclease 1-like 2	0.9478673	0.6974	0.8327
24018	Rngtt	RNA guanylyltransferase and 5'-phosphatase	0.9478673	0.7027	0.8357
216739	Acsl6	acyl-CoA synthetase long-chain family member 6	0.9478673	0.7165	0.8451
78670	Plekhj1	pleckstrin homology domain containing, family J member 1	0.9478673	0.7392	0.8589
74182	Gpcpd1	glycerophosphocholine phosphodiesterase GDE1 homolog (S. cerevisiae)	0.9478673	0.7478	0.8641
209683	Ttc28	tetratricopeptide repeat domain 28	0.9478673	0.7508	0.8661
243308	A430033K04Rik	RIKEN cDNA A430033K04 gene	0.9478673	0.7726	0.8789
21371	Tbca	tubulin cofactor A	0.9478673	0.784	0.8869
230376	Haus6	HAUS augmin-like complex, subunit 6	0.9478673	0.8267	0.9104
114666	Krtap5-5	keratin associated protein 5-5	0.9478673	0.8372	0.9158
234699	Edc4	enhancer of mRNA decapping 4	0.9469697	0.1996	NA
13871	Ercc2	excision repair cross-complementing rodent repair deficiency, complementation group 2	0.9469697	0.2208	NA
56637	Gsk3b	glycogen synthase kinase 3 beta	0.9469697	0.2467	NA
69064	1810014F10Rik	RIKEN cDNA 1810014F10 gene	0.9469697	0.262	NA
67804	Snx2	sorting nexin 2	0.9469697	0.2767	NA
328234	Rnf182	ring finger protein 182	0.9469697	0.3241	NA
218294	Cdc14b	CDC14 cell division cycle 14 homolog B (S. cerevisiae)	0.9469697	0.3385	NA
218989	6720456H20Rik	RIKEN cDNA 6720456H20 gene	0.9469697	0.3438	NA
330780	E130110O22Rik	RIKEN cDNA E130110O22 gene	0.9469697	0.3557	NA
66632	Atpbd4	ATP binding domain 4	0.9469697	0.361	NA
66118	Sarnp	SAP domain containing ribonucleoprotein	0.9469697	0.3647	NA
14479	Usp15	ubiquitin specific peptidase 15	0.9469697	0.3689	NA
56445	Dnaja2	DnaJ (Hsp40) homolog, subfamily A, member 2	0.9469697	0.3704	NA
11364	Acadm	acyl-Coenzyme A dehydrogenase, medium chain	0.9469697	0.3789	NA
72284	Oraov1	oral cancer overexpressed 1	0.9469697	0.3812	NA
94091	Trim11	tripartite motif-containing 11	0.9469697	0.4172	NA
237782	Smcr8	Smith-Magenis syndrome chromosome region, candidate 8 homolog (human)	0.9469697	0.4198	NA
18763	Pkd1	polycystic kidney disease 1 homolog	0.9469697	0.4312	NA
74577	Glb1l	galactosidase, beta 1-like	0.9469697	0.4426	NA
67006	Cisd2	CDGSH iron sulfur domain 2	0.9469697	0.4435	NA
216439	Agap2	ArfGAP with GTPase domain, ankyrin repeat and PH domain 2	0.9469697	0.4477	NA
54170	Rragc	Ras-related GTP binding C	0.9469697	0.4555	NA
68058	Chd1l	chromodomain helicase DNA binding protein 1-like	0.9469697	0.4714	NA
13180	Pcbd1	pterin 4 alpha carbinolamine dehydratase/dimerization cofactor of hepatocyte nuclear factor 1 alpha (TCF1) 1	0.9469697	0.4815	NA
319415	Hs3st5	heparan sulfate (glucosamine) 3-O-sulfotransferase 5	0.9469697	0.4848	NA
320234	Ccdc66	coiled-coil domain containing 66	0.9469697	0.4879	NA
434249	Gm5602	predicted gene 5602	0.9469697	0.4924	NA
225655	Slmo1	slowmo homolog 1 (Drosophila)	0.9469697	0.4972	NA
100043732	Gm4613	60S ribosomal protein L37a pseudogene	0.9469697	0.4977	NA
233865	D430042O09Rik	RIKEN cDNA D430042O09 gene	0.9469697	0.5123	NA
330409	Cecr2	cat eye syndrome chromosome region, candidate 2 homolog (human)	0.9469697	0.5133	NA
640502	LOC640502	UDP-N-acetylhexosamine pyrophosphorylase-like	0.9469697	0.5134	NA
12695	Inadl	InaD-like (Drosophila)	0.9469697	0.5289	0.7148
239364	Tspyl5	testis-specific protein, Y-encoded-like 5	0.9469697	0.5373	0.7215
108888	Atad3a	ATPase family, AAA domain containing 3A	0.9469697	0.54	0.7233
239570	Ttc38	tetratricopeptide repeat domain 38	0.9469697	0.5737	0.7479
105835	Sgsm3	small G protein signaling modulator 3	0.9469697	0.5792	0.7516
320860	B130021B11Rik	RIKEN cDNA B130021B11 gene	0.9469697	0.5882	0.7584
208643	Eif4g1	eukaryotic translation initiation factor 4, gamma 1	0.9469697	0.6037	0.7706

442803	A830005F24Rik	RIKEN cDNA A830005F24 gene	0.9469697	0.6178	0.78
170771	Khdrbs2	KH domain containing, RNA binding, signal transduction associated 2	0.9469697	0.6387	0.7954
399566	Btbd6	BTB (POZ) domain containing 6	0.9469697	0.6408	0.7968
14534	Kat2a	K(lysine) acetyltransferase 2A	0.9469697	0.6477	0.8012
245537	Nlgn3	neuroligin 3	0.9469697	0.653	0.8041
94187	Zfp423	zinc finger protein 423	0.9469697	0.6537	0.8045
66268	Pigyl	phosphatidylinositol glycan anchor biosynthesis, class Y-like	0.9469697	0.6609	0.809
75173	4930544O15Rik	RIKEN cDNA 4930544O15 gene	0.9469697	0.6682	0.8135
83922	Tsga14	testis specific gene A14	0.9469697	0.6689	0.8139
58869	Pex5l	peroxisomal biogenesis factor 5-like	0.9469697	0.6697	0.8146
71997	1500002O20Rik	RIKEN cDNA 1500002O20 gene	0.9469697	0.6988	0.8333
224111	Ubxn7	UBX domain protein 7	0.9469697	0.6999	0.8337
15257	Hipk1	homeodomain interacting protein kinase 1	0.9469697	0.723	0.849
381346	Gm13194	predicted gene 13194	0.9469697	0.7458	0.8631
232791	Cnot3	CCR4-NOT transcription complex, subunit 3	0.9469697	0.763	0.8742
627788	Gm6788	predicted gene 6788	0.9469697	0.7765	0.8812
209456	Trp53bp2	transformation related protein 53 binding protein 2	0.9469697	0.7954	0.8927
233833	Tnrc6a	trinucleotide repeat containing 6a	0.94607379	0.1887	NA
14000	Drosha	drosha, ribonuclease type III	0.94607379	0.1949	NA
54712	Plxnc1	plexin C1	0.94607379	0.2074	NA
68194	Ndufb4	NADH dehydrogenase (ubiquinone) 1 beta subcomplex 4	0.94607379	0.2171	NA
14050	Eya3	eyes absent 3 homolog (Drosophila)	0.94607379	0.2429	NA
30059	Timm10	translocase of inner mitochondrial membrane 10 homolog (yeast)	0.94607379	0.2669	NA
53328	Pgrmc1	progesterone receptor membrane component 1	0.94607379	0.2733	NA
29877	Hdgfrp3	hepatoma-derived growth factor, related protein 3	0.94607379	0.3022	NA
240087	Mdc1	mediator of DNA damage checkpoint 1	0.94607379	0.3123	NA
328530	D030063E12	hypothetical protein D030063E12	0.94607379	0.3141	NA
26413	Mapk1	mitogen-activated protein kinase 1	0.94607379	0.3201	NA
21823	Th	tyrosine hydroxylase	0.94607379	0.3238	NA
338359	Supv3l1	suppressor of var1, 3-like 1 (S. cerevisiae)	0.94607379	0.346	NA
11950	Atp5f1	ATP synthase, H+ transporting, mitochondrial F0 complex, subunit B1	0.94607379	0.3506	NA
347740	2900097C17Rik	RIKEN cDNA 2900097C17 gene	0.94607379	0.3826	NA
215707	Ccdc92	coiled-coil domain containing 92	0.94607379	0.384	NA
67676	Rpp21	ribonuclease P 21 subunit (human)	0.94607379	0.3853	NA
619441	BC096441	cDNA sequence BC096441	0.94607379	0.3861	NA
54418	Fmn2	formin 2	0.94607379	0.3881	NA
108156	Mthfd1	methylenetetrahydrofolate dehydrogenase (NADP+ dependent), methenyltetrahydrofolate cyclohydrolase, formyltetrahydrofolate synthase	0.94607379	0.4041	NA
30050	Fbxw2	F-box and WD-40 domain protein 2	0.94607379	0.4363	NA
66432	Slc7a6os	solute carrier family 7, member 6 opposite strand	0.94607379	0.4379	NA
12861	Cox6a1	cytochrome c oxidase, subunit VI a, polypeptide 1	0.94607379	0.5205	0.7089
319352	C530028O21Rik	RIKEN cDNA C530028O21 gene	0.94607379	0.5274	0.7138
271457	Rab5a	RAB5A, member RAS oncogene family	0.94607379	0.5377	0.7216
20411	Sorbs1	sorbin and SH3 domain containing 1	0.94607379	0.5385	0.7222
67998	Fam134c	family with sequence similarity 134, member C	0.94607379	0.545	0.727
69536	Hemk1	HemK methyltransferase family member 1	0.94607379	0.556	0.7358
217030	Synrg	synergin, gamma	0.94607379	0.56	0.7386
101867	Rrp8	ribosomal RNA processing 8, methyltransferase, homolog (yeast)	0.94607379	0.5641	0.7416
14600	Ghr	growth hormone receptor	0.94607379	0.5658	0.7423
241296	Lrrc8a	leucine rich repeat containing 8A	0.94607379	0.5719	0.7468
211712	Pcdh9	protocadherin 9	0.94607379	0.5755	0.7491

11843	Arf4	ADP-ribosylation factor 4	0.94607379	0.5838	0.755
11518	Add1	adducin 1 (alpha)	0.94607379	0.6	0.7679
217951	Tmem196	transmembrane protein 196	0.94607379	0.6004	0.7682
243833	Zfp128	zinc finger protein 128	0.94607379	0.6169	0.7795
238384	Slc24a4	solute carrier family 24 (sodium/potassium/calcium exchanger), member 4	0.94607379	0.6342	0.7921
333088	Kcp	kielin/chordin-like protein	0.94607379	0.6475	0.8012
226539	Dars2	aspartyl-tRNA synthetase 2 (mitochondrial)	0.94607379	0.6531	0.8042
209497	Tmem164	transmembrane protein 164	0.94607379	0.6971	0.8326
12519	Cd80	CD80 antigen	0.94607379	0.6981	0.8328
621239	Nhlrc4	NHL repeat containing 4	0.94607379	0.7255	0.8506
15366	Hmmr	hyaluronan mediated motility receptor (RHAMM)	0.94607379	0.7413	0.86
110084	Dnahc1	dynein, axonemal, heavy chain 1	0.94607379	0.7658	0.8756
76187	Adhfe1	alcohol dehydrogenase, iron containing, 1	0.94607379	0.7815	0.885
97683	C76332	expressed sequence C76332	0.94607379	0.8388	0.9169
71745	Cul2	cullin 2	0.94517958	0.1703	NA
243923	Rgs9bp	regulator of G-protein signalling 9 binding protein	0.94517958	0.2087	NA
68041	Mid1ip1	Mid1 interacting protein 1 (gastrulation specific G12-like (zebrafish))	0.94517958	0.221	NA
228858	Gdap1l1	ganglioside-induced differentiation-associated protein 1-like 1	0.94517958	0.2574	NA
76089	Rapgef2	Rap guanine nucleotide exchange factor (GEF) 2	0.94517958	0.2576	NA
18557	Cdk18	cyclin-dependent kinase 18	0.94517958	0.3047	NA
66359	Fam36a	family with sequence similarity 36, member A	0.94517958	0.3151	NA
29864	Rnf11	ring finger protein 11	0.94517958	0.3314	NA
76167	Snrnp35	small nuclear ribonucleoprotein 35 (U11/U12)	0.94517958	0.336	NA
70967	4931408A02Rik	RIKEN cDNA 4931408A02 gene	0.94517958	0.347	NA
194237	Rimkla	ribosomal modification protein rimK-like family member A	0.94517958	0.3487	NA
14160	Lgr5	leucine rich repeat containing G protein coupled receptor 5	0.94517958	0.3602	NA
20688	Sp4	trans-acting transcription factor 4	0.94517958	0.3778	NA
223649	Nrbp2	nuclear receptor binding protein 2	0.94517958	0.3814	NA
110033	Kif22	kinesin family member 22	0.94517958	0.3869	NA
56468	Socs5	suppressor of cytokine signaling 5	0.94517958	0.387	NA
56292	Naa10	N(alpha)-acetyltransferase 10, NatA catalytic subunitNalpha acetyltransferase 10	0.94517958	0.3951	NA
269587	Epb4.1	erythrocyte protein band 4.1	0.94517958	0.3959	NA
67544	Fam120b	family with sequence similarity 120, member B	0.94517958	0.4105	NA
382014	Ano8	anoctamin 8	0.94517958	0.4248	NA
23950	Dnajb6	DnaJ (Hsp40) homolog, subfamily B, member 6	0.94517958	0.4381	NA
77305	Wdr82	WD repeat domain containing 82	0.94517958	0.4395	NA
19211	Pten	phosphatase and tensin homolog	0.94517958	0.4402	NA
13429	Dnm1	dynamin 1	0.94517958	0.4633	NA
20773	Sptlc2	serine palmitoyltransferase, long chain base subunit 2	0.94517958	0.4753	NA
216134	Pdxk	pyridoxal (pyridoxine, vitamin B6) kinase	0.94517958	0.4766	NA
66881	Pcyox1	prenylcysteine oxidase 1	0.94517958	0.4774	NA
72195	Supt7l	suppressor of Ty 7 (S. cerevisiae)-like	0.94517958	0.4807	NA
114642	Brdt	bromodomain, testis-specific	0.94517958	0.5326	0.7176
13010	Cst3	cystatin C	0.94517958	0.5347	0.7194
21353	Tank	TRAF family member-associated Nf-kappa B activator	0.94517958	0.5465	0.7283
68910	Zfp467	zinc finger protein 467	0.94517958	0.5509	0.7319
14381	G6pdx	glucose-6-phosphate dehydrogenase X-linked	0.94517958	0.5637	0.7412
68981	Snrpa1	small nuclear ribonucleoprotein polypeptide A'	0.94517958	0.5648	0.742
67930	1700101I11Rik	RIKEN cDNA 1700101I11 gene	0.94517958	0.5739	0.7479
17855	Mvk	mevalonate kinase	0.94517958	0.5817	0.7535

67231	Tbc1d20	TBC1 domain family, member 20	0.94517958	0.5923	0.7618
71128	4933417C20Rik	RIKEN cDNA 4933417C20 gene	0.94517958	0.5979	0.7665
54673	Sh3glb1	SH3-domain GRB2-like B1 (endophilin)	0.94517958	0.5989	0.7672
69188	Mll5	myeloid/lymphoid or mixed-lineage leukemia 5	0.94517958	0.6196	0.7816
18984	Por	P450 (cytochrome) oxidoreductase	0.94517958	0.6274	0.7877
16648	Kpna3	karyopherin (importin) alpha 3	0.94517958	0.6453	0.7995
50721	Sirt6	sirtuin 6 (silent mating type information regulation 2, homolog) 6 (S. cerevisiae)	0.94517958	0.6478	0.8013
66687	Tbc1d15	TBC1 domain family, member 15	0.94517958	0.6517	0.8036
228359	Arhgap1	Rho GTPase activating protein 1	0.94517958	0.6675	0.8128
19231	Ptma	prothymosin alpha	0.94517958	0.68	0.8212
319953	Ttll1	tubulin tyrosine ligase-like 1	0.94517958	0.6802	0.8213
67048	Vma21	VMA21 vacuolar H+-ATPase homolog (S. cerevisiae)	0.94517958	0.7108	0.8412
18559	Pctp	phosphatidylcholine transfer protein	0.94517958	0.7277	0.8519
81840	Sorcs2	sortilin-related VPS10 domain containing receptor 2	0.94517958	0.7474	0.8639
20981	Syt3	synaptotagmin III	0.94517958	0.7477	0.864
73490	Mipol1	mirror-image polydactyly gene 1 homolog (human)	0.94517958	0.7497	0.8654
27204	Syn3	synapsin III	0.94517958	0.7529	0.8673
20855	Stc1	stanniocalcin 1	0.94517958	0.7571	0.8701
227800	Rabgap1	RAB GTPase activating protein 1	0.94517958	0.7643	0.875
78655	Eif3j	eukaryotic translation initiation factor 3, subunit J	0.94517958	0.8021	0.8961
21854	Timm17a	translocase of inner mitochondrial membrane 17a	0.94428706	0.2015	NA
213990	Agap3	ArfGAP with GTPase domain, ankyrin repeat and PH domain 3	0.94428706	0.2194	NA
13682	Eif4a2	eukaryotic translation initiation factor 4A2	0.94428706	0.2281	NA
68147	Gar1	GAR1 ribonucleoprotein homolog (yeast)	0.94428706	0.2355	NA
28169	Agpat3	1-acylglycerol-3-phosphate O-acyltransferase 3	0.94428706	0.2407	NA
66950	Tmem206	transmembrane protein 206	0.94428706	0.2792	NA
78266	Zfp687	zinc finger protein 687	0.94428706	0.2973	NA
73886	4930431P19Rik	RIKEN cDNA 4930431P19 gene	0.94428706	0.3078	NA
67755	Ddx47	DEAD (Asp-Glu-Ala-Asp) box polypeptide 47	0.94428706	0.3101	NA
52846	D1Bwg0212e	DNA segment, Chr 1, Brigham & Women's Genetics 0212 expressed	0.94428706	0.3116	NA
56874	Rnf32	ring finger protein 32	0.94428706	0.3166	NA
192232	Hps4	Hermansky-Pudlak syndrome 4 homolog (human)	0.94428706	0.3226	NA
11797	Birc2	baculoviral IAP repeat-containing 2	0.94428706	0.3277	NA
225372	Apbb3	amyloid beta (A4) precursor protein-binding, family B, member 3	0.94428706	0.346	NA
67471	Gpatch1	G patch domain containing 1	0.94428706	0.3558	NA
74383	Ubap2l	ubiquitin associated protein 2-like	0.94428706	0.3692	NA
432508	Cpsf6	cleavage and polyadenylation specific factor 6	0.94428706	0.376	NA
14169	Fgf14	fibroblast growth factor 14	0.94428706	0.3764	NA
227699	Nup188	nucleoporin 188	0.94428706	0.3839	NA
11841	Arf2	ADP-ribosylation factor 2	0.94428706	0.3856	NA
108043	Chrnb3	cholinergic receptor, nicotinic, beta polypeptide 3	0.94428706	0.3889	NA
73389	Hbp1	high mobility group box transcription factor 1	0.94428706	0.3898	NA
67254	2900011O08Rik	RIKEN cDNA 2900011O08 gene	0.94428706	0.3941	NA
100317	AU040320	expressed sequence AU040320	0.94428706	0.3942	NA
101476	Plekha1	pleckstrin homology domain containing, family A (phosphoinositide binding specific) member 1	0.94428706	0.3947	NA
66531	2310061C15Rik	RIKEN cDNA 2310061C15 gene	0.94428706	0.4004	NA
219103	Cenpj	centromere protein J	0.94428706	0.4087	NA
22260	Nr1h2	nuclear receptor subfamily 1, group H, member 2	0.94428706	0.413	NA
269639	Zfp512	zinc finger protein 512	0.94428706	0.4142	NA
69372	Mocs3	molybdenum cofactor synthesis 3	0.94428706	0.4169	NA

75007	Fam63a	family with sequence similarity 63, member A	0.94428706	0.4216	NA
18986	Pou2f1	POU domain, class 2, transcription factor 1	0.94428706	0.4288	NA
105445	Dock9	dedicator of cytokinesis 9	0.94428706	0.4326	NA
19289	Igdcc3	immunoglobulin superfamily, DCC subclass, member 3	0.94428706	0.4412	NA
78885	Coro7	coronin 7	0.94428706	0.4461	NA
72720	Zfp248	zinc finger protein 248	0.94428706	0.4604	NA
12569	Cdk5r1	cyclin-dependent kinase 5, regulatory subunit 1 (p35)	0.94428706	0.4637	NA
67030	Fancl	Fanconi anemia, complementation group L	0.94428706	0.4683	NA
16923	Sh2b3	SH2B adaptor protein 3	0.94428706	0.47	NA
13819	Epas1	endothelial PAS domain protein 1	0.94428706	0.4835	NA
84653	Hes7	hairy and enhancer of split 7 (Drosophila)	0.94428706	0.5026	0.6945
54127	Rps28	ribosomal protein S28	0.94428706	0.5035	0.695
192775	Kcnh6	potassium voltage-gated channel, subfamily H (eag-related), member 6	0.94428706	0.5439	0.7261
52626	Cdkn2aipnl	CDKN2A interacting protein N-terminal like	0.94428706	0.5474	0.7291
66089	Rmnd5b	required for meiotic nuclear division 5 homolog B (S. cerevisiae)	0.94428706	0.5601	0.7386
56738	Mocs1	molybdenum cofactor synthesis 1	0.94428706	0.5659	0.7423
18519	Kat2b	K(lysine) acetyltransferase 2B	0.94428706	0.5725	0.7473
72774	Neil1	nei endonuclease VIII-like 1 (E. coli)	0.94428706	0.5791	0.7515
269180	Inpp4a	inositol polyphosphate-4-phosphatase, type I	0.94428706	0.5904	0.7603
231602	P2rx2	purinergic receptor P2X, ligand-gated ion channel, 2	0.94428706	0.6027	0.7696
69312	Lrrc67	leucine rich repeat containing 67	0.94428706	0.6133	0.7771
26385	Grk6	G protein-coupled receptor kinase 6	0.94428706	0.6183	0.7804
243771	Parp12	poly (ADP-ribose) polymerase family, member 12	0.94428706	0.6198	0.7816
106264	0610012G03Rik	RIKEN cDNA 0610012G03 gene	0.94428706	0.6384	0.7952
12896	Cpt2	carnitine palmitoyltransferase 2	0.94428706	0.6421	0.7976
17939	Naga	N-acetyl galactosaminidase, alpha	0.94428706	0.661	0.809
140546	Eri3	exoribonuclease 3	0.94428706	0.6628	0.8099
67062	Mcart6	mitochondrial carrier triple repeat 6	0.94428706	0.6651	0.8114
20473	Six3	sine oculis-related homeobox 3 homolog (Drosophila)	0.94428706	0.6713	0.8158
69714	Tfpt	TCF3 (E2A) fusion partner	0.94428706	0.6846	0.8238
21763	Tex2	testis expressed gene 2	0.94428706	0.7078	0.8392
229694	AI504432	expressed sequence AI504432	0.94428706	0.7151	0.8441
234695	Rltpr	RGD motif, leucine rich repeats, tropomodulin domain and proline-rich containing	0.94428706	0.7194	0.8467
331487	Uprt	uracil phosphoribosyltransferase (FUR1) homolog (S. cerevisiae)	0.94428706	0.7261	0.8512
241070	Gpr1	G protein-coupled receptor 1	0.94428706	0.7608	0.8726
80901	Cxcr6	chemokine (C-X-C motif) receptor 6	0.94428706	0.8103	0.9016
100042947	Gm14816	predicted gene 14816	0.94428706	0.8533	0.9246
67219	Med18	mediator of RNA polymerase II transcription, subunit 18 homolog (yeast)	0.94339623	0.1937	NA
28113	Tinf2	Terf1 (TRF1)-interacting nuclear factor 2	0.94339623	0.225	NA
320024	Nceh1	arylacetamide deacetylase-like 1	0.94339623	0.2434	NA
320162	Ccdc45	coiled-coil domain containing 45	0.94339623	0.2579	NA
71330	Rcbtb1	regulator of chromosome condensation (RCC1) and BTB (POZ) domain containing protein 1	0.94339623	0.2623	NA
20463	Cox7a2l	cytochrome c oxidase subunit VIIa polypeptide 2-like	0.94339623	0.2628	NA
212647	Aldh4a1	aldehyde dehydrogenase 4 family, member A1	0.94339623	0.2969	NA
68767	ORF19	open reading frame 19	0.94339623	0.301	NA
102334	Ankrd10	ankyrin repeat domain 10	0.94339623	0.3049	NA
83431	Ndel1	nuclear distribution gene E-like homolog 1 (A. nidulans)	0.94339623	0.3121	NA
276846	Pigs	phosphatidylinositol glycan anchor biosynthesis, class S	0.94339623	0.3125	NA
170762	Nup155	nucleoporin 155	0.94339623	0.3532	NA
21408	Zfp354a	zinc finger protein 354A	0.94339623	0.3539	NA

68421	Lmbrd1	LMBR1 domain containing 1	0.94339623	0.3716	NA
66335	Atp6v1c1	ATPase, H+ transporting, lysosomal V1 subunit C1	0.94339623	0.3749	NA
223753	Cerk	ceramide kinase	0.94339623	0.3873	NA
80707	Wwox	WW domain-containing oxidoreductase	0.94339623	0.3904	NA
625249	Gpx4	glutathione peroxidase 4	0.94339623	0.4061	NA
216516	Ccdc157	coiled-coil domain containing 157	0.94339623	0.4101	NA
22652	Mkrn3	makorin, ring finger protein, 3	0.94339623	0.4209	NA
668272	Gm9079	transmembrane emp24 domain trafficking protein 2 pseudogene	0.94339623	0.4252	NA
109186	6720427I07Rik	RIKEN cDNA 6720427I07 gene	0.94339623	0.4388	NA
100038587	Gm10278	predicted gene 10278	0.94339623	0.4446	NA
66962	2310047B19Rik	RIKEN cDNA 2310047B19 gene	0.94339623	0.4642	NA
75541	1700019G17Rik	RIKEN cDNA 1700019G17 gene	0.94339623	0.4847	NA
22698	Zfp39	zinc finger protein 39	0.94339623	0.5235	0.7105
209003	Rbmx2	RNA binding motif protein, X-linked 2	0.94339623	0.533	0.7178
224093	Fam43a	family with sequence similarity 43, member A	0.94339623	0.5349	0.7196
101861	Ints4	integrator complex subunit 4	0.94339623	0.5957	0.7646
75616	2810008M24Rik	RIKEN cDNA 2810008M24 gene	0.94339623	0.6022	0.7693
104836	Cbll1	Casitas B-lineage lymphoma-like 1	0.94339623	0.6062	0.7725
69227	2810407C02Rik	RIKEN cDNA 2810407C02 gene	0.94339623	0.608	0.7732
56404	Trip4	thyroid hormone receptor interactor 4	0.94339623	0.6092	0.7741
75906	Fam184a	family with sequence similarity 184, member A	0.94339623	0.6249	0.7856
227094	Tmem194b	transmembrane protein 194B	0.94339623	0.6275	0.7877
269623	C030048B08Rik	RIKEN cDNA C030048B08 gene	0.94339623	0.6373	0.7946
244550	Podnl1	podocan-like 1	0.94339623	0.654	0.8045
109202	A930024E05Rik	RIKEN cDNA A930024E05 gene	0.94339623	0.6633	0.8104
223989	4921513D23Rik	RIKEN cDNA 4921513D23 gene	0.94339623	0.6638	0.8106
100041879	Gm3561	predicted gene 3561	0.94339623	0.6872	0.8259
105859	Csdc2	cold shock domain containing C2, RNA binding	0.94339623	0.7295	0.853
433791	Gm13251	predicted gene 13251	0.94339623	0.7324	0.8555
70408	Polr3f	polymerase (RNA) III (DNA directed) polypeptide F	0.94339623	0.783	0.8861
545192	Baiap3	BAI1-associated protein 3	0.94339623	0.7899	0.8901
381405	Zfp663	zinc finger protein 663	0.94339623	0.8153	0.9045
266459	Gm5039	eukaryotic translation initiation factor 1A pseudogene	0.94339623	0.9349	0.9668
67533	Ppfibp1	PTPRF interacting protein, binding protein 1 (liprin beta 1)	0.94250707	0.2167	NA
13855	Epn2	epsin 2	0.94250707	0.2308	NA
51897	Atg13	ATG13 autophagy related 13 homolog (S. cerevisiae)	0.94250707	0.2312	NA
14645	Glul	glutamate-ammonia ligase (glutamine synthetase)	0.94250707	0.2454	NA
225010	Lclat1	lysocardiolipin acyltransferase 1	0.94250707	0.2564	NA
104871	Spata7	spermatogenesis associated 7	0.94250707	0.2749	NA
13207	Ddx5	DEAD (Asp-Glu-Ala-Asp) box polypeptide 5	0.94250707	0.2769	NA
219105	Zmym5	zinc finger, MYM-type 5	0.94250707	0.2846	NA
18583	Pde7a	phosphodiesterase 7A	0.94250707	0.2854	NA
52120	Hgsnat	heparan-alpha-glucosaminide N-acetyltransferase	0.94250707	0.2908	NA
12892	Cpox	coproporphyrinogen oxidase	0.94250707	0.3013	NA
74370	Rptor	regulatory associated protein of MTOR, complex 1	0.94250707	0.3092	NA
21974	Top2b	topoisomerase (DNA) II beta	0.94250707	0.3175	NA
15982	Ifrd1	interferon-related developmental regulator 1	0.94250707	0.3177	NA
114863	Prosc	proline synthetase co-transcribed	0.94250707	0.3192	NA
56426	Pdcd10	programmed cell death 10	0.94250707	0.3196	NA
20479	Vps4b	vacuolar protein sorting 4b (yeast)	0.94250707	0.3351	NA

67285	Cwc27	CWC27 spliceosome-associated protein homolog (S. cerevisiae)	0.94250707	0.3498	NA
54204	Sep-01	septin 1	0.94250707	0.3501	NA
66114	Dnajc30	DnaJ (Hsp40) homolog, subfamily C, member 30	0.94250707	0.3667	NA
93683	Glce	glucuronyl C5-epimerase	0.94250707	0.3765	NA
19934	Rpl22	ribosomal protein L22	0.94250707	0.3812	NA
97287	Mtmr14	myotubularin related protein 14	0.94250707	0.3864	NA
381979	Brsk1	BR serine/threonine kinase 1	0.94250707	0.3888	NA
76650	Srxn1	sulfiredoxin 1 homolog (S. cerevisiae)	0.94250707	0.4056	NA
107701	Sf3b4	splicing factor 3b, subunit 4	0.94250707	0.4119	NA
18521	Pcbp2	poly(rC) binding protein 2	0.94250707	0.4252	NA
654788	4732440D04Rik	RIKEN cDNA 4732440D04 gene	0.94250707	0.4317	NA
100504423	LOC100504423	hypothetical protein LOC100504423	0.94250707	0.4363	NA
77573	Vps33a	vacuolar protein sorting 33A (yeast)	0.94250707	0.4614	NA
209815	Tbc1d25	TBC1 domain family, member 25	0.94250707	0.467	NA
240023	Pnldc1	poly(A)-specific ribonuclease (PARN)-like domain containing 1	0.94250707	0.5034	0.695
107239	Carns1	carnosine synthase 1	0.94250707	0.52	0.7087
70207	Taco1	translational activator of mitochondrially encoded cytochrome c oxidase I	0.94250707	0.5258	0.7125
72296	Rusc1	RUN and SH3 domain containing 1	0.94250707	0.5426	0.7253
71835	Lancl2	LanC (bacterial lantibiotic synthetase component C)-like 2	0.94250707	0.5548	0.7348
14595	B4galt1	UDP-Gal:betaGlcNAc beta 1,4- galactosyltransferase, polypeptide 1	0.94250707	0.5577	0.7372
18993	Pou3f3	POU domain, class 3, transcription factor 3	0.94250707	0.569	0.7446
19775	Xpr1	xenotropic and polytropic retrovirus receptor 1	0.94250707	0.5733	0.7476
23805	Apc2	adenomatosis polyposis coli 2	0.94250707	0.5867	0.7572
99011	Pomt1	protein-O-mannosyltransferase 1	0.94250707	0.5898	0.7597
212986	Scfd2	Sec1 family domain containing 2	0.94250707	0.5992	0.7674
11432	Acp2	acid phosphatase 2, lysosomal	0.94250707	0.6005	0.7682
74443	P4htm	prolyl 4-hydroxylase, transmembrane (endoplasmic reticulum)	0.94250707	0.614	0.7776
103844	Inca1	inhibitor of CDK, cyclin A1 interacting protein 1	0.94250707	0.6176	0.7799
214345	Lrrc1	leucine rich repeat containing 1	0.94250707	0.6224	0.7834
234384	Mpv17l2	MPV17 mitochondrial membrane protein-like 2	0.94250707	0.6233	0.7841
67263	Zswim6	zinc finger, SWIM domain containing 6	0.94250707	0.63	0.7896
75660	Lin37	lin-37 homolog (C. elegans)	0.94250707	0.6305	0.7901
19726	Rfx3	regulatory factor X, 3 (influences HLA class II expression)	0.94250707	0.6336	0.7918
66139	Tmem8c	transmembrane protein 8C	0.94250707	0.6439	0.7987
26456	Sema4g	sema domain, immunoglobulin domain (Ig), transmembrane domain (TM) and short cytoplasmic domain, (semaphorin) 4G	0.94250707	0.6513	0.8034
17690	Msi1	Musashi homolog 1(Drosophila)	0.94250707	0.6552	0.8056
52398	Sep-11	septin 11	0.94250707	0.6563	0.806
57434	Xrcc2	X-ray repair complementing defective repair in Chinese hamster cells 2	0.94250707	0.6636	0.8105
238161	Akap6	A kinase (PRKA) anchor protein 6	0.94250707	0.6649	0.8113
432611	Dnaic2	dynein, axonemal, intermediate chain 2	0.94250707	0.7029	0.8357
230775	Bai2	brain-specific angiogenesis inhibitor 2	0.94250707	0.7127	0.8425
100505017	LOC100505017	putative Pol polyprotein-like	0.94250707	0.7129	0.8426
225845	Pla2g16	phospholipase A2, group XVI	0.94250707	0.7332	0.8561
74159	Acbd5	acyl-Coenzyme A binding domain containing 5	0.94250707	0.7639	0.8748
100039086	Gm15032	predicted gene 15032	0.94250707	0.8507	0.9235
19881	Rom1	rod outer segment membrane protein 1	0.94161959	0.1426	NA
68964	1500010J02Rik	RIKEN cDNA 1500010J02 gene	0.94161959	0.1466	NA
60596	Gucy1a3	guanylate cyclase 1, soluble, alpha 3	0.94161959	0.1738	NA
16882	Lig3	ligase III, DNA, ATP-dependent	0.94161959	0.1974	NA
78912	Sp2	Sp2 transcription factor	0.94161959	0.2012	NA

68512	Tomm5	translocase of outer mitochondrial membrane 5 homolog (yeast)	0.94161959	0.2088	NA
233103	4931406P16Rik	RIKEN cDNA 4931406P16 gene	0.94161959	0.2162	NA
80883	Ntng1	netrin G1	0.94161959	0.229	NA
56282	Mrpl12	mitochondrial ribosomal protein L12	0.94161959	0.2381	NA
56748	Nfu1	NFU1 iron-sulfur cluster scaffold homolog (S. cerevisiae)	0.94161959	0.2488	NA
52592	Brms1l	breast cancer metastasis-suppressor 1-like	0.94161959	0.2533	NA
13430	Dnm2	dynamin 2	0.94161959	0.2765	NA
20301	Ccl27a	chemokine (C-C motif) ligand 27A	0.94161959	0.2781	NA
67500	Ccar1	cell division cycle and apoptosis regulator 1	0.94161959	0.2875	NA
106298	Rrn3	RRN3 RNA polymerase I transcription factor homolog (yeast)	0.94161959	0.2981	NA
18637	Pfdn2	prefoldin 2	0.94161959	0.3053	NA
106326	Osbpl11	oxysterol binding protein-like 11	0.94161959	0.3159	NA
97114	Hist2h3c2-ps	histone cluster 2, H3c2, pseudogene	0.94161959	0.3592	NA
74438	Clvs1	clavesin 1	0.94161959	0.3756	NA
216869	Arrb2	arrestin, beta 2	0.94161959	0.3813	NA
21413	Tcf4	transcription factor 4	0.94161959	0.3855	NA
319448	Fndc3a	fibronectin type III domain containing 3A	0.94161959	0.3981	NA
74244	Atg7	autophagy-related 7 (yeast)	0.94161959	0.4007	NA
73447	Wdr13	WD repeat domain 13	0.94161959	0.4246	NA
19157	Cyth1	cytohesin 1	0.94161959	0.4356	NA
21423	Tcf3	transcription factor 3	0.94161959	0.4385	NA
19365	Rad52	RAD52 homolog (S. cerevisiae)	0.94161959	0.4642	NA
12426	Cckbr	cholecystokinin B receptor	0.94161959	0.4679	NA
227449	Zcchc2	zinc finger, CCHC domain containing 2	0.94161959	0.4718	NA
16443	Itsn1	intersectin 1 (SH3 domain protein 1A)	0.94161959	0.4841	0.6795
66315	Senp7	SUMO1/sentrin specific peptidase 7	0.94161959	0.4895	0.6838
226977	Actr1b	ARP1 actin-related protein 1 homolog B, centractin beta (yeast)	0.94161959	0.4989	0.6914
21885	Tle1	transducin-like enhancer of split 1, homolog of Drosophila E(spl)	0.94161959	0.5141	0.7042
230991	B930041F14Rik	RIKEN cDNA B930041F14 gene	0.94161959	0.5156	0.7051
18293	Ogdh	oxoglutarate dehydrogenase (lipoamide)	0.94161959	0.5241	0.7109
53357	Pla2g6	phospholipase A2, group VI	0.94161959	0.533	0.7178
666060	Frmpd1	FERM and PDZ domain containing 1	0.94161959	0.5413	0.7243
214663	Slc25a29	solute carrier family 25 (mitochondrial carrier, palmitoylcarnitine transporter), member 29	0.94161959	0.55	0.7313
402724	A330106F07Rik	RIKEN cDNA A330106F07 gene	0.94161959	0.5599	0.7386
66225	Llph	LLP homolog, long-term synaptic facilitation (Aplysia)	0.94161959	0.5995	0.7676
13855	Epn2	epsin 2	0.94161959	0.6538	0.8045
12416	Cbx2	chromobox homolog 2 (Drosophila Pc class)	0.94161959	0.6903	0.8283
97715	C79015	expressed sequence C79015	0.94161959	0.7143	0.8436
12153	Bmp1	bone morphogenetic protein 1	0.94161959	0.7483	0.8644
319934	Sbf2	SET binding factor 2	0.94161959	0.7499	0.8654
83679	Pde4dip	phosphodiesterase 4D interacting protein (myomegalin)	0.94161959	0.7557	0.8692
93871	Brwd1	bromodomain and WD repeat domain containing 1	0.94073377	0.2012	NA
16330	Inpp5b	inositol polyphosphate-5-phosphatase B	0.94073377	0.2033	NA
232906	Grlf1	glucocorticoid receptor DNA binding factor 1	0.94073377	0.2498	NA
17850	Mut	methylmalonyl-Coenzyme A mutase	0.94073377	0.2504	NA
338371	A730011L01Rik	RIKEN cDNA A730011L01 gene	0.94073377	0.2598	NA
109785	Pgm3	phosphoglucomutase 3	0.94073377	0.2645	NA
67966	Zcchc10	zinc finger, CCHC domain containing 10	0.94073377	0.2702	NA
54484	Mkrn1	makorin, ring finger protein, 1	0.94073377	0.2717	NA
52858	Cdipt	CDP-diacylglycerol--inositol 3-phosphatidyltransferase (phosphatidylinositol synthase)	0.94073377	0.2719	NA

66689	Klhl28	kelch-like 28 (Drosophila)	0.94073377	0.2819	NA
50754	Fbxw7	F-box and WD-40 domain protein 7	0.94073377	0.2836	NA
78232	Trappc6b	trafficking protein particle complex 6B	0.94073377	0.2863	NA
26569	Slc27a4	solute carrier family 27 (fatty acid transporter), member 4	0.94073377	0.3047	NA
12651	Chkb	choline kinase beta	0.94073377	0.3071	NA
21750	Terf2	telomeric repeat binding factor 2	0.94073377	0.3118	NA
53356	Eif3g	eukaryotic translation initiation factor 3, subunit G	0.94073377	0.3119	NA
236732	Rbm10	RNA binding motif protein 10	0.94073377	0.3145	NA
105239	Rnf44	ring finger protein 44	0.94073377	0.3341	NA
11519	Add2	adducin 2 (beta)	0.94073377	0.3755	NA
110109	Nop2	NOP2 nucleolar protein homolog (yeast)	0.94073377	0.3772	NA
69162	Sec31a	Sec31 homolog A (S. cerevisiae)	0.94073377	0.3897	NA
20346	Sema3a	sema domain, immunoglobulin domain (Ig), short basic domain, secreted, (semaphorin) 3A	0.94073377	0.3942	NA
74570	Zkscan1	zinc finger with KRAB and SCAN domains 1	0.94073377	0.3945	NA
64337	Gng13	guanine nucleotide binding protein (G protein), gamma 13	0.94073377	0.3961	NA
100042453	Gm3852	predicted gene 3852	0.94073377	0.3974	NA
67416	Armcx2	armadillo repeat containing, X-linked 2	0.94073377	0.3999	NA
241568	Lrrc4c	leucine rich repeat containing 4C	0.94073377	0.4016	NA
226154	Lzts2	leucine zipper, putative tumor suppressor 2	0.94073377	0.4017	NA
28071	Twistnb	TWIST neighbor	0.94073377	0.4092	NA
170760	Acbd3	acyl-Coenzyme A binding domain containing 3	0.94073377	0.4112	NA
13382	Dld	dihydrolipoamide dehydrogenase	0.94073377	0.4144	NA
53325	Banp	BTG3 associated nuclear protein	0.94073377	0.4156	NA
11964	Atp6v1a	ATPase, H+ transporting, lysosomal V1 subunit A	0.94073377	0.4238	NA
68737	Angel1	angel homolog 1 (Drosophila)	0.94073377	0.4324	NA
229279	Hnrnpa3	heterogeneous nuclear ribonucleoprotein A3	0.94073377	0.4334	NA
93679	Trim8	tripartite motif-containing 8	0.94073377	0.4361	NA
214459	Fnbp1l	formin binding protein 1-like	0.94073377	0.4429	NA
320722	A330050F15Rik	RIKEN cDNA A330050F15 gene	0.94073377	0.4622	NA
13492	Drd5	dopamine receptor D5	0.94073377	0.4649	NA
100047183	LOC100047183	protein AHNAK2-like	0.94073377	0.4664	0.6662
76608	Hectd3	HECT domain containing 3	0.94073377	0.4691	0.6686
66897	Naa16	N(alpha)-acetyltransferase 16, NatA auxiliary subunit	0.94073377	0.4811	0.6773
237847	Rtn4rl1	reticulon 4 receptor-like 1	0.94073377	0.483	0.6786
76527	Il34	interleukin 34	0.94073377	0.4872	0.6823
192289	Tmlhe	trimethyllysine hydroxylase, epsilon	0.94073377	0.489	0.6836
245670	Rragb	Ras-related GTP binding B	0.94073377	0.5131	0.7037
216825	Usp22	ubiquitin specific peptidase 22	0.94073377	0.5364	0.721
74189	Phactr3	phosphatase and actin regulator 3	0.94073377	0.5436	0.7261
59057	Zfp191	zinc finger protein 191	0.94073377	0.5641	0.7416
19762	Rit2	Ras-like without CAAX 2	0.94073377	0.5732	0.7476
66931	1700010I14Rik	RIKEN cDNA 1700010I14 gene	0.94073377	0.5734	0.7476
26409	Map3k7	mitogen-activated protein kinase kinase kinase 7	0.94073377	0.5739	0.7479
20972	Syngr1	synaptogyrin 1	0.94073377	0.5803	0.7525
140500	Acap3	ArfGAP with coiled-coil, ankyrin repeat and PH domains 3	0.94073377	0.5938	0.7631
100039138	Gm2061	predicted gene 2061	0.94073377	0.5958	0.7646
225895	Taf6l	TAF6-like RNA polymerase II, p300/CBP-associated factor (PCAF)-associated factor	0.94073377	0.6001	0.7679
66315	Senp7	SUMO1/sentrin specific peptidase 7	0.94073377	0.6388	0.7954
19704	Upf1	UPF1 regulator of nonsense transcripts homolog (yeast)	0.94073377	0.6487	0.802
16598	Klf2	Kruppel-like factor 2 (lung)	0.94073377	0.6577	0.8066

78514	Arhgap10	Rho GTPase activating protein 10	0.94073377	0.7182	0.846
381073	Npw	neuropeptide W	0.94073377	0.7188	0.8464
16775	Lama4	laminin, alpha 4	0.94073377	0.7222	0.8485
103968	Plin1	perilipin 1	0.94073377	0.7561	0.8694
433416	Gm13547	predicted gene 13547	0.94073377	0.7641	0.875
230126	Shb	src homology 2 domain-containing transforming protein B	0.94073377	0.771	0.8785
12180	Smyd1	SET and MYND domain containing 1	0.94073377	0.7894	0.8899
621852	Rhox3f	reproductive homeobox 3F	0.94073377	0.8138	0.9035
18415	Hspa4l	heat shock protein 4 like	0.93984962	0.1367	NA
225849	Ppp2r5b	protein phosphatase 2, regulatory subunit B (B56), beta isoform	0.93984962	0.1494	NA
74157	Ftsjd2	FtsJ methyltransferase domain containing 2	0.93984962	0.1529	NA
69875	Ndufa11	NADH dehydrogenase (ubiquinone) 1 alpha subcomplex 11	0.93984962	0.1633	NA
22334	Vdac2	voltage-dependent anion channel 2	0.93984962	0.1701	NA
12593	Cdyl	chromodomain protein, Y chromosome-like	0.93984962	0.1857	NA
71673	Rnf215	ring finger protein 215	0.93984962	0.2017	NA
213993	A630007B06Rik	RIKEN cDNA A630007B06 gene	0.93984962	0.2058	NA
100047292	LOC100047292	pleckstrin homology domain-containing family A member 7-like	0.93984962	0.2192	NA
19056	Ppp3cb	protein phosphatase 3, catalytic subunit, beta isoform	0.93984962	0.2282	NA
211401	Mtss1	metastasis suppressor 1	0.93984962	0.2312	NA
246179	Fktn	fukutin	0.93984962	0.2398	NA
71382	Pex1	peroxisomal biogenesis factor 1	0.93984962	0.2487	NA
229714	Gpr61	G protein-coupled receptor 61	0.93984962	0.2494	NA
237943	Gpatch8	G patch domain containing 8	0.93984962	0.2655	NA
633385	Gm7111	predicted gene 7111	0.93984962	0.2812	NA
56196	Tdp2	tyrosyl-DNA phosphodiesterase 2	0.93984962	0.2884	NA
235339	Dlat	dihydrolipoamide S-acetyltransferase (E2 component of pyruvate dehydrogenase complex)	0.93984962	0.2905	NA
216860	Neurl4	neuralized homolog 4 (Drosophila)	0.93984962	0.3202	NA
20617	Snca	synuclein, alpha	0.93984962	0.3282	NA
68953	Chmp2a	chromatin modifying protein 2A	0.93984962	0.3414	NA
56382	Rab9	RAB9, member RAS oncogene family	0.93984962	0.3443	NA
20597	Smpd1	sphingomyelin phosphodiesterase 1, acid lysosomal	0.93984962	0.346	NA
13063	Cycs	cytochrome c, somatic	0.93984962	0.3559	NA
214627	Papd5	PAP associated domain containing 5	0.93984962	0.3591	NA
14615	Gjc1	gap junction protein, gamma 1	0.93984962	0.3715	NA
240215	Slc4a9	solute carrier family 4, sodium bicarbonate cotransporter, member 9	0.93984962	0.3963	NA
21787	Tfg	Trk-fused gene	0.93984962	0.3965	NA
22668	Sf1	splicing factor 1	0.93984962	0.4036	NA
234463	Tmem184c	transmembrane protein 184C	0.93984962	0.4054	NA
231386	Ythdc1	YTH domain containing 1	0.93984962	0.4077	NA
621580	Gm13308	predicted gene 13308	0.93984962	0.4247	NA
56743	Lat2	linker for activation of T cells family, member 2	0.93984962	0.428	NA
68666	Svop	SV2 related protein	0.93984962	0.4333	NA
67713	Dnajc19	DnaJ (Hsp40) homolog, subfamily C, member 19	0.93984962	0.436	NA
68777	Tmem53	transmembrane protein 53	0.93984962	0.4608	0.6615
53970	Rfx5	regulatory factor X, 5 (influences HLA class II expression)	0.93984962	0.4659	0.6658
67027	Mkrn2	makorin, ring finger protein, 2	0.93984962	0.4677	0.6675
208691	Eif5a2	eukaryotic translation initiation factor 5A2	0.93984962	0.4882	0.6829
72776	Sass6	spindle assembly 6 homolog (C. elegans)	0.93984962	0.5238	0.7108
77578	Bcl9	B-cell CLL/lymphoma 9	0.93984962	0.5276	0.7138
109900	Asl	argininosuccinate lyase	0.93984962	0.5381	0.722

75723	Amotl1	angiomotin-like 1	0.93984962	0.5393	0.7228
11744	Anxa11	annexin A11	0.93984962	0.5441	0.7262
268319	BC025920	zinc finger protein pseudogene	0.93984962	0.5498	0.7313
669149	Vmn2r88	vomeronasal 2, receptor 88	0.93984962	0.5602	0.7386
67016	Tbc1d2b	TBC1 domain family, member 2B	0.93984962	0.5659	0.7423
116905	Dph1	DPH1 homolog (S. cerevisiae)	0.93984962	0.5772	0.7502
80292	Zxdc	ZXD family zinc finger C	0.93984962	0.5774	0.7503
18577	Pde4a	phosphodiesterase 4A, cAMP specific	0.93984962	0.5785	0.7512
76380	Ccdc46	coiled-coil domain containing 46	0.93984962	0.5848	0.7556
70047	Trnt1	tRNA nucleotidyl transferase, CCA-adding, 1	0.93984962	0.5855	0.7563
75387	Sirt4	sirtuin 4 (silent mating type information regulation 2 homolog) 4 (S. cerevisiae)	0.93984962	0.5908	0.7605
70113	Odf3b	outer dense fiber of sperm tails 3B	0.93984962	0.595	0.7638
12497	Entpd6	ectonucleoside triphosphate diphosphohydrolase 6	0.93984962	0.5965	0.7652
18578	Pde4b	phosphodiesterase 4B, cAMP specific	0.93984962	0.6123	0.7764
192287	Slc25a36	solute carrier family 25, member 36	0.93984962	0.6418	0.7974
432964	K230010J24Rik	RIKEN cDNA K230010J24 gene	0.93984962	0.6735	0.8171
170624	Dep1	diabetic embryopathy 1	0.93984962	0.7017	0.8349
229279	Hnrnpa3	heterogeneous nuclear ribonucleoprotein A3	0.93984962	0.7335	0.8561
259086	Olfr609	olfactory receptor 609	0.93984962	0.7344	0.8565
20981	Syt3	synaptotagmin III	0.93984962	0.7395	0.8591
387343	Tas2r109	taste receptor, type 2, member 109	0.93984962	0.7503	0.8657
171261	Vmn1r87	vomeronasal 1 receptor 87	0.93984962	0.8879	0.9423
66663	Uba5	ubiquitin-like modifier activating enzyme 5	0.93896714	0.1357	NA
83435	Plekha3	pleckstrin homology domain-containing, family A (phosphoinositide binding specific) member 3	0.93896714	0.1442	NA
68734	Smek1	SMEK homolog 1, suppressor of mek1 (Dictyostelium)	0.93896714	0.1605	NA
28185	Tomm70a	translocase of outer mitochondrial membrane 70 homolog A (yeast)	0.93896714	0.1789	NA
75778	Them4	thioesterase superfamily member 4	0.93896714	0.181	NA
208080	Gm514	predicted gene 514	0.93896714	0.1864	NA
68524	Wipf2	WAS/WASL interacting protein family, member 2	0.93896714	0.205	NA
21386	Tbx3	T-box 3	0.93896714	0.2213	NA
66401	Nudt2	nudix (nucleoside diphosphate linked moiety X)-type motif 2	0.93896714	0.2367	NA
68815	Btbd10	BTB (POZ) domain containing 10	0.93896714	0.2411	NA
69163	Mrpl44	mitochondrial ribosomal protein L44	0.93896714	0.2529	NA
105504	Exoc5	exocyst complex component 5	0.93896714	0.2647	NA
14853	Gspt2	G1 to S phase transition 2	0.93896714	0.2673	NA
22040	Trex1	three prime repair exonuclease 1	0.93896714	0.2727	NA
72699	Lime1	Lck interacting transmembrane adaptor 1	0.93896714	0.2825	NA
17151	Ccndbp1	cyclin D-type binding-protein 1	0.93896714	0.2873	NA
21753	Tes	testis derived transcript	0.93896714	0.2999	NA
76614	Immt	inner membrane protein, mitochondrial	0.93896714	0.2999	NA
71336	Rbks	ribokinase	0.93896714	0.3099	NA
22762	Zfpm2	zinc finger protein, multitype 2	0.93896714	0.3116	NA
11416	Slc33a1	solute carrier family 33 (acetyl-CoA transporter), member 1	0.93896714	0.3172	NA
106504	Stk38	serine/threonine kinase 38	0.93896714	0.3279	NA
237943	Gpatch8	G patch domain containing 8	0.93896714	0.33	NA
219249	Tdrd3	tudor domain containing 3	0.93896714	0.3377	NA
93739	Gabarapl2	gamma-aminobutyric acid (GABA) A receptor-associated protein-like 2	0.93896714	0.3412	NA
628779	Hs3st4	heparan sulfate (glucosamine) 3-O-sulfotransferase 4	0.93896714	0.3458	NA
54633	Pqbp1	polyglutamine binding protein 1	0.93896714	0.3762	NA
27393	Mrpl39	mitochondrial ribosomal protein L39	0.93896714	0.3849	NA

70675	Vcpip1	valosin containing protein (p97)/p47 complex interacting protein 1	0.93896714	0.4001	NA
19896	Rpl10a	ribosomal protein L10A	0.93896714	0.4014	NA
108807	4933415E08Rik	RIKEN cDNA 4933415E08 gene	0.93896714	0.419	NA
15975	Ifnar1	interferon (alpha and beta) receptor 1	0.93896714	0.4202	NA
70036	2700023E23Rik	RIKEN cDNA 2700023E23 gene	0.93896714	0.4224	NA
18148	Npm1	nucleophosmin 1	0.93896714	0.4229	NA
235611	Plxnb1	plexin B1	0.93896714	0.4268	NA
320940	Atp11c	ATPase, class VI, type 11C	0.93896714	0.4309	NA
66171	Pgls	6-phosphogluconolactonase	0.93896714	0.4332	NA
230796	Wdtc1	WD and tetratricopeptide repeats 1	0.93896714	0.4364	NA
13528	Dtnb	dystrobrevin, beta	0.93896714	0.4424	NA
14869	Gstp2	glutathione S-transferase, pi 2	0.93896714	0.4591	0.6601
66366	Ergic3	ERGIC and golgi 3	0.93896714	0.4656	0.6656
75914	Exoc6b	exocyst complex component 6B	0.93896714	0.467	0.6669
100504965	LOC100504965	hypothetical LOC100504965	0.93896714	0.4707	0.6697
22694	Zfp35	zinc finger protein 35	0.93896714	0.4717	0.6701
235661	Dync1li1	dynein cytoplasmic 1 light intermediate chain 1	0.93896714	0.488	0.6828
69656	Pir	pirin	0.93896714	0.5196	0.7086
22330	Vcl	vinculin	0.93896714	0.5238	0.7108
74015	Fcho1	FCH domain only 1	0.93896714	0.5276	0.7138
226856	Lpgat1	lysophosphatidylglycerol acyltransferase 1	0.93896714	0.5645	0.7418
230098	E130306D19Rik	RIKEN cDNA E130306D19 gene	0.93896714	0.5677	0.7435
27784	Commd8	COMM domain containing 8	0.93896714	0.5737	0.7479
52898	Rnasek	ribonuclease, RNase K	0.93896714	0.5738	0.7479
381410	Zfp408	zinc finger protein 408	0.93896714	0.605	0.7717
94187	Zfp423	zinc finger protein 423	0.93896714	0.6169	0.7795
67911	Zfp169	zinc finger protein 169	0.93896714	0.6315	0.7905
69297	Lrrc46	leucine rich repeat containing 46	0.93896714	0.64	0.7964
11637	Ak2	adenylate kinase 2	0.93896714	0.6661	0.8118
15257	Hipk1	homeodomain interacting protein kinase 1	0.93896714	0.6673	0.8127
50721	Sirt6	sirtuin 6 (silent mating type information regulation 2, homolog) 6 (S. cerevisiae)	0.93896714	0.7024	0.8356
68235	2410066E13Rik	RIKEN cDNA 2410066E13 gene	0.93896714	0.7084	0.8397
12419	Cbx5	chromobox homolog 5 (Drosophila HP1a)	0.93896714	0.7191	0.8466
22764	Zfx	zinc finger protein X-linked	0.93896714	0.7405	0.8596
319148	Hist1h3c	histone cluster 1, H3c	0.93896714	0.8144	0.904
26940	Ecsit	ECSIT homolog (Drosophila)	0.9380863	0.1526	NA
12454	Ccnk	cyclin K	0.9380863	0.171	NA
66405	Mcts2	malignant T cell amplified sequence 2	0.9380863	0.2018	NA
54563	Nup210	nucleoporin 210	0.9380863	0.2026	NA
71701	Pnpt1	polyribonucleotide nucleotidyltransferase 1	0.9380863	0.2051	NA
18099	Nlk	nemo like kinase	0.9380863	0.2261	NA
214290	Zcchc6	zinc finger, CCHC domain containing 6	0.9380863	0.2342	NA
75497	Fabp12	fatty acid binding protein 12	0.9380863	0.2353	NA
16880	Lifr	leukemia inhibitory factor receptor	0.9380863	0.2514	NA
75782	Lca5	Leber congenital amaurosis 5 (human)	0.9380863	0.2515	NA
76793	Snip1	Smad nuclear interacting protein 1	0.9380863	0.2769	NA
105559	Mbnl2	muscleblind-like 2	0.9380863	0.2909	NA
28028	Mrpl50	mitochondrial ribosomal protein L50	0.9380863	0.2913	NA
71900	Tmem106b	transmembrane protein 106B	0.9380863	0.2975	NA
55988	Snx12	sorting nexin 12	0.9380863	0.303	NA

94187	Zfp423	zinc finger protein 423	0.9380863	0.3051	NA
16401	Itga4	integrin alpha 4	0.9380863	0.3544	NA
14567	Gdi1	guanosine diphosphate (GDP) dissociation inhibitor 1	0.9380863	0.3551	NA
70503	Ddo	D-aspartate oxidase	0.9380863	0.3653	NA
56705	Ranbp9	RAN binding protein 9	0.9380863	0.3662	NA
12314	Calm2	calmodulin 2	0.9380863	0.3807	NA
68708	Rabl2	RAB, member of RAS oncogene family-like 2	0.9380863	0.3828	NA
68832	1110057K04Rik	RIKEN cDNA 1110057K04 gene	0.9380863	0.3834	NA
72930	Ppp2r2b	protein phosphatase 2 (formerly 2A), regulatory subunit B (PR 52), beta isoform	0.9380863	0.4023	NA
74206	Sipa1l3	signal-induced proliferation-associated 1 like 3	0.9380863	0.4033	NA
56436	Adrm1	adhesion regulating molecule 1	0.9380863	0.4091	NA
671641	Gm10063	predicted gene 10063	0.9380863	0.4216	NA
67088	Cand2	cullin-associated and neddylation-dissociated 2 (putative)	0.9380863	0.4219	NA
27886	Dgcr14	DiGeorge syndrome critical region gene 14	0.9380863	0.4329	NA
474156	Zbtb9	zinc finger and BTB domain containing 9	0.9380863	0.4402	NA
72825	Mon1a	MON1 homolog A (yeast)	0.9380863	0.4456	NA
56284	Mrpl19	mitochondrial ribosomal protein L19	0.9380863	0.4534	0.6552
50780	Rgs3	regulator of G-protein signaling 3	0.9380863	0.4546	0.6562
24012	Rgs7	regulator of G protein signaling 7	0.9380863	0.4682	0.6679
18639	Pfkfb1	6-phosphofructo-2-kinase/fructose-2,6-biphosphatase 1	0.9380863	0.4773	0.6744
320208	Tmem91	transmembrane protein 91	0.9380863	0.4835	0.6792
66681	Pgm1	phosphoglucomutase 1	0.9380863	0.4899	0.6843
71151	Eri2	exoribonuclease 2	0.9380863	0.4976	0.6905
15932	Idua	iduronidase, alpha-L-	0.9380863	0.5075	0.6983
67199	Pfdn1	prefoldin 1	0.9380863	0.5144	0.7045
104248	Cabin1	calcineurin binding protein 1	0.9380863	0.5163	0.706
225049	Ttc7	tetratricopeptide repeat domain 7	0.9380863	0.5369	0.7213
58212	Srrm3	serine/arginine repetitive matrix 3	0.9380863	0.5581	0.7377
68034	Fam122a	family with sequence similarity 122, member A	0.9380863	0.5719	0.7468
106251	D230034L24Rik	RIKEN cDNA D230034L24 gene	0.9380863	0.5775	0.7503
67790	Rab39b	RAB39B, member RAS oncogene family	0.9380863	0.5944	0.7636
74996	Usp47	ubiquitin specific peptidase 47	0.9380863	0.6266	0.7869
212377	Mms22l	MMS22-like, DNA repair protein	0.9380863	0.6427	0.7978
75729	4933432B09Rik	RIKEN cDNA 4933432B09 gene	0.9380863	0.645	0.7995
70230	3300002P13Rik	RIKEN cDNA 3300002P13 gene	0.9380863	0.6505	0.8033
71373	Prr16	proline rich 16	0.9380863	0.6601	0.8086
15257	Hipk1	homeodomain interacting protein kinase 1	0.9380863	0.6796	0.8209
74634	4930423D22Rik	RIKEN cDNA 4930423D22 gene	0.9380863	0.691	0.8286
66774	4933439G12Rik	RIKEN cDNA 4933439G12 gene	0.9380863	0.7159	0.8446
11864	Arnt2	aryl hydrocarbon receptor nuclear translocator 2	0.9380863	0.729	0.8528
20445	St6galnac1	ST6 (alpha-N-acetyl-neuraminyl-2,3-beta-galactosyl-1,3)-N-acetylgalactosaminide alpha-2,6-sialyltransferase 1	0.9380863	0.7923	0.8911
14917	Gucy2c	guanylate cyclase 2c	0.9380863	0.8322	0.9135
13011	Cst7	cystatin F (leukocystatin)	0.9380863	0.8395	0.9173
239134	Gucy1b2	guanylate cyclase 1, soluble, beta 2	0.9380863	0.8411	0.918
67968	Ooep	oocyte expressed protein homolog (dog)	0.9380863	0.8699	0.9327
110196	Fdps	farnesyl diphosphate synthetase	0.93720712	0.1621	NA
67427	Rps20	ribosomal protein S20	0.93720712	0.2008	NA
16521	Kcnj5	potassium inwardly-rectifying channel, subfamily J, member 5	0.93720712	0.2038	NA
97130	C77080	expressed sequence C77080	0.93720712	0.211	NA
13196	Asap1	ArfGAP with SH# domain, ankyrin repeat and PH domain1	0.93720712	0.2137	NA

73046	Glrx5	glutaredoxin 5 homolog (S. cerevisiae)	0.93720712	0.2145	NA
68209	Rnaseh2c	ribonuclease H2, subunit C	0.93720712	0.2149	NA
12537	Cdk11b	cyclin-dependent kinase 11B	0.93720712	0.219	NA
68721	1110032A03Rik	RIKEN cDNA 1110032A03 gene	0.93720712	0.2205	NA
11490	Adam15	a disintegrin and metallopeptidase domain 15 (metargidin)	0.93720712	0.2283	NA
58994	Smpd3	sphingomyelin phosphodiesterase 3, neutral	0.93720712	0.2477	NA
66422	Dctpp1	dCTP pyrophosphatase 1	0.93720712	0.2608	NA
192786	Rapgef6	Rap guanine nucleotide exchange factor (GEF) 6	0.93720712	0.3035	NA
213827	Arcn1	archain 1	0.93720712	0.3063	NA
74108	Parn	poly(A)-specific ribonuclease (deadenylation nuclease)	0.93720712	0.3261	NA
268512	Slc26a11	solute carrier family 26, member 11	0.93720712	0.3265	NA
227674	Ddx31	DEAD/H (Asp-Glu-Ala-Asp/His) box polypeptide 31	0.93720712	0.3355	NA
217366	Lrrc45	leucine rich repeat containing 45	0.93720712	0.3408	NA
19133	Prph2	peripherin 2	0.93720712	0.3455	NA
75099	Lysmd4	LysM, putative peptidoglycan-binding, domain containing 4	0.93720712	0.3518	NA
50753	Fbxo8	F-box protein 8	0.93720712	0.3554	NA
21915	Dtymk	deoxythymidylate kinase	0.93720712	0.3731	NA
57431	Dnajc4	DnaJ (Hsp40) homolog, subfamily C, member 4	0.93720712	0.3772	NA
229279	Hnrnpa3	heterogeneous nuclear ribonucleoprotein A3	0.93720712	0.408	NA
16564	Kif21a	kinesin family member 21A	0.93720712	0.4284	NA
75141	Rasd2	RASD family, member 2	0.93720712	0.4284	NA
66877	Crnkl1	Crn, crooked neck-like 1 (Drosophila)	0.93720712	0.433	NA
109910	Zfp91	zinc finger protein 91	0.93720712	0.4792	0.6757
18720	Pip5k1a	phosphatidylinositol-4-phosphate 5-kinase, type 1 alpha	0.93720712	0.4905	0.6848
233011	Itpkc	inositol 1,4,5-trisphosphate 3-kinase C	0.93720712	0.4939	0.6879
18548	Pcsk1	proprotein convertase subtilisin/kexin type 1	0.93720712	0.4972	0.69
241311	Zbtb34	zinc finger and BTB domain containing 34	0.93720712	0.5258	0.7125
60534	Fancg	Fanconi anemia, complementation group G	0.93720712	0.539	0.7227
230996	9430015G10Rik	RIKEN cDNA 9430015G10 gene	0.93720712	0.5438	0.7261
71833	Dcaf7	DDB1 and CUL4 associated factor 7	0.93720712	0.5565	0.7361
78283	Mtap7d2	MAP7 domain containing 2	0.93720712	0.5591	0.7382
18263	Odc1	ornithine decarboxylase, structural 1	0.93720712	0.5596	0.7385
226777	C130074G19Rik	RIKEN cDNA C130074G19 gene	0.93720712	0.5679	0.7437
432582	E130309D14Rik	RIKEN cDNA E130309D14 gene	0.93720712	0.5705	0.7457
100302432	Gm14435	predicted gene 14435	0.93720712	0.5729	0.7474
20672	Sox18	SRY-box containing gene 18	0.93720712	0.599	0.7673
30938	Fgd3	FYVE, RhoGEF and PH domain containing 3	0.93720712	0.6385	0.7953
12361	Cask	calcium/calmodulin-dependent serine protein kinase (MAGUK family)	0.93720712	0.678	0.82
215476	C330019G07Rik	RIKEN cDNA C330019G07 gene	0.93720712	0.7339	0.8564
19052	Ppp2ca	protein phosphatase 2 (formerly 2A), catalytic subunit, alpha isoform	0.93632959	0.1556	NA
72503	2610507B11Rik	RIKEN cDNA 2610507B11 gene	0.93632959	0.1587	NA
70779	Prdm5	PR domain containing 5	0.93632959	0.1599	NA
106618	Wdr90	WD repeat domain 90	0.93632959	0.1847	NA
240068	Zfp563	zinc finger protein 563	0.93632959	0.1931	NA
214469	Fam168b	family with sequence similarity 168, member B	0.93632959	0.1991	NA
217692	Sipa1l1	signal-induced proliferation-associated 1 like 1	0.93632959	0.2069	NA
74187	Katnb1	katanin p80 (WD40-containing) subunit B 1	0.93632959	0.2208	NA
68002	1110058L19Rik	RIKEN cDNA 1110058L19 gene	0.93632959	0.2246	NA
20912	Stxbp3a	syntaxin binding protein 3A	0.93632959	0.2436	NA
59057	Zfp191	zinc finger protein 191	0.93632959	0.2537	NA

94065	Mrpl34	mitochondrial ribosomal protein L34	0.93632959	0.2544	NA
242736	Pramef8	PRAME family member 8	0.93632959	0.2584	NA
76007	Zmym2	zinc finger, MYM-type 2	0.93632959	0.2672	NA
68033	Cox19	COX19 cytochrome c oxidase assembly homolog (S. cerevisiae)	0.93632959	0.2868	NA
16485	Kcna1	potassium voltage-gated channel, shaker-related subfamily, member 1	0.93632959	0.292	NA
381979	Brsk1	BR serine/threonine kinase 1	0.93632959	0.3086	NA
14870	Gstp1	glutathione S-transferase, pi 1	0.93632959	0.315	NA
217980	Larp4b	La ribonucleoprotein domain family, member 4B	0.93632959	0.3179	NA
57294	Rps27	ribosomal protein S27	0.93632959	0.3266	NA
22276	Uros	uroporphyrinogen III synthase	0.93632959	0.3271	NA
76551	Ccdc6	coiled-coil domain containing 6	0.93632959	0.3311	NA
54131	Irf3	interferon regulatory factor 3	0.93632959	0.3314	NA
231803	Mepce	methylphosphate capping enzyme	0.93632959	0.3386	NA
12660	Chka	choline kinase alpha	0.93632959	0.347	NA
19266	Ptprd	protein tyrosine phosphatase, receptor type, D	0.93632959	0.3602	NA
69077	Psmd11	proteasome (prosome, macropain) 26S subunit, non-ATPase, 11	0.93632959	0.3704	NA
11790	Speg	SPEG complex locus	0.93632959	0.3932	NA
22248	Unc119	unc-119 homolog (C. elegans)	0.93632959	0.4046	NA
56433	Vps29	vacuolar protein sorting 29 (S. pombe)	0.93632959	0.4057	NA
236792	Mmgt1	membrane magnesium transporter 1	0.93632959	0.4061	NA
70430	Tbce	tubulin-specific chaperone E	0.93632959	0.4093	NA
72190	2510009E07Rik	RIKEN cDNA 2510009E07 gene	0.93632959	0.4167	NA
72719	2810040C05Rik	RIKEN cDNA 2810040C05 gene	0.93632959	0.4282	NA
67286	Rabl5	RAB, member of RAS oncogene family-like 5	0.93632959	0.438	0.6412
98985	Clp1	CLP1, cleavage and polyadenylation factor I subunit, homolog (S. cerevisiae)	0.93632959	0.453	0.655
66634	Mcm8	minichromosome maintenance deficient 8 (S. cerevisiae)	0.93632959	0.4531	0.655
93681	Zfp192	zinc finger protein 192	0.93632959	0.4549	0.6564
18294	Ogg1	8-oxoguanine DNA-glycosylase 1	0.93632959	0.4715	0.67
26413	Mapk1	mitogen-activated protein kinase 1	0.93632959	0.4794	0.6758
239618	Pdzrn4	PDZ domain containing RING finger 4	0.93632959	0.4798	0.6759
67200	Ccdc77	coiled-coil domain containing 77	0.93632959	0.4881	0.6829
67426	Adck3	aarF domain containing kinase 3	0.93632959	0.4952	0.6886
56469	Pias1	protein inhibitor of activated STAT 1	0.93632959	0.5215	0.7097
107528	Magee1	melanoma antigen, family E, 1	0.93632959	0.529	0.7149
192192	Shkbp1	Sh3kbp1 binding protein 1	0.93632959	0.5376	0.7216
18213	Ntrk3	neurotrophic tyrosine kinase, receptor, type 3	0.93632959	0.5423	0.725
27362	Dnajb9	DnaJ (Hsp40) homolog, subfamily B, member 9	0.93632959	0.5532	0.7336
100043272	5430417L22Rik	RIKEN cDNA 5430417L22 gene	0.93632959	0.562	0.7399
11465	Actg1	actin, gamma, cytoplasmic 1	0.93632959	0.5891	0.7593
70828	4633401B06Rik	RIKEN cDNA 4633401B06 gene	0.93632959	0.611	0.7753
71624	4833411C07Rik	RIKEN cDNA 4833411C07 gene	0.93632959	0.6239	0.7846
216131	Trappc10	trafficking protein particle complex 10	0.93632959	0.6348	0.7926
12326	Camk4	calcium/calmodulin-dependent protein kinase IV	0.93632959	0.6415	0.7973
12921	Crhr1	corticotropin releasing hormone receptor 1	0.93632959	0.6451	0.7995
56697	Akap10	A kinase (PRKA) anchor protein 10	0.93632959	0.6488	0.802
54525	Syt7	synaptotagmin VII	0.93632959	0.6905	0.8284
22631	Ywhaz	tyrosine 3-monooxygenase/tryptophan 5-monooxygenase activation protein, zeta polypeptide	0.93632959	0.7146	0.844
13728	Mark2	MAP/microtubule affinity-regulating kinase 2	0.93632959	0.7198	0.8469
244694	Kdm4d	lysine (K)-specific demethylase 4D	0.93632959	0.7448	0.8624
258507	Olfr96	olfactory receptor 96	0.93632959	0.8641	0.9296

74455	Nsun6	NOL1/NOP2/Sun domain family member 6	0.9354537	0.1601	NA
223648	2410075B13Rik	RIKEN cDNA 2410075B13 gene	0.9354537	0.1605	NA
68192	Leprotl1	leptin receptor overlapping transcript-like 1	0.9354537	0.1988	NA
18975	Polg	polymerase (DNA directed), gamma	0.9354537	0.1993	NA
227723	Prrc2b	proline-rich coiled-coil 2B	0.9354537	0.2072	NA
98403	Zfp451	zinc finger protein 451	0.9354537	0.2127	NA
20740	Spna2	spectrin alpha 2	0.9354537	0.231	NA
110391	Qdpr	quinoid dihydropteridine reductase	0.9354537	0.2351	NA
72194	Fbxl20	F-box and leucine-rich repeat protein 20	0.9354537	0.2372	NA
237422	Ric8b	resistance to inhibitors of cholinesterase 8 homolog B (C. elegans)	0.9354537	0.2575	NA
73516	1700086O06Rik	RIKEN cDNA 1700086O06 gene	0.9354537	0.2595	NA
51813	Ccnc	cyclin C	0.9354537	0.2656	NA
210711	Mcmbp	MCM (minichromosome maintenance deficient) binding protein	0.9354537	0.2686	NA
13508	Dscam	Down syndrome cell adhesion molecule	0.9354537	0.2825	NA
15463	Agfg1	ArfGAP with FG repeats 1	0.9354537	0.2986	NA
66867	Hmg20a	high mobility group 20A	0.9354537	0.3028	NA
338354	Zfp780b	zinc finger protein 780B	0.9354537	0.3089	NA
12974	Cs	citrate synthase	0.9354537	0.3109	NA
22758	Zscan12	zinc finger and SCAN domain containing 12	0.9354537	0.3292	NA
67116	Cuedc2	CUE domain containing 2	0.9354537	0.3432	NA
67881	Mdp1	magnesium-dependent phosphatase 1	0.9354537	0.348	NA
66456	2810001G20Rik	RIKEN cDNA 2810001G20 gene	0.9354537	0.3492	NA
230088	B230312A22Rik	RIKEN cDNA B230312A22 gene	0.9354537	0.3667	NA
99650	4933434E20Rik	RIKEN cDNA 4933434E20 gene	0.9354537	0.3726	NA
387314	Tmtc1	transmembrane and tetratricopeptide repeat containing 1	0.9354537	0.3771	NA
17999	Nedd4	neural precursor cell expressed, developmentally down-regulated 4	0.9354537	0.3889	NA
74018	Als2	amyotrophic lateral sclerosis 2 (juvenile) homolog (human)	0.9354537	0.3904	NA
72421	Ttc30b	tetratricopeptide repeat domain 30B	0.9354537	0.3914	NA
100039199	Gm2095	predicted gene 2095	0.9354537	0.3925	NA
22346	Vhl	von Hippel-Lindau tumor suppressor	0.9354537	0.393	NA
384281	Gatc	glutamyl-tRNA(Gln) amidotransferase, subunit C homolog (bacterial)	0.9354537	0.3941	NA
23988	Pin1	protein (peptidyl-prolyl cis/trans isomerase) NIMA-interacting 1	0.9354537	0.4074	NA
103551	E130012A19Rik	RIKEN cDNA E130012A19 gene	0.9354537	0.4201	NA
66408	Aptx	aprataxin	0.9354537	0.4229	NA
64379	Irx6	Iroquois related homeobox 6 (Drosophila)	0.9354537	0.4235	NA
102193	Zdhhc7	zinc finger, DHHC domain containing 7	0.9354537	0.4261	NA
18854	Pml	promyelocytic leukemia	0.9354537	0.4438	0.6467
229504	Isg20l2	interferon stimulated exonuclease gene 20-like 2	0.9354537	0.446	0.6487
69179	Tmem110	transmembrane protein 110	0.9354537	0.4591	0.6601
22129	Ttc3	tetratricopeptide repeat domain 3	0.9354537	0.4712	0.67
226517	Smg7	Smg-7 homolog, nonsense mediated mRNA decay factor (C. elegans)	0.9354537	0.4826	0.6783
57138	Slc12a5	solute carrier family 12, member 5	0.9354537	0.504	0.6955
12728	Clcn5	chloride channel 5	0.9354537	0.5156	0.7051
14562	Gdf3	growth differentiation factor 3	0.9354537	0.5242	0.711
192197	Bcas3	breast carcinoma amplified sequence 3	0.9354537	0.5365	0.7211
83703	Dbr1	debranching enzyme homolog 1 (S. cerevisiae)	0.9354537	0.5395	0.723
78373	Nudt17	nudix (nucleoside diphosphate linked moiety X)-type motif 17	0.9354537	0.5944	0.7636
13199	Ddn	dendrin	0.9354537	0.5981	0.7665
224902	Safb2	scaffold attachment factor B2	0.9354537	0.5981	0.7665
245007	Zbtb38	zinc finger and BTB domain containing 38	0.9354537	0.6485	0.8019

381570	Oog2	oogenesin 2	0.9354537	0.6664	0.8119
67070	Lsm14a	LSM14 homolog A (SCD6, S. cerevisiae)	0.9354537	0.6722	0.8165
67579	Cpeb4	cytoplasmic polyadenylation element binding protein 4	0.9354537	0.6905	0.8284
233187	Lim2	lens intrinsic membrane protein 2	0.9354537	0.7525	0.8669
320862	A730054J21Rik	RIKEN cDNA A730054J21 gene	0.9354537	0.7617	0.8734
20724	Serpinb5	serine (or cysteine) peptidase inhibitor, clade B, member 5	0.9354537	0.7712	0.8785
74304	1700094M24Rik	RIKEN cDNA 1700094M24 gene	0.9354537	0.8275	0.9109
72252	1700022A21Rik	glycerol-3-phosphate dehydrogenase 1-like pseudogene	0.9354537	0.9075	0.9529
80517	Herpud2	HERPUD family member 2	0.93457944	0.1201	NA
101023	Zfp513	zinc finger protein 513	0.93457944	0.127	NA
22110	Tspyl1	testis-specific protein, Y-encoded-like 1	0.93457944	0.129	NA
83965	Enpp5	ectonucleotide pyrophosphatase/phosphodiesterase 5	0.93457944	0.1334	NA
66822	Fbxo25	F-box protein 25	0.93457944	0.1529	NA
217143	Gpr179	G protein-coupled receptor 179	0.93457944	0.1598	NA
72278	Ccpg1	cell cycle progression 1	0.93457944	0.1684	NA
237253	Lrp11	low density lipoprotein receptor-related protein 11	0.93457944	0.187	NA
68626	Elac2	elaC homolog 2 (E. coli)	0.93457944	0.2052	NA
217127	Myst2	MYST histone acetyltransferase 2	0.93457944	0.2407	NA
67043	Syap1	synapse associated protein 1	0.93457944	0.246	NA
22630	Ywhaq	tyrosine 3-monooxygenase/tryptophan 5-monooxygenase activation protein, theta polypeptide	0.93457944	0.255	NA
215351	Senp6	SUMO/sentrin specific peptidase 6	0.93457944	0.26	NA
233977	Ppfia1	protein tyrosine phosphatase, receptor type, f polypeptide (PTPRF), interacting protein (liprin), alpha 1	0.93457944	0.2623	NA
66736	Ttc35	tetratricopeptide repeat domain 35	0.93457944	0.2627	NA
15469	Prmt1	protein arginine N-methyltransferase 1	0.93457944	0.2803	NA
72416	Lrpprc	leucine-rich PPR-motif containing	0.93457944	0.311	NA
330361	AW146020	expressed sequence AW146020	0.93457944	0.3148	NA
67892	1810063B05Rik	RIKEN cDNA 1810063B05 gene	0.93457944	0.3249	NA
104859	Tecpr2	tectonin beta-propeller repeat containing 2	0.93457944	0.3286	NA
26896	Med14	mediator complex subunit 14	0.93457944	0.3366	NA
69085	Zcchc9	zinc finger, CCHC domain containing 9	0.93457944	0.3373	NA
56307	Metap2	methionine aminopeptidase 2	0.93457944	0.3377	NA
66105	Ube2d3	ubiquitin-conjugating enzyme E2D 3 (UBC4/5 homolog, yeast)	0.93457944	0.3466	NA
67959	Puf60	poly-U binding splicing factor 60	0.93457944	0.3485	NA
28080	Atp5o	ATP synthase, H+ transporting, mitochondrial F1 complex, O subunit	0.93457944	0.3495	NA
14719	Got2	glutamate oxaloacetate transaminase 2, mitochondrial	0.93457944	0.3498	NA
26397	Map2k3	mitogen-activated protein kinase kinase 3	0.93457944	0.3631	NA
80902	Zfp202	zinc finger protein 202	0.93457944	0.3639	NA
18777	Lypla1	lysophospholipase 1	0.93457944	0.3959	NA
64050	Yeats4	YEATS domain containing 4	0.93457944	0.3972	NA
232210	8430410A17Rik	RIKEN cDNA 8430410A17 gene	0.93457944	0.3999	NA
57247	Zfp276	zinc finger protein (C2H2 type) 276	0.93457944	0.4014	NA
108083	Pip4k2b	phosphatidylinositol-5-phosphate 4-kinase, type II, beta	0.93457944	0.4041	NA
94254	Wbscr16	Williams-Beuren syndrome chromosome region 16 homolog (human)	0.93457944	0.4303	0.6344
30841	Kdm2b	lysine (K)-specific demethylase 2B	0.93457944	0.4332	0.6368
80877	Lrba	LPS-responsive beige-like anchor	0.93457944	0.4423	0.6451
17330	Minpp1	multiple inositol polyphosphate histidine phosphatase 1	0.93457944	0.4532	0.6551
15270	H2afx	H2A histone family, member X	0.93457944	0.4721	0.6704
72665	2810039B14Rik	RIKEN cDNA 2810039B14 gene	0.93457944	0.4825	0.6783
22278	Usf1	upstream transcription factor 1	0.93457944	0.4841	0.6795
80892	Zfhx4	zinc finger homeodomain 4	0.93457944	0.4953	0.6886

414077	BC056474	cDNA sequence BC056474	0.93457944	0.4982	0.6911
11975	Atp6v0a1	ATPase, H+ transporting, lysosomal V0 subunit A1	0.93457944	0.532	0.7171
54141	Spag5	sperm associated antigen 5	0.93457944	0.5557	0.7355
70762	Dclk2	doublecortin-like kinase 2	0.93457944	0.5613	0.7395
223672	Apol9a	apolipoprotein L 9a	0.93457944	0.5658	0.7423
242667	Dlgap3	discs, large (Drosophila) homolog-associated protein 3	0.93457944	0.5667	0.7428
20401	Sh3bp1	SH3-domain binding protein 1	0.93457944	0.5734	0.7476
378430	Nanos2	nanos homolog 2 (Drosophila)	0.93457944	0.587	0.7575
383770	Gm11449	predicted gene 11449	0.93457944	0.6184	0.7805
17069	Ly6e	lymphocyte antigen 6 complex, locus E	0.93457944	0.6276	0.7879
76251	0610007P08Rik	RIKEN cDNA 0610007P08 gene	0.93457944	0.6332	0.7916
208198	Btbd2	BTB (POZ) domain containing 2	0.93457944	0.6573	0.8065
77469	C030034E14Rik	RIKEN cDNA C030034E14 gene	0.93457944	0.6982	0.8328
239122	Setdb2	SET domain, bifurcated 2	0.93457944	0.756	0.8694
66201	Vta1	Vps20-associated 1 homolog (S. cerevisiae)	0.93457944	0.7922	0.8911
16409	Itgam	integrin alpha M	0.93457944	0.7957	0.8927
258319	Olfr187	olfactory receptor 187	0.93457944	0.824	0.9093
12396	Cbfa2t2	core-binding factor, runt domain, alpha subunit 2, translocated to, 2 (human)	0.93370682	0.09133	NA
236899	Pcyt1b	phosphate cytidylyltransferase 1, choline, beta isoform	0.93370682	0.1481	NA
77987	Ascc3	activating signal cointegrator 1 complex subunit 3	0.93370682	0.1553	NA
270162	Elmod1	ELMO domain containing 1	0.93370682	0.1554	NA
66592	Stoml2	stomatin (Epb7.2)-like 2	0.93370682	0.1723	NA
72325	1300018I17Rik	RIKEN cDNA 1300018I17 gene	0.93370682	0.1737	NA
18201	Nsmaf	neutral sphingomyelinase (N-SMase) activation associated factor	0.93370682	0.1806	NA
73158	Larp1	La ribonucleoprotein domain family, member 1	0.93370682	0.2058	NA
23918	Impdh2	inosine 5'-phosphate dehydrogenase 2	0.93370682	0.2083	NA
12308	Calb2	calbindin 2	0.93370682	0.2331	NA
109346	Ankrd39	ankyrin repeat domain 39	0.93370682	0.2386	NA
99045	Mrps26	mitochondrial ribosomal protein S26	0.93370682	0.2427	NA
76563	Qrsl1	glutaminyl-tRNA synthase (glutamine-hydrolyzing)-like 1	0.93370682	0.2534	NA
243813	Leng9	leukocyte receptor cluster (LRC) member 9	0.93370682	0.2618	NA
100494	Zfand2a	zinc finger, AN1-type domain 2A	0.93370682	0.2635	NA
19267	Ptpre	protein tyrosine phosphatase, receptor type, E	0.93370682	0.2716	NA
72425	2410042D21Rik	RIKEN cDNA 2410042D21 gene	0.93370682	0.2769	NA
18577	Pde4a	phosphodiesterase 4A, cAMP specific	0.93370682	0.3038	NA
55951	Brp44l	brain protein 44-like	0.93370682	0.3077	NA
74239	Iqce	IQ motif containing E	0.93370682	0.3115	NA
13006	Smc3	structural maintenace of chromosomes 3	0.93370682	0.3189	NA
668178	Gm10035	predicted gene 10035	0.93370682	0.3285	NA
64378	Gpr88	G-protein coupled receptor 88	0.93370682	0.3292	NA
15193	Hdgfrp2	hepatoma-derived growth factor, related protein 2	0.93370682	0.339	NA
54364	Rpp30	ribonuclease P/MRP 30 subunit (human)	0.93370682	0.3425	NA
380684	Nefh	neurofilament, heavy polypeptide	0.93370682	0.3431	NA
224860	Plcl2	phospholipase C-like 2	0.93370682	0.3463	NA
75560	Ep400	E1A binding protein p400	0.93370682	0.347	NA
14924	Magi1	membrane associated guanylate kinase, WW and PDZ domain containing 1	0.93370682	0.3678	NA
18212	Ntrk2	neurotrophic tyrosine kinase, receptor, type 2	0.93370682	0.3747	NA
433256	Acsl5	acyl-CoA synthetase long-chain family member 5	0.93370682	0.3765	NA
67134	Nop56	NOP56 ribonucleoprotein homolog (yeast)	0.93370682	0.3772	NA
12876	Cpe	carboxypeptidase E	0.93370682	0.3807	NA

26396	Map2k2	mitogen-activated protein kinase kinase 2	0.93370682	0.3824	NA
69798	1810044D09Rik	RIKEN cDNA 1810044D09 gene	0.93370682	0.3864	NA
71704	Arhgef3	Rho guanine nucleotide exchange factor (GEF) 3	0.93370682	0.3976	NA
18706	Pik3ca	phosphatidylinositol 3-kinase, catalytic, alpha polypeptide	0.93370682	0.411	NA
52478	D6Ertd131e	DNA segment, Chr 6, ERATO Doi 131, expressed	0.93370682	0.4177	0.6239
75547	Akap13	A kinase (PRKA) anchor protein 13	0.93370682	0.4221	0.6279
213498	Arhgef11	Rho guanine nucleotide exchange factor (GEF) 11	0.93370682	0.4234	0.6291
75764	Slx1b	SLX1 structure-specific endonuclease subunit homolog B (S. cerevisiae)	0.93370682	0.4272	0.632
74007	Btbd11	BTB (POZ) domain containing 11	0.93370682	0.4276	0.6322
14870	Gstp1	glutathione S-transferase, pi 1	0.93370682	0.4284	0.6329
218699	Pxk	PX domain containing serine/threonine kinase	0.93370682	0.4309	0.6349
235907	Zfp71-rs1	zinc finger protein 71, related sequence	0.93370682	0.4352	0.639
433486	Gm14151	predicted gene 14151	0.93370682	0.4655	0.6656
434778	Ccdc160	coiled-coil domain containing 160	0.93370682	0.4875	0.6824
235584	Dusp7	dual specificity phosphatase 7	0.93370682	0.489	0.6836
353282	Sfmbt2	Scm-like with four mbt domains 2	0.93370682	0.5058	0.6969
319211	Nol4	nucleolar protein 4	0.93370682	0.5068	0.6977
19243	Ptp4a1	protein tyrosine phosphatase 4a1	0.93370682	0.5152	0.705
12373	Casq2	calsequestrin 2	0.93370682	0.5166	0.706
19009	Pou6f1	POU domain, class 6, transcription factor 1	0.93370682	0.5206	0.709
73449	1700066B19Rik	RIKEN cDNA 1700066B19 gene	0.93370682	0.5232	0.7103
69743	Casz1	castor homolog 1, zinc finger (Drosophila)	0.93370682	0.569	0.7446
208666	Diras1	DIRAS family, GTP-binding RAS-like 1	0.93370682	0.5824	0.7541
236451	Gm4902	predicted gene 4902	0.93370682	0.5981	0.7665
68082	Dusp19	dual specificity phosphatase 19	0.93370682	0.6191	0.7812
67972	Atp2b1	ATPase, Ca++ transporting, plasma membrane 1	0.93370682	0.6309	0.7903
13389	Dll3	delta-like 3 (Drosophila)	0.93370682	0.6471	0.801
242519	Ifna12	interferon alpha 12	0.93370682	0.6554	0.8056
12867	Cox7c	cytochrome c oxidase, subunit VIIc	0.93370682	0.6978	0.8327
12825	Col3a1	collagen, type III, alpha 1	0.93370682	0.6983	0.8328
243725	Ppp1r9a	protein phosphatase 1, regulatory (inhibitor) subunit 9A	0.93370682	0.6997	0.8337
268663	Cdhr2	cadherin-related family member 2	0.93370682	0.7092	0.8401
75610	2010109A12Rik	RIKEN cDNA 2010109A12 gene	0.93370682	0.7102	0.8408
329918	Skint9	selection and upkeep of intraepithelial T cells 9	0.93370682	0.7855	0.8877
320731	A530030E21Rik	RIKEN cDNA A530030E21 gene	0.93370682	0.7883	0.8891
627927	Gm6812	predicted gene 6812	0.93370682	0.8075	0.9001
622446	Gm6323	predicted gene 6323	0.93370682	0.8307	0.9126
245877	Mtap7d1	microtubule-associated protein 7 domain containing 1	0.93283582	0.1217	NA
12328	Caml	calcium modulating ligand	0.93283582	0.1328	NA
68614	Letmd1	LETM1 domain containing 1	0.93283582	0.1343	NA
233802	Thumpd1	THUMP domain containing 1	0.93283582	0.1396	NA
78757	Rictor	RPTOR independent companion of MTOR, complex 2	0.93283582	0.1556	NA
66882	Bzw1	basic leucine zipper and W2 domains 1	0.93283582	0.1841	NA
66618	Snrnp27	small nuclear ribonucleoprotein 27 (U4/U6.U5)	0.93283582	0.1877	NA
69934	Rg9mtd3	RNA (guanine-9-) methyltransferase domain containing 3	0.93283582	0.1884	NA
19360	Rad50	RAD50 homolog (S. cerevisiae)	0.93283582	0.1974	NA
30853	Mlf2	myeloid leukemia factor 2	0.93283582	0.2032	NA
73373	Phospho2	phosphatase, orphan 2	0.93283582	0.2325	NA
81004	Tbl1xr1	transducin (beta)-like 1X-linked receptor 1	0.93283582	0.235	NA
17169	Mark3	MAP/microtubule affinity-regulating kinase 3	0.93283582	0.2353	NA

52633	Nit2	nitrilase family, member 2	0.93283582	0.2365	NA
238328	Vash1	vasohibin 1	0.93283582	0.2378	NA
216028	Lrrtm3	leucine rich repeat transmembrane neuronal 3	0.93283582	0.2463	NA
381820	2700089E24Rik	RIKEN cDNA 2700089E24 gene	0.93283582	0.2543	NA
69159	Rhebl1	Ras homolog enriched in brain like 1	0.93283582	0.265	NA
22172	Tyms-ps	thymidylate synthase, pseudogene	0.93283582	0.2673	NA
67921	Ube2f	ubiquitin-conjugating enzyme E2F (putative)	0.93283582	0.2686	NA
433941	Gm5561	ribosomal protein L29 pseudogene	0.93283582	0.2763	NA
18632	Pex11b	peroxisomal biogenesis factor 11 beta	0.93283582	0.2812	NA
64291	Osbpl1a	oxysterol binding protein-like 1A	0.93283582	0.2877	NA
13480	Dpm1	dolichol-phosphate (beta-D) mannosyltransferase 1	0.93283582	0.298	NA
192232	Hps4	Hermansky-Pudlak syndrome 4 homolog (human)	0.93283582	0.2987	NA
72469	Plcd3	phospholipase C, delta 3	0.93283582	0.3192	NA
18682	Phkg1	phosphorylase kinase gamma 1	0.93283582	0.3254	NA
68202	Ndufa5	NADH dehydrogenase (ubiquinone) 1 alpha subcomplex, 5	0.93283582	0.3255	NA
67077	1700019N12Rik	RIKEN cDNA 1700019N12 gene	0.93283582	0.3379	NA
15902	Id2	inhibitor of DNA binding 2	0.93283582	0.3539	NA
231841	AA881470	EST AA881470	0.93283582	0.3615	NA
109161	Ube2q2	ubiquitin-conjugating enzyme E2Q (putative) 2	0.93283582	0.3655	NA
15893	Ica1	islet cell autoantigen 1	0.93283582	0.3792	NA
21423	Tcf3	transcription factor 3	0.93283582	0.3831	NA
12669	Chrm1	cholinergic receptor, muscarinic 1, CNS	0.93283582	0.4005	NA
66271	Tmem126a	transmembrane protein 126A	0.93283582	0.4113	0.6176
71752	Gtf3c2	general transcription factor IIIC, polypeptide 2, beta	0.93283582	0.4302	0.6344
216971	BC017647	cDNA sequence BC017647	0.93283582	0.4353	0.6391
230751	Oscp1	organic solute carrier partner 1	0.93283582	0.4454	0.648
52430	Echdc2	enoyl Coenzyme A hydratase domain containing 2	0.93283582	0.4527	0.6549
83673	Snhg1	small nucleolar RNA host gene (non-protein coding) 1	0.93283582	0.4607	0.6614
12842	Col1a1	collagen, type I, alpha 1	0.93283582	0.4624	0.6629
65098	Zfand6	zinc finger, AN1-type domain 6	0.93283582	0.4765	0.6736
74102	Slc35a5	solute carrier family 35, member A5	0.93283582	0.477	0.6742
56334	Tmed2	transmembrane emp24 domain trafficking protein 2	0.93283582	0.4784	0.6752
20350	Sema3f	sema domain, immunoglobulin domain (Ig), short basic domain, secreted, (semaphorin) 3F	0.93283582	0.4794	0.6758
16491	Kcna3	potassium voltage-gated channel, shaker-related subfamily, member 3	0.93283582	0.4828	0.6784
218503	Fcho2	FCH domain only 2	0.93283582	0.4856	0.681
13807	Eno2	enolase 2, gamma neuronal	0.93283582	0.5248	0.7116
18195	Nsf	N-ethylmaleimide sensitive fusion protein	0.93283582	0.5325	0.7176
23947	Mid2	midline 2	0.93283582	0.5506	0.7317
243923	Rgs9bp	regulator of G-protein signalling 9 binding protein	0.93283582	0.5552	0.7351
228355	Madd	MAP-kinase activating death domain	0.93283582	0.5619	0.7398
242667	Dlgap3	discs, large (Drosophila) homolog-associated protein 3	0.93283582	0.5628	0.7404
243846	Ccdc9	coiled-coil domain containing 9	0.93283582	0.5677	0.7435
109054	Pfdn4	prefoldin 4	0.93283582	0.5683	0.744
11305	Abca2	ATP-binding cassette, sub-family A (ABC1), member 2	0.93283582	0.584	0.7551
21849	Trim28	tripartite motif-containing 28	0.93283582	0.6067	0.7727
20168	Rtn3	reticulon 3	0.93283582	0.6077	0.7731
320429	Trank1	tetratricopeptide repeat and ankyrin repeat containing 1	0.93283582	0.6148	0.7781
432768	Gm5451	predicted gene 5451	0.93283582	0.6328	0.7914
319586	Celf5	CUGBP, Elav-like family member 5	0.93283582	0.6524	0.804
22384	Eif4h	eukaryotic translation initiation factor 4H	0.93196645	0.1581	NA

56469	Pias1	protein inhibitor of activated STAT 1	0.93196645	0.1765	NA
234854	Cdk10	cyclin-dependent kinase 10	0.93196645	0.1906	NA
269113	Nup54	nucleoporin 54	0.93196645	0.1957	NA
107182	Btaf1	BTAF1 RNA polymerase II, B-TFIID transcription factor-associated, (Mot1 homolog, S. cerevisiae)	0.93196645	0.2209	NA
360216	Zranb1	zinc finger, RAN-binding domain containing 1	0.93196645	0.2242	NA
74148	1300001I01Rik	RIKEN cDNA 1300001I01 gene	0.93196645	0.2314	NA
16976	Lrpap1	low density lipoprotein receptor-related protein associated protein 1	0.93196645	0.2342	NA
71529	9030409G11Rik	RIKEN cDNA 9030409G11 gene	0.93196645	0.2416	NA
66246	Osgep	O-sialoglycoprotein endopeptidase	0.93196645	0.2459	NA
622404	Ccdc107	coiled-coil domain containing 107	0.93196645	0.2465	NA
80915	Dusp12	dual specificity phosphatase 12	0.93196645	0.2548	NA
56442	Serinc1	serine incorporator 1	0.93196645	0.2759	NA
12748	Clk2	CDC-like kinase 2	0.93196645	0.2806	NA
51786	Cpsf2	cleavage and polyadenylation specific factor 2	0.93196645	0.281	NA
100504508	LOC100504508	protein SET-like	0.93196645	0.2988	NA
67163	Ccdc47	coiled-coil domain containing 47	0.93196645	0.3153	NA
228966	Ppp1r3d	protein phosphatase 1, regulatory subunit 3D	0.93196645	0.3165	NA
74178	Stk40	serine/threonine kinase 40	0.93196645	0.3204	NA
74412	Gle1	GLE1 RNA export mediator (yeast)	0.93196645	0.3209	NA
27281	Hrasls	HRAS-like suppressor	0.93196645	0.329	NA
71982	Snx10	sorting nexin 10	0.93196645	0.3579	NA
71835	Lancl2	LanC (bacterial lantibiotic synthetase component C)-like 2	0.93196645	0.3759	NA
259302	Srgap3	SLIT-ROBO Rho GTPase activating protein 3	0.93196645	0.3819	NA
101883	Tmem149	transmembrane protein 149	0.93196645	0.389	NA
22083	Ctr9	Ctr9, Paf1/RNA polymerase II complex component, homolog (S. cerevisiae)	0.93196645	0.3894	NA
227377	Farp2	FERM, RhoGEF and pleckstrin domain protein 2	0.93196645	0.3968	NA
140904	Caln1	calneuron 1	0.93196645	0.4035	0.6109
75869	Arl5b	ADP-ribosylation factor-like 5B	0.93196645	0.4141	0.6205
207304	Hectd1	HECT domain containing 1	0.93196645	0.4226	0.6282
170753	Zfp704	zinc finger protein 704	0.93196645	0.4393	0.6422
192195	Ash1l	ash1 (absent, small, or homeotic)-like (Drosophila)	0.93196645	0.4465	0.649
268510	Mgat5b	mannoside acetylglucosaminyltransferase 5, isoenzyme B	0.93196645	0.4504	0.6527
235610	Atrip	ATR interacting protein	0.93196645	0.452	0.6542
269788	Lhfpl4	lipoma HMGIC fusion partner-like protein 4	0.93196645	0.4529	0.6549
54369	Nme6	non-metastatic cells 6, protein expressed in (nucleoside-diphosphate kinase)	0.93196645	0.4735	0.6715
100233175	AK010878	cDNA sequence AK010878	0.93196645	0.4807	0.6768
100764	1110008J03Rik	RIKEN cDNA 1110008J03 gene	0.93196645	0.5001	0.6922
15182	Hdac2	histone deacetylase 2	0.93196645	0.5146	0.7045
17769	Mthfr	5,10-methylenetetrahydrofolate reductase	0.93196645	0.5175	0.7068
67453	Slc25a46	solute carrier family 25, member 46	0.93196645	0.5189	0.7083
17183	Matn4	matrilin 4	0.93196645	0.5204	0.7089
100046223	LOC100046223	40S ribosomal protein S15-like	0.93196645	0.5268	0.7133
627004	Gm12770	predicted gene 12770	0.93196645	0.576	0.7493
12890	Cplx2	complexin 2	0.93196645	0.5849	0.7556
68198	Ndufb2	NADH dehydrogenase (ubiquinone) 1 beta subcomplex, 2	0.93196645	0.5949	0.7638
74600	Mrpl47	mitochondrial ribosomal protein L47	0.93196645	0.5966	0.7653
16648	Kpna3	karyopherin (importin) alpha 3	0.93196645	0.6041	0.7709
241112	Gm216	predicted gene 216	0.93196645	0.6077	0.7731
56736	Rnf14	ring finger protein 14	0.93196645	0.608	0.7732
104709	Pik3r6	phosphoinositide-3-kinase, regulatory subunit 6	0.93196645	0.621	0.7825

15201	Hells	helicase, lymphoid specific	0.93196645	0.6219	0.7831
13383	Dlg1	discs, large homolog 1 (Drosophila)	0.93196645	0.6353	0.7929
13638	Efna3	ephrin A3	0.93196645	0.653	0.8041
101739	Psip1	PC4 and SFRS1 interacting protein 1	0.93196645	0.6657	0.8117
78906	9130017N09Rik	RIKEN cDNA 9130017N09 gene	0.93196645	0.6727	0.8167
71863	1700019O17Rik	RIKEN cDNA 1700019O17 gene	0.93196645	0.6735	0.8171
237979	Sdk2	sidekick homolog 2 (chicken)	0.93196645	0.6907	0.8285
100505024	LOC100505024	homeobox protein TGIF2-like	0.93196645	0.7631	0.8742
93696	Chrac1	chromatin accessibility complex 1	0.9310987	0.09259	NA
22193	Ube2e3	ubiquitin-conjugating enzyme E2E 3, UBC4/5 homolog (yeast)	0.9310987	0.138	NA
72657	2700094K13Rik	RIKEN cDNA 2700094K13 gene	0.9310987	0.1381	NA
11907	Ate1	arginyltransferase 1	0.9310987	0.1496	NA
17685	Msh2	mutS homolog 2 (E. coli)	0.9310987	0.2006	NA
16563	Kif2a	kinesin family member 2A	0.9310987	0.2176	NA
68423	Ankrd13d	ankyrin repeat domain 13 family, member D	0.9310987	0.235	NA
118451	Mrps2	mitochondrial ribosomal protein S2	0.9310987	0.2402	NA
67967	Pold3	polymerase (DNA-directed), delta 3, accessory subunit	0.9310987	0.2405	NA
21969	Top1	topoisomerase (DNA) I	0.9310987	0.2582	NA
241694	Ralgapa2	Ral GTPase activating protein, alpha subunit 2 (catalytic)	0.9310987	0.2606	NA
71041	Pcgf6	polycomb group ring finger 6	0.9310987	0.2991	NA
214489	BC003965	cDNA sequence BC003965	0.9310987	0.3197	NA
22247	Umps	uridine monophosphate synthetase	0.9310987	0.3256	NA
11677	Akr1b3	aldo-keto reductase family 1, member B3 (aldose reductase)	0.9310987	0.3287	NA
27015	Polk	polymerase (DNA directed), kappa	0.9310987	0.3391	NA
170749	Mtmr4	myotubularin related protein 4	0.9310987	0.3488	NA
69938	Scrn1	secernin 1	0.9310987	0.3544	NA
14738	Gpr12	G-protein coupled receptor 12	0.9310987	0.373	NA
74453	Ccdc11	coiled-coil domain containing 11	0.9310987	0.3881	NA
243961	Shank1	SH3/ankyrin domain gene 1	0.9310987	0.3978	0.6057
56706	Ccnl1	cyclin L1	0.9310987	0.3986	0.6064
18718	Pip4k2a	phosphatidylinositol-5-phosphate 4-kinase, type II, alpha	0.9310987	0.3998	0.6076
619307	9430078G10Rik	RIKEN cDNA 9430078G10 gene	0.9310987	0.4085	0.615
233879	Asphd1	aspartate beta-hydroxylase domain containing 1	0.9310987	0.4118	0.6182
69232	Qrich1	glutamine-rich 1	0.9310987	0.4371	0.6406
13002	Dnajc5	DnaJ (Hsp40) homolog, subfamily C, member 5	0.9310987	0.444	0.6468
15568	Elavl1	ELAV (embryonic lethal, abnormal vision, Drosophila)-like 1 (Hu antigen R)	0.9310987	0.4656	0.6656
319984	Jph4	junctophilin 4	0.9310987	0.4689	0.6685
381668	Fbrsl1	fibrosin-like 1	0.9310987	0.4757	0.673
100039043	Gm10731	predicted gene 10731	0.9310987	0.4912	0.6852
19883	Rora	RAR-related orphan receptor alpha	0.9310987	0.4933	0.6873
71449	Mettl13	methyltransferase like 13	0.9310987	0.52	0.7087
226470	Zbtb41	zinc finger and BTB domain containing 41 homolog	0.9310987	0.5307	0.7161
171209	Accn3	amiloride-sensitive cation channel 3	0.9310987	0.531	0.7164
18591	Pdgfb	platelet derived growth factor, B polypeptide	0.9310987	0.5372	0.7214
70357	Kcnip1	Kv channel-interacting protein 1	0.9310987	0.5411	0.7242
53614	Reck	reversion-inducing-cysteine-rich protein with kazal motifs	0.9310987	0.5524	0.733
56310	Gps2	G protein pathway suppressor 2	0.9310987	0.5653	0.742
74376	Myo18b	myosin XVIIIb	0.9310987	0.7268	0.8514
627782	Gm6787	proteasome (prosome, macropain) subunit, beta type 7 pseudogene	0.9310987	0.7357	0.8572
81601	Kat5	K(lysine) acetyltransferase 5	0.93023256	0.1112	NA

244608	Ccdc113	coiled-coil domain containing 113	0.93023256	0.122	NA
231600	Chfr	checkpoint with forkhead and ring finger domains	0.93023256	0.1492	NA
110816	Pwp2	PWP2 periodic tryptophan protein homolog (yeast)	0.93023256	0.1515	NA
319586	Celf5	CUGBP, Elav-like family member 5	0.93023256	0.1515	NA
104346	Gas8	growth arrest specific 8	0.93023256	0.1588	NA
100040298	Gm15501	predicted pseudogene 15501	0.93023256	0.1687	NA
67434	Ankrd33b	ankyrin repeat domain 33B	0.93023256	0.1823	NA
381990	Zbtb2	zinc finger and BTB domain containing 2	0.93023256	0.1954	NA
67694	Ift74	intraflagellar transport 74 homolog (Chlamydomonas)	0.93023256	0.2255	NA
52615	Suz12	suppressor of zeste 12 homolog (Drosophila)	0.93023256	0.2348	NA
17846	Commd1	COMM domain containing 1	0.93023256	0.2372	NA
140489	Bhlhe23	basic helix-loop-helix family, member e23	0.93023256	0.2384	NA
64450	Gpr85	G protein-coupled receptor 85	0.93023256	0.2474	NA
67759	5033414D02Rik	RIKEN cDNA 5033414D02 gene	0.93023256	0.2679	NA
21781	Tfdp1	transcription factor Dp 1	0.93023256	0.2773	NA
68966	Ngdn	neuroguidin, EIF4E binding protein	0.93023256	0.2775	NA
76454	Fbxo31	F-box protein 31	0.93023256	0.2794	NA
66603	Sip1	survival of motor neuron protein interacting protein 1	0.93023256	0.2909	NA
53857	Tuba8	tubulin, alpha 8	0.93023256	0.2945	NA
74766	Yipf2	Yip1 domain family, member 2	0.93023256	0.2972	NA
66935	Cir1	corepressor interacting with RBPJ, 1	0.93023256	0.3016	NA
94093	Trim33	tripartite motif-containing 33	0.93023256	0.3166	NA
229801	Tram1l1	translocation associated membrane protein 1-like 1	0.93023256	0.317	NA
243634	Ano2	anoctamin 2	0.93023256	0.3185	NA
320384	B230334L07Rik	RIKEN cDNA B230334L07 gene	0.93023256	0.3199	NA
83814	Nedd4l	neural precursor cell expressed, developmentally down-regulated gene 4-like	0.93023256	0.3388	NA
56550	Ube2d2	ubiquitin-conjugating enzyme E2D 2	0.93023256	0.3402	NA
20528	Slc2a4	solute carrier family 2 (facilitated glucose transporter), member 4	0.93023256	0.3433	NA
66586	Crls1	cardiolipin synthase 1	0.93023256	0.3506	NA
56788	Scube2	signal peptide, CUB domain, EGF-like 2	0.93023256	0.3528	NA
381305	Rc3h1	RING CCCH (C3H) domains 1	0.93023256	0.3544	NA
14167	Fgf12	fibroblast growth factor 12	0.93023256	0.3641	NA
57785	Rangrf	RAN guanine nucleotide release factor	0.93023256	0.3666	NA
73381	Cmtm2a	CKLF-like MARVEL transmembrane domain containing 2A	0.93023256	0.3678	NA
93686	Rbfox2	RNA binding protein, fox-1 homolog (C. elegans) 2	0.93023256	0.3691	NA
22019	Tpp2	tripeptidyl peptidase II	0.93023256	0.3761	NA
109161	Ube2q2	ubiquitin-conjugating enzyme E2Q (putative) 2	0.93023256	0.384	0.5931
79059	Nme3	non-metastatic cells 3, protein expressed in	0.93023256	0.3978	0.6057
17524	Mpp1	membrane protein, palmitoylated	0.93023256	0.3985	0.6064
66629	Golph3	golgi phosphoprotein 3	0.93023256	0.4079	0.6144
67695	Ost4	oligosaccharyltransferase 4 homolog (S. cerevisiae)	0.93023256	0.4274	0.6321
74204	Xpo6	exportin 6	0.93023256	0.4419	0.6449
68628	Fbxw9	F-box and WD-40 domain protein 9	0.93023256	0.4426	0.6454
66704	Rbm4b	RNA binding motif protein 4B	0.93023256	0.4505	0.6527
382007	Adam26b	a disintegrin and metallopeptidase domain 26B	0.93023256	0.4527	0.6549
56223	Fscn3	fascin homolog 3, actin-bundling protein, testicular (Strongylocentrotus purpuratus)	0.93023256	0.4794	0.6758
98388	Chst10	carbohydrate sulfotransferase 10	0.93023256	0.4863	0.6814
16069	Igj	immunoglobulin joining chain	0.93023256	0.4879	0.6828
241447	Lass6	LAG1 homolog, ceramide synthase 6	0.93023256	0.4897	0.6841
66700	Vps24	vacuolar protein sorting 24 (yeast)	0.93023256	0.5205	0.7089

12421	Rb1cc1	RB1-inducible coiled-coil 1	0.93023256	0.5225	0.7102
16569	Kif3b	kinesin family member 3B	0.93023256	0.5286	0.7146
51799	Rundc3a	RUN domain containing 3A	0.93023256	0.5319	0.717
381085	Tbc1d22b	TBC1 domain family, member 22B	0.93023256	0.573	0.7475
640382	LOC640382	hypothetical protein LOC640382	0.93023256	0.5805	0.7526
17245	Mdm1	transformed mouse 3T3 cell double minute 1	0.93023256	0.5875	0.7578
77391	9530003O04Rik	RIKEN cDNA 9530003O04 gene	0.93023256	0.5923	0.7618
399599	Ccdc87	coiled-coil domain containing 87	0.93023256	0.626	0.7863
13118	Cyp4a12b	cytochrome P450, family 4, subfamily a, polypeptide 12B	0.93023256	0.6306	0.7901
72320	2510003E04Rik	RIKEN cDNA 2510003E04 gene	0.93023256	0.6354	0.793
22791	Dnajc2	DnaJ (Hsp40) homolog, subfamily C, member 2	0.93023256	0.6947	0.831
214968	Sema6d	sema domain, transmembrane domain (TM), and cytoplasmic domain, (semaphorin) 6D	0.93023256	0.7655	0.8755
77908	9230113P08Rik	RIKEN cDNA 9230113P08 gene	0.93023256	0.8537	0.9249
18771	Pknox1	Pbx/knotted 1 homeobox	0.92936803	0.07628	NA
66396	Ccdc82	coiled-coil domain containing 82	0.92936803	0.1034	NA
216805	Flcn	folliculin	0.92936803	0.1105	NA
70456	Brp44	brain protein 44	0.92936803	0.1157	NA
67295	Rab3c	RAB3C, member RAS oncogene family	0.92936803	0.1432	NA
216154	Med16	mediator complex subunit 16	0.92936803	0.152	NA
12190	Brca2	breast cancer 2	0.92936803	0.1899	NA
71238	Acn9	ACN9 homolog (S. cerevisiae)	0.92936803	0.1976	NA
66423	2410022L05Rik	RIKEN cDNA 2410022L05 gene	0.92936803	0.2023	NA
107939	Pom121	nuclear pore membrane protein 121	0.92936803	0.2115	NA
74763	Nat15	N-acetyltransferase 15 (GCN5-related, putative)	0.92936803	0.2152	NA
79221	Hdac9	histone deacetylase 9	0.92936803	0.2228	NA
228136	Zdhhc5	zinc finger, DHHC domain containing 5	0.92936803	0.2294	NA
213012	Abhd10	abhydrolase domain containing 10	0.92936803	0.2372	NA
226139	Cox15	COX15 homolog, cytochrome c oxidase assembly protein (yeast)	0.92936803	0.2389	NA
268930	Pkmyt1	protein kinase, membrane associated tyrosine/threonine 1	0.92936803	0.2449	NA
26383	Fto	fat mass and obesity associated	0.92936803	0.2538	NA
243382	Ppm1k	protein phosphatase 1K (PP2C domain containing)	0.92936803	0.2625	NA
56405	Dusp14	dual specificity phosphatase 14	0.92936803	0.2646	NA
269424	Phf17	PHD finger protein 17	0.92936803	0.2663	NA
17281	Fyco1	FYVE and coiled-coil domain containing 1	0.92936803	0.2861	NA
71982	Snx10	sorting nexin 10	0.92936803	0.2949	NA
237611	Stac3	SH3 and cysteine rich domain 3	0.92936803	0.3136	NA
22378	Wbp2	WW domain binding protein 2	0.92936803	0.3242	NA
19250	Ptpn14	protein tyrosine phosphatase, non-receptor type 14	0.92936803	0.3294	NA
20924	Supt5h	suppressor of Ty 5 homolog (S. cerevisiae)	0.92936803	0.3362	NA
14677	Gnai1	guanine nucleotide binding protein (G protein), alpha inhibiting 1	0.92936803	0.3392	NA
67661	Ift172	intraflagellar transport 172 homolog (Chlamydomonas)	0.92936803	0.3403	NA
16785	Rpsa	ribosomal protein SA	0.92936803	0.3555	NA
75029	Purg	purine-rich element binding protein G	0.92936803	0.3557	NA
23830	Capn10	calpain 10	0.92936803	0.359	NA
26385	Grk6	G protein-coupled receptor kinase 6	0.92936803	0.3599	NA
27062	Cadps	Ca2+-dependent secretion activator	0.92936803	0.3712	NA
228356	1110051M20Rik	RIKEN cDNA 1110051M20 gene	0.92936803	0.3744	NA
54650	Sfmbt1	Scm-like with four mbt domains 1	0.92936803	0.3786	0.5875
216148	Shc2	SHC (Src homology 2 domain containing) transforming protein 2	0.92936803	0.3822	0.5913
235633	Als2cl	ALS2 C-terminal like	0.92936803	0.3945	0.6028

211064	Alkbh1	alkB, alkylation repair homolog 1 (E. coli)	0.92936803	0.3959	0.6039
52915	Zmiz2	zinc finger, MIZ-type containing 2	0.92936803	0.4024	0.61
235627	Nbeal2	neurobeachin-like 2	0.92936803	0.4381	0.6412
26356	Ing1	inhibitor of growth family, member 1	0.92936803	0.4639	0.6642
56504	Srpk3	serine/arginine-rich protein specific kinase 3	0.92936803	0.4764	0.6736
76257	Slc38a3	solute carrier family 38, member 3	0.92936803	0.4842	0.6796
50767	Pnpla6	patatin-like phospholipase domain containing 6	0.92936803	0.4848	0.6803
67842	2610027L16Rik	RIKEN cDNA 2610027L16 gene	0.92936803	0.4891	0.6837
329559	Zfp335	zinc finger protein 335	0.92936803	0.5004	0.6924
226519	Lamc1	laminin, gamma 1	0.92936803	0.5072	0.6981
71623	Krtap5-2	keratin associated protein 5-2	0.92936803	0.5292	0.7151
18191	Nrxn3	neurexin III	0.92936803	0.5497	0.7312
432939	Gm5468	predicted gene 5468	0.92936803	0.5849	0.7556
15560	Htr2c	5-hydroxytryptamine (serotonin) receptor 2C	0.92936803	0.5874	0.7578
18599	Padi1	peptidyl arginine deiminase, type I	0.92936803	0.6098	0.7744
12890	Cplx2	complexin 2	0.92936803	0.642	0.7976
70472	Atad2	ATPase family, AAA domain containing 2	0.92936803	0.6785	0.8202
338366	Mia3	melanoma inhibitory activity 3	0.92936803	0.693	0.83
195646	Hs3st2	heparan sulfate (glucosamine) 3-O-sulfotransferase 2	0.92936803	0.6944	0.8308
74276	Cldnd2	claudin domain containing 2	0.92936803	0.7674	0.8765
408058	BC048507	cDNA sequence BC048507	0.92936803	0.8325	0.9137
27425	Atp5l	ATP synthase, H+ transporting, mitochondrial F0 complex, subunit g	0.92850511	0.08767	NA
72946	Lrrc47	leucine rich repeat containing 47	0.92850511	0.1213	NA
67383	2410127L17Rik	RIKEN cDNA 2410127L17 gene	0.92850511	0.1302	NA
231915	Uspl1	ubiquitin specific peptidase like 1	0.92850511	0.1609	NA
353342	Peg13	paternally expressed 13	0.92850511	0.1698	NA
20931	Surf2	surfeit gene 2	0.92850511	0.1952	NA
66125	Sf3b5	splicing factor 3b, subunit 5	0.92850511	0.2053	NA
269397	Ss18l1	synovial sarcoma translocation gene on chromosome 18-like 1	0.92850511	0.2059	NA
56741	Igdcc4	immunoglobulin superfamily, DCC subclass, member 4	0.92850511	0.2154	NA
210106	Papd7	PAP associated domain containing 7	0.92850511	0.2195	NA
77116	Mtmr2	myotubularin related protein 2	0.92850511	0.226	NA
76042	5830427D02Rik	RIKEN cDNA 5830427D02 gene	0.92850511	0.2278	NA
27362	Dnajb9	DnaJ (Hsp40) homolog, subfamily B, member 9	0.92850511	0.2394	NA
242418	Dcaf10	DDB1 and CUL4 associated factor 10	0.92850511	0.2406	NA
66272	Cox16	COX16 cytochrome c oxidase assembly homolog (S. cerevisiae)	0.92850511	0.2438	NA
67666	Hapln3	hyaluronan and proteoglycan link protein 3	0.92850511	0.2462	NA
14026	Evl	Ena-vasodilator stimulated phosphoprotein	0.92850511	0.2555	NA
66921	Prpf38b	PRP38 pre-mRNA processing factor 38 (yeast) domain containing B	0.92850511	0.2656	NA
16508	Kcnd2	potassium voltage-gated channel, Shal-related family, member 2	0.92850511	0.2665	NA
71691	Pnmal1	PNMA-like 1	0.92850511	0.2678	NA
12663	Chml	choroideremia-like	0.92850511	0.2716	NA
217708	Lin52	lin-52 homolog (C. elegans)	0.92850511	0.2754	NA
71787	Trnau1ap	tRNA selenocysteine 1 associated protein 1	0.92850511	0.3177	NA
240641	Kif20b	kinesin family member 20B	0.92850511	0.323	NA
22187	Ubb	ubiquitin B	0.92850511	0.3237	NA
63953	Dusp10	dual specificity phosphatase 10	0.92850511	0.3296	NA
67187	Zmynd19	zinc finger, MYND domain containing 19	0.92850511	0.3436	NA
13841	Epha7	Eph receptor A7	0.92850511	0.3438	NA
80905	Polh	polymerase (DNA directed), eta (RAD 30 related)	0.92850511	0.3443	NA

13488	Drd1a	dopamine receptor D1A	0.92850511	0.3478	NA
100040297	Gm2695	predicted gene 2695	0.92850511	0.3581	NA
225215	Rsl24d1	ribosomal L24 domain containing 1	0.92850511	0.3631	NA
11306	Abcb7	ATP-binding cassette, sub-family B (MDR/TAP), member 7	0.92850511	0.3816	0.5908
71710	Lrrcc1	leucine rich repeat and coiled-coil domain containing 1	0.92850511	0.3875	0.5961
207182	Ggt7	gamma-glutamyltransferase 7	0.92850511	0.3915	0.5997
50849	Rnf10	ring finger protein 10	0.92850511	0.3986	0.6064
218975	Mapk1ip1l	mitogen-activated protein kinase 1 interacting protein 1-like	0.92850511	0.4281	0.6326
57080	Gtf2ird1	general transcription factor II I repeat domain-containing 1	0.92850511	0.4321	0.6361
75620	2810422J05Rik	RIKEN cDNA 2810422J05 gene	0.92850511	0.4328	0.6366
73261	1700037C18Rik	RIKEN cDNA 1700037C18 gene	0.92850511	0.4388	0.6417
101540	Prkd2	protein kinase D2	0.92850511	0.4408	0.6437
11630	Aim1	absent in melanoma 1	0.92850511	0.4507	0.6529
13004	Ncan	neurocan	0.92850511	0.4611	0.6616
13548	Dyrk1a	dual-specificity tyrosine-(Y)-phosphorylation regulated kinase 1a	0.92850511	0.4692	0.6686
269356	Slc4a11	solute carrier family 4, sodium bicarbonate transporter-like, member 11	0.92850511	0.4938	0.6878
230991	B930041F14Rik	RIKEN cDNA B930041F14 gene	0.92850511	0.4986	0.6913
66384	Srp19	signal recognition particle 19	0.92850511	0.5139	0.7041
17395	Mmp9	matrix metallopeptidase 9	0.92850511	0.5153	0.705
11514	Adcy8	adenylate cyclase 8	0.92850511	0.5467	0.7286
244859	Ankk1	ankyrin repeat and kinase domain containing 1	0.92850511	0.5544	0.7345
666173	Vps13b	vacuolar protein sorting 13B (yeast)	0.92850511	0.5548	0.7348
232341	Wnk1	WNK lysine deficient protein kinase 1	0.92850511	0.5642	0.7416
195522	Zfp691	zinc finger protein 691	0.92850511	0.5671	0.7431
207740	Fam100a	family with sequence similarity 100, member A	0.92850511	0.5752	0.749
223626	4930572J05Rik	RIKEN cDNA 4930572J05 gene	0.92850511	0.6427	0.7978
102595	Plekho2	pleckstrin homology domain containing, family O member 2	0.92850511	0.6535	0.8044
69226	Snx24	sorting nexing 24	0.92850511	0.6881	0.8266
75320	Etnk1	ethanolamine kinase 1	0.92850511	0.6993	0.8336
78887	Sfi1	Sfi1 homolog, spindle assembly associated (yeast)	0.92850511	0.7173	0.8456
13207	Ddx5	DEAD (Asp-Glu-Ala-Asp) box polypeptide 5	0.92850511	0.7252	0.8505
12399	Runx3	runt related transcription factor 3	0.92850511	0.7429	0.861
414075	BC049265	cDNA sequence BC049265	0.92850511	0.7441	0.8618
245877	Mtap7d1	microtubule-associated protein 7 domain containing 1	0.92764378	0.0831	NA
19181	Psmc2	proteasome (prosome, macropain) 26S subunit, ATPase 2	0.92764378	0.109	NA
66094	Lsm7	LSM7 homolog, U6 small nuclear RNA associated (S. cerevisiae)	0.92764378	0.1314	NA
232670	Tspan33	tetraspanin 33	0.92764378	0.1321	NA
72393	Faim2	Fas apoptotic inhibitory molecule 2	0.92764378	0.1786	NA
231506	Lin54	lin-54 homolog (C. elegans)	0.92764378	0.1807	NA
108829	Jmjd1c	jumonji domain containing 1C	0.92764378	0.1887	NA
17268	Meis1	Meis homeobox 1	0.92764378	0.1967	NA
67266	Fam69a	family with sequence similarity 69, member A	0.92764378	0.2058	NA
16392	Isl1	ISL1 transcription factor, LIM/homeodomain	0.92764378	0.2159	NA
54604	Pcnx	pecanex homolog (Drosophila)	0.92764378	0.2241	NA
20872	Stk16	serine/threonine kinase 16	0.92764378	0.2297	NA
16924	Lnx1	ligand of numb-protein X 1	0.92764378	0.2341	NA
545611	Gm13298	predicted gene 13298	0.92764378	0.2384	NA
100087	Kti12	KTI12 homolog, chromatin associated (S. cerevisiae)	0.92764378	0.242	NA
56525	Zfp235	zinc finger protein 235	0.92764378	0.26	NA
207352	Sec23ip	Sec23 interacting protein	0.92764378	0.2696	NA

26374	Rfwd2	ring finger and WD repeat domain 2	0.92764378	0.2774	NA
73242	Atat1	alpha tubulin acetyltransferase 1	0.92764378	0.3032	NA
19655	Rbmx	RNA binding motif protein, X chromosome	0.92764378	0.3129	NA
109093	Rars2	arginyl-tRNA synthetase 2, mitochondrial	0.92764378	0.3211	NA
29859	Sult4a1	sulfotransferase family 4A, member 1	0.92764378	0.3217	NA
234593	Ndrg4	N-myc downstream regulated gene 4	0.92764378	0.3236	NA
259302	Srgap3	SLIT-ROBO Rho GTPase activating protein 3	0.92764378	0.3339	NA
320080	Zbtb39	zinc finger and BTB domain containing 39	0.92764378	0.3351	NA
100504473	LOC100504473	RNA-binding protein Musashi homolog 2-like	0.92764378	0.358	NA
74355	Smchd1	SMC hinge domain containing 1	0.92764378	0.3689	0.578
235047	Zfp809	zinc finger protein 809	0.92764378	0.3703	0.5793
71732	Vps11	vacuolar protein sorting 11 (yeast)	0.92764378	0.3737	0.5824
234023	Arglu1	arginine and glutamate rich 1	0.92764378	0.3917	0.5999
103836	Zfp692	zinc finger protein 692	0.92764378	0.3965	0.6043
320060	B230308N11Rik	RIKEN cDNA B230308N11 gene	0.92764378	0.3977	0.6057
97440	B3gnt9-ps	UDP-GlcNAc:betaGal beta-1,3-N-acetylglucosaminyltransferase 9, pseudogene	0.92764378	0.4006	0.6083
545975	Lass3	LAG1 homolog, ceramide synthase 3	0.92764378	0.4237	0.6293
231549	Lrrc8d	leucine rich repeat containing 8D	0.92764378	0.4263	0.6314
108863	4833420G11Rik	RIKEN cDNA 4833420G11 gene	0.92764378	0.427	0.6319
269870	Zfp446	zinc finger protein 446	0.92764378	0.4299	0.6341
330836	Slc7a6	solute carrier family 7 (cationic amino acid transporter, y+ system), member 6	0.92764378	0.4327	0.6366
63958	Ube4b	ubiquitination factor E4B, UFD2 homolog (S. cerevisiae)	0.92764378	0.4332	0.6368
260305	Nphp4	nephronophthisis 4 (juvenile) homolog (human)	0.92764378	0.4361	0.6396
29849	Olfr159	olfactory receptor 159	0.92764378	0.4413	0.6443
100043468	Zfp955b	zinc finger protein 955B	0.92764378	0.4442	0.647
104401	Pcnxl3	pecanex-like 3 (Drosophila)	0.92764378	0.457	0.6584
75985	Rab30	RAB30, member RAS oncogene family	0.92764378	0.4658	0.6657
11941	Atp2b2	ATPase, Ca++ transporting, plasma membrane 2	0.92764378	0.4861	0.6813
110886	Gabra5	gamma-aminobutyric acid (GABA) A receptor, subunit alpha 5	0.92764378	0.5045	0.6957
80744	Cwc22	CWC22 spliceosome-associated protein homolog (S. cerevisiae)	0.92764378	0.5226	0.7102
22317	Vamp1	vesicle-associated membrane protein 1	0.92764378	0.5366	0.7211
102153	C230098O21Rik	RIKEN cDNA C230098O21 gene	0.92764378	0.541	0.7241
69718	Ipmk	inositol polyphosphate multikinase	0.92764378	0.5779	0.7507
226751	Cdc42bpa	CDC42 binding protein kinase alpha	0.92764378	0.5946	0.7637
231042	Nupl2	nucleoporin like 2	0.92764378	0.6239	0.7846
329324	Syt14	synaptotagmin XIV	0.92764378	0.6326	0.7914
277854	Depdc5	DEP domain containing 5	0.92764378	0.6333	0.7916
552880	LOC552880	hypothetical LOC552880	0.92764378	0.6516	0.8034
67530	Uqcrb	ubiquinol-cytochrome c reductase binding protein	0.92678406	0.1097	NA
13434	Trdmt1	tRNA aspartic acid methyltransferase 1	0.92678406	0.1223	NA
74479	Snx11	sorting nexin 11	0.92678406	0.1228	NA
14056	Ezh2	enhancer of zeste homolog 2 (Drosophila)	0.92678406	0.1258	NA
100041585	Amd2	S-adenosylmethionine decarboxylase 2	0.92678406	0.1375	NA
240916	Vsig8	V-set and immunoglobulin domain containing 8	0.92678406	0.1611	NA
110380	Shroom2	shroom family member 2	0.92678406	0.1819	NA
15185	Hdac6	histone deacetylase 6	0.92678406	0.1851	NA
75826	Senp2	SUMO/sentrin specific peptidase 2	0.92678406	0.1886	NA
319554	Idi1	isopentenyl-diphosphate delta isomerase	0.92678406	0.2074	NA
14430	Galt	galactose-1-phosphate uridyl transferase	0.92678406	0.212	NA
268932	Caskin1	CASK interacting protein 1	0.92678406	0.2325	NA

66612	Ormdl3	ORM1-like 3 (S. cerevisiae)	0.92678406	0.235	NA
106025	Sharpin	SHANK-associated RH domain interacting protein	0.92678406	0.2512	NA
22201	Uba1	ubiquitin-like modifier activating enzyme 1	0.92678406	0.2612	NA
79233	Zfp319	zinc finger protein 319	0.92678406	0.2694	NA
66882	Bzw1	basic leucine zipper and W2 domains 1	0.92678406	0.2764	NA
93885	Pcdhb14	protocadherin beta 14	0.92678406	0.296	NA
27425	Atp5l	ATP synthase, H+ transporting, mitochondrial F0 complex, subunit g	0.92678406	0.3032	NA
77521	Mtus2	microtubule associated tumor suppressor candidate 2	0.92678406	0.3105	NA
217893	Pacs2	phosphofurin acidic cluster sorting protein 2	0.92678406	0.322	NA
67976	Trabd	TraB domain containing	0.92678406	0.3232	NA
108013	Celf4	CUGBP, Elav-like family member 4	0.92678406	0.3263	NA
68742	Tmem219	transmembrane protein 219	0.92678406	0.3278	NA
74493	Tnks2	tankyrase, TRF1-interacting ankyrin-related ADP-ribose polymerase 2	0.92678406	0.33	NA
71206	Katnal2	katanin p60 subunit A-like 2	0.92678406	0.3599	NA
76073	Pcgf5	polycomb group ring finger 5	0.92678406	0.3788	0.5878
70680	3021401N23Rik	RIKEN cDNA 3021401N23 gene	0.92678406	0.3821	0.5913
29866	Cabp2	calcium binding protein 2	0.92678406	0.4197	0.6259
110355	Adrbk1	adrenergic receptor kinase, beta 1	0.92678406	0.421	0.6271
71816	Rnf180	ring finger protein 180	0.92678406	0.4545	0.6562
268319	BC025920	zinc finger protein pseudogene	0.92678406	0.4546	0.6562
100044171	LOC100044171	hypothetical LOC100044171	0.92678406	0.4578	0.6591
104836	Cbll1	Casitas B-lineage lymphoma-like 1	0.92678406	0.4679	0.6677
59053	Brp16	brain protein 16	0.92678406	0.4944	0.688
56459	Sae1	SUMO1 activating enzyme subunit 1	0.92678406	0.4967	0.6899
77361	9430085M18Rik	RIKEN cDNA 9430085M18 gene	0.92678406	0.5027	0.6945
70729	Nos1ap	nitric oxide synthase 1 (neuronal) adaptor protein	0.92678406	0.5192	0.7085
57314	Th1l	TH1-like homolog (Drosophila)	0.92678406	0.5295	0.7153
67672	0610040B10Rik	RIKEN cDNA 0610040B10 gene	0.92678406	0.5303	0.7159
19159	Cyth3	cytohesin 3	0.92678406	0.5408	0.724
20585	Hltf	helicase-like transcription factor	0.92678406	0.5524	0.733
234967	Slc36a4	solute carrier family 36 (proton/amino acid symporter), member 4	0.92678406	0.5664	0.7427
19344	Rab5b	RAB5B, member RAS oncogene family	0.92678406	0.5984	0.7668
54712	Plxnc1	plexin C1	0.92678406	0.6786	0.8202
219181	Akap11	A kinase (PRKA) anchor protein 11	0.92678406	0.6989	0.8333
69694	Tatdn1	TatD DNase domain containing 1	0.92678406	0.7304	0.8538
621699	Gm6252	predicted gene 6252	0.92678406	0.7754	0.8805
18390	Oprm1	opioid receptor, mu 1	0.92678406	0.7897	0.8901
103067	AA522020	expressed sequence AA522020	0.92678406	0.7958	0.8927
110532	Adarb1	adenosine deaminase, RNA-specific, B1	0.92592593	0.06623	NA
14595	B4galt1	UDP-Gal:betaGlcNAc beta 1,4- galactosyltransferase, polypeptide 1	0.92592593	0.09174	NA
11352	Abl2	v-abl Abelson murine leukemia viral oncogene homolog 2 (arg, Abelson-related gene)	0.92592593	0.09359	NA
56066	Cxcl11	chemokine (C-X-C motif) ligand 11	0.92592593	0.1071	NA
109331	Rnf20	ring finger protein 20	0.92592593	0.1077	NA
231086	Hadhb	hydroxyacyl-Coenzyme A dehydrogenase/3-ketoacyl-Coenzyme A thiolase/enoyl-Coenzyme A hydratase (trifunctional protein), beta subunit	0.92592593	0.1112	NA
11765	Ap1g1	adaptor protein complex AP-1, gamma 1 subunit	0.92592593	0.1178	NA
212880	Ddx46	DEAD (Asp-Glu-Ala-Asp) box polypeptide 46	0.92592593	0.1205	NA
66576	Uqcrh	ubiquinol-cytochrome c reductase hinge protein	0.92592593	0.128	NA
67738	Ppid	peptidylprolyl isomerase D (cyclophilin D)	0.92592593	0.137	NA
69956	Ptcd3	pentatricopeptide repeat domain 3	0.92592593	0.1704	NA
233410	Zfp592	zinc finger protein 592	0.92592593	0.1835	NA

66349	Atp5sl	ATP5S-like	0.92592593	0.1846	NA
54170	Rragc	Ras-related GTP binding C	0.92592593	0.1988	NA
269113	Nup54	nucleoporin 54	0.92592593	0.2123	NA
67157	2610301B20Rik	RIKEN cDNA 2610301B20 gene	0.92592593	0.2217	NA
213027	Evi5l	ecotropic viral integration site 5 like	0.92592593	0.222	NA
53618	Fut8	fucosyltransferase 8	0.92592593	0.2253	NA
238323	Rps6kl1	ribosomal protein S6 kinase-like 1	0.92592593	0.228	NA
50912	Exosc10	exosome component 10	0.92592593	0.2287	NA
67529	Fgfr1op2	FGFR1 oncogene partner 2	0.92592593	0.2295	NA
12868	Cox8a	cytochrome c oxidase, subunit VIIIa	0.92592593	0.2343	NA
75641	1700029I15Rik	RIKEN cDNA 1700029I15 gene	0.92592593	0.2467	NA
68194	Ndufb4	NADH dehydrogenase (ubiquinone) 1 beta subcomplex 4	0.92592593	0.2476	NA
66427	Cyb5b	cytochrome b5 type B	0.92592593	0.2542	NA
68114	Mum1	melanoma associated antigen (mutated) 1	0.92592593	0.268	NA
15115	Hars	histidyl-tRNA synthetase	0.92592593	0.279	NA
70480	5730407M17Rik	RIKEN cDNA 5730407M17 gene	0.92592593	0.283	NA
237758	Zfp454	zinc finger protein 454	0.92592593	0.2908	NA
66209	1110054O05Rik	RIKEN cDNA 1110054O05 gene	0.92592593	0.2944	NA
332993	Gm5129	predicted gene 5129	0.92592593	0.3041	NA
14805	Grik1	glutamate receptor, ionotropic, kainate 1	0.92592593	0.3056	NA
27367	Rpl3	ribosomal protein L3	0.92592593	0.3171	NA
17193	Mbd4	methyl-CpG binding domain protein 4	0.92592593	0.3213	NA
17524	Mpp1	membrane protein, palmitoylated	0.92592593	0.3227	NA
232223	Txnrd3	thioredoxin reductase 3	0.92592593	0.3308	NA
66254	Dimt1	DIM1 dimethyladenosine transferase 1-like (S. cerevisiae)	0.92592593	0.3327	NA
331461	Il1rapl1	interleukin 1 receptor accessory protein-like 1	0.92592593	0.3351	NA
66923	Pbrm1	polybromo 1	0.92592593	0.3552	0.5648
245174	Zfp937	zinc finger protein 937	0.92592593	0.3653	0.5743
12561	Cdh4	cadherin 4	0.92592593	0.37	0.579
56009	Refbp2	RNA and export factor binding protein 2	0.92592593	0.3711	0.5799
218271	B4galt7	xylosylprotein beta1,4-galactosyltransferase, polypeptide 7 (galactosyltransferase I)	0.92592593	0.3854	0.5945
55994	Smad9	MAD homolog 9 (Drosophila)	0.92592593	0.3866	0.5952
64898	Lpin2	lipin 2	0.92592593	0.3985	0.6064
23792	Adam23	a disintegrin and metallopeptidase domain 23	0.92592593	0.4097	0.6161
216766	Gemin5	gem (nuclear organelle) associated protein 5	0.92592593	0.4221	0.6279
67878	Tmem33	transmembrane protein 33	0.92592593	0.4417	0.6446
328505	Skint7	selection and upkeep of intraepithelial T cells 7	0.92592593	0.4648	0.665
218397	Rasa1	RAS p21 protein activator 1	0.92592593	0.4678	0.6676
71073	4933421O10Rik	RIKEN cDNA 4933421O10 gene	0.92592593	0.4814	0.6775
380714	Rph3al	rabphilin 3A-like (without C2 domains)	0.92592593	0.4889	0.6836
11836	Araf	v-raf murine sarcoma 3611 viral oncogene homolog	0.92592593	0.4907	0.6849
26406	Map3k3	mitogen-activated protein kinase kinase kinase 3	0.92592593	0.5028	0.6945
12212	Chic1	cysteine-rich hydrophobic domain 1	0.92592593	0.5043	0.6956
21804	Tgfb1i1	transforming growth factor beta 1 induced transcript 1	0.92592593	0.5305	0.716
78294	Rps27a	ribosomal protein S27A	0.92592593	0.539	0.7227
75782	Lca5	Leber congenital amaurosis 5 (human)	0.92592593	0.547	0.7288
240066	Zfp870	zinc finger protein 870	0.92592593	0.5474	0.7291
15465	Hrh1	histamine receptor H1	0.92592593	0.6405	0.7967
19131	Prh1	proline rich protein HaeIII subfamily 1	0.92592593	0.657	0.8063
15932	Idua	iduronidase, alpha-L-	0.92592593	0.7655	0.8755

57439	Tmem183a	transmembrane protein 183A	0.92506938	0.06289	NA
69940	Exoc1	exocyst complex component 1	0.92506938	0.07849	NA
76306	1110021L09Rik	RIKEN cDNA 1110021L09 gene	0.92506938	0.08722	NA
66980	Zdhhc6	zinc finger, DHHC domain containing 6	0.92506938	0.1017	NA
52335	Atxn1l	ataxin 1-like	0.92506938	0.1055	NA
219135	Mtmr6	myotubularin related protein 6	0.92506938	0.1093	NA
68972	Tatdn3	TatD DNase domain containing 3	0.92506938	0.1137	NA
66050	0610009B22Rik	RIKEN cDNA 0610009B22 gene	0.92506938	0.1244	NA
66433	Chchd7	coiled-coil-helix-coiled-coil-helix domain containing 7	0.92506938	0.125	NA
16834	Cog1	component of oligomeric golgi complex 1	0.92506938	0.1401	NA
75625	Mageh1	melanoma antigen, family H, 1	0.92506938	0.153	NA
73173	Pcdh18	protocadherin 18	0.92506938	0.1532	NA
320472	Ppm1e	protein phosphatase 1E (PP2C domain containing)	0.92506938	0.1728	NA
12386	Ctnna2	catenin (cadherin associated protein), alpha 2	0.92506938	0.1759	NA
278304	Zfp385c	zinc finger protein 385C	0.92506938	0.1793	NA
224647	D17Wsu92e	DNA segment, Chr 17, Wayne State University 92, expressed	0.92506938	0.1978	NA
102607	Snx19	sorting nexin 19	0.92506938	0.1982	NA
208908	Ccdc62	coiled-coil domain containing 62	0.92506938	0.2025	NA
17119	Mxd1	MAX dimerization protein 1	0.92506938	0.2091	NA
72170	Chchd4	coiled-coil-helix-coiled-coil-helix domain containing 4	0.92506938	0.2098	NA
102098	Arhgef18	rho/rac guanine nucleotide exchange factor (GEF) 18	0.92506938	0.2242	NA
19212	Pter	phosphotriesterase related	0.92506938	0.2297	NA
108123	Napg	N-ethylmaleimide sensitive fusion protein attachment protein gamma	0.92506938	0.2502	NA
227622	BC029214	cDNA sequence BC029214	0.92506938	0.2577	NA
74112	Usp16	ubiquitin specific peptidase 16	0.92506938	0.2602	NA
75689	Higd1b	HIG1 domain family, member 1B	0.92506938	0.282	NA
244958	Mrap2	melanocortin 2 receptor accessory protein 2	0.92506938	0.2963	NA
19716	Bex1	brain expressed gene 1	0.92506938	0.3022	NA
70604	Dnajb14	DnaJ (Hsp40) homolog, subfamily B, member 14	0.92506938	0.3024	NA
14245	Lpin1	lipin 1	0.92506938	0.3091	NA
20927	Abcc8	ATP-binding cassette, sub-family C (CFTR/MRP), member 8	0.92506938	0.3133	NA
19735	Rgs2	regulator of G-protein signaling 2	0.92506938	0.3186	NA
56200	Ddx21	DEAD (Asp-Glu-Ala-Asp) box polypeptide 21	0.92506938	0.3273	NA
26896	Med14	mediator complex subunit 14	0.92506938	0.3307	NA
399548	Scn4b	sodium channel, type IV, beta	0.92506938	0.3387	NA
21338	Tacr3	tachykinin receptor 3	0.92506938	0.34	NA
12824	Col2a1	collagen, type II, alpha 1	0.92506938	0.3407	NA
97827	Exd2	exonuclease 3'-5' domain containing 2	0.92506938	0.3421	NA
21406	Tcf12	transcription factor 12	0.92506938	0.3507	0.5609
22003	Tpm1	tropomyosin 1, alpha	0.92506938	0.3603	0.5701
544696	D630037F22Rik	RIKEN cDNA D630037F22 gene	0.92506938	0.3888	0.5971
211556	Ap1ar	adaptor-related protein complex 1 associated regulatory protein	0.92506938	0.3974	0.6054
13628	Eef1a2	eukaryotic translation elongation factor 1 alpha 2	0.92506938	0.4063	0.6135
117229	Stk33	serine/threonine kinase 33	0.92506938	0.4104	0.6168
213012	Abhd10	abhydrolase domain containing 10	0.92506938	0.4213	0.6273
69519	Rwdd2a	RWD domain containing 2A	0.92506938	0.434	0.6378
27364	Srr	serine racemase	0.92506938	0.4385	0.6416
23900	Hcst	hematopoietic cell signal transducer	0.92506938	0.4393	0.6422
244810	AW551984	expressed sequence AW551984	0.92506938	0.4675	0.6674
20823	Ssb	Sjogren syndrome antigen B	0.92506938	0.5003	0.6924

56441	Nat6	N-acetyltransferase 6	0.92506938	0.5224	0.7102
100038641	Gm10452	predicted gene 10452	0.92506938	0.523	0.7102
208366	Rpp40	ribonuclease P 40 subunit (human)	0.92506938	0.5729	0.7474
382066	Prdm10	PR domain containing 10	0.92506938	0.5774	0.7503
320460	Vwc2l	von Willebrand factor C domain-containing protein 2-like	0.92506938	0.5922	0.7617
14169	Fgf14	fibroblast growth factor 14	0.92506938	0.6075	0.773
668311	Gm9099	predicted gene 9099	0.92506938	0.654	0.8045
100503757	LOC100503757	putative transposase element L1Md-A101/L1Md-A102/L1Md-A2-like	0.92506938	0.6911	0.8287
100044832	LOC100044832	mucosal pentraxin-like	0.92506938	0.693	0.83
57264	Retn	resistin	0.92506938	0.7093	0.8401
233812	BC030336	cDNA sequence BC030336	0.92421442	0.09138	NA
54616	Extl3	exostoses (multiple)-like 3	0.92421442	0.1204	NA
212706	N4bp3	NEDD4 binding protein 3	0.92421442	0.1286	NA
20463	Cox7a2l	cytochrome c oxidase subunit VIIa polypeptide 2-like	0.92421442	0.1306	NA
320632	Snrnp200	small nuclear ribonucleoprotein 200 (U5)	0.92421442	0.1519	NA
11732	Ank	progressive ankylosis	0.92421442	0.1555	NA
72502	Cwf19l1	CWF19-like 1, cell cycle control (S. pombe)	0.92421442	0.1748	NA
22084	Tsc2	tuberous sclerosis 2	0.92421442	0.1768	NA
78618	Acap2	ArfGAP with coiled-coil, ankyrin repeat and PH domains 2	0.92421442	0.1967	NA
240063	Zfp811	zinc finger protein 811	0.92421442	0.2025	NA
56214	Scamp4	secretory carrier membrane protein 4	0.92421442	0.2039	NA
19356	Rad17	RAD17 homolog (S. pombe)	0.92421442	0.2069	NA
73274	Gpbp1	GC-rich promoter binding protein 1	0.92421442	0.2126	NA
216810	Tom1l2	target of myb1-like 2 (chicken)	0.92421442	0.2201	NA
77965	A930033C23Rik	RIKEN cDNA A930033C23 gene	0.92421442	0.2268	NA
21745	Tep1	telomerase associated protein 1	0.92421442	0.2348	NA
69028	Mitd1	MIT, microtubule interacting and transport, domain containing 1	0.92421442	0.2377	NA
69727	Usp46	ubiquitin specific peptidase 46	0.92421442	0.2378	NA
77853	Msl2	male-specific lethal 2 homolog (Drosophila)	0.92421442	0.2436	NA
230393	BC057079	cDNA sequence BC057079	0.92421442	0.2568	NA
16653	Kras	v-Ki-ras2 Kirsten rat sarcoma viral oncogene homolog	0.92421442	0.2572	NA
68585	Rtn4	reticulon 4	0.92421442	0.2574	NA
81000	Rad54l2	RAD54 like 2 (S. cerevisiae)	0.92421442	0.2649	NA
11773	Ap2m1	adaptor protein complex AP-2, mu1	0.92421442	0.2654	NA
215693	Zmat1	zinc finger, matrin type 1	0.92421442	0.2754	NA
240332	Slc6a7	solute carrier family 6 (neurotransmitter transporter, L-proline), member 7	0.92421442	0.3153	NA
67988	Tmx3	thioredoxin-related transmembrane protein 3	0.92421442	0.3328	NA
268859	Rbfox1	RNA binding protein, fox-1 homolog (C. elegans) 1	0.92421442	0.3356	NA
18530	Pcdh8	protocadherin 8	0.92421442	0.3432	0.5542
52686	Mettl2	methyltransferase like 2	0.92421442	0.3535	0.5634
67252	Cap2	CAP, adenylate cyclase-associated protein, 2 (yeast)	0.92421442	0.3716	0.5803
69309	Slc16a13	solute carrier family 16 (monocarboxylic acid transporters), member 13	0.92421442	0.3876	0.5961
140483	Hnmt	histamine N-methyltransferase	0.92421442	0.3954	0.6033
56878	Rbms1	RNA binding motif, single stranded interacting protein 1	0.92421442	0.4018	0.6094
118451	Mrps2	mitochondrial ribosomal protein S2	0.92421442	0.4168	0.623
102243	AU024581	expressed sequence AU024581	0.92421442	0.4227	0.6283
74334	Ranbp10	RAN binding protein 10	0.92421442	0.4328	0.6366
50793	Orc3	origin recognition complex, subunit 3	0.92421442	0.4441	0.6468
58188	Vstm2b	V-set and transmembrane domain containing 2B	0.92421442	0.445	0.6478
69713	Pin4	protein (peptidyl-prolyl cis/trans isomerase) NIMA-interacting, 4 (parvulin)	0.92421442	0.4453	0.648

100039284	Gm2137	predicted gene 2137	0.92421442	0.4603	0.6611
234740	Tmem231	transmembrane protein 231	0.92421442	0.4605	0.6613
280287	Kiss1	KiSS-1 metastasis-suppressor	0.92421442	0.4627	0.6632
24086	Tlk2	tousled-like kinase 2 (Arabidopsis)	0.92421442	0.4643	0.6645
26412	Map4k2	mitogen-activated protein kinase kinase kinase kinase 2	0.92421442	0.4755	0.6729
76022	Gon4l	gon-4-like (C.elegans)	0.92421442	0.4821	0.6781
52014	Nus1	nuclear undecaprenyl pyrophosphate synthase 1 homolog (S. cerevisiae)	0.92421442	0.4977	0.6905
104252	Cdc42ep2	CDC42 effector protein (Rho GTPase binding) 2	0.92421442	0.4987	0.6913
66934	Dsn1	DSN1, MIND kinetochore complex component, homolog (S. cerevisiae)	0.92421442	0.5199	0.7087
15168	Hcn3	hyperpolarization-activated, cyclic nucleotide-gated K+ 3	0.92421442	0.52	0.7087
210789	Tbc1d4	TBC1 domain family, member 4	0.92421442	0.5318	0.717
21985	Tpd52	tumor protein D52	0.92421442	0.5711	0.7461
215798	Gpr126	G protein-coupled receptor 126	0.92421442	0.5753	0.749
72181	Nsun4	NOL1/NOP2/Sun domain family, member 4	0.92421442	0.593	0.7624
73192	Xpot	exportin, tRNA (nuclear export receptor for tRNAs)	0.92421442	0.6503	0.8031
234135	Whsc1l1	Wolf-Hirschhorn syndrome candidate 1-like 1 (human)	0.92421442	0.6785	0.8202
244199	Ovch2	ovochymase 2	0.92421442	0.6825	0.8228
69587	Pcgf3	polycomb group ring finger 3	0.92421442	0.7253	0.8505
320538	Ubn2	ubinuclein 2	0.92336103	0.08141	NA
67388	1110008F13Rik	RIKEN cDNA 1110008F13 gene	0.92336103	0.09682	NA
234728	Ftsjd1	FtsJ methyltransferase domain containing 1	0.92336103	0.1083	NA
28040	D6Wsu163e	DNA segment, Chr 6, Wayne State University 163, expressed	0.92336103	0.1387	NA
77781	Epm2aip1	EPM2A (laforin) interacting protein 1	0.92336103	0.1449	NA
227541	Camk1d	calcium/calmodulin-dependent protein kinase ID	0.92336103	0.1871	NA
14586	Gfra2	glial cell line derived neurotrophic factor family receptor alpha 2	0.92336103	0.2191	NA
209131	Snx30	sorting nexin family member 30	0.92336103	0.2235	NA
15893	Ica1	islet cell autoantigen 1	0.92336103	0.2313	NA
235604	Camkv	CaM kinase-like vesicle-associated	0.92336103	0.232	NA
19293	Pvalb	parvalbumin	0.92336103	0.2368	NA
105727	Slc38a1	solute carrier family 38, member 1	0.92336103	0.2393	NA
73766	4833420D23Rik	RIKEN cDNA 4833420D23 gene	0.92336103	0.2443	NA
229004	Gmeb2	glucocorticoid modulatory element binding protein 2	0.92336103	0.2631	NA
13063	Cycs	cytochrome c, somatic	0.92336103	0.2659	NA
224893	Zfp959	zinc finger protein 959	0.92336103	0.2688	NA
21771	Cirh1a	cirrhosis, autosomal recessive 1A (human)	0.92336103	0.2724	NA
20768	Sephs2	selenophosphate synthetase 2	0.92336103	0.2792	NA
103466	Nt5dc3	5'-nucleotidase domain containing 3	0.92336103	0.2877	NA
329178	Unc80	unc-80 homolog (C. elegans)	0.92336103	0.2957	NA
109637	Upk1a	uroplakin 1A	0.92336103	0.3122	NA
226251	Ablim1	actin-binding LIM protein 1	0.92336103	0.3314	NA
214901	Chtf18	CTF18, chromosome transmission fidelity factor 18 homolog (S. cerevisiae)	0.92336103	0.3315	NA
66194	Pycrl	pyrroline-5-carboxylate reductase-like	0.92336103	0.3466	0.5572
218035	Vps41	vacuolar protein sorting 41 (yeast)	0.92336103	0.3666	0.5757
21838	Thy1	thymus cell antigen 1, theta	0.92336103	0.389	0.5973
67619	Nob1	NIN1/RPN12 binding protein 1 homolog (S. cerevisiae)	0.92336103	0.3896	0.5978
442821	9330175M20Rik	RIKEN cDNA 9330175M20 gene	0.92336103	0.3911	0.5993
18004	Nek1	NIMA (never in mitosis gene a)-related expressed kinase 1	0.92336103	0.409	0.6154
226791	Lyplal1	lysophospholipase-like 1	0.92336103	0.4156	0.6219
228788	BC020535	cDNA sequence BC020535	0.92336103	0.4225	0.6282
67671	Rpl38	ribosomal protein L38	0.92336103	0.4332	0.6368

27221	Chaf1a	chromatin assembly factor 1, subunit A (p150)	0.92336103	0.4353	0.6391
79555	BC005537	cDNA sequence BC005537	0.92336103	0.4359	0.6395
14347	Fut7	fucosyltransferase 7	0.92336103	0.4529	0.6549
230866	C230096C10Rik	RIKEN cDNA C230096C10 gene	0.92336103	0.4594	0.6604
208595	Gm9897	predicted gene 9897	0.92336103	0.5094	0.7001
100040654	Gm2885	predicted gene 2885	0.92336103	0.5274	0.7138
20183	Rxrg	retinoid X receptor gamma	0.92336103	0.5347	0.7194
53761	Prrc2a	proline-rich coiled-coil 2A	0.92336103	0.5557	0.7355
229791	D3Bwg0562e	DNA segment, Chr 3, Brigham & Women's Genetics 0562 expressed	0.92336103	0.5908	0.7605
268729	Gm626	predicted gene 626	0.92336103	0.5983	0.7667
16421	Itgb7	integrin beta 7	0.92336103	0.6157	0.7787
664883	Nova1	neuro-oncological ventral antigen 1	0.92336103	0.6259	0.7862
381199	Tmem151a	transmembrane protein 151A	0.92336103	0.6291	0.789
229571	Gm4858	predicted gene 4858	0.92336103	0.7686	0.8772
68708	Rabl2	RAB, member of RAS oncogene family-like 2	0.92250923	0.06799	NA
16832	Ldhb	lactate dehydrogenase B	0.92250923	0.09092	NA
16004	Igf2r	insulin-like growth factor 2 receptor	0.92250923	0.09129	NA
65112	Pmepa1	prostate transmembrane protein, androgen induced 1	0.92250923	0.1026	NA
17713	Grpel1	GrpE-like 1, mitochondrial	0.92250923	0.1264	NA
66039	D14Ertd449e	DNA segment, Chr 14, ERATO Doi 449, expressed	0.92250923	0.135	NA
56772	Mllt11	myeloid/lymphoid or mixed-lineage leukemia (trithorax homolog, Drosophila); translocated to, 11	0.92250923	0.1375	NA
22779	Ikzf2	IKAROS family zinc finger 2	0.92250923	0.1415	NA
224440	Setd4	SET domain containing 4	0.92250923	0.1474	NA
216805	Flcn	folliculin	0.92250923	0.1499	NA
52708	Zfp410	zinc finger protein 410	0.92250923	0.1517	NA
59009	Sh3rf1	SH3 domain containing ring finger 1	0.92250923	0.1537	NA
229709	Ahcyl1	S-adenosylhomocysteine hydrolase-like 1	0.92250923	0.1641	NA
16648	Kpna3	karyopherin (importin) alpha 3	0.92250923	0.171	NA
16561	Kif1b	kinesin family member 1B	0.92250923	0.1782	NA
67864	Yipf4	Yip1 domain family, member 4	0.92250923	0.1927	NA
101923	BB212172	expressed sequence BB212172	0.92250923	0.1935	NA
97820	4833439L19Rik	RIKEN cDNA 4833439L19 gene	0.92250923	0.194	NA
69721	Nkiras1	NFKB inhibitor interacting Ras-like protein 1	0.92250923	0.2077	NA
67049	Pus3	pseudouridine synthase 3	0.92250923	0.2097	NA
66320	Tmem208	transmembrane protein 208	0.92250923	0.2189	NA
268373	Ppia	peptidylprolyl isomerase A	0.92250923	0.2408	NA
68184	Denr	density-regulated protein	0.92250923	0.2425	NA
64085	Clstn2	calsyntenin 2	0.92250923	0.2448	NA
66686	Dcbld1	discoidin, CUB and LCCL domain containing 1	0.92250923	0.2531	NA
13368	Dffb	DNA fragmentation factor, beta subunit	0.92250923	0.2573	NA
68364	0610030E20Rik	RIKEN cDNA 0610030E20 gene	0.92250923	0.2624	NA
232941	Ppm1n	protein phosphatase, Mg2+/Mn2+ dependent, 1N (putative)	0.92250923	0.266	NA
68316	Apoo	apolipoprotein O	0.92250923	0.2826	NA
70640	Dcp2	DCP2 decapping enzyme homolog (S. cerevisiae)	0.92250923	0.2933	NA
20383	Srsf3	serine/arginine-rich splicing factor 3	0.92250923	0.2941	NA
434437	Amt	aminomethyltransferase	0.92250923	0.3006	NA
19651	Rbl2	retinoblastoma-like 2	0.92250923	0.3246	NA
216154	Med16	mediator complex subunit 16	0.92250923	0.3304	NA
18762	Prkcz	protein kinase C, zeta	0.92250923	0.3434	0.5545
57295	Icmt	isoprenylcysteine carboxyl methyltransferase	0.92250923	0.3435	0.5546

353170	Txlng	taxilin gamma	0.92250923	0.3435	0.5546
330998	Ankrd34c	ankyrin repeat domain 34C	0.92250923	0.3495	0.5599
107769	Tm6sf1	transmembrane 6 superfamily member 1	0.92250923	0.3695	0.5785
70144	Lrch3	leucine-rich repeats and calponin homology (CH) domain containing 3	0.92250923	0.3798	0.5889
22666	Zfp161	zinc finger protein 161	0.92250923	0.4006	0.6083
211255	Kbtbd7	kelch repeat and BTB (POZ) domain containing 7	0.92250923	0.4033	0.6108
69906	Slc25a32	solute carrier family 25, member 32	0.92250923	0.4034	0.6108
56292	Naa10	N(alpha)-acetyltransferase 10, NatA catalytic subunitNalpha acetyltransferase 10	0.92250923	0.4419	0.6449
224903	Safb	scaffold attachment factor B	0.92250923	0.4484	0.6505
19877	Rock1	Rho-associated coiled-coil containing protein kinase 1	0.92250923	0.4651	0.6653
67547	Slc39a8	solute carrier family 39 (metal ion transporter), member 8	0.92250923	0.4659	0.6658
269630	5031425E22Rik	RIKEN cDNA 5031425E22 gene	0.92250923	0.4685	0.6682
16716	Ky	kyphoscoliosis peptidase	0.92250923	0.4858	0.6811
380928	Lmo7	LIM domain only 7	0.92250923	0.4985	0.6912
320206	A730028G07Rik	RIKEN cDNA A730028G07 gene	0.92250923	0.5211	0.7094
211948	Pde12	phosphodiesterase 12	0.92250923	0.522	0.7099
72935	Ddx41	DEAD (Asp-Glu-Ala-Asp) box polypeptide 41	0.92250923	0.5292	0.7151
73852	D3Ertd751e	DNA segment, Chr 3, ERATO Doi 751, expressed	0.92250923	0.5344	0.7191
23969	Pacsin1	protein kinase C and casein kinase substrate in neurons 1	0.92250923	0.5452	0.7272
11556	Adrb3	adrenergic receptor, beta 3	0.92250923	0.551	0.732
50817	Solh	small optic lobes homolog (Drosophila)	0.92250923	0.5839	0.755
234353	Psd3	pleckstrin and Sec7 domain containing 3	0.92250923	0.5971	0.7657
272158	Poln	DNA polymerase N	0.92250923	0.7267	0.8514
216739	Acsl6	acyl-CoA synthetase long-chain family member 6	0.92165899	0.07608	NA
16319	Incenp	inner centromere protein	0.92165899	0.07926	NA
235036	Ppan	peter pan homolog (Drosophila)	0.92165899	0.08142	NA
226747	Ahctf1	AT hook containing transcription factor 1	0.92165899	0.1121	NA
381760	Ssbp1	single-stranded DNA binding protein 1	0.92165899	0.117	NA
77264	Zfp142	zinc finger protein 142	0.92165899	0.1277	NA
22381	Wbp5	WW domain binding protein 5	0.92165899	0.1401	NA
12859	Cox5b	cytochrome c oxidase, subunit Vb	0.92165899	0.1428	NA
66084	Rmnd1	required for meiotic nuclear division 1 homolog (S. cerevisiae)	0.92165899	0.1495	NA
225655	Slmo1	slowmo homolog 1 (Drosophila)	0.92165899	0.1598	NA
213011	Zfp583	zinc finger protein 583	0.92165899	0.1758	NA
68910	Zfp467	zinc finger protein 467	0.92165899	0.1782	NA
216292	BC067068	cDNA sequence BC067068	0.92165899	0.1812	NA
80732	Mynn	myoneurin	0.92165899	0.1822	NA
226123	Morn4	MORN repeat containing 4	0.92165899	0.1889	NA
19082	Prkag1	protein kinase, AMP-activated, gamma 1 non-catalytic subunit	0.92165899	0.1947	NA
16434	Itpa	inosine triphosphatase (nucleoside triphosphate pyrophosphatase)	0.92165899	0.2053	NA
239393	Lrp12	low density lipoprotein-related protein 12	0.92165899	0.2145	NA
329152	Hecw2	HECT, C2 and WW domain containing E3 ubiquitin protein ligase 2	0.92165899	0.2165	NA
246133	Kcne2	potassium voltage-gated channel, Isk-related subfamily, gene 2	0.92165899	0.2294	NA
59126	Nek6	NIMA (never in mitosis gene a)-related expressed kinase 6	0.92165899	0.2452	NA
11989	Slc7a3	solute carrier family 7 (cationic amino acid transporter, y+ system), member 3	0.92165899	0.2477	NA
69161	Manbal	mannosidase, beta A, lysosomal-like	0.92165899	0.2641	NA
117146	Ube3b	ubiquitin protein ligase E3B	0.92165899	0.2669	NA
230119	Zbtb5	zinc finger and BTB domain containing 5	0.92165899	0.2706	NA
54189	Rabep1	rabaptin, RAB GTPase binding effector protein 1	0.92165899	0.2814	NA
57837	Eral1	Era (G-protein)-like 1 (E. coli)	0.92165899	0.3004	NA

17207	Mcf2l	mcf.2 transforming sequence-like	0.92165899	0.3019	NA
18751	Prkcb	protein kinase C, beta	0.92165899	0.3063	NA
14884	Gtf2h1	general transcription factor II H, polypeptide 1	0.92165899	0.3083	NA
330267	Thsd7a	thrombospondin, type I, domain containing 7A	0.92165899	0.3091	NA
13131	Dab1	disabled homolog 1 (Drosophila)	0.92165899	0.3246	NA
12721	Coro1a	coronin, actin binding protein 1A	0.92165899	0.3498	0.5602
231861	Tnrc18	trinucleotide repeat containing 18	0.92165899	0.359	0.5688
77300	Raph1	Ras association (RalGDS/AF-6) and pleckstrin homology domains 1	0.92165899	0.3842	0.5933
675578	Gm9648	predicted gene 9648	0.92165899	0.3853	0.5945
64009	Syne1	synaptic nuclear envelope 1	0.92165899	0.4062	0.6135
16010	Igfbp4	insulin-like growth factor binding protein 4	0.92165899	0.4268	0.6318
11991	Hnrnpd	heterogeneous nuclear ribonucleoprotein D	0.92165899	0.4657	0.6657
320982	Arl4c	ADP-ribosylation factor-like 4C	0.92165899	0.4691	0.6686
214899	Kdm5a	lysine (K)-specific demethylase 5A	0.92165899	0.4697	0.6689
14232	Fkbp8	FK506 binding protein 8	0.92165899	0.505	0.6962
78912	Sp2	Sp2 transcription factor	0.92165899	0.5319	0.717
16009	Igfbp3	insulin-like growth factor binding protein 3	0.92165899	0.5393	0.7228
258593	Olfr700	olfactory receptor 700	0.92165899	0.5419	0.7247
14472	Gbx2	gastrulation brain homeobox 2	0.92165899	0.5622	0.74
58208	Bcl11b	B-cell leukemia/lymphoma 11B	0.92165899	0.5877	0.7579
16563	Kif2a	kinesin family member 2A	0.92165899	0.5925	0.762
69824	Glod5	glyoxalase domain containing 5	0.92165899	0.6023	0.7693
53975	Ddx20	DEAD (Asp-Glu-Ala-Asp) box polypeptide 20	0.92165899	0.6198	0.7816
56744	Pf4	platelet factor 4	0.92165899	0.7412	0.8599
432572	Specc1	sperm antigen with calponin homology and coiled-coil domains 1	0.92165899	0.7496	0.8653
109575	Tbx10	T-box 10	0.92165899	0.7611	0.8729
216453	Rdh19	retinol dehydrogenase 19	0.92165899	0.7956	0.8927
16878	Lif	leukemia inhibitory factor	0.92165899	0.831	0.9126
72661	Serp2	stress-associated endoplasmic reticulum protein family member 2	0.92081031	0.07546	NA
23924	Katna1	katanin p60 (ATPase-containing) subunit A1	0.92081031	0.07702	NA
52705	Krr1	KRR1, small subunit (SSU) processome component, homolog (yeast)	0.92081031	0.08121	NA
15500	Hsf2	heat shock factor 2	0.92081031	0.09649	NA
207615	Wdr37	WD repeat domain 37	0.92081031	0.1012	NA
231042	Nupl2	nucleoporin like 2	0.92081031	0.1209	NA
13382	Dld	dihydrolipoamide dehydrogenase	0.92081031	0.1223	NA
74776	Ppa2	pyrophosphatase (inorganic) 2	0.92081031	0.1306	NA
75624	Metap1	methionyl aminopeptidase 1	0.92081031	0.1472	NA
75734	Mff	mitochondrial fission factor	0.92081031	0.1567	NA
69860	Eif1ad	eukaryotic translation initiation factor 1A domain containing	0.92081031	0.1651	NA
74256	Cyld	cylindromatosis (turban tumor syndrome)	0.92081031	0.1662	NA
94282	Sfxn5	sideroflexin 5	0.92081031	0.1704	NA
227707	BC005624	cDNA sequence BC005624	0.92081031	0.1766	NA
27681	Snf8	SNF8, ESCRT-II complex subunit, homolog (S. cerevisiae)	0.92081031	0.1769	NA
16211	Kpnb1	karyopherin (importin) beta 1	0.92081031	0.2219	NA
246257	Ovca2	candidate tumor suppressor in ovarian cancer 2	0.92081031	0.2511	NA
17756	Mtap2	microtubule-associated protein 2	0.92081031	0.258	NA
272027	Tstd2	thiosulfate sulfurtransferase (rhodanese)-like domain containing 2	0.92081031	0.287	NA
66297	2610017I09Rik	RIKEN cDNA 2610017I09 gene	0.92081031	0.2998	NA
212996	Wbscr17	Williams-Beuren syndrome chromosome region 17 homolog (human)	0.92081031	0.3134	NA
54214	Golga4	golgi autoantigen, golgin subfamily a, 4	0.92081031	0.3192	0.529

20778	Scarb1	scavenger receptor class B, member 1	0.92081031	0.3562	0.566
230789	Fam76a	family with sequence similarity 76, member A	0.92081031	0.3643	0.5733
628110	LOC628110	similar to gonadotropin inducible ovarian transcription factor 1	0.92081031	0.3698	0.5788
56692	Mapksp1	MAPK scaffold protein 1	0.92081031	0.3879	0.5964
104111	Adcy3	adenylate cyclase 3	0.92081031	0.4146	0.6209
18987	Pou2f2	POU domain, class 2, transcription factor 2	0.92081031	0.4222	0.6279
60597	Mapk8ip2	mitogen-activated protein kinase 8 interacting protein 2	0.92081031	0.4707	0.6697
219189	1300010F03Rik	RIKEN cDNA 1300010F03 gene	0.92081031	0.4822	0.6782
327951	Cyb5d1	cytochrome b5 domain containing 1	0.92081031	0.4849	0.6803
52647	D15Ertd509e	DNA segment, Chr 15, ERATO Doi 509, expressed	0.92081031	0.5108	0.7013
11736	Ankfy1	ankyrin repeat and FYVE domain containing 1	0.92081031	0.5165	0.706
74018	Als2	amyotrophic lateral sclerosis 2 (juvenile) homolog (human)	0.92081031	0.5218	0.7098
17184	Matr3	matrin 3	0.92081031	0.5226	0.7102
14809	Grik5	glutamate receptor, ionotropic, kainate 5 (gamma 2)	0.92081031	0.5521	0.7328
243612	D630042P16Rik	RIKEN cDNA D630042P16 gene	0.92081031	0.5748	0.7486
83762	Otof	otoferlin	0.92081031	0.6414	0.7973
77629	Sphkap	SPHK1 interactor, AKAP domain containing	0.92081031	0.6572	0.8065
238662	Gm4934	predicted gene 4934	0.92081031	0.679	0.8206
54638	Ccdc22	coiled-coil domain containing 22	0.9199632	0.06682	NA
192159	Prpf8	pre-mRNA processing factor 8	0.9199632	0.07268	NA
223435	Trio	triple functional domain (PTPRF interacting)	0.9199632	0.0768	NA
280411	Lix1l	Lix1-like	0.9199632	0.08875	NA
66912	Bzw2	basic leucine zipper and W2 domains 2	0.9199632	0.1173	NA
15962	Ifna1	interferon alpha 1	0.9199632	0.1292	NA
67667	Alkbh8	alkB, alkylation repair homolog 8 (E. coli)	0.9199632	0.1386	NA
207214	Larp4	La ribonucleoprotein domain family, member 4	0.9199632	0.1409	NA
51797	Ctps	cytidine 5'-triphosphate synthase	0.9199632	0.1426	NA
108062	Cstf2	cleavage stimulation factor, 3' pre-RNA subunit 2	0.9199632	0.1439	NA
13663	Ei24	etoposide induced 2.4 mRNA	0.9199632	0.1526	NA
13191	Dctn1	dynactin 1	0.9199632	0.1551	NA
77038	Arfgap2	ADP-ribosylation factor GTPase activating protein 2	0.9199632	0.1562	NA
16826	Ldb2	LIM domain binding 2	0.9199632	0.1681	NA
212439	AA986860	expressed sequence AA986860	0.9199632	0.1835	NA
77006	Ddrgk1	DDRGK domain containing 1	0.9199632	0.2114	NA
108073	Grm7	glutamate receptor, metabotropic 7	0.9199632	0.2129	NA
78016	Ccdc150	coiled-coil domain containing 150	0.9199632	0.2143	NA
244059	Chd2	chromodomain helicase DNA binding protein 2	0.9199632	0.2283	NA
106877	Afap1l1	actin filament associated protein 1-like 1	0.9199632	0.2532	NA
319817	Rc3h2	ring finger and CCCH-type zinc finger domains 2	0.9199632	0.2541	NA
67059	Ola1	Obg-like ATPase 1	0.9199632	0.2566	NA
17912	Myo1b	myosin IB	0.9199632	0.2584	NA
67797	Snrnp48	small nuclear ribonucleoprotein 48 (U11/U12)	0.9199632	0.2689	NA
170763	Zfp87	zinc finger protein 87	0.9199632	0.2978	NA
386750	Slitrk3	SLIT and NTRK-like family, member 3	0.9199632	0.2985	NA
68832	1110057K04Rik	RIKEN cDNA 1110057K04 gene	0.9199632	0.3221	0.5318
100039286	Gm2138	predicted gene 2138	0.9199632	0.3276	0.5376
67731	Fbxo32	F-box protein 32	0.9199632	0.3323	0.5424
66291	1810030N24Rik	RIKEN cDNA 1810030N24 gene	0.9199632	0.3363	0.5467
53382	Txnl1	thioredoxin-like 1	0.9199632	0.3459	0.5567
213464	Rbbp5	retinoblastoma binding protein 5	0.9199632	0.3474	0.5578

67188	2700046G09Rik	RIKEN cDNA 2700046G09 gene	0.9199632	0.3743	0.5831
20266	Scn1b	sodium channel, voltage-gated, type I, beta	0.9199632	0.3999	0.6077
544782	Gm12620	poly(A) binding protein, cytoplasmic 4 pseudogene	0.9199632	0.4158	0.622
68874	Klhdc9	kelch domain containing 9	0.9199632	0.4799	0.6759
241066	Carf	calcium response factor	0.9199632	0.4976	0.6905
13858	Eps15	epidermal growth factor receptor pathway substrate 15	0.9199632	0.4996	0.692
13360	Dhcr7	7-dehydrocholesterol reductase	0.9199632	0.5296	0.7154
72338	Wdr89	WD repeat domain 89	0.9199632	0.5402	0.7234
12340	Capza1	capping protein (actin filament) muscle Z-line, alpha 1	0.9199632	0.5662	0.7425
100043699	Gm4593	predicted gene 4593	0.9199632	0.63	0.7896
76055	Mgea5	meningioma expressed antigen 5 (hyaluronidase)	0.9199632	0.6424	0.7978
619941	Gm13770	predicted gene 13770	0.9199632	0.6488	0.802
71996	1600014K23Rik	RIKEN cDNA 1600014K23 gene	0.9199632	0.6722	0.8165
244049	Mctp2	multiple C2 domains, transmembrane 2	0.9199632	0.7065	0.8383
14673	Gna12	guanine nucleotide binding protein, alpha 12	0.9199632	0.7336	0.8561
258290	Olfr1143	olfactory receptor 1143	0.9199632	0.8248	0.9097
257973	Olfr207	olfactory receptor 207	0.9199632	0.8546	0.925
71177	4933424B01Rik	RIKEN cDNA 4933424B01 gene	0.91911765	0.06308	NA
80889	Mesdc1	mesoderm development candidate 1	0.91911765	0.06858	NA
666945	Gm10638	predicted gene 10638	0.91911765	0.07526	NA
68291	Mto1	mitochondrial translation optimization 1 homolog (S. cerevisiae)	0.91911765	0.07636	NA
71707	Ubiad1	UbiA prenyltransferase domain containing 1	0.91911765	0.08075	NA
239546	Zfp647	zinc finger protein 647	0.91911765	0.0818	NA
19384	Ran	RAN, member RAS oncogene family	0.91911765	0.08973	NA
106957	Slc39a6	solute carrier family 39 (metal ion transporter), member 6	0.91911765	0.09169	NA
20656	Sod2	superoxide dismutase 2, mitochondrial	0.91911765	0.1034	NA
11776	Ap3d1	adaptor-related protein complex 3, delta 1 subunit	0.91911765	0.1152	NA
66083	Setd6	SET domain containing 6	0.91911765	0.1309	NA
76863	Dcun1d5	DCN1, defective in cullin neddylation 1, domain containing 5 (S. cerevisiae)	0.91911765	0.1337	NA
26404	Map3k12	mitogen-activated protein kinase kinase kinase 12	0.91911765	0.1338	NA
100503774	LOC100503774	hypothetical LOC100503774	0.91911765	0.1338	NA
66869	Zfp869	zinc finger protein 869	0.91911765	0.1397	NA
15331	Hmgn2	high mobility group nucleosomal binding domain 2	0.91911765	0.1451	NA
17169	Mark3	MAP/microtubule affinity-regulating kinase 3	0.91911765	0.1485	NA
225875	Lrfn4	leucine rich repeat and fibronectin type III domain containing 4	0.91911765	0.1494	NA
74384	4932433N03Rik	RIKEN cDNA 4932433N03 gene	0.91911765	0.1564	NA
22025	Nr2c1	nuclear receptor subfamily 2, group C, member 1	0.91911765	0.1683	NA
68316	Apoo	apolipoprotein O	0.91911765	0.1696	NA
217463	Snx13	sorting nexin 13	0.91911765	0.1802	NA
67207	Lsm1	LSM1 homolog, U6 small nuclear RNA associated (S. cerevisiae)	0.91911765	0.1879	NA
12695	Inadl	InaD-like (Drosophila)	0.91911765	0.1897	NA
74493	Tnks2	tankyrase, TRF1-interacting ankyrin-related ADP-ribose polymerase 2	0.91911765	0.1944	NA
71978	Ppp2r2a	protein phosphatase 2 (formerly 2A), regulatory subunit B (PR 52), alpha isoform	0.91911765	0.2014	NA
13047	Cux1	cut-like homeobox 1	0.91911765	0.2073	NA
70248	Dazap1	DAZ associated protein 1	0.91911765	0.2103	NA
74616	Scrn3	secernin 3	0.91911765	0.2113	NA
17257	Mecp2	methyl CpG binding protein 2	0.91911765	0.2245	NA
327885	C030046G05	hypothetical protein C030046G05	0.91911765	0.233	NA
68626	Elac2	elaC homolog 2 (E. coli)	0.91911765	0.2475	NA
12326	Camk4	calcium/calmodulin-dependent protein kinase IV	0.91911765	0.26	NA

71941	Cars2	cysteinyl-tRNA synthetase 2 (mitochondrial)(putative)	0.91911765	0.2638	NA
104625	Cnot6	CCR4-NOT transcription complex, subunit 6	0.91911765	0.275	NA
13680	Ddx19a	DEAD (Asp-Glu-Ala-Asp) box polypeptide 19a	0.91911765	0.2781	NA
140630	Ube4a	ubiquitination factor E4A, UFD2 homolog (S. cerevisiae)	0.91911765	0.2796	NA
58237	Nkain4	Na+/K+ transporting ATPase interacting 4	0.91911765	0.2827	NA
72981	Prkrir	protein-kinase, interferon-inducible double stranded RNA dependent inhibitor, repressor of (P58 repressor)	0.91911765	0.2905	NA
67534	Ttll4	tubulin tyrosine ligase-like family, member 4	0.91911765	0.3006	NA
50795	Sh3bgr	SH3-binding domain glutamic acid-rich protein	0.91911765	0.3056	NA
74053	Grip1	glutamate receptor interacting protein 1	0.91911765	0.3097	0.5195
791358	Gm10065	predicted gene 10065	0.91911765	0.3178	0.5274
68795	Ubr3	ubiquitin protein ligase E3 component n-recognin 3	0.91911765	0.3207	0.5306
22608	Ybx1	Y box protein 1	0.91911765	0.3336	0.5441
14105	Srsf10	serine/arginine-rich splicing factor 10	0.91911765	0.3391	0.5494
70209	Tmem143	transmembrane protein 143	0.91911765	0.3709	0.5796
238988	Erc2	ELKS/RAB6-interacting/CAST family member 2	0.91911765	0.3849	0.5941
69549	2310009B15Rik	RIKEN cDNA 2310009B15 gene	0.91911765	0.4078	0.6144
66734	Map1lc3a	microtubule-associated protein 1 light chain 3 alpha	0.91911765	0.4204	0.6266
320495	Ipcef1	interaction protein for cytohesin exchange factors 1	0.91911765	0.4306	0.6345
13486	Dr1	down-regulator of transcription 1	0.91911765	0.4395	0.6423
270049	Galntl6	UDP-N-acetyl-alpha-D-galactosamine:polypeptide N-acetylgalactosaminyltransferase-like 6	0.91911765	0.4663	0.6661
17261	Mef2d	myocyte enhancer factor 2D	0.91911765	0.4797	0.6759
54357	Epb4.1l4b	erythrocyte protein band 4.1-like 4b	0.91911765	0.4818	0.6779
67157	2610301B20Rik	RIKEN cDNA 2610301B20 gene	0.91911765	0.4863	0.6814
22719	Zfp61	zinc finger protein 61	0.91911765	0.5145	0.7045
71900	Tmem106b	transmembrane protein 106B	0.91911765	0.5307	0.7161
320604	A730037C10Rik	RIKEN cDNA A730037C10 gene	0.91911765	0.5652	0.742
382823	D13Mgi7	DNA Segment, Chr 13, Mouse Genome Informatics 7	0.91911765	0.5813	0.7533
19418	Rasgrf2	RAS protein-specific guanine nucleotide-releasing factor 2	0.91911765	0.6113	0.7756
12458	Ccr6	chemokine (C-C motif) receptor 6	0.91911765	0.6887	0.827
11994	Pcdh15	protocadherin 15	0.91911765	0.6996	0.8337
19744	Rheb	Ras homolog enriched in brain	0.91911765	0.7426	0.8608
66306	Fam53c	family with sequence similarity 53, member C	0.91827365	0.03962	NA
210573	Tmem151b	transmembrane protein 151B	0.91827365	0.05719	NA
66660	Sltm	SAFB-like, transcription modulator	0.91827365	0.09061	NA
22017	Tpmt	thiopurine methyltransferase	0.91827365	0.1298	NA
330485	Tmem145	transmembrane protein 145	0.91827365	0.1347	NA
74051	Steap2	six transmembrane epithelial antigen of prostate 2	0.91827365	0.15	NA
18751	Prkcb	protein kinase C, beta	0.91827365	0.18	NA
244551	Nanos3	nanos homolog 3 (Drosophila)	0.91827365	0.1945	NA
22202	Ube1y1	ubiquitin-activating enzyme E1, Chr Y 1	0.91827365	0.2039	NA
71710	Lrrcc1	leucine rich repeat and coiled-coil domain containing 1	0.91827365	0.2084	NA
68032	Tmem85	transmembrane protein 85	0.91827365	0.2199	NA
22360	Nrsn1	neurensin 1	0.91827365	0.2289	NA
231003	Klhl17	kelch-like 17 (Drosophila)	0.91827365	0.2394	NA
66143	Eef1e1	eukaryotic translation elongation factor 1 epsilon 1	0.91827365	0.2514	NA
230753	Thrap3	thyroid hormone receptor associated protein 3	0.91827365	0.2569	NA
16597	Klf12	Kruppel-like factor 12	0.91827365	0.2662	NA
19736	Rgs4	regulator of G-protein signaling 4	0.91827365	0.29	NA
20926	Supt6h	suppressor of Ty 6 homolog (S. cerevisiae)	0.91827365	0.2922	NA
320394	Cenpt	centromere protein T	0.91827365	0.3166	0.526

228005	Ppig	peptidyl-prolyl isomerase G (cyclophilin G)	0.91827365	0.3313	0.5417
13170	Dbp	D site albumin promoter binding protein	0.91827365	0.3408	0.5513
231380	Uba6	ubiquitin-like modifier activating enzyme 6	0.91827365	0.3546	0.5645
100608	Noc4l	nucleolar complex associated 4 homolog (S. cerevisiae)	0.91827365	0.3886	0.597
80912	Pum1	pumilio 1 (Drosophila)	0.91827365	0.3948	0.6029
12009	Azi1	5-azacytidine induced gene 1	0.91827365	0.4191	0.6253
56335	Mettl3	methyltransferase like 3	0.91827365	0.4305	0.6345
552912	LOC552912	hypothetical LOC552912	0.91827365	0.4315	0.6355
99526	Usp53	ubiquitin specific peptidase 53	0.91827365	0.4337	0.6374
66776	Pisd-ps3	phosphatidylserine decarboxylase, pseudogene 3	0.91827365	0.4464	0.6489
230257	Rod1	ROD1 regulator of differentiation 1 (S. pombe)	0.91827365	0.4621	0.6627
227620	Uap1l1	UDP-N-acteylglucosamine pyrophosphorylase 1-like 1	0.91827365	0.4673	0.6672
17134	Mafg	v-maf musculoaponeurotic fibrosarcoma oncogene family, protein G (avian)	0.91827365	0.486	0.6812
72054	Cyp4f18	cytochrome P450, family 4, subfamily f, polypeptide 18	0.91827365	0.4949	0.6883
66156	Anapc11	anaphase promoting complex subunit 11	0.91827365	0.5377	0.7216
13383	Dlg1	discs, large homolog 1 (Drosophila)	0.91827365	0.5584	0.7378
81004	Tbl1xr1	transducin (beta)-like 1X-linked receptor 1	0.91827365	0.5967	0.7653
76383	1700012L04Rik	RIKEN cDNA 1700012L04 gene	0.91827365	0.5999	0.7678
140557	Smc1b	structural maintenance of chromosomes 1B	0.91827365	0.6649	0.8113
19116	Prlr	prolactin receptor	0.91827365	0.6815	0.8221
13106	Cyp2e1	cytochrome P450, family 2, subfamily e, polypeptide 1	0.91827365	0.7797	0.8835
235493	BC031353	cDNA sequence BC031353	0.91743119	0.05538	NA
18582	Pde6d	phosphodiesterase 6D, cGMP-specific, rod, delta	0.91743119	0.06003	NA
99512	Wdr47	WD repeat domain 47	0.91743119	0.06652	NA
233865	D430042O09Rik	RIKEN cDNA D430042O09 gene	0.91743119	0.08003	NA
66291	1810030N24Rik	RIKEN cDNA 1810030N24 gene	0.91743119	0.08486	NA
77827	Krba1	KRAB-A domain containing 1	0.91743119	0.08735	NA
56030	Tmem131	transmembrane protein 131	0.91743119	0.09107	NA
67792	Rgs8	regulator of G-protein signaling 8	0.91743119	0.09161	NA
12913	Creb3	cAMP responsive element binding protein 3	0.91743119	0.1068	NA
240756	Klhl12	kelch-like 12 (Drosophila)	0.91743119	0.1293	NA
67878	Tmem33	transmembrane protein 33	0.91743119	0.1368	NA
70497	Arhgap17	Rho GTPase activating protein 17	0.91743119	0.1411	NA
52626	Cdkn2aipnl	CDKN2A interacting protein N-terminal like	0.91743119	0.1424	NA
100504534	LOC100504534	hypothetical LOC100504534	0.91743119	0.1427	NA
68050	Akirin1	akirin 1	0.91743119	0.1792	NA
68241	Fam195a	family with sequence similarity 195, member A	0.91743119	0.1965	NA
108841	Rdh13	retinol dehydrogenase 13 (all-trans and 9-cis)	0.91743119	0.1991	NA
58248	1700123O20Rik	RIKEN cDNA 1700123O20 gene	0.91743119	0.1997	NA
218544	Sgtb	small glutamine-rich tetratricopeptide repeat (TPR)-containing, beta	0.91743119	0.2439	NA
100039442	Gm2238	predicted gene 2238	0.91743119	0.2459	NA
223828	Pphln1	periphilin 1	0.91743119	0.2742	NA
219094	Khnyn	KH and NYN domain containing	0.91743119	0.2755	NA
21968	Tom1	target of myb1 homolog (chicken)	0.91743119	0.2782	NA
231659	Gcn1l1	GCN1 general control of amino-acid synthesis 1-like 1 (yeast)	0.91743119	0.2942	NA
77744	6720463M24Rik	RIKEN cDNA 6720463M24 gene	0.91743119	0.3092	0.519
100383	Bsdc1	BSD domain containing 1	0.91743119	0.3312	0.5417
56428	Mtch2	mitochondrial carrier homolog 2 (C. elegans)	0.91743119	0.3313	0.5417
72925	Mar-01	membrane-associated ring finger (C3HC4) 1	0.91743119	0.3317	0.5421
76701	Ctrc	chymotrypsin C (caldecrin)	0.91743119	0.3398	0.5501

319791	9430011C21Rik	RIKEN cDNA 9430011C21 gene	0.91743119	0.3595	0.5692
11513	Adcy7	adenylate cyclase 7	0.91743119	0.3599	0.5696
74111	Rbm19	RNA binding motif protein 19	0.91743119	0.3818	0.591
107371	Exoc6	exocyst complex component 6	0.91743119	0.3872	0.5958
69536	Hemk1	HemK methyltransferase family member 1	0.91743119	0.3873	0.596
12988	Csk	c-src tyrosine kinase	0.91743119	0.424	0.6295
69944	2810021J22Rik	RIKEN cDNA 2810021J22 gene	0.91743119	0.4293	0.6336
52139	D5Ertd560e	DNA segment, Chr 5, ERATO Doi 560, expressed	0.91743119	0.4325	0.6365
74376	Myo18b	myosin XVIIIb	0.91743119	0.5753	0.749
19309	Pygm	muscle glycogen phosphorylase	0.91743119	0.5837	0.755
229905	Ccbl2	cysteine conjugate-beta lyase 2	0.91743119	0.6487	0.802
94190	Ophn1	oligophrenin 1	0.91743119	0.6515	0.8034
380711	Rap1gap2	RAP1 GTPase activating protein 2	0.91743119	0.6552	0.8056
15130	Hbb-b2	hemoglobin, beta adult minor chain	0.91743119	0.7879	0.8888
76179	Usp31	ubiquitin specific peptidase 31	0.91659028	0.03616	NA
73694	2410091C18Rik	RIKEN cDNA 2410091C18 gene	0.91659028	0.05011	NA
105428	Fam149b	family with sequence similarity 149, member B	0.91659028	0.1044	NA
20742	Spnb2	spectrin beta 2	0.91659028	0.1066	NA
68926	Ubap2	ubiquitin-associated protein 2	0.91659028	0.1221	NA
77040	Atg16l1	autophagy-related 16-like 1 (yeast)	0.91659028	0.1317	NA
66917	Chordc1	cysteine and histidine-rich domain (CHORD)-containing, zinc-binding protein 1	0.91659028	0.137	NA
232811	Suv420h2	suppressor of variegation 4-20 homolog 2 (Drosophila)	0.91659028	0.1411	NA
242584	Wdr78	WD repeat domain 78	0.91659028	0.1439	NA
108927	Lhfp	lipoma HMGIC fusion partner	0.91659028	0.1768	NA
319266	A130010J15Rik	RIKEN cDNA A130010J15 gene	0.91659028	0.1891	NA
11545	Parp1	poly (ADP-ribose) polymerase family, member 1	0.91659028	0.1954	NA
74375	Gcc1	golgi coiled coil 1	0.91659028	0.2013	NA
69035	Zdhhc3	zinc finger, DHHC domain containing 3	0.91659028	0.211	NA
77721	Mrps5	mitochondrial ribosomal protein S5	0.91659028	0.211	NA
269878	Megf8	multiple EGF-like-domains 8	0.91659028	0.2129	NA
100134990	LOC100134990	selenoprotein K pseudogene	0.91659028	0.2294	NA
19341	Rab4a	RAB4A, member RAS oncogene family	0.91659028	0.2295	NA
229055	Zbtb10	zinc finger and BTB domain containing 10	0.91659028	0.2303	NA
14660	Gls	glutaminase	0.91659028	0.2389	NA
19647	Rbbp6	retinoblastoma binding protein 6	0.91659028	0.2448	NA
99371	Arfgef2	ADP-ribosylation factor guanine nucleotide-exchange factor 2 (brefeldin A-inhibited)	0.91659028	0.2471	NA
381511	Pdp1	pyruvate dehyrogenase phosphatase catalytic subunit 1	0.91659028	0.2502	NA
76205	Stard3nl	STARD3 N-terminal like	0.91659028	0.2519	NA
216134	Pdxk	pyridoxal (pyridoxine, vitamin B6) kinase	0.91659028	0.2712	NA
245386	Fam70a	family with sequence similarity 70, member A	0.91659028	0.2776	NA
68910	Zfp467	zinc finger protein 467	0.91659028	0.2809	NA
73942	Fam151b	family with sequence similarity 151, member B	0.91659028	0.2854	NA
14387	Gaa	glucosidase, alpha, acid	0.91659028	0.2962	0.5052
100504262	LOC100504262	hypothetical LOC100504262	0.91659028	0.305	0.5141
404580	AL117821	expressed sequence AL117821	0.91659028	0.3391	0.5494
26874	Abcd2	ATP-binding cassette, sub-family D (ALD), member 2	0.91659028	0.3518	0.5618
72046	Urgcp	upregulator of cell proliferation	0.91659028	0.3567	0.5665
70683	Utp20	UTP20, small subunit (SSU) processome component, homolog (yeast)	0.91659028	0.3604	0.5701
213084	Cdkl3	cyclin-dependent kinase-like 3	0.91659028	0.3624	0.5716
72429	Dnajc25	DnaJ (Hsp40) homolog, subfamily C, member 25	0.91659028	0.3705	0.5794

22083	Ctr9	Ctr9, Paf1/RNA polymerase II complex component, homolog (S. cerevisiae)	0.91659028	0.3766	0.5855
71770	Ap2b1	adaptor-related protein complex 2, beta 1 subunit	0.91659028	0.3778	0.5867
70315	Hdac8	histone deacetylase 8	0.91659028	0.3783	0.5873
74455	Nsun6	NOL1/NOP2/Sun domain family member 6	0.91659028	0.395	0.6029
29858	Pmm1	phosphomannomutase 1	0.91659028	0.4038	0.6112
67738	Ppid	peptidylprolyl isomerase D (cyclophilin D)	0.91659028	0.4089	0.6154
319263	Pcmtd1	protein-L-isoaspartate (D-aspartate) O-methyltransferase domain containing 1	0.91659028	0.426	0.6311
208258	Ankrd33	ankyrin repeat domain 33	0.91659028	0.4269	0.6319
18798	Plcb4	phospholipase C, beta 4	0.91659028	0.4292	0.6336
209446	Tcfe3	transcription factor E3	0.91659028	0.4606	0.6613
27028	Ermap	erythroblast membrane-associated protein	0.91659028	0.4778	0.6748
212390	Klhl32	kelch-like 32 (Drosophila)	0.91659028	0.4969	0.6899
105193	Nhlrc1	NHL repeat containing 1	0.91659028	0.5631	0.7406
259058	Olfr646	olfactory receptor 646	0.91659028	0.7562	0.8694
52468	Ctdsp2	CTD (carboxy-terminal domain, RNA polymerase II, polypeptide A) small phosphatase 2	0.91659028	0.8199	0.9069
625276	Gm6569	predicted gene 6569	0.91659028	0.824	0.9093
330031	Gm5106	predicted gene 5106	0.91659028	0.8473	0.921
68729	Trim37	tripartite motif-containing 37	0.91575092	0.04889	NA
21985	Tpd52	tumor protein D52	0.91575092	0.06251	NA
67011	Mettl6	methyltransferase like 6	0.91575092	0.07731	NA
102058	Exoc8	exocyst complex component 8	0.91575092	0.0942	NA
15387	Hnrnpk	heterogeneous nuclear ribonucleoprotein K	0.91575092	0.09931	NA
193838	Eme2	essential meiotic endonuclease 1 homolog 2 (S. pombe)	0.91575092	0.1042	NA
64384	Sirt3	sirtuin 3 (silent mating type information regulation 2, homolog) 3 (S. cerevisiae)	0.91575092	0.1059	NA
11515	Adcy9	adenylate cyclase 9	0.91575092	0.1131	NA
381633	Gm1673	predicted gene 1673	0.91575092	0.1134	NA
216831	Arhgap44	Rho GTPase activating protein 44	0.91575092	0.1142	NA
226744	Cnst	consortin, connexin sorting protein	0.91575092	0.1406	NA
17933	Myt1l	myelin transcription factor 1-like	0.91575092	0.1483	NA
76976	2900062L11Rik	RIKEN cDNA 2900062L11 gene	0.91575092	0.1548	NA
236844	Gm4910	predicted pseudogene 4910	0.91575092	0.1561	NA
70247	Psmd1	proteasome (prosome, macropain) 26S subunit, non-ATPase, 1	0.91575092	0.1693	NA
235072	Sep-07	septin 7	0.91575092	0.1771	NA
407812	Zfp941	zinc finger protein 941	0.91575092	0.1861	NA
18190	Nrxn2	neurexin II	0.91575092	0.1985	NA
233529	Kctd14	potassium channel tetramerisation domain containing 14	0.91575092	0.2243	NA
17428	Mnt	max binding protein	0.91575092	0.2274	NA
67224	Med29	mediator complex subunit 29	0.91575092	0.2325	NA
71331	5430411C19Rik	RIKEN cDNA 5430411C19 gene	0.91575092	0.2364	NA
68379	Ciz1	CDKN1A interacting zinc finger protein 1	0.91575092	0.2389	NA
15531	Ndst1	N-deacetylase/N-sulfotransferase (heparan glucosaminyl) 1	0.91575092	0.2609	NA
230967	BC046331	cDNA sequence BC046331	0.91575092	0.2766	NA
218975	Mapk1ip1l	mitogen-activated protein kinase 1 interacting protein 1-like	0.91575092	0.2838	0.4924
53959	AA914427	EST AA914427	0.91575092	0.2902	0.4987
269702	Mphosph9	M-phase phosphoprotein 9	0.91575092	0.3015	0.5109
13497	Drp2	dystrophin related protein 2	0.91575092	0.3358	0.5461
15259	Hipk3	homeodomain interacting protein kinase 3	0.91575092	0.336	0.5464
104175	Sbk1	SH3-binding kinase 1	0.91575092	0.3369	0.5472
16337	Insr	insulin receptor	0.91575092	0.3726	0.5814
53321	Cntnap1	contactin associated protein-like 1	0.91575092	0.3733	0.5821

100048345	LOC100048345	hypothetical protein LOC100048345	0.91575092	0.3735	0.5822
76719	1700081L11Rik	RIKEN cDNA 1700081L11 gene	0.91575092	0.3943	0.6026
209584	Tyw3	tRNA-yW synthesizing protein 3 homolog (S. cerevisiae)	0.91575092	0.3972	0.6052
67781	Ilf2	interleukin enhancer binding factor 2	0.91575092	0.4047	0.6124
20467	Sin3b	transcriptional regulator, SIN3B (yeast)	0.91575092	0.4052	0.6129
338374	Il28b	interleukin 28B	0.91575092	0.407	0.6139
664883	Nova1	neuro-oncological ventral antigen 1	0.91575092	0.4246	0.6301
239364	Tspyl5	testis-specific protein, Y-encoded-like 5	0.91575092	0.4256	0.6308
100039861	Gm2464	predicted gene 2464	0.91575092	0.4797	0.6759
320817	Atad2b	ATPase family, AAA domain containing 2B	0.91575092	0.5214	0.7096
241576	Ldlrad3	low density lipoprotein receptor class A domain containing 3	0.91575092	0.549	0.7308
212168	Zswim4	zinc finger, SWIM domain containing 4	0.91575092	0.5886	0.7588
66793	Efcab1	EF hand calcium binding domain 1	0.91575092	0.6057	0.7722
76740	Efr3a	EFR3 homolog A (S. cerevisiae)	0.91575092	0.632	0.7909
217847	Serpina10	serine (or cysteine) peptidase inhibitor, clade A (alpha-1 antiproteinase, antitrypsin), member 10	0.91575092	0.6619	0.8095
224833	AI661453	expressed sequence AI661453	0.91575092	0.7556	0.8691
66641	Sike1	suppressor of IKBKE 1	0.91491308	0.05471	NA
76916	4930455C21Rik	RIKEN cDNA 4930455C21 gene	0.91491308	0.06743	NA
224656	Zfp523	zinc finger protein 523	0.91491308	0.0683	NA
66086	0610037P05Rik	RIKEN cDNA 0610037P05 gene	0.91491308	0.08606	NA
83410	Cstf2t	cleavage stimulation factor, 3' pre-RNA subunit 2, tau	0.91491308	0.0931	NA
16650	Kpna6	karyopherin (importin) alpha 6	0.91491308	0.1067	NA
22697	Zscan21	zinc finger and SCAN domain containing 21	0.91491308	0.1131	NA
224092	Lsg1	large subunit GTPase 1 homolog (S. cerevisiae)	0.91491308	0.1138	NA
93834	Peli2	pellino 2	0.91491308	0.1206	NA
108143	Taf9	TAF9 RNA polymerase II, TATA box binding protein (TBP)-associated factor	0.91491308	0.1215	NA
231855	C330006K01Rik	RIKEN cDNA C330006K01 gene	0.91491308	0.1514	NA
17122	Mxd4	Max dimerization protein 4	0.91491308	0.1697	NA
71916	Dus4l	dihydrouridine synthase 4-like (S. cerevisiae)	0.91491308	0.1732	NA
243725	Ppp1r9a	protein phosphatase 1, regulatory (inhibitor) subunit 9A	0.91491308	0.1868	NA
67770	5830433M19Rik	RIKEN cDNA 5830433M19 gene	0.91491308	0.1902	NA
16956	Lpl	lipoprotein lipase	0.91491308	0.2217	NA
13859	Eps15l1	epidermal growth factor receptor pathway substrate 15-like 1	0.91491308	0.2219	NA
74342	Lrrtm1	leucine rich repeat transmembrane neuronal 1	0.91491308	0.2436	NA
15289	Hmgb1	high mobility group box 1	0.91491308	0.2859	0.4944
21429	Ubtf	upstream binding transcription factor, RNA polymerase I	0.91491308	0.2968	0.5059
270084	Lpcat2	lysophosphatidylcholine acyltransferase 2	0.91491308	0.3055	0.5147
22401	Zmat3	zinc finger matrin type 3	0.91491308	0.3088	0.5185
23893	Grem2	gremlin 2 homolog, cysteine knot superfamily (Xenopus laevis)	0.91491308	0.3096	0.5193
18213	Ntrk3	neurotrophic tyrosine kinase, receptor, type 3	0.91491308	0.3156	0.525
108857	Ankhd1	ankyrin repeat and KH domain containing 1	0.91491308	0.3287	0.5389
74213	Rbm26	RNA binding motif protein 26	0.91491308	0.3302	0.5405
240613	9930021J03Rik	RIKEN cDNA 9930021J03 gene	0.91491308	0.3712	0.5799
13619	Phc1	polyhomeotic-like 1 (Drosophila)	0.91491308	0.4018	0.6094
77031	Slc9a8	solute carrier family 9 (sodium/hydrogen exchanger), member 8	0.91491308	0.4028	0.6103
77264	Zfp142	zinc finger protein 142	0.91491308	0.4064	0.6136
230235	6430704M03Rik	RIKEN cDNA 6430704M03 gene	0.91491308	0.4191	0.6253
71398	5430427O19Rik	RIKEN cDNA 5430427O19 gene	0.91491308	0.4446	0.6474
67538	Zswim3	zinc finger, SWIM domain containing 3	0.91491308	0.4713	0.67
69085	Zcchc9	zinc finger, CCHC domain containing 9	0.91491308	0.4826	0.6783

93840	Vangl2	vang-like 2 (van gogh, Drosophila)	0.91491308	0.49	0.6843
20928	Abcc9	ATP-binding cassette, sub-family C (CFTR/MRP), member 9	0.91491308	0.5062	0.6973
331392	Gm5124	predicted pseudogene 5124	0.91491308	0.5193	0.7085
66874	1200014J11Rik	RIKEN cDNA 1200014J11 gene	0.91491308	0.653	0.8041
245695	Tceanc	transcription elongation factor A (SII) N-terminal and central domain containing	0.91491308	0.6546	0.8051
269799	Clec4a1	C-type lectin domain family 4, member a1	0.91491308	0.7064	0.8383
433804	Gm13154	predicted gene 13154	0.91491308	0.7901	0.8901
52850	Sgsm1	small G protein signaling modulator 1	0.91407678	0.04468	NA
18197	Nsg2	neuron specific gene family member 2	0.91407678	0.05707	NA
214931	Fbxl16	F-box and leucine-rich repeat protein 16	0.91407678	0.07495	NA
19775	Xpr1	xenotropic and polytropic retrovirus receptor 1	0.91407678	0.07953	NA
170439	Elovl6	ELOVL family member 6, elongation of long chain fatty acids (yeast)	0.91407678	0.0883	NA
67150	Rnf141	ring finger protein 141	0.91407678	0.09704	NA
19691	Recql	RecQ protein-like	0.91407678	0.1077	NA
109676	Ank2	ankyrin 2, brain	0.91407678	0.1137	NA
19359	Rad23b	RAD23b homolog (S. cerevisiae)	0.91407678	0.12	NA
74252	Armc1	armadillo repeat containing 1	0.91407678	0.1342	NA
226043	Cbwd1	COBW domain containing 1	0.91407678	0.1379	NA
67469	Abhd5	abhydrolase domain containing 5	0.91407678	0.1382	NA
235169	Foxred1	FAD-dependent oxidoreductase domain containing 1	0.91407678	0.1647	NA
56177	Olfm1	olfactomedin 1	0.91407678	0.1653	NA
78454	9530077C14Rik	RIKEN cDNA 9530077C14 gene	0.91407678	0.176	NA
80890	Trim2	tripartite motif-containing 2	0.91407678	0.1774	NA
77045	Bcl7a	B-cell CLL/lymphoma 7A	0.91407678	0.1886	NA
26757	Dpysl4	dihydropyrimidinase-like 4	0.91407678	0.1948	NA
69690	2310057B04Rik	RIKEN cDNA 2310057B04 gene	0.91407678	0.1967	NA
22088	Tsg101	tumor susceptibility gene 101	0.91407678	0.2111	NA
12933	Crmp1	collapsin response mediator protein 1	0.91407678	0.2117	NA
68865	Arv1	ARV1 homolog (yeast)	0.91407678	0.2128	NA
75705	Eif4b	eukaryotic translation initiation factor 4B	0.91407678	0.2191	NA
20713	Serpini1	serine (or cysteine) peptidase inhibitor, clade I, member 1	0.91407678	0.2423	NA
12631	Cfl1	cofilin 1, non-muscle	0.91407678	0.2567	NA
18671	Abcb1a	ATP-binding cassette, sub-family B (MDR/TAP), member 1A	0.91407678	0.2801	0.4882
66274	1810012P15Rik	RIKEN cDNA 1810012P15 gene	0.91407678	0.289	0.4975
227721	Ppapdc3	phosphatidic acid phosphatase type 2 domain containing 3	0.91407678	0.2924	0.5012
21340	Taf1b	TATA box binding protein (Tbp)-associated factor, RNA polymerase I, B	0.91407678	0.2925	0.5013
245474	Dkc1	dyskeratosis congenita 1, dyskerin homolog (human)	0.91407678	0.3054	0.5147
20265	Scn1a	sodium channel, voltage-gated, type I, alpha	0.91407678	0.3064	0.5158
72621	Pdzd11	PDZ domain containing 11	0.91407678	0.3182	0.5278
75288	Slc35f4	solute carrier family 35, member F4	0.91407678	0.3248	0.5346
24086	Tlk2	tousled-like kinase 2 (Arabidopsis)	0.91407678	0.3269	0.537
230649	Atpaf1	ATP synthase mitochondrial F1 complex assembly factor 1	0.91407678	0.3319	0.5423
52710	Gpr172b	G protein-coupled receptor 172B	0.91407678	0.335	0.5456
16334	Ins2	insulin II	0.91407678	0.3439	0.5547
368202	Prss48	protease, serine, 48	0.91407678	0.3523	0.5624
70387	Ttc9c	tetratricopeptide repeat domain 9C	0.91407678	0.355	0.5648
78935	Saal1	serum amyloid A-like 1	0.91407678	0.3619	0.5714
19716	Bex1	brain expressed gene 1	0.91407678	0.3833	0.5923
11775	Ap3b2	adaptor-related protein complex 3, beta 2 subunit	0.91407678	0.4372	0.6406
320717	Pptc7	PTC7 protein phosphatase homolog (S. cerevisiae)	0.91407678	0.4532	0.6551

56839	Lgi1	leucine-rich repeat LGI family, member 1	0.91407678	0.4555	0.6571
74201	Cep97	centrosomal protein 97	0.91407678	0.464	0.6643
76775	Slc10a7	solute carrier family 10 (sodium/bile acid cotransporter family), member 7	0.91407678	0.4694	0.6687
100040599	Gm15319	predicted gene 15319	0.91407678	0.4968	0.6899
320150	Zdhhc17	zinc finger, DHHC domain containing 17	0.91407678	0.5133	0.7038
69309	Slc16a13	solute carrier family 16 (monocarboxylic acid transporters), member 13	0.91407678	0.5262	0.7128
195727	Nhs	Nance-Horan syndrome (human)	0.91407678	0.5293	0.7151
217732	2310044G17Rik	RIKEN cDNA 2310044G17 gene	0.91407678	0.5326	0.7176
13367	Diap1	diaphanous homolog 1 (Drosophila)	0.91407678	0.5465	0.7283
20348	Sema3c	sema domain, immunoglobulin domain (Ig), short basic domain, secreted, (semaphorin) 3C	0.91407678	0.5487	0.7304
433091	Pnpla1	patatin-like phospholipase domain containing 1	0.91407678	0.5534	0.7337
52040	Ppp1r10	protein phosphatase 1, regulatory subunit 10	0.91407678	0.5625	0.7402
17155	Man1a	mannosidase 1, alpha	0.91407678	0.5762	0.7495
109270	Prr5	proline rich 5 (renal)	0.91407678	0.5771	0.7502
66570	Cenpm	centromere protein M	0.91407678	0.5847	0.7556
217558	G2e3	G2/M-phase specific E3 ubiquitin ligase	0.91407678	0.5863	0.7569
56531	Ylpm1	YLP motif containing 1	0.91407678	0.5903	0.7601
269702	Mphosph9	M-phase phosphoprotein 9	0.91407678	0.6702	0.815
67926	Spert	spermatid associated	0.91407678	0.7164	0.8451
71146	Golga7b	golgi autoantigen, golgin subfamily a, 7B	0.91407678	0.7343	0.8565
17441	Mog	myelin oligodendrocyte glycoprotein	0.91407678	0.8164	0.9047
17758	Mtap4	microtubule-associated protein 4	0.91324201	0.04575	NA
228788	BC020535	cDNA sequence BC020535	0.91324201	0.05178	NA
12050	Bcl2l2	BCL2-like 2	0.91324201	0.07142	NA
53375	Mtx2	metaxin 2	0.91324201	0.07556	NA
76577	Faf2	Fas associated factor family member 2	0.91324201	0.07643	NA
497652	Acd	adrenocortical dysplasia	0.91324201	0.08152	NA
319801	9630033F20Rik	RIKEN cDNA 9630033F20 gene	0.91324201	0.09865	NA
72795	Ttc19	tetratricopeptide repeat domain 19	0.91324201	0.1148	NA
16396	Itch	itchy, E3 ubiquitin protein ligase	0.91324201	0.1487	NA
74718	Snx16	sorting nexin 16	0.91324201	0.1933	NA
66096	0910001L09Rik	RIKEN cDNA 0910001L09 gene	0.91324201	0.2093	NA
382038	Urb2	URB2 ribosome biogenesis 2 homolog (S. cerevisiae)	0.91324201	0.2847	0.4932
71745	Cul2	cullin 2	0.91324201	0.3014	0.5108
56453	Mbtps1	membrane-bound transcription factor peptidase, site 1	0.91324201	0.3046	0.5136
27081	Zfp275	zinc finger protein 275	0.91324201	0.3114	0.5213
20430	Cyfip1	cytoplasmic FMR1 interacting protein 1	0.91324201	0.3117	0.5214
53604	Zpbp	zona pellucida binding protein	0.91324201	0.3324	0.5425
67046	Tbc1d7	TBC1 domain family, member 7	0.91324201	0.3454	0.5561
72865	Cxx1c	CAAX box 1 homolog C (human)	0.91324201	0.3613	0.5709
330814	Lphn1	latrophilin 1	0.91324201	0.3702	0.5792
504193	Npcd	neuronal pentraxin chromo domain	0.91324201	0.4007	0.6083
65019	Rpl23	ribosomal protein L23	0.91324201	0.4068	0.6139
104418	Dgkz	diacylglycerol kinase zeta	0.91324201	0.4077	0.6144
67569	Mgat4c	mannosyl (alpha-1,3-)-glycoprotein beta-1,4-N-acetylglucosaminyltransferase, isozyme C (putative)	0.91324201	0.4111	0.6174
78943	Ern1	endoplasmic reticulum (ER) to nucleus signalling 1	0.91324201	0.4123	0.6186
71323	Rassf8	Ras association (RalGDS/AF-6) domain family (N-terminal) member 8	0.91324201	0.4238	0.6293
26405	Map3k2	mitogen-activated protein kinase kinase kinase 2	0.91324201	0.4272	0.632
18646	Prf1	perforin 1 (pore forming protein)	0.91324201	0.4745	0.6722
211446	Exoc3	exocyst complex component 3	0.91324201	0.537	0.7214

93717	Pcdhga9	protocadherin gamma subfamily A, 9	0.91324201	0.6398	0.7962
52512	D14Ertd670e	DNA segment, Chr 14, ERATO Doi 670, expressed	0.91324201	0.6732	0.817
66356	2310008H09Rik	RIKEN cDNA 2310008H09 gene	0.91324201	0.694	0.8305
12355	Nr1i3	nuclear receptor subfamily 1, group I, member 3	0.91324201	0.7309	0.8543
232236	C130022K22Rik	RIKEN cDNA C130022K22 gene	0.91240876	0.04769	NA
233895	Prr14	proline rich 14	0.91240876	0.0585	NA
77683	Ehmt1	euchromatic histone methyltransferase 1	0.91240876	0.07717	NA
76485	Glt8d1	glycosyltransferase 8 domain containing 1	0.91240876	0.0987	NA
216134	Pdxk	pyridoxal (pyridoxine, vitamin B6) kinase	0.91240876	0.1015	NA
77116	Mtmr2	myotubularin related protein 2	0.91240876	0.1027	NA
329068	A330044H09	hypothetical protein A330044H09	0.91240876	0.105	NA
16493	Kcna5	potassium voltage-gated channel, shaker-related subfamily, member 5	0.91240876	0.1151	NA
22232	Slc35a2	solute carrier family 35 (UDP-galactose transporter), member A2	0.91240876	0.1192	NA
66960	Fam188a	family with sequence similarity 188, member A	0.91240876	0.1214	NA
621381	Gm6222	predicted pseudogene 6222	0.91240876	0.1246	NA
328417	Parp4	poly (ADP-ribose) polymerase family, member 4	0.91240876	0.1264	NA
28113	Tinf2	Terf1 (TRF1)-interacting nuclear factor 2	0.91240876	0.1319	NA
71865	Fbxo30	F-box protein 30	0.91240876	0.1476	NA
58800	Trpm7	transient receptor potential cation channel, subfamily M, member 7	0.91240876	0.1561	NA
66970	Ssbp2	single-stranded DNA binding protein 2	0.91240876	0.1619	NA
330941	AI593442	expressed sequence AI593442	0.91240876	0.1631	NA
59090	Midn	midnolin	0.91240876	0.1658	NA
52530	Nhp2	NHP2 ribonucleoprotein homolog (yeast)	0.91240876	0.1714	NA
320191	Hook3	hook homolog 3 (Drosophila)	0.91240876	0.1758	NA
76628	1700112J16Rik	RIKEN cDNA 1700112J16 gene	0.91240876	0.1978	NA
70925	Cdkn2aip	CDKN2A interacting protein	0.91240876	0.2022	NA
66437	Fis1	fission 1 (mitochondrial outer membrane) homolog (yeast)	0.91240876	0.2026	NA
269061	Cpsf7	cleavage and polyadenylation specific factor 7	0.91240876	0.2102	NA
100978	Nfxl1	nuclear transcription factor, X-box binding-like 1	0.91240876	0.2145	NA
27965	Spg21	spastic paraplegia 21 homolog (human)	0.91240876	0.2478	NA
20747	Spop	speckle-type POZ protein	0.91240876	0.2484	NA
13350	Dgat1	diacylglycerol O-acyltransferase 1	0.91240876	0.2706	0.4782
231868	E130309D02Rik	RIKEN cDNA E130309D02 gene	0.91240876	0.285	0.4935
72759	Tmem135	transmembrane protein 135	0.91240876	0.2911	0.4996
22036	Traip	TRAF-interacting protein	0.91240876	0.3134	0.5231
231699	Oas1e	2'-5' oligoadenylate synthetase 1E	0.91240876	0.3316	0.542
245305	B230307C23Rik	RIKEN cDNA B230307C23 gene	0.91240876	0.3367	0.5471
208748	Prrg3	proline rich Gla (G-carboxyglutamic acid) 3 (transmembrane)	0.91240876	0.3478	0.5583
70461	Crtc3	CREB regulated transcription coactivator 3	0.91240876	0.3572	0.5672
14165	Fgf10	fibroblast growth factor 10	0.91240876	0.3799	0.589
319675	5830418K08Rik	RIKEN cDNA 5830418K08 gene	0.91240876	0.4068	0.6139
244058	Rgma	RGM domain family, member A	0.91240876	0.4192	0.6254
23853	Def6	differentially expressed in FDCP 6	0.91240876	0.4267	0.6317
20355	Sema4f	sema domain, immunoglobulin domain (Ig), TM domain, and short cytoplasmic domain	0.91240876	0.4452	0.6479
433966	5730422E09Rik	RIKEN cDNA 5730422E09 gene	0.91240876	0.4528	0.6549
13209	Ddx6	DEAD (Asp-Glu-Ala-Asp) box polypeptide 6	0.91240876	0.4563	0.6579
230316	Megf9	multiple EGF-like-domains 9	0.91240876	0.4756	0.6729
68652	Tab2	TGF-beta activated kinase 1/MAP3K7 binding protein 2	0.91240876	0.4788	0.6754
74694	Tbc1d30	TBC1 domain family, member 30	0.91240876	0.4945	0.688
108686	Ccdc88a	coiled coil domain containing 88A	0.91240876	0.5089	0.6997

237758	Zfp454	zinc finger protein 454	0.91240876	0.5157	0.7053
170757	Eltd1	EGF, latrophilin seven transmembrane domain containing 1	0.91240876	0.5183	0.7078
52076	Tmem38b	transmembrane protein 38B	0.91240876	0.5202	0.7089
12850	Coq7	demethyl-Q 7	0.91240876	0.5203	0.7089
72114	Zbed3	zinc finger, BED domain containing 3	0.91240876	0.5249	0.7117
12212	Chic1	cysteine-rich hydrophobic domain 1	0.91240876	0.5267	0.7132
98685	1190005F20Rik	RIKEN cDNA 1190005F20 gene	0.91240876	0.5276	0.7138
218543	Srsf12	serine/arginine-rich splicing factor 12	0.91240876	0.5316	0.7168
192174	Rwdd4a	RWD domain containing 4A	0.91240876	0.5602	0.7386
224132	Dirc2	disrupted in renal carcinoma 2 (human)	0.91240876	0.5719	0.7468
13002	Dnajc5	DnaJ (Hsp40) homolog, subfamily C, member 5	0.91240876	0.6427	0.7978
239337	Adamts12	a disintegrin-like and metallopeptidase (reprolysin type) with thrombospondin type 1 motif, 12	0.91240876	0.66	0.8086
12564	Cdh8	cadherin 8	0.91240876	0.6658	0.8117
333193	BC053749	cDNA sequence BC053749	0.91240876	0.6903	0.8283
84505	Setdb1	SET domain, bifurcated 1	0.91157703	0.06583	NA
321008	6330408A02Rik	RIKEN cDNA 6330408A02 gene	0.91157703	0.0735	NA
56705	Ranbp9	RAN binding protein 9	0.91157703	0.07987	NA
17210	Mcl1	myeloid cell leukemia sequence 1	0.91157703	0.09312	NA
224619	Traf7	TNF receptor-associated factor 7	0.91157703	0.09377	NA
16978	Lrrfip1	leucine rich repeat (in FLII) interacting protein 1	0.91157703	0.1093	NA
72175	Mfsd8	major facilitator superfamily domain containing 8	0.91157703	0.1112	NA
227331	Gigyf2	GRB10 interacting GYF protein 2	0.91157703	0.1227	NA
60364	Donson	downstream neighbor of SON	0.91157703	0.1286	NA
97884	B3galnt2	UDP-GalNAc:betaGlcNAc beta 1,3-galactosaminyltransferase, polypeptide 2	0.91157703	0.1308	NA
69178	Snx5	sorting nexin 5	0.91157703	0.1524	NA
192193	Edem1	ER degradation enhancer, mannosidase alpha-like 1	0.91157703	0.1552	NA
66998	Psmd5	proteasome (prosome, macropain) 26S subunit, non-ATPase, 5	0.91157703	0.1633	NA
109079	Sephs1	selenophosphate synthetase 1	0.91157703	0.1701	NA
242735	Lrrc38	leucine rich repeat containing 38	0.91157703	0.1714	NA
69932	2810004I08Rik	RIKEN cDNA 2810004I08 gene	0.91157703	0.1772	NA
11946	Atp5a1	ATP synthase, H+ transporting, mitochondrial F1 complex, alpha subunit 1	0.91157703	0.1881	NA
73721	1110017D15Rik	RIKEN cDNA 1110017D15 gene	0.91157703	0.2013	NA
668303	Kif26a	kinesin family member 26A	0.91157703	0.2056	NA
27406	Abcf3	ATP-binding cassette, sub-family F (GCN20), member 3	0.91157703	0.2074	NA
17977	Ncoa1	nuclear receptor coactivator 1	0.91157703	0.2234	NA
19184	Psmc5	protease (prosome, macropain) 26S subunit, ATPase 5	0.91157703	0.2566	0.4617
269695	Rnft2	ring finger protein, transmembrane 2	0.91157703	0.273	0.4805
235086	Igsf9b	immunoglobulin superfamily, member 9B	0.91157703	0.2787	0.4868
14172	Fgf18	fibroblast growth factor 18	0.91157703	0.2815	0.4895
73893	Tmem202	transmembrane protein 202	0.91157703	0.2865	0.4949
231630	Ficd	FIC domain containing	0.91157703	0.304	0.5132
74931	4930481A15Rik	RIKEN cDNA 4930481A15 gene	0.91157703	0.3059	0.5153
81840	Sorcs2	sortilin-related VPS10 domain containing receptor 2	0.91157703	0.3139	0.5235
71436	Flrt3	fibronectin leucine rich transmembrane protein 3	0.91157703	0.3192	0.529
77428	9430083A17Rik	RIKEN cDNA 9430083A17 gene	0.91157703	0.3209	0.5309
29859	Sult4a1	sulfotransferase family 4A, member 1	0.91157703	0.3335	0.544
242748	Ptchd2	patched domain containing 2	0.91157703	0.3355	0.5458
433766	Trim63	tripartite motif-containing 63	0.91157703	0.3365	0.5469
78755	Fam122b	family with sequence similarity 122, member B	0.91157703	0.3392	0.5494
15354	Hmgb3	high mobility group box 3	0.91157703	0.3398	0.5501

246746	Cd300lf	CD300 antigen like family member F	0.91157703	0.3596	0.5692
53872	Caprin1	cell cycle associated protein 1	0.91157703	0.3733	0.5821
16582	Kifc3	kinesin family member C3	0.91157703	0.3911	0.5993
240067	Zfp952	zinc finger protein 952	0.91157703	0.426	0.6311
68020	2810002N01Rik	RIKEN cDNA 2810002N01 gene	0.91157703	0.4322	0.6362
381305	Rc3h1	RING CCCH (C3H) domains 1	0.91157703	0.4358	0.6395
13498	Atn1	atrophin 1	0.91157703	0.4743	0.672
77480	Kidins220	kinase D-interacting substrate 220	0.91157703	0.4757	0.673
73728	Psd	pleckstrin and Sec7 domain containing	0.91157703	0.5185	0.708
239650	AI836003	expressed sequence AI836003	0.91157703	0.5186	0.708
214763	E330016A19Rik	RIKEN cDNA E330016A19 gene	0.91157703	0.5507	0.7317
77463	C030014O09Rik	RIKEN cDNA C030014O09 gene	0.91157703	0.5759	0.7493
66923	Pbrm1	polybromo 1	0.91157703	0.5915	0.7612
14562	Gdf3	growth differentiation factor 3	0.91157703	0.6075	0.773
620709	Lcn6	lipocalin 6	0.91157703	0.7091	0.84
109036	6230415J03Rik	RIKEN cDNA 6230415J03 gene	0.91157703	0.7197	0.8469
211673	Arfgef1	ADP-ribosylation factor guanine nucleotide-exchange factor 1(brefeldin A-inhibited)	0.91074681	0.05266	NA
72301	1810041L15Rik	RIKEN cDNA 1810041L15 gene	0.91074681	0.06525	NA
77733	Rnf170	ring finger protein 170	0.91074681	0.06865	NA
73218	3110056O03Rik	RIKEN cDNA 3110056O03 gene	0.91074681	0.08388	NA
269614	Pank4	pantothenate kinase 4	0.91074681	0.1156	NA
269788	Lhfpl4	lipoma HMGIC fusion partner-like protein 4	0.91074681	0.1156	NA
67890	Ufm1	ubiquitin-fold modifier 1	0.91074681	0.1179	NA
80281	Cttnbp2nl	CTTNBP2 N-terminal like	0.91074681	0.1251	NA
75744	Svip	small VCP/p97-interacting protein	0.91074681	0.127	NA
30056	Timm9	translocase of inner mitochondrial membrane 9 homolog (yeast)	0.91074681	0.1434	NA
99183	AU018505	expressed sequence AU018505	0.91074681	0.1541	NA
12142	Prdm1	PR domain containing 1, with ZNF domain	0.91074681	0.1544	NA
74203	Eif4enif1	eukaryotic translation initiation factor 4E nuclear import factor 1	0.91074681	0.1596	NA
218333	BC018507	cDNA sequence BC018507	0.91074681	0.167	NA
224691	Zfp472	zinc finger protein 472	0.91074681	0.1696	NA
20927	Abcc8	ATP-binding cassette, sub-family C (CFTR/MRP), member 8	0.91074681	0.1802	NA
140810	Ttbk2	tau tubulin kinase 2	0.91074681	0.1812	NA
245666	Iqsec2	IQ motif and Sec7 domain 2	0.91074681	0.1831	NA
320679	Samd12	sterile alpha motif domain containing 12	0.91074681	0.1873	NA
320158	Zmat4	zinc finger, matrin type 4	0.91074681	0.1951	NA
13800	Enah	enabled homolog (Drosophila)	0.91074681	0.2051	NA
110948	Hlcs	holocarboxylase synthetase (biotin- [propriony-Coenzyme A-carboxylase (ATP-hydrolysing)] ligase)	0.91074681	0.2156	NA
66362	Exosc3	exosome component 3	0.91074681	0.229	NA
19126	Prom1	prominin 1	0.91074681	0.2365	NA
66849	Ppp1r2	protein phosphatase 1, regulatory (inhibitor) subunit 2	0.91074681	0.2436	NA
53334	Gosr1	golgi SNAP receptor complex member 1	0.91074681	0.2473	NA
19158	Cyth2	cytohesin 2	0.91074681	0.2531	0.458
79264	Krit1	KRIT1, ankyrin repeat containing	0.91074681	0.2559	0.4609
14228	Fkbp4	FK506 binding protein 4	0.91074681	0.2633	0.4694
234814	Mthfsd	methenyltetrahydrofolate synthetase domain containing	0.91074681	0.2832	0.4917
328580	Tubgcp6	tubulin, gamma complex associated protein 6	0.91074681	0.2873	0.4958
73825	Klraq1	KLRAQ motif containing 1	0.91074681	0.29	0.4985
415115	Neurl2	neuralized-like 2 (Drosophila)	0.91074681	0.3089	0.5187
53902	Rcan3	regulator of calcineurin 3	0.91074681	0.3215	0.5312

414095	B130034C11Rik		RIKEN cDNA B130034C11 gene	0.91074681	0.3227	0.5325
140629	Ubox5		U box domain containing 5	0.91074681	0.3268	0.537
114602	Zmynd10		zinc finger, MYND domain containing 10	0.91074681	0.3472	0.5576
108062	Cstf2		cleavage stimulation factor, 3' pre-RNA subunit 2	0.91074681	0.3483	0.5587
76373	Zfp773		zinc finger protein 773	0.91074681	0.3644	0.5733
100042588	Gm3916		predicted gene 3916	0.91074681	0.3662	0.5752
76217	Jakmip2		janus kinase and microtubule interacting protein 2	0.91074681	0.417	0.6232
74491	5430420F09Rik		RIKEN cDNA 5430420F09 gene	0.91074681	0.4226	0.6282
71911	Bdh1		3-hydroxybutyrate dehydrogenase, type 1	0.91074681	0.4326	0.6365
67015	Ccdc91		coiled-coil domain containing 91	0.91074681	0.4365	0.6401
69823	Fyttd1		forty-two-three domain containing 1	0.91074681	0.4423	0.6452
12224	Klf5		Kruppel-like factor 5	0.91074681	0.4631	0.6635
21892	Tll1		tolloid-like	0.91074681	0.4821	0.6781
100044509	Gm14378		predicted gene 14378	0.91074681	0.5	0.6922
84585	Rnf123		ring finger protein 123	0.91074681	0.5193	0.7085
240174	Thada		thyroid adenoma associated	0.91074681	0.6041	0.7709
320808	Dcaf5		DDB1 and CUL4 associated factor 5	0.91074681	0.6298	0.7895
69459	Ubl7		ubiquitin-like 7 (bone marrow stromal cell-derived)	0.91074681	0.6387	0.7954
24047	Ccl19		chemokine (C-C motif) ligand 19	0.91074681	0.7075	0.839
100043225	Gm16378		predicted gene 16378	0.91074681	0.7267	0.8514
330474	Zc3h4		zinc finger CCCH-type containing 4	0.90991811	0.04801	NA
67278	2900092E17Rik		RIKEN cDNA 2900092E17 gene	0.90991811	0.04976	NA
68268	Zdhhc21		zinc finger, DHHC domain containing 21	0.90991811	0.06126	NA
14406	Gabrg2		gamma-aminobutyric acid (GABA) A receptor, subunit gamma 2	0.90991811	0.09469	NA
71779		Mar-08	membrane-associated ring finger (C3HC4) 8	0.90991811	0.0972	NA
75398	Mrpl32		mitochondrial ribosomal protein L32	0.90991811	0.09878	NA
56205	Ensa		endosulfine alpha	0.90991811	0.09905	NA
319801	9630033F20Rik		RIKEN cDNA 9630033F20 gene	0.90991811	0.1022	NA
15384	Hnrnpab		heterogeneous nuclear ribonucleoprotein A/B	0.90991811	0.1138	NA
74411	Ppapdc2		phosphatidic acid phosphatase type 2 domain containing 2	0.90991811	0.1196	NA
53323	Ube2k		ubiquitin-conjugating enzyme E2K (UBC1 homolog, yeast)	0.90991811	0.1283	NA
195733	Grhl1		grainyhead-like 1 (Drosophila)	0.90991811	0.1318	NA
319582	6430573F11Rik		RIKEN cDNA 6430573F11 gene	0.90991811	0.1398	NA
14924	Magi1		membrane associated guanylate kinase, WW and PDZ domain containing 1	0.90991811	0.1581	NA
52186	D8Ertd158e		DNA segment, Chr 8, ERATO Doi 158, expressed	0.90991811	0.1628	NA
29858	Pmm1		phosphomannomutase 1	0.90991811	0.1637	NA
319211	Nol4		nucleolar protein 4	0.90991811	0.1656	NA
71512	9030419F21Rik		RIKEN cDNA 9030419F21 gene	0.90991811	0.1758	NA
24136	Zeb2		zinc finger E-box binding homeobox 2	0.90991811	0.1799	NA
192167	Nlgn1		neuroligin 1	0.90991811	0.1803	NA
22402	Wisp1		WNT1 inducible signaling pathway protein 1	0.90991811	0.2138	NA
54006	Deaf1		deformed epidermal autoregulatory factor 1 (Drosophila)	0.90991811	0.232	NA
59028	Rcl1		RNA terminal phosphate cyclase-like 1	0.90991811	0.2361	NA
216166	Plk5		polo-like kinase 5 (Drosophila)	0.90991811	0.2514	0.4561
22390	Wee1		WEE 1 homolog 1 (S. pombe)	0.90991811	0.2784	0.4866
17309	Mgat3		mannoside acetylglucosaminyltransferase 3	0.90991811	0.2793	0.4874
67749	4930583H14Rik		RIKEN cDNA 4930583H14 gene	0.90991811	0.2927	0.5015
14186	Fgfr4		fibroblast growth factor receptor 4	0.90991811	0.2941	0.503
22196	Ube2i		ubiquitin-conjugating enzyme E2I	0.90991811	0.302	0.5111
72649	Tmem209		transmembrane protein 209	0.90991811	0.3269	0.537

14654	Glra1	glycine receptor, alpha 1 subunit	0.90991811	0.336	0.5464
70458	2610318N02Rik	RIKEN cDNA 2610318N02 gene	0.90991811	0.3368	0.5472
18573	Pde1a	phosphodiesterase 1A, calmodulin-dependent	0.90991811	0.3505	0.5609
67015	Ccdc91	coiled-coil domain containing 91	0.90991811	0.3595	0.5692
230935	Dnajc11	DnaJ (Hsp40) homolog, subfamily C, member 11	0.90991811	0.3627	0.5719
54153	Rasa4	RAS p21 protein activator 4	0.90991811	0.3661	0.5751
83925	Trps1	trichorhinophalangeal syndrome I (human)	0.90991811	0.3801	0.5892
83557	Lin28a	lin-28 homolog A (C. elegans)	0.90991811	0.4801	0.6761
14601	Ghrh	growth hormone releasing hormone	0.90991811	0.5067	0.6977
29813	Zfp385a	zinc finger protein 385A	0.90991811	0.5847	0.7556
75914	Exoc6b	exocyst complex component 6B	0.90991811	0.587	0.7575
77951	Cyp20a1	cytochrome P450, family 20, subfamily A, polypeptide 1	0.90991811	0.6096	0.7744
227094	Tmem194b	transmembrane protein 194B	0.90991811	0.6172	0.7797
30951	Cbx8	chromobox homolog 8 (Drosophila Pc class)	0.90991811	0.6995	0.8337
100043885	Gm4710	predicted gene 4710	0.90991811	0.701	0.8344
102787	AW552889	expressed sequence AW552889	0.90991811	0.7437	0.8615
212528	Trmt1	TRM1 tRNA methyltransferase 1 homolog (S. cerevisiae)	0.90909091	0.04962	NA
13001	Csnk2b	casein kinase 2, beta polypeptide	0.90909091	0.0533	NA
13593	Ebf3	early B-cell factor 3	0.90909091	0.07033	NA
208768	BC031781	cDNA sequence BC031781	0.90909091	0.07436	NA
22272	Uqcrq	ubiquinol-cytochrome c reductase, complex III subunit VII	0.90909091	0.08348	NA
214585	Spg11	spastic paraplegia 11	0.90909091	0.08398	NA
15925	Ide	insulin degrading enzyme	0.90909091	0.08807	NA
13669	Eif3a	eukaryotic translation initiation factor 3, subunit A	0.90909091	0.09788	NA
226412	R3hdm1	R3H domain 1 (binds single-stranded nucleic acids)	0.90909091	0.1014	NA
16574	Kif5c	kinesin family member 5C	0.90909091	0.1045	NA
23806	Arih1	ariadne ubiquitin-conjugating enzyme E2 binding protein homolog 1 (Drosophila)	0.90909091	0.1175	NA
11566	Adss	adenylosuccinate synthetase, non muscle	0.90909091	0.1301	NA
26414	Mapk10	mitogen-activated protein kinase 10	0.90909091	0.1556	NA
52397	Zfp644	zinc finger protein 644	0.90909091	0.159	NA
66970	Ssbp2	single-stranded DNA binding protein 2	0.90909091	0.1686	NA
210356	Nckap5	NCK-associated protein 5	0.90909091	0.1922	NA
232314	Ppp4r2	protein phosphatase 4, regulatory subunit 2	0.90909091	0.2214	NA
106794	Dhx57	DEAH (Asp-Glu-Ala-Asp/His) box polypeptide 57	0.90909091	0.2232	NA
66671	Ccnh	cyclin H	0.90909091	0.232	NA
68490	Zfp579	zinc finger protein 579	0.90909091	0.2328	NA
212483	Fam193b	family with sequence similarity 193, member B	0.90909091	0.2353	NA
108911	Rcc2	regulator of chromosome condensation 2	0.90909091	0.2368	NA
12291	Cacna1g	calcium channel, voltage-dependent, T type, alpha 1G subunit	0.90909091	0.2512	0.456
100382	AW011738	expressed sequence AW011738	0.90909091	0.2525	0.4574
12032	Bcan	brevican	0.90909091	0.2532	0.458
56370	Tagln3	transgelin 3	0.90909091	0.2587	0.4643
402752	C130068B02Rik	RIKEN cDNA C130068B02 gene	0.90909091	0.2808	0.489
73750	Whrn	whirlin	0.90909091	0.2864	0.4949
268980	Strn	striatin, calmodulin binding protein	0.90909091	0.2864	0.4949
12307	Calb1	calbindin 1	0.90909091	0.2883	0.4967
67235	Zkscan14	zinc finger with KRAB and SCAN domains 14	0.90909091	0.3087	0.5185
675812	Zfp605	zinc finger protein 605	0.90909091	0.3366	0.547
67200	Ccdc77	coiled-coil domain containing 77	0.90909091	0.348	0.5585
76044	Ncapg2	non-SMC condensin II complex, subunit G2	0.90909091	0.3496	0.56

381404	Pabpc1l	poly(A) binding protein, cytoplasmic 1-like	0.90909091	0.3499	0.5602
237175	Gpr64	G protein-coupled receptor 64	0.90909091	0.3528	0.5629
93713	Pcdhga5	protocadherin gamma subfamily A, 5	0.90909091	0.3549	0.5647
216156	Wdr18	WD repeat domain 18	0.90909091	0.3784	0.5873
13614	Edn1	endothelin 1	0.90909091	0.3989	0.6066
20677	Sox4	SRY-box containing gene 4	0.90909091	0.4182	0.6244
103554	Psme4	proteasome (prosome, macropain) activator subunit 4	0.90909091	0.4289	0.6335
54122	Uevld	UEV and lactate/malate dehyrogenase domains	0.90909091	0.4332	0.6368
232989	Hnrnpul1	heterogeneous nuclear ribonucleoprotein U-like 1	0.90909091	0.4476	0.6498
67074	Mon2	MON2 homolog (yeast)	0.90909091	0.4528	0.6549
67746	4930577N17Rik	RIKEN cDNA 4930577N17 gene	0.90909091	0.4529	0.6549
73739	Cby1	chibby homolog 1 (Drosophila)	0.90909091	0.4641	0.6644
258527	Olfr1368	olfactory receptor 1368	0.90909091	0.4963	0.6895
15901	Id1	inhibitor of DNA binding 1	0.90909091	0.5143	0.7044
214968	Sema6d	sema domain, transmembrane domain (TM), and cytoplasmic domain, (semaphorin) 6D	0.90909091	0.5167	0.7061
71065	4933407O12Rik	RIKEN cDNA 4933407O12 gene	0.90909091	0.5482	0.7299
106059	A430088P11Rik	RIKEN cDNA A430088P11 gene	0.90909091	0.5486	0.7304
68031	Rnf146	ring finger protein 146	0.90909091	0.5709	0.746
94111	Mepe	matrix extracellular phosphoglycoprotein with ASARM motif (bone)	0.90909091	0.593	0.7624
320541	Slc35e2	solute carrier family 35, member E2	0.90909091	0.5935	0.7629
19744	Rheb	Ras homolog enriched in brain	0.90909091	0.6099	0.7744
70088	Meaf6	MYST/Esa1-associated factor 6	0.90826521	0.05727	NA
55978	Ift20	intraflagellar transport 20 homolog (Chlamydomonas)	0.90826521	0.05941	NA
23854	Def8	differentially expressed in FDCP 8	0.90826521	0.06487	NA
234724	Tat	tyrosine aminotransferase	0.90826521	0.06748	NA
216543	Cep68	centrosomal protein 68	0.90826521	0.07467	NA
18572	Pdcd11	programmed cell death 11	0.90826521	0.07954	NA
330485	Tmem145	transmembrane protein 145	0.90826521	0.08293	NA
433940	BC057022	cDNA sequence BC057022	0.90826521	0.08311	NA
68346	Sirt5	sirtuin 5 (silent mating type information regulation 2 homolog) 5 (S. cerevisiae)	0.90826521	0.08462	NA
76245	6530415H11Rik	RIKEN cDNA 6530415H11 gene	0.90826521	0.08527	NA
76804	Kdm4c	lysine (K)-specific demethylase 4C	0.90826521	0.08964	NA
71452	Ankrd40	ankyrin repeat domain 40	0.90826521	0.09387	NA
74386	Rmi1	RMI1, RecQ mediated genome instability 1, homolog (S. cerevisiae)	0.90826521	0.09808	NA
52830	Pnrc2	proline-rich nuclear receptor coactivator 2	0.90826521	0.1002	NA
18516	Pbx3	pre B-cell leukemia transcription factor 3	0.90826521	0.122	NA
66711	Sbds	Shwachman-Bodian-Diamond syndrome homolog (human)	0.90826521	0.1362	NA
67815	Sec14l2	SEC14-like 2 (S. cerevisiae)	0.90826521	0.2056	NA
102423	Hinfp	histone H4 transcription factor	0.90826521	0.2114	NA
229615	Pias3	protein inhibitor of activated STAT 3	0.90826521	0.2138	NA
14571	Gpd2	glycerol phosphate dehydrogenase 2, mitochondrial	0.90826521	0.2216	NA
72167	Thumpd2	THUMP domain containing 2	0.90826521	0.2217	NA
381269	Mreg	melanoregulin	0.90826521	0.2387	0.4411
104015	Synj1	synaptojanin 1	0.90826521	0.2674	0.4744
225348	Wdr36	WD repeat domain 36	0.90826521	0.2921	0.5009
74159	Acbd5	acyl-Coenzyme A binding domain containing 5	0.90826521	0.2942	0.5032
72023	Cyb561d1	cytochrome b-561 domain containing 1	0.90826521	0.3014	0.5109
72873	2900006K08Rik	RIKEN cDNA 2900006K08 gene	0.90826521	0.3184	0.528
17687	Msh5	mutS homolog 5 (E. coli)	0.90826521	0.3374	0.5477
319574	9330133O14Rik	RIKEN cDNA 9330133O14 gene	0.90826521	0.3535	0.5634

14593	Ggps1	geranylgeranyl diphosphate synthase 1	0.90826521	0.3596	0.5692
225115	Svil	supervillin	0.90826521	0.3668	0.5759
433004	B830017H08Rik	RIKEN cDNA B830017H08 gene	0.90826521	0.4012	0.6089
22411	Wnt11	wingless-related MMTV integration site 11	0.90826521	0.4149	0.6211
228765	Sdcbp2	syndecan binding protein (syntenin) 2	0.90826521	0.4539	0.6555
19051	Gsbs	G substrate	0.90826521	0.4771	0.6743
52023	Pibf1	progesterone immunomodulatory binding factor 1	0.90826521	0.4995	0.692
70425	Csnk1g3	casein kinase 1, gamma 3	0.90826521	0.5183	0.7078
68112	Sdccag3	serologically defined colon cancer antigen 3	0.90826521	0.5188	0.7082
246179	Fktn	fukutin	0.90826521	0.5218	0.7098
18071	Nhlh1	nescient helix loop helix 1	0.90826521	0.5401	0.7233
67197	Zcrb1	zinc finger CCHC-type and RNA binding motif 1	0.90826521	0.6311	0.7903
71774	Shroom1	shroom family member 1	0.90826521	0.6505	0.8033
328825	Gm5093	predicted gene 5093	0.90826521	0.6842	0.8236
16988	Lst1	leukocyte specific transcript 1	0.90826521	0.7061	0.838
208501	1810043H04Rik	RIKEN cDNA 1810043H04 gene	0.90826521	0.721	0.8475
20704	Serpina1e	serine (or cysteine) peptidase inhibitor, clade A, member 1E	0.90826521	0.7233	0.8491
56620	Clec4n	C-type lectin domain family 4, member n	0.90826521	0.7954	0.8927
74025	Nphp3	nephronophthisis 3 (adolescent)	0.90826521	0.8134	0.9033
66383	Iscu	IscU iron-sulfur cluster scaffold homolog (E. coli)	0.90744102	0.05181	NA
70536	Qpct	glutaminyl-peptide cyclotransferase (glutaminyl cyclase)	0.90744102	0.06585	NA
75339	Mphosph8	M-phase phosphoprotein 8	0.90744102	0.07658	NA
436230	BC065397	cDNA sequence BC065397	0.90744102	0.08336	NA
71385	5430434G16Rik	RIKEN cDNA 5430434G16 gene	0.90744102	0.09644	NA
68310	Zmym1	zinc finger, MYM domain containing 1	0.90744102	0.1022	NA
18710	Pik3r3	phosphatidylinositol 3 kinase, regulatory subunit, polypeptide 3 (p55)	0.90744102	0.1043	NA
72345	Fam123b	family with sequence similarity 123, member B	0.90744102	0.1061	NA
12929	Crkl	v-crk sarcoma virus CT10 oncogene homolog (avian)-like	0.90744102	0.1101	NA
20482	Skil	SKI-like	0.90744102	0.1206	NA
320714	D030016E14Rik	RIKEN cDNA D030016E14 gene	0.90744102	0.1281	NA
69912	Nup43	nucleoporin 43	0.90744102	0.1444	NA
55947	Dclre1a	DNA cross-link repair 1A, PSO2 homolog (S. cerevisiae)	0.90744102	0.1447	NA
71750	R3hdm2	R3H domain containing 2	0.90744102	0.1455	NA
13139	Dgka	diacylglycerol kinase, alpha	0.90744102	0.1621	NA
20935	Surf6	surfeit gene 6	0.90744102	0.1804	NA
72865	Cxx1c	CAAX box 1 homolog C (human)	0.90744102	0.1864	NA
59049	Slc22a17	solute carrier family 22 (organic cation transporter), member 17	0.90744102	0.1996	NA
74173	1700012B15Rik	RIKEN cDNA 1700012B15 gene	0.90744102	0.2166	NA
18107	Nmt1	N-myristoyltransferase 1	0.90744102	0.2365	0.4383
191578	Helq	helicase, POLQ-like	0.90744102	0.2522	0.4573
66664	Tmem41a	transmembrane protein 41a	0.90744102	0.2982	0.5076
70078	Nol7	nucleolar protein 7	0.90744102	0.3063	0.5158
12794	Cnih2	cornichon homolog 2 (Drosophila)	0.90744102	0.3358	0.5461
19240	Tmsb10	thymosin, beta 10	0.90744102	0.3538	0.5635
70571	Tcerg1l	transcription elongation regulator 1-like	0.90744102	0.3625	0.5717
19085	Prkar1b	protein kinase, cAMP dependent regulatory, type I beta	0.90744102	0.393	0.6013
18508	Pax6	paired box gene 6	0.90744102	0.3948	0.6029
68473	Mobkl1a	MOB1, Mps One Binder kinase activator-like 1A (yeast)	0.90744102	0.4121	0.6185
69786	Tprkb	Tp53rk binding protein	0.90744102	0.4333	0.6369
66840	Wdr45l	Wdr45 like	0.90744102	0.4422	0.6451

27401	Skp2	S-phase kinase-associated protein 2 (p45)	0.90744102	0.4609	0.6615
71804	Fam54a	family with sequence similarity 54, member A	0.90744102	0.463	0.6634
100041114	Gm3146	predicted gene 3146	0.90744102	0.5277	0.7139
72236	Tsnaxip1	translin-associated factor X (Tsnax) interacting protein 1	0.90744102	0.5459	0.7278
105833	Ccdc65	coiled-coil domain containing 65	0.90744102	0.5537	0.7339
80893	Tmprss5	transmembrane protease, serine 5 (spinesin)	0.90744102	0.5763	0.7495
227800	Rabgap1	RAB GTPase activating protein 1	0.90744102	0.6596	0.8084
53975	Ddx20	DEAD (Asp-Glu-Ala-Asp) box polypeptide 20	0.90661831	0.03711	NA
66223	Mrpl35	mitochondrial ribosomal protein L35	0.90661831	0.08587	NA
20320	Nptn	neuroplastin	0.90661831	0.1127	NA
11947	Atp5b	ATP synthase, H+ transporting mitochondrial F1 complex, beta subunit	0.90661831	0.1167	NA
12293	Cacna2d1	calcium channel, voltage-dependent, alpha2/delta subunit 1	0.90661831	0.1488	NA
241520	Fam171b	family with sequence similarity 171, member B	0.90661831	0.1591	NA
73683	Atg16l2	autophagy related 16 like 2 (S. cerevisiae)	0.90661831	0.1678	NA
666185	Gm7969	predicted gene 7969	0.90661831	0.1711	NA
56737	Alg2	asparagine-linked glycosylation 2 homolog (yeast, alpha-1,3-mannosyltransferase)	0.90661831	0.1742	NA
320560	Dennd5b	DENN/MADD domain containing 5B	0.90661831	0.1829	NA
19325	Rab10	RAB10, member RAS oncogene family	0.90661831	0.1931	NA
73919	Lyrm1	LYR motif containing 1	0.90661831	0.1963	NA
66206	1110059E24Rik	RIKEN cDNA 1110059E24 gene	0.90661831	0.1983	NA
78913	Ltn1	listerin E3 ubiquitin protein ligase 1	0.90661831	0.2336	0.4352
234463	Tmem184c	transmembrane protein 184C	0.90661831	0.2383	0.4406
229055	Zbtb10	zinc finger and BTB domain containing 10	0.90661831	0.265	0.4714
73720	Cst6	cystatin E/M	0.90661831	0.3069	0.5163
76781	Mettl4	methyltransferase like 4	0.90661831	0.3139	0.5235
17718	ND3	NADH dehydrogenase subunit 3	0.90661831	0.3142	0.5236
19240	Tmsb10	thymosin, beta 10	0.90661831	0.3286	0.5387
15560	Htr2c	5-hydroxytryptamine (serotonin) receptor 2C	0.90661831	0.332	0.5423
66880	Rsrc1	arginine/serine-rich coiled-coil 1	0.90661831	0.3518	0.5618
72852	Mblac2	metallo-beta-lactamase domain containing 2	0.90661831	0.3922	0.6004
69454	Clic3	chloride intracellular channel 3	0.90661831	0.4836	0.6792
15444	Hpca	hippocalcin	0.90661831	0.4986	0.6913
106064	AW549877	expressed sequence AW549877	0.90661831	0.5058	0.6969
57780	Fxyd7	FXYD domain-containing ion transport regulator 7	0.90661831	0.5395	0.723
78547	E130304I02Rik	RIKEN cDNA E130304I02 gene	0.90661831	0.5745	0.7484
22135	Tgoln2	trans-golgi network protein 2	0.90661831	0.588	0.7583
620306	LOC620306	similar to Glyceraldehyde-3-phosphate dehydrogenase (GAPDH)	0.90661831	0.7456	0.8629
18521	Pcbp2	poly(rC) binding protein 2	0.9057971	0.03689	NA
433022	Plcxd2	phosphatidylinositol-specific phospholipase C, X domain containing 2	0.9057971	0.04949	NA
56017	Slc2a8	solute carrier family 2, (facilitated glucose transporter), member 8	0.9057971	0.07136	NA
70495	Atp6ap2	ATPase, H+ transporting, lysosomal accessory protein 2	0.9057971	0.07611	NA
65114	Vps35	vacuolar protein sorting 35	0.9057971	0.07615	NA
83674	Cnnm1	cyclin M1	0.9057971	0.08234	NA
67948	Fbxo28	F-box protein 28	0.9057971	0.08592	NA
18514	Pbx1	pre B-cell leukemia transcription factor 1	0.9057971	0.08716	NA
67263	Zswim6	zinc finger, SWIM domain containing 6	0.9057971	0.09755	NA
18045	Nfyb	nuclear transcription factor-Y beta	0.9057971	0.1119	NA
110809	Srsf1	serine/arginine-rich splicing factor 1	0.9057971	0.1152	NA
83679	Pde4dip	phosphodiesterase 4D interacting protein (myomegalin)	0.9057971	0.1214	NA
50493	Txnrd1	thioredoxin reductase 1	0.9057971	0.142	NA

69886	2610002J23Rik	RIKEN cDNA 2610002J23 gene	0.9057971	0.1489	NA
26939	Polr3e	polymerase (RNA) III (DNA directed) polypeptide E	0.9057971	0.168	NA
240058	Cpne5	copine V	0.9057971	0.1711	NA
22385	Baz1b	bromodomain adjacent to zinc finger domain, 1B	0.9057971	0.172	NA
56632	Sphk2	sphingosine kinase 2	0.9057971	0.1829	NA
71436	Flrt3	fibronectin leucine rich transmembrane protein 3	0.9057971	0.2003	NA
269295	Rtn4rl2	reticulon 4 receptor-like 2	0.9057971	0.2279	0.428
16341	Eif3e	eukaryotic translation initiation factor 3, subunit E	0.9057971	0.2368	0.4387
57895	Ccdc126	coiled-coil domain containing 126	0.9057971	0.2453	0.4489
17937	Nab2	Ngfi-A binding protein 2	0.9057971	0.269	0.4761
74020	Cpne4	copine IV	0.9057971	0.2881	0.4966
23918	Impdh2	inosine 5'-phosphate dehydrogenase 2	0.9057971	0.2891	0.4975
14766	Gpr56	G protein-coupled receptor 56	0.9057971	0.2917	0.5005
12894	Cpt1a	carnitine palmitoyltransferase 1a, liver	0.9057971	0.3147	0.5241
320163	4930525G20Rik	RIKEN cDNA 4930525G20 gene	0.9057971	0.3155	0.5249
56390	Sssca1	Sjogren's syndrome/scleroderma autoantigen 1 homolog (human)	0.9057971	0.3197	0.5296
20620	Plk2	polo-like kinase 2 (Drosophila)	0.9057971	0.3301	0.5404
104732	4930427A07Rik	RIKEN cDNA 4930427A07 gene	0.9057971	0.347	0.5575
76686	Clip3	CAP-GLY domain containing linker protein 3	0.9057971	0.3527	0.5628
117229	Stk33	serine/threonine kinase 33	0.9057971	0.3572	0.5672
20536	Slc4a3	solute carrier family 4 (anion exchanger), member 3	0.9057971	0.4014	0.609
13131	Dab1	disabled homolog 1 (Drosophila)	0.9057971	0.4502	0.6525
230594	Zcchc11	zinc finger, CCHC domain containing 11	0.9057971	0.4636	0.6639
77329	C030010C08Rik	RIKEN cDNA C030010C08 gene	0.9057971	0.4647	0.6649
104582	Rprml	reprimo-like	0.9057971	0.4719	0.6702
16801	Arhgef1	Rho guanine nucleotide exchange factor (GEF) 1	0.9057971	0.4741	0.6718
76223	Agbl3	ATP/GTP binding protein-like 3	0.9057971	0.5221	0.71
242800	Ttc34	tetratricopeptide repeat domain 34	0.9057971	0.5228	0.7102
12231	Btn1a1	butyrophilin, subfamily 1, member A1	0.9057971	0.5414	0.7243
67769	Gpatch2	G patch domain containing 2	0.9057971	0.5415	0.7244
20534	Slc4a1ap	solute carrier family 4 (anion exchanger), member 1, adaptor protein	0.9057971	0.5801	0.7524
11949	Atp5c1	ATP synthase, H+ transporting, mitochondrial F1 complex, gamma polypeptide 1	0.9057971	0.7253	0.8505
50755	Fbxo18	F-box protein 18	0.90497738	0.03596	NA
234875	Ttc13	tetratricopeptide repeat domain 13	0.90497738	0.03974	NA
12069	Bex2	brain expressed X-linked 2	0.90497738	0.04287	NA
231915	Uspl1	ubiquitin specific peptidase like 1	0.90497738	0.05521	NA
74360	Cep57	centrosomal protein 57	0.90497738	0.07574	NA
231580	Gak	cyclin G associated kinase	0.90497738	0.07824	NA
216742	Fnip1	folliculin interacting protein 1	0.90497738	0.08749	NA
320634	Ocrl	oculocerebrorenal syndrome of Lowe	0.90497738	0.09189	NA
69663	Ddx51	DEAD (Asp-Glu-Ala-Asp) box polypeptide 51	0.90497738	0.09366	NA
66730	4921531C22Rik	RIKEN cDNA 4921531C22 gene	0.90497738	0.104	NA
69718	Ipmk	inositol polyphosphate multikinase	0.90497738	0.1051	NA
72654	Ccdc12	coiled-coil domain containing 12	0.90497738	0.1165	NA
18647	Cdk14	cyclin-dependent kinase 14	0.90497738	0.1352	NA
414093	A830082N09Rik	RIKEN cDNA A830082N09 gene	0.90497738	0.1409	NA
16497	Kcnab1	potassium voltage-gated channel, shaker-related subfamily, beta member 1	0.90497738	0.1619	NA
14456	Gas6	growth arrest specific 6	0.90497738	0.166	NA
227659	Slc2a6	solute carrier family 2 (facilitated glucose transporter), member 6	0.90497738	0.1684	NA
434156	Eid2b	EP300 interacting inhibitor of differentiation 2B	0.90497738	0.1783	NA

19025	Ctsa	cathepsin A	0.90497738	0.1785	NA
544752	Tug1	taurine upregulated gene 1	0.90497738	0.1864	NA
110351	Rap1gap	Rap1 GTPase-activating protein	0.90497738	0.2071	NA
68166	Spire1	spire homolog 1 (Drosophila)	0.90497738	0.2086	NA
214764	2700050L05Rik	RIKEN cDNA 2700050L05 gene	0.90497738	0.2191	0.4177
20853	Stau1	staufen (RNA binding protein) homolog 1 (Drosophila)	0.90497738	0.2511	0.4559
14585	Gfra1	glial cell line derived neurotrophic factor family receptor alpha 1	0.90497738	0.2542	0.4592
442829	Ccin	calicin	0.90497738	0.2805	0.4887
20874	Slk	STE20-like kinase (yeast)	0.90497738	0.3041	0.5132
18607	Pdpk1	3-phosphoinositide dependent protein kinase 1	0.90497738	0.3156	0.525
264134	Ttc26	tetratricopeptide repeat domain 26	0.90497738	0.3352	0.5457
14123	Fbrs	fibrosin	0.90497738	0.3693	0.5783
107586	Ovol2	ovo-like 2 (Drosophila)	0.90497738	0.3706	0.5794
22222	Ubr1	ubiquitin protein ligase E3 component n-recognin 1	0.90497738	0.3824	0.5915
234967	Slc36a4	solute carrier family 36 (proton/amino acid symporter), member 4	0.90497738	0.3949	0.6029
79263	Trim39	tripartite motif-containing 39	0.90497738	0.3959	0.6039
72568	Lin9	lin-9 homolog (C. elegans)	0.90497738	0.4271	0.6319
19275	Ptprn	protein tyrosine phosphatase, receptor type, N	0.90497738	0.4373	0.6407
54447	Asah2	N-acylsphingosine amidohydrolase 2	0.90497738	0.4435	0.6465
76179	Usp31	ubiquitin specific peptidase 31	0.90497738	0.4471	0.6493
74481	Batf2	basic leucine zipper transcription factor, ATF-like 2	0.90497738	0.479	0.6755
19879	Slc22a8	solute carrier family 22 (organic anion transporter), member 8	0.90497738	0.5107	0.7012
399568	BC052040	cDNA sequence BC052040	0.90497738	0.5476	0.7292
328789	Lhfpl5	lipoma HMGIC fusion partner-like 5	0.90497738	0.5778	0.7506
56191	Tro	trophinin	0.90497738	0.5887	0.7589
29807	Tpk1	thiamine pyrophosphokinase	0.90497738	0.6376	0.7948
16492	Kcna4	potassium voltage-gated channel, shaker-related subfamily, member 4	0.90497738	0.6402	0.7964
16504	Kcnc3	potassium voltage gated channel, Shaw-related subfamily, member 3	0.90497738	0.6474	0.8012
66481	Rps21	ribosomal protein S21	0.90497738	0.7288	0.8526
66965	Ctu2	cytosolic thiouridylase subunit 2 homolog (S. pombe)	0.90415913	0.04347	NA
50780	Rgs3	regulator of G-protein signaling 3	0.90415913	0.07001	NA
52398	Sep-11	septin 11	0.90415913	0.07141	NA
13063	Cycs	cytochrome c, somatic	0.90415913	0.07255	NA
17760	Mtap6	microtubule-associated protein 6	0.90415913	0.07692	NA
214897	Csnk1g1	casein kinase 1, gamma 1	0.90415913	0.08271	NA
19385	Ranbp1	RAN binding protein 1	0.90415913	0.09302	NA
68379	Ciz1	CDKN1A interacting zinc finger protein 1	0.90415913	0.09405	NA
271508	4933408B17Rik	RIKEN cDNA 4933408B17 gene	0.90415913	0.09489	NA
228836	Dlgap4	discs, large homolog-associated protein 4 (Drosophila)	0.90415913	0.09922	NA
18046	Nfyc	nuclear transcription factor-Y gamma	0.90415913	0.09923	NA
68097	Dynll2	dynein light chain LC8-type 2	0.90415913	0.1048	NA
11432	Acp2	acid phosphatase 2, lysosomal	0.90415913	0.1084	NA
108760	Galntl1	UDP-N-acetyl-alpha-D-galactosamine:polypeptide N-acetylgalactosaminyltransferase-like 1	0.90415913	0.1084	NA
12395	Runx1t1	runt-related transcription factor 1; translocated to, 1 (cyclin D-related)	0.90415913	0.1351	NA
72308	Brf1	BRF1 homolog, subunit of RNA polymerase III transcription initiation factor IIIB (S. cerevisiae)	0.90415913	0.1389	NA
319583	Lig4	ligase IV, DNA, ATP-dependent	0.90415913	0.1408	NA
66865	Pmpca	peptidase (mitochondrial processing) alpha	0.90415913	0.1584	NA
16591	Kl	klotho	0.90415913	0.1607	NA
19277	Ptpro	protein tyrosine phosphatase, receptor type, O	0.90415913	0.1654	NA
100043685	Gm4588	predicted gene 4588	0.90415913	0.1732	NA

56364	Zmym3	zinc finger, MYM-type 3	0.90415913	0.1749	NA
68197	Ndufc2	NADH dehydrogenase (ubiquinone) 1, subcomplex unknown, 2	0.90415913	0.1783	NA
52575	Rg9mtd1	RNA (guanine-9-) methyltransferase domain containing 1	0.90415913	0.1849	NA
229279	Hnrnpa3	heterogeneous nuclear ribonucleoprotein A3	0.90415913	0.1894	NA
53625	B3gnt2	UDP-GlcNAc:betaGal beta-1,3-N-acetylglucosaminyltransferase 2	0.90415913	0.1933	NA
84095	Pi4k2a	phosphatidylinositol 4-kinase type 2 alpha	0.90415913	0.1933	NA
213760	Prepl	prolyl endopeptidase-like	0.90415913	0.1934	NA
319675	5830418K08Rik	RIKEN cDNA 5830418K08 gene	0.90415913	0.1988	NA
68644	Abhd14a	abhydrolase domain containing 14A	0.90415913	0.2069	NA
103210	D630004K10Rik	RIKEN cDNA D630004K10 gene	0.90415913	0.2443	0.4481
239985	Arid1b	AT rich interactive domain 1B (SWI-like)	0.90415913	0.2627	0.4687
68420	Ankrd13a	ankyrin repeat domain 13a	0.90415913	0.2638	0.4698
12300	Cacng2	calcium channel, voltage-dependent, gamma subunit 2	0.90415913	0.2683	0.4754
12291	Cacna1g	calcium channel, voltage-dependent, T type, alpha 1G subunit	0.90415913	0.3219	0.5317
14169	Fgf14	fibroblast growth factor 14	0.90415913	0.3364	0.5467
12562	Cdh5	cadherin 5	0.90415913	0.3383	0.5485
246317	Neto1	neuropilin (NRP) and tolloid (TLL)-like 1	0.90415913	0.3444	0.5549
22042	Tfrc	transferrin receptor	0.90415913	0.3535	0.5634
100710	Pds5b	PDS5, regulator of cohesion maintenance, homolog B (S. cerevisiae)	0.90415913	0.3861	0.5947
20273	Scn8a	sodium channel, voltage-gated, type VIII, alpha	0.90415913	0.421	0.6271
239857	Cadm2	cell adhesion molecule 2	0.90415913	0.4754	0.6729
75050	Kif27	kinesin family member 27	0.90415913	0.5075	0.6983
20319	Sfrp2	secreted frizzled-related protein 2	0.90415913	0.5336	0.7184
338355	Fkbp15	FK506 binding protein 15	0.90415913	0.5381	0.722
68255	Tmem86b	transmembrane protein 86B	0.90415913	0.5468	0.7287
75754	9030607L02Rik	RIKEN cDNA 9030607L02 gene	0.90415913	0.5564	0.7361
20515	Slc20a1	solute carrier family 20, member 1	0.90415913	0.5645	0.7418
100042014	Gm3625	predicted gene 3625	0.90415913	0.5925	0.762
83382	Siglece	sialic acid binding Ig-like lectin E	0.90415913	0.6045	0.7712
327987	Med13	mediator complex subunit 13	0.90415913	0.6131	0.7771
218440	Ankrd34b	ankyrin repeat domain 34B	0.90415913	0.6165	0.7793
69699	2310079G19Rik	RIKEN cDNA 2310079G19 gene	0.90415913	0.7328	0.8557
12569	Cdk5r1	cyclin-dependent kinase 5, regulatory subunit 1 (p35)	0.90334237	0.04356	NA
52864	Slx4	SLX4 structure-specific endonuclease subunit homolog (S. cerevisiae)	0.90334237	0.05469	NA
20286	Zc3h7b	zinc finger CCCH type containing 7B	0.90334237	0.06136	NA
67418	Ppil4	peptidylprolyl isomerase (cyclophilin)-like 4	0.90334237	0.06391	NA
66167	Ccdc72	coiled-coil domain containing 72	0.90334237	0.06952	NA
19735	Rgs2	regulator of G-protein signaling 2	0.90334237	0.07167	NA
78697	Pus7	pseudouridylate synthase 7 homolog (S. cerevisiae)	0.90334237	0.07649	NA
30059	Timm10	translocase of inner mitochondrial membrane 10 homolog (yeast)	0.90334237	0.09862	NA
320213	Senp5	SUMO/sentrin specific peptidase 5	0.90334237	0.1003	NA
14559	Gdf1	growth differentiation factor 1	0.90334237	0.1185	NA
209011	Sirt7	sirtuin 7 (silent mating type information regulation 2, homolog) 7 (S. cerevisiae)	0.90334237	0.1185	NA
30057	Timm8b	translocase of inner mitochondrial membrane 8 homolog b (yeast)	0.90334237	0.1193	NA
20239	Atxn2	ataxin 2	0.90334237	0.1287	NA
73447	Wdr13	WD repeat domain 13	0.90334237	0.1501	NA
217026	Heatr6	HEAT repeat containing 6	0.90334237	0.1531	NA
14859	Gsta3	glutathione S-transferase, alpha 3	0.90334237	0.1547	NA
140475	Bsnd	Bartter syndrome, infantile, with sensorineural deafness (Barttin)	0.90334237	0.1738	NA
50795	Sh3bgr	SH3-binding domain glutamic acid-rich protein	0.90334237	0.1838	NA

71566	9030425E11Rik	RIKEN cDNA 9030425E11 gene	0.90334237	0.2057	NA
53420	Syt5	synaptotagmin V	0.90334237	0.2126	0.4085
93697	Narg2	NMDA receptor-regulated gene 2	0.90334237	0.228	0.428
71751	Map3k13	mitogen-activated protein kinase kinase kinase 13	0.90334237	0.2444	0.4482
236848	BC023829	cDNA sequence BC023829	0.90334237	0.254	0.4591
106338	Nsun3	NOL1/NOP2/Sun domain family member 3	0.90334237	0.2704	0.4779
99939	BB085087	expressed sequence BB085087	0.90334237	0.296	0.5049
12310	Calca	calcitonin/calcitonin-related polypeptide, alpha	0.90334237	0.2993	0.5086
243328	Slc29a4	solute carrier family 29 (nucleoside transporters), member 4	0.90334237	0.3119	0.5216
216724	Rufy1	RUN and FYVE domain containing 1	0.90334237	0.3147	0.5241
219189	1300010F03Rik	RIKEN cDNA 1300010F03 gene	0.90334237	0.3339	0.5443
232035	Fam190a	family with sequence similarity 190, member A	0.90334237	0.3351	0.5456
14218	Sh3pxd2a	SH3 and PX domains 2A	0.90334237	0.354	0.5639
53861	Zranb2	zinc finger, RAN-binding domain containing 2	0.90334237	0.3592	0.569
117160	Ttyh2	tweety homolog 2 (Drosophila)	0.90334237	0.3811	0.5903
19158	Cyth2	cytohesin 2	0.90334237	0.3859	0.5947
73178	Wasl	Wiskott-Aldrich syndrome-like (human)	0.90334237	0.4141	0.6205
14708	Gng7	guanine nucleotide binding protein (G protein), gamma 7	0.90334237	0.438	0.6412
269060	Dagla	diacylglycerol lipase, alpha	0.90334237	0.4844	0.6798
100038604	3426406K10Rik	RIKEN cDNA 3426406K10 gene	0.90334237	0.5207	0.7091
12491	Cd36	CD36 antigen	0.90334237	0.5262	0.7128
24047	Ccl19	chemokine (C-C motif) ligand 19	0.90334237	0.5593	0.7383
100042480	Nhsl2	NHS-like 2	0.90334237	0.6389	0.7955
382384	Odf3l2	outer dense fiber of sperm tails 3-like 2	0.90334237	0.6525	0.804
78211	4930558N11Rik	RIKEN cDNA 4930558N11 gene	0.90334237	0.6529	0.8041
14563	Gdf5	growth differentiation factor 5	0.90334237	0.7929	0.8914
74369	Mei1	meiosis defective 1	0.90252708	0.04928	NA
17769	Mthfr	5,10-methylenetetrahydrofolate reductase	0.90252708	0.05917	NA
66645	Pspc1	paraspeckle protein 1	0.90252708	0.05972	NA
27416	Abcc5	ATP-binding cassette, sub-family C (CFTR/MRP), member 5	0.90252708	0.06305	NA
54375	Azin1	antizyme inhibitor 1	0.90252708	0.06962	NA
227634	Camsap1	calmodulin regulated spectrin-associated protein 1	0.90252708	0.1113	NA
52897	Rbfox3	RNA binding protein, fox-1 homolog (C. elegans) 3	0.90252708	0.1265	NA
60365	Rbm8a	RNA binding motif protein 8a	0.90252708	0.1563	NA
66445	Cyc1	cytochrome c-1	0.90252708	0.1567	NA
271377	Zbtb11	zinc finger and BTB domain containing 11	0.90252708	0.1595	NA
72899	Macrod2	MACRO domain containing 2	0.90252708	0.1604	NA
19090	Prkdc	protein kinase, DNA activated, catalytic polypeptide	0.90252708	0.165	NA
497255	LOC497255	hypothetical LOC497255	0.90252708	0.1753	NA
19708	Dpf2	D4, zinc and double PHD fingers family 2	0.90252708	0.1764	NA
333605	Frmpd4	FERM and PDZ domain containing 4	0.90252708	0.1777	NA
20563	Slit2	slit homolog 2 (Drosophila)	0.90252708	0.1795	NA
109135	Plekha5	pleckstrin homology domain containing, family A member 5	0.90252708	0.2169	0.4143
215114	Hip1	huntingtin interacting protein 1	0.90252708	0.2398	0.4422
232016	Ccdc129	coiled-coil domain containing 129	0.90252708	0.2443	0.4481
235180	Fez1	fasciculation and elongation protein zeta 1 (zygin I)	0.90252708	0.2464	0.4504
17931	Ppp1r12a	protein phosphatase 1, regulatory (inhibitor) subunit 12A	0.90252708	0.2478	0.4519
27425	Atp5l	ATP synthase, H+ transporting, mitochondrial F0 complex, subunit g	0.90252708	0.2651	0.4715
14270	Srgap2	SLIT-ROBO Rho GTPase activating protein 2	0.90252708	0.2904	0.4987
59035	Carm1	coactivator-associated arginine methyltransferase 1	0.90252708	0.2916	0.5002

20208	Saa1	serum amyloid A 1	0.90252708	0.3438	0.5547
69179	Tmem110	transmembrane protein 110	0.90252708	0.366	0.5751
18996	Pou4f1	POU domain, class 4, transcription factor 1	0.90252708	0.3714	0.5801
26556	Homer1	homer homolog 1 (Drosophila)	0.90252708	0.4719	0.6702
74490	Mamstr	MEF2 activating motif and SAP domain containing transcriptional regulator	0.90252708	0.4741	0.6718
380732	Gm885	predicted gene 885	0.90252708	0.4798	0.6759
380969	Nckap5l	NCK-associated protein 5-like	0.90252708	0.551	0.732
238130	Dock4	dedicator of cytokinesis 4	0.90252708	0.5683	0.744
408192	Gm9839	predicted gene 9839	0.90252708	0.6117	0.7758
319660	Tmem195	transmembrane protein 195	0.90252708	0.7109	0.8413
75859	4930568D16Rik	RIKEN cDNA 4930568D16 gene	0.90252708	0.793	0.8914
260297	Prrt1	proline-rich transmembrane protein 1	0.90171326	0.05512	NA
20912	Stxbp3a	syntaxin binding protein 3A	0.90171326	0.05732	NA
67155	Smarca2	SWI/SNF related, matrix associated, actin dependent regulator of chromatin, subfamily a, member 2	0.90171326	0.06175	NA
18472	Pafah1b1	platelet-activating factor acetylhydrolase, isoform 1b, subunit 1	0.90171326	0.0694	NA
12035	Bcat1	branched chain aminotransferase 1, cytosolic	0.90171326	0.07548	NA
17777	Mttp	microsomal triglyceride transfer protein	0.90171326	0.07686	NA
66074	Tmem167	transmembrane protein 167	0.90171326	0.07986	NA
50790	Acsl4	acyl-CoA synthetase long-chain family member 4	0.90171326	0.08488	NA
57750	Wdr12	WD repeat domain 12	0.90171326	0.08675	NA
11909	Atf2	activating transcription factor 2	0.90171326	0.09708	NA
20713	Serpini1	serine (or cysteine) peptidase inhibitor, clade I, member 1	0.90171326	0.1184	NA
225004	BC027072	cDNA sequence BC027072	0.90171326	0.1225	NA
76969	Chst1	carbohydrate (keratan sulfate Gal-6) sulfotransferase 1	0.90171326	0.128	NA
14664	Slc6a9	solute carrier family 6 (neurotransmitter transporter, glycine), member 9	0.90171326	0.1356	NA
74366	4932422M17Rik	RIKEN cDNA 4932422M17 gene	0.90171326	0.1513	NA
212307	Mapre2	microtubule-associated protein, RP/EB family, member 2	0.90171326	0.176	NA
22223	Uchl1	ubiquitin carboxy-terminal hydrolase L1	0.90171326	0.1827	NA
67053	Rpp14	ribonuclease P 14 subunit (human)	0.90171326	0.1888	NA
58996	Arhgap23	Rho GTPase activating protein 23	0.90171326	0.1919	NA
170719	Oxr1	oxidation resistance 1	0.90171326	0.1936	NA
105203	BC016423	cDNA sequence BC016423	0.90171326	0.224	0.4237
11782	Ap4s1	adaptor-related protein complex AP-4, sigma 1	0.90171326	0.2488	0.4529
230767	Iqcc	IQ motif containing C	0.90171326	0.2623	0.4683
497097	Xkr4	X Kell blood group precursor related family member 4	0.90171326	0.2636	0.4696
12921	Crhr1	corticotropin releasing hormone receptor 1	0.90171326	0.2969	0.506
210673	Prrt3	proline-rich transmembrane protein 3	0.90171326	0.3082	0.5179
233033	Samd4b	sterile alpha motif domain containing 4B	0.90171326	0.3179	0.5274
75881	4930579K19Rik	RIKEN cDNA 4930579K19 gene	0.90171326	0.351	0.5611
107522	Ece2	endothelin converting enzyme 2	0.90171326	0.3652	0.5743
212531	Sh3bgrl2	SH3 domain binding glutamic acid-rich protein like 2	0.90171326	0.4374	0.6408
104248	Cabin1	calcineurin binding protein 1	0.90171326	0.4578	0.6591
269593	Luzp1	leucine zipper protein 1	0.90171326	0.483	0.6786
382793	Mtx3	metaxin 3	0.90171326	0.5669	0.7429
20190	Ryr1	ryanodine receptor 1, skeletal muscle	0.90171326	0.5877	0.7579
26931	Ppp2r5c	protein phosphatase 2, regulatory subunit B (B56), gamma isoform	0.90171326	0.6391	0.7956
259032	Olfr1134	olfactory receptor 1134	0.90171326	0.755	0.8686
66146	Tmem57	transmembrane protein 57	0.9009009	0.03533	NA
68955	Srrm4	serine/arginine repetitive matrix 4	0.9009009	0.04934	NA
26379	Esrra	estrogen related receptor, alpha	0.9009009	0.0575	NA

21843	Tial1	Tia1 cytotoxic granule-associated RNA binding protein-like 1	0.9009009	0.05884	NA
110172	Slc35b1	solute carrier family 35, member B1	0.9009009	0.06479	NA
228850	Ralgapb	Ral GTPase activating protein, beta subunit (non-catalytic)	0.9009009	0.08287	NA
77980	Sbf1	SET binding factor 1	0.9009009	0.09126	NA
68165	Fdx1l	ferredoxin 1-like	0.9009009	0.1	NA
107065	Lrrtm2	leucine rich repeat transmembrane neuronal 2	0.9009009	0.115	NA
67414	Mfn1	mitofusin 1	0.9009009	0.1159	NA
18575	Pde1c	phosphodiesterase 1C	0.9009009	0.1256	NA
14319	Fth1	ferritin heavy chain 1	0.9009009	0.1325	NA
332175	Zdhhc23	zinc finger, DHHC domain containing 23	0.9009009	0.1355	NA
243743	Plxna4	plexin A4	0.9009009	0.1465	NA
14664	Slc6a9	solute carrier family 6 (neurotransmitter transporter, glycine), member 9	0.9009009	0.1466	NA
224650	Anks1	ankyrin repeat and SAM domain containing 1	0.9009009	0.1485	NA
18044	Nfya	nuclear transcription factor-Y alpha	0.9009009	0.1847	NA
623661	Lipt1	lipoyltransferase 1	0.9009009	0.2059	0.4
21417	Zeb1	zinc finger E-box binding homeobox 1	0.9009009	0.2103	0.4058
67144	Lrrc40	leucine rich repeat containing 40	0.9009009	0.2231	0.4226
381352	Mamdc4	MAM domain containing 4	0.9009009	0.2254	0.4252
320360	Ric3	resistance to inhibitors of cholinesterase 3 homolog (C. elegans)	0.9009009	0.2844	0.4928
60507	Qtrt1	queuine tRNA-ribosyltransferase 1	0.9009009	0.2855	0.494
320616	B130006D01Rik	RIKEN cDNA B130006D01 gene	0.9009009	0.2959	0.5048
243897	Ggn	gametogenetin	0.9009009	0.303	0.512
109652	Acy1	aminoacylase 1	0.9009009	0.3071	0.5166
214498	Cdc73	cell division cycle 73, Paf1/RNA polymerase II complex component, homolog (S. cerevisiae)	0.9009009	0.3672	0.5762
94090	Trim9	tripartite motif-containing 9	0.9009009	0.3788	0.5878
100042784	Prdm11	PR domain containing 11	0.9009009	0.3937	0.6018
414066	BC037032	cDNA Sequence BC037032	0.9009009	0.3989	0.6066
71970	Scand3	SCAN domain containing 3	0.9009009	0.4005	0.6083
237558	Gm239	predicted gene 239	0.9009009	0.4291	0.6336
69564	Itgb1bp3	integrin beta 1 binding protein 3	0.9009009	0.4294	0.6337
30052	Pcsk1n	proprotein convertase subtilisin/kexin type 1 inhibitor	0.9009009	0.4596	0.6605
13839	Epha5	Eph receptor A5	0.9009009	0.5234	0.7104
100039631	Gm2347	predicted gene 2347	0.9009009	0.5493	0.7311
215335	Slc36a1	solute carrier family 36 (proton/amino acid symporter), member 1	0.9009009	0.5927	0.7621
15116	Has1	hyaluronan synthase1	0.9009009	0.5998	0.7678
66605	1700017N19Rik	RIKEN cDNA 1700017N19 gene	0.9009009	0.6453	0.7995
258272	Olfr1402	olfactory receptor 1402	0.9009009	0.6727	0.8167
57782	Rbak	RB-associated KRAB repressor	0.90009001	0.02996	NA
69215	Sat2	spermidine/spermine N1-acetyl transferase 2	0.90009001	0.03441	NA
260409	Cdc42ep3	CDC42 effector protein (Rho GTPase binding) 3	0.90009001	0.04085	NA
29812	Ndrg3	N-myc downstream regulated gene 3	0.90009001	0.04196	NA
117171	1110038F14Rik	RIKEN cDNA 1110038F14 gene	0.90009001	0.04369	NA
67414	Mfn1	mitofusin 1	0.90009001	0.048	NA
269523	Vcp	valosin containing protein	0.90009001	0.05937	NA
71901	2310028H24Rik	RIKEN cDNA 2310028H24 gene	0.90009001	0.07247	NA
235344	Sik2	salt inducible kinase 2	0.90009001	0.08799	NA
66419	Mrpl11	mitochondrial ribosomal protein L11	0.90009001	0.09027	NA
233904	Setd1a	SET domain containing 1A	0.90009001	0.09385	NA
269643	Ppp2r2c	protein phosphatase 2 (formerly 2A), regulatory subunit B (PR 52), gamma isoform	0.90009001	0.1027	NA
214597	Sidt2	SID1 transmembrane family, member 2	0.90009001	0.105	NA

231225	Tapt1	transmembrane anterior posterior transformation 1	0.90009001	0.1061	NA
544922	Zkscan4	zinc finger with KRAB and SCAN domains 4	0.90009001	0.1139	NA
68564	Nufip2	nuclear fragile X mental retardation protein interacting protein 2	0.90009001	0.1246	NA
319518	Pdpr	pyruvate dehydrogenase phosphatase regulatory subunit	0.90009001	0.1284	NA
12632	Cfl2	cofilin 2, muscle	0.90009001	0.1424	NA
75316	Taf1d	TATA box binding protein (Tbp)-associated factor, RNA polymerase I, D	0.90009001	0.1531	NA
217887	BC022687	cDNA sequence BC022687	0.90009001	0.1671	NA
68493	Ndufaf4	NADH dehydrogenase (ubiquinone) 1 alpha subcomplex, assembly factor 4	0.90009001	0.1677	NA
208967	Thnsl1	threonine synthase-like 1 (bacterial)	0.90009001	0.1776	NA
16467	Atcay	ataxia, cerebellar, Cayman type homolog (human)	0.90009001	0.1811	NA
329628	Fat4	FAT tumor suppressor homolog 4 (Drosophila)	0.90009001	0.1899	NA
71375	Foxn3	forkhead box N3	0.90009001	0.2029	0.3959
66743	Rnf220	ring finger protein 220	0.90009001	0.2078	0.4027
24084	Tekt2	tektin 2	0.90009001	0.2197	0.4186
23964	Odz2	odd Oz/ten-m homolog 2 (Drosophila)	0.90009001	0.2278	0.428
23965	Odz3	odd Oz/ten-m homolog 3 (Drosophila)	0.90009001	0.2543	0.4593
27412	Peg12	paternally expressed 12	0.90009001	0.2997	0.5091
71519	Cyp2u1	cytochrome P450, family 2, subfamily u, polypeptide 1	0.90009001	0.3301	0.5404
242362	Manea	mannosidase, endo-alpha	0.90009001	0.3411	0.5516
50772	Mapk6	mitogen-activated protein kinase 6	0.90009001	0.3907	0.599
14245	Lpin1	lipin 1	0.90009001	0.4014	0.6091
102058	Exoc8	exocyst complex component 8	0.90009001	0.4514	0.6536
217734	Pomt2	protein-O-mannosyltransferase 2	0.90009001	0.4584	0.6595
239556	Cacna1i	calcium channel, voltage-dependent, alpha 1I subunit	0.90009001	0.4798	0.6759
100102	Pcsk9	proprotein convertase subtilisin/kexin type 9	0.90009001	0.5012	0.6931
52245	Commd2	COMM domain containing 2	0.90009001	0.5097	0.7003
246738	Dnajc28	DnaJ (Hsp40) homolog, subfamily C, member 28	0.90009001	0.5107	0.7013
100038734	Gm10845	predicted gene 10845	0.90009001	0.5125	0.7031
380780	Serpina11	serine (or cysteine) peptidase inhibitor, clade A (alpha-1 antiproteinase, antitrypsin), member 11	0.90009001	0.5405	0.7237
19668	Rbpjl	recombination signal binding protein for immunoglobulin kappa J region-like	0.90009001	0.5515	0.7323
18707	Pik3cd	phosphatidylinositol 3-kinase catalytic delta polypeptide	0.90009001	0.5621	0.74
667452	Dcdc2b	doublecortin domain containing 2b	0.90009001	0.5833	0.7546
93760	Arid1a	AT rich interactive domain 1A (SWI-like)	0.90009001	0.6073	0.7728
13717	Eln	elastin	0.90009001	0.6386	0.7953
14783	Grb10	growth factor receptor bound protein 10	0.90009001	0.6436	0.7984
545055	Cma2	chymase 2, mast cell	0.90009001	0.7654	0.8755
68767	ORF19	open reading frame 19	0.89928058	0.03804	NA
54380	Smarcal1	SWI/SNF related matrix associated, actin dependent regulator of chromatin, subfamily a-like 1	0.89928058	0.04027	NA
17977	Ncoa1	nuclear receptor coactivator 1	0.89928058	0.04373	NA
74570	Zkscan1	zinc finger with KRAB and SCAN domains 1	0.89928058	0.04433	NA
73834	Atp6v1d	ATPase, H+ transporting, lysosomal V1 subunit D	0.89928058	0.06855	NA
213056	Fam126b	family with sequence similarity 126, member B	0.89928058	0.06891	NA
13854	Epn1	epsin 1	0.89928058	0.07381	NA
76252	Atp6v0e2	ATPase, H+ transporting, lysosomal V0 subunit E2	0.89928058	0.08518	NA
19367	Rad9	RAD9 homolog (S. pombe)	0.89928058	0.086	NA
104681	Slc16a6	solute carrier family 16 (monocarboxylic acid transporters), member 6	0.89928058	0.0895	NA
67166	Arl8b	ADP-ribosylation factor-like 8B	0.89928058	0.09432	NA
116848	Baz2a	bromodomain adjacent to zinc finger domain, 2A	0.89928058	0.1027	NA
74267	Iqcf1	IQ motif containing F1	0.89928058	0.1044	NA
56697	Akap10	A kinase (PRKA) anchor protein 10	0.89928058	0.1147	NA

77808	A930015G24Rik	RIKEN cDNA A930015G24 gene	0.89928058	0.1158	NA
433752	AA415398	expressed sequence AA415398	0.89928058	0.1165	NA
327826	Frs2	fibroblast growth factor receptor substrate 2	0.89928058	0.1175	NA
12724	Clcn2	chloride channel 2	0.89928058	0.1317	NA
19088	Prkar2b	protein kinase, cAMP dependent regulatory, type II beta	0.89928058	0.1416	NA
75220	4930535I16Rik	RIKEN cDNA 4930535I16 gene	0.89928058	0.1482	NA
74246	Gale	galactose-4-epimerase, UDP	0.89928058	0.1506	NA
99890	Prmt6	protein arginine N-methyltransferase 6	0.89928058	0.1615	NA
101994	Zfp828	zinc finger protein 828	0.89928058	0.1748	NA
109284	C030046I01Rik	RIKEN cDNA C030046I01 gene	0.89928058	0.1832	NA
71910	Ppapdc1b	phosphatidic acid phosphatase type 2 domain containing 1B	0.89928058	0.1879	0.3761
71175	Nipbl	Nipped-B homolog (Drosophila)	0.89928058	0.259	0.4646
18973	Pole	polymerase (DNA directed), epsilon	0.89928058	0.2632	0.4693
66971	Cdk5rap1	CDK5 regulatory subunit associated protein 1	0.89928058	0.3489	0.5593
73737	1110008P14Rik	RIKEN cDNA 1110008P14 gene	0.89928058	0.3723	0.5811
110542	Amhr2	anti-Mullerian hormone type 2 receptor	0.89928058	0.3811	0.5903
53965	9430099O15Rik	RIKEN cDNA 9430099O15 gene	0.89928058	0.3828	0.5918
18647	Cdk14	cyclin-dependent kinase 14	0.89928058	0.4116	0.6179
14396	Gabra3	gamma-aminobutyric acid (GABA) A receptor, subunit alpha 3	0.89928058	0.4136	0.62
217265	Abca5	ATP-binding cassette, sub-family A (ABC1), member 5	0.89928058	0.4487	0.6508
263406	Plekhg3	pleckstrin homology domain containing, family G (with RhoGef domain) member 3	0.89928058	0.4598	0.6607
547150	6820431F20Rik	cadherin 11 pseudogene	0.89928058	0.4707	0.6697
229279	Hnrnpa3	heterogeneous nuclear ribonucleoprotein A3	0.89928058	0.5305	0.716
330908	Opcml	opioid binding protein/cell adhesion molecule-like	0.89928058	0.698	0.8328
100042786	Gm16381	predicted gene 16381	0.89928058	0.7155	0.8444
494468	Armcx5	armadillo repeat containing, X-linked 5	0.8984726	0.06305	NA
74194	Rnd3	Rho family GTPase 3	0.8984726	0.07433	NA
67382	Brd3	bromodomain containing 3	0.8984726	0.07828	NA
77127	A930001A20Rik	RIKEN cDNA A930001A20 gene	0.8984726	0.0872	NA
101631	Pwwp2b	PWWP domain containing 2B	0.8984726	0.08846	NA
319944	Taf2	TAF2 RNA polymerase II, TATA box binding protein (TBP)-associated factor	0.8984726	0.09526	NA
228545	Vps18	vacuolar protein sorting 18 (yeast)	0.8984726	0.1074	NA
230777	Hcrtr1	hypocretin (orexin) receptor 1	0.8984726	0.1233	NA
319594	Hif1an	hypoxia-inducible factor 1, alpha subunit inhibitor	0.8984726	0.1531	NA
71865	Fbxo30	F-box protein 30	0.8984726	0.1612	NA
22685	Zfp239	zinc finger protein 239	0.8984726	0.1624	NA
12724	Clcn2	chloride channel 2	0.8984726	0.173	NA
69315	1700001L19Rik	RIKEN cDNA 1700001L19 gene	0.8984726	0.1937	0.3837
14226	Fkbp1b	FK506 binding protein 1b	0.8984726	0.1954	0.386
13497	Drp2	dystrophin related protein 2	0.8984726	0.2614	0.4674
100504758	LOC100504758	hypothetical LOC100504758	0.8984726	0.2799	0.4881
242377	Pm20d2	peptidase M20 domain containing 2	0.8984726	0.3038	0.513
232227	Iqsec1	IQ motif and Sec7 domain 1	0.8984726	0.3143	0.5238
22658	Pcgf2	polycomb group ring finger 2	0.8984726	0.3222	0.532
16519	Kcnj3	potassium inwardly-rectifying channel, subfamily J, member 3	0.8984726	0.401	0.6086
241627	Wdr76	WD repeat domain 76	0.8984726	0.4088	0.6153
208076	Pknox2	Pbx/knotted 1 homeobox 2	0.8984726	0.4195	0.6257
70025	Acot7	acyl-CoA thioesterase 7	0.8984726	0.4226	0.6282
26556	Homer1	homer homolog 1 (Drosophila)	0.8984726	0.4301	0.6342
110920	Hspa13	heat shock protein 70 family, member 13	0.8984726	0.4581	0.6593

407788	BC051142	cDNA sequence BC051142	0.8984726	0.4788	0.6754
230257	Rod1	ROD1 regulator of differentiation 1 (S. pombe)	0.8984726	0.5029	0.6946
664608	Rhox4g	reproductive homeobox 4G	0.8984726	0.5138	0.7041
237716	Gpr75	G protein-coupled receptor 75	0.8984726	0.5139	0.7041
66384	Srp19	signal recognition particle 19	0.8984726	0.5307	0.7161
77574	Fam115a	family with sequence similarity 115, member A	0.8984726	0.5312	0.7165
547160	Gm14484	predicted gene 14484	0.8984726	0.5329	0.7178
70310	Plscr3	phospholipid scramblase 3	0.8984726	0.5587	0.7379
106369	Ypel1	yippee-like 1 (Drosophila)	0.8984726	0.5727	0.7473
73699	Ppp2r1b	protein phosphatase 2 (formerly 2A), regulatory subunit A (PR 65), beta isoform	0.8984726	0.6431	0.798
50929	Il22	interleukin 22	0.8984726	0.6632	0.8102
107932	Chd4	chromodomain helicase DNA binding protein 4	0.89766607	0.03642	NA
110789	Gpr98	G protein-coupled receptor 98	0.89766607	0.03987	NA
69886	2610002J23Rik	RIKEN cDNA 2610002J23 gene	0.89766607	0.03992	NA
319740	Zfyve27	zinc finger, FYVE domain containing 27	0.89766607	0.04071	NA
268706	Slc38a9	solute carrier family 38, member 9	0.89766607	0.04836	NA
16210	Impact	imprinted and ancient	0.89766607	0.04985	NA
67618	Aasdhppt	aminoadipate-semialdehyde dehydrogenase-phosphopantetheinyl transferase	0.89766607	0.07028	NA
14204	Il4i1	interleukin 4 induced 1	0.89766607	0.07359	NA
70615	Ankrd24	ankyrin repeat domain 24	0.89766607	0.088	NA
216892	Spns2	spinster homolog 2 (Drosophila)	0.89766607	0.09228	NA
72747	Ttc39c	tetratricopeptide repeat domain 39C	0.89766607	0.09449	NA
271209	Rp1l1	retinitis pigmentosa 1 homolog (human)-like 1	0.89766607	0.09753	NA
21821	Ift88	intraflagellar transport 88 homolog (Chlamydomonas)	0.89766607	0.09995	NA
26419	Mapk8	mitogen-activated protein kinase 8	0.89766607	0.1001	NA
50762	Fbxo6	F-box protein 6	0.89766607	0.1017	NA
66505	Zmynd11	zinc finger, MYND domain containing 11	0.89766607	0.112	NA
71116	Stx18	syntaxin 18	0.89766607	0.1145	NA
233900	Rnf40	ring finger protein 40	0.89766607	0.1255	NA
68273	Pomgnt1	protein O-linked mannose beta1,2-N-acetylglucosaminyltransferase	0.89766607	0.1297	NA
56177	Olfm1	olfactomedin 1	0.89766607	0.1634	NA
381644	Cep135	centrosomal protein 135	0.89766607	0.1646	NA
217733	Tmem63c	transmembrane protein 63c	0.89766607	0.1698	NA
80515	A030009H04Rik	RIKEN cDNA A030009H04 gene	0.89766607	0.1964	0.3873
14299	Ncs1	neuronal calcium sensor 1	0.89766607	0.2085	0.4034
383436	Gm5244	ribosomal protein, large, P1 pseudogene	0.89766607	0.2103	0.4058
213262	Fstl5	follistatin-like 5	0.89766607	0.2208	0.4199
67846	Tmem39a	transmembrane protein 39a	0.89766607	0.2394	0.4419
57330	Gigyf1	GRB10 interacting GYF protein 1	0.89766607	0.2672	0.4742
29867	Cabp1	calcium binding protein 1	0.89766607	0.2992	0.5084
17931	Ppp1r12a	protein phosphatase 1, regulatory (inhibitor) subunit 12A	0.89766607	0.3309	0.5414
66648	5730494M16Rik	RIKEN cDNA 5730494M16 gene	0.89766607	0.3438	0.5547
229542	Gatad2b	GATA zinc finger domain containing 2B	0.89766607	0.3507	0.5609
54161	Copg	coatomer protein complex, subunit gamma	0.89766607	0.3584	0.5683
229722	5330417C22Rik	RIKEN cDNA 5330417C22 gene	0.89766607	0.3622	0.5716
53886	Cdkl2	cyclin-dependent kinase-like 2 (CDC2-related kinase)	0.89766607	0.3683	0.5773
216344	Rab21	RAB21, member RAS oncogene family	0.89766607	0.3986	0.6064
18508	Pax6	paired box gene 6	0.89766607	0.4027	0.6102
211652	Wwc1	WW, C2 and coiled-coil domain containing 1	0.89766607	0.421	0.6271
269855	A430110N23Rik	RIKEN cDNA A430110N23 gene	0.89766607	0.4458	0.6485

20823	Ssb	Sjogren syndrome antigen B	0.89766607	0.4523	0.6546
235542	Ppp2r3a	protein phosphatase 2, regulatory subunit B'', alpha	0.89766607	0.4631	0.6635
26423	Nr5a1	nuclear receptor subfamily 5, group A, member 1	0.89766607	0.4963	0.6895
13549	Dyrk1b	dual-specificity tyrosine-(Y)-phosphorylation regulated kinase 1b	0.89766607	0.5	0.6922
22067	Trpc5	transient receptor potential cation channel, subfamily C, member 5	0.89766607	0.5145	0.7045
12398	Cbfa2t3	core-binding factor, runt domain, alpha subunit 2, translocated to, 3 (human)	0.89766607	0.5264	0.7129
381650	Thap6	THAP domain containing 6	0.89766607	0.5265	0.713
29820	Tnfrsf19	tumor necrosis factor receptor superfamily, member 19	0.89766607	0.5674	0.7433
68232	1700120K04Rik	RIKEN cDNA 1700120K04 gene	0.89766607	0.5858	0.7564
14534	Kat2a	K(lysine) acetyltransferase 2A	0.89766607	0.6068	0.7727
100041352	Gm9880	predicted gene 9880	0.89766607	0.7676	0.8767
628100	Fbxo39	F-box protein 39	0.89766607	0.8452	0.92
74120	Zfp263	zinc finger protein 263	0.89686099	0.03625	NA
109151	Chd9	chromodomain helicase DNA binding protein 9	0.89686099	0.05799	NA
211986	Tmem18	transmembrane protein 18	0.89686099	0.06159	NA
12632	Cfl2	cofilin 2, muscle	0.89686099	0.06216	NA
214133	Tet2	tet oncogene family member 2	0.89686099	0.06315	NA
78267	Klhdc8b	kelch domain containing 8B	0.89686099	0.07427	NA
67224	Med29	mediator complex subunit 29	0.89686099	0.1001	NA
212569	Zfp273	zinc finger protein 273	0.89686099	0.1023	NA
16373	Irx3	Iroquois related homeobox 3 (Drosophila)	0.89686099	0.1069	NA
100042970	Kifc1	kinesin family member C1	0.89686099	0.1163	NA
102954	Nudt10	nudix (nucleoside diphosphate linked moiety X)-type motif 10	0.89686099	0.1228	NA
218441	Zfyve16	zinc finger, FYVE domain containing 16	0.89686099	0.1248	NA
240832	Tor1aip2	torsin A interacting protein 2	0.89686099	0.133	NA
11834	Aqr	aquarius	0.89686099	0.1366	NA
238505	Mtr	5-methyltetrahydrofolate-homocysteine methyltransferase	0.89686099	0.1417	NA
269180	Inpp4a	inositol polyphosphate-4-phosphatase, type I	0.89686099	0.1545	NA
319653	Slc25a40	solute carrier family 25, member 40	0.89686099	0.1733	NA
76626	Msi2	Musashi homolog 2 (Drosophila)	0.89686099	0.1839	0.3709
66361	Zfand1	zinc finger, AN1-type domain 1	0.89686099	0.184	0.3709
667214	9930111J21Rik1	RIKEN cDNA 9930111J21 gene 1	0.89686099	0.2054	0.3992
67095	Trak1	trafficking protein, kinesin binding 1	0.89686099	0.2393	0.4419
73681	Trmt11	tRNA methyltransferase 11 homolog (S. cerevisiae)	0.89686099	0.2398	0.4422
170791	Rbm39	RNA binding motif protein 39	0.89686099	0.2506	0.4555
227634	Camsap1	calmodulin regulated spectrin-associated protein 1	0.89686099	0.2528	0.4578
22201	Uba1	ubiquitin-like modifier activating enzyme 1	0.89686099	0.2539	0.459
246103	Atxn7	ataxin 7	0.89686099	0.2982	0.5076
20358	Sema6a	sema domain, transmembrane domain (TM), and cytoplasmic domain, (semaphorin) 6A	0.89686099	0.3349	0.5455
233752	Insc	inscuteable homolog (Drosophila)	0.89686099	0.3395	0.5498
67439	Xab2	XPA binding protein 2	0.89686099	0.3487	0.5591
72554	Utp14a	UTP14, U3 small nucleolar ribonucleoprotein, homolog A (yeast)	0.89686099	0.351	0.5611
214812	Zfp609	zinc finger protein 609	0.89686099	0.3583	0.5683
320333	D830030K20Rik	RIKEN cDNA D830030K20 gene	0.89686099	0.3637	0.5727
22750	Zfp9	zinc finger protein 9	0.89686099	0.385	0.5941
212114	Nhlrc3	NHL repeat containing 3	0.89686099	0.3939	0.602
230657	Tmem69	transmembrane protein 69	0.89686099	0.4928	0.6868
100503168	LOC100503168	hypothetical LOC100503168	0.89686099	0.6106	0.7751
13041	Ctsw	cathepsin W	0.89686099	0.6267	0.787
20740	Spna2	spectrin alpha 2	0.89686099	0.6487	0.802

433003	Gm5481	predicted gene 5481	0.89686099	0.7179	0.8459
171166	Mcoln3	mucolipin 3	0.89686099	0.7433	0.8613
69389	1700014N06Rik	RIKEN cDNA 1700014N06 gene	0.89686099	0.7451	0.8626
15502	Dnaja1	DnaJ (Hsp40) homolog, subfamily A, member 1	0.89605735	0.039	NA
69155	1810030O07Rik	RIKEN cDNA 1810030O07 gene	0.89605735	0.05384	NA
56205	Ensa	endosulfine alpha	0.89605735	0.05495	NA
75746	Morc4	microrchidia 4	0.89605735	0.06337	NA
26987	Eif4e2	eukaryotic translation initiation factor 4E member 2	0.89605735	0.06671	NA
75533	Nme5	non-metastatic cells 5, protein expressed in (nucleoside-diphosphate kinase)	0.89605735	0.08156	NA
320799	Zhx3	zinc fingers and homeoboxes 3	0.89605735	0.08292	NA
16494	Kcna6	potassium voltage-gated channel, shaker-related, subfamily, member 6	0.89605735	0.08469	NA
12151	Bmi1	Bmi1 polycomb ring finger oncogene	0.89605735	0.09934	NA
66826	Taz	tafazzin	0.89605735	0.1054	NA
29867	Cabp1	calcium binding protein 1	0.89605735	0.1069	NA
75420	Secisbp2	SECIS binding protein 2	0.89605735	0.1283	NA
57261	Brd4	bromodomain containing 4	0.89605735	0.1448	NA
228983	Osbpl2	oxysterol binding protein-like 2	0.89605735	0.15	NA
20399	Sh2b1	SH2B adaptor protein 1	0.89605735	0.1771	0.3621
14732	Gpam	glycerol-3-phosphate acyltransferase, mitochondrial	0.89605735	0.1779	0.363
272031	E130309F12Rik	RIKEN cDNA E130309F12 gene	0.89605735	0.187	0.3751
51810	Hnrnpu	heterogeneous nuclear ribonucleoprotein U	0.89605735	0.1881	0.3763
12868	Cox8a	cytochrome c oxidase, subunit VIIIa	0.89605735	0.1981	0.3895
67824	Nmral1	NmrA-like family domain containing 1	0.89605735	0.2002	0.3923
22025	Nr2c1	nuclear receptor subfamily 2, group C, member 1	0.89605735	0.2076	0.4024
22592	Ercc5	excision repair cross-complementing rodent repair deficiency, complementation group 5	0.89605735	0.2086	0.4034
234686	Fhod1	formin homology 2 domain containing 1	0.89605735	0.2142	0.4107
170788	Crb1	crumbs homolog 1 (Drosophila)	0.89605735	0.2248	0.4245
74405	Efhc2	EF-hand domain (C-terminal) containing 2	0.89605735	0.2704	0.4779
277939	C2cd3	C2 calcium-dependent domain containing 3	0.89605735	0.2874	0.4958
381352	Mamdc4	MAM domain containing 4	0.89605735	0.2896	0.498
211484	Tsga10	testis specific 10	0.89605735	0.299	0.5084
244631	Pskh1	protein serine kinase H1	0.89605735	0.3101	0.52
16798	Lats1	large tumor suppressor	0.89605735	0.3197	0.5297
69528	1700030J22Rik	RIKEN cDNA 1700030J22 gene	0.89605735	0.3357	0.546
72519	Tmem55a	transmembrane protein 55A	0.89605735	0.3369	0.5472
12296	Cacnb2	calcium channel, voltage-dependent, beta 2 subunit	0.89605735	0.3643	0.5733
320943	B230213L16Rik	RIKEN cDNA B230213L16 gene	0.89605735	0.3901	0.5984
327655	Ppip5k1	diphosphoinositol pentakisphosphate kinase 1	0.89605735	0.3948	0.6029
69605	Lnp	limb and neural patterns	0.89605735	0.3983	0.6064
100041694	Gm10451	predicted gene 10451	0.89605735	0.4306	0.6345
110834	Chrna3	cholinergic receptor, nicotinic, alpha polypeptide 3	0.89605735	0.4707	0.6697
240892	Dusp27	dual specificity phosphatase 27 (putative)	0.89605735	0.4709	0.6698
13822	Epb4.1l2	erythrocyte protein band 4.1-like 2	0.89605735	0.478	0.6749
104082	Wdr7	WD repeat domain 7	0.89605735	0.5035	0.695
71751	Map3k13	mitogen-activated protein kinase kinase kinase 13	0.89605735	0.522	0.7099
269587	Epb4.1	erythrocyte protein band 4.1	0.89605735	0.5305	0.716
277939	C2cd3	C2 calcium-dependent domain containing 3	0.89605735	0.5432	0.7259
14531	Gcm1	glial cells missing homolog 1 (Drosophila)	0.89605735	0.6068	0.7727
26448	Stk30	serine/threonine kinase 30	0.89525515	0.01928	NA
72162	Dhx36	DEAH (Asp-Glu-Ala-His) box polypeptide 36	0.89525515	0.03448	NA

72972	Gcap14	granule cell antiserum positive 14	0.89525515	0.04756	NA
269437	Plch1	phospholipase C, eta 1	0.89525515	0.05409	NA
226016	Fam108b	family with sequence similarity 108, member B	0.89525515	0.06091	NA
51796	Srrm1	serine/arginine repetitive matrix 1	0.89525515	0.06253	NA
59014	Rrs1	RRS1 ribosome biogenesis regulator homolog (S. cerevisiae)	0.89525515	0.06438	NA
72515	Wdr43	WD repeat domain 43	0.89525515	0.07348	NA
26951	Zw10	ZW10 homolog (Drosophila), centromere/kinetochore protein	0.89525515	0.07992	NA
56878	Rbms1	RNA binding motif, single stranded interacting protein 1	0.89525515	0.08229	NA
22594	Xrcc1	X-ray repair complementing defective repair in Chinese hamster cells 1	0.89525515	0.09163	NA
14612	Gja4	gap junction protein, alpha 4	0.89525515	0.0969	NA
235439	Herc1	hect (homologous to the E6-AP (UBE3A) carboxyl terminus) domain and RCC1 (CHC1)-like domain (RLD) 1	0.89525515	0.1048	NA
13424	Dync1h1	dynein cytoplasmic 1 heavy chain 1	0.89525515	0.1115	NA
13998	Fgd6	FYVE, RhoGEF and PH domain containing 6	0.89525515	0.1152	NA
73991	Atl1	atlastin GTPase 1	0.89525515	0.1244	NA
15931	Ids	iduronate 2-sulfatase	0.89525515	0.1322	NA
279653	Pcdh19	protocadherin 19	0.89525515	0.143	NA
11977	Atp7a	ATPase, Cu++ transporting, alpha polypeptide	0.89525515	0.152	NA
223669	Zfp7	zinc finger protein 7	0.89525515	0.1545	NA
63830	Kcnq1ot1	KCNQ1 overlapping transcript 1	0.89525515	0.1629	NA
20437	Siah1a	seven in absentia 1A	0.89525515	0.1659	0.3464
52202	Rbm34	RNA binding motif protein 34	0.89525515	0.1674	NA
12151	Bmi1	Bmi1 polycomb ring finger oncogene	0.89525515	0.1729	0.3564
21366	Slc6a6	solute carrier family 6 (neurotransmitter transporter, taurine), member 6	0.89525515	0.1938	0.3838
232878	Zscan22	zinc finger and SCAN domain containing 22	0.89525515	0.2093	0.4046
67333	Stk35	serine/threonine kinase 35	0.89525515	0.2208	0.4199
214742	Rcor3	REST corepressor 3	0.89525515	0.225	0.4246
22122	Tsta3	tissue specific transplantation antigen P35B	0.89525515	0.2411	0.4437
74464	Zswim5	zinc finger, SWIM domain containing 5	0.89525515	0.2748	0.4827
227622	BC029214	cDNA sequence BC029214	0.89525515	0.2756	0.4835
68146	Arl13b	ADP-ribosylation factor-like 13B	0.89525515	0.3038	0.5129
54648	Ccdc120	coiled-coil domain containing 120	0.89525515	0.3251	0.5349
239618	Pdzrn4	PDZ domain containing RING finger 4	0.89525515	0.3391	0.5494
100503311	1700027A23Rik	RIKEN cDNA 1700027A23 gene	0.89525515	0.3511	0.5612
14000	Drosha	drosha, ribonuclease type III	0.89525515	0.3513	0.5612
329506	Ctdspl2	CTD (carboxy-terminal domain, RNA polymerase II, polypeptide A) small phosphatase like 2	0.89525515	0.3702	0.5792
232975	Atp1a3	ATPase, Na+/K+ transporting, alpha 3 polypeptide	0.89525515	0.3708	0.5795
71096	Sntg1	syntrophin, gamma 1	0.89525515	0.3721	0.5809
353170	Txlng	taxilin gamma	0.89525515	0.3884	0.5969
545611	Gm13298	predicted gene 13298	0.89525515	0.3977	0.6057
211383	Fam123c	family with sequence similarity 123, member C	0.89525515	0.4074	0.6142
234595	Slc38a7	solute carrier family 38, member 7	0.89525515	0.4291	0.6336
212163	8030462N17Rik	RIKEN cDNA 8030462N17 gene	0.89525515	0.4355	0.6392
246103	Atxn7	ataxin 7	0.89525515	0.4902	0.6845
100041481	Gm3364	predicted gene 3364	0.89525515	0.55	0.7313
667370	I830012O16Rik	RIKEN cDNA I830012O16 gene	0.89525515	0.642	0.7976
71834	Zbtb43	zinc finger and BTB domain containing 43	0.89445438	0.01622	NA
75894	Adal	adenosine deaminase-like	0.89445438	0.03537	NA
19133	Prph2	peripherin 2	0.89445438	0.0426	NA
666185	Gm7969	predicted gene 7969	0.89445438	0.05812	NA
13557	E2f3	E2F transcription factor 3	0.89445438	0.0615	NA

20965	Syn2	synapsin II	0.89445438	0.09541	NA
332359	Tigd3	tigger transposable element derived 3	0.89445438	0.098	NA
209683	Ttc28	tetratricopeptide repeat domain 28	0.89445438	0.1109	NA
104001	Rtn1	reticulon 1	0.89445438	0.1141	NA
56150	Mad2l1	MAD2 mitotic arrest deficient-like 1 (yeast)	0.89445438	0.1171	NA
227399	Ppip5k2	diphosphoinositol pentakisphosphate kinase 2	0.89445438	0.1293	NA
67292	Pigc	phosphatidylinositol glycan anchor biosynthesis, class C	0.89445438	0.132	NA
66226	Trappc2	trafficking protein particle complex 2	0.89445438	0.1419	NA
71955	2400003C14Rik	RIKEN cDNA 2400003C14 gene	0.89445438	0.1834	0.3706
170731	Mfn2	mitofusin 2	0.89445438	0.2076	0.4024
70802	Pwwp2a	PWWP domain containing 2A	0.89445438	0.2305	0.4314
14699	Gngt1	guanine nucleotide binding protein (G protein), gamma transducing activity polypeptide 1	0.89445438	0.2513	0.4561
108154	Adamts6	a disintegrin-like and metallopeptidase (reprolysin type) with thrombospondin type 1 motif, 6	0.89445438	0.2673	0.4743
270190	Ephb1	Eph receptor B1	0.89445438	0.2686	0.4757
65111	Dap3	death associated protein 3	0.89445438	0.2728	0.4803
105844	Card10	caspase recruitment domain family, member 10	0.89445438	0.2808	0.489
14312	Brd2	bromodomain containing 2	0.89445438	0.3018	0.511
98741	Kcnb2	potassium voltage gated channel, Shab-related subfamily, member 2	0.89445438	0.3332	0.5436
55985	Cxcl13	chemokine (C-X-C motif) ligand 13	0.89445438	0.401	0.6086
11752	Anxa8	annexin A8	0.89445438	0.4365	0.6401
320633	Zbtb26	zinc finger and BTB domain containing 26	0.89445438	0.4467	0.6491
435791	Gm13271	predicted gene 13271	0.89445438	0.5193	0.7085
229776	Cdc14a	CDC14 cell division cycle 14 homolog A (S. cerevisiae)	0.89445438	0.5597	0.7385
113849	Vmn1r52	vomeronasal 1 receptor 52	0.89445438	0.58	0.7523
66966	Trit1	tRNA isopentenyltransferase 1	0.89365505	0.02308	NA
55984	Camkk1	calcium/calmodulin-dependent protein kinase kinase 1, alpha	0.89365505	0.03166	NA
105827	Amigo2	adhesion molecule with Ig like domain 2	0.89365505	0.04432	NA
67014	Mina	myc induced nuclear antigen	0.89365505	0.05189	NA
67300	Cltc	clathrin, heavy polypeptide (Hc)	0.89365505	0.05497	NA
239037	Lrit1	leucine-rich repeat, immunoglobulin-like and transmembrane domains 1	0.89365505	0.0618	NA
103573	Xpo1	exportin 1, CRM1 homolog (yeast)	0.89365505	0.07362	NA
18537	Pcmt1	protein-L-isoaspartate (D-aspartate) O-methyltransferase 1	0.89365505	0.07367	NA
67952	Tomm20	translocase of outer mitochondrial membrane 20 homolog (yeast)	0.89365505	0.07828	NA
30877	Gnl3	guanine nucleotide binding protein-like 3 (nucleolar)	0.89365505	0.08084	NA
381903	Alg8	asparagine-linked glycosylation 8 homolog (yeast, alpha-1,3-glucosyltransferase)	0.89365505	0.08669	NA
56533	Rgs17	regulator of G-protein signaling 17	0.89365505	0.0939	NA
72469	Plcd3	phospholipase C, delta 3	0.89365505	0.1036	NA
380718	Mks1	Meckel syndrome, type 1	0.89365505	0.1038	NA
14154	Fem1a	feminization 1 homolog a (C. elegans)	0.89365505	0.1101	NA
140723	Cacng5	calcium channel, voltage-dependent, gamma subunit 5	0.89365505	0.1152	NA
13018	Ctcf	CCCTC-binding factor	0.89365505	0.1198	NA
320129	Adrbk2	adrenergic receptor kinase, beta 2	0.89365505	0.1257	NA
76781	Mettl4	methyltransferase like 4	0.89365505	0.1376	NA
107368	Pdzd8	PDZ domain containing 8	0.89365505	0.1581	NA
320827	C530008M17Rik	RIKEN cDNA C530008M17 gene	0.89365505	0.1602	0.3383
73683	Atg16l2	autophagy related 16 like 2 (S. cerevisiae)	0.89365505	0.1785	0.3636
78899	4932443L11Rik	RIKEN cDNA 4932443L11 gene	0.89365505	0.1806	0.3666
73288	Ccdc132	coiled-coil domain containing 132	0.89365505	0.1832	0.3704
629378	Dact3	dapper homolog 3, antagonist of beta-catenin (xenopus)	0.89365505	0.185	0.3721
29869	Ulk2	Unc-51 like kinase 2 (C. elegans)	0.89365505	0.1861	0.3736

22368	Trpv2	transient receptor potential cation channel, subfamily V, member 2	0.89365505	0.191	0.3803
79456	Recql4	RecQ protein-like 4	0.89365505	0.2242	0.424
219149	Xkr6	X Kell blood group precursor related family member 6 homolog	0.89365505	0.2311	0.432
22718	Zfp60	zinc finger protein 60	0.89365505	0.2382	0.4406
228998	Arfgap1	ADP-ribosylation factor GTPase activating protein 1	0.89365505	0.2602	0.4659
230235	6430704M03Rik	RIKEN cDNA 6430704M03 gene	0.89365505	0.272	0.4794
213773	Tbl3	transducin (beta)-like 3	0.89365505	0.2724	0.4798
17035	Lxn	latexin	0.89365505	0.2908	0.4993
69046	Isca1	iron-sulfur cluster assembly 1 homolog (S. cerevisiae)	0.89365505	0.298	0.5074
56542	Ick	intestinal cell kinase	0.89365505	0.318	0.5276
16520	Kcnj4	potassium inwardly-rectifying channel, subfamily J, member 4	0.89365505	0.3536	0.5634
13800	Enah	enabled homolog (Drosophila)	0.89365505	0.3678	0.5768
69207	Srsf11	serine/arginine-rich splicing factor 11	0.89365505	0.4247	0.6301
74330	Dnajc14	DnaJ (Hsp40) homolog, subfamily C, member 14	0.89365505	0.4506	0.6528
381065	Gm5145	small nuclear ribonucleoprotein polypeptide A pseudogene	0.89365505	0.4634	0.6637
66817	Tmem170	transmembrane protein 170	0.89365505	0.4859	0.6811
22064	Trpc2	transient receptor potential cation channel, subfamily C, member 2	0.89365505	0.6912	0.8287
12992	Csn1s2b	casein alpha s2-like B	0.89365505	0.7638	0.8748
544881	BB287469	expressed sequence BB287469	0.89365505	0.7955	0.8927
19647	Rbbp6	retinoblastoma binding protein 6	0.89285714	0.0294	NA
18010	Neu1	neuraminidase 1	0.89285714	0.03225	NA
12858	Cox5a	cytochrome c oxidase, subunit Va	0.89285714	0.0383	NA
239038	Lrit2	leucine-rich repeat, immunoglobulin-like and transmembrane domains 2	0.89285714	0.04155	NA
211488	Ado	2-aminoethanethiol (cysteamine) dioxygenase	0.89285714	0.06383	NA
66310	Dpy30	dpy-30 homolog (C. elegans)	0.89285714	0.06488	NA
20615	Snapin	SNAP-associated protein	0.89285714	0.06894	NA
217364	Engase	endo-beta-N-acetylglucosaminidase	0.89285714	0.07167	NA
14680	Gnal	guanine nucleotide binding protein, alpha stimulating, olfactory type	0.89285714	0.07738	NA
11790	Speg	SPEG complex locus	0.89285714	0.09426	NA
64176	Sv2b	synaptic vesicle glycoprotein 2 b	0.89285714	0.1023	NA
94280	Sfxn3	sideroflexin 3	0.89285714	0.1053	NA
93759	Sirt1	sirtuin 1 (silent mating type information regulation 2, homolog) 1 (S. cerevisiae)	0.89285714	0.1094	NA
114128	Laptm4b	lysosomal-associated protein transmembrane 4B	0.89285714	0.1244	NA
319478	Cxxc4	CXXC finger 4	0.89285714	0.1275	NA
675851	LOC675851	NADH dehydrogenase [ubiquinone] 1 subunit C2-like	0.89285714	0.1415	NA
78787	Usp54	ubiquitin specific peptidase 54	0.89285714	0.1426	NA
19691	Recql	RecQ protein-like	0.89285714	0.1579	0.3352
77976	Nuak1	NUAK family, SNF1-like kinase, 1	0.89285714	0.1651	0.3451
69151	Lzic	leucine zipper and CTNNBIP1 domain containing	0.89285714	0.1672	0.3481
18749	Prkacb	protein kinase, cAMP dependent, catalytic, beta	0.89285714	0.1731	0.3567
229672	Bcl2l15	BCLl2-like 15	0.89285714	0.1886	0.3768
75735	Pank1	pantothenate kinase 1	0.89285714	0.1957	0.3864
217379	Ubxn2a	UBX domain protein 2A	0.89285714	0.214	0.4104
140580	Elmo1	engulfment and cell motility 1, ced-12 homolog (C. elegans)	0.89285714	0.2379	0.4402
68732	Lrrc16a	leucine rich repeat containing 16A	0.89285714	0.2448	0.4484
232023	Vopp1	vesicular, overexpressed in cancer, prosurvival protein 1	0.89285714	0.2706	0.4782
235132	Zbtb44	zinc finger and BTB domain containing 44	0.89285714	0.2788	0.4868
320951	Pisd	phosphatidylserine decarboxylase	0.89285714	0.284	0.4926
57912	Cdc42se1	CDC42 small effector 1	0.89285714	0.2874	0.4958
23928	Lamc3	laminin gamma 3	0.89285714	0.3022	0.5113

68566	Caly	calcyon neuron-specific vesicular protein	0.89285714	0.3594	0.5692
72098	Tmem68	transmembrane protein 68	0.89285714	0.3622	0.5716
19256	Ptpn20	protein tyrosine phosphatase, non-receptor type 20	0.89285714	0.389	0.5973
18125	Nos1	nitric oxide synthase 1, neuronal	0.89285714	0.4144	0.6207
231724	Rad9b	RAD9 homolog B (S. cerevisiae)	0.89285714	0.43	0.6342
236915	Arhgef9	CDC42 guanine nucleotide exchange factor (GEF) 9	0.89285714	0.5517	0.7325
258955	Olfr531	olfactory receptor 531	0.89285714	0.7004	0.8339
27274	Zfp354b	zinc finger protein 354B	0.89206066	0.03991	NA
68332	Sdhaf1	succinate dehydrogenase complex assembly factor 1	0.89206066	0.04114	NA
75951	4930578M01Rik	RIKEN cDNA 4930578M01 gene	0.89206066	0.04461	NA
67238	2810453I06Rik	RIKEN cDNA 2810453I06 gene	0.89206066	0.04599	NA
70791	Hars2	histidyl-tRNA synthetase 2, mitochondrial (putative)	0.89206066	0.0531	NA
66061	Tctex1d2	Tctex1 domain containing 2	0.89206066	0.05924	NA
110809	Srsf1	serine/arginine-rich splicing factor 1	0.89206066	0.06552	NA
229584	Pogz	pogo transposable element with ZNF domain	0.89206066	0.07741	NA
22065	Trpc3	transient receptor potential cation channel, subfamily C, member 3	0.89206066	0.0835	NA
77971	A930037O16Rik	RIKEN cDNA A930037O16 gene	0.89206066	0.08684	NA
16568	Kif3a	kinesin family member 3A	0.89206066	0.1159	NA
226144	Erlin1	ER lipid raft associated 1	0.89206066	0.1178	NA
105663	Thtpa	thiamine triphosphatase	0.89206066	0.1263	NA
224694	Zfp81	zinc finger protein 81	0.89206066	0.1284	NA
67845	Rnf115	ring finger protein 115	0.89206066	0.1327	NA
17876	Myef2	myelin basic protein expression factor 2, repressor	0.89206066	0.1429	NA
98710	Rabif	RAB interacting factor	0.89206066	0.146	NA
108100	Baiap2	brain-specific angiogenesis inhibitor 1-associated protein 2	0.89206066	0.1769	0.3618
320495	Ipcef1	interaction protein for cytohesin exchange factors 1	0.89206066	0.1771	0.3621
228140	Tnks1bp1	tankyrase 1 binding protein 1	0.89206066	0.1939	0.3839
67972	Atp2b1	ATPase, Ca++ transporting, plasma membrane 1	0.89206066	0.2086	0.4034
72899	Macrod2	MACRO domain containing 2	0.89206066	0.2206	0.4196
14419	Gal	galanin	0.89206066	0.2214	0.4204
170823	Glmn	glomulin, FKBP associated protein	0.89206066	0.2341	0.4356
71592	Pogk	pogo transposable element with KRAB domain	0.89206066	0.2395	0.4419
14313	Fst	follistatin	0.89206066	0.2396	0.4421
81497	Btnl5	butyrophilin-like 5	0.89206066	0.253	0.458
11938	Atp2a2	ATPase, Ca++ transporting, cardiac muscle, slow twitch 2	0.89206066	0.258	0.4633
140481	Man2a2	mannosidase 2, alpha 2	0.89206066	0.3045	0.5136
242669	Adc	arginine decarboxylase	0.89206066	0.3319	0.5423
13996	Etohd2	ethanol decreased 2	0.89206066	0.3392	0.5494
329831	Fam166b	family with sequence similarity 166, member B	0.89206066	0.3511	0.5611
75677	Cldn22	claudin 22	0.89206066	0.3835	0.5926
54369	Nme6	non-metastatic cells 6, protein expressed in (nucleoside-diphosphate kinase)	0.89206066	0.386	0.5947
100043726	Gm4610	predicted gene 4610	0.89206066	0.3985	0.6064
276920	Ccdc42	coiled-coil domain containing 42	0.89206066	0.3992	0.6069
109181	Trip11	thyroid hormone receptor interactor 11	0.89206066	0.4101	0.6165
80838	Hist1h1a	histone cluster 1, H1a	0.89206066	0.4208	0.6271
56711	Plag1	pleiomorphic adenoma gene 1	0.89206066	0.4595	0.6604
67434	Ankrd33b	ankyrin repeat domain 33B	0.89206066	0.5106	0.7012
328019	4933400C05Rik	RIKEN cDNA 4933400C05 gene	0.89206066	0.5236	0.7106
22288	Utrn	utrophin	0.89206066	0.5316	0.7168
13117	Cyp4a10	cytochrome P450, family 4, subfamily a, polypeptide 10	0.89206066	0.5805	0.7526

234663	Dync1li2	dynein, cytoplasmic 1 light intermediate chain 2	0.8912656	0.02019	NA
212127	2810046L04Rik	RIKEN cDNA 2810046L04 gene	0.8912656	0.02822	NA
170930	Sumo2	SMT3 suppressor of mif two 3 homolog 2 (yeast)	0.8912656	0.03915	NA
269870	Zfp446	zinc finger protein 446	0.8912656	0.04221	NA
235606	Apeh	acylpeptide hydrolase	0.8912656	0.05432	NA
69612	2310037I24Rik	RIKEN cDNA 2310037I24 gene	0.8912656	0.05674	NA
72480	Tspyl4	TSPY-like 4	0.8912656	0.08144	NA
227333	Dgkd	diacylglycerol kinase, delta	0.8912656	0.08239	NA
68099	Fam92a	family with sequence similarity 92, member A	0.8912656	0.08948	NA
215008	Vezt	vezatin, adherens junctions transmembrane protein	0.8912656	0.09086	NA
11800	Api5	apoptosis inhibitor 5	0.8912656	0.1047	NA
217410	Trib2	tribbles homolog 2 (Drosophila)	0.8912656	0.1112	NA
56075	Pdss1	prenyl (solanesyl) diphosphate synthase, subunit 1	0.8912656	0.1137	NA
107513	Ssr1	signal sequence receptor, alpha	0.8912656	0.1196	NA
59058	Bhlhe22	basic helix-loop-helix family, member e22	0.8912656	0.1202	NA
108943	Rg9mtd2	RNA (guanine-9-) methyltransferase domain containing 2	0.8912656	0.1298	NA
329731	Fam19a3	family with sequence similarity 19, member A3	0.8912656	0.1375	NA
29819	Stau2	staufen (RNA binding protein) homolog 2 (Drosophila)	0.8912656	0.1468	NA
19395	Rasgrp2	RAS, guanyl releasing protein 2	0.8912656	0.1599	0.338
69893	2010305A19Rik	RIKEN cDNA 2010305A19 gene	0.8912656	0.1639	0.3436
70985	4931406E20Rik	RIKEN cDNA 4931406E20 gene	0.8912656	0.1814	0.3677
219072	Haus4	HAUS augmin-like complex, subunit 4	0.8912656	0.1828	0.3699
66049	Rogdi	rogdi homolog (Drosophila)	0.8912656	0.2139	0.4104
66236	1500011B03Rik	RIKEN cDNA 1500011B03 gene	0.8912656	0.249	0.4531
66282	1810029B16Rik	RIKEN cDNA 1810029B16 gene	0.8912656	0.2549	0.4599
71063	Zfp597	zinc finger protein 597	0.8912656	0.2664	0.4732
20420	Shd	src homology 2 domain-containing transforming protein D	0.8912656	0.268	0.4749
237782	Smcr8	Smith-Magenis syndrome chromosome region, candidate 8 homolog (human)	0.8912656	0.281	0.4892
269378	Ahcy	S-adenosylhomocysteine hydrolase	0.8912656	0.2832	0.4917
73174	Tbkbp1	TBK1 binding protein 1	0.8912656	0.3066	0.5159
67549	Gpr89	G protein-coupled receptor 89	0.8912656	0.3169	0.5264
26556	Homer1	homer homolog 1 (Drosophila)	0.8912656	0.3351	0.5456
229011	Samd10	sterile alpha motif domain containing 10	0.8912656	0.3557	0.5655
665596	Hist1h2bq	histone cluster 1, H2bq	0.8912656	0.3777	0.5867
239102	Zfhx2	zinc finger homeobox 2	0.8912656	0.4741	0.6718
320949	D830039M14Rik	RIKEN cDNA D830039M14 gene	0.8912656	0.49	0.6843
223989	4921513D23Rik	RIKEN cDNA 4921513D23 gene	0.8912656	0.5247	0.7114
210982	BC032203	cDNA sequence BC032203	0.8912656	0.5364	0.721
19192	Psme3	proteaseome (prosome, macropain) 28 subunit, 3	0.8912656	0.5776	0.7504
71240	Osbpl7	oxysterol binding protein-like 7	0.89047195	0.02309	NA
108958	Fam73b	family with sequence similarity 73, member B	0.89047195	0.02658	NA
328110	Prpf39	PRP39 pre-mRNA processing factor 39 homolog (yeast)	0.89047195	0.02731	NA
109181	Trip11	thyroid hormone receptor interactor 11	0.89047195	0.03572	NA
30843	Fbxl12	F-box and leucine-rich repeat protein 12	0.89047195	0.04012	NA
236915	Arhgef9	CDC42 guanine nucleotide exchange factor (GEF) 9	0.89047195	0.04055	NA
19300	Abcd4	ATP-binding cassette, sub-family D (ALD), member 4	0.89047195	0.06172	NA
27425	Atp5l	ATP synthase, H+ transporting, mitochondrial F0 complex, subunit g	0.89047195	0.07358	NA
66790	Grtp1	GH regulated TBC protein 1	0.89047195	0.07547	NA
19341	Rab4a	RAB4A, member RAS oncogene family	0.89047195	0.08043	NA
235956	Zfp825	zinc finger protein 825	0.89047195	0.08355	NA

14256	Flt3l	FMS-like tyrosine kinase 3 ligand	0.89047195	0.08567	NA
69635	Dapk1	death associated protein kinase 1	0.89047195	0.08593	NA
66147	Necap2	NECAP endocytosis associated 2	0.89047195	0.09812	NA
15444	Hpca	hippocalcin	0.89047195	0.09837	NA
224938	Pja2	praja 2, RING-H2 motif containing	0.89047195	0.107	NA
70047	Trnt1	tRNA nucleotidyl transferase, CCA-adding, 1	0.89047195	0.1161	NA
22775	Zik1	zinc finger protein interacting with K protein 1	0.89047195	0.1174	NA
100039133	Gm2058	predicted gene 2058	0.89047195	0.1218	NA
108946	Zzz3	zinc finger, ZZ domain containing 3	0.89047195	0.1267	NA
207920	Esrp1	epithelial splicing regulatory protein 1	0.89047195	0.1674	0.3483
208111	C330019L16Rik	RIKEN cDNA C330019L16 gene	0.89047195	0.1743	0.3581
18514	Pbx1	pre B-cell leukemia transcription factor 1	0.89047195	0.1808	0.3669
66403	Asf1a	ASF1 anti-silencing function 1 homolog A (S. cerevisiae)	0.89047195	0.1846	0.3716
233056	Zfp790	zinc finger protein 790	0.89047195	0.1848	0.372
74998	Rab11fip2	RAB11 family interacting protein 2 (class I)	0.89047195	0.1858	0.3732
233168	AI987944	expressed sequence AI987944	0.89047195	0.1935	0.3835
107515	Lgr4	leucine-rich repeat-containing G protein-coupled receptor 4	0.89047195	0.1997	0.3915
214137	Arhgap29	Rho GTPase activating protein 29	0.89047195	0.2094	0.4047
16987	Lss	lanosterol synthase	0.89047195	0.2227	0.4221
78894	Aacs	acetoacetyl-CoA synthetase	0.89047195	0.2244	0.4241
226352	Epb4.1l5	erythrocyte protein band 4.1-like 5	0.89047195	0.2362	0.4381
277432	Vstm2l	V-set and transmembrane domain containing 2-like	0.89047195	0.2448	0.4485
100043697	Gm9959	predicted gene 9959	0.89047195	0.2541	0.4592
59092	Pcbp4	poly(rC) binding protein 4	0.89047195	0.261	0.467
212632	Iffo2	intermediate filament family orphan 2	0.89047195	0.2744	0.4823
232934	Mypop	Myb-related transcription factor, partner of profilin	0.89047195	0.297	0.506
235584	Dusp7	dual specificity phosphatase 7	0.89047195	0.3118	0.5215
20977	Syp	synaptophysin	0.89047195	0.3241	0.5338
23984	Pde10a	phosphodiesterase 10A	0.89047195	0.3312	0.5416
11838	Arc	activity regulated cytoskeletal-associated protein	0.89047195	0.3483	0.5587
223881	Rnd1	Rho family GTPase 1	0.89047195	0.3611	0.5708
12323	Camk2b	calcium/calmodulin-dependent protein kinase II, beta	0.89047195	0.3689	0.578
320560	Dennd5b	DENN/MADD domain containing 5B	0.89047195	0.3779	0.5868
69794	1600027J07Rik	RIKEN cDNA 1600027J07 gene	0.89047195	0.4111	0.6174
240843	Fam5b	family with sequence similarity 5, member B	0.89047195	0.4117	0.6181
52679	E2f7	E2F transcription factor 7	0.89047195	0.4423	0.6451
22648	Zfp11	zinc finger protein 11	0.89047195	0.4569	0.6583
435965	Lrp3	low density lipoprotein receptor-related protein 3	0.89047195	0.4816	0.6776
80911	Acox3	acyl-Coenzyme A oxidase 3, pristanoyl	0.88967972	0.01661	NA
64294	Itm2c	integral membrane protein 2C	0.88967972	0.01796	NA
20403	Itsn2	intersectin 2	0.88967972	0.0198	NA
320436	C030009J22Rik	RIKEN cDNA C030009J22 gene	0.88967972	0.02318	NA
53892	Ppm1d	protein phosphatase 1D magnesium-dependent, delta isoform	0.88967972	0.02897	NA
75387	Sirt4	sirtuin 4 (silent mating type information regulation 2 homolog) 4 (S. cerevisiae)	0.88967972	0.04958	NA
22670	Trim26	tripartite motif-containing 26	0.88967972	0.05066	NA
66983	Zfp830	zinc finger protein 830	0.88967972	0.05097	NA
11538	Adnp	activity-dependent neuroprotective protein	0.88967972	0.06005	NA
19231	Ptma	prothymosin alpha	0.88967972	0.06139	NA
223435	Trio	triple functional domain (PTPRF interacting)	0.88967972	0.06513	NA
215705	Arrdc1	arrestin domain containing 1	0.88967972	0.06687	NA

100042480	Nhsl2	NHS-like 2	0.88967972	0.07698	NA
331532	Tceal5	transcription elongation factor A (SII)-like 5	0.88967972	0.08805	NA
70099	Smc4	structural maintenance of chromosomes 4	0.88967972	0.09786	NA
237761	Ankrd43	ankyrin repeat domain 43	0.88967972	0.1055	NA
13712	Elk1	ELK1, member of ETS oncogene family	0.88967972	0.1349	NA
73242	Atat1	alpha tubulin acetyltransferase 1	0.88967972	0.1372	NA
320720	Fastkd1	FAST kinase domains 1	0.88967972	0.1465	0.3191
75579	2310034G01Rik	RIKEN cDNA 2310034G01 gene	0.88967972	0.158	0.3352
71472	Usp19	ubiquitin specific peptidase 19	0.88967972	0.1598	0.338
13591	Ebf1	early B-cell factor 1	0.88967972	0.1715	0.3544
22230	Ufd1l	ubiquitin fusion degradation 1 like	0.88967972	0.1775	0.3624
434234	2610020H08Rik	RIKEN cDNA 2610020H08 gene	0.88967972	0.1973	0.3885
66537	Pomp	proteasome maturation protein	0.88967972	0.2097	0.4051
67471	Gpatch1	G patch domain containing 1	0.88967972	0.2249	0.4246
68260	Trmt12	tRNA methyltranferase 12 homolog (S. cerevisiae)	0.88967972	0.2255	0.4252
384261	Gm5296	predicted gene 5296	0.88967972	0.2455	0.449
212999	Tnpo2	transportin 2 (importin 3, karyopherin beta 2b)	0.88967972	0.2566	0.4617
14180	Fgf9	fibroblast growth factor 9	0.88967972	0.2634	0.4695
75763	Dcaf17	DDB1 and CUL4 associated factor 17	0.88967972	0.2738	0.4817
245860	Atg9a	autophagy-related 9A (yeast)	0.88967972	0.301	0.5104
319476	Lrtm1	leucine-rich repeats and transmembrane domains 1	0.88967972	0.3027	0.5117
97820	4833439L19Rik	RIKEN cDNA 4833439L19 gene	0.88967972	0.3501	0.5605
22367	Vrk1	vaccinia related kinase 1	0.88967972	0.3795	0.5886
28006	D6Wsu116e	DNA segment, Chr 6, Wayne State University 116, expressed	0.88967972	0.3821	0.5913
232975	Atp1a3	ATPase, Na+/K+ transporting, alpha 3 polypeptide	0.88967972	0.3821	0.5913
268729	Gm626	predicted gene 626	0.88967972	0.4181	0.6244
19134	Prpf4b	PRP4 pre-mRNA processing factor 4 homolog B (yeast)	0.88967972	0.4405	0.6434
67163	Ccdc47	coiled-coil domain containing 47	0.88967972	0.4875	0.6824
72672	Zfp518a	zinc finger protein 518A	0.88967972	0.5172	0.7067
623503	Prlh	prolactin releasing hormone	0.88967972	0.5405	0.7237
68671	Pcyt2	phosphate cytidylyltransferase 2, ethanolamine	0.88888889	0.02538	NA
12752	Cln3	ceroid lipofuscinosis, neuronal 3, juvenile (Batten, Spielmeyer-Vogt disease)	0.88888889	0.03091	NA
11773	Ap2m1	adaptor protein complex AP-2, mu1	0.88888889	0.03968	NA
233724	Tmem41b	transmembrane protein 41B	0.88888889	0.04002	NA
19819	Rnaseh1	ribonuclease H1	0.88888889	0.04252	NA
100121	Tdrd7	tudor domain containing 7	0.88888889	0.04403	NA
114896	Afg3l1	AFG3(ATPase family gene 3)-like 1 (yeast)	0.88888889	0.04955	NA
69482	Nup35	nucleoporin 35	0.88888889	0.05217	NA
246694	Hps5	Hermansky-Pudlak syndrome 5 homolog (human)	0.88888889	0.05669	NA
17423	Ndst2	N-deacetylase/N-sulfotransferase (heparan glucosaminyl) 2	0.88888889	0.08587	NA
70052	Prpf4	PRP4 pre-mRNA processing factor 4 homolog (yeast)	0.88888889	0.09093	NA
66293	1810032O08Rik	RIKEN cDNA 1810032O08 gene	0.88888889	0.09262	NA
72607	Usp13	ubiquitin specific peptidase 13 (isopeptidase T-3)	0.88888889	0.1208	NA
212073	4831426I19Rik	RIKEN cDNA 4831426I19 gene	0.88888889	0.1225	NA
53602	Hpcal1	hippocalcin-like 1	0.88888889	0.1266	NA
214254	Nudt15	nudix (nucleoside diphosphate linked moiety X)-type motif 15	0.88888889	0.1289	NA
19244	Ptp4a2	protein tyrosine phosphatase 4a2	0.88888889	0.1421	0.3128
11831	Aqp6	aquaporin 6	0.88888889	0.1464	0.319
627626	3110082D06Rik	RIKEN cDNA 3110082D06 gene	0.88888889	0.1485	0.3223
20090	Rps29	ribosomal protein S29	0.88888889	0.1528	0.3281

67553	Gstcd	glutathione S-transferase, C-terminal domain containing	0.88888889	0.1572	0.3343
226562	Prrc2c	proline-rich coiled-coil 2C	0.88888889	0.1743	0.3581
66532	Rep15	RAB15 effector protein	0.88888889	0.1754	0.3599
231991	Creb5	cAMP responsive element binding protein 5	0.88888889	0.1849	0.3721
54387	Mcm3ap	minichromosome maintenance deficient 3 (S. cerevisiae) associated protein	0.88888889	0.1872	0.3753
54650	Sfmbt1	Scm-like with four mbt domains 1	0.88888889	0.204	0.3975
24074	Taf7	TAF7 RNA polymerase II, TATA box binding protein (TBP)-associated factor	0.88888889	0.2335	0.435
22318	Vamp2	vesicle-associated membrane protein 2	0.88888889	0.2364	0.4383
50771	Atp9b	ATPase, class II, type 9B	0.88888889	0.2853	0.4937
230379	Acer2	alkaline ceramidase 2	0.88888889	0.3128	0.5225
71330	Rcbtb1	regulator of chromosome condensation (RCC1) and BTB (POZ) domain containing protein 1	0.88888889	0.3256	0.5355
100043335	Gm4371	eukaryotic translation initiation factor 3, subunit I pseudogene	0.88888889	0.3522	0.5623
18707	Pik3cd	phosphatidylinositol 3-kinase catalytic delta polypeptide	0.88888889	0.3858	0.5946
71766	Raver1	ribonucleoprotein, PTB-binding 1	0.88888889	0.4054	0.613
74211	1700017B05Rik	RIKEN cDNA 1700017B05 gene	0.88888889	0.4185	0.6249
13821	Epb4.1l1	erythrocyte protein band 4.1-like 1	0.88888889	0.4219	0.6278
75086	4930520P13Rik	RIKEN cDNA 4930520P13 gene	0.88888889	0.4305	0.6345
214444	Cdk5rap2	CDK5 regulatory subunit associated protein 2	0.88888889	0.444	0.6468
109359	Fam175b	family with sequence similarity 175, member B	0.88888889	0.4491	0.6513
72961	Slc17a7	solute carrier family 17 (sodium-dependent inorganic phosphate cotransporter), member 7	0.88888889	0.4916	0.6857
69352	Necab1	N-terminal EF-hand calcium binding protein 1	0.88888889	0.5	0.6922
83397	Akap12	A kinase (PRKA) anchor protein (gravin) 12	0.88888889	0.5647	0.7419
69601	Dab2ip	disabled homolog 2 (Drosophila) interacting protein	0.88888889	0.574	0.748
16536	Kcnq2	potassium voltage-gated channel, subfamily Q, member 2	0.88809947	0.01315	NA
17207	Mcf2l	mcf.2 transforming sequence-like	0.88809947	0.01727	NA
66337	1700025K23Rik	RIKEN cDNA 1700025K23 gene	0.88809947	0.04361	NA
17954	Nap1l2	nucleosome assembly protein 1-like 2	0.88809947	0.05324	NA
66967	Edem3	ER degradation enhancer, mannosidase alpha-like 3	0.88809947	0.08212	NA
218756	Slc4a7	solute carrier family 4, sodium bicarbonate cotransporter, member 7	0.88809947	0.08391	NA
52055	Rab11fip5	RAB11 family interacting protein 5 (class I)	0.88809947	0.0889	NA
66075	Chchd3	coiled-coil-helix-coiled-coil-helix domain containing 3	0.88809947	0.1	NA
11444	Chrnb2	cholinergic receptor, nicotinic, beta polypeptide 2 (neuronal)	0.88809947	0.1077	NA
78425	9530053H05Rik	RIKEN cDNA 9530053H05 gene	0.88809947	0.1359	0.3034
54645	Gripap1	GRIP1 associated protein 1	0.88809947	0.1417	0.3121
13842	Epha8	Eph receptor A8	0.88809947	0.1427	0.3136
320742	A230072C01Rik	RIKEN cDNA A230072C01 gene	0.88809947	0.1511	0.3258
12891	Cpne6	copine VI	0.88809947	0.1514	0.3261
16151	Ikbkg	inhibitor of kappaB kinase gamma	0.88809947	0.1643	0.3441
76898	B3gat1	beta-1,3-glucuronyltransferase 1 (glucuronosyltransferase P)	0.88809947	0.1668	0.3476
71371	Arid5b	AT rich interactive domain 5B (MRF1-like)	0.88809947	0.18	0.3656
277414	Trp53i11	transformation related protein 53 inducible protein 11	0.88809947	0.1905	0.3795
67952	Tomm20	translocase of outer mitochondrial membrane 20 homolog (yeast)	0.88809947	0.201	0.3935
72275	2200002D01Rik	RIKEN cDNA 2200002D01 gene	0.88809947	0.2026	0.3956
29807	Tpk1	thiamine pyrophosphokinase	0.88809947	0.2165	0.4139
270110	Irf2bp2	interferon regulatory factor 2 binding protein 2	0.88809947	0.2198	0.4188
327762	Dna2	DNA replication helicase 2 homolog (yeast)	0.88809947	0.2269	0.4267
629147	Ctxn3	cortexin 3	0.88809947	0.2532	0.458
234407	Glt25d1	glycosyltransferase 25 domain containing 1	0.88809947	0.2608	0.4667
14084	Faf1	Fas-associated factor 1	0.88809947	0.2634	0.4695
228421	Kif18a	kinesin family member 18A	0.88809947	0.3001	0.5094

106068	Slc45a4	solute carrier family 45, member 4	0.88809947	0.3037	0.5128
233274	Siglech	sialic acid binding Ig-like lectin H	0.88809947	0.3898	0.5981
20273	Scn8a	sodium channel, voltage-gated, type VIII, alpha	0.88809947	0.4389	0.6417
73545	1700094D03Rik	RIKEN cDNA 1700094D03 gene	0.88731145	0.01864	NA
70999	Naa40	N(alpha)-acetyltransferase 40, NatD catalytic subunit, homolog (S. cerevisiae)	0.88731145	0.02287	NA
20254	Scg2	secretogranin II	0.88731145	0.03137	NA
81702	Ankrd17	ankyrin repeat domain 17	0.88731145	0.03733	NA
60525	Acss2	acyl-CoA synthetase short-chain family member 2	0.88731145	0.04089	NA
246317	Neto1	neuropilin (NRP) and tolloid (TLL)-like 1	0.88731145	0.04089	NA
17925	Myo9b	myosin IXb	0.88731145	0.05372	NA
79560	Ublcp1	ubiquitin-like domain containing CTD phosphatase 1	0.88731145	0.05545	NA
66994	1500031L02Rik	RIKEN cDNA 1500031L02 gene	0.88731145	0.06406	NA
76219	6530401D17Rik	RIKEN cDNA 6530401D17 gene	0.88731145	0.07995	NA
234353	Psd3	pleckstrin and Sec7 domain containing 3	0.88731145	0.08272	NA
20218	Khdrbs1	KH domain containing, RNA binding, signal transduction associated 1	0.88731145	0.08419	NA
230895	Vps13d	vacuolar protein sorting 13 D (yeast)	0.88731145	0.09356	NA
66995	Zcchc18	zinc finger, CCHC domain containing 18	0.88731145	0.09826	NA
94063	Mrpl16	mitochondrial ribosomal protein L16	0.88731145	0.1029	NA
67422	Dhdds	dehydrodolichyl diphosphate synthase	0.88731145	0.1405	0.3103
381438	Gm5148	predicted gene 5148	0.88731145	0.1411	0.3113
15531	Ndst1	N-deacetylase/N-sulfotransferase (heparan glucosaminyl) 1	0.88731145	0.1454	0.3178
98740	BB116930	expressed sequence BB116930	0.88731145	0.1489	0.3227
17112	Tm4sf1	transmembrane 4 superfamily member 1	0.88731145	0.153	0.3283
74525	8430419L09Rik	RIKEN cDNA 8430419L09 gene	0.88731145	0.16	0.3381
319285	A430061O12Rik	RIKEN cDNA A430061O12 gene	0.88731145	0.1717	0.3545
18045	Nfyb	nuclear transcription factor-Y beta	0.88731145	0.1755	0.3601
225631	Onecut2	one cut domain, family member 2	0.88731145	0.1803	0.3662
68299	Vps53	vacuolar protein sorting 53 (yeast)	0.88731145	0.2015	0.3941
229512	Smg5	Smg-5 homolog, nonsense mediated mRNA decay factor (C. elegans)	0.88731145	0.2233	0.4228
233878	Sez6l2	seizure related 6 homolog like 2	0.88731145	0.2489	0.4531
67048	Vma21	VMA21 vacuolar H+-ATPase homolog (S. cerevisiae)	0.88731145	0.2557	0.4607
18245	Oaz1	ornithine decarboxylase antizyme 1	0.88731145	0.2866	0.495
67952	Tomm20	translocase of outer mitochondrial membrane 20 homolog (yeast)	0.88731145	0.2994	0.5088
53325	Banp	BTG3 associated nuclear protein	0.88731145	0.3227	0.5325
116837	Rims1	regulating synaptic membrane exocytosis 1	0.88731145	0.3574	0.5672
210108	D130043K22Rik	RIKEN cDNA D130043K22 gene	0.88731145	0.3593	0.5691
16924	Lnx1	ligand of numb-protein X 1	0.88731145	0.367	0.576
329506	Ctdspl2	CTD (carboxy-terminal domain, RNA polymerase II, polypeptide A) small phosphatase like 2	0.88731145	0.3702	0.5792
11803	Aplp1	amyloid beta (A4) precursor-like protein 1	0.88731145	0.4079	0.6145
217698	Acot5	acyl-CoA thioesterase 5	0.88731145	0.4257	0.6309
666190	Gm7972	predicted gene 7972	0.88731145	0.4271	0.6319
26561	Mmp23	matrix metallopeptidase 23	0.88731145	0.428	0.6326
68484	Krtap16-8	keratin associated protein 16-8	0.88731145	0.4482	0.6504
17341	Bhlha15	basic helix-loop-helix family, member a15	0.88731145	0.4537	0.6553
74256	Cyld	cylindromatosis (turban tumor syndrome)	0.88731145	0.5802	0.7524
100045736	LOC100045736	hypothetical protein LOC100045736	0.88731145	0.7207	0.8475
93760	Arid1a	AT rich interactive domain 1A (SWI-like)	0.88652482	0.03423	NA
100223	9630041G16Rik	RIKEN cDNA 9630041G16 gene	0.88652482	0.03869	NA
245944	Vps54	vacuolar protein sorting 54 (yeast)	0.88652482	0.04241	NA
53602	Hpcal1	hippocalcin-like 1	0.88652482	0.04665	NA

81601	Kat5	K(lysine) acetyltransferase 5	0.88652482	0.04796	NA
24116	Whsc2	Wolf-Hirschhorn syndrome candidate 2 (human)	0.88652482	0.05027	NA
16179	Irak1	interleukin-1 receptor-associated kinase 1	0.88652482	0.06638	NA
72039	Mccc1	methylcrotonoyl-Coenzyme A carboxylase 1 (alpha)	0.88652482	0.0693	NA
69612	2310037I24Rik	RIKEN cDNA 2310037I24 gene	0.88652482	0.07963	NA
245446	Slitrk4	SLIT and NTRK-like family, member 4	0.88652482	0.08097	NA
214952	Rhot2	ras homolog gene family, member T2	0.88652482	0.08132	NA
57908	Zfp318	zinc finger protein 318	0.88652482	0.09678	NA
26885	Casp8ap2	caspase 8 associated protein 2	0.88652482	0.1061	NA
244672	Cwf19l2	CWF19-like 2, cell cycle control (S. pombe)	0.88652482	0.1219	NA
107449	Unc5b	unc-5 homolog B (C. elegans)	0.88652482	0.1224	NA
19882	Mst1r	macrophage stimulating 1 receptor (c-met-related tyrosine kinase)	0.88652482	0.1322	0.2987
621239	Nhlrc4	NHL repeat containing 4	0.88652482	0.1404	0.3102
17967	Ncam1	neural cell adhesion molecule 1	0.88652482	0.1596	0.3377
16658	Mafb	v-maf musculoaponeurotic fibrosarcoma oncogene family, protein B (avian)	0.88652482	0.1675	0.3483
69456	Commd10	COMM domain containing 10	0.88652482	0.1734	0.357
213006	Mfsd4	major facilitator superfamily domain containing 4	0.88652482	0.1762	0.3609
67996	Srsf6	serine/arginine-rich splicing factor 6	0.88652482	0.178	0.3631
76539	D19Ertd737e	DNA segment, Chr 19, ERATO Doi 737, expressed	0.88652482	0.1783	0.3634
11363	Acadl	acyl-Coenzyme A dehydrogenase, long-chain	0.88652482	0.1791	0.3646
13813	Eomes	eomesodermin homolog (Xenopus laevis)	0.88652482	0.2206	0.4196
402733	A930009L07Rik	RIKEN cDNA A930009L07 gene	0.88652482	0.2719	0.4793
17196	Mbp	myelin basic protein	0.88652482	0.2944	0.5033
245671	Klf8	Kruppel-like factor 8	0.88652482	0.3017	0.511
225861	Snx32	sorting nexin 32	0.88652482	0.3354	0.5458
100504089	LOC100504089	calcium-binding protein p22-like	0.88652482	0.3375	0.5478
11771	Ap2a1	adaptor protein complex AP-2, alpha 1 subunit	0.88652482	0.375	0.5839
53897	Gal3st1	galactose-3-O-sulfotransferase 1	0.88652482	0.3893	0.5975
56013	Srcin1	SRC kinase signaling inhibitor 1	0.88652482	0.391	0.5992
431706	Zfp457	zinc finger protein 457	0.88652482	0.5504	0.7316
71718	Telo2	TEL2, telomere maintenance 2, homolog (S. cerevisiae)	0.88573959	0.03235	NA
21408	Zfp354a	zinc finger protein 354A	0.88573959	0.03664	NA
22751	Zfp90	zinc finger protein 90	0.88573959	0.04458	NA
23856	Dido1	death inducer-obliterator 1	0.88573959	0.04735	NA
103573	Xpo1	exportin 1, CRM1 homolog (yeast)	0.88573959	0.06368	NA
76501	Commd9	COMM domain containing 9	0.88573959	0.07207	NA
15183	Hdac3	histone deacetylase 3	0.88573959	0.0767	NA
66505	Zmynd11	zinc finger, MYND domain containing 11	0.88573959	0.0823	NA
71675	0610010F05Rik	RIKEN cDNA 0610010F05 gene	0.88573959	0.08908	NA
56258	Hnrnph2	heterogeneous nuclear ribonucleoprotein H2	0.88573959	0.1004	NA
66433	Chchd7	coiled-coil-helix-coiled-coil-helix domain containing 7	0.88573959	0.1011	NA
626596	Rgs22	regulator of G-protein signalling 22	0.88573959	0.1019	NA
226970	Arhgef4	Rho guanine nucleotide exchange factor (GEF) 4	0.88573959	0.1181	NA
50706	Postn	periostin, osteoblast specific factor	0.88573959	0.1567	0.3336
23912	Rhof	ras homolog gene family, member f	0.88573959	0.1796	0.3653
66049	Rogdi	rogdi homolog (Drosophila)	0.88573959	0.1815	0.368
77110	Gpbp1l1	GC-rich promoter binding protein 1-like 1	0.88573959	0.197	0.3883
68226	Efcab2	EF-hand calcium binding domain 2	0.88573959	0.1973	0.3885
11540	Adora2a	adenosine A2a receptor	0.88573959	0.2153	0.4124
21413	Tcf4	transcription factor 4	0.88573959	0.2769	0.4849

27425	Atp5l	ATP synthase, H+ transporting, mitochondrial F0 complex, subunit g	0.88573959	0.2769	0.4849
66138	Wbscr22	Williams Beuren syndrome chromosome region 22	0.88573959	0.4088	0.6153
16490	Kcna2	potassium voltage-gated channel, shaker-related subfamily, member 2	0.88573959	0.4145	0.6207
12340	Capza1	capping protein (actin filament) muscle Z-line, alpha 1	0.88573959	0.415	0.6213
16494	Kcna6	potassium voltage-gated channel, shaker-related, subfamily, member 6	0.88573959	0.4296	0.6339
68152	Fam133b	family with sequence similarity 133, member B	0.88573959	0.4722	0.6705
12006	Axin2	axin2	0.88573959	0.4772	0.6743
239510	Phf20l1	PHD finger protein 20-like 1	0.88573959	0.4786	0.6754
622480	Spocd1	SPOC domain containing 1	0.88573959	0.5136	0.704
18033	Nfkb1	nuclear factor of kappa light polypeptide gene enhancer in B-cells 1, p105	0.88573959	0.595	0.7638
75578	Fggy	FGGY carbohydrate kinase domain containing	0.88573959	0.6218	0.783
55988	Snx12	sorting nexin 12	0.88495575	0.02635	NA
213109	Phf3	PHD finger protein 3	0.88495575	0.04041	NA
18546	Pcp4	Purkinje cell protein 4	0.88495575	0.04072	NA
70118	Srrd	SRR1 domain containing	0.88495575	0.04187	NA
67728	Dph2	DPH2 homolog (S. cerevisiae)	0.88495575	0.04965	NA
72125	Fam123a	family with sequence similarity 123, member A	0.88495575	0.05747	NA
22700	Zfp40	zinc finger protein 40	0.88495575	0.05968	NA
74112	Usp16	ubiquitin specific peptidase 16	0.88495575	0.08732	NA
57258	Xpo4	exportin 4	0.88495575	0.09754	NA
20320	Nptn	neuroplastin	0.88495575	0.1055	NA
78070	Cpt1c	carnitine palmitoyltransferase 1c	0.88495575	0.1441	0.3158
242384	Lingo2	leucine rich repeat and Ig domain containing 2	0.88495575	0.1578	0.335
114640	Pth2	parathyroid hormone 2	0.88495575	0.1797	0.3654
74148	1300001I01Rik	RIKEN cDNA 1300001I01 gene	0.88495575	0.1867	0.3745
74596	Cds1	CDP-diacylglycerol synthase 1	0.88495575	0.1928	0.3828
269019	Stk32a	serine/threonine kinase 32A	0.88495575	0.2127	0.4086
109181	Trip11	thyroid hormone receptor interactor 11	0.88495575	0.2163	0.4136
382867	Zfp488	zinc finger protein 488	0.88495575	0.2449	0.4486
228875	Slc35c2	solute carrier family 35, member C2	0.88495575	0.2653	0.4717
12121	Bicd1	bicaudal D homolog 1 (Drosophila)	0.88495575	0.277	0.4851
75697	C2cd4b	C2 calcium-dependent domain containing 4B	0.88495575	0.3636	0.5726
12168	Bmpr2	bone morphogenic protein receptor, type II (serine/threonine kinase)	0.88495575	0.4122	0.6185
207375	Fam120c	family with sequence similarity 120, member C	0.88495575	0.4377	0.6409
78695	C030005K06Rik	RIKEN cDNA C030005K06 gene	0.88495575	0.5515	0.7323
70008	Ace2	angiotensin I converting enzyme (peptidyl-dipeptidase A) 2	0.88495575	0.5569	0.7365
59038	Pxmp4	peroxisomal membrane protein 4	0.88495575	0.5813	0.7533
237178	Ppef1	protein phosphatase with EF hand calcium-binding domain 1	0.88495575	0.6316	0.7905
231329	Polr2b	polymerase (RNA) II (DNA directed) polypeptide B	0.8841733	0.01174	NA
69482	Nup35	nucleoporin 35	0.8841733	0.02456	NA
195040	Tmem199	transmembrane protein 199	0.8841733	0.02868	NA
74168	Zdhhc16	zinc finger, DHHC domain containing 16	0.8841733	0.03712	NA
70834	Spag9	sperm associated antigen 9	0.8841733	0.03909	NA
67254	2900011O08Rik	RIKEN cDNA 2900011O08 gene	0.8841733	0.05554	NA
12727	Clcn4-2	chloride channel 4-2	0.8841733	0.05677	NA
67040	Ddx17	DEAD (Asp-Glu-Ala-Asp) box polypeptide 17	0.8841733	0.05883	NA
72993	Appl1	adaptor protein, phosphotyrosine interaction, PH domain and leucine zipper containing 1	0.8841733	0.06239	NA
233532	Rsf1	remodeling and spacing factor 1	0.8841733	0.07621	NA
338337	Cog3	component of oligomeric golgi complex 3	0.8841733	0.0774	NA
66724	Tab3	TGF-beta activated kinase 1/MAP3K7 binding protein 3	0.8841733	0.08709	NA

208146	Yeats2	YEATS domain containing 2	0.8841733	0.09039	NA
209630	Frmd4a	FERM domain containing 4A	0.8841733	0.09426	NA
66631	Hiatl1	hippocampus abundant transcript-like 1	0.8841733	0.09432	NA
214505	Gnptg	N-acetylglucosamine-1-phosphotransferase, gamma subunit	0.8841733	0.09625	NA
21652	Phf1	PHD finger protein 1	0.8841733	0.09965	NA
66202	1110059G10Rik	RIKEN cDNA 1110059G10 gene	0.8841733	0.102	NA
55946	Ap3m1	adaptor-related protein complex 3, mu 1 subunit	0.8841733	0.1193	0.2792
26939	Polr3e	polymerase (RNA) III (DNA directed) polypeptide E	0.8841733	0.1311	0.2971
18189	Nrxn1	neurexin I	0.8841733	0.1317	0.298
432842	LOC432842	hypothetical LOC432842	0.8841733	0.133	0.3
72775	Fance	Fanconi anemia, complementation group E	0.8841733	0.1567	0.3336
59041	Stk25	serine/threonine kinase 25 (yeast)	0.8841733	0.1603	0.3383
68053	Ubxn2b	UBX domain protein 2B	0.8841733	0.1649	0.3449
13645	Egf	epidermal growth factor	0.8841733	0.1676	0.3485
69539	Trnp1	TMF1-regulated nuclear protein 1	0.8841733	0.1687	0.3497
67574	Alg13	asparagine-linked glycosylation 13 homolog (S. cerevisiae)	0.8841733	0.1712	0.354
19261	Sirpa	signal-regulatory protein alpha	0.8841733	0.1783	0.3634
16201	Ilf3	interleukin enhancer binding factor 3	0.8841733	0.2106	0.4061
330267	Thsd7a	thrombospondin, type I, domain containing 7A	0.8841733	0.2173	0.415
244654	Mtss1l	metastasis suppressor 1-like	0.8841733	0.2835	0.4921
11658	Alcam	activated leukocyte cell adhesion molecule	0.8841733	0.2959	0.5048
55993	Msh4	mutS homolog 4 (E. coli)	0.8841733	0.3274	0.5375
228003	Kbtbd10	kelch repeat and BTB (POZ) domain containing 10	0.8841733	0.3613	0.5709
219105	Zmym5	zinc finger, MYM-type 5	0.8841733	0.3752	0.5841
215789	Phactr2	phosphatase and actin regulator 2	0.8841733	0.4088	0.6153
622629	Gm10318	predicted gene 10318	0.8841733	0.4585	0.6595
16519	Kcnj3	potassium inwardly-rectifying channel, subfamily J, member 3	0.8841733	0.507	0.6979
80904	Dtx3	deltex 3 homolog (Drosophila)	0.8841733	0.5289	0.7148
268752	Wdfy2	WD repeat and FYVE domain containing 2	0.8841733	0.5779	0.7507
71301	4930593A02Rik	RIKEN cDNA 4930593A02 gene	0.8841733	0.5797	0.7521
433490	Defb45	defensin beta 45	0.8841733	0.7121	0.842
218629	Dhx29	DEAH (Asp-Glu-Ala-His) box polypeptide 29	0.88339223	0.01555	NA
14077	Fabp3	fatty acid binding protein 3, muscle and heart	0.88339223	0.01978	NA
78656	Brd8	bromodomain containing 8	0.88339223	0.02346	NA
11804	Aplp2	amyloid beta (A4) precursor-like protein 2	0.88339223	0.02384	NA
76824	Fam54b	family with sequence similarity 54, member B	0.88339223	0.03358	NA
67465	Sf3a1	splicing factor 3a, subunit 1	0.88339223	0.03508	NA
229700	Rbm15	RNA binding motif protein 15	0.88339223	0.0359	NA
72948	Tppp	tubulin polymerization promoting protein	0.88339223	0.0393	NA
319885	Zcchc7	zinc finger, CCHC domain containing 7	0.88339223	0.04425	NA
329165	Abi2	abl-interactor 2	0.88339223	0.04625	NA
78786	4833439F03Rik	RIKEN cDNA 4833439F03 gene	0.88339223	0.06209	NA
17761	Mtap7	microtubule-associated protein 7	0.88339223	0.07384	NA
11990	Atrn	attractin	0.88339223	0.08415	NA
22348	Slc32a1	solute carrier family 32 (GABA vesicular transporter), member 1	0.88339223	0.08992	NA
14593	Ggps1	geranylgeranyl diphosphate synthase 1	0.88339223	0.105	NA
54473	Tollip	toll interacting protein	0.88339223	0.1286	0.2937
382620	Tmed8	transmembrane emp24 domain containing 8	0.88339223	0.1347	0.3016
11554	Adrb1	adrenergic receptor, beta 1	0.88339223	0.1353	0.3025
140780	Bmp2k	BMP2 inducible kinase	0.88339223	0.1386	0.3073

380664	Lemd3	LEM domain containing 3	0.88339223	0.1386	0.3073
14058	F10	coagulation factor X	0.88339223	0.1393	0.3085
11857	Arhgdib	Rho, GDP dissociation inhibitor (GDI) beta	0.88339223	0.1466	0.3193
96979	Ptges2	prostaglandin E synthase 2	0.88339223	0.1476	0.3208
70458	2610318N02Rik	RIKEN cDNA 2610318N02 gene	0.88339223	0.1586	0.3362
60532	Wtap	Wilms' tumour 1-associating protein	0.88339223	0.1615	0.3399
56384	Letm1	leucine zipper-EF-hand containing transmembrane protein 1	0.88339223	0.1662	0.3469
110911	Cds2	CDP-diacylglycerol synthase (phosphatidate cytidylyltransferase) 2	0.88339223	0.176	0.3606
77958	A930023M06Rik	RIKEN cDNA A930023M06 gene	0.88339223	0.1782	0.3632
106906	AI450241	expressed sequence AI450241	0.88339223	0.182	0.3687
20227	Sart1	squamous cell carcinoma antigen recognized by T-cells 1	0.88339223	0.1927	0.3826
171429	Slc26a6	solute carrier family 26, member 6	0.88339223	0.1948	0.3851
272027	Tstd2	thiosulfate sulfurtransferase (rhodanese)-like domain containing 2	0.88339223	0.2227	0.4221
229709	Ahcyl1	S-adenosylhomocysteine hydrolase-like 1	0.88339223	0.2594	0.4651
71833	Dcaf7	DDB1 and CUL4 associated factor 7	0.88339223	0.2815	0.4895
72133	Trub1	TruB pseudouridine (psi) synthase homolog 1 (E. coli)	0.88339223	0.2908	0.4993
75986	Agmat	agmatine ureohydrolase (agmatinase)	0.88339223	0.3064	0.5158
68477	Rmnd5a	required for meiotic nuclear division 5 homolog A (S. cerevisiae)	0.88339223	0.3211	0.5311
83603	Elovl4	elongation of very long chain fatty acids (FEN1/Elo2, SUR4/Elo3, yeast)-like 4	0.88339223	0.3603	0.5701
72194	Fbxl20	F-box and leucine-rich repeat protein 20	0.88339223	0.3794	0.5885
330941	AI593442	expressed sequence AI593442	0.88339223	0.461	0.6615
544752	Tug1	taurine upregulated gene 1	0.88339223	0.5302	0.7158
71099	Tssk4	testis-specific serine kinase 4	0.88339223	0.5651	0.742
385354	Frmd7	FERM domain containing 7	0.88339223	0.5676	0.7435
66277	Klf15	Kruppel-like factor 15	0.88339223	0.651	0.8034
217351	Tnrc6c	trinucleotide repeat containing 6C	0.88261253	0.01477	NA
83486	Rbm5	RNA binding motif protein 5	0.88261253	0.01846	NA
67604	Get4	golgi to ER traffic protein 4 homolog (S. cerevisiae)	0.88261253	0.0241	NA
20394	Scg5	secretogranin V	0.88261253	0.02608	NA
212390	Klhl32	kelch-like 32 (Drosophila)	0.88261253	0.02983	NA
107321	Lpxn	leupaxin	0.88261253	0.03825	NA
101142	Itfg2	integrin alpha FG-GAP repeat containing 2	0.88261253	0.04322	NA
13831	Epc1	enhancer of polycomb homolog 1 (Drosophila)	0.88261253	0.0441	NA
11434	Acr	acrosin prepropeptide	0.88261253	0.06344	NA
12339	Capn7	calpain 7	0.88261253	0.06516	NA
74385	Mudeng	MU-2/AP1M2 domain containing, death-inducing	0.88261253	0.06531	NA
217039	Ggnbp2	gametogenetin binding protein 2	0.88261253	0.06563	NA
170731	Mfn2	mitofusin 2	0.88261253	0.07012	NA
245386	Fam70a	family with sequence similarity 70, member A	0.88261253	0.07101	NA
71810	Ranbp3	RAN binding protein 3	0.88261253	0.07181	NA
231999	Plekha8	pleckstrin homology domain containing, family A (phosphoinositide binding specific) member 8	0.88261253	0.0755	NA
72199	Mms19	MMS19 (MET18 S. cerevisiae)	0.88261253	0.07971	NA
100502767	LOC100502767	hypothetical LOC100502767	0.88261253	0.1069	NA
234854	Cdk10	cyclin-dependent kinase 10	0.88261253	0.1565	0.3334
70930	Nol8	nucleolar protein 8	0.88261253	0.1566	0.3334
382985	Rrm2b	ribonucleotide reductase M2 B (TP53 inducible)	0.88261253	0.1726	0.3559
78249	Gpr115	G protein-coupled receptor 115	0.88261253	0.1789	0.3642
330323	Fam188b	family with sequence similarity 188, member B	0.88261253	0.1825	0.3693
319822	Smyd4	SET and MYND domain containing 4	0.88261253	0.2105	0.4061
320709	Tmem117	transmembrane protein 117	0.88261253	0.2269	0.4268

231801	Agfg2	ArfGAP with FG repeats 2	0.88261253	0.2365	0.4383
105833	Ccdc65	coiled-coil domain containing 65	0.88261253	0.2412	0.4438
100737	Dcun1d4	DCN1, defective in cullin neddylation 1, domain containing 4 (S. cerevisiae)	0.88261253	0.2421	0.445
320473	Heatr5b	HEAT repeat containing 5B	0.88261253	0.2482	0.4525
217882	AW555464	expressed sequence AW555464	0.88261253	0.2552	0.4602
360013	Myo18a	myosin XVIIIA	0.88261253	0.2632	0.4693
319953	Ttll1	tubulin tyrosine ligase-like 1	0.88261253	0.2641	0.4703
233649	Cnga4	cyclic nucleotide gated channel alpha 4	0.88261253	0.2944	0.5033
12313	Calm1	calmodulin 1	0.88261253	0.2998	0.5092
78779	Spata2L	spermatogenesis associated 2-like	0.88261253	0.3019	0.511
242474	D730040F13Rik	RIKEN cDNA D730040F13 gene	0.88261253	0.3046	0.5136
66526	2210012G02Rik	RIKEN cDNA 2210012G02 gene	0.88261253	0.3111	0.5211
20308	Ccl9	chemokine (C-C motif) ligand 9	0.88261253	0.3217	0.5315
76022	Gon4l	gon-4-like (C.elegans)	0.88261253	0.3372	0.5475
12576	Cdkn1b	cyclin-dependent kinase inhibitor 1B	0.88261253	0.3573	0.5672
20965	Syn2	synapsin II	0.88261253	0.3701	0.5792
75813	4930484H19Rik	RIKEN cDNA 4930484H19 gene	0.88261253	0.3839	0.5931
544696	D630037F22Rik	RIKEN cDNA D630037F22 gene	0.88261253	0.4001	0.6079
236428	BC026762	cDNA sequence BC026762	0.88261253	0.4434	0.6464
243168	Hsd17b13	hydroxysteroid (17-beta) dehydrogenase 13	0.88261253	0.4723	0.6705
97230	C78228	expressed sequence C78228	0.88261253	0.5755	0.7491
210982	BC032203	cDNA sequence BC032203	0.88183422	0.01629	NA
241944	D3Ertd254e	DNA segment, Chr 3, ERATO Doi 254, expressed	0.88183422	0.01639	NA
55951	Brp44l	brain protein 44-like	0.88183422	0.02197	NA
24015	Abce1	ATP-binding cassette, sub-family E (OABP), member 1	0.88183422	0.02274	NA
208258	Ankrd33	ankyrin repeat domain 33	0.88183422	0.03615	NA
69546	Mapk1ip1	mitogen-activated protein kinase 1 interacting protein 1	0.88183422	0.04198	NA
108857	Ankhd1	ankyrin repeat and KH domain containing 1	0.88183422	0.04558	NA
101772	Ano1	anoctamin 1, calcium activated chloride channel	0.88183422	0.04571	NA
26373	Clcn7	chloride channel 7	0.88183422	0.05651	NA
72821	Scn2b	sodium channel, voltage-gated, type II, beta	0.88183422	0.0631	NA
14272	Fnta	farnesyltransferase, CAAX box, alpha	0.88183422	0.06738	NA
71435	Arhgap21	Rho GTPase activating protein 21	0.88183422	0.0678	NA
208650	Cblb	Casitas B-lineage lymphoma b	0.88183422	0.09284	NA
234594	Cnot1	CCR4-NOT transcription complex, subunit 1	0.88183422	0.09739	NA
67923	Tceb1	transcription elongation factor B (SIII), polypeptide 1	0.88183422	0.1013	NA
13047	Cux1	cut-like homeobox 1	0.88183422	0.1112	0.266
232807	Ppp1r12c	protein phosphatase 1, regulatory (inhibitor) subunit 12C	0.88183422	0.1286	0.2937
14104	Fasn	fatty acid synthase	0.88183422	0.1361	0.3036
229517	Slc25a44	solute carrier family 25, member 44	0.88183422	0.1538	0.3294
105525	AI480461	expressed sequence AI480461	0.88183422	0.1551	0.3314
66373	Lsm5	LSM5 homolog, U6 small nuclear RNA associated (S. cerevisiae)	0.88183422	0.1599	0.338
64436	Inpp5e	inositol polyphosphate-5-phosphatase E	0.88183422	0.1722	0.3552
100910	Chpf2	chondroitin polymerizing factor 2	0.88183422	0.2027	0.3957
68904	Abhd13	abhydrolase domain containing 13	0.88183422	0.2714	0.4789
12728	Clcn5	chloride channel 5	0.88183422	0.2818	0.4899
20360	Sema6c	sema domain, transmembrane domain (TM), and cytoplasmic domain, (semaphorin) 6C	0.88183422	0.3081	0.5179
15361	Hmga1	high mobility group AT-hook 1	0.88183422	0.3094	0.5193
64242	Ngb	neuroglobin	0.88183422	0.3134	0.5231
23937	Mab21l2	mab-21-like 2 (C. elegans)	0.88183422	0.3339	0.5442

18612	Etv4	ets variant gene 4 (E1A enhancer binding protein, E1AF)	0.88183422	0.4056	0.6131
77371	Sec24a	Sec24 related gene family, member A (S. cerevisiae)	0.88183422	0.4564	0.6579
76510	Trappc9	trafficking protein particle complex 9	0.88183422	0.5302	0.7158
74376	Myo18b	myosin XVIIIb	0.88183422	0.5329	0.7178
381832	Prpmp5	proline-rich protein MP5	0.88183422	0.5496	0.7312
99889	Arfip1	ADP-ribosylation factor interacting protein 1	0.88183422	0.5591	0.7382
75845	4930567K12Rik	RIKEN cDNA 4930567K12 gene	0.88183422	0.666	0.8118
546711	LOC546711	40S ribosomal protein S25-like	0.88183422	0.6936	0.8302
622665	Ccdc17	coiled-coil domain containing 17	0.88105727	0.01563	NA
170643	Kirrel	kin of IRRE like (Drosophila)	0.88105727	0.01661	NA
17918	Myo5a	myosin VA	0.88105727	0.02011	NA
320717	Pptc7	PTC7 protein phosphatase homolog (S. cerevisiae)	0.88105727	0.03887	NA
80976	Syt13	synaptotagmin XIII	0.88105727	0.04041	NA
381626	Rbm33	RNA binding motif protein 33	0.88105727	0.05527	NA
74467	Pus10	pseudouridylate synthase 10	0.88105727	0.05846	NA
69834	Rab43	RAB43, member RAS oncogene family	0.88105727	0.06391	NA
320840	Negr1	neuronal growth regulator 1	0.88105727	0.06715	NA
68842	Tulp4	tubby like protein 4	0.88105727	0.06805	NA
98956	Nat10	N-acetyltransferase 10	0.88105727	0.07247	NA
245886	Ankrd27	ankyrin repeat domain 27 (VPS9 domain)	0.88105727	0.09398	NA
76505	1500009C09Rik	RIKEN cDNA 1500009C09 gene	0.88105727	0.1007	NA
192196	Luc7l2	LUC7-like 2 (S. cerevisiae)	0.88105727	0.1122	0.2677
544971	Bdp1	B double prime 1, subunit of RNA polymerase III transcription initiation factor IIIB	0.88105727	0.1331	0.3
17179	Matk	megakaryocyte-associated tyrosine kinase	0.88105727	0.1528	0.3281
54194	Akap8l	A kinase (PRKA) anchor protein 8-like	0.88105727	0.1625	0.3414
68263	Pdhb	pyruvate dehydrogenase (lipoamide) beta	0.88105727	0.1703	0.3524
104662	Tsr1	TSR1, 20S rRNA accumulation, homolog (yeast)	0.88105727	0.1769	0.3618
19130	Prox1	prospero-related homeobox 1	0.88105727	0.1904	0.3793
51938	Ccdc39	coiled-coil domain containing 39	0.88105727	0.2445	0.4483
19661	Rbp3	retinol binding protein 3, interstitial	0.88105727	0.2694	0.4767
26558	Homer3	homer homolog 3 (Drosophila)	0.88105727	0.2713	0.4789
13429	Dnm1	dynamin 1	0.88105727	0.2752	0.483
11305	Abca2	ATP-binding cassette, sub-family A (ABC1), member 2	0.88105727	0.2768	0.4848
319615	Zfp944	zinc finger protein 944	0.88105727	0.284	0.4926
71950	Nanog	Nanog homeobox	0.88105727	0.309	0.5188
50915	Grb14	growth factor receptor bound protein 14	0.88105727	0.3174	0.527
77059	4931408D14Rik	RIKEN cDNA 4931408D14 gene	0.88105727	0.3813	0.5906
54216	Pcdh7	protocadherin 7	0.88105727	0.3936	0.6017
234214	Sorbs2	sorbin and SH3 domain containing 2	0.88105727	0.3979	0.6058
110094	Phka2	phosphorylase kinase alpha 2	0.88105727	0.4637	0.664
19366	Rad54l	RAD54 like (S. cerevisiae)	0.88105727	0.47	0.6692
240514	Ccdc85b	coiled-coil domain containing 85B	0.88105727	0.5083	0.6991
50701	Elane	elastase, neutrophil expressed	0.88105727	0.5605	0.7389
320333	D830030K20Rik	RIKEN cDNA D830030K20 gene	0.88105727	0.5868	0.7573
74154	Unkl	unkempt-like (Drosophila)	0.88105727	0.617	0.7796
12643	Chad	chondroadherin	0.88105727	0.7348	0.8567
105351	AW209491	expressed sequence AW209491	0.88028169	0.02731	NA
103405	4932439E07Rik	RIKEN cDNA 4932439E07 gene	0.88028169	0.02807	NA
100763	Ube3c	ubiquitin protein ligase E3C	0.88028169	0.02946	NA
227696	Phyhd1	phytanoyl-CoA dioxygenase domain containing 1	0.88028169	0.03211	NA

72635	Lins	lines homolog (Drosophila)	0.88028169	0.03699	NA
241075	Plekhm3	pleckstrin homology domain containing, family M, member 3	0.88028169	0.04434	NA
68722	1110029L17Rik	RIKEN cDNA 1110029L17 gene	0.88028169	0.04898	NA
237052	Tceal1	transcription elongation factor A (SII)-like 1	0.88028169	0.05466	NA
320713	Mysm1	myb-like, SWIRM and MPN domains 1	0.88028169	0.06841	NA
110446	Acat1	acetyl-Coenzyme A acetyltransferase 1	0.88028169	0.07399	NA
55989	Nop58	NOP58 ribonucleoprotein homolog (yeast)	0.88028169	0.07869	NA
232878	Zscan22	zinc finger and SCAN domain containing 22	0.88028169	0.08205	NA
78658	Ncapd3	non-SMC condensin II complex, subunit D3	0.88028169	0.08346	NA
76505	1500009C09Rik	RIKEN cDNA 1500009C09 gene	0.88028169	0.08743	NA
216860	Neurl4	neuralized homolog 4 (Drosophila)	0.88028169	0.09478	NA
75974	Dock11	dedicator of cytokinesis 11	0.88028169	0.1333	0.3001
268345	Kcnc2	potassium voltage gated channel, Shaw-related subfamily, member 2	0.88028169	0.1444	0.3163
234678	D230025D16Rik	RIKEN cDNA D230025D16 gene	0.88028169	0.1451	0.3173
22034	Traf6	TNF receptor-associated factor 6	0.88028169	0.1454	0.3178
72017	Cyb5r1	cytochrome b5 reductase 1	0.88028169	0.154	0.3297
241694	Ralgapa2	Ral GTPase activating protein, alpha subunit 2 (catalytic)	0.88028169	0.1586	0.3362
108071	Grm5	glutamate receptor, metabotropic 5	0.88028169	0.1601	0.3382
207792	BC034090	cDNA sequence BC034090	0.88028169	0.1716	0.3545
434768	Rhox8	reproductive homeobox 8	0.88028169	0.1953	0.3859
66930	Fank1	fibronectin type 3 and ankyrin repeat domains 1	0.88028169	0.2068	0.4015
73432	1700061N14Rik	RIKEN cDNA 1700061N14 gene	0.88028169	0.2233	0.4228
63859	Impg1	interphotoreceptor matrix proteoglycan 1	0.88028169	0.2282	0.4283
320208	Tmem91	transmembrane protein 91	0.88028169	0.2374	0.4395
99334	Zscan29	zinc finger SCAN domains 29	0.88028169	0.2707	0.4783
56631	Trim17	tripartite motif-containing 17	0.88028169	0.2787	0.4868
100038595	Gm16287	predicted gene 16287	0.88028169	0.3017	0.511
65256	Asb2	ankyrin repeat and SOCS box-containing 2	0.88028169	0.3063	0.5158
78651	Lsm6	LSM6 homolog, U6 small nuclear RNA associated (S. cerevisiae)	0.88028169	0.3079	0.5176
19016	Pparg	peroxisome proliferator activated receptor gamma	0.88028169	0.3139	0.5235
100036525	LOC100036525	hypothetical LOC100036525	0.88028169	0.3219	0.5317
676974	LOC676974	glucose-6-phosphate isomerase-like	0.88028169	0.3464	0.557
791333	Gm10050	predicted gene 10050	0.88028169	0.3472	0.5576
74330	Dnajc14	DnaJ (Hsp40) homolog, subfamily C, member 14	0.88028169	0.3498	0.5602
114714	Rad51c	RAD51 homolog c (S. cerevisiae)	0.88028169	0.3628	0.5719
58804	Cdc42ep5	CDC42 effector protein (Rho GTPase binding) 5	0.88028169	0.3769	0.5858
71291	4933435E02Rik	RIKEN cDNA 4933435E02 gene	0.88028169	0.4281	0.6326
207212	Arhgef17	Rho guanine nucleotide exchange factor (GEF) 17	0.87950748	0.01945	NA
72584	Cul4b	cullin 4B	0.87950748	0.02807	NA
17763	Mtcp1	mature T-cell proliferation 1	0.87950748	0.03753	NA
22033	Traf5	TNF receptor-associated factor 5	0.87950748	0.06708	NA
68044	Chac2	ChaC, cation transport regulator homolog 2 (E. coli)	0.87950748	0.08124	NA
245902	Ccdc15	coiled-coil domain containing 15	0.87950748	0.1013	0.2508
58194	Sh3kbp1	SH3-domain kinase binding protein 1	0.87950748	0.1016	0.2511
74166	Tmem38a	transmembrane protein 38A	0.87950748	0.1241	0.2868
70551	Tmtc4	transmembrane and tetratricopeptide repeat containing 4	0.87950748	0.125	0.2881
381067	Zfp229	zinc finger protein	0.87950748	0.1283	0.2933
229503	BC023814	cDNA sequence BC023814	0.87950748	0.1323	0.2988
56469	Pias1	protein inhibitor of activated STAT 1	0.87950748	0.1438	0.3154
59008	Anapc5	anaphase-promoting complex subunit 5	0.87950748	0.1562	0.3331

68808	1110046J04Rik	RIKEN cDNA 1110046J04 gene	0.87950748	0.1836	0.3708
383563	Gpr25	G protein-coupled receptor 25	0.87950748	0.2116	0.4074
11941	Atp2b2	ATPase, Ca++ transporting, plasma membrane 2	0.87950748	0.2286	0.4289
74322	Cxxc1	CXXC finger 1 (PHD domain)	0.87950748	0.2725	0.4799
234267	Gpm6a	glycoprotein m6a	0.87950748	0.3185	0.5281
242607	Slc1a7	solute carrier family 1 (glutamate transporter), member 7	0.87950748	0.3793	0.5884
78876	B230214N19Rik	RIKEN cDNA B230214N19 gene	0.87950748	0.3933	0.6016
70363	Fam135b	family with sequence similarity 135, member B	0.87950748	0.4079	0.6144
11428	Aco1	aconitase 1	0.87950748	0.4292	0.6336
83492	Gsdmc	gasdermin C	0.87950748	0.525	0.7117
56706	Ccnl1	cyclin L1	0.87950748	0.5439	0.7261
270163	Myo9a	myosin IXa	0.87950748	0.575	0.7489
216011	Lrrc20	leucine rich repeat containing 20	0.87873462	0.02134	NA
75612	Gns	glucosamine (N-acetyl)-6-sulfatase	0.87873462	0.02668	NA
330064	Slc5a6	solute carrier family 5 (sodium-dependent vitamin transporter), member 6	0.87873462	0.03412	NA
107477	Guca1b	guanylate cyclase activator 1B	0.87873462	0.03434	NA
83946	Phip	pleckstrin homology domain interacting protein	0.87873462	0.0359	NA
107566	Arl2bp	ADP-ribosylation factor-like 2 binding protein	0.87873462	0.03994	NA
22770	Zhx1	zinc fingers and homeoboxes 1	0.87873462	0.04091	NA
72545	2700008G24Rik	RIKEN cDNA 2700008G24 gene	0.87873462	0.04651	NA
18760	Prkd1	protein kinase D1	0.87873462	0.05433	NA
238673	Zfp367	zinc finger protein 367	0.87873462	0.05612	NA
230376	Haus6	HAUS augmin-like complex, subunit 6	0.87873462	0.06497	NA
108699	Chn1	chimerin (chimaerin) 1	0.87873462	0.06894	NA
68477	Rmnd5a	required for meiotic nuclear division 5 homolog A (S. cerevisiae)	0.87873462	0.07124	NA
14105	Srsf10	serine/arginine-rich splicing factor 10	0.87873462	0.0717	NA
209586	Nudcd3	NudC domain containing 3	0.87873462	0.08291	NA
66559	Metap1d	methionyl aminopeptidase type 1D (mitochondrial)	0.87873462	0.0945	NA
16319	Incenp	inner centromere protein	0.87873462	0.1045	0.2555
56216	Stx1b	syntaxin 1B	0.87873462	0.1099	0.2639
70461	Crtc3	CREB regulated transcription coactivator 3	0.87873462	0.1227	0.2847
20661	Sort1	sortilin 1	0.87873462	0.1244	0.2871
70603	Mutyh	mutY homolog (E. coli)	0.87873462	0.1672	0.3481
66447	Mgst3	microsomal glutathione S-transferase 3	0.87873462	0.1674	0.3482
12794	Cnih2	cornichon homolog 2 (Drosophila)	0.87873462	0.2072	0.4019
11994	Pcdh15	protocadherin 15	0.87873462	0.2257	0.4256
77093	A330094K24Rik	RIKEN cDNA A330094K24 gene	0.87873462	0.2262	0.426
19338	Rab33b	RAB33B, member of RAS oncogene family	0.87873462	0.2267	0.4265
12315	Calm3	calmodulin 3	0.87873462	0.2447	0.4484
100042497	Gm3871	predicted gene 3871	0.87873462	0.2475	0.4517
74648	S100pbp	S100P binding protein	0.87873462	0.3	0.5093
20222	Sf3a2	splicing factor 3a, subunit 2	0.87873462	0.3146	0.524
69952	2810011L19Rik	RIKEN cDNA 2810011L19 gene	0.87873462	0.3277	0.5377
11733	Ank1	ankyrin 1, erythroid	0.87873462	0.3322	0.5423
66065	Hsd17b14	hydroxysteroid (17-beta) dehydrogenase 14	0.87873462	0.3335	0.544
226255	Atrnl1	attractin like 1	0.87873462	0.3448	0.5554
27059	Sh3d19	SH3 domain protein D19	0.87873462	0.4218	0.6278
69655	Cd164l2	CD164 sialomucin-like 2	0.87873462	0.4228	0.6283
22059	Trp53	transformation related protein 53	0.87873462	0.4239	0.6294
21885	Tle1	transducin-like enhancer of split 1, homolog of Drosophila E(spl)	0.87873462	0.4282	0.6327

66646	Rpe	ribulose-5-phosphate-3-epimerase	0.87873462	0.4298	0.634
239673	4732456N10Rik	RIKEN cDNA 4732456N10 gene	0.87873462	0.4786	0.6753
234797	6430548M08Rik	RIKEN cDNA 6430548M08 gene	0.87873462	0.5524	0.7331
620949	Dph3b-ps	DPH3B, KTI11 homolog B (S. cerevisiae), pseudogene	0.87873462	0.6656	0.8117
319845	Bbs9	Bardet-Biedl syndrome 9 (human)	0.87796313	0.005498	NA
227693	Zer1	zer-1 homolog (C. elegans)	0.87796313	0.01737	NA
68364	0610030E20Rik	RIKEN cDNA 0610030E20 gene	0.87796313	0.01774	NA
223669	Zfp7	zinc finger protein 7	0.87796313	0.02473	NA
230233	Ikbkap	inhibitor of kappa light polypeptide enhancer in B-cells, kinase complex-associated protein	0.87796313	0.02479	NA
269424	Phf17	PHD finger protein 17	0.87796313	0.03249	NA
64113	Moap1	modulator of apoptosis 1	0.87796313	0.03252	NA
14149	Fdxr	ferredoxin reductase	0.87796313	0.03567	NA
17978	Ncoa2	nuclear receptor coactivator 2	0.87796313	0.06236	NA
545859	Vax2os2	Vax2 opposite strand transcript 2	0.87796313	0.07042	NA
69617	Pitrm1	pitrilysin metallepetidase 1	0.87796313	0.07605	NA
215085	Slc35f1	solute carrier family 35, member F1	0.87796313	0.0773	NA
71027	Tmem30c	transmembrane protein 30C	0.87796313	0.07738	NA
58226	Cacna1h	calcium channel, voltage-dependent, T type, alpha 1H subunit	0.87796313	0.07779	NA
59026	Huwe1	HECT, UBA and WWE domain containing 1	0.87796313	0.08095	NA
57295	Icmt	isoprenylcysteine carboxyl methyltransferase	0.87796313	0.08557	NA
239554	Foxred2	FAD-dependent oxidoreductase domain containing 2	0.87796313	0.0864	NA
234725	Zfp612	zinc finger protein 612	0.87796313	0.09058	NA
56089	Ramp3	receptor (calcitonin) activity modifying protein 3	0.87796313	0.09131	NA
101214	Tra2a	transformer 2 alpha homolog (Drosophila)	0.87796313	0.09457	0.2395
12380	Cast	calpastatin	0.87796313	0.1054	0.2568
56490	Zbtb20	zinc finger and BTB domain containing 20	0.87796313	0.1159	0.2734
13207	Ddx5	DEAD (Asp-Glu-Ala-Asp) box polypeptide 5	0.87796313	0.1448	0.3169
269209	Stk36	serine/threonine kinase 36 (fused homolog, Drosophila)	0.87796313	0.1563	0.3331
237759	Col23a1	collagen, type XXIII, alpha 1	0.87796313	0.1595	0.3376
20747	Spop	speckle-type POZ protein	0.87796313	0.1701	0.3522
100710	Pds5b	PDS5, regulator of cohesion maintenance, homolog B (S. cerevisiae)	0.87796313	0.1882	0.3764
54524	Syt6	synaptotagmin VI	0.87796313	0.2085	0.4034
226841	Vash2	vasohibin 2	0.87796313	0.2501	0.4547
56515	Rnf138	ring finger protein 138	0.87796313	0.2617	0.4675
192786	Rapgef6	Rap guanine nucleotide exchange factor (GEF) 6	0.87796313	0.2636	0.4697
268902	Robo2	roundabout homolog 2 (Drosophila)	0.87796313	0.2667	0.4736
259302	Srgap3	SLIT-ROBO Rho GTPase activating protein 3	0.87796313	0.3122	0.5219
432989	D930007P13Rik	Riken cDNA D930007P13 gene	0.87796313	0.3163	0.5257
72795	Ttc19	tetratricopeptide repeat domain 19	0.87796313	0.3521	0.5622
103012	6720401G13Rik	RIKEN cDNA 6720401G13 gene	0.87796313	0.3737	0.5824
13824	Epb4.1l4a	erythrocyte protein band 4.1-like 4a	0.87796313	0.419	0.6253
244059	Chd2	chromodomain helicase DNA binding protein 2	0.87796313	0.4253	0.6306
72349	Dusp3	dual specificity phosphatase 3 (vaccinia virus phosphatase VH1-related)	0.87796313	0.4558	0.6573
319211	Nol4	nucleolar protein 4	0.87796313	0.4998	0.6922
98758	Hnrnpf	heterogeneous nuclear ribonucleoprotein F	0.87796313	0.5471	0.7289
100041799	Gm3515	predicted gene 3515	0.87796313	0.7461	0.8632
74252	Armc1	armadillo repeat containing 1	0.87719298	0.009172	NA
233040	Fbxo27	F-box protein 27	0.87719298	0.03364	NA
207304	Hectd1	HECT domain containing 1	0.87719298	0.0447	NA
225027	Srsf7	serine/arginine-rich splicing factor 7	0.87719298	0.06832	NA

22648	Zfp11	zinc finger protein 11	0.87719298	0.0724	NA
11899	Astn1	astrotactin 1	0.87719298	0.07393	NA
24086	Tlk2	tousled-like kinase 2 (Arabidopsis)	0.87719298	0.08244	NA
213452	Dstyk	dual serine/threonine and tyrosine protein kinase	0.87719298	0.1054	0.2568
21374	Tbp	TATA box binding protein	0.87719298	0.1058	0.2573
16876	Lhx9	LIM homeobox protein 9	0.87719298	0.116	0.2735
17995	Ndufv1	NADH dehydrogenase (ubiquinone) flavoprotein 1	0.87719298	0.1263	0.2899
72960	Top1mt	DNA topoisomerase 1, mitochondrial	0.87719298	0.1409	0.3108
74551	Pck2	phosphoenolpyruvate carboxykinase 2 (mitochondrial)	0.87719298	0.1485	0.3222
224836	Usp49	ubiquitin specific peptidase 49	0.87719298	0.1516	0.3264
64051	Sv2a	synaptic vesicle glycoprotein 2 a	0.87719298	0.1694	0.3508
66801	Prkrip1	Prkr interacting protein 1 (IL11 inducible)	0.87719298	0.1839	0.3709
106039	Gga1	golgi associated, gamma adaptin ear containing, ARF binding protein 1	0.87719298	0.2006	0.3928
121021	Cspg4	chondroitin sulfate proteoglycan 4	0.87719298	0.2044	0.3981
20964	Syn1	synapsin I	0.87719298	0.2314	0.4323
75302	Asxl2	additional sex combs like 2 (Drosophila)	0.87719298	0.3893	0.5975
54003	Nell2	NEL-like 2 (chicken)	0.87719298	0.4093	0.6156
71840	Tekt4	tektin 4	0.87719298	0.4166	0.6227
71828	Gtf2a1l	general transcription factor IIA, 1-like	0.87719298	0.4252	0.6305
67540	4931428A05Rik	RIKEN cDNA 4931428A05 gene	0.87719298	0.4367	0.6401
11814	Apoc3	apolipoprotein C-III	0.87719298	0.6329	0.7915
252876	Gin1	gypsy retrotransposon integrase 1	0.87719298	0.6472	0.8011
18768	Pkib	protein kinase inhibitor beta, cAMP dependent, testis specific	0.87719298	0.6964	0.8324
227648	Sec16a	SEC16 homolog A (S. cerevisiae)	0.87642419	0.00723	NA
66425	Pcp4l1	Purkinje cell protein 4-like 1	0.87642419	0.007434	NA
224020	Pi4ka	phosphatidylinositol 4-kinase, catalytic, alpha polypeptide	0.87642419	0.01658	NA
13716	Ell	elongation factor RNA polymerase II	0.87642419	0.0291	NA
68164	A430106A12Rik	RIKEN cDNA A430106A12 gene	0.87642419	0.03297	NA
67072	Cdc37l1	cell division cycle 37 homolog (S. cerevisiae)-like 1	0.87642419	0.04598	NA
13000	Csnk2a2	casein kinase 2, alpha prime polypeptide	0.87642419	0.07131	NA
114641	Rpl31	ribosomal protein L31	0.87642419	0.07193	NA
360213	Trim46	tripartite motif-containing 46	0.87642419	0.0832	NA
18457	Pldn	pallidin	0.87642419	0.0873	NA
68177	Ebpl	emopamil binding protein-like	0.87642419	0.09323	0.2371
235033	Rdh8	retinol dehydrogenase 8	0.87642419	0.09628	0.2422
269630	5031425E22Rik	RIKEN cDNA 5031425E22 gene	0.87642419	0.1013	0.2509
60364	Donson	downstream neighbor of SON	0.87642419	0.1036	0.2542
22036	Traip	TRAF-interacting protein	0.87642419	0.104	0.2547
320747	Lingo4	leucine rich repeat and Ig domain containing 4	0.87642419	0.1128	0.2687
21335	Tacc3	transforming, acidic coiled-coil containing protein 3	0.87642419	0.1264	0.2901
170729	Scrt1	scratch homolog 1, zinc finger protein (Drosophila)	0.87642419	0.1295	0.295
54217	Rpl36	ribosomal protein L36	0.87642419	0.1406	0.3104
28105	Trim36	tripartite motif-containing 36	0.87642419	0.1664	0.3471
21944	Tnfsf12	tumor necrosis factor (ligand) superfamily, member 12	0.87642419	0.1859	0.3733
244059	Chd2	chromodomain helicase DNA binding protein 2	0.87642419	0.2127	0.4086
74189	Phactr3	phosphatase and actin regulator 3	0.87642419	0.2171	0.4147
242669	Adc	arginine decarboxylase	0.87642419	0.2415	0.4442
17761	Mtap7	microtubule-associated protein 7	0.87642419	0.2501	0.4547
64602	Ireb2	iron responsive element binding protein 2	0.87642419	0.3211	0.5311
14186	Fgfr4	fibroblast growth factor receptor 4	0.87642419	0.357	0.567

13557	E2f3	E2F transcription factor 3	0.87642419	0.3784	0.5873
100505153	LOC100505153	heterogeneous nuclear ribonucleoprotein A3-like	0.87642419	0.4277	0.6322
546849	Gm13178	predicted gene 13178	0.87642419	0.4663	0.6661
14618	Gjb1	gap junction protein, beta 1	0.87642419	0.4789	0.6755
264064	Cdk8	cyclin-dependent kinase 8	0.87642419	0.4887	0.6834
15132	Hbb-bh1	hemoglobin Z, beta-like embryonic chain	0.87642419	0.657	0.8063
73712	Dmkn	dermokine	0.87642419	0.6578	0.8066
54378	Cacng6	calcium channel, voltage-dependent, gamma subunit 6	0.87642419	0.6643	0.8111
319965	Cc2d1b	coiled-coil and C2 domain containing 1B	0.87565674	0.008711	NA
75089	Uhrf1bp1l	UHRF1 (ICBP90) binding protein 1-like	0.87565674	0.009386	NA
320150	Zdhhc17	zinc finger, DHHC domain containing 17	0.87565674	0.012	NA
68404	Nrn1	neuritin 1	0.87565674	0.02313	NA
67384	Bag4	BCL2-associated athanogene 4	0.87565674	0.02434	NA
207165	Bptf	bromodomain PHD finger transcription factor	0.87565674	0.03457	NA
106205	Zc3h7a	zinc finger CCCH type containing 7 A	0.87565674	0.03662	NA
83922	Tsga14	testis specific gene A14	0.87565674	0.04377	NA
212448	9330159F19Rik	RIKEN cDNA 9330159F19 gene	0.87565674	0.04638	NA
18597	Pdha1	pyruvate dehydrogenase E1 alpha 1	0.87565674	0.04782	NA
67772	Chd8	chromodomain helicase DNA binding protein 8	0.87565674	0.05364	NA
110876	Scn2a1	sodium channel, voltage-gated, type II, alpha 1	0.87565674	0.05516	NA
19302	Pxmp3	peroxisomal membrane protein 3	0.87565674	0.06126	NA
192198	Lrrc4	leucine rich repeat containing 4	0.87565674	0.06227	NA
77110	Gpbp1l1	GC-rich promoter binding protein 1-like 1	0.87565674	0.06752	NA
319807	3110047P20Rik	RIKEN cDNA 3110047P20 gene	0.87565674	0.07347	NA
69876	Thap3	THAP domain containing, apoptosis associated protein 3	0.87565674	0.08665	0.226
114642	Brdt	bromodomain, testis-specific	0.87565674	0.1015	0.2511
77771	Csrnp3	cysteine-serine-rich nuclear protein 3	0.87565674	0.1275	0.292
20530	Slc31a2	solute carrier family 31, member 2	0.87565674	0.1473	0.3203
26398	Map2k4	mitogen-activated protein kinase kinase 4	0.87565674	0.1609	0.3391
110094	Phka2	phosphorylase kinase alpha 2	0.87565674	0.1838	0.3709
67149	Nkain1	Na+/K+ transporting ATPase interacting 1	0.87565674	0.188	0.3762
238271	Kcnh5	potassium voltage-gated channel, subfamily H (eag-related), member 5	0.87565674	0.1961	0.3869
76972	2810008D09Rik	RIKEN cDNA 2810008D09 gene	0.87565674	0.234	0.4356
11488	Adam11	a disintegrin and metallopeptidase domain 11	0.87565674	0.248	0.4522
271377	Zbtb11	zinc finger and BTB domain containing 11	0.87565674	0.2546	0.4596
269784	Cntn4	contactin 4	0.87565674	0.2552	0.4602
100033459	Pydc3	pyrin domain containing 3	0.87565674	0.26	0.4657
71932	Ephx3	epoxide hydrolase 3	0.87565674	0.289	0.4975
56194	Prpf40a	PRP40 pre-mRNA processing factor 40 homolog A (yeast)	0.87565674	0.2988	0.5082
70290	2310079F09Rik	RIKEN cDNA 2310079F09 gene	0.87565674	0.3141	0.5236
18572	Pdcd11	programmed cell death 11	0.87565674	0.3247	0.5345
67311	Nanp	N-acetylneuraminic acid phosphatase	0.87565674	0.3293	0.5395
384724	Cyp2t4	cytochrome P450, family 2, subfamily t, polypeptide 4	0.87565674	0.3429	0.5538
74035	Nol9	nucleolar protein 9	0.87565674	0.4497	0.6519
78428	Wibg	within bgcn homolog (Drosophila)	0.87565674	0.4912	0.6852
237782	Smcr8	Smith-Magenis syndrome chromosome region, candidate 8 homolog (human)	0.87565674	0.506	0.697
14527	Gcgr	glucagon receptor	0.87565674	0.5438	0.7261
269536	Tex10	testis expressed gene 10	0.87489064	0.01455	NA
228356	1110051M20Rik	RIKEN cDNA 1110051M20 gene	0.87489064	0.01731	NA
72149	Strada	STE20-related kinase adaptor alpha	0.87489064	0.01802	NA

66540	Fam107b	family with sequence similarity 107, member B	0.87489064	0.02035	NA
70317	Arl16	ADP-ribosylation factor-like 16	0.87489064	0.04735	NA
244548	Elmod2	ELMO domain containing 2	0.87489064	0.04779	NA
56403	Syncrip	synaptotagmin binding, cytoplasmic RNA interacting protein	0.87489064	0.05682	NA
12313	Calm1	calmodulin 1	0.87489064	0.05743	NA
70757	Ptplb	protein tyrosine phosphatase-like (proline instead of catalytic arginine), member b	0.87489064	0.06755	NA
79555	BC005537	cDNA sequence BC005537	0.87489064	0.0685	NA
52653	Nudcd2	NudC domain containing 2	0.87489064	0.06935	NA
29808	Mga	MAX gene associated	0.87489064	0.07477	NA
69116	Ubr4	ubiquitin protein ligase E3 component n-recognin 4	0.87489064	0.08373	0.2209
606497	Otx2os1	Otx2 opposite strand transcript 1	0.87489064	0.08713	0.2269
66262	Ing5	inhibitor of growth family, member 5	0.87489064	0.09383	0.2381
107448	Unc5a	unc-5 homolog A (C. elegans)	0.87489064	0.111	0.2657
72168	Aifm3	apoptosis-inducing factor, mitochondrion-associated 3	0.87489064	0.1362	0.3036
56220	Zfp386	zinc finger protein 386 (Kruppel-like)	0.87489064	0.1573	0.3344
280621	BC089491	cDNA sequence BC089491	0.87489064	0.179	0.3643
68020	2810002N01Rik	RIKEN cDNA 2810002N01 gene	0.87489064	0.1818	0.3683
233529	Kctd14	potassium channel tetramerisation domain containing 14	0.87489064	0.193	0.383
60525	Acss2	acyl-CoA synthetase short-chain family member 2	0.87489064	0.1973	0.3885
66388	Cutc	cutC copper transporter homolog (E.coli)	0.87489064	0.2029	0.396
66849	Ppp1r2	protein phosphatase 1, regulatory (inhibitor) subunit 2	0.87489064	0.212	0.4078
12953	Cry2	cryptochrome 2 (photolyase-like)	0.87489064	0.2297	0.4304
15159	Hccs	holocytochrome c synthetase	0.87489064	0.2321	0.4331
80913	Pum2	pumilio 2 (Drosophila)	0.87489064	0.2549	0.4599
68243	A930018P22Rik	RIKEN cDNA A930018P22 gene	0.87489064	0.2696	0.4769
100090	Zbtb48	zinc finger and BTB domain containing 48	0.87489064	0.2724	0.4799
243910	Nfkbid	nuclear factor of kappa light polypeptide gene enhancer in B-cells inhibitor, delta	0.87489064	0.2765	0.4845
16372	Irx2	Iroquois related homeobox 2 (Drosophila)	0.87489064	0.288	0.4966
22355	Vipr2	vasoactive intestinal peptide receptor 2	0.87489064	0.3376	0.5478
84004	Mcam	melanoma cell adhesion molecule	0.87489064	0.3498	0.5602
232816	Zfp628	zinc finger protein 628	0.87489064	0.395	0.6029
242409	Tmem8b	transmembrane protein 8B	0.87489064	0.4377	0.6409
27029	Sgsh	N-sulfoglucosamine sulfohydrolase (sulfamidase)	0.87489064	0.4614	0.6619
320506	Lmbrd2	LMBR1 domain containing 2	0.87489064	0.4646	0.6648
110651	Rps6ka3	ribosomal protein S6 kinase polypeptide 3	0.87489064	0.4948	0.6883
13490	Drd3	dopamine receptor D3	0.87489064	0.504	0.6955
18263	Odc1	ornithine decarboxylase, structural 1	0.87489064	0.519	0.7084
668917	LOC668917	hypothetical LOC668917	0.87489064	0.6258	0.7862
64453	Zfp280b	zinc finger protein 280B	0.87412587	0.01967	NA
67298	Gprasp1	G protein-coupled receptor associated sorting protein 1	0.87412587	0.02342	NA
67230	Zfp329	zinc finger protein 329	0.87412587	0.02452	NA
76890	Memo1	mediator of cell motility 1	0.87412587	0.03003	NA
18472	Pafah1b1	platelet-activating factor acetylhydrolase, isoform 1b, subunit 1	0.87412587	0.04554	NA
105853	Mal2	mal, T-cell differentiation protein 2	0.87412587	0.049	NA
207278	Fchsd2	FCH and double SH3 domains 2	0.87412587	0.05722	NA
12296	Cacnb2	calcium channel, voltage-dependent, beta 2 subunit	0.87412587	0.08584	0.2246
17714	Grpel2	GrpE-like 2, mitochondrial	0.87412587	0.09653	0.2426
71745	Cul2	cullin 2	0.87412587	0.1022	0.2521
66686	Dcbld1	discoidin, CUB and LCCL domain containing 1	0.87412587	0.1101	0.2642
19335	Rab23	RAB23, member RAS oncogene family	0.87412587	0.116	0.2735

227699	Nup188	nucleoporin 188	0.87412587	0.1195	0.2797
14348	Fut9	fucosyltransferase 9	0.87412587	0.1835	0.3708
13830	Stom	stomatin	0.87412587	0.1838	0.3709
17156	Man1a2	mannosidase, alpha, class 1A, member 2	0.87412587	0.1961	0.3869
381695	N4bp2l2	NEDD4 binding protein 2-like 2	0.87412587	0.1977	0.3889
17122	Mxd4	Max dimerization protein 4	0.87412587	0.2186	0.4169
211347	Pank3	pantothenate kinase 3	0.87412587	0.2402	0.4427
93876	Pcdhb5	protocadherin beta 5	0.87412587	0.2412	0.4438
52389	Gpr123	G protein-coupled receptor 123	0.87412587	0.2565	0.4617
330812	Rnf150	ring finger protein 150	0.87412587	0.2779	0.4861
20618	Sncg	synuclein, gamma	0.87412587	0.2893	0.4976
109032	Sp110	Sp110 nuclear body protein	0.87412587	0.331	0.5414
14810	Grin1	glutamate receptor, ionotropic, NMDA1 (zeta 1)	0.87412587	0.4403	0.6432
215335	Slc36a1	solute carrier family 36 (proton/amino acid symporter), member 1	0.87412587	0.4694	0.6687
72767	2810427A07Rik	RIKEN cDNA 2810427A07 gene	0.87412587	0.5151	0.705
100043580	Gm4532	predicted gene 4532	0.87412587	0.6433	0.7982
56045	Samhd1	SAM domain and HD domain, 1	0.87336245	0.008683	NA
228005	Ppig	peptidyl-prolyl isomerase G (cyclophilin G)	0.87336245	0.01645	NA
19386	Ranbp2	RAN binding protein 2	0.87336245	0.01778	NA
381626	Rbm33	RNA binding motif protein 33	0.87336245	0.02095	NA
195018	Zzef1	zinc finger, ZZ-type with EF hand domain 1	0.87336245	0.02235	NA
108058	Camk2d	calcium/calmodulin-dependent protein kinase II, delta	0.87336245	0.02304	NA
207565	Camkk2	calcium/calmodulin-dependent protein kinase kinase 2, beta	0.87336245	0.03292	NA
69276	Sec62	SEC62 homolog (S. cerevisiae)	0.87336245	0.0339	NA
66661	Srp72	signal recognition particle 72	0.87336245	0.03737	NA
69046	Isca1	iron-sulfur cluster assembly 1 homolog (S. cerevisiae)	0.87336245	0.06451	NA
102941	B630019K06Rik	RIKEN cDNA B630019K06 gene	0.87336245	0.06807	NA
68512	Tomm5	translocase of outer mitochondrial membrane 5 homolog (yeast)	0.87336245	0.06844	NA
23996	Psmc4	proteasome (prosome, macropain) 26S subunit, ATPase, 4	0.87336245	0.07369	NA
103012	6720401G13Rik	RIKEN cDNA 6720401G13 gene	0.87336245	0.07565	NA
54610	Tbc1d8	TBC1 domain family, member 8	0.87336245	0.08864	0.2293
11931	Atp1b1	ATPase, Na+/K+ transporting, beta 1 polypeptide	0.87336245	0.09028	0.2321
59040	Rhot1	ras homolog gene family, member T1	0.87336245	0.1104	0.2647
74105	Gga2	golgi associated, gamma adaptin ear containing, ARF binding protein 2	0.87336245	0.1111	0.2658
16531	Kcnma1	potassium large conductance calcium-activated channel, subfamily M, alpha member 1	0.87336245	0.1196	0.2797
20841	Zfp143	zinc finger protein 143	0.87336245	0.1309	0.2969
11418	Accn1	amiloride-sensitive cation channel 1, neuronal (degenerin)	0.87336245	0.1323	0.2988
17161	Maoa	monoamine oxidase A	0.87336245	0.1359	0.3033
22364	Vpreb3	pre-B lymphocyte gene 3	0.87336245	0.1511	0.3258
57434	Xrcc2	X-ray repair complementing defective repair in Chinese hamster cells 2	0.87336245	0.1517	0.3265
380856	AA987161	expressed sequence AA987161	0.87336245	0.1879	0.3761
319934	Sbf2	SET binding factor 2	0.87336245	0.2038	0.3974
54607	Socs6	suppressor of cytokine signaling 6	0.87336245	0.2438	0.4475
243872	Rpl7a-ps8	ribosomal protein L7A, pseudogene 8	0.87336245	0.2599	0.4656
100045653	LOC100045653	hypothetical LOC100045653	0.87336245	0.3294	0.5395
20602	Ncor2	nuclear receptor co-repressor 2	0.87336245	0.3681	0.5771
20912	Stxbp3a	syntaxin binding protein 3A	0.87336245	0.5391	0.7227
108989	Tpr	translocated promoter region	0.87336245	0.5456	0.7276
67713	Dnajc19	DnaJ (Hsp40) homolog, subfamily C, member 19	0.87336245	0.5536	0.7339
211586	Tfdp2	transcription factor Dp 2	0.87260035	0.01222	NA

70297	Gcc2	GRIP and coiled-coil domain containing 2	0.87260035	0.03193	NA
70974	Pgm2l1	phosphoglucomutase 2-like 1	0.87260035	0.03255	NA
214580	Pstk	phosphoseryl-tRNA kinase	0.87260035	0.03293	NA
76630	Stambpl1	STAM binding protein like 1	0.87260035	0.03921	NA
56079	Astn2	astrotactin 2	0.87260035	0.0475	NA
16882	Lig3	ligase III, DNA, ATP-dependent	0.87260035	0.05376	NA
100043489	1300002E11Rik	RIKEN cDNA 1300002E11 gene	0.87260035	0.06308	NA
71448	Tmem80	transmembrane protein 80	0.87260035	0.06599	NA
329274	Fam163a	family with sequence similarity 163, member A	0.87260035	0.07453	NA
244585	Rpgrip1l	Rpgrip1-like	0.87260035	0.07974	0.214
110829	Lims1	LIM and senescent cell antigen-like domains 1	0.87260035	0.1084	0.2614
18021	Nfatc3	nuclear factor of activated T-cells, cytoplasmic, calcineurin-dependent 3	0.87260035	0.1111	0.2658
12447	Ccne1	cyclin E1	0.87260035	0.1302	0.2959
100502723	Tsga8	testis specific gene A8	0.87260035	0.1415	0.3119
108058	Camk2d	calcium/calmodulin-dependent protein kinase II, delta	0.87260035	0.191	0.3803
17710	COX3	cytochrome c oxidase subunit III	0.87260035	0.1915	0.3811
319477	6030419C18Rik	RIKEN cDNA 6030419C18 gene	0.87260035	0.2137	0.4101
228598	Ebf4	early B-cell factor 4	0.87260035	0.2205	0.4196
20492	Slbp	stem-loop binding protein	0.87260035	0.2233	0.4228
227210	Ccnyl1	cyclin Y-like 1	0.87260035	0.2407	0.4433
12532	Cdc25c	cell division cycle 25 homolog C (S. pombe)	0.87260035	0.248	0.4521
106585	Ankrd12	ankyrin repeat domain 12	0.87260035	0.276	0.4841
56198	Heyl	hairy/enhancer-of-split related with YRPW motif-like	0.87260035	0.2987	0.5082
218756	Slc4a7	solute carrier family 4, sodium bicarbonate cotransporter, member 7	0.87260035	0.3047	0.5138
22764	Zfx	zinc finger protein X-linked	0.87260035	0.3102	0.52
100042202	Gm3718	predicted gene 3718	0.87260035	0.3275	0.5376
19326	Rab11b	RAB11B, member RAS oncogene family	0.87260035	0.3625	0.5716
22141	Tub	tubby candidate gene	0.87260035	0.3955	0.6035
226518	Nmnat2	nicotinamide nucleotide adenylyltransferase 2	0.87260035	0.4161	0.6222
381914	B230311B06Rik	RIKEN cDNA B230311B06 gene	0.87260035	0.4161	0.6222
27962	D9Wsu90e	DNA segment, Chr 9, Wayne State University 90, expressed	0.87260035	0.5035	0.695
140743	Rem2	rad and gem related GTP binding protein 2	0.87260035	0.5394	0.7229
237886	Slfn9	schlafen 9	0.87260035	0.5844	0.7553
100177	Zmym6	zinc finger, MYM-type 6	0.87183958	0.008141	NA
16572	Kif5a	kinesin family member 5A	0.87183958	0.02041	NA
83679	Pde4dip	phosphodiesterase 4D interacting protein (myomegalin)	0.87183958	0.02407	NA
17765	Mtf2	metal response element binding transcription factor 2	0.87183958	0.02514	NA
69191	Pdia2	protein disulfide isomerase associated 2	0.87183958	0.02699	NA
170677	Cdhr1	cadherin-related family member 1	0.87183958	0.03286	NA
72607	Usp13	ubiquitin specific peptidase 13 (isopeptidase T-3)	0.87183958	0.03698	NA
101563	AI426330	expressed sequence AI426330	0.87183958	0.03945	NA
245944	Vps54	vacuolar protein sorting 54 (yeast)	0.87183958	0.05295	NA
24057	Sh3yl1	Sh3 domain YSC-like 1	0.87183958	0.06374	NA
68040	Zfp593	zinc finger protein 593	0.87183958	0.06565	NA
67530	Uqcrb	ubiquinol-cytochrome c reductase binding protein	0.87183958	0.07448	0.2041
94281	Sfxn4	sideroflexin 4	0.87183958	0.08746	0.2274
227526	Cdnf	cerebral dopamine neurotrophic factor	0.87183958	0.08746	0.2274
67379	Dedd2	death effector domain-containing DNA binding protein 2	0.87183958	0.09276	0.2364
16012	Igfbp6	insulin-like growth factor binding protein 6	0.87183958	0.1019	0.2517
628431	Hmgb1-rs17	high mobility group box 1, related sequence 17	0.87183958	0.1152	0.2725

109904	Mcf2	mcf.2 transforming sequence	0.87183958	0.1856	0.3731
74734	Rhoh	ras homolog gene family, member H	0.87183958	0.1939	0.384
18108	Nmt2	N-myristoyltransferase 2	0.87183958	0.1969	0.3881
117150	Pip4k2c	phosphatidylinositol-5-phosphate 4-kinase, type II, gamma	0.87183958	0.2067	0.4013
243548	Prickle2	prickle homolog 2 (Drosophila)	0.87183958	0.2075	0.4023
21834	Thrb	thyroid hormone receptor beta	0.87183958	0.2242	0.424
22327	Vbp1	von Hippel-Lindau binding protein 1	0.87183958	0.2733	0.4809
27059	Sh3d19	SH3 domain protein D19	0.87183958	0.2872	0.4957
76156	Fam131b	family with sequence similarity 131, member B	0.87183958	0.3122	0.5218
77480	Kidins220	kinase D-interacting substrate 220	0.87183958	0.3408	0.5513
12351	Car4	carbonic anhydrase 4	0.87183958	0.3526	0.5627
20539	Slc7a5	solute carrier family 7 (cationic amino acid transporter, y+ system), member 5	0.87183958	0.3827	0.5918
21942	Tnfrsf9	tumor necrosis factor receptor superfamily, member 9	0.87183958	0.4794	0.6758
107476	Acaca	acetyl-Coenzyme A carboxylase alpha	0.87183958	0.5172	0.7066
16353	Ipw	imprinted gene in the Prader-Willi syndrome region	0.87183958	0.5176	0.707
78790	4930414F18Rik	RIKEN cDNA 4930414F18 gene	0.87183958	0.6568	0.8063
14417	Gad2	glutamic acid decarboxylase 2	0.87108014	0.008513	NA
59288	Dctn5	dynactin 5	0.87108014	0.01099	NA
432467	Hnrnph3	heterogeneous nuclear ribonucleoprotein H3	0.87108014	0.01579	NA
21812	Tgfbr1	transforming growth factor, beta receptor I	0.87108014	0.01809	NA
18949	Pnn	pinin	0.87108014	0.02798	NA
56177	Olfm1	olfactomedin 1	0.87108014	0.04266	NA
20016	Polr1c	polymerase (RNA) I polypeptide C	0.87108014	0.04782	NA
104662	Tsr1	TSR1, 20S rRNA accumulation, homolog (yeast)	0.87108014	0.05184	NA
20586	Smarca4	SWI/SNF related, matrix associated, actin dependent regulator of chromatin, subfamily a, member 4	0.87108014	0.05722	NA
114565	Zfp295	zinc finger protein 295	0.87108014	0.05883	NA
320311	Rnf152	ring finger protein 152	0.87108014	0.06035	NA
223601	Fam49b	family with sequence similarity 49, member B	0.87108014	0.06058	NA
319521	A730056I06Rik	RIKEN cDNA A730056I06 gene	0.87108014	0.1036	0.2543
97961	Nol12	nucleolar protein 12	0.87108014	0.1562	0.3331
57444	Isg20	interferon-stimulated protein	0.87108014	0.1571	0.3342
226413	Lct	lactase	0.87108014	0.1712	0.354
75561	1700016J18Rik	RIKEN cDNA 1700016J18 gene	0.87108014	0.1721	0.3551
76376	Slc24a2	solute carrier family 24 (sodium/potassium/calcium exchanger), member 2	0.87108014	0.221	0.4201
83669	Wdr6	WD repeat domain 6	0.87108014	0.2381	0.4405
70603	Mutyh	mutY homolog (E. coli)	0.87108014	0.2553	0.4602
68162	A930003A15Rik	RIKEN cDNA A930003A15 gene	0.87108014	0.2563	0.4615
277939	C2cd3	C2 calcium-dependent domain containing 3	0.87108014	0.2645	0.4708
381067	Zfp229	zinc finger protein	0.87108014	0.2896	0.498
17951	Naip5	NLR family, apoptosis inhibitory protein 5	0.87108014	0.3288	0.5389
269623	C030048B08Rik	RIKEN cDNA C030048B08 gene	0.87108014	0.3335	0.544
622175	E430024I08Rik	RIKEN cDNA E430024I08 gene	0.87108014	0.3568	0.5667
228662	Btbd3	BTB (POZ) domain containing 3	0.87108014	0.3874	0.596
627191	Tmem90a	transmembrane protein 90a	0.87108014	0.3898	0.598
16494	Kcna6	potassium voltage-gated channel, shaker-related, subfamily, member 6	0.87108014	0.4238	0.6294
83797	Smarcd1	SWI/SNF related, matrix associated, actin dependent regulator of chromatin, subfamily d, member 1	0.87108014	0.4328	0.6366
100040852	Gm3002	alpha-takusan pseudogene	0.87108014	0.4423	0.6451
211468	Kcnh8	potassium voltage-gated channel, subfamily H (eag-related), member 8	0.87108014	0.4597	0.6606
319466	9330199G10Rik	RIKEN cDNA 9330199G10 gene	0.87108014	0.6251	0.7857
234814	Mthfsd	methenyltetrahydrofolate synthetase domain containing	0.87032202	0.006086	NA

67834	Idh3a	isocitrate dehydrogenase 3 (NAD+) alpha	0.87032202	0.01085	NA
64213	St7	suppression of tumorigenicity 7	0.87032202	0.01658	NA
20393	Sgk1	serum/glucocorticoid regulated kinase 1	0.87032202	0.03506	NA
57775	Usp29	ubiquitin specific peptidase 29	0.87032202	0.03768	NA
66875	1200016B10Rik	RIKEN cDNA 1200016B10 gene	0.87032202	0.04189	NA
14402	Gabrb3	gamma-aminobutyric acid (GABA) A receptor, subunit beta 3	0.87032202	0.04248	NA
11994	Pcdh15	protocadherin 15	0.87032202	0.04719	NA
20262	Stmn3	stathmin-like 3	0.87032202	0.06744	NA
14057	Sfxn1	sideroflexin 1	0.87032202	0.06964	0.1946
269198	Nbeal1	neurobeachin like 1	0.87032202	0.07008	0.1953
19713	Ret	ret proto-oncogene	0.87032202	0.07547	0.2057
67276	Eri1	exoribonuclease 1	0.87032202	0.0761	0.2071
381760	Ssbp1	single-stranded DNA binding protein 1	0.87032202	0.09449	0.2394
12314	Calm2	calmodulin 2	0.87032202	0.09845	0.246
71000	4931440J10Rik	RIKEN cDNA 4931440J10 gene	0.87032202	0.105	0.2562
80915	Dusp12	dual specificity phosphatase 12	0.87032202	0.1169	0.275
107477	Guca1b	guanylate cyclase activator 1B	0.87032202	0.1195	0.2797
67058	2810428J06Rik	RIKEN cDNA 2810428J06 gene	0.87032202	0.1465	0.3191
54403	Slc4a4	solute carrier family 4 (anion exchanger), member 4	0.87032202	0.1817	0.3683
68510	Ints1	integrator complex subunit 1	0.87032202	0.1848	0.3719
621976	Tmem170b	transmembrane protein 170B	0.87032202	0.1867	0.3745
270120	Fat3	FAT tumor suppressor homolog 3 (Drosophila)	0.87032202	0.1988	0.3903
16876	Lhx9	LIM homeobox protein 9	0.87032202	0.2612	0.4672
212531	Sh3bgrl2	SH3 domain binding glutamic acid-rich protein like 2	0.87032202	0.3141	0.5236
100041479	Gm3363	predicted gene 3363	0.87032202	0.4608	0.6615
333467	B020031M17Rik	RIKEN cDNA B020031M17 gene	0.87032202	0.5348	0.7195
442801	Arhgef15	Rho guanine nucleotide exchange factor (GEF) 15	0.87032202	0.5384	0.7222
225288	Fhod3	formin homology 2 domain containing 3	0.86956522	0.008818	NA
70285	Rpf1	ribosome production factor 1 homolog (S. cerevisiae)	0.86956522	0.01262	NA
215193	AA408296	expressed sequence AA408296	0.86956522	0.01555	NA
331021	9630017O17	hypothetical protein 9630017O17	0.86956522	0.02048	NA
66264	Ccdc28b	coiled coil domain containing 28B	0.86956522	0.02431	NA
210105	Zfp719	zinc finger protein 719	0.86956522	0.02445	NA
329165	Abi2	abl-interactor 2	0.86956522	0.03344	NA
545938	Zfp607	zinc finger proten 607	0.86956522	0.03636	NA
70450	Unc13d	unc-13 homolog D (C. elegans)	0.86956522	0.04977	NA
69743	Casz1	castor homolog 1, zinc finger (Drosophila)	0.86956522	0.05477	NA
75607	Wnk2	WNK lysine deficient protein kinase 2	0.86956522	0.05529	NA
272009	Srsf13b	serine/arginine-rich splicing factor 13B	0.86956522	0.05796	NA
50784	Ppap2c	phosphatidic acid phosphatase type 2C	0.86956522	0.0602	NA
16885	Limk1	LIM-domain containing, protein kinase	0.86956522	0.07	0.1951
15289	Hmgb1	high mobility group box 1	0.86956522	0.07093	0.1969
380840	Lyrm4	LYR motif containing 4	0.86956522	0.07681	0.2084
74521	Ppp4r4	protein phosphatase 4, regulatory subunit 4	0.86956522	0.08173	0.2174
69137	2200002K05Rik	RIKEN cDNA 2200002K05 gene	0.86956522	0.08945	0.2304
66373	Lsm5	LSM5 homolog, U6 small nuclear RNA associated (S. cerevisiae)	0.86956522	0.1015	0.2511
73945	Otud4	OTU domain containing 4	0.86956522	0.1068	0.2587
54614	Prpf40b	PRP40 pre-mRNA processing factor 40 homolog B (yeast)	0.86956522	0.1571	0.3341
27993	Imp4	IMP4, U3 small nucleolar ribonucleoprotein, homolog (yeast)	0.86956522	0.1581	0.3353
29856	Smtn	smoothelin	0.86956522	0.1842	0.371

67307	Pbld2	phenazine biosynthesis-like protein domain containing 2	0.86956522	0.2044	0.3981
380684	Nefh	neurofilament, heavy polypeptide	0.86956522	0.2145	0.4112
13845	Ephb3	Eph receptor B3	0.86956522	0.2159	0.4131
12449	Ccnf	cyclin F	0.86956522	0.2299	0.4307
18190	Nrxn2	neurexin II	0.86956522	0.2356	0.4374
100033459	Pydc3	pyrin domain containing 3	0.86956522	0.2509	0.4557
70432	Rufy2	RUN and FYVE domain-containing 2	0.86956522	0.254	0.4591
65103	Arl6ip6	ADP-ribosylation factor-like 6 interacting protein 6	0.86956522	0.257	0.4621
77799	Sla2	Src-like-adaptor 2	0.86956522	0.2586	0.4642
270672	Map3k15	mitogen-activated protein kinase kinase kinase 15	0.86956522	0.26	0.4657
20671	Sox17	SRY-box containing gene 17	0.86956522	0.2643	0.4706
667055	Gm9992	predicted gene 9992	0.86956522	0.2951	0.5041
104458	Rars	arginyl-tRNA synthetase	0.86956522	0.296	0.5049
64011	Nrgn	neurogranin	0.86956522	0.3414	0.5517
14421	B4galnt1	beta-1,4-N-acetyl-galactosaminyl transferase 1	0.86956522	0.4032	0.6107
22661	Zfp148	zinc finger protein 148	0.86956522	0.4249	0.6302
19784	Rprl2	ribonuclease P RNA-like 2	0.86956522	0.4594	0.6604
224763	Gm4831	predicted gene 4831	0.86956522	0.5105	0.7012
140765	Tmprss3	transmembrane protease, serine 3	0.86956522	0.5781	0.7508
70208	Med23	mediator complex subunit 23	0.86880973	0.02298	NA
56805	Zbtb33	zinc finger and BTB domain containing 33	0.86880973	0.03689	NA
235132	Zbtb44	zinc finger and BTB domain containing 44	0.86880973	0.03746	NA
229584	Pogz	pogo transposable element with ZNF domain	0.86880973	0.04339	NA
103136	Pwp1	PWP1 homolog (S. cerevisiae)	0.86880973	0.0492	NA
66241	Tmem9	transmembrane protein 9	0.86880973	0.06939	0.1941
67958	2610101N10Rik	RIKEN cDNA 2610101N10 gene	0.86880973	0.09107	0.2336
56403	Syncrip	synaptotagmin binding, cytoplasmic RNA interacting protein	0.86880973	0.09337	0.2373
244867	Arhgap20	Rho GTPase activating protein 20	0.86880973	0.1246	0.2874
78797	Ndor1	NADPH dependent diflavin oxidoreductase 1	0.86880973	0.1253	0.2887
97112	Nmd3	NMD3 homolog (S. cerevisiae)	0.86880973	0.1264	0.2902
66975	2410002O22Rik	RIKEN cDNA 2410002O22 gene	0.86880973	0.1429	0.3138
209601	4922501L14Rik	RIKEN cDNA 4922501L14 gene	0.86880973	0.1483	0.322
77922	A030005L19Rik	RIKEN cDNA A030005L19 gene	0.86880973	0.1682	0.3489
140481	Man2a2	mannosidase 2, alpha 2	0.86880973	0.1808	0.3669
117148	Necab2	N-terminal EF-hand calcium binding protein 2	0.86880973	0.1849	0.3721
16150	Ikbkb	inhibitor of kappaB kinase beta	0.86880973	0.1994	0.3912
12418	Cbx4	chromobox homolog 4 (Drosophila Pc class)	0.86880973	0.2016	0.3941
269198	Nbeal1	neurobeachin like 1	0.86880973	0.2167	0.4141
20776	Tmie	transmembrane inner ear	0.86880973	0.2259	0.4256
321006	Vprbp	Vpr (HIV-1) binding protein	0.86880973	0.228	0.428
71693	Colec11	collectin sub-family member 11	0.86880973	0.3546	0.5645
78552	E130201H02Rik	Y box protein 1 pseudogene	0.86880973	0.4468	0.6491
632971	Rergl	RERG/RAS-like	0.86880973	0.645	0.7995
72775	Fance	Fanconi anemia, complementation group E	0.86805556	0.00851	NA
74143	Opa1	optic atrophy 1 homolog (human)	0.86805556	0.008538	NA
15081	H3f3b	H3 histone, family 3B	0.86805556	0.0164	NA
170835	Inpp5j	inositol polyphosphate 5-phosphatase J	0.86805556	0.01937	NA
19765	Ralbp1	ralA binding protein 1	0.86805556	0.02486	NA
116891	Derl2	Der1-like domain family, member 2	0.86805556	0.02918	NA
245607	Gprasp2	G protein-coupled receptor associated sorting protein 2	0.86805556	0.03925	NA

328108	Fam179b	family with sequence similarity 179, member B	0.86805556	0.0582	NA
226089	C030046E11Rik	RIKEN cDNA C030046E11 gene	0.86805556	0.06314	0.1818
76332	Cog2	component of oligomeric golgi complex 2	0.86805556	0.07195	0.1991
71059	Hexim2	hexamthylene bis-acetamide inducible 2	0.86805556	0.08305	0.2196
268281	Shprh	SNF2 histone linker PHD RING helicase	0.86805556	0.08622	0.2253
237940	Aoc2	amine oxidase, copper containing 2 (retina-specific)	0.86805556	0.09621	0.2421
16173	Il18	interleukin 18	0.86805556	0.0982	0.2455
14455	Gas5	growth arrest specific 5	0.86805556	0.1109	0.2655
269642	Nat8l	N-acetyltransferase 8-like	0.86805556	0.1196	0.2797
56089	Ramp3	receptor (calcitonin) activity modifying protein 3	0.86805556	0.1205	0.2811
53870	Cntn6	contactin 6	0.86805556	0.1233	0.2856
218460	Wdr41	WD repeat domain 41	0.86805556	0.1333	0.3001
210710	Gab3	growth factor receptor bound protein 2-associated protein 3	0.86805556	0.1369	0.3044
66410	Mterfd1	MTERF domain containing 1	0.86805556	0.1387	0.3076
74006	Dnm1l	dynamin 1-like	0.86805556	0.1542	0.33
13082	Cyp26a1	cytochrome P450, family 26, subfamily a, polypeptide 1	0.86805556	0.1565	0.3334
69816	2010001M09Rik	RIKEN cDNA 2010001M09 gene	0.86805556	0.1569	0.3338
67845	Rnf115	ring finger protein 115	0.86805556	0.1595	0.3376
330490	Nlrp9c	NLR family, pyrin domain containing 9C	0.86805556	0.1713	0.354
66168	Grina	glutamate receptor, ionotropic, N-methyl D-aspartate-associated protein 1 (glutamate binding)	0.86805556	0.1892	0.3776
67120	Ttc14	tetratricopeptide repeat domain 14	0.86805556	0.2046	0.3983
12322	Camk2a	calcium/calmodulin-dependent protein kinase II alpha	0.86805556	0.2528	0.4578
26932	Ppp2r5e	protein phosphatase 2, regulatory subunit B (B56), epsilon isoform	0.86805556	0.3757	0.5846
208104	Mlxip	MLX interacting protein	0.86805556	0.4093	0.6157
77560	9330198I05Rik	RIKEN cDNA 9330198I05 gene	0.86805556	0.4359	0.6395
69671	Tmem52	transmembrane protein 52	0.86805556	0.4852	0.6805
14069	F8	coagulation factor VIII	0.86805556	0.6259	0.7862
12876	Cpe	carboxypeptidase E	0.86730269	0.01454	NA
14950	H13	histocompatibility 13	0.86730269	0.0186	NA
228714	Csrp2bp	cysteine and glycine-rich protein 2 binding protein	0.86730269	0.02187	NA
408067	Zfp874b	zinc finger protein 874b	0.86730269	0.02724	NA
407823	Baz2b	bromodomain adjacent to zinc finger domain, 2B	0.86730269	0.04796	NA
73327	1700040I03Rik	RIKEN cDNA 1700040I03 gene	0.86730269	0.05194	NA
545156	Kalrn	kalirin, RhoGEF kinase	0.86730269	0.05308	NA
77519	Zfp266	zinc finger protein 266	0.86730269	0.0651	0.1857
18227	Nr4a2	nuclear receptor subfamily 4, group A, member 2	0.86730269	0.06722	0.1895
18377	Omg	oligodendrocyte myelin glycoprotein	0.86730269	0.06858	0.1923
13048	Cux2	cut-like homeobox 2	0.86730269	0.07042	0.1959
235542	Ppp2r3a	protein phosphatase 2, regulatory subunit B'', alpha	0.86730269	0.07736	0.2095
66078	Tsen34	tRNA splicing endonuclease 34 homolog (S. cerevisiae)	0.86730269	0.08055	0.2153
21357	Tarbp2	TAR (HIV) RNA binding protein 2	0.86730269	0.08266	0.2191
67460	Decr1	2,4-dienoyl CoA reductase 1, mitochondrial	0.86730269	0.09138	0.234
226982	Eif5b	eukaryotic translation initiation factor 5B	0.86730269	0.1062	0.2579
215243	Traf3ip3	TRAF3 interacting protein 3	0.86730269	0.1121	0.2675
75394	0610040F04Rik	RIKEN cDNA 0610040F04 gene	0.86730269	0.1149	0.2722
112419	2010002M12Rik	RIKEN cDNA 2010002M12 gene	0.86730269	0.1176	0.2761
230753	Thrap3	thyroid hormone receptor associated protein 3	0.86730269	0.124	0.2866
320854	9030203C11Rik	RIKEN cDNA 9030203C11 gene	0.86730269	0.1359	0.3033
66674	6330409N04Rik	RIKEN cDNA 6330409N04 gene	0.86730269	0.1672	0.3481
68792	Srpx2	sushi-repeat-containing protein, X-linked 2	0.86730269	0.1673	0.3481

331480	Gm5126	predicted pseudogene 5126	0.86730269	0.1681	0.3489
72145	Wdfy3	WD repeat and FYVE domain containing 3	0.86730269	0.1726	0.356
271127	Adamts16	a disintegrin-like and metallopeptidase (reprolysin type) with thrombospondin type 1 motif, 16	0.86730269	0.1758	0.3603
72258	Kcnk10	potassium channel, subfamily K, member 10	0.86730269	0.1832	0.3703
76157	Slc35d3	solute carrier family 35, member D3	0.86730269	0.1957	0.3863
21341	Taf1c	TATA box binding protein (Tbp)-associated factor, RNA polymerase I, C	0.86730269	0.21	0.4054
54338	Slc23a2	solute carrier family 23 (nucleobase transporters), member 2	0.86730269	0.213	0.4091
50754	Fbxw7	F-box and WD-40 domain protein 7	0.86730269	0.2352	0.4369
14802	Gria4	glutamate receptor, ionotropic, AMPA4 (alpha 4)	0.86730269	0.2366	0.4384
11891	Rab27a	RAB27A, member RAS oncogene family	0.86730269	0.285	0.4935
50774	Krtap5-1	keratin associated protein 5-1	0.86730269	0.313	0.5227
71947	2310067B10Rik	RIKEN cDNA 2310067B10 gene	0.86730269	0.3375	0.5478
50911	Exosc9	exosome component 9	0.86730269	0.3958	0.6037
104401	Pcnxl3	pecanex-like 3 (Drosophila)	0.86730269	0.467	0.6669
77900	6720473M11Rik	RIKEN cDNA 6720473M11 gene	0.86730269	0.5331	0.7179
217718	Nek9	NIMA (never in mitosis gene a)-related expressed kinase 9	0.86655113	0.007013	NA
16451	Jak1	Janus kinase 1	0.86655113	0.02191	NA
107986	Ddb2	damage specific DNA binding protein 2	0.86655113	0.02262	NA
70432	Rufy2	RUN and FYVE domain-containing 2	0.86655113	0.02483	NA
104831	Ptpn23	protein tyrosine phosphatase, non-receptor type 23	0.86655113	0.03705	NA
193736	Zbtb12	zinc finger and BTB domain containing 12	0.86655113	0.03843	NA
140740	Sec63	SEC63-like (S. cerevisiae)	0.86655113	0.04638	NA
72108	Ddhd2	DDHD domain containing 2	0.86655113	0.05057	NA
20230	Satb1	special AT-rich sequence binding protein 1	0.86655113	0.06329	0.1821
107746	Rapgef1	Rap guanine nucleotide exchange factor (GEF) 1	0.86655113	0.06673	0.1888
13121	Cyp51	cytochrome P450, family 51	0.86655113	0.09678	0.243
239552	Apol8	apolipoprotein L 8	0.86655113	0.1162	0.2738
56715	Rabgef1	RAB guanine nucleotide exchange factor (GEF) 1	0.86655113	0.1295	0.295
109349	Fam163b	family with sequence similarity 163, member B	0.86655113	0.1366	0.3041
226026	Smc5	structural maintenance of chromosomes 5	0.86655113	0.1849	0.3721
74290	1700102J08Rik	RIKEN cDNA 1700102J08 gene	0.86655113	0.1944	0.3847
629016	Zfp953	zinc finger protein 953	0.86655113	0.211	0.4065
19679	Pitpnm2	phosphatidylinositol transfer protein, membrane-associated 2	0.86655113	0.2279	0.428
70954	4922502B01Rik	RIKEN cDNA 4922502B01 gene	0.86655113	0.2653	0.4717
434350	9430091E24Rik	RIKEN cDNA 9430091E24 gene	0.86655113	0.2762	0.4841
100041194	Ahnak2	AHNAK nucleoprotein 2	0.86655113	0.3515	0.5615
100040599	Gm15319	predicted gene 15319	0.86655113	0.4065	0.6137
50926	Hnrpdl	heterogeneous nuclear ribonucleoprotein D-like	0.86580087	0.009139	NA
433771	2310028O11Rik	RIKEN cDNA 2310028O11 gene	0.86580087	0.01245	NA
19084	Prkar1a	protein kinase, cAMP dependent regulatory, type I, alpha	0.86580087	0.01291	NA
18541	Pcnt	pericentrin (kendrin)	0.86580087	0.03377	NA
78038	Mccc2	methylcrotonoyl-Coenzyme A carboxylase 2 (beta)	0.86580087	0.06582	0.187
19647	Rbbp6	retinoblastoma binding protein 6	0.86580087	0.07806	0.2108
76965	Slitrk1	SLIT and NTRK-like family, member 1	0.86580087	0.1041	0.2548
73020	2900073C17Rik	RIKEN cDNA 2900073C17 gene	0.86580087	0.1052	0.2565
12563	Cdh6	cadherin 6	0.86580087	0.1101	0.2641
69391	1700018A14Rik	RIKEN cDNA 1700018A14 gene	0.86580087	0.1275	0.292
16493	Kcna5	potassium voltage-gated channel, shaker-related subfamily, member 5	0.86580087	0.1665	0.3472
382427	Best3	bestrophin 3	0.86580087	0.1678	0.3485
192197	Bcas3	breast carcinoma amplified sequence 3	0.86580087	0.1681	0.3489

17540	Mrvi1	MRV integration site 1	0.86580087	0.202	0.3948
230822	A330049M08Rik	RIKEN cDNA A330049M08 gene	0.86580087	0.2382	0.4406
29876	Clic4	chloride intracellular channel 4 (mitochondrial)	0.86580087	0.2409	0.4435
319240	9330159N05Rik	RIKEN cDNA 9330159N05 gene	0.86580087	0.2631	0.4692
242425	Gabbr2	gamma-aminobutyric acid (GABA) B receptor, 2	0.86580087	0.3048	0.5138
330267	Thsd7a	thrombospondin, type I, domain containing 7A	0.86580087	0.3198	0.5298
211499	Tmem87a	transmembrane protein 87A	0.86580087	0.3396	0.5499
71148	Mier1	mesoderm induction early response 1 homolog (Xenopus laevis	0.86580087	0.3408	0.5513
675812	Zfp605	zinc finger protein 605	0.86580087	0.352	0.5621
194231	Cnksr1	connector enhancer of kinase suppressor of Ras 1	0.86580087	0.3811	0.5903
241794	Kcng1	potassium voltage-gated channel, subfamily G, member 1	0.86580087	0.4469	0.6491
244198	Olfml1	olfactomedin-like 1	0.86580087	0.6008	0.7684
234421	Cib3	calcium and integrin binding family member 3	0.86580087	0.6062	0.7725
67134	Nop56	NOP56 ribonucleoprotein homolog (yeast)	0.8650519	0.007043	NA
52028	Bbs1	Bardet-Biedl syndrome 1 (human)	0.8650519	0.01104	NA
78658	Ncapd3	non-SMC condensin II complex, subunit D3	0.8650519	0.01285	NA
50783	Lsm4	LSM4 homolog, U6 small nuclear RNA associated (S. cerevisiae)	0.8650519	0.01401	NA
170772	Glcci1	glucocorticoid induced transcript 1	0.8650519	0.01679	NA
319613	Sybu	syntabulin (syntaxin-interacting)	0.8650519	0.01991	NA
29812	Ndrg3	N-myc downstream regulated gene 3	0.8650519	0.02442	NA
72650	2810006K23Rik	RIKEN cDNA 2810006K23 gene	0.8650519	0.04186	NA
170752	Bco2	beta-carotene oxygenase 2	0.8650519	0.04211	NA
227731	Slc25a25	solute carrier family 25 (mitochondrial carrier, phosphate carrier), member 25	0.8650519	0.04591	NA
70381	Tecpr1	tectonin beta-propeller repeat containing 1	0.8650519	0.04985	NA
70579	Zc3h11a	zinc finger CCCH type containing 11A	0.8650519	0.05478	0.1653
14672	Gna11	guanine nucleotide binding protein, alpha 11	0.8650519	0.0588	0.1732
227334	Usp40	ubiquitin specific peptidase 40	0.8650519	0.07369	0.2026
19272	Ptprk	protein tyrosine phosphatase, receptor type, K	0.8650519	0.07664	0.2082
545527	Fam194a	family with sequence similarity 194, member A	0.8650519	0.07862	0.212
244886	AI118078	expressed sequence AI118078	0.8650519	0.1114	0.2662
100043911	Ppp4r1l-ps	protein phosphatase 4, regulatory subunit 1-like, pseudogene	0.8650519	0.12	0.2803
272465	Fam70b	family with sequence similarity 70, member B	0.8650519	0.1651	0.3452
14030	Ewsr1	Ewing sarcoma breakpoint region 1	0.8650519	0.1673	0.3481
71984	Sars2	seryl-aminoacyl-tRNA synthetase 2	0.8650519	0.2523	0.4573
71240	Osbpl7	oxysterol binding protein-like 7	0.8650519	0.2786	0.4868
70885	Ints10	integrator complex subunit 10	0.8650519	0.315	0.5245
76832	Hyls1	hydrolethalus syndrome 1	0.8650519	0.339	0.5493
103554	Psme4	proteasome (prosome, macropain) activator subunit 4	0.8650519	0.3591	0.5689
214253	Etnk2	ethanolamine kinase 2	0.8650519	0.369	0.578
319933	E230024E03Rik	RIKEN cDNA E230024E03 gene	0.8650519	0.4106	0.617
223473	Nipal2	NIPA-like domain containing 2	0.8650519	0.4218	0.6278
106583	Scaf8	SR-related CTD-associated factor 8	0.86430424	0.008692	NA
17763	Mtcp1	mature T-cell proliferation 1	0.86430424	0.0192	NA
382793	Mtx3	metaxin 3	0.86430424	0.02197	NA
67800	Dgat2	diacylglycerol O-acyltransferase 2	0.86430424	0.03228	NA
54126	Arhgef7	Rho guanine nucleotide exchange factor (GEF7)	0.86430424	0.0374	NA
210293	Dock10	dedicator of cytokinesis 10	0.86430424	0.03947	NA
231327	Ppat	phosphoribosyl pyrophosphate amidotransferase	0.86430424	0.05519	0.1661
234515	Inpp4b	inositol polyphosphate-4-phosphatase, type II	0.86430424	0.05749	0.1707
74094	Tjap1	tight junction associated protein 1	0.86430424	0.06539	0.1862

66874	1200014J11Rik	RIKEN cDNA 1200014J11 gene	0.86430424	0.08141	0.2169
242297	Fam110b	family with sequence similarity 110, member B	0.86430424	0.1034	0.2539
28109	D10Wsu102e	DNA segment, Chr 10, Wayne State University 102, expressed	0.86430424	0.1129	0.2688
22689	Zfp27	zinc finger protein 27	0.86430424	0.1348	0.3016
18004	Nek1	NIMA (never in mitosis gene a)-related expressed kinase 1	0.86430424	0.1784	0.3635
18209	Ntn3	netrin 3	0.86430424	0.1809	0.3669
54137	Acrbp	proacrosin binding protein	0.86430424	0.1987	0.3902
18185	Nrl	neural retina leucine zipper gene	0.86430424	0.2158	0.413
106763	Ttbk1	tau tubulin kinase 1	0.86430424	0.2219	0.4211
70454	Cenpl	centromere protein L	0.86430424	0.2334	0.4349
320362	9530085L11Rik	RIKEN cDNA 9530085L11 gene	0.86430424	0.2497	0.4542
30853	Mlf2	myeloid leukemia factor 2	0.86430424	0.2814	0.4895
75956	Srrm2	serine/arginine repetitive matrix 2	0.86430424	0.3406	0.551
76873	4930447F24Rik	RIKEN cDNA 4930447F24 gene	0.86430424	0.5236	0.7106
320632	Snrnp200	small nuclear ribonucleoprotein 200 (U5)	0.86355786	0.007008	NA
207565	Camkk2	calcium/calmodulin-dependent protein kinase kinase 2, beta	0.86355786	0.01961	NA
77604	C430048L16Rik	RIKEN cDNA C430048L16 gene	0.86355786	0.0217	NA
12323	Camk2b	calcium/calmodulin-dependent protein kinase II, beta	0.86355786	0.02256	NA
103135	Pan2	PAN2 polyA specific ribonuclease subunit homolog (S. cerevisiae)	0.86355786	0.02403	NA
67102	D16Ertd472e	DNA segment, Chr 16, ERATO Doi 472, expressed	0.86355786	0.02553	NA
330177	Taok3	TAO kinase 3	0.86355786	0.0338	NA
239554	Foxred2	FAD-dependent oxidoreductase domain containing 2	0.86355786	0.03511	NA
110119	Mpi	mannose phosphate isomerase	0.86355786	0.04404	NA
15519	Hsp90aa1	heat shock protein 90, alpha (cytosolic), class A member 1	0.86355786	0.04414	NA
100502705	LOC100502705	hypothetical LOC100502705	0.86355786	0.04524	NA
71310	Tbc1d9	TBC1 domain family, member 9	0.86355786	0.05314	0.1618
70524	5730414N17Rik	RIKEN cDNA 5730414N17 gene	0.86355786	0.05792	0.1716
235130	Adamts15	a disintegrin-like and metallopeptidase (reprolysin type) with thrombospondin type 1 motif, 15	0.86355786	0.05887	0.1733
74665	Lrrc48	leucine rich repeat containing 48	0.86355786	0.07052	0.1961
11787	Apbb2	amyloid beta (A4) precursor protein-binding, family B, member 2	0.86355786	0.07292	0.2012
76117	Arhgap15	Rho GTPase activating protein 15	0.86355786	0.08451	0.2223
22630	Ywhaq	tyrosine 3-monooxygenase/tryptophan 5-monooxygenase activation protein, theta polypeptide	0.86355786	0.08987	0.2312
69277	3300002I08Rik	RIKEN cDNA 3300002I08 gene	0.86355786	0.1096	0.2634
19094	Mapk11	mitogen-activated protein kinase 11	0.86355786	0.1209	0.282
72739	Zkscan3	zinc finger with KRAB and SCAN domains 3	0.86355786	0.1276	0.2922
217082	Hlf	hepatic leukemia factor	0.86355786	0.1426	0.3135
72634	Tdrkh	tudor and KH domain containing protein	0.86355786	0.1842	0.371
81003	Trim23	tripartite motif-containing 23	0.86355786	0.2117	0.4074
71971	Zswim1	zinc finger, SWIM domain containing 1	0.86355786	0.226	0.4257
100039757	Gm10153	predicted gene 10153	0.86355786	0.3275	0.5376
30944	Zfp354c	zinc finger protein 354C	0.86355786	0.3819	0.5911
14248	Flii	flightless I homolog (Drosophila)	0.86355786	0.4215	0.6276
55963	Slc1a4	solute carrier family 1 (glutamate/neutral amino acid transporter), member 4	0.86355786	0.5689	0.7446
64451	Dip2a	DIP2 disco-interacting protein 2 homolog A (Drosophila)	0.86281277	0.004411	NA
57296	Psmd8	proteasome (prosome, macropain) 26S subunit, non-ATPase, 8	0.86281277	0.01374	NA
21419	Tcfap2b	transcription factor AP-2 beta	0.86281277	0.01424	NA
140484	Pofut1	protein O-fucosyltransferase 1	0.86281277	0.01816	NA
71973	Rbpms2	RNA binding protein with multiple splicing 2	0.86281277	0.02898	NA
270163	Myo9a	myosin IXa	0.86281277	0.03552	NA
319642	Rab9b	RAB9B, member RAS oncogene family	0.86281277	0.05716	0.17

208440	Dip2c	DIP2 disco-interacting protein 2 homolog C (Drosophila)	0.86281277	0.06301	0.1815
54137	Acrbp	proacrosin binding protein	0.86281277	0.06851	0.1922
69732	2410018L13Rik	RIKEN cDNA 2410018L13 gene	0.86281277	0.08046	0.2152
108079	Prkaa2	protein kinase, AMP-activated, alpha 2 catalytic subunit	0.86281277	0.09612	0.242
17173	Ascl2	achaete-scute complex homolog 2 (Drosophila)	0.86281277	0.1101	0.2641
72505	2610319H10Rik	RIKEN cDNA 2610319H10 gene	0.86281277	0.1106	0.2649
28071	Twistnb	TWIST neighbor	0.86281277	0.1117	0.2669
58799	Crbn	cereblon	0.86281277	0.1456	0.3182
329908	Usp24	ubiquitin specific peptidase 24	0.86281277	0.1501	0.3246
382236	Brwd3	bromodomain and WD repeat domain containing 3	0.86281277	0.1948	0.3851
70359	Gtpbp3	GTP binding protein 3	0.86281277	0.2079	0.4027
21885	Tle1	transducin-like enhancer of split 1, homolog of Drosophila E(spl)	0.86281277	0.2081	0.4028
100038598	Gm10386	predicted gene 10386	0.86281277	0.2752	0.483
74478	Snx29	sorting nexin 29	0.86281277	0.3337	0.5441
99922	AW552393	expressed sequence AW552393	0.86281277	0.4477	0.6498
238803	Zfp366	zinc finger protein 366	0.86281277	0.4569	0.6583
434128	Pnmal2	PNMA-like 2	0.86281277	0.5153	0.705
17425	Foxk1	forkhead box K1	0.86206897	0.006564	NA
16594	Klc2	kinesin light chain 2	0.86206897	0.008004	NA
100072	Camta1	calmodulin binding transcription activator 1	0.86206897	0.01238	NA
216578	Papolg	poly(A) polymerase gamma	0.86206897	0.02244	NA
16581	Kifc2	kinesin family member C2	0.86206897	0.02463	NA
22355	Vipr2	vasoactive intestinal peptide receptor 2	0.86206897	0.0301	NA
208518	Cep78	centrosomal protein 78	0.86206897	0.03729	NA
321000	4933421E11Rik	RIKEN cDNA 4933421E11 gene	0.86206897	0.04123	NA
17535	Mre11a	meiotic recombination 11 homolog A (S. cerevisiae)	0.86206897	0.05002	0.1552
18711	Pikfyve	phosphoinositide kinase, FYVE finger containing	0.86206897	0.05615	0.1681
237831	Slc13a5	solute carrier family 13 (sodium-dependent citrate transporter), member 5	0.86206897	0.05914	0.1738
235380	Dmxl2	Dmx-like 2	0.86206897	0.05973	0.1748
236604	Pisd-ps1	phosphatidylserine decarboxylase, pseudogene 1	0.86206897	0.0626	0.1808
237500	Tmtc3	transmembrane and tetratricopeptide repeat containing 3	0.86206897	0.06599	0.1872
14563	Gdf5	growth differentiation factor 5	0.86206897	0.08367	0.2208
330286	D630045J12Rik	RIKEN cDNA D630045J12 gene	0.86206897	0.08566	0.2244
208727	Hdac4	histone deacetylase 4	0.86206897	0.1132	0.2694
232943	Klc3	kinesin light chain 3	0.86206897	0.1341	0.3011
67302	Zc3h13	zinc finger CCCH type containing 13	0.86206897	0.154	0.3297
18584	Pde8a	phosphodiesterase 8A	0.86206897	0.1648	0.3449
100041781	Gm3510	predicted gene 3510	0.86206897	0.1666	0.3473
23859	Dlg2	discs, large homolog 2 (Drosophila)	0.86206897	0.1698	0.3517
329260	Dennd1b	DENN/MADD domain containing 1B	0.86206897	0.1811	0.3672
14806	Grik2	glutamate receptor, ionotropic, kainate 2 (beta 2)	0.86206897	0.2061	0.4003
20423	Shh	sonic hedgehog	0.86206897	0.2189	0.4175
77215	Krtap5-3	keratin associated protein 5-3	0.86206897	0.2235	0.423
107895	Mgat5	mannoside acetylglucosaminyltransferase 5	0.86206897	0.2447	0.4484
102747	Lrrc49	leucine rich repeat containing 49	0.86206897	0.2549	0.4599
50932	Mink1	misshapen-like kinase 1 (zebrafish)	0.86206897	0.2557	0.4607
216274	Cep290	centrosomal protein 290	0.86206897	0.2955	0.5044
100504362	Gm1987	predicted gene 1987	0.86206897	0.3133	0.5231
66011	Ranbp17	RAN binding protein 17	0.86206897	0.3855	0.5945
170720	Card14	caspase recruitment domain family, member 14	0.86206897	0.4448	0.6476

258384	Olfr1350	olfactory receptor 1350	0.86206897	0.4936	0.6876
230753	Thrap3	thyroid hormone receptor associated protein 3	0.86206897	0.4996	0.692
14270	Srgap2	SLIT-ROBO Rho GTPase activating protein 2	0.86206897	0.5361	0.7207
243621	Iqsec3	IQ motif and Sec7 domain 3	0.86132644	0.004451	NA
108937	Rnf169	ring finger protein 169	0.86132644	0.004927	NA
74252	Armc1	armadillo repeat containing 1	0.86132644	0.01333	NA
140742	Sesn1	sestrin 1	0.86132644	0.01766	NA
53609	Clasrp	CLK4-associating serine/arginine rich protein	0.86132644	0.02351	NA
230676	Szt2	seizure threshold 2	0.86132644	0.02415	NA
17760	Mtap6	microtubule-associated protein 6	0.86132644	0.02637	NA
207785	Csrnp2	cysteine-serine-rich nuclear protein 2	0.86132644	0.03215	NA
382056	Crtc1	CREB regulated transcription coactivator 1	0.86132644	0.0366	NA
320268	B930095G15Rik	RIKEN cDNA B930095G15 gene	0.86132644	0.0457	NA
260302	Gga3	golgi associated, gamma adaptin ear containing, ARF binding protein 3	0.86132644	0.0539	0.1635
233532	Rsf1	remodeling and spacing factor 1	0.86132644	0.09493	0.24
216227	Slc17a8	solute carrier family 17 (sodium-dependent inorganic phosphate cotransporter), member 8	0.86132644	0.1116	0.2666
16469	Jrk	jerky	0.86132644	0.1204	0.2809
12289	Cacna1d	calcium channel, voltage-dependent, L type, alpha 1D subunit	0.86132644	0.124	0.2866
76792	2410131K14Rik	RIKEN cDNA 2410131K14 gene	0.86132644	0.1342	0.3013
56422	Hbs1l	Hbs1-like (S. cerevisiae)	0.86132644	0.1465	0.3191
333433	Gpd1l	glycerol-3-phosphate dehydrogenase 1-like	0.86132644	0.1954	0.386
320127	Dgki	diacylglycerol kinase, iota	0.86132644	0.2049	0.3986
216441	Slc26a10	solute carrier family 26, member 10	0.86132644	0.2079	0.4027
239017	Ogdhl	oxoglutarate dehydrogenase-like	0.86132644	0.2196	0.4185
18578	Pde4b	phosphodiesterase 4B, cAMP specific	0.86132644	0.2619	0.4677
229697	Cym	chymosin	0.86132644	0.2836	0.4922
628951	Gm6936	predicted gene 6936	0.86132644	0.2946	0.5035
109979	Art3	ADP-ribosyltransferase 3	0.86132644	0.3507	0.5609
69576	2310010M20Rik	RIKEN cDNA 2310010M20 gene	0.86132644	0.3991	0.6069
217194	Klhl11	kelch-like 11 (Drosophila)	0.86132644	0.5532	0.7336
69443	1700027J07Rik	RIKEN cDNA 1700027J07 gene	0.86132644	0.5794	0.7518
12283	Cab39	calcium binding protein 39	0.8605852	0.004578	NA
233908	Fus	fusion, derived from t(12;16) malignant liposarcoma (human)	0.8605852	0.01905	NA
14394	Gabra1	gamma-aminobutyric acid (GABA) A receptor, subunit alpha 1	0.8605852	0.02086	NA
69217	Plekha4	pleckstrin homology domain containing, family A (phosphoinositide binding specific) member 4	0.8605852	0.02103	NA
75547	Akap13	A kinase (PRKA) anchor protein 13	0.8605852	0.02414	NA
12447	Ccne1	cyclin E1	0.8605852	0.0265	NA
67095	Trak1	trafficking protein, kinesin binding 1	0.8605852	0.03148	NA
54196	Pabpn1	poly(A) binding protein, nuclear 1	0.8605852	0.03527	NA
20589	Ighmbp2	immunoglobulin mu binding protein 2	0.8605852	0.03584	NA
72041	Alkbh4	alkB, alkylation repair homolog 4 (E. coli)	0.8605852	0.04761	0.1495
15289	Hmgb1	high mobility group box 1	0.8605852	0.05021	0.1555
327814	Ppfia2	protein tyrosine phosphatase, receptor type, f polypeptide (PTPRF), interacting protein (liprin), alpha 2	0.8605852	0.05222	0.1598
208117	Aph1b	anterior pharynx defective 1b homolog (C. elegans)	0.8605852	0.05468	0.1652
66302	Fam82b	family with sequence similarity 82, member B	0.8605852	0.06447	0.1844
319622	Itpripl2	inositol 1,4,5-triphosphate receptor interacting protein-like 2	0.8605852	0.07207	0.1993
320961	A630026N12Rik	RIKEN cDNA A630026N12 gene	0.8605852	0.08091	0.2159
108653	Rimklb	ribosomal modification protein rimK-like family member B	0.8605852	0.08312	0.2198
20911	Stxbp2	syntaxin binding protein 2	0.8605852	0.1096	0.2635
319737	D130051D11Rik	RIKEN cDNA D130051D11 gene	0.8605852	0.1153	0.2725

65020	Zfp110	zinc finger protein 110	0.8605852	0.1913	0.3808
16569	Kif3b	kinesin family member 3B	0.8605852	0.2081	0.4028
75547	Akap13	A kinase (PRKA) anchor protein 13	0.8605852	0.2155	0.4127
22589	Atrx	alpha thalassemia/mental retardation syndrome X-linked homolog (human)	0.8605852	0.2244	0.4241
69462	2300005B03Rik	RIKEN cDNA 2300005B03 gene	0.8605852	0.3104	0.5203
230657	Tmem69	transmembrane protein 69	0.8605852	0.3279	0.5379
24013	Grk1	G protein-coupled receptor kinase 1	0.8605852	0.3413	0.5517
17329	Cxcl9	chemokine (C-X-C motif) ligand 9	0.8605852	0.4027	0.6102
638517	LOC638517	cyclic AMP-responsive element-binding protein 3-like protein 2-like	0.8605852	0.433	0.6368
217328	Myo15b	myosin XVB	0.8605852	0.4711	0.6699
71302	Arhgap26	Rho GTPase activating protein 26	0.8605852	0.5441	0.7262
17355	Aff1	AF4/FMR2 family, member 1	0.85984523	0.01351	NA
108902	B3gnt1	UDP-GlcNAc:betaGal beta-1,3-N-acetylglucosaminyltransferase 1	0.85984523	0.03068	NA
108745	2310051F07Rik	RIKEN cDNA 2310051F07 gene	0.85984523	0.03375	NA
58235	Pvrl1	poliovirus receptor-related 1	0.85984523	0.03894	NA
67102	D16Ertd472e	DNA segment, Chr 16, ERATO Doi 472, expressed	0.85984523	0.05068	0.1564
18013	Neurod2	neurogenic differentiation 2	0.85984523	0.05421	0.1642
20383	Srsf3	serine/arginine-rich splicing factor 3	0.85984523	0.05593	0.1678
71354	Wdr31	WD repeat domain 31	0.85984523	0.07069	0.1965
320165	Tacc1	transforming, acidic coiled-coil containing protein 1	0.85984523	0.07345	0.2023
70465	Wdr77	WD repeat domain 77	0.85984523	0.07357	0.2024
474160	BC033916	cDNA sequence BC033916	0.85984523	0.07374	0.2027
245446	Slitrk4	SLIT and NTRK-like family, member 4	0.85984523	0.07471	0.2043
272322	Arntl2	aryl hydrocarbon receptor nuclear translocator-like 2	0.85984523	0.08436	0.2221
14365	Fzd3	frizzled homolog 3 (Drosophila)	0.85984523	0.08925	0.2303
77781	Epm2aip1	EPM2A (laforin) interacting protein 1	0.85984523	0.1124	0.268
319304	A730081D07Rik	RIKEN cDNA A730081D07 gene	0.85984523	0.1136	0.27
105670	Rcbtb2	regulator of chromosome condensation (RCC1) and BTB (POZ) domain containing protein 2	0.85984523	0.1215	0.2829
100043309	4732414G09Rik	RIKEN cDNA 4732414G09 gene	0.85984523	0.1405	0.3103
18676	Phf2	PHD finger protein 2	0.85984523	0.166	0.3466
71132	Cabyr	calcium-binding tyrosine-(Y)-phosphorylation regulated (fibrousheathin 2)	0.85984523	0.1723	0.3555
224694	Zfp81	zinc finger protein 81	0.85984523	0.175	0.3591
56695	Pnkd	paroxysmal nonkinesiogenic dyskinesia	0.85984523	0.1763	0.3609
100504389	LOC100504389	hypothetical LOC100504389	0.85984523	0.204	0.3975
57743	Sec61a2	Sec61, alpha subunit 2 (S. cerevisiae)	0.85984523	0.3122	0.5218
68760	Synpo2l	synaptopodin 2-like	0.85984523	0.341	0.5516
23857	Dmtf1	cyclin D binding myb-like transcription factor 1	0.85984523	0.3707	0.5794
219181	Akap11	A kinase (PRKA) anchor protein 11	0.85910653	0.007272	NA
12751	Tpp1	tripeptidyl peptidase I	0.85910653	0.01055	NA
18738	Pitpna	phosphatidylinositol transfer protein, alpha	0.85910653	0.01441	NA
75296	Fgfr1op	Fgfr1 oncogene partner	0.85910653	0.01581	NA
69089	Oxa1l	oxidase assembly 1-like	0.85910653	0.0176	NA
78777	2410002I01Rik	RIKEN cDNA 2410002I01 gene	0.85910653	0.01918	NA
17933	Myt1l	myelin transcription factor 1-like	0.85910653	0.03524	NA
224432	Scaf4	SR-related CTD-associated factor 4	0.85910653	0.03652	NA
70650	Zcchc8	zinc finger, CCHC domain containing 8	0.85910653	0.03672	NA
72795	Ttc19	tetratricopeptide repeat domain 19	0.85910653	0.03927	NA
21334	Tac2	tachykinin 2	0.85910653	0.04321	0.1396
73132	Slc25a16	solute carrier family 25 (mitochondrial carrier, Graves disease autoantigen), member 16	0.85910653	0.04602	0.146
233876	Hirip3	HIRA interacting protein 3	0.85910653	0.04738	0.149

71340	Riok1	RIO kinase 1 (yeast)	0.85910653	0.04804	0.1504
216705	Clint1	clathrin interactor 1	0.85910653	0.06075	0.1771
98445	AI844869	expressed sequence AI844869	0.85910653	0.07717	0.2091
14172	Fgf18	fibroblast growth factor 18	0.85910653	0.08305	0.2196
212555	Pqlc2	PQ loop repeat containing 2	0.85910653	0.08744	0.2274
212541	Rho	rhodopsin	0.85910653	0.09237	0.2358
270028	Fam155a	family with sequence similarity 155, member A	0.85910653	0.1028	0.253
12662	Chm	choroidermia	0.85910653	0.1053	0.2567
224019	Tmem191c	transmembrane protein 191C	0.85910653	0.1365	0.304
209707	Lcorl	ligand dependent nuclear receptor corepressor-like	0.85910653	0.139	0.3081
243385	Gprin3	GPRIN family member 3	0.85910653	0.1422	0.3129
320840	Negr1	neuronal growth regulator 1	0.85910653	0.1832	0.3704
170737	Znrf1	zinc and ring finger 1	0.85910653	0.2394	0.4419
78286	Nav2	neuron navigator 2	0.85910653	0.2488	0.453
319974	Auts2	autism susceptibility candidate 2	0.85910653	0.4111	0.6174
382118	Zfp167	zinc finger protein 167	0.85910653	0.4199	0.626
225028	Map4k3	mitogen-activated protein kinase kinase kinase kinase 3	0.8583691	0.004968	NA
224139	Golgb1	golgi autoantigen, golgin subfamily b, macrogolgin 1	0.8583691	0.006296	NA
26950	Vsnl1	visinin-like 1	0.8583691	0.008429	NA
27215	Azi2	5-azacytidine induced gene 2	0.8583691	0.01263	NA
58909	Fam13a	family with sequence similarity 13, member A	0.8583691	0.01717	NA
17719	ND4	NADH dehydrogenase subunit 4	0.8583691	0.02119	NA
241919	Slc7a14	solute carrier family 7 (cationic amino acid transporter, y+ system), member 14	0.8583691	0.02699	NA
216867	Slc16a11	solute carrier family 16 (monocarboxylic acid transporters), member 11	0.8583691	0.03159	NA
326622	Upf2	UPF2 regulator of nonsense transcripts homolog (yeast)	0.8583691	0.03261	NA
51886	Fubp1	far upstream element (FUSE) binding protein 1	0.8583691	0.04083	0.1347
170823	Glmn	glomulin, FKBP associated protein	0.8583691	0.04779	0.15
22652	Mkrn3	makorin, ring finger protein, 3	0.8583691	0.05343	0.1625
228536	Bahd1	bromo adjacent homology domain containing 1	0.8583691	0.05801	0.1716
244891	Scaper	S phase cyclin A-associated protein in the ER	0.8583691	0.06836	0.1919
330222	Sdk1	sidekick homolog 1 (chicken)	0.8583691	0.06883	0.1929
18185	Nrl	neural retina leucine zipper gene	0.8583691	0.07148	0.1981
20935	Surf6	surfeit gene 6	0.8583691	0.09878	0.2464
280645	B3gat2	beta-1,3-glucuronyltransferase 2 (glucuronosyltransferase S)	0.8583691	0.1248	0.2878
75605	Kdm5b	lysine (K)-specific demethylase 5B	0.8583691	0.1277	0.2923
402771	C130090I23Rik	RIKEN cDNA C130090I23 gene	0.8583691	0.1419	0.3125
106131	AA589532	expressed sequence AA589532	0.8583691	0.1459	0.3185
56149	Grasp	GRP1 (general receptor for phosphoinositides 1)-associated scaffold protein	0.8583691	0.1483	0.322
72098	Tmem68	transmembrane protein 68	0.8583691	0.1632	0.3423
78233	6330522J23Rik	RIKEN cDNA 6330522J23 gene	0.8583691	0.1677	0.3485
50926	Hnrpdl	heterogeneous nuclear ribonucleoprotein D-like	0.8583691	0.1851	0.3723
243385	Gprin3	GPRIN family member 3	0.8583691	0.1952	0.3857
30806	Adamts8	a disintegrin-like and metallopeptidase (reprolysin type) with thrombospondin type 1 motif, 8	0.8583691	0.204	0.3975
12942	Pcdha11	protocadherin alpha 11	0.8583691	0.2178	0.4157
24057	Sh3yl1	Sh3 domain YSC-like 1	0.8583691	0.2232	0.4228
50932	Mink1	misshapen-like kinase 1 (zebrafish)	0.8583691	0.2575	0.4629
320473	Heatr5b	HEAT repeat containing 5B	0.8583691	0.2848	0.4932
327992	Hsf5	heat shock transcription factor family member 5	0.8583691	0.3441	0.5548
50787	Hs6st3	heparan sulfate 6-O-sulfotransferase 3	0.8583691	0.348	0.5585
20975	Synj2	synaptojanin 2	0.8583691	0.3481	0.5586

12914	Crebbp	CREB binding protein	0.8583691	0.3534	0.5634
239319	Card6	caspase recruitment domain family, member 6	0.8583691	0.3944	0.6026
67412	6330407J23Rik	RIKEN cDNA 6330407J23 gene	0.85763293	0.01043	NA
234023	Arglu1	arginine and glutamate rich 1	0.85763293	0.01292	NA
80907	Lactb	lactamase, beta	0.85763293	0.01365	NA
66477	Usmg5	upregulated during skeletal muscle growth 5	0.85763293	0.01777	NA
72193	Scaf11	SR-related CTD-associated factor 11	0.85763293	0.02383	NA
57276	Vsig2	V-set and immunoglobulin domain containing 2	0.85763293	0.02595	NA
216151	Polrmt	polymerase (RNA) mitochondrial (DNA directed)	0.85763293	0.03737	0.1273
26556	Homer1	homer homolog 1 (Drosophila)	0.85763293	0.04153	0.1363
68817	Ddi2	DNA-damage inducible protein 2	0.85763293	0.06287	0.1814
50769	Atp8a2	ATPase, aminophospholipid transporter-like, class I, type 8A, member 2	0.85763293	0.06439	0.1843
54607	Socs6	suppressor of cytokine signaling 6	0.85763293	0.076	0.2069
228787	Xkr7	X Kell blood group precursor related family member 7 homolog	0.85763293	0.09208	0.2353
54006	Deaf1	deformed epidermal autoregulatory factor 1 (Drosophila)	0.85763293	0.1095	0.2633
98514	AU015232	expressed sequence AU015232	0.85763293	0.1247	0.2876
20492	Slbp	stem-loop binding protein	0.85763293	0.1531	0.3283
78283	Mtap7d2	MAP7 domain containing 2	0.85763293	0.1535	0.3289
432995	Gm5480	predicted gene 5480	0.85763293	0.1702	0.3522
54607	Socs6	suppressor of cytokine signaling 6	0.85763293	0.1734	0.357
72313	Fryl	furry homolog-like (Drosophila)	0.85763293	0.1798	0.3654
319758	Rfx7	regulatory factor X, 7	0.85763293	0.1883	0.3765
80883	Ntng1	netrin G1	0.85763293	0.2046	0.3984
11908	Atf1	activating transcription factor 1	0.85763293	0.2124	0.4084
244885	Sh2d7	SH2 domain containing 7	0.85763293	0.2191	0.4177
231042	Nupl2	nucleoporin like 2	0.85763293	0.2231	0.4226
101190	AI853106	expressed sequence AI853106	0.85763293	0.237	0.4389
84506	Hamp	hepcidin antimicrobial peptide	0.85763293	0.2609	0.4669
231380	Uba6	ubiquitin-like modifier activating enzyme 6	0.85763293	0.262	0.4679
15467	Eif2ak1	eukaryotic translation initiation factor 2 alpha kinase 1	0.85763293	0.2634	0.4695
75607	Wnk2	WNK lysine deficient protein kinase 2	0.85763293	0.2703	0.4778
69719	Cad	carbamoyl-phosphate synthetase 2, aspartate transcarbamylase, and dihydroorotase	0.85763293	0.2778	0.486
20199	S100a5	S100 calcium binding protein A5	0.85763293	0.2902	0.4987
239556	Cacna1i	calcium channel, voltage-dependent, alpha 1I subunit	0.85763293	0.2904	0.4987
235533	Gk5	glycerol kinase 5 (putative)	0.85763293	0.2932	0.502
71101	4933407H18Rik	RIKEN cDNA 4933407H18 gene	0.85763293	0.3216	0.5313
56222	Cited4	Cbp/p300-interacting transactivator, with Glu/Asp-rich carboxy-terminal domain, 4	0.85763293	0.3552	0.5649
16886	Limk2	LIM motif-containing protein kinase 2	0.85763293	0.3726	0.5814
94246	Arid4b	AT rich interactive domain 4B (RBP1-like)	0.85763293	0.3912	0.5994
24069	Sufu	suppressor of fused homolog (Drosophila)	0.85763293	0.5133	0.7038
108989	Tpr	translocated promoter region	0.85763293	0.5205	0.7089
666023	Gm7894	predicted gene 7894	0.85763293	0.5214	0.7096
56364	Zmym3	zinc finger, MYM-type 3	0.85689803	0.004666	NA
237711	Eml6	echinoderm microtubule associated protein like 6	0.85689803	0.01492	NA
19023	Ppef2	protein phosphatase, EF hand calcium-binding domain 2	0.85689803	0.01819	NA
70727	Rasgef1a	RasGEF domain family, member 1A	0.85689803	0.01934	NA
239667	Dip2b	DIP2 disco-interacting protein 2 homolog B (Drosophila)	0.85689803	0.03301	NA
74213	Rbm26	RNA binding motif protein 26	0.85689803	0.03513	NA
319278	A230050P20Rik	RIKEN cDNA A230050P20 gene	0.85689803	0.04377	0.1409
57257	Vav3	vav 3 oncogene	0.85689803	0.0447	0.1429

228889	Ddx27	DEAD (Asp-Glu-Ala-Asp) box polypeptide 27	0.85689803	0.04782	0.15
210503	Zfp677	zinc finger protein 677	0.85689803	0.06253	0.1807
12839	Col9a1	collagen, type IX, alpha 1	0.85689803	0.06301	0.1815
20983	Syt4	synaptotagmin IV	0.85689803	0.07191	0.199
399591	Tmsb15l	thymosin beta 15b like	0.85689803	0.08904	0.23
22696	Zfp37	zinc finger protein 37	0.85689803	0.1103	0.2646
219228	Pcdh17	protocadherin 17	0.85689803	0.1141	0.2708
56095	Ftsj3	FtsJ homolog 3 (E. coli)	0.85689803	0.1255	0.2887
245537	Nlgn3	neuroligin 3	0.85689803	0.1378	0.3059
76183	Celf6	CUGBP, Elav-like family member 6	0.85689803	0.1627	0.3416
218877	Sema3g	sema domain, immunoglobulin domain (Ig), short basic domain, secreted, (semaphorin) 3G	0.85689803	0.3043	0.5134
75424	Zfp820	zinc finger protein 820	0.85689803	0.3573	0.5672
53619	Blcap	bladder cancer associated protein homolog (human)	0.85689803	0.4219	0.6278
402729	9330184L24Rik	RIKEN cDNA 9330184L24 gene	0.85689803	0.4306	0.6345
112414	4930540M05Rik	RIKEN cDNA 4930540M05 gene	0.85689803	0.4704	0.6696
68515	Myadml2	myeloid-associated differentiation marker-like 2	0.85689803	0.4713	0.67
269713	Clip2	CAP-GLY domain containing linker protein 2	0.85689803	0.4959	0.6892
100535	Oas1d	2'-5' oligoadenylate synthetase 1D	0.85689803	0.6116	0.7758
24131	Ldb3	LIM domain binding 3	0.85689803	0.6374	0.7946
100039672	Msmp	microseminoprotein, prostate associated	0.85689803	0.6882	0.8266
76559	Atg2b	ATG2 autophagy related 2 homolog B (S. cerevisiae)	0.85616438	0.009521	NA
110809	Srsf1	serine/arginine-rich splicing factor 1	0.85616438	0.01642	NA
71017	4933407E14Rik	RIKEN cDNA 4933407E14 gene	0.85616438	0.02938	NA
66569	Gdpd1	glycerophosphodiester phosphodiesterase domain containing 1	0.85616438	0.03641	0.1251
19293	Pvalb	parvalbumin	0.85616438	0.04409	0.1416
268902	Robo2	roundabout homolog 2 (Drosophila)	0.85616438	0.05474	0.1653
12877	Cpeb1	cytoplasmic polyadenylation element binding protein 1	0.85616438	0.05755	0.1708
116905	Dph1	DPH1 homolog (S. cerevisiae)	0.85616438	0.06362	0.1827
333329	Cngb1	cyclic nucleotide gated channel beta 1	0.85616438	0.09898	0.2468
208263	Tor1aip1	torsin A interacting protein 1	0.85616438	0.1026	0.2526
73389	Hbp1	high mobility group box transcription factor 1	0.85616438	0.1366	0.3042
67618	Aasdhppt	aminoadipate-semialdehyde dehydrogenase-phosphopantetheinyl transferase	0.85616438	0.1374	0.3053
170738	Kcnh7	potassium voltage-gated channel, subfamily H (eag-related), member 7	0.85616438	0.1424	0.3131
329015	Atg2a	ATG2 autophagy related 2 homolog A (S. cerevisiae)	0.85616438	0.1642	0.344
18987	Pou2f2	POU domain, class 2, transcription factor 2	0.85616438	0.2177	0.4156
58988	Rps6kb2	ribosomal protein S6 kinase, polypeptide 2	0.85616438	0.2313	0.4322
69546	Mapk1ip1	mitogen-activated protein kinase 1 interacting protein 1	0.85616438	0.233	0.4344
271944	C2cd4d	C2 calcium-dependent domain containing 4D	0.85616438	0.2564	0.4615
237615	Ankrd52	ankyrin repeat domain 52	0.85616438	0.2703	0.4778
109711	Actn1	actinin, alpha 1	0.85616438	0.3714	0.5801
100042480	Nhsl2	NHS-like 2	0.85616438	0.3778	0.5867
170740	Zfp287	zinc finger protein 287	0.85616438	0.3847	0.5939
53313	Atp2a3	ATPase, Ca++ transporting, ubiquitous	0.85616438	0.4083	0.6148
65254	Dpysl5	dihydropyrimidinase-like 5	0.85616438	0.469	0.6686
66451	2610528J11Rik	RIKEN cDNA 2610528J11 gene	0.85616438	0.5373	0.7215
74376	Myo18b	myosin XVIIIb	0.85616438	0.7038	0.8365
74197	Gtf2e1	general transcription factor II E, polypeptide 1 (alpha subunit)	0.85543199	0.008848	NA
224019	Tmem191c	transmembrane protein 191C	0.85543199	0.01439	NA
50760	Fbxo17	F-box protein 17	0.85543199	0.01918	NA
14179	Fgf8	fibroblast growth factor 8	0.85543199	0.0194	NA

20515	Slc20a1	solute carrier family 20, member 1	0.85543199	0.02021	NA
108138	Xrcc4	X-ray repair complementing defective repair in Chinese hamster cells 4	0.85543199	0.02975	NA
22682	Zfand5	zinc finger, AN1-type domain 5	0.85543199	0.03044	NA
66674	6330409N04Rik	RIKEN cDNA 6330409N04 gene	0.85543199	0.03301	0.1174
319780	D030002E05Rik	RIKEN cDNA D030002E05 gene	0.85543199	0.03522	0.1225
14913	Guca1a	guanylate cyclase activator 1a (retina)	0.85543199	0.04088	0.1347
15184	Hdac5	histone deacetylase 5	0.85543199	0.05005	0.1552
13872	Ercc3	excision repair cross-complementing rodent repair deficiency, complementation group 3	0.85543199	0.06072	0.177
224014	Fgd4	FYVE, RhoGEF and PH domain containing 4	0.85543199	0.06702	0.1892
67657	Rabl3	RAB, member of RAS oncogene family-like 3	0.85543199	0.07708	0.2089
16600	Klf4	Kruppel-like factor 4 (gut)	0.85543199	0.08368	0.2208
22222	Ubr1	ubiquitin protein ligase E3 component n-recognin 1	0.85543199	0.08587	0.2246
67048	Vma21	VMA21 vacuolar H+-ATPase homolog (S. cerevisiae)	0.85543199	0.09291	0.2367
233871	Atxn2l	ataxin 2-like	0.85543199	0.09597	0.2419
380694	Ccnjl	cyclin J-like	0.85543199	0.1134	0.2697
12336	Capns1	calpain, small subunit 1	0.85543199	0.1216	0.283
213084	Cdkl3	cyclin-dependent kinase-like 3	0.85543199	0.1284	0.2935
13483	Dpp6	dipeptidylpeptidase 6	0.85543199	0.1463	0.319
14697	Gnb5	guanine nucleotide binding protein (G protein), beta 5	0.85543199	0.1508	0.3258
239336	Rxfp3	relaxin family peptide receptor 3	0.85543199	0.1662	0.3469
320007	Sidt1	SID1 transmembrane family, member 1	0.85543199	0.1773	0.3623
106707	Rpusd1	RNA pseudouridylate synthase domain containing 1	0.85543199	0.2041	0.3976
17125	Smad1	MAD homolog 1 (Drosophila)	0.85543199	0.2182	0.4164
68725	1110032F04Rik	RIKEN cDNA 1110032F04 gene	0.85543199	0.2331	0.4346
69349	1700008O03Rik	RIKEN cDNA 1700008O03 gene	0.85543199	0.2636	0.4697
67596	5830405N20Rik	RIKEN cDNA 5830405N20 gene	0.85543199	0.2764	0.4844
622629	Gm10318	predicted gene 10318	0.85543199	0.4436	0.6465
18711	Pikfyve	phosphoinositide kinase, FYVE finger containing	0.85543199	0.454	0.6555
22272	Uqcrq	ubiquinol-cytochrome c reductase, complex III subunit VII	0.85543199	0.5008	0.6928
75538	Fam71e1	family with sequence similarity 71, member E1	0.85470085	0.008545	NA
67900	Mtfp1	mitochondrial fission process 1	0.85470085	0.01043	NA
107566	Arl2bp	ADP-ribosylation factor-like 2 binding protein	0.85470085	0.0108	NA
51799	Rundc3a	RUN domain containing 3A	0.85470085	0.01423	NA
269615	Plch2	phospholipase C, eta 2	0.85470085	0.01712	NA
18536	Pcm1	pericentriolar material 1	0.85470085	0.02879	NA
545156	Kalrn	kalirin, RhoGEF kinase	0.85470085	0.03076	NA
75124	Nxnl2	nucleoredoxin-like 2	0.85470085	0.05329	0.1622
20841	Zfp143	zinc finger protein 143	0.85470085	0.07193	0.199
67768	N6amt1	N-6 adenine-specific DNA methyltransferase 1 (putative)	0.85470085	0.07462	0.2042
13618	Ednrb	endothelin receptor type B	0.85470085	0.1014	0.251
29862	Ninj2	ninjurin 2	0.85470085	0.1056	0.257
234542	Rtbdn	retbindin	0.85470085	0.1063	0.258
330401	Tmcc1	transmembrane and coiled coil domains 1	0.85470085	0.1174	0.2758
215999	Ccdc109a	coiled-coil domain containing 109A	0.85470085	0.1208	0.2819
17968	Ncam2	neural cell adhesion molecule 2	0.85470085	0.122	0.2837
20667	Sox12	SRY-box containing gene 12	0.85470085	0.1247	0.2876
56316	Ggcx	gamma-glutamyl carboxylase	0.85470085	0.1345	0.3015
100047123	LOC100047123	hypothetical protein LOC100047123	0.85470085	0.1368	0.3044
67620	Lrp2bp	Lrp2 binding protein	0.85470085	0.1403	0.31
320452	P4ha3	procollagen-proline, 2-oxoglutarate 4-dioxygenase (proline 4-hydroxylase), alpha polypeptide III	0.85470085	0.1449	0.3172

14007	Celf2	CUGBP, Elav-like family member 2	0.85470085	0.1501	0.3246
232943	Klc3	kinesin light chain 3	0.85470085	0.1769	0.3618
66185	1110037F02Rik	RIKEN cDNA 1110037F02 gene	0.85470085	0.1856	0.3731
214932	Cecr5	cat eye syndrome chromosome region, candidate 5 homolog (human)	0.85470085	0.2159	0.4131
15572	Elavl4	ELAV (embryonic lethal, abnormal vision, Drosophila)-like 4 (Hu antigen D)	0.85470085	0.222	0.4212
17537	Meis3	Meis homeobox 3	0.85470085	0.2243	0.424
81600	Chia	chitinase, acidic	0.85470085	0.2317	0.4326
69837	Pcgf1	polycomb group ring finger 1	0.85470085	0.2678	0.4746
18087	Nktr	natural killer tumor recognition sequence	0.85470085	0.2679	0.4748
269784	Cntn4	contactin 4	0.85470085	0.3065	0.5159
73610	Zfp433	RIKEN cDNA 1700123A16 gene	0.85470085	0.327	0.5372
622935	Krtap20-2	keratin associated protein 20-2	0.85470085	0.3448	0.5554
330409	Cecr2	cat eye syndrome chromosome region, candidate 2 homolog (human)	0.85470085	0.3517	0.5618
105596	AU020094	expressed sequence AU020094	0.85470085	0.5168	0.7062
258718	Olfr513	olfactory receptor 513	0.85470085	0.6658	0.8117
20833	Ssrp1	structure specific recognition protein 1	0.85470085	0.7059	0.8379
11938	Atp2a2	ATPase, Ca++ transporting, cardiac muscle, slow twitch 2	0.85397096	0.003943	NA
269400	Rtel1	regulator of telomere elongation helicase 1	0.85397096	0.01726	NA
12753	Clock	circadian locomotor output cycles kaput	0.85397096	0.01801	NA
235134	Nfrkb	nuclear factor related to kappa B binding protein	0.85397096	0.02772	NA
100038746	LOC100038746	hypothetical LOC100038746	0.85397096	0.02941	NA
243853	Fkrp	fukutin related protein	0.85397096	0.03049	NA
77036	1700109H08Rik	RIKEN cDNA 1700109H08 gene	0.85397096	0.03187	0.1147
75415	Arhgap12	Rho GTPase activating protein 12	0.85397096	0.04088	0.1347
78754	Galntl2	UDP-N-acetyl-alpha-D-galactosamine:polypeptide N-acetylgalactosaminyltransferase-like 2	0.85397096	0.04795	0.1503
668173	Pex10	peroxisomal biogenesis factor 10	0.85397096	0.05054	0.1561
12014	Bach2	BTB and CNC homology 2	0.85397096	0.05108	0.1573
223527	Eny2	enhancer of yellow 2 homolog (Drosophila)	0.85397096	0.05356	0.1627
71807	Tars2	threonyl-tRNA synthetase 2, mitochondrial (putative)	0.85397096	0.06466	0.1849
436230	BC065397	cDNA sequence BC065397	0.85397096	0.0765	0.208
20536	Slc4a3	solute carrier family 4 (anion exchanger), member 3	0.85397096	0.08553	0.2242
56210	Rev1	REV1 homolog (S. cerevisiae)	0.85397096	0.106	0.2576
224674	Slc37a1	solute carrier family 37 (glycerol-3-phosphate transporter), member 1	0.85397096	0.1281	0.2929
270086	Ogfod1	2-oxoglutarate and iron-dependent oxygenase domain containing 1	0.85397096	0.1377	0.3059
17929	Myom1	myomesin 1	0.85397096	0.1602	0.3383
20733	Spint2	serine protease inhibitor, Kunitz type 2	0.85397096	0.1686	0.3497
240614	Ranbp6	RAN binding protein 6	0.85397096	0.1855	0.3729
67684	Luc7l3	LUC7-like 3 (S. cerevisiae)	0.85397096	0.1986	0.3902
100039855	Gm2461	predicted gene 2461	0.85397096	0.2365	0.4384
435616	Gm5699	predicted gene 5699	0.85397096	0.2456	0.4492
209707	Lcorl	ligand dependent nuclear receptor corepressor-like	0.85397096	0.283	0.4915
73158	Larp1	La ribonucleoprotein domain family, member 1	0.85397096	0.2871	0.4956
66231	Thoc7	THO complex 7 homolog (Drosophila)	0.85397096	0.2886	0.497
72508	Rps6kb1	ribosomal protein S6 kinase, polypeptide 1	0.85397096	0.3124	0.522
381654	C87414	expressed sequence C87414	0.85397096	0.4301	0.6342
234479	Gm4890	predicted gene 4890	0.85397096	0.4603	0.6611
66132	1110008L16Rik	RIKEN cDNA 1110008L16 gene	0.85397096	0.4956	0.6889
227210	Ccnyl1	cyclin Y-like 1	0.85324232	0.002276	NA
210933	Bai3	brain-specific angiogenesis inhibitor 3	0.85324232	0.01755	NA
102293	AW111846	expressed sequence AW111846	0.85324232	0.03324	0.1179

13859	Eps15l1	epidermal growth factor receptor pathway substrate 15-like 1	0.85324232	0.03468	0.1211
353237	Pcdhac2	protocadherin alpha subfamily C, 2	0.85324232	0.0389	0.1305
224938	Pja2	praja 2, RING-H2 motif containing	0.85324232	0.04279	0.1388
106565	Dlk2	delta-like 2 homolog (Drosophila)	0.85324232	0.06552	0.1865
78455	Helz	helicase with zinc finger domain	0.85324232	0.06728	0.1897
12337	Capn5	calpain 5	0.85324232	0.07014	0.1953
58991	Ghrl	ghrelin	0.85324232	0.09646	0.2426
207181	Rbms3	RNA binding motif, single stranded interacting protein	0.85324232	0.1006	0.2496
12544	Cdc45	cell division cycle 45 homolog (S. cerevisiae)	0.85324232	0.1229	0.2851
243547	Grip2	glutamate receptor interacting protein 2	0.85324232	0.1402	0.3099
110351	Rap1gap	Rap1 GTPase-activating protein	0.85324232	0.1501	0.3246
236511	Eif2c1	eukaryotic translation initiation factor 2C, 1	0.85324232	0.2609	0.4669
15478	Hs3st3a1	heparan sulfate (glucosamine) 3-O-sulfotransferase 3A1	0.85324232	0.2863	0.4949
24071	Synj2bp	synaptojanin 2 binding protein	0.85324232	0.2924	0.5012
70827	Trak2	trafficking protein, kinesin binding 2	0.85324232	0.3017	0.511
347740	2900097C17Rik	RIKEN cDNA 2900097C17 gene	0.85324232	0.3027	0.5118
73094	Sgip1	SH3-domain GRB2-like (endophilin) interacting protein 1	0.85324232	0.3121	0.5218
552909	LOC552909	hypothetical LOC552909	0.85324232	0.3607	0.5703
629678	Gm6994	predicted gene 6994	0.85324232	0.3676	0.5766
19652	Rbm3	RNA binding motif protein 3	0.85324232	0.4054	0.613
11863	Arnt	aryl hydrocarbon receptor nuclear translocator	0.85251492	0.009277	NA
231051	Mll3	myeloid/lymphoid or mixed-lineage leukemia 3	0.85251492	0.009612	NA
171429	Slc26a6	solute carrier family 26, member 6	0.85251492	0.01423	NA
226442	Zfp281	zinc finger protein 281	0.85251492	0.01841	NA
68080	Gpn3	GPN-loop GTPase 3	0.85251492	0.02028	NA
279766	Rhbdd3	rhomboid domain containing 3	0.85251492	0.02661	NA
232333	Slc6a1	solute carrier family 6 (neurotransmitter transporter, GABA), member 1	0.85251492	0.02855	0.1065
72016	1600002H07Rik	RIKEN cDNA 1600002H07 gene	0.85251492	0.03315	0.1177
14583	Gfpt1	glutamine fructose-6-phosphate transaminase 1	0.85251492	0.03374	0.119
56417	Adar	adenosine deaminase, RNA-specific	0.85251492	0.03459	0.1208
71354	Wdr31	WD repeat domain 31	0.85251492	0.03847	0.1294
77480	Kidins220	kinase D-interacting substrate 220	0.85251492	0.04983	0.1548
68750	Rreb1	ras responsive element binding protein 1	0.85251492	0.05045	0.156
76897	Ralyl	RALY RNA binding protein-like	0.85251492	0.0529	0.1614
237213	Glra2	glycine receptor, alpha 2 subunit	0.85251492	0.0649	0.1854
244871	Zc3h12c	zinc finger CCCH type containing 12C	0.85251492	0.07329	0.202
100502641	LOC100502641	60S acidic ribosomal protein P1-like	0.85251492	0.07968	0.2139
20744	Strbp	spermatid perinuclear RNA binding protein	0.85251492	0.08078	0.2157
105246	Brd9	bromodomain containing 9	0.85251492	0.0866	0.2259
20666	Sox11	SRY-box containing gene 11	0.85251492	0.09594	0.2419
211712	Pcdh9	protocadherin 9	0.85251492	0.09966	0.2478
69784	1500009L16Rik	RIKEN cDNA 1500009L16 gene	0.85251492	0.1015	0.2511
329877	Dennd4c	DENN/MADD domain containing 4C	0.85251492	0.1321	0.2987
22710	Zfp52	zinc finger protein 52	0.85251492	0.1323	0.2988
226180	Ina	internexin neuronal intermediate filament protein, alpha	0.85251492	0.1332	0.3001
56150	Mad2l1	MAD2 mitotic arrest deficient-like 1 (yeast)	0.85251492	0.1383	0.3068
228432	Ano3	anoctamin 3	0.85251492	0.1609	0.3391
14461	Gata2	GATA binding protein 2	0.85251492	0.176	0.3606
225432	Rbm27	RNA binding motif protein 27	0.85251492	0.1791	0.3645
19270	Ptprg	protein tyrosine phosphatase, receptor type, G	0.85251492	0.1878	0.3761

73692	2410089E03Rik	RIKEN cDNA 2410089E03 gene	0.85251492	0.1902	0.3791
228880	Zmynd8	zinc finger, MYND-type containing 8	0.85251492	0.1967	0.3877
22284	Usp9x	ubiquitin specific peptidase 9, X chromosome	0.85251492	0.2054	0.3992
330788	Zfp866	zinc finger protein 866	0.85251492	0.2126	0.4085
17202	Mc4r	melanocortin 4 receptor	0.85251492	0.2211	0.4201
237412	Gm4924	predicted gene 4924	0.85251492	0.2719	0.4793
268448	Phf12	PHD finger protein 12	0.85251492	0.2982	0.5076
226265	Eno4	enolase 4	0.85251492	0.3349	0.5455
207495	Baiap2l2	BAI1-associated protein 2-like 2	0.85251492	0.3622	0.5716
22061	Trp63	transformation related protein 63	0.85251492	0.3846	0.5937
12455	Ccnt1	cyclin T1	0.85251492	0.3926	0.6009
17864	Mybl1	myeloblastosis oncogene-like 1	0.85251492	0.4035	0.6109
11552	Adra2b	adrenergic receptor, alpha 2b	0.85251492	0.4439	0.6468
15165	Hcn1	hyperpolarization-activated, cyclic nucleotide-gated K+ 1	0.85251492	0.6313	0.7905
14077	Fabp3	fatty acid binding protein 3, muscle and heart	0.85178876	0.01081	NA
12583	Cdo1	cysteine dioxygenase 1, cytosolic	0.85178876	0.01195	NA
67260	Lass4	LAG1 homolog, ceramide synthase 4	0.85178876	0.01504	NA
26896	Med14	mediator complex subunit 14	0.85178876	0.01587	NA
66880	Rsrc1	arginine/serine-rich coiled-coil 1	0.85178876	0.01688	NA
105787	Prkaa1	protein kinase, AMP-activated, alpha 1 catalytic subunit	0.85178876	0.02043	NA
76707	Clasp1	CLIP associating protein 1	0.85178876	0.02348	NA
69149	Kbtbd3	kelch repeat and BTB (POZ) domain containing 3	0.85178876	0.03111	0.1129
78937	Avl9	AVL9 homolog (S. cerevisiase)	0.85178876	0.03592	0.124
235050	Zfp810	zinc finger protein 810	0.85178876	0.03968	0.132
109079	Sephs1	selenophosphate synthetase 1	0.85178876	0.04287	0.1389
20382	Srsf2	serine/arginine-rich splicing factor 2	0.85178876	0.04652	0.147
18286	Odf2	outer dense fiber of sperm tails 2	0.85178876	0.05206	0.1595
70747	Tspan2	tetraspanin 2	0.85178876	0.08164	0.2172
29856	Smtn	smoothelin	0.85178876	0.09204	0.2353
56542	Ick	intestinal cell kinase	0.85178876	0.09966	0.2478
13483	Dpp6	dipeptidylpeptidase 6	0.85178876	0.1136	0.27
232664	Ccdc136	coiled-coil domain containing 136	0.85178876	0.1169	0.275
223473	Nipal2	NIPA-like domain containing 2	0.85178876	0.1282	0.293
432442	Akap7	A kinase (PRKA) anchor protein 7	0.85178876	0.1345	0.3015
76233	Dnttip1	deoxynucleotidyltransferase, terminal, interacting protein 1	0.85178876	0.1447	0.3168
13171	Dbt	dihydrolipoamide branched chain transacylase E2	0.85178876	0.1677	0.3485
636931	Trim71	tripartite motif-containing 71	0.85178876	0.1707	0.3532
67569	Mgat4c	mannosyl (alpha-1,3-)-glycoprotein beta-1,4-N-acetylglucosaminyltransferase, isozyme C (putative)	0.85178876	0.1888	0.377
15569	Elavl2	ELAV (embryonic lethal, abnormal vision, Drosophila)-like 2 (Hu antigen B)	0.85178876	0.2131	0.4091
403345	BC037438	cDNA sequence BC037438	0.85178876	0.228	0.4281
217232	Cdc27	cell division cycle 27 homolog (S. cerevisiae)	0.85178876	0.245	0.4486
19157	Cyth1	cytohesin 1	0.85178876	0.2777	0.486
17311	Kitl	kit ligand	0.85178876	0.2857	0.4943
74852	4930402D18Rik	RIKEN cDNA 4930402D18 gene	0.85178876	0.2909	0.4993
100502966	LOC100502966	zinc finger protein 717-like	0.85178876	0.3107	0.5205
76020	5830409B07Rik	RIKEN cDNA 5830409B07 gene	0.85178876	0.3314	0.5417
19276	Ptprn2	protein tyrosine phosphatase, receptor type, N polypeptide 2	0.85178876	0.4733	0.6713
13221	Defa-rs12	defensin, alpha, related sequence 12	0.85178876	0.5094	0.7001
17258	Mef2a	myocyte enhancer factor 2A	0.85178876	0.5356	0.7201
228355	Madd	MAP-kinase activating death domain	0.85178876	0.5734	0.7476

381511	Pdp1	pyruvate dehyrogenase phosphatase catalytic subunit 1	0.85106383	0.01238	NA
19248	Ptpn12	protein tyrosine phosphatase, non-receptor type 12	0.85106383	0.01781	NA
338349	Cntln	centlein, centrosomal protein	0.85106383	0.0207	NA
100040834	Gm2990	predicted gene 2990	0.85106383	0.02555	0.09898
194590	Reps2	RALBP1 associated Eps domain containing protein 2	0.85106383	0.0269	0.1024
15364	Hmga2	high mobility group AT-hook 2	0.85106383	0.02723	0.1031
320563	Islr2	immunoglobulin superfamily containing leucine-rich repeat 2	0.85106383	0.02723	0.1031
269700	Gm15800	predicted gene 15800	0.85106383	0.02835	0.1061
20658	Son	Son DNA binding protein	0.85106383	0.03061	0.1117
103967	Dnm3	dynamin 3	0.85106383	0.03674	0.1257
11932	Atp1b2	ATPase, Na+/K+ transporting, beta 2 polypeptide	0.85106383	0.03736	0.1272
319909	Ism1	isthmin 1 homolog (zebrafish)	0.85106383	0.04626	0.1465
18191	Nrxn3	neurexin III	0.85106383	0.0557	0.1672
12561	Cdh4	cadherin 4	0.85106383	0.06594	0.1872
18393	Orc2	origin recognition complex, subunit 2	0.85106383	0.1047	0.2559
170707	Usp48	ubiquitin specific peptidase 48	0.85106383	0.1229	0.2851
51796	Srrm1	serine/arginine repetitive matrix 1	0.85106383	0.1236	0.2861
100129	Gpr153	G protein-coupled receptor 153	0.85106383	0.1356	0.303
21892	Tll1	tolloid-like	0.85106383	0.1466	0.3193
23943	Esyt1	extended synaptotagmin-like protein 1	0.85106383	0.2052	0.3991
226251	Ablim1	actin-binding LIM protein 1	0.85106383	0.2213	0.4204
67296	Socs4	suppressor of cytokine signaling 4	0.85106383	0.2523	0.4573
100502641	LOC100502641	60S acidic ribosomal protein P1-like	0.85106383	0.2571	0.4622
338349	Cntln	centlein, centrosomal protein	0.85106383	0.2739	0.4818
236904	Klhl15	kelch-like 15 (Drosophila)	0.85106383	0.3932	0.6015
229096	Ythdf3	YTH domain family 3	0.85106383	0.4111	0.6174
57264	Retn	resistin	0.85106383	0.4256	0.6308
18378	Omp	olfactory marker protein	0.85106383	0.4714	0.67
74473	4933433H22Rik	RIKEN cDNA 4933433H22 gene	0.85106383	0.4932	0.6872
11307	Abcg1	ATP-binding cassette, sub-family G (WHITE), member 1	0.85034014	0.002041	NA
17764	Mtf1	metal response element binding transcription factor 1	0.85034014	0.01072	NA
66244	Sdccag1	serologically defined colon cancer antigen 1	0.85034014	0.01225	NA
11990	Atrn	attractin	0.85034014	0.01308	NA
52635	Esyt2	extended synaptotagmin-like protein 2	0.85034014	0.01394	NA
12750	Clk4	CDC like kinase 4	0.85034014	0.01694	NA
216238	Eea1	early endosome antigen 1	0.85034014	0.02001	NA
16524	Kcnj9	potassium inwardly-rectifying channel, subfamily J, member 9	0.85034014	0.02309	NA
54394	Crlf3	cytokine receptor-like factor 3	0.85034014	0.02401	NA
56495	Asna1	arsA arsenite transporter, ATP-binding, homolog 1 (bacterial)	0.85034014	0.0442	0.1419
101739	Psip1	PC4 and SFRS1 interacting protein 1	0.85034014	0.04857	0.1518
26380	Esrrb	estrogen related receptor, beta	0.85034014	0.06981	0.1949
72129	Pex13	peroxisomal biogenesis factor 13	0.85034014	0.07078	0.1966
26938	St6galnac5	ST6 (alpha-N-acetyl-neuraminyl-2,3-beta-galactosyl-1,3)-N-acetylgalactosaminide alpha-2,6-sialyltransferase 5	0.85034014	0.07925	0.2131
66447	Mgst3	microsomal glutathione S-transferase 3	0.85034014	0.07949	0.2136
54614	Prpf40b	PRP40 pre-mRNA processing factor 40 homolog B (yeast)	0.85034014	0.08972	0.231
18514	Pbx1	pre B-cell leukemia transcription factor 1	0.85034014	0.1025	0.2525
71648	Optn	optineurin	0.85034014	0.1029	0.2531
623169	Gm6402	ribosomal protein S17 pseudogene	0.85034014	0.1174	0.2758
103098	Slc6a15	solute carrier family 6 (neurotransmitter transporter), member 15	0.85034014	0.1181	0.277
320556	C230085N15Rik	RIKEN cDNA C230085N15 gene	0.85034014	0.1198	0.2799

230598	Nrd1		nardilysin, N-arginine dibasic convertase, NRD convertase 1	0.85034014	0.1248	0.2877
52906	Ahi1		Abelson helper integration site 1	0.85034014	0.1258	0.289
215378	Fam5c		family with sequence similarity 5, member C	0.85034014	0.1359	0.3033
73451	Zfp763		zinc finger protein 763	0.85034014	0.1387	0.3076
225913	Dak		dihydroxyacetone kinase 2 homolog (yeast)	0.85034014	0.1455	0.3181
108058	Camk2d		calcium/calmodulin-dependent protein kinase II, delta	0.85034014	0.1583	0.3357
104103	Airn		antisense Igf2r RNA	0.85034014	0.1743	0.3581
58226	Cacna1h		calcium channel, voltage-dependent, T type, alpha 1H subunit	0.85034014	0.184	0.3709
68971	1500001M20Rik		RIKEN cDNA 1500001M20 gene	0.85034014	0.2801	0.4883
93842	Igsf9		immunoglobulin superfamily, member 9	0.85034014	0.2922	0.501
213993	A630007B06Rik		RIKEN cDNA A630007B06 gene	0.85034014	0.3138	0.5235
224703		Mar-02	membrane-associated ring finger (C3HC4) 2	0.85034014	0.4154	0.6216
52841	D2Bwg0886e		DNA segment, Chr 2, Brigham & Women's Genetics 0886 expressed	0.85034014	0.6065	0.7727
54367	Zfp326		zinc finger protein 326	0.84961767	0.004046	NA
66690	Tmem186		transmembrane protein 186	0.84961767	0.00962	NA
14660	Gls		glutaminase	0.84961767	0.01556	NA
12301	Cacybp		calcyclin binding protein	0.84961767	0.0196	NA
94249	Slc24a3		solute carrier family 24 (sodium/potassium/calcium exchanger), member 3	0.84961767	0.02175	NA
18087	Nktr		natural killer tumor recognition sequence	0.84961767	0.0241	0.09508
330959	Snapc5		small nuclear RNA activating complex, polypeptide 5	0.84961767	0.02525	0.09804
68724	Arl8a		ADP-ribosylation factor-like 8A	0.84961767	0.03277	0.1169
225055	Fbxo11		F-box protein 11	0.84961767	0.03591	0.124
74149	Zfp946		zinc finger protein 946	0.84961767	0.03998	0.1328
66310	Dpy30		dpy-30 homolog (C. elegans)	0.84961767	0.0436	0.1405
21841	Tia1		cytotoxic granule-associated RNA binding protein 1	0.84961767	0.04659	0.1472
15571	Elavl3		ELAV (embryonic lethal, abnormal vision, Drosophila)-like 3 (Hu antigen C)	0.84961767	0.05084	0.1568
23874	Farsb		phenylalanyl-tRNA synthetase, beta subunit	0.84961767	0.06997	0.1951
268721	2310021P13Rik		RIKEN cDNA 2310021P13 gene	0.84961767	0.09041	0.2324
26445	Psmb2		proteasome (prosome, macropain) subunit, beta type 2	0.84961767	0.09118	0.2337
16502	Kcnc1		potassium voltage gated channel, Shaw-related subfamily, member 1	0.84961767	0.09325	0.2371
207932	Urb1		URB1 ribosome biogenesis 1 homolog (S. cerevisiae)	0.84961767	0.1037	0.2544
77733	Rnf170		ring finger protein 170	0.84961767	0.1088	0.2621
73582	1700106N22Rik		RIKEN cDNA 1700106N22 gene	0.84961767	0.1214	0.2827
65115	Bean1		brain expressed, associated with Nedd4, 1	0.84961767	0.1237	0.2861
23888	Gpc6		glypican 6	0.84961767	0.1514	0.3261
22215	Ube3a		ubiquitin protein ligase E3A	0.84961767	0.2204	0.4196
12175	Bnip2		BCL2/adenovirus E1B interacting protein 2	0.84961767	0.2568	0.462
70028	Dopey2		dopey family member 2	0.84961767	0.2634	0.4695
229504	Isg20l2		interferon stimulated exonuclease gene 20-like 2	0.84961767	0.2758	0.4838
74004	Jakmip3		janus kinase and microtubule interacting protein 3	0.84961767	0.2829	0.4913
54611	Pde3a		phosphodiesterase 3A, cGMP inhibited	0.84961767	0.3226	0.5324
71392	5430401F13Rik		RIKEN cDNA 5430401F13 gene	0.84961767	0.3595	0.5692
16691	Krt8		keratin 8	0.84961767	0.4168	0.6229
16495	Kcna7		potassium voltage-gated channel, shaker-related subfamily, member 7	0.84961767	0.4463	0.6489
74525	8430419L09Rik		RIKEN cDNA 8430419L09 gene	0.84961767	0.4839	0.6794
50997	Mpp2		membrane protein, palmitoylated 2 (MAGUK p55 subfamily member 2)	0.84961767	0.5038	0.6953
231876	Lmtk2		lemur tyrosine kinase 2	0.84961767	0.555	0.7349
73712	Dmkn		dermokine	0.84961767	0.5896	0.7596
233532	Rsf1		remodeling and spacing factor 1	0.84889643	0.007443	NA
230661	Tesk2		testis-specific kinase 2	0.84889643	0.01271	NA

67574	Alg13	asparagine-linked glycosylation 13 homolog (S. cerevisiae)	0.84889643	0.01411	NA
11836	Araf	v-raf murine sarcoma 3611 viral oncogene homolog	0.84889643	0.02125	NA
235567	Dnajc13	DnaJ (Hsp40) homolog, subfamily C, member 13	0.84889643	0.02242	0.09076
18020	Nfatc2ip	nuclear factor of activated T-cells, cytoplasmic, calcineurin-dependent 2 interacting protein	0.84889643	0.02427	0.09533
67283	Slc25a19	solute carrier family 25 (mitochondrial thiamine pyrophosphate carrier), member 19	0.84889643	0.0305	0.1115
320105	D030036P13Rik	RIKEN cDNA D030036P13 gene	0.84889643	0.03496	0.1218
18772	Pkp1	plakophilin 1	0.84889643	0.03804	0.1287
67230	Zfp329	zinc finger protein 329	0.84889643	0.05685	0.1695
117592	B3galt6	UDP-Gal:betaGal beta 1,3-galactosyltransferase, polypeptide 6	0.84889643	0.08	0.2145
14545	Gdap1	ganglioside-induced differentiation-associated-protein 1	0.84889643	0.08702	0.2267
54208	Arl6ip1	ADP-ribosylation factor-like 6 interacting protein 1	0.84889643	0.0882	0.2285
71735	Lrwd1	leucine-rich repeats and WD repeat domain containing 1	0.84889643	0.09618	0.2421
22121	Rpl13a	ribosomal protein L13A	0.84889643	0.1021	0.2521
223227	Sox21	SRY-box containing gene 21	0.84889643	0.1238	0.2864
109242	Kif24	kinesin family member 24	0.84889643	0.1539	0.3296
78933	Agbl4	ATP/GTP binding protein-like 4	0.84889643	0.2155	0.4127
240869	Zbtb37	zinc finger and BTB domain containing 37	0.84889643	0.2162	0.4135
20419	Shcbp1	Shc SH2-domain binding protein 1	0.84889643	0.2342	0.4357
75137	Rprd2	regulation of nuclear pre-mRNA domain containing 2	0.84889643	0.2346	0.4363
240057	Syngap1	synaptic Ras GTPase activating protein 1 homolog (rat)	0.84889643	0.2824	0.4906
56873	Lmbr1	limb region 1	0.84889643	0.3511	0.5611
11694	Alx3	aristaless-like homeobox 3	0.84889643	0.5044	0.6957
83395	Sp6	trans-acting transcription factor 6	0.84889643	0.5446	0.7266
677113	LOC677113	40S ribosomal protein S24-like	0.84889643	0.6922	0.8295
230157	Tmeff1	transmembrane protein with EGF-like and two follistatin-like domains 1	0.84817642	0.02611	0.1004
69847	Wnk4	WNK lysine deficient protein kinase 4	0.84817642	0.02686	0.1022
57784	Bin3	bridging integrator 3	0.84817642	0.05139	0.158
320650	9530056K15Rik	RIKEN cDNA 9530056K15 gene	0.84817642	0.0536	0.1628
286940	Flnb	filamin, beta	0.84817642	0.08806	0.2284
57895	Ccdc126	coiled-coil domain containing 126	0.84817642	0.09889	0.2466
99100	Cep152	centrosomal protein 152	0.84817642	0.09903	0.2468
214084	Slc18a2	solute carrier family 18 (vesicular monoamine), member 2	0.84817642	0.1026	0.2526
107568	Wwp1	WW domain containing E3 ubiquitin protein ligase 1	0.84817642	0.1384	0.3071
18754	Prkce	protein kinase C, epsilon	0.84817642	0.175	0.3591
17763	Mtcp1	mature T-cell proliferation 1	0.84817642	0.1973	0.3885
77987	Ascc3	activating signal cointegrator 1 complex subunit 3	0.84817642	0.2086	0.4034
56695	Pnkd	paroxysmal nonkinesiogenic dyskinesia	0.84817642	0.2197	0.4186
30044	Opn4	opsin 4 (melanopsin)	0.84817642	0.2318	0.4326
233987	Zfp958	zinc finger protein 958	0.84817642	0.2712	0.4788
94253	Hecw1	HECT, C2 and WW domain containing E3 ubiquitin protein ligase 1	0.84817642	0.3003	0.5097
12511	Cd6	CD6 antigen	0.84817642	0.3013	0.5108
320150	Zdhhc17	zinc finger, DHHC domain containing 17	0.84817642	0.3101	0.52
70892	Ttll7	tubulin tyrosine ligase-like family, member 7	0.84817642	0.3206	0.5305
338351	Akap17b	A kinase (PRKA) anchor protein 17B	0.84817642	0.321	0.531
19131	Prh1	proline rich protein HaeIII subfamily 1	0.84817642	0.3997	0.6075
19276	Ptprn2	protein tyrosine phosphatase, receptor type, N polypeptide 2	0.84817642	0.4078	0.6144
14269	Fnbp1	formin binding protein 1	0.84817642	0.4251	0.6305
217666	L2hgdh	L-2-hydroxyglutarate dehydrogenase	0.84817642	0.4748	0.6724
435766	Tnni3k	TNNI3 interacting kinase	0.84817642	0.5582	0.7377
58184	Rqcd1	rcd1 (required for cell differentiation) homolog 1 (S. pombe)	0.84745763	0.006911	NA

140492	Kcnn2	potassium intermediate/small conductance calcium-activated channel, subfamily N, member 2	0.84745763	0.01119	NA
217648	Gm527	predicted gene 527	0.84745763	0.01696	NA
80892	Zfhx4	zinc finger homeodomain 4	0.84745763	0.0211	0.08719
320806	Gfm2	G elongation factor, mitochondrial 2	0.84745763	0.02541	0.09853
59013	Hnrnph1	heterogeneous nuclear ribonucleoprotein H1	0.84745763	0.0335	0.1184
245684	Cnksr2	connector enhancer of kinase suppressor of Ras 2	0.84745763	0.04696	0.148
15499	Hsf1	heat shock factor 1	0.84745763	0.05733	0.1703
56398	1500003O03Rik	RIKEN cDNA 1500003O03 gene	0.84745763	0.09605	0.2419
226896	Tcfap2d	transcription factor AP-2, delta	0.84745763	0.1241	0.2868
14812	Grin2b	glutamate receptor, ionotropic, NMDA2B (epsilon 2)	0.84745763	0.1907	0.3799
71452	Ankrd40	ankyrin repeat domain 40	0.84745763	0.2035	0.3969
399603	Fam84b	family with sequence similarity 84, member B	0.84745763	0.2102	0.4058
432769	Zfp708	zinc finger protein 708	0.84745763	0.2155	0.4127
15234	Hgf	hepatocyte growth factor	0.84745763	0.2158	0.413
218030	Pou6f2	POU domain, class 6, transcription factor 2	0.84745763	0.2844	0.4928
27376	Slc25a10	solute carrier family 25 (mitochondrial carrier, dicarboxylate transporter), member 10	0.84745763	0.3944	0.6026
319615	Zfp944	zinc finger protein 944	0.84674005	0.003703	NA
68097	Dynll2	dynein light chain LC8-type 2	0.84674005	0.005687	NA
23806	Arih1	ariadne ubiquitin-conjugating enzyme E2 binding protein homolog 1 (Drosophila)	0.84674005	0.007014	NA
269966	Nup98	nucleoporin 98	0.84674005	0.01257	NA
668212	Efr3b	EFR3 homolog B (S. cerevisiae)	0.84674005	0.0132	NA
52715	Ccdc43	coiled-coil domain containing 43	0.84674005	0.01918	NA
107723	Slc12a6	solute carrier family 12, member 6	0.84674005	0.02051	0.08534
14007	Celf2	CUGBP, Elav-like family member 2	0.84674005	0.0378	0.1282
100038651	D130062J21Rik	RIKEN cDNA D130062J21 gene	0.84674005	0.06706	0.1892
211978	Zfyve26	zinc finger, FYVE domain containing 26	0.84674005	0.07125	0.1976
23873	Faim	Fas apoptotic inhibitory molecule	0.84674005	0.07424	0.2036
72371	2210408I21Rik	RIKEN cDNA 2210408I21 gene	0.84674005	0.07635	0.2077
116939	Pnpla3	patatin-like phospholipase domain containing 3	0.84674005	0.09541	0.2409
29875	Iqgap1	IQ motif containing GTPase activating protein 1	0.84674005	0.1256	0.2889
63993	Slc5a7	solute carrier family 5 (choline transporter), member 7	0.84674005	0.13	0.2957
14348	Fut9	fucosyltransferase 9	0.84674005	0.1303	0.296
100732	Mapre3	microtubule-associated protein, RP/EB family, member 3	0.84674005	0.1336	0.3005
76897	Ralyl	RALY RNA binding protein-like	0.84674005	0.1552	0.3314
74016	Phf19	PHD finger protein 19	0.84674005	0.1599	0.338
58988	Rps6kb2	ribosomal protein S6 kinase, polypeptide 2	0.84674005	0.1649	0.3449
105372	Utp15	UTP15, U3 small nucleolar ribonucleoprotein, homolog (yeast)	0.84674005	0.1658	0.3462
219132	D14Ertd668e	DNA segment, Chr 14, ERATO Doi 668, expressed	0.84674005	0.1879	0.3761
668763	Gm9340	predicted gene 9340	0.84674005	0.2161	0.4135
76897	Ralyl	RALY RNA binding protein-like	0.84674005	0.2226	0.4221
232217	4933427D06Rik	RIKEN cDNA 4933427D06 gene	0.84674005	0.279	0.487
13982	Esr1	estrogen receptor 1 (alpha)	0.84674005	0.2952	0.5042
22057	Tob1	transducer of ErbB-2.1	0.84674005	0.3259	0.5359
77951	Cyp20a1	cytochrome P450, family 20, subfamily A, polypeptide 1	0.84674005	0.3852	0.5944
54427	Dnmt3l	DNA (cytosine-5-)-methyltransferase 3-like	0.84674005	0.3889	0.5972
668382	Pabpc1l2b-ps	poly(A) binding protein, cytoplasmic 1-like 2B, pseudogene	0.84674005	0.4054	0.613
73761	4833415N18Rik	RIKEN cDNA 4833415N18 gene	0.84674005	0.4755	0.6729
54384	Mtmr7	myotubularin related protein 7	0.84602369	0.003835	NA
51885	Tubgcp4	tubulin, gamma complex associated protein 4	0.84602369	0.01194	NA
20018	Polr1d	polymerase (RNA) I polypeptide D	0.84602369	0.031	0.1127

20807	Srf	serum response factor	0.84602369	0.04406	0.1415
230861	Eif4g3	eukaryotic translation initiation factor 4 gamma, 3	0.84602369	0.06672	0.1888
53621	Cnot4	CCR4-NOT transcription complex, subunit 4	0.84602369	0.08105	0.2161
83925	Trps1	trichorhinophalangeal syndrome I (human)	0.84602369	0.08571	0.2244
75785	Klhl24	kelch-like 24 (Drosophila)	0.84602369	0.1016	0.2512
100043364	Gm4392	predicted gene 4392	0.84602369	0.1023	0.2522
76073	Pcgf5	polycomb group ring finger 5	0.84602369	0.1093	0.263
215001	Wfikkn1	WAP, FS, Ig, KU, and NTR-containing protein 1	0.84602369	0.1294	0.2949
216080	Ube2d1	ubiquitin-conjugating enzyme E2D 1, UBC4/5 homolog (yeast)	0.84602369	0.142	0.3127
15109	Hal	histidine ammonia lyase	0.84602369	0.1947	0.3851
100043305	Gm4349	SET domain, bifurcated 1 pseudogene	0.84602369	0.2523	0.4574
240168	Rasgrp3	RAS, guanyl releasing protein 3	0.84602369	0.2762	0.4841
12902	Cr2	complement receptor 2	0.84602369	0.3275	0.5376
258693	Olfr1443	olfactory receptor 1443	0.84602369	0.3359	0.5463
17880	Myh11	myosin, heavy polypeptide 11, smooth muscle	0.84602369	0.471	0.6698
100009609	Vmn2r65	vomeronasal 2, receptor 65	0.84602369	0.6609	0.809
432486	Gnptab	N-acetylglucosamine-1-phosphate transferase, alpha and beta subunits	0.84530854	0.00992	NA
75901	Dcp1a	DCP1 decapping enzyme homolog A (S. cerevisiae)	0.84530854	0.01724	NA
12894	Cpt1a	carnitine palmitoyltransferase 1a, liver	0.84530854	0.01799	0.07808
67143	Ikzf5	IKAROS family zinc finger 5	0.84530854	0.02126	0.08763
56622	Adam21	a disintegrin and metallopeptidase domain 21	0.84530854	0.02966	0.1092
100039684	5031434O11Rik	RIKEN cDNA 5031434O11 gene	0.84530854	0.03114	0.1129
16709	Ktn1	kinectin 1	0.84530854	0.03401	0.1194
100038570	Gm11744	predicted gene 11744	0.84530854	0.04036	0.1336
73225	Fam118a	family with sequence similarity 118, member A	0.84530854	0.04072	0.1344
67071	Rps6ka6	ribosomal protein S6 kinase polypeptide 6	0.84530854	0.07451	0.2041
170731	Mfn2	mitofusin 2	0.84530854	0.07645	0.2079
15248	Hic1	hypermethylated in cancer 1	0.84530854	0.08012	0.2147
14325	Ftl1	ferritin light chain 1	0.84530854	0.08133	0.2167
72289	Malat1	metastasis associated lung adenocarcinoma transcript 1 (non-coding RNA)	0.84530854	0.09489	0.24
11861	Arl4a	ADP-ribosylation factor-like 4A	0.84530854	0.1214	0.2827
20508	Slc18a3	solute carrier family 18 (vesicular monoamine), member 3	0.84530854	0.1225	0.2844
80877	Lrba	LPS-responsive beige-like anchor	0.84530854	0.1254	0.2887
19820	Rlim	ring finger protein, LIM domain interacting	0.84530854	0.1517	0.3265
77929	Yipf6	Yip1 domain family, member 6	0.84530854	0.1618	0.3403
11920	Atm	ataxia telangiectasia mutated homolog (human)	0.84530854	0.2183	0.4166
74534	8430428J23Rik	RIKEN cDNA 8430428J23 gene	0.84530854	0.2207	0.4197
20265	Scn1a	sodium channel, voltage-gated, type I, alpha	0.84530854	0.2616	0.4675
26556	Homer1	homer homolog 1 (Drosophila)	0.84530854	0.3097	0.5195
224598	Zfp758	zinc finger protein 758	0.84530854	0.3283	0.5383
244666	Gm505	predicted gene 505	0.84530854	0.3624	0.5716
22746	Zfp85-rs1	zinc finger protein 85, related sequence 1	0.84530854	0.3766	0.5855
20742	Spnb2	spectrin beta 2	0.84530854	0.3839	0.5931
21676	Tead1	TEA domain family member 1	0.84530854	0.4305	0.6345
16679	Krt86	keratin 86	0.84530854	0.6024	0.7693
30948	Bin1	bridging integrator 1	0.84459459	0.007856	NA
56036	Ccnl2	cyclin L2	0.84459459	0.007952	NA
18526	Pcdh10	protocadherin 10	0.84459459	0.01927	0.08174
30944	Zfp354c	zinc finger protein 354C	0.84459459	0.03125	0.1132
18997	Pou4f2	POU domain, class 4, transcription factor 2	0.84459459	0.03219	0.1154

14159	Fes	feline sarcoma oncogene	0.84459459	0.03317	0.1178
26938	St6galnac5	ST6 (alpha-N-acetyl-neuraminyl-2,3-beta-galactosyl-1,3)-N-acetylgalactosaminide alpha-2,6-sialyltransferase 5	0.84459459	0.03715	0.1267
245867	Pcmtd2	protein-L-isoaspartate (D-aspartate) O-methyltransferase domain containing 2	0.84459459	0.05086	0.1568
15161	Hcfc1	host cell factor C1	0.84459459	0.05195	0.1594
69726	Smyd3	SET and MYND domain containing 3	0.84459459	0.05294	0.1615
668572	2610021A01Rik	RIKEN cDNA 2610021A01 gene	0.84459459	0.06187	0.1794
80292	Zxdc	ZXD family zinc finger C	0.84459459	0.07979	0.2141
81907	Tmem108	transmembrane protein 108	0.84459459	0.08053	0.2153
23917	Impdh1	inosine 5'-phosphate dehydrogenase 1	0.84459459	0.1016	0.2512
18191	Nrxn3	neurexin III	0.84459459	0.106	0.2575
51944	D2Ertd750e	DNA segment, Chr 2, ERATO Doi 750, expressed	0.84459459	0.1607	0.3389
209692	Dhtkd1	dehydrogenase E1 and transketolase domain containing 1	0.84459459	0.1738	0.3574
246104	Rhbdl3	rhomboid, veinlet-like 3 (Drosophila)	0.84459459	0.1773	0.3622
330671	B4galnt4	beta-1,4-N-acetyl-galactosaminyl transferase 4	0.84459459	0.2841	0.4927
93725	Ear10	eosinophil-associated, ribonuclease A family, member 10	0.84459459	0.3091	0.519
258866	Olfr1008	olfactory receptor 1008	0.84459459	0.3356	0.546
260301	Otos	otospiralin	0.84459459	0.3653	0.5743
56045	Samhd1	SAM domain and HD domain, 1	0.84388186	0.003564	NA
211914	Asap2	ArfGAP with SH3 domain, ankyrin repeat and PH domain 2	0.84388186	0.01164	NA
75801	4930447C04Rik	RIKEN cDNA 4930447C04 gene	0.84388186	0.01671	0.0741
116940	Tgs1	trimethylguanosine synthase homolog (S. cerevisiae)	0.84388186	0.01829	0.07892
71703	Armcx3	armadillo repeat containing, X-linked 3	0.84388186	0.01967	0.08286
116837	Rims1	regulating synaptic membrane exocytosis 1	0.84388186	0.01969	0.08289
110175	Ggct	gamma-glutamyl cyclotransferase	0.84388186	0.02082	0.08635
13204	Dhx15	DEAH (Asp-Glu-Ala-His) box polypeptide 15	0.84388186	0.0212	0.08744
623474	Rad54b	RAD54 homolog B (S. cerevisiae)	0.84388186	0.02189	0.08936
73744	Man2c1	mannosidase, alpha, class 2C, member 1	0.84388186	0.02807	0.1053
224907	Dus3l	dihydrouridine synthase 3-like (S. cerevisiae)	0.84388186	0.03546	0.123
231855	C330006K01Rik	RIKEN cDNA C330006K01 gene	0.84388186	0.0365	0.1252
240442	Adnp2	ADNP homeobox 2	0.84388186	0.03855	0.1296
56070	Tcerg1	transcription elongation regulator 1 (CA150)	0.84388186	0.04294	0.1391
330301	Zfp786	zinc finger protein 786	0.84388186	0.04389	0.1412
225929	Patl1	protein associated with topoisomerase II homolog 1 (yeast)	0.84388186	0.04928	0.1534
269774	Aak1	AP2 associated kinase 1	0.84388186	0.05022	0.1555
229949	Ak5	adenylate kinase 5	0.84388186	0.05685	0.1695
72039	Mccc1	methylcrotonoyl-Coenzyme A carboxylase 1 (alpha)	0.84388186	0.07348	0.2023
18636	Cfp	complement factor properdin	0.84388186	0.07765	0.21
70891	Spdya	speedy homolog A (Xenopus laevis)	0.84388186	0.1127	0.2685
23984	Pde10a	phosphodiesterase 10A	0.84388186	0.1611	0.3393
71263	Mro	maestro	0.84388186	0.1942	0.3845
20680	Sox7	SRY-box containing gene 7	0.84388186	0.2478	0.4519
407821	Znrf3	zinc and ring finger 3	0.84388186	0.2852	0.4936
320560	Dennd5b	DENN/MADD domain containing 5B	0.84388186	0.3321	0.5423
18605	Enpp1	ectonucleotide pyrophosphatase/phosphodiesterase 1	0.84388186	0.3876	0.5961
13211	Dhx9	DEAH (Asp-Glu-Ala-His) box polypeptide 9	0.84388186	0.4063	0.6135
232889	Pla2g4c	phospholipase A2, group IVC (cytosolic, calcium-independent)	0.84388186	0.4637	0.664
19933	Rpl21	ribosomal protein L21	0.84317032	0.004933	NA
270198	Pfkfb4	6-phosphofructo-2-kinase/fructose-2,6-biphosphatase 4	0.84317032	0.01072	NA
19395	Rasgrp2	RAS, guanyl releasing protein 2	0.84317032	0.0131	NA
68276	Toe1	target of EGR1, member 1 (nuclear)	0.84317032	0.01869	0.08012

215090	Maneal	mannosidase, endo-alpha-like	0.84317032	0.0189	0.08063
207227	Stxbp5l	syntaxin binding protein 5-like	0.84317032	0.02961	0.1092
102442	Dennd4a	DENN/MADD domain containing 4A	0.84317032	0.03513	0.1222
235184	BC024479	cDNA sequence BC024479	0.84317032	0.03637	0.125
11308	Abi1	abl-interactor 1	0.84317032	0.03772	0.128
216873	Spag7	sperm associated antigen 7	0.84317032	0.04665	0.1473
244310	Dlgap2	discs, large (Drosophila) homolog-associated protein 2	0.84317032	0.05461	0.165
244484	Wdr17	WD repeat domain 17	0.84317032	0.07334	0.2021
225392	Rell2	RELT-like 2	0.84317032	0.08061	0.2154
321007	Serac1	serine active site containing 1	0.84317032	0.08148	0.217
213895	Bms1	BMS1 homolog, ribosome assembly protein (yeast)	0.84317032	0.08526	0.2237
69743	Casz1	castor homolog 1, zinc finger (Drosophila)	0.84317032	0.08736	0.2273
18952	Sep-04	septin 4	0.84317032	0.09936	0.2473
15258	Hipk2	homeodomain interacting protein kinase 2	0.84317032	0.1029	0.2531
56401	Lepre1	leprecan 1	0.84317032	0.1237	0.2861
546024	Crxos1	Crx opposite strand transcript 1	0.84317032	0.1734	0.357
666183	Gm7968	predicted gene 7968	0.84317032	0.1757	0.3602
27373	Csnk1e	casein kinase 1, epsilon	0.84317032	0.3174	0.527
76295	Atp11b	ATPase, class VI, type 11B	0.84317032	0.3698	0.5788
76080	Ttpal	tocopherol (alpha) transfer protein-like	0.84317032	0.4024	0.61
12834	Col6a2	collagen, type VI, alpha 2	0.84317032	0.4059	0.6134
218314	Zfp595	zinc finger protein 595	0.84317032	0.4446	0.6474
78341	2900016J10Rik	RIKEN cDNA 2900016J10 gene	0.84245998	0.00696	NA
76338	Rab2b	RAB2B, member RAS oncogene family	0.84245998	0.01166	NA
320705	Bend6	BEN domain containing 6	0.84245998	0.01777	0.07741
77626	Smpd4	sphingomyelin phosphodiesterase 4	0.84245998	0.01924	0.08162
12193	Zfp36l2	zinc finger protein 36, C3H type-like 2	0.84245998	0.01959	0.08263
76484	Kndc1	kinase non-catalytic C-lobe domain (KIND) containing 1	0.84245998	0.03395	0.1193
67062	Mcart6	mitochondrial carrier triple repeat 6	0.84245998	0.03658	0.1254
19335	Rab23	RAB23, member RAS oncogene family	0.84245998	0.06976	0.1948
73168	3110027N22Rik	RIKEN cDNA 3110027N22 gene	0.84245998	0.07068	0.1965
27371	Sh2d2a	SH2 domain protein 2A	0.84245998	0.1119	0.267
54609	Ubqln2	ubiquilin 2	0.84245998	0.1121	0.2675
68897	Disp1	dispatched homolog 1 (Drosophila)	0.84245998	0.1269	0.2909
240261	Ccdc112	coiled-coil domain containing 112	0.84245998	0.1526	0.3278
27389	Dusp13	dual specificity phosphatase 13	0.84245998	0.1686	0.3497
54169	Myst4	MYST histone acetyltransferase monocytic leukemia 4	0.84245998	0.174	0.3577
72148	2610019F03Rik	RIKEN cDNA 2610019F03 gene	0.84245998	0.1768	0.3616
97547	C81489	expressed sequence C81489	0.84245998	0.2412	0.4438
98258	Txndc9	thioredoxin domain containing 9	0.84245998	0.2843	0.4928
78103	8430431K14Rik	RIKEN cDNA 8430431K14 gene	0.84245998	0.295	0.5039
30878	Apln	apelin	0.84245998	0.3171	0.5266
100043468	Zfp955b	zinc finger protein 955B	0.84245998	0.3442	0.5548
100504346	Gm13304	predicted gene 13304	0.84245998	0.403	0.6106
16199	Il9r	interleukin 9 receptor	0.84245998	0.4858	0.6811
383619	Aim2	absent in melanoma 2	0.84245998	0.5783	0.751
229317	Eif2a	eukaryotic translation initiation factor 2A	0.84175084	0.008666	NA
79263	Trim39	tripartite motif-containing 39	0.84175084	0.0101	NA
18203	Ntan1	N-terminal Asn amidase	0.84175084	0.01107	NA
16647	Kpna2	karyopherin (importin) alpha 2	0.84175084	0.01207	NA

110876	Scn2a1	sodium channel, voltage-gated, type II, alpha 1	0.84175084	0.01588	0.07195
77832	Tchp	trichoplein, keratin filament binding	0.84175084	0.01668	0.07401
76210	6430597G12Rik	RIKEN cDNA 6430597G12 gene	0.84175084	0.01717	0.07555
58243	Nap1l5	nucleosome assembly protein 1-like 5	0.84175084	0.01881	0.08037
269717	Orai2	ORAI calcium release-activated calcium modulator 2	0.84175084	0.02675	0.1021
22690	Zfp28	zinc finger protein 28	0.84175084	0.03134	0.1134
12745	Clgn	calmegin	0.84175084	0.0325	0.1163
70028	Dopey2	dopey family member 2	0.84175084	0.0335	0.1184
232680	Cpa2	carboxypeptidase A2, pancreatic	0.84175084	0.03759	0.1278
69368	Wdfy1	WD repeat and FYVE domain containing 1	0.84175084	0.03894	0.1305
12671	Chrm3	cholinergic receptor, muscarinic 3, cardiac	0.84175084	0.05157	0.1585
104349	Zfp119a	zinc finger protein 119a	0.84175084	0.052	0.1594
100182	Akna	AT-hook transcription factor	0.84175084	0.06092	0.1773
17116	Mab21l1	mab-21-like 1 (C. elegans)	0.84175084	0.06784	0.1908
791275	Gm9885	predicted gene 9885	0.84175084	0.07722	0.2092
21841	Tia1	cytotoxic granule-associated RNA binding protein 1	0.84175084	0.09123	0.2338
231637	Ssh1	slingshot homolog 1 (Drosophila)	0.84175084	0.1006	0.2496
245468	Pnma3	paraneoplastic antigen MA3	0.84175084	0.1029	0.2531
17391	Mmp24	matrix metallopeptidase 24	0.84175084	0.1218	0.2834
12728	Clcn5	chloride channel 5	0.84175084	0.1857	0.3731
68564	Nufip2	nuclear fragile X mental retardation protein interacting protein 2	0.84175084	0.2224	0.4218
234353	Psd3	pleckstrin and Sec7 domain containing 3	0.84175084	0.2305	0.4314
71972	Dnmbp	dynamin binding protein	0.84175084	0.2445	0.4483
414069	BC024978	cDNA sequence BC024978	0.84175084	0.2679	0.4748
12504	Cd4	CD4 antigen	0.84175084	0.2978	0.5072
624219	Gm6484	predicted gene 6484	0.84175084	0.3291	0.5394
13654	Egr2	early growth response 2	0.84175084	0.7461	0.8632
56200	Ddx21	DEAD (Asp-Glu-Ala-Asp) box polypeptide 21	0.84104289	0.006703	NA
18040	Nefm	neurofilament, medium polypeptide	0.84104289	0.008796	NA
78523	Mrpl9	mitochondrial ribosomal protein L9	0.84104289	0.01063	NA
329504	Lcmt2	leucine carboxyl methyltransferase 2	0.84104289	0.01151	NA
107934	Celsr3	cadherin, EGF LAG seven-pass G-type receptor 3 (flamingo homolog, Drosophila)	0.84104289	0.02564	0.09917
72656	Ints8	integrator complex subunit 8	0.84104289	0.02677	0.1021
27390	Mmel1	membrane metallo-endopeptidase-like 1	0.84104289	0.04177	0.1367
170716	Cyp4f13	cytochrome P450, family 4, subfamily f, polypeptide 13	0.84104289	0.05902	0.1736
72139	2610044O15Rik	RIKEN cDNA 2610044O15 gene	0.84104289	0.06165	0.179
75678	Ippk	inositol 1,3,4,5,6-pentakisphosphate 2-kinase	0.84104289	0.07045	0.196
26557	Homer2	homer homolog 2 (Drosophila)	0.84104289	0.08227	0.2184
110332	4921523A10Rik	RIKEN cDNA 4921523A10 gene	0.84104289	0.08404	0.2214
14400	Gabrb1	gamma-aminobutyric acid (GABA) A receptor, subunit beta 1	0.84104289	0.08978	0.231
239318	Plcxd3	phosphatidylinositol-specific phospholipase C, X domain containing 3	0.84104289	0.1294	0.2949
66776	Pisd-ps3	phosphatidylserine decarboxylase, pseudogene 3	0.84104289	0.131	0.2969
60345	Nrip2	nuclear receptor interacting protein 2	0.84104289	0.1507	0.3255
73122	Tgfbrap1	transforming growth factor, beta receptor associated protein 1	0.84104289	0.1804	0.3662
268729	Gm626	predicted gene 626	0.84104289	0.1956	0.3862
207592	Tbc1d16	TBC1 domain family, member 16	0.84104289	0.1974	0.3886
102423	Hinfp	histone H4 transcription factor	0.84104289	0.2579	0.4632
330812	Rnf150	ring finger protein 150	0.84104289	0.3029	0.5119
14583	Gfpt1	glutamine fructose-6-phosphate transaminase 1	0.84104289	0.3881	0.5966
94089	Trim7	tripartite motif-containing 7	0.84104289	0.4687	0.6684

320429	Trank1	tetratricopeptide repeat and ankyrin repeat containing 1	0.84104289	0.4777	0.6747
237500	Tmtc3	transmembrane and tetratricopeptide repeat containing 3	0.84104289	0.4814	0.6775
619310	Zfp872	zinc finger protein 872	0.84104289	0.6199	0.7816
66204	Acyp1	acylphosphatase 1, erythrocyte (common) type	0.84104289	0.7251	0.8505
641376	Tomm40l	translocase of outer mitochondrial membrane 40 homolog-like (yeast)	0.84033613	0.002958	NA
19014	Med1	mediator complex subunit 1	0.84033613	0.004962	NA
74126	Syvn1	synovial apoptosis inhibitor 1, synoviolin	0.84033613	0.008608	NA
72128	2610008E11Rik	RIKEN cDNA 2610008E11 gene	0.84033613	0.01033	NA
666244	Tmsb15b1	thymosin beta 15b1	0.84033613	0.01851	0.07962
14680	Gnal	guanine nucleotide binding protein, alpha stimulating, olfactory type	0.84033613	0.01873	0.08022
67727	Stx17	syntaxin 17	0.84033613	0.02412	0.09508
104015	Synj1	synaptojanin 1	0.84033613	0.02512	0.09771
19895	Rpia	ribose 5-phosphate isomerase A	0.84033613	0.02611	0.1004
14287	Fpgs	folylpolyglutamyl synthetase	0.84033613	0.0633	0.1822
56752	Aldh9a1	aldehyde dehydrogenase 9, subfamily A1	0.84033613	0.1024	0.2524
16764	Aff3	AF4/FMR2 family, member 3	0.84033613	0.1172	0.2754
74103	Nebl	nebulette	0.84033613	0.1877	0.376
14536	Nr6a1	nuclear receptor subfamily 6, group A, member 1	0.84033613	0.2447	0.4484
17131	Smad7	MAD homolog 7 (Drosophila)	0.84033613	0.3605	0.5701
73713	Rbm20	RNA binding motif protein 20	0.84033613	0.3721	0.5809
258652	Olfr1131	olfactory receptor 1131	0.84033613	0.5753	0.749
433458	Gm13880	predicted gene 13880	0.84033613	0.7345	0.8565
67785	Zmym4	zinc finger, MYM-type 4	0.83963056	0.001672	NA
72693	Zcchc12	zinc finger, CCHC domain containing 12	0.83963056	0.008774	NA
69499	Tsr2	TSR2, 20S rRNA accumulation, homolog (S. cerevisiae)	0.83963056	0.008791	NA
227644	Snapc4	small nuclear RNA activating complex, polypeptide 4	0.83963056	0.009046	NA
237831	Slc13a5	solute carrier family 13 (sodium-dependent citrate transporter), member 5	0.83963056	0.01016	NA
228607	Mavs	mitochondrial antiviral signaling protein	0.83963056	0.02365	0.0939
170772	Glcci1	glucocorticoid induced transcript 1	0.83963056	0.03366	0.1188
171167	Fut10	fucosyltransferase 10	0.83963056	0.03384	0.1191
11836	Araf	v-raf murine sarcoma 3611 viral oncogene homolog	0.83963056	0.04324	0.1397
55982	Paxip1	PAX interacting (with transcription-activation domain) protein 1	0.83963056	0.04602	0.146
545758	Gm5868	predicted gene 5868	0.83963056	0.04645	0.1469
225888	Suv420h1	suppressor of variegation 4-20 homolog 1 (Drosophila)	0.83963056	0.04673	0.1475
11740	Slc25a5	solute carrier family 25 (mitochondrial carrier, adenine nucleotide translocator), member 5	0.83963056	0.05259	0.1606
68055	Atp5s	ATP synthase, H+ transporting, mitochondrial F0 complex, subunit s	0.83963056	0.05519	0.1661
69780	Smap2	stromal membrane-associated GTPase-activating protein 2	0.83963056	0.06102	0.1774
215890	Clvs2	clavesin 2	0.83963056	0.06495	0.1854
74691	Tdrd9	tudor domain containing 9	0.83963056	0.0732	0.2019
215113	Slc43a2	solute carrier family 43, member 2	0.83963056	0.1064	0.2581
77318	Ankrd55	ankyrin repeat domain 55	0.83963056	0.1301	0.2958
71592	Pogk	pogo transposable element with KRAB domain	0.83963056	0.1346	0.3015
75170	4930529F24Rik	RIKEN cDNA 4930529F24 gene	0.83963056	0.2248	0.4245
69256	Zfp397	zinc finger protein 397	0.83963056	0.2358	0.4376
71409	Fmnl2	formin-like 2	0.83963056	0.2495	0.4539
72108	Ddhd2	DDHD domain containing 2	0.83963056	0.2511	0.4559
56367	Scoc	short coiled-coil protein	0.83963056	0.2613	0.4673
94282	Sfxn5	sideroflexin 5	0.83963056	0.2839	0.4924
233726	Ipo7	importin 7	0.83963056	0.3319	0.5423
277854	Depdc5	DEP domain containing 5	0.83963056	0.4295	0.6338

75687	Fam65a	family with sequence similarity 65, member A	0.83892617	0.009255	NA
70292	Afap1	actin filament associated protein 1	0.83892617	0.02112	0.08721
83431	Ndel1	nuclear distribution gene E-like homolog 1 (A. nidulans)	0.83892617	0.02243	0.09076
321007	Serac1	serine active site containing 1	0.83892617	0.02314	0.09252
14897	Trip12	thyroid hormone receptor interactor 12	0.83892617	0.04152	0.1363
26422	Nbea	neurobeachin	0.83892617	0.05812	0.1719
14658	Glrb	glycine receptor, beta subunit	0.83892617	0.05905	0.1736
22717	Zfp59	zinc finger protein 59	0.83892617	0.06348	0.1825
20361	Sema7a	sema domain, immunoglobulin domain (Ig), and GPI membrane anchor, (semaphorin) 7A	0.83892617	0.06544	0.1863
320714	D030016E14Rik	RIKEN cDNA D030016E14 gene	0.83892617	0.06741	0.1899
14787	Rhpn1	rhophilin, Rho GTPase binding protein 1	0.83892617	0.08086	0.2158
57444	Isg20	interferon-stimulated protein	0.83892617	0.08397	0.2213
74288	Spem1	sperm maturation 1	0.83892617	0.08621	0.2253
15558	Htr2a	5-hydroxytryptamine (serotonin) receptor 2A	0.83892617	0.1023	0.2522
20648	Snta1	syntrophin, acidic 1	0.83892617	0.1043	0.2552
75740	Egfem1	EGF-like and EMI domain containing 1	0.83892617	0.1183	0.2772
230379	Acer2	alkaline ceramidase 2	0.83892617	0.1293	0.2949
667635	Trav7n-5	T cell receptor alpha variable 7N-5	0.83892617	0.2048	0.3984
105245	Txndc5	thioredoxin domain containing 5	0.83892617	0.2206	0.4196
231207	Cpeb2	cytoplasmic polyadenylation element binding protein 2	0.83892617	0.2206	0.4196
277414	Trp53i11	transformation related protein 53 inducible protein 11	0.83892617	0.2299	0.4308
331401	Thoc2	THO complex 2	0.83892617	0.2924	0.5012
319613	Sybu	syntabulin (syntaxin-interacting)	0.83892617	0.3666	0.5757
224829	Trerf1	transcriptional regulating factor 1	0.83892617	0.3827	0.5918
57785	Rangrf	RAN guanine nucleotide release factor	0.83892617	0.4071	0.6139
194735	4930430D24Rik	RIKEN cDNA 4930430D24 gene	0.83892617	0.5529	0.7333
217600	Rpl26-ps2	ribosomal protein L26, pseudogene 2	0.83892617	0.6165	0.7793
768252	Foxl2os	forkhead box L2 opposite strand transcript	0.83892617	0.6339	0.792
192160	Casc3	cancer susceptibility candidate 3	0.83822297	0.001214	NA
109050	6530418L21Rik	RIKEN cDNA 6530418L21 gene	0.83822297	0.004668	NA
98910	Usp6nl	USP6 N-terminal like	0.83822297	0.005949	NA
26420	Mapk9	mitogen-activated protein kinase 9	0.83822297	0.007366	NA
329540	8430427H17Rik	RIKEN cDNA 8430427H17 gene	0.83822297	0.01267	0.06219
78785	Clip4	CAP-GLY domain containing linker protein family, member 4	0.83822297	0.01479	0.06875
69562	Cdk13	cyclin-dependent kinase 13	0.83822297	0.02179	0.08915
53330	Vamp4	vesicle-associated membrane protein 4	0.83822297	0.0219	0.0894
94275	Maged1	melanoma antigen, family D, 1	0.83822297	0.025	0.09745
207781	C2cd2	C2 calcium-dependent domain containing 2	0.83822297	0.02838	0.1062
66308	2810021B07Rik	RIKEN cDNA 2810021B07 gene	0.83822297	0.02977	0.1096
27390	Mmel1	membrane metallo-endopeptidase-like 1	0.83822297	0.04115	0.1353
94045	P2rx5	purinergic receptor P2X, ligand-gated ion channel, 5	0.83822297	0.05511	0.166
241201	Cdh7	cadherin 7, type 2	0.83822297	0.06189	0.1795
80289	Lysmd3	LysM, putative peptidoglycan-binding, domain containing 3	0.83822297	0.0625	0.1807
80707	Wwox	WW domain-containing oxidoreductase	0.83822297	0.06893	0.1931
665934	Gm7854	predicted gene 7854	0.83822297	0.0848	0.2228
238871	Pde4d	phosphodiesterase 4D, cAMP specific	0.83822297	0.145	0.3172
675812	Zfp605	zinc finger protein 605	0.83822297	0.2085	0.4034
270118	Maml2	mastermind like 2 (Drosophila)	0.83822297	0.2211	0.4201
74359	4931414P19Rik	RIKEN cDNA 4931414P19 gene	0.83822297	0.2226	0.4221
14660	Gls	glutaminase	0.83822297	0.2538	0.4589

71901	2310028H24Rik	RIKEN cDNA 2310028H24 gene	0.83822297	0.2844	0.4928
12361	Cask	calcium/calmodulin-dependent serine protein kinase (MAGUK family)	0.83822297	0.2926	0.5014
16691	Krt8	keratin 8	0.83822297	0.3656	0.5747
244556	Zfp791	zinc finger protein 791	0.83822297	0.3855	0.5945
109658	Txlna	taxilin alpha	0.83822297	0.4704	0.6696
18762	Prkcz	protein kinase C, zeta	0.83752094	0.00199	NA
52840	Dbndd2	dysbindin (dystrobrevin binding protein 1) domain containing 2	0.83752094	0.01379	0.06572
54343	Atf7ip	activating transcription factor 7 interacting protein	0.83752094	0.0176	0.07687
66583	Exosc1	exosome component 1	0.83752094	0.0307	0.1119
66950	Tmem206	transmembrane protein 206	0.83752094	0.03376	0.119
268936	Brpf3	bromodomain and PHD finger containing, 3	0.83752094	0.03399	0.1194
69770	1600002K03Rik	RIKEN cDNA 1600002K03 gene	0.83752094	0.03542	0.123
319475	Zfp672	zinc finger protein 672	0.83752094	0.04018	0.1332
19699	Reln	reelin	0.83752094	0.04459	0.1427
16869	Lhx1	LIM homeobox protein 1	0.83752094	0.04745	0.1492
320727	Ipo8	importin 8	0.83752094	0.05087	0.1568
56177	Olfm1	olfactomedin 1	0.83752094	0.05619	0.1682
73660	Cabp4	calcium binding protein 4	0.83752094	0.05879	0.1732
71564	Izumo4	IZUMO family member 4	0.83752094	0.06498	0.1855
13446	Doc2a	double C2, alpha	0.83752094	0.08754	0.2276
12815	Col11a2	collagen, type XI, alpha 2	0.83752094	0.1074	0.2596
71703	Armcx3	armadillo repeat containing, X-linked 3	0.83752094	0.1473	0.3203
239985	Arid1b	AT rich interactive domain 1B (SWI-like)	0.83752094	0.1605	0.3387
229706	Slc6a17	solute carrier family 6 (neurotransmitter transporter), member 17	0.83752094	0.1744	0.3583
219257	Pcdh20	protocadherin 20	0.83752094	0.193	0.383
64292	Ptges	prostaglandin E synthase	0.83752094	0.2865	0.4949
16204	Fabp6	fatty acid binding protein 6, ileal (gastrotropin)	0.83752094	0.3987	0.6064
268706	Slc38a9	solute carrier family 38, member 9	0.83682008	0.00255	NA
244721	Zfp846	zinc finger protein 846	0.83682008	0.0144	0.06768
218460	Wdr41	WD repeat domain 41	0.83682008	0.01571	0.07149
233410	Zfp592	zinc finger protein 592	0.83682008	0.01789	0.07771
12404	Cbln1	cerebellin 1 precursor protein	0.83682008	0.0188	0.08036
217325	Llgl2	lethal giant larvae homolog 2 (Drosophila)	0.83682008	0.029	0.1077
16647	Kpna2	karyopherin (importin) alpha 2	0.83682008	0.03904	0.1307
78619	Zfp449	zinc finger protein 449	0.83682008	0.07758	0.2099
237858	Tusc5	tumor suppressor candidate 5	0.83682008	0.1193	0.2792
66559	Metap1d	methionyl aminopeptidase type 1D (mitochondrial)	0.83682008	0.1229	0.285
19014	Med1	mediator complex subunit 1	0.83682008	0.1319	0.2983
381270	Mar-04	membrane-associated ring finger (C3HC4) 4	0.83682008	0.1655	0.3458
72371	2210408I21Rik	RIKEN cDNA 2210408I21 gene	0.83682008	0.2155	0.4127
11306	Abcb7	ATP-binding cassette, sub-family B (MDR/TAP), member 7	0.83682008	0.2254	0.4252
74352	Zfp84	zinc finger protein 84	0.83682008	0.2295	0.4301
78739	9530062K07Rik	RIKEN cDNA 9530062K07 gene	0.83682008	0.2394	0.4419
66861	Dnajc10	DnaJ (Hsp40) homolog, subfamily C, member 10	0.83682008	0.2553	0.4602
546049	C330021F23Rik	RIKEN cDNA C330021F23 gene	0.83682008	0.2654	0.4719
620709	Lcn6	lipocalin 6	0.83682008	0.2689	0.476
24014	Rnasel	ribonuclease L (2', 5'-oligoisoadenylate synthetase-dependent)	0.83682008	0.2755	0.4834
670912	Gm9510	predicted gene 9510	0.83682008	0.2755	0.4834
330070	Gm5107	predicted gene 5107	0.83682008	0.3022	0.5113
71682	Wdr27	WD repeat domain 27	0.83682008	0.3086	0.5184

12648	Chd1	chromodomain helicase DNA binding protein 1	0.83682008	0.3631	0.5722
216021	Stox1	storkhead box 1	0.83682008	0.4696	0.6689
27277	Golga5	golgi autoantigen, golgin subfamily a, 5	0.8361204	0.001584	NA
11501	Adam8	a disintegrin and metallopeptidase domain 8	0.8361204	0.03242	0.1161
17716	ND1	NADH dehydrogenase subunit 1	0.8361204	0.04464	0.1428
320737	4732416N19Rik	RIKEN cDNA 4732416N19 gene	0.8361204	0.06056	0.1766
241943	Ccdc144b	coiled-coil domain containing 144B	0.8361204	0.06757	0.1903
211468	Kcnh8	potassium voltage-gated channel, subfamily H (eag-related), member 8	0.8361204	0.06968	0.1947
72017	Cyb5r1	cytochrome b5 reductase 1	0.8361204	0.07418	0.2035
16661	Krt10	keratin 10	0.8361204	0.1289	0.2942
77316	C030011L09Rik	RIKEN cDNA C030011L09 gene	0.8361204	0.1358	0.3033
22634	Plagl1	pleiomorphic adenoma gene-like 1	0.8361204	0.1378	0.306
73874	4930419G24Rik	RIKEN cDNA 4930419G24 gene	0.8361204	0.1486	0.3224
320983	B130019D13Rik	RIKEN cDNA B130019D13 gene	0.8361204	0.1627	0.3416
13176	Dcc	deleted in colorectal carcinoma	0.8361204	0.1932	0.3832
14816	Grm1	glutamate receptor, metabotropic 1	0.8361204	0.2604	0.4662
667214	9930111J21Rik1	RIKEN cDNA 9930111J21 gene 1	0.8361204	0.2893	0.4976
110593	Prdm2	PR domain containing 2, with ZNF domain	0.8361204	0.3234	0.533
100041870	Gm3556	predicted gene 3556	0.83542189	0.00502	NA
58180	Hic2	hypermethylated in cancer 2	0.83542189	0.009665	0.05214
73178	Wasl	Wiskott-Aldrich syndrome-like (human)	0.83542189	0.02795	0.105
75209	Sv2c	synaptic vesicle glycoprotein 2c	0.83542189	0.03374	0.119
14702	Gng2	guanine nucleotide binding protein (G protein), gamma 2	0.83542189	0.03456	0.1207
75605	Kdm5b	lysine (K)-specific demethylase 5B	0.83542189	0.04551	0.1448
672098	Gm9548	predicted gene 9548	0.83542189	0.05837	0.1723
100045292	LOC100045292	hypothetical protein LOC100045292	0.83542189	0.07408	0.2033
72716	2810047C21Rik1	RIKEN cDNA 2810047C21 gene 1	0.83542189	0.07517	0.2051
319758	Rfx7	regulatory factor X, 7	0.83542189	0.08071	0.2156
75974	Dock11	dedicator of cytokinesis 11	0.83542189	0.1066	0.2585
11909	Atf2	activating transcription factor 2	0.83542189	0.1177	0.2763
432731	Zfp187	zinc finger protein 187	0.83542189	0.118	0.2769
229672	Bcl2l15	BCLl2-like 15	0.83542189	0.1501	0.3246
53817	Bat1a	HLA-B-associated transcript 1A	0.83542189	0.2015	0.394
26420	Mapk9	mitogen-activated protein kinase 9	0.83542189	0.2373	0.4393
329152	Hecw2	HECT, C2 and WW domain containing E3 ubiquitin protein ligase 2	0.83542189	0.2487	0.4529
270757	Bpil2	bactericidal/permeability-increasing protein-like 2	0.83542189	0.3094	0.5193
233805	Dcun1d3	DCN1, defective in cullin neddylation 1, domain containing 3 (S. cerevisiae)	0.83542189	0.3131	0.5228
241308	Ralgps1	Ral GEF with PH domain and SH3 binding motif 1	0.83542189	0.3886	0.597
228980	Taf4a	TAF4A RNA polymerase II, TATA box binding protein (TBP)-associated factor	0.83472454	0.002358	NA
71640	Zfp949	zinc finger protein 949	0.83472454	0.007176	NA
12234	Btrc	beta-transducin repeat containing protein	0.83472454	0.009495	0.05149
20910	Stxbp1	syntaxin binding protein 1	0.83472454	0.0118	0.0596
15490	Hsd17b7	hydroxysteroid (17-beta) dehydrogenase 7	0.83472454	0.03057	0.1116
100043072	A230070E04Rik	RIKEN cDNA A230070E04 gene	0.83472454	0.0429	0.139
74711	Ttll9	tubulin tyrosine ligase-like family, member 9	0.83472454	0.05015	0.1555
19362	Rad51ap1	RAD51 associated protein 1	0.83472454	0.05198	0.1594
432628	Mfsd2b	major facilitator superfamily domain containing 2B	0.83472454	0.05537	0.1666
224617	Tbc1d24	TBC1 domain family, member 24	0.83472454	0.06386	0.1833
319263	Pcmtd1	protein-L-isoaspartate (D-aspartate) O-methyltransferase domain containing 1	0.83472454	0.06677	0.1888
269784	Cntn4	contactin 4	0.83472454	0.06738	0.1899

320586	A630089N07Rik	RIKEN cDNA A630089N07 gene	0.83472454	0.07609	0.2071
382090	4922501C03Rik	RIKEN cDNA 4922501C03 gene	0.83472454	0.0762	0.2073
105590	Zfp957	zinc finger protein 957	0.83472454	0.08227	0.2184
100041799	Gm3515	predicted gene 3515	0.83472454	0.09211	0.2353
215751	BC013529	cDNA sequence BC013529	0.83472454	0.1039	0.2547
14167	Fgf12	fibroblast growth factor 12	0.83472454	0.1209	0.282
74100	Arpp21	cyclic AMP-regulated phosphoprotein, 21	0.83472454	0.1297	0.2952
67939	Prorsd1	prolyl-tRNA synthetase domain containing 1	0.83472454	0.1397	0.3092
27278	Clnk	cytokine-dependent hematopoietic cell linker	0.83472454	0.1803	0.3662
319535	Zfp182	zinc finger protein 182	0.83472454	0.2021	0.3948
11438	Chrna4	cholinergic receptor, nicotinic, alpha polypeptide 4	0.83472454	0.2214	0.4205
72400	Pinx1	PIN2/TERF1 interacting, telomerase inhibitor 1	0.83472454	0.3265	0.5367
102623	AI414108	expressed sequence AI414108	0.83472454	0.4071	0.6139
70753	6330415B21Rik	RIKEN cDNA 6330415B21 gene	0.83472454	0.5229	0.7102
19206	Ptch1	patched homolog 1	0.83472454	0.7672	0.8764
70556	Slc25a33	solute carrier family 25, member 33	0.83402836	0.002588	NA
27981	D4Wsu53e	DNA segment, Chr 4, Wayne State University 53, expressed	0.83402836	0.008857	0.04914
53418	B4galt2	UDP-Gal:betaGlcNAc beta 1,4- galactosyltransferase, polypeptide 2	0.83402836	0.02121	0.08747
22370	Vtn	vitronectin	0.83402836	0.03529	0.1227
237542	Osbpl8	oxysterol binding protein-like 8	0.83402836	0.03613	0.1245
96957	Tmem62	transmembrane protein 62	0.83402836	0.04957	0.1541
100042554	Gm3902	predicted gene 3902	0.83402836	0.07658	0.2081
16531	Kcnma1	potassium large conductance calcium-activated channel, subfamily M, alpha member 1	0.83402836	0.07713	0.209
77246	9330177L23Rik	RIKEN cDNA 9330177L23 gene	0.83402836	0.09651	0.2426
69181	Dyrk2	dual-specificity tyrosine-(Y)-phosphorylation regulated kinase 2	0.83402836	0.1004	0.2493
212528	Trmt1	TRM1 tRNA methyltransferase 1 homolog (S. cerevisiae)	0.83402836	0.105	0.2562
232288	Frmd4b	FERM domain containing 4B	0.83402836	0.1145	0.2716
78797	Ndor1	NADPH dependent diflavin oxidoreductase 1	0.83402836	0.1148	0.272
241694	Ralgapa2	Ral GTPase activating protein, alpha subunit 2 (catalytic)	0.83402836	0.1225	0.2844
110521	Hivep1	human immunodeficiency virus type I enhancer binding protein 1	0.83402836	0.1509	0.3258
14337	Ftl2	ferritin light chain 2	0.83402836	0.1522	0.3272
243905	Zfp568	zinc finger protein 568	0.83402836	0.1548	0.3309
232790	Oscar	osteoclast associated receptor	0.83402836	0.1691	0.3503
544971	Bdp1	B double prime 1, subunit of RNA polymerase III transcription initiation factor IIIB	0.83402836	0.2014	0.3938
271711	Tmem169	transmembrane protein 169	0.83402836	0.246	0.4498
109050	6530418L21Rik	RIKEN cDNA 6530418L21 gene	0.83402836	0.2775	0.4858
12236	Bub1b	budding uninhibited by benzimidazoles 1 homolog, beta (S. cerevisiae)	0.83402836	0.2881	0.4966
232400	BC048546	cDNA sequence BC048546	0.83402836	0.3135	0.5232
77963	Hook1	hook homolog 1 (Drosophila)	0.83402836	0.4052	0.6129
101185	Pot1a	protection of telomeres 1A	0.83402836	0.435	0.6389
19060	Ppp5c	protein phosphatase 5, catalytic subunit	0.83402836	0.6585	0.8074
637053	Vmn2r4	vomeronasal 2, receptor 4	0.83402836	0.6814	0.8221
19088	Prkar2b	protein kinase, cAMP dependent regulatory, type II beta	0.83333333	0.003819	NA
99031	Osbpl6	oxysterol binding protein-like 6	0.83333333	0.01322	0.06405
270109	Pcnxl2	pecanex-like 2 (Drosophila)	0.83333333	0.01459	0.06819
238831	Ppwd1	peptidylprolyl isomerase domain and WD repeat containing 1	0.83333333	0.03556	0.1233
18587	Pde6b	phosphodiesterase 6B, cGMP, rod receptor, beta polypeptide	0.83333333	0.05082	0.1568
68121	Cep70	centrosomal protein 70	0.83333333	0.05625	0.1683
114715	Spred1	sprouty protein with EVH-1 domain 1, related sequence	0.83333333	0.07467	0.2043
67466	Pdcl	phosducin-like	0.83333333	0.1012	0.2507

170936	Zfp369	zinc finger protein 369	0.83333333	0.1493	0.3233
26987	Eif4e2	eukaryotic translation initiation factor 4E member 2	0.83333333	0.152	0.3268
58194	Sh3kbp1	SH3-domain kinase binding protein 1	0.83333333	0.1637	0.3431
51998	D17Ertd191e	DNA segment, Chr 17, ERATO Doi 191, expressed	0.83333333	0.2487	0.4529
53970	Rfx5	regulatory factor X, 5 (influences HLA class II expression)	0.83333333	0.2619	0.4678
74635	4930425O10Rik	RIKEN cDNA 4930425O10 gene	0.83333333	0.2983	0.5078
194388	Tet3	tet oncogene family member 3	0.83333333	0.3881	0.5966
211660	Cspp1	centrosome and spindle pole associated protein 1	0.83263947	0.002387	NA
76884	Cyfip2	cytoplasmic FMR1 interacting protein 2	0.83263947	0.008414	0.04762
68999	Anapc10	anaphase promoting complex subunit 10	0.83263947	0.01531	0.07053
68040	Zfp593	zinc finger protein 593	0.83263947	0.01564	0.07134
16647	Kpna2	karyopherin (importin) alpha 2	0.83263947	0.02187	0.08933
66459	Pigy	phosphatidylinositol glycan anchor biosynthesis, class Y	0.83263947	0.02826	0.1058
171429	Slc26a6	solute carrier family 26, member 6	0.83263947	0.02832	0.106
53886	Cdkl2	cyclin-dependent kinase-like 2 (CDC2-related kinase)	0.83263947	0.04093	0.1348
67708	1810048J11Rik	RIKEN cDNA 1810048J11 gene	0.83263947	0.04431	0.1421
76071	Jakmip1	janus kinase and microtubule interacting protein 1	0.83263947	0.0452	0.1443
319998	Tmem198	transmembrane protein 198	0.83263947	0.04584	0.1456
12828	Col4a3	collagen, type IV, alpha 3	0.83263947	0.04836	0.1512
378435	Mafa	v-maf musculoaponeurotic fibrosarcoma oncogene family, protein A (avian)	0.83263947	0.0522	0.1598
192786	Rapgef6	Rap guanine nucleotide exchange factor (GEF) 6	0.83263947	0.05547	0.1667
80910	Gpr84	G protein-coupled receptor 84	0.83263947	0.06147	0.1786
209707	Lcorl	ligand dependent nuclear receptor corepressor-like	0.83263947	0.06215	0.1799
100993	AW549542	expressed sequence AW549542	0.83263947	0.06285	0.1813
271457	Rab5a	RAB5A, member RAS oncogene family	0.83263947	0.1062	0.2579
77593	Usp45	ubiquitin specific petidase 45	0.83263947	0.1133	0.2695
328329	Mast4	microtubule associated serine/threonine kinase family member 4	0.83263947	0.1316	0.2979
26942	Spag1	sperm associated antigen 1	0.83263947	0.1625	0.3414
70012	Ccdc21	coiled-coil domain containing 21	0.83263947	0.2017	0.3942
319565	Syne2	synaptic nuclear envelope 2	0.83263947	0.2643	0.4706
635702	Naaladl2	N-acetylated alpha-linked acidic dipeptidase-like 2	0.83263947	0.3129	0.5226
664609	Rhox4a	reproductive homeobox 4A	0.83263947	0.3443	0.5549
224014	Fgd4	FYVE, RhoGEF and PH domain containing 4	0.83263947	0.3483	0.5587
72293	Nkd2	naked cuticle 2 homolog (Drosophila)	0.83263947	0.3514	0.5614
66854	Trim35	tripartite motif-containing 35	0.83263947	0.531	0.7164
627924	Gm6811	predicted gene 6811	0.83263947	0.547	0.7288
67300	Cltc	clathrin, heavy polypeptide (Hc)	0.83194676	0.002455	NA
72599	Pdia5	protein disulfide isomerase associated 5	0.83194676	0.00377	NA
14385	Slc37a4	solute carrier family 37 (glucose-6-phosphate transporter), member 4	0.83194676	0.003909	NA
76901	Phf15	PHD finger protein 15	0.83194676	0.006537	0.04051
381835	Gm1078	predicted gene 1078	0.83194676	0.01259	0.06201
20910	Stxbp1	syntaxin binding protein 1	0.83194676	0.01783	0.07758
19279	Ptprr	protein tyrosine phosphatase, receptor type, R	0.83194676	0.02322	0.09265
74440	4933407C03Rik	RIKEN cDNA 4933407C03 gene	0.83194676	0.02353	0.09354
78617	Cstad	CSA-conditional, T cell activation-dependent protein	0.83194676	0.02404	0.09494
104718	Ttc7b	tetratricopeptide repeat domain 7B	0.83194676	0.03113	0.1129
104806	Fancm	Fanconi anemia, complementation group M	0.83194676	0.04715	0.1485
67153	Rnaseh2b	ribonuclease H2, subunit B	0.83194676	0.05912	0.1737
320938	Tnpo3	transportin 3	0.83194676	0.06004	0.1754
15388	Hnrnpl	heterogeneous nuclear ribonucleoprotein L	0.83194676	0.07096	0.1969

99045	Mrps26	mitochondrial ribosomal protein S26	0.83194676	0.09057	0.2326
19726	Rfx3	regulatory factor X, 3 (influences HLA class II expression)	0.83194676	0.1533	0.3287
107823	Whsc1	Wolf-Hirschhorn syndrome candidate 1 (human)	0.83194676	0.1691	0.3503
26549	Itgb1bp2	integrin beta 1 binding protein 2	0.83194676	0.1904	0.3793
225651	Mppe1	metallophosphoesterase 1	0.83194676	0.2579	0.4632
105203	BC016423	cDNA sequence BC016423	0.83194676	0.3067	0.5161
624582	A730098P11Rik	RIKEN cDNA A730098P11 gene	0.83194676	0.4584	0.6595
101401	Adamts9	a disintegrin-like and metallopeptidase (reprolysin type) with thrombospondin type 1 motif, 9	0.83194676	0.4664	0.6661
101122	Rpusd3	RNA pseudouridylate synthase domain containing 3	0.8312552	0.004986	NA
100034363	Tmsb15b2	thymosin beta 15b2	0.8312552	0.008015	0.0462
503690	A930005G22Rik	RIKEN cDNA A930005G22 gene	0.8312552	0.01134	0.05791
69237	Gtpbp4	GTP binding protein 4	0.8312552	0.01249	0.06156
381353	Gm996	predicted gene 996	0.8312552	0.01266	0.06219
18720	Pip5k1a	phosphatidylinositol-4-phosphate 5-kinase, type 1 alpha	0.8312552	0.01477	0.06874
55946	Ap3m1	adaptor-related protein complex 3, mu 1 subunit	0.8312552	0.01535	0.07056
100038570	Gm11744	predicted gene 11744	0.8312552	0.01708	0.07524
320320	B230343J05Rik	RIKEN cDNA B230343J05 gene	0.8312552	0.01842	0.07933
13196	Asap1	ArfGAP with SH# domain, ankyrin repeat and PH domain1	0.8312552	0.02036	0.08497
56527	Mast1	microtubule associated serine/threonine kinase 1	0.8312552	0.02209	0.08993
66999	Med28	mediator of RNA polymerase II transcription, subunit 28 homolog (yeast)	0.8312552	0.02227	0.09047
58212	Srrm3	serine/arginine repetitive matrix 3	0.8312552	0.02297	0.09213
18588	Pde6g	phosphodiesterase 6G, cGMP-specific, rod, gamma	0.8312552	0.04304	0.1393
73130	Tmed5	transmembrane emp24 protein transport domain containing 5	0.8312552	0.05129	0.1578
13401	Dmwd	dystrophia myotonica-containing WD repeat motif	0.8312552	0.06438	0.1843
76508	2210015D19Rik	RIKEN cDNA 2210015D19 gene	0.8312552	0.07984	0.2141
17690	Msi1	Musashi homolog 1(Drosophila)	0.8312552	0.08788	0.2281
108995	Tbc1d10c	TBC1 domain family, member 10c	0.8312552	0.09282	0.2365
64383	Sirt2	sirtuin 2 (silent mating type information regulation 2, homolog) 2 (S. cerevisiae)	0.8312552	0.1315	0.2978
70620	Ube2v2	ubiquitin-conjugating enzyme E2 variant 2	0.8312552	0.1398	0.3094
223870	Senp1	SUMO1/sentrin specific peptidase 1	0.8312552	0.2279	0.428
14811	Grin2a	glutamate receptor, ionotropic, NMDA2A (epsilon 1)	0.8312552	0.2688	0.4759
78774	4930529M08Rik	RIKEN cDNA 4930529M08 gene	0.8312552	0.2977	0.5071
73331	1700034P13Rik	RIKEN cDNA 1700034P13 gene	0.8312552	0.3224	0.5322
237029	4932411N23Rik	RIKEN cDNA 4932411N23 gene	0.8312552	0.6447	0.7993
234847	Spg7	spastic paraplegia 7 homolog (human)	0.83056478	0.007	0.04254
67263	Zswim6	zinc finger, SWIM domain containing 6	0.83056478	0.0122	0.06066
12293	Cacna2d1	calcium channel, voltage-dependent, alpha2/delta subunit 1	0.83056478	0.01231	0.06102
74011	Slc25a27	solute carrier family 25, member 27	0.83056478	0.01688	0.07459
226856	Lpgat1	lysophosphatidylglycerol acyltransferase 1	0.83056478	0.01837	0.0792
382137	Fdxacb1	ferredoxin-fold anticodon binding domain containing 1	0.83056478	0.01864	0.08005
77832	Tchp	trichoplein, keratin filament binding	0.83056478	0.03097	0.1127
67134	Nop56	NOP56 ribonucleoprotein homolog (yeast)	0.83056478	0.03249	0.1163
227525	Dclre1c	DNA cross-link repair 1C, PSO2 homolog (S. cerevisiae)	0.83056478	0.03278	0.1169
235674	Acaa1b	acetyl-Coenzyme A acyltransferase 1B	0.83056478	0.03953	0.1318
105439	Slain1	SLAIN motif family, member 1	0.83056478	0.0406	0.1341
230777	Hcrtr1	hypocretin (orexin) receptor 1	0.83056478	0.04536	0.1445
227960	Gca	grancalcin	0.83056478	0.04746	0.1492
22034	Traf6	TNF receptor-associated factor 6	0.83056478	0.05448	0.1648
12889	Cplx1	complexin 1	0.83056478	0.08216	0.2183
20185	Ncor1	nuclear receptor co-repressor 1	0.83056478	0.09936	0.2473

207181	Rbms3	RNA binding motif, single stranded interacting protein	0.83056478	0.1039	0.2547
103268	Cep57l1	centrosomal protein 57-like 1	0.83056478	0.1305	0.2962
12723	Clcn1	chloride channel 1	0.83056478	0.1523	0.3274
18383	Tnfrsf11b	tumor necrosis factor receptor superfamily, member 11b (osteoprotegerin)	0.83056478	0.2234	0.4229
242425	Gabbr2	gamma-aminobutyric acid (GABA) B receptor, 2	0.83056478	0.2349	0.4365
214162	Mll1	myeloid/lymphoid or mixed-lineage leukemia 1	0.83056478	0.2778	0.486
20300	Ccl25	chemokine (C-C motif) ligand 25	0.83056478	0.2801	0.4882
16666	Krt16	keratin 16	0.83056478	0.3241	0.5338
234072	Adprhl1	ADP-ribosylhydrolase like 1	0.83056478	0.3538	0.5635
12340	Capza1	capping protein (actin filament) muscle Z-line, alpha 1	0.83056478	0.4249	0.6302
100043123	Gm11710	predicted gene 11710	0.83056478	0.5944	0.7636
230908	Tardbp	TAR DNA binding protein	0.82987552	0.006015	0.03834
624918	Gm6537	predicted gene 6537	0.82987552	0.007172	0.04324
245468	Pnma3	paraneoplastic antigen MA3	0.82987552	0.01695	0.07479
108089	Rnf144a	ring finger protein 144A	0.82987552	0.01908	0.08124
15483	Hsd11b1	hydroxysteroid 11-beta dehydrogenase 1	0.82987552	0.0324	0.1161
19244	Ptp4a2	protein tyrosine phosphatase 4a2	0.82987552	0.04622	0.1464
218832	Polr3a	polymerase (RNA) III (DNA directed) polypeptide A	0.82987552	0.05331	0.1623
67420	Far1	fatty acyl CoA reductase 1	0.82987552	0.08017	0.2148
66237	Atp6v1g2	ATPase, H+ transporting, lysosomal V1 subunit G2	0.82987552	0.1014	0.251
16801	Arhgef1	Rho guanine nucleotide exchange factor (GEF) 1	0.82987552	0.1779	0.363
269589	Sytl1	synaptotagmin-like 1	0.82987552	0.1857	0.3731
319804	Glt1d1	glycosyltransferase 1 domain containing 1	0.82987552	0.2047	0.3984
347740	2900097C17Rik	RIKEN cDNA 2900097C17 gene	0.82987552	0.2071	0.4018
229227	4932438A13Rik	RIKEN cDNA 4932438A13 gene	0.82987552	0.2413	0.4439
319991	Kif6	kinesin family member 6	0.82987552	0.2938	0.5029
212281	A530054K11Rik	RIKEN cDNA A530054K11 gene	0.82987552	0.2959	0.5048
21416	Tcf7l2	transcription factor 7-like 2, T-cell specific, HMG-box	0.82987552	0.4014	0.6091
16616	Klk1b21	kallikrein 1-related peptidase b21	0.82987552	0.4507	0.6529
94191	Adarb2	adenosine deaminase, RNA-specific, B2	0.82987552	0.4622	0.6627
547349	LOC547349	similar to MHC class I antigen precursor	0.82987552	0.7127	0.8425
57344	As3mt	arsenic (+3 oxidation state) methyltransferase	0.8291874	0.002109	NA
65111	Dap3	death associated protein 3	0.8291874	0.002983	NA
52822	Rufy3	RUN and FYVE domain containing 3	0.8291874	0.004784	0.0333
66246	Osgep	O-sialoglycoprotein endopeptidase	0.8291874	0.008884	0.04922
241525	Ypel4	yippee-like 4 (Drosophila)	0.8291874	0.01196	0.06006
211480	Kcnj14	potassium inwardly-rectifying channel, subfamily J, member 14	0.8291874	0.02142	0.08809
16560	Kif1a	kinesin family member 1A	0.8291874	0.02228	0.09047
78784	Celf3	VCUGBP, Elav-like family member 3	0.8291874	0.02624	0.1007
67417	Ears2	glutamyl-tRNA synthetase 2 (mitochondrial)(putative)	0.8291874	0.02807	0.1053
269717	Orai2	ORAI calcium release-activated calcium modulator 2	0.8291874	0.03395	0.1193
80286	Tusc3	tumor suppressor candidate 3	0.8291874	0.04174	0.1366
72244	1600014C10Rik	RIKEN cDNA 1600014C10 gene	0.8291874	0.04781	0.15
78749	Filip1l	filamin A interacting protein 1-like	0.8291874	0.04842	0.1514
245007	Zbtb38	zinc finger and BTB domain containing 38	0.8291874	0.05642	0.1686
208117	Aph1b	anterior pharynx defective 1b homolog (C. elegans)	0.8291874	0.07345	0.2023
11839	Areg	amphiregulin	0.8291874	0.09531	0.2408
75873	4930568B11Rik	RIKEN cDNA 4930568B11 gene	0.8291874	0.09762	0.2444
208144	Dhx37	DEAH (Asp-Glu-Ala-His) box polypeptide 37	0.8291874	0.1518	0.3266
11754	Aoc3	amine oxidase, copper containing 3	0.8291874	0.1613	0.3398

75444	1700011I03Rik	RIKEN cDNA 1700011I03 gene	0.8291874	0.2182	0.4163
76500	Ip6k2	inositol hexaphosphate kinase 2	0.8291874	0.2991	0.5084
18021	Nfatc3	nuclear factor of activated T-cells, cytoplasmic, calcineurin-dependent 3	0.8291874	0.3241	0.5338
320595	Phf8	PHD finger protein 8	0.8291874	0.3414	0.5517
208084	Pif1	PIF1 5'-to-3' DNA helicase homolog (S. cerevisiae)	0.8291874	0.3615	0.5711
14772	Grk4	G protein-coupled receptor kinase 4	0.82850041	0.006056	0.03851
18039	Nefl	neurofilament, light polypeptide	0.82850041	0.006934	0.04228
18203	Ntan1	N-terminal Asn amidase	0.82850041	0.01746	0.07644
20616	Snap91	synaptosomal-associated protein 91	0.82850041	0.02097	0.08677
212326	Fam149a	family with sequence similarity 149, member A	0.82850041	0.02352	0.09353
207615	Wdr37	WD repeat domain 37	0.82850041	0.0332	0.1178
11496	Adam22	a disintegrin and metallopeptidase domain 22	0.82850041	0.04434	0.1422
212531	Sh3bgrl2	SH3 domain binding glutamic acid-rich protein like 2	0.82850041	0.04752	0.1493
319713	Ablim3	actin binding LIM protein family, member 3	0.82850041	0.06469	0.1849
71091	Cdkl1	cyclin-dependent kinase-like 1 (CDC2-related kinase)	0.82850041	0.07452	0.2041
319783	A730056A06Rik	RIKEN cDNA A730056A06 gene	0.82850041	0.07839	0.2114
277250	Kdm3b	KDM3B lysine (K)-specific demethylase 3B	0.82850041	0.1003	0.249
104923	Adi1	acireductone dioxygenase 1	0.82850041	0.1056	0.257
22671	Rnf112	ring finger protein 112	0.82850041	0.121	0.282
19266	Ptprd	protein tyrosine phosphatase, receptor type, D	0.82850041	0.1249	0.288
545554	Ankrd34a	ankyrin repeat domain 34A	0.82850041	0.1625	0.3413
244061	Gm4971	predicted gene 4971	0.82850041	0.208	0.4028
16518	Kcnj2	potassium inwardly-rectifying channel, subfamily J, member 2	0.82850041	0.2267	0.4265
70715	6330405D24Rik	RIKEN cDNA 6330405D24 gene	0.82850041	0.2486	0.4529
71713	Cdc40	cell division cycle 40 homolog (yeast)	0.82850041	0.2518	0.4566
100503353	Gm14440	predicted gene 14440	0.82850041	0.2722	0.4796
66471	Anp32e	acidic (leucine-rich) nuclear phosphoprotein 32 family, member E	0.82850041	0.2766	0.4846
238205	Lrfn5	leucine rich repeat and fibronectin type III domain containing 5	0.82850041	0.325	0.5348
13876	Erg	avian erythroblastosis virus E-26 (v-ets) oncogene related	0.82850041	0.3639	0.5729
16169	Il15ra	interleukin 15 receptor, alpha chain	0.82850041	0.3864	0.5951
13829	Epb4.9	erythrocyte protein band 4.9	0.82850041	0.4375	0.6408
11980	Atp8a1	ATPase, aminophospholipid transporter (APLT), class I, type 8A, member 1	0.82850041	0.463	0.6634
432731	Zfp187	zinc finger protein 187	0.82781457	0.002919	NA
100101806	Srp54c	signal recognition particle 54C	0.82781457	0.01481	0.06879
100041585	Amd2	S-adenosylmethionine decarboxylase 2	0.82781457	0.02206	0.08984
67832	Brix1	BRX1, biogenesis of ribosomes, homolog (S. cerevisiae)	0.82781457	0.02351	0.09352
56036	Ccnl2	cyclin L2	0.82781457	0.03889	0.1305
23963	Odz1	odd Oz/ten-m homolog 1 (Drosophila)	0.82781457	0.04055	0.134
104348	Zfp120	zinc finger protein 120	0.82781457	0.06502	0.1856
76303	Osbp	oxysterol binding protein	0.82781457	0.06697	0.1891
227325	Dner	delta/notch-like EGF-related receptor	0.82781457	0.0926	0.2362
22256	Ung	uracil DNA glycosylase	0.82781457	0.09715	0.2434
67727	Stx17	syntaxin 17	0.82781457	0.1555	0.332
628779	Hs3st4	heparan sulfate (glucosamine) 3-O-sulfotransferase 4	0.82781457	0.1602	0.3383
76484	Kndc1	kinase non-catalytic C-lobe domain (KIND) containing 1	0.82781457	0.1985	0.39
68732	Lrrc16a	leucine rich repeat containing 16A	0.82781457	0.2313	0.4322
192196	Luc7l2	LUC7-like 2 (S. cerevisiae)	0.82781457	0.2987	0.5082
12638	Cftr	cystic fibrosis transmembrane conductance regulator homolog	0.82781457	0.3354	0.5458
16790	Anpep	alanyl (membrane) aminopeptidase	0.82781457	0.3452	0.556
211535	Ccdc114	coiled-coil domain containing 114	0.82781457	0.3559	0.5656

19134	Prpf4b	PRP4 pre-mRNA processing factor 4 homolog B (yeast)	0.82712986	0.003988	0.02969
67374	Jam2	junction adhesion molecule 2	0.82712986	0.004834	0.03346
228564	Frmd5	FERM domain containing 5	0.82712986	0.004969	0.03398
54561	Nap1l3	nucleosome assembly protein 1-like 3	0.82712986	0.005436	0.03602
272347	Zfp398	zinc finger protein 398	0.82712986	0.005447	0.03604
23885	Gmcl1	germ cell-less homolog 1 (Drosophila)	0.82712986	0.009729	0.05234
19714	Rev3l	REV3-like, catalytic subunit of DNA polymerase zeta RAD54 like (S. cerevisiae)	0.82712986	0.0106	0.05522
20671	Sox17	SRY-box containing gene 17	0.82712986	0.01913	0.0813
102234	AU022229	expressed sequence AU022229	0.82712986	0.03031	0.111
230235	6430704M03Rik	RIKEN cDNA 6430704M03 gene	0.82712986	0.03374	0.119
208968	Zfp280c	zinc finger protein 280C	0.82712986	0.03725	0.1269
216177	AU041133	expressed sequence AU041133	0.82712986	0.03943	0.1316
338370	Nalcn	sodium leak channel, non-selective	0.82712986	0.05028	0.1556
53623	Gria3	glutamate receptor, ionotropic, AMPA3 (alpha 3)	0.82712986	0.05564	0.1671
74309	Osbp2	oxysterol binding protein 2	0.82712986	0.05865	0.1729
26875	Pclo	piccolo (presynaptic cytomatrix protein)	0.82712986	0.06803	0.1911
225027	Srsf7	serine/arginine-rich splicing factor 7	0.82712986	0.07761	0.21
64380	Ms4a4c	membrane-spanning 4-domains, subfamily A, member 4C	0.82712986	0.09716	0.2434
70081	2210404O09Rik	RIKEN cDNA 2210404O09 gene	0.82712986	0.09767	0.2445
227736	1700019L03Rik	RIKEN cDNA 1700019L03 gene	0.82712986	0.1026	0.2526
71599	Senp8	SUMO/sentrin specific peptidase 8	0.82712986	0.1069	0.2588
71823	3300002A11Rik	RIKEN cDNA 3300002A11 gene	0.82712986	0.1213	0.2826
83380	Prp2	proline rich protein 2	0.82712986	0.1271	0.2912
17933	Myt1l	myelin transcription factor 1-like	0.82712986	0.1958	0.3865
237940	Aoc2	amine oxidase, copper containing 2 (retina-specific)	0.82712986	0.2159	0.4131
76665	1700123M08Rik	RIKEN cDNA 1700123M08 gene	0.82712986	0.219	0.4176
116733	Vps4a	vacuolar protein sorting 4a (yeast)	0.82712986	0.2227	0.4221
14265	Fmr1	fragile X mental retardation syndrome 1 homolog	0.82712986	0.2277	0.4279
70717	6330406I15Rik	RIKEN cDNA 6330406I15 gene	0.82712986	0.2399	0.4422
381921	Taok2	TAO kinase 2	0.82712986	0.3043	0.5134
74215	Prss51	protease, serine, 51	0.82712986	0.3299	0.5401
12021	Bard1	BRCA1 associated RING domain 1	0.82712986	0.495	0.6883
11787	Apbb2	amyloid beta (A4) precursor protein-binding, family B, member 2	0.82644628	0.001724	NA
13197	Gadd45a	growth arrest and DNA-damage-inducible 45 alpha	0.82644628	0.005059	0.0344
103537	Mbtd1	mbt domain containing 1	0.82644628	0.007448	0.04417
67475	Ero1lb	ERO1-like beta (S. cerevisiae)	0.82644628	0.02624	0.1007
57266	Cxcl14	chemokine (C-X-C motif) ligand 14	0.82644628	0.03381	0.1191
380614	Intu	inturned planar cell polarity effector homolog (Drosophila)	0.82644628	0.03648	0.1252
70556	Slc25a33	solute carrier family 25, member 33	0.82644628	0.04245	0.1382
16500	Kcnb1	potassium voltage gated channel, Shab-related subfamily, member 1	0.82644628	0.04351	0.1402
245865	Spag4	sperm associated antigen 4	0.82644628	0.04865	0.152
213988	Tnrc6b	trinucleotide repeat containing 6b	0.82644628	0.05372	0.1632
77524	C030017B01Rik	RIKEN cDNA C030017B01 gene	0.82644628	0.05992	0.1752
20650	Sntb2	syntrophin, basic 2	0.82644628	0.08728	0.2271
107769	Tm6sf1	transmembrane 6 superfamily member 1	0.82644628	0.1072	0.2594
13603	Opn3	opsin 3	0.82644628	0.1144	0.2713
74482	Ifitm7	interferon induced transmembrane protein 7	0.82644628	0.1902	0.3791
240261	Ccdc112	coiled-coil domain containing 112	0.82644628	0.1991	0.3908
66799	Ube2w	ubiquitin-conjugating enzyme E2W (putative)	0.82644628	0.2746	0.4826
100504017	LOC100504017	hypothetical LOC100504017	0.82644628	0.3378	0.548

50774	Krtap5-1	keratin associated protein 5-1	0.82644628	0.3384	0.5486
21958	Tnp1	transition protein 1	0.82644628	0.3434	0.5545
16443	Itsn1	intersectin 1 (SH3 domain protein 1A)	0.82644628	0.3503	0.5607
12776	Ccr8	chemokine (C-C motif) receptor 8	0.82644628	0.639	0.7955
74022	Glyr1	glyoxylate reductase 1 homolog (Arabidopsis)	0.82576383	0.001541	NA
338467	Morc3	microrchidia 3	0.82576383	0.002525	NA
22719	Zfp61	zinc finger protein 61	0.82576383	0.004883	0.03364
11834	Aqr	aquarius	0.82576383	0.009383	0.05103
12696	Cirbp	cold inducible RNA binding protein	0.82576383	0.01047	0.05485
56717	Mtor	mechanistic target of rapamycin (serine/threonine kinase)	0.82576383	0.01477	0.06874
238693	Zfp58	zinc finger protein 58	0.82576383	0.02531	0.09819
217944	Rapgef5	Rap guanine nucleotide exchange factor (GEF) 5	0.82576383	0.02597	0.09993
17936	Nab1	Ngfi-A binding protein 1	0.82576383	0.02756	0.104
67010	Rbm7	RNA binding motif protein 7	0.82576383	0.03087	0.1124
22712	Zfp54	zinc finger protein 54	0.82576383	0.03543	0.123
56048	Lgals8	lectin, galactose binding, soluble 8	0.82576383	0.04092	0.1348
100503209	LOC100503209	hypothetical LOC100503209	0.82576383	0.04161	0.1364
56298	Atl2	atlastin GTPase 2	0.82576383	0.05096	0.157
229227	4932438A13Rik	RIKEN cDNA 4932438A13 gene	0.82576383	0.06221	0.18
56449	Csda	cold shock domain protein A	0.82576383	0.06553	0.1865
116972	Fam57a	family with sequence similarity 57, member A	0.82576383	0.08115	0.2164
19799	Rn4.5s	4.5S RNA	0.82576383	0.08655	0.2259
75533	Nme5	non-metastatic cells 5, protein expressed in (nucleoside-diphosphate kinase)	0.82576383	0.1142	0.271
407831	Tmem204	transmembrane protein 204	0.82576383	0.1244	0.2871
208990	Npb	neuropeptide B	0.82576383	0.1293	0.2949
240119	St6gal2	beta galactoside alpha 2,6 sialyltransferase 2	0.82576383	0.1443	0.3161
24074	Taf7	TAF7 RNA polymerase II, TATA box binding protein (TBP)-associated factor	0.82576383	0.1517	0.3265
14405	Gabrg1	gamma-aminobutyric acid (GABA) A receptor, subunit gamma 1	0.82576383	0.1922	0.3818
72139	2610044O15Rik	RIKEN cDNA 2610044O15 gene	0.82576383	0.2891	0.4975
102920	Cenpi	centromere protein I	0.82576383	0.337	0.5473
228839	Tgif2	TGFB-induced factor homeobox 2	0.82576383	0.387	0.5957
19739	Rgs9	regulator of G-protein signaling 9	0.82576383	0.4247	0.6301
230753	Thrap3	thyroid hormone receptor associated protein 3	0.82576383	0.4553	0.6568
244209	Cyp2r1	cytochrome P450, family 2, subfamily r, polypeptide 1	0.82576383	0.6239	0.7846
271564	Vps13a	vacuolar protein sorting 13A (yeast)	0.82508251	0.004117	0.03032
320351	D230037D09Rik	RIKEN cDNA D230037D09 gene	0.82508251	0.007211	0.04341
50770	Atp11a	ATPase, class VI, type 11A	0.82508251	0.01056	0.05513
72674	Adipor1	adiponectin receptor 1	0.82508251	0.01197	0.06006
70710	6330407I18Rik	RIKEN cDNA 6330407I18 gene	0.82508251	0.03194	0.1148
328977	Zfp532	zinc finger protein 532	0.82508251	0.03738	0.1273
11640	Akap1	A kinase (PRKA) anchor protein 1	0.82508251	0.04202	0.1373
16372	Irx2	Iroquois related homeobox 2 (Drosophila)	0.82508251	0.04674	0.1475
232286	Tmf1	TATA element modulatory factor 1	0.82508251	0.0581	0.1718
320816	Ankrd16	ankyrin repeat domain 16	0.82508251	0.05814	0.1719
100042862	Gm4076	predicted gene 4076	0.82508251	0.05976	0.1748
228602	4930402H24Rik	RIKEN cDNA 4930402H24 gene	0.82508251	0.06407	0.1838
21916	Tmod1	tropomodulin 1	0.82508251	0.06486	0.1853
215654	Cdh12	cadherin 12	0.82508251	0.07945	0.2135
74407	Ttc25	tetratricopeptide repeat domain 25	0.82508251	0.08276	0.2192
242253	Wdr63	WD repeat domain 63	0.82508251	0.09148	0.2342

216987	Utp6	UTP6, small subunit (SSU) processome component, homolog (yeast)	0.82508251	0.09649	0.2426
71876	Mlf1ip	myeloid leukemia factor 1 interacting protein	0.82508251	0.09805	0.2452
100036521	Gm16039	predicted gene 16039	0.82508251	0.1161	0.2737
72899	Macrod2	MACRO domain containing 2	0.82508251	0.131	0.2969
85030	Tnfrsf25	tumor necrosis factor receptor superfamily, member 25	0.82508251	0.1427	0.3136
319580	9630055L06Rik	RIKEN cDNA 9630055L06 gene	0.82508251	0.2054	0.3992
16988	Lst1	leukocyte specific transcript 1	0.82508251	0.2347	0.4363
93879	Pcdhb8	protocadherin beta 8	0.82508251	0.2721	0.4795
20541	Slc8a1	solute carrier family 8 (sodium/calcium exchanger), member 1	0.82508251	0.4629	0.6634
546954	Gm6003	predicted gene 6003	0.82508251	0.6832	0.8231
30910	C230037E05Rik	RIKEN cDNA C230037E05 gene	0.82440231	0.002427	0.02168
78795	Armc9	armadillo repeat containing 9	0.82440231	0.003021	0.02493
68162	A930003A15Rik	RIKEN cDNA A930003A15 gene	0.82440231	0.003392	0.02691
19888	Rp1	retinitis pigmentosa 1 (human)	0.82440231	0.006872	0.04197
217265	Abca5	ATP-binding cassette, sub-family A (ABC1), member 5	0.82440231	0.01037	0.05448
216233	Socs2	suppressor of cytokine signaling 2	0.82440231	0.01088	0.05612
67466	Pdcl	phosducin-like	0.82440231	0.01098	0.05644
73953	4930421J07Rik	RIKEN cDNA 4930421J07 gene	0.82440231	0.01295	0.06305
77072	6720420G18Rik	RIKEN cDNA 6720420G18 gene	0.82440231	0.03157	0.1139
14296	Frat1	frequently rearranged in advanced T-cell lymphomas	0.82440231	0.0388	0.1303
57911	Gsdma	gasdermin A	0.82440231	0.04441	0.1424
24127	Xrn1	5'-3' exoribonuclease 1	0.82440231	0.05306	0.1617
22327	Vbp1	von Hippel-Lindau binding protein 1	0.82440231	0.05399	0.1637
66283	Gkn1	gastrokine 1	0.82440231	0.05965	0.1747
66810	Rbm22	RNA binding motif protein 22	0.82440231	0.06678	0.1888
217217	Asb16	ankyrin repeat and SOCS box-containing 16	0.82440231	0.07479	0.2045
54525	Syt7	synaptotagmin VII	0.82440231	0.08499	0.2231
76886	Fam81a	family with sequence similarity 81, member A	0.82440231	0.1425	0.3133
17101	Lyst	lysosomal trafficking regulator	0.82440231	0.1458	0.3184
231327	Ppat	phosphoribosyl pyrophosphate amidotransferase	0.82440231	0.1597	0.3379
13730	Emp1	epithelial membrane protein 1	0.82440231	0.1755	0.36
20926	Supt6h	suppressor of Ty 6 homolog (S. cerevisiae)	0.82440231	0.2841	0.4927
76613	1700069B07Rik	RIKEN cDNA 1700069B07 gene	0.82440231	0.3245	0.5343
211134	Lzts1	leucine zipper, putative tumor suppressor 1	0.82440231	0.3829	0.5919
50787	Hs6st3	heparan sulfate 6-O-sulfotransferase 3	0.82440231	0.4254	0.6307
110524	Dgkq	diacylglycerol kinase, theta	0.82372323	0.005982	0.03824
22355	Vipr2	vasoactive intestinal peptide receptor 2	0.82372323	0.015	0.0694
408062	Zfp873	zinc finger protein 873	0.82372323	0.01623	0.07292
76220	6530402F18Rik	RIKEN cDNA 6530402F18 gene	0.82372323	0.0224	0.09076
17436	Me1	malic enzyme 1, NADP(+)-dependent, cytosolic	0.82372323	0.04495	0.1436
233490	Crebzf	CREB/ATF bZIP transcription factor	0.82372323	0.0522	0.1598
234857	Spire2	spire homolog 2 (Drosophila)	0.82372323	0.08797	0.2283
16431	Itm2a	integral membrane protein 2A	0.82372323	0.08927	0.2303
22145	Tuba4a	tubulin, alpha 4A	0.82372323	0.09961	0.2478
214575	Tdrd5	tudor domain containing 5	0.82372323	0.1105	0.2648
22688	Zfp26	zinc finger protein 26	0.82372323	0.1137	0.27
240064	Zfp799	zinc finger protein 799	0.82372323	0.1254	0.2887
76872	Ccdc116	coiled-coil domain containing 116	0.82372323	0.1738	0.3573
208650	Cblb	Casitas B-lineage lymphoma b	0.82372323	0.243	0.4462
224826	Ubr2	ubiquitin protein ligase E3 component n-recognin 2	0.82372323	0.3269	0.537

20273	Scn8a	sodium channel, voltage-gated, type VIII, alpha	0.82372323	0.3861	0.5947
232371	C1rl	complement component 1, r subcomponent-like	0.82372323	0.5285	0.7146
232087	Mat2a	methionine adenosyltransferase II, alpha	0.82304527	0.001676	NA
665113	Tnik	TRAF2 and NCK interacting kinase	0.82304527	0.001908	0.01908
229780	Ccdc76	coiled-coil domain containing 76	0.82304527	0.008682	0.04863
234915	AK129341	cDNA sequence AK129341	0.82304527	0.03003	0.1103
246709	Rgs13	regulator of G-protein signaling 13	0.82304527	0.0399	0.1326
402728	Pax6os1	Pax6 opposite strand transcript 1	0.82304527	0.05988	0.1751
279029	Gm711	predicted gene 711	0.82304527	0.06078	0.1771
638247	9530082P21Rik	RIKEN cDNA 9530082P21 gene	0.82304527	0.06633	0.1879
54611	Pde3a	phosphodiesterase 3A, cGMP inhibited	0.82304527	0.06875	0.1927
69017	Prrt2	proline-rich transmembrane protein 2	0.82304527	0.09434	0.239
67225	Rnpc3	RNA-binding region (RNP1, RRM) containing 3	0.82304527	0.09577	0.2417
207214	Larp4	La ribonucleoprotein domain family, member 4	0.82304527	0.1064	0.2581
238693	Zfp58	zinc finger protein 58	0.82304527	0.1309	0.2969
140484	Pofut1	protein O-fucosyltransferase 1	0.82304527	0.1359	0.3034
231646	Myo1h	myosin 1H	0.82304527	0.1515	0.3263
59056	Evc	Ellis van Creveld gene homolog (human)	0.82304527	0.16	0.3381
20259	Scin	scinderin	0.82304527	0.2106	0.4061
271377	Zbtb11	zinc finger and BTB domain containing 11	0.82304527	0.2484	0.4526
108155	Ogt	O-linked N-acetylglucosamine (GlcNAc) transferase (UDP-N-acetylglucosamine:polypeptide-N-acetylglucosaminyl transferase)	0.82304527	0.2746	0.4825
494519	Abpd	androgen binding protein delta	0.82304527	0.505	0.6962
68146	Arl13b	ADP-ribosylation factor-like 13B	0.82236842	0.004812	0.0334
217843	Unc79	unc-79 homolog (C. elegans)	0.82236842	0.01697	0.07485
18795	Plcb1	phospholipase C, beta 1	0.82236842	0.01784	0.07758
99326	Garnl3	GTPase activating RANGAP domain-like 3	0.82236842	0.01797	0.07803
77531	Anks1b	ankyrin repeat and sterile alpha motif domain containing 1B	0.82236842	0.0282	0.1057
21929	Tnfaip3	tumor necrosis factor, alpha-induced protein 3	0.82236842	0.03741	0.1273
83691	Crispld1	cysteine-rich secretory protein LCCL domain containing 1	0.82236842	0.04279	0.1388
15572	Elavl4	ELAV (embryonic lethal, abnormal vision, Drosophila)-like 4 (Hu antigen D)	0.82236842	0.04794	0.1503
56790	Fam48a	family with sequence similarity 48, member A	0.82236842	0.07406	0.2033
239528	Eif2c2	eukaryotic translation initiation factor 2C, 2	0.82236842	0.1021	0.2521
77290	9430078K24Rik	RIKEN cDNA 9430078K24 gene	0.82236842	0.1061	0.2577
100043902	Six3os1	Six3 opposite strand transcript 1	0.82236842	0.1062	0.2579
20662	Sos1	son of sevenless homolog 1 (Drosophila)	0.82236842	0.1731	0.3567
55948	Sfn	stratifin	0.82236842	0.2304	0.4313
68473	Mobkl1a	MOB1, Mps One Binder kinase activator-like 1A (yeast)	0.82236842	0.4321	0.6361
75939	4930579G24Rik	RIKEN cDNA 4930579G24 gene	0.82169269	0.01183	0.05966
14772	Grk4	G protein-coupled receptor kinase 4	0.82169269	0.02756	0.104
21375	Tbr1	T-box brain gene 1	0.82169269	0.05672	0.1692
100042218	Gm3728	predicted gene 3728	0.82169269	0.05706	0.1699
320865	Cdh18	cadherin 18	0.82169269	0.06557	0.1865
225131	Wac	WW domain containing adaptor with coiled-coil	0.82169269	0.08032	0.215
19888	Rp1	retinitis pigmentosa 1 (human)	0.82169269	0.08396	0.2213
320110	B230369F24Rik	RIKEN cDNA B230369F24 gene	0.82169269	0.09206	0.2353
20020	Polr2a	polymerase (RNA) II (DNA directed) polypeptide A	0.82169269	0.0992	0.247
53893	Nudt5	nudix (nucleoside diphosphate linked moiety X)-type motif 5	0.82169269	0.1029	0.2531
12833	Col6a1	collagen, type VI, alpha 1	0.82169269	0.1678	0.3486
235283	Gramd1b	GRAM domain containing 1B	0.82169269	0.2124	0.4084
72685	Dnajc6	DnaJ (Hsp40) homolog, subfamily C, member 6	0.82169269	0.2282	0.4282

230861	Eif4g3	eukaryotic translation initiation factor 4 gamma, 3	0.82169269	0.2324	0.4336
235044	BC018242	cDNA sequence BC018242	0.82169269	0.299	0.5084
76007	Zmym2	zinc finger, MYM-type 2	0.82169269	0.3134	0.5231
271842	Rpusd2	RNA pseudouridylate synthase domain containing 2	0.82169269	0.3974	0.6054
217698	Acot5	acyl-CoA thioesterase 5	0.82169269	0.4599	0.6607
26875	Pclo	piccolo (presynaptic cytomatrix protein)	0.82101806	0.007231	0.04349
22341	Vegfc	vascular endothelial growth factor C	0.82101806	0.01451	0.068
380916	Lrch1	leucine-rich repeats and calponin homology (CH) domain containing 1	0.82101806	0.0172	0.07559
14617	Gjd2	gap junction protein, delta 2	0.82101806	0.01843	0.07934
16000	Igf1	insulin-like growth factor 1	0.82101806	0.01886	0.08051
54377	Cacng4	calcium channel, voltage-dependent, gamma subunit 4	0.82101806	0.0323	0.1158
218232	Ptpdc1	protein tyrosine phosphatase domain containing 1	0.82101806	0.03761	0.1278
73095	Slc25a42	solute carrier family 25, member 42	0.82101806	0.04041	0.1337
22152	Tubb3	tubulin, beta 3	0.82101806	0.04618	0.1463
76186	6330563C09Rik	RIKEN cDNA 6330563C09 gene	0.82101806	0.04772	0.1498
67426	Adck3	aarF domain containing kinase 3	0.82101806	0.04978	0.1546
11304	Abca4	ATP-binding cassette, sub-family A (ABC1), member 4	0.82101806	0.05903	0.1736
78796	Zcchc4	zinc finger, CCHC domain containing 4	0.82101806	0.1028	0.253
117600	Srgap1	SLIT-ROBO Rho GTPase activating protein 1	0.82101806	0.1186	0.2779
71217	4933431I19Rik	RIKEN cDNA 4933431I19 gene	0.82101806	0.139	0.3081
231769	Sfswap	splicing factor, suppressor of white-apricot homolog (Drosophila)	0.82101806	0.1462	0.3188
209239	Gan	giant axonal neuropathy	0.82101806	0.1631	0.3422
17717	ND2	NADH dehydrogenase subunit 2	0.82101806	0.1637	0.3431
114677	4930590A17Rik	RIKEN cDNA 4930590A17 gene	0.82101806	0.1982	0.3897
233071	Arhgap33	Rho GTPase activating protein 33	0.82101806	0.3363	0.5467
368203	Gm5136	predicted gene 5136	0.82101806	0.3944	0.6026
100040529	Gm2824	predicted gene 2824	0.82101806	0.4559	0.6573
258728	Olfr482	olfactory receptor 482	0.82101806	0.4922	0.6863
671564	Rnf212	ring finger protein 212	0.82101806	0.5659	0.7423
211151	Churc1	churchill domain containing 1	0.82034454	0.01078	0.05576
243272	Sbno1	sno, strawberry notch homolog 1 (Drosophila)	0.82034454	0.01122	0.05743
71096	Sntg1	syntrophin, gamma 1	0.82034454	0.02038	0.08503
353047	Plekhm1	pleckstrin homology domain containing, family M (with RUN domain) member 1	0.82034454	0.02853	0.1065
54137	Acrbp	proacrosin binding protein	0.82034454	0.03035	0.1111
21844	Tiam1	T-cell lymphoma invasion and metastasis 1	0.82034454	0.04833	0.1512
237823	Pfas	phosphoribosylformylglycinamidine synthase (FGAR amidotransferase)	0.82034454	0.06067	0.1769
72672	Zfp518a	zinc finger protein 518A	0.82034454	0.07154	0.1982
328949	Mcc	mutated in colorectal cancers	0.82034454	0.08294	0.2195
72027	Slc39a4	solute carrier family 39 (zinc transporter), member 4	0.82034454	0.08307	0.2197
74471	4933440N22Rik	RIKEN cDNA 4933440N22 gene	0.82034454	0.1626	0.3415
653016	Gm7325	predicted gene 7325	0.82034454	0.178	0.363
170707	Usp48	ubiquitin specific peptidase 48	0.82034454	0.2242	0.424
11522	Adh1	alcohol dehydrogenase 1 (class I)	0.82034454	0.2332	0.4346
23920	Insrr	insulin receptor-related receptor	0.82034454	0.2441	0.4479
384382	A430108E01Rik	RIKEN cDNA A430108E01 gene	0.82034454	0.2836	0.4922
100505237	LOC100505237	calcium-binding and coiled-coil domain-containing protein 2-like	0.82034454	0.3643	0.5733
66849	Ppp1r2	protein phosphatase 1, regulatory (inhibitor) subunit 2	0.82034454	0.3989	0.6066
16918	Mycl1	v-myc myelocytomatosis viral oncogene homolog 1, lung carcinoma derived (avian)	0.82034454	0.4161	0.6222
237886	Slfn9	schlafen 9	0.82034454	0.4506	0.6528
226255	Atrnl1	attractin like 1	0.81967213	0.01037	0.05448

69156	Comtd1	catechol-O-methyltransferase domain containing 1	0.81967213	0.01324	0.06408
320571	Atp8b5	ATPase, class I, type 8B, member 5	0.81967213	0.01682	0.07444
270091	Lrrc36	leucine rich repeat containing 36	0.81967213	0.01934	0.0819
234353	Psd3	pleckstrin and Sec7 domain containing 3	0.81967213	0.0539	0.1635
232035	Fam190a	family with sequence similarity 190, member A	0.81967213	0.05591	0.1678
78433	A930006K02Rik	RIKEN cDNA A930006K02 gene	0.81967213	0.05867	0.1729
330427	9330102E08Rik	RIKEN cDNA 9330102E08 gene	0.81967213	0.06055	0.1766
229759	Olfm3	olfactomedin 3	0.81967213	0.07704	0.2088
12523	Cd84	CD84 antigen	0.81967213	0.08276	0.2192
268481	Krt222	keratin 222	0.81967213	0.08645	0.2257
75884	4930572J10Rik	RIKEN cDNA 4930572J10 gene	0.81967213	0.1023	0.2522
12874	Cpd	carboxypeptidase D	0.81967213	0.1129	0.2688
114863	Prosc	proline synthetase co-transcribed	0.81967213	0.1142	0.271
20448	St6galnac4	ST6 (alpha-N-acetyl-neuraminyl-2,3-beta-galactosyl-1,3)-N-acetylgalactosaminide alpha-2,6-sialyltransferase 4	0.81967213	0.2104	0.4059
381598	2610005L07Rik	cadherin 11 pseudogene	0.81967213	0.2206	0.4196
74081	Cep350	centrosomal protein 350	0.81967213	0.2673	0.4743
668825	Gm13931	predicted gene 13931	0.81967213	0.5654	0.7421
59015	Nup160	nucleoporin 160	0.81900082	0.001658	0.01748
77300	Raph1	Ras association (RalGDS/AF-6) and pleckstrin homology domains 1	0.81900082	0.002148	0.02023
13690	Eif4g2	eukaryotic translation initiation factor 4, gamma 2	0.81900082	0.008417	0.04762
70465	Wdr77	WD repeat domain 77	0.81900082	0.01124	0.05752
72307	2510002D24Rik	RIKEN cDNA 2510002D24 gene	0.81900082	0.03219	0.1154
75578	Fggy	FGGY carbohydrate kinase domain containing	0.81900082	0.05427	0.1644
14772	Grk4	G protein-coupled receptor kinase 4	0.81900082	0.1055	0.2569
52036	Ppp6r3	protein phosphatase 6, regulatory subunit 3	0.81900082	0.3305	0.5408
380728	Kcnh4	potassium voltage-gated channel, subfamily H (eag-related), member 4	0.81900082	0.3364	0.5467
268281	Shprh	SNF2 histone linker PHD RING helicase	0.81900082	0.3858	0.5946
97954	C77847	expressed sequence C77847	0.81900082	0.3918	0.6
103288	AI836737	expressed sequence AI836737	0.81900082	0.4719	0.6702
70373	1700020O03Rik	RIKEN cDNA 1700020O03 gene	0.81833061	0.002343	0.02134
76080	Ttpal	tocopherol (alpha) transfer protein-like	0.81833061	0.002371	0.02141
217588	Mbip	MAP3K12 binding inhibitory protein 1	0.81833061	0.004868	0.03358
319535	Zfp182	zinc finger protein 182	0.81833061	0.009232	0.05052
213119	Itga10	integrin, alpha 10	0.81833061	0.01076	0.05573
433319	Gm5528	predicted gene 5528	0.81833061	0.01602	0.07226
12801	Cnr1	cannabinoid receptor 1 (brain)	0.81833061	0.0196	0.08263
619441	BC096441	cDNA sequence BC096441	0.81833061	0.02211	0.09002
228410	Cstf3	cleavage stimulation factor, 3' pre-RNA, subunit 3	0.81833061	0.02507	0.09759
69445	1700023D09Rik	RIKEN cDNA 1700023D09 gene	0.81833061	0.04031	0.1335
29857	Mapk12	mitogen-activated protein kinase 12	0.81833061	0.04104	0.135
104360	Isl2	insulin related protein 2 (islet 2)	0.81833061	0.04877	0.1522
66661	Srp72	signal recognition particle 72	0.81833061	0.05406	0.1639
19017	Ppargc1a	peroxisome proliferative activated receptor, gamma, coactivator 1 alpha	0.81833061	0.06626	0.1878
54004	Diap2	diaphanous homolog 2 (Drosophila)	0.81833061	0.07576	0.2064
52882	Rgs7bp	regulator of G-protein signalling 7 binding protein	0.81833061	0.1336	0.3005
66970	Ssbp2	single-stranded DNA binding protein 2	0.81833061	0.1782	0.3632
382014	Ano8	anoctamin 8	0.81833061	0.2007	0.3929
77574	Fam115a	family with sequence similarity 115, member A	0.81833061	0.2366	0.4384
100040872	Gm3014	predicted gene 3014	0.81833061	0.4022	0.6098
637776	Gm7221	predicted gene 7221	0.81833061	0.4359	0.6395

15235	Mst1	macrophage stimulating 1 (hepatocyte growth factor-like)	0.81833061	0.4881	0.6829
237943	Gpatch8	G patch domain containing 8	0.81766149	0.001336	0.01543
71492	Bbs7	Bardet-Biedl syndrome 7 (human)	0.81766149	0.001925	0.01917
100502627	LOC100502627	hypothetical LOC100502627	0.81766149	0.004993	0.03411
66276	1810009A15Rik	RIKEN cDNA 1810009A15 gene	0.81766149	0.01564	0.07134
66995	Zcchc18	zinc finger, CCHC domain containing 18	0.81766149	0.01588	0.07195
233065	Alkbh6	alkB, alkylation repair homolog 6 (E. coli)	0.81766149	0.01652	0.0736
319955	Ercc6	excision repair cross-complementing rodent repair deficiency, complementation group 6	0.81766149	0.02302	0.0923
237761	Ankrd43	ankyrin repeat domain 43	0.81766149	0.03993	0.1327
74015	Fcho1	FCH domain only 1	0.81766149	0.05252	0.1605
67222	Srfbp1	serum response factor binding protein 1	0.81766149	0.09044	0.2324
207425	Wdr11	WD repeat domain 11	0.81766149	0.1117	0.2669
69060	Pnlip	pancreatic lipase	0.81766149	0.1277	0.2922
100036572	LOC100036572	hypothetical LOC100036572	0.81766149	0.1325	0.299
107065	Lrrtm2	leucine rich repeat transmembrane neuronal 2	0.81766149	0.1537	0.3292
77794	Adamtsl2	ADAMTS-like 2	0.81766149	0.1551	0.3314
67367	Gcfc1	GC-rich sequence DNA-binding factor 1	0.81766149	0.1603	0.3384
217151	Arl5c	ADP-ribosylation factor-like 5C	0.81766149	0.1677	0.3485
67847	Sncaip	synuclein, alpha interacting protein (synphilin)	0.81766149	0.1703	0.3524
73961	4930447J18Rik	RIKEN cDNA 4930447J18 gene	0.81766149	0.4589	0.6599
233789	Smg1	SMG1 homolog, phosphatidylinositol 3-kinase-related kinase (C. elegans)	0.81699346	0.004052	0.03
75965	Zdhhc20	zinc finger, DHHC domain containing 20	0.81699346	0.005892	0.03785
67201	Glod4	glyoxalase domain containing 4	0.81699346	0.005939	0.03802
100040086	Gm2590	predicted gene 2590	0.81699346	0.009311	0.05078
320497	A630065K11Rik	RIKEN cDNA A630065K11 gene	0.81699346	0.0157	0.07148
317717	Sec22a	SEC22 vesicle trafficking protein homologe A (S. cerevisiae)	0.81699346	0.01598	0.07221
53379	Hnrnpa2b1	heterogeneous nuclear ribonucleoprotein A2/B1	0.81699346	0.02382	0.09434
18952	Sep-04	septin 4	0.81699346	0.02641	0.1011
100046290	LOC100046290	60S ribosomal protein L21-like	0.81699346	0.02706	0.1027
239410	A930017M01Rik	Smg-5 homolog, nonsense mediated mRNA decay factor pseudogene	0.81699346	0.02711	0.1028
107815	Scml2	sex comb on midleg-like 2 (Drosophila)	0.81699346	0.04056	0.134
14236	Foxn2	forkhead box N2	0.81699346	0.04626	0.1465
74613	4833412C15Rik	RIKEN cDNA 4833412C15 gene	0.81699346	0.0572	0.1701
69017	Prrt2	proline-rich transmembrane protein 2	0.81699346	0.07257	0.2004
12821	Col17a1	collagen, type XVII, alpha 1	0.81699346	0.07508	0.205
77521	Mtus2	microtubule associated tumor suppressor candidate 2	0.81699346	0.08376	0.221
69440	Fam116b	family with sequence similarity 116, member B	0.81699346	0.08548	0.2241
17721	ND5	NADH dehydrogenase subunit 5	0.81699346	0.08656	0.2259
22634	Plagl1	pleiomorphic adenoma gene-like 1	0.81699346	0.1012	0.2507
66648	5730494M16Rik	RIKEN cDNA 5730494M16 gene	0.81699346	0.1484	0.3221
100073351	Yy2	Yy2 transcription factor	0.81699346	0.1669	0.3477
24052	Sgcd	sarcoglycan, delta (dystrophin-associated glycoprotein)	0.81699346	0.2463	0.4502
226105	Cyp2c70	cytochrome P450, family 2, subfamily c, polypeptide 70	0.81699346	0.347	0.5575
407243	Tmem189	transmembrane protein 189	0.81699346	0.6808	0.8218
66113	Apoa5	apolipoprotein A-V	0.81699346	0.7169	0.8454
116873	Stim2	stromal interaction molecule 2	0.81632653	0.001509	0.01665
110350	Dync2h1	dynein cytoplasmic 2 heavy chain 1	0.81632653	0.001682	0.01766
225523	Cep120	centrosomal protein 120	0.81632653	0.01068	0.05546
76217	Jakmip2	janus kinase and microtubule interacting protein 2	0.81632653	0.01197	0.06006
18613	Pecam1	platelet/endothelial cell adhesion molecule 1	0.81632653	0.0207	0.08598

245174	Zfp937	zinc finger protein 937	0.81632653	0.02221	0.0903
110593	Prdm2	PR domain containing 2, with ZNF domain	0.81632653	0.02309	0.09245
68108	9430008C03Rik	RIKEN cDNA 9430008C03 gene	0.81632653	0.02842	0.1062
67890	Ufm1	ubiquitin-fold modifier 1	0.81632653	0.02996	0.1101
70546	Zdhhc2	zinc finger, DHHC domain containing 2	0.81632653	0.03999	0.1328
230996	9430015G10Rik	RIKEN cDNA 9430015G10 gene	0.81632653	0.07761	0.21
54710	Hs3st3b1	heparan sulfate (glucosamine) 3-O-sulfotransferase 3B1	0.81632653	0.08761	0.2277
320772	Mdga2	MAM domain containing glycosylphosphatidylinositol anchor 2	0.81632653	0.0887	0.2294
239857	Cadm2	cell adhesion molecule 2	0.81632653	0.1153	0.2725
56309	Mycbp	c-myc binding protein	0.81632653	0.1419	0.3125
27416	Abcc5	ATP-binding cassette, sub-family C (CFTR/MRP), member 5	0.81632653	0.2133	0.4094
23908	Hs2st1	heparan sulfate 2-O-sulfotransferase 1	0.81632653	0.3672	0.5761
13179	Dcn	decorin	0.81632653	0.3886	0.597
237362	Npffr1	neuropeptide FF receptor 1	0.81632653	0.5155	0.7051
665635	LOC665635	hypothetical protein LOC665635	0.81566069	0.008321	0.04728
227157	Mpp4	membrane protein, palmitoylated 4 (MAGUK p55 subfamily member 4)	0.81566069	0.008706	0.04873
93692	Glrx	glutaredoxin	0.81566069	0.01342	0.06455
17119	Mxd1	MAX dimerization protein 1	0.81566069	0.01822	0.07869
67391	Fundc2	FUN14 domain containing 2	0.81566069	0.02192	0.08942
383103	Fam18a	family with sequence similarity 18, member A	0.81566069	0.02923	0.1082
70387	Ttc9c	tetratricopeptide repeat domain 9C	0.81566069	0.04397	0.1414
242691	Gpatch3	G patch domain containing 3	0.81566069	0.09231	0.2357
11909	Atf2	activating transcription factor 2	0.81566069	0.1163	0.2739
26920	Cep110	centrosomal protein 110	0.81566069	0.1197	0.2797
93966	Hemgn	hemogen	0.81566069	0.1342	0.3012
76784	Mtif2	mitochondrial translational initiation factor 2	0.81566069	0.1949	0.3852
328516	D430034N21	hypothetical protein D430034N21	0.81566069	0.3192	0.529
383295	Ypel5	yippee-like 5 (Drosophila)	0.81499593	0.007083	0.0429
78245	Acbd7	acyl-Coenzyme A binding domain containing 7	0.81499593	0.008506	0.04795
23959	Nt5e	5' nucleotidase, ecto	0.81499593	0.01197	0.06006
319807	3110047P20Rik	RIKEN cDNA 3110047P20 gene	0.81499593	0.01413	0.06685
12405	Cbln2	cerebellin 2 precursor protein	0.81499593	0.0175	0.0765
71902	Cand1	cullin associated and neddylation disassociated 1	0.81499593	0.02907	0.1078
14049	Eya2	eyes absent 2 homolog (Drosophila)	0.81499593	0.02988	0.1099
109959	Amy2a5	amylase 2a5	0.81499593	0.03581	0.1238
20269	Scn3a	sodium channel, voltage-gated, type III, alpha	0.81499593	0.03611	0.1244
20530	Slc31a2	solute carrier family 31, member 2	0.81499593	0.03833	0.1293
228564	Frmd5	FERM domain containing 5	0.81499593	0.04926	0.1534
110460	Acat2	acetyl-Coenzyme A acetyltransferase 2	0.81499593	0.05176	0.1589
75429	Fam183b	family with sequence similarity 183, member B	0.81499593	0.1122	0.2675
338521	Fa2h	fatty acid 2-hydroxylase	0.81499593	0.1642	0.3441
71392	5430401F13Rik	RIKEN cDNA 5430401F13 gene	0.81499593	0.1994	0.3912
100040972	Tceal7	transcription elongation factor A (SII)-like 7	0.81499593	0.2474	0.4517
319196	Ankrd5	ankyrin repeat domain 5	0.81499593	0.2484	0.4526
244416	Ppp1r3b	protein phosphatase 1, regulatory (inhibitor) subunit 3B	0.81499593	0.2853	0.4938
54446	Nfat5	nuclear factor of activated T-cells 5	0.81499593	0.3224	0.5322
100041639	Gm3448	predicted gene 3448	0.81499593	0.3453	0.5561
242594	1700024P16Rik	RIKEN cDNA 1700024P16 gene	0.81499593	0.4713	0.67
245403	Dcaf12l2	DDB1 and CUL4 associated factor 12-like 2	0.81499593	0.474	0.6718
234395	Ushbp1	Usher syndrome 1C binding protein 1	0.81433225	0.002127	0.02015

71389	Chd6	chromodomain helicase DNA binding protein 6	0.81433225	0.01642	0.07344
59025	Usp14	ubiquitin specific peptidase 14	0.81433225	0.02792	0.1049
433520	Gm14403	predicted gene 14403	0.81433225	0.03154	0.1139
83603	Elovl4	elongation of very long chain fatty acids (FEN1/Elo2, SUR4/Elo3, yeast)-like 4	0.81433225	0.03601	0.1242
13837	Epha3	Eph receptor A3	0.81433225	0.04329	0.1397
230082	Nol6	nucleolar protein family 6 (RNA-associated)	0.81433225	0.05879	0.1732
215705	Arrdc1	arrestin domain containing 1	0.81433225	0.06984	0.1949
12545	Cdc7	cell division cycle 7 (S. cerevisiae)	0.81433225	0.08351	0.2206
70584	Pak4	p21 protein (Cdc42/Rac)-activated kinase 4	0.81433225	0.1403	0.31
100048658	Ddx43	DEAD (Asp-Glu-Ala-Asp) box polypeptide 43	0.81433225	0.1873	0.3755
106565	Dlk2	delta-like 2 homolog (Drosophila)	0.81433225	0.1933	0.3832
107508	Eprs	glutamyl-prolyl-tRNA synthetase	0.81433225	0.1933	0.3832
268297	Scml4	sex comb on midleg-like 4 (Drosophila)	0.81433225	0.2798	0.488
269587	Epb4.1	erythrocyte protein band 4.1	0.81433225	0.4009	0.6086
60440	Iigp1	interferon inducible GTPase 1	0.81433225	0.4829	0.6785
11426	Macf1	microtubule-actin crosslinking factor 1	0.81366965	0.0005446	0.009025
269682	Golga3	golgi autoantigen, golgin subfamily a, 3	0.81366965	0.005518	0.03635
100637	N4bp2l1	NEDD4 binding protein 2-like 1	0.81366965	0.006352	0.03984
75770	Brsk2	BR serine/threonine kinase 2	0.81366965	0.01198	0.06007
14859	Gsta3	glutathione S-transferase, alpha 3	0.81366965	0.0129	0.06293
75710	Rbm12	RNA binding motif protein 12	0.81366965	0.01493	0.06916
243961	Shank1	SH3/ankyrin domain gene 1	0.81366965	0.0158	0.07178
14688	Gnb1	guanine nucleotide binding protein (G protein), beta 1	0.81366965	0.01585	0.07193
232174	Cyp26b1	cytochrome P450, family 26, subfamily b, polypeptide 1	0.81366965	0.02315	0.09252
11304	Abca4	ATP-binding cassette, sub-family A (ABC1), member 4	0.81366965	0.03845	0.1294
13349	Darc	Duffy blood group, chemokine receptor	0.81366965	0.06343	0.1824
17364	Trpm1	transient receptor potential cation channel, subfamily M, member 1	0.81366965	0.07088	0.1969
320213	Senp5	SUMO/sentrin specific peptidase 5	0.81366965	0.1218	0.2834
100040322	3830408C21Rik	RIKEN cDNA 3830408C21 gene	0.81366965	0.1323	0.2988
68026	2810417H13Rik	RIKEN cDNA 2810417H13 gene	0.81366965	0.1981	0.3895
56508	Rapgef4	Rap guanine nucleotide exchange factor (GEF) 4	0.81366965	0.2128	0.4088
64707	Suv39h2	suppressor of variegation 3-9 homolog 2 (Drosophila)	0.81366965	0.2425	0.4454
77683	Ehmt1	euchromatic histone methyltransferase 1	0.81366965	0.3137	0.5233
216799	Nlrp3	NLR family, pyrin domain containing 3	0.81366965	0.3579	0.5679
219150	Hmbox1	homeobox containing 1	0.81366965	0.4474	0.6496
52323	Klhl7	kelch-like 7 (Drosophila)	0.81300813	0.002439	0.02177
68693	Hnrnpul2	heterogeneous nuclear ribonucleoprotein U-like 2	0.81300813	0.005782	0.03743
93871	Brwd1	bromodomain and WD repeat domain containing 1	0.81300813	0.007277	0.0436
108115	Slco4a1	solute carrier organic anion transporter family, member 4a1	0.81300813	0.008287	0.04721
19014	Med1	mediator complex subunit 1	0.81300813	0.01059	0.05517
93871	Brwd1	bromodomain and WD repeat domain containing 1	0.81300813	0.012	0.06013
243906	Zfp14	zinc finger protein 14	0.81300813	0.016	0.07222
227634	Camsap1	calmodulin regulated spectrin-associated protein 1	0.81300813	0.01635	0.07326
77519	Zfp266	zinc finger protein 266	0.81300813	0.0165	0.07359
227731	Slc25a25	solute carrier family 25 (mitochondrial carrier, phosphate carrier), member 25	0.81300813	0.02409	0.09508
227525	Dclre1c	DNA cross-link repair 1C, PSO2 homolog (S. cerevisiae)	0.81300813	0.03491	0.1217
241263	Gpr158	G protein-coupled receptor 158	0.81300813	0.03899	0.1306
241846	Lsm14b	LSM14 homolog B (SCD6, S. cerevisiae)	0.81300813	0.07299	0.2014
11484	Aspa	aspartoacylase	0.81300813	0.1153	0.2725
78751	Zc3h6	zinc finger CCCH type containing 6	0.81300813	0.1421	0.3128

78286	Nav2	neuron navigator 2	0.81300813	0.1459	0.3185
241915	Phc3	polyhomeotic-like 3 (Drosophila)	0.81300813	0.1619	0.3404
50769	Atp8a2	ATPase, aminophospholipid transporter-like, class I, type 8A, member 2	0.81300813	0.3528	0.5629
208158	Map6d1	MAP6 domain containing 1	0.81300813	0.3853	0.5945
67367	Gcfc1	GC-rich sequence DNA-binding factor 1	0.81234768	0.001557	0.01698
213053	Slc39a14	solute carrier family 39 (zinc transporter), member 14	0.81234768	0.004917	0.03379
69641	Wdr20a	WD repeat domain 20A	0.81234768	0.009612	0.05191
69930	Zfp715	zinc finger protein 715	0.81234768	0.009992	0.05329
16563	Kif2a	kinesin family member 2A	0.81234768	0.0116	0.05892
233575	Pgap2	post-GPI attachment to proteins 2	0.81234768	0.02999	0.1102
68397	0610039H22Rik	RIKEN cDNA 0610039H22 gene	0.81234768	0.03927	0.1312
545085	Wdr70	WD repeat domain 70	0.81234768	0.06619	0.1876
18551	Pcsk4	proprotein convertase subtilisin/kexin type 4	0.81234768	0.07177	0.1987
77766	Elp4	elongation protein 4 homolog (S. cerevisiae)	0.81234768	0.07972	0.214
70227	Zfp619	zinc finger protein 619	0.81234768	0.1126	0.2684
67488	Calcoco1	calcium binding and coiled coil domain 1	0.81234768	0.1175	0.2758
381107	Tmem232	transmembrane protein 232	0.81234768	0.1313	0.2976
67639	4930474N09Rik	RIKEN cDNA 4930474N09 gene	0.81234768	0.1462	0.3189
12478	Cd19	CD19 antigen	0.81234768	0.2576	0.4629
574418	Serinc4	serine incorporator 4	0.81168831	0.001549	0.01695
319974	Auts2	autism susceptibility candidate 2	0.81168831	0.002762	0.02362
12121	Bicd1	bicaudal D homolog 1 (Drosophila)	0.81168831	0.005182	0.03495
53414	Bysl	bystin-like	0.81168831	0.007686	0.0451
15114	Hap1	huntingtin-associated protein 1	0.81168831	0.02434	0.09552
54524	Syt6	synaptotagmin VI	0.81168831	0.0368	0.1259
12662	Chm	choroidermia	0.81168831	0.04202	0.1373
78703	C330013J21Rik	RIKEN cDNA C330013J21 gene	0.81168831	0.1415	0.3119
19894	Rph3a	rabphilin 3A	0.81168831	0.1665	0.3473
78506	Efha2	EF-hand domain family, member A2	0.81168831	0.316	0.5255
107831	Bai1	brain-specific angiogenesis inhibitor 1	0.81103001	0.0009112	0.01227
20658	Son	Son DNA binding protein	0.81103001	0.003928	0.02942
53963	D630030B22Rik	RIKEN cDNA D630030B22 gene	0.81103001	0.003991	0.02969
13437	Dnpep	aspartyl aminopeptidase	0.81103001	0.004604	0.03247
73202	3110067C02Rik	RIKEN cDNA 3110067C02 gene	0.81103001	0.01082	0.05587
20215	Sag	retinal S-antigen	0.81103001	0.01104	0.05663
218236	Fam120a	family with sequence similarity 120, member A	0.81103001	0.02009	0.08403
20648	Snta1	syntrophin, acidic 1	0.81103001	0.03206	0.1151
77889	Lbh	limb-bud and heart	0.81103001	0.04867	0.152
19017	Ppargc1a	peroxisome proliferative activated receptor, gamma, coactivator 1 alpha	0.81103001	0.05238	0.1602
216874	Camta2	calmodulin binding transcription activator 2	0.81103001	0.08388	0.2212
71702	Cdc5l	cell division cycle 5-like (S. pombe)	0.81103001	0.08587	0.2246
100038734	Gm10845	predicted gene 10845	0.81103001	0.08873	0.2294
268747	Lrrc16b	leucine rich repeat containing 16B	0.81103001	0.09307	0.237
72344	Usp36	ubiquitin specific peptidase 36	0.81103001	0.1007	0.2497
75693	3010001F23Rik	RIKEN cDNA 3010001F23 gene	0.81103001	0.1109	0.2655
77188	A430105D02Rik	RIKEN cDNA A430105D02 gene	0.81103001	0.1213	0.2826
207227	Stxbp5l	syntaxin binding protein 5-like	0.81103001	0.1533	0.3286
670496	Gm11564	predicted gene 11564	0.81103001	0.1928	0.3827
14065	F2rl3	coagulation factor II (thrombin) receptor-like 3	0.81103001	0.2152	0.4124
26405	Map3k2	mitogen-activated protein kinase kinase kinase 2	0.81103001	0.3467	0.5573

433882	Gm16223	predicted gene 16223	0.81103001	0.582	0.7537
27403	Abca7	ATP-binding cassette, sub-family A (ABC1), member 7	0.81037277	0.001994	0.01949
235300	Tmem136	transmembrane protein 136	0.81037277	0.002422	0.02168
619317	D930026N18Rik	RIKEN cDNA D930026N18 gene	0.81037277	0.002765	0.02362
71997	1500002O20Rik	RIKEN cDNA 1500002O20 gene	0.81037277	0.006442	0.04015
74320	Wdr33	WD repeat domain 33	0.81037277	0.007244	0.04353
233575	Pgap2	post-GPI attachment to proteins 2	0.81037277	0.01181	0.05962
12343	Capza2	capping protein (actin filament) muscle Z-line, alpha 2	0.81037277	0.01219	0.06063
241322	Zbtb6	zinc finger and BTB domain containing 6	0.81037277	0.01252	0.06169
235048	Zfp599	zinc finger protein 599	0.81037277	0.01303	0.06334
19263	Ptprb	protein tyrosine phosphatase, receptor type, B	0.81037277	0.015	0.0694
12402	Cbl	Casitas B-lineage lymphoma	0.81037277	0.02432	0.0955
12890	Cplx2	complexin 2	0.81037277	0.03517	0.1223
100041576	Gm3414	predicted gene 3414	0.81037277	0.03815	0.1289
66329	Susd3	sushi domain containing 3	0.81037277	0.03838	0.1294
20745	Spock1	sparc/osteonectin, cwcv and kazal-like domains proteoglycan 1	0.81037277	0.06084	0.1771
69449	1700027A15Rik	RIKEN cDNA 1700027A15 gene	0.81037277	0.08096	0.216
260305	Nphp4	nephronophthisis 4 (juvenile) homolog (human)	0.81037277	0.1244	0.2871
217558	G2e3	G2/M-phase specific E3 ubiquitin ligase	0.81037277	0.1577	0.335
268970	Arhgap28	Rho GTPase activating protein 28	0.81037277	0.2347	0.4363
224694	Zfp81	zinc finger protein 81	0.81037277	0.252	0.457
664858	BB094273	expressed sequence BB094273	0.81037277	0.4331	0.6368
545554	Ankrd34a	ankyrin repeat domain 34A	0.8097166	0.0005289	0.008883
227446	2310035C23Rik	RIKEN cDNA 2310035C23 gene	0.8097166	0.002663	0.02308
19711	Resp18	regulated endocrine-specific protein 18	0.8097166	0.005011	0.03419
75458	Cklf	chemokine-like factor	0.8097166	0.007289	0.04364
72477	Tmem87b	transmembrane protein 87B	0.8097166	0.01293	0.06301
70616	Sugp1	SURP and G patch domain containing 1	0.8097166	0.01948	0.08234
380850	Gm5141	predicted gene 5141	0.8097166	0.03822	0.129
18196	Nsg1	neuron specific gene family member 1	0.8097166	0.04468	0.1429
210035	Tmem194	transmembrane protein 194	0.8097166	0.04544	0.1447
22171	Tyms	thymidylate synthase	0.8097166	0.0827	0.2191
239667	Dip2b	DIP2 disco-interacting protein 2 homolog B (Drosophila)	0.8097166	0.109	0.2624
71514	Sfpq	splicing factor proline/glutamine rich (polypyrimidine tract binding protein associated)	0.8097166	0.1783	0.3634
109323	C1qtnf7	C1q and tumor necrosis factor related protein 7	0.8097166	0.3705	0.5794
14106	Foxh1	forkhead box H1	0.8097166	0.453	0.655
66779	4933432I09Rik	RIKEN cDNA 4933432I09 gene	0.8097166	0.4541	0.6556
76376	Slc24a2	solute carrier family 24 (sodium/potassium/calcium exchanger), member 2	0.80906149	0.006526	0.04048
18526	Pcdh10	protocadherin 10	0.80906149	0.01156	0.05873
107684	Coro2a	coronin, actin binding protein 2A	0.80906149	0.01597	0.07219
218850	D14Abb1e	DNA segment, Chr 14, Abbott 1 expressed	0.80906149	0.01812	0.07841
59032	Ppp2r3c	protein phosphatase 2, regulatory subunit B'', gamma	0.80906149	0.02307	0.09241
66598	3110001I22Rik	RIKEN cDNA 3110001I22 gene	0.80906149	0.03352	0.1184
72054	Cyp4f18	cytochrome P450, family 4, subfamily f, polypeptide 18	0.80906149	0.03951	0.1318
51960	Kctd18	potassium channel tetramerisation domain containing 18	0.80906149	0.05024	0.1556
16580	Kifc5b	kinesin family member C5B	0.80906149	0.07249	0.2003
218203	Mylip	myosin regulatory light chain interacting protein	0.80906149	0.1048	0.2559
80720	Pbx4	pre-B-cell leukemia homeobox 4	0.80906149	0.1141	0.2709
215280	Wipf1	WAS/WASL interacting protein family, member 1	0.80906149	0.1575	0.3347
71059	Hexim2	hexamthylene bis-acetamide inducible 2	0.80906149	0.186	0.3735

69926	Dnahc17	dynein, axonemal, heavy chain 17	0.80906149	0.1989	0.3904
70497	Arhgap17	Rho GTPase activating protein 17	0.80906149	0.2479	0.452
70405	Calml3	calmodulin-like 3	0.80906149	0.275	0.4829
258750	Olfr551	olfactory receptor 551	0.80906149	0.2811	0.4892
75424	Zfp820	zinc finger protein 820	0.80906149	0.3428	0.5538
107656	Krt9	keratin 9	0.80906149	0.4583	0.6594
330863	Trim67	tripartite motif-containing 67	0.80840744	0.001653	0.01746
14800	Gria2	glutamate receptor, ionotropic, AMPA2 (alpha 2)	0.80840744	0.0103	0.0543
53951	Ccdc75	coiled-coil domain containing 75	0.80840744	0.01183	0.05966
381411	Accsl	1-aminocyclopropane-1-carboxylate synthase homolog (Arabidopsis)(non-functional)-like	0.80840744	0.01466	0.06841
80297	Spnb4	spectrin beta 4	0.80840744	0.0268	0.1021
26372	Clcn6	chloride channel 6	0.80840744	0.03256	0.1165
18545	Pcp2	Purkinje cell protein 2 (L7)	0.80840744	0.03626	0.1247
11772	Ap2a2	adaptor protein complex AP-2, alpha 2 subunit	0.80840744	0.0372	0.1268
105727	Slc38a1	solute carrier family 38, member 1	0.80840744	0.04936	0.1536
72507	Dzip1l	DAZ interacting protein 1-like	0.80840744	0.0502	0.1555
30051	Spdef	SAM pointed domain containing ets transcription factor	0.80840744	0.06214	0.1799
75507	Pou5f2	POU domain class 5, transcription factor 2	0.80840744	0.07198	0.1991
228942	Cbln4	cerebellin 4 precursor protein	0.80840744	0.1439	0.3155
53621	Cnot4	CCR4-NOT transcription complex, subunit 4	0.80840744	0.1832	0.3703
664783	Dux	double homeobox	0.80840744	0.3577	0.5676
381974	Mrgprg	MAS-related GPR, member G	0.80840744	0.5696	0.745
14199	Fhl1	four and a half LIM domains 1	0.80775444	0.00475	0.03315
239510	Phf20l1	PHD finger protein 20-like 1	0.80775444	0.01236	0.06117
71474	Ppp6r2	protein phosphatase 6, regulatory subunit 2	0.80775444	0.01444	0.0678
78785	Clip4	CAP-GLY domain containing linker protein family, member 4	0.80775444	0.0396	0.1319
19069	Nup88	nucleoporin 88	0.80775444	0.04218	0.1377
238988	Erc2	ELKS/RAB6-interacting/CAST family member 2	0.80775444	0.06057	0.1766
52857	Gramd1a	GRAM domain containing 1A	0.80775444	0.06292	0.1814
71517	9030624J02Rik	RIKEN cDNA 9030624J02 gene	0.80775444	0.06416	0.1839
104348	Zfp120	zinc finger protein 120	0.80775444	0.1213	0.2826
17975	Ncl	nucleolin	0.80775444	0.1368	0.3044
56376	Pdlim5	PDZ and LIM domain 5	0.80775444	0.1542	0.3299
194401	Mical3	microtubule associated monoxygenase, calponin and LIM domain containing 3	0.80775444	0.1738	0.3574
11605	Gla	galactosidase, alpha	0.80775444	0.1821	0.3688
67118	Bfar	bifunctional apoptosis regulator	0.80775444	0.2546	0.4596
12952	Cry1	cryptochrome 1 (photolyase-like)	0.80775444	0.4656	0.6656
56784	Ralgapa1	Ral GTPase activating protein, alpha subunit 1	0.8071025	0.0013	0.01516
12750	Clk4	CDC like kinase 4	0.8071025	0.001436	0.01609
12876	Cpe	carboxypeptidase E	0.8071025	0.001899	0.01904
66797	Cntnap2	contactin associated protein-like 2	0.8071025	0.002482	0.02201
407818	BC055308	cDNA sequence BC055308	0.8071025	0.002815	0.02387
12816	Col12a1	collagen, type XII, alpha 1	0.8071025	0.01143	0.05824
100637	N4bp2l1	NEDD4 binding protein 2-like 1	0.8071025	0.01998	0.08375
74309	Osbp2	oxysterol binding protein 2	0.8071025	0.0232	0.09262
99334	Zscan29	zinc finger SCAN domains 29	0.8071025	0.04717	0.1485
654424	A330050B17Rik	RIKEN cDNA A330050B17 gene	0.8071025	0.07212	0.1994
544752	Tug1	taurine upregulated gene 1	0.8071025	0.07424	0.2036
66514	Asrgl1	asparaginase like 1	0.8071025	0.09703	0.2433
434674	Gm5631	predicted gene 5631	0.8071025	0.09917	0.247

239170	Fam160b2	family with sequence similarity 160, member B2	0.8071025	0.1336	0.3005
224997	Dlgap1	discs, large (Drosophila) homolog-associated protein 1	0.8071025	0.1531	0.3283
67037	Pmf1	polyamine-modulated factor 1	0.8071025	0.157	0.3341
67704	1810037I17Rik	RIKEN cDNA 1810037I17 gene	0.8071025	0.1653	0.3453
240216	E230025N22Rik	Riken cDNA E230025N22 gene	0.8071025	0.3637	0.5727
23969	Pacsin1	protein kinase C and casein kinase substrate in neurons 1	0.80645161	0.004546	0.03225
67590	Tctn3	tectonic family member 3	0.80645161	0.005142	0.03476
13870	Ercc1	excision repair cross-complementing rodent repair deficiency, complementation group 1	0.80645161	0.02472	0.0966
72899	Macrod2	MACRO domain containing 2	0.80645161	0.03378	0.1191
75266	Tomm20l	translocase of outer mitochondrial membrane 20 homolog (yeast)-like	0.80645161	0.0399	0.1326
100038441	Gm10419	predicted gene 10419	0.80645161	0.04028	0.1334
242509	Bnc2	basonuclin 2	0.80645161	0.04037	0.1336
383348	Kctd16	potassium channel tetramerisation domain containing 16	0.80645161	0.05244	0.1603
227707	BC005624	cDNA sequence BC005624	0.80645161	0.08247	0.2188
76654	Upp2	uridine phosphorylase 2	0.80645161	0.08797	0.2283
19055	Ppp3ca	protein phosphatase 3, catalytic subunit, alpha isoform	0.80645161	0.1064	0.2581
231464	Cnot6l	CCR4-NOT transcription complex, subunit 6-like	0.80645161	0.1331	0.3
14657	Glra4	glycine receptor, alpha 4 subunit	0.80645161	0.1344	0.3015
234549	Heatr3	HEAT repeat containing 3	0.80645161	0.1664	0.3471
71517	9030624J02Rik	RIKEN cDNA 9030624J02 gene	0.80645161	0.1671	0.3479
170571	Cntnap4	contactin associated protein-like 4	0.80645161	0.1947	0.3851
11789	Apc	adenomatosis polyposis coli	0.80645161	0.2508	0.4557
100042761	Gm10697	predicted gene 10697	0.80645161	0.2899	0.4983
208884	Zdhhc9	zinc finger, DHHC domain containing 9	0.80645161	0.3248	0.5346
232313	Gxylt2	glucoside xylosyltransferase 2	0.80645161	0.3385	0.5486
628100	Fbxo39	F-box protein 39	0.80645161	0.3974	0.6054
232077	Foxi3	forkhead box I3	0.80645161	0.5994	0.7675
99371	Arfgef2	ADP-ribosylation factor guanine nucleotide-exchange factor 2 (brefeldin A-inhibited)	0.80580177	0.001532	0.01682
100072	Camta1	calmodulin binding transcription activator 1	0.80580177	0.002669	0.02312
53626	Insm1	insulinoma-associated 1	0.80580177	0.01246	0.06146
11863	Arnt	aryl hydrocarbon receptor nuclear translocator	0.80580177	0.01383	0.06583
72508	Rps6kb1	ribosomal protein S6 kinase, polypeptide 1	0.80580177	0.01383	0.06583
216613	Ccdc85a	coiled-coil domain containing 85A	0.80580177	0.01508	0.06972
338351	Akap17b	A kinase (PRKA) anchor protein 17B	0.80580177	0.01586	0.07193
77286	Nkrf	NF-kappaB repressing factor	0.80580177	0.02882	0.1072
78752	Csgalnact2	chondroitin sulfate N-acetylgalactosaminyltransferase 2	0.80580177	0.03642	0.1251
12890	Cplx2	complexin 2	0.80580177	0.04164	0.1364
19263	Ptprb	protein tyrosine phosphatase, receptor type, B	0.80580177	0.06629	0.1879
104681	Slc16a6	solute carrier family 16 (monocarboxylic acid transporters), member 6	0.80580177	0.08647	0.2257
13560	E4f1	E4F transcription factor 1	0.80580177	0.1664	0.3471
67859	2310002J15Rik	RIKEN cDNA 2310002J15 gene	0.80580177	0.2453	0.4489
93890	Pcdhb19	protocadherin beta 19	0.80580177	0.2651	0.4715
68134	Upf3b	UPF3 regulator of nonsense transcripts homolog B (yeast)	0.80515298	0.0006538	0.01003
51902	Rnf24	ring finger protein 24	0.80515298	0.001637	0.01738
78885	Coro7	coronin 7	0.80515298	0.001933	0.01921
212281	A530054K11Rik	RIKEN cDNA A530054K11 gene	0.80515298	0.002265	0.02091
73473	Iws1	IWS1 homolog (S. cerevisiae)	0.80515298	0.008374	0.04745
78785	Clip4	CAP-GLY domain containing linker protein family, member 4	0.80515298	0.01178	0.05955
76890	Memo1	mediator of cell motility 1	0.80515298	0.01183	0.05966
232813	Shisa7	shisa homolog 7 (Xenopus laevis)	0.80515298	0.02112	0.08721

407812	Zfp941	zinc finger protein 941	0.80515298	0.0273	0.1033
66978	Luc7l	Luc7 homolog (S. cerevisiae)-like	0.80515298	0.02732	0.1034
12286	Cacna1a	calcium channel, voltage-dependent, P/Q type, alpha 1A subunit	0.80515298	0.03322	0.1178
76132	6230409E13Rik	RIKEN cDNA 6230409E13 gene	0.80515298	0.03686	0.126
320046	F730043M19Rik	RIKEN cDNA F730043M19 gene	0.80515298	0.04255	0.1384
20689	Sall3	sal-like 3 (Drosophila)	0.80515298	0.1042	0.255
71805	Nup93	nucleoporin 93	0.80515298	0.1107	0.2653
59031	Chst12	carbohydrate sulfotransferase 12	0.80515298	0.1645	0.3443
257632	Nod2	nucleotide-binding oligomerization domain containing 2	0.80515298	0.1742	0.3581
105653	Phyhip	phytanoyl-CoA hydroxylase interacting protein	0.80515298	0.193	0.383
74626	Tmem81	transmembrane protein 81	0.80515298	0.2012	0.3936
12311	Calcr	calcitonin receptor	0.80515298	0.2703	0.4778
244431	Sgcz	sarcoglycan zeta	0.80515298	0.2729	0.4804
14459	Gast	gastrin	0.80515298	0.2909	0.4993
211712	Pcdh9	protocadherin 9	0.80450523	0.01202	0.06015
78655	Eif3j	eukaryotic translation initiation factor 3, subunit J	0.80450523	0.0131	0.06362
14681	Gnao1	guanine nucleotide binding protein, alpha O	0.80450523	0.02356	0.09363
140919	Slc17a6	solute carrier family 17 (sodium-dependent inorganic phosphate cotransporter), member 6	0.80450523	0.03311	0.1176
28250	Slco1a4	solute carrier organic anion transporter family, member 1a4	0.80450523	0.05078	0.1567
230872	Crocc	ciliary rootlet coiled-coil, rootletin	0.80450523	0.05164	0.1586
26554	Cul3	cullin 3	0.80450523	0.13	0.2957
12212	Chic1	cysteine-rich hydrophobic domain 1	0.80450523	0.1918	0.3814
382793	Mtx3	metaxin 3	0.80450523	0.2365	0.4383
23821	Bace1	beta-site APP cleaving enzyme 1	0.80385852	0.001451	0.0162
77531	Anks1b	ankyrin repeat and sterile alpha motif domain containing 1B	0.80385852	0.002075	0.01983
27999	Fam3c	family with sequence similarity 3, member C	0.80385852	0.002582	0.02258
381511	Pdp1	pyruvate dehyrogenase phosphatase catalytic subunit 1	0.80385852	0.005746	0.03729
64930	Tsc1	tuberous sclerosis 1	0.80385852	0.008994	0.04962
319734	Cacna2d4	calcium channel, voltage-dependent, alpha 2/delta subunit 4	0.80385852	0.01457	0.06819
99458	BB166591	expressed sequence BB166591	0.80385852	0.05454	0.1649
73813	Fam83e	family with sequence similarity 83, member E	0.80385852	0.05522	0.1662
26428	Orc4	origin recognition complex, subunit 4	0.80385852	0.07397	0.2031
386454	Rnf39	ring finger protein 39	0.80385852	0.1472	0.3202
71200	Dydc2	DPY30 domain containing 2	0.80385852	0.2311	0.432
547176	Zc3h12b	zinc finger CCCH-type containing 12B	0.80385852	0.2851	0.4935
12487	Cd28	CD28 antigen	0.80385852	0.313	0.5228
16210	Impact	imprinted and ancient	0.80385852	0.4142	0.6206
19127	Prop1	paired like homeodomain factor 1	0.80385852	0.5068	0.6977
381310	6330403A02Rik	RIKEN cDNA 6330403A02 gene	0.80385852	0.6202	0.7819
20451	St8sia3	ST8 alpha-N-acetyl-neuraminide alpha-2,8-sialyltransferase 3	0.80321285	0.002424	0.02168
20602	Ncor2	nuclear receptor co-repressor 2	0.80321285	0.003319	0.0265
113868	Acaa1a	acetyl-Coenzyme A acyltransferase 1A	0.80321285	0.006027	0.0384
112415	C030039L03Rik	RIKEN cDNA C030039L03 gene	0.80321285	0.01352	0.06476
75710	Rbm12	RNA binding motif protein 12	0.80321285	0.03331	0.118
23856	Dido1	death inducer-obliterator 1	0.80321285	0.03964	0.1319
11909	Atf2	activating transcription factor 2	0.80321285	0.0563	0.1683
13831	Epc1	enhancer of polycomb homolog 1 (Drosophila)	0.80321285	0.06566	0.1867
13809	Enpep	glutamyl aminopeptidase	0.80321285	0.08756	0.2276
118449	Synpo2	synaptopodin 2	0.80321285	0.08794	0.2282
77128	A930001N09Rik	RIKEN cDNA A930001N09 gene	0.80321285	0.2457	0.4494

66921	Prpf38b	PRP38 pre-mRNA processing factor 38 (yeast) domain containing B	0.80321285	0.2507	0.4556
23856	Dido1	death inducer-obliterator 1	0.80321285	0.2648	0.4713
382985	Rrm2b	ribonucleotide reductase M2 B (TP53 inducible)	0.80321285	0.4984	0.6912
386400	LOC386400	carbonyl reductase [NADPH] 1-like	0.80321285	0.5791	0.7515
75410	Wbp7	WW domain binding protein 7	0.80256822	0.003295	0.02638
18803	Plcg1	phospholipase C, gamma 1	0.80256822	0.004072	0.03007
212531	Sh3bgrl2	SH3 domain binding glutamic acid-rich protein like 2	0.80256822	0.005244	0.03524
65257	Asb3	ankyrin repeat and SOCS box-containing 3	0.80256822	0.01204	0.06021
100041995	Gm3613	predicted gene 3613	0.80256822	0.01436	0.06758
20269	Scn3a	sodium channel, voltage-gated, type III, alpha	0.80256822	0.02047	0.08528
215303	Camk1g	calcium/calmodulin-dependent protein kinase I gamma	0.80256822	0.03211	0.1153
320011	Uggt1	UDP-glucose glycoprotein glucosyltransferase 1	0.80256822	0.04434	0.1422
20901	Strap	serine/threonine kinase receptor associated protein	0.80256822	0.04526	0.1443
78834	Zfp623	zinc finger protein 623	0.80256822	0.05163	0.1586
71361	Aifm2	apoptosis-inducing factor, mitochondrion-associated 2	0.80256822	0.05229	0.16
217069	Trim25	tripartite motif-containing 25	0.80256822	0.08251	0.2188
258532	Olfr373	olfactory receptor 373	0.80256822	0.1055	0.2569
107094	Rrp12	ribosomal RNA processing 12 homolog (S. cerevisiae)	0.80256822	0.1153	0.2725
100048452	LOC100048452	hypothetical protein LOC100048452	0.80256822	0.2047	0.3984
20725	Serpinb8	serine (or cysteine) peptidase inhibitor, clade B, member 8	0.80256822	0.366	0.5751
53612	Vti1b	vesicle transport through interaction with t-SNAREs 1B homolog	0.80192462	0.000736	0.01088
216190	Appl2	adaptor protein, phosphotyrosine interaction, PH domain and leucine zipper containing 2	0.80192462	0.0009975	0.01302
77652	Zfp955a	zinc finger protein 955A	0.80192462	0.001692	0.0177
380921	Dgkh	diacylglycerol kinase, eta	0.80192462	0.007513	0.04441
170483	Grin3b	glutamate receptor, ionotropic, NMDA3B	0.80192462	0.01382	0.06583
194268	9930104L06Rik	RIKEN cDNA 9930104L06 gene	0.80192462	0.02142	0.08808
11492	Adam19	a disintegrin and metallopeptidase domain 19 (meltrin beta)	0.80192462	0.03032	0.111
244349	Myst3	MYST histone acetyltransferase (monocytic leukemia) 3	0.80192462	0.0387	0.13
210162	Zkscan2	zinc finger with KRAB and SCAN domains 2	0.80192462	0.04293	0.1391
100042498	2210403K04Rik	RIKEN cDNA 2210403K04 gene	0.80192462	0.1022	0.2522
268747	Lrrc16b	leucine rich repeat containing 16B	0.80192462	0.1077	0.2602
99633	Lphn2	latrophilin 2	0.80192462	0.1345	0.3015
73032	Ttc9b	tetratricopeptide repeat domain 9B	0.80192462	0.196	0.3867
244713	Zfp317	zinc finger protein 317	0.80192462	0.2135	0.4096
15983	Ifrd2	interferon-related developmental regulator 2	0.80192462	0.2165	0.4139
72780	Rspo3	R-spondin 3 homolog (Xenopus laevis)	0.80192462	0.2377	0.4399
13983	Esr2	estrogen receptor 2 (beta)	0.80192462	0.2591	0.4646
76877	Rab36	RAB36, member RAS oncogene family	0.80192462	0.281	0.4892
239188	Enox1	ecto-NOX disulfide-thiol exchanger 1	0.80192462	0.6445	0.7992
216874	Camta2	calmodulin binding transcription activator 2	0.80128205	0.0005764	0.009315
72102	Dusp11	dual specificity phosphatase 11 (RNA/RNP complex 1-interacting)	0.80128205	0.0008476	0.01177
75957	Mir17hg	MIR17 host gene 1 (non-protein coding)	0.80128205	0.00177	0.01823
109880	Braf	Braf transforming gene	0.80128205	0.001811	0.01845
218203	Mylip	myosin regulatory light chain interacting protein	0.80128205	0.002046	0.0197
72662	Dis3	DIS3 mitotic control homolog (S. cerevisiae)	0.80128205	0.003279	0.02633
93873	Pcdhb2	protocadherin beta 2	0.80128205	0.01022	0.05407
14265	Fmr1	fragile X mental retardation syndrome 1 homolog	0.80128205	0.01558	0.07122
238331	Zdhhc22	zinc finger, DHHC-type containing 22	0.80128205	0.0158	0.07178
69002	1500026H17Rik	RIKEN cDNA 1500026H17 gene	0.80128205	0.02382	0.09434
211323	Nrg1	neuregulin 1	0.80128205	0.02503	0.09752

12298	Cacnb4	calcium channel, voltage-dependent, beta 4 subunit	0.80128205	0.02765	0.1042
319922	Vwc2	von Willebrand factor C domain containing 2	0.80128205	0.03564	0.1235
69487	2310003L22Rik	RIKEN cDNA 2310003L22 gene	0.80128205	0.04138	0.136
18187	Nrp2	neuropilin 2	0.80128205	0.04797	0.1503
229487	Pet112l	PET112-like (yeast)	0.80128205	0.08956	0.2306
67522	1700097N02Rik	RIKEN cDNA 1700097N02 gene	0.80128205	0.09849	0.246
74542	9030625N01Rik	RIKEN cDNA 9030625N01 gene	0.80128205	0.121	0.2821
80901	Cxcr6	chemokine (C-X-C motif) receptor 6	0.80128205	0.1377	0.3059
67586	Ubxn11	UBX domain protein 11	0.80128205	0.1436	0.3149
319387	Lphn3	latrophilin 3	0.80128205	0.1521	0.327
100041351	Gm3285	predicted gene 3285	0.80128205	0.1671	0.348
106459	BB163080	expressed sequence BB163080	0.80128205	0.1735	0.357
320798	4831440D22Rik	RIKEN cDNA 4831440D22 gene	0.80128205	0.2013	0.3937
12014	Bach2	BTB and CNC homology 2	0.80128205	0.2155	0.4127
383592	Gm1305	predicted gene 1305	0.80128205	0.2615	0.4675
77018	Col25a1	collagen, type XXV, alpha 1	0.80128205	0.5397	0.7231
15109	Hal	histidine ammonia lyase	0.80128205	0.57	0.7453
67102	D16Ertd472e	DNA segment, Chr 16, ERATO Doi 472, expressed	0.80064051	0.001396	0.01581
319885	Zcchc7	zinc finger, CCHC domain containing 7	0.80064051	0.005802	0.03752
268706	Slc38a9	solute carrier family 38, member 9	0.80064051	0.00923	0.05052
12850	Coq7	demethyl-Q 7	0.80064051	0.01236	0.06117
103765	Tmem17	transmembrane protein 17	0.80064051	0.01434	0.06754
108797	Mex3b	mex3 homolog B (C. elegans)	0.80064051	0.0262	0.1006
20778	Scarb1	scavenger receptor class B, member 1	0.80064051	0.02979	0.1096
381217	Fam189a2	family with sequence similarity 189, member A2	0.80064051	0.04235	0.138
18795	Plcb1	phospholipase C, beta 1	0.80064051	0.08453	0.2223
70661	Sik3	SIK family kinase 3	0.80064051	0.09463	0.2395
399607	D230019N24Rik	RIKEN cDNA D230019N24 gene	0.80064051	0.1628	0.3417
20404	Sh3gl2	SH3-domain GRB2-like 2	0.80064051	0.179	0.3644
58234	Shank3	SH3/ankyrin domain gene 3	0.80064051	0.2153	0.4124
225192	Hrh4	histamine receptor H4	0.80064051	0.236	0.4379
76469	Cmya5	cardiomyopathy associated 5	0.80064051	0.2894	0.4977
333050	Ksr2	kinase suppressor of ras 2	0.80064051	0.3399	0.5502
54672	Gpr97	G protein-coupled receptor 97	0.80064051	0.3986	0.6064
71233	Enkur	enkurin, TRPC channel interacting protein	0.8	0.00384	0.02905
216856	Nlgn2	neuroligin 2	0.8	0.003918	0.02938
239570	Ttc38	tetratricopeptide repeat domain 38	0.8	0.007385	0.04394
226844	Mfsd7b	major facilitator superfamily domain containing 7B	0.8	0.008311	0.04725
100502841	5430411K18Rik	RIKEN cDNA 5430411K18 gene	0.8	0.009335	0.05084
229363	Gmps	guanine monophosphate synthetase	0.8	0.01202	0.06015
50786	Hs6st2	heparan sulfate 6-O-sulfotransferase 2	0.8	0.03233	0.1159
212398	Frat2	frequently rearranged in advanced T-cell lymphomas 2	0.8	0.05738	0.1704
100041585	Amd2	S-adenosylmethionine decarboxylase 2	0.8	0.07506	0.205
17720	ND4L	NADH dehydrogenase subunit 4L	0.8	0.132	0.2984
68564	Nufip2	nuclear fragile X mental retardation protein interacting protein 2	0.8	0.1345	0.3015
320534	Tmem104	transmembrane protein 104	0.8	0.285	0.4935
24067	Srp54a	signal recognition particle 54A	0.8	0.3002	0.5095
320661	D5Ertd579e	DNA segment, Chr 5, ERATO Doi 579, expressed	0.8	0.4064	0.6136
100503357	LOC100503357	hypothetical LOC100503357	0.8	0.4067	0.6139
18729	Pira6	paired-Ig-like receptor A6	0.8	0.4359	0.6395

76051	Ganc	glucosidase, alpha; neutral C	0.79936051	0.002438	0.02177
328113	A230055C15	hypothetical protein A230055C15	0.79936051	0.00448	0.03198
245049	Myrip	myosin VIIA and Rab interacting protein	0.79936051	0.009887	0.05291
240690	St18	suppression of tumorigenicity 18	0.79936051	0.0136	0.06508
71492	Bbs7	Bardet-Biedl syndrome 7 (human)	0.79936051	0.01467	0.06841
108124	Napa	N-ethylmaleimide sensitive fusion protein attachment protein alpha	0.79936051	0.01597	0.07219
70127	Dpf3	D4, zinc and double PHD fingers, family 3	0.79936051	0.01806	0.07825
16000	Igf1	insulin-like growth factor 1	0.79936051	0.02216	0.09015
16506	Kcnd1	potassium voltage-gated channel, Shal-related family, member 1	0.79936051	0.02412	0.09508
99003	Qser1	glutamine and serine rich 1	0.79936051	0.02578	0.0995
268448	Phf12	PHD finger protein 12	0.79936051	0.0646	0.1847
19877	Rock1	Rho-associated coiled-coil containing protein kinase 1	0.79936051	0.09197	0.2352
11752	Anxa8	annexin A8	0.79936051	0.13	0.2957
223922	Atf7	activating transcription factor 7	0.79936051	0.1489	0.3227
14048	Eya1	eyes absent 1 homolog (Drosophila)	0.79936051	0.1663	0.3471
217593	Slc25a21	solute carrier family 25 (mitochondrial oxodicarboxylate carrier), member 21	0.79936051	0.2836	0.4922
402769	LOC402769	hypothetical LOC402769	0.79936051	0.3474	0.5578
18475	Pafah1b2	platelet-activating factor acetylhydrolase, isoform 1b, subunit 2	0.79872204	0.004014	0.02981
223642	Zc3h3	zinc finger CCCH type containing 3	0.79872204	0.02073	0.08604
18166	Npy1r	neuropeptide Y receptor Y1	0.79872204	0.0231	0.09245
442834	D830031N03Rik	RIKEN cDNA D830031N03 gene	0.79872204	0.0371	0.1266
22688	Zfp26	zinc finger protein 26	0.79872204	0.1222	0.2841
67525	Trim62	tripartite motif-containing 62	0.79872204	0.1409	0.3108
100504289	LOC100504289	hypothetical LOC100504289	0.79872204	0.2627	0.4687
622473	Ripply1	ripply1 homolog (zebrafish)	0.79872204	0.3854	0.5945
94229	Slc4a10	solute carrier family 4, sodium bicarbonate cotransporter-like, member 10	0.79872204	0.3858	0.5946
432725	Gm5445	predicted gene 5445	0.7980846	0.001264	0.01497
11777	Ap3s1	adaptor-related protein complex 3, sigma 1 subunit	0.7980846	0.001369	0.01564
12617	Cenpc1	centromere protein C1	0.7980846	0.001978	0.0194
12807	Hps3	Hermansky-Pudlak syndrome 3 homolog (human)	0.7980846	0.002504	0.02214
18578	Pde4b	phosphodiesterase 4B, cAMP specific	0.7980846	0.005415	0.03596
26918	Ern2	endoplasmic reticulum (ER) to nucleus signalling 2	0.7980846	0.006005	0.03832
268903	Nrip1	nuclear receptor interacting protein 1	0.7980846	0.009862	0.0529
74337	Palm3	paralemmin 3	0.7980846	0.0122	0.06066
100662	D930016D06Rik	RIKEN cDNA D930016D06 gene	0.7980846	0.01429	0.06739
76367	Trp53rk	transformation related protein 53 regulating kinase	0.7980846	0.02861	0.1067
208285	Cyp4f17	cytochrome P450, family 4, subfamily f, polypeptide 17	0.7980846	0.03749	0.1275
71132	Cabyr	calcium-binding tyrosine-(Y)-phosphorylation regulated (fibrousheathin 2)	0.7980846	0.04731	0.1489
228859	Fitm2	fat storage-inducing transmembrane protein 2	0.7980846	0.04912	0.1531
210004	B3gntl1	UDP-GlcNAc:betaGal beta-1,3-N-acetylglucosaminyltransferase-like 1	0.7980846	0.05476	0.1653
67958	2610101N10Rik	RIKEN cDNA 2610101N10 gene	0.7980846	0.07376	0.2027
627648	Klhl3	kelch-like 3 (Drosophila)	0.7980846	0.07962	0.2138
66816	Thap2	THAP domain containing, apoptosis associated protein 2	0.7980846	0.08782	0.2281
12994	Csn3	casein kappa	0.7980846	0.0949	0.24
100210	Gpn2	GPN-loop GTPase 2	0.7980846	0.102	0.252
17537	Meis3	Meis homeobox 3	0.7980846	0.1299	0.2957
78177	Ninl	ninein-like	0.7980846	0.2667	0.4735
70449	2610209C05Rik	RIKEN cDNA 2610209C05 gene	0.7980846	0.4068	0.6139
57753	Noc3l	nucleolar complex associated 3 homolog (S. cerevisiae)	0.79744817	0.007256	0.04355
64074	Smoc2	SPARC related modular calcium binding 2	0.79744817	0.02776	0.1044

224585	Zfp160	zinc finger protein 160	0.79744817	0.03535	0.1228
218343	Ttc37	tetratricopeptide repeat domain 37	0.79744817	0.05161	0.1586
66885	Acadsb	acyl-Coenzyme A dehydrogenase, short/branched chain	0.79744817	0.05375	0.1632
14685	Gnat1	guanine nucleotide binding protein, alpha transducing 1	0.79744817	0.07372	0.2027
210710	Gab3	growth factor receptor bound protein 2-associated protein 3	0.79744817	0.1884	0.3765
78934	4930581F22Rik	RIKEN cDNA 4930581F22 gene	0.79744817	0.4132	0.6195
170740	Zfp287	zinc finger protein 287	0.79681275	0.003449	0.02713
237560	Lrrc10	leucine rich repeat containing 10	0.79681275	0.00381	0.02893
16068	Il18bp	interleukin 18 binding protein	0.79681275	0.009592	0.05186
234353	Psd3	pleckstrin and Sec7 domain containing 3	0.79681275	0.01085	0.05603
268882	Fbxo45	F-box protein 45	0.79681275	0.0135	0.06474
50907	Preb	prolactin regulatory element binding	0.79681275	0.01716	0.07551
66411	Tbcb	tubulin folding cofactor B	0.79681275	0.01811	0.07838
67920	Mak16	MAK16 homolog (S. cerevisiae)	0.79681275	0.0238	0.0943
106557	Ldhal6b	lactate dehydrogenase A-like 6B	0.79681275	0.03055	0.1115
238247	Arid4a	AT rich interactive domain 4A (RBP1-like)	0.79681275	0.03574	0.1236
268890	Lsamp	limbic system-associated membrane protein	0.79681275	0.03642	0.1251
381983	Lmtk3	lemur tyrosine kinase 3	0.79681275	0.04227	0.1379
59012	Moxd1	monooxygenase, DBH-like 1	0.79681275	0.0692	0.1938
231452	Sdad1	SDA1 domain containing 1	0.79681275	0.4009	0.6085
11370	Acadvl	acyl-Coenzyme A dehydrogenase, very long chain	0.79617834	0.0003918	0.007292
20148	Dhrs3	dehydrogenase/reductase (SDR family) member 3	0.79617834	0.001645	0.0174
329795	Tmem67	transmembrane protein 67	0.79617834	0.002754	0.02359
240880	Scyl3	SCY1-like 3 (S. cerevisiae)	0.79617834	0.004698	0.03293
77068	5930436O19Rik	RIKEN cDNA 5930436O19 gene	0.79617834	0.004974	0.03401
209131	Snx30	sorting nexin family member 30	0.79617834	0.007574	0.04463
13175	Dclk1	doublecortin-like kinase 1	0.79617834	0.01562	0.07134
77189	A430105J06Rik	RIKEN cDNA A430105J06 gene	0.79617834	0.01677	0.07427
74522	Morc2a	microrchidia 2A	0.79617834	0.01753	0.07657
224814	Abcc10	ATP-binding cassette, sub-family C (CFTR/MRP), member 10	0.79617834	0.02067	0.08591
21886	Tle2	transducin-like enhancer of split 2, homolog of Drosophila E(spl)	0.79617834	0.02707	0.1028
70557	5730416O20Rik	RIKEN cDNA 5730416O20 gene	0.79617834	0.04785	0.1501
16536	Kcnq2	potassium voltage-gated channel, subfamily Q, member 2	0.79617834	0.07169	0.1985
66799	Ube2w	ubiquitin-conjugating enzyme E2W (putative)	0.79617834	0.07449	0.2041
66385	Ppp1r7	protein phosphatase 1, regulatory (inhibitor) subunit 7	0.79617834	0.08052	0.2153
434624	LOC434624	ferritin light chain 1-like	0.79617834	0.1611	0.3393
331401	Thoc2	THO complex 2	0.79617834	0.1998	0.3916
30959	Ddx25	DEAD (Asp-Glu-Ala-Asp) box polypeptide 25	0.79617834	0.2807	0.489
74727	4930517G19Rik	RIKEN cDNA 4930517G19 gene	0.79617834	0.3796	0.5887
70802	Pwwp2a	PWWP domain containing 2A	0.79554495	0.001248	0.01485
215436	Slc35e3	solute carrier family 35, member E3	0.79554495	0.001757	0.01819
231238	Sel1l3	sel-1 suppressor of lin-12-like 3 (C. elegans)	0.79554495	0.001806	0.01843
327655	Ppip5k1	diphosphoinositol pentakisphosphate kinase 1	0.79554495	0.004789	0.0333
319739	B230303O12Rik	RIKEN cDNA B230303O12 gene	0.79554495	0.01065	0.05538
14256	Flt3l	FMS-like tyrosine kinase 3 ligand	0.79554495	0.01323	0.06406
12716	Ckmt1	creatine kinase, mitochondrial 1, ubiquitous	0.79554495	0.01535	0.07056
70884	Ccdc81	coiled-coil domain containing 81	0.79554495	0.03101	0.1127
17184	Matr3	matrin 3	0.79554495	0.03483	0.1214
20353	Sema4c	sema domain, immunoglobulin domain (Ig), transmembrane domain (TM) and short cytoplasmic domain, (semaphorin) 4C	0.79554495	0.03795	0.1285
213121	Ankrd35	ankyrin repeat domain 35	0.79554495	0.06284	0.1813

114741	Supt16h	suppressor of Ty 16 homolog (S. cerevisiae)	0.79554495	0.08415	0.2217
232087	Mat2a	methionine adenosyltransferase II, alpha	0.79554495	0.09013	0.2318
11735	Ank3	ankyrin 3, epithelial	0.79554495	0.1179	0.2767
14681	Gnao1	guanine nucleotide binding protein, alpha O	0.79554495	0.1765	0.3612
235497	Leo1	Leo1, Paf1/RNA polymerase II complex component, homolog (S. cerevisiae)	0.79554495	0.2689	0.476
66226	Trappc2	trafficking protein particle complex 2	0.79554495	0.5966	0.7653
102731	4833445I07Rik	RIKEN cDNA 4833445I07 gene	0.79491256	0.003397	0.02692
101565	6330503K22Rik	RIKEN cDNA 6330503K22 gene	0.79491256	0.004493	0.03202
66625	Sfrs18	serine/arginine-rich splicing factor 18	0.79491256	0.01389	0.06603
74020	Cpne4	copine IV	0.79491256	0.01606	0.07239
71909	Haus5	HAUS augmin-like complex, subunit 5	0.79491256	0.02771	0.1044
241656	Pak7	p21 protein (Cdc42/Rac)-activated kinase 7	0.79491256	0.0314	0.1135
26562	Ncdn	neurochondrin	0.79491256	0.03558	0.1233
241589	D430041D05Rik	RIKEN cDNA D430041D05 gene	0.79491256	0.03648	0.1252
14004	Chchd2	coiled-coil-helix-coiled-coil-helix domain containing 2	0.79491256	0.05717	0.17
17754	Mtap1a	microtubule-associated protein 1 A	0.79491256	0.06343	0.1824
232339	Ankrd26	ankyrin repeat domain 26	0.79491256	0.06972	0.1947
22221	Ubp1	upstream binding protein 1	0.79491256	0.07861	0.212
26878	B3galt2	UDP-Gal:betaGlcNAc beta 1,3-galactosyltransferase, polypeptide 2	0.79491256	0.08791	0.2282
100504720	LOC100504720	hypothetical LOC100504720	0.79491256	0.1364	0.3038
242202	Pde5a	phosphodiesterase 5A, cGMP-specific	0.79491256	0.2294	0.4299
75299	4930547M16Rik	RIKEN cDNA 4930547M16 gene	0.79491256	0.5171	0.7066
192119	Dicer1	Dicer1, Dcr-1 homolog (Drosophila)	0.79428118	0.00208	0.01984
231712	Trafd1	TRAF type zinc finger domain containing 1	0.79428118	0.007292	0.04364
216345	Zfc3h1	zinc finger, C3H1-type containing	0.79428118	0.01655	0.07368
67911	Zfp169	zinc finger protein 169	0.79428118	0.01857	0.07976
16917	Lmx1b	LIM homeobox transcription factor 1 beta	0.79428118	0.02313	0.09249
12950	Hapln1	hyaluronan and proteoglycan link protein 1	0.79428118	0.02339	0.0931
60599	Trp53inp1	transformation related protein 53 inducible nuclear protein 1	0.79428118	0.03212	0.1153
73724	Mcee	methylmalonyl CoA epimerase	0.79428118	0.05297	0.1615
234889	Gucy1a2	guanylate cyclase 1, soluble, alpha 2	0.79428118	0.09712	0.2434
67704	1810037I17Rik	RIKEN cDNA 1810037I17 gene	0.79428118	0.1578	0.335
16663	Krt13	keratin 13	0.79428118	0.1756	0.3601
214384	Myocd	myocardin	0.79428118	0.2484	0.4526
73225	Fam118a	family with sequence similarity 118, member A	0.79428118	0.2966	0.5056
18128	Notch1	Notch gene homolog 1 (Drosophila)	0.79428118	0.363	0.5721
75173	4930544O15Rik	RIKEN cDNA 4930544O15 gene	0.79428118	0.3694	0.5784
93736	Aff4	AF4/FMR2 family, member 4	0.79428118	0.5554	0.7352
99138	Stard7	START domain containing 7	0.79365079	0.0003976	0.007349
77938	Fam53b	family with sequence similarity 53, member B	0.79365079	0.002113	0.02006
66848	Fuca2	fucosidase, alpha-L- 2, plasma	0.79365079	0.005359	0.03573
433809	Rnf207	ring finger protein 207	0.79365079	0.01938	0.08202
12615	Cenpa	centromere protein A	0.79365079	0.0233	0.09289
20742	Spnb2	spectrin beta 2	0.79365079	0.02507	0.09759
68014	Zwilch	Zwilch, kinetochore associated, homolog (Drosophila)	0.79365079	0.02681	0.1021
100041677	Gm13157	predicted gene 13157	0.79365079	0.1113	0.2661
74552	Nipal3	NIPA-like domain containing 3	0.79365079	0.1128	0.2687
279499	Kctd19	potassium channel tetramerisation domain containing 19	0.79365079	0.2569	0.462
58992	F12	coagulation factor XII (Hageman factor)	0.79365079	0.2955	0.5044
16424	Itih1	inter-alpha trypsin inhibitor, heavy chain 1	0.79365079	0.6111	0.7754

12369	Casp7	caspase 7	0.79302141	0.0009527	0.01262
226541	Klhl20	kelch-like 20 (Drosophila)	0.79302141	0.003022	0.02493
69232	Qrich1	glutamine-rich 1	0.79302141	0.004584	0.0324
235106	Ntm	neurotrimin	0.79302141	0.006382	0.03993
71302	Arhgap26	Rho GTPase activating protein 26	0.79302141	0.01779	0.07747
74597	4833418N02Rik	RIKEN cDNA 4833418N02 gene	0.79302141	0.03412	0.1197
381356	5930434B04Rik	RIKEN cDNA 5930434B04 gene	0.79302141	0.06483	0.1853
110651	Rps6ka3	ribosomal protein S6 kinase polypeptide 3	0.79302141	0.07774	0.2102
224908	Prr22	proline rich 22	0.79302141	0.07969	0.2139
71752	Gtf3c2	general transcription factor IIIC, polypeptide 2, beta	0.79302141	0.1036	0.2542
101809	Spred3	sprouty-related, EVH1 domain containing 3	0.79302141	0.1493	0.3233
236511	Eif2c1	eukaryotic translation initiation factor 2C, 1	0.79302141	0.1666	0.3473
170936	Zfp369	zinc finger protein 369	0.79302141	0.6085	0.7734
228714	Csrp2bp	cysteine and glycine-rich protein 2 binding protein	0.79239303	0.000434	0.007809
13518	Dst	dystonin	0.79239303	0.0008299	0.01162
240354	Malt1	mucosa associated lymphoid tissue lymphoma translocation gene 1	0.79239303	0.002913	0.02433
239845	Gpr156	G protein-coupled receptor 156	0.79239303	0.007813	0.04554
67065	Polr3d	polymerase (RNA) III (DNA directed) polypeptide D	0.79239303	0.02003	0.08385
548102	LOC548102	hypothetical LOC548102	0.79239303	0.02845	0.1063
66673	Sorcs3	sortilin-related VPS10 domain containing receptor 3	0.79239303	0.05605	0.168
105442	B130052P14Rik	RIKEN cDNA B130052P14 gene	0.79239303	0.06263	0.1809
24050	Sep-03	septin 3	0.79239303	0.07115	0.1974
217558	G2e3	G2/M-phase specific E3 ubiquitin ligase	0.79239303	0.1531	0.3283
14121	Fbp1	fructose bisphosphatase 1	0.79239303	0.1533	0.3287
75352	4930550L24Rik	RIKEN cDNA 4930550L24 gene	0.79239303	0.2117	0.4075
668357	Dmrtc1c2	DMRT-like family C1c2	0.79239303	0.2763	0.4844
245440	Gm4988	predicted gene 4988	0.79176564	0.0004885	0.00842
330908	Opcml	opioid binding protein/cell adhesion molecule-like	0.79176564	0.003812	0.02893
66119	Tomm6	translocase of outer mitochondrial membrane 6 homolog (yeast)	0.79176564	0.004933	0.03385
66375	Dhrs7	dehydrogenase/reductase (SDR family) member 7	0.79176564	0.00545	0.03605
19253	Ptpn18	protein tyrosine phosphatase, non-receptor type 18	0.79176564	0.005577	0.03661
66634	Mcm8	minichromosome maintenance deficient 8 (S. cerevisiae)	0.79176564	0.01838	0.07921
18628	Per3	period homolog 3 (Drosophila)	0.79176564	0.02624	0.1007
70335	Reep6	receptor accessory protein 6	0.79176564	0.04027	0.1334
100042165	BC005561	cDNA sequence BC005561	0.79176564	0.04097	0.1349
56220	Zfp386	zinc finger protein 386 (Kruppel-like)	0.79176564	0.044	0.1414
232566	Amn1	antagonist of mitotic exit network 1 homolog (S. cerevisiae)	0.79176564	0.05297	0.1615
22717	Zfp59	zinc finger protein 59	0.79176564	0.05465	0.1651
70335	Reep6	receptor accessory protein 6	0.79176564	0.07357	0.2024
18227	Nr4a2	nuclear receptor subfamily 4, group A, member 2	0.79176564	0.09235	0.2358
24000	Ptpn21	protein tyrosine phosphatase, non-receptor type 21	0.79176564	0.104	0.2547
17168	Nprl3	nitrogen permease regulator-like 3 (S. cerevisiae)	0.79176564	0.2528	0.4578
67344	Tctex1d1	Tctex1 domain containing 1	0.79176564	0.2913	0.4997
434168	Gm5590	predicted gene 5590	0.79176564	0.426	0.6311
22249	Unc13b	unc-13 homolog B (C. elegans)	0.79113924	0.0007681	0.01106
18223	Numbl	numb-like	0.79113924	0.005048	0.03436
105710	BB086117	expressed sequence BB086117	0.79113924	0.005706	0.03713
74160	1300014J16Rik	RIKEN cDNA 1300014J16 gene	0.79113924	0.03869	0.13
552890	LOC552890	hypothetical LOC552890	0.79113924	0.0406	0.1341
75578	Fggy	FGGY carbohydrate kinase domain containing	0.79113924	0.06097	0.1774

22174	Tyro3	TYRO3 protein tyrosine kinase 3	0.79113924	0.06523	0.1859
333182	Cox6b2	cytochrome c oxidase subunit VIb polypeptide 2	0.79113924	0.1151	0.2723
665186	Gm7534	predicted gene 7534	0.79113924	0.1264	0.2901
11717	Ampd3	adenosine monophosphate deaminase 3	0.79113924	0.1409	0.3108
12986	Csf3r	colony stimulating factor 3 receptor (granulocyte)	0.79113924	0.1431	0.3142
74549	Mau2	MAU2 chromatid cohesion factor homolog (C. elegans)	0.79113924	0.1464	0.319
22592	Ercc5	excision repair cross-complementing rodent repair deficiency, complementation group 5	0.79113924	0.1531	0.3283
14182	Fgfr1	fibroblast growth factor receptor 1	0.79113924	0.229	0.4293
69504	Zfp932	zinc finger protein 932	0.79051383	0.001198	0.01447
100040724	Mirg	miRNA containing gene	0.79051383	0.005187	0.03495
56055	Gtpbp2	GTP binding protein 2	0.79051383	0.02422	0.09527
16000	Igf1	insulin-like growth factor 1	0.79051383	0.03965	0.132
75276	Ppp1r1c	protein phosphatase 1, regulatory (inhibitor) subunit 1C	0.79051383	0.1075	0.2598
72633	2810019C22Rik	RIKEN cDNA 2810019C22 gene	0.79051383	0.1242	0.2869
71389	Chd6	chromodomain helicase DNA binding protein 6	0.79051383	0.1886	0.3768
269774	Aak1	AP2 associated kinase 1	0.79051383	0.2143	0.4109
26431	Git2	G protein-coupled receptor kinase-interactor 2	0.79051383	0.2202	0.4194
21944	Tnfsf12	tumor necrosis factor (ligand) superfamily, member 12	0.79051383	0.2286	0.4288
624860	Gm12253	predicted gene 12253	0.79051383	0.2564	0.4616
74722	4930517J16Rik	RIKEN cDNA 4930517J16 gene	0.78988942	0.002987	0.02475
99029	AI596198	expressed sequence AI596198	0.78988942	0.01329	0.06418
52028	Bbs1	Bardet-Biedl syndrome 1 (human)	0.78988942	0.0156	0.07128
73094	Sgip1	SH3-domain GRB2-like (endophilin) interacting protein 1	0.78988942	0.0216	0.08862
228876	Zfp334	zinc finger protein 334	0.78988942	0.04453	0.1427
667933	Gm8884	predicted gene 8884	0.78988942	0.06926	0.1938
242425	Gabbr2	gamma-aminobutyric acid (GABA) B receptor, 2	0.78988942	0.09395	0.2383
11480	Acvr2a	activin receptor IIA	0.78988942	0.1113	0.2661
14265	Fmr1	fragile X mental retardation syndrome 1 homolog	0.78988942	0.1375	0.3056
380654	4930485B16Rik	RIKEN cDNA 4930485B16 gene	0.78988942	0.524	0.7109
170938	Zfp617	zinc finger protein 617	0.78926598	0.0005608	0.009177
29820	Tnfrsf19	tumor necrosis factor receptor superfamily, member 19	0.78926598	0.01637	0.07331
16409	Itgam	integrin alpha M	0.78926598	0.03339	0.1182
72097	2010300C02Rik	RIKEN cDNA 2010300C02 gene	0.78926598	0.03717	0.1268
552873	LOC552873	hypothetical LOC552873	0.78926598	0.05134	0.1579
71169	Nbas	neuroblastoma amplified sequence	0.78926598	0.1115	0.2664
16443	Itsn1	intersectin 1 (SH3 domain protein 1A)	0.78926598	0.1531	0.3283
320253	Mar-03	membrane-associated ring finger (C3HC4) 3	0.78926598	0.3231	0.5329
237754	Btnl9	butyrophilin-like 9	0.78926598	0.5456	0.7276
56291	Styx	serine/threonine/tyrosine interaction protein	0.78864353	0.0009629	0.01267
319924	Apba1	amyloid beta (A4) precursor protein binding, family A, member 1	0.78864353	0.002136	0.02017
234413	Zfp961	zinc finger protein 961	0.78864353	0.002679	0.02317
67302	Zc3h13	zinc finger CCCH type containing 13	0.78864353	0.007342	0.04379
101100	Ttll3	tubulin tyrosine ligase-like family, member 3	0.78864353	0.01165	0.05906
231279	Guf1	GUF1 GTPase homolog (S. cerevisiae)	0.78864353	0.03022	0.1109
228880	Zmynd8	zinc finger, MYND-type containing 8	0.78864353	0.06446	0.1844
18667	Pgr	progesterone receptor	0.78864353	0.06773	0.1906
241656	Pak7	p21 protein (Cdc42/Rac)-activated kinase 7	0.78864353	0.07009	0.1953
18583	Pde7a	phosphodiesterase 7A	0.78864353	0.0726	0.2004
67848	Ddx55	DEAD (Asp-Glu-Ala-Asp) box polypeptide 55	0.78864353	0.1285	0.2937
53620	Vamp5	vesicle-associated membrane protein 5	0.78864353	0.1882	0.3764

26382	Fgd2	FYVE, RhoGEF and PH domain containing 2	0.78864353	0.3876	0.5961
277973	Slc9a5	solute carrier family 9 (sodium/hydrogen exchanger), member 5	0.78802206	0.007455	0.04417
13605	Ect2	ect2 oncogene	0.78802206	0.01052	0.05502
329178	Unc80	unc-80 homolog (C. elegans)	0.78802206	0.03407	0.1196
19933	Rpl21	ribosomal protein L21	0.78802206	0.03855	0.1296
20479	Vps4b	vacuolar protein sorting 4b (yeast)	0.78802206	0.1099	0.2639
20338	Sel1l	sel-1 suppressor of lin-12-like (C. elegans)	0.78802206	0.114	0.2706
71536	8430437O03Rik	RIKEN cDNA 8430437O03 gene	0.78740157	0.001936	0.01922
107522	Ece2	endothelin converting enzyme 2	0.78740157	0.00531	0.03551
100041979	Gm3604	predicted gene 3604	0.78740157	0.01014	0.05374
26879	B3galnt1	UDP-GalNAc:betaGlcNAc beta 1,3-galactosaminyltransferase, polypeptide 1	0.78740157	0.02033	0.08487
319430	Gpr77	G protein-coupled receptor 77	0.78740157	0.02368	0.09393
13612	Edil3	EGF-like repeats and discoidin I-like domains 3	0.78740157	0.02561	0.09912
320929	4732460I02Rik	RIKEN cDNA 4732460I02 gene	0.78740157	0.05608	0.168
319636	Fsd1l	fibronectin type III and SPRY domain containing 1-like	0.78740157	0.1146	0.2716
23856	Dido1	death inducer-obliterator 1	0.78740157	0.1304	0.2962
12163	Bmp8a	bone morphogenetic protein 8a	0.78740157	0.2421	0.445
210789	Tbc1d4	TBC1 domain family, member 4	0.78678206	0.004877	0.03361
170707	Usp48	ubiquitin specific peptidase 48	0.78678206	0.01025	0.05415
230163	Aldob	aldolase B, fructose-bisphosphate	0.78678206	0.0163	0.07314
114332	Lyve1	lymphatic vessel endothelial hyaluronan receptor 1	0.78678206	0.02001	0.0838
14811	Grin2a	glutamate receptor, ionotropic, NMDA2A (epsilon 1)	0.78678206	0.06547	0.1864
110695	Aldh7a1	aldehyde dehydrogenase family 7, member A1	0.78678206	0.08914	0.2302
100038585	F830223B06Rik	RIKEN cDNA F830223B06 gene	0.78678206	0.0903	0.2321
24132	Zfp53	zinc finger protein 53	0.78678206	0.09411	0.2386
67855	Asprv1	aspartic peptidase, retroviral-like 1	0.78678206	0.2008	0.3931
18846	Plxna3	plexin A3	0.78616352	0.003602	0.02787
18739	Pitpnm1	phosphatidylinositol transfer protein, membrane-associated 1	0.78616352	0.005224	0.03515
101122	Rpusd3	RNA pseudouridylate synthase domain containing 3	0.78616352	0.005812	0.03755
53376	Usp2	ubiquitin specific peptidase 2	0.78616352	0.008911	0.04929
67480	Cwc25	CWC25 spliceosome-associated protein homolog (S. cerevisiae)	0.78616352	0.01342	0.06455
19724	Rfx1	regulatory factor X, 1 (influences HLA class II expression)	0.78616352	0.01803	0.07819
217325	Llgl2	lethal giant larvae homolog 2 (Drosophila)	0.78616352	0.04227	0.1379
328365	Zmiz1	zinc finger, MIZ-type containing 1	0.78616352	0.04796	0.1503
15953	Ifi47	interferon gamma inducible protein 47	0.78616352	0.06528	0.186
73229	3110052M02Rik	RIKEN cDNA 3110052M02 gene	0.78616352	0.07823	0.2111
77085	3010027C24Rik	RIKEN cDNA 3010027C24 gene	0.78616352	0.1539	0.3296
676882	Gm9693	predicted gene 9693	0.78616352	0.1944	0.3847
56790	Fam48a	family with sequence similarity 48, member A	0.78616352	0.2873	0.4958
105000	Dnalc1	dynein, axonemal, light chain 1	0.78616352	0.5294	0.7152
353235	Pcdha8	protocadherin alpha 8	0.78554595	0.0005801	0.009333
56176	Pigp	phosphatidylinositol glycan anchor biosynthesis, class P	0.78554595	0.02368	0.09393
12585	Cdr2	cerebellar degeneration-related 2	0.78554595	0.02426	0.09533
69025	1500032P08Rik	RIKEN cDNA 1500032P08 gene	0.78554595	0.02617	0.1005
193003	Pirt	phosphoinositide-interacting regulator of transient receptor potential channels	0.78554595	0.05055	0.1561
50887	Hmgn5	high-mobility group nucleosome binding domain 5	0.78554595	0.1097	0.2636
74195	Elp3	elongation protein 3 homolog (S. cerevisiae)	0.78492936	0.001267	0.01497
68393	Mogat1	monoacylglycerol O-acyltransferase 1	0.78492936	0.002139	0.02017
240034	Zfp760	zinc finger protein 760	0.78492936	0.002763	0.02362
16647	Kpna2	karyopherin (importin) alpha 2	0.78492936	0.008217	0.04697

214133	Tet2	tet oncogene family member 2	0.78492936	0.009339	0.05085
320817	Atad2b	ATPase family, AAA domain containing 2B	0.78492936	0.009875	0.0529
224813	Gm88	predicted gene 88	0.78492936	0.01625	0.07298
56323	Dnajb5	DnaJ (Hsp40) homolog, subfamily B, member 5	0.78492936	0.02523	0.09795
51929	D1Ertd704e	DNA segment, Chr 1, ERATO Doi 704, expressed	0.78492936	0.03303	0.1175
230770	Tmem39b	transmembrane protein 39b	0.78492936	0.06967	0.1946
19281	Ptprt	protein tyrosine phosphatase, receptor type, T	0.78492936	0.08677	0.2262
260315	Nav3	neuron navigator 3	0.78492936	0.1152	0.2725
78787	Usp54	ubiquitin specific peptidase 54	0.78492936	0.1427	0.3135
353130	Prss33	protease, serine, 33	0.78492936	0.1579	0.3352
109700	Itga1	integrin alpha 1	0.78492936	0.2081	0.4029
114564	Csprs	component of Sp100-rs	0.78492936	0.3042	0.5133
68721	1110032A03Rik	RIKEN cDNA 1110032A03 gene	0.78492936	0.4378	0.6409
56808	Cacna2d2	calcium channel, voltage-dependent, alpha 2/delta subunit 2	0.78492936	0.556	0.7358
553096	LOC553096	hypothetical LOC553096	0.78431373	0.001554	0.01696
330788	Zfp866	zinc finger protein 866	0.78431373	0.003947	0.02949
106967	4732423E21Rik	RIKEN cDNA 4732423E21 gene	0.78431373	0.005856	0.03771
67996	Srsf6	serine/arginine-rich splicing factor 6	0.78431373	0.007522	0.04442
74665	Lrrc48	leucine rich repeat containing 48	0.78431373	0.008629	0.04844
71764	C2cd2l	C2 calcium-dependent domain containing 2-like	0.78431373	0.008635	0.04846
236727	Slc9a7	solute carrier family 9 (sodium/hydrogen exchanger), member 7	0.78431373	0.014	0.06642
11512	Adcy6	adenylate cyclase 6	0.78431373	0.02467	0.09652
230751	Oscp1	organic solute carrier partner 1	0.78431373	0.03019	0.1108
11923	Neurod4	neurogenic differentiation 4	0.78431373	0.08033	0.215
319391	D230014I24Rik	RIKEN cDNA D230014I24 gene	0.78431373	0.08355	0.2206
434825	LOC434825	hypothetical LOC434825	0.78431373	0.1125	0.2682
393082	Mettl7a2	methyltransferase like 7A2	0.78431373	0.3214	0.5312
232875	Zscan18	zinc finger and SCAN domain containing 18	0.78369906	0.002535	0.02229
74675	Ptchd3	patched domain containing 3	0.78369906	0.02982	0.1097
215690	Nav1	neuron navigator 1	0.78369906	0.04153	0.1363
73692	2410089E03Rik	RIKEN cDNA 2410089E03 gene	0.78369906	0.05257	0.1606
71766	Raver1	ribonucleoprotein, PTB-binding 1	0.78369906	0.06884	0.1929
384763	Zfp667	zinc finger protein 667	0.78369906	0.09631	0.2423
212391	Lcor	ligand dependent nuclear receptor corepressor	0.78369906	0.09798	0.2451
224694	Zfp81	zinc finger protein 81	0.78369906	0.134	0.3011
27416	Abcc5	ATP-binding cassette, sub-family C (CFTR/MRP), member 5	0.78369906	0.183	0.3702
104394	E2f4	E2F transcription factor 4	0.78369906	0.4596	0.6605
14768	Lancl1	LanC (bacterial lantibiotic synthetase component C)-like 1	0.78308536	0.0001941	0.004863
12675	Chuk	conserved helix-loop-helix ubiquitous kinase	0.78308536	0.001111	0.0139
192164	Pcdha12	protocadherin alpha 12	0.78308536	0.007743	0.04529
432442	Akap7	A kinase (PRKA) anchor protein 7	0.78308536	0.009143	0.0502
21749	Terf1	telomeric repeat binding factor 1	0.78308536	0.009975	0.05321
73848	5430406J06Rik	RIKEN cDNA 5430406J06 gene	0.78308536	0.01197	0.06006
18777	Lypla1	lysophospholipase 1	0.78308536	0.01589	0.07195
66648	5730494M16Rik	RIKEN cDNA 5730494M16 gene	0.78308536	0.02614	0.1005
214575	Tdrd5	tudor domain containing 5	0.78308536	0.0393	0.1313
56527	Mast1	microtubule associated serine/threonine kinase 1	0.78308536	0.04431	0.1421
74718	Snx16	sorting nexin 16	0.78308536	0.07863	0.212
211429	Pla2g4b	phospholipase A2, group IVB (cytosolic)	0.78308536	0.08459	0.2224
83672	Sytl3	synaptotagmin-like 3	0.78308536	0.1497	0.324

17168	Nprl3	nitrogen permease regulator-like 3 (S. cerevisiae)	0.78308536	0.1718	0.3547
15974	Ifnab	interferon alpha B	0.78308536	0.5501	0.7314
15259	Hipk3	homeodomain interacting protein kinase 3	0.78247261	0.001069	0.01361
13813	Eomes	eomesodermin homolog (Xenopus laevis)	0.78247261	0.006372	0.03989
68728	Trp53inp2	transformation related protein 53 inducible nuclear protein 2	0.78247261	0.006679	0.04113
14069	F8	coagulation factor VIII	0.78247261	0.01281	0.06264
329679	Fnip2	folliculin interacting protein 2	0.78247261	0.01594	0.07212
214058	Megf11	multiple EGF-like-domains 11	0.78247261	0.03374	0.119
67039	Rbm25	RNA binding motif protein 25	0.78247261	0.03815	0.1289
16363	Irf2	interferon regulatory factor 2	0.78247261	0.05203	0.1595
63993	Slc5a7	solute carrier family 5 (choline transporter), member 7	0.78247261	0.06312	0.1818
14812	Grin2b	glutamate receptor, ionotropic, NMDA2B (epsilon 2)	0.78247261	0.178	0.3632
239170	Fam160b2	family with sequence similarity 160, member B2	0.78247261	0.2248	0.4245
320615	Dopey1	dopey family member 1	0.78247261	0.2279	0.428
258581	Olfr1030	olfactory receptor 1030	0.78247261	0.3001	0.5094
54426	Hgfac	hepatocyte growth factor activator	0.78247261	0.3653	0.5743
100043231	Gm4303	predicted gene 4303	0.78247261	0.5561	0.7358
83398	Ndst3	N-deacetylase/N-sulfotransferase (heparan glucosaminyl) 3	0.78186083	0.0003812	0.0072
20384	Srsf5	serine/arginine-rich splicing factor 5	0.78186083	0.001715	0.01789
18750	Prkca	protein kinase C, alpha	0.78186083	0.002774	0.02366
17930	Myom2	myomesin 2	0.78186083	0.01107	0.05676
56642	Ankrd2	ankyrin repeat domain 2 (stretch responsive muscle)	0.78186083	0.01602	0.07226
235106	Ntm	neurotrimin	0.78186083	0.0358	0.1237
213011	Zfp583	zinc finger protein 583	0.78186083	0.08069	0.2156
667103	Gm13570	predicted gene 13570	0.78186083	0.1299	0.2957
103079	AA408251	expressed sequence AA408251	0.78186083	0.1567	0.3336
74200	2810403A07Rik	RIKEN cDNA 2810403A07 gene	0.78125	0.001405	0.01587
77015	Mpped2	metallophosphoesterase domain containing 2	0.78125	0.001641	0.01739
26381	Esrrg	estrogen-related receptor gamma	0.78125	0.008286	0.04721
233913	BC017158	cDNA sequence BC017158	0.78125	0.008444	0.04769
78330	Ndufv3	NADH dehydrogenase (ubiquinone) flavoprotein 3	0.78125	0.01467	0.06841
93961	B3galt5	UDP-Gal:betaGlcNAc beta 1,3-galactosyltransferase, polypeptide 5	0.78125	0.01693	0.07476
320772	Mdga2	MAM domain containing glycosylphosphatidylinositol anchor 2	0.78125	0.02228	0.09047
328440	Npm2	nucleophosmin/nucleoplasmin 2	0.78125	0.05105	0.1572
26558	Homer3	homer homolog 3 (Drosophila)	0.78125	0.0543	0.1644
225160	Thoc1	THO complex 1	0.78125	0.06185	0.1794
13433	Dnmt1	DNA methyltransferase (cytosine-5) 1	0.78125	0.1107	0.2653
11789	Apc	adenomatosis polyposis coli	0.78125	0.1162	0.2739
54562	Lrrc6	leucine rich repeat containing 6 (testis)	0.78125	0.1473	0.3203
210853	Zfp947	zinc finger protein 947	0.78125	0.1974	0.3885
74469	Taf7l	TAF7-like RNA polymerase II, TATA box binding protein (TBP)-associated factor	0.78125	0.1979	0.3892
110385	Pde4c	phosphodiesterase 4C, cAMP specific	0.78125	0.3351	0.5456
72508	Rps6kb1	ribosomal protein S6 kinase, polypeptide 1	0.78064012	0.001204	0.01451
76130	Las1l	LAS1-like (S. cerevisiae)	0.78064012	0.003508	0.02745
225600	Pde6a	phosphodiesterase 6A, cGMP-specific, rod, alpha	0.78064012	0.004521	0.03213
66965	Ctu2	cytosolic thiouridylase subunit 2 homolog (S. pombe)	0.78064012	0.01007	0.05361
100929	Tyw1	tRNA-yW synthesizing protein 1 homolog (S. cerevisiae)	0.78064012	0.01217	0.06059
21960	Tnr	tenascin R	0.78064012	0.02667	0.1018
333789	N4bp2	NEDD4 binding protein 2	0.78064012	0.06612	0.1875
18767	Pkia	protein kinase inhibitor, alpha	0.78064012	0.07138	0.1979

13549	Dyrk1b	dual-specificity tyrosine-(Y)-phosphorylation regulated kinase 1b	0.78064012	0.1258	0.289
77779	A930007I19Rik	RIKEN cDNA A930007I19 gene	0.78064012	0.248	0.4521
217705	Fam161b	family with sequence similarity 161, member B	0.7800312	0.002332	0.02131
16514	Kcnj11	potassium inwardly rectifying channel, subfamily J, member 11	0.7800312	0.01263	0.06211
100040305	Gm2701	predicted gene 2701	0.7800312	0.04234	0.138
26457	Slc27a1	solute carrier family 27 (fatty acid transporter), member 1	0.7800312	0.1146	0.2716
76491	Abhd14b	abhydrolase domain containing 14b	0.77942323	0.001915	0.01913
19893	Rpgr	retinitis pigmentosa GTPase regulator	0.77942323	0.01724	0.07574
242259	Slc44a5	solute carrier family 44, member 5	0.77942323	0.03509	0.1222
14531	Gcm1	glial cells missing homolog 1 (Drosophila)	0.77942323	0.05119	0.1575
235682	Zfp445	zinc finger protein 445	0.77942323	0.05949	0.1744
18191	Nrxn3	neurexin III	0.77942323	0.07253	0.2003
66204	Acyp1	acylphosphatase 1, erythrocyte (common) type	0.77942323	0.1046	0.2557
16621	Klkb1	kallikrein B, plasma 1	0.7788162	0.001486	0.01642
67370	Zfp606	zinc finger protein 606	0.7788162	0.001569	0.01704
216233	Socs2	suppressor of cytokine signaling 2	0.7788162	0.003276	0.02633
56195	Ptbp2	polypyrimidine tract binding protein 2	0.7788162	0.004113	0.03031
69608	Sec24d	Sec24 related gene family, member D (S. cerevisiae)	0.7788162	0.008603	0.04832
223642	Zc3h3	zinc finger CCCH type containing 3	0.7788162	0.009242	0.05056
331524	Xkrx	X Kell blood group precursor related X linked	0.7788162	0.01291	0.06293
328572	Ep300	E1A binding protein p300	0.7788162	0.03377	0.1191
211378	6720489N17Rik	RIKEN cDNA 6720489N17 gene	0.7788162	0.06729	0.1897
209707	Lcorl	ligand dependent nuclear receptor corepressor-like	0.7788162	0.07457	0.2042
104943	Fam110c	family with sequence similarity 110, member C	0.7788162	0.1029	0.2531
17706	ATP8	ATP synthase F0 subunit 8	0.7788162	0.1058	0.2573
238247	Arid4a	AT rich interactive domain 4A (RBP1-like)	0.7788162	0.1145	0.2715
239420	Csmd3	CUB and Sushi multiple domains 3	0.7788162	0.151	0.3258
12672	Chrm4	cholinergic receptor, muscarinic 4	0.7788162	0.2662	0.4731
13086	Cyp2a4	cytochrome P450, family 2, subfamily a, polypeptide 4	0.7788162	0.3064	0.5158
12747	Clk1	CDC-like kinase 1	0.77821012	0.001343	0.01549
20504	Slc17a1	solute carrier family 17 (sodium phosphate), member 1	0.77821012	0.001357	0.01557
320095	6430550D23Rik	RIKEN cDNA 6430550D23 gene	0.77821012	0.05314	0.1618
100038666	Gm12298	predicted gene 12298	0.77821012	0.05386	0.1634
76421	1700028K03Rik	RIKEN cDNA 1700028K03 gene	0.77821012	0.08341	0.2204
57259	Tob2	transducer of ERBB2, 2	0.77821012	0.1367	0.3042
264895	Acsf2	acyl-CoA synthetase family member 2	0.77821012	0.1519	0.3267
14201	Fhl3	four and a half LIM domains 3	0.77821012	0.2165	0.4139
99980	AA408865	expressed sequence AA408865	0.77821012	0.2531	0.458
98662	AW061147	expressed sequence AW061147	0.77821012	0.4876	0.6825
19428	Rasl2-9-ps	RAS-like, family 2, locus 9, pseudogene	0.77821012	0.5771	0.7502
19885	Rorc	RAR-related orphan receptor gamma	0.77760498	0.00483	0.03346
353188	Adam32	a disintegrin and metallopeptidase domain 32	0.77760498	0.007651	0.04495
76199	Med13l	mediator complex subunit 13-like	0.77760498	0.007951	0.04596
237411	Zfp938	zinc finger protein 938	0.77760498	0.02294	0.09207
245827	Fat2	FAT tumor suppressor homolog 2 (Drosophila)	0.77760498	0.02375	0.09418
70408	Polr3f	polymerase (RNA) III (DNA directed) polypeptide F	0.77760498	0.02684	0.1022
77741	6720483E21Rik	RIKEN cDNA 6720483E21 gene	0.77760498	0.05165	0.1586
329986	BC080695	cDNA sequence BC080695	0.77760498	0.05797	0.1716
19646	Rbbp4	retinoblastoma binding protein 4	0.77760498	0.08496	0.2231
217431	Nol10	nucleolar protein 10	0.77760498	0.08497	0.2231

233987	Zfp958	zinc finger protein 958	0.77760498	0.105	0.2562
13370	Dio1	deiodinase, iodothyronine, type I	0.77760498	0.1367	0.3042
76056	5830443J22Rik	RIKEN cDNA 5830443J22 gene	0.77760498	0.1489	0.3227
11551	Adra2a	adrenergic receptor, alpha 2a	0.77700078	0.001578	0.01708
664949	Gm7420	predicted gene 7420	0.77700078	0.002781	0.0237
216233	Socs2	suppressor of cytokine signaling 2	0.77700078	0.008891	0.04922
20744	Strbp	spermatid perinuclear RNA binding protein	0.77700078	0.03069	0.1119
228602	4930402H24Rik	RIKEN cDNA 4930402H24 gene	0.77700078	0.1032	0.2536
76080	Ttpal	tocopherol (alpha) transfer protein-like	0.77700078	0.1376	0.3057
328580	Tubgcp6	tubulin, gamma complex associated protein 6	0.77700078	0.1692	0.3504
258673	Olfr1428	olfactory receptor 1428	0.77700078	0.3425	0.5534
53623	Gria3	glutamate receptor, ionotropic, AMPA3 (alpha 3)	0.77639752	0.0002647	0.005839
234366	Gatad2a	GATA zinc finger domain containing 2A	0.77639752	0.0002874	0.006071
13601	Ecm1	extracellular matrix protein 1	0.77639752	0.002658	0.02307
55935	Fnbp4	formin binding protein 4	0.77639752	0.0046	0.03247
18072	Nhlh2	nescient helix loop helix 2	0.77639752	0.01057	0.05514
70549	Tln2	talin 2	0.77639752	0.0116	0.05892
12788	Cnga1	cyclic nucleotide gated channel alpha 1	0.77639752	0.01543	0.07086
19258	Ptpn4	protein tyrosine phosphatase, non-receptor type 4	0.77639752	0.06979	0.1949
66101	Ppih	peptidyl prolyl isomerase H	0.77639752	0.1009	0.2502
17968	Ncam2	neural cell adhesion molecule 2	0.77639752	0.1033	0.2537
67272	Cmtm5	CKLF-like MARVEL transmembrane domain containing 5	0.77639752	0.2391	0.4416
12819	Col15a1	collagen, type XV, alpha 1	0.77639752	0.4912	0.6852
11798	Xiap	X-linked inhibitor of apoptosis	0.77579519	0.0003594	0.006916
60409	Trappc4	trafficking protein particle complex 4	0.77579519	0.0005128	0.008671
77877	6030458C11Rik	RIKEN cDNA 6030458C11 gene	0.77579519	0.0006476	0.009989
12810	Coch	coagulation factor C homolog (Limulus polyphemus)	0.77579519	0.001297	0.01514
106248	Qtrtd1	queuine tRNA-ribosyltransferase domain containing 1	0.77579519	0.0024	0.02155
77963	Hook1	hook homolog 1 (Drosophila)	0.77579519	0.01194	0.06005
16000	Igf1	insulin-like growth factor 1	0.77579519	0.01241	0.06135
68952	Fam57b	family with sequence similarity 57, member B	0.77579519	0.01465	0.06841
18191	Nrxn3	neurexin III	0.77579519	0.01905	0.08112
17248	Mdm4	transformed mouse 3T3 cell double minute 4	0.77579519	0.05337	0.1624
665270	Plb1	phospholipase B1	0.77579519	0.08499	0.2231
16994	Ltb	lymphotoxin B	0.77579519	0.08847	0.2291
69593	2310026I22Rik	RIKEN cDNA 2310026I22 gene	0.77579519	0.09714	0.2434
100503794	LOC100503794	hypothetical LOC100503794	0.77579519	0.1627	0.3416
29863	Pde7b	phosphodiesterase 7B	0.77579519	0.1716	0.3545
50525	Spag6	sperm associated antigen 6	0.77579519	0.2739	0.4818
67036	Mrpl45	mitochondrial ribosomal protein L45	0.7751938	0.001969	0.01937
73998	Herc3	hect domain and RLD 3	0.7751938	0.005112	0.03464
16561	Kif1b	kinesin family member 1B	0.7751938	0.01322	0.06403
629756	Wfdc10	WAP four-disulfide core domain 10	0.7751938	0.03307	0.1175
242557	Atg4c	autophagy-related 4C (yeast)	0.7751938	0.09321	0.2371
622935	Krtap20-2	keratin associated protein 20-2	0.7751938	0.2716	0.4791
12211	Birc6	baculoviral IAP repeat-containing 6	0.77459334	0.003031	0.02499
59033	Slc4a8	solute carrier family 4 (anion exchanger), member 8	0.77459334	0.004155	0.03048
20028	Pdc	phosducin	0.77459334	0.008021	0.0462
11722	Amy1	amylase 1, salivary	0.77459334	0.01214	0.06053
76357	Trmt5	TRM5 tRNA methyltransferase 5 homolog (S. cerevisiae)	0.77459334	0.01651	0.07359

74838	Naa15		N(alpha)-acetyltransferase 15, NatA auxiliary subunit	0.77459334	0.03572	0.1236
241656	Pak7		p21 protein (Cdc42/Rac)-activated kinase 7	0.77459334	0.05521	0.1662
100310809	Gm10509		zinc finger protein 51 pseudogene	0.77459334	0.09078	0.233
272538	Tmco7		transmembrane and coiled-coil domains 7	0.77459334	0.1092	0.2629
207806	Gm608		predicted gene 608	0.77459334	0.1327	0.2994
66166	S100a14		S100 calcium binding protein A14	0.77459334	0.1449	0.3172
17101	Lyst		lysosomal trafficking regulator	0.77459334	0.1715	0.3544
100038620	Gm10631		predicted gene 10631	0.77459334	0.2829	0.4913
57442	Kcne3		potassium voltage-gated channel, Isk-related subfamily, gene 3	0.77459334	0.3837	0.5928
211147		Mar-11	membrane-associated ring finger (C3HC4) 11	0.77399381	0.006374	0.03989
72358	2210411A11Rik		RIKEN cDNA 2210411A11 gene	0.77399381	0.006437	0.04013
102913	6330509M05Rik		RIKEN cDNA 6330509M05 gene	0.77399381	0.008178	0.04683
59024	Med12		mediator of RNA polymerase II transcription, subunit 12 homolog (yeast)	0.77399381	0.01103	0.05663
100040724	Mirg		miRNA containing gene	0.77399381	0.01458	0.06819
73332	Ccdc30		coiled-coil domain containing 30	0.77399381	0.01515	0.06995
30952	Cngb3		cyclic nucleotide gated channel beta 3	0.77399381	0.01781	0.07752
320609	Fam40b		family with sequence similarity 40, member B	0.77399381	0.01999	0.08377
72949	Ccnt2		cyclin T2	0.77399381	0.02373	0.09412
228602	4930402H24Rik		RIKEN cDNA 4930402H24 gene	0.77399381	0.04388	0.1412
77255	9430014F16Rik		RIKEN cDNA 9430014F16 gene	0.77399381	0.1178	0.2764
19378	Aldh1a2		aldehyde dehydrogenase family 1, subfamily A2	0.77399381	0.15	0.3245
381290	Atp2b4		ATPase, Ca++ transporting, plasma membrane 4	0.77399381	0.1688	0.3499
67071	Rps6ka6		ribosomal protein S6 kinase polypeptide 6	0.77399381	0.1862	0.3738
26943	Serinc3		serine incorporator 3	0.7733952	0.001188	0.01439
208606	Rsrc2		arginine/serine-rich coiled-coil 2	0.7733952	0.004959	0.03395
20028	Pdc		phosducin	0.7733952	0.02635	0.1009
242860	Rsbn1l		round spermatid basic protein 1-like	0.7733952	0.02965	0.1092
66169	Tomm7		translocase of outer mitochondrial membrane 7 homolog (yeast)	0.7733952	0.03102	0.1127
27223	Trp53bp1		transformation related protein 53 binding protein 1	0.7733952	0.03589	0.124
76963	2310076G05Rik		RIKEN cDNA 2310076G05 gene	0.7733952	0.03931	0.1313
72020	Zfp654		zinc finger protein 654	0.7733952	0.1064	0.2581
18183	Nrg3		neuregulin 3	0.7733952	0.1443	0.3161
319587	4930539J05Rik		RIKEN cDNA 4930539J05 gene	0.7733952	0.149	0.3229
73722	Lce1a2		late cornified envelope 1A2	0.7733952	0.2476	0.4518
235281	Scn3b		sodium channel, voltage-gated, type III, beta	0.7733952	0.4375	0.6408
21374	Tbp		TATA box binding protein	0.77279753	0.0009071	0.01223
56758	Mbnl1		muscleblind-like 1 (Drosophila)	0.77279753	0.003563	0.02773
269336	Ccdc32		coiled-coil domain containing 32	0.77279753	0.00834	0.04737
21951	Tnks		tankyrase, TRF1-interacting ankyrin-related ADP-ribose polymerase	0.77279753	0.0341	0.1197
56523	Pmfbp1		polyamine modulated factor 1 binding protein 1	0.77279753	0.1064	0.2581
76654	Upp2		uridine phosphorylase 2	0.77279753	0.2335	0.435
100126203	6820426E19Rik		RIKEN cDNA 6820426E19 gene	0.77279753	0.2523	0.4573
654469	LOC654469		hypothetical LOC654469	0.77279753	0.2964	0.5054
233080	Ffar3		free fatty acid receptor 3	0.77279753	0.3203	0.5303
69318	1700007K09Rik		RIKEN cDNA 1700007K09 gene	0.77279753	0.5149	0.7048
235293	Sc5d		sterol-C5-desaturase (fungal ERG3, delta-5-desaturase) homolog (S. cerevisae)	0.77220077	0.0006078	0.009602
72461	Prcp		prolylcarboxypeptidase (angiotensinase C)	0.77220077	0.003856	0.02914
218103	Slc17a2		solute carrier family 17 (sodium phosphate), member 2	0.77220077	0.01474	0.06867
56324	Stam2		signal transducing adaptor molecule (SH3 domain and ITAM motif) 2	0.77220077	0.02247	0.09081
72179	Fbxl2		F-box and leucine-rich repeat protein 2	0.77220077	0.06213	0.1798

217378	Dnajc27	DnaJ (Hsp40) homolog, subfamily C, member 27	0.77220077	0.0834	0.2204
223827	Gxylt1	glucoside xylosyltransferase 1	0.77220077	0.1257	0.289
258611	Olfr297	olfactory receptor 297	0.77220077	0.1425	0.3133
72135	Pygo1	pygopus 1	0.77220077	0.3112	0.5212
100113398	Adat3	adenosine deaminase, tRNA-specific 3, TAD2 homolog (S. cerevisiae)	0.77160494	0.005637	0.03686
14816	Grm1	glutamate receptor, metabotropic 1	0.77160494	0.00672	0.04133
226182	Taf5	TAF5 RNA polymerase II, TATA box binding protein (TBP)-associated factor	0.77160494	0.01092	0.05624
100042480	Nhsl2	NHS-like 2	0.77160494	0.01563	0.07134
433408	Gm13375	predicted gene 13375	0.77160494	0.039	0.1306
110876	Scn2a1	sodium channel, voltage-gated, type II, alpha 1	0.77160494	0.044	0.1414
654439	D430004P15Rik	Riken cDNA D430004P15 gene	0.77160494	0.08953	0.2306
28135	Cep63	centrosomal protein 63	0.77160494	0.1211	0.2822
665211	Gm14326	predicted gene 14326	0.77160494	0.1618	0.3403
407795	BC030870	cDNA sequence BC030870	0.77160494	0.2344	0.436
71721	Fam13c	family with sequence similarity 13, member C	0.77101002	0.001359	0.01558
225998	Rorb	RAR-related orphan receptor beta	0.77101002	0.002938	0.02445
20562	Slit1	slit homolog 1 (Drosophila)	0.77101002	0.002987	0.02475
75725	Phf14	PHD finger protein 14	0.77101002	0.01471	0.06856
78309	Cul9	cullin 9	0.77101002	0.02163	0.08865
27361	Sepx1	selenoprotein X 1	0.77101002	0.02745	0.1037
72179	Fbxl2	F-box and leucine-rich repeat protein 2	0.77101002	0.02902	0.1077
12804	Cntfr	ciliary neurotrophic factor receptor	0.77101002	0.05846	0.1724
105559	Mbnl2	muscleblind-like 2	0.77101002	0.07381	0.2028
76184	Abca6	ATP-binding cassette, sub-family A (ABC1), member 6	0.77101002	0.0819	0.2178
74762	Mdga1	MAM domain containing glycosylphosphatidylinositol anchor 1	0.77101002	0.08934	0.2304
258464	Olfr1384	olfactory receptor 1384	0.77101002	0.09943	0.2475
71519	Cyp2u1	cytochrome P450, family 2, subfamily u, polypeptide 1	0.77101002	0.1492	0.3232
237880	1700071K01Rik	RIKEN cDNA 1700071K01 gene	0.77101002	0.167	0.3479
100041639	Gm3448	predicted gene 3448	0.77101002	0.1781	0.3632
13423	Dnase2a	deoxyribonuclease II alpha	0.77101002	0.3462	0.5568
545172	Rpl21-ps5	ribosomal protein L21, pseudogene 5	0.77041602	0.0002884	0.006073
70103	Znhit1	zinc finger, HIT domain containing 1	0.77041602	0.002423	0.02168
66435	Uggt2	UDP-glucose glycoprotein glucosyltransferase 2	0.77041602	0.002482	0.02201
241324	Crb2	crumbs homolog 2 (Drosophila)	0.77041602	0.003281	0.02634
68187	Fam135a	family with sequence similarity 135, member A	0.77041602	0.00432	0.03128
26372	Clcn6	chloride channel 6	0.77041602	0.004956	0.03395
77328	C030009O12Rik	RIKEN cDNA C030009O12 gene	0.77041602	0.01114	0.05707
213469	Lgi3	leucine-rich repeat LGI family, member 3	0.77041602	0.01419	0.06706
240753	Plekha6	pleckstrin homology domain containing, family A member 6	0.77041602	0.02559	0.0991
77974	Rdh12	retinol dehydrogenase 12	0.77041602	0.03443	0.1204
13176	Dcc	deleted in colorectal carcinoma	0.77041602	0.06583	0.187
13009	Csrp3	cysteine and glycine-rich protein 3	0.77041602	0.1576	0.3349
72145	Wdfy3	WD repeat and FYVE domain containing 3	0.77041602	0.1942	0.3845
381409	Cdh26	cadherin-like 26	0.77041602	0.2598	0.4655
238871	Pde4d	phosphodiesterase 4D, cAMP specific	0.76982294	0.003356	0.02671
320846	A530058N18Rik	RIKEN cDNA A530058N18 gene	0.76982294	0.007421	0.0441
68014	Zwilch	Zwilch, kinetochore associated, homolog (Drosophila)	0.76982294	0.008049	0.04631
66717	Ccdc96	coiled-coil domain containing 96	0.76982294	0.02417	0.09514
21339	Taf1a	TATA box binding protein (Tbp)-associated factor, RNA polymerase I, A	0.76982294	0.03888	0.1305
11480	Acvr2a	activin receptor IIA	0.76982294	0.04636	0.1467

100039681	Zcwpw2	zinc finger, CW type with PWWP domain 2	0.76982294	0.05602	0.168
100041261	Gm3238	predicted gene 3238	0.76982294	0.09462	0.2395
245174	Zfp937	zinc finger protein 937	0.76982294	0.09478	0.2398
72777	2810455B08Rik	RIKEN cDNA 2810455B08 gene	0.76923077	0.007002	0.04254
15331	Hmgn2	high mobility group nucleosomal binding domain 2	0.76923077	0.009895	0.05291
240505	Cdc42bpg	CDC42 binding protein kinase gamma (DMPK-like)	0.76923077	0.01878	0.08031
231290	Slc10a4	solute carrier family 10 (sodium/bile acid cotransporter family), member 4	0.76923077	0.04045	0.1338
328734	Pisd-ps2	phosphatidylserine decarboxylase, pseudogene 2	0.76923077	0.1006	0.2495
13840	Epha6	Eph receptor A6	0.76923077	0.1259	0.2892
114661	Prss28	protease, serine, 28	0.76923077	0.1877	0.376
74081	Cep350	centrosomal protein 350	0.76863951	0.0004357	0.007831
27223	Trp53bp1	transformation related protein 53 binding protein 1	0.76863951	0.0006554	0.01004
15357	Hmgcr	3-hydroxy-3-methylglutaryl-Coenzyme A reductase	0.76863951	0.001932	0.0192
110962	Mbd6	methyl-CpG binding domain protein 6	0.76863951	0.005703	0.03713
56229	Thsd1	thrombospondin, type I, domain 1	0.76863951	0.01401	0.06646
110829	Lims1	LIM and senescent cell antigen-like domains 1	0.76863951	0.0438	0.1409
71675	0610010F05Rik	RIKEN cDNA 0610010F05 gene	0.76863951	0.0996	0.2478
329251	Ppp1r12b	protein phosphatase 1, regulatory (inhibitor) subunit 12B	0.76863951	0.142	0.3126
75645	1700011F14Rik	RIKEN cDNA 1700011F14 gene	0.76863951	0.1483	0.322
77097	Tanc2	tetratricopeptide repeat, ankyrin repeat and coiled-coil containing 2	0.76863951	0.1843	0.3713
69851	2010007E15Rik	RIKEN cDNA 2010007E15 gene	0.76863951	0.1966	0.3876
14945	Gzmk	granzyme K	0.76863951	0.2226	0.4221
380714	Rph3al	rabphilin 3A-like (without C2 domains)	0.76863951	0.4753	0.6728
77656	C430045I18Rik	RIKEN cDNA C430045I18 gene	0.76804916	0.0002811	0.005976
552906	LOC552906	hypothetical LOC552906	0.76804916	0.001861	0.01878
627214	Fam196a	family with sequence similarity 196, member A	0.76804916	0.003815	0.02893
217664	Mgat2	mannoside acetylglucosaminyltransferase 2	0.76804916	0.007843	0.04561
70857	4921509J17Rik	RIKEN cDNA 4921509J17 gene	0.76804916	0.01547	0.07096
70549	Tln2	talin 2	0.76804916	0.02321	0.09265
332934	Zmynd12	zinc finger, MYND domain containing 12	0.76804916	0.04203	0.1373
74303	1700109K24Rik	RIKEN cDNA 1700109K24 gene	0.76804916	0.05737	0.1704
329251	Ppp1r12b	protein phosphatase 1, regulatory (inhibitor) subunit 12B	0.76804916	0.0668	0.1888
19417	Rasgrf1	RAS protein-specific guanine nucleotide-releasing factor 1	0.76804916	0.08634	0.2256
72987	2900057C01Rik	RIKEN cDNA 2900057C01 gene	0.76804916	0.09224	0.2356
239157	Pnma2	paraneoplastic antigen MA2	0.76804916	0.184	0.3709
75202	Ncrna00085	non-protein coding RNA 85	0.76804916	0.2192	0.4178
18102	Nme1	non-metastatic cells 1, protein (NM23A) expressed in	0.76745971	0.001036	0.01334
238831	Ppwd1	peptidylprolyl isomerase domain and WD repeat containing 1	0.76745971	0.006181	0.03907
59024	Med12	mediator of RNA polymerase II transcription, subunit 12 homolog (yeast)	0.76745971	0.00658	0.0407
74558	Gvin1	GTPase, very large interferon inducible 1	0.76745971	0.01342	0.06455
70924	4921511C10Rik	RIKEN cDNA 4921511C10 gene	0.76745971	0.0196	0.08263
72029	Cnpy3	canopy 3 homolog (zebrafish)	0.76745971	0.02131	0.08775
225998	Rorb	RAR-related orphan receptor beta	0.76745971	0.02906	0.1078
665005	Gm7444	ring finger protein 19A pseudogene	0.76745971	0.1596	0.3377
319603	A730009E18Rik	RIKEN cDNA A730009E18 gene	0.76745971	0.1613	0.3396
76560	Prss8	protease, serine, 8 (prostasin)	0.76745971	0.266	0.4727
19259	Ptpn5	protein tyrosine phosphatase, non-receptor type 5	0.76687117	0.0008956	0.01215
68750	Rreb1	ras responsive element binding protein 1	0.76687117	0.009432	0.05126
238161	Akap6	A kinase (PRKA) anchor protein 6	0.76687117	0.01924	0.08162
216622	4931440F15Rik	RIKEN cDNA 4931440F15 gene	0.76687117	0.03829	0.1292

100040529	Gm2824	predicted gene 2824	0.76687117	0.05793	0.1716
76376	Slc24a2	solute carrier family 24 (sodium/potassium/calcium exchanger), member 2	0.76687117	0.06703	0.1892
66399	Tsfm	Ts translation elongation factor, mitochondrial	0.76687117	0.3276	0.5376
100505283	LOC100505283	60S ribosomal protein L21-like	0.76628352	0.0009562	0.01265
244713	Zfp317	zinc finger protein 317	0.76628352	0.002314	0.02123
50887	Hmgn5	high-mobility group nucleosome binding domain 5	0.76628352	0.003341	0.02662
19260	Ptpn22	protein tyrosine phosphatase, non-receptor type 22 (lymphoid)	0.76628352	0.004935	0.03385
52410	D8Ertd769e	DNA segment, Chr 8, ERATO Doi 769, expressed	0.76628352	0.03825	0.1291
270086	Ogfod1	2-oxoglutarate and iron-dependent oxygenase domain containing 1	0.76628352	0.0682	0.1916
264134	Ttc26	tetratricopeptide repeat domain 26	0.76628352	0.08144	0.2169
210104	Zfp658	zinc finger protein 658	0.76628352	0.1938	0.3838
76478	Haus8	4HAUS augmin-like complex, subunit 8	0.76569678	0.000547	0.009042
14688	Gnb1	guanine nucleotide binding protein (G protein), beta 1	0.76569678	0.000723	0.01076
268670	Zfp759	zinc finger protein 759	0.76569678	0.001132	0.014
328087	D930001B02	hypothetical protein D930001B02	0.76569678	0.00164	0.01738
108112	Eif4ebp3	eukaryotic translation initiation factor 4E binding protein 3	0.76569678	0.004685	0.03286
106064	AW549877	expressed sequence AW549877	0.76569678	0.007329	0.04377
208982	Hmgcll1	3-hydroxymethyl-3-methylglutaryl-Coenzyme A lyase-like 1	0.76569678	0.007329	0.04377
77789	A930007D18Rik	RIKEN cDNA A930007D18 gene	0.76569678	0.008853	0.04914
69116	Ubr4	ubiquitin protein ligase E3 component n-recognin 4	0.76569678	0.009318	0.05078
80794	Cblc	Casitas B-lineage lymphoma c	0.76569678	0.09071	0.2329
66326	Dnajc5b	DnaJ (Hsp40) homolog, subfamily C, member 5 beta	0.76569678	0.5138	0.7041
13447	Doc2b	double C2, beta	0.76511094	0.0008453	0.01176
19777	C80913	expressed sequence C80913	0.76511094	0.003657	0.02817
231279	Guf1	GUF1 GTPase homolog (S. cerevisiae)	0.76511094	0.006978	0.04245
233058	Zfp420	zinc finger protein 420	0.76511094	0.007107	0.04298
14776	Gpx2	glutathione peroxidase 2	0.76511094	0.0141	0.06675
20913	Stxbp4	syntaxin binding protein 4	0.76511094	0.0188	0.08036
72153	2610020P09Rik	RIKEN cDNA 2610020P09 gene	0.76511094	0.07355	0.2024
67220	Plekho1	pleckstrin homology domain containing, family O member 1	0.76452599	0.0005639	0.009194
320365	Fry	furry homolog (Drosophila)	0.76452599	0.0007966	0.01136
225898	Eml3	echinoderm microtubule associated protein like 3	0.76452599	0.0009192	0.01234
67894	Fam45a	family with sequence similarity 45, member A	0.76452599	0.001268	0.01497
22589	Atrx	alpha thalassemia/mental retardation syndrome X-linked homolog (human)	0.76452599	0.03054	0.1115
216810	Tom1l2	target of myb1-like 2 (chicken)	0.76452599	0.0465	0.147
77552	Shisa4	shisa homolog 4 (Xenopus laevis)	0.76452599	0.05542	0.1667
574519	Vax2os1	Vax2 opposite strand transcript 1	0.76452599	0.06353	0.1826
17355	Aff1	AF4/FMR2 family, member 1	0.76452599	0.1224	0.2844
56380	Arid3b	AT rich interactive domain 3B (BRIGHT-like)	0.76452599	0.3189	0.5287
66204	Acyp1	acylphosphatase 1, erythrocyte (common) type	0.76452599	0.5008	0.6928
14823	Grm8	glutamate receptor, metabotropic 8	0.76394194	0.0004086	0.007506
67937	Tmem59l	transmembrane protein 59-like	0.76394194	0.002975	0.02467
320111	Prr18	proline rich region 18	0.76394194	0.003796	0.02889
320478	Sox2ot	SOX2 overlapping transcript (non-protein coding)	0.76394194	0.004215	0.03075
20660	Sorl1	sortilin-related receptor, LDLR class A repeats-containing	0.76394194	0.01436	0.06757
58175	Rgs20	regulator of G-protein signaling 20	0.76394194	0.02134	0.08785
68905	1110065H08Rik	RIKEN cDNA 1110065H08 gene	0.76394194	0.02973	0.1095
17996	Neb	nebulin	0.76394194	0.05767	0.171
75668	Rasl10a	RAS-like, family 10, member A	0.76394194	0.06417	0.1839
12409	Cbr2	carbonyl reductase 2	0.76394194	0.1874	0.3757

103762	AI503301	expressed sequence AI503301	0.76335878	0.0002394	0.005541
17992	Ndufa4	NADH dehydrogenase (ubiquinone) 1 alpha subcomplex, 4	0.76335878	0.0007214	0.01074
320487	Heatr5a	HEAT repeat containing 5A	0.76335878	0.001386	0.01574
402775	D130095D21Rik	RIKEN cDNA D130095D21 gene	0.76335878	0.002389	0.0215
66787	4933433P14Rik	RIKEN cDNA 4933433P14 gene	0.76335878	0.008286	0.04721
353187	Nr1d2	nuclear receptor subfamily 1, group D, member 2	0.76335878	0.00855	0.04815
13417	Dnahc8	dynein, axonemal, heavy chain 8	0.76335878	0.02908	0.1078
16559	Kif17	kinesin family member 17	0.76335878	0.03624	0.1247
211232	Cpne9	copine family member IX	0.76335878	0.07011	0.1953
619719	6430590A07Rik	RIKEN cDNA 6430590A07 gene	0.76335878	0.09599	0.2419
83814	Nedd4l	neural precursor cell expressed, developmentally down-regulated gene 4-like	0.76335878	0.1069	0.2588
18703	Pigr	polymeric immunoglobulin receptor	0.76335878	0.1164	0.2742
327760	4930565A17	hypothetical protein 4930565A17	0.76335878	0.2184	0.4167
320191	Hook3	hook homolog 3 (Drosophila)	0.76277651	0.001187	0.01439
17135	Mafk	v-maf musculoaponeurotic fibrosarcoma oncogene family, protein K (avian)	0.76277651	0.004958	0.03395
70827	Trak2	trafficking protein, kinesin binding 2	0.76277651	0.005386	0.03583
227632	Kcnt1	potassium channel, subfamily T, member 1	0.76277651	0.006505	0.04036
320216	D230040J21Rik	RIKEN cDNA D230040J21 gene	0.76277651	0.01012	0.05371
232664	Ccdc136	coiled-coil domain containing 136	0.76277651	0.01533	0.07056
51886	Fubp1	far upstream element (FUSE) binding protein 1	0.76277651	0.02898	0.1076
109959	Amy2a5	amylase 2a5	0.76277651	0.04147	0.1363
100037262	Gm12359	predicted gene 12359	0.76277651	0.04247	0.1382
20606	Sstr2	somatostatin receptor 2	0.76277651	0.05797	0.1716
72404	Wdr44	WD repeat domain 44	0.76277651	0.06087	0.1772
109314	Prr9	proline rich 9	0.76277651	0.07479	0.2045
68672	1110035E04Rik	RIKEN cDNA 1110035E04 gene	0.76277651	0.1045	0.2555
217149	Cisd3	CDGSH iron sulfur domain 3	0.76277651	0.1217	0.2832
100043767	Gm4633	predicted gene 4633	0.76277651	0.5928	0.7622
74200	2810403A07Rik	RIKEN cDNA 2810403A07 gene	0.76219512	0.000602	0.009543
21461	Tcp10b	t-complex protein 10b	0.76219512	0.005446	0.03604
209645	Bend7	BEN domain containing 7	0.76219512	0.01334	0.06433
68854	Asb11	ankyrin repeat and SOCS box-containing 11	0.76219512	0.1598	0.338
13518	Dst	dystonin	0.76219512	0.2475	0.4517
66140	Fam33a	family with sequence similarity 33, member A	0.76219512	0.2589	0.4645
383563	Gpr25	G protein-coupled receptor 25	0.76219512	0.5412	0.7242
320678	Iffo1	intermediate filament family orphan 1	0.76161462	0.0006471	0.009989
665251	Gm7555	predicted gene 7555	0.76161462	0.0008848	0.01209
66526	2210012G02Rik	RIKEN cDNA 2210012G02 gene	0.76161462	0.007667	0.04502
54384	Mtmr7	myotubularin related protein 7	0.76161462	0.01869	0.08012
67917	Zcchc3	zinc finger, CCHC domain containing 3	0.76161462	0.02229	0.09047
76138	Ccdc138	coiled-coil domain containing 138	0.76161462	0.02715	0.1029
59036	Dact1	dapper homolog 1, antagonist of beta-catenin (xenopus)	0.76161462	0.02722	0.1031
78774	4930529M08Rik	RIKEN cDNA 4930529M08 gene	0.76161462	0.04562	0.1451
252876	Gin1	gypsy retrotransposon integrase 1	0.76161462	0.05203	0.1595
19283	Ptprz1	protein tyrosine phosphatase, receptor type Z, polypeptide 1	0.76161462	0.1021	0.2521
328424	Kcnrg	potassium channel regulator	0.76161462	0.1659	0.3464
231570	A830010M20Rik	RIKEN cDNA A830010M20 gene	0.76161462	0.2383	0.4407
71434	5530400B01Rik	RIKEN cDNA 5530400B01 gene	0.76161462	0.4101	0.6165
22141	Tub	tubby candidate gene	0.76103501	0.0002255	0.005341
68925	Rpap1	RNA polymerase II associated protein 1	0.76103501	0.001379	0.0157

627939	Gm6813	predicted gene 6813	0.76103501	0.002016	0.01961
26409	Map3k7	mitogen-activated protein kinase kinase kinase 7	0.76103501	0.0654	0.1862
100043316	Gm4354	predicted gene 4354	0.76103501	0.07831	0.2113
14415	Gad1	glutamic acid decarboxylase 1	0.76045627	0.002129	0.02016
114873	Dscaml1	Down syndrome cell adhesion molecule-like 1	0.76045627	0.01236	0.06117
69239	2610034M16Rik	RIKEN cDNA 2610034M16 gene	0.76045627	0.01522	0.07022
239435	Aard	alanine and arginine rich domain containing protein	0.76045627	0.01592	0.07207
17776	Mast2	microtubule associated serine/threonine kinase 2	0.76045627	0.01606	0.07239
69256	Zfp397	zinc finger protein 397	0.76045627	0.01684	0.07446
70599	Ssfa2	sperm specific antigen 2	0.76045627	0.03672	0.1257
24136	Zeb2	zinc finger E-box binding homeobox 2	0.76045627	0.04225	0.1379
223828	Pphln1	periphilin 1	0.76045627	0.04523	0.1443
60367	Il1rapl2	interleukin 1 receptor accessory protein-like 2	0.76045627	0.05133	0.1579
93884	Pcdhb13	protocadherin beta 13	0.76045627	0.05844	0.1724
73873	Fam161a	family with sequence similarity 161, member A	0.76045627	0.09426	0.2389
545156	Kalrn	kalirin, RhoGEF kinase	0.76045627	0.09776	0.2446
24102	Trex2	three prime repair exonuclease 2	0.76045627	0.1562	0.3331
110308	Krt5	keratin 5	0.76045627	0.2105	0.406
83813	Tnk1	tyrosine kinase, non-receptor, 1	0.76045627	0.3245	0.5343
399629	9530029O12Rik	RIKEN cDNA 9530029O12 gene	0.75987842	0.001446	0.01616
72899	Macrod2	MACRO domain containing 2	0.75987842	0.008118	0.04661
70769	Nolc1	nucleolar and coiled-body phosphoprotein 1	0.75987842	0.01225	0.06079
211147	Mar-11	membrane-associated ring finger (C3HC4) 11	0.75987842	0.05007	0.1553
170643	Kirrel	kin of IRRE like (Drosophila)	0.75987842	0.1294	0.2949
101179	6430519N07Rik	RIKEN cDNA 6430519N07 gene	0.75987842	0.2711	0.4788
108155	Ogt	O-linked N-acetylglucosamine (GlcNAc) transferase (UDP-N-acetylglucosamine:polypeptide-N-acetylglucosaminyl transferase)	0.75930144	0.001078	0.01361
105689	Mycbp2	MYC binding protein 2	0.75930144	0.001727	0.01797
226419	Dyrk3	dual-specificity tyrosine-(Y)-phosphorylation regulated kinase 3	0.75930144	0.002359	0.02138
104601	Mycbpap	MYCBP associated protein	0.75930144	0.006054	0.03851
11735	Ank3	ankyrin 3, epithelial	0.75930144	0.02562	0.09912
27377	Yme1l1	YME1-like 1 (S. cerevisiae)	0.75930144	0.07909	0.2129
100041377	Gm3300	predicted gene 3300	0.75930144	0.1605	0.3386
17444	Grap2	GRB2-related adaptor protein 2	0.75930144	0.2778	0.486
791373	B930078G14Rik	RIKEN cDNA B930078G14 gene	0.75930144	0.5026	0.6945
320095	6430550D23Rik	RIKEN cDNA 6430550D23 gene	0.75872534	0.0006414	0.009969
18554	Pcsk7	proprotein convertase subtilisin/kexin type 7	0.75872534	0.008841	0.04914
100019	Mdn1	midasin homolog (yeast)	0.75872534	0.02246	0.09081
234889	Gucy1a2	guanylate cyclase 1, soluble, alpha 2	0.75872534	0.03659	0.1254
100503813	LOC100503813	hypothetical protein LOC100503813	0.75872534	0.04927	0.1534
67102	D16Ertd472e	DNA segment, Chr 16, ERATO Doi 472, expressed	0.75872534	0.09353	0.2376
227800	Rabgap1	RAB GTPase activating protein 1	0.75872534	0.3915	0.5997
207686	A330021E22Rik	RIKEN cDNA A330021E22 gene	0.75815011	0.03028	0.111
56551	Txn2	thioredoxin 2	0.75815011	0.0365	0.1252
64293	Stk32b	serine/threonine kinase 32B	0.75815011	0.0402	0.1332
224833	AI661453	expressed sequence AI661453	0.75815011	0.08276	0.2192
71508	Zfp935	zinc finger protein 935	0.75815011	0.09233	0.2358
223917	Krt79	keratin 79	0.75815011	0.1617	0.3402
18802	Plcd4	phospholipase C, delta 4	0.75815011	0.2505	0.4553
11546	Parp2	poly (ADP-ribose) polymerase family, member 2	0.75757576	0.0007612	0.01102
666244	Tmsb15b1	thymosin beta 15b1	0.75757576	0.003635	0.02808

320124	B930095M22Rik	RIKEN cDNA B930095M22 gene	0.75757576	0.01553	0.07111
270163	Myo9a	myosin IXa	0.75757576	0.01732	0.07591
100042510	Gm3877	predicted gene 3877	0.75757576	0.02248	0.09081
12217	Bsn	bassoon	0.75757576	0.06294	0.1814
22589	Atrx	alpha thalassemia/mental retardation syndrome X-linked homolog (human)	0.75757576	0.0639	0.1834
23937	Mab21l2	mab-21-like 2 (C. elegans)	0.75757576	0.06762	0.1904
213989	Tmem82	transmembrane protein 82	0.75757576	0.07244	0.2002
54611	Pde3a	phosphodiesterase 3A, cGMP inhibited	0.75757576	0.1175	0.2758
107723	Slc12a6	solute carrier family 12, member 6	0.75757576	0.1333	0.3001
234135	Whsc1l1	Wolf-Hirschhorn syndrome candidate 1-like 1 (human)	0.75757576	0.1734	0.357
77864	Ypel2	yippee-like 2 (Drosophila)	0.75700227	0.0005855	0.009376
66196	Myo19	myosin XIX	0.75700227	0.001175	0.01433
280668	Adam1a	a disintegrin and metallopeptidase domain 1a	0.75700227	0.002067	0.01982
72611	Zfp655	zinc finger protein 655	0.75700227	0.002323	0.02126
319366	C920008N22Rik	RIKEN cDNA C920008N22 gene	0.75700227	0.004284	0.03112
17755	Mtap1b	microtubule-associated protein 1B	0.75700227	0.01182	0.05963
22756	Zfp94	zinc finger protein 94	0.75700227	0.014	0.06642
232086	Tmem150a	transmembrane protein 150A	0.75700227	0.01445	0.0678
26371	Ciao1	cytosolic iron-sulfur protein assembly 1 homolog (S. cerevisiae)	0.75700227	0.02294	0.09209
320775	B230319C09Rik	RIKEN cDNA B230319C09 gene	0.75700227	0.02383	0.09437
224116	Muc20	mucin 20	0.75700227	0.06802	0.1911
71907	Serpina9	serine (or cysteine) peptidase inhibitor, clade A (alpha-1 antiproteinase, antitrypsin), member 9	0.75700227	0.2384	0.4407
664787	LOC664787	similar to Sp110 nuclear body protein	0.75700227	0.2899	0.4983
68501	Nsmce2	non-SMC element 2 homolog (MMS21, S. cerevisiae)	0.75700227	0.3173	0.527
14428	Galr2	galanin receptor 2	0.75700227	0.4751	0.6727
270201	Klhl18	kelch-like 18 (Drosophila)	0.75642965	0.0003332	0.006628
69606	Mtfmt	mitochondrial methionyl-tRNA formyltransferase	0.75642965	0.0008659	0.01194
80334	Kcnip4	Kv channel interacting protein 4	0.75642965	0.001383	0.01572
631323	Gm12250	predicted gene 12250	0.75642965	0.004165	0.03052
14544	Gda	guanine deaminase	0.75642965	0.007413	0.04407
66039	D14Ertd449e	DNA segment, Chr 14, ERATO Doi 449, expressed	0.75642965	0.03004	0.1104
237625	Pla2g3	phospholipase A2, group III	0.75642965	0.04163	0.1364
75029	Purg	purine-rich element binding protein G	0.75642965	0.05891	0.1733
66610	Abi3	ABI gene family, member 3	0.75642965	0.1588	0.3364
12307	Calb1	calbindin 1	0.7558579	0.001135	0.01401
104886	Rab15	RAB15, member RAS oncogene family	0.7558579	0.001782	0.0183
574403	Fam196b	family with sequence similarity 196, member B	0.7558579	0.001857	0.01877
13860	Eps8	epidermal growth factor receptor pathway substrate 8	0.7558579	0.1111	0.2659
100504944	LOC100504944	hypothetical protein LOC100504944	0.7558579	0.3708	0.5795
76498	Paqr4	progestin and adipoQ receptor family member IV	0.75528701	0.001395	0.0158
246086	Onecut3	one cut domain, family member 3	0.75528701	0.006237	0.03933
240869	Zbtb37	zinc finger and BTB domain containing 37	0.75528701	0.01652	0.0736
13347	Dffa	DNA fragmentation factor, alpha subunit	0.75528701	0.03686	0.126
14255	Flt3	FMS-like tyrosine kinase 3	0.75528701	0.06334	0.1822
233919	Gpr26	G protein-coupled receptor 26	0.75528701	0.07973	0.214
381022	Mll2	myeloid/lymphoid or mixed-lineage leukemia 2	0.75528701	0.09519	0.2406
210741	Kcnk12	potassium channel, subfamily K, member 12	0.75528701	0.1025	0.2526
239731	Rimbp3	RIMS binding protein 3	0.75471698	0.001911	0.01911
100683	Trrap	transformation/transcription domain-associated protein	0.75471698	0.004245	0.03092
385674	Zfp174	zinc finger protein 174	0.75471698	0.007514	0.04441

268534	Sntg2	syntrophin, gamma 2	0.75471698	0.01115	0.05709
328606	4731420N21	hypothetical protein 4731420N21	0.75471698	0.01472	0.06857
70285	Rpf1	ribosome production factor 1 homolog (S. cerevisiae)	0.75471698	0.01711	0.07532
73346	1700039I01Rik	RIKEN cDNA 1700039I01 gene	0.75471698	0.1708	0.3532
74760	Rab3il1	RAB3A interacting protein (rabin3)-like 1	0.75414781	0.02939	0.1086
100504462	LOC100504462	hypothetical LOC100504462	0.75414781	0.04661	0.1472
75373	4930597O21Rik	RIKEN cDNA 4930597O21 gene	0.75414781	0.5271	0.7135
58243	Nap1l5	nucleosome assembly protein 1-like 5	0.7535795	0.0001492	0.004238
12659	Ovgp1	oviductal glycoprotein 1	0.7535795	0.002526	0.02226
229227	4932438A13Rik	RIKEN cDNA 4932438A13 gene	0.7535795	0.003518	0.0275
266692	Cpne1	copine I	0.7535795	0.00548	0.03618
18286	Odf2	outer dense fiber of sperm tails 2	0.7535795	0.01346	0.0646
192161	Pcdha9	protocadherin alpha 9	0.7535795	0.02561	0.09912
100101807	1700047I17Rik2	RIKEN cDNA 1700047I17 gene 2	0.7535795	0.03066	0.1118
193385	Fam65b	family with sequence similarity 65, member B	0.7535795	0.03695	0.1262
14531	Gcm1	glial cells missing homolog 1 (Drosophila)	0.7535795	0.08912	0.2302
100504004	LOC100504004	putative transposase element L1Md-A101/L1Md-A102/L1Md-A2-like	0.7535795	0.1194	0.2794
67420	Far1	fatty acyl CoA reductase 1	0.7535795	0.1236	0.2861
108058	Camk2d	calcium/calmodulin-dependent protein kinase II, delta	0.7535795	0.1333	0.3001
231855	C330006K01Rik	RIKEN cDNA C330006K01 gene	0.7535795	0.1349	0.3019
67225	Rnpc3	RNA-binding region (RNP1, RRM) containing 3	0.7535795	0.1463	0.3189
94216	Col4a6	collagen, type IV, alpha 6	0.7535795	0.1531	0.3283
52857	Gramd1a	GRAM domain containing 1A	0.75301205	0.0002848	0.006027
330577	Fam154b	family with sequence similarity 154, member B	0.75301205	0.0003898	0.007282
227157	Mpp4	membrane protein, palmitoylated 4 (MAGUK p55 subfamily member 4)	0.75301205	0.0005341	0.008931
223827	Gxylt1	glucoside xylosyltransferase 1	0.75301205	0.001455	0.0162
14163	Fgd1	FYVE, RhoGEF and PH domain containing 1	0.75301205	0.004051	0.03
11997	Akr1b7	aldo-keto reductase family 1, member B7	0.75301205	0.01092	0.05624
269344	Ell3	elongation factor RNA polymerase II-like 3	0.75301205	0.01649	0.07358
319893	A230057D06Rik	RIKEN cDNA A230057D06 gene	0.75301205	0.01886	0.08052
11601	Angpt2	angiopoietin 2	0.75301205	0.01948	0.08234
541456	LOC541456	hypothetical LOC541456	0.75301205	0.01993	0.08364
58245	Gpr180	G protein-coupled receptor 180	0.75301205	0.027	0.1026
70385	Ccdc99	coiled-coil domain containing 99	0.75301205	0.04582	0.1455
70626	5730522E02Rik	RIKEN cDNA 5730522E02 gene	0.75301205	0.04942	0.1537
76279	Cyp2d26	cytochrome P450, family 2, subfamily d, polypeptide 26	0.75301205	0.08496	0.2231
105727	Slc38a1	solute carrier family 38, member 1	0.75301205	0.1166	0.2745
224648	Uhrf1bp1	UHRF1 (ICBP90) binding protein 1	0.75301205	0.2102	0.4058
56066	Cxcl11	chemokine (C-X-C motif) ligand 11	0.75301205	0.3347	0.5454
102075	Plekhg4	pleckstrin homology domain containing, family G (with RhoGef domain) member 4	0.75244545	0.0003345	0.006637
20191	Ryr2	ryanodine receptor 2, cardiac	0.75244545	0.008004	0.04618
228410	Cstf3	cleavage stimulation factor, 3' pre-RNA, subunit 3	0.75244545	0.008211	0.04694
268903	Nrip1	nuclear receptor interacting protein 1	0.75244545	0.009139	0.05019
240614	Ranbp6	RAN binding protein 6	0.75244545	0.01549	0.071
76081	5830458C19Rik	RIKEN cDNA 5830458C19 gene	0.75244545	0.02292	0.09202
225467	Pggt1b	protein geranylgeranyltransferase type I, beta subunit	0.75244545	0.02396	0.09474
54711	Plagl2	pleiomorphic adenoma gene-like 2	0.75244545	0.05677	0.1693
26943	Serinc3	serine incorporator 3	0.7518797	0.0005325	0.008922
67564	Tmem35	transmembrane protein 35	0.7518797	0.001626	0.01733
20887	Sult1a1	sulfotransferase family 1A, phenol-preferring, member 1	0.7518797	0.003716	0.02845

231214	Cc2d2a	coiled-coil and C2 domain containing 2A	0.7518797	0.01329	0.06418
102916	AI451458	expressed sequence AI451458	0.7518797	0.01361	0.06511
207952	Klhl25	kelch-like 25 (Drosophila)	0.7518797	0.01372	0.0655
330409	Cecr2	cat eye syndrome chromosome region, candidate 2 homolog (human)	0.7518797	0.02632	0.1009
243262	Oas1f	2'-5' oligoadenylate synthetase 1F	0.7518797	0.02703	0.1027
68799	Rgmb	RGM domain family, member B	0.7518797	0.1153	0.2725
12409	Cbr2	carbonyl reductase 2	0.7518797	0.1668	0.3475
100503563	LOC100503563	60S ribosomal protein L21-like	0.7513148	0.0006337	0.00989
71601	Ceacam20	carcinoembryonic antigen-related cell adhesion molecule 20	0.7513148	0.004585	0.0324
19771	Rlbp1	retinaldehyde binding protein 1	0.7513148	0.01997	0.08372
333329	Cngb1	cyclic nucleotide gated channel beta 1	0.7513148	0.02471	0.09658
329954	Catsper4	cation channel, sperm associated 4	0.7513148	0.06187	0.1794
68744	Zfp740	zinc finger protein 740	0.7513148	0.09179	0.2348
641387	1700120B22Rik	RIKEN cDNA 1700120B22 gene	0.7513148	0.1876	0.3759
217303	Cd300a	CD300A antigen	0.7513148	0.4318	0.6358
20401	Sh3bp1	SH3-domain binding protein 1	0.75075075	0.0004728	0.008239
24030	Mrps12	mitochondrial ribosomal protein S12	0.75075075	0.00133	0.01541
98314	D2hgdh	D-2-hydroxyglutarate dehydrogenase	0.75075075	0.003358	0.02671
114874	Ddhd1	DDHD domain containing 1	0.75075075	0.004147	0.03047
20887	Sult1a1	sulfotransferase family 1A, phenol-preferring, member 1	0.75075075	0.004505	0.03208
242642	Hpdl	4-hydroxyphenylpyruvate dioxygenase-like	0.75075075	0.007111	0.04299
432478	Tmprss9	transmembrane protease, serine 9	0.75075075	0.01233	0.0611
214150	Eif2c3	eukaryotic translation initiation factor 2C, 3	0.75075075	0.03803	0.1286
53868	Rab25	RAB25, member RAS oncogene family	0.75075075	0.09321	0.2371
227058	Dnahc7b	dynein, axonemal, heavy chain 7B	0.75075075	0.1622	0.3409
20147	Rs1	retinoschisis (X-linked, juvenile) 1 (human)	0.75075075	0.1839	0.3709
225638	Alpk2	alpha-kinase 2	0.75018755	0.002366	0.0214
12836	Col7a1	collagen, type VII, alpha 1	0.75018755	0.007776	0.0454
330463	Zfp78	zinc finger protein 78	0.74962519	0.0002914	0.006115
19419	Rasgrp1	RAS guanyl releasing protein 1	0.74962519	0.0008876	0.01209
27220	Cartpt	CART prepropeptide	0.74962519	0.002067	0.01982
100042584	Gm16523	mitochondrial ribosomal protein L40 pseudogene	0.74962519	0.01049	0.05491
100303744	Sprr2a2	small proline-rich protein 2A2	0.74962519	0.01364	0.06521
71163	Zfp626	zinc finger protein 626	0.74962519	0.03761	0.1278
17364	Trpm1	transient receptor potential cation channel, subfamily M, member 1	0.74962519	0.1278	0.2924
620760	2900079G21Rik	RIKEN cDNA 2900079G21 gene	0.74962519	0.1546	0.3306
404583	AL118220	expressed sequence AL118220	0.74906367	0.003428	0.02703
56715	Rabgef1	RAB guanine nucleotide exchange factor (GEF) 1	0.74906367	0.2112	0.4068
16565	Kif21b	kinesin family member 21B	0.74850299	0.0001368	0.00406
320609	Fam40b	family with sequence similarity 40, member B	0.74850299	0.0004945	0.008478
56077	Dgke	diacylglycerol kinase, epsilon	0.74850299	0.002354	0.02135
639653	Gm7276	predicted gene 7276	0.74850299	0.006612	0.04084
211378	6720489N17Rik	RIKEN cDNA 6720489N17 gene	0.74850299	0.02926	0.1083
383196	Gm5222	predicted gene 5222	0.74850299	0.04152	0.1363
231470	Fras1	Fraser syndrome 1 homolog (human)	0.74850299	0.04271	0.1387
69863	Ttc39b	tetratricopeptide repeat domain 39B	0.74850299	0.04859	0.1518
751865	Sap25	sin3 associated polypeptide	0.74850299	0.05219	0.1598
72792	2810459M11Rik	RIKEN cDNA 2810459M11 gene	0.74850299	0.05818	0.1719
241041	Gm4956	predicted gene 4956	0.74850299	0.3018	0.511
223773	Zbed4	zinc finger, BED domain containing 4	0.74850299	0.3094	0.5193

213948	Atg9b	ATG9 autophagy related 9 homolog B (S. cerevisiae)	0.74850299	0.4711	0.67
244721	Zfp846	zinc finger protein 846	0.74794316	0.001621	0.01731
75479	1700012D14Rik	RIKEN cDNA 1700012D14 gene	0.74794316	0.004786	0.0333
14105	Srsf10	serine/arginine-rich splicing factor 10	0.74794316	0.01728	0.07583
22776	Zim1	zinc finger, imprinted 1	0.74794316	0.03599	0.1242
328092	6530401N04Rik	RIKEN cDNA 6530401N04 gene	0.74794316	0.03783	0.1282
71643	4930422G04Rik	RIKEN cDNA 4930422G04 gene	0.74794316	0.2391	0.4416
381072	Abca17	ATP-binding cassette, sub-family A (ABC1), member 17	0.74794316	0.3502	0.5605
67196	Ube2t	ubiquitin-conjugating enzyme E2T (putative)	0.74794316	0.4077	0.6144
69181	Dyrk2	dual-specificity tyrosine-(Y)-phosphorylation regulated kinase 2	0.74738416	0.01061	0.05523
53422	Ybx2	Y box protein 2	0.74738416	0.02458	0.09625
226151	Fam178a	family with sequence similarity 178, member A	0.74738416	0.1067	0.2586
72343	2600002B07Rik	RIKEN cDNA 2600002B07 gene	0.74738416	0.1933	0.3832
56809	Gmeb1	glucocorticoid modulatory element binding protein 1	0.74682599	0.01392	0.06613
232855	Zfp772	zinc finger protein 772	0.74682599	0.03335	0.1181
654494	Gm7337	DAZ interacting protein 1 pseudogene	0.74682599	0.09264	0.2362
77940	A930004D18Rik	RIKEN cDNA A930004D18 gene	0.74682599	0.1275	0.292
21665	Tdg	thymine DNA glycosylase	0.74682599	0.1932	0.3832
11735	Ank3	ankyrin 3, epithelial	0.74626866	8.08E-05	0.003088
433956	Heatr2	HEAT repeat containing 2	0.74626866	0.0002039	0.005024
209018	Vps8	vacuolar protein sorting 8 homolog (S. cerevisiae)	0.74626866	0.00228	0.02098
271981	Tbck	TBC1 domain containing kinase	0.74626866	0.002892	0.02426
11777	Ap3s1	adaptor-related protein complex 3, sigma 1 subunit	0.74626866	0.00542	0.03596
78815	5031420N21Rik	RIKEN cDNA 5031420N21 gene	0.74626866	0.007227	0.04349
77795	A930010G16Rik	RIKEN cDNA A930010G16 gene	0.74626866	0.02658	0.1015
76964	2610028H24Rik	RIKEN cDNA 2610028H24 gene	0.74626866	0.2202	0.4194
72397	Rbm12b	RNA binding motif protein 12B	0.74571216	0.0003555	0.006863
17263	Meg3	maternally expressed 3	0.74571216	0.001787	0.0183
72834	2810468N07Rik	RIKEN cDNA 2810468N07 gene	0.74571216	0.008707	0.04873
403174	A930005I04Rik	RIKEN cDNA A930005I04 gene	0.74571216	0.01671	0.0741
19417	Rasgrf1	RAS protein-specific guanine nucleotide-releasing factor 1	0.74571216	0.02698	0.1025
27214	Dbf4	DBF4 homolog (S. cerevisiae)	0.74571216	0.03197	0.1149
207806	Gm608	predicted gene 608	0.74571216	0.07514	0.2051
18150	Npm3	nucleoplasmin 3	0.74571216	0.4024	0.61
13193	Dcx	doublecortin	0.74515648	0.01433	0.06749
70405	Calml3	calmodulin-like 3	0.74515648	0.0164	0.07339
237636	Npc1l1	NPC1-like 1	0.74515648	0.02565	0.09921
68018	Col4a3bp	collagen, type IV, alpha 3 (Goodpasture antigen) binding protein	0.74515648	0.04035	0.1336
319984	Jph4	junctophilin 4	0.74515648	0.06863	0.1925
664987	LOC664987	novel KRAB box and zinc finger, C2H2 type domain containing protein	0.74515648	0.1214	0.2827
231727	B3gnt4	UDP-GlcNAc:betaGal beta-1,3-N-acetylglucosaminyltransferase 4	0.74515648	0.1402	0.3099
17926	Myoc	myocilin	0.74515648	0.2015	0.394
100038875	Gm15450	predicted gene 15450	0.74460164	0.0002233	0.005328
56381	Spen	SPEN homolog, transcriptional regulator (Drosophila)	0.74460164	0.0002995	0.006215
333329	Cngb1	cyclic nucleotide gated channel beta 1	0.74460164	0.0006641	0.01011
22643	Zfp101	zinc finger protein 101	0.74460164	0.0009904	0.01295
240595	Kcnv2	potassium channel, subfamily V, member 2	0.74460164	0.00171	0.01785
66208	Nenf	neuron derived neurotrophic factor	0.74460164	0.005746	0.03729
12647	Chat	choline acetyltransferase	0.74460164	0.009677	0.05216
320858	L3mbtl4	l(3)mbt-like 4 (Drosophila)	0.74460164	0.0821	0.2182

100043450	AA387883	expressed sequence AA387883	0.74460164	0.1035	0.2541
194227	Gm13023	predicted gene 13023	0.74460164	0.2403	0.4428
666340	Gm8054	Rpl21 pseudogene	0.74404762	0.0007579	0.01098
18739	Pitpnm1	phosphatidylinositol transfer protein, membrane-associated 1	0.74404762	0.009251	0.05059
74076	4933406C10Rik	RIKEN cDNA 4933406C10 gene	0.74404762	0.09282	0.2365
381022	Mll2	myeloid/lymphoid or mixed-lineage leukemia 2	0.74404762	0.1012	0.2507
100505283	LOC100505283	60S ribosomal protein L21-like	0.74349442	5.89E-05	0.002619
72930	Ppp2r2b	protein phosphatase 2 (formerly 2A), regulatory subunit B (PR 52), beta isoform	0.74349442	0.0003208	0.006458
69815	Krtcap3	keratinocyte associated protein 3	0.74349442	0.0004672	0.008156
73207	3110068a07rik	RIKEN cDNA 3110068A07 gene	0.74349442	0.005707	0.03713
93835	Amn	amnionless	0.74349442	0.01656	0.07372
77531	Anks1b	ankyrin repeat and sterile alpha motif domain containing 1B	0.74349442	0.01793	0.07788
98682	Mfsd6	major facilitator superfamily domain containing 6	0.74349442	0.03301	0.1174
237868	Sarm1	sterile alpha and HEAT/Armadillo motif containing 1	0.74349442	0.04279	0.1388
18536	Pcm1	pericentriolar material 1	0.74349442	0.08218	0.2183
16687	Krt6a	keratin 6A	0.74349442	0.1876	0.3759
20185	Ncor1	nuclear receptor co-repressor 1	0.74294205	0.0004858	0.0084
229776	Cdc14a	CDC14 cell division cycle 14 homolog A (S. cerevisiae)	0.74294205	0.002231	0.02067
77938	Fam53b	family with sequence similarity 53, member B	0.74294205	0.01877	0.08028
330635	3830612M24	hypothetical protein 3830612M24	0.74294205	0.02199	0.0896
77018	Col25a1	collagen, type XXV, alpha 1	0.74294205	0.02583	0.09955
320091	Ano4	anoctamin 4	0.74294205	0.02736	0.1035
229096	Ythdf3	YTH domain family 3	0.74294205	0.03528	0.1227
320319	E330018D03Rik	RIKEN cDNA E330018D03 gene	0.74294205	0.03994	0.1327
666182	Gm7967	predicted gene 7967	0.74294205	0.05842	0.1724
320277	Spef2	sperm flagellar 2	0.74294205	0.07408	0.2033
13405	Dmd	dystrophin, muscular dystrophy	0.74294205	0.08195	0.2179
227933	Ccdc148	coiled-coil domain containing 148	0.74294205	0.1082	0.261
57425	U90926	cDNA sequence U90926	0.74294205	0.1679	0.3486
100046456	LOC100046456	zinc finger protein 91-like	0.74294205	0.2464	0.4503
99003	Qser1	glutamine and serine rich 1	0.7423905	0.001298	0.01515
20147	Rs1	retinoschisis (X-linked, juvenile) 1 (human)	0.7423905	0.001794	0.01834
67374	Jam2	junction adhesion molecule 2	0.7423905	0.001821	0.01852
240753	Plekha6	pleckstrin homology domain containing, family A member 6	0.7423905	0.003165	0.0258
331513	Gm9927	predicted gene 9927	0.7423905	0.006598	0.04077
69269	Scnm1	sodium channel modifier 1	0.7423905	0.006981	0.04245
70656	5730526G10Rik	RIKEN cDNA 5730526G10 gene	0.7423905	0.007601	0.04477
12874	Cpd	carboxypeptidase D	0.7423905	0.007766	0.04537
71870	Ccdc19	coiled-coil domain containing 19	0.7423905	0.04002	0.1328
213753	Zfp598	zinc finger protein 598	0.7423905	0.09622	0.2421
242235	Lrit3	leucine-rich repeat, immunoglobulin-like and transmembrane domains 3	0.7423905	0.1398	0.3094
630579	Zfp808	zinc finger protein 80	0.7423905	0.161	0.3392
27412	Peg12	paternally expressed 12	0.7423905	0.1758	0.3603
26381	Esrrg	estrogen-related receptor gamma	0.74183976	0.0003213	0.006461
629059	Fam124a	family with sequence similarity 124, member A	0.74183976	0.004267	0.03103
216848	Chd3	chromodomain helicase DNA binding protein 3	0.74183976	0.007783	0.04542
17436	Me1	malic enzyme 1, NADP(+)-dependent, cytosolic	0.74183976	0.04534	0.1445
319317	Snhg11	small nucleolar RNA host gene 11	0.74183976	0.06042	0.1763
17897	Myl3	myosin, light polypeptide 3	0.74183976	0.2678	0.4746
240665	Ccnj	cyclin J	0.74128984	0.0001246	0.003851

239652	Zfp641	zinc finger protein 641	0.74128984	0.0001443	0.004169
665251	Gm7555	predicted gene 7555	0.74128984	0.0005635	0.009194
14426	Galnt4	UDP-N-acetyl-alpha-D-galactosamine:polypeptide N-acetylgalactosaminyltransferase 4	0.74128984	0.001259	0.01493
103712	6330403K07Rik	RIKEN cDNA 6330403K07 gene	0.74128984	0.001354	0.01555
212943	Fam46a	family with sequence similarity 46, member A	0.74128984	0.007937	0.04591
231570	A830010M20Rik	RIKEN cDNA A830010M20 gene	0.74128984	0.01149	0.05846
23919	Insl5	insulin-like 5	0.74128984	0.01162	0.05897
17005	Ltk	leukocyte tyrosine kinase	0.74128984	0.03511	0.1222
76918	3110001N23Rik	RIKEN cDNA 3110001N23 gene	0.74128984	0.03606	0.1243
73884	Zdbf2	zinc finger, DBF-type containing 2	0.74128984	0.03781	0.1282
230822	A330049M08Rik	RIKEN cDNA A330049M08 gene	0.74128984	0.05095	0.157
622139	Gm6289	predicted gene 6289	0.74128984	0.09368	0.2379
17537	Meis3	Meis homeobox 3	0.74128984	0.1124	0.268
654438	A630031M23Rik	Riken cDNA A630031M23 gene	0.74128984	0.1292	0.2946
100043232	3110099E03Rik	RIKEN cDNA 3110099E03 gene	0.74128984	0.1561	0.333
668319	Gm9104	predicted gene 9104	0.74074074	0.0001622	0.004382
94040	Clmn	calmin	0.74074074	0.0002392	0.005541
668110	Syce1l	synaptonemal complex central element protein 1 like	0.74074074	0.001285	0.01507
70646	Naa30	N(alpha)-acetyltransferase 30, NatC catalytic subunit	0.74074074	0.001822	0.01852
100043100	Zfp534	zinc finger protein 534	0.74074074	0.1988	0.3903
70090	2310005E17Rik	RIKEN cDNA 2310005E17 gene	0.74074074	0.3272	0.5374
80334	Kcnip4	Kv channel interacting protein 4	0.74019245	0.004833	0.03346
207777	Bzrap1	benzodiazepine receptor associated protein 1	0.74019245	0.00837	0.04744
20541	Slc8a1	solute carrier family 8 (sodium/calcium exchanger), member 1	0.74019245	0.02113	0.08721
227292	Ctdsp1	CTD (carboxy-terminal domain, RNA polymerase II, polypeptide A) small phosphatase 1	0.74019245	0.05391	0.1635
12545	Cdc7	cell division cycle 7 (S. cerevisiae)	0.74019245	0.09309	0.237
227058	Dnahc7b	dynein, axonemal, heavy chain 7B	0.74019245	0.09913	0.2469
105005	Fam84a	family with sequence similarity 84, member A	0.73964497	0.01822	0.07869
50753	Fbxo8	F-box protein 8	0.73964497	0.01945	0.08224
67531	5730408K05Rik	RIKEN cDNA 5730408K05 gene	0.73964497	0.06986	0.1949
212712	Satb2	special AT-rich sequence binding protein 2	0.73964497	0.09665	0.2428
16589	Uhmk1	U2AF homology motif (UHM) kinase 1	0.73964497	0.133	0.3
18169	Npy6r	neuropeptide Y receptor Y6	0.73964497	0.1339	0.3009
414115	D330050I16Rik	RIKEN cDNA D330050I16 gene	0.73964497	0.1457	0.3184
239420	Csmd3	CUB and Sushi multiple domains 3	0.73964497	0.1634	0.3427
28129	D11Wsu173e	DNA segment, Chr 11, Wayne State University 173, expressed	0.7390983	0.001838	0.01863
71003	Prss41	protease, serine, 41	0.7390983	0.04096	0.1349
245886	Ankrd27	ankyrin repeat domain 27 (VPS9 domain)	0.7390983	0.08929	0.2303
353499	Tmc4	transmembrane channel-like gene family 4	0.73855244	0.003441	0.02711
552901	LOC552901	hypothetical LOC552901	0.73855244	0.0061	0.03869
72745	Tmem161b	transmembrane protein 161B	0.73855244	0.01438	0.06761
432987	Gm5478	predicted pseudogene 5478	0.73855244	0.03275	0.1169
677168	Gm9706	predicted gene 9706	0.73855244	0.1449	0.3172
78088	Ankrd56	ankyrin repeat domain 56	0.73855244	0.4396	0.6423
503851	B930025B16Rik	RIKEN cDNA B930025B16 gene	0.73800738	0.005393	0.03584
72344	Usp36	ubiquitin specific peptidase 36	0.73800738	0.008441	0.04769
11736	Ankfy1	ankyrin repeat and FYVE domain containing 1	0.73800738	0.01399	0.0664
56150	Mad2l1	MAD2 mitotic arrest deficient-like 1 (yeast)	0.73800738	0.04887	0.1525
73852	D3Ertd751e	DNA segment, Chr 3, ERATO Doi 751, expressed	0.73800738	0.1473	0.3203
100044236	Copg2as2	coatomer protein complex, subunit gamma 2, antisense 2	0.73800738	0.1562	0.3331

242721	Klhdc7a	kelch domain containing 7A	0.73800738	0.2414	0.4441
70319	2600006K01Rik	RIKEN cDNA 2600006K01 gene	0.73800738	0.2752	0.483
13618	Ednrb	endothelin receptor type B	0.73746313	0.0001171	0.003744
21418	Tcfap2a	transcription factor AP-2, alpha	0.73746313	0.003994	0.02969
399617	A130019P10Rik	RIKEN cDNA A130019P10 gene	0.73746313	0.004386	0.03154
75426	Igfbpl1	insulin-like growth factor binding protein-like 1	0.73746313	0.004416	0.03165
240255	Ythdc2	YTH domain containing 2	0.73746313	0.009816	0.05273
14806	Grik2	glutamate receptor, ionotropic, kainate 2 (beta 2)	0.73746313	0.01628	0.07306
233406	Prc1	protein regulator of cytokinesis 1	0.73746313	0.03749	0.1275
240726	Slco5a1	solute carrier organic anion transporter family, member 5A1	0.73746313	0.08646	0.2257
97149	C78893	expressed sequence C78893	0.73746313	0.1021	0.2521
622434	Arhgef26	Rho guanine nucleotide exchange factor (GEF) 26	0.73691968	0.000971	0.01273
54161	Copg	coatomer protein complex, subunit gamma	0.73691968	0.001801	0.0184
11766	Ap1g2	adaptor protein complex AP-1, gamma 2 subunit	0.73691968	0.009959	0.05315
224656	Zfp523	zinc finger protein 523	0.73691968	0.03475	0.1213
232409	Clec2e	C-type lectin domain family 2, member e	0.73691968	0.05621	0.1682
20452	St8sia4	ST8 alpha-N-acetyl-neuraminide alpha-2,8-sialyltransferase 4	0.73691968	0.06236	0.1804
77492	8030456M14Rik	RIKEN cDNA 8030456M14 gene	0.73691968	0.1174	0.2758
16922	Phyh	phytanoyl-CoA hydroxylase	0.73637703	0.0003068	0.006295
13411	Dnahc11	dynein, axonemal, heavy chain 11	0.73637703	0.006479	0.04027
16180	Il1rap	interleukin 1 receptor accessory protein	0.73637703	0.01079	0.05579
234138	BC019943	cDNA sequence BC019943	0.73637703	0.01313	0.06376
224792	Gpr116	G protein-coupled receptor 116	0.73637703	0.0257	0.09932
12326	Camk4	calcium/calmodulin-dependent protein kinase IV	0.73637703	0.03709	0.1266
213006	Mfsd4	major facilitator superfamily domain containing 4	0.73583517	0.001054	0.0135
66978	Luc7l	Luc7 homolog (S. cerevisiae)-like	0.73583517	0.001322	0.01536
214084	Slc18a2	solute carrier family 18 (vesicular monoamine), member 2	0.73583517	0.01023	0.05407
232288	Frmd4b	FERM domain containing 4B	0.73583517	0.01037	0.05448
26875	Pclo	piccolo (presynaptic cytomatrix protein)	0.73583517	0.0181	0.07838
100040990	Gm3081	predicted gene 3081	0.73583517	0.02485	0.09695
56399	Akap8	A kinase (PRKA) anchor protein 8	0.73583517	0.03531	0.1227
233038	Nccrp1	non-specific cytotoxic cell receptor protein 1 homolog (zebrafish)	0.73583517	0.1357	0.3033
238057	Gdf7	growth differentiation factor 7	0.73583517	0.2033	0.3966
320271	Scai	suppressor of cancer cell invasion	0.73583517	0.2859	0.4944
19893	Rpgr	retinitis pigmentosa GTPase regulator	0.73529412	0.0007538	0.01098
320840	Negr1	neuronal growth regulator 1	0.73529412	0.005764	0.03734
51922	D2Ertd612e	DNA segment, Chr 2, ERATO Doi 612, expressed	0.73529412	0.008442	0.04769
260315	Nav3	neuron navigator 3	0.73529412	0.0571	0.17
667118	Zbed6	zinc finger, BED domain containing 6	0.73529412	0.08554	0.2242
320360	Ric3	resistance to inhibitors of cholinesterase 3 homolog (C. elegans)	0.73529412	0.0898	0.231
100093715	Gm10404	predicted gene 10404	0.73529412	0.1353	0.3025
319610	9530083O12Rik	RIKEN cDNA 9530083O12 gene	0.73529412	0.3063	0.5158
21778	Tex9	testis expressed gene 9	0.73475386	0.002491	0.02208
170707	Usp48	ubiquitin specific peptidase 48	0.73475386	0.00285	0.02405
15552	Htr1d	5-hydroxytryptamine (serotonin) receptor 1D	0.73475386	0.01444	0.0678
93699	Pcdhgb1	protocadherin gamma subfamily B, 1	0.73475386	0.01957	0.0826
231002	Plekhn1	pleckstrin homology domain containing, family N member 1	0.73475386	0.03285	0.1169
245622	Fam199x	family with sequence similarity 199, X-linked	0.73475386	0.05238	0.1602
230576	Ttc22	tetratricopeptide repeat domain 22	0.73475386	0.06375	0.183
213119	Itga10	integrin, alpha 10	0.73421439	0.0001476	0.004212

320238	A830054O07Rik	RIKEN cDNA A830054O07 gene	0.73421439	0.0007374	0.01089
72413	Kcnmb2	potassium large conductance calcium-activated channel, subfamily M, beta member 2	0.73421439	0.008322	0.04728
71998	Slc25a35	solute carrier family 25, member 35	0.73421439	0.008718	0.04874
227357	Espnl	espin-like	0.73421439	0.02874	0.107
21846	Tie1	tyrosine kinase with immunoglobulin-like and EGF-like domains 1	0.73421439	0.04734	0.1489
240120	Zfp119b	zinc finger protein 119b	0.73421439	0.09277	0.2364
17258	Mef2a	myocyte enhancer factor 2A	0.73421439	0.123	0.2851
67887	Tmem66	transmembrane protein 66	0.73367572	0.0002782	0.00595
240665	Ccnj	cyclin J	0.73367572	0.00355	0.02765
23958	Nr2e3	nuclear receptor subfamily 2, group E, member 3	0.73367572	0.004032	0.02993
192897	Itgb4	integrin beta 4	0.73367572	0.008047	0.04631
269629	Lhfpl3	lipoma HMGIC fusion partner-like 3	0.73367572	0.02583	0.09955
235048	Zfp599	zinc finger protein 599	0.73367572	0.08953	0.2306
77767	Ermn	ermin, ERM-like protein	0.73367572	0.2166	0.4139
18102	Nme1	non-metastatic cells 1, protein (NM23A) expressed in	0.73313783	0.0003016	0.006245
23859	Dlg2	discs, large homolog 2 (Drosophila)	0.73313783	0.0003113	0.006337
77604	C430048L16Rik	RIKEN cDNA C430048L16 gene	0.73313783	0.00148	0.01638
235854	Mrgpra4	MAS-related GPR, member A4	0.73313783	0.007318	0.04376
18164	Nptx1	neuronal pentraxin 1	0.73313783	0.03495	0.1217
73386	1700048M11Rik	RIKEN cDNA 1700048M11 gene	0.73313783	0.04548	0.1448
230249	AI314180	expressed sequence AI314180	0.73313783	0.04764	0.1496
258661	Olfr740	olfactory receptor 740	0.73313783	0.1097	0.2636
22780	Ikzf3	IKAROS family zinc finger 3	0.73313783	0.1304	0.2962
100303732	Gm14431	predicted gene 14431	0.73313783	0.1948	0.3851
319931	A330032B11Rik	RIKEN cDNA A330032B11 gene	0.73313783	0.3015	0.5109
236727	Slc9a7	solute carrier family 9 (sodium/hydrogen exchanger), member 7	0.73260073	0.01068	0.05545
69350	1700003G18Rik	RIKEN cDNA 1700003G18 gene	0.73260073	0.02094	0.08669
64580	Ndst4	N-deacetylase/N-sulfotransferase (heparin glucosaminyl) 4	0.73260073	0.02505	0.09752
13134	Dach1	dachshund 1 (Drosophila)	0.73206442	0.00504	0.03431
330177	Taok3	TAO kinase 3	0.73206442	0.006901	0.04212
320615	Dopey1	dopey family member 1	0.73206442	0.008889	0.04922
71566	9030425E11Rik	RIKEN cDNA 9030425E11 gene	0.73206442	0.03779	0.1282
218693	Paip1	polyadenylate binding protein-interacting protein 1	0.73206442	0.0396	0.1319
76421	1700028K03Rik	RIKEN cDNA 1700028K03 gene	0.73206442	0.1033	0.2538
71876	Mlf1ip	myeloid leukemia factor 1 interacting protein	0.73206442	0.1092	0.2629
210541	Gm581	predicted gene 581	0.73206442	0.1336	0.3005
382209	Rhox3a	reproductive homeobox 3A	0.73206442	0.4875	0.6824
22353	Vip	vasoactive intestinal polypeptide	0.7315289	0.003143	0.0257
72832	Crtac1	cartilage acidic protein 1	0.7315289	0.01043	0.05471
16536	Kcnq2	potassium voltage-gated channel, subfamily Q, member 2	0.7315289	0.01898	0.08092
56427	Tubd1	tubulin, delta 1	0.7315289	0.01922	0.0816
17691	Sik1	salt inducible kinase 1	0.7315289	0.0501	0.1553
16408	Itgal	integrin alpha L	0.7315289	0.06201	0.1797
329509	1810024B03Rik	RIKEN cDNA 1810024B03 gene	0.7315289	0.2645	0.4709
12802	Cnr2	cannabinoid receptor 2 (macrophage)	0.7315289	0.3546	0.5645
72632	2700090O03Rik	RIKEN cDNA 2700090O03 gene	0.73099415	0.003855	0.02913
14573	Gdnf	glial cell line derived neurotrophic factor	0.73099415	0.1494	0.3235
627939	Gm6813	predicted gene 6813	0.73046019	0.0005111	0.008665
107368	Pdzd8	PDZ domain containing 8	0.73046019	0.007843	0.04561
67768	N6amt1	N-6 adenine-specific DNA methyltransferase 1 (putative)	0.73046019	0.01602	0.07226

320116	C030019I05Rik	RIKEN cDNA C030019I05 gene	0.73046019	0.0724	0.2001
235527	Plscr4	phospholipid scramblase 4	0.73046019	0.287	0.4955
271849	Shc4	SHC (Src homology 2 domain containing) family, member 4	0.73046019	0.3178	0.5274
18195	Nsf	N-ethylmaleimide sensitive fusion protein	0.72992701	0.001548	0.01695
231801	Agfg2	ArfGAP with FG repeats 2	0.72992701	0.00311	0.02551
100045796	LOC100045796	cysteine desulfurase, mitochondrial-like	0.72992701	0.007916	0.04585
67640	4930465K10Rik	RIKEN cDNA 4930465K10 gene	0.72992701	0.01782	0.07757
18830	Pltp	phospholipid transfer protein	0.72992701	0.04503	0.1438
100039307	Gm2149	predicted gene 2149	0.72992701	0.07095	0.1969
623279	Dok6	docking protein 6	0.72992701	0.0854	0.2239
67647	4930523C07Rik	RIKEN cDNA 4930523C07 gene	0.72992701	0.221	0.4201
636187	LOC636187	60S ribosomal protein L21-like	0.7293946	0.0001336	0.004003
50796	Dmrt1	doublesex and mab-3 related transcription factor 1	0.7293946	0.003907	0.02936
68168	A930009E08Rik	RIKEN cDNA A930009E08 gene	0.7293946	0.004169	0.03053
100038486	Gm10742	predicted gene 10742	0.7293946	0.02464	0.09642
668030	OTTMUSG00000016609	predicted gene, OTTMUSG00000016609	0.7293946	0.05707	0.1699
73473	Iws1	IWS1 homolog (S. cerevisiae)	0.7293946	0.08096	0.216
233905	Zfp646	zinc finger protein 646	0.7293946	0.1233	0.2856
67809	Fam82a2	family with sequence similarity 82, member A2	0.72886297	0.004272	0.03106
74463	Exoc3l2	exocyst complex component 3-like 2	0.72886297	0.1822	0.3688
76683	1500002F19Rik	RIKEN cDNA 1500002F19 gene	0.72886297	0.2596	0.4653
12337	Capn5	calpain 5	0.72886297	0.3869	0.5955
432838	Gm5460	predicted gene 5460	0.72833212	0.000849	0.01178
338363	6030446N20Rik	RIKEN cDNA 6030446N20 gene	0.72833212	0.003102	0.02547
69504	Zfp932	zinc finger protein 932	0.72833212	0.01648	0.07356
67529	Fgfr1op2	FGFR1 oncogene partner 2	0.72833212	0.06039	0.1763
319386	D130062J10Rik	RIKEN cDNA D130062J10 gene	0.72833212	0.1147	0.2717
17932	Myt1	myelin transcription factor 1	0.72833212	0.2637	0.4698
18106	Cd244	CD244 natural killer cell receptor 2B4	0.72833212	0.3693	0.5783
235599	6430571L13Rik	RIKEN cDNA 6430571L13 gene	0.72780204	0.001861	0.01878
14268	Fn1	fibronectin 1	0.72780204	0.00202	0.01961
330602	Gm5115	predicted gene 5115	0.72780204	0.006015	0.03834
78124	4930458L03Rik	RIKEN cDNA 4930458L03 gene	0.72780204	0.03853	0.1296
67658	4930563F15Rik	RIKEN cDNA 4930563F15 gene	0.72780204	0.09598	0.2419
207596	Thsd4	thrombospondin, type I, domain containing 4	0.72780204	0.13	0.2957
109332	Cdcp1	CUB domain containing protein 1	0.72780204	0.4706	0.6697
327958	Pitpnm3	PITPNM family member 3	0.72727273	0.00577	0.03736
29863	Pde7b	phosphodiesterase 7B	0.72727273	0.005933	0.03801
75718	Vwa5b1	von Willebrand factor A domain containing 5B1	0.72727273	0.05832	0.1722
258436	Olfr458	olfactory receptor 458	0.72727273	0.1491	0.323
225115	Svil	supervillin	0.72674419	0.0001857	0.004709
77371	Sec24a	Sec24 related gene family, member A (S. cerevisiae)	0.72674419	0.008251	0.04713
319385	D130061D10Rik	RIKEN cDNA D130061D10 gene	0.72674419	0.009474	0.05143
13340	Slc29a2	solute carrier family 29 (nucleoside transporters), member 2	0.72674419	0.01743	0.07633
234413	Zfp961	zinc finger protein 961	0.72674419	0.02562	0.09912
435791	Gm13271	predicted gene 13271	0.72674419	0.03892	0.1305
67492	Anubl1	AN1, ubiquitin-like, homolog (Xenopus laevis)	0.72674419	0.03955	0.1318
434232	Iqck	IQ motif containing K	0.72674419	0.1054	0.2568
18071	Nhlh1	nescient helix loop helix 1	0.72674419	0.2882	0.4966
319190	Hist2h2be	histone cluster 2, H2be	0.72621641	0.001279	0.01502

225994	BC016495	cDNA sequence BC016495	0.72621641	0.028	0.1051
70974	Pgm2l1	phosphoglucomutase 2-like 1	0.72621641	0.03094	0.1126
76295	Atp11b	ATPase, class VI, type 11B	0.72621641	0.03324	0.1179
320015	A130082J08Rik	RIKEN cDNA A130082J08 gene	0.72621641	0.1323	0.2988
76196	6330575P09Rik	RIKEN cDNA 6330575P09 gene	0.72621641	0.1786	0.3638
237246	BC022960	cDNA sequence BC022960	0.7256894	0.0001825	0.004665
70799	Cep192	centrosomal protein 192	0.7256894	0.000931	0.01245
208292	Zfp871	zinc finger protein 871	0.7256894	0.002782	0.0237
26875	Pclo	piccolo (presynaptic cytomatrix protein)	0.7256894	0.003705	0.02845
319862	E230008O15Rik	RIKEN cDNA E230008O15 gene	0.7256894	0.004866	0.03358
24046	Scn11a	sodium channel, voltage-gated, type XI, alpha	0.7256894	0.01501	0.06941
70835	Prss22	protease, serine, 22	0.7256894	0.2103	0.4058
434423	Dppa5a	developmental pluripotency associated 5A	0.7256894	0.2515	0.4563
26920	Cep110	centrosomal protein 110	0.72516316	0.0001604	0.004382
330355	Dnahc6	dynein, axonemal, heavy chain 6	0.72516316	0.00174	0.01808
626082	LOC626082	hypothetical protein LOC626082	0.72516316	0.022	0.08962
432460	Gm5423	predicted gene 5423	0.72516316	0.03815	0.1289
19281	Ptprt	protein tyrosine phosphatase, receptor type, T	0.72516316	0.04202	0.1373
66204	Acyp1	acylphosphatase 1, erythrocyte (common) type	0.72516316	0.1683	0.3492
19893	Rpgr	retinitis pigmentosa GTPase regulator	0.72463768	0.001464	0.01626
68428	Steap3	STEAP family member 3	0.72463768	0.003212	0.02608
666529	Gm8149	predicted gene 8149	0.72463768	0.01308	0.06356
194126	Mtmr11	myotubularin related protein 11	0.72463768	0.01726	0.0758
269610	Chd5	chromodomain helicase DNA binding protein 5	0.72411296	0.001021	0.01322
116838	Rims2	regulating synaptic membrane exocytosis 2	0.72411296	0.001105	0.01386
23850	Pappa2	pappalysin 2	0.72411296	0.001236	0.01476
22174	Tyro3	TYRO3 protein tyrosine kinase 3	0.72411296	0.001866	0.01881
12227	Btg2	B-cell translocation gene 2, anti-proliferative	0.72411296	0.01028	0.05422
77799	Sla2	Src-like-adaptor 2	0.72411296	0.0804	0.2151
240041	Zfp945	zinc finger protein 945	0.723589	0.01152	0.05858
319332	B930096F20Rik	RIKEN cDNA B930096F20 gene	0.723589	0.01458	0.06819
319457	C130045F17Rik	RIKEN cDNA C130045F17 gene	0.723589	0.03276	0.1169
544888	Fam181a	family with sequence similarity 181, member A	0.723589	0.03349	0.1184
59308	Emcn	endomucin	0.723589	0.0499	0.1549
19252	Dusp1	dual specificity phosphatase 1	0.723589	0.08162	0.2172
53417	Hif3a	hypoxia inducible factor 3, alpha subunit	0.723589	0.1775	0.3624
381434	C230057A21Rik	RIKEN cDNA C230057A21 gene	0.723589	0.4548	0.6563
241633	Atp8b4	ATPase, class I, type 8B, member 4	0.723589	0.5019	0.6937
171273	Vmn1r217	vomeronasal 1 receptor 217	0.723589	0.5569	0.7366
99003	Qser1	glutamine and serine rich 1	0.7230658	0.000699	0.01051
240038	Gm4944	predicted gene 4944	0.7230658	0.003942	0.02946
66949	Trim59	tripartite motif-containing 59	0.7230658	0.007769	0.04537
18708	Pik3r1	phosphatidylinositol 3-kinase, regulatory subunit, polypeptide 1 (p85 alpha)	0.7230658	0.0364	0.1251
110789	Gpr98	G protein-coupled receptor 98	0.7230658	0.06789	0.1908
74100	Arpp21	cyclic AMP-regulated phosphoprotein, 21	0.7230658	0.1336	0.3005
50720	Sacs	sacsin	0.7230658	0.3287	0.5389
116939	Pnpla3	patatin-like phospholipase domain containing 3	0.72254335	0.001143	0.01408
83924	Gpr137b	G protein-coupled receptor 137B	0.72254335	0.00894	0.04941
233274	Siglech	sialic acid binding Ig-like lectin H	0.72254335	0.07398	0.2031
71535	9030411M13Rik	RIKEN cDNA 9030411M13 gene	0.72254335	0.08929	0.2303

271209	Rp1l1	retinitis pigmentosa 1 homolog (human)-like 1	0.72254335	0.1367	0.3043
100019	Mdn1	midasin homolog (yeast)	0.72254335	0.1557	0.3322
434797	Gm5640	predicted gene 5640	0.72254335	0.2525	0.4574
670833	Gm11213	predicted gene 11213	0.72254335	0.479	0.6755
320870	E330013P08Rik	RIKEN cDNA E330013P08 gene	0.72202166	0.0002989	0.00621
329906	C030014L02	hypothetical protein C030014L02	0.72202166	0.0004741	0.008245
71511	9030601B04Rik	RIKEN cDNA 9030601B04 gene	0.72202166	0.006542	0.04052
231858	Radil	Ras association and DIL domains	0.72202166	0.04617	0.1463
14710	Gngt2	guanine nucleotide binding protein (G protein), gamma transducing activity polypeptide 2	0.72150072	0.001868	0.01883
238247	Arid4a	AT rich interactive domain 4A (RBP1-like)	0.72150072	0.009879	0.0529
77241	9430013L14Rik	RIKEN cDNA 9430013L14 gene	0.72150072	0.01537	0.07062
64075	Smoc1	SPARC related modular calcium binding 1	0.72150072	0.02174	0.08899
69893	2010305A19Rik	RIKEN cDNA 2010305A19 gene	0.72098053	0.004411	0.03164
15898	Icam5	intercellular adhesion molecule 5, telencephalin	0.72098053	0.004906	0.03374
67876	Coq10b	coenzyme Q10 homolog B (S. cerevisiae)	0.72098053	0.004911	0.03377
319254	9630039A02Rik	RIKEN cDNA 9630039A02 gene	0.72098053	0.009151	0.05023
72392	Tmem175	transmembrane protein 175	0.72098053	0.03894	0.1305
208836	Fanci	Fanconi anemia, complementation group I	0.72098053	0.1048	0.2559
66904	Pccb	propionyl Coenzyme A carboxylase, beta polypeptide	0.7204611	0.00371	0.02845
72386	2610035D17Rik	RIKEN cDNA 2610035D17 gene	0.7204611	0.006726	0.04135
242253	Wdr63	WD repeat domain 63	0.7204611	0.0197	0.08293
668319	Gm9104	predicted gene 9104	0.7204611	0.03419	0.1198
100039227	Gm10394	predicted gene 10394	0.7204611	0.05425	0.1643
102142	AI662245	expressed sequence AI662245	0.7204611	0.07581	0.2065
19054	Ppp2r3d	protein phosphatase 2 (formerly 2A), regulatory subunit B'', delta	0.7199424	0.0001164	0.003741
20822	Trove2	TROVE domain family, member 2	0.7199424	0.00131	0.01523
230863	Sh2d5	SH2 domain containing 5	0.7199424	0.002361	0.02139
226418	Yod1	YOD1 OTU deubiquitinating enzyme 1 homologue (S. cerevisiae)	0.7199424	0.003246	0.02623
373864	Col27a1	collagen, type XXVII, alpha 1	0.7199424	0.005609	0.03675
77938	Fam53b	family with sequence similarity 53, member B	0.7199424	0.008796	0.04897
320469	9930014A18Rik	RIKEN cDNA 9930014A18 gene	0.7199424	0.02845	0.1063
353187	Nr1d2	nuclear receptor subfamily 1, group D, member 2	0.7199424	0.04883	0.1524
107338	Gbf1	golgi-specific brefeldin A-resistance factor 1	0.7199424	0.05471	0.1652
433632	Gm5544	predicted gene 5544	0.7199424	0.2475	0.4517
331045	A530083I20Rik	RIKEN cDNA A530083I20 gene	0.71942446	0.0008443	0.01175
77996	D730039F16Rik	RIKEN cDNA D730039F16 gene	0.71942446	0.001034	0.01332
50759	Fbxo16	F-box protein 16	0.71942446	0.01201	0.06015
78672	9530057J20Rik	RIKEN cDNA 9530057J20 gene	0.71942446	0.01494	0.06916
268379	Abca13	ATP-binding cassette, sub-family A (ABC1), member 13	0.71942446	0.02362	0.09384
14205	Figf	c-fos induced growth factor	0.71942446	0.0332	0.1178
381218	4430402I18Rik	RIKEN cDNA 4430402I18 gene	0.71942446	0.06164	0.179
244495	Gm4975	predicted gene 4975	0.71942446	0.1106	0.2649
241638	Prosapip1	ProSAPiP1 protein	0.71942446	0.219	0.4176
12359	Cat	catalase	0.71890726	0.004299	0.03116
68947	Chst8	carbohydrate (N-acetylgalactosamine 4-0) sulfotransferase 8	0.71890726	0.005155	0.03482
53422	Ybx2	Y box protein 2	0.71890726	0.01297	0.06315
78751	Zc3h6	zinc finger CCCH type containing 6	0.71890726	0.04244	0.1382
69863	Ttc39b	tetratricopeptide repeat domain 39B	0.71890726	0.1254	0.2887
14938	Gzma	granzyme A	0.71890726	0.4082	0.6147
241688	6330439K17Rik	RIKEN cDNA 6330439K17 gene	0.7183908	0.002889	0.02426

408067	Zfp874b	zinc finger protein 874b	0.7183908	0.01376	0.06563
56742	Psrc1	proline/serine-rich coiled-coil 1	0.7183908	0.01626	0.07299
70832	4921504A21Rik	RIKEN cDNA 4921504A21 gene	0.7183908	0.02459	0.09629
17125	Smad1	MAD homolog 1 (Drosophila)	0.7183908	0.09388	0.2382
93842	Igsf9	immunoglobulin superfamily, member 9	0.71787509	0.001218	0.01459
399635	D230044B12Rik	RIKEN cDNA D230044B12 gene	0.71787509	0.002317	0.02124
320141	9330156P08Rik	RIKEN cDNA 9330156P08 gene	0.71787509	0.005883	0.0378
381522	E230008N13Rik	RIKEN cDNA E230008N13 gene	0.71787509	0.00737	0.04389
100434	Slc44a1	solute carrier family 44, member 1	0.71787509	0.01029	0.05426
67085	1700024G13Rik	RIKEN cDNA 1700024G13 gene	0.71787509	0.01125	0.05754
235439	Herc1	hect (homologous to the E6-AP (UBE3A) carboxyl terminus) domain and RCC1 (CHC1)-like domain (RLD) 1	0.71787509	0.06411	0.1838
99151	Cercam	cerebral endothelial cell adhesion molecule	0.71787509	0.1434	0.3147
380669	Lin28b	lin-28 homolog B (C. elegans)	0.71736011	0.000548	0.009048
214111	Slc24a1	solute carrier family 24 (sodium/potassium/calcium exchanger), member 1	0.71736011	0.002992	0.02477
235067	Cypt4	cysteine-rich perinuclear theca 4	0.71736011	0.02174	0.08898
67272	Cmtm5	CKLF-like MARVEL transmembrane domain containing 5	0.71736011	0.04822	0.1509
628822	Gm6919	predicted gene 6919	0.71736011	0.08324	0.22
626848	Etohi1	ethanol induced 1	0.71736011	0.09488	0.24
76469	Cmya5	cardiomyopathy associated 5	0.71736011	0.1257	0.289
13384	Mpp3	membrane protein, palmitoylated 3 (MAGUK p55 subfamily member 3)	0.71736011	0.2554	0.4603
19339	Rab3a	RAB3A, member RAS oncogene family	0.71684588	7.00E-05	0.002856
19652	Rbm3	RNA binding motif protein 3	0.71684588	0.0004012	0.0074
73862	4930415F15Rik	RIKEN cDNA 4930415F15 gene	0.71684588	0.005649	0.03692
320644	C130030K03Rik	RIKEN cDNA C130030K03 gene	0.71684588	0.04401	0.1414
232798	Leng8	leukocyte receptor cluster (LRC) member 8	0.71684588	0.05088	0.1568
14201	Fhl3	four and a half LIM domains 3	0.71684588	0.05555	0.1669
22147	Tuba3b	tubulin, alpha 3B	0.71684588	0.05922	0.1739
19270	Ptprg	protein tyrosine phosphatase, receptor type, G	0.71684588	0.06116	0.1778
240869	Zbtb37	zinc finger and BTB domain containing 37	0.71684588	0.07922	0.2131
20646	Snrpn	small nuclear ribonucleoprotein N	0.71633238	0.0007877	0.01127
78656	Brd8	bromodomain containing 8	0.71633238	0.001012	0.01313
11994	Pcdh15	protocadherin 15	0.71633238	0.002954	0.02455
654493	8030494B02Rik	Riken cDNA 8030494B02 gene	0.71633238	0.03852	0.1296
434232	Iqck	IQ motif containing K	0.71633238	0.1962	0.3869
228139	P2rx3	purinergic receptor P2X, ligand-gated ion channel, 3	0.71633238	0.3376	0.5478
20192	Ryr3	ryanodine receptor 3	0.71581961	0.0006913	0.01045
241494	Zfp385b	zinc finger protein 385B	0.71581961	0.0228	0.09167
15216	Hfe	hemochromatosis	0.71581961	0.02657	0.1015
67337	Cstf1	cleavage stimulation factor, 3' pre-RNA, subunit 1	0.71581961	0.07929	0.2132
214230	Pak6	p21 protein (Cdc42/Rac)-activated kinase 6	0.71581961	0.09873	0.2464
170942	Erdr1	erythroid differentiation regulator 1	0.71581961	0.1311	0.2972
68263	Pdhb	pyruvate dehydrogenase (lipoamide) beta	0.71530758	0.005161	0.03484
100294583	Fam150b	family with sequence similarity 150, member B	0.71530758	0.008484	0.04786
28022	D5Wsu152e	DNA segment, Chr 5, Wayne State University 152, expressed	0.71530758	0.2869	0.4954
68267	Slc25a22	solute carrier family 25 (mitochondrial carrier, glutamate), member 22	0.71530758	0.3624	0.5716
75787	4930471M09Rik	RIKEN cDNA 4930471M09 gene	0.71479628	0.03284	0.1169
665155	Srp54b	signal recognition particle 54B	0.71479628	0.1182	0.2772
242851	Gnat3	guanine nucleotide binding protein, alpha transducing 3	0.71479628	0.1917	0.3813
11770	Fabp4	fatty acid binding protein 4, adipocyte	0.71428571	0.002822	0.02391
242443	Grin3a	glutamate receptor ionotropic, NMDA3A	0.71428571	0.00727	0.0436

100503726	LOC100503726	hypothetical protein LOC100503726	0.71428571	0.01592	0.07207
108069	Grm3	glutamate receptor, metabotropic 3	0.71428571	0.02503	0.09752
16428	Itk	IL2-inducible T-cell kinase	0.71428571	0.03132	0.1134
75504	1700013F07Rik	RIKEN cDNA 1700013F07 gene	0.71428571	0.05027	0.1556
16538	Kcns1	K+ voltage-gated channel, subfamily S, 1	0.71428571	0.1531	0.3283
404549	Ifna14	interferon, alpha 14	0.71428571	0.3607	0.5703
12032	Bcan	brevican	0.71377587	0.00376	0.0287
53958	AU035318	EST AU035318	0.71377587	0.00404	0.02996
399623	5930433N17Rik	RIKEN cDNA 5930433N17 gene	0.71377587	0.006887	0.04205
11906	Zfhx3	zinc finger homeobox 3	0.71377587	0.009051	0.04981
71874	2310007B03Rik	RIKEN cDNA 2310007B03 gene	0.71377587	0.02378	0.09422
54216	Pcdh7	protocadherin 7	0.71377587	0.0309	0.1124
258484	Olfr1410	olfactory receptor 1410	0.71377587	0.07499	0.2049
330554	Fan1	FANCD2/FANCI-associated nuclease 1	0.71377587	0.07647	0.2079
241175	Cntnap5b	contactin associated protein-like 5B	0.71377587	0.1841	0.371
21854	Timm17a	translocase of inner mitochondrial membrane 17a	0.71326676	0.0004402	0.007865
11994	Pcdh15	protocadherin 15	0.71326676	0.02111	0.08721
99031	Osbpl6	oxysterol binding protein-like 6	0.71326676	0.04032	0.1335
381546	Ccdc24	coiled-coil domain containing 24	0.71326676	0.05266	0.1607
59069	Tpm3	tropomyosin 3, gamma	0.71326676	0.05432	0.1645
238722	Zfp72	zinc finger protein 72	0.71326676	0.1201	0.2805
19652	Rbm3	RNA binding motif protein 3	0.71275837	0.003463	0.0272
100233208	Gm10778	predicted gene 10778	0.71275837	0.1666	0.3473
56640	Klk4	kallikrein related-peptidase 4 (prostase, enamel matrix, prostate)	0.71275837	0.3773	0.5864
80718	Rab27b	RAB27b, member RAS oncogene family	0.71225071	0.004406	0.03164
433387	Rpl21-ps1	ribosomal protein 21, pseudogene 1	0.71225071	0.02745	0.1038
223770	Brd1	bromodomain containing 1	0.71225071	0.07701	0.2088
56522	Papolb	poly (A) polymerase beta (testis specific)	0.71225071	0.09885	0.2465
320693	C130021H21Rik	RIKEN cDNA C130021H21 gene	0.71225071	0.3025	0.5116
73407	Tepp	testis, prostate and placenta expressed	0.71174377	0.001439	0.01611
215418	Csrnp1	cysteine-serine-rich nuclear protein 1	0.71174377	0.001767	0.01823
11640	Akap1	A kinase (PRKA) anchor protein 1	0.71174377	0.008795	0.04897
76421	1700028K03Rik	RIKEN cDNA 1700028K03 gene	0.71174377	0.01278	0.06258
328643	Vwa5b2	von Willebrand factor A domain containing 5B2	0.71174377	0.04679	0.1475
52682	D11Ertd726e	DNA segment, Chr 11, ERATO Doi 726, expressed	0.71174377	0.1059	0.2573
100042948	Gm15760	mitochondrial ribosomal protein S18B pseudogene	0.71174377	0.2932	0.502
53972	Ngef	neuronal guanine nucleotide exchange factor	0.71123755	0.0002879	0.006071
237860	Ssh2	slingshot homolog 2 (Drosophila)	0.71123755	0.04507	0.1439
12561	Cdh4	cadherin 4	0.71123755	0.09053	0.2326
27493	A230006K03Rik	RIKEN cDNA A230006K03 gene	0.71123755	0.1884	0.3765
257917	Olfr314	olfactory receptor 314	0.71123755	0.4527	0.6549
70103	Znhit1	zinc finger, HIT domain containing 1	0.71073205	0.006727	0.04135
381290	Atp2b4	ATPase, Ca++ transporting, plasma membrane 4	0.71073205	0.03171	0.1143
67419	3632451O06Rik	RIKEN cDNA 3632451O06 gene	0.71073205	0.05181	0.1591
217116	Spata20	spermatogenesis associated 20	0.71073205	0.1897	0.3784
68233	1700125D06Rik	RIKEN cDNA 1700125D06 gene	0.71073205	0.282	0.49
105243	Slc9a3	solute carrier family 9 (sodium/hydrogen exchanger), member 3	0.71022727	0.01215	0.06055
320595	Phf8	PHD finger protein 8	0.71022727	0.02338	0.0931
100040929	Gm10362	predicted gene 10362	0.71022727	0.3533	0.5633
16939	Lor	loricrin	0.70972321	0.00121	0.01454

20686	Spa17	sperm autoantigenic protein 17	0.70972321	0.003195	0.02602
78088	Ankrd56	ankyrin repeat domain 56	0.70972321	0.06565	0.1867
75180	4930538K18Rik	RIKEN cDNA 4930538K18 gene	0.70972321	0.09304	0.2369
75572	Acyp2	acylphosphatase 2, muscle type	0.70972321	0.4074	0.6142
243911	Kirrel2	kin of IRRE like 2 (Drosophila)	0.70921986	0.003828	0.02898
319454	A430071A18Rik	RIKEN cDNA A430071A18 gene	0.70921986	0.02281	0.09167
100504289	LOC100504289	hypothetical LOC100504289	0.70921986	0.0255	0.09882
637900	Gm12618	predicted gene 12618	0.70921986	0.03273	0.1168
18704	Pik3c2a	phosphatidylinositol 3-kinase, C2 domain containing, alpha polypeptide	0.70921986	0.03281	0.1169
21946	Pglyrp1	peptidoglycan recognition protein 1	0.70921986	0.1136	0.27
546336	Prrg1	proline rich Gla (G-carboxyglutamic acid) 1	0.70921986	0.2108	0.4063
675440	Gm13430	predicted gene 13430	0.70921986	0.3827	0.5918
100038680	Gm1971	predicted gene 1971	0.70871722	0.009048	0.04981
100039239	Gm2115	predicted gene 2115	0.70871722	0.01142	0.05823
72580	Zufsp	zinc finger with UFM1-specific peptidase domain	0.70871722	0.02062	0.08572
76357	Trmt5	TRM5 tRNA methyltransferase 5 homolog (S. cerevisiae)	0.7082153	6.89E-05	0.002836
76093	5830469G19Rik	RIKEN cDNA 5830469G19 gene	0.7082153	0.00215	0.02024
215821	D10Bwg1379e	DNA segment, Chr 10, Brigham & Women's Genetics 1379 expressed	0.7082153	0.03555	0.1233
67620	Lrp2bp	Lrp2 binding protein	0.7082153	0.05818	0.1719
211329	Ncoa7	nuclear receptor coactivator 7	0.7082153	0.07441	0.204
216810	Tom1l2	target of myb1-like 2 (chicken)	0.7082153	0.0786	0.212
16627	Klra1	killer cell lectin-like receptor, subfamily A, member 1	0.7082153	0.3461	0.5567
20462	Tra2b	transformer 2 beta homolog (Drosophila)	0.7082153	0.4901	0.6844
17910	Myo15	myosin XV	0.70771408	0.008874	0.04919
69207	Srsf11	serine/arginine-rich splicing factor 11	0.70771408	0.02795	0.105
140477	Dmbx1	diencephalon/mesencephalon homeobox 1	0.70771408	0.03085	0.1123
72413	Kcnmb2	potassium large conductance calcium-activated channel, subfamily M, beta member 2	0.70771408	0.03672	0.1257
11554	Adrb1	adrenergic receptor, beta 1	0.70721358	0.0007207	0.01074
18549	Pcsk2	proprotein convertase subtilisin/kexin type 2	0.70721358	0.003304	0.02641
77481	C030048H21Rik	RIKEN cDNA C030048H21 gene	0.70721358	0.04103	0.135
83671	Sytl2	synaptotagmin-like 2	0.70671378	0.0008506	0.01179
11847	Arg2	arginase type II	0.70671378	0.001293	0.01513
57908	Zfp318	zinc finger protein 318	0.70671378	0.001611	0.01724
99650	4933434E20Rik	RIKEN cDNA 4933434E20 gene	0.70671378	0.01129	0.0577
230594	Zcchc11	zinc finger, CCHC domain containing 11	0.70671378	0.03997	0.1328
667127	Gm15847	predicted gene 15847	0.70671378	0.1266	0.2905
73349	1700042G15Rik	RIKEN cDNA 1700042G15 gene	0.70671378	0.2398	0.4422
58181	Il20	interleukin 20	0.70671378	0.3844	0.5936
330503	Gm5113	predicted gene 5113	0.70621469	5.92E-05	0.002619
269053	Gpr152	G protein-coupled receptor 152	0.70621469	0.001582	0.01708
101100	Ttll3	tubulin tyrosine ligase-like family, member 3	0.70621469	0.01569	0.07144
19270	Ptprg	protein tyrosine phosphatase, receptor type, G	0.70621469	0.01929	0.08177
22349	Vil1	villin 1	0.7057163	2.91E-05	0.001747
211623	Plac9	placenta specific 9	0.7057163	0.0008094	0.01147
231214	Cc2d2a	coiled-coil and C2 domain containing 2A	0.7057163	0.00206	0.01978
237859	Ccdc55	coiled-coil domain containing 55	0.7057163	0.006636	0.04092
399612	9630010G10Rik	RIKEN cDNA 9630010G10 gene	0.7057163	0.01557	0.07122
231858	Radil	Ras association and DIL domains	0.7057163	0.02686	0.1022
269198	Nbeal1	neurobeachin like 1	0.7057163	0.08779	0.2281
67295	Rab3c	RAB3C, member RAS oncogene family	0.7057163	0.1311	0.2971

16688	Krt6b	keratin 6B	0.7057163	0.1738	0.3574
67703	Kirrel3	kin of IRRE like 3 (Drosophila)	0.7057163	0.4452	0.6479
319670	Eml5	echinoderm microtubule associated protein like 5	0.70521862	0.001344	0.01549
93882	Pcdhb11	protocadherin beta 11	0.70521862	0.00217	0.02032
52850	Sgsm1	small G protein signaling modulator 1	0.70521862	0.0885	0.2291
331188	Zfp781	zinc finger protein 781	0.70521862	0.08861	0.2293
15528	Hspe1	heat shock protein 1 (chaperonin 10)	0.70472163	0.0002581	0.005776
21463	Tcp11	t-complex protein 11	0.70472163	0.01484	0.06888
212943	Fam46a	family with sequence similarity 46, member A	0.70472163	0.02285	0.09179
381406	2810408M09Rik	RIKEN cDNA 2810408M09 gene	0.70472163	0.0551	0.166
51966	D11Bhm181e	DNA segment, Chr 11, Boehm 181, expressed	0.70472163	0.07094	0.1969
432769	Zfp708	zinc finger protein 708	0.70472163	0.08977	0.231
74762	Mdga1	MAM domain containing glycosylphosphatidylinositol anchor 1	0.70422535	0.004567	0.03233
233405	Vps33b	vacuolar protein sorting 33B (yeast)	0.70422535	0.00899	0.04962
330119	Adamts3	a disintegrin-like and metallopeptidase (reprolysin type) with thrombospondin type 1 motif, 3	0.70422535	0.01355	0.06487
14406	Gabrg2	gamma-aminobutyric acid (GABA) A receptor, subunit gamma 2	0.70372977	0.0009057	0.01222
320878	Mical2	microtubule associated monoxygenase, calponin and LIM domain containing 2	0.70372977	0.007387	0.04394
13521	Slc26a2	solute carrier family 26 (sulfate transporter), member 2	0.70372977	0.009053	0.04981
54120	Gipc2	GIPC PDZ domain containing family, member 2	0.70372977	0.01609	0.07241
73010	Gpr22	G protein-coupled receptor 22	0.70372977	0.02198	0.08959
21954	Tnni3	troponin I, cardiac 3	0.70372977	0.05491	0.1657
69211	2310081J21Rik	RIKEN cDNA 2310081J21 gene	0.70323488	0.1549	0.331
338352	Nell1	NEL-like 1 (chicken)	0.70274069	0.003396	0.02692
100043772	Gm4636	predicted gene 4636	0.70274069	0.008802	0.04898
94090	Trim9	tripartite motif-containing 9	0.70274069	0.01578	0.0717
245650	Gucy2f	guanylate cyclase 2f	0.70274069	0.01675	0.07422
235344	Sik2	salt inducible kinase 2	0.70274069	0.2865	0.4949
71801	Plekhf2	pleckstrin homology domain containing, family F (with FYVE domain) member 2	0.70224719	1.96E-05	0.001424
328456	6720482D04	hypothetical protein 6720482D04	0.70224719	0.002749	0.02359
233057	Zfp940	zinc finger protein 940	0.70224719	0.005228	0.03515
75209	Sv2c	synaptic vesicle glycoprotein 2c	0.70224719	0.01009	0.05366
22192	Ube2m	ubiquitin-conjugating enzyme E2M (UBC12 homolog, yeast)	0.70224719	0.01767	0.07712
13592	Ebf2	early B-cell factor 2	0.70224719	0.3226	0.5323
100042679	Gm16386	predicted gene 16386	0.70224719	0.3367	0.5471
70432	Rufy2	RUN and FYVE domain-containing 2	0.70175439	0.002073	0.01983
233060	Zfp382	zinc finger protein 382	0.70175439	0.00885	0.04914
100505352	LOC100505352	hypothetical LOC100505352	0.70175439	0.02107	0.08712
29865	Cabp5	calcium binding protein 5	0.70175439	0.165	0.3451
69131	Cdk12	cyclin-dependent kinase 12	0.70126227	0.01476	0.06874
70592	5730480H06Rik	RIKEN cDNA 5730480H06 gene	0.70077085	0.002214	0.02056
243574	Kbtbd8	kelch repeat and BTB (POZ) domain containing 8	0.70077085	0.003963	0.02957
241230	St8sia6	ST8 alpha-N-acetyl-neuraminide alpha-2,8-sialyltransferase 6	0.70077085	0.01369	0.0654
233781	Xylt1	xylosyltransferase 1	0.70077085	0.03529	0.1227
104362	Meig1	meiosis expressed gene 1	0.70028011	0.0003474	0.006763
24067	Srp54a	signal recognition particle 54A	0.70028011	0.000883	0.01208
319711	E230029C05Rik	RIKEN cDNA E230029C05 gene	0.70028011	0.00517	0.03489
23827	Bpnt1	bisphosphate 3'-nucleotidase 1	0.70028011	0.01232	0.06104
239857	Cadm2	cell adhesion molecule 2	0.70028011	0.05716	0.17
22363	Vpreb2	pre-B lymphocyte gene 2	0.70028011	0.3628	0.5719
328949	Mcc	mutated in colorectal cancers	0.69979006	0.007732	0.04528

319615	Zfp944	zinc finger protein 944	0.69979006	0.009497	0.05149
100039210	Gm2102	predicted gene 2102	0.69979006	0.01951	0.08243
19649	Robo3	roundabout homolog 3 (Drosophila)	0.69979006	0.03327	0.1179
320358	C430003N24Rik	RIKEN cDNA C430003N24 gene	0.6993007	0.001689	0.0177
71369	Krtap16-10	keratin associated protein 16-10	0.6993007	0.01928	0.08176
66793	Efcab1	EF hand calcium binding domain 1	0.6993007	0.1287	0.2938
76798	2410137F16Rik	RIKEN cDNA 2410137F16 gene	0.69881202	0.0008009	0.01139
69327	1700007K13Rik	RIKEN cDNA 1700007K13 gene	0.69881202	0.0128	0.06262
19222	Ptgir	prostaglandin I receptor (IP)	0.69881202	0.01741	0.07626
68556	Uckl1	uridine-cytidine kinase 1-like 1	0.69881202	0.01973	0.08303
52145	D9Ertd720e	DNA segment, Chr 9, ERATO Doi 720, expressed	0.69881202	0.1538	0.3294
76251	0610007P08Rik	RIKEN cDNA 0610007P08 gene	0.69881202	0.2086	0.4034
74264	1700045I19Rik	ring finger protein 138 pseudogene	0.69832402	0.01978	0.08318
68189	5330431K02Rik	RIKEN cDNA 5330431K02 gene	0.69783671	0.0002342	0.005471
98878	Ehd4	EH-domain containing 4	0.69783671	0.0003355	0.006637
12865	Cox7a1	cytochrome c oxidase, subunit VIIa 1	0.69783671	0.002511	0.0222
76173	6330531I01Rik	RIKEN cDNA 6330531I01 gene	0.69783671	0.005573	0.03661
56419	Diap3	diaphanous homolog 3 (Drosophila)	0.69783671	0.01215	0.06055
381112	Arhgef33	Rho guanine nucleotide exchange factor (GEF) 33	0.69783671	0.02474	0.09664
403344	C230006B20	hypothetical LOC403344	0.69783671	0.04286	0.1389
170676	Peg10	paternally expressed 10	0.69783671	0.2214	0.4204
73884	Zdbf2	zinc finger, DBF-type containing 2	0.69783671	0.2315	0.4324
16502	Kcnc1	potassium voltage gated channel, Shaw-related subfamily, member 1	0.69735007	0.001045	0.01342
20017	Polr1b	polymerase (RNA) I polypeptide B	0.69735007	0.001122	0.01394
71799	Ptcd1	pentatricopeptide repeat domain 1	0.69735007	0.003932	0.02944
231760	Rimbp2	RIMS binding protein 2	0.69735007	0.02446	0.09592
353208	Zfp931	zinc finger protein 931	0.69735007	0.03955	0.1318
14255	Flt3	FMS-like tyrosine kinase 3	0.69686411	0.0005638	0.009194
78553	E130118H10Rik	RIKEN cDNA E130118H10 gene	0.69686411	0.004377	0.03151
319636	Fsd1l	fibronectin type III and SPRY domain containing 1-like	0.69686411	0.01465	0.06841
94109	Csmd1	CUB and Sushi multiple domains 1	0.69686411	0.0166	0.07383
216971	BC017647	cDNA sequence BC017647	0.69637883	0.03469	0.1211
258525	Olfr1170	olfactory receptor 1170	0.69637883	0.1734	0.357
170735	Arr3	arrestin 3, retinal	0.69589422	0.0005133	0.008671
244281	Myo16	myosin XVI	0.69589422	0.001077	0.01361
19893	Rpgr	retinitis pigmentosa GTPase regulator	0.69589422	0.01885	0.08051
208583	Nek11	NIMA (never in mitosis gene a)-related expressed kinase 11	0.69589422	0.1071	0.2591
619309	A630081J09Rik	RIKEN cDNA A630081J09 gene	0.69541029	0.0008749	0.01202
233066	AI428936	expressed sequence AI428936	0.69541029	0.001875	0.01887
18037	Nfkbie	nuclear factor of kappa light polypeptide gene enhancer in B-cells inhibitor, epsilon	0.69541029	0.006818	0.04177
620401	Gm16442	predicted gene 16442	0.69541029	0.0392	0.131
18315	Olfr18	olfactory receptor 18	0.69541029	0.1819	0.3684
53379	Hnrnpa2b1	heterogeneous nuclear ribonucleoprotein A2/B1	0.69541029	0.1866	0.3744
67225	Rnpc3	RNA-binding region (RNP1, RRM) containing 3	0.69492703	0.04229	0.1379
75619	Fastkd2	FAST kinase domains 2	0.69492703	0.05821	0.1719
269615	Plch2	phospholipase C, eta 2	0.69492703	0.08268	0.2191
57390	Psors1c2	psoriasis susceptibility 1 candidate 2 (human)	0.69492703	0.09063	0.2327
403171	Banf2	barrier to autointegration factor 2	0.69492703	0.3087	0.5185
279610	E530001F21Rik	RIKEN cDNA E530001F21 gene	0.69444444	0.006996	0.04253
100504857	LOC100504857	hypothetical LOC100504857	0.69444444	0.007975	0.04605

215474	Sec22c	SEC22 vesicle trafficking protein homolog C (S. cerevisiae)	0.69444444	0.01286	0.06282
626123	9130004C02Rik	RIKEN cDNA 9130004C02 gene	0.69444444	0.02471	0.09658
16449	Jag1	jagged 1	0.69396253	0.0001454	0.004169
54711	Plagl2	pleiomorphic adenoma gene-like 2	0.69348128	0.01774	0.07733
331461	Il1rapl1	interleukin 1 receptor accessory protein-like 1	0.69348128	0.08936	0.2304
50770	Atp11a	ATPase, class VI, type 11A	0.69348128	0.137	0.3046
104362	Meig1	meiosis expressed gene 1	0.69300069	0.0004129	0.007561
665211	Gm14326	predicted gene 14326	0.69300069	0.05644	0.1686
17082	Il1rl1	interleukin 1 receptor-like 1	0.69300069	0.1346	0.3015
381318	Nsl1	NSL1, MIND kinetochore complex component, homolog (S. cerevisiae)	0.69252078	0.0006166	0.009693
18784	Pla2g5	phospholipase A2, group V	0.69252078	0.003405	0.02694
71780	Isyna1	myo-inositol 1-phosphate synthase A1	0.69252078	0.005581	0.03662
224997	Dlgap1	discs, large (Drosophila) homolog-associated protein 1	0.69252078	0.01364	0.06521
77929	Yipf6	Yip1 domain family, member 6	0.69252078	0.05842	0.1724
382843	Rpl21-ps4	ribosomal protein L21, pseudogene 4	0.69204152	0.0003112	0.006337
17847	Usp34	ubiquitin specific peptidase 34	0.69204152	0.0205	0.08534
72883	2900035I09Rik	RIKEN cDNA 2900035I09 gene	0.69204152	0.03153	0.1139
66573	Dzip1	DAZ interacting protein 1	0.69204152	0.05557	0.1669
408067	Zfp874b	zinc finger protein 874b	0.69204152	0.0764	0.2078
170826	Ppargc1b	peroxisome proliferative activated receptor, gamma, coactivator 1 beta	0.69204152	0.09207	0.2353
66926	Trmt6	tRNA methyltransferase 6 homolog (S. cerevisiae)	0.69204152	0.1038	0.2544
214552	Cep164	centrosomal protein 164	0.69156293	0.004092	0.0302
214106	4933430I17Rik	RIKEN cDNA 4933430I17 gene	0.69156293	0.01142	0.05823
100038413	D130050E23Rik	RIKEN cDNA D130050E23 gene	0.69156293	0.01426	0.0673
57778	Fmnl1	formin-like 1	0.69156293	0.2206	0.4196
239102	Zfhx2	zinc finger homeobox 2	0.69156293	0.2786	0.4868
93969	Klra22	killer cell lectin-like receptor subfamily A, member 22	0.69156293	0.3674	0.5765
106622	AI605517	expressed sequence AI605517	0.691085	0.001609	0.01723
17708	COX1	cytochrome c oxidase subunit I	0.691085	0.004408	0.03164
76022	Gon4l	gon-4-like (C.elegans)	0.691085	0.01375	0.0656
22144	Tuba3a	tubulin, alpha 3A	0.691085	0.01967	0.08286
78833	Gins3	GINS complex subunit 3 (Psf3 homolog)	0.691085	0.02189	0.08936
100505360	LOC100505360	putative transposase element L1Md-A101/L1Md-A102/L1Md-A2-like	0.691085	0.04943	0.1537
240174	Thada	thyroid adenoma associated	0.691085	0.09866	0.2463
66797	Cntnap2	contactin associated protein-like 2	0.691085	0.2265	0.4262
252864	Dusp15	dual specificity phosphatase-like 15	0.691085	0.4822	0.6782
627939	Gm6813	predicted gene 6813	0.69060773	0.0003857	0.007246
226245	9930023K05Rik	RIKEN cDNA 9930023K05 gene	0.69060773	0.09671	0.2429
545861	Gm5878	predicted gene 5878	0.69060773	0.2798	0.488
20503	Slc16a7	solute carrier family 16 (monocarboxylic acid transporters), member 7	0.69060773	0.3213	0.5311
30056	Timm9	translocase of inner mitochondrial membrane 9 homolog (yeast)	0.69013112	7.97E-05	0.003065
71972	Dnmbp	dynamin binding protein	0.69013112	0.002651	0.02302
545861	Gm5878	predicted gene 5878	0.69013112	0.3115	0.5214
100502931	LOC100502931	hypothetical protein LOC100502931	0.69013112	0.3592	0.569
235048	Zfp599	zinc finger protein 599	0.68965517	0.04916	0.1532
230779	Serinc2	serine incorporator 2	0.68965517	0.06297	0.1815
109032	Sp110	Sp110 nuclear body protein	0.68917988	0.004542	0.03225
12428	Ccna2	cyclin A2	0.68870523	0.0003388	0.006681
71781	Slc16a14	solute carrier family 16 (monocarboxylic acid transporters), member 14	0.68870523	0.0005663	0.009215
16765	Stmn1	stathmin 1	0.68870523	0.0005714	0.009267

625175	1700028E10Rik	RIKEN cDNA 1700028E10 gene	0.68870523	0.0007314	0.01084
52882	Rgs7bp	regulator of G-protein signalling 7 binding protein	0.68870523	0.001822	0.01852
20400	Sh2d1a	SH2 domain protein 1A	0.68870523	0.002529	0.02226
319622	Itpripl2	inositol 1,4,5-triphosphate receptor interacting protein-like 2	0.68870523	0.00335	0.02668
56397	Morf4l2	mortality factor 4 like 2	0.68870523	0.07269	0.2006
328232	Gfod1	glucose-fructose oxidoreductase domain containing 1	0.68870523	0.5528	0.7333
667230	Gm8526	predicted gene 8526	0.68823125	0.0002533	0.005731
433215	BC048609	cDNA sequence BC048609	0.68823125	0.04152	0.1363
319377	D130012P04Rik	RIKEN cDNA D130012P04 gene	0.68823125	0.08676	0.2262
76862	4833405L11Rik	RIKEN cDNA 4833405L11 gene	0.68775791	0.0146	0.06821
74554	9130002K18Rik	RIKEN cDNA 9130002K18 gene	0.68775791	0.0394	0.1315
382090	4922501C03Rik	RIKEN cDNA 4922501C03 gene	0.68775791	0.04844	0.1514
102991	AU022751	expressed sequence AU022751	0.68775791	0.1202	0.2806
213819	Casd1	CAS1 domain containing 1	0.68775791	0.3152	0.5246
243753	2010107G12Rik	RIKEN cDNA 2010107G12 gene	0.68728522	0.0008071	0.01144
15207	Hes3	hairy and enhancer of split 3 (Drosophila)	0.68728522	0.006004	0.03832
271697	Cdk15	cyclin-dependent kinase 15	0.68728522	0.007462	0.0442
234413	Zfp961	zinc finger protein 961	0.68728522	0.0088	0.04898
12814	Col11a1	collagen, type XI, alpha 1	0.68728522	0.05515	0.1661
320262	A830073O21Rik	RIKEN cDNA A830073O21 gene	0.68728522	0.1984	0.3898
15959	Ifit3	interferon-induced protein with tetratricopeptide repeats 3	0.68681319	0.005849	0.03769
60532	Wtap	Wilms' tumour 1-associating protein	0.68681319	0.006774	0.04156
66573	Dzip1	DAZ interacting protein 1	0.68681319	0.007079	0.04289
100036524	C430017C20Rik	RIKEN cDNA C430017C20 gene	0.68681319	0.0123	0.06099
16665	Krt15	keratin 15	0.68681319	0.0225	0.09084
545238	G430049J08Rik	RIKEN cDNA G430049J08 gene	0.68681319	0.04149	0.1363
320669	A230092J17Rik	RIKEN cDNA A230092J17 gene	0.6863418	0.04073	0.1344
70956	Tex19.2	testis expressed gene 19.2	0.6863418	0.2742	0.4821
29806	Limd1	LIM domains containing 1	0.68587106	0.0006427	0.009975
11790	Speg	SPEG complex locus	0.68587106	0.0007347	0.01087
224840	Treml4	triggering receptor expressed on myeloid cells-like 4	0.68587106	0.003782	0.02881
233064	Wdr62	WD repeat domain 62	0.68587106	0.03531	0.1227
67429	Nudcd1	NudC domain containing 1	0.68587106	0.4852	0.6805
98256	Kmo	kynurenine 3-monooxygenase (kynurenine 3-hydroxylase)	0.68540096	0.0001759	0.004568
77035	Jmjd5	jumonji domain containing 5	0.68493151	0.0002081	0.005074
12936	Pcdha4	protocadherin alpha 4	0.68493151	0.004803	0.03336
94246	Arid4b	AT rich interactive domain 4B (RBP1-like)	0.68493151	0.01021	0.05407
74183	2310042D19Rik	RIKEN cDNA 2310042D19 gene	0.68493151	0.02925	0.1083
60345	Nrip2	nuclear receptor interacting protein 2	0.6844627	0.003186	0.02596
237987	Otop2	otopetrin 2	0.6844627	0.1569	0.3339
100041412	Gm11938	predicted gene 11938	0.6844627	0.2065	0.4009
383032	Gm5215	predicted gene 5215	0.6844627	0.2613	0.4672
269109	Dpp10	dipeptidylpeptidase 10	0.68399453	0.001487	0.01642
381591	L1td1	LINE-1 type transposase domain containing 1	0.68399453	0.01339	0.06449
238386	Btbd7	BTB (POZ) domain containing 7	0.68399453	0.03919	0.131
67472	Mtfr1	mitochondrial fission regulator 1	0.683527	7.70E-05	0.003014
73130	Tmed5	transmembrane emp24 protein transport domain containing 5	0.683527	0.00109	0.01374
68817	Ddi2	DNA-damage inducible protein 2	0.683527	0.02799	0.1051
319317	Snhg11	small nucleolar RNA host gene 11	0.68306011	0.006977	0.04245
170651	Krtap16-1	keratin associated protein 16-1	0.68306011	0.02939	0.1086

100040462	Mndal	myeloid nuclear differentiation antigen like	0.68259386	0.006354	0.03984
239170	Fam160b2	family with sequence similarity 160, member B2	0.68259386	0.007324	0.04377
328092	6530401N04Rik	RIKEN cDNA 6530401N04 gene	0.68259386	0.06941	0.1941
224023	Klhl22	kelch-like 22 (Drosophila)	0.68259386	0.07813	0.211
227699	Nup188	nucleoporin 188	0.68259386	0.1689	0.35
75795	4930455D15Rik	RIKEN cDNA 4930455D15 gene	0.68259386	0.4015	0.6091
192663	Abcg4	ATP-binding cassette, sub-family G (WHITE), member 4	0.68212824	0.001179	0.01434
54652	Cacna1f	calcium channel, voltage-dependent, alpha 1F subunit	0.68212824	0.01054	0.05506
13405	Dmd	dystrophin, muscular dystrophy	0.68212824	0.01103	0.05663
93681	Zfp192	zinc finger protein 192	0.68212824	0.0155	0.07105
16526	Kcnk2	potassium channel, subfamily K, member 2	0.68212824	0.0233	0.09289
17248	Mdm4	transformed mouse 3T3 cell double minute 4	0.68212824	0.04678	0.1475
70567	5730455O13Rik	RIKEN cDNA 5730455O13 gene	0.68166326	0.0006459	0.009989
414326	D930050J11	hypothetical D930050J11	0.68166326	0.0008884	0.01209
237010	Klhl4	kelch-like 4 (Drosophila)	0.68166326	0.01912	0.08126
71334	5430416G10Rik	RIKEN cDNA 5430416G10 gene	0.68166326	0.03908	0.1307
668923	Zfp442	zinc finger protein 442	0.68166326	0.06557	0.1865
26877	B3galt1	UDP-Gal:betaGlcNAc beta 1,3-galactosyltransferase, polypeptide 1	0.68119891	0.0001224	0.003816
244853	Fam55d	family with sequence similarity 55, member D	0.68119891	0.0001357	0.004044
212326	Fam149a	family with sequence similarity 149, member A	0.68119891	0.0001615	0.004382
66457	2810002D19Rik	RIKEN cDNA 2810002D19 gene	0.68119891	0.01163	0.05901
76022	Gon4l	gon-4-like (C.elegans)	0.68119891	0.08466	0.2226
14281	Fos	FBJ osteosarcoma oncogene	0.68073519	0.01844	0.07936
69131	Cdk12	cyclin-dependent kinase 12	0.68073519	0.02862	0.1067
109820	Pgc	progastricsin (pepsinogen C)	0.68073519	0.02944	0.1087
70918	Nsun7	NOL1/NOP2/Sun domain family, member 7	0.68073519	0.04936	0.1536
67161	Sclt1	sodium channel and clathrin linker 1	0.68027211	0.003713	0.02845
56220	Zfp386	zinc finger protein 386 (Kruppel-like)	0.68027211	0.01327	0.06416
319592	D030020J04Rik	RIKEN cDNA D030020J04 gene	0.68027211	0.03592	0.124
230779	Serinc2	serine incorporator 2	0.67980965	0.08158	0.2172
320557	Fam169a	family with sequence similarity 169, member A	0.67934783	6.74E-05	0.002797
20607	Sstr3	somatostatin receptor 3	0.67934783	0.01449	0.06792
333050	Ksr2	kinase suppressor of ras 2	0.67934783	0.01489	0.06901
20505	Slc34a1	solute carrier family 34 (sodium phosphate), member 1	0.67934783	0.3139	0.5235
18640	Pfkfb2	6-phosphofructo-2-kinase/fructose-2,6-biphosphatase 2	0.67888663	4.99E-05	0.002397
236790	Ddx26b	DEAD/H (Asp-Glu-Ala-Asp/His) box polypeptide 26B	0.67888663	7.39E-05	0.002964
381022	Mll2	myeloid/lymphoid or mixed-lineage leukemia 2	0.67888663	0.000158	0.004382
624710	Gm6524	katanin p60 (ATPase-containing) subunit A1 pseudogene	0.67888663	0.0006982	0.01051
18627	Per2	period homolog 2 (Drosophila)	0.67888663	0.007822	0.04555
210172	Zfp526	zinc finger protein 526	0.67888663	0.0817	0.2174
67472	Mtfr1	mitochondrial fission regulator 1	0.67842605	8.98E-05	0.00327
53961	9530053J19Rik	RIKEN cDNA 9530053J19 gene	0.67842605	0.0002399	0.005546
320202	Lefty2	left-right determination factor 2	0.67842605	0.02109	0.08719
171543	Bmf	BCL2 modifying factor	0.67842605	0.09815	0.2454
320438	Alg6	asparagine-linked glycosylation 6 homolog (yeast, alpha-1,3,-glucosyltransferase)	0.6779661	0.0006356	0.009907
639396	Gm7265	predicted gene 7265	0.6779661	0.001203	0.01451
140917	Dclre1b	DNA cross-link repair 1B, PSO2 homolog (S. cerevisiae)	0.6779661	0.01752	0.07657
67040	Ddx17	DEAD (Asp-Glu-Ala-Asp) box polypeptide 17	0.6779661	0.07358	0.2024
100040962	Gm3065	predicted gene 3065	0.67750678	0.001523	0.01675
552875	AU015680	expressed sequence AU015680	0.67750678	0.006969	0.04244

13347	Dffa	DNA fragmentation factor, alpha subunit	0.67750678	0.008359	0.0474
68127	B230217C12Rik	RIKEN cDNA B230217C12 gene	0.67704807	0.002706	0.02329
217305	Cd300ld	CD300 molecule-like family member d	0.67704807	0.2714	0.4789
192663	Abcg4	ATP-binding cassette, sub-family G (WHITE), member 4	0.67658999	0.0003083	0.006311
66296	Haus2	HAUS augmin-like complex, subunit 2	0.67658999	0.01134	0.05792
625175	1700028E10Rik	RIKEN cDNA 1700028E10 gene	0.67658999	0.05847	0.1724
435366	Gm5665	predicted gene 5665	0.67658999	0.06843	0.192
381476	B930007M17Rik	RIKEN cDNA B930007M17 gene	0.67658999	0.4366	0.6401
667284	Gm8556	predicted gene 8556	0.67658999	0.5449	0.7269
107166	AI415730	expressed sequence AI415730	0.67613252	0.0001093	0.003637
328329	Mast4	microtubule associated serine/threonine kinase family member 4	0.67613252	0.0009379	0.01248
51873	D2Ertd127e	DNA segment, Chr 2, ERATO Doi 127, expressed	0.67613252	0.002022	0.01962
77875	Cyp4f41-ps	cytochrome P450, family 4, subfamily f, polypeptide 41 pseudogene	0.67613252	0.06161	0.1789
68642	Tmem216	transmembrane protein 216	0.67613252	0.4909	0.6851
227058	Dnahc7b	dynein, axonemal, heavy chain 7B	0.67567568	0.000218	0.005232
17350	Mlh1	mutL homolog 1 (E. coli)	0.67567568	0.003909	0.02936
75956	Srrm2	serine/arginine repetitive matrix 2	0.67567568	0.03132	0.1134
319556	A330041J22Rik	RIKEN cDNA A330041J22 gene	0.67567568	0.08975	0.231
73898	4930429E23Rik	RIKEN cDNA 4930429E23 gene	0.67521945	0.001532	0.01682
105260	D930043N17Rik	RIKEN cDNA D930043N17 gene	0.67521945	0.001761	0.0182
100041677	Gm13157	predicted gene 13157	0.67521945	0.08217	0.2183
23942	Mta2	metastasis-associated gene family, member 2	0.67476383	0.002372	0.02141
98541	AU019278	expressed sequence AU019278	0.67476383	0.007648	0.04495
268890	Lsamp	limbic system-associated membrane protein	0.67476383	0.0356	0.1234
100038759	LOC100038759	hypothetical LOC100038759	0.67476383	0.04677	0.1475
229937	Znhit6	zinc finger, HIT type 6	0.67476383	0.04982	0.1547
102220	E330037G11Rik	RIKEN cDNA E330037G11 gene	0.67476383	0.05022	0.1555
140474	Muc4	mucin 4	0.67476383	0.1128	0.2687
224090	Tmem44	transmembrane protein 44	0.67476383	0.1244	0.2871
18823	Plp1	proteolipid protein (myelin) 1	0.67430883	0.0001588	0.004382
170735	Arr3	arrestin 3, retinal	0.67430883	0.0001663	0.004452
382019	Zfp882	zinc finger protein 882	0.67385445	0.0001207	0.003807
227648	Sec16a	SEC16 homolog A (S. cerevisiae)	0.67385445	0.005587	0.03663
13612	Edil3	EGF-like repeats and discoidin I-like domains 3	0.67385445	0.008951	0.04944
58188	Vstm2b	V-set and transmembrane domain containing 2B	0.67385445	0.339	0.5493
74057	4933405D12Rik	RIKEN cDNA 4933405D12 gene	0.67385445	0.3461	0.5567
19645	Rb1	retinoblastoma 1	0.67340067	0.01471	0.06856
258186	Olfr75-ps1	olfactory receptor 75, pseudogene 1	0.67340067	0.01626	0.07299
320082	Fbxw21	F-box and WD-40 domain protein 21	0.67340067	0.02684	0.1022
108903	Tbcd	tubulin-specific chaperone d	0.67294751	0.001788	0.0183
52704	D11Ertd717e	DNA segment, Chr 11, ERATO Doi 717, expressed	0.67294751	0.003545	0.02764
69189	1810033B17Rik	RIKEN cDNA 1810033B17 gene	0.67294751	0.02567	0.09924
15051	H2-T9	histocompatibility 2, T region locus 9	0.67294751	0.07668	0.2082
14915	Guca2a	guanylate cyclase activator 2a (guanylin)	0.67294751	0.2599	0.4655
15528	Hspe1	heat shock protein 1 (chaperonin 10)	0.67294751	0.3563	0.5661
71494	8430406P12Rik	RIKEN cDNA 8430406P12 gene	0.67249496	0.003789	0.02884
100505360	LOC100505360	putative transposase element L1Md-A101/L1Md-A102/L1Md-A2-like	0.67249496	0.03969	0.132
233529	Kctd14	potassium channel tetramerisation domain containing 14	0.67249496	0.09669	0.2429
67375	Qprt	quinolinate phosphoribosyltransferase	0.67249496	0.2134	0.4096
17260	Mef2c	myocyte enhancer factor 2C	0.67204301	0.001387	0.01574

69329	1700003M02Rik	RIKEN cDNA 1700003M02 gene	0.67204301	0.002112	0.02006
100046290	LOC100046290	60S ribosomal protein L21-like	0.67204301	0.01187	0.05975
269952	D330012F22Rik	RIKEN cDNA D330012F22 gene	0.67204301	0.01324	0.06406
73673	2410076I21Rik	RIKEN cDNA 2410076I21 gene	0.67159167	0.001783	0.0183
11647	Alpl	alkaline phosphatase, liver/bone/kidney	0.67159167	0.01336	0.06442
26367	Ceacam2	carcinoembryonic antigen-related cell adhesion molecule 2	0.67159167	0.03419	0.1198
328329	Mast4	microtubule associated serine/threonine kinase family member 4	0.67159167	0.04944	0.1537
328110	Prpf39	PRP39 pre-mRNA processing factor 39 homolog (yeast)	0.67114094	0.0009846	0.01289
230735	Epha10	Eph receptor A10	0.67114094	0.007252	0.04355
327780	D430050E20Rik	RIKEN cDNA D430050E20 gene	0.67114094	0.00757	0.04462
22114	Tssk1	testis-specific serine kinase 1	0.67114094	0.01657	0.07372
16506	Kcnd1	potassium voltage-gated channel, Shal-related family, member 1	0.67114094	0.1976	0.3887
53322	Nucb2	nucleobindin 2	0.67069081	0.004677	0.03283
245578	Pcdh11x	protocadherin 11 X-linked	0.67069081	0.06656	0.1884
100043381	Gm14308	predicted gene 14308	0.67069081	0.125	0.2881
319229	Sctr	secretin receptor	0.67069081	0.1334	0.3003
52680	D13Ertd787e	DNA segment, Chr 13, ERATO Doi 787, expressed	0.67024129	0.000315	0.006371
629591	Gm6987	predicted pseudogene 6987	0.66979236	0.3414	0.5517
29809	Rabgap1l	RAB GTPase activating protein 1-like	0.66934404	0.0003848	0.007246
13347	Dffa	DNA fragmentation factor, alpha subunit	0.66934404	0.004926	0.03383
238123	Cog5	component of oligomeric golgi complex 5	0.66934404	0.05888	0.1733
545276	Gal3st3	galactose-3-O-sulfotransferase 3	0.66934404	0.06921	0.1938
110616	Atxn3	ataxin 3	0.66889632	0.002899	0.02427
50933	Uchl3	ubiquitin carboxyl-terminal esterase L3 (ubiquitin thiolesterase)	0.66889632	0.5124	0.7029
228998	Arfgap1	ADP-ribosylation factor GTPase activating protein 1	0.6684492	0.0002063	0.005051
78282	5330439K02Rik	RIKEN cDNA 5330439K02 gene	0.6684492	0.0008209	0.01153
210417	Thsd7b	thrombospondin, type I, domain containing 7B	0.6684492	0.007812	0.04554
70981	4931423N10Rik	RIKEN cDNA 4931423N10 gene	0.6684492	0.01161	0.05892
628239	LOC628239	zinc finger protein 14-like	0.6684492	0.08717	0.2269
330554	Fan1	FANCD2/FANCI-associated nuclease 1	0.66800267	0.002712	0.02333
12351	Car4	carbonic anhydrase 4	0.66800267	0.2761	0.4841
399579	6720462K09Rik	RIKEN cDNA 6720462K09 gene	0.66755674	7.84E-05	0.003046
109821	F11	coagulation factor XI	0.66755674	0.0008461	0.01176
67120	Ttc14	tetratricopeptide repeat domain 14	0.66755674	0.01942	0.08217
72154	Zfp157	zinc finger protein 157	0.66711141	0.009587	0.05186
98763	AA407782	expressed sequence AA407782	0.66711141	0.01315	0.06385
85031	Pla1a	phospholipase A1 member A	0.66711141	0.1423	0.3131
67295	Rab3c	RAB3C, member RAS oncogene family	0.66711141	0.1727	0.3561
20744	Strbp	spermatid perinuclear RNA binding protein	0.66666667	0.0001132	0.003699
208659	Fam20a	family with sequence similarity 20, member A	0.66666667	0.004671	0.03282
22157	Tulp1	tubby like protein 1	0.66666667	0.01195	0.06006
19211	Pten	phosphatase and tensin homolog	0.66666667	0.02413	0.09508
101694	AI854517	expressed sequence AI854517	0.66622252	0.0005823	0.009352
98401	AI594674	expressed sequence AI594674	0.66622252	0.0007976	0.01137
72383	Cnfn	cornifelin	0.66622252	0.01938	0.08202
240041	Zfp945	zinc finger protein 945	0.66622252	0.04688	0.1478
70948	Wdr20b	WD repeat domain 20b	0.66622252	0.1722	0.3552
320977	A330023F24Rik	RIKEN cDNA A330023F24 gene	0.66577896	0.005028	0.03428
665211	Gm14326	predicted gene 14326	0.66577896	0.07027	0.1956
100505360	LOC100505360	putative transposase element L1Md-A101/L1Md-A102/L1Md-A2-like	0.66577896	0.1196	0.2797

665466	Gm7644	predicted gene 7644	0.66577896	0.1515	0.3263
15204	Herc2	hect (homologous to the E6-AP (UBE3A) carboxyl terminus) domain and RCC1 (CHC1)-like domain (RLD) 2	0.66577896	0.1681	0.3489
27385	Magel2	melanoma antigen, family L, 2	0.66533599	0.0002459	0.005612
11775	Ap3b2	adaptor-related protein complex 3, beta 2 subunit	0.66489362	0.00512	0.03465
11994	Pcdh15	protocadherin 15	0.66489362	0.09981	0.248
15251	Hif1a	hypoxia inducible factor 1, alpha subunit	0.66401062	0.002574	0.02254
70470	Rprd1b	regulation of nuclear pre-mRNA domain containing 1B	0.66401062	0.02243	0.09076
97455	C78859	expressed sequence C78859	0.66357001	9.07E-05	0.003288
54447	Asah2	N-acylsphingosine amidohydrolase 2	0.66357001	0.03619	0.1246
433759	Hdac1	histone deacetylase 1	0.66357001	0.04699	0.148
228357	Lrp4	low density lipoprotein receptor-related protein 4	0.66312997	2.19E-05	0.001501
56357	Ivd	isovaleryl coenzyme A dehydrogenase	0.66312997	3.43E-05	0.001924
75972	5033423O07Rik	RIKEN cDNA 5033423O07 gene	0.66269052	0.005602	0.03672
76072	Rnf183	ring finger protein 183	0.66269052	0.03847	0.1294
246746	Cd300lf	CD300 antigen like family member F	0.66269052	0.2848	0.4932
100101046	D230018H15Rik	RIKEN cDNA D230018H15 gene	0.66225166	0.006328	0.03974
16664	Krt14	keratin 14	0.66225166	0.03285	0.1169
52480	D7Ertd715e	DNA segment, Chr 7, ERATO Doi 715, expressed	0.66225166	0.05769	0.171
212391	Lcor	ligand dependent nuclear receptor corepressor	0.66137566	0.005678	0.03705
94118	Kifc5c-ps	kinesin family member C5C, pseudogene	0.66137566	0.2111	0.4066
246048	Chodl	chondrolectin	0.66093853	0.2005	0.3927
243906	Zfp14	zinc finger protein 14	0.66006601	0.00129	0.01512
665211	Gm14326	predicted gene 14326	0.66006601	0.08007	0.2147
18168	Npy5r	neuropeptide Y receptor Y5	0.65963061	0.00434	0.03135
414113	D230040A04Rik	RIKEN cDNA D230040A04 gene	0.65919578	0.001091	0.01374
267019	Rps15a	ribosomal protein S15A	0.65919578	0.003449	0.02713
353211	Prune2	prune homolog 2 (Drosophila)	0.65919578	0.04053	0.134
381677	Vgf	VGF nerve growth factor inducible	0.65832785	0.0007826	0.01123
329908	Usp24	ubiquitin specific peptidase 24	0.65832785	0.001153	0.01416
15951	Ifi204	interferon activated gene 204	0.65832785	0.03038	0.1111
52902	D10Bwg1070e	DNA segment, Chr 10, Brigham & Women's Genetics 1070 expressed	0.65789474	0.002038	0.01967
77596	Gpr110	G protein-coupled receptor 110	0.65789474	0.09592	0.2419
234912	9230110C19Rik	RIKEN cDNA 9230110C19 gene	0.65789474	0.1634	0.3427
76815	Calcoco2	calcium binding and coiled-coil domain 2	0.65789474	0.1772	0.3622
20687	Sp3	trans-acting transcription factor 3	0.65789474	0.3796	0.5887
241494	Zfp385b	zinc finger protein 385B	0.6574622	0.02197	0.08959
215384	Fcgbp	Fc fragment of IgG binding protein	0.6574622	0.03132	0.1134
668620	Zfp936	zinc finger protein 936	0.6574622	0.1014	0.251
72351	Ptar1	protein prenyltransferase alpha subunit repeat containing 1	0.65703022	0.001042	0.0134
320438	Alg6	asparagine-linked glycosylation 6 homolog (yeast, alpha-1,3,-glucosyltransferase)	0.65703022	0.0103	0.0543
665001	Gm14391	predicted gene 14391	0.65703022	0.08578	0.2246
76719	1700081L11Rik	RIKEN cDNA 1700081L11 gene	0.65703022	0.1986	0.3901
80877	Lrba	LPS-responsive beige-like anchor	0.65659882	0.001465	0.01626
68895	Rasl11a	RAS-like, family 11, member A	0.65659882	0.009161	0.05027
109700	Itga1	integrin alpha 1	0.65659882	0.4226	0.6282
232685	AB041803	cDNA sequence AB041803	0.65616798	0.005026	0.03427
319800	C730048C13Rik	RIKEN cDNA C730048C13 gene	0.6557377	0.005106	0.03461
667414	Gm8618	predicted gene 8618	0.6557377	0.3159	0.5255
242126	Slc22a15	solute carrier family 22 (organic anion/cation transporter), member 15	0.65530799	0.0807	0.2156
74561	Nkx6-3	NK6 homeobox 3	0.65487885	0.0001795	0.004623

15331	Hmgn2	high mobility group nucleosomal binding domain 2	0.65487885	0.0006224	0.009748
23854	Def8	differentially expressed in FDCP 8	0.65487885	0.04152	0.1363
100037396	D030047H15Rik	RIKEN cDNA D030047H15 gene	0.65487885	0.113	0.2689
13860	Eps8	epidermal growth factor receptor pathway substrate 8	0.65402224	0.0001414	0.004139
75880	4930592A05Rik	RIKEN cDNA 4930592A05 gene	0.65402224	0.0503	0.1556
270058	Mtap1s	microtubule-associated protein 1S	0.65359477	0.002251	0.02079
77881	6030458E02Rik	RIKEN cDNA 6030458E02 gene	0.65359477	0.0259	0.09974
14051	Eya4	eyes absent 4 homolog (Drosophila)	0.65359477	0.3955	0.6035
15331	Hmgn2	high mobility group nucleosomal binding domain 2	0.65316786	0.002405	0.02158
98884	AI225934	expressed sequence AI225934	0.65316786	0.005724	0.0372
100043089	Gm4221	predicted gene 4221	0.65316786	0.0161	0.07245
99229	AU041474	expressed sequence AU041474	0.65316786	0.05664	0.169
70701	Nipal1	NIPA-like domain containing 1	0.65274151	0.00049	0.008433
57443	Fbxo3	F-box protein 3	0.65274151	0.0007257	0.01078
353208	Zfp931	zinc finger protein 931	0.65274151	0.07516	0.2051
76286	1110006E14Rik	RIKEN cDNA 1110006E14 gene	0.65231572	0.0008072	0.01144
60599	Trp53inp1	transformation related protein 53 inducible nuclear protein 1	0.65231572	0.0121	0.06043
74180	Muc5b	mucin 5, subtype B, tracheobronchial	0.65189048	0.1544	0.3302
19361	Rad51	RAD51 homolog (S. cerevisiae)	0.6514658	0.0003351	0.006637
12795	Plk3	polo-like kinase 3 (Drosophila)	0.6514658	0.0005595	0.009165
232685	AB041803	cDNA sequence AB041803	0.6514658	0.003681	0.0283
70897	Fam71d	family with sequence similarity 71, member D	0.6514658	0.0531	0.1618
76880	6430411K18Rik	RIKEN cDNA 6430411K18 gen	0.65104167	0.01147	0.05837
320129	Adrbk2	adrenergic receptor kinase, beta 2	0.65061809	0.0002603	0.0058
14813	Grin2c	glutamate receptor, ionotropic, NMDA2C (epsilon 3)	0.65061809	0.001573	0.01706
15559	Htr2b	5-hydroxytryptamine (serotonin) receptor 2B	0.65061809	0.002404	0.02158
73430	1700049G17Rik	RIKEN cDNA 1700049G17 gene	0.65019506	0.002362	0.02139
319319	B230214O09Rik	RIKEN cDNA B230214O09 gene	0.65019506	0.03197	0.1149
232836	Galp	galanin-like peptide	0.65019506	0.07211	0.1994
100039888	Gm11223	stathmin 1 pseudogene	0.64977258	0.0004888	0.00842
12006	Axin2	axin2	0.64977258	0.4467	0.6491
12823	Col19a1	collagen, type XIX, alpha 1	0.64935065	0.004543	0.03225
72350	Fam164c	family with sequence similarity 164, member C	0.64935065	0.01202	0.06015
68338	Golt1a	golgi transport 1 homolog A (S. cerevisiae)	0.64935065	0.1491	0.3229
100038662	D130046C19Rik	RIKEN cDNA D130046C19 gene	0.64892927	0.008886	0.04922
76789	Mzt1	mitotic spindle organizing protein 1	0.64892927	0.04006	0.1329
20913	Stxbp4	syntaxin binding protein 4	0.64850843	0.0005818	0.009352
74448	Arl13a	ADP-ribosylation factor-like 13A	0.64850843	0.001214	0.01457
68052	Rps13	ribosomal protein S13	0.64808814	9.93E-05	0.003415
68142	Ino80	INO80 homolog (S. cerevisiae)	0.64808814	0.0002955	0.006162
66300	Prr24	proline rich 24	0.64766839	6.52E-06	0.0007681
75788	Smurf1	SMAD specific E3 ubiquitin protein ligase 1	0.64766839	0.00039	0.007282
319998	Tmem198	transmembrane protein 198	0.64766839	0.001453	0.0162
12286	Cacna1a	calcium channel, voltage-dependent, P/Q type, alpha 1A subunit	0.64766839	0.00176	0.0182
73757	4833421G17Rik	RIKEN cDNA 4833421G17 gene	0.64766839	0.00539	0.03584
20861	Stfa1	stefin A1	0.64724919	0.06785	0.1908
331188	Zfp781	zinc finger protein 781	0.64599483	0.01047	0.05485
20333	Sec22b	SEC22 vesicle trafficking protein homolog B (S. cerevisiae)	0.64557779	0.0002555	0.005746
20348	Sema3c	sema domain, immunoglobulin domain (Ig), short basic domain, secreted, (semaphorin) 3C	0.64557779	0.2239	0.4237
12660	Chka	choline kinase alpha	0.64516129	0.001114	0.0139

434484	Sp140	Sp140 nuclear body protein	0.64516129	0.002228	0.02065
18036	Nfkbib	nuclear factor of kappa light polypeptide gene enhancer in B-cells inhibitor, beta	0.64516129	0.08818	0.2285
71764	C2cd2l	C2 calcium-dependent domain containing 2-like	0.6443299	0.0001403	0.004114
399614	C130009A20Rik	RIKEN cDNA C130009A20 gene	0.6443299	0.0001426	0.004152
14219	Ctgf	connective tissue growth factor	0.6443299	0.016	0.07222
245596	Hdx	highly divergent homeobox	0.6443299	0.02454	0.09618
319626	9530059O14Rik	RIKEN cDNA 9530059O14 gene	0.6443299	0.02749	0.1038
109200	A430102J17Rik	RIKEN cDNA A430102J17 gene	0.643915	0.0005001	0.008535
52108	D9Ertd115e	DNA segment, Chr 9, ERATO Doi 115, expressed	0.64350064	0.0189	0.08063
242960	Fbxl5	F-box and leucine-rich repeat protein 5	0.64308682	0.002855	0.02407
545475	Defb28	defensin beta 28	0.64267352	0.353	0.563
320793	A330072L02Rik	RIKEN cDNA A330072L02 gene	0.64267352	0.4294	0.6338
77200	5430403G16Rik	RIKEN cDNA 5430403G16 gene	0.64226076	0.003427	0.02703
22018	Tpo	thyroid peroxidase	0.64226076	0.005386	0.03583
99094	AI849538	expressed sequence AI849538	0.64226076	0.02397	0.09474
18610	Pdyn	prodynorphin	0.64184852	0.0008913	0.0121
320209	Ddx11	DEAD/H (Asp-Glu-Ala-Asp/His) box polypeptide 11 (CHL1-like helicase homolog, S. cerevisiae)	0.64184852	0.009319	0.05078
102371	Gcom1	GRINL1A complex locus	0.64184852	0.02387	0.0945
18616	Peg3	paternally expressed 3	0.64143682	0.0188	0.08036
21667	Tdgf1	teratocarcinoma-derived growth factor 1	0.64143682	0.3037	0.5129
81898	Sf3b1	splicing factor 3b, subunit 1	0.64102564	0.001194	0.01444
544848	Gm5784	predicted gene 5784	0.64102564	0.02323	0.09266
29809	Rabgap1l	RAB GTPase activating protein 1-like	0.64061499	0.0001221	0.003816
76560	Prss8	protease, serine, 8 (prostasin)	0.64020487	0.05021	0.1555
18510	Pax8	paired box gene 8	0.63979527	0.04157	0.1364
433759	Hdac1	histone deacetylase 1	0.63938619	0.0118	0.05961
20315	Cxcl12	chemokine (C-X-C motif) ligand 12	0.63897764	0.0003404	0.006698
12412	Cbx1	chromobox homolog 1 (Drosophila HP1 beta)	0.63897764	0.0009649	0.01269
23956	Neu2	neuraminidase 2	0.63897764	0.002238	0.0207
338349	Cntln	centlein, centrosomal protein	0.63897764	0.04263	0.1386
76671	5330406M23Rik	RIKEN cDNA 5330406M23 gene	0.6385696	0.000265	0.005839
18779	Pla2r1	phospholipase A2 receptor 1	0.63816209	0.002834	0.02399
80288	Bcl9l	B-cell CLL/lymphoma 9-like	0.63816209	0.004546	0.03225
18019	Nfatc2	nuclear factor of activated T-cells, cytoplasmic, calcineurin-dependent 2	0.6377551	4.74E-05	0.002322
320171	D130019J16Rik	RIKEN cDNA D130019J16 gene	0.6377551	0.004118	0.03032
68070	Pdzd2	PDZ domain containing 2	0.6377551	0.04092	0.1348
71263	Mro	maestro	0.6377551	0.1093	0.2629
75555	1700020D05Rik	RIKEN cDNA 1700020D05 gene	0.63734863	0.0002546	0.005743
74363	4931430N09Rik	RIKEN cDNA 4931430N09 gene	0.63734863	0.002729	0.02346
381853	Gipr	gastric inhibitory polypeptide receptor	0.63734863	0.003274	0.02633
78672	9530057J20Rik	RIKEN cDNA 9530057J20 gene	0.63734863	0.01772	0.07726
26447	Poli	polymerase (DNA directed), iota	0.63734863	0.04572	0.1454
228714	Csrp2bp	cysteine and glycine-rich protein 2 binding protein	0.63734863	0.1764	0.361
20445	St6galnac1	ST6 (alpha-N-acetyl-neuraminyl-2,3-beta-galactosyl-1,3)-N-acetylgalactosaminide alpha-2,6-sialyltransferase 1	0.63734863	0.2181	0.4162
328309	Gm9776	predicted gene 9776	0.63734863	0.2421	0.445
17263	Meg3	maternally expressed 3	0.63694268	0.002004	0.01955
269629	Lhfpl3	lipoma HMGIC fusion partner-like 3	0.63653724	0.0001116	0.003683
330149	Hfm1	HFM1, ATP-dependent DNA helicase homolog (S. cerevisiae)	0.63653724	0.0002281	0.005382
73002	2900076A13Rik	RIKEN cDNA 2900076A13 gene	0.63653724	0.0006463	0.009989
271970	Arsj	arylsulfatase J	0.63613232	0.02039	0.08505

382111	Susd5	sushi domain containing 5	0.63613232	0.1894	0.3781
245572	Tbx22	T-box 22	0.63613232	0.2671	0.4742
230863	Sh2d5	SH2 domain containing 5	0.63572791	0.002687	0.02321
100036538	BC028777	cDNA sequence BC028777	0.63572791	0.08271	0.2191
15042	H2-T24	histocompatibility 2, T region locus 24	0.63572791	0.1432	0.3143
66203	Lce1m	late cornified envelope 1M	0.63532402	0.14	0.3095
73332	Ccdc30	coiled-coil domain containing 30	0.63411541	0.003506	0.02744
338349	Cntln	centlein, centrosomal protein	0.63411541	0.007603	0.04477
77117	Zfp934	zinc finger protein 934	0.63371356	0.001251	0.01487
546160	Fbxw25	F-box and WD-40 domain protein 25	0.63371356	0.2447	0.4484
15186	Hdc	histidine decarboxylase	0.63331222	0.04748	0.1492
80718	Rab27b	RAB27b, member RAS oncogene family	0.63291139	0.006846	0.04187
52679	E2f7	E2F transcription factor 7	0.63291139	0.08261	0.219
70973	4931431B13Rik	RIKEN cDNA 4931431B13 gene	0.63291139	0.3244	0.5342
100504699	LOC100504699	hypothetical LOC100504699	0.63251107	0.002756	0.0236
546144	Wdr72	WD repeat domain 72	0.63251107	0.02313	0.09249
13371	Dio2	deiodinase, iodothyronine, type II	0.63211125	0.004474	0.03197
26366	Ceacam10	carcinoembryonic antigen-related cell adhesion molecule 10	0.63211125	0.01404	0.06654
100504146	LOC100504146	zinc finger protein 709-like	0.63211125	0.05061	0.1562
16656	Hivep3	human immunodeficiency virus type I enhancer binding protein 3	0.63171194	0.05725	0.1702
668239	Gm12270	predicted gene 12270	0.63131313	0.0001077	0.003608
100381258	D930001M23Rik	Riken cDNA D930001M23 gene	0.63131313	0.0009632	0.01267
668809	Gm9372	predicted gene 9372	0.63091483	0.2873	0.4958
50720	Sacs	sacsin	0.63051702	0.008603	0.04832
107350	AW112010	expressed sequence AW112010	0.63051702	0.01339	0.0645
24135	Zfp68	zinc finger protein 68	0.63051702	0.01852	0.07964
234358	Zfp930	zinc finger protein 930	0.63051702	0.0629	0.1814
17263	Meg3	maternally expressed 3	0.63011972	0.004154	0.03048
13052	Cxadr	coxsackie virus and adenovirus receptor	0.62972292	0.003887	0.02924
78202	4930557K07Rik	RIKEN cDNA 4930557K07 gene	0.62972292	0.06707	0.1892
241514	Zfp804a	zinc finger protein 804A	0.62893082	0.4006	0.6083
223915	Krt73	keratin 73	0.6281407	0.03013	0.1107
244757	Glb1l2	galactosidase, beta 1-like 2	0.62774639	0.0002041	0.005024
13653	Egr1	early growth response 1	0.62774639	0.05676	0.1693
100303732	Gm14431	predicted gene 14431	0.62774639	0.08143	0.2169
12565	Cdh9	cadherin 9	0.62735257	0.0002081	0.005074
327781	A130033P14	hypothetical protein A130033P14	0.62735257	0.008071	0.04641
17475	Mpdz	multiple PDZ domain protein	0.62695925	0.0005838	0.009369
78871	B230117O15Rik	RIKEN cDNA B230117O15 gene	0.62656642	0.0003024	0.006254
11486	Ada	adenosine deaminase	0.62656642	0.0005995	0.009529
76286	1110006E14Rik	RIKEN cDNA 1110006E14 gene	0.62656642	0.0008833	0.01208
68943	Pink1	PTEN induced putative kinase 1	0.62578223	0.01865	0.08005
69236	2610034E01Rik	RIKEN cDNA 2610034E01 gene	0.62578223	0.02272	0.09146
545548	Lce3a	late cornified envelope 3A	0.62539087	0.1068	0.2588
15950	Ifi203	interferon activated gene 203	0.62539087	0.1234	0.2857
78547	E130304I02Rik	RIKEN cDNA E130304I02 gene	0.625	0.001645	0.0174
432628	Mfsd2b	major facilitator superfamily domain containing 2B	0.625	0.007696	0.04511
207921	A830093I24Rik	RIKEN cDNA A830093I24 gene	0.62460962	0.001333	0.01542
240334	Pcyox1l	prenylcysteine oxidase 1 like	0.62460962	0.004887	0.03365
77528	C030014A21Rik	RIKEN cDNA C030014A21 gene	0.62460962	0.004937	0.03386

13405	Dmd	dystrophin, muscular dystrophy	0.62421973	4.70E-05	0.002311
17199	Mc1r	melanocortin 1 receptor	0.62421973	0.0001625	0.004382
76872	Ccdc116	coiled-coil domain containing 116	0.62421973	0.02076	0.08614
22190	Ubc	ubiquitin C	0.62383032	1.10E-05	0.001039
382571	Kcnf1	potassium voltage-gated channel, subfamily F, member 1	0.62383032	0.02626	0.1007
414872	Zyg11b	zyg-ll homolog B (C. elegans)	0.6234414	9.93E-05	0.003415
66395	Ahnak	AHNAK nucleoprotein (desmoyokin)	0.62305296	0.2284	0.4286
239036	4930596D02Rik	RIKEN cDNA 4930596D02 gene	0.62266501	0.4383	0.6414
78279	5330421C15Rik	RIKEN cDNA 5330421C15 gene	0.62227754	0.0005502	0.009067
381650	Thap6	THAP domain containing 6	0.62189055	0.000153	0.004299
170822	Usp33	ubiquitin specific peptidase 33	0.62111801	6.91E-05	0.002836
15528	Hspe1	heat shock protein 1 (chaperonin 10)	0.62034739	2.23E-05	0.001507
319520	Dusp4	dual specificity phosphatase 4	0.6199628	0.001629	0.01733
22215	Ube3a	ubiquitin protein ligase E3A	0.6199628	0.003083	0.02535
170731	Mfn2	mitofusin 2	0.61881188	0.008261	0.04714
243842	Gltscr1	glioma tumor suppressor candidate region gene 1	0.61881188	0.01052	0.05502
320583	C530030K21Rik	RIKEN cDNA C530030K21 gene	0.61842919	0.01908	0.08123
70831	Krtap31-1	keratin associated protein 31-1	0.61842919	0.02938	0.1086
108816	4933409K07Rik	RIKEN cDNA 4933409K07 gene	0.61804697	0.02669	0.1019
69294	Cst13	cystatin 13	0.61804697	0.3587	0.5686
66522	Pgpep1	pyroglutamyl-peptidase I	0.61766523	0.001617	0.01727
12050	Bcl2l2	BCL2-like 2	0.61766523	0.4748	0.6724
67876	Coq10b	coenzyme Q10 homolog B (S. cerevisiae)	0.61728395	1.20E-05	0.001077
320327	A430106G13Rik	RIKEN cDNA A430106G13 gene	0.61728395	1.35E-05	0.001142
16329	Inpp1	inositol polyphosphate-1-phosphatase	0.61728395	0.001044	0.01342
219131	Phf11	PHD finger protein 11	0.61728395	0.02697	0.1025
242747	Zfp933	zinc finger protein 933	0.61690315	0.05917	0.1738
101100	Ttll3	tubulin tyrosine ligase-like family, member 3	0.61652281	0.002464	0.02189
24053	Sgcg	sarcoglycan, gamma (dystrophin-associated glycoprotein)	0.61652281	0.03576	0.1237
20589	Ighmbp2	immunoglobulin mu binding protein 2	0.61614295	0.005264	0.03533
433215	BC048609	cDNA sequence BC048609	0.61576355	0.003428	0.02703
544678	2010015L04Rik	RIKEN cDNA 2010015L04 gene	0.61538462	0.001022	0.01322
17245	Mdm1	transformed mouse 3T3 cell double minute 1	0.61538462	0.003121	0.02558
69698	2310046K01Rik	RIKEN cDNA 2310046K01 gene	0.61538462	0.1276	0.2922
382161	Gm5165	predicted gene 5165	0.61500615	0.003869	0.02919
73852	D3Ertd751e	DNA segment, Chr 3, ERATO Doi 751, expressed	0.61500615	0.007227	0.04349
21462	Tcp10c	t-complex protein 10c	0.61500615	0.0268	0.1021
320927	6720474J12Rik	RIKEN cDNA 6720474J12 gene	0.61462815	0.001583	0.01708
73722	Lce1a2	late cornified envelope 1A2	0.61462815	0.04646	0.1469
77599	5830420C07Rik	RIKEN cDNA 5830420C07 gene	0.61425061	0.00118	0.01434
97874	B430203I24Rik	RIKEN cDNA B430203I24 gene	0.61387354	0.001553	0.01696
54632	Ftsj1	FtsJ homolog 1 (E. coli)	0.61387354	0.001754	0.01817
259002	Olfr173	olfactory receptor 173	0.61349693	0.03179	0.1145
245555	C77370	expressed sequence C77370	0.61349693	0.1578	0.335
74855	4930417G10Rik	RIKEN cDNA 4930417G10 gene	0.61312078	0.002514	0.0222
97868	C78704	expressed sequence C78704	0.61312078	0.01011	0.05371
192236	Hps1	Hermansky-Pudlak syndrome 1 homolog (human)	0.61236987	0.006595	0.04077
11811	Apobec2	apolipoprotein B mRNA editing enzyme, catalytic polypeptide 2	0.61236987	0.008721	0.04874
75739	Mpp7	membrane protein, palmitoylated 7 (MAGUK p55 subfamily member 7)	0.6119951	0.0003748	0.007108
243078	Tecrl	trans-2,3-enoyl-CoA reductase-like	0.6119951	0.001745	0.0181

56219	Extl1	exostoses (multiple)-like 1	0.6119951	0.0175	0.07653
547103	Gm10674	predicted gene 10674	0.6116208	0.01485	0.0689
208111	C330019L16Rik	RIKEN cDNA C330019L16 gene	0.61124694	0.04302	0.1393
51795	Srpx	sushi-repeat-containing protein	0.61087355	1.60E-05	0.001266
58807	Slco1c1	solute carrier organic anion transporter family, member 1c1	0.61087355	0.002814	0.02387
216859	Acap1	ArfGAP with coiled-coil, ankyrin repeat and PH domains 1	0.61050061	0.03036	0.1111
18108	Nmt2	N-myristoyltransferase 2	0.61012813	0.007056	0.0428
319424	E030004H24Rik	RIKEN cDNA E030004H24 gene	0.60938452	0.0008417	0.01175
19259	Ptpn5	protein tyrosine phosphatase, non-receptor type 5	0.60938452	0.009358	0.05092
226025	Trpm3	transient receptor potential cation channel, subfamily M, member 3	0.60938452	0.07086	0.1968
545428	Ccdc141	coiled-coil domain containing 141	0.60938452	0.1	0.2485
245403	Dcaf12l2	DDB1 and CUL4 associated factor 12-like 2	0.60938452	0.1494	0.3235
22283	Ush2a	Usher syndrome 2A (autosomal recessive, mild) homolog (human)	0.6090134	0.0004867	0.008408
22715	Zfp57	zinc finger protein 57	0.6090134	0.01417	0.067
67886	Camsap1l1	calmodulin regulated spectrin-associated protein 1-like 1	0.60864273	0.00549	0.03623
402762	A630039O03Rik	RIKEN cDNA A630039O03 gene	0.60790274	0.001334	0.01542
193217	BC018473	cDNA sequence BC018473	0.60790274	0.04274	0.1388
433323	Sgpp2	sphingosine-1-phosphate phosphotase 2	0.60753341	0.000243	0.005581
329160	9130024F11Rik	RIKEN cDNA 9130024F11 gene	0.60753341	0.003005	0.02484
233230	Mrgprb4	MAS-related GPR, member B4	0.60753341	0.3093	0.5193
69849	2010007H06Rik	RIKEN cDNA 2010007H06 gene	0.60679612	0.002287	0.02102
100040346	Gm2724	predicted gene 2724	0.60679612	0.01486	0.06893
100043188	Gm10696	predicted gene 10696	0.60679612	0.01857	0.07977
269378	Ahcy	S-adenosylhomocysteine hydrolase	0.60642814	0.0008143	0.01148
320152	4930412C18Rik	RIKEN cDNA 4930412C18 gene	0.60606061	0.07702	0.2088
239133	Dleu7	deleted in lymphocytic leukemia, 7	0.60569352	0.0008971	0.01215
67687	1700011L22Rik	RIKEN cDNA 1700011L22 gene	0.60569352	0.3583	0.5683
30785	Cttnbp2	cortactin binding protein 2	0.60532688	0.001181	0.01434
192289	Tmlhe	trimethyllysine hydroxylase, epsilon	0.60532688	0.002606	0.02277
109905	Rap1a	RAS-related protein-1a	0.60532688	0.5197	0.7087
329650	Med12l	mediator of RNA polymerase II transcription, subunit 12 homolog (yeast)-like	0.60496068	0.05981	0.175
13113	Cyp3a13	cytochrome P450, family 3, subfamily a, polypeptide 13	0.60422961	0.02004	0.08387
234915	AK129341	cDNA sequence AK129341	0.6035003	0.2055	0.3993
214240	Disp2	dispatched homolog 2 (Drosophila)	0.60313631	0.0001218	0.003815
76619	1700087I21Rik	RIKEN cDNA 1700087I21 gene	0.60240964	0.00234	0.02133
237806	Dnahc9	dynein, axonemal, heavy chain 9	0.60240964	0.008394	0.04754
140795	P2ry14	purinergic receptor P2Y, G-protein coupled, 14	0.60168472	0.009831	0.05278
66805	Tspan1	tetraspanin 1	0.60168472	0.05632	0.1684
103775	Slc25a41	solute carrier family 25, member 41	0.60132291	0.007257	0.04355
628709	Gm10324	predicted gene 10324	0.6006006	0.1151	0.2723
635253	Usp51	ubiquitin specific protease 51	0.59988002	8.28E-05	0.003125
75706	Krt24	keratin 24	0.59988002	0.008094	0.0465
69367	Glrx2	glutaredoxin 2 (thioltransferase)	0.59988002	0.442	0.645
216961	Coro6	coronin 6	0.59952038	0.002139	0.02017
791388	AY512938	cDNA sequence AY512938	0.59916117	0.002168	0.02032
20514	Slc1a5	solute carrier family 1 (neutral amino acid transporter), member 5	0.59844405	0.0002558	0.005746
76073	Pcgf5	polycomb group ring finger 5	0.59844405	0.0005331	0.008923
240041	Zfp945	zinc finger protein 945	0.59844405	0.00404	0.02996
213393	8430408G22Rik	RIKEN cDNA 8430408G22 gene	0.59808612	0.02415	0.09511
98878	Ehd4	EH-domain containing 4	0.59737157	2.66E-05	0.001661

75763	Dcaf17	DDB1 and CUL4 associated factor 17	0.59737157	0.0002151	0.00519
67182	Pdzk1ip1	PDZK1 interacting protein 1	0.59737157	0.00386	0.02915
27204	Syn3	synapsin III	0.59737157	0.2475	0.4517
100702	Mpa2l	macrophage activation 2 like	0.59665871	0.2277	0.4279
544678	2010015L04Rik	RIKEN cDNA 2010015L04 gene	0.59630292	0.0003732	0.007103
225872	Npas4	neuronal PAS domain protein 4	0.59594756	0.003643	0.02812
77938	Fam53b	family with sequence similarity 53, member B	0.59559261	5.40E-05	0.002503
381062	2210404J11Rik	RIKEN cDNA 2210404J11 gene	0.59559261	0.0007633	0.01103
74041	4632434I11Rik	RIKEN cDNA 4632434I11 gene	0.5952381	0.0009001	0.01218
12294	Cacna2d3	calcium channel, voltage-dependent, alpha2/delta subunit 3	0.59453032	1.86E-05	0.001397
77979	B230104C14Rik	RIKEN cDNA B230104C14 gene	0.59417706	0.0006583	0.01007
212684	Pdzd7	PDZ domain containing 7	0.59417706	0.004765	0.03323
22144	Tuba3a	tubulin, alpha 3A	0.59382423	0.009098	0.05
433466	Jmjd7	jumonji domain containing 7	0.59311981	1.07E-05	0.001026
628709	Gm10324	predicted gene 10324	0.59311981	0.1406	0.3104
224671	Btbd9	BTB (POZ) domain containing 9	0.59276823	0.0005248	0.008832
100316870	LOC100316870	DDB1 and CUL4 associated factor 5 pseudogene	0.59276823	0.009763	0.05252
100034684	BC100530	cDNA sequence BC100530	0.59276823	0.03182	0.1145
73941	4930412L05Rik	RIKEN cDNA 4930412L05 gene	0.59241706	8.55E-05	0.00317
24135	Zfp68	zinc finger protein 68	0.59136606	1.20E-05	0.001077
107351	Kank1	KN motif and ankyrin repeat domains 1	0.59101655	0.000219	0.005248
268567	Tmem229b	transmembrane protein 229B	0.59066745	0.0007567	0.01098
93728	Pabpc5	poly(A) binding protein, cytoplasmic 5	0.59031877	0.1118	0.267
68416	Sycn	syncollin	0.59031877	0.1336	0.3005
71413	5430410E06Rik	RIKEN cDNA 5430410E06 gene	0.59031877	0.2882	0.4966
320719	6030443J06Rik	RIKEN cDNA 6030443J06 gene	0.58962264	0.008509	0.04796
245128	AU018091	expressed sequence AU018091	0.58927519	0.1793	0.3648
66161	Pop4	processing of precursor 4, ribonuclease P/MRP family, (S. cerevisiae)	0.58892815	0.002004	0.01955
553089	LOC553089	hypothetical LOC553089	0.58858152	0.000474	0.008245
68888	Gkn3	gastrokine 3	0.58858152	0.008676	0.04861
100043387	100043387	predicted gene, 100043387	0.58823529	0.02791	0.1049
107684	Coro2a	coronin, actin binding protein 2A	0.58823529	0.0529	0.1614
20753	Sprr1a	small proline-rich protein 1A	0.58788948	0.0608	0.1771
329504	Lcmt2	leucine carboxyl methyltransferase 2	0.58754407	0.0002682	0.005858
667666	Zfp600	zinc finger protein 600	0.58651026	0.03031	0.111
65246	Xpo7	exportin 7	0.58616647	6.08E-06	0.0007456
18616	Peg3	paternally expressed 3	0.58616647	0.003356	0.02671
319814	E130106K03Rik	RIKEN cDNA E130106K03 gene	0.58616647	0.005898	0.03786
94180	Acsbg1	acyl-CoA synthetase bubblegum family member 1	0.58616647	0.02791	0.1049
14237	Foxd4	forkhead box D4	0.58616647	0.0431	0.1394
20269	Scn3a	sodium channel, voltage-gated, type III, alpha	0.58582308	0.002684	0.02319
69311	1700008K24Rik	RIKEN cDNA 1700008K24 gene	0.58582308	0.08976	0.231
22283	Ush2a	Usher syndrome 2A (autosomal recessive, mild) homolog (human)	0.58548009	0.003257	0.02625
75763	Dcaf17	DDB1 and CUL4 associated factor 17	0.58513751	0.01876	0.08028
258392	Olfr190	olfactory receptor 190	0.58479532	0.001632	0.01734
52473	D11Ertd80e	DNA segment, Chr 11, ERATO Doi 80, expressed	0.58479532	0.01178	0.05954
18770	Pklr	pyruvate kinase liver and red blood cell	0.58445354	0.1245	0.2874
16688	Krt6b	keratin 6B	0.58411215	0.1373	0.3051
14178	Fgf7	fibroblast growth factor 7	0.58377116	0.001927	0.01917
320032	C130093G08Rik	RIKEN cDNA C130093G08 gene	0.58343057	0.0003555	0.006863

110257	Hba-a2	hemoglobin alpha, adult chain 2	0.58343057	0.0882	0.2285
236193	Zfp709	zinc finger protein 709	0.58309038	0.0004515	0.007966
664994	Isoc2a	isochorismatase domain containing 2a	0.58309038	0.004228	0.03082
93730	Lztfl1	leucine zipper transcription factor-like 1	0.58275058	9.19E-05	0.003297
27413	Abcb11	ATP-binding cassette, sub-family B (MDR/TAP), member 11	0.58275058	0.0003955	0.007334
320692	9430037G07Rik	RIKEN cDNA 9430037G07 gene	0.58241118	0.003217	0.02608
16571	Kif4	kinesin family member 4	0.58207218	0.0229	0.09197
224139	Golgb1	golgi autoantigen, golgin subfamily b, macrogolgin 1	0.58072009	0.008757	0.04883
27049	Etv3	ets variant gene 3	0.58038305	0.0003502	0.006801
654803	B130055M24Rik	RIKEN cDNA B130055M24 gene	0.5800464	0.0001703	0.004488
22436	Xdh	xanthine dehydrogenase	0.5800464	0.01442	0.06775
407828	BC023969	cDNA sequence BC023969	0.57971014	0.0003492	0.006792
15379	Onecut1	one cut domain, family member 1	0.57937428	0.01699	0.07489
70308	2610005M20Rik	RIKEN cDNA 2610005M20 gene	0.5790388	0.01006	0.0536
399602	9930024M15Rik	RIKEN cDNA 9930024M15 gene	0.5787037	0.0002633	0.005839
319347	C230094B09Rik	RIKEN cDNA C230094B09 gene	0.57803468	0.0005323	0.008922
75739	Mpp7	membrane protein, palmitoylated 7 (MAGUK p55 subfamily member 7)	0.57803468	0.001175	0.01433
50875	Tmod3	tropomodulin 3	0.57770075	0.0007541	0.01098
207798	Gramd1c	GRAM domain containing 1C	0.57770075	0.006145	0.03889
20715	Serpina3g	serine (or cysteine) peptidase inhibitor, clade A, member 3G	0.57736721	0.0009293	0.01244
107589	Mylk	myosin, light polypeptide kinase	0.57670127	0.00128	0.01502
209012	Ulk4	unc-51-like kinase 4 (C. elegans)	0.57636888	0.0007655	0.01105
635253	Usp51	ubiquitin specific protease 51	0.57603687	0.0001342	0.004009
66259	Camk2n1	calcium/calmodulin-dependent protein kinase II inhibitor 1	0.57603687	0.001002	0.01307
239839	Ccdc14	coiled-coil domain containing 14	0.57603687	0.02434	0.09552
65112	Pmepa1	prostate transmembrane protein, androgen induced 1	0.57537399	1.88E-05	0.001403
170740	Zfp287	zinc finger protein 287	0.57537399	0.03379	0.1191
68268	Zdhhc21	zinc finger, DHHC domain containing 21	0.57471264	0.01424	0.06725
74558	Gvin1	GTPase, very large interferon inducible 1	0.57471264	0.08739	0.2274
12064	Bdnf	brain derived neurotrophic factor	0.57438254	6.66E-05	0.00279
100042951	Gm4123	predicted gene 4123	0.57438254	0.00734	0.04379
18600	Padi2	peptidyl arginine deiminase, type II	0.57438254	0.02661	0.1016
100126243	A030001D20Rik	RIKEN cDNA A030001D20 gene	0.57372347	5.09E-05	0.002425
544848	Gm5784	predicted gene 5784	0.57372347	0.009033	0.04976
56705	Ranbp9	RAN binding protein 9	0.5733945	0.1683	0.3491
668253	Dleu2	deleted in lymphocytic leukemia, 2	0.57240985	0.004927	0.03383
320873	Cdh10	cadherin 10	0.57208238	0.0002693	0.005858
71960	Myh14	myosin, heavy polypeptide 14	0.57208238	0.03105	0.1128
83453	Chrdl1	chordin-like 1	0.57175529	0.0003606	0.006931
72373	Psca	prostate stem cell antigen	0.57142857	0.01057	0.05516
64452	Slc5a4a	solute carrier family 5, member 4a	0.57110223	0.263	0.4691
243308	A430033K04Rik	RIKEN cDNA A430033K04 gene	0.57045066	4.79E-05	0.002328
26388	Ifi202b	interferon activated gene 202B	0.57045066	0.1213	0.2826
100302730	5830417I10Rik	RIKEN cDNA 5830417I10 gene	0.56980057	0.000746	0.01095
226407	Rab3gap1	RAB3 GTPase activating protein subunit 1	0.56947608	9.89E-05	0.003415
12683	Cidea	cell death-inducing DNA fragmentation factor, alpha subunit-like effector A	0.56947608	0.003401	0.02693
54651	Usp27x	ubiquitin specific peptidase 27, X chromosome	0.56915196	0.1331	0.3
93837	Dach2	dachshund 2 (Drosophila)	0.56882821	0.01546	0.07093
72090	Entpd8	ectonucleoside triphosphate diphosphohydrolase 8	0.56882821	0.1134	0.2696
229595	Adamtsl4	ADAMTS-like 4	0.56850483	2.71E-05	0.001664

66255	Hsbp1l1	heat shock factor binding protein 1-like 1	0.56850483	0.001679	0.01765
67973	Mphosph10	M-phase phosphoprotein 10 (U3 small nucleolar ribonucleoprotein)	0.56850483	0.004194	0.03065
73719	Lce1c	late cornified envelope 1C	0.56850483	0.03801	0.1286
629581	Gm6985	predicted pseudogene 6985	0.56850483	0.1511	0.3258
69363	Spaca4	sperm acrosome associated 4	0.56850483	0.2378	0.44
72269	Cda	cytidine deaminase	0.56818182	0.1088	0.262
98661	AW061096	expressed sequence AW061096	0.56785917	5.63E-05	0.002567
93837	Dach2	dachshund 2 (Drosophila)	0.56753689	0.0001337	0.004003
70918	Nsun7	NOL1/NOP2/Sun domain family, member 7	0.56753689	0.003881	0.02923
66329	Susd3	sushi domain containing 3	0.56753689	0.4684	0.6681
277854	Depdc5	DEP domain containing 5	0.56721497	0.01072	0.05557
15122	Hba-a1	hemoglobin alpha, adult chain 1	0.56721497	0.1078	0.2604
665389	Gm7616	predicted gene 7616	0.56689342	0.006106	0.0387
108803	4933402P03Rik	RIKEN cDNA 4933402P03 gene	0.56625142	0.1055	0.257
52728	D17Zt10e	DNA segment, Chr 17, Zdenek Trachtulec 10 expressed	0.56593096	0.01334	0.06434
76415	Fam187b	family with sequence similarity 187, member B	0.56593096	0.02428	0.09537
100041420	Gm3325	predicted gene 3325	0.56561086	0.003998	0.02971
18600	Padi2	peptidyl arginine deiminase, type II	0.56529112	0.0198	0.08322
16858	Lgals7	lectin, galactose binding, soluble 7	0.56401579	0.06934	0.194
108075	Ltbp4	latent transforming growth factor beta binding protein 4	0.56179775	0.0003583	0.00691
100215	Gm11266	predicted gene 11266	0.56148231	0.2405	0.443
100042727	Gm3993	predicted gene 3993	0.56116723	0.009028	0.04976
226438	Igfn1	immunoglobulin-like and fibronectin type III domain containing 1	0.5608525	0.001117	0.01392
207686	A330021E22Rik	RIKEN cDNA A330021E22 gene	0.56053812	9.67E-05	0.003408
319864	D230035N22Rik	RIKEN cDNA D230035N22 gene	0.56053812	0.1841	0.371
237926	Rsad1	radical S-adenosyl methionine domain containing 1	0.56022409	0.0003251	0.006503
216225	Slc5a8	solute carrier family 5 (iodide transporter), member 8	0.56022409	0.06292	0.1814
100038417	Spink13	serine peptidase inhibitor, Kazal type 13	0.55928412	0.0007572	0.01098
114873	Dscaml1	Down syndrome cell adhesion molecule-like 1	0.55928412	0.004249	0.03092
209012	Ulk4	unc-51-like kinase 4 (C. elegans)	0.55928412	0.04904	0.1529
53415	Htatip2	HIV-1 tat interactive protein 2, homolog (human)	0.55897149	0.01846	0.07944
216797	Prss38	protease, serine, 38	0.55803571	2.02E-05	0.001424
12695	Inadl	InaD-like (Drosophila)	0.5574136	0.01303	0.06335
51885	Tubgcp4	tubulin, gamma complex associated protein 4	0.55710306	0.007287	0.04364
320658	D430040L24Rik	RIKEN cDNA D430040L24 gene	0.55679287	0.0005536	0.009099
66044	Dtd1	D-tyrosyl-tRNA deacylase 1 homolog (S. cerevisiae)	0.55648303	0.001563	0.01702
69282	1700001J03Rik	RIKEN cDNA 1700001J03 gene	0.55617353	4.18E-05	0.002162
100503605	Beta-s	hemoglobin subunit beta-1-like	0.55617353	0.08817	0.2285
100042840	Gm16501	predicted gene 16501	0.55524708	0.002188	0.02041
11496	Adam22	a disintegrin and metallopeptidase domain 22	0.55493896	0.04165	0.1364
380698	Obscn	obscurin, cytoskeletal calmodulin and titin-interacting RhoGEF	0.55493896	0.2874	0.4958
79202	Tnfrsf22	tumor necrosis factor receptor superfamily, member 22	0.55463117	0.2741	0.482
72504	Taf4b	TAF4B RNA polymerase II, TATA box binding protein (TBP)-associated factor	0.55432373	0.001485	0.01642
544763	Hbq1b	hemoglobin, theta 1B	0.55401662	0.1112	0.266
76658	1700123K08Rik	RIKEN cDNA 1700123K08 gene	0.55340343	0.0002715	0.005889
636931	Trim71	tripartite motif-containing 71	0.5527916	0.01329	0.06418
75894	Adal	adenosine deaminase-like	0.5527916	0.02956	0.1091
93837	Dach2	dachshund 2 (Drosophila)	0.55248619	0.009703	0.05225
18781	Pla2g2c	phospholipase A2, group IIC	0.55248619	0.02267	0.09137
20304	Ccl5	chemokine (C-C motif) ligand 5	0.55187638	0.005851	0.03769

17472	Gbp4	guanylate binding protein 4	0.55066079	0.0007053	0.01058
626854	Gm6712	predicted gene 6712	0.54945055	0.003823	0.02895
75136	Rsph10b2	radial spoke head 10 homolog B (Chlamydomonas)	0.54914882	0.0004399	0.007865
100038882	Isg15	ISG15 ubiquitin-like modifier	0.54914882	0.0006975	0.01051
353211	Prune2	prune homolog 2 (Drosophila)	0.54884742	0.002322	0.02126
73709	2410125D13Rik	RIKEN cDNA 2410125D13 gene	0.54794521	0.0003956	0.007334
320086	E430022K19Rik	RIKEN cDNA E430022K19 gene	0.54794521	0.0006194	0.009718
67020	Tmem88	transmembrane protein 88	0.54794521	0.001798	0.01838
15370	Nr4a1	nuclear receptor subfamily 4, group A, member 1	0.54794521	0.002834	0.02399
76509	1600029D21Rik	RIKEN cDNA 1600029D21 gene	0.54734537	0.02532	0.09823
12096	Bglap	bone gamma carboxyglutamate protein	0.54704595	0.369	0.578
224826	Ubr2	ubiquitin protein ligase E3 component n-recognin 2	0.54644809	0.3427	0.5536
22301	Vmn2r89	vomeronasal 2, receptor 89	0.54614965	0.03111	0.1129
319742	Mpzl3	myelin protein zero-like 3	0.54614965	0.03245	0.1162
628709	Gm10324	predicted gene 10324	0.54585153	0.009576	0.05183
434179	Gm5595	predicted gene 5595	0.54585153	0.06354	0.1826
70725	6330411D24Rik	RIKEN cDNA 6330411D24 gene	0.54525627	0.0002584	0.005777
14800	Gria2	glutamate receptor, ionotropic, AMPA2 (alpha 2)	0.54466231	0.0003098	0.006329
16663	Krt13	keratin 13	0.54466231	0.01042	0.05467
58807	Slco1c1	solute carrier organic anion transporter family, member 1c1	0.54347826	0.0003055	0.00629
327984	E130101M22	hypothetical protein E130101M22	0.54288817	9.19E-05	0.003297
399101	Snhg3	small nucleolar RNA host gene (non-protein coding) 3	0.54229935	0.0005775	0.009324
22239	Ugt8a	UDP galactosyltransferase 8A	0.54229935	0.08924	0.2303
319893	A230057D06Rik	RIKEN cDNA A230057D06 gene	0.54171181	0.001458	0.01622
320654	E330024J20Rik	RIKEN cDNA E330024J20 gene	0.54141852	0.009887	0.05291
320057	A630057N01Rik	RIKEN cDNA A630057N01 gene	0.54112554	0.002098	0.01999
208606	Rsrc2	arginine/serine-rich coiled-coil 2	0.54083288	0.04602	0.146
404318	Olfr681	olfactory receptor 681	0.54083288	0.1797	0.3654
93730	Lztfl1	leucine zipper transcription factor-like 1	0.54054054	2.55E-06	0.0004573
114672	1700007E05Rik	RIKEN cDNA 1700007E05 gene	0.54024851	0.001451	0.0162
69976	Galk2	galactokinase 2	0.54024851	0.002661	0.02307
668605	Gm9265	predicted pseudogene 9265	0.53908356	6.97E-05	0.002852
72585	Lypd1	Ly6/Plaur domain containing 1	0.5387931	0.0002764	0.005939
170643	Kirrel	kin of IRRE like (Drosophila)	0.53821313	0.001171	0.01432
74050	4921525O09Rik	RIKEN cDNA 4921525O09 gene	0.53763441	0.2859	0.4944
214552	Cep164	centrosomal protein 164	0.53705693	0.0006728	0.01022
18813	Pa2g4	proliferation-associated 2G4	0.53676865	0.003147	0.0257
100504146	LOC100504146	zinc finger protein 709-like	0.53648069	0.06184	0.1794
69489	2310007J06Rik	RIKEN cDNA 2310007J06 gene	0.53619303	2.70E-05	0.001664
381062	2210404J11Rik	RIKEN cDNA 2210404J11 gene	0.53619303	5.98E-05	0.002628
75761	Apol7a	apolipoprotein L 7a	0.53590568	0.0446	0.1427
101458	AA536887	expressed sequence AA536887	0.53418803	0.001243	0.01483
381413	Gpr176	G protein-coupled receptor 176	0.53304904	7.22E-05	0.002915
227525	Dclre1c	DNA cross-link repair 1C, PSO2 homolog (S. cerevisiae)	0.53304904	0.0002707	0.005877
100502632	LOC100502632	zinc finger protein 709-like	0.53304904	0.006683	0.04115
12823	Col19a1	collagen, type XIX, alpha 1	0.53276505	0.0258	0.0995
227618	Lrrc26	leucine rich repeat containing 26	0.53219798	0.1048	0.2559
100038882	Isg15	ISG15 ubiquitin-like modifier	0.53134963	0.001361	0.01559
63828	Fn3k	fructosamine 3 kinase	0.53022269	0.0003412	0.006706
246787	Slc5a2	solute carrier family 5 (sodium/glucose cotransporter), member 2	0.53022269	0.03051	0.1115

213171	Prss27	protease, serine, 27	0.53022269	0.1392	0.3083
15129	Hbb-b1	hemoglobin, beta adult major chain	0.52742616	0.03264	0.1166
100041864	Gm3552	predicted gene 3552	0.52687039	0.005902	0.03786
212190	Ubxn10	UBX domain protein 10	0.52631579	0.01032	0.05439
69700	Col22a1	collagen, type XXII, alpha 1	0.52603893	6.24E-05	0.0027
20514	Slc1a5	solute carrier family 1 (neutral amino acid transporter), member 5	0.52576236	0.0001454	0.004169
74571	Kcnk16	potassium channel, subfamily K, member 16	0.52493438	0.09347	0.2375
24066	Spry4	sprouty homolog 4 (Drosophila)	0.52438385	0.2881	0.4966
353371	Oxct2b	3-oxoacid CoA transferase 2B	0.52328624	0.4484	0.6505
667712	Gm8777	predicted gene 8777	0.52301255	0.2545	0.4594
243834	Zfp324	zinc finger protein 324	0.52273915	1.27E-05	0.001112
329636	Gm5103	predicted gene 5103	0.52273915	0.03028	0.111
26388	Ifi202b	interferon activated gene 202B	0.52219321	0.0968	0.243
71908	Cldn23	claudin 23	0.52137643	9.17E-05	0.003297
68789	Trmt61b	tRNA methyltransferase 61 homolog B (S. cerevisiae)	0.52110474	0.0007788	0.0112
100042899	Gm4098	predicted gene 4098	0.52110474	0.01162	0.05897
20614	Snap25	synaptosomal-associated protein 25	0.5192108	0.006388	0.03995
100041574	9030025P20Rik	RIKEN cDNA 9030025P20 gene	0.51894136	4.40E-05	0.002225
234290	BC030500	cDNA sequence BC030500	0.51840332	1.12E-05	0.001048
100042057	Gm3643	predicted gene 3643	0.51786639	0.000814	0.01148
320440	9530091C08Rik	RIKEN cDNA 9530091C08 gene	0.51759834	0.08412	0.2216
207921	A830093I24Rik	RIKEN cDNA A830093I24 gene	0.51652893	0.000696	0.0105
21784	Tff1	trefoil factor 1	0.51652893	0.1056	0.257
328643	Vwa5b2	von Willebrand factor A domain containing 5B2	0.51626226	0.1062	0.2579
97783	C76336	expressed sequence C76336	0.51546392	0.001514	0.0167
13711	Elf5	E74-like factor 5	0.51546392	0.005515	0.03634
110310	Krt7	keratin 7	0.51440329	0.0002945	0.006161
63830	Kcnq1ot1	KCNQ1 overlapping transcript 1	0.51440329	0.0421	0.1374
238130	Dock4	dedicator of cytokinesis 4	0.51387461	3.84E-05	0.002066
77883	6030498E09Rik	RIKEN cDNA 6030498E09 gene	0.51387461	0.05747	0.1706
99946	6720422M22Rik	RIKEN cDNA 6720422M22 gene	0.51308363	1.73E-05	0.00133
102219	AU018794	expressed sequence AU018794	0.51282051	0.0004498	0.007952
16992	Lta	lymphotoxin A	0.51229508	0.00724	0.04352
21784	Tff1	trefoil factor 1	0.51203277	0.1309	0.2969
319830	1500004A13Rik	RIKEN cDNA 1500004A13 gene	0.51177073	0.001812	0.01846
22021	Tpst1	protein-tyrosine sulfotransferase 1	0.51177073	0.3666	0.5757
77193	A930030B08Rik	RIKEN cDNA A930030B08 gene	0.51150895	0.00117	0.01432
94179	Krt23	keratin 23	0.51124744	0.0003094	0.006328
17700	Mstn	myostatin	0.51020408	0.00647	0.04023
76968	2310046K23Rik	RIKEN cDNA 2310046K23 gene	0.50994391	0.006433	0.04012
52882	Rgs7bp	regulator of G-protein signalling 7 binding protein	0.50994391	0.0682	0.1916
101807	AV356131	expressed sequence AV356131	0.50942435	3.59E-06	0.0005503
100434	Slc44a1	solute carrier family 44, member 1	0.50942435	0.0001739	0.004549
71981	Tdrd12	tudor domain containing 12	0.50916497	0.0002676	0.005855
170765	Ripply3	ripply3 homolog (zebrafish)	0.50890585	0.000206	0.00505
214552	Cep164	centrosomal protein 164	0.50890585	0.0004471	0.007919
12767	Cxcr4	chemokine (C-X-C motif) receptor 4	0.50813008	0.03231	0.1158
668110	Syce1l	synaptonemal complex central element protein 1 like	0.50735667	0.0001385	0.004072
16638	Klra7	killer cell lectin-like receptor, subfamily A, member 7	0.50735667	0.09189	0.2351
78685	B430105A11Rik	RIKEN cDNA B430105A11 gene	0.50658561	0.0007259	0.01078

59036	Dact1	dapper homolog 1, antagonist of beta-catenin (xenopus)	0.50581689	0.0007179	0.01071
16328	Cep250	centrosomal protein 250	0.50556117	0.0001381	0.004072
70992	4931407E12Rik	RIKEN cDNA 4931407E12 gene	0.50505051	0.07398	0.2031
74221	1700011F03Rik	RIKEN cDNA 1700011F03 gene	0.50479556	0.008183	0.04684
117147	Acsm1	acyl-CoA synthetase medium-chain family member 1	0.50428643	0.01174	0.05935
320415	Gchfr	GTP cyclohydrolase I feedback regulator	0.50403226	8.73E-06	0.0009269
108075	Ltbp4	latent transforming growth factor beta binding protein 4	0.50403226	0.003022	0.02493
73103	3110009E18Rik	RIKEN cDNA 3110009E18 gene	0.50403226	0.003992	0.02969
627871	Gm6802	predicted gene 6802	0.50327126	0.1363	0.3038
76509	1600029D21Rik	RIKEN cDNA 1600029D21 gene	0.50226017	0.006777	0.04157
320064	D130017N08Rik	RIKEN cDNA D130017N08 gene	0.50200803	0.0008427	0.01175
56546	Sec1	secretory blood group 1	0.50125313	0.0001896	0.004775
671132	Gm9519	predicted gene 9519	0.49975012	0.01842	0.07934
320456	B330016D10Rik	RIKEN cDNA B330016D10 gene	0.49925112	0.003806	0.02892
54200	Sult2b1	sulfotransferase family, cytosolic, 2B, member 1	0.49850449	0.0008128	0.01148
638580	Gm7244	predicted gene 7244	0.49800797	4.21E-06	0.0006048
100043034	Gm13138	predicted gene 13138	0.49776008	1.28E-05	0.001113
216227	Slc17a8	solute carrier family 17 (sodium-dependent inorganic phosphate cotransporter), member 8	0.49701789	0.0004457	0.007902
13599	Ecel1	endothelin converting enzyme-like 1	0.49701789	0.00509	0.03455
100216455	Gm14124	predicted gene 14124	0.49701789	0.04168	0.1365
319430	Gpr77	G protein-coupled receptor 77	0.49603175	0.01764	0.077
69368	Wdfy1	WD repeat and FYVE domain containing 1	0.49578582	8.79E-06	0.0009274
75818	4930478L05Rik	RIKEN cDNA 4930478L05 gene	0.49407115	0.0003187	0.006424
14349	Fv1	Friend virus susceptibility 1	0.49188392	0.001997	0.0195
546896	Olfr455	olfactory receptor 455	0.49115914	0.002038	0.01967
74424	Tmc5	transmembrane channel-like gene family 5	0.49115914	0.06361	0.1827
12490	Cd34	CD34 antigen	0.49067713	0.001007	0.01312
67812	Ubxn4	UBX domain protein 4	0.49019608	0.1918	0.3815
208982	Hmgcll1	3-hydroxymethyl-3-methylglutaryl-Coenzyme A lyase-like 1	0.48923679	0.0001682	0.004465
171530	Ucn2	urocortin 2	0.48923679	0.09902	0.2468
269033	4930503L19Rik	RIKEN cDNA 4930503L19 gene	0.48875855	0.001719	0.01791
328191	B230303A05Rik	RIKEN cDNA B230303A05 gene	0.48875855	0.01634	0.07324
227058	Dnahc7b	dynein, axonemal, heavy chain 7B	0.48875855	0.02081	0.08633
78391	2610524J22Rik	RIKEN cDNA 2610524J22 gene	0.48875855	0.1303	0.296
75617	Rps25	ribosomal protein S25	0.48828125	2.10E-05	0.001458
242773	Slc45a1	solute carrier family 45, member 1	0.48804295	0.001955	0.01932
228576	Mall	mal, T-cell differentiation protein-like	0.48804295	0.004223	0.0308
319691	D430022A14Rik	RIKEN cDNA D430022A14 gene	0.48685492	0.001072	0.01361
214253	Etnk2	ethanolamine kinase 2	0.48685492	0.001324	0.01537
56365	Clcnkb	chloride channel Kb	0.48567266	0.0009519	0.01262
74978	Lrriq1	leucine-rich repeats and IQ motif containing 1	0.48543689	0.3543	0.5642
15397	Hoxa11as	HOXA11 antisense RNA (non-protein coding)	0.48473097	0.006449	0.04015
66344	Lce3b	late cornified envelope 3B	0.48473097	0.06202	0.1797
68231	H2afb1	H2A histone family, member B1	0.48379294	0.0004798	0.008329
16517	Kcnj16	potassium inwardly-rectifying channel, subfamily J, member 16	0.48285852	0.005426	0.03599
76933	Ifi27l2a	interferon, alpha-inducible protein 27 like 2A	0.48239267	0.00186	0.01878
239083	Ccnb1ip1	cyclin B1 interacting protein 1	0.48216008	0.006395	0.03996
76265	Tsen54	tRNA splicing endonuclease 54 homolog (S. cerevisiae)	0.48146365	6.85E-05	0.00283
18101	Nmbr	neuromedin B receptor	0.48146365	0.0008682	0.01197
76426	1700029J11Rik	RIKEN cDNA 1700029J11 gene	0.48123195	0.05598	0.1679

231004	Samd11	sterile alpha motif domain containing 11	0.48007681	1.62E-05	0.001278
71898	Apol9b	apolipoprotein L 9b	0.4784689	0.0002605	0.0058
328399	A930018M24Rik	RIKEN cDNA A930018M24 gene	0.47687172	4.79E-05	0.002328
17833	Muc5ac	mucin 5, subtypes A and C, tracheobronchial/gastric	0.47551117	0.1151	0.2723
18626	Per1	period homolog 1 (Drosophila)	0.47483381	0.0005868	0.009381
58801	Pmaip1	phorbol-12-myristate-13-acetate-induced protein 1	0.4743833	0.0003039	0.006271
19763	Ring1	ring finger protein 1	0.47415837	0.000148	0.004215
668643	EG668643	predicted gene, EG668643	0.47326077	0.008555	0.04816
73463	Als2cr11	amyotrophic lateral sclerosis 2 (juvenile) chromosome region, candidate 11 (human)	0.47281324	0.001344	0.01549
214531	Tmprss13	transmembrane protease, serine 13	0.47258979	0.004874	0.03361
258964	Olfr541	olfactory receptor 541	0.47214353	0.3054	0.5146
26971	Pla2g2f	phospholipase A2, group IIF	0.47014575	0.08474	0.2228
100303645	LOC100303645	hypothetical LOC100303645	0.46926326	0.0296	0.1092
73617	1700122H20Rik	RIKEN cDNA 1700122H20 gene	0.46926326	0.3239	0.5337
13214	Defb1	defensin beta 1	0.46904315	0.0006633	0.01011
11656	Alas2	aminolevulinic acid synthase 2, erythroid	0.46882325	0.05848	0.1724
18553	Pcsk6	proprotein convertase subtilisin/kexin type 6	0.46838407	7.05E-06	0.0008047
67607	Zfp788	zinc finger protein 788	0.46838407	0.1937	0.3837
13852	Stx2	syntaxin 2	0.46772685	0.0002315	0.005434
16869	Lhx1	LIM homeobox protein 1	0.46685341	6.62E-05	0.00279
17285	Meox1	mesenchyme homeobox 1	0.46446818	0.00312	0.02558
434166	Gm5589	predicted gene 5589	0.46403712	0.004831	0.03346
20471	Six1	sine oculis-related homeobox 1 homolog (Drosophila)	0.46382189	2.07E-06	0.0004063
258379	Olfr1284	olfactory receptor 1284	0.46232085	0.2353	0.4371
272322	Arntl2	aryl hydrocarbon receptor nuclear translocator-like 2	0.46125461	0.007937	0.04591
232813	Shisa7	shisa homolog 7 (Xenopus laevis)	0.456621	0.002578	0.02257
100043332	Gm4368	predicted gene 4368	0.45310376	0.0007116	0.01065
66042	Sostdc1	sclerostin domain containing 1	0.45269353	0.005741	0.03728
14873	Gsto1	glutathione S-transferase omega 1	0.45248869	0.0004817	0.008346
232983	Cxcl17	chemokine (C-X-C motif) ligand 17	0.45248869	0.04079	0.1346
195434	Utp14b	UTP14, U3 small nucleolar ribonucleoprotein, homolog B (yeast)	0.45187528	0.007534	0.04444
100043706	Gm4598	predicted gene 4598	0.45065345	0.2073	0.402
329253	6530439I21	hypothetical protein 6530439I21	0.44964029	0.001084	0.01368
58185	Rsad2	radical S-adenosyl methionine domain containing 2	0.44903458	0.01246	0.06146
217109	Utp18	UTP18, small subunit (SSU) processome component, homolog (yeast)	0.4454343	0.162	0.3404
13107	Cyp2f2	cytochrome P450, family 2, subfamily f, polypeptide 2	0.44483986	0.0634	0.1824
384817	4930448N21Rik	RIKEN cDNA 4930448N21 gene	0.44483986	0.1774	0.3624
16612	Klk1	kallikrein 1	0.444247	0.1071	0.2592
330734	B930018H19	hypothetical protein B930018H19	0.44247788	6.84E-05	0.00283
228770	Rspo4	R-spondin family, member 4	0.4415011	0.006406	0.03998
74176	Tgm5	transglutaminase 5	0.44072279	0.001472	0.01631
68070	Pdzd2	PDZ domain containing 2	0.44052863	8.07E-05	0.003088
234366	Gatad2a	GATA zinc finger domain containing 2A	0.43975374	3.40E-05	0.001912
664788	Gm7339	predicted gene 7339	0.43821209	0.02378	0.09422
114479	Slc5a5	solute carrier family 5 (sodium iodide symporter), member 5	0.43802015	0.001655	0.01747
70893	Glb1l3	galactosidase, beta 1 like 3	0.43782837	0.005434	0.03602
81879	Tcfcp2l1	transcription factor CP2-like 1	0.43572985	0.02911	0.1079
59095	Fxyd6	FXYD domain-containing ion transport regulator 6	0.43497173	2.36E-06	0.0004324
74354	Lrguk	leucine-rich repeats and guanylate kinase domain containing	0.43440487	0.001022	0.01322
320082	Fbxw21	F-box and WD-40 domain protein 21	0.43327556	0.0003229	0.006475

74648	S100pbp	S100P binding protein	0.43308792	0.0004934	0.008468
664788	Gm7339	predicted gene 7339	0.43252595	0.01687	0.07455
13710	Elf3	E74-like factor 3	0.43159258	0.0001223	0.003816
114564	Csprs	component of Sp100-rs	0.43010753	0.01682	0.07444
77883	6030498E09Rik	RIKEN cDNA 6030498E09 gene	0.42955326	0.008892	0.04922
94094	Trim34a	tripartite motif-containing 34A	0.42936883	0.03389	0.1192
546524	Gm5953	predicted gene 5953	0.42936883	0.04007	0.133
100040049	1810010D01Rik	RIKEN cDNA 1810010D01 gene	0.42844901	0.000131	0.003976
246730	Oas1a	2'-5' oligoadenylate synthetase 1A	0.42680324	8.85E-05	0.003239
71141	4933407L21Rik	RIKEN cDNA 4933407L21 gene	0.42535091	0.0005218	0.008798
26465	Zfp146	zinc finger protein 146	0.42517007	0.0005525	0.009089
94222	Olig3	oligodendrocyte transcription factor 3	0.42517007	0.0005969	0.009502
320365	Fry	furry homolog (Drosophila)	0.42480884	0.02248	0.09081
76561	Snx7	sorting nexin 7	0.42462845	0.111	0.2657
12555	Cdh15	cadherin 15	0.42301184	3.40E-05	0.001912
11684	Alox12	arachidonate 12-lipoxygenase	0.41911148	0.07697	0.2087
66583	Exosc1	exosome component 1	0.41823505	0.0008765	0.01202
101028	BB070754	expressed sequence BB070754	0.41649313	0.06534	0.1862
241134	9430031J16Rik	RIKEN cDNA 9430031J16 gene	0.41442188	4.46E-06	0.0006223
14842	Gsx1	GS homeobox 1	0.41254125	0.005825	0.03759
71449	Mettl13	methyltransferase like 13	0.41169205	0.002005	0.01955
216725	Adamts2	a disintegrin-like and metallopeptidase (reprolysin type) with thrombospondin type 1 motif, 2	0.40983607	0.01445	0.0678
319615	Zfp944	zinc finger protein 944	0.40950041	0.04209	0.1374
258744	Olfr875	olfactory receptor 875	0.40816327	0.1553	0.3314
11846	Arg1	arginase, liver	0.40700041	0.0007005	0.01053
320715	C530043K16Rik	RIKEN cDNA C530043K16 gene	0.40567951	0.0001182	0.003766
20202	S100a9	S100 calcium binding protein A9 (calgranulin B)	0.40387722	0.2123	0.4082
18261	Ocm	oncomodulin	0.40306328	4.41E-06	0.0006223
72556	Zfp566	zinc finger protein 566	0.40176778	9.27E-06	0.0009567
320226	4930473A06Rik	RIKEN cDNA 4930473A06 gene	0.4014452	4.08E-05	0.002124
66353	Riiad1	regulatory subunit of type II PKA R-subunit (RIIa) domain containing 1	0.40096231	0.0001029	0.003493
67718	Lce1h	late cornified envelope 1H	0.40096231	0.08559	0.2243
231004	Samd11	sterile alpha motif domain containing 11	0.39666799	0.0003045	0.006275
72785	2810474C18Rik	RIKEN cDNA 2810474C18 gene	0.39666799	0.02161	0.08862
17002	Ltf	lactotransferrin	0.39588282	0.006284	0.03955
104103	Airn	antisense Igf2r RNA	0.39572616	6.12E-05	0.002659
76265	Tsen54	tRNA splicing endonuclease 54 homolog (S. cerevisiae)	0.39308176	0.000204	0.005024
234358	Zfp930	zinc finger protein 930	0.39047247	5.25E-05	0.002456
71831	1700007B14Rik	RIKEN cDNA 1700007B14 gene	0.38880249	0.1009	0.2501
272636	Esyt3	extended synaptotagmin-like protein 3	0.3875969	0.07677	0.2083
72461	Prcp	prolylcarboxypeptidase (angiotensinase C)	0.38491147	9.28E-05	0.003316
100039796	Tgtp2	T-cell specific GTPase 2	0.38491147	0.0004982	0.008526
380669	Lin28b	lin-28 homolog B (C. elegans)	0.38270188	0.08846	0.2291
231507	Plac8	placenta-specific 8	0.38255547	0.001926	0.01917
210274	Shank2	SH3/ankyrin domain gene 2	0.38124285	0.03134	0.1134
67446	Dusp28	dual specificity phosphatase 28	0.38037277	0.003411	0.02698
625353	Vmn2r35	vomeronasal 2, receptor 35	0.37893141	0.003939	0.02946
18782	Pla2g2d	phospholipase A2, group IID	0.37807183	0.01287	0.06284
68655	Fndc1	fibronectin type III domain containing 1	0.37481259	5.53E-06	0.000723
80907	Lactb	lactamase, beta	0.37453184	9.70E-06	0.0009814

70227	Zfp619	zinc finger protein 619	0.37383178	7.88E-05	0.003048
211135	D130040H23Rik	RIKEN cDNA D130040H23 gene	0.37230082	0.000805	0.01144
64661	Krtdap	keratinocyte differentiation associated protein	0.37230082	0.007847	0.04562
21873	Tjp2	tight junction protein 2	0.3705076	2.78E-06	0.0004794
67374	Jam2	junction adhesion molecule 2	0.37009623	0.001947	0.01926
99171	AU015584	expressed sequence AU015584	0.37009623	0.01066	0.05539
100038605	E030047D23Rik	RIKEN cDNA E030047D23 gene	0.36900369	0.0006015	0.009543
20863	Stfa3	stefin A3	0.36845984	0.02849	0.1064
666021	2300003K06Rik	RIKEN cDNA 2300003K06 gene	0.368053	0.1359	0.3033
71064	4933406B15Rik	RIKEN cDNA 4933406B15 gene	0.36683786	0.06989	0.195
53324	Nptx2	neuronal pentraxin 2	0.36536354	0.0004045	0.007453
667373	Gm14446	predicted gene 14446	0.36509675	0.0003182	0.006424
223672	Apol9a	apolipoprotein L 9a	0.36483035	0.004896	0.0337
68655	Fndc1	fibronectin type III domain containing 1	0.36350418	3.39E-05	0.001912
12234	Btrc	beta-transducin repeat containing protein	0.36192544	5.38E-07	0.0001559
109685	Hyal3	hyaluronoglucosaminidase 3	0.36192544	0.09651	0.2426
76229	Vmn2r29	vomeronasal 2, receptor 29	0.36101083	0.005663	0.03698
67014	Mina	myc induced nuclear antigen	0.36062027	0.0001652	0.004429
545471	Zfp345	zinc finger protein 345	0.35958288	0.04922	0.1533
353211	Prune2	prune homolog 2 (Drosophila)	0.35880875	9.12E-05	0.003297
77090	Ocel1	occludin/ELL domain containing 1	0.35829452	2.72E-06	0.0004772
18159	Nppc	natriuretic peptide type C	0.35803795	1.55E-05	0.001256
12509	Cd59a	CD59a antigen	0.35790981	4.26E-07	0.0001317
100043407	Gm4419	predicted gene 4419	0.35561878	0.001662	0.01751
100043332	Gm4368	predicted gene 4368	0.35549236	0.001838	0.01863
84112	Sucnr1	succinate receptor 1	0.3534818	0.01538	0.07062
58203	Zbp1	Z-DNA binding protein 1	0.35248502	7.02E-06	0.0008047
238024	Fn3krp	fructosamine 3 kinase related protein	0.35087719	0.00194	0.01924
66809	Krt20	keratin 20	0.34566194	0.000274	0.005915
381157	Greb1l	growth regulation by estrogen in breast cancer-like	0.34340659	5.42E-06	0.0007224
66195	Lce1g	late cornified envelope 1G	0.34281796	0.02422	0.09527
237890	Slfn14-ps	schlafen family member 14, pseudogene	0.34153005	0.1675	0.3483
16867	Lhcgr	luteinizing hormone/choriogonadotropin receptor	0.34141345	0.1004	0.2493
672682	Gm9573	predicted gene 9573	0.33726813	0.0005725	0.009267
23795	Agr2	anterior gradient 2 (Xenopus laevis)	0.33101622	0.0002752	0.005927
21940	Cd27	CD27 antigen	0.32948929	0.03018	0.1108
433470	AA467197	expressed sequence AA467197	0.3288392	0.002365	0.0214
13656	Egr4	early growth response 4	0.32776139	0.0007165	0.0107
73906	4833417C18Rik	RIKEN cDNA 4833417C18 gene	0.32615786	0.03257	0.1165
56092	Cts7	cathepsin 7	0.32552083	0.03098	0.1127
100038492	F630048H11Rik	RIKEN cDNA F630048H11 gene	0.3254149	0.0002677	0.005855
68307	Lrriq4	leucine-rich repeats and IQ motif containing 4	0.32478077	0.0008346	0.01166
93689	Lmod1	leiomodin 1 (smooth muscle)	0.3227889	0.03248	0.1163
108124	Napa	N-ethylmaleimide sensitive fusion protein attachment protein alpha	0.32268474	1.25E-06	0.0003029
17219	Mcm6	minichromosome maintenance deficient 6 (MIS5 homolog, S. pombe) (S. cerevisiae)	0.31989763	5.14E-07	0.0001538
241490	Rbm45	RNA binding motif protein 45	0.31948882	0.0001878	0.004749
84111	Gpr87	G protein-coupled receptor 87	0.31948882	0.002877	0.02421
26905	Eif2s3x	eukaryotic translation initiation factor 2, subunit 3, structural gene X-linked	0.31938678	0.01488	0.06901
100042166	Gm3704	predicted gene 3704	0.3187759	9.72E-05	0.003408
110855	Pde6c	phosphodiesterase 6C, cGMP specific, cone, alpha prime	0.31715826	3.32E-07	0.0001124

66757	Adat2	adenosine deaminase, tRNA-specific 2, TAD2 homolog (S. cerevisiae)	0.31585597	7.28E-06	0.000821
319271	A130072N09Rik	RIKEN cDNA A130072N09 gene	0.31505986	0.0004271	0.007719
449000	Zfp960	zinc finger protein 960	0.31456433	3.77E-05	0.002047
73252	1600022D10Rik	RIKEN cDNA 1600022D10 gene	0.31065548	0.007532	0.04444
110855	Pde6c	phosphodiesterase 6C, cGMP specific, cone, alpha prime	0.30854674	5.36E-07	0.0001559
319707	C430002N11Rik	RIKEN cDNA C430002N11 gene	0.30835646	0.02411	0.09508
269593	Luzp1	leucine zipper protein 1	0.30590395	0.01151	0.05852
171167	Fut10	fucosyltransferase 10	0.30506406	8.04E-06	0.0008786
21684	Tectb	tectorin beta	0.30395137	1.40E-05	0.001178
55936	Ctps2	cytidine 5'-triphosphate synthase 2	0.30138638	1.58E-06	0.0003464
109820	Pgc	progastricsin (pepsinogen C)	0.29949087	0.0006876	0.01041
620253	Dcpp3	demilune cell and parotid protein 3	0.29770765	0.1274	0.2918
73719	Lce1c	late cornified envelope 1C	0.2919708	0.04312	0.1395
67127	Lce1a1	late cornified envelope 1A1	0.2895194	0.009717	0.05231
100503910	LOC100503910	hypothetical LOC100503910	0.28901734	0.001155	0.01418
69134	Fam25c	family with sequence similarity 25, member C	0.28810141	0.01129	0.05773
76464	Casc5	cancer susceptibility candidate 5	0.28669725	0.03534	0.1228
243659	Styk1	serine/threonine/tyrosine kinase 1	0.28579594	0.09688	0.243
434223	Gm1966	predicted gene 1966	0.285144	0.1914	0.3809
77394	9530025H10Rik	RIKEN cDNA 9530025H10 gene	0.27824151	0.1083	0.2612
668814	Gm9376	predicted gene 9376	0.27800945	1.63E-07	7.98E-05
207854	Fmr1nb	fragile X mental retardation 1 neighbor	0.27570995	0.03793	0.1285
231655	Oasl1	2'-5' oligoadenylate synthetase-like 1	0.27151778	0.0001607	0.004382
68789	Trmt61b	tRNA methyltransferase 61 homolog B (S. cerevisiae)	0.2708559	0.09503	0.2402
76808	Rpl18a	ribosomal protein L18A	0.269324	0.02085	0.0864
74175	Crct1	cysteine-rich C-terminal 1	0.26752274	0.01301	0.0633
68720	Lce1b	late cornified envelope 1B	0.2664535	0.007276	0.0436
258097	Olfr1500	olfactory receptor 1500	0.26441036	0.03333	0.118
381680	BC055004	cDNA sequence BC055004	0.26434047	0.06099	0.1774
625530	Dub3	deubiquitinating enzyme 3	0.26226069	0.09452	0.2394
224552	Vmn2r98	vomeronasal 2, receptor 98	0.26143791	0.1572	0.3344
107047	Psmg2	proteasome (prosome, macropain) assembly chaperone 2	0.26096033	0.08569	0.2244
67441	Isoc2b	isochorismatase domain containing 2b	0.25940337	0.0002829	0.005997
22226	Ucn	urocortin	0.2593361	0.001519	0.01672
67588	Rnf41	ring finger protein 41	0.25753284	4.38E-05	0.002225
19223	Ptgis	prostaglandin I2 (prostacyclin) synthase	0.24943876	1.39E-06	0.0003151
12319	Car8	carbonic anhydrase 8	0.24919013	0.000825	0.01157
52020	Umodl1	uromodulin-like 1	0.24727992	0.000756	0.01098
234138	BC019943	cDNA sequence BC019943	0.24697456	6.39E-07	0.0001795
67483	1700028P14Rik	RIKEN cDNA 1700028P14 gene	0.24563989	1.17E-05	0.00107
72902	Spock3	sparc/osteonectin, cwcv and kazal-like domains proteoglycan 3	0.24455857	0.02633	0.1009
67133	Gp2	glycoprotein 2 (zymogen granule membrane)	0.24449878	0.04266	0.1386
20908	Stx3	syntaxin 3	0.24295432	2.51E-07	9.54E-05
72723	Zfp74	zinc finger protein 74	0.24003841	5.78E-06	0.0007388
76359	2610028D06Rik	RIKEN cDNA 2610028D06 gene	0.2399808	5.53E-06	0.000723
16181	Il1rn	interleukin 1 receptor antagonist	0.23975066	0.07522	0.2052
80796	Calm4	calmodulin 4	0.23894863	0.01787	0.07768
108812	Als2cr12	amyotrophic lateral sclerosis 2 (juvenile) chromosome region, candidate 12 (human)	0.23826543	2.24E-05	0.001507
76568	Ift46	intraflagellar transport 46 homolog (Chlamydomonas)	0.23792529	2.28E-05	0.001521
12319	Car8	carbonic anhydrase 8	0.23293734	0.0001227	0.003818

234404	Nxnl1	nucleoredoxin-like 1	0.23004371	1.98E-07	8.79E-05
75080	4930518C09Rik	RIKEN cDNA 4930518C09 gene	0.22825839	0.02161	0.08862
11808	Apoa4	apolipoprotein A-IV	0.22441652	0.1308	0.2968
234138	BC019943	cDNA sequence BC019943	0.21815009	7.35E-08	5.26E-05
100041290	D4Ertd617e	DNA segment, Chr 4, ERATO Doi 617, expressed	0.21786492	0.0003467	0.006763
98736	1700034H15Rik	RIKEN cDNA 1700034H15 gene	0.21673169	0.09105	0.2336
67483	1700028P14Rik	RIKEN cDNA 1700028P14 gene	0.21663778	6.13E-06	0.0007462
22290	Uty	ubiquitously transcribed tetratricopeptide repeat gene, Y chromosome	0.20990764	0.01989	0.08353
231507	Plac8	placenta-specific 8	0.20881186	2.71E-05	0.001664
21412	Tcf21	transcription factor 21	0.20768432	0.0621	0.1798
76585	Lce1i	late cornified envelope 1I	0.20725389	0.01088	0.05612
14246	Flg	filaggrin	0.20669698	0.02712	0.1028
100043034	Gm13138	predicted gene 13138	0.20193861	0.0003366	0.006653
16061	Igh-VJ558	immunoglobulin heavy chain (J558 family)	0.1973165	3.86E-05	0.002066
22290	Uty	ubiquitously transcribed tetratricopeptide repeat gene, Y chromosome	0.19665683	0.04838	0.1512
319742	Mpzl3	myelin protein zero-like 3	0.18754689	0.006575	0.04069
209760	Tmc7	transmembrane channel-like gene family 7	0.18716077	4.51E-05	0.002249
271221	5031414D18Rik	RIKEN cDNA 5031414D18 gene	0.18521949	0.09344	0.2375
73061	3110007F17Rik	RIKEN cDNA 3110007F17 gene	0.18467221	0.01026	0.05418
74528	8430406I07Rik	RIKEN cDNA 8430406I07 gene	0.18224895	0.008492	0.04789
76681	Trim12a	tripartite motif-containing 12A	0.18018018	0.0004647	0.008135
68694	Lce1e	late cornified envelope 1E	0.180018	0.0036	0.02787
67828	Lce1f	late cornified envelope 1F	0.17522341	0.00443	0.03173
67133	Gp2	glycoprotein 2 (zymogen granule membrane)	0.17461149	0.02909	0.1078
66442	Spc25	SPC25, NDC80 kinetochore complex component, homolog (S. cerevisiae)	0.16801075	7.49E-05	0.00298
229613	6330549D23Rik	RIKEN cDNA 6330549D23 gene	0.1552795	8.57E-07	0.0002301
71687	Tmem25	transmembrane protein 25	0.15441631	6.43E-08	4.89E-05
20592	Kdm5d	lysine (K)-specific demethylase 5D	0.15003751	0.05474	0.1653
69611	Lce1d	late cornified envelope 1D	0.14766686	0.01599	0.07222
78609	8030411F24Rik	RIKEN cDNA 8030411F24 gene	0.1459002	1.46E-05	0.001213
94216	Col4a6	collagen, type IV, alpha 6	0.12906557	0.0003292	0.00657
17921	Myo7a	myosin VIIA	0.124471	1.77E-07	8.07E-05
140709	Emid2	EMI domain containing 2	0.12014898	1.01E-06	0.0002638
73162	Otud3	OTU domain containing 3	0.11830119	0.06001	0.1754
16061	Igh-VJ558	immunoglobulin heavy chain (J558 family)	0.11682243	0.0004181	0.00762
72461	Prcp	prolylcarboxypeptidase (angiotensinase C)	0.11543345	1.35E-08	2.46E-05
100040412	LOC100040412	lysine-specific demethylase 5D-like	0.11405109	0.05389	0.1635
100040410	Gm14957	predicted gene 14957	0.10751532	7.81E-08	5.26E-05
17427	Mns1	meiosis-specific nuclear structural protein 1	0.10049241	1.01E-06	0.0002638
214523	Tmprss4	transmembrane protease, serine 4	0.09514748	2.39E-08	3.05E-05
269209	Stk36	serine/threonine kinase 36 (fused homolog, Drosophila)	0.09140768	4.27E-06	0.0006091
67705	1810058I24Rik	RIKEN cDNA 1810058I24 gene	0.08244023	4.14E-05	0.002149
433100	AA388235	expressed sequence AA388235	0.08084074	0.0134	0.06454
26450	Rbbp9	retinoblastoma binding protein 9	0.07674597	0.06533	0.1861
74602	4833428L15Rik	RIKEN cDNA 4833428L15 gene	0.06381621	5.37E-08	4.88E-05
73061	3110007F17Rik	RIKEN cDNA 3110007F17 gene	0.06038647	2.97E-08	3.32E-05
72154	Zfp157	zinc finger protein 157	0.05790388	4.90E-07	0.0001492
75764	Slx1b	SLX1 structure-specific endonuclease subunit homolog B (S. cerevisiae)	0.0507872	2.71E-09	9.89E-06
26900	Ddx3y	DEAD (Asp-Glu-Ala-Asp) box polypeptide 3, Y-linked	0.04436557	0.05882	0.1732
69301	1700008P20Rik	RIKEN cDNA 1700008P20 gene	0.04393673	1.02E-05	0.0009941

30939	Pttg1	pituitary tumor-transforming gene 1	0.04244482	1.84E-06	0.0003729
14537	Gcnt1	glucosaminyl (N-acetyl) transferase 1, core 2	0.03949447	1.14E-09	5.20E-06
69301	1700008P20Rik	RIKEN cDNA 1700008P20 gene	0.03585515	1.07E-06	0.0002746
54631	Nphs1	nephrosis 1 homolog, nephrin (human)	0.03220612	1.02E-07	5.80E-05
77936	BC065403	cDNA sequence BC065403	0.03111388	1.52E-07	7.70E-05
26908	Eif2s3y	eukaryotic translation initiation factor 2, subunit 3, structural gene Y-linked	0.03098853	0.05194	0.1594
30939	Pttg1	pituitary tumor-transforming gene 1	0.02600104	1.03E-09	5.20E-06
76681	Trim12a	tripartite motif-containing 12A	0.0237023	2.23E-08	3.05E-05
80509	Med8	mediator of RNA polymerase II transcription, subunit 8 homolog (yeast)	0.01467567	3.21E-07	0.0001114
258198	Olfr224	olfactory receptor 224	0.01461775	2.21E-06	0.0004235
329478	C130078N14	hypothetical protein C130078N14	0.00588235	3.04E-11	5.56E-07
