# Supplementary material for: Hif1a inactivation rescues photoreceptor degeneration induced by a chronic hypoxia-like stress
Source: Cell Death Differ. 2018 Apr 17;25(12):2071–85. doi: 10.1038/s41418-018-0094-7 (PMC6261999; doi:10.1038/s41418-018-0094-7)
Supplement: Supplementary file 3 — Supplemental File S2 [file 41418_2018_94_MOESM3_ESM.pdf]

Suppl. Table S2: Comparison: Average expression in *rod*$^{\Delta Vhl;Hif1a}$ divided by average expression in *Vhl*$^{f/f}$*;Hif1a*$^{f/f}$ *(control)*

Entrez Gene ID [Agilent]	Gene Symbol [Agilent]	Description [Agilent]	ratio	pValue	fdr
70045	2610528A11Rik	RIKEN cDNA 2610528A11 gene	24.04	2.38E-09	1.88E-05
213742	Xist	inactive X specific transcripts	13.42	0.08964	0.5224
17996	Neb	nebulin	13.39	0.1965	0.6313
213742	Xist	inactive X specific transcripts	7.028	0.05035	0.4516
109685	Hyal3	hyaluronoglucosaminidase 3	6.959	0.01261	0.3277
70979	4931417G12Rik	RIKEN cDNA 4931417G12 gene	6.84	1.02E-06	0.003238
19076	Prim2	DNA primase, p58 subunit	5.255	7.60E-09	4.01E-05
232801	Lilra5	leukocyte immunoglobulin-like receptor, subfamily A (with TM domain), member 5	4.874	0.09846	0.5367
272643	Prss43	protease, serine, 43	4.814	0.04997	0.451
77044	Arid2	AT rich interactive domain 2 (ARID, RFX-like)	4.807	0.03884	0.4213
382109	Fbxw26	F-box and WD-40 domain protein 26	4.708	0.09601	0.5344
434794	Xlr4a	X-linked lymphocyte-regulated 4A	4.484	0.00859	0.3069
331529	Gm5128	predicted gene 5128	4.13	0.02661	0.3809
106610	AI504002	expressed sequence AI504002	4.01	0.2894	0.7004
223332	Ranbp3l	RAN binding protein 3-like	3.692	0.1048	0.5453
16816	Lcat	lecithin cholesterol acyltransferase	3.592	0.2167	0.6482
434540	Psg20	pregnancy-specific glycoprotein 20	3.536	0.1571	0.6016
12040	Bckdhb	branched chain ketoacid dehydrogenase E1, beta polypeptide	3.503	0.2697	0.6878
18216	Ntsr1	neurotensin receptor 1	3.475	0.1094	0.5529
50500	Ttpa	tocopherol (alpha) transfer protein	3.417	0.1324	0.5768
67722	4921517D21Rik	RIKEN cDNA 4921517D21 gene	3.386	0.022	0.3694
73748	Gadl1	glutamate decarboxylase-like 1	3.326	0.01482	0.3369
71078	Adam30	a disintegrin and metallopeptidase domain 30	3.298	0.08237	0.5144
100042761	Gm10697	predicted gene 10697	3.258	0.169	0.6098
246229	Bivm	basic, immunoglobulin-like variable motif containing	3.25	0.2965	0.7051
329972	Spata21	spermatogenesis associated 21	3.172	0.08715	0.5184
258757	Olfr1013	olfactory receptor 1013	3.152	0.06975	0.4884
114662	Prss29	protease, serine, 29	2.968	0.1561	0.5991
433100	AA388235	expressed sequence AA388235	2.956	0.2096	0.6412
258408	Olfr463	olfactory receptor 463	2.934	0.1849	0.6223
545548	Lce3a	late cornified envelope 3A	2.933	0.003649	0.2427
277345	Wfdc16	WAP four-disulfide core domain 16	2.924	0.09921	0.5367
244071	Agbl1	ATP/GTP binding protein-like 1	2.899	0.08761	0.5191
98763	AA407782	expressed sequence AA407782	2.895	0.4335	0.7872
258354	Olfr168	olfactory receptor 168	2.86	0.2075	0.6398
27083	Xlr4b	X-linked lymphocyte-regulated 4B	2.853	0.03772	0.417
56773	Chst5	carbohydrate (N-acetylglucosamine 6-O) sulfotransferase 5	2.844	0.05652	0.4638
268482	Krt12	keratin 12	2.833	0.06684	0.4851
268482	Krt12	keratin 12	2.812	0.06741	0.4855
57277	Slurp1	secreted Ly6/Plaur domain containing 1	2.808	0.02823	0.3883
68668	Klk5	kallikrein related-peptidase 5	2.802	0.203	0.6356
17748	Mt1	metallothionein 1	2.745	6.82E-08	0.0002699

53311	Mybph	myosin binding protein H	2.724	0.03581	0.4125
667977	Gm8909	predicted gene 8909	2.66	0.2198	0.6513
74725	4930524B17Rik	RIKEN cDNA 4930524B17 gene	2.633	0.1311	0.5751
100505078	LOC100505078	hypothetical protein LOC100505078	2.623	0.04709	0.4435
69036	Zg16	zymogen granule protein 16	2.582	0.1806	0.6198
790956	LOC790956	5.8S ribosomal RNA	2.548	0.09268	0.5273
408061	BC051408	cDNA sequence BC051408	2.547	0.1219	0.5652
239151	Gm600	predicted gene 600	2.479	0.246	0.6733
100174921	4930505N22Rik	RIKEN cDNA 4930505N22 gene	2.396	0.1378	0.5815
623166	Prr23a	proline rich 23A	2.367	0.1208	0.5643
212989	Best2	bestrophin 2	2.323	0.3498	0.7388
14428	Galr2	galanin receptor 2	2.312	0.01654	0.3416
238393	Serpina3f	serine (or cysteine) peptidase inhibitor, clade A, member 3F	2.297	2.42E-05	0.03314
107581	Col16a1	collagen, type XVI, alpha 1	2.295	0.1534	0.5974
379043	Raet1e	retinoic acid early transcript 1E	2.29	0.2539	0.6787
631105	Gm7056	predicted gene 7056	2.272	0.1806	0.6198
18295	Ogn	osteoglycin	2.248	0.3511	0.7393
654818	C030030A07Rik	RIKEN cDNA C030030A07 gene	2.222	0.07306	0.4952
338417	Scgb1c1	secretoglobin, family 1C, member 1	2.208	0.136	0.5794
100382	AW011738	expressed sequence AW011738	2.208	0.08646	0.5176
69930	Zfp715	zinc finger protein 715	2.206	0.02043	0.3609
72061	2010111I01Rik	RIKEN cDNA 2010111I01 gene	2.203	0.2774	0.6925
18205	Ntf3	neurotrophin 3	2.194	0.05114	0.4519
12288	Cacna1c	calcium channel, voltage-dependent, L type, alpha 1C subunit	2.188	0.1836	0.6217
109978	Art4	ADP-ribosyltransferase 4	2.186	0.2253	0.654
18979	Pon1	paraoxonase 1	2.179	0.00896	0.3086
214301	Crygn	crystallin, gamma N	2.171	0.4863	0.8127
233670	Olfr6	olfactory receptor 6	2.17	0.2256	0.654
75571	Spata9	spermatogenesis associated 9	2.159	2.89E-06	0.007628
64435	Fcamr	Fc receptor, IgA, IgM, high affinity	2.151	0.1897	0.6273
78458	1700064N11Rik	RIKEN cDNA 1700064N11 gene	2.144	0.0584	0.4703
79235	Lrat	lecithin-retinol acyltransferase (phosphatidylcholine-retinol-O-acyltransferase)	2.137	0.02696	0.3841
71355	Col24a1	collagen, type XXIV, alpha 1	2.091	0.04717	0.4435
13067	Cyct	cytochrome c, testis	2.086	0.02279	0.3702
98267	Stk17b	serine/threonine kinase 17b (apoptosis-inducing)	2.083	0.0001024	0.0829
16573	Kif5b	kinesin family member 5B	2.073	0.003071	0.2228
328561	Apol10b	apolipoprotein L 10b	2.072	0.06062	0.4741
234199	Fgl1	fibrinogen-like protein 1	2.05	0.0148	0.3369
20702	Serpina1c	serine (or cysteine) peptidase inhibitor, clade A, member 1C	2.046	0.01134	0.3181
12738	Cldn2	claudin 2	2.045	0.4427	0.7928
68311	Lypd2	Ly6/Plaur domain containing 2	2.013	0.1007	0.5394
14089	Fap	fibroblast activation protein	2.008	0.01679	0.3424
105332	AW011956	expressed sequence AW011956	2.001	0.009106	0.3086
106757	Tmem146	transmembrane protein 146	1.991	0.04896	0.4469
16774	Lama3	laminin, alpha 3	1.99	0.0252	0.3786
14778	Gpx3	glutathione peroxidase 3	1.986	0.1982	0.6331
22774	Zic4	zinc finger protein of the cerebellum 4	1.981	0.2327	0.6622
19746	Rhd	Rh blood group, D antigen	1.981	0.02427	0.3771
74249	Lrrc2	leucine rich repeat containing 2	1.974	0.002656	0.2224

106648	Cyp4f15	cytochrome P450, family 4, subfamily f, polypeptide 15	1.961	0.1938	0.6299
385343	Rhox1	reproductive homeobox 1	1.957	0.0516	0.4524
108816	4933409K07Rik	RIKEN cDNA 4933409K07 gene	1.957	0.1231	0.5672
52670	Cpsf4l	cleavage and polyadenylation specific factor 4-like	1.956	0.05126	0.4519
329941	Col8a2	collagen, type VIII, alpha 2	1.953	0.01848	0.3515
73333	Slc25a31	solute carrier family 25 (mitochondrial carrier; adenine nucleotide translocator), member 31	1.952	0.3822	0.7596
258689	Olfr1466	olfactory receptor 1466	1.943	0.06203	0.4771
100038666	Gm12298	predicted gene 12298	1.942	0.02008	0.3604
14663	Glycam1	glycosylation dependent cell adhesion molecule 1	1.94	0.3041	0.709
14999	H2-DMb1	histocompatibility 2, class II, locus Mb1	1.932	0.04802	0.4443
66402	Sln	sarcolipin	1.931	0.2451	0.6727
627367	Vmn2r97	vomeronasal 2, receptor 97	1.924	0.2385	0.667
57742	Abhd1	abhydrolase domain containing 1	1.918	0.0007889	0.148
69665	2310043J07Rik	RIKEN cDNA 2310043J07 gene	1.916	0.02514	0.3786
12954	Cryaa	crystallin, alpha A	1.914	0.5639	0.8465
192199	Rspo1	R-spondin homolog (Xenopus laevis)	1.912	0.5919	0.8584
18241	Gpr143	G protein-coupled receptor 143	1.911	0.05662	0.4638
18787	Serpine1	serine (or cysteine) peptidase inhibitor, clade E, member 1	1.91	0.2463	0.6734
319545	D430020J02Rik	RIKEN cDNA D430020J02 gene	1.907	0.1106	0.5546
114671	4930444G20Rik	RIKEN cDNA 4930444G20 gene	1.907	0.1997	0.6343
67425	Eps8l1	EPS8-like 1	1.907	0.009907	0.3112
547349	LOC547349	similar to MHC class I antigen precursor	1.906	0.2287	0.658
68355	2010204K13Rik	RIKEN cDNA 2010204K13 gene	1.905	0.002332	0.2166
385380	Tex28	testis expressed 28	1.904	0.08344	0.5144
69121	Chrdl2	chordin-like 2	1.889	0.004533	0.2618
628900	Serpina3i	serine (or cysteine) peptidase inhibitor, clade A, member 3I	1.885	0.003333	0.2294
76459	Car12	carbonic anyhydrase 12	1.885	0.01889	0.353
73009	2900057B20Rik	RIKEN cDNA 2900057B20 gene	1.883	0.01424	0.3347
14012	Mpzl2	myelin protein zero-like 2	1.882	0.06135	0.4763
20969	Sdc1	syndecan 1	1.88	0.1803	0.6197
17287	Mep1a	meprin 1 alpha	1.875	0.1815	0.6206
22139	Ttr	transthyretin	1.862	0.02536	0.3786
69665	2310043J07Rik	RIKEN cDNA 2310043J07 gene	1.855	0.02919	0.3911
77998	Grifin	galectin-related inter-fiber protein	1.854	0.5494	0.8414
14284	Fosl2	fos-like antigen 2	1.85	0.1675	0.6092
53868	Rab25	RAB25, member RAS oncogene family	1.845	0.05832	0.4703
22268	Upk1b	uroplakin 1B	1.833	0.03283	0.4035
269120	Optc	opticin	1.832	0.5043	0.8221
110877	Slc18a1	solute carrier family 18 (vesicular monoamine), member 1	1.828	0.07681	0.5014
60531	Npvf	neuropeptide VF precursor	1.821	0.4997	0.8197
72373	Psca	prostate stem cell antigen	1.819	0.07243	0.4941
54672	Gpr97	G protein-coupled receptor 97	1.819	0.09003	0.5229
22774	Zic4	zinc finger protein of the cerebellum 4	1.812	0.2698	0.6878
57811	Rgr	retinal G protein coupled receptor	1.809	0.03326	0.4053
74579	4833419E13Rik	RIKEN cDNA 4833419E13 gene	1.791	0.1253	0.5703
654818	C030030A07Rik	RIKEN cDNA C030030A07 gene	1.789	0.1764	0.6156
624855	Gm6531	predicted gene 6531	1.788	0.004109	0.255
71413	5430410E06Rik	RIKEN cDNA 5430410E06 gene	1.788	0.2754	0.6925
546886	Ccdc42b	coiled-coil domain containing 42B	1.778	0.2646	0.6845

76722	Ckmt2	creatine kinase, mitochondrial 2	1.778	0.1604	0.6055
21948	Cd70	CD70 antigen	1.778	0.01628	0.3414
622657	Gm6340	predicted pseudogene 6340	1.777	0.00479	0.2647
110308	Krt5	keratin 5	1.77	0.1357	0.5794
100040591	Kcnj13	potassium inwardly-rectifying channel, subfamily J, member 13	1.768	0.1017	0.5397
238393	Serpina3f	serine (or cysteine) peptidase inhibitor, clade A, member 3F	1.763	0.0004491	0.1365
56175	Bace2	beta-site APP-cleaving enzyme 2	1.763	0.1733	0.6147
114644	Slc13a3	solute carrier family 13 (sodium-dependent dicarboxylate transporter), member 3	1.759	0.08604	0.517
57811	Rgr	retinal G protein coupled receptor	1.759	0.009344	0.3107
18074	Nid2	nidogen 2	1.758	0.1073	0.5498
27029	Sgsh	N-sulfoglucosamine sulfohydrolase (sulfamidase)	1.755	0.002691	0.2224
246079	Defb9	defensin beta 9	1.754	0.4244	0.7819
17921	Myo7a	myosin VIIA	1.748	0.0001238	0.08906
14863	Gstm2	glutathione S-transferase, mu 2	1.747	0.4417	0.7921
13733	Emr1	EGF-like module containing, mucin-like, hormone receptor-like sequence 1	1.746	0.2272	0.6561
12869	Cox8b	cytochrome c oxidase, subunit VIIIb	1.745	0.07409	0.4962
208634	Tspan10	tetraspanin 10	1.738	0.07043	0.4915
53896	Slc7a10	solute carrier family 7 (cationic amino acid transporter, y+ system), member 10	1.738	0.17	0.6112
22772	Zic2	zinc finger protein of the cerebellum 2	1.737	0.03917	0.4218
55987	Cpxm2	carboxypeptidase X 2 (M14 family)	1.729	0.01484	0.3369
57274	Slc16a8	solute carrier family 16 (monocarboxylic acid transporters), member 8	1.727	0.01057	0.3126
11639	Ak4	adenylate kinase 4	1.724	3.37E-05	0.03809
242705	E2f2	E2F transcription factor 2	1.722	0.007459	0.2934
20657	Sod3	superoxide dismutase 3, extracellular	1.718	0.1281	0.5716
19892	Rpe65	retinal pigment epithelium 65	1.717	0.0208	0.3609
21828	Thbs4	thrombospondin 4	1.715	0.03869	0.4213
11830	Aqp5	aquaporin 5	1.715	0.1214	0.5651
243084	Tmprss11e	transmembrane protease, serine 11e	1.714	0.1911	0.6278
13003	Vcan	versican	1.711	0.4095	0.7739
223732	Ldoc1l	leucine zipper, down-regulated in cancer 1-like	1.71	0.031	0.3964
12957	Cryba1	crystallin, beta A1	1.71	0.5721	0.8499
67855	Asprv1	aspartic peptidase, retroviral-like 1	1.706	0.09936	0.5367
14858	Gsta2	glutathione S-transferase, alpha 2 (Yc2)	1.702	0.1296	0.5745
12630	Cfi	complement component factor i	1.7	0.0008128	0.148
15492	Hsd3b1	hydroxy-delta-5-steroid dehydrogenase, 3 beta- and steroid delta-isomerase 1	1.699	0.5303	0.8333
224833	AI661453	expressed sequence AI661453	1.695	0.0334	0.4063
12159	Bmp4	bone morphogenetic protein 4	1.693	0.2559	0.68
72169	Trim29	tripartite motif-containing 29	1.691	0.05104	0.4519
107585	Dio3	deiodinase, iodothyronine type III	1.69	0.1766	0.6159
54124	Cks1b	CDC28 protein kinase 1b	1.69	0.004007	0.2497
20431	Pmel	premelanosome protein	1.687	0.03272	0.4034
57740	Stk32c	serine/threonine kinase 32C	1.686	0.0228	0.3702
11670	Aldh3a1	aldehyde dehydrogenase family 3, subfamily A1	1.683	0.4287	0.7838
76681	Trim12a	tripartite motif-containing 12A	1.677	0.003774	0.2433
19215	Ptgds	prostaglandin D2 synthase (brain)	1.676	0.1161	0.558
633093	Gm7104	REX1, RNA exonuclease 1 homolog pseudogene	1.674	0.1302	0.5747
14863	Gstm2	glutathione S-transferase, mu 2	1.674	0.4184	0.7787
574437	Xlr3b	X-linked lymphocyte-regulated 3B	1.673	0.08819	0.5202
574437	Xlr3b	X-linked lymphocyte-regulated 3B	1.669	0.1377	0.5815

218763	Lrrc3b	leucine rich repeat containing 3B	1.669	0.0242	0.3771
67935	Ces5a	carboxylesterase 5A	1.667	0.0888	0.5209
19744	Rheb	Ras homolog enriched in brain	1.662	0.1548	0.5975
71683	Gypc	glycophorin C	1.661	0.04499	0.4353
353504	Dio3os	deiodinase, iodothyronine type III, opposite strand	1.66	0.1279	0.5716
74202	Fblim1	filamin binding LIM protein 1	1.658	0.02246	0.3702
12959	Cryba4	crystallin, beta A4	1.656	0.5753	0.8504
11830	Aqp5	aquaporin 5	1.653	0.1187	0.5618
94346	Tmem40	transmembrane protein 40	1.651	0.5618	0.8465
387345	Tas2r113	taste receptor, type 2, member 113	1.65	0.02888	0.3903
100039948	Rhox2c	reproductive homeobox 2C	1.645	0.04452	0.4349
235435	Lctl	lactase-like	1.644	0.5469	0.8398
243755	Slc13a4	solute carrier family 13 (sodium/sulfate symporters), member 4	1.642	0.4189	0.7791
94094	Trim34a	tripartite motif-containing 34A	1.642	0.000552	0.1365
14264	Fmod	fibromodulin	1.642	0.3922	0.7647
100038452	Gm13372	predicted gene 13372	1.641	0.08024	0.5098
14114	Fbln1	fibulin 1	1.641	0.5029	0.8214
14412	Slc6a13	solute carrier family 6 (neurotransmitter transporter, GABA), member 13	1.64	0.2884	0.6996
546144	Wdr72	WD repeat domain 72	1.637	0.001164	0.1645
56485	Slc2a5	solute carrier family 2 (facilitated glucose transporter), member 5	1.637	0.1066	0.5477
77558	9330179D12Rik	RIKEN cDNA 9330179D12 gene	1.636	0.4031	0.7708
67434	Ankrd33b	ankyrin repeat domain 33B	1.636	0.2174	0.6497
111975	Igf2as	insulin-like growth factor 2, antisense	1.634	0.000506	0.1365
76640	1700113H08Rik	RIKEN cDNA 1700113H08 gene	1.633	0.01629	0.3414
64929	Scel	sciellin	1.633	0.1443	0.589
225642	Grp	gastrin releasing peptide	1.632	0.000137	0.09032
402767	A830052D11Rik	RIKEN cDNA A830052D11 gene	1.631	0.002894	0.2228
244198	Olfml1	olfactomedin-like 1	1.631	0.2604	0.6815
76293	Mfap4	microfibrillar-associated protein 4	1.63	0.5574	0.8454
21810	Tgfbi	transforming growth factor, beta induced	1.63	0.0615	0.477
230099	Car9	carbonic anhydrase 9	1.629	0.3477	0.7383
77836	Mlana	melan-A	1.627	0.1257	0.5703
71874	2310007B03Rik	RIKEN cDNA 2310007B03 gene	1.627	0.05221	0.4535
353504	Dio3os	deiodinase, iodothyronine type III, opposite strand	1.624	0.1359	0.5794
242484	D630039A03Rik	RIKEN cDNA D630039A03 gene	1.617	0.1269	0.5712
68854	Asb11	ankyrin repeat and SOCS box-containing 11	1.617	0.01399	0.3347
16780	Lamb3	laminin, beta 3	1.617	0.01074	0.3148
27219	Sgk2	serum/glucocorticoid regulated kinase 2	1.614	0.001212	0.1654
12310	Calca	calcitonin/calcitonin-related polypeptide, alpha	1.614	0.004139	0.2551
11522	Adh1	alcohol dehydrogenase 1 (class I)	1.614	0.08611	0.517
252967	Ropn1l	ropporin 1-like	1.61	0.00699	0.2924
75058	4930519H02Rik	RIKEN cDNA 4930519H02 gene	1.61	0.05883	0.4712
54601	Foxo4	forkhead box O4	1.605	0.0628	0.4772
69055	1810007D17Rik	RIKEN cDNA 1810007D17 gene	1.604	0.1566	0.6002
408190	Wfdc13	WAP four-disulfide core domain 13	1.603	0.1389	0.5829
16333	Ins1	insulin I	1.603	0.009496	0.3112
242939	Cpz	carboxypeptidase Z	1.602	0.1009	0.5394
11945	Atp4b	ATPase, H+/K+ exchanging, beta polypeptide	1.602	0.1785	0.6181
11689	Alox5	arachidonate 5-lipoxygenase	1.601	0.0001038	0.0829

54357	Epb4.1l4b	erythrocyte protein band 4.1-like 4b	1.599	0.2078	0.6401
14863	Gstm2	glutathione S-transferase, mu 2	1.598	0.5038	0.8221
664994	Isoc2a	isochorismatase domain containing 2a	1.594	0.05419	0.4596
79565	Wbscr27	Williams Beuren syndrome chromosome region 27 (human)	1.594	0.3114	0.7136
12960	Crybb1	crystallin, beta B1	1.594	0.6191	0.8709
20700	Serpina1a	serine (or cysteine) peptidase inhibitor, clade A, member 1A	1.592	0.02529	0.3786
78896	1500015O10Rik	RIKEN cDNA 1500015O10 gene	1.586	0.4288	0.784
12959	Cryba4	crystallin, beta A4	1.586	0.5887	0.8572
11639	Ak4	adenylate kinase 4	1.585	0.00156	0.1903
74249	Lrrc2	leucine rich repeat containing 2	1.584	0.002012	0.2121
14114	Fbln1	fibulin 1	1.584	0.6172	0.8698
22041	Trf	transferrin	1.583	0.03013	0.3947
12961	Crybb2	crystallin, beta B2	1.579	0.6237	0.8724
71721	Fam13c	family with sequence similarity 13, member C	1.578	0.2582	0.6815
20377	Sfrp1	secreted frizzled-related protein 1	1.578	0.5001	0.8197
12958	Cryba2	crystallin, beta A2	1.578	0.6461	0.8809
22178	Tyrp1	tyrosinase-related protein 1	1.576	0.1853	0.6223
319875	Tmprss11bnl	transmembrane protease, serine 11b N terminal like	1.575	0.04172	0.4282
140494	Atp6v0a4	ATPase, H+ transporting, lysosomal V0 subunit A4	1.575	0.01904	0.3531
70370	Fbln7	fibulin 7	1.575	0.04502	0.4353
12960	Crybb1	crystallin, beta B1	1.575	0.6206	0.8718
69367	Glrx2	glutaredoxin 2 (thioltransferase)	1.574	0.02745	0.3862
16997	Ltbp2	latent transforming growth factor beta binding protein 2	1.573	0.6102	0.8667
100038415	F830034J09Rik	RIKEN cDNA F830034J09 gene	1.572	0.09408	0.5311
100040591	Kcnj13	potassium inwardly-rectifying channel, subfamily J, member 13	1.571	0.1751	0.6156
229905	Ccbl2	cysteine conjugate-beta lyase 2	1.57	0.2213	0.6525
16688	Krt6b	keratin 6B	1.57	0.1301	0.5747
24064	Spry2	sprouty homolog 2 (Drosophila)	1.569	0.04992	0.4509
18121	Nog	noggin	1.567	0.1471	0.591
13730	Emp1	epithelial membrane protein 1	1.567	0.05321	0.4565
94346	Tmem40	transmembrane protein 40	1.566	0.5921	0.8584
13590	Lefty1	left right determination factor 1	1.566	0.1517	0.5948
76509	1600029D21Rik	RIKEN cDNA 1600029D21 gene	1.565	0.108	0.551
21391	Tbxas1	thromboxane A synthase 1, platelet	1.564	0.0414	0.4282
13648	Klk1b9	kallikrein 1-related peptidase b9	1.559	0.4401	0.7909
69681	Cdk3-ps	cyclin-dependent kinase 3, pseudogene	1.558	0.1101	0.5535
106407	Osta	organic solute transporter alpha	1.555	0.01789	0.3475
20887	Sult1a1	sulfotransferase family 1A, phenol-preferring, member 1	1.554	0.0003623	0.1365
21825	Thbs1	thrombospondin 1	1.552	0.0364	0.4154
78465	1700084C01Rik	RIKEN cDNA 1700084C01 gene	1.551	0.02465	0.3786
100043380	Gm4402	predicted gene 4402	1.548	0.09077	0.5229
272636	Esyt3	extended synaptotagmin-like protein 3	1.548	0.4456	0.7938
56615	Mgst1	microsomal glutathione S-transferase 1	1.548	0.04466	0.4353
13710	Elf3	E74-like factor 3	1.548	0.07592	0.5007
211623	Plac9	placenta specific 9	1.547	0.002428	0.2179
93695	Gpnmb	glycoprotein (transmembrane) nmb	1.543	0.1235	0.568
22351	Vill	villin-like	1.543	0.06496	0.4816
18641	Pfkl	phosphofructokinase, liver, B-type	1.543	0.03516	0.4105
22041	Trf	transferrin	1.542	0.05195	0.4525

12970	Crygs	crystallin, gamma S	1.542	0.5694	0.8491
75098	4930515G13Rik	RIKEN cDNA 4930515G13 gene	1.54	0.02389	0.377
67859	2310002J15Rik	RIKEN cDNA 2310002J15 gene	1.54	0.0547	0.4614
54634	Magix	MAGI family member, X-linked	1.54	0.1672	0.6085
664876	Gm7385	predicted gene 7385	1.539	0.2463	0.6734
99681	Tchh	trichohyalin	1.539	0.108	0.551
18155	Pnoc	prepronociceptin	1.539	0.002709	0.2224
71760	Agxt2l1	alanine-glyoxylate aminotransferase 2-like 1	1.538	0.01619	0.3414
233406	Prc1	protein regulator of cytokinesis 1	1.537	0.01879	0.353
58864	Tssk3	testis-specific serine kinase 3	1.537	0.09393	0.5308
98660	Atp1a2	ATPase, Na+/K+ transporting, alpha 2 polypeptide	1.536	0.1297	0.5746
57435	Plin4	perilipin 4	1.535	0.1347	0.5779
545253	Gm5820	predicted gene 5820	1.533	0.08836	0.5202
66788	5430414B12Rik	RIKEN cDNA 5430414B12 gene	1.532	0.0004432	0.1365
381175	Ccdc68	coiled-coil domain containing 68	1.53	0.1049	0.5453
22417	Wnt4	wingless-related MMTV integration site 4	1.529	0.005983	0.2803
15360	Hmgcs2	3-hydroxy-3-methylglutaryl-Coenzyme A synthase 2	1.529	0.3257	0.7222
12961	Crybb2	crystallin, beta B2	1.528	0.6671	0.8904
100503619	LOC100503619	hypothetical LOC100503619	1.527	0.001438	0.185
56615	Mgst1	microsomal glutathione S-transferase 1	1.527	0.0864	0.5176
15160	Serpind1	serine (or cysteine) peptidase inhibitor, clade D, member 1	1.527	0.5147	0.8273
13190	Dct	dopachrome tautomerase	1.524	0.1955	0.6304
58187	Cldn10	claudin 10	1.52	0.03012	0.3947
74463	Exoc3l2	exocyst complex component 3-like 2	1.518	0.0961	0.5344
217830	9030617O03Rik	RIKEN cDNA 9030617O03 gene	1.517	0.0004762	0.1365
23985	Slc26a4	solute carrier family 26, member 4	1.517	0.6245	0.8728
66395	Ahnak	AHNAK nucleoprotein (desmoyokin)	1.515	0.1583	0.6029
57741	Noc2l	nucleolar complex associated 2 homolog (S. cerevisiae)	1.515	0.001093	0.1616
54601	Foxo4	forkhead box O4	1.515	0.1485	0.5921
381822	1190002F15Rik	RIKEN cDNA 1190002F15 gene	1.513	0.01775	0.3475
225187	Ankrd29	ankyrin repeat domain 29	1.513	0.0003511	0.1365
21816	Tgm1	transglutaminase 1, K polypeptide	1.511	0.2454	0.6732
192188	Stab2	stabilin 2	1.509	0.0001546	0.09489
18400	Slc22a18	solute carrier family 22 (organic cation transporter), member 18	1.509	0.3643	0.7491
69982	Spink2	serine peptidase inhibitor, Kazal type 2	1.508	0.06937	0.4874
632793	Vmn1r204	vomeronasal 1 receptor 204	1.507	0.03126	0.3964
233038	Nccrp1	non-specific cytotoxic cell receptor protein 1 homolog (zebrafish)	1.504	0.07755	0.5026
72112	Ppp1r14d	protein phosphatase 1, regulatory (inhibitor) subunit 14D	1.503	0.008901	0.3086
14580	Gfap	glial fibrillary acidic protein	1.503	0.01911	0.3534
73863	4930415O20Rik	RIKEN cDNA 4930415O20 gene	1.502	0.04346	0.4329
27411	Slc14a2	solute carrier family 14 (urea transporter), member 2	1.502	0.6211	0.8719
26968	Islr	immunoglobulin superfamily containing leucine-rich repeat	1.502	0.3935	0.7656
329977	Fhad1	forkhead-associated (FHA) phosphopeptide binding domain 1	1.5	0.002877	0.2228
75563	Dnali1	dynein, axonemal, light intermediate polypeptide 1	1.5	0.004368	0.2609
56175	Bace2	beta-site APP-cleaving enzyme 2	1.5	0.1521	0.5954
66113	Apoa5	apolipoprotein A-V	1.498	0.3805	0.7594
14857	Gsta1	glutathione S-transferase, alpha 1 (Ya)	1.498	0.1125	0.5549
231832	Tmem184a	transmembrane protein 184a	1.497	0.2411	0.6688
170743	Tlr7	toll-like receptor 7	1.496	0.1645	0.6076

75764	Slx1b	SLX1 structure-specific endonuclease subunit homolog B (S. cerevisiae)	1.495	0.009704	0.3112
71862	Gpr160	G protein-coupled receptor 160	1.494	0.4345	0.7878
70918	Nsun7	NOL1/NOP2/Sun domain family, member 7	1.494	0.1541	0.5975
380712	Tlcd2	TLC domain containing 2	1.493	0.04916	0.4469
12458	Ccr6	chemokine (C-C motif) receptor 6	1.493	0.004481	0.2618
545260	Arsi	arylsulfatase i	1.492	0.3727	0.7537
107526	Gimap4	GTPase, IMAP family member 4	1.492	0.09094	0.5229
69301	1700008P20Rik	RIKEN cDNA 1700008P20 gene	1.492	0.19	0.6274
64058	Perp	PERP, TP53 apoptosis effector	1.492	0.149	0.5934
19223	Ptgis	prostaglandin I2 (prostacyclin) synthase	1.492	0.00972	0.3112
246787	Slc5a2	solute carrier family 5 (sodium/glucose cotransporter), member 2	1.491	0.1144	0.5569
13190	Dct	dopachrome tautomerase	1.491	0.1911	0.6278
19682	Rdh5	retinol dehydrogenase 5	1.49	0.05594	0.4631
12955	Cryab	crystallin, alpha B	1.49	0.6399	0.8784
107029	Me2	malic enzyme 2, NAD(+)-dependent, mitochondrial	1.487	6.54E-05	0.06467
791383	Gm9962	predicted gene 9962	1.486	0.01597	0.3408
240638	Slc16a12	solute carrier family 16 (monocarboxylic acid transporters), member 12	1.486	0.4958	0.8182
17444	Grap2	GRB2-related adaptor protein 2	1.486	0.414	0.7757
54124	Cks1b	CDC28 protein kinase 1b	1.485	0.0002646	0.1196
622139	Gm6289	predicted gene 6289	1.484	0.04652	0.4407
20887	Sult1a1	sulfotransferase family 1A, phenol-preferring, member 1	1.482	0.0005208	0.1365
20732	Spint1	serine protease inhibitor, Kunitz type 1	1.482	0.03875	0.4213
73061	3110007F17Rik	RIKEN cDNA 3110007F17 gene	1.479	0.1657	0.6077
23880	Fyb	FYN binding protein	1.479	0.1256	0.5703
329554	Gm826	predicted gene 826	1.478	0.008566	0.3068
100504710	LOC100504710	camello-like 3-like	1.477	0.02627	0.3791
100043335	Gm4371	eukaryotic translation initiation factor 3, subunit I pseudogene	1.477	0.02779	0.3875
235312	C1qtnf5	C1q and tumor necrosis factor related protein 5	1.477	0.05475	0.4614
546546	Serpina3h	serine (or cysteine) peptidase inhibitor, clade A, member 3H	1.476	0.2331	0.6622
229697	Cym	chymosin	1.476	0.006459	0.288
16364	Irf4	interferon regulatory factor 4	1.475	0.06233	0.4771
71145	Scara5	scavenger receptor class A, member 5 (putative)	1.474	0.03717	0.4163
109901	Cela1	chymotrypsin-like elastase family, member 1	1.473	0.3404	0.7328
11828	Aqp3	aquaporin 3	1.473	0.1276	0.5716
66857	Plbd1	phospholipase B domain containing 1	1.472	0.2163	0.6479
12739	Cldn3	claudin 3	1.472	0.2067	0.6393
230145	Galnt12	UDP-N-acetyl-alpha-D-galactosamine:polypeptide N-acetylgalactosaminyltransferase 12	1.471	0.434	0.7875
11857	Arhgdib	Rho, GDP dissociation inhibitor (GDI) beta	1.47	0.007636	0.2948
384071	Slc25a34	solute carrier family 25, member 34	1.468	0.1454	0.5906
268807	Klhl38	kelch-like 38 (Drosophila)	1.468	0.001659	0.1903
170748	BC017612	cDNA sequence BC017612	1.467	0.0001128	0.08504
100042314	Gm10639	predicted gene 10639	1.466	0.08857	0.5203
258248	Olfr576	olfactory receptor 576	1.466	0.06543	0.4816
497106	Rnase12	ribonuclease, RNase A family, 12 (non-active)	1.465	0.04711	0.4435
212933	Pm20d1	peptidase M20 domain containing 1	1.465	0.02092	0.3618
22702	Zfp42	zinc finger protein 42	1.465	0.6778	0.8931
12501	Cd3e	CD3 antigen, epsilon polypeptide	1.465	0.1745	0.6148
12266	C3	complement component 3	1.465	0.03576	0.4123
19013	Ppara	peroxisome proliferator activated receptor alpha	1.464	0.1178	0.5609

14555	Gpd1	glycerol-3-phosphate dehydrogenase 1 (soluble)	1.464	0.2628	0.6838
72293	Nkd2	naked cuticle 2 homolog (Drosophila)	1.462	0.04413	0.4338
19065	Ppyr1	pancreatic polypeptide receptor 1	1.461	0.1021	0.5409
16149	Cd74	CD74 antigen (invariant polypeptide of major histocompatibility complex, class II antigen-associated)	1.461	0.1886	0.6263
171167	Fut10	fucosyltransferase 10	1.46	0.002209	0.2121
257632	Nod2	nucleotide-binding oligomerization domain containing 2	1.459	0.2245	0.654
71660	Rarres2	retinoic acid receptor responder (tazarotene induced) 2	1.459	0.2297	0.6596
12983	Csf2rb	colony stimulating factor 2 receptor, beta, low-affinity (granulocyte-macrophage)	1.459	0.02603	0.3786
75677	Cldn22	claudin 22	1.457	0.008264	0.3025
17201	Mc3r	melanocortin 3 receptor	1.456	0.001296	0.1724
18742	Pitx3	paired-like homeodomain transcription factor 3	1.455	0.6479	0.8816
246691	Prok1	prokineticin 1	1.454	0.00213	0.2121
12444	Ccnd2	cyclin D2	1.454	0.3553	0.7434
381218	4430402I18Rik	RIKEN cDNA 4430402I18 gene	1.453	0.3192	0.7184
269633	Wdr86	WD repeat domain 86	1.453	0.2034	0.6362
209760	Tmc7	transmembrane channel-like gene family 7	1.453	0.002988	0.2228
15235	Mst1	macrophage stimulating 1 (hepatocyte growth factor-like)	1.453	0.06503	0.4816
68458	Ppp1r14a	protein phosphatase 1, regulatory (inhibitor) subunit 14A	1.451	0.009501	0.3112
20901	Strap	serine/threonine kinase receptor associated protein	1.451	0.006733	0.288
217430	Pqlc3	PQ loop repeat containing	1.45	0.1207	0.5643
107993	Bfsp2	beaded filament structural protein 2, phakinin	1.45	0.6108	0.8671
386454	Rnf39	ring finger protein 39	1.449	0.1386	0.5825
69382	1700024P04Rik	RIKEN cDNA 1700024P04 gene	1.449	0.02892	0.3903
69301	1700008P20Rik	RIKEN cDNA 1700008P20 gene	1.449	0.1313	0.5751
72434	Lypd3	Ly6/Plaur domain containing 3	1.448	0.2554	0.6798
19329	Rab17	RAB17, member RAS oncogene family	1.448	0.335	0.73
210321	BC048679	cDNA sequence BC048679	1.447	0.7266	0.9129
17147	Mageb3	melanoma antigen, family B, 3	1.447	0.3979	0.7684
80876	Ifitm2	interferon induced transmembrane protein 2	1.446	0.1689	0.6096
12919	Crhbp	corticotropin releasing hormone binding protein	1.445	0.3132	0.7152
20708	Serpinb6b	serine (or cysteine) peptidase inhibitor, clade B, member 6b	1.443	0.09375	0.53
74286	Tbc1d21	TBC1 domain family, member 21	1.442	0.2059	0.6386
69571	2310034O05Rik	RIKEN cDNA 2310034O05 gene	1.442	0.1129	0.5554
11936	Fxyd2	FXYD domain-containing ion transport regulator 2	1.442	0.01294	0.3304
50909	C1ra	complement component 1, r subcomponent A	1.441	0.0633	0.478
14609	Gja1	gap junction protein, alpha 1	1.441	0.5312	0.8339
666048	Gm12824	predicted gene 12824	1.44	0.1985	0.6334
76509	1600029D21Rik	RIKEN cDNA 1600029D21 gene	1.44	0.1843	0.6217
75578	Fggy	FGGY carbohydrate kinase domain containing	1.439	0.06	0.4728
22673	Zfp185	zinc finger protein 185	1.439	0.2393	0.6679
18016	Nf2	neurofibromatosis 2	1.439	0.04849	0.445
654810	D630032N06Rik	RIKEN cDNA D630032N06 gene	1.438	0.2025	0.6356
630146	Cd101	CD101 antigen	1.438	0.2931	0.7028
12954	Cryaa	crystallin, alpha A	1.438	0.6016	0.8636
76560	Prss8	protease, serine, 8 (prostasin)	1.437	0.1357	0.5794
19662	Rbp4	retinol binding protein 4, plasma	1.437	8.05E-05	0.07491
100503359	LOC100503359	hypothetical LOC100503359	1.436	0.5139	0.8271
24102	Trex2	three prime repair exonuclease 2	1.436	0.1668	0.6083
12962	Crybb3	crystallin, beta B3	1.436	0.6835	0.8956

209550	Rad51ap2	RAD51 associated protein 2	1.435	0.0004459	0.1365
20604	Sst	somatostatin	1.435	0.02797	0.3875
14177	Fgf6	fibroblast growth factor 6	1.435	0.1008	0.5394
12350	Car3	carbonic anhydrase 3	1.434	0.0852	0.5169
70370	Fbln7	fibulin 7	1.433	0.2026	0.6356
545648	Gm13272	predicted gene 13272	1.432	0.02523	0.3786
234199	Fgl1	fibrinogen-like protein 1	1.432	0.1112	0.5549
69253	Hspb2	heat shock protein 2	1.432	0.5101	0.8251
74199	Vit	vitrin	1.431	0.4785	0.81
20623	Snrk	SNF related kinase	1.431	0.001847	0.2019
67705	1810058I24Rik	RIKEN cDNA 1810058I24 gene	1.43	0.1327	0.5771
434130	Ccdc8	coiled-coil domain containing 8	1.429	0.03462	0.4091
241303	Fam78a	family with sequence similarity 78, member A	1.429	0.1186	0.5618
170765	Ripply3	ripply3 homolog (zebrafish)	1.429	0.007375	0.2933
78600	Pde6h	phosphodiesterase 6H, cGMP-specific, cone, gamma	1.428	0.001114	0.1616
237038	Nox1	NADPH oxidase 1	1.427	0.1546	0.5975
232400	BC048546	cDNA sequence BC048546	1.427	0.05532	0.4623
114304	Slc28a3	solute carrier family 28 (sodium-coupled nucleoside transporter), member 3	1.427	0.0632	0.478
19378	Aldh1a2	aldehyde dehydrogenase family 1, subfamily A2	1.427	0.07456	0.4974
12977	Csf1	colony stimulating factor 1 (macrophage)	1.427	0.3442	0.7358
20198	S100a4	S100 calcium binding protein A4	1.426	0.5606	0.8462
17771	Mtl5	metallothionein-like 5, testis-specific (tesmin)	1.426	0.1409	0.5853
213019	Pdlim2	PDZ and LIM domain 2	1.425	0.007074	0.2929
67182	Pdzk1ip1	PDZK1 interacting protein 1	1.425	0.1795	0.6187
50527	Ero1l	ERO1-like (S. cerevisiae)	1.425	0.004243	0.2553
54139	Irf6	interferon regulatory factor 6	1.424	0.006494	0.288
21928	Tnfaip2	tumor necrosis factor, alpha-induced protein 2	1.423	0.09489	0.5323
11818	Apoh	apolipoprotein H	1.423	0.01639	0.3414
277468	Slc39a12	solute carrier family 39 (zinc transporter), member 12	1.422	0.02097	0.3619
269356	Slc4a11	solute carrier family 4, sodium bicarbonate transporter-like, member 11	1.422	0.06158	0.477
233744	Spon1	spondin 1, (f-spondin) extracellular matrix protein	1.421	0.02849	0.3896
619842	Gm12633	zinc finger protein 352 pseudogene	1.42	0.401	0.7703
100038637	Gm10134	predicted gene 10134	1.419	0.0476	0.4442
76681	Trim12a	tripartite motif-containing 12A	1.419	0.04514	0.4353
16181	Il1rn	interleukin 1 receptor antagonist	1.419	0.7709	0.9257
11475	Acta2	actin, alpha 2, smooth muscle, aorta	1.419	0.4167	0.7772
14867	Gstm6	glutathione S-transferase, mu 6	1.418	0.009759	0.3112
329828	AI464131	expressed sequence AI464131	1.417	0.01927	0.3548
98660	Atp1a2	ATPase, Na+/K+ transporting, alpha 2 polypeptide	1.417	0.175	0.6153
11670	Aldh3a1	aldehyde dehydrogenase family 3, subfamily A1	1.417	0.4945	0.8171
327768	A330049N07Rik	RIKEN cDNA A330049N07 gene	1.416	0.2244	0.654
18261	Ocm	oncomodulin	1.416	0.03633	0.4154
13665	Eif2s1	eukaryotic translation initiation factor 2, subunit 1 alpha	1.416	0.02587	0.3786
14969	H2-Eb1	histocompatibility 2, class II antigen E beta	1.415	0.1946	0.6303
72789	Veph1	ventricular zone expressed PH domain homolog 1 (zebrafish)	1.414	0.2151	0.6475
16002	Igf2	insulin-like growth factor 2	1.414	0.02586	0.3786
12955	Cryab	crystallin, alpha B	1.414	0.6994	0.9031
69454	Clic3	chloride intracellular channel 3	1.413	0.05994	0.4728
11752	Anxa8	annexin A8	1.413	0.1498	0.5938

100038417	Spink13	serine peptidase inhibitor, Kazal type 13	1.412	0.006266	0.2842
263876	Spata2	spermatogenesis associated 2	1.412	0.01796	0.3475
20200	S100a6	S100 calcium binding protein A6 (calcyclin)	1.412	0.2916	0.7018
11534	Adk	adenosine kinase	1.412	0.000834	0.148
19369	Raet1b	retinoic acid early transcript beta	1.411	0.08369	0.5144
20720	Serpine2	serine (or cysteine) peptidase inhibitor, clade E, member 2	1.41	0.09404	0.5311
17339	Mip	major intrinsic protein of eye lens fiber	1.409	0.7911	0.9333
12683	Cidea	cell death-inducing DNA fragmentation factor, alpha subunit-like effector A	1.409	0.006989	0.2924
107047	Psmg2	proteasome (prosome, macropain) assembly chaperone 2	1.408	0.000974	0.1524
13117	Cyp4a10	cytochrome P450, family 4, subfamily a, polypeptide 10	1.408	0.05084	0.4519
72080	2010317E24Rik	RIKEN cDNA 2010317E24 gene	1.407	0.02009	0.3604
24053	Sgcg	sarcoglycan, gamma (dystrophin-associated glycoprotein)	1.407	0.1045	0.5445
14867	Gstm6	glutathione S-transferase, mu 6	1.407	0.0219	0.3691
238377	Gpr68	G protein-coupled receptor 68	1.405	0.008205	0.3023
72049	Tnfrsf13c	tumor necrosis factor receptor superfamily, member 13c	1.405	0.01099	0.3175
57248	Ly6i	lymphocyte antigen 6 complex, locus I	1.405	0.03763	0.417
68233	1700125D06Rik	RIKEN cDNA 1700125D06 gene	1.404	0.2509	0.6767
78403	2900041M22Rik	RIKEN cDNA 2900041M22 gene	1.403	0.09619	0.5345
20348	Sema3c	sema domain, immunoglobulin domain (Ig), short basic domain, secreted, (semaphorin) 3C	1.402	0.01318	0.3319
14587	Gfra3	glial cell line derived neurotrophic factor family receptor alpha 3	1.402	0.06018	0.4728
14862	Gstm1	glutathione S-transferase, mu 1	1.4	0.289	0.7
252967	Ropn1l	ropporin 1-like	1.398	0.05664	0.4638
67085	1700024G13Rik	RIKEN cDNA 1700024G13 gene	1.398	0.1917	0.6278
105355	Slc17a3	solute carrier family 17 (sodium phosphate), member 3	1.397	0.04193	0.4285
77481	C030048H21Rik	RIKEN cDNA C030048H21 gene	1.397	0.01564	0.3403
18173	Slc11a1	solute carrier family 11 (proton-coupled divalent metal ion transporters), member 1	1.397	0.004007	0.2497
17301	Foxd2	forkhead box D2	1.397	0.03474	0.4092
17175	Masp2	mannan-binding lectin serine peptidase 2	1.397	0.3182	0.7182
13649	Egfr	epidermal growth factor receptor	1.397	0.4967	0.8192
11752	Anxa8	annexin A8	1.397	0.1854	0.6225
66859	Slc16a9	solute carrier family 16 (monocarboxylic acid transporters), member 9	1.396	0.05751	0.4687
114664	Hsd17b11	hydroxysteroid (17-beta) dehydrogenase 11	1.395	0.04172	0.4282
224833	AI661453	expressed sequence AI661453	1.394	0.1204	0.5643
20897	Stra6	stimulated by retinoic acid gene 6	1.393	0.03447	0.4091
13732	Emp3	epithelial membrane protein 3	1.393	0.09087	0.5229
12409	Cbr2	carbonyl reductase 2	1.393	0.1511	0.5942
12176	Bnip3	BCL2/adenovirus E1B interacting protein 3	1.393	0.0006967	0.145
72219	Fam75d3	family with sequence similarity 75, member D3	1.392	0.3514	0.7395
18431	Oca2	oculocutaneous albinism II	1.391	0.3356	0.7302
16906	Lmnb1	lamin B1	1.391	0.003893	0.2456
15478	Hs3st3a1	heparan sulfate (glucosamine) 3-O-sulfotransferase 3A1	1.39	0.08566	0.517
574428	Zmynd15	zinc finger, MYND-type containing 15	1.389	0.05455	0.4612
193385	Fam65b	family with sequence similarity 65, member B	1.389	0.2261	0.6545
72282	1810062G17Rik	RIKEN cDNA 1810062G17 gene	1.389	0.1654	0.6077
12156	Bmp2	bone morphogenetic protein 2	1.389	0.4245	0.7819
100039227	Gm10394	predicted gene 10394	1.388	0.03693	0.4161
546134	Gramd2	GRAM domain containing 2	1.388	0.0004515	0.1365
68468	Ly6g6c	lymphocyte antigen 6 complex, locus G6C	1.388	0.1369	0.5807
54698	Crtam	cytotoxic and regulatory T cell molecule	1.388	0.01216	0.3236

26918	Ern2	endoplasmic reticulum (ER) to nucleus signalling 2	1.388	0.3156	0.7165
320152	4930412C18Rik	RIKEN cDNA 4930412C18 gene	1.387	0.195	0.6304
108105	B3gnt5	UDP-GlcNAc:betaGal beta-1,3-N-acetylglucosaminyltransferase 5	1.387	0.6637	0.8896
17240	Mdfi	MyoD family inhibitor	1.387	0.1206	0.5643
15564	Htr5b	5-hydroxytryptamine (serotonin) receptor 5B	1.387	0.1472	0.591
77596	Gpr110	G protein-coupled receptor 110	1.386	0.2048	0.6376
14584	Gfpt2	glutamine fructose-6-phosphate transaminase 2	1.384	0.1667	0.6083
13179	Dcn	decorin	1.384	0.1352	0.5788
217353	Tmc6	transmembrane channel-like gene family 6	1.383	0.1398	0.5834
109225	Ms4a7	membrane-spanning 4-domains, subfamily A, member 7	1.383	0.04017	0.4248
76974	1190003J15Rik	RIKEN cDNA 1190003J15 gene	1.383	0.1626	0.607
20704	Serpina1e	serine (or cysteine) peptidase inhibitor, clade A, member 1E	1.382	0.1125	0.5549
73332	Ccdc30	coiled-coil domain containing 30	1.381	0.1293	0.5743
50490	Nox4	NADPH oxidase 4	1.381	0.2548	0.6792
434768	Rhox8	reproductive homeobox 8	1.38	0.1308	0.5751
101488	Slco2b1	solute carrier organic anion transporter family, member 2b1	1.38	0.01309	0.3305
66438	Hamp2	hepcidin antimicrobial peptide 2	1.379	0.001091	0.1616
12837	Col8a1	collagen, type VIII, alpha 1	1.379	0.0967	0.5345
320876	D930002L09Rik	RIKEN cDNA D930002L09 gene	1.378	0.3119	0.7139
230073	Ddx58	DEAD (Asp-Glu-Ala-Asp) box polypeptide 58	1.378	0.237	0.6659
20202	S100a9	S100 calcium binding protein A9 (calgranulin B)	1.378	0.2639	0.6845
12560	Cdh3	cadherin 3	1.378	0.2641	0.6845
75040	Efcab10	EF-hand calcium binding domain 10	1.377	0.06956	0.4877
66197	Cks2	CDC28 protein kinase regulatory subunit 2	1.377	0.0009167	0.1513
54630	Prickle3	prickle homolog 3 (Drosophila)	1.377	0.03684	0.4161
11474	Actn3	actinin alpha 3	1.377	0.1445	0.5892
71279	Slc29a3	solute carrier family 29 (nucleoside transporters), member 3	1.376	0.07906	0.5062
213522	Plekhg6	pleckstrin homology domain containing, family G (with RhoGef domain) member 6	1.375	0.09666	0.5345
56484	Foxo3	forkhead box O3	1.375	0.00948	0.3112
30053	Reg3d	regenerating islet-derived 3 delta	1.375	0.1257	0.5703
12176	Bnip3	BCL2/adenovirus E1B interacting protein 3	1.375	0.005517	0.2775
666105	Gm7932	predicted gene 7932	1.374	0.1598	0.6042
243362	Stard13	StAR-related lipid transfer (START) domain containing 13	1.374	0.1651	0.6077
328092	6530401N04Rik	RIKEN cDNA 6530401N04 gene	1.373	0.1402	0.5834
252830	Obox6	oocyte specific homeobox 6	1.372	0.005749	0.2775
75761	Apol7a	apolipoprotein L 7a	1.371	0.2594	0.6815
20776	Tmie	transmembrane inner ear	1.37	0.006192	0.2833
19415	Rasal1	RAS protein activator like 1 (GAP1 like)	1.369	0.1879	0.626
11936	Fxyd2	FXYD domain-containing ion transport regulator 2	1.369	0.03005	0.3947
320021	C430042M11Rik	RIKEN cDNA C430042M11 gene	1.368	0.003523	0.2383
14345	Fut4	fucosyltransferase 4	1.368	0.01379	0.3347
110084	Dnahc1	dynein, axonemal, heavy chain 1	1.367	0.09217	0.5263
19041	Ppl	periplakin	1.367	0.3779	0.7571
12122	Bid	BH3 interacting domain death agonist	1.367	0.08957	0.5224
415115	Neurl2	neuralized-like 2 (Drosophila)	1.366	0.006907	0.2915
16498	Kcnab2	potassium voltage-gated channel, shaker-related subfamily, beta member 2	1.366	0.2462	0.6734
20341	Selenbp1	selenium binding protein 1	1.365	0.002403	0.2173
17110	Lyz1	lysozyme 1	1.365	0.05934	0.4728
232156	Slc4a5	solute carrier family 4, sodium bicarbonate cotransporter, member 5	1.364	0.1003	0.5393

215819	Nhsl1	NHS-like 1	1.364	0.1125	0.5549
79202	Tnfrsf22	tumor necrosis factor receptor superfamily, member 22	1.364	0.3628	0.7483
51801	Ramp1	receptor (calcitonin) activity modifying protein 1	1.364	0.2061	0.6388
331461	Il1rapl1	interleukin 1 receptor accessory protein-like 1	1.363	0.2123	0.6449
327959	Xaf1	XIAP associated factor 1	1.363	0.02066	0.3609
234788	Slc38a8	solute carrier family 38, member 8	1.362	0.417	0.7776
230796	Wdtc1	WD and tetratricopeptide repeats 1	1.362	0.08372	0.5144
195531	Gm13152	predicted gene 13152	1.362	0.3854	0.7607
106572	Rab31	RAB31, member RAS oncogene family	1.362	0.03138	0.3964
77803	Fam159b	family with sequence similarity 159, member B	1.362	0.1549	0.5975
71962	Gatsl3	GATS protein-like 3	1.362	0.05787	0.469
17314	Mgmt	O-6-methylguanine-DNA methyltransferase	1.362	0.2636	0.6843
12797	Cnn1	calponin 1	1.362	0.3404	0.7328
12162	Bmp7	bone morphogenetic protein 7	1.362	0.1954	0.6304
277089	Gm5068	predicted gene 5068	1.361	0.1301	0.5747
71839	Osgin1	oxidative stress induced growth inhibitor 1	1.361	0.1634	0.6074
70847	4733401D01Rik	RIKEN cDNA 4733401D01 gene	1.361	0.2168	0.6482
17259	Mef2b	myocyte enhancer factor 2B	1.361	0.08577	0.517
66662	5730577I03Rik	zinc finger protein pseudogene	1.36	0.006219	0.2837
21939	Cd40	CD40 antigen	1.36	0.003728	0.2432
242481	Palm2	paralemmin 2	1.359	0.259	0.6815
75209	Sv2c	synaptic vesicle glycoprotein 2c	1.359	0.266	0.6856
68738	Acss1	acyl-CoA synthetase short-chain family member 1	1.359	0.1541	0.5975
98970	Fibcd1	fibrinogen C domain containing 1	1.358	0.0135	0.3343
66326	Dnajc5b	DnaJ (Hsp40) homolog, subfamily C, member 5 beta	1.358	0.00885	0.3086
14263	Fmo5	flavin containing monooxygenase 5	1.358	0.07132	0.4933
75578	Fggy	FGGY carbohydrate kinase domain containing	1.357	0.01154	0.3208
74743	5830403F22Rik	RIKEN cDNA 5830403F22 gene	1.357	0.0396	0.4222
98365	Slamf9	SLAM family member 9	1.356	0.02061	0.3609
76974	1190003J15Rik	RIKEN cDNA 1190003J15 gene	1.356	0.1776	0.6167
27276	Plekhb1	pleckstrin homology domain containing, family B (evectins) member 1	1.356	0.02211	0.3702
226791	Lyplal1	lysophospholipase-like 1	1.355	0.003764	0.2433
214531	Tmprss13	transmembrane protease, serine 13	1.355	0.2211	0.6525
66968	Plin5	perilipin 5	1.355	0.1411	0.5856
20305	Ccl6	chemokine (C-C motif) ligand 6	1.355	0.2701	0.6883
448987	Fbxl7	F-box and leucine-rich repeat protein 7	1.353	0.2317	0.6613
217116	Spata20	spermatogenesis associated 20	1.353	0.1762	0.6156
668158	Ccdc85c	coiled-coil domain containing 85C	1.352	0.09658	0.5345
100041244	Gm3230	predicted gene 3230	1.351	0.6739	0.8924
244859	Ankk1	ankyrin repeat and kinase domain containing 1	1.351	0.1263	0.5703
70358	Steap1	six transmembrane epithelial antigen of the prostate 1	1.351	0.1865	0.6239
20198	S100a4	S100 calcium binding protein A4	1.351	0.6247	0.8728
14017	Evi2a	ecotropic viral integration site 2a	1.351	0.006168	0.2833
98845	Eps8l2	EPS8-like 2	1.35	0.1655	0.6077
80718	Rab27b	RAB27b, member RAS oncogene family	1.35	0.006721	0.288
76487	Ppp1r3g	protein phosphatase 1, regulatory (inhibitor) subunit 3G	1.35	0.4312	0.7851
14184	Fgfr3	fibroblast growth factor receptor 3	1.35	0.7514	0.9213
317755	Zar1	zygote arrest 1	1.349	0.009962	0.3112
77805	Esco1	establishment of cohesion 1 homolog 1 (S. cerevisiae)	1.349	0.00245	0.2179

74075	Syce1	synaptonemal complex central element protein 1	1.349	0.2073	0.6398
50709	Hist1h1e	histone cluster 1, H1e	1.349	0.04926	0.4469
230145	Galnt12	UDP-N-acetyl-alpha-D-galactosamine:polypeptide N-acetylgalactosaminyltransferase 12	1.348	0.3866	0.761
97640	C78653	expressed sequence C78653	1.348	0.2187	0.6507
72287	Plekhf1	pleckstrin homology domain containing, family F (with FYVE domain) member 1	1.348	0.01127	0.3181
16543	Mdfic	MyoD family inhibitor domain containing	1.348	0.2202	0.6518
227753	Gsn	gelsolin	1.347	0.4213	0.7798
100039672	Msmp	microseminoprotein, prostate associated	1.346	0.6385	0.8779
223433	Fam105a	family with sequence similarity 105, member A	1.346	0.03401	0.4091
109267	Srcrb4d	scavenger receptor cysteine rich domain containing, group B (4 domains)	1.346	0.009063	0.3086
76406	1700019B03Rik	RIKEN cDNA 1700019B03 gene	1.346	0.182	0.6206
14864	Gstm3	glutathione S-transferase, mu 3	1.346	0.3929	0.7651
71790	Anxa9	annexin A9	1.345	0.2709	0.689
16619	Klk1b27	kallikrein 1-related peptidase b27	1.345	0.0846	0.5154
320700	A930033H14Rik	RIKEN cDNA A930033H14 gene	1.344	0.06515	0.4816
240025	Dact2	dapper homolog 2, antagonist of beta-catenin (xenopus)	1.344	0.09156	0.5241
230674	Kdm4a	lysine (K)-specific demethylase 4A	1.344	0.008093	0.302
269378	Ahcy	S-adenosylhomocysteine hydrolase	1.343	0.06579	0.4823
66039	D14Ertd449e	DNA segment, Chr 14, ERATO Doi 449, expressed	1.343	0.009874	0.3112
14067	F5	coagulation factor V	1.343	0.5948	0.8599
270151	Nlrx1	NLR family member X1	1.341	0.03911	0.4217
94180	Acsbg1	acyl-CoA synthetase bubblegum family member 1	1.34	0.02065	0.3609
15206	Hes2	hairy and enhancer of split 2 (Drosophila)	1.34	0.2141	0.6467
140474	Muc4	mucin 4	1.339	0.2715	0.6891
11997	Akr1b7	aldo-keto reductase family 1, member B7	1.339	0.04051	0.4257
11815	Apod	apolipoprotein D	1.339	0.003244	0.227
100042342	Gm10375	predicted gene 10375	1.338	0.2495	0.6767
112422	2610305D13Rik	RIKEN cDNA 2610305D13 gene	1.338	0.1831	0.6217
19218	Ptger3	prostaglandin E receptor 3 (subtype EP3)	1.338	0.2362	0.6657
18542	Pcolce	procollagen C-endopeptidase enhancer protein	1.338	0.4181	0.7785
15442	Hpse	heparanase	1.338	0.0008602	0.148
243025	Tmem156	transmembrane protein 156	1.337	0.09094	0.5229
171382	Trpm8	transient receptor potential cation channel, subfamily M, member 8	1.337	0.1713	0.6121
73961	4930447J18Rik	RIKEN cDNA 4930447J18 gene	1.337	0.1943	0.6299
30806	Adamts8	a disintegrin-like and metallopeptidase (reprolysin type) with thrombospondin type 1 motif, 8	1.337	0.04451	0.4349
15983	Ifrd2	interferon-related developmental regulator 2	1.337	0.08245	0.5144
14601	Ghrh	growth hormone releasing hormone	1.337	0.04294	0.4315
228576	Mall	mal, T-cell differentiation protein-like	1.336	0.1854	0.6225
71138	Tmem217	transmembrane protein 217	1.336	0.08694	0.5184
70083	Metrn	meteorin, glial cell differentiation regulator	1.336	0.004726	0.2643
68396	Nat8	N-acetyltransferase 8 (GCN5-related, putative)	1.336	0.1495	0.5938
66042	Sostdc1	sclerostin domain containing 1	1.336	0.1615	0.607
619289	Rfx8	regulatory factor X 8	1.335	0.347	0.7371
68713	Ifitm1	interferon induced transmembrane protein 1	1.335	0.09709	0.5347
12822	Col18a1	collagen, type XVIII, alpha 1	1.335	0.4368	0.7888
246707	Emilin2	elastin microfibril interfacer 2	1.334	0.08621	0.517
12176	Bnip3	BCL2/adenovirus E1B interacting protein 3	1.334	0.005728	0.2775
434438	Ccdc36	coiled-coil domain containing 36	1.333	0.1469	0.591
258642	Olfr1165-ps	olfactory receptor 1165, pseudogene	1.333	0.3917	0.7645

67182	Pdzk1ip1	PDZK1 interacting protein 1	1.333	0.1399	0.5834
75234	Rnf19b	ring finger protein 19B	1.332	0.04769	0.4442
71914	Antxr2	anthrax toxin receptor 2	1.332	0.005576	0.2775
71413	5430410E06Rik	RIKEN cDNA 5430410E06 gene	1.332	0.04387	0.4336
56421	Pfkp	phosphofructokinase, platelet	1.332	0.004201	0.2553
19358	Rad23a	RAD23a homolog (S. cerevisiae)	1.332	0.03047	0.3964
17181	Matn2	matrilin 2	1.332	0.4403	0.7911
12223	Btc	betacellulin, epidermal growth factor family member	1.332	0.04031	0.4253
665536	Gm7676	interferon induced transmembrane protein 1 pseudogene	1.331	0.122	0.5652
75578	Fggy	FGGY carbohydrate kinase domain containing	1.331	0.01716	0.3452
57394	Tmem27	transmembrane protein 27	1.329	0.006321	0.2859
23886	Gdf15	growth differentiation factor 15	1.329	0.003708	0.2432
14726	Pdpn	podoplanin	1.329	0.06378	0.4798
546519	Gm12581	predicted gene 12581	1.328	0.056	0.4631
214704	Iqub	IQ motif and ubiquitin domain containing	1.328	0.2757	0.6925
64817	Svep1	sushi, von Willebrand factor type A, EGF and pentraxin domain containing 1	1.328	0.04597	0.4381
21813	Tgfbr2	transforming growth factor, beta receptor II	1.328	0.01011	0.3112
17060	Blnk	B-cell linker	1.328	0.1638	0.6074
12505	Cd44	CD44 antigen	1.328	0.3183	0.7182
11303	Abca1	ATP-binding cassette, sub-family A (ABC1), member 1	1.328	0.01352	0.3343
330554	Fan1	FANCD2/FANCI-associated nuclease 1	1.327	0.0787	0.5052
20259	Scin	scinderin	1.327	0.03369	0.408
227541	Camk1d	calcium/calmodulin-dependent protein kinase ID	1.326	0.08774	0.5191
110749	Chaf1b	chromatin assembly factor 1, subunit B (p60)	1.326	0.04773	0.4442
23876	Fbln5	fibulin 5	1.326	0.2898	0.7004
11876	Artn	artemin	1.326	0.03238	0.4017
74692	4930442P07Rik	RIKEN cDNA 4930442P07 gene	1.325	0.1241	0.5693
11433	Acp5	acid phosphatase 5, tartrate resistant	1.325	0.1434	0.5879
13214	Defb1	defensin beta 1	1.324	0.006831	0.2906
12902	Cr2	complement receptor 2	1.324	0.1688	0.6096
231633	Tmem119	transmembrane protein 119	1.323	0.04468	0.4353
230098	E130306D19Rik	RIKEN cDNA E130306D19 gene	1.323	0.01482	0.3369
54519	Apbb1ip	amyloid beta (A4) precursor protein-binding, family B, member 1 interacting protein	1.323	0.1689	0.6096
18619	Penk	preproenkephalin	1.323	0.4698	0.8065
17153	Mal	myelin and lymphocyte protein, T-cell differentiation protein	1.323	0.1257	0.5703
12258	Serping1	serine (or cysteine) peptidase inhibitor, clade G, member 1	1.323	0.2582	0.6815
234138	BC019943	cDNA sequence BC019943	1.322	0.01285	0.3301
16852	Lgals1	lectin, galactose binding, soluble 1	1.322	0.1898	0.6273
269823	Pon3	paraoxonase 3	1.321	0.2591	0.6815
242747	Zfp933	zinc finger protein 933	1.321	0.265	0.6849
94217	Lrp1b	low density lipoprotein-related protein 1B (deleted in tumors)	1.32	0.7768	0.9272
74048	4632428N05Rik	RIKEN cDNA 4632428N05 gene	1.32	0.006155	0.2833
22350	Ezr	ezrin	1.32	0.2441	0.6719
18654	Pgf	placental growth factor	1.32	0.2159	0.6479
269994	Gsg1l	GSG1-like	1.319	0.2512	0.6767
244666	Gm505	predicted gene 505	1.319	0.1624	0.607
76166	6330545A04Rik	RIKEN cDNA 6330545A04 gene	1.319	0.2086	0.6401
433470	AA467197	expressed sequence AA467197	1.318	0.06262	0.4771
233045	C130030J05	hypothetical protein C130030J05	1.318	0.1331	0.5772

216445	Arhgap9	Rho GTPase activating protein 9	1.318	0.007229	0.2929
94179	Krt23	keratin 23	1.318	0.08304	0.5144
19229	Ptk2b	PTK2 protein tyrosine kinase 2 beta	1.318	0.1498	0.5938
110911	Cds2	CDP-diacylglycerol synthase (phosphatidate cytidylyltransferase) 2	1.317	0.002154	0.2121
73822	F630110N24Rik	RIKEN cDNA F630110N24 gene	1.317	0.3539	0.7414
22420	Wnt6	wingless-related MMTV integration site 6	1.317	0.03954	0.4222
227541	Camk1d	calcium/calmodulin-dependent protein kinase ID	1.316	0.0008443	0.148
56274	Stk3	serine/threonine kinase 3 (Ste20, yeast homolog)	1.316	0.2328	0.6622
21824	Thbd	thrombomodulin	1.316	0.009442	0.3112
74405	Efhc2	EF-hand domain (C-terminal) containing 2	1.315	0.03994	0.424
56363	Tmeff2	transmembrane protein with EGF-like and two follistatin-like domains 2	1.315	0.009882	0.3112
27276	Plekhb1	pleckstrin homology domain containing, family B (evectins) member 1	1.315	0.004741	0.2643
268709	Fam107a	family with sequence similarity 107, member A	1.314	0.05005	0.451
70405	Calml3	calmodulin-like 3	1.314	0.1093	0.5529
66042	Sostdc1	sclerostin domain containing 1	1.314	0.195	0.6304
50909	C1ra	complement component 1, r subcomponent A	1.314	0.2238	0.654
12345	Capzb	capping protein (actin filament) muscle Z-line, beta	1.314	0.3358	0.7303
244418	D8Ertd82e	DNA segment, Chr 8, ERATO Doi 82, expressed	1.313	0.07977	0.5091
74270	Usp20	ubiquitin specific peptidase 20	1.313	0.2272	0.6561
70377	Derl3	Der1-like domain family, member 3	1.313	0.09214	0.5263
12759	Clu	clusterin	1.313	0.005765	0.2775
227099	Pms1	postmeiotic segregation increased 1 (S. cerevisiae)	1.312	0.0008423	0.148
104816	Aspg	asparaginase homolog (S. cerevisiae)	1.312	0.2828	0.6969
68151	Wls	wntless homolog (Drosophila)	1.312	0.4096	0.7739
11409	Acads	acyl-Coenzyme A dehydrogenase, short chain	1.312	0.05682	0.4643
230971	Megf6	multiple EGF-like-domains 6	1.311	0.1913	0.6278
277743	Fam131c	family with sequence similarity 131, member C	1.309	0.3689	0.7521
16848	Lfng	LFNG O-fucosylpeptide 3-beta-N-acetylglucosaminyltransferase	1.309	0.1442	0.589
14873	Gsto1	glutathione S-transferase omega 1	1.309	0.08619	0.517
245945	Rbm47	RNA binding motif protein 47	1.308	0.2108	0.6426
170720	Card14	caspase recruitment domain family, member 14	1.308	0.09849	0.5367
74720	Tmem114	transmembrane protein 114	1.308	0.04305	0.4316
329934	Foxo6	forkhead box O6	1.307	0.2634	0.6842
227720	Nup214	nucleoporin 214	1.307	0.07933	0.5074
107435	Hat1	histone aminotransferase 1	1.307	0.03091	0.3964
102680	Slc6a20a	solute carrier family 6 (neurotransmitter transporter), member 20A	1.307	0.1933	0.6294
66166	S100a14	S100 calcium binding protein A14	1.307	0.06053	0.4741
59009	Sh3rf1	SH3 domain containing ring finger 1	1.307	0.399	0.7697
23936	Lynx1	Ly6/neurotoxin 1	1.307	0.1345	0.5779
21803	Tgfb1	transforming growth factor, beta 1	1.307	0.204	0.6368
20446	St6galnac2	ST6 (alpha-N-acetyl-neuraminyl-2,3-beta-galactosyl-1,3)-N-acetylgalactosaminide alpha-2,6-sialyltransferase 2	1.307	0.0305	0.3964
16168	Il15	interleukin 15	1.307	0.1051	0.5453
14654	Glra1	glycine receptor, alpha 1 subunit	1.307	0.01884	0.353
244049	Mctp2	multiple C2 domains, transmembrane 2	1.306	0.1852	0.6223
239083	Ccnb1ip1	cyclin B1 interacting protein 1	1.306	0.315	0.7161
72014	Btbd17	BTB (POZ) domain containing 17	1.306	0.06163	0.477
67432	Hoga1	4-hydroxy-2-oxoglutarate aldolase 1	1.306	0.1353	0.5789
22771	Zic1	zinc finger protein of the cerebellum 1	1.306	0.2341	0.6633
104271	Tex15	testis expressed gene 15	1.305	0.1741	0.6148

80292	Zxdc	ZXD family zinc finger C	1.305	0.3565	0.7443
66039	D14Ertd449e	DNA segment, Chr 14, ERATO Doi 449, expressed	1.305	0.1176	0.5607
57265	Fzd2	frizzled homolog 2 (Drosophila)	1.305	0.2007	0.6345
414116	D630024D03Rik	RIKEN cDNA D630024D03 gene	1.304	0.1594	0.6042
67073	Pi4k2b	phosphatidylinositol 4-kinase type 2 beta	1.304	0.146	0.591
27222	Atp1a4	ATPase, Na+/K+ transporting, alpha 4 polypeptide	1.304	0.03964	0.4222
69540	Klk10	kallikrein related-peptidase 10	1.303	0.05954	0.4728
14183	Fgfr2	fibroblast growth factor receptor 2	1.303	0.1038	0.5441
75355	4930553P18Rik	RIKEN cDNA 4930553P18 gene	1.302	0.07666	0.5014
68802	Mypn	myopalladin	1.302	0.425	0.7821
13482	Dpp4	dipeptidylpeptidase 4	1.302	0.07745	0.5026
12928	Crk	v-crk sarcoma virus CT10 oncogene homolog (avian)	1.302	0.5358	0.835
12259	C1qa	complement component 1, q subcomponent, alpha polypeptide	1.302	0.01386	0.3347
268782	Agxt2	alanine-glyoxylate aminotransferase 2	1.301	0.4056	0.7717
73005	2900072G11Rik	RIKEN cDNA 2900072G11 gene	1.301	0.3873	0.7611
68312	Gstm7	glutathione S-transferase, mu 7	1.301	0.09011	0.5229
30060	Mfi2	antigen p97 (melanoma associated) identified by monoclonal antibodies 133.2 and 96.5	1.301	0.01225	0.3236
20544	Slc9a1	solute carrier family 9 (sodium/hydrogen exchanger), member 1	1.301	0.164	0.6075
12335	Capn3	calpain 3	1.301	0.0407	0.4258
100042916	Gm4107	predicted gene 4107	1.3	0.05604	0.4631
234138	BC019943	cDNA sequence BC019943	1.3	0.03177	0.3991
75080	4930518C09Rik	RIKEN cDNA 4930518C09 gene	1.3	0.6402	0.8785
56523	Pmfbp1	polyamine modulated factor 1 binding protein 1	1.3	0.07158	0.4935
20348	Sema3c	sema domain, immunoglobulin domain (Ig), short basic domain, secreted, (semaphorin) 3C	1.3	0.4973	0.8193
104886	Rab15	RAB15, member RAS oncogene family	1.299	0.05551	0.4626
14425	Galnt3	UDP-N-acetyl-alpha-D-galactosamine:polypeptide N-acetylgalactosaminyltransferase 3	1.299	0.03997	0.424
246221	Mpst	mercaptopyruvate sulfurtransferase	1.298	0.06006	0.4728
66112	Mosc1	MOCO sulphurase C-terminal domain containing 1	1.298	0.04712	0.4435
23827	Bpnt1	bisphosphate 3'-nucleotidase 1	1.298	0.01107	0.3177
18655	Pgk1	phosphoglycerate kinase 1	1.298	0.01008	0.3112
320085	B830012L14Rik	RIKEN cDNA B830012L14 gene	1.297	0.2129	0.6454
110253	Triobp	TRIO and F-actin binding protein	1.297	0.2726	0.6897
101488	Slco2b1	solute carrier organic anion transporter family, member 2b1	1.297	0.02983	0.3942
67588	Rnf41	ring finger protein 41	1.297	0.08339	0.5144
56847	Aldh1a3	aldehyde dehydrogenase family 1, subfamily A3	1.297	0.4877	0.8135
19011	Endou	endonuclease, polyU-specific	1.297	0.03189	0.3992
328778	Rab26	RAB26, member RAS oncogene family	1.296	0.007127	0.2929
16332	Inppl1	inositol polyphosphate phosphatase-like 1	1.296	0.002623	0.2224
100039239	Gm2115	predicted gene 2115	1.295	0.0361	0.414
666168	Cyp4a31	cytochrome P450, family 4, subfamily a, polypeptide 31	1.295	0.4433	0.7931
338320	Mia2	melanoma inhibitory activity 2	1.295	0.08377	0.5144
14786	Grb7	growth factor receptor bound protein 7	1.295	0.166	0.6079
13544	Dvl3	dishevelled 3, dsh homolog (Drosophila)	1.295	0.2503	0.6767
436188	Gm5751	predicted gene 5751	1.294	0.02135	0.365
268977	Ltbp1	latent transforming growth factor beta binding protein 1	1.294	0.2849	0.6978
67896	Ccdc80	coiled-coil domain containing 80	1.294	0.4708	0.8076
67654	4930558C23Rik	RIKEN cDNA 4930558C23 gene	1.294	0.08312	0.5144
26408	Map3k5	mitogen-activated protein kinase kinase kinase 5	1.294	0.118	0.5609
21788	Tfpi	tissue factor pathway inhibitor	1.294	0.2862	0.6982

81600	Chia	chitinase, acidic	1.293	0.07511	0.4989
71602	Myo1e	myosin IE	1.293	0.03999	0.424
54324	Arhgef5	Rho guanine nucleotide exchange factor (GEF) 5	1.293	0.1335	0.5774
14708	Gng7	guanine nucleotide binding protein (G protein), gamma 7	1.293	0.05693	0.4647
407802	BC028789	cDNA sequence BC028789	1.292	0.3765	0.7559
216725	Adamts2	a disintegrin-like and metallopeptidase (reprolysin type) with thrombospondin type 1 motif, 2	1.292	0.6313	0.8751
66898	Baiap2l1	BAI1-associated protein 2-like 1	1.292	0.1796	0.6187
58200	Ppp1r1a	protein phosphatase 1, regulatory (inhibitor) subunit 1A	1.292	0.03305	0.4039
319622	Itpripl2	inositol 1,4,5-triphosphate receptor interacting protein-like 2	1.291	0.02576	0.3786
235330	Ttc12	tetratricopeptide repeat domain 12	1.291	0.01022	0.3117
23972	Papss2	3'-phosphoadenosine 5'-phosphosulfate synthase 2	1.291	0.03902	0.4217
14676	Gna15	guanine nucleotide binding protein, alpha 15	1.291	0.2241	0.654
12484	Cd24a	CD24a antigen	1.291	0.7045	0.9049
319711	E230029C05Rik	RIKEN cDNA E230029C05 gene	1.29	0.03118	0.3964
218639	Arl15	ADP-ribosylation factor-like 15	1.29	0.1112	0.5549
109731	Maob	monoamine oxidase B	1.29	0.1372	0.5808
71893	Noxo1	NADPH oxidase organizer 1	1.29	0.01463	0.3369
70134	2210011C24Rik	RIKEN cDNA 2210011C24 gene	1.29	0.1431	0.5879
21909	Tlx2	T-cell leukemia, homeobox 2	1.29	0.04168	0.4282
14675	Gna14	guanine nucleotide binding protein, alpha 14	1.29	0.1496	0.5938
329636	Gm5103	predicted gene 5103	1.289	0.1095	0.5531
268934	Grm4	glutamate receptor, metabotropic 4	1.289	0.5838	0.8544
232016	Ccdc129	coiled-coil domain containing 129	1.289	0.01942	0.3557
171504	Apob48r	apolipoprotein B48 receptor	1.289	0.2109	0.6426
80982	9930013L23Rik	RIKEN cDNA 9930013L23 gene	1.289	0.237	0.6659
70405	Calml3	calmodulin-like 3	1.289	0.1034	0.5433
12075	Bfsp1	beaded filament structural protein 1, in lens-CP94	1.289	0.7096	0.9069
436022	6030429G01Rik	RIKEN cDNA 6030429G01 gene	1.288	0.2101	0.6414
271047	Serpina3b	serine (or cysteine) peptidase inhibitor, clade A, member 3B	1.288	0.08423	0.5154
22146	Tuba1c	tubulin, alpha 1C	1.288	0.02163	0.367
19228	Pth1r	parathyroid hormone 1 receptor	1.288	0.009742	0.3112
18049	Ngf	nerve growth factor	1.288	0.1655	0.6077
16592	Fabp5	fatty acid binding protein 5, epidermal	1.288	0.4523	0.7955
14182	Fgfr1	fibroblast growth factor receptor 1	1.288	0.08448	0.5154
544717	1190007I07Rik	RIKEN cDNA 1190007I07 gene	1.287	0.07659	0.5014
387131	Ssxb9	synovial sarcoma, X member B, breakpoint 9	1.287	0.06752	0.4855
320802	6330512M04Rik	RIKEN cDNA 6330512M04 gene	1.287	0.05827	0.4703
319555	Nwd1	NACHT and WD repeat domain containing 1	1.287	0.6059	0.8645
319197	Gpr4	G protein-coupled receptor 4	1.287	0.1738	0.6148
276829	Smtnl2	smoothelin-like 2	1.287	0.01343	0.3343
77883	6030498E09Rik	RIKEN cDNA 6030498E09 gene	1.287	0.4453	0.7938
72318	Cyth4	cytohesin 4	1.287	0.06012	0.4728
654805	F930015N05Rik	RIKEN cDNA F930015N05 gene	1.286	0.0673	0.4855
381693	Wdr95	WD40 repeat domain 95	1.286	0.07221	0.4941
72368	2310045N01Rik	RIKEN cDNA 2310045N01 gene	1.286	0.0174	0.3468
69602	Otop3	otopetrin 3	1.286	0.02218	0.3702
58222	Rab37	RAB37, member of RAS oncogene family	1.286	0.08063	0.5102
21857	Timp1	tissue inhibitor of metalloproteinase 1	1.286	0.1642	0.6076
14027	Evpl	envoplakin	1.286	0.08711	0.5184

676894	Gm9694	predicted gene 9694	1.285	0.06913	0.4872
217294	BC006965	cDNA sequence BC006965	1.285	0.06788	0.4855
100342	Fam46b	family with sequence similarity 46, member B	1.285	0.2667	0.6862
76995	1700095A13Rik	RIKEN cDNA 1700095A13 gene	1.285	0.2198	0.6513
71738	Mamdc2	MAM domain containing 2	1.285	0.06329	0.478
69640	Fam83g	family with sequence similarity 83, member G	1.285	0.128	0.5716
22174	Tyro3	TYRO3 protein tyrosine kinase 3	1.285	0.3175	0.7179
16995	Ltb4r1	leukotriene B4 receptor 1	1.285	0.05156	0.4524
328789	Lhfpl5	lipoma HMGIC fusion partner-like 5	1.284	0.0157	0.3403
67968	Ooep	oocyte expressed protein homolog (dog)	1.284	0.682	0.8951
22146	Tuba1c	tubulin, alpha 1C	1.284	0.03062	0.3964
16774	Lama3	laminin, alpha 3	1.284	0.05731	0.4675
105976	AU022793	expressed sequence AU022793	1.283	0.2257	0.654
54381	Pgcp	plasma glutamate carboxypeptidase	1.283	0.04921	0.4469
21390	Tbxa2r	thromboxane A2 receptor	1.283	0.001198	0.1654
16687	Krt6a	keratin 6A	1.283	0.4122	0.7747
13096	Cyp2c37	cytochrome P450, family 2. subfamily c, polypeptide 37	1.283	0.1081	0.5513
319259	9930021D14Rik	RIKEN cDNA 9930021D14 gene	1.282	0.1974	0.6324
238405	Adam6b	a disintegrin and metallopeptidase domain 6B	1.282	0.3237	0.721
210029	Metrnl	meteorin, glial cell differentiation regulator-like	1.282	0.3836	0.7599
76915	Mnd1	meiotic nuclear divisions 1 homolog (S. cerevisiae)	1.282	0.04503	0.4353
15361	Hmga1	high mobility group AT-hook 1	1.282	0.02745	0.3862
242406	Rgp1	RGP1 retrograde golgi transport homolog (S. cerevisiae)	1.281	0.2947	0.7044
192970	Dhrs11	dehydrogenase/reductase (SDR family) member 11	1.281	0.1402	0.5834
67866	Wfdc1	WAP four-disulfide core domain 1	1.281	0.2838	0.6974
66720	Klhl10	kelch-like 10 (Drosophila)	1.281	0.06193	0.4771
112405	Egln1	EGL nine homolog 1 (C. elegans)	1.28	0.04085	0.4263
58222	Rab37	RAB37, member of RAS oncogene family	1.28	0.08313	0.5144
52377	Rcn3	reticulocalbin 3, EF-hand calcium binding domain	1.28	0.112	0.5549
26358	Aldh1a7	aldehyde dehydrogenase family 1, subfamily A7	1.28	0.06855	0.4867
320095	6430550D23Rik	RIKEN cDNA 6430550D23 gene	1.279	0.1357	0.5794
66605	1700017N19Rik	RIKEN cDNA 1700017N19 gene	1.279	0.2406	0.6687
19049	Ppp1r1b	protein phosphatase 1, regulatory (inhibitor) subunit 1B	1.279	0.1424	0.5872
331474	Rgag4	retrotransposon gag domain containing 4	1.278	0.03293	0.4038
328035	Fads6	fatty acid desaturase domain family, member 6	1.278	0.005817	0.2775
102545	Cmtm7	CKLF-like MARVEL transmembrane domain containing 7	1.278	0.06927	0.4874
77675	5033406O09Rik	RIKEN cDNA 5033406O09 gene	1.278	0.02806	0.3875
72097	2010300C02Rik	RIKEN cDNA 2010300C02 gene	1.278	0.0661	0.483
16402	Itga5	integrin alpha 5 (fibronectin receptor alpha)	1.278	0.3962	0.7671
13162	Slc6a3	solute carrier family 6 (neurotransmitter transporter, dopamine), member 3	1.278	0.4199	0.7795
11799	Birc5	baculoviral IAP repeat-containing 5	1.278	0.08613	0.517
108907	Nusap1	nucleolar and spindle associated protein 1	1.277	0.1192	0.5628
108017	Fxyd4	FXYD domain-containing ion transport regulator 4	1.277	0.1231	0.5672
12843	Col1a2	collagen, type I, alpha 2	1.277	0.3306	0.726
100169878	Gm10941	predicted gene 10941	1.276	0.05271	0.4554
192120	Bspry	B-box and SPRY domain containing	1.276	0.1671	0.6084
69987	1700026L06Rik	RIKEN cDNA 1700026L06 gene	1.276	0.398	0.7684
66205	Cd302	CD302 antigen	1.276	0.05348	0.4578
19659	Rbp1	retinol binding protein 1, cellular	1.276	0.0141	0.3347

18216	Ntsr1	neurotensin receptor 1	1.276	0.046	0.4381
381073	Npw	neuropeptide W	1.275	0.05287	0.4558
57875	Angptl4	angiopoietin-like 4	1.275	0.08399	0.5151
14865	Gstm4	glutathione S-transferase, mu 4	1.275	0.3201	0.7191
13052	Cxadr	coxsackie virus and adenovirus receptor	1.275	0.1008	0.5394
666539	Gm8154	predicted gene 8154	1.274	0.3448	0.736
320368	A730063M14Rik	RIKEN cDNA A730063M14 gene	1.274	0.005798	0.2775
74525	8430419L09Rik	RIKEN cDNA 8430419L09 gene	1.274	0.1024	0.5418
67331	Atp8b3	ATPase, class I, type 8B, member 3	1.274	0.1451	0.5896
20347	Sema3b	sema domain, immunoglobulin domain (Ig), short basic domain, secreted, (semaphorin) 3B	1.274	0.03773	0.417
19046	Ppp1cb	protein phosphatase 1, catalytic subunit, beta isoform	1.274	0.01344	0.3343
639653	Gm7276	predicted gene 7276	1.273	0.002874	0.2228
328121	Abhd12b	abhydrolase domain containing 12B	1.273	0.1643	0.6076
207798	Gramd1c	GRAM domain containing 1C	1.273	0.2029	0.6356
75136	Rsph10b2	radial spoke head 10 homolog B (Chlamydomonas)	1.273	0.2633	0.6841
381922	D830044I16Rik	RIKEN cDNA D830044I16 gene	1.272	0.01892	0.353
332579	Card9	caspase recruitment domain family, member 9	1.272	0.2029	0.6356
319179	Hist1h2be	histone cluster 1, H2be	1.272	0.02318	0.3732
239570	Ttc38	tetratricopeptide repeat domain 38	1.272	0.3794	0.7584
239368	BC030476	cDNA sequence BC030476	1.272	0.1272	0.5715
233187	Lim2	lens intrinsic membrane protein 2	1.272	0.8379	0.948
170829	Tram2	translocating chain-associating membrane protein 2	1.272	0.04931	0.4469
114654	Ly6g6d	lymphocyte antigen 6 complex, locus G6D	1.272	0.006467	0.288
114643	Oas1c	2'-5' oligoadenylate synthetase 1C	1.272	0.03099	0.3964
80915	Dusp12	dual specificity phosphatase 12	1.272	0.009625	0.3112
76142	Ppp1r14c	protein phosphatase 1, regulatory (inhibitor) subunit 14c	1.272	0.09901	0.5367
76117	Arhgap15	Rho GTPase activating protein 15	1.272	0.0109	0.317
56846	Necab3	N-terminal EF-hand calcium binding protein 3	1.272	0.1172	0.5599
20724	Serpinb5	serine (or cysteine) peptidase inhibitor, clade B, member 5	1.272	0.09649	0.5345
13685	Eif4ebp1	eukaryotic translation initiation factor 4E binding protein 1	1.272	0.09099	0.5229
245865	Spag4	sperm associated antigen 4	1.271	0.007394	0.2933
216987	Utp6	UTP6, small subunit (SSU) processome component, homolog (yeast)	1.271	0.09959	0.537
77318	Ankrd55	ankyrin repeat domain 55	1.271	0.05596	0.4631
72461	Prcp	prolylcarboxypeptidase (angiotensinase C)	1.271	0.06464	0.4816
67317	1700022I11Rik	RIKEN cDNA 1700022I11 gene	1.271	0.06532	0.4816
12798	Cnn2	calponin 2	1.271	0.0626	0.4771
12411	Cbs	cystathionine beta-synthase	1.271	0.2094	0.641
268759	9930012K11Rik	RIKEN cDNA 9930012K11 gene	1.27	0.06031	0.4733
236428	BC026762	cDNA sequence BC026762	1.27	0.2401	0.6685
234757	BC024137	cDNA sequence BC024137	1.27	0.2239	0.654
94226	S1pr5	sphingosine-1-phosphate receptor 5	1.27	0.07087	0.4923
68991	Ssu72	Ssu72 RNA polymerase II CTD phosphatase homolog (yeast)	1.27	0.001203	0.1654
15525	Hspa4	heat shock protein 4	1.27	0.004656	0.2642
100040353	2810416G20Rik	RIKEN cDNA 2810416G20 gene	1.269	0.07913	0.5063
666422	Gm8096	3-phosphoglycerate dehydrogenase pseudogene	1.269	0.5531	0.8439
78685	B430105A11Rik	RIKEN cDNA B430105A11 gene	1.269	0.07466	0.4974
76633	1700112E06Rik	RIKEN cDNA 1700112E06 gene	1.269	0.005085	0.2706
22228	Ucp2	uncoupling protein 2 (mitochondrial, proton carrier)	1.269	0.2504	0.6767
19733	Rgn	regucalcin	1.269	0.01411	0.3347

16578	Kif9	kinesin family member 9	1.269	0.1329	0.5772
13195	Ddc	dopa decarboxylase	1.269	0.05088	0.4519
235330	Ttc12	tetratricopeptide repeat domain 12	1.268	0.00728	0.2933
69699	2310079G19Rik	RIKEN cDNA 2310079G19 gene	1.268	0.2831	0.697
69117	Adh6a	alcohol dehydrogenase 6A (class V)	1.268	0.2429	0.6706
67774	Loh12cr1	loss of heterozygosity, 12, chromosomal region 1 homolog (human)	1.268	0.02198	0.3694
56787	Ascl3	achaete-scute complex homolog 3 (Drosophila)	1.268	0.1153	0.5578
56358	Copz2	coatomer protein complex, subunit zeta 2	1.268	0.07348	0.4957
17364	Trpm1	transient receptor potential cation channel, subfamily M, member 1	1.268	0.2437	0.6715
12966	Crygc	crystallin, gamma C	1.268	0.6931	0.8999
11464	Actc1	actin, alpha, cardiac muscle 1	1.268	0.07052	0.4917
319508	Syt15	synaptotagmin XV	1.267	0.01471	0.3369
236539	Phgdh	3-phosphoglycerate dehydrogenase	1.267	0.5731	0.8501
225651	Mppe1	metallophosphoesterase 1	1.267	0.072	0.4941
212996	Wbscr17	Williams-Beuren syndrome chromosome region 17 homolog (human)	1.267	0.0344	0.4091
107377	AW492981	expressed sequence AW492981	1.267	0.3142	0.7155
76808	Rpl18a	ribosomal protein L18A	1.267	0.3862	0.761
70355	Gprc5c	G protein-coupled receptor, family C, group 5, member C	1.267	0.2752	0.6925
56491	Vapb	vesicle-associated membrane protein, associated protein B and C	1.267	0.01021	0.3117
26447	Poli	polymerase (DNA directed), iota	1.267	0.01172	0.3214
14867	Gstm6	glutathione S-transferase, mu 6	1.267	0.04014	0.4248
14121	Fbp1	fructose bisphosphatase 1	1.267	0.1002	0.5389
620949	Dph3b-ps	DPH3B, KTI11 homolog B (S. cerevisiae), pseudogene	1.266	0.5048	0.8224
230868	Igsf21	immunoglobin superfamily, member 21	1.266	0.00423	0.2553
56012	Pgam2	phosphoglycerate mutase 2	1.266	0.1625	0.607
17087	Ly96	lymphocyte antigen 96	1.266	0.06628	0.4837
14739	S1pr2	sphingosine-1-phosphate receptor 2	1.266	0.02902	0.3903
12165	Gdf2	growth differentiation factor 2	1.266	0.06976	0.4884
66922	Rras2	related RAS viral (r-ras) oncogene homolog 2	1.265	0.05634	0.4632
56421	Pfkp	phosphofructokinase, platelet	1.265	0.002888	0.2228
100038941	Vmn2r121	vomeronasal 2, receptor 121	1.264	0.08498	0.5166
230866	C230096C10Rik	RIKEN cDNA C230096C10 gene	1.264	0.03672	0.4161
101807	AV356131	expressed sequence AV356131	1.264	0.001642	0.1903
17318	Mid1	midline 1	1.264	0.005271	0.2718
16949	Loxl1	lysyl oxidase-like 1	1.264	0.2698	0.6878
112407	Egln3	EGL nine homolog 3 (C. elegans)	1.263	0.01448	0.335
101533	Klk9	kallikrein related-peptidase 9	1.263	0.1013	0.5395
100198	H6pd	hexose-6-phosphate dehydrogenase (glucose 1-dehydrogenase)	1.263	0.2563	0.6803
50722	Dkkl1	dickkopf-like 1	1.263	0.08429	0.5154
30806	Adamts8	a disintegrin-like and metallopeptidase (reprolysin type) with thrombospondin type 1 motif, 8	1.263	0.009973	0.3112
28240	Trpm2	transient receptor potential cation channel, subfamily M, member 2	1.263	0.02992	0.3944
20195	S100a11	S100 calcium binding protein A11 (calgizzarin)	1.263	0.2226	0.654
19773	Rln1	relaxin 1	1.263	0.1153	0.5578
17150	Mfap2	microfibrillar-associated protein 2	1.263	0.0645	0.4816
13853	Epm2a	epilepsy, progressive myoclonic epilepsy, type 2 gene alpha	1.263	0.1631	0.6074
13118	Cyp4a12b	cytochrome P450, family 4, subfamily a, polypeptide 12B	1.263	0.1885	0.6263
12123	Hrk	harakiri, BCL2 interacting protein (contains only BH3 domain)	1.263	0.2268	0.6558
381570	Oog2	oogenesin 2	1.262	0.4471	0.7938
230917	Tmem201	transmembrane protein 201	1.262	0.08924	0.522

78929	Polr3h	polymerase (RNA) III (DNA directed) polypeptide H	1.262	0.077	0.5016
69784	1500009L16Rik	RIKEN cDNA 1500009L16 gene	1.262	0.03462	0.4091
18430	Oxtr	oxytocin receptor	1.262	0.1545	0.5975
14407	Gabrg3	gamma-aminobutyric acid (GABA) A receptor, subunit gamma 3	1.262	0.3418	0.7338
11601	Angpt2	angiopoietin 2	1.262	0.03413	0.4091
11496	Adam22	a disintegrin and metallopeptidase domain 22	1.262	0.5417	0.8379
234878	BC021891	cDNA sequence BC021891	1.261	0.3245	0.7213
232440	H2afj	H2A histone family, member J	1.261	0.01498	0.3382
22626	Slc23a3	solute carrier family 23 (nucleobase transporters), member 3	1.261	0.104	0.5444
22156	Tuft1	tuftelin 1	1.261	0.002765	0.2224
399568	BC052040	cDNA sequence BC052040	1.26	0.1042	0.5444
224079	Atp13a4	ATPase type 13A4	1.26	0.4401	0.7909
217353	Tmc6	transmembrane channel-like gene family 6	1.26	0.2965	0.7051
71093	Atoh8	atonal homolog 8 (Drosophila)	1.26	0.1477	0.5911
69065	Chac1	ChaC, cation transport regulator-like 1 (E. coli)	1.26	0.1821	0.6206
66873	Tril	TLR4 interactor with leucine-rich repeats	1.26	0.07399	0.4961
57278	Bcam	basal cell adhesion molecule	1.26	0.2893	0.7004
23928	Lamc3	laminin gamma 3	1.26	0.0584	0.4703
17318	Mid1	midline 1	1.26	0.007074	0.2929
11556	Adrb3	adrenergic receptor, beta 3	1.26	0.03479	0.4094
100040049	1810010D01Rik	RIKEN cDNA 1810010D01 gene	1.259	0.09128	0.5237
327956	Vmo1	vitelline membrane outer layer 1 homolog (chicken)	1.259	0.02589	0.3786
170721	Papln	papilin, proteoglycan-like sulfated glycoprotein	1.259	0.2727	0.6897
102626	Mapkapk3	mitogen-activated protein kinase-activated protein kinase 3	1.259	0.08252	0.5144
66775	Ptplad2	protein tyrosine phosphatase-like A domain containing 2	1.259	0.2278	0.6569
12331	Cap1	CAP, adenylate cyclase-associated protein 1 (yeast)	1.259	0.02655	0.3809
640502	LOC640502	UDP-N-acetylhexosamine pyrophosphorylase-like	1.258	0.01163	0.3214
629756	Wfdc10	WAP four-disulfide core domain 10	1.258	0.04864	0.4461
385668	Lca5l	Leber congenital amaurosis 5-like	1.258	0.09336	0.5293
225997	Trpm6	transient receptor potential cation channel, subfamily M, member 6	1.258	0.2454	0.6731
83436	Plekha2	pleckstrin homology domain-containing, family A (phosphoinositide binding specific) member 2	1.258	0.00093	0.1517
74490	Mamstr	MEF2 activating motif and SAP domain containing transcriptional regulator	1.258	0.1828	0.6215
14067	F5	coagulation factor V	1.258	0.6107	0.8671
320522	Bhlha9	basic helix-loop-helix family, member a9	1.257	0.002359	0.2166
319765	Igf2bp2	insulin-like growth factor 2 mRNA binding protein 2	1.257	0.006508	0.288
59010	Sqrdl	sulfide quinone reductase-like (yeast)	1.257	0.2976	0.7051
27060	Tcirg1	T-cell, immune regulator 1, ATPase, H+ transporting, lysosomal V0 protein A3	1.257	0.04734	0.4442
14735	Gpc4	glypican 4	1.257	0.09058	0.5229
13222	Defa-rs2	defensin, alpha, related sequence 2	1.257	0.07213	0.4941
12038	Bche	butyrylcholinesterase	1.257	0.03726	0.4163
320435	Rinl	Ras and Rab interactor-like	1.256	0.2332	0.6622
258623	Olfr123	olfactory receptor 123	1.256	0.1941	0.6299
110596	Rgnef	Rho-guanine nucleotide exchange factor	1.256	0.06452	0.4816
73121	Fam101a	family with sequence similarity 101, member A	1.256	0.09485	0.5323
18442	P2ry2	purinergic receptor P2Y, G-protein coupled 2	1.256	0.3065	0.7108
14961	H2-Ab1	histocompatibility 2, class II antigen A, beta 1	1.256	0.1745	0.6148
100038433	9130213A22Rik	RIKEN cDNA 9130213A22 gene	1.255	0.2226	0.654
622384	Fabp5l2	fatty acid binding protein 5-like 2	1.255	0.5003	0.8197
231830	Micall2	MICAL-like 2	1.255	0.2285	0.6577

192136	5033411D12Rik	RIKEN cDNA 5033411D12 gene	1.255	0.6192	0.8709
26386	Hsf4	heat shock transcription factor 4	1.255	0.3152	0.7162
12164	Bmp8b	bone morphogenetic protein 8b	1.255	0.15	0.5938
667572	Gm8709	glyceraldehyde-3-phosphate dehydrogenase pseudogene	1.254	0.132	0.576
338368	Fam109b	family with sequence similarity 109, member B	1.254	0.5146	0.8273
14862	Gstm1	glutathione S-transferase, mu 1	1.254	0.5041	0.8221
100217450	Snora47	small nucleolar RNA, H/ACA box 47	1.253	0.1464	0.591
226245	9930023K05Rik	RIKEN cDNA 9930023K05 gene	1.253	0.1336	0.5774
215387	Ncaph	non-SMC condensin I complex, subunit H	1.253	0.04171	0.4282
93694	Clec2d	C-type lectin domain family 2, member d	1.253	0.08295	0.5144
69694	Tatdn1	TatD DNase domain containing 1	1.253	0.09827	0.5365
22177	Tyrobp	TYRO protein tyrosine kinase binding protein	1.253	0.008984	0.3086
16796	Lasp1	LIM and SH3 protein 1	1.253	0.09422	0.5311
14373	G0s2	G0/G1 switch gene 2	1.253	0.01181	0.3223
14368	Fzd6	frizzled homolog 6 (Drosophila)	1.253	0.1347	0.5779
233571	P2ry6	pyrimidinergic receptor P2Y, G-protein coupled, 6	1.252	0.2057	0.6386
216343	Tph2	tryptophan hydroxylase 2	1.252	0.01553	0.3403
106759	Ticam1	toll-like receptor adaptor molecule 1	1.252	0.2159	0.6479
83433	Trem2	triggering receptor expressed on myeloid cells 2	1.252	0.03676	0.4161
78600	Pde6h	phosphodiesterase 6H, cGMP-specific, cone, gamma	1.252	0.000696	0.145
68180	Hyi	hydroxypyruvate isomerase homolog (E. coli)	1.252	0.02737	0.3861
56312	Nupr1	nuclear protein 1	1.252	0.7435	0.919
20324	Sdpr	serum deprivation response	1.252	0.2658	0.6856
14961	H2-Ab1	histocompatibility 2, class II antigen A, beta 1	1.252	0.1799	0.6189
436583	Snora74a	small nucleolar RNA, H/ACA box 74A	1.251	0.2602	0.6815
269604	Gpr157	G protein-coupled receptor 157	1.251	0.3226	0.7202
114249	Npnt	nephronectin	1.251	0.3919	0.7645
68939	Rasl11b	RAS-like, family 11, member B	1.251	0.05194	0.4525
66473	Ctrb1	chymotrypsinogen B1	1.251	0.04909	0.4469
18552	Pcsk5	proprotein convertase subtilisin/kexin type 5	1.251	0.1367	0.5807
17380	Mme	membrane metallo endopeptidase	1.251	0.7256	0.9126
16204	Fabp6	fatty acid binding protein 6, ileal (gastrotropin)	1.251	0.2815	0.6967
320178	4921529L05Rik	RIKEN cDNA 4921529L05 gene	1.25	0.2159	0.6479
192166	Sardh	sarcosine dehydrogenase	1.25	0.0767	0.5014
94047	Cecr6	cat eye syndrome chromosome region, candidate 6 homolog (human)	1.25	0.2203	0.6518
70884	Ccdc81	coiled-coil domain containing 81	1.25	0.03677	0.4161
70785	Dennd1c	DENN/MADD domain containing 1C	1.25	0.1635	0.6074
69376	Zpbp2	zona pellucida binding protein 2	1.25	0.5265	0.8323
56856	Insm2	insulinoma-associated 2	1.25	0.02496	0.3786
16538	Kcns1	K+ voltage-gated channel, subfamily S, 1	1.25	0.1667	0.6083
14751	Gpi1	glucose phosphate isomerase 1	1.25	0.02253	0.3702
13057	Cyba	cytochrome b-245, alpha polypeptide	1.25	0.125	0.5703
11486	Ada	adenosine deaminase	1.25	0.2209	0.6525
217071	Gm525	predicted gene 525	1.249	0.01164	0.3214
73721	1110017D15Rik	RIKEN cDNA 1110017D15 gene	1.249	0.1135	0.5564
69765	1500004F05Rik	RIKEN cDNA 1500004F05 gene	1.249	0.1043	0.5444
13601	Ecm1	extracellular matrix protein 1	1.249	0.2948	0.7044
271842	Rpusd2	RNA pseudouridylate synthase domain containing 2	1.248	0.1035	0.5436
269952	D330012F22Rik	RIKEN cDNA D330012F22 gene	1.248	0.07401	0.4961

217826	Kcnk13	potassium channel, subfamily K, member 13	1.248	0.1578	0.6019
69379	C8g	complement component 8, gamma polypeptide	1.248	0.01003	0.3112
66253	Aig1	androgen-induced 1	1.248	0.1122	0.5549
20318	Sdf4	stromal cell derived factor 4	1.248	0.006057	0.2828
19296	Pvt1	plasmacytoma variant translocation 1	1.248	0.01578	0.3403
11981	Atp9a	ATPase, class II, type 9A	1.248	0.1189	0.5622
11600	Angpt1	angiopoietin 1	1.248	0.06641	0.4842
230577	Pars2	prolyl-tRNA synthetase (mitochondrial)(putative)	1.247	0.04309	0.4316
109934	Abr	active BCR-related gene	1.247	0.04806	0.4443
74191	P2ry13	purinergic receptor P2Y, G-protein coupled 13	1.247	0.2234	0.654
22156	Tuft1	tuftelin 1	1.247	0.004404	0.2611
18746	Pkm2	pyruvate kinase, muscle	1.247	0.08446	0.5154
18261	Ocm	oncomodulin	1.247	0.06822	0.486
18188	Nrtn	neurturin	1.247	0.02519	0.3786
16949	Loxl1	lysyl oxidase-like 1	1.247	0.2849	0.6978
629557	Gm6981	glyceraldehyde-3-phosphate dehydrogenase pseudogene	1.246	0.1711	0.6118
223726	Mpped1	metallophosphoesterase domain containing 1	1.246	0.03587	0.413
223658	Heatr7a	HEAT repeat containing 7A	1.246	0.1754	0.6156
73239	3110054G05Rik	RIKEN cDNA 3110054G05 gene	1.246	0.332	0.7269
16691	Krt8	keratin 8	1.246	0.4164	0.7771
16543	Mdfic	MyoD family inhibitor domain containing	1.246	0.3738	0.7544
12840	Col9a2	collagen, type IX, alpha 2	1.246	0.1062	0.5468
432879	Gm5465	predicted gene 5465	1.245	0.04048	0.4257
320309	1520401A03Rik	RIKEN cDNA 1520401A03 gene	1.245	0.5366	0.8354
242608	Podn	podocan	1.245	0.5842	0.8546
70426	Tekt5	tektin 5	1.245	0.2679	0.6871
54132	Pdlim1	PDZ and LIM domain 1 (elfin)	1.245	0.1941	0.6299
51801	Ramp1	receptor (calcitonin) activity modifying protein 1	1.245	0.01075	0.3148
19039	Lgals3bp	lectin, galactoside-binding, soluble, 3 binding protein	1.245	0.05938	0.4728
18552	Pcsk5	proprotein convertase subtilisin/kexin type 5	1.245	0.09426	0.5311
13537	Dusp2	dual specificity phosphatase 2	1.245	0.05071	0.4519
11629	Aif1	allograft inflammatory factor 1	1.245	0.01628	0.3414
103743	Tmem98	transmembrane protein 98	1.244	0.126	0.5703
75578	Fggy	FGGY carbohydrate kinase domain containing	1.244	0.08437	0.5154
63913	Fam129a	family with sequence similarity 129, member A	1.244	0.3756	0.7556
57890	Il17re	interleukin 17 receptor E	1.244	0.1804	0.6198
56016	Hebp2	heme binding protein 2	1.244	0.2577	0.6815
12580	Cdkn2c	cyclin-dependent kinase inhibitor 2C (p18, inhibits CDK4)	1.244	0.002023	0.2121
12316	Aspm	asp (abnormal spindle)-like, microcephaly associated (Drosophila)	1.244	0.1131	0.5556
353287	Clec18a	C-type lectin domain family 18, member A	1.243	0.3347	0.73
114479	Slc5a5	solute carrier family 5 (sodium iodide symporter), member 5	1.243	0.2697	0.6878
109042	Prkcdbp	protein kinase C, delta binding protein	1.243	0.07782	0.5028
75690	Vsig10l	ZV-set and immunoglobulin domain containing 10 like	1.243	0.1148	0.5569
12224	Klf5	Kruppel-like factor 5	1.243	0.1998	0.6343
320907	B430105G09Rik	RIKEN cDNA B430105G09 gene	1.242	0.004503	0.2618
224344	Rbm11	RNA binding motif protein 11	1.242	0.05169	0.4525
74634	4930423D22Rik	RIKEN cDNA 4930423D22 gene	1.242	0.241	0.6687
66809	Krt20	keratin 20	1.242	0.2499	0.6767
12021	Bard1	BRCA1 associated RING domain 1	1.242	0.3224	0.7202

11668	Aldh1a1	aldehyde dehydrogenase family 1, subfamily A1	1.242	0.2133	0.6459
319455	Pld5	phospholipase D family, member 5	1.241	0.2422	0.6704
24059	Slco2a1	solute carrier organic anion transporter family, member 2a1	1.241	0.08939	0.5223
22255	Uncx	UNC homeobox	1.241	0.1592	0.6038
16453	Jak3	Janus kinase 3	1.241	0.1084	0.5521
12841	Col9a3	collagen, type IX, alpha 3	1.241	0.2544	0.6792
11604	Agrp	agouti related protein	1.241	0.1376	0.5815
11433	Acp5	acid phosphatase 5, tartrate resistant	1.241	0.2459	0.6733
100040736	9130206I24Rik	RIKEN cDNA 9130206I24 gene	1.24	0.2194	0.6513
329934	Foxo6	forkhead box O6	1.24	0.05006	0.451
100041724	Gm15217	predicted gene 15217	1.239	0.1745	0.6148
320502	Lmod3	leiomodin 3 (fetal)	1.239	0.02052	0.3609
78369	Icam4	intercellular adhesion molecule 4, Landsteiner-Wiener blood group	1.239	0.01599	0.3408
74215	Prss51	protease, serine, 51	1.239	0.3795	0.7586
18707	Pik3cd	phosphatidylinositol 3-kinase catalytic delta polypeptide	1.239	0.3938	0.7656
17687	Msh5	mutS homolog 5 (E. coli)	1.239	0.01502	0.3383
12628	Cfh	complement component factor h	1.239	0.01997	0.3604
12522	Cd83	CD83 antigen	1.239	0.04448	0.4349
668239	Gm12270	predicted gene 12270	1.238	0.012	0.3235
433766	Trim63	tripartite motif-containing 63	1.238	0.01328	0.3331
433594	Gm5537	phosphoglycerate kinase 1-like	1.238	0.02108	0.3626
432779	Lrrc14b	leucine rich repeat containing 14B	1.238	0.0057	0.2775
170756	Slc24a6	solute carrier family 24 (sodium/potassium/calcium exchanger), member 6	1.238	0.1944	0.6299
76905	Lrg1	leucine-rich alpha-2-glycoprotein 1	1.238	0.3116	0.7137
75396	Spp2	secreted phosphoprotein 2	1.238	0.05025	0.4514
21922	Clec3b	C-type lectin domain family 3, member b	1.238	0.2965	0.7051
14204	Il4i1	interleukin 4 induced 1	1.238	0.2544	0.6792
621893	Hist2h2ab	histone cluster 2, H2ab	1.237	0.3806	0.7594
246085	Defb10	defensin beta 10	1.237	0.2322	0.6621
218440	Ankrd34b	ankyrin repeat domain 34B	1.237	0.14	0.5834
70713	Gpr137c	G protein-coupled receptor 137C	1.237	0.1309	0.5751
22004	Tpm2	tropomyosin 2, beta	1.237	0.03527	0.4109
17293	Mesp2	mesoderm posterior 2	1.237	0.2834	0.6974
14275	Folr1	folate receptor 1 (adult)	1.237	0.3536	0.7414
14245	Lpin1	lipin 1	1.237	0.08267	0.5144
170706	Tmem37	transmembrane protein 37	1.236	0.539	0.8366
72902	Spock3	sparc/osteonectin, cwcv and kazal-like domains proteoglycan 3	1.236	0.6626	0.8895
71699	Slc41a3	solute carrier family 41, member 3	1.236	0.09847	0.5367
329763	Gm5105	predicted gene 5105	1.235	0.1488	0.5928
237010	Klhl4	kelch-like 4 (Drosophila)	1.235	0.07227	0.4941
71562	Afmid	arylformamidase	1.235	0.08343	0.5144
56338	Txnip	thioredoxin interacting protein	1.235	0.01702	0.3443
22761	Zfpm1	zinc finger protein, multitype 1	1.235	0.04776	0.4442
19119	Prm2	protamine 2	1.235	0.5551	0.8444
16414	Itgb2	integrin beta 2	1.235	0.07874	0.5052
15482	Hspa1l	heat shock protein 1-like	1.235	0.1559	0.599
14431	Gamt	guanidinoacetate methyltransferase	1.235	0.1158	0.558
667373	Gm14446	predicted gene 14446	1.234	0.2509	0.6767
257926	Olfr544	olfactory receptor 544	1.234	0.2959	0.705

114570	Crip3	cysteine-rich protein 3	1.234	0.09521	0.5332
83984	Tssk6	testis-specific serine kinase 6	1.234	0.2693	0.6878
72160	Tmem163	transmembrane protein 163	1.234	0.08045	0.5098
381694	B3galtl	beta 1,3-galactosyltransferase-like	1.233	0.05243	0.4547
333182	Cox6b2	cytochrome c oxidase subunit VIb polypeptide 2	1.233	0.176	0.6156
269774	Aak1	AP2 associated kinase 1	1.233	0.3496	0.7388
228801	U46068	cDNA sequence U46068	1.233	0.2214	0.6525
69060	Pnlip	pancreatic lipase	1.233	0.05601	0.4631
56695	Pnkd	paroxysmal nonkinesiogenic dyskinesia	1.233	0.1301	0.5747
20425	Shmt1	serine hydroxymethyltransferase 1 (soluble)	1.233	0.4035	0.7708
15559	Htr2b	5-hydroxytryptamine (serotonin) receptor 2B	1.233	0.0732	0.4957
545366	BC026782	cDNA sequence BC026782	1.232	0.07392	0.4961
227620	Uap1l1	UDP-N-acteylglucosamine pyrophosphorylase 1-like 1	1.232	0.1506	0.5938
224109	Lrrc33	leucine rich repeat containing 33	1.232	0.02574	0.3786
171207	Arhgap4	Rho GTPase activating protein 4	1.232	0.0477	0.4442
76832	Hyls1	hydrolethalus syndrome 1	1.232	0.007469	0.2934
75465	Dynlrb2	dynein light chain roadblock-type 2	1.232	0.01707	0.3443
71334	5430416G10Rik	RIKEN cDNA 5430416G10 gene	1.232	0.197	0.632
66952	2310030G06Rik	RIKEN cDNA 2310030G06 gene	1.232	0.3061	0.7106
30935	Tor3a	torsin family 3, member A	1.232	0.1078	0.5509
20448	St6galnac4	ST6 (alpha-N-acetyl-neuraminyl-2,3-beta-galactosyl-1,3)-N-acetylgalactosaminide alpha-2,6-sialyltransferase 4	1.232	0.109	0.5529
14917	Gucy2c	guanylate cyclase 2c	1.232	0.2021	0.6356
435766	Tnni3k	TNNI3 interacting kinase	1.231	0.2929	0.7027
244911	C2cd4a	C2 calcium-dependent domain containing 4A	1.231	0.1593	0.6042
234577	Cpne2	copine II	1.231	0.1177	0.5609
233733	Galntl4	UDP-N-acetyl-alpha-D-galactosamine:polypeptide N-acetylgalactosaminyltransferase-like 4	1.231	0.05319	0.4565
100017	Ldlrap1	low density lipoprotein receptor adaptor protein 1	1.231	0.1157	0.558
22241	Ulk1	Unc-51 like kinase 1 (C. elegans)	1.231	0.003311	0.2288
19703	Renbp	renin binding protein	1.231	0.2973	0.7051
14009	Etv1	ets variant gene 1	1.231	0.08781	0.5191
329641	6030405A18Rik	RIKEN cDNA 6030405A18 gene	1.23	0.01657	0.3416
243362	Stard13	StAR-related lipid transfer (START) domain containing 13	1.23	0.03695	0.4161
83924	Gpr137b	G protein-coupled receptor 137B	1.23	0.08793	0.5194
77446	Heg1	HEG homolog 1 (zebrafish)	1.23	0.3054	0.7101
76486	Ly6k	lymphocyte antigen 6 complex, locus K	1.23	0.2378	0.6664
73847	Fam110a	family with sequence similarity 110, member A	1.23	0.01478	0.3369
69270	Gins1	GINS complex subunit 1 (Psf1 homolog)	1.23	0.01152	0.3208
27222	Atp1a4	ATPase, Na+/K+ transporting, alpha 4 polypeptide	1.23	0.3169	0.7174
20698	Sphk1	sphingosine kinase 1	1.23	0.1102	0.5535
17175	Masp2	mannan-binding lectin serine peptidase 2	1.23	0.253	0.6778
12409	Cbr2	carbonyl reductase 2	1.23	0.3604	0.7467
320844	Amigo3	adhesion molecule with Ig like domain 3	1.229	0.1844	0.6217
320775	B230319C09Rik	RIKEN cDNA B230319C09 gene	1.229	0.1355	0.5793
245631	Mum1l1	melanoma associated antigen (mutated) 1-like 1	1.229	0.01168	0.3214
103767	A530047J11Rik	RIKEN cDNA A530047J11 gene	1.229	0.09096	0.5229
71846	Syce2	synaptonemal complex central element protein 2	1.229	0.02078	0.3609
57319	Smpdl3a	sphingomyelin phosphodiesterase, acid-like 3A	1.229	0.084	0.5151
16770	Lalba	lactalbumin, alpha	1.229	0.1368	0.5807
15507	Hspb1	heat shock protein 1	1.229	0.02857	0.3896

15461	Hras1	Harvey rat sarcoma virus oncogene 1	1.229	0.1501	0.5938
14058	F10	coagulation factor X	1.229	0.3599	0.7465
100041489	Gm3367	predicted gene 3367	1.228	0.05137	0.4519
116701	Fgfrl1	fibroblast growth factor receptor-like 1	1.228	0.1496	0.5938
68968	Cdan1	congenital dyserythropoietic anemia, type I (human)	1.228	0.1469	0.591
22419	Wnt5b	wingless-related MMTV integration site 5B	1.228	0.4155	0.7767
18612	Etv4	ets variant gene 4 (E1A enhancer binding protein, E1AF)	1.228	0.2593	0.6815
16704	Krtap8-2	keratin associated protein 8-2	1.228	0.2459	0.6733
15162	Hck	hemopoietic cell kinase	1.228	0.3029	0.7087
246746	Cd300lf	CD300 antigen like family member F	1.227	0.03834	0.4199
210622	Pamr1	peptidase domain containing associated with muscle regeneration 1	1.227	0.009613	0.3112
208151	Tmem132b	transmembrane protein 132B	1.227	0.1015	0.5395
170952	Prima1	proline rich membrane anchor 1	1.227	0.04374	0.4336
67128	Ube2g1	ubiquitin-conjugating enzyme E2G 1 (UBC7 homolog, C. elegans)	1.227	0.03257	0.4018
19858	Rnu3b1	U3B small nuclear RNA 1	1.227	0.3389	0.732
328699	Gabrr3	gamma-aminobutyric acid (GABA) receptor, rho 3	1.226	0.2344	0.6636
282663	Serpinb1b	serine (or cysteine) peptidase inhibitor, clade B, member 1b	1.226	0.06397	0.4803
171273	Vmn1r217	vomeronasal 1 receptor 217	1.226	0.293	0.7027
76453	Prss23	protease, serine, 23	1.226	0.01038	0.3117
66058	Tmem176a	transmembrane protein 176A	1.226	0.161	0.6067
23921	Sh2b2	SH2B adaptor protein 2	1.226	0.05364	0.4589
19331	Rab19	RAB19, member RAS oncogene family	1.226	0.02564	0.3786
17750	Mt2	metallothionein 2	1.226	0.004797	0.2647
17202	Mc4r	melanocortin 4 receptor	1.226	0.06531	0.4816
14814	Grin2d	glutamate receptor, ionotropic, NMDA2D (epsilon 4)	1.226	0.3985	0.769
14433	Gapdh	glyceraldehyde-3-phosphate dehydrogenase	1.226	0.1798	0.6187
14411	Slc6a12	solute carrier family 6 (neurotransmitter transporter, betaine/GABA), member 12	1.226	0.2006	0.6345
12258	Serping1	serine (or cysteine) peptidase inhibitor, clade G, member 1	1.226	0.4475	0.7938
11607	Agtr1a	angiotensin II receptor, type 1a	1.226	0.3569	0.7443
225266	Klhl14	kelch-like 14 (Drosophila)	1.225	0.2099	0.6413
76898	B3gat1	beta-1,3-glucuronyltransferase 1 (glucuronosyltransferase P)	1.225	0.08164	0.5122
20322	Sord	sorbitol dehydrogenase	1.225	0.08083	0.5104
17002	Ltf	lactotransferrin	1.225	0.4015	0.7703
12977	Csf1	colony stimulating factor 1 (macrophage)	1.225	0.6438	0.8802
234396	Ankle1	ankyrin repeat and LEM domain containing 1	1.224	0.08592	0.517
104174	Gldc	glycine decarboxylase	1.224	0.105	0.5453
72621	Pdzd11	PDZ domain containing 11	1.224	0.06275	0.4771
71693	Colec11	collectin sub-family member 11	1.224	0.2645	0.6845
71670	Acy3	aspartoacylase (aminoacylase) 3	1.224	0.194	0.6299
66864	Clec14a	C-type lectin domain family 14, member a	1.224	0.2473	0.6743
66090	Ypel3	yippee-like 3 (Drosophila)	1.224	0.04555	0.4367
63955	Cables1	CDK5 and Abl enzyme substrate 1	1.224	0.02253	0.3702
18429	Oxt	oxytocin	1.224	0.1258	0.5703
17701	Msx1	homeobox, msh-like 1	1.224	0.3292	0.7254
382139	Gm1715	predicted gene 1715	1.223	0.1681	0.6095
271944	C2cd4d	C2 calcium-dependent domain containing 4D	1.223	0.2085	0.6401
94332	Cadm3	cell adhesion molecule 3	1.223	0.096	0.5344
76477	Pcolce2	procollagen C-endopeptidase enhancer 2	1.223	0.03775	0.417
73673	2410076I21Rik	RIKEN cDNA 2410076I21 gene	1.223	0.03133	0.3964

70904	4921515G04Rik	RIKEN cDNA 4921515G04 gene	1.223	0.6378	0.8776
56338	Txnip	thioredoxin interacting protein	1.223	0.03614	0.4142
52276	Cdca8	cell division cycle associated 8	1.223	0.05161	0.4524
17341	Bhlha15	basic helix-loop-helix family, member a15	1.223	0.2113	0.6435
13166	Dbh	dopamine beta hydroxylase	1.223	0.1519	0.595
268756	Gulo	gulonolactone (L-) oxidase	1.222	0.04549	0.4363
257902	Olfr704	olfactory receptor 704	1.222	0.5975	0.8611
246709	Rgs13	regulator of G-protein signaling 13	1.222	0.01054	0.3125
235406	Snx33	sorting nexin 33	1.222	0.08295	0.5144
217830	9030617O03Rik	RIKEN cDNA 9030617O03 gene	1.222	0.03852	0.4207
110891	Slc8a2	solute carrier family 8 (sodium/calcium exchanger), member 2	1.222	0.09761	0.5353
70031	Cmtm8	CKLF-like MARVEL transmembrane domain containing 8	1.222	0.05604	0.4631
66902	Mtap	methylthioadenosine phosphorylase	1.222	0.2891	0.7001
65971	Tbata	thymus, brain and testes associated	1.222	0.033	0.4038
52430	Echdc2	enoyl Coenzyme A hydratase domain containing 2	1.222	0.04925	0.4469
28010	Miip	migration and invasion inhibitory protein	1.222	0.3027	0.7087
18249	Obp1a	odorant binding protein Ia	1.222	0.2505	0.6767
18071	Nhlh1	nescient helix loop helix 1	1.222	0.2879	0.699
16582	Kifc3	kinesin family member C3	1.222	0.1034	0.5433
629091	LOC629091	hypothetical protein LOC629091	1.221	0.3355	0.7302
245269	E130304F04Rik	RIKEN cDNA E130304F04 gene	1.221	0.4179	0.7784
241528	Lrrc55	leucine rich repeat containing 55	1.221	0.06225	0.4771
231287	Atp10d	ATPase, class V, type 10D	1.221	0.3324	0.7273
74645	Fam46c	family with sequence similarity 46, member C	1.221	0.6426	0.8794
27055	Fkbp9	FK506 binding protein 9	1.221	0.08165	0.5122
21991	Tpi1	triosephosphate isomerase 1	1.221	0.0817	0.5123
18477	Prdx1	peroxiredoxin 1	1.221	0.01816	0.3486
14751	Gpi1	glucose phosphate isomerase 1	1.221	0.008926	0.3086
385138	BC061237	cDNA sequence BC061237	1.22	0.04522	0.4353
246746	Cd300lf	CD300 antigen like family member F	1.22	0.01022	0.3117
104445	Cdc42ep1	CDC42 effector protein (Rho GTPase binding) 1	1.22	0.0442	0.4339
52685	Cd300lg	CD300 antigen like family member G	1.22	0.09748	0.5352
21990	Tph1	tryptophan hydroxylase 1	1.22	0.1088	0.5527
19153	Prx	periaxin	1.22	0.7815	0.929
18212	Ntrk2	neurotrophic tyrosine kinase, receptor, type 2	1.22	0.06901	0.487
12140	Fabp7	fatty acid binding protein 7, brain	1.22	0.03069	0.3964
11548	Adra1b	adrenergic receptor, alpha 1b	1.22	0.1864	0.6239
320858	L3mbtl4	l(3)mbt-like 4 (Drosophila)	1.219	0.0426	0.4301
209086	Samd9l	sterile alpha motif domain containing 9-like	1.219	0.03249	0.4017
105853	Mal2	mal, T-cell differentiation protein 2	1.219	0.005545	0.2775
76950	2900001G08Rik	RIKEN cDNA 2900001G08 gene	1.219	0.175	0.6153
67106	Zbtb8os	zinc finger and BTB domain containing 8 opposite strand	1.219	0.06091	0.4741
56735	Krt71	keratin 71	1.219	0.1043	0.5444
52502	Carhsp1	calcium regulated heat stable protein 1	1.219	0.4186	0.7787
30794	Pdlim4	PDZ and LIM domain 4	1.219	0.1761	0.6156
22004	Tpm2	tropomyosin 2, beta	1.219	0.006862	0.2912
19106	Eif2ak2	eukaryotic translation initiation factor 2-alpha kinase 2	1.219	0.08945	0.5223
17161	Maoa	monoamine oxidase A	1.219	0.01171	0.3214
12684	Cideb	cell death-inducing DNA fragmentation factor, alpha subunit-like effector B	1.219	0.1417	0.5863

381406	2810408M09Rik	RIKEN cDNA 2810408M09 gene	1.218	0.1914	0.6278
276952	Rasl10b	RAS-like, family 10, member B	1.218	0.08782	0.5191
231602	P2rx2	purinergic receptor P2X, ligand-gated ion channel, 2	1.218	0.1008	0.5394
227399	Ppip5k2	diphosphoinositol pentakisphosphate kinase 2	1.218	0.282	0.6967
114741	Supt16h	suppressor of Ty 16 homolog (S. cerevisiae)	1.218	0.08082	0.5104
73847	Fam110a	family with sequence similarity 110, member A	1.218	0.04804	0.4443
70059	Degs2	degenerative spermatocyte homolog 2 (Drosophila), lipid desaturase	1.218	0.06835	0.4863
67576	4930429B21Rik	RIKEN cDNA 4930429B21 gene	1.218	0.1216	0.5651
22092	Rsph1	radial spoke head 1 homolog (Chlamydomonas)	1.218	0.3489	0.7388
19734	Rgs16	regulator of G-protein signaling 16	1.218	0.007888	0.3002
18639	Pfkfb1	6-phosphofructo-2-kinase/fructose-2,6-biphosphatase 1	1.218	0.01349	0.3343
13614	Edn1	endothelin 1	1.218	0.02807	0.3875
11833	Aqp8	aquaporin 8	1.218	0.4356	0.7884
81013	Vmn1r65	vomeronasal 1 receptor 65	1.217	0.5108	0.8254
78754	Galntl2	UDP-N-acetyl-alpha-D-galactosamine:polypeptide N-acetylgalactosaminyltransferase-like 2	1.217	0.1472	0.591
70113	Odf3b	outer dense fiber of sperm tails 3B	1.217	0.1646	0.6076
67880	Dcxr	dicarbonyl L-xylulose reductase	1.217	0.2163	0.6479
66255	Hsbp1l1	heat shock factor binding protein 1-like 1	1.217	0.1256	0.5703
16515	Kcnj12	potassium inwardly-rectifying channel, subfamily J, member 12	1.217	0.03548	0.4117
434797	Gm5640	predicted gene 5640	1.216	0.366	0.7501
228778	6820408C15Rik	RIKEN cDNA 6820408C15 gene	1.216	0.1953	0.6304
107995	Cdc20	cell division cycle 20 homolog (S. cerevisiae)	1.216	0.1942	0.6299
97165	Hmgb2	high mobility group box 2	1.216	0.02877	0.3903
81879	Tcfcp2l1	transcription factor CP2-like 1	1.216	0.5822	0.8533
75564	Rsph9	radial spoke head 9 homolog (Chlamydomonas)	1.216	0.07634	0.5013
72184	Klhl35	kelch-like 35 (Drosophila)	1.216	0.08227	0.5144
67512	Agpat2	1-acylglycerol-3-phosphate O-acyltransferase 2 (lysophosphatidic acid acyltransferase, beta)	1.216	0.1707	0.6115
66832	Rsph3a	radial spoke 3A homolog (Chlamydomonas)	1.216	0.06484	0.4816
30924	Angptl3	angiopoietin-like 3	1.216	0.05311	0.4565
21849	Trim28	tripartite motif-containing 28	1.216	0.07669	0.5014
20965	Syn2	synapsin II	1.216	0.06719	0.4855
17896	Myl4	myosin, light polypeptide 4	1.216	0.09856	0.5367
13086	Cyp2a4	cytochrome P450, family 2, subfamily a, polypeptide 4	1.216	0.5396	0.8367
12633	Cflar	CASP8 and FADD-like apoptosis regulator	1.216	0.01092	0.317
100043609	Gm14207	predicted gene 14207	1.215	0.766	0.9251
632764	5730471H19Rik	RIKEN cDNA 5730471H19 gene	1.215	0.2594	0.6815
433485	Tmem90b	transmembrane protein 90B	1.215	0.1138	0.5569
433215	BC048609	cDNA sequence BC048609	1.215	0.08944	0.5223
114229	Kiss1r	KISS1 receptor	1.215	0.02297	0.3708
109305	Orai1	ORAI calcium release-activated calcium modulator 1	1.215	0.05285	0.4558
108069	Grm3	glutamate receptor, metabotropic 3	1.215	0.05945	0.4728
73671	Sult6b1	sulfotransferase family, cytosolic, 6B, member 1	1.215	0.06729	0.4855
68075	1520402A15Rik	RIKEN cDNA 1520402A15 gene	1.215	0.09774	0.5358
21877	Tk1	thymidine kinase 1	1.215	0.5448	0.839
20500	Slc13a2	solute carrier family 13 (sodium-dependent dicarboxylate transporter), member 2	1.215	0.3884	0.7616
20276	Scnn1a	sodium channel, nonvoltage-gated 1 alpha	1.215	0.3604	0.7467
17242	Mdk	midkine	1.215	0.1259	0.5703
12490	Cd34	CD34 antigen	1.215	0.1589	0.6035
381306	BC055324	cDNA sequence BC055324	1.214	0.06702	0.4855

209584	Tyw3	tRNA-yW synthesizing protein 3 homolog (S. cerevisiae)	1.214	0.01759	0.3469
77312	C030010L15Rik	RIKEN cDNA C030010L15 gene	1.214	0.04277	0.4312
77072	6720420G18Rik	RIKEN cDNA 6720420G18 gene	1.214	0.5089	0.8244
76915	Mnd1	meiotic nuclear divisions 1 homolog (S. cerevisiae)	1.214	0.07562	0.4998
72893	2900040C04Rik	RIKEN cDNA 2900040C04 gene	1.214	0.3232	0.7205
72296	Rusc1	RUN and SH3 domain containing 1	1.214	0.08506	0.5166
69903	Rasip1	Ras interacting protein 1	1.214	0.02952	0.392
68440	Dusp23	dual specificity phosphatase 23	1.214	0.04366	0.4336
67269	Agtpbp1	ATP/GTP binding protein 1	1.214	0.02603	0.3786
66824	Pycard	PYD and CARD domain containing	1.214	0.05283	0.4558
22784	Slc30a3	solute carrier family 30 (zinc transporter), member 3	1.214	0.05599	0.4631
12306	Anxa2	annexin A2	1.214	0.1264	0.5703
12041	Bckdk	branched chain ketoacid dehydrogenase kinase	1.214	0.09104	0.523
627035	Gm6729	predicted gene 6729	1.213	0.2372	0.6659
621304	Gm6209	predicted gene 6209	1.213	0.6915	0.8991
554327	2610042L04Rik	RIKEN cDNA 2610042L04 gene	1.213	0.006132	0.2833
381801	Tatdn2	TatD DNase domain containing 2	1.213	0.01005	0.3112
320302	Glt28d2	glycosyltransferase 28 domain containing 2	1.213	0.1071	0.5493
236193	Zfp709	zinc finger protein 709	1.213	0.05611	0.4631
230126	Shb	src homology 2 domain-containing transforming protein B	1.213	0.3256	0.7222
211798	Mfsd9	major facilitator superfamily domain containing 9	1.213	0.1153	0.5578
65254	Dpysl5	dihydropyrimidinase-like 5	1.213	0.2784	0.6934
56213	Htra1	HtrA serine peptidase 1	1.213	0.04909	0.4469
27984	Efhd2	EF hand domain containing 2	1.213	0.03238	0.4017
20845	Star	steroidogenic acute regulatory protein	1.213	0.2353	0.6654
20345	Selplg	selectin, platelet (p-selectin) ligand	1.213	0.1941	0.6299
20318	Sdf4	stromal cell derived factor 4	1.213	0.01	0.3112
17175	Masp2	mannan-binding lectin serine peptidase 2	1.213	0.2187	0.6507
14560	Gdf10	growth differentiation factor 10	1.213	0.1978	0.6329
12447	Ccne1	cyclin E1	1.213	0.02327	0.3739
380654	4930485B16Rik	RIKEN cDNA 4930485B16 gene	1.212	0.5375	0.8358
331480	Gm5126	predicted pseudogene 5126	1.212	0.03033	0.3954
240261	Ccdc112	coiled-coil domain containing 112	1.212	0.2482	0.6755
232078	Thnsl2	threonine synthase-like 2 (bacterial)	1.212	0.05039	0.4516
171463	Il17rd	interleukin 17 receptor D	1.212	0.08082	0.5104
66624	Spcs2	signal peptidase complex subunit 2 homolog (S. cerevisiae)	1.212	0.2378	0.6664
19332	Rab20	RAB20, member RAS oncogene family	1.212	0.02917	0.3911
15064	Mr1	major histocompatibility complex, class I-related	1.212	0.1657	0.6077
332937	Tcfap2e	transcription factor AP-2, epsilon	1.211	0.0805	0.5098
71887	Ppm1j	protein phosphatase 1J	1.211	0.09533	0.5333
70719	Hmha1	histocompatibility (minor) HA-1	1.211	0.002628	0.2224
67887	Tmem66	transmembrane protein 66	1.211	0.04203	0.4285
67747	Ribc2	RIB43A domain with coiled-coils 2	1.211	0.1288	0.5733
17216	Mcm2	minichromosome maintenance deficient 2 mitotin (S. cerevisiae)	1.211	0.09919	0.5367
16562	Kif1c	kinesin family member 1C	1.211	0.2358	0.6655
14127	Fcer1g	Fc receptor, IgE, high affinity I, gamma polypeptide	1.211	0.1589	0.6035
11812	Apoc1	apolipoprotein C-I	1.211	0.02291	0.3708
100034684	BC100530	cDNA sequence BC100530	1.21	0.2019	0.6356
236539	Phgdh	3-phosphoglycerate dehydrogenase	1.21	0.6471	0.8812

230903	Fbxo44	F-box protein 44	1.21	0.2496	0.6767
217369	Uts2r	urotensin 2 receptor	1.21	0.2266	0.6554
102595	Plekho2	pleckstrin homology domain containing, family O member 2	1.21	0.2972	0.7051
94090	Trim9	tripartite motif-containing 9	1.21	0.1371	0.5807
83554	Fstl3	follistatin-like 3	1.21	0.1437	0.5881
74351	Ddx23	DEAD (Asp-Glu-Ala-Asp) box polypeptide 23	1.21	0.1379	0.5816
70057	2210008F06Rik	RIKEN cDNA 2210008F06 gene	1.21	0.1135	0.5564
69642	2310046A06Rik	RIKEN cDNA 2310046A06 gene	1.21	0.4145	0.776
65103	Arl6ip6	ADP-ribosylation factor-like 6 interacting protein 6	1.21	0.2702	0.6884
18974	Pole2	polymerase (DNA directed), epsilon 2 (p59 subunit)	1.21	0.109	0.5529
16012	Igfbp6	insulin-like growth factor binding protein 6	1.21	0.04935	0.4469
14132	Fcgrt	Fc receptor, IgG, alpha chain transporter	1.21	0.1385	0.5825
14061	F2	coagulation factor II	1.21	0.1229	0.5672
12918	Crh	corticotropin releasing hormone	1.21	0.04794	0.4443
654472	Gm12070	glyceraldehyde-3-phosphate dehydrogenase pseudogene	1.209	0.04739	0.4442
385668	Lca5l	Leber congenital amaurosis 5-like	1.209	0.0945	0.5314
320207	Pik3r5	phosphoinositide-3-kinase, regulatory subunit 5, p101	1.209	0.06067	0.4741
269033	4930503L19Rik	RIKEN cDNA 4930503L19 gene	1.209	0.1395	0.5834
224671	Btbd9	BTB (POZ) domain containing 9	1.209	0.005716	0.2775
73182	Pear1	platelet endothelial aggregation receptor 1	1.209	0.3198	0.719
56226	Espn	espin	1.209	0.4619	0.8022
27368	Tbl2	transducin (beta)-like 2	1.209	0.007934	0.3002
24088	Tlr2	toll-like receptor 2	1.209	0.4976	0.8193
22761	Zfpm1	zinc finger protein, multitype 1	1.209	0.0336	0.4076
22286	Utf1	undifferentiated embryonic cell transcription factor 1	1.209	0.1244	0.5694
16161	Il12rb1	interleukin 12 receptor, beta 1	1.209	0.4304	0.7845
14431	Gamt	guanidinoacetate methyltransferase	1.209	0.2098	0.6412
13733	Emr1	EGF-like module containing, mucin-like, hormone receptor-like sequence 1	1.209	0.3093	0.7127
227612	A830007P12Rik	RIKEN cDNA A830007P12 gene	1.208	0.3064	0.7108
227545	5430407P10Rik	RIKEN cDNA 5430407P10 gene	1.208	0.2595	0.6815
213649	Arhgef19	Rho guanine nucleotide exchange factor (GEF) 19	1.208	0.1847	0.6222
107324	AV313155	expressed sequence AV313155	1.208	0.08324	0.5144
76670	Ttc18	tetratricopeptide repeat domain 18	1.208	0.165	0.6077
76573	1700027D21Rik	RIKEN cDNA 1700027D21 gene	1.208	0.02403	0.377
75614	2610019E17Rik	RIKEN cDNA 2610019E17 gene	1.208	0.3511	0.7393
72512	Tmem173	transmembrane protein 173	1.208	0.2272	0.6561
72148	2610019F03Rik	RIKEN cDNA 2610019F03 gene	1.208	0.03067	0.3964
72076	Mospd4	motile sperm domain containing 4	1.208	0.4084	0.7731
72055	Slc38a10	solute carrier family 38, member 10	1.208	0.1178	0.5609
67801	Pllp	plasma membrane proteolipid	1.208	0.2925	0.7024
66985	Rassf7	Ras association (RalGDS/AF-6) domain family (N-terminal) member 7	1.208	0.01496	0.3382
20878	Aurka	aurora kinase A	1.208	0.1394	0.5834
12778	Cxcr7	chemokine (C-X-C motif) receptor 7	1.208	0.09037	0.5229
12654	Chi3l1	chitinase 3-like 1	1.208	0.1378	0.5815
100039258	Gm10290	glyceraldehyde-3-phosphate dehydrogenase pseudogene	1.207	0.2245	0.654
653016	Gm7325	predicted gene 7325	1.207	0.04597	0.4381
234684	Lrrc29	leucine rich repeat containing 29	1.207	0.1252	0.5703
83436	Plekha2	pleckstrin homology domain-containing, family A (phosphoinositide binding specific) member 2	1.207	0.1073	0.5496
72535	Aldh1b1	aldehyde dehydrogenase 1 family, member B1	1.207	0.07745	0.5026

16678	Krt1	keratin 1	1.207	0.4486	0.7942
12702	Socs3	suppressor of cytokine signaling 3	1.207	0.02352	0.375
12161	Bmp6	bone morphogenetic protein 6	1.207	0.2443	0.6723
100042371	Gm3807	predicted gene 3807	1.206	0.3117	0.7138
328779	Hs3st6	heparan sulfate (glucosamine) 3-O-sulfotransferase 6	1.206	0.02888	0.3903
227095	Hibch	3-hydroxyisobutyryl-Coenzyme A hydrolase	1.206	0.009758	0.3112
217340	Rnf157	ring finger protein 157	1.206	0.06503	0.4816
99738	Kcnc4	potassium voltage gated channel, Shaw-related subfamily, member 4	1.206	0.1904	0.6275
70355	Gprc5c	G protein-coupled receptor, family C, group 5, member C	1.206	0.3613	0.7476
56373	Cpb2	carboxypeptidase B2 (plasma)	1.206	0.04152	0.4282
53883	Celsr2	cadherin, EGF LAG seven-pass G-type receptor 2 (flamingo homolog, Drosophila)	1.206	0.4219	0.7801
20723	Serpinb9	serine (or cysteine) peptidase inhibitor, clade B, member 9	1.206	0.1078	0.5509
20450	St8sia2	ST8 alpha-N-acetyl-neuraminide alpha-2,8-sialyltransferase 2	1.206	0.2862	0.6982
19701	Ren1	renin 1 structural	1.206	0.1006	0.5394
16145	Igtp	interferon gamma induced GTPase	1.206	0.06255	0.4771
791379	C030009H01Rik	RIKEN cDNA C030009H01 gene	1.205	0.3851	0.7607
625342	Gm13315	lactate dehydrogenase A pseudogene	1.205	0.1839	0.6217
503610	Zdhhc18	zinc finger, DHHC domain containing 18	1.205	0.0609	0.4741
435145	AI848285	expressed sequence AI848285	1.205	0.1467	0.591
383592	Gm1305	predicted gene 1305	1.205	0.7393	0.9177
272790	Magee2	melanoma antigen, family E, 2	1.205	0.1833	0.6217
226527	BC026585	cDNA sequence BC026585	1.205	0.0473	0.4442
217310	C630004H02Rik	RIKEN cDNA C630004H02 gene	1.205	0.04174	0.4282
194655	Klf11	Kruppel-like factor 11	1.205	0.1882	0.6261
73571	1700096K18Rik	RIKEN cDNA 1700096K18 gene	1.205	0.1368	0.5807
71897	Lypd6b	LY6/PLAUR domain containing 6B	1.205	0.02804	0.3875
71751	Map3k13	mitogen-activated protein kinase kinase kinase 13	1.205	0.138	0.5816
70530	Lrfn2	leucine rich repeat and fibronectin type III domain containing 2	1.205	0.1979	0.6329
68701	Dysfip1	dysferlin interacting protein 1	1.205	0.1255	0.5703
58198	Sall1	sal-like 1 (Drosophila)	1.205	0.5447	0.839
21682	Tec	tec protein tyrosine kinase	1.205	0.1011	0.5395
16828	Ldha	lactate dehydrogenase A	1.205	0.07137	0.4933
13638	Efna3	ephrin A3	1.205	0.2918	0.7019
100169874	Gm11110	predicted gene 11110	1.204	0.04617	0.4386
664862	Gpr137b-ps	G protein-coupled receptor 137B, pseudogene	1.204	0.01582	0.3404
328977	Zfp532	zinc finger protein 532	1.204	0.009083	0.3086
232146	Fam176a	family with sequence similarity 176, member A	1.204	0.006657	0.288
229595	Adamtsl4	ADAMTS-like 4	1.204	0.05413	0.4596
228770	Rspo4	R-spondin family, member 4	1.204	0.2125	0.6451
105833	Ccdc65	coiled-coil domain containing 65	1.204	0.06457	0.4816
80893	Tmprss5	transmembrane protease, serine 5 (spinesin)	1.204	0.032	0.3992
76263	Gstk1	glutathione S-transferase kappa 1	1.204	0.03967	0.4222
72785	2810474C18Rik	RIKEN cDNA 2810474C18 gene	1.204	0.4033	0.7708
71137	Rfx4	regulatory factor X, 4 (influences HLA class II expression)	1.204	0.3894	0.7624
69072	Ebna1bp2	EBNA1 binding protein 2	1.204	0.02811	0.3875
64292	Ptges	prostaglandin E synthase	1.204	0.1062	0.5468
22701	Zfp41	zinc finger protein 41	1.204	0.08661	0.5178
21898	Tlr4	toll-like receptor 4	1.204	0.3284	0.7252
20840	Stac	src homology three (SH3) and cysteine rich domain	1.204	0.258	0.6815

18993	Pou3f3	POU domain, class 3, transcription factor 3	1.204	0.2762	0.6925
18005	Nek2	NIMA (never in mitosis gene a)-related expressed kinase 2	1.204	0.1352	0.5788
16416	Itgb3	integrin beta 3	1.204	0.3845	0.7606
547349	LOC547349	similar to MHC class I antigen precursor	1.203	0.1645	0.6076
381560	Xkr8	X Kell blood group precursor related family member 8 homolog	1.203	0.03105	0.3964
280287	Kiss1	KiSS-1 metastasis-suppressor	1.203	0.1776	0.6167
56534	Hspb3	heat shock protein 3	1.203	0.1451	0.5896
16818	Lck	lymphocyte protein tyrosine kinase	1.203	0.02197	0.3694
14119	Fbn2	fibrillin 2	1.203	0.3943	0.7659
13448	Dok1	docking protein 1	1.203	0.03945	0.4222
13051	Cx3cr1	chemokine (C-X3-C) receptor 1	1.203	0.1339	0.5777
380714	Rph3al	rabphilin 3A-like (without C2 domains)	1.202	0.1179	0.5609
353025	Caps2	calcyphosphine 2	1.202	0.2133	0.6459
320405	Cadps2	Ca2+-dependent activator protein for secretion 2	1.202	0.01157	0.3208
269132	Glt25d2	glycosyltransferase 25 domain containing 2	1.202	0.002844	NA
244958	Mrap2	melanocortin 2 receptor accessory protein 2	1.202	0.01845	0.3513
239435	Aard	alanine and arginine rich domain containing protein	1.202	0.01319	0.3319
171167	Fut10	fucosyltransferase 10	1.202	0.007988	0.3003
106407	Osta	organic solute transporter alpha	1.202	0.1148	0.5569
70355	Gprc5c	G protein-coupled receptor, family C, group 5, member C	1.202	0.3748	0.755
66447	Mgst3	microsomal glutathione S-transferase 3	1.202	0.02347	0.3748
58234	Shank3	SH3/ankyrin domain gene 3	1.202	0.2587	0.6815
19132	Prph	peripherin	1.202	0.2685	0.6875
18799	Plcd1	phospholipase C, delta 1	1.202	0.005661	0.2775
18096	Nkx6-1	NK6 homeobox 1	1.202	0.1071	0.5493
16988	Lst1	leukocyte specific transcript 1	1.202	0.227	0.656
14865	Gstm4	glutathione S-transferase, mu 4	1.202	0.1147	0.5569
14561	Gdf11	growth differentiation factor 11	1.202	0.2159	0.6479
12606	Cebpa	CCAAT/enhancer binding protein (C/EBP), alpha	1.202	0.2127	0.6452
11745	Anxa3	annexin A3	1.202	0.549	0.8411
11350	Abl1	c-abl oncogene 1, non-receptor tyrosine kinase	1.202	0.2841	0.6977
100039781	Hrct1	histidine rich carboxyl terminus 1	1.201	0.1685	0.6096
230500	Efcab7	EF-hand calcium binding domain 7	1.201	0.3768	0.7563
224116	Muc20	mucin 20	1.201	0.4127	0.7748
109342	Slc5a10	solute carrier family 5 (sodium/glucose cotransporter), member 10	1.201	0.5388	0.8366
68312	Gstm7	glutathione S-transferase, mu 7	1.201	0.2211	0.6525
56190	Rbm38	RNA binding motif protein 38	1.201	0.3164	0.7172
52892	Sco1	SCO cytochrome oxidase deficient homolog 1 (yeast)	1.201	0.08498	0.5166
52187	Rragd	Ras-related GTP binding D	1.201	0.002459	NA
18162	Npr3	natriuretic peptide receptor 3	1.201	0.05553	0.4626
16980	Lrrn2	leucine rich repeat protein 2, neuronal	1.201	0.09012	0.5229
12048	Bcl2l1	BCL2-like 1	1.201	0.2436	0.6714
100505352	LOC100505352	hypothetical LOC100505352	1.2	0.03836	0.4199
100038850	A130082M07Rik	RIKEN cDNA A130082M07 gene	1.2	0.2141	0.6467
667370	I830012O16Rik	RIKEN cDNA I830012O16 gene	1.2	0.1114	0.5549
329993	Gm438	predicted gene 438	1.2	0.2884	0.6996
319755	6330566A10Rik	RIKEN cDNA 6330566A10 gene	1.2	0.3541	0.7418
269344	Ell3	elongation factor RNA polymerase II-like 3	1.2	0.0179	0.3475
234779	Plcg2	phospholipase C, gamma 2	1.2	0.05514	0.4616

229011	Samd10	sterile alpha motif domain containing 10	1.2	0.4177	0.7783
208677	Creb3l3	cAMP responsive element binding protein 3-like 3	1.2	0.04758	0.4442
117160	Ttyh2	tweety homolog 2 (Drosophila)	1.2	0.1516	0.5948
104252	Cdc42ep2	CDC42 effector protein (Rho GTPase binding) 2	1.2	0.1646	0.6076
66222	Serpinb1a	serine (or cysteine) peptidase inhibitor, clade B, member 1a	1.2	0.3186	0.7182
14859	Gsta3	glutathione S-transferase, alpha 3	1.2	0.1307	0.5751
14115	Fbln2	fibulin 2	1.2	0.1009	0.5394
13555	E2f1	E2F transcription factor 1	1.2	0.08576	0.517
13491	Drd4	dopamine receptor D4	1.2	0.02269	0.3702
545156	Kalrn	kalirin, RhoGEF kinase	1.199	0.1056	0.5468
244864	Layn	layilin	1.199	0.1147	0.5569
109305	Orai1	ORAI calcium release-activated calcium modulator 1	1.199	0.1378	0.5815
109272	Mybpc1	myosin binding protein C, slow-type	1.199	0.2973	0.7051
100434	Slc44a1	solute carrier family 44, member 1	1.199	0.1168	0.559
76366	Mtif3	mitochondrial translational initiation factor 3	1.199	0.09606	0.5344
68920	1110065P20Rik	RIKEN cDNA 1110065P20 gene	1.199	0.07346	0.4957
68243	A930018P22Rik	RIKEN cDNA A930018P22 gene	1.199	0.1081	0.5513
67103	Ptgr1	prostaglandin reductase 1	1.199	0.1598	0.6042
57394	Tmem27	transmembrane protein 27	1.199	0.02624	0.3791
26914	H2afy	H2A histone family, member Y	1.199	0.2336	0.6626
239029	Antxrl	anthrax toxin receptor-like	1.198	0.2107	0.6426
225341	Lims2	LIM and senescent cell antigen like domains 2	1.198	0.2873	0.6986
217306	Cd300e	CD300e antigen	1.198	0.2093	0.6409
110596	Rgnef	Rho-guanine nucleotide exchange factor	1.198	0.1502	0.5938
102657	Cd276	CD276 antigen	1.198	0.04594	0.4381
73230	Bmper	BMP-binding endothelial regulator	1.198	0.2856	0.6982
73086	Rps6ka5	ribosomal protein S6 kinase, polypeptide 5	1.198	0.01399	0.3347
72119	Tpx2	TPX2, microtubule-associated protein homolog (Xenopus laevis)	1.198	0.128	0.5716
71059	Hexim2	hexamthylene bis-acetamide inducible 2	1.198	0.1921	0.6282
70355	Gprc5c	G protein-coupled receptor, family C, group 5, member C	1.198	0.3869	0.761
70024	Mcm10	minichromosome maintenance deficient 10 (S. cerevisiae)	1.198	0.08733	0.5187
68957	Paqr6	progestin and adipoQ receptor family member VI	1.198	0.1494	0.5938
66153	Fbxo36	F-box protein 36	1.198	0.1448	0.5896
56722	Litaf	LPS-induced TN factor	1.198	0.1198	0.5633
56460	Pkp3	plakophilin 3	1.198	0.2663	0.6862
20271	Scn5a	sodium channel, voltage-gated, type V, alpha	1.198	0.4124	0.7747
16651	Sspn	sarcospan	1.198	0.1878	0.626
14581	Gfi1	growth factor independent 1	1.198	0.1465	0.591
13711	Elf5	E74-like factor 5	1.198	0.125	0.5703
668592	Gm9258	predicted gene 9258	1.197	0.2677	0.687
230899	Nppa	natriuretic peptide type A	1.197	0.5771	0.8512
107173	Gpr137	G protein-coupled receptor 137	1.197	0.206	0.6386
73301	Ttc29	tetratricopeptide repeat domain 29	1.197	0.5889	0.8575
69726	Smyd3	SET and MYND domain containing 3	1.197	0.1194	0.5628
68304	Kdelc2	KDEL (Lys-Asp-Glu-Leu) containing 2	1.197	0.2977	0.7051
66447	Mgst3	microsomal glutathione S-transferase 3	1.197	0.04302	0.4315
56693	Crtap	cartilage associated protein	1.197	0.145	0.5896
54204	Sep-01	septin 1	1.197	0.3214	0.7199
14025	Bcl11a	B-cell CLL/lymphoma 11A (zinc finger protein)	1.197	0.04651	0.4407

552880	LOC552880	hypothetical LOC552880	1.196	0.2185	0.6506
381549	Zfp69	zinc finger protein 69	1.196	0.484	0.8123
228765	Sdcbp2	syndecan binding protein (syntenin) 2	1.196	0.1476	0.591
208777	Sned1	sushi, nidogen and EGF-like domains 1	1.196	0.5446	0.839
75827	4930542N06Rik	RIKEN cDNA 4930542N06 gene	1.196	0.425	0.7821
74525	8430419L09Rik	RIKEN cDNA 8430419L09 gene	1.196	0.03732	0.4163
66548	Adamtsl5	ADAMTS-like 5	1.196	0.2637	0.6843
14859	Gsta3	glutathione S-transferase, alpha 3	1.196	0.08795	0.5194
14761	Gpr27	G protein-coupled receptor 27	1.196	0.4731	0.8088
14701	Gng12	guanine nucleotide binding protein (G protein), gamma 12	1.196	0.03893	0.4215
545013	Gm5797	predicted gene 5797	1.195	0.1405	0.584
381418	Ctxn2	cortexin 2	1.195	0.03665	0.4161
235534	Acpl2	acid phosphatase-like 2	1.195	0.03769	0.417
231842	Amz1	archaelysin family metallopeptidase 1	1.195	0.1208	0.5643
78928	Pigt	phosphatidylinositol glycan anchor biosynthesis, class T	1.195	0.2902	0.7006
76184	Abca6	ATP-binding cassette, sub-family A (ABC1), member 6	1.195	0.2373	0.666
74343	Crtc2	CREB regulated transcription coactivator 2	1.195	0.1344	0.5779
74174	Gtsf1	gametocyte specific factor 1	1.195	0.5435	0.839
67733	Itgb3bp	integrin beta 3 binding protein (beta3-endonexin)	1.195	0.05315	0.4565
66975	2410002O22Rik	RIKEN cDNA 2410002O22 gene	1.195	0.04525	0.4353
66953	Cdca7	cell division cycle associated 7	1.195	0.1954	0.6304
56307	Metap2	methionine aminopeptidase 2	1.195	0.06295	0.4779
21415	Tcf7l1	transcription factor 7-like 1 (T-cell specific, HMG box)	1.195	0.1703	0.6115
17364	Trpm1	transient receptor potential cation channel, subfamily M, member 1	1.195	0.2774	0.6925
16591	Kl	klotho	1.195	0.008993	0.3086
12587	Mia1	melanoma inhibitory activity 1	1.195	0.3233	0.7205
384701	Dub2a	deubiquitinating enzyme 2a	1.194	0.5631	0.8465
242700	Il28ra	interleukin 28 receptor alpha	1.194	0.2004	0.6345
208628	Kntc1	kinetochore associated 1	1.194	0.3558	0.744
77113	Klhl2	kelch-like 2, Mayven (Drosophila)	1.194	0.00533	NA
74123	Foxp4	forkhead box P4	1.194	0.416	0.7768
71296	4933436C20Rik	RIKEN cDNA 4933436C20 gene	1.194	0.1574	0.6016
69073	1810019J16Rik	RIKEN cDNA 1810019J16 gene	1.194	0.2358	0.6655
67037	Pmf1	polyamine-modulated factor 1	1.194	0.3758	0.7556
60345	Nrip2	nuclear receptor interacting protein 2	1.194	0.1074	0.5498
21937	Tnfrsf1a	tumor necrosis factor receptor superfamily, member 1a	1.194	0.09365	0.5296
20788	Srebf2	sterol regulatory element binding factor 2	1.194	0.438	0.7894
20475	Six5	sine oculis-related homeobox 5 homolog (Drosophila)	1.194	0.1913	0.6278
19981	Rpl37a	ribosomal protein L37a	1.194	0.07324	0.4957
228413	Prrg4	proline rich Gla (G-carboxyglutamic acid) 4 (transmembrane)	1.193	0.5893	0.8575
219151	Scara3	scavenger receptor class A, member 3	1.193	0.2085	0.6401
110542	Amhr2	anti-Mullerian hormone type 2 receptor	1.193	0.08379	0.5144
70274	Ly6g6e	lymphocyte antigen 6 complex, locus G6E	1.193	0.2956	0.7047
66340	Psenen	presenilin enhancer 2 homolog (C. elegans)	1.193	0.1024	0.5419
58175	Rgs20	regulator of G-protein signaling 20	1.193	0.293	0.7028
50908	C1s	complement component 1, s subcomponent	1.193	0.2382	0.667
19876	Robo1	roundabout homolog 1 (Drosophila)	1.193	0.08869	0.5207
14068	F7	coagulation factor VII	1.193	0.0558	0.4631
100041639	Gm3448	predicted gene 3448	1.192	0.2376	0.6663

242506	Frmd3	FERM domain containing 3	1.192	0.04632	0.4395
232156	Slc4a5	solute carrier family 4, sodium bicarbonate cotransporter, member 5	1.192	0.1187	0.5618
215280	Wipf1	WAS/WASL interacting protein family, member 1	1.192	0.05662	0.4638
103220	BC030307	cDNA sequence BC030307	1.192	0.2059	0.6386
78593	Nrip3	nuclear receptor interacting protein 3	1.192	0.01577	0.3403
69034	4930579G22Rik	RIKEN cDNA 4930579G22 gene	1.192	0.0279	0.3875
53860	Sep-09	septin 9	1.192	0.1597	0.6042
19725	Rfx2	regulatory factor X, 2 (influences HLA class II expression)	1.192	0.3912	0.7643
18736	Pou1f1	POU domain, class 1, transcription factor 1	1.192	0.262	0.6831
16691	Krt8	keratin 8	1.192	0.4117	0.7747
16494	Kcna6	potassium voltage-gated channel, shaker-related, subfamily, member 6	1.192	0.348	0.7385
14559	Gdf1	growth differentiation factor 1	1.192	0.2562	0.6801
12984	Csf2rb2	colony stimulating factor 2 receptor, beta 2, low-affinity (granulocyte-macrophage)	1.192	0.04776	0.4442
672682	Gm9573	predicted gene 9573	1.191	0.5619	0.8465
381476	B930007M17Rik	RIKEN cDNA B930007M17 gene	1.191	0.6931	0.8999
381280	Hjurp	Holliday junction recognition protein	1.191	0.0227	0.3702
380912	Zfp395	zinc finger protein 395	1.191	0.3032	0.7089
353287	Clec18a	C-type lectin domain family 18, member A	1.191	0.4424	0.7928
320736	E130203B14Rik	RIKEN cDNA E130203B14 gene	1.191	0.3305	0.726
109979	Art3	ADP-ribosyltransferase 3	1.191	0.06092	0.4741
109820	Pgc	progastricsin (pepsinogen C)	1.191	0.2719	0.6891
67856	Echdc3	enoyl Coenzyme A hydratase domain containing 3	1.191	0.1274	0.5716
67149	Nkain1	Na+/K+ transporting ATPase interacting 1	1.191	0.1492	0.5937
56296	Dmrtb1	DMRT-like family B with proline-rich C-terminal, 1	1.191	0.04679	0.4424
56188	Fxyd1	FXYD domain-containing ion transport regulator 1	1.191	0.1165	0.5581
52538	Acaa2	acetyl-Coenzyme A acyltransferase 2 (mitochondrial 3-oxoacyl-Coenzyme A thiolase)	1.191	0.01928	0.3548
22689	Zfp27	zinc finger protein 27	1.191	0.1314	0.5751
20315	Cxcl12	chemokine (C-X-C motif) ligand 12	1.191	0.06337	0.478
18451	P4ha1	procollagen-proline, 2-oxoglutarate 4-dioxygenase (proline 4-hydroxylase), alpha 1 polypeptide	1.191	0.01256	0.327
17970	Ncf2	neutrophil cytosolic factor 2	1.191	0.4302	0.7844
17196	Mbp	myelin basic protein	1.191	0.02898	0.3903
12799	Cnp	2',3'-cyclic nucleotide 3' phosphodiesterase	1.191	0.2418	0.6697
380712	Tlcd2	TLC domain containing 2	1.19	0.03638	0.4154
280287	Kiss1	KiSS-1 metastasis-suppressor	1.19	0.3567	0.7443
213211	Rnf26	ring finger protein 26	1.19	0.1528	0.5965
170756	Slc24a6	solute carrier family 24 (sodium/potassium/calcium exchanger), member 6	1.19	0.4585	0.8
94242	Tinagl1	tubulointerstitial nephritis antigen-like 1	1.19	0.2686	0.6876
74347	4632415K11Rik	RIKEN cDNA 4632415K11 gene	1.19	0.08599	0.517
72898	Asphd2	aspartate beta-hydroxylase domain containing 2	1.19	0.02101	0.3619
72691	Calhm2	calcium homeostasis modulator 2	1.19	0.2581	0.6815
72614	Pih1d2	PIH1 domain containing 2	1.19	0.00631	NA
72324	Plxdc1	plexin domain containing 1	1.19	0.01631	0.3414
52150	Kcnk6	potassium inwardly-rectifying channel, subfamily K, member 6	1.19	0.1314	0.5751
27368	Tbl2	transducin (beta)-like 2	1.19	0.08776	0.5191
26987	Eif4e2	eukaryotic translation initiation factor 4E member 2	1.19	0.1691	0.61
22117	Tst	thiosulfate sulfurtransferase, mitochondrial	1.19	0.3821	0.7596
12452	Ccng2	cyclin G2	1.19	0.1267	0.5709
666317	Prl2c1	Prolactin family 2, subfamily c, member 1	1.189	0.01635	0.3414
244694	Kdm4d	lysine (K)-specific demethylase 4D	1.189	0.3689	0.7521

244550	Podnl1	podocan-like 1	1.189	0.1789	0.6181
239650	AI836003	expressed sequence AI836003	1.189	0.261	0.682
216810	Tom1l2	target of myb1-like 2 (chicken)	1.189	0.233	0.6622
109032	Sp110	Sp110 nuclear body protein	1.189	0.009008	NA
74189	Phactr3	phosphatase and actin regulator 3	1.189	0.1616	0.607
70839	P2ry12	purinergic receptor P2Y, G-protein coupled 12	1.189	0.09699	0.5346
70574	Cpm	carboxypeptidase M	1.189	0.2161	0.6479
69794	1600027J07Rik	RIKEN cDNA 1600027J07 gene	1.189	0.2877	0.6988
20272	Scn7a	sodium channel, voltage-gated, type VII, alpha	1.189	0.1932	0.6294
17084	Ly86	lymphocyte antigen 86	1.189	0.1461	0.591
353188	Adam32	a disintegrin and metallopeptidase domain 32	1.188	0.2954	0.7046
269181	Mgat4a	mannoside acetylglucosaminyltransferase 4, isoenzyme A	1.188	0.02729	0.386
228608	Smox	spermine oxidase	1.188	0.02763	0.387
104709	Pik3r6	phosphoinositide-3-kinase, regulatory subunit 6	1.188	0.1966	0.6315
77090	Ocel1	occludin/ELL domain containing 1	1.188	0.07841	0.5045
74487	5430405H02Rik	RIKEN cDNA 5430405H02 gene	1.188	0.05438	0.4601
74330	Dnajc14	DnaJ (Hsp40) homolog, subfamily C, member 14	1.188	0.1464	0.591
68591	Mocos	molybdenum cofactor sulfurase	1.188	0.1295	0.5745
66926	Trmt6	tRNA methyltransferase 6 homolog (S. cerevisiae)	1.188	0.114	0.5569
66161	Pop4	processing of precursor 4, ribonuclease P/MRP family, (S. cerevisiae)	1.188	0.114	0.5569
14866	Gstm5	glutathione S-transferase, mu 5	1.188	0.04386	0.4336
12909	Crcp	calcitonin gene-related peptide-receptor component protein	1.188	0.0274	0.3862
12182	Bst1	bone marrow stromal cell antigen 1	1.188	0.3829	0.7599
11737	Anp32a	acidic (leucine-rich) nuclear phosphoprotein 32 family, member A	1.188	0.00889	NA
100043060	Gm4200	predicted gene 4200	1.187	0.387	0.761
320701	Fam19a4	family with sequence similarity 19, member A4	1.187	0.06276	0.4771
223697	Sun2	Sad1 and UNC84 domain containing 2	1.187	0.02539	0.3786
212167	Pion	pigeon homolog (Drosophila)	1.187	0.1137	0.5569
110078	Pygb	brain glycogen phosphorylase	1.187	0.06752	0.4855
94352	Loxl2	lysyl oxidase-like 2	1.187	0.2607	0.6816
71176	Fbxo24	F-box protein 24	1.187	0.03753	0.417
68292	Stt3b	STT3, subunit of the oligosaccharyltransferase complex, homolog B (S. cerevisiae)	1.187	0.03286	0.4035
67198	Spats2l	spermatogenesis associated, serine-rich 2-like	1.187	0.1645	0.6076
50799	Slc25a13	solute carrier family 25 (mitochondrial carrier, adenine nucleotide translocator), member 13	1.187	0.1748	0.6151
20181	Rxra	retinoid X receptor alpha	1.187	0.2731	0.6904
19210	Ptdss1	phosphatidylserine synthase 1	1.187	0.04764	0.4442
17773	Mtnr1a	melatonin receptor 1A	1.187	0.2095	0.6411
17766	Nudt1	nudix (nucleoside diphosphate linked moiety X)-type motif 1	1.187	0.1028	0.5424
16194	Il6ra	interleukin 6 receptor, alpha	1.187	0.1704	0.6115
15277	Hk2	hexokinase 2	1.187	0.008017	NA
258866	Olfr1008	olfactory receptor 1008	1.186	0.2979	0.7051
258830	Olfr103	olfactory receptor 103	1.186	0.537	0.8354
229699	Slc16a4	solute carrier family 16 (monocarboxylic acid transporters), member 4	1.186	0.1735	0.6147
227659	Slc2a6	solute carrier family 2 (facilitated glucose transporter), member 6	1.186	0.3112	0.7135
170461	Stard6	StAR-related lipid transfer (START) domain containing 6	1.186	0.1713	0.6121
103511	Fam26e	family with sequence similarity 26, member E	1.186	0.291	0.7013
69397	1700019A02Rik	RIKEN cDNA 1700019A02 gene	1.186	0.1184	0.5612
68667	Trpm4	transient receptor potential cation channel, subfamily M, member 4	1.186	0.161	0.6067
68527	Ucma	upper zone of growth plate and cartilage matrix associated	1.186	0.2191	0.6511

65963	Tmem176b	transmembrane protein 176B	1.186	0.1063	0.547
65079	Rtn4r	reticulon 4 receptor	1.186	0.1161	0.558
60613	Kcnq4	potassium voltage-gated channel, subfamily Q, member 4	1.186	0.0755	0.4997
52466	Slc46a1	solute carrier family 46, member 1	1.186	0.206	0.6387
20588	Smarcc1	SWI/SNF related, matrix associated, actin dependent regulator of chromatin, subfamily c, member 1	1.186	0.3169	0.7174
20472	Six2	sine oculis-related homeobox 2 homolog (Drosophila)	1.186	0.2659	0.6856
14397	Gabra4	gamma-aminobutyric acid (GABA) A receptor, subunit alpha 4	1.186	0.06249	0.4771
13589	Mapre1	microtubule-associated protein, RP/EB family, member 1	1.186	0.08156	0.5122
11550	Adra1d	adrenergic receptor, alpha 1d	1.186	0.4467	0.7938
668311	Gm9099	predicted gene 9099	1.185	0.2475	0.6746
338362	Ust	uronyl-2-sulfotransferase	1.185	0.1719	0.6126
229279	Hnrnpa3	heterogeneous nuclear ribonucleoprotein A3	1.185	0.1349	0.5782
77707	9130604C24Rik	RIKEN cDNA 9130604C24 gene	1.185	0.123	0.5672
69035	Zdhhc3	zinc finger, DHHC domain containing 3	1.185	0.1381	0.5817
66757	Adat2	adenosine deaminase, tRNA-specific 2, TAD2 homolog (S. cerevisiae)	1.185	0.3301	0.726
66340	Psenen	presenilin enhancer 2 homolog (C. elegans)	1.185	0.1009	0.5394
64297	Gprc5b	G protein-coupled receptor, family C, group 5, member B	1.185	0.3936	0.7656
56486	Gabarap	gamma-aminobutyric acid receptor associated protein	1.185	0.05022	0.4514
20510	Slc1a1	solute carrier family 1 (neuronal/epithelial high affinity glutamate transporter, system Xag), member 1	1.185	0.3138	0.7152
12505	Cd44	CD44 antigen	1.185	0.4102	0.7739
11443	Chrnb1	cholinergic receptor, nicotinic, beta polypeptide 1 (muscle)	1.185	0.02767	0.3872
100233175	AK010878	cDNA sequence AK010878	1.184	0.1131	0.5556
326623	Tnfsf15	tumor necrosis factor (ligand) superfamily, member 15	1.184	0.1717	0.6126
319508	Syt15	synaptotagmin XV	1.184	0.03256	0.4018
278507	Wfikkn2	WAP, follistatin/kazal, immunoglobulin, kunitz and netrin domain containing 2	1.184	0.1873	0.6255
270058	Mtap1s	microtubule-associated protein 1S	1.184	0.1396	0.5834
232989	Hnrnpul1	heterogeneous nuclear ribonucleoprotein U-like 1	1.184	0.0459	0.4381
232798	Leng8	leukocyte receptor cluster (LRC) member 8	1.184	0.1115	0.5549
93879	Pcdhb8	protocadherin beta 8	1.184	0.2897	0.7004
64380	Ms4a4c	membrane-spanning 4-domains, subfamily A, member 4C	1.184	0.09905	0.5367
56248	Ak3	adenylate kinase 3	1.184	0.03311	0.404
21748	Terc	telomerase RNA component	1.184	0.334	0.7292
18612	Etv4	ets variant gene 4 (E1A enhancer binding protein, E1AF)	1.184	0.3309	0.7264
18053	Ngfr	nerve growth factor receptor (TNFR superfamily, member 16)	1.184	0.2477	0.6748
14088	Fancc	Fanconi anemia, complementation group C	1.184	0.1809	0.6199
12424	Cck	cholecystokinin	1.184	0.008627	NA
11666	Abcd1	ATP-binding cassette, sub-family D (ALD), member 1	1.184	0.3508	0.7393
623172	Gm6403	predicted gene 6403	1.183	0.3509	0.7393
380753	Atxn7l1	ataxin 7-like 1	1.183	0.09169	0.5245
242505	Rasef	RAS and EF hand domain containing	1.183	0.2533	0.6778
236598	LOC236598	28S ribosomal RNA	1.183	0.1401	0.5834
215798	Gpr126	G protein-coupled receptor 126	1.183	0.2517	0.6767
108096	Slco1a5	solute carrier organic anion transporter family, member 1a5	1.183	0.3538	0.7414
106878	2010002N04Rik	RIKEN cDNA 2010002N04 gene	1.183	0.171	0.6118
77041	Arsk	arylsulfatase K	1.183	0.02414	0.3771
73368	Col20a1	collagen, type XX, alpha 1	1.183	0.1137	0.5569
70296	Tbc1d13	TBC1 domain family, member 13	1.183	0.2561	0.6801
67690	Prss37	protease, serine, 37	1.183	0.1718	0.6126
56455	Dynll1	dynein light chain LC8-type 1	1.183	0.0381	0.4193

27399	Ip6k1	inositol hexaphosphate kinase 1	1.183	0.02178	0.3688
22364	Vpreb3	pre-B lymphocyte gene 3	1.183	0.0609	0.4741
20111	Rps6ka1	ribosomal protein S6 kinase polypeptide 1	1.183	0.1542	0.5975
16514	Kcnj11	potassium inwardly rectifying channel, subfamily J, member 11	1.183	0.07423	0.4962
12903	Crabp1	cellular retinoic acid binding protein I	1.183	0.1842	0.6217
242083	Ppm1l	protein phosphatase 1 (formerly 2C)-like	1.182	0.06283	0.4772
103534	Mgat4b	mannoside acetylglucosaminyltransferase 4, isoenzyme B	1.182	0.06481	0.4816
76787	Ppfia3	protein tyrosine phosphatase, receptor type, f polypeptide (PTPRF), interacting protein (liprin), alpha 3	1.182	0.196	0.6308
72129	Pex13	peroxisomal biogenesis factor 13	1.182	0.02455	0.3786
71764	C2cd2l	C2 calcium-dependent domain containing 2-like	1.182	0.0672	0.4855
71384	5430440L12Rik	RIKEN cDNA 5430440L12 gene	1.182	0.2674	0.6867
68588	Cthrc1	collagen triple helix repeat containing 1	1.182	0.5204	0.8286
56193	Plek	pleckstrin	1.182	0.02075	0.3609
54485	Dll4	delta-like 4 (Drosophila)	1.182	0.2145	0.6467
52430	Echdc2	enoyl Coenzyme A hydratase domain containing 2	1.182	0.07838	0.5045
22117	Tst	thiosulfate sulfurtransferase, mitochondrial	1.182	0.4231	0.7809
14069	F8	coagulation factor VIII	1.182	0.4576	0.7991
12752	Cln3	ceroid lipofuscinosis, neuronal 3, juvenile (Batten, Spielmeyer-Vogt disease)	1.182	0.1332	0.5772
667370	I830012O16Rik	RIKEN cDNA I830012O16 gene	1.181	0.2719	0.6891
622512	Gm6328	predicted gene 6328	1.181	0.2822	0.6967
320946	A930035D04Rik	RIKEN cDNA A930035D04 gene	1.181	0.06197	0.4771
266744	Lgsn	lengsin, lens protein with glutamine synthetase domain	1.181	0.6796	0.8944
252864	Dusp15	dual specificity phosphatase-like 15	1.181	0.2362	0.6657
231605	Galnt9	UDP-N-acetyl-alpha-D-galactosamine:polypeptide N-acetylgalactosaminyltransferase 9	1.181	0.2937	0.7033
230576	Ttc22	tetratricopeptide repeat domain 22	1.181	0.3562	0.7443
224824	Pex6	peroxisomal biogenesis factor 6	1.181	0.2176	0.6499
208228	Mobkl2a	MOB1, Mps One Binder kinase activator-like 2A (yeast)	1.181	0.1059	0.5468
109648	Npy	neuropeptide Y	1.181	0.2141	0.6467
80748	BC004004	cDNA sequence BC004004	1.181	0.08064	0.5102
75216	4930534B04Rik	RIKEN cDNA 4930534B04 gene	1.181	0.2965	0.7051
74777	Sepn1	selenoprotein N, 1	1.181	0.1597	0.6042
74354	Lrguk	leucine-rich repeats and guanylate kinase domain containing	1.181	0.4484	0.7942
67689	Aldh3b1	aldehyde dehydrogenase 3 family, member B1	1.181	0.03351	0.4071
67621	Bend5	BEN domain containing 5	1.181	0.08813	0.5202
66391	2310061J03Rik	RIKEN cDNA 2310061J03 gene	1.181	0.04189	0.4285
54393	Gabbr1	gamma-aminobutyric acid (GABA) B receptor, 1	1.181	0.04041	0.4257
50759	Fbxo16	F-box protein 16	1.181	0.3696	0.7521
22668	Sf1	splicing factor 1	1.181	0.0501	0.451
22259	Nr1h3	nuclear receptor subfamily 1, group H, member 3	1.181	0.168	0.6095
18606	Enpp2	ectonucleotide pyrophosphatase/phosphodiesterase 2	1.181	0.03463	0.4091
15170	Ptpn6	protein tyrosine phosphatase, non-receptor type 6	1.181	0.5356	0.835
14664	Slc6a9	solute carrier family 6 (neurotransmitter transporter, glycine), member 9	1.181	0.1058	0.5468
100040016	Rhox2e	reproductive homeobox 2E	1.18	0.0915	0.5241
544817	Arhgap27	Rho GTPase activating protein 27	1.18	0.3111	0.7135
385109	Gm1499	predicted gene 1499	1.18	0.1469	0.591
338372	Map3k9	mitogen-activated protein kinase kinase kinase 9	1.18	0.2372	0.6659
236266	Alms1	Alstrom syndrome 1 homolog (human)	1.18	0.3292	0.7254
217721	Mfsd7c	major facilitator superfamily domain containing 7C	1.18	0.1811	0.6203
106522	Pkdcc	protein kinase domain containing, cytoplasmic	1.18	0.1236	0.5681

65256	Asb2	ankyrin repeat and SOCS box-containing 2	1.18	0.1018	0.5404
56222	Cited4	Cbp/p300-interacting transactivator, with Glu/Asp-rich carboxy-terminal domain, 4	1.18	0.3727	0.7537
53324	Nptx2	neuronal pentraxin 2	1.18	0.2146	0.6467
29811	Ndrg2	N-myc downstream regulated gene 2	1.18	0.00303	NA
16170	Il16	interleukin 16	1.18	0.313	0.715
16156	Il11	interleukin 11	1.18	0.1784	0.6181
791373	B930078G14Rik	RIKEN cDNA B930078G14 gene	1.179	0.5219	0.829
382014	Ano8	anoctamin 8	1.179	0.2322	0.6621
381868	Gm1082	predicted gene 1082	1.179	0.5576	0.8454
320116	C030019I05Rik	RIKEN cDNA C030019I05 gene	1.179	0.3784	0.7574
243780	E330009J07Rik	RIKEN cDNA E330009J07 gene	1.179	0.03495	0.4096
243328	Slc29a4	solute carrier family 29 (nucleoside transporters), member 4	1.179	0.1475	0.591
240479	Fam69c	family with sequence similarity 69, member C	1.179	0.2652	0.6849
240058	Cpne5	copine V	1.179	0.138	0.5816
238988	Erc2	ELKS/RAB6-interacting/CAST family member 2	1.179	0.04109	0.4273
232431	Gprc5a	G protein-coupled receptor, family C, group 5, member A	1.179	0.2774	0.6925
216795	Wnt9a	wingless-type MMTV integration site 9A	1.179	0.04395	0.4336
101359	Prrt4	proline-rich transmembrane protein 4	1.179	0.2815	0.6967
75941	4930570B17Rik	RIKEN cDNA 4930570B17 gene	1.179	0.1214	0.5651
68922	Dnaic1	dynein, axonemal, intermediate chain 1	1.179	0.1511	0.5942
59126	Nek6	NIMA (never in mitosis gene a)-related expressed kinase 6	1.179	0.03244	0.4017
56018	Stard10	START domain containing 10	1.179	0.04324	0.4321
52163	Camk1	calcium/calmodulin-dependent protein kinase I	1.179	0.2774	0.6925
21673	Dntt	deoxynucleotidyltransferase, terminal	1.179	0.1279	0.5716
21336	Tacr1	tachykinin receptor 1	1.179	0.05773	0.469
20665	Sox10	SRY-box containing gene 10	1.179	0.2461	0.6733
18991	Pou3f1	POU domain, class 3, transcription factor 1	1.179	0.02934	0.3911
17150	Mfap2	microfibrillar-associated protein 2	1.179	0.2559	0.68
791344	E330037I15Rik	RIKEN cDNA E330037I15 gene	1.178	0.5866	0.856
381310	6330403A02Rik	RIKEN cDNA 6330403A02 gene	1.178	0.09438	0.5311
278097	Armcx6	armadillo repeat containing, X-linked 6	1.178	0.02162	0.367
234094	Arhgef10	Rho guanine nucleotide exchange factor (GEF) 10	1.178	0.2915	0.7018
170461	Stard6	StAR-related lipid transfer (START) domain containing 6	1.178	0.09547	0.5334
97423	R74862	expressed sequence R74862	1.178	0.03187	0.3992
80718	Rab27b	RAB27b, member RAS oncogene family	1.178	0.01338	NA
78004	Prr15	proline rich 15	1.178	0.1005	0.5394
76982	3110035E14Rik	RIKEN cDNA 3110035E14 gene	1.178	0.04205	0.4285
69563	2310015B20Rik	RIKEN cDNA 2310015B20 gene	1.178	0.03384	0.4086
69101	Ydjc	YdjC homolog (bacterial)	1.178	0.02716	0.3854
68777	Tmem53	transmembrane protein 53	1.178	0.02721	0.3854
67443	Map1lc3b	microtubule-associated protein 1 light chain 3 beta	1.178	0.07032	0.4912
66412	Arrdc4	arrestin domain containing 4	1.178	0.04449	0.4349
54208	Arl6ip1	ADP-ribosylation factor-like 6 interacting protein 1	1.178	0.06942	0.4874
26886	Cenph	centromere protein H	1.178	0.3364	0.7305
22771	Zic1	zinc finger protein of the cerebellum 1	1.178	0.1349	0.5782
21991	Tpi1	triosephosphate isomerase 1	1.178	0.05504	0.4614
20535	Slc4a2	solute carrier family 4 (anion exchanger), member 2	1.178	0.3022	0.7083
18212	Ntrk2	neurotrophic tyrosine kinase, receptor, type 2	1.178	0.2259	0.6543
17532	Mras	muscle and microspikes RAS	1.178	0.1995	0.6343

16324	Inhbb	inhibin beta-B	1.178	0.1609	0.6067
16172	Il17ra	interleukin 17 receptor A	1.178	0.3112	0.7135
15235	Mst1	macrophage stimulating 1 (hepatocyte growth factor-like)	1.178	0.4386	0.7897
13849	Ephx1	epoxide hydrolase 1, microsomal	1.178	0.08378	0.5144
241770	Rims4	regulating synaptic membrane exocytosis 4	1.177	0.4295	0.7843
239673	4732456N10Rik	RIKEN cDNA 4732456N10 gene	1.177	0.6415	0.8789
232983	Cxcl17	chemokine (C-X-C motif) ligand 17	1.177	0.5356	0.835
228866	Pcif1	PDX1 C-terminal inhibiting factor 1	1.177	0.2493	0.6767
226861	Hhat	hedgehog acyltransferase	1.177	0.06007	0.4728
225182	Rbbp8	retinoblastoma binding protein 8	1.177	0.05102	0.4519
107271	Yars	tyrosyl-tRNA synthetase	1.177	0.1297	0.5746
106869	Tnfaip8	tumor necrosis factor, alpha-induced protein 8	1.177	0.1267	0.5708
76987	Hdhd2	haloacid dehalogenase-like hydrolase domain containing 2	1.177	0.1446	0.5893
71853	Pdia6	protein disulfide isomerase associated 6	1.177	0.01209	NA
71461	Ptk7	PTK7 protein tyrosine kinase 7	1.177	0.2361	0.6657
70911	Phyhipl	phytanoyl-CoA hydroxylase interacting protein-like	1.177	0.1231	0.5672
67893	Tmem86a	transmembrane protein 86A	1.177	0.06206	0.4771
67873	Mri1	methylthioribose-1-phosphate isomerase homolog (S. cerevisiae)	1.177	0.07551	0.4997
66278	1810013D10Rik	RIKEN cDNA 1810013D10 gene	1.177	0.09239	0.5264
55985	Cxcl13	chemokine (C-X-C motif) ligand 13	1.177	0.07227	0.4941
20861	Stfa1	stefin A1	1.177	0.3584	0.7457
20852	Stat6	signal transducer and activator of transcription 6	1.177	0.2602	0.6815
20821	Trim21	tripartite motif-containing 21	1.177	0.04841	0.445
20725	Serpinb8	serine (or cysteine) peptidase inhibitor, clade B, member 8	1.177	0.3798	0.7592
12047	Bcl2a1d	B-cell leukemia/lymphoma 2 related protein A1d	1.177	0.07	0.4896
11758	Prdx6	peroxiredoxin 6	1.177	0.02925	0.3911
100042545	Gm3896	predicted gene 3896	1.176	0.4372	0.789
639281	Gm10382	predicted gene 10382	1.176	0.2556	0.6798
621239	Nhlrc4	NHL repeat containing 4	1.176	0.3489	0.7388
233752	Insc	inscuteable homolog (Drosophila)	1.176	0.04932	0.4469
232969	Zfp428	zinc finger protein 428	1.176	0.02515	0.3786
114663	Impa2	inositol (myo)-1(or 4)-monophosphatase 2	1.176	0.1842	0.6217
108802	Calr4	calreticulin 4	1.176	0.1588	0.6033
75813	4930484H19Rik	RIKEN cDNA 4930484H19 gene	1.176	0.2682	0.6871
75104	Mmd2	monocyte to macrophage differentiation-associated 2	1.176	0.06012	0.4728
66972	Slc25a23	solute carrier family 25 (mitochondrial carrier; phosphate carrier), member 23	1.176	0.5097	0.825
57441	Gmnn	geminin	1.176	0.07748	0.5026
20975	Synj2	synaptojanin 2	1.176	0.04844	0.445
20818	Srprb	signal recognition particle receptor, B subunit	1.176	0.08505	0.5166
18633	Pex16	peroxisomal biogenesis factor 16	1.176	0.1919	0.6281
14709	Gng8	guanine nucleotide binding protein (G protein), gamma 8	1.176	0.4505	0.7945
14630	Gclm	glutamate-cysteine ligase, modifier subunit	1.176	0.1474	0.591
100040294	Gm2694	predicted gene 2694	1.175	0.03854	0.4207
100040259	Gm16379	predicted pseudogene 16379	1.175	0.07968	0.5088
623279	Dok6	docking protein 6	1.175	0.3758	0.7556
320206	A730028G07Rik	RIKEN cDNA A730028G07 gene	1.175	0.2138	0.6467
239405	Rspo2	R-spondin 2 homolog (Xenopus laevis)	1.175	0.3824	0.7596
235627	Nbeal2	neurobeachin-like 2	1.175	0.146	0.591
233271	Luzp2	leucine zipper protein 2	1.175	0.0149	NA

230837	Asap3	ArfGAP with SH3 domain, ankyrin repeat and PH domain 3	1.175	0.4043	0.7713
227659	Slc2a6	solute carrier family 2 (facilitated glucose transporter), member 6	1.175	0.1215	0.5651
208846	Daam1	dishevelled associated activator of morphogenesis 1	1.175	0.09663	0.5345
106840	Unc119b	unc-119 homolog B (C. elegans)	1.175	0.1169	0.5592
105005	Fam84a	family with sequence similarity 84, member A	1.175	0.06023	0.4729
103988	Gck	glucokinase	1.175	0.3664	0.7504
84112	Sucnr1	succinate receptor 1	1.175	0.1741	0.6148
74127	Krt80	keratin 80	1.175	0.2741	0.6919
68839	Ankrd46	ankyrin repeat domain 46	1.175	0.1464	0.591
67776	Vwa5a	von Willebrand factor A domain containing 5A	1.175	0.1269	0.5712
22724	Zbtb7b	zinc finger and BTB domain containing 7B	1.175	0.1649	0.6077
17300	Foxc1	forkhead box C1	1.175	0.1092	0.5529
15525	Hspa4	heat shock protein 4	1.175	0.05463	0.4614
14539	Opn1mw	opsin 1 (cone pigments), medium-wave-sensitive (color blindness, deutan)	1.175	0.06913	0.4872
14062	F2r	coagulation factor II (thrombin) receptor	1.175	0.1832	0.6217
12319	Car8	carbonic anhydrase 8	1.175	0.4038	0.7711
383563	Gpr25	G protein-coupled receptor 25	1.174	0.1285	0.5729
270160	Rab39	RAB39, member RAS oncogene family	1.174	0.02161	NA
233913	BC017158	cDNA sequence BC017158	1.174	0.03668	0.4161
225724	Mapk4	mitogen-activated protein kinase 4	1.174	0.01728	NA
217217	Asb16	ankyrin repeat and SOCS box-containing 16	1.174	0.02756	0.3868
215653	Rassf2	Ras association (RalGDS/AF-6) domain family member 2	1.174	0.3301	0.726
104923	Adi1	acireductone dioxygenase 1	1.174	0.1636	0.6074
75697	C2cd4b	C2 calcium-dependent domain containing 4B	1.174	0.2396	0.6681
73191	Fezf1	Fez family zinc finger 1	1.174	0.2419	0.67
71729	Rgs12	regulator of G-protein signaling 12	1.174	0.03115	0.3964
71567	Mcm9	minichromosome maintenance complex component 9	1.174	0.06166	0.477
71529	9030409G11Rik	RIKEN cDNA 9030409G11 gene	1.174	0.1075	0.5499
26549	Itgb1bp2	integrin beta 1 binding protein 2	1.174	0.1903	0.6275
22030	Traf2	TNF receptor-associated factor 2	1.174	0.01009	NA
20981	Syt3	synaptotagmin III	1.174	0.3766	0.7559
17760	Mtap6	microtubule-associated protein 6	1.174	0.02849	0.3896
16188	Il3ra	interleukin 3 receptor, alpha chain	1.174	0.1716	0.6126
100040766	Heatr7b1	HEAT repeat containing 7B1	1.173	0.6031	0.8643
100039307	Gm2149	predicted gene 2149	1.173	0.3868	0.761
613254	AA465934	expressed sequence AA465934	1.173	0.4495	0.7943
225049	Ttc7	tetratricopeptide repeat domain 7	1.173	0.1641	0.6076
223473	Nipal2	NIPA-like domain containing 2	1.173	0.0653	0.4816
117229	Stk33	serine/threonine kinase 33	1.173	0.1531	0.5967
116914	Slc19a2	solute carrier family 19 (thiamine transporter), member 2	1.173	0.01069	NA
108832	5430405G05Rik	RIKEN cDNA 5430405G05 gene	1.173	0.1492	0.5938
105855	Nckap1l	NCK associated protein 1 like	1.173	0.1086	0.5521
84506	Hamp	hepcidin antimicrobial peptide	1.173	0.2244	0.654
72183	Snx6	sorting nexin 6	1.173	0.06736	0.4855
69034	4930579G22Rik	RIKEN cDNA 4930579G22 gene	1.173	0.01799	NA
68507	Ppfia4	protein tyrosine phosphatase, receptor type, f polypeptide (PTPRF), interacting protein (liprin), alpha 4	1.173	0.1686	0.6096
67711	Nsmce1	non-SMC element 1 homolog (S. cerevisiae)	1.173	0.0208	NA
67015	Ccdc91	coiled-coil domain containing 91	1.173	0.03328	0.4053
66617	Mettl11a	methyltransferase like 11A	1.173	0.09018	0.5229

64177	Trpv6	transient receptor potential cation channel, subfamily V, member 6	1.173	0.2368	0.6659
56699	Cdc42ep4	CDC42 effector protein (Rho GTPase binding) 4	1.173	0.07773	0.5026
56317	Anapc7	anaphase promoting complex subunit 7	1.173	0.243	0.6706
50767	Pnpla6	patatin-like phospholipase domain containing 6	1.173	0.1261	0.5703
27393	Mrpl39	mitochondrial ribosomal protein L39	1.173	0.08709	0.5184
19879	Slc22a8	solute carrier family 22 (organic anion transporter), member 8	1.173	0.5288	0.8332
15893	Ica1	islet cell autoantigen 1	1.173	0.04371	0.4336
14725	Lrp2	low density lipoprotein receptor-related protein 2	1.173	0.0428	0.4312
13215	Defb2	defensin beta 2	1.173	0.8224	0.9435
545758	Gm5868	predicted gene 5868	1.172	0.03011	0.3947
435684	Shf	Src homology 2 domain containing F	1.172	0.3338	0.729
338349	Cntln	centlein, centrosomal protein	1.172	0.1235	0.568
239618	Pdzrn4	PDZ domain containing RING finger 4	1.172	0.2009	0.6348
236733	Usp11	ubiquitin specific peptidase 11	1.172	0.2381	0.6668
114601	Ehbp1l1	EH domain binding protein 1-like 1	1.172	0.1755	0.6156
107650	Pi4kb	phosphatidylinositol 4-kinase, catalytic, beta polypeptide	1.172	0.128	0.5716
107449	Unc5b	unc-5 homolog B (C. elegans)	1.172	0.1141	0.5569
75475	Oplah	5-oxoprolinase (ATP-hydrolysing)	1.172	0.1067	0.5482
72275	2200002D01Rik	RIKEN cDNA 2200002D01 gene	1.172	0.07017	0.4904
26362	Axl	AXL receptor tyrosine kinase	1.172	0.09592	0.5344
20293	Ccl12	chemokine (C-C motif) ligand 12	1.172	0.3467	0.7369
15939	Ier5	immediate early response 5	1.172	0.1721	0.6126
15504	Dnajb3	DnaJ (Hsp40) homolog, subfamily B, member 3	1.172	0.1333	0.5772
12661	Chl1	cell adhesion molecule with homology to L1CAM	1.172	0.1377	0.5815
12295	Cacnb1	calcium channel, voltage-dependent, beta 1 subunit	1.172	0.1743	0.6148
11867	Arpc1b	actin related protein 2/3 complex, subunit 1B	1.172	0.3421	0.7338
320712	Abi3bp	ABI gene family, member 3 (NESH) binding protein	1.171	0.5393	0.8366
240216	E230025N22Rik	Riken cDNA E230025N22 gene	1.171	0.4542	0.7973
225742	St8sia5	ST8 alpha-N-acetyl-neuraminide alpha-2,8-sialyltransferase 5	1.171	0.02326	NA
140475	Bsnd	Bartter syndrome, infantile, with sensorineural deafness (Barttin)	1.171	0.2141	0.6467
107239	Carns1	carnosine synthase 1	1.171	0.1243	0.5694
74915	Atp6v1e2	ATPase, H+ transporting, lysosomal V1 subunit E2	1.171	0.3519	0.7399
71367	Chst9	carbohydrate (N-acetylgalactosamine 4-0) sulfotransferase 9	1.171	0.03191	0.3992
70122	Mllt3	myeloid/lymphoid or mixed-lineage leukemia (trithorax homolog, Drosophila); translocated to, 3	1.171	0.0681	0.4858
69376	Zpbp2	zona pellucida binding protein 2	1.171	0.2275	0.6566
67800	Dgat2	diacylglycerol O-acyltransferase 2	1.171	0.01567	NA
64138	Ctsz	cathepsin Z	1.171	0.1075	0.5499
18667	Pgr	progesterone receptor	1.171	0.1196	0.5629
16998	Ltbp3	latent transforming growth factor beta binding protein 3	1.171	0.2022	0.6356
14635	Galk1	galactokinase 1	1.171	0.1368	0.5807
12550	Cdh1	cadherin 1	1.171	0.7743	0.9266
11686	Alox12b	arachidonate 12-lipoxygenase, 12R type	1.171	0.2572	0.681
434178	Zfp141	zinc finger protein 141	1.17	0.1107	0.5546
269881	Map3k10	mitogen-activated protein kinase kinase kinase 10	1.17	0.3129	0.715
259053	Olfr362	olfactory receptor 362	1.17	0.5304	0.8334
241070	Gpr1	G protein-coupled receptor 1	1.17	0.2629	0.6838
239706	BC024814	cDNA sequence BC024814	1.17	0.1097	0.5535
238205	Lrfn5	leucine rich repeat and fibronectin type III domain containing 5	1.17	0.3294	0.7257
231510	Agpat9	1-acylglycerol-3-phosphate O-acyltransferase 9	1.17	0.03774	0.417

219228	Pcdh17	protocadherin 17	1.17	0.1809	0.6199
209086	Samd9l	sterile alpha motif domain containing 9-like	1.17	0.1482	0.5917
102566	Ano10	anoctamin 10	1.17	0.2253	0.654
70835	Prss22	protease, serine, 22	1.17	0.4641	0.8036
68792	Srpx2	sushi-repeat-containing protein, X-linked 2	1.17	0.297	0.7051
57230	Sap30bp	SAP30 binding protein	1.17	0.1184	0.5612
53412	Ppp1r3c	protein phosphatase 1, regulatory (inhibitor) subunit 3C	1.17	0.02654	NA
20404	Sh3gl2	SH3-domain GRB2-like 2	1.17	0.2846	0.6977
16880	Lifr	leukemia inhibitory factor receptor	1.17	0.1182	0.5609
16413	Itgb1bp1	integrin beta 1 binding protein 1	1.17	0.015	NA
14069	F8	coagulation factor VIII	1.17	0.03448	0.4091
12966	Crygc	crystallin, gamma C	1.17	0.7821	0.9291
11758	Prdx6	peroxiredoxin 6	1.17	0.08247	0.5144
100503238	LOC100503238	hypothetical LOC100503238	1.169	0.05416	0.4596
434782	Gm5637	predicted pseudogene 5637	1.169	0.2006	0.6345
384261	Gm5296	predicted gene 5296	1.169	0.1033	0.5433
240444	Kcng2	potassium voltage-gated channel, subfamily G, member 2	1.169	0.5285	0.8332
235415	Cplx3	complexin 3	1.169	0.2063	0.639
224090	Tmem44	transmembrane protein 44	1.169	0.09017	0.5229
211666	Mgst2	microsomal glutathione S-transferase 2	1.169	0.2121	0.6448
116733	Vps4a	vacuolar protein sorting 4a (yeast)	1.169	0.2425	0.6705
74777	Sepn1	selenoprotein N, 1	1.169	0.1843	0.6217
74134	Cyp2s1	cytochrome P450, family 2, subfamily s, polypeptide 1	1.169	0.1797	0.6187
71733	Susd2	sushi domain containing 2	1.169	0.1431	0.5879
70784	Rasl12	RAS-like, family 12	1.169	0.33	0.726
70762	Dclk2	doublecortin-like kinase 2	1.169	0.1881	0.6261
70235	Poc1a	POC1 centriolar protein homolog A (Chlamydomonas)	1.169	0.09913	0.5367
68428	Steap3	STEAP family member 3	1.169	0.1879	0.626
67951	Tubb6	tubulin, beta 6	1.169	0.7638	0.9248
54631	Nphs1	nephrosis 1 homolog, nephrin (human)	1.169	0.371	0.7532
27380	Tcl1b4	T-cell leukemia/lymphoma 1B, 4	1.169	0.4283	0.7838
22051	Trip6	thyroid hormone receptor interactor 6	1.169	0.179	0.6181
16205	Gimap1	GTPase, IMAP family member 1	1.169	0.1625	0.607
13226	Defa-rs7	defensin, alpha, related sequence 7	1.169	0.03474	0.4092
434446	Ccdc13	coiled-coil domain containing 13	1.168	0.2041	0.6368
380930	9330188P03Rik	RIKEN cDNA 9330188P03 gene	1.168	0.0646	0.4816
319625	Galm	galactose mutarotase	1.168	0.1446	0.5892
319610	9530083O12Rik	RIKEN cDNA 9530083O12 gene	1.168	0.4693	0.8063
277343	Wfdc8	WAP four-disulfide core domain 8	1.168	0.338	0.7314
241732	Tspyl3	TSPY-like 3	1.168	0.02978	NA
212518	Sprn	shadow of prion protein	1.168	0.1317	0.5758
207175	Cetn4	centrin 4	1.168	0.02229	NA
170768	Pfkfb3	6-phosphofructo-2-kinase/fructose-2,6-biphosphatase 3	1.168	0.007484	NA
109349	Fam163b	family with sequence similarity 163, member B	1.168	0.1123	0.5549
108832	5430405G05Rik	RIKEN cDNA 5430405G05 gene	1.168	0.009962	NA
75341	4930564C03Rik	RIKEN cDNA 4930564C03 gene	1.168	0.323	0.7204
74770	Hhatl	hedgehog acyltransferase-like	1.168	0.2836	0.6974
74318	Hopx	HOP homeobox	1.168	0.07394	0.4961
69564	Itgb1bp3	integrin beta 1 binding protein 3	1.168	0.2788	0.6937

67196	Ube2t	ubiquitin-conjugating enzyme E2T (putative)	1.168	0.2426	0.6705
56839	Lgi1	leucine-rich repeat LGI family, member 1	1.168	0.1112	0.5549
56504	Srpk3	serine/arginine-rich protein specific kinase 3	1.168	0.08435	0.5154
21389	Tbx6	T-box 6	1.168	0.05114	0.4519
19434	Rax	retina and anterior neural fold homeobox	1.168	0.09909	0.5367
19025	Ctsa	cathepsin A	1.168	0.04828	0.4448
17534	Mrc2	mannose receptor, C type 2	1.168	0.5821	0.8533
15976	Ifnar2	interferon (alpha and beta) receptor 2	1.168	0.1253	0.5703
14079	Fabp2	fatty acid binding protein 2, intestinal	1.168	0.5224	0.8293
13885	Esd	esterase D/formylglutathione hydrolase	1.168	0.06229	0.4771
13040	Ctss	cathepsin S	1.168	0.05216	0.4535
626231	Gm6658	predicted gene 6658	1.167	0.2725	0.6897
403185	4932443I19Rik	RIKEN cDNA 4932443I19 gene	1.167	0.1777	0.6168
329502	Pla2g4e	phospholipase A2, group IVE	1.167	0.06962	0.4878
321022	Cdv3	carnitine deficiency-associated gene expressed in ventricle 3	1.167	0.06263	0.4771
268445	Ankrd13b	ankyrin repeat domain 13b	1.167	0.482	0.8108
230279	6330416G13Rik	RIKEN cDNA 6330416G13 gene	1.167	0.09625	0.5345
217734	Pomt2	protein-O-mannosyltransferase 2	1.167	0.275	0.6925
171095	Il17rc	interleukin 17 receptor C	1.167	0.3313	0.7265
93896	Glp2r	glucagon-like peptide 2 receptor	1.167	0.02086	NA
80838	Hist1h1a	histone cluster 1, H1a	1.167	0.3458	0.7361
69922	Vrk2	vaccinia related kinase 2	1.167	0.2659	0.6856
69697	2310057J16Rik	RIKEN cDNA 2310057J16 gene	1.167	0.1501	0.5938
69634	Clybl	citrate lyase beta like	1.167	0.2167	0.6482
67784	Plxnd1	plexin D1	1.167	0.1683	0.6095
67509	1810063B07Rik	RIKEN cDNA 1810063B07 gene	1.167	0.01486	NA
67272	Cmtm5	CKLF-like MARVEL transmembrane domain containing 5	1.167	0.1052	0.5455
66905	Plin3	perilipin 3	1.167	0.2214	0.6525
66634	Mcm8	minichromosome maintenance deficient 8 (S. cerevisiae)	1.167	0.01857	NA
56702	Hist1h1b	histone cluster 1, H1b	1.167	0.1498	0.5938
53322	Nucb2	nucleobindin 2	1.167	0.1231	0.5672
50788	Fbxl8	F-box and leucine-rich repeat protein 8	1.167	0.2058	0.6386
24109	Ubl3	ubiquitin-like 3	1.167	0.02562	NA
21463	Tcp11	t-complex protein 11	1.167	0.1272	0.5715
18822	Plod1	procollagen-lysine, 2-oxoglutarate 5-dioxygenase 1	1.167	0.1535	0.5975
17069	Ly6e	lymphocyte antigen 6 complex, locus E	1.167	0.3819	0.7596
14205	Figf	c-fos induced growth factor	1.167	0.172	0.6126
14073	Faah	fatty acid amide hydrolase	1.167	0.07911	0.5063
319655	Podxl2	podocalyxin-like 2	1.166	0.25	0.6767
319651	Usp37	ubiquitin specific peptidase 37	1.166	0.1027	0.5424
233744	Spon1	spondin 1, (f-spondin) extracellular matrix protein	1.166	0.05785	0.469
216964	Trp53i13	transformation related protein 53 inducible protein 13	1.166	0.01994	NA
195046	Nlrp1a	NLR family, pyrin domain containing 1A	1.166	0.03442	0.4091
99151	Cercam	cerebral endothelial cell adhesion molecule	1.166	0.3345	0.7298
75784	1700007G11Rik	RIKEN cDNA 1700007G11 gene	1.166	0.1119	0.5549
72027	Slc39a4	solute carrier family 39 (zinc transporter), member 4	1.166	0.3081	0.7112
71086	4933412E12Rik	RIKEN cDNA 4933412E12 gene	1.166	0.2485	0.6761
66671	Ccnh	cyclin H	1.166	0.04056	0.4257
23992	Prkra	protein kinase, interferon inducible double stranded RNA dependent activator	1.166	0.04609	0.4382

22724	Zbtb7b	zinc finger and BTB domain containing 7B	1.166	0.3563	0.7443
22321	Vars	valyl-tRNA synthetase	1.166	0.3586	0.7457
18488	Cntn3	contactin 3	1.166	0.1492	0.5937
17476	Mpeg1	macrophage expressed gene 1	1.166	0.05566	0.4631
17178	Fxyd3	FXYD domain-containing ion transport regulator 3	1.166	0.07173	0.4939
15483	Hsd11b1	hydroxysteroid 11-beta dehydrogenase 1	1.166	0.02904	NA
15476	Hs3st1	heparan sulfate (glucosamine) 3-O-sulfotransferase 1	1.166	0.07643	0.5014
12705	Cited1	Cbp/p300-interacting transactivator with Glu/Asp-rich carboxy-terminal domain 1	1.166	0.03189	NA
435684	Shf	Src homology 2 domain containing F	1.165	0.123	0.5672
244329	Mcph1	microcephaly, primary autosomal recessive 1	1.165	0.0795	0.5082
233875	Ino80e	INO80 complex subunit E	1.165	0.195	0.6304
217216	BC030867	cDNA sequence BC030867	1.165	0.2621	0.6831
78249	Gpr115	G protein-coupled receptor 115	1.165	0.1523	0.5958
75659	Wdr54	WD repeat domain 54	1.165	0.3836	0.7599
75647	1700025E21Rik	RIKEN cDNA 1700025E21 gene	1.165	0.3224	0.7202
72433	Rab38	RAB38, member of RAS oncogene family	1.165	0.4876	0.8135
71912	Jsrp1	junctional sarcoplasmic reticulum protein 1	1.165	0.03503	NA
69459	Ubl7	ubiquitin-like 7 (bone marrow stromal cell-derived)	1.165	0.5342	0.8348
68632	Myct1	myc target 1	1.165	0.4738	0.8088
67344	Tctex1d1	Tctex1 domain containing 1	1.165	0.3333	0.7282
56401	Lepre1	leprecan 1	1.165	0.2443	0.6722
56371	Fzr1	fizzy/cell division cycle 20 related 1 (Drosophila)	1.165	0.1962	0.6308
12978	Csf1r	colony stimulating factor 1 receptor	1.165	0.1201	0.564
100042150	Nrg2	neuregulin 2	1.164	0.06774	0.4855
100038402	BC025933	cDNA sequence BC025933	1.164	0.4904	0.815
629557	Gm6981	glyceraldehyde-3-phosphate dehydrogenase pseudogene	1.164	0.2154	0.6479
258778	Olfr921	olfactory receptor 921	1.164	0.4176	0.7783
252973	Grhl2	grainyhead-like 2 (Drosophila)	1.164	0.4435	0.7931
213391	Rassf4	Ras association (RalGDS/AF-6) domain family member 4	1.164	0.2652	0.6849
78376	Ng23	Ng23 protein	1.164	0.6301	0.8748
68846	Rnf208	ring finger protein 208	1.164	0.1724	0.6133
68393	Mogat1	monoacylglycerol O-acyltransferase 1	1.164	0.1003	0.5393
63986	Gmfg	glia maturation factor, gamma	1.164	0.4483	0.7942
56226	Espn	espin	1.164	0.4816	0.8108
55927	Hes6	hairy and enhancer of split 6 (Drosophila)	1.164	0.02353	NA
54130	Actr1a	ARP1 actin-related protein 1 homolog A, centractin alpha (yeast)	1.164	0.1635	0.6074
53320	Folh1	folate hydrolase	1.164	0.02641	NA
27376	Slc25a10	solute carrier family 25 (mitochondrial carrier, dicarboxylate transporter), member 10	1.164	0.3423	0.734
22173	Tyr	tyrosinase	1.164	0.3248	0.7218
20972	Syngr1	synaptogyrin 1	1.164	0.02972	NA
17035	Lxn	latexin	1.164	0.232	0.6618
16994	Ltb	lymphotoxin B	1.164	0.1974	0.6324
15502	Dnaja1	DnaJ (Hsp40) homolog, subfamily A, member 1	1.164	0.2629	0.6838
14807	Grik3	glutamate receptor, ionotropic, kainate 3	1.164	0.2658	0.6856
14082	Fadd	Fas (TNFRSF6)-associated via death domain	1.164	0.1896	0.6272
12491	Cd36	CD36 antigen	1.164	0.1389	0.5829
11541	Adora2b	adenosine A2b receptor	1.164	0.05295	0.4562
11438	Chrna4	cholinergic receptor, nicotinic, alpha polypeptide 4	1.164	0.2007	0.6345
627872	Dnahc7a	dynein, axonemal, heavy chain 7A	1.163	0.07461	0.4974

383619	Aim2	absent in melanoma 2	1.163	0.3028	0.7087
381199	Tmem151a	transmembrane protein 151A	1.163	0.3074	0.711
242125	BC037703	cDNA sequence BC037703	1.163	0.5227	0.8294
76156	Fam131b	family with sequence similarity 131, member B	1.163	0.2813	0.6967
74007	Btbd11	BTB (POZ) domain containing 11	1.163	0.07385	0.4961
73296	Rhobtb3	Rho-related BTB domain containing 3	1.163	0.05852	0.4706
71918	Zcchc24	zinc finger, CCHC domain containing 24	1.163	0.05527	0.4621
70227	Zfp619	zinc finger protein 619	1.163	0.2963	0.7051
66959	Dusp26	dual specificity phosphatase 26 (putative)	1.163	0.01487	NA
26943	Serinc3	serine incorporator 3	1.163	0.009142	NA
16334	Ins2	insulin II	1.163	0.1013	0.5395
100039861	Gm2464	predicted gene 2464	1.162	0.06647	0.4842
100039128	LOC100039128	nuclear pore complex protein Nup93-like	1.162	0.1762	0.6156
434057	Nat8b	N-acetyltransferase 8B	1.162	0.2828	0.6969
268420	Alkbh5	alkB, alkylation repair homolog 5 (E. coli)	1.162	0.02914	NA
243529	H1fx	H1 histone family, member X	1.162	0.3492	0.7388
232748	Fam115c	family with sequence similarity 115, member C	1.162	0.1896	0.6272
227094	Tmem194b	transmembrane protein 194B	1.162	0.5826	0.8537
140580	Elmo1	engulfment and cell motility 1, ced-12 homolog (C. elegans)	1.162	0.1195	0.5628
110326	Tas1r1	taste receptor, type 1, member 1	1.162	0.2097	0.6412
107227	Macrod1	MACRO domain containing 1	1.162	0.1814	0.6206
71709	Syde1	synapse defective 1, Rho GTPase, homolog 1 (C. elegans)	1.162	0.2619	0.6831
68808	1110046J04Rik	RIKEN cDNA 1110046J04 gene	1.162	0.1435	0.5879
67860	S100a16	S100 calcium binding protein A16	1.162	0.2386	0.6671
66835	Snord123	small nucleolar RNA, C/D box 123	1.162	0.07996	0.5096
66773	Gm17019	predicted gene	1.162	0.6481	0.8816
65963	Tmem176b	transmembrane protein 176B	1.162	0.236	0.6657
16782	Lamc2	laminin, gamma 2	1.162	0.1541	0.5975
14464	Gata5	GATA binding protein 5	1.162	0.2624	0.6835
12492	Scarb2	scavenger receptor class B, member 2	1.162	0.01171	NA
100042784	Prdm11	PR domain containing 11	1.161	0.5794	0.8522
399591	Tmsb15l	thymosin beta 15b like	1.161	0.03291	NA
381229	Ccdc147	coiled-coil domain containing 147	1.161	0.1113	0.5549
319188	Hist1h2bp	histone cluster 1, H2bp	1.161	0.1355	0.5793
245469	Pdzd4	PDZ domain containing 4	1.161	0.1665	0.6083
245190	Gm4980	predicted gene 4980	1.161	0.3518	0.7399
195046	Nlrp1a	NLR family, pyrin domain containing 1A	1.161	0.7339	0.916
117589	Asb7	ankyrin repeat and SOCS box-containing 7	1.161	0.1125	0.5549
100061	Lrrc19	leucine rich repeat containing 19	1.161	0.3618	0.7477
83766	Actl6b	actin-like 6B	1.161	0.5217	0.829
80733	Car15	carbonic anhydrase 15	1.161	0.08188	0.513
74744	5830408C22Rik	RIKEN cDNA 5830408C22 gene	1.161	0.1652	0.6077
68201	Ccdc34	coiled-coil domain containing 34	1.161	0.04073	NA
66795	Atg10	autophagy-related 10 (yeast)	1.161	0.1506	0.5938
58804	Cdc42ep5	CDC42 effector protein (Rho GTPase binding) 5	1.161	0.4017	0.7703
56744	Pf4	platelet factor 4	1.161	0.6037	0.8644
30049	Scd3	stearoyl-coenzyme A desaturase 3	1.161	0.1474	0.591
29873	Cspg5	chondroitin sulfate proteoglycan 5	1.161	0.05467	0.4614
27356	Insl6	insulin-like 6	1.161	0.2879	0.699

27280	Phlda3	pleckstrin homology-like domain, family A, member 3	1.161	0.0656	0.4818
22365	Vps45	vacuolar protein sorting 45 (yeast)	1.161	0.07344	0.4957
20055	Rps16	ribosomal protein S16	1.161	0.1163	0.558
19062	Inpp5k	inositol polyphosphate 5-phosphatase K	1.161	0.1958	0.6306
16905	Lmna	lamin A	1.161	0.2108	0.6426
15108	Hsd17b10	hydroxysteroid (17-beta) dehydrogenase 10	1.161	0.1771	0.6165
14539	Opn1mw	opsin 1 (cone pigments), medium-wave-sensitive (color blindness, deutan)	1.161	0.1038	0.5441
14325	Ftl1	ferritin light chain 1	1.161	0.02863	NA
14118	Fbn1	fibrillin 1	1.161	0.5959	0.8607
13864	Nr2f6	nuclear receptor subfamily 2, group F, member 6	1.161	0.1646	0.6076
13219	Defa-rs10	defensin, alpha, related sequence 10	1.161	0.08486	0.5164
546336	Prrg1	proline rich Gla (G-carboxyglutamic acid) 1	1.16	0.2021	0.6356
435376	Atp6ap1l	ATPase, H+ transporting, lysosomal accessory protein 1-like	1.16	0.1214	0.5651
333050	Ksr2	kinase suppressor of ras 2	1.16	0.1556	0.599
270084	Lpcat2	lysophosphatidylcholine acyltransferase 2	1.16	0.03676	NA
244418	D8Ertd82e	DNA segment, Chr 8, ERATO Doi 82, expressed	1.16	0.165	0.6077
231430	Cox18	COX18 cytochrome c oxidase assembly homolog (S. cerevisiae)	1.16	0.09093	0.5229
210741	Kcnk12	potassium channel, subfamily K, member 12	1.16	0.4183	0.7787
207818	Smagp	small cell adhesion glycoprotein	1.16	0.2693	0.6878
97998	Deptor	DEP domain containing MTOR-interacting protein	1.16	0.2939	0.7036
93840	Vangl2	vang-like 2 (van gogh, Drosophila)	1.16	0.2764	0.6925
78892	Crispld2	cysteine-rich secretory protein LCCL domain containing 2	1.16	0.09894	0.5367
73167	Arhgap8	Rho GTPase activating protein 8	1.16	0.1904	0.6275
72273	2210404O07Rik	RIKEN cDNA 2210404O07 gene	1.16	0.2361	0.6657
68275	Rpa1	replication protein A1	1.16	0.04366	0.4336
66522	Pgpep1	pyroglutamyl-peptidase I	1.16	0.1017	0.5397
56212	Rhog	ras homolog gene family, member G	1.16	0.2112	0.6431
54371	Chst2	carbohydrate sulfotransferase 2	1.16	0.4043	0.7713
52504	Cenpo	centromere protein O	1.16	0.01514	NA
26383	Fto	fat mass and obesity associated	1.16	0.1039	0.5444
22637	Zap70	zeta-chain (TCR) associated protein kinase	1.16	0.1115	0.5549
22439	Xk	Kell blood group precursor (McLeod phenotype) homolog	1.16	0.05757	0.4687
19293	Pvalb	parvalbumin	1.16	0.01138	NA
19094	Mapk11	mitogen-activated protein kinase 11	1.16	0.09869	0.5367
18391	Sigmar1	sigma non-opioid intracellular receptor 1	1.16	0.09831	0.5366
15467	Eif2ak1	eukaryotic translation initiation factor 2 alpha kinase 1	1.16	0.25	0.6767
14547	Gdap2	ganglioside-induced differentiation-associated-protein 2	1.16	0.04505	0.4353
13135	Dad1	defender against cell death 1	1.16	0.1292	0.5741
12521	Cd82	CD82 antigen	1.16	0.1564	0.5996
12517	Cd72	CD72 antigen	1.16	0.1833	0.6217
436062	Fam92b	family with sequence similarity 92, member B	1.159	0.1333	0.5773
208990	Npb	neuropeptide B	1.159	0.2304	0.6601
106393	Srl	sarcalumenin	1.159	0.376	0.7556
102153	C230098O21Rik	RIKEN cDNA C230098O21 gene	1.159	0.2194	0.6513
97848	Serpinb6c	serine (or cysteine) peptidase inhibitor, clade B, member 6c	1.159	0.1347	0.5779
84653	Hes7	hairy and enhancer of split 7 (Drosophila)	1.159	0.1703	0.6115
74048	4632428N05Rik	RIKEN cDNA 4632428N05 gene	1.159	0.1371	0.5807
73368	Col20a1	collagen, type XX, alpha 1	1.159	0.06446	0.4816
70445	Cd248	CD248 antigen, endosialin	1.159	0.3101	0.7131

70266	Ccbl1	cysteine conjugate-beta lyase 1	1.159	0.155	0.5975
68731	Rbfa	ribosome binding factor A	1.159	0.1362	0.5798
68090	Yif1a	Yip1 interacting factor homolog A (S. cerevisiae)	1.159	0.2194	0.6513
67675	Cuta	cutA divalent cation tolerance homolog (E. coli)	1.159	0.3772	0.7563
60411	Cenpk	centromere protein K	1.159	0.3447	0.7359
54612	Sfrp5	secreted frizzled-related sequence protein 5	1.159	0.2568	0.6809
52331	Stbd1	starch binding domain 1	1.159	0.04513	0.4353
51960	Kctd18	potassium channel tetramerisation domain containing 18	1.159	0.01325	NA
50789	Fbxl3	F-box and leucine-rich repeat protein 3	1.159	0.09078	0.5229
21950	Tnfsf9	tumor necrosis factor (ligand) superfamily, member 9	1.159	0.07649	0.5014
21425	Tcfeb	transcription factor EB	1.159	0.4	0.7702
16918	Mycl1	v-myc myelocytomatosis viral oncogene homolog 1, lung carcinoma derived (avian)	1.159	0.4425	0.7928
16765	Stmn1	stathmin 1	1.159	0.0604	0.4738
12268	C4b	complement component 4B (Childo blood group)	1.159	0.5797	0.8524
11651	Akt1	thymoma viral proto-oncogene 1	1.159	0.1367	0.5807
399566	Btbd6	BTB (POZ) domain containing 6	1.158	0.2468	0.6739
239273	Abcc4	ATP-binding cassette, sub-family C (CFTR/MRP), member 4	1.158	0.1542	0.5975
237987	Otop2	otopetrin 2	1.158	0.648	0.8816
212439	AA986860	expressed sequence AA986860	1.158	0.05757	0.4687
209039	Tenc1	tensin like C1 domain-containing phosphatase	1.158	0.3216	0.7199
192196	Luc7l2	LUC7-like 2 (S. cerevisiae)	1.158	0.1059	0.5468
73720	Cst6	cystatin E/M	1.158	0.1876	0.6259
72046	Urgcp	upregulator of cell proliferation	1.158	0.1787	0.6181
70807	Arrdc2	arrestin domain containing 2	1.158	0.05256	0.4549
68231	H2afb1	H2A histone family, member B1	1.158	0.2289	0.6581
68197	Ndufc2	NADH dehydrogenase (ubiquinone) 1, subcomplex unknown, 2	1.158	0.004896	NA
67784	Plxnd1	plexin D1	1.158	0.1768	0.6164
57745	Zfp112	zinc finger protein 112	1.158	0.5872	0.8564
52466	Slc46a1	solute carrier family 46, member 1	1.158	0.2331	0.6622
20689	Sall3	sal-like 3 (Drosophila)	1.158	0.1931	0.6294
20104	Rps6	ribosomal protein S6	1.158	0.0904	0.5229
18211	Ntrk1	neurotrophic tyrosine kinase, receptor, type 1	1.158	0.2301	0.6599
18003	Nedd9	neural precursor cell expressed, developmentally down-regulated gene 9	1.158	0.1212	0.5649
17874	Myd88	myeloid differentiation primary response gene 88	1.158	0.07751	0.5026
17127	Smad3	MAD homolog 3 (Drosophila)	1.158	0.01124	NA
14955	H19	H19 fetal liver mRNA	1.158	0.2384	0.667
13857	Epor	erythropoietin receptor	1.158	0.1573	0.6016
12566	Cdk2	cyclin-dependent kinase 2	1.158	0.3883	0.7616
12263	C2	complement component 2 (within H-2S)	1.158	0.1568	0.601
11479	Acvr1b	activin A receptor, type 1B	1.158	0.3601	0.7466
100042693	Gm3970	predicted gene 3970	1.157	0.257	0.681
545975	Lass3	LAG1 homolog, ceramide synthase 3	1.157	0.2035	0.6362
245537	Nlgn3	neuroligin 3	1.157	0.4072	0.7726
226352	Epb4.1l5	erythrocyte protein band 4.1-like 5	1.157	0.09076	0.5229
215446	Entpd3	ectonucleoside triphosphate diphosphohydrolase 3	1.157	0.1665	0.6083
214779	Zfp879	zinc figer protein 879	1.157	0.1217	0.5652
208624	Alg3	asparagine-linked glycosylation 3 homolog (yeast, alpha-1,3-mannosyltransferase)	1.157	0.3491	0.7388
171506	H1foo	H1 histone family, member O, oocyte-specific	1.157	0.2316	0.6612
114716	Spred2	sprouty-related, EVH1 domain containing 2	1.157	0.1962	0.6308

110902	Chrna2	cholinergic receptor, nicotinic, alpha polypeptide 2 (neuronal)	1.157	0.1815	0.6206
73061	3110007F17Rik	RIKEN cDNA 3110007F17 gene	1.157	0.2982	0.7052
68938	Aspscr1	alveolar soft part sarcoma chromosome region, candidate 1 (human)	1.157	0.3885	0.7616
66815	Ccdc109b	coiled-coil domain containing 109B	1.157	0.1341	0.5779
65967	Eefsec	eukaryotic elongation factor, selenocysteine-tRNA-specific	1.157	0.2631	0.6838
20981	Syt3	synaptotagmin III	1.157	0.3885	0.7616
18676	Phf2	PHD finger protein 2	1.157	0.15	0.5938
17444	Grap2	GRB2-related adaptor protein 2	1.157	0.1281	0.5716
17319	Mif	macrophage migration inhibitory factor	1.157	0.1238	0.5685
14842	Gsx1	GS homeobox 1	1.157	0.5162	0.8277
100042342	Gm10375	predicted gene 10375	1.156	0.3307	0.726
627191	Tmem90a	transmembrane protein 90a	1.156	0.4143	0.7759
233328	Lrrk1	leucine-rich repeat kinase 1	1.156	0.387	0.761
231602	P2rx2	purinergic receptor P2X, ligand-gated ion channel, 2	1.156	0.5766	0.8509
229722	5330417C22Rik	RIKEN cDNA 5330417C22 gene	1.156	0.2355	0.6654
212398	Frat2	frequently rearranged in advanced T-cell lymphomas 2	1.156	0.1429	0.5879
118445	Klf16	Kruppel-like factor 16	1.156	0.1791	0.6184
114679	Selm	selenoprotein M	1.156	0.1259	0.5703
77794	Adamtsl2	ADAMTS-like 2	1.156	0.137	0.5807
76916	4930455C21Rik	RIKEN cDNA 4930455C21 gene	1.156	0.1681	0.6095
74123	Foxp4	forkhead box P4	1.156	0.2306	0.6601
73043	2900064F13Rik	RIKEN cDNA 2900064F13 gene	1.156	0.122	0.5652
68970	Dcaf12	DDB1 and CUL4 associated factor 12	1.156	0.02006	NA
68846	Rnf208	ring finger protein 208	1.156	0.2387	0.6671
68813	Dock5	dedicator of cytokinesis 5	1.156	0.7128	0.9075
59046	Arpp19	cAMP-regulated phosphoprotein 19	1.156	0.2497	0.6767
21917	Tmpo	thymopoietin	1.156	0.1231	0.5672
20907	Stx1a	syntaxin 1A (brain)	1.156	0.3302	0.726
20408	Sh3gl3	SH3-domain GRB2-like 3	1.156	0.01622	NA
19125	Prodh	proline dehydrogenase	1.156	0.2728	0.69
18604	Pdk2	pyruvate dehydrogenase kinase, isoenzyme 2	1.156	0.1908	0.6276
14570	Arhgdig	Rho GDP dissociation inhibitor (GDI) gamma	1.156	0.2394	0.6679
12521	Cd82	CD82 antigen	1.156	0.1634	0.6074
11747	Anxa5	annexin A5	1.156	0.07379	0.4961
333315	Frem3	Fras1 related extracellular matrix protein 3	1.155	0.1019	0.5404
240084	Cchcr1	coiled-coil alpha-helical rod protein 1	1.155	0.1955	0.6304
234155	Mboat4	membrane bound O-acyltransferase domain containing 4	1.155	0.4542	0.7973
230073	Ddx58	DEAD (Asp-Glu-Ala-Asp) box polypeptide 58	1.155	0.1312	0.5751
226255	Atrnl1	attractin like 1	1.155	0.1263	0.5703
214359	Tmem51	transmembrane protein 51	1.155	0.1935	0.6295
211949	Spsb4	splA/ryanodine receptor domain and SOCS box containing 4	1.155	0.02595	NA
110173	Manba	mannosidase, beta A, lysosomal	1.155	0.1033	0.5433
106795	Tcf19	transcription factor 19	1.155	0.162	0.607
105847	Lmf2	lipase maturation factor 2	1.155	0.2895	0.7004
97387	Strn4	striatin, calmodulin binding protein 4	1.155	0.2117	0.6443
80837	Rhoj	ras homolog gene family, member J	1.155	0.5088	0.8244
76183	Celf6	CUGBP, Elav-like family member 6	1.155	0.08232	0.5144
70821	4921507P07Rik	RIKEN cDNA 4921507P07 gene	1.155	0.4108	0.7742
70804	Pgrmc2	progesterone receptor membrane component 2	1.155	0.174	0.6148

68184	Denr	density-regulated protein	1.155	0.06683	0.4851
68161	A930005H10Rik	RIKEN cDNA A930005H10 gene	1.155	0.05405	0.4596
66343	Tmem177	transmembrane protein 177	1.155	0.1829	0.6215
66206	1110059E24Rik	RIKEN cDNA 1110059E24 gene	1.155	0.05108	NA
66112	Mosc1	MOCO sulphurase C-terminal domain containing 1	1.155	0.313	0.715
28017	D7Wsu130e	DNA segment, Chr 7, Wayne State University 130, expressed	1.155	0.1991	0.6343
22229	Ucp3	uncoupling protein 3 (mitochondrial, proton carrier)	1.155	0.4142	0.7758
19848	Rnu2	U2 small nuclear RNA	1.155	0.4596	0.801
19401	Rara	retinoic acid receptor, alpha	1.155	0.125	0.5703
19294	Pvrl2	poliovirus receptor-related 2	1.155	0.1749	0.6152
19051	Gsbs	G substrate	1.155	0.06345	0.478
18733	Lilrb3	leukocyte immunoglobulin-like receptor, subfamily B (with TM and ITIM domains), member 3	1.155	0.4816	0.8108
18510	Pax8	paired box gene 8	1.155	0.4007	0.7703
14570	Arhgdig	Rho GDP dissociation inhibitor (GDI) gamma	1.155	0.2162	0.6479
14367	Fzd5	frizzled homolog 5 (Drosophila)	1.155	0.5784	0.8519
12904	Crabp2	cellular retinoic acid binding protein II	1.155	0.04452	NA
12014	Bach2	BTB and CNC homology 2	1.155	0.1545	0.5975
11831	Aqp6	aquaporin 6	1.155	0.0462	NA
11431	Acp1	acid phosphatase 1, soluble	1.155	0.352	0.7401
622459	Gm12216	predicted gene 12216	1.154	0.2962	0.7051
545136	Fam186b	family with sequence similarity 186, member B	1.154	0.372	0.7535
387314	Tmtc1	transmembrane and tetratricopeptide repeat containing 1	1.154	0.02532	NA
381716	1700015F17Rik	RIKEN cDNA 1700015F17 gene	1.154	0.2027	0.6356
381270	Mar-04	membrane-associated ring finger (C3HC4) 4	1.154	0.1206	0.5643
328092	6530401N04Rik	RIKEN cDNA 6530401N04 gene	1.154	0.2639	0.6845
319574	9330133O14Rik	RIKEN cDNA 9330133O14 gene	1.154	0.2511	0.6767
268595	D430019H16Rik	RIKEN cDNA D430019H16 gene	1.154	0.02554	NA
232879	Zbtb45	zinc finger and BTB domain containing 45	1.154	0.272	0.6891
230917	Tmem201	transmembrane protein 201	1.154	0.06019	0.4728
226422	Rab7l1	RAB7, member RAS oncogene family-like 1	1.154	0.04916	NA
223672	Apol9a	apolipoprotein L 9a	1.154	0.4707	0.8076
212198	Wdr25	WD repeat domain 25	1.154	0.2624	0.6835
80288	Bcl9l	B-cell CLL/lymphoma 9-like	1.154	0.3282	0.7251
73723	Sh3bgrl3	SH3 domain binding glutamic acid-rich protein-like 3	1.154	0.2768	0.6925
72046	Urgcp	upregulator of cell proliferation	1.154	0.2561	0.6801
72041	Alkbh4	alkB, alkylation repair homolog 4 (E. coli)	1.154	0.01135	NA
67753	4930579C15Rik	RIKEN cDNA 4930579C15 gene	1.154	0.5919	0.8584
66715	4921515J06Rik	RIKEN cDNA 4921515J06 gene	1.154	0.4598	0.801
66167	Ccdc72	coiled-coil domain containing 72	1.154	0.0127	NA
54384	Mtmr7	myotubularin related protein 7	1.154	0.01068	NA
27386	Npas3	neuronal PAS domain protein 3	1.154	0.01461	NA
26561	Mmp23	matrix metallopeptidase 23	1.154	0.3063	0.7108
21788	Tfpi	tissue factor pathway inhibitor	1.154	0.2966	0.7051
20733	Spint2	serine protease inhibitor, Kunitz type 2	1.154	0.2081	0.6401
19054	Ppp2r3d	protein phosphatase 2 (formerly 2A), regulatory subunit B'', delta	1.154	0.3417	0.7338
17766	Nudt1	nudix (nucleoside diphosphate linked moiety X)-type motif 1	1.154	0.1153	0.5578
16681	Krt2	keratin 2	1.154	0.5632	0.8465
14421	B4galnt1	beta-1,4-N-acetyl-galactosaminyl transferase 1	1.154	0.4032	0.7708
14362	Fzd1	frizzled homolog 1 (Drosophila)	1.154	0.223	0.654

14049	Eya2	eyes absent 2 homolog (Drosophila)	1.154	0.05134	NA
13167	Dbi	diazepam binding inhibitor	1.154	0.1756	0.6156
13063	Cycs	cytochrome c, somatic	1.154	0.06317	0.478
12964	Cryga	crystallin, gamma A	1.154	0.7785	0.9276
12445	Ccnd3	cyclin D3	1.154	0.08053	0.5098
100045396	Gm9908	predicted gene 9908	1.153	0.8401	0.9485
652925	4930420K17Rik	RIKEN cDNA 4930420K17 gene	1.153	0.01388	NA
545030	Wdfy4	WD repeat and FYVE domain containing 4	1.153	0.3154	0.7165
268933	Wdr24	WD repeat domain 24	1.153	0.2886	0.6996
268291	Rnf217	ring finger protein 217	1.153	0.02266	NA
233575	Pgap2	post-GPI attachment to proteins 2	1.153	0.03346	NA
230903	Fbxo44	F-box protein 44	1.153	0.2525	0.6776
230810	Slc30a2	solute carrier family 30 (zinc transporter), member 2	1.153	0.1145	0.5569
224008	2310008H04Rik	RIKEN cDNA 2310008H04 gene	1.153	0.09221	0.5263
217779	Lysmd1	LysM, putative peptidoglycan-binding, domain containing 1	1.153	0.07077	0.4923
215999	Ccdc109a	coiled-coil domain containing 109A	1.153	0.1851	0.6223
110749	Chaf1b	chromatin assembly factor 1, subunit B (p60)	1.153	0.1379	0.5816
108121	U2af1	U2 small nuclear ribonucleoprotein auxiliary factor (U2AF) 1	1.153	0.153	0.5966
104130	Ndufb11	NADH dehydrogenase (ubiquinone) 1 beta subcomplex, 11	1.153	0.08664	0.5178
102791	Tcta	T-cell leukemia translocation altered gene	1.153	0.09075	0.5229
99382	Abtb2	ankyrin repeat and BTB (POZ) domain containing 2	1.153	0.07802	0.503
76629	Wbscr28	Williams-Beuren syndrome chromosome region 28 (human)	1.153	0.507	0.8234
76438	Rftn1	raftlin lipid raft linker 1	1.153	0.0581	0.4703
74318	Hopx	HOP homeobox	1.153	0.2392	0.6679
72901	2900011F02Rik	RIKEN cDNA 2900011F02 gene	1.153	0.1731	0.6145
71956	Rnf135	ring finger protein 135	1.153	0.2275	0.6566
70807	Arrdc2	arrestin domain containing 2	1.153	0.05953	0.4728
69195	Tmem121	transmembrane protein 121	1.153	0.06241	0.4771
66734	Map1lc3a	microtubule-associated protein 1 light chain 3 alpha	1.153	0.2078	0.6401
57340	Jph3	junctophilin 3	1.153	0.1691	0.6098
56727	Miox	myo-inositol oxygenase	1.153	0.2578	0.6815
53378	Sdcbp	syndecan binding protein	1.153	0.07984	0.5091
22271	Upp1	uridine phosphorylase 1	1.153	0.45	0.7944
22045	Trhr	thyrotropin releasing hormone receptor	1.153	0.1102	0.5535
21954	Tnni3	troponin I, cardiac 3	1.153	0.2994	0.7054
17540	Mrvi1	MRV integration site 1	1.153	0.06897	0.487
14701	Gng12	guanine nucleotide binding protein (G protein), gamma 12	1.153	0.09688	0.5346
13731	Emp2	epithelial membrane protein 2	1.153	0.5787	0.8519
13033	Ctsd	cathepsin D	1.153	0.09935	0.5367
12517	Cd72	CD72 antigen	1.153	0.07508	0.4989
11536	Gpr182	G protein-coupled receptor 182	1.153	0.2135	0.6463
11520	Plin2	perilipin 2	1.153	0.1289	0.5736
100504017	LOC100504017	hypothetical LOC100504017	1.152	0.5479	0.8406
100041019	Gm3099	predicted gene 3099	1.152	0.08323	0.5144
100039284	Gm2137	predicted gene 2137	1.152	0.04216	NA
668763	Gm9340	predicted gene 9340	1.152	0.1638	0.6074
320020	6330415G19Rik	RIKEN cDNA 6330415G19 gene	1.152	0.6277	0.8738
276919	Gemin4	gem (nuclear organelle) associated protein 4	1.152	0.2253	0.654
266692	Cpne1	copine I	1.152	0.1527	0.5964

246048	Chodl	chondrolectin	1.152	0.3776	0.7567
241303	Fam78a	family with sequence similarity 78, member A	1.152	0.1889	0.6266
240514	Ccdc85b	coiled-coil domain containing 85B	1.152	0.362	0.7477
230779	Serinc2	serine incorporator 2	1.152	0.4583	0.7997
226777	C130074G19Rik	RIKEN cDNA C130074G19 gene	1.152	0.3564	0.7443
105785	Kdelr3	KDEL (Lys-Asp-Glu-Leu) endoplasmic reticulum protein retention receptor 3	1.152	0.4452	0.7938
72230	Zfp558	zinc finger protein 558	1.152	0.5928	0.8585
71685	Galnt14	UDP-N-acetyl-alpha-D-galactosamine:polypeptide N-acetylgalactosaminyltransferase 14	1.152	0.06758	0.4855
67528	Nudt7	nudix (nucleoside diphosphate linked moiety X)-type motif 7	1.152	0.05601	NA
66733	Kcng4	potassium voltage-gated channel, subfamily G, member 4	1.152	0.1899	0.6273
64385	Cyp4f14	cytochrome P450, family 4, subfamily f, polypeptide 14	1.152	0.07092	0.4923
58998	Pvrl3	poliovirus receptor-related 3	1.152	0.1052	0.5457
30944	Zfp354c	zinc finger protein 354C	1.152	0.0502	NA
21785	Tff2	trefoil factor 2 (spasmolytic protein 1)	1.152	0.5065	0.8233
19400	Rapsn	receptor-associated protein of the synapse	1.152	0.3695	0.7521
19186	Psme1	proteasome (prosome, macropain) 28 subunit, alpha	1.152	0.2267	0.6554
18158	Nppb	natriuretic peptide type B	1.152	0.424	0.7818
18104	Nqo1	NAD(P)H dehydrogenase, quinone 1	1.152	0.06784	0.4855
18016	Nf2	neurofibromatosis 2	1.152	0.1281	0.5716
16998	Ltbp3	latent transforming growth factor beta binding protein 3	1.152	0.2002	0.6345
16814	Lbx1	ladybird homeobox homolog 1 (Drosophila)	1.152	0.4972	0.8193
14824	Grn	granulin	1.152	0.1172	0.5599
14469	Gbp2	guanylate binding protein 2	1.152	0.2038	0.6362
14251	Flot1	flotillin 1	1.152	0.06667	0.4848
13661	Ehf	ets homologous factor	1.152	0.09227	0.5264
11674	Aldoa	aldolase A, fructose-bisphosphate	1.152	0.08724	0.5185
100294583	Fam150b	family with sequence similarity 150, member B	1.151	0.2591	0.6815
100101457	A830035O19Rik	RIKEN cDNA A830035O19 gene	1.151	0.1219	0.5652
100043309	4732414G09Rik	RIKEN cDNA 4732414G09 gene	1.151	0.1403	0.5834
545085	Wdr70	WD repeat domain 70	1.151	0.05058	NA
338374	Il28b	interleukin 28B	1.151	0.238	0.6668
329421	Myo3b	myosin IIIB	1.151	0.2203	0.6518
242915	Fam59b	family with sequence similarity 59, member B	1.151	0.2145	0.6467
215378	Fam5c	family with sequence similarity 5, member C	1.151	0.08454	0.5154
102941	B630019K06Rik	RIKEN cDNA B630019K06 gene	1.151	0.05028	NA
72650	2810006K23Rik	RIKEN cDNA 2810006K23 gene	1.151	0.2226	0.654
72590	Ppme1	protein phosphatase methylesterase 1	1.151	0.277	0.6925
72415	Sgol1	shugoshin-like 1 (S. pombe)	1.151	0.3225	0.7202
70005	1700029I01Rik	RIKEN cDNA 1700029I01 gene	1.151	0.3833	0.7599
69952	2810011L19Rik	RIKEN cDNA 2810011L19 gene	1.151	0.3238	0.721
69547	Nkpd1	NTPase, KAP family P-loop domain containing 1	1.151	0.08605	0.517
69071	Tmem97	transmembrane protein 97	1.151	0.06547	0.4816
67180	Yipf5	Yip1 domain family, member 5	1.151	0.03183	NA
22195	Ube2l3	ubiquitin-conjugating enzyme E2L 3	1.151	0.07117	0.4932
20199	S100a5	S100 calcium binding protein A5	1.151	0.3023	0.7084
20163	Rsu1	Ras suppressor protein 1	1.151	0.09988	0.5379
19288	Ptx3	pentraxin related gene	1.151	0.1814	0.6206
18775	Prl3d1	prolactin family 3, subfamily d, member 1	1.151	0.5482	0.8406
17961	Nat2	N-acetyltransferase 2 (arylamine N-acetyltransferase)	1.151	0.04228	NA

17259	Mef2b	myocyte enhancer factor 2B	1.151	0.4015	0.7703
16912	Psmb9	proteasome (prosome, macropain) subunit, beta type 9 (large multifunctional peptidase 2)	1.151	0.2937	0.7033
16190	Il4ra	interleukin 4 receptor, alpha	1.151	0.3165	0.7173
13358	Slc25a1	solute carrier family 25 (mitochondrial carrier, citrate transporter), member 1	1.151	0.1856	0.6227
12966	Crygc	crystallin, gamma C	1.151	0.7946	0.9341
12570	Cdk5r2	cyclin-dependent kinase 5, regulatory subunit 2 (p39)	1.151	0.0738	0.4961
11568	Aebp1	AE binding protein 1	1.151	0.2145	0.6467
434249	Gm5602	predicted gene 5602	1.15	0.04362	NA
258186	Olfr75-ps1	olfactory receptor 75, pseudogene 1	1.15	0.282	0.6967
242037	Gm410	predicted gene 410	1.15	0.4723	0.8083
224024	Scarf2	scavenger receptor class F, member 2	1.15	0.2176	0.6499
212898	Dse	dermatan sulfate epimerase	1.15	0.1692	0.61
208777	Sned1	sushi, nidogen and EGF-like domains 1	1.15	0.652	0.8837
140629	Ubox5	U box domain containing 5	1.15	0.2315	0.6612
109979	Art3	ADP-ribosyltransferase 3	1.15	0.3649	0.7493
98733	Obsl1	obscurin-like 1	1.15	0.112	0.5549
94216	Col4a6	collagen, type IV, alpha 6	1.15	0.7752	0.9266
80290	Gpr146	G protein-coupled receptor 146	1.15	0.09233	0.5264
74096	Hvcn1	hydrogen voltage-gated channel 1	1.15	0.2306	0.6601
71617	9130011E15Rik	RIKEN cDNA 9130011E15 gene	1.15	0.3999	0.7702
69551	2310022B05Rik	RIKEN cDNA 2310022B05 gene	1.15	0.1447	0.5893
56376	Pdlim5	PDZ and LIM domain 5	1.15	0.01047	NA
54375	Azin1	antizyme inhibitor 1	1.15	0.05719	NA
24099	Tnfsf13b	tumor necrosis factor (ligand) superfamily, member 13b	1.15	0.2092	0.6409
24074	Taf7	TAF7 RNA polymerase II, TATA box binding protein (TBP)-associated factor	1.15	0.3447	0.7359
22143	Tuba1b	tubulin, alpha 1B	1.15	0.09524	0.5332
20364	Sepw1	selenoprotein W, muscle 1	1.15	0.2535	0.678
18826	Lcp1	lymphocyte cytosolic protein 1	1.15	0.1699	0.6112
18207	Nthl1	nth (endonuclease III)-like 1 (E.coli)	1.15	0.1477	0.5911
15223	Foxj1	forkhead box J1	1.15	0.0683	0.4861
13848	Ephb6	Eph receptor B6	1.15	0.2045	0.637
12752	Cln3	ceroid lipofuscinosis, neuronal 3, juvenile (Batten, Spielmeyer-Vogt disease)	1.15	0.1921	0.6281
12614	Celsr1	cadherin, EGF LAG seven-pass G-type receptor 1 (flamingo homolog, Drosophila)	1.15	0.7649	0.9251
667572	Gm8709	glyceraldehyde-3-phosphate dehydrogenase pseudogene	1.149	0.3416	0.7337
629732	Rps8-ps1	ribosomal protein S8, pseudogene 1	1.149	0.06261	NA
381629	0610007C21Rik	RIKEN cDNA 0610007C21 gene	1.149	0.05281	NA
338350	9330129D05Rik	RIKEN cDNA 9330129D05 gene	1.149	0.2905	0.7007
242773	Slc45a1	solute carrier family 45, member 1	1.149	0.1323	0.5767
230597	Zfyve9	zinc finger, FYVE domain containing 9	1.149	0.02025	NA
227733	Pip5kl1	phosphatidylinositol-4-phosphate 5-kinase-like 1	1.149	0.39	0.7629
225655	Slmo1	slowmo homolog 1 (Drosophila)	1.149	0.1473	0.591
224997	Dlgap1	discs, large (Drosophila) homolog-associated protein 1	1.149	0.1091	0.5529
216011	Lrrc20	leucine rich repeat containing 20	1.149	0.2258	0.6543
103742	1810046J19Rik	RIKEN cDNA 1810046J19 gene	1.149	0.03898	NA
81896	Ift122	intraflagellar transport 122 homolog (Chlamydomonas)	1.149	0.04134	NA
76701	Ctrc	chymotrypsin C (caldecrin)	1.149	0.4898	0.8145
75805	Nln	neurolysin (metallopeptidase M3 family)	1.149	0.4492	0.7943
75600	Calml4	calmodulin-like 4	1.149	0.3652	0.7495
72244	1600014C10Rik	RIKEN cDNA 1600014C10 gene	1.149	0.143	0.5879

68778	1110038D17Rik	RIKEN cDNA 1110038D17 gene	1.149	0.006399	NA
66658	Ccdc51	coiled-coil domain containing 51	1.149	0.05912	NA
57916	Tnfrsf13b	tumor necrosis factor receptor superfamily, member 13b	1.149	0.4474	0.7938
56176	Pigp	phosphatidylinositol glycan anchor biosynthesis, class P	1.149	0.08749	0.5189
20933	Med22	mediator complex subunit 22	1.149	0.1527	0.5964
20130	Rras	Harvey rat sarcoma oncogene, subgroup R	1.149	0.1665	0.6083
16658	Mafb	v-maf musculoaponeurotic fibrosarcoma oncogene family, protein B (avian)	1.149	0.08912	0.5217
12803	Cntf	ciliary neurotrophic factor	1.149	0.4582	0.7997
100040972	Tceal7	transcription elongation factor A (SII)-like 7	1.148	0.1364	0.5802
664808	Gm7349	predicted gene 7349	1.148	0.1633	0.6074
319172	Hist1h2ab	histone cluster 1, H2ab	1.148	0.3141	0.7155
232493	Gys2	glycogen synthase 2	1.148	0.4004	0.7703
230125	Mcart1	mitochondrial carrier triple repeat 1	1.148	0.01171	NA
218341	Rfesd	Rieske (Fe-S) domain containing	1.148	0.1668	0.6083
215456	Gpat2	glycerol-3-phosphate acyltransferase 2, mitochondrial	1.148	0.3651	0.7495
208076	Pknox2	Pbx/knotted 1 homeobox 2	1.148	0.2923	0.7024
192192	Shkbp1	Sh3kbp1 binding protein 1	1.148	0.2291	0.6587
140795	P2ry14	purinergic receptor P2Y, G-protein coupled, 14	1.148	0.03737	NA
106766	Stap2	signal transducing adaptor family member 2	1.148	0.1083	0.552
106581	Itfg3	integrin alpha FG-GAP repeat containing 3	1.148	0.2006	0.6345
103406	Zfr2	zinc finger RNA binding protein 2	1.148	0.2415	0.6694
76257	Slc38a3	solute carrier family 38, member 3	1.148	0.2532	0.6778
71711	Mus81	MUS81 endonuclease homolog (yeast)	1.148	0.2136	0.6464
70725	6330411D24Rik	RIKEN cDNA 6330411D24 gene	1.148	0.5924	0.8584
66578	2610039C10Rik	RIKEN cDNA 2610039C10 gene	1.148	0.07443	0.4971
59007	Ngly1	N-glycanase 1	1.148	0.1402	0.5834
54635	Pdgfc	platelet-derived growth factor, C polypeptide	1.148	0.2047	0.6375
52443	Mrpl48	mitochondrial ribosomal protein L48	1.148	0.03426	NA
29862	Ninj2	ninjurin 2	1.148	0.08714	0.5184
21788	Tfpi	tissue factor pathway inhibitor	1.148	0.5097	0.825
20181	Rxra	retinoid X receptor alpha	1.148	0.01201	NA
18682	Phkg1	phosphorylase kinase gamma 1	1.148	0.3316	0.7266
18167	Npy2r	neuropeptide Y receptor Y2	1.148	0.8785	0.9628
18034	Nfkb2	nuclear factor of kappa light polypeptide gene enhancer in B-cells 2, p49/p100	1.148	0.4363	0.7888
16548	Khk	ketohexokinase	1.148	0.1947	0.6304
14403	Gabrd	gamma-aminobutyric acid (GABA) A receptor, subunit delta	1.148	0.1014	0.5395
13836	Epha2	Eph receptor A2	1.148	0.4042	0.7713
13180	Pcbd1	pterin 4 alpha carbinolamine dehydratase/dimerization cofactor of hepatocyte nuclear factor 1 alpha (TCF1) 1	1.148	0.05145	NA
12017	Bag1	BCL2-associated athanogene 1	1.148	0.1506	0.5938
11853	Rhoc	ras homolog gene family, member C	1.148	0.249	0.6767
11746	Anxa4	annexin A4	1.148	0.182	0.6206
664805	Skor2	SKI family transcriptional corepressor 2	1.147	0.103	0.5426
229644	Trim45	tripartite motif-containing 45	1.147	0.1762	0.6156
228003	Kbtbd10	kelch repeat and BTB (POZ) domain containing 10	1.147	0.3112	0.7135
213012	Abhd10	abhydrolase domain containing 10	1.147	0.01859	NA
76653	Cby3	chibby homolog 3 (Drosophila)	1.147	0.2498	0.6767
76527	Il34	interleukin 34	1.147	0.1326	0.5771
74563	Rasgef1c	RasGEF domain family, member 1C	1.147	0.1398	0.5834
70419	2810408A11Rik	RIKEN cDNA 2810408A11 gene	1.147	0.324	0.7211

65962	Slc9a3r2	solute carrier family 9 (sodium/hydrogen exchanger), member 3 regulator 2	1.147	0.3616	0.7477
57279	Slc25a20	solute carrier family 25 (mitochondrial carnitine/acylcarnitine translocase), member 20	1.147	0.09465	0.5318
54135	Lsr	lipolysis stimulated lipoprotein receptor	1.147	0.1895	0.6272
50997	Mpp2	membrane protein, palmitoylated 2 (MAGUK p55 subfamily member 2)	1.147	0.5974	0.8611
22259	Nr1h3	nuclear receptor subfamily 1, group H, member 3	1.147	0.1823	0.6207
20732	Spint1	serine protease inhibitor, Kunitz type 1	1.147	0.306	0.7106
20355	Sema4f	sema domain, immunoglobulin domain (Ig), TM domain, and short cytoplasmic domain	1.147	0.1288	0.5733
18483	Palm	paralemmin	1.147	0.3904	0.7633
17000	Ltbr	lymphotoxin B receptor	1.147	0.1667	0.6083
14854	Gss	glutathione synthetase	1.147	0.4211	0.7798
12091	Glb1	galactosidase, beta 1	1.147	0.1279	0.5716
100503637	LOC100503637	envelope glycoprotein-like	1.146	0.1243	0.5694
625638	Fam43b	family with sequence similarity 43, member B	1.146	0.1983	0.6331
434778	Ccdc160	coiled-coil domain containing 160	1.146	0.1074	0.5499
381813	Prmt8	protein arginine N-methyltransferase 8	1.146	0.0388	NA
279499	Kctd19	potassium channel tetramerisation domain containing 19	1.146	0.3363	0.7305
269233	Fam171a1	family with sequence similarity 171, member A1	1.146	0.0414	NA
239167	D930020E02Rik	RIKEN cDNA D930020E02 gene	1.146	0.3737	0.7543
237926	Rsad1	radical S-adenosyl methionine domain containing 1	1.146	0.219	0.6511
235610	Atrip	ATR interacting protein	1.146	0.2014	0.635
230577	Pars2	prolyl-tRNA synthetase (mitochondrial)(putative)	1.146	0.05185	NA
227800	Rabgap1	RAB GTPase activating protein 1	1.146	0.4991	0.8197
217779	Lysmd1	LysM, putative peptidoglycan-binding, domain containing 1	1.146	0.03903	NA
217149	Cisd3	CDGSH iron sulfur domain 3	1.146	0.4053	0.7717
213208	Il20rb	interleukin 20 receptor beta	1.146	0.3449	0.736
140497	AF251705	cDNA sequence AF251705	1.146	0.0855	0.517
99929	Tiparp	TCDD-inducible poly(ADP-ribose) polymerase	1.146	0.004087	NA
74376	Myo18b	myosin XVIIIb	1.146	0.5206	0.8286
73047	Camk2n2	calcium/calmodulin-dependent protein kinase II inhibitor 2	1.146	0.1308	0.5751
71776	Tha1	threonine aldolase 1	1.146	0.2035	0.6362
69379	C8g	complement component 8, gamma polypeptide	1.146	0.1967	0.6316
68051	Nutf2	nuclear transport factor 2	1.146	0.2074	0.6398
57908	Zfp318	zinc finger protein 318	1.146	0.01792	NA
19038	Ppic	peptidylprolyl isomerase C	1.146	0.1372	0.5807
18441	P2ry1	purinergic receptor P2Y, G-protein coupled 1	1.146	0.1264	0.5705
17388	Mmp15	matrix metallopeptidase 15	1.146	0.2045	0.637
14734	Gpc3	glypican 3	1.146	0.1472	0.591
12870	Cp	ceruloplasmin	1.146	0.1043	0.5444
668178	Gm10035	predicted gene 10035	1.145	0.103	0.5426
619301	G630016D24Rik	RIKEN cDNA G630016D24 gene	1.145	0.2868	0.6982
320709	Tmem117	transmembrane protein 117	1.145	0.05706	NA
245886	Ankrd27	ankyrin repeat domain 27 (VPS9 domain)	1.145	0.08102	0.5105
242864	Napepld	N-acyl phosphatidylethanolamine phospholipase D	1.145	0.04855	NA
233186	Siglec5	sialic acid binding Ig-like lectin 5	1.145	0.2307	0.6601
225895	Taf6l	TAF6-like RNA polymerase II, p300/CBP-associated factor (PCAF)-associated factor	1.145	0.2864	0.6982
223646	Naprt1	nicotinate phosphoribosyltransferase domain containing 1	1.145	0.259	0.6815
218734	3830406C13Rik	RIKEN cDNA 3830406C13 gene	1.145	0.04364	NA
192657	Ell2	elongation factor RNA polymerase II 2	1.145	0.2426	0.6705
110454	Ly6a	lymphocyte antigen 6 complex, locus A	1.145	0.3044	0.7091

76747	Dapl1	death associated protein-like 1	1.145	0.7417	0.918
76260	Ttc8	tetratricopeptide repeat domain 8	1.145	0.03426	NA
72022	Slc35f2	solute carrier family 35, member F2	1.145	0.197	0.632
70382	Kctd2	potassium channel tetramerisation domain containing 2	1.145	0.01314	NA
69716	Trip13	thyroid hormone receptor interactor 13	1.145	0.05509	NA
68961	Phkg2	phosphorylase kinase, gamma 2 (testis)	1.145	0.1976	0.6328
68888	Gkn3	gastrokine 3	1.145	0.1616	0.607
66375	Dhrs7	dehydrogenase/reductase (SDR family) member 7	1.145	0.03004	NA
56760	Clec1b	C-type lectin domain family 1, member b	1.145	0.5082	0.8242
56462	Mtch1	mitochondrial carrier homolog 1 (C. elegans)	1.145	0.1823	0.6207
27801	Zdhhc8	zinc finger, DHHC domain containing 8	1.145	0.3135	0.7152
27355	X99384	cDNA sequence X99384	1.145	0.1469	0.591
24131	Ldb3	LIM domain binding 3	1.145	0.04234	NA
15568	Elavl1	ELAV (embryonic lethal, abnormal vision, Drosophila)-like 1 (Hu antigen R)	1.145	0.1794	0.6187
14683	Gnas	GNAS (guanine nucleotide binding protein, alpha stimulating) complex locus	1.145	0.018	NA
14226	Fkbp1b	FK506 binding protein 1b	1.145	0.09791	0.5361
13829	Epb4.9	erythrocyte protein band 4.9	1.145	0.1101	0.5535
100041677	Gm13157	predicted gene 13157	1.144	0.387	0.761
328424	Kcnrg	potassium channel regulator	1.144	0.4604	0.8014
272381	Lrrc4b	leucine rich repeat containing 4B	1.144	0.2773	0.6925
244723	Olfm2	olfactomedin 2	1.144	0.5598	0.8459
242297	Fam110b	family with sequence similarity 110, member B	1.144	0.1156	0.558
233020	Hipk4	homeodomain interacting protein kinase 4	1.144	0.2605	0.6815
218314	Zfp595	zinc finger protein 595	1.144	0.1683	0.6095
216881	Wscd1	WSC domain containing 1	1.144	0.3356	0.7302
216443	Mars	methionine-tRNA synthetase	1.144	0.0552	NA
208990	Npb	neuropeptide B	1.144	0.1316	0.5758
208117	Aph1b	anterior pharynx defective 1b homolog (C. elegans)	1.144	0.08117	0.5107
80285	Parp9	poly (ADP-ribose) polymerase family, member 9	1.144	0.02049	NA
77889	Lbh	limb-bud and heart	1.144	0.02702	NA
77254	Yif1b	Yip1 interacting factor homolog B (S. cerevisiae)	1.144	0.2323	0.6621
72461	Prcp	prolylcarboxypeptidase (angiotensinase C)	1.144	0.02829	NA
71918	Zcchc24	zinc finger, CCHC domain containing 24	1.144	0.1163	0.558
71704	Arhgef3	Rho guanine nucleotide exchange factor (GEF) 3	1.144	0.121	0.5647
68050	Akirin1	akirin 1	1.144	0.2026	0.6356
67529	Fgfr1op2	FGFR1 oncogene partner 2	1.144	0.4453	0.7938
67177	Cdt1	chromatin licensing and DNA replication factor 1	1.144	0.0959	0.5344
67151	Psmd9	proteasome (prosome, macropain) 26S subunit, non-ATPase, 9	1.144	0.03318	NA
66995	Zcchc18	zinc finger, CCHC domain containing 18	1.144	0.1894	0.6272
65112	Pmepa1	prostate transmembrane protein, androgen induced 1	1.144	0.02579	NA
58998	Pvrl3	poliovirus receptor-related 3	1.144	0.3271	0.7237
57373	D930014E17Rik	RIKEN cDNA D930014E17 gene	1.144	0.1064	0.5471
21350	Tal2	T-cell acute lymphocytic leukemia 2	1.144	0.3851	0.7607
17218	Mcm5	minichromosome maintenance deficient 5, cell division cycle 46 (S. cerevisiae)	1.144	0.1242	0.5693
16969	Zbtb7a	zinc finger and BTB domain containing 7a	1.144	0.1653	0.6077
13170	Dbp	D site albumin promoter binding protein	1.144	0.1582	0.6029
11513	Adcy7	adenylate cyclase 7	1.144	0.1086	0.5521
546071	Mast3	microtubule associated serine/threonine kinase 3	1.143	0.2142	0.6467
320951	Pisd	phosphatidylserine decarboxylase	1.143	0.05598	NA

268420	Alkbh5	alkB, alkylation repair homolog 5 (E. coli)	1.143	0.01065	NA
231946	D330028D13Rik	RIKEN cDNA D330028D13 gene	1.143	0.04309	NA
228993	Slc17a9	solute carrier family 17, member 9	1.143	0.05061	NA
228961	Npepl1	aminopeptidase-like 1	1.143	0.1222	0.5656
217700	Acot6	acyl-CoA thioesterase 6	1.143	0.2606	0.6815
171210	Acot2	acyl-CoA thioesterase 2	1.143	0.37	0.7523
170654	Krtap16-4	keratin associated protein 16-4	1.143	0.4616	0.8021
105511	Fam170b	family with sequence similarity 170, member B	1.143	0.3525	0.7406
100877	AV074028	expressed sequence AV074028	1.143	0.3321	0.7269
78372	Snrnp25	small nuclear ribonucleoprotein 25 (U11/U12)	1.143	0.04062	NA
78353	2500002B13Rik	RIKEN cDNA 2500002B13 gene	1.143	0.46	0.8011
75430	3200002M19Rik	RIKEN cDNA 3200002M19 gene	1.143	0.06534	NA
71971	Zswim1	zinc finger, SWIM domain containing 1	1.143	0.0485	NA
71885	2310003H01Rik	RIKEN cDNA 2310003H01 gene	1.143	0.4179	0.7784
71096	Sntg1	syntrophin, gamma 1	1.143	0.2253	0.654
68453	Gpihbp1	GPI-anchored HDL-binding protein 1	1.143	0.3752	0.7555
67732	Iah1	isoamyl acetate-hydrolyzing esterase 1 homolog (S. cerevisiae)	1.143	0.03284	NA
22153	Tubb4	tubulin, beta 4	1.143	0.2746	0.6924
21818	Tgm3	transglutaminase 3, E polypeptide	1.143	0.5537	0.844
20909	Stx4a	syntaxin 4A (placental)	1.143	0.01978	NA
18604	Pdk2	pyruvate dehydrogenase kinase, isoenzyme 2	1.143	0.4207	0.7798
18595	Pdgfra	platelet derived growth factor receptor, alpha polypeptide	1.143	0.3619	0.7477
15018	H2-Q7	histocompatibility 2, Q region locus 7	1.143	0.2091	0.6409
14119	Fbn2	fibrillin 2	1.143	0.5628	0.8465
13132	Dab2	disabled homolog 2 (Drosophila)	1.143	0.4569	0.7987
12294	Cacna2d3	calcium channel, voltage-dependent, alpha2/delta subunit 3	1.143	0.05	NA
503692	Aym1	activator of yeast meiotic promoters 1	1.142	0.2848	0.6977
381792	2310040G24Rik	RIKEN cDNA 2310040G24 gene	1.142	0.08072	NA
267019	Rps15a	ribosomal protein S15A	1.142	0.1194	0.5628
258818	Olfr629	olfactory receptor 629	1.142	0.2513	0.6767
229658	Vangl1	vang-like 1 (van gogh, Drosophila)	1.142	0.08158	0.5122
104010	Cdh22	cadherin 22	1.142	0.0446	NA
100102	Pcsk9	proprotein convertase subtilisin/kexin type 9	1.142	0.2357	0.6654
93840	Vangl2	vang-like 2 (van gogh, Drosophila)	1.142	0.2833	0.6972
83672	Sytl3	synaptotagmin-like 3	1.142	0.1736	0.6148
77254	Yif1b	Yip1 interacting factor homolog B (S. cerevisiae)	1.142	0.1235	0.568
75394	0610040F04Rik	RIKEN cDNA 0610040F04 gene	1.142	0.05496	NA
73139	Cenpv	centromere protein V	1.142	0.1125	0.5549
70021	Nt5dc2	5'-nucleotidase domain containing 2	1.142	0.292	0.7023
69709	2410017P09Rik	RIKEN cDNA 2410017P09 gene	1.142	0.168	0.6095
67252	Cap2	CAP, adenylate cyclase-associated protein, 2 (yeast)	1.142	0.06415	NA
67235	Zkscan14	zinc finger with KRAB and SCAN domains 14	1.142	0.1165	0.5581
67042	Ift27	intraflagellar transport 27 homolog (Chlamydomonas)	1.142	0.05244	NA
66141	Ifitm3	interferon induced transmembrane protein 3	1.142	0.457	0.7987
56264	Cpxm1	carboxypeptidase X 1 (M14 family)	1.142	0.2604	0.6815
22143	Tuba1b	tubulin, alpha 1B	1.142	0.1352	0.5788
21814	Tgfbr3	transforming growth factor, beta receptor III	1.142	0.1951	0.6304
20681	Sox8	SRY-box containing gene 8	1.142	0.2048	0.6375
17357	Marcksl1	MARCKS-like 1	1.142	0.2019	0.6356

17312	Clec10a	C-type lectin domain family 10, member A	1.142	0.1816	0.6206
17001	Ltc4s	leukotriene C4 synthase	1.142	0.2752	0.6925
16764	Aff3	AF4/FMR2 family, member 3	1.142	0.4842	0.8123
14409	Gabrr2	gamma-aminobutyric acid (GABA) C receptor, subunit rho 2	1.142	0.38	0.7592
13384	Mpp3	membrane protein, palmitoylated 3 (MAGUK p55 subfamily member 3)	1.142	0.03868	NA
12908	Crat	carnitine acetyltransferase	1.142	0.1322	0.5767
12575	Cdkn1a	cyclin-dependent kinase inhibitor 1A (P21)	1.142	0.4093	0.7737
12310	Calca	calcitonin/calcitonin-related polypeptide, alpha	1.142	0.1032	0.5433
100043899	R3hdml	R3H domain containing-like	1.141	0.8483	0.9522
621998	Gm6277	predicted gene 6277	1.141	0.2675	0.6867
433804	Gm13154	predicted gene 13154	1.141	0.6379	0.8776
382111	Susd5	sushi domain containing 5	1.141	0.7072	0.9063
382083	Snx22	sorting nexin 22	1.141	0.375	0.7552
381582	Gm5151	predicted gene 5151	1.141	0.05264	NA
380787	A230065H16Rik	RIKEN cDNA A230065H16 gene	1.141	0.1956	0.6304
338368	Fam109b	family with sequence similarity 109, member B	1.141	0.3723	0.7536
320869	4732415M23Rik	RIKEN cDNA 4732415M23 gene	1.141	0.2242	0.654
230603	Ttc39a	tetratricopeptide repeat domain 39A	1.141	0.2668	0.6863
208990	Npb	neuropeptide B	1.141	0.1294	0.5743
108978	4930555G01Rik	RIKEN cDNA 4930555G01 gene	1.141	0.08848	0.5202
78921	9130019O22Rik	RIKEN cDNA 9130019O22 gene	1.141	0.3864	0.761
76133	6230400D17Rik	RIKEN cDNA 6230400D17 gene	1.141	0.1844	0.6217
73293	Ccdc103	coiled-coil domain containing 103	1.141	0.412	0.7747
72614	Pih1d2	PIH1 domain containing 2	1.141	0.01607	NA
71956	Rnf135	ring finger protein 135	1.141	0.2439	0.6718
71904	Paqr7	progestin and adipoQ receptor family member VII	1.141	0.1049	0.5453
69871	2010007H12Rik	RIKEN cDNA 2010007H12 gene	1.141	0.4518	0.7951
69071	Tmem97	transmembrane protein 97	1.141	0.167	0.6083
68810	Nexn	nexilin	1.141	0.2894	0.7004
67263	Zswim6	zinc finger, SWIM domain containing 6	1.141	0.3967	0.7675
66569	Gdpd1	glycerophosphodiester phosphodiesterase domain containing 1	1.141	0.1206	0.5643
56424	Stub1	STIP1 homology and U-Box containing protein 1	1.141	0.09752	0.5352
54353	Skap2	src family associated phosphoprotein 2	1.141	0.05549	NA
26941	Slc9a3r1	solute carrier family 9 (sodium/hydrogen exchanger), member 3 regulator 1	1.141	0.3712	0.7532
20499	Slc12a7	solute carrier family 12, member 7	1.141	0.2423	0.6704
19339	Rab3a	RAB3A, member RAS oncogene family	1.141	0.0862	0.517
18260	Ocln	occludin	1.141	0.2202	0.6518
17702	Msx2	homeobox, msh-like 2	1.141	0.6788	0.894
17136	Mag	myelin-associated glycoprotein	1.141	0.374	0.7545
16418	Eif6	eukaryotic translation initiation factor 6	1.141	0.1771	0.6165
14528	Gch1	GTP cyclohydrolase 1	1.141	0.2844	0.6977
12266	C3	complement component 3	1.141	0.4412	0.7921
11852	Rhob	ras homolog gene family, member B	1.141	0.5829	0.8538
100073351	Yy2	Yy2 transcription factor	1.14	0.1485	0.592
619441	BC096441	cDNA sequence BC096441	1.14	0.07534	NA
319748	Zfp865	zinc finger protein 865	1.14	0.2157	0.6479
244958	Mrap2	melanocortin 2 receptor accessory protein 2	1.14	0.2742	0.692
234582	Ccdc102a	coiled-coil domain containing 102A	1.14	0.203	0.6356
230775	Bai2	brain-specific angiogenesis inhibitor 2	1.14	0.2629	0.6838

230767	Iqcc	IQ motif containing C	1.14	0.155	0.5975
209601	4922501L14Rik	RIKEN cDNA 4922501L14 gene	1.14	0.1395	0.5834
118452	Baalc	brain and acute leukemia, cytoplasmic	1.14	0.2241	0.654
108147	Atic	5-aminoimidazole-4-carboxamide ribonucleotide formyltransferase/IMP cyclohydrolase	1.14	0.1259	0.5703
107976	Bre	brain and reproductive organ-expressed protein	1.14	0.2926	0.7024
101602	AI467606	expressed sequence AI467606	1.14	0.3227	0.7202
67399	Pdlim7	PDZ and LIM domain 7	1.14	0.2983	0.7052
66793	Efcab1	EF hand calcium binding domain 1	1.14	0.5362	0.8351
56356	Gltp	glycolipid transfer protein	1.14	0.2759	0.6925
50529	Mrps7	mitchondrial ribosomal protein S7	1.14	0.07869	NA
22174	Tyro3	TYRO3 protein tyrosine kinase 3	1.14	0.04615	NA
22062	Trp73	transformation related protein 73	1.14	0.4805	0.8101
20516	Slc20a2	solute carrier family 20, member 2	1.14	0.1884	0.6263
19334	Rab22a	RAB22A, member RAS oncogene family	1.14	0.2319	0.6618
17319	Mif	macrophage migration inhibitory factor	1.14	0.07835	NA
17164	Mapkapk2	MAP kinase-activated protein kinase 2	1.14	0.1876	0.6259
16419	Itgb5	integrin beta 5	1.14	0.2321	0.662
15959	Ifit3	interferon-induced protein with tetratricopeptide repeats 3	1.14	0.1218	0.5652
15529	Sdc2	syndecan 2	1.14	0.02801	NA
15458	Hpx	hemopexin	1.14	0.3976	0.7683
14998	H2-DMa	histocompatibility 2, class II, locus DMa	1.14	0.276	0.6925
14871	Gstt1	glutathione S-transferase, theta 1	1.14	0.07885	NA
14472	Gbx2	gastrulation brain homeobox 2	1.14	0.3189	0.7183
14466	Gba	glucosidase, beta, acid	1.14	0.1075	0.5499
12822	Col18a1	collagen, type XVIII, alpha 1	1.14	0.6205	0.8718
12057	Opn1sw	opsin 1 (cone pigments), short-wave-sensitive (color blindness, tritan)	1.14	0.05313	NA
11363	Acadl	acyl-Coenzyme A dehydrogenase, long-chain	1.14	0.01818	NA
100048345	LOC100048345	hypothetical protein LOC100048345	1.139	0.1723	0.6133
100043474	Gm4461	predicted gene 4461	1.139	0.4415	0.7921
664787	LOC664787	similar to Sp110 nuclear body protein	1.139	0.6705	0.8912
235584	Dusp7	dual specificity phosphatase 7	1.139	0.2645	0.6845
233824	Cog7	component of oligomeric golgi complex 7	1.139	0.01745	NA
212111	Inpp5a	inositol polyphosphate-5-phosphatase A	1.139	0.02955	NA
109552	Sri	sorcin	1.139	0.1184	0.5612
100669	9930105H17Rik	RIKEN cDNA 9930105H17 gene	1.139	0.1954	0.6304
71870	Ccdc19	coiled-coil domain containing 19	1.139	0.1733	0.6147
71637	4930413F20Rik	RIKEN cDNA 4930413F20 gene	1.139	0.351	0.7393
69024	Snx15	sorting nexin 15	1.139	0.2424	0.6704
68947	Chst8	carbohydrate (N-acetylgalactosamine 4-0) sulfotransferase 8	1.139	0.1996	0.6343
68082	Dusp19	dual specificity phosphatase 19	1.139	0.3411	0.7334
67729	Mansc1	MANSC domain containing 1	1.139	0.3406	0.7328
67623	Tm7sf3	transmembrane 7 superfamily member 3	1.139	0.4036	0.771
30930	Vps26a	vacuolar protein sorting 26 homolog A (yeast)	1.139	0.1295	0.5745
20359	Sema6b	sema domain, transmembrane domain (TM), and cytoplasmic domain, (semaphorin) 6B	1.139	0.3606	0.7467
20300	Ccl25	chemokine (C-C motif) ligand 25	1.139	0.6081	0.8655
19344	Rab5b	RAB5B, member RAS oncogene family	1.139	0.3012	0.7073
19279	Ptprr	protein tyrosine phosphatase, receptor type, R	1.139	0.0621	NA
18378	Omp	olfactory marker protein	1.139	0.5814	0.8529
18127	Nos3	nitric oxide synthase 3, endothelial cell	1.139	0.04603	NA

17215	Mcm3	minichromosome maintenance deficient 3 (S. cerevisiae)	1.139	0.008234	NA
14872	Gstt2	glutathione S-transferase, theta 2	1.139	0.1009	0.5394
14198	Fhit	fragile histidine triad gene	1.139	0.08759	NA
13824	Epb4.1l4a	erythrocyte protein band 4.1-like 4a	1.139	0.4428	0.7928
13478	Dpagt1	dolichyl-phosphate (UDP-N-acetylglucosamine) acetylglucosaminephosphotransferase 1 (GlcNAc-1-P transferase)	1.139	0.09346	0.5294
11807	Apoa2	apolipoprotein A-II	1.139	0.3369	0.7307
11517	Adcyap1r1	adenylate cyclase activating polypeptide 1 receptor 1	1.139	0.3003	0.706
319613	Sybu	syntabulin (syntaxin-interacting)	1.138	0.4688	0.8059
244238	Mrgpre	MAS-related GPR, member E	1.138	0.2759	0.6925
242800	Ttc34	tetratricopeptide repeat domain 34	1.138	0.2686	0.6876
240725	Sulf1	sulfatase 1	1.138	0.02453	NA
234404	Nxnl1	nucleoredoxin-like 1	1.138	0.2248	0.654
234384	Mpv17l2	MPV17 mitochondrial membrane protein-like 2	1.138	0.3165	0.7174
233075	Gm4883	predicted gene 4883	1.138	0.1463	0.591
219189	1300010F03Rik	RIKEN cDNA 1300010F03 gene	1.138	0.1688	0.6096
214779	Zfp879	zinc figer protein 879	1.138	0.07258	NA
207740	Fam100a	family with sequence similarity 100, member A	1.138	0.3937	0.7656
171095	Il17rc	interleukin 17 receptor C	1.138	0.5807	0.8527
72446	Prr5l	proline rich 5 like	1.138	0.2416	0.6694
72106	Jmjd8	jumonji domain containing 8	1.138	0.2558	0.68
71213	Cage1	cancer antigen 1	1.138	0.1725	0.6135
67683	2610029G23Rik	RIKEN cDNA 2610029G23 gene	1.138	0.05191	NA
59126	Nek6	NIMA (never in mitosis gene a)-related expressed kinase 6	1.138	0.09053	NA
57355	BC051019	cDNA sequence BC051019	1.138	0.479	0.81
56473	Fads2	fatty acid desaturase 2	1.138	0.07067	NA
53404	Atoh7	atonal homolog 7 (Drosophila)	1.138	0.4458	0.7938
22637	Zap70	zeta-chain (TCR) associated protein kinase	1.138	0.1622	0.607
20664	Sox1	SRY-box containing gene 1	1.138	0.7648	0.9251
20193	S100a1	S100 calcium binding protein A1	1.138	0.2309	0.6601
18607	Pdpk1	3-phosphoinositide dependent protein kinase 1	1.138	0.2448	0.6726
13497	Drp2	dystrophin related protein 2	1.138	0.207	0.6395
12453	Ccni	cyclin I	1.138	0.04244	NA
12406	Serpinh1	serine (or cysteine) peptidase inhibitor, clade H, member 1	1.138	0.407	0.7726
258851	Olfr1339	olfactory receptor 1339	1.137	0.3569	0.7443
233067	Lrfn3	leucine rich repeat and fibronectin type III domain containing 3	1.137	0.2124	0.6449
229731	Slc25a24	solute carrier family 25 (mitochondrial carrier, phosphate carrier), member 24	1.137	0.1786	0.6181
213121	Ankrd35	ankyrin repeat domain 35	1.137	0.1473	0.591
113849	Vmn1r52	vomeronasal 1 receptor 52	1.137	0.5198	0.8284
109077	Ints5	integrator complex subunit 5	1.137	0.1462	0.591
107503	Atf5	activating transcription factor 5	1.137	0.08032	NA
106512	Gpsm3	G-protein signalling modulator 3 (AGS3-like, C. elegans)	1.137	0.2249	0.654
80752	Fam20c	family with sequence similarity 20, member C	1.137	0.2177	0.6499
70957	4921530L18Rik	RIKEN cDNA 4921530L18 gene	1.137	0.2681	0.6871
70788	Klhl30	kelch-like 30 (Drosophila)	1.137	0.1905	0.6275
70673	Prdm16	PR domain containing 16	1.137	0.1745	0.6148
69732	2410018L13Rik	RIKEN cDNA 2410018L13 gene	1.137	0.159	0.6036
63958	Ube4b	ubiquitination factor E4B, UFD2 homolog (S. cerevisiae)	1.137	0.2051	0.638
18617	Rhox5	reproductive homeobox 5	1.137	0.1388	0.5829
16828	Ldha	lactate dehydrogenase A	1.137	0.08663	NA

16773	Lama2	laminin, alpha 2	1.137	0.6466	0.8812
14726	Pdpn	podoplanin	1.137	0.1942	0.6299
14198	Fhit	fragile histidine triad gene	1.137	0.2344	0.6636
11744	Anxa11	annexin A11	1.137	0.3747	0.755
100505195	LOC100505195	hypothetical LOC100505195	1.136	0.5212	0.8286
665563	Mthfd2l	methylenetetrahydrofolate dehydrogenase (NADP+ dependent) 2-like	1.136	0.05831	NA
664862	Gpr137b-ps	G protein-coupled receptor 137B, pseudogene	1.136	0.4344	0.7878
629557	Gm6981	glyceraldehyde-3-phosphate dehydrogenase pseudogene	1.136	0.1955	0.6304
319899	Dock6	dedicator of cytokinesis 6	1.136	0.1566	0.6003
234358	Zfp930	zinc finger protein 930	1.136	0.344	0.7357
230584	Yipf1	Yip1 domain family, member 1	1.136	0.05179	NA
226422	Rab7l1	RAB7, member RAS oncogene family-like 1	1.136	0.112	0.5549
223626	4930572J05Rik	RIKEN cDNA 4930572J05 gene	1.136	0.5454	0.839
216001	Cbara1	calcium binding atopy-related autoantigen 1	1.136	0.09876	0.5367
214523	Tmprss4	transmembrane protease, serine 4	1.136	0.05979	NA
107375	Slc25a45	solute carrier family 25, member 45	1.136	0.4035	0.7708
106073	Mfsd5	major facilitator superfamily domain containing 5	1.136	0.1538	0.5975
100317	AU040320	expressed sequence AU040320	1.136	0.406	0.7721
83395	Sp6	trans-acting transcription factor 6	1.136	0.5797	0.8524
76560	Prss8	protease, serine, 8 (prostasin)	1.136	0.4708	0.8076
73598	1700001O22Rik	RIKEN cDNA 1700001O22 gene	1.136	0.3279	0.7248
69993	Chn2	chimerin (chimaerin) 2	1.136	0.1344	0.5779
69863	Ttc39b	tetratricopeptide repeat domain 39B	1.136	0.6284	0.8744
69291	1700001L05Rik	RIKEN cDNA 1700001L05 gene	1.136	0.06087	NA
68597	1110021J02Rik	RIKEN cDNA 1110021J02 gene	1.136	0.1969	0.632
57908	Zfp318	zinc finger protein 318	1.136	0.03478	NA
52858	Cdipt	CDP-diacylglycerol--inositol 3-phosphatidyltransferase (phosphatidylinositol synthase)	1.136	0.02207	NA
27029	Sgsh	N-sulfoglucosamine sulfohydrolase (sulfamidase)	1.136	0.4537	0.797
26943	Serinc3	serine incorporator 3	1.136	0.055	NA
22352	Vim	vimentin	1.136	0.4977	0.8193
20439	Siah2	seven in absentia 2	1.136	0.01872	NA
20295	Ccl17	chemokine (C-C motif) ligand 17	1.136	0.4567	0.7987
19298	Pex19	peroxisomal biogenesis factor 19	1.136	0.2145	0.6467
18302	Oit3	oncoprotein induced transcript 3	1.136	0.08231	NA
18036	Nfkbib	nuclear factor of kappa light polypeptide gene enhancer in B-cells inhibitor, beta	1.136	0.2042	0.6369
16669	Krt19	keratin 19	1.136	0.6495	0.8822
15275	Hk1	hexokinase 1	1.136	0.3666	0.7504
13644	Efs	embryonal Fyn-associated substrate	1.136	0.2551	0.6796
13489	Drd2	dopamine receptor D2	1.136	0.2861	0.6982
319622	Itpripl2	inositol 1,4,5-triphosphate receptor interacting protein-like 2	1.135	0.08152	NA
277414	Trp53i11	transformation related protein 53 inducible protein 11	1.135	0.2065	0.6392
268816	Gm628	predicted gene 628	1.135	0.434	0.7875
239552	Apol8	apolipoprotein L 8	1.135	0.05049	NA
226139	Cox15	COX15 homolog, cytochrome c oxidase assembly protein (yeast)	1.135	0.2921	0.7024
207212	Arhgef17	Rho guanine nucleotide exchange factor (GEF) 17	1.135	0.03566	NA
192986	Cyb5d2	cytochrome b5 domain containing 2	1.135	0.1769	0.6164
108699	Chn1	chimerin (chimaerin) 1	1.135	0.04378	NA
104156	Etv5	ets variant gene 5	1.135	0.1945	0.6303
94214	Spock2	sparc/osteonectin, cwcv and kazal-like domains proteoglycan 2	1.135	0.145	0.5896

77035	Jmjd5	jumonji domain containing 5	1.135	0.08872	NA
76294	Asb5	ankyrin repeat and SOCs box-containing 5	1.135	0.1558	0.599
75552	Paqr9	progestin and adipoQ receptor family member IX	1.135	0.2231	0.654
74729	Setmar	SET domain and mariner transposase fusion gene	1.135	0.1778	0.6168
74015	Fcho1	FCH domain only 1	1.135	0.154	0.5975
71994	Cnn3	calponin 3, acidic	1.135	0.06168	NA
69890	Zfp219	zinc finger protein 219	1.135	0.4564	0.7987
69710	Arap1	ArfGAP with RhoGAP domain, ankyrin repeat and PH domain 1	1.135	0.311	0.7135
69572	Mfsd3	major facilitator superfamily domain containing 3	1.135	0.1182	0.5609
66958	Tmx2	thioredoxin-related transmembrane protein 2	1.135	0.218	0.6502
66011	Ranbp17	RAN binding protein 17	1.135	0.3803	0.7594
64378	Gpr88	G-protein coupled receptor 88	1.135	0.0971	NA
21925	Tnnc2	troponin C2, fast	1.135	0.07814	NA
18549	Pcsk2	proprotein convertase subtilisin/kexin type 2	1.135	0.3109	0.7135
17698	Msn	moesin	1.135	0.1052	0.5455
17304	Mfge8	milk fat globule-EGF factor 8 protein	1.135	0.1119	0.5549
16869	Lhx1	LIM homeobox protein 1	1.135	0.2243	0.654
16491	Kcna3	potassium voltage-gated channel, shaker-related subfamily, member 3	1.135	0.2008	0.6345
16470	Ush1g	Usher syndrome 1G homolog (human)	1.135	0.1518	0.5949
14860	Gsta4	glutathione S-transferase, alpha 4	1.135	0.1081	0.5513
14544	Gda	guanine deaminase	1.135	0.2226	0.654
14055	Ezh1	enhancer of zeste homolog 1 (Drosophila)	1.135	0.3995	0.7699
13197	Gadd45a	growth arrest and DNA-damage-inducible 45 alpha	1.135	0.06066	NA
12796	Camp	cathelicidin antimicrobial peptide	1.135	0.2758	0.6925
12531	Cdc25b	cell division cycle 25 homolog B (S. pombe)	1.135	0.04949	NA
12228	Btg3	B-cell translocation gene 3	1.135	0.04515	NA
11749	Anxa6	annexin A6	1.135	0.4076	0.7726
100042198	Gm3716	predicted gene 3716	1.134	0.4017	0.7703
320707	Atp2b3	ATPase, Ca++ transporting, plasma membrane 3	1.134	0.2847	0.6977
320616	B130006D01Rik	RIKEN cDNA B130006D01 gene	1.134	0.6457	0.8809
319493	A430078G23Rik	RIKEN cDNA A430078G23 gene	1.134	0.1044	0.5444
242050	Igsf10	immunoglobulin superfamily, member 10	1.134	0.2753	0.6925
229801	Tram1l1	translocation associated membrane protein 1-like 1	1.134	0.04997	NA
224523	4732491K20Rik	RIKEN cDNA 4732491K20 gene	1.134	0.07378	NA
93742	Pard3	par-3 (partitioning defective 3) homolog (C. elegans)	1.134	0.1539	0.5975
83672	Sytl3	synaptotagmin-like 3	1.134	0.4797	0.8101
74424	Tmc5	transmembrane channel-like gene family 5	1.134	0.6884	0.8976
67487	Dhx40	DEAH (Asp-Glu-Ala-His) box polypeptide 40	1.134	0.1547	0.5975
66171	Pgls	6-phosphogluconolactonase	1.134	0.1619	0.607
66158	Cxx1a	CAAX box 1 homolog A (human)	1.134	0.3702	0.7525
56405	Dusp14	dual specificity phosphatase 14	1.134	0.05806	NA
28075	Pppde2	PPPDE peptidase domain containing 2	1.134	0.1635	0.6074
22264	Prap1	proline-rich acidic protein 1	1.134	0.1292	0.5741
21767	Tex264	testis expressed gene 264	1.134	0.1038	NA
20467	Sin3b	transcriptional regulator, SIN3B (yeast)	1.134	0.04921	NA
19246	Ptpn1	protein tyrosine phosphatase, non-receptor type 1	1.134	0.05437	NA
15007	H2-Q10	histocompatibility 2, Q region locus 10	1.134	0.2795	0.6949
14296	Frat1	frequently rearranged in advanced T-cell lymphomas	1.134	0.1401	0.5834
12609	Cebpd	CCAAT/enhancer binding protein (C/EBP), delta	1.134	0.04786	NA

12181	Bop1	block of proliferation 1	1.134	0.3289	0.7253
100233208	Gm10778	predicted gene 10778	1.133	0.06665	NA
320127	Dgki	diacylglycerol kinase, iota	1.133	0.2888	0.6998
286942	Kif19a	kinesin family member 19A	1.133	0.3927	0.7651
278097	Armcx6	armadillo repeat containing, X-linked 6	1.133	0.1864	0.6239
269784	Cntn4	contactin 4	1.133	0.09144	NA
269608	Plekhg5	pleckstrin homology domain containing, family G (with RhoGef domain) member 5	1.133	0.3576	0.7451
213417	Klhdc8a	kelch domain containing 8A	1.133	0.1741	0.6148
212996	Wbscr17	Williams-Beuren syndrome chromosome region 17 homolog (human)	1.133	0.1505	0.5938
170571	Cntnap4	contactin associated protein-like 4	1.133	0.3004	0.7062
107242	AI837181	expressed sequence AI837181	1.133	0.4069	0.7726
93960	Nkd1	naked cuticle 1 homolog (Drosophila)	1.133	0.3928	0.7651
84111	Gpr87	G protein-coupled receptor 87	1.133	0.6406	0.8788
78365	1500016L03Rik	RIKEN cDNA 1500016L03 gene	1.133	0.4712	0.8076
76025	Cant1	calcium activated nucleotidase 1	1.133	0.1148	0.5569
74629	4930426L09Rik	RIKEN cDNA 4930426L09 gene	1.133	0.3954	0.7669
74123	Foxp4	forkhead box P4	1.133	0.2236	0.654
73736	Fcf1	FCF1 small subunit (SSU) processome component homolog (S. cerevisiae)	1.133	0.06163	NA
73420	1700054N08Rik	RIKEN cDNA 1700054N08 gene	1.133	0.09054	NA
72729	Cdc42se2	CDC42 small effector 2	1.133	0.06313	NA
67063	2810432L12Rik	RIKEN cDNA 2810432L12 gene	1.133	0.01494	NA
66839	0610009O20Rik	RIKEN cDNA 0610009O20 gene	1.133	0.1296	0.5745
66294	Fam3a	family with sequence similarity 3, member A	1.133	0.06167	NA
63954	Rbp7	retinol binding protein 7, cellular	1.133	0.1103	0.5537
54393	Gabbr1	gamma-aminobutyric acid (GABA) B receptor, 1	1.133	0.06596	NA
53871	Pkd2l2	polycystic kidney disease 2-like 2	1.133	0.3091	0.7126
52502	Carhsp1	calcium regulated heat stable protein 1	1.133	0.6534	0.8848
50917	Galns	galactosamine (N-acetyl)-6-sulfate sulfatase	1.133	0.3704	0.7525
22433	Xbp1	X-box binding protein 1	1.133	0.1216	0.5651
22380	Wbp4	WW domain binding protein 4	1.133	0.01556	NA
21462	Tcp10c	t-complex protein 10c	1.133	0.4007	0.7703
21393	Tcap	titin-cap	1.133	0.1619	0.607
20917	Suclg2	succinate-Coenzyme A ligase, GDP-forming, beta subunit	1.133	0.3084	0.7115
19345	Rab5c	RAB5C, member RAS oncogene family	1.133	0.051	NA
17385	Mmp11	matrix metallopeptidase 11	1.133	0.3731	0.7538
16453	Jak3	Janus kinase 3	1.133	0.692	0.8991
15560	Htr2c	5-hydroxytryptamine (serotonin) receptor 2C	1.133	0.1186	0.5618
14841	Gsg2	germ cell-specific gene 2	1.133	0.4504	0.7945
14788	Gpr162	G protein-coupled receptor 162	1.133	0.2988	0.7052
14704	Gng3	guanine nucleotide binding protein (G protein), gamma 3	1.133	0.3037	0.709
14681	Gnao1	guanine nucleotide binding protein, alpha O	1.133	0.151	0.5942
14537	Gcnt1	glucosaminyl (N-acetyl) transferase 1, core 2	1.133	0.2359	0.6657
13200	Ddost	dolichyl-di-phosphooligosaccharide-protein glycotransferase	1.133	0.203	0.6356
13007	Csrp1	cysteine and glycine-rich protein 1	1.133	0.02356	NA
12017	Bag1	BCL2-associated athanogene 1	1.133	0.05228	NA
100043585	Gm4535	predicted gene 4535	1.132	0.02506	NA
381338	Lonrf2	LON peptidase N-terminal domain and ring finger 2	1.132	0.2857	0.6982
245884	Fam71f2	family with sequence similarity 71, member F2	1.132	0.2482	0.6755
233877	Kctd13	potassium channel tetramerisation domain containing 13	1.132	0.395	0.7666

231672	Fbxw8	F-box and WD-40 domain protein 8	1.132	0.3496	0.7388
228875	Slc35c2	solute carrier family 35, member C2	1.132	0.3218	0.72
225896	Ubxn1	UBX domain protein 1	1.132	0.07401	NA
217353	Tmc6	transmembrane channel-like gene family 6	1.132	0.3509	0.7393
214058	Megf11	multiple EGF-like-domains 11	1.132	0.2722	0.6895
170460	Stard5	StAR-related lipid transfer (START) domain containing 5	1.132	0.4294	0.7843
110006	Gusb	glucuronidase, beta	1.132	0.07755	NA
99543	Olfml3	olfactomedin-like 3	1.132	0.5504	0.8423
94315	Prcc	papillary renal cell carcinoma (translocation-associated)	1.132	0.2254	0.654
68992	Zfp580	zinc finger protein 580	1.132	0.3684	0.752
68859	1190007F08Rik	RIKEN cDNA 1190007F08 gene	1.132	0.422	0.7801
68770	Phtf2	putative homeodomain transcription factor 2	1.132	0.5383	0.8362
68566	Caly	calcyon neuron-specific vesicular protein	1.132	0.3452	0.736
68027	Tmem178	transmembrane protein 178	1.132	0.05136	NA
67871	Mrrf	mitochondrial ribosome recycling factor	1.132	0.2312	0.6607
66610	Abi3	ABI gene family, member 3	1.132	0.4921	0.8157
56745	C1qtnf1	C1q and tumor necrosis factor related protein 1	1.132	0.4434	0.7931
56177	Olfm1	olfactomedin 1	1.132	0.08251	NA
55943	Stx8	syntaxin 8	1.132	0.0358	NA
52897	Rbfox3	RNA binding protein, fox-1 homolog (C. elegans) 3	1.132	0.4989	0.8197
52808	Tspyl2	TSPY-like 2	1.132	0.3571	0.7444
52585	Dhrs1	dehydrogenase/reductase (SDR family) member 1	1.132	0.1195	0.5628
50776	Polg2	polymerase (DNA directed), gamma 2, accessory subunit	1.132	0.1916	0.6278
30050	Fbxw2	F-box and WD-40 domain protein 2	1.132	0.07693	NA
29857	Mapk12	mitogen-activated protein kinase 12	1.132	0.1635	0.6074
27364	Srr	serine racemase	1.132	0.08238	NA
26949	Vat1	vesicle amine transport protein 1 homolog (T californica)	1.132	0.3792	0.7583
21418	Tcfap2a	transcription factor AP-2, alpha	1.132	0.06912	NA
20498	Slc12a4	solute carrier family 12, member 4	1.132	0.2199	0.6515
20440	St6gal1	beta galactoside alpha 2,6 sialyltransferase 1	1.132	0.05685	NA
19210	Ptdss1	phosphatidylserine synthase 1	1.132	0.1798	0.6187
17330	Minpp1	multiple inositol polyphosphate histidine phosphatase 1	1.132	0.1163	0.558
15212	Hexb	hexosaminidase B	1.132	0.06001	NA
14793	Cdca3	cell division cycle associated 3	1.132	0.1627	0.6073
13144	Dapk3	death-associated protein kinase 3	1.132	0.4469	0.7938
12870	Cp	ceruloplasmin	1.132	0.05138	NA
11418	Accn1	amiloride-sensitive cation channel 1, neuronal (degenerin)	1.132	0.1267	0.5709
626231	Gm6658	predicted gene 6658	1.131	0.6062	0.8645
432713	Gm5441	predicted gene 5441	1.131	0.2904	0.7006
320469	9930014A18Rik	RIKEN cDNA 9930014A18 gene	1.131	0.2804	0.6954
269338	Vps39	vacuolar protein sorting 39 (yeast)	1.131	0.2155	0.6479
243867	Fbxo46	F-box protein 46	1.131	0.06086	NA
239570	Ttc38	tetratricopeptide repeat domain 38	1.131	0.2246	0.654
239017	Ogdhl	oxoglutarate dehydrogenase-like	1.131	0.38	0.7592
231672	Fbxw8	F-box and WD-40 domain protein 8	1.131	0.388	0.7614
230654	Lrrc41	leucine rich repeat containing 41	1.131	0.4922	0.8157
217344	Rhbdf2	rhomboid 5 homolog 2 (Drosophila)	1.131	0.1703	0.6115
216560	AV249152	expressed sequence AV249152	1.131	0.1239	0.5685
215001	Wfikkn1	WAP, FS, Ig, KU, and NTR-containing protein 1	1.131	0.3178	0.7179

76252	Atp6v0e2	ATPase, H+ transporting, lysosomal V0 subunit E2	1.131	0.05732	NA
75541	1700019G17Rik	RIKEN cDNA 1700019G17 gene	1.131	0.1017	NA
74530	9030612E09Rik	RIKEN cDNA 9030612E09 gene	1.131	0.488	0.8138
72123	2010109K11Rik	RIKEN cDNA 2010109K11 gene	1.131	0.04375	NA
69900	Mfsd11	major facilitator superfamily domain containing 11	1.131	0.05465	NA
66214	1190002H23Rik	RIKEN cDNA 1190002H23 gene	1.131	0.00843	NA
28018	Ubfd1	ubiquitin family domain containing 1	1.131	0.05366	NA
26417	Mapk3	mitogen-activated protein kinase 3	1.131	0.1561	0.5991
21407	Tcf15	transcription factor 15	1.131	0.1302	0.5747
20319	Sfrp2	secreted frizzled-related protein 2	1.131	0.4453	0.7938
19739	Rgs9	regulator of G-protein signaling 9	1.131	0.2236	0.654
17855	Mvk	mevalonate kinase	1.131	0.2714	0.6891
17775	Laptm4a	lysosomal-associated protein transmembrane 4A	1.131	0.2474	0.6745
16532	Kcnu1	potassium channel, subfamily U, member 1	1.131	0.2014	0.635
14566	Gdf9	growth differentiation factor 9	1.131	0.278	0.693
14226	Fkbp1b	FK506 binding protein 1b	1.131	0.1394	0.5834
12039	Bckdha	branched chain ketoacid dehydrogenase E1, alpha polypeptide	1.131	0.226	0.6545
12007	Azgp1	alpha-2-glycoprotein 1, zinc	1.131	0.3579	0.7451
100043074	Gm4211	predicted gene 4211	1.13	0.1636	0.6074
100038347	Fam174b	family with sequence similarity 174, member B	1.13	0.03606	NA
234076	Tmco3	transmembrane and coiled-coil domains 3	1.13	0.06759	NA
233724	Tmem41b	transmembrane protein 41B	1.13	0.06208	NA
225020	Fez2	fasciculation and elongation protein zeta 2 (zygin II)	1.13	0.04679	NA
224055	Rtp2	receptor transporter protein 2	1.13	0.7109	0.907
217682	3830431G21Rik	RIKEN cDNA 3830431G21 gene	1.13	0.03568	NA
214489	BC003965	cDNA sequence BC003965	1.13	0.1256	0.5703
97458	C80012	expressed sequence C80012	1.13	0.2469	0.674
93734	Mpv17l	Mpv17 transgene, kidney disease mutant-like	1.13	0.1993	0.6343
78388	Mvp	major vault protein	1.13	0.235	0.665
74666	4930432K21Rik	RIKEN cDNA 4930432K21 gene	1.13	0.1652	0.6077
72899	Macrod2	MACRO domain containing 2	1.13	0.0323	NA
72404	Wdr44	WD repeat domain 44	1.13	0.1858	0.623
71923	2310047M10Rik	RIKEN cDNA 2310047M10 gene	1.13	0.1772	0.6166
71133	4933422A05Rik	RIKEN cDNA 4933422A05 gene	1.13	0.3409	0.7333
70101	Cyp4f16	cytochrome P450, family 4, subfamily f, polypeptide 16	1.13	0.2591	0.6815
68166	Spire1	spire homolog 1 (Drosophila)	1.13	0.08216	NA
67446	Dusp28	dual specificity phosphatase 28	1.13	0.0244	NA
56700	0610031J06Rik	RIKEN cDNA 0610031J06 gene	1.13	0.283	0.697
54218	B3galt4	UDP-Gal:betaGlcNAc beta 1,3-galactosyltransferase, polypeptide 4	1.13	0.3148	0.7159
52897	Rbfox3	RNA binding protein, fox-1 homolog (C. elegans) 3	1.13	0.4208	0.7798
50797	Copb2	coatomer protein complex, subunit beta 2 (beta prime)	1.13	0.05613	NA
50768	Dlc1	deleted in liver cancer 1	1.13	0.0602	NA
29858	Pmm1	phosphomannomutase 1	1.13	0.1756	0.6156
20527	Slc2a3	solute carrier family 2 (facilitated glucose transporter), member 3	1.13	0.2412	0.669
20345	Selplg	selectin, platelet (p-selectin) ligand	1.13	0.2601	0.6815
20308	Ccl9	chemokine (C-C motif) ligand 9	1.13	0.2501	0.6767
20190	Ryr1	ryanodine receptor 1, skeletal muscle	1.13	0.3081	0.7112
18709	Pik3r2	phosphatidylinositol 3-kinase, regulatory subunit, polypeptide 2 (p85 beta)	1.13	0.3693	0.7521
18221	Nudc	nuclear distribution gene C homolog (Aspergillus)	1.13	0.06957	NA

18030	Nfil3	nuclear factor, interleukin 3, regulated	1.13	0.04685	NA
17436	Me1	malic enzyme 1, NADP(+)-dependent, cytosolic	1.13	0.0262	NA
17390	Mmp2	matrix metallopeptidase 2	1.13	0.1881	0.6261
17183	Matn4	matrilin 4	1.13	0.1174	0.5606
14415	Gad1	glutamic acid decarboxylase 1	1.13	0.06912	NA
13830	Stom	stomatin	1.13	0.4503	0.7945
11853	Rhoc	ras homolog gene family, member C	1.13	0.1358	0.5794
666704	Samd1	sterile alpha motif domain containing 1	1.129	0.164	0.6075
433375	Creg1	cellular repressor of E1A-stimulated genes 1	1.129	0.1303	0.5747
319953	Ttll1	tubulin tyrosine ligase-like 1	1.129	0.369	0.7521
319477	6030419C18Rik	RIKEN cDNA 6030419C18 gene	1.129	0.2533	0.6778
246694	Hps5	Hermansky-Pudlak syndrome 5 homolog (human)	1.129	0.6478	0.8816
242721	Klhdc7a	kelch domain containing 7A	1.129	0.6401	0.8785
230935	Dnajc11	DnaJ (Hsp40) homolog, subfamily C, member 11	1.129	0.1934	0.6295
229759	Olfm3	olfactomedin 3	1.129	0.07115	NA
226970	Arhgef4	Rho guanine nucleotide exchange factor (GEF) 4	1.129	0.06848	NA
218820	Zfp503	zinc finger protein 503	1.129	0.2547	0.6792
213262	Fstl5	follistatin-like 5	1.129	0.06692	NA
140557	Smc1b	structural maintenance of chromosomes 1B	1.129	0.3875	0.7611
110835	Chrna5	cholinergic receptor, nicotinic, alpha polypeptide 5	1.129	0.1243	0.5694
110751	Adam33	a disintegrin and metallopeptidase domain 33	1.129	0.5284	0.8332
110351	Rap1gap	Rap1 GTPase-activating protein	1.129	0.06842	NA
97848	Serpinb6c	serine (or cysteine) peptidase inhibitor, clade B, member 6c	1.129	0.476	0.8097
75202	Ncrna00085	non-protein coding RNA 85	1.129	0.2981	0.7051
74589	Kbtbd12	kelch repeat and BTB (POZ) domain containing 12	1.129	0.3822	0.7596
69094	Tmem160	transmembrane protein 160	1.129	0.05501	NA
68067	3010026O09Rik	RIKEN cDNA 3010026O09 gene	1.129	0.09089	NA
67305	Gpx7	glutathione peroxidase 7	1.129	0.2751	0.6925
66797	Cntnap2	contactin associated protein-like 2	1.129	0.05377	NA
60406	Sap30	sin3 associated polypeptide	1.129	0.3162	0.7171
54006	Deaf1	deformed epidermal autoregulatory factor 1 (Drosophila)	1.129	0.08042	NA
52815	Ldhd	lactate dehydrogenase D	1.129	0.1727	0.6136
50760	Fbxo17	F-box protein 17	1.129	0.2235	0.654
26564	Ror2	receptor tyrosine kinase-like orphan receptor 2	1.129	0.4499	0.7943
22754	Zfp92	zinc finger protein 92	1.129	0.1511	0.5942
22390	Wee1	WEE 1 homolog 1 (S. pombe)	1.129	0.1428	0.5879
22340	Vegfb	vascular endothelial growth factor B	1.129	0.1423	0.5871
21770	Ppp2r5d	protein phosphatase 2, regulatory subunit B (B56), delta isoform	1.129	0.4096	0.7739
20810	Srm	spermidine synthase	1.129	0.283	0.697
19698	Relb	avian reticuloendotheliosis viral (v-rel) oncogene related B	1.129	0.2621	0.6831
18107	Nmt1	N-myristoyltransferase 1	1.129	0.08326	NA
16427	Itih4	inter alpha-trypsin inhibitor, heavy chain 4	1.129	0.2459	0.6733
13168	Dbil5	diazepam binding inhibitor-like 5	1.129	0.126	0.5703
11737	Anp32a	acidic (leucine-rich) nuclear phosphoprotein 32 family, member A	1.129	0.02615	NA
100043407	Gm4419	predicted gene 4419	1.128	0.5563	0.8448
381280	Hjurp	Holliday junction recognition protein	1.128	0.08651	NA
320910	Itgb8	integrin beta 8	1.128	0.7239	0.9126
276905	Armc7	armadillo repeat containing 7	1.128	0.2825	0.6969
234069	Pcid2	PCI domain containing 2	1.128	0.3532	0.741

233649	Cnga4	cyclic nucleotide gated channel alpha 4	1.128	0.1506	0.5938
230784	Sesn2	sestrin 2	1.128	0.2914	0.7018
228807	Zfp341	zinc finger protein 341	1.128	0.1538	0.5975
216453	Rdh19	retinol dehydrogenase 19	1.128	0.6141	0.869
94218	Cnnm3	cyclin M3	1.128	0.1793	0.6186
80286	Tusc3	tumor suppressor candidate 3	1.128	0.2776	0.6925
76832	Hyls1	hydrolethalus syndrome 1	1.128	0.2646	0.6845
71772	Plbd2	phospholipase B domain containing 2	1.128	0.2402	0.6685
70396	Asnsd1	asparagine synthetase domain containing 1	1.128	0.08324	NA
69920	Polr2i	polymerase (RNA) II (DNA directed) polypeptide I	1.128	0.05628	NA
67453	Slc25a46	solute carrier family 25, member 46	1.128	0.08882	NA
66437	Fis1	fission 1 (mitochondrial outer membrane) homolog (yeast)	1.128	0.07559	NA
58234	Shank3	SH3/ankyrin domain gene 3	1.128	0.5318	0.8339
54006	Deaf1	deformed epidermal autoregulatory factor 1 (Drosophila)	1.128	0.1857	0.6227
26568	Slc27a3	solute carrier family 27 (fatty acid transporter), member 3	1.128	0.08189	NA
22339	Vegfa	vascular endothelial growth factor A	1.128	0.3045	0.7091
22215	Ube3a	ubiquitin protein ligase E3A	1.128	0.246	0.6733
20418	Shc3	src homology 2 domain-containing transforming protein C3	1.128	0.3256	0.7222
19055	Ppp3ca	protein phosphatase 3, catalytic subunit, alpha isoform	1.128	0.1681	0.6095
17120	Mad1l1	mitotic arrest deficient 1-like 1	1.128	0.315	0.7161
16418	Eif6	eukaryotic translation initiation factor 6	1.128	0.3697	0.7521
12049	Bcl2l10	Bcl2-like 10	1.128	0.2644	0.6845
11792	Apex1	apurinic/apyrimidinic endonuclease 1	1.128	0.2462	0.6734
100043072	A230070E04Rik	RIKEN cDNA A230070E04 gene	1.127	0.05381	NA
381925	Ppapdc1a	phosphatidic acid phosphatase type 2 domain containing 1A	1.127	0.4067	0.7726
277854	Depdc5	DEP domain containing 5	1.127	0.4805	0.8101
272381	Lrrc4b	leucine rich repeat containing 4B	1.127	0.3237	0.721
259277	Klk8	kallikrein related-peptidase 8	1.127	0.5446	0.839
227737	Fam129b	family with sequence similarity 129, member B	1.127	0.09638	NA
216616	Efemp1	epidermal growth factor-containing fibulin-like extracellular matrix protein 1	1.127	0.325	0.7219
170947	Myoz3	myozenin 3	1.127	0.3645	0.7492
109904	Mcf2	mcf.2 transforming sequence	1.127	0.1156	NA
106931	Kctd1	potassium channel tetramerisation domain containing 1	1.127	0.1794	0.6187
104080	Nxph4	neurexophilin 4	1.127	0.4398	0.7908
97031	Tprn	taperin	1.127	0.401	0.7703
75705	Eif4b	eukaryotic translation initiation factor 4B	1.127	0.1473	0.591
74614	4833422F24Rik	RIKEN cDNA 4833422F24 gene	1.127	0.6866	0.8966
74211	1700017B05Rik	RIKEN cDNA 1700017B05 gene	1.127	0.2466	0.6736
69574	Cmbl	carboxymethylenebutenolidase-like (Pseudomonas)	1.127	0.2299	0.6596
67916	Ppap2b	phosphatidic acid phosphatase type 2B	1.127	0.1057	NA
65019	Rpl23	ribosomal protein L23	1.127	0.0331	NA
60533	Cd274	CD274 antigen	1.127	0.219	0.6511
27375	Tjp3	tight junction protein 3	1.127	0.3588	0.7457
22068	Trpc6	transient receptor potential cation channel, subfamily C, member 6	1.127	0.1894	0.6272
20508	Slc18a3	solute carrier family 18 (vesicular monoamine), member 3	1.127	0.2333	0.6622
20005	Rpl9	ribosomal protein L9	1.127	0.08989	NA
18457	Pldn	pallidin	1.127	0.1194	NA
16904	Gzmm	granzyme M (lymphocyte met-ase 1)	1.127	0.0819	NA
16665	Krt15	keratin 15	1.127	0.4741	0.8089

14396	Gabra3	gamma-aminobutyric acid (GABA) A receptor, subunit alpha 3	1.127	0.009818 NA	
13852	Stx2	syntaxin 2	1.127	0.1279	0.5716
13813	Eomes	eomesodermin homolog (Xenopus laevis)	1.127	0.1137 NA	
12608	Cebpb	CCAAT/enhancer binding protein (C/EBP), beta	1.127	0.1474	0.591
12257	Tspo	translocator protein	1.127	0.2579	0.6815
11622	Ahr	aryl-hydrocarbon receptor	1.127	0.07421 NA	
100042510	Gm3877	predicted gene 3877	1.126	0.2579	0.6815
665635	LOC665635	hypothetical protein LOC665635	1.126	0.1615	0.607
432989	D930007P13Rik	Riken cDNA D930007P13 gene	1.126	0.1606	0.6058
381933	6430531B16Rik	RIKEN cDNA 6430531B16 gene	1.126	0.2542	0.6789
378954	3000002C10Rik	glyceraldehyde-3-phosphate dehydrogenase pseudogene	1.126	0.3631	0.7483
319582	6430573F11Rik	RIKEN cDNA 6430573F11 gene	1.126	0.01577 NA	
319262	Fchsd1	FCH and double SH3 domains 1	1.126	0.2631	0.6838
279618	Gm715	predicted pseudogene 715	1.126	0.1251 NA	
269642	Nat8l	N-acetyltransferase 8-like	1.126	0.07489 NA	
246177	Myo1g	myosin IG	1.126	0.2278	0.6569
243510	Ccdc142	coiled-coil domain containing 142	1.126	0.4265	0.7823
228796	Bpil3	bactericidal/permeability-increasing protein-like 3	1.126	0.5535	0.844
219249	Tdrd3	tudor domain containing 3	1.126	0.02625 NA	
211586	Tfdp2	transcription factor Dp 2	1.126	0.1422	0.5871
192775	Kcnh6	potassium voltage-gated channel, subfamily H (eag-related), member 6	1.126	0.2433	0.6713
140570	Plxnb2	plexin B2	1.126	0.2631	0.6838
109218	Tmem139	transmembrane protein 139	1.126	0.5615	0.8465
105675	Ppif	peptidylprolyl isomerase F (cyclophilin F)	1.126	0.2756	0.6925
105298	Epdr1	ependymin related protein 1 (zebrafish)	1.126	0.1925	0.6283
104522	AU040972	expressed sequence AU040972	1.126	0.3553	0.7434
98388	Chst10	carbohydrate sulfotransferase 10	1.126	0.1703	0.6115
78134	Lpar4	lysophosphatidic acid receptor 4	1.126	0.4946	0.8173
76366	Mtif3	mitochondrial translational initiation factor 3	1.126	0.08486 NA	
75570	Nhej1	nonhomologous end-joining factor 1	1.126	0.2088	0.6401
74519	Cyp2j9	cytochrome P450, family 2, subfamily j, polypeptide 9	1.126	0.02439 NA	
74268	Aven	apoptosis, caspase activation inhibitor	1.126	0.1117 NA	
71665	Fuca1	fucosidase, alpha-L- 1, tissue	1.126	0.1184 NA	
70810	Krt25	keratin 25	1.126	0.3838	0.76
69315	1700001L19Rik	RIKEN cDNA 1700001L19 gene	1.126	0.03847 NA	
68031	Rnf146	ring finger protein 146	1.126	0.1595	0.6042
67458	Ergic1	endoplasmic reticulum-golgi intermediate compartment (ERGIC) 1	1.126	0.07051 NA	
66526	2210012G02Rik	RIKEN cDNA 2210012G02 gene	1.126	0.351	0.7393
54125	Polm	polymerase (DNA directed), mu	1.126	0.4525	0.7958
26372	Clcn6	chloride channel 6	1.126	0.4123	0.7747
19762	Rit2	Ras-like without CAAX 2	1.126	0.2404	0.6687
18753	Prkcd	protein kinase C, delta	1.126	0.1286 NA	
18553	Pcsk6	proprotein convertase subtilisin/kexin type 6	1.126	0.08384 NA	
18036	Nfkbib	nuclear factor of kappa light polypeptide gene enhancer in B-cells inhibitor, beta	1.126	0.3223	0.7202
17196	Mbp	myelin basic protein	1.126	0.044 NA	
16527	Kcnk3	potassium channel, subfamily K, member 3	1.126	0.3136	0.7152
14391	Gabpb1	GA repeat binding protein, beta 1	1.126	0.3272	0.7239
12450	Ccng1	cyclin G1	1.126	0.05285 NA	
12315	Calm3	calmodulin 3	1.126	0.1163 NA	

100039528	1110002E22Rik	RIKEN cDNA 1110002E22 gene	1.125	0.3187	0.7182
791286	Gm9886	predicted gene 9886	1.125	0.4709	0.8076
433297	Gm5526	predicted pseudogene 5526	1.125	0.07162	NA
319636	Fsd1l	fibronectin type III and SPRY domain containing 1-like	1.125	0.279	0.694
319477	6030419C18Rik	RIKEN cDNA 6030419C18 gene	1.125	0.4312	0.7851
246747	Adig	adipogenin	1.125	0.2308	0.6601
239217	Kctd12	potassium channel tetramerisation domain containing 12	1.125	0.1797	0.6187
237339	L3mbtl3	l(3)mbt-like 3 (Drosophila)	1.125	0.06288	NA
231871	Daglb	diacylglycerol lipase, beta	1.125	0.2151	0.6475
227737	Fam129b	family with sequence similarity 129, member B	1.125	0.08815	NA
223186	Gm4822	predicted pseudogene 4822	1.125	0.3943	0.7659
194309	Vps37d	vacuolar protein sorting 37D (yeast)	1.125	0.06235	NA
109801	Glo1	glyoxalase 1	1.125	0.02954	NA
99152	Anapc2	anaphase promoting complex subunit 2	1.125	0.2023	0.6356
76889	Adck4	aarF domain containing kinase 4	1.125	0.1574	0.6016
75660	Lin37	lin-37 homolog (C. elegans)	1.125	0.3913	0.7643
73919	Lyrm1	LYR motif containing 1	1.125	0.02716	NA
72649	Tmem209	transmembrane protein 209	1.125	0.1705	0.6115
72284	Oraov1	oral cancer overexpressed 1	1.125	0.2989	0.7052
72000	1600016N20Rik	RIKEN cDNA 1600016N20 gene	1.125	0.4797	0.8101
71943	Tom1l1	target of myb1-like 1 (chicken)	1.125	0.5154	0.8277
70450	Unc13d	unc-13 homolog D (C. elegans)	1.125	0.05978	NA
70003	1700028I16Rik	RIKEN cDNA 1700028I16 gene	1.125	0.5637	0.8465
68431	Fbxl15	F-box and leucine-rich repeat protein 15	1.125	0.2518	0.6767
68043	N6amt2	N-6 adenine-specific DNA methyltransferase 2 (putative)	1.125	0.07207	NA
66183	1110032A04Rik	RIKEN cDNA 1110032A04 gene	1.125	0.1386	0.5825
66169	Tomm7	translocase of outer mitochondrial membrane 7 homolog (yeast)	1.125	0.09199	NA
56526	Sep-06	septin 6	1.125	0.1065	NA
53602	Hpcal1	hippocalcin-like 1	1.125	0.1853	0.6223
50994	Mtag2	metastasis associated gene 2	1.125	0.1543	0.5975
29861	Dpf1	D4, zinc and double PHD fingers family 1	1.125	0.1682	0.6095
23920	Insrr	insulin receptor-related receptor	1.125	0.2245	0.654
22668	Sf1	splicing factor 1	1.125	0.1439	0.5884
21937	Tnfrsf1a	tumor necrosis factor receptor superfamily, member 1a	1.125	0.1531	0.5966
21454	Tcp1	t-complex protein 1	1.125	0.0652	NA
20437	Siah1a	seven in absentia 1A	1.125	0.08162	NA
20167	Rtn2	reticulon 2 (Z-band associated protein)	1.125	0.1719	0.6126
19340	Rab3d	RAB3D, member RAS oncogene family	1.125	0.2778	0.6928
18813	Pa2g4	proliferation-associated 2G4	1.125	0.1573	0.6016
17192	Mbd3	methyl-CpG binding domain protein 3	1.125	0.1837	0.6217
16779	Lamb2	laminin, beta 2	1.125	0.2713	0.6891
16520	Kcnj4	potassium inwardly-rectifying channel, subfamily J, member 4	1.125	0.1618	0.607
16328	Cep250	centrosomal protein 250	1.125	0.3639	0.7485
14169	Fgf14	fibroblast growth factor 14	1.125	0.1024	NA
12443	Ccnd1	cyclin D1	1.125	0.4637	0.8033
12035	Bcat1	branched chain aminotransferase 1, cytosolic	1.125	0.08844	NA
433256	Acsl5	acyl-CoA synthetase long-chain family member 5	1.124	0.02637	NA
385138	BC061237	cDNA sequence BC061237	1.124	0.237	0.6659
319659	A730091E23Rik	RIKEN cDNA A730091E23 gene	1.124	0.2988	0.7052

243833	Zfp128	zinc finger protein 128	1.124	0.1768	0.6164
238317	C130039O16Rik	RIKEN cDNA C130039O16 gene	1.124	0.2408	0.6687
233575	Pgap2	post-GPI attachment to proteins 2	1.124	0.1185	NA
228550	Itpka	inositol 1,4,5-trisphosphate 3-kinase A	1.124	0.1587	0.6033
212307	Mapre2	microtubule-associated protein, RP/EB family, member 2	1.124	0.2517	0.6767
210417	Thsd7b	thrombospondin, type I, domain containing 7B	1.124	0.1684	0.6095
104215	Rhoq	ras homolog gene family, member Q	1.124	0.07686	NA
103784	Wdr92	WD repeat domain 92	1.124	0.3637	0.7483
83965	Enpp5	ectonucleotide pyrophosphatase/phosphodiesterase 5	1.124	0.05025	NA
76157	Slc35d3	solute carrier family 35, member D3	1.124	0.1581	0.6028
75387	Sirt4	sirtuin 4 (silent mating type information regulation 2 homolog) 4 (S. cerevisiae)	1.124	0.3356	0.7302
73681	Trmt11	tRNA methyltransferase 11 homolog (S. cerevisiae)	1.124	0.07478	NA
70796	Zdhhc1	zinc finger, DHHC domain containing 1	1.124	0.463	0.8031
70281	2310068J16Rik	RIKEN cDNA 2310068J16 gene	1.124	0.1439	0.5884
69146	Gsdmd	gasdermin D	1.124	0.2723	0.6895
69009	Thap7	THAP domain containing 7	1.124	0.2987	0.7052
68794	Flnc	filamin C, gamma	1.124	0.3696	0.7521
67847	Sncaip	synuclein, alpha interacting protein (synphilin)	1.124	0.3021	0.7082
67326	1700037H04Rik	RIKEN cDNA 1700037H04 gene	1.124	0.5286	0.8332
66821	Bcs1l	BCS1-like (yeast)	1.124	0.117	NA
66437	Fis1	fission 1 (mitochondrial outer membrane) homolog (yeast)	1.124	0.06693	NA
66292	Mrps21	mitochondrial ribosomal protein S21	1.124	0.04837	NA
57266	Cxcl14	chemokine (C-X-C motif) ligand 14	1.124	0.1163	NA
56468	Socs5	suppressor of cytokine signaling 5	1.124	0.04524	NA
54607	Socs6	suppressor of cytokine signaling 6	1.124	0.05938	NA
54397	Ppt2	palmitoyl-protein thioesterase 2	1.124	0.4519	0.7951
52020	Umodl1	uromodulin-like 1	1.124	0.4635	0.8032
27390	Mmel1	membrane metallo-endopeptidase-like 1	1.124	0.1621	0.607
27373	Csnk1e	casein kinase 1, epsilon	1.124	0.4381	0.7894
22320	Vamp8	vesicle-associated membrane protein 8	1.124	0.08175	NA
22200	Uba3	ubiquitin-like modifier activating enzyme 3	1.124	0.08957	NA
20370	Sez6	seizure related gene 6	1.124	0.4223	0.7802
19092	Prkg2	protein kinase, cGMP-dependent, type II	1.124	0.07874	NA
18973	Pole	polymerase (DNA directed), epsilon	1.124	0.03132	NA
18642	Pfkm	phosphofructokinase, muscle	1.124	0.3189	0.7183
18220	Nucb1	nucleobindin 1	1.124	0.361	0.7471
17930	Myom2	myomesin 2	1.124	0.1623	0.607
16617	Klk1b24	kallikrein 1-related peptidase b24	1.124	0.3298	0.726
16008	Igfbp2	insulin-like growth factor binding protein 2	1.124	0.3399	0.7326
15465	Hrh1	histamine receptor H1	1.124	0.0855	NA
12183	Bpgm	2,3-bisphosphoglycerate mutase	1.124	0.03175	NA
627967	Gm6816	predicted gene 6816	1.123	0.3241	0.7212
382051	Pdp2	pyruvate dehyrogenase phosphatase catalytic subunit 2	1.123	0.2771	0.6925
319876	Cobll1	Cobl-like 1	1.123	0.3536	0.7414
239114	Il17d	interleukin 17D	1.123	0.07159	NA
239099	Homez	homeodomain leucine zipper-encoding gene	1.123	0.1012	NA
225362	Reep2	receptor accessory protein 2	1.123	0.2239	0.654
218461	Pde8b	phosphodiesterase 8B	1.123	0.01932	NA
207686	A330021E22Rik	RIKEN cDNA A330021E22 gene	1.123	0.4171	0.7777

170763	Zfp87	zinc finger protein 87	1.123	0.1246	NA
74288	Spem1	sperm maturation 1	1.123	0.2366	0.6659
74196	Ttc27	tetratricopeptide repeat domain 27	1.123	0.09756	NA
71472	Usp19	ubiquitin specific peptidase 19	1.123	0.2168	0.6482
71242	Spata24	spermatogenesis associated 24	1.123	0.1036	NA
70561	Txndc16	thioredoxin domain containing 16	1.123	0.06078	NA
68841	1110054M08Rik	RIKEN cDNA 1110054M08 gene	1.123	0.4214	0.7798
68525	Evc2	Ellis van Creveld syndrome 2 homolog (human)	1.123	0.5679	0.8485
68095	Ociad1	OCIA domain containing 1	1.123	0.02807	NA
67884	1810043G02Rik	RIKEN cDNA 1810043G02 gene	1.123	0.242	0.6702
67763	Prpsap1	phosphoribosyl pyrophosphate synthetase-associated protein 1	1.123	0.06937	NA
67503	1700001G17Rik	RIKEN cDNA 1700001G17 gene	1.123	0.08556	NA
67115	Rpl14	ribosomal protein L14	1.123	0.1723	0.6131
66884	Appbp2	amyloid beta precursor protein (cytoplasmic tail) binding protein 2	1.123	0.1128	NA
66849	Ppp1r2	protein phosphatase 1, regulatory (inhibitor) subunit 2	1.123	0.05966	NA
66116	Cml1	camello-like 1	1.123	0.07325	NA
58801	Pmaip1	phorbol-12-myristate-13-acetate-induced protein 1	1.123	0.2182	0.6502
53857	Tuba8	tubulin, alpha 8	1.123	0.04428	NA
50782	Rgs11	regulator of G-protein signaling 11	1.123	0.1912	0.6278
27412	Peg12	paternally expressed 12	1.123	0.5364	0.8352
26961	Rpl8	ribosomal protein L8	1.123	0.153	0.5966
24086	Tlk2	tousled-like kinase 2 (Arabidopsis)	1.123	0.09527	NA
22186	Uba52	ubiquitin A-52 residue ribosomal protein fusion product 1	1.123	0.103	NA
20441	St3gal3	ST3 beta-galactoside alpha-2,3-sialyltransferase 3	1.123	0.2535	0.678
19766	Ripk1	receptor (TNFRSF)-interacting serine-threonine kinase 1	1.123	0.03683	NA
19012	Ppap2a	phosphatidic acid phosphatase type 2A	1.123	0.1937	0.6299
18805	Pld1	phospholipase D1	1.123	0.556	0.8448
17067	Ly6c1	lymphocyte antigen 6 complex, locus C1	1.123	0.05395	NA
16819	Lcn2	lipocalin 2	1.123	0.466	0.8049
16418	Eif6	eukaryotic translation initiation factor 6	1.123	0.3912	0.7643
15932	Idua	iduronidase, alpha-L-	1.123	0.2587	0.6815
15451	Hpn	hepsin	1.123	0.1212	NA
15081	H3f3b	H3 histone, family 3B	1.123	0.1428	0.5879
14707	Gng5	guanine nucleotide binding protein (G protein), gamma 5	1.123	0.1477	0.5911
13014	Cstb	cystatin B	1.123	0.03294	NA
11632	Aip	aryl-hydrocarbon receptor-interacting protein	1.123	0.4182	0.7786
545005	LOC545005	hypothetical protein LOC545005	1.122	0.1204	NA
380997	Cyp2d12	cytochrome P450, family 2, subfamily d, polypeptide 12	1.122	0.3753	0.7555
276952	Rasl10b	RAS-like, family 10, member B	1.122	0.4863	0.8127
268512	Slc26a11	solute carrier family 26, member 11	1.122	0.02255	NA
245638	Tbc1d8b	TBC1 domain family, member 8B	1.122	0.2262	0.6547
230673	Ipo13	importin 13	1.122	0.4926	0.8157
224598	Zfp758	zinc finger protein 758	1.122	0.3618	0.7477
114642	Brdt	bromodomain, testis-specific	1.122	0.2087	0.6401
94185	Tnfrsf21	tumor necrosis factor receptor superfamily, member 21	1.122	0.2828	0.6969
78899	4932443L11Rik	RIKEN cDNA 4932443L11 gene	1.122	0.0845	NA
76866	Morn1	MORN repeat containing 1	1.122	0.3761	0.7556
75409	Slitrk5	SLIT and NTRK-like family, member 5	1.122	0.1665	0.6083
72632	2700090O03Rik	RIKEN cDNA 2700090O03 gene	1.122	0.1047	NA

72507	Dzip1l	DAZ interacting protein 1-like	1.122	0.4551	0.7978
71881	2310001A20Rik	RIKEN cDNA 2310001A20 gene	1.122	0.03894	NA
71828	Gtf2a1l	general transcription factor IIA, 1-like	1.122	0.3283	0.7251
66700	Vps24	vacuolar protein sorting 24 (yeast)	1.122	0.2194	0.6513
66532	Rep15	RAB15 effector protein	1.122	0.2816	0.6967
66497	2610528E23Rik	RIKEN cDNA 2610528E23 gene	1.122	0.1024	NA
66124	Josd2	Josephin domain containing 2	1.122	0.4605	0.8014
58992	F12	coagulation factor XII (Hageman factor)	1.122	0.7094	0.9069
54376	Cacng3	calcium channel, voltage-dependent, gamma subunit 3	1.122	0.4494	0.7943
52040	Ppp1r10	protein phosphatase 1, regulatory subunit 10	1.122	0.2194	0.6513
24075	Taf10	TAF10 RNA polymerase II, TATA box binding protein (TBP)-associated factor	1.122	0.3065	0.7108
23991	Cib1	calcium and integrin binding 1 (calmyrin)	1.122	0.05291	NA
23948	Mmp17	matrix metallopeptidase 17	1.122	0.2548	0.6792
20649	Sntb1	syntrophin, basic 1	1.122	0.2776	0.6925
19363	Rad51l1	RAD51-like 1 (S. cerevisiae)	1.122	0.3037	0.709
18230	Nxn	nucleoredoxin	1.122	0.2368	0.6659
18109	Mycn	v-myc myelocytomatosis viral related oncogene, neuroblastoma derived (avian)	1.122	0.02212	NA
17356	Mllt4	myeloid/lymphoid or mixed-lineage leukemia (trithorax homolog, Drosophila); translocated to, 4	1.122	0.1403	NA
14731	Gpaa1	GPI anchor attachment protein 1	1.122	0.2977	0.7051
14009	Etv1	ets variant gene 1	1.122	0.2399	0.6685
13804	Endog	endonuclease G	1.122	0.06337	NA
13497	Drp2	dystrophin related protein 2	1.122	0.2208	0.6524
12166	Bmpr1a	bone morphogenetic protein receptor, type 1A	1.122	0.01811	NA
545192	Baiap3	BAI1-associated protein 3	1.121	0.48	0.8101
384185	Arl9	ADP-ribosylation factor-like 9	1.121	0.4073	0.7726
317677	Gm5077	predicted gene 5077	1.121	0.4666	0.8049
237782	Smcr8	Smith-Magenis syndrome chromosome region, candidate 8 homolog (human)	1.121	0.2219	0.6533
230991	B930041F14Rik	RIKEN cDNA B930041F14 gene	1.121	0.2875	0.6986
230972	Arhgef16	Rho guanine nucleotide exchange factor (GEF) 16	1.121	0.4042	0.7713
217371	Rab40b	Rab40b, member RAS oncogene family	1.121	0.03192	NA
216820	Dhrs7b	dehydrogenase/reductase (SDR family) member 7B	1.121	0.04141	NA
209378	Itih5	inter-alpha (globulin) inhibitor H5	1.121	0.3174	0.7178
192662	Arhgdia	Rho GDP dissociation inhibitor (GDI) alpha	1.121	0.3366	0.7305
170472	Recql5	RecQ protein-like 5	1.121	0.4192	0.7791
109820	Pgc	progastricsin (pepsinogen C)	1.121	0.6414	0.8788
108073	Grm7	glutamate receptor, metabotropic 7	1.121	0.06187	NA
107260	Otub1	OTU domain, ubiquitin aldehyde binding 1	1.121	0.2288	0.6581
104175	Sbk1	SH3-binding kinase 1	1.121	0.315	0.7161
104079	Nxph3	neurexophilin 3	1.121	0.1994	0.6343
97287	Mtmr14	myotubularin related protein 14	1.121	0.1625	0.607
74883	4930461C15Rik	RIKEN cDNA 4930461C15 gene	1.121	0.2422	0.6703
72244	1600014C10Rik	RIKEN cDNA 1600014C10 gene	1.121	0.2143	0.6467
69399	1700025G04Rik	RIKEN cDNA 1700025G04 gene	1.121	0.2762	0.6925
69195	Tmem121	transmembrane protein 121	1.121	0.08796	NA
68910	Zfp467	zinc finger protein 467	1.121	0.1674	0.6091
67666	Hapln3	hyaluronan and proteoglycan link protein 3	1.121	0.05485	NA
67364	1700121C10Rik	RIKEN cDNA 1700121C10 gene	1.121	0.372	0.7535
66686	Dcbld1	discoidin, CUB and LCCL domain containing 1	1.121	0.1262	NA
66496	Ppdpf	pancreatic progenitor cell differentiation and proliferation factor homolog (zebrafish)RIKEN cDNA 2700038C09 gene	1.121	0.07402	NA

66082	Abhd6	abhydrolase domain containing 6	1.121	0.4146	0.776
58521	Eid1	EP300 interacting inhibitor of differentiation 1	1.121	0.1672	0.6085
52898	Rnasek	ribonuclease, RNase K	1.121	0.3772	0.7563
22064	Trpc2	transient receptor potential cation channel, subfamily C, member 2	1.121	0.06461	NA
20652	Soat1	sterol O-acyltransferase 1	1.121	0.3961	0.7671
20350	Sema3f	sema domain, immunoglobulin domain (Ig), short basic domain, secreted, (semaphorin) 3F	1.121	0.208	0.6401
19933	Rpl21	ribosomal protein L21	1.121	0.1427	NA
19719	Rfng	RFNG O-fucosylpeptide 3-beta-N-acetylglucosaminyltransferase	1.121	0.3483	0.7385
18111	Nnat	neuronatin	1.121	0.5322	0.8339
18000	Sep-02	septin 2	1.121	0.106	NA
17869	Myc	myelocytomatosis oncogene	1.121	0.2607	0.6816
16502	Kcnc1	potassium voltage gated channel, Shaw-related subfamily, member 1	1.121	0.1102	NA
14165	Fgf10	fibroblast growth factor 10	1.121	0.1511	0.5942
12988	Csk	c-src tyrosine kinase	1.121	0.111	NA
12398	Cbfa2t3	core-binding factor, runt domain, alpha subunit 2, translocated to, 3 (human)	1.121	0.5714	0.8495
100042480	Nhsl2	NHS-like 2	1.12	0.5535	0.844
319748	Zfp865	zinc finger protein 865	1.12	0.2075	0.6398
243897	Ggn	gametogenetin	1.12	0.1779	0.6169
228876	Zfp334	zinc finger protein 334	1.12	0.3071	0.7108
219072	Haus4	HAUS augmin-like complex, subunit 4	1.12	0.1455	NA
218952	Fermt2	fermitin family homolog 2 (Drosophila)	1.12	0.1222	NA
216859	Acap1	ArfGAP with coiled-coil, ankyrin repeat and PH domains 1	1.12	0.5417	0.8379
213464	Rbbp5	retinoblastoma binding protein 5	1.12	0.03153	NA
208691	Eif5a2	eukaryotic translation initiation factor 5A2	1.12	0.2333	0.6622
109270	Prr5	proline rich 5 (renal)	1.12	0.3418	0.7338
105653	Phyhip	phytanoyl-CoA hydroxylase interacting protein	1.12	0.531	0.8339
103775	Slc25a41	solute carrier family 25, member 41	1.12	0.5607	0.8462
103199	Fig4	FIG4 homolog (S. cerevisiae)	1.12	0.296	0.705
93835	Amn	amnionless	1.12	0.2096	0.6412
77209	8030453O22Rik	RIKEN cDNA 8030453O22 gene	1.12	0.269	0.6878
75735	Pank1	pantothenate kinase 1	1.12	0.1308	NA
73112	3110003A17Rik	RIKEN cDNA 3110003A17 gene	1.12	0.01686	NA
72552	Hsdl1	hydroxysteroid dehydrogenase like 1	1.12	0.2886	0.6996
71436	Flrt3	fibronectin leucine rich transmembrane protein 3	1.12	0.2649	0.6848
67226	Tmem19	transmembrane protein 19	1.12	0.09771	NA
66460	Sys1	SYS1 Golgi-localized integral membrane protein homolog (S. cerevisiae)	1.12	0.08669	NA
66404	2410001C21Rik	RIKEN cDNA 2410001C21 gene	1.12	0.1516	0.5948
66087	Tmem111	transmembrane protein 111	1.12	0.0772	NA
59029	Psmd14	proteasome (prosome, macropain) 26S subunit, non-ATPase, 14	1.12	0.2867	0.6982
58250	Chst11	carbohydrate sulfotransferase 11	1.12	0.3733	0.7539
53417	Hif3a	hypoxia inducible factor 3, alpha subunit	1.12	0.2054	0.6386
52838	Dnlz	DNL-type zinc finger	1.12	0.1557	0.599
29871	Scmh1	sex comb on midleg homolog 1	1.12	0.3154	0.7165
23828	Bves	blood vessel epicardial substance	1.12	0.03656	NA
22301	Vmn2r89	vomeronasal 2, receptor 89	1.12	0.3244	0.7213
22294	Uxt	ubiquitously expressed transcript	1.12	0.3601	0.7466
22294	Uxt	ubiquitously expressed transcript	1.12	0.3704	0.7525
21981	Ppp1r13b	protein phosphatase 1, regulatory (inhibitor) subunit 13B	1.12	0.1713	0.6121
21809	Tgfb3	transforming growth factor, beta 3	1.12	0.1659	0.6078

21334	Tac2	tachykinin 2	1.12	0.03052	NA
20044	Rps14	ribosomal protein S14	1.12	0.1087	NA
19326	Rab11b	RAB11B, member RAS oncogene family	1.12	0.3289	0.7253
18041	Nfs1	nitrogen fixation gene 1 (S. cerevisiae)	1.12	0.0959	NA
17387	Mmp14	matrix metallopeptidase 14 (membrane-inserted)	1.12	0.3077	0.7112
15211	Hexa	hexosaminidase A	1.12	0.03901	NA
14571	Gpd2	glycerol phosphate dehydrogenase 2, mitochondrial	1.12	0.07794	NA
13074	Cyp17a1	cytochrome P450, family 17, subfamily a, polypeptide 1	1.12	0.7	0.9033
13056	Cyb561	cytochrome b-561	1.12	0.2028	0.6356
12978	Csf1r	colony stimulating factor 1 receptor	1.12	0.1857	0.6228
12922	Crhr2	corticotropin releasing hormone receptor 2	1.12	0.3097	0.713
11465	Actg1	actin, gamma, cytoplasmic 1	1.12	0.1717	0.6126
11423	Ache	acetylcholinesterase	1.12	0.513	0.8268
669149	Vmn2r88	vomeronasal 2, receptor 88	1.119	0.4458	0.7938
381979	Brsk1	BR serine/threonine kinase 1	1.119	0.1225	NA
330554	Fan1	FANCD2/FANCI-associated nuclease 1	1.119	0.1225	NA
320237	Ncrna00086	non-protein coding RNA 86	1.119	0.3101	0.713
280645	B3gat2	beta-1,3-glucuronyltransferase 2 (glucuronosyltransferase S)	1.119	0.1889	0.6266
231042	Nupl2	nucleoporin like 2	1.119	0.2822	0.6967
230991	B930041F14Rik	RIKEN cDNA B930041F14 gene	1.119	0.3157	0.7165
229011	Samd10	sterile alpha motif domain containing 10	1.119	0.4382	0.7894
224090	Tmem44	transmembrane protein 44	1.119	0.6176	0.8701
213389	Prdm9	PR domain containing 9	1.119	0.1087	NA
208194	Exog	endo/exonuclease (5'-3'), endonuclease G-like	1.119	0.2257	0.654
192654	Pla2g15	phospholipase A2, group XV	1.119	0.1838	0.6217
102060	Gadd45gip1	growth arrest and DNA-damage-inducible, gamma interacting protein 1	1.119	0.2816	0.6967
94242	Tinagl1	tubulointerstitial nephritis antigen-like 1	1.119	0.5905	0.8579
78317	Ccdc88b	coiled-coil domain containing 88B	1.119	0.1017	NA
76898	B3gat1	beta-1,3-glucuronyltransferase 1 (glucuronosyltransferase P)	1.119	0.2666	0.6862
74096	Hvcn1	hydrogen voltage-gated channel 1	1.119	0.2248	0.654
72749	Tonsl	tonsoku-like, DNA repair protein	1.119	0.3112	0.7135
72371	2210408I21Rik	RIKEN cDNA 2210408I21 gene	1.119	0.3754	0.7555
71833	Dcaf7	DDB1 and CUL4 associated factor 7	1.119	0.3346	0.73
70693	Gpr125	G protein-coupled receptor 125	1.119	0.3692	0.7521
69310	Pacrg	PARK2 co-regulated	1.119	0.1816	0.6206
69009	Thap7	THAP domain containing 7	1.119	0.208	0.6401
67469	Abhd5	abhydrolase domain containing 5	1.119	0.09231	NA
66144	Atp6v1f	ATPase, H+ transporting, lysosomal V1 subunit F	1.119	0.01923	NA
56530	Cnpy2	canopy 2 homolog (zebrafish)	1.119	0.08896	NA
56087	Dnahc10	dynein, axonemal, heavy chain 10	1.119	0.5162	0.8277
27061	Bcap31	B-cell receptor-associated protein 31	1.119	0.09658	NA
26562	Ncdn	neurochondrin	1.119	0.3121	0.7139
23997	Psmd13	proteasome (prosome, macropain) 26S subunit, non-ATPase, 13	1.119	0.03538	NA
22757	Zkscan5	zinc finger with KRAB and SCAN domains 5	1.119	0.1306	NA
22247	Umps	uridine monophosphate synthetase	1.119	0.05906	NA
20480	Clpb	ClpB caseinolytic peptidase B homolog (E. coli)	1.119	0.3956	0.7671
19739	Rgs9	regulator of G-protein signaling 9	1.119	0.2518	0.6767
19143	St14	suppression of tumorigenicity 14 (colon carcinoma)	1.119	0.1307	NA
14695	Gnb3	guanine nucleotide binding protein (G protein), beta 3	1.119	0.335	0.73

13841	Epha7	Eph receptor A7	1.119	0.03839	NA
13429	Dnm1	dynamin 1	1.119	0.3262	0.7227
13401	Dmwd	dystrophia myotonica-containing WD repeat motif	1.119	0.2741	0.6919
12803	Cntf	ciliary neurotrophic factor	1.119	0.5204	0.8286
12018	Bak1	BCL2-antagonist/killer 1	1.119	0.1336	NA
11877	Arvcf	armadillo repeat gene deleted in velo-cardio-facial syndrome	1.119	0.5418	0.8379
11854	Rhod	ras homolog gene family, member D	1.119	0.0279	NA
11685	Alox12e	arachidonate lipoxygenase, epidermal	1.119	0.517	0.8277
100042659	Gm3952	predicted gene 3952	1.118	0.7714	0.9258
768252	Foxl2os	forkhead box L2 opposite strand transcript	1.118	0.6856	0.8963
320301	E530011L22Rik	RIKEN cDNA E530011L22 gene	1.118	0.1282	NA
282619	Sbsn	suprabasin	1.118	0.3453	0.736
269109	Dpp10	dipeptidylpeptidase 10	1.118	0.1332	NA
218194	Phactr1	phosphatase and actin regulator 1	1.118	0.07453	NA
216792	A230051G13Rik	RIKEN cDNA A230051G13 gene	1.118	0.1164	NA
170758	Rac3	RAS-related C3 botulinum substrate 3	1.118	0.2236	0.654
106565	Dlk2	delta-like 2 homolog (Drosophila)	1.118	0.1084	NA
104130	Ndufb11	NADH dehydrogenase (ubiquinone) 1 beta subcomplex, 11	1.118	0.2024	0.6356
76373	Zfp773	zinc finger protein 773	1.118	0.1314	NA
75224	4930528J11Rik	RIKEN cDNA 4930528J11 gene	1.118	0.2782	0.6933
70616	Sugp1	SURP and G patch domain containing 1	1.118	0.03879	NA
69714	Tfpt	TCF3 (E2A) fusion partner	1.118	0.4555	0.7981
67864	Yipf4	Yip1 domain family, member 4	1.118	0.0645	NA
67017	2010011I20Rik	RIKEN cDNA 2010011I20 gene	1.118	0.03707	NA
66995	Zcchc18	zinc finger, CCHC domain containing 18	1.118	0.03145	NA
58810	Akr1a4	aldo-keto reductase family 1, member A4 (aldehyde reductase)	1.118	0.1518	NA
58249	Fibp	fibroblast growth factor (acidic) intracellular binding protein	1.118	0.1909	0.6277
52055	Rab11fip5	RAB11 family interacting protein 5 (class I)	1.118	0.07057	NA
30956	Aass	aminoadipate-semialdehyde synthase	1.118	0.2294	0.6592
27412	Peg12	paternally expressed 12	1.118	0.1911	0.6278
21956	Tnnt2	troponin T2, cardiac	1.118	0.3331	0.728
18548	Pcsk1	proprotein convertase subtilisin/kexin type 1	1.118	0.1935	0.6295
18174	Slc11a2	solute carrier family 11 (proton-coupled divalent metal ion transporters), member 2	1.118	0.271	0.689
17972	Ncf4	neutrophil cytosolic factor 4	1.118	0.2987	0.7052
17751	Mt3	metallothionein 3	1.118	0.3917	0.7645
14408	Gabrr1	gamma-aminobutyric acid (GABA) C receptor, subunit rho 1	1.118	0.1638	0.6074
13047	Cux1	cut-like homeobox 1	1.118	0.1761	0.6156
12889	Cplx1	complexin 1	1.118	0.3068	0.7108
666938	Bend4	BEN domain containing 4	1.117	0.2978	0.7051
353282	Sfmbt2	Scm-like with four mbt domains 2	1.117	0.3968	0.7675
333329	Cngb1	cyclic nucleotide gated channel beta 1	1.117	0.1524	NA
319758	Rfx7	regulatory factor X, 7	1.117	0.2007	0.6345
277333	Gm5069	glyceraldehyde-3-phosphate dehydrogenase pseudogene	1.117	0.4446	0.7936
272031	E130309F12Rik	RIKEN cDNA E130309F12 gene	1.117	0.2732	0.6905
269854	Nat14	N-acetyltransferase 14	1.117	0.1562	NA
238247	Arid4a	AT rich interactive domain 4A (RBP1-like)	1.117	0.3114	0.7136
233826	Palb2	partner and localizer of BRCA2	1.117	0.3773	0.7563
233056	Zfp790	zinc finger protein 790	1.117	0.3971	0.7677
230935	Dnajc11	DnaJ (Hsp40) homolog, subfamily C, member 11	1.117	0.3214	0.7199

228026	Pdk1	pyruvate dehydrogenase kinase, isoenzyme 1	1.117	0.1597	NA
217827	BC002230	cDNA sequence BC002230	1.117	0.2215	0.6526
117148	Necab2	N-terminal EF-hand calcium binding protein 2	1.117	0.3357	0.7302
108903	Tbcd	tubulin-specific chaperone d	1.117	0.2493	0.6767
101513	2700078K21Rik	RIKEN cDNA 2700078K21 gene	1.117	0.08331	NA
83924	Gpr137b	G protein-coupled receptor 137B	1.117	0.168	0.6095
76820	Fam49a	family with sequence similarity 49, member A	1.117	0.06463	NA
75619	Fastkd2	FAST kinase domains 2	1.117	0.2817	0.6967
74597	4833418N02Rik	RIKEN cDNA 4833418N02 gene	1.117	0.2167	0.6482
74498	Gorasp1	golgi reassembly stacking protein 1	1.117	0.04481	NA
73828	Dcaf4	DDB1 and CUL4 associated factor 4	1.117	0.1959	0.6307
72729	Cdc42se2	CDC42 small effector 2	1.117	0.08136	NA
72543	Fam125b	family with sequence similarity 125, member B	1.117	0.1429	NA
71004	4931440P22Rik	RIKEN cDNA 4931440P22 gene	1.117	0.02354	NA
70676	Gulp1	GULP, engulfment adaptor PTB domain containing 1	1.117	0.1634	0.6074
68519	Eml1	echinoderm microtubule associated protein like 1	1.117	0.2828	0.6969
68089	Arpc4	actin related protein 2/3 complex, subunit 4	1.117	0.5413	0.8379
64082	Popdc2	popeye domain containing 2	1.117	0.4892	0.8143
59022	Edf1	endothelial differentiation-related factor 1	1.117	0.06003	NA
27387	Sh2d3c	SH2 domain containing 3C	1.117	0.1269	NA
26388	Ifi202b	interferon activated gene 202B	1.117	0.6797	0.8944
24030	Mrps12	mitochondrial ribosomal protein S12	1.117	0.06462	NA
20818	Srprb	signal recognition particle receptor, B subunit	1.117	0.3713	0.7532
20481	Ski	ski sarcoma viral oncogene homolog (avian)	1.117	0.6535	0.8848
19271	Ptprj	protein tyrosine phosphatase, receptor type, J	1.117	0.6312	0.8751
19085	Prkar1b	protein kinase, cAMP dependent regulatory, type I beta	1.117	0.2548	0.6792
18761	Prkcq	protein kinase C, theta	1.117	0.07643	NA
16593	Klc1	kinesin light chain 1	1.117	0.1364	NA
16421	Itgb7	integrin beta 7	1.117	0.5069	0.8234
14687	Gnaz	guanine nucleotide binding protein, alpha z subunit	1.117	0.2135	0.6463
14677	Gnai1	guanine nucleotide binding protein (G protein), alpha inhibiting 1	1.117	0.131	NA
12390	Cav2	caveolin 2	1.117	0.2	0.6345
432754	LOC432754	similar to glyceraldehyde-3-phosphate dehydrogenase	1.116	0.3298	0.726
406218	Panx2	pannexin 2	1.116	0.3855	0.7608
380780	Serpina11	serine (or cysteine) peptidase inhibitor, clade A (alpha-1 antiproteinase, antitrypsin), member 11	1.116	0.3963	0.7674
378937	Lrrc24	leucine rich repeat containing 24	1.116	0.338	0.7314
236690	Nyx	nyctalopin	1.116	0.03446	NA
223774	Alg12	asparagine-linked glycosylation 12 homolog (yeast, alpha-1,6-mannosyltransferase)	1.116	0.4056	0.7717
218030	Pou6f2	POU domain, class 6, transcription factor 2	1.116	0.4733	0.8088
170711	Otud7a	OTU domain containing 7A	1.116	0.1763	0.6156
116891	Derl2	Der1-like domain family, member 2	1.116	0.02648	NA
110033	Kif22	kinesin family member 22	1.116	0.01818	NA
102580	Alg9	asparagine-linked glycosylation 9 homolog (yeast, alpha 1,2 mannosyltransferase)	1.116	0.09825	NA
80794	Cblc	Casitas B-lineage lymphoma c	1.116	0.4939	0.8167
69638	Enho	energy homeostasis associated	1.116	0.1679	0.6095
68515	Myadml2	myeloid-associated differentiation marker-like 2	1.116	0.1912	0.6278
67819	Derl1	Der1-like domain family, member 1	1.116	0.01761	NA
64337	Gng13	guanine nucleotide binding protein (G protein), gamma 13	1.116	0.1761	0.6156
60597	Mapk8ip2	mitogen-activated protein kinase 8 interacting protein 2	1.116	0.3913	0.7643

58244	Stx6	syntaxin 6	1.116	0.08737	NA	
57764	Ntn4	netrin 4	1.116	0.5399		0.8369
56371	Fzr1	fizzy/cell division cycle 20 related 1 (Drosophila)	1.116	0.2036		0.6362
56079	Astn2	astrotactin 2	1.116	0.5051		0.8226
55991	Panx1	pannexin 1	1.116	0.09796	NA	
54369	Nme6	non-metastatic cells 6, protein expressed in (nucleoside-diphosphate kinase)	1.116	0.2004		0.6345
51799	Rundc3a	RUN domain containing 3A	1.116	0.3944		0.7659
50753	Fbxo8	F-box protein 8	1.116	0.4766		0.8099
26371	Ciao1	cytosolic iron-sulfur protein assembly 1 homolog (S. cerevisiae)	1.116	0.1312	NA	
22240	Dpysl3	dihydropyrimidinase-like 3	1.116	0.264		0.6845
21968	Tom1	target of myb1 homolog (chicken)	1.116	0.5363		0.8352
20502	Slc16a2	solute carrier family 16 (monocarboxylic acid transporters), member 2	1.116	0.09812	NA	
17342	Mitf	microphthalmia-associated transcription factor	1.116	0.06651	NA	
16373	Irx3	Iroquois related homeobox 3 (Drosophila)	1.116	0.0675	NA	
15213	Hey1	hairy/enhancer-of-split related with YRPW motif 1	1.116	0.1034	NA	
14991	H2-M3	histocompatibility 2, M region locus 3	1.116	0.3738		0.7544
14634	Gli3	GLI-Kruppel family member GLI3	1.116	0.3503		0.739
12558	Cdh2	cadherin 2	1.116	0.3208		0.7196
12021	Bard1	BRCA1 associated RING domain 1	1.116	0.2038		0.6362
11984	Atp6v0c	ATPase, H+ transporting, lysosomal V0 subunit C	1.116	0.298		0.7051
622301	Rhox2h	reproductive homeobox 2H	1.115	0.02331	NA	
574403	Fam196b	family with sequence similarity 196, member B	1.115	0.08911	NA	
381353	Gm996	predicted gene 996	1.115	0.615		0.8695
353190	Edc3	enhancer of mRNA decapping 3 homolog (S. cerevisiae)	1.115	0.09016	NA	
330189	Tmem120b	transmembrane protein 120B	1.115	0.2255		0.654
330108	4732457N14	hypothetical protein 4732457N14	1.115	0.7811		0.9289
235302	D630033O11Rik	RIKEN cDNA D630033O11 gene	1.115	0.2427		0.6705
230815	Man1c1	mannosidase, alpha, class 1C, member 1	1.115	0.3668		0.7504
228858	Gdap1l1	ganglioside-induced differentiation-associated protein 1-like 1	1.115	0.3221		0.7202
219140	Spata13	spermatogenesis associated 13	1.115	0.2477		0.6749
213211	Rnf26	ring finger protein 26	1.115	0.5482		0.8406
102414	Clk3	CDC-like kinase 3	1.115	0.0746	NA	
101113	Snx21	sorting nexin family member 21	1.115	0.1335	NA	
93884	Pcdhb13	protocadherin beta 13	1.115	0.2629		0.6838
78668	E130112N10Rik	RIKEN cDNA E130112N10 gene	1.115	0.376		0.7556
71302	Arhgap26	Rho GTPase activating protein 26	1.115	0.548		0.8406
69539	Trnp1	TMF1-regulated nuclear protein 1	1.115	0.3217		0.7199
69408	Dnajc17	DnaJ (Hsp40) homolog, subfamily C, member 17	1.115	0.5061		0.823
69215	Sat2	spermidine/spermine N1-acetyl transferase 2	1.115	0.06092	NA	
68874	Klhdc9	kelch domain containing 9	1.115	0.2586		0.6815
67455	Klhl13	kelch-like 13 (Drosophila)	1.115	0.1229	NA	
67268	2900073G15Rik	RIKEN cDNA 2900073G15 gene	1.115	0.1819		0.6206
58172	Sertad2	SERTA domain containing 2	1.115	0.331		0.7264
56378	Arpc3	actin related protein 2/3 complex, subunit 3	1.115	0.05609	NA	
20677	Sox4	SRY-box containing gene 4	1.115	0.2904		0.7006
19866	Rnu7	U7 small nuclear RNA	1.115	0.05303	NA	
19052	Ppp2ca	protein phosphatase 2 (formerly 2A), catalytic subunit, alpha isoform	1.115	0.05687	NA	
17524	Mpp1	membrane protein, palmitoylated	1.115	0.1573	NA	
15903	Id3	inhibitor of DNA binding 3	1.115	0.2866		0.6982

13476	Reep5	receptor accessory protein 5	1.115	0.1133	NA
100038595	Gm16287	predicted gene 16287	1.114	0.3563	0.7443
668224	Gm9054	predicted gene 9054	1.114	0.4849	0.8126
381605	Tbc1d2	TBC1 domain family, member 2	1.114	0.2562	0.6801
246277	Csad	cysteine sulfinic acid decarboxylase	1.114	0.2898	0.7004
244653	Hydin	hydrocephalus inducing	1.114	0.7095	0.9069
243371	Lrrc61	leucine rich repeat containing 61	1.114	0.2979	0.7051
240121	Fsd1	fibronectin type 3 and SPRY domain-containing protein	1.114	0.2719	0.6891
234290	BC030500	cDNA sequence BC030500	1.114	0.1228	NA
233529	Kctd14	potassium channel tetramerisation domain containing 14	1.114	0.2371	0.6659
225392	Rell2	RELT-like 2	1.114	0.2557	0.68
223723	Ttll12	tubulin tyrosine ligase-like family, member 12	1.114	0.07767	NA
217119	Xylt2	xylosyltransferase II	1.114	0.28	0.6952
192174	Rwdd4a	RWD domain containing 4A	1.114	0.5295	0.8332
171469	Gpr37l1	G protein-coupled receptor 37-like 1	1.114	0.5299	0.8332
114875	Plcz1	phospholipase C, zeta 1	1.114	0.4455	0.7938
105892	9030619P08Rik	RIKEN cDNA 9030619P08 gene	1.114	0.4554	0.798
104086	Cyp27a1	cytochrome P450, family 27, subfamily a, polypeptide 1	1.114	0.3303	0.726
71835	Lancl2	LanC (bacterial lantibiotic synthetase component C)-like 2	1.114	0.1925	0.6283
68758	Abhd11	abhydrolase domain containing 11	1.114	0.2649	0.6848
68505	1110014N23Rik	RIKEN cDNA 1110014N23 gene	1.114	0.3493	0.7388
67973	Mphosph10	M-phase phosphoprotein 10 (U3 small nucleolar ribonucleoprotein)	1.114	0.04217	NA
66566	Ntpcr	nucleoside-triphosphatase, cancer-related	1.114	0.04906	NA
65106	Arl6ip5	ADP-ribosylation factor-like 6 interacting protein 5	1.114	0.03302	NA
60527	Fads3	fatty acid desaturase 3	1.114	0.2536	0.6783
57028	Pdxp	pyridoxal (pyridoxine, vitamin B6) phosphatase	1.114	0.4486	0.7942
56470	Rgs19	regulator of G-protein signaling 19	1.114	0.2037	0.6362
56459	Sae1	SUMO1 activating enzyme subunit 1	1.114	0.2361	0.6657
56325	Abcb9	ATP-binding cassette, sub-family B (MDR/TAP), member 9	1.114	0.2692	0.6878
52513	Ddx56	DEAD (Asp-Glu-Ala-Asp) box polypeptide 56	1.114	0.2889	0.6998
28075	Pppde2	PPPDE peptidase domain containing 2	1.114	0.1923	0.6283
27414	Sergef	secretion regulating guanine nucleotide exchange factor	1.114	0.1769	0.6164
27357	Gyg	glycogenin	1.114	0.1104	NA
22324	Vav1	vav 1 oncogene	1.114	0.8419	0.9487
21975	Top3a	topoisomerase (DNA) III alpha	1.114	0.1974	0.6324
20419	Shcbp1	Shc SH2-domain binding protein 1	1.114	0.1911	0.6278
20269	Scn3a	sodium channel, voltage-gated, type III, alpha	1.114	0.6189	0.8709
19338	Rab33b	RAB33B, member of RAS oncogene family	1.114	0.144	NA
19261	Sirpa	signal-regulatory protein alpha	1.114	0.1785	0.6181
16400	Itga3	integrin alpha 3	1.114	0.504	0.8221
14628	Ostm1	osteopetrosis associated transmembrane protein 1	1.114	0.0784	NA
13483	Dpp6	dipeptidylpeptidase 6	1.114	0.2038	0.6362
12539	Cdc37	cell division cycle 37 homolog (S. cerevisiae)	1.114	0.228	0.6569
12292	Cacna1s	calcium channel, voltage-dependent, L type, alpha 1S subunit	1.114	0.2136	0.6464
11983	Atpif1	ATPase inhibitory factor 1	1.114	0.1018	NA
11758	Prdx6	peroxiredoxin 6	1.114	0.06286	NA
100303744	Sprr2a2	small proline-rich protein 2A2	1.113	0.182	0.6206
621580	Gm13308	predicted gene 13308	1.113	0.1899	0.6273
613117	4930571B16Rik	RIKEN cDNA 4930571B16 gene	1.113	0.4858	0.8127

553127	Cxx1b	CAAX box 1 homolog B (human)	1.113	0.2801	0.6952
432516	Myo1a	myosin IA	1.113	0.2988	0.7052
353499	Tmc4	transmembrane channel-like gene family 4	1.113	0.1346	NA
246081	Defb11	defensin beta 11	1.113	0.6634	0.8896
235441	Usp3	ubiquitin specific peptidase 3	1.113	0.1269	NA
232680	Cpa2	carboxypeptidase A2, pancreatic	1.113	0.1798	0.6187
216028	Lrrtm3	leucine rich repeat transmembrane neuronal 3	1.113	0.09322	NA
214189	Scgn	secretagogin, EF-hand calcium binding protein	1.113	0.1489	NA
207667	Skor1	SKI family transcriptional corepressor 1	1.113	0.2402	0.6685
170767	Rfxap	regulatory factor X-associated protein	1.113	0.1659	NA
105988	Espl1	extra spindle poles-like 1 (S. cerevisiae)	1.113	0.4686	0.8057
103836	Zfp692	zinc finger protein 692	1.113	0.3679	0.7518
103140	Gstt3	glutathione S-transferase, theta 3	1.113	0.2	0.6345
74580	Pyroxd2	pyridine nucleotide-disulphide oxidoreductase domain 2	1.113	0.5962	0.8609
71745	Cul2	cullin 2	1.113	0.2864	0.6982
71586	Ifih1	interferon induced with helicase C domain 1	1.113	0.0564	NA
69091	Vps26b	vacuolar protein sorting 26 homolog B (yeast)	1.113	0.06416	NA
67248	Rpl39	ribosomal protein L39	1.113	0.3055	0.7101
66101	Ppih	peptidyl prolyl isomerase H	1.113	0.3262	0.7227
60525	Acss2	acyl-CoA synthetase short-chain family member 2	1.113	0.2448	0.6726
59022	Edf1	endothelial differentiation-related factor 1	1.113	0.1431	NA
26934	Racgap1	Rac GTPase-activating protein 1	1.113	0.2531	0.6778
20787	Srebf1	sterol regulatory element binding transcription factor 1	1.113	0.4909	0.8155
20668	Sox13	SRY-box containing gene 13	1.113	0.2991	0.7052
19156	Psap	prosaposin	1.113	0.1805	0.6198
16456	F11r	F11 receptor	1.113	0.3126	0.7145
16371	Irx1	Iroquois related homeobox 1 (Drosophila)	1.113	0.2357	0.6654
14972	H2-K1	histocompatibility 2, K1, K region	1.113	0.08587	NA
13799	En2	engrailed 2	1.113	0.01617	NA
11883	Arsa	arylsulfatase A	1.113	0.09127	NA
11655	Alas1	aminolevulinic acid synthase 1	1.113	0.116	NA
545123	Cyp2d11	cytochrome P450, family 2, subfamily d, polypeptide 11	1.112	0.3626	0.7483
432611	Dnaic2	dynein, axonemal, intermediate chain 2	1.112	0.4485	0.7942
380993	Zfat	zinc finger and AT hook domain containing	1.112	0.3964	0.7674
330908	Opcml	opioid binding protein/cell adhesion molecule-like	1.112	0.6303	0.8749
328971	Spink10	serine peptidase inhibitor, Kazal type 10	1.112	0.4802	0.8101
320078	Olfml2b	olfactomedin-like 2B	1.112	0.135	NA
239114	Il17d	interleukin 17D	1.112	0.3217	0.7199
234695	Rltpr	RGD motif, leucine rich repeats, tropomodulin domain and proline-rich containing	1.112	0.5759	0.8506
231946	D330028D13Rik	RIKEN cDNA D330028D13 gene	1.112	0.1627	NA
228913	Zfp217	zinc finger protein 217	1.112	0.2566	0.6807
219158	2610301G19Rik	RIKEN cDNA 2610301G19 gene	1.112	0.1097	NA
218294	Cdc14b	CDC14 cell division cycle 14 homolog B (S. cerevisiae)	1.112	0.1758	NA
217151	Arl5c	ADP-ribosylation factor-like 5C	1.112	0.4367	0.7888
140483	Hnmt	histamine N-methyltransferase	1.112	0.2427	0.6705
118449	Synpo2	synaptopodin 2	1.112	0.3853	0.7607
114674	Gtf2ird2	GTF2I repeat domain containing 2	1.112	0.1554	NA
114642	Brdt	bromodomain, testis-specific	1.112	0.05847	NA
108052	Slc14a1	solute carrier family 14 (urea transporter), member 1	1.112	0.1919	0.6281

107522	Ece2	endothelin converting enzyme 2	1.112	0.2546	0.6792
78779	Spata2L	spermatogenesis associated 2-like	1.112	0.38	0.7592
76654	Upp2	uridine phosphorylase 2	1.112	0.3815	0.7596
74166	Tmem38a	transmembrane protein 38A	1.112	0.1437	NA
73723	Sh3bgrl3	SH3 domain binding glutamic acid-rich protein-like 3	1.112	0.4208	0.7798
72825	Mon1a	MON1 homolog A (yeast)	1.112	0.1976	0.6328
70478	Mipep	mitochondrial intermediate peptidase	1.112	0.2163	0.6479
70252	2010000I03Rik	RIKEN cDNA 2010000I03 gene	1.112	0.4559	0.7984
69269	Scnm1	sodium channel modifier 1	1.112	0.3304	0.726
69250	2610029K11Rik	RIKEN cDNA 2610029K11 gene	1.112	0.312	0.7139
68999	Anapc10	anaphase promoting complex subunit 10	1.112	0.1335	NA
68618	1110012L19Rik	RIKEN cDNA 1110012L19 gene	1.112	0.09394	NA
67746	4930577N17Rik	RIKEN cDNA 4930577N17 gene	1.112	0.2029	0.6356
67538	Zswim3	zinc finger, SWIM domain containing 3	1.112	0.2569	0.6809
66648	5730494M16Rik	RIKEN cDNA 5730494M16 gene	1.112	0.4637	0.8033
56613	Rps6ka4	ribosomal protein S6 kinase, polypeptide 4	1.112	0.2309	0.6602
56374	Tmem59	transmembrane protein 59	1.112	0.1353	NA
56185	Hao2	hydroxyacid oxidase 2	1.112	0.2254	0.654
29866	Cabp2	calcium binding protein 2	1.112	0.1471	NA
27984	Efhd2	EF hand domain containing 2	1.112	0.3978	0.7684
27784	Commd8	COMM domain containing 8	1.112	0.3329	0.728
23801	Aloxe3	arachidonate lipoxygenase 3	1.112	0.2207	0.6524
21689	Tekt1	tektin 1	1.112	0.1995	0.6343
20846	Stat1	signal transducer and activator of transcription 1	1.112	0.3303	0.726
19934	Rpl22	ribosomal protein L22	1.112	0.1052	NA
19895	Rpia	ribose 5-phosphate isomerase A	1.112	0.1284	NA
19182	Psmc3	proteasome (prosome, macropain) 26S subunit, ATPase 3	1.112	0.2682	0.6871
18858	Pmp22	peripheral myelin protein 22	1.112	0.5324	0.8339
18828	Plscr2	phospholipid scramblase 2	1.112	0.4435	0.7931
18611	Pea15a	phosphoprotein enriched in astrocytes 15A	1.112	0.1664	NA
18476	Pafah1b3	platelet-activating factor acetylhydrolase, isoform 1b, subunit 3	1.112	0.3818	0.7596
18230	Nxn	nucleoredoxin	1.112	0.2087	0.6401
16688	Krt6b	keratin 6B	1.112	0.5883	0.8571
16663	Krt13	keratin 13	1.112	0.6257	0.8728
14275	Folr1	folate receptor 1 (adult)	1.112	0.4711	0.8076
14269	Fnbp1	formin binding protein 1	1.112	0.3289	0.7253
12789	Cnga2	cyclic nucleotide gated channel alpha 2	1.112	0.6216	0.8719
12336	Capns1	calpain, small subunit 1	1.112	0.353	0.7409
11994	Pcdh15	protocadherin 15	1.112	0.07577	NA
11754	Aoc3	amine oxidase, copper containing 3	1.112	0.3606	0.7468
11553	Adra2c	adrenergic receptor, alpha 2c	1.112	0.3276	0.7244
11432	Acp2	acid phosphatase 2, lysosomal	1.112	0.3768	0.7562
665389	Gm7616	predicted gene 7616	1.111	0.7007	0.9033
403346	A730062M13Rik	RIKEN cDNA A730062M13 gene	1.111	0.7564	0.9223
383295	Ypel5	yippee-like 5 (Drosophila)	1.111	0.07363	NA
381979	Brsk1	BR serine/threonine kinase 1	1.111	0.2023	0.6356
360216	Zranb1	zinc finger, RAN-binding domain containing 1	1.111	0.05275	NA
277432	Vstm2l	V-set and transmembrane domain containing 2-like	1.111	0.2644	0.6845
242506	Frmd3	FERM domain containing 3	1.111	0.1321	NA

231296	Lrrc66	leucine rich repeat containing 66	1.111	0.1346	NA
227721	Ppapdc3	phosphatidic acid phosphatase type 2 domain containing 3	1.111	0.1472	NA
226594	Rcsd1	RCSD domain containing 1	1.111	0.4657	0.8049
225655	Slmo1	slowmo homolog 1 (Drosophila)	1.111	0.05686	NA
217700	Acot6	acyl-CoA thioesterase 6	1.111	0.2985	0.7052
216864	Mgl2	macrophage galactose N-acetyl-galactosamine specific lectin 2	1.111	0.03214	NA
108037	Shmt2	serine hydroxymethyltransferase 2 (mitochondrial)	1.111	0.3086	0.7118
104582	Rprml	reprimo-like	1.111	0.2836	0.6974
102614	Rpp25	ribonuclease P 25 subunit (human)	1.111	0.4777	0.81
77697	Mmab	methylmalonic aciduria (cobalamin deficiency) type B homolog (human)	1.111	0.107	NA
73032	Ttc9b	tetratricopeptide repeat domain 9B	1.111	0.4982	0.8194
71766	Raver1	ribonucleoprotein, PTB-binding 1	1.111	0.4818	0.8108
71667	0610007L01Rik	RIKEN cDNA 0610007L01 gene	1.111	0.03237	NA
71445	5530601H04Rik	RIKEN cDNA 5530601H04 gene	1.111	0.1929	0.6291
68877	Maf1	MAF1 homolog (S. cerevisiae)	1.111	0.2765	0.6925
68347	0610011F06Rik	RIKEN cDNA 0610011F06 gene	1.111	0.2003	0.6345
67391	Fundc2	FUN14 domain containing 2	1.111	0.08968	NA
66443	Tnfaip8l1	tumor necrosis factor, alpha-induced protein 8-like 1	1.111	0.1082	NA
66245	Hspbp1	HSPA (heat shock 70kDa) binding protein, cytoplasmic cochaperone 1	1.111	0.4321	0.7859
59044	Rnf130	ring finger protein 130	1.111	0.134	NA
57816	Tesc	tescalcin	1.111	0.08591	NA
57275	Lenep	lens epithelial protein	1.111	0.5849	0.855
56811	Dkk2	dickkopf homolog 2 (Xenopus laevis)	1.111	0.5601	0.846
56447	Copz1	coatomer protein complex, subunit zeta 1	1.111	0.113	NA
26445	Psmb2	proteasome (prosome, macropain) subunit, beta type 2	1.111	0.347	0.7371
26396	Map2k2	mitogen-activated protein kinase kinase 2	1.111	0.1744	NA
22273	Uqcrc1	ubiquinol-cytochrome c reductase core protein 1	1.111	0.2935	0.703
19383	Raly	hnRNP-associated with lethal yellow	1.111	0.09337	NA
19088	Prkar2b	protein kinase, cAMP dependent regulatory, type II beta	1.111	0.06834	NA
18753	Prkcd	protein kinase C, delta	1.111	0.1591	NA
16409	Itgam	integrin alpha M	1.111	0.6565	0.8859
14664	Slc6a9	solute carrier family 6 (neurotransmitter transporter, glycine), member 9	1.111	0.109	NA
14085	Fah	fumarylacetoacetate hydrolase	1.111	0.1059	NA
12576	Cdkn1b	cyclin-dependent kinase inhibitor 1B	1.111	0.1031	NA
12337	Capn5	calpain 5	1.111	0.2934	0.703
11889	Asgr1	asialoglycoprotein receptor 1	1.111	0.1045	NA
319748	Zfp865	zinc finger protein 865	1.11	0.2953	0.7046
319415	Hs3st5	heparan sulfate (glucosamine) 3-O-sulfotransferase 5	1.11	0.05147	NA
243219	2900026A02Rik	RIKEN cDNA 2900026A02 gene	1.11	0.5391	0.8366
242481	Palm2	paralemmin 2	1.11	0.7449	0.9192
232816	Zfp628	zinc finger protein 628	1.11	0.3729	0.7538
231571	Rpap2	RNA polymerase II associated protein 2	1.11	0.02268	NA
224630	Bnip1	BCL2/adenovirus E1B interacting protein 1	1.11	0.3462	0.7365
209018	Vps8	vacuolar protein sorting 8 homolog (S. cerevisiae)	1.11	0.2607	0.6816
192976	BC046404	cDNA sequence BC046404	1.11	0.06876	NA
93747	Echs1	enoyl Coenzyme A hydratase, short chain, 1, mitochondrial	1.11	0.1236	NA
75482	Hspb9	heat shock protein, alpha-crystallin-related, B9	1.11	0.376	0.7556
74347	4632415K11Rik	RIKEN cDNA 4632415K11 gene	1.11	0.2888	0.6998
72103	Aplf	aprataxin and PNKP like factor	1.11	0.1571	NA

72043	Sulf2	sulfatase 2	1.11	0.2428	0.6705
71799	Ptcd1	pentatricopeptide repeat domain 1	1.11	0.2693	0.6878
71770	Ap2b1	adaptor-related protein complex 2, beta 1 subunit	1.11	0.3079	0.7112
69358	Lrrc51	leucine rich repeat containing 51	1.11	0.2896	0.7004
69219	Ddah1	dimethylarginine dimethylaminohydrolase 1	1.11	0.09343	NA
69090	Ascc1	activating signal cointegrator 1 complex subunit 1	1.11	0.3665	0.7504
68682	Slc44a2	solute carrier family 44, member 2	1.11	0.1768	NA
67669	l7Rn6	lethal, Chr 7, Rinchik 6	1.11	0.03384	NA
67606	Fibin	fin bud initiation factor homolog (zebrafish)	1.11	0.4115	0.7747
67439	Xab2	XPA binding protein 2	1.11	0.4313	0.7852
67126	Atp5e	ATP synthase, H+ transporting, mitochondrial F1 complex, epsilon subunit	1.11	0.1743	NA
64654	Fgf23	fibroblast growth factor 23	1.11	0.5241	0.8302
56358	Copz2	coatomer protein complex, subunit zeta 2	1.11	0.2503	0.6767
53317	Plrg1	pleiotropic regulator 1, PRL1 homolog (Arabidopsis)	1.11	0.09008	NA
26921	Map4k4	mitogen-activated protein kinase kinase kinase kinase 4	1.11	0.156	NA
23829	C1ql1	complement component 1, q subcomponent-like 1	1.11	0.5557	0.8448
21955	Tnnt1	troponin T1, skeletal, slow	1.11	0.1219	NA
20116	Rps8	ribosomal protein S8	1.11	0.2784	0.6934
19039	Lgals3bp	lectin, galactoside-binding, soluble, 3 binding protein	1.11	0.1928	0.629
18769	Pkig	protein kinase inhibitor, gamma	1.11	0.1243	NA
18643	Pfn1	profilin 1	1.11	0.5266	0.8324
17344	Pias2	protein inhibitor of activated STAT 2	1.11	0.06156	NA
17283	Men1	multiple endocrine neoplasia 1	1.11	0.3649	0.7493
17160	Man2b2	mannosidase 2, alpha B2	1.11	0.5012	0.8204
16155	Il10rb	interleukin 10 receptor, beta	1.11	0.09391	NA
15166	Hcn2	hyperpolarization-activated, cyclic nucleotide-gated K+ 2	1.11	0.4808	0.8103
14528	Gch1	GTP cyclohydrolase 1	1.11	0.1591	NA
13205	Ddx3x	DEAD/H (Asp-Glu-Ala-Asp/His) box polypeptide 3, X-linked	1.11	0.1147	NA
13198	Ddit3	DNA-damage inducible transcript 3	1.11	0.1254	NA
13000	Csnk2a2	casein kinase 2, alpha prime polypeptide	1.11	0.2555	0.6798
12304	Pdia4	protein disulfide isomerase associated 4	1.11	0.528	0.8332
12155	Bmp15	bone morphogenetic protein 15	1.11	0.4903	0.815
330814	Lphn1	latrophilin 1	1.109	0.5275	0.8332
319455	Pld5	phospholipase D family, member 5	1.109	0.405	0.7716
269713	Clip2	CAP-GLY domain containing linker protein 2	1.109	0.6573	0.8863
269152	Kif26b	kinesin family member 26B	1.109	0.3861	0.761
258353	Olfr521	olfactory receptor 521	1.109	0.2857	0.6982
239845	Gpr156	G protein-coupled receptor 156	1.109	0.1553	NA
239559	A4galt	alpha 1,4-galactosyltransferase	1.109	0.6909	0.8989
235330	Ttc12	tetratricopeptide repeat domain 12	1.109	0.2352	0.6653
212862	Chpt1	choline phosphotransferase 1	1.109	0.4825	0.8112
110854	Ppp2r4	protein phosphatase 2A, regulatory subunit B (PR 53)	1.109	0.4534	0.7968
106952	Arap3	ArfGAP with RhoGAP domain, ankyrin repeat and PH domain 3	1.109	0.1409	NA
101694	AI854517	expressed sequence AI854517	1.109	0.1213	NA
100929	Tyw1	tRNA-yW synthesizing protein 1 homolog (S. cerevisiae)	1.109	0.2621	0.6831
98682	Mfsd6	major facilitator superfamily domain containing 6	1.109	0.03987	NA
78892	Crispld2	cysteine-rich secretory protein LCCL domain containing 2	1.109	0.3916	0.7643
78834	Zfp623	zinc finger protein 623	1.109	0.2207	0.6524
77857	9430065F17Rik	RIKEN cDNA 9430065F17 gene	1.109	0.4209	0.7798

75930	4930567H12Rik	RIKEN cDNA 4930567H12 gene	1.109	0.746	0.9193
75620	2810422J05Rik	RIKEN cDNA 2810422J05 gene	1.109	0.2605	0.6815
75141	Rasd2	RASD family, member 2	1.109	0.3312	0.7265
74919	4930471M23Rik	RIKEN cDNA 4930471M23 gene	1.109	0.3672	0.7508
73242	Atat1	alpha tubulin acetyltransferase 1	1.109	0.5618	0.8465
72392	Tmem175	transmembrane protein 175	1.109	0.1493	NA
71003	Prss41	protease, serine, 41	1.109	0.3919	0.7645
70726	Angptl6	angiopoietin-like 6	1.109	0.1607	NA
70551	Tmtc4	transmembrane and tetratricopeptide repeat containing 4	1.109	0.1254	NA
68481	Mpzl1	myelin protein zero-like 1	1.109	0.1315	NA
67445	C1qtnf4	C1q and tumor necrosis factor related protein 4	1.109	0.3686	0.7521
67106	Zbtb8os	zinc finger and BTB domain containing 8 opposite strand	1.109	0.2415	0.6694
65116	Prrg2	proline-rich Gla (G-carboxyglutamic acid) polypeptide 2	1.109	0.1282	NA
56812	Dnajb2	DnaJ (Hsp40) homolog, subfamily B, member 2	1.109	0.02447	NA
53858	Rwdd2b	RWD domain containing 2B	1.109	0.14	NA
53317	Plrg1	pleiotropic regulator 1, PRL1 homolog (Arabidopsis)	1.109	0.1072	NA
51812	Mcrs1	microspherule protein 1	1.109	0.4084	0.7731
28064	Yipf3	Yip1 domain family, member 3	1.109	0.3047	0.7093
26357	Abcg2	ATP-binding cassette, sub-family G (WHITE), member 2	1.109	0.05703	NA
24100	Tpra1	transmembrane protein, adipocyte asscociated 1	1.109	0.3844	0.7606
20317	Serpinf1	serine (or cysteine) peptidase inhibitor, clade F, member 1	1.109	0.4767	0.8099
19271	Ptprj	protein tyrosine phosphatase, receptor type, J	1.109	0.2079	0.6401
18011	Neurl1a	neuralized homolog 1A (Drosophila)	1.109	0.1261	NA
16536	Kcnq2	potassium voltage-gated channel, subfamily Q, member 2	1.109	0.5408	0.8375
14945	Gzmk	granzyme K	1.109	0.6191	0.8709
14405	Gabrg1	gamma-aminobutyric acid (GABA) A receptor, subunit gamma 1	1.109	0.05275	NA
13650	Rhbdf1	rhomboid family 1 (Drosophila)	1.109	0.3927	0.7651
12854	Cort	cortistatin	1.109	0.4082	0.7731
12015	Bad	BCL2-associated agonist of cell death	1.109	0.2293	0.6591
433904	Ociad2	OCIA domain containing 2	1.108	0.3006	0.7065
269261	Rpl12	ribosomal protein L12	1.108	0.1668	NA
246104	Rhbdl3	rhomboid, veinlet-like 3 (Drosophila)	1.108	0.3452	0.736
234729	Vac14	Vac14 homolog (S. cerevisiae)	1.108	0.3437	0.7356
226744	Cnst	consortin, connexin sorting protein	1.108	0.08178	NA
224023	Klhl22	kelch-like 22 (Drosophila)	1.108	0.2122	0.6449
223473	Nipal2	NIPA-like domain containing 2	1.108	0.4594	0.8009
223453	Dap	death-associated protein	1.108	0.4358	0.7885
217219	Fam171a2	family with sequence similarity 171, member A2	1.108	0.2732	0.6905
216119	Ybey	ybeY metallopeptidase	1.108	0.2456	0.6733
110095	Pygl	liver glycogen phosphorylase	1.108	0.7035	0.9046
108121	U2af1	U2 small nuclear ribonucleoprotein auxiliary factor (U2AF) 1	1.108	0.04256	NA
108116	Slco3a1	solute carrier organic anion transporter family, member 3a1	1.108	0.1909	NA
107581	Col16a1	collagen, type XVI, alpha 1	1.108	0.3366	0.7305
77066	5930409G06Rik	RIKEN cDNA 5930409G06 gene	1.108	0.3459	0.7362
76483	Lmf1	lipase maturation factor 1	1.108	0.2688	0.6876
76482	3110002H16Rik	RIKEN cDNA 3110002H16 gene	1.108	0.181	NA
76429	Lhpp	phospholysine phosphohistidine inorganic pyrophosphate phosphatase	1.108	0.5394	0.8366
74778	Rrp7a	ribosomal RNA processing 7 homolog A (S. cerevisiae)	1.108	0.3404	0.7328
74359	4931414P19Rik	RIKEN cDNA 4931414P19 gene	1.108	0.2901	0.7006

74013	Rftn2	raftlin family member 2	1.108	0.1961	NA
73833	Fam98c	family with sequence similarity 98, member C	1.108	0.2164	0.6479
71667	0610007L01Rik	RIKEN cDNA 0610007L01 gene	1.108	0.09298	NA
70025	Acot7	acyl-CoA thioesterase 7	1.108	0.4076	0.7726
69953	2810025M15Rik	RIKEN cDNA 2810025M15 gene	1.108	0.06094	NA
69080	Gmppa	GDP-mannose pyrophosphorylase A	1.108	0.3875	0.7611
68149	Otub2	OTU domain, ubiquitin aldehyde binding 2	1.108	0.1331	NA
67704	1810037I17Rik	RIKEN cDNA 1810037I17 gene	1.108	0.1604	NA
67422	Dhdds	dehydrodolichyl diphosphate synthase	1.108	0.2492	0.6767
67203	Nde1	nuclear distribution gene E homolog 1 (A nidulans)	1.108	0.07934	NA
66526	2210012G02Rik	RIKEN cDNA 2210012G02 gene	1.108	0.2184	0.6506
66496	Ppdpf	pancreatic progenitor cell differentiation and proliferation factor homolog (zebrafish)RIKEN cDNA 2700038C09 gene	1.108	0.1388	NA
57357	Srd5a3	steroid 5 alpha-reductase 3	1.108	0.1872	NA
56436	Adrm1	adhesion regulating molecule 1	1.108	0.2058	0.6386
50758	Fbxl17	F-box and leucine-rich repeat protein 17	1.108	0.1618	NA
26417	Mapk3	mitogen-activated protein kinase 3	1.108	0.3373	0.731
26363	Btd	biotinidase	1.108	0.2247	0.654
23837	Cfdp1	craniofacial development protein 1	1.108	0.03871	NA
21401	Tcea3	transcription elongation factor A (SII), 3	1.108	0.6734	0.8924
20516	Slc20a2	solute carrier family 20, member 2	1.108	0.2195	0.6513
20257	Stmn2	stathmin-like 2	1.108	0.06366	NA
20220	Sap18	Sin3-associated polypeptide 18	1.108	0.09421	NA
20196	S100a13	S100 calcium binding protein A13	1.108	0.2363	0.6657
14810	Grin1	glutamate receptor, ionotropic, NMDA1 (zeta 1)	1.108	0.5705	0.8492
14809	Grik5	glutamate receptor, ionotropic, kainate 5 (gamma 2)	1.108	0.4263	0.7823
14705	Bscl2	Bernardinelli-Seip congenital lipodystrophy 2 homolog (human)	1.108	0.3176	0.7179
14230	Fkbp10	FK506 binding protein 10	1.108	0.445	0.7938
13990	Smarcad1	SWI/SNF-related, matrix-associated actin-dependent regulator of chromatin, subfamily a, containing DEAD/H box 1	1.108	0.05237	NA
13865	Nr2f1	nuclear receptor subfamily 2, group F, member 1	1.108	0.1552	NA
11669	Aldh2	aldehyde dehydrogenase 2, mitochondrial	1.108	0.148	NA
11419	Accn2	amiloride-sensitive cation channel 2, neuronal	1.108	0.1813	NA
100503637	LOC100503637	envelope glycoprotein-like	1.107	0.2235	0.654
100044193	LOC100044193	hypothetical protein LOC100044193	1.107	0.3469	0.7371
100038991	Gm2000	predicted gene 2000	1.107	0.2697	0.6878
626578	Gbp10	guanylate-binding protein 10	1.107	0.2132	0.6459
544678	2010015L04Rik	RIKEN cDNA 2010015L04 gene	1.107	0.06207	NA
408063	BC062258	cDNA sequence BC062258	1.107	0.1606	NA
381914	B230311B06Rik	RIKEN cDNA B230311B06 gene	1.107	0.3865	0.761
332397	Nanos1	nanos homolog 1 (Drosophila)	1.107	0.3228	0.7203
331474	Rgag4	retrotransposon gag domain containing 4	1.107	0.4848	0.8126
244958	Mrap2	melanocortin 2 receptor accessory protein 2	1.107	0.2844	0.6977
244551	Nanos3	nanos homolog 3 (Drosophila)	1.107	0.2479	0.675
234734	Aars	alanyl-tRNA synthetase	1.107	0.2792	0.6944
230959	Ajap1	adherens junction associated protein 1	1.107	0.234	0.6633
223665	C030006K11Rik	RIKEN cDNA C030006K11 gene	1.107	0.4272	0.7827
209446	Tcfe3	transcription factor E3	1.107	0.4406	0.7913
207792	BC034090	cDNA sequence BC034090	1.107	0.02395	NA
114143	Atp6v0b	ATPase, H+ transporting, lysosomal V0 subunit B	1.107	0.3567	0.7443
94282	Sfxn5	sideroflexin 5	1.107	0.3975	0.7683

81703	Jdp2	Jun dimerization protein 2	1.107	0.434	0.7875
78749	Filip1l	filamin A interacting protein 1-like	1.107	0.172	NA
78334	Cdk19	cyclin-dependent kinase 19	1.107	0.05768	NA
76781	Mettl4	methyltransferase like 4	1.107	0.2331	0.6622
75838	4930560O18Rik	RIKEN cDNA 4930560O18 gene	1.107	0.4207	0.7798
72669	2810032G03Rik	RIKEN cDNA 2810032G03 gene	1.107	0.4298	0.7843
71913	Tmem79	transmembrane protein 79	1.107	0.4517	0.7951
70266	Ccbl1	cysteine conjugate-beta lyase 1	1.107	0.05797	NA
68531	1110020A21Rik	RIKEN cDNA 1110020A21 gene	1.107	0.1713	NA
68135	Eif3h	eukaryotic translation initiation factor 3, subunit H	1.107	0.1259	NA
67763	Prpsap1	phosphoribosyl pyrophosphate synthetase-associated protein 1	1.107	0.1475	NA
66541	Immp1l	IMP1 inner mitochondrial membrane peptidase-like (S. cerevisiae)	1.107	0.5484	0.8407
66487	2010107H07Rik	RIKEN cDNA 2010107H07 gene	1.107	0.1228	NA
64660	Mrps24	mitochondrial ribosomal protein S24	1.107	0.07538	NA
64242	Ngb	neuroglobin	1.107	0.1208	NA
50783	Lsm4	LSM4 homolog, U6 small nuclear RNA associated (S. cerevisiae)	1.107	0.01527	NA
28000	Prpf19	PRP19/PSO4 pre-mRNA processing factor 19 homolog (S. cerevisiae)	1.107	0.3098	0.713
27357	Gyg	glycogenin	1.107	0.1007	NA
26874	Abcd2	ATP-binding cassette, sub-family D (ALD), member 2	1.107	0.1718	NA
26459	Slc27a5	solute carrier family 27 (fatty acid transporter), member 5	1.107	0.2306	0.6601
22590	Xpa	xeroderma pigmentosum, complementation group A	1.107	0.03965	NA
20605	Sstr1	somatostatin receptor 1	1.107	0.3357	0.7302
20430	Cyfip1	cytoplasmic FMR1 interacting protein 1	1.107	0.2719	0.6891
16785	Rpsa	ribosomal protein SA	1.107	0.3851	0.7607
16768	Lag3	lymphocyte-activation gene 3	1.107	0.4684	0.8056
16668	Krt18	keratin 18	1.107	0.02347	NA
14775	Gpx1	glutathione peroxidase 1	1.107	0.3075	0.7112
14057	Sfxn1	sideroflexin 1	1.107	0.1376	NA
12921	Crhr1	corticotropin releasing hormone receptor 1	1.107	0.3564	0.7443
12700	Cish	cytokine inducible SH2-containing protein	1.107	0.2056	0.6386
11972	Atp6v0d1	ATPase, H+ transporting, lysosomal V0 subunit D1	1.107	0.3243	0.7213
100038604	3426406K10Rik	RIKEN cDNA 3426406K10 gene	1.106	0.6812	0.8949
654472	Gm12070	glyceraldehyde-3-phosphate dehydrogenase pseudogene	1.106	0.3897	0.7626
329274	Fam163a	family with sequence similarity 163, member A	1.106	0.04575	NA
319901	Dsel	dermatan sulfate epimerase-like	1.106	0.0952	NA
257932	Olfr332	olfactory receptor 332	1.106	0.8092	0.9396
252838	Tox	thymocyte selection-associated high mobility group box	1.106	0.1052	NA
234396	Ankle1	ankyrin repeat and LEM domain containing 1	1.106	0.6604	0.888
192662	Arhgdia	Rho GDP dissociation inhibitor (GDI) alpha	1.106	0.2928	0.7026
171171	Ntng2	netrin G2	1.106	0.121	NA
104184	Blmh	bleomycin hydrolase	1.106	0.07528	NA
76573	1700027D21Rik	RIKEN cDNA 1700027D21 gene	1.106	0.2456	0.6733
75805	Nln	neurolysin (metallopeptidase M3 family)	1.106	0.1559	NA
69917	Obfc2b	oligonucleotide/oligosaccharide-binding fold containing 2B	1.106	0.4749	0.8094
69824	Glod5	glyoxalase domain containing 5	1.106	0.2598	0.6815
69656	Pir	pirin	1.106	0.1325	NA
69186	1810027O10Rik	RIKEN cDNA 1810027O10 gene	1.106	0.04943	NA
68910	Zfp467	zinc finger protein 467	1.106	0.07051	NA
68852	Lrrn4cl	LRRN4 C-terminal like	1.106	0.1593	NA

67557	Larp6	La ribonucleoprotein domain family, member 6	1.106	0.07979	NA	
66911	Nudt16l1	nudix (nucleoside diphosphate linked moiety X)-type motif 16-like 1	1.106	0.09472	NA	
60455	Tmem8	transmembrane protein 8 (five membrane-spanning domains)	1.106	0.2034	NA	
56454	Aldh18a1	aldehyde dehydrogenase 18 family, member A1	1.106	0.5717		0.8497
54218	B3galt4	UDP-Gal:betaGlcNAc beta 1,3-galactosyltransferase, polypeptide 4	1.106	0.4236		0.7816
50929	Il22	interleukin 22	1.106	0.5619		0.8465
50780	Rgs3	regulator of G-protein signaling 3	1.106	0.05151	NA	
30838	Fbxw4	F-box and WD-40 domain protein 4	1.106	0.2156		0.6479
27416	Abcc5	ATP-binding cassette, sub-family C (CFTR/MRP), member 5	1.106	0.4315		0.7855
23893	Grem2	gremlin 2 homolog, cysteine knot superfamily (Xenopus laevis)	1.106	0.08493	NA	
20194	S100a10	S100 calcium binding protein A10 (calpactin)	1.106	0.1515	NA	
19981	Rpl37a	ribosomal protein L37a	1.106	0.3463		0.7366
17063	Muc13	mucin 13, epithelial transmembrane	1.106	0.09811	NA	
16913	Psmb8	proteasome (prosome, macropain) subunit, beta type 8 (large multifunctional peptidase 7)	1.106	0.3144		0.7157
16855	Lgals4	lectin, galactose binding, soluble 4	1.106	0.4398		0.7908
16650	Kpna6	karyopherin (importin) alpha 6	1.106	0.2463		0.6734
16590	Kit	kit oncogene	1.106	0.3557		0.7439
15530	Hspg2	perlecan (heparan sulfate proteoglycan 2)	1.106	0.8212		0.9433
15013	H2-Q2	histocompatibility 2, Q region locus 2	1.106	0.2454		0.6731
13713	Elk3	ELK3, member of ETS oncogene family	1.106	0.3413		0.7334
12497	Entpd6	ectonucleoside triphosphate diphosphohydrolase 6	1.106	0.4015		0.7703
12475	Cd14	CD14 antigen	1.106	0.1909	NA	
12332	Capg	capping protein (actin filament), gelsolin-like	1.106	0.5022		0.8214
381835	Gm1078	predicted gene 1078	1.105	0.3359		0.7303
331004	Slc9a9	solute carrier family 9 (sodium/hydrogen exchanger), member 9	1.105	0.4778		0.81
269630	5031425E22Rik	RIKEN cDNA 5031425E22 gene	1.105	0.5257		0.832
258028	Olfr901	olfactory receptor 901	1.105	0.8204		0.9432
245532	Awat2	acyl-CoA wax alcohol acyltransferase 2	1.105	0.5437		0.839
238076	Kcns3	potassium voltage-gated channel, delayed-rectifier, subfamily S, member 3	1.105	0.3169		0.7174
226413	Lct	lactase	1.105	0.2739		0.6918
219022	Ttc5	tetratricopeptide repeat domain 5	1.105	0.2284		0.6577
140483	Hnmt	histamine N-methyltransferase	1.105	0.2995		0.7054
102294	Cyp4v3	cytochrome P450, family 4, subfamily v, polypeptide 3	1.105	0.1729	NA	
93875	Pcdhb4	protocadherin beta 4	1.105	0.377		0.7563
77767	Ermn	ermin, ERM-like protein	1.105	0.5303		0.8333
77359	9430063H18Rik	RIKEN cDNA 9430063H18 gene	1.105	0.4215		0.7798
76820	Fam49a	family with sequence similarity 49, member A	1.105	0.05081	NA	
73721	1110017D15Rik	RIKEN cDNA 1110017D15 gene	1.105	0.2265		0.6553
72511	2610316D01Rik	RIKEN cDNA 2610316D01 gene	1.105	0.3205		0.7196
72388	Ripk4	receptor-interacting serine-threonine kinase 4	1.105	0.2695		0.6878
71745	Cul2	cullin 2	1.105	0.1123	NA	
70527	Stambp	STAM binding protein	1.105	0.05811	NA	
69683	2310044H10Rik	RIKEN cDNA 2310044H10 gene	1.105	0.5221		0.8293
69297	Lrrc46	leucine rich repeat containing 46	1.105	0.4931		0.8159
68420	Ankrd13a	ankyrin repeat domain 13a	1.105	0.1534	NA	
68052	Rps13	ribosomal protein S13	1.105	0.2981		0.7051
67681	Mrpl18	mitochondrial ribosomal protein L18	1.105	0.1225	NA	
67574	Alg13	asparagine-linked glycosylation 13 homolog (S. cerevisiae)	1.105	0.07924	NA	
67247	Mosc2	MOCO sulphurase C-terminal domain containing 2	1.105	0.05875	NA	

66241	Tmem9	transmembrane protein 9	1.105	0.2744	0.6922
66066	Gng11	guanine nucleotide binding protein (G protein), gamma 11	1.105	0.3778	0.7569
63873	Trpv4	transient receptor potential cation channel, subfamily V, member 4	1.105	0.5708	0.8492
57080	Gtf2ird1	general transcription factor II I repeat domain-containing 1	1.105	0.2558	0.68
56738	Mocs1	molybdenum cofactor synthesis 1	1.105	0.4954	0.8179
56513	Pard6a	par-6 (partitioning defective 6,) homolog alpha (C. elegans)	1.105	0.2126	0.6451
56177	Olfm1	olfactomedin 1	1.105	0.09159	NA
54411	Atp6ap1	ATPase, H+ transporting, lysosomal accessory protein 1	1.105	0.3451	0.736
53413	Exoc7	exocyst complex component 7	1.105	0.1139	NA
26897	Acot1	acyl-CoA thioesterase 1	1.105	0.3148	0.7159
26559	Hunk	hormonally upregulated Neu-associated kinase	1.105	0.4227	0.7804
20287	Sct	secretin	1.105	0.4257	0.7823
18984	Por	P450 (cytochrome) oxidoreductase	1.105	0.1874	NA
17532	Mras	muscle and microspikes RAS	1.105	0.5	0.8197
17434	Mocs2	molybdenum cofactor synthesis 2	1.105	0.1358	NA
17391	Mmp24	matrix metallopeptidase 24	1.105	0.4195	0.7793
15464	Hrc	histidine rich calcium binding protein	1.105	0.06181	NA
14430	Galt	galactose-1-phosphate uridyl transferase	1.105	0.2937	0.7033
14225	Fkbp1a	FK506 binding protein 1a	1.105	0.1014	NA
12845	Comp	cartilage oligomeric matrix protein	1.105	0.5205	0.8286
12804	Cntfr	ciliary neurotrophic factor receptor	1.105	0.4142	0.7758
622320	Kctd21	potassium channel tetramerisation domain containing 21	1.104	0.03861	NA
407243	Tmem189	transmembrane protein 189	1.104	0.5455	0.839
353502	Hcfc1r1	host cell factor C1 regulator 1 (XPO1-dependent)	1.104	0.3196	0.7189
329659	E130311K13Rik	RIKEN cDNA E130311K13 gene	1.104	0.4048	0.7716
319953	Ttll1	tubulin tyrosine ligase-like 1	1.104	0.3869	0.761
258662	Olfr738	olfactory receptor 738	1.104	0.7354	0.9169
245049	Myrip	myosin VIIA and Rab interacting protein	1.104	0.1102	NA
241556	Tspan18	tetraspanin 18	1.104	0.358	0.7454
225724	Mapk4	mitogen-activated protein kinase 4	1.104	0.2328	0.6622
214575	Tdrd5	tudor domain containing 5	1.104	0.1789	NA
170935	Grid2ip	glutamate receptor, ionotropic, delta 2 (Grid2) interacting protein 1	1.104	0.4211	0.7798
117160	Ttyh2	tweety homolog 2 (Drosophila)	1.104	0.4248	0.7819
108114	Slc22a7	solute carrier family 22 (organic anion transporter), member 7	1.104	0.521	0.8286
103551	E130012A19Rik	RIKEN cDNA E130012A19 gene	1.104	0.1372	NA
83558	Tex11	testis expressed gene 11	1.104	0.2214	0.6525
77573	Vps33a	vacuolar protein sorting 33A (yeast)	1.104	0.1326	NA
76687	Spcs3	signal peptidase complex subunit 3 homolog (S. cerevisiae)	1.104	0.08599	NA
72992	2900076A07Rik	RIKEN cDNA 2900076A07 gene	1.104	0.258	0.6815
72121	Dennd2d	DENN/MADD domain containing 2D	1.104	0.1854	NA
71983	Tmco6	transmembrane and coiled-coil domains 6	1.104	0.3746	0.755
71609	Tradd	TNFRSF1A-associated via death domain	1.104	0.2684	0.6873
70617	5730508B09Rik	RIKEN cDNA 5730508B09 gene	1.104	0.2044	NA
69792	Med6	mediator of RNA polymerase II transcription, subunit 6 homolog (yeast)	1.104	0.05847	NA
69187	Erp27	endoplasmic reticulum protein 27	1.104	0.4754	0.8097
68002	1110058L19Rik	RIKEN cDNA 1110058L19 gene	1.104	0.1499	NA
67180	Yipf5	Yip1 domain family, member 5	1.104	0.152	NA
67109	Zfp787	zinc finger protein 787	1.104	0.1929	NA
64136	Sdf2l1	stromal cell-derived factor 2-like 1	1.104	0.3003	0.706

58246	Slc35b4	solute carrier family 35, member B4	1.104	0.279	0.694
56471	Stmn4	stathmin-like 4	1.104	0.09816	NA
56448	Cyp2d22	cytochrome P450, family 2, subfamily d, polypeptide 22	1.104	0.4298	0.7843
56395	Tmem115	transmembrane protein 115	1.104	0.4377	0.7894
56279	Fam69b	family with sequence similarity 69, member B	1.104	0.156	NA
54006	Deaf1	deformed epidermal autoregulatory factor 1 (Drosophila)	1.104	0.1932	NA
53625	B3gnt2	UDP-GlcNAc:betaGal beta-1,3-N-acetylglucosaminyltransferase 2	1.104	0.08781	NA
50781	Dkk3	dickkopf homolog 3 (Xenopus laevis)	1.104	0.171	NA
27361	Sepx1	selenoprotein X 1	1.104	0.05457	NA
26562	Ncdn	neurochondrin	1.104	0.5018	0.821
22201	Uba1	ubiquitin-like modifier activating enzyme 1	1.104	0.1142	NA
20810	Srm	spermidine synthase	1.104	0.3628	0.7483
20055	Rps16	ribosomal protein S16	1.104	0.2069	NA
18673	Phb	prohibitin	1.104	0.2021	NA
18286	Odf2	outer dense fiber of sperm tails 2	1.104	0.1107	NA
18127	Nos3	nitric oxide synthase 3, endothelial cell	1.104	0.1944	NA
18081	Ninj1	ninjurin 1	1.104	0.2195	NA
16969	Zbtb7a	zinc finger and BTB domain containing 7a	1.104	0.0502	NA
16190	Il4ra	interleukin 4 receptor, alpha	1.104	0.2772	0.6925
15114	Hap1	huntingtin-associated protein 1	1.104	0.3976	0.7683
14633	Gli2	GLI-Kruppel family member GLI2	1.104	0.6546	0.8854
11519	Add2	adducin 2 (beta)	1.104	0.1823	NA
630499	H2-K2	histocompatibility 2, K region locus 2	1.103	0.2752	0.6925
432825	Gm5458	predicted gene 5458	1.103	0.1786	NA
407803	BC051226	cDNA sequence BC051226	1.103	0.357	0.7444
279029	Gm711	predicted gene 711	1.103	0.4926	0.8157
270893	Tmem132e	transmembrane protein 132E	1.103	0.3833	0.7599
270028	Fam155a	family with sequence similarity 155, member A	1.103	0.1622	NA
234388	Ccdc124	coiled-coil domain containing 124	1.103	0.3682	0.7518
230126	Shb	src homology 2 domain-containing transforming protein B	1.103	0.2698	0.6878
223776	1300018J18Rik	RIKEN cDNA 1300018J18 gene	1.103	0.1285	NA
212555	Pqlc2	PQ loop repeat containing 2	1.103	0.1643	NA
117229	Stk33	serine/threonine kinase 33	1.103	0.4106	0.7742
109161	Ube2q2	ubiquitin-conjugating enzyme E2Q (putative) 2	1.103	0.2354	0.6654
103694	Tmed4	transmembrane emp24 protein transport domain containing 4	1.103	0.05808	NA
79362	Bhlhe41	basic helix-loop-helix family, member e41	1.103	0.5084	0.8242
77220	Tmem200a	transmembrane protein 200A	1.103	0.2727	0.6897
76773	Wdyhv1	WDYHV motif containing 1	1.103	0.04066	NA
76580	Mib2	mindbomb homolog 2 (Drosophila)	1.103	0.1691	NA
76505	1500009C09Rik	RIKEN cDNA 1500009C09 gene	1.103	0.1152	NA
74513	Neto2	neuropilin (NRP) and tolloid (TLL)-like 2	1.103	0.2502	0.6767
74513	Neto2	neuropilin (NRP) and tolloid (TLL)-like 2	1.103	0.2695	0.6878
72136	Chst14	carbohydrate (N-acetylgalactosamine 4-0) sulfotransferase 14	1.103	0.4918	0.8156
72084	Pigx	phosphatidylinositol glycan anchor biosynthesis, class X	1.103	0.07436	NA
72050	Kdelc1	KDEL (Lys-Asp-Glu-Leu) containing 1	1.103	0.6673	0.8904
70420	2610034B18Rik	RIKEN cDNA 2610034B18 gene	1.103	0.3157	0.7165
69724	Rnaseh2a	ribonuclease H2, large subunit	1.103	0.3461	0.7364
67672	0610040B10Rik	RIKEN cDNA 0610040B10 gene	1.103	0.1157	NA
67655	Ctdp1	CTD (carboxy-terminal domain, RNA polymerase II, polypeptide A) phosphatase, subunit 1	1.103	0.2299	0.6596

67393	Cxxc5	CXXC finger 5	1.103	0.04983	NA
67106	Zbtb8os	zinc finger and BTB domain containing 8 opposite strand	1.103	0.2924	0.7024
66464	Taf12	TAF12 RNA polymerase II, TATA box binding protein (TBP)-associated factor	1.103	0.1492	NA
66102	Cxcl16	chemokine (C-X-C motif) ligand 16	1.103	0.3729	0.7538
64379	Irx6	Iroquois related homeobox 6 (Drosophila)	1.103	0.2672	0.6865
56550	Ube2d2	ubiquitin-conjugating enzyme E2D 2	1.103	0.1718	NA
54351	Rai12	retinoic acid induced 12	1.103	0.06643	NA
52626	Cdkn2aipnl	CDKN2A interacting protein N-terminal like	1.103	0.0248	NA
51793	Ddah2	dimethylarginine dimethylaminohydrolase 2	1.103	0.02439	NA
26426	Nubp2	nucleotide binding protein 2	1.103	0.2212	NA
23986	Peci	peroxisomal delta3, delta2-enoyl-Coenzyme A isomerase	1.103	0.2036	NA
23945	Mgll	monoglyceride lipase	1.103	0.3239	0.7211
22282	Usf2	upstream transcription factor 2	1.103	0.2025	NA
22163	Tnfrsf4	tumor necrosis factor receptor superfamily, member 4	1.103	0.2801	0.6952
21930	Tnfaip6	tumor necrosis factor alpha induced protein 6	1.103	0.1114	NA
20927	Abcc8	ATP-binding cassette, sub-family C (CFTR/MRP), member 8	1.103	0.1879	NA
18168	Npy5r	neuropeptide Y receptor Y5	1.103	0.4516	0.7951
16784	Lamp2	lysosomal-associated membrane protein 2	1.103	0.1937	NA
14178	Fgf7	fibroblast growth factor 7	1.103	0.3217	0.7199
13057	Cyba	cytochrome b-245, alpha polypeptide	1.103	0.2371	0.6659
12876	Cpe	carboxypeptidase E	1.103	0.0818	NA
12036	Bcat2	branched chain aminotransferase 2, mitochondrial	1.103	0.6243	0.8728
11793	Atg5	autophagy-related 5 (yeast)	1.103	0.06503	NA
100126824	Sco2	SCO cytochrome oxidase deficient homolog 2 (yeast)	1.102	0.3701	0.7524
435518	Pdxk-ps	pyridoxal (pyridoxine, vitamin B6) kinase, pseudogene	1.102	0.71	0.9069
381085	Tbc1d22b	TBC1 domain family, member 22B	1.102	0.4474	0.7938
332110	Mapk15	mitogen-activated protein kinase 15	1.102	0.569	0.8488
330734	B930018H19	hypothetical protein B930018H19	1.102	0.4154	0.7767
320795	Pkn1	protein kinase N1	1.102	0.2912	0.7016
319737	D130051D11Rik	RIKEN cDNA D130051D11 gene	1.102	0.6879	0.8975
230857	Ece1	endothelin converting enzyme 1	1.102	0.3507	0.7393
230603	Ttc39a	tetratricopeptide repeat domain 39A	1.102	0.3697	0.7521
228777	Nrsn2	neurensin 2	1.102	0.4966	0.819
227835	Gtdc1	glycosyltransferase-like domain containing 1	1.102	0.05424	NA
227622	BC029214	cDNA sequence BC029214	1.102	0.2148	NA
218506	Mrps27	mitochondrial ribosomal protein S27	1.102	0.5351	0.835
215210	Tmem120a	transmembrane protein 120A	1.102	0.1066	NA
214575	Tdrd5	tudor domain containing 5	1.102	0.189	NA
212986	Scfd2	Sec1 family domain containing 2	1.102	0.1226	NA
207175	Cetn4	centrin 4	1.102	0.1045	NA
118454	Gjc2	gap junction protein, gamma 2	1.102	0.04767	NA
110606	Fntb	farnesyltransferase, CAAX box, beta	1.102	0.4863	0.8127
105446	Gmpr2	guanosine monophosphate reductase 2	1.102	0.2112	NA
101568	Vrk3	vaccinia related kinase 3	1.102	0.09595	NA
76267	Fads1	fatty acid desaturase 1	1.102	0.1084	NA
74476	4933439C10Rik	RIKEN cDNA 4933439C10 gene	1.102	0.3716	0.7535
74164	Nfx1	nuclear transcription factor, X-box binding 1	1.102	0.3822	0.7596
73736	Fcf1	FCF1 small subunit (SSU) processome component homolog (S. cerevisiae)	1.102	0.09066	NA
72446	Prr5l	proline rich 5 like	1.102	0.315	0.7161

71911	Bdh1	3-hydroxybutyrate dehydrogenase, type 1	1.102	0.3463	0.7366
70853	Vwa3b	von Willebrand factor A domain containing 3B	1.102	0.4345	0.7878
70478	Mipep	mitochondrial intermediate peptidase	1.102	0.2461	0.6733
70396	Asnsd1	asparagine synthetase domain containing 1	1.102	0.1061	NA
69736	Nup37	nucleoporin 37	1.102	0.1336	NA
69723	Rpain	RPA interacting protein	1.102	0.1609	NA
68916	Cdkal1	CDK5 regulatory subunit associated protein 1-like 1	1.102	0.08114	NA
68066	Slc25a39	solute carrier family 25, member 39	1.102	0.3679	0.7518
58208	Bcl11b	B-cell leukemia/lymphoma 11B	1.102	0.6815	0.8949
57785	Rangrf	RAN guanine nucleotide release factor	1.102	0.08044	NA
56445	Dnaja2	DnaJ (Hsp40) homolog, subfamily A, member 2	1.102	0.1528	NA
27384	Akr1c13	aldo-keto reductase family 1, member C13	1.102	0.4635	0.8032
26913	Gprin1	G protein-regulated inducer of neurite outgrowth 1	1.102	0.3706	0.7527
22360	Nrsn1	neurensin 1	1.102	0.08077	NA
20955	Vamp7	vesicle-associated membrane protein 7	1.102	0.07304	NA
20379	Sfrp4	secreted frizzled-related protein 4	1.102	0.2598	0.6815
20317	Serpinf1	serine (or cysteine) peptidase inhibitor, clade F, member 1	1.102	0.5855	0.8552
18073	Nid1	nidogen 1	1.102	0.9227	0.9776
16362	Irf1	interferon regulatory factor 1	1.102	0.1134	NA
15929	Idh3g	isocitrate dehydrogenase 3 (NAD+), gamma	1.102	0.1718	NA
15371	Hmx1	H6 homeobox 1	1.102	0.342	0.7338
14964	H2-D1	histocompatibility 2, D region locus 1	1.102	0.3225	0.7202
14773	Grk5	G protein-coupled receptor kinase 5	1.102	0.1121	NA
14169	Fgf14	fibroblast growth factor 14	1.102	0.1464	NA
14057	Sfxn1	sideroflexin 1	1.102	0.2508	0.6767
13660	Ehd1	EH-domain containing 1	1.102	0.1282	NA
13004	Ncan	neurocan	1.102	0.1522	NA
12891	Cpne6	copine VI	1.102	0.1989	NA
12125	Bcl2l11	BCL2-like 11 (apoptosis facilitator)	1.102	0.5176	0.8278
11534	Adk	adenosine kinase	1.102	0.4462	0.7938
100041639	Gm3448	predicted gene 3448	1.101	0.144	NA
675851	LOC675851	NADH dehydrogenase [ubiquinone] 1 subunit C2-like	1.101	0.09635	NA
665180	Clec2l	C-type lectin domain family, member L	1.101	0.4353	0.7883
320609	Fam40b	family with sequence similarity 40, member B	1.101	0.1795	NA
276920	Ccdc42	coiled-coil domain containing 42	1.101	0.4787	0.81
232232	Hdac11	histone deacetylase 11	1.101	0.2606	0.6815
227682	Trub2	TruB pseudouridine (psi) synthase homolog 2 (E. coli)	1.101	0.04116	NA
226041	Pgm5	phosphoglucomutase 5	1.101	0.8177	0.9421
219134	Shisa2	shisa homolog 2 (Xenopus laevis)	1.101	0.5308	0.8338
217473	Ankmy2	ankyrin repeat and MYND domain containing 2	1.101	0.2368	0.6659
217125	Samd14	sterile alpha motif domain containing 14	1.101	0.3018	0.708
216527	Ccm2	cerebral cavernous malformation 2 homolog (human)	1.101	0.05065	NA
214791	Sertad4	SERTA domain containing 4	1.101	0.2441	0.672
214158	Trim38	tripartite motif-containing 38	1.101	0.2514	0.6767
213993	A630007B06Rik	RIKEN cDNA A630007B06 gene	1.101	0.09635	NA
210719	Mkx	mohawk homeobox	1.101	0.02997	NA
209497	Tmem164	transmembrane protein 164	1.101	0.1788	NA
171508	Creld1	cysteine-rich with EGF-like domains 1	1.101	0.3074	0.711
140703	Emid1	EMI domain containing 1	1.101	0.4503	0.7945

140482	Zfp358	zinc finger protein 358	1.101	0.1603	NA
109689	Arrb1	arrestin, beta 1	1.101	0.2037	NA
109246	Tspan9	tetraspanin 9	1.101	0.5162	0.8277
106046	AW413774	expressed sequence AW413774	1.101	0.4104	0.774
105859	Csdc2	cold shock domain containing C2, RNA binding	1.101	0.5989	0.862
104910	Slc25a47	solute carrier family 25, member 47	1.101	0.413	0.7751
101772	Ano1	anoctamin 1, calcium activated chloride channel	1.101	0.3157	0.7165
99890	Prmt6	protein arginine N-methyltransferase 6	1.101	0.1297	NA
93837	Dach2	dachshund 2 (Drosophila)	1.101	0.4786	0.81
83454	Nxf2	nuclear RNA export factor 2	1.101	0.5003	0.8197
81904	Cacng7	calcium channel, voltage-dependent, gamma subunit 7	1.101	0.1112	NA
76795	Tbc1d9b	TBC1 domain family, member 9B	1.101	0.2975	0.7051
76611	1700071A11Rik	RIKEN cDNA 1700071A11 gene	1.101	0.34	0.7326
75608	Chmp4b	chromatin modifying protein 4B	1.101	0.1051	NA
73390	Msl3l2	male-specific lethal 3-like 2 (Drosophila)	1.101	0.1967	NA
72962	Tymp	thymidine phosphorylase	1.101	0.3306	0.726
70382	Kctd2	potassium channel tetramerisation domain containing 2	1.101	0.1967	NA
69537	Dnase1l1	deoxyribonuclease 1-like 1	1.101	0.5048	0.8224
69161	Manbal	mannosidase, beta A, lysosomal-like	1.101	0.4509	0.7946
68275	Rpa1	replication protein A1	1.101	0.076	NA
68047	Mpnd	MPN domain containing	1.101	0.3531	0.7409
67948	Fbxo28	F-box protein 28	1.101	0.0878	NA
67077	1700019N12Rik	RIKEN cDNA 1700019N12 gene	1.101	0.2496	0.6767
66894	Wwp2	WW domain containing E3 ubiquitin protein ligase 2	1.101	0.1607	NA
66743	Rnf220	ring finger protein 220	1.101	0.2396	0.6681
64450	Gpr85	G protein-coupled receptor 85	1.101	0.08436	NA
57780	Fxyd7	FXYD domain-containing ion transport regulator 7	1.101	0.5094	0.8247
56496	Tspan6	tetraspanin 6	1.101	0.04907	NA
56401	Lepre1	leprecan 1	1.101	0.1761	NA
56363	Tmeff2	transmembrane protein with EGF-like and two follistatin-like domains 2	1.101	0.2239	NA
53893	Nudt5	nudix (nucleoside diphosphate linked moiety X)-type motif 5	1.101	0.2344	0.6636
27643	Ubl4	ubiquitin-like 4	1.101	0.1052	NA
23988	Pin1	protein (peptidyl-prolyl cis/trans isomerase) NIMA-interacting 1	1.101	0.4498	0.7943
22247	Umps	uridine monophosphate synthetase	1.101	0.2366	0.6659
20378	Frzb	frizzled-related protein	1.101	0.1264	NA
18514	Pbx1	pre B-cell leukemia transcription factor 1	1.101	0.3783	0.7573
16992	Lta	lymphotoxin A	1.101	0.5819	0.8533
16783	Lamp1	lysosomal-associated membrane protein 1	1.101	0.1398	NA
15531	Ndst1	N-deacetylase/N-sulfotransferase (heparan glucosaminyl) 1	1.101	0.4366	0.7888
15374	Hn1	hematological and neurological expressed sequence 1	1.101	0.2192	NA
13835	Epha1	Eph receptor A1	1.101	0.6088	0.8658
13713	Elk3	ELK3, member of ETS oncogene family	1.101	0.1843	NA
13167	Dbi	diazepam binding inhibitor	1.101	0.4092	0.7737
11800	Api5	apoptosis inhibitor 5	1.101	0.2324	NA
11479	Acvr1b	activin A receptor, type 1B	1.101	0.3224	0.7202
629016	Zfp953	zinc finger protein 953	1.1	0.1969	NA
381438	Gm5148	predicted gene 5148	1.1	0.1493	NA
381413	Gpr176	G protein-coupled receptor 176	1.1	0.1498	NA
338352	Nell1	NEL-like 1 (chicken)	1.1	0.07006	NA

331532	Tceal5	transcription elongation factor A (SII)-like 5	1.1	0.1448	NA
320563	Islr2	immunoglobulin superfamily containing leucine-rich repeat 2	1.1	0.0599	NA
320376	Bcorl1	BCL6 co-repressor-like 1	1.1	0.4475	0.7938
270086	Ogfod1	2-oxoglutarate and iron-dependent oxygenase domain containing 1	1.1	0.1914	NA
244417	Gm501	predicted gene 501	1.1	0.34	0.7326
242736	Pramef8	PRAME family member 8	1.1	0.2184	NA
234736	Rfwd3	ring finger and WD repeat domain 3	1.1	0.02306	NA
232970	Phldb3	pleckstrin homology-like domain, family B, member 3	1.1	0.3528	0.7408
228598	Ebf4	early B-cell factor 4	1.1	0.3632	0.7483
211232	Cpne9	copine family member IX	1.1	0.4837	0.8123
192976	BC046404	cDNA sequence BC046404	1.1	0.1855	NA
101197	Zfp956	zinc finger protein 956	1.1	0.1742	NA
98314	D2hgdh	D-2-hydroxyglutarate dehydrogenase	1.1	0.3719	0.7535
77766	Elp4	elongation protein 4 homolog (S. cerevisiae)	1.1	0.2851	0.6979
76282	Gpt	glutamic pyruvic transaminase, soluble	1.1	0.07916	NA
73884	Zdbf2	zinc finger, DBF-type containing 2	1.1	0.3578	0.7451
71908	Cldn23	claudin 23	1.1	0.4578	0.7993
67874	Rprm	reprimo, TP53 dependent G2 arrest mediator candidate	1.1	0.1179	NA
67281	Rpl37	ribosomal protein L37	1.1	0.1129	NA
66993	Smarcd3	SWI/SNF related, matrix associated, actin dependent regulator of chromatin, subfamily d, member 3	1.1	0.363	0.7483
66964	Golt1b	golgi transport 1 homolog B (S. cerevisiae)	1.1	0.2029	NA
66706	Ndufaf3	NADH dehydrogenase (ubiquinone) 1 alpha subcomplex, assembly factor 3	1.1	0.282	0.6967
66603	Sip1	survival of motor neuron protein interacting protein 1	1.1	0.2771	0.6925
66294	Fam3a	family with sequence similarity 3, member A	1.1	0.1601	NA
65962	Slc9a3r2	solute carrier family 9 (sodium/hydrogen exchanger), member 3 regulator 2	1.1	0.4012	0.7703
59043	Wsb2	WD repeat and SOCS box-containing 2	1.1	0.1051	NA
59041	Stk25	serine/threonine kinase 25 (yeast)	1.1	0.2173	NA
57837	Eral1	Era (G-protein)-like 1 (E. coli)	1.1	0.4488	0.7943
54411	Atp6ap1	ATPase, H+ transporting, lysosomal accessory protein 1	1.1	0.3217	0.7199
51944	D2Ertd750e	DNA segment, Chr 2, ERATO Doi 750, expressed	1.1	0.6292	0.8745
28035	Usp39	ubiquitin specific peptidase 39	1.1	0.2124	NA
27965	Spg21	spastic paraplegia 21 homolog (human)	1.1	0.3528	0.7408
22084	Tsc2	tuberous sclerosis 2	1.1	0.1621	NA
21817	Tgm2	transglutaminase 2, C polypeptide	1.1	0.4009	0.7703
20867	Stip1	stress-induced phosphoprotein 1	1.1	0.4186	0.7787
20116	Rps8	ribosomal protein S8	1.1	0.2713	0.6891
19354	Rac2	RAS-related C3 botulinum substrate 2	1.1	0.4364	0.7888
19085	Prkar1b	protein kinase, cAMP dependent regulatory, type I beta	1.1	0.2797	0.6951
18822	Plod1	procollagen-lysine, 2-oxoglutarate 5-dioxygenase 1	1.1	0.4051	0.7716
18293	Ogdh	oxoglutarate dehydrogenase (lipoamide)	1.1	0.421	0.7798
18263	Odc1	ornithine decarboxylase, structural 1	1.1	0.2221	NA
18008	Nes	nestin	1.1	0.4075	0.7726
17319	Mif	macrophage migration inhibitory factor	1.1	0.1604	NA
17199	Mc1r	melanocortin 1 receptor	1.1	0.144	NA
14567	Gdi1	guanosine diphosphate (GDP) dissociation inhibitor 1	1.1	0.4157	0.7768
13972	Gnb1l	guanine nucleotide binding protein (G protein), beta polypeptide 1-like	1.1	0.2882	0.6993
13492	Drd5	dopamine receptor D5	1.1	0.3034	0.709
12333	Capn1	calpain 1	1.1	0.4647	0.8042
12121	Bicd1	bicaudal D homolog 1 (Drosophila)	1.1	0.4416	0.7921

504193	Npcd	neuronal pentraxin chromo domain	1.099	0.3447	0.7359
381199	Tmem151a	transmembrane protein 151A	1.099	0.5593	0.8459
327900	Ubtd2	ubiquitin domain containing 2	1.099	0.07356	NA
320106	Slc38a11	solute carrier family 38, member 11	1.099	0.7187	0.9102
243914	Lgi4	leucine-rich repeat LGI family, member 4	1.099	0.3003	0.706
232223	Txnrd3	thioredoxin reductase 3	1.099	0.1296	NA
226971	Plekhb2	pleckstrin homology domain containing, family B (evectins) member 2	1.099	0.3615	0.7477
213539	Bag2	BCL2-associated athanogene 2	1.099	0.2613	0.6825
140546	Eri3	exoribonuclease 3	1.099	0.4644	0.8038
108723	Card11	caspase recruitment domain family, member 11	1.099	0.4783	0.81
106763	Ttbk1	tau tubulin kinase 1	1.099	0.5169	0.8277
97440	B3gnt9-ps	UDP-GlcNAc:betaGal beta-1,3-N-acetylglucosaminyltransferase 9, pseudogene	1.099	0.3647	0.7493
77974	Rdh12	retinol dehydrogenase 12	1.099	0.3861	0.761
75221	Dpp3	dipeptidylpeptidase 3	1.099	0.3365	0.7305
74482	Ifitm7	interferon induced transmembrane protein 7	1.099	0.05427	NA
74340	Ahcyl2	S-adenosylhomocysteine hydrolase-like 2	1.099	0.3758	0.7556
74044	Ttf2	transcription termination factor, RNA polymerase II	1.099	0.1504	NA
72084	Pigx	phosphatidylinositol glycan anchor biosynthesis, class X	1.099	0.1043	NA
71295	4933431K14Rik	RIKEN cDNA 4933431K14 gene	1.099	0.1538	NA
70451	Dhrs13	dehydrogenase/reductase (SDR family) member 13	1.099	0.06711	NA
69329	1700003M02Rik	RIKEN cDNA 1700003M02 gene	1.099	0.247	0.674
69185	Dtwd1	DTW domain containing 1	1.099	0.1606	NA
67657	Rabl3	RAB, member of RAS oncogene family-like 3	1.099	0.2096	NA
67474	Snap29	synaptosomal-associated protein 29	1.099	0.08841	NA
67458	Ergic1	endoplasmic reticulum-golgi intermediate compartment (ERGIC) 1	1.099	0.4102	0.7739
66597	Trim13	tripartite motif-containing 13	1.099	0.1665	NA
66576	Uqcrh	ubiquinol-cytochrome c reductase hinge protein	1.099	0.1276	NA
58244	Stx6	syntaxin 6	1.099	0.2366	NA
56420	Ppp4c	protein phosphatase 4, catalytic subunit	1.099	0.5397	0.8367
56375	B4galt4	UDP-Gal:betaGlcNAc beta 1,4-galactosyltransferase, polypeptide 4	1.099	0.1108	NA
54445	Unc93b1	unc-93 homolog B1 (C. elegans)	1.099	0.4494	0.7943
52683	Ncaph2	non-SMC condensin II complex, subunit H2	1.099	0.4282	0.7837
28199	Dcaf11	DDB1 and CUL4 associated factor 11	1.099	0.06397	NA
26415	Mapk13	mitogen-activated protein kinase 13	1.099	0.6427	0.8794
22275	Urod	uroporphyrinogen decarboxylase	1.099	0.07581	NA
21987	Tpd52l1	tumor protein D52-like 1	1.099	0.2573	0.6811
21402	Skp1a	S-phase kinase-associated protein 1A	1.099	0.04765	NA
19729	Slc50a1	solute carrier family 50 (sugar transporter), member 1	1.099	0.2929	0.7027
18802	Plcd4	phospholipase C, delta 4	1.099	0.3663	0.7504
17395	Mmp9	matrix metallopeptidase 9	1.099	0.3944	0.7659
14588	Gfra4	glial cell line derived neurotrophic factor family receptor alpha 4	1.099	0.71	0.9069
14371	Fzd9	frizzled homolog 9 (Drosophila)	1.099	0.2756	0.6925
14066	F3	coagulation factor III	1.099	0.1164	NA
13803	Enc1	ectodermal-neural cortex 1	1.099	0.1708	NA
13143	Dapk2	death-associated protein kinase 2	1.099	0.427	0.7825
13043	Cttn	cortactin	1.099	0.3637	0.7483
13036	Ctsh	cathepsin H	1.099	0.3245	0.7213
12124	Bik	BCL2-interacting killer	1.099	0.5055	0.8229
545007	ENSMUSG00000068790	predicted gene, ENSMUSG00000068790	1.098	0.328	0.7248

432478	Tmprss9	transmembrane protease, serine 9	1.098	0.3763	0.7557
394435	Ugt1a6b	UDP glucuronosyltransferase 1 family, polypeptide A6B	1.098	0.5172	0.8277
333088	Kcp	kielin/chordin-like protein	1.098	0.4561	0.7986
320100	Relt	RELT tumor necrosis factor receptor	1.098	0.3847	0.7606
319263	Pcmtd1	protein-L-isoaspartate (D-aspartate) O-methyltransferase domain containing 1	1.098	0.2605	0.6815
269862	Olfr1349	olfactory receptor 1349	1.098	0.4637	0.8033
243963	Zfp473	zinc finger protein 473	1.098	0.2147	NA
242702	Myom3	myomesin family, member 3	1.098	0.2825	0.6969
242506	Frmd3	FERM domain containing 3	1.098	0.3045	0.7091
236920	Stard8	START domain containing 8	1.098	0.1908	NA
228775	Trib3	tribbles homolog 3 (Drosophila)	1.098	0.2697	0.6878
227325	Dner	delta/notch-like EGF-related receptor	1.098	0.3682	0.7518
170787	Hdac10	histone deacetylase 10	1.098	0.2692	0.6878
108807	4933415E08Rik	RIKEN cDNA 4933415E08 gene	1.098	0.2511	0.6767
98932	Myl9	myosin, light polypeptide 9, regulatory	1.098	0.07162	NA
93871	Brwd1	bromodomain and WD repeat domain containing 1	1.098	0.1146	NA
78933	Agbl4	ATP/GTP binding protein-like 4	1.098	0.6623	0.8895
75986	Agmat	agmatine ureohydrolase (agmatinase)	1.098	0.2162	NA
75695	Rilpl1	Rab interacting lysosomal protein-like 1	1.098	0.1666	NA
74760	Rab3il1	RAB3A interacting protein (rabin3)-like 1	1.098	0.3514	0.7395
74617	Scpep1	serine carboxypeptidase 1	1.098	0.1318	NA
70550	5730416F02Rik	RIKEN cDNA 5730416F02 gene	1.098	0.6416	0.8789
69190	Dym	dymeclin	1.098	0.2316	NA
68631	Cryl1	crystallin, lambda 1	1.098	0.3351	0.73
68299	Vps53	vacuolar protein sorting 53 (yeast)	1.098	0.1932	NA
67442	Retsat	retinol saturase (all trans retinol 13,14 reductase)	1.098	0.299	0.7052
67282	Ccdc53	coiled-coil domain containing 53	1.098	0.09669	NA
65972	Ifi30	interferon gamma inducible protein 30	1.098	0.4706	0.8076
60532	Wtap	Wilms' tumour 1-associating protein	1.098	0.08704	NA
52840	Dbndd2	dysbindin (dystrobrevin binding protein 1) domain containing 2	1.098	0.112	NA
52535	Mett11d1	methyltransferase 11 domain containing 1	1.098	0.2889	0.6998
27059	Sh3d19	SH3 domain protein D19	1.098	0.2917	0.7019
26893	Cops6	COP9 (constitutive photomorphogenic) homolog, subunit 6 (Arabidopsis thaliana)	1.098	0.2517	0.6767
24075	Taf10	TAF10 RNA polymerase II, TATA box binding protein (TBP)-associated factor	1.098	0.2809	0.6963
23790	Coro1c	coronin, actin binding protein 1C	1.098	0.07275	NA
22235	Ugdh	UDP-glucose dehydrogenase	1.098	0.04533	NA
20170	Hps6	Hermansky-Pudlak syndrome 6	1.098	0.1955	NA
19246	Ptpn1	protein tyrosine phosphatase, non-receptor type 1	1.098	0.4446	0.7936
18555	Cdk16	cyclin-dependent kinase 16	1.098	0.4767	0.8099
18232	Nxph2	neurexophilin 2	1.098	0.2873	0.6986
18107	Nmt1	N-myristoyltransferase 1	1.098	0.1395	NA
14419	Gal	galanin	1.098	0.2222	NA
14299	Ncs1	neuronal calcium sensor 1	1.098	0.2193	NA
13646	Klk1b22	kallikrein 1-related peptidase b22	1.098	0.7293	0.9143
665669	Gm7742	predicted gene 7742	1.097	0.2904	0.7006
545527	Fam194a	family with sequence similarity 194, member A	1.097	0.2009	NA
277333	Gm5069	glyceraldehyde-3-phosphate dehydrogenase pseudogene	1.097	0.4565	0.7987
245578	Pcdh11x	protocadherin 11 X-linked	1.097	0.4378	0.7894
241846	Lsm14b	LSM14 homolog B (SCD6, S. cerevisiae)	1.097	0.33	0.726

240186	Zfp438	zinc finger protein 438	1.097	0.05382	NA
234678	D230025D16Rik	RIKEN cDNA D230025D16 gene	1.097	0.2406	NA
231148	Ablim2	actin-binding LIM protein 2	1.097	0.3744	0.7548
224454	Zdhhc14	zinc finger, DHHC domain containing 14	1.097	0.2187	NA
216558	Ugp2	UDP-glucose pyrophosphorylase 2	1.097	0.04731	NA
215015	Fam20b	family with sequence similarity 20, member B	1.097	0.3079	0.7112
208795	Tmem63a	transmembrane protein 63a	1.097	0.4998	0.8197
207952	Klhl25	kelch-like 25 (Drosophila)	1.097	0.2155	NA
104885	Tmem179	transmembrane protein 179	1.097	0.2252	NA
104799	Vipar	VPS33B interacting protein, apical-basolateral polarity regulator	1.097	0.2951	0.7045
94061	Mrpl1	mitochondrial ribosomal protein L1	1.097	0.07108	NA
78929	Polr3h	polymerase (RNA) III (DNA directed) polypeptide H	1.097	0.1464	NA
77252	9430038I01Rik	RIKEN cDNA 9430038I01 gene	1.097	0.2342	NA
76809	Bri3bp	Bri3 binding protein	1.097	0.1057	NA
76421	1700028K03Rik	RIKEN cDNA 1700028K03 gene	1.097	0.1997	NA
74270	Usp20	ubiquitin specific peptidase 20	1.097	0.1095	NA
74189	Phactr3	phosphatase and actin regulator 3	1.097	0.2525	NA
73754	Thap1	THAP domain containing, apoptosis associated protein 1	1.097	0.07671	NA
71941	Cars2	cysteinyl-tRNA synthetase 2 (mitochondrial)(putative)	1.097	0.2616	0.6831
71141	4933407L21Rik	RIKEN cDNA 4933407L21 gene	1.097	0.25	NA
70110	Ifi35	interferon-induced protein 35	1.097	0.2305	NA
68957	Paqr6	progestin and adipoQ receptor family member VI	1.097	0.346	0.7363
68929	Mospd3	motile sperm domain containing 3	1.097	0.4032	0.7708
68328	Rab13	RAB13, member RAS oncogene family	1.097	0.3647	0.7493
67811	Poldip2	polymerase (DNA-directed), delta interacting protein 2	1.097	0.3492	0.7388
67057	Yaf2	YY1 associated factor 2	1.097	0.1642	NA
57756	Fhl5	four and a half LIM domains 5	1.097	0.6308	0.8751
53945	Slc40a1	solute carrier family 40 (iron-regulated transporter), member 1	1.097	0.08508	NA
53611	Vti1a	vesicle transport through interaction with t-SNAREs homolog 1A (yeast)	1.097	0.07568	NA
23945	Mgll	monoglyceride lipase	1.097	0.2329	NA
22693	Zfp30	zinc finger protein 30	1.097	0.1895	NA
21881	Tkt	transketolase	1.097	0.1532	NA
20539	Slc7a5	solute carrier family 7 (cationic amino acid transporter, y+ system), member 5	1.097	0.2557	0.68
17159	Man2b1	mannosidase 2, alpha B1	1.097	0.3041	0.709
16952	Anxa1	annexin A1	1.097	0.7496	0.9208
16796	Lasp1	LIM and SH3 protein 1	1.097	0.089	NA
14724	Gp1bb	glycoprotein Ib, beta polypeptide	1.097	0.4328	0.7866
14347	Fut7	fucosyltransferase 7	1.097	0.3185	0.7182
12905	Cradd	CASP2 and RIPK1 domain containing adaptor with death domain	1.097	0.1079	NA
11846	Arg1	arginase, liver	1.097	0.5239	0.83
11840	Arf1	ADP-ribosylation factor 1	1.097	0.19	NA
100039258	Gm10290	glyceraldehyde-3-phosphate dehydrogenase pseudogene	1.096	0.4733	0.8088
432582	E130309D14Rik	RIKEN cDNA E130309D14 gene	1.096	0.5971	0.8611
381605	Tbc1d2	TBC1 domain family, member 2	1.096	0.4098	0.7739
319322	Sf3b2	splicing factor 3b, subunit 2	1.096	0.09758	NA
277396	Klhl23	kelch-like 23 (Drosophila)	1.096	0.3092	0.7126
245944	Vps54	vacuolar protein sorting 54 (yeast)	1.096	0.1855	NA
238123	Cog5	component of oligomeric golgi complex 5	1.096	0.4222	0.7801
232947	Lrrc68	leucine rich repeat containing 68	1.096	0.2563	0.6803

232853	Zfp954	zinc finger protein 954	1.096	0.06785	NA	
226123	Morn4	MORN repeat containing 4	1.096	0.08578	NA	
224796	Clic5	chloride intracellular channel 5	1.096	0.3837		0.7599
224045	Eif2b5	eukaryotic translation initiation factor 2B, subunit 5 epsilon	1.096	0.2006	NA	
217708	Lin52	lin-52 homolog (C. elegans)	1.096	0.2786		0.6936
209195	Clic6	chloride intracellular channel 6	1.096	0.6397		0.8783
208198	Btbd2	BTB (POZ) domain containing 2	1.096	0.5777		0.8514
208092	Chmp6	chromatin modifying protein 6	1.096	0.1769	NA	
114142	Foxp2	forkhead box P2	1.096	0.491		0.8155
109594	Lmo1	LIM domain only 1	1.096	0.2096	NA	
108912	Cdca2	cell division cycle associated 2	1.096	0.5556		0.8448
83924	Gpr137b	G protein-coupled receptor 137B	1.096	0.2988		0.7052
75573	2310007L24Rik	RIKEN cDNA 2310007L24 gene	1.096	0.4709		0.8076
75007	Fam63a	family with sequence similarity 63, member A	1.096	0.2553	NA	
74653	4930444A02Rik	RIKEN cDNA 4930444A02 gene	1.096	0.4204		0.7798
74551	Pck2	phosphoenolpyruvate carboxykinase 2 (mitochondrial)	1.096	0.3216		0.7199
73166	Tm7sf2	transmembrane 7 superfamily member 2	1.096	0.3001		0.706
72754	Arhgef10l	Rho guanine nucleotide exchange factor (GEF) 10-like	1.096	0.093	NA	
72355	2210021J22Rik	RIKEN cDNA 2210021J22 gene	1.096	0.2429	NA	
72151	Rfc5	replication factor C (activator 1) 5	1.096	0.1075	NA	
71909	Haus5	HAUS augmin-like complex, subunit 5	1.096	0.2954		0.7046
70387	Ttc9c	tetratricopeptide repeat domain 9C	1.096	0.1767	NA	
69269	Scnm1	sodium channel modifier 1	1.096	0.2973		0.7051
67704	1810037I17Rik	RIKEN cDNA 1810037I17 gene	1.096	0.1605	NA	
67678	Lsm3	LSM3 homolog, U6 small nuclear RNA associated (S. cerevisiae)	1.096	0.2184	NA	
67483	1700028P14Rik	RIKEN cDNA 1700028P14 gene	1.096	0.3225		0.7202
67068	Dynlrb1	dynein light chain roadblock-type 1	1.096	0.2056	NA	
66774	4933439G12Rik	RIKEN cDNA 4933439G12 gene	1.096	0.3138		0.7152
66404	2410001C21Rik	RIKEN cDNA 2410001C21 gene	1.096	0.3478		0.7384
66071	Ethe1	ethylmalonic encephalopathy 1	1.096	0.2808		0.6962
60315	Myg1	melanocyte proliferating gene 1	1.096	0.307		0.7108
57869	Adck2	aarF domain containing kinase 2	1.096	0.3168		0.7174
56351	Ptges3	prostaglandin E synthase 3 (cytosolic)	1.096	0.1404	NA	
56294	Ptpn9	protein tyrosine phosphatase, non-receptor type 9	1.096	0.4014		0.7703
55942	Sertad1	SERTA domain containing 1	1.096	0.1239	NA	
50723	Icosl	icos ligand	1.096	0.4589		0.8006
23912	Rhof	ras homolog gene family, member f	1.096	0.3983		0.7688
22750	Zfp9	zinc finger protein 9	1.096	0.3503		0.739
20972	Syngr1	synaptogyrin 1	1.096	0.3761		0.7556
19718	Rfc2	replication factor C (activator 1) 2	1.096	0.287		0.6983
19079	Prkab1	protein kinase, AMP-activated, beta 1 non-catalytic subunit	1.096	0.4726		0.8084
18484	Pam	peptidylglycine alpha-amidating monooxygenase	1.096	0.2114	NA	
15369	Hmox2	heme oxygenase (decycling) 2	1.096	0.362		0.7477
14840	Gsg1	germ cell-specific gene 1	1.096	0.08242	NA	
14381	G6pdx	glucose-6-phosphate dehydrogenase X-linked	1.096	0.4072		0.7726
13388	Dll1	delta-like 1 (Drosophila)	1.096	0.3681		0.7518
12297	Cacnb3	calcium channel, voltage-dependent, beta 3 subunit	1.096	0.442		0.7926
11677	Akr1b3	aldo-keto reductase family 1, member B3 (aldose reductase)	1.096	0.0295	NA	
100039043	Gm10731	predicted gene 10731	1.095	0.2325	NA	

434624	LOC434624	ferritin light chain 1-like	1.095	0.3168	0.7174
320208	Tmem91	transmembrane protein 91	1.095	0.3668	0.7504
319229	Sctr	secretin receptor	1.095	0.4044	0.7713
233335	Synm	synemin, intermediate filament protein	1.095	0.2337	NA
232217	4933427D06Rik	RIKEN cDNA 4933427D06 gene	1.095	0.4472	0.7938
231134	Dok7	docking protein 7	1.095	0.2669	0.6864
230696	AU022252	expressed sequence AU022252	1.095	0.03404	NA
226591	Tiprl	TIP41, TOR signalling pathway regulator-like (S. cerevisiae)	1.095	0.2173	NA
226548	Aph1a	anterior pharynx defective 1a homolog (C. elegans)	1.095	0.3568	0.7443
225372	Apbb3	amyloid beta (A4) precursor protein-binding, family B, member 3	1.095	0.05975	NA
224697	Adamts10	a disintegrin-like and metallopeptidase (reprolysin type) with thrombospondin type 1 motif, 10	1.095	0.1881	NA
224022	Slc7a4	solute carrier family 7 (cationic amino acid transporter, y+ system), member 4	1.095	0.3753	0.7555
223664	Lrrc14	leucine rich repeat containing 14	1.095	0.3921	0.7647
218035	Vps41	vacuolar protein sorting 41 (yeast)	1.095	0.291	0.7013
215384	Fcgbp	Fc fragment of IgG binding protein	1.095	0.5345	0.8349
209018	Vps8	vacuolar protein sorting 8 homolog (S. cerevisiae)	1.095	0.405	0.7716
192187	Stab1	stabilin 1	1.095	0.5572	0.8454
108900	Fam72a	family with sequence similarity 72, member A	1.095	0.1634	NA
105670	Rcbtb2	regulator of chromosome condensation (RCC1) and BTB (POZ) domain containing protein 2	1.095	0.331	0.7264
98766	Ubac1	ubiquitin associated domain containing 1	1.095	0.2676	0.6867
78908	Igsf3	immunoglobulin superfamily, member 3	1.095	0.4599	0.801
77219	Ptgr2	prostaglandin reductase 2	1.095	0.1628	NA
76943	Psapl1	prosaposin-like 1	1.095	0.261	NA
76505	1500009C09Rik	RIKEN cDNA 1500009C09 gene	1.095	0.148	NA
74910	4930480E11Rik	RIKEN cDNA 4930480E11 gene	1.095	0.5954	0.8605
74840	Manf	mesencephalic astrocyte-derived neurotrophic factor	1.095	0.05784	NA
73893	Tmem202	transmembrane protein 202	1.095	0.1179	NA
73728	Psd	pleckstrin and Sec7 domain containing	1.095	0.56	0.8459
73533	1700080G18Rik	RIKEN cDNA 1700080G18 gene	1.095	0.204	NA
72716	2810047C21Rik1	RIKEN cDNA 2810047C21 gene 1	1.095	0.154	NA
71684	Rbm43	RNA binding motif protein 43	1.095	0.2184	NA
71519	Cyp2u1	cytochrome P450, family 2, subfamily u, polypeptide 1	1.095	0.2543	NA
69068	1810011O10Rik	RIKEN cDNA 1810011O10 gene	1.095	0.4768	0.8099
68957	Paqr6	progestin and adipoQ receptor family member VI	1.095	0.2048	NA
67582	Slc25a26	solute carrier family 25 (mitochondrial carrier, phosphate carrier), member 26	1.095	0.1999	NA
66355	Gmpr	guanosine monophosphate reductase	1.095	0.32	0.7191
66343	Tmem177	transmembrane protein 177	1.095	0.3516	0.7397
66180	Leprel4	leprecan-like 4	1.095	0.2334	NA
66174	Nudt14	nudix (nucleoside diphosphate linked moiety X)-type motif 14	1.095	0.3823	0.7596
66104	Tceal6	transcription elongation factor A (SII)-like 6	1.095	0.1084	NA
59031	Chst12	carbohydrate sulfotransferase 12	1.095	0.4657	0.8049
56464	Ctsf	cathepsin F	1.095	0.2358	NA
55938	Apom	apolipoprotein M	1.095	0.5477	0.8405
54633	Pqbp1	polyglutamine binding protein 1	1.095	0.3791	0.7583
54608	Abhd2	abhydrolase domain containing 2	1.095	0.5431	0.8389
27401	Skp2	S-phase kinase-associated protein 2 (p45)	1.095	0.2386	NA
27056	Irf5	interferon regulatory factor 5	1.095	0.4675	0.8056
26992	Brd7	bromodomain containing 7	1.095	0.03098	NA
21665	Tdg	thymine DNA glycosylase	1.095	0.2513	NA

20909	Stx4a	syntaxin 4A (placental)	1.095	0.08855	NA
19784	Rprl2	ribonuclease P RNA-like 2	1.095	0.6443	0.8802
19294	Pvrl2	poliovirus receptor-related 2	1.095	0.2774	0.6925
16560	Kif1a	kinesin family member 1A	1.095	0.6465	0.8812
14388	Gab1	growth factor receptor bound protein 2-associated protein 1	1.095	0.2596	NA
14314	Fstl1	follistatin-like 1	1.095	0.4804	0.8101
14158	Fert2	fer (fms/fps related) protein kinase, testis specific 2	1.095	0.2861	0.6982
13866	Erbb2	v-erb-b2 erythroblastic leukemia viral oncogene homolog 2, neuro/glioblastoma derived oncogene homolog (avian)	1.095	0.5044	0.8221
12879	Cys1	cystin 1	1.095	0.3111	0.7135
12684	Cideb	cell death-inducing DNA fragmentation factor, alpha subunit-like effector B	1.095	0.6461	0.8809
12555	Cdh15	cadherin 15	1.095	0.4569	0.7987
11775	Ap3b2	adaptor-related protein complex 3, beta 2 subunit	1.095	0.4245	0.7819
100046223	LOC100046223	40S ribosomal protein S15-like	1.094	0.4635	0.8032
625054	Gm6548	eukaryotic translation elongation factor 1 alpha 1 pseudogene	1.094	0.1991	NA
278507	Wfikkn2	WAP, follistatin/kazal, immunoglobulin, kunitz and netrin domain containing 2	1.094	0.3605	0.7467
246257	Ovca2	candidate tumor suppressor in ovarian cancer 2	1.094	0.2514	NA
239985	Arid1b	AT rich interactive domain 1B (SWI-like)	1.094	0.3625	0.7483
239408	Tmem74	transmembrane protein 74	1.094	0.422	0.7801
237403	Lingo3	leucine rich repeat and Ig domain containing 3	1.094	0.4405	0.7913
237082	Nxt2	nuclear transport factor 2-like export factor 2	1.094	0.1218	NA
235130	Adamts15	a disintegrin-like and metallopeptidase (reprolysin type) with thrombospondin type 1 motif, 15	1.094	0.1596	NA
234086	Erich1	glutamate-rich 1	1.094	0.05142	NA
232227	Iqsec1	IQ motif and Sec7 domain 1	1.094	0.3508	0.7393
232035	Fam190a	family with sequence similarity 190, member A	1.094	0.1876	NA
226421	5430435G22Rik	RIKEN cDNA 5430435G22 gene	1.094	0.323	0.7204
224650	Anks1	ankyrin repeat and SAM domain containing 1	1.094	0.2149	NA
217119	Xylt2	xylosyltransferase II	1.094	0.1359	NA
216344	Rab21	RAB21, member RAS oncogene family	1.094	0.4246	0.7819
211550	Tifa	TRAF-interacting protein with forkhead-associated domain	1.094	0.1219	NA
142688	Asb13	ankyrin repeat and SOCS box-containing 13	1.094	0.115	NA
110265	Msra	methionine sulfoxide reductase A	1.094	0.06961	NA
108803	4933402P03Rik	RIKEN cDNA 4933402P03 gene	1.094	0.6948	0.9009
107371	Exoc6	exocyst complex component 6	1.094	0.08484	NA
99890	Prmt6	protein arginine N-methyltransferase 6	1.094	0.3066	0.7108
99586	Dpyd	dihydropyrimidine dehydrogenase	1.094	0.2959	0.705
98496	Pid1	phosphotyrosine interaction domain containing 1	1.094	0.07035	NA
97351	E230006M18Rik	RIKEN cDNA E230006M18 gene	1.094	0.1262	NA
97212	Hadha	hydroxyacyl-Coenzyme A dehydrogenase/3-ketoacyl-Coenzyme A thiolase/enoyl-Coenzyme A hydratase (trifunctional protein), alpha subunit	1.094	0.5331	0.8341
78894	Aacs	acetoacetyl-CoA synthetase	1.094	0.365	0.7495
76137	Ccdc90a	coiled-coil domain containing 90A	1.094	0.4292	0.7843
75736	Bcl2l12	BCL2-like 12 (proline rich)	1.094	0.5172	0.8277
75430	3200002M19Rik	RIKEN cDNA 3200002M19 gene	1.094	0.3318	0.7268
74251	Ankrd9	ankyrin repeat domain 9	1.094	0.4794	0.8101
72720	Zfp248	zinc finger protein 248	1.094	0.3962	0.7671
72338	Wdr89	WD repeat domain 89	1.094	0.3381	0.7314
71091	Cdkl1	cyclin-dependent kinase-like 1 (CDC2-related kinase)	1.094	0.2242	NA
70887	Dmrtc1a	DMRT-like family C1a	1.094	0.3453	0.736
70239	Gtf3c5	general transcription factor IIIC, polypeptide 5	1.094	0.03481	NA
69890	Zfp219	zinc finger protein 219	1.094	0.4613	0.802

69234	Zfp688	zinc finger protein 688	1.094	0.2688	0.6876
69179	Tmem110	transmembrane protein 110	1.094	0.3617	0.7477
68292	Stt3b	STT3, subunit of the oligosaccharyltransferase complex, homolog B (S. cerevisiae)	1.094	0.1069	NA
67605	Akt1s1	AKT1 substrate 1 (proline-rich)	1.094	0.3746	0.755
67563	Narfl	nuclear prelamin A recognition factor-like	1.094	0.3207	0.7196
67239	Rpf2	ribosome production factor 2 homolog (S. cerevisiae)	1.094	0.05859	NA
66973	Mrps18b	mitochondrial ribosomal protein S18B	1.094	0.2303	NA
66860	Tanc1	tetratricopeptide repeat, ankyrin repeat and coiled-coil containing 1	1.094	0.432	0.7859
66366	Ergic3	ERGIC and golgi 3	1.094	0.2561	NA
64657	Mrps10	mitochondrial ribosomal protein S10	1.094	0.3137	0.7152
63993	Slc5a7	solute carrier family 5 (choline transporter), member 7	1.094	0.2579	NA
56626	Poll	polymerase (DNA directed), lambda	1.094	0.3805	0.7594
56089	Ramp3	receptor (calcitonin) activity modifying protein 3	1.094	0.1439	NA
27883	D16H22S680E	DNA segment, Chr 16, human D22S680E, expressed	1.094	0.1183	NA
26961	Rpl8	ribosomal protein L8	1.094	0.1886	NA
23971	Papss1	3'-phosphoadenosine 5'-phosphosulfate synthase 1	1.094	0.2056	NA
20729	Spin1	spindlin 1	1.094	0.191	NA
20370	Sez6	seizure related gene 6	1.094	0.5073	0.8234
19894	Rph3a	rabphilin 3A	1.094	0.638	0.8776
19401	Rara	retinoic acid receptor, alpha	1.094	0.4395	0.7908
18559	Pctp	phosphatidylcholine transfer protein	1.094	0.5788	0.8519
18223	Numbl	numb-like	1.094	0.1266	NA
18222	Numb	numb gene homolog (Drosophila)	1.094	0.2264	NA
18011	Neurl1a	neuralized homolog 1A (Drosophila)	1.094	0.1067	NA
15444	Hpca	hippocalcin	1.094	0.256	NA
15289	Hmgb1	high mobility group box 1	1.094	0.0638	NA
14708	Gng7	guanine nucleotide binding protein (G protein), gamma 7	1.094	0.4614	0.802
14348	Fut9	fucosyltransferase 9	1.094	0.259	NA
14083	Ptk2	PTK2 protein tyrosine kinase 2	1.094	0.1934	NA
13003	Vcan	versican	1.094	0.5975	0.8611
12653	Chgb	chromogranin B	1.094	0.05791	NA
12055	Bcl7c	B-cell CLL/lymphoma 7C	1.094	0.1669	NA
673094	Cd99	CD99 antigen	1.093	0.4199	0.7795
613262	BC029722	cDNA sequence BC029722	1.093	0.1465	NA
546849	Gm13178	predicted gene 13178	1.093	0.622	0.8719
432442	Akap7	A kinase (PRKA) anchor protein 7	1.093	0.3037	0.709
330070	Gm5107	predicted gene 5107	1.093	0.5413	0.8379
320091	Ano4	anoctamin 4	1.093	0.2391	NA
319996	Casc4	cancer susceptibility candidate 4	1.093	0.1457	NA
268935	Scube3	signal peptide, CUB domain, EGF-like 3	1.093	0.5286	0.8332
240479	Fam69c	family with sequence similarity 69, member C	1.093	0.465	0.8044
237988	Cdr2l	cerebellar degeneration-related protein 2-like	1.093	0.1894	NA
233208	Scaf1	SR-related CTD-associated factor 1	1.093	0.4478	0.7938
232946	Bloc1s3	biogenesis of lysosome-related organelles complex-1, subunit 3	1.093	0.4018	0.7703
232855	Zfp772	zinc finger protein 772	1.093	0.3713	0.7532
231668	Vsig10	V-set and immunoglobulin domain containing 10	1.093	0.4335	0.7872
229613	6330549D23Rik	RIKEN cDNA 6330549D23 gene	1.093	0.275	0.6925
224807	Tmem63b	transmembrane protein 63b	1.093	0.1472	NA
216549	Aftph	aftiphilin	1.093	0.1746	NA

140792	Colec12	collectin sub-family member 12	1.093	0.6516	0.8834
110695	Aldh7a1	aldehyde dehydrogenase family 7, member A1	1.093	0.3656	0.7497
110639	Prps2	phosphoribosyl pyrophosphate synthetase 2	1.093	0.4942	0.8169
110157	Raf1	v-raf-leukemia viral oncogene 1	1.093	0.1258	NA
106014	Fam19a5	family with sequence similarity 19, member A5	1.093	0.2069	NA
105670	Rcbtb2	regulator of chromosome condensation (RCC1) and BTB (POZ) domain containing protein 2	1.093	0.1996	NA
103768	Tubg2	tubulin, gamma 2	1.093	0.2631	NA
99031	Osbpl6	oxysterol binding protein-like 6	1.093	0.4642	0.8037
78246	Phf23	PHD finger protein 23	1.093	0.151	NA
77531	Anks1b	ankyrin repeat and sterile alpha motif domain containing 1B	1.093	0.1436	NA
74039	Nfam1	Nfat activating molecule with ITAM motif 1	1.093	0.6738	0.8924
72154	Zfp157	zinc finger protein 157	1.093	0.3413	0.7334
72114	Zbed3	zinc finger, BED domain containing 3	1.093	0.4505	0.7945
71517	9030624J02Rik	RIKEN cDNA 9030624J02 gene	1.093	0.1632	NA
70239	Gtf3c5	general transcription factor IIIC, polypeptide 5	1.093	0.2105	NA
69660	Tmbim1	transmembrane BAX inhibitor motif containing 1	1.093	0.1816	NA
68861	1190002N15Rik	RIKEN cDNA 1190002N15 gene	1.093	0.3576	0.7451
66202	1110059G10Rik	RIKEN cDNA 1110059G10 gene	1.093	0.1789	NA
66131	Tipin	timeless interacting protein	1.093	0.1371	NA
59049	Slc22a17	solute carrier family 22 (organic cation transporter), member 17	1.093	0.3166	0.7174
59045	Stard3	START domain containing 3	1.093	0.05979	NA
28084	Vps25	vacuolar protein sorting 25 (yeast)	1.093	0.3232	0.7205
28018	Ubfd1	ubiquitin family domain containing 1	1.093	0.1812	NA
27410	Abca3	ATP-binding cassette, sub-family A (ABC1), member 3	1.093	0.07644	NA
24056	Sh3bp5	SH3-domain binding protein 5 (BTK-associated)	1.093	0.4271	0.7827
23945	Mgll	monoglyceride lipase	1.093	0.3589	0.7458
22793	Zyx	zyxin	1.093	0.32	0.7191
22436	Xdh	xanthine dehydrogenase	1.093	0.5151	0.8276
22409	Wnt10a	wingless related MMTV integration site 10a	1.093	0.2871	0.6984
21858	Timp2	tissue inhibitor of metalloproteinase 2	1.093	0.5454	0.839
21815	Tgif1	TGFB-induced factor homeobox 1	1.093	0.4914	0.8156
20842	Stag1	stromal antigen 1	1.093	0.04452	NA
20598	Smpd2	sphingomyelin phosphodiesterase 2, neutral	1.093	0.2388	NA
20256	Clec11a	C-type lectin domain family 11, member a	1.093	0.3055	0.7101
20186	Nr1h4	nuclear receptor subfamily 1, group H, member 4	1.093	0.5846	0.8549
19341	Rab4a	RAB4A, member RAS oncogene family	1.093	0.2587	NA
19179	Psmc1	protease (prosome, macropain) 26S subunit, ATPase 1	1.093	0.2475	NA
19099	Mapk8ip1	mitogen-activated protein kinase 8 interacting protein 1	1.093	0.4141	0.7758
17929	Myom1	myomesin 1	1.093	0.4076	0.7726
17768	Mthfd2	methylenetetrahydrofolate dehydrogenase (NAD+ dependent), methenyltetrahydrofolate cyclohydrolase	1.093	0.2923	0.7024
14380	G6pd2	glucose-6-phosphate dehydrogenase 2	1.093	0.6359	0.8772
12426	Cckbr	cholecystokinin B receptor	1.093	0.348	0.7385
11606	Agt	angiotensinogen (serpin peptidase inhibitor, clade A, member 8)	1.093	0.6296	0.8745
723988	A330062J17Rik	RIKEN cDNA A330062J17 gene	1.092	0.2123	NA
386612	Thoc6	THO complex 6 homolog (Drosophila)	1.092	0.2386	NA
382867	Zfp488	zinc finger protein 488	1.092	0.4112	0.7746
329504	Lcmt2	leucine carboxyl methyltransferase 2	1.092	0.1387	NA
320343	Lypd6	LY6/PLAUR domain containing 6	1.092	0.3744	0.7548
237175	Gpr64	G protein-coupled receptor 64	1.092	0.5536	0.844

229004	Gmeb2	glucocorticoid modulatory element binding protein 2	1.092	0.2085	NA
226025	Trpm3	transient receptor potential cation channel, subfamily M, member 3	1.092	0.3887	0.7616
217365	Nploc4	nuclear protein localization 4 homolog (S. cerevisiae)	1.092	0.4689	0.8059
210094	Iglon5	IgLON family member 5	1.092	0.2433	NA
192734	AI646023	expressed sequence AI646023	1.092	0.2749	NA
111175	Pecr	peroxisomal trans-2-enoyl-CoA reductase	1.092	0.2597	NA
106369	Ypel1	yippee-like 1 (Drosophila)	1.092	0.5808	0.8527
102595	Plekho2	pleckstrin homology domain containing, family O member 2	1.092	0.1558	NA
94187	Zfp423	zinc finger protein 423	1.092	0.4336	0.7872
77590	Chst15	carbohydrate (N-acetylgalactosamine 4-sulfate 6-O) sulfotransferase 15	1.092	0.4245	0.7819
75697	C2cd4b	C2 calcium-dependent domain containing 4B	1.092	0.2948	0.7044
75686	Nudt16	nudix (nucleoside diphosphate linked moiety X)-type motif 16	1.092	0.1954	NA
75668	Rasl10a	RAS-like, family 10, member A	1.092	0.4625	0.8026
74769	Pik3cb	phosphatidylinositol 3-kinase, catalytic, beta polypeptide	1.092	0.09953	NA
74585	Sppl3	signal peptide peptidase 3	1.092	0.2506	NA
74195	Elp3	elongation protein 3 homolog (S. cerevisiae)	1.092	0.1634	NA
74173	1700012B15Rik	RIKEN cDNA 1700012B15 gene	1.092	0.1134	NA
71954	Suds3	suppressor of defective silencing 3 homolog (S. cerevisiae)	1.092	0.3068	0.7108
70310	Plscr3	phospholipid scramblase 3	1.092	0.381	0.7594
70233	Cd2bp2	CD2 antigen (cytoplasmic tail) binding protein 2	1.092	0.1008	NA
69926	Dnahc17	dynein, axonemal, heavy chain 17	1.092	0.5175	0.8278
69191	Pdia2	protein disulfide isomerase associated 2	1.092	0.3061	0.7106
68295	0610011L14Rik	RIKEN cDNA 0610011L14 gene	1.092	0.2991	0.7052
68045	2700060E02Rik	RIKEN cDNA 2700060E02 gene	1.092	0.0333	NA
66680	3230401D17Rik	RIKEN cDNA 3230401D17 gene	1.092	0.06658	NA
66585	Snrnp40	small nuclear ribonucleoprotein 40 (U5)	1.092	0.1587	NA
66373	Lsm5	LSM5 homolog, U6 small nuclear RNA associated (S. cerevisiae)	1.092	0.2818	0.6967
66078	Tsen34	tRNA splicing endonuclease 34 homolog (S. cerevisiae)	1.092	0.2049	NA
54200	Sult2b1	sulfotransferase family, cytosolic, 2B, member 1	1.092	0.3658	0.7499
53624	Cldn7	claudin 7	1.092	0.3246	0.7216
53622	Krt85	keratin 85	1.092	0.7056	0.9057
53614	Reck	reversion-inducing-cysteine-rich protein with kazal motifs	1.092	0.2001	NA
30949	Lcmt1	leucine carboxyl methyltransferase 1	1.092	0.06699	NA
24105	Rbck1	RanBP-type and C3HC4-type zinc finger containing 1	1.092	0.385	0.7607
24086	Tlk2	tousled-like kinase 2 (Arabidopsis)	1.092	0.4477	0.7938
22066	Trpc4	transient receptor potential cation channel, subfamily C, member 4	1.092	0.1302	NA
17865	Mybl2	myeloblastosis oncogene-like 2	1.092	0.2737	NA
15505	Hsph1	heat shock 105kDa/110kDa protein 1	1.092	0.1405	NA
14586	Gfra2	glial cell line derived neurotrophic factor family receptor alpha 2	1.092	0.07285	NA
12950	Hapln1	hyaluronan and proteoglycan link protein 1	1.092	0.2542	NA
12554	Cdh13	cadherin 13	1.092	0.4578	0.7993
12461	Cct2	chaperonin containing Tcp1, subunit 2 (beta)	1.092	0.04666	NA
100504389	LOC100504389	hypothetical LOC100504389	1.091	0.4374	0.7892
100038474	K230015D01Rik	RIKEN cDNA K230015D01 gene	1.091	0.07975	NA
435965	Lrp3	low density lipoprotein receptor-related protein 3	1.091	0.5822	0.8533
319370	Fam100b	family with sequence similarity 100, member B	1.091	0.1725	NA
270162	Elmod1	ELMO domain containing 1	1.091	0.1006	NA
241263	Gpr158	G protein-coupled receptor 158	1.091	0.255	NA
239647	Fam113b	family with sequence similarity 113, member B	1.091	0.4925	0.8157

234366 Gatad2a	GATA zinc finger domain containing 2A	1.091	0.5007		0.82
234159 Gm4889	predicted gene 4889	1.091	0.1601	NA	
232679 Zc3hc1	zinc finger, C3HC type 1	1.091	0.3094		0.7127
232664 Ccdc136	coiled-coil domain containing 136	1.091	0.261	NA	
228942 Cbln4	cerebellin 4 precursor protein	1.091	0.3874		0.7611
225875 Lrfn4	leucine rich repeat and fibronectin type III domain containing 4	1.091	0.2172	NA	
225861 Snx32	sorting nexin 32	1.091	0.5059		0.823
213350 Pddc1	Parkinson disease 7 domain containing 1	1.091	0.5637		0.8465
114641 Rpl31	ribosomal protein L31	1.091	0.09282	NA	
107895 Mgat5	mannoside acetylglucosaminyltransferase 5	1.091	0.2846	NA	
106298 Rrn3	RRN3 RNA polymerase I transcription factor homolog (yeast)	1.091	0.1855	NA	
105835 Sgsm3	small G protein signaling modulator 3	1.091	0.3373		0.731
103711 Pnpo	pyridoxine 5'-phosphate oxidase	1.091	0.1651	NA	
103149 Upb1	ureidopropionase, beta	1.091	0.3965		0.7674
99377 Sall4	sal-like 4 (Drosophila)	1.091	0.4319		0.7859
94245 Dtnbp1	dystrobrevin binding protein 1	1.091	0.2109	NA	
83675 Bicc1	bicaudal C homolog 1 (Drosophila)	1.091	0.4324		0.7863
76916 4930455C21Rik	RIKEN cDNA 4930455C21 gene	1.091	0.2425	NA	
76850 Eif2c4	eukaryotic translation initiation factor 2C, 4	1.091	0.09061	NA	
72938 Hspb11	heat shock protein family B (small), member 11	1.091	0.1635	NA	
72865 Cxx1c	CAAX box 1 homolog C (human)	1.091	0.3026		0.7087
72828 Ubash3b	ubiquitin associated and SH3 domain containing, B	1.091	0.2033	NA	
72350 Fam164c	family with sequence similarity 164, member C	1.091	0.5212		0.8286
72273 2210404O07Rik	RIKEN cDNA 2210404O07 gene	1.091	0.4841		0.8123
71998 Slc25a35	solute carrier family 25, member 35	1.091	0.2826	NA	
71732 Vps11	vacuolar protein sorting 11 (yeast)	1.091	0.3636		0.7483
71336 Rbks	ribokinase	1.091	0.178	NA	
70310 Plscr3	phospholipid scramblase 3	1.091	0.3759		0.7556
70178 Fam108c	family with sequence similarity 108, member C	1.091	0.1755	NA	
69748 Aldh16a1	aldehyde dehydrogenase 16 family, member A1	1.091	0.1232	NA	
67836 Wdr83	WD repeat domain containing 83	1.091	0.2363	NA	
67266 Fam69a	family with sequence similarity 69, member A	1.091	0.2199	NA	
67149 Nkain1	Na+/K+ transporting ATPase interacting 1	1.091	0.2704	NA	
67123 Ubap1	ubiquitin-associated protein 1	1.091	0.07925	NA	
66513 Tab1	TGF-beta activated kinase 1/MAP3K7 binding protein 1	1.091	0.3125		0.7145
66505 Zmynd11	zinc finger, MYND domain containing 11	1.091	0.2686	NA	
57437 Golga7	golgi autoantigen, golgin subfamily a, 7	1.091	0.09067	NA	
56873 Lmbr1	limb region 1	1.091	0.4889		0.8142
30938 Fgd3	FYVE, RhoGEF and PH domain containing 3	1.091	0.4384		0.7896
29856 Smtn	smoothelin	1.091	0.4031		0.7708
23954 Nek3	NIMA (never in mitosis gene a)-related expressed kinase 3	1.091	0.2986		0.7052
22630 Ywhaq	tyrosine 3-monooxygenase/tryptophan 5-monooxygenase activation protein, theta polypeptide	1.091	0.1182	NA	
21823 Th	tyrosine hydroxylase	1.091	0.2787	NA	
20643 Snrpe	small nuclear ribonucleoprotein E	1.091	0.09938	NA	
19727 Rfxank	regulatory factor X-associated ankyrin-containing protein	1.091	0.2687	NA	
19012 Ppap2a	phosphatidic acid phosphatase type 2A	1.091	0.1222	NA	
18826 Lcp1	lymphocyte cytosolic protein 1	1.091	0.3416		0.7337
18203 Ntan1	N-terminal Asn amidase	1.091	0.1614	NA	
17524 Mpp1	membrane protein, palmitoylated	1.091	0.15	NA	

15019	H2-Q8	histocompatibility 2, Q region locus 8	1.091	0.5053	0.8228
14260	Fmn1	formin 1	1.091	0.3664	0.7504
12053	Bcl6	B-cell leukemia/lymphoma 6	1.091	0.1703	NA
11898	Ass1	argininosuccinate synthetase 1	1.091	0.5131	0.8268
330369	Fbxo41	F-box protein 41	1.09	0.341	0.7334
329360	Gm757	predicted gene 757	1.09	0.7499	0.921
327992	Hsf5	heat shock transcription factor family member 5	1.09	0.6331	0.8758
320528	Vps13c	vacuolar protein sorting 13C (yeast)	1.09	0.3442	0.7358
243339	Tmem130	transmembrane protein 130	1.09	0.2063	NA
232943	Klc3	kinesin light chain 3	1.09	0.4132	0.7753
232910	Ap2s1	adaptor-related protein complex 2, sigma 1 subunit	1.09	0.241	NA
228608	Smox	spermine oxidase	1.09	0.2814	NA
228410	Cstf3	cleavage stimulation factor, 3' pre-RNA, subunit 3	1.09	0.1687	NA
215114	Hip1	huntingtin interacting protein 1	1.09	0.3234	0.7206
209683	Ttc28	tetratricopeptide repeat domain 28	1.09	0.1367	NA
192174	Rwdd4a	RWD domain containing 4A	1.09	0.3037	0.709
109232	Sccpdh	saccharopine dehydrogenase (putative)	1.09	0.1153	NA
109115	Supt3h	suppressor of Ty 3 homolog (S. cerevisiae)	1.09	0.1135	NA
99296	Hrh3	histamine receptor H3	1.09	0.3879	0.7614
97485	C88045	expressed sequence C88045	1.09	0.2806	NA
84585	Rnf123	ring finger protein 123	1.09	0.4866	0.8129
78514	Arhgap10	Rho GTPase activating protein 10	1.09	0.5967	0.8611
76308	Rab1b	RAB1B, member RAS oncogene family	1.09	0.2876	NA
75691	Anks6	ankyrin repeat and sterile alpha motif domain containing 6	1.09	0.4	0.7702
74781	Wipi2	WD repeat domain, phosphoinositide interacting 2	1.09	0.2239	NA
71949	Lass5	LAG1 homolog, ceramide synthase 5	1.09	0.1027	NA
71837	1700003E16Rik	RIKEN cDNA 1700003E16 gene	1.09	0.4775	0.81
71728	Stk11ip	serine/threonine kinase 11 interacting protein	1.09	0.4544	0.7973
71648	Optn	optineurin	1.09	0.4302	0.7844
71474	Ppp6r2	protein phosphatase 6, regulatory subunit 2	1.09	0.4327	0.7866
70458	2610318N02Rik	RIKEN cDNA 2610318N02 gene	1.09	0.363	0.7483
68975	Med27	mediator complex subunit 27	1.09	0.3348	0.73
68857	Dtwd2	DTW domain containing 2	1.09	0.2807	NA
68603	Pmvk	phosphomevalonate kinase	1.09	0.1156	NA
68038	Chid1	chitinase domain containing 1	1.09	0.2959	0.705
67188	2700046G09Rik	RIKEN cDNA 2700046G09 gene	1.09	0.1138	NA
67151	Psmd9	proteasome (prosome, macropain) 26S subunit, non-ATPase, 9	1.09	0.3179	0.718
66634	Mcm8	minichromosome maintenance deficient 8 (S. cerevisiae)	1.09	0.2488	NA
66611	Ribc1	RIB43A domain with coiled-coils 1	1.09	0.3514	0.7395
66445	Cyc1	cytochrome c-1	1.09	0.217	NA
66136	Znrd1	zinc ribbon domain containing, 1	1.09	0.1447	NA
56527	Mast1	microtubule associated serine/threonine kinase 1	1.09	0.1984	NA
54399	Bet1l	blocked early in transport 1 homolog (S. cerevisiae)-like	1.09	0.2975	0.7051
54123	Irf7	interferon regulatory factor 7	1.09	0.3426	0.7344
29816	Hip1r	huntingtin interacting protein 1 related	1.09	0.3628	0.7483
22022	Tpst2	protein-tyrosine sulfotransferase 2	1.09	0.1008	NA
21769	Zfand3	zinc finger, AN1-type domain 3	1.09	0.03613	NA
21754	Tesk1	testis specific protein kinase 1	1.09	0.05509	NA
20813	Srp14	signal recognition particle 14	1.09	0.1292	NA

20719	Serpinb6a	serine (or cysteine) peptidase inhibitor, clade B, member 6a	1.09	0.466	0.8049
19222	Ptgir	prostaglandin I receptor (IP)	1.09	0.1314	NA
19015	Ppard	peroxisome proliferator activator receptor delta	1.09	0.4741	0.8089
18858	Pmp22	peripheral myelin protein 22	1.09	0.4281	0.7837
18536	Pcm1	pericentriolar material 1	1.09	0.04829	NA
18221	Nudc	nuclear distribution gene C homolog (Aspergillus)	1.09	0.2478	NA
18050	Klk1b3	kallikrein 1-related peptidase b3	1.09	0.705	0.9052
16855	Lgals4	lectin, galactose binding, soluble 4	1.09	0.4627	0.8028
16195	Il6st	interleukin 6 signal transducer	1.09	0.1127	NA
14433	Gapdh	glyceraldehyde-3-phosphate dehydrogenase	1.09	0.4916	0.8156
14086	Fscn1	fascin homolog 1, actin bundling protein (Strongylocentrotus purpuratus)	1.09	0.4975	0.8193
11857	Arhgdib	Rho, GDP dissociation inhibitor (GDI) beta	1.09	0.4952	0.8177
11773	Ap2m1	adaptor protein complex AP-2, mu1	1.09	0.2101	NA
100042720	Gm3988	predicted gene 3988	1.089	0.1842	NA
434179	Gm5595	predicted gene 5595	1.089	0.2005	NA
380856	AA987161	expressed sequence AA987161	1.089	0.2666	NA
329152	Hecw2	HECT, C2 and WW domain containing E3 ubiquitin protein ligase 2	1.089	0.7416	0.918
320404	Itpkb	inositol 1,4,5-trisphosphate 3-kinase B	1.089	0.5725	0.8501
319215	4932413F04Rik	RIKEN cDNA 4932413F04 gene	1.089	0.5026	0.8214
258661	Olfr740	olfactory receptor 740	1.089	0.6294	0.8745
245404	Dcaf12l1	DDB1 and CUL4 associated factor 12-like 1	1.089	0.2264	NA
226971	Plekhb2	pleckstrin homology domain containing, family B (evectins) member 2	1.089	0.4613	0.802
214579	Aldh5a1	aldhehyde dehydrogenase family 5, subfamily A1	1.089	0.192	NA
214084	Slc18a2	solute carrier family 18 (vesicular monoamine), member 2	1.089	0.3992	0.7698
211378	6720489N17Rik	RIKEN cDNA 6720489N17 gene	1.089	0.6002	0.8635
208869	Dock3	dedicator of cyto-kinesis 3	1.089	0.4978	0.8193
171286	Slc12a8	solute carrier family 12 (potassium/chloride transporters), member 8	1.089	0.6384	0.8779
170759	Atp13a1	ATPase type 13A1	1.089	0.4396	0.7908
170574	Sp7	Sp7 transcription factor 7	1.089	0.6388	0.8779
114889	Vsx1	visual system homeobox 1 homolog (zebrafish)	1.089	0.2355	NA
112403	Dom3z	DOM-3 homolog Z (C. elegans)	1.089	0.2734	NA
109006	Ciapin1	cytokine induced apoptosis inhibitor 1	1.089	0.3586	0.7457
108897	Aif1l	allograft inflammatory factor 1-like	1.089	0.242	NA
108097	Prkab2	protein kinase, AMP-activated, beta 2 non-catalytic subunit	1.089	0.2889	NA
103425	Ncln	nicalin homolog (zebrafish)	1.089	0.3682	0.7518
100763	Ube3c	ubiquitin protein ligase E3C	1.089	0.1386	NA
80911	Acox3	acyl-Coenzyme A oxidase 3, pristanoyl	1.089	0.5761	0.8506
76547	Tmem101	transmembrane protein 101	1.089	0.2451	NA
75533	Nme5	non-metastatic cells 5, protein expressed in (nucleoside-diphosphate kinase)	1.089	0.174	NA
74626	Tmem81	transmembrane protein 81	1.089	0.4341	0.7875
74257	Tspan17	tetraspanin 17	1.089	0.2491	NA
71990	Ddx54	DEAD (Asp-Glu-Ala-Asp) box polypeptide 54	1.089	0.4023	0.7706
69713	Pin4	protein (peptidyl-prolyl cis/trans isomerase) NIMA-interacting, 4 (parvulin)	1.089	0.139	NA
69019	Spcs1	signal peptidase complex subunit 1 homolog (S. cerevisiae)	1.089	0.06085	NA
68845	Pih1d1	PIH1 domain containing 1	1.089	0.2255	NA
68709	Cilp2	cartilage intermediate layer protein 2	1.089	0.1478	NA
68118	9430023L20Rik	RIKEN cDNA 9430023L20 gene	1.089	0.09685	NA
66790	Grtp1	GH regulated TBC protein 1	1.089	0.09638	NA
66373	Lsm5	LSM5 homolog, U6 small nuclear RNA associated (S. cerevisiae)	1.089	0.3995	0.7699

66054	Cndp2	CNDP dipeptidase 2 (metallopeptidase M20 family)	1.089	0.3184	0.7182
65111	Dap3	death associated protein 3	1.089	0.2221	NA
64540	Tspan4	tetraspanin 4	1.089	0.2474	NA
57911	Gsdma	gasdermin A	1.089	0.2903	NA
57390	Psors1c2	psoriasis susceptibility 1 candidate 2 (human)	1.089	0.6493	0.8821
52897	Rbfox3	RNA binding protein, fox-1 homolog (C. elegans) 3	1.089	0.169	NA
52468	Ctdsp2	CTD (carboxy-terminal domain, RNA polymerase II, polypeptide A) small phosphatase 2	1.089	0.3135	0.7152
27390	Mmel1	membrane metallo-endopeptidase-like 1	1.089	0.2071	NA
26458	Slc27a2	solute carrier family 27 (fatty acid transporter), member 2	1.089	0.4996	0.8197
22166	Txn1	thioredoxin 1	1.089	0.1963	NA
21349	Tal1	T-cell acute lymphocytic leukemia 1	1.089	0.542	0.8379
19684	Rdx	radixin	1.089	0.2257	NA
19164	Psen1	presenilin 1	1.089	0.2073	NA
19108	Prkx	protein kinase, X-linked	1.089	0.3289	0.7253
18105	Nqo2	NAD(P)H dehydrogenase, quinone 2	1.089	0.1377	NA
17992	Ndufa4	NADH dehydrogenase (ubiquinone) 1 alpha subcomplex, 4	1.089	0.2146	NA
17330	Minpp1	multiple inositol polyphosphate histidine phosphatase 1	1.089	0.1276	NA
16985	Lsp1	lymphocyte specific 1	1.089	0.2454	NA
16834	Cog1	component of oligomeric golgi complex 1	1.089	0.3121	0.7139
14805	Grik1	glutamate receptor, ionotropic, kainate 1	1.089	0.4276	0.7831
13244	Degs1	degenerative spermatocyte homolog 1 (Drosophila)	1.089	0.08147	NA
12857	Cox4i1	cytochrome c oxidase subunit IV isoform 1	1.089	0.3199	0.7191
12540	Cdc42	cell division cycle 42 homolog (S. cerevisiae)	1.089	0.1282	NA
503690	A930005G22Rik	RIKEN cDNA A930005G22 gene	1.088	0.2059	NA
434179	Gm5595	predicted gene 5595	1.088	0.1322	NA
407790	Ndufa4l2	NADH dehydrogenase (ubiquinone) 1 alpha subcomplex, 4-like 2	1.088	0.532	0.8339
381511	Pdp1	pyruvate dehyrogenase phosphatase catalytic subunit 1	1.088	0.09174	NA
380918	Siah3	seven in absentia homolog 3 (Drosophila)	1.088	0.3338	0.729
333605	Frmpd4	FERM and PDZ domain containing 4	1.088	0.1218	NA
330721	Nek5	NIMA (never in mitosis gene a)-related expressed kinase 5	1.088	0.6865	0.8966
330319	Wipf3	WAS/WASL interacting protein family, member 3	1.088	0.3366	0.7305
327987	Med13	mediator complex subunit 13	1.088	0.5468	0.8397
320328	B130024M06Rik	RIKEN cDNA B130024M06 gene	1.088	0.5277	0.8332
319939	Tns3	tensin 3	1.088	0.3495	0.7388
268420	Alkbh5	alkB, alkylation repair homolog 5 (E. coli)	1.088	0.4411	0.792
259105	Olfr549	olfactory receptor 549	1.088	0.8124	0.9403
245468	Pnma3	paraneoplastic antigen MA3	1.088	0.3466	0.7369
239618	Pdzrn4	PDZ domain containing RING finger 4	1.088	0.3422	0.7339
231868	E130309D02Rik	RIKEN cDNA E130309D02 gene	1.088	0.3673	0.7509
229672	Bcl2l15	BCLl2-like 15	1.088	0.6837	0.8956
225743	Rnf165	ring finger protein 165	1.088	0.4022	0.7706
224860	Plcl2	phospholipase C-like 2	1.088	0.1984	NA
224705	Vps52	vacuolar protein sorting 52 (yeast)	1.088	0.6452	0.8806
218121	Mboat1	membrane bound O-acyltransferase domain containing 1	1.088	0.6661	0.8904
209773	Dennd2a	DENN/MADD domain containing 2A	1.088	0.179	NA
116891	Derl2	Der1-like domain family, member 2	1.088	0.2708	NA
109674	Ampd2	adenosine monophosphate deaminase 2	1.088	0.3869	0.761
109323	C1qtnf7	C1q and tumor necrosis factor related protein 7	1.088	0.6398	0.8783
108735	Sft2d2	SFT2 domain containing 2	1.088	0.2064	NA

106039	Gga1	golgi associated, gamma adaptin ear containing, ARF binding protein 1	1.088	0.4755	0.8097
103978	Gpc5	glypican 5	1.088	0.1985	NA
103844	Inca1	inhibitor of CDK, cyclin A1 interacting protein 1	1.088	0.3756	0.7556
98238	Lrrc59	leucine rich repeat containing 59	1.088	0.5139	0.8271
80884	Maged2	melanoma antigen, family D, 2	1.088	0.3603	0.7467
78372	Snrnp25	small nuclear ribonucleoprotein 25 (U11/U12)	1.088	0.1139	NA
77980	Sbf1	SET binding factor 1	1.088	0.4171	0.7777
77560	9330198I05Rik	RIKEN cDNA 9330198I05 gene	1.088	0.6021	0.8638
76429	Lhpp	phospholysine phosphohistidine inorganic pyrophosphate phosphatase	1.088	0.2901	NA
75602	1810062O18Rik	RIKEN cDNA 1810062O18 gene	1.088	0.2948	NA
75430	3200002M19Rik	RIKEN cDNA 3200002M19 gene	1.088	0.3039	0.709
74764	Klc4	kinesin light chain 4	1.088	0.4495	0.7943
74090	Paqr5	progestin and adipoQ receptor family member V	1.088	0.4216	0.7798
73649	Cybrd1	cytochrome b reductase 1	1.088	0.6589	0.8871
72657	2700094K13Rik	RIKEN cDNA 2700094K13 gene	1.088	0.04948	NA
72413	Kcnmb2	potassium large conductance calcium-activated channel, subfamily M, beta member 2	1.088	0.09922	NA
72123	2010109K11Rik	RIKEN cDNA 2010109K11 gene	1.088	0.249	NA
71520	Grap	GRB2-related adaptor protein	1.088	0.6398	0.8783
70603	Mutyh	mutY homolog (E. coli)	1.088	0.3378	0.7314
69847	Wnk4	WNK lysine deficient protein kinase 4	1.088	0.1761	NA
68611	Mrpl28	mitochondrial ribosomal protein L28	1.088	0.1716	NA
68563	Dpm3	dolichyl-phosphate mannosyltransferase polypeptide 3	1.088	0.12	NA
67337	Cstf1	cleavage stimulation factor, 3' pre-RNA, subunit 1	1.088	0.2956	NA
67272	Cmtm5	CKLF-like MARVEL transmembrane domain containing 5	1.088	0.2388	NA
67168	Lpar6	lysophosphatidic acid receptor 6	1.088	0.467	0.8053
66975	2410002O22Rik	RIKEN cDNA 2410002O22 gene	1.088	0.2229	NA
66043	Atp5d	ATP synthase, H+ transporting, mitochondrial F1 complex, delta subunit	1.088	0.4068	0.7726
63985	Gmfb	glia maturation factor, beta	1.088	0.196	NA
59050	Nsa2	NSA2 ribosome biogenesis homolog (S. cerevisiae)	1.088	0.173	NA
57357	Srd5a3	steroid 5 alpha-reductase 3	1.088	0.2765	NA
56546	Sec1	secretory blood group 1	1.088	0.3883	0.7616
56473	Fads2	fatty acid desaturase 2	1.088	0.2264	NA
56438	Rbx1	ring-box 1	1.088	0.024	NA
54525	Syt7	synaptotagmin VII	1.088	0.3875	0.7611
54160	Copg2	coatomer protein complex, subunit gamma 2	1.088	0.1621	NA
51789	Tnk2	tyrosine kinase, non-receptor, 2	1.088	0.4145	0.776
50877	Neu3	neuraminidase 3	1.088	0.1239	NA
29858	Pmm1	phosphomannomutase 1	1.088	0.09181	NA
26943	Serinc3	serine incorporator 3	1.088	0.3303	0.726
26403	Map3k11	mitogen-activated protein kinase kinase kinase 11	1.088	0.599	0.862
24050	Sep-03	septin 3	1.088	0.4245	0.7819
23956	Neu2	neuraminidase 2	1.088	0.3191	0.7184
22337	Vdr	vitamin D receptor	1.088	0.7294	0.9143
19941	Rpl26	ribosomal protein L26	1.088	0.07609	NA
19769	Rit1	Ras-like without CAAX 1	1.088	0.09659	NA
18605	Enpp1	ectonucleotide pyrophosphatase/phosphodiesterase 1	1.088	0.3668	0.7504
17850	Mut	methylmalonyl-Coenzyme A mutase	1.088	0.09966	NA
17480	Mpl	myeloproliferative leukemia virus oncogene	1.088	0.2575	NA
16523	Kcnj8	potassium inwardly-rectifying channel, subfamily J, member 8	1.088	0.2775	NA

16519	Kcnj3	potassium inwardly-rectifying channel, subfamily J, member 3	1.088	0.5867	0.856
16469	Jrk	jerky	1.088	0.4164	0.7771
15461	Hras1	Harvey rat sarcoma virus oncogene 1	1.088	0.2393	NA
15331	Hmgn2	high mobility group nucleosomal binding domain 2	1.088	0.1358	NA
13446	Doc2a	double C2, alpha	1.088	0.3592	0.7461
13131	Dab1	disabled homolog 1 (Drosophila)	1.088	0.2163	NA
12721	Coro1a	coronin, actin binding protein 1A	1.088	0.199	NA
12238	Commd3	COMM domain containing 3	1.088	0.1015	NA
11761	Aox1	aldehyde oxidase 1	1.088	0.4918	0.8156
100040632	AA684185	expressed sequence AA684185	1.087	0.4787	0.81
621239	Nhlrc4	NHL repeat containing 4	1.087	0.2169	NA
433408	Gm13375	predicted gene 13375	1.087	0.4981	0.8194
403347	F830014O18Rik	RIKEN cDNA F830014O18 gene	1.087	0.2208	NA
329470	Accs	1-aminocyclopropane-1-carboxylate synthase homolog (Arabidopsis)(non-functional)	1.087	0.4862	0.8127
319586	Celf5	CUGBP, Elav-like family member 5	1.087	0.3606	0.7467
277333	Gm5069	glyceraldehyde-3-phosphate dehydrogenase pseudogene	1.087	0.4736	0.8088
266781	Snx17	sorting nexin 17	1.087	0.2416	NA
245828	Trappc1	trafficking protein particle complex 1	1.087	0.1281	NA
243274	Tmem132d	transmembrane protein 132D	1.087	0.3544	0.7422
239827	Pigz	phosphatidylinositol glycan anchor biosynthesis, class Z	1.087	0.3707	0.7528
234309	Cbr4	carbonyl reductase 4	1.087	0.2755	NA
229905	Ccbl2	cysteine conjugate-beta lyase 2	1.087	0.1856	NA
227715	Exosc2	exosome component 2	1.087	0.2045	NA
226180	Ina	internexin neuronal intermediate filament protein, alpha	1.087	0.5727	0.8501
218460	Wdr41	WD repeat domain 41	1.087	0.2644	NA
217218	Atxn7l3	ataxin 7-like 3	1.087	0.5727	0.8501
217124	Ppp1r9b	protein phosphatase 1, regulatory subunit 9B	1.087	0.4779	0.81
170787	Hdac10	histone deacetylase 10	1.087	0.4013	0.7703
109754	Cyb5r3	cytochrome b5 reductase 3	1.087	0.3849	0.7607
107747	Aldh1l1	aldehyde dehydrogenase 1 family, member L1	1.087	0.3524	0.7404
102566	Ano10	anoctamin 10	1.087	0.209	NA
94065	Mrpl34	mitochondrial ribosomal protein L34	1.087	0.07965	NA
76709	Arpc2	actin related protein 2/3 complex, subunit 2	1.087	0.2541	NA
75665	Ccdc64	coiled-coil domain containing 64	1.087	0.4416	0.7921
75429	Fam183b	family with sequence similarity 183, member B	1.087	0.4792	0.8101
72029	Cnpy3	canopy 3 homolog (zebrafish)	1.087	0.4051	0.7716
70686	Dusp16	dual specificity phosphatase 16	1.087	0.08588	NA
69697	2310057J16Rik	RIKEN cDNA 2310057J16 gene	1.087	0.3099	0.713
68479	Phf5a	PHD finger protein 5A	1.087	0.3659	0.75
68338	Golt1a	golgi transport 1 homolog A (S. cerevisiae)	1.087	0.7666	0.9251
68133	Gcsh	glycine cleavage system protein H (aminomethyl carrier)	1.087	0.1642	NA
68021	Bphl	biphenyl hydrolase-like (serine hydrolase, breast epithelial mucin-associated antigen)	1.087	0.09902	NA
67767	Jagn1	jagunal homolog 1 (Drosophila)	1.087	0.0752	NA
67671	Rpl38	ribosomal protein L38	1.087	0.2675	NA
67397	Erp29	endoplasmic reticulum protein 29	1.087	0.08394	NA
67170	2610306M01Rik	RIKEN cDNA 2610306M01 gene	1.087	0.5046	0.8221
67067	Romo1	reactive oxygen species modulator 1	1.087	0.3324	0.7273
66949	Trim59	tripartite motif-containing 59	1.087	0.195	NA
66914	Vps28	vacuolar protein sorting 28 (yeast)	1.087	0.1518	NA

59290	Gpa33	glycoprotein A33 (transmembrane)	1.087	0.6752	0.8926
59056	Evc	Ellis van Creveld gene homolog (human)	1.087	0.5406	0.8375
52137	D7Ertd595e	DNA segment, Chr 7, ERATO Doi 595, expressed	1.087	0.3748	0.755
27784	Commd8	COMM domain containing 8	1.087	0.2534	NA
26433	Plod3	procollagen-lysine, 2-oxoglutarate 5-dioxygenase 3	1.087	0.4147	0.776
24058	Sigirr	single immunoglobulin and toll-interleukin 1 receptor (TIR) domain	1.087	0.5136	0.8268
22145	Tuba4a	tubulin, alpha 4A	1.087	0.2947	NA
21388	Tbx5	T-box 5	1.087	0.5301	0.8333
20869	Stk11	serine/threonine kinase 11	1.087	0.25	NA
20463	Cox7a2l	cytochrome c oxidase subunit VIIa polypeptide 2-like	1.087	0.0632	NA
20312	Cx3cl1	chemokine (C-X3-C motif) ligand 1	1.087	0.3506	0.7393
19731	Rgl1	ral guanine nucleotide dissociation stimulator,-like 1	1.087	0.2336	NA
16871	Lhx3	LIM homeobox protein 3	1.087	0.1642	NA
16865	Eif2d	eukaryotic translation initiation factor 2D	1.087	0.393	0.7652
16468	Jarid2	jumonji, AT rich interactive domain 2	1.087	0.3071	0.7108
16323	Inhba	inhibin beta-A	1.087	0.6659	0.8904
15898	Icam5	intercellular adhesion molecule 5, telencephalin	1.087	0.2966	NA
14357	Dtx1	deltex 1 homolog (Drosophila)	1.087	0.4304	0.7846
14269	Fnbp1	formin binding protein 1	1.087	0.6016	0.8636
13417	Dnahc8	dynein, axonemal, heavy chain 8	1.087	0.559	0.8459
13123	Cyp7b1	cytochrome P450, family 7, subfamily b, polypeptide 1	1.087	0.2037	NA
12971	Crym	crystallin, mu	1.087	0.2782	NA
12631	Cfl1	cofilin 1, non-muscle	1.087	0.4963	0.8188
12262	C1qc	complement component 1, q subcomponent, C chain	1.087	0.5514	0.843
12177	Bnip3l	BCL2/adenovirus E1B interacting protein 3-like	1.087	0.1905	NA
12023	Barx2	BarH-like homeobox 2	1.087	0.5614	0.8465
11480	Acvr2a	activin receptor IIA	1.087	0.1931	NA
100041294	Gm3258	predicted gene 3258	1.086	0.06577	NA
624860	Gm12253	predicted gene 12253	1.086	0.6643	0.8899
384619	Ccdc155	coiled-coil domain containing 155	1.086	0.3392	0.7321
381375	Dfnb59	deafness, autosomal recessive 59 (human)	1.086	0.3457	0.7361
320394	Cenpt	centromere protein T	1.086	0.284	NA
241035	Pkhd1	polycystic kidney and hepatic disease 1	1.086	0.5	0.8197
240916	Vsig8	V-set and immunoglobulin domain containing 8	1.086	0.1132	NA
230979	Tnfrsf14	tumor necrosis factor receptor superfamily, member 14 (herpesvirus entry mediator)	1.086	0.7359	0.9169
226641	Atf6	activating transcription factor 6	1.086	0.2498	NA
223752	Gramd4	GRAM domain containing 4	1.086	0.1329	NA
218335	Clptm1l	CLPTM1-like	1.086	0.3865	0.761
216169	Fam108a	family with sequence similarity 108, member A	1.086	0.447	0.7938
213469	Lgi3	leucine-rich repeat LGI family, member 3	1.086	0.4192	0.7791
212647	Aldh4a1	aldehyde dehydrogenase 4 family, member A1	1.086	0.1198	NA
192652	Wdr81	WD repeat domain 81	1.086	0.1984	NA
116748	Lsm10	U7 snRNP-specific Sm-like protein LSM10	1.086	0.1736	NA
94066	Mrpl36	mitochondrial ribosomal protein L36	1.086	0.1713	NA
75495	Morn5	MORN repeat containing 5	1.086	0.3888	0.7616
74600	Mrpl47	mitochondrial ribosomal protein L47	1.086	0.22	NA
72393	Faim2	Fas apoptotic inhibitory molecule 2	1.086	0.1539	NA
71994	Cnn3	calponin 3, acidic	1.086	0.3434	0.7354
70231	Gorasp2	golgi reassembly stacking protein 2	1.086	0.3188	0.7182

69938	Scrn1	secernin 1	1.086	0.1121	NA
67119	Ccdc159	coiled-coil domain containing 159	1.086	0.3979	0.7684
67059	Ola1	Obg-like ATPase 1	1.086	0.1732	NA
66869	Zfp869	zinc finger protein 869	1.086	0.1876	NA
66606	Lrrc57	leucine rich repeat containing 57	1.086	0.07871	NA
66596	Gtf3a	general transcription factor III A	1.086	0.2332	NA
66510	Rnf181	ring finger protein 181	1.086	0.2016	NA
66487	2010107H07Rik	RIKEN cDNA 2010107H07 gene	1.086	0.2017	NA
66350	Pla2g12a	phospholipase A2, group XIIA	1.086	0.2748	NA
66193	1110049F12Rik	RIKEN cDNA 1110049F12 gene	1.086	0.07359	NA
66170	Chchd5	coiled-coil-helix-coiled-coil-helix domain containing 5	1.086	0.1014	NA
64242	Ngb	neuroglobin	1.086	0.2439	NA
64011	Nrgn	neurogranin	1.086	0.5983	0.8615
57329	Otor	otoraplin	1.086	0.1368	NA
54723	Tfip11	tuftelin interacting protein 11	1.086	0.4449	0.7938
54139	Irf6	interferon regulatory factor 6	1.086	0.6816	0.8949
53323	Ube2k	ubiquitin-conjugating enzyme E2K (UBC1 homolog, yeast)	1.086	0.04709	NA
27275	Nufip1	nuclear fragile X mental retardation protein interacting protein 1	1.086	0.2541	NA
23989	Med24	mediator complex subunit 24	1.086	0.3981	0.7686
23936	Lynx1	Ly6/neurotoxin 1	1.086	0.4784	0.81
22644	Rnf103	ring finger protein 103	1.086	0.2389	NA
22341	Vegfc	vascular endothelial growth factor C	1.086	0.1898	NA
21946	Pglyrp1	peptidoglycan recognition protein 1	1.086	0.7113	0.907
21345	Tagln	transgelin	1.086	0.4216	0.7798
20405	Sh3gl1	SH3-domain GRB2-like 1	1.086	0.542	0.8379
20090	Rps29	ribosomal protein S29	1.086	0.3561	0.7443
19027	Sypl	synaptophysin-like protein	1.086	0.1656	NA
18749	Prkacb	protein kinase, cAMP dependent, catalytic, beta	1.086	0.09136	NA
18738	Pitpna	phosphatidylinositol transfer protein, alpha	1.086	0.0724	NA
16924	Lnx1	ligand of numb-protein X 1	1.086	0.1627	NA
16497	Kcnab1	potassium voltage-gated channel, shaker-related subfamily, beta member 1	1.086	0.1316	NA
15239	Hgs	HGF-regulated tyrosine kinase substrate	1.086	0.2534	NA
14562	Gdf3	growth differentiation factor 3	1.086	0.5209	0.8286
14407	Gabrg3	gamma-aminobutyric acid (GABA) A receptor, subunit gamma 3	1.086	0.2158	NA
13712	Elk1	ELK1, member of ETS oncogene family	1.086	0.4324	0.7863
12506	Cd48	CD48 antigen	1.086	0.3374	0.7311
12334	Capn2	calpain 2	1.086	0.1821	NA
12282	Hyou1	hypoxia up-regulated 1	1.086	0.5125	0.8262
12013	Bach1	BTB and CNC homology 1	1.086	0.3123	NA
100504473	LOC100504473	RNA-binding protein Musashi homolog 2-like	1.085	0.337	0.7308
382245	Tmem29	transmembrane protein 29	1.085	0.1939	NA
338350	9330129D05Rik	RIKEN cDNA 9330129D05 gene	1.085	0.3642	0.7488
269378	Ahcy	S-adenosylhomocysteine hydrolase	1.085	0.3162	NA
241118	Accn4	amiloride-sensitive cation channel 4, pituitary	1.085	0.2258	NA
226123	Morn4	MORN repeat containing 4	1.085	0.1962	NA
216198	Tcp11l2	t-complex 11 (mouse) like 2	1.085	0.2651	NA
212123	Dcaf15	DDB1 and CUL4 associated factor 15	1.085	0.4079	0.7729
108124	Napa	N-ethylmaleimide sensitive fusion protein attachment protein alpha	1.085	0.2017	NA
108071	Grm5	glutamate receptor, metabotropic 5	1.085	0.413	0.7751

104027	Synpo	synaptopodin	1.085	0.4494	0.7943
100206	Adprhl2	ADP-ribosylhydrolase like 2	1.085	0.1826	NA
81904	Cacng7	calcium channel, voltage-dependent, gamma subunit 7	1.085	0.4864	0.8127
76568	Ift46	intraflagellar transport 46 homolog (Chlamydomonas)	1.085	0.2981	NA
76524	Cln6	ceroid-lipofuscinosis, neuronal 6	1.085	0.452	0.7951
75860	4930588N13Rik	RIKEN cDNA 4930588N13 gene	1.085	0.5438	0.839
75234	Rnf19b	ring finger protein 19B	1.085	0.08964	NA
74012	Rap2b	RAP2B, member of RAS oncogene family	1.085	0.6098	0.8665
72831	Dhx30	DEAH (Asp-Glu-Ala-His) box polypeptide 30	1.085	0.297	NA
71131	Zfp689	zinc finger protein 689	1.085	0.2017	NA
70603	Mutyh	mutY homolog (E. coli)	1.085	0.5197	0.8284
70575	Gfod2	glucose-fructose oxidoreductase domain containing 2	1.085	0.3658	0.7499
69860	Eif1ad	eukaryotic translation initiation factor 1A domain containing	1.085	0.3317	0.7267
68303	Fam114a1	family with sequence similarity 114, member A1	1.085	0.4916	0.8156
68251	5430437P03Rik	RIKEN cDNA 5430437P03 gene	1.085	0.04771	NA
67905	Ppm1m	protein phosphatase 1M	1.085	0.2976	NA
67803	Limd2	LIM domain containing 2	1.085	0.2053	NA
67135	2310021H06Rik	RIKEN cDNA 2310021H06 gene	1.085	0.7344	0.9162
67115	Rpl14	ribosomal protein L14	1.085	0.4335	0.7872
66626	5730403B10Rik	RIKEN cDNA 5730403B10 gene	1.085	0.3404	0.7328
66349	Atp5sl	ATP5S-like	1.085	0.2089	NA
66286	Sec11c	SEC11 homolog C (S. cerevisiae)	1.085	0.2426	NA
59014	Rrs1	RRS1 ribosome biogenesis regulator homolog (S. cerevisiae)	1.085	0.1429	NA
59005	Trappc2l	trafficking protein particle complex 2-like	1.085	0.3411	0.7334
56844	Tssc4	tumor-suppressing subchromosomal transferable fragment 4	1.085	0.2391	NA
56428	Mtch2	mitochondrial carrier homolog 2 (C. elegans)	1.085	0.3817	0.7596
54338	Slc23a2	solute carrier family 23 (nucleobase transporters), member 2	1.085	0.4356	0.7884
30935	Tor3a	torsin family 3, member A	1.085	0.03098	NA
26394	Lypla2	lysophospholipase 2	1.085	0.102	NA
23934	Ly6h	lymphocyte antigen 6 complex, locus H	1.085	0.2073	NA
22782	Slc30a1	solute carrier family 30 (zinc transporter), member 1	1.085	0.3135	NA
22746	Zfp85-rs1	zinc finger protein 85, related sequence 1	1.085	0.2263	NA
22690	Zfp28	zinc finger protein 28	1.085	0.5194	0.8282
20964	Syn1	synapsin I	1.085	0.444	0.7931
19267	Ptpre	protein tyrosine phosphatase, receptor type, E	1.085	0.1554	NA
19018	Scand1	SCAN domain-containing 1	1.085	0.3287	0.7253
18704	Pik3c2a	phosphatidylinositol 3-kinase, C2 domain containing, alpha polypeptide	1.085	0.6041	0.8644
18033	Nfkb1	nuclear factor of kappa light polypeptide gene enhancer in B-cells 1, p105	1.085	0.4412	0.7921
18002	Nedd8	neural precursor cell expressed, developmentally down-regulated gene 8	1.085	0.188	NA
17686	Msh3	mutS homolog 3 (E. coli)	1.085	0.3294	0.7257
17192	Mbd3	methyl-CpG binding domain protein 3	1.085	0.203	NA
16979	Lrrn1	leucine rich repeat protein 1, neuronal	1.085	0.1037	NA
16866	Lhb	luteinizing hormone beta	1.085	0.7502	0.921
16859	Lgals9	lectin, galactose binding, soluble 9	1.085	0.6154	0.8696
16660	Krt31	keratin 31	1.085	0.5086	0.8242
16493	Kcna5	potassium voltage-gated channel, shaker-related subfamily, member 5	1.085	0.4705	0.8074
15166	Hcn2	hyperpolarization-activated, cyclic nucleotide-gated K+ 2	1.085	0.5597	0.8459
14751	Gpi1	glucose phosphate isomerase 1	1.085	0.2235	NA
12671	Chrm3	cholinergic receptor, muscarinic 3, cardiac	1.085	0.1849	NA

12368	Casp6	caspase 6	1.085	0.2725	NA
432987	Gm5478	predicted pseudogene 5478	1.084	0.1818	NA
380977	A330009N23Rik	RIKEN cDNA A330009N23 gene	1.084	0.3695	0.7521
380840	Lyrm4	LYR motif containing 4	1.084	0.2374	NA
380608	Tagap1	T-cell activation GTPase activating protein 1	1.084	0.4248	0.7819
277463	Gpr107	G protein-coupled receptor 107	1.084	0.2347	NA
263876	Spata2	spermatogenesis associated 2	1.084	0.1763	NA
243078	Tecrl	trans-2,3-enoyl-CoA reductase-like	1.084	0.2804	NA
242667	Dlgap3	discs, large (Drosophila) homolog-associated protein 3	1.084	0.5562	0.8448
241576	Ldlrad3	low density lipoprotein receptor class A domain containing 3	1.084	0.5112	0.8258
240064	Zfp799	zinc finger protein 799	1.084	0.2527	NA
233016	Blvrb	biliverdin reductase B (flavin reductase (NADPH))	1.084	0.2141	NA
227624	B230208H17Rik	RIKEN cDNA B230208H17 gene	1.084	0.4963	0.8187
227377	Farp2	FERM, RhoGEF and pleckstrin domain protein 2	1.084	0.3619	0.7477
227298	Fam134a	family with sequence similarity 134, member A	1.084	0.3271	NA
226180	Ina	internexin neuronal intermediate filament protein, alpha	1.084	0.4099	0.7739
170938	Zfp617	zinc finger protein 617	1.084	0.09342	NA
140703	Emid1	EMI domain containing 1	1.084	0.4124	0.7747
109079	Sephs1	selenophosphate synthetase 1	1.084	0.2147	NA
107999	Gtpbp6	GTP binding protein 6 (putative)	1.084	0.4013	0.7703
102098	Arhgef18	rho/rac guanine nucleotide exchange factor (GEF) 18	1.084	0.202	NA
81909	Zfpl1	zinc finger like protein 1	1.084	0.5144	0.8272
76380	Ccdc46	coiled-coil domain containing 46	1.084	0.3445	0.7359
74772	Atp13a2	ATPase type 13A2	1.084	0.3161	NA
74549	Mau2	MAU2 chromatid cohesion factor homolog (C. elegans)	1.084	0.5873	0.8564
74304	1700094M24Rik	RIKEN cDNA 1700094M24 gene	1.084	0.6053	0.8645
72759	Tmem135	transmembrane protein 135	1.084	0.1852	NA
72736	Tmx1	thioredoxin-related transmembrane protein 1	1.084	0.2573	NA
72605	Car10	carbonic anhydrase 10	1.084	0.2236	NA
71929	Tmem123	transmembrane protein 123	1.084	0.1148	NA
70383	Cox10	COX10 homolog, cytochrome c oxidase assembly protein, heme A: farnesyltransferase (yeast)	1.084	0.5379	0.8359
68666	Svop	SV2 related protein	1.084	0.3302	0.726
68603	Pmvk	phosphomevalonate kinase	1.084	0.1014	NA
68229	AI846148	expressed sequence AI846148	1.084	0.3809	0.7594
68174	4930534H18Rik	RIKEN cDNA 4930534H18 gene	1.084	0.6842	0.8956
68045	2700060E02Rik	RIKEN cDNA 2700060E02 gene	1.084	0.4441	0.7931
67884	1810043G02Rik	RIKEN cDNA 1810043G02 gene	1.084	0.3528	0.7408
67775	Rtp4	receptor transporter protein 4	1.084	0.5438	0.839
67369	Qpctl	glutaminyl-peptide cyclotransferase-like	1.084	0.3092	NA
66830	Nacc1	nucleus accumbens associated 1, BEN and BTB (POZ) domain containing	1.084	0.6611	0.8886
66743	Rnf220	ring finger protein 220	1.084	0.09612	NA
66576	Uqcrh	ubiquinol-cytochrome c reductase hinge protein	1.084	0.08112	NA
66482	Exoc2	exocyst complex component 2	1.084	0.1881	NA
66297	2610017I09Rik	RIKEN cDNA 2610017I09 gene	1.084	0.3118	NA
66229	Rpl7l1	ribosomal protein L7-like 1	1.084	0.3146	NA
66091	Ndufa3	NADH dehydrogenase (ubiquinone) 1 alpha subcomplex, 3	1.084	0.2714	NA
66060	0610010O12Rik	RIKEN cDNA 0610010O12 gene	1.084	0.4132	0.7753
60525	Acss2	acyl-CoA synthetase short-chain family member 2	1.084	0.08688	NA
57320	Park7	Parkinson disease (autosomal recessive, early onset) 7	1.084	0.2579	NA

56305	Pitpnb	phosphatidylinositol transfer protein, beta	1.084	0.298	NA
52389	Gpr123	G protein-coupled receptor 123	1.084	0.5386	0.8366
20745	Spock1	sparc/osteonectin, cwcv and kazal-like domains proteoglycan 1	1.084	0.3881	0.7615
18574	Pde1b	phosphodiesterase 1B, Ca2+-calmodulin dependent	1.084	0.3372	0.731
18181	Nrf1	nuclear respiratory factor 1	1.084	0.2467	NA
17436	Me1	malic enzyme 1, NADP(+)-dependent, cytosolic	1.084	0.1276	NA
17385	Mmp11	matrix metallopeptidase 11	1.084	0.4532	0.7966
17219	Mcm6	minichromosome maintenance deficient 6 (MIS5 homolog, S. pombe) (S. cerevisiae)	1.084	0.2248	NA
16775	Lama4	laminin, alpha 4	1.084	0.4953	0.8178
14686	Gnat2	guanine nucleotide binding protein, alpha transducing 2	1.084	0.2224	NA
13385	Dlg4	discs, large homolog 4 (Drosophila)	1.084	0.4114	0.7747
13078	Cyp1b1	cytochrome P450, family 1, subfamily b, polypeptide 1	1.084	0.444	0.7931
13033	Ctsd	cathepsin D	1.084	0.4821	0.8108
12521	Cd82	CD82 antigen	1.084	0.5063	0.8231
12010	B2m	beta-2 microglobulin	1.084	0.3492	0.7388
11803	Aplp1	amyloid beta (A4) precursor-like protein 1	1.084	0.5986	0.8617
791318	Gm10125	predicted gene 10125	1.083	0.4564	0.7987
434215	Lrrc32	leucine rich repeat containing 32	1.083	0.2901	NA
381633	Gm1673	predicted gene 1673	1.083	0.2387	NA
320404	Itpkb	inositol 1,4,5-trisphosphate 3-kinase B	1.083	0.4103	0.7739
319830	1500004A13Rik	RIKEN cDNA 1500004A13 gene	1.083	0.3016	NA
242418	Dcaf10	DDB1 and CUL4 associated factor 10	1.083	0.1694	NA
233870	Tufm	Tu translation elongation factor, mitochondrial	1.083	0.3583	0.7457
233057	Zfp940	zinc finger protein 940	1.083	0.3928	0.7651
232670	Tspan33	tetraspanin 33	1.083	0.1846	NA
231874	Ccz1	CCZ1 vacuolar protein trafficking and biogenesis associated homolog (S. cerevisiae)	1.083	0.1186	NA
231861	Tnrc18	trinucleotide repeat containing 18	1.083	0.5803	0.8527
230824	Grhl3	grainyhead-like 3 (Drosophila)	1.083	0.3211	NA
215890	Clvs2	clavesin 2	1.083	0.2495	NA
114893	Dcun1d1	DCN1, defective in cullin neddylation 1, domain containing 1 (S. cerevisiae)	1.083	0.09896	NA
108013	Celf4	CUGBP, Elav-like family member 4	1.083	0.2058	NA
107951	Cdk9	cyclin-dependent kinase 9 (CDC2-related kinase)	1.083	0.3184	NA
101502	Hsd3b7	hydroxy-delta-5-steroid dehydrogenase, 3 beta- and steroid delta-isomerase 7	1.083	0.3809	0.7594
93717	Pcdhga9	protocadherin gamma subfamily A, 9	1.083	0.4716	0.8077
76080	Ttpal	tocopherol (alpha) transfer protein-like	1.083	0.6569	0.8862
76080	Ttpal	tocopherol (alpha) transfer protein-like	1.083	0.08002	NA
74443	P4htm	prolyl 4-hydroxylase, transmembrane (endoplasmic reticulum)	1.083	0.4949	0.8175
73316	Calr3	calreticulin 3	1.083	0.4549	0.7977
72630	Hspa12b	heat shock protein 12B	1.083	0.1739	NA
72003	Synpr	synaptoporin	1.083	0.2978	NA
70503	Ddo	D-aspartate oxidase	1.083	0.3163	NA
70235	Poc1a	POC1 centriolar protein homolog A (Chlamydomonas)	1.083	0.189	NA
70153	2210016F16Rik	RIKEN cDNA 2210016F16 gene	1.083	0.1026	NA
69449	1700027A15Rik	RIKEN cDNA 1700027A15 gene	1.083	0.3005	NA
69051	Pycr2	pyrroline-5-carboxylate reductase family, member 2	1.083	0.1516	NA
68544	2310036O22Rik	RIKEN cDNA 2310036O22 gene	1.083	0.3363	0.7305
68487	Tmem140	transmembrane protein 140	1.083	0.6115	0.8673
67630	Samd8	sterile alpha motif domain containing 8	1.083	0.2471	NA
67525	Trim62	tripartite motif-containing 62	1.083	0.4994	0.8197

66240	Kcne1l	potassium voltage-gated channel, Isk-related family, member 1-like, pseudogene	1.083	0.3636	0.7483
60530	Fignl1	fidgetin-like 1	1.083	0.305	NA
58998	Pvrl3	poliovirus receptor-related 3	1.083	0.4896	0.8145
57915	Tbc1d1	TBC1 domain family, member 1	1.083	0.2395	NA
54354	Rassf5	Ras association (RalGDS/AF-6) domain family member 5	1.083	0.1746	NA
52250	Reep1	receptor accessory protein 1	1.083	0.09021	NA
50876	Tmod2	tropomodulin 2	1.083	0.3088	NA
28193	Reep3	receptor accessory protein 3	1.083	0.1711	NA
26914	H2afy	H2A histone family, member Y	1.083	0.4644	0.8038
24067	Srp54a	signal recognition particle 54A	1.083	0.2989	NA
23908	Hs2st1	heparan sulfate 2-O-sulfotransferase 1	1.083	0.6327	0.8758
22003	Tpm1	tropomyosin 1, alpha	1.083	0.3348	0.73
21422	Tcfcp2	transcription factor CP2	1.083	0.05779	NA
20977	Syp	synaptophysin	1.083	0.5092	0.8247
20729	Spin1	spindlin 1	1.083	0.1113	NA
20698	Sphk1	sphingosine kinase 1	1.083	0.4059	0.7719
20618	Sncg	synuclein, gamma	1.083	0.552	0.8432
20420	Shd	src homology 2 domain-containing transforming protein D	1.083	0.4357	0.7884
19411	Rarg	retinoic acid receptor, gamma	1.083	0.4264	0.7823
18389	Oprl1	opioid receptor-like 1	1.083	0.2685	NA
18377	Omg	oligodendrocyte myelin glycoprotein	1.083	0.248	NA
17313	Mgp	matrix Gla protein	1.083	0.3209	NA
16815	Lbx2	ladybird homeobox homolog 2 (Drosophila)	1.083	0.4851	0.8126
16664	Krt14	keratin 14	1.083	0.6728	0.8922
16372	Irx2	Iroquois related homeobox 2 (Drosophila)	1.083	0.2659	NA
15267	Hist2h2aa1	histone cluster 2, H2aa1	1.083	0.09016	NA
14886	Gtf2i	general transcription factor II I	1.083	0.4089	0.7734
14598	Ggt1	gamma-glutamyltransferase 1	1.083	0.7419	0.918
14585	Gfra1	glial cell line derived neurotrophic factor family receptor alpha 1	1.083	0.2879	NA
14433	Gapdh	glyceraldehyde-3-phosphate dehydrogenase	1.083	0.5639	0.8465
13038	Ctsk	cathepsin K	1.083	0.3231	NA
12371	Casp9	caspase 9	1.083	0.2707	NA
11655	Alas1	aminolevulinic acid synthase 1	1.083	0.1937	NA
100505098	LOC100505098	60S ribosomal protein L19-like	1.082	0.1196	NA
434234	2610020H08Rik	RIKEN cDNA 2610020H08 gene	1.082	0.08341	NA
353170	Txlng	taxilin gamma	1.082	0.5021	0.8214
269994	Gsg1l	GSG1-like	1.082	0.2293	NA
269952	D330012F22Rik	RIKEN cDNA D330012F22 gene	1.082	0.4186	0.7787
242384	Lingo2	leucine rich repeat and Ig domain containing 2	1.082	0.1984	NA
239157	Pnma2	paraneoplastic antigen MA2	1.082	0.1189	NA
195646	Hs3st2	heparan sulfate (glucosamine) 3-O-sulfotransferase 2	1.082	0.6578	0.8865
193796	Kdm4b	lysine (K)-specific demethylase 4B	1.082	0.5088	0.8244
140559	Igsf8	immunoglobulin superfamily, member 8	1.082	0.3062	NA
110198	Akr7a5	aldo-keto reductase family 7, member A5 (aflatoxin aldehyde reductase)	1.082	0.3126	NA
104416	Bap1	Brca1 associated protein 1	1.082	0.3584	0.7457
94332	Cadm3	cell adhesion molecule 3	1.082	0.4787	0.81
80749	Lrfn1	leucine rich repeat and fibronectin type III domain containing 1	1.082	0.7258	0.9126
77085	3010027C24Rik	RIKEN cDNA 3010027C24 gene	1.082	0.6953	0.901
76448	2310014H01Rik	RIKEN cDNA 2310014H01 gene	1.082	0.7713	0.9258

76432	2310001H17Rik	RIKEN cDNA 2310001H17 gene	1.082	0.7017	0.9038
75284	Bcdin3d	BCDIN3 domain containing	1.082	0.321	NA
74901	Kbtbd11	kelch repeat and BTB (POZ) domain containing 11	1.082	0.1595	NA
73666	Thoc3	THO complex 3	1.082	0.2712	NA
73130	Tmed5	transmembrane emp24 protein transport domain containing 5	1.082	0.5606	0.8462
71890	Mad2l2	MAD2 mitotic arrest deficient-like 2 (yeast)	1.082	0.1952	NA
70004	1700028J19Rik	RIKEN cDNA 1700028J19 gene	1.082	0.4478	0.7938
69942	Rnf113a1	ring finger protein 113A1	1.082	0.09166	NA
68350	Mul1	mitochondrial ubiquitin ligase activator of NFKB 1	1.082	0.1725	NA
67872	Nsmce4a	non-SMC element 4 homolog A (S. cerevisiae)	1.082	0.07263	NA
67844	Rab32	RAB32, member RAS oncogene family	1.082	0.3286	NA
67752	4930579J09Rik	RIKEN cDNA 4930579J09 gene	1.082	0.5359	0.835
67742	Samsn1	SAM domain, SH3 domain and nuclear localization signals, 1	1.082	0.07509	NA
67184	Ndufa13	NADH dehydrogenase (ubiquinone) 1 alpha subcomplex, 13	1.082	0.3497	0.7388
66459	Pigy	phosphatidylinositol glycan anchor biosynthesis, class Y	1.082	0.1311	NA
66335	Atp6v1c1	ATPase, H+ transporting, lysosomal V1 subunit C1	1.082	0.1728	NA
59054	Mrps30	mitochondrial ribosomal protein S30	1.082	0.1041	NA
58245	Gpr180	G protein-coupled receptor 180	1.082	0.3003	NA
56550	Ube2d2	ubiquitin-conjugating enzyme E2D 2	1.082	0.08038	NA
56486	Gabarap	gamma-aminobutyric acid receptor associated protein	1.082	0.2087	NA
56441	Nat6	N-acetyltransferase 6	1.082	0.5636	0.8465
55990	Fmo2	flavin containing monooxygenase 2	1.082	0.5514	0.843
23999	Twf2	twinfilin, actin-binding protein, homolog 2 (Drosophila)	1.082	0.455	0.7977
23874	Farsb	phenylalanyl-tRNA synthetase, beta subunit	1.082	0.2164	NA
22121	Rpl13a	ribosomal protein L13A	1.082	0.3012	NA
21420	Tcfap2c	transcription factor AP-2, gamma	1.082	0.144	NA
20672	Sox18	SRY-box containing gene 18	1.082	0.5218	0.829
20363	Sepp1	selenoprotein P, plasma, 1	1.082	0.343	0.7347
19702	Ren2	renin 2 tandem duplication of Ren1	1.082	0.604	0.8644
18830	Pltp	phospholipid transfer protein	1.082	0.5964	0.861
18618	Pemt	phosphatidylethanolamine N-methyltransferase	1.082	0.6017	0.8636
18578	Pde4b	phosphodiesterase 4B, cAMP specific	1.082	0.2877	NA
17828	Muted	muted	1.082	0.1001	NA
17470	Cd200	CD200 antigen	1.082	0.1927	NA
16950	Loxl3	lysyl oxidase-like 3	1.082	0.4442	0.7932
14782	Gsr	glutathione reductase	1.082	0.2025	NA
14687	Gnaz	guanine nucleotide binding protein, alpha z subunit	1.082	0.5226	0.8294
14470	Rabac1	Rab acceptor 1 (prenylated)	1.082	0.2066	NA
14377	G6pc	glucose-6-phosphatase, catalytic	1.082	0.6411	0.8788
14129	Fcgr1	Fc receptor, IgG, high affinity I	1.082	0.5878	0.857
13495	Drg2	developmentally regulated GTP binding protein 2	1.082	0.3834	0.7599
12177	Bnip3l	BCL2/adenovirus E1B interacting protein 3-like	1.082	0.2123	NA
666945	Gm10638	predicted gene 10638	1.081	0.4873	0.8134
381738	Gm1060	predicted gene 1060	1.081	0.4451	0.7938
380752	Tssc1	tumor suppressing subtransferable candidate 1	1.081	0.3197	NA
330192	Vps37b	vacuolar protein sorting 37B (yeast)	1.081	0.3596	0.7464
269881	Map3k10	mitogen-activated protein kinase kinase kinase 10	1.081	0.4114	0.7747
268291	Rnf217	ring finger protein 217	1.081	0.4498	0.7943
237759	Col23a1	collagen, type XXIII, alpha 1	1.081	0.2872	NA

237213	Glra2	glycine receptor, alpha 2 subunit	1.081	0.2323	NA
236792	Mmgt1	membrane magnesium transporter 1	1.081	0.1937	NA
234353	Psd3	pleckstrin and Sec7 domain containing 3	1.081	0.6294	0.8745
233064	Wdr62	WD repeat domain 62	1.081	0.2424	NA
231821	Adap1	ArfGAP with dual PH domains 1	1.081	0.4166	0.7772
229707	Fam40a	family with sequence similarity 40, member A	1.081	0.1086	NA
229474	Fhdc1	FH2 domain containing 1	1.081	0.4898	0.8145
228852	Ppp1r16b	protein phosphatase 1, regulatory (inhibitor) subunit 16B	1.081	0.6548	0.8854
224824	Pex6	peroxisomal biogenesis factor 6	1.081	0.4511	0.7948
224792	Gpr116	G protein-coupled receptor 116	1.081	0.3748	0.755
218194	Phactr1	phosphatase and actin regulator 1	1.081	0.07586	NA
216858	Kctd11	potassium channel tetramerisation domain containing 11	1.081	0.4367	0.7888
211548	Nomo1	nodal modulator 1	1.081	0.367	0.7505
210673	Prrt3	proline-rich transmembrane protein 3	1.081	0.5407	0.8375
194908	Pld6	phospholipase D family, member 6	1.081	0.5707	0.8492
114641	Rpl31	ribosomal protein L31	1.081	0.1177	NA
109154	Mlec	malectin	1.081	0.2235	NA
107767	Scamp1	secretory carrier membrane protein 1	1.081	0.0884	NA
107513	Ssr1	signal sequence receptor, alpha	1.081	0.2961	NA
103963	Rpn1	ribophorin I	1.081	0.2889	NA
97613	C85181	expressed sequence C85181	1.081	0.07109	NA
79196	Osbpl5	oxysterol binding protein-like 5	1.081	0.09172	NA
78938	Fbxo34	F-box protein 34	1.081	0.09679	NA
78255	Ralgps2	Ral GEF with PH domain and SH3 binding motif 2	1.081	0.2488	NA
77574	Fam115a	family with sequence similarity 115, member A	1.081	0.7256	0.9126
76566	Fam101b	family with sequence similarity 101, member B	1.081	0.5755	0.8505
74464	Zswim5	zinc finger, SWIM domain containing 5	1.081	0.4256	0.7823
74176	Tgm5	transglutaminase 5	1.081	0.5174	0.8278
73710	Tubb2b	tubulin, beta 2B	1.081	0.2661	NA
71978	Ppp2r2a	protein phosphatase 2 (formerly 2A), regulatory subunit B (PR 52), alpha isoform	1.081	0.3007	NA
70209	Tmem143	transmembrane protein 143	1.081	0.5299	0.8332
69908	Rab3b	RAB3B, member RAS oncogene family	1.081	0.3746	0.755
69368	Wdfy1	WD repeat and FYVE domain containing 1	1.081	0.3997	0.77
69080	Gmppa	GDP-mannose pyrophosphorylase A	1.081	0.4473	0.7938
68525	Evc2	Ellis van Creveld syndrome 2 homolog (human)	1.081	0.2308	NA
68520	Zfyve21	zinc finger, FYVE domain containing 21	1.081	0.2044	NA
68295	0610011L14Rik	RIKEN cDNA 0610011L14 gene	1.081	0.2085	NA
68214	Gsto2	glutathione S-transferase omega 2	1.081	0.3694	0.7521
68115	9430016H08Rik	RIKEN cDNA 9430016H08 gene	1.081	0.1896	NA
68050	Akirin1	akirin 1	1.081	0.4877	0.8135
67673	Tceb2	transcription elongation factor B (SIII), polypeptide 2	1.081	0.1585	NA
66935	Cir1	corepressor interacting with RBPJ, 1	1.081	0.4432	0.7931
66525	Timm50	translocase of inner mitochondrial membrane 50 homolog (yeast)	1.081	0.4426	0.7928
66491	Polr2l	polymerase (RNA) II (DNA directed) polypeptide L	1.081	0.5122	0.8262
66333	Aqp11	aquaporin 11	1.081	0.2445	NA
66204	Acyp1	acylphosphatase 1, erythrocyte (common) type	1.081	0.5336	0.8343
58242	Nudt11	nudix (nucleoside diphosphate linked moiety X)-type motif 11	1.081	0.2199	NA
56494	Gosr2	golgi SNAP receptor complex member 2	1.081	0.2306	NA
50785	Hs6st1	heparan sulfate 6-O-sulfotransferase 1	1.081	0.5636	0.8465

29859	Sult4a1	sulfotransferase family 4A, member 1	1.081	0.4299	0.7843
26893	Cops6	COP9 (constitutive photomorphogenic) homolog, subunit 6 (Arabidopsis thaliana)	1.081	0.3492	0.7388
23989	Med24	mediator complex subunit 24	1.081	0.4757	0.8097
22781	Ikzf4	IKAROS family zinc finger 4	1.081	0.7991	0.9361
22038	Plscr1	phospholipid scramblase 1	1.081	0.2257	NA
21385	Tbx2	T-box 2	1.081	0.1272	NA
21338	Tacr3	tachykinin receptor 3	1.081	0.1819	NA
20454	St3gal5	ST3 beta-galactoside alpha-2,3-sialyltransferase 5	1.081	0.05105	NA
20447	St6galnac3	ST6 (alpha-N-acetyl-neuraminyl-2,3-beta-galactosyl-1,3)-N-acetylgalactosaminide alpha-2,6-sialyltransferase 3	1.081	0.5727	0.8501
20336	Exoc4	exocyst complex component 4	1.081	0.5008	0.8201
19646	Rbbp4	retinoblastoma binding protein 4	1.081	0.3818	0.7596
19344	Rab5b	RAB5B, member RAS oncogene family	1.081	0.3249	NA
16885	Limk1	LIM-domain containing, protein kinase	1.081	0.2417	NA
16525	Kcnk1	potassium channel, subfamily K, member 1	1.081	0.3483	0.7385
15902	Id2	inhibitor of DNA binding 2	1.081	0.1236	NA
14433	Gapdh	glyceraldehyde-3-phosphate dehydrogenase	1.081	0.3791	0.7583
13494	Drg1	developmentally regulated GTP binding protein 1	1.081	0.192	NA
12577	Cdkn1c	cyclin-dependent kinase inhibitor 1C (P57)	1.081	0.3659	0.7499
12419	Cbx5	chromobox homolog 5 (Drosophila HP1a)	1.081	0.1993	NA
12404	Cbln1	cerebellin 1 precursor protein	1.081	0.1538	NA
11848	Rhoa	ras homolog gene family, member A	1.081	0.2749	NA
11306	Abcb7	ATP-binding cassette, sub-family B (MDR/TAP), member 7	1.081	0.5465	0.8396
674321	LOC674321	glycine cleavage system H protein, mitochondrial-like	1.08	0.1294	NA
625249	Gpx4	glutathione peroxidase 4	1.08	0.464	0.8035
544707	Gm5779	predicted gene 5779	1.08	0.2334	NA
433941	Gm5561	ribosomal protein L29 pseudogene	1.08	0.2379	NA
330401	Tmcc1	transmembrane and coiled coil domains 1	1.08	0.1031	NA
320039	A030010E16Rik	RIKEN cDNA A030010E16 gene	1.08	0.385	0.7607
242662	Rims3	regulating synaptic membrane exocytosis 3	1.08	0.5778	0.8514
240334	Pcyox1l	prenylcysteine oxidase 1 like	1.08	0.5942	0.8596
240058	Cpne5	copine V	1.08	0.289	NA
235028	Zfp426	zinc finger protein 426	1.08	0.3841	0.7603
230584	Yipf1	Yip1 domain family, member 1	1.08	0.2287	NA
230582	Cyb5rl	cytochrome b5 reductase-like	1.08	0.5045	0.8221
211488	Ado	2-aminoethanethiol (cysteamine) dioxygenase	1.08	0.14	NA
192167	Nlgn1	neuroligin 1	1.08	0.2708	NA
110351	Rap1gap	Rap1 GTPase-activating protein	1.08	0.2842	NA
107586	Ovol2	ovo-like 2 (Drosophila)	1.08	0.5375	0.8358
107448	Unc5a	unc-5 homolog A (C. elegans)	1.08	0.2555	NA
106529	Tecr	trans-2,3-enoyl-CoA reductase	1.08	0.4852	0.8127
101923	BB212172	expressed sequence BB212172	1.08	0.2495	NA
75604	Tm4sf5	transmembrane 4 superfamily member 5	1.08	0.5103	0.8253
75544	1700016K05Rik	RIKEN cDNA 1700016K05 gene	1.08	0.4528	0.7961
75033	Mei4	meiosis-specific, MEI4 homolog (S. cerevisiae)	1.08	0.7665	0.9251
74696	4930519A11Rik	RIKEN cDNA 4930519A11 gene	1.08	0.7305	0.915
74147	Ehhadh	enoyl-Coenzyme A, hydratase/3-hydroxyacyl Coenzyme A dehydrogenase	1.08	0.3039	NA
74122	Tmem43	transmembrane protein 43	1.08	0.1665	NA
73699	Ppp2r1b	protein phosphatase 2 (formerly 2A), regulatory subunit A (PR 65), beta isoform	1.08	0.6967	0.9018
73296	Rhobtb3	Rho-related BTB domain containing 3	1.08	0.2368	NA

73205	3110043O21Rik	RIKEN cDNA 3110043O21 gene	1.08	0.2555	NA
73062	Ppp1r16a	protein phosphatase 1, regulatory (inhibitor) subunit 16A	1.08	0.3305	NA
70611	Fbxo33	F-box protein 33	1.08	0.4905	0.8151
69714	Tfpt	TCF3 (E2A) fusion partner	1.08	0.6468	0.8812
69104	Mar-05	membrane-associated ring finger (C3HC4) 5	1.08	0.5381	0.8362
69082	Zc3h15	zinc finger CCCH-type containing 15	1.08	0.2608	NA
69002	1500026H17Rik	RIKEN cDNA 1500026H17 gene	1.08	0.2025	NA
67602	Necap1	NECAP endocytosis associated 1	1.08	0.2302	NA
67460	Decr1	2,4-dienoyl CoA reductase 1, mitochondrial	1.08	0.2553	NA
67278	2900092E17Rik	RIKEN cDNA 2900092E17 gene	1.08	0.1153	NA
66889	Rnf128	ring finger protein 128	1.08	0.5286	0.8332
66530	Ubxn6	UBX domain protein 6	1.08	0.299	NA
66489	Rpl35	ribosomal protein L35	1.08	0.3505	0.7392
66192	Lage3	L antigen family, member 3	1.08	0.2771	NA
66148	Dnajc15	DnaJ (Hsp40) homolog, subfamily C, member 15	1.08	0.0596	NA
66053	Ppil2	peptidylprolyl isomerase (cyclophilin)-like 2	1.08	0.2757	NA
56438	Rbx1	ring-box 1	1.08	0.1495	NA
56298	Atl2	atlastin GTPase 2	1.08	0.2451	NA
53602	Hpcal1	hippocalcin-like 1	1.08	0.2947	NA
28019	Ing4	inhibitor of growth family, member 4	1.08	0.1296	NA
27377	Yme1l1	YME1-like 1 (S. cerevisiae)	1.08	0.5119	0.8262
26919	Zfp346	zinc finger protein 346	1.08	0.3766	0.7559
26456	Sema4g	sema domain, immunoglobulin domain (Ig), transmembrane domain (TM) and short cytoplasmic domain, (semaphorin) 4G	1.08	0.497	0.8193
22630	Ywhaq	tyrosine 3-monooxygenase/tryptophan 5-monooxygenase activation protein, theta polypeptide	1.08	0.302	NA
22142	Tuba1a	tubulin, alpha 1A	1.08	0.5157	0.8277
20262	Stmn3	stathmin-like 3	1.08	0.145	NA
20226	Sars	seryl-aminoacyl-tRNA synthetase	1.08	0.5624	0.8465
20018	Polr1d	polymerase (RNA) I polypeptide D	1.08	0.1054	NA
19649	Robo3	roundabout homolog 3 (Drosophila)	1.08	0.4892	0.8143
19346	Rab6	RAB6, member RAS oncogene family	1.08	0.2906	NA
19179	Psmc1	protease (prosome, macropain) 26S subunit, ATPase 1	1.08	0.136	NA
18824	Plp2	proteolipid protein 2	1.08	0.4712	0.8076
18563	Pcx	pyruvate carboxylase	1.08	0.426	0.7823
17156	Man1a2	mannosidase, alpha, class 1A, member 2	1.08	0.08595	NA
16494	Kcna6	potassium voltage-gated channel, shaker-related, subfamily, member 6	1.08	0.08986	NA
14615	Gjc1	gap junction protein, gamma 1	1.08	0.2365	NA
13837	Epha3	Eph receptor A3	1.08	0.229	NA
13607	Eda	ectodysplasin-A	1.08	0.5808	0.8527
13202	Ddt	D-dopachrome tautomerase	1.08	0.117	NA
12972	Cryz	crystallin, zeta	1.08	0.2906	NA
12569	Cdk5r1	cyclin-dependent kinase 5, regulatory subunit 1 (p35)	1.08	0.3016	NA
12416	Cbx2	chromobox homolog 2 (Drosophila Pc class)	1.08	0.6071	0.8648
11544	Adprh	ADP-ribosylarginine hydrolase	1.08	0.1302	NA
633387	LOC633387	nucleophosmin-like	1.079	0.2156	NA
320701	Fam19a4	family with sequence similarity 19, member A4	1.079	0.4057	0.7717
319822	Smyd4	SET and MYND domain containing 4	1.079	0.4395	0.7908
229715	Amigo1	adhesion molecule with Ig like domain 1	1.079	0.5117	0.826
224727	Bat3	HLA-B-associated transcript 3	1.079	0.5639	0.8465
224624	Rab40c	Rab40c, member RAS oncogene family	1.079	0.2032	NA

216810	Tom1l2	target of myb1-like 2 (chicken)	1.079	0.1915	NA
209003	Rbmx2	RNA binding motif protein, X-linked 2	1.079	0.3629	0.7483
110954	Rpl10	ribosomal protein 10	1.079	0.249	NA
110784	Nr3c2	nuclear receptor subfamily 3, group C, member 2	1.079	0.2744	NA
105638	Dph3	DPH3 homolog (KTI11, S. cerevisiae)	1.079	0.2324	NA
103220	BC030307	cDNA sequence BC030307	1.079	0.5321	0.8339
94111	Mepe	matrix extracellular phosphoglycoprotein with ASARM motif (bone)	1.079	0.5927	0.8585
85305	Kars	lysyl-tRNA synthetase	1.079	0.4017	0.7703
78108	4930414L22Rik	RIKEN cDNA 4930414L22 gene	1.079	0.4724	0.8084
76917	Flywch2	FLYWCH family member 2	1.079	0.3365	NA
76376	Slc24a2	solute carrier family 24 (sodium/potassium/calcium exchanger), member 2	1.079	0.4582	0.7997
74762	Mdga1	MAM domain containing glycosylphosphatidylinositol anchor 1	1.079	0.6006	0.8636
74430	4930452B06Rik	RIKEN cDNA 4930452B06 gene	1.079	0.267	NA
72981	Prkrir	protein-kinase, interferon-inducible double stranded RNA dependent inhibitor, repressor of (P58 repressor)	1.079	0.2498	NA
72661	Serp2	stress-associated endoplasmic reticulum protein family member 2	1.079	0.05325	NA
71990	Ddx54	DEAD (Asp-Glu-Ala-Asp) box polypeptide 54	1.079	0.4025	0.7708
70598	Filip1	filamin A interacting protein 1	1.079	0.4351	0.7883
70316	Ndufab1	NADH dehydrogenase (ubiquinone) 1, alpha/beta subcomplex, 1	1.079	0.284	NA
69833	Polr2f	polymerase (RNA) II (DNA directed) polypeptide F	1.079	0.08623	NA
69724	Rnaseh2a	ribonuclease H2, large subunit	1.079	0.2053	NA
68736	1110034B05Rik	RIKEN cDNA 1110034B05 gene	1.079	0.5659	0.8478
67895	Ppa1	pyrophosphatase (inorganic) 1	1.079	0.2528	NA
67724	Pop1	processing of precursor 1, ribonuclease P/MRP family, (S. cerevisiae)	1.079	0.2386	NA
67528	Nudt7	nudix (nucleoside diphosphate linked moiety X)-type motif 7	1.079	0.3037	NA
67112	Fgf22	fibroblast growth factor 22	1.079	0.4932	0.816
66929	Asf1b	ASF1 anti-silencing function 1 homolog B (S. cerevisiae)	1.079	0.3571	0.7444
66682	Trappc5	trafficking protein particle complex 5	1.079	0.2187	NA
66642	Ctnnbl1	catenin, beta like 1	1.079	0.09161	NA
66337	1700025K23Rik	RIKEN cDNA 1700025K23 gene	1.079	0.07576	NA
66146	Tmem57	transmembrane protein 57	1.079	0.1022	NA
60532	Wtap	Wilms' tumour 1-associating protein	1.079	0.1427	NA
59012	Moxd1	monooxygenase, DBH-like 1	1.079	0.4286	0.7838
58875	Hibadh	3-hydroxyisobutyrate dehydrogenase	1.079	0.4022	0.7706
57294	Rps27	ribosomal protein S27	1.079	0.2183	NA
56697	Akap10	A kinase (PRKA) anchor protein 10	1.079	0.5045	0.8221
56535	Pex3	peroxisomal biogenesis factor 3	1.079	0.2025	NA
56378	Arpc3	actin related protein 2/3 complex, subunit 3	1.079	0.1358	NA
56205	Ensa	endosulfine alpha	1.079	0.2709	NA
54720	Rcan1	regulator of calcineurin 1	1.079	0.1288	NA
29867	Cabp1	calcium binding protein 1	1.079	0.5095	0.8249
29819	Stau2	staufen (RNA binding protein) homolog 2 (Drosophila)	1.079	0.2555	NA
26934	Racgap1	Rac GTPase-activating protein 1	1.079	0.1868	NA
26385	Grk6	G protein-coupled receptor kinase 6	1.079	0.4592	0.8007
22035	Tnfsf10	tumor necrosis factor (ligand) superfamily, member 10	1.079	0.7689	0.9255
21916	Tmod1	tropomodulin 1	1.079	0.2618	NA
21763	Tex2	testis expressed gene 2	1.079	0.518	0.828
20091	Rps3a	ribosomal protein S3A	1.079	0.05664	NA
18972	Pold2	polymerase (DNA directed), delta 2, regulatory subunit	1.079	0.517	0.8277
16865	Eif2d	eukaryotic translation initiation factor 2D	1.079	0.3408	NA

16493	Kcna5	potassium voltage-gated channel, shaker-related subfamily, member 5	1.079	0.1198	NA
16431	Itm2a	integral membrane protein 2A	1.079	0.1255	NA
16008	Igfbp2	insulin-like growth factor binding protein 2	1.079	0.3996	0.7699
15168	Hcn3	hyperpolarization-activated, cyclic nucleotide-gated K+ 3	1.079	0.5024	0.8214
11848	Rhoa	ras homolog gene family, member A	1.079	0.2187	NA
11641	Akap2	A kinase (PRKA) anchor protein 2	1.079	0.4914	0.8156
11632	Aip	aryl-hydrocarbon receptor-interacting protein	1.079	0.506	0.823
11539	Adora1	adenosine A1 receptor	1.079	0.1914	NA
11532	Adh5	alcohol dehydrogenase 5 (class III), chi polypeptide	1.079	0.2212	NA
654472	Gm12070	glyceraldehyde-3-phosphate dehydrogenase pseudogene	1.078	0.5151	0.8276
545007	ENSMUSG00000068790	predicted gene, ENSMUSG00000068790	1.078	0.3511	NA
434402	Gm5617	predicted gene 5617	1.078	0.5164	0.8277
432995	Gm5480	predicted gene 5480	1.078	0.6219	0.8719
414872	Zyg11b	zyg-ll homolog B (C. elegans)	1.078	0.477	0.8099
382062	AB124611	cDNA sequence AB124611	1.078	0.5301	0.8333
353237	Pcdhac2	protocadherin alpha subfamily C, 2	1.078	0.3172	NA
330171	Kctd10	potassium channel tetramerisation domain containing 10	1.078	0.5665	0.8481
320159	Fam179a	family with sequence similarity 179, member A	1.078	0.5779	0.8514
319535	Zfp182	zinc finger protein 182	1.078	0.2307	NA
269336	Ccdc32	coiled-coil domain containing 32	1.078	0.3146	NA
268930	Pkmyt1	protein kinase, membrane associated tyrosine/threonine 1	1.078	0.1811	NA
245828	Trappc1	trafficking protein particle complex 1	1.078	0.1574	NA
234683	Elmo3	engulfment and cell motility 3, ced-12 homolog (C. elegans)	1.078	0.4639	0.8034
232944	Mark4	MAP/microtubule affinity-regulating kinase 4	1.078	0.516	0.8277
231503	Tmem150c	transmembrane protein 150C	1.078	0.4102	0.7739
231290	Slc10a4	solute carrier family 10 (sodium/bile acid cotransporter family), member 4	1.078	0.3113	NA
229488	Fam160a1	family with sequence similarity 160, member A1	1.078	0.4374	0.7892
223267	A2ld1	AIG2-like domain 1	1.078	0.318	NA
209018	Vps8	vacuolar protein sorting 8 homolog (S. cerevisiae)	1.078	0.2711	NA
116871	Mta3	metastasis associated 3	1.078	0.373	0.7538
110954	Rpl10	ribosomal protein 10	1.078	0.3432	NA
108655	Foxp1	forkhead box P1	1.078	0.5249	0.8312
107272	Psat1	phosphoserine aminotransferase 1	1.078	0.2454	NA
105732	Fam83h	family with sequence similarity 83, member H	1.078	0.4178	0.7784
105663	Thtpa	thiamine triphosphatase	1.078	0.3673	0.7509
83885	Slc25a2	solute carrier family 25 (mitochondrial carrier, ornithine transporter) member 2	1.078	0.6575	0.8863
77044	Arid2	AT rich interactive domain 2 (ARID, RFX-like)	1.078	0.2578	NA
75617	Rps25	ribosomal protein S25	1.078	0.1003	NA
74895	4930455F23Rik	RIKEN cDNA 4930455F23 gene	1.078	0.1079	NA
73181	Nfatc4	nuclear factor of activated T-cells, cytoplasmic, calcineurin-dependent 4	1.078	0.5213	0.8286
71890	Mad2l2	MAD2 mitotic arrest deficient-like 2 (yeast)	1.078	0.3637	0.7483
70387	Ttc9c	tetratricopeptide repeat domain 9C	1.078	0.2813	NA
70357	Kcnip1	Kv channel-interacting protein 1	1.078	0.4881	0.8138
69772	Bdh2	3-hydroxybutyrate dehydrogenase, type 2	1.078	0.3828	0.7599
69535	2310004N24Rik	RIKEN cDNA 2310004N24 gene	1.078	0.1598	NA
68799	Rgmb	RGM domain family, member B	1.078	0.6653	0.8904
68598	Dnajc8	DnaJ (Hsp40) homolog, subfamily C, member 8	1.078	0.2871	NA
68212	Tmbim4	transmembrane BAX inhibitor motif containing 4	1.078	0.1309	NA
67871	Mrrf	mitochondrial ribosome recycling factor	1.078	0.3783	0.7573

67586	Ubxn11	UBX domain protein 11	1.078	0.5958	0.8606
67399	Pdlim7	PDZ and LIM domain 7	1.078	0.5075	0.8235
67071	Rps6ka6	ribosomal protein S6 kinase polypeptide 6	1.078	0.1173	NA
66427	Cyb5b	cytochrome b5 type B	1.078	0.2419	NA
58239	Dexi	dexamethasone-induced transcript	1.078	0.144	NA
57765	Tbx21	T-box 21	1.078	0.5071	0.8234
56695	Pnkd	paroxysmal nonkinesiogenic dyskinesia	1.078	0.5452	0.839
56491	Vapb	vesicle-associated membrane protein, associated protein B and C	1.078	0.09284	NA
56321	Aatf	apoptosis antagonizing transcription factor	1.078	0.3433	NA
54636	Wdr45	WD repeat domain 45	1.078	0.1942	NA
52551	Sgta	small glutamine-rich tetratricopeptide repeat (TPR)-containing, alpha	1.078	0.2022	NA
52014	Nus1	nuclear undecaprenyl pyrophosphate synthase 1 homolog (S. cerevisiae)	1.078	0.4995	0.8197
50525	Spag6	sperm associated antigen 6	1.078	0.5981	0.8613
27388	Ptdss2	phosphatidylserine synthase 2	1.078	0.4575	0.7991
26388	Ifi202b	interferon activated gene 202B	1.078	0.7248	0.9126
24061	Smc1a	structural maintenance of chromosomes 1A	1.078	0.3184	NA
23877	Fiz1	Flt3 interacting zinc finger protein 1	1.078	0.5966	0.8611
20935	Surf6	surfeit gene 6	1.078	0.1595	NA
20536	Slc4a3	solute carrier family 4 (anion exchanger), member 3	1.078	0.5031	0.8215
20410	Sorbs3	sorbin and SH3 domain containing 3	1.078	0.6051	0.8645
19703	Renbp	renin binding protein	1.078	0.4633	0.8032
19243	Ptp4a1	protein tyrosine phosphatase 4a1	1.078	0.1374	NA
18166	Npy1r	neuropeptide Y receptor Y1	1.078	0.2503	NA
18019	Nfatc2	nuclear factor of activated T-cells, cytoplasmic, calcineurin-dependent 2	1.078	0.448	0.7938
15931	Ids	iduronate 2-sulfatase	1.078	0.1943	NA
15560	Htr2c	5-hydroxytryptamine (serotonin) receptor 2C	1.078	0.7608	0.9236
14450	Gart	phosphoribosylglycinamide formyltransferase	1.078	0.2164	NA
13844	Ephb2	Eph receptor B2	1.078	0.565	0.8472
13603	Opn3	opsin 3	1.078	0.4824	0.8112
13393	Dlx3	distal-less homeobox 3	1.078	0.4989	0.8197
13131	Dab1	disabled homolog 1 (Drosophila)	1.078	0.32	NA
13097	Cyp2c38	cytochrome P450, family 2, subfamily c, polypeptide 38	1.078	0.6525	0.8842
12751	Tpp1	tripeptidyl peptidase I	1.078	0.09608	NA
11864	Arnt2	aryl hydrocarbon receptor nuclear translocator 2	1.078	0.4777	0.81
100503659	Dos	downstream of Stk11	1.077	0.4725	0.8084
100503215	LOC100503215	hypothetical protein LOC100503215	1.077	0.6682	0.8904
635504	Gm7160	predicted gene 7160	1.077	0.3442	NA
545487	Gm14439	predicted gene 14439	1.077	0.1162	NA
434246	Trim72	tripartite motif-containing 72	1.077	0.7246	0.9126
408070	B930036G03Rik	RIKEN cDNA B930036G03 gene	1.077	0.1419	NA
407821	Znrf3	zinc and ring finger 3	1.077	0.6275	0.8737
380629	Heca	headcase homolog (Drosophila)	1.077	0.2709	NA
328440	Npm2	nucleophosmin/nucleoplasmin 2	1.077	0.5368	0.8354
328133	Slc39a9	solute carrier family 39 (zinc transporter), member 9	1.077	0.4797	0.8101
242681	Rab42-ps	RAB42, member RAS oncogene family, pseudogene	1.077	0.4136	0.7754
237211	Fancb	Fanconi anemia, complementation group B	1.077	0.1795	NA
234854	Cdk10	cyclin-dependent kinase 10	1.077	0.295	NA
230279	6330416G13Rik	RIKEN cDNA 6330416G13 gene	1.077	0.5966	0.8611
230163	Aldob	aldolase B, fructose-bisphosphate	1.077	0.2865	NA

230157	Tmeff1	transmembrane protein with EGF-like and two follistatin-like domains 1	1.077	0.1621	NA
225888	Suv420h1	suppressor of variegation 4-20 homolog 1 (Drosophila)	1.077	0.231	NA
225870	Rin1	Ras and Rab interactor 1	1.077	0.7113	0.907
224247	E330017A01Rik	RIKEN cDNA E330017A01 gene	1.077	0.6592	0.8873
216818	Gm4802	predicted gene 4802	1.077	0.2148	NA
216441	Slc26a10	solute carrier family 26, member 10	1.077	0.6459	0.8809
213573	Efcab4a	EF-hand calcium binding domain 4A	1.077	0.5295	0.8332
107607	Nod1	nucleotide-binding oligomerization domain containing 1	1.077	0.5556	0.8448
102632	Acad11	acyl-Coenzyme A dehydrogenase family, member 11	1.077	0.1775	NA
100910	Chpf2	chondroitin polymerizing factor 2	1.077	0.4177	0.7783
98766	Ubac1	ubiquitin associated domain containing 1	1.077	0.184	NA
93874	Pcdhb3	protocadherin beta 3	1.077	0.2821	NA
93843	Pnck	pregnancy upregulated non-ubiquitously expressed CaM kinase	1.077	0.3936	0.7656
80281	Cttnbp2nl	CTTNBP2 N-terminal like	1.077	0.2671	NA
78541	Asb8	ankyrin repeat and SOCS box-containing 8	1.077	0.1843	NA
74934	Armc4	armadillo repeat containing 4	1.077	0.6713	0.8915
74386	Rmi1	RMI1, RecQ mediated genome instability 1, homolog (S. cerevisiae)	1.077	0.1527	NA
74337	Palm3	paralemmin 3	1.077	0.3207	NA
71532	9030418K01Rik	RIKEN cDNA 9030418K01 gene	1.077	0.5159	0.8277
71436	Flrt3	fibronectin leucine rich transmembrane protein 3	1.077	0.4104	0.774
69215	Sat2	spermidine/spermine N1-acetyl transferase 2	1.077	0.1929	NA
69161	Manbal	mannosidase, beta A, lysosomal-like	1.077	0.1428	NA
68832	1110057K04Rik	RIKEN cDNA 1110057K04 gene	1.077	0.2899	NA
67903	Gipc1	GIPC PDZ domain containing family, member 1	1.077	0.5933	0.8588
67468	Mmd	monocyte to macrophage differentiation-associated	1.077	0.09914	NA
67311	Nanp	N-acetylneuraminic acid phosphatase	1.077	0.5685	0.8487
66849	Ppp1r2	protein phosphatase 1, regulatory (inhibitor) subunit 2	1.077	0.1836	NA
66821	Bcs1l	BCS1-like (yeast)	1.077	0.3626	NA
66714	4921524J17Rik	RIKEN cDNA 4921524J17 gene	1.077	0.3654	NA
66179	1110031I02Rik	RIKEN cDNA 1110031I02 gene	1.077	0.1122	NA
64340	Dhx38	DEAH (Asp-Glu-Ala-His) box polypeptide 38	1.077	0.2772	NA
57295	Icmt	isoprenylcysteine carboxyl methyltransferase	1.077	0.4503	0.7945
56622	Adam21	a disintegrin and metallopeptidase domain 21	1.077	0.1877	NA
54607	Socs6	suppressor of cytokine signaling 6	1.077	0.2057	NA
54391	Rfk	riboflavin kinase	1.077	0.2281	NA
52915	Zmiz2	zinc finger, MIZ-type containing 2	1.077	0.4344	0.7878
26931	Ppp2r5c	protein phosphatase 2, regulatory subunit B (B56), gamma isoform	1.077	0.3857	0.761
22095	Tshr	thyroid stimulating hormone receptor	1.077	0.5116	0.826
21769	Zfand3	zinc finger, AN1-type domain 3	1.077	0.3865	0.761
20597	Smpd1	sphingomyelin phosphodiesterase 1, acid lysosomal	1.077	0.3754	0.7555
20438	Siah1b	seven in absentia 1B	1.077	0.09098	NA
20255	Scg3	secretogranin III	1.077	0.2925	NA
19185	Psmd4	proteasome (prosome, macropain) 26S subunit, non-ATPase, 4	1.077	0.3075	NA
18549	Pcsk2	proprotein convertase subtilisin/kexin type 2	1.077	0.2568	NA
18018	Nfatc1	nuclear factor of activated T-cells, cytoplasmic, calcineurin-dependent 1	1.077	0.5685	0.8487
17826	Mtvr2	mammary tumor virus receptor 2	1.077	0.4504	0.7945
17203	Mc5r	melanocortin 5 receptor	1.077	0.7501	0.921
16826	Ldb2	LIM domain binding 2	1.077	0.2851	NA
15531	Ndst1	N-deacetylase/N-sulfotransferase (heparan glucosaminyl) 1	1.077	0.2442	NA

13726	Emd	emerin	1.077	0.5093	0.8247
13506	Dsc2	desmocollin 2	1.077	0.654	0.8851
13389	Dll3	delta-like 3 (Drosophila)	1.077	0.272	NA
13039	Ctsl	cathepsin L	1.077	0.5294	0.8332
13030	Ctsb	cathepsin B	1.077	0.2441	NA
12511	Cd6	CD6 antigen	1.077	0.6103	0.8668
12468	Cct7	chaperonin containing Tcp1, subunit 7 (eta)	1.077	0.241	NA
12400	Cbfb	core binding factor beta	1.077	0.2782	NA
11837	Rplp0	ribosomal protein, large, P0	1.077	0.1593	NA
100503670	Rpl5	ribosomal protein L5	1.076	0.4567	0.7987
100043431	Gm4430	predicted gene 4430	1.076	0.4509	0.7946
100039181	LOC100039181	uncharacterized protein C4orf3 homolog	1.076	0.1279	NA
667682	Gm8759	predicted gene 8759	1.076	0.1925	NA
666676	Gm8230	predicted gene 8230	1.076	0.2784	NA
434179	Gm5595	predicted gene 5595	1.076	0.2805	NA
406218	Panx2	pannexin 2	1.076	0.5287	0.8332
381269	Mreg	melanoregulin	1.076	0.2637	NA
328633	4930515I15	hypothetical protein 4930515I15	1.076	0.5736	0.8501
328274	Zfp459	zinc finger protein 459	1.076	0.3462	NA
319757	Smo	smoothened homolog (Drosophila)	1.076	0.43	0.7843
239364	Tspyl5	testis-specific protein, Y-encoded-like 5	1.076	0.2462	NA
233073	U2af1l4	U2 small nuclear RNA auxiliary factor 1-like 4	1.076	0.4466	0.7938
233065	Alkbh6	alkB, alkylation repair homolog 6 (E. coli)	1.076	0.378	0.7572
228859	Fitm2	fat storage-inducing transmembrane protein 2	1.076	0.2829	NA
227622	BC029214	cDNA sequence BC029214	1.076	0.5161	0.8277
226841	Vash2	vasohibin 2	1.076	0.1122	NA
223255	Stk24	serine/threonine kinase 24 (STE20 homolog, yeast)	1.076	0.2737	NA
217887	BC022687	cDNA sequence BC022687	1.076	0.2757	NA
216438	Mar-09	membrane-associated ring finger (C3HC4) 9	1.076	0.4474	0.7938
212390	Klhl32	kelch-like 32 (Drosophila)	1.076	0.4812	0.8106
211922	Fam116a	family with sequence similarity 116, member A	1.076	0.202	NA
170942	Erdr1	erythroid differentiation regulator 1	1.076	0.4593	0.8007
106877	Afap1l1	actin filament associated protein 1-like 1	1.076	0.2301	NA
106869	Tnfaip8	tumor necrosis factor, alpha-induced protein 8	1.076	0.2126	NA
105348	Golm1	golgi membrane protein 1	1.076	0.3101	NA
102442	Dennd4a	DENN/MADD domain containing 4A	1.076	0.4977	0.8193
101197	Zfp956	zinc finger protein 956	1.076	0.2714	NA
100910	Chpf2	chondroitin polymerizing factor 2	1.076	0.4619	0.8022
98314	D2hgdh	D-2-hydroxyglutarate dehydrogenase	1.076	0.3725	NA
83396	Glis2	GLIS family zinc finger 2	1.076	0.3107	NA
71986	Ddx28	DEAD (Asp-Glu-Ala-Asp) box polypeptide 28	1.076	0.4279	0.7834
71891	Cdadc1	cytidine and dCMP deaminase domain containing 1	1.076	0.05938	NA
71701	Pnpt1	polyribonucleotide nucleotidyltransferase 1	1.076	0.2213	NA
71675	0610010F05Rik	RIKEN cDNA 0610010F05 gene	1.076	0.2181	NA
69974	2810405F15Rik	RIKEN cDNA 2810405F15 gene	1.076	0.5405	0.8375
69953	2810025M15Rik	RIKEN cDNA 2810025M15 gene	1.076	0.1093	NA
69318	1700007K09Rik	RIKEN cDNA 1700007K09 gene	1.076	0.4871	0.8133
69046	Isca1	iron-sulfur cluster assembly 1 homolog (S. cerevisiae)	1.076	0.09112	NA
68724	Arl8a	ADP-ribosylation factor-like 8A	1.076	0.2911	NA

67338	Rffl	ring finger and FYVE like domain containing protein	1.076	0.3473	NA
67267	2900010M23Rik	RIKEN cDNA 2900010M23 gene	1.076	0.3172	NA
66394	Nosip	nitric oxide synthase interacting protein	1.076	0.3681	NA
66268	Pigyl	phosphatidylinositol glycan anchor biosynthesis, class Y-like	1.076	0.1442	NA
57784	Bin3	bridging integrator 3	1.076	0.327	NA
56868	Psg23	pregnancy-specific glycoprotein 23	1.076	0.3641	NA
56527	Mast1	microtubule associated serine/threonine kinase 1	1.076	0.3836	0.7599
27096	Trappc3	trafficking protein particle complex 3	1.076	0.132	NA
23836	Cdh20	cadherin 20	1.076	0.3183	NA
22724	Zbtb7b	zinc finger and BTB domain containing 7B	1.076	0.237	NA
22342	Lin7b	lin-7 homolog B (C. elegans)	1.076	0.4328	0.7866
20916	Sucla2	succinate-Coenzyme A ligase, ADP-forming, beta subunit	1.076	0.1826	NA
20851	Stat5b	signal transducer and activator of transcription 5B	1.076	0.4608	0.8018
20528	Slc2a4	solute carrier family 2 (facilitated glucose transporter), member 4	1.076	0.4157	0.7768
19293	Pvalb	parvalbumin	1.076	0.3211	NA
17855	Mvk	mevalonate kinase	1.076	0.228	NA
17692	Msl3	male-specific lethal 3 homolog (Drosophila)	1.076	0.203	NA
16351	Ipp	IAP promoted placental gene	1.076	0.2156	NA
15114	Hap1	huntingtin-associated protein 1	1.076	0.1971	NA
14885	Gtf2h4	general transcription factor II H, polypeptide 4	1.076	0.2942	NA
14738	Gpr12	G-protein coupled receptor 12	1.076	0.3106	NA
14055	Ezh1	enhancer of zeste homolog 1 (Drosophila)	1.076	0.2124	NA
13992	Khdrbs3	KH domain containing, RNA binding, signal transduction associated 3	1.076	0.2343	NA
13082	Cyp26a1	cytochrome P450, family 26, subfamily a, polypeptide 1	1.076	0.446	0.7938
12442	Ccnb2	cyclin B2	1.076	0.2601	NA
12385	Ctnna1	catenin (cadherin associated protein), alpha 1	1.076	0.1713	NA
100043450	AA387883	expressed sequence AA387883	1.075	0.388	0.7614
433771	2310028O11Rik	RIKEN cDNA 2310028O11 gene	1.075	0.1424	NA
382253	Cdkl5	cyclin-dependent kinase-like 5	1.075	0.5286	0.8332
380785	Begain	brain-enriched guanylate kinase-associated	1.075	0.5458	0.8391
353211	Prune2	prune homolog 2 (Drosophila)	1.075	0.4668	0.805
244216	Zfp771	zinc finger protein 771	1.075	0.3718	NA
237759	Col23a1	collagen, type XXIII, alpha 1	1.075	0.4047	0.7716
234797	6430548M08Rik	RIKEN cDNA 6430548M08 gene	1.075	0.4486	0.7942
232089	Elmod3	ELMO/CED-12 domain containing 3	1.075	0.3127	NA
231507	Plac8	placenta-specific 8	1.075	0.6998	0.9033
229706	Slc6a17	solute carrier family 6 (neurotransmitter transporter), member 17	1.075	0.5321	0.8339
224908	Prr22	proline rich 22	1.075	0.559	0.8459
218442	Serinc5	serine incorporator 5	1.075	0.1227	NA
215494	C85492	expressed sequence C85492	1.075	0.2214	NA
207742	Rnf43	ring finger protein 43	1.075	0.5073	0.8234
110750	Cse1l	chromosome segregation 1-like (S. cerevisiae)	1.075	0.4428	0.7928
108664	Atp6v1h	ATPase, H+ transporting, lysosomal V1 subunit H	1.075	0.3196	NA
108100	Baiap2	brain-specific angiogenesis inhibitor 1-associated protein 2	1.075	0.4124	0.7747
98710	Rabif	RAB interacting factor	1.075	0.286	NA
94064	Mrpl27	mitochondrial ribosomal protein L27	1.075	0.1769	NA
93790	Nipa2	non imprinted in Prader-Willi/Angelman syndrome 2 homolog (human)	1.075	0.138	NA
76467	Msrb2	methionine sulfoxide reductase B2	1.075	0.1353	NA
74919	4930471M23Rik	RIKEN cDNA 4930471M23 gene	1.075	0.3651	NA

74105	Gga2	golgi associated, gamma adaptin ear containing, ARF binding protein 2	1.075	0.3229	NA
73363	1700056E22Rik	RIKEN cDNA 1700056E22 gene	1.075	0.1915	NA
72502	Cwf19l1	CWF19-like 1, cell cycle control (S. pombe)	1.075	0.102	NA
72310	Nkg7	natural killer cell group 7 sequence	1.075	0.5764	0.8508
71769	Bbs10	Bardet-Biedl syndrome 10 (human)	1.075	0.3306	NA
71765	Klhdc3	kelch domain containing 3	1.075	0.4903	0.815
70082	Lysmd2	LysM, putative peptidoglycan-binding, domain containing 2	1.075	0.1921	NA
68606	Ppm1f	protein phosphatase 1F (PP2C domain containing)	1.075	0.2705	NA
68559	Pdrg1	p53 and DNA damage regulated 1	1.075	0.201	NA
68137	Kdelr1	KDEL (Lys-Asp-Glu-Leu) endoplasmic reticulum protein retention receptor 1	1.075	0.1736	NA
67792	Rgs8	regulator of G-protein signaling 8	1.075	0.4592	0.8007
67733	Itgb3bp	integrin beta 3 binding protein (beta3-endonexin)	1.075	0.3815	0.7596
67473	Slc47a1	solute carrier family 47, member 1	1.075	0.5452	0.839
67181	Ctdnep1	CTD nuclear envelope phosphatase 1	1.075	0.5687	0.8488
66114	Dnajc30	DnaJ (Hsp40) homolog, subfamily C, member 30	1.075	0.2129	NA
65019	Rpl23	ribosomal protein L23	1.075	0.1143	NA
60363	Cldn15	claudin 15	1.075	0.261	NA
58809	Rnase4	ribonuclease, RNase A family 4	1.075	0.4356	0.7884
56724	Cript	cysteine-rich PDZ-binding protein	1.075	0.3837	0.7599
56698	Phax	phosphorylated adaptor for RNA export	1.075	0.4634	0.8032
56524	Mpp6	membrane protein, palmitoylated 6 (MAGUK p55 subfamily member 6)	1.075	0.364	NA
54393	Gabbr1	gamma-aminobutyric acid (GABA) B receptor, 1	1.075	0.2214	NA
53619	Blcap	bladder cancer associated protein homolog (human)	1.075	0.6266	0.873
28114	Nsun2	NOL1/NOP2/Sun domain family member 2	1.075	0.08168	NA
26459	Slc27a5	solute carrier family 27 (fatty acid transporter), member 5	1.075	0.4667	0.8049
22147	Tuba3b	tubulin, alpha 3B	1.075	0.4352	0.7883
21885	Tle1	transducin-like enhancer of split 1, homolog of Drosophila E(spl)	1.075	0.3114	NA
20334	Sec23a	SEC23A (S. cerevisiae)	1.075	0.2686	NA
20266	Scn1b	sodium channel, voltage-gated, type I, beta	1.075	0.5446	0.839
20193	S100a1	S100 calcium binding protein A1	1.075	0.3922	0.7647
19419	Rasgrp1	RAS guanyl releasing protein 1	1.075	0.155	NA
19193	Pipox	pipecolic acid oxidase	1.075	0.4719	0.8079
19172	Psmb4	proteasome (prosome, macropain) subunit, beta type 4	1.075	0.2695	NA
19043	Ppm1b	protein phosphatase 1B, magnesium dependent, beta isoform	1.075	0.2117	NA
18412	Sqstm1	sequestosome 1	1.075	0.6011	0.8636
16528	Kcnk4	potassium channel, subfamily K, member 4	1.075	0.5586	0.8459
16499	Kcnab3	potassium voltage-gated channel, shaker-related subfamily, beta member 3	1.075	0.07998	NA
15254	Hint1	histidine triad nucleotide binding protein 1	1.075	0.4191	0.7791
14706	Gng4	guanine nucleotide binding protein (G protein), gamma 4	1.075	0.6255	0.8728
13728	Mark2	MAP/microtubule affinity-regulating kinase 2	1.075	0.7224	0.9121
13481	Dpm2	dolichol-phosphate (beta-D) mannosyltransferase 2	1.075	0.2068	NA
12292	Cacna1s	calcium channel, voltage-dependent, L type, alpha 1S subunit	1.075	0.308	NA
100047133	LOC100047133	hypothetical LOC100047133	1.074	0.4951	0.8177
402735	B230114P17Rik	RIKEN cDNA B230114P17 gene	1.074	0.5556	0.8448
381582	Gm5151	predicted gene 5151	1.074	0.4854	0.8127
330355	Dnahc6	dynein, axonemal, heavy chain 6	1.074	0.4041	0.7713
320295	C920006O11Rik	RIKEN cDNA C920006O11 gene	1.074	0.6528	0.8843
320265	Fam19a1	family with sequence similarity 19, member A1	1.074	0.4805	0.8101
269999	Orai3	ORAI calcium release-activated calcium modulator 3	1.074	0.6453	0.8806

245537	Nlgn3	neuroligin 3	1.074	0.5718	0.8497
242362	Manea	mannosidase, endo-alpha	1.074	0.5685	0.8487
234366	Gatad2a	GATA zinc finger domain containing 2A	1.074	0.1677	NA
231717	Fam109a	family with sequence similarity 109, member A	1.074	0.1622	NA
230752	Fam176b	family with sequence similarity 176, member B	1.074	0.3646	NA
229707	Fam40a	family with sequence similarity 40, member A	1.074	0.399	0.7697
223754	Tbc1d22a	TBC1 domain family, member 22a	1.074	0.1431	NA
214048	Larp1b	La ribonucleoprotein domain family, member 1B	1.074	0.1875	NA
211945	Plekhh1	pleckstrin homology domain containing, family H (with MyTH4 domain) member 1	1.074	0.3215	NA
210172	Zfp526	zinc finger protein 526	1.074	0.6816	0.8949
171567	Nme7	non-metastatic cells 7, protein expressed in (nucleoside-diphosphate kinase)	1.074	0.3871	NA
117606	Boc	biregional cell adhesion molecule-related/down-regulated by oncogenes (Cdon) binding protein	1.074	0.5236	0.83
104069	Sncb	synuclein, beta	1.074	0.7135	0.9077
81004	Tbl1xr1	transducin (beta)-like 1X-linked receptor 1	1.074	0.1676	NA
80915	Dusp12	dual specificity phosphatase 12	1.074	0.3812	NA
78754	Galntl2	UDP-N-acetyl-alpha-D-galactosamine:polypeptide N-acetylgalactosaminyltransferase-like 2	1.074	0.294	NA
78330	Ndufv3	NADH dehydrogenase (ubiquinone) flavoprotein 3	1.074	0.4124	0.7747
78317	Ccdc88b	coiled-coil domain containing 88B	1.074	0.2221	NA
77032	2610029I01Rik	RIKEN cDNA 2610029I01 gene	1.074	0.1575	NA
76960	Bcas1	breast carcinoma amplified sequence 1	1.074	0.3209	NA
76789	Mzt1	mitotic spindle organizing protein 1	1.074	0.4697	0.8064
76551	Ccdc6	coiled-coil domain containing 6	1.074	0.5132	0.8268
75873	4930568B11Rik	RIKEN cDNA 4930568B11 gene	1.074	0.5323	0.8339
74542	9030625N01Rik	RIKEN cDNA 9030625N01 gene	1.074	0.5677	0.8485
72388	Ripk4	receptor-interacting serine-threonine kinase 4	1.074	0.3513	NA
71354	Wdr31	WD repeat domain 31	1.074	0.4538	0.797
68816	Ppil1	peptidylprolyl isomerase (cyclophilin)-like 1	1.074	0.1077	NA
67808	Tprgl	transformation related protein 63 regulated like	1.074	0.1212	NA
67739	Slc48a1	solute carrier family 48 (heme transporter), member 1	1.074	0.2892	NA
67255	Zfp422	zinc finger protein 422	1.074	0.3071	NA
67044	Higd2a	HIG1 domain family, member 2A	1.074	0.3371	NA
67028	2610002M06Rik	RIKEN cDNA 2610002M06 gene	1.074	0.4098	0.7739
66938	1700029G01Rik	RIKEN cDNA 1700029G01 gene	1.074	0.258	NA
60530	Fignl1	fidgetin-like 1	1.074	0.4277	0.7832
56292	Naa10	N(alpha)-acetyltransferase 10, NatA catalytic subunitNalpha acetyltransferase 10	1.074	0.5427	0.8386
56275	Rbm14	RNA binding motif protein 14	1.074	0.5554	0.8447
54396	Irgm2	immunity-related GTPase family M member 2	1.074	0.5002	0.8197
54138	Atxn10	ataxin 10	1.074	0.3775	NA
30805	Slc22a4	solute carrier family 22 (organic cation transporter), member 4	1.074	0.3914	0.7643
26914	H2afy	H2A histone family, member Y	1.074	0.5215	0.8289
26451	Rpl27a	ribosomal protein L27A	1.074	0.2777	NA
21817	Tgm2	transglutaminase 2, C polypeptide	1.074	0.604	0.8644
20423	Shh	sonic hedgehog	1.074	0.4592	0.8007
19720	Trim27	tripartite motif-containing 27	1.074	0.157	NA
18976	Pomc	pro-opiomelanocortin-alpha	1.074	0.4678	0.8056
18292	Sebox	SEBOX homeobox	1.074	0.1843	NA
16579	Kifap3	kinesin-associated protein 3	1.074	0.1009	NA
14613	Gja5	gap junction protein, alpha 5	1.074	0.6223	0.872
14066	F3	coagulation factor III	1.074	0.6039	0.8644

13139	Dgka	diacylglycerol kinase, alpha	1.074	0.1977	NA
12865	Cox7a1	cytochrome c oxidase, subunit VIIa 1	1.074	0.2585	NA
12496	Entpd2	ectonucleoside triphosphate diphosphohydrolase 2	1.074	0.4805	0.8101
12457	Ccrn4l	CCR4 carbon catabolite repression 4-like (S. cerevisiae)	1.074	0.2347	NA
12169	Bmx	BMX non-receptor tyrosine kinase	1.074	0.4051	0.7716
11773	Ap2m1	adaptor protein complex AP-2, mu1	1.074	0.3624	NA
100216474	Ttll2	tubulin tyrosine ligase-like family, member 2	1.073	0.6898	0.8984
402728	Pax6os1	Pax6 opposite strand transcript 1	1.073	0.1321	NA
277010	Marveld1	MARVEL (membrane-associating) domain containing 1	1.073	0.4489	0.7943
271424	Ip6k3	inositol hexaphosphate kinase 3	1.073	0.4943	0.8169
269629	Lhfpl3	lipoma HMGIC fusion partner-like 3	1.073	0.2496	NA
263406	Plekhg3	pleckstrin homology domain containing, family G (with RhoGef domain) member 3	1.073	0.5316	0.8339
242960	Fbxl5	F-box and leucine-rich repeat protein 5	1.073	0.4667	0.8049
237558	Gm239	predicted gene 239	1.073	0.4871	0.8133
235559	Topbp1	topoisomerase (DNA) II binding protein 1	1.073	0.1439	NA
232566	Amn1	antagonist of mitotic exit network 1 homolog (S. cerevisiae)	1.073	0.2235	NA
232286	Tmf1	TATA element modulatory factor 1	1.073	0.3923	NA
230775	Bai2	brain-specific angiogenesis inhibitor 2	1.073	0.1713	NA
228769	Psmf1	proteasome (prosome, macropain) inhibitor subunit 1	1.073	0.4167	0.7772
223701	Mkl1	MKL (megakaryoblastic leukemia)/myocardin-like 1	1.073	0.452	0.7951
217737	Ahsa1	AHA1, activator of heat shock protein ATPase homolog 1 (yeast)	1.073	0.218	NA
216049	Zfp365	zinc finger protein 365	1.073	0.249	NA
192161	Pcdha9	protocadherin alpha 9	1.073	0.4914	0.8156
114641	Rpl31	ribosomal protein L31	1.073	0.1086	NA
112406	Egln2	EGL nine homolog 2 (C. elegans)	1.073	0.1201	NA
110175	Ggct	gamma-glutamyl cyclotransferase	1.073	0.0842	NA
109075	Exosc4	exosome component 4	1.073	0.325	NA
103724	Tbc1d10a	TBC1 domain family, member 10a	1.073	0.2275	NA
103142	Rdh9	retinol dehydrogenase 9	1.073	0.4616	0.8021
102142	AI662245	expressed sequence AI662245	1.073	0.2189	NA
100317	AU040320	expressed sequence AU040320	1.073	0.3084	NA
80287	Apobec3	apolipoprotein B mRNA editing enzyme, catalytic polypeptide 3	1.073	0.6144	0.8691
78912	Sp2	Sp2 transcription factor	1.073	0.5773	0.8514
73419	1700052N19Rik	RIKEN cDNA 1700052N19 gene	1.073	0.2323	NA
72585	Lypd1	Ly6/Plaur domain containing 1	1.073	0.3184	NA
71981	Tdrd12	tudor domain containing 12	1.073	0.5362	0.8351
71860	Wdr16	WD repeat domain 16	1.073	0.6174	0.87
71853	Pdia6	protein disulfide isomerase associated 6	1.073	0.2939	NA
71146	Golga7b	golgi autoantigen, golgin subfamily a, 7B	1.073	0.5062	0.823
69806	Slc39a11	solute carrier family 39 (metal ion transporter), member 11	1.073	0.1391	NA
69608	Sec24d	Sec24 related gene family, member D (S. cerevisiae)	1.073	0.3353	NA
69549	2310009B15Rik	RIKEN cDNA 2310009B15 gene	1.073	0.09587	NA
69538	Antxr1	anthrax toxin receptor 1	1.073	0.4434	0.7931
68758	Abhd11	abhydrolase domain containing 11	1.073	0.475	0.8095
67943	Mesdc2	mesoderm development candidate 2	1.073	0.162	NA
67388	1110008F13Rik	RIKEN cDNA 1110008F13 gene	1.073	0.1004	NA
67046	Tbc1d7	TBC1 domain family, member 7	1.073	0.4117	0.7747
66916	Ndufb7	NADH dehydrogenase (ubiquinone) 1 beta subcomplex, 7	1.073	0.4133	0.7753
66855	Tcf25	transcription factor 25 (basic helix-loop-helix)	1.073	0.3979	0.7684

66653	Brf2	BRF2, subunit of RNA polymerase III transcription initiation factor, BRF1-like	1.073	0.5125	0.8262
57913	Lrdd	leucine-rich and death domain containing	1.073	0.3706	NA
56398	1500003O03Rik	RIKEN cDNA 1500003O03 gene	1.073	0.2464	NA
54650	Sfmbt1	Scm-like with four mbt domains 1	1.073	0.3624	NA
54161	Copg	coatomer protein complex, subunit gamma	1.073	0.5359	0.835
53357	Pla2g6	phospholipase A2, group VI	1.073	0.581	0.8527
51875	Tmem141	transmembrane protein 141	1.073	0.34	NA
30957	Mapk8ip3	mitogen-activated protein kinase 8 interacting protein 3	1.073	0.6017	0.8636
29859	Sult4a1	sulfotransferase family 4A, member 1	1.073	0.572	0.8499
27660	1700088E04Rik	RIKEN cDNA 1700088E04 gene	1.073	0.3312	NA
26457	Slc27a1	solute carrier family 27 (fatty acid transporter), member 1	1.073	0.6868	0.8966
26451	Rpl27a	ribosomal protein L27A	1.073	0.2567	NA
26412	Map4k2	mitogen-activated protein kinase kinase kinase kinase 2	1.073	0.4655	0.8047
22694	Zfp35	zinc finger protein 35	1.073	0.1694	NA
22370	Vtn	vitronectin	1.073	0.3235	NA
22070	Tpt1	tumor protein, translationally-controlled 1	1.073	0.2099	NA
21341	Taf1c	TATA box binding protein (Tbp)-associated factor, RNA polymerase I, C	1.073	0.3634	NA
20667	Sox12	SRY-box containing gene 12	1.073	0.2279	NA
20482	Skil	SKI-like	1.073	0.6988	0.903
20203	S100b	S100 protein, beta polypeptide, neural	1.073	0.3411	NA
19942	Rpl27	ribosomal protein L27	1.073	0.3689	NA
19299	Abcd3	ATP-binding cassette, sub-family D (ALD), member 3	1.073	0.1297	NA
19207	Ptch2	patched homolog 2	1.073	0.7517	0.9213
18938	Ppp1r14b	protein phosphatase 1, regulatory (inhibitor) subunit 14B	1.073	0.4393	0.7906
18477	Prdx1	peroxiredoxin 1	1.073	0.2076	NA
18293	Ogdh	oxoglutarate dehydrogenase (lipoamide)	1.073	0.5704	0.8492
16006	Igfbp1	insulin-like growth factor binding protein 1	1.073	0.3408	NA
13008	Csrp2	cysteine and glycine-rich protein 2	1.073	0.6507	0.883
12868	Cox8a	cytochrome c oxidase, subunit VIIIa	1.073	0.3731	NA
12408	Cbr1	carbonyl reductase 1	1.073	0.3349	NA
12160	Bmp5	bone morphogenetic protein 5	1.073	0.4776	0.81
12033	Bcap29	B-cell receptor-associated protein 29	1.073	0.3667	NA
11821	Aprt	adenine phosphoribosyl transferase	1.073	0.3616	NA
11785	Apbb1	amyloid beta (A4) precursor protein-binding, family B, member 1	1.073	0.5579	0.8456
11732	Ank	progressive ankylosis	1.073	0.2229	NA
668661	2410002F23Rik	RIKEN cDNA 2410002F23 gene	1.072	0.313	NA
667766	Gm8801	protein phosphatase 1, regulatory subunit 10 pseudogene	1.072	0.4815	0.8108
628705	Gm6907	predicted gene 6907	1.072	0.4997	0.8197
621381	Gm6222	predicted pseudogene 6222	1.072	0.1894	NA
446101	Xrra1	X-ray radiation resistance associated 1	1.072	0.5767	0.8509
383766	Gm1332	predicted gene 1332	1.072	0.5293	0.8332
319564	C230012O17Rik	RIKEN cDNA C230012O17 gene	1.072	0.6967	0.9018
237553	Trhde	TRH-degrading enzyme	1.072	0.2229	NA
234378	Klhl26	kelch-like 26 (Drosophila)	1.072	0.4202	0.7797
230459	Cyp2j13	cytochrome P450, family 2, subfamily j, polypeptide 13	1.072	0.6665	0.8904
223601	Fam49b	family with sequence similarity 49, member B	1.072	0.1428	NA
218793	Ube2e2	ubiquitin-conjugating enzyme E2E 2 (UBC4/5 homolog, yeast)	1.072	0.2516	NA
217038	Mrm1	mitochondrial rRNA methyltransferase 1 homolog (S. cerevisiae)	1.072	0.3904	NA
216363	Rab3ip	RAB3A interacting protein	1.072	0.14	NA

194952	Jmjd4	jumonji domain containing 4	1.072	0.3681	NA
110826	Etfb	electron transferring flavoprotein, beta polypeptide	1.072	0.3818	NA
109019	Obfc2a	oligonucleotide/oligosaccharide-binding fold containing 2A	1.072	0.5116	0.826
108837	Ibtk	inhibitor of Bruton agammaglobulinemia tyrosine kinase	1.072	0.3996	0.7699
103236	Csnk1g2	casein kinase 1, gamma 2	1.072	0.2101	NA
94219	Cnnm2	cyclin M2	1.072	0.2194	NA
84113	Ptov1	prostate tumor over expressed gene 1	1.072	0.3424	NA
75642	1700020C07Rik	RIKEN cDNA 1700020C07 gene	1.072	0.7939	0.9336
74901	Kbtbd11	kelch repeat and BTB (POZ) domain containing 11	1.072	0.4477	0.7938
74257	Tspan17	tetraspanin 17	1.072	0.2551	NA
72171	Shq1	SHQ1 homolog (S. cerevisiae)	1.072	0.2341	NA
72053	Tmub2	transmembrane and ubiquitin-like domain containing 2	1.072	0.2266	NA
71901	2310028H24Rik	RIKEN cDNA 2310028H24 gene	1.072	0.6409	0.8788
71729	Rgs12	regulator of G-protein signaling 12	1.072	0.5526	0.8436
70397	Tmem70	transmembrane protein 70	1.072	0.1219	NA
69743	Casz1	castor homolog 1, zinc finger (Drosophila)	1.072	0.3005	NA
69581	Rhou	ras homolog gene family, member U	1.072	0.3587	NA
67771	Arpc5	actin related protein 2/3 complex, subunit 5	1.072	0.2058	NA
67416	Armcx2	armadillo repeat containing, X-linked 2	1.072	0.3555	NA
67059	Ola1	Obg-like ATPase 1	1.072	0.1595	NA
67054	Paics	phosphoribosylaminoimidazole carboxylase, phosphoribosylaminoribosylaminoimidazole, succinocarboxamide synthetase	1.072	0.2352	NA
66940	Shisa5	shisa homolog 5 (Xenopus laevis)	1.072	0.4039	0.7711
66890	Lman2	lectin, mannose-binding 2	1.072	0.2657	NA
66700	Vps24	vacuolar protein sorting 24 (yeast)	1.072	0.3792	NA
66536	Nipsnap3b	nipsnap homolog 3B (C. elegans)	1.072	0.1751	NA
66480	Rpl15	ribosomal protein L15	1.072	0.2831	NA
66449	Pam16	presequence translocase-asssociated motor 16 homolog (S. cerevisiae)	1.072	0.1964	NA
66177	Ubl5	ubiquitin-like 5	1.072	0.3192	NA
66089	Rmnd5b	required for meiotic nuclear division 5 homolog B (S. cerevisiae)	1.072	0.461	0.8018
56516	Rbms2	RNA binding motif, single stranded interacting protein 2	1.072	0.3572	NA
56418	Ykt6	YKT6 homolog (S. Cerevisiae)	1.072	0.2324	NA
56089	Ramp3	receptor (calcitonin) activity modifying protein 3	1.072	0.2193	NA
55992	Trim3	tripartite motif-containing 3	1.072	0.6145	0.8693
54473	Tollip	toll interacting protein	1.072	0.2769	NA
54201	Zfp316	zinc finger protein 316	1.072	0.43	0.7843
53605	Nap1l1	nucleosome assembly protein 1-like 1	1.072	0.29	NA
53380	Psmd10	proteasome (prosome, macropain) 26S subunit, non-ATPase, 10	1.072	0.3233	NA
52846	D1Bwg0212e	DNA segment, Chr 1, Brigham & Women's Genetics 0212 expressed	1.072	0.275	NA
50873	Park2	Parkinson disease (autosomal recessive, juvenile) 2, parkin	1.072	0.3107	NA
50795	Sh3bgr	SH3-binding domain glutamic acid-rich protein	1.072	0.3797	NA
26754	Cops5	COP9 (constitutive photomorphogenic) homolog, subunit 5 (Arabidopsis thaliana)	1.072	0.1057	NA
26559	Hunk	hormonally upregulated Neu-associated kinase	1.072	0.4057	0.7717
26429	Orc5	origin recognition complex, subunit 5	1.072	0.5816	0.853
24113	Vax2	ventral anterior homeobox containing gene 2	1.072	0.246	NA
22225	Usp5	ubiquitin specific peptidase 5 (isopeptidase T)	1.072	0.3284	NA
22201	Uba1	ubiquitin-like modifier activating enzyme 1	1.072	0.4794	0.8101
22169	Cmpk2	cytidine monophosphate (UMP-CMP) kinase 2, mitochondrial	1.072	0.523	0.8295
20927	Abcc8	ATP-binding cassette, sub-family C (CFTR/MRP), member 8	1.072	0.389	NA
19171	Psmb10	proteasome (prosome, macropain) subunit, beta type 10	1.072	0.1061	NA

18453	P4hb	prolyl 4-hydroxylase, beta polypeptide	1.072	0.2497	NA
17988	Ndrg1	N-myc downstream regulated gene 1	1.072	0.1187	NA
17254	Slc3a2	solute carrier family 3 (activators of dibasic and neutral amino acid transport), member 2	1.072	0.4782	0.81
17158	Man2a1	mannosidase 2, alpha 1	1.072	0.2136	NA
17132	Maf	avian musculoaponeurotic fibrosarcoma (v-maf) AS42 oncogene homolog	1.072	0.6684	0.8904
16173	Il18	interleukin 18	1.072	0.4048	0.7716
15944	Irgm1	immunity-related GTPase family M member 1	1.072	0.2984	NA
14803	Grid1	glutamate receptor, ionotropic, delta 1	1.072	0.7591	0.9229
14724	Gp1bb	glycoprotein Ib, beta polypeptide	1.072	0.4759	0.8097
14605	Tsc22d3	TSC22 domain family, member 3	1.072	0.125	NA
13629	Eef2	eukaryotic translation elongation factor 2	1.072	0.3681	NA
12177	Bnip3l	BCL2/adenovirus E1B interacting protein 3-like	1.072	0.2999	NA
11898	Ass1	argininosuccinate synthetase 1	1.072	0.5606	0.8462
11861	Arl4a	ADP-ribosylation factor-like 4A	1.072	0.2556	NA
11477	Acvr1	activin A receptor, type 1	1.072	0.4198	0.7795
666060	Frmpd1	FERM and PDZ domain containing 1	1.071	0.489	0.8142
665934	Gm7854	predicted gene 7854	1.071	0.3475	NA
620592	Tmem28	transmembrane protein 28	1.071	0.3285	NA
544710	Krtap10-10	keratin associated protein 10-10	1.071	0.7862	0.9311
434204	Whamm	WAS protein homolog associated with actin, golgi membranes and microtubules	1.071	0.7865	0.9313
414093	A830082N09Rik	RIKEN cDNA A830082N09 gene	1.071	0.2357	NA
382562	Pfn4	profilin family, member 4	1.071	0.4431	0.7931
320014	B930025P03Rik	RIKEN cDNA B930025P03 gene	1.071	0.336	NA
319865	E130114P18Rik	RIKEN cDNA E130114P18 gene	1.071	0.2405	NA
319158	Hist1h4i	histone cluster 1, H4i	1.071	0.3724	NA
317717	Sec22a	SEC22 vesicle trafficking protein homologe A (S. cerevisiae)	1.071	0.1512	NA
269799	Clec4a1	C-type lectin domain family 4, member a1	1.071	0.7395	0.9178
246703	Apoa1bp	apolipoprotein A-I binding protein	1.071	0.4076	0.7726
245020	Tmem22	transmembrane protein 22	1.071	0.1714	NA
244654	Mtss1l	metastasis suppressor 1-like	1.071	0.5779	0.8514
241118	Accn4	amiloride-sensitive cation channel 4, pituitary	1.071	0.2815	NA
234404	Nxnl1	nucleoredoxin-like 1	1.071	0.5729	0.8501
230459	Cyp2j13	cytochrome P450, family 2, subfamily j, polypeptide 13	1.071	0.2104	NA
229534	Pbxip1	pre-B-cell leukemia transcription factor interacting protein 1	1.071	0.3543	NA
229487	Pet112l	PET112-like (yeast)	1.071	0.3669	NA
228858	Gdap1l1	ganglioside-induced differentiation-associated protein 1-like 1	1.071	0.1866	NA
228139	P2rx3	purinergic receptor P2X, ligand-gated ion channel, 3	1.071	0.3345	NA
226646	Ndufs2	NADH dehydrogenase (ubiquinone) Fe-S protein 2	1.071	0.3231	NA
226351	Tmem185b	transmembrane protein 185B	1.071	0.2051	NA
226090	Ermp1	endoplasmic reticulum metallopeptidase 1	1.071	0.2902	NA
224671	Btbd9	BTB (POZ) domain containing 9	1.071	0.1115	NA
215615	Rnpep	arginyl aminopeptidase (aminopeptidase B)	1.071	0.5497	0.8417
213056	Fam126b	family with sequence similarity 126, member B	1.071	0.2624	NA
211151	Churc1	churchill domain containing 1	1.071	0.1799	NA
207615	Wdr37	WD repeat domain 37	1.071	0.1999	NA
207182	Ggt7	gamma-glutamyltransferase 7	1.071	0.4973	0.8193
116870	Mta1	metastasis associated 1	1.071	0.5527	0.8436
108671	Dnajc9	DnaJ (Hsp40) homolog, subfamily C, member 9	1.071	0.134	NA
107732	Mrpl10	mitochondrial ribosomal protein L10	1.071	0.4973	0.8193

105193	Nhlrc1	NHL repeat containing 1	1.071	0.4413	0.7921
104884	Tdp1	tyrosyl-DNA phosphodiesterase 1	1.071	0.4199	0.7795
101122	Rpusd3	RNA pseudouridylate synthase domain containing 3	1.071	0.3762	NA
100182	Akna	AT-hook transcription factor	1.071	0.3752	NA
100169	Phactr4	phosphatase and actin regulator 4	1.071	0.2721	NA
76954	St5	suppression of tumorigenicity 5	1.071	0.2973	NA
76808	Rpl18a	ribosomal protein L18A	1.071	0.2567	NA
76407	Sun5	Sad1 and UNC84 domain containing 5	1.071	0.6051	0.8645
76281	Tax1bp3	Tax1 (human T-cell leukemia virus type I) binding protein 3	1.071	0.2666	NA
76178	6330578E17Rik	RIKEN cDNA 6330578E17 gene	1.071	0.3662	NA
73046	Glrx5	glutaredoxin 5 homolog (S. cerevisiae)	1.071	0.09882	NA
71994	Cnn3	calponin 3, acidic	1.071	0.4357	0.7884
69944	2810021J22Rik	RIKEN cDNA 2810021J22 gene	1.071	0.5203	0.8286
69306	Efcab9	EF-hand calcium binding domain 9	1.071	0.7282	0.9136
68910	Zfp467	zinc finger protein 467	1.071	0.4102	0.7739
68737	Angel1	angel homolog 1 (Drosophila)	1.071	0.4351	0.7883
68512	Tomm5	translocase of outer mitochondrial membrane 5 homolog (yeast)	1.071	0.2683	NA
68273	Pomgnt1	protein O-linked mannose beta1,2-N-acetylglucosaminyltransferase	1.071	0.2477	NA
68239	Krt42	keratin 42	1.071	0.7234	0.9124
68126	Fahd2a	fumarylacetoacetate hydrolase domain containing 2A	1.071	0.2147	NA
67861	Akr1b10	aldo-keto reductase family 1, member B10 (aldose reductase)	1.071	0.3984	NA
67276	Eri1	exoribonuclease 1	1.071	0.3236	NA
66877	Crnkl1	Crn, crooked neck-like 1 (Drosophila)	1.071	0.2171	NA
66431	1810049H13Rik	RIKEN cDNA 1810049H13 gene	1.071	0.4711	0.8076
66233	Dmap1	DNA methyltransferase 1-associated protein 1	1.071	0.2287	NA
66184	Rps4y2	ribosomal protein S4, Y-linked 2	1.071	0.2888	NA
66119	Tomm6	translocase of outer mitochondrial membrane 6 homolog (yeast)	1.071	0.2652	NA
57439	Tmem183a	transmembrane protein 183A	1.071	0.1371	NA
54188	Cpsf4	cleavage and polyadenylation specific factor 4	1.071	0.5201	0.8286
53872	Caprin1	cell cycle associated protein 1	1.071	0.2299	NA
53420	Syt5	synaptotagmin V	1.071	0.4238	0.7817
50529	Mrps7	mitchondrial ribosomal protein S7	1.071	0.252	NA
50524	Sall2	sal-like 2 (Drosophila)	1.071	0.2723	NA
27407	Abcf2	ATP-binding cassette, sub-family F (GCN20), member 2	1.071	0.3785	NA
27225	Ddx24	DEAD (Asp-Glu-Ala-Asp) box polypeptide 24	1.071	0.2075	NA
26411	Map4k1	mitogen-activated protein kinase kinase kinase kinase 1	1.071	0.5125	0.8262
23863	Dand5	DAN domain family, member 5	1.071	0.4057	0.7717
22329	Vcam1	vascular cell adhesion molecule 1	1.071	0.3573	NA
20540	Slc7a7	solute carrier family 7 (cationic amino acid transporter, y+ system), member 7	1.071	0.4716	0.8077
19826	Rnps1	ribonucleic acid binding protein S1	1.071	0.4088	0.7734
19418	Rasgrf2	RAS protein-specific guanine nucleotide-releasing factor 2	1.071	0.1315	NA
19056	Ppp3cb	protein phosphatase 3, catalytic subunit, beta isoform	1.071	0.2545	NA
18769	Pkig	protein kinase inhibitor, gamma	1.071	0.3735	NA
17285	Meox1	mesenchyme homeobox 1	1.071	0.6176	0.8701
17025	Alad	aminolevulinate, delta-, dehydratase	1.071	0.5459	0.8392
16177	Il1r1	interleukin 1 receptor, type I	1.071	0.2151	NA
16154	Il10ra	interleukin 10 receptor, alpha	1.071	0.3872	NA
15468	Prmt2	protein arginine N-methyltransferase 2	1.071	0.1187	NA
15239	Hgs	HGF-regulated tyrosine kinase substrate	1.071	0.4155	0.7767

14468	Gbp1	guanylate binding protein 1	1.071	0.5351	0.835
14227	Fkbp2	FK506 binding protein 2	1.071	0.1083	NA
14159	Fes	feline sarcoma oncogene	1.071	0.5454	0.839
14159	Fes	feline sarcoma oncogene	1.071	0.3411	NA
13400	Dmpk	dystrophia myotonica-protein kinase	1.071	0.6163	0.8698
12182	Bst1	bone marrow stromal cell antigen 1	1.071	0.7793	0.9278
11767	Ap1m1	adaptor-related protein complex AP-1, mu subunit 1	1.071	0.4298	0.7843
11514	Adcy8	adenylate cyclase 8	1.071	0.3709	NA
11504	Adamts1	a disintegrin-like and metallopeptidase (reprolysin type) with thrombospondin type 1 motif, 1	1.071	0.4227	0.7804
100040834	Gm2990	predicted gene 2990	1.07	0.187	NA
387524	Znrf2	zinc and ring finger 2	1.07	0.2598	NA
329251	Ppp1r12b	protein phosphatase 1, regulatory (inhibitor) subunit 12B	1.07	0.6168	0.8698
319998	Tmem198	transmembrane protein 198	1.07	0.3536	NA
319804	Glt1d1	glycosyltransferase 1 domain containing 1	1.07	0.5454	0.839
252972	Tpcn1	two pore channel 1	1.07	0.5159	0.8277
246228	Vwa1	von Willebrand factor A domain containing 1	1.07	0.4926	0.8157
245638	Tbc1d8b	TBC1 domain family, member 8B	1.07	0.5682	0.8486
244059	Chd2	chromodomain helicase DNA binding protein 2	1.07	0.703	0.9042
243923	Rgs9bp	regulator of G-protein signalling 9 binding protein	1.07	0.5443	0.839
233890	Zfp768	zinc finger protein 768	1.07	0.2471	NA
231440	Parm1	prostate androgen-regulated mucin-like protein 1	1.07	0.5024	0.8214
229949	Ak5	adenylate kinase 5	1.07	0.5728	0.8501
223918	Spryd3	SPRY domain containing 3	1.07	0.627	0.8733
223732	Ldoc1l	leucine zipper, down-regulated in cancer 1-like	1.07	0.3349	NA
218877	Sema3g	sema domain, immunoglobulin domain (Ig), short basic domain, secreted, (semaphorin) 3G	1.07	0.1414	NA
214812	Zfp609	zinc finger protein 609	1.07	0.5565	0.8448
207683	Igsf11	immunoglobulin superfamily, member 11	1.07	0.3723	NA
170729	Scrt1	scratch homolog 1, zinc finger protein (Drosophila)	1.07	0.4438	0.7931
170638	Hpcal4	hippocalcin-like 4	1.07	0.4378	0.7894
107769	Tm6sf1	transmembrane 6 superfamily member 1	1.07	0.4455	0.7938
107569	Nt5c3	5'-nucleotidase, cytosolic III	1.07	0.1571	NA
107321	Lpxn	leupaxin	1.07	0.2683	NA
100900	Hscb	HscB iron-sulfur cluster co-chaperone homolog (E. coli)	1.07	0.3088	NA
100072	Camta1	calmodulin binding transcription activator 1	1.07	0.2464	NA
93686	Rbfox2	RNA binding protein, fox-1 homolog (C. elegans) 2	1.07	0.2526	NA
78781	Zc3hav1	zinc finger CCCH type, antiviral 1	1.07	0.2728	NA
76927	1700021C14Rik	RIKEN cDNA 1700021C14 gene	1.07	0.4603	0.8012
75530	Lyrm7	LYR motif containing 7	1.07	0.6459	0.8809
75044	4930506A18Rik	RIKEN cDNA 4930506A18 gene	1.07	0.6233	0.8724
74187	Katnb1	katanin p80 (WD40-containing) subunit B 1	1.07	0.2941	NA
74098	0610037L13Rik	RIKEN cDNA 0610037L13 gene	1.07	0.4062	NA
73737	1110008P14Rik	RIKEN cDNA 1110008P14 gene	1.07	0.5582	0.8458
72780	Rspo3	R-spondin 3 homolog (Xenopus laevis)	1.07	0.7911	0.9333
70626	5730522E02Rik	RIKEN cDNA 5730522E02 gene	1.07	0.4263	0.7823
70470	Rprd1b	regulation of nuclear pre-mRNA domain containing 1B	1.07	0.6788	0.894
70233	Cd2bp2	CD2 antigen (cytoplasmic tail) binding protein 2	1.07	0.1633	NA
69171	1810031K17Rik	RIKEN cDNA 1810031K17 gene	1.07	0.2706	NA
68889	Ubac2	ubiquitin associated domain containing 2	1.07	0.3377	NA
68729	Trim37	tripartite motif-containing 37	1.07	0.1626	NA

68119	Cmtm3	CKLF-like MARVEL transmembrane domain containing 3	1.07	0.4737	0.8088
67803	Limd2	LIM domain containing 2	1.07	0.5892	0.8575
67168	Lpar6	lysophosphatidic acid receptor 6	1.07	0.4999	0.8197
66853	Pnpla2	patatin-like phospholipase domain containing 2	1.07	0.3474	NA
66469	2810405K02Rik	RIKEN cDNA 2810405K02 gene	1.07	0.3589	NA
66462	2810428I15Rik	RIKEN cDNA 2810428I15 gene	1.07	0.4884	0.8138
58865	Tdh	L-threonine dehydrogenase	1.07	0.7098	0.9069
56442	Serinc1	serine incorporator 1	1.07	0.1563	NA
56347	Eif3c	eukaryotic translation initiation factor 3, subunit C	1.07	0.4635	0.8032
56323	Dnajb5	DnaJ (Hsp40) homolog, subfamily B, member 5	1.07	0.5453	0.839
55979	Agpat1	1-acylglycerol-3-phosphate O-acyltransferase 1 (lysophosphatidic acid acyltransferase, alpha)	1.07	0.1731	NA
55963	Slc1a4	solute carrier family 1 (glutamate/neutral amino acid transporter), member 4	1.07	0.7207	0.9111
54524	Syt6	synaptotagmin VI	1.07	0.5297	0.8332
51789	Tnk2	tyrosine kinase, non-receptor, 2	1.07	0.2279	NA
50927	Nasp	nuclear autoantigenic sperm protein (histone-binding)	1.07	0.724	0.9126
27979	Eif3b	eukaryotic translation initiation factor 3, subunit B	1.07	0.1249	NA
27973	Vkorc1	vitamin K epoxide reductase complex, subunit 1	1.07	0.2615	NA
27406	Abcf3	ATP-binding cassette, sub-family F (GCN20), member 3	1.07	0.4233	0.7813
24057	Sh3yl1	Sh3 domain YSC-like 1	1.07	0.5577	0.8454
23789	Coro1b	coronin, actin binding protein 1B	1.07	0.4884	0.8138
21886	Tle2	transducin-like enhancer of split 2, homolog of Drosophila E(spl)	1.07	0.3328	NA
21677	Tead2	TEA domain family member 2	1.07	0.4793	0.8101
20682	Sox9	SRY-box containing gene 9	1.07	0.5551	0.8444
20286	Zc3h7b	zinc finger CCCH type containing 7B	1.07	0.2296	NA
19340	Rab3d	RAB3D, member RAS oncogene family	1.07	0.6602	0.888
18693	Pick1	protein interacting with C kinase 1	1.07	0.405	NA
18440	P2rx6	purinergic receptor P2X, ligand-gated ion channel, 6	1.07	0.8389	0.9482
16443	Itsn1	intersectin 1 (SH3 domain protein 1A)	1.07	0.2123	NA
14756	Gpld1	glycosylphosphatidylinositol specific phospholipase D1	1.07	0.601	0.8636
14245	Lpin1	lipin 1	1.07	0.2563	NA
14148	Fdx1	ferredoxin 1	1.07	0.1702	NA
13549	Dyrk1b	dual-specificity tyrosine-(Y)-phosphorylation regulated kinase 1b	1.07	0.7231	0.9121
12404	Cbln1	cerebellin 1 precursor protein	1.07	0.601	0.8636
12336	Capns1	calpain, small subunit 1	1.07	0.5144	0.8272
12313	Calm1	calmodulin 1	1.07	0.4589	0.8006
12032	Bcan	brevican	1.07	0.2436	NA
100503659	Dos	downstream of Stk11	1.069	0.4355	0.7884
100043332	Gm4368	predicted gene 4368	1.069	0.7631	0.9247
664883	Nova1	neuro-oncological ventral antigen 1	1.069	0.4882	0.8138
630499	H2-K2	histocompatibility 2, K region locus 2	1.069	0.5027	0.8214
620592	Tmem28	transmembrane protein 28	1.069	0.4826	0.8112
545474	Scrt2	scratch homolog 2, zinc finger protein (Drosophila)	1.069	0.6536	0.8848
504193	Npcd	neuronal pentraxin chromo domain	1.069	0.5823	0.8534
435965	Lrp3	low density lipoprotein receptor-related protein 3	1.069	0.6835	0.8956
381259	Als2cr4	amyotrophic lateral sclerosis 2 (juvenile) chromosome region, candidate 4	1.069	0.6401	0.8785
319463	C230057M02Rik	RIKEN cDNA C230057M02 gene	1.069	0.5859	0.8555
258611	Olfr297	olfactory receptor 297	1.069	0.3475	NA
240261	Ccdc112	coiled-coil domain containing 112	1.069	0.2532	NA
235044	BC018242	cDNA sequence BC018242	1.069	0.7104	0.9069

229725	Clcc1		chloride channel CLIC-like 1	1.069	0.7413	0.918
219132	D14Ertd668e		DNA segment, Chr 14, ERATO Doi 668, expressed	1.069	0.56	0.8459
215627	Zbtb8b		zinc finger and BTB domain containing 8b	1.069	0.6687	0.8904
214063	Dnajc16		DnaJ (Hsp40) homolog, subfamily C, member 16	1.069	0.373	NA
212139	Cc2d1a		coiled-coil and C2 domain containing 1A	1.069	0.5521	0.8433
211147		Mar-11	membrane-associated ring finger (C3HC4) 11	1.069	0.3284	NA
108098	Med21		mediator complex subunit 21	1.069	0.3236	NA
105243	Slc9a3		solute carrier family 9 (sodium/hydrogen exchanger), member 3	1.069	0.4382	0.7894
75812	Tasp1		taspase, threonine aspartase 1	1.069	0.4392	0.7906
74849	4930412F12Rik		RIKEN cDNA 4930412F12 gene	1.069	0.3177	NA
74585	Sppl3		signal peptide peptidase 3	1.069	0.2733	NA
74552	Nipal3		NIPA-like domain containing 3	1.069	0.21	NA
73634	1700125H20Rik		RIKEN cDNA 1700125H20 gene	1.069	0.6483	0.8816
71544	Arhgap42		Rho GTPase activating protein 42	1.069	0.4088	NA
70573	Tbccd1		TBCC domain containing 1	1.069	0.2984	NA
69066	1810010H24Rik		RIKEN cDNA 1810010H24 gene	1.069	0.3557	NA
68576	Hbxip		hepatitis B virus x interacting protein	1.069	0.189	NA
67881	Mdp1		magnesium-dependent phosphatase 1	1.069	0.3388	NA
67622	Mxra7		matrix-remodelling associated 7	1.069	0.4542	0.7973
67398	Srpr		signal recognition particle receptor ('docking protein')	1.069	0.4369	0.7888
67048	Vma21		VMA21 vacuolar H+-ATPase homolog (S. cerevisiae)	1.069	0.4121	NA
66990	Tmem134		transmembrane protein 134	1.069	0.2317	NA
66707	Nkapl		NFKB activating protein-like	1.069	0.5315	0.8339
66498	Dda1		DET1 and DDB1 associated 1	1.069	0.3768	NA
57276	Vsig2		V-set and immunoglobulin domain containing 2	1.069	0.8888	0.9676
56807	Scamp5		secretory carrier membrane protein 5	1.069	0.4622	0.8024
50916	Irx4		Iroquois related homeobox 4 (Drosophila)	1.069	0.4622	0.8024
50772	Mapk6		mitogen-activated protein kinase 6	1.069	0.5445	0.839
30055	Timm13		translocase of inner mitochondrial membrane 13 homolog (yeast)	1.069	0.2375	NA
27878	Tada1		transcriptional adaptor 1	1.069	0.2062	NA
27207	Rps11		ribosomal protein S11	1.069	0.2126	NA
22775	Zik1		zinc finger protein interacting with K protein 1	1.069	0.1924	NA
22685	Zfp239		zinc finger protein 239	1.069	0.385	NA
21835	Thrsp		thyroid hormone responsive SPOT14 homolog (Rattus)	1.069	0.3339	NA
21356	Tapbp		TAP binding protein	1.069	0.4915	0.8156
20924	Supt5h		suppressor of Ty 5 homolog (S. cerevisiae)	1.069	0.3795	NA
20788	Srebf2		sterol regulatory element binding factor 2	1.069	0.4272	0.7827
20529	Slc31a1		solute carrier family 31, member 1	1.069	0.1458	NA
18747	Prkaca		protein kinase, cAMP dependent, catalytic, alpha	1.069	0.6614	0.8888
18148	Npm1		nucleophosmin 1	1.069	0.1594	NA
18038	Nfkbil1		nuclear factor of kappa light polypeptide gene enhancer in B-cells inhibitor-like 1	1.069	0.2942	NA
17939	Naga		N-acetyl galactosaminidase, alpha	1.069	0.6051	0.8645
15245	Hhip		Hedgehog-interacting protein	1.069	0.6584	0.8869
14579	Gem		GTP binding protein (gene overexpressed in skeletal muscle)	1.069	0.1805	NA
14406	Gabrg2		gamma-aminobutyric acid (GABA) A receptor, subunit gamma 2	1.069	0.1676	NA
14395	Gabra2		gamma-aminobutyric acid (GABA) A receptor, subunit alpha 2	1.069	0.555	0.8444
14255	Flt3		FMS-like tyrosine kinase 3	1.069	0.3081	NA
13136	Cd55		CD55 antigen	1.069	0.5688	0.8488
13116	Cyp46a1		cytochrome P450, family 46, subfamily a, polypeptide 1	1.069	0.6456	0.8809

12977	Csf1	colony stimulating factor 1 (macrophage)	1.069	0.4872	0.8133
12009	Azi1	5-azacytidine induced gene 1	1.069	0.4112	NA
11569	Aebp2	AE binding protein 2	1.069	0.2486	NA
100046302	LOC100046302	protein disulfide-isomerase A6-like	1.068	0.5191	0.8282
627939	Gm6813	predicted gene 6813	1.068	0.3179	NA
624367	Gm6498	glyceraldehyde-3-phosphate dehydrogenase pseudogene	1.068	0.6251	0.8728
623131	Prr19	proline rich 19	1.068	0.7614	0.9237
545611	Gm13298	predicted gene 13298	1.068	0.4767	0.8099
386612	Thoc6	THO complex 6 homolog (Drosophila)	1.068	0.3316	NA
327951	Cyb5d1	cytochrome b5 domain containing 1	1.068	0.4879	0.8138
269788	Lhfpl4	lipoma HMGIC fusion partner-like protein 4	1.068	0.6171	0.8698
245866	Ift52	intraflagellar transport 52 homolog (Chlamydomonas)	1.068	0.2391	NA
245386	Fam70a	family with sequence similarity 70, member A	1.068	0.2158	NA
240613	9930021J03Rik	RIKEN cDNA 9930021J03 gene	1.068	0.3011	NA
234797	6430548M08Rik	RIKEN cDNA 6430548M08 gene	1.068	0.4556	0.7982
232943	Klc3	kinesin light chain 3	1.068	0.5762	0.8506
232784	Zfp212	Zinc finger protein 212	1.068	0.1526	NA
229534	Pbxip1	pre-B-cell leukemia transcription factor interacting protein 1	1.068	0.4265	NA
228543	Rhov	ras homolog gene family, member V	1.068	0.5057	0.823
216019	Hkdc1	hexokinase domain containing 1	1.068	0.5619	0.8465
207667	Skor1	SKI family transcriptional corepressor 1	1.068	0.5029	0.8214
140709	Emid2	EMI domain containing 2	1.068	0.5501	0.8421
110611	Hdlbp	high density lipoprotein (HDL) binding protein	1.068	0.5944	0.8597
107522	Ece2	endothelin converting enzyme 2	1.068	0.3988	NA
104457	0610010K14Rik	RIKEN cDNA 0610010K14 gene	1.068	0.1412	NA
100383	Bsdc1	BSD domain containing 1	1.068	0.3728	NA
98682	Mfsd6	major facilitator superfamily domain containing 6	1.068	0.477	0.8099
97998	Deptor	DEP domain containing MTOR-interacting protein	1.068	0.5739	0.8503
96957	Tmem62	transmembrane protein 62	1.068	0.3901	NA
80752	Fam20c	family with sequence similarity 20, member C	1.068	0.5847	0.8549
78586	Srbd1	S1 RNA binding domain 1	1.068	0.2894	NA
76980	Ube2ql1	ubiquitin-conjugating enzyme E2Q family-like 1	1.068	0.5325	0.8339
75871	Zfp821	zinc finger protein 821	1.068	0.3146	NA
75740	Egfem1	EGF-like and EMI domain containing 1	1.068	0.4558	0.7983
75617	Rps25	ribosomal protein S25	1.068	0.2002	NA
74325	Cltb	clathrin, light polypeptide (Lcb)	1.068	0.1306	NA
74111	Rbm19	RNA binding motif protein 19	1.068	0.4278	NA
72475	Ssbp3	single-stranded DNA binding protein 3	1.068	0.1204	NA
71743	Coasy	Coenzyme A synthase	1.068	0.1955	NA
70441	2610100L16Rik	RIKEN cDNA 2610100L16 gene	1.068	0.2836	NA
70103	Znhit1	zinc finger, HIT domain containing 1	1.068	0.477	0.8099
69724	Rnaseh2a	ribonuclease H2, large subunit	1.068	0.4187	NA
69590	Gpx8	glutathione peroxidase 8 (putative)	1.068	0.4098	NA
69217	Plekha4	pleckstrin homology domain containing, family A (phosphoinositide binding specific) member 4	1.068	0.397	NA
68250	Fam96a	family with sequence similarity 96, member A	1.068	0.182	NA
68230	1700102H20Rik	RIKEN cDNA 1700102H20 gene	1.068	0.3699	NA
67861	Akr1b10	aldo-keto reductase family 1, member B10 (aldose reductase)	1.068	0.2506	NA
67819	Derl1	Der1-like domain family, member 1	1.068	0.2661	NA
67705	1810058I24Rik	RIKEN cDNA 1810058I24 gene	1.068	0.7687	0.9255

67484	Eepd1	endonuclease/exonuclease/phosphatase family domain containing 1	1.068	0.1198	NA
67381	Med4	mediator of RNA polymerase II transcription, subunit 4 homolog (yeast)	1.068	0.2123	NA
66700	Vps24	vacuolar protein sorting 24 (yeast)	1.068	0.168	NA
66194	Pycrl	pyrroline-5-carboxylate reductase-like	1.068	0.3522	NA
66125	Sf3b5	splicing factor 3b, subunit 5	1.068	0.2635	NA
59043	Wsb2	WD repeat and SOCS box-containing 2	1.068	0.5298	0.8332
58240	Hs1bp3	HCLS1 binding protein 3	1.068	0.5458	0.8391
58238	Fam181b	family with sequence similarity 181, member B	1.068	0.5277	0.8332
56788	Scube2	signal peptide, CUB domain, EGF-like 2	1.068	0.6688	0.8904
56470	Rgs19	regulator of G-protein signaling 19	1.068	0.4911	0.8155
51800	Bok	BCL2-related ovarian killer protein	1.068	0.3144	NA
30853	Mlf2	myeloid leukemia factor 2	1.068	0.272	NA
27221	Chaf1a	chromatin assembly factor 1, subunit A (p150)	1.068	0.4988	0.8197
26611	Rcn2	reticulocalbin 2	1.068	0.4112	NA
22348	Slc32a1	solute carrier family 32 (GABA vesicular transporter), member 1	1.068	0.5886	0.8572
21855	Timm17b	translocase of inner mitochondrial membrane 17b	1.068	0.2211	NA
20224	Sar1a	SAR1 gene homolog A (S. cerevisiae)	1.068	0.2771	NA
19256	Ptpn20	protein tyrosine phosphatase, non-receptor type 20	1.068	0.583	0.8538
19184	Psmc5	protease (prosome, macropain) 26S subunit, ATPase 5	1.068	0.3648	NA
18163	Ctnnd2	catenin (cadherin associated protein), delta 2	1.068	0.4461	0.7938
18132	Notch4	Notch gene homolog 4 (Drosophila)	1.068	0.4404	0.7911
17965	Nbl1	neuroblastoma, suppression of tumorigenicity 1	1.068	0.7138	0.9078
17168	Nprl3	nitrogen permease regulator-like 3 (S. cerevisiae)	1.068	0.7642	0.925
16776	Lama5	laminin, alpha 5	1.068	0.6357	0.8771
16574	Kif5c	kinesin family member 5C	1.068	0.2528	NA
16404	Itga7	integrin alpha 7	1.068	0.4252	NA
14904	Gtpbp1	GTP binding protein 1	1.068	0.5575	0.8454
14712	Gnpat	glyceronephosphate O-acyltransferase	1.068	0.1396	NA
14533	Bloc1s1	biogenesis of lysosome-related organelles complex-1, subunit 1	1.068	0.08667	NA
14270	Srgap2	SLIT-ROBO Rho GTPase activating protein 2	1.068	0.4026	NA
13885	Esd	esterase D/formylglutathione hydrolase	1.068	0.2531	NA
12757	Clta	clathrin, light polypeptide (Lca)	1.068	0.3755	NA
12661	Chl1	cell adhesion molecule with homology to L1CAM	1.068	0.285	NA
382089	Ripply2	ripply2 homolog (zebrafish)	1.067	0.2545	NA
381201	Gm962	predicted gene 962	1.067	0.3724	NA
329509	1810024B03Rik	RIKEN cDNA 1810024B03 gene	1.067	0.7766	0.9271
320333	D830030K20Rik	RIKEN cDNA D830030K20 gene	1.067	0.6163	0.8698
320292	Rasgef1b	RasGEF domain family, member 1B	1.067	0.3387	NA
320208	Tmem91	transmembrane protein 91	1.067	0.5167	0.8277
279572	Tlr13	toll-like receptor 13	1.067	0.7123	0.9075
269954	Ttll13	tubulin tyrosine ligase-like family, member 13	1.067	0.5926	0.8585
269831	Tspan12	tetraspanin 12	1.067	0.4103	NA
269514	Fbxl4	F-box and leucine-rich repeat protein 4	1.067	0.3767	NA
264064	Cdk8	cyclin-dependent kinase 8	1.067	0.3617	NA
242584	Wdr78	WD repeat domain 78	1.067	0.6067	0.8646
239528	Eif2c2	eukaryotic translation initiation factor 2C, 2	1.067	0.7429	0.9187
239027	Arhgap22	Rho GTPase activating protein 22	1.067	0.6213	0.8719
236082	Dhrsx	dehydrogenase/reductase (SDR family) X chromosome	1.067	0.5293	0.8332
234852	Chmp1a	chromatin modifying protein 1A	1.067	0.1167	NA

232089	Elmod3	ELMO/CED-12 domain containing 3	1.067	0.3342	NA
226041	Pgm5	phosphoglucomutase 5	1.067	0.8554	0.9552
224023	Klhl22	kelch-like 22 (Drosophila)	1.067	0.2688	NA
216799	Nlrp3	NLR family, pyrin domain containing 3	1.067	0.7366	0.9169
209012	Ulk4	unc-51-like kinase 4 (C. elegans)	1.067	0.5227	0.8294
208618	Etl4	enhancer trap locus 4	1.067	0.5335	0.8343
207474	Kctd12b	potassium channel tetramerisation domain containing 12b	1.067	0.2658	NA
192156	Mvd	mevalonate (diphospho) decarboxylase	1.067	0.6458	0.8809
170789	Acot8	acyl-CoA thioesterase 8	1.067	0.3031	NA
110119	Mpi	mannose phosphate isomerase	1.067	0.167	NA
109910	Zfp91	zinc finger protein 91	1.067	0.2436	NA
109815	H47	histocompatibility 47	1.067	0.1012	NA
103844	Inca1	inhibitor of CDK, cyclin A1 interacting protein 1	1.067	0.4756	0.8097
98238	Lrrc59	leucine rich repeat containing 59	1.067	0.3982	NA
98193	Dcaf8	DDB1 and CUL4 associated factor 8	1.067	0.3997	NA
78977	Popdc3	popeye domain containing 3	1.067	0.4134	NA
77996	D730039F16Rik	RIKEN cDNA D730039F16 gene	1.067	0.5784	0.8519
76854	Gpr30	G protein-coupled receptor 30	1.067	0.4275	NA
76721	1700085B13Rik	RIKEN cDNA 1700085B13 gene	1.067	0.6636	0.8896
76646	Wdr38	WD repeat domain 38	1.067	0.6331	0.8758
73910	Arhgap18	Rho GTPase activating protein 18	1.067	0.3183	NA
72349	Dusp3	dual specificity phosphatase 3 (vaccinia virus phosphatase VH1-related)	1.067	0.3134	NA
72183	Snx6	sorting nexin 6	1.067	0.4667	0.8049
72088	Ush1c	Usher syndrome 1C homolog (human)	1.067	0.7405	0.9179
71838	Phf7	PHD finger protein 7	1.067	0.4932	0.816
71147	Oxsm	3-oxoacyl-ACP synthase, mitochondrial	1.067	0.4128	NA
70829	Ccdc93	coiled-coil domain containing 93	1.067	0.3087	NA
68001	1110004E09Rik	RIKEN cDNA 1110004E09 gene	1.067	0.2382	NA
67489	Ap4b1	adaptor-related protein complex AP-4, beta 1	1.067	0.1435	NA
67374	Jam2	junction adhesion molecule 2	1.067	0.148	NA
67326	1700037H04Rik	RIKEN cDNA 1700037H04 gene	1.067	0.3476	NA
67166	Arl8b	ADP-ribosylation factor-like 8B	1.067	0.2667	NA
66930	Fank1	fibronectin type 3 and ankyrin repeat domains 1	1.067	0.3606	NA
66686	Dcbld1	discoidin, CUB and LCCL domain containing 1	1.067	0.1365	NA
66236	1500011B03Rik	RIKEN cDNA 1500011B03 gene	1.067	0.2296	NA
66096	0910001L09Rik	RIKEN cDNA 0910001L09 gene	1.067	0.3824	NA
57437	Golga7	golgi autoantigen, golgin subfamily a, 7	1.067	0.1803	NA
56327	Arl2	ADP-ribosylation factor-like 2	1.067	0.1995	NA
54351	Rai12	retinoic acid induced 12	1.067	0.2278	NA
53902	Rcan3	regulator of calcineurin 3	1.067	0.4882	0.8138
52040	Ppp1r10	protein phosphatase 1, regulatory subunit 10	1.067	0.5133	0.8268
27370	Rps26	ribosomal protein S26	1.067	0.2099	NA
26446	Psmb3	proteasome (prosome, macropain) subunit, beta type 3	1.067	0.4817	0.8108
21945	Dedd	death effector domain-containing	1.067	0.4036	NA
20807	Srf	serum response factor	1.067	0.2415	NA
20687	Sp3	trans-acting transcription factor 3	1.067	0.8885	0.9676
20536	Slc4a3	solute carrier family 4 (anion exchanger), member 3	1.067	0.5061	0.823
20534	Slc4a1ap	solute carrier family 4 (anion exchanger), member 1, adaptor protein	1.067	0.3248	NA
20480	Clpb	ClpB caseinolytic peptidase B homolog (E. coli)	1.067	0.617	0.8698

20185	Ncor1	nuclear receptor co-repressor 1	1.067	0.3692	NA
19941	Rpl26	ribosomal protein L26	1.067	0.1676	NA
19921	Rpl19	ribosomal protein L19	1.067	0.2712	NA
19377	Rai1	retinoic acid induced 1	1.067	0.4061	NA
19342	Rab4b	RAB4B, member RAS oncogene family	1.067	0.5688	0.8488
19072	Prep	prolyl endopeptidase	1.067	0.3393	NA
18762	Prkcz	protein kinase C, zeta	1.067	0.3994	NA
16331	Inpp5d	inositol polyphosphate-5-phosphatase D	1.067	0.3549	NA
14148	Fdx1	ferredoxin 1	1.067	0.1338	NA
12933	Crmp1	collapsin response mediator protein 1	1.067	0.3757	NA
11992	Auh	AU RNA binding protein/enoyl-coenzyme A hydratase	1.067	0.1598	NA
11515	Adcy9	adenylate cyclase 9	1.067	0.3195	NA
100042480	Nhsl2	NHS-like 2	1.066	0.5656	0.8478
402729	9330184L24Rik	RIKEN cDNA 9330184L24 gene	1.066	0.7149	0.9083
338351	Akap17b	A kinase (PRKA) anchor protein 17B	1.066	0.2303	NA
246154	Vasn	vasorin	1.066	0.3717	NA
243616	Slc6a11	solute carrier family 6 (neurotransmitter transporter, GABA), member 11	1.066	0.3652	NA
241656	Pak7	p21 protein (Cdc42/Rac)-activated kinase 7	1.066	0.3352	NA
241062	Pgap1	post-GPI attachment to proteins 1	1.066	0.5638	0.8465
240753	Plekha6	pleckstrin homology domain containing, family A member 6	1.066	0.4605	0.8014
232976	Zfp574	zinc finger protein 574	1.066	0.608	0.8655
230904	Fbxo2	F-box protein 2	1.066	0.3304	NA
228998	Arfgap1	ADP-ribosylation factor GTPase activating protein 1	1.066	0.3084	NA
228983	Osbpl2	oxysterol binding protein-like 2	1.066	0.2297	NA
228536	Bahd1	bromo adjacent homology domain containing 1	1.066	0.259	NA
227545	5430407P10Rik	RIKEN cDNA 5430407P10 gene	1.066	0.2125	NA
224093	Fam43a	family with sequence similarity 43, member A	1.066	0.1481	NA
216136	Ilvbl	ilvB (bacterial acetolactate synthase)-like	1.066	0.2196	NA
214895	Lman2l	lectin, mannose-binding 2-like	1.066	0.1641	NA
110834	Chrna3	cholinergic receptor, nicotinic, alpha polypeptide 3	1.066	0.3761	NA
110074	Dut	deoxyuridine triphosphatase	1.066	0.4366	NA
109032	Sp110	Sp110 nuclear body protein	1.066	0.1096	NA
108124	Napa	N-ethylmaleimide sensitive fusion protein attachment protein alpha	1.066	0.482	0.8108
104418	Dgkz	diacylglycerol kinase zeta	1.066	0.5638	0.8465
101148	B630005N14Rik	RIKEN cDNA B630005N14 gene	1.066	0.4818	0.8108
99890	Prmt6	protein arginine N-methyltransferase 6	1.066	0.2207	NA
98396	Slc41a1	solute carrier family 41, member 1	1.066	0.4151	NA
80280	Cdk5rap3	CDK5 regulatory subunit associated protein 3	1.066	0.4249	NA
77741	6720483E21Rik	RIKEN cDNA 6720483E21 gene	1.066	0.5625	0.8465
76612	Lrrc27	leucine rich repeat containing 27	1.066	0.4515	0.7951
76303	Osbp	oxysterol binding protein	1.066	0.4118	NA
76192	Abhd12	abhydrolase domain containing 12	1.066	0.5501	0.8421
74596	Cds1	CDP-diacylglycerol synthase 1	1.066	0.4351	NA
74342	Lrrtm1	leucine rich repeat transmembrane neuronal 1	1.066	0.3952	NA
73836	Slc35b2	solute carrier family 35, member B2	1.066	0.1749	NA
72018	Fundc1	FUN14 domain containing 1	1.066	0.2016	NA
71996	1600014K23Rik	RIKEN cDNA 1600014K23 gene	1.066	0.67	0.8912
71960	Myh14	myosin, heavy polypeptide 14	1.066	0.467	0.8053
71901	2310028H24Rik	RIKEN cDNA 2310028H24 gene	1.066	0.2456	NA

69556	Bod1	biorientation of chromosomes in cell division 1	1.066	0.1016	NA
68597	1110021J02Rik	RIKEN cDNA 1110021J02 gene	1.066	0.2127	NA
68480	1110007C09Rik	RIKEN cDNA 1110007C09 gene	1.066	0.5148	0.8274
68047	Mpnd	MPN domain containing	1.066	0.4943	0.8169
66863	Lztr1	leucine-zipper-like transcriptional regulator, 1	1.066	0.376	NA
66270	Fam134b	family with sequence similarity 134, member B	1.066	0.3552	NA
66070	Cwc15	CWC15 homolog (S. cerevisiae)	1.066	0.2044	NA
64293	Stk32b	serine/threonine kinase 32B	1.066	0.6066	0.8645
63872	Zfp296	zinc finger protein 296	1.066	0.6828	0.8956
59016	Thap11	THAP domain containing 11	1.066	0.1903	NA
58185	Rsad2	radical S-adenosyl methionine domain containing 2	1.066	0.5907	0.858
57314	Th1l	TH1-like homolog (Drosophila)	1.066	0.4399	0.7908
56632	Sphk2	sphingosine kinase 2	1.066	0.215	NA
56369	Apip	APAF1 interacting protein	1.066	0.3351	NA
56224	Tspan5	tetraspanin 5	1.066	0.5636	0.8465
56149	Grasp	GRP1 (general receptor for phosphoinositides 1)-associated scaffold protein	1.066	0.2286	NA
56013	Srcin1	SRC kinase signaling inhibitor 1	1.066	0.6289	0.8745
55984	Camkk1	calcium/calmodulin-dependent protein kinase kinase 1, alpha	1.066	0.1136	NA
54384	Mtmr7	myotubularin related protein 7	1.066	0.5339	0.8344
53972	Ngef	neuronal guanine nucleotide exchange factor	1.066	0.2811	NA
52504	Cenpo	centromere protein O	1.066	0.4784	0.81
51792	Ppp2r1a	protein phosphatase 2 (formerly 2A), regulatory subunit A (PR 65), alpha isoform	1.066	0.6644	0.8899
50505	Ercc4	excision repair cross-complementing rodent repair deficiency, complementation group 4	1.066	0.2789	NA
30941	Usp21	ubiquitin specific peptidase 21	1.066	0.6236	0.8724
27966	Rrp9	RRP9, small subunit (SSU) processome component, homolog (yeast)	1.066	0.6014	0.8636
27362	Dnajb9	DnaJ (Hsp40) homolog, subfamily B, member 9	1.066	0.2713	NA
27050	Rps3	ribosomal protein S3	1.066	0.5134	0.8268
23897	Hax1	HCLS1 associated X-1	1.066	0.2142	NA
23806	Arih1	ariadne ubiquitin-conjugating enzyme E2 binding protein homolog 1 (Drosophila)	1.066	0.2895	NA
23802	Amfr	autocrine motility factor receptor	1.066	0.484	0.8123
22282	Usf2	upstream transcription factor 2	1.066	0.3236	NA
22129	Ttc3	tetratricopeptide repeat domain 3	1.066	0.4935	0.8163
22038	Plscr1	phospholipid scramblase 1	1.066	0.3473	NA
20872	Stk16	serine/threonine kinase 16	1.066	0.3216	NA
20729	Spin1	spindlin 1	1.066	0.204	NA
20583	Snai2	snail homolog 2 (Drosophila)	1.066	0.6003	0.8635
20084	Rps18	ribosomal protein S18	1.066	0.313	NA
19777	C80913	expressed sequence C80913	1.066	0.3447	NA
19277	Ptpro	protein tyrosine phosphatase, receptor type, O	1.066	0.2574	NA
19224	Ptgs1	prostaglandin-endoperoxide synthase 1	1.066	0.7247	0.9126
19013	Ppara	peroxisome proliferator activated receptor alpha	1.066	0.5648	0.8471
17764	Mtf1	metal response element binding transcription factor 1	1.066	0.6821	0.8951
15937	Ier3	immediate early response 3	1.066	0.3986	NA
14733	Gpc1	glypican 1	1.066	0.3199	NA
14433	Gapdh	glyceraldehyde-3-phosphate dehydrogenase	1.066	0.355	NA
14128	Fcer2a	Fc receptor, IgE, low affinity II, alpha polypeptide	1.066	0.7474	0.9199
13723	Emb	embigin	1.066	0.13	NA
12974	Cs	citrate synthase	1.066	0.2811	NA
12890	Cplx2	complexin 2	1.066	0.3953	NA

12850	Coq7	demethyl-Q 7	1.066	0.3799	NA
12349	Car2	carbonic anhydrase 2	1.066	0.4321	NA
100036538	BC028777	cDNA sequence BC028777	1.065	0.6758	0.8928
666794	Rbm24	RNA binding motif protein 24	1.065	0.7815	0.929
382571	Kcnf1	potassium voltage-gated channel, subfamily F, member 1	1.065	0.6025	0.8639
320604	A730037C10Rik	RIKEN cDNA A730037C10 gene	1.065	0.6668	0.8904
320504	5930403N24Rik	RIKEN cDNA 5930403N24 gene	1.065	0.2611	NA
319807	3110047P20Rik	RIKEN cDNA 3110047P20 gene	1.065	0.7288	0.914
269514	Fbxl4	F-box and leucine-rich repeat protein 4	1.065	0.1797	NA
268709	Fam107a	family with sequence similarity 107, member A	1.065	0.3223	NA
260302	Gga3	golgi associated, gamma adaptin ear containing, ARF binding protein 3	1.065	0.2119	NA
260297	Prrt1	proline-rich transmembrane protein 1	1.065	0.2716	NA
245555	C77370	expressed sequence C77370	1.065	0.8302	0.9452
241494	Zfp385b	zinc finger protein 385B	1.065	0.561	0.8465
240913	Adamts4	a disintegrin-like and metallopeptidase (reprolysin type) with thrombospondin type 1 motif, 4	1.065	0.8394	0.9484
234857	Spire2	spire homolog 2 (Drosophila)	1.065	0.5617	0.8465
234595	Slc38a7	solute carrier family 38, member 7	1.065	0.6796	0.8944
232944	Mark4	MAP/microtubule affinity-regulating kinase 4	1.065	0.6476	0.8816
230709	Zmpste24	zinc metallopeptidase, STE24 homolog (S. cerevisiae)	1.065	0.2177	NA
230648	4732418C07Rik	RIKEN cDNA 4732418C07 gene	1.065	0.3524	NA
229541	Dennd4b	DENN/MADD domain containing 4B	1.065	0.501	0.8202
229228	Nudt6	nudix (nucleoside diphosphate linked moiety X)-type motif 6	1.065	0.3458	NA
228714	Csrp2bp	cysteine and glycine-rich protein 2 binding protein	1.065	0.2243	NA
228356	1110051M20Rik	RIKEN cDNA 1110051M20 gene	1.065	0.4023	NA
223499	Dcaf13	DDB1 and CUL4 associated factor 13	1.065	0.4648	0.8042
219024	Tmem55b	transmembrane protein 55b	1.065	0.5	0.8197
216829	Mmgt2	membrane magnesium transporter 2	1.065	0.3978	NA
216161	Sbno2	strawberry notch homolog 2 (Drosophila)	1.065	0.6302	0.8748
213582	Mtap9	microtubule-associated protein 9	1.065	0.4497	0.7943
170735	Arr3	arrestin 3, retinal	1.065	0.2556	NA
112418	1700102P08Rik	RIKEN cDNA 1700102P08 gene	1.065	0.5826	0.8537
108655	Foxp1	forkhead box P1	1.065	0.7116	0.9072
108148	Galnt2	UDP-N-acetyl-alpha-D-galactosamine:polypeptide N-acetylgalactosaminyltransferase 2	1.065	0.4198	NA
107723	Slc12a6	solute carrier family 12, member 6	1.065	0.2586	NA
102247	Agpat6	1-acylglycerol-3-phosphate O-acyltransferase 6 (lysophosphatidic acid acyltransferase, zeta)	1.065	0.2762	NA
80515	A030009H04Rik	RIKEN cDNA A030009H04 gene	1.065	0.1792	NA
78285	5330426L24Rik	RIKEN cDNA 5330426L24 gene	1.065	0.6371	0.8776
76787	Ppfia3	protein tyrosine phosphatase, receptor type, f polypeptide (PTPRF), interacting protein (liprin), alpha 3	1.065	0.5695	0.8491
76478	Haus8	4HAUS augmin-like complex, subunit 8	1.065	0.2009	NA
74868	Tmem65	transmembrane protein 65	1.065	0.3322	NA
74479	Snx11	sorting nexin 11	1.065	0.6488	0.8818
74414	Polr3c	polymerase (RNA) III (DNA directed) polypeptide C	1.065	0.4144	NA
74244	Atg7	autophagy-related 7 (yeast)	1.065	0.5774	0.8514
73332	Ccdc30	coiled-coil domain containing 30	1.065	0.1484	NA
71617	9130011E15Rik	RIKEN cDNA 9130011E15 gene	1.065	0.7751	0.9266
69136	Tusc1	tumor suppressor candidate 1	1.065	0.1191	NA
67843	Slc35a4	solute carrier family 35, member A4	1.065	0.4109	NA
67826	Snap47	synaptosomal-associated protein, 47	1.065	0.5136	0.8268
67206	2810013P06Rik	RIKEN cDNA 2810013P06 gene	1.065	0.3398	NA

66664	Tmem41a	transmembrane protein 41a	1.065	0.4366	NA
66314	Tpd52l2	tumor protein D52-like 2	1.065	0.379	NA
66059	Krtcap2	keratinocyte associated protein 2	1.065	0.1764	NA
65973	Asph	aspartate-beta-hydroxylase	1.065	0.2506	NA
59025	Usp14	ubiquitin specific peptidase 14	1.065	0.3314	NA
56524	Mpp6	membrane protein, palmitoylated 6 (MAGUK p55 subfamily member 6)	1.065	0.3945	NA
53418	B4galt2	UDP-Gal:betaGlcNAc beta 1,4- galactosyltransferase, polypeptide 2	1.065	0.2735	NA
51788	H2afz	H2A histone family, member Z	1.065	0.1663	NA
30937	Lmcd1	LIM and cysteine-rich domains 1	1.065	0.6832	0.8956
27419	Naglu	alpha-N-acetylglucosaminidase (Sanfilippo disease IIIB)	1.065	0.4988	0.8197
27053	Asns	asparagine synthetase	1.065	0.4266	NA
23856	Dido1	death inducer-obliterator 1	1.065	0.6735	0.8924
22717	Zfp59	zinc finger protein 59	1.065	0.3075	NA
22353	Vip	vasoactive intestinal polypeptide	1.065	0.4308	NA
22223	Uchl1	ubiquitin carboxy-terminal hydrolase L1	1.065	0.3555	NA
21853	Timeless	timeless homolog (Drosophila)	1.065	0.2194	NA
21677	Tead2	TEA domain family member 2	1.065	0.5333	0.8343
21375	Tbr1	T-box brain gene 1	1.065	0.3789	NA
20204	Prrx2	paired related homeobox 2	1.065	0.7322	0.9157
19365	Rad52	RAD52 homolog (S. cerevisiae)	1.065	0.437	NA
18938	Ppp1r14b	protein phosphatase 1, regulatory (inhibitor) subunit 14B	1.065	0.4899	0.8146
18438	P2rx4	purinergic receptor P2X, ligand-gated ion channel 4	1.065	0.273	NA
16549	Khsrp	KH-type splicing regulatory protein	1.065	0.6651	0.8903
16322	Inha	inhibin alpha	1.065	0.4853	0.8127
15473	Hrsp12	heat-responsive protein 12	1.065	0.375	NA
14232	Fkbp8	FK506 binding protein 8	1.065	0.5879	0.857
13640	Efna5	ephrin A5	1.065	0.2365	NA
12373	Casq2	calsequestrin 2	1.065	0.6117	0.8673
12362	Casp1	caspase 1	1.065	0.4739	0.8088
12322	Camk2a	calcium/calmodulin-dependent protein kinase II alpha	1.065	0.5977	0.8611
11461	Actb	actin, beta	1.065	0.4776	0.81
100504462	LOC100504462	hypothetical LOC100504462	1.064	0.7673	0.9255
100044874	LOC100044874	h-2 class I histocompatibility antigen, K-W28 alpha chain-like	1.064	0.6772	0.893
636931	Trim71	tripartite motif-containing 71	1.064	0.7114	0.907
623661	Lipt1	lipoyltransferase 1	1.064	0.4592	0.8007
432768	Gm5451	predicted gene 5451	1.064	0.6901	0.8987
353047	Plekhm1	pleckstrin homology domain containing, family M (with RUN domain) member 1	1.064	0.1645	NA
328643	Vwa5b2	von Willebrand factor A domain containing 5B2	1.064	0.6341	0.8765
320799	Zhx3	zinc fingers and homeoboxes 3	1.064	0.3862	NA
320103	A730080H06Rik	RIKEN cDNA A730080H06 gene	1.064	0.5594	0.8459
319468	Ppm1h	protein phosphatase 1H (PP2C domain containing)	1.064	0.2482	NA
270066	Slc35e1	solute carrier family 35, member E1	1.064	0.3871	NA
268449	Rpl23a	ribosomal protein L23A	1.064	0.2171	NA
237775	Zfp867	zinc finger protein 867	1.064	0.3499	NA
230761	Zfp362	zinc finger protein 362	1.064	0.604	0.8644
229473	D930015E06Rik	RIKEN cDNA D930015E06 gene	1.064	0.281	NA
227738	Lrsam1	leucine rich repeat and sterile alpha motif containing 1	1.064	0.448	NA
227707	BC005624	cDNA sequence BC005624	1.064	0.2782	NA
227683	Coq4	coenzyme Q4 homolog (yeast)	1.064	0.5337	0.8343

224650	Anks1	ankyrin repeat and SAM domain containing 1	1.064	0.3234	NA
223921	Aaas	achalasia, adrenocortical insufficiency, alacrimia	1.064	0.6231	0.8724
217847	Serpina10	serine (or cysteine) peptidase inhibitor, clade A (alpha-1 antiproteinase, antitrypsin), member 10	1.064	0.7167	0.9094
216974	Proca1	protein interacting with cyclin A1	1.064	0.6284	0.8744
216974	Proca1	protein interacting with cyclin A1	1.064	0.6564	0.8859
215951	Lace1	lactation elevated 1	1.064	0.5606	0.8462
211712	Pcdh9	protocadherin 9	1.064	0.2267	NA
210293	Dock10	dedicator of cytokinesis 10	1.064	0.2644	NA
109113	Uhrf2	ubiquitin-like, containing PHD and RING finger domains 2	1.064	0.2432	NA
106757	Tmem146	transmembrane protein 146	1.064	0.6441	0.8802
104885	Tmem179	transmembrane protein 179	1.064	0.3427	NA
96979	Ptges2	prostaglandin E synthase 2	1.064	0.4941	0.8168
80981	Arl4d	ADP-ribosylation factor-like 4D	1.064	0.2944	NA
80909	Gatsl2	GATS protein-like 2	1.064	0.3145	NA
80888	Hspb8	heat shock protein 8	1.064	0.239	NA
76965	Slitrk1	SLIT and NTRK-like family, member 1	1.064	0.4155	NA
75565	Ccdc101	coiled-coil domain containing 101	1.064	0.3616	NA
74140	Tm9sf1	transmembrane 9 superfamily member 1	1.064	0.5198	0.8284
74005	6330412A17Rik	RIKEN cDNA 6330412A17 gene	1.064	0.5719	0.8499
73430	1700049G17Rik	RIKEN cDNA 1700049G17 gene	1.064	0.4536	0.797
72568	Lin9	lin-9 homolog (C. elegans)	1.064	0.2185	NA
71876	Mlf1ip	myeloid leukemia factor 1 interacting protein	1.064	0.3423	NA
71743	Coasy	Coenzyme A synthase	1.064	0.284	NA
71706	Slc46a3	solute carrier family 46, member 3	1.064	0.348	NA
69724	Rnaseh2a	ribonuclease H2, large subunit	1.064	0.5443	0.839
69655	Cd164l2	CD164 sialomucin-like 2	1.064	0.4661	0.8049
67939	Prorsd1	prolyl-tRNA synthetase domain containing 1	1.064	0.4648	0.8042
67838	Dnajb11	DnaJ (Hsp40) homolog, subfamily B, member 11	1.064	0.2186	NA
67666	Hapln3	hyaluronan and proteoglycan link protein 3	1.064	0.4276	NA
67552	H2afy3	H2A histone family, member Y3	1.064	0.1999	NA
67125	Tspan31	tetraspanin 31	1.064	0.4919	0.8156
66868	Mfsd1	major facilitator superfamily domain containing 1	1.064	0.3523	NA
66756	4933411K20Rik	RIKEN cDNA 4933411K20 gene	1.064	0.1834	NA
66586	Crls1	cardiolipin synthase 1	1.064	0.4247	NA
66570	Cenpm	centromere protein M	1.064	0.7896	0.9329
66568	Rwdd3	RWD domain containing 3	1.064	0.3536	NA
66537	Pomp	proteasome maturation protein	1.064	0.225	NA
66496	Ppdpf	pancreatic progenitor cell differentiation and proliferation factor homolog (zebrafish)RIKEN cDNA 2700038C09 gene	1.064	0.4858	0.8127
66477	Usmg5	upregulated during skeletal muscle growth 5	1.064	0.1798	NA
66268	Pigyl	phosphatidylinositol glycan anchor biosynthesis, class Y-like	1.064	0.4917	0.8156
57425	U90926	cDNA sequence U90926	1.064	0.5811	0.8527
56314	Zfp113	zinc finger protein 113	1.064	0.4127	NA
53970	Rfx5	regulatory factor X, 5 (influences HLA class II expression)	1.064	0.324	NA
52588	Tspan14	tetraspanin 14	1.064	0.4545	NA
50492	Thop1	thimet oligopeptidase 1	1.064	0.5549	0.8444
27204	Syn3	synapsin III	1.064	0.7372	0.9171
23831	Car14	carbonic anhydrase 14	1.064	0.5292	0.8332
22196	Ube2i	ubiquitin-conjugating enzyme E2I	1.064	0.2302	NA
21969	Top1	topoisomerase (DNA) I	1.064	0.4925	0.8157

21357	Tarbp2	TAR (HIV) RNA binding protein 2	1.064	0.3797	NA
20466	Sin3a	transcriptional regulator, SIN3A (yeast)	1.064	0.4898	0.8145
20222	Sf3a2	splicing factor 3a, subunit 2	1.064	0.6835	0.8956
19763	Ring1	ring finger protein 1	1.064	0.6808	0.8949
18971	Pold1	polymerase (DNA directed), delta 1, catalytic subunit	1.064	0.4749	0.8094
17993	Ndufs4	NADH dehydrogenase (ubiquinone) Fe-S protein 4	1.064	0.1159	NA
16857	Lgals6	lectin, galactose binding, soluble 6	1.064	0.4686	0.8057
14651	Hagh	hydroxyacyl glutathione hydrolase	1.064	0.3631	NA
14600	Ghr	growth hormone receptor	1.064	0.4086	NA
13549	Dyrk1b	dual-specificity tyrosine-(Y)-phosphorylation regulated kinase 1b	1.064	0.7084	0.9066
12921	Crhr1	corticotropin releasing hormone receptor 1	1.064	0.5565	0.8448
12662	Chm	choroidermia	1.064	0.3405	NA
11924	Neurog2	neurogenin 2	1.064	0.4191	NA
11842	Arf3	ADP-ribosylation factor 3	1.064	0.1984	NA
11820	App	amyloid beta (A4) precursor protein	1.064	0.2976	NA
622404	Ccdc107	coiled-coil domain containing 107	1.063	0.3353	NA
384525	Gm5321	predicted gene 5321	1.063	0.1163	NA
268451	Rab11fip4	RAB11 family interacting protein 4 (class II)	1.063	0.5946	0.8598
246293	Klhl8	kelch-like 8 (Drosophila)	1.063	0.1917	NA
242819	Rundc3b	RUN domain containing 3B	1.063	0.3027	NA
238328	Vash1	vasohibin 1	1.063	0.2083	NA
234700	Nrn1l	neuritin 1-like	1.063	0.4109	NA
233168	AI987944	expressed sequence AI987944	1.063	0.4634	NA
231670	Fbxo21	F-box protein 21	1.063	0.3984	NA
231148	Ablim2	actin-binding LIM protein 2	1.063	0.431	NA
230996	9430015G10Rik	RIKEN cDNA 9430015G10 gene	1.063	0.5844	0.8547
229524	Msto1	misato homolog 1 (Drosophila)	1.063	0.4262	NA
226359	C1ql2	complement component 1, q subcomponent-like 2	1.063	0.6329	0.8758
223752	Gramd4	GRAM domain containing 4	1.063	0.4804	0.8101
219189	1300010F03Rik	RIKEN cDNA 1300010F03 gene	1.063	0.6217	0.8719
216963	Git1	G protein-coupled receptor kinase-interactor 1	1.063	0.366	NA
216724	Rufy1	RUN and FYVE domain containing 1	1.063	0.438	NA
209200	Dtx3l	deltex 3-like (Drosophila)	1.063	0.6469	0.8812
208258	Ankrd33	ankyrin repeat domain 33	1.063	0.1899	NA
106582	Nrm	nurim (nuclear envelope membrane protein)	1.063	0.5319	0.8339
102293	AW111846	expressed sequence AW111846	1.063	0.1992	NA
101612	Grwd1	glutamate-rich WD repeat containing 1	1.063	0.5679	0.8485
78688	Nol3	nucleolar protein 3 (apoptosis repressor with CARD domain)	1.063	0.55	0.8421
77521	Mtus2	microtubule associated tumor suppressor candidate 2	1.063	0.5189	0.8282
77463	C030014O09Rik	RIKEN cDNA C030014O09 gene	1.063	0.6769	0.893
77065	Ints7	integrator complex subunit 7	1.063	0.5056	0.823
76983	Scfd1	Sec1 family domain containing 1	1.063	0.2824	NA
76820	Fam49a	family with sequence similarity 49, member A	1.063	0.5169	0.8277
75718	Vwa5b1	von Willebrand factor A domain containing 5B1	1.063	0.5229	0.8294
74103	Nebl	nebulette	1.063	0.5909	0.858
73284	Ddit4l	DNA-damage-inducible transcript 4-like	1.063	0.3133	NA
72344	Usp36	ubiquitin specific peptidase 36	1.063	0.3862	NA
71728	Stk11ip	serine/threonine kinase 11 interacting protein	1.063	0.5593	0.8459
70047	Trnt1	tRNA nucleotidyl transferase, CCA-adding, 1	1.063	0.2057	NA

69638	Enho	energy homeostasis associated	1.063	0.2456	NA
68725	1110032F04Rik	RIKEN cDNA 1110032F04 gene	1.063	0.4862	0.8127
68581	Tmed10	transmembrane emp24-like trafficking protein 10 (yeast)	1.063	0.3858	NA
68552	1110003E01Rik	RIKEN cDNA 1110003E01 gene	1.063	0.2759	NA
68209	Rnaseh2c	ribonuclease H2, subunit C	1.063	0.4634	NA
68205	Urm1	ubiquitin related modifier 1 homolog (S. cerevisiae)	1.063	0.4342	NA
68133	Gcsh	glycine cleavage system protein H (aminomethyl carrier)	1.063	0.3306	NA
67511	Tmed9	transmembrane emp24 protein transport domain containing 9	1.063	0.2427	NA
67308	Mrpl46	mitochondrial ribosomal protein L46	1.063	0.2542	NA
66970	Ssbp2	single-stranded DNA binding protein 2	1.063	0.2537	NA
66797	Cntnap2	contactin associated protein-like 2	1.063	0.4508	NA
66536	Nipsnap3b	nipsnap homolog 3B (C. elegans)	1.063	0.5658	0.8478
66407	Mrps15	mitochondrial ribosomal protein S15	1.063	0.2473	NA
64209	Herpud1	homocysteine-inducible, endoplasmic reticulum stress-inducible, ubiquitin-like domain member 1	1.063	0.3964	NA
56471	Stmn4	stathmin-like 4	1.063	0.3541	NA
54648	Ccdc120	coiled-coil domain containing 120	1.063	0.4413	NA
54610	Tbc1d8	TBC1 domain family, member 8	1.063	0.1672	NA
54217	Rpl36	ribosomal protein L36	1.063	0.4207	NA
30045	Dnajc12	DnaJ (Hsp40) homolog, subfamily C, member 12	1.063	0.4071	NA
28081	D11Wsu99e	DNA segment, Chr 11, Wayne State University 99, expressed	1.063	0.2864	NA
23939	Mapk7	mitogen-activated protein kinase 7	1.063	0.5185	0.8282
22344	Vezf1	vascular endothelial zinc finger 1	1.063	0.2834	NA
22183	Zrsr1	zinc finger (CCCH type), RNA binding motif and serine/arginine rich 1	1.063	0.3516	NA
21632	Tcrg-V1	T-cell receptor gamma, variable 1	1.063	0.7772	0.9272
21333	Tac1	tachykinin 1	1.063	0.2205	NA
20905	Sts	steroid sulfatase	1.063	0.7079	0.9064
20729	Spin1	spindlin 1	1.063	0.2506	NA
20449	St8sia1	ST8 alpha-N-acetyl-neuraminide alpha-2,8-sialyltransferase 1	1.063	0.5895	0.8575
19943	Rpl28	ribosomal protein L28	1.063	0.479	0.81
19359	Rad23b	RAD23b homolog (S. cerevisiae)	1.063	0.1263	NA
19182	Psmc3	proteasome (prosome, macropain) 26S subunit, ATPase 3	1.063	0.2892	NA
18717	Pip5k1c	phosphatidylinositol-4-phosphate 5-kinase, type 1 gamma	1.063	0.5061	0.823
17168	Nprl3	nitrogen permease regulator-like 3 (S. cerevisiae)	1.063	0.7651	0.9251
16624	Klk1b8	kallikrein 1-related peptidase b8	1.063	0.6935	0.9001
16412	Itgb1	integrin beta 1 (fibronectin receptor beta)	1.063	0.1684	NA
15980	Ifngr2	interferon gamma receptor 2	1.063	0.5492	0.8412
13807	Eno2	enolase 2, gamma neuronal	1.063	0.5636	0.8465
12695	Inadl	InaD-like (Drosophila)	1.063	0.349	NA
12493	Cd37	CD37 antigen	1.063	0.4025	NA
668303	Kif26a	kinesin family member 26A	1.062	0.4608	NA
667338	Gm8580	ribosomal protein L29 pseudogene	1.062	0.5009	0.8202
594844	Tceal3	transcription elongation factor A (SII)-like 3	1.062	0.2257	NA
399603	Fam84b	family with sequence similarity 84, member B	1.062	0.4978	0.8193
330780	E130110O22Rik	RIKEN cDNA E130110O22 gene	1.062	0.3616	NA
320234	Ccdc66	coiled-coil domain containing 66	1.062	0.3196	NA
252870	Usp7	ubiquitin specific peptidase 7	1.062	0.4285	NA
239796	1600021P15Rik	RIKEN cDNA 1600021P15 gene	1.062	0.6896	0.8984
235606	Apeh	acylpeptide hydrolase	1.062	0.2769	NA
234407	Glt25d1	glycosyltransferase 25 domain containing 1	1.062	0.5077	0.8238

233651	Dchs1	dachsous 1 (Drosophila)	1.062	0.6023	0.8639
229524	Msto1	misato homolog 1 (Drosophila)	1.062	0.3877	NA
217449	Ttc15	tetratricopeptide repeat domain 15	1.062	0.2059	NA
216148	Shc2	SHC (Src homology 2 domain containing) transforming protein 2	1.062	0.3341	NA
208718	Dis3l2	DIS3 mitotic control homolog (S. cerevisiae)-like 2	1.062	0.5319	0.8339
207920	Esrp1	epithelial splicing regulatory protein 1	1.062	0.392	NA
192285	Phf21a	PHD finger protein 21A	1.062	0.4637	NA
140500	Acap3	ArfGAP with coiled-coil, ankyrin repeat and PH domains 3	1.062	0.6254	0.8728
110920	Hspa13	heat shock protein 70 family, member 13	1.062	0.3308	NA
110323	Cox6b1	cytochrome c oxidase, subunit VIb polypeptide 1	1.062	0.2899	NA
107372	C030016D13Rik	RIKEN cDNA C030016D13 gene	1.062	0.4867	0.8129
93757	Immp2l	IMP2 inner mitochondrial membrane peptidase-like (S. cerevisiae)	1.062	0.2802	NA
78829	Tsc22d4	TSC22 domain family, member 4	1.062	0.7645	0.9251
77717	6030408B16Rik	RIKEN cDNA 6030408B16 gene	1.062	0.5763	0.8508
77219	Ptgr2	prostaglandin reductase 2	1.062	0.2778	NA
76829	Dok5	docking protein 5	1.062	0.8021	0.9373
75698	Fam35a	family with sequence similarity 35, member A	1.062	0.311	NA
74617	Scpep1	serine carboxypeptidase 1	1.062	0.3788	NA
74320	Wdr33	WD repeat domain 33	1.062	0.4593	NA
74117	Actr3	ARP3 actin-related protein 3 homolog (yeast)	1.062	0.1571	NA
74104	Abcb6	ATP-binding cassette, sub-family B (MDR/TAP), member 6	1.062	0.4449	NA
72185	Dbndd1	dysbindin (dystrobrevin binding protein 1) domain containing 1	1.062	0.4176	NA
71856	Wfdc3	WAP four-disulfide core domain 3	1.062	0.738	0.9173
71448	Tmem80	transmembrane protein 80	1.062	0.2359	NA
71448	Tmem80	transmembrane protein 80	1.062	0.3264	NA
71446	Wrb	tryptophan rich basic protein	1.062	0.4864	0.8127
70423	Tspan15	tetraspanin 15	1.062	0.5208	0.8286
69150	Snx4	sorting nexin 4	1.062	0.3393	NA
68977	Haghl	hydroxyacylglutathione hydrolase-like	1.062	0.384	NA
68401	G6pc3	glucose 6 phosphatase, catalytic, 3	1.062	0.5251	0.8312
67615	Ube2r2	ubiquitin-conjugating enzyme E2R 2	1.062	0.1509	NA
67451	Pkp2	plakophilin 2	1.062	0.3991	NA
67417	Ears2	glutamyl-tRNA synthetase 2 (mitochondrial)(putative)	1.062	0.3838	NA
66880	Rsrc1	arginine/serine-rich coiled-coil 1	1.062	0.5423	0.8381
66704	Rbm4b	RNA binding motif protein 4B	1.062	0.4978	0.8193
66508	2400001E08Rik	RIKEN cDNA 2400001E08 gene	1.062	0.4949	0.8175
66354	Snw1	SNW domain containing 1	1.062	0.2336	NA
66191	Ier3ip1	immediate early response 3 interacting protein 1	1.062	0.2578	NA
64945	Cldn12	claudin 12	1.062	0.2245	NA
56876	Nelf	nasal embryonic LHRH factor	1.062	0.3415	NA
56351	Ptges3	prostaglandin E synthase 3 (cytosolic)	1.062	0.2884	NA
56273	Pex14	peroxisomal biogenesis factor 14	1.062	0.6132	0.8683
56228	Ube2j1	ubiquitin-conjugating enzyme E2, J1	1.062	0.3225	NA
52712	Zkscan6	zinc finger with KRAB and SCAN domains 6	1.062	0.2497	NA
52708	Zfp410	zinc finger protein 410	1.062	0.2102	NA
29805	Znhit2-ps	zinc finger, HIT domain containing 2, pseudogene	1.062	0.371	NA
27366	Txnl4a	thioredoxin-like 4A	1.062	0.133	NA
26891	Cops4	COP9 (constitutive photomorphogenic) homolog, subunit 4 (Arabidopsis thaliana)	1.062	0.1533	NA
23890	Gpr34	G protein-coupled receptor 34	1.062	0.5776	0.8514

22192	Ube2m	ubiquitin-conjugating enzyme E2M (UBC12 homolog, yeast)	1.062	0.6053	0.8645
21961	Tns1	tensin 1	1.062	0.346	NA
21807	Tsc22d1	TSC22 domain family, member 1	1.062	0.3793	NA
21371	Tbca	tubulin cofactor A	1.062	0.1249	NA
20346	Sema3a	sema domain, immunoglobulin domain (Ig), short basic domain, secreted, (semaphorin) 3A	1.062	0.3524	NA
18491	Pappa	pregnancy-associated plasma protein A	1.062	0.4069	NA
18174	Slc11a2	solute carrier family 11 (proton-coupled divalent metal ion transporters), member 2	1.062	0.4339	NA
18028	Nfib	nuclear factor I/B	1.062	0.3533	NA
15016	H2-Q5	histocompatibility 2, Q region locus 5	1.062	0.3597	NA
11933	Atp1b3	ATPase, Na+/K+ transporting, beta 3 polypeptide	1.062	0.5894	0.8575
640636	Gm7303	predicted gene 7303	1.061	0.5931	0.8587
547160	Gm14484	predicted gene 14484	1.061	0.7324	0.9157
329154	Ankrd44	ankyrin repeat domain 44	1.061	0.4083	NA
319945	Flad1	RFad1, flavin adenine dinucleotide synthetase, homolog (yeast)	1.061	0.5385	0.8364
319475	Zfp672	zinc finger protein 672	1.061	0.2557	NA
278240	Spin2	spindlin family, member 2	1.061	0.2562	NA
277414	Trp53i11	transformation related protein 53 inducible protein 11	1.061	0.5418	0.8379
242406	Rgp1	RGP1 retrograde golgi transport homolog (S. cerevisiae)	1.061	0.5465	0.8396
234267	Gpm6a	glycoprotein m6a	1.061	0.4838	0.8123
230908	Tardbp	TAR DNA binding protein	1.061	0.6441	0.8802
227674	Ddx31	DEAD/H (Asp-Glu-Ala-Asp/His) box polypeptide 31	1.061	0.1843	NA
218543	Srsf12	serine/arginine-rich splicing factor 12	1.061	0.5669	0.8481
216825	Usp22	ubiquitin specific peptidase 22	1.061	0.5702	0.8492
192191	Med9	mediator of RNA polymerase II transcription, subunit 9 homolog (yeast)	1.061	0.1862	NA
171580	Mical1	microtubule associated monoxygenase, calponin and LIM domain containing 1	1.061	0.5399	0.8369
140741	Gpr6	G protein-coupled receptor 6	1.061	0.6482	0.8816
140721	Caskin2	CASK-interacting protein 2	1.061	0.2429	NA
121022	Mrps6	mitochondrial ribosomal protein S6	1.061	0.3295	NA
114641	Rpl31	ribosomal protein L31	1.061	0.2346	NA
109672	Cyb5	cytochrome b-5	1.061	0.2979	NA
108934	BC024659	cDNA sequence BC024659	1.061	0.2488	NA
101095	Zfp282	zinc finger protein 282	1.061	0.6692	0.8904
99167	Ssx2ip	synovial sarcoma, X breakpoint 2 interacting protein	1.061	0.1731	NA
94190	Ophn1	oligophrenin 1	1.061	0.7787	0.9276
84094	Plvap	plasmalemma vesicle associated protein	1.061	0.6294	0.8745
79059	Nme3	non-metastatic cells 3, protein expressed in	1.061	0.5206	0.8286
78934	4930581F22Rik	RIKEN cDNA 4930581F22 gene	1.061	0.6484	0.8816
78829	Tsc22d4	TSC22 domain family, member 4	1.061	0.5882	0.8571
78284	Creb3l4	cAMP responsive element binding protein 3-like 4	1.061	0.3792	NA
77634	Snapc3	small nuclear RNA activating complex, polypeptide 3	1.061	0.6084	0.8657
76792	2410131K14Rik	RIKEN cDNA 2410131K14 gene	1.061	0.1655	NA
75089	Uhrf1bp1l	UHRF1 (ICBP90) binding protein 1-like	1.061	0.1339	NA
74374	Clec16a	C-type lectin domain family 16, member A	1.061	0.1448	NA
73112	3110003A17Rik	RIKEN cDNA 3110003A17 gene	1.061	0.385	NA
71984	Sars2	seryl-aminoacyl-tRNA synthetase 2	1.061	0.6333	0.8758
70532	5730433K22Rik	RIKEN cDNA 5730433K22 gene	1.061	0.5315	0.8339
70319	2600006K01Rik	RIKEN cDNA 2600006K01 gene	1.061	0.8287	0.945
69962	2810422O20Rik	RIKEN cDNA 2810422O20 gene	1.061	0.5066	0.8233
69745	Pold4	polymerase (DNA-directed), delta 4	1.061	0.5153	0.8277

69332	Lelp1	late cornified envelope-like proline-rich 1	1.061	0.7201	0.9106
69008	Cab39l	calcium binding protein 39-like	1.061	0.4978	0.8193
69008	Cab39l	calcium binding protein 39-like	1.061	0.2812	NA
68033	Cox19	COX19 cytochrome c oxidase assembly homolog (S. cerevisiae)	1.061	0.3705	NA
67263	Zswim6	zinc finger, SWIM domain containing 6	1.061	0.4983	0.8194
67145	Tomm34	translocase of outer mitochondrial membrane 34	1.061	0.3315	NA
66455	Cnpy4	canopy 4 homolog (zebrafish)	1.061	0.2086	NA
66101	Ppih	peptidyl prolyl isomerase H	1.061	0.2101	NA
66077	Aurkaip1	aurora kinase A interacting protein 1	1.061	0.338	NA
58859	Efemp2	epidermal growth factor-containing fibulin-like extracellular matrix protein 2	1.061	0.4423	NA
56612	Pfdn5	prefoldin 5	1.061	0.1517	NA
56457	Clptm1	cleft lip and palate associated transmembrane protein 1	1.061	0.6225	0.872
54151	Cyhr1	cysteine and histidine rich 1	1.061	0.416	NA
52076	Tmem38b	transmembrane protein 38B	1.061	0.7501	0.921
50798	Gne	glucosamine	1.061	0.4917	0.8156
30052	Pcsk1n	proprotein convertase subtilisin/kexin type 1 inhibitor	1.061	0.6681	0.8904
26427	Creb3l1	cAMP responsive element binding protein 3-like 1	1.061	0.4988	0.8197
23969	Pacsin1	protein kinase C and casein kinase substrate in neurons 1	1.061	0.6954	0.901
22388	Wdr1	WD repeat domain 1	1.061	0.4094	NA
22033	Traf5	TNF receptor-associated factor 5	1.061	0.3534	NA
21682	Tec	tec protein tyrosine kinase	1.061	0.4114	NA
20778	Scarb1	scavenger receptor class B, member 1	1.061	0.4169	NA
20692	Sparc	secreted acidic cysteine rich glycoprotein	1.061	0.5217	0.829
19847	Rnu1b6	U1b6 small nuclear RNA	1.061	0.2636	NA
19791	Rn18s	18S ribosomal RNA	1.061	0.575	0.8504
19305	Pex5	peroxisomal biogenesis factor 5	1.061	0.5759	0.8506
18242	Oat	ornithine aminotransferase	1.061	0.3117	NA
18196	Nsg1	neuron specific gene family member 1	1.061	0.2496	NA
18190	Nrxn2	neurexin II	1.061	0.6672	0.8904
18037	Nfkbie	nuclear factor of kappa light polypeptide gene enhancer in B-cells inhibitor, epsilon	1.061	0.2832	NA
17991	Ndufa2	NADH dehydrogenase (ubiquinone) 1 alpha subcomplex, 2	1.061	0.2672	NA
17187	Max	Max protein	1.061	0.1767	NA
16855	Lgals4	lectin, galactose binding, soluble 4	1.061	0.6524	0.8842
16539	Kcns2	K+ voltage-gated channel, subfamily S, 2	1.061	0.3249	NA
16408	Itgal	integrin alpha L	1.061	0.6914	0.8991
15566	Htr7	5-hydroxytryptamine (serotonin) receptor 7	1.061	0.363	NA
14252	Flot2	flotillin 2	1.061	0.3989	NA
14026	Evl	Ena-vasodilator stimulated phosphoprotein	1.061	0.2951	NA
13846	Ephb4	Eph receptor B4	1.061	0.7245	0.9126
13170	Dbp	D site albumin promoter binding protein	1.061	0.6098	0.8665
12558	Cdh2	cadherin 2	1.061	0.5693	0.8491
12361	Cask	calcium/calmodulin-dependent serine protein kinase (MAGUK family)	1.061	0.7065	0.9059
11671	Aldh3a2	aldehyde dehydrogenase family 3, subfamily A2	1.061	0.4869	0.8132
672214	Gm10136	predicted pseudogene 10136	1.06	0.2644	NA
629159	1700008J07Rik	RIKEN cDNA 1700008J07 gene	1.06	0.5293	0.8332
628431	Hmgb1-rs17	high mobility group box 1, related sequence 17	1.06	0.4182	NA
545013	Gm5797	predicted gene 5797	1.06	0.5156	0.8277
407812	Zfp941	zinc finger protein 941	1.06	0.349	NA
399548	Scn4b	sodium channel, type IV, beta	1.06	0.4688	NA

319207	Pgbd1	piggyBac transposable element derived 1	1.06	0.567	0.8482
276919	Gemin4	gem (nuclear organelle) associated protein 4	1.06	0.5102	0.8252
268739	Arhgef40	Rho guanine nucleotide exchange factor (GEF) 40	1.06	0.5941	0.8596
268449	Rpl23a	ribosomal protein L23A	1.06	0.3465	NA
257635	Sdsl	serine dehydratase-like	1.06	0.5123	0.8262
242126	Slc22a15	solute carrier family 22 (organic anion/cation transporter), member 15	1.06	0.3595	NA
240725	Sulf1	sulfatase 1	1.06	0.5023	0.8214
234740	Tmem231	transmembrane protein 231	1.06	0.7177	0.9096
231125	Zfyve28	zinc finger, FYVE domain containing 28	1.06	0.5259	0.832
229003	BC006779	cDNA sequence BC006779	1.06	0.444	NA
225631	Onecut2	one cut domain, family member 2	1.06	0.4125	NA
223690	Ankrd54	ankyrin repeat domain 54	1.06	0.413	NA
218440	Ankrd34b	ankyrin repeat domain 34B	1.06	0.7659	0.9251
218138	Gmds	GDP-mannose 4, 6-dehydratase	1.06	0.3246	NA
213350	Pddc1	Parkinson disease 7 domain containing 1	1.06	0.7028	0.9042
212974	Athl1	ATH1, acid trehalase-like 1 (yeast)	1.06	0.6735	0.8924
211151	Churc1	churchill domain containing 1	1.06	0.2221	NA
207728	Pde2a	phosphodiesterase 2A, cGMP-stimulated	1.06	0.4875	0.8135
207704	Gtpbp10	GTP-binding protein 10 (putative)	1.06	0.4837	NA
193043	Zfp3	zinc finger protein 3	1.06	0.3937	NA
114640	Pth2	parathyroid hormone 2	1.06	0.6265	0.873
109900	Asl	argininosuccinate lyase	1.06	0.5344	0.8349
108083	Pip4k2b	phosphatidylinositol-5-phosphate 4-kinase, type II, beta	1.06	0.4881	0.8138
108030	Lin7a	lin-7 homolog A (C. elegans)	1.06	0.2798	NA
103517	BB187676	expressed sequence BB187676	1.06	0.4866	0.8129
102339	Cog4	component of oligomeric golgi complex 4	1.06	0.291	NA
101631	Pwwp2b	PWWP domain containing 2B	1.06	0.4854	0.8127
99887	Tmem56	transmembrane protein 56	1.06	0.3378	NA
97908	Hist1h3g	histone cluster 1, H3g	1.06	0.6469	0.8812
80294	Pofut2	protein O-fucosyltransferase 2	1.06	0.7144	0.908
78651	Lsm6	LSM6 homolog, U6 small nuclear RNA associated (S. cerevisiae)	1.06	0.1465	NA
77701	Lcn12	lipocalin 12	1.06	0.649	0.882
76936	Hnrnpm	heterogeneous nuclear ribonucleoprotein M	1.06	0.5486	0.8408
76813	Armc6	armadillo repeat containing 6	1.06	0.5864	0.8558
75914	Exoc6b	exocyst complex component 6B	1.06	0.5202	0.8286
75516	Ttc32	tetratricopeptide repeat domain 32	1.06	0.3654	NA
75212	Rnf121	ring finger protein 121	1.06	0.2742	NA
74648	S100pbp	S100P binding protein	1.06	0.7004	0.9033
72168	Aifm3	apoptosis-inducing factor, mitochondrion-associated 3	1.06	0.5766	0.8509
72155	Cenpn	centromere protein N	1.06	0.5706	0.8492
70892	Ttll7	tubulin tyrosine ligase-like family, member 7	1.06	0.6341	0.8765
70638	Fam189a1	family with sequence similarity 189, member A1	1.06	0.4648	NA
69228	Zfp746	zinc finger protein 746	1.06	0.4562	NA
68634	Tm2d3	TM2 domain containing 3	1.06	0.2762	NA
68262	Agpat4	1-acylglycerol-3-phosphate O-acyltransferase 4 (lysophosphatidic acid acyltransferase, delta)	1.06	0.2724	NA
67997	Ddx59	DEAD (Asp-Glu-Ala-Asp) box polypeptide 59	1.06	0.2497	NA
67930	1700101I11Rik	RIKEN cDNA 1700101I11 gene	1.06	0.5171	0.8277
67886	Camsap1l1	calmodulin regulated spectrin-associated protein 1-like 1	1.06	0.2075	NA
67788	6330577E15Rik	RIKEN cDNA 6330577E15 gene	1.06	0.2623	NA

67781	Ilf2	interleukin enhancer binding factor 2	1.06	0.2275	NA
67510	Fam18b	family with sequence similarity 18, member B	1.06	0.5854	0.8551
67489	Ap4b1	adaptor-related protein complex AP-4, beta 1	1.06	0.1363	NA
67414	Mfn1	mitofusin 1	1.06	0.2803	NA
66999	Med28	mediator of RNA polymerase II transcription, subunit 28 homolog (yeast)	1.06	0.1284	NA
66949	Trim59	tripartite motif-containing 59	1.06	0.2528	NA
66878	Riok3	RIO kinase 3 (yeast)	1.06	0.2536	NA
66589	Ube2v1	ubiquitin-conjugating enzyme E2 variant 1	1.06	0.2085	NA
66552	2010106G01Rik	RIKEN cDNA 2010106G01 gene	1.06	0.3081	NA
59287	Ncstn	nicastrin	1.06	0.7231	0.9121
57914	Crlf2	cytokine receptor-like factor 2	1.06	0.2493	NA
56433	Vps29	vacuolar protein sorting 29 (S. pombe)	1.06	0.2484	NA
54683	Prdx5	peroxiredoxin 5	1.06	0.3991	NA
54325	Elovl1	elongation of very long chain fatty acids (FEN1/Elo2, SUR4/Elo3, yeast)-like 1	1.06	0.6524	0.8842
22042	Tfrc	transferrin receptor	1.06	0.4672	NA
21912	Tspan7	tetraspanin 7	1.06	0.4496	NA
20939	Sva	seminal vesicle antigen	1.06	0.7146	0.9081
20852	Stat6	signal transducer and activator of transcription 6	1.06	0.4809	NA
19044	Ppox	protoporphyrinogen oxidase	1.06	0.6087	0.8658
18950	Pnp	purine-nucleoside phosphorylase	1.06	0.1548	NA
16490	Kcna2	potassium voltage-gated channel, shaker-related subfamily, member 2	1.06	0.6256	0.8728
16452	Jak2	Janus kinase 2	1.06	0.4373	NA
16370	Irs4	insulin receptor substrate 4	1.06	0.7007	0.9033
14874	Gstz1	glutathione transferase zeta 1 (maleylacetoacetate isomerase)	1.06	0.527	0.8328
14766	Gpr56	G protein-coupled receptor 56	1.06	0.3503	NA
14470	Rabac1	Rab acceptor 1 (prenylated)	1.06	0.2998	NA
14468	Gbp1	guanylate binding protein 1	1.06	0.5913	0.8582
13809	Enpep	glutamyl aminopeptidase	1.06	0.4802	NA
13805	Eng	endoglin	1.06	0.4454	NA
13016	Ctbp1	C-terminal binding protein 1	1.06	0.2963	NA
12965	Crygb	crystallin, gamma B	1.06	0.9035	0.9717
12794	Cnih2	cornichon homolog 2 (Drosophila)	1.06	0.5912	0.8582
12330	Canx	calnexin	1.06	0.3269	NA
12283	Cab39	calcium binding protein 39	1.06	0.2452	NA
11982	Atp10a	ATPase, class V, type 10A	1.06	0.6158	0.8698
11459	Acta1	actin, alpha 1, skeletal muscle	1.06	0.578	0.8514
100042958	Gm4129	predicted gene 4129	1.059	0.5263	0.8323
100042786	Gm16381	predicted gene 16381	1.059	0.3797	NA
408067	Zfp874b	zinc finger protein 874b	1.059	0.2854	NA
380701	Slc47a2	solute carrier family 47, member 2	1.059	0.6316	0.8751
380684	Nefh	neurofilament, heavy polypeptide	1.059	0.4501	NA
330324	6430584L05	hypothetical protein 6430584L05	1.059	0.6706	0.8912
329384	Ptrh1	peptidyl-tRNA hydrolase 1 homolog (S. cerevisiae)	1.059	0.5298	0.8332
327749	Gm5079	predicted gene 5079	1.059	0.7023	0.9039
320226	4930473A06Rik	RIKEN cDNA 4930473A06 gene	1.059	0.7428	0.9186
269999	Orai3	ORAI calcium release-activated calcium modulator 3	1.059	0.5017	0.8208
252864	Dusp15	dual specificity phosphatase-like 15	1.059	0.518	0.828
243910	Nfkbid	nuclear factor of kappa light polypeptide gene enhancer in B-cells inhibitor, delta	1.059	0.6165	0.8698
242202	Pde5a	phosphodiesterase 5A, cGMP-specific	1.059	0.725	0.9126

237636	Npc1l1	NPC1-like 1	1.059	0.6276	0.8737
235379	Gldn	gliomedin	1.059	0.4583	NA
231014	9330182L06Rik	RIKEN cDNA 9330182L06 gene	1.059	0.2219	NA
230082	Nol6	nucleolar protein family 6 (RNA-associated)	1.059	0.5547	0.8444
228788	BC020535	cDNA sequence BC020535	1.059	0.1806	NA
228714	Csrp2bp	cysteine and glycine-rich protein 2 binding protein	1.059	0.2746	NA
225416	Gm4838	predicted gene 4838	1.059	0.315	NA
216505	Pik3ip1	phosphoinositide-3-kinase interacting protein 1	1.059	0.4739	NA
210582	Coq10a	coenzyme Q10 homolog A (yeast)	1.059	0.3163	NA
207425	Wdr11	WD repeat domain 11	1.059	0.4817	NA
108657	Rnpepl1	arginyl aminopeptidase (aminopeptidase B)-like 1	1.059	0.4572	NA
108068	Grm2	glutamate receptor, metabotropic 2	1.059	0.6942	0.9006
108067	Eif2b3	eukaryotic translation initiation factor 2B, subunit 3	1.059	0.3528	NA
104886	Rab15	RAB15, member RAS oncogene family	1.059	0.3107	NA
101831	C230052I12Rik	RIKEN cDNA C230052I12 gene	1.059	0.6108	0.8671
81877	Tnxb	tenascin XB	1.059	0.4472	NA
78920	Dlst	dihydrolipoamide S-succinyltransferase (E2 component of 2-oxo-glutarate complex)	1.059	0.3625	NA
77582	Mboat7	membrane bound O-acyltransferase domain containing 7	1.059	0.4722	NA
76246	Rtf1	Rtf1, Paf1/RNA polymerase II complex component, homolog (S. cerevisiae)	1.059	0.4661	NA
76178	6330578E17Rik	RIKEN cDNA 6330578E17 gene	1.059	0.4153	NA
75497	Fabp12	fatty acid binding protein 12	1.059	0.2204	NA
75430	3200002M19Rik	RIKEN cDNA 3200002M19 gene	1.059	0.1572	NA
74528	8430406I07Rik	RIKEN cDNA 8430406I07 gene	1.059	0.6752	0.8926
74330	Dnajc14	DnaJ (Hsp40) homolog, subfamily C, member 14	1.059	0.6648	0.8903
72357	2210016L21Rik	RIKEN cDNA 2210016L21 gene	1.059	0.4043	NA
71803	Slc25a18	solute carrier family 25 (mitochondrial carrier), member 18	1.059	0.6755	0.8928
70458	2610318N02Rik	RIKEN cDNA 2610318N02 gene	1.059	0.4996	0.8197
70434	2610201A13Rik	RIKEN cDNA 2610201A13 gene	1.059	0.2711	NA
69955	Fars2	phenylalanine-tRNA synthetase 2 (mitochondrial)	1.059	0.2088	NA
69816	2010001M09Rik	RIKEN cDNA 2010001M09 gene	1.059	0.5319	0.8339
68152	Fam133b	family with sequence similarity 133, member B	1.059	0.2885	NA
67959	Puf60	poly-U binding splicing factor 60	1.059	0.4307	NA
66874	1200014J11Rik	RIKEN cDNA 1200014J11 gene	1.059	0.5232	0.8298
66398	Commd5	COMM domain containing 5	1.059	0.1444	NA
66248	Alg5	asparagine-linked glycosylation 5 homolog (yeast, dolichyl-phosphate beta-glucosyltransferase)	1.059	0.2014	NA
66194	Pycrl	pyrroline-5-carboxylate reductase-like	1.059	0.4428	NA
66175	Mustn1	musculoskeletal, embryonic nuclear protein 1	1.059	0.8057	0.9386
64075	Smoc1	SPARC related modular calcium binding 1	1.059	0.5101	0.8251
56422	Hbs1l	Hbs1-like (S. cerevisiae)	1.059	0.3547	NA
54646	Ppp1r3f	protein phosphatase 1, regulatory (inhibitor) subunit 3F	1.059	0.2753	NA
52132	Ccdc97	coiled-coil domain containing 97	1.059	0.3907	NA
27058	Srp9	signal recognition particle 9	1.059	0.5005	0.8199
23934	Ly6h	lymphocyte antigen 6 complex, locus H	1.059	0.3136	NA
23872	Ets2	E26 avian leukemia oncogene 2, 3' domain	1.059	0.1751	NA
22224	Usp10	ubiquitin specific peptidase 10	1.059	0.4515	NA
22134	Tgoln1	trans-golgi network protein	1.059	0.4052	NA
22116	Tsks	testis-specific serine kinase substrate	1.059	0.5854	0.8551
22029	Traf1	TNF receptor-associated factor 1	1.059	0.5806	0.8527
21846	Tie1	tyrosine kinase with immunoglobulin-like and EGF-like domains 1	1.059	0.6549	0.8854

19337	Rab33a		RAB33A, member of RAS oncogene family	1.059	0.3395	NA
18998	Pou4f3		POU domain, class 4, transcription factor 3	1.059	0.7205	0.9109
18952		Sep-04	septin 4	1.059	0.3321	NA
18530	Pcdh8		protocadherin 8	1.059	0.3969	NA
15289	Hmgb1		high mobility group box 1	1.059	0.3755	NA
14706	Gng4		guanine nucleotide binding protein (G protein), gamma 4	1.059	0.689	0.898
13885	Esd		esterase D/formylglutathione hydrolase	1.059	0.4873	NA
13860	Eps8		epidermal growth factor receptor pathway substrate 8	1.059	0.2499	NA
13849	Ephx1		epoxide hydrolase 1, microsomal	1.059	0.6302	0.8748
13169	Dbnl		drebrin-like	1.059	0.4013	NA
12810	Coch		coagulation factor C homolog (Limulus polyphemus)	1.059	0.216	NA
12476	Cd151		CD151 antigen	1.059	0.4347	NA
12455	Ccnt1		cyclin T1	1.059	0.8024	0.9374
11881	Arsb		arylsulfatase B	1.059	0.3116	NA
100504362	Gm1987		predicted gene 1987	1.058	0.5659	0.8478
100043133	9130023H24Rik		RIKEN cDNA 9130023H24 gene	1.058	0.2604	NA
100041294	Gm3258		predicted gene 3258	1.058	0.2987	NA
627049	Zfp800		zinc finger protein 800	1.058	0.3704	NA
546052	Gm5908		predicted gene 5908	1.058	0.1972	NA
432825	Gm5458		predicted gene 5458	1.058	0.6166	0.8698
381334	Gal3st2		galactose-3-O-sulfotransferase 2	1.058	0.7823	0.9292
381126	Fam59a		family with sequence similarity 59, member A	1.058	0.359	NA
333315	Frem3		Fras1 related extracellular matrix protein 3	1.058	0.6519	0.8836
330474	Zc3h4		zinc finger CCCH-type containing 4	1.058	0.2339	NA
319945	Flad1		RFad1, flavin adenine dinucleotide synthetase, homolog (yeast)	1.058	0.5708	0.8492
319158	Hist1h4i		histone cluster 1, H4i	1.058	0.6201	0.8716
242669	Adc		arginine decarboxylase	1.058	0.6127	0.8681
242667	Dlgap3		discs, large (Drosophila) homolog-associated protein 3	1.058	0.7367	0.9169
241547	Harbi1		harbinger transposase derived 1	1.058	0.2368	NA
241112	Gm216		predicted gene 216	1.058	0.678	0.8932
238673	Zfp367		zinc finger protein 367	1.058	0.3165	NA
237615	Ankrd52		ankyrin repeat domain 52	1.058	0.611	0.8672
235604	Camkv		CaM kinase-like vesicle-associated	1.058	0.3649	NA
235441	Usp3		ubiquitin specific peptidase 3	1.058	0.1438	NA
232816	Zfp628		zinc finger protein 628	1.058	0.6444	0.8802
232157	Mobkl1b		MOB1, Mps One Binder kinase activator-like 1B (yeast)	1.058	0.5908	0.858
230025	Prdm13		PR domain containing 13	1.058	0.312	NA
226757	Wdr26		WD repeat domain 26	1.058	0.2477	NA
226122	Ubtd1		ubiquitin domain containing 1	1.058	0.6555	0.8858
223753	Cerk		ceramide kinase	1.058	0.354	NA
216157	ORF61		open reading frame 61	1.058	0.4055	NA
210146	Irgq		immunity-related GTPase family, Q	1.058	0.2199	NA
209837	Slc38a5		solute carrier family 38, member 5	1.058	0.6388	0.8779
110446	Acat1		acetyl-Coenzyme A acetyltransferase 1	1.058	0.2572	NA
102913	6330509M05Rik		RIKEN cDNA 6330509M05 gene	1.058	0.3978	NA
94191	Adarb2		adenosine deaminase, RNA-specific, B2	1.058	0.8375	0.9479
93881	Pcdhb10		protocadherin beta 10	1.058	0.4122	NA
80886	Senp3		SUMO/sentrin specific peptidase 3	1.058	0.4495	NA
78751	Zc3h6		zinc finger CCCH type containing 6	1.058	0.4439	NA

78070	Cpt1c	carnitine palmitoyltransferase 1c	1.058	0.4703	NA
75956	Srrm2	serine/arginine repetitive matrix 2	1.058	0.6233	0.8724
75565	Ccdc101	coiled-coil domain containing 101	1.058	0.4787	NA
75406	Ndufs7	NADH dehydrogenase (ubiquinone) Fe-S protein 7	1.058	0.4924	NA
75079	Zbtb49	zinc finger and BTB domain containing 49	1.058	0.571	0.8493
73825	Klraq1	KLRAQ motif containing 1	1.058	0.5414	0.8379
72978	Cnih3	cornichon homolog 3 (Drosophila)	1.058	0.5735	0.8501
72960	Top1mt	DNA topoisomerase 1, mitochondrial	1.058	0.4929	NA
72900	Ndufv2	NADH dehydrogenase (ubiquinone) flavoprotein 2	1.058	0.2177	NA
72479	Hsdl2	hydroxysteroid dehydrogenase like 2	1.058	0.2705	NA
71607	Snx20	sorting nexin 20	1.058	0.4759	NA
70419	2810408A11Rik	RIKEN cDNA 2810408A11 gene	1.058	0.4304	NA
69882	2010321M09Rik	RIKEN cDNA 2010321M09 gene	1.058	0.3121	NA
69788	1600023N17Rik	RIKEN cDNA 1600023N17 gene	1.058	0.4834	NA
69634	Clybl	citrate lyase beta like	1.058	0.5017	0.8208
69099	1810009N02Rik	RIKEN cDNA 1810009N02 gene	1.058	0.4113	NA
69038	1810006K21Rik	RIKEN cDNA 1810006K21 gene	1.058	0.2705	NA
68352	Aspdh	aspartate dehydrogenase domain containing	1.058	0.655	0.8854
68188	Sympk	symplekin	1.058	0.5726	0.8501
67851	1700021F05Rik	RIKEN cDNA 1700021F05 gene	1.058	0.2413	NA
67695	Ost4	oligosaccharyltransferase 4 homolog (S. cerevisiae)	1.058	0.5235	0.83
67020	Tmem88	transmembrane protein 88	1.058	0.6676	0.8904
66725	Lrrk2	leucine-rich repeat kinase 2	1.058	0.3884	NA
66108	Ndufa9	NADH dehydrogenase (ubiquinone) 1 alpha subcomplex, 9	1.058	0.3546	NA
59036	Dact1	dapper homolog 1, antagonist of beta-catenin (xenopus)	1.058	0.4702	NA
56741	Igdcc4	immunoglobulin superfamily, DCC subclass, member 4	1.058	0.4874	NA
56372	1110004F10Rik	RIKEN cDNA 1110004F10 gene	1.058	0.4481	NA
54128	Pmm2	phosphomannomutase 2	1.058	0.7387	0.9176
52855	Lair1	leukocyte-associated Ig-like receptor 1	1.058	0.3498	NA
52389	Gpr123	G protein-coupled receptor 123	1.058	0.6849	0.8959
50795	Sh3bgr	SH3-binding domain glutamic acid-rich protein	1.058	0.463	NA
50753	Fbxo8	F-box protein 8	1.058	0.3093	NA
27050	Rps3	ribosomal protein S3	1.058	0.4437	NA
20935	Surf6	surfeit gene 6	1.058	0.283	NA
20648	Snta1	syntrophin, acidic 1	1.058	0.5403	0.8374
20102	Rps4x	ribosomal protein S4, X-linked	1.058	0.3284	NA
19173	Psmb5	proteasome (prosome, macropain) subunit, beta type 5	1.058	0.4639	NA
19141	Lgmn	legumain	1.058	0.4355	NA
18103	Nme2	non-metastatic cells 2, protein (NM23B) expressed in	1.058	0.4165	NA
16578	Kif9	kinesin family member 9	1.058	0.5868	0.856
16403	Itga6	integrin alpha 6	1.058	0.8627	0.9574
15953	Ifi47	interferon gamma inducible protein 47	1.058	0.6299	0.8747
15229	Foxd1	forkhead box D1	1.058	0.5263	0.8323
15013	H2-Q2	histocompatibility 2, Q region locus 2	1.058	0.2418	NA
12315	Calm3	calmodulin 3	1.058	0.4237	NA
11927	Atox1	ATX1 (antioxidant protein 1) homolog 1 (yeast)	1.058	0.3686	NA
11816	Apoe	apolipoprotein E	1.058	0.5146	0.8273
11676	Aldoc	aldolase C, fructose-bisphosphate	1.058	0.435	NA
11596	Ager	advanced glycosylation end product-specific receptor	1.058	0.6141	0.869

666676	Gm8230	predicted gene 8230	1.057	0.5199	0.8285
380614	Intu	inturned planar cell polarity effector homolog (Drosophila)	1.057	0.2836	NA
353172	Gars	glycyl-tRNA synthetase	1.057	0.2808	NA
330050	Fam185a	family with sequence similarity 185, member A	1.057	0.4258	NA
320429	Trank1	tetratricopeptide repeat and ankyrin repeat containing 1	1.057	0.6398	0.8783
320183	Msrb3	methionine sulfoxide reductase B3	1.057	0.4645	NA
319466	9330199G10Rik	RIKEN cDNA 9330199G10 gene	1.057	0.8353	0.9466
270066	Slc35e1	solute carrier family 35, member E1	1.057	0.71	0.9069
237625	Pla2g3	phospholipase A2, group III	1.057	0.5641	0.8466
235907	Zfp71-rs1	zinc finger protein 71, related sequence	1.057	0.4444	NA
235633	Als2cl	ALS2 C-terminal like	1.057	0.7162	0.909
233071	Arhgap33	Rho GTPase activating protein 33	1.057	0.7763	0.9269
232201	Arhgap25	Rho GTPase activating protein 25	1.057	0.5902	0.8578
231549	Lrrc8d	leucine rich repeat containing 8D	1.057	0.4106	NA
230801	Pigv	phosphatidylinositol glycan anchor biosynthesis, class V	1.057	0.4739	NA
230103	Npr2	natriuretic peptide receptor 2	1.057	0.5106	0.8254
229512	Smg5	Smg-5 homolog, nonsense mediated mRNA decay factor (C. elegans)	1.057	0.5867	0.856
214239	A430105I19Rik	RIKEN cDNA A430105I19 gene	1.057	0.5628	0.8465
210933	Bai3	brain-specific angiogenesis inhibitor 3	1.057	0.278	NA
210573	Tmem151b	transmembrane protein 151B	1.057	0.1771	NA
210503	Zfp677	zinc finger protein 677	1.057	0.3694	NA
208258	Ankrd33	ankyrin repeat domain 33	1.057	0.6795	0.8944
192678	Rassf3	Ras association (RalGDS/AF-6) domain family member 3	1.057	0.5424	0.8382
171212	Galnt10	UDP-N-acetyl-alpha-D-galactosamine:polypeptide N-acetylgalactosaminyltransferase 10	1.057	0.4742	NA
114666	Krtap5-5	keratin associated protein 5-5	1.057	0.5116	0.826
108123	Napg	N-ethylmaleimide sensitive fusion protein attachment protein gamma	1.057	0.3317	NA
108077	Skiv2l	superkiller viralicidic activity 2-like (S. cerevisiae)	1.057	0.2108	NA
107817	Jmjd6	jumonji domain containing 6	1.057	0.4529	NA
102032	AI316807	expressed sequence AI316807	1.057	0.3172	NA
101604	E430018J23Rik	RIKEN cDNA E430018J23 gene	1.057	0.3822	NA
100535	Oas1d	2'-5' oligoadenylate synthetase 1D	1.057	0.8253	0.9444
99031	Osbpl6	oxysterol binding protein-like 6	1.057	0.375	NA
80913	Pum2	pumilio 2 (Drosophila)	1.057	0.7142	0.908
77630	Prdm8	PR domain containing 8	1.057	0.268	NA
76688	Arfrp1	ADP-ribosylation factor related protein 1	1.057	0.2951	NA
76411	1700019E19Rik	RIKEN cDNA 1700019E19 gene	1.057	0.2982	NA
76205	Stard3nl	STARD3 N-terminal like	1.057	0.3779	NA
76022	Gon4l	gon-4-like (C.elegans)	1.057	0.4827	NA
75561	1700016J18Rik	RIKEN cDNA 1700016J18 gene	1.057	0.4866	NA
74558	Gvin1	GTPase, very large interferon inducible 1	1.057	0.5861	0.8556
74116	Pi16	peptidase inhibitor 16	1.057	0.6185	0.8707
73813	Fam83e	family with sequence similarity 83, member E	1.057	0.7217	0.9118
73168	3110027N22Rik	RIKEN cDNA 3110027N22 gene	1.057	0.529	0.8332
72572	Spats2	spermatogenesis associated, serine-rich 2	1.057	0.3781	NA
72075	Ogfr	opioid growth factor receptor	1.057	0.5435	0.839
71957	Cpsf3l	cleavage and polyadenylation specific factor 3-like	1.057	0.3662	NA
71729	Rgs12	regulator of G-protein signaling 12	1.057	0.6342	0.8765
71472	Usp19	ubiquitin specific peptidase 19	1.057	0.4065	NA
70625	Med26	mediator complex subunit 26	1.057	0.4039	NA

70123	2210013O21Rik	RIKEN cDNA 2210013O21 gene	1.057	0.2881	NA
69480	Ttc9	tetratricopeptide repeat domain 9	1.057	0.2895	NA
68918	1190005I06Rik	RIKEN cDNA 1190005I06 gene	1.057	0.4458	NA
68366	Tmem129	transmembrane protein 129	1.057	0.2893	NA
68283	9530077C05Rik	RIKEN cDNA 9530077C05 gene	1.057	0.337	NA
68190	5330426P16Rik	RIKEN cDNA 5330426P16 gene	1.057	0.7213	0.9115
67978	Tctn2	tectonic family member 2	1.057	0.2975	NA
67770	5830433M19Rik	RIKEN cDNA 5830433M19 gene	1.057	0.3985	NA
67426	Adck3	aarF domain containing kinase 3	1.057	0.5037	NA
66965	Ctu2	cytosolic thiouridylase subunit 2 homolog (S. pombe)	1.057	0.563	0.8465
66849	Ppp1r2	protein phosphatase 1, regulatory (inhibitor) subunit 2	1.057	0.4004	NA
66251	Arfgap3	ADP-ribosylation factor GTPase activating protein 3	1.057	0.5629	0.8465
66082	Abhd6	abhydrolase domain containing 6	1.057	0.182	NA
66056	Zfp524	zinc finger protein 524	1.057	0.5162	0.8277
58799	Crbn	cereblon	1.057	0.2917	NA
56737	Alg2	asparagine-linked glycosylation 2 homolog (yeast, alpha-1,3-mannosyltransferase)	1.057	0.5192	0.8282
56461	Kcnip3	Kv channel interacting protein 3, calsenilin	1.057	0.4634	NA
56374	Tmem59	transmembrane protein 59	1.057	0.4687	NA
55949	Eef1b2	eukaryotic translation elongation factor 1 beta 2	1.057	0.3486	NA
54563	Nup210	nucleoporin 210	1.057	0.2542	NA
51798	Ech1	enoyl coenzyme A hydratase 1, peroxisomal	1.057	0.3685	NA
50927	Nasp	nuclear autoantigenic sperm protein (histone-binding)	1.057	0.2839	NA
50794	Klf13	Kruppel-like factor 13	1.057	0.6581	0.8867
30044	Opn4	opsin 4 (melanopsin)	1.057	0.6102	0.8667
24055	Sh3bp2	SH3-domain binding protein 2	1.057	0.4982	NA
24017	Rnf13	ring finger protein 13	1.057	0.2828	NA
23980	Pebp1	phosphatidylethanolamine binding protein 1	1.057	0.651	0.8831
23938	Map2k5	mitogen-activated protein kinase kinase 5	1.057	0.5309	0.8339
23830	Capn10	calpain 10	1.057	0.397	NA
22682	Zfand5	zinc finger, AN1-type domain 5	1.057	0.4522	NA
21968	Tom1	target of myb1 homolog (chicken)	1.057	0.3772	NA
21838	Thy1	thymus cell antigen 1, theta	1.057	0.5626	0.8465
20750	Spp1	secreted phosphoprotein 1	1.057	0.5054	NA
20607	Sstr3	somatostatin receptor 3	1.057	0.532	0.8339
19691	Recql	RecQ protein-like	1.057	0.3871	NA
18125	Nos1	nitric oxide synthase 1, neuronal	1.057	0.6757	0.8928
17117	Amacr	alpha-methylacyl-CoA racemase	1.057	0.4213	NA
16911	Lmo4	LIM domain only 4	1.057	0.6556	0.8858
13713	Elk3	ELK3, member of ETS oncogene family	1.057	0.8185	0.9425
13048	Cux2	cut-like homeobox 2	1.057	0.5098	0.825
12848	Cops2	COP9 (constitutive photomorphogenic) homolog, subunit 2 (Arabidopsis thaliana)	1.057	0.384	NA
12794	Cnih2	cornichon homolog 2 (Drosophila)	1.057	0.579	0.8519
12662	Chm	choroidermia	1.057	0.5179	0.828
12540	Cdc42	cell division cycle 42 homolog (S. cerevisiae)	1.057	0.2578	NA
12527	Cd9	CD9 antigen	1.057	0.148	NA
12326	Camk4	calcium/calmodulin-dependent protein kinase IV	1.057	0.3664	NA
100043726	Gm4610	predicted gene 4610	1.056	0.6762	0.8928
668605	Gm9265	predicted pseudogene 9265	1.056	0.5335	0.8343
626575	Gm6687	predicted gene 6687	1.056	0.7398	0.9179

433926	Lrrc8b	leucine rich repeat containing 8 family, member B	1.056	0.6066	0.8645
381802	Tsen2	tRNA splicing endonuclease 2 homolog (S. cerevisiae)	1.056	0.2278	NA
381236	Lipo1	lipase, member O1	1.056	0.2155	NA
381217	Fam189a2	family with sequence similarity 189, member A2	1.056	0.514	0.8271
330836	Slc7a6	solute carrier family 7 (cationic amino acid transporter, y+ system), member 6	1.056	0.4909	NA
320398	Lrig3	leucine-rich repeats and immunoglobulin-like domains 3	1.056	0.5068	NA
319931	A330032B11Rik	RIKEN cDNA A330032B11 gene	1.056	0.8843	0.9656
319924	Apba1	amyloid beta (A4) precursor protein binding, family A, member 1	1.056	0.3246	NA
271377	Zbtb11	zinc finger and BTB domain containing 11	1.056	0.7707	0.9257
269233	Fam171a1	family with sequence similarity 171, member A1	1.056	0.5402	0.8372
245468	Pnma3	paraneoplastic antigen MA3	1.056	0.1861	NA
244579	Tox3	TOX high mobility group box family member 3	1.056	0.303	NA
243376	Doxl2	diamine oxidase-like protein 2	1.056	0.749	0.9207
239102	Zfhx2	zinc finger homeobox 2	1.056	0.7641	0.925
237436	Gas2l3	growth arrest-specific 2 like 3	1.056	0.5536	0.844
235047	Zfp809	zinc finger protein 809	1.056	0.5292	0.8332
231997	Fkbp14	FK506 binding protein 14	1.056	0.542	0.8379
231713	Naa25	N(alpha)-acetyltransferase 25, NatB auxiliary subunit	1.056	0.4533	NA
229681	St7l	suppression of tumorigenicity 7-like	1.056	0.2383	NA
217201	Rundc1	RUN domain containing 1	1.056	0.2923	NA
216558	Ugp2	UDP-glucose pyrophosphorylase 2	1.056	0.2779	NA
214812	Zfp609	zinc finger protein 609	1.056	0.7133	0.9076
210853	Zfp947	zinc finger protein 947	1.056	0.3185	NA
210541	Gm581	predicted gene 581	1.056	0.744	0.9191
209268	Igsf1	immunoglobulin superfamily, member 1	1.056	0.6803	0.8948
170472	Recql5	RecQ protein-like 5	1.056	0.7915	0.9333
109242	Kif24	kinesin family member 24	1.056	0.5198	0.8284
104130	Ndufb11	NADH dehydrogenase (ubiquinone) 1 beta subcomplex, 11	1.056	0.5873	0.8564
99375	Cul4a	cullin 4A	1.056	0.3025	NA
93760	Arid1a	AT rich interactive domain 1A (SWI-like)	1.056	0.8039	0.938
81535	Sgpp1	sphingosine-1-phosphate phosphatase 1	1.056	0.2483	NA
77883	6030498E09Rik	RIKEN cDNA 6030498E09 gene	1.056	0.5708	0.8492
76952	Nt5c2	5'-nucleotidase, cytosolic II	1.056	0.3373	NA
76846	Rps9	ribosomal protein S9	1.056	0.2109	NA
76788	Klhdc10	kelch domain containing 10	1.056	0.4059	NA
76000	5033430I15Rik	RIKEN cDNA 5033430I15 gene	1.056	0.3058	NA
75608	Chmp4b	chromatin modifying protein 4B	1.056	0.4319	NA
72144	Slc37a3	solute carrier family 37 (glycerol-3-phosphate transporter), member 3	1.056	0.2026	NA
70552	Lrrc56	leucine rich repeat containing 56	1.056	0.5352	0.835
70394	Kptn	kaptin	1.056	0.51	NA
69726	Smyd3	SET and MYND domain containing 3	1.056	0.2776	NA
69104	Mar-05	membrane-associated ring finger (C3HC4) 5	1.056	0.2206	NA
69072	Ebna1bp2	EBNA1 binding protein 2	1.056	0.2862	NA
69046	Isca1	iron-sulfur cluster assembly 1 homolog (S. cerevisiae)	1.056	0.4763	NA
69035	Zdhhc3	zinc finger, DHHC domain containing 3	1.056	0.4194	NA
68904	Abhd13	abhydrolase domain containing 13	1.056	0.5029	NA
68137	Kdelr1	KDEL (Lys-Asp-Glu-Leu) endoplasmic reticulum protein retention receptor 1	1.056	0.3646	NA
67949	Mki67ip	Mki67 (FHA domain) interacting nucleolar phosphoprotein	1.056	0.1822	NA
67878	Tmem33	transmembrane protein 33	1.056	0.2966	NA

67702	Rnf149	ring finger protein 149	1.056	0.3965	NA
67701	Wfdc2	WAP four-disulfide core domain 2	1.056	0.7109	0.907
67131	Acbd4	acyl-Coenzyme A binding domain containing 4	1.056	0.3086	NA
66588	Cmpk1	cytidine monophosphate (UMP-CMP) kinase 1	1.056	0.438	NA
66511	2500003M10Rik	RIKEN cDNA 2500003M10 gene	1.056	0.3584	NA
66480	Rpl15	ribosomal protein L15	1.056	0.2933	NA
66385	Ppp1r7	protein phosphatase 1, regulatory (inhibitor) subunit 7	1.056	0.3269	NA
66151	Prr13	proline rich 13	1.056	0.4305	NA
66086	0610037P05Rik	RIKEN cDNA 0610037P05 gene	1.056	0.2028	NA
64658	Mrps25	mitochondrial ribosomal protein S25	1.056	0.333	NA
64051	Sv2a	synaptic vesicle glycoprotein 2 a	1.056	0.6064	0.8645
58186	Rad18	RAD18 homolog (S. cerevisiae)	1.056	0.5756	0.8506
57776	Ttyh1	tweety homolog 1 (Drosophila)	1.056	0.3334	NA
56334	Tmed2	transmembrane emp24 domain trafficking protein 2	1.056	0.3271	NA
56207	Uchl5	ubiquitin carboxyl-terminal esterase L5	1.056	0.217	NA
55946	Ap3m1	adaptor-related protein complex 3, mu 1 subunit	1.056	0.35	NA
53605	Nap1l1	nucleosome assembly protein 1-like 1	1.056	0.3091	NA
52892	Sco1	SCO cytochrome oxidase deficient homolog 1 (yeast)	1.056	0.3844	NA
30947	Adat1	adenosine deaminase, tRNA-specific 1	1.056	0.6958	0.9013
26936	Mprip	myosin phosphatase Rho interacting protein	1.056	0.4729	NA
24047	Ccl19	chemokine (C-C motif) ligand 19	1.056	0.7496	0.9209
22185	U2af2	U2 small nuclear ribonucleoprotein auxiliary factor (U2AF) 2	1.056	0.7007	0.9033
20851	Stat5b	signal transducer and activator of transcription 5B	1.056	0.4752	NA
20463	Cox7a2l	cytochrome c oxidase subunit VIIa polypeptide 2-like	1.056	0.35	NA
20409	Ostf1	osteoclast stimulating factor 1	1.056	0.5259	0.832
20055	Rps16	ribosomal protein S16	1.056	0.4665	NA
19384	Ran	RAN, member RAS oncogene family	1.056	0.2119	NA
19348	Kif20a	kinesin family member 20A	1.056	0.4647	NA
19025	Ctsa	cathepsin A	1.056	0.2068	NA
18674	Slc25a3	solute carrier family 25 (mitochondrial carrier, phosphate carrier), member 3	1.056	0.5183	0.8282
16648	Kpna3	karyopherin (importin) alpha 3	1.056	0.2682	NA
16211	Kpnb1	karyopherin (importin) beta 1	1.056	0.3731	NA
12896	Cpt2	carnitine palmitoyltransferase 2	1.056	0.784	0.9302
12831	Col5a1	collagen, type V, alpha 1	1.056	0.6957	0.9013
12616	Cenpb	centromere protein B	1.056	0.3106	NA
11306	Abcb7	ATP-binding cassette, sub-family B (MDR/TAP), member 7	1.056	0.5455	0.839
100046282	LOC100046282	selenoprotein K pseudogene	1.055	0.2176	NA
100039757	Gm10153	predicted gene 10153	1.055	0.745	0.9192
100039316	Gm9843	predicted gene 9843	1.055	0.4588	NA
668110	Syce1l	synaptonemal complex central element protein 1 like	1.055	0.461	NA
636931	Trim71	tripartite motif-containing 71	1.055	0.6671	0.8904
625929	Gm6636	predicted gene 6636	1.055	0.5778	0.8514
434437	Amt	aminomethyltransferase	1.055	0.6631	0.8895
380686	Cnrip1	cannabinoid receptor interacting protein 1	1.055	0.3013	NA
332175	Zdhhc23	zinc finger, DHHC domain containing 23	1.055	0.3969	NA
328788	Gm749	predicted gene 749	1.055	0.8175	0.942
320080	Zbtb39	zinc finger and BTB domain containing 39	1.055	0.4159	NA
319370	Fam100b	family with sequence similarity 100, member B	1.055	0.356	NA
277744	Gm694	predicted gene 694	1.055	0.582	0.8533

270190	Ephb1	Eph receptor B1	1.055	0.3603	NA
269788	Lhfpl4	lipoma HMGIC fusion partner-like protein 4	1.055	0.1531	NA
243634	Ano2	anoctamin 2	1.055	0.4325	NA
225929	Patl1	protein associated with topoisomerase II homolog 1 (yeast)	1.055	0.4835	NA
224671	Btbd9	BTB (POZ) domain containing 9	1.055	0.4225	NA
223881	Rnd1	Rho family GTPase 1	1.055	0.2707	NA
223732	Ldoc1l	leucine zipper, down-regulated in cancer 1-like	1.055	0.4907	NA
217715	Eif2b2	eukaryotic translation initiation factor 2B, subunit 2 beta	1.055	0.3594	NA
215707	Ccdc92	coiled-coil domain containing 92	1.055	0.4427	NA
214917	Fam173a	family with sequence similarity 173, member A	1.055	0.2902	NA
212276	Zfp748	zinc finger protein 748	1.055	0.4478	NA
192652	Wdr81	WD repeat domain 81	1.055	0.3787	NA
192173	Fam195b	family with sequence similarity 195, member B	1.055	0.5284	0.8332
110821	Pcca	propionyl-Coenzyme A carboxylase, alpha polypeptide	1.055	0.424	NA
107976	Bre	brain and reproductive organ-expressed protein	1.055	0.3114	NA
107702	Rnh1	ribonuclease/angiogenin inhibitor 1	1.055	0.3729	NA
105559	Mbnl2	muscleblind-like 2	1.055	0.5819	0.8533
100223	9630041G16Rik	RIKEN cDNA 9630041G16 gene	1.055	0.1857	NA
94190	Ophn1	oligophrenin 1	1.055	0.3351	NA
80987	Nckipsd	NCK interacting protein with SH3 domain	1.055	0.6557	0.8858
76630	Stambpl1	STAM binding protein like 1	1.055	0.339	NA
76100	5830454E08Rik	RIKEN cDNA 5830454E08 gene	1.055	0.4741	NA
75599	Pcdh1	protocadherin 1	1.055	0.6766	0.8929
75472	1700009P17Rik	RIKEN cDNA 1700009P17 gene	1.055	0.4675	NA
75086	4930520P13Rik	RIKEN cDNA 4930520P13 gene	1.055	0.6206	0.8718
74194	Rnd3	Rho family GTPase 3	1.055	0.3313	NA
72748	Hdhd3	haloacid dehalogenase-like hydrolase domain containing 3	1.055	0.5509	0.8427
71710	Lrrcc1	leucine rich repeat and coiled-coil domain containing 1	1.055	0.4502	NA
71310	Tbc1d9	TBC1 domain family, member 9	1.055	0.4937	NA
70967	4931408A02Rik	RIKEN cDNA 4931408A02 gene	1.055	0.2605	NA
69234	Zfp688	zinc finger protein 688	1.055	0.2849	NA
68675	Fam172a	family with sequence similarity 172, member A	1.055	0.194	NA
67923	Tceb1	transcription elongation factor B (SIII), polypeptide 1	1.055	0.2415	NA
67419	3632451O06Rik	RIKEN cDNA 3632451O06 gene	1.055	0.2656	NA
67287	Parp6	poly (ADP-ribose) polymerase family, member 6	1.055	0.4693	NA
67286	Rabl5	RAB, member of RAS oncogene family-like 5	1.055	0.5593	0.8459
67059	Ola1	Obg-like ATPase 1	1.055	0.5568	0.845
66044	Dtd1	D-tyrosyl-tRNA deacylase 1 homolog (S. cerevisiae)	1.055	0.3931	NA
59020	Pdzk1	PDZ domain containing 1	1.055	0.5835	0.854
56443	Arpc1a	actin related protein 2/3 complex, subunit 1A	1.055	0.3757	NA
56386	B4galt6	UDP-Gal:betaGlcNAc beta 1,4-galactosyltransferase, polypeptide 6	1.055	0.4288	NA
56249	Actr8	ARP8 actin-related protein 8 homolog (S. cerevisiae)	1.055	0.2995	NA
55992	Trim3	tripartite motif-containing 3	1.055	0.6453	0.8806
52468	Ctdsp2	CTD (carboxy-terminal domain, RNA polymerase II, polypeptide A) small phosphatase 2	1.055	0.4282	NA
28015	Grinl1a	glutamate receptor, ionotropic, N-methyl D-aspartate-like 1A	1.055	0.1493	NA
27373	Csnk1e	casein kinase 1, epsilon	1.055	0.3505	NA
26374	Rfwd2	ring finger and WD repeat domain 2	1.055	0.255	NA
22059	Trp53	transformation related protein 53	1.055	0.7146	0.9081
21917	Tmpo	thymopoietin	1.055	0.2897	NA

21849	Trim28	tripartite motif-containing 28	1.055	0.6168	0.8698
21770	Ppp2r5d	protein phosphatase 2, regulatory subunit B (B56), delta isoform	1.055	0.5709	0.8492
21389	Tbx6	T-box 6	1.055	0.5731	0.8501
20913	Stxbp4	syntaxin binding protein 4	1.055	0.5584	0.8459
20913	Stxbp4	syntaxin binding protein 4	1.055	0.5942	0.8596
20832	Ssr4	signal sequence receptor, delta	1.055	0.2096	NA
20351	Sema4a	sema domain, immunoglobulin domain (Ig), transmembrane domain (TM) and short cytoplasmic domain, (semaphorin) 4A	1.055	0.2536	NA
20182	Rxrb	retinoid X receptor beta	1.055	0.5112	NA
19944	Rpl29	ribosomal protein L29	1.055	0.4693	NA
19245	Ptp4a3	protein tyrosine phosphatase 4a3	1.055	0.6592	0.8873
18771	Pknox1	Pbx/knotted 1 homeobox	1.055	0.579	0.8519
18599	Padi1	peptidyl arginine deiminase, type I	1.055	0.726	0.9126
18115	Nnt	nicotinamide nucleotide transhydrogenase	1.055	0.4527	NA
16977	Lrrc23	leucine rich repeat containing 23	1.055	0.6439	0.8802
15900	Irf8	interferon regulatory factor 8	1.055	0.5709	0.8492
15199	Hebp1	heme binding protein 1	1.055	0.4291	NA
14600	Ghr	growth hormone receptor	1.055	0.3937	NA
12121	Bicd1	bicaudal D homolog 1 (Drosophila)	1.055	0.5759	0.8506
12042	Bcl10	B-cell leukemia/lymphoma 10	1.055	0.4178	NA
11856	Arhgap6	Rho GTPase activating protein 6	1.055	0.5686	0.8487
100047292	LOC100047292	pleckstrin homology domain-containing family A member 7-like	1.054	0.3686	NA
100041686	Gm15427	predicted pseudogene 15427	1.054	0.5338	0.8343
100041479	Gm3363	predicted gene 3363	1.054	0.7544	0.9216
100039252	Gm12693	predicted gene 12693	1.054	0.1889	NA
667572	Gm8709	glyceraldehyde-3-phosphate dehydrogenase pseudogene	1.054	0.6763	0.8928
638833	Gm7251	glyceraldehyde-3-phosphate dehydrogenase pseudogene	1.054	0.7569	0.9225
383712	Gm13637	predicted gene 13637	1.054	0.3291	NA
360013	Myo18a	myosin XVIIIA	1.054	0.6357	0.8771
353170	Txlng	taxilin gamma	1.054	0.5036	NA
331461	Il1rapl1	interleukin 1 receptor accessory protein-like 1	1.054	0.3232	NA
319832	Tmem229a	transmembrane protein 229A	1.054	0.6037	0.8644
319469	A230056J06Rik	RIKEN cDNA A230056J06 gene	1.054	0.6446	0.8802
270757	Bpil2	bactericidal/permeability-increasing protein-like 2	1.054	0.7019	0.9039
268480	Rapgefl1	Rap guanine nucleotide exchange factor (GEF)-like 1	1.054	0.4963	NA
252870	Usp7	ubiquitin specific peptidase 7	1.054	0.2902	NA
243961	Shank1	SH3/ankyrin domain gene 1	1.054	0.3317	NA
243771	Parp12	poly (ADP-ribose) polymerase family, member 12	1.054	0.3914	NA
243373	AI854703	expressed sequence AI854703	1.054	0.6033	0.8643
242748	Ptchd2	patched domain containing 2	1.054	0.5924	0.8584
235041	Kank2	KN motif and ankyrin repeat domains 2	1.054	0.2647	NA
234309	Cbr4	carbonyl reductase 4	1.054	0.4048	NA
234159	Gm4889	predicted gene 4889	1.054	0.4018	NA
231841	AA881470	EST AA881470	1.054	0.6116	0.8673
228807	Zfp341	zinc finger protein 341	1.054	0.3329	NA
227700	Sh3glb2	SH3-domain GRB2-like endophilin B2	1.054	0.3088	NA
226356	Gm101	predicted gene 101	1.054	0.7704	0.9257
217866	Cdc42bpb	CDC42 binding protein kinase beta	1.054	0.6979	0.9025
211739	Vstm2a	V-set and transmembrane domain containing 2A	1.054	0.3662	NA
211286	Cln5	ceroid-lipofuscinosis, neuronal 5	1.054	0.2325	NA

192236	Hps1	Hermansky-Pudlak syndrome 1 homolog (human)	1.054	0.74	0.9179
109929	Zbtb25	zinc finger and BTB domain containing 25	1.054	0.3011	NA
107605	Rdh1	retinol dehydrogenase 1 (all trans)	1.054	0.6952	0.901
106564	Ppcs	phosphopantothenoylcysteine synthetase	1.054	0.3126	NA
105083	Pelo	pelota homolog (Drosophila)	1.054	0.3756	NA
100764	1110008J03Rik	RIKEN cDNA 1110008J03 gene	1.054	0.6004	0.8635
93742	Pard3	par-3 (partitioning defective 3) homolog (C. elegans)	1.054	0.6043	0.8644
84004	Mcam	melanoma cell adhesion molecule	1.054	0.6428	0.8795
78771	Mctp1	multiple C2 domains, transmembrane 1	1.054	0.2163	NA
78541	Asb8	ankyrin repeat and SOCS box-containing 8	1.054	0.3385	NA
74637	Shpk	sedoheptulokinase	1.054	0.5078	NA
73610	Zfp433	RIKEN cDNA 1700123A16 gene	1.054	0.1818	NA
72935	Ddx41	DEAD (Asp-Glu-Ala-Asp) box polypeptide 41	1.054	0.6865	0.8966
72549	Reep4	receptor accessory protein 4	1.054	0.7289	0.9141
72042	Cotl1	coactosin-like 1 (Dictyostelium)	1.054	0.5394	0.8366
72007	Fndc3b	fibronectin type III domain containing 3B	1.054	0.4182	NA
69723	Rpain	RPA interacting protein	1.054	0.281	NA
69382	1700024P04Rik	RIKEN cDNA 1700024P04 gene	1.054	0.7329	0.9157
69104	Mar-05	membrane-associated ring finger (C3HC4) 5	1.054	0.5092	NA
68975	Med27	mediator complex subunit 27	1.054	0.4186	NA
68971	1500001M20Rik	RIKEN cDNA 1500001M20 gene	1.054	0.1398	NA
68969	Eif1b	eukaryotic translation initiation factor 1B	1.054	0.2969	NA
68034	Fam122a	family with sequence similarity 122, member A	1.054	0.692	0.8991
67671	Rpl38	ribosomal protein L38	1.054	0.5741	0.8503
67242	Gemin6	gem (nuclear organelle) associated protein 6	1.054	0.5022	NA
67062	Mcart6	mitochondrial carrier triple repeat 6	1.054	0.7251	0.9126
66943	Pqlc1	PQ loop repeat containing 1	1.054	0.2623	NA
64707	Suv39h2	suppressor of variegation 3-9 homolog 2 (Drosophila)	1.054	0.5761	0.8506
59047	Pnkp	polynucleotide kinase 3'- phosphatase	1.054	0.4167	NA
56458	Foxo1	forkhead box O1	1.054	0.3169	NA
56422	Hbs1l	Hbs1-like (S. cerevisiae)	1.054	0.3764	NA
54645	Gripap1	GRIP1 associated protein 1	1.054	0.4791	NA
54369	Nme6	non-metastatic cells 6, protein expressed in (nucleoside-diphosphate kinase)	1.054	0.4938	NA
54151	Cyhr1	cysteine and histidine rich 1	1.054	0.193	NA
52696	Zwint	ZW10 interactor	1.054	0.415	NA
26444	Psma7	proteasome (prosome, macropain) subunit, alpha type 7	1.054	0.4166	NA
24004	Rai2	retinoic acid induced 2	1.054	0.4405	NA
23849	Klf6	Kruppel-like factor 6	1.054	0.4141	NA
23805	Apc2	adenomatosis polyposis coli 2	1.054	0.665	0.8903
22687	Zfp259	zinc finger protein 259	1.054	0.3056	NA
22184	Zrsr2	zinc finger (CCCH type), RNA binding motif and serine/arginine rich 2	1.054	0.3619	NA
22003	Tpm1	tropomyosin 1, alpha	1.054	0.4696	NA
20677	Sox4	SRY-box containing gene 4	1.054	0.6678	0.8904
20091	Rps3a	ribosomal protein S3A	1.054	0.7652	0.9251
20016	Polr1c	polymerase (RNA) I polypeptide C	1.054	0.1398	NA
19357	Rad21	RAD21 homolog (S. pombe)	1.054	0.2982	NA
19317	Qk	quaking	1.054	0.3486	NA
19167	Psma3	proteasome (prosome, macropain) subunit, alpha type 3	1.054	0.2176	NA
19139	Prps1	phosphoribosyl pyrophosphate synthetase 1	1.054	0.2872	NA

19024	Ppfibp2	PTPRF interacting protein, binding protein 2 (liprin beta 2)	1.054	0.7143	0.908
18583	Pde7a	phosphodiesterase 7A	1.054	0.2433	NA
18577	Pde4a	phosphodiesterase 4A, cAMP specific	1.054	0.5196	NA
18526	Pcdh10	protocadherin 10	1.054	0.3523	NA
17193	Mbd4	methyl-CpG binding domain protein 4	1.054	0.5434	0.839
16440	Itpr3	inositol 1,4,5-triphosphate receptor 3	1.054	0.5309	0.8339
15353	Hmg20b	high mobility group 20 B	1.054	0.2656	NA
14169	Fgf14	fibroblast growth factor 14	1.054	0.5242	NA
13543	Dvl2	dishevelled 2, dsh homolog (Drosophila)	1.054	0.629	0.8745
12815	Col11a2	collagen, type XI, alpha 2	1.054	0.7046	0.9049
12487	Cd28	CD28 antigen	1.054	0.7708	0.9257
100504872	LOC100504872	60S ribosomal protein L32-like	1.053	0.3988	NA
666113	Gm7935	predicted pseudogene 7935	1.053	0.805	0.9381
404634	H2afy2	H2A histone family, member Y2	1.053	0.3114	NA
387343	Tas2r109	taste receptor, type 2, member 109	1.053	0.779	0.9278
382423	Atxn7l3b	ataxin 7-like 3B	1.053	0.3458	NA
380753	Atxn7l1	ataxin 7-like 1	1.053	0.7392	0.9177
330941	AI593442	expressed sequence AI593442	1.053	0.3893	NA
320827	C530008M17Rik	RIKEN cDNA C530008M17 gene	1.053	0.4589	NA
320127	Dgki	diacylglycerol kinase, iota	1.053	0.6255	0.8728
319613	Sybu	syntabulin (syntaxin-interacting)	1.053	0.3358	NA
244144	Usp35	ubiquitin specific peptidase 35	1.053	0.6778	0.8931
243382	Ppm1k	protein phosphatase 1K (PP2C domain containing)	1.053	0.3467	NA
239122	Setdb2	SET domain, bifurcated 2	1.053	0.8161	0.9416
228869	Ncoa5	nuclear receptor coactivator 5	1.053	0.3959	NA
225115	Svil	supervillin	1.053	0.5769	0.851
224613	Flywch1	FLYWCH-type zinc finger 1	1.053	0.6536	0.8848
214951	Rhbdl1	rhomboid, veinlet-like 1 (Drosophila)	1.053	0.5869	0.8561
212980	Slc45a3	solute carrier family 45, member 3	1.053	0.7013	0.9037
212503	Paox	polyamine oxidase (exo-N4-amino)	1.053	0.5329	NA
211383	Fam123c	family with sequence similarity 123, member C	1.053	0.6703	0.8912
210530	Leprel1	leprecan-like 1	1.053	0.5952	0.8603
208104	Mlxip	MLX interacting protein	1.053	0.6621	0.8894
109857	Cbr3	carbonyl reductase 3	1.053	0.2587	NA
109054	Pfdn4	prefoldin 4	1.053	0.5665	0.8481
101985	AA960436	expressed sequence AA960436	1.053	0.6527	0.8842
97550	C130081A10Rik	RIKEN cDNA C130081A10 gene	1.053	0.4154	NA
83679	Pde4dip	phosphodiesterase 4D interacting protein (myomegalin)	1.053	0.259	NA
80795	Selk	selenoprotein K	1.053	0.298	NA
78926	Gas2l1	growth arrest-specific 2 like 1	1.053	0.4461	NA
78925	Srd5a1	steroid 5 alpha-reductase 1	1.053	0.4551	NA
77407	Rab35	RAB35, member RAS oncogene family	1.053	0.302	NA
75036	4930488B01Rik	RIKEN cDNA 4930488B01 gene	1.053	0.2735	NA
74392	Specc1l	sperm antigen with calponin homology and coiled-coil domains 1-like	1.053	0.534	NA
74363	4931430N09Rik	RIKEN cDNA 4931430N09 gene	1.053	0.5136	NA
72865	Cxx1c	CAAX box 1 homolog C (human)	1.053	0.4871	NA
71778	Klhl5	kelch-like 5 (Drosophila)	1.053	0.1664	NA
71766	Raver1	ribonucleoprotein, PTB-binding 1	1.053	0.7262	0.9126
71365	Pdss2	prenyl (solanesyl) diphosphate synthase, subunit 2	1.053	0.5098	NA

69875	Ndufa11	NADH dehydrogenase (ubiquinone) 1 alpha subcomplex 11	1.053	0.3469	NA
69431	1700022N22Rik	RIKEN cDNA 1700022N22 gene	1.053	0.683	0.8956
68653	Samm50	sorting and assembly machinery component 50 homolog (S. cerevisiae)	1.053	0.3071	NA
68644	Abhd14a	abhydrolase domain containing 14A	1.053	0.6896	0.8984
68567	Cgref1	cell growth regulator with EF hand domain 1	1.053	0.3947	NA
68083	Pak1ip1	PAK1 interacting protein 1	1.053	0.3642	NA
67815	Sec14l2	SEC14-like 2 (S. cerevisiae)	1.053	0.5367	0.8354
67737	Ttc39d	tetratricopeptide repeat domain 39D	1.053	0.8004	0.9366
67706	Tmem179b	transmembrane protein 179B	1.053	0.6596	0.8876
67468	Mmd	monocyte to macrophage differentiation-associated	1.053	0.2858	NA
67453	Slc25a46	solute carrier family 25, member 46	1.053	0.566	0.8478
67087	Ctnnbip1	catenin beta interacting protein 1	1.053	0.2571	NA
66854	Trim35	tripartite motif-containing 35	1.053	0.2092	NA
66367	2310022A10Rik	RIKEN cDNA 2310022A10 gene	1.053	0.4478	NA
66212	Sec61b	Sec61 beta subunit	1.053	0.3102	NA
59042	Cope	coatomer protein complex, subunit epsilon	1.053	0.5445	0.839
58994	Smpd3	sphingomyelin phosphodiesterase 3, neutral	1.053	0.4303	NA
57808	Rpl35a	ribosomal protein L35A	1.053	0.4143	NA
57750	Wdr12	WD repeat domain 12	1.053	0.2647	NA
57320	Park7	Parkinson disease (autosomal recessive, early onset) 7	1.053	0.2328	NA
56444	Actr10	ARP10 actin-related protein 10 homolog (S. cerevisiae)	1.053	0.2042	NA
56364	Zmym3	zinc finger, MYM-type 3	1.053	0.4441	NA
52589	Ncald	neurocalcin delta	1.053	0.3025	NA
50887	Hmgn5	high-mobility group nucleosome binding domain 5	1.053	0.3309	NA
26377	Dapp1	dual adaptor for phosphotyrosine and 3-phosphoinositides 1	1.053	0.515	NA
23965	Odz3	odd Oz/ten-m homolog 3 (Drosophila)	1.053	0.6717	0.8918
22379	Fmnl3	formin-like 3	1.053	0.673	0.8924
21834	Thrb	thyroid hormone receptor beta	1.053	0.592	0.8584
21664	Phlda1	pleckstrin homology-like domain, family A, member 1	1.053	0.6154	0.8696
19655	Rbmx	RNA binding motif protein, X chromosome	1.053	0.5131	NA
19173	Psmb5	proteasome (prosome, macropain) subunit, beta type 5	1.053	0.5543	0.8443
18806	Pld2	phospholipase D2	1.053	0.3871	NA
18784	Pla2g5	phospholipase A2, group V	1.053	0.6421	0.8792
18749	Prkacb	protein kinase, cAMP dependent, catalytic, beta	1.053	0.5463	0.8395
18546	Pcp4	Purkinje cell protein 4	1.053	0.1552	NA
17220	Mcm7	minichromosome maintenance deficient 7 (S. cerevisiae)	1.053	0.4255	NA
16001	Igf1r	insulin-like growth factor I receptor	1.053	0.4855	NA
15384	Hnrnpab	heterogeneous nuclear ribonucleoprotein A/B	1.053	0.4237	NA
14559	Gdf1	growth differentiation factor 1	1.053	0.3736	NA
14164	Fgf1	fibroblast growth factor 1	1.053	0.6893	0.8982
13205	Ddx3x	DEAD/H (Asp-Glu-Ala-Asp/His) box polypeptide 3, X-linked	1.053	0.3563	NA
12847	Copa	coatomer protein complex subunit alpha	1.053	0.4503	NA
12663	Chml	choroideremia-like	1.053	0.4039	NA
12319	Car8	carbonic anhydrase 8	1.053	0.769	0.9255
11863	Arnt	aryl hydrocarbon receptor nuclear translocator	1.053	0.5694	0.8491
11695	Alx4	aristaless-like homeobox 4	1.053	0.7771	0.9272
11490	Adam15	a disintegrin and metallopeptidase domain 15 (metargidin)	1.053	0.3735	NA
100306953	4122401K19Rik	RIKEN cDNA 4122401K19 gene	1.052	0.5413	NA
100039286	Gm2138	predicted gene 2138	1.052	0.5364	NA

433375	Creg1	cellular repressor of E1A-stimulated genes 1	1.052	0.439	NA
332359	Tigd3	tigger transposable element derived 3	1.052	0.5811	0.8527
330286	D630045J12Rik	RIKEN cDNA D630045J12 gene	1.052	0.4084	NA
329910	Acot11	acyl-CoA thioesterase 11	1.052	0.3461	NA
329739	Fam102b	family with sequence similarity 102, member B	1.052	0.6235	0.8724
326618	Tpm4	tropomyosin 4	1.052	0.3432	NA
320683	Zfp629	zinc finger protein 629	1.052	0.2148	NA
258319	Olfr187	olfactory receptor 187	1.052	0.8325	0.9457
234839	Fam38a	family with sequence similarity 38, member A	1.052	0.7004	0.9033
234723	Txnl4b	thioredoxin-like 4B	1.052	0.5527	0.8436
234366	Gatad2a	GATA zinc finger domain containing 2A	1.052	0.5596	0.8459
233806	Tmem159	transmembrane protein 159	1.052	0.4439	NA
224019	Tmem191c	transmembrane protein 191C	1.052	0.3331	NA
208440	Dip2c	DIP2 disco-interacting protein 2 homolog C (Drosophila)	1.052	0.4042	NA
171209	Accn3	amiloride-sensitive cation channel 3	1.052	0.5138	NA
108687	Edem2	ER degradation enhancer, mannosidase alpha-like 2	1.052	0.6025	0.8639
107823	Whsc1	Wolf-Hirschhorn syndrome candidate 1 (human)	1.052	0.4176	NA
107477	Guca1b	guanylate cyclase activator 1B	1.052	0.4812	NA
104923	Adi1	acireductone dioxygenase 1	1.052	0.4218	NA
93730	Lztfl1	leucine zipper transcription factor-like 1	1.052	0.444	NA
76479	Smndc1	survival motor neuron domain containing 1	1.052	0.7937	0.9336
74665	Lrrc48	leucine rich repeat containing 48	1.052	0.3368	NA
74153	Uba7	ubiquitin-like modifier activating enzyme 7	1.052	0.469	NA
72157	Pgm2	phosphoglucomutase 2	1.052	0.4954	NA
71997	1500002O20Rik	RIKEN cDNA 1500002O20 gene	1.052	0.4227	NA
69536	Hemk1	HemK methyltransferase family member 1	1.052	0.5421	NA
68537	Mrpl13	mitochondrial ribosomal protein L13	1.052	0.2879	NA
68133	Gcsh	glycine cleavage system protein H (aminomethyl carrier)	1.052	0.2947	NA
68035	Rbm42	RNA binding motif protein 42	1.052	0.5561	0.8448
67460	Decr1	2,4-dienoyl CoA reductase 1, mitochondrial	1.052	0.5529	0.8438
67041	Oxct1	3-oxoacid CoA transferase 1	1.052	0.3234	NA
67027	Mkrn2	makorin, ring finger protein, 2	1.052	0.4801	NA
67009	Ttc23	tetratricopeptide repeat domain 23	1.052	0.5854	0.8551
66913	Kdelr2	KDEL (Lys-Asp-Glu-Leu) endoplasmic reticulum protein retention receptor 2	1.052	0.4584	NA
66665	5730528L13Rik	RIKEN cDNA 5730528L13 gene	1.052	0.3215	NA
56760	Clec1b	C-type lectin domain family 1, member b	1.052	0.7359	0.9169
50780	Rgs3	regulator of G-protein signaling 3	1.052	0.268	NA
30791	Slc39a1	solute carrier family 39 (zinc transporter), member 1	1.052	0.4425	NA
27425	Atp5l	ATP synthase, H+ transporting, mitochondrial F0 complex, subunit g	1.052	0.2063	NA
26466	Zfp260	zinc finger protein 260	1.052	0.3149	NA
26450	Rbbp9	retinoblastoma binding protein 9	1.052	0.2686	NA
24068	Sra1	steroid receptor RNA activator 1	1.052	0.4474	NA
21942	Tnfrsf9	tumor necrosis factor receptor superfamily, member 9	1.052	0.7362	0.9169
21685	Tef	thyrotroph embryonic factor	1.052	0.3552	NA
20112	Rps6ka2	ribosomal protein S6 kinase, polypeptide 2	1.052	0.4749	NA
19988	Rpl6	ribosomal protein L6	1.052	0.6666	0.8904
19944	Rpl29	ribosomal protein L29	1.052	0.5681	0.8486
19417	Rasgrf1	RAS protein-specific guanine nucleotide-releasing factor 1	1.052	0.6863	0.8966
19324	Rab1	RAB1, member RAS oncogene family	1.052	0.3094	NA

19073	Srgn	serglycin	1.052	0.4942	NA
18102	Nme1	non-metastatic cells 1, protein (NM23A) expressed in	1.052	0.3192	NA
16854	Lgals3	lectin, galactose binding, soluble 3	1.052	0.8199	0.9429
14950	H13	histocompatibility 13	1.052	0.4717	NA
14151	Fech	ferrochelatase	1.052	0.2267	NA
13595	Ebp	phenylalkylamine Ca2+ antagonist (emopamil) binding protein	1.052	0.5586	0.8459
12412	Cbx1	chromobox homolog 1 (Drosophila HP1 beta)	1.052	0.6018	0.8636
12311	Calcr	calcitonin receptor	1.052	0.6805	0.8949
12226	Btg1	B-cell translocation gene 1, anti-proliferative	1.052	0.2804	NA
12015	Bad	BCL2-associated agonist of cell death	1.052	0.5703	0.8492
11841	Arf2	ADP-ribosylation factor 2	1.052	0.3832	NA
100044951	LOC100044951	hypothetical LOC100044951	1.051	0.3749	NA
100041231	Gm3219	B-cell CLL/lymphoma 7C pseudogene	1.051	0.7204	0.9109
640374	Gm7293	glyceraldehyde-3-phosphate dehydrogenase pseudogene	1.051	0.691	0.8989
637004	Vmn2r3	vomeronasal 2, receptor 3	1.051	0.7657	0.9251
627110	Tubb2a-ps2	tubulin, beta 2a, pseudogene 2	1.051	0.3198	NA
432940	Fam105b	family with sequence similarity 105, member B	1.051	0.231	NA
385658	Fam55c	family with sequence similarity 55, member C	1.051	0.3618	NA
241944	D3Ertd254e	DNA segment, Chr 3, ERATO Doi 254, expressed	1.051	0.2673	NA
241621	Gm13981	predicted gene 13981	1.051	0.2615	NA
235043	Tmem205	transmembrane protein 205	1.051	0.4317	NA
233899	Gm166	predicted gene 166	1.051	0.5547	0.8444
226778	Mark1	MAP/microtubule affinity-regulating kinase 1	1.051	0.3864	NA
213575	Dync2li1	dynein cytoplasmic 2 light intermediate chain 1	1.051	0.4993	NA
208922	Cpeb3	cytoplasmic polyadenylation element binding protein 3	1.051	0.4934	NA
192651	Zfp286	zinc finger protein 286	1.051	0.4413	NA
171486	Cd99l2	CD99 antigen-like 2	1.051	0.4076	NA
170721	Papln	papilin, proteoglycan-like sulfated glycoprotein	1.051	0.782	0.9291
117146	Ube3b	ubiquitin protein ligase E3B	1.051	0.4892	NA
117005	Olfr74	olfactory receptor 74	1.051	0.7858	0.9309
110380	Shroom2	shroom family member 2	1.051	0.3711	NA
108072	Grm6	glutamate receptor, metabotropic 6	1.051	0.3361	NA
104600	AW125324	expressed sequence AW125324	1.051	0.6735	0.8924
101966	D8Ertd738e	DNA segment, Chr 8, ERATO Doi 738, expressed	1.051	0.2698	NA
93834	Peli2	pellino 2	1.051	0.2988	NA
81630	Zbtb22	zinc finger and BTB domain containing 22	1.051	0.4879	NA
80907	Lactb	lactamase, beta	1.051	0.3599	NA
76510	Trappc9	trafficking protein particle complex 9	1.051	0.6442	0.8802
76281	Tax1bp3	Tax1 (human T-cell leukemia virus type I) binding protein 3	1.051	0.5396	NA
75692	Nr2c2ap	nuclear receptor 2C2-associated protein	1.051	0.581	0.8527
75173	4930544O15Rik	RIKEN cDNA 4930544O15 gene	1.051	0.691	0.8989
74440	4933407C03Rik	RIKEN cDNA 4933407C03 gene	1.051	0.3856	NA
74192	Arpc5l	actin related protein 2/3 complex, subunit 5-like	1.051	0.2496	NA
73078	Pmpcb	peptidase (mitochondrial processing) beta	1.051	0.3796	NA
72135	Pygo1	pygopus 1	1.051	0.809	0.9395
70186	Fam162a	family with sequence similarity 162, member A	1.051	0.4566	NA
69226	Snx24	sorting nexing 24	1.051	0.6993	0.9031
69191	Pdia2	protein disulfide isomerase associated 2	1.051	0.5591	0.8459
67902	Sumf2	sulfatase modifying factor 2	1.051	0.6551	0.8854

67254	2900011O08Rik	RIKEN cDNA 2900011O08 gene	1.051	0.4454	NA
67070	Lsm14a	LSM14 homolog A (SCD6, S. cerevisiae)	1.051	0.7325	0.9157
66874	1200014J11Rik	RIKEN cDNA 1200014J11 gene	1.051	0.3224	NA
66776	Pisd-ps3	phosphatidylserine decarboxylase, pseudogene 3	1.051	0.3599	NA
66446	Exosc7	exosome component 7	1.051	0.3932	NA
66425	Pcp4l1	Purkinje cell protein 4-like 1	1.051	0.349	NA
66085	Eif3f	eukaryotic translation initiation factor 3, subunit F	1.051	0.4055	NA
65111	Dap3	death associated protein 3	1.051	0.3197	NA
57423	Atp5j2	ATP synthase, H+ transporting, mitochondrial F0 complex, subunit F2	1.051	0.3038	NA
56812	Dnajb2	DnaJ (Hsp40) homolog, subfamily B, member 2	1.051	0.4119	NA
56734	Tulp2	tubby-like protein 2	1.051	0.7758	0.9266
56295	Higd1a	HIG1 domain family, member 1A	1.051	0.237	NA
53618	Fut8	fucosyltransferase 8	1.051	0.3248	NA
52829	D4Bwg0951e	DNA segment, Chr 4, Brigham & Women's Genetics 0951 expressed	1.051	0.36	NA
52690	Setd3	SET domain containing 3	1.051	0.3028	NA
52120	Hgsnat	heparan-alpha-glucosaminide N-acetyltransferase	1.051	0.2968	NA
30953	Schip1	schwannomin interacting protein 1	1.051	0.524	NA
27632	Rdbp	RD RNA-binding protein	1.051	0.5076	NA
27392	Pign	phosphatidylinositol glycan anchor biosynthesis, class N	1.051	0.8469	0.9517
22378	Wbp2	WW domain binding protein 2	1.051	0.4295	NA
22232	Slc35a2	solute carrier family 35 (UDP-galactose transporter), member A2	1.051	0.5498	NA
22066	Trpc4	transient receptor potential cation channel, subfamily C, member 4	1.051	0.4791	NA
21780	Tfam	transcription factor A, mitochondrial	1.051	0.5952	0.8603
19300	Abcd4	ATP-binding cassette, sub-family D (ALD), member 4	1.051	0.247	NA
18952	Sep-04	septin 4	1.051	0.4222	NA
18739	Pitpnm1	phosphatidylinositol transfer protein, membrane-associated 1	1.051	0.5829	0.8538
18032	Nfix	nuclear factor I/X	1.051	0.7821	0.9291
14790	Grcc10	gene rich cluster, C10 gene	1.051	0.3689	NA
14694	Gnb2l1	guanine nucleotide binding protein (G protein), beta polypeptide 2 like 1	1.051	0.6158	0.8698
14533	Bloc1s1	biogenesis of lysosome-related organelles complex-1, subunit 1	1.051	0.1512	NA
14084	Faf1	Fas-associated factor 1	1.051	0.5662	0.848
13480	Dpm1	dolichol-phosphate (beta-D) mannosyltransferase 1	1.051	0.4028	NA
13178	Dck	deoxycytidine kinase	1.051	0.6933	0.9
12821	Col17a1	collagen, type XVII, alpha 1	1.051	0.5911	0.8582
12793	Cnih	cornichon homolog (Drosophila)	1.051	0.4124	NA
11636	Ak1	adenylate kinase 1	1.051	0.4158	NA
100040608	Fancf	Fanconi anemia, complementation group F	1.05	0.4762	NA
100040410	Gm14957	predicted gene 14957	1.05	0.7654	0.9251
100039027	Gm2011	predicted gene 2011	1.05	0.5497	NA
666372	Gm8066	predicted gene 8066	1.05	0.6024	0.8639
625123	Gm6557	predicted gene 6557	1.05	0.6011	0.8636
622640	Gm6337	predicted gene 6337	1.05	0.2338	NA
433702	Ncbp1	nuclear cap binding protein subunit 1	1.05	0.3207	NA
382014	Ano8	anoctamin 8	1.05	0.5791	0.8519
319880	Tmcc3	transmembrane and coiled coil domains 3	1.05	0.6536	0.8848
319478	Cxxc4	CXXC finger 4	1.05	0.425	NA
269954	Ttll13	tubulin tyrosine ligase-like family, member 13	1.05	0.5931	0.8587
269529	Fbxo10	F-box protein 10	1.05	0.3046	NA
268527	Greb1	gene regulated by estrogen in breast cancer protein	1.05	0.5501	NA

246278	Cd207	CD207 antigen	1.05	0.7061	0.9058
246196	Zfp277	zinc finger protein 277	1.05	0.3665	NA
246154	Vasn	vasorin	1.05	0.4746	NA
241274	Pnpla7	patatin-like phospholipase domain containing 7	1.05	0.6262	0.873
237400	Mex3d	mex3 homolog D (C. elegans)	1.05	0.4699	NA
234515	Inpp4b	inositol polyphosphate-4-phosphatase, type II	1.05	0.412	NA
231464	Cnot6l	CCR4-NOT transcription complex, subunit 6-like	1.05	0.2543	NA
213211	Rnf26	ring finger protein 26	1.05	0.3358	NA
117150	Pip4k2c	phosphatidylinositol-5-phosphate 4-kinase, type II, gamma	1.05	0.5883	0.8571
114128	Laptm4b	lysosomal-associated protein transmembrane 4B	1.05	0.4162	NA
110593	Prdm2	PR domain containing 2, with ZNF domain	1.05	0.4014	NA
99349	Dnajc24	DnaJ (Hsp40) homolog, subfamily C, member 24	1.05	0.4844	NA
93889	Pcdhb18	protocadherin beta 18	1.05	0.3389	NA
81018	Rnf114	ring finger protein 114	1.05	0.6626	0.8895
80880	Kank3	KN motif and ankyrin repeat domains 3	1.05	0.5858	0.8555
78653	Bola3	bolA-like 3 (E. coli)	1.05	0.3821	NA
78408	Fam131a	family with sequence similarity 131, member A	1.05	0.2997	NA
74309	Osbp2	oxysterol binding protein 2	1.05	0.4036	NA
73212	3110082I17Rik	RIKEN cDNA 3110082I17 gene	1.05	0.422	NA
72181	Nsun4	NOL1/NOP2/Sun domain family, member 4	1.05	0.5807	0.8527
72103	Aplf	aprataxin and PNKP like factor	1.05	0.3479	NA
72098	Tmem68	transmembrane protein 68	1.05	0.4947	NA
72083	Mzt2	mitotic spindle organizing protein 2	1.05	0.4844	NA
72023	Cyb561d1	cytochrome b-561 domain containing 1	1.05	0.4266	NA
72008	Zfyve19	zinc finger, FYVE domain containing 19	1.05	0.2593	NA
71691	Pnmal1	PNMA-like 1	1.05	0.4791	NA
70454	Cenpl	centromere protein L	1.05	0.4086	NA
70425	Csnk1g3	casein kinase 1, gamma 3	1.05	0.4104	NA
70240	Ufsp1	UFM1-specific peptidase 1	1.05	0.4412	NA
69219	Ddah1	dimethylarginine dimethylaminohydrolase 1	1.05	0.6264	0.873
68521	Fam189b	family with sequence similarity 189, member B	1.05	0.3738	NA
68449	Tbc1d10b	TBC1 domain family, member 10b	1.05	0.26	NA
68250	Fam96a	family with sequence similarity 96, member A	1.05	0.3715	NA
67899	Cmc1	COX assembly mitochondrial protein homolog (S. cerevisiae)	1.05	0.3631	NA
66844	Ormdl2	ORM1-like 2 (S. cerevisiae)	1.05	0.4841	NA
66736	Ttc35	tetratricopeptide repeat domain 35	1.05	0.2326	NA
66665	5730528L13Rik	RIKEN cDNA 5730528L13 gene	1.05	0.3643	NA
66346	1700029P11Rik	RIKEN cDNA 1700029P11 gene	1.05	0.7262	0.9126
60409	Trappc4	trafficking protein particle complex 4	1.05	0.3222	NA
58988	Rps6kb2	ribosomal protein S6 kinase, polypeptide 2	1.05	0.6418	0.879
56533	Rgs17	regulator of G-protein signaling 17	1.05	0.4546	NA
56463	Snd1	staphylococcal nuclease and tudor domain containing 1	1.05	0.6636	0.8896
56369	Apip	APAF1 interacting protein	1.05	0.3959	NA
56323	Dnajb5	DnaJ (Hsp40) homolog, subfamily B, member 5	1.05	0.5915	0.8582
56209	Gde1	glycerophosphodiester phosphodiesterase 1	1.05	0.4464	NA
53901	Rcan2	regulator of calcineurin 2	1.05	0.3272	NA
26398	Map2k4	mitogen-activated protein kinase kinase 4	1.05	0.4392	NA
26371	Ciao1	cytosolic iron-sulfur protein assembly 1 homolog (S. cerevisiae)	1.05	0.4645	NA
24045	Scamp3	secretory carrier membrane protein 3	1.05	0.3453	NA

22123	Psmd3	proteasome (prosome, macropain) 26S subunit, non-ATPase, 3	1.05	0.4321	NA
21924	Tnnc1	troponin C, cardiac/slow skeletal	1.05	0.6095	0.8664
20843	Stag2	stromal antigen 2	1.05	0.4719	NA
20532	Slc3a1	solute carrier family 3, member 1	1.05	0.4463	NA
20353	Sema4c	sema domain, immunoglobulin domain (Ig), transmembrane domain (TM) and short cytoplasmic domain, (semaphorin) 4C	1.05	0.554	NA
18750	Prkca	protein kinase C, alpha	1.05	0.4102	NA
18717	Pip5k1c	phosphatidylinositol-4-phosphate 5-kinase, type 1 gamma	1.05	0.6843	0.8956
15957	Ifit1	interferon-induced protein with tetratricopeptide repeats 1	1.05	0.7259	0.9126
15257	Hipk1	homeodomain interacting protein kinase 1	1.05	0.6811	0.8949
14797	Aes	amino-terminal enhancer of split	1.05	0.6883	0.8976
14700	Gng10	guanine nucleotide binding protein (G protein), gamma 10	1.05	0.3337	NA
14661	Glud1	glutamate dehydrogenase 1	1.05	0.4081	NA
14406	Gabrg2	gamma-aminobutyric acid (GABA) A receptor, subunit gamma 2	1.05	0.2377	NA
13360	Dhcr7	7-dehydrocholesterol reductase	1.05	0.7376	0.9172
12967	Crygd	crystallin, gamma D	1.05	0.9117	0.9743
12847	Copa	coatomer protein complex subunit alpha	1.05	0.6821	0.8951
12833	Col6a1	collagen, type VI, alpha 1	1.05	0.6712	0.8915
12616	Cenpb	centromere protein B	1.05	0.6434	0.88
12398	Cbfa2t3	core-binding factor, runt domain, alpha subunit 2, translocated to, 3 (human)	1.05	0.6176	0.8701
12314	Calm2	calmodulin 2	1.05	0.2738	NA
11890	Asgr2	asialoglycoprotein receptor 2	1.05	0.8101	0.94
11484	Aspa	aspartoacylase	1.05	0.6224	0.872
11431	Acp1	acid phosphatase 1, soluble	1.05	0.585	0.855
100037283	Rnaset2a	ribonuclease T2A	1.049	0.6062	0.8645
637916	LOC637916	midline-1-like	1.049	0.7063	0.9059
622645	Tmem200c	transmembrane protein 200C	1.049	0.7454	0.9192
432763	Prr7	proline rich 7 (synaptic)	1.049	0.5153	NA
407816	BC023202	cDNA sequence BC023202	1.049	0.5649	NA
338363	6030446N20Rik	RIKEN cDNA 6030446N20 gene	1.049	0.588	0.857
270118	Maml2	mastermind like 2 (Drosophila)	1.049	0.7222	0.9121
243385	Gprin3	GPRIN family member 3	1.049	0.6447	0.8802
237859	Ccdc55	coiled-coil domain containing 55	1.049	0.6086	0.8658
236848	BC023829	cDNA sequence BC023829	1.049	0.4761	NA
233879	Asphd1	aspartate beta-hydroxylase domain containing 1	1.049	0.6014	0.8636
232975	Atp1a3	ATPase, Na+/K+ transporting, alpha 3 polypeptide	1.049	0.7098	0.9069
232196	C87436	expressed sequence C87436	1.049	0.3077	NA
226518	Nmnat2	nicotinamide nucleotide adenylyltransferase 2	1.049	0.7538	0.9216
217364	Engase	endo-beta-N-acetylglucosaminidase	1.049	0.2411	NA
217127	Myst2	MYST histone acetyltransferase 2	1.049	0.5309	NA
216767	Mrpl22	mitochondrial ribosomal protein L22	1.049	0.4009	NA
208188	Ghsr	growth hormone secretagogue receptor	1.049	0.5286	NA
208177	Phldb2	pleckstrin homology-like domain, family B, member 2	1.049	0.7351	0.9167
107869	Cth	cystathionase (cystathionine gamma-lyase)	1.049	0.3589	NA
107823	Whsc1	Wolf-Hirschhorn syndrome candidate 1 (human)	1.049	0.6114	0.8673
106957	Slc39a6	solute carrier family 39 (metal ion transporter), member 6	1.049	0.2653	NA
103288	AI836737	expressed sequence AI836737	1.049	0.8408	0.9486
99011	Pomt1	protein-O-mannosyltransferase 1	1.049	0.6531	0.8846
98363	Efhd1	EF hand domain containing 1	1.049	0.5398	NA
97064	Wwtr1	WW domain containing transcription regulator 1	1.049	0.4002	NA

96957	Tmem62	transmembrane protein 62	1.049	0.6344	0.8767
93686	Rbfox2	RNA binding protein, fox-1 homolog (C. elegans) 2	1.049	0.6143	0.8691
80976	Syt13	synaptotagmin XIII	1.049	0.3907	NA
78755	Fam122b	family with sequence similarity 122, member B	1.049	0.5692	NA
76916	4930455C21Rik	RIKEN cDNA 4930455C21 gene	1.049	0.5737	0.8501
75623	1700029F09Rik	RIKEN cDNA 1700029F09 gene	1.049	0.6543	0.8852
75580	Zbtb4	zinc finger and BTB domain containing 4	1.049	0.6092	0.8662
74498	Gorasp1	golgi reassembly stacking protein 1	1.049	0.4645	NA
73845	Ankrd42	ankyrin repeat domain 42	1.049	0.3542	NA
72238	Tbc1d5	TBC1 domain family, member 5	1.049	0.4751	NA
71162	4933421I07Rik	RIKEN cDNA 4933421I07 gene	1.049	0.7572	0.9226
69807	Trim32	tripartite motif-containing 32	1.049	0.2531	NA
69786	Tprkb	Tp53rk binding protein	1.049	0.3528	NA
69327	1700007K13Rik	RIKEN cDNA 1700007K13 gene	1.049	0.6582	0.8868
69305	Dcps	decapping enzyme, scavenger	1.049	0.2393	NA
69089	Oxa1l	oxidase assembly 1-like	1.049	0.4161	NA
68875	Tmcc2	transmembrane and coiled-coil domains 2	1.049	0.4553	NA
68797	Pdgfrl	platelet-derived growth factor receptor-like	1.049	0.681	0.8949
67889	Rbm18	RNA binding motif protein 18	1.049	0.2006	NA
67621	Bend5	BEN domain containing 5	1.049	0.3741	NA
67371	Gtf3c6	general transcription factor IIIC, polypeptide 6, alpha	1.049	0.3699	NA
67287	Parp6	poly (ADP-ribose) polymerase family, member 6	1.049	0.509	NA
67204	Eif2s2	eukaryotic translation initiation factor 2, subunit 2 (beta)	1.049	0.5897	0.8576
67067	Romo1	reactive oxygen species modulator 1	1.049	0.4474	NA
66266	Eapp	E2F-associated phosphoprotein	1.049	0.224	NA
66199	Commd4	COMM domain containing 4	1.049	0.5039	NA
66163	Mrpl4	mitochondrial ribosomal protein L4	1.049	0.6529	0.8844
64292	Ptges	prostaglandin E synthase	1.049	0.3165	NA
58887	Repin1	replication initiator 1	1.049	0.2539	NA
58207	Slc43a3	solute carrier family 43, member 3	1.049	0.731	0.9152
56187	Rabggta	Rab geranylgeranyl transferase, a subunit	1.049	0.4277	NA
27207	Rps11	ribosomal protein S11	1.049	0.3051	NA
27045	Nit1	nitrilase 1	1.049	0.299	NA
26413	Mapk1	mitogen-activated protein kinase 1	1.049	0.1981	NA
24070	Mpdu1	mannose-P-dolichol utilization defect 1	1.049	0.5362	NA
23797	Akt3	thymoma viral proto-oncogene 3	1.049	0.3785	NA
22689	Zfp27	zinc finger protein 27	1.049	0.5692	NA
22213	Ube2g2	ubiquitin-conjugating enzyme E2G 2	1.049	0.71	0.9069
21917	Tmpo	thymopoietin	1.049	0.4799	NA
21853	Timeless	timeless homolog (Drosophila)	1.049	0.2283	NA
20639	Snrpb2	U2 small nuclear ribonucleoprotein B	1.049	0.4461	NA
20541	Slc8a1	solute carrier family 8 (sodium/calcium exchanger), member 1	1.049	0.6706	0.8912
19262	Ptpra	protein tyrosine phosphatase, receptor type, A	1.049	0.2949	NA
18783	Pla2g4a	phospholipase A2, group IVA (cytosolic, calcium-dependent)	1.049	0.6295	0.8745
17955	Nap1l4	nucleosome assembly protein 1-like 4	1.049	0.3703	NA
14164	Fgf1	fibroblast growth factor 1	1.049	0.529	NA
13543	Dvl2	dishevelled 2, dsh homolog (Drosophila)	1.049	0.3107	NA
13488	Drd1a	dopamine receptor D1A	1.049	0.5296	NA
12927	Bcar1	breast cancer anti-estrogen resistance 1	1.049	0.2688	NA

11989	Slc7a3	solute carrier family 7 (cationic amino acid transporter, y+ system), member 3	1.049	0.5035	NA
11981	Atp9a	ATPase, class II, type 9A	1.049	0.588	0.857
100042332	2810410L24Rik	RIKEN cDNA 2810410L24 gene	1.048	0.4779	NA
100038570	Gm11744	predicted gene 11744	1.048	0.4523	NA
544881	BB287469	expressed sequence BB287469	1.048	0.9064	0.9725
382384	Odf3l2	outer dense fiber of sperm tails 3-like 2	1.048	0.836	0.9474
381933	6430531B16Rik	RIKEN cDNA 6430531B16 gene	1.048	0.5189	NA
360216	Zranb1	zinc finger, RAN-binding domain containing 1	1.048	0.4638	NA
321007	Serac1	serine active site containing 1	1.048	0.4623	NA
320938	Tnpo3	transportin 3	1.048	0.6493	0.8821
269109	Dpp10	dipeptidylpeptidase 10	1.048	0.3409	NA
246317	Neto1	neuropilin (NRP) and tolloid (TLL)-like 1	1.048	0.3328	NA
241589	D430041D05Rik	RIKEN cDNA D430041D05 gene	1.048	0.677	0.893
232878	Zscan22	zinc finger and SCAN domain containing 22	1.048	0.5474	NA
232337	Zfp637	zinc finger protein 637	1.048	0.4479	NA
226139	Cox15	COX15 homolog, cytochrome c oxidase assembly protein (yeast)	1.048	0.402	NA
223722	Mcat	malonyl CoA:ACP acyltransferase (mitochondrial)	1.048	0.5746	NA
208080	Gm514	predicted gene 514	1.048	0.3011	NA
207521	Dtx4	deltex 4 homolog (Drosophila)	1.048	0.3855	NA
170799	Rtkn2	rhotekin 2	1.048	0.6701	0.8912
108012	Ap1s2	adaptor-related protein complex 1, sigma 2 subunit	1.048	0.4478	NA
105844	Card10	caspase recruitment domain family, member 10	1.048	0.688	0.8975
104457	0610010K14Rik	RIKEN cDNA 0610010K14 gene	1.048	0.3935	NA
103537	Mbtd1	mbt domain containing 1	1.048	0.407	NA
99167	Ssx2ip	synovial sarcoma, X breakpoint 2 interacting protein	1.048	0.5774	0.8514
94186	Strn3	striatin, calmodulin binding protein 3	1.048	0.4365	NA
84035	Kremen1	kringle containing transmembrane protein 1	1.048	0.5346	NA
80906	Kcnip2	Kv channel-interacting protein 2	1.048	0.7433	0.9189
80880	Kank3	KN motif and ankyrin repeat domains 3	1.048	0.5264	NA
80877	Lrba	LPS-responsive beige-like anchor	1.048	0.5613	NA
77552	Shisa4	shisa homolog 4 (Xenopus laevis)	1.048	0.5733	NA
76497	Ppp1r11	protein phosphatase 1, regulatory (inhibitor) subunit 11	1.048	0.6374	0.8776
75991	Slain2	SLAIN motif family, member 2	1.048	0.3918	NA
74600	Mrpl47	mitochondrial ribosomal protein L47	1.048	0.456	NA
71863	1700019O17Rik	RIKEN cDNA 1700019O17 gene	1.048	0.7273	0.9134
70997	Spef1	sperm flagellar 1	1.048	0.455	NA
70873	4921517L17Rik	RIKEN cDNA 4921517L17 gene	1.048	0.7219	0.912
70772	Ggnbp1	gametogenetin binding protein 1	1.048	0.624	0.8726
70317	Arl16	ADP-ribosylation factor-like 16	1.048	0.5734	NA
70123	2210013O21Rik	RIKEN cDNA 2210013O21 gene	1.048	0.378	NA
69601	Dab2ip	disabled homolog 2 (Drosophila) interacting protein	1.048	0.4661	NA
69549	2310009B15Rik	RIKEN cDNA 2310009B15 gene	1.048	0.5246	NA
68927	Ptcd2	pentatricopeptide repeat domain 2	1.048	0.3429	NA
68861	1190002N15Rik	RIKEN cDNA 1190002N15 gene	1.048	0.4354	NA
68705	Gtf2f2	general transcription factor IIF, polypeptide 2	1.048	0.3241	NA
68298	Ncapd2	non-SMC condensin I complex, subunit D2	1.048	0.5405	NA
68229	AI846148	expressed sequence AI846148	1.048	0.505	NA
67803	Limd2	LIM domain containing 2	1.048	0.6396	0.8783
67050	Nkap	NFKB activating protein	1.048	0.4533	NA

66899	Fip1l1	FIP1 like 1 (S. cerevisiae)	1.048	0.4306	NA
66882	Bzw1	basic leucine zipper and W2 domains 1	1.048	0.4041	NA
66266	Eapp	E2F-associated phosphoprotein	1.048	0.3666	NA
66092	Ghitm	growth hormone inducible transmembrane protein	1.048	0.4314	NA
64099	Parvg	parvin, gamma	1.048	0.8623	0.9574
64074	Smoc2	SPARC related modular calcium binding 2	1.048	0.4509	NA
60425	Doc2g	double C2, gamma	1.048	0.4958	NA
58193	Extl2	exostoses (multiple)-like 2	1.048	0.3262	NA
56710	Dbc1	deleted in bladder cancer 1 (human)	1.048	0.5892	0.8575
56690	Mlycd	malonyl-CoA decarboxylase	1.048	0.3483	NA
56045	Samhd1	SAM domain and HD domain, 1	1.048	0.2343	NA
54613	St3gal6	ST3 beta-galactoside alpha-2,3-sialyltransferase 6	1.048	0.6182	0.8703
29856	Smtn	smoothelin	1.048	0.5358	NA
23844	Clca3	chloride channel calcium activated 3	1.048	0.5449	NA
22652	Mkrn3	makorin, ring finger protein, 3	1.048	0.4819	NA
21933	Tnfrsf10b	tumor necrosis factor receptor superfamily, member 10b	1.048	0.7956	0.9346
20740	Spna2	spectrin alpha 2	1.048	0.4125	NA
20397	Sgpl1	sphingosine phosphate lyase 1	1.048	0.2575	NA
20354	Sema4d	sema domain, immunoglobulin domain (Ig), transmembrane domain (TM) and short cytoplasmic domain, (semaphorin) 4D	1.048	0.6542	0.8852
19417	Rasgrf1	RAS protein-specific guanine nucleotide-releasing factor 1	1.048	0.7789	0.9278
18772	Pkp1	plakophilin 1	1.048	0.4072	NA
18755	Prkch	protein kinase C, eta	1.048	0.7131	0.9076
18637	Pfdn2	prefoldin 2	1.048	0.6117	0.8673
18632	Pex11b	peroxisomal biogenesis factor 11 beta	1.048	0.4491	NA
18231	Nxph1	neurexophilin 1	1.048	0.676	0.8928
17897	Myl3	myosin, light polypeptide 3	1.048	0.9041	0.9717
17305	Mfng	MFNG O-fucosylpeptide 3-beta-N-acetylglucosaminyltransferase	1.048	0.5435	NA
16369	Irs3	insulin receptor substrate 3	1.048	0.6647	0.8902
15369	Hmox2	heme oxygenase (decycling) 2	1.048	0.6471	0.8812
14911	Thumpd3	THUMP domain containing 3	1.048	0.3834	NA
14580	Gfap	glial fibrillary acidic protein	1.048	0.8412	0.9487
13639	Efna4	ephrin A4	1.048	0.6336	0.8761
13449	Dok2	docking protein 2	1.048	0.594	0.8596
13110	Cyp2j6	cytochrome P450, family 2, subfamily j, polypeptide 6	1.048	0.5967	0.8611
12995	Csnk2a1	casein kinase 2, alpha 1 polypeptide	1.048	0.3114	NA
12866	Cox7a2	cytochrome c oxidase, subunit VIIa 2	1.048	0.5207	NA
12151	Bmi1	Bmi1 polycomb ring finger oncogene	1.048	0.4331	NA
11973	Atp6v1e1	ATPase, H+ transporting, lysosomal V1 subunit E1	1.048	0.3842	NA
11810	Apobec1	apolipoprotein B mRNA editing enzyme, catalytic polypeptide 1	1.048	0.7454	0.9192
11652	Akt2	thymoma viral proto-oncogene 2	1.048	0.376	NA
11630	Aim1	absent in melanoma 1	1.048	0.6862	0.8966
668166	Zxdb	zinc finger, X-linked, duplicated B	1.047	0.3403	NA
629499	4922505G16Rik	RIKEN cDNA 4922505G16 gene	1.047	0.7684	0.9255
622534	Gm13611	predicted gene 13611	1.047	0.6005	0.8636
434282	Gm5608	predicted gene 5608	1.047	0.6259	0.8728
434232	Iqck	IQ motif containing K	1.047	0.6117	0.8673
319998	Tmem198	transmembrane protein 198	1.047	0.6013	0.8636
269473	Lrig2	leucine-rich repeats and immunoglobulin-like domains 2	1.047	0.7076	0.9064
268749	Rnf31	ring finger protein 31	1.047	0.5597	NA

243369	Sspo	SCO-spondin	1.047	0.7711	0.9258
239099	Homez	homeodomain leucine zipper-encoding gene	1.047	0.3223	NA
237775	Zfp867	zinc finger protein 867	1.047	0.6317	0.8751
234847	Spg7	spastic paraplegia 7 homolog (human)	1.047	0.2648	NA
230936	Phf13	PHD finger protein 13	1.047	0.3697	NA
230890	Gm436	predicted gene 436	1.047	0.7706	0.9257
229589	Prune	prune homolog (Drosophila)	1.047	0.7376	0.9172
229534	Pbxip1	pre-B-cell leukemia transcription factor interacting protein 1	1.047	0.5901	0.8578
227333	Dgkd	diacylglycerol kinase, delta	1.047	0.3195	NA
224912	Crb3	crumbs homolog 3 (Drosophila)	1.047	0.6032	0.8643
223917	Krt79	keratin 79	1.047	0.8001	0.9364
140489	Bhlhe23	basic helix-loop-helix family, member e23	1.047	0.716	0.9089
107975	Pacs1	phosphofurin acidic cluster sorting protein 1	1.047	0.7065	0.9059
106952	Arap3	ArfGAP with RhoGAP domain, ankyrin repeat and PH domain 3	1.047	0.4017	NA
104570	Smek2	SMEK homolog 2, suppressor of mek1 (Dictyostelium)	1.047	0.4355	NA
94249	Slc24a3	solute carrier family 24 (sodium/potassium/calcium exchanger), member 3	1.047	0.4664	NA
94187	Zfp423	zinc finger protein 423	1.047	0.6046	0.8644
81896	Ift122	intraflagellar transport 122 homolog (Chlamydomonas)	1.047	0.5404	NA
76571	Styxl1	serine/threonine/tyrosine interacting-like 1	1.047	0.3745	NA
75740	Egfem1	EGF-like and EMI domain containing 1	1.047	0.5994	0.8626
75425	Tti1	Tel2 interacting protein 1 homolog (S. pombe)	1.047	0.4214	NA
74694	Tbc1d30	TBC1 domain family, member 30	1.047	0.7402	0.9179
74167	Nudt9	nudix (nucleoside diphosphate linked moiety X)-type motif 9	1.047	0.427	NA
73075	Ppil6	peptidylprolyl isomerase (cyclophilin)-like 6	1.047	0.4272	NA
72522	Atxn7l2	ataxin 7-like 2	1.047	0.7756	0.9266
72023	Cyb561d1	cytochrome b-561 domain containing 1	1.047	0.5458	NA
71972	Dnmbp	dynamin binding protein	1.047	0.7032	0.9044
71966	Nkiras2	NFKB inhibitor interacting Ras-like protein 2	1.047	0.2637	NA
68259	Ift80	intraflagellar transport 80 homolog (Chlamydomonas)	1.047	0.3129	NA
67942	Atp5g2	ATP synthase, H+ transporting, mitochondrial F0 complex, subunit C2 (subunit 9)	1.047	0.2802	NA
67465	Sf3a1	splicing factor 3a, subunit 1	1.047	0.2677	NA
67383	2410127L17Rik	RIKEN cDNA 2410127L17 gene	1.047	0.4274	NA
66713	Actr2	ARP2 actin-related protein 2 homolog (yeast)	1.047	0.3029	NA
66400	Alkbh7	alkB, alkylation repair homolog 7 (E. coli)	1.047	0.4	NA
57316	C1d	C1D nuclear receptor co-repressor	1.047	0.6861	0.8966
57296	Psmd8	proteasome (prosome, macropain) 26S subunit, non-ATPase, 8	1.047	0.389	NA
56707	Zfp111	zinc finger protein 111	1.047	0.7318	0.9154
56541	Habp4	hyaluronic acid binding protein 4	1.047	0.5039	NA
56330	Pdcd5	programmed cell death 5	1.047	0.391	NA
29806	Limd1	LIM domains containing 1	1.047	0.4196	NA
26394	Lypla2	lysophospholipase 2	1.047	0.3708	NA
22230	Ufd1l	ubiquitin fusion degradation 1 like	1.047	0.6296	0.8745
20713	Serpini1	serine (or cysteine) peptidase inhibitor, clade I, member 1	1.047	0.3862	NA
20289	Scx	scleraxis	1.047	0.5287	NA
19240	Tmsb10	thymosin, beta 10	1.047	0.6689	0.8904
19035	Ppib	peptidylprolyl isomerase B	1.047	0.5224	NA
17938	Naca	nascent polypeptide-associated complex alpha polypeptide	1.047	0.3309	NA
17128	Smad4	MAD homolog 4 (Drosophila)	1.047	0.2012	NA
16535	Kcnq1	potassium voltage-gated channel, subfamily Q, member 1	1.047	0.8135	0.9406

16521	Kcnj5	potassium inwardly-rectifying channel, subfamily J, member 5	1.047	0.2642	NA
15171	Hcrt	hypocretin	1.047	0.6563	0.8859
14710	Gngt2	guanine nucleotide binding protein (G protein), gamma transducing activity polypeptide 2	1.047	0.3624	NA
13649	Egfr	epidermal growth factor receptor	1.047	0.6318	0.8751
13589	Mapre1	microtubule-associated protein, RP/EB family, member 1	1.047	0.4644	NA
13430	Dnm2	dynamin 2	1.047	0.5144	NA
12672	Chrm4	cholinergic receptor, muscarinic 4	1.047	0.5876	NA
12035	Bcat1	branched chain aminotransferase 1, cytosolic	1.047	0.5488	NA
11421	Ace	angiotensin I converting enzyme (peptidyl-dipeptidase A) 1	1.047	0.7783	0.9274
100039317	Gm2155	predicted gene 2155	1.046	0.69	0.8986
668661	2410002F23Rik	RIKEN cDNA 2410002F23 gene	1.046	0.5292	NA
433693	Akirin2	akirin 2	1.046	0.414	NA
329260	Dennd1b	DENN/MADD domain containing 1B	1.046	0.6445	0.8802
321022	Cdv3	carnitine deficiency-associated gene expressed in ventricle 3	1.046	0.4004	NA
320469	9930014A18Rik	RIKEN cDNA 9930014A18 gene	1.046	0.6977	0.9024
320320	B230343J05Rik	RIKEN cDNA B230343J05 gene	1.046	0.2117	NA
319176	Hist2h2ac	histone cluster 2, H2ac	1.046	0.4734	NA
271209	Rp1l1	retinitis pigmentosa 1 homolog (human)-like 1	1.046	0.8438	0.9501
269878	Megf8	multiple EGF-like-domains 8	1.046	0.558	NA
252966	Cables2	CDK5 and Abl enzyme substrate 2	1.046	0.5098	NA
246710	Rhobtb2	Rho-related BTB domain containing 2	1.046	0.6248	0.8728
245424	Gpr101	G protein-coupled receptor 101	1.046	0.3135	NA
244579	Tox3	TOX high mobility group box family member 3	1.046	0.5537	NA
242687	Wasf2	WAS protein family, member 2	1.046	0.523	NA
240613	9930021J03Rik	RIKEN cDNA 9930021J03 gene	1.046	0.5678	NA
237730	Fbll1	fibrillarin-like 1	1.046	0.6132	0.8683
235584	Dusp7	dual specificity phosphatase 7	1.046	0.6634	0.8896
235461	Fam63b	family with sequence similarity 63, member B	1.046	0.7167	0.9094
234825	Klhdc4	kelch domain containing 4	1.046	0.4937	NA
231834	Snx8	sorting nexin 8	1.046	0.4517	NA
231003	Klhl17	kelch-like 17 (Drosophila)	1.046	0.4505	NA
228019	Mettl8	methyltransferase like 8	1.046	0.4691	NA
227638	Qsox2	quiescin Q6 sulfhydryl oxidase 2	1.046	0.5911	NA
226162	Dpcd	deleted in primary ciliary dyskinesia	1.046	0.3232	NA
212442	Lactb2	lactamase, beta 2	1.046	0.5787	NA
209131	Snx30	sorting nexin family member 30	1.046	0.5862	NA
208666	Diras1	DIRAS family, GTP-binding RAS-like 1	1.046	0.725	0.9126
207785	Csrnp2	cysteine-serine-rich nuclear protein 2	1.046	0.4729	NA
193116	Slu7	SLU7 splicing factor homolog (S. cerevisiae)	1.046	0.4146	NA
192170	Eif4a3	eukaryotic translation initiation factor 4A3	1.046	0.6312	0.8751
140904	Caln1	calneuron 1	1.046	0.6054	0.8645
109145	Gins4	GINS complex subunit 4 (Sld5 homolog)	1.046	0.427	NA
108705	Pttg1ip	pituitary tumor-transforming 1 interacting protein	1.046	0.4187	NA
107869	Cth	cystathionase (cystathionine gamma-lyase)	1.046	0.3191	NA
107392	Brms1	breast cancer metastasis-suppressor 1	1.046	0.4586	NA
94280	Sfxn3	sideroflexin 3	1.046	0.5743	NA
94112	Med15	mediator complex subunit 15	1.046	0.4461	NA
93897	Fzd10	frizzled homolog 10 (Drosophila)	1.046	0.5945	0.8598
93841	Uchl4	ubiquitin carboxyl-terminal esterase L4	1.046	0.4734	NA

93683	Glce	glucuronyl C5-epimerase	1.046	0.2809	NA
89867	Sec16b	SEC16 homolog B (S. cerevisiae)	1.046	0.7201	0.9106
77766	Elp4	elongation protein 4 homolog (S. cerevisiae)	1.046	0.5152	NA
76872	Ccdc116	coiled-coil domain containing 116	1.046	0.8008	0.9368
75416	Nop14	NOP14 nucleolar protein homolog (yeast)	1.046	0.6024	0.8639
74533	Gzf1	GDNF-inducible zinc finger protein 1	1.046	0.3993	NA
74383	Ubap2l	ubiquitin associated protein 2-like	1.046	0.6065	0.8645
74254	Gpn1	GPN-loop GTPase 1	1.046	0.2888	NA
73451	Zfp763	zinc finger protein 763	1.046	0.4414	NA
72844	Kctd17	potassium channel tetramerisation domain containing 17	1.046	0.6656	0.8904
71974	Prmt3	protein arginine N-methyltransferase 3	1.046	0.6467	0.8812
70533	Btf3l4	basic transcription factor 3-like 4	1.046	0.408	NA
69962	2810422O20Rik	RIKEN cDNA 2810422O20 gene	1.046	0.6222	0.872
68944	Tmco1	transmembrane and coiled-coil domains 1	1.046	0.2031	NA
68507	Ppfia4	protein tyrosine phosphatase, receptor type, f polypeptide (PTPRF), interacting protein (liprin), alpha 4	1.046	0.3983	NA
67963	Npc2	Niemann Pick type C2	1.046	0.3981	NA
67701	Wfdc2	WAP four-disulfide core domain 2	1.046	0.753	0.9214
66989	Kctd20	potassium channel tetramerisation domain containing 20	1.046	0.3919	NA
66200	Commd6	COMM domain containing 6	1.046	0.5184	NA
66129	1110018J18Rik	RIKEN cDNA 1110018J18 gene	1.046	0.3983	NA
65107	Lrp10	low-density lipoprotein receptor-related protein 10	1.046	0.5556	NA
64706	Scube1	signal peptide, CUB domain, EGF-like 1	1.046	0.6366	0.8776
58233	Dnaja4	DnaJ (Hsp40) homolog, subfamily A, member 4	1.046	0.4505	NA
57080	Gtf2ird1	general transcription factor II I repeat domain-containing 1	1.046	0.3785	NA
56748	Nfu1	NFU1 iron-sulfur cluster scaffold homolog (S. cerevisiae)	1.046	0.3267	NA
56370	Tagln3	transgelin 3	1.046	0.5639	NA
56317	Anapc7	anaphase promoting complex subunit 7	1.046	0.5206	NA
53331	Stx7	syntaxin 7	1.046	0.4216	NA
50765	Trfr2	transferrin receptor 2	1.046	0.4533	NA
27386	Npas3	neuronal PAS domain protein 3	1.046	0.4894	NA
24116	Whsc2	Wolf-Hirschhorn syndrome candidate 2 (human)	1.046	0.2346	NA
24051	Sgcb	sarcoglycan, beta (dystrophin-associated glycoprotein)	1.046	0.4342	NA
24045	Scamp3	secretory carrier membrane protein 3	1.046	0.4889	NA
22196	Ube2i	ubiquitin-conjugating enzyme E2I	1.046	0.5379	NA
21934	Tnfrsf11a	tumor necrosis factor receptor superfamily, member 11a	1.046	0.6903	0.8988
20230	Satb1	special AT-rich sequence binding protein 1	1.046	0.5129	NA
20174	Ruvbl2	RuvB-like protein 2	1.046	0.5258	NA
19698	Relb	avian reticuloendotheliosis viral (v-rel) oncogene related B	1.046	0.6727	0.8922
19359	Rad23b	RAD23b homolog (S. cerevisiae)	1.046	0.3015	NA
18807	Pld3	phospholipase D family, member 3	1.046	0.6569	0.8862
18618	Pemt	phosphatidylethanolamine N-methyltransferase	1.046	0.7757	0.9266
18515	Pbx2	pre B-cell leukemia transcription factor 2	1.046	0.6628	0.8895
17126	Smad2	MAD homolog 2 (Drosophila)	1.046	0.4933	NA
16706	Ksr1	kinase suppressor of ras 1	1.046	0.7181	0.91
16011	Igfbp5	insulin-like growth factor binding protein 5	1.046	0.4388	NA
14359	Fxr1	fragile X mental retardation gene 1, autosomal homolog	1.046	0.2431	NA
13875	Erf	Ets2 repressor factor	1.046	0.3704	NA
13854	Epn1	epsin 1	1.046	0.3565	NA
12808	Cobl	cordon-bleu	1.046	0.6111	0.8672

12055	Bcl7c	B-cell CLL/lymphoma 7C	1.046	0.6227	0.872
100049077	LOC100049077	hypothetical protein LOC100049077	1.045	0.6865	0.8966
100039795	Ildr2	immunoglobulin-like domain containing receptor 2	1.045	0.6572	0.8863
636901	LOC636901	40S ribosomal protein SA-like	1.045	0.4647	NA
606496	Gsk3a	glycogen synthase kinase 3 alpha	1.045	0.6573	0.8863
545007	ENSMUSG00000068790	predicted gene, ENSMUSG00000068790	1.045	0.3611	NA
494448	Cbx6	chromobox homolog 6	1.045	0.6945	0.9007
414073	BC040756	cDNA sequence BC040756	1.045	0.6264	0.873
347740	2900097C17Rik	RIKEN cDNA 2900097C17 gene	1.045	0.3722	NA
329693	Fcrl5	Fc receptor-like 5	1.045	0.7389	0.9177
320982	Arl4c	ADP-ribosylation factor-like 4C	1.045	0.7478	0.9202
320184	Lrrc58	leucine rich repeat containing 58	1.045	0.4543	NA
269252	Gtf3c4	general transcription factor IIIC, polypeptide 4	1.045	0.6056	0.8645
269019	Stk32a	serine/threonine kinase 32A	1.045	0.4685	NA
259302	Srgap3	SLIT-ROBO Rho GTPase activating protein 3	1.045	0.6861	0.8966
241727	Snph	syntaphilin	1.045	0.6341	0.8765
241201	Cdh7	cadherin 7, type 2	1.045	0.4805	NA
237300	Gm4922	predicted gene 4922	1.045	0.7522	0.9213
235169	Foxred1	FAD-dependent oxidoreductase domain containing 1	1.045	0.5039	NA
233335	Synm	synemin, intermediate filament protein	1.045	0.5502	NA
229504	Isg20l2	interferon stimulated exonuclease gene 20-like 2	1.045	0.4715	NA
223775	Pim3	proviral integration site 3	1.045	0.6743	0.8924
223770	Brd1	bromodomain containing 1	1.045	0.5667	NA
217837	Itpk1	inositol 1,3,4-triphosphate 5/6 kinase	1.045	0.7124	0.9075
217166	Nr1d1	nuclear receptor subfamily 1, group D, member 1	1.045	0.7442	0.9192
216150	Cdc34	cell division cycle 34 homolog (S. cerevisiae)	1.045	0.4147	NA
213171	Prss27	protease, serine, 27	1.045	0.874	0.9616
208211	Alg1	asparagine-linked glycosylation 1 homolog (yeast, beta-1,4-mannosyltransferase)	1.045	0.5287	NA
194388	Tet3	tet oncogene family member 3	1.045	0.8136	0.9406
194237	Rimkla	ribosomal modification protein rimK-like family member A	1.045	0.454	NA
170755	Sgk3	serum/glucocorticoid regulated kinase 3	1.045	0.696	0.9013
170735	Arr3	arrestin 3, retinal	1.045	0.4502	NA
117109	Pop5	processing of precursor 5, ribonuclease P/MRP family (S. cerevisiae)	1.045	0.8059	0.9386
103850	Nt5m	5',3'-nucleotidase, mitochondrial	1.045	0.4965	NA
103710	Slc35e4	solute carrier family 35, member E4	1.045	0.6462	0.881
84682	Cox4i2	cytochrome c oxidase subunit IV isoform 2	1.045	0.7903	0.9331
78830	Slc25a12	solute carrier family 25 (mitochondrial carrier, Aralar), member 12	1.045	0.3581	NA
78406	2900041H08Rik	RIKEN cDNA 2900041H08 gene	1.045	0.6927	0.8997
77036	1700109H08Rik	RIKEN cDNA 1700109H08 gene	1.045	0.5354	NA
76872	Ccdc116	coiled-coil domain containing 116	1.045	0.7402	0.9179
76302	Pcnp	PEST proteolytic signal containing nuclear protein	1.045	0.3782	NA
75292	Prkd3	protein kinase D3	1.045	0.2603	NA
74255	Smu1	smu-1 suppressor of mec-8 and unc-52 homolog (C. elegans)	1.045	0.4263	NA
73122	Tgfbrap1	transforming growth factor, beta receptor associated protein 1	1.045	0.6747	0.8925
72902	Spock3	sparc/osteonectin, cwcv and kazal-like domains proteoglycan 3	1.045	0.896	0.9699
72795	Ttc19	tetratricopeptide repeat domain 19	1.045	0.5036	NA
72522	Atxn7l2	ataxin 7-like 2	1.045	0.7486	0.9204
71991	Ercc8	excision repaiross-complementing rodent repair deficiency, complementation group 8	1.045	0.6328	0.8758
71653	4930506M07Rik	RIKEN cDNA 4930506M07 gene	1.045	0.4851	NA

71367	Chst9		carbohydrate (N-acetylgalactosamine 4-0) sulfotransferase 9	1.045	0.6971	0.902
71361	Aifm2		apoptosis-inducing factor, mitochondrion-associated 2	1.045	0.4584	NA
69274	Ctdspl		CTD (carboxy-terminal domain, RNA polymerase II, polypeptide A) small phosphatase-like	1.045	0.5923	NA
69162	Sec31a		Sec31 homolog A (S. cerevisiae)	1.045	0.543	NA
68917	Hint2		histidine triad nucleotide binding protein 2	1.045	0.3355	NA
68524	Wipf2		WAS/WASL interacting protein family, member 2	1.045	0.43	NA
67826	Snap47		synaptosomal-associated protein, 47	1.045	0.5891	NA
67713	Dnajc19		DnaJ (Hsp40) homolog, subfamily C, member 19	1.045	0.8066	0.9388
67422	Dhdds		dehydrodolichyl diphosphate synthase	1.045	0.5782	NA
67397	Erp29		endoplasmic reticulum protein 29	1.045	0.4886	NA
66492	Zmat2		zinc finger, matrin type 2	1.045	0.5593	NA
66311	Cenpw		centromere protein W	1.045	0.4446	NA
66241	Tmem9		transmembrane protein 9	1.045	0.5538	NA
66218	Ndufb9		NADH dehydrogenase (ubiquinone) 1 beta subcomplex, 9	1.045	0.3055	NA
66111	Tmed3		transmembrane emp24 domain containing 3	1.045	0.4775	NA
59032	Ppp2r3c		protein phosphatase 2, regulatory subunit B'', gamma	1.045	0.1809	NA
57808	Rpl35a		ribosomal protein L35A	1.045	0.2623	NA
57438		Mar-07	membrane-associated ring finger (C3HC4) 7	1.045	0.5856	NA
57258	Xpo4		exportin 4	1.045	0.5035	NA
56709	Dnajb12		DnaJ (Hsp40) homolog, subfamily B, member 12	1.045	0.3605	NA
56150	Mad2l1		MAD2 mitotic arrest deficient-like 1 (yeast)	1.045	0.5066	NA
53424	Tsnax		translin-associated factor X	1.045	0.3509	NA
52717	Anapc16		anaphase promoting complex subunit 16	1.045	0.4248	NA
50880	Scly		selenocysteine lyase	1.045	0.6739	0.8924
50721	Sirt6		sirtuin 6 (silent mating type information regulation 2, homolog) 6 (S. cerevisiae)	1.045	0.7563	0.9223
50497	Hspa14		heat shock protein 14	1.045	0.4202	NA
28126	Nop16		NOP16 nucleolar protein homolog (yeast)	1.045	0.6101	0.8667
26451	Rpl27a		ribosomal protein L27A	1.045	0.2866	NA
23994	Dazap2		DAZ associated protein 2	1.045	0.352	NA
22195	Ube2l3		ubiquitin-conjugating enzyme E2L 3	1.045	0.3105	NA
22142	Tuba1a		tubulin, alpha 1A	1.045	0.5481	NA
20610	Sumo3		SMT3 suppressor of mif two 3 homolog 3 (yeast)	1.045	0.6884	0.8976
20524	Slc25a17		solute carrier family 25 (mitochondrial carrier, peroxisomal membrane protein), member 17	1.045	0.2457	NA
20335	Sec61g		SEC61, gamma subunit	1.045	0.534	NA
20005	Rpl9		ribosomal protein L9	1.045	0.4896	NA
19988	Rpl6		ribosomal protein L6	1.045	0.4431	NA
19982	Rpl36a		ribosomal protein L36A	1.045	0.3443	NA
19265	Ptprcap		protein tyrosine phosphatase, receptor type, C polypeptide-associated protein	1.045	0.5081	NA
18591	Pdgfb		platelet derived growth factor, B polypeptide	1.045	0.6424	0.8793
18585	Pde9a		phosphodiesterase 9A	1.045	0.5768	NA
16716	Ky		kyphoscoliosis peptidase	1.045	0.7267	0.9129
14936	Gys1		glycogen synthase 1, muscle	1.045	0.5668	NA
14724	Gp1bb		glycoprotein Ib, beta polypeptide	1.045	0.6629	0.8895
14391	Gabpb1		GA repeat binding protein, beta 1	1.045	0.7218	0.9119
14004	Chchd2		coiled-coil-helix-coiled-coil-helix domain containing 2	1.045	0.6083	0.8656
13199	Ddn		dendrin	1.045	0.753	0.9214
12518	Cd79a		CD79A antigen (immunoglobulin-associated alpha)	1.045	0.7688	0.9255
11844	Arf5		ADP-ribosylation factor 5	1.045	0.4748	NA
11757	Prdx3		peroxiredoxin 3	1.045	0.6422	0.8793

11750	Anxa7	annexin A7	1.045	0.5387	NA
11491	Adam17	a disintegrin and metallopeptidase domain 17	1.045	0.4368	NA
100134990	LOC100134990	selenoprotein K pseudogene	1.044	0.6124	0.8679
665533	Gm13004	60S ribosomal protein L31 pseudogene	1.044	0.317	NA
547008	Gm6010	predicted gene 6010	1.044	0.5502	NA
434156	Eid2b	EP300 interacting inhibitor of differentiation 2B	1.044	0.4326	NA
384179	Gm5292	predicted gene 5292	1.044	0.3215	NA
382019	Zfp882	zinc finger protein 882	1.044	0.4505	NA
353155	Gjd3	gap junction protein, delta 3	1.044	0.8043	0.9381
329165	Abi2	abl-interactor 2	1.044	0.2764	NA
320534	Tmem104	transmembrane protein 104	1.044	0.6604	0.888
319880	Tmcc3	transmembrane and coiled coil domains 3	1.044	0.3315	NA
319195	Rpl17	ribosomal protein L17	1.044	0.3811	NA
270685	Mthfd1l	methylenetetrahydrofolate dehydrogenase (NADP+ dependent) 1-like	1.044	0.5493	NA
270091	Lrrc36	leucine rich repeat containing 36	1.044	0.3416	NA
259302	Srgap3	SLIT-ROBO Rho GTPase activating protein 3	1.044	0.6352	0.877
243923	Rgs9bp	regulator of G-protein signalling 9 binding protein	1.044	0.6011	NA
238205	Lrfn5	leucine rich repeat and fibronectin type III domain containing 5	1.044	0.7346	0.9163
236576	Spry3	sprouty homolog 3 (Drosophila)	1.044	0.5158	NA
234353	Psd3	pleckstrin and Sec7 domain containing 3	1.044	0.5985	NA
231999	Plekha8	pleckstrin homology domain containing, family A (phosphoinositide binding specific) member 8	1.044	0.5277	NA
230936	Phf13	PHD finger protein 13	1.044	0.7421	0.9182
230718	Nt5c1a	5'-nucleotidase, cytosolic IA	1.044	0.8991	0.9704
228911	Tshz2	teashirt zinc finger family member 2	1.044	0.4666	NA
226610	Fam78b	family with sequence similarity 78, member B	1.044	0.4135	NA
225058	Gm4832	predicted gene 4832	1.044	0.853	0.9544
223593	E430025E21Rik	RIKEN cDNA E430025E21 gene	1.044	0.4615	NA
216831	Arhgap44	Rho GTPase activating protein 44	1.044	0.2604	NA
215085	Slc35f1	solute carrier family 35, member F1	1.044	0.7642	0.925
211484	Tsga10	testis specific 10	1.044	0.6159	0.8698
208638	Slc25a38	solute carrier family 25, member 38	1.044	0.4713	NA
114641	Rpl31	ribosomal protein L31	1.044	0.2759	NA
110109	Nop2	NOP2 nucleolar protein homolog (yeast)	1.044	0.5187	NA
106389	Eaf2	ELL associated factor 2	1.044	0.5739	NA
100532	Rell1	RELT-like 1	1.044	0.4581	NA
99311	Commd7	COMM domain containing 7	1.044	0.5126	NA
99045	Mrps26	mitochondrial ribosomal protein S26	1.044	0.5993	NA
94184	Pdxdc1	pyridoxal-dependent decarboxylase domain containing 1	1.044	0.6152	0.8696
78287	Zfyve20	zinc finger, FYVE domain containing 20	1.044	0.2647	NA
77045	Bcl7a	B-cell CLL/lymphoma 7A	1.044	0.5167	NA
76886	Fam81a	family with sequence similarity 81, member A	1.044	0.6908	0.8989
76884	Cyfip2	cytoplasmic FMR1 interacting protein 2	1.044	0.4885	NA
76737	Creld2	cysteine-rich with EGF-like domains 2	1.044	0.4404	NA
75273	Pelp1	proline, glutamic acid and leucine rich protein 1	1.044	0.7279	0.9135
74721	4930519P11Rik	RIKEN cDNA 4930519P11 gene	1.044	0.7931	0.9335
73689	Bloc1s2	biogenesis of lysosome-related organelles complex-1, subunit 2	1.044	0.3224	NA
72832	Crtac1	cartilage acidic protein 1	1.044	0.4556	NA
72056	1810055G02Rik	RIKEN cDNA 1810055G02 gene	1.044	0.6307	0.8751
71742	Ulk3	unc-51-like kinase 3 (C. elegans)	1.044	0.5939	NA

71723	Dhx34	DEAH (Asp-Glu-Ala-His) box polypeptide 34	1.044	0.5951	NA
70601	Ecd	ecdysoneless homolog (Drosophila)	1.044	0.5548	NA
70349	Copb1	coatomer protein complex, subunit beta 1	1.044	0.487	NA
70292	Afap1	actin filament associated protein 1	1.044	0.4787	NA
68626	Elac2	elaC homolog 2 (E. coli)	1.044	0.487	NA
68572	Ict1	immature colon carcinoma transcript 1	1.044	0.6277	0.8737
68421	Lmbrd1	LMBR1 domain containing 1	1.044	0.3486	NA
68127	B230217C12Rik	RIKEN cDNA B230217C12 gene	1.044	0.6377	0.8776
68017	Ftsj2	FtsJ homolog 2 (E. coli)	1.044	0.4662	NA
67958	2610101N10Rik	RIKEN cDNA 2610101N10 gene	1.044	0.6375	0.8776
67937	Tmem59l	transmembrane protein 59-like	1.044	0.5638	NA
67768	N6amt1	N-6 adenine-specific DNA methyltransferase 1 (putative)	1.044	0.4869	NA
66999	Med28	mediator of RNA polymerase II transcription, subunit 28 homolog (yeast)	1.044	0.4302	NA
66849	Ppp1r2	protein phosphatase 1, regulatory (inhibitor) subunit 2	1.044	0.4868	NA
66848	Fuca2	fucosidase, alpha-L- 2, plasma	1.044	0.4237	NA
66629	Golph3	golgi phosphoprotein 3	1.044	0.3849	NA
66416	Ndufa7	NADH dehydrogenase (ubiquinone) 1 alpha subcomplex, 7 (B14.5a)	1.044	0.4984	NA
66374	2310011J03Rik	RIKEN cDNA 2310011J03 gene	1.044	0.4633	NA
66291	1810030N24Rik	RIKEN cDNA 1810030N24 gene	1.044	0.3237	NA
64424	Polr1e	polymerase (RNA) I polypeptide E	1.044	0.5226	NA
64424	Polr1e	polymerase (RNA) I polypeptide E	1.044	0.5569	NA
58867	Syngr4	synaptogyrin 4	1.044	0.7934	0.9335
57258	Xpo4	exportin 4	1.044	0.7126	0.9075
56715	Rabgef1	RAB guanine nucleotide exchange factor (GEF) 1	1.044	0.5921	NA
55980	Impa1	inositol (myo)-1(or 4)-monophosphatase 1	1.044	0.5733	NA
55944	Eif3d	eukaryotic translation initiation factor 3, subunit D	1.044	0.5221	NA
54447	Asah2	N-acylsphingosine amidohydrolase 2	1.044	0.7121	0.9075
53599	Cd164	CD164 antigen	1.044	0.4149	NA
50915	Grb14	growth factor receptor bound protein 14	1.044	0.6559	0.8859
27886	Dgcr14	DiGeorge syndrome critical region gene 14	1.044	0.659	0.8872
27059	Sh3d19	SH3 domain protein D19	1.044	0.6667	0.8904
26396	Map2k2	mitogen-activated protein kinase kinase 2	1.044	0.5859	NA
23983	Pcbp1	poly(rC) binding protein 1	1.044	0.4506	NA
22666	Zfp161	zinc finger protein 161	1.044	0.5525	NA
20965	Syn2	synapsin II	1.044	0.3614	NA
20661	Sort1	sortilin 1	1.044	0.619	0.8709
20132	Rrh	retinal pigment epithelium derived rhodopsin homolog	1.044	0.6208	0.8718
19708	Dpf2	D4, zinc and double PHD fingers family 2	1.044	0.5361	NA
19177	Psmb7	proteasome (prosome, macropain) subunit, beta type 7	1.044	0.7004	0.9033
19063	Ppt1	palmitoyl-protein thioesterase 1	1.044	0.4972	NA
19047	Ppp1cc	protein phosphatase 1, catalytic subunit, gamma isoform	1.044	0.6413	0.8788
18969	Pola2	polymerase (DNA directed), alpha 2	1.044	0.7929	0.9334
18846	Plxna3	plexin A3	1.044	0.6696	0.8908
18762	Prkcz	protein kinase C, zeta	1.044	0.4727	NA
18020	Nfatc2ip	nuclear factor of activated T-cells, cytoplasmic, calcineurin-dependent 2 interacting protein	1.044	0.4303	NA
17997	Nedd1	neural precursor cell expressed, developmentally down-regulated gene 1	1.044	0.6346	0.8768
14782	Gsr	glutathione reductase	1.044	0.4453	NA
14667	Gm2a	GM2 ganglioside activator protein	1.044	0.5981	NA
13722	Aimp1	aminoacyl tRNA synthetase complex-interacting multifunctional protein 1	1.044	0.2251	NA

13032	Ctsc	cathepsin C	1.044	0.4496	NA
12796	Camp	cathelicidin antimicrobial peptide	1.044	0.7723	0.926
12745	Clgn	calmegin	1.044	0.4913	NA
12567	Cdk4	cyclin-dependent kinase 4	1.044	0.5745	NA
12386	Ctnna2	catenin (cadherin associated protein), alpha 2	1.044	0.4278	NA
12051	Bcl3	B-cell leukemia/lymphoma 3	1.044	0.7493	0.9207
11302	Aatk	apoptosis-associated tyrosine kinase	1.044	0.5438	NA
100504876	LOC100504876	60S ribosomal protein L7a-like	1.043	0.4742	NA
547267	Gm6030	predicted gene 6030	1.043	0.4689	NA
494504	Apcdd1	adenomatosis polyposis coli down-regulated 1	1.043	0.4828	NA
433938	Mn1	meningioma 1	1.043	0.4697	NA
403178	Plcxd1	phosphatidylinositol-specific phospholipase C, X domain containing 1	1.043	0.5584	NA
320004	A930002H24Rik	RIKEN cDNA A930002H24 gene	1.043	0.645	0.8806
319934	Sbf2	SET binding factor 2	1.043	0.6748	0.8925
319887	E030030I06Rik	RIKEN cDNA E030030I06 gene	1.043	0.4635	NA
319594	Hif1an	hypoxia-inducible factor 1, alpha subunit inhibitor	1.043	0.4779	NA
270110	Irf2bp2	interferon regulatory factor 2 binding protein 2	1.043	0.5634	NA
269695	Rnft2	ring finger protein, transmembrane 2	1.043	0.6497	0.8823
269587	Epb4.1	erythrocyte protein band 4.1	1.043	0.4649	NA
268780	Egflam	EGF-like, fibronectin type III and laminin G domains	1.043	0.6048	NA
268510	Mgat5b	mannoside acetylglucosaminyltransferase 5, isoenzyme B	1.043	0.7415	0.918
245522	Zc4h2	zinc finger, C4H2 domain containing	1.043	0.5793	NA
238323	Rps6kl1	ribosomal protein S6 kinase-like 1	1.043	0.4116	NA
237847	Rtn4rl1	reticulon 4 receptor-like 1	1.043	0.7261	0.9126
230376	Haus6	HAUS augmin-like complex, subunit 6	1.043	0.8555	0.9552
225995	D030056L22Rik	RIKEN cDNA D030056L22 gene	1.043	0.3731	NA
225887	Ndufs8	NADH dehydrogenase (ubiquinone) Fe-S protein 8	1.043	0.4439	NA
225876	Kdm2a	lysine (K)-specific demethylase 2A	1.043	0.7884	0.9321
217517	Stxbp6	syntaxin binding protein 6 (amisyn)	1.043	0.3643	NA
217351	Tnrc6c	trinucleotide repeat containing 6C	1.043	0.5494	NA
209815	Tbc1d25	TBC1 domain family, member 25	1.043	0.5774	NA
192897	Itgb4	integrin beta 4	1.043	0.4325	NA
117599	Helb	helicase (DNA) B	1.043	0.4382	NA
114615	Elac1	elaC homolog 1 (E. coli)	1.043	0.4897	NA
109280	9330176C04Rik	solute carrier family 22 (organic cation transporter), member 13 gene:pseudogene	1.043	0.5247	NA
108138	Xrcc4	X-ray repair complementing defective repair in Chinese hamster cells 4	1.043	0.4128	NA
107071	Wdr74	WD repeat domain 74	1.043	0.5441	NA
106672	AI413582	expressed sequence AI413582	1.043	0.3719	NA
105501	Abhd4	abhydrolase domain containing 4	1.043	0.5062	NA
94245	Dtnbp1	dystrobrevin binding protein 1	1.043	0.3513	NA
94063	Mrpl16	mitochondrial ribosomal protein L16	1.043	0.3515	NA
93842	Igsf9	immunoglobulin superfamily, member 9	1.043	0.6806	0.8949
93739	Gabarapl2	gamma-aminobutyric acid (GABA) A receptor-associated protein-like 2	1.043	0.5424	NA
93730	Lztfl1	leucine zipper transcription factor-like 1	1.043	0.2908	NA
78796	Zcchc4	zinc finger, CCHC domain containing 4	1.043	0.6893	0.8982
78792	4930432F04Rik	RIKEN cDNA 4930432F04 gene	1.043	0.4823	NA
77644	C330007P06Rik	RIKEN cDNA C330007P06 gene	1.043	0.5646	NA
77605	H2afv	H2A histone family, member V	1.043	0.4571	NA
77125	Il33	interleukin 33	1.043	0.5792	NA

74268	Aven	apoptosis, caspase activation inhibitor	1.043	0.6237	0.8724
74198	Dtx2	deltex 2 homolog (Drosophila)	1.043	0.3163	NA
74167	Nudt9	nudix (nucleoside diphosphate linked moiety X)-type motif 9	1.043	0.5686	NA
72108	Ddhd2	DDHD domain containing 2	1.043	0.6488	0.8818
71963	Cdca4	cell division cycle associated 4	1.043	0.4868	NA
71927	Itfg1	integrin alpha FG-GAP repeat containing 1	1.043	0.3076	NA
70710	6330407I18Rik	RIKEN cDNA 6330407I18 gene	1.043	0.5235	NA
70375	Ica1l	islet cell autoantigen 1-like	1.043	0.5127	NA
69837	Pcgf1	polycomb group ring finger 1	1.043	0.7505	0.921
69655	Cd164l2	CD164 sialomucin-like 2	1.043	0.8089	0.9395
69202	Ptms	parathymosin	1.043	0.7689	0.9255
68743	Anln	anillin, actin binding protein	1.043	0.7508	0.9211
68051	Nutf2	nuclear transport factor 2	1.043	0.2516	NA
67994	Mrps11	mitochondrial ribosomal protein S11	1.043	0.4481	NA
67914	Coq9	coenzyme Q9 homolog (yeast)	1.043	0.4481	NA
67669	l7Rn6	lethal, Chr 7, Rinchik 6	1.043	0.4808	NA
67389	Fam132a	family with sequence similarity 132, member A	1.043	0.4752	NA
67338	Rffl	ring finger and FYVE like domain containing protein	1.043	0.6125	NA
66911	Nudt16l1	nudix (nucleoside diphosphate linked moiety X)-type motif 16-like 1	1.043	0.5313	NA
66830	Nacc1	nucleus accumbens associated 1, BEN and BTB (POZ) domain containing	1.043	0.6686	0.8904
66176	Nat9	N-acetyltransferase 9 (GCN5-related, putative)	1.043	0.4924	NA
65247	Asb1	ankyrin repeat and SOCS box-containing 1	1.043	0.3725	NA
60441	Mrpl38	mitochondrial ribosomal protein L38	1.043	0.7078	0.9064
57431	Dnajc4	DnaJ (Hsp40) homolog, subfamily C, member 4	1.043	0.5722	NA
56739	Rec8	REC8 homolog (yeast)	1.043	0.7622	0.9241
54401	Ywhab	tyrosine 3-monooxygenase/tryptophan 5-monooxygenase activation protein, beta polypeptide	1.043	0.5975	NA
54380	Smarcal1	SWI/SNF related matrix associated, actin dependent regulator of chromatin, subfamily a-like 1	1.043	0.4251	NA
53422	Ybx2	Y box protein 2	1.043	0.6565	0.8859
27207	Rps11	ribosomal protein S11	1.043	0.4106	NA
23988	Pin1	protein (peptidyl-prolyl cis/trans isomerase) NIMA-interacting 1	1.043	0.3085	NA
22627	Ywhae	tyrosine 3-monooxygenase/tryptophan 5-monooxygenase activation protein, epsilon polypeptide	1.043	0.5094	NA
20980	Syt2	synaptotagmin II	1.043	0.6867	0.8966
20912	Stxbp3a	syntaxin binding protein 3A	1.043	0.367	NA
20893	Bhlhe40	basic helix-loop-helix family, member e40	1.043	0.56	NA
20646	Snrpn	small nuclear ribonucleoprotein N	1.043	0.3902	NA
20091	Rps3a	ribosomal protein S3A	1.043	0.3065	NA
19943	Rpl28	ribosomal protein L28	1.043	0.4249	NA
19341	Rab4a	RAB4A, member RAS oncogene family	1.043	0.4906	NA
18301	Fxyd5	FXYD domain-containing ion transport regulator 5	1.043	0.7728	0.9262
18148	Npm1	nucleophosmin 1	1.043	0.6134	NA
16790	Anpep	alanyl (membrane) aminopeptidase	1.043	0.8096	0.94
15488	Hsd17b4	hydroxysteroid (17-beta) dehydrogenase 4	1.043	0.4083	NA
14588	Gfra4	glial cell line derived neurotrophic factor family receptor alpha 4	1.043	0.6668	0.8904
14337	Ftl2	ferritin light chain 2	1.043	0.6355	0.8771
14194	Fh1	fumarate hydratase 1	1.043	0.3633	NA
13368	Dffb	DNA fragmentation factor, beta subunit	1.043	0.5702	NA
13356	Dgcr2	DiGeorge syndrome critical region gene 2	1.043	0.7212	0.9115
12741	Cldn5	claudin 5	1.043	0.6832	0.8956
12348	Car11	carbonic anhydrase 11	1.043	0.6491	0.8821

12307	Calb1	calbindin 1	1.043	0.5154	NA
100043404	Gm4416	predicted gene 4416	1.042	0.5459	NA
100042332	2810410L24Rik	RIKEN cDNA 2810410L24 gene	1.042	0.4854	NA
432582	E130309D14Rik	RIKEN cDNA E130309D14 gene	1.042	0.6691	0.8904
380967	Tmem106c	transmembrane protein 106C	1.042	0.7312	0.9153
320706	9830001H06Rik	RIKEN cDNA 9830001H06 gene	1.042	0.6628	0.8895
320634	Ocrl	oculocerebrorenal syndrome of Lowe	1.042	0.5304	NA
319638	Nt5dc1	5'-nucleotidase domain containing 1	1.042	0.5522	NA
319480	Itga11	integrin alpha 11	1.042	0.7521	0.9213
268857	Nlrc3	NLR family, CARD domain containing 3	1.042	0.8099	0.94
259302	Srgap3	SLIT-ROBO Rho GTPase activating protein 3	1.042	0.5634	NA
241490	Rbm45	RNA binding motif protein 45	1.042	0.7721	0.9259
241230	St8sia6	ST8 alpha-N-acetyl-neuraminide alpha-2,8-sialyltransferase 6	1.042	0.5278	NA
240174	Thada	thyroid adenoma associated	1.042	0.8393	0.9484
239760	Gm4943	predicted pseudogene 4943	1.042	0.5702	NA
239691	AU021092	expressed sequence AU021092	1.042	0.67	0.8912
235627	Nbeal2	neurobeachin-like 2	1.042	0.5468	NA
232785	Zfp783	zinc finger protein 783	1.042	0.633	0.8758
232430	Crebl2	cAMP responsive element binding protein-like 2	1.042	0.7256	0.9126
232288	Frmd4b	FERM domain containing 4B	1.042	0.5649	NA
231801	Agfg2	ArfGAP with FG repeats 2	1.042	0.5595	NA
227615	Tmem203	transmembrane protein 203	1.042	0.2519	NA
223918	Spryd3	SPRY domain containing 3	1.042	0.5434	NA
223650	Eppk1	epiplakin 1	1.042	0.7139	0.9078
217882	AW555464	expressed sequence AW555464	1.042	0.6067	NA
216440	Os9	amplified in osteosarcoma	1.042	0.5639	NA
214932	Cecr5	cat eye syndrome chromosome region, candidate 5 homolog (human)	1.042	0.7034	0.9046
210998	D15Ertd621e	DNA segment, Chr 15, ERATO Doi 621, expressed	1.042	0.4078	NA
208439	Klhl29	kelch-like 29 (Drosophila)	1.042	0.3884	NA
192775	Kcnh6	potassium voltage-gated channel, subfamily H (eag-related), member 6	1.042	0.6644	0.8899
170458	Gpha2	glycoprotein hormone alpha 2	1.042	0.8614	0.9573
113865	Vmn1r25	vomeronasal 1 receptor 25	1.042	0.7453	0.9192
105352	Dusp22	dual specificity phosphatase 22	1.042	0.5874	NA
98488	Gtf3c3	general transcription factor IIIC, polypeptide 3	1.042	0.3594	NA
80292	Zxdc	ZXD family zinc finger C	1.042	0.4485	NA
80291	Rilpl2	Rab interacting lysosomal protein-like 2	1.042	0.4278	NA
78937	Avl9	AVL9 homolog (S. cerevisiase)	1.042	0.4511	NA
78656	Brd8	bromodomain containing 8	1.042	0.3816	NA
78294	Rps27a	ribosomal protein S27A	1.042	0.595	NA
77524	C030017B01Rik	RIKEN cDNA C030017B01 gene	1.042	0.4612	NA
77038	Arfgap2	ADP-ribosylation factor GTPase activating protein 2	1.042	0.6413	0.8788
76942	Lypd5	Ly6/Plaur domain containing 5	1.042	0.6686	0.8904
76850	Eif2c4	eukaryotic translation initiation factor 2C, 4	1.042	0.5282	NA
76628	1700112J16Rik	RIKEN cDNA 1700112J16 gene	1.042	0.3668	NA
76073	Pcgf5	polycomb group ring finger 5	1.042	0.5981	NA
75731	5133401N09Rik	RIKEN cDNA 5133401N09 gene	1.042	0.639	0.878
74616	Scrn3	secernin 3	1.042	0.6042	NA
72454	Ccdc71	coiled-coil domain containing 71	1.042	0.4184	NA
71263	Mro	maestro	1.042	0.8796	0.9631

71207	Nudt4	nudix (nucleoside diphosphate linked moiety X)-type motif 4	1.042	0.4705	NA
71131	Zfp689	zinc finger protein 689	1.042	0.7135	0.9077
70640	Dcp2	DCP2 decapping enzyme homolog (S. cerevisiae)	1.042	0.5499	NA
70454	Cenpl	centromere protein L	1.042	0.6317	0.8751
70223	Nars	asparaginyl-tRNA synthetase	1.042	0.5451	NA
68732	Lrrc16a	leucine rich repeat containing 16A	1.042	0.7119	0.9073
68051	Nutf2	nuclear transport factor 2	1.042	0.5503	NA
67877	Naa20	N(alpha)-acetyltransferase 20, NatB catalytic subunit	1.042	0.4079	NA
67673	Tceb2	transcription elongation factor B (SIII), polypeptide 2	1.042	0.4952	NA
67467	1200011I18Rik	RIKEN cDNA 1200011I18 gene	1.042	0.3061	NA
67399	Pdlim7	PDZ and LIM domain 7	1.042	0.6486	0.8818
67269	Agtpbp1	ATP/GTP binding protein 1	1.042	0.5074	NA
67169	Nradd	neurotrophin receptor associated death domain	1.042	0.5838	NA
67091	Trappc6a	trafficking protein particle complex 6A	1.042	0.4847	NA
66724	Tab3	TGF-beta activated kinase 1/MAP3K7 binding protein 3	1.042	0.4363	NA
66661	Srp72	signal recognition particle 72	1.042	0.258	NA
66242	Mrps16	mitochondrial ribosomal protein S16	1.042	0.4094	NA
66078	Tsen34	tRNA splicing endonuclease 34 homolog (S. cerevisiae)	1.042	0.4267	NA
66049	Rogdi	rogdi homolog (Drosophila)	1.042	0.5465	NA
64933	Ap3m2	adaptor-related protein complex 3, mu 2 subunit	1.042	0.4346	NA
58180	Hic2	hypermethylated in cancer 2	1.042	0.739	0.9177
57813	Tk2	thymidine kinase 2, mitochondrial	1.042	0.4459	NA
56838	Ccl28	chemokine (C-C motif) ligand 28	1.042	0.8732	0.9612
56198	Heyl	hairy/enhancer-of-split related with YRPW motif-like	1.042	0.765	0.9251
54709	Eif3i	eukaryotic translation initiation factor 3, subunit I	1.042	0.5474	NA
53867	Col5a3	collagen, type V, alpha 3	1.042	0.8047	0.9381
52123	Agpat5	1-acylglycerol-3-phosphate O-acyltransferase 5 (lysophosphatidic acid acyltransferase, epsilon)	1.042	0.4714	NA
30059	Timm10	translocase of inner mitochondrial membrane 10 homolog (yeast)	1.042	0.4072	NA
28084	Vps25	vacuolar protein sorting 25 (yeast)	1.042	0.6108	NA
27015	Polk	polymerase (DNA directed), kappa	1.042	0.6527	0.8842
23922	Jtb	jumping translocation breakpoint	1.042	0.4305	NA
22376	Was	Wiskott-Aldrich syndrome homolog (human)	1.042	0.819	0.9426
20068	Rps17	ribosomal protein S17	1.042	0.3687	NA
19428	Rasl2-9-ps	RAS-like, family 2, locus 9, pseudogene	1.042	0.5067	NA
19302	Pxmp3	peroxisomal membrane protein 3	1.042	0.3237	NA
18951	Sep-05	septin 5	1.042	0.7943	0.9339
18726	Lilra6	leukocyte immunoglobulin-like receptor, subfamily A (with TM domain), member 6	1.042	0.8117	0.9403
18479	Pak1	p21 protein (Cdc42/Rac)-activated kinase 1	1.042	0.4862	NA
16513	Kcnj10	potassium inwardly-rectifying channel, subfamily J, member 10	1.042	0.4266	NA
14683	Gnas	GNAS (guanine nucleotide binding protein, alpha stimulating) complex locus	1.042	0.6002	NA
13714	Elk4	ELK4, member of ETS oncogene family	1.042	0.6841	0.8956
13602	Sparcl1	SPARC-like 1	1.042	0.5669	NA
13429	Dnm1	dynamin 1	1.042	0.765	0.9251
13353	Dgcr6	DiGeorge syndrome critical region gene 6	1.042	0.6186	NA
13193	Dcx	doublecortin	1.042	0.5681	NA
100038999	Gm13552	predicted gene 13552	1.041	0.5805	NA
100038760	LOC100038760	hypothetical LOC100038760	1.041	0.6211	NA
545428	Ccdc141	coiled-coil domain containing 141	1.041	0.8289	0.945
434394	Gm5614	predicted gene 5614	1.041	0.5872	NA

386649	Nsfl1c	NSFL1 (p97) cofactor (p47)	1.041	0.4324	NA
330173	2610524H06Rik	RIKEN cDNA 2610524H06 gene	1.041	0.5745	NA
272350	Gm5065	predicted gene 5065	1.041	0.8282	0.945
269951	Idh2	isocitrate dehydrogenase 2 (NADP+), mitochondrial	1.041	0.6118	NA
268390	Ahsa2	AHA1, activator of heat shock protein ATPase homolog 2 (yeast)	1.041	0.446	NA
242521	Klhl9	kelch-like 9 (Drosophila)	1.041	0.3815	NA
237253	Lrp11	low density lipoprotein receptor-related protein 11	1.041	0.435	NA
236604	Pisd-ps1	phosphatidylserine decarboxylase, pseudogene 1	1.041	0.4286	NA
235416	Lman1l	lectin, mannose-binding 1 like	1.041	0.6445	0.8802
234854	Cdk10	cyclin-dependent kinase 10	1.041	0.5287	NA
233902	Fbxl19	F-box and leucine-rich repeat protein 19	1.041	0.4624	NA
230657	Tmem69	transmembrane protein 69	1.041	0.7284	0.9137
226977	Actr1b	ARP1 actin-related protein 1 homolog B, centractin beta (yeast)	1.041	0.6983	0.9028
225600	Pde6a	phosphodiesterase 6A, cGMP-specific, rod, alpha	1.041	0.6765	0.8929
225348	Wdr36	WD repeat domain 36	1.041	0.6281	NA
211389	Suox	sulfite oxidase	1.041	0.5853	NA
170736	Parvb	parvin, beta	1.041	0.5975	NA
140723	Cacng5	calcium channel, voltage-dependent, gamma subunit 5	1.041	0.6055	NA
108100	Baiap2	brain-specific angiogenesis inhibitor 1-associated protein 2	1.041	0.6711	0.8914
106264	0610012G03Rik	RIKEN cDNA 0610012G03 gene	1.041	0.5992	NA
103765	Tmem17	transmembrane protein 17	1.041	0.3918	NA
102866	Pls3	plastin 3 (T-isoform)	1.041	0.4233	NA
102866	Pls3	plastin 3 (T-isoform)	1.041	0.4793	NA
83674	Cnnm1	cyclin M1	1.041	0.5347	NA
80886	Senp3	SUMO/sentrin specific peptidase 3	1.041	0.4709	NA
80877	Lrba	LPS-responsive beige-like anchor	1.041	0.3713	NA
80748	BC004004	cDNA sequence BC004004	1.041	0.4485	NA
78829	Tsc22d4	TSC22 domain family, member 4	1.041	0.7362	0.9169
78581	Utp23	UTP23, small subunit (SSU) processome component, homolog (yeast)	1.041	0.4607	NA
77569	Limch1	LIM and calponin homology domains 1	1.041	0.519	NA
76688	Arfrp1	ADP-ribosylation factor related protein 1	1.041	0.296	NA
76311	1110019D14Rik	RIKEN cDNA 1110019D14 gene	1.041	0.666	0.8904
74722	4930517J16Rik	RIKEN cDNA 4930517J16 gene	1.041	0.6056	NA
74627	4930413E15Rik	RIKEN cDNA 4930413E15 gene	1.041	0.8647	0.9582
74412	Gle1	GLE1 RNA export mediator (yeast)	1.041	0.5948	NA
74407	Ttc25	tetratricopeptide repeat domain 25	1.041	0.6821	0.8951
73016	Kremen2	kringle containing transmembrane protein 2	1.041	0.7411	0.918
72834	2810468N07Rik	RIKEN cDNA 2810468N07 gene	1.041	0.6198	NA
72831	Dhx30	DEAH (Asp-Glu-Ala-His) box polypeptide 30	1.041	0.4796	NA
72085	Osgepl1	O-sialoglycoprotein endopeptidase-like 1	1.041	0.3964	NA
71393	Kctd6	potassium channel tetramerisation domain containing 6	1.041	0.39	NA
71207	Nudt4	nudix (nucleoside diphosphate linked moiety X)-type motif 4	1.041	0.5362	NA
71151	Eri2	exoribonuclease 2	1.041	0.5469	NA
70646	Naa30	N(alpha)-acetyltransferase 30, NatC catalytic subunit	1.041	0.3577	NA
69259	Kctd5	potassium channel tetramerisation domain containing 5	1.041	0.6369	NA
69179	Tmem110	transmembrane protein 110	1.041	0.6243	NA
69126	1810022K09Rik	RIKEN cDNA 1810022K09 gene	1.041	0.5064	NA
68895	Rasl11a	RAS-like, family 11, member A	1.041	0.699	0.903
68644	Abhd14a	abhydrolase domain containing 14A	1.041	0.5119	NA

68170	B230118H07Rik	RIKEN cDNA B230118H07 gene	1.041	0.4427	NA
68079	Pdcd2l	programmed cell death 2-like	1.041	0.3884	NA
68015	Trap1	TNF receptor-associated protein 1	1.041	0.5259	NA
67898	Pef1	penta-EF hand domain containing 1	1.041	0.481	NA
67824	Nmral1	NmrA-like family domain containing 1	1.041	0.6916	0.8991
67095	Trak1	trafficking protein, kinesin binding 1	1.041	0.7384	0.9174
66988	Lap3	leucine aminopeptidase 3	1.041	0.3856	NA
66975	2410002O22Rik	RIKEN cDNA 2410002O22 gene	1.041	0.4387	NA
66701	Spryd4	SPRY domain containing 4	1.041	0.3185	NA
64934	Pes1	pescadillo homolog 1, containing BRCT domain (zebrafish)	1.041	0.4229	NA
63828	Fn3k	fructosamine 3 kinase	1.041	0.4731	NA
57912	Cdc42se1	CDC42 small effector 1	1.041	0.6346	0.8768
56878	Rbms1	RNA binding motif, single stranded interacting protein 1	1.041	0.512	NA
56275	Rbm14	RNA binding motif protein 14	1.041	0.4086	NA
56191	Tro	trophinin	1.041	0.4876	NA
52710	Gpr172b	G protein-coupled receptor 172B	1.041	0.6196	NA
28169	Agpat3	1-acylglycerol-3-phosphate O-acyltransferase 3	1.041	0.4361	NA
22756	Zfp94	zinc finger protein 94	1.041	0.6517	0.8834
22619	Siae	sialic acid acetylesterase	1.041	0.677	0.893
21848	Trim24	tripartite motif-containing 24	1.041	0.5485	NA
19364	Rad51l3	RAD51-like 3 (S. cerevisiae)	1.041	0.6709	0.8914
19328	Rab12	RAB12, member RAS oncogene family	1.041	0.397	NA
18451	P4ha1	procollagen-proline, 2-oxoglutarate 4-dioxygenase (proline 4-hydroxylase), alpha 1 polypeptide	1.041	0.6496	0.8823
17932	Myt1	myelin transcription factor 1	1.041	0.8704	0.9604
16443	Itsn1	intersectin 1 (SH3 domain protein 1A)	1.041	0.4505	NA
14828	Hspa5	heat shock protein 5	1.041	0.5014	NA
14784	Grb2	growth factor receptor bound protein 2	1.041	0.7852	0.9307
14102	Fas	Fas (TNF receptor superfamily member 6)	1.041	0.7627	0.9244
13714	Elk4	ELK4, member of ETS oncogene family	1.041	0.7376	0.9172
11911	Atf4	activating transcription factor 4	1.041	0.4275	NA
11804	Aplp2	amyloid beta (A4) precursor-like protein 2	1.041	0.3856	NA
100529082	Gm11127	predicted gene 11127	1.04	0.5284	NA
100045653	LOC100045653	hypothetical LOC100045653	1.04	0.8225	0.9435
100043732	Gm4613	60S ribosomal protein L37a pseudogene	1.04	0.553	NA
100041481	Gm3364	predicted gene 3364	1.04	0.8764	0.962
619605	Zcchc17	zinc finger, CCHC domain containing 17	1.04	0.4434	NA
432768	Gm5451	predicted gene 5451	1.04	0.6362	NA
407819	BC031181	cDNA sequence BC031181	1.04	0.3974	NA
381113	Cdkl4	cyclin-dependent kinase-like 4	1.04	0.791	0.9333
320095	6430550D23Rik	RIKEN cDNA 6430550D23 gene	1.04	0.6633	0.8896
319934	Sbf2	SET binding factor 2	1.04	0.6783	0.8936
268783	Mtmr12	myotubularin related protein 12	1.04	0.4285	NA
266614	Ly6g5b	lymphocyte antigen 6 complex, locus G5B	1.04	0.7173	0.9096
244556	Zfp791	zinc finger protein 791	1.04	0.802	0.9373
243853	Fkrp	fukutin related protein	1.04	0.6003	NA
239546	Zfp647	zinc finger protein 647	1.04	0.4448	NA
237313	Il20ra	interleukin 20 receptor, alpha	1.04	0.5482	NA
235442	Rab8b	RAB8B, member RAS oncogene family	1.04	0.5287	NA
232089	Elmod3	ELMO/CED-12 domain containing 3	1.04	0.5989	NA

231807	BC037034	cDNA sequence BC037034	1.04	0.7123	0.9075
231630	Ficd	FIC domain containing	1.04	0.668	0.8904
228994	Ythdf1	YTH domain family 1	1.04	0.4776	NA
227197	Ndufs1	NADH dehydrogenase (ubiquinone) Fe-S protein 1	1.04	0.4952	NA
226982	Eif5b	eukaryotic translation initiation factor 5B	1.04	0.5704	NA
224813	Gm88	predicted gene 88	1.04	0.5638	NA
217874	BC048943	cDNA sequence BC048943	1.04	0.4629	NA
214597	Sidt2	SID1 transmembrane family, member 2	1.04	0.4342	NA
211978	Zfyve26	zinc finger, FYVE domain containing 26	1.04	0.6149	NA
170930	Sumo2	SMT3 suppressor of mif two 3 homolog 2 (yeast)	1.04	0.5971	NA
114255	Dok4	docking protein 4	1.04	0.5876	NA
110855	Pde6c	phosphodiesterase 6C, cGMP specific, cone, alpha prime	1.04	0.7046	0.9049
109065	1110034A24Rik	RIKEN cDNA 1110034A24 gene	1.04	0.6307	NA
104263	Kdm3a	lysine (K)-specific demethylase 3A	1.04	0.5467	NA
103210	D630004K10Rik	RIKEN cDNA D630004K10 gene	1.04	0.5582	NA
98170	Tmem132a	transmembrane protein 132A	1.04	0.5217	NA
94281	Sfxn4	sideroflexin 4	1.04	0.5869	NA
83767	Wasf1	WASP family 1	1.04	0.425	NA
80509	Med8	mediator of RNA polymerase II transcription, subunit 8 homolog (yeast)	1.04	0.5792	NA
78906	9130017N09Rik	RIKEN cDNA 9130017N09 gene	1.04	0.797	0.9351
78321	Ankrd23	ankyrin repeat domain 23	1.04	0.6124	NA
76686	Clip3	CAP-GLY domain containing linker protein 3	1.04	0.7201	0.9106
73447	Wdr13	WD repeat domain 13	1.04	0.6375	NA
72584	Cul4b	cullin 4B	1.04	0.4936	NA
72486	Rnf219	ring finger protein 219	1.04	0.6831	0.8956
72308	Brf1	BRF1 homolog, subunit of RNA polymerase III transcription initiation factor IIIB (S. cerevisiae)	1.04	0.2565	NA
72108	Ddhd2	DDHD domain containing 2	1.04	0.4687	NA
71952	2410016O06Rik	RIKEN cDNA 2410016O06 gene	1.04	0.4754	NA
71785	Pdgfd	platelet-derived growth factor, D polypeptide	1.04	0.5777	NA
71769	Bbs10	Bardet-Biedl syndrome 10 (human)	1.04	0.4661	NA
71746	Rgl3	ral guanine nucleotide dissociation stimulator-like 3	1.04	0.6801	0.8947
71354	Wdr31	WD repeat domain 31	1.04	0.6268	NA
69662	2310061I04Rik	RIKEN cDNA 2310061I04 gene	1.04	0.6642	0.8899
68833	Pdcl3	phosducin-like 3	1.04	0.7189	0.9102
68565	Mrps18a	mitochondrial ribosomal protein S18A	1.04	0.5427	NA
67840	Mrp63	mitochondrial ribosomal protein 63	1.04	0.3971	NA
67203	Nde1	nuclear distribution gene E homolog 1 (A nidulans)	1.04	0.4412	NA
67150	Rnf141	ring finger protein 141	1.04	0.4032	NA
67130	Ndufa6	NADH dehydrogenase (ubiquinone) 1 alpha subcomplex, 6 (B14)	1.04	0.4004	NA
66973	Mrps18b	mitochondrial ribosomal protein S18B	1.04	0.5526	NA
66818	9130011J15Rik	RIKEN cDNA 9130011J15 gene	1.04	0.5723	NA
66627	Ogfod2	2-oxoglutarate and iron-dependent oxygenase domain containing 2	1.04	0.3298	NA
66383	Iscu	IscU iron-sulfur cluster scaffold homolog (E. coli)	1.04	0.3298	NA
66256	Ssr2	signal sequence receptor, beta	1.04	0.6143	NA
66154	Tmem14c	transmembrane protein 14C	1.04	0.5041	NA
65254	Dpysl5	dihydropyrimidinase-like 5	1.04	0.8033	0.9377
64452	Slc5a4a	solute carrier family 5, member 4a	1.04	0.929	0.9789
64296	Abhd8	abhydrolase domain containing 8	1.04	0.5928	NA
59027	Nampt	nicotinamide phosphoribosyltransferase	1.04	0.573	NA

56174	Nagk	N-acetylglucosamine kinase	1.04	0.7103	0.9069
53872	Caprin1	cell cycle associated protein 1	1.04	0.4399	NA
53607	Snrpa	small nuclear ribonucleoprotein polypeptide A	1.04	0.7018	0.9038
53414	Bysl	bystin-like	1.04	0.511	NA
50498	Ebi3	Epstein-Barr virus induced gene 3	1.04	0.6145	NA
30938	Fgd3	FYVE, RhoGEF and PH domain containing 3	1.04	0.649	0.882
27214	Dbf4	DBF4 homolog (S. cerevisiae)	1.04	0.7705	0.9257
26950	Vsnl1	visinin-like 1	1.04	0.5851	NA
22773	Zic3	zinc finger protein of the cerebellum 3	1.04	0.8079	0.9391
22608	Ybx1	Y box protein 1	1.04	0.5937	NA
22375	Wars	tryptophanyl-tRNA synthetase	1.04	0.7568	0.9225
22256	Ung	uracil DNA glycosylase	1.04	0.6238	NA
22193	Ube2e3	ubiquitin-conjugating enzyme E2E 3, UBC4/5 homolog (yeast)	1.04	0.3145	NA
22017	Tpmt	thiopurine methyltransferase	1.04	0.5712	NA
21968	Tom1	target of myb1 homolog (chicken)	1.04	0.5017	NA
21452	Tcn2	transcobalamin 2	1.04	0.5848	NA
21400	Tcea2	transcription elongation factor A (SII), 2	1.04	0.6165	NA
20974	Syngr3	synaptogyrin 3	1.04	0.734	0.9161
20662	Sos1	son of sevenless homolog 1 (Drosophila)	1.04	0.518	NA
20646	Snrpn	small nuclear ribonucleoprotein N	1.04	0.608	NA
20451	St8sia3	ST8 alpha-N-acetyl-neuraminide alpha-2,8-sialyltransferase 3	1.04	0.4699	NA
19166	Psma2	proteasome (prosome, macropain) subunit, alpha type 2	1.04	0.2824	NA
18669	Abcb1b	ATP-binding cassette, sub-family B (MDR/TAP), member 1B	1.04	0.5902	NA
18516	Pbx3	pre B-cell leukemia transcription factor 3	1.04	0.4348	NA
18194	Nsdhl	NAD(P) dependent steroid dehydrogenase-like	1.04	0.6817	0.8949
18032	Nfix	nuclear factor I/X	1.04	0.7415	0.918
17916	Myo1f	myosin IF	1.04	0.8538	0.9544
16828	Ldha	lactate dehydrogenase A	1.04	0.5957	NA
15901	Id1	inhibitor of DNA binding 1	1.04	0.7251	0.9126
15381	Hnrnpc	heterogeneous nuclear ribonucleoprotein C	1.04	0.6278	NA
14972	H2-K1	histocompatibility 2, K1, K region	1.04	0.4966	NA
14137	Fdft1	farnesyl diphosphate farnesyl transferase 1	1.04	0.5562	NA
13121	Cyp51	cytochrome P450, family 51	1.04	0.5165	NA
13117	Cyp4a10	cytochrome P450, family 4, subfamily a, polypeptide 10	1.04	0.7812	0.9289
12912	Creb1	cAMP responsive element binding protein 1	1.04	0.5926	NA
12677	Vsx2	visual system homeobox 2	1.04	0.6313	NA
11950	Atp5f1	ATP synthase, H+ transporting, mitochondrial F0 complex, subunit B1	1.04	0.3974	NA
11907	Ate1	arginyltransferase 1	1.04	0.4514	NA
100042959	Gm4130	predicted gene 4130	1.039	0.7324	0.9157
626683	Gm11362	predicted gene 11362	1.039	0.8233	0.9439
606497	Otx2os1	Otx2 opposite strand transcript 1	1.039	0.703	0.9042
432769	Zfp708	zinc finger protein 708	1.039	0.612	NA
402737	A130014A01Rik	RIKEN cDNA A130014A01 gene	1.039	0.6332	NA
382823	D13Mgi7	DNA Segment, Chr 13, Mouse Genome Informatics 7	1.039	0.8119	0.9403
319939	Tns3	tensin 3	1.039	0.5783	NA
319430	Gpr77	G protein-coupled receptor 77	1.039	0.8375	0.9479
319195	Rpl17	ribosomal protein L17	1.039	0.5472	NA
268970	Arhgap28	Rho GTPase activating protein 28	1.039	0.6691	0.8904
233058	Zfp420	zinc finger protein 420	1.039	0.7535	0.9216

231872	Aimp2	aminoacyl tRNA synthetase complex-interacting multifunctional protein 2	1.039	0.4513	NA
230959	Ajap1	adherens junction associated protein 1	1.039	0.6817	0.8949
227298	Fam134a	family with sequence similarity 134, member A	1.039	0.6012	NA
224829	Trerf1	transcriptional regulating factor 1	1.039	0.7416	0.918
224129	Adcy5	adenylate cyclase 5	1.039	0.5414	NA
215351	Senp6	SUMO/sentrin specific peptidase 6	1.039	0.434	NA
212503	Paox	polyamine oxidase (exo-N4-amino)	1.039	0.7061	0.9058
212377	Mms22l	MMS22-like, DNA repair protein	1.039	0.7592	0.9229
193003	Pirt	phosphoinositide-interacting regulator of transient receptor potential channels	1.039	0.6817	0.8949
192231	Hexim1	hexamethylene bis-acetamide inducible 1	1.039	0.5468	NA
116871	Mta3	metastasis associated 3	1.039	0.673	0.8924
110012	Gm16517	predicted gene, Gm16517	1.039	0.68	0.8946
107566	Arl2bp	ADP-ribosylation factor-like 2 binding protein	1.039	0.4704	NA
107435	Hat1	histone aminotransferase 1	1.039	0.6808	0.8949
103266	AI597468	expressed sequence AI597468	1.039	0.3431	NA
100710	Pds5b	PDS5, regulator of cohesion maintenance, homolog B (S. cerevisiae)	1.039	0.6372	NA
94064	Mrpl27	mitochondrial ribosomal protein L27	1.039	0.4584	NA
93765	Ube2n	ubiquitin-conjugating enzyme E2N	1.039	0.336	NA
81500	Sil1	endoplasmic reticulum chaperone SIL1 homolog (S. cerevisiae)	1.039	0.5519	NA
78610	Uvrag	UV radiation resistance associated gene	1.039	0.5206	NA
78070	Cpt1c	carnitine palmitoyltransferase 1c	1.039	0.6173	NA
77697	Mmab	methylmalonic aciduria (cobalamin deficiency) type B homolog (human)	1.039	0.5406	NA
76454	Fbxo31	F-box protein 31	1.039	0.4722	NA
75146	Tmem180	transmembrane protein 180	1.039	0.7652	0.9251
73711	Fam125a	family with sequence similarity 125, member A	1.039	0.6691	0.8904
71883	Coq2	coenzyme Q2 homolog, prenyltransferase (yeast)	1.039	0.4469	NA
71517	9030624J02Rik	RIKEN cDNA 9030624J02 gene	1.039	0.6281	NA
71338	Tprg	transformation related protein 63 regulated	1.039	0.9264	0.9785
71323	Rassf8	Ras association (RalGDS/AF-6) domain family (N-terminal) member 8	1.039	0.7197	0.9106
71000	4931440J10Rik	RIKEN cDNA 4931440J10 gene	1.039	0.9204	0.9768
70435	Inf2	inverted formin, FH2 and WH2 domain containing	1.039	0.6689	0.8904
69511	Klk12	kallikrein related-peptidase 12	1.039	0.7952	0.9343
69125	Cnot8	CCR4-NOT transcription complex, subunit 8	1.039	0.3264	NA
68117	Apool	apolipoprotein O-like	1.039	0.4761	NA
67993	Nudt12	nudix (nucleoside diphosphate linked moiety X)-type motif 12	1.039	0.7006	0.9033
67922	Fam32a	family with sequence similarity 32, member A	1.039	0.6032	NA
67917	Zcchc3	zinc finger, CCHC domain containing 3	1.039	0.6716	0.8917
67092	Gatm	glycine amidinotransferase (L-arginine:glycine amidinotransferase)	1.039	0.7015	0.9038
67003	Uqcrc2	ubiquinol cytochrome c reductase core protein 2	1.039	0.3794	NA
66776	Pisd-ps3	phosphatidylserine decarboxylase, pseudogene 3	1.039	0.7376	0.9172
66646	Rpe	ribulose-5-phosphate-3-epimerase	1.039	0.7922	0.9333
66403	Asf1a	ASF1 anti-silencing function 1 homolog A (S. cerevisiae)	1.039	0.5358	NA
58248	1700123O20Rik	RIKEN cDNA 1700123O20 gene	1.039	0.497	NA
56409	Nudt3	nudix (nucleotide diphosphate linked moiety X)-type motif 3	1.039	0.336	NA
56360	Acot9	acyl-CoA thioesterase 9	1.039	0.5573	NA
56284	Mrpl19	mitochondrial ribosomal protein L19	1.039	0.6003	NA
53422	Ybx2	Y box protein 2	1.039	0.7497	0.9209
52857	Gramd1a	GRAM domain containing 1A	1.039	0.4269	NA
30839	Fbxw5	F-box and WD-40 domain protein 5	1.039	0.6906	0.8988

28084	Vps25	vacuolar protein sorting 25 (yeast)	1.039	0.6293	NA
27361	Sepx1	selenoprotein X 1	1.039	0.6672	0.8904
26374	Rfwd2	ring finger and WD repeat domain 2	1.039	0.3903	NA
23897	Hax1	HCLS1 associated X-1	1.039	0.6471	NA
22209	Ube2a	ubiquitin-conjugating enzyme E2A, RAD6 homolog (S. cerevisiae)	1.039	0.3248	NA
22110	Tspyl1	testis-specific protein, Y-encoded-like 1	1.039	0.3659	NA
20848	Stat3	signal transducer and activator of transcription 3	1.039	0.7473	0.9199
20842	Stag1	stromal antigen 1	1.039	0.3499	NA
20603	Sms	spermine synthase	1.039	0.3866	NA
20280	Scp2	sterol carrier protein 2, liver	1.039	0.3468	NA
19711	Resp18	regulated endocrine-specific protein 18	1.039	0.3383	NA
18771	Pknox1	Pbx/knotted 1 homeobox	1.039	0.248	NA
18046	Nfyc	nuclear transcription factor-Y gamma	1.039	0.5757	NA
17713	Grpel1	GrpE-like 1, mitochondrial	1.039	0.3412	NA
17347	Mknk2	MAP kinase-interacting serine/threonine kinase 2	1.039	0.5081	NA
16157	Il11ra1	interleukin 11 receptor, alpha chain 1	1.039	0.5194	NA
15257	Hipk1	homeodomain interacting protein kinase 1	1.039	0.7316	0.9153
14870	Gstp1	glutathione S-transferase, pi 1	1.039	0.6466	NA
14527	Gcgr	glucagon receptor	1.039	0.8436	0.95
13680	Ddx19a	DEAD (Asp-Glu-Ala-Asp) box polypeptide 19a	1.039	0.8077	0.939
13019	Ctf1	cardiotrophin 1	1.039	0.6763	0.8928
12834	Col6a2	collagen, type VI, alpha 2	1.039	0.8449	0.9506
12777	Ccr10	chemokine (C-C motif) receptor 10	1.039	0.7458	0.9192
12579	Cdkn2b	cyclin-dependent kinase inhibitor 2B (p15, inhibits CDK4)	1.039	0.7796	0.928
12322	Camk2a	calcium/calmodulin-dependent protein kinase II alpha	1.039	0.658	0.8867
11489	Adam12	a disintegrin and metallopeptidase domain 12 (meltrin alpha)	1.039	0.6562	0.8859
100042539	Gm3893	predicted gene 3893	1.038	0.827	0.9449
100009600	Zglp1	zinc finger, GATA-like protein 1	1.038	0.6747	0.8925
625730	Gm6616	predicted gene 6616	1.038	0.6358	NA
620306	LOC620306	similar to Glyceraldehyde-3-phosphate dehydrogenase (GAPDH)	1.038	0.8925	0.9691
547103	Gm10674	predicted gene 10674	1.038	0.8348	0.9466
384214	Ephx4	epoxide hydrolase 4	1.038	0.3541	NA
382221	Gm1141	predicted gene 1141	1.038	0.8678	0.9594
381820	2700089E24Rik	RIKEN cDNA 2700089E24 gene	1.038	0.4679	NA
277203	Tm4sf19	transmembrane 4 L six family member 19	1.038	0.8875	0.9671
269999	Orai3	ORAI calcium release-activated calcium modulator 3	1.038	0.8367	0.9478
260409	Cdc42ep3	CDC42 effector protein (Rho GTPase binding) 3	1.038	0.4038	NA
257633	Acsf3	acyl-CoA synthetase family member 3	1.038	0.4381	NA
244672	Cwf19l2	CWF19-like 2, cell cycle control (S. pombe)	1.038	0.6063	NA
242785	Klhl21	kelch-like 21 (Drosophila)	1.038	0.7323	0.9157
240843	Fam5b	family with sequence similarity 5, member B	1.038	0.7231	0.9121
229675	Rsbn1	rosbin, round spermatid basic protein 1	1.038	0.542	NA
229055	Zbtb10	zinc finger and BTB domain containing 10	1.038	0.4611	NA
228714	Csrp2bp	cysteine and glycine-rich protein 2 binding protein	1.038	0.8019	0.9373
228366	Gyltl1b	glycosyltransferase-like 1B	1.038	0.7457	0.9192
224694	Zfp81	zinc finger protein 81	1.038	0.4127	NA
213980	Fbxw10	F-box and WD-40 domain protein 10	1.038	0.7595	0.9229
193670	Rnf185	ring finger protein 185	1.038	0.3839	NA
109136	Mmaa	methylmalonic aciduria (cobalamin deficiency) type A	1.038	0.6294	NA

108679	Cops8	COP9 (constitutive photomorphogenic) homolog, subunit 8 (Arabidopsis thaliana)	1.038	0.6602	NA
107513	Ssr1	signal sequence receptor, alpha	1.038	0.4027	NA
107328	Trpt1	tRNA phosphotransferase 1	1.038	0.5188	NA
101489	Ric8	resistance to inhibitors of cholinesterase 8 homolog (C. elegans)	1.038	0.5676	NA
94187	Zfp423	zinc finger protein 423	1.038	0.7593	0.9229
93692	Glrx	glutaredoxin	1.038	0.4228	NA
80708	Pacsin3	protein kinase C and casein kinase substrate in neurons 3	1.038	0.5241	NA
80284	BC003266	cDNA sequence BC003266	1.038	0.5131	NA
78938	Fbxo34	F-box protein 34	1.038	0.7226	0.9121
76650	Srxn1	sulfiredoxin 1 homolog (S. cerevisiae)	1.038	0.4841	NA
74267	Iqcf1	IQ motif containing F1	1.038	0.6152	NA
73834	Atp6v1d	ATPase, H+ transporting, lysosomal V1 subunit D	1.038	0.2913	NA
73316	Calr3	calreticulin 3	1.038	0.6838	0.8956
73049	2900054C01Rik	RIKEN cDNA 2900054C01 gene	1.038	0.5646	NA
72290	Lsm11	U7 snRNP-specific Sm-like protein LSM11	1.038	0.5621	NA
72181	Nsun4	NOL1/NOP2/Sun domain family, member 4	1.038	0.5899	NA
71529	9030409G11Rik	RIKEN cDNA 9030409G11 gene	1.038	0.5802	NA
70713	Gpr137c	G protein-coupled receptor 137C	1.038	0.7934	0.9335
70508	Bbx	bobby sox homolog (Drosophila)	1.038	0.3996	NA
69860	Eif1ad	eukaryotic translation initiation factor 1A domain containing	1.038	0.4137	NA
69804	Tmem147	transmembrane protein 147	1.038	0.5825	NA
69008	Cab39l	calcium binding protein 39-like	1.038	0.6555	NA
67980	Gnpda2	glucosamine-6-phosphate deaminase 2	1.038	0.4854	NA
67936	Wdr55	WD repeat domain 55	1.038	0.3035	NA
67710	Polr2g	polymerase (RNA) II (DNA directed) polypeptide G	1.038	0.3633	NA
67582	Slc25a26	solute carrier family 25 (mitochondrial carrier, phosphate carrier), member 26	1.038	0.6203	NA
67495	Tmem167b	transmembrane protein 167B	1.038	0.6347	NA
67437	Ssr3	signal sequence receptor, gamma	1.038	0.3705	NA
67282	Ccdc53	coiled-coil domain containing 53	1.038	0.4933	NA
67236	Cinp	cyclin-dependent kinase 2 interacting protein	1.038	0.6063	NA
67228	Wdr85	WD repeat domain 85	1.038	0.533	NA
66940	Shisa5	shisa homolog 5 (Xenopus laevis)	1.038	0.775	0.9266
66855	Tcf25	transcription factor 25 (basic helix-loop-helix)	1.038	0.2976	NA
66449	Pam16	presequence translocase-asssociated motor 16 homolog (S. cerevisiae)	1.038	0.4724	NA
66231	Thoc7	THO complex 7 homolog (Drosophila)	1.038	0.4728	NA
64656	Mrps23	mitochondrial ribosomal protein S23	1.038	0.5056	NA
59003	Maea	macrophage erythroblast attacher	1.038	0.4176	NA
56458	Foxo1	forkhead box O1	1.038	0.7703	0.9257
56433	Vps29	vacuolar protein sorting 29 (S. pombe)	1.038	0.3748	NA
54607	Socs6	suppressor of cytokine signaling 6	1.038	0.4554	NA
54484	Mkrn1	makorin, ring finger protein, 1	1.038	0.4661	NA
52639	Wipi1	WD repeat domain, phosphoinositide interacting 1	1.038	0.6138	NA
50874	Tmod4	tropomodulin 4	1.038	0.7465	0.9196
27389	Dusp13	dual specificity phosphatase 13	1.038	0.744	0.9191
22704	Zfp46	zinc finger protein 46	1.038	0.4958	NA
22630	Ywhaq	tyrosine 3-monooxygenase/tryptophan 5-monooxygenase activation protein, theta polypeptide	1.038	0.4205	NA
20476	Six6	sine oculis-related homeobox 6 homolog (Drosophila)	1.038	0.4407	NA
20273	Scn8a	sodium channel, voltage-gated, type VIII, alpha	1.038	0.8341	0.9463
20104	Rps6	ribosomal protein S6	1.038	0.5676	NA

19362	Rad51ap1	RAD51 associated protein 1	1.038	0.553	NA
19170	Psmb1	proteasome (prosome, macropain) subunit, beta type 1	1.038	0.5686	NA
18673	Phb	prohibitin	1.038	0.7364	0.9169
18647	Cdk14	cyclin-dependent kinase 14	1.038	0.7594	0.9229
18209	Ntn3	netrin 3	1.038	0.7535	0.9216
18114	Rrp1	ribosomal RNA processing 1 homolog (S. cerevisiae)	1.038	0.5814	NA
17527	Mpv17	MpV17 mitochondrial inner membrane protein	1.038	0.6202	NA
17391	Mmp24	matrix metallopeptidase 24	1.038	0.7002	0.9033
17258	Mef2a	myocyte enhancer factor 2A	1.038	0.8519	0.9537
16578	Kif9	kinesin family member 9	1.038	0.7607	0.9236
16512	Kcnh3	potassium voltage-gated channel, subfamily H (eag-related), member 3	1.038	0.8702	0.9602
15365	Hmga2-ps1	high mobility group AT-hook 2, pseudogene 1	1.038	0.6214	NA
14791	Emg1	EMG1 nucleolar protein homolog (S. cerevisiae)	1.038	0.4698	NA
14672	Gna11	guanine nucleotide binding protein, alpha 11	1.038	0.4216	NA
14221	Fjx1	four jointed box 1 (Drosophila)	1.038	0.6375	NA
13709	Elf1	E74-like factor 1	1.038	0.4098	NA
13397	Dlx6as	distal-less homeobox 6, antisense	1.038	0.8377	0.9479
13051	Cx3cr1	chemokine (C-X3-C) receptor 1	1.038	0.8216	0.9434
12874	Cpd	carboxypeptidase D	1.038	0.6663	0.8904
12729	Clns1a	chloride channel, nucleotide-sensitive, 1A	1.038	0.5483	NA
12631	Cfl1	cofilin 1, non-muscle	1.038	0.6218	NA
12569	Cdk5r1	cyclin-dependent kinase 5, regulatory subunit 1 (p35)	1.038	0.3953	NA
12460	Ccs	copper chaperone for superoxide dismutase	1.038	0.4889	NA
11950	Atp5f1	ATP synthase, H+ transporting, mitochondrial F0 complex, subunit B1	1.038	0.4967	NA
11946	Atp5a1	ATP synthase, H+ transporting, mitochondrial F1 complex, alpha subunit 1	1.038	0.549	NA
11434	Acr	acrosin prepropeptide	1.038	0.6739	0.8924
11350	Abl1	c-abl oncogene 1, non-receptor tyrosine kinase	1.038	0.6474	NA
100505017	LOC100505017	putative Pol polyprotein-like	1.037	0.7583	0.9227
675578	Gm9648	predicted gene 9648	1.037	0.7359	0.9169
407243	Tmem189	transmembrane protein 189	1.037	0.925	0.9783
396184	Flrt1	fibronectin leucine rich transmembrane protein 1	1.037	0.778	0.9273
320878	Mical2	microtubule associated monoxygenase, calponin and LIM domain containing 2	1.037	0.6097	NA
319195	Rpl17	ribosomal protein L17	1.037	0.5305	NA
268395	Mpg	N-methylpurine-DNA glycosylase	1.037	0.4425	NA
246696	Slc25a28	solute carrier family 25, member 28	1.037	0.6749	0.8925
246086	Onecut3	one cut domain, family member 3	1.037	0.6119	NA
240041	Zfp945	zinc finger protein 945	1.037	0.7214	0.9115
239606	Slc2a13	solute carrier family 2 (facilitated glucose transporter), member 13	1.037	0.4387	NA
238690	Zfp458	zinc finger protein 458	1.037	0.4706	NA
235416	Lman1l	lectin, mannose-binding 1 like	1.037	0.7046	0.9049
233908	Fus	fusion, derived from t(12;16) malignant liposarcoma (human)	1.037	0.4665	NA
233878	Sez6l2	seizure related 6 homolog like 2	1.037	0.7336	0.9159
230753	Thrap3	thyroid hormone receptor associated protein 3	1.037	0.5586	NA
226654	Tstd1	thiosulfate sulfurtransferase (rhodanese)-like domain containing 1	1.037	0.6224	NA
218952	Fermt2	fermitin family homolog 2 (Drosophila)	1.037	0.6953	0.901
213550	Dis3l	DIS3 mitotic control homolog (S. cerevisiae)-like	1.037	0.5473	NA
212514	Spice1	spindle and centriole associated protein 1	1.037	0.4947	NA
208117	Aph1b	anterior pharynx defective 1b homolog (C. elegans)	1.037	0.6479	NA
171257	Vmn1r195	vomeronasal 1 receptor 195	1.037	0.8979	0.9699

107951	Cdk9	cyclin-dependent kinase 9 (CDC2-related kinase)	1.037	0.6778	0.8931
103268	Cep57l1	centrosomal protein 57-like 1	1.037	0.6397	NA
101122	Rpusd3	RNA pseudouridylate synthase domain containing 3	1.037	0.6552	NA
98386	Lbr	lamin B receptor	1.037	0.5659	NA
93961	B3galt5	UDP-Gal:betaGlcNAc beta 1,3-galactosyltransferase, polypeptide 5	1.037	0.584	NA
84095	Pi4k2a	phosphatidylinositol 4-kinase type 2 alpha	1.037	0.4584	NA
83921	Tmem2	transmembrane protein 2	1.037	0.635	NA
80981	Arl4d	ADP-ribosylation factor-like 4D	1.037	0.5586	NA
78412	3110062M04Rik	RIKEN cDNA 3110062M04 gene	1.037	0.6254	NA
75613	Med25	mediator of RNA polymerase II transcription, subunit 25 homolog (yeast)	1.037	0.7431	0.9188
74711	Ttll9	tubulin tyrosine ligase-like family, member 9	1.037	0.8033	0.9377
74504	Fam53a	family with sequence similarity 53, member A	1.037	0.4698	NA
73363	1700056E22Rik	RIKEN cDNA 1700056E22 gene	1.037	0.7566	0.9225
70572	Ipo5	importin 5	1.037	0.6851	0.8959
70511	Fam86	family with sequence similarity 86	1.037	0.603	NA
69601	Dab2ip	disabled homolog 2 (Drosophila) interacting protein	1.037	0.8726	0.9608
69276	Sec62	SEC62 homolog (S. cerevisiae)	1.037	0.5422	NA
69227	2810407C02Rik	RIKEN cDNA 2810407C02 gene	1.037	0.5436	NA
68949	1500012F01Rik	RIKEN cDNA 1500012F01 gene	1.037	0.51	NA
68879	Prpf6	PRP6 pre-mRNA splicing factor 6 homolog (yeast)	1.037	0.407	NA
68501	Nsmce2	non-SMC element 2 homolog (MMS21, S. cerevisiae)	1.037	0.6214	NA
68375	Ndufa8	NADH dehydrogenase (ubiquinone) 1 alpha subcomplex, 8	1.037	0.6931	0.8999
67414	Mfn1	mitofusin 1	1.037	0.399	NA
67249	Tbc1d19	TBC1 domain family, member 19	1.037	0.4864	NA
67048	Vma21	VMA21 vacuolar H+-ATPase homolog (S. cerevisiae)	1.037	0.5884	NA
66994	1500031L02Rik	RIKEN cDNA 1500031L02 gene	1.037	0.4543	NA
66827	Ttc1	tetratricopeptide repeat domain 1	1.037	0.5113	NA
66377	Ndufc1	NADH dehydrogenase (ubiquinone) 1, subcomplex unknown, 1	1.037	0.5173	NA
59038	Pxmp4	peroxisomal membrane protein 4	1.037	0.7637	0.9248
59033	Slc4a8	solute carrier family 4 (anion exchanger), member 8	1.037	0.5005	NA
59008	Anapc5	anaphase-promoting complex subunit 5	1.037	0.6565	NA
58172	Sertad2	SERTA domain containing 2	1.037	0.4808	NA
57230	Sap30bp	SAP30 binding protein	1.037	0.6857	0.8964
56620	Clec4n	C-type lectin domain family 4, member n	1.037	0.8798	0.9631
56515	Rnf138	ring finger protein 138	1.037	0.5616	NA
56392	Shoc2	soc-2 (suppressor of clear) homolog (C. elegans)	1.037	0.8026	0.9376
56389	Stx5a	syntaxin 5A	1.037	0.4883	NA
54375	Azin1	antizyme inhibitor 1	1.037	0.4298	NA
54160	Copg2	coatomer protein complex, subunit gamma 2	1.037	0.427	NA
52850	Sgsm1	small G protein signaling modulator 1	1.037	0.517	NA
52639	Wipi1	WD repeat domain, phosphoinositide interacting 1	1.037	0.6091	NA
52421	D4Ertd681e	DNA segment, Chr 4, ERATO Doi 681, expressed	1.037	0.8534	0.9544
29815	Bcar3	breast cancer anti-estrogen resistance 3	1.037	0.4292	NA
27397	Mrpl17	mitochondrial ribosomal protein L17	1.037	0.5325	NA
26420	Mapk9	mitogen-activated protein kinase 9	1.037	0.4124	NA
24057	Sh3yl1	Sh3 domain YSC-like 1	1.037	0.5269	NA
22629	Ywhah	tyrosine 3-monooxygenase/tryptophan 5-monooxygenase activation protein, eta polypeptide	1.037	0.4425	NA
22594	Xrcc1	X-ray repair complementing defective repair in Chinese hamster cells 1	1.037	0.6253	NA
22258	Usp4	ubiquitin specific peptidase 4 (proto-oncogene)	1.037	0.3134	NA

21983	Tpbg	trophoblast glycoprotein	1.037	0.6403	NA
20675	Sox3	SRY-box containing gene 3	1.037	0.7455	0.9192
20238	Atxn1	ataxin 1	1.037	0.6286	NA
20090	Rps29	ribosomal protein S29	1.037	0.6218	NA
19052	Ppp2ca	protein phosphatase 2 (formerly 2A), catalytic subunit, alpha isoform	1.037	0.4699	NA
18948	Pnmt	phenylethanolamine-N-methyltransferase	1.037	0.7559	0.9223
18701	Pigf	phosphatidylinositol glycan anchor biosynthesis, class F	1.037	0.6565	NA
18648	Pgam1	phosphoglycerate mutase 1	1.037	0.6704	0.8912
18148	Npm1	nucleophosmin 1	1.037	0.6429	NA
18103	Nme2	non-metastatic cells 2, protein (NM23B) expressed in	1.037	0.655	NA
17986	Ndp	Norrie disease (pseudoglioma) (human)	1.037	0.4234	NA
17846	Commd1	COMM domain containing 1	1.037	0.34	NA
16924	Lnx1	ligand of numb-protein X 1	1.037	0.7576	0.9227
16562	Kif1c	kinesin family member 1C	1.037	0.6244	NA
16478	Jund	Jun proto-oncogene related gene d	1.037	0.8135	0.9406
16423	Cd47	CD47 antigen (Rh-related antigen, integrin-associated signal transducer)	1.037	0.3475	NA
15289	Hmgb1	high mobility group box 1	1.037	0.6347	NA
15200	Hbegf	heparin-binding EGF-like growth factor	1.037	0.4379	NA
14763	Gpr37	G protein-coupled receptor 37	1.037	0.5826	NA
14043	Ext2	exostoses (multiple) 2	1.037	0.385	NA
12727	Clcn4-2	chloride channel 4-2	1.037	0.4029	NA
12695	Inadl	InaD-like (Drosophila)	1.037	0.4821	NA
12395	Runx1t1	runt-related transcription factor 1; translocated to, 1 (cyclin D-related)	1.037	0.4867	NA
12286	Cacna1a	calcium channel, voltage-dependent, P/Q type, alpha 1A subunit	1.037	0.7225	0.9121
11496	Adam22	a disintegrin and metallopeptidase domain 22	1.037	0.5772	NA
100044339	LOC100044339	zinc finger protein 764-like	1.036	0.6488	NA
100038485	Gm10398	predicted gene 10398	1.036	0.9335	0.9805
668940	Myh7b	myosin, heavy chain 7B, cardiac muscle, beta	1.036	0.8495	0.953
667055	Gm9992	predicted gene 9992	1.036	0.7992	0.9361
631470	Gm7065	predicted gene 7065	1.036	0.6093	NA
629557	Gm6981	glyceraldehyde-3-phosphate dehydrogenase pseudogene	1.036	0.7929	0.9334
434325	Tmem221	transmembrane protein 221	1.036	0.8514	0.9537
381404	Pabpc1l	poly(A) binding protein, cytoplasmic 1-like	1.036	0.7792	0.9278
330171	Kctd10	potassium channel tetramerisation domain containing 10	1.036	0.551	NA
328505	Skint7	selection and upkeep of intraepithelial T cells 7	1.036	0.6019	NA
320534	Tmem104	transmembrane protein 104	1.036	0.7174	0.9096
268294	Zbtb24	zinc finger and BTB domain containing 24	1.036	0.334	NA
260297	Prrt1	proline-rich transmembrane protein 1	1.036	0.733	0.9157
243963	Zfp473	zinc finger protein 473	1.036	0.6246	NA
240185	9430020K01Rik	RIKEN cDNA 9430020K01 gene	1.036	0.6674	NA
235623	Scap	SREBF chaperone	1.036	0.4125	NA
235459	Gtf2a2	general transcription factor II A, 2	1.036	0.5709	NA
233724	Tmem41b	transmembrane protein 41B	1.036	0.4618	NA
231931	Gimap6	GTPase, IMAP family member 6	1.036	0.7522	0.9213
230751	Oscp1	organic solute carrier partner 1	1.036	0.4274	NA
228812	Pigu	phosphatidylinositol glycan anchor biosynthesis, class U	1.036	0.697	0.902
228788	BC020535	cDNA sequence BC020535	1.036	0.663	NA
216148	Shc2	SHC (Src homology 2 domain containing) transforming protein 2	1.036	0.7169	0.9095
209416	Gpkow	G patch domain and KOW motifs	1.036	0.7127	0.9075

208144	Dhx37	DEAH (Asp-Glu-Ala-His) box polypeptide 37	1.036	0.8029	0.9376
170755	Sgk3	serum/glucocorticoid regulated kinase 3	1.036	0.3884	NA
110557	H2-Q6	histocompatibility 2, Q region locus 6	1.036	0.6638	NA
108682	Gpt2	glutamic pyruvate transaminase (alanine aminotransferase) 2	1.036	0.3678	NA
108660	Rnf187	ring finger protein 187	1.036	0.4951	NA
108645	Mat2b	methionine adenosyltransferase II, beta	1.036	0.4426	NA
107746	Rapgef1	Rap guanine nucleotide exchange factor (GEF) 1	1.036	0.4406	NA
105727	Slc38a1	solute carrier family 38, member 1	1.036	0.6329	NA
104112	Acly	ATP citrate lyase	1.036	0.383	NA
81910	Rrbp1	ribosome binding protein 1	1.036	0.6014	NA
79554	Gltpd1	glycolipid transfer protein domain containing 1	1.036	0.6074	NA
77697	Mmab	methylmalonic aciduria (cobalamin deficiency) type B homolog (human)	1.036	0.7012	0.9036
77462	Tmem116	transmembrane protein 116	1.036	0.6716	NA
76793	Snip1	Smad nuclear interacting protein 1	1.036	0.5831	NA
76138	Ccdc138	coiled-coil domain containing 138	1.036	0.6141	NA
74150	Slc35f5	solute carrier family 35, member F5	1.036	0.5842	NA
74125	Armc8	armadillo repeat containing 8	1.036	0.6199	NA
74026	Msl1	male-specific lethal 1 homolog (Drosophila)	1.036	0.4095	NA
73293	Ccdc103	coiled-coil domain containing 103	1.036	0.6743	0.8924
73062	Ppp1r16a	protein phosphatase 1, regulatory (inhibitor) subunit 16A	1.036	0.6848	0.8959
72265	Tram1	translocating chain-associating membrane protein 1	1.036	0.7173	0.9096
72124	Seh1l	SEH1-like (S. cerevisiae	1.036	0.6509	NA
71955	2400003C14Rik	RIKEN cDNA 2400003C14 gene	1.036	0.5697	NA
71911	Bdh1	3-hydroxybutyrate dehydrogenase, type 1	1.036	0.6285	NA
71900	Tmem106b	transmembrane protein 106B	1.036	0.3907	NA
71720	Osbpl3	oxysterol binding protein-like 3	1.036	0.6996	0.9031
71702	Cdc5l	cell division cycle 5-like (S. pombe)	1.036	0.5355	NA
69274	Ctdspl	CTD (carboxy-terminal domain, RNA polymerase II, polypeptide A) small phosphatase-like	1.036	0.7444	0.9192
69106	Stoml1	stomatin-like 1	1.036	0.5279	NA
68915	Vars2	valyl-tRNA synthetase 2, mitochondrial (putative)	1.036	0.6353	NA
68140	Tigd2	tigger transposable element derived 2	1.036	0.4333	NA
68087	Dcakd	dephospho-CoA kinase domain containing	1.036	0.7839	0.9302
68055	Atp5s	ATP synthase, H+ transporting, mitochondrial F0 complex, subunit s	1.036	0.5042	NA
67941	Rps27l	ribosomal protein S27-like	1.036	0.537	NA
67865	Rgs10	regulator of G-protein signalling 10	1.036	0.4257	NA
67667	Alkbh8	alkB, alkylation repair homolog 8 (E. coli)	1.036	0.6556	NA
66868	Mfsd1	major facilitator superfamily domain containing 1	1.036	0.6538	NA
66865	Pmpca	peptidase (mitochondrial processing) alpha	1.036	0.4657	NA
66500	Slc30a7	solute carrier family 30 (zinc transporter), member 7	1.036	0.665	NA
66273	1810020D17Rik	RIKEN cDNA 1810020D17 gene	1.036	0.5819	NA
66055	0610009D07Rik	RIKEN cDNA 0610009D07 gene	1.036	0.4213	NA
63873	Trpv4	transient receptor potential cation channel, subfamily V, member 4	1.036	0.863	0.9575
59040	Rhot1	ras homolog gene family, member T1	1.036	0.5221	NA
58996	Arhgap23	Rho GTPase activating protein 23	1.036	0.5674	NA
56772	Mllt11	myeloid/lymphoid or mixed-lineage leukemia (trithorax homolog, Drosophila); translocated to, 11	1.036	0.4626	NA
56199	Abcb10	ATP-binding cassette, sub-family B (MDR/TAP), member 10	1.036	0.3883	NA
54633	Pqbp1	polyglutamine binding protein 1	1.036	0.6679	NA
52202	Rbm34	RNA binding motif protein 34	1.036	0.5895	NA
51885	Tubgcp4	tubulin, gamma complex associated protein 4	1.036	0.5557	NA

50935	St6galnac6	ST6 (alpha-N-acetyl-neuraminyl-2,3-beta-galactosyl-1,3)-N-acetylgalactosaminide alpha-2,6-sialyltransferase 6	1.036	0.5077	NA
50780	Rgs3	regulator of G-protein signaling 3	1.036	0.6542	NA
27395	Mrpl15	mitochondrial ribosomal protein L15	1.036	0.5159	NA
26413	Mapk1	mitogen-activated protein kinase 1	1.036	0.5056	NA
26379	Esrra	estrogen related receptor, alpha	1.036	0.5534	NA
24070	Mpdu1	mannose-P-dolichol utilization defect 1	1.036	0.3487	NA
24053	Sgcg	sarcoglycan, gamma (dystrophin-associated glycoprotein)	1.036	0.9048	0.9717
22418	Wnt5a	wingless-related MMTV integration site 5A	1.036	0.6792	0.8944
22375	Wars	tryptophanyl-tRNA synthetase	1.036	0.3691	NA
22122	Tsta3	tissue specific transplantation antigen P35B	1.036	0.685	0.8959
22027	Hsp90b1	heat shock protein 90, beta (Grp94), member 1	1.036	0.3546	NA
20892	Stra13	stimulated by retinoic acid 13	1.036	0.6245	NA
20648	Snta1	syntrophin, acidic 1	1.036	0.6235	NA
19275	Ptprn	protein tyrosine phosphatase, receptor type, N	1.036	0.7864	0.9312
19158	Cyth2	cytohesin 2	1.036	0.6877	0.8974
18596	Pdgfrb	platelet derived growth factor receptor, beta polypeptide	1.036	0.7458	0.9192
18590	Pdgfa	platelet derived growth factor, alpha	1.036	0.663	NA
18169	Npy6r	neuropeptide Y receptor Y6	1.036	0.7637	0.9248
16890	Lipe	lipase, hormone sensitive	1.036	0.7305	0.915
14588	Gfra4	glial cell line derived neurotrophic factor family receptor alpha 4	1.036	0.7675	0.9255
13858	Eps15	epidermal growth factor receptor pathway substrate 15	1.036	0.6759	0.8928
12757	Clta	clathrin, light polypeptide (Lca)	1.036	0.3782	NA
12520	Cd81	CD81 antigen	1.036	0.444	NA
12494	Cd38	CD38 antigen	1.036	0.8109	0.9401
12467	Cct6b	chaperonin containing Tcp1, subunit 6b (zeta)	1.036	0.7505	0.921
12462	Cct3	chaperonin containing Tcp1, subunit 3 (gamma)	1.036	0.4105	NA
12323	Camk2b	calcium/calmodulin-dependent protein kinase II, beta	1.036	0.5193	NA
11987	Slc7a1	solute carrier family 7 (cationic amino acid transporter, y+ system), member 1	1.036	0.557	NA
11909	Atf2	activating transcription factor 2	1.036	0.4099	NA
11899	Astn1	astrotactin 1	1.036	0.5458	NA
11832	Aqp7	aquaporin 7	1.036	0.8252	0.9444
11610	Agtrap	angiotensin II, type I receptor-associated protein	1.036	0.756	0.9223
668489	Gm9199	glycine cleavage system protein H (aminomethyl carrier) pseudogene	1.035	0.8362	0.9474
654409	4932416H05Rik	RIKEN cDNA 4932416H05 gene	1.035	0.7066	0.9059
546164	Gm5921	predicted gene 5921	1.035	0.7281	0.9136
432731	Zfp187	zinc finger protein 187	1.035	0.7742	0.9266
330790	Hapln4	hyaluronan and proteoglycan link protein 4	1.035	0.8334	0.9459
329152	Hecw2	HECT, C2 and WW domain containing E3 ubiquitin protein ligase 2	1.035	0.7341	0.9161
277360	Prex1	phosphatidylinositol-3,4,5-trisphosphate-dependent Rac exchange factor 1	1.035	0.6168	NA
245403	Dcaf12l2	DDB1 and CUL4 associated factor 12-like 2	1.035	0.8829	0.9648
244349	Myst3	MYST histone acetyltransferase (monocytic leukemia) 3	1.035	0.6842	0.8956
244152	Tsku	tsukushin	1.035	0.7149	0.9083
242653	Cldn19	claudin 19	1.035	0.9265	0.9785
241593	Pin1-ps1	peptidylprolyl cis/trans isomerase, NIMA-interacting 1, pseudogene 1	1.035	0.4021	NA
233115	Dpy19l3	dpy-19-like 3 (C. elegans)	1.035	0.5868	NA
232337	Zfp637	zinc finger protein 637	1.035	0.5127	NA
231655	Oasl1	2'-5' oligoadenylate synthetase-like 1	1.035	0.8935	0.9693
225326	Pik3c3	phosphoinositide-3-kinase, class 3	1.035	0.7051	0.9052
218194	Phactr1	phosphatase and actin regulator 1	1.035	0.5099	NA

216156	Wdr18	WD repeat domain 18	1.035	0.7686	0.9255
170771	Khdrbs2	KH domain containing, RNA binding, signal transduction associated 2	1.035	0.7721	0.9259
170460	Stard5	StAR-related lipid transfer (START) domain containing 5	1.035	0.5203	NA
110208	Pgd	phosphogluconate dehydrogenase	1.035	0.5716	NA
107686	Snrpd2	small nuclear ribonucleoprotein D2	1.035	0.6581	NA
106639	Vmac	vimentin-type intermediate filament associated coiled-coil protein	1.035	0.7395	0.9178
106489	Sft2d1	SFT2 domain containing 1	1.035	0.6465	NA
102954	Nudt10	nudix (nucleoside diphosphate linked moiety X)-type motif 10	1.035	0.5616	NA
102462	Imp3	IMP3, U3 small nucleolar ribonucleoprotein, homolog (yeast)	1.035	0.554	NA
101543	Wtip	WT1-interacting protein	1.035	0.4619	NA
99922	AW552393	expressed sequence AW552393	1.035	0.828	0.945
83964	Jam3	junction adhesion molecule 3	1.035	0.5139	NA
78912	Sp2	Sp2 transcription factor	1.035	0.6727	NA
77583	Notum	notum pectinacetylesterase homolog (Drosophila)	1.035	0.8487	0.9524
76338	Rab2b	RAB2B, member RAS oncogene family	1.035	0.4899	NA
75612	Gns	glucosamine (N-acetyl)-6-sulfatase	1.035	0.5126	NA
75593	2410003K15Rik	RIKEN cDNA 2410003K15 gene	1.035	0.57	NA
75352	4930550L24Rik	RIKEN cDNA 4930550L24 gene	1.035	0.723	0.9121
74734	Rhoh	ras homolog gene family, member H	1.035	0.7274	0.9134
74032	Sdr42e1	short chain dehydrogenase/reductase family 42E, member 1	1.035	0.6484	NA
72083	Mzt2	mitotic spindle organizing protein 2	1.035	0.4786	NA
71989	Rpusd4	RNA pseudouridylate synthase domain containing 4	1.035	0.3232	NA
71928	2310047K21Rik	RIKEN cDNA 2310047K21 gene	1.035	0.6664	NA
71059	Hexim2	hexamthylene bis-acetamide inducible 2	1.035	0.5882	NA
67890	Ufm1	ubiquitin-fold modifier 1	1.035	0.5256	NA
67628	Anp32b	acidic (leucine-rich) nuclear phosphoprotein 32 family, member B	1.035	0.4252	NA
67341	Ascl4	achaete-scute complex homolog 4 (Drosophila)	1.035	0.8258	0.9446
66768	Pacrgl	PARK2 co-regulated-like	1.035	0.4387	NA
66406	Sac3d1	SAC3 domain containing 1	1.035	0.3408	NA
66152	Uqcr10	ubiquinol-cytochrome c reductase, complex III subunit X	1.035	0.5222	NA
65115	Bean1	brain expressed, associated with Nedd4, 1	1.035	0.7139	0.9078
58208	Bcl11b	B-cell leukemia/lymphoma 11B	1.035	0.7722	0.9259
58194	Sh3kbp1	SH3-domain kinase binding protein 1	1.035	0.7403	0.9179
58184	Rqcd1	rcd1 (required for cell differentiation) homolog 1 (S. pombe)	1.035	0.489	NA
57376	Smarce1	SWI/SNF related, matrix associated, actin dependent regulator of chromatin, subfamily e, member 1	1.035	0.5037	NA
56529	Sec11a	SEC11 homolog A (S. cerevisiae)	1.035	0.582	NA
56398	1500003O03Rik	RIKEN cDNA 1500003O03 gene	1.035	0.4868	NA
56017	Slc2a8	solute carrier family 2, (facilitated glucose transporter), member 8	1.035	0.49	NA
28113	Tinf2	Terf1 (TRF1)-interacting nuclear factor 2	1.035	0.4935	NA
27681	Snf8	SNF8, ESCRT-II complex subunit, homolog (S. cerevisiae)	1.035	0.4784	NA
22781	Ikzf4	IKAROS family zinc finger 4	1.035	0.6837	0.8956
22764	Zfx	zinc finger protein X-linked	1.035	0.4668	NA
22671	Rnf112	ring finger protein 112	1.035	0.7293	0.9143
22381	Wbp5	WW domain binding protein 5	1.035	0.5416	NA
21944	Tnfsf12	tumor necrosis factor (ligand) superfamily, member 12	1.035	0.7579	0.9227
20850	Stat5a	signal transducer and activator of transcription 5A	1.035	0.6021	NA
19347	Dennd5a	DENN/MADD domain containing 5A	1.035	0.4701	NA
18986	Pou2f1	POU domain, class 2, transcription factor 1	1.035	0.7776	0.9272
18854	Pml	promyelocytic leukemia	1.035	0.6259	NA

18802	Plcd4	phospholipase C, delta 4	1.035	0.6915	0.8991
18750	Prkca	protein kinase C, alpha	1.035	0.5368	NA
18393	Orc2	origin recognition complex, subunit 2	1.035	0.7069	0.9061
18218	Dusp8	dual specificity phosphatase 8	1.035	0.7775	0.9272
18163	Ctnnd2	catenin (cadherin associated protein), delta 2	1.035	0.6664	NA
18019	Nfatc2	nuclear factor of activated T-cells, cytoplasmic, calcineurin-dependent 2	1.035	0.8105	0.94
17769	Mthfr	5,10-methylenetetrahydrofolate reductase	1.035	0.656	NA
17537	Meis3	Meis homeobox 3	1.035	0.8003	0.9365
17274	Rab8a	RAB8A, member RAS oncogene family	1.035	0.6375	NA
16880	Lifr	leukemia inhibitory factor receptor	1.035	0.5984	NA
16568	Kif3a	kinesin family member 3A	1.035	0.5641	NA
16560	Kif1a	kinesin family member 1A	1.035	0.5646	NA
15288	Hmbs	hydroxymethylbilane synthase	1.035	0.6272	NA
14799	Gria1	glutamate receptor, ionotropic, AMPA1 (alpha 1)	1.035	0.5156	NA
14536	Nr6a1	nuclear receptor subfamily 6, group A, member 1	1.035	0.746	0.9193
13842	Epha8	Eph receptor A8	1.035	0.7224	0.9121
13427	Dync1i2	dynein cytoplasmic 1 intermediate chain 2	1.035	0.5221	NA
13002	Dnajc5	DnaJ (Hsp40) homolog, subfamily C, member 5	1.035	0.4907	NA
12728	Clcn5	chloride channel 5	1.035	0.6168	NA
12504	Cd4	CD4 antigen	1.035	0.8323	0.9456
11765	Ap1g1	adaptor protein complex AP-1, gamma 1 subunit	1.035	0.404	NA
100502683	LOC100502683	40S ribosomal protein S29-like	1.034	0.6645	NA
665618	Gm7715	predicted gene 7715	1.034	0.7212	0.9115
664968	2210411K11Rik	RIKEN cDNA 2210411K11 gene	1.034	0.7796	0.928
432530	Adcy1	adenylate cyclase 1	1.034	0.7327	0.9157
338365	Slc41a2	solute carrier family 41, member 2	1.034	0.4062	NA
330177	Taok3	TAO kinase 3	1.034	0.702	0.9039
280411	Lix1l	Lix1-like	1.034	0.4086	NA
276852	D11Wsu47e	DNA segment, Chr 11, Wayne State University 47, expressed	1.034	0.5871	NA
268747	Lrrc16b	leucine rich repeat containing 16B	1.034	0.7578	0.9227
245877	Mtap7d1	microtubule-associated protein 7 domain containing 1	1.034	0.4907	NA
243373	AI854703	expressed sequence AI854703	1.034	0.7192	0.9104
242022	Frem2	Fras1 related extracellular matrix protein 2	1.034	0.5744	NA
233405	Vps33b	vacuolar protein sorting 33B (yeast)	1.034	0.7991	0.9361
233189	Ctu1	cytosolic thiouridylase subunit 1 homolog (S. pombe)	1.034	0.6268	NA
232934	Mypop	Myb-related transcription factor, partner of profilin	1.034	0.7681	0.9255
227695	D2Wsu81e	DNA segment, Chr 2, Wayne State University 81, expressed	1.034	0.6568	NA
226517	Smg7	Smg-7 homolog, nonsense mediated mRNA decay factor (C. elegans)	1.034	0.6691	NA
219105	Zmym5	zinc finger, MYM-type 5	1.034	0.6211	NA
218734	3830406C13Rik	RIKEN cDNA 3830406C13 gene	1.034	0.5828	NA
218215	Rnf144b	ring finger protein 144B	1.034	0.6737	NA
217140	Scrn2	secernin 2	1.034	0.6474	NA
214547	She	src homology 2 domain-containing transforming protein E	1.034	0.6531	NA
171543	Bmf	BCL2 modifying factor	1.034	0.7921	0.9333
117147	Acsm1	acyl-CoA synthetase medium-chain family member 1	1.034	0.8888	0.9676
107701	Sf3b4	splicing factor 3b, subunit 4	1.034	0.5005	NA
101314	6720456B07Rik	RIKEN cDNA 6720456B07 gene	1.034	0.5303	NA
99929	Tiparp	TCDD-inducible poly(ADP-ribose) polymerase	1.034	0.667	NA
98376	Gorab	golgin, RAB6-interacting	1.034	0.5288	NA

97114	Hist2h3c2-ps	histone cluster 2, H3c2, pseudogene	1.034	0.7524	0.9213
83962	Btbd1	BTB (POZ) domain containing 1	1.034	0.398	NA
81840	Sorcs2	sortilin-related VPS10 domain containing receptor 2	1.034	0.7545	0.9216
80883	Ntng1	netrin G1	1.034	0.477	NA
80795	Selk	selenoprotein K	1.034	0.5023	NA
79233	Zfp319	zinc finger protein 319	1.034	0.6387	NA
78893	Cnot10	CCR4-NOT transcription complex, subunit 10	1.034	0.6284	NA
77891	Ube2s	ubiquitin-conjugating enzyme E2S	1.034	0.6046	NA
77683	Ehmt1	euchromatic histone methyltransferase 1	1.034	0.8331	0.9458
77569	Limch1	LIM and calponin homology domains 1	1.034	0.4971	NA
77559	Agl	amylo-1,6-glucosidase, 4-alpha-glucanotransferase	1.034	0.4552	NA
75288	Slc35f4	solute carrier family 35, member F4	1.034	0.6415	NA
75016	4930480K23Rik	RIKEN cDNA 4930480K23 gene	1.034	0.8036	0.9378
74427	Eaf1	ELL associated factor 1	1.034	0.6849	NA
72821	Scn2b	sodium channel, voltage-gated, type II, beta	1.034	0.5727	NA
72240	1600014C23Rik	RIKEN cDNA 1600014C23 gene	1.034	0.6989	0.903
71816	Rnf180	ring finger protein 180	1.034	0.7826	0.9293
69834	Rab43	RAB43, member RAS oncogene family	1.034	0.5476	NA
68202	Ndufa5	NADH dehydrogenase (ubiquinone) 1 alpha subcomplex, 5	1.034	0.5638	NA
68196	Hsbp1	heat shock factor binding protein 1	1.034	0.5116	NA
67966	Zcchc10	zinc finger, CCHC domain containing 10	1.034	0.3152	NA
67724	Pop1	processing of precursor 1, ribonuclease P/MRP family, (S. cerevisiae)	1.034	0.6463	NA
67568	Mrfap1	Morf4 family associated protein 1	1.034	0.6119	NA
67067	Romo1	reactive oxygen species modulator 1	1.034	0.6438	NA
66933	1700025L06Rik	RIKEN cDNA 1700025L06 gene	1.034	0.8715	0.9608
66705	Dnase1l2	deoxyribonuclease 1-like 2	1.034	0.6853	NA
66656	Eef1d	eukaryotic translation elongation factor 1 delta (guanine nucleotide exchange protein)	1.034	0.3741	NA
66411	Tbcb	tubulin folding cofactor B	1.034	0.4987	NA
66390	Slmo2	slowmo homolog 2 (Drosophila)	1.034	0.5846	NA
66368	Rtcd1	RNA terminal phosphate cyclase domain 1	1.034	0.6429	NA
66164	Nip7	nuclear import 7 homolog (S. cerevisiae)	1.034	0.7653	0.9251
64436	Inpp5e	inositol polyphosphate-5-phosphatase E	1.034	0.6708	NA
56486	Gabarap	gamma-aminobutyric acid receptor associated protein	1.034	0.3698	NA
54195	Gucy1b3	guanylate cyclase 1, soluble, beta 3	1.034	0.5228	NA
53890	Sart3	squamous cell carcinoma antigen recognized by T-cells 3	1.034	0.5643	NA
52882	Rgs7bp	regulator of G-protein signalling 7 binding protein	1.034	0.555	NA
52615	Suz12	suppressor of zeste 12 homolog (Drosophila)	1.034	0.5394	NA
52469	Ccdc56	coiled-coil domain containing 56	1.034	0.5279	NA
30795	Fkbp3	FK506 binding protein 3	1.034	0.4044	NA
27425	Atp5l	ATP synthase, H+ transporting, mitochondrial F0 complex, subunit g	1.034	0.5627	NA
26885	Casp8ap2	caspase 8 associated protein 2	1.034	0.6111	NA
26416	Mapk14	mitogen-activated protein kinase 14	1.034	0.5445	NA
23882	Gadd45g	growth arrest and DNA-damage-inducible 45 gamma	1.034	0.535	NA
23792	Adam23	a disintegrin and metallopeptidase domain 23	1.034	0.6502	NA
22687	Zfp259	zinc finger protein 259	1.034	0.3742	NA
21652	Phf1	PHD finger protein 1	1.034	0.4358	NA
21429	Ubtf	upstream binding transcription factor, RNA polymerase I	1.034	0.5549	NA
20589	Ighmbp2	immunoglobulin mu binding protein 2	1.034	0.5129	NA
20442	St3gal1	ST3 beta-galactoside alpha-2,3-sialyltransferase 1	1.034	0.6014	NA

19988	Rpl6	ribosomal protein L6	1.034	0.6459	NA
19935	Mrpl23	mitochondrial ribosomal protein L23	1.034	0.6207	NA
19826	Rnps1	ribonucleic acid binding protein S1	1.034	0.7047	0.9049
19240	Tmsb10	thymosin, beta 10	1.034	0.7594	0.9229
19047	Ppp1cc	protein phosphatase 1, catalytic subunit, gamma isoform	1.034	0.6187	NA
17904	Myl6	myosin, light polypeptide 6, alkali, smooth muscle and non-muscle	1.034	0.6754	NA
16911	Lmo4	LIM domain only 4	1.034	0.44	NA
16876	Lhx9	LIM homeobox protein 9	1.034	0.6655	NA
13726	Emd	emerin	1.034	0.5858	NA
13667	Eif2b4	eukaryotic translation initiation factor 2B, subunit 4 delta	1.034	0.611	NA
12953	Cry2	cryptochrome 2 (photolyase-like)	1.034	0.4237	NA
12801	Cnr1	cannabinoid receptor 1 (brain)	1.034	0.4779	NA
11837	Rplp0	ribosomal protein, large, P0	1.034	0.5796	NA
11797	Birc2	baculoviral IAP repeat-containing 2	1.034	0.5583	NA
11686	Alox12b	arachidonate 12-lipoxygenase, 12R type	1.034	0.8298	0.945
11477	Acvr1	activin A receptor, type 1	1.034	0.5479	NA
100504871	LOC100504871	ATP synthase lipid-binding protein, mitochondrial-like	1.033	0.7867	0.9314
100041585	Amd2	S-adenosylmethionine decarboxylase 2	1.033	0.5085	NA
100039815	Gm2436	predicted gene 2436	1.033	0.3228	NA
100038847	Gm10406	predicted gene 10406	1.033	0.4907	NA
677073	Gm16477	ribosomal protein L7a pseudogene	1.033	0.4101	NA
619941	Gm13770	predicted gene 13770	1.033	0.873	0.9611
404580	AL117821	expressed sequence AL117821	1.033	0.7749	0.9266
320858	L3mbtl4	l(3)mbt-like 4 (Drosophila)	1.033	0.6744	NA
320165	Tacc1	transforming, acidic coiled-coil containing protein 1	1.033	0.5845	NA
319513	Fam113a	family with sequence similarity 113, member A	1.033	0.5873	NA
319304	A730081D07Rik	RIKEN cDNA A730081D07 gene	1.033	0.7117	0.9072
319278	A230050P20Rik	RIKEN cDNA A230050P20 gene	1.033	0.6889	NA
270802	BC048403	cDNA sequence BC048403	1.033	0.7248	0.9126
269019	Stk32a	serine/threonine kinase 32A	1.033	0.7595	0.9229
259300	Ehd2	EH-domain containing 2	1.033	0.8299	0.945
245676	Gm4997	predicted gene 4997	1.033	0.5212	NA
232023	Vopp1	vesicular, overexpressed in cancer, prosurvival protein 1	1.033	0.6205	NA
230649	Atpaf1	ATP synthase mitochondrial F1 complex assembly factor 1	1.033	0.8065	0.9388
229214	Qrfpr	pyroglutamylated RFamide peptide receptor	1.033	0.7138	0.9078
228140	Tnks1bp1	tankyrase 1 binding protein 1	1.033	0.6981	NA
227290	Aamp	angio-associated migratory protein	1.033	0.4543	NA
224912	Crb3	crumbs homolog 3 (Drosophila)	1.033	0.7537	0.9216
217716	Mlh3	mutL homolog 3 (E coli)	1.033	0.5617	NA
217069	Trim25	tripartite motif-containing 25	1.033	0.5785	NA
210106	Papd7	PAP associated domain containing 7	1.033	0.491	NA
210009	Mtrr	5-methyltetrahydrofolate-homocysteine methyltransferase reductase	1.033	0.5699	NA
208650	Cblb	Casitas B-lineage lymphoma b	1.033	0.6405	NA
207565	Camkk2	calcium/calmodulin-dependent protein kinase kinase 2, beta	1.033	0.4674	NA
195522	Zfp691	zinc finger protein 691	1.033	0.8108	0.9401
170790	Mlc1	megalencephalic leukoencephalopathy with subcortical cysts 1 homolog (human)	1.033	0.4149	NA
140919	Slc17a6	solute carrier family 17 (sodium-dependent inorganic phosphate cotransporter), member 6	1.033	0.6878	NA
112407	Egln3	EGL nine homolog 3 (C. elegans)	1.033	0.7471	0.9199
110094	Phka2	phosphorylase kinase alpha 2	1.033	0.721	0.9113

109785	Pgm3	phosphoglucomutase 3	1.033	0.7346	0.9163
107566	Arl2bp	ADP-ribosylation factor-like 2 binding protein	1.033	0.533	NA
107035	Fbxo38	F-box protein 38	1.033	0.41	NA
101604	E430018J23Rik	RIKEN cDNA E430018J23 gene	1.033	0.4963	NA
80283	Abtb1	ankyrin repeat and BTB (POZ) domain containing 1	1.033	0.4643	NA
77975	Tmem50b	transmembrane protein 50B	1.033	0.4966	NA
76897	Ralyl	RALY RNA binding protein-like	1.033	0.5285	NA
76577	Faf2	Fas associated factor family member 2	1.033	0.5713	NA
76561	Snx7	sorting nexin 7	1.033	0.689	NA
76246	Rtf1	Rtf1, Paf1/RNA polymerase II complex component, homolog (S. cerevisiae)	1.033	0.4341	NA
75540	Fpgt	fucose-1-phosphate guanylyltransferase	1.033	0.6309	NA
74610	Abcb8	ATP-binding cassette, sub-family B (MDR/TAP), member 8	1.033	0.6919	NA
74335	Xrcc3	X-ray repair complementing defective repair in Chinese hamster cells 3	1.033	0.719	0.9103
74159	Acbd5	acyl-Coenzyme A binding domain containing 5	1.033	0.6992	NA
72946	Lrrc47	leucine rich repeat containing 47	1.033	0.4317	NA
72701	Zfp618	zinc fingerprotein 618	1.033	0.5874	NA
72654	Ccdc12	coiled-coil domain containing 12	1.033	0.5	NA
71865	Fbxo30	F-box protein 30	1.033	0.4045	NA
70312	2510012J08Rik	RIKEN cDNA 2510012J08 gene	1.033	0.4559	NA
69747	Zswim7	zinc finger, SWIM-type containing 7	1.033	0.5567	NA
69702	Ndufaf1	NADH dehydrogenase (ubiquinone) 1 alpha subcomplex, assembly factor 1	1.033	0.6535	NA
69368	Wdfy1	WD repeat and FYVE domain containing 1	1.033	0.5707	NA
68972	Tatdn3	TatD DNase domain containing 3	1.033	0.4364	NA
68721	1110032A03Rik	RIKEN cDNA 1110032A03 gene	1.033	0.527	NA
67824	Nmral1	NmrA-like family domain containing 1	1.033	0.5919	NA
67569	Mgat4c	mannosyl (alpha-1,3-)-glycoprotein beta-1,4-N-acetylglucosaminyltransferase, isozyme C (putative)	1.033	0.7104	0.9069
67513	2610002J02Rik	RIKEN cDNA 2610002J02 gene	1.033	0.6283	NA
67483	1700028P14Rik	RIKEN cDNA 1700028P14 gene	1.033	0.6728	NA
67434	Ankrd33b	ankyrin repeat domain 33B	1.033	0.8301	0.9452
66970	Ssbp2	single-stranded DNA binding protein 2	1.033	0.5265	NA
66587	Fastk	Fas-activated serine/threonine kinase	1.033	0.7383	0.9174
66313	Smurf2	SMAD specific E3 ubiquitin protein ligase 2	1.033	0.6813	NA
66282	1810029B16Rik	RIKEN cDNA 1810029B16 gene	1.033	0.6316	NA
66156	Anapc11	anaphase promoting complex subunit 11	1.033	0.514	NA
59030	Mkks	McKusick-Kaufman syndrome protein	1.033	0.52	NA
56543	Kcnd3	potassium voltage-gated channel, Shal-related family, member 3	1.033	0.8159	0.9416
56543	Kcnd3	potassium voltage-gated channel, Shal-related family, member 3	1.033	0.5555	NA
56451	Suclg1	succinate-CoA ligase, GDP-forming, alpha subunit	1.033	0.5427	NA
56431	Dstn	destrin	1.033	0.6717	NA
56422	Hbs1l	Hbs1-like (S. cerevisiae)	1.033	0.6528	NA
56386	B4galt6	UDP-Gal:betaGlcNAc beta 1,4-galactosyltransferase, polypeptide 6	1.033	0.7656	0.9251
56299	Fkbpl	FK506 binding protein-like	1.033	0.5461	NA
56050	Cyp39a1	cytochrome P450, family 39, subfamily a, polypeptide 1	1.033	0.7715	0.9258
55951	Brp44l	brain protein 44-like	1.033	0.5948	NA
53897	Gal3st1	galactose-3-O-sulfotransferase 1	1.033	0.796	0.9346
50790	Acsl4	acyl-CoA synthetase long-chain family member 4	1.033	0.4762	NA
27226	Pla2g7	phospholipase A2, group VII (platelet-activating factor acetylhydrolase, plasma)	1.033	0.5926	NA
26938	St6galnac5	ST6 (alpha-N-acetyl-neuraminyl-2,3-beta-galactosyl-1,3)-N-acetylgalactosaminide alpha-2,6-sialyltransferase 5	1.033	0.7418	0.918
26373	Clcn7	chloride channel 7	1.033	0.5524	NA

22245	Uck1	uridine-cytidine kinase 1	1.033	0.5885	NA
22209	Ube2a	ubiquitin-conjugating enzyme E2A, RAD6 homolog (S. cerevisiae)	1.033	0.5806	NA
22067	Trpc5	transient receptor potential cation channel, subfamily C, member 5	1.033	0.8086	0.9395
20926	Supt6h	suppressor of Ty 6 homolog (S. cerevisiae)	1.033	0.8313	0.9455
20751	Spr	sepiapterin reductase	1.033	0.5041	NA
20218	Khdrbs1	KH domain containing, RNA binding, signal transduction associated 1	1.033	0.71	0.9069
19687	Rfc1	replication factor C (activator 1) 1	1.033	0.3623	NA
19355	Rad1	RAD1 homolog (S. pombe)	1.033	0.6017	NA
19296	Pvt1	plasmacytoma variant translocation 1	1.033	0.6283	NA
18610	Pdyn	prodynorphin	1.033	0.5145	NA
18119	Nodal	nodal	1.033	0.8991	0.9704
17926	Myoc	myocilin	1.033	0.8588	0.9564
17261	Mef2d	myocyte enhancer factor 2D	1.033	0.7425	0.9185
15469	Prmt1	protein arginine N-methyltransferase 1	1.033	0.6017	NA
15275	Hk1	hexokinase 1	1.033	0.686	NA
14797	Aes	amino-terminal enhancer of split	1.033	0.7414	0.918
14167	Fgf12	fibroblast growth factor 12	1.033	0.6485	NA
12931	Crlf1	cytokine receptor-like factor 1	1.033	0.7401	0.9179
12905	Cradd	CASP2 and RIPK1 domain containing adaptor with death domain	1.033	0.7145	0.908
12827	Col4a2	collagen, type IV, alpha 2	1.033	0.8964	0.9699
12716	Ckmt1	creatine kinase, mitochondrial 1, ubiquitous	1.033	0.643	NA
11957	Atp5j	ATP synthase, H+ transporting, mitochondrial F0 complex, subunit F	1.033	0.5833	NA
11865	Arntl	aryl hydrocarbon receptor nuclear translocator-like	1.033	0.6418	NA
667452	Dcdc2b	doublecortin domain containing 2b	1.032	0.8684	0.9596
606735	A330069E16Rik	RIKEN cDNA A330069E16 gene	1.032	0.5909	NA
544922	Zkscan4	zinc finger with KRAB and SCAN domains 4	1.032	0.578	NA
381823	Apold1	apolipoprotein L domain containing 1	1.032	0.618	NA
381337	Fam178b	family with sequence similarity 178, member B	1.032	0.804	0.938
320916	Wscd2	WSC domain containing 2	1.032	0.7093	0.9069
320747	Lingo4	leucine rich repeat and Ig domain containing 4	1.032	0.763	0.9246
319229	Sctr	secretin receptor	1.032	0.9102	0.9738
319192	Hist2h2aa2	histone cluster 2, H2aa2	1.032	0.5919	NA
271849	Shc4	SHC (Src homology 2 domain containing) family, member 4	1.032	0.5962	NA
270151	Nlrx1	NLR family member X1	1.032	0.7053	NA
245405	Gm4987	predicted gene 4987	1.032	0.6188	NA
240660	Tmem20	transmembrane protein 20	1.032	0.5144	NA
232536	Mrps35	mitochondrial ribosomal protein S35	1.032	0.5763	NA
229588	Gm128	predicted gene 128	1.032	0.7364	0.9169
229521	Syt11	synaptotagmin XI	1.032	0.7251	0.9126
228368	Slc35c1	solute carrier family 35, member C1	1.032	0.5854	NA
228071	Sestd1	SEC14 and spectrin domains 1	1.032	0.7142	0.908
226043	Cbwd1	COBW domain containing 1	1.032	0.4907	NA
217593	Slc25a21	solute carrier family 25 (mitochondrial oxodicarboxylate carrier), member 21	1.032	0.8813	0.9637
214763	E330016A19Rik	RIKEN cDNA E330016A19 gene	1.032	0.7462	0.9193
213233	Tapbpl	TAP binding protein-like	1.032	0.7744	0.9266
211556	Ap1ar	adaptor-related protein complex 1 associated regulatory protein	1.032	0.5751	NA
210044	Adcy2	adenylate cyclase 2	1.032	0.5457	NA
208836	Fanci	Fanconi anemia, complementation group I	1.032	0.8585	0.9564
109778	Blvra	biliverdin reductase A	1.032	0.4374	NA

108909	Aida	axin interactor, dorsalization associated	1.032	0.6099	NA
108737	Oxsr1	oxidative-stress responsive 1	1.032	0.7343	0.9162
108099	Prkag2	protein kinase, AMP-activated, gamma 2 non-catalytic subunit	1.032	0.6786	NA
106344	Rfc4	replication factor C (activator 1) 4	1.032	0.6168	NA
104307	Rnu12	RNA U12, small nuclear	1.032	0.6962	NA
100434	Slc44a1	solute carrier family 44, member 1	1.032	0.7409	0.918
100336	Ppp1r8	protein phosphatase 1, regulatory (inhibitor) subunit 8	1.032	0.7713	0.9258
97863	C78339	expressed sequence C78339	1.032	0.8172	0.942
78689	Naa35	N(alpha)-acetyltransferase 35, NatC auxiliary subunit	1.032	0.4687	NA
75607	Wnk2	WNK lysine deficient protein kinase 2	1.032	0.7826	0.9293
75426	Igfbpl1	insulin-like growth factor binding protein-like 1	1.032	0.5811	NA
74168	Zdhhc16	zinc finger, DHHC domain containing 16	1.032	0.5168	NA
73724	Mcee	methylmalonyl CoA epimerase	1.032	0.5671	NA
73486	1700084J12Rik	RIKEN cDNA 1700084J12 gene	1.032	0.871	0.9607
72205	Eml2	echinoderm microtubule associated protein like 2	1.032	0.6689	NA
71263	Mro	maestro	1.032	0.8058	0.9386
70951	Spata1	spermatogenesis associated 1	1.032	0.86	0.957
70757	Ptplb	protein tyrosine phosphatase-like (proline instead of catalytic arginine), member b	1.032	0.4499	NA
70005	1700029I01Rik	RIKEN cDNA 1700029I01 gene	1.032	0.7702	0.9257
69478	2300009A05Rik	RIKEN cDNA 2300009A05 gene	1.032	0.6092	NA
68598	Dnajc8	DnaJ (Hsp40) homolog, subfamily C, member 8	1.032	0.8089	0.9395
68427	Slc39a13	solute carrier family 39 (metal ion transporter), member 13	1.032	0.6655	NA
67832	Brix1	BRX1, biogenesis of ribosomes, homolog (S. cerevisiae)	1.032	0.4893	NA
67680	Sdhb	succinate dehydrogenase complex, subunit B, iron sulfur (Ip)	1.032	0.4777	NA
67512	Agpat2	1-acylglycerol-3-phosphate O-acyltransferase 2 (lysophosphatidic acid acyltransferase, beta)	1.032	0.6574	NA
67241	Smc6	structural maintenance of chromosomes 6	1.032	0.5571	NA
67157	2610301B20Rik	RIKEN cDNA 2610301B20 gene	1.032	0.5296	NA
67112	Fgf22	fibroblast growth factor 22	1.032	0.8551	0.9551
66397	Sar1b	SAR1 gene homolog B (S. cerevisiae)	1.032	0.6499	NA
66384	Srp19	signal recognition particle 19	1.032	0.8303	0.9453
66314	Tpd52l2	tumor protein D52-like 2	1.032	0.6578	NA
66143	Eef1e1	eukaryotic translation elongation factor 1 epsilon 1	1.032	0.6512	NA
66092	Ghitm	growth hormone inducible transmembrane protein	1.032	0.4981	NA
65112	Pmepa1	prostate transmembrane protein, androgen induced 1	1.032	0.7022	NA
58802	Kcnmb4	potassium large conductance calcium-activated channel, subfamily M, beta member 4	1.032	0.5433	NA
57916	Tnfrsf13b	tumor necrosis factor receptor superfamily, member 13b	1.032	0.8435	0.95
57785	Rangrf	RAN guanine nucleotide release factor	1.032	0.8551	0.9551
57330	Gigyf1	GRB10 interacting GYF protein 1	1.032	0.7046	NA
57295	Icmt	isoprenylcysteine carboxyl methyltransferase	1.032	0.7683	0.9255
56349	Net1	neuroepithelial cell transforming gene 1	1.032	0.6935	NA
56289	Rassf1	Ras association (RalGDS/AF-6) domain family member 1	1.032	0.6123	NA
53627	Porcn	porcupine homolog (Drosophila)	1.032	0.4241	NA
52837	Tmx4	thioredoxin-related transmembrane protein 4	1.032	0.5832	NA
50932	Mink1	misshapen-like kinase 1 (zebrafish)	1.032	0.7736	0.9266
50523	Lats2	large tumor suppressor 2	1.032	0.8344	0.9464
27993	Imp4	IMP4, U3 small nucleolar ribonucleoprotein, homolog (yeast)	1.032	0.5278	NA
23969	Pacsin1	protein kinase C and casein kinase substrate in neurons 1	1.032	0.6511	NA
23794	Adamts5	a disintegrin-like and metallopeptidase (reprolysin type) with thrombospondin type 1 motif, 5 (aggrecanase-2)	1.032	0.7692	0.9255
22348	Slc32a1	solute carrier family 32 (GABA vesicular transporter), member 1	1.032	0.7028	NA

22034	Traf6	TNF receptor-associated factor 6	1.032	0.3934	NA	
21855	Timm17b	translocase of inner mitochondrial membrane 17b	1.032	0.4852	NA	
21780	Tfam	transcription factor A, mitochondrial	1.032	0.5787	NA	
20926	Supt6h	suppressor of Ty 6 homolog (S. cerevisiae)	1.032	0.7279		0.9135
20643	Snrpe	small nuclear ribonucleoprotein E	1.032	0.5797	NA	
20362	Sep-08	septin 8	1.032	0.6961	NA	
20360	Sema6c	sema domain, transmembrane domain (TM), and cytoplasmic domain, (semaphorin) 6C	1.032	0.7691		0.9255
20300	Ccl25	chemokine (C-C motif) ligand 25	1.032	0.6303	NA	
20115	Rps7	ribosomal protein S7	1.032	0.6626	NA	
20103	Rps5	ribosomal protein S5	1.032	0.7189		0.9102
19023	Ppef2	protein phosphatase, EF hand calcium-binding domain 2	1.032	0.5259	NA	
18131	Notch3	Notch gene homolog 3 (Drosophila)	1.032	0.7719		0.9259
18102	Nme1	non-metastatic cells 1, protein (NM23A) expressed in	1.032	0.5272	NA	
18048	Klk1b4	kallikrein 1-related pepidase b4	1.032	0.7752		0.9266
17718	ND3	NADH dehydrogenase subunit 3	1.032	0.5051	NA	
16202	Ilk	integrin linked kinase	1.032	0.7691		0.9255
15368	Hmox1	heme oxygenase (decycling) 1	1.032	0.8576		0.9562
14950	H13	histocompatibility 13	1.032	0.5368	NA	
14802	Gria4	glutamate receptor, ionotropic, AMPA4 (alpha 4)	1.032	0.6769	NA	
14548	Mrps33	mitochondrial ribosomal protein S33	1.032	0.563	NA	
14547	Gdap2	ganglioside-induced differentiation-associated-protein 2	1.032	0.7406		0.9179
14239	Foxs1	forkhead box S1	1.032	0.7076	NA	
13865	Nr2f1	nuclear receptor subfamily 2, group F, member 1	1.032	0.4644	NA	
13717	Eln	elastin	1.032	0.8636		0.9578
13643	Efnb3	ephrin B3	1.032	0.671	NA	
12696	Cirbp	cold inducible RNA binding protein	1.032	0.4455	NA	
12552	Cdh11	cadherin 11	1.032	0.6894	NA	
11886	Asah1	N-acylsphingosine amidohydrolase 1	1.032	0.7857		0.9309
11781	Ap4m1	adaptor-related protein complex AP-4, mu 1	1.032	0.6366	NA	
671535	Parp10	poly (ADP-ribose) polymerase family, member 10	1.031	0.6451	NA	
634012	LOC634012	ubiquitin-conjugating enzyme E2 Q2-like	1.031	0.535	NA	
494448	Cbx6	chromobox homolog 6	1.031	0.5689	NA	
433022	Plcxd2	phosphatidylinositol-specific phospholipase C, X domain containing 2	1.031	0.6627	NA	
403344	C230006B20	hypothetical LOC403344	1.031	0.8309		0.9455
383815	Rps24-ps2	ribosomal protein S24, pseudogene 2	1.031	0.7181	NA	
353237	Pcdhac2	protocadherin alpha subfamily C, 2	1.031	0.8021		0.9373
328580	Tubgcp6	tubulin, gamma complex associated protein 6	1.031	0.6859	NA	
320840	Negr1	neuronal growth regulator 1	1.031	0.5115	NA	
319801	9630033F20Rik	RIKEN cDNA 9630033F20 gene	1.031	0.5088	NA	
319468	Ppm1h	protein phosphatase 1H (PP2C domain containing)	1.031	0.6175	NA	
246103	Atxn7	ataxin 7	1.031	0.7858		0.9309
244667	Disc1	disrupted in schizophrenia 1	1.031	0.4737	NA	
241514	Zfp804a	zinc finger protein 804A	1.031	0.9191		0.9764
240880	Scyl3	SCY1-like 3 (S. cerevisiae)	1.031	0.5695	NA	
237107	Gnl3l	guanine nucleotide binding protein-like 3 (nucleolar)-like	1.031	0.782		0.9291
236915	Arhgef9	CDC42 guanine nucleotide exchange factor (GEF) 9	1.031	0.5397	NA	
234683	Elmo3	engulfment and cell motility 3, ced-12 homolog (C. elegans)	1.031	0.8328		0.9457
234624	A330008L17Rik	RIKEN cDNA A330008L17 gene	1.031	0.7325		0.9157
231915	Uspl1	ubiquitin specific peptidase like 1	1.031	0.529	NA	

223696	Tomm22	translocase of outer mitochondrial membrane 22 homolog (yeast)	1.031	0.6824	NA	
216021	Stox1	storkhead box 1	1.031	0.9043		0.9717
214106	4933430I17Rik	RIKEN cDNA 4933430I17 gene	1.031	0.6717	NA	
170789	Acot8	acyl-CoA thioesterase 8	1.031	0.6113	NA	
117592	B3galt6	UDP-Gal:betaGal beta 1,3-galactosyltransferase, polypeptide 6	1.031	0.6926	NA	
114774	Pawr	PRKC, apoptosis, WT1, regulator	1.031	0.6699	NA	
110075	Bmp3	bone morphogenetic protein 3	1.031	0.5883	NA	
109202	A930024E05Rik	RIKEN cDNA A930024E05 gene	1.031	0.758		0.9227
103737	Pex12	peroxisomal biogenesis factor 12	1.031	0.4962	NA	
102162	Taf5l	TAF5-like RNA polymerase II, p300/CBP-associated factor (PCAF)-associated factor	1.031	0.5254	NA	
101206	Tada3	transcriptional adaptor 3	1.031	0.5008	NA	
100561	Slc15a4	solute carrier family 15, member 4	1.031	0.4747	NA	
98417	Cnih4	cornichon homolog 4 (Drosophila)	1.031	0.7231		0.9121
98415	Nucks1	nuclear casein kinase and cyclin-dependent kinase substrate 1	1.031	0.7115	NA	
94230	Cpsf1	cleavage and polyadenylation specific factor 1	1.031	0.6784	NA	
94223	Dgcr8	DiGeorge syndrome critical region gene 8	1.031	0.69	NA	
93722	Pcdhga10	protocadherin gamma subfamily A, 10	1.031	0.8259		0.9446
83703	Dbr1	debranching enzyme homolog 1 (S. cerevisiae)	1.031	0.7551		0.922
80861	Dhx58	DEXH (Asp-Glu-X-His) box polypeptide 58	1.031	0.6791	NA	
76568	Ift46	intraflagellar transport 46 homolog (Chlamydomonas)	1.031	0.5505	NA	
75422	Mettl5	methyltransferase like 5	1.031	0.3911	NA	
74097	Pop7	processing of precursor 7, ribonuclease P family, (S. cerevisiae)	1.031	0.4941	NA	
73826	Poldip3	polymerase (DNA-directed), delta interacting protein 3	1.031	0.6248	NA	
73825	Klraq1	KLRAQ motif containing 1	1.031	0.7503		0.921
73431	1700052K11Rik	RIKEN cDNA 1700052K11 gene	1.031	0.6101	NA	
72634	Tdrkh	tudor and KH domain containing protein	1.031	0.6917	NA	
72567	Bclaf1	BCL2-associated transcription factor 1	1.031	0.6502	NA	
72523	2700005E23Rik	RIKEN cDNA 2700005E23 gene	1.031	0.8721		0.9608
70568	Cpne3	copine III	1.031	0.6347	NA	
69834	Rab43	RAB43, member RAS oncogene family	1.031	0.5724	NA	
69820	1810059H22Rik	RIKEN cDNA 1810059H22 gene	1.031	0.7495		0.9208
68050	Akirin1	akirin 1	1.031	0.6408	NA	
67860	S100a16	S100 calcium binding protein A16	1.031	0.7444		0.9192
67704	1810037I17Rik	RIKEN cDNA 1810037I17 gene	1.031	0.617	NA	
67288	Srek1ip1	splicing regulatory glutamine/lysine-rich protein 1interacting protein 1	1.031	0.5075	NA	
66890	Lman2	lectin, mannose-binding 2	1.031	0.5141	NA	
66333	Aqp11	aquaporin 11	1.031	0.596	NA	
66181	Nop10	NOP10 ribonucleoprotein homolog (yeast)	1.031	0.6625	NA	
59050	Nsa2	NSA2 ribosome biogenesis homolog (S. cerevisiae)	1.031	0.4451	NA	
56736	Rnf14	ring finger protein 14	1.031	0.4856	NA	
56506	Cib2	calcium and integrin binding family member 2	1.031	0.515	NA	
54624	Paf1	Paf1, RNA polymerase II associated factor, homolog (S. cerevisiae)	1.031	0.5257	NA	
54217	Rpl36	ribosomal protein L36	1.031	0.7025	NA	
53869	Rab11a	RAB11a, member RAS oncogene family	1.031	0.6346	NA	
52840	Dbndd2	dysbindin (dystrobrevin binding protein 1) domain containing 2	1.031	0.7255		0.9126
52683	Ncaph2	non-SMC condensin II complex, subunit H2	1.031	0.5575	NA	
28088	D10Wsu52e	DNA segment, Chr 10, Wayne State University 52, expressed	1.031	0.5212	NA	
27494	Amot	angiomotin	1.031	0.5581	NA	
27425	Atp5l	ATP synthase, H+ transporting, mitochondrial F0 complex, subunit g	1.031	0.6308	NA	

27224	Tceb3	transcription elongation factor B (SIII), polypeptide 3	1.031	0.6777	NA
23943	Esyt1	extended synaptotagmin-like protein 1	1.031	0.7633	0.9247
23900	Hcst	hematopoietic cell signal transducer	1.031	0.8155	0.9416
23808	Ash2l	ash2 (absent, small, or homeotic)-like (Drosophila)	1.031	0.4289	NA
22195	Ube2l3	ubiquitin-conjugating enzyme E2L 3	1.031	0.5922	NA
20609	Sstr5	somatostatin receptor 5	1.031	0.847	0.9517
20463	Cox7a2l	cytochrome c oxidase subunit VIIa polypeptide 2-like	1.031	0.3929	NA
20336	Exoc4	exocyst complex component 4	1.031	0.671	NA
20183	Rxrg	retinoid X receptor gamma	1.031	0.7516	0.9213
20166	Rtkn	rhotekin	1.031	0.6622	NA
19988	Rpl6	ribosomal protein L6	1.031	0.6715	NA
19765	Ralbp1	ralA binding protein 1	1.031	0.5469	NA
19353	Rac1	RAS-related C3 botulinum substrate 1	1.031	0.7436	0.919
19259	Ptpn5	protein tyrosine phosphatase, non-receptor type 5	1.031	0.5783	NA
19241	Tmsb4x	thymosin, beta 4, X chromosome	1.031	0.5315	NA
19175	Psmb6	proteasome (prosome, macropain) subunit, beta type 6	1.031	0.4734	NA
19089	Prkcsh	protein kinase C substrate 80K-H	1.031	0.4478	NA
18759	Prkci	protein kinase C, iota	1.031	0.4481	NA
18197	Nsg2	neuron specific gene family member 2	1.031	0.4423	NA
17973	Nck1	non-catalytic region of tyrosine kinase adaptor protein 1	1.031	0.5121	NA
17246	Mdm2	transformed mouse 3T3 cell double minute 2	1.031	0.3193	NA
16956	Lpl	lipoprotein lipase	1.031	0.7479	0.9202
16467	Atcay	ataxia, cerebellar, Cayman type homolog (human)	1.031	0.7089	NA
15512	Hspa2	heat shock protein 2	1.031	0.6292	NA
15193	Hdgfrp2	hepatoma-derived growth factor, related protein 2	1.031	0.7068	NA
13642	Efnb2	ephrin B2	1.031	0.8446	0.9504
13627	Eef1a1	eukaryotic translation elongation factor 1 alpha 1	1.031	0.8227	0.9436
13048	Cux2	cut-like homeobox 2	1.031	0.6791	NA
12858	Cox5a	cytochrome c oxidase, subunit Va	1.031	0.4878	NA
12830	Col4a5	collagen, type IV, alpha 5	1.031	0.8992	0.9704
12752	Cln3	ceroid lipofuscinosis, neuronal 3, juvenile (Batten, Spielmeyer-Vogt disease)	1.031	0.6405	NA
12300	Cacng2	calcium channel, voltage-dependent, gamma subunit 2	1.031	0.7608	0.9236
11516	Adcyap1	adenylate cyclase activating polypeptide 1	1.031	0.684	NA
11432	Acp2	acid phosphatase 2, lysosomal	1.031	0.6112	NA
100038734	Gm10845	predicted gene 10845	1.03	0.5935	NA
433004	B830017H08Rik	RIKEN cDNA B830017H08 gene	1.03	0.7116	NA
414085	9330151L19Rik	RIKEN cDNA 9330151L19 gene	1.03	0.559	NA
382056	Crtc1	CREB regulated transcription coactivator 1	1.03	0.5729	NA
330260	Pon2	paraoxonase 2	1.03	0.6949	NA
328162	Trmt61a	tRNA methyltransferase 61 homolog A (S. cerevisiae)	1.03	0.7504	0.921
320705	Bend6	BEN domain containing 6	1.03	0.5083	NA
279766	Rhbdd3	rhomboid domain containing 3	1.03	0.6263	NA
272551	Gins2	GINS complex subunit 2 (Psf2 homolog)	1.03	0.7333	0.9159
268354	Fam19a2	family with sequence similarity 19, member A2	1.03	0.5466	NA
236900	Pdk3	pyruvate dehydrogenase kinase, isoenzyme 3	1.03	0.816	0.9416
234395	Ushbp1	Usher syndrome 1C binding protein 1	1.03	0.5746	NA
232313	Gxylt2	glucoside xylosyltransferase 2	1.03	0.8654	0.9584
231915	Uspl1	ubiquitin specific peptidase like 1	1.03	0.4384	NA
230809	Pdik1l	PDLIM1 interacting kinase 1 like	1.03	0.8039	0.938

230075	Ndufb6	NADH dehydrogenase (ubiquinone) 1 beta subcomplex, 6	1.03	0.5574	NA
227210	Ccnyl1	cyclin Y-like 1	1.03	0.7872	0.9314
227054	Rpl23a-ps1	ribosomal protein 23A, pseudogene 1	1.03	0.4834	NA
226409	Zranb3	zinc finger, RAN-binding domain containing 3	1.03	0.6046	NA
224674	Slc37a1	solute carrier family 37 (glycerol-3-phosphate transporter), member 1	1.03	0.8076	0.939
224224	Impg2	interphotoreceptor matrix proteoglycan 2	1.03	0.5547	NA
218311	Zfp455	zinc finger protein 455	1.03	0.7104	NA
215051	Bud13	BUD13 homolog (yeast)	1.03	0.7869	0.9314
213006	Mfsd4	major facilitator superfamily domain containing 4	1.03	0.8205	0.9433
212541	Rho	rhodopsin	1.03	0.6365	NA
211446	Exoc3	exocyst complex component 3	1.03	0.8251	0.9444
114564	Csprs	component of Sp100-rs	1.03	0.8845	0.9657
109652	Acy1	aminoacylase 1	1.03	0.6088	NA
109168	Atl3	atlastin GTPase 3	1.03	0.5813	NA
107734	Mrpl30	mitochondrial ribosomal protein L30	1.03	0.616	NA
105014	Rdh14	retinol dehydrogenase 14 (all-trans and 9-cis)	1.03	0.5209	NA
102115	Dohh	deoxyhypusine hydroxylase/monooxygenase	1.03	0.6311	NA
101685	Spty2d1	SPT2, Suppressor of Ty, domain containing 1 (S. cerevisiae)	1.03	0.8206	0.9433
94213	Ddx50	DEAD (Asp-Glu-Ala-Asp) box polypeptide 50	1.03	0.625	NA
76987	Hdhd2	haloacid dehalogenase-like hydrolase domain containing 2	1.03	0.8626	0.9574
74775	Lmbr1l	limb region 1 like	1.03	0.6456	NA
74569	Ttc17	tetratricopeptide repeat domain 17	1.03	0.8458	0.9511
72865	Cxx1c	CAAX box 1 homolog C (human)	1.03	0.84	0.9485
72775	Fance	Fanconi anemia, complementation group E	1.03	0.6136	NA
72113	Adck1	aarF domain containing kinase 1	1.03	0.4828	NA
71787	Trnau1ap	tRNA selenocysteine 1 associated protein 1	1.03	0.3796	NA
71566	9030425E11Rik	RIKEN cDNA 9030425E11 gene	1.03	0.8478	0.9521
71389	Chd6	chromodomain helicase DNA binding protein 6	1.03	0.5929	NA
69823	Fyttd1	forty-two-three domain containing 1	1.03	0.5993	NA
69786	Tprkb	Tp53rk binding protein	1.03	0.7946	0.9341
68526	Gpr155	G protein-coupled receptor 155	1.03	0.7885	0.9322
67870	Enoph1	enolase-phosphatase 1	1.03	0.493	NA
66998	Psmd5	proteasome (prosome, macropain) 26S subunit, non-ATPase, 5	1.03	0.546	NA
66225	Llph	LLP homolog, long-term synaptic facilitation (Aplysia)	1.03	0.4882	NA
66163	Mrpl4	mitochondrial ribosomal protein L4	1.03	0.4912	NA
66049	Rogdi	rogdi homolog (Drosophila)	1.03	0.7316	0.9153
59057	Zfp191	zinc finger protein 191	1.03	0.4612	NA
57276	Vsig2	V-set and immunoglobulin domain containing 2	1.03	0.7396	0.9178
56310	Gps2	G protein pathway suppressor 2	1.03	0.7612	0.9236
54219	Cd320	CD320 antigen	1.03	0.7187	NA
53870	Cntn6	contactin 6	1.03	0.5884	NA
51960	Kctd18	potassium channel tetramerisation domain containing 18	1.03	0.692	NA
27050	Rps3	ribosomal protein S3	1.03	0.7843	0.9303
26912	Gcat	glycine C-acetyltransferase (2-amino-3-ketobutyrate-coenzyme A ligase)	1.03	0.7495	0.9208
26448	Stk30	serine/threonine kinase 30	1.03	0.7961	0.9346
22632	Yy1	YY1 transcription factor	1.03	0.4194	NA
22601	Yap1	yes-associated protein 1	1.03	0.66	NA
22169	Cmpk2	cytidine monophosphate (UMP-CMP) kinase 2, mitochondrial	1.03	0.6393	NA
22083	Ctr9	Ctr9, Paf1/RNA polymerase II complex component, homolog (S. cerevisiae)	1.03	0.7243	NA

20970	Sdc3	syndecan 3	1.03	0.6667	NA
19896	Rpl10a	ribosomal protein L10A	1.03	0.6038	NA
19731	Rgl1	ral guanine nucleotide dissociation stimulator,-like 1	1.03	0.5805	NA
18472	Pafah1b1	platelet-activating factor acetylhydrolase, isoform 1b, subunit 1	1.03	0.4986	NA
18452	P4ha2	procollagen-proline, 2-oxoglutarate 4-dioxygenase (proline 4-hydroxylase), alpha II polypeptide	1.03	0.7164	NA
18247	Oaz2-ps	ornithine decarboxylase antizyme 2, pseudogene	1.03	0.534	NA
18245	Oaz1	ornithine decarboxylase antizyme 1	1.03	0.6786	NA
18227	Nr4a2	nuclear receptor subfamily 4, group A, member 2	1.03	0.7605	0.9235
17827	Mtx1	metaxin 1	1.03	0.7042	NA
17237	Mgrn1	mahogunin, ring finger 1	1.03	0.6491	NA
16840	Lect1	leukocyte cell derived chemotaxin 1	1.03	0.7256	0.9126
16423	Cd47	CD47 antigen (Rh-related antigen, integrin-associated signal transducer)	1.03	0.7037	NA
14658	Glrb	glycine receptor, beta subunit	1.03	0.5884	NA
14360	Fyn	Fyn proto-oncogene	1.03	0.5615	NA
14088	Fancc	Fanconi anemia, complementation group C	1.03	0.6588	NA
12861	Cox6a1	cytochrome c oxidase, subunit VI a, polypeptide 1	1.03	0.761	0.9236
12389	Cav1	caveolin 1, caveolae protein	1.03	0.7717	0.9259
11974	Atp6v0e	ATPase, H+ transporting, lysosomal V0 subunit E	1.03	0.547	NA
11964	Atp6v1a	ATPase, H+ transporting, lysosomal V1 subunit A	1.03	0.676	NA
100046290	LOC100046290	60S ribosomal protein L21-like	1.029	0.802	0.9373
100038538	Gm10767	predicted gene 10767	1.029	0.6271	NA
668809	Gm9372	predicted gene 9372	1.029	0.9451	0.9844
665095	Cyp2j8-ps	cytochrome P450, family 2, subfamily j, polypeptide 8, pseudogene	1.029	0.5518	NA
626082	LOC626082	hypothetical protein LOC626082	1.029	0.7662	0.9251
497210	Tpt1p	tumor protein, translationally-controlled 1 pseudogene	1.029	0.7224	NA
448850	Znhit3	zinc finger, HIT type 3	1.029	0.615	NA
384719	Gm5341	predicted pseudogene 5341	1.029	0.8398	0.9485
381350	BC061194	cDNA sequence BC061194	1.029	0.8274	0.9449
353342	Peg13	paternally expressed 13	1.029	0.6256	NA
270685	Mthfd1l	methylenetetrahydrofolate dehydrogenase (NADP+ dependent) 1-like	1.029	0.6445	NA
268373	Ppia	peptidylprolyl isomerase A	1.029	0.6291	NA
236069	Gm13238	predicted gene 13238	1.029	0.7331	NA
231866	Zfp12	zinc finger protein 12	1.029	0.8666	0.9587
231580	Gak	cyclin G associated kinase	1.029	0.5639	NA
229644	Trim45	tripartite motif-containing 45	1.029	0.6664	NA
228355	Madd	MAP-kinase activating death domain	1.029	0.7614	0.9237
226757	Wdr26	WD repeat domain 26	1.029	0.6168	NA
226151	Fam178a	family with sequence similarity 178, member A	1.029	0.5875	NA
223691	Eif3l	eukaryotic translation initiation factor 3, subunit L	1.029	0.5585	NA
217733	Tmem63c	transmembrane protein 63c	1.029	0.7582	0.9227
215654	Cdh12	cadherin 12	1.029	0.7251	NA
215335	Slc36a1	solute carrier family 36 (proton/amino acid symporter), member 1	1.029	0.853	0.9544
212163	8030462N17Rik	RIKEN cDNA 8030462N17 gene	1.029	0.7989	0.9361
209225	Zfp710	zinc finger protein 710	1.029	0.6287	NA
170677	Cdhr1	cadherin-related family member 1	1.029	0.5694	NA
170638	Hpcal4	hippocalcin-like 4	1.029	0.8247	0.9443
116905	Dph1	DPH1 homolog (S. cerevisiae)	1.029	0.7786	0.9276
104662	Tsr1	TSR1, 20S rRNA accumulation, homolog (yeast)	1.029	0.7112	NA
104360	Isl2	insulin related protein 2 (islet 2)	1.029	0.4427	NA

104009	Qsox1	quiescin Q6 sulfhydryl oxidase 1	1.029	0.704	NA
94181	Nans	N-acetylneuraminic acid synthase (sialic acid synthase)	1.029	0.6571	NA
80707	Wwox	WW domain-containing oxidoreductase	1.029	0.7623	0.9241
78651	Lsm6	LSM6 homolog, U6 small nuclear RNA associated (S. cerevisiae)	1.029	0.4729	NA
77074	4930426I24Rik	RIKEN cDNA 4930426I24 gene	1.029	0.9039	0.9717
76608	Hectd3	HECT domain containing 3	1.029	0.6888	NA
76367	Trp53rk	transformation related protein 53 regulating kinase	1.029	0.6641	NA
75995	5033417F24Rik	RIKEN cDNA 5033417F24 gene	1.029	0.7564	0.9223
75705	Eif4b	eukaryotic translation initiation factor 4B	1.029	0.659	NA
74107	Cep55	centrosomal protein 55	1.029	0.8337	0.946
74015	Fcho1	FCH domain only 1	1.029	0.8552	0.9551
72320	2510003E04Rik	RIKEN cDNA 2510003E04 gene	1.029	0.5624	NA
72193	Scaf11	SR-related CTD-associated factor 11	1.029	0.585	NA
72124	Seh1l	SEH1-like (S. cerevisiae	1.029	0.6093	NA
71832	Csl	citrate synthase like	1.029	0.6432	NA
71566	9030425E11Rik	RIKEN cDNA 9030425E11 gene	1.029	0.7168	NA
71481	Alpk1	alpha-kinase 1	1.029	0.6827	NA
70620	Ube2v2	ubiquitin-conjugating enzyme E2 variant 2	1.029	0.6573	NA
70604	Dnajb14	DnaJ (Hsp40) homolog, subfamily B, member 14	1.029	0.6458	NA
69743	Casz1	castor homolog 1, zinc finger (Drosophila)	1.029	0.6624	NA
69684	Aarsd1	alanyl-tRNA synthetase domain containing 1	1.029	0.6473	NA
69217	Plekha4	pleckstrin homology domain containing, family A (phosphoinositide binding specific) member 4	1.029	0.7121	NA
68203	Diras2	DIRAS family, GTP-binding RAS-like 2	1.029	0.6209	NA
67703	Kirrel3	kin of IRRE like 3 (Drosophila)	1.029	0.7247	NA
67026	Thap4	THAP domain containing 4	1.029	0.6658	NA
66691	Gapvd1	GTPase activating protein and VPS9 domains 1	1.029	0.5939	NA
66510	Rnf181	ring finger protein 181	1.029	0.6701	NA
66491	Polr2l	polymerase (RNA) II (DNA directed) polypeptide L	1.029	0.6771	NA
66419	Mrpl11	mitochondrial ribosomal protein L11	1.029	0.6099	NA
66300	Prr24	proline rich 24	1.029	0.5148	NA
66121	Chchd1	coiled-coil-helix-coiled-coil-helix domain containing 1	1.029	0.601	NA
59058	Bhlhe22	basic helix-loop-helix family, member e22	1.029	0.7289	NA
56631	Trim17	tripartite motif-containing 17	1.029	0.8613	0.9573
56517	Slc22a21	solute carrier family 22 (organic cation transporter), member 21	1.029	0.7377	0.9172
56310	Gps2	G protein pathway suppressor 2	1.029	0.8234	0.9439
54375	Azin1	antizyme inhibitor 1	1.029	0.7695	0.9255
52838	Dnlz	DNL-type zinc finger	1.029	0.6967	NA
52668	Ifi27l1	interferon, alpha-inducible protein 27 like 1	1.029	0.7404	0.9179
30960	Vapa	vesicle-associated membrane protein, associated protein A	1.029	0.4849	NA
30952	Cngb3	cyclic nucleotide gated channel beta 3	1.029	0.7141	NA
24063	Spry1	sprouty homolog 1 (Drosophila)	1.029	0.755	0.922
22368	Trpv2	transient receptor potential cation channel, subfamily V, member 2	1.029	0.737	0.9171
21927	Tnfaip1	tumor necrosis factor, alpha-induced protein 1 (endothelial)	1.029	0.5835	NA
20872	Stk16	serine/threonine kinase 16	1.029	0.6234	NA
20362	Sep-08	septin 8	1.029	0.8011	0.937
19822	Rnf4	ring finger protein 4	1.029	0.6073	NA
19664	Rbpj	recombination signal binding protein for immunoglobulin kappa J region	1.029	0.7588	0.9229
19158	Cyth2	cytohesin 2	1.029	0.7107	NA
19016	Pparg	peroxisome proliferator activated receptor gamma	1.029	0.6732	NA

18590	Pdgfa	platelet derived growth factor, alpha	1.029	0.67	NA
18414	Osmr	oncostatin M receptor	1.029	0.6742	NA
18399	Slc22a6	solute carrier family 22 (organic anion transporter), member 6	1.029	0.907	0.9728
18194	Nsdhl	NAD(P) dependent steroid dehydrogenase-like	1.029	0.8071	0.9388
18148	Npm1	nucleophosmin 1	1.029	0.7059	NA
18045	Nfyb	nuclear transcription factor-Y beta	1.029	0.4812	NA
18012	Neurod1	neurogenic differentiation 1	1.029	0.6233	NA
16777	Lamb1	laminin B1	1.029	0.66	NA
14869	Gstp2	glutathione S-transferase, pi 2	1.029	0.6706	NA
14395	Gabra2	gamma-aminobutyric acid (GABA) A receptor, subunit alpha 2	1.029	0.7985	0.9361
14186	Fgfr4	fibroblast growth factor receptor 4	1.029	0.8132	0.9406
14007	Celf2	CUGBP, Elav-like family member 2	1.029	0.6132	NA
13808	Eno3	enolase 3, beta muscle	1.029	0.7449	0.9192
13018	Ctcf	CCCTC-binding factor	1.029	0.4597	NA
12669	Chrm1	cholinergic receptor, muscarinic 1, CNS	1.029	0.7593	0.9229
11692	Gfer	growth factor, erv1 (S. cerevisiae)-like (augmenter of liver regeneration)	1.029	0.6334	NA
11416	Slc33a1	solute carrier family 33 (acetyl-CoA transporter), member 1	1.029	0.6278	NA
100137727	9130230N09Rik	RIKEN cDNA 9130230N09 gene	1.028	0.6547	NA
100040322	3830408C21Rik	RIKEN cDNA 3830408C21 gene	1.028	0.59	NA
665434	Gm7634	predicted gene 7634	1.028	0.6717	NA
545989	Gm5901	predicted gene 5901	1.028	0.8931	0.9693
545056	Gm5801	ubiquitin-conjugating enzyme E2, J2 homolog pseudogene	1.028	0.9148	0.9751
449521	Zfp213	zinc finger protein 213	1.028	0.6333	NA
433273	Gm5523	glyceraldehyde-3-phosphate dehydrogenase pseudogene	1.028	0.7639	0.9249
432572	Specc1	sperm antigen with calponin homology and coiled-coil domains 1	1.028	0.6401	NA
414801	Itprip	inositol 1,4,5-triphosphate receptor interacting protein	1.028	0.8651	0.9584
383103	Fam18a	family with sequence similarity 18, member A	1.028	0.6831	NA
382090	4922501C03Rik	RIKEN cDNA 4922501C03 gene	1.028	0.5621	NA
381352	Mamdc4	MAM domain containing 4	1.028	0.764	0.9249
330863	Trim67	tripartite motif-containing 67	1.028	0.8413	0.9487
319807	3110047P20Rik	RIKEN cDNA 3110047P20 gene	1.028	0.5387	NA
276852	D11Wsu47e	DNA segment, Chr 11, Wayne State University 47, expressed	1.028	0.6035	NA
268932	Caskin1	CASK interacting protein 1	1.028	0.7322	NA
240068	Zfp563	zinc finger protein 563	1.028	0.5226	NA
236451	Gm4902	predicted gene 4902	1.028	0.85	0.9531
232440	H2afj	H2A histone family, member J	1.028	0.6485	NA
231699	Oas1e	2'-5' oligoadenylate synthetase 1E	1.028	0.7526	0.9214
228019	Mettl8	methyltransferase like 8	1.028	0.5539	NA
227619	Man1b1	mannosidase, alpha, class 1B, member 1	1.028	0.6855	NA
227154	Stradb	STE20-related kinase adaptor beta	1.028	0.5725	NA
225791	Zadh2	zinc binding alcohol dehydrogenase, domain containing 2	1.028	0.5828	NA
224742	Abcf1	ATP-binding cassette, sub-family F (GCN20), member 1	1.028	0.5995	NA
224143	Poglut1	protein O-glucosyltransferase 1	1.028	0.7484	0.9204
216028	Lrrtm3	leucine rich repeat transmembrane neuronal 3	1.028	0.7223	NA
214424	Parp16	poly (ADP-ribose) polymerase family, member 16	1.028	0.6875	NA
171429	Slc26a6	solute carrier family 26, member 6	1.028	0.4715	NA
170768	Pfkfb3	6-phosphofructo-2-kinase/fructose-2,6-biphosphatase 3	1.028	0.8576	0.9562
110355	Adrbk1	adrenergic receptor kinase, beta 1	1.028	0.708	NA
108707	1810008A18Rik	RIKEN cDNA 1810008A18 gene	1.028	0.4817	NA

108673	Ccdc86	coiled-coil domain containing 86	1.028	0.7319	NA	
107701	Sf3b4	splicing factor 3b, subunit 4	1.028	0.8505		0.9532
103220	BC030307	cDNA sequence BC030307	1.028	0.8193		0.9427
102193	Zdhhc7	zinc finger, DHHC domain containing 7	1.028	0.7064	NA	
99349	Dnajc24	DnaJ (Hsp40) homolog, subfamily C, member 24	1.028	0.6289	NA	
94186	Strn3	striatin, calmodulin binding protein 3	1.028	0.6391	NA	
83815	Cenpq	centromere protein Q	1.028	0.5605	NA	
79566	Sh3bp5l	SH3 binding domain protein 5 like	1.028	0.472	NA	
78797	Ndor1	NADPH dependent diflavin oxidoreductase 1	1.028	0.7766		0.9271
78100	8430410K20Rik	RIKEN cDNA 8430410K20 gene	1.028	0.5885	NA	
76826	Nubpl	nucleotide binding protein-like	1.028	0.5279	NA	
76425	2310003C23Rik	RIKEN cDNA 2310003C23 gene	1.028	0.5086	NA	
73761	4833415N18Rik	RIKEN cDNA 4833415N18 gene	1.028	0.8946		0.9698
73341	Arhgef6	Rac/Cdc42 guanine nucleotide exchange factor (GEF) 6	1.028	0.6698	NA	
73327	1700040I03Rik	RIKEN cDNA 1700040I03 gene	1.028	0.678	NA	
71982	Snx10	sorting nexin 10	1.028	0.7092	NA	
71302	Arhgap26	Rho GTPase activating protein 26	1.028	0.6909	NA	
70615	Ankrd24	ankyrin repeat domain 24	1.028	0.5095	NA	
70238	Rnf168	ring finger protein 168	1.028	0.6087	NA	
69534	Avpi1	arginine vasopressin-induced 1	1.028	0.6676	NA	
68972	Tatdn3	TatD DNase domain containing 3	1.028	0.6587	NA	
68473	Mobkl1a	MOB1, Mps One Binder kinase activator-like 1A (yeast)	1.028	0.7449		0.9192
68276	Toe1	target of EGR1, member 1 (nuclear)	1.028	0.8029		0.9376
68241	Fam195a	family with sequence similarity 195, member A	1.028	0.6376	NA	
67830	Rer1	RER1 retention in endoplasmic reticulum 1 homolog (S. cerevisiae)	1.028	0.4191	NA	
67795	Rnls	renalase, FAD-dependent amine oxidase	1.028	0.7374	NA	
67665	Dctn4	dynactin 4	1.028	0.4887	NA	
67620	Lrp2bp	Lrp2 binding protein	1.028	0.7437	NA	
67216	Mboat2	membrane bound O-acyltransferase domain containing 2	1.028	0.6541	NA	
67078	Pgp	phosphoglycolate phosphatase	1.028	0.7031	NA	
67065	Polr3d	polymerase (RNA) III (DNA directed) polypeptide D	1.028	0.7191	NA	
66822	Fbxo25	F-box protein 25	1.028	0.4312	NA	
66673	Sorcs3	sortilin-related VPS10 domain containing receptor 3	1.028	0.7836		0.9301
66622	Ubr7	ubiquitin protein ligase E3 component n-recognin 7 (putative)	1.028	0.6134	NA	
66618	Snrnp27	small nuclear ribonucleoprotein 27 (U4/U6.U5)	1.028	0.4515	NA	
66589	Ube2v1	ubiquitin-conjugating enzyme E2 variant 1	1.028	0.7959		0.9346
66541	Immp1l	IMP1 inner mitochondrial membrane peptidase-like (S. cerevisiae)	1.028	0.7026	NA	
66360	Bbip1	BBSome interacting protein 1	1.028	0.6957	NA	
66359	Fam36a	family with sequence similarity 36, member A	1.028	0.6545	NA	
66251	Arfgap3	ADP-ribosylation factor GTPase activating protein 3	1.028	0.5648	NA	
66156	Anapc11	anaphase promoting complex subunit 11	1.028	0.7648		0.9251
66155	Ufc1	ubiquitin-fold modifier conjugating enzyme 1	1.028	0.5374	NA	
66098	Chchd6	coiled-coil-helix-coiled-coil-helix domain containing 6	1.028	0.5381	NA	
65246	Xpo7	exportin 7	1.028	0.5851	NA	
65114	Vps35	vacuolar protein sorting 35	1.028	0.6688	NA	
59093	Pcbp3	poly(rC) binding protein 3	1.028	0.682	NA	
58188	Vstm2b	V-set and transmembrane domain containing 2B	1.028	0.7281	NA	
57905	Isy1	ISY1 splicing factor homolog (S. cerevisiae)	1.028	0.5497	NA	
56529	Sec11a	SEC11 homolog A (S. cerevisiae)	1.028	0.6194	NA	

56488	Nxt1	NTF2-related export protein 1	1.028	0.5608	NA
56378	Arpc3	actin related protein 2/3 complex, subunit 3	1.028	0.6993	NA
56320	Dbn1	drebrin 1	1.028	0.7852	0.9307
56233	Hdac7	histone deacetylase 7	1.028	0.7061	NA
53892	Ppm1d	protein phosphatase 1D magnesium-dependent, delta isoform	1.028	0.4959	NA
52637	Cisd1	CDGSH iron sulfur domain 1	1.028	0.568	NA
52174	Tmem222	transmembrane protein 222	1.028	0.6408	NA
51966	D11Bhm181e	DNA segment, Chr 11, Boehm 181, expressed	1.028	0.8783	0.9628
51799	Rundc3a	RUN domain containing 3A	1.028	0.6773	NA
50755	Fbxo18	F-box protein 18	1.028	0.5689	NA
27413	Abcb11	ATP-binding cassette, sub-family B (MDR/TAP), member 11	1.028	0.7325	NA
26931	Ppp2r5c	protein phosphatase 2, regulatory subunit B (B56), gamma isoform	1.028	0.9036	0.9717
26399	Map2k6	mitogen-activated protein kinase kinase 6	1.028	0.7546	0.9217
26377	Dapp1	dual adaptor for phosphotyrosine and 3-phosphoinositides 1	1.028	0.8285	0.945
22678	Zfp2	zinc finger protein 2	1.028	0.7086	NA
22428	Dctn6	dynactin 6	1.028	0.6059	NA
21399	Tcea1	transcription elongation factor A (SII) 1	1.028	0.8296	0.945
21354	Tap1	transporter 1, ATP-binding cassette, sub-family B (MDR/TAP)	1.028	0.8446	0.9504
20912	Stxbp3a	syntaxin binding protein 3A	1.028	0.5342	NA
20729	Spin1	spindlin 1	1.028	0.7606	0.9235
20523	Slc25a14	solute carrier family 25 (mitochondrial carrier, brain), member 14	1.028	0.5822	NA
20333	Sec22b	SEC22 vesicle trafficking protein homolog B (S. cerevisiae)	1.028	0.4373	NA
20028	Pdc	phosducin	1.028	0.6394	NA
19713	Ret	ret proto-oncogene	1.028	0.5401	NA
19414	Rasa3	RAS p21 protein activator 3	1.028	0.5357	NA
19366	Rad54l	RAD54 like (S. cerevisiae)	1.028	0.8031	0.9377
19361	Rad51	RAD51 homolog (S. cerevisiae)	1.028	0.6254	NA
17165	Mapkapk5	MAP kinase-activated protein kinase 5	1.028	0.5034	NA
16917	Lmx1b	LIM homeobox transcription factor 1 beta	1.028	0.6396	NA
16795	Large	like-glycosyltransferase	1.028	0.601	NA
14590	Ggh	gamma-glutamyl hydrolase	1.028	0.5686	NA
14319	Fth1	ferritin heavy chain 1	1.028	0.6166	NA
14260	Fmn1	formin 1	1.028	0.8767	0.962
14176	Fgf5	fibroblast growth factor 5	1.028	0.7451	NA
14137	Fdft1	farnesyl diphosphate farnesyl transferase 1	1.028	0.8626	0.9574
13496	Arid3a	AT rich interactive domain 3A (BRIGHT-like)	1.028	0.7344	NA
13426	Dync1i1	dynein cytoplasmic 1 intermediate chain 1	1.028	0.7859	0.9309
12507	Cd5	CD5 antigen	1.028	0.8478	0.9521
12369	Casp7	caspase 7	1.028	0.468	NA
12328	Caml	calcium modulating ligand	1.028	0.6288	NA
414077	BC056474	cDNA sequence BC056474	1.027	0.8029	0.9376
385674	Zfp174	zinc finger protein 174	1.027	0.7579	0.9227
383341	Gm5239	ubiquitin A-52 residue ribosomal protein fusion product 1 pseudogene	1.027	0.7597	0.9229
382252	A830080D01Rik	RIKEN cDNA A830080D01 gene	1.027	0.648	NA
380768	Gm1568	predicted gene 1568	1.027	0.6514	NA
378462	Morn2	MORN repeat containing 2	1.027	0.5574	NA
353170	Txlng	taxilin gamma	1.027	0.7598	0.9229
333654	Ppp1r13l	protein phosphatase 1, regulatory (inhibitor) subunit 13 like	1.027	0.6283	NA
330635	3830612M24	hypothetical protein 3830612M24	1.027	0.7988	0.9361

330217	Gal3st4	galactose-3-O-sulfotransferase 4	1.027	0.844	0.9502
270097	Vat1l	vesicle amine transport protein 1 homolog-like (T. californica)	1.027	0.6286	NA
260298	Fev	FEV (ETS oncogene family)	1.027	0.7729	0.9262
243262	Oas1f	2'-5' oligoadenylate synthetase 1F	1.027	0.8075	0.939
233865	D430042O09Rik	RIKEN cDNA D430042O09 gene	1.027	0.7399	NA
233066	AI428936	expressed sequence AI428936	1.027	0.7684	0.9255
232975	Atp1a3	ATPase, Na+/K+ transporting, alpha 3 polypeptide	1.027	0.848	0.9521
229543	Ints3	integrator complex subunit 3	1.027	0.6833	NA
218454	Lhfpl2	lipoma HMGIC fusion partner-like 2	1.027	0.6365	NA
217695	Zfyve1	zinc finger, FYVE domain containing 1	1.027	0.7871	0.9314
217370	BC017643	cDNA sequence BC017643	1.027	0.4959	NA
217154	Stac2	SH3 and cysteine rich domain 2	1.027	0.8218	0.9435
215445	Rab11fip3	RAB11 family interacting protein 3 (class II)	1.027	0.7587	0.9229
213673	9530068E07Rik	RIKEN cDNA 9530068E07 gene	1.027	0.4892	NA
209212	Osgin2	oxidative stress induced growth inhibitor family member 2	1.027	0.5697	NA
140859	Nek8	NIMA (never in mitosis gene a)-related expressed kinase 8	1.027	0.6722	NA
109889	Mzf1	myeloid zinc finger 1	1.027	0.7994	0.9361
108888	Atad3a	ATPase family, AAA domain containing 3A	1.027	0.7732	0.9263
107476	Acaca	acetyl-Coenzyme A carboxylase alpha	1.027	0.8752	0.9618
99939	BB085087	expressed sequence BB085087	1.027	0.8089	0.9395
98733	Obsl1	obscurin-like 1	1.027	0.8799	0.9631
98221	Eif3m	eukaryotic translation initiation factor 3, subunit M	1.027	0.6998	NA
83679	Pde4dip	phosphodiesterase 4D interacting protein (myomegalin)	1.027	0.5582	NA
83669	Wdr6	WD repeat domain 6	1.027	0.8061	0.9387
80907	Lactb	lactamase, beta	1.027	0.5556	NA
78903	Wrnip1	Werner helicase interacting protein 1	1.027	0.6427	NA
78651	Lsm6	LSM6 homolog, U6 small nuclear RNA associated (S. cerevisiae)	1.027	0.7884	0.9321
77605	H2afv	H2A histone family, member V	1.027	0.5879	NA
75454	Phpt1	phosphohistidine phosphatase 1	1.027	0.6833	NA
75209	Sv2c	synaptic vesicle glycoprotein 2c	1.027	0.8001	0.9364
74237	Tubgcp2	tubulin, gamma complex associated protein 2	1.027	0.6936	NA
74137	Nuak2	NUAK family, SNF1-like kinase, 2	1.027	0.767	0.9253
72776	Sass6	spindle assembly 6 homolog (C. elegans)	1.027	0.7561	0.9223
71972	Dnmbp	dynamin binding protein	1.027	0.7718	0.9259
69821	Mterfd2	MTERF domain containing 2	1.027	0.5864	NA
69802	Cox11	COX11 homolog, cytochrome c oxidase assembly protein (yeast)	1.027	0.5112	NA
68796	Tmem214	transmembrane protein 214	1.027	0.6636	NA
68332	Sdhaf1	succinate dehydrogenase complex assembly factor 1	1.027	0.5391	NA
68193	Rpl24	ribosomal protein L24	1.027	0.6466	NA
68089	Arpc4	actin related protein 2/3 complex, subunit 4	1.027	0.7951	0.9343
67938	Myl12b	myosin, light chain 12B, regulatory	1.027	0.7662	0.9251
67834	Idh3a	isocitrate dehydrogenase 3 (NAD+) alpha	1.027	0.6249	NA
67703	Kirrel3	kin of IRRE like 3 (Drosophila)	1.027	0.7756	0.9266
67547	Slc39a8	solute carrier family 39 (metal ion transporter), member 8	1.027	0.731	NA
67383	2410127L17Rik	RIKEN cDNA 2410127L17 gene	1.027	0.5827	NA
67238	2810453I06Rik	RIKEN cDNA 2810453I06 gene	1.027	0.7216	NA
67155	Smarca2	SWI/SNF related, matrix associated, actin dependent regulator of chromatin, subfamily a, member 2	1.027	0.6358	NA
66589	Ube2v1	ubiquitin-conjugating enzyme E2 variant 1	1.027	0.8484	0.9522
66531	2310061C15Rik	RIKEN cDNA 2310061C15 gene	1.027	0.5123	NA

66460	Sys1	SYS1 Golgi-localized integral membrane protein homolog (S. cerevisiae)	1.027	0.7295	NA
64707	Suv39h2	suppressor of variegation 3-9 homolog 2 (Drosophila)	1.027	0.8563	0.9554
64339	Fndc4	fibronectin type III domain containing 4	1.027	0.6799	NA
56707	Zfp111	zinc finger protein 111	1.027	0.5236	NA
56353	Rybp	RING1 and YY1 binding protein	1.027	0.6009	NA
56041	Uso1	USO1 homolog, vesicle docking protein (yeast)	1.027	0.5402	NA
53608	Map3k6	mitogen-activated protein kinase kinase kinase 6	1.027	0.7363	NA
53328	Pgrmc1	progesterone receptor membrane component 1	1.027	0.6304	NA
52004	Cdk2ap2	CDK2-associated protein 2	1.027	0.7462	NA
50794	Klf13	Kruppel-like factor 13	1.027	0.8529	0.9544
26927	Foxl2	forkhead box L2	1.027	0.8102	0.94
26414	Mapk10	mitogen-activated protein kinase 10	1.027	0.7706	0.9257
21750	Terf2	telomeric repeat binding factor 2	1.027	0.7525	NA
20743	Spnb3	spectrin beta 3	1.027	0.7694	0.9255
20621	Snn	stannin	1.027	0.5103	NA
20544	Slc9a1	solute carrier family 9 (sodium/hydrogen exchanger), member 1	1.027	0.6294	NA
19933	Rpl21	ribosomal protein L21	1.027	0.6003	NA
19336	Rab24	RAB24, member RAS oncogene family	1.027	0.512	NA
18046	Nfyc	nuclear transcription factor-Y gamma	1.027	0.7147	NA
18010	Neu1	neuraminidase 1	1.027	0.5095	NA
16976	Lrpap1	low density lipoprotein receptor-related protein associated protein 1	1.027	0.6054	NA
16909	Lmo2	LIM domain only 2	1.027	0.7131	NA
15460	Hr	hairless	1.027	0.8377	0.9479
15444	Hpca	hippocalcin	1.027	0.8298	0.945
15275	Hk1	hexokinase 1	1.027	0.6664	NA
14369	Fzd7	frizzled homolog 7 (Drosophila)	1.027	0.6634	NA
14269	Fnbp1	formin binding protein 1	1.027	0.6623	NA
14154	Fem1a	feminization 1 homolog a (C. elegans)	1.027	0.562	NA
13628	Eef1a2	eukaryotic translation elongation factor 1 alpha 2	1.027	0.837	0.9479
13134	Dach1	dachshund 1 (Drosophila)	1.027	0.6735	NA
12895	Cpt1b	carnitine palmitoyltransferase 1b, muscle	1.027	0.8266	0.9448
12583	Cdo1	cysteine dioxygenase 1, cytosolic	1.027	0.6201	NA
11958	Atp5k	ATP synthase, H+ transporting, mitochondrial F1F0 complex, subunit e	1.027	0.6091	NA
11771	Ap2a1	adaptor protein complex AP-2, alpha 1 subunit	1.027	0.8401	0.9485
11542	Adora3	adenosine A3 receptor	1.027	0.879	0.9629
675440	Gm13430	predicted gene 13430	1.026	0.9493	0.9856
633640	Gm7120	predicted gene 7120	1.026	0.9032	0.9717
625540	Gm6598	predicted gene 6598	1.026	0.6664	NA
545055	Cma2	chymase 2, mast cell	1.026	0.9278	0.9789
407786	Taf9b	TAF9B RNA polymerase II, TATA box binding protein (TBP)-associated factor	1.026	0.6357	NA
384482	Gm5316	predicted gene 5316	1.026	0.7447	NA
382985	Rrm2b	ribonucleotide reductase M2 B (TP53 inducible)	1.026	0.7324	NA
381306	BC055324	cDNA sequence BC055324	1.026	0.6871	NA
380912	Zfp395	zinc finger protein 395	1.026	0.8211	0.9433
338355	Fkbp15	FK506 binding protein 15	1.026	0.8138	0.9406
272009	Srsf13b	serine/arginine-rich splicing factor 13B	1.026	0.5528	NA
244548	Elmod2	ELMO domain containing 2	1.026	0.6543	NA
244281	Myo16	myosin XVI	1.026	0.7369	NA
240023	Pnldc1	poly(A)-specific ribonuclease (PARN)-like domain containing 1	1.026	0.7598	NA

239170	Fam160b2	family with sequence similarity 160, member B2	1.026	0.679	NA
234353	Psd3	pleckstrin and Sec7 domain containing 3	1.026	0.6789	NA
234344	Naf1	nuclear assembly factor 1 homolog (S. cerevisiae)	1.026	0.6757	NA
232187	Smyd5	SET and MYND domain containing 5	1.026	0.8089	0.9395
230866	C230096C10Rik	RIKEN cDNA C230096C10 gene	1.026	0.7944	0.9339
229517	Slc25a44	solute carrier family 25, member 44	1.026	0.7207	NA
228998	Arfgap1	ADP-ribosylation factor GTPase activating protein 1	1.026	0.7595	NA
228730	Plk1s1	polo-like kinase 1 substrate 1	1.026	0.5237	NA
226823	Kctd3	potassium channel tetramerisation domain containing 3	1.026	0.5053	NA
225283	Rprd1a	regulation of nuclear pre-mRNA domain containing 1A	1.026	0.4607	NA
216134	Pdxk	pyridoxal (pyridoxine, vitamin B6) kinase	1.026	0.6145	NA
215472	Gm4792	predicted gene 4792	1.026	0.8931	0.9693
213491	D4Ertd22e	DNA segment, Chr 4, ERATO Doi 22, expressed	1.026	0.7979	0.9357
213484	Nudt18	nudix (nucleoside diphosphate linked moiety X)-type motif 18	1.026	0.9163	0.9757
212679	Mars2	methionine-tRNA synthetase 2 (mitochondrial)	1.026	0.7573	NA
194231	Cnksr1	connector enhancer of kinase suppressor of Ras 1	1.026	0.8909	0.9687
116905	Dph1	DPH1 homolog (S. cerevisiae)	1.026	0.7101	NA
110196	Fdps	farnesyl diphosphate synthetase	1.026	0.5947	NA
109050	6530418L21Rik	RIKEN cDNA 6530418L21 gene	1.026	0.5416	NA
100637	N4bp2l1	NEDD4 binding protein 2-like 1	1.026	0.733	NA
99412	Golga2	golgi autoantigen, golgin subfamily a, 2	1.026	0.5349	NA
83768	Dpp7	dipeptidylpeptidase 7	1.026	0.7136	NA
80879	Slc16a3	solute carrier family 16 (monocarboxylic acid transporters), member 3	1.026	0.9046	0.9717
78670	Plekhj1	pleckstrin homology domain containing, family J member 1	1.026	0.8814	0.9637
78552	E130201H02Rik	Y box protein 1 pseudogene	1.026	0.7754	0.9266
77480	Kidins220	kinase D-interacting substrate 220	1.026	0.8579	0.9562
75841	Rnf139	ring finger protein 139	1.026	0.5801	NA
74479	Snx11	sorting nexin 11	1.026	0.5022	NA
74149	Zfp946	zinc finger protein 946	1.026	0.6131	NA
73094	Sgip1	SH3-domain GRB2-like (endophilin) interacting protein 1	1.026	0.8198	0.9429
72999	Insig2	insulin induced gene 2	1.026	0.6027	NA
72555	Shisa9	shisa homolog 9 (Xenopus laevis)	1.026	0.7501	NA
72536	Tagap	T-cell activation Rho GTPase-activating protein	1.026	0.7517	NA
72278	Ccpg1	cell cycle progression 1	1.026	0.6155	NA
71910	Ppapdc1b	phosphatidic acid phosphatase type 2 domain containing 1B	1.026	0.6918	NA
71640	Zfp949	zinc finger protein 949	1.026	0.5759	NA
69790	Med30	mediator complex subunit 30	1.026	0.5363	NA
69527	Mrps9	mitochondrial ribosomal protein S9	1.026	0.5591	NA
69227	2810407C02Rik	RIKEN cDNA 2810407C02 gene	1.026	0.8107	0.9401
69216	Ccdc23	coiled-coil domain containing 23	1.026	0.6193	NA
68607	Serhl	serine hydrolase-like	1.026	0.7496	NA
68193	Rpl24	ribosomal protein L24	1.026	0.7727	0.9262
67673	Tceb2	transcription elongation factor B (SIII), polypeptide 2	1.026	0.5942	NA
67628	Anp32b	acidic (leucine-rich) nuclear phosphoprotein 32 family, member B	1.026	0.5756	NA
67515	Ttc33	tetratricopeptide repeat domain 33	1.026	0.5928	NA
67475	Ero1lb	ERO1-like beta (S. cerevisiae)	1.026	0.5357	NA
66072	Sdhaf2	succinate dehydrogenase complex assembly factor 2	1.026	0.7105	NA
57434	Xrcc2	X-ray repair complementing defective repair in Chinese hamster cells 2	1.026	0.7383	NA
56438	Rbx1	ring-box 1	1.026	0.6648	NA

54418	Fmn2	formin 2	1.026	0.6786	NA
50791	Magi2	membrane associated guanylate kinase, WW and PDZ domain containing 2	1.026	0.741	NA
28071	Twistnb	TWIST neighbor	1.026	0.7217	NA
26879	B3galnt1	UDP-GalNAc:betaGlcNAc beta 1,3-galactosaminyltransferase, polypeptide 1	1.026	0.5821	NA
26413	Mapk1	mitogen-activated protein kinase 1	1.026	0.6152	NA
20661	Sort1	sortilin 1	1.026	0.7326	NA
20402	Zfp106	zinc finger protein 106	1.026	0.5854	NA
20316	Sdf2	stromal cell derived factor 2	1.026	0.5386	NA
19395	Rasgrp2	RAS, guanyl releasing protein 2	1.026	0.7186	NA
19285	Ptrf	polymerase I and transcript release factor	1.026	0.8393	0.9484
19116	Prlr	prolactin receptor	1.026	0.898	0.9699
18618	Pemt	phosphatidylethanolamine N-methyltransferase	1.026	0.7995	0.9362
16773	Lama2	laminin, alpha 2	1.026	0.9338	0.9806
16697	LOC16697	keratin associated protein LOC16697	1.026	0.7288	NA
16679	Krt86	keratin 86	1.026	0.9351	0.981
15382	Hnrnpa1	heterogeneous nuclear ribonucleoprotein A1	1.026	0.5768	NA
15257	Hipk1	homeodomain interacting protein kinase 1	1.026	0.8175	0.942
14228	Fkbp4	FK506 binding protein 4	1.026	0.7115	NA
11829	Aqp4	aquaporin 4	1.026	0.6807	NA
11750	Anxa7	annexin A7	1.026	0.5252	NA
11465	Actg1	actin, gamma, cytoplasmic 1	1.026	0.851	0.9536
100502641	LOC100502641	60S acidic ribosomal protein P1-like	1.025	0.6614	NA
629378	Dact3	dapper homolog 3, antagonist of beta-catenin (xenopus)	1.025	0.7441	NA
622208	Gm6297	predicted gene 6297	1.025	0.7456	NA
320060	B230308N11Rik	RIKEN cDNA B230308N11 gene	1.025	0.7054	NA
242553	Kank4	KN motif and ankyrin repeat domains 4	1.025	0.8814	0.9637
242316	Gdf6	growth differentiation factor 6	1.025	0.8562	0.9554
241490	Rbm45	RNA binding motif protein 45	1.025	0.6827	NA
240832	Tor1aip2	torsin A interacting protein 2	1.025	0.5916	NA
234796	Klhl36	kelch-like 36 (Drosophila)	1.025	0.82	0.9429
234740	Tmem231	transmembrane protein 231	1.025	0.8293	0.945
232813	Shisa7	shisa homolog 7 (Xenopus laevis)	1.025	0.8285	0.945
230587	Glis1	GLIS family zinc finger 1	1.025	0.7159	NA
229615	Pias3	protein inhibitor of activated STAT 3	1.025	0.8068	0.9388
227743	Mapkap1	mitogen-activated protein kinase associated protein 1	1.025	0.6795	NA
214424	Parp16	poly (ADP-ribose) polymerase family, member 16	1.025	0.7844	0.9304
214253	Etnk2	ethanolamine kinase 2	1.025	0.7083	NA
211401	Mtss1	metastasis suppressor 1	1.025	0.6519	NA
171429	Slc26a6	solute carrier family 26, member 6	1.025	0.7027	NA
108899	2700081O15Rik	RIKEN cDNA 2700081O15 gene	1.025	0.8373	0.9479
103712	6330403K07Rik	RIKEN cDNA 6330403K07 gene	1.025	0.6645	NA
102693	Phldb1	pleckstrin homology-like domain, family B, member 1	1.025	0.7327	NA
98878	Ehd4	EH-domain containing 4	1.025	0.6558	NA
98417	Cnih4	cornichon homolog 4 (Drosophila)	1.025	0.6031	NA
93842	Igsf9	immunoglobulin superfamily, member 9	1.025	0.7175	NA
84652	Fam126a	family with sequence similarity 126, member A	1.025	0.843	0.9497
78827	5830426C09Rik	RIKEN cDNA 5830426C09 gene	1.025	0.8244	0.9443
78825	Pppde1	PPPDE peptidase domain containing 1	1.025	0.7832	0.9298
77305	Wdr82	WD repeat domain containing 82	1.025	0.6953	NA

76900	Ssbp4	single stranded DNA binding protein 4	1.025	0.7535	NA
75769	4833424O15Rik	RIKEN cDNA 4833424O15 gene	1.025	0.707	NA
75729	4933432B09Rik	RIKEN cDNA 4933432B09 gene	1.025	0.7349	NA
75712	Tmem14a	transmembrane protein 14A	1.025	0.5802	NA
74766	Yipf2	Yip1 domain family, member 2	1.025	0.4896	NA
74412	Gle1	GLE1 RNA export mediator (yeast)	1.025	0.7202	NA
74203	Eif4enif1	eukaryotic translation initiation factor 4E nuclear import factor 1	1.025	0.6113	NA
70767	Prpf3	PRP3 pre-mRNA processing factor 3 homolog (yeast)	1.025	0.6773	NA
70428	Polr3b	polymerase (RNA) III (DNA directed) polypeptide B	1.025	0.629	NA
69276	Sec62	SEC62 homolog (S. cerevisiae)	1.025	0.6072	NA
68051	Nutf2	nuclear transport factor 2	1.025	0.5704	NA
67160	Eef1g	eukaryotic translation elongation factor 1 gamma	1.025	0.6467	NA
67031	Upf3a	UPF3 regulator of nonsense transcripts homolog A (yeast)	1.025	0.6587	NA
66958	Tmx2	thioredoxin-related transmembrane protein 2	1.025	0.7705	0.9257
66805	Tspan1	tetraspanin 1	1.025	0.9059	0.9722
66643	Lix1	limb expression 1 homolog (chicken)	1.025	0.9594	0.9883
65113	Ndfip1	Nedd4 family interacting protein 1	1.025	0.7245	NA
59052	Mettl9	methyltransferase like 9	1.025	0.5516	NA
59009	Sh3rf1	SH3 domain containing ring finger 1	1.025	0.7084	NA
57750	Wdr12	WD repeat domain 12	1.025	0.5758	NA
56390	Sssca1	Sjogren's syndrome/scleroderma autoantigen 1 homolog (human)	1.025	0.7513	NA
56044	Rala	v-ral simian leukemia viral oncogene homolog A (ras related)	1.025	0.707	NA
54199	Ccrl2	chemokine (C-C motif) receptor-like 2	1.025	0.6393	NA
52822	Rufy3	RUN and FYVE domain containing 3	1.025	0.5894	NA
52708	Zfp410	zinc finger protein 410	1.025	0.7024	NA
51902	Rnf24	ring finger protein 24	1.025	0.4808	NA
27398	Mrpl2	mitochondrial ribosomal protein L2	1.025	0.6876	NA
27367	Rpl3	ribosomal protein L3	1.025	0.778	0.9273
27054	Sec23b	SEC23B (S. cerevisiae)	1.025	0.7188	NA
26942	Spag1	sperm associated antigen 1	1.025	0.7646	NA
22385	Baz1b	bromodomain adjacent to zinc finger domain, 1B	1.025	0.7527	NA
21417	Zeb1	zinc finger E-box binding homeobox 1	1.025	0.6651	NA
20713	Serpini1	serine (or cysteine) peptidase inhibitor, clade I, member 1	1.025	0.8136	0.9406
20666	Sox11	SRY-box containing gene 11	1.025	0.7051	NA
20479	Vps4b	vacuolar protein sorting 4b (yeast)	1.025	0.8224	0.9435
19719	Rfng	RFNG O-fucosylpeptide 3-beta-N-acetylglucosaminyltransferase	1.025	0.6958	NA
18817	Plk1	polo-like kinase 1 (Drosophila)	1.025	0.6754	NA
18634	Pex7	peroxisomal biogenesis factor 7	1.025	0.6853	NA
18111	Nnat	neuronatin	1.025	0.8789	0.9629
17979	Ncoa3	nuclear receptor coactivator 3	1.025	0.8353	0.9466
17974	Nck2	non-catalytic region of tyrosine kinase adaptor protein 2	1.025	0.7329	NA
16923	Sh2b3	SH2B adaptor protein 3	1.025	0.8057	0.9386
16907	Lmnb2	lamin B2	1.025	0.7718	0.9259
16870	Lhx2	LIM homeobox protein 2	1.025	0.6401	NA
16363	Irf2	interferon regulatory factor 2	1.025	0.7565	NA
15361	Hmga1	high mobility group AT-hook 1	1.025	0.7014	NA
14417	Gad2	glutamic acid decarboxylase 2	1.025	0.5932	NA
14211	Smc2	structural maintenance of chromosomes 2	1.025	0.702	NA
14055	Ezh1	enhancer of zeste homolog 1 (Drosophila)	1.025	0.5872	NA

13340	Slc29a2	solute carrier family 29 (nucleoside transporters), member 2	1.025	0.7323	NA
13163	Daxx	Fas death domain-associated protein	1.025	0.7908	0.9333
13010	Cst3	cystatin C	1.025	0.6874	NA
11858	Rnd2	Rho family GTPase 2	1.025	0.7697	0.9256
11551	Adra2a	adrenergic receptor, alpha 2a	1.025	0.5172	NA
100041953	Gm10094	predicted gene 10094	1.024	0.6027	NA
100040297	Gm2695	predicted gene 2695	1.024	0.7976	0.9355
665189	Gm7536	predicted gene 7536	1.024	0.8394	0.9484
628324	S100a2	S100 calcium binding protein A2	1.024	0.8949	0.9698
624713	Gm6525	ribosomal protein L36a pseudogene	1.024	0.6003	NA
436332	Gm5766	ribosomal protein L7a pseudogene	1.024	0.5674	NA
403187	Opa3	optic atrophy 3 (human)	1.024	0.7553	NA
381353	Gm996	predicted gene 996	1.024	0.6357	NA
381107	Tmem232	transmembrane protein 232	1.024	0.788	0.9319
380773	1810035L17Rik	RIKEN cDNA 1810035L17 gene	1.024	0.7176	NA
330064	Slc5a6	solute carrier family 5 (sodium-dependent vitamin transporter), member 6	1.024	0.5824	NA
320840	Negr1	neuronal growth regulator 1	1.024	0.6745	NA
319984	Jph4	junctophilin 4	1.024	0.8068	0.9388
269424	Phf17	PHD finger protein 17	1.024	0.6441	NA
259300	Ehd2	EH-domain containing 2	1.024	0.8288	0.945
244421	Lonrf1	LON peptidase N-terminal domain and ring finger 1	1.024	0.6218	NA
243983	Zdhhc13	zinc finger, DHHC domain containing 13	1.024	0.5839	NA
241308	Ralgps1	Ral GEF with PH domain and SH3 binding motif 1	1.024	0.8954	0.9698
237831	Slc13a5	solute carrier family 13 (sodium-dependent citrate transporter), member 5	1.024	0.7936	0.9336
233902	Fbxl19	F-box and leucine-rich repeat protein 19	1.024	0.7051	NA
231413	Grsf1	G-rich RNA sequence binding factor 1	1.024	0.6619	NA
231086	Hadhb	hydroxyacyl-Coenzyme A dehydrogenase/3-ketoacyl-Coenzyme A thiolase/enoyl-Coenzyme A hydratase (trifunctional protein), beta subunit	1.024	0.6059	NA
229663	Csde1	cold shock domain containing E1, RNA binding	1.024	0.715	NA
227094	Tmem194b	transmembrane protein 194B	1.024	0.7392	NA
226025	Trpm3	transient receptor potential cation channel, subfamily M, member 3	1.024	0.6668	NA
213499	Fbxo42	F-box protein 42	1.024	0.7686	NA
207352	Sec23ip	Sec23 interacting protein	1.024	0.6729	NA
171429	Slc26a6	solute carrier family 26, member 6	1.024	0.6547	NA
108089	Rnf144a	ring finger protein 144A	1.024	0.6716	NA
104112	Acly	ATP citrate lyase	1.024	0.6601	NA
102502	Pls1	plastin 1 (I-isoform)	1.024	0.8541	0.9546
101240	Wdr91	WD repeat domain 91	1.024	0.7715	NA
100201	Tmem64	transmembrane protein 64	1.024	0.6847	NA
94045	P2rx5	purinergic receptor P2X, ligand-gated ion channel, 5	1.024	0.7584	NA
78309	Cul9	cullin 9	1.024	0.6585	NA
76376	Slc24a2	solute carrier family 24 (sodium/potassium/calcium exchanger), member 2	1.024	0.5153	NA
76375	Det1	de-etiolated homolog 1 (Arabidopsis)	1.024	0.6694	NA
75758	9130401M01Rik	RIKEN cDNA 9130401M01 gene	1.024	0.7047	NA
75212	Rnf121	ring finger protein 121	1.024	0.6899	NA
74376	Myo18b	myosin XVIIIb	1.024	0.8969	0.9699
74143	Opa1	optic atrophy 1 homolog (human)	1.024	0.5498	NA
73010	Gpr22	G protein-coupled receptor 22	1.024	0.7137	NA
72961	Slc17a7	solute carrier family 17 (sodium-dependent inorganic phosphate cotransporter), member 7	1.024	0.8981	0.9699
72745	Tmem161b	transmembrane protein 161B	1.024	0.7263	NA

72514	Fgfbp3	fibroblast growth factor binding protein 3	1.024	0.7291	NA
68988	Prpf31	PRP31 pre-mRNA processing factor 31 homolog (yeast)	1.024	0.7291	NA
68954	1500012K07Rik	RIKEN cDNA 1500012K07 gene	1.024	0.7429	NA
68936	Fam165b	family with sequence similarity 165, member B	1.024	0.7231	NA
67736	Ccdc130	coiled-coil domain containing 130	1.024	0.7349	NA
67095	Trak1	trafficking protein, kinesin binding 1	1.024	0.6279	NA
67080	1700019D03Rik	RIKEN cDNA 1700019D03 gene	1.024	0.7038	NA
67030	Fancl	Fanconi anemia, complementation group L	1.024	0.6939	NA
66942	Ddx18	DEAD (Asp-Glu-Ala-Asp) box polypeptide 18	1.024	0.7666	NA
66408	Aptx	aprataxin	1.024	0.7457	NA
66176	Nat9	N-acetyltransferase 9 (GCN5-related, putative)	1.024	0.6505	NA
63958	Ube4b	ubiquitination factor E4B, UFD2 homolog (S. cerevisiae)	1.024	0.5999	NA
57775	Usp29	ubiquitin specific peptidase 29	1.024	0.6194	NA
57294	Rps27	ribosomal protein S27	1.024	0.7219	NA
56282	Mrpl12	mitochondrial ribosomal protein L12	1.024	0.5128	NA
56278	Gkap1	G kinase anchoring protein 1	1.024	0.6015	NA
55936	Ctps2	cytidine 5'-triphosphate synthase 2	1.024	0.6577	NA
54616	Extl3	exostoses (multiple)-like 3	1.024	0.6049	NA
50769	Atp8a2	ATPase, aminophospholipid transporter-like, class I, type 8A, member 2	1.024	0.6353	NA
50724	Sap30l	SAP30-like	1.024	0.6449	NA
28075	Pppde2	PPPDE peptidase domain containing 2	1.024	0.8061	0.9387
26903	Dysf	dysferlin	1.024	0.837	0.9479
26757	Dpysl4	dihydropyrimidinase-like 4	1.024	0.54	NA
22785	Slc30a4	solute carrier family 30 (zinc transporter), member 4	1.024	0.6089	NA
22393	Wfs1	Wolfram syndrome 1 homolog (human)	1.024	0.7401	NA
22384	Eif4h	eukaryotic translation initiation factor 4H	1.024	0.6172	NA
22186	Uba52	ubiquitin A-52 residue ribosomal protein fusion product 1	1.024	0.8109	0.9401
22025	Nr2c1	nuclear receptor subfamily 2, group C, member 1	1.024	0.7727	NA
20617	Snca	synuclein, alpha	1.024	0.7003	NA
20513	Slc1a6	solute carrier family 1 (high affinity aspartate/glutamate transporter), member 6	1.024	0.6589	NA
19384	Ran	RAN, member RAS oncogene family	1.024	0.6753	NA
17977	Ncoa1	nuclear receptor coactivator 1	1.024	0.7178	NA
17119	Mxd1	MAX dimerization protein 1	1.024	0.7119	NA
14247	Fli1	Friend leukemia integration 1	1.024	0.6721	NA
13709	Elf1	E74-like factor 1	1.024	0.8099	0.94
13106	Cyp2e1	cytochrome P450, family 2, subfamily e, polypeptide 1	1.024	0.9162	0.9757
12321	Calu	calumenin	1.024	0.7892	0.9327
12227	Btg2	B-cell translocation gene 2, anti-proliferative	1.024	0.7728	NA
12095	Bglap-rs1	bone gamma-carboxyglutamate protein, related sequence 1	1.024	0.812	0.9403
11790	Speg	SPEG complex locus	1.024	0.7153	NA
11603	Agrn	agrin	1.024	0.9369	0.9816
11465	Actg1	actin, gamma, cytoplasmic 1	1.024	0.7971	0.9351
11298	Aanat	arylalkylamine N-acetyltransferase	1.024	0.8562	0.9554
100039864	Snhg12	small nucleolar RNA host gene 12	1.023	0.7777	NA
626858	Gm6713	predicted gene 6713	1.023	0.9381	0.9823
381827	1700073E17Rik	ribosomal protein L7 pseudogene	1.023	0.622	NA
381287	A530032D15Rik	RIKEN cDNA A530032D15Rik gene	1.023	0.9053	0.9718
378702	Serf2	small EDRK-rich factor 2	1.023	0.8085	0.9395
328066	C920021A13	hypothetical protein C920021A13	1.023	0.9128	0.9743

320500	Tmem215	transmembrane protein 215	1.023	0.8323	0.9456
320163	4930525G20Rik	RIKEN cDNA 4930525G20 gene	1.023	0.7383	NA
244886	AI118078	expressed sequence AI118078	1.023	0.7384	NA
244668	Sipa1l2	signal-induced proliferation-associated 1 like 2	1.023	0.5108	NA
243813	Leng9	leukocyte receptor cluster (LRC) member 9	1.023	0.6067	NA
243302	Gm4963	predicted gene 4963	1.023	0.6729	NA
240690	St18	suppression of tumorigenicity 18	1.023	0.7447	NA
238333	Samd15	sterile alpha motif domain containing 15	1.023	0.8926	0.9692
237759	Col23a1	collagen, type XXIII, alpha 1	1.023	0.758	NA
235956	Zfp825	zinc finger protein 825	1.023	0.6466	NA
230661	Tesk2	testis-specific kinase 2	1.023	0.5806	NA
230119	Zbtb5	zinc finger and BTB domain containing 5	1.023	0.6537	NA
227613	Tubb2c	tubulin, beta 2C	1.023	0.6449	NA
226539	Dars2	aspartyl-tRNA synthetase 2 (mitochondrial)	1.023	0.8498	0.9531
225913	Dak	dihydroxyacetone kinase 2 homolog (yeast)	1.023	0.8551	0.9551
225523	Cep120	centrosomal protein 120	1.023	0.7042	NA
219094	Khnyn	KH and NYN domain containing	1.023	0.7286	NA
218877	Sema3g	sema domain, immunoglobulin domain (Ig), short basic domain, secreted, (semaphorin) 3G	1.023	0.8676	0.9593
216869	Arrb2	arrestin, beta 2	1.023	0.7424	NA
215114	Hip1	huntingtin interacting protein 1	1.023	0.8209	0.9433
212627	Prpsap2	phosphoribosyl pyrophosphate synthetase-associated protein 2	1.023	0.5775	NA
207704	Gtpbp10	GTP-binding protein 10 (putative)	1.023	0.6624	NA
207683	Igsf11	immunoglobulin superfamily, member 11	1.023	0.7845	NA
107371	Exoc6	exocyst complex component 6	1.023	0.8046	0.9381
77286	Nkrf	NF-kappaB repressing factor	1.023	0.6801	NA
77038	Arfgap2	ADP-ribosylation factor GTPase activating protein 2	1.023	0.6593	NA
76426	1700029J11Rik	RIKEN cDNA 1700029J11 gene	1.023	0.8772	0.9623
76229	Vmn2r29	vomeronasal 2, receptor 29	1.023	0.7921	0.9333
75669	Pik3r4	phosphatidylinositol 3 kinase, regulatory subunit, polypeptide 4, p150	1.023	0.6052	NA
74369	Mei1	meiosis defective 1	1.023	0.741	NA
73024	2900064A13Rik	RIKEN cDNA 2900064A13 gene	1.023	0.617	NA
72075	Ogfr	opioid growth factor receptor	1.023	0.5852	NA
71968	Wdr73	WD repeat domain 73	1.023	0.6115	NA
71966	Nkiras2	NFKB inhibitor interacting Ras-like protein 2	1.023	0.8034	0.9377
71564	Izumo4	IZUMO family member 4	1.023	0.7911	0.9333
70564	5730469M10Rik	RIKEN cDNA 5730469M10 gene	1.023	0.717	NA
69780	Smap2	stromal membrane-associated GTPase-activating protein 2	1.023	0.7546	NA
69221	2410006H16Rik	RIKEN cDNA 2410006H16 gene	1.023	0.7188	NA
68514	Efha1	EF hand domain family A1	1.023	0.6546	NA
67760	Slc38a2	solute carrier family 38, member 2	1.023	0.7487	NA
67290	3110040N11Rik	RIKEN cDNA 3110040N11 gene	1.023	0.7257	NA
67099	Fam119a	family with sequence similarity 119, member A	1.023	0.6765	NA
66881	Pcyox1	prenylcysteine oxidase 1	1.023	0.6521	NA
66427	Cyb5b	cytochrome b5 type B	1.023	0.6365	NA
66406	Sac3d1	SAC3 domain containing 1	1.023	0.606	NA
66356	2310008H09Rik	RIKEN cDNA 2310008H09 gene	1.023	0.9131	0.9743
66132	1110008L16Rik	RIKEN cDNA 1110008L16 gene	1.023	0.851	0.9536
66061	Tctex1d2	Tctex1 domain containing 2	1.023	0.7181	NA
64704	Htra2	HtrA serine peptidase 2	1.023	0.6491	NA

57247	Zfp276	zinc finger protein (C2H2 type) 276	1.023	0.758	NA
56794	Hacl1	2-hydroxyacyl-CoA lyase 1	1.023	0.6213	NA
56336	B4galt5	UDP-Gal:betaGlcNAc beta 1,4-galactosyltransferase, polypeptide 5	1.023	0.7357	NA
54409	Ramp2	receptor (calcitonin) activity modifying protein 2	1.023	0.7126	NA
54197	Rnf5	ring finger protein 5	1.023	0.7328	NA
53333	Tomm40	translocase of outer mitochondrial membrane 40 homolog (yeast)	1.023	0.6871	NA
30957	Mapk8ip3	mitogen-activated protein kinase 8 interacting protein 3	1.023	0.7888	NA
30925	Slamf6	SLAM family member 6	1.023	0.9264	0.9785
28295	D10Jhu81e	DNA segment, Chr 10, Johns Hopkins University 81 expressed	1.023	0.5665	NA
24044	Scamp2	secretory carrier membrane protein 2	1.023	0.7192	NA
23980	Pebp1	phosphatidylethanolamine binding protein 1	1.023	0.7254	NA
23808	Ash2l	ash2 (absent, small, or homeotic)-like (Drosophila)	1.023	0.6137	NA
22719	Zfp61	zinc finger protein 61	1.023	0.6387	NA
21808	Tgfb2	transforming growth factor, beta 2	1.023	0.6864	NA
21672	Prdx2	peroxiredoxin 2	1.023	0.6629	NA
20911	Stxbp2	syntaxin binding protein 2	1.023	0.9105	0.9739
20562	Slit1	slit homolog 1 (Drosophila)	1.023	0.7522	NA
20361	Sema7a	sema domain, immunoglobulin domain (Ig), and GPI membrane anchor, (semaphorin) 7A	1.023	0.8265	0.9448
20356	Sema5a	sema domain, seven thrombospondin repeats (type 1 and type 1-like), transmembrane domain (TM) and short cytoplasmic domain, (semaphorin) 5A	1.023	0.6409	NA
19823	Rnf7	ring finger protein 7	1.023	0.662	NA
18987	Pou2f2	POU domain, class 2, transcription factor 2	1.023	0.8747	0.9617
18175	Nrap	nebulin-related anchoring protein	1.023	0.7937	0.9336
17904	Myl6	myosin, light polypeptide 6, alkali, smooth muscle and non-muscle	1.023	0.6763	NA
16201	Ilf3	interleukin enhancer binding factor 3	1.023	0.814	0.9408
14802	Gria4	glutamate receptor, ionotropic, AMPA4 (alpha 4)	1.023	0.8534	0.9544
14579	Gem	GTP binding protein (gene overexpressed in skeletal muscle)	1.023	0.772	NA
13859	Eps15l1	epidermal growth factor receptor pathway substrate 15-like 1	1.023	0.8484	0.9522
12952	Cry1	cryptochrome 1 (photolyase-like)	1.023	0.9299	0.979
11922	Neurod6	neurogenic differentiation 6	1.023	0.838	0.948
100045796	LOC100045796	cysteine desulfurase, mitochondrial-like	1.022	0.7949	NA
100037282	Rsph3b	radial spoke 3B homolog (Chlamydomonas)	1.022	0.5714	NA
668272	Gm9079	transmembrane emp24 domain trafficking protein 2 pseudogene	1.022	0.6448	NA
666182	Gm7967	predicted gene 7967	1.022	0.8783	0.9628
622282	Gm6306	predicted gene 6306	1.022	0.7392	NA
544888	Fam181a	family with sequence similarity 181, member A	1.022	0.8952	0.9698
414077	BC056474	cDNA sequence BC056474	1.022	0.8249	0.9443
320865	Cdh18	cadherin 18	1.022	0.8122	0.9403
320213	Senp5	SUMO/sentrin specific peptidase 5	1.022	0.59	NA
270906	Prr11	proline rich 11	1.022	0.6752	NA
270624	Spin4	spindlin family, member 4	1.022	0.7735	NA
241489	Pde11a	phosphodiesterase 11A	1.022	0.8832	0.965
233977	Ppfia1	protein tyrosine phosphatase, receptor type, f polypeptide (PTPRF), interacting protein (liprin), alpha 1	1.022	0.6703	NA
232854	Zfp418	zinc finger protein 418	1.022	0.8102	0.94
231912	Katnal1	katanin p60 subunit A-like 1	1.022	0.7227	NA
231798	Lrch4	leucine-rich repeats and calponin homology (CH) domain containing 4	1.022	0.8748	0.9617
230848	Zbtb40	zinc finger and BTB domain containing 40	1.022	0.7498	NA
227736	1700019L03Rik	RIKEN cDNA 1700019L03 gene	1.022	0.7232	NA
224640	Lemd2	LEM domain containing 2	1.022	0.6728	NA
216766	Gemin5	gem (nuclear organelle) associated protein 5	1.022	0.8314	0.9455

212531	Sh3bgrl2	SH3 domain binding glutamic acid-rich protein like 2	1.022	0.6403	NA
210108	D130043K22Rik	RIKEN cDNA D130043K22 gene	1.022	0.8641	0.9579
209318	Gps1	G protein pathway suppressor 1	1.022	0.6373	NA
193813	Mcfd2	multiple coagulation factor deficiency 2	1.022	0.6042	NA
192173	Fam195b	family with sequence similarity 195, member B	1.022	0.7456	NA
108911	Rcc2	regulator of chromosome condensation 2	1.022	0.7429	NA
106565	Dlk2	delta-like 2 homolog (Drosophila)	1.022	0.81	0.94
102058	Exoc8	exocyst complex component 8	1.022	0.712	NA
100210	Gpn2	GPN-loop GTPase 2	1.022	0.7269	NA
100129	Gpr153	G protein-coupled receptor 153	1.022	0.817	0.942
98432	Phlpp1	PH domain and leucine rich repeat protein phosphatase 1	1.022	0.7781	NA
98258	Txndc9	thioredoxin domain containing 9	1.022	0.6992	NA
97244	C80140	expressed sequence C80140	1.022	0.8616	0.9573
78653	Bola3	bolA-like 3 (E. coli)	1.022	0.6552	NA
77938	Fam53b	family with sequence similarity 53, member B	1.022	0.6689	NA
77766	Elp4	elongation protein 4 homolog (S. cerevisiae)	1.022	0.763	NA
77428	9430083A17Rik	RIKEN cDNA 9430083A17 gene	1.022	0.8017	0.9373
74670	Zfp943	zinc finger prtoein 943	1.022	0.6507	NA
74365	Lonrf3	LON peptidase N-terminal domain and ring finger 3	1.022	0.7227	NA
73739	Cby1	chibby homolog 1 (Drosophila)	1.022	0.8639	0.9579
71291	4933435E02Rik	RIKEN cDNA 4933435E02 gene	1.022	0.9133	0.9743
70257	2010107E04Rik	RIKEN cDNA 2010107E04 gene	1.022	0.7774	NA
70208	Med23	mediator complex subunit 23	1.022	0.7394	NA
70001	1700028B04Rik	RIKEN cDNA 1700028B04 gene	1.022	0.7535	NA
69961	2810432D09Rik	RIKEN cDNA 2810432D09 gene	1.022	0.6111	NA
69577	Fastkd3	FAST kinase domains 3	1.022	0.6894	NA
68193	Rpl24	ribosomal protein L24	1.022	0.8008	0.9368
67979	Atad1	ATPase family, AAA domain containing 1	1.022	0.6626	NA
67933	Hcfc2	host cell factor C2	1.022	0.8155	0.9416
67618	Aasdhppt	aminoadipate-semialdehyde dehydrogenase-phosphopantetheinyl transferase	1.022	0.7091	NA
67291	Ccdc137	coiled-coil domain containing 137	1.022	0.831	0.9455
67088	Cand2	cullin-associated and neddylation-dissociated 2 (putative)	1.022	0.7762	NA
66848	Fuca2	fucosidase, alpha-L- 2, plasma	1.022	0.7523	NA
66400	Alkbh7	alkB, alkylation repair homolog 7 (E. coli)	1.022	0.7087	NA
66320	Tmem208	transmembrane protein 208	1.022	0.6812	NA
66293	1810032O08Rik	RIKEN cDNA 1810032O08 gene	1.022	0.7139	NA
56878	Rbms1	RNA binding motif, single stranded interacting protein 1	1.022	0.8271	0.9449
56527	Mast1	microtubule associated serine/threonine kinase 1	1.022	0.8554	0.9552
56229	Thsd1	thrombospondin, type I, domain 1	1.022	0.7599	NA
54422	Barhl1	BarH-like 1 (Drosophila)	1.022	0.917	0.9759
51812	Mcrs1	microspherule protein 1	1.022	0.8195	0.9427
50878	Stag3	stromal antigen 3	1.022	0.7441	NA
28113	Tinf2	Terf1 (TRF1)-interacting nuclear factor 2	1.022	0.6716	NA
27081	Zfp275	zinc finger protein 275	1.022	0.7748	NA
26889	Cln8	ceroid-lipofuscinosis, neuronal 8	1.022	0.5693	NA
26757	Dpysl4	dihydropyrimidinase-like 4	1.022	0.6664	NA
23853	Def6	differentially expressed in FDCP 6	1.022	0.8574	0.9561
21944	Tnfsf12	tumor necrosis factor (ligand) superfamily, member 12	1.022	0.8967	0.9699
21917	Tmpo	thymopoietin	1.022	0.5751	NA

21750	Terf2	telomeric repeat binding factor 2	1.022	0.7798	NA
20371	Foxp3	forkhead box P3	1.022	0.9529	0.9869
20286	Zc3h7b	zinc finger CCCH type containing 7B	1.022	0.7485	NA
20148	Dhrs3	dehydrogenase/reductase (SDR family) member 3	1.022	0.7063	NA
19946	Rpl30	ribosomal protein L30	1.022	0.5289	NA
19732	Rgl2	ral guanine nucleotide dissociation stimulator-like 2	1.022	0.6962	NA
19376	Rab34	RAB34, member of RAS oncogene family	1.022	0.7198	NA
19347	Dennd5a	DENN/MADD domain containing 5A	1.022	0.6834	NA
19188	Psme2	proteasome (prosome, macropain) 28 subunit, beta	1.022	0.6281	NA
19082	Prkag1	protein kinase, AMP-activated, gamma 1 non-catalytic subunit	1.022	0.6208	NA
18707	Pik3cd	phosphatidylinositol 3-kinase catalytic delta polypeptide	1.022	0.813	0.9405
18245	Oaz1	ornithine decarboxylase antizyme 1	1.022	0.7632	NA
16881	Lig1	ligase I, DNA, ATP-dependent	1.022	0.513	NA
16872	Lhx4	LIM homeobox protein 4	1.022	0.8564	0.9554
16559	Kif17	kinesin family member 17	1.022	0.8406	0.9486
15191	Hdgf	hepatoma-derived growth factor	1.022	0.8386	0.9482
14569	Gdi2	guanosine diphosphate (GDP) dissociation inhibitor 2	1.022	0.6287	NA
14000	Drosha	drosha, ribonuclease type III	1.022	0.5588	NA
13684	Eif4e	eukaryotic translation initiation factor 4E	1.022	0.6798	NA
13665	Eif2s1	eukaryotic translation initiation factor 2, subunit 1 alpha	1.022	0.5804	NA
13649	Egfr	epidermal growth factor receptor	1.022	0.8266	0.9448
11938	Atp2a2	ATPase, Ca++ transporting, cardiac muscle, slow twitch 2	1.022	0.621	NA
11550	Adra1d	adrenergic receptor, alpha 1d	1.022	0.9198	0.9765
11440	Chrna6	cholinergic receptor, nicotinic, alpha polypeptide 6	1.022	0.7318	NA
403187	Opa3	optic atrophy 3 (human)	1.021	0.8802	0.9631
386655	Eid2	EP300 interacting inhibitor of differentiation 2	1.021	0.7897	NA
382035	Pabpn1l	poly(A)binding protein nuclear 1-like	1.021	0.7513	NA
330788	Zfp866	zinc finger protein 866	1.021	0.7055	NA
319352	C530028O21Rik	RIKEN cDNA C530028O21 gene	1.021	0.8407	0.9486
268739	Arhgef40	Rho guanine nucleotide exchange factor (GEF) 40	1.021	0.8061	0.9387
268319	BC025920	zinc finger protein pseudogene	1.021	0.835	0.9466
246782	Atpaf2	ATP synthase mitochondrial F1 complex assembly factor 2	1.021	0.7546	NA
244373	Erlin2	ER lipid raft associated 2	1.021	0.6686	NA
240756	Klhl12	kelch-like 12 (Drosophila)	1.021	0.6918	NA
240476	Zfp407	zinc finger protein 407	1.021	0.6398	NA
232791	Cnot3	CCR4-NOT transcription complex, subunit 3	1.021	0.9102	0.9738
231506	Lin54	lin-54 homolog (C. elegans)	1.021	0.6102	NA
229228	Nudt6	nudix (nucleoside diphosphate linked moiety X)-type motif 6	1.021	0.7116	NA
223978	Cpped1	calcineurin-like phosphoesterase domain containing 1	1.021	0.6583	NA
217869	Eif5	eukaryotic translation initiation factor 5	1.021	0.6641	NA
216892	Spns2	spinster homolog 2 (Drosophila)	1.021	0.7268	NA
216867	Slc16a11	solute carrier family 16 (monocarboxylic acid transporters), member 11	1.021	0.6282	NA
216292	BC067068	cDNA sequence BC067068	1.021	0.6956	NA
213819	Casd1	CAS1 domain containing 1	1.021	0.7121	NA
210035	Tmem194	transmembrane protein 194	1.021	0.8382	0.9481
114565	Zfp295	zinc finger protein 295	1.021	0.6338	NA
108015	Chrnb4	cholinergic receptor, nicotinic, beta polypeptide 4	1.021	0.7503	NA
107684	Coro2a	coronin, actin binding protein 2A	1.021	0.8114	0.9403
103806	Maml1	mastermind like 1 (Drosophila)	1.021	0.6591	NA

100090	Zbtb48	zinc finger and BTB domain containing 48	1.021	0.7758	NA
100072	Camta1	calmodulin binding transcription activator 1	1.021	0.6906	NA
98366	Smap1	stromal membrane-associated protein 1	1.021	0.7061	NA
98053	Gtf2f1	general transcription factor IIF, polypeptide 1	1.021	0.6879	NA
93896	Glp2r	glucagon-like peptide 2 receptor	1.021	0.916	0.9756
80879	Slc16a3	solute carrier family 16 (monocarboxylic acid transporters), member 3	1.021	0.9189	0.9764
77634	Snapc3	small nuclear RNA activating complex, polypeptide 3	1.021	0.8153	0.9416
76522	Naa38	N(alpha)-acetyltransferase 38, NatC auxiliary subunit	1.021	0.7331	NA
76072	Rnf183	ring finger protein 183	1.021	0.8599	0.957
75939	4930579G24Rik	RIKEN cDNA 4930579G24 gene	1.021	0.7732	NA
75458	Cklf	chemokine-like factor	1.021	0.8072	NA
74020	Cpne4	copine IV	1.021	0.826	0.9446
73668	Ttc21b	tetratricopeptide repeat domain 21B	1.021	0.7102	NA
73582	1700106N22Rik	RIKEN cDNA 1700106N22 gene	1.021	0.8376	0.9479
72542	Pgam5	phosphoglycerate mutase family member 5	1.021	0.6013	NA
72309	Tmem158	transmembrane protein 158	1.021	0.6865	NA
72301	1810041L15Rik	RIKEN cDNA 1810041L15 gene	1.021	0.903	0.9717
70605	Zdhhc24	zinc finger, DHHC domain containing 24	1.021	0.8667	0.9587
69635	Dapk1	death associated protein kinase 1	1.021	0.7725	NA
69318	1700007K09Rik	RIKEN cDNA 1700007K09 gene	1.021	0.8433	0.9499
69020	Zfp707	zinc finger protein 707	1.021	0.7948	NA
68135	Eif3h	eukaryotic translation initiation factor 3, subunit H	1.021	0.7066	NA
68053	Ubxn2b	UBX domain protein 2B	1.021	0.7643	NA
67694	Ift74	intraflagellar transport 74 homolog (Chlamydomonas)	1.021	0.6677	NA
67307	Pbld2	phenazine biosynthesis-like protein domain containing 2	1.021	0.8108	0.9401
67186	Rplp2	ribosomal protein, large P2	1.021	0.839	0.9483
67037	Pmf1	polyamine-modulated factor 1	1.021	0.9172	0.9759
67013	Oma1	OMA1 homolog, zinc metallopeptidase (S. cerevisiae)	1.021	0.7702	NA
66362	Exosc3	exosome component 3	1.021	0.777	NA
66330	1700020L24Rik	RIKEN cDNA 1700020L24 gene	1.021	0.8113	0.9403
66226	Trappc2	trafficking protein particle complex 2	1.021	0.9607	0.9886
66162	Bola2	bolA-like 2 (E. coli)	1.021	0.7245	NA
66078	Tsen34	tRNA splicing endonuclease 34 homolog (S. cerevisiae)	1.021	0.6454	NA
58202	Cobra1	cofactor of BRCA1	1.021	0.6682	NA
57423	Atp5j2	ATP synthase, H+ transporting, mitochondrial F0 complex, subunit F2	1.021	0.5895	NA
56382	Rab9	RAB9, member RAS oncogene family	1.021	0.7354	NA
56208	Becn1	beclin 1, autophagy related	1.021	0.7609	NA
54405	Ndufa1	NADH dehydrogenase (ubiquinone) 1 alpha subcomplex, 1	1.021	0.6944	NA
53332	Mtmr1	myotubularin related protein 1	1.021	0.8324	0.9456
53332	Mtmr1	myotubularin related protein 1	1.021	0.66	NA
52563	Cdc23	CDC23 (cell division cycle 23, yeast, homolog)	1.021	0.8335	0.9459
52512	D14Ertd670e	DNA segment, Chr 14, ERATO Doi 670, expressed	1.021	0.9149	0.9751
27402	Pdhx	pyruvate dehydrogenase complex, component X	1.021	0.6968	NA
26404	Map3k12	mitogen-activated protein kinase kinase kinase 12	1.021	0.7799	NA
23797	Akt3	thymoma viral proto-oncogene 3	1.021	0.8909	0.9687
22324	Vav1	vav 1 oncogene	1.021	0.8601	0.957
21859	Timp3	tissue inhibitor of metalloproteinase 3	1.021	0.7735	NA
20586	Smarca4	SWI/SNF related, matrix associated, actin dependent regulator of chromatin, subfamily a, member 4	1.021	0.7564	NA
19335	Rab23	RAB23, member RAS oncogene family	1.021	0.7852	NA

19271	Ptprj	protein tyrosine phosphatase, receptor type, J	1.021	0.9029	0.9717
18861	Pms2	postmeiotic segregation increased 2 (S. cerevisiae)	1.021	0.6562	NA
18557	Cdk18	cyclin-dependent kinase 18	1.021	0.6645	NA
18181	Nrf1	nuclear respiratory factor 1	1.021	0.7741	NA
17688	Msh6	mutS homolog 6 (E. coli)	1.021	0.6355	NA
17149	Magoh	mago-nashi homolog, proliferation-associated (Drosophila)	1.021	0.6565	NA
17145	Mageb1	melanoma antigen, family B, 1	1.021	0.818	0.9422
16704	Krtap8-2	keratin associated protein 8-2	1.021	0.8847	0.9658
14489	Mtpn	myotrophin	1.021	0.7321	NA
14420	Galc	galactosylceramidase	1.021	0.8417	0.9487
14231	Fkbp7	FK506 binding protein 7	1.021	0.6839	NA
14208	Ppm1g	protein phosphatase 1G (formerly 2C), magnesium-dependent, gamma isoform	1.021	0.7204	NA
13617	Ednra	endothelin receptor type A	1.021	0.8236	0.944
13430	Dnm2	dynamin 2	1.021	0.8242	0.9443
13185	Dscr3	Down syndrome critical region gene 3	1.021	0.7045	NA
12633	Cflar	CASP8 and FADD-like apoptosis regulator	1.021	0.8064	0.9388
12345	Capzb	capping protein (actin filament) muscle Z-line, beta	1.021	0.8103	0.94
12153	Bmp1	bone morphogenetic protein 1	1.021	0.7849	NA
11932	Atp1b2	ATPase, Na+/K+ transporting, beta 2 polypeptide	1.021	0.7992	NA
100046456	LOC100046456	zinc finger protein 91-like	1.02	0.9465	0.9847
100043699	Gm4593	predicted gene 4593	1.02	0.9033	0.9717
621542	Gm6238	predicted pseudogene 6238	1.02	0.6521	NA
384179	Gm5292	predicted gene 5292	1.02	0.8183	0.9423
381409	Cdh26	cadherin-like 26	1.02	0.9188	0.9764
320720	Fastkd1	FAST kinase domains 1	1.02	0.7543	NA
319653	Slc25a40	solute carrier family 25, member 40	1.02	0.716	NA
319504	Nrcam	neuron-glia-CAM-related cell adhesion molecule	1.02	0.8008	NA
246133	Kcne2	potassium voltage-gated channel, Isk-related subfamily, gene 2	1.02	0.7163	NA
244608	Ccdc113	coiled-coil domain containing 113	1.02	0.7847	NA
242960	Fbxl5	F-box and leucine-rich repeat protein 5	1.02	0.7773	NA
242409	Tmem8b	transmembrane protein 8B	1.02	0.8763	0.9619
240063	Zfp811	zinc finger protein 811	1.02	0.7942	NA
235339	Dlat	dihydrolipoamide S-acetyltransferase (E2 component of pyruvate dehydrogenase complex)	1.02	0.6832	NA
235180	Fez1	fasciculation and elongation protein zeta 1 (zygin I)	1.02	0.803	NA
234964	Ccdc67	coiled-coil domain containing 67	1.02	0.8613	0.9573
231803	Mepce	methylphosphate capping enzyme	1.02	0.689	NA
231570	A830010M20Rik	RIKEN cDNA A830010M20 gene	1.02	0.6427	NA
229603	Otud7b	OTU domain containing 7B	1.02	0.8161	0.9416
229214	Qrfpr	pyroglutamylated RFamide peptide receptor	1.02	0.7599	NA
224092	Lsg1	large subunit GTPase 1 homolog (S. cerevisiae)	1.02	0.6834	NA
217026	Heatr6	HEAT repeat containing 6	1.02	0.7558	NA
209354	Eif2b1	eukaryotic translation initiation factor 2B, subunit 1 (alpha)	1.02	0.7645	NA
192197	Bcas3	breast carcinoma amplified sequence 3	1.02	0.8691	0.9597
170728	Rtn4ip1	reticulon 4 interacting protein 1	1.02	0.7598	NA
114896	Afg3l1	AFG3(ATPase family gene 3)-like 1 (yeast)	1.02	0.5671	NA
109658	Txlna	taxilin alpha	1.02	0.8573	0.9561
108903	Tbcd	tubulin-specific chaperone d	1.02	0.8334	0.9459
107686	Snrpd2	small nuclear ribonucleoprotein D2	1.02	0.7506	NA
107272	Psat1	phosphoserine aminotransferase 1	1.02	0.8273	0.9449

103583	Fbxw11	F-box and WD-40 domain protein 11	1.02	0.7063	NA
101612	Grwd1	glutamate-rich WD repeat containing 1	1.02	0.8819	0.9639
93679	Trim8	tripartite motif-containing 8	1.02	0.8219	0.9435
78670	Plekhj1	pleckstrin homology domain containing, family J member 1	1.02	0.901	0.9712
77134	Hnrnpa0	heterogeneous nuclear ribonucleoprotein A0	1.02	0.6125	NA
76383	1700012L04Rik	RIKEN cDNA 1700012L04 gene	1.02	0.808	NA
76041	Ccdc125	coiled-coil domain containing 125	1.02	0.7989	NA
75758	9130401M01Rik	RIKEN cDNA 9130401M01 gene	1.02	0.6374	NA
75705	Eif4b	eukaryotic translation initiation factor 4B	1.02	0.8995	0.9706
74375	Gcc1	golgi coiled coil 1	1.02	0.5974	NA
74114	Crot	carnitine O-octanoyltransferase	1.02	0.6756	NA
73635	1700113I22Rik	RIKEN cDNA 1700113I22 gene	1.02	0.8003	NA
73067	Tmem192	transmembrane protein 192	1.02	0.7579	NA
72515	Wdr43	WD repeat domain 43	1.02	0.6559	NA
72236	Tsnaxip1	translin-associated factor X (Tsnax) interacting protein 1	1.02	0.7829	NA
72026	Trmu	tRNA 5-methylaminomethyl-2-thiouridylate methyltransferase	1.02	0.6754	NA
71751	Map3k13	mitogen-activated protein kinase kinase kinase 13	1.02	0.8195	0.9427
71721	Fam13c	family with sequence similarity 13, member C	1.02	0.7817	NA
70960	4921531P14Rik	RIKEN cDNA 4921531P14 gene	1.02	0.9196	0.9765
69672	Txndc15	thioredoxin domain containing 15	1.02	0.6396	NA
67726	Fam114a2	family with sequence similarity 114, member A2	1.02	0.699	NA
67219	Med18	mediator of RNA polymerase II transcription, subunit 18 homolog (yeast)	1.02	0.6725	NA
66441	Magohb	mago-nashi homolog B (Drosophila)	1.02	0.7106	NA
66317	Wdr61	WD repeat domain 61	1.02	0.6762	NA
63993	Slc5a7	solute carrier family 5 (choline transporter), member 7	1.02	0.873	0.9611
59095	Fxyd6	FXYD domain-containing ion transport regulator 6	1.02	0.8155	0.9416
59050	Nsa2	NSA2 ribosome biogenesis homolog (S. cerevisiae)	1.02	0.9146	0.9749
56748	Nfu1	NFU1 iron-sulfur cluster scaffold homolog (S. cerevisiae)	1.02	0.5866	NA
56470	Rgs19	regulator of G-protein signaling 19	1.02	0.7126	NA
54217	Rpl36	ribosomal protein L36	1.02	0.8054	NA
53321	Cntnap1	contactin associated protein-like 1	1.02	0.8603	0.9571
50762	Fbxo6	F-box protein 6	1.02	0.7203	NA
23996	Psmc4	proteasome (prosome, macropain) 26S subunit, ATPase, 4	1.02	0.7491	NA
21346	Tagln2	transgelin 2	1.02	0.8782	0.9628
20591	Kdm5c	lysine (K)-specific demethylase 5C	1.02	0.9222	0.9776
19227	Pthlh	parathyroid hormone-like peptide	1.02	0.8621	0.9574
19122	Prnp	prion protein	1.02	0.706	NA
18567	Pdcd2	programmed cell death 2	1.02	0.7058	NA
17919	Myo5b	myosin VB	1.02	0.8245	0.9443
17067	Ly6c1	lymphocyte antigen 6 complex, locus C1	1.02	0.7887	NA
16825	Ldb1	LIM domain binding 1	1.02	0.7638	NA
16508	Kcnd2	potassium voltage-gated channel, Shal-related family, member 2	1.02	0.7296	NA
14376	Ganab	alpha glucosidase 2 alpha neutral subunit	1.02	0.6075	NA
14235	Foxm1	forkhead box M1	1.02	0.927	0.9787
14042	Ext1	exostoses (multiple) 1	1.02	0.8018	NA
13823	Epb4.1l3	erythrocyte protein band 4.1-like 3	1.02	0.7742	NA
13682	Eif4a2	eukaryotic translation initiation factor 4A2	1.02	0.5959	NA
12894	Cpt1a	carnitine palmitoyltransferase 1a, liver	1.02	0.6809	NA
11429	Aco2	aconitase 2, mitochondrial	1.02	0.8178	0.9421

100505122	LOC100505122	hypothetical LOC100505122	1.019	0.6626	NA
100503330	LOC100503330	hypothetical LOC100503330	1.019	0.8312	0.9455
100041352	Gm9880	predicted gene 9880	1.019	0.9552	0.9872
100039138	Gm2061	predicted gene 2061	1.019	0.9006	0.971
672284	Nkx1-1	NK1 transcription factor related, locus 1 (Drosophila)	1.019	0.8035	NA
668144	Gm9000	predicted gene 9000	1.019	0.7715	NA
546100	Gm5914	predicted gene 5914	1.019	0.7978	NA
544752	Tug1	taurine upregulated gene 1	1.019	0.841	0.9487
494468	Armcx5	armadillo repeat containing, X-linked 5	1.019	0.6243	NA
433050	Rpl26-ps4	ribosomal protein L26, pseudogene 4	1.019	0.7326	NA
381921	Taok2	TAO kinase 2	1.019	0.8154	NA
381110	Fam82a1	family with sequence similarity 82, member A1	1.019	0.7987	NA
338354	Zfp780b	zinc finger protein 780B	1.019	0.871	0.9607
327655	Ppip5k1	diphosphoinositol pentakisphosphate kinase 1	1.019	0.8617	0.9573
319168	Hist1h2ah	histone cluster 1, H2ah	1.019	0.7313	NA
269630	5031425E22Rik	RIKEN cDNA 5031425E22 gene	1.019	0.7981	NA
245688	Rbbp7	retinoblastoma binding protein 7	1.019	0.629	NA
244895	C230081A13Rik	RIKEN cDNA C230081A13 gene	1.019	0.765	NA
243168	Hsd17b13	hydroxysteroid (17-beta) dehydrogenase 13	1.019	0.8979	0.9699
241846	Lsm14b	LSM14 homolog B (SCD6, S. cerevisiae)	1.019	0.8183	NA
237940	Aoc2	amine oxidase, copper containing 2 (retina-specific)	1.019	0.8279	0.945
232853	Zfp954	zinc finger protein 954	1.019	0.7862	NA
231889	Bud31	BUD31 homolog (yeast)	1.019	0.809	NA
231123	Haus3	HAUS augmin-like complex, subunit 3	1.019	0.6767	NA
228715	Gm561	predicted gene 561	1.019	0.698	NA
227697	Dolk	dolichol kinase	1.019	0.7314	NA
227632	Kcnt1	potassium channel, subfamily T, member 1	1.019	0.7538	NA
227526	Cdnf	cerebral dopamine neurotrophic factor	1.019	0.8174	NA
226265	Eno4	enolase 4	1.019	0.8496	0.953
226098	Hectd2	HECT domain containing 2	1.019	0.7379	NA
207278	Fchsd2	FCH and double SH3 domains 2	1.019	0.7387	NA
192232	Hps4	Hermansky-Pudlak syndrome 4 homolog (human)	1.019	0.7982	NA
108101	Fermt3	fermitin family homolog 3 (Drosophila)	1.019	0.9004	0.9709
107350	AW112010	expressed sequence AW112010	1.019	0.8906	0.9687
105428	Fam149b	family with sequence similarity 149, member B	1.019	0.8689	0.9596
103733	Tubg1	tubulin, gamma 1	1.019	0.8333	0.9459
101685	Spty2d1	SPT2, Suppressor of Ty, domain containing 1 (S. cerevisiae)	1.019	0.6738	NA
99730	Taf13	TAF13 RNA polymerase II, TATA box binding protein (TBP)-associated factor	1.019	0.9088	0.9733
93765	Ube2n	ubiquitin-conjugating enzyme E2N	1.019	0.9048	0.9717
78321	Ankrd23	ankyrin repeat domain 23	1.019	0.8151	NA
77809	Lrrc42	leucine rich repeat containing 42	1.019	0.74	NA
76969	Chst1	carbohydrate (keratan sulfate Gal-6) sulfotransferase 1	1.019	0.7665	NA
76959	Chmp5	chromatin modifying protein 5	1.019	0.7435	NA
76688	Arfrp1	ADP-ribosylation factor related protein 1	1.019	0.7357	NA
76612	Lrrc27	leucine rich repeat containing 27	1.019	0.8197	NA
76167	Snrnp35	small nuclear ribonucleoprotein 35 (U11/U12)	1.019	0.7213	NA
76071	Jakmip1	janus kinase and microtubule interacting protein 1	1.019	0.7772	NA
75770	Brsk2	BR serine/threonine kinase 2	1.019	0.8716	0.9608
74691	Tdrd9	tudor domain containing 9	1.019	0.7557	NA

74370	Rptor	regulatory associated protein of MTOR, complex 1	1.019	0.7457	NA
73713	Rbm20	RNA binding motif protein 20	1.019	0.91	0.9738
73130	Tmed5	transmembrane emp24 protein transport domain containing 5	1.019	0.7594	NA
72096	Mettl10	methyltransferase like 10	1.019	0.6961	NA
71151	Eri2	exoribonuclease 2	1.019	0.8675	0.9593
70495	Atp6ap2	ATPase, H+ transporting, lysosomal accessory protein 2	1.019	0.7691	NA
70472	Atad2	ATPase family, AAA domain containing 2	1.019	0.9294	0.9789
70427	Mier2	mesoderm induction early response 1, family member 2	1.019	0.8714	0.9608
69434	Snhg10	small nucleolar RNA host gene (non-protein coding) 10	1.019	0.7685	NA
69368	Wdfy1	WD repeat and FYVE domain containing 1	1.019	0.7361	NA
68948	1500011H22Rik	RIKEN cDNA 1500011H22 gene	1.019	0.5698	NA
68598	Dnajc8	DnaJ (Hsp40) homolog, subfamily C, member 8	1.019	0.7589	NA
68490	Zfp579	zinc finger protein 579	1.019	0.7802	NA
68342	Ndufb10	NADH dehydrogenase (ubiquinone) 1 beta subcomplex, 10	1.019	0.7424	NA
67792	Rgs8	regulator of G-protein signaling 8	1.019	0.6911	NA
67440	Mtpap	mitochondrial poly(A) polymerase	1.019	0.5668	NA
67412	6330407J23Rik	RIKEN cDNA 6330407J23 gene	1.019	0.6537	NA
67236	Cinp	cyclin-dependent kinase 2 interacting protein	1.019	0.8515	0.9537
67224	Med29	mediator complex subunit 29	1.019	0.7863	NA
66978	Luc7l	Luc7 homolog (S. cerevisiae)-like	1.019	0.6673	NA
66663	Uba5	ubiquitin-like modifier activating enzyme 5	1.019	0.6607	NA
66493	Mrpl51	mitochondrial ribosomal protein L51	1.019	0.781	NA
66309	Tmem128	transmembrane protein 128	1.019	0.708	NA
66065	Hsd17b14	hydroxysteroid (17-beta) dehydrogenase 14	1.019	0.8668	0.9587
58194	Sh3kbp1	SH3-domain kinase binding protein 1	1.019	0.739	NA
56771	Med20	mediator complex subunit 20	1.019	0.6656	NA
56692	Mapksp1	MAPK scaffold protein 1	1.019	0.6789	NA
54364	Rpp30	ribonuclease P/MRP 30 subunit (human)	1.019	0.8025	NA
53598	Dctn3	dynactin 3	1.019	0.6465	NA
53416	Stk39	serine/threonine kinase 39, STE20/SPS1 homolog (yeast)	1.019	0.733	NA
53330	Vamp4	vesicle-associated membrane protein 4	1.019	0.8194	NA
53313	Atp2a3	ATPase, Ca++ transporting, ubiquitous	1.019	0.9214	0.977
27425	Atp5l	ATP synthase, H+ transporting, mitochondrial F0 complex, subunit g	1.019	0.8239	NA
26894	Cops7a	COP9 (constitutive photomorphogenic) homolog, subunit 7a (Arabidopsis thaliana)	1.019	0.8602	0.957
23879	Fxr2	fragile X mental retardation, autosomal homolog 2	1.019	0.7162	NA
19821	Rnf2	ring finger protein 2	1.019	0.6531	NA
19309	Pygm	muscle glycogen phosphorylase	1.019	0.8981	0.9699
18389	Oprl1	opioid receptor-like 1	1.019	0.8992	0.9704
18226	Nup62	nucleoporin 62	1.019	0.6795	NA
18176	Nras	neuroblastoma ras oncogene	1.019	0.7393	NA
17703	Msx3	homeobox, msh-like 3	1.019	0.8191	NA
17684	Cited2	Cbp/p300-interacting transactivator, with Glu/Asp-rich carboxy-terminal domain, 2	1.019	0.9105	0.9739
17210	Mcl1	myeloid cell leukemia sequence 1	1.019	0.6529	NA
15926	Idh1	isocitrate dehydrogenase 1 (NADP+), soluble	1.019	0.8537	0.9544
15182	Hdac2	histone deacetylase 2	1.019	0.8968	0.9699
14370	Fzd8	frizzled homolog 8 (Drosophila)	1.019	0.8401	0.9485
14272	Fnta	farnesyltransferase, CAAX box, alpha	1.019	0.6244	NA
13445	Cdk2ap1	CDK2 (cyclin-dependent kinase 2)-associated protein 1	1.019	0.5939	NA
12563	Cdh6	cadherin 6	1.019	0.8094	NA

100040851	Gm9767	predicted gene 9767	1.018	0.7199	NA
100038683	Gm10775	predicted gene 10775	1.018	0.6924	NA
626903	Gm6718	predicted gene 6718	1.018	0.9133	0.9743
626055	Gm15645	predicted gene 15645	1.018	0.832	NA
624086	A230045G11Rik	RIKEN cDNA A230045G11 gene	1.018	0.8011	NA
433215	BC048609	cDNA sequence BC048609	1.018	0.8459	0.9511
408022	Ccdc111	coiled-coil domain containing 111	1.018	0.8657	0.9585
330817	Dhps	deoxyhypusine synthase	1.018	0.6531	NA
330427	9330102E08Rik	RIKEN cDNA 9330102E08 gene	1.018	0.8537	0.9544
269585	Zscan20	zinc finger and SCAN domains 20	1.018	0.7708	NA
268395	Mpg	N-methylpurine-DNA glycosylase	1.018	0.8338	NA
268345	Kcnc2	potassium voltage gated channel, Shaw-related subfamily, member 2	1.018	0.7604	NA
245944	Vps54	vacuolar protein sorting 54 (yeast)	1.018	0.8039	NA
243834	Zfp324	zinc finger protein 324	1.018	0.8415	0.9487
241627	Wdr76	WD repeat domain 76	1.018	0.9345	0.981
233073	U2af1l4	U2 small nuclear RNA auxiliary factor 1-like 4	1.018	0.7999	NA
231887	Pdap1	PDGFA associated protein 1	1.018	0.6165	NA
230752	Fam176b	family with sequence similarity 176, member B	1.018	0.7356	NA
225160	Thoc1	THO complex 1	1.018	0.8404	0.9486
224129	Adcy5	adenylate cyclase 5	1.018	0.8371	0.9479
218038	Amph	amphiphysin	1.018	0.6529	NA
213989	Tmem82	transmembrane protein 82	1.018	0.8699	0.9601
210035	Tmem194	transmembrane protein 194	1.018	0.8078	NA
209318	Gps1	G protein pathway suppressor 1	1.018	0.7136	NA
209224	Enox2	ecto-NOX disulfide-thiol exchanger 2	1.018	0.7718	NA
207181	Rbms3	RNA binding motif, single stranded interacting protein	1.018	0.7794	NA
192950	Nacad	NAC alpha domain containing	1.018	0.7046	NA
108058	Camk2d	calcium/calmodulin-dependent protein kinase II, delta	1.018	0.7383	NA
107508	Eprs	glutamyl-prolyl-tRNA synthetase	1.018	0.8917	0.9688
106338	Nsun3	NOL1/NOP2/Sun domain family member 3	1.018	0.7495	NA
104771	Jkamp	JNK1/MAPK8-associated membrane protein	1.018	0.6589	NA
100637	N4bp2l1	NEDD4 binding protein 2-like 1	1.018	0.8164	NA
99526	Usp53	ubiquitin specific peptidase 53	1.018	0.8725	0.9608
99371	Arfgef2	ADP-ribosylation factor guanine nucleotide-exchange factor 2 (brefeldin A-inhibited)	1.018	0.7544	NA
97159	A430005L14Rik	RIKEN cDNA A430005L14 gene	1.018	0.6413	NA
94216	Col4a6	collagen, type IV, alpha 6	1.018	0.8954	0.9698
83997	Slmap	sarcolemma associated protein	1.018	0.6803	NA
80720	Pbx4	pre-B-cell leukemia homeobox 4	1.018	0.8759	0.9618
79362	Bhlhe41	basic helix-loop-helix family, member e41	1.018	0.8451	0.9507
79201	Tnfrsf23	tumor necrosis factor receptor superfamily, member 23	1.018	0.9126	0.9743
78611	Btbd19	BTB (POZ) domain containing 19	1.018	0.8577	0.9562
78506	Efha2	EF-hand domain family, member A2	1.018	0.9082	0.9733
78394	Ddx52	DEAD (Asp-Glu-Ala-Asp) box polypeptide 52	1.018	0.7671	NA
78339	Ttyh3	tweety homolog 3 (Drosophila)	1.018	0.7199	NA
77134	Hnrnpa0	heterogeneous nuclear ribonucleoprotein A0	1.018	0.8759	0.9618
77031	Slc9a8	solute carrier family 9 (sodium/hydrogen exchanger), member 8	1.018	0.8005	NA
76952	Nt5c2	5'-nucleotidase, cytosolic II	1.018	0.8012	NA
76408	Abcc3	ATP-binding cassette, sub-family C (CFTR/MRP), member 3	1.018	0.7792	NA
75782	Lca5	Leber congenital amaurosis 5 (human)	1.018	0.8317	0.9456

75608	Chmp4b	chromatin modifying protein 4B	1.018	0.6596	NA
74376	Myo18b	myosin XVIIIb	1.018	0.9185	0.9764
74302	Mtmr3	myotubularin related protein 3	1.018	0.865	0.9584
73103	3110009E18Rik	RIKEN cDNA 3110009E18 gene	1.018	0.7534	NA
71742	Ulk3	unc-51-like kinase 3 (C. elegans)	1.018	0.8817	0.9638
69934	Rg9mtd3	RNA (guanine-9-) methyltransferase domain containing 3	1.018	0.8149	NA
69573	2310016C08Rik	RIKEN cDNA 2310016C08 gene	1.018	0.6901	NA
69064	1810014F10Rik	RIKEN cDNA 1810014F10 gene	1.018	0.7912	NA
67759	5033414D02Rik	RIKEN cDNA 5033414D02 gene	1.018	0.6598	NA
66917	Chordc1	cysteine and histidine-rich domain (CHORD)-containing, zinc-binding protein 1	1.018	0.6888	NA
66356	2310008H09Rik	RIKEN cDNA 2310008H09 gene	1.018	0.6672	NA
66220	Zdhhc12	zinc finger, DHHC domain containing 12	1.018	0.7788	NA
66128	Mrps36	mitochondrial ribosomal protein S36	1.018	0.6959	NA
64295	Tmub1	transmembrane and ubiquitin-like domain containing 1	1.018	0.7157	NA
58233	Dnaja4	DnaJ (Hsp40) homolog, subfamily A, member 4	1.018	0.923	0.9776
53610	Nono	non-POU-domain-containing, octamer binding protein	1.018	0.7374	NA
52856	Gtpbp5	GTP binding protein 5	1.018	0.817	NA
52700	Txndc17	thioredoxin domain containing 17	1.018	0.8006	NA
50907	Preb	prolactin regulatory element binding	1.018	0.8204	NA
26390	Mapkbp1	mitogen-activated protein kinase binding protein 1	1.018	0.7754	NA
23806	Arih1	ariadne ubiquitin-conjugating enzyme E2 binding protein homolog 1 (Drosophila)	1.018	0.7272	NA
22362	Vpreb1	pre-B lymphocyte gene 1	1.018	0.8807	0.9635
22334	Vdac2	voltage-dependent anion channel 2	1.018	0.7166	NA
21761	Morf4l1	mortality factor 4 like 1	1.018	0.7424	NA
20683	Sp1	trans-acting transcription factor 1	1.018	0.7771	NA
20680	Sox7	SRY-box containing gene 7	1.018	0.8913	0.9687
20392	Sgce	sarcoglycan, epsilon	1.018	0.7547	NA
20183	Rxrg	retinoid X receptor gamma	1.018	0.8574	0.9561
19353	Rac1	RAS-related C3 botulinum substrate 1	1.018	0.6564	NA
19181	Psmc2	proteasome (prosome, macropain) 26S subunit, ATPase 2	1.018	0.7078	NA
18508	Pax6	paired box gene 6	1.018	0.8206	NA
18181	Nrf1	nuclear respiratory factor 1	1.018	0.8201	NA
18029	Nfic	nuclear factor I/C	1.018	0.8907	0.9687
17758	Mtap4	microtubule-associated protein 4	1.018	0.7205	NA
14678	Gnai2	guanine nucleotide binding protein (G protein), alpha inhibiting 2	1.018	0.8013	NA
13134	Dach1	dachshund 1 (Drosophila)	1.018	0.7637	NA
13047	Cux1	cut-like homeobox 1	1.018	0.8874	0.9671
12946	Cr1l	complement component (3b/4b) receptor 1-like	1.018	0.838	0.948
12212	Chic1	cysteine-rich hydrophobic domain 1	1.018	0.8596	0.9567
12167	Bmpr1b	bone morphogenetic protein receptor, type 1B	1.018	0.8116	NA
100502982	LOC100502982	hypothetical LOC100502982	1.017	0.8175	NA
100041154	Gm3167	predicted gene 3167	1.017	0.6309	NA
100038969	Gm14958	predicted gene 14958	1.017	0.872	0.9608
100038875	Gm15450	predicted gene 15450	1.017	0.8518	0.9537
654801	Zfp784	zinc finger protein 784	1.017	0.7178	NA
353310	Zfp703	zinc finger protein 703	1.017	0.8051	NA
353258	Ltv1	LTV1 homolog (S. cerevisiae)	1.017	0.6534	NA
272465	Fam70b	family with sequence similarity 70, member B	1.017	0.6706	NA
270096	Mon1b	MON1 homolog b (yeast)	1.017	0.7072	NA

269855	A430110N23Rik	RIKEN cDNA A430110N23 gene	1.017	0.9026	0.9717
246728	Oas2	2'-5' oligoadenylate synthetase 2	1.017	0.9238	0.978
246317	Neto1	neuropilin (NRP) and tolloid (TLL)-like 1	1.017	0.8531	0.9544
244144	Usp35	ubiquitin specific peptidase 35	1.017	0.8616	0.9573
243510	Ccdc142	coiled-coil domain containing 142	1.017	0.7223	NA
242642	Hpdl	4-hydroxyphenylpyruvate dioxygenase-like	1.017	0.8226	NA
233056	Zfp790	zinc finger protein 790	1.017	0.7773	NA
231380	Uba6	ubiquitin-like modifier activating enzyme 6	1.017	0.8267	NA
230085	N28178	expressed sequence N28178	1.017	0.8361	NA
229709	Ahcyl1	S-adenosylhomocysteine hydrolase-like 1	1.017	0.7972	NA
229096	Ythdf3	YTH domain family 3	1.017	0.765	NA
228019	Mettl8	methyltransferase like 8	1.017	0.7695	NA
217463	Snx13	sorting nexin 13	1.017	0.7665	NA
212880	Ddx46	DEAD (Asp-Glu-Ala-Asp) box polypeptide 46	1.017	0.6745	NA
212706	N4bp3	NEDD4 binding protein 3	1.017	0.7577	NA
211914	Asap2	ArfGAP with SH3 domain, ankyrin repeat and PH domain 2	1.017	0.7631	NA
211446	Exoc3	exocyst complex component 3	1.017	0.7874	NA
211187	Lrtm2	leucine-rich repeats and transmembrane domains 2	1.017	0.7562	NA
170930	Sumo2	SMT3 suppressor of mif two 3 homolog 2 (yeast)	1.017	0.7422	NA
110595	Timp4	tissue inhibitor of metalloproteinase 4	1.017	0.8802	0.9631
108653	Rimklb	ribosomal modification protein rimK-like family member B	1.017	0.7076	NA
105351	AW209491	expressed sequence AW209491	1.017	0.7756	NA
103573	Xpo1	exportin 1, CRM1 homolog (yeast)	1.017	0.7967	NA
101867	Rrp8	ribosomal RNA processing 8, methyltransferase, homolog (yeast)	1.017	0.7529	NA
100163	Pafah2	platelet-activating factor acetylhydrolase 2	1.017	0.8045	NA
98258	Txndc9	thioredoxin domain containing 9	1.017	0.7847	NA
98193	Dcaf8	DDB1 and CUL4 associated factor 8	1.017	0.7153	NA
97761	Sgsm2	small G protein signaling modulator 2	1.017	0.8269	NA
78547	E130304I02Rik	RIKEN cDNA E130304I02 gene	1.017	0.9357	0.9814
75974	Dock11	dedicator of cytokinesis 11	1.017	0.7608	NA
75734	Mff	mitochondrial fission factor	1.017	0.686	NA
75051	4930578N16Rik	RIKEN cDNA 4930578N16 gene	1.017	0.8389	NA
74711	Ttll9	tubulin tyrosine ligase-like family, member 9	1.017	0.8692	0.9597
73103	3110009E18Rik	RIKEN cDNA 3110009E18 gene	1.017	0.781	NA
72050	Kdelc1	KDEL (Lys-Asp-Glu-Leu) containing 1	1.017	0.6925	NA
71946	Endod1	endonuclease domain containing 1	1.017	0.7588	NA
70925	Cdkn2aip	CDKN2A interacting protein	1.017	0.7563	NA
70247	Psmd1	proteasome (prosome, macropain) 26S subunit, non-ATPase, 1	1.017	0.7091	NA
69886	2610002J23Rik	RIKEN cDNA 2610002J23 gene	1.017	0.6973	NA
69539	Trnp1	TMF1-regulated nuclear protein 1	1.017	0.7384	NA
69519	Rwdd2a	RWD domain containing 2A	1.017	0.8254	NA
69392	1700024P12Rik	RIKEN cDNA 1700024P12 gene	1.017	0.9171	0.9759
69064	1810014F10Rik	RIKEN cDNA 1810014F10 gene	1.017	0.732	NA
68801	Elovl5	ELOVL family member 5, elongation of long chain fatty acids (yeast)	1.017	0.6965	NA
68539	Tmem109	transmembrane protein 109	1.017	0.6735	NA
68263	Pdhb	pyruvate dehydrogenase (lipoamide) beta	1.017	0.7887	NA
68077	Gltscr2	glioma tumor suppressor candidate region gene 2	1.017	0.8343	NA
67188	2700046G09Rik	RIKEN cDNA 2700046G09 gene	1.017	0.8336	NA
67187	Zmynd19	zinc finger, MYND domain containing 19	1.017	0.7591	NA

67102	D16Ertd472e	DNA segment, Chr 16, ERATO Doi 472, expressed	1.017	0.7839	NA
67028	2610002M06Rik	RIKEN cDNA 2610002M06 gene	1.017	0.731	NA
66915	Myeov2	myeloma overexpressed 2	1.017	0.8306	NA
66637	Tsen15	tRNA splicing endonuclease 15 homolog (S. cerevisiae)	1.017	0.7556	NA
66409	Rsl1d1	ribosomal L1 domain containing 1	1.017	0.7412	NA
59024	Med12	mediator of RNA polymerase II transcription, subunit 12 homolog (yeast)	1.017	0.8806	0.9635
58227	Fam184b	family with sequence similarity 184, member B	1.017	0.8394	NA
57783	Tnip1	TNFAIP3 interacting protein 1	1.017	0.757	NA
56716	Mlst8	MTOR associated protein, LST8 homolog (S. cerevisiae)	1.017	0.8441	NA
56428	Mtch2	mitochondrial carrier homolog 2 (C. elegans)	1.017	0.7249	NA
56403	Syncrip	synaptotagmin binding, cytoplasmic RNA interacting protein	1.017	0.739	NA
56228	Ube2j1	ubiquitin-conjugating enzyme E2, J1	1.017	0.672	NA
54632	Ftsj1	FtsJ homolog 1 (E. coli)	1.017	0.7943	NA
54604	Pcnx	pecanex homolog (Drosophila)	1.017	0.8018	NA
54170	Rragc	Ras-related GTP binding C	1.017	0.8124	NA
53312	Nub1	negative regulator of ubiquitin-like proteins 1	1.017	0.8573	0.9561
52428	Rhpn2	rhophilin, Rho GTPase binding protein 2	1.017	0.7399	NA
52357	Wwc2	WW, C2 and coiled-coil domain containing 2	1.017	0.8333	NA
30933	Tor2a	torsin family 2, member A	1.017	0.7556	NA
27261	Dok3	docking protein 3	1.017	0.88	0.9631
24069	Sufu	suppressor of fused homolog (Drosophila)	1.017	0.8688	0.9596
23937	Mab21l2	mab-21-like 2 (C. elegans)	1.017	0.8654	0.9584
21355	Tap2	transporter 2, ATP-binding cassette, sub-family B (MDR/TAP)	1.017	0.8157	NA
20844	Stam	signal transducing adaptor molecule (SH3 domain and ITAM motif) 1	1.017	0.8319	NA
20666	Sox11	SRY-box containing gene 11	1.017	0.8402	NA
19255	Ptpn2	protein tyrosine phosphatase, non-receptor type 2	1.017	0.7137	NA
18968	Pola1	polymerase (DNA directed), alpha 1	1.017	0.8285	NA
18738	Pitpna	phosphatidylinositol transfer protein, alpha	1.017	0.6652	NA
17957	Napb	N-ethylmaleimide sensitive fusion protein attachment protein beta	1.017	0.8364	NA
16450	Jag2	jagged 2	1.017	0.8392	NA
16432	Itm2b	integral membrane protein 2B	1.017	0.7604	NA
16201	Ilf3	interleukin enhancer binding factor 3	1.017	0.7571	NA
15950	Ifi203	interferon activated gene 203	1.017	0.8406	NA
15500	Hsf2	heat shock factor 2	1.017	0.7628	NA
14958	H1f0	H1 histone family, member 0	1.017	0.7969	NA
14784	Grb2	growth factor receptor bound protein 2	1.017	0.7522	NA
14772	Grk4	G protein-coupled receptor kinase 4	1.017	0.8734	0.9613
14401	Gabrb2	gamma-aminobutyric acid (GABA) A receptor, subunit beta 2	1.017	0.7852	NA
13528	Dtnb	dystrobrevin, beta	1.017	0.7965	NA
13498	Atn1	atrophin 1	1.017	0.9023	0.9716
12995	Csnk2a1	casein kinase 2, alpha 1 polypeptide	1.017	0.6895	NA
12846	Comt	catechol-O-methyltransferase	1.017	0.815	NA
12660	Chka	choline kinase alpha	1.017	0.7117	NA
12483	Cd22	CD22 antigen	1.017	0.9339	0.9806
12293	Cacna2d1	calcium channel, voltage-dependent, alpha2/delta subunit 1	1.017	0.8092	NA
12068	Bet1	blocked early in transport 1 homolog (S. cerevisiae)	1.017	0.8122	NA
11993	Aup1	ancient ubiquitous protein 1	1.017	0.8417	NA
100504663	Atg14	VATG14 autophagy related 14 homolog (S. cerevisiae)	1.016	0.7289	NA
620709	Lcn6	lipocalin 6	1.016	0.9121	0.9743

382007	Adam26b	a disintegrin and metallopeptidase domain 26B	1.016	0.9046	0.9717
381990	Zbtb2	zinc finger and BTB domain containing 2	1.016	0.756	NA
381067	Zfp229	zinc finger protein	1.016	0.7644	NA
378702	Serf2	small EDRK-rich factor 2	1.016	0.7581	NA
329918	Skint9	selection and upkeep of intraepithelial T cells 9	1.016	0.9538	0.9871
328092	6530401N04Rik	RIKEN cDNA 6530401N04 gene	1.016	0.7912	NA
320024	Nceh1	arylacetamide deacetylase-like 1	1.016	0.7199	NA
319642	Rab9b	RAB9B, member RAS oncogene family	1.016	0.8075	NA
246229	Bivm	basic, immunoglobulin-like variable motif containing	1.016	0.7909	NA
245474	Dkc1	dyskeratosis congenita 1, dyskerin homolog (human)	1.016	0.8018	NA
241296	Lrrc8a	leucine rich repeat containing 8A	1.016	0.79	NA
234542	Rtbdn	retbindin	1.016	0.8615	0.9573
230125	Mcart1	mitochondrial carrier triple repeat 1	1.016	0.7819	NA
229317	Eif2a	eukaryotic translation initiation factor 2A	1.016	0.8399	NA
227738	Lrsam1	leucine rich repeat and sterile alpha motif containing 1	1.016	0.8358	NA
227656	Rexo4	REX4, RNA exonuclease 4 homolog (S. cerevisiae)	1.016	0.7738	NA
224893	Zfp959	zinc finger protein 959	1.016	0.8029	NA
217674	Gphb5	glycoprotein hormone beta 5	1.016	0.937	0.9816
214459	Fnbp1l	formin binding protein 1-like	1.016	0.8262	NA
210982	BC032203	cDNA sequence BC032203	1.016	0.9258	0.9784
210148	Slc30a6	solute carrier family 30 (zinc transporter), member 6	1.016	0.6839	NA
114584	Clic1	chloride intracellular channel 1	1.016	0.7853	NA
106707	Rpusd1	RNA pseudouridylate synthase domain containing 1	1.016	0.8332	NA
106338	Nsun3	NOL1/NOP2/Sun domain family member 3	1.016	0.9469	0.9849
105439	Slain1	SLAIN motif family, member 1	1.016	0.8273	NA
103583	Fbxw11	F-box and WD-40 domain protein 11	1.016	0.7054	NA
102644	Oaf	OAF homolog (Drosophila)	1.016	0.902	0.9716
102209	Snapc2	small nuclear RNA activating complex, polypeptide 2	1.016	0.8982	0.9699
79221	Hdac9	histone deacetylase 9	1.016	0.7988	NA
77929	Yipf6	Yip1 domain family, member 6	1.016	0.8367	NA
75641	1700029I15Rik	RIKEN cDNA 1700029I15 gene	1.016	0.8631	0.9575
73822	F630110N24Rik	RIKEN cDNA F630110N24 gene	1.016	0.8295	NA
73766	4833420D23Rik	RIKEN cDNA 4833420D23 gene	1.016	0.7764	NA
72587	Pan3	PAN3 polyA specific ribonuclease subunit homolog (S. cerevisiae)	1.016	0.7115	NA
72429	Dnajc25	DnaJ (Hsp40) homolog, subfamily C, member 25	1.016	0.8143	NA
72129	Pex13	peroxisomal biogenesis factor 13	1.016	0.7485	NA
71991	Ercc8	excision repaiross-complementing rodent repair deficiency, complementation group 8	1.016	0.6726	NA
71735	Lrwd1	leucine-rich repeats and WD repeat domain containing 1	1.016	0.8672	0.959
71436	Flrt3	fibronectin leucine rich transmembrane protein 3	1.016	0.7773	NA
71213	Cage1	cancer antigen 1	1.016	0.876	0.9618
70769	Nolc1	nucleolar and coiled-body phosphoprotein 1	1.016	0.8163	NA
70584	Pak4	p21 protein (Cdc42/Rac)-activated kinase 4	1.016	0.8955	0.9698
69920	Polr2i	polymerase (RNA) II (DNA directed) polypeptide I	1.016	0.8087	NA
69906	Slc25a32	solute carrier family 25, member 32	1.016	0.775	NA
69731	Gemin7	gem (nuclear organelle) associated protein 7	1.016	0.8167	NA
69713	Pin4	protein (peptidyl-prolyl cis/trans isomerase) NIMA-interacting, 4 (parvulin)	1.016	0.868	0.9595
69537	Dnase1l1	deoxyribonuclease 1-like 1	1.016	0.8813	0.9637
68073	Fam173b	family with sequence similarity 173, member B	1.016	0.7312	NA
68052	Rps13	ribosomal protein S13	1.016	0.8035	NA

68011	Snrpg	small nuclear ribonucleoprotein polypeptide G	1.016	0.7165	NA
67922	Fam32a	family with sequence similarity 32, member A	1.016	0.7962	NA
67665	Dctn4	dynactin 4	1.016	0.687	NA
67211	Armc10	armadillo repeat containing 10	1.016	0.7883	NA
67115	Rpl14	ribosomal protein L14	1.016	0.8034	NA
66848	Fuca2	fucosidase, alpha-L- 2, plasma	1.016	0.8391	NA
66313	Smurf2	SMAD specific E3 ubiquitin protein ligase 2	1.016	0.8628	0.9574
66054	Cndp2	CNDP dipeptidase 2 (metallopeptidase M20 family)	1.016	0.8664	0.9587
59032	Ppp2r3c	protein phosphatase 2, regulatory subunit B'', gamma	1.016	0.7931	NA
57896	Krcc1	lysine-rich coiled-coil 1	1.016	0.8583	0.9564
56407	Trpc4ap	transient receptor potential cation channel, subfamily C, member 4 associated protein	1.016	0.7797	NA
52064	Coq5	coenzyme Q5 homolog, methyltransferase (yeast)	1.016	0.8478	NA
27385	Magel2	melanoma antigen, family L, 2	1.016	0.7499	NA
26557	Homer2	homer homolog 2 (Drosophila)	1.016	0.8435	NA
26434	Prnd	prion protein dublet	1.016	0.9016	0.9715
23802	Amfr	autocrine motility factor receptor	1.016	0.736	NA
22065	Trpc3	transient receptor potential cation channel, subfamily C, member 3	1.016	0.7224	NA
20815	Srpk1	serine/arginine-rich protein specific kinase 1	1.016	0.776	NA
20525	Slc2a1	solute carrier family 2 (facilitated glucose transporter), member 1	1.016	0.779	NA
20133	Rrm1	ribonucleotide reductase M1	1.016	0.6754	NA
20132	Rrh	retinal pigment epithelium derived rhodopsin homolog	1.016	0.7874	NA
19387	Rangap1	RAN GTPase activating protein 1	1.016	0.8642	0.9579
19286	Pts	6-pyruvoyl-tetrahydropterin synthase	1.016	0.8789	0.9629
19108	Prkx	protein kinase, X-linked	1.016	0.8123	NA
18996	Pou4f1	POU domain, class 4, transcription factor 1	1.016	0.9044	0.9717
17537	Meis3	Meis homeobox 3	1.016	0.8975	0.9699
17129	Smad5	MAD homolog 5 (Drosophila)	1.016	0.7025	NA
16649	Kpna4	karyopherin (importin) alpha 4	1.016	0.847	NA
16396	Itch	itchy, E3 ubiquitin protein ligase	1.016	0.801	NA
16169	Il15ra	interleukin 15 receptor, alpha chain	1.016	0.9216	0.9771
15191	Hdgf	hepatoma-derived growth factor	1.016	0.8291	NA
14387	Gaa	glucosidase, alpha, acid	1.016	0.9215	0.977
14123	Fbrs	fibrosin	1.016	0.8866	0.9669
13870	Ercc1	excision repair cross-complementing rodent repair deficiency, complementation group 1	1.016	0.848	NA
13175	Dclk1	doublecortin-like kinase 1	1.016	0.7829	NA
13024	Ctla2a	cytotoxic T lymphocyte-associated protein 2 alpha	1.016	0.7624	NA
13002	Dnajc5	DnaJ (Hsp40) homolog, subfamily C, member 5	1.016	0.8473	NA
12660	Chka	choline kinase alpha	1.016	0.66	NA
12144	Blm	Bloom syndrome, RecQ helicase-like	1.016	0.7437	NA
11908	Atf1	activating transcription factor 1	1.016	0.79	NA
11852	Rhob	ras homolog gene family, member B	1.016	0.763	NA
11769	Ap1s1	adaptor protein complex AP-1, sigma 1	1.016	0.7804	NA
11769	Ap1s1	adaptor protein complex AP-1, sigma 1	1.016	0.7849	NA
100503302	LOC100503302	40S ribosomal protein S19-like	1.015	0.8488	NA
100042786	Gm16381	predicted gene 16381	1.015	0.9549	0.9872
545600	Gm12372	predicted gene 12372	1.015	0.7554	NA
436332	Gm5766	ribosomal protein L7a pseudogene	1.015	0.6739	NA
403174	A930005I04Rik	RIKEN cDNA A930005I04 gene	1.015	0.8222	NA
382985	Rrm2b	ribonucleotide reductase M2 B (TP53 inducible)	1.015	0.9586	0.9881

382620	Tmed8	transmembrane emp24 domain containing 8	1.015	0.742	NA	
381511	Pdp1	pyruvate dehyrogenase phosphatase catalytic subunit 1	1.015	0.7717	NA	
328949	Mcc	mutated in colorectal cancers	1.015	0.8757		0.9618
320717	Pptc7	PTC7 protein phosphatase homolog (S. cerevisiae)	1.015	0.909		0.9733
320360	Ric3	resistance to inhibitors of cholinesterase 3 homolog (C. elegans)	1.015	0.8716		0.9608
320244	Ttll5	tubulin tyrosine ligase-like family, member 5	1.015	0.8556	NA	
319195	Rpl17	ribosomal protein L17	1.015	0.7875	NA	
319169	Hist1h2ak	histone cluster 1, H2ak	1.015	0.8355	NA	
269593	Luzp1	leucine zipper protein 1	1.015	0.8	NA	
244853	Fam55d	family with sequence similarity 55, member D	1.015	0.7688	NA	
241066	Carf	calcium response factor	1.015	0.8418	NA	
238331	Zdhhc22	zinc finger, DHHC-type containing 22	1.015	0.8874		0.9671
230594	Zcchc11	zinc finger, CCHC domain containing 11	1.015	0.893		0.9693
230484	Usp1	ubiquitin specific peptidase 1	1.015	0.8128	NA	
228966	Ppp1r3d	protein phosphatase 1, regulatory subunit 3D	1.015	0.7729	NA	
228911	Tshz2	teashirt zinc finger family member 2	1.015	0.7551	NA	
226252	Fam160b1	family with sequence similarity 160, member B1	1.015	0.827	NA	
223669	Zfp7	zinc finger protein 7	1.015	0.8224	NA	
223527	Eny2	enhancer of yellow 2 homolog (Drosophila)	1.015	0.7866	NA	
217615	Ctage5	CTAGE family, member 5	1.015	0.8119	NA	
215201	Trmt2b	TRM2 tRNA methyltransferase 2 homolog B (S. cerevisiae)	1.015	0.832	NA	
213012	Abhd10	abhydrolase domain containing 10	1.015	0.8515	NA	
208449	Sgms1	sphingomyelin synthase 1	1.015	0.8645		0.958
195727	Nhs	Nance-Horan syndrome (human)	1.015	0.9023		0.9716
109229	Fam118b	family with sequence similarity 118, member B	1.015	0.7095	NA	
107476	Acaca	acetyl-Coenzyme A carboxylase alpha	1.015	0.7603	NA	
100705	Acacb	acetyl-Coenzyme A carboxylase beta	1.015	0.8663		0.9587
100088	Rcc1	regulator of chromosome condensation 1	1.015	0.7702	NA	
93670	Tac4	tachykinin 4	1.015	0.8581	NA	
80732	Mynn	myoneurin	1.015	0.7942	NA	
76233	Dnttip1	deoxynucleotidyltransferase, terminal, interacting protein 1	1.015	0.7527	NA	
75424	Zfp820	zinc finger protein 820	1.015	0.9026		0.9717
74244	Atg7	autophagy-related 7 (yeast)	1.015	0.8543	NA	
74158	Josd1	Josephin domain containing 1	1.015	0.759	NA	
73379	Dcbld2	discoidin, CUB and LCCL domain containing 2	1.015	0.8939		0.9694
73174	Tbkbp1	TBK1 binding protein 1	1.015	0.9108		0.9741
73166	Tm7sf2	transmembrane 7 superfamily member 2	1.015	0.862		0.9573
72017	Cyb5r1	cytochrome b5 reductase 1	1.015	0.8345	NA	
72016	1600002H07Rik	RIKEN cDNA 1600002H07 gene	1.015	0.731	NA	
71703	Armcx3	armadillo repeat containing, X-linked 3	1.015	0.8759		0.9618
70870	4921516I12Rik	RIKEN cDNA 4921516I12 gene	1.015	0.7672	NA	
70497	Arhgap17	Rho GTPase activating protein 17	1.015	0.7672	NA	
69126	1810022K09Rik	RIKEN cDNA 1810022K09 gene	1.015	0.7716	NA	
69048	Slc30a5	solute carrier family 30 (zinc transporter), member 5	1.015	0.7548	NA	
68966	Ngdn	neuroguidin, EIF4E binding protein	1.015	0.7266	NA	
68519	Eml1	echinoderm microtubule associated protein like 1	1.015	0.7697	NA	
68493	Ndufaf4	NADH dehydrogenase (ubiquinone) 1 alpha subcomplex, assembly factor 4	1.015	0.8058	NA	
68192	Leprotl1	leptin receptor overlapping transcript-like 1	1.015	0.7562	NA	
67619	Nob1	NIN1/RPN12 binding protein 1 homolog (S. cerevisiae)	1.015	0.8834		0.9652

67454	Ikbip	IKBKB interacting protein	1.015	0.7986	NA
66887	Lonp2	lon peptidase 2, peroxisomal	1.015	0.829	NA
66645	Pspc1	paraspeckle protein 1	1.015	0.8005	NA
66597	Trim13	tripartite motif-containing 13	1.015	0.8043	NA
66225	Llph	LLP homolog, long-term synaptic facilitation (Aplysia)	1.015	0.8717	0.9608
66170	Chchd5	coiled-coil-helix-coiled-coil-helix domain containing 5	1.015	0.8226	NA
66125	Sf3b5	splicing factor 3b, subunit 5	1.015	0.7979	NA
66117	1110001J03Rik	RIKEN cDNA 1110001J03 gene	1.015	0.7676	NA
64685	Nmi	N-myc (and STAT) interactor	1.015	0.871	0.9607
63859	Impg1	interphotoreceptor matrix proteoglycan 1	1.015	0.9204	0.9768
59028	Rcl1	RNA terminal phosphate cyclase-like 1	1.015	0.7916	NA
57267	Apba3	amyloid beta (A4) precursor protein-binding, family A, member 3	1.015	0.914	0.9746
56692	Mapksp1	MAPK scaffold protein 1	1.015	0.7266	NA
55988	Snx12	sorting nexin 12	1.015	0.7738	NA
54451	Cpsf3	cleavage and polyadenylation specificity factor 3	1.015	0.7717	NA
54196	Pabpn1	poly(A) binding protein, nuclear 1	1.015	0.7876	NA
54131	Irf3	interferon regulatory factor 3	1.015	0.7754	NA
52397	Zfp644	zinc finger protein 644	1.015	0.7711	NA
52118	Pvr	poliovirus receptor	1.015	0.8584	NA
50911	Exosc9	exosome component 9	1.015	0.9055	0.9718
29877	Hdgfrp3	hepatoma-derived growth factor, related protein 3	1.015	0.8256	NA
28040	D6Wsu163e	DNA segment, Chr 6, Wayne State University 163, expressed	1.015	0.7368	NA
26406	Map3k3	mitogen-activated protein kinase kinase kinase 3	1.015	0.8275	NA
22715	Zfp57	zinc finger protein 57	1.015	0.9144	0.9749
22193	Ube2e3	ubiquitin-conjugating enzyme E2E 3, UBC4/5 homolog (yeast)	1.015	0.7926	NA
21833	Thra	thyroid hormone receptor alpha	1.015	0.785	NA
19259	Ptpn5	protein tyrosine phosphatase, non-receptor type 5	1.015	0.9132	0.9743
19053	Ppp2cb	protein phosphatase 2 (formerly 2A), catalytic subunit, beta isoform	1.015	0.7352	NA
18791	Plat	plasminogen activator, tissue	1.015	0.8525	NA
18203	Ntan1	N-terminal Asn amidase	1.015	0.792	NA
18186	Nrp1	neuropilin 1	1.015	0.8421	NA
18164	Nptx1	neuronal pentraxin 1	1.015	0.9006	0.971
18071	Nhlh1	nescient helix loop helix 1	1.015	0.9509	0.9865
17826	Mtvr2	mammary tumor virus receptor 2	1.015	0.837	NA
17463	Psmd7	proteasome (prosome, macropain) 26S subunit, non-ATPase, 7	1.015	0.7624	NA
17256	Mea1	male enhanced antigen 1	1.015	0.8509	NA
16661	Krt10	keratin 10	1.015	0.8325	NA
15510	Hspd1	heat shock protein 1 (chaperonin)	1.015	0.7458	NA
15207	Hes3	hairy and enhancer of split 3 (Drosophila)	1.015	0.8627	0.9574
13350	Dgat1	diacylglycerol O-acyltransferase 1	1.015	0.8488	NA
11564	Adsl	adenylosuccinate lyase	1.015	0.8702	0.9602
100043404	Gm4416	predicted gene 4416	1.014	0.8498	NA
100038712	Gm10516	predicted gene 10516	1.014	0.876	0.9618
100034363	Tmsb15b2	thymosin beta 15b2	1.014	0.767	NA
545718	Gm13141	predicted gene 13141	1.014	0.8304	NA
434858	Gm5643	heterogeneous nuclear ribonucleoprotein A1 pseudogene	1.014	0.7589	NA
433424	Gm13476	predicted gene 13476	1.014	0.9031	0.9717
381280	Hjurp	Holliday junction recognition protein	1.014	0.9543	0.9872
380928	Lmo7	LIM domain only 7	1.014	0.8974	0.9699

244416	Ppp1r3b	protein phosphatase 1, regulatory (inhibitor) subunit 3B	1.014	0.929	0.9789
242570	Raver2	ribonucleoprotein, PTB-binding 2	1.014	0.758	NA
229593	Golph3l	golgi phosphoprotein 3-like	1.014	0.7794	NA
229320	Clrn1	clarin 1	1.014	0.7531	NA
227699	Nup188	nucleoporin 188	1.014	0.8651	NA
226252	Fam160b1	family with sequence similarity 160, member B1	1.014	0.773	NA
224105	Pak2	p21 protein (Cdc42/Rac)-activated kinase 2	1.014	0.8267	NA
216739	Acsl6	acyl-CoA synthetase long-chain family member 6	1.014	0.6964	NA
213006	Mfsd4	major facilitator superfamily domain containing 4	1.014	0.7632	NA
209318	Gps1	G protein pathway suppressor 1	1.014	0.7688	NA
208092	Chmp6	chromatin modifying protein 6	1.014	0.8583	NA
194126	Mtmr11	myotubularin related protein 11	1.014	0.771	NA
114332	Lyve1	lymphatic vessel endothelial hyaluronan receptor 1	1.014	0.7826	NA
110147	Ehmt2	euchromatic histone lysine N-methyltransferase 2	1.014	0.7857	NA
109093	Rars2	arginyl-tRNA synthetase 2, mitochondrial	1.014	0.8418	NA
107476	Acaca	acetyl-Coenzyme A carboxylase alpha	1.014	0.7856	NA
104776	Aldh6a1	aldehyde dehydrogenase family 6, subfamily A1	1.014	0.7835	NA
101471	Phrf1	PHD and ring finger domains 1	1.014	0.8299	NA
99696	Ankrd50	ankyrin repeat domain 50	1.014	0.8024	NA
83925	Trps1	trichorhinophalangeal syndrome I (human)	1.014	0.8452	NA
80860	Ghdc	GH3 domain containing	1.014	0.9189	0.9764
78785	Clip4	CAP-GLY domain containing linker protein family, member 4	1.014	0.8064	NA
78303	Hist3h2ba	histone cluster 3, H2ba	1.014	0.8106	NA
77644	C330007P06Rik	RIKEN cDNA C330007P06 gene	1.014	0.8029	NA
76857	Spopl	speckle-type POZ protein-like	1.014	0.8631	NA
76132	6230409E13Rik	RIKEN cDNA 6230409E13 gene	1.014	0.8801	0.9631
74325	Cltb	clathrin, light polypeptide (Lcb)	1.014	0.8259	NA
73830	Eif3k	eukaryotic translation initiation factor 3, subunit K	1.014	0.7797	NA
73747	1110034G24Rik	RIKEN cDNA 1110034G24 gene	1.014	0.785	NA
73381	Cmtm2a	CKLF-like MARVEL transmembrane domain containing 2A	1.014	0.806	NA
73068	Fut11	fucosyltransferase 11	1.014	0.8664	NA
72399	Brap	BRCA1 associated protein	1.014	0.7676	NA
72306	Zfp777	zinc finger protein 777	1.014	0.7952	NA
72068	Cnot2	CCR4-NOT transcription complex, subunit 2	1.014	0.7478	NA
72061	2010111I01Rik	RIKEN cDNA 2010111I01 gene	1.014	0.8339	NA
71999	Fbxo22	F-box protein 22	1.014	0.7635	NA
71365	Pdss2	prenyl (solanesyl) diphosphate synthase, subunit 2	1.014	0.7664	NA
69938	Scrn1	secernin 1	1.014	0.7326	NA
69786	Tprkb	Tp53rk binding protein	1.014	0.7645	NA
69654	Dctn2	dynactin 2	1.014	0.8286	NA
68671	Pcyt2	phosphate cytidylyltransferase 2, ethanolamine	1.014	0.7675	NA
68512	Tomm5	translocase of outer mitochondrial membrane 5 homolog (yeast)	1.014	0.7322	NA
68401	G6pc3	glucose 6 phosphatase, catalytic, 3	1.014	0.8775	0.9625
67661	Ift172	intraflagellar transport 172 homolog (Chlamydomonas)	1.014	0.7723	NA
67513	2610002J02Rik	RIKEN cDNA 2610002J02 gene	1.014	0.7724	NA
67464	Entpd4	ectonucleoside triphosphate diphosphohydrolase 4	1.014	0.8261	NA
67306	Fam164a	family with sequence similarity 164, member A	1.014	0.8222	NA
67201	Glod4	glyoxalase domain containing 4	1.014	0.7038	NA
67150	Rnf141	ring finger protein 141	1.014	0.885	0.966

66881	Pcyox1	prenylcysteine oxidase 1	1.014	0.746	NA	
66615	Atg4b	autophagy-related 4B (yeast)	1.014	0.8154	NA	
66467	Gtf2h5	general transcription factor IIH, polypeptide 5	1.014	0.8682		0.9596
66191	Ier3ip1	immediate early response 3 interacting protein 1	1.014	0.8358	NA	
64602	Ireb2	iron responsive element binding protein 2	1.014	0.892		0.9689
56805	Zbtb33	zinc finger and BTB domain containing 33	1.014	0.768	NA	
56480	Tbk1	TANK-binding kinase 1	1.014	0.8224	NA	
56417	Adar	adenosine deaminase, RNA-specific	1.014	0.8373	NA	
56368	Cyb561d2	cytochrome b-561 domain containing 2	1.014	0.7875	NA	
56307	Metap2	methionine aminopeptidase 2	1.014	0.8118	NA	
56085	Ubqln1	ubiquilin 1	1.014	0.81	NA	
54615	Npff	neuropeptide FF-amide peptide precursor	1.014	0.8846		0.9657
53323	Ube2k	ubiquitin-conjugating enzyme E2K (UBC1 homolog, yeast)	1.014	0.8299	NA	
52245	Commd2	COMM domain containing 2	1.014	0.9234		0.9777
50996	Pdcd7	programmed cell death 7	1.014	0.7681	NA	
50755	Fbxo18	F-box protein 18	1.014	0.7371	NA	
30841	Kdm2b	lysine (K)-specific demethylase 2B	1.014	0.7556	NA	
24083	Gm16515	predicted gene, Gm16515	1.014	0.7995	NA	
22654	Zfp13	zinc finger protein 13	1.014	0.8237	NA	
22253	Unc5c	unc-5 homolog C (C. elegans)	1.014	0.8905		0.9687
22129	Ttc3	tetratricopeptide repeat domain 3	1.014	0.9131		0.9743
21854	Timm17a	translocase of inner mitochondrial membrane 17a	1.014	0.6497	NA	
20729	Spin1	spindlin 1	1.014	0.8718		0.9608
20469	Sipa1	signal-induced proliferation associated gene 1	1.014	0.7128	NA	
19303	Pxn	paxillin	1.014	0.9125		0.9743
18975	Polg	polymerase (DNA directed), gamma	1.014	0.7923	NA	
17309	Mgat3	mannoside acetylglucosaminyltransferase 3	1.014	0.7901	NA	
17207	Mcf2l	mcf.2 transforming sequence-like	1.014	0.8837		0.9652
17165	Mapkapk5	MAP kinase-activated protein kinase 5	1.014	0.7826	NA	
17119	Mxd1	MAX dimerization protein 1	1.014	0.759	NA	
16952	Anxa1	annexin A1	1.014	0.9656		0.9901
16834	Cog1	component of oligomeric golgi complex 1	1.014	0.7528	NA	
14297	Fxn	frataxin	1.014	0.8741		0.9616
14237	Foxd4	forkhead box D4	1.014	0.9212		0.977
13660	Ehd1	EH-domain containing 1	1.014	0.711	NA	
13389	Dll3	delta-like 3 (Drosophila)	1.014	0.9248		0.9783
12317	Calr	calreticulin	1.014	0.8895		0.9681
11787	Apbb2	amyloid beta (A4) precursor protein-binding, family B, member 2	1.014	0.8163	NA	
100039478	Gm11810	predicted gene 11810	1.013	0.8969		0.9699
100037283	Rnaset2a	ribonuclease T2A	1.013	0.8592	NA	
654795	Sdr39u1	short chain dehydrogenase/reductase family 39U, member 1	1.013	0.7447	NA	
629557	Gm6981	glyceraldehyde-3-phosphate dehydrogenase pseudogene	1.013	0.93		0.979
628779	Hs3st4	heparan sulfate (glucosamine) 3-O-sulfotransferase 4	1.013	0.7915	NA	
544696	D630037F22Rik	RIKEN cDNA D630037F22 gene	1.013	0.8722	NA	
497652	Acd	adrenocortical dysplasia	1.013	0.7472	NA	
414075	BC049265	cDNA sequence BC049265	1.013	0.9481		0.9854
320554	Tcp11l1	t-complex 11 like 1	1.013	0.8975		0.9699
320438	Alg6	asparagine-linked glycosylation 6 homolog (yeast, alpha-1,3,-glucosyltransferase)	1.013	0.8105	NA	
270669	Mbtps2	membrane-bound transcription factor peptidase, site 2	1.013	0.8495	NA	

240041	Zfp945	zinc finger protein 945	1.013	0.8456	NA
238831	Ppwd1	peptidylprolyl isomerase domain and WD repeat containing 1	1.013	0.7876	NA
234865	Nup133	nucleoporin 133	1.013	0.8111	NA
234374	Ddx49	DEAD (Asp-Glu-Ala-Asp) box polypeptide 49	1.013	0.792	NA
233204	Tbc1d17	TBC1 domain family, member 17	1.013	0.8522	NA
232164	Paip2b	poly(A) binding protein interacting protein 2B	1.013	0.7831	NA
224619	Traf7	TNF receptor-associated factor 7	1.013	0.7163	NA
217864	Rcor1	REST corepressor 1	1.013	0.8355	NA
217431	Nol10	nucleolar protein 10	1.013	0.9274	0.9788
217149	Cisd3	CDGSH iron sulfur domain 3	1.013	0.8067	NA
216152	BC005764	cDNA sequence BC005764	1.013	0.869	NA
215474	Sec22c	SEC22 vesicle trafficking protein homolog C (S. cerevisiae)	1.013	0.8507	NA
110842	Etfa	electron transferring flavoprotein, alpha polypeptide	1.013	0.7745	NA
101476	Plekha1	pleckstrin homology domain containing, family A (phosphoinositide binding specific) member 1	1.013	0.8666	NA
98985	Clp1	CLP1, cleavage and polyadenylation factor I subunit, homolog (S. cerevisiae)	1.013	0.8375	NA
83962	Btbd1	BTB (POZ) domain containing 1	1.013	0.8159	NA
83410	Cstf2t	cleavage stimulation factor, 3' pre-RNA subunit 2, tau	1.013	0.7755	NA
80914	Uck2	uridine-cytidine kinase 2	1.013	0.7981	NA
78550	E130119H09Rik	RIKEN cDNA E130119H09 gene	1.013	0.8857	0.9664
76742	Snx27	sorting nexin family member 27	1.013	0.8169	NA
76469	Cmya5	cardiomyopathy associated 5	1.013	0.9339	0.9806
75553	Zc3h14	zinc finger CCCH type containing 14	1.013	0.8771	NA
75458	Cklf	chemokine-like factor	1.013	0.8175	NA
75452	Ascc2	activating signal cointegrator 1 complex subunit 2	1.013	0.8278	NA
74665	Lrrc48	leucine rich repeat containing 48	1.013	0.9255	0.9784
74340	Ahcyl2	S-adenosylhomocysteine hydrolase-like 2	1.013	0.8377	NA
74229	Paqr8	progestin and adipoQ receptor family member VIII	1.013	0.8048	NA
73174	Tbkbp1	TBK1 binding protein 1	1.013	0.9452	0.9844
72843	Prdm4	PR domain containing 4	1.013	0.772	NA
72787	Tmem48	transmembrane protein 48	1.013	0.8714	NA
72341	2610002I17Rik	RIKEN cDNA 2610002I17	1.013	0.87	NA
71836	1700012A16Rik	RIKEN cDNA 1700012A16 gene	1.013	0.7305	NA
71834	Zbtb43	zinc finger and BTB domain containing 43	1.013	0.7177	NA
71805	Nup93	nucleoporin 93	1.013	0.7554	NA
70456	Brp44	brain protein 44	1.013	0.7312	NA
70052	Prpf4	PRP4 pre-mRNA processing factor 4 homolog (yeast)	1.013	0.8658	NA
69470	Tmem127	transmembrane protein 127	1.013	0.8231	NA
69309	Slc16a13	solute carrier family 16 (monocarboxylic acid transporters), member 13	1.013	0.9044	0.9717
69241	Polr2d	polymerase (RNA) II (DNA directed) polypeptide D	1.013	0.8487	NA
69089	Oxa1l	oxidase assembly 1-like	1.013	0.8251	NA
68612	Ube2c	ubiquitin-conjugating enzyme E2C	1.013	0.9567	0.9877
68346	Sirt5	sirtuin 5 (silent mating type information regulation 2 homolog) 5 (S. cerevisiae)	1.013	0.8237	NA
67382	Brd3	bromodomain containing 3	1.013	0.8171	NA
67370	Zfp606	zinc finger protein 606	1.013	0.8332	NA
66999	Med28	mediator of RNA polymerase II transcription, subunit 28 homolog (yeast)	1.013	0.8304	NA
66540	Fam107b	family with sequence similarity 107, member B	1.013	0.7451	NA
66101	Ppih	peptidyl prolyl isomerase H	1.013	0.9085	0.9733
64339	Fndc4	fibronectin type III domain containing 4	1.013	0.8049	NA
57257	Vav3	vav 3 oncogene	1.013	0.8386	NA

50868	Keap1	kelch-like ECH-associated protein 1	1.013	0.8674	NA
30963	Ptpla	protein tyrosine phosphatase-like (proline instead of catalytic arginine), member a	1.013	0.8303	NA
30945	Rnf19a	ring finger protein 19A	1.013	0.7868	NA
28295	D10Jhu81e	DNA segment, Chr 10, Johns Hopkins University 81 expressed	1.013	0.8253	NA
24010	Ik	IK cytokine	1.013	0.7703	NA
23937	Mab21l2	mab-21-like 2 (C. elegans)	1.013	0.9362	0.9814
22709	Zfp51	zinc finger protein 51	1.013	0.8183	NA
22260	Nr1h2	nuclear receptor subfamily 1, group H, member 2	1.013	0.8742	NA
21763	Tex2	testis expressed gene 2	1.013	0.7489	NA
20826	Nhp2l1	NHP2 non-histone chromosome protein 2-like 1 (S. cerevisiae)	1.013	0.8031	NA
20462	Tra2b	transformer 2 beta homolog (Drosophila)	1.013	0.9781	0.9932
19989	Rpl7	ribosomal protein L7	1.013	0.812	NA
19674	Rcvrn	recoverin	1.013	0.9018	0.9715
19183	Psmc3ip	proteasome (prosome, macropain) 26S subunit, ATPase 3, interacting protein	1.013	0.856	NA
18844	Plxna1	plexin A1	1.013	0.9365	0.9814
18762	Prkcz	protein kinase C, zeta	1.013	0.7958	NA
18227	Nr4a2	nuclear receptor subfamily 4, group A, member 2	1.013	0.8652	NA
18141	Nup50	nucleoporin 50	1.013	0.8404	NA
17863	Myb	myeloblastosis oncogene	1.013	0.87	NA
17760	Mtap6	microtubule-associated protein 6	1.013	0.8743	NA
17305	Mfng	MFNG O-fucosylpeptide 3-beta-N-acetylglucosaminyltransferase	1.013	0.8646	NA
17130	Smad6	MAD homolog 6 (Drosophila)	1.013	0.9136	0.9745
16705	Krtap9-1	keratin associated protein 9-1	1.013	0.935	0.981
15526	Hspa9	heat shock protein 9	1.013	0.7654	NA
15248	Hic1	hypermethylated in cancer 1	1.013	0.8838	0.9653
15203	Heph	hephaestin	1.013	0.91	0.9738
14963	H2-Bl	histocompatibility 2, blastocyst	1.013	0.8274	NA
14645	Glul	glutamate-ammonia ligase (glutamine synthetase)	1.013	0.8057	NA
14000	Drosha	drosha, ribonuclease type III	1.013	0.9089	0.9733
13636	Efna1	ephrin A1	1.013	0.8706	NA
12805	Cntn1	contactin 1	1.013	0.7445	NA
12652	Chga	chromogranin A	1.013	0.8688	NA
12479	Cd1d1	CD1d1 antigen	1.013	0.8439	NA
12464	Cct4	chaperonin containing Tcp1, subunit 4 (delta)	1.013	0.7989	NA
11774	Ap3b1	adaptor-related protein complex 3, beta 1 subunit	1.013	0.7951	NA
100503563	LOC100503563	60S ribosomal protein L21-like	1.012	0.8663	NA
100503168	LOC100503168	hypothetical LOC100503168	1.012	0.9531	0.987
629678	Gm6994	predicted gene 6994	1.012	0.9295	0.9789
621407	Gm9970	predicted gene 9970	1.012	0.9029	0.9717
382018	Unc13a	unc-13 homolog A (C. elegans)	1.012	0.9264	0.9785
353188	Adam32	a disintegrin and metallopeptidase domain 32	1.012	0.91	0.9738
333473	Zfp36l3	zinc finger protein 36, C3H type-like 3	1.012	0.9475	0.9852
319586	Celf5	CUGBP, Elav-like family member 5	1.012	0.9388	0.9824
270086	Ogfod1	2-oxoglutarate and iron-dependent oxygenase domain containing 1	1.012	0.9046	0.9717
268417	Zkscan17	zinc finger with KRAB and SCAN domains 17	1.012	0.8582	NA
252907	Vmn1r192	vomeronasal 1 receptor 192	1.012	0.8776	NA
232821	Ccdc106	coiled-coil domain containing 106	1.012	0.8476	NA
230777	Hcrtr1	hypocretin (orexin) receptor 1	1.012	0.9111	0.9741
230753	Thrap3	thyroid hormone receptor associated protein 3	1.012	0.8614	NA

229877	Rap1gds1	RAP1, GTP-GDP dissociation stimulator 1	1.012	0.7878	NA	
229487	Pet112l	PET112-like (yeast)	1.012	0.8599	NA	
225583	A730017C20Rik	RIKEN cDNA A730017C20 gene	1.012	0.9172		0.9759
223701	Mkl1	MKL (megakaryoblastic leukemia)/myocardin-like 1	1.012	0.8402	NA	
216456	Gls2	glutaminase 2 (liver, mitochondrial)	1.012	0.7891	NA	
213402	Armc2	armadillo repeat containing 2	1.012	0.8397	NA	
213119	Itga10	integrin, alpha 10	1.012	0.8606	NA	
211652	Wwc1	WW, C2 and coiled-coil domain containing 1	1.012	0.8978		0.9699
192198	Lrrc4	leucine rich repeat containing 4	1.012	0.8499	NA	
170731	Mfn2	mitofusin 2	1.012	0.8554	NA	
114713	Rasa2	RAS p21 protein activator 2	1.012	0.8242	NA	
108960	Irak2	interleukin-1 receptor-associated kinase 2	1.012	0.8973		0.9699
108888	Atad3a	ATPase family, AAA domain containing 3A	1.012	0.7885	NA	
107686	Snrpd2	small nuclear ribonucleoprotein D2	1.012	0.871	NA	
99712	Cept1	choline/ethanolaminephosphotransferase 1	1.012	0.795	NA	
98828	Cdc123	cell division cycle 123 homolog (S. cerevisiae)	1.012	0.7095	NA	
94184	Pdxdc1	pyridoxal-dependent decarboxylase domain containing 1	1.012	0.8459	NA	
79464	Lias	lipoic acid synthetase	1.012	0.8092	NA	
77889	Lbh	limb-bud and heart	1.012	0.9045		0.9717
76932	Arfip2	ADP-ribosylation factor interacting protein 2	1.012	0.8604	NA	
76577	Faf2	Fas associated factor family member 2	1.012	0.843	NA	
75901	Dcp1a	DCP1 decapping enzyme homolog A (S. cerevisiae)	1.012	0.7771	NA	
75880	4930592A05Rik	RIKEN cDNA 4930592A05 gene	1.012	0.934		0.9806
75099	Lysmd4	LysM, putative peptidoglycan-binding, domain containing 4	1.012	0.8175	NA	
74753	5830415F09Rik	RIKEN cDNA 5830415F09 gene	1.012	0.9461		0.9846
74570	Zkscan1	zinc finger with KRAB and SCAN domains 1	1.012	0.8435	NA	
74108	Parn	poly(A)-specific ribonuclease (deadenylation nuclease)	1.012	0.839	NA	
73072	BC068157	cDNA sequence BC068157	1.012	0.9302		0.979
71877	Efhc1	EF-hand domain (C-terminal) containing 1	1.012	0.932		0.9795
71817	Tmem50a	transmembrane protein 50A	1.012	0.8571	NA	
71382	Pex1	peroxisomal biogenesis factor 1	1.012	0.8299	NA	
71027	Tmem30c	transmembrane protein 30C	1.012	0.9065		0.9725
70008	Ace2	angiotensin I converting enzyme (peptidyl-dipeptidase A) 2	1.012	0.955		0.9872
69573	2310016C08Rik	RIKEN cDNA 2310016C08 gene	1.012	0.8124	NA	
68040	Zfp593	zinc finger protein 593	1.012	0.8035	NA	
67712	Slc25a37	solute carrier family 25, member 37	1.012	0.8389	NA	
67304	3110070M22Rik	RIKEN cDNA 3110070M22 gene	1.012	0.9383		0.9823
67295	Rab3c	RAB3C, member RAS oncogene family	1.012	0.8367	NA	
67057	Yaf2	YY1 associated factor 2	1.012	0.786	NA	
66932	Rexo1	REX1, RNA exonuclease 1 homolog (S. cerevisiae)	1.012	0.9382		0.9823
66832	Rsph3a	radial spoke 3A homolog (Chlamydomonas)	1.012	0.904		0.9717
66771	4933439F18Rik	RIKEN cDNA 4933439F18 gene	1.012	0.7791	NA	
66691	Gapvd1	GTPase activating protein and VPS9 domains 1	1.012	0.798	NA	
66323	1700001K19Rik	RIKEN cDNA 1700001K19 gene	1.012	0.9316		0.9795
66048	Tmem93	transmembrane protein 93	1.012	0.8066	NA	
57752	Tacc2	transforming, acidic coiled-coil containing protein 2	1.012	0.7954	NA	
56375	B4galt4	UDP-Gal:betaGlcNAc beta 1,4-galactosyltransferase, polypeptide 4	1.012	0.8909		0.9687
56196	Tdp2	tyrosyl-DNA phosphodiesterase 2	1.012	0.7456	NA	
28042	Ept1	ethanolaminephosphotransferase 1 (CDP-ethanolamine-specific)	1.012	0.7621	NA	

27176	Rpl7a	ribosomal protein L7A	1.012	0.8	NA
26936	Mprip	myosin phosphatase Rho interacting protein	1.012	0.8869	NA
26931	Ppp2r5c	protein phosphatase 2, regulatory subunit B (B56), gamma isoform	1.012	0.7936	NA
24018	Rngtt	RNA guanylyltransferase and 5'-phosphatase	1.012	0.7889	NA
20841	Zfp143	zinc finger protein 143	1.012	0.8499	NA
20338	Sel1l	sel-1 suppressor of lin-12-like (C. elegans)	1.012	0.8415	NA
18754	Prkce	protein kinase C, epsilon	1.012	0.8735	NA
18551	Pcsk4	proprotein convertase subtilisin/kexin type 4	1.012	0.9139	0.9746
17532	Mras	muscle and microspikes RAS	1.012	0.884	0.9654
17112	Tm4sf1	transmembrane 4 superfamily member 1	1.012	0.8497	NA
16832	Ldhb	lactate dehydrogenase B	1.012	0.8057	NA
16502	Kcnc1	potassium voltage gated channel, Shaw-related subfamily, member 1	1.012	0.9108	0.9741
15040	H2-T23	histocompatibility 2, T region locus 23	1.012	0.8542	NA
14394	Gabra1	gamma-aminobutyric acid (GABA) A receptor, subunit alpha 1	1.012	0.8839	NA
13645	Egf	epidermal growth factor	1.012	0.8633	NA
11975	Atp6v0a1	ATPase, H+ transporting, lysosomal V0 subunit A1	1.012	0.9137	0.9745
100040880	Gm3020	predicted gene 3020	1.011	0.9288	0.9789
100038580	6820445E23Rik	RIKEN cDNA 6820445E23 gene	1.011	0.8931	NA
791275	Gm9885	predicted gene 9885	1.011	0.8891	NA
434423	Dppa5a	developmental pluripotency associated 5A	1.011	0.9643	0.9898
384281	Gatc	glutamyl-tRNA(Gln) amidotransferase, subunit C homolog (bacterial)	1.011	0.8817	NA
381853	Gipr	gastric inhibitory polypeptide receptor	1.011	0.8906	NA
338367	Myo1d	myosin ID	1.011	0.8707	NA
332397	Nanos1	nanos homolog 1 (Drosophila)	1.011	0.8469	NA
319974	Auts2	autism susceptibility candidate 2	1.011	0.956	0.9876
241624	Exd1	exonuclease 3'-5' domain containing 1	1.011	0.879	NA
241520	Fam171b	family with sequence similarity 171, member B	1.011	0.895	NA
241075	Plekhm3	pleckstrin homology domain containing, family M, member 3	1.011	0.8329	NA
233893	Zfp764	zinc finger protein 764	1.011	0.8022	NA
233315	Mtmr10	myotubularin related protein 10	1.011	0.8319	NA
232337	Zfp637	zinc finger protein 637	1.011	0.8115	NA
226541	Klhl20	kelch-like 20 (Drosophila)	1.011	0.8372	NA
226016	Fam108b	family with sequence similarity 108, member B	1.011	0.8751	NA
214444	Cdk5rap2	CDK5 regulatory subunit associated protein 2	1.011	0.9351	0.981
212090	Tmem60	transmembrane protein 60	1.011	0.8453	NA
210789	Tbc1d4	TBC1 domain family, member 4	1.011	0.889	NA
210376	Mtmr9	myotubularin related protein 9	1.011	0.8899	NA
208285	Cyp4f17	cytochrome P450, family 4, subfamily f, polypeptide 17	1.011	0.8674	NA
170483	Grin3b	glutamate receptor, ionotropic, NMDA3B	1.011	0.8848	NA
110954	Rpl10	ribosomal protein 10	1.011	0.8569	NA
109359	Fam175b	family with sequence similarity 175, member B	1.011	0.8209	NA
109205	Sobp	sine oculis-binding protein homolog (Drosophila)	1.011	0.824	NA
108160	Fam50a	family with sequence similarity 50, member A	1.011	0.7615	NA
104394	E2f4	E2F transcription factor 4	1.011	0.972	0.992
104383	Rcor2	REST corepressor 2	1.011	0.8973	0.9699
102122	Fam192a	family with sequence similarity 192, member A	1.011	0.8353	NA
100678	Psph	phosphoserine phosphatase	1.011	0.8806	NA
79566	Sh3bp5l	SH3 binding domain protein 5 like	1.011	0.7745	NA
78102	8430426J06Rik	RIKEN cDNA 8430426J06 gene	1.011	0.9548	0.9872

77629	Sphkap	SPHK1 interactor, AKAP domain containing	1.011	0.9484	0.9854
77219	Ptgr2	prostaglandin reductase 2	1.011	0.8557	NA
75062	Sf3a3	splicing factor 3a, subunit 3	1.011	0.8222	NA
73827	1110012D08Rik	RIKEN cDNA 1110012D08 gene	1.011	0.8824	NA
73130	Tmed5	transmembrane emp24 protein transport domain containing 5	1.011	0.8927	NA
72301	1810041L15Rik	RIKEN cDNA 1810041L15 gene	1.011	0.7835	NA
72278	Ccpg1	cell cycle progression 1	1.011	0.8785	NA
71835	Lancl2	LanC (bacterial lantibiotic synthetase component C)-like 2	1.011	0.8485	NA
70873	4921517L17Rik	RIKEN cDNA 4921517L17 gene	1.011	0.9018	0.9715
70757	Ptplb	protein tyrosine phosphatase-like (proline instead of catalytic arginine), member b	1.011	0.8673	NA
69727	Usp46	ubiquitin specific peptidase 46	1.011	0.8106	NA
69596	2310035K24Rik	RIKEN cDNA 2310035K24 gene	1.011	0.8566	NA
69109	Fam58b	family with sequence similarity 58, member B	1.011	0.8192	NA
69047	Atp2c2	ATPase, Ca++ transporting, type 2C, member 2	1.011	0.9466	0.9848
68865	Arv1	ARV1 homolog (yeast)	1.011	0.7819	NA
68323	Nudt22	nudix (nucleoside diphosphate linked moiety X)-type motif 22	1.011	0.8892	NA
67869	Paip2	polyadenylate-binding protein-interacting protein 2	1.011	0.8689	NA
67845	Rnf115	ring finger protein 115	1.011	0.8577	NA
66925	Sdhd	succinate dehydrogenase complex, subunit D, integral membrane protein	1.011	0.8403	NA
66390	Slmo2	slowmo homolog 2 (Drosophila)	1.011	0.8618	NA
66230	Mrps28	mitochondrial ribosomal protein S28	1.011	0.8629	NA
64010	Sav1	salvador homolog 1 (Drosophila)	1.011	0.848	NA
59288	Dctn5	dynactin 5	1.011	0.8212	NA
50907	Preb	prolactin regulatory element binding	1.011	0.833	NA
27366	Txnl4a	thioredoxin-like 4A	1.011	0.833	NA
27267	Cars	cysteinyl-tRNA synthetase	1.011	0.8837	NA
23892	Grem1	gremlin 1	1.011	0.9133	0.9743
22698	Zfp39	zinc finger protein 39	1.011	0.903	0.9717
22661	Zfp148	zinc finger protein 148	1.011	0.8991	0.9704
22630	Ywhaq	tyrosine 3-monooxygenase/tryptophan 5-monooxygenase activation protein, theta polypeptide	1.011	0.8325	NA
22354	Vipr1	vasoactive intestinal peptide receptor 1	1.011	0.8858	NA
22194	Ube2e1	ubiquitin-conjugating enzyme E2E 1, UBC4/5 homolog (yeast)	1.011	0.9022	0.9716
22018	Tpo	thyroid peroxidase	1.011	0.908	0.9733
20664	Sox1	SRY-box containing gene 1	1.011	0.9649	0.99
20624	Eftud2	elongation factor Tu GTP binding domain containing 2	1.011	0.8649	NA
20084	Rps18	ribosomal protein S18	1.011	0.8328	NA
19730	Ralgds	ral guanine nucleotide dissociation stimulator	1.011	0.8693	NA
19188	Psme2	proteasome (prosome, macropain) 28 subunit, beta	1.011	0.8096	NA
18824	Plp2	proteolipid protein 2	1.011	0.9536	0.987
18720	Pip5k1a	phosphatidylinositol-4-phosphate 5-kinase, type 1 alpha	1.011	0.8314	NA
18645	Pfn2	profilin 2	1.011	0.8427	NA
18481	Pak3	p21 protein (Cdc42/Rac)-activated kinase 3	1.011	0.8563	NA
17954	Nap1l2	nucleosome assembly protein 1-like 2	1.011	0.8194	NA
16976	Lrpap1	low density lipoprotein receptor-related protein associated protein 1	1.011	0.8224	NA
15569	Elavl2	ELAV (embryonic lethal, abnormal vision, Drosophila)-like 2 (Hu antigen B)	1.011	0.8864	NA
14688	Gnb1	guanine nucleotide binding protein (G protein), beta 1	1.011	0.8793	NA
14256	Flt3l	FMS-like tyrosine kinase 3 ligand	1.011	0.9188	0.9764
13619	Phc1	polyhomeotic-like 1 (Drosophila)	1.011	0.9088	0.9733
13548	Dyrk1a	dual-specificity tyrosine-(Y)-phosphorylation regulated kinase 1a	1.011	0.8848	NA

12445	Ccnd3	cyclin D3	1.011	0.7641	NA
12237	Bub3	budding uninhibited by benzimidazoles 3 homolog (S. cerevisiae)	1.011	0.8382	NA
12153	Bmp1	bone morphogenetic protein 1	1.011	0.9551	0.9872
12151	Bmi1	Bmi1 polycomb ring finger oncogene	1.011	0.8541	NA
11966	Atp6v1b2	ATPase, H+ transporting, lysosomal V1 subunit B2	1.011	0.8562	NA
11836	Araf	v-raf murine sarcoma 3611 viral oncogene homolog	1.011	0.8496	NA
11652	Akt2	thymoma viral proto-oncogene 2	1.011	0.8769	NA
100505330	LOC100505330	NHP2-like protein 1-like	1.01	0.8035	NA
100033459	Pydc3	pyrin domain containing 3	1.01	0.934	0.9806
619441	BC096441	cDNA sequence BC096441	1.01	0.8783	NA
432842	LOC432842	hypothetical LOC432842	1.01	0.919	0.9764
319195	Rpl17	ribosomal protein L17	1.01	0.9274	0.9788
268379	Abca13	ATP-binding cassette, sub-family A (ABC1), member 13	1.01	0.9305	0.9791
245578	Pcdh11x	protocadherin 11 X-linked	1.01	0.9302	0.979
243912	Hspb6	heat shock protein, alpha-crystallin-related, B6	1.01	0.9349	0.981
237781	Smcr7	Smith-Magenis syndrome chromosome region, candidate 7 homolog (human)	1.01	0.8486	NA
237504	Rassf9	Ras association (RalGDS/AF-6) domain family (N-terminal) member 9	1.01	0.9416	0.9835
235315	Rnf214	ring finger protein 214	1.01	0.8766	NA
232236	C130022K22Rik	RIKEN cDNA C130022K22 gene	1.01	0.8114	NA
230779	Serinc2	serine incorporator 2	1.01	0.9568	0.9877
230514	Leprot	leptin receptor overlapping transcript	1.01	0.8048	NA
230233	Ikbkap	inhibitor of kappa light polypeptide enhancer in B-cells, kinase complex-associated protein	1.01	0.8335	NA
224647	D17Wsu92e	DNA segment, Chr 17, Wayne State University 92, expressed	1.01	0.8447	NA
219181	Akap11	A kinase (PRKA) anchor protein 11	1.01	0.8375	NA
218693	Paip1	polyadenylate binding protein-interacting protein 1	1.01	0.9388	0.9824
218271	B4galt7	xylosylprotein beta1,4-galactosyltransferase, polypeptide 7 (galactosyltransferase I)	1.01	0.884	NA
214764	2700050L05Rik	RIKEN cDNA 2700050L05 gene	1.01	0.7867	NA
213990	Agap3	ArfGAP with GTPase domain, ankyrin repeat and PH domain 3	1.01	0.8016	NA
213119	Itga10	integrin, alpha 10	1.01	0.8906	NA
212986	Scfd2	Sec1 family domain containing 2	1.01	0.8613	NA
208982	Hmgcll1	3-hydroxymethyl-3-methylglutaryl-Coenzyme A lyase-like 1	1.01	0.9005	NA
192196	Luc7l2	LUC7-like 2 (S. cerevisiae)	1.01	0.8756	NA
170757	Eltd1	EGF, latrophilin seven transmembrane domain containing 1	1.01	0.9087	NA
170731	Mfn2	mitofusin 2	1.01	0.8962	NA
140489	Bhlhe23	basic helix-loop-helix family, member e23	1.01	0.8446	NA
114874	Ddhd1	DDHD domain containing 1	1.01	0.8774	NA
110637	Grik4	glutamate receptor, ionotropic, kainate 4	1.01	0.9041	NA
107733	Mrpl41	mitochondrial ribosomal protein L41	1.01	0.823	NA
105638	Dph3	DPH3 homolog (KTI11, S. cerevisiae)	1.01	0.7844	NA
103468	Nup107	nucleoporin 107	1.01	0.8019	NA
98258	Txndc9	thioredoxin domain containing 9	1.01	0.9451	0.9844
98221	Eif3m	eukaryotic translation initiation factor 3, subunit M	1.01	0.8792	NA
94218	Cnnm3	cyclin M3	1.01	0.9253	0.9783
93883	Pcdhb12	protocadherin beta 12	1.01	0.875	NA
81000	Rad54l2	RAD54 like 2 (S. cerevisiae)	1.01	0.8671	NA
80883	Ntng1	netrin G1	1.01	0.8982	NA
78808	Stxbp5	syntaxin binding protein 5 (tomosyn)	1.01	0.9229	0.9776
78038	Mccc2	methylcrotonoyl-Coenzyme A carboxylase 2 (beta)	1.01	0.8686	NA
77721	Mrps5	mitochondrial ribosomal protein S5	1.01	0.8683	NA

75605	Kdm5b	lysine (K)-specific demethylase 5B	1.01	0.8072	NA
74386	Rmi1	RMI1, RecQ mediated genome instability 1, homolog (S. cerevisiae)	1.01	0.8531	NA
73389	Hbp1	high mobility group box transcription factor 1	1.01	0.8933	NA
73192	Xpot	exportin, tRNA (nuclear export receptor for tRNAs)	1.01	0.8299	NA
71452	Ankrd40	ankyrin repeat domain 40	1.01	0.8435	NA
70681	Fam175a	family with sequence similarity 175, member A	1.01	0.8279	NA
69710	Arap1	ArfGAP with RhoGAP domain, ankyrin repeat and PH domain 1	1.01	0.9033	NA
69674	Mif4gd	MIF4G domain containing	1.01	0.8794	NA
69263	Rfc3	replication factor C (activator 1) 3	1.01	0.9147	0.9751
69113	Alkbh3	alkB, alkylation repair homolog 3 (E. coli)	1.01	0.8156	NA
68014	Zwilch	Zwilch, kinetochore associated, homolog (Drosophila)	1.01	0.8999	NA
67976	Trabd	TraB domain containing	1.01	0.8697	NA
67680	Sdhb	succinate dehydrogenase complex, subunit B, iron sulfur (Ip)	1.01	0.832	NA
67590	Tctn3	tectonic family member 3	1.01	0.8287	NA
67298	Gprasp1	G protein-coupled receptor associated sorting protein 1	1.01	0.7748	NA
67204	Eif2s2	eukaryotic translation initiation factor 2, subunit 2 (beta)	1.01	0.8385	NA
66801	Prkrip1	Prkr interacting protein 1 (IL11 inducible)	1.01	0.8654	NA
66573	Dzip1	DAZ interacting protein 1	1.01	0.914	0.9746
66258	Mrps17	mitochondrial ribosomal protein S17	1.01	0.769	NA
66201	Vta1	Vps20-associated 1 homolog (S. cerevisiae)	1.01	0.8883	NA
66170	Chchd5	coiled-coil-helix-coiled-coil-helix domain containing 5	1.01	0.786	NA
66105	Ube2d3	ubiquitin-conjugating enzyme E2D 3 (UBC4/5 homolog, yeast)	1.01	0.8232	NA
59004	Pias4	protein inhibitor of activated STAT 4	1.01	0.9527	0.9869
57895	Ccdc126	coiled-coil domain containing 126	1.01	0.8906	NA
57028	Pdxp	pyridoxal (pyridoxine, vitamin B6) phosphatase	1.01	0.9129	0.9743
56494	Gosr2	golgi SNAP receptor complex member 2	1.01	0.7559	NA
54712	Plxnc1	plexin C1	1.01	0.7918	NA
53356	Eif3g	eukaryotic translation initiation factor 3, subunit G	1.01	0.8436	NA
52013	D19Ertd386e	DNA segment, Chr 19, ERATO Doi 386, expressed	1.01	0.8814	NA
30947	Adat1	adenosine deaminase, tRNA-specific 1	1.01	0.9248	0.9783
29813	Zfp385a	zinc finger protein 385A	1.01	0.9582	0.9881
27057	Ncoa4	nuclear receptor coactivator 4	1.01	0.8161	NA
26572	Cops3	COP9 (constitutive photomorphogenic) homolog, subunit 3 (Arabidopsis thaliana)	1.01	0.8845	NA
22770	Zhx1	zinc fingers and homeoboxes 1	1.01	0.8293	NA
22628	Ywhag	tyrosine 3-monooxygenase/tryptophan 5-monooxygenase activation protein, gamma polypeptide	1.01	0.8734	NA
21753	Tes	testis derived transcript	1.01	0.8421	NA
21678	Tead3	TEA domain family member 3	1.01	0.931	0.9792
21335	Tacc3	transforming, acidic coiled-coil containing protein 3	1.01	0.8992	NA
20918	Eif1	eukaryotic translation initiation factor 1	1.01	0.891	NA
20588	Smarcc1	SWI/SNF related, matrix associated, actin dependent regulator of chromatin, subfamily c, member 1	1.01	0.9528	0.9869
20515	Slc20a1	solute carrier family 20, member 1	1.01	0.8384	NA
19942	Rpl27	ribosomal protein L27	1.01	0.835	NA
19192	Psme3	proteaseome (prosome, macropain) 28 subunit, 3	1.01	0.9383	0.9823
17083	Tmed1	transmembrane emp24 domain containing 1	1.01	0.8523	NA
16428	Itk	IL2-inducible T-cell kinase	1.01	0.9273	0.9788
15469	Prmt1	protein arginine N-methyltransferase 1	1.01	0.8919	NA
15278	Tfb2m	transcription factor B2, mitochondrial	1.01	0.8546	NA
15270	H2afx	H2A histone family, member X	1.01	0.8972	NA
14719	Got2	glutamate oxaloacetate transaminase 2, mitochondrial	1.01	0.8789	NA

14451	Gas1	growth arrest specific 1	1.01	0.8832	NA	
14433	Gapdh	glyceraldehyde-3-phosphate dehydrogenase	1.01	0.9079		0.9733
12417	Cbx3	chromobox homolog 3 (Drosophila HP1 gamma)	1.01	0.8218	NA	
11565	Adssl1	adenylosuccinate synthetase like 1	1.01	0.867	NA	
100040220	Gm9892	predicted gene 9892	1.009	0.8644	NA	
100038371	Zfp389	zinc finger protein 389	1.009	0.9388		0.9824
433813	Pusl1	pseudouridylate synthase-like 1	1.009	0.8893	NA	
381884	Slc6a16	solute carrier family 6, member 16	1.009	0.9419		0.9835
320714	D030016E14Rik	RIKEN cDNA D030016E14 gene	1.009	0.8907	NA	
320376	Bcorl1	BCL6 co-repressor-like 1	1.009	0.8713	NA	
320268	B930095G15Rik	RIKEN cDNA B930095G15 gene	1.009	0.8952	NA	
269717	Orai2	ORAI calcium release-activated calcium modulator 2	1.009	0.9051	NA	
268902	Robo2	roundabout homolog 2 (Drosophila)	1.009	0.872	NA	
268448	Phf12	PHD finger protein 12	1.009	0.9529		0.9869
260305	Nphp4	nephronophthisis 4 (juvenile) homolog (human)	1.009	0.9362		0.9814
244310	Dlgap2	discs, large (Drosophila) homolog-associated protein 2	1.009	0.8516	NA	
243743	Plxna4	plexin A4	1.009	0.8896	NA	
236643	Sytl5	synaptotagmin-like 5	1.009	0.9481		0.9854
235459	Gtf2a2	general transcription factor II A, 2	1.009	0.8453	NA	
234135	Whsc1l1	Wolf-Hirschhorn syndrome candidate 1-like 1 (human)	1.009	0.8139	NA	
230796	Wdtc1	WD and tetratricopeptide repeats 1	1.009	0.9057	NA	
227102	Ormdl1	ORM1-like 1 (S. cerevisiae)	1.009	0.7889	NA	
226154	Lzts2	leucine zipper, putative tumor suppressor 2	1.009	0.8925	NA	
224703	Mar-02	membrane-associated ring finger (C3HC4) 2	1.009	0.8978	NA	
223648	2410075B13Rik	RIKEN cDNA 2410075B13 gene	1.009	0.8796	NA	
216197	Ckap4	cytoskeleton-associated protein 4	1.009	0.9638		0.9898
215418	Csrnp1	cysteine-serine-rich nuclear protein 1	1.009	0.9143	NA	
212569	Zfp273	zinc finger protein 273	1.009	0.8241	NA	
212427	A730008H23Rik	RIKEN cDNA A730008H23 gene	1.009	0.8708	NA	
212114	Nhlrc3	NHL repeat containing 3	1.009	0.9398		0.9827
209707	Lcorl	ligand dependent nuclear receptor corepressor-like	1.009	0.9363		0.9814
170750	Xpnpep1	X-prolyl aminopeptidase (aminopeptidase P) 1, soluble	1.009	0.8657	NA	
108159	Ubxn8	UBX domain protein 8	1.009	0.9062	NA	
106021	Topors	topoisomerase I binding, arginine/serine-rich	1.009	0.8555	NA	
101861	Ints4	integrator complex subunit 4	1.009	0.9244		0.9783
101476	Plekha1	pleckstrin homology domain containing, family A (phosphoinositide binding specific) member 1	1.009	0.8549	NA	
100737	Dcun1d4	DCN1, defective in cullin neddylation 1, domain containing 4 (S. cerevisiae)	1.009	0.9134	NA	
97086	Nhedc2	Na+/H+ exchanger domain containing 2	1.009	0.9249		0.9783
83796	Smarcd2	SWI/SNF related, matrix associated, actin dependent regulator of chromatin, subfamily d, member 2	1.009	0.8964	NA	
80748	BC004004	cDNA sequence BC004004	1.009	0.8797	NA	
78651	Lsm6	LSM6 homolog, U6 small nuclear RNA associated (S. cerevisiae)	1.009	0.8454	NA	
75616	2810008M24Rik	RIKEN cDNA 2810008M24 gene	1.009	0.8335	NA	
74718	Snx16	sorting nexin 16	1.009	0.9172		0.9759
74589	Kbtbd12	kelch repeat and BTB (POZ) domain containing 12	1.009	0.9118	NA	
74525	8430419L09Rik	RIKEN cDNA 8430419L09 gene	1.009	0.9657		0.9901
73389	Hbp1	high mobility group box transcription factor 1	1.009	0.8788	NA	
72482	Acbd6	acyl-Coenzyme A binding domain containing 6	1.009	0.8171	NA	
71889	Epn3	epsin 3	1.009	0.9464		0.9847
71198	Otud1	OTU domain containing 1	1.009	0.9178		0.9761

70579	Zc3h11a	zinc finger CCCH type containing 11A	1.009	0.9159	NA
70571	Tcerg1l	transcription elongation regulator 1-like	1.009	0.9109	NA
70544	5730437N04Rik	RIKEN cDNA 5730437N04 gene	1.009	0.822	NA
70380	Mospd1	motile sperm domain containing 1	1.009	0.8297	NA
70040	2610037D02Rik	RIKEN cDNA 2610037D02 gene	1.009	0.9807	0.994
69129	Pex11c	peroxisomal biogenesis factor 11 gamma	1.009	0.9288	0.9789
68235	2410066E13Rik	RIKEN cDNA 2410066E13 gene	1.009	0.9527	0.9869
68170	B230118H07Rik	RIKEN cDNA B230118H07 gene	1.009	0.8889	NA
68146	Arl13b	ADP-ribosylation factor-like 13B	1.009	0.853	NA
67974	Ccny	cyclin Y	1.009	0.959	0.9882
67955	Sugt1	SGT1, suppressor of G2 allele of SKP1 (S. cerevisiae)	1.009	0.8379	NA
67603	Dusp6	dual specificity phosphatase 6	1.009	0.8799	NA
67464	Entpd4	ectonucleoside triphosphate diphosphohydrolase 4	1.009	0.8937	NA
67452	Pnpla8	patatin-like phospholipase domain containing 8	1.009	0.8814	NA
66961	Neat1	nuclear paraspeckle assembly transcript 1 (non-protein coding)	1.009	0.9507	0.9864
66880	Rsrc1	arginine/serine-rich coiled-coil 1	1.009	0.86	NA
66488	Fam136a	family with sequence similarity 136, member A	1.009	0.8015	NA
66456	2810001G20Rik	RIKEN cDNA 2810001G20 gene	1.009	0.8949	NA
66279	Tmem218	transmembrane protein 218	1.009	0.8196	NA
66046	Ndufb5	NADH dehydrogenase (ubiquinone) 1 beta subcomplex, 5	1.009	0.8731	NA
65971	Tbata	thymus, brain and testes associated	1.009	0.9643	0.9898
58911	Sumf1	sulfatase modifying factor 1	1.009	0.8738	NA
56453	Mbtps1	membrane-bound transcription factor peptidase, site 1	1.009	0.9096	NA
56293	Amac1	acyl-malonyl condensing enzyme 1	1.009	0.9544	0.9872
56088	Psmg1	proteasome (prosome, macropain) assembly chaperone 1	1.009	0.8802	NA
53610	Nono	non-POU-domain-containing, octamer binding protein	1.009	0.8706	NA
52398	Sep-11	septin 11	1.009	0.8646	NA
29869	Ulk2	Unc-51 like kinase 2 (C. elegans)	1.009	0.923	0.9776
27756	Lsm2	LSM2 homolog, U6 small nuclear RNA associated (S. cerevisiae)	1.009	0.9701	0.9914
26905	Eif2s3x	eukaryotic translation initiation factor 2, subunit 3, structural gene X-linked	1.009	0.9132	NA
26448	Stk30	serine/threonine kinase 30	1.009	0.8185	NA
26419	Mapk8	mitogen-activated protein kinase 8	1.009	0.8809	NA
26356	Ing1	inhibitor of growth family, member 1	1.009	0.9278	0.9789
22779	Ikzf2	IKAROS family zinc finger 2	1.009	0.8901	NA
22408	Wnt1	wingless-related MMTV integration site 1	1.009	0.9483	0.9854
22195	Ube2l3	ubiquitin-conjugating enzyme E2L 3	1.009	0.9387	0.9824
21380	Tbx1	T-box 1	1.009	0.9419	0.9835
20595	Smn1	survival motor neuron 1	1.009	0.8969	NA
20520	Slc22a5	solute carrier family 22 (organic cation transporter), member 5	1.009	0.8875	NA
20357	Sema5b	sema domain, seven thrombospondin repeats (type 1 and type 1-like), transmembrane domain (TM) and short cytoplasmic domain, (semaphorin) 5B	1.009	0.9433	0.9839
19707	Reps1	RalBP1 associated Eps domain containing protein	1.009	0.8728	NA
19679	Pitpnm2	phosphatidylinositol transfer protein, membrane-associated 2	1.009	0.9511	0.9865
18797	Plcb3	phospholipase C, beta 3	1.009	0.9247	0.9783
18576	Pde3b	phosphodiesterase 3B, cGMP-inhibited	1.009	0.889	NA
18475	Pafah1b2	platelet-activating factor acetylhydrolase, isoform 1b, subunit 2	1.009	0.8714	NA
16835	Ldlr	low density lipoprotein receptor	1.009	0.8747	NA
14158	Fert2	fer (fms/fps related) protein kinase, testis specific 2	1.009	0.947	0.9849
14149	Fdxr	ferredoxin reductase	1.009	0.8731	NA
14104	Fasn	fatty acid synthase	1.009	0.8739	NA

11826	Aqp1	aquaporin 1	1.009	0.9248	0.9783
671641	Gm10063	predicted gene 10063	1.008	0.8415	NA
667338	Gm8580	ribosomal protein L29 pseudogene	1.008	0.9239	0.978
623474	Rad54b	RAD54 homolog B (S. cerevisiae)	1.008	0.8854	NA
432767	Gm5450	predicted gene 5450	1.008	0.8846	NA
384309	Trim56	tripartite motif-containing 56	1.008	0.9239	NA
338359	Supv3l1	suppressor of var1, 3-like 1 (S. cerevisiae)	1.008	0.8393	NA
330577	Fam154b	family with sequence similarity 154, member B	1.008	0.8589	NA
327655	Ppip5k1	diphosphoinositol pentakisphosphate kinase 1	1.008	0.8365	NA
320271	Scai	suppressor of cancer cell invasion	1.008	0.9768	0.9928
319887	E030030I06Rik	RIKEN cDNA E030030I06 gene	1.008	0.9672	0.9904
270106	Rpl13	ribosomal protein L13	1.008	0.914	NA
269397	Ss18l1	synovial sarcoma translocation gene on chromosome 18-like 1	1.008	0.8495	NA
268319	BC025920	zinc finger protein pseudogene	1.008	0.9387	0.9824
252903	Ap1s3	adaptor-related protein complex AP-1, sigma 3	1.008	0.9313	0.9795
243725	Ppp1r9a	protein phosphatase 1, regulatory (inhibitor) subunit 9A	1.008	0.9124	NA
243302	Gm4963	predicted gene 4963	1.008	0.8421	NA
242425	Gabbr2	gamma-aminobutyric acid (GABA) B receptor, 2	1.008	0.9492	0.9856
238266	Syt16	synaptotagmin XVI	1.008	0.8706	NA
234814	Mthfsd	methenyltetrahydrofolate synthetase domain containing	1.008	0.9226	0.9776
231727	B3gnt4	UDP-GlcNAc:betaGal beta-1,3-N-acetylglucosaminyltransferase 4	1.008	0.9692	0.9911
231329	Polr2b	polymerase (RNA) II (DNA directed) polypeptide B	1.008	0.8987	NA
230822	A330049M08Rik	RIKEN cDNA A330049M08 gene	1.008	0.9539	0.9871
228359	Arhgap1	Rho GTPase activating protein 1	1.008	0.9553	0.9872
226118	AI606181	expressed sequence AI606181	1.008	0.9153	NA
224805	Aars2	alanyl-tRNA synthetase 2, mitochondrial (putative)	1.008	0.9612	0.9889
218544	Sgtb	small glutamine-rich tetratricopeptide repeat (TPR)-containing, beta	1.008	0.9351	0.981
217684	4933426M11Rik	RIKEN cDNA 4933426M11 gene	1.008	0.8668	NA
216134	Pdxk	pyridoxal (pyridoxine, vitamin B6) kinase	1.008	0.9318	0.9795
213389	Prdm9	PR domain containing 9	1.008	0.9203	NA
210992	Lpcat1	lysophosphatidylcholine acyltransferase 1	1.008	0.9458	0.9846
210027	Slc35f3	solute carrier family 35, member F3	1.008	0.9523	0.9869
209011	Sirt7	sirtuin 7 (silent mating type information regulation 2, homolog) 7 (S. cerevisiae)	1.008	0.8523	NA
208820	Gm11818	predicted gene 11818	1.008	0.8931	NA
110172	Slc35b1	solute carrier family 35, member B1	1.008	0.8433	NA
108960	Irak2	interleukin-1 receptor-associated kinase 2	1.008	0.9371	0.9816
108800	Ston2	stonin 2	1.008	0.927	NA
105245	Txndc5	thioredoxin domain containing 5	1.008	0.9463	0.9847
99650	4933434E20Rik	RIKEN cDNA 4933434E20 gene	1.008	0.873	NA
94067	Mrpl43	mitochondrial ribosomal protein L43	1.008	0.8588	NA
83485	Ngrn	neugrin, neurite outgrowth associated	1.008	0.893	NA
83453	Chrdl1	chordin-like 1	1.008	0.9227	NA
78308	Gpr108	G protein-coupled receptor 108	1.008	0.8226	NA
76892	Rnft1	ring finger protein, transmembrane 1	1.008	0.9128	NA
76187	Adhfe1	alcohol dehydrogenase, iron containing, 1	1.008	0.9668	0.9903
75995	5033417F24Rik	RIKEN cDNA 5033417F24 gene	1.008	0.9538	0.9871
75847	Ispd	isoprenoid synthase domain containing	1.008	0.9158	NA
75296	Fgfr1op	Fgfr1 oncogene partner	1.008	0.8768	NA
72895	Setd5	SET domain containing 5	1.008	0.8892	NA

72805	Zfp839	zinc finger protein 839	1.008	0.9391	0.9824
72658	2700097O09Rik	RIKEN cDNA 2700097O09 gene	1.008	0.853	NA
72515	Wdr43	WD repeat domain 43	1.008	0.857	NA
71843	R3hcc1	R3H domain and coiled-coil containing 1	1.008	0.9067	NA
71678	Brox	BRO1 domain and CAAX motif containing	1.008	0.9302	0.979
71524	8430432A02Rik	RIKEN cDNA 8430432A02 gene	1.008	0.8962	NA
71131	Zfp689	zinc finger protein 689	1.008	0.9297	0.9789
70036	2700023E23Rik	RIKEN cDNA 2700023E23 gene	1.008	0.911	NA
70028	Dopey2	dopey family member 2	1.008	0.9154	NA
69976	Galk2	galactokinase 2	1.008	0.8908	NA
69792	Med6	mediator of RNA polymerase II transcription, subunit 6 homolog (yeast)	1.008	0.8239	NA
69358	Lrrc51	leucine rich repeat containing 51	1.008	0.918	NA
69178	Snx5	sorting nexin 5	1.008	0.8667	NA
68953	Chmp2a	chromatin modifying protein 2A	1.008	0.8871	NA
67971	Tppp3	tubulin polymerization-promoting protein family member 3	1.008	0.9441	0.9842
67876	Coq10b	coenzyme Q10 homolog B (S. cerevisiae)	1.008	0.9388	0.9824
67544	Fam120b	family with sequence similarity 120, member B	1.008	0.8977	NA
66492	Zmat2	zinc finger, matrin type 2	1.008	0.8675	NA
66413	Psmd6	proteasome (prosome, macropain) 26S subunit, non-ATPase, 6	1.008	0.8785	NA
65960	Twsg1	twisted gastrulation homolog 1 (Drosophila)	1.008	0.925	0.9783
65103	Arl6ip6	ADP-ribosylation factor-like 6 interacting protein 6	1.008	0.9413	0.9835
60510	Syt9	synaptotagmin IX	1.008	0.8821	NA
60345	Nrip2	nuclear receptor interacting protein 2	1.008	0.9405	0.9829
59289	Ccbp2	chemokine binding protein 2	1.008	0.9273	0.9788
58909	Fam13a	family with sequence similarity 13, member A	1.008	0.874	NA
56382	Rab9	RAB9, member RAS oncogene family	1.008	0.8786	NA
56214	Scamp4	secretory carrier membrane protein 4	1.008	0.8266	NA
51788	H2afz	H2A histone family, member Z	1.008	0.8501	NA
50995	Uba2	ubiquitin-like modifier activating enzyme 2	1.008	0.8711	NA
29864	Rnf11	ring finger protein 11	1.008	0.9073	NA
27998	Exosc5	exosome component 5	1.008	0.9102	NA
26987	Eif4e2	eukaryotic translation initiation factor 4E member 2	1.008	0.8951	NA
26370	Cetn2	centrin 2	1.008	0.8007	NA
22401	Zmat3	zinc finger matrin type 3	1.008	0.8988	NA
20868	Stk10	serine/threonine kinase 10	1.008	0.9172	NA
20509	Slc19a1	solute carrier family 19 (sodium/hydrogen exchanger), member 1	1.008	0.8829	NA
20185	Ncor1	nuclear receptor co-repressor 1	1.008	0.9477	0.9853
20042	Rps12	ribosomal protein S12	1.008	0.9201	NA
19982	Rpl36a	ribosomal protein L36A	1.008	0.8993	NA
19395	Rasgrp2	RAS, guanyl releasing protein 2	1.008	0.8957	NA
19024	Ppfibp2	PTPRF interacting protein, binding protein 2 (liprin beta 2)	1.008	0.9449	0.9844
14886	Gtf2i	general transcription factor II I	1.008	0.865	NA
14456	Gas6	growth arrest specific 6	1.008	0.8895	NA
12951	Crx	cone-rod homeobox containing gene	1.008	0.935	0.981
12633	Cflar	CASP8 and FADD-like apoptosis regulator	1.008	0.9118	NA
12615	Cenpa	centromere protein A	1.008	0.8999	NA
12478	Cd19	CD19 antigen	1.008	0.9262	NA
12387	Ctnnb1	catenin (cadherin associated protein), beta 1	1.008	0.8949	NA
11535	Adm	adrenomedullin	1.008	0.9284	0.9789

632883	LOC632883	protein transport protein Sec61 subunit gamma-like	1.007	0.9553	0.9872
442801	Arhgef15	Rho guanine nucleotide exchange factor (GEF) 15	1.007	0.9682	0.9906
382034	Gse1	genetic suppressor element 1	1.007	0.9614	0.9889
320923	Mtap7d3	MAP7 domain containing 3	1.007	0.9619	0.9889
320799	Zhx3	zinc fingers and homeoboxes 3	1.007	0.8744	NA
320405	Cadps2	Ca2+-dependent activator protein for secretion 2	1.007	0.9423	0.9837
320105	D030036P13Rik	RIKEN cDNA D030036P13 gene	1.007	0.9435	0.9839
319604	Fam168a	family with sequence similarity 168, member A	1.007	0.8923	NA
268749	Rnf31	ring finger protein 31	1.007	0.9117	NA
245440	Gm4988	predicted gene 4988	1.007	0.8652	NA
244666	Gm505	predicted gene 505	1.007	0.9239	NA
236732	Rbm10	RNA binding motif protein 10	1.007	0.9098	NA
230777	Hcrtr1	hypocretin (orexin) receptor 1	1.007	0.9433	0.9839
228790	Asxl1	additional sex combs like 1 (Drosophila)	1.007	0.889	NA
228361	Ambra1	autophagy/beclin 1 regulator 1	1.007	0.9183	NA
226548	Aph1a	anterior pharynx defective 1a homolog (C. elegans)	1.007	0.9025	NA
224897	Dpp9	dipeptidylpeptidase 9	1.007	0.9399	0.9827
223864	Rapgef3	Rap guanine nucleotide exchange factor (GEF) 3	1.007	0.9418	0.9835
217708	Lin52	lin-52 homolog (C. elegans)	1.007	0.8761	NA
214685	Chadl	chondroadherin-like	1.007	0.9258	NA
213027	Evi5l	ecotropic viral integration site 5 like	1.007	0.897	NA
212999	Tnpo2	transportin 2 (importin 3, karyopherin beta 2b)	1.007	0.9453	0.9844
212127	2810046L04Rik	RIKEN cDNA 2810046L04 gene	1.007	0.9858	0.9949
209586	Nudcd3	NudC domain containing 3	1.007	0.8887	NA
192292	Nrbp1	nuclear receptor binding protein 1	1.007	0.9428	0.9838
110524	Dgkq	diacylglycerol kinase, theta	1.007	0.9282	NA
109821	F11	coagulation factor XI	1.007	0.9437	0.9841
108075	Ltbp4	latent transforming growth factor beta binding protein 4	1.007	0.9535	0.987
107734	Mrpl30	mitochondrial ribosomal protein L30	1.007	0.9131	NA
105372	Utp15	UTP15, U3 small nucleolar ribonucleoprotein, homolog (yeast)	1.007	0.9057	NA
105239	Rnf44	ring finger protein 44	1.007	0.8905	NA
101646	B830008H07Rik	RIKEN cDNA B830008H07 gene	1.007	0.9091	NA
96935	Susd4	sushi domain containing 4	1.007	0.9189	NA
94275	Maged1	melanoma antigen, family D, 1	1.007	0.9063	NA
93690	Gpr45	G protein-coupled receptor 45	1.007	0.9653	0.99
80891	Fcrls	Fc receptor-like S, scavenger receptor	1.007	0.9235	NA
78454	9530077C14Rik	RIKEN cDNA 9530077C14 gene	1.007	0.8737	NA
78329	2310010J17Rik	RIKEN cDNA 2310010J17 gene	1.007	0.9295	NA
77870	E130116L18Rik	RIKEN cDNA E130116L18 gene	1.007	0.9572	0.9879
77795	A930010G16Rik	RIKEN cDNA A930010G16 gene	1.007	0.9252	NA
77775	A430103D13Rik	RIKEN cDNA A430103D13 gene	1.007	0.9168	NA
77652	Zfp955a	zinc finger protein 955A	1.007	0.8952	NA
76245	6530415H11Rik	RIKEN cDNA 6530415H11 gene	1.007	0.8979	NA
74479	Snx11	sorting nexin 11	1.007	0.9327	NA
73823	4930401B11Rik	RIKEN cDNA 4930401B11 gene	1.007	0.891	NA
73442	Hspa12a	heat shock protein 12A	1.007	0.9694	0.9911
72162	Dhx36	DEAH (Asp-Glu-Ala-His) box polypeptide 36	1.007	0.8565	NA
71720	Osbpl3	oxysterol binding protein-like 3	1.007	0.9396	0.9827
71449	Mettl13	methyltransferase like 13	1.007	0.9211	NA

69675	Pxdn	peroxidasin homolog (Drosophila)	1.007	0.9584	0.9881
69499	Tsr2	TSR2, 20S rRNA accumulation, homolog (S. cerevisiae)	1.007	0.8872	NA
69211	2310081J21Rik	RIKEN cDNA 2310081J21 gene	1.007	0.9722	0.992
69085	Zcchc9	zinc finger, CCHC domain containing 9	1.007	0.9431	0.9838
68736	1110034B05Rik	RIKEN cDNA 1110034B05 gene	1.007	0.9281	NA
67273	Ndufa10	NADH dehydrogenase (ubiquinone) 1 alpha subcomplex 10	1.007	0.9304	NA
67075	Magt1	magnesium transporter 1	1.007	0.9406	0.983
67045	Riok2	RIO kinase 2 (yeast)	1.007	0.891	NA
67036	Mrpl45	mitochondrial ribosomal protein L45	1.007	0.8754	NA
66870	Serbp1	serpine1 mRNA binding protein 1	1.007	0.9274	NA
64656	Mrps23	mitochondrial ribosomal protein S23	1.007	0.9072	NA
56322	Timm22	translocase of inner mitochondrial membrane 22 homolog (yeast)	1.007	0.8649	NA
52592	Brms1l	breast cancer metastasis-suppressor 1-like	1.007	0.865	NA
52028	Bbs1	Bardet-Biedl syndrome 1 (human)	1.007	0.879	NA
27281	Hrasls	HRAS-like suppressor	1.007	0.9161	NA
26896	Med14	mediator complex subunit 14	1.007	0.8951	NA
26430	Parg	poly (ADP-ribose) glycohydrolase	1.007	0.8684	NA
22355	Vipr2	vasoactive intestinal peptide receptor 2	1.007	0.9124	NA
22151	Tubb2a	tubulin, beta 2A	1.007	0.9317	NA
22031	Traf3	TNF receptor-associated factor 3	1.007	0.887	NA
21847	Klf10	Kruppel-like factor 10	1.007	0.8574	NA
21652	Phf1	PHD finger protein 1	1.007	0.9318	NA
21428	Mlx	MAX-like protein X	1.007	0.9009	NA
21419	Tcfap2b	transcription factor AP-2 beta	1.007	0.8696	NA
21371	Tbca	tubulin cofactor A	1.007	0.9656	0.9901
20930	Surf1	surfeit gene 1	1.007	0.9248	NA
20910	Stxbp1	syntaxin binding protein 1	1.007	0.9158	NA
20411	Sorbs1	sorbin and SH3 domain containing 1	1.007	0.9399	0.9827
19982	Rpl36a	ribosomal protein L36A	1.007	0.9039	NA
19891	Rpa2	replication protein A2	1.007	0.8878	NA
19877	Rock1	Rho-associated coiled-coil containing protein kinase 1	1.007	0.9342	NA
19823	Rnf7	ring finger protein 7	1.007	0.8949	NA
19330	Rab18	RAB18, member RAS oncogene family	1.007	0.8665	NA
19231	Ptma	prothymosin alpha	1.007	0.9207	NA
19230	Twf1	twinfilin, actin-binding protein, homolog 1 (Drosophila)	1.007	0.8581	NA
19084	Prkar1a	protein kinase, cAMP dependent regulatory, type I, alpha	1.007	0.9284	NA
17995	Ndufv1	NADH dehydrogenase (ubiquinone) flavoprotein 1	1.007	0.9028	NA
15525	Hspa4	heat shock protein 4	1.007	0.8335	NA
15356	Hmgcl	3-hydroxy-3-methylglutaryl-Coenzyme A lyase	1.007	0.8741	NA
14084	Faf1	Fas-associated factor 1	1.007	0.9274	NA
11770	Fabp4	fatty acid binding protein 4, adipocyte	1.007	0.9347	NA
100126243	A030001D20Rik	RIKEN cDNA A030001D20 gene	1.006	0.9379	NA
100101807	1700047I17Rik2	RIKEN cDNA 1700047I17 gene 2	1.006	0.9409	NA
100036521	Gm16039	predicted gene 16039	1.006	0.9584	0.9881
629147	Ctxn3	cortexin 3	1.006	0.9349	NA
329650	Med12l	mediator of RNA polymerase II transcription, subunit 12 homolog (yeast)-like	1.006	0.9535	0.987
328309	Gm9776	predicted gene 9776	1.006	0.9335	NA
320793	A330072L02Rik	RIKEN cDNA A330072L02 gene	1.006	0.99	0.9964
319740	Zfyve27	zinc finger, FYVE domain containing 27	1.006	0.9225	NA

270192	Rab6b	RAB6B, member RAS oncogene family	1.006	0.9089	NA
238831	Ppwd1	peptidylprolyl isomerase domain and WD repeat containing 1	1.006	0.9327	NA
234463	Tmem184c	transmembrane protein 184C	1.006	0.9205	NA
232236	C130022K22Rik	RIKEN cDNA C130022K22 gene	1.006	0.8692	NA
231014	9330182L06Rik	RIKEN cDNA 9330182L06 gene	1.006	0.9039	NA
226432	Ipo9	importin 9	1.006	0.8862	NA
225845	Pla2g16	phospholipase A2, group XVI	1.006	0.9703	0.9915
225049	Ttc7	tetratricopeptide repeat domain 7	1.006	0.96	0.9886
223978	Cpped1	calcineurin-like phosphoesterase domain containing 1	1.006	0.9113	NA
223753	Cerk	ceramide kinase	1.006	0.9141	NA
223435	Trio	triple functional domain (PTPRF interacting)	1.006	0.9084	NA
209446	Tcfe3	transcription factor E3	1.006	0.919	NA
195733	Grhl1	grainyhead-like 1 (Drosophila)	1.006	0.9149	NA
108760	Galntl1	UDP-N-acetyl-alpha-D-galactosamine:polypeptide N-acetylgalactosaminyltransferase-like 1	1.006	0.9408	NA
108735	Sft2d2	SFT2 domain containing 2	1.006	0.8743	NA
108151	Sema3d	sema domain, immunoglobulin domain (Ig), short basic domain, secreted, (semaphorin) 3D	1.006	0.9721	0.992
107305	Vps37c	vacuolar protein sorting 37C (yeast)	1.006	0.9291	NA
106894	Hmgxb3	HMG box domain containing 3	1.006	0.9083	NA
103677	Smg6	Smg-6 homolog, nonsense mediated mRNA decay factor (C. elegans)	1.006	0.9419	NA
98386	Lbr	lamin B receptor	1.006	0.9245	NA
98303	D630023F18Rik	RIKEN cDNA D630023F18 gene	1.006	0.9072	NA
93735	Wnt16	wingless-related MMTV integration site 16	1.006	0.9814	0.9942
84094	Plvap	plasmalemma vesicle associated protein	1.006	0.9336	NA
78895	Pus7l	pseudouridylate synthase 7 homolog (S. cerevisiae)-like	1.006	0.9346	NA
76890	Memo1	mediator of cell motility 1	1.006	0.8931	NA
76594	Dnajc18	DnaJ (Hsp40) homolog, subfamily C, member 18	1.006	0.8942	NA
76485	Glt8d1	glycosyltransferase 8 domain containing 1	1.006	0.8961	NA
74018	Als2	amyotrophic lateral sclerosis 2 (juvenile) homolog (human)	1.006	0.9501	0.9859
72726	Tbcc	tubulin-specific chaperone C	1.006	0.8993	NA
71891	Cdadc1	cytidine and dCMP deaminase domain containing 1	1.006	0.9315	NA
71116	Stx18	syntaxin 18	1.006	0.9309	NA
70620	Ube2v2	ubiquitin-conjugating enzyme E2 variant 2	1.006	0.8925	NA
70369	Bag5	BCL2-associated athanogene 5	1.006	0.9071	NA
70120	Yars2	tyrosyl-tRNA synthetase 2 (mitochondrial)	1.006	0.9069	NA
69627	Fam89a	family with sequence similarity 89, member A	1.006	0.9425	NA
69386	Hist1h4h	histone cluster 1, H4h	1.006	0.9603	0.9886
68533	Mphosph6	M phase phosphoprotein 6	1.006	0.8847	NA
68202	Ndufa5	NADH dehydrogenase (ubiquinone) 1 alpha subcomplex, 5	1.006	0.9103	NA
68036	Zfp706	zinc finger protein 706	1.006	0.8735	NA
67889	Rbm18	RNA binding motif protein 18	1.006	0.9642	0.9898
67116	Cuedc2	CUE domain containing 2	1.006	0.9335	NA
66834	Acot13	acyl-CoA thioesterase 13	1.006	0.9097	NA
66660	Sltm	SAFB-like, transcription modulator	1.006	0.8921	NA
66612	Ormdl3	ORM1-like 3 (S. cerevisiae)	1.006	0.8761	NA
66388	Cutc	cutC copper transporter homolog (E.coli)	1.006	0.9527	0.9869
66315	Senp7	SUMO1/sentrin specific peptidase 7	1.006	0.9275	NA
66302	Fam82b	family with sequence similarity 82, member B	1.006	0.8972	NA
64659	Mrps14	mitochondrial ribosomal protein S14	1.006	0.9445	0.9842
64144	Mllt1	myeloid/lymphoid or mixed-lineage leukemia (trithorax homolog, Drosophila); translocated to, 1	1.006	0.9453	0.9844

59025	Usp14	ubiquitin specific peptidase 14	1.006	0.898	NA
56505	Ruvbl1	RuvB-like protein 1	1.006	0.9096	NA
56426	Pdcd10	programmed cell death 10	1.006	0.9031	NA
56361	Pus1	pseudouridine synthase 1	1.006	0.9308	NA
55934	rp9	retinitis pigmentosa 9 (human)	1.006	0.9156	NA
54722	Dfna5	deafness, autosomal dominant 5 (human)	1.006	0.9069	NA
54638	Ccdc22	coiled-coil domain containing 22	1.006	0.9191	NA
54153	Rasa4	RAS p21 protein activator 4	1.006	0.9526	0.9869
53330	Vamp4	vesicle-associated membrane protein 4	1.006	0.9281	NA
53310	Dlg3	discs, large homolog 3 (Drosophila)	1.006	0.9026	NA
52662	D18Ertd653e	DNA segment, Chr 18, ERATO Doi 653, expressed	1.006	0.9469	0.9849
50773	Nt5c	5',3'-nucleotidase, cytosolic	1.006	0.9152	NA
30853	Mlf2	myeloid leukemia factor 2	1.006	0.9525	0.9869
23881	G3bp2	GTPase activating protein (SH3 domain) binding protein 2	1.006	0.8863	NA
22025	Nr2c1	nuclear receptor subfamily 2, group C, member 1	1.006	0.9014	NA
21780	Tfam	transcription factor A, mitochondrial	1.006	0.9229	NA
21665	Tdg	thymine DNA glycosylase	1.006	0.9733	0.9922
21416	Tcf7l2	transcription factor 7-like 2, T-cell specific, HMG-box	1.006	0.9468	0.9849
20975	Synj2	synaptojanin 2	1.006	0.9724	0.992
20088	Rps24	ribosomal protein S24	1.006	0.9516	0.9867
20042	Rps12	ribosomal protein S12	1.006	0.9224	NA
19045	Ppp1ca	protein phosphatase 1, catalytic subunit, alpha isoform	1.006	0.9421	NA
18744	Pja1	praja1, RING-H2 motif containing	1.006	0.8765	NA
18190	Nrxn2	neurexin II	1.006	0.9317	NA
17350	Mlh1	mutL homolog 1 (E. coli)	1.006	0.9039	NA
16531	Kcnma1	potassium large conductance calcium-activated channel, subfamily M, alpha member 1	1.006	0.9319	NA
14618	Gjb1	gap junction protein, beta 1	1.006	0.9724	0.992
14467	Gbas	glioblastoma amplified sequence	1.006	0.909	NA
13591	Ebf1	early B-cell factor 1	1.006	0.9513	0.9866
13445	Cdk2ap1	CDK2 (cyclin-dependent kinase 2)-associated protein 1	1.006	0.9213	NA
12479	Cd1d1	CD1d1 antigen	1.006	0.9269	NA
11843	Arf4	ADP-ribosylation factor 4	1.006	0.9511	0.9865
11836	Araf	v-raf murine sarcoma 3611 viral oncogene homolog	1.006	0.9355	NA
11487	Adam10	a disintegrin and metallopeptidase domain 10	1.006	0.9083	NA
11430	Acox1	acyl-Coenzyme A oxidase 1, palmitoyl	1.006	0.901	NA
100504876	LOC100504876	60S ribosomal protein L7a-like	1.005	0.9047	NA
545861	Gm5878	predicted gene 5878	1.005	0.9341	NA
414115	D330050I16Rik	RIKEN cDNA D330050I16 gene	1.005	0.9832	0.9947
381038	Parl	presenilin associated, rhomboid-like	1.005	0.8986	NA
380702	Shisa6	shisa homolog 6 (Xenopus laevis)	1.005	0.9366	NA
320923	Mtap7d3	MAP7 domain containing 3	1.005	0.9758	0.9928
317750	Slc24a5	solute carrier family 24, member 5	1.005	0.9559	NA
270109	Pcnxl2	pecanex-like 2 (Drosophila)	1.005	0.9542	NA
243374	Gimap8	GTPase, IMAP family member 8	1.005	0.9573	0.9879
234371	Tmem161a	transmembrane protein 161A	1.005	0.9336	NA
233912	Armc5	armadillo repeat containing 5	1.005	0.9471	NA
223648	2410075B13Rik	RIKEN cDNA 2410075B13 gene	1.005	0.9262	NA
217864	Rcor1	REST corepressor 1	1.005	0.9183	NA
217031	Tada2a	transcriptional adaptor 2A	1.005	0.911	NA

211480	Kcnj14	potassium inwardly-rectifying channel, subfamily J, member 14	1.005	0.9135	NA
192185	Nadk	NAD kinase	1.005	0.9451	NA
109151	Chd9	chromodomain helicase DNA binding protein 9	1.005	0.9155	NA
105827	Amigo2	adhesion molecule with Ig like domain 2	1.005	0.9319	NA
99326	Garnl3	GTPase activating RANGAP domain-like 3	1.005	0.9033	NA
98256	Kmo	kynurenine 3-monooxygenase (kynurenine 3-hydroxylase)	1.005	0.941	NA
93790	Nipa2	non imprinted in Prader-Willi/Angelman syndrome 2 homolog (human)	1.005	0.9014	NA
93737	Pard6g	par-6 partitioning defective 6 homolog gamma (C. elegans)	1.005	0.9514	NA
83797	Smarcd1	SWI/SNF related, matrix associated, actin dependent regulator of chromatin, subfamily d, member 1	1.005	0.9759	0.9928
83673	Snhg1	small nucleolar RNA host gene (non-protein coding) 1	1.005	0.9301	NA
77056	Tmco4	transmembrane and coiled-coil domains 4	1.005	0.9678	0.9906
76281	Tax1bp3	Tax1 (human T-cell leukemia virus type I) binding protein 3	1.005	0.9534	NA
76014	Zc3h18	zinc finger CCCH-type containing 18	1.005	0.9616	0.9889
75624	Metap1	methionyl aminopeptidase 1	1.005	0.9505	NA
74026	Msl1	male-specific lethal 1 homolog (Drosophila)	1.005	0.9571	0.9879
73680	Zbtb8a	zinc finger and BTB domain containing 8a	1.005	0.9803	0.9939
73432	1700061N14Rik	RIKEN cDNA 1700061N14 gene	1.005	0.9531	NA
73251	Setd7	SET domain containing (lysine methyltransferase) 7	1.005	0.9384	NA
72667	Zfp444	zinc finger protein 444	1.005	0.9608	0.9886
72665	2810039B14Rik	RIKEN cDNA 2810039B14 gene	1.005	0.9595	0.9883
72003	Synpr	synaptoporin	1.005	0.9232	NA
71910	Ppapdc1b	phosphatidic acid phosphatase type 2 domain containing 1B	1.005	0.9468	NA
71810	Ranbp3	RAN binding protein 3	1.005	0.914	NA
70524	5730414N17Rik	RIKEN cDNA 5730414N17 gene	1.005	0.9623	0.989
69740	Dph5	DPH5 homolog (S. cerevisiae)	1.005	0.9366	NA
69731	Gemin7	gem (nuclear organelle) associated protein 7	1.005	0.8797	NA
69225	Carkd	carbohydrate kinase domain containing	1.005	0.913	NA
69156	Comtd1	catechol-O-methyltransferase domain containing 1	1.005	0.9227	NA
68837	Foxk2	forkhead box K2	1.005	0.9157	NA
68475	Ssna1	Sjogren's syndrome nuclear autoantigen 1	1.005	0.9164	NA
68044	Chac2	ChaC, cation transport regulator homolog 2 (E. coli)	1.005	0.9278	NA
67511	Tmed9	transmembrane emp24 protein transport domain containing 9	1.005	0.9653	0.99
67466	Pdcl	phosducin-like	1.005	0.9504	NA
67264	Ndufb8	NADH dehydrogenase (ubiquinone) 1 beta subcomplex 8	1.005	0.9118	NA
67179	Ccdc25	coiled-coil domain containing 25	1.005	0.9462	NA
66970	Ssbp2	single-stranded DNA binding protein 2	1.005	0.963	0.9893
66590	Farsa	phenylalanyl-tRNA synthetase, alpha subunit	1.005	0.9119	NA
66448	Mrpl20	mitochondrial ribosomal protein L20	1.005	0.9338	NA
66365	Ccdc90b	coiled-coil domain containing 90B	1.005	0.9411	NA
66074	Tmem167	transmembrane protein 167	1.005	0.9196	NA
66070	Cwc15	CWC15 homolog (S. cerevisiae)	1.005	0.9516	NA
66069	Snupn	snurportin 1	1.005	0.9121	NA
65257	Asb3	ankyrin repeat and SOCS box-containing 3	1.005	0.9781	0.9932
64931	Folr4	folate receptor 4 (delta)	1.005	0.9766	0.9928
57317	Srsf4	serine/arginine-rich splicing factor 4	1.005	0.9386	NA
56786	Tmem9b	TMEM9 domain family, member B	1.005	0.9432	NA
54636	Wdr45	WD repeat domain 45	1.005	0.9304	NA
54352	Irx5	Iroquois related homeobox 5 (Drosophila)	1.005	0.9685	0.9908
54342	Gnpnat1	glucosamine-phosphate N-acetyltransferase 1	1.005	0.939	NA

53965	9430099O15Rik	RIKEN cDNA 9430099O15 gene	1.005	0.9664	0.9901
50850	Spast	spastin	1.005	0.943	NA
50760	Fbxo17	F-box protein 17	1.005	0.9478	NA
26372	Clcn6	chloride channel 6	1.005	0.9593	0.9883
23970	Pacsin2	protein kinase C and casein kinase substrate in neurons 2	1.005	0.9195	NA
22215	Ube3a	ubiquitin protein ligase E3A	1.005	0.9626	0.9892
21892	Tll1	tolloid-like	1.005	0.9574	0.9879
20655	Sod1	superoxide dismutase 1, soluble	1.005	0.947	NA
20399	Sh2b1	SH2B adaptor protein 1	1.005	0.9471	NA
20394	Scg5	secretogranin V	1.005	0.9089	NA
20250	Scd2	stearoyl-Coenzyme A desaturase 2	1.005	0.9304	NA
20090	Rps29	ribosomal protein S29	1.005	0.9381	NA
18101	Nmbr	neuromedin B receptor	1.005	0.971	0.9916
18007	Neo1	neogenin	1.005	0.9383	NA
17967	Ncam1	neural cell adhesion molecule 1	1.005	0.9168	NA
17217	Mcm4	minichromosome maintenance deficient 4 homolog (S. cerevisiae)	1.005	0.8984	NA
17210	Mcl1	myeloid cell leukemia sequence 1	1.005	0.9251	NA
16939	Lor	loricrin	1.005	0.938	NA
14654	Glra1	glycine receptor, alpha 1 subunit	1.005	0.9565	0.9877
14077	Fabp3	fatty acid binding protein 3, muscle and heart	1.005	0.8948	NA
14048	Eya1	eyes absent 1 homolog (Drosophila)	1.005	0.964	0.9898
13349	Darc	Duffy blood group, chemokine receptor	1.005	0.9626	0.9892
12915	Atf6b	activating transcription factor 6 beta	1.005	0.9615	0.9889
12890	Cplx2	complexin 2	1.005	0.9355	NA
12795	Plk3	polo-like kinase 3 (Drosophila)	1.005	0.9285	NA
12567	Cdk4	cyclin-dependent kinase 4	1.005	0.9554	0.9872
12050	Bcl2l2	BCL2-like 2	1.005	0.9324	NA
12005	Axin1	axin 1	1.005	0.9235	NA
100504965	LOC100504965	hypothetical LOC100504965	1.004	0.9673	0.9904
666938	Bend4	BEN domain containing 4	1.004	0.9562	NA
627626	3110082D06Rik	RIKEN cDNA 3110082D06 gene	1.004	0.9569	NA
627004	Gm12770	predicted gene 12770	1.004	0.9729	0.9921
625603	Gm6607	40S ribosomal protein S20 pseudogene	1.004	0.9621	0.9889
552877	LOC552877	hypothetical LOC552877	1.004	0.9419	NA
545366	BC026782	cDNA sequence BC026782	1.004	0.9753	0.9926
381356	5930434B04Rik	RIKEN cDNA 5930434B04 gene	1.004	0.9398	NA
381352	Mamdc4	MAM domain containing 4	1.004	0.9653	NA
381085	Tbc1d22b	TBC1 domain family, member 22B	1.004	0.9738	0.9924
360213	Trim46	tripartite motif-containing 46	1.004	0.964	0.9898
330474	Zc3h4	zinc finger CCCH-type containing 4	1.004	0.9472	NA
320926	C730029A08Rik	RIKEN cDNA C730029A08 gene	1.004	0.9649	0.99
319893	A230057D06Rik	RIKEN cDNA A230057D06 gene	1.004	0.9734	0.9922
241289	Gm347	predicted gene 347	1.004	0.9419	NA
236904	Klhl15	kelch-like 15 (Drosophila)	1.004	0.962	NA
233011	Itpkc	inositol 1,4,5-trisphosphate 3-kinase C	1.004	0.9594	NA
228960	Stx16	syntaxin 16	1.004	0.9212	NA
228850	Ralgapb	Ral GTPase activating protein, beta subunit (non-catalytic)	1.004	0.941	NA
225745	Haus1	HAUS augmin-like complex, subunit 1	1.004	0.9621	NA
223739	5031439G07Rik	RIKEN cDNA 5031439G07 gene	1.004	0.9474	NA

219135	Mtmr6	myotubularin related protein 6	1.004	0.944	NA
216156	Wdr18	WD repeat domain 18	1.004	0.9532	NA
213827	Arcn1	archain 1	1.004	0.938	NA
213326	Scyl2	SCY1-like 2 (S. cerevisiae)	1.004	0.9403	NA
170759	Atp13a1	ATPase type 13A1	1.004	0.9532	NA
112419	2010002M12Rik	RIKEN cDNA 2010002M12 gene	1.004	0.9617	0.9889
110593	Prdm2	PR domain containing 2, with ZNF domain	1.004	0.9844	0.9949
109674	Ampd2	adenosine monophosphate deaminase 2	1.004	0.9292	NA
108863	4833420G11Rik	RIKEN cDNA 4833420G11 gene	1.004	0.9613	NA
105727	Slc38a1	solute carrier family 38, member 1	1.004	0.9753	0.9926
104725	1110002B05Rik	RIKEN cDNA 1110002B05 gene	1.004	0.9573	NA
104444	Rexo2	REX2, RNA exonuclease 2 homolog (S. cerevisiae)	1.004	0.9511	NA
94254	Wbscr16	Williams-Beuren syndrome chromosome region 16 homolog (human)	1.004	0.9458	NA
78739	9530062K07Rik	RIKEN cDNA 9530062K07 gene	1.004	0.9754	0.9926
77862	Thyn1	thymocyte nuclear protein 1	1.004	0.947	NA
75909	Tmem49	transmembrane protein 49	1.004	0.9219	NA
75770	Brsk2	BR serine/threonine kinase 2	1.004	0.9598	NA
75415	Arhgap12	Rho GTPase activating protein 12	1.004	0.9506	NA
74434	Sohlh2	spermatogenesis and oogenesis specific basic helix-loop-helix 2	1.004	0.9808	0.994
74334	Ranbp10	RAN binding protein 10	1.004	0.9662	0.9901
74157	Ftsjd2	FtsJ methyltransferase domain containing 2	1.004	0.9282	NA
74018	Als2	amyotrophic lateral sclerosis 2 (juvenile) homolog (human)	1.004	0.9555	NA
73942	Fam151b	family with sequence similarity 151, member B	1.004	0.9273	NA
73161	3110035C09Rik	RIKEN cDNA 3110035C09 gene	1.004	0.9781	0.9932
72711	2810037O22Rik	RIKEN cDNA 2810037O22 gene	1.004	0.9196	NA
72544	Exosc6	exosome component 6	1.004	0.9276	NA
72170	Chchd4	coiled-coil-helix-coiled-coil-helix domain containing 4	1.004	0.949	NA
72096	Mettl10	methyltransferase like 10	1.004	0.9246	NA
71911	Bdh1	3-hydroxybutyrate dehydrogenase, type 1	1.004	0.9266	NA
71679	Atp5h	ATP synthase, H+ transporting, mitochondrial F0 complex, subunit d	1.004	0.917	NA
70248	Dazap1	DAZ associated protein 1	1.004	0.9446	NA
69976	Galk2	galactokinase 2	1.004	0.9436	NA
69726	Smyd3	SET and MYND domain containing 3	1.004	0.9508	NA
68926	Ubap2	ubiquitin-associated protein 2	1.004	0.9479	NA
68215	Fam98b	family with sequence similarity 98, member B	1.004	0.9593	NA
66867	Hmg20a	high mobility group 20A	1.004	0.9476	NA
66861	Dnajc10	DnaJ (Hsp40) homolog, subfamily C, member 10	1.004	0.9264	NA
66817	Tmem170	transmembrane protein 170	1.004	0.9794	0.9935
66775	Ptplad2	protein tyrosine phosphatase-like A domain containing 2	1.004	0.9838	0.9949
66520	2610001J05Rik	RIKEN cDNA 2610001J05 gene	1.004	0.936	NA
66420	Polr2e	polymerase (RNA) II (DNA directed) polypeptide E	1.004	0.9539	NA
64659	Mrps14	mitochondrial ribosomal protein S14	1.004	0.9415	NA
59021	Rab2a	RAB2A, member RAS oncogene family	1.004	0.944	NA
57912	Cdc42se1	CDC42 small effector 1	1.004	0.9595	NA
53872	Caprin1	cell cycle associated protein 1	1.004	0.9623	0.989
52679	E2f7	E2F transcription factor 7	1.004	0.9744	0.9926
52023	Pibf1	progesterone immunomodulatory binding factor 1	1.004	0.9767	0.9928
27426	Nagpa	N-acetylglucosamine-1-phosphodiester alpha-N-acetylglucosaminidase	1.004	0.9709	0.9916
27207	Rps11	ribosomal protein S11	1.004	0.9086	NA

26401	Map3k1	mitogen-activated protein kinase kinase kinase 1	1.004	0.9311	NA
26397	Map2k3	mitogen-activated protein kinase kinase 3	1.004	0.9311	NA
26381	Esrrg	estrogen-related receptor gamma	1.004	0.9521	NA
26356	Ing1	inhibitor of growth family, member 1	1.004	0.9629	0.9893
22648	Zfp11	zinc finger protein 11	1.004	0.9621	NA
22232	Slc35a2	solute carrier family 35 (UDP-galactose transporter), member A2	1.004	0.9422	NA
22187	Ubb	ubiquitin B	1.004	0.9647	0.99
20686	Spa17	sperm autoantigenic protein 17	1.004	0.9566	NA
20671	Sox17	SRY-box containing gene 17	1.004	0.9597	NA
20352	Sema4b	sema domain, immunoglobulin domain (Ig), transmembrane domain (TM) and short cytoplasmic domain, (semaphorin) 4B	1.004	0.9653	0.99
19744	Rheb	Ras homolog enriched in brain	1.004	0.9866	0.9949
19344	Rab5b	RAB5B, member RAS oncogene family	1.004	0.9455	NA
19271	Ptprj	protein tyrosine phosphatase, receptor type, J	1.004	0.9781	0.9932
18126	Nos2	nitric oxide synthase 2, inducible	1.004	0.9741	0.9925
17719	ND4	NADH dehydrogenase subunit 4	1.004	0.9499	NA
17258	Mef2a	myocyte enhancer factor 2A	1.004	0.9854	0.9949
17155	Man1a	mannosidase 1, alpha	1.004	0.9695	0.9911
17118	Marcks	myristoylated alanine rich protein kinase C substrate	1.004	0.9548	NA
16475	Jub	ajuba	1.004	0.9785	0.9933
16319	Incenp	inner centromere protein	1.004	0.951	NA
16162	Il12rb2	interleukin 12 receptor, beta 2	1.004	0.9664	0.9901
14714	Gnrh1	gonadotropin releasing hormone 1	1.004	0.9715	0.9919
14432	Gap43	growth associated protein 43	1.004	0.9557	NA
14020	Evi5	ecotropic viral integration site 5	1.004	0.9326	NA
11991	Hnrnpd	heterogeneous nuclear ribonucleoprotein D	1.004	0.9705	0.9915
11819	Nr2f2	nuclear receptor subfamily 2, group F, member 2	1.004	0.9277	NA
100502698	1700021K19Rik	RIKEN cDNA 1700021K19 gene	1.003	0.964	NA
100040599	Gm15319	predicted gene 15319	1.003	0.9829	0.9947
620986	Gm6195	predicted pseudogene 6195	1.003	0.9661	NA
404331	Olfr1252	olfactory receptor 1252	1.003	0.9884	0.9957
380969	Nckap5l	NCK-associated protein 5-like	1.003	0.985	0.9949
338366	Mia3	melanoma inhibitory activity 3	1.003	0.9706	NA
320678	Iffo1	intermediate filament family orphan 1	1.003	0.9361	NA
320279	C630007K24Rik	RIKEN cDNA C630007K24 gene	1.003	0.9719	NA
319601	Zfp653	zinc finger protein 653	1.003	0.9498	NA
319169	Hist1h2ak	histone cluster 1, H2ak	1.003	0.9585	NA
270106	Rpl13	ribosomal protein L13	1.003	0.9605	NA
269401	Znf512b	zinc finger protein 512B	1.003	0.9472	NA
269003	Sap130	Sin3A associated protein	1.003	0.9679	0.9906
244219	Zfp668	zinc finger protein 668	1.003	0.9764	0.9928
237858	Tusc5	tumor suppressor candidate 5	1.003	0.9773	0.993
233826	Palb2	partner and localizer of BRCA2	1.003	0.9727	NA
228889	Ddx27	DEAD (Asp-Glu-Ala-Asp) box polypeptide 27	1.003	0.9658	NA
223870	Senp1	SUMO1/sentrin specific peptidase 1	1.003	0.9825	0.9947
223745	Gm4825	predicted pseudogene 4825	1.003	0.9399	NA
218613	Mier3	mesoderm induction early response 1, family member 3	1.003	0.9665	NA
211401	Mtss1	metastasis suppressor 1	1.003	0.9749	NA
105246	Brd9	bromodomain containing 9	1.003	0.9696	NA
94229	Slc4a10	solute carrier family 4, sodium bicarbonate cotransporter-like, member 10	1.003	0.9917	0.9969

83922	Tsga14	testis specific gene A14	1.003	0.9557	NA
78935	Saal1	serum amyloid A-like 1	1.003	0.9593	NA
78795	Armc9	armadillo repeat containing 9	1.003	0.9364	NA
77462	Tmem116	transmembrane protein 116	1.003	0.9645	NA
76893	Lass2	LAG1 homolog, ceramide synthase 2	1.003	0.9601	NA
76108	Rap2a	RAS related protein 2a	1.003	0.9341	NA
75552	Paqr9	progestin and adipoQ receptor family member IX	1.003	0.9734	0.9922
75444	1700011I03Rik	RIKEN cDNA 1700011I03 gene	1.003	0.9806	0.994
74213	Rbm26	RNA binding motif protein 26	1.003	0.9553	NA
74062	Speer8-ps1	spermatogenesis associated glutamate (E)-rich protein 8, pseudogene 1	1.003	0.9804	0.9939
73941	4930412L05Rik	RIKEN cDNA 4930412L05 gene	1.003	0.973	0.9921
72947	Agxt2l2	alanine-glyoxylate aminotransferase 2-like 2	1.003	0.9566	NA
71752	Gtf3c2	general transcription factor IIIC, polypeptide 2, beta	1.003	0.9763	0.9928
71041	Pcgf6	polycomb group ring finger 6	1.003	0.965	NA
70155	Ogfrl1	opioid growth factor receptor-like 1	1.003	0.975	NA
69256	Zfp397	zinc finger protein 397	1.003	0.9862	0.9949
68327	0610007P22Rik	RIKEN cDNA 0610007P22 gene	1.003	0.9585	NA
67923	Tceb1	transcription elongation factor B (SIII), polypeptide 1	1.003	0.9414	NA
67639	4930474N09Rik	RIKEN cDNA 4930474N09 gene	1.003	0.9812	0.9942
66966	Trit1	tRNA isopentenyltransferase 1	1.003	0.9617	NA
66840	Wdr45l	Wdr45 like	1.003	0.9653	NA
66810	Rbm22	RNA binding motif protein 22	1.003	0.9647	NA
66314	Tpd52l2	tumor protein D52-like 2	1.003	0.9528	NA
66237	Atp6v1g2	ATPase, H+ transporting, lysosomal V1 subunit G2	1.003	0.9768	0.9928
58867	Syngr4	synaptogyrin 4	1.003	0.9811	0.9942
58807	Slco1c1	solute carrier organic anion transporter family, member 1c1	1.003	0.9725	NA
58230	Rnf8	ring finger protein 8	1.003	0.9328	NA
58212	Srrm3	serine/arginine repetitive matrix 3	1.003	0.9721	NA
56397	Morf4l2	mortality factor 4 like 2	1.003	0.9412	NA
56292	Naa10	N(alpha)-acetyltransferase 10, NatA catalytic subunitNalpha acetyltransferase 10	1.003	0.9574	NA
56177	Olfm1	olfactomedin 1	1.003	0.9757	NA
54198	Snx3	sorting nexin 3	1.003	0.968	NA
54126	Arhgef7	Rho guanine nucleotide exchange factor (GEF7)	1.003	0.9629	NA
27965	Spg21	spastic paraplegia 21 homolog (human)	1.003	0.9699	NA
27425	Atp5l	ATP synthase, H+ transporting, mitochondrial F0 complex, subunit g	1.003	0.9758	0.9928
27008	Micall1	microtubule associated monoxygenase, calponin and LIM domain containing -like 1	1.003	0.9545	NA
26378	Decr2	2-4-dienoyl-Coenzyme A reductase 2, peroxisomal	1.003	0.9623	NA
26374	Rfwd2	ring finger and WD repeat domain 2	1.003	0.9509	NA
23950	Dnajb6	DnaJ (Hsp40) homolog, subfamily B, member 6	1.003	0.9812	0.9942
23825	Banf1	barrier to autointegration factor 1	1.003	0.966	NA
22755	Zfp93	zinc finger protein 93	1.003	0.9561	NA
22184	Zrsr2	zinc finger (CCCH type), RNA binding motif and serine/arginine rich 2	1.003	0.9549	NA
21888	Tle4	transducin-like enhancer of split 4, homolog of Drosophila E(spl)	1.003	0.967	NA
20853	Stau1	staufen (RNA binding protein) homolog 1 (Drosophila)	1.003	0.9646	NA
20684	Sp100	nuclear antigen Sp100	1.003	0.9815	0.9943
20444	St3gal2	ST3 beta-galactoside alpha-2,3-sialyltransferase 2	1.003	0.9364	NA
19899	Rpl18	ribosomal protein L18	1.003	0.9519	NA
19782	Rmrp	RNA component of mitochondrial RNAase P	1.003	0.9884	0.9957
19729	Slc50a1	solute carrier family 50 (sugar transporter), member 1	1.003	0.9777	0.9932

19651	Rbl2	retinoblastoma-like 2	1.003	0.9753	0.9926
19349	Rab7	RAB7, member RAS oncogene family	1.003	0.9478	NA
19325	Rab10	RAB10, member RAS oncogene family	1.003	0.9468	NA
19205	Ptbp1	polypyrimidine tract binding protein 1	1.003	0.9676	NA
19060	Ppp5c	protein phosphatase 5, catalytic subunit	1.003	0.9928	0.9975
18711	Pikfyve	phosphoinositide kinase, FYVE finger containing	1.003	0.9668	NA
18679	Phka1	phosphorylase kinase alpha 1	1.003	0.9708	0.9916
18597	Pdha1	pyruvate dehydrogenase E1 alpha 1	1.003	0.9631	NA
17436	Me1	malic enzyme 1, NADP(+)-dependent, cytosolic	1.003	0.9722	0.992
17173	Ascl2	achaete-scute complex homolog 2 (Drosophila)	1.003	0.9651	NA
16970	Lrmp	lymphoid-restricted membrane protein	1.003	0.971	NA
16412	Itgb1	integrin beta 1 (fibronectin receptor beta)	1.003	0.9822	0.9946
14936	Gys1	glycogen synthase 1, muscle	1.003	0.9796	0.9935
14248	Flii	flightless I homolog (Drosophila)	1.003	0.9776	0.9932
14229	Fkbp5	FK506 binding protein 5	1.003	0.9498	NA
14158	Fert2	fer (fms/fps related) protein kinase, testis specific 2	1.003	0.9559	NA
13855	Epn2	epsin 2	1.003	0.9632	NA
12994	Csn3	casein kappa	1.003	0.9795	0.9935
12296	Cacnb2	calcium channel, voltage-dependent, beta 2 subunit	1.003	0.9707	0.9916
11877	Arvcf	armadillo repeat gene deleted in velo-cardio-facial syndrome	1.003	0.9613	NA
100125931	A130049A11Rik	RIKEN cDNA A130049A11 gene	1.002	0.9844	0.9949
100042583	Gm10268	predicted gene 10268	1.002	0.9716	NA
100040298	Gm15501	predicted pseudogene 15501	1.002	0.9829	0.9947
666244	Tmsb15b1	thymosin beta 15b1	1.002	0.9521	NA
619883	Gm6109	predicted gene 6109	1.002	0.9485	NA
329559	Zfp335	zinc finger protein 335	1.002	0.9854	0.9949
320473	Heatr5b	HEAT repeat containing 5B	1.002	0.9861	0.9949
320111	Prr18	proline rich region 18	1.002	0.9829	0.9947
319475	Zfp672	zinc finger protein 672	1.002	0.9741	NA
278279	Tmtc2	transmembrane and tetratricopeptide repeat containing 2	1.002	0.9675	NA
276846	Pigs	phosphatidylinositol glycan anchor biosynthesis, class S	1.002	0.973	NA
269295	Rtn4rl2	reticulon 4 receptor-like 2	1.002	0.9842	0.9949
240055	Neurl1b	neuralized homolog 1b (Drosophila)	1.002	0.9872	0.9951
239393	Lrp12	low density lipoprotein-related protein 12	1.002	0.9722	NA
234959	Med17	mediator complex subunit 17	1.002	0.9657	NA
234776	Atmin	ATM interactor	1.002	0.9762	NA
231858	Radil	Ras association and DIL domains	1.002	0.9842	0.9949
231642	Alkbh2	alkB, alkylation repair homolog 2 (E. coli)	1.002	0.975	NA
231571	Rpap2	RNA polymerase II associated protein 2	1.002	0.9592	NA
230822	A330049M08Rik	RIKEN cDNA A330049M08 gene	1.002	0.9911	0.9966
230700	Foxj3	forkhead box J3	1.002	0.9693	NA
230259	E130308A19Rik	RIKEN cDNA E130308A19 gene	1.002	0.976	NA
229725	Clcc1	chloride channel CLIC-like 1	1.002	0.9663	NA
227634	Camsap1	calmodulin regulated spectrin-associated protein 1	1.002	0.9657	NA
226856	Lpgat1	lysophosphatidylglycerol acyltransferase 1	1.002	0.9783	NA
224019	Tmem191c	transmembrane protein 191C	1.002	0.9803	NA
212390	Klhl32	kelch-like 32 (Drosophila)	1.002	0.9837	0.9948
209456	Trp53bp2	transformation related protein 53 binding protein 2	1.002	0.9921	0.9971
192232	Hps4	Hermansky-Pudlak syndrome 4 homolog (human)	1.002	0.9814	NA

109129	Mmadhc	methylmalonic aciduria (cobalamin deficiency) cblD type, with homocystinuria	1.002	0.9727	NA
107971	Frs3	fibroblast growth factor receptor substrate 3	1.002	0.9969	0.9989
102436	Lars2	leucyl-tRNA synthetase, mitochondrial	1.002	0.9688	NA
100756	Usp30	ubiquitin specific peptidase 30	1.002	0.9652	NA
94282	Sfxn5	sideroflexin 5	1.002	0.9694	NA
83603	Elovl4	elongation of very long chain fatty acids (FEN1/Elo2, SUR4/Elo3, yeast)-like 4	1.002	0.974	NA
80281	Cttnbp2nl	CTTNBP2 N-terminal like	1.002	0.9629	NA
79043	Spsb3	splA/ryanodine receptor domain and SOCS box containing 3	1.002	0.9669	NA
76880	6430411K18Rik	RIKEN cDNA 6430411K18 gen	1.002	0.984	0.9949
76357	Trmt5	TRM5 tRNA methyltransferase 5 homolog (S. cerevisiae)	1.002	0.9721	NA
75625	Mageh1	melanoma antigen, family H, 1	1.002	0.9733	NA
75580	Zbtb4	zinc finger and BTB domain containing 4	1.002	0.9499	NA
75497	Fabp12	fatty acid binding protein 12	1.002	0.9625	NA
74255	Smu1	smu-1 suppressor of mec-8 and unc-52 homolog (C. elegans)	1.002	0.9729	NA
74164	Nfx1	nuclear transcription factor, X-box binding 1	1.002	0.9571	NA
73178	Wasl	Wiskott-Aldrich syndrome-like (human)	1.002	0.9808	NA
72674	Adipor1	adiponectin receptor 1	1.002	0.9779	NA
72480	Tspyl4	TSPY-like 4	1.002	0.9833	NA
72345	Fam123b	family with sequence similarity 123, member B	1.002	0.9785	NA
72054	Cyp4f18	cytochrome P450, family 4, subfamily f, polypeptide 18	1.002	0.9867	0.995
71805	Nup93	nucleoporin 93	1.002	0.9829	0.9947
70974	Pgm2l1	phosphoglucomutase 2-like 1	1.002	0.9637	NA
70797	Ankib1	ankyrin repeat and IBR domain containing 1	1.002	0.9555	NA
70737	Cgn	cingulin	1.002	0.982	0.9946
70675	Vcpip1	valosin containing protein (p97)/p47 complex interacting protein 1	1.002	0.9671	NA
69163	Mrpl44	mitochondrial ribosomal protein L44	1.002	0.9591	NA
68980	Wdr53	WD repeat domain 53	1.002	0.971	NA
68735	Mrps18c	mitochondrial ribosomal protein S18C	1.002	0.9677	NA
68365	Rab14	RAB14, member RAS oncogene family	1.002	0.9602	NA
68273	Pomgnt1	protein O-linked mannose beta1,2-N-acetylglucosaminyltransferase	1.002	0.9755	NA
68219	Nudt21	nudix (nucleoside diphosphate linked moiety X)-type motif 21	1.002	0.968	NA
67841	Atg3	autophagy-related 3 (yeast)	1.002	0.9798	NA
67158	Sft2d3	SFT2 domain containing 3	1.002	0.9827	0.9947
67043	Syap1	synapse associated protein 1	1.002	0.9646	NA
67016	Tbc1d2b	TBC1 domain family, member 2B	1.002	0.9861	0.9949
66401	Nudt2	nudix (nucleoside diphosphate linked moiety X)-type motif 2	1.002	0.9704	NA
66308	2810021B07Rik	RIKEN cDNA 2810021B07 gene	1.002	0.9667	NA
66152	Uqcr10	ubiquinol-cytochrome c reductase, complex III subunit X	1.002	0.9641	NA
66046	Ndufb5	NADH dehydrogenase (ubiquinone) 1 beta subcomplex, 5	1.002	0.9608	NA
65105	Arl6ip4	ADP-ribosylation factor-like 6 interacting protein 4	1.002	0.9576	NA
64704	Htra2	HtrA serine peptidase 2	1.002	0.9734	NA
64383	Sirt2	sirtuin 2 (silent mating type information regulation 2, homolog) 2 (S. cerevisiae)	1.002	0.985	0.9949
57440	Ehd3	EH-domain containing 3	1.002	0.9779	NA
56749	Dhodh	dihydroorotate dehydrogenase	1.002	0.9571	NA
53761	Prrc2a	proline-rich coiled-coil 2A	1.002	0.9868	0.995
50787	Hs6st3	heparan sulfate 6-O-sulfotransferase 3	1.002	0.9916	0.9969
27393	Mrpl39	mitochondrial ribosomal protein L39	1.002	0.9757	NA
27374	Prmt5	protein arginine N-methyltransferase 5	1.002	0.9843	NA
23794	Adamts5	a disintegrin-like and metallopeptidase (reprolysin type) with thrombospondin type 1 motif, 5 (aggrecanase-2)	1.002	0.9785	NA

22412	Wnt9b	wingless-type MMTV integration site 9B	1.002	0.9909	0.9965
22278	Usf1	upstream transcription factor 1	1.002	0.9853	0.9949
22032	Traf4	TNF receptor associated factor 4	1.002	0.9814	0.9942
19941	Rpl26	ribosomal protein L26	1.002	0.9813	NA
19697	Rela	v-rel reticuloendotheliosis viral oncogene homolog A (avian)	1.002	0.9798	NA
19043	Ppm1b	protein phosphatase 1B, magnesium dependent, beta isoform	1.002	0.9762	NA
19027	Sypl	synaptophysin-like protein	1.002	0.9737	NA
18631	Pex11a	peroxisomal biogenesis factor 11 alpha	1.002	0.9725	NA
17762	Mapt	microtubule-associated protein tau	1.002	0.9764	NA
16451	Jak1	Janus kinase 1	1.002	0.9747	NA
15288	Hmbs	hydroxymethylbilane synthase	1.002	0.9728	NA
15081	H3f3b	H3 histone, family 3B	1.002	0.9623	NA
14612	Gja4	gap junction protein, alpha 4	1.002	0.9904	0.9964
13527	Dtna	dystrobrevin alpha	1.002	0.9657	NA
13000	Csnk2a2	casein kinase 2, alpha prime polypeptide	1.002	0.9739	NA
12916	Crem	cAMP responsive element modulator	1.002	0.9706	NA
12390	Cav2	caveolin 2	1.002	0.9883	0.9957
12351	Car4	carbonic anhydrase 4	1.002	0.9882	0.9957
12153	Bmp1	bone morphogenetic protein 1	1.002	0.9885	0.9957
11441	Chrna7	cholinergic receptor, nicotinic, alpha polypeptide 7	1.002	0.9859	0.9949
380836	Mrs2	MRS2 magnesium homeostasis factor homolog (S. cerevisiae)	1.001	0.9919	0.9971
338349	Cntln	centlein, centrosomal protein	1.001	0.9868	NA
269116	Nfasc	neurofascin	1.001	0.9961	0.9986
268935	Scube3	signal peptide, CUB domain, EGF-like 3	1.001	0.9975	0.9991
245174	Zfp937	zinc finger protein 937	1.001	0.983	NA
243621	Iqsec3	IQ motif and Sec7 domain 3	1.001	0.9807	NA
240034	Zfp760	zinc finger protein 760	1.001	0.987	NA
239759	Liph	lipase, member H	1.001	0.9952	0.998
236899	Pcyt1b	phosphate cytidylyltransferase 1, choline, beta isoform	1.001	0.9927	NA
234356	Csgalnact1	chondroitin sulfate N-acetylgalactosaminyltransferase 1	1.001	0.9791	NA
233332	Adamts17	a disintegrin-like and metallopeptidase (reprolysin type) with thrombospondin type 1 motif, 17	1.001	0.9931	0.9975
233274	Siglech	sialic acid binding Ig-like lectin H	1.001	0.9941	0.9979
232491	Pyroxd1	pyridine nucleotide-disulphide oxidoreductase domain 1	1.001	0.9829	NA
226178	D19Wsu162e	DNA segment, Chr 19, Wayne State University 162, expressed	1.001	0.9925	NA
225912	Cybasc3	cytochrome b, ascorbate dependent 3	1.001	0.9891	0.996
216705	Clint1	clathrin interactor 1	1.001	0.985	NA
215449	Rap1b	RAS related protein 1b	1.001	0.9797	NA
212862	Chpt1	choline phosphotransferase 1	1.001	0.9836	NA
208718	Dis3l2	DIS3 mitotic control homolog (S. cerevisiae)-like 2	1.001	0.9906	NA
208501	1810043H04Rik	RIKEN cDNA 1810043H04 gene	1.001	0.9977	0.9991
207615	Wdr37	WD repeat domain 37	1.001	0.9836	NA
193286	BC049762	cDNA sequence BC049762	1.001	0.9935	NA
110332	4921523A10Rik	RIKEN cDNA 4921523A10 gene	1.001	0.992	0.9971
109241	Mbd5	methyl-CpG binding domain protein 5	1.001	0.9848	NA
109079	Sephs1	selenophosphate synthetase 1	1.001	0.9757	NA
108067	Eif2b3	eukaryotic translation initiation factor 2B, subunit 3	1.001	0.9914	NA
106059	A430088P11Rik	RIKEN cDNA A430088P11 gene	1.001	0.9969	0.9989
105734	Tigd5	tigger transposable element derived 5	1.001	0.9968	0.9989
104318	Csnk1d	casein kinase 1, delta	1.001	0.9895	NA

100608	Noc4l	nucleolar complex associated 4 homolog (S. cerevisiae)	1.001	0.9937	0.9978
98999	Znfx1	zinc finger, NFX1-type containing 1	1.001	0.9848	NA
94253	Hecw1	HECT, C2 and WW domain containing E3 ubiquitin protein ligase 1	1.001	0.9952	0.998
85308	Fam158a	family with sequence similarity 158, member A	1.001	0.9845	NA
78786	4833439F03Rik	RIKEN cDNA 4833439F03 gene	1.001	0.9858	NA
77771	Csrnp3	cysteine-serine-rich nuclear protein 3	1.001	0.9949	NA
77579	Myh10	myosin, heavy polypeptide 10, non-muscle	1.001	0.9889	NA
76826	Nubpl	nucleotide binding protein-like	1.001	0.9881	NA
76740	Efr3a	EFR3 homolog A (S. cerevisiae)	1.001	0.9852	NA
76457	Ccdc134	coiled-coil domain containing 134	1.001	0.9879	NA
75764	Slx1b	SLX1 structure-specific endonuclease subunit homolog B (S. cerevisiae)	1.001	0.9909	NA
74235	1700020G03Rik	RIKEN cDNA 1700020G03 gene	1.001	0.9923	0.9972
73916	Ift57	intraflagellar transport 57 homolog (Chlamydomonas)	1.001	0.9784	NA
73173	Pcdh18	protocadherin 18	1.001	0.9888	NA
72881	Zdhhc4	zinc finger, DHHC domain containing 4	1.001	0.9944	NA
72699	Lime1	Lck interacting transmembrane adaptor 1	1.001	0.986	NA
72545	2700008G24Rik	RIKEN cDNA 2700008G24 gene	1.001	0.9817	NA
72454	Ccdc71	coiled-coil domain containing 71	1.001	0.9945	0.9979
70552	Lrrc56	leucine rich repeat containing 56	1.001	0.995	0.998
69934	Rg9mtd3	RNA (guanine-9-) methyltransferase domain containing 3	1.001	0.9787	NA
68836	Mrpl52	mitochondrial ribosomal protein L52	1.001	0.9884	NA
68316	Apoo	apolipoprotein O	1.001	0.99	NA
68079	Pdcd2l	programmed cell death 2-like	1.001	0.9838	NA
67898	Pef1	penta-EF hand domain containing 1	1.001	0.9917	NA
66836	Tmem223	transmembrane protein 223	1.001	0.9818	NA
66730	4921531C22Rik	RIKEN cDNA 4921531C22 gene	1.001	0.9837	NA
66277	Klf15	Kruppel-like factor 15	1.001	0.9961	0.9986
66236	1500011B03Rik	RIKEN cDNA 1500011B03 gene	1.001	0.985	NA
66223	Mrpl35	mitochondrial ribosomal protein L35	1.001	0.9906	NA
66077	Aurkaip1	aurora kinase A interacting protein 1	1.001	0.9877	NA
58237	Nkain4	Na+/K+ transporting ATPase interacting 4	1.001	0.9863	NA
56551	Txn2	thioredoxin 2	1.001	0.9845	NA
56503	Ankrd49	ankyrin repeat domain 49	1.001	0.9853	NA
56365	Clcnkb	chloride channel Kb	1.001	0.9941	0.9979
54188	Cpsf4	cleavage and polyadenylation specific factor 4	1.001	0.994	0.9979
51885	Tubgcp4	tubulin, gamma complex associated protein 4	1.001	0.9881	NA
50754	Fbxw7	F-box and WD-40 domain protein 7	1.001	0.9824	NA
21892	Tll1	tolloid-like	1.001	0.985	NA
20775	Sqle	squalene epoxidase	1.001	0.9865	NA
20022	Polr2j	polymerase (RNA) II (DNA directed) polypeptide J	1.001	0.9742	NA
19946	Rpl30	ribosomal protein L30	1.001	0.9878	NA
19663	Rbpms	RNA binding protein gene with multiple splicing	1.001	0.9934	0.9977
19201	Pstpip2	proline-serine-threonine phosphatase-interacting protein 2	1.001	0.9914	NA
18751	Prkcb	protein kinase C, beta	1.001	0.979	NA
18710	Pik3r3	phosphatidylinositol 3 kinase, regulatory subunit, polypeptide 3 (p55)	1.001	0.9835	NA
18035	Nfkbia	nuclear factor of kappa light polypeptide gene enhancer in B-cells inhibitor, alpha	1.001	0.9807	NA
17967	Ncam1	neural cell adhesion molecule 1	1.001	0.9953	0.998
17283	Men1	multiple endocrine neoplasia 1	1.001	0.9941	NA
17215	Mcm3	minichromosome maintenance deficient 3 (S. cerevisiae)	1.001	0.9848	NA

17151	Ccndbp1	cyclin D-type binding-protein 1	1.001	0.9826	NA
16869	Lhx1	LIM homeobox protein 1	1.001	0.9883	NA
16847	Lepr	leptin receptor	1.001	0.9976	0.9991
16510	Kcnh1	potassium voltage-gated channel, subfamily H (eag-related), member 1	1.001	0.9935	0.9977
16000	Igf1	insulin-like growth factor 1	1.001	0.9897	NA
15081	H3f3b	H3 histone, family 3B	1.001	0.9921	NA
13982	Esr1	estrogen receptor 1 (alpha)	1.001	0.9943	0.9979
13841	Epha7	Eph receptor A7	1.001	0.9857	NA
13194	Ddb1	damage specific DNA binding protein 1	1.001	0.9829	NA
12839	Col9a1	collagen, type IX, alpha 1	1.001	0.9969	0.9989
12709	Ckb	creatine kinase, brain	1.001	0.9878	NA
12631	Cfl1	cofilin 1, non-muscle	1.001	0.9953	0.998
12465	Cct5	chaperonin containing Tcp1, subunit 5 (epsilon)	1.001	0.9884	NA
100505024	LOC100505024	homeobox protein TGIF2-like	1	0.9993	0.9997
100041143	Gm3161	predicted gene 3161	1	0.9998	1
677368	LOC677368	hypothetical protein LOC677368	1	0.9989	0.9997
624710	Gm6524	katanin p60 (ATPase-containing) subunit A1 pseudogene	1	0.9997	1
433486	Gm14151	predicted gene 14151	1	0.999	0.9997
269717	Orai2	ORAI calcium release-activated calcium modulator 2	1	0.995	NA
269060	Dagla	diacylglycerol lipase, alpha	1	0.9993	0.9997
268469	Zfp652	zinc finger protein 652	1	0.9957	NA
246779	Il27	interleukin 27	1	1	1
242083	Ppm1l	protein phosphatase 1 (formerly 2C)-like	1	0.9936	NA
235582	Glyctk	glycerate kinase	1	0.9994	NA
231855	C330006K01Rik	RIKEN cDNA C330006K01 gene	1	0.9989	NA
225898	Eml3	echinoderm microtubule associated protein like 3	1	0.9994	NA
225876	Kdm2a	lysine (K)-specific demethylase 2A	1	0.9974	0.9991
214987	Chtf8	CTF8, chromosome transmission fidelity factor 8 homolog (S. cerevisiae)	1	0.9987	NA
214290	Zcchc6	zinc finger, CCHC domain containing 6	1	0.9941	NA
213760	Prepl	prolyl endopeptidase-like	1	0.9998	NA
209966	Pgbd5	piggyBac transposable element derived 5	1	0.9994	NA
191578	Helq	helicase, POLQ-like	1	0.9975	NA
108071	Grm5	glutamate receptor, metabotropic 5	1	0.9992	0.9997
104318	Csnk1d	casein kinase 1, delta	1	0.989	NA
78798	Eml4	echinoderm microtubule associated protein like 4	1	0.998	NA
78658	Ncapd3	non-SMC condensin II complex, subunit D3	1	0.9997	NA
78655	Eif3j	eukaryotic translation initiation factor 3, subunit J	1	0.9956	NA
74316	Isca2	iron-sulfur cluster assembly 2 homolog (S. cerevisiae)	1	0.9994	NA
73608	Marveld3	MARVEL (membrane-associating) domain containing 3	1	0.9985	NA
73261	1700037C18Rik	RIKEN cDNA 1700037C18 gene	1	0.998	NA
72440	5930416I19Rik	RIKEN cDNA 5930416I19 gene	1	0.9951	NA
71745	Cul2	cullin 2	1	0.9926	NA
71132	Cabyr	calcium-binding tyrosine-(Y)-phosphorylation regulated (fibrousheathin 2)	1	0.9997	1
70829	Ccdc93	coiled-coil domain containing 93	1	0.9989	0.9997
69975	2810405F17Rik	RIKEN cDNA 2810405F17 gene	1	0.9964	NA
69757	Leng1	leukocyte receptor cluster (LRC) member 1	1	0.9952	NA
69536	Hemk1	HemK methyltransferase family member 1	1	0.9975	NA
68463	Mrpl14	mitochondrial ribosomal protein L14	1	0.9978	NA
68436	Rpl34	ribosomal protein L34	1	0.9948	NA

67674	Trmt112	tRNA methyltransferase 11-2 homolog (S. cerevisiae)	1	0.9994	NA
67487	Dhx40	DEAH (Asp-Glu-Ala-His) box polypeptide 40	1	0.998	NA
67210	Gatad1	GATA zinc finger domain containing 1	1	0.9924	NA
67131	Acbd4	acyl-Coenzyme A binding domain containing 4	1	0.9979	NA
67099	Fam119a	family with sequence similarity 119, member A	1	0.9981	NA
66511	2500003M10Rik	RIKEN cDNA 2500003M10 gene	1	0.9926	NA
66273	1810020D17Rik	RIKEN cDNA 1810020D17 gene	1	0.9974	NA
66169	Tomm7	translocase of outer mitochondrial membrane 7 homolog (yeast)	1	0.9991	NA
66152	Uqcr10	ubiquinol-cytochrome c reductase, complex III subunit X	1	0.9939	NA
64451	Dip2a	DIP2 disco-interacting protein 2 homolog A (Drosophila)	1	0.9986	NA
59008	Anapc5	anaphase-promoting complex subunit 5	1	0.9951	NA
57783	Tnip1	TNFAIP3 interacting protein 1	1	0.9996	NA
57754	Cend1	cell cycle exit and neuronal differentiation 1	1	0.9999	NA
54637	Praf2	PRA1 domain family 2	1	0.9992	NA
52874	D19Bwg1357e	DNA segment, Chr 19, Brigham & Women's Genetics 1357 expressed	1	0.9984	0.9996
30058	Timm8a1	translocase of inner mitochondrial membrane 8 homolog a1 (yeast)	1	0.9916	NA
27416	Abcc5	ATP-binding cassette, sub-family C (CFTR/MRP), member 5	1	0.9971	NA
22359	Vldlr	very low density lipoprotein receptor	1	0.9996	NA
17152	Mak	male germ cell-associated kinase	1	0.999	NA
15904	Id4	inhibitor of DNA binding 4	1	0.9952	NA
14768	Lancl1	LanC (bacterial lantibiotic synthetase component C)-like 1	1	0.9908	NA
13839	Epha5	Eph receptor A5	1	0.997	NA
12709	Ckb	creatine kinase, brain	1	0.999	NA
100040632	AA684185	expressed sequence AA684185	0.9999	0.9989	NA
330657	Prss53	protease, serine, 53	0.9999	0.9995	0.9998
244962	Snx14	sorting nexin 14	0.9999	0.9978	NA
140858	Wdr5	WD repeat domain 5	0.9999	0.998	NA
71373	Prr16	proline rich 16	0.9999	0.9983	NA
68742	Tmem219	transmembrane protein 219	0.9999	0.9984	NA
20932	Surf4	surfeit gene 4	0.9999	0.9983	NA
432768	Gm5451	predicted gene 5451	0.9998	0.9987	0.9997
330671	B4galnt4	beta-1,4-N-acetyl-galactosaminyl transferase 4	0.9998	0.9985	0.9996
224530	Acat3	acetyl-Coenzyme A acetyltransferase 3	0.9998	0.9979	NA
211253	Mtrf1	mitochondrial translational release factor 1	0.9998	0.9978	NA
170760	Acbd3	acyl-Coenzyme A binding domain containing 3	0.9998	0.9974	NA
13830	Stom	stomatin	0.9998	0.998	NA
270076	Gcdh	glutaryl-Coenzyme A dehydrogenase	0.9997	0.9972	NA
214968	Sema6d	sema domain, transmembrane domain (TM), and cytoplasmic domain, (semaphorin) 6D	0.9997	0.9985	0.9996
99237	Tm9sf4	transmembrane 9 superfamily protein member 4	0.9997	0.9974	0.9991
78617	Cstad	CSA-conditional, T cell activation-dependent protein	0.9997	0.9959	NA
74008	Arsg	arylsulfatase G	0.9997	0.9925	NA
69137	2200002K05Rik	RIKEN cDNA 2200002K05 gene	0.9997	0.9964	NA
67832	Brix1	BRX1, biogenesis of ribosomes, homolog (S. cerevisiae)	0.9997	0.9957	NA
66231	Thoc7	THO complex 7 homolog (Drosophila)	0.9997	0.9977	0.9991
21762	Psmd2	proteasome (prosome, macropain) 26S subunit, non-ATPase, 2	0.9997	0.9967	NA
17769	Mthfr	5,10-methylenetetrahydrofolate reductase	0.9997	0.9955	NA
407812	Zfp941	zinc finger protein 941	0.9996	0.9965	0.9988
330463	Zfp78	zinc finger protein 78	0.9996	0.9897	NA
229007	Zgpat	zinc finger, CCCH-type with G patch domain	0.9996	0.9955	NA

226182	Taf5	TAF5 RNA polymerase II, TATA box binding protein (TBP)-associated factor	0.9996	0.9936	NA
225207	Zfp521	zinc finger protein 521	0.9996	0.9947	NA
72591	2700022O18Rik	RIKEN cDNA 2700022O18 gene	0.9996	0.9975	0.9991
56278	Gkap1	G kinase anchoring protein 1	0.9996	0.9925	NA
14453	Gas2	growth arrest specific 2	0.9996	0.9936	NA
12728	Clcn5	chloride channel 5	0.9996	0.9963	0.9986
268859	Rbfox1	RNA binding protein, fox-1 homolog (C. elegans) 1	0.9995	0.9946	NA
258272	Olfr1402	olfactory receptor 1402	0.9995	0.998	0.9992
239554	Foxred2	FAD-dependent oxidoreductase domain containing 2	0.9995	0.9923	NA
73830	Eif3k	eukaryotic translation initiation factor 3, subunit K	0.9995	0.9924	NA
72325	1300018I17Rik	RIKEN cDNA 1300018I17 gene	0.9995	0.9924	NA
66766	4933425O20Rik	RIKEN cDNA 4933425O20 gene	0.9995	0.9964	0.9986
52036	Ppp6r3	protein phosphatase 6, regulatory subunit 3	0.9995	0.9949	NA
26436	Psg16	pregnancy specific glycoprotein 16	0.9995	0.9923	NA
22196	Ube2i	ubiquitin-conjugating enzyme E2I	0.9995	0.9928	NA
20085	Rps19	ribosomal protein S19	0.9995	0.9924	NA
20018	Polr1d	polymerase (RNA) I polypeptide D	0.9995	0.9934	NA
319211	Nol4	nucleolar protein 4	0.9994	0.9911	NA
210982	BC032203	cDNA sequence BC032203	0.9994	0.9881	NA
76186	6330563C09Rik	RIKEN cDNA 6330563C09 gene	0.9994	0.9882	NA
74043	Pex26	peroxisomal biogenesis factor 26	0.9994	0.9894	NA
67713	Dnajc19	DnaJ (Hsp40) homolog, subfamily C, member 19	0.9994	0.993	NA
66578	2610039C10Rik	RIKEN cDNA 2610039C10 gene	0.9994	0.995	0.998
50932	Mink1	misshapen-like kinase 1 (zebrafish)	0.9994	0.9955	0.9981
20301	Ccl27a	chemokine (C-C motif) ligand 27A	0.9994	0.9903	NA
17095	Lyl1	lymphoblastomic leukemia 1	0.9994	0.9962	0.9986
15077	Hist2h3c1	histone cluster 2, H3c1	0.9994	0.9946	NA
11910	Atf3	activating transcription factor 3	0.9994	0.9891	NA
231050	Galnt11	UDP-N-acetyl-alpha-D-galactosamine:polypeptide N-acetylgalactosaminyltransferase 11	0.9993	0.9943	0.9979
71361	Aifm2	apoptosis-inducing factor, mitochondrion-associated 2	0.9993	0.9924	NA
67053	Rpp14	ribonuclease P 14 subunit (human)	0.9993	0.9916	NA
66591	Mad2l1bp	MAD2L1 binding protein	0.9993	0.9897	NA
64453	Zfp280b	zinc finger protein 280B	0.9993	0.9884	NA
22591	Xpc	xeroderma pigmentosum, complementation group C	0.9993	0.9845	NA
22213	Ube2g2	ubiquitin-conjugating enzyme E2G 2	0.9993	0.9901	NA
20215	Sag	retinal S-antigen	0.9993	0.9895	NA
14385	Slc37a4	solute carrier family 37 (glucose-6-phosphate transporter), member 4	0.9993	0.9905	NA
215693	Zmat1	zinc finger, matrin type 1	0.9992	0.9865	NA
83771	Tas1r3	taste receptor, type 1, member 3	0.9992	0.9947	0.998
66192	Lage3	L antigen family, member 3	0.9992	0.9914	NA
22317	Vamp1	vesicle-associated membrane protein 1	0.9992	0.9951	0.998
20931	Surf2	surfeit gene 2	0.9992	0.9886	NA
19243	Ptp4a1	protein tyrosine phosphatase 4a1	0.9992	0.9864	NA
18975	Polg	polymerase (DNA directed), gamma	0.9992	0.9841	NA
12231	Btn1a1	butyrophilin, subfamily 1, member A1	0.9992	0.9943	0.9979
433313	Rpl17-ps1	ribosomal protein L17, pseudogene 1	0.9991	0.9901	NA
382117	D9Ertd402e	DNA segment, Chr 9, ERATO Doi 402, expressed	0.9991	0.9896	NA
102141	Snx25	sorting nexin 25	0.9991	0.9828	NA
93966	Hemgn	hemogen	0.9991	0.9925	0.9972

83398	Ndst3	N-deacetylase/N-sulfotransferase (heparan glucosaminyl) 3	0.9991	0.9855	NA
30931	Tor1a	torsin family 1, member A (torsin A)	0.9991	0.9886	NA
18970	Polb	polymerase (DNA directed), beta	0.9991	0.9873	NA
100044509	Gm14378	predicted gene 14378	0.999	0.9949	0.998
791088	C630016N16Rik	RIKEN cDNA C630016N16 gene	0.999	0.9931	0.9975
245877	Mtap7d1	microtubule-associated protein 7 domain containing 1	0.999	0.9858	NA
217893	Pacs2	phosphofurin acidic cluster sorting protein 2	0.999	0.9921	0.9971
77593	Usp45	ubiquitin specific petidase 45	0.999	0.9831	NA
67773	Myst1	MYST histone acetyltransferase 1	0.999	0.981	NA
67288	Srek1ip1	splicing regulatory glutamine/lysine-rich protein 1interacting protein 1	0.999	0.9908	0.9965
54194	Akap8l	A kinase (PRKA) anchor protein 8-like	0.999	0.9864	NA
100038570	Gm11744	predicted gene 11744	0.9989	0.98	NA
75607	Wnk2	WNK lysine deficient protein kinase 2	0.9989	0.9862	NA
74521	Ppp4r4	protein phosphatase 4, regulatory subunit 4	0.9989	0.9835	NA
30937	Lmcd1	LIM and cysteine-rich domains 1	0.9989	0.9906	0.9965
22194	Ube2e1	ubiquitin-conjugating enzyme E2E 1, UBC4/5 homolog (yeast)	0.9989	0.9837	NA
21854	Timm17a	translocase of inner mitochondrial membrane 17a	0.9989	0.9799	NA
20021	Polr2c	polymerase (RNA) II (DNA directed) polypeptide C	0.9989	0.9909	0.9965
14755	Pigq	phosphatidylinositol glycan anchor biosynthesis, class Q	0.9989	0.9841	NA
12447	Ccne1	cyclin E1	0.9989	0.984	NA
12352	Car5a	carbonic anhydrase 5a, mitochondrial	0.9989	0.9955	0.9981
100042265	Gm9790	predicted gene 9790	0.9988	0.9801	NA
319807	3110047P20Rik	RIKEN cDNA 3110047P20 gene	0.9988	0.9862	NA
236844	Gm4910	predicted pseudogene 4910	0.9988	0.9826	NA
216856	Nlgn2	neuroligin 2	0.9988	0.9873	NA
207958	Alg11	asparagine-linked glycosylation 11 homolog (yeast, alpha-1,2-mannosyltransferase)	0.9988	0.99	0.9964
78267	Klhdc8b	kelch domain containing 8B	0.9988	0.9735	NA
72852	Mblac2	metallo-beta-lactamase domain containing 2	0.9988	0.9872	NA
70747	Tspan2	tetraspanin 2	0.9988	0.9793	NA
17261	Mef2d	myocyte enhancer factor 2D	0.9988	0.9905	0.9965
16956	Lpl	lipoprotein lipase	0.9988	0.9904	0.9964
14718	Got1	glutamate oxaloacetate transaminase 1, soluble	0.9988	0.9795	NA
235050	Zfp810	zinc finger protein 810	0.9987	0.9774	NA
111175	Pecr	peroxisomal trans-2-enoyl-CoA reductase	0.9987	0.9891	0.996
104896	AI852580	expressed sequence AI852580	0.9987	0.9885	0.9957
83397	Akap12	A kinase (PRKA) anchor protein (gravin) 12	0.9987	0.9825	NA
70891	Spdya	speedy homolog A (Xenopus laevis)	0.9987	0.989	0.996
54126	Arhgef7	Rho guanine nucleotide exchange factor (GEF7)	0.9987	0.9781	NA
17999	Nedd4	neural precursor cell expressed, developmentally down-regulated 4	0.9987	0.9877	NA
17268	Meis1	Meis homeobox 1	0.9987	0.9799	NA
100415785	Gm11559	predicted gene 11559	0.9986	0.9839	NA
170738	Kcnh7	potassium voltage-gated channel, subfamily H (eag-related), member 7	0.9986	0.9894	0.9962
140499	Ube2j2	ubiquitin-conjugating enzyme E2, J2 homolog (yeast)	0.9986	0.9809	NA
94043	Tm2d1	TM2 domain containing 1	0.9986	0.9805	NA
67466	Pdcl	phosducin-like	0.9986	0.9789	NA
19377	Rai1	retinoic acid induced 1	0.9986	0.9766	NA
12177	Bnip3l	BCL2/adenovirus E1B interacting protein 3-like	0.9986	0.9821	NA
474160	BC033916	cDNA sequence BC033916	0.9985	0.9773	NA
243872	Rpl7a-ps8	ribosomal protein L7A, pseudogene 8	0.9985	0.9683	NA

234463	Tmem184c	transmembrane protein 184C	0.9985	0.9771	NA
80334	Kcnip4	Kv channel interacting protein 4	0.9985	0.9791	NA
76332	Cog2	component of oligomeric golgi complex 2	0.9985	0.9794	NA
71306	Mfap3l	microfibrillar-associated protein 3-like	0.9985	0.9852	NA
66213	Med7	mediator complex subunit 7	0.9985	0.9822	NA
66050	0610009B22Rik	RIKEN cDNA 0610009B22 gene	0.9985	0.9767	NA
60364	Donson	downstream neighbor of SON	0.9985	0.9807	NA
12859	Cox5b	cytochrome c oxidase, subunit Vb	0.9985	0.9686	NA
269023	Zfp608	zinc finger protein 608	0.9984	0.9863	0.9949
239170	Fam160b2	family with sequence similarity 160, member B2	0.9984	0.9886	0.9957
218629	Dhx29	DEAH (Asp-Glu-Ala-His) box polypeptide 29	0.9984	0.9639	NA
216846	Cntrob	centrobin, centrosomal BRCA2 interacting protein	0.9984	0.9847	NA
192289	Tmlhe	trimethyllysine hydroxylase, epsilon	0.9984	0.9761	NA
78177	Ninl	ninein-like	0.9984	0.9929	0.9975
74747	Ddit4	DNA-damage-inducible transcript 4	0.9984	0.9698	NA
74352	Zfp84	zinc finger protein 84	0.9984	0.9784	NA
72148	2610019F03Rik	RIKEN cDNA 2610019F03 gene	0.9984	0.9832	NA
72141	Adpgk	ADP-dependent glucokinase	0.9984	0.9732	NA
71685	Galnt14	UDP-N-acetyl-alpha-D-galactosamine:polypeptide N-acetylgalactosaminyltransferase 14	0.9984	0.9869	0.9951
68626	Elac2	elaC homolog 2 (E. coli)	0.9984	0.972	NA
68294	Mfsd10	major facilitator superfamily domain containing 10	0.9984	0.9861	0.9949
68080	Gpn3	GPN-loop GTPase 3	0.9984	0.9755	NA
55951	Brp44l	brain protein 44-like	0.9984	0.9727	NA
28105	Trim36	tripartite motif-containing 36	0.9984	0.9844	NA
22670	Trim26	tripartite motif-containing 26	0.9984	0.9726	NA
18436	P2rx1	purinergic receptor P2X, ligand-gated ion channel, 1	0.9984	0.9949	0.998
18263	Odc1	ornithine decarboxylase, structural 1	0.9984	0.981	NA
320556	C230085N15Rik	RIKEN cDNA C230085N15 gene	0.9983	0.9789	NA
109778	Blvra	biliverdin reductase A	0.9983	0.9828	NA
107771	Bmyc	brain expressed myelocytomatosis oncogene	0.9983	0.9901	0.9964
71755	Dhdh	dihydrodiol dehydrogenase (dimeric)	0.9983	0.9836	NA
67581	Tbc1d23	TBC1 domain family, member 23	0.9983	0.9667	NA
57810	Cdon	cell adhesion molecule-related/down-regulated by oncogenes	0.9983	0.9777	NA
50926	Hnrpdl	heterogeneous nuclear ribonucleoprotein D-like	0.9983	0.9859	0.9949
20135	Rrm2	ribonucleotide reductase M2	0.9983	0.9941	0.9979
16443	Itsn1	intersectin 1 (SH3 domain protein 1A)	0.9983	0.9832	NA
13001	Csnk2b	casein kinase 2, beta polypeptide	0.9983	0.9708	NA
320609	Fam40b	family with sequence similarity 40, member B	0.9982	0.979	NA
241128	Fam124b	family with sequence similarity 124, member B	0.9982	0.9876	0.9954
109108	Slc30a9	solute carrier family 30 (zinc transporter), member 9	0.9982	0.9865	0.9949
99889	Arfip1	ADP-ribosylation factor interacting protein 1	0.9982	0.993	0.9975
66379	2310016M24Rik	RIKEN cDNA 2310016M24 gene	0.9982	0.9808	NA
66235	Eif1ax	eukaryotic translation initiation factor 1A, X-linked	0.9982	0.9756	NA
52477	Angel2	angel homolog 2 (Drosophila)	0.9982	0.9631	NA
16518	Kcnj2	potassium inwardly-rectifying channel, subfamily J, member 2	0.9982	0.9871	0.9951
320007	Sidt1	SID1 transmembrane family, member 1	0.9981	0.9876	0.9954
239096	Cdh24	cadherin-like 24	0.9981	0.9833	0.9947
216874	Camta2	calmodulin binding transcription activator 2	0.9981	0.9849	0.9949
98758	Hnrnpf	heterogeneous nuclear ribonucleoprotein F	0.9981	0.9694	NA

77329	C030010C08Rik	RIKEN cDNA C030010C08 gene	0.9981	0.9855	0.9949
74055	Plce1	phospholipase C, epsilon 1	0.9981	0.9849	0.9949
59092	Pcbp4	poly(rC) binding protein 4	0.9981	0.9852	0.9949
58217	Trem1	triggering receptor expressed on myeloid cells 1	0.9981	0.9849	0.9949
29820	Tnfrsf19	tumor necrosis factor receptor superfamily, member 19	0.9981	0.9693	NA
627214	Fam196a	family with sequence similarity 196, member A	0.998	0.9692	NA
258852	Olfr1341	olfactory receptor 1341	0.998	0.9945	0.9979
15975	Ifnar1	interferon (alpha and beta) receptor 1	0.998	0.9761	NA
474156	Zbtb9	zinc finger and BTB domain containing 9	0.9979	0.9847	0.9949
272322	Arntl2	aryl hydrocarbon receptor nuclear translocator-like 2	0.9979	0.9832	0.9947
225280	Ino80c	INO80 complex subunit C	0.9979	0.9726	NA
223435	Trio	triple functional domain (PTPRF interacting)	0.9979	0.968	NA
102607	Snx19	sorting nexin 19	0.9979	0.9662	NA
71240	Osbpl7	oxysterol binding protein-like 7	0.9979	0.9677	NA
66271	Tmem126a	transmembrane protein 126A	0.9979	0.972	NA
18715	Pim2	proviral integration site 2	0.9979	0.9792	NA
18187	Nrp2	neuropilin 2	0.9979	0.968	NA
17308	Mgat1	mannoside acetylglucosaminyltransferase 1	0.9979	0.9695	NA
14787	Rhpn1	rhophilin, Rho GTPase binding protein 1	0.9979	0.9797	NA
381059	Gm1604b	predicted gene 1604b	0.9978	0.9848	0.9949
106628	Trip10	thyroid hormone receptor interactor 10	0.9978	0.9734	NA
78755	Fam122b	family with sequence similarity 122, member B	0.9978	0.9851	0.9949
66483	Rpl36al	ribosomal protein L36A-like	0.9978	0.9643	NA
18189	Nrxn1	neurexin I	0.9978	0.9803	0.9939
17420	Mnat1	menage a trois 1	0.9978	0.9684	NA
17005	Ltk	leukocyte tyrosine kinase	0.9978	0.986	0.9949
319170	Hist1h2an	histone cluster 1, H2an	0.9977	0.9632	NA
269997	Zfp747	zinc finger protein 747	0.9977	0.9599	NA
72248	1700014B07Rik	RIKEN cDNA 1700014B07 gene	0.9977	0.9731	NA
269523	Vcp	valosin containing protein	0.9976	0.9614	NA
242607	Slc1a7	solute carrier family 1 (glutamate transporter), member 7	0.9976	0.9737	NA
227648	Sec16a	SEC16 homolog A (S. cerevisiae)	0.9976	0.9602	NA
77683	Ehmt1	euchromatic histone methyltransferase 1	0.9976	0.962	NA
68023	Pdf	peptide deformylase (mitochondrial)	0.9976	0.9543	NA
66185	1110037F02Rik	RIKEN cDNA 1110037F02 gene	0.9976	0.9804	0.9939
66052	Sdhc	succinate dehydrogenase complex, subunit C, integral membrane protein	0.9976	0.9714	NA
26922	Mecr	mitochondrial trans-2-enoyl-CoA reductase	0.9976	0.9621	NA
668917	LOC668917	hypothetical LOC668917	0.9975	0.9909	0.9965
320119	Rps6kc1	ribosomal protein S6 kinase polypeptide 1	0.9975	0.9882	0.9957
319801	9630033F20Rik	RIKEN cDNA 9630033F20 gene	0.9975	0.9533	NA
114896	Afg3l1	AFG3(ATPase family gene 3)-like 1 (yeast)	0.9975	0.9691	NA
75209	Sv2c	synaptic vesicle glycoprotein 2c	0.9975	0.9843	0.9949
68385	Tlcd1	TLC domain containing 1	0.9975	0.9735	NA
67213	Cmtm6	CKLF-like MARVEL transmembrane domain containing 6	0.9975	0.9677	NA
53600	Timm23	translocase of inner mitochondrial membrane 23 homolog (yeast)	0.9975	0.9485	NA
20630	Snrpc	U1 small nuclear ribonucleoprotein C	0.9975	0.9659	NA
233905	Zfp646	zinc finger protein 646	0.9974	0.989	0.996
107358	Tm9sf3	transmembrane 9 superfamily member 3	0.9974	0.9731	NA
74148	1300001I01Rik	RIKEN cDNA 1300001I01 gene	0.9974	0.966	NA

71233	Enkur	enkurin, TRPC channel interacting protein	0.9974	0.9664	NA
66590	Farsa	phenylalanyl-tRNA synthetase, alpha subunit	0.9974	0.9681	NA
66423	2410022L05Rik	RIKEN cDNA 2410022L05 gene	0.9974	0.9433	NA
15958	Ifit2	interferon-induced protein with tetratricopeptide repeats 2	0.9974	0.9624	NA
11778	Ap3s2	adaptor-related protein complex 3, sigma 2 subunit	0.9974	0.9627	NA
226976	4632411B12Rik	RIKEN cDNA 4632411B12 gene	0.9973	0.9515	NA
225289	AW554918	expressed sequence AW554918	0.9973	0.944	NA
192651	Zfp286	zinc finger protein 286	0.9973	0.9457	NA
68337	Crip2	cysteine rich protein 2	0.9973	0.9487	NA
68283	9530077C05Rik	RIKEN cDNA 9530077C05 gene	0.9973	0.9598	NA
66274	1810012P15Rik	RIKEN cDNA 1810012P15 gene	0.9973	0.9727	NA
58523	Elp2	elongation protein 2 homolog (S. cerevisiae)	0.9973	0.9624	NA
55950	Bri3	brain protein I3	0.9973	0.9708	NA
654470	Tctn1	tectonic family member 1	0.9972	0.9746	0.9926
433956	Heatr2	HEAT repeat containing 2	0.9972	0.9445	NA
414066	BC037032	cDNA Sequence BC037032	0.9972	0.9841	0.9949
114715	Spred1	sprouty protein with EVH-1 domain 1, related sequence	0.9972	0.9551	NA
109331	Rnf20	ring finger protein 20	0.9972	0.9558	NA
75974	Dock11	dedicator of cytokinesis 11	0.9972	0.9736	NA
71715	Dhx35	DEAH (Asp-Glu-Ala-His) box polypeptide 35	0.9972	0.971	NA
22339	Vegfa	vascular endothelial growth factor A	0.9972	0.9664	NA
17828	Muted	muted	0.9972	0.9667	NA
11844	Arf5	ADP-ribosylation factor 5	0.9972	0.9655	NA
246198	Mllt6	myeloid/lymphoid or mixed-lineage leukemia (trithorax homolog, Drosophila); translocated to, 6	0.9971	0.9561	NA
216618	Ccdc104	coiled-coil domain containing 104	0.9971	0.9465	NA
75665	Ccdc64	coiled-coil domain containing 64	0.9971	0.9527	NA
75291	Zbtb3	zinc finger and BTB domain containing 3	0.9971	0.9692	NA
72322	Xpo5	exportin 5	0.9971	0.9652	NA
22335	Vdac3	voltage-dependent anion channel 3	0.9971	0.9493	NA
16570	Kif3c	kinesin family member 3C	0.9971	0.9366	NA
15312	Hmgn1	high mobility group nucleosomal binding domain 1	0.9971	0.9751	0.9926
100505291	LOC100505291	60S ribosomal protein L36-like	0.997	0.9795	0.9935
381626	Rbm33	RNA binding motif protein 33	0.997	0.9639	NA
268670	Zfp759	zinc finger protein 759	0.997	0.9513	NA
268301	Ankrd57	ankyrin repeat domain 57	0.997	0.9391	NA
69020	Zfp707	zinc finger protein 707	0.997	0.9432	NA
66845	Mrpl33	mitochondrial ribosomal protein L33	0.997	0.9583	NA
52206	Anapc4	anaphase promoting complex subunit 4	0.997	0.9471	NA
22218	Sumo1	SMT3 suppressor of mif two 3 homolog 1 (yeast)	0.997	0.9453	NA
12616	Cenpb	centromere protein B	0.997	0.9785	0.9934
384569	Nova2	neuro-oncological ventral antigen 2	0.9969	0.9794	0.9935
380694	Ccnjl	cyclin J-like	0.9969	0.9716	0.9919
245666	Iqsec2	IQ motif and Sec7 domain 2	0.9969	0.9602	NA
240427	Setbp1	SET binding protein 1	0.9969	0.9773	0.993
140499	Ube2j2	ubiquitin-conjugating enzyme E2, J2 homolog (yeast)	0.9969	0.9638	NA
107732	Mrpl10	mitochondrial ribosomal protein L10	0.9969	0.9659	NA
67469	Abhd5	abhydrolase domain containing 5	0.9969	0.9791	0.9935
20744	Strbp	spermatid perinuclear RNA binding protein	0.9969	0.9447	NA
20365	Serf1	small EDRK-rich factor 1	0.9969	0.9628	NA

17936	Nab1	Ngfi-A binding protein 1	0.9969	0.9739	0.9925
14400	Gabrb1	gamma-aminobutyric acid (GABA) A receptor, subunit beta 1	0.9969	0.9692	NA
12982	Csf2ra	colony stimulating factor 2 receptor, alpha, low-affinity (granulocyte-macrophage)	0.9969	0.935	NA
328329	Mast4	microtubule associated serine/threonine kinase family member 4	0.9968	0.976	0.9928
228012	Tlk1	tousled-like kinase 1	0.9968	0.9548	NA
216853	Wrap53	WD repeat containing, antisense to TP53	0.9968	0.9481	NA
207565	Camkk2	calcium/calmodulin-dependent protein kinase kinase 2, beta	0.9968	0.9512	NA
108958	Fam73b	family with sequence similarity 73, member B	0.9968	0.9323	NA
56210	Rev1	REV1 homolog (S. cerevisiae)	0.9968	0.9715	0.9919
18570	Pdcd6	programmed cell death 6	0.9968	0.942	NA
18514	Pbx1	pre B-cell leukemia transcription factor 1	0.9968	0.9637	NA
17906	Myl2	myosin, light polypeptide 2, regulatory, cardiac, slow	0.9968	0.9811	0.9942
330409	Cecr2	cat eye syndrome chromosome region, candidate 2 homolog (human)	0.9967	0.967	NA
233552	Gdpd5	glycerophosphodiester phosphodiesterase domain containing 5	0.9967	0.969	0.991
216767	Mrpl22	mitochondrial ribosomal protein L22	0.9967	0.9353	NA
211986	Tmem18	transmembrane protein 18	0.9967	0.9474	NA
98952	Fam102a	family with sequence similarity 102, member A	0.9967	0.9452	NA
70258	1500035N22Rik	RIKEN cDNA 1500035N22 gene	0.9967	0.9723	0.992
69932	2810004I08Rik	RIKEN cDNA 2810004I08 gene	0.9967	0.9575	NA
67207	Lsm1	LSM1 homolog, U6 small nuclear RNA associated (S. cerevisiae)	0.9967	0.9587	NA
23854	Def8	differentially expressed in FDCP 8	0.9967	0.979	0.9935
21374	Tbp	TATA box binding protein	0.9967	0.9228	NA
20443	St3gal4	ST3 beta-galactoside alpha-2,3-sialyltransferase 4	0.9967	0.9618	NA
13929	Amz2	archaelysin family metallopeptidase 2	0.9967	0.9586	NA
11305	Abca2	ATP-binding cassette, sub-family A (ABC1), member 2	0.9967	0.9767	0.9928
641387	1700120B22Rik	RIKEN cDNA 1700120B22 gene	0.9966	0.9783	0.9933
382864	Colq	collagen-like tail subunit (single strand of homotrimer) of asymmetric acetylcholinesterase	0.9966	0.9719	0.992
104732	4930427A07Rik	RIKEN cDNA 4930427A07 gene	0.9966	0.9752	0.9926
71017	4933407E14Rik	RIKEN cDNA 4933407E14 gene	0.9966	0.9479	NA
56330	Pdcd5	programmed cell death 5	0.9966	0.9365	NA
52633	Nit2	nitrilase family, member 2	0.9966	0.9654	NA
23859	Dlg2	discs, large homolog 2 (Drosophila)	0.9966	0.9651	NA
434484	Sp140	Sp140 nuclear body protein	0.9965	0.9725	0.9921
276770	Eif5a	eukaryotic translation initiation factor 5A	0.9965	0.9681	0.9906
107581	Col16a1	collagen, type XVI, alpha 1	0.9965	0.9729	0.9921
73373	Phospho2	phosphatase, orphan 2	0.9965	0.9535	NA
72803	2810454L23Rik	RIKEN cDNA 2810454L23 gene	0.9965	0.9558	NA
70417	Megf10	multiple EGF-like-domains 10	0.9965	0.9453	NA
69627	Fam89a	family with sequence similarity 89, member A	0.9965	0.9623	NA
26951	Zw10	ZW10 homolog (Drosophila), centromere/kinetochore protein	0.9965	0.9368	NA
26894	Cops7a	COP9 (constitutive photomorphogenic) homolog, subunit 7a (Arabidopsis thaliana)	0.9965	0.9451	NA
24082	Gm16516	predicted gene, Gm16516	0.9965	0.9571	NA
19647	Rbbp6	retinoblastoma binding protein 6	0.9965	0.9471	NA
11783	Apaf1	apoptotic peptidase activating factor 1	0.9965	0.9566	NA
245880	Wasf3	WAS protein family, member 3	0.9964	0.9594	NA
232906	Grlf1	glucocorticoid receptor DNA binding factor 1	0.9964	0.9407	NA
73407	Tepp	testis, prostate and placenta expressed	0.9964	0.9422	NA
66921	Prpf38b	PRP38 pre-mRNA processing factor 38 (yeast) domain containing B	0.9964	0.9534	NA
66132	1110008L16Rik	RIKEN cDNA 1110008L16 gene	0.9964	0.9868	0.995

56520	Nme4	non-metastatic cells 4, protein expressed in	0.9964	0.9556	NA
24051	Sgcb	sarcoglycan, beta (dystrophin-associated glycoprotein)	0.9964	0.963	NA
22682	Zfand5	zinc finger, AN1-type domain 5	0.9964	0.9351	NA
19211	Pten	phosphatase and tensin homolog	0.9964	0.9584	NA
14682	Gnaq	guanine nucleotide binding protein, alpha q polypeptide	0.9964	0.9513	NA
14163	Fgd1	FYVE, RhoGEF and PH domain containing 1	0.9964	0.9763	0.9928
13839	Epha5	Eph receptor A5	0.9964	0.9769	0.9928
12568	Cdk5	cyclin-dependent kinase 5	0.9964	0.9466	NA
12308	Calb2	calbindin 2	0.9964	0.9679	0.9906
277010	Marveld1	MARVEL (membrane-associating) domain containing 1	0.9963	0.9533	NA
240066	Zfp870	zinc finger protein 870	0.9963	0.966	0.9901
170930	Sumo2	SMT3 suppressor of mif two 3 homolog 2 (yeast)	0.9963	0.9568	NA
79264	Krit1	KRIT1, ankyrin repeat containing	0.9963	0.926	NA
70699	Nup205	nucleoporin 205	0.9963	0.9277	NA
67109	Zfp787	zinc finger protein 787	0.9963	0.9787	0.9935
66844	Ormdl2	ORM1-like 2 (S. cerevisiae)	0.9963	0.9531	NA
50884	Nckap1	NCK-associated protein 1	0.9963	0.9573	NA
19128	Pros1	protein S (alpha)	0.9963	0.9323	NA
18571	Pdcd6ip	programmed cell death 6 interacting protein	0.9963	0.9234	NA
17686	Msh3	mutS homolog 3 (E. coli)	0.9963	0.9286	NA
14816	Grm1	glutamate receptor, metabotropic 1	0.9963	0.9705	0.9915
66991	2410004A20Rik	RIKEN cDNA 2410004A20 gene	0.9962	0.9697	0.9912
66433	Chchd7	coiled-coil-helix-coiled-coil-helix domain containing 7	0.9962	0.9504	NA
20482	Skil	SKI-like	0.9962	0.946	NA
20320	Nptn	neuroplastin	0.9962	0.9461	NA
321008	6330408A02Rik	RIKEN cDNA 6330408A02 gene	0.9961	0.9474	NA
320129	Adrbk2	adrenergic receptor kinase, beta 2	0.9961	0.9447	NA
75579	2310034G01Rik	RIKEN cDNA 2310034G01 gene	0.9961	0.9666	0.9902
67197	Zcrb1	zinc finger CCHC-type and RNA binding motif 1	0.9961	0.9821	0.9946
17722	ND6	NADH dehydrogenase subunit 6	0.9961	0.985	0.9949
16210	Impact	imprinted and ancient	0.9961	0.9392	NA
666945	Gm10638	predicted gene 10638	0.996	0.959	NA
267019	Rps15a	ribosomal protein S15A	0.996	0.927	NA
211488	Ado	2-aminoethanethiol (cysteamine) dioxygenase	0.996	0.9745	0.9926
104721	Ddx1	DEAD (Asp-Glu-Ala-Asp) box polypeptide 1	0.996	0.9381	NA
80883	Ntng1	netrin G1	0.996	0.9658	0.9901
73844	Ankrd45	ankyrin repeat domain 45	0.996	0.9689	0.991
69554	Klhdc2	kelch domain containing 2	0.996	0.96	NA
66594	Uqcr11	ubiquinol-cytochrome c reductase, complex III subunit XI	0.996	0.9424	NA
52432	Ppp2r2d	protein phosphatase 2, regulatory subunit B, delta isoform	0.996	0.9408	NA
23917	Impdh1	inosine 5'-phosphate dehydrogenase 1	0.996	0.9576	NA
22230	Ufd1l	ubiquitin fusion degradation 1 like	0.996	0.952	NA
12405	Cbln2	cerebellin 2 precursor protein	0.996	0.9547	NA
321022	Cdv3	carnitine deficiency-associated gene expressed in ventricle 3	0.9959	0.9603	NA
268566	Gphn	gephyrin	0.9959	0.9168	NA
105727	Slc38a1	solute carrier family 38, member 1	0.9959	0.9527	NA
105171	Arrdc3	arrestin domain containing 3	0.9959	0.9574	NA
77631	4930554H23Rik	RIKEN cDNA 4930554H23 gene	0.9959	0.9632	0.9894
76559	Atg2b	ATG2 autophagy related 2 homolog B (S. cerevisiae)	0.9959	0.9161	NA

73040	2900052N01Rik	RIKEN cDNA 2900052N01 gene	0.9959	0.9699	0.9913
54394	Crlf3	cytokine receptor-like factor 3	0.9959	0.9331	NA
53381	Prdx4	peroxiredoxin 4	0.9959	0.95	NA
26462	Txnrd2	thioredoxin reductase 2	0.9959	0.9379	NA
11502	Adam9	a disintegrin and metallopeptidase domain 9 (meltrin gamma)	0.9959	0.9324	NA
68307	Lrriq4	leucine-rich repeats and IQ motif containing 4	0.9958	0.9924	0.9972
65961	Utp3	UTP3, small subunit (SSU) processome component, homolog (S. cerevisiae)	0.9958	0.9401	NA
57342	Parva	parvin, alpha	0.9958	0.9214	NA
29867	Cabp1	calcium binding protein 1	0.9958	0.9615	0.9889
17749	Polr2k	polymerase (RNA) II (DNA directed) polypeptide K	0.9958	0.9212	NA
12359	Cat	catalase	0.9958	0.9701	0.9914
232370	Clstn3	calsyntenin 3	0.9957	0.9303	NA
105710	BB086117	expressed sequence BB086117	0.9957	0.9139	NA
74411	Ppapdc2	phosphatidic acid phosphatase type 2 domain containing 2	0.9957	0.9029	NA
74205	Acsl3	acyl-CoA synthetase long-chain family member 3	0.9957	0.9743	0.9926
71718	Telo2	TEL2, telomere maintenance 2, homolog (S. cerevisiae)	0.9957	0.948	NA
68032	Tmem85	transmembrane protein 85	0.9957	0.9442	NA
66293	1810032O08Rik	RIKEN cDNA 1810032O08 gene	0.9957	0.9252	NA
56350	Arl3	ADP-ribosylation factor-like 3	0.9957	0.9657	0.9901
56219	Extl1	exostoses (multiple)-like 1	0.9957	0.9809	0.9941
30954	Siva1	SIVA1, apoptosis-inducing factor	0.9957	0.9524	NA
12291	Cacna1g	calcium channel, voltage-dependent, T type, alpha 1G subunit	0.9957	0.9643	0.9898
11881	Arsb	arylsulfatase B	0.9957	0.9717	0.9919
100044832	LOC100044832	mucosal pentraxin-like	0.9956	0.9829	0.9947
237877	Atad5	ATPase family, AAA domain containing 5	0.9956	0.9348	NA
225058	Gm4832	predicted gene 4832	0.9956	0.9599	0.9886
216080	Ube2d1	ubiquitin-conjugating enzyme E2D 1, UBC4/5 homolog (yeast)	0.9956	0.9738	0.9924
215303	Camk1g	calcium/calmodulin-dependent protein kinase I gamma	0.9956	0.9466	NA
207181	Rbms3	RNA binding motif, single stranded interacting protein	0.9956	0.9727	0.9921
117109	Pop5	processing of precursor 5, ribonuclease P/MRP family (S. cerevisiae)	0.9956	0.9216	NA
72649	Tmem209	transmembrane protein 209	0.9956	0.9578	NA
71902	Cand1	cullin associated and neddylation disassociated 1	0.9956	0.94	NA
66592	Stoml2	stomatin (Epb7.2)-like 2	0.9956	0.9205	NA
20603	Sms	spermine synthase	0.9956	0.9264	NA
20187	Ryk	receptor-like tyrosine kinase	0.9956	0.9443	NA
19716	Bex1	brain expressed gene 1	0.9956	0.9448	NA
19046	Ppp1cb	protein phosphatase 1, catalytic subunit, beta isoform	0.9956	0.9512	NA
100041799	Gm3515	predicted gene 3515	0.9955	0.9891	0.996
268396	Sh3pxd2b	SH3 and PX domains 2B	0.9955	0.9692	0.9911
227933	Ccdc148	coiled-coil domain containing 148	0.9955	0.9705	0.9915
214585	Spg11	spastic paraplegia 11	0.9955	0.9274	NA
195209	Gm22	predicted gene 22	0.9955	0.9761	0.9928
76376	Slc24a2	solute carrier family 24 (sodium/potassium/calcium exchanger), member 2	0.9955	0.9833	0.9947
73274	Gpbp1	GC-rich promoter binding protein 1	0.9955	0.9314	NA
71707	Ubiad1	UbiA prenyltransferase domain containing 1	0.9955	0.9046	NA
68818	Zfand2b	zinc finger, AN1 type domain 2B	0.9955	0.9484	NA
67493	Mett10d	methyltransferase 10 domain containing	0.9955	0.9489	NA
64294	Itm2c	integral membrane protein 2C	0.9955	0.9145	NA
50770	Atp11a	ATPase, class VI, type 11A	0.9955	0.935	NA

28193	Reep3	receptor accessory protein 3	0.9955	0.9497	NA
14348	Fut9	fucosyltransferase 9	0.9955	0.9633	0.9894
192285	Phf21a	PHD finger protein 21A	0.9954	0.9153	NA
70097	Sash1	SAM and SH3 domain containing 1	0.9954	0.9117	NA
54342	Gnpnat1	glucosamine-phosphate N-acetyltransferase 1	0.9954	0.9539	NA
50721	Sirt6	sirtuin 6 (silent mating type information regulation 2, homolog) 6 (S. cerevisiae)	0.9954	0.9794	0.9935
18514	Pbx1	pre B-cell leukemia transcription factor 1	0.9954	0.9337	NA
13176	Dcc	deleted in colorectal carcinoma	0.9954	0.9619	0.9889
213084	Cdkl3	cyclin-dependent kinase-like 3	0.9953	0.9574	0.9879
76894	Mett5d1	methyltransferase 5 domain containing 1	0.9953	0.9372	NA
74410	Ttll11	tubulin tyrosine ligase-like family, member 11	0.9953	0.9669	0.9903
71777	Ing3	inhibitor of growth family, member 3	0.9953	0.9277	NA
67609	4930453N24Rik	RIKEN cDNA 4930453N24 gene	0.9953	0.9384	NA
26384	Gnpda1	glucosamine-6-phosphate deaminase 1	0.9953	0.9407	NA
26381	Esrrg	estrogen-related receptor gamma	0.9953	0.9285	NA
20591	Kdm5c	lysine (K)-specific demethylase 5C	0.9953	0.9653	0.99
100039220	Gm12751	predicted gene 12751	0.9952	0.9252	NA
330222	Sdk1	sidekick homolog 1 (chicken)	0.9952	0.9546	NA
225187	Ankrd29	ankyrin repeat domain 29	0.9952	0.9392	NA
106143	Cggbp1	CGG triplet repeat binding protein 1	0.9952	0.9488	NA
68982	1500015A07Rik	RIKEN cDNA 1500015A07 gene	0.9952	0.9672	0.9904
67789	Dalrd3	DALR anticodon binding domain containing 3	0.9952	0.9675	0.9905
67019	Actr6	ARP6 actin-related protein 6 homolog (yeast)	0.9952	0.922	NA
54161	Copg	coatomer protein complex, subunit gamma	0.9952	0.9501	NA
18045	Nfyb	nuclear transcription factor-Y beta	0.9952	0.9242	NA
627626	3110082D06Rik	RIKEN cDNA 3110082D06 gene	0.9951	0.9832	0.9947
245607	Gprasp2	G protein-coupled receptor associated sorting protein 2	0.9951	0.921	NA
218820	Zfp503	zinc finger protein 503	0.9951	0.9647	0.99
70350	Basp1	brain abundant, membrane attached signal protein 1	0.9951	0.9528	NA
20185	Ncor1	nuclear receptor co-repressor 1	0.9951	0.9326	NA
639606	LOC639606	40S ribosomal protein S2-like	0.995	0.9605	0.9886
268934	Grm4	glutamate receptor, metabotropic 4	0.995	0.9662	0.9901
209462	Hace1	HECT domain and ankyrin repeat containing, E3 ubiquitin protein ligase 1	0.995	0.9288	NA
208213	Tmem132c	transmembrane protein 132C	0.995	0.9565	0.9877
114606	Tle6	transducin-like enhancer of split 6, homolog of Drosophila E(spl)	0.995	0.9219	NA
106025	Sharpin	SHANK-associated RH domain interacting protein	0.995	0.9414	NA
98878	Ehd4	EH-domain containing 4	0.995	0.9081	NA
74438	Clvs1	clavesin 1	0.995	0.9523	NA
67471	Gpatch1	G patch domain containing 1	0.995	0.9259	NA
27041	G3bp1	Ras-GTPase-activating protein SH3-domain binding protein 1	0.995	0.9589	0.9882
23918	Impdh2	inosine 5'-phosphate dehydrogenase 2	0.995	0.9501	NA
16443	Itsn1	intersectin 1 (SH3 domain protein 1A)	0.995	0.9342	NA
230908	Tardbp	TAR DNA binding protein	0.9949	0.915	NA
229211	Acad9	acyl-Coenzyme A dehydrogenase family, member 9	0.9949	0.9056	NA
170749	Mtmr4	myotubularin related protein 4	0.9949	0.9265	NA
111173	Erc1	ELKS/RAB6-interacting/CAST family member 1	0.9949	0.9769	0.9928
104303	Arl1	ADP-ribosylation factor-like 1	0.9949	0.8866	NA
72611	Zfp655	zinc finger protein 655	0.9949	0.9486	NA
19205	Ptbp1	polypyrimidine tract binding protein 1	0.9949	0.9304	NA

19084	Prkar1a	protein kinase, cAMP dependent regulatory, type I, alpha	0.9949	0.9092	NA
12499	Entpd5	ectonucleoside triphosphate diphosphohydrolase 5	0.9949	0.9507	NA
654796	9530036O11Rik	RIKEN cDNA 9530036O11Rik	0.9948	0.9443	NA
80911	Acox3	acyl-Coenzyme A oxidase 3, pristanoyl	0.9948	0.9095	NA
66168	Grina	glutamate receptor, ionotropic, N-methyl D-aspartate-associated protein 1 (glutamate binding)	0.9948	0.9603	0.9886
59079	Erbb2ip	Erbb2 interacting protein	0.9948	0.9112	NA
56747	Sez6l	seizure related 6 homolog like	0.9948	0.9551	0.9872
17113	M6pr	mannose-6-phosphate receptor, cation dependent	0.9948	0.8997	NA
14670	Gnl1	guanine nucleotide binding protein-like 1	0.9948	0.9173	NA
12340	Capza1	capping protein (actin filament) muscle Z-line, alpha 1	0.9948	0.9568	0.9877
12048	Bcl2l1	BCL2-like 1	0.9948	0.9535	0.987
626391	Zfp951	zinc finger protein 951	0.9947	0.9832	0.9947
227334	Usp40	ubiquitin specific peptidase 40	0.9947	0.9368	NA
22319	Vamp3	vesicle-associated membrane protein 3	0.9947	0.9328	NA
18100	Mrpl40	mitochondrial ribosomal protein L40	0.9947	0.9102	NA
11790	Speg	SPEG complex locus	0.9947	0.9361	NA
218232	Ptpdc1	protein tyrosine phosphatase domain containing 1	0.9946	0.9341	NA
97112	Nmd3	NMD3 homolog (S. cerevisiae)	0.9946	0.9421	NA
83435	Plekha3	pleckstrin homology domain-containing, family A (phosphoinositide binding specific) member 3	0.9946	0.8935	NA
65973	Asph	aspartate-beta-hydroxylase	0.9946	0.9409	NA
106389	Eaf2	ELL associated factor 2	0.9945	0.9491	0.9856
75751	Ipo4	importin 4	0.9945	0.9559	0.9876
233280	Nipa1	non imprinted in Prader-Willi/Angelman syndrome 1 homolog (human)	0.9944	0.9211	NA
231093	Agbl5	ATP/GTP binding protein-like 5	0.9944	0.9404	NA
227937	Pkp4	plakophilin 4	0.9944	0.9193	NA
207607	Ccdc40	coiled-coil domain containing 40	0.9944	0.978	0.9932
85031	Pla1a	phospholipase A1 member A	0.9944	0.9835	0.9947
85029	Rpph1	ribonuclease P RNA component H1	0.9944	0.9834	0.9947
68499	Mrpl53	mitochondrial ribosomal protein L53	0.9944	0.8973	NA
66687	Tbc1d15	TBC1 domain family, member 15	0.9944	0.9356	NA
59035	Carm1	coactivator-associated arginine methyltransferase 1	0.9944	0.9481	0.9854
19280	Ptprs	protein tyrosine phosphatase, receptor type, S	0.9944	0.9019	NA
12562	Cdh5	cadherin 5	0.9944	0.9594	0.9883
75292	Prkd3	protein kinase D3	0.9943	0.9386	NA
67727	Stx17	syntaxin 17	0.9943	0.8876	NA
53622	Krt85	keratin 85	0.9943	0.9698	0.9913
26558	Homer3	homer homolog 3 (Drosophila)	0.9943	0.9681	0.9906
18518	Igbp1	immunoglobulin (CD79A) binding protein 1	0.9943	0.9375	NA
271457	Rab5a	RAB5A, member RAS oncogene family	0.9942	0.923	NA
105722	Ano6	anoctamin 6	0.9942	0.9588	0.9881
103836	Zfp692	zinc finger protein 692	0.9942	0.921	NA
93886	Pcdhb15	protocadherin beta 15	0.9942	0.9227	NA
76416	Znrd1as	ZNRD1 antisense RNA	0.9942	0.9083	NA
72883	2900035I09Rik	RIKEN cDNA 2900035I09 gene	0.9942	0.9603	0.9886
71517	9030624J02Rik	RIKEN cDNA 9030624J02 gene	0.9942	0.9373	NA
67072	Cdc37l1	cell division cycle 37 homolog (S. cerevisiae)-like 1	0.9942	0.8886	NA
19882	Mst1r	macrophage stimulating 1 receptor (c-met-related tyrosine kinase)	0.9942	0.9217	NA
19724	Rfx1	regulatory factor X, 1 (influences HLA class II expression)	0.9942	0.9377	NA
14910	Gt(ROSA)26Sor	gene trap ROSA 26, Philippe Soriano	0.9942	0.9267	NA

574428	Zmynd15	zinc finger, MYND-type containing 15	0.9941	0.9416	NA
232210	8430410A17Rik	RIKEN cDNA 8430410A17 gene	0.9941	0.9265	NA
76816	Sdccag8	serologically defined colon cancer antigen 8	0.9941	0.9759	0.9928
70533	Btf3l4	basic transcription factor 3-like 4	0.9941	0.8896	NA
68523	Fam96b	family with sequence similarity 96, member B	0.9941	0.8893	NA
65099	Irak1bp1	interleukin-1 receptor-associated kinase 1 binding protein 1	0.9941	0.8914	NA
18571	Pdcd6ip	programmed cell death 6 interacting protein	0.9941	0.9443	0.9842
100038846	Gm1973	predicted gene 1973	0.994	0.9597	0.9884
381654	C87414	expressed sequence C87414	0.994	0.9596	0.9884
331026	Gmppb	GDP-mannose pyrophosphorylase B	0.994	0.9116	NA
320299	Iqcb1	IQ calmodulin-binding motif containing 1	0.994	0.906	NA
216154	Med16	mediator complex subunit 16	0.994	0.9327	NA
79565	Wbscr27	Williams Beuren syndrome chromosome region 27 (human)	0.994	0.9427	NA
66632	Atpbd4	ATP binding domain 4	0.994	0.9312	NA
66336	Cenpp	centromere protein P	0.994	0.9194	NA
54366	Ctnnal1	catenin (cadherin associated protein), alpha-like 1	0.994	0.9562	0.9876
52064	Coq5	coenzyme Q5 homolog, methyltransferase (yeast)	0.994	0.9306	NA
19133	Prph2	peripherin 2	0.994	0.9019	NA
18193	Nsd1	nuclear receptor-binding SET-domain protein 1	0.994	0.9042	NA
140740	Sec63	SEC63-like (S. cerevisiae)	0.9939	0.9455	0.9844
104348	Zfp120	zinc finger protein 120	0.9939	0.9301	NA
66258	Mrps17	mitochondrial ribosomal protein S17	0.9939	0.858	NA
57436	Gabarapl1	gamma-aminobutyric acid (GABA) A receptor-associated protein-like 1	0.9939	0.9302	NA
12226	Btg1	B-cell translocation gene 1, anti-proliferative	0.9939	0.945	0.9844
329540	8430427H17Rik	RIKEN cDNA 8430427H17 gene	0.9938	0.9042	NA
235072	Sep-07	septin 7	0.9938	0.8941	NA
227522	Rpp38	ribonuclease P/MRP 38 subunit (human)	0.9938	0.886	NA
192157	Socs7	suppressor of cytokine signaling 7	0.9938	0.8951	NA
114714	Rad51c	RAD51 homolog c (S. cerevisiae)	0.9938	0.9653	0.99
107045	Lars	leucyl-tRNA synthetase	0.9938	0.9055	NA
104346	Gas8	growth arrest specific 8	0.9938	0.9196	NA
102060	Gadd45gip1	growth arrest and DNA-damage-inducible, gamma interacting protein 1	0.9938	0.9439	0.9842
101142	Itfg2	integrin alpha FG-GAP repeat containing 2	0.9938	0.9203	NA
75796	Cdyl2	chromodomain protein, Y chromosome-like 2	0.9938	0.9361	NA
73067	Tmem192	transmembrane protein 192	0.9938	0.9074	NA
67553	Gstcd	glutathione S-transferase, C-terminal domain containing	0.9938	0.9495	0.9856
66172	Med11	mediator of RNA polymerase II transcription, subunit 11 homolog (S. cerevisiae)	0.9938	0.9139	NA
21953	Tnni2	troponin I, skeletal, fast 2	0.9938	0.9689	0.991
20638	Snrpb	small nuclear ribonucleoprotein B	0.9938	0.9318	NA
14894	Gtl3	gene trap locus 3	0.9938	0.892	NA
640530	Gm7298	predicted gene 7298	0.9937	0.9834	0.9947
625281	Gm6570	predicted gene 6570	0.9937	0.9586	0.9881
240444	Kcng2	potassium voltage-gated channel, subfamily G, member 2	0.9937	0.9733	0.9922
238693	Zfp58	zinc finger protein 58	0.9937	0.9016	NA
68776	Taf11	TAF11 RNA polymerase II, TATA box binding protein (TBP)-associated factor	0.9937	0.8837	NA
66789	Alg14	asparagine-linked glycosylation 14 homolog (yeast)	0.9937	0.8972	NA
21812	Tgfbr1	transforming growth factor, beta receptor I	0.9937	0.9072	NA
18036	Nfkbib	nuclear factor of kappa light polypeptide gene enhancer in B-cells inhibitor, beta	0.9937	0.966	0.9901
384281	Gatc	glutamyl-tRNA(Gln) amidotransferase, subunit C homolog (bacterial)	0.9936	0.9522	0.9869

330222	Sdk1	sidekick homolog 1 (chicken)	0.9936	0.9435	0.9839
319476	Lrtm1	leucine-rich repeats and transmembrane domains 1	0.9936	0.9502	0.9859
319167	Hist1h2ag	histone cluster 1, H2ag	0.9936	0.9015	NA
243872	Rpl7a-ps8	ribosomal protein L7A, pseudogene 8	0.9936	0.9328	NA
217333	Trim47	tripartite motif-containing 47	0.9936	0.9206	NA
210973	Kbtbd2	kelch repeat and BTB (POZ) domain containing 2	0.9936	0.8961	NA
71745	Cul2	cullin 2	0.9936	0.9032	NA
68112	Sdccag3	serologically defined colon cancer antigen 3	0.9936	0.9454	0.9844
56839	Lgi1	leucine-rich repeat LGI family, member 1	0.9936	0.9588	0.9881
20208	Saa1	serum amyloid A 1	0.9936	0.9614	0.9889
319583	Lig4	ligase IV, DNA, ATP-dependent	0.9935	0.8996	NA
239410	A930017M01Rik	Smg-5 homolog, nonsense mediated mRNA decay factor pseudogene	0.9935	0.9291	NA
76582	Ipo11	importin 11	0.9935	0.89	NA
76355	Tgds	TDP-glucose 4,6-dehydratase	0.9935	0.9297	NA
76089	Rapgef2	Rap guanine nucleotide exchange factor (GEF) 2	0.9935	0.8742	NA
72171	Shq1	SHQ1 homolog (S. cerevisiae)	0.9935	0.9586	0.9881
56449	Csda	cold shock domain protein A	0.9935	0.8847	NA
17242	Mdk	midkine	0.9935	0.9169	NA
269437	Plch1	phospholipase C, eta 1	0.9934	0.891	NA
231225	Tapt1	transmembrane anterior posterior transformation 1	0.9934	0.9194	NA
217303	Cd300a	CD300A antigen	0.9934	0.9827	0.9947
77591	Ddx10	DEAD (Asp-Glu-Ala-Asp) box polypeptide 10	0.9934	0.9136	NA
74051	Steap2	six transmembrane epithelial antigen of prostate 2	0.9934	0.893	NA
67433	Ccdc127	coiled-coil domain containing 127	0.9934	0.9303	NA
12386	Ctnna2	catenin (cadherin associated protein), alpha 2	0.9934	0.9014	NA
11841	Arf2	ADP-ribosylation factor 2	0.9934	0.8822	NA
628100	Fbxo39	F-box protein 39	0.9933	0.9861	0.9949
229776	Cdc14a	CDC14 cell division cycle 14 homolog A (S. cerevisiae)	0.9933	0.8848	NA
214742	Rcor3	REST corepressor 3	0.9933	0.9031	NA
209630	Frmd4a	FERM domain containing 4A	0.9933	0.9482	0.9854
108014	Srsf9	serine/arginine-rich splicing factor 9	0.9933	0.9143	NA
83431	Ndel1	nuclear distribution gene E-like homolog 1 (A. nidulans)	0.9933	0.9084	NA
77031	Slc9a8	solute carrier family 9 (sodium/hydrogen exchanger), member 8	0.9933	0.9764	0.9928
67839	Gpsm1	G-protein signalling modulator 1 (AGS3-like, C. elegans)	0.9933	0.9663	0.9901
66405	Mcts2	malignant T cell amplified sequence 2	0.9933	0.8701	NA
22349	Vil1	villin 1	0.9933	0.8696	NA
21885	Tle1	transducin-like enhancer of split 1, homolog of Drosophila E(spl)	0.9933	0.9492	0.9856
20054	Rps15	ribosomal protein S15	0.9933	0.9536	0.987
12234	Btrc	beta-transducin repeat containing protein	0.9933	0.9192	NA
321006	Vprbp	Vpr (HIV-1) binding protein	0.9932	0.9587	0.9881
245038	Dclk3	doublecortin-like kinase 3	0.9932	0.9551	0.9872
237387	Lrrc3	leucine rich repeat containing 3	0.9932	0.9483	0.9854
234889	Gucy1a2	guanylate cyclase 1, soluble, alpha 2	0.9932	0.9526	0.9869
229007	Zgpat	zinc finger, CCCH-type with G patch domain	0.9932	0.9555	0.9874
67804	Snx2	sorting nexin 2	0.9932	0.8639	NA
66257	Nicn1	nicolin 1	0.9932	0.9073	NA
11891	Rab27a	RAB27A, member RAS oncogene family	0.9932	0.9553	0.9872
100226	Stx12	syntaxin 12	0.9931	0.9187	NA
68441	Rraga	Ras-related GTP binding A	0.9931	0.9024	NA

66128	Mrps36	mitochondrial ribosomal protein S36	0.9931	0.8699	NA
20773	Sptlc2	serine palmitoyltransferase, long chain base subunit 2	0.9931	0.9317	NA
20383	Srsf3	serine/arginine-rich splicing factor 3	0.9931	0.9063	NA
19989	Rpl7	ribosomal protein L7	0.9931	0.8911	NA
14534	Kat2a	K(lysine) acetyltransferase 2A	0.9931	0.9718	0.992
11843	Arf4	ADP-ribosylation factor 4	0.9931	0.9188	NA
216848	Chd3	chromodomain helicase DNA binding protein 3	0.993	0.9348	0.981
78751	Zc3h6	zinc finger CCCH type containing 6	0.993	0.948	0.9854
72701	Zfp618	zinc fingerprotein 618	0.993	0.9598	0.9885
68114	Mum1	melanoma associated antigen (mutated) 1	0.993	0.9258	NA
66729	Ankrd61	ankyrin repeat domain 61	0.993	0.9238	NA
17762	Mapt	microtubule-associated protein tau	0.993	0.9187	NA
100043736	Gm10762	predicted gene 10762	0.9929	0.9757	0.9928
231712	Trafd1	TRAF type zinc finger domain containing 1	0.9929	0.8662	NA
80751	Rnf34	ring finger protein 34	0.9929	0.8939	NA
77264	Zfp142	zinc finger protein 142	0.9929	0.8931	NA
72612	2700029M09Rik	RIKEN cDNA 2700029M09 gene	0.9929	0.8985	NA
71898	Apol9b	apolipoprotein L 9b	0.9929	0.9742	0.9926
70093	Ube2q1	ubiquitin-conjugating enzyme E2Q (putative) 1	0.9929	0.8542	NA
67048	Vma21	VMA21 vacuolar H+-ATPase homolog (S. cerevisiae)	0.9929	0.9577	0.988
52065	Mfhas1	malignant fibrous histiocytoma amplified sequence 1	0.9929	0.9081	NA
20833	Ssrp1	structure specific recognition protein 1	0.9929	0.858	NA
17841	Mup2	major urinary protein 2	0.9929	0.9722	0.992
627049	Zfp800	zinc finger protein 800	0.9928	0.9202	NA
381072	Abca17	ATP-binding cassette, sub-family A (ABC1), member 17	0.9928	0.9748	0.9926
66138	Wbscr22	Williams Beuren syndrome chromosome region 22	0.9928	0.9577	0.988
64113	Moap1	modulator of apoptosis 1	0.9928	0.9285	NA
21961	Tns1	tensin 1	0.9928	0.9228	NA
17978	Ncoa2	nuclear receptor coactivator 2	0.9928	0.9004	NA
14225	Fkbp1a	FK506 binding protein 1a	0.9928	0.9511	0.9865
13641	Efnb1	ephrin B1	0.9928	0.9294	NA
11566	Adss	adenylosuccinate synthetase, non muscle	0.9928	0.8967	NA
626391	Zfp951	zinc finger protein 951	0.9927	0.9544	0.9872
228829	Phf20	PHD finger protein 20	0.9927	0.8398	NA
109660	Ctrl	chymotrypsin-like	0.9927	0.9244	NA
101437	Dhx32	DEAH (Asp-Glu-Ala-His) box polypeptide 32	0.9927	0.9231	NA
93888	Pcdhb17	protocadherin beta 17	0.9927	0.8903	NA
72140	Ccdc123	coiled-coil domain containing 123	0.9927	0.9328	0.9801
69632	Arhgef12	Rho guanine nucleotide exchange factor (GEF) 12	0.9927	0.9157	NA
68048	Aen	apoptosis enhancing nuclease	0.9927	0.8921	NA
66983	Zfp830	zinc finger protein 830	0.9927	0.8694	NA
66052	Sdhc	succinate dehydrogenase complex, subunit C, integral membrane protein	0.9927	0.8972	NA
27062	Cadps	Ca2+-dependent secretion activator	0.9927	0.9258	NA
17252	Rdh11	retinol dehydrogenase 11	0.9927	0.8737	NA
16151	Ikbkg	inhibitor of kappaB kinase gamma	0.9927	0.9319	0.9795
628012	Gm16441	predicted pseudogene 16441	0.9926	0.9788	0.9935
544752	Tug1	taurine upregulated gene 1	0.9926	0.9131	NA
266690	Cyb5r4	cytochrome b5 reductase 4	0.9926	0.9158	NA
231659	Gcn1l1	GCN1 general control of amino-acid synthesis 1-like 1 (yeast)	0.9926	0.9276	NA

103236	Csnk1g2	casein kinase 1, gamma 2	0.9926	0.9297	NA
77127	A930001A20Rik	RIKEN cDNA A930001A20 gene	0.9926	0.8913	NA
76217	Jakmip2	janus kinase and microtubule interacting protein 2	0.9926	0.9358	0.9814
75734	Mff	mitochondrial fission factor	0.9926	0.9048	NA
71591	Zfp251	zinc finger protein 251	0.9926	0.8871	NA
68046	2700062C07Rik	RIKEN cDNA 2700062C07 gene	0.9926	0.8642	NA
67390	Rnmtl1	RNA methyltransferase like 1	0.9926	0.8908	NA
66498	Dda1	DET1 and DDB1 associated 1	0.9926	0.9161	NA
67883	Uxs1	UDP-glucuronate decarboxylase 1	0.9925	0.962	0.9889
30957	Mapk8ip3	mitogen-activated protein kinase 8 interacting protein 3	0.9925	0.8911	NA
20688	Sp4	trans-acting transcription factor 4	0.9925	0.9361	0.9814
20014	Rpn2	ribophorin II	0.9925	0.9399	0.9827
229782	Slc35a3	solute carrier family 35 (UDP-N-acetylglucosamine (UDP-GlcNAc) transporter), member 3	0.9924	0.8905	NA
223527	Eny2	enhancer of yellow 2 homolog (Drosophila)	0.9924	0.8865	NA
78560	Gpr124	G protein-coupled receptor 124	0.9924	0.9095	NA
77531	Anks1b	ankyrin repeat and sterile alpha motif domain containing 1B	0.9924	0.9082	NA
70448	2610204G22Rik	RIKEN cDNA 2610204G22 gene	0.9924	0.9024	NA
69288	Rhobtb1	Rho-related BTB domain containing 1	0.9924	0.9525	0.9869
68106	Nt5c3l	5'-nucleotidase, cytosolic III-like	0.9924	0.8803	NA
66523	2810004N23Rik	RIKEN cDNA 2810004N23 gene	0.9924	0.8678	NA
19087	Prkar2a	protein kinase, cAMP dependent regulatory, type II alpha	0.9924	0.9496	0.9856
665992	Krtap4-8	keratin associated protein 4-8	0.9923	0.9486	0.9855
319481	Wdr59	WD repeat domain 59	0.9923	0.8756	NA
224705	Vps52	vacuolar protein sorting 52 (yeast)	0.9923	0.9377	0.9823
75732	Iqcd	IQ motif containing D	0.9923	0.9034	NA
59047	Pnkp	polynucleotide kinase 3'- phosphatase	0.9923	0.8638	NA
15568	Elavl1	ELAV (embryonic lethal, abnormal vision, Drosophila)-like 1 (Hu antigen R)	0.9923	0.9338	0.9806
12978	Csf1r	colony stimulating factor 1 receptor	0.9923	0.9563	0.9876
11858	Rnd2	Rho family GTPase 2	0.9923	0.9151	NA
641340	Nrbf2	nuclear receptor binding factor 2	0.9922	0.8836	NA
407785	Ndufs6	NADH dehydrogenase (ubiquinone) Fe-S protein 6	0.9922	0.9297	0.9789
270049	Galntl6	UDP-N-acetyl-alpha-D-galactosamine:polypeptide N-acetylgalactosaminyltransferase-like 6	0.9922	0.9285	0.9789
243300	6430598A04Rik	RIKEN cDNA 6430598A04 gene	0.9922	0.9009	NA
76602	1700040D17Rik	RIKEN cDNA 1700040D17 gene	0.9922	0.8932	NA
74356	4931428F04Rik	RIKEN cDNA 4931428F04 gene	0.9922	0.8833	NA
72692	Hnrpll	heterogeneous nuclear ribonucleoprotein L-like	0.9922	0.888	NA
68870	Ak8	adenylate kinase 8	0.9922	0.9426	0.9838
68240	Rpa3	replication protein A3	0.9922	0.9077	NA
56045	Samhd1	SAM domain and HD domain, 1	0.9922	0.882	NA
21376	Tbrg1	transforming growth factor beta regulated gene 1	0.9922	0.9183	NA
13426	Dync1i1	dynein cytoplasmic 1 intermediate chain 1	0.9922	0.8805	NA
545428	Ccdc141	coiled-coil domain containing 141	0.9921	0.986	0.9949
231042	Nupl2	nucleoporin like 2	0.9921	0.8853	NA
77531	Anks1b	ankyrin repeat and sterile alpha motif domain containing 1B	0.9921	0.9364	0.9814
67025	Rpl11	ribosomal protein L11	0.9921	0.9177	NA
22017	Tpmt	thiopurine methyltransferase	0.9921	0.8984	NA
14270	Srgap2	SLIT-ROBO Rho GTPase activating protein 2	0.9921	0.9737	0.9924
13859	Eps15l1	epidermal growth factor receptor pathway substrate 15-like 1	0.9921	0.9136	NA
100503774	LOC100503774	hypothetical LOC100503774	0.992	0.8602	NA

100046692	LOC100046692	40S ribosomal protein S10-like	0.992	0.9608	0.9886
231327	Ppat	phosphoribosyl pyrophosphate amidotransferase	0.992	0.8639	NA
228366	Gyltl1b	glycosyltransferase-like 1B	0.992	0.9679	0.9906
223649	Nrbp2	nuclear receptor binding protein 2	0.992	0.8717	NA
217732	2310044G17Rik	RIKEN cDNA 2310044G17 gene	0.992	0.943	0.9838
216850	Kdm6b	KDM1 lysine (K)-specific demethylase 6B	0.992	0.9127	NA
107351	Kank1	KN motif and ankyrin repeat domains 1	0.992	0.9087	NA
83945	Dnaja3	DnaJ (Hsp40) homolog, subfamily A, member 3	0.992	0.8968	NA
76799	2510006D16Rik	RIKEN cDNA 2510006D16 gene	0.992	0.8796	NA
75299	4930547M16Rik	RIKEN cDNA 4930547M16 gene	0.992	0.9383	0.9823
68316	Apoo	apolipoprotein O	0.992	0.8274	NA
66945	Sdha	succinate dehydrogenase complex, subunit A, flavoprotein (Fp)	0.992	0.8809	NA
56205	Ensa	endosulfine alpha	0.992	0.8774	NA
12296	Cacnb2	calcium channel, voltage-dependent, beta 2 subunit	0.992	0.9249	0.9783
108689	Obfc1	oligonucleotide/oligosaccharide-binding fold containing 1	0.9919	0.9001	NA
105148	Iars	isoleucine-tRNA synthetase	0.9919	0.8578	NA
68059	Tm9sf2	transmembrane 9 superfamily member 2	0.9919	0.8563	NA
22404	Wiz	widely-interspaced zinc finger motifs	0.9919	0.8975	NA
17535	Mre11a	meiotic recombination 11 homolog A (S. cerevisiae)	0.9919	0.8677	NA
14238	Foxf2	forkhead box F2	0.9919	0.9276	0.9789
13433	Dnmt1	DNA methyltransferase (cytosine-5) 1	0.9919	0.9515	0.9866
13063	Cycs	cytochrome c, somatic	0.9919	0.9014	NA
100043772	Gm4636	predicted gene 4636	0.9918	0.899	NA
240263	Fem1c	fem-1 homolog c (C.elegans)	0.9918	0.8695	NA
65247	Asb1	ankyrin repeat and SOCS box-containing 1	0.9918	0.9379	0.9823
55978	Ift20	intraflagellar transport 20 homolog (Chlamydomonas)	0.9918	0.9229	0.9776
17762	Mapt	microtubule-associated protein tau	0.9918	0.9193	NA
16519	Kcnj3	potassium inwardly-rectifying channel, subfamily J, member 3	0.9918	0.9525	0.9869
15051	H2-T9	histocompatibility 2, T region locus 9	0.9918	0.95	0.9859
384061	Fndc5	fibronectin type III domain containing 5	0.9917	0.9121	NA
216169	Fam108a	family with sequence similarity 108, member A	0.9917	0.919	NA
117600	Srgap1	SLIT-ROBO Rho GTPase activating protein 1	0.9917	0.945	0.9844
12916	Crem	cAMP responsive element modulator	0.9917	0.9086	NA
666731	Trim43c	tripartite motif-containing 43C	0.9916	0.9252	0.9783
319974	Auts2	autism susceptibility candidate 2	0.9916	0.8539	NA
232664	Ccdc136	coiled-coil domain containing 136	0.9916	0.9415	0.9835
224273	Crybg3	beta-gamma crystallin domain containing 3	0.9916	0.8958	NA
116940	Tgs1	trimethylguanosine synthase homolog (S. cerevisiae)	0.9916	0.8537	NA
108013	Celf4	CUGBP, Elav-like family member 4	0.9916	0.9369	0.9816
66538	Rps19bp1	ribosomal protein S19 binding protein 1	0.9916	0.9046	NA
66126	Elof1	elongation factor 1 homolog (ELF1, S. cerevisiae)	0.9916	0.8926	NA
237400	Mex3d	mex3 homolog D (C. elegans)	0.9915	0.8713	NA
217325	Llgl2	lethal giant larvae homolog 2 (Drosophila)	0.9915	0.9404	0.9829
73795	4930405D01Rik	RIKEN cDNA 4930405D01 gene	0.9915	0.9098	NA
70652	Tmem144	transmembrane protein 144	0.9915	0.9085	NA
69849	2010007H06Rik	RIKEN cDNA 2010007H06 gene	0.9915	0.9474	0.9852
51813	Ccnc	cyclin C	0.9915	0.8898	NA
19181	Psmc2	proteasome (prosome, macropain) 26S subunit, ATPase 2	0.9915	0.868	NA
16165	Il13ra2	interleukin 13 receptor, alpha 2	0.9915	0.9281	0.9789

233781	Xylt1	xylosyltransferase 1	0.9914	0.9177	NA
217143	Gpr179	G protein-coupled receptor 179	0.9914	0.8844	NA
212281	A530054K11Rik	RIKEN cDNA A530054K11 gene	0.9914	0.861	NA
69597	Afg3l2	AFG3(ATPase family gene 3)-like 2 (yeast)	0.9914	0.8244	NA
18707	Pik3cd	phosphatidylinositol 3-kinase catalytic delta polypeptide	0.9914	0.9124	NA
12848	Cops2	COP9 (constitutive photomorphogenic) homolog, subunit 2 (Arabidopsis thaliana)	0.9914	0.8605	NA
229517	Slc25a44	solute carrier family 25, member 44	0.9913	0.9389	0.9824
108652	Slc35b3	solute carrier family 35, member B3	0.9913	0.8269	NA
70297	Gcc2	GRIP and coiled-coil domain containing 2	0.9913	0.8207	NA
69876	Thap3	THAP domain containing, apoptosis associated protein 3	0.9913	0.9148	NA
68760	Synpo2l	synaptopodin 2-like	0.9913	0.9383	0.9823
66525	Timm50	translocase of inner mitochondrial membrane 50 homolog (yeast)	0.9913	0.9207	0.9768
18029	Nfic	nuclear factor I/C	0.9913	0.9306	0.9791
15208	Hes5	hairy and enhancer of split 5 (Drosophila)	0.9913	0.9294	0.9789
237320	Aldh8a1	aldehyde dehydrogenase 8 family, member A1	0.9912	0.9502	0.9859
215708	Fam73a	family with sequence similarity 73, member A	0.9912	0.8504	NA
106504	Stk38	serine/threonine kinase 38	0.9912	0.8732	NA
103172	Chchd10	coiled-coil-helix-coiled-coil-helix domain containing 10	0.9912	0.8726	NA
74522	Morc2a	microrchidia 2A	0.9912	0.9069	NA
72333	Palld	palladin, cytoskeletal associated protein	0.9912	0.9306	0.9791
70373	1700020O03Rik	RIKEN cDNA 1700020O03 gene	0.9912	0.8515	NA
68036	Zfp706	zinc finger protein 706	0.9912	0.9087	NA
20479	Vps4b	vacuolar protein sorting 4b (yeast)	0.9912	0.8689	NA
14688	Gnb1	guanine nucleotide binding protein (G protein), beta 1	0.9912	0.8969	NA
11886	Asah1	N-acylsphingosine amidohydrolase 1	0.9912	0.9108	NA
382867	Zfp488	zinc finger protein 488	0.9911	0.9253	0.9783
331524	Xkrx	X Kell blood group precursor related X linked	0.9911	0.9333	0.9805
319159	Hist1h4j	histone cluster 1, H4j	0.9911	0.9562	0.9876
242687	Wasf2	WAS protein family, member 2	0.9911	0.8907	NA
223227	Sox21	SRY-box containing gene 21	0.9911	0.9392	0.9824
74718	Snx16	sorting nexin 16	0.9911	0.8554	NA
72899	Macrod2	MACRO domain containing 2	0.9911	0.9177	0.9761
69902	Mrto4	MRT4, mRNA turnover 4, homolog (S. cerevisiae)	0.9911	0.8641	NA
22158	Tulp3	tubby-like protein 3	0.9911	0.9055	NA
11845	Arf6	ADP-ribosylation factor 6	0.9911	0.9261	0.9785
76441	Daam2	dishevelled associated activator of morphogenesis 2	0.991	0.9071	NA
76044	Ncapg2	non-SMC condensin II complex, subunit G2	0.991	0.8775	NA
72993	Appl1	adaptor protein, phosphotyrosine interaction, PH domain and leucine zipper containing 1	0.991	0.8551	NA
66609	Cryzl1	crystallin, zeta (quinone reductase)-like 1	0.991	0.8219	NA
66164	Nip7	nuclear import 7 homolog (S. cerevisiae)	0.991	0.8469	NA
20692	Sparc	secreted acidic cysteine rich glycoprotein	0.991	0.9769	0.9928
16007	Cyr61	cysteine rich protein 61	0.991	0.8755	NA
13877	Erh	enhancer of rudimentary homolog (Drosophila)	0.991	0.8345	NA
11739	Slc25a4	solute carrier family 25 (mitochondrial carrier, adenine nucleotide translocator), member 4	0.991	0.8436	NA
666185	Gm7969	predicted gene 7969	0.9909	0.8308	NA
66626	5730403B10Rik	RIKEN cDNA 5730403B10 gene	0.9909	0.9287	0.9789
29864	Rnf11	ring finger protein 11	0.9909	0.8719	NA
20826	Nhp2l1	NHP2 non-histone chromosome protein 2-like 1 (S. cerevisiae)	0.9909	0.8309	NA
13542	Dvl1	dishevelled, dsh homolog 1 (Drosophila)	0.9909	0.8664	NA

225131	Wac	WW domain containing adaptor with coiled-coil	0.9908	0.8418	NA
68910	Zfp467	zinc finger protein 467	0.9908	0.912	NA
14356	Fxc1	fractured callus expressed transcript 1	0.9908	0.8515	NA
384009	Glipr2	GLI pathogenesis-related 2	0.9907	0.9346	0.981
105504	Exoc5	exocyst complex component 5	0.9907	0.834	NA
67862	2310033P09Rik	RIKEN cDNA 2310033P09 gene	0.9907	0.8874	NA
50530	Mfap5	microfibrillar associated protein 5	0.9907	0.9455	0.9844
13601	Ecm1	extracellular matrix protein 1	0.9907	0.9178	0.9761
244058	Rgma	RGM domain family, member A	0.9906	0.9289	0.9789
110310	Krt7	keratin 7	0.9906	0.946	0.9846
103968	Plin1	perilipin 1	0.9906	0.9546	0.9872
16434	Itpa	inosine triphosphatase (nucleoside triphosphate pyrophosphatase)	0.9906	0.8527	NA
11908	Atf1	activating transcription factor 1	0.9906	0.8948	NA
381314	Iars2	isoleucine-tRNA synthetase 2, mitochondrial	0.9905	0.9075	NA
209630	Frmd4a	FERM domain containing 4A	0.9905	0.8641	NA
104458	Rars	arginyl-tRNA synthetase	0.9905	0.9514	0.9866
70315	Hdac8	histone deacetylase 8	0.9905	0.8922	NA
22596	Xrcc5	X-ray repair complementing defective repair in Chinese hamster cells 5	0.9905	0.7919	NA
13637	Efna2	ephrin A2	0.9905	0.916	0.9756
433771	2310028O11Rik	RIKEN cDNA 2310028O11 gene	0.9904	0.8862	NA
319587	4930539J05Rik	RIKEN cDNA 4930539J05 gene	0.9904	0.9415	0.9835
269941	Chsy1	chondroitin sulfate synthase 1	0.9904	0.8267	NA
268469	Zfp652	zinc finger protein 652	0.9904	0.8277	NA
225215	Rsl24d1	ribosomal L24 domain containing 1	0.9904	0.9017	NA
225010	Lclat1	lysocardiolipin acyltransferase 1	0.9904	0.8311	NA
170625	Snx18	sorting nexin 18	0.9904	0.8349	NA
100494	Zfand2a	zinc finger, AN1-type domain 2A	0.9904	0.8644	NA
66585	Snrnp40	small nuclear ribonucleoprotein 40 (U5)	0.9904	0.8509	NA
66260	Tmem54	transmembrane protein 54	0.9904	0.9664	0.9901
54377	Cacng4	calcium channel, voltage-dependent, gamma subunit 4	0.9904	0.9113	0.9742
50926	Hnrpdl	heterogeneous nuclear ribonucleoprotein D-like	0.9904	0.8992	NA
24136	Zeb2	zinc finger E-box binding homeobox 2	0.9904	0.8398	NA
24051	Sgcb	sarcoglycan, beta (dystrophin-associated glycoprotein)	0.9904	0.8936	NA
19247	Ptpn11	protein tyrosine phosphatase, non-receptor type 11	0.9904	0.8114	NA
664894	Gm13215	predicted gene 13215	0.9903	0.9176	0.9761
433287	Gm15455	predicted gene 15455	0.9903	0.8436	NA
214944	Mobkl2b	MOB1, Mps One Binder kinase activator-like 2B (yeast)	0.9903	0.8875	NA
140742	Sesn1	sestrin 1	0.9903	0.7923	NA
102857	Slc6a8	solute carrier family 6 (neurotransmitter transporter, creatine), member 8	0.9903	0.8327	NA
93688	Klhl1	kelch-like 1 (Drosophila)	0.9903	0.8493	NA
66641	Sike1	suppressor of IKBKE 1	0.9903	0.871	NA
57444	Isg20	interferon-stimulated protein	0.9903	0.9064	NA
52521	Zfp622	zinc finger protein 622	0.9903	0.8258	NA
30939	Pttg1	pituitary tumor-transforming gene 1	0.9903	0.8971	NA
18670	Abcb4	ATP-binding cassette, sub-family B (MDR/TAP), member 4	0.9903	0.8464	NA
11847	Arg2	arginase type II	0.9903	0.8743	NA
328699	Gabrr3	gamma-aminobutyric acid (GABA) receptor, rho 3	0.9902	0.9238	0.978
328265	A530001N23Rik	RIKEN cDNA A530001N23 gene	0.9902	0.9445	0.9842
218490	Btf3	basic transcription factor 3	0.9902	0.7848	NA

215193	AA408296	expressed sequence AA408296	0.9902	0.8144	NA
74100	Arpp21	cyclic AMP-regulated phosphoprotein, 21	0.9902	0.9321	0.9796
70823	Hmgxb4	HMG box domain containing 4	0.9902	0.7905	NA
69773	1810026J23Rik	RIKEN cDNA 1810026J23 gene	0.9902	0.8707	NA
67530	Uqcrb	ubiquinol-cytochrome c reductase binding protein	0.9902	0.8777	NA
107951	Cdk9	cyclin-dependent kinase 9 (CDC2-related kinase)	0.9901	0.8668	NA
93762	Smarca5	SWI/SNF related, matrix associated, actin dependent regulator of chromatin, subfamily a, member 5	0.9901	0.8957	NA
59053	Brp16	brain protein 16	0.9901	0.9121	0.9743
21915	Dtymk	deoxythymidylate kinase	0.9901	0.8599	NA
20467	Sin3b	transcriptional regulator, SIN3B (yeast)	0.9901	0.8485	NA
233274	Siglech	sialic acid binding Ig-like lectin H	0.99	0.9037	NA
229599	Gm129	predicted gene 129	0.99	0.925	0.9783
225215	Rsl24d1	ribosomal L24 domain containing 1	0.99	0.9225	0.9776
217069	Trim25	tripartite motif-containing 25	0.99	0.8975	NA
212516	BC060267	cDNA sequence BC060267	0.99	0.944	0.9842
97122	Hist2h4	histone cluster 2, H4	0.99	0.9352	0.981
74020	Cpne4	copine IV	0.99	0.8995	NA
73750	Whrn	whirlin	0.99	0.9093	0.9733
71687	Tmem25	transmembrane protein 25	0.99	0.8857	NA
70650	Zcchc8	zinc finger, CCHC domain containing 8	0.99	0.8521	NA
68194	Ndufb4	NADH dehydrogenase (ubiquinone) 1 beta subcomplex 4	0.99	0.8621	NA
67542	Cog6	component of oligomeric golgi complex 6	0.99	0.8534	NA
50701	Elane	elastase, neutrophil expressed	0.99	0.9638	0.9898
230234	BC026590	cDNA sequence BC026590	0.9899	0.8509	NA
208439	Klhl29	kelch-like 29 (Drosophila)	0.9899	0.8033	NA
78248	Armcx1	armadillo repeat containing, X-linked 1	0.9899	0.8608	NA
76217	Jakmip2	janus kinase and microtubule interacting protein 2	0.9899	0.8709	NA
74781	Wipi2	WD repeat domain, phosphoinositide interacting 2	0.9899	0.87	NA
72747	Ttc39c	tetratricopeptide repeat domain 39C	0.9899	0.8275	NA
67426	Adck3	aarF domain containing kinase 3	0.9899	0.9194	0.9765
26896	Med14	mediator complex subunit 14	0.9899	0.8603	NA
18521	Pcbp2	poly(rC) binding protein 2	0.9899	0.8335	NA
16401	Itga4	integrin alpha 4	0.9899	0.845	NA
623503	Prlh	prolactin releasing hormone	0.9898	0.9497	0.9857
433466	Jmjd7	jumonji domain containing 7	0.9898	0.8246	NA
333050	Ksr2	kinase suppressor of ras 2	0.9898	0.9783	0.9933
245695	Tceanc	transcription elongation factor A (SII) N-terminal and central domain containing	0.9898	0.9547	0.9872
107770	Tm6sf2	transmembrane 6 superfamily member 2	0.9898	0.9017	NA
54402	Stk19	serine/threonine kinase 19	0.9898	0.8479	NA
12867	Cox7c	cytochrome c oxidase, subunit VIIc	0.9898	0.9428	0.9838
11782	Ap4s1	adaptor-related protein complex AP-4, sigma 1	0.9898	0.9089	0.9733
208924	A730045E13Rik	RIKEN cDNA A730045E13 gene	0.9897	0.9443	0.9842
109108	Slc30a9	solute carrier family 30 (zinc transporter), member 9	0.9897	0.8923	NA
74761	Mxra8	matrix-remodelling associated 8	0.9897	0.9481	0.9854
71275	4933437F05Rik	RIKEN cDNA 4933437F05 gene	0.9897	0.9025	NA
20416	Shc1	src homology 2 domain-containing transforming protein C1	0.9897	0.9129	0.9743
11923	Neurod4	neurogenic differentiation 4	0.9897	0.9169	0.9759
414072	BC031361	cDNA sequence BC031361	0.9896	0.8632	NA
384864	Gm1943	WD repeat domain 70 pseudogene	0.9896	0.8414	NA

109264	Me3	malic enzyme 3, NADP(+)-dependent, mitochondrial	0.9896	0.8889	NA
79264	Krit1	KRIT1, ankyrin repeat containing	0.9896	0.8944	NA
74838	Naa15	N(alpha)-acetyltransferase 15, NatA auxiliary subunit	0.9896	0.9038	0.9717
71973	Rbpms2	RNA binding protein with multiple splicing 2	0.9896	0.8242	NA
66395	Ahnak	AHNAK nucleoprotein (desmoyokin)	0.9896	0.9492	0.9856
66142	Cox7b	cytochrome c oxidase subunit VIIb	0.9896	0.8767	NA
18044	Nfya	nuclear transcription factor-Y alpha	0.9896	0.8947	NA
433752	AA415398	expressed sequence AA415398	0.9895	0.8232	NA
93696	Chrac1	chromatin accessibility complex 1	0.9895	0.8143	NA
79456	Recql4	RecQ protein-like 4	0.9895	0.9058	0.972
68165	Fdx1l	ferredoxin 1-like	0.9895	0.8811	NA
57908	Zfp318	zinc finger protein 318	0.9895	0.8376	NA
50927	Nasp	nuclear autoantigenic sperm protein (histone-binding)	0.9895	0.8669	NA
26440	Psma1	proteasome (prosome, macropain) subunit, alpha type 1	0.9895	0.8043	NA
18231	Nxph1	neurexophilin 1	0.9895	0.9296	0.9789
68094	Smarcc2	SWI/SNF related, matrix associated, actin dependent regulator of chromatin, subfamily c, member 2	0.9894	0.9154	0.9753
30938	Fgd3	FYVE, RhoGEF and PH domain containing 3	0.9894	0.9292	0.9789
21843	Tial1	Tia1 cytotoxic granule-associated RNA binding protein-like 1	0.9894	0.799	NA
17179	Matk	megakaryocyte-associated tyrosine kinase	0.9894	0.892	NA
140887	Lnx2	ligand of numb-protein X 2	0.9893	0.8026	NA
108156	Mthfd1	methylenetetrahydrofolate dehydrogenase (NADP+ dependent), methenyltetrahydrofolate cyclohydrolase, formyltetrahydrofolate synthase	0.9893	0.8004	NA
73542	Tssk5	testis-specific serine kinase 5	0.9893	0.9639	0.9898
67867	Lrrc28	leucine rich repeat containing 28	0.9893	0.8672	NA
57138	Slc12a5	solute carrier family 12, member 5	0.9893	0.8605	NA
22003	Tpm1	tropomyosin 1, alpha	0.9893	0.891	NA
21985	Tpd52	tumor protein D52	0.9893	0.7904	NA
18526	Pcdh10	protocadherin 10	0.9893	0.845	NA
12035	Bcat1	branched chain aminotransferase 1, cytosolic	0.9893	0.8724	NA
434460	Gm5623	predicted gene 5623	0.9892	0.8671	NA
381903	Alg8	asparagine-linked glycosylation 8 homolog (yeast, alpha-1,3-glucosyltransferase)	0.9892	0.8269	NA
272396	Tarsl2	threonyl-tRNA synthetase-like 2	0.9892	0.8137	NA
104110	Adcy4	adenylate cyclase 4	0.9892	0.9352	0.981
98711	Rdh10	retinol dehydrogenase 10 (all-trans)	0.9892	0.8563	NA
70930	Nol8	nucleolar protein 8	0.9892	0.8017	NA
67222	Srfbp1	serum response factor binding protein 1	0.9892	0.844	NA
66147	Necap2	NECAP endocytosis associated 2	0.9892	0.8652	NA
64436	Inpp5e	inositol polyphosphate-5-phosphatase E	0.9892	0.9322	0.9796
54201	Zfp316	zinc finger protein 316	0.9892	0.9187	0.9764
14385	Slc37a4	solute carrier family 37 (glucose-6-phosphate transporter), member 4	0.9892	0.8316	NA
12343	Capza2	capping protein (actin filament) muscle Z-line, alpha 2	0.9892	0.8654	NA
628596	Gm6900	predicted gene 6900	0.9891	0.8384	NA
236537	Zfp352	zinc finger protein 352	0.9891	0.8479	NA
112406	Egln2	EGL nine homolog 2 (C. elegans)	0.9891	0.8606	NA
101835	AW146154	expressed sequence AW146154	0.9891	0.8431	NA
74185	Gbe1	glucan (1,4-alpha-), branching enzyme 1	0.9891	0.8884	NA
68423	Ankrd13d	ankyrin repeat domain 13 family, member D	0.9891	0.8664	NA
67269	Agtpbp1	ATP/GTP binding protein 1	0.9891	0.8269	NA
51944	D2Ertd750e	DNA segment, Chr 2, ERATO Doi 750, expressed	0.9891	0.8632	NA
20741	Spnb1	spectrin beta 1	0.9891	0.875	NA

15441	Hp1bp3	heterochromatin protein 1, binding protein 3	0.9891	0.833	NA
15247	Hiat1	hippocampus abundant gene transcript 1	0.9891	0.7979	NA
14313	Fst	follistatin	0.9891	0.9167	0.9759
319173	Hist1h2af	histone cluster 1, H2af	0.989	0.8568	NA
232933	Ccdc61	coiled-coil domain containing 61	0.989	0.9089	0.9733
106042	Prickle1	prickle homolog 1 (Drosophila)	0.989	0.8447	NA
74315	Rnf145	ring finger protein 145	0.989	0.7918	NA
74102	Slc35a5	solute carrier family 35, member A5	0.989	0.8924	NA
56705	Ranbp9	RAN binding protein 9	0.989	0.8659	NA
26364	Cd97	CD97 antigen	0.989	0.8606	NA
17690	Msi1	Musashi homolog 1(Drosophila)	0.989	0.9316	0.9795
219132	D14Ertd668e	DNA segment, Chr 14, ERATO Doi 668, expressed	0.9889	0.9392	0.9824
218914	Wapal	wings apart-like homolog (Drosophila)	0.9889	0.893	NA
102570	Slc22a13	solute carrier family 22 (organic cation transporter), member 13	0.9889	0.8995	0.9706
78913	Ltn1	listerin E3 ubiquitin protein ligase 1	0.9889	0.8635	NA
72421	Ttc30b	tetratricopeptide repeat domain 30B	0.9889	0.8831	NA
68646	1110020G09Rik	RIKEN cDNA 1110020G09 gene	0.9889	0.7644	NA
66885	Acadsb	acyl-Coenzyme A dehydrogenase, short/branched chain	0.9889	0.9422	0.9837
66593	Diablo	diablo homolog (Drosophila)	0.9889	0.8236	NA
56364	Zmym3	zinc finger, MYM-type 3	0.9889	0.8403	NA
19367	Rad9	RAD9 homolog (S. pombe)	0.9889	0.8549	NA
18146	Npdc1	neural proliferation, differentiation and control gene 1	0.9889	0.8509	NA
15950	Ifi203	interferon activated gene 203	0.9889	0.9319	0.9795
100504720	LOC100504720	hypothetical LOC100504720	0.9888	0.9305	0.9791
619331	Zfp551	zinc fingr protein 551	0.9888	0.8846	NA
83925	Trps1	trichorhinophalangeal syndrome I (human)	0.9888	0.8866	NA
76947	2310030N02Rik	RIKEN cDNA 2310030N02 gene	0.9888	0.7944	NA
74778	Rrp7a	ribosomal RNA processing 7 homolog A (S. cerevisiae)	0.9888	0.7889	NA
73710	Tubb2b	tubulin, beta 2B	0.9888	0.963	0.9893
18519	Kat2b	K(lysine) acetyltransferase 2B	0.9888	0.8962	0.9699
209707	Lcorl	ligand dependent nuclear receptor corepressor-like	0.9887	0.8722	NA
109264	Me3	malic enzyme 3, NADP(+)-dependent, mitochondrial	0.9887	0.8236	NA
104001	Rtn1	reticulon 1	0.9887	0.826	NA
101497	Plekhg2	pleckstrin homology domain containing, family G (with RhoGef domain) member 2	0.9887	0.9095	0.9735
83704	Slc12a9	solute carrier family 12 (potassium/chloride transporters), member 9	0.9887	0.9207	0.9768
67122	Nrarp	Notch-regulated ankyrin repeat protein	0.9887	0.9135	0.9745
65105	Arl6ip4	ADP-ribosylation factor-like 6 interacting protein 4	0.9887	0.7855	NA
14155	Fem1b	feminization 1 homolog b (C. elegans)	0.9887	0.8303	NA
545554	Ankrd34a	ankyrin repeat domain 34A	0.9886	0.9085	0.9733
330323	Fam188b	family with sequence similarity 188, member B	0.9886	0.9495	0.9856
329160	9130024F11Rik	RIKEN cDNA 9130024F11 gene	0.9886	0.8613	NA
320046	F730043M19Rik	RIKEN cDNA F730043M19 gene	0.9886	0.8911	NA
272359	Irf2bp1	interferon regulatory factor 2 binding protein 1	0.9886	0.9028	0.9717
214505	Gnptg	N-acetylglucosamine-1-phosphotransferase, gamma subunit	0.9886	0.8642	NA
109791	Clps	colipase, pancreatic	0.9886	0.9447	0.9844
108116	Slco3a1	solute carrier organic anion transporter family, member 3a1	0.9886	0.8161	NA
103136	Pwp1	PWP1 homolog (S. cerevisiae)	0.9886	0.8185	NA
76251	0610007P08Rik	RIKEN cDNA 0610007P08 gene	0.9886	0.9335	0.9805
66412	Arrdc4	arrestin domain containing 4	0.9886	0.8925	NA

20641	Snrpd1	small nuclear ribonucleoprotein D1	0.9886	0.864	NA
14976	H2-Ke2	H2-K region expressed gene 2	0.9886	0.8643	NA
638532	Gm7241	predicted pseudogene 7241	0.9885	0.8199	NA
237221	Gemin8	gem (nuclear organelle) associated protein 8	0.9885	0.7893	NA
209707	Lcorl	ligand dependent nuclear receptor corepressor-like	0.9885	0.919	0.9764
108143	Taf9	TAF9 RNA polymerase II, TATA box binding protein (TBP)-associated factor	0.9885	0.8936	0.9693
101700	Trim68	tripartite motif-containing 68	0.9885	0.9084	0.9733
100710	Pds5b	PDS5, regulator of cohesion maintenance, homolog B (S. cerevisiae)	0.9885	0.9209	0.9768
78655	Eif3j	eukaryotic translation initiation factor 3, subunit J	0.9885	0.8076	NA
66826	Taz	tafazzin	0.9885	0.8272	NA
58802	Kcnmb4	potassium large conductance calcium-activated channel, subfamily M, beta member 4	0.9885	0.8987	0.9703
22154	Tubb5	tubulin, beta 5	0.9885	0.9045	0.9717
12928	Crk	v-crk sarcoma virus CT10 oncogene homolog (avian)	0.9885	0.9268	0.9787
240518	Peli3	pellino 3	0.9884	0.9176	0.9761
230596	Prpf38a	PRP38 pre-mRNA processing factor 38 (yeast) domain containing A	0.9884	0.8037	NA
226414	Dars	aspartyl-tRNA synthetase	0.9884	0.9194	0.9765
215705	Arrdc1	arrestin domain containing 1	0.9884	0.8727	NA
208618	Etl4	enhancer trap locus 4	0.9884	0.9248	0.9783
98404	AI597479	expressed sequence AI597479	0.9884	0.7225	NA
77480	Kidins220	kinase D-interacting substrate 220	0.9884	0.8693	NA
76179	Usp31	ubiquitin specific peptidase 31	0.9884	0.8767	NA
72383	Cnfn	cornifelin	0.9884	0.9434	0.9839
72323	Asb6	ankyrin repeat and SOCS box-containing 6	0.9884	0.9124	0.9743
54713	Fezf2	Fez family zinc finger 2	0.9884	0.9048	0.9717
100042480	Nhsl2	NHS-like 2	0.9883	0.7774	NA
257921	Olfr1229	olfactory receptor 1229	0.9883	0.9261	0.9785
225207	Zfp521	zinc finger protein 521	0.9883	0.8919	0.9689
216459	Myl6b	myosin, light polypeptide 6B	0.9883	0.9212	0.977
105000	Dnalc1	dynein, axonemal, light chain 1	0.9883	0.9714	0.9919
77093	A330094K24Rik	RIKEN cDNA A330094K24 gene	0.9883	0.8094	NA
74136	Sec14l1	SEC14-like 1 (S. cerevisiae)	0.9883	0.919	0.9764
66262	Ing5	inhibitor of growth family, member 5	0.9883	0.8176	NA
56697	Akap10	A kinase (PRKA) anchor protein 10	0.9883	0.8342	NA
53951	Ccdc75	coiled-coil domain containing 75	0.9883	0.8672	NA
20301	Ccl27a	chemokine (C-C motif) ligand 27A	0.9883	0.8065	NA
100043902	Six3os1	Six3 opposite strand transcript 1	0.9882	0.9038	0.9717
106326	Osbpl11	oxysterol binding protein-like 11	0.9882	0.8474	NA
84652	Fam126a	family with sequence similarity 126, member A	0.9882	0.8201	NA
73137	Prrc1	proline-rich coiled-coil 1	0.9882	0.851	NA
65019	Rpl23	ribosomal protein L23	0.9882	0.8675	NA
64655	Mrps22	mitochondrial ribosomal protein S22	0.9882	0.8288	NA
59040	Rhot1	ras homolog gene family, member T1	0.9882	0.8632	NA
338355	Fkbp15	FK506 binding protein 15	0.9881	0.9347	0.981
212391	Lcor	ligand dependent nuclear receptor corepressor	0.9881	0.8771	NA
100213	Rusc2	RUN and SH3 domain containing 2	0.9881	0.8621	NA
72774	Neil1	nei endonuclease VIII-like 1 (E. coli)	0.9881	0.9082	0.9733
69721	Nkiras1	NFKB inhibitor interacting Ras-like protein 1	0.9881	0.8948	0.9698
66849	Ppp1r2	protein phosphatase 1, regulatory (inhibitor) subunit 2	0.9881	0.9603	0.9886
20610	Sumo3	SMT3 suppressor of mif two 3 homolog 3 (yeast)	0.9881	0.8769	NA

497255	LOC497255	hypothetical LOC497255	0.988	0.9076	0.9733
192176	Flna	filamin, alpha	0.988	0.8663	NA
170716	Cyp4f13	cytochrome P450, family 4, subfamily f, polypeptide 13	0.988	0.8625	NA
69544	Wdr5b	WD repeat domain 5B	0.988	0.7439	NA
56297	Arl6	ADP-ribosylation factor-like 6	0.988	0.8472	NA
21781	Tfdp1	transcription factor Dp 1	0.988	0.884	NA
20511	Slc1a2	solute carrier family 1 (glial high affinity glutamate transporter), member 2	0.988	0.8933	0.9693
12581	Cdkn2d	cyclin-dependent kinase inhibitor 2D (p19, inhibits CDK4)	0.988	0.7547	NA
12576	Cdkn1b	cyclin-dependent kinase inhibitor 1B	0.988	0.8792	NA
238024	Fn3krp	fructosamine 3 kinase related protein	0.9879	0.9084	0.9733
230075	Ndufb6	NADH dehydrogenase (ubiquinone) 1 beta subcomplex, 6	0.9879	0.813	NA
229937	Znhit6	zinc finger, HIT type 6	0.9879	0.8281	NA
228545	Vps18	vacuolar protein sorting 18 (yeast)	0.9879	0.7453	NA
170752	Bco2	beta-carotene oxygenase 2	0.9879	0.8406	NA
114600	Gm4736	predicted gene 4736	0.9879	0.9296	0.9789
74091	Npl	N-acetylneuraminate pyruvate lyase	0.9879	0.8982	0.9699
50793	Orc3	origin recognition complex, subunit 3	0.9879	0.7872	NA
22646	Zfp105	zinc finger protein 105	0.9879	0.7865	NA
18415	Hspa4l	heat shock protein 4 like	0.9879	0.7636	NA
214254	Nudt15	nudix (nucleoside diphosphate linked moiety X)-type motif 15	0.9878	0.9257	0.9784
106200	Txndc11	thioredoxin domain containing 11	0.9878	0.7511	NA
74756	5830408B19Rik	RIKEN cDNA 5830408B19 gene	0.9878	0.9101	0.9738
68039	Nmb	neuromedin B	0.9878	0.8852	0.9661
223881	Rnd1	Rho family GTPase 1	0.9877	0.8769	NA
77976	Nuak1	NUAK family, SNF1-like kinase, 1	0.9877	0.9009	0.9712
73261	1700037C18Rik	RIKEN cDNA 1700037C18 gene	0.9877	0.9257	0.9784
66494	Prelid1	PRELI domain containing 1	0.9877	0.8158	NA
66212	Sec61b	Sec61 beta subunit	0.9877	0.9089	0.9733
53859	Map3k14	mitogen-activated protein kinase kinase kinase 14	0.9877	0.8334	NA
17196	Mbp	myelin basic protein	0.9877	0.9618	0.9889
17155	Man1a	mannosidase 1, alpha	0.9877	0.9152	0.9753
330863	Trim67	tripartite motif-containing 67	0.9876	0.8035	NA
237806	Dnahc9	dynein, axonemal, heavy chain 9	0.9876	0.9223	0.9776
213827	Arcn1	archain 1	0.9876	0.9223	0.9776
192169	Ufsp2	UFM1-specific peptidase 2	0.9876	0.8293	NA
105833	Ccdc65	coiled-coil domain containing 65	0.9876	0.9225	0.9776
55960	Ebag9	estrogen receptor-binding fragment-associated gene 9	0.9876	0.7988	NA
53376	Usp2	ubiquitin specific peptidase 2	0.9876	0.8326	NA
22758	Zscan12	zinc finger and SCAN domain containing 12	0.9876	0.7554	NA
20024	Sub1	SUB1 homolog (S. cerevisiae)	0.9876	0.7643	NA
16981	Lrrn3	leucine rich repeat protein 3, neuronal	0.9876	0.7427	NA
13486	Dr1	down-regulator of transcription 1	0.9876	0.8852	0.9661
319740	Zfyve27	zinc finger, FYVE domain containing 27	0.9875	0.7783	NA
233046	Rasgrp4	RAS guanyl releasing protein 4	0.9875	0.9391	0.9824
215814	Ccdc28a	coiled-coil domain containing 28A	0.9875	0.8443	NA
72828	Ubash3b	ubiquitin associated and SH3 domain containing, B	0.9875	0.8199	NA
72068	Cnot2	CCR4-NOT transcription complex, subunit 2	0.9875	0.8224	NA
68260	Trmt12	tRNA methyltranferase 12 homolog (S. cerevisiae)	0.9875	0.8175	NA
67869	Paip2	polyadenylate-binding protein-interacting protein 2	0.9875	0.8248	NA

53421	Sec61a1	Sec61 alpha 1 subunit (S. cerevisiae)	0.9875	0.7142	NA
19216	Ptger1	prostaglandin E receptor 1 (subtype EP1)	0.9875	0.9347	0.981
14109	Fau	Finkel-Biskis-Reilly murine sarcoma virus (FBR-MuSV) ubiquitously expressed (fox derived)	0.9875	0.8802	NA
242691	Gpatch3	G patch domain containing 3	0.9874	0.8629	NA
72117	Naa50	N(alpha)-acetyltransferase 50, NatE catalytic subunit	0.9874	0.8764	NA
67890	Ufm1	ubiquitin-fold modifier 1	0.9874	0.8384	NA
30951	Cbx8	chromobox homolog 8 (Drosophila Pc class)	0.9874	0.954	0.9871
631906	Gm7075	predicted gene 7075	0.9873	0.9325	0.9798
435392	Gm5670	predicted gene 5670	0.9873	0.9568	0.9877
319915	A830049F12Rik	RIKEN cDNA A830049F12 gene	0.9873	0.8888	0.9676
252876	Gin1	gypsy retrotransposon integrase 1	0.9873	0.9628	0.9893
214345	Lrrc1	leucine rich repeat containing 1	0.9873	0.7978	NA
193452	Zfp184	zinc finger protein 184 (Kruppel-like)	0.9873	0.8702	NA
73490	Mipol1	mirror-image polydactyly gene 1 homolog (human)	0.9873	0.9488	0.9855
72198	Skiv2l2	superkiller viralicidic activity 2-like 2 (S. cerevisiae)	0.9873	0.7291	NA
69349	1700008O03Rik	RIKEN cDNA 1700008O03 gene	0.9873	0.927	0.9787
26380	Esrrb	estrogen related receptor, beta	0.9873	0.8767	NA
13819	Epas1	endothelial PAS domain protein 1	0.9873	0.8922	0.969
244757	Glb1l2	galactosidase, beta 1-like 2	0.9872	0.7613	NA
210105	Zfp719	zinc finger protein 719	0.9872	0.7586	NA
76804	Kdm4c	lysine (K)-specific demethylase 4C	0.9872	0.8336	NA
74388	Dpp8	dipeptidylpeptidase 8	0.9872	0.8182	NA
70510	Rnf167	ring finger protein 167	0.9872	0.8297	NA
67698	Fam174a	family with sequence similarity 174, member A	0.9872	0.7994	NA
67569	Mgat4c	mannosyl (alpha-1,3-)-glycoprotein beta-1,4-N-acetylglucosaminyltransferase, isozyme C (putative)	0.9872	0.8838	0.9652
66594	Uqcr11	ubiquinol-cytochrome c reductase, complex III subunit XI	0.9872	0.79	NA
57295	Icmt	isoprenylcysteine carboxyl methyltransferase	0.9872	0.8669	NA
17685	Msh2	mutS homolog 2 (E. coli)	0.9872	0.8057	NA
16367	Irs1	insulin receptor substrate 1	0.9872	0.8344	NA
13593	Ebf3	early B-cell factor 3	0.9872	0.7134	NA
12896	Cpt2	carnitine palmitoyltransferase 2	0.9872	0.8838	0.9652
668110	Syce1l	synaptonemal complex central element protein 1 like	0.9871	0.8613	NA
330812	Rnf150	ring finger protein 150	0.9871	0.9111	0.9741
78151	4930404H24Rik	RIKEN cDNA 4930404H24 gene	0.9871	0.9204	0.9768
69582	Plekhm2	pleckstrin homology domain containing, family M (with RUN domain) member 2	0.9871	0.7908	NA
66841	Etfdh	electron transferring flavoprotein, dehydrogenase	0.9871	0.8169	NA
15258	Hipk2	homeodomain interacting protein kinase 2	0.9871	0.8499	NA
407831	Tmem204	transmembrane protein 204	0.987	0.8445	NA
101358	Fbxl14	F-box and leucine-rich repeat protein 14	0.987	0.873	NA
80889	Mesdc1	mesoderm development candidate 1	0.987	0.8431	NA
67878	Tmem33	transmembrane protein 33	0.987	0.9086	0.9733
67212	Mrpl55	mitochondrial ribosomal protein L55	0.987	0.8639	NA
57913	Lrdd	leucine-rich and death domain containing	0.987	0.9286	0.9789
21923	Tnc	tenascin C	0.987	0.9493	0.9856
18760	Prkd1	protein kinase D1	0.987	0.7665	NA
100504346	Gm13304	predicted gene 13304	0.9869	0.9434	0.9839
321008	6330408A02Rik	RIKEN cDNA 6330408A02 gene	0.9869	0.8133	NA
268958	Capn11	calpain 11	0.9869	0.9284	0.9789
244864	Layn	layilin	0.9869	0.9398	0.9827

210356	Nckap5	NCK-associated protein 5	0.9869	0.8603	NA
110876	Scn2a1	sodium channel, voltage-gated, type II, alpha 1	0.9869	0.8349	NA
99045	Mrps26	mitochondrial ribosomal protein S26	0.9869	0.7467	NA
72508	Rps6kb1	ribosomal protein S6 kinase, polypeptide 1	0.9869	0.7379	NA
59008	Anapc5	anaphase-promoting complex subunit 5	0.9869	0.8368	NA
30944	Zfp354c	zinc finger protein 354C	0.9869	0.9227	0.9776
13207	Ddx5	DEAD (Asp-Glu-Ala-Asp) box polypeptide 5	0.9869	0.8001	NA
13025	Ctla2b	cytotoxic T lymphocyte-associated protein 2 beta	0.9869	0.8137	NA
386750	Slitrk3	SLIT and NTRK-like family, member 3	0.9868	0.8529	NA
232989	Hnrnpul1	heterogeneous nuclear ribonucleoprotein U-like 1	0.9868	0.9125	0.9743
216821	Tmem11	transmembrane protein 11	0.9868	0.761	NA
114886	Cygb	cytoglobin	0.9868	0.8509	NA
69155	1810030O07Rik	RIKEN cDNA 1810030O07 gene	0.9868	0.7913	NA
56705	Ranbp9	RAN binding protein 9	0.9868	0.8331	NA
21413	Tcf4	transcription factor 4	0.9868	0.8797	0.9631
16578	Kif9	kinesin family member 9	0.9868	0.8962	0.9699
12798	Cnn2	calponin 2	0.9868	0.9197	0.9765
11690	Alox5ap	arachidonate 5-lipoxygenase activating protein	0.9868	0.7602	NA
100043697	Gm9959	predicted gene 9959	0.9867	0.8595	NA
380664	Lemd3	LEM domain containing 3	0.9867	0.8607	NA
319991	Kif6	kinesin family member 6	0.9867	0.8997	0.9706
272031	E130309F12Rik	RIKEN cDNA E130309F12 gene	0.9867	0.8877	0.9672
268747	Lrrc16b	leucine rich repeat containing 16B	0.9867	0.8913	0.9687
107522	Ece2	endothelin converting enzyme 2	0.9867	0.8778	0.9626
70572	Ipo5	importin 5	0.9867	0.7518	NA
66997	Psmd12	proteasome (prosome, macropain) 26S subunit, non-ATPase, 12	0.9867	0.6991	NA
66094	Lsm7	LSM7 homolog, U6 small nuclear RNA associated (S. cerevisiae)	0.9867	0.7388	NA
30840	Fbxl6	F-box and leucine-rich repeat protein 6	0.9867	0.9284	0.9789
26436	Psg16	pregnancy specific glycoprotein 16	0.9867	0.9138	0.9745
20733	Spint2	serine protease inhibitor, Kunitz type 2	0.9867	0.7924	NA
17979	Ncoa3	nuclear receptor coactivator 3	0.9867	0.8255	NA
383563	Gpr25	G protein-coupled receptor 25	0.9866	0.9673	0.9904
260305	Nphp4	nephronophthisis 4 (juvenile) homolog (human)	0.9866	0.8472	NA
225028	Map4k3	mitogen-activated protein kinase kinase kinase kinase 3	0.9866	0.77	NA
170731	Mfn2	mitofusin 2	0.9866	0.8664	NA
67997	Ddx59	DEAD (Asp-Glu-Ala-Asp) box polypeptide 59	0.9866	0.8427	NA
67379	Dedd2	death effector domain-containing DNA binding protein 2	0.9866	0.7752	NA
66573	Dzip1	DAZ interacting protein 1	0.9866	0.9184	0.9764
66556	Drap1	Dr1 associated protein 1 (negative cofactor 2 alpha)	0.9866	0.787	NA
24013	Grk1	G protein-coupled receptor kinase 1	0.9866	0.8041	NA
20656	Sod2	superoxide dismutase 2, mitochondrial	0.9866	0.7579	NA
11938	Atp2a2	ATPase, Ca++ transporting, cardiac muscle, slow twitch 2	0.9866	0.8406	NA
231238	Sel1l3	sel-1 suppressor of lin-12-like 3 (C. elegans)	0.9865	0.8246	NA
170770	Bbc3	BCL2 binding component 3	0.9865	0.8421	NA
69349	1700008O03Rik	RIKEN cDNA 1700008O03 gene	0.9865	0.831	NA
26940	Ecsit	ECSIT homolog (Drosophila)	0.9865	0.7388	NA
16917	Lmx1b	LIM homeobox transcription factor 1 beta	0.9865	0.9483	0.9854
15259	Hipk3	homeodomain interacting protein kinase 3	0.9865	0.7911	NA
268996	Ss18	synovial sarcoma translocation, Chromosome 18	0.9864	0.7592	NA

104871	Spata7	spermatogenesis associated 7	0.9864	0.8327	NA
73886	4930431P19Rik	RIKEN cDNA 4930431P19 gene	0.9864	0.8413	NA
66440	Cdc26	cell division cycle 26	0.9864	0.8276	NA
53817	Bat1a	HLA-B-associated transcript 1A	0.9864	0.8247	NA
50708	Hist1h1c	histone cluster 1, H1c	0.9864	0.8025	NA
269615	Plch2	phospholipase C, eta 2	0.9863	0.8118	NA
217666	L2hgdh	L-2-hydroxyglutarate dehydrogenase	0.9863	0.8018	NA
212483	Fam193b	family with sequence similarity 193, member B	0.9863	0.7981	NA
101943	Sf3b3	splicing factor 3b, subunit 3	0.9863	0.86	NA
83429	Ctns	cystinosis, nephropathic	0.9863	0.8182	NA
56335	Mettl3	methyltransferase like 3	0.9863	0.8586	NA
53951	Ccdc75	coiled-coil domain containing 75	0.9863	0.8397	NA
51897	Atg13	ATG13 autophagy related 13 homolog (S. cerevisiae)	0.9863	0.8006	NA
22781	Ikzf4	IKAROS family zinc finger 4	0.9863	0.8936	0.9693
14699	Gngt1	guanine nucleotide binding protein (G protein), gamma transducing activity polypeptide 1	0.9863	0.8926	0.9692
12493	Cd37	CD37 antigen	0.9863	0.955	0.9872
215493	A3galt2	alpha 1,3-galactosyltransferase 2 (isoglobotriaosylceramide synthase)	0.9862	0.8782	0.9628
108911	Rcc2	regulator of chromosome condensation 2	0.9862	0.7342	NA
69430	1700048O20Rik	RIKEN cDNA 1700048O20 gene	0.9862	0.9498	0.9858
67695	Ost4	oligosaccharyltransferase 4 homolog (S. cerevisiae)	0.9862	0.8325	NA
28071	Twistnb	TWIST neighbor	0.9862	0.8754	0.9618
19356	Rad17	RAD17 homolog (S. pombe)	0.9862	0.8438	NA
15260	Hira	histone cell cycle regulation defective homolog A (S. cerevisiae)	0.9862	0.8267	NA
110651	Rps6ka3	ribosomal protein S6 kinase polypeptide 3	0.9861	0.8534	NA
101565	6330503K22Rik	RIKEN cDNA 6330503K22 gene	0.9861	0.7244	NA
74630	4930432N10Rik	RIKEN cDNA 4930432N10 gene	0.9861	0.8787	0.9628
69568	Vkorc1l1	vitamin K epoxide reductase complex, subunit 1-like 1	0.9861	0.8447	NA
67296	Socs4	suppressor of cytokine signaling 4	0.9861	0.8366	NA
14077	Fabp3	fatty acid binding protein 3, muscle and heart	0.9861	0.7662	NA
675985	Rps6-ps2	ribosomal protein S6, pseudogene 2	0.986	0.862	NA
320492	A830018L16Rik	RIKEN cDNA A830018L16 gene	0.986	0.8548	NA
212326	Fam149a	family with sequence similarity 149, member A	0.986	0.795	NA
103213	Traf3ip2	TRAF3 interacting protein 2	0.986	0.8608	NA
68770	Phtf2	putative homeodomain transcription factor 2	0.986	0.8198	NA
66648	5730494M16Rik	RIKEN cDNA 5730494M16 gene	0.986	0.9024	0.9716
26442	Psma5	proteasome (prosome, macropain) subunit, alpha type 5	0.986	0.7178	NA
22427	Wrn	Werner syndrome homolog (human)	0.986	0.8738	0.9616
17257	Mecp2	methyl CpG binding protein 2	0.986	0.8208	NA
13666	Eif2ak3	eukaryotic translation initiation factor 2 alpha kinase 3	0.986	0.7657	NA
100042757	Gm4013	predicted gene 4013	0.9859	0.9088	0.9733
214084	Slc18a2	solute carrier family 18 (vesicular monoamine), member 2	0.9859	0.8506	NA
110796	Tshz1	teashirt zinc finger family member 1	0.9859	0.8368	NA
67410	4930449I24Rik	RIKEN cDNA 4930449I24 gene	0.9859	0.9042	0.9717
66101	Ppih	peptidyl prolyl isomerase H	0.9859	0.6874	NA
28028	Mrpl50	mitochondrial ribosomal protein L50	0.9859	0.745	NA
26425	Nubp1	nucleotide binding protein 1	0.9859	0.7224	NA
19878	Rock2	Rho-associated coiled-coil containing protein kinase 2	0.9859	0.8063	NA
546663	Gm5963	predicted pseudogene 5963	0.9858	0.8676	0.9593
378430	Nanos2	nanos homolog 2 (Drosophila)	0.9858	0.9089	0.9733

242557	Atg4c	autophagy-related 4C (yeast)	0.9858	0.764	NA
224014	Fgd4	FYVE, RhoGEF and PH domain containing 4	0.9858	0.8715	0.9608
116939	Pnpla3	patatin-like phospholipase domain containing 3	0.9858	0.8157	NA
77041	Arsk	arylsulfatase K	0.9858	0.8422	NA
76871	4930422N03Rik	RIKEN cDNA 4930422N03 gene	0.9858	0.8733	0.9612
21982	Tmem165	transmembrane protein 165	0.9858	0.7733	NA
20512	Slc1a3	solute carrier family 1 (glial high affinity glutamate transporter), member 3	0.9858	0.7493	NA
18247	Oaz2-ps	ornithine decarboxylase antizyme 2, pseudogene	0.9858	0.8422	NA
14182	Fgfr1	fibroblast growth factor receptor 1	0.9858	0.911	0.9741
100042092	Gm3662	predicted gene 3662	0.9857	0.8742	0.9616
234664	Nae1	NEDD8 activating enzyme E1 subunit 1	0.9857	0.8859	0.9664
140629	Ubox5	U box domain containing 5	0.9857	0.8271	NA
74155	Errfi1	ERBB receptor feedback inhibitor 1	0.9857	0.7783	NA
68281	4930430F08Rik	RIKEN cDNA 4930430F08 gene	0.9857	0.8063	NA
67618	Aasdhppt	aminoadipate-semialdehyde dehydrogenase-phosphopantetheinyl transferase	0.9857	0.8973	0.9699
67501	Ccdc50	coiled-coil domain containing 50	0.9857	0.827	NA
64009	Syne1	synaptic nuclear envelope 1	0.9857	0.8263	NA
18185	Nrl	neural retina leucine zipper gene	0.9857	0.8266	NA
16922	Phyh	phytanoyl-CoA hydroxylase	0.9857	0.6727	NA
623174	Gm6404	predicted gene 6404	0.9856	0.8297	NA
621312	Gm6211	predicted gene 6211	0.9856	0.7761	NA
237353	Sh3rf3	SH3 domain containing ring finger 3	0.9856	0.8081	NA
233870	Tufm	Tu translation elongation factor, mitochondrial	0.9856	0.8193	NA
227699	Nup188	nucleoporin 188	0.9856	0.8866	0.9669
208884	Zdhhc9	zinc finger, DHHC domain containing 9	0.9856	0.9348	0.981
117171	1110038F14Rik	RIKEN cDNA 1110038F14 gene	0.9856	0.7889	NA
97487	Cmtm4	CKLF-like MARVEL transmembrane domain containing 4	0.9856	0.8424	NA
72425	2410042D21Rik	RIKEN cDNA 2410042D21 gene	0.9856	0.8498	NA
68581	Tmed10	transmembrane emp24-like trafficking protein 10 (yeast)	0.9856	0.8203	NA
53886	Cdkl2	cyclin-dependent kinase-like 2 (CDC2-related kinase)	0.9856	0.7586	NA
30948	Bin1	bridging integrator 1	0.9856	0.7707	NA
12892	Cpox	coproporphyrinogen oxidase	0.9856	0.8266	NA
100038759	LOC100038759	hypothetical LOC100038759	0.9855	0.9093	0.9733
625353	Vmn2r35	vomeronasal 2, receptor 35	0.9855	0.9051	0.9718
223527	Eny2	enhancer of yellow 2 homolog (Drosophila)	0.9855	0.7791	NA
116837	Rims1	regulating synaptic membrane exocytosis 1	0.9855	0.8854	0.9663
113868	Acaa1a	acetyl-Coenzyme A acyltransferase 1A	0.9855	0.7908	NA
60596	Gucy1a3	guanylate cyclase 1, soluble, alpha 3	0.9855	0.7264	NA
30059	Timm10	translocase of inner mitochondrial membrane 10 homolog (yeast)	0.9855	0.806	NA
19653	Rbm4	RNA binding motif protein 4	0.9855	0.7122	NA
13110	Cyp2j6	cytochrome P450, family 2, subfamily j, polypeptide 6	0.9855	0.8277	NA
328019	4933400C05Rik	RIKEN cDNA 4933400C05 gene	0.9854	0.9303	0.979
218215	Rnf144b	ring finger protein 144B	0.9854	0.8609	NA
216613	Ccdc85a	coiled-coil domain containing 85A	0.9854	0.8493	NA
80904	Dtx3	deltex 3 homolog (Drosophila)	0.9854	0.9353	0.981
78832	2700078E11Rik	RIKEN cDNA 2700078E11 gene	0.9854	0.8354	NA
68981	Snrpa1	small nuclear ribonucleoprotein polypeptide A'	0.9854	0.8107	NA
58175	Rgs20	regulator of G-protein signaling 20	0.9854	0.8333	NA
17912	Myo1b	myosin IB	0.9854	0.8272	NA

12850	Coq7	demethyl-Q 7	0.9854	0.9158	0.9756
12396	Cbfa2t2	core-binding factor, runt domain, alpha subunit 2, translocated to, 2 (human)	0.9854	0.6636	NA
269643	Ppp2r2c	protein phosphatase 2 (formerly 2A), regulatory subunit B (PR 52), gamma isoform	0.9853	0.7793	NA
235587	Parp3	poly (ADP-ribose) polymerase family, member 3	0.9853	0.9053	0.9718
101883	Tmem149	transmembrane protein 149	0.9853	0.845	NA
75403	1010001B22Rik	RIKEN cDNA 1010001B22 gene	0.9853	0.942	0.9835
74252	Armc1	armadillo repeat containing 1	0.9853	0.7701	NA
69444	Lyzl6	lysozyme-like 6	0.9853	0.8933	0.9693
69307	Pxt1	peroxisomal, testis specific 1	0.9853	0.8916	0.9688
66861	Dnajc10	DnaJ (Hsp40) homolog, subfamily C, member 10	0.9853	0.8955	0.9698
66193	1110049F12Rik	RIKEN cDNA 1110049F12 gene	0.9853	0.8419	NA
56044	Rala	v-ral simian leukemia viral oncogene homolog A (ras related)	0.9853	0.743	NA
11652	Akt2	thymoma viral proto-oncogene 2	0.9853	0.8937	0.9693
100502841	5430411K18Rik	RIKEN cDNA 5430411K18 gene	0.9852	0.8034	NA
381434	C230057A21Rik	RIKEN cDNA C230057A21 gene	0.9852	0.971	0.9916
93840	Vangl2	vang-like 2 (van gogh, Drosophila)	0.9852	0.9233	0.9777
69162	Sec31a	Sec31 homolog A (S. cerevisiae)	0.9852	0.8458	NA
57778	Fmnl1	formin-like 1	0.9852	0.9578	0.9881
56088	Psmg1	proteasome (prosome, macropain) assembly chaperone 1	0.9852	0.7274	NA
53605	Nap1l1	nucleosome assembly protein 1-like 1	0.9852	0.7326	NA
19291	Purb	purine rich element binding protein B	0.9852	0.9129	0.9743
17873	Gadd45b	growth arrest and DNA-damage-inducible 45 beta	0.9852	0.8629	0.9574
14813	Grin2c	glutamate receptor, ionotropic, NMDA2C (epsilon 3)	0.9852	0.8267	NA
100043885	Gm4710	predicted gene 4710	0.9851	0.9477	0.9853
319263	Pcmtd1	protein-L-isoaspartate (D-aspartate) O-methyltransferase domain containing 1	0.9851	0.9003	0.9709
232807	Ppp1r12c	protein phosphatase 1, regulatory (inhibitor) subunit 12C	0.9851	0.8201	NA
103284	Zc3h10	zinc finger CCCH type containing 10	0.9851	0.7776	NA
77864	Ypel2	yippee-like 2 (Drosophila)	0.9851	0.7236	NA
69957	Cdc16	CDC16 cell division cycle 16 homolog (S. cerevisiae)	0.9851	0.7471	NA
67248	Rpl39	ribosomal protein L39	0.9851	0.8098	NA
19775	Xpr1	xenotropic and polytropic retrovirus receptor 1	0.9851	0.8586	NA
19124	Procr	protein C receptor, endothelial	0.9851	0.866	0.9586
18139	Zfml	zinc finger, matrin-like	0.9851	0.7267	NA
217410	Trib2	tribbles homolog 2 (Drosophila)	0.985	0.8231	NA
94067	Mrpl43	mitochondrial ribosomal protein L43	0.985	0.6925	NA
74120	Zfp263	zinc finger protein 263	0.985	0.77	NA
70294	Rnf126	ring finger protein 126	0.985	0.9129	0.9743
57815	Spata5	spermatogenesis associated 5	0.985	0.8304	NA
20254	Scg2	secretogranin II	0.985	0.6982	NA
14853	Gspt2	G1 to S phase transition 2	0.985	0.7106	NA
14595	B4galt1	UDP-Gal:betaGlcNAc beta 1,4- galactosyltransferase, polypeptide 1	0.985	0.7468	NA
14312	Brd2	bromodomain containing 2	0.985	0.7915	NA
13929	Amz2	archaelysin family metallopeptidase 2	0.985	0.8626	0.9574
234686	Fhod1	formin homology 2 domain containing 1	0.9849	0.8358	NA
226407	Rab3gap1	RAB3 GTPase activating protein subunit 1	0.9849	0.8769	0.9622
66647	Ndnl2	necdin-like 2	0.9849	0.7538	NA
66599	Rdm1	RAD52 motif 1	0.9849	0.9105	0.9739
66361	Zfand1	zinc finger, AN1-type domain 1	0.9849	0.8091	NA
24135	Zfp68	zinc finger protein 68	0.9849	0.8116	NA

19325	Rab10	RAB10, member RAS oncogene family	0.9849	0.8623	0.9574
16647	Kpna2	karyopherin (importin) alpha 2	0.9849	0.7946	NA
13869	Erbb4	v-erb-a erythroblastic leukemia viral oncogene homolog 4 (avian)	0.9849	0.8581	0.9562
269470	Wdr3	WD repeat domain 3	0.9848	0.7385	NA
232966	Zfp114	zinc finger protein 114	0.9848	0.866	0.9586
101471	Phrf1	PHD and ring finger domains 1	0.9848	0.8134	NA
73658	Spns1	spinster homolog 1 (Drosophila)	0.9848	0.7035	NA
67676	Rpp21	ribonuclease P 21 subunit (human)	0.9848	0.7047	NA
19650	Rbl1	retinoblastoma-like 1 (p107)	0.9848	0.8229	NA
18575	Pde1c	phosphodiesterase 1C	0.9848	0.9254	0.9783
14919	Gucy2e	guanylate cyclase 2e	0.9848	0.8092	NA
433416	Gm13547	predicted gene 13547	0.9847	0.8934	0.9693
97484	Cog8	component of oligomeric golgi complex 8	0.9847	0.8762	0.9619
73738	Haus7	HAUS augmin-like complex, subunit 7	0.9847	0.8121	NA
70335	Reep6	receptor accessory protein 6	0.9847	0.8875	0.9671
70026	Tspo2	translocator protein 2	0.9847	0.9365	0.9814
66129	1110018J18Rik	RIKEN cDNA 1110018J18 gene	0.9847	0.8747	0.9617
58220	Pard6b	par-6 (partitioning defective 6) homolog beta (C. elegans)	0.9847	0.9065	0.9725
56406	Ncoa6	nuclear receptor coactivator 6	0.9847	0.8564	0.9554
50493	Txnrd1	thioredoxin reductase 1	0.9847	0.7447	NA
14156	Fen1	flap structure specific endonuclease 1	0.9847	0.7594	NA
320655	Pgap3	post-GPI attachment to proteins 3	0.9846	0.8878	0.9673
244585	Rpgrip1l	Rpgrip1-like	0.9846	0.7292	NA
208748	Prrg3	proline rich Gla (G-carboxyglutamic acid) 3 (transmembrane)	0.9846	0.8214	NA
102093	Phkb	phosphorylase kinase beta	0.9846	0.8326	NA
75646	Rai14	retinoic acid induced 14	0.9846	0.8281	NA
72931	2900010J23Rik	RIKEN cDNA 2900010J23 gene	0.9846	0.7109	NA
70998	Phf6	PHD finger protein 6	0.9846	0.7156	NA
58243	Nap1l5	nucleosome assembly protein 1-like 5	0.9846	0.8044	NA
55994	Smad9	MAD homolog 9 (Drosophila)	0.9846	0.8591	0.9565
55936	Ctps2	cytidine 5'-triphosphate synthase 2	0.9846	0.7976	NA
26965	Cul1	cullin 1	0.9846	0.8589	0.9564
19346	Rab6	RAB6, member RAS oncogene family	0.9846	0.7022	NA
237898	Usp32	ubiquitin specific peptidase 32	0.9845	0.7227	NA
233410	Zfp592	zinc finger protein 592	0.9845	0.7893	NA
231997	Fkbp14	FK506 binding protein 14	0.9845	0.8	NA
218630	Ccno	cyclin O	0.9845	0.88	0.9631
218397	Rasa1	RAS p21 protein activator 1	0.9845	0.8635	0.9577
209683	Ttc28	tetratricopeptide repeat domain 28	0.9845	0.849	NA
108937	Rnf169	ring finger protein 169	0.9845	0.6637	NA
56309	Mycbp	c-myc binding protein	0.9845	0.8048	NA
12215	Bsg	basigin	0.9845	0.8875	0.9671
545554	Ankrd34a	ankyrin repeat domain 34A	0.9844	0.785	NA
320343	Lypd6	LY6/PLAUR domain containing 6	0.9844	0.7922	NA
234593	Ndrg4	N-myc downstream regulated gene 4	0.9844	0.8749	0.9617
230908	Tardbp	TAR DNA binding protein	0.9844	0.7448	NA
217698	Acot5	acyl-CoA thioesterase 5	0.9844	0.9172	0.9759
116891	Derl2	Der1-like domain family, member 2	0.9844	0.7551	NA
107065	Lrrtm2	leucine rich repeat transmembrane neuronal 2	0.9844	0.887	0.9671

103466	Nt5dc3	5'-nucleotidase domain containing 3	0.9844	0.8274	NA
73830	Eif3k	eukaryotic translation initiation factor 3, subunit K	0.9844	0.7791	NA
70984	4931406C07Rik	RIKEN cDNA 4931406C07 gene	0.9844	0.7721	NA
67454	Ikbip	IKBKB interacting protein	0.9844	0.8229	NA
11949	Atp5c1	ATP synthase, H+ transporting, mitochondrial F1 complex, gamma polypeptide 1	0.9844	0.8274	NA
11777	Ap3s1	adaptor-related protein complex 3, sigma 1 subunit	0.9844	0.7706	NA
68554	1110001A16Rik	RIKEN cDNA 1110001A16 gene	0.9843	0.7729	NA
68510	Ints1	integrator complex subunit 1	0.9843	0.8723	0.9608
56469	Pias1	protein inhibitor of activated STAT 1	0.9843	0.9067	0.9725
11980	Atp8a1	ATPase, aminophospholipid transporter (APLT), class I, type 8A, member 1	0.9843	0.9361	0.9814
320873	Cdh10	cadherin 10	0.9842	0.8585	0.9564
271786	Galnt13	UDP-N-acetyl-alpha-D-galactosamine:polypeptide N-acetylgalactosaminyltransferase 13	0.9842	0.7317	NA
263876	Spata2	spermatogenesis associated 2	0.9842	0.829	NA
245446	Slitrk4	SLIT and NTRK-like family, member 4	0.9842	0.7799	NA
229791	D3Bwg0562e	DNA segment, Chr 3, Brigham & Women's Genetics 0562 expressed	0.9842	0.7156	NA
212670	Catsper2	cation channel, sperm associated 2	0.9842	0.8487	NA
70300	Fuz	fuzzy homolog (Drosophila)	0.9842	0.782	NA
69675	Pxdn	peroxidasin homolog (Drosophila)	0.9842	0.942	0.9835
66580	Esf1	ESF1, nucleolar pre-rRNA processing protein, homolog (S. cerevisiae)	0.9842	0.7159	NA
17294	Mest	mesoderm specific transcript	0.9842	0.7646	NA
433759	Hdac1	histone deacetylase 1	0.9841	0.6551	NA
380732	Gm885	predicted gene 885	0.9841	0.7567	NA
241915	Phc3	polyhomeotic-like 3 (Drosophila)	0.9841	0.7527	NA
224823	Rrp36	ribosomal RNA processing 36 homolog (S. cerevisiae)	0.9841	0.8237	NA
116972	Fam57a	family with sequence similarity 57, member A	0.9841	0.8447	NA
104248	Cabin1	calcineurin binding protein 1	0.9841	0.8661	0.9586
68310	Zmym1	zinc finger, MYM domain containing 1	0.9841	0.7386	NA
53895	Clpp	caseinolytic peptidase, ATP-dependent, proteolytic subunit homolog (E. coli)	0.9841	0.7849	NA
12626	Cetn3	centrin 3	0.9841	0.8775	0.9625
11518	Add1	adducin 1 (alpha)	0.9841	0.6996	NA
627939	Gm6813	predicted gene 6813	0.984	0.849	NA
432486	Gnptab	N-acetylglucosamine-1-phosphate transferase, alpha and beta subunits	0.984	0.7775	NA
353169	Slc2a12	solute carrier family 2 (facilitated glucose transporter), member 12	0.984	0.7264	NA
239037	Lrit1	leucine-rich repeat, immunoglobulin-like and transmembrane domains 1	0.984	0.7422	NA
106947	Slc39a3	solute carrier family 39 (zinc transporter), member 3	0.984	0.7681	NA
93691	Klf7	Kruppel-like factor 7 (ubiquitous)	0.984	0.8235	NA
78914	Nadsyn1	NAD synthetase 1	0.984	0.7954	NA
78381	2310047N11Rik	RIKEN cDNA 2310047N11 gene	0.984	0.9281	0.9789
58799	Crbn	cereblon	0.984	0.8794	0.9631
12925	Crip1	cysteine-rich protein 1 (intestinal)	0.984	0.8586	0.9564
574519	Vax2os1	Vax2 opposite strand transcript 1	0.9839	0.8901	0.9684
232227	Iqsec1	IQ motif and Sec7 domain 1	0.9839	0.8859	0.9664
224997	Dlgap1	discs, large (Drosophila) homolog-associated protein 1	0.9839	0.8882	0.9675
224904	2410015M20Rik	RIKEN cDNA 2410015M20 gene	0.9839	0.7737	NA
102442	Dennd4a	DENN/MADD domain containing 4A	0.9839	0.7914	NA
77116	Mtmr2	myotubularin related protein 2	0.9839	0.7583	NA
75710	Rbm12	RNA binding motif protein 12	0.9839	0.827	NA
72313	Fryl	furry homolog-like (Drosophila)	0.9839	0.8754	0.9618
69440	Fam116b	family with sequence similarity 116, member B	0.9839	0.8836	0.9652

59048	C1galt1c1	C1GALT1-specific chaperone 1	0.9839	0.762	NA
20663	Sos2	son of sevenless homolog 2 (Drosophila)	0.9839	0.7197	NA
19157	Cyth1	cytohesin 1	0.9839	0.8341	NA
654795	Sdr39u1	short chain dehydrogenase/reductase family 39U, member 1	0.9838	0.9128	0.9743
241919	Slc7a14	solute carrier family 7 (cationic amino acid transporter, y+ system), member 14	0.9838	0.7888	NA
170740	Zfp287	zinc finger protein 287	0.9838	0.6835	NA
114873	Dscaml1	Down syndrome cell adhesion molecule-like 1	0.9838	0.9207	0.9768
104362	Meig1	meiosis expressed gene 1	0.9838	0.7901	NA
69757	Leng1	leukocyte receptor cluster (LRC) member 1	0.9838	0.7206	NA
66912	Bzw2	basic leucine zipper and W2 domains 2	0.9838	0.7978	NA
66725	Lrrk2	leucine-rich repeat kinase 2	0.9838	0.7426	NA
66515	Cul7	cullin 7	0.9838	0.7621	NA
16653	Kras	v-Ki-ras2 Kirsten rat sarcoma viral oncogene homolog	0.9838	0.7862	NA
12936	Pcdha4	protocadherin alpha 4	0.9838	0.871	0.9607
270110	Irf2bp2	interferon regulatory factor 2 binding protein 2	0.9837	0.8223	NA
236794	Slc9a6	solute carrier family 9 (sodium/hydrogen exchanger), member 6	0.9837	0.7556	NA
103537	Mbtd1	mbt domain containing 1	0.9837	0.7634	NA
73261	1700037C18Rik	RIKEN cDNA 1700037C18 gene	0.9837	0.8383	NA
72690	Grrp1	glycine/arginine rich protein 1	0.9837	0.8033	NA
67945	Rpl41	ribosomal protein L41	0.9837	0.7628	NA
67048	Vma21	VMA21 vacuolar H+-ATPase homolog (S. cerevisiae)	0.9837	0.7454	NA
66511	2500003M10Rik	RIKEN cDNA 2500003M10 gene	0.9837	0.7778	NA
66314	Tpd52l2	tumor protein D52-like 2	0.9837	0.7655	NA
12767	Cxcr4	chemokine (C-X-C motif) receptor 4	0.9837	0.8963	0.9699
234730	Fuk	fucokinase	0.9836	0.7323	NA
110829	Lims1	LIM and senescent cell antigen-like domains 1	0.9836	0.8135	NA
108079	Prkaa2	protein kinase, AMP-activated, alpha 2 catalytic subunit	0.9836	0.845	NA
105559	Mbnl2	muscleblind-like 2	0.9836	0.8517	0.9537
76824	Fam54b	family with sequence similarity 54, member B	0.9836	0.8245	NA
74450	Pank2	pantothenate kinase 2	0.9836	0.74	NA
74245	Ctbs	chitobiase, di-N-acetyl-	0.9836	0.7891	NA
67153	Rnaseh2b	ribonuclease H2, subunit B	0.9836	0.7193	NA
20715	Serpina3g	serine (or cysteine) peptidase inhibitor, clade A, member 3G	0.9836	0.9081	0.9733
18550	Furin	furin (paired basic amino acid cleaving enzyme)	0.9836	0.799	NA
14461	Gata2	GATA binding protein 2	0.9836	0.8858	0.9664
12307	Calb1	calbindin 1	0.9836	0.7668	NA
100041979	Gm3604	predicted gene 3604	0.9835	0.7572	NA
223669	Zfp7	zinc finger protein 7	0.9835	0.6967	NA
192663	Abcg4	ATP-binding cassette, sub-family G (WHITE), member 4	0.9835	0.8208	NA
77422	C330018D20Rik	RIKEN cDNA C330018D20 gene	0.9835	0.7177	NA
75723	Amotl1	angiomotin-like 1	0.9835	0.7249	NA
75533	Nme5	non-metastatic cells 5, protein expressed in (nucleoside-diphosphate kinase)	0.9835	0.8416	NA
72205	Eml2	echinoderm microtubule associated protein like 2	0.9835	0.7846	NA
68634	Tm2d3	TM2 domain containing 3	0.9835	0.7362	NA
67738	Ppid	peptidylprolyl isomerase D (cyclophilin D)	0.9835	0.8132	NA
66454	Nmnat1	nicotinamide nucleotide adenylyltransferase 1	0.9835	0.7534	NA
57743	Sec61a2	Sec61, alpha subunit 2 (S. cerevisiae)	0.9835	0.9008	0.9711
23854	Def8	differentially expressed in FDCP 8	0.9835	0.6934	NA
22758	Zscan12	zinc finger and SCAN domain containing 12	0.9835	0.7364	NA

19989	Rpl7	ribosomal protein L7	0.9835	0.8515	0.9537
18578	Pde4b	phosphodiesterase 4B, cAMP specific	0.9835	0.8811	0.9636
15446	Hpgd	hydroxyprostaglandin dehydrogenase 15 (NAD)	0.9835	0.8011	NA
269513	Nkain3	Na+/K+ transporting ATPase interacting 3	0.9834	0.8388	NA
235431	Coro2b	coronin, actin binding protein, 2B	0.9834	0.8481	0.9522
233107	Kctd15	potassium channel tetramerisation domain containing 15	0.9834	0.7193	NA
224794	Enpp4	ectonucleotide pyrophosphatase/phosphodiesterase 4	0.9834	0.7836	NA
74022	Glyr1	glyoxylate reductase 1 homolog (Arabidopsis)	0.9834	0.7385	NA
72569	Bbs5	Bardet-Biedl syndrome 5 (human)	0.9834	0.7504	NA
67144	Lrrc40	leucine rich repeat containing 40	0.9834	0.7964	NA
22099	Tsn	translin	0.9834	0.7597	NA
18217	Ntsr2	neurotensin receptor 2	0.9834	0.8901	0.9684
12842	Col1a1	collagen, type I, alpha 1	0.9834	0.8937	0.9693
263803	Pkn3	protein kinase N3	0.9833	0.9557	0.9875
76303	Osbp	oxysterol binding protein	0.9833	0.8483	0.9522
30926	Glrx3	glutaredoxin 3	0.9833	0.8088	NA
19823	Rnf7	ring finger protein 7	0.9833	0.7828	NA
17937	Nab2	Ngfi-A binding protein 2	0.9833	0.9194	0.9765
14794	Spsb2	splA/ryanodine receptor domain and SOCS box containing 2	0.9833	0.8246	NA
238130	Dock4	dedicator of cytokinesis 4	0.9832	0.8083	NA
75420	Secisbp2	SECIS binding protein 2	0.9832	0.798	NA
70961	4921532D01Rik	RIKEN cDNA 4921532D01 gene	0.9832	0.9008	0.9711
16842	Lef1	lymphoid enhancer binding factor 1	0.9832	0.8794	0.9631
100048658	Ddx43	DEAD (Asp-Glu-Ala-Asp) box polypeptide 43	0.9831	0.8999	0.9707
100040631	Dynlt1e	dynein light chain Tctex-type 1E	0.9831	0.7372	NA
245688	Rbbp7	retinoblastoma binding protein 7	0.9831	0.8498	0.9531
108927	Lhfp	lipoma HMGIC fusion partner	0.9831	0.8105	NA
79044	Mrps34	mitochondrial ribosomal protein S34	0.9831	0.6813	NA
74754	Dhcr24	24-dehydrocholesterol reductase	0.9831	0.8695	0.9598
74570	Zkscan1	zinc finger with KRAB and SCAN domains 1	0.9831	0.7613	NA
71592	Pogk	pogo transposable element with KRAB domain	0.9831	0.8139	NA
67529	Fgfr1op2	FGFR1 oncogene partner 2	0.9831	0.7953	NA
100042049	LOC100042049	ribosomal protein L22 like 1 pseudogene	0.983	0.8	NA
216395	Tmem5	transmembrane protein 5	0.983	0.8174	NA
216166	Plk5	polo-like kinase 5 (Drosophila)	0.983	0.7332	NA
75746	Morc4	microrchidia 4	0.983	0.8065	NA
74197	Gtf2e1	general transcription factor II E, polypeptide 1 (alpha subunit)	0.983	0.751	NA
68979	Nol11	nucleolar protein 11	0.983	0.7059	NA
57354	Cramp1l	Crm, cramped-like (Drosophila)	0.983	0.7534	NA
54721	Tyk2	tyrosine kinase 2	0.983	0.7946	NA
21427	Vps72	vacuolar protein sorting 72 (yeast)	0.983	0.9263	0.9785
19885	Rorc	RAR-related orphan receptor gamma	0.983	0.7971	NA
14897	Trip12	thyroid hormone receptor interactor 12	0.983	0.8025	NA
12549	Arhgap31	Rho GTPase activating protein 31	0.983	0.8251	NA
227696	Phyhd1	phytanoyl-CoA dioxygenase domain containing 1	0.9829	0.7453	NA
109275	Actr5	ARP5 actin-related protein 5 homolog (yeast)	0.9829	0.8611	0.9573
93687	Csnk1a1	casein kinase 1, alpha 1	0.9829	0.715	NA
76863	Dcun1d5	DCN1, defective in cullin neddylation 1, domain containing 5 (S. cerevisiae)	0.9829	0.7615	NA
71876	Mlf1ip	myeloid leukemia factor 1 interacting protein	0.9829	0.9029	0.9717

22190	Ubc	ubiquitin C	0.9829	0.8633	0.9577
21873	Tjp2	tight junction protein 2	0.9829	0.7778	NA
14923	Guk1	guanylate kinase 1	0.9829	0.7718	NA
104479	Ccdc117	coiled-coil domain containing 117	0.9828	0.7909	NA
102278	Cpne7	copine VII	0.9828	0.7595	NA
73824	Snhg6	small nucleolar RNA host gene (non-protein coding) 6	0.9828	0.8475	0.952
66494	Prelid1	PRELI domain containing 1	0.9828	0.7399	NA
18854	Pml	promyelocytic leukemia	0.9828	0.7395	NA
17423	Ndst2	N-deacetylase/N-sulfotransferase (heparan glucosaminyl) 2	0.9828	0.7787	NA
433182	Gm5506	predicted gene 5506	0.9827	0.8658	0.9585
269639	Zfp512	zinc finger protein 512	0.9827	0.7934	NA
67674	Trmt112	tRNA methyltransferase 11-2 homolog (S. cerevisiae)	0.9827	0.7813	NA
29849	Olfr159	olfactory receptor 159	0.9827	0.895	0.9698
12260	C1qb	complement component 1, q subcomponent, beta polypeptide	0.9827	0.9002	0.9709
100732	Mapre3	microtubule-associated protein, RP/EB family, member 3	0.9826	0.8685	0.9596
52463	Tet1	tet oncogene 1	0.9826	0.8664	0.9587
20619	Snap23	synaptosomal-associated protein 23	0.9826	0.775	NA
18082	Nipsnap1	4-nitrophenylphosphatase domain and non-neuronal SNAP25-like protein homolog 1 (C. elegans)	0.9826	0.846	0.9512
17763	Mtcp1	mature T-cell proliferation 1	0.9826	0.711	NA
17156	Man1a2	mannosidase, alpha, class 1A, member 2	0.9826	0.8514	0.9537
13063	Cycs	cytochrome c, somatic	0.9826	0.6332	NA
100042202	Gm3718	predicted gene 3718	0.9825	0.9036	0.9717
81845	Gpank1	G patch domain and ankyrin repeats 1	0.9825	0.7362	NA
72354	Ttc4	tetratricopeptide repeat domain 4	0.9825	0.7811	NA
67477	Abhd15	abhydrolase domain containing 15	0.9825	0.9296	0.9789
50789	Fbxl3	F-box and leucine-rich repeat protein 3	0.9825	0.708	NA
19142	Prss12	protease, serine, 12 neurotrypsin (motopsin)	0.9825	0.864	0.9579
14451	Gas1	growth arrest specific 1	0.9825	0.8267	NA
11488	Adam11	a disintegrin and metallopeptidase domain 11	0.9825	0.8694	0.9598
326622	Upf2	UPF2 regulator of nonsense transcripts homolog (yeast)	0.9824	0.7461	NA
272465	Fam70b	family with sequence similarity 70, member B	0.9824	0.7644	NA
242894	Actr3b	ARP3 actin-related protein 3 homolog B (yeast)	0.9824	0.8168	NA
233863	Gtf3c1	general transcription factor III C 1	0.9824	0.8515	0.9537
108812	Als2cr12	amyotrophic lateral sclerosis 2 (juvenile) chromosome region, candidate 12 (human)	0.9824	0.8394	0.9484
70361	Lman1	lectin, mannose-binding, 1	0.9824	0.624	NA
67556	Pigm	phosphatidylinositol glycan anchor biosynthesis, class M	0.9824	0.7773	NA
57294	Rps27	ribosomal protein S27	0.9824	0.7984	NA
56695	Pnkd	paroxysmal nonkinesiogenic dyskinesia	0.9824	0.6812	NA
27220	Cartpt	CART prepropeptide	0.9824	0.8306	NA
20088	Rps24	ribosomal protein S24	0.9824	0.8793	0.9631
11838	Arc	activity regulated cytoskeletal-associated protein	0.9824	0.8832	0.965
11307	Abcg1	ATP-binding cassette, sub-family G (WHITE), member 1	0.9824	0.5993	NA
76963	2310076G05Rik	RIKEN cDNA 2310076G05 gene	0.9823	0.7959	NA
72850	2900024J01Rik	RIKEN cDNA 2900024J01 gene	0.9823	0.9049	0.9717
69641	Wdr20a	WD repeat domain 20A	0.9823	0.6476	NA
68911	Pygo2	pygopus 2	0.9823	0.7746	NA
68184	Denr	density-regulated protein	0.9823	0.7842	NA
57810	Cdon	cell adhesion molecule-related/down-regulated by oncogenes	0.9823	0.8959	0.9699
22697	Zscan21	zinc finger and SCAN domain containing 21	0.9823	0.6573	NA

19360	Rad50	RAD50 homolog (S. cerevisiae)	0.9823	0.7083	NA
17190	Mbd1	methyl-CpG binding domain protein 1	0.9823	0.8963	0.9699
16842	Lef1	lymphoid enhancer binding factor 1	0.9823	0.8925	0.9691
16576	Kif7	kinesin family member 7	0.9823	0.8954	0.9698
11540	Adora2a	adenosine A2a receptor	0.9823	0.8169	NA
110391	Qdpr	quinoid dihydropteridine reductase	0.9822	0.8186	NA
76219	6530401D17Rik	RIKEN cDNA 6530401D17 gene	0.9822	0.7125	NA
70381	Tecpr1	tectonin beta-propeller repeat containing 1	0.9822	0.7653	NA
68815	Btbd10	BTB (POZ) domain containing 10	0.9822	0.6986	NA
52715	Ccdc43	coiled-coil domain containing 43	0.9822	0.8262	NA
26398	Map2k4	mitogen-activated protein kinase kinase 4	0.9822	0.7954	NA
100502627	LOC100502627	hypothetical LOC100502627	0.9821	0.7634	NA
76498	Paqr4	progestin and adipoQ receptor family member IV	0.9821	0.7131	NA
76022	Gon4l	gon-4-like (C.elegans)	0.9821	0.943	0.9838
70285	Rpf1	ribosome production factor 1 homolog (S. cerevisiae)	0.9821	0.799	NA
20422	Shfm1	split hand/foot malformation (ectrodactyly) type 1	0.9821	0.6691	NA
18424	Otx2	orthodenticle homolog 2 (Drosophila)	0.9821	0.8395	0.9485
11556	Adrb3	adrenergic receptor, beta 3	0.9821	0.8547	0.9551
381062	2210404J11Rik	RIKEN cDNA 2210404J11 gene	0.982	0.8244	NA
227059	Slc39a10	solute carrier family 39 (zinc transporter), member 10	0.982	0.8526	0.9542
216705	Clint1	clathrin interactor 1	0.982	0.8668	0.9587
81601	Kat5	K(lysine) acetyltransferase 5	0.982	0.7194	NA
78885	Coro7	coronin 7	0.982	0.7751	NA
77116	Mtmr2	myotubularin related protein 2	0.982	0.7751	NA
67246	2810474O19Rik	RIKEN cDNA 2810474O19 gene	0.982	0.8581	0.9562
27360	Add3	adducin 3 (gamma)	0.982	0.7775	NA
20606	Sstr2	somatostatin receptor 2	0.982	0.8787	0.9628
20088	Rps24	ribosomal protein S24	0.982	0.8724	0.9608
18458	Pabpc1	poly(A) binding protein, cytoplasmic 1	0.982	0.7864	NA
217843	Unc79	unc-79 homolog (C. elegans)	0.9819	0.8307	0.9455
107831	Bai1	brain-specific angiogenesis inhibitor 1	0.9819	0.766	NA
107065	Lrrtm2	leucine rich repeat transmembrane neuronal 2	0.9819	0.7711	NA
66461	Ptpmt1	protein tyrosine phosphatase, mitochondrial 1	0.9819	0.6043	NA
56703	Pigo	phosphatidylinositol glycan anchor biosynthesis, class O	0.9819	0.7245	NA
620709	Lcn6	lipocalin 6	0.9818	0.9493	0.9856
237782	Smcr8	Smith-Magenis syndrome chromosome region, candidate 8 homolog (human)	0.9818	0.779	NA
68981	Snrpa1	small nuclear ribonucleoprotein polypeptide A'	0.9818	0.8294	0.945
16772	Lama1	laminin, alpha 1	0.9818	0.8505	0.9532
100038554	Gm15348	predicted gene 15348	0.9817	0.8051	NA
269941	Chsy1	chondroitin sulfate synthase 1	0.9817	0.6232	NA
212398	Frat2	frequently rearranged in advanced T-cell lymphomas 2	0.9817	0.8288	0.945
75624	Metap1	methionyl aminopeptidase 1	0.9817	0.7303	NA
74393	4933403G14Rik	RIKEN cDNA 4933403G14 gene	0.9817	0.7253	NA
71902	Cand1	cullin associated and neddylation disassociated 1	0.9817	0.5911	NA
71538	Fbxo9	f-box protein 9	0.9817	0.6694	NA
71348	5430439M09Rik	RIKEN cDNA 5430439M09 gene	0.9817	0.7162	NA
65102	Nif3l1	Ngg1 interacting factor 3-like 1 (S. pombe)	0.9817	0.657	NA
50779	Rgs6	regulator of G-protein signaling 6	0.9817	0.7955	NA
20509	Slc19a1	solute carrier family 19 (sodium/hydrogen exchanger), member 1	0.9817	0.862	0.9573

331513	Gm9927	predicted gene 9927	0.9816	0.8397	0.9485
268567	Tmem229b	transmembrane protein 229B	0.9816	0.8558	0.9553
233887	Zfp553	zinc finger protein 553	0.9816	0.6891	NA
217364	Engase	endo-beta-N-acetylglucosaminidase	0.9816	0.78	NA
574418	Serinc4	serine incorporator 4	0.9815	0.7812	NA
546164	Gm5921	predicted gene 5921	0.9815	0.806	NA
433586	Maml3	mastermind like 3 (Drosophila)	0.9815	0.8955	0.9698
320415	Gchfr	GTP cyclohydrolase I feedback regulator	0.9815	0.8258	NA
241226	Itga8	integrin alpha 8	0.9815	0.8161	NA
226438	Igfn1	immunoglobulin-like and fibronectin type III domain containing 1	0.9815	0.7117	NA
224648	Uhrf1bp1	UHRF1 (ICBP90) binding protein 1	0.9815	0.8982	0.9699
211577	Mrgprf	MAS-related GPR, member F	0.9815	0.9122	0.9743
76484	Kndc1	kinase non-catalytic C-lobe domain (KIND) containing 1	0.9815	0.7402	NA
71146	Golga7b	golgi autoantigen, golgin subfamily a, 7B	0.9815	0.929	0.9789
13829	Epb4.9	erythrocyte protein band 4.9	0.9815	0.9201	0.9767
619547	Rpl34-ps1	ribosomal protein L34, pseudogene 1	0.9814	0.7725	NA
381511	Pdp1	pyruvate dehyrogenase phosphatase catalytic subunit 1	0.9814	0.7968	NA
330788	Zfp866	zinc finger protein 866	0.9814	0.8348	0.9466
240832	Tor1aip2	torsin A interacting protein 2	0.9814	0.8043	NA
217057	Ptrh2	peptidyl-tRNA hydrolase 2	0.9814	0.7617	NA
69863	Ttc39b	tetratricopeptide repeat domain 39B	0.9814	0.876	0.9618
69072	Ebna1bp2	EBNA1 binding protein 2	0.9814	0.8765	0.962
67738	Ppid	peptidylprolyl isomerase D (cyclophilin D)	0.9814	0.6497	NA
22289	Kdm6a	4lysine (K)-specific demethylase 6A	0.9814	0.784	NA
670211	Gm12508	predicted gene 12508	0.9813	0.774	NA
380664	Lemd3	LEM domain containing 3	0.9813	0.8226	NA
102103	Mtus1	mitochondrial tumor suppressor 1	0.9813	0.8503	0.9531
66246	Osgep	O-sialoglycoprotein endopeptidase	0.9813	0.7303	NA
56176	Pigp	phosphatidylinositol glycan anchor biosynthesis, class P	0.9813	0.6611	NA
26409	Map3k7	mitogen-activated protein kinase kinase kinase 7	0.9813	0.8009	NA
22330	Vcl	vinculin	0.9813	0.7201	NA
19152	Prtn3	proteinase 3	0.9813	0.8362	0.9474
100502705	LOC100502705	hypothetical LOC100502705	0.9812	0.7468	NA
668459	Gm9182	predicted gene 9182	0.9812	0.7948	NA
241694	Ralgapa2	Ral GTPase activating protein, alpha subunit 2 (catalytic)	0.9812	0.741	NA
100705	Acacb	acetyl-Coenzyme A carboxylase beta	0.9812	0.9077	0.9733
74841	Usp38	ubiquitin specific peptidase 38	0.9812	0.7419	NA
70544	5730437N04Rik	RIKEN cDNA 5730437N04 gene	0.9812	0.6802	NA
67863	Slc25a11	solute carrier family 25 (mitochondrial carrier oxoglutarate carrier), member 11	0.9812	0.6719	NA
13716	Ell	elongation factor RNA polymerase II	0.9812	0.6134	NA
245670	Rragb	Ras-related GTP binding B	0.9811	0.8139	NA
238330	6430527G18Rik	RIKEN cDNA 6430527G18 gene	0.9811	0.7596	NA
73102	Slc22a23	solute carrier family 22, member 23	0.9811	0.6799	NA
70118	Srrd	SRR1 domain containing	0.9811	0.7353	NA
66965	Ctu2	cytosolic thiouridylase subunit 2 homolog (S. pombe)	0.9811	0.6447	NA
12421	Rb1cc1	RB1-inducible coiled-coil 1	0.9811	0.7115	NA
100043468	Zfp955b	zinc finger protein 955B	0.981	0.8436	0.95
544848	Gm5784	predicted gene 5784	0.981	0.9054	0.9718
381175	Ccdc68	coiled-coil domain containing 68	0.981	0.9571	0.9879

332934	Zmynd12	zinc finger, MYND domain containing 12	0.981	0.8617	0.9573
26414	Mapk10	mitogen-activated protein kinase 10	0.981	0.8213	0.9433
18795	Plcb1	phospholipase C, beta 1	0.981	0.8809	0.9635
16524	Kcnj9	potassium inwardly-rectifying channel, subfamily J, member 9	0.981	0.7038	NA
12913	Creb3	cAMP responsive element binding protein 3	0.981	0.6695	NA
19819	Rnaseh1	ribonuclease H1	0.9809	0.702	NA
18803	Plcg1	phospholipase C, gamma 1	0.9809	0.8455	0.951
235134	Nfrkb	nuclear factor related to kappa B binding protein	0.9808	0.6665	NA
110379	Sec13	SEC13 homolog (S. cerevisiae)	0.9808	0.813	NA
108853	Mtrf1l	mitochondrial translational release factor 1-like	0.9808	0.7436	NA
67885	1500011K16Rik	RIKEN cDNA 1500011K16 gene	0.9808	0.8324	0.9456
67381	Med4	mediator of RNA polymerase II transcription, subunit 4 homolog (yeast)	0.9808	0.8538	0.9544
11465	Actg1	actin, gamma, cytoplasmic 1	0.9808	0.7835	NA
100503357	LOC100503357	hypothetical LOC100503357	0.9807	0.9142	0.9747
105428	Fam149b	family with sequence similarity 149, member B	0.9807	0.7621	NA
104601	Mycbpap	MYCBP associated protein	0.9807	0.824	0.9443
72981	Prkrir	protein-kinase, interferon-inducible double stranded RNA dependent inhibitor, repressor of (P58 repressor)	0.9807	0.8046	NA
68436	Rpl34	ribosomal protein L34	0.9807	0.7469	NA
58805	Mlxipl	MLX interacting protein-like	0.9807	0.9208	0.9768
21976	Top3b	topoisomerase (DNA) III beta	0.9807	0.7908	NA
380855	Rsl1	regulator of sex limited protein 1	0.9806	0.7286	NA
268481	Krt222	keratin 222	0.9806	0.8771	0.9623
68936	Fam165b	family with sequence similarity 165, member B	0.9806	0.8928	0.9693
67008	1600012F09Rik	RIKEN cDNA 1600012F09 gene	0.9806	0.7138	NA
19317	Qk	quaking	0.9806	0.7946	NA
17772	Mtm1	X-linked myotubular myopathy gene 1	0.9806	0.7002	NA
16621	Klkb1	kallikrein B, plasma 1	0.9806	0.6798	NA
13612	Edil3	EGF-like repeats and discoidin I-like domains 3	0.9806	0.7626	NA
385380	Tex28	testis expressed 28	0.9805	0.9018	0.9715
234353	Psd3	pleckstrin and Sec7 domain containing 3	0.9805	0.7314	NA
215748	Cnksr3	Cnksr family member 3	0.9805	0.6916	NA
105278	Cdk20	cyclin-dependent kinase 20	0.9805	0.7733	NA
77958	A930023M06Rik	RIKEN cDNA A930023M06 gene	0.9805	0.8303	0.9453
68011	Snrpg	small nuclear ribonucleoprotein polypeptide G	0.9805	0.6728	NA
56863	Cldn9	claudin 9	0.9805	0.8644	0.958
52686	Mettl2	methyltransferase like 2	0.9805	0.8739	0.9616
27403	Abca7	ATP-binding cassette, sub-family A (ABC1), member 7	0.9805	0.8221	0.9435
26443	Psma6	proteasome (prosome, macropain) subunit, alpha type 6	0.9805	0.7796	NA
21681	Thoc4	THO complex 4	0.9805	0.7417	NA
12069	Bex2	brain expressed X-linked 2	0.9805	0.7035	NA
100101806	Srp54c	signal recognition particle 54C	0.9804	0.7415	NA
100043534	Gm11686	predicted gene 11686	0.9804	0.7755	NA
100038941	Vmn2r121	vomeronasal 2, receptor 121	0.9804	0.9368	0.9816
329068	A330044H09	hypothetical protein A330044H09	0.9804	0.7303	NA
108755	Lyrm2	LYR motif containing 2	0.9804	0.7526	NA
93761	Smarca1	SWI/SNF related, matrix associated, actin dependent regulator of chromatin, subfamily a, member 1	0.9804	0.7277	NA
72947	Agxt2l2	alanine-glyoxylate aminotransferase 2-like 2	0.9804	0.7674	NA
72607	Usp13	ubiquitin specific peptidase 13 (isopeptidase T-3)	0.9804	0.7506	NA
71330	Rcbtb1	regulator of chromosome condensation (RCC1) and BTB (POZ) domain containing protein 1	0.9804	0.6908	NA

70088	Meaf6	MYST/Esa1-associated factor 6	0.9804	0.6337	NA
67608	Narf	nuclear prelamin A recognition factor	0.9804	0.8283	0.945
67553	Gstcd	glutathione S-transferase, C-terminal domain containing	0.9804	0.7853	NA
53970	Rfx5	regulatory factor X, 5 (influences HLA class II expression)	0.9804	0.8808	0.9635
13361	Dhfr	dihydrofolate reductase	0.9804	0.7309	NA
12476	Cd151	CD151 antigen	0.9804	0.7096	NA
266690	Cyb5r4	cytochrome b5 reductase 4	0.9803	0.6719	NA
230393	BC057079	cDNA sequence BC057079	0.9803	0.7188	NA
209361	Taf3	TAF3 RNA polymerase II, TATA box binding protein (TBP)-associated factor	0.9803	0.8724	0.9608
76779	Cluap1	clusterin associated protein 1	0.9803	0.6412	NA
74252	Armc1	armadillo repeat containing 1	0.9803	0.6679	NA
330485	Tmem145	transmembrane protein 145	0.9802	0.7146	NA
231876	Lmtk2	lemur tyrosine kinase 2	0.9802	0.9319	0.9795
193838	Eme2	essential meiotic endonuclease 1 homolog 2 (S. pombe)	0.9802	0.7777	NA
67534	Ttll4	tubulin tyrosine ligase-like family, member 4	0.9802	0.8387	0.9482
22590	Xpa	xeroderma pigmentosum, complementation group A	0.9802	0.7807	NA
19042	Ppm1a	protein phosphatase 1A, magnesium dependent, alpha isoform	0.9802	0.6721	NA
12812	Coil	coilin	0.9802	0.7268	NA
100039199	Gm2095	predicted gene 2095	0.9801	0.8068	NA
230582	Cyb5rl	cytochrome b5 reductase-like	0.9801	0.8641	0.9579
217379	Ubxn2a	UBX domain protein 2A	0.9801	0.8289	0.945
71073	4933421O10Rik	RIKEN cDNA 4933421O10 gene	0.9801	0.8129	0.9405
67755	Ddx47	DEAD (Asp-Glu-Ala-Asp) box polypeptide 47	0.9801	0.7842	NA
18033	Nfkb1	nuclear factor of kappa light polypeptide gene enhancer in B-cells 1, p105	0.9801	0.922	0.9775
77015	Mpped2	metallophosphoesterase domain containing 2	0.98	0.7771	NA
67921	Ube2f	ubiquitin-conjugating enzyme E2F (putative)	0.98	0.6791	NA
67674	Trmt112	tRNA methyltransferase 11-2 homolog (S. cerevisiae)	0.98	0.7471	NA
67374	Jam2	junction adhesion molecule 2	0.98	0.7788	NA
320332	Hist4h4	histone cluster 4, H4	0.9799	0.876	0.9618
94246	Arid4b	AT rich interactive domain 4B (RBP1-like)	0.9799	0.8965	0.9699
78294	Rps27a	ribosomal protein S27A	0.9799	0.8321	0.9456
78251	Zfp712	zinc finger protein 712	0.9799	0.6095	NA
77965	A930033C23Rik	RIKEN cDNA A930033C23 gene	0.9799	0.7763	NA
77110	Gpbp1l1	GC-rich promoter binding protein 1-like 1	0.9799	0.7524	NA
75786	Ckap5	cytoskeleton associated protein 5	0.9799	0.6136	NA
69462	2300005B03Rik	RIKEN cDNA 2300005B03 gene	0.9799	0.8889	0.9676
17764	Mtf1	metal response element binding transcription factor 1	0.9799	0.6451	NA
214901	Chtf18	CTF18, chromosome transmission fidelity factor 18 homolog (S. cerevisiae)	0.9798	0.6735	NA
99982	Kdm1a	lysine (K)-specific demethylase 1A	0.9798	0.6725	NA
71722	Cic	capicua homolog (Drosophila)	0.9798	0.7119	NA
66495	Ndufb3	NADH dehydrogenase (ubiquinone) 1 beta subcomplex 3	0.9798	0.6896	NA
50753	Fbxo8	F-box protein 8	0.9798	0.7608	NA
17872	Ppp1r15a	protein phosphatase 1, regulatory (inhibitor) subunit 15A	0.9798	0.8073	NA
11798	Xiap	X-linked inhibitor of apoptosis	0.9798	0.735	NA
319615	Zfp944	zinc finger protein 944	0.9797	0.6692	NA
107515	Lgr4	leucine-rich repeat-containing G protein-coupled receptor 4	0.9797	0.7794	NA
104662	Tsr1	TSR1, 20S rRNA accumulation, homolog (yeast)	0.9797	0.6505	NA
75547	Akap13	A kinase (PRKA) anchor protein 13	0.9797	0.6917	NA
67150	Rnf141	ring finger protein 141	0.9797	0.6146	NA

66514	Asrgl1	asparaginase like 1	0.9797	0.8882	0.9675
59001	Pole3	polymerase (DNA directed), epsilon 3 (p17 subunit)	0.9797	0.8457	0.951
20615	Snapin	SNAP-associated protein	0.9797	0.6937	NA
14617	Gjd2	gap junction protein, delta 2	0.9797	0.8173	0.942
12509	Cd59a	CD59a antigen	0.9797	0.7193	NA
12193	Zfp36l2	zinc finger protein 36, C3H type-like 2	0.9797	0.7615	NA
445007	Nup85	nucleoporin 85	0.9796	0.727	NA
406217	Bex4	brain expressed gene 4	0.9796	0.6966	NA
328424	Kcnrg	potassium channel regulator	0.9796	0.9017	0.9715
211556	Ap1ar	adaptor-related protein complex 1 associated regulatory protein	0.9796	0.8912	0.9687
211135	D130040H23Rik	RIKEN cDNA D130040H23 gene	0.9796	0.8286	0.945
74241	Chpf	chondroitin polymerizing factor	0.9796	0.7769	NA
66204	Acyp1	acylphosphatase 1, erythrocyte (common) type	0.9796	0.9517	0.9867
20615	Snapin	SNAP-associated protein	0.9796	0.659	NA
20197	S100a3	S100 calcium binding protein A3	0.9796	0.7985	NA
19070	Mobkl3	MOB1, Mps One Binder kinase activator-like 3 (yeast)	0.9796	0.8117	0.9403
12537	Cdk11b	cyclin-dependent kinase 11B	0.9796	0.6501	NA
252875	Mios	missing oocyte, meiosis regulator, homolog (Drosophila)	0.9795	0.8208	0.9433
74384	4932433N03Rik	RIKEN cDNA 4932433N03 gene	0.9795	0.6883	NA
72284	Oraov1	oral cancer overexpressed 1	0.9795	0.6543	NA
22040	Trex1	three prime repair exonuclease 1	0.9795	0.727	NA
20912	Stxbp3a	syntaxin binding protein 3A	0.9795	0.9195	0.9765
15552	Htr1d	5-hydroxytryptamine (serotonin) receptor 1D	0.9795	0.8288	0.945
13819	Epas1	endothelial PAS domain protein 1	0.9795	0.718	NA
432939	Gm5468	predicted gene 5468	0.9794	0.8685	0.9596
327814	Ppfia2	protein tyrosine phosphatase, receptor type, f polypeptide (PTPRF), interacting protein (liprin), alpha 2	0.9794	0.7221	NA
320234	Ccdc66	coiled-coil domain containing 66	0.9794	0.6512	NA
26556	Homer1	homer homolog 1 (Drosophila)	0.9794	0.7186	NA
19736	Rgs4	regulator of G-protein signaling 4	0.9794	0.716	NA
12785	Cnbp	cellular nucleic acid binding protein	0.9794	0.7868	NA
100043685	Gm4588	predicted gene 4588	0.9793	0.7393	NA
433091	Pnpla1	patatin-like phospholipase domain containing 1	0.9793	0.8797	0.9631
268822	Adck5	aarF domain containing kinase 5	0.9793	0.7586	NA
231659	Gcn1l1	GCN1 general control of amino-acid synthesis 1-like 1 (yeast)	0.9793	0.7409	NA
78891	Scyl1	SCY1-like 1 (S. cerevisiae)	0.9793	0.739	NA
73750	Whrn	whirlin	0.9793	0.8753	0.9618
72093	2010320M18Rik	RIKEN cDNA 2010320M18 gene	0.9793	0.8329	0.9457
66435	Uggt2	UDP-glucose glycoprotein glucosyltransferase 2	0.9793	0.7015	NA
54125	Polm	polymerase (DNA directed), mu	0.9793	0.7921	NA
17349	Mlf1	myeloid leukemia factor 1	0.9793	0.6926	NA
16978	Lrrfip1	leucine rich repeat (in FLII) interacting protein 1	0.9793	0.756	NA
14457	Gas7	growth arrest specific 7	0.9793	0.8958	0.9699
100039359	Gm10399	predicted gene 10399	0.9792	0.8292	0.945
268741	Tox4	TOX high mobility group box family member 4	0.9792	0.8156	0.9416
237222	Ofd1	oral-facial-digital syndrome 1 gene homolog (human)	0.9792	0.7107	NA
208968	Zfp280c	zinc finger protein 280C	0.9792	0.7827	NA
69893	2010305A19Rik	RIKEN cDNA 2010305A19 gene	0.9792	0.7375	NA
69010	Anapc13	anaphase promoting complex subunit 13	0.9792	0.7374	NA
66234	Sc4mol	sterol-C4-methyl oxidase-like	0.9792	0.6094	NA

17975	Ncl	nucleolin	0.9792	0.7677	NA
14919	Gucy2e	guanylate cyclase 2e	0.9792	0.7386	NA
69232	Qrich1	glutamine-rich 1	0.9791	0.7793	NA
68767	ORF19	open reading frame 19	0.9791	0.6771	NA
17919	Myo5b	myosin VB	0.9791	0.762	NA
13447	Doc2b	double C2, beta	0.9791	0.6508	NA
11677	Akr1b3	aldo-keto reductase family 1, member B3 (aldose reductase)	0.9791	0.7829	NA
432555	Gm5431	predicted gene 5431	0.979	0.7508	NA
378460	Pram1	PML-RAR alpha-regulated adaptor molecule 1	0.979	0.8132	0.9406
228607	Mavs	mitochondrial antiviral signaling protein	0.979	0.643	NA
76375	Det1	de-etiolated homolog 1 (Arabidopsis)	0.979	0.6675	NA
71452	Ankrd40	ankyrin repeat domain 40	0.979	0.6704	NA
68795	Ubr3	ubiquitin protein ligase E3 component n-recognin 3	0.979	0.8097	0.94
56314	Zfp113	zinc finger protein 113	0.979	0.8298	0.945
55950	Bri3	brain protein I3	0.979	0.6601	NA
28080	Atp5o	ATP synthase, H+ transporting, mitochondrial F1 complex, O subunit	0.979	0.7025	NA
21761	Morf4l1	mortality factor 4 like 1	0.979	0.6095	NA
16531	Kcnma1	potassium large conductance calcium-activated channel, subfamily M, alpha member 1	0.979	0.582	NA
14187	Akr1b8	aldo-keto reductase family 1, member B8	0.979	0.825	0.9443
13138	Dag1	dystroglycan 1	0.979	0.8283	0.945
666244	Tmsb15b1	thymosin beta 15b1	0.9789	0.7653	NA
268729	Gm626	predicted gene 626	0.9789	0.8234	0.9439
217011	Nle1	notchless homolog 1 (Drosophila)	0.9789	0.8015	0.9373
117197	Cno	cappuccino	0.9789	0.7499	NA
74763	Nat15	N-acetyltransferase 15 (GCN5-related, putative)	0.9789	0.6081	NA
53380	Psmd10	proteasome (prosome, macropain) 26S subunit, non-ATPase, 10	0.9789	0.7692	NA
23986	Peci	peroxisomal delta3, delta2-enoyl-Coenzyme A isomerase	0.9789	0.9015	0.9715
20641	Snrpd1	small nuclear ribonucleoprotein D1	0.9789	0.6738	NA
20452	St8sia4	ST8 alpha-N-acetyl-neuraminide alpha-2,8-sialyltransferase 4	0.9789	0.8258	0.9446
17977	Ncoa1	nuclear receptor coactivator 1	0.9789	0.6567	NA
17777	Mttp	microsomal triglyceride transfer protein	0.9789	0.6411	NA
16492	Kcna4	potassium voltage-gated channel, shaker-related subfamily, member 4	0.9789	0.8999	0.9707
13638	Efna3	ephrin A3	0.9789	0.9042	0.9717
100505283	LOC100505283	60S ribosomal protein L21-like	0.9788	0.695	NA
239038	Lrit2	leucine-rich repeat, immunoglobulin-like and transmembrane domains 2	0.9788	0.7122	NA
212153	2610015P09Rik	RIKEN cDNA 2610015P09 gene	0.9788	0.7518	NA
69482	Nup35	nucleoporin 35	0.9788	0.6363	NA
66310	Dpy30	dpy-30 homolog (C. elegans)	0.9788	0.7238	NA
20192	Ryr3	ryanodine receptor 3	0.9788	0.774	NA
18537	Pcmt1	protein-L-isoaspartate (D-aspartate) O-methyltransferase 1	0.9788	0.7797	NA
15939	Ier5	immediate early response 5	0.9788	0.7652	NA
217648	Gm527	predicted gene 527	0.9787	0.6938	NA
104806	Fancm	Fanconi anemia, complementation group M	0.9787	0.7497	NA
77087	Ankrd11	ankyrin repeat domain 11	0.9787	0.6019	NA
66352	Blzf1	basic leucine zipper nuclear factor 1	0.9787	0.642	NA
18777	Lypla1	lysophospholipase 1	0.9787	0.8023	0.9374
620760	2900079G21Rik	RIKEN cDNA 2900079G21 gene	0.9786	0.907	0.9728
382097	Gm1123	predicted gene 1123	0.9786	0.8964	0.9699
68276	Toe1	target of EGR1, member 1 (nuclear)	0.9786	0.6126	NA

53893	Nudt5	nudix (nucleoside diphosphate linked moiety X)-type motif 5	0.9786	0.8614	0.9573
319618	Dcp1b	DCP1 decapping enzyme homolog b (S. cerevisiae)	0.9785	0.6283	NA
268882	Fbxo45	F-box protein 45	0.9785	0.7868	NA
243272	Sbno1	sno, strawberry notch homolog 1 (Drosophila)	0.9785	0.8119	0.9403
110391	Qdpr	quinoid dihydropteridine reductase	0.9785	0.6683	NA
67890	Ufm1	ubiquitin-fold modifier 1	0.9785	0.699	NA
66067	Gtpbp8	GTP-binding protein 8 (putative)	0.9785	0.6751	NA
52552	Parp8	poly (ADP-ribose) polymerase family, member 8	0.9785	0.7701	NA
26901	Deb1	differentially expressed in B16F10 1	0.9785	0.6539	NA
21351	Taldo1	transaldolase 1	0.9785	0.6063	NA
380684	Nefh	neurofilament, heavy polypeptide	0.9784	0.8174	0.942
218975	Mapk1ip1l	mitogen-activated protein kinase 1 interacting protein 1-like	0.9784	0.8077	0.939
67337	Cstf1	cleavage stimulation factor, 3' pre-RNA, subunit 1	0.9784	0.8943	0.9697
67236	Cinp	cyclin-dependent kinase 2 interacting protein	0.9784	0.921	0.9769
30056	Timm9	translocase of inner mitochondrial membrane 9 homolog (yeast)	0.9784	0.7684	NA
16563	Kif2a	kinesin family member 2A	0.9784	0.7075	NA
12725	Clcn3	chloride channel 3	0.9784	0.8077	0.939
94093	Trim33	tripartite motif-containing 33	0.9783	0.7157	NA
76483	Lmf1	lipase maturation factor 1	0.9783	0.8749	0.9617
66874	1200014J11Rik	RIKEN cDNA 1200014J11 gene	0.9783	0.9159	0.9756
64176	Sv2b	synaptic vesicle glycoprotein 2 b	0.9783	0.7525	NA
22318	Vamp2	vesicle-associated membrane protein 2	0.9783	0.8195	0.9427
13972	Gnb1l	guanine nucleotide binding protein (G protein), beta polypeptide 1-like	0.9783	0.7125	NA
192663	Abcg4	ATP-binding cassette, sub-family G (WHITE), member 4	0.9782	0.7109	NA
106821	AI314976	expressed sequence AI314976	0.9782	0.6835	NA
77938	Fam53b	family with sequence similarity 53, member B	0.9782	0.7998	0.9364
60365	Rbm8a	RNA binding motif protein 8a	0.9782	0.8151	0.9413
27041	G3bp1	Ras-GTPase-activating protein SH3-domain binding protein 1	0.9782	0.6433	NA
20227	Sart1	squamous cell carcinoma antigen recognized by T-cells 1	0.9782	0.782	NA
19661	Rbp3	retinol binding protein 3, interstitial	0.9782	0.8563	0.9554
14680	Gnal	guanine nucleotide binding protein, alpha stimulating, olfactory type	0.9782	0.8327	0.9457
13831	Epc1	enhancer of polycomb homolog 1 (Drosophila)	0.9782	0.6611	NA
13490	Drd3	dopamine receptor D3	0.9782	0.9115	0.9743
399558	Flrt2	fibronectin leucine rich transmembrane protein 2	0.9781	0.6824	NA
353187	Nr1d2	nuclear receptor subfamily 1, group D, member 2	0.9781	0.7929	NA
347740	2900097C17Rik	RIKEN cDNA 2900097C17 gene	0.9781	0.9003	0.9709
104362	Meig1	meiosis expressed gene 1	0.9781	0.682	NA
69123	1810022C23Rik	RIKEN cDNA 1810022C23 gene	0.9781	0.7058	NA
67532	Mfap1a	microfibrillar-associated protein 1A	0.9781	0.57	NA
19047	Ppp1cc	protein phosphatase 1, catalytic subunit, gamma isoform	0.9781	0.8069	0.9388
74246	Gale	galactose-4-epimerase, UDP	0.978	0.6757	NA
26401	Map3k1	mitogen-activated protein kinase kinase kinase 1	0.978	0.6402	NA
18099	Nlk	nemo like kinase	0.978	0.6261	NA
16601	Klf9	Kruppel-like factor 9	0.978	0.7375	NA
15979	Ifngr1	interferon gamma receptor 1	0.978	0.6538	NA
628919	Gm6934	predicted gene 6934	0.9779	0.8779	0.9627
331045	A530083I20Rik	RIKEN cDNA A530083I20 gene	0.9779	0.6952	NA
319728	E030042N06Rik	RIKEN cDNA E030042N06 gene	0.9779	0.8343	0.9464
233904	Setd1a	SET domain containing 1A	0.9779	0.6579	NA

216622	4931440F15Rik	RIKEN cDNA 4931440F15 gene	0.9779	0.8295	0.945
214572	Prmt7	protein arginine N-methyltransferase 7	0.9779	0.6897	NA
212670	Catsper2	cation channel, sperm associated 2	0.9779	0.823	0.9438
93742	Pard3	par-3 (partitioning defective 3) homolog (C. elegans)	0.9779	0.7848	NA
77519	Zfp266	zinc finger protein 266	0.9779	0.7305	NA
59036	Dact1	dapper homolog 1, antagonist of beta-catenin (xenopus)	0.9779	0.767	NA
54216	Pcdh7	protocadherin 7	0.9779	0.8963	0.9699
27369	Dguok	deoxyguanosine kinase	0.9779	0.7816	NA
83493	Sacm1l	SAC1 (suppressor of actin mutations 1, homolog)-like (S. cerevisiae)	0.9778	0.7594	NA
81018	Rnf114	ring finger protein 114	0.9778	0.6677	NA
73744	Man2c1	mannosidase, alpha, class 2C, member 1	0.9778	0.6592	NA
15078	H3f3a	H3 histone, family 3A	0.9778	0.6357	NA
12361	Cask	calcium/calmodulin-dependent serine protein kinase (MAGUK family)	0.9778	0.6837	NA
79560	Ublcp1	ubiquitin-like domain containing CTD phosphatase 1	0.9777	0.5577	NA
70536	Qpct	glutaminyl-peptide cyclotransferase (glutaminyl cyclase)	0.9777	0.7008	NA
66506	Psmg3	proteasome (prosome, macropain) assembly chaperone 3	0.9777	0.7592	NA
23918	Impdh2	inosine 5'-phosphate dehydrogenase 2	0.9777	0.6146	NA
18700	Piga	phosphatidylinositol glycan anchor biosynthesis, class A	0.9777	0.6855	NA
16801	Arhgef1	Rho guanine nucleotide exchange factor (GEF) 1	0.9777	0.8215	0.9434
13690	Eif4g2	eukaryotic translation initiation factor 4, gamma 2	0.9777	0.7562	NA
13043	Cttn	cortactin	0.9777	0.5745	NA
269105	Gm5048	predicted gene 5048	0.9776	0.7441	NA
235040	Atg4d	autophagy-related 4D (yeast)	0.9776	0.7126	NA
234072	Adprhl1	ADP-ribosylhydrolase like 1	0.9776	0.8686	0.9596
215201	Trmt2b	TRM2 tRNA methyltransferase 2 homolog B (S. cerevisiae)	0.9776	0.7716	NA
170718	Idh3b	isocitrate dehydrogenase 3 (NAD+) beta	0.9776	0.7526	NA
76901	Phf15	PHD finger protein 15	0.9776	0.6737	NA
73288	Ccdc132	coiled-coil domain containing 132	0.9776	0.6702	NA
69482	Nup35	nucleoporin 35	0.9776	0.6101	NA
68477	Rmnd5a	required for meiotic nuclear division 5 homolog A (S. cerevisiae)	0.9776	0.7498	NA
56742	Psrc1	proline/serine-rich coiled-coil 1	0.9776	0.8171	0.942
22187	Ubb	ubiquitin B	0.9776	0.7918	0.9333
319713	Ablim3	actin binding LIM protein family, member 3	0.9775	0.7586	NA
72722	Fam98a	family with sequence similarity 98, member A	0.9775	0.6481	NA
67749	4930583H14Rik	RIKEN cDNA 4930583H14 gene	0.9775	0.666	NA
67676	Rpp21	ribonuclease P 21 subunit (human)	0.9775	0.747	NA
66882	Bzw1	basic leucine zipper and W2 domains 1	0.9775	0.6603	NA
20742	Spnb2	spectrin beta 2	0.9775	0.6132	NA
19744	Rheb	Ras homolog enriched in brain	0.9775	0.8989	0.9704
14534	Kat2a	K(lysine) acetyltransferase 2A	0.9775	0.8297	0.945
12428	Ccna2	cyclin A2	0.9775	0.7783	NA
100040377	LOC100040377	hypothetical protein LOC100040377	0.9774	0.8274	0.9449
74482	Ifitm7	interferon induced transmembrane protein 7	0.9774	0.8092	0.9396
70103	Znhit1	zinc finger, HIT domain containing 1	0.9774	0.8308	0.9455
23795	Agr2	anterior gradient 2 (Xenopus laevis)	0.9774	0.8314	0.9455
18013	Neurod2	neurogenic differentiation 2	0.9774	0.7665	NA
17448	Mdh2	malate dehydrogenase 2, NAD (mitochondrial)	0.9774	0.774	NA
11776	Ap3d1	adaptor-related protein complex 3, delta 1 subunit	0.9774	0.6329	NA
170439	Elovl6	ELOVL family member 6, elongation of long chain fatty acids (yeast)	0.9773	0.6627	NA

75958	5033403F01Rik	RIKEN cDNA 5033403F01 gene	0.9773	0.861	0.9573
75627	Snapc1	small nuclear RNA activating complex, polypeptide 1	0.9773	0.7184	NA
69456	Commd10	COMM domain containing 10	0.9773	0.7153	NA
58988	Rps6kb2	ribosomal protein S6 kinase, polypeptide 2	0.9773	0.8258	0.9446
52864	Slx4	SLX4 structure-specific endonuclease subunit homolog (S. cerevisiae)	0.9773	0.59	NA
22688	Zfp26	zinc finger protein 26	0.9773	0.6996	NA
17765	Mtf2	metal response element binding transcription factor 2	0.9773	0.5968	NA
14674	Gna13	guanine nucleotide binding protein, alpha 13	0.9773	0.6154	NA
13437	Dnpep	aspartyl aminopeptidase	0.9773	0.6546	NA
11836	Araf	v-raf murine sarcoma 3611 viral oncogene homolog	0.9773	0.7331	NA
327762	Dna2	DNA replication helicase 2 homolog (yeast)	0.9772	0.712	NA
267019	Rps15a	ribosomal protein S15A	0.9772	0.6537	NA
217207	Dhx8	DEAH (Asp-Glu-Ala-His) box polypeptide 8	0.9772	0.7766	NA
210529	Mettl14	methyltransferase like 14	0.9772	0.5801	NA
81489	Dnajb1	DnaJ (Hsp40) homolog, subfamily B, member 1	0.9772	0.6039	NA
72693	Zcchc12	zinc finger, CCHC domain containing 12	0.9772	0.52	NA
54127	Rps28	ribosomal protein S28	0.9772	0.7384	NA
53333	Tomm40	translocase of outer mitochondrial membrane 40 homolog (yeast)	0.9772	0.8351	0.9466
19291	Purb	purine rich element binding protein B	0.9772	0.6079	NA
237433	Gm4925	predicted gene 4925	0.9771	0.8556	0.9552
230863	Sh2d5	SH2 domain containing 5	0.9771	0.7853	0.9307
225994	BC016495	cDNA sequence BC016495	0.9771	0.8418	0.9487
72175	Mfsd8	major facilitator superfamily domain containing 8	0.9771	0.6409	NA
70230	3300002P13Rik	RIKEN cDNA 3300002P13 gene	0.9771	0.812	0.9403
23924	Katna1	katanin p60 (ATPase-containing) subunit A1	0.9771	0.6802	NA
18347	Olfr48	olfactory receptor 48	0.9771	0.9526	0.9869
170835	Inpp5j	inositol polyphosphate 5-phosphatase J	0.977	0.6981	NA
77059	4931408D14Rik	RIKEN cDNA 4931408D14 gene	0.977	0.8485	0.9522
74343	Crtc2	CREB regulated transcription coactivator 2	0.977	0.6524	NA
67062	Mcart6	mitochondrial carrier triple repeat 6	0.977	0.7914	0.9333
64652	Nisch	nischarin	0.977	0.6802	NA
56399	Akap8	A kinase (PRKA) anchor protein 8	0.977	0.8551	0.9551
52530	Nhp2	NHP2 ribonucleoprotein homolog (yeast)	0.977	0.7476	NA
11863	Arnt	aryl hydrocarbon receptor nuclear translocator	0.977	0.6752	NA
68472	Tmem126b	transmembrane protein 126B	0.9769	0.7588	NA
66934	Dsn1	DSN1, MIND kinetochore complex component, homolog (S. cerevisiae)	0.9769	0.832	0.9456
18213	Ntrk3	neurotrophic tyrosine kinase, receptor, type 3	0.9769	0.774	NA
228545	Vps18	vacuolar protein sorting 18 (yeast)	0.9768	0.5526	NA
140492	Kcnn2	potassium intermediate/small conductance calcium-activated channel, subfamily N, member 2	0.9768	0.6552	NA
109346	Ankrd39	ankyrin repeat domain 39	0.9768	0.737	NA
77629	Sphkap	SPHK1 interactor, AKAP domain containing	0.9768	0.625	NA
69257	Elf2	E74-like factor 2	0.9768	0.5949	NA
22152	Tubb3	tubulin, beta 3	0.9768	0.7595	NA
16572	Kif5a	kinesin family member 5A	0.9768	0.6015	NA
16558	Kif16b	kinesin family member 16B	0.9768	0.7763	NA
11979	Atp7b	ATPase, Cu++ transporting, beta polypeptide	0.9768	0.8477	0.9521
320333	D830030K20Rik	RIKEN cDNA D830030K20 gene	0.9767	0.8235	0.9439
272347	Zfp398	zinc finger protein 398	0.9767	0.6285	NA
212448	9330159F19Rik	RIKEN cDNA 9330159F19 gene	0.9767	0.6297	NA

109082	Fbxw17	F-box and WD-40 domain protein 17	0.9767	0.8045	0.9381
70314	Rabep2	rabaptin, RAB GTPase binding effector protein 2	0.9767	0.7278	NA
66598	3110001I22Rik	RIKEN cDNA 3110001I22 gene	0.9767	0.6856	NA
55934	rp9	retinitis pigmentosa 9 (human)	0.9767	0.7154	NA
622434	Arhgef26	Rho guanine nucleotide exchange factor (GEF) 26	0.9766	0.6837	NA
231841	AA881470	EST AA881470	0.9766	0.832	0.9456
72615	Anks3	ankyrin repeat and sterile alpha motif domain containing 3	0.9766	0.651	NA
66309	Tmem128	transmembrane protein 128	0.9766	0.8585	0.9564
56048	Lgals8	lectin, galactose binding, soluble 8	0.9766	0.759	NA
21343	Taf6	TAF6 RNA polymerase II, TATA box binding protein (TBP)-associated factor	0.9766	0.6047	NA
329152	Hecw2	HECT, C2 and WW domain containing E3 ubiquitin protein ligase 2	0.9765	0.6763	NA
102323	Dcun1d2	DCN1, defective in cullin neddylation 1, domain containing 2 (S. cerevisiae)	0.9765	0.5127	NA
21884	Fabp9	fatty acid binding protein 9, testis	0.9765	0.8138	0.9406
14081	Acsl1	acyl-CoA synthetase long-chain family member 1	0.9765	0.8016	0.9373
11727	Ang	angiogenin, ribonuclease, RNase A family, 5	0.9765	0.8125	0.9403
330662	Dock1	dedicator of cytokinesis 1	0.9764	0.727	NA
100972	Rab28	RAB28, member RAS oncogene family	0.9764	0.7046	NA
80297	Spnb4	spectrin beta 4	0.9764	0.7774	NA
66212	Sec61b	Sec61 beta subunit	0.9764	0.7181	NA
20677	Sox4	SRY-box containing gene 4	0.9764	0.7538	NA
16495	Kcna7	potassium voltage-gated channel, shaker-related subfamily, member 7	0.9764	0.9054	0.9718
328530	D030063E12	hypothetical protein D030063E12	0.9763	0.7797	0.928
229672	Bcl2l15	BCLl2-like 15	0.9763	0.7306	NA
224814	Abcc10	ATP-binding cassette, sub-family C (CFTR/MRP), member 10	0.9763	0.777	0.9272
81907	Tmem108	transmembrane protein 108	0.9763	0.8748	0.9617
80517	Herpud2	HERPUD family member 2	0.9763	0.5686	NA
76658	1700123K08Rik	RIKEN cDNA 1700123K08 gene	0.9763	0.72	NA
69666	Psmg4	proteasome (prosome, macropain) assembly chaperone 4	0.9763	0.7077	NA
66966	Trit1	tRNA isopentenyltransferase 1	0.9763	0.7338	NA
11764	Ap1b1	adaptor protein complex AP-1, beta 1 subunit	0.9763	0.5964	NA
217692	Sipa1l1	signal-induced proliferation-associated 1 like 1	0.9762	0.6185	NA
68905	1110065H08Rik	RIKEN cDNA 1110065H08 gene	0.9762	0.8278	0.945
67988	Tmx3	thioredoxin-related transmembrane protein 3	0.9762	0.7572	NA
67025	Rpl11	ribosomal protein L11	0.9762	0.729	NA
22327	Vbp1	von Hippel-Lindau binding protein 1	0.9762	0.7367	NA
20658	Son	Son DNA binding protein	0.9762	0.679	NA
100039210	Gm2102	predicted gene 2102	0.9761	0.8656	0.9585
211612	Ptchd1	patched domain containing 1	0.9761	0.7522	NA
98445	AI844869	expressed sequence AI844869	0.9761	0.7564	NA
76895	Bicd2	bicaudal D homolog 2 (Drosophila)	0.9761	0.5288	NA
71392	5430401F13Rik	RIKEN cDNA 5430401F13 gene	0.9761	0.8705	0.9604
18472	Pafah1b1	platelet-activating factor acetylhydrolase, isoform 1b, subunit 1	0.9761	0.6683	NA
18117	Cox4nb	COX4 neighbor	0.9761	0.5302	NA
277978	Exoc3l	exocyst complex component 3-like	0.976	0.8428	0.9496
75398	Mrpl32	mitochondrial ribosomal protein L32	0.976	0.6826	NA
67089	Psmc6	proteasome (prosome, macropain) 26S subunit, ATPase, 6	0.976	0.6083	NA
67023	Use1	unconventional SNARE in the ER 1 homolog (S. cerevisiae)	0.976	0.7106	NA
57434	Xrcc2	X-ray repair complementing defective repair in Chinese hamster cells 2	0.976	0.7674	NA
13436	Dnmt3b	DNA methyltransferase 3B	0.976	0.8223	0.9435

101540	Prkd2	protein kinase D2	0.9759	0.8183	0.9423
70779	Prdm5	PR domain containing 5	0.9759	0.6852	NA
66967	Edem3	ER degradation enhancer, mannosidase alpha-like 3	0.9759	0.6366	NA
54525	Syt7	synaptotagmin VII	0.9759	0.905	0.9718
24017	Rnf13	ring finger protein 13	0.9759	0.6748	NA
13642	Efnb2	ephrin B2	0.9759	0.7051	NA
215476	C330019G07Rik	RIKEN cDNA C330019G07 gene	0.9758	0.7564	NA
72117	Naa50	N(alpha)-acetyltransferase 50, NatE catalytic subunit	0.9758	0.7197	NA
70546	Zdhhc2	zinc finger, DHHC domain containing 2	0.9758	0.7352	NA
68394	Ccdc163	coiled-coil domain containing 163	0.9758	0.8008	0.9368
68198	Ndufb2	NADH dehydrogenase (ubiquinone) 1 beta subcomplex, 2	0.9758	0.6339	NA
66396	Ccdc82	coiled-coil domain containing 82	0.9758	0.5903	NA
20847	Stat2	signal transducer and activator of transcription 2	0.9758	0.6137	NA
19230	Twf1	twinfilin, actin-binding protein, homolog 1 (Drosophila)	0.9758	0.4697	NA
19057	Ppp3cc	protein phosphatase 3, catalytic subunit, gamma isoform	0.9758	0.6571	NA
14977	Slc39a7	solute carrier family 39 (zinc transporter), member 7	0.9758	0.7776	0.9272
14772	Grk4	G protein-coupled receptor kinase 4	0.9758	0.7238	NA
14693	Gnb2	guanine nucleotide binding protein (G protein), beta 2	0.9758	0.823	0.9438
13631	Eef2k	eukaryotic elongation factor-2 kinase	0.9758	0.847	0.9517
100504262	LOC100504262	hypothetical LOC100504262	0.9757	0.7104	NA
71159	4933416I08Rik	RIKEN cDNA 4933416I08 gene	0.9757	0.8883	0.9675
22288	Utrn	utrophin	0.9757	0.8758	0.9618
19014	Med1	mediator complex subunit 1	0.9757	0.6798	NA
16000	Igf1	insulin-like growth factor 1	0.9757	0.8753	0.9618
14661	Glud1	glutamate dehydrogenase 1	0.9757	0.7307	NA
435285	Krtap4-16	keratin associated protein 4-16	0.9756	0.8503	0.9531
319909	Ism1	isthmin 1 homolog (zebrafish)	0.9756	0.7664	NA
101095	Zfp282	zinc finger protein 282	0.9756	0.7966	0.935
93719	Ear6	eosinophil-associated, ribonuclease A family, member 6	0.9756	0.8695	0.9598
75304	4930563E22Rik	RIKEN cDNA 4930563E22 gene	0.9756	0.7714	0.9258
70291	2510049J12Rik	RIKEN cDNA 2510049J12 gene	0.9756	0.7631	NA
50776	Polg2	polymerase (DNA directed), gamma 2, accessory subunit	0.9756	0.6847	NA
20910	Stxbp1	syntaxin binding protein 1	0.9756	0.7288	NA
17536	Meis2	Meis homeobox 2	0.9756	0.7644	NA
14433	Gapdh	glyceraldehyde-3-phosphate dehydrogenase	0.9756	0.8124	0.9403
319317	Snhg11	small nucleolar RNA host gene 11	0.9755	0.7327	NA
68039	Nmb	neuromedin B	0.9755	0.7838	0.9301
67457	Frmd8	FERM domain containing 8	0.9755	0.7579	NA
66048	Tmem93	transmembrane protein 93	0.9755	0.6731	NA
20603	Sms	spermine synthase	0.9755	0.7417	NA
15184	Hdac5	histone deacetylase 5	0.9755	0.7099	NA
14950	H13	histocompatibility 13	0.9755	0.5308	NA
100039895	Gm2479	predicted gene 2479	0.9754	0.9143	0.9747
279653	Pcdh19	protocadherin 19	0.9754	0.542	NA
224111	Ubxn7	UBX domain protein 7	0.9754	0.8613	0.9573
73218	3110056O03Rik	RIKEN cDNA 3110056O03 gene	0.9754	0.5506	NA
72201	Otud6b	OTU domain containing 6B	0.9754	0.6198	NA
22377	Wbp1	WW domain binding protein 1	0.9754	0.7192	NA
15574	Hus1	Hus1 homolog (S. pombe)	0.9754	0.5879	NA

14852	Gspt1	G1 to S phase transition 1	0.9754	0.7081	NA
14160	Lgr5	leucine rich repeat containing G protein coupled receptor 5	0.9754	0.6914	NA
103819	AI663975	expressed sequence AI663975	0.9753	0.6791	NA
77095	D330022H12Rik	RIKEN cDNA D330022H12 gene	0.9753	0.751	NA
18191	Nrxn3	neurexin III	0.9753	0.7368	NA
380959	Alg10b	asparagine-linked glycosylation 10 homolog B (yeast, alpha-1,2-glucosyltransferase)	0.9752	0.7085	NA
319675	5830418K08Rik	RIKEN cDNA 5830418K08 gene	0.9752	0.6775	NA
214663	Slc25a29	solute carrier family 25 (mitochondrial carrier, palmitoylcarnitine transporter), member 29	0.9752	0.7738	0.9266
170753	Zfp704	zinc finger protein 704	0.9752	0.5989	NA
22652	Mkrn3	makorin, ring finger protein, 3	0.9752	0.714	NA
22194	Ube2e1	ubiquitin-conjugating enzyme E2E 1, UBC4/5 homolog (yeast)	0.9752	0.7493	NA
21843	Tial1	Tia1 cytotoxic granule-associated RNA binding protein-like 1	0.9752	0.6397	NA
791358	Gm10065	predicted gene 10065	0.9751	0.7587	NA
239134	Gucy1b2	guanylate cyclase 1, soluble, beta 2	0.9751	0.9362	0.9814
229906	Gtf2b	general transcription factor IIB	0.9751	0.6867	NA
74094	Tjap1	tight junction associated protein 1	0.9751	0.7012	NA
28250	Slco1a4	solute carrier organic anion transporter family, member 1a4	0.9751	0.7372	NA
19656	Rbmxrt	RNA binding motif protein, X chromosome retrogene	0.9751	0.6826	NA
17428	Mnt	max binding protein	0.9751	0.7128	NA
100039684	5031434O11Rik	RIKEN cDNA 5031434O11 gene	0.975	0.7775	0.9272
330485	Tmem145	transmembrane protein 145	0.975	0.664	NA
226830	Smyd2	SET and MYND domain containing 2	0.975	0.7147	NA
216134	Pdxk	pyridoxal (pyridoxine, vitamin B6) kinase	0.975	0.7754	0.9266
99683	Sec24b	Sec24 related gene family, member B (S. cerevisiae)	0.975	0.5958	NA
98910	Usp6nl	USP6 N-terminal like	0.975	0.6961	NA
77987	Ascc3	activating signal cointegrator 1 complex subunit 3	0.975	0.6102	NA
68796	Tmem214	transmembrane protein 214	0.975	0.76	NA
24136	Zeb2	zinc finger E-box binding homeobox 2	0.975	0.7894	0.9329
11736	Ankfy1	ankyrin repeat and FYVE domain containing 1	0.975	0.8091	0.9396
628847	Gm6921	predicted pseudogene 6921	0.9749	0.8427	0.9496
245880	Wasf3	WAS protein family, member 3	0.9749	0.8187	0.9425
229672	Bcl2l15	BCLl2-like 15	0.9749	0.8057	0.9386
207393	Elfn2	leucine rich repeat and fibronectin type III, extracellular 2	0.9749	0.8444	0.9504
67040	Ddx17	DEAD (Asp-Glu-Ala-Asp) box polypeptide 17	0.9749	0.5465	NA
66381	Rnf113a2	ring finger protein 113A2	0.9749	0.5119	NA
16784	Lamp2	lysosomal-associated membrane protein 2	0.9749	0.7058	NA
381983	Lmtk3	lemur tyrosine kinase 3	0.9748	0.7738	0.9266
217356	Tmc8	transmembrane channel-like gene family 8	0.9748	0.867	0.9588
106633	Ift140	intraflagellar transport 140 homolog (Chlamydomonas)	0.9748	0.7772	0.9272
78752	Csgalnact2	chondroitin sulfate N-acetylgalactosaminyltransferase 2	0.9748	0.6423	NA
74144	Robo4	roundabout homolog 4 (Drosophila)	0.9748	0.7621	NA
73754	Thap1	THAP domain containing, apoptosis associated protein 1	0.9748	0.8006	0.9367
71767	Tysnd1	trypsin domain containing 1	0.9748	0.683	NA
69151	Lzic	leucine zipper and CTNNBIP1 domain containing	0.9748	0.703	NA
69034	4930579G22Rik	RIKEN cDNA 4930579G22 gene	0.9748	0.8327	0.9457
60534	Fancg	Fanconi anemia, complementation group G	0.9748	0.7878	0.9317
22631	Ywhaz	tyrosine 3-monooxygenase/tryptophan 5-monooxygenase activation protein, zeta polypeptide	0.9748	0.6776	NA
20768	Sephs2	selenophosphate synthetase 2	0.9748	0.7911	0.9333
19896	Rpl10a	ribosomal protein L10A	0.9748	0.6068	NA

16443	Itsn1	intersectin 1 (SH3 domain protein 1A)	0.9748	0.9125	0.9743
11518	Add1	adducin 1 (alpha)	0.9748	0.7251	NA
620695	Gm13889	predicted gene 13889	0.9747	0.491	NA
434179	Gm5595	predicted gene 5595	0.9747	0.7929	0.9334
193742	Abhd16a	abhydrolase domain containing 16A	0.9747	0.7403	NA
66931	1700010I14Rik	RIKEN cDNA 1700010I14 gene	0.9747	0.7439	NA
52335	Atxn1l	ataxin 1-like	0.9747	0.5906	NA
17169	Mark3	MAP/microtubule affinity-regulating kinase 3	0.9747	0.6368	NA
15185	Hdac6	histone deacetylase 6	0.9747	0.6143	NA
237831	Slc13a5	solute carrier family 13 (sodium-dependent citrate transporter), member 5	0.9746	0.7634	0.9247
73247	1600027N09Rik	RIKEN cDNA 1600027N09 gene	0.9746	0.6952	NA
71997	1500002O20Rik	RIKEN cDNA 1500002O20 gene	0.9746	0.7958	0.9346
66962	2310047B19Rik	RIKEN cDNA 2310047B19 gene	0.9746	0.7028	NA
56434	Tspan3	tetraspanin 3	0.9746	0.6549	NA
19877	Rock1	Rho-associated coiled-coil containing protein kinase 1	0.9746	0.8304	0.9453
16485	Kcna1	potassium voltage-gated channel, shaker-related subfamily, member 1	0.9746	0.6745	NA
232286	Tmf1	TATA element modulatory factor 1	0.9745	0.5616	NA
225339	Ammecr1l	AMME chromosomal region gene 1-like	0.9745	0.6398	NA
213773	Tbl3	transducin (beta)-like 3	0.9745	0.8248	0.9443
83946	Phip	pleckstrin homology domain interacting protein	0.9745	0.4994	NA
72699	Lime1	Lck interacting transmembrane adaptor 1	0.9745	0.6831	NA
57370	B4galt3	UDP-Gal:betaGlcNAc beta 1,4-galactosyltransferase, polypeptide 3	0.9745	0.72	NA
18538	Pcna	proliferating cell nuclear antigen	0.9745	0.6542	NA
13663	Ei24	etoposide induced 2.4 mRNA	0.9745	0.75	NA
320595	Phf8	PHD finger protein 8	0.9744	0.5173	NA
230917	Tmem201	transmembrane protein 201	0.9744	0.796	0.9346
72633	2810019C22Rik	RIKEN cDNA 2810019C22 gene	0.9744	0.8604	0.9571
70081	2210404O09Rik	RIKEN cDNA 2210404O09 gene	0.9744	0.749	NA
69894	2010107G23Rik	RIKEN cDNA 2010107G23 gene	0.9744	0.6179	NA
66942	Ddx18	DEAD (Asp-Glu-Ala-Asp) box polypeptide 18	0.9744	0.7285	NA
233900	Rnf40	ring finger protein 40	0.9743	0.7216	NA
225743	Rnf165	ring finger protein 165	0.9743	0.7206	NA
194590	Reps2	RALBP1 associated Eps domain containing protein 2	0.9743	0.5707	NA
101206	Tada3	transcriptional adaptor 3	0.9743	0.7234	NA
78890	2310079F23Rik	RIKEN cDNA 2310079F23 gene	0.9743	0.5752	NA
58180	Hic2	hypermethylated in cancer 2	0.9743	0.505	NA
13559	E2f5	E2F transcription factor 5	0.9743	0.7364	NA
242509	Bnc2	basonuclin 2	0.9742	0.767	0.9253
216439	Agap2	ArfGAP with GTPase domain, ankyrin repeat and PH domain 2	0.9742	0.6997	NA
216131	Trappc10	trafficking protein particle complex 10	0.9742	0.8233	0.9439
94062	Mrpl3	mitochondrial ribosomal protein L3	0.9742	0.5158	NA
93836	Rnf111	ring finger 111	0.9742	0.6615	NA
67026	Thap4	THAP domain containing 4	0.9742	0.7442	NA
239122	Setdb2	SET domain, bifurcated 2	0.9741	0.5958	NA
231571	Rpap2	RNA polymerase II associated protein 2	0.9741	0.5475	NA
94221	Gopc	golgi associated PDZ and coiled-coil motif containing	0.9741	0.6169	NA
68048	Aen	apoptosis enhancing nuclease	0.9741	0.6336	NA
56469	Pias1	protein inhibitor of activated STAT 1	0.9741	0.7729	0.9262
56430	Clip1	CAP-GLY domain containing linker protein 1	0.9741	0.6394	NA

19317	Qk	quaking	0.9741	0.714	NA
381510	Dpy19l4	dpy-19-like 4 (C. elegans)	0.974	0.7748	0.9266
380732	Gm885	predicted gene 885	0.974	0.8975	0.9699
320436	C030009J22Rik	RIKEN cDNA C030009J22 gene	0.974	0.6142	NA
233802	Thumpd1	THUMP domain containing 1	0.974	0.4948	NA
110532	Adarb1	adenosine deaminase, RNA-specific, B1	0.974	0.4546	NA
104303	Arl1	ADP-ribosylation factor-like 1	0.974	0.8157	0.9416
65116	Prrg2	proline-rich Gla (G-carboxyglutamic acid) polypeptide 2	0.974	0.7699	0.9257
56642	Ankrd2	ankyrin repeat domain 2 (stretch responsive muscle)	0.974	0.627	NA
30051	Spdef	SAM pointed domain containing ets transcription factor	0.974	0.7821	0.9291
26926	Aifm1	apoptosis-inducing factor, mitochondrion-associated 1	0.974	0.5495	NA
18514	Pbx1	pre B-cell leukemia transcription factor 1	0.974	0.8253	0.9444
17925	Myo9b	myosin IXb	0.974	0.6491	NA
13850	Ephx2	epoxide hydrolase 2, cytoplasmic	0.974	0.7841	0.9302
11423	Ache	acetylcholinesterase	0.974	0.8269	0.9449
620631	Ttc30a2	tetratricopeptide repeat domain 30A2	0.9739	0.6888	NA
239719	Mkl2	MKL/myocardin-like 2	0.9739	0.5605	NA
225326	Pik3c3	phosphoinositide-3-kinase, class 3	0.9739	0.6354	NA
110279	Bcr	breakpoint cluster region	0.9739	0.7329	NA
76800	Usp42	ubiquitin specific peptidase 42	0.9739	0.5925	NA
74762	Mdga1	MAM domain containing glycosylphosphatidylinositol anchor 1	0.9739	0.7961	0.9346
69893	2010305A19Rik	RIKEN cDNA 2010305A19 gene	0.9739	0.678	NA
68971	1500001M20Rik	RIKEN cDNA 1500001M20 gene	0.9739	0.8891	0.9677
68642	Tmem216	transmembrane protein 216	0.9739	0.4391	NA
67920	Mak16	MAK16 homolog (S. cerevisiae)	0.9739	0.8087	0.9395
67885	1500011K16Rik	RIKEN cDNA 1500011K16 gene	0.9739	0.6409	NA
67333	Stk35	serine/threonine kinase 35	0.9739	0.6393	NA
21339	Taf1a	TATA box binding protein (Tbp)-associated factor, RNA polymerase I, A	0.9739	0.8191	0.9426
16391	Irf9	interferon regulatory factor 9	0.9739	0.528	NA
414095	B130034C11Rik	RIKEN cDNA B130034C11 gene	0.9738	0.6822	NA
228662	Btbd3	BTB (POZ) domain containing 3	0.9738	0.8222	0.9435
212307	Mapre2	microtubule-associated protein, RP/EB family, member 2	0.9738	0.5673	NA
192786	Rapgef6	Rap guanine nucleotide exchange factor (GEF) 6	0.9738	0.6534	NA
54120	Gipc2	GIPC PDZ domain containing family, member 2	0.9738	0.492	NA
26356	Ing1	inhibitor of growth family, member 1	0.9738	0.6424	NA
17425	Foxk1	forkhead box K1	0.9738	0.4531	NA
232813	Shisa7	shisa homolog 7 (Xenopus laevis)	0.9737	0.6809	NA
107769	Tm6sf1	transmembrane 6 superfamily member 1	0.9737	0.7842	0.9303
70099	Smc4	structural maintenance of chromosomes 4	0.9737	0.6777	NA
69878	Snrpf	small nuclear ribonucleoprotein polypeptide F	0.9737	0.7475	NA
16549	Khsrp	KH-type splicing regulatory protein	0.9737	0.7973	0.9353
13859	Eps15l1	epidermal growth factor receptor pathway substrate 15-like 1	0.9737	0.5987	NA
12632	Cfl2	cofilin 2, muscle	0.9737	0.622	NA
240853	Gm4953	ATP synthase, H+ transporting, mitochondrial F0 complex, subunit d pseudogene	0.9736	0.7476	NA
68349	Ndufs3	NADH dehydrogenase (ubiquinone) Fe-S protein 3	0.9736	0.5854	NA
67134	Nop56	NOP56 ribonucleoprotein homolog (yeast)	0.9736	0.664	NA
30926	Glrx3	glutaredoxin 3	0.9736	0.755	0.922
26395	Map2k1	mitogen-activated protein kinase kinase 1	0.9736	0.7159	NA
22402	Wisp1	WNT1 inducible signaling pathway protein 1	0.9736	0.5839	NA

20588 Smarcc1	SWI/SNF related, matrix associated, actin dependent regulator of chromatin, subfamily c, member 1	0.9736	0.8163	0.9417
19730 Ralgds	ral guanine nucleotide dissociation stimulator	0.9736	0.8681	0.9596
320534 Tmem104	transmembrane protein 104	0.9735	0.8903	0.9685
209131 Snx30	sorting nexin family member 30	0.9735	0.6155	NA
108653 Rimklb	ribosomal modification protein rimK-like family member B	0.9735	0.8516	0.9537
75689 Higd1b	HIG1 domain family, member 1B	0.9735	0.6076	NA
67387 Unc50	unc-50 homolog (C. elegans)	0.9735	0.6517	NA
56531 Ylpm1	YLP motif containing 1	0.9735	0.729	NA
27681 Snf8	SNF8, ESCRT-II complex subunit, homolog (S. cerevisiae)	0.9735	0.7307	NA
22276 Uros	uroporphyrinogen III synthase	0.9735	0.8595	0.9567
13855 Epn2	epsin 2	0.9735	0.8119	0.9403
319618 Dcp1b	DCP1 decapping enzyme homolog b (S. cerevisiae)	0.9734	0.5647	NA
227867 Epc2	enhancer of polycomb homolog 2 (Drosophila)	0.9734	0.436	NA
210789 Tbc1d4	TBC1 domain family, member 4	0.9734	0.8245	0.9443
75785 Klhl24	kelch-like 24 (Drosophila)	0.9734	0.8009	0.9368
69159 Rhebl1	Ras homolog enriched in brain like 1	0.9734	0.6638	NA
55932 Gbp3	guanylate binding protein 3	0.9734	0.7569	0.9225
20116 Rps8	ribosomal protein S8	0.9734	0.8147	0.9412
18777 Lypla1	lysophospholipase 1	0.9734	0.8068	0.9388
18588 Pde6g	phosphodiesterase 6G, cGMP-specific, rod, gamma	0.9734	0.6883	NA
18100 Mrpl40	mitochondrial ribosomal protein L40	0.9734	0.6064	NA
16553 Kif13a	kinesin family member 13A	0.9734	0.452	NA
14007 Celf2	CUGBP, Elav-like family member 2	0.9734	0.6803	NA
258285 Olfr122	olfactory receptor 122	0.9733	0.9418	0.9835
240817 5830403L16Rik	RIKEN cDNA 5830403L16 gene	0.9733	0.8324	0.9456
233812 BC030336	cDNA sequence BC030336	0.9733	0.7465	NA
230676 Szt2	seizure threshold 2	0.9733	0.6645	NA
218811 Sec24c	Sec24 related gene family, member C (S. cerevisiae)	0.9733	0.57	NA
140765 Tmprss3	transmembrane protease, serine 3	0.9733	0.8836	0.9652
60344 Fign	fidgetin	0.9733	0.8616	0.9573
28036 Larp7	La ribonucleoprotein domain family, member 7	0.9733	0.6992	NA
19823 Rnf7	ring finger protein 7	0.9733	0.6817	NA
19088 Prkar2b	protein kinase, cAMP dependent regulatory, type II beta	0.9733	0.6848	NA
622629 Gm10318	predicted gene 10318	0.9732	0.8653	0.9584
353156 Egfl7	EGF-like domain 7	0.9732	0.5971	NA
240396 Mex3c	mex3 homolog C (C. elegans)	0.9732	0.6611	NA
93728 Pabpc5	poly(A) binding protein, cytoplasmic 5	0.9732	0.6874	NA
56046 Uqcc	ubiquinol-cytochrome c reductase complex chaperone, CBP3 homolog (yeast)	0.9732	0.6491	NA
545652 Gm13275	predicted gene 13275	0.9731	0.8578	0.9562
320244 Ttll5	tubulin tyrosine ligase-like family, member 5	0.9731	0.8537	0.9544
320011 Uggt1	UDP-glucose glycoprotein glucosyltransferase 1	0.9731	0.531	NA
319615 Zfp944	zinc finger protein 944	0.9731	0.9423	0.9837
170930 Sumo2	SMT3 suppressor of mif two 3 homolog 2 (yeast)	0.9731	0.8101	0.94
74143 Opa1	optic atrophy 1 homolog (human)	0.9731	0.6537	NA
67332 Snrpd3	small nuclear ribonucleoprotein D3	0.9731	0.656	NA
67160 Eef1g	eukaryotic translation elongation factor 1 gamma	0.9731	0.7395	NA
58520 0610007P14Rik	RIKEN cDNA 0610007P14 gene	0.9731	0.5198	NA
56280 Mrpl37	mitochondrial ribosomal protein L37	0.9731	0.647	NA
56216 Stx1b	syntaxin 1B	0.9731	0.7759	0.9266

19385	Ranbp1	RAN binding protein 1	0.9731	0.7037	NA
665268	1600029O15Rik	ribosomal protein L17 pseudogene	0.973	0.6046	NA
232333	Slc6a1	solute carrier family 6 (neurotransmitter transporter, GABA), member 1	0.973	0.707	NA
75137	Rprd2	regulation of nuclear pre-mRNA domain containing 2	0.973	0.7683	0.9255
72584	Cul4b	cullin 4B	0.973	0.6001	NA
71924	Tube1	epsilon-tubulin 1	0.973	0.5196	NA
66306	Fam53c	family with sequence similarity 53, member C	0.973	0.5844	NA
56200	Ddx21	DEAD (Asp-Glu-Ala-Asp) box polypeptide 21	0.973	0.6363	NA
20660	Sorl1	sortilin-related receptor, LDLR class A repeats-containing	0.973	0.7255	NA
18088	Nkx2-2	NK2 transcription factor related, locus 2 (Drosophila)	0.973	0.885	0.966
12346	Car1	carbonic anhydrase 1	0.973	0.8711	0.9607
69886	2610002J23Rik	RIKEN cDNA 2610002J23 gene	0.9729	0.7008	NA
69550	Bst2	bone marrow stromal cell antigen 2	0.9729	0.7444	NA
55947	Dclre1a	DNA cross-link repair 1A, PSO2 homolog (S. cerevisiae)	0.9729	0.5621	NA
21771	Cirh1a	cirrhosis, autosomal recessive 1A (human)	0.9729	0.692	NA
16330	Inpp5b	inositol polyphosphate-5-phosphatase B	0.9729	0.6134	NA
271305	Phf21b	PHD finger protein 21B	0.9728	0.5067	NA
230162	Zfp189	zinc finger protein 189	0.9728	0.6676	NA
214105	Sox30	SRY-box containing gene 30	0.9728	0.7622	0.9241
77533	C030034I22Rik	RIKEN cDNA C030034I22 gene	0.9728	0.7552	0.922
77264	Zfp142	zinc finger protein 142	0.9728	0.6911	NA
74252	Armc1	armadillo repeat containing 1	0.9728	0.56	NA
68767	ORF19	open reading frame 19	0.9728	0.7163	NA
66290	Atp6v1g1	ATPase, H+ transporting, lysosomal V1 subunit G1	0.9728	0.5215	NA
56445	Dnaja2	DnaJ (Hsp40) homolog, subfamily A, member 2	0.9728	0.7219	NA
19088	Prkar2b	protein kinase, cAMP dependent regulatory, type II beta	0.9728	0.8022	0.9373
11482	Acvrl1	activin A receptor, type II-like 1	0.9728	0.7076	NA
100033459	Pydc3	pyrin domain containing 3	0.9727	0.8068	0.9388
268534	Sntg2	syntrophin, gamma 2	0.9727	0.5762	NA
216151	Polrmt	polymerase (RNA) mitochondrial (DNA directed)	0.9727	0.4986	NA
76938	Rbm17	RNA binding motif protein 17	0.9727	0.6464	NA
18472	Pafah1b1	platelet-activating factor acetylhydrolase, isoform 1b, subunit 1	0.9727	0.679	NA
17131	Smad7	MAD homolog 7 (Drosophila)	0.9727	0.85	0.9531
17113	M6pr	mannose-6-phosphate receptor, cation dependent	0.9727	0.564	NA
16319	Incenp	inner centromere protein	0.9727	0.5688	NA
14065	F2rl3	coagulation factor II (thrombin) receptor-like 3	0.9727	0.8476	0.9521
11906	Zfhx3	zinc finger homeobox 3	0.9727	0.7363	NA
100040671	Gm2897	predicted gene 2897	0.9726	0.8418	0.9487
226751	Cdc42bpa	CDC42 binding protein kinase alpha	0.9726	0.6993	NA
74646	Spsb1	splA/ryanodine receptor domain and SOCS box containing 1	0.9726	0.87	0.9601
72899	Macrod2	MACRO domain containing 2	0.9726	0.8279	0.945
71750	R3hdm2	R3H domain containing 2	0.9726	0.621	NA
69524	Esam	endothelial cell-specific adhesion molecule	0.9726	0.7047	NA
64143	Ralb	v-ral simian leukemia viral oncogene homolog B (ras related)	0.9726	0.8021	0.9373
57267	Apba3	amyloid beta (A4) precursor protein-binding, family A, member 3	0.9726	0.8482	0.9522
28077	Med10	mediator of RNA polymerase II transcription, subunit 10 homolog (NUT2, S. cerevisiae)	0.9726	0.7107	NA
22428	Dctn6	dynactin 6	0.9726	0.5451	NA
22088	Tsg101	tumor susceptibility gene 101	0.9726	0.612	NA
20301	Ccl27a	chemokine (C-C motif) ligand 27A	0.9726	0.6647	NA

20054	Rps15	ribosomal protein S15	0.9726	0.7404	NA
140499	Ube2j2	ubiquitin-conjugating enzyme E2, J2 homolog (yeast)	0.9725	0.6491	NA
103573	Xpo1	exportin 1, CRM1 homolog (yeast)	0.9725	0.556	NA
81897	Tlr9	toll-like receptor 9	0.9725	0.716	NA
67480	Cwc25	CWC25 spliceosome-associated protein homolog (S. cerevisiae)	0.9725	0.7013	NA
67330	1700047M11Rik	RIKEN cDNA 1700047M11 gene	0.9725	0.6734	NA
66866	Nhlrc2	NHL repeat containing 2	0.9725	0.5129	NA
268936	Brpf3	bromodomain and PHD finger containing, 3	0.9724	0.7357	NA
217664	Mgat2	mannoside acetylglucosaminyltransferase 2	0.9724	0.7689	0.9255
72008	Zfyve19	zinc finger, FYVE domain containing 19	0.9724	0.5939	NA
56291	Styx	serine/threonine/tyrosine interaction protein	0.9724	0.582	NA
15185	Hdac6	histone deacetylase 6	0.9724	0.5959	NA
12995	Csnk2a1	casein kinase 2, alpha 1 polypeptide	0.9724	0.8539	0.9545
381833	Prb1	proline-rich protein BstNI subfamily 1	0.9723	0.8229	0.9438
226861	Hhat	hedgehog acyltransferase	0.9723	0.9292	0.9789
213053	Slc39a14	solute carrier family 39 (zinc transporter), member 14	0.9723	0.7435	0.919
67725	Nudt13	nudix (nucleoside diphosphate linked moiety X)-type motif 13	0.9723	0.672	NA
66414	Ndufa12	NADH dehydrogenase (ubiquinone) 1 alpha subcomplex, 12	0.9723	0.4213	NA
66353	Riiad1	regulatory subunit of type II PKA R-subunit (RIIa) domain containing 1	0.9723	0.687	NA
50706	Postn	periostin, osteoblast specific factor	0.9723	0.7683	0.9255
20465	Sim2	single-minded homolog 2 (Drosophila)	0.9723	0.8321	0.9456
17151	Ccndbp1	cyclin D-type binding-protein 1	0.9723	0.6003	NA
13664	Eif1a	eukaryotic translation initiation factor 1A	0.9723	0.4992	NA
12567	Cdk4	cyclin-dependent kinase 4	0.9723	0.6713	NA
12301	Cacybp	calcyclin binding protein	0.9723	0.6577	NA
333329	Cngb1	cyclic nucleotide gated channel beta 1	0.9722	0.6126	NA
80898	Erap1	endoplasmic reticulum aminopeptidase 1	0.9722	0.7368	NA
67141	Fbxo5	F-box protein 5	0.9722	0.7116	NA
53975	Ddx20	DEAD (Asp-Glu-Ala-Asp) box polypeptide 20	0.9722	0.4808	NA
13196	Asap1	ArfGAP with SH# domain, ankyrin repeat and PH domain1	0.9722	0.4951	NA
236573	Gbp9	guanylate-binding protein 9	0.9721	0.629	NA
216233	Socs2	suppressor of cytokine signaling 2	0.9721	0.6578	NA
105837	Mtbp	Mdm2, transformed 3T3 cell double minute p53 binding protein	0.9721	0.5369	NA
76976	2900062L11Rik	RIKEN cDNA 2900062L11 gene	0.9721	0.5475	NA
76332	Cog2	component of oligomeric golgi complex 2	0.9721	0.469	NA
73202	3110067C02Rik	RIKEN cDNA 3110067C02 gene	0.9721	0.7493	0.9207
12651	Chkb	choline kinase beta	0.9721	0.6158	NA
100040382	Gm2745	predicted gene 2745	0.972	0.8776	0.9625
268451	Rab11fip4	RAB11 family interacting protein 4 (class II)	0.972	0.6447	NA
230737	Gnl2	guanine nucleotide binding protein-like 2 (nucleolar)	0.972	0.3955	NA
223827	Gxylt1	glucoside xylosyltransferase 1	0.972	0.6921	NA
217732	2310044G17Rik	RIKEN cDNA 2310044G17 gene	0.972	0.6903	NA
76642	1700113A16Rik	RIKEN cDNA 1700113A16 gene	0.972	0.7846	0.9304
73683	Atg16l2	autophagy related 16 like 2 (S. cerevisiae)	0.972	0.6466	NA
54387	Mcm3ap	minichromosome maintenance deficient 3 (S. cerevisiae) associated protein	0.972	0.7578	0.9227
23859	Dlg2	discs, large homolog 2 (Drosophila)	0.972	0.5368	NA
20496	Slc12a2	solute carrier family 12, member 2	0.972	0.676	NA
17918	Myo5a	myosin VA	0.972	0.545	NA
641376	Tomm40l	translocase of outer mitochondrial membrane 40 homolog-like (yeast)	0.9719	0.5966	NA

383196	Gm5222	predicted gene 5222	0.9719	0.8749	0.9617
269113	Nup54	nucleoporin 54	0.9719	0.4749	NA
235344	Sik2	salt inducible kinase 2	0.9719	0.7159	NA
233532	Rsf1	remodeling and spacing factor 1	0.9719	0.6361	NA
227449	Zcchc2	zinc finger, CCHC domain containing 2	0.9719	0.7192	NA
118451	Mrps2	mitochondrial ribosomal protein S2	0.9719	0.5826	NA
666752	Gm8273	predicted gene 8273	0.9718	0.7269	NA
239336	Rxfp3	relaxin family peptide receptor 3	0.9718	0.8306	0.9455
233802	Thumpd1	THUMP domain containing 1	0.9718	0.5587	NA
109095	Rbm15b	RNA binding motif protein 15B	0.9718	0.763	0.9246
69718	Ipmk	inositol polyphosphate multikinase	0.9718	0.8144	0.9411
14201	Fhl3	four and a half LIM domains 3	0.9718	0.7398	0.9179
100302730	5830417I10Rik	RIKEN cDNA 5830417I10 gene	0.9717	0.5975	NA
100041799	Gm3515	predicted gene 3515	0.9717	0.6981	NA
214240	Disp2	dispatched homolog 2 (Drosophila)	0.9717	0.6884	NA
108075	Ltbp4	latent transforming growth factor beta binding protein 4	0.9717	0.7681	0.9255
72254	1700030K09Rik	RIKEN cDNA 1700030K09 gene	0.9717	0.7102	NA
64213	St7	suppression of tumorigenicity 7	0.9717	0.4785	NA
26921	Map4k4	mitogen-activated protein kinase kinase kinase kinase 4	0.9717	0.8241	0.9443
23850	Pappa2	pappalysin 2	0.9717	0.5385	NA
19775	Xpr1	xenotropic and polytropic retrovirus receptor 1	0.9717	0.4077	NA
14732	Gpam	glycerol-3-phosphate acyltransferase, mitochondrial	0.9717	0.7702	0.9257
13661	Ehf	ets homologous factor	0.9717	0.8785	0.9628
272589	Tbcel	tubulin folding cofactor E-like	0.9716	0.59	NA
269037	Gm672	predicted gene 672	0.9716	0.7633	0.9247
224694	Zfp81	zinc finger protein 81	0.9716	0.5332	NA
100855	Tbc1d14	TBC1 domain family, member 14	0.9716	0.3675	NA
76138	Ccdc138	coiled-coil domain containing 138	0.9716	0.8293	0.945
72750	Fam117b	family with sequence similarity 117, member B	0.9716	0.5278	NA
67229	Prpf18	PRP18 pre-mRNA processing factor 18 homolog (yeast)	0.9716	0.431	NA
20401	Sh3bp1	SH3-domain binding protein 1	0.9716	0.7439	0.9191
19272	Ptprk	protein tyrosine phosphatase, receptor type, K	0.9716	0.6764	NA
100047123	LOC100047123	hypothetical protein LOC100047123	0.9715	0.8036	0.9378
269955	Rccd1	RCC1 domain containing 1	0.9715	0.6792	NA
268706	Slc38a9	solute carrier family 38, member 9	0.9715	0.573	NA
226089	C030046E11Rik	RIKEN cDNA C030046E11 gene	0.9715	0.6945	NA
94090	Trim9	tripartite motif-containing 9	0.9715	0.6657	NA
76179	Usp31	ubiquitin specific peptidase 31	0.9715	0.4127	NA
20249	Scd1	stearoyl-Coenzyme A desaturase 1	0.9715	0.8313	0.9455
16179	Irak1	interleukin-1 receptor-associated kinase 1	0.9715	0.6405	NA
669393	Gm9457	predicted gene 9457	0.9714	0.5616	NA
230657	Tmem69	transmembrane protein 69	0.9714	0.7802	0.9283
214804	Syde2	synapse defective 1, Rho GTPase, homolog 2 (C. elegans)	0.9714	0.6998	NA
73463	Als2cr11	amyotrophic lateral sclerosis 2 (juvenile) chromosome region, candidate 11 (human)	0.9714	0.8537	0.9544
67454	Ikbip	IKBKB interacting protein	0.9714	0.623	NA
58178	Sorcs1	VPS10 domain receptor protein SORCS 1	0.9714	0.7085	NA
17968	Ncam2	neural cell adhesion molecule 2	0.9714	0.6613	NA
237336	Tbpl1	TATA box binding protein-like 1	0.9713	0.5576	NA
74600	Mrpl47	mitochondrial ribosomal protein L47	0.9713	0.8501	0.9531

74006	Dnm1l	dynamin 1-like	0.9713	0.7152	NA
28200	Dhrs4	dehydrogenase/reductase (SDR family) member 4	0.9713	0.7369	0.9171
320714	D030016E14Rik	RIKEN cDNA D030016E14 gene	0.9712	0.8276	0.9449
112422	2610305D13Rik	RIKEN cDNA 2610305D13 gene	0.9712	0.8149	0.9412
76619	1700087I21Rik	RIKEN cDNA 1700087I21 gene	0.9712	0.5843	NA
57815	Spata5	spermatogenesis associated 5	0.9712	0.6714	NA
19155	Npepps	aminopeptidase puromycin sensitive	0.9712	0.6332	NA
16650	Kpna6	karyopherin (importin) alpha 6	0.9712	0.4593	NA
13191	Dctn1	dynactin 1	0.9712	0.6	NA
211147	Mar-11	membrane-associated ring finger (C3HC4) 11	0.9711	0.7951	0.9343
101185	Pot1a	protection of telomeres 1A	0.9711	0.8753	0.9618
77963	Hook1	hook homolog 1 (Drosophila)	0.9711	0.8873	0.9671
66421	2410004B18Rik	RIKEN cDNA 2410004B18 gene	0.9711	0.6921	NA
26896	Med14	mediator complex subunit 14	0.9711	0.5222	NA
24071	Synj2bp	synaptojanin 2 binding protein	0.9711	0.7456	0.9192
22019	Tpp2	tripeptidyl peptidase II	0.9711	0.7227	NA
20602	Ncor2	nuclear receptor co-repressor 2	0.9711	0.5807	NA
20511	Slc1a2	solute carrier family 1 (glial high affinity glutamate transporter), member 2	0.9711	0.6305	NA
20357	Sema5b	sema domain, seven thrombospondin repeats (type 1 and type 1-like), transmembrane domain (TM) and short cytoplasmic domain, (semaphorin) 5B	0.9711	0.7968	0.935
13411	Dnahc11	dynein, axonemal, heavy chain 11	0.9711	0.8158	0.9416
628596	Gm6900	predicted gene 6900	0.971	0.5974	NA
320634	Ocrl	oculocerebrorenal syndrome of Lowe	0.971	0.5481	NA
231474	Paqr3	progestin and adipoQ receptor family member III	0.971	0.7797	0.928
27416	Abcc5	ATP-binding cassette, sub-family C (CFTR/MRP), member 5	0.971	0.5952	NA
21676	Tead1	TEA domain family member 1	0.971	0.7241	NA
12261	C1qbp	complement component 1, q subcomponent binding protein	0.971	0.5537	NA
268656	Sptlc1	serine palmitoyltransferase, long chain base subunit 1	0.9709	0.6039	NA
226255	Atrnl1	attractin like 1	0.9709	0.7336	0.9159
102774	Bbs4	Bardet-Biedl syndrome 4 (human)	0.9709	0.567	NA
73225	Fam118a	family with sequence similarity 118, member A	0.9709	0.6388	NA
68763	1110038B12Rik	RIKEN cDNA 1110038B12 gene	0.9709	0.6562	NA
18024	Nfe2l2	nuclear factor, erythroid derived 2, like 2	0.9709	0.5299	NA
100039086	Gm15032	predicted gene 15032	0.9708	0.9247	0.9783
240119	St6gal2	beta galactoside alpha 2,6 sialyltransferase 2	0.9708	0.7776	0.9272
226849	Ppp2r5a	protein phosphatase 2, regulatory subunit B (B56), alpha isoform	0.9708	0.5582	NA
70951	Spata1	spermatogenesis associated 1	0.9708	0.5534	NA
27756	Lsm2	LSM2 homolog, U6 small nuclear RNA associated (S. cerevisiae)	0.9708	0.7018	NA
56743	Lat2	linker for activation of T cells family, member 2	0.9707	0.7749	0.9266
56637	Gsk3b	glycogen synthase kinase 3 beta	0.9707	0.5367	NA
320961	A630026N12Rik	RIKEN cDNA A630026N12 gene	0.9706	0.5764	NA
217331	Unk	unkempt homolog (Drosophila)	0.9706	0.6999	NA
216225	Slc5a8	solute carrier family 5 (iodide transporter), member 8	0.9706	0.7074	NA
102093	Phkb	phosphorylase kinase beta	0.9706	0.8146	0.9412
76967	2700049A03Rik	RIKEN cDNA 2700049A03 gene	0.9706	0.6359	NA
76161	6330527O06Rik	RIKEN cDNA 6330527O06 gene	0.9706	0.6965	NA
69698	2310046K01Rik	RIKEN cDNA 2310046K01 gene	0.9706	0.8985	0.9702
68628	Fbxw9	F-box and WD-40 domain protein 9	0.9706	0.6551	NA
21858	Timp2	tissue inhibitor of metalloproteinase 2	0.9706	0.6226	NA
13175	Dclk1	doublecortin-like kinase 1	0.9706	0.6079	NA

383770	Gm11449	predicted gene 11449	0.9705	0.8125	0.9403
243931	Tshz3	teashirt zinc finger family member 3	0.9705	0.5721	NA
236749	Gm4907	predicted gene 4907	0.9705	0.72	NA
234577	Cpne2	copine II	0.9705	0.7531	0.9214
219170	AU021034	expressed sequence AU021034	0.9705	0.9113	0.9742
104681	Slc16a6	solute carrier family 16 (monocarboxylic acid transporters), member 6	0.9705	0.8368	0.9479
100609	Nsun5	NOL1/NOP2/Sun domain family, member 5	0.9705	0.6419	NA
99650	4933434E20Rik	RIKEN cDNA 4933434E20 gene	0.9705	0.7534	0.9216
68014	Zwilch	Zwilch, kinetochore associated, homolog (Drosophila)	0.9705	0.5764	NA
67708	1810048J11Rik	RIKEN cDNA 1810048J11 gene	0.9705	0.6697	NA
57782	Rbak	RB-associated KRAB repressor	0.9705	0.6292	NA
20973	Syngr2	synaptogyrin 2	0.9705	0.7557	0.9223
19888	Rp1	retinitis pigmentosa 1 (human)	0.9705	0.6327	NA
626359	Wdr93	WD repeat domain 93	0.9704	0.79	0.933
268281	Shprh	SNF2 histone linker PHD RING helicase	0.9704	0.7231	0.9121
235256	Olfr149	olfactory receptor 149	0.9704	0.9253	0.9783
218832	Polr3a	polymerase (RNA) III (DNA directed) polypeptide A	0.9704	0.6084	NA
74455	Nsun6	NOL1/NOP2/Sun domain family member 6	0.9704	0.6593	NA
74375	Gcc1	golgi coiled coil 1	0.9704	0.7506	0.921
23807	Arih2	ariadne homolog 2 (Drosophila)	0.9704	0.463	NA
21646	Tcte2	t-complex-associated testis expressed 2	0.9704	0.7456	0.9192
75597	Ndufaf2	NADH dehydrogenase (ubiquinone) 1 alpha subcomplex, assembly factor 2	0.9703	0.5984	NA
19335	Rab23	RAB23, member RAS oncogene family	0.9703	0.7931	0.9335
227800	Rabgap1	RAB GTPase activating protein 1	0.9702	0.8389	0.9482
109754	Cyb5r3	cytochrome b5 reductase 3	0.9702	0.617	NA
68194	Ndufb4	NADH dehydrogenase (ubiquinone) 1 beta subcomplex 4	0.9702	0.5363	NA
22712	Zfp54	zinc finger protein 54	0.9702	0.6362	NA
433864	Nom1	nucleolar protein with MIF4G domain 1	0.9701	0.6164	NA
228136	Zdhhc5	zinc finger, DHHC domain containing 5	0.9701	0.7684	0.9255
56335	Mettl3	methyltransferase like 3	0.9701	0.5374	NA
20901	Strap	serine/threonine kinase receptor associated protein	0.9701	0.682	NA
18986	Pou2f1	POU domain, class 2, transcription factor 1	0.9701	0.6596	NA
546954	Gm6003	predicted gene 6003	0.97	0.9425	0.9838
216233	Socs2	suppressor of cytokine signaling 2	0.97	0.5979	NA
211961	Asxl3	additional sex combs like 3 (Drosophila)	0.97	0.6581	NA
21749	Terf1	telomeric repeat binding factor 1	0.97	0.5811	NA
241275	Noxa1	NADPH oxidase activator 1	0.9699	0.7012	NA
216565	Ehbp1	EH domain binding protein 1	0.9699	0.8187	0.9425
69587	Pcgf3	polycomb group ring finger 3	0.9699	0.8501	0.9531
57342	Parva	parvin, alpha	0.9699	0.5779	NA
52276	Cdca8	cell division cycle associated 8	0.9699	0.7531	0.9214
17865	Mybl2	myeloblastosis oncogene-like 2	0.9699	0.7693	0.9255
17132	Maf	avian musculoaponeurotic fibrosarcoma (v-maf) AS42 oncogene homolog	0.9699	0.8814	0.9637
263764	Creg2	cellular repressor of E1A-stimulated genes 2	0.9698	0.7486	0.9204
101023	Zfp513	zinc finger protein 513	0.9698	0.4961	NA
94232	Ubqln4	ubiquilin 4	0.9698	0.7189	0.9102
68041	Mid1ip1	Mid1 interacting protein 1 (gastrulation specific G12-like (zebrafish))	0.9698	0.594	NA
56055	Gtpbp2	GTP binding protein 2	0.9698	0.6562	NA
432731	Zfp187	zinc finger protein 187	0.9697	0.4976	NA

83679	Pde4dip	phosphodiesterase 4D interacting protein (myomegalin)	0.9697	0.7221	0.9121
77971	A930037O16Rik	RIKEN cDNA A930037O16 gene	0.9697	0.5631	NA
76113	Lpo	lactoperoxidase	0.9697	0.8593	0.9566
69189	1810033B17Rik	RIKEN cDNA 1810033B17 gene	0.9697	0.7903	0.933
68897	Disp1	dispatched homolog 1 (Drosophila)	0.9697	0.7411	0.918
52665	Echdc1	enoyl Coenzyme A hydratase domain containing 1	0.9697	0.7455	0.9192
269224	Pask	PAS domain containing serine/threonine kinase	0.9696	0.5247	NA
77034	2510039O18Rik	RIKEN cDNA 2510039O18 gene	0.9696	0.4681	NA
60507	Qtrt1	queuine tRNA-ribosyltransferase 1	0.9696	0.6141	NA
57370	B4galt3	UDP-Gal:betaGlcNAc beta 1,4-galactosyltransferase, polypeptide 3	0.9696	0.6204	NA
14105	Srsf10	serine/arginine-rich splicing factor 10	0.9696	0.7767	0.9271
13557	E2f3	E2F transcription factor 3	0.9696	0.6309	NA
12593	Cdyl	chromodomain protein, Y chromosome-like	0.9696	0.4905	NA
228836	Dlgap4	discs, large homolog-associated protein 4 (Drosophila)	0.9695	0.5401	NA
94353	Hmgn3	high mobility group nucleosomal binding domain 3	0.9695	0.6199	NA
74366	4932422M17Rik	RIKEN cDNA 4932422M17 gene	0.9695	0.6429	NA
71132	Cabyr	calcium-binding tyrosine-(Y)-phosphorylation regulated (fibrousheathin 2)	0.9695	0.805	0.9381
66997	Psmd12	proteasome (prosome, macropain) 26S subunit, non-ATPase, 12	0.9695	0.8105	0.94
56412	2610024G14Rik	RIKEN cDNA 2610024G14 gene	0.9695	0.5274	NA
56384	Letm1	leucine zipper-EF-hand containing transmembrane protein 1	0.9695	0.602	NA
14679	Gnai3	guanine nucleotide binding protein (G protein), alpha inhibiting 3	0.9695	0.5464	NA
381305	Rc3h1	RING CCCH (C3H) domains 1	0.9694	0.6666	NA
329416	Nostrin	nitric oxide synthase trafficker	0.9694	0.7953	0.9344
328108	Fam179b	family with sequence similarity 179, member B	0.9694	0.7517	0.9213
17207	Mcf2l	mcf.2 transforming sequence-like	0.9694	0.4893	NA
246316	Lgi2	leucine-rich repeat LGI family, member 2	0.9693	0.6474	NA
234699	Edc4	enhancer of mRNA decapping 4	0.9693	0.513	NA
227157	Mpp4	membrane protein, palmitoylated 4 (MAGUK p55 subfamily member 4)	0.9693	0.5794	NA
110893	Slc8a3	solute carrier family 8 (sodium/calcium exchanger), member 3	0.9693	0.7986	0.9361
107684	Coro2a	coronin, actin binding protein 2A	0.9693	0.9318	0.9795
72607	Usp13	ubiquitin specific peptidase 13 (isopeptidase T-3)	0.9693	0.5269	NA
23856	Dido1	death inducer-obliterator 1	0.9693	0.542	NA
16728	L1cam	L1 cell adhesion molecule	0.9693	0.8219	0.9435
12724	Clcn2	chloride channel 2	0.9693	0.6808	NA
399603	Fam84b	family with sequence similarity 84, member B	0.9692	0.7738	0.9266
320806	Gfm2	G elongation factor, mitochondrial 2	0.9692	0.6035	NA
75706	Krt24	keratin 24	0.9692	0.8735	0.9614
74019	Traf3ip1	TRAF3 interacting protein 1	0.9692	0.8169	0.942
69737	Ttl	tubulin tyrosine ligase	0.9692	0.609	NA
21888	Tle4	transducin-like enhancer of split 4, homolog of Drosophila E(spl)	0.9692	0.73	0.9148
320742	A230072C01Rik	RIKEN cDNA A230072C01 gene	0.9691	0.7464	0.9196
270166	Clpx	caseinolytic peptidase X (E.coli)	0.9691	0.6796	NA
76614	Immt	inner membrane protein, mitochondrial	0.9691	0.5791	NA
69577	Fastkd3	FAST kinase domains 3	0.9691	0.5482	NA
67707	Mrpl24	mitochondrial ribosomal protein L24	0.9691	0.5369	NA
66240	Kcne1l	potassium voltage-gated channel, Isk-related family, member 1-like, pseudogene	0.9691	0.7348	0.9164
58231	Stk4	serine/threonine kinase 4	0.9691	0.8361	0.9474
24131	Ldb3	LIM domain binding 3	0.9691	0.9091	0.9733
21386	Tbx3	T-box 3	0.9691	0.5878	NA

11837	Rplp0	ribosomal protein, large, P0	0.9691	0.7009	NA
627798	Gm6790	tumor protein, translationally-controlled 1 pseudogene	0.969	0.7253	0.9126
547176	Zc3h12b	zinc finger CCCH-type containing 12B	0.969	0.8861	0.9665
245405	Gm4987	predicted gene 4987	0.969	0.6697	NA
218441	Zfyve16	zinc finger, FYVE domain containing 16	0.969	0.61	NA
218100	Zfp322a	zinc finger protein 322A	0.969	0.6234	NA
70843	Krt28	keratin 28	0.969	0.8074	0.939
70567	5730455O13Rik	RIKEN cDNA 5730455O13 gene	0.969	0.4769	NA
58991	Ghrl	ghrelin	0.969	0.6952	NA
22323	Vasp	vasodilator-stimulated phosphoprotein	0.969	0.6268	NA
380916	Lrch1	leucine-rich repeats and calponin homology (CH) domain containing 1	0.9689	0.5012	NA
320965	4831440E17Rik	RIKEN cDNA 4831440E17 gene	0.9689	0.799	0.9361
228788	BC020535	cDNA sequence BC020535	0.9689	0.7598	0.9229
104836	Cbll1	Casitas B-lineage lymphoma-like 1	0.9689	0.6465	NA
102334	Ankrd10	ankyrin repeat domain 10	0.9689	0.4887	NA
66549	Aggf1	angiogenic factor with G patch and FHA domains 1	0.9689	0.5234	NA
16563	Kif2a	kinesin family member 2A	0.9689	0.8058	0.9386
13204	Dhx15	DEAH (Asp-Glu-Ala-His) box polypeptide 15	0.9689	0.7456	0.9192
100503304	LOC100503304	STAT3-interacting protein as a repressor-like	0.9688	0.7108	0.907
239554	Foxred2	FAD-dependent oxidoreductase domain containing 2	0.9688	0.494	NA
208643	Eif4g1	eukaryotic translation initiation factor 4, gamma 1	0.9688	0.7559	0.9223
79263	Trim39	tripartite motif-containing 39	0.9688	0.419	NA
68465	Adipor2	adiponectin receptor 2	0.9688	0.546	NA
53817	Bat1a	HLA-B-associated transcript 1A	0.9688	0.6886	NA
22333	Vdac1	voltage-dependent anion channel 1	0.9688	0.6725	NA
14545	Gdap1	ganglioside-induced differentiation-associated-protein 1	0.9688	0.6031	NA
214669	L3mbtl2	l(3)mbt-like 2 (Drosophila)	0.9687	0.725	0.9126
194388	Tet3	tet oncogene family member 3	0.9687	0.8513	0.9537
67486	Polr3g	polymerase (RNA) III (DNA directed) polypeptide G	0.9687	0.6698	NA
13046	Celf1	CUGBP, Elav-like family member 1	0.9687	0.7334	0.9159
105203	BC016423	cDNA sequence BC016423	0.9686	0.7367	0.9169
93880	Pcdhb9	protocadherin beta 9	0.9686	0.8323	0.9456
74776	Ppa2	pyrophosphatase (inorganic) 2	0.9686	0.573	NA
74302	Mtmr3	myotubularin related protein 3	0.9686	0.5156	NA
70503	Ddo	D-aspartate oxidase	0.9686	0.6841	NA
15388	Hnrnpl	heterogeneous nuclear ribonucleoprotein L	0.9686	0.6861	NA
140740	Sec63	SEC63-like (S. cerevisiae)	0.9685	0.6025	NA
68198	Ndufb2	NADH dehydrogenase (ubiquinone) 1 beta subcomplex, 2	0.9685	0.7579	0.9227
66282	1810029B16Rik	RIKEN cDNA 1810029B16 gene	0.9685	0.5566	NA
23950	Dnajb6	DnaJ (Hsp40) homolog, subfamily B, member 6	0.9685	0.5777	NA
16430	Stt3a	STT3, subunit of the oligosaccharyltransferase complex, homolog A (S. cerevisiae)	0.9685	0.7382	0.9173
11431	Acp1	acid phosphatase 1, soluble	0.9685	0.6947	NA
667250	Gm12657	predicted gene 12657	0.9684	0.5348	NA
215705	Arrdc1	arrestin domain containing 1	0.9684	0.766	0.9251
77832	Tchp	trichoplein, keratin filament binding	0.9684	0.6117	NA
72195	Supt7l	suppressor of Ty 7 (S. cerevisiae)-like	0.9684	0.708	0.9064
22222	Ubr1	ubiquitin protein ligase E3 component n-recognin 1	0.9684	0.6409	NA
11928	Atp1a1	ATPase, Na+/K+ transporting, alpha 1 polypeptide	0.9684	0.6286	NA
226153	Peo1	progressive external ophthalmoplegia 1 (human)	0.9683	0.6075	NA

218699	Pxk	PX domain containing serine/threonine kinase	0.9683	0.7427	0.9186
83701	Srrt	serrate RNA effector molecule homolog (Arabidopsis)	0.9683	0.6181	NA
78304	Lsmd1	LSM domain containing 1	0.9683	0.4634	NA
77189	A430105J06Rik	RIKEN cDNA A430105J06 gene	0.9683	0.7626	0.9244
72823	Pard3b	par-3 partitioning defective 3 homolog B (C. elegans)	0.9683	0.8159	0.9416
52850	Sgsm1	small G protein signaling modulator 1	0.9683	0.7155	0.9087
14390	Gabpa	GA repeat binding protein, alpha	0.9683	0.6473	NA
635470	Gm14407	predicted gene 14407	0.9682	0.5696	NA
93960	Nkd1	naked cuticle 1 homolog (Drosophila)	0.9682	0.5551	NA
74239	Iqce	IQ motif containing E	0.9682	0.7517	0.9213
69639	Exosc8	exosome component 8	0.9682	0.5256	NA
68695	Hddc3	HD domain containing 3	0.9682	0.3774	NA
67501	Ccdc50	coiled-coil domain containing 50	0.9682	0.8441	0.9502
67049	Pus3	pseudouridine synthase 3	0.9682	0.5747	NA
66592	Stoml2	stomatin (Epb7.2)-like 2	0.9682	0.5367	NA
51869	Rif1	Rap1 interacting factor 1 homolog (yeast)	0.9682	0.6132	NA
28185	Tomm70a	translocase of outer mitochondrial membrane 70 homolog A (yeast)	0.9682	0.7571	0.9226
26885	Casp8ap2	caspase 8 associated protein 2	0.9682	0.5081	NA
20637	Snrnp70	small nuclear ribonucleoprotein 70 (U1)	0.9682	0.8166	0.9419
100043597	Srcap	Snf2-related CREBBP activator protein	0.9681	0.7383	0.9174
225849	Ppp2r5b	protein phosphatase 2, regulatory subunit B (B56), beta isoform	0.9681	0.3747	NA
217325	Llgl2	lethal giant larvae homolog 2 (Drosophila)	0.9681	0.5827	NA
108954	Ppp1r15b	protein phosphatase 1, regulatory (inhibitor) subunit 15b	0.9681	0.616	NA
84505	Setdb1	SET domain, bifurcated 1	0.9681	0.4041	NA
66847	Hint3	histidine triad nucleotide binding protein 3	0.9681	0.6778	NA
23966	Odz4	odd Oz/ten-m homolog 4 (Drosophila)	0.9681	0.7113	0.907
18028	Nfib	nuclear factor I/B	0.9681	0.6817	NA
17967	Ncam1	neural cell adhesion molecule 1	0.9681	0.6828	NA
330119	Adamts3	a disintegrin-like and metallopeptidase (reprolysin type) with thrombospondin type 1 motif, 3	0.968	0.7367	0.9169
269997	Zfp747	zinc finger protein 747	0.968	0.5948	NA
223828	Pphln1	periphilin 1	0.968	0.6569	NA
170762	Nup155	nucleoporin 155	0.968	0.5738	NA
71240	Osbpl7	oxysterol binding protein-like 7	0.968	0.7791	0.9278
67693	2310003F16Rik	RIKEN cDNA 2310003F16 gene	0.968	0.5764	NA
67025	Rpl11	ribosomal protein L11	0.968	0.5981	NA
60611	Foxj2	forkhead box J2	0.968	0.7325	0.9157
22722	Zfp64	zinc finger protein 64	0.968	0.5686	NA
17713	Grpel1	GrpE-like 1, mitochondrial	0.968	0.5172	NA
13131	Dab1	disabled homolog 1 (Drosophila)	0.968	0.7856	0.9309
327954	Dnahc2	dynein, axonemal, heavy chain 2	0.9679	0.8273	0.9449
236848	BC023829	cDNA sequence BC023829	0.9679	0.6976	NA
233545	2210018M11Rik	RIKEN cDNA 2210018M11 gene	0.9679	0.5768	NA
226525	Rasal2	RAS protein activator like 2	0.9679	0.7652	0.9251
108112	Eif4ebp3	eukaryotic translation initiation factor 4E binding protein 3	0.9679	0.6083	NA
74451	Pgs1	phosphatidylglycerophosphate synthase 1	0.9679	0.5013	NA
66717	Ccdc96	coiled-coil domain containing 96	0.9679	0.6304	NA
21411	Tcf20	transcription factor 20	0.9679	0.5725	NA
16151	Ikbkg	inhibitor of kappaB kinase gamma	0.9679	0.5759	NA
13347	Dffa	DNA fragmentation factor, alpha subunit	0.9679	0.774	0.9266

269774	Aak1	AP2 associated kinase 1	0.9678	0.6405	NA
245305	B230307C23Rik	RIKEN cDNA B230307C23 gene	0.9678	0.7558	0.9223
245000	Atr	ataxia telangiectasia and Rad3 related	0.9678	0.5698	NA
76563	Qrsl1	glutaminyl-tRNA synthase (glutamine-hydrolyzing)-like 1	0.9678	0.4122	NA
70078	Nol7	nucleolar protein 7	0.9678	0.5839	NA
69956	Ptcd3	pentatricopeptide repeat domain 3	0.9678	0.5228	NA
67245	Peli1	pellino 1	0.9678	0.4531	NA
320124	B930095M22Rik	RIKEN cDNA B930095M22 gene	0.9677	0.7197	0.9106
193736	Zbtb12	zinc finger and BTB domain containing 12	0.9677	0.5171	NA
70686	Dusp16	dual specificity phosphatase 16	0.9677	0.8047	0.9381
66246	Osgep	O-sialoglycoprotein endopeptidase	0.9677	0.5087	NA
24071	Synj2bp	synaptojanin 2 binding protein	0.9677	0.4417	NA
15289	Hmgb1	high mobility group box 1	0.9677	0.6779	NA
15220	Foxq1	forkhead box Q1	0.9677	0.8035	0.9378
665055	Gm7467	predicted gene 7467	0.9676	0.7127	0.9075
433273	Gm5523	glyceraldehyde-3-phosphate dehydrogenase pseudogene	0.9676	0.7993	0.9361
170459	Stard4	StAR-related lipid transfer (START) domain containing 4	0.9676	0.7915	0.9333
108150	Galnt7	UDP-N-acetyl-alpha-D-galactosamine: polypeptide N-acetylgalactosaminyltransferase 7	0.9676	0.4524	NA
71807	Tars2	threonyl-tRNA synthetase 2, mitochondrial (putative)	0.9676	0.5212	NA
68477	Rmnd5a	required for meiotic nuclear division 5 homolog A (S. cerevisiae)	0.9676	0.743	0.9187
60595	Actn4	actinin alpha 4	0.9676	0.5968	NA
15925	Ide	insulin degrading enzyme	0.9676	0.5023	NA
12286	Cacna1a	calcium channel, voltage-dependent, P/Q type, alpha 1A subunit	0.9676	0.6924	NA
100040563	Dynlt1c	dynein light chain Tctex-type 1C	0.9675	0.6053	NA
621967	Gm6272	predicted pseudogene 6272	0.9675	0.8742	0.9616
246735	AY074887	cDNA sequence AY074887	0.9675	0.8082	0.9393
240038	Gm4944	predicted gene 4944	0.9675	0.6736	NA
231279	Guf1	GUF1 GTPase homolog (S. cerevisiae)	0.9675	0.5669	NA
212684	Pdzd7	PDZ domain containing 7	0.9675	0.8166	0.9419
72611	Zfp655	zinc finger protein 655	0.9675	0.5285	NA
66537	Pomp	proteasome maturation protein	0.9675	0.5716	NA
667847	Gm8842	predicted gene 8842	0.9674	0.7381	0.9173
213311	Fbxl21	F-box and leucine-rich repeat protein 21	0.9674	0.7863	0.9312
71673	Rnf215	ring finger protein 215	0.9674	0.37	NA
68328	Rab13	RAB13, member RAS oncogene family	0.9674	0.6929	NA
66073	Txndc12	thioredoxin domain containing 12 (endoplasmic reticulum)	0.9674	0.5496	NA
20024	Sub1	SUB1 homolog (S. cerevisiae)	0.9674	0.5778	NA
332993	Gm5129	predicted gene 5129	0.9673	0.7763	0.9269
237711	Eml6	echinoderm microtubule associated protein like 6	0.9673	0.5988	NA
233410	Zfp592	zinc finger protein 592	0.9673	0.3661	NA
76261	0610040J01Rik	RIKEN cDNA 0610040J01 gene	0.9673	0.7204	0.9109
74466	4933427G17Rik	RIKEN cDNA 4933427G17 gene	0.9673	0.7924	0.9333
19014	Med1	mediator complex subunit 1	0.9673	0.7777	0.9273
14356	Fxc1	fractured callus expressed transcript 1	0.9673	0.6197	NA
327826	Frs2	fibroblast growth factor receptor substrate 2	0.9672	0.5564	NA
117600	Srgap1	SLIT-ROBO Rho GTPase activating protein 1	0.9672	0.726	0.9126
78286	Nav2	neuron navigator 2	0.9672	0.7932	0.9335
67434	Ankrd33b	ankyrin repeat domain 33B	0.9672	0.4611	NA
16987	Lss	lanosterol synthase	0.9672	0.7836	0.9301

545938	Zfp607	zinc finger proten 607	0.9671	0.48	NA
228421	Kif18a	kinesin family member 18A	0.9671	0.7184	0.9102
214627	Papd5	PAP associated domain containing 5	0.9671	0.5721	NA
76824	Fam54b	family with sequence similarity 54, member B	0.9671	0.4908	NA
67345	Herc4	hect domain and RLD 4	0.9671	0.5422	NA
29869	Ulk2	Unc-51 like kinase 2 (C. elegans)	0.9671	0.6391	NA
20848	Stat3	signal transducer and activator of transcription 3	0.9671	0.5547	NA
20501	Slc16a1	solute carrier family 16 (monocarboxylic acid transporters), member 1	0.9671	0.8267	0.9449
11305	Abca2	ATP-binding cassette, sub-family A (ABC1), member 2	0.9671	0.7527	0.9214
668137	Gm8994	predicted gene 8994	0.967	0.5782	NA
224742	Abcf1	ATP-binding cassette, sub-family F (GCN20), member 1	0.967	0.7071	0.9062
22290	Uty	ubiquitously transcribed tetratricopeptide repeat gene, Y chromosome	0.967	0.904	0.9717
433940	BC057022	cDNA sequence BC057022	0.9669	0.6239	NA
237073	Rbm41	RNA binding motif protein 41	0.9669	0.7906	0.9333
218214	Kdm1b	lysine (K)-specific demethylase 1B	0.9669	0.5276	NA
209357	Gtf2h3	general transcription factor IIH, polypeptide 3	0.9669	0.5856	NA
107934	Celsr3	cadherin, EGF LAG seven-pass G-type receptor 3 (flamingo homolog, Drosophila)	0.9669	0.5048	NA
71494	8430406P12Rik	RIKEN cDNA 8430406P12 gene	0.9669	0.6451	NA
66118	Sarnp	SAP domain containing ribonucleoprotein	0.9669	0.5704	NA
59308	Emcn	endomucin	0.9669	0.7556	0.9223
277939	C2cd3	C2 calcium-dependent domain containing 3	0.9668	0.5434	NA
104111	Adcy3	adenylate cyclase 3	0.9668	0.699	0.903
241053	Rpl12-ps1	ribosomal protein L12, pseudogene 1	0.9667	0.5763	NA
215090	Maneal	mannosidase, endo-alpha-like	0.9667	0.5518	NA
212772	2700007P21Rik	RIKEN cDNA 2700007P21 gene	0.9667	0.5679	NA
100087	Kti12	KTI12 homolog, chromatin associated (S. cerevisiae)	0.9667	0.5588	NA
76574	Mfsd2a	major facilitator superfamily domain containing 2A	0.9667	0.6059	NA
69752	Zfp511	zinc finger protein 511	0.9667	0.6301	NA
66272	Cox16	COX16 cytochrome c oxidase assembly homolog (S. cerevisiae)	0.9667	0.663	NA
28081	D11Wsu99e	DNA segment, Chr 11, Wayne State University 99, expressed	0.9667	0.6679	NA
17933	Myt1l	myelin transcription factor 1-like	0.9667	0.5013	NA
319236	9230105E10Rik	RIKEN cDNA 9230105E10 gene	0.9666	0.8118	0.9403
239570	Ttc38	tetratricopeptide repeat domain 38	0.9666	0.8052	0.9382
235028	Zfp426	zinc finger protein 426	0.9666	0.6694	NA
232878	Zscan22	zinc finger and SCAN domain containing 22	0.9666	0.5927	NA
231889	Bud31	BUD31 homolog (yeast)	0.9666	0.5479	NA
218460	Wdr41	WD repeat domain 41	0.9666	0.4182	NA
217328	Myo15b	myosin XVB	0.9666	0.7943	0.9339
108655	Foxp1	forkhead box P1	0.9666	0.6189	NA
22003	Tpm1	tropomyosin 1, alpha	0.9666	0.7058	0.9057
19253	Ptpn18	protein tyrosine phosphatase, non-receptor type 18	0.9666	0.6415	NA
381626	Rbm33	RNA binding motif protein 33	0.9665	0.4674	NA
239667	Dip2b	DIP2 disco-interacting protein 2 homolog B (Drosophila)	0.9665	0.5204	NA
212943	Fam46a	family with sequence similarity 46, member A	0.9665	0.6739	NA
192185	Nadk	NAD kinase	0.9665	0.7861	0.9311
104943	Fam110c	family with sequence similarity 110, member C	0.9665	0.7593	0.9229
55946	Ap3m1	adaptor-related protein complex 3, mu 1 subunit	0.9665	0.5526	NA
22343	Lin7c	lin-7 homolog C (C. elegans)	0.9665	0.6935	0.9001
18810	Plec	plectin	0.9665	0.6851	NA

240332	Slc6a7	solute carrier family 6 (neurotransmitter transporter, L-proline), member 7	0.9664	0.5863	NA
100715	Papd4	PAP associated domain containing 4	0.9664	0.5652	NA
73467	1700066M21Rik	RIKEN cDNA 1700066M21 gene	0.9664	0.6245	NA
69282	1700001J03Rik	RIKEN cDNA 1700001J03 gene	0.9664	0.7107	0.9069
68106	Nt5c3l	5'-nucleotidase, cytosolic III-like	0.9664	0.5474	NA
22700	Zfp40	zinc finger protein 40	0.9664	0.6251	NA
18685	Phtf1	putative homeodomain transcription factor 1	0.9664	0.4812	NA
14760	Gpr19	G protein-coupled receptor 19	0.9664	0.5583	NA
13845	Ephb3	Eph receptor B3	0.9664	0.7872	0.9314
732482	Gm9731	Fam58a pseudogene	0.9663	0.582	NA
223666	Arhgap39	Rho GTPase activating protein 39	0.9663	0.5997	NA
68755	Cgrrf1	cell growth regulator with ring finger domain 1	0.9663	0.7132	0.9076
68187	Fam135a	family with sequence similarity 135, member A	0.9663	0.5754	NA
56542	Ick	intestinal cell kinase	0.9663	0.611	NA
26423	Nr5a1	nuclear receptor subfamily 5, group A, member 1	0.9663	0.8724	0.9608
16889	Lipa	lysosomal acid lipase A	0.9663	0.5343	NA
236266	Alms1	Alstrom syndrome 1 homolog (human)	0.9662	0.796	0.9346
235459	Gtf2a2	general transcription factor II A, 2	0.9662	0.5309	NA
71678	Brox	BRO1 domain and CAAX motif containing	0.9662	0.7996	0.9363
68099	Fam92a	family with sequence similarity 92, member A	0.9662	0.6964	0.9016
67469	Abhd5	abhydrolase domain containing 5	0.9662	0.5103	NA
28036	Larp7	La ribonucleoprotein domain family, member 7	0.9662	0.4623	NA
270163	Myo9a	myosin IXa	0.9661	0.5919	NA
242425	Gabbr2	gamma-aminobutyric acid (GABA) B receptor, 2	0.9661	0.7378	0.9172
74309	Osbp2	oxysterol binding protein 2	0.9661	0.6563	NA
71795	Pitpnc1	phosphatidylinositol transfer protein, cytoplasmic 1	0.9661	0.6457	NA
66387	Nudt8	nudix (nucleoside diphosphate linked moiety X)-type motif 8	0.9661	0.47	NA
14265	Fmr1	fragile X mental retardation syndrome 1 homolog	0.9661	0.6361	NA
100504758	LOC100504758	hypothetical LOC100504758	0.966	0.7874	0.9314
433375	Creg1	cellular repressor of E1A-stimulated genes 1	0.966	0.6784	NA
319149	Hist1h3d	histone cluster 1, H3d	0.966	0.3229	NA
239420	Csmd3	CUB and Sushi multiple domains 3	0.966	0.869	0.9597
59009	Sh3rf1	SH3 domain containing ring finger 1	0.966	0.638	NA
15107	Hadh	hydroxyacyl-Coenzyme A dehydrogenase	0.966	0.592	NA
14827	Pdia3	protein disulfide isomerase associated 3	0.966	0.7081	0.9064
14657	Glra4	glycine receptor, alpha 4 subunit	0.966	0.7895	0.9329
100502854	LOC100502854	hypothetical LOC100502854	0.9659	0.445	NA
668212	Efr3b	EFR3 homolog B (S. cerevisiae)	0.9659	0.4652	NA
442829	Ccin	calicin	0.9659	0.5513	NA
268996	Ss18	synovial sarcoma translocation, Chromosome 18	0.9659	0.3925	NA
239337	Adamts12	a disintegrin-like and metallopeptidase (reprolysin type) with thrombospondin type 1 motif, 12	0.9659	0.8079	0.9391
109161	Ube2q2	ubiquitin-conjugating enzyme E2Q (putative) 2	0.9659	0.4645	NA
70047	Trnt1	tRNA nucleotidyl transferase, CCA-adding, 1	0.9659	0.7392	0.9177
26419	Mapk8	mitogen-activated protein kinase 8	0.9659	0.8253	0.9444
22248	Unc119	unc-119 homolog (C. elegans)	0.9659	0.7365	0.9169
13841	Epha7	Eph receptor A7	0.9659	0.6844	0.8956
277250	Kdm3b	KDM3B lysine (K)-specific demethylase 3B	0.9658	0.734	0.9161
239250	Slitrk6	SLIT and NTRK-like family, member 6	0.9658	0.4077	NA
229285	Spg20	spastic paraplegia 20, spartin (Troyer syndrome) homolog (human)	0.9658	0.7185	0.9102

76222	Magef1	melanoma antigen family F, 1	0.9658	0.4324	NA
72931	2900010J23Rik	RIKEN cDNA 2900010J23 gene	0.9658	0.4583	NA
66358	2310004I24Rik	RIKEN cDNA 2310004I24 gene	0.9658	0.5538	NA
21807	Tsc22d1	TSC22 domain family, member 1	0.9658	0.5137	NA
19364	Rad51l3	RAD51-like 3 (S. cerevisiae)	0.9658	0.6116	NA
14375	Xrcc6	X-ray repair complementing defective repair in Chinese hamster cells 6	0.9658	0.6667	NA
338521	Fa2h	fatty acid 2-hydroxylase	0.9657	0.8313	0.9455
320615	Dopey1	dopey family member 1	0.9657	0.7186	0.9102
213056	Fam126b	family with sequence similarity 126, member B	0.9657	0.575	NA
77619	Prelid2	PRELI domain containing 2	0.9657	0.5266	NA
75064	Zcchc13	zinc finger, CCHC domain containing 13	0.9657	0.8213	0.9433
71787	Trnau1ap	tRNA selenocysteine 1 associated protein 1	0.9657	0.5665	NA
71116	Stx18	syntaxin 18	0.9657	0.4674	NA
50912	Exosc10	exosome component 10	0.9657	0.4396	NA
20586	Smarca4	SWI/SNF related, matrix associated, actin dependent regulator of chromatin, subfamily a, member 4	0.9657	0.6581	NA
19255	Ptpn2	protein tyrosine phosphatase, non-receptor type 2	0.9657	0.7637	0.9248
16206	Lrig1	leucine-rich repeats and immunoglobulin-like domains 1	0.9657	0.6234	NA
14702	Gng2	guanine nucleotide binding protein (G protein), gamma 2	0.9657	0.4452	NA
625054	Gm6548	eukaryotic translation elongation factor 1 alpha 1 pseudogene	0.9656	0.7104	0.9069
231570	A830010M20Rik	RIKEN cDNA A830010M20 gene	0.9656	0.8859	0.9664
116838	Rims2	regulating synaptic membrane exocytosis 2	0.9656	0.7021	0.9039
107733	Mrpl41	mitochondrial ribosomal protein L41	0.9656	0.6203	NA
94044	Bcl2l13	BCL2-like 13 (apoptosis facilitator)	0.9656	0.6659	NA
93837	Dach2	dachshund 2 (Drosophila)	0.9656	0.7608	0.9236
71883	Coq2	coenzyme Q2 homolog, prenyltransferase (yeast)	0.9656	0.5426	NA
28106	D17Wsu104e	DNA segment, Chr 17, Wayne State University 104, expressed	0.9656	0.694	0.9004
27277	Golga5	golgi autoantigen, golgin subfamily a, 5	0.9656	0.3914	NA
17172	Ascl1	achaete-scute complex homolog 1 (Drosophila)	0.9656	0.6573	NA
13877	Erh	enhancer of rudimentary homolog (Drosophila)	0.9656	0.7316	0.9153
633640	Gm7120	predicted gene 7120	0.9655	0.8952	0.9698
235533	Gk5	glycerol kinase 5 (putative)	0.9655	0.751	0.9213
101437	Dhx32	DEAH (Asp-Glu-Ala-His) box polypeptide 32	0.9655	0.689	0.898
13205	Ddx3x	DEAD/H (Asp-Glu-Ala-Asp/His) box polypeptide 3, X-linked	0.9655	0.6625	NA
382038	Urb2	URB2 ribosome biogenesis 2 homolog (S. cerevisiae)	0.9654	0.538	NA
105559	Mbnl2	muscleblind-like 2	0.9654	0.6502	NA
102423	Hinfp	histone H4 transcription factor	0.9654	0.4112	NA
71169	Nbas	neuroblastoma amplified sequence	0.9654	0.512	NA
69528	1700030J22Rik	RIKEN cDNA 1700030J22 gene	0.9654	0.6337	NA
69487	2310003L22Rik	RIKEN cDNA 2310003L22 gene	0.9654	0.6415	NA
28077	Med10	mediator of RNA polymerase II transcription, subunit 10 homolog (NUT2, S. cerevisiae)	0.9654	0.7092	0.9069
14376	Ganab	alpha glucosidase 2 alpha neutral subunit	0.9654	0.4287	NA
319535	Zfp182	zinc finger protein 182	0.9653	0.6254	NA
74204	Xpo6	exportin 6	0.9653	0.6327	NA
70028	Dopey2	dopey family member 2	0.9653	0.7983	0.9361
69754	Fbxo7	F-box protein 7	0.9653	0.51	NA
67530	Uqcrb	ubiquinol-cytochrome c reductase binding protein	0.9653	0.47	NA
66084	Rmnd1	required for meiotic nuclear division 1 homolog (S. cerevisiae)	0.9653	0.5132	NA
57315	Wdr46	WD repeat domain 46	0.9653	0.561	NA
26420	Mapk9	mitogen-activated protein kinase 9	0.9653	0.7289	0.9141

16438	Itpr1	inositol 1,4,5-triphosphate receptor 1	0.9653	0.6305	NA
100502723	Tsga8	testis specific gene A8	0.9652	0.7891	0.9327
102448	Xylb	xylulokinase homolog (H. influenzae)	0.9652	0.543	NA
81601	Kat5	K(lysine) acetyltransferase 5	0.9652	0.4859	NA
68114	Mum1	melanoma associated antigen (mutated) 1	0.9652	0.7805	0.9285
68108	9430008C03Rik	RIKEN cDNA 9430008C03 gene	0.9652	0.703	0.9042
67900	Mtfp1	mitochondrial fission process 1	0.9652	0.5704	NA
58807	Slco1c1	solute carrier organic anion transporter family, member 1c1	0.9652	0.6373	NA
58186	Rad18	RAD18 homolog (S. cerevisiae)	0.9652	0.6652	NA
19126	Prom1	prominin 1	0.9652	0.7304	0.915
15896	Icam2	intercellular adhesion molecule 2	0.9652	0.6763	0.8928
13838	Epha4	Eph receptor A4	0.9652	0.5783	NA
331623	Bend3	BEN domain containing 3	0.9651	0.675	0.8925
319160	Hist1h4k	histone cluster 1, H4k	0.9651	0.79	0.933
101592	Eftud1	elongation factor Tu GTP binding domain containing 1	0.9651	0.446	NA
72948	Tppp	tubulin polymerization promoting protein	0.9651	0.5893	NA
67367	Gcfc1	GC-rich sequence DNA-binding factor 1	0.9651	0.605	NA
19663	Rbpms	RNA binding protein gene with multiple splicing	0.9651	0.6506	NA
16882	Lig3	ligase III, DNA, ATP-dependent	0.9651	0.6736	NA
11787	Apbb2	amyloid beta (A4) precursor protein-binding, family B, member 2	0.9651	0.3955	NA
667433	Gm8630	predicted gene 8630	0.965	0.8298	0.945
214931	Fbxl16	F-box and leucine-rich repeat protein 16	0.965	0.5017	NA
50918	Myadm	myeloid-associated differentiation marker	0.965	0.6685	NA
20102	Rps4x	ribosomal protein S4, X-linked	0.965	0.6808	0.8949
244721	Zfp846	zinc finger protein 846	0.9649	0.5237	NA
99029	AI596198	expressed sequence AI596198	0.9649	0.607	NA
72500	Ier5l	immediate early response 5-like	0.9649	0.5156	NA
67738	Ppid	peptidylprolyl isomerase D (cyclophilin D)	0.9649	0.4165	NA
19735	Rgs2	regulator of G-protein signaling 2	0.9649	0.6009	NA
19671	Rce1	RCE1 homolog, prenyl protein peptidase (S. cerevisiae)	0.9649	0.5989	NA
107022	Gramd3	GRAM domain containing 3	0.9648	0.4899	NA
74243	2210009G21Rik	RIKEN cDNA 2210009G21 gene	0.9648	0.5739	NA
67143	Ikzf5	IKAROS family zinc finger 5	0.9648	0.3433	NA
16372	Irx2	Iroquois related homeobox 2 (Drosophila)	0.9648	0.7828	0.9295
15561	Htr3a	5-hydroxytryptamine (serotonin) receptor 3A	0.9648	0.7907	0.9333
347722	Agap1	ArfGAP with GTPase domain, ankyrin repeat and PH domain 1	0.9647	0.685	0.8959
319155	Hist1h4c	histone cluster 1, H4c	0.9647	0.7342	0.9162
224902	Safb2	scaffold attachment factor B2	0.9647	0.7762	0.9269
223773	Zbed4	zinc finger, BED domain containing 4	0.9647	0.4457	NA
70225	Ppil3	peptidylprolyl isomerase (cyclophilin)-like 3	0.9647	0.3022	NA
69912	Nup43	nucleoporin 43	0.9647	0.5435	NA
67991	Nacc2	nucleus accumbens associated 2, BEN and BTB (POZ) domain containing	0.9647	0.5997	NA
66396	Ccdc82	coiled-coil domain containing 82	0.9647	0.5018	NA
60321	Wbp11	WW domain binding protein 11	0.9647	0.5214	NA
54725	Cadm1	cell adhesion molecule 1	0.9647	0.6722	0.8922
381921	Taok2	TAO kinase 2	0.9646	0.8418	0.9487
208715	Hmgcs1	3-hydroxy-3-methylglutaryl-Coenzyme A synthase 1	0.9646	0.5695	NA
75725	Phf14	PHD finger protein 14	0.9646	0.4714	NA
74025	Nphp3	nephronophthisis 3 (adolescent)	0.9646	0.9281	0.9789

59002	Wdr8	WD repeat domain 8	0.9646	0.5798	NA
16506	Kcnd1	potassium voltage-gated channel, Shal-related family, member 1	0.9646	0.6985	0.9029
14107	Fat1	FAT tumor suppressor homolog 1 (Drosophila)	0.9646	0.9187	0.9764
234138	BC019943	cDNA sequence BC019943	0.9645	0.7308	0.915
107766	Haao	3-hydroxyanthranilate 3,4-dioxygenase	0.9645	0.7926	0.9333
98403	Zfp451	zinc finger protein 451	0.9644	0.5331	NA
56043	Akr1e1	aldo-keto reductase family 1, member E1	0.9644	0.4831	NA
12095	Bglap-rs1	bone gamma-carboxyglutamate protein, related sequence 1	0.9644	0.6621	NA
11496	Adam22	a disintegrin and metallopeptidase domain 22	0.9644	0.5902	NA
381411	Accsl	1-aminocyclopropane-1-carboxylate synthase homolog (Arabidopsis)(non-functional)-like	0.9643	0.6454	NA
56452	Orc6	origin recognition complex, subunit 6	0.9643	0.5216	NA
51786	Cpsf2	cleavage and polyadenylation specific factor 2	0.9643	0.5815	NA
18536	Pcm1	pericentriolar material 1	0.9643	0.8336	0.946
14402	Gabrb3	gamma-aminobutyric acid (GABA) A receptor, subunit beta 3	0.9643	0.5542	NA
665225	Gm7544	predicted gene 7544	0.9642	0.8796	0.9631
218975	Mapk1ip1l	mitogen-activated protein kinase 1 interacting protein 1-like	0.9642	0.6275	NA
107939	Pom121	nuclear pore membrane protein 121	0.9642	0.4637	NA
73845	Ankrd42	ankyrin repeat domain 42	0.9642	0.7043	0.9049
68380	0610042G04Rik	RIKEN cDNA 0610042G04 gene	0.9642	0.8146	0.9412
54004	Diap2	diaphanous homolog 2 (Drosophila)	0.9642	0.6161	NA
53324	Nptx2	neuronal pentraxin 2	0.9642	0.8286	0.945
29812	Ndrg3	N-myc downstream regulated gene 3	0.9642	0.51	NA
26992	Brd7	bromodomain containing 7	0.9642	0.5657	NA
15183	Hdac3	histone deacetylase 3	0.9642	0.4993	NA
15116	Has1	hyaluronan synthase1	0.9642	0.8616	0.9573
100045292	LOC100045292	hypothetical protein LOC100045292	0.9641	0.6479	NA
100039968	Gm12942	predicted gene 12942	0.9641	0.5577	NA
624219	Gm6484	predicted gene 6484	0.9641	0.8523	0.954
269800	Zfp384	zinc finger protein 384	0.9641	0.6835	0.8956
231326	Aasdh	aminoadipate-semialdehyde dehydrogenase	0.9641	0.574	NA
227693	Zer1	zer-1 homolog (C. elegans)	0.9641	0.5895	NA
116940	Tgs1	trimethylguanosine synthase homolog (S. cerevisiae)	0.9641	0.6555	NA
78816	Gmip	Gem-interacting protein	0.9641	0.7702	0.9257
21374	Tbp	TATA box binding protein	0.9641	0.6876	0.8974
21340	Taf1b	TATA box binding protein (Tbp)-associated factor, RNA polymerase I, B	0.9641	0.7603	0.9233
13017	Ctbp2	C-terminal binding protein 2	0.9641	0.3961	NA
100044171	LOC100044171	hypothetical LOC100044171	0.964	0.4283	NA
621580	Gm13308	predicted gene 13308	0.964	0.5629	NA
238328	Vash1	vasohibin 1	0.964	0.6617	NA
231986	Jazf1	JAZF zinc finger 1	0.964	0.711	0.907
211770	Trib1	tribbles homolog 1 (Drosophila)	0.964	0.4722	NA
68404	Nrn1	neuritin 1	0.964	0.5783	NA
67279	Med31	mediator of RNA polymerase II transcription, subunit 31 homolog (yeast)	0.964	0.3664	NA
66855	Tcf25	transcription factor 25 (basic helix-loop-helix)	0.964	0.5831	NA
66793	Efcab1	EF hand calcium binding domain 1	0.964	0.8748	0.9617
20947	Swap70	SWA-70 protein	0.964	0.5945	NA
12642	Ch25h	cholesterol 25-hydroxylase	0.964	0.9563	0.9876
100037396	D030047H15Rik	RIKEN cDNA D030047H15 gene	0.9639	0.7221	0.9121
216578	Papolg	poly(A) polymerase gamma	0.9639	0.3621	NA

211922	Fam116a	family with sequence similarity 116, member A	0.9639	0.5885	NA
110809	Srsf1	serine/arginine-rich splicing factor 1	0.9639	0.491	NA
67891	Rpl4	ribosomal protein L4	0.9639	0.7359	0.9169
60599	Trp53inp1	transformation related protein 53 inducible nuclear protein 1	0.9639	0.6096	NA
56177	Olfm1	olfactomedin 1	0.9639	0.5219	NA
16697	LOC16697	keratin associated protein LOC16697	0.9639	0.7897	0.933
15558	Htr2a	5-hydroxytryptamine (serotonin) receptor 2A	0.9639	0.6342	NA
215160	Rhbdd2	rhomboid domain containing 2	0.9638	0.6038	NA
83671	Sytl2	synaptotagmin-like 2	0.9638	0.4698	NA
70325	Pigw	phosphatidylinositol glycan anchor biosynthesis, class W	0.9638	0.7793	0.9278
56070	Tcerg1	transcription elongation regulator 1 (CA150)	0.9638	0.655	NA
18028	Nfib	nuclear factor I/B	0.9638	0.551	NA
14562	Gdf3	growth differentiation factor 3	0.9638	0.778	0.9273
768271	BE949265	cDNA sequence BE949265	0.9637	0.7302	0.9149
625347	Gm6578	mitochondrial ribosomal protein L32 pseudogene	0.9637	0.3794	NA
245841	Polr2h	polymerase (RNA) II (DNA directed) polypeptide H	0.9637	0.5554	NA
241311	Zbtb34	zinc finger and BTB domain containing 34	0.9637	0.6161	NA
68059	Tm9sf2	transmembrane 9 superfamily member 2	0.9637	0.616	NA
66320	Tmem208	transmembrane protein 208	0.9637	0.4219	NA
53319	Nxf1	nuclear RNA export factor 1 homolog (S. cerevisiae)	0.9637	0.507	NA
18263	Odc1	ornithine decarboxylase, structural 1	0.9637	0.8347	0.9466
14748	Gpr3	G-protein coupled receptor 3	0.9637	0.738	0.9173
12032	Bcan	brevican	0.9637	0.6972	0.902
225579	Slc27a6	solute carrier family 27 (fatty acid transporter), member 6	0.9636	0.8163	0.9417
212442	Lactb2	lactamase, beta 2	0.9636	0.5362	NA
170644	Ubn1	ubinuclein 1	0.9636	0.7087	0.9066
76719	1700081L11Rik	RIKEN cDNA 1700081L11 gene	0.9636	0.4982	NA
68316	Apoo	apolipoprotein O	0.9636	0.4228	NA
55982	Paxip1	PAX interacting (with transcription-activation domain) protein 1	0.9636	0.5498	NA
53600	Timm23	translocase of inner mitochondrial membrane 23 homolog (yeast)	0.9636	0.6654	0.8904
18607	Pdpk1	3-phosphoinositide dependent protein kinase 1	0.9636	0.5471	NA
18212	Ntrk2	neurotrophic tyrosine kinase, receptor, type 2	0.9636	0.6837	0.8956
381410	Zfp408	zinc finger protein 408	0.9635	0.6678	0.8904
277854	Depdc5	DEP domain containing 5	0.9635	0.7805	0.9285
240215	Slc4a9	solute carrier family 4, sodium bicarbonate cotransporter, member 9	0.9635	0.5767	NA
232974	Gm4881	predicted gene 4881	0.9635	0.8137	0.9406
68028	Rpl22l1	ribosomal protein L22 like 1	0.9635	0.5464	NA
17966	Nbr1	neighbor of Brca1 gene 1	0.9635	0.5957	NA
223435	Trio	triple functional domain (PTPRF interacting)	0.9634	0.8912	0.9687
217980	Larp4b	La ribonucleoprotein domain family, member 4B	0.9634	0.5338	NA
212531	Sh3bgrl2	SH3 domain binding glutamic acid-rich protein like 2	0.9634	0.799	0.9361
171262	Vmn1r70	vomeronasal 1 receptor 70	0.9633	0.8248	0.9443
97287	Mtmr14	myotubularin related protein 14	0.9633	0.4736	NA
68558	Ankra2	ankyrin repeat, family A (RFXANK-like), 2	0.9633	0.6473	NA
18624	Pepd	peptidase D	0.9633	0.484	NA
17754	Mtap1a	microtubule-associated protein 1 A	0.9633	0.7176	0.9096
100047044	LOC100047044	hypothetical protein LOC100047044	0.9632	0.486	NA
100042834	Gm4055	predicted gene 4055	0.9632	0.4731	NA
381455	6430573P05Rik	RIKEN cDNA 6430573P05 gene	0.9632	0.581	NA

330059	A430017K17	hypothetical protein A430017K17	0.9632	0.6813	0.8949
320674	4921534A09Rik	RIKEN cDNA 4921534A09 gene	0.9632	0.7406	0.9179
231991	Creb5	cAMP responsive element binding protein 5	0.9632	0.7356	0.9169
20404	Sh3gl2	SH3-domain GRB2-like 2	0.9632	0.7644	0.9251
19243	Ptp4a1	protein tyrosine phosphatase 4a1	0.9632	0.4537	NA
319211	Nol4	nucleolar protein 4	0.9631	0.8311	0.9455
97213	C77137	expressed sequence C77137	0.9631	0.7711	0.9258
67911	Zfp169	zinc finger protein 169	0.9631	0.6044	NA
57170	Dolpp1	dolichyl pyrophosphate phosphatase 1	0.9631	0.5435	NA
21405	Hnf1a	HNF1 homeobox A	0.9631	0.7167	0.9094
18627	Per2	period homolog 2 (Drosophila)	0.9631	0.5141	NA
433966	5730422E09Rik	RIKEN cDNA 5730422E09 gene	0.963	0.6723	0.8922
230770	Tmem39b	transmembrane protein 39b	0.963	0.7505	0.921
66201	Vta1	Vps20-associated 1 homolog (S. cerevisiae)	0.963	0.8549	0.9551
22411	Wnt11	wingless-related MMTV integration site 11	0.963	0.6574	0.8863
17880	Myh11	myosin, heavy polypeptide 11, smooth muscle	0.963	0.8247	0.9443
11682	Alk	anaplastic lymphoma kinase	0.963	0.4696	NA
215512	Fam117a	family with sequence similarity 117, memberA	0.9629	0.7282	0.9136
74296	1700093J21Rik	RIKEN cDNA 1700093J21 gene	0.9629	0.6026	NA
66795	Atg10	autophagy-related 10 (yeast)	0.9629	0.5248	NA
57773	Wdr4	WD repeat domain 4	0.9629	0.4227	NA
21411	Tcf20	transcription factor 20	0.9629	0.6048	NA
14194	Fh1	fumarate hydratase 1	0.9629	0.3877	NA
12895	Cpt1b	carnitine palmitoyltransferase 1b, muscle	0.9629	0.7298	0.9147
330409	Cecr2	cat eye syndrome chromosome region, candidate 2 homolog (human)	0.9628	0.5854	NA
214058	Megf11	multiple EGF-like-domains 11	0.9628	0.7367	0.9169
66799	Ube2w	ubiquitin-conjugating enzyme E2W (putative)	0.9628	0.5435	NA
23950	Dnajb6	DnaJ (Hsp40) homolog, subfamily B, member 6	0.9628	0.7452	0.9192
545725	Mterf	mitochondrial transcription termination factor	0.9627	0.498	NA
243961	Shank1	SH3/ankyrin domain gene 1	0.9627	0.7469	0.9198
98732	Rab3gap2	RAB3 GTPase activating protein subunit 2	0.9627	0.6614	0.8888
76551	Ccdc6	coiled-coil domain containing 6	0.9627	0.6321	NA
69742	Tm2d2	TM2 domain containing 2	0.9627	0.4837	NA
328234	Rnf182	ring finger protein 182	0.9626	0.6346	NA
320541	Slc35e2	solute carrier family 35, member E2	0.9626	0.6117	NA
216805	Flcn	folliculin	0.9626	0.3565	NA
107368	Pdzd8	PDZ domain containing 8	0.9626	0.5214	NA
13806	Eno1	enolase 1, alpha non-neuron	0.9626	0.5756	NA
545172	Rpl21-ps5	ribosomal protein L21, pseudogene 5	0.9625	0.4874	NA
67967	Pold3	polymerase (DNA-directed), delta 3, accessory subunit	0.9625	0.512	NA
67285	Cwc27	CWC27 spliceosome-associated protein homolog (S. cerevisiae)	0.9625	0.6435	NA
66928	3110001D03Rik	RIKEN cDNA 3110001D03 gene	0.9625	0.4183	NA
66787	4933433P14Rik	RIKEN cDNA 4933433P14 gene	0.9625	0.5543	NA
65098	Zfand6	zinc finger, AN1-type domain 6	0.9625	0.6893	0.8982
19647	Rbbp6	retinoblastoma binding protein 6	0.9625	0.4871	NA
18795	Plcb1	phospholipase C, beta 1	0.9625	0.5433	NA
12667	Chrd	chordin	0.9625	0.7926	0.9333
11766	Ap1g2	adaptor protein complex AP-1, gamma 2 subunit	0.9625	0.5162	NA
268445	Ankrd13b	ankyrin repeat domain 13b	0.9624	0.6	NA

55988	Snx12	sorting nexin 12	0.9624	0.6384	NA
23961	Oas1b	2'-5' oligoadenylate synthetase 1B	0.9624	0.4329	NA
331401	Thoc2	THO complex 2	0.9623	0.6848	0.8959
214505	Gnptg	N-acetylglucosamine-1-phosphotransferase, gamma subunit	0.9623	0.342	NA
106583	Scaf8	SR-related CTD-associated factor 8	0.9623	0.4598	NA
83382	Siglece	sialic acid binding Ig-like lectin E	0.9623	0.8486	0.9524
22634	Plagl1	pleiomorphic adenoma gene-like 1	0.9623	0.6669	0.8904
20280	Scp2	sterol carrier protein 2, liver	0.9623	0.5776	NA
14680	Gnal	guanine nucleotide binding protein, alpha stimulating, olfactory type	0.9623	0.2932	NA
11488	Adam11	a disintegrin and metallopeptidase domain 11	0.9623	0.7347	0.9163
619310	Zfp872	zinc finger protein 872	0.9622	0.9192	0.9765
320662	Casc1	cancer susceptibility candidate 1	0.9622	0.7144	0.908
230101	Gba2	glucosidase beta 2	0.9622	0.2498	NA
83602	Gtf2a1	general transcription factor II A, 1	0.9622	0.4458	NA
78232	Trappc6b	trafficking protein particle complex 6B	0.9622	0.6214	NA
68925	Rpap1	RNA polymerase II associated protein 1	0.9622	0.5204	NA
16581	Kifc2	kinesin family member C2	0.9622	0.5856	NA
100041114	Gm3146	predicted gene 3146	0.9621	0.7493	0.9207
208884	Zdhhc9	zinc finger, DHHC domain containing 9	0.9621	0.5922	NA
97827	Exd2	exonuclease 3'-5' domain containing 2	0.9621	0.5789	NA
78885	Coro7	coronin 7	0.9621	0.6711	0.8914
67892	1810063B05Rik	RIKEN cDNA 1810063B05 gene	0.9621	0.5084	NA
67564	Tmem35	transmembrane protein 35	0.9621	0.725	0.9126
67117	Dynlt3	dynein light chain Tctex-type 3	0.9621	0.6726	0.8922
22690	Zfp28	zinc finger protein 28	0.9621	0.5313	NA
13480	Dpm1	dolichol-phosphate (beta-D) mannosyltransferase 1	0.9621	0.4404	NA
12724	Clcn2	chloride channel 2	0.9621	0.5667	NA
117198	Ivns1abp	influenza virus NS1A binding protein	0.962	0.6732	0.8924
66676	Tmed7	transmembrane emp24 protein transport domain containing 7	0.962	0.7359	0.9169
56349	Net1	neuroepithelial cell transforming gene 1	0.962	0.6447	NA
623483	LOC623483	60S ribosomal protein L22-like	0.9619	0.5012	NA
380601	Fastkd5	FAST kinase domains 5	0.9619	0.4983	NA
266690	Cyb5r4	cytochrome b5 reductase 4	0.9619	0.6869	0.8967
72807	Zfp429	zinc finger protein 429	0.9619	0.5764	NA
68728	Trp53inp2	transformation related protein 53 inducible nuclear protein 2	0.9619	0.5752	NA
66684	Tceal8	transcription elongation factor A (SII)-like 8	0.9619	0.6638	0.8896
56550	Ube2d2	ubiquitin-conjugating enzyme E2D 2	0.9619	0.2831	NA
338348	Ttc16	tetratricopeptide repeat domain 16	0.9618	0.4289	NA
216742	Fnip1	folliculin interacting protein 1	0.9618	0.4204	NA
170772	Glcci1	glucocorticoid induced transcript 1	0.9618	0.7447	0.9192
140579	Elmo2	engulfment and cell motility 2, ced-12 homolog (C. elegans)	0.9618	0.5235	NA
70533	Btf3l4	basic transcription factor 3-like 4	0.9618	0.3653	NA
68021	Bphl	biphenyl hydrolase-like (serine hydrolase, breast epithelial mucin-associated antigen)	0.9618	0.4863	NA
67220	Plekho1	pleckstrin homology domain containing, family O member 1	0.9618	0.4625	NA
24012	Rgs7	regulator of G protein signaling 7	0.9618	0.6725	0.8922
19889	Rp2h	retinitis pigmentosa 2 homolog (human)	0.9618	0.6713	0.8915
16648	Kpna3	karyopherin (importin) alpha 3	0.9618	0.7104	0.9069
435784	Gm12471	predicted gene 12471	0.9617	0.7799	0.9281
245595	Zfp711	zinc finger protein 711	0.9617	0.4802	NA

54216	Pcdh7	protocadherin 7	0.9617	0.6948	0.9009
20021	Polr2c	polymerase (RNA) II (DNA directed) polypeptide C	0.9617	0.4992	NA
16569	Kif3b	kinesin family member 3B	0.9617	0.7337	0.9159
13627	Eef1a1	eukaryotic translation elongation factor 1 alpha 1	0.9617	0.6505	0.8829
240028	Lnpep	leucyl/cystinyl aminopeptidase	0.9616	0.6393	NA
74121	Acoxl	acyl-Coenzyme A oxidase-like	0.9616	0.6981	0.9027
50849	Rnf10	ring finger protein 10	0.9616	0.5873	NA
20866	Stim1	stromal interaction molecule 1	0.9616	0.5966	NA
20492	Slbp	stem-loop binding protein	0.9616	0.7183	0.9102
246782	Atpaf2	ATP synthase mitochondrial F1 complex assembly factor 2	0.9615	0.6462	0.881
244913	Gm4978	ribosomal protein L7A pseudogene	0.9615	0.5897	NA
108012	Ap1s2	adaptor-related protein complex 1, sigma 2 subunit	0.9615	0.7086	0.9066
74164	Nfx1	nuclear transcription factor, X-box binding 1	0.9615	0.5108	NA
71624	4833411C07Rik	RIKEN cDNA 4833411C07 gene	0.9615	0.7612	0.9236
22717	Zfp59	zinc finger protein 59	0.9615	0.7436	0.919
20383	Srsf3	serine/arginine-rich splicing factor 3	0.9615	0.7002	0.9033
14030	Ewsr1	Ewing sarcoma breakpoint region 1	0.9615	0.6186	NA
110920	Hspa13	heat shock protein 70 family, member 13	0.9614	0.8069	0.9388
97820	4833439L19Rik	RIKEN cDNA 4833439L19 gene	0.9614	0.4609	NA
78309	Cul9	cullin 9	0.9614	0.552	NA
74112	Usp16	ubiquitin specific peptidase 16	0.9614	0.4732	NA
67998	Fam134c	family with sequence similarity 134, member C	0.9614	0.6767	0.8929
66631	Hiatl1	hippocampus abundant transcript-like 1	0.9614	0.6319	NA
230753	Thrap3	thyroid hormone receptor associated protein 3	0.9613	0.8378	0.948
67912	1600012H06Rik	RIKEN cDNA 1600012H06 gene	0.9613	0.6611	0.8886
56075	Pdss1	prenyl (solanesyl) diphosphate synthase, subunit 1	0.9613	0.4963	NA
56040	Rplp1	ribosomal protein, large, P1	0.9613	0.6014	NA
17191	Mbd2	methyl-CpG binding domain protein 2	0.9613	0.5897	NA
433882	Gm16223	predicted gene 16223	0.9612	0.9271	0.9787
269682	Golga3	golgi autoantigen, golgin subfamily a, 3	0.9612	0.4902	NA
72307	2510002D24Rik	RIKEN cDNA 2510002D24 gene	0.9612	0.393	NA
19301	Pxmp2	peroxisomal membrane protein 2	0.9612	0.7644	0.9251
18263	Odc1	ornithine decarboxylase, structural 1	0.9612	0.7047	0.9049
13047	Cux1	cut-like homeobox 1	0.9612	0.6325	NA
100039024	LOC100039024	arf-GAP with SH3 domain, ANK repeat and PH domain-containing protein 1-like	0.9611	0.6071	NA
277854	Depdc5	DEP domain containing 5	0.9611	0.8404	0.9486
242735	Lrrc38	leucine rich repeat containing 38	0.9611	0.5688	NA
77328	C030009O12Rik	RIKEN cDNA C030009O12 gene	0.9611	0.5477	NA
75472	1700009P17Rik	RIKEN cDNA 1700009P17 gene	0.9611	0.7071	0.9062
67030	Fancl	Fanconi anemia, complementation group L	0.9611	0.6882	0.8976
59069	Tpm3	tropomyosin 3, gamma	0.9611	0.2477	NA
15259	Hipk3	homeodomain interacting protein kinase 3	0.9611	0.6201	NA
13631	Eef2k	eukaryotic elongation factor-2 kinase	0.9611	0.5149	NA
13528	Dtnb	dystrobrevin, beta	0.9611	0.6056	NA
100382	AW011738	expressed sequence AW011738	0.961	0.3668	NA
77578	Bcl9	B-cell CLL/lymphoma 9	0.961	0.6723	0.8922
70727	Rasgef1a	RasGEF domain family, member 1A	0.961	0.4528	NA
69870	Polr3gl	polymerase (RNA) III (DNA directed) polypeptide G like	0.961	0.5016	NA
67105	1700034H14Rik	RIKEN cDNA 1700034H14 gene	0.961	0.4404	NA

30057	Timm8b	translocase of inner mitochondrial membrane 8 homolog b (yeast)	0.961	0.3972	NA
20679	Sox6	SRY-box containing gene 6	0.961	0.6672	0.8904
268880	AI480653	expressed sequence AI480653	0.9609	0.6766	0.8929
232314	Ppp4r2	protein phosphatase 4, regulatory subunit 2	0.9609	0.5501	NA
109959	Amy2a5	amylase 2a5	0.9609	0.688	0.8975
108653	Rimklb	ribosomal modification protein rimK-like family member B	0.9609	0.6135	NA
28028	Mrpl50	mitochondrial ribosomal protein L50	0.9609	0.6508	0.8831
19009	Pou6f1	POU domain, class 6, transcription factor 1	0.9609	0.6946	0.9008
330914	Arhgap32	Rho GTPase activating protein 32	0.9608	0.7455	0.9192
268860	Abat	4-aminobutyrate aminotransferase	0.9608	0.3807	NA
268706	Slc38a9	solute carrier family 38, member 9	0.9608	0.4783	NA
228852	Ppp1r16b	protein phosphatase 1, regulatory (inhibitor) subunit 16B	0.9608	0.7742	0.9266
226251	Ablim1	actin-binding LIM protein 1	0.9608	0.5362	NA
216874	Camta2	calmodulin binding transcription activator 2	0.9608	0.4592	NA
213498	Arhgef11	Rho guanine nucleotide exchange factor (GEF) 11	0.9608	0.5714	NA
74931	4930481A15Rik	RIKEN cDNA 4930481A15 gene	0.9608	0.4397	NA
73473	Iws1	IWS1 homolog (S. cerevisiae)	0.9608	0.6512	0.8833
17125	Smad1	MAD homolog 1 (Drosophila)	0.9608	0.8141	0.9408
329679	Fnip2	folliculin interacting protein 2	0.9607	0.4192	NA
241694	Ralgapa2	Ral GTPase activating protein, alpha subunit 2 (catalytic)	0.9607	0.5776	NA
70834	Spag9	sperm associated antigen 9	0.9607	0.3942	NA
100042982	Gm4146	predicted gene 4146	0.9606	0.7664	0.9251
226751	Cdc42bpa	CDC42 binding protein kinase alpha	0.9606	0.4303	NA
78774	4930529M08Rik	RIKEN cDNA 4930529M08 gene	0.9606	0.5762	NA
67238	2810453I06Rik	RIKEN cDNA 2810453I06 gene	0.9606	0.3243	NA
13548	Dyrk1a	dual-specificity tyrosine-(Y)-phosphorylation regulated kinase 1a	0.9606	0.5486	NA
11990	Atrn	attractin	0.9606	0.4471	NA
399599	Ccdc87	coiled-coil domain containing 87	0.9605	0.7395	0.9178
269587	Epb4.1	erythrocyte protein band 4.1	0.9605	0.7319	0.9154
231861	Tnrc18	trinucleotide repeat containing 18	0.9605	0.6724	0.8922
227723	Prrc2b	proline-rich coiled-coil 2B	0.9605	0.3379	NA
223915	Krt73	keratin 73	0.9605	0.7696	0.9255
208967	Thnsl1	threonine synthase-like 1 (bacterial)	0.9605	0.5149	NA
72097	2010300C02Rik	RIKEN cDNA 2010300C02 gene	0.9605	0.6627	0.8895
71238	Acn9	ACN9 homolog (S. cerevisiae)	0.9605	0.4474	NA
68255	Tmem86b	transmembrane protein 86B	0.9605	0.79	0.933
14758	Gpm6b	glycoprotein m6b	0.9605	0.3077	NA
207495	Baiap2l2	BAI1-associated protein 2-like 2	0.9604	0.8087	0.9395
107823	Whsc1	Wolf-Hirschhorn syndrome candidate 1 (human)	0.9604	0.5511	NA
83962	Btbd1	BTB (POZ) domain containing 1	0.9604	0.7757	0.9266
20112	Rps6ka2	ribosomal protein S6 kinase, polypeptide 2	0.9604	0.6194	NA
16506	Kcnd1	potassium voltage-gated channel, Shal-related family, member 1	0.9604	0.8726	0.9608
15024	H2-T10	histocompatibility 2, T region locus 10	0.9604	0.7198	0.9106
234967	Slc36a4	solute carrier family 36 (proton/amino acid symporter), member 4	0.9603	0.7813	0.9289
230088	B230312A22Rik	RIKEN cDNA B230312A22 gene	0.9603	0.6283	NA
108086	Rnf216	ring finger protein 216	0.9603	0.5972	NA
99371	Arfgef2	ADP-ribosylation factor guanine nucleotide-exchange factor 2 (brefeldin A-inhibited)	0.9603	0.5578	NA
69692	Hddc2	HD domain containing 2	0.9603	0.4245	NA
67178	Zmat5	zinc finger, matrin type 5	0.9603	0.6834	0.8956

20588	Smarcc1	SWI/SNF related, matrix associated, actin dependent regulator of chromatin, subfamily c, member 1	0.9603	0.6566	0.886
12289	Cacna1d	calcium channel, voltage-dependent, L type, alpha 1D subunit	0.9603	0.596	NA
436230	BC065397	cDNA sequence BC065397	0.9602	0.4864	NA
224903	Safb	scaffold attachment factor B	0.9602	0.6637	0.8896
224090	Tmem44	transmembrane protein 44	0.9602	0.8612	0.9573
75339	Mphosph8	M-phase phosphoprotein 8	0.9602	0.4233	NA
73516	1700086O06Rik	RIKEN cDNA 1700086O06 gene	0.9602	0.5344	NA
17968	Ncam2	neural cell adhesion molecule 2	0.9602	0.7619	0.924
16068	Il18bp	interleukin 18 binding protein	0.9602	0.5613	NA
13626	Eed	embryonic ectoderm development	0.9602	0.3854	NA
217198	Plekhh3	pleckstrin homology domain containing, family H (with MyTH4 domain) member 3	0.9601	0.3776	NA
105450	Mmrn2	multimerin 2	0.9601	0.4912	NA
101490	Inpp5f	inositol polyphosphate-5-phosphatase F	0.9601	0.7956	0.9346
67500	Ccar1	cell division cycle and apoptosis regulator 1	0.9601	0.3968	NA
27274	Zfp354b	zinc finger protein 354B	0.9601	0.4536	NA
17984	Ndn	necdin	0.9601	0.6011	NA
11554	Adrb1	adrenergic receptor, beta 1	0.9601	0.5562	NA
328108	Fam179b	family with sequence similarity 179, member B	0.96	0.3236	NA
75129	4930524J08Rik	RIKEN cDNA 4930524J08 gene	0.96	0.5685	NA
74102	Slc35a5	solute carrier family 35, member A5	0.96	0.695	0.9009
27078	B9d1	B9 protein domain 1	0.96	0.2739	NA
19206	Ptch1	patched homolog 1	0.96	0.5977	NA
11352	Abl2	v-abl Abelson murine leukemia viral oncogene homolog 2 (arg, Abelson-related gene)	0.96	0.262	NA
385354	Frmd7	FERM domain containing 7	0.9599	0.8464	0.9515
235048	Zfp599	zinc finger protein 599	0.9599	0.4242	NA
218232	Ptpdc1	protein tyrosine phosphatase domain containing 1	0.9599	0.5467	NA
110960	Tars	threonyl-tRNA synthetase	0.9599	0.4598	NA
53881	Slc5a3	solute carrier family 5 (inositol transporters), member 3	0.9599	0.4935	NA
24018	Rngtt	RNA guanylyltransferase and 5'-phosphatase	0.9599	0.7022	0.9039
20602	Ncor2	nuclear receptor co-repressor 2	0.9599	0.7541	0.9216
271564	Vps13a	vacuolar protein sorting 13A (yeast)	0.9598	0.445	NA
67097	Rps10	ribosomal protein S10	0.9598	0.5376	NA
56306	Fam60a	family with sequence similarity 60, member A	0.9598	0.7082	0.9066
56031	Ppie	peptidylprolyl isomerase E (cyclophilin E)	0.9598	0.7491	0.9207
22070	Tpt1	tumor protein, translationally-controlled 1	0.9598	0.5881	NA
242669	Adc	arginine decarboxylase	0.9597	0.7314	0.9153
229584	Pogz	pogo transposable element with ZNF domain	0.9597	0.475	NA
216860	Neurl4	neuralized homolog 4 (Drosophila)	0.9597	0.5107	NA
77053	Sun1	Sad1 and UNC84 domain containing 1	0.9597	0.623	NA
76073	Pcgf5	polycomb group ring finger 5	0.9597	0.6218	NA
74011	Slc25a27	solute carrier family 25, member 27	0.9597	0.4907	NA
73738	Haus7	HAUS augmin-like complex, subunit 7	0.9597	0.4402	NA
18751	Prkcb	protein kinase C, beta	0.9597	0.5254	NA
17690	Msi1	Musashi homolog 1(Drosophila)	0.9597	0.5613	NA
381379	Med19	mediator of RNA polymerase II transcription, subunit 19 homolog (yeast)	0.9596	0.5006	NA
229722	5330417C22Rik	RIKEN cDNA 5330417C22 gene	0.9596	0.6687	0.8904
27215	Azi2	5-azacytidine induced gene 2	0.9596	0.3733	NA
15452	Hprt	hypoxanthine guanine phosphoribosyl transferase	0.9596	0.4749	NA
381356	5930434B04Rik	RIKEN cDNA 5930434B04 gene	0.9595	0.4833	NA

239555	Smcr7l	Smith-Magenis syndrome chromosome region, candidate 7-like (human)	0.9595	0.369	NA
237221	Gemin8	gem (nuclear organelle) associated protein 8	0.9595	0.7156	0.9087
227120	Plcl1	phospholipase C-like 1	0.9595	0.6193	NA
77128	A930001N09Rik	RIKEN cDNA A930001N09 gene	0.9595	0.7315	0.9153
22402	Wisp1	WNT1 inducible signaling pathway protein 1	0.9595	0.6213	NA
17537	Meis3	Meis homeobox 3	0.9595	0.7453	0.9192
100041879	Gm3561	predicted gene 3561	0.9594	0.6925	0.8995
545551	BC021767	cingulin-like	0.9594	0.7414	0.918
105513	Chmp7	CHMP family, member 7	0.9594	0.6655	0.8904
102124	E130303B06Rik	RIKEN cDNA E130303B06 gene	0.9594	0.4366	NA
74635	4930425O10Rik	RIKEN cDNA 4930425O10 gene	0.9594	0.7874	0.9314
14168	Fgf13	fibroblast growth factor 13	0.9594	0.7372	0.9171
320360	Ric3	resistance to inhibitors of cholinesterase 3 homolog (C. elegans)	0.9593	0.7849	0.9306
224807	Tmem63b	transmembrane protein 63b	0.9593	0.8128	0.9405
70351	Ppp4r1	protein phosphatase 4, regulatory subunit 1	0.9593	0.431	NA
432479	4930404N11Rik	RIKEN cDNA 4930404N11 gene	0.9592	0.6311	0.8751
320661	D5Ertd579e	DNA segment, Chr 5, ERATO Doi 579, expressed	0.9592	0.8771	0.9623
74178	Stk40	serine/threonine kinase 40	0.9592	0.4485	NA
71276	Ccdc57	coiled-coil domain containing 57	0.9592	0.6749	0.8925
57344	As3mt	arsenic (+3 oxidation state) methyltransferase	0.9592	0.3242	NA
22249	Unc13b	unc-13 homolog B (C. elegans)	0.9592	0.3963	NA
17169	Mark3	MAP/microtubule affinity-regulating kinase 3	0.9592	0.4108	NA
16647	Kpna2	karyopherin (importin) alpha 2	0.9592	0.5364	NA
627908	LOC627908	hypothetical LOC627908	0.9591	0.5034	NA
227580	C1ql3	C1q-like 3	0.9591	0.5475	NA
224938	Pja2	praja 2, RING-H2 motif containing	0.9591	0.5576	NA
74256	Cyld	cylindromatosis (turban tumor syndrome)	0.9591	0.5103	NA
30934	Tor1b	torsin family 1, member B	0.9591	0.6736	0.8924
16413	Itgb1bp1	integrin beta 1 binding protein 1	0.9591	0.7636	0.9248
15289	Hmgb1	high mobility group box 1	0.9591	0.5181	NA
319719	4732471D19Rik	RIKEN cDNA 4732471D19 gene	0.959	0.3737	NA
243833	Zfp128	zinc finger protein 128	0.959	0.4799	NA
218885	Oxnad1	oxidoreductase NAD-binding domain containing 1	0.959	0.4238	NA
72650	2810006K23Rik	RIKEN cDNA 2810006K23 gene	0.959	0.385	NA
72386	2610035D17Rik	RIKEN cDNA 2610035D17 gene	0.959	0.5706	NA
71919	Rpap3	RNA polymerase II associated protein 3	0.959	0.3676	NA
545276	Gal3st3	galactose-3-O-sulfotransferase 3	0.9589	0.847	0.9517
320737	4732416N19Rik	RIKEN cDNA 4732416N19 gene	0.9589	0.6213	0.8719
229584	Pogz	pogo transposable element with ZNF domain	0.9589	0.5251	NA
53626	Insm1	insulinoma-associated 1	0.9589	0.3984	NA
100042497	Gm3871	predicted gene 3871	0.9588	0.8194	0.9427
232430	Crebl2	cAMP responsive element binding protein-like 2	0.9588	0.4048	NA
211323	Nrg1	neuregulin 1	0.9588	0.6079	NA
102787	AW552889	expressed sequence AW552889	0.9588	0.8554	0.9552
101739	Psip1	PC4 and SFRS1 interacting protein 1	0.9588	0.5549	NA
78305	1500032F14Rik	RIKEN cDNA 1500032F14 gene	0.9588	0.6774	0.8931
74126	Syvn1	synovial apoptosis inhibitor 1, synoviolin	0.9588	0.3144	NA
70556	Slc25a33	solute carrier family 25, member 33	0.9588	0.6675	0.8904
70088	Meaf6	MYST/Esa1-associated factor 6	0.9588	0.5174	NA

23961	Oas1b	2'-5' oligoadenylate synthetase 1B	0.9588	0.6438	0.8802
21781	Tfdp1	transcription factor Dp 1	0.9588	0.6267	0.8731
19211	Pten	phosphatase and tensin homolog	0.9588	0.5715	NA
12790	Cnga3	cyclic nucleotide gated channel alpha 3	0.9588	0.6136	NA
245007	Zbtb38	zinc finger and BTB domain containing 38	0.9587	0.6104	NA
110213	Tmbim6	transmembrane BAX inhibitor motif containing 6	0.9587	0.6108	NA
72477	Tmem87b	transmembrane protein 87B	0.9587	0.5556	NA
64050	Yeats4	YEATS domain containing 4	0.9587	0.715	0.9083
27967	Cherp	calcium homeostasis endoplasmic reticulum protein	0.9587	0.4297	NA
14459	Gast	gastrin	0.9587	0.8453	0.9508
76501	Commd9	COMM domain containing 9	0.9586	0.5502	NA
22030	Traf2	TNF receptor-associated factor 2	0.9586	0.5303	NA
329795	Tmem67	transmembrane protein 67	0.9585	0.418	NA
320557	Fam169a	family with sequence similarity 169, member A	0.9585	0.6907	0.8988
268980	Strn	striatin, calmodulin binding protein	0.9585	0.5485	NA
108767	Pnrc1	proline-rich nuclear receptor coactivator 1	0.9585	0.6744	0.8924
20168	Rtn3	reticulon 3	0.9585	0.7573	0.9226
12864	Cox6c	cytochrome c oxidase, subunit VIc	0.9585	0.6577	0.8865
665434	Gm7634	predicted gene 7634	0.9584	0.7226	0.9121
627788	Gm6788	predicted gene 6788	0.9584	0.8125	0.9403
217082	Hlf	hepatic leukemia factor	0.9584	0.6727	0.8922
72147	Zbtb46	zinc finger and BTB domain containing 46	0.9584	0.6325	0.8756
66971	Cdk5rap1	CDK5 regulatory subunit associated protein 1	0.9584	0.5904	NA
54325	Elovl1	elongation of very long chain fatty acids (FEN1/Elo2, SUR4/Elo3, yeast)-like 1	0.9584	0.5812	NA
19726	Rfx3	regulatory factor X, 3 (influences HLA class II expression)	0.9584	0.6919	0.8991
433667	Ankrd13c	ankyrin repeat domain 13c	0.9583	0.6582	0.8868
214137	Arhgap29	Rho GTPase activating protein 29	0.9583	0.5537	NA
105193	Nhlrc1	NHL repeat containing 1	0.9583	0.7677	0.9255
78783	Brpf1	bromodomain and PHD finger containing, 1	0.9583	0.4914	NA
18712	Pim1	proviral integration site 1	0.9583	0.7348	0.9164
13871	Ercc2	excision repair cross-complementing rodent repair deficiency, complementation group 2	0.9583	0.388	NA
243547	Grip2	glutamate receptor interacting protein 2	0.9582	0.7107	0.9069
224902	Safb2	scaffold attachment factor B2	0.9582	0.5932	NA
78771	Mctp1	multiple C2 domains, transmembrane 1	0.9582	0.7259	0.9126
77877	6030458C11Rik	RIKEN cDNA 6030458C11 gene	0.9582	0.4137	NA
26441	Psma4	proteasome (prosome, macropain) subunit, alpha type 4	0.9582	0.5761	NA
21766	Tex261	testis expressed gene 261	0.9582	0.4809	NA
382843	Rpl21-ps4	ribosomal protein L21, pseudogene 4	0.9581	0.5453	NA
76965	Slitrk1	SLIT and NTRK-like family, member 1	0.9581	0.8561	0.9554
54484	Mkrn1	makorin, ring finger protein, 1	0.9581	0.638	0.8776
13557	E2f3	E2F transcription factor 3	0.9581	0.7043	0.9049
338337	Cog3	component of oligomeric golgi complex 3	0.958	0.3399	NA
234889	Gucy1a2	guanylate cyclase 1, soluble, alpha 2	0.958	0.6166	0.8698
207686	A330021E22Rik	RIKEN cDNA A330021E22 gene	0.958	0.7069	0.9061
77609	Ccdc151	coiled-coil domain containing 151	0.958	0.4944	NA
16580	Kifc5b	kinesin family member C5B	0.958	0.7816	0.929
11784	Apba2	amyloid beta (A4) precursor protein-binding, family A, member 2	0.958	0.7013	0.9037
408062	Zfp873	zinc finger protein 873	0.9579	0.5663	NA
243819	Ppp6r1	protein phosphatase 6, regulatory subunit 1	0.9579	0.7992	0.9361

227289	Gpbar1	G protein-coupled bile acid receptor 1	0.9579	0.6288	0.8745
69168	Bola1	bolA-like 1 (E. coli)	0.9579	0.5024	NA
29876	Clic4	chloride intracellular channel 4 (mitochondrial)	0.9579	0.7659	0.9251
18045	Nfyb	nuclear transcription factor-Y beta	0.9579	0.6459	0.8809
15312	Hmgn1	high mobility group nucleosomal binding domain 1	0.9579	0.6665	0.8904
235599	6430571L13Rik	RIKEN cDNA 6430571L13 gene	0.9578	0.3093	NA
227634	Camsap1	calmodulin regulated spectrin-associated protein 1	0.9578	0.6605	0.8881
72667	Zfp444	zinc finger protein 444	0.9578	0.516	NA
66047	Mrpl54	mitochondrial ribosomal protein L54	0.9578	0.5828	NA
56195	Ptbp2	polypyrimidine tract binding protein 2	0.9578	0.6656	0.8904
12695	Inadl	InaD-like (Drosophila)	0.9578	0.7541	0.9216
100037260	9430060I03Rik	RIKEN cDNA 9430060I03 gene	0.9577	0.552	NA
240411	Loxhd1	lipoxygenase homology domains 1	0.9577	0.8557	0.9553
104401	Pcnxl3	pecanex-like 3 (Drosophila)	0.9577	0.8001	0.9364
74570	Zkscan1	zinc finger with KRAB and SCAN domains 1	0.9577	0.775	0.9266
72930	Ppp2r2b	protein phosphatase 2 (formerly 2A), regulatory subunit B (PR 52), beta isoform	0.9577	0.4374	NA
20444	St3gal2	ST3 beta-galactoside alpha-2,3-sialyltransferase 2	0.9577	0.7088	0.9066
14938	Gzma	granzyme A	0.9577	0.8021	0.9373
442803	A830005F24Rik	RIKEN cDNA A830005F24 gene	0.9576	0.7262	0.9126
217692	Sipa1l1	signal-induced proliferation-associated 1 like 1	0.9576	0.4737	NA
57738	Slc15a2	solute carrier family 15 (H+/peptide transporter), member 2	0.9576	0.622	0.8719
53375	Mtx2	metaxin 2	0.9576	0.6256	0.8728
13356	Dgcr2	DiGeorge syndrome critical region gene 2	0.9576	0.325	NA
12097	Bglap2	bone gamma-carboxyglutamate protein 2	0.9576	0.7988	0.9361
434203	Slc28a1	solute carrier family 28 (sodium-coupled nucleoside transporter), member 1	0.9575	0.8122	0.9403
320323	A930038B10Rik	RIKEN cDNA A930038B10 gene	0.9575	0.7739	0.9266
225115	Svil	supervillin	0.9575	0.4769	NA
210544	Wdr67	WD repeat domain 67	0.9575	0.4508	NA
72727	B3gat3	beta-1,3-glucuronyltransferase 3 (glucuronosyltransferase I)	0.9575	0.5563	NA
50786	Hs6st2	heparan sulfate 6-O-sulfotransferase 2	0.9575	0.4817	NA
22668	Sf1	splicing factor 1	0.9575	0.4495	NA
100121	Tdrd7	tudor domain containing 7	0.9574	0.4337	NA
68428	Steap3	STEAP family member 3	0.9574	0.5577	NA
27176	Rpl7a	ribosomal protein L7A	0.9574	0.8535	0.9544
22083	Ctr9	Ctr9, Paf1/RNA polymerase II complex component, homolog (S. cerevisiae)	0.9574	0.5382	NA
16785	Rpsa	ribosomal protein SA	0.9574	0.6122	0.8678
11416	Slc33a1	solute carrier family 33 (acetyl-CoA transporter), member 1	0.9574	0.6506	0.883
100043902	Six3os1	Six3 opposite strand transcript 1	0.9573	0.5733	NA
320311	Rnf152	ring finger protein 152	0.9573	0.408	NA
226499	BC003331	cDNA sequence BC003331	0.9573	0.408	NA
218210	Nup153	nucleoporin 153	0.9573	0.3601	NA
19712	Rest	RE1-silencing transcription factor	0.9573	0.5115	NA
108654	4933403F05Rik	RIKEN cDNA 4933403F05 gene	0.9572	0.6451	0.8806
104718	Ttc7b	tetratricopeptide repeat domain 7B	0.9572	0.5356	NA
71779	Mar-08	membrane-associated ring finger (C3HC4) 8	0.9572	0.483	NA
67809	Fam82a2	family with sequence similarity 82, member A2	0.9572	0.4239	NA
56297	Arl6	ADP-ribosylation factor-like 6	0.9572	0.6219	0.8719
229714	Gpr61	G protein-coupled receptor 61	0.9571	0.3921	NA
100689	Spon2	spondin 2, extracellular matrix protein	0.9571	0.9067	0.9725

76932	Arfip2	ADP-ribosylation factor interacting protein 2	0.9571	0.366	NA
70954	4922502B01Rik	RIKEN cDNA 4922502B01 gene	0.9571	0.6684	0.8904
20382	Srsf2	serine/arginine-rich splicing factor 2	0.9571	0.4994	NA
13996	Etohd2	ethanol decreased 2	0.9571	0.6977	0.9024
13383	Dlg1	discs, large homolog 1 (Drosophila)	0.9571	0.6833	0.8956
240067	Zfp952	zinc finger protein 952	0.957	0.5997	NA
230861	Eif4g3	eukaryotic translation initiation factor 4 gamma, 3	0.957	0.5455	NA
30058	Timm8a1	translocase of inner mitochondrial membrane 8 homolog a1 (yeast)	0.957	0.6734	0.8924
237422	Ric8b	resistance to inhibitors of cholinesterase 8 homolog B (C. elegans)	0.9569	0.434	NA
77832	Tchp	trichoplein, keratin filament binding	0.9569	0.3701	NA
67392	4833420G17Rik	RIKEN cDNA 4833420G17 gene	0.9569	0.7599	0.923
56150	Mad2l1	MAD2 mitotic arrest deficient-like 1 (yeast)	0.9569	0.6674	0.8904
23950	Dnajb6	DnaJ (Hsp40) homolog, subfamily B, member 6	0.9569	0.7913	0.9333
19338	Rab33b	RAB33B, member of RAS oncogene family	0.9569	0.7285	0.9137
14081	Acsl1	acyl-CoA synthetase long-chain family member 1	0.9569	0.5663	NA
666340	Gm8054	Rpl21 pseudogene	0.9568	0.4659	NA
268859	Rbfox1	RNA binding protein, fox-1 homolog (C. elegans) 1	0.9568	0.5642	NA
64898	Lpin2	lipin 2	0.9568	0.5557	NA
338523	Jhdm1d	jumonji C domain-containing histone demethylase 1 homolog D (S. cerevisiae)	0.9567	0.6744	0.8924
102182	Prmt10	protein arginine methyltransferase 10 (putative)	0.9567	0.3395	NA
101869	Unc45a	unc-45 homolog A (C. elegans)	0.9567	0.3913	NA
18997	Pou4f2	POU domain, class 4, transcription factor 2	0.9567	0.327	NA
16898	Rps2	ribosomal protein S2	0.9567	0.4922	NA
14919	Gucy2e	guanylate cyclase 2e	0.9567	0.6041	0.8644
14811	Grin2a	glutamate receptor, ionotropic, NMDA2A (epsilon 1)	0.9567	0.6607	0.8883
13872	Ercc3	excision repair cross-complementing rodent repair deficiency, complementation group 3	0.9567	0.494	NA
12589	Ift81	intraflagellar transport 81 homolog (Chlamydomonas)	0.9567	0.6319	0.8751
68164	A430106A12Rik	RIKEN cDNA A430106A12 gene	0.9566	0.5243	NA
67925	1700066D14Rik	RIKEN cDNA 1700066D14 gene	0.9566	0.8927	0.9692
666185	Gm7969	predicted gene 7969	0.9565	0.371	NA
228564	Frmd5	FERM domain containing 5	0.9565	0.2762	NA
97884	B3galnt2	UDP-GalNAc:betaGlcNAc beta 1,3-galactosaminyltransferase, polypeptide 2	0.9565	0.4356	NA
16647	Kpna2	karyopherin (importin) alpha 2	0.9565	0.4344	NA
15384	Hnrnpab	heterogeneous nuclear ribonucleoprotein A/B	0.9565	0.4204	NA
15161	Hcfc1	host cell factor C1	0.9565	0.5301	NA
668253	Dleu2	deleted in lymphocytic leukemia, 2	0.9564	0.3681	NA
99696	Ankrd50	ankyrin repeat domain 50	0.9564	0.7246	0.9126
69171	1810031K17Rik	RIKEN cDNA 1810031K17 gene	0.9564	0.4056	NA
238023	Hexdc	hexosaminidase (glycosyl hydrolase family 20, catalytic domain) containing	0.9563	0.6042	0.8644
97961	Nol12	nucleolar protein 12	0.9563	0.6705	0.8912
94178	Mcoln1	mucolipin 1	0.9563	0.42	NA
77980	Sbf1	SET binding factor 1	0.9563	0.2908	NA
66074	Tmem167	transmembrane protein 167	0.9563	0.5469	NA
20539	Slc7a5	solute carrier family 7 (cationic amino acid transporter, y+ system), member 5	0.9563	0.7391	0.9177
11444	Chrnb2	cholinergic receptor, nicotinic, beta polypeptide 2 (neuronal)	0.9563	0.4688	NA
230751	Oscp1	organic solute carrier partner 1	0.9562	0.5455	NA
72145	Wdfy3	WD repeat and FYVE domain containing 3	0.9562	0.7957	0.9346
58235	Pvrl1	poliovirus receptor-related 1	0.9562	0.4112	NA
22272	Uqcrq	ubiquinol-cytochrome c reductase, complex III subunit VII	0.9562	0.5694	NA

20218	Khdrbs1	KH domain containing, RNA binding, signal transduction associated 1	0.9562	0.2352	NA
100042150	Nrg2	neuregulin 2	0.9561	0.5595	NA
233187	Lim2	lens intrinsic membrane protein 2	0.9561	0.7782	0.9274
212880	Ddx46	DEAD (Asp-Glu-Ala-Asp) box polypeptide 46	0.9561	0.5662	NA
109136	Mmaa	methylmalonic aciduria (cobalamin deficiency) type A	0.9561	0.5499	NA
104303	Arl1	ADP-ribosylation factor-like 1	0.9561	0.6315	0.8751
22608	Ybx1	Y box protein 1	0.9561	0.5357	NA
11958	Atp5k	ATP synthase, H+ transporting, mitochondrial F1F0 complex, subunit e	0.9561	0.4277	NA
100038847	Gm10406	predicted gene 10406	0.956	0.7176	0.9096
233489	Picalm	phosphatidylinositol binding clathrin assembly protein	0.956	0.5556	NA
76539	D19Ertd737e	DNA segment, Chr 19, ERATO Doi 737, expressed	0.956	0.6089	0.8659
71679	Atp5h	ATP synthase, H+ transporting, mitochondrial F0 complex, subunit d	0.956	0.5364	NA
71268	Lrrfip2	leucine rich repeat (in FLII) interacting protein 2	0.956	0.7791	0.9278
68097	Dynll2	dynein light chain LC8-type 2	0.956	0.4467	NA
17342	Mitf	microphthalmia-associated transcription factor	0.956	0.608	0.8655
12695	Inadl	InaD-like (Drosophila)	0.956	0.6362	0.8774
382769	Gm5196	predicted gene 5196	0.9559	0.5438	NA
239556	Cacna1i	calcium channel, voltage-dependent, alpha 1I subunit	0.9559	0.6372	0.8776
237052	Tceal1	transcription elongation factor A (SII)-like 1	0.9559	0.6708	0.8913
214133	Tet2	tet oncogene family member 2	0.9559	0.3613	NA
93681	Zfp192	zinc finger protein 192	0.9559	0.6813	0.8949
81907	Tmem108	transmembrane protein 108	0.9559	0.5376	NA
69641	Wdr20a	WD repeat domain 20A	0.9559	0.6231	0.8724
56525	Zfp235	zinc finger protein 235	0.9559	0.2557	NA
329908	Usp24	ubiquitin specific peptidase 24	0.9558	0.6072	0.8648
224897	Dpp9	dipeptidylpeptidase 9	0.9558	0.4074	NA
110855	Pde6c	phosphodiesterase 6C, cGMP specific, cone, alpha prime	0.9558	0.2972	NA
77940	A930004D18Rik	RIKEN cDNA A930004D18 gene	0.9558	0.7058	0.9057
68592	Syf2	SYF2 homolog, RNA splicing factor (S. cerevisiae)	0.9558	0.7586	0.9229
68394	Ccdc163	coiled-coil domain containing 163	0.9558	0.5629	NA
68379	Ciz1	CDKN1A interacting zinc finger protein 1	0.9558	0.4428	NA
23821	Bace1	beta-site APP cleaving enzyme 1	0.9558	0.3022	NA
19243	Ptp4a1	protein tyrosine phosphatase 4a1	0.9558	0.513	NA
99633	Lphn2	latrophilin 2	0.9557	0.7538	0.9216
69076	Triap1	TP53 regulated inhibitor of apoptosis 1	0.9557	0.2593	NA
68344	Tmem174	transmembrane protein 174	0.9557	0.8262	0.9447
56357	Ivd	isovaleryl coenzyme A dehydrogenase	0.9557	0.4367	NA
53614	Reck	reversion-inducing-cysteine-rich protein with kazal motifs	0.9557	0.6259	0.8728
20511	Slc1a2	solute carrier family 1 (glial high affinity glutamate transporter), member 2	0.9557	0.7041	0.9049
17754	Mtap1a	microtubule-associated protein 1 A	0.9557	0.5566	NA
320411	A730089K16Rik	RIKEN cDNA A730089K16 gene	0.9556	0.7231	0.9121
195040	Tmem199	transmembrane protein 199	0.9556	0.3663	NA
101809	Spred3	sprouty-related, EVH1 domain containing 3	0.9556	0.7582	0.9227
74534	8430428J23Rik	RIKEN cDNA 8430428J23 gene	0.9556	0.6422	0.8793
319845	Bbs9	Bardet-Biedl syndrome 9 (human)	0.9555	0.2515	NA
214469	Fam168b	family with sequence similarity 168, member B	0.9555	0.3763	NA
100702	Mpa2l	macrophage activation 2 like	0.9555	0.6336	0.8761
74016	Phf19	PHD finger protein 19	0.9555	0.5598	NA
68291	Mto1	mitochondrial translation optimization 1 homolog (S. cerevisiae)	0.9555	0.2948	NA

67005	Polr3k	polymerase (RNA) III (DNA directed) polypeptide K	0.9555	0.4294	NA
56795	Arl10	ADP-ribosylation factor-like 10	0.9555	0.8226	0.9436
14789	Leprel2	leprecan-like 2	0.9555	0.4409	NA
67463	Poc5	POC5 centriolar protein homolog (Chlamydomonas)	0.9554	0.3702	NA
66420	Polr2e	polymerase (RNA) II (DNA directed) polypeptide E	0.9554	0.3695	NA
60599	Trp53inp1	transformation related protein 53 inducible nuclear protein 1	0.9554	0.6671	0.8904
59125	Nek7	NIMA (never in mitosis gene a)-related expressed kinase 7	0.9554	0.4628	NA
12293	Cacna2d1	calcium channel, voltage-dependent, alpha2/delta subunit 1	0.9554	0.3089	NA
621818	Gm9785	BMI1-like	0.9553	0.7529	0.9214
228491	Zfp770	zinc finger protein 770	0.9553	0.3602	NA
72972	Gcap14	granule cell antiserum positive 14	0.9553	0.2467	NA
68585	Rtn4	reticulon 4	0.9553	0.7037	0.9046
67157	2610301B20Rik	RIKEN cDNA 2610301B20 gene	0.9553	0.6843	0.8956
54380	Smarcal1	SWI/SNF related matrix associated, actin dependent regulator of chromatin, subfamily a-like 1	0.9553	0.4125	NA
53334	Gosr1	golgi SNAP receptor complex member 1	0.9553	0.5976	0.8611
50817	Solh	small optic lobes homolog (Drosophila)	0.9553	0.7824	0.9293
29861	Dpf1	D4, zinc and double PHD fingers family 1	0.9553	0.6266	0.873
14573	Gdnf	glial cell line derived neurotrophic factor	0.9553	0.7304	0.915
210766	Brcc3	BRCA1/BRCA2-containing complex, subunit 3	0.9552	0.5783	NA
71804	Fam54a	family with sequence similarity 54, member A	0.9552	0.6703	0.8912
67357	1700092C02Rik	RIKEN cDNA 1700092C02 gene	0.9552	0.7615	0.9238
19283	Ptprz1	protein tyrosine phosphatase, receptor type Z, polypeptide 1	0.9552	0.4827	NA
67952	Tomm20	translocase of outer mitochondrial membrane 20 homolog (yeast)	0.9551	0.6008	0.8636
22036	Traip	TRAF-interacting protein	0.9551	0.703	0.9042
228005	Ppig	peptidyl-prolyl isomerase G (cyclophilin G)	0.955	0.2824	NA
219181	Akap11	A kinase (PRKA) anchor protein 11	0.955	0.3304	NA
72203	2610507I01Rik	RIKEN cDNA 2610507I01 gene	0.955	0.6073	0.8648
69372	Mocs3	molybdenum cofactor synthesis 3	0.955	0.5839	0.8544
22217	Usp12	ubiquitin specific peptidase 12	0.955	0.4524	NA
19075	Prim1	DNA primase, p49 subunit	0.955	0.4617	NA
13435	Dnmt3a	DNA methyltransferase 3A	0.955	0.3487	NA
67490	1810074P20Rik	RIKEN cDNA 1810074P20 gene	0.9549	0.4076	NA
56469	Pias1	protein inhibitor of activated STAT 1	0.9549	0.4059	NA
17776	Mast2	microtubule associated serine/threonine kinase 2	0.9549	0.6036	0.8644
14548	Mrps33	mitochondrial ribosomal protein S33	0.9549	0.7386	0.9175
230793	Ahdc1	AT hook, DNA binding motif, containing 1	0.9548	0.5545	NA
69841	2010002M09Rik	RIKEN cDNA 2010002M09 gene	0.9548	0.5176	NA
544696	D630037F22Rik	RIKEN cDNA D630037F22 gene	0.9547	0.5042	NA
72568	Lin9	lin-9 homolog (C. elegans)	0.9547	0.727	0.9131
72351	Ptar1	protein prenyltransferase alpha subunit repeat containing 1	0.9547	0.6315	0.8751
30785	Cttnbp2	cortactin binding protein 2	0.9547	0.6627	0.8895
20269	Scn3a	sodium channel, voltage-gated, type III, alpha	0.9547	0.5016	NA
16588	Kin	antigenic determinant of rec-A protein	0.9547	0.4406	NA
12512	Cd63	CD63 antigen	0.9547	0.3657	NA
545198	LOC545198	hypothetical LOC545198	0.9546	0.5533	NA
214952	Rhot2	ras homolog gene family, member T2	0.9546	0.5147	NA
192656	Ripk2	receptor (TNFRSF)-interacting serine-threonine kinase 2	0.9546	0.683	0.8956
140477	Dmbx1	diencephalon/mesencephalon homeobox 1	0.9546	0.6138	0.8688
83602	Gtf2a1	general transcription factor II A, 1	0.9546	0.6989	0.903

71900	Tmem106b	transmembrane protein 106B	0.9546	0.6197	0.8711
54357	Epb4.1l4b	erythrocyte protein band 4.1-like 4b	0.9546	0.6973	0.9021
22121	Rpl13a	ribosomal protein L13A	0.9546	0.4913	NA
12426	Cckbr	cholecystokinin B receptor	0.9546	0.7064	0.9059
235086	Igsf9b	immunoglobulin superfamily, member 9B	0.9545	0.7812	0.9289
231125	Zfyve28	zinc finger, FYVE domain containing 28	0.9545	0.409	NA
216177	AU041133	expressed sequence AU041133	0.9545	0.5551	NA
110809	Srsf1	serine/arginine-rich splicing factor 1	0.9545	0.3856	NA
107829	Thoc5	THO complex 5	0.9545	0.3634	NA
72992	2900076A07Rik	RIKEN cDNA 2900076A07 gene	0.9545	0.7867	0.9314
72133	Trub1	TruB pseudouridine (psi) synthase homolog 1 (E. coli)	0.9545	0.6194	0.8709
69192	Dhx16	DEAH (Asp-Glu-Ala-His) box polypeptide 16	0.9545	0.5116	NA
67845	Rnf115	ring finger protein 115	0.9545	0.6409	0.8788
67444	Ilkap	integrin-linked kinase-associated serine/threonine phosphatase 2C	0.9545	0.4132	NA
17876	Myef2	myelin basic protein expression factor 2, repressor	0.9545	0.4454	NA
16898	Rps2	ribosomal protein S2	0.9545	0.747	0.9199
12725	Clcn3	chloride channel 3	0.9545	0.3289	NA
12305	Ddr1	discoidin domain receptor family, member 1	0.9545	0.5526	NA
108841	Rdh13	retinol dehydrogenase 13 (all-trans and 9-cis)	0.9544	0.4688	NA
73490	Mipol1	mirror-image polydactyly gene 1 homolog (human)	0.9544	0.5255	NA
67687	1700011L22Rik	RIKEN cDNA 1700011L22 gene	0.9544	0.9154	0.9753
58869	Pex5l	peroxisomal biogenesis factor 5-like	0.9544	0.7053	0.9054
29812	Ndrg3	N-myc downstream regulated gene 3	0.9544	0.5836	0.854
16562	Kif1c	kinesin family member 1C	0.9544	0.5274	NA
623483	LOC623483	60S ribosomal protein L22-like	0.9543	0.6271	0.8734
434377	Zfp560	zinc finger protein 560	0.9543	0.4552	NA
382427	Best3	bestrophin 3	0.9543	0.7024	0.904
235293	Sc5d	sterol-C5-desaturase (fungal ERG3, delta-5-desaturase) homolog (S. cerevisae)	0.9543	0.3243	NA
225164	Mib1	mindbomb homolog 1 (Drosophila)	0.9543	0.454	NA
72149	Strada	STE20-related kinase adaptor alpha	0.9543	0.2724	NA
72054	Cyp4f18	cytochrome P450, family 4, subfamily f, polypeptide 18	0.9543	0.7253	0.9126
66793	Efcab1	EF hand calcium binding domain 1	0.9543	0.8429	0.9497
22234	Ugcg	UDP-glucose ceramide glucosyltransferase	0.9543	0.368	NA
16494	Kcna6	potassium voltage-gated channel, shaker-related, subfamily, member 6	0.9543	0.7541	0.9216
241520	Fam171b	family with sequence similarity 171, member B	0.9542	0.4757	NA
71182	4933417G07Rik	RIKEN cDNA 4933417G07 gene	0.9542	0.6315	0.8751
18108	Nmt2	N-myristoyltransferase 2	0.9542	0.6919	0.8991
16330	Inpp5b	inositol polyphosphate-5-phosphatase B	0.9542	0.5461	NA
218203	Mylip	myosin regulatory light chain interacting protein	0.9541	0.5788	0.8519
67588	Rnf41	ring finger protein 41	0.9541	0.6047	0.8644
54003	Nell2	NEL-like 2 (chicken)	0.9541	0.7815	0.929
12704	Cit	citron	0.9541	0.6914	0.8991
434758	Rhox3h	reproductive homeobox 3H	0.954	0.8294	0.945
81701	Egfl8	EGF-like domain 8	0.954	0.3081	NA
56078	Car5b	carbonic anhydrase 5b, mitochondrial	0.954	0.6601	0.888
21353	Tank	TRAF family member-associated Nf-kappa B activator	0.954	0.6153	0.8696
244646	Pkd1l3	polycystic kidney disease 1 like 3	0.9539	0.8815	0.9637
73158	Larp1	La ribonucleoprotein domain family, member 1	0.9539	0.7417	0.918
67785	Zmym4	zinc finger, MYM-type 4	0.9539	0.4175	NA

24015	Abce1	ATP-binding cassette, sub-family E (OABP), member 1	0.9539	0.303	NA
20514	Slc1a5	solute carrier family 1 (neutral amino acid transporter), member 5	0.9539	0.5801	0.8527
12929	Crkl	v-crk sarcoma virus CT10 oncogene homolog (avian)-like	0.9539	0.533	NA
234413	Zfp961	zinc finger protein 961	0.9538	0.4191	NA
67996	Srsf6	serine/arginine-rich splicing factor 6	0.9538	0.5576	NA
13680	Ddx19a	DEAD (Asp-Glu-Ala-Asp) box polypeptide 19a	0.9538	0.4419	NA
13382	Dld	dihydrolipoamide dehydrogenase	0.9538	0.402	NA
11481	Acvr2b	activin receptor IIB	0.9538	0.5436	NA
235033	Rdh8	retinol dehydrogenase 8	0.9537	0.5133	NA
72039	Mccc1	methylcrotonoyl-Coenzyme A carboxylase 1 (alpha)	0.9537	0.4922	NA
71217	4933431I19Rik	RIKEN cDNA 4933431I19 gene	0.9537	0.6542	0.8852
66682	Trappc5	trafficking protein particle complex 5	0.9537	0.6128	0.8681
15587	Hyal2	hyaluronoglucosaminidase 2	0.9537	0.6862	0.8966
12894	Cpt1a	carnitine palmitoyltransferase 1a, liver	0.9537	0.5969	0.8611
100503813	LOC100503813	hypothetical protein LOC100503813	0.9536	0.6319	0.8751
100042784	Prdm11	PR domain containing 11	0.9536	0.6949	0.9009
103098	Slc6a15	solute carrier family 6 (neurotransmitter transporter), member 15	0.9536	0.3871	NA
77044	Arid2	AT rich interactive domain 2 (ARID, RFX-like)	0.9536	0.6062	0.8645
60411	Cenpk	centromere protein K	0.9536	0.6376	0.8776
16594	Klc2	kinesin light chain 2	0.9536	0.3175	NA
16542	Kdr	kinase insert domain protein receptor	0.9536	0.7257	0.9126
226250	Afap1l2	actin filament associated protein 1-like 2	0.9535	0.7682	0.9255
223989	4921513D23Rik	RIKEN cDNA 4921513D23 gene	0.9535	0.7177	0.9096
218989	6720456H20Rik	RIKEN cDNA 6720456H20 gene	0.9535	0.2808	NA
170791	Rbm39	RNA binding motif protein 39	0.9535	0.5062	NA
71702	Cdc5l	cell division cycle 5-like (S. pombe)	0.9535	0.6556	0.8858
67231	Tbc1d20	TBC1 domain family, member 20	0.9535	0.5527	NA
54141	Spag5	sperm associated antigen 5	0.9535	0.603	0.8643
20587	Smarcb1	SWI/SNF related, matrix associated, actin dependent regulator of chromatin, subfamily b, member 1	0.9535	0.4204	NA
15944	Irgm1	immunity-related GTPase family M member 1	0.9535	0.608	0.8655
11964	Atp6v1a	ATPase, H+ transporting, lysosomal V1 subunit A	0.9535	0.4592	NA
328801	Zfp414	zinc finger protein 414	0.9534	0.575	0.8504
110052	Dek	DEK oncogene (DNA binding)	0.9534	0.2637	NA
72569	Bbs5	Bardet-Biedl syndrome 5 (human)	0.9534	0.6246	0.8728
63828	Fn3k	fructosamine 3 kinase	0.9534	0.5003	NA
12826	Col4a1	collagen, type IV, alpha 1	0.9534	0.8415	0.9487
627939	Gm6813	predicted gene 6813	0.9533	0.4503	NA
260296	Trim61	tripartite motif-containing 61	0.9533	0.7616	0.9238
243308	A430033K04Rik	RIKEN cDNA A430033K04 gene	0.9533	0.7517	0.9213
229615	Pias3	protein inhibitor of activated STAT 3	0.9533	0.6866	0.8966
226169	Pprc1	peroxisome proliferative activated receptor, gamma, coactivator-related 1	0.9533	0.4504	NA
69605	Lnp	limb and neural patterns	0.9533	0.741	0.918
229363	Gmps	guanine monophosphate synthetase	0.9532	0.5146	NA
106064	AW549877	expressed sequence AW549877	0.9532	0.3189	NA
68097	Dynll2	dynein light chain LC8-type 2	0.9532	0.3532	NA
15982	Ifrd1	interferon-related developmental regulator 1	0.9532	0.5105	NA
11827	Aqp2	aquaporin 2	0.9532	0.7588	0.9229
270156	AU019823	expressed sequence AU019823	0.9531	0.5399	NA
242291	Impad1	inositol monophosphatase domain containing 1	0.9531	0.3265	NA

113845	Vmn1r48	vomeronasal 1 receptor 48	0.9531	0.7157	0.9087
71592	Pogk	pogo transposable element with KRAB domain	0.9531	0.7055	0.9055
22718	Zfp60	zinc finger protein 60	0.9531	0.5968	0.8611
328365	Zmiz1	zinc finger, MIZ-type containing 1	0.953	0.6502	0.8828
109785	Pgm3	phosphoglucomutase 3	0.953	0.315	NA
74453	Ccdc11	coiled-coil domain containing 11	0.953	0.6061	0.8645
54611	Pde3a	phosphodiesterase 3A, cGMP inhibited	0.953	0.7749	0.9266
665270	Plb1	phospholipase B1	0.9529	0.5637	NA
235036	Ppan	peter pan homolog (Drosophila)	0.9529	0.4295	NA
224481	Tfb1m	transcription factor B1, mitochondrial	0.9529	0.6266	0.873
110948	Hlcs	holocarboxylase synthetase (biotin- [propriony-Coenzyme A-carboxylase (ATP-hydrolysing)] ligase)	0.9529	0.5026	NA
77480	Kidins220	kinase D-interacting substrate 220	0.9529	0.6014	0.8636
72873	2900006K08Rik	RIKEN cDNA 2900006K08 gene	0.9529	0.7008	0.9034
72503	2610507B11Rik	RIKEN cDNA 2610507B11 gene	0.9529	0.3049	NA
22329	Vcam1	vascular cell adhesion molecule 1	0.9529	0.6837	0.8956
13731	Emp2	epithelial membrane protein 2	0.9529	0.8527	0.9543
225131	Wac	WW domain containing adaptor with coiled-coil	0.9528	0.5547	NA
224020	Pi4ka	phosphatidylinositol 4-kinase, catalytic, alpha polypeptide	0.9528	0.3252	NA
74493	Tnks2	tankyrase, TRF1-interacting ankyrin-related ADP-ribose polymerase 2	0.9528	0.299	NA
74287	Kcmf1	potassium channel modulatory factor 1	0.9528	0.3767	NA
65103	Arl6ip6	ADP-ribosylation factor-like 6 interacting protein 6	0.9528	0.6133	0.8684
28030	Gfm1	G elongation factor, mitochondrial 1	0.9528	0.4083	NA
12703	Socs1	suppressor of cytokine signaling 1	0.9528	0.5524	NA
665113	Tnik	TRAF2 and NCK interacting kinase	0.9527	0.3449	NA
545363	Gm5833	predicted gene 5833	0.9527	0.8372	0.9479
269209	Stk36	serine/threonine kinase 36 (fused homolog, Drosophila)	0.9527	0.3797	NA
231002	Plekhn1	pleckstrin homology domain containing, family N member 1	0.9527	0.6746	0.8924
208518	Cep78	centrosomal protein 78	0.9527	0.4764	NA
67778	Zfp639	zinc finger protein 639	0.9527	0.3021	NA
20273	Scn8a	sodium channel, voltage-gated, type VIII, alpha	0.9527	0.5668	0.8481
381644	Cep135	centrosomal protein 135	0.9526	0.5389	NA
227157	Mpp4	membrane protein, palmitoylated 4 (MAGUK p55 subfamily member 4)	0.9526	0.5905	0.8579
72792	2810459M11Rik	RIKEN cDNA 2810459M11 gene	0.9526	0.7519	0.9213
70827	Trak2	trafficking protein, kinesin binding 2	0.9526	0.5007	NA
13615	Edn2	endothelin 2	0.9526	0.7588	0.9229
353235	Pcdha8	protocadherin alpha 8	0.9525	0.4575	NA
320717	Pptc7	PTC7 protein phosphatase homolog (S. cerevisiae)	0.9525	0.4336	NA
228432	Ano3	anoctamin 3	0.9525	0.4364	NA
106740	LOC106740	hypothetical LOC106740	0.9525	0.4971	NA
66648	5730494M16Rik	RIKEN cDNA 5730494M16 gene	0.9525	0.5734	0.8501
14200	Fhl2	four and a half LIM domains 2	0.9525	0.2612	NA
244199	Ovch2	ovochymase 2	0.9524	0.8049	0.9381
223978	Cpped1	calcineurin-like phosphoesterase domain containing 1	0.9524	0.58	0.8527
110842	Etfa	electron transferring flavoprotein, alpha polypeptide	0.9524	0.598	0.8613
66365	Ccdc90b	coiled-coil domain containing 90B	0.9524	0.5361	NA
54189	Rabep1	rabaptin, RAB GTPase binding effector protein 1	0.9524	0.4865	NA
30932	Zfp330	zinc finger protein 330	0.9524	0.5555	NA
21894	Tln1	talin 1	0.9524	0.4495	NA
12490	Cd34	CD34 antigen	0.9524	0.3685	NA

474156	Zbtb9	zinc finger and BTB domain containing 9	0.9523	0.3413	NA
432467	Hnrnph3	heterogeneous nuclear ribonucleoprotein H3	0.9523	0.4128	NA
74383	Ubap2l	ubiquitin associated protein 2-like	0.9523	0.5404	NA
72416	Lrpprc	leucine-rich PPR-motif containing	0.9523	0.4838	NA
12867	Cox7c	cytochrome c oxidase, subunit VIIc	0.9523	0.8381	0.948
11593	Aga	aspartylglucosaminidase	0.9523	0.6178	0.8701
320679	Samd12	sterile alpha motif domain containing 12	0.9522	0.4424	NA
30050	Fbxw2	F-box and WD-40 domain protein 2	0.9522	0.619	0.8709
232341	Wnk1	WNK lysine deficient protein kinase 1	0.9521	0.3861	NA
230779	Serinc2	serine incorporator 2	0.9521	0.82	0.9429
72047	Ddx42	DEAD (Asp-Glu-Ala-Asp) box polypeptide 42	0.9521	0.2375	NA
17346	Mknk1	MAP kinase-interacting serine/threonine kinase 1	0.9521	0.6528	0.8843
264134	Ttc26	tetratricopeptide repeat domain 26	0.952	0.6018	0.8636
243272	Sbno1	sno, strawberry notch homolog 1 (Drosophila)	0.952	0.365	NA
233812	BC030336	cDNA sequence BC030336	0.952	0.4871	NA
219103	Cenpj	centromere protein J	0.952	0.4743	NA
71985	Acad10	acyl-Coenzyme A dehydrogenase family, member 10	0.952	0.6253	0.8728
70127	Dpf3	D4, zinc and double PHD fingers, family 3	0.952	0.43	NA
66897	Naa16	N(alpha)-acetyltransferase 16, NatA auxiliary subunit	0.952	0.4285	NA
66871	Cpne8	copine VIII	0.952	0.4753	NA
64384	Sirt3	sirtuin 3 (silent mating type information regulation 2, homolog) 3 (S. cerevisiae)	0.952	0.3197	NA
54152	Dnalc4	dynein, axonemal, light chain 4	0.952	0.4253	NA
67526	Atg12	autophagy-related 12 (yeast)	0.9519	0.3816	NA
17250	Abcc1	ATP-binding cassette, sub-family C (CFTR/MRP), member 1	0.9519	0.2877	NA
381832	Prpmp5	proline-rich protein MP5	0.9518	0.6914	0.8991
239556	Cacna1i	calcium channel, voltage-dependent, alpha 1I subunit	0.9518	0.7095	0.9069
233210	Prr12	proline rich 12	0.9518	0.7085	0.9066
78802	Ttc30a1	tetratricopeptide repeat domain 30A1	0.9518	0.3182	NA
23894	Gtf2h2	general transcription factor II H, polypeptide 2	0.9518	0.5152	NA
13041	Ctsw	cathepsin W	0.9518	0.7838	0.9301
329731	Fam19a3	family with sequence similarity 19, member A3	0.9517	0.5364	NA
193740	Hspa1a	heat shock protein 1A	0.9517	0.4401	NA
78425	9530053H05Rik	RIKEN cDNA 9530053H05 gene	0.9517	0.4828	NA
76306	1110021L09Rik	RIKEN cDNA 1110021L09 gene	0.9517	0.3075	NA
70612	5730494N06Rik	RIKEN cDNA 5730494N06 gene	0.9517	0.2718	NA
66882	Bzw1	basic leucine zipper and W2 domains 1	0.9517	0.6796	0.8944
330817	Dhps	deoxyhypusine synthase	0.9516	0.4661	NA
227154	Stradb	STE20-related kinase adaptor beta	0.9516	0.4961	NA
93736	Aff4	AF4/FMR2 family, member 4	0.9516	0.8974	0.9699
93684	Sep-15	selenoprotein	0.9516	0.2936	NA
77300	Raph1	Ras association (RalGDS/AF-6) and pleckstrin homology domains 1	0.9516	0.3658	NA
74360	Cep57	centrosomal protein 57	0.9516	0.2661	NA
69690	2310057B04Rik	RIKEN cDNA 2310057B04 gene	0.9516	0.5509	NA
12354	Car7	carbonic anhydrase 7	0.9516	0.827	0.9449
258384	Olfr1350	olfactory receptor 1350	0.9515	0.7963	0.9347
107056	AW742475	expressed sequence AW742475	0.9515	0.6683	0.8904
72320	2510003E04Rik	RIKEN cDNA 2510003E04 gene	0.9515	0.7412	0.918
67448	Plxdc2	plexin domain containing 2	0.9515	0.602	0.8638
66357	Ostc	oligosaccharyltransferase complex subunit	0.9515	0.4824	NA

667414	Gm8618	predicted gene 8618	0.9514	0.896	0.9699
320806	Gfm2	G elongation factor, mitochondrial 2	0.9514	0.4767	NA
227446	2310035C23Rik	RIKEN cDNA 2310035C23 gene	0.9514	0.5829	0.8538
102920	Cenpi	centromere protein I	0.9514	0.7586	0.9229
83453	Chrdl1	chordin-like 1	0.9514	0.3686	NA
77462	Tmem116	transmembrane protein 116	0.9514	0.6009	0.8636
74385	Mudeng	MU-2/AP1M2 domain containing, death-inducing	0.9514	0.3633	NA
72635	Lins	lines homolog (Drosophila)	0.9514	0.4762	NA
67738	Ppid	peptidylprolyl isomerase D (cyclophilin D)	0.9514	0.7193	0.9104
28135	Cep63	centrosomal protein 63	0.9514	0.2855	NA
27176	Rpl7a	ribosomal protein L7A	0.9514	0.321	NA
22644	Rnf103	ring finger protein 103	0.9514	0.7563	0.9223
234730	Fuk	fucokinase	0.9513	0.3504	NA
233876	Hirip3	HIRA interacting protein 3	0.9513	0.4231	NA
73694	2410091C18Rik	RIKEN cDNA 2410091C18 gene	0.9513	0.3223	NA
21827	Thbs3	thrombospondin 3	0.9513	0.5935	0.8589
14812	Grin2b	glutamate receptor, ionotropic, NMDA2B (epsilon 2)	0.9513	0.71	0.9069
328353	D030051J21Rik	RIKEN cDNA D030051J21 gene	0.9512	0.585	0.855
239408	Tmem74	transmembrane protein 74	0.9512	0.6919	0.8991
216233	Socs2	suppressor of cytokine signaling 2	0.9512	0.5372	NA
211535	Ccdc114	coiled-coil domain containing 114	0.9512	0.7952	0.9343
18080	Nin	ninein	0.9512	0.5435	NA
224132	Dirc2	disrupted in renal carcinoma 2 (human)	0.9511	0.748	0.9202
70549	Tln2	talin 2	0.9511	0.5286	NA
21808	Tgfb2	transforming growth factor, beta 2	0.9511	0.5118	NA
16179	Irak1	interleukin-1 receptor-associated kinase 1	0.9511	0.381	NA
13800	Enah	enabled homolog (Drosophila)	0.9511	0.4064	NA
214897	Csnk1g1	casein kinase 1, gamma 1	0.951	0.3809	NA
75687	Fam65a	family with sequence similarity 65, member A	0.951	0.3064	NA
56351	Ptges3	prostaglandin E synthase 3 (cytosolic)	0.951	0.4569	NA
17184	Matr3	matrin 3	0.951	0.5751	0.8504
433323	Sgpp2	sphingosine-1-phosphate phosphotase 2	0.9509	0.6434	0.88
75420	Secisbp2	SECIS binding protein 2	0.9509	0.5611	0.8465
68730	Dus1l	dihydrouridine synthase 1-like (S. cerevisiae)	0.9509	0.3738	NA
63856	Taf8	TAF8 RNA polymerase II, TATA box binding protein (TBP)-associated factorq	0.9509	0.29	NA
19946	Rpl30	ribosomal protein L30	0.9509	0.5304	NA
11789	Apc	adenomatosis polyposis coli	0.9509	0.7296	0.9145
69178	Snx5	sorting nexin 5	0.9508	0.6558	0.8858
56086	Set	SET nuclear oncogene	0.9508	0.6065	0.8645
50784	Ppap2c	phosphatidic acid phosphatase type 2C	0.9508	0.4257	NA
100046950	LOC100046950	hypothetical LOC100046950	0.9507	0.7086	0.9066
217265	Abca5	ATP-binding cassette, sub-family A (ABC1), member 5	0.9507	0.3073	NA
217109	Utp18	UTP18, small subunit (SSU) processome component, homolog (yeast)	0.9507	0.8951	0.9698
74355	Smchd1	SMC hinge domain containing 1	0.9507	0.4187	NA
67857	Ppp6c	protein phosphatase 6, catalytic subunit	0.9507	0.5454	NA
67596	5830405N20Rik	RIKEN cDNA 5830405N20 gene	0.9507	0.6412	0.8788
22222	Ubr1	ubiquitin protein ligase E3 component n-recognin 1	0.9507	0.4573	NA
319156	Hist1h4d	histone cluster 1, H4d	0.9506	0.6903	0.8988
270201	Klhl18	kelch-like 18 (Drosophila)	0.9506	0.5061	NA

114230	Aipl1	aryl hydrocarbon receptor-interacting protein-like 1	0.9506	0.6088	0.8658
105352	Dusp22	dual specificity phosphatase 22	0.9506	0.424	NA
14711	Gnmt	glycine N-methyltransferase	0.9506	0.3281	NA
433319	Gm5528	predicted gene 5528	0.9505	0.5276	NA
272158	Poln	DNA polymerase N	0.9505	0.5502	0.8421
269870	Zfp446	zinc finger protein 446	0.9505	0.5052	NA
83560	Tex14	testis expressed gene 14	0.9505	0.5423	NA
72179	Fbxl2	F-box and leucine-rich repeat protein 2	0.9505	0.3386	NA
67201	Glod4	glyoxalase domain containing 4	0.9505	0.5353	NA
15465	Hrh1	histamine receptor H1	0.9505	0.7047	0.9049
12561	Cdh4	cadherin 4	0.9505	0.4513	NA
328855	E330032C10Rik	RIKEN cDNA E330032C10 gene	0.9504	0.3288	NA
71599	Senp8	SUMO/sentrin specific peptidase 8	0.9504	0.6054	0.8645
18577	Pde4a	phosphodiesterase 4A, cAMP specific	0.9504	0.7582	0.9227
14823	Grm8	glutamate receptor, metabotropic 8	0.9504	0.2951	NA
77994	2810055G20Rik	RIKEN cDNA 2810055G20 gene	0.9503	0.6064	0.8645
18201	Nsmaf	neutral sphingomyelinase (N-SMase) activation associated factor	0.9503	0.3916	NA
667250	Gm12657	predicted gene 12657	0.9502	0.5913	0.8582
320351	D230037D09Rik	RIKEN cDNA D230037D09 gene	0.9502	0.3865	NA
211329	Ncoa7	nuclear receptor coactivator 7	0.9502	0.5458	0.8391
209361	Taf3	TAF3 RNA polymerase II, TATA box binding protein (TBP)-associated factor	0.9502	0.3995	NA
80902	Zfp202	zinc finger protein 202	0.9502	0.3124	NA
77644	C330007P06Rik	RIKEN cDNA C330007P06 gene	0.9502	0.6939	0.9004
67101	2310039H08Rik	RIKEN cDNA 2310039H08 gene	0.9502	0.2605	NA
56692	Mapksp1	MAPK scaffold protein 1	0.9502	0.6778	0.8931
544872	Gm5786	predicted pseudogene 5786	0.9501	0.6899	0.8985
231162	Cytl1	cytokine-like 1	0.9501	0.6512	0.8833
103012	6720401G13Rik	RIKEN cDNA 6720401G13 gene	0.9501	0.2615	NA
74004	Jakmip3	janus kinase and microtubule interacting protein 3	0.9501	0.7195	0.9106
52014	Nus1	nuclear undecaprenyl pyrophosphate synthase 1 homolog (S. cerevisiae)	0.9501	0.4949	NA
26569	Slc27a4	solute carrier family 27 (fatty acid transporter), member 4	0.9501	0.4475	NA
26384	Gnpda1	glucosamine-6-phosphate deaminase 1	0.9501	0.6414	0.8788
18021	Nfatc3	nuclear factor of activated T-cells, cytoplasmic, calcineurin-dependent 3	0.9501	0.4433	NA
268470	Ube2z	ubiquitin-conjugating enzyme E2Z (putative)	0.95	0.2853	NA
245857	Ssh3	slingshot homolog 3 (Drosophila)	0.95	0.2724	NA
99311	Commd7	COMM domain containing 7	0.95	0.4322	NA
68040	Zfp593	zinc finger protein 593	0.95	0.3423	NA
66711	Sbds	Shwachman-Bodian-Diamond syndrome homolog (human)	0.95	0.4156	NA
22634	Plagl1	pleiomorphic adenoma gene-like 1	0.95	0.5448	0.839
16978	Lrrfip1	leucine rich repeat (in FLII) interacting protein 1	0.95	0.4826	NA
239170	Fam160b2	family with sequence similarity 160, member B2	0.9499	0.7274	0.9134
223642	Zc3h3	zinc finger CCCH type containing 3	0.9499	0.4444	NA
207165	Bptf	bromodomain PHD finger transcription factor	0.9499	0.3168	NA
110880	Scn4a	sodium channel, voltage-gated, type IV, alpha	0.9499	0.7327	0.9157
78777	2410002I01Rik	RIKEN cDNA 2410002I01 gene	0.9499	0.4247	NA
71340	Riok1	RIO kinase 1 (yeast)	0.9499	0.4936	NA
30928	Zfp238	zinc finger protein 238	0.9499	0.36	NA
28071	Twistnb	TWIST neighbor	0.9499	0.5623	0.8465
19249	Ptpn13	protein tyrosine phosphatase, non-receptor type 13	0.9499	0.4834	NA

18767	Pkia	protein kinase inhibitor, alpha	0.9499	0.4966	NA
14455	Gas5	growth arrest specific 5	0.9499	0.5181	NA
12934	Dpysl2	dihydropyrimidinase-like 2	0.9499	0.2932	NA
330959	Snapc5	small nuclear RNA activating complex, polypeptide 5	0.9498	0.5466	0.8396
329504	Lcmt2	leucine carboxyl methyltransferase 2	0.9498	0.5587	0.8459
240753	Plekha6	pleckstrin homology domain containing, family A member 6	0.9498	0.49	NA
67039	Rbm25	RNA binding motif protein 25	0.9498	0.4932	NA
54170	Rragc	Ras-related GTP binding C	0.9498	0.5297	NA
12764	Cmas	cytidine monophospho-N-acetylneuraminic acid synthetase	0.9498	0.6227	0.872
244059	Chd2	chromodomain helicase DNA binding protein 2	0.9497	0.4445	NA
192159	Prpf8	pre-mRNA processing factor 8	0.9497	0.3856	NA
80890	Trim2	tripartite motif-containing 2	0.9497	0.3643	NA
72795	Ttc19	tetratricopeptide repeat domain 19	0.9497	0.3948	NA
320472	Ppm1e	protein phosphatase 1E (PP2C domain containing)	0.9496	0.3474	NA
259279	Tubgcp3	tubulin, gamma complex associated protein 3	0.9496	0.2193	NA
238252	Gpr135	G protein-coupled receptor 135	0.9496	0.6681	0.8904
140484	Pofut1	protein O-fucosyltransferase 1	0.9496	0.442	NA
77006	Ddrgk1	DDRGK domain containing 1	0.9496	0.6365	0.8776
66935	Cir1	corepressor interacting with RBPJ, 1	0.9496	0.558	0.8456
15505	Hsph1	heat shock 105kDa/110kDa protein 1	0.9496	0.3799	NA
677044	Gm10653	ribsomal protein S2 pseudogene	0.9495	0.5678	0.8485
667230	Gm8526	predicted gene 8526	0.9495	0.4472	NA
242860	Rsbn1l	round spermatid basic protein 1-like	0.9495	0.4697	NA
68379	Ciz1	CDKN1A interacting zinc finger protein 1	0.9495	0.3539	NA
66494	Prelid1	PRELI domain containing 1	0.9495	0.5077	NA
56874	Rnf32	ring finger protein 32	0.9495	0.3655	NA
56542	Ick	intestinal cell kinase	0.9495	0.456	NA
22144	Tuba3a	tubulin, alpha 3A	0.9495	0.6331	0.8758
17191	Mbd2	methyl-CpG binding domain protein 2	0.9495	0.2337	NA
16709	Ktn1	kinectin 1	0.9495	0.4288	NA
15968	Ifna5	interferon alpha 5	0.9495	0.8439	0.9502
269702	Mphosph9	M-phase phosphoprotein 9	0.9494	0.4394	NA
217232	Cdc27	cell division cycle 27 homolog (S. cerevisiae)	0.9494	0.6248	0.8728
207952	Klhl25	kelch-like 25 (Drosophila)	0.9494	0.2566	NA
114715	Spred1	sprouty protein with EVH-1 domain 1, related sequence	0.9494	0.4765	NA
18028	Nfib	nuclear factor I/B	0.9494	0.5218	NA
218772	Rarb	retinoic acid receptor, beta	0.9493	0.3732	NA
104681	Slc16a6	solute carrier family 16 (monocarboxylic acid transporters), member 6	0.9493	0.3353	NA
71409	Fmnl2	formin-like 2	0.9493	0.762	0.924
67223	Rrp15	ribosomal RNA processing 15 homolog (S. cerevisiae)	0.9493	0.3414	NA
66399	Tsfm	Ts translation elongation factor, mitochondrial	0.9493	0.5912	0.8582
56228	Ube2j1	ubiquitin-conjugating enzyme E2, J1	0.9493	0.6408	0.8788
14359	Fxr1	fragile X mental retardation gene 1, autosomal homolog	0.9493	0.6024	0.8639
319739	B230303O12Rik	RIKEN cDNA B230303O12 gene	0.9492	0.3519	NA
258505	Olfr97	olfactory receptor 97	0.9492	0.6816	0.8949
215476	C330019G07Rik	RIKEN cDNA C330019G07 gene	0.9492	0.7675	0.9255
67604	Get4	golgi to ER traffic protein 4 homolog (S. cerevisiae)	0.9492	0.3822	NA
67374	Jam2	junction adhesion molecule 2	0.9492	0.6766	0.8929
17772	Mtm1	X-linked myotubular myopathy gene 1	0.9492	0.6177	0.8701

11958	Atp5k	ATP synthase, H+ transporting, mitochondrial F1F0 complex, subunit e	0.9492	0.3139	NA
330502	Zfp82	zinc finger protein 82	0.9491	0.4367	NA
328825	Gm5093	predicted gene 5093	0.9491	0.7673	0.9255
79560	Ublcp1	ubiquitin-like domain containing CTD phosphatase 1	0.9491	0.4036	NA
78512	3300005D01Rik	RIKEN cDNA 3300005D01 gene	0.9491	0.8478	0.9521
54152	Dnalc4	dynein, axonemal, light chain 4	0.9491	0.3337	NA
19989	Rpl7	ribosomal protein L7	0.9491	0.5742	0.8503
19260	Ptpn22	protein tyrosine phosphatase, non-receptor type 22 (lymphoid)	0.9491	0.3806	NA
16443	Itsn1	intersectin 1 (SH3 domain protein 1A)	0.9491	0.1967	NA
75744	Svip	small VCP/p97-interacting protein	0.949	0.3111	NA
60367	Il1rapl2	interleukin 1 receptor accessory protein-like 2	0.949	0.6433	0.88
30954	Siva1	SIVA1, apoptosis-inducing factor	0.949	0.469	NA
16536	Kcnq2	potassium voltage-gated channel, subfamily Q, member 2	0.949	0.2704	NA
16392	Isl1	ISL1 transcription factor, LIM/homeodomain	0.949	0.5389	0.8366
12839	Col9a1	collagen, type IX, alpha 1	0.949	0.427	NA
668173	Pex10	peroxisomal biogenesis factor 10	0.9489	0.4933	NA
382137	Fdxacb1	ferredoxin-fold anticodon binding domain containing 1	0.9489	0.2871	NA
320808	Dcaf5	DDB1 and CUL4 associated factor 5	0.9489	0.8122	0.9403
320632	Snrnp200	small nuclear ribonucleoprotein 200 (U5)	0.9489	0.1863	NA
271127	Adamts16	a disintegrin-like and metallopeptidase (reprolysin type) with thrombospondin type 1 motif, 16	0.9489	0.686	0.8966
233103	4931406P16Rik	RIKEN cDNA 4931406P16 gene	0.9489	0.2544	NA
225192	Hrh4	histamine receptor H4	0.9489	0.7736	0.9266
209462	Hace1	HECT domain and ankyrin repeat containing, E3 ubiquitin protein ligase 1	0.9489	0.5393	0.8366
74148	1300001I01Rik	RIKEN cDNA 1300001I01 gene	0.9489	0.3832	NA
68145	Etaa1	Ewing's tumor-associated antigen 1	0.9489	0.4986	NA
26405	Map3k2	mitogen-activated protein kinase kinase kinase 2	0.9489	0.5942	0.8596
18706	Pik3ca	phosphatidylinositol 3-kinase, catalytic, alpha polypeptide	0.9489	0.6701	0.8912
18432	Mybbp1a	MYB binding protein (P160) 1a	0.9489	0.4194	NA
12723	Clcn1	chloride channel 1	0.9489	0.4693	NA
11941	Atp2b2	ATPase, Ca++ transporting, plasma membrane 2	0.9489	0.6213	0.8719
230767	Iqcc	IQ motif containing C	0.9488	0.6456	0.8809
216188	Aldh1l2	aldehyde dehydrogenase 1 family, member L2	0.9488	0.4614	NA
170719	Oxr1	oxidation resistance 1	0.9488	0.429	NA
72640	Mex3a	mex3 homolog A (C. elegans)	0.9488	0.4624	NA
70757	Ptplb	protein tyrosine phosphatase-like (proline instead of catalytic arginine), member b	0.9488	0.3876	NA
68185	Chchd8	coiled-coil-helix-coiled-coil-helix domain containing 8	0.9488	0.4265	NA
67205	Utp11l	UTP11-like, U3 small nucleolar ribonucleoprotein, (yeast)	0.9488	0.3086	NA
21894	Tln1	talin 1	0.9488	0.637	0.8776
74341	G630025P09Rik	RIKEN cDNA G630025P09 gene	0.9487	0.475	NA
68226	Efcab2	EF-hand calcium binding domain 2	0.9487	0.5719	0.8499
68183	Bcas2	breast carcinoma amplified sequence 2	0.9487	0.341	NA
19691	Recql	RecQ protein-like	0.9487	0.326	NA
16663	Krt13	keratin 13	0.9487	0.7695	0.9255
15516	Hsp90ab1	heat shock protein 90 alpha (cytosolic), class B member 1	0.9487	0.5807	0.8527
100041261	Gm3238	predicted gene 3238	0.9486	0.6792	0.8944
230459	Cyp2j13	cytochrome P450, family 2, subfamily j, polypeptide 13	0.9486	0.5565	0.8448
217039	Ggnbp2	gametogenetin binding protein 2	0.9486	0.3852	NA
109711	Actn1	actinin, alpha 1	0.9486	0.7895	0.9329
71228	Dlg5	discs, large homolog 5 (Drosophila)	0.9486	0.3326	NA

67554	Slc25a30	solute carrier family 25, member 30	0.9486	0.6073	0.8648
29808	Mga	MAX gene associated	0.9486	0.2381	NA
27425	Atp5l	ATP synthase, H+ transporting, mitochondrial F0 complex, subunit g	0.9486	0.8298	0.945
22141	Tub	tubby candidate gene	0.9486	0.6999	0.9033
21408	Zfp354a	zinc finger protein 354A	0.9486	0.5276	NA
16506	Kcnd1	potassium voltage-gated channel, Shal-related family, member 1	0.9486	0.6993	0.9031
11364	Acadm	acyl-Coenzyme A dehydrogenase, medium chain	0.9486	0.1877	NA
230157	Tmeff1	transmembrane protein with EGF-like and two follistatin-like domains 1	0.9485	0.4433	NA
228839	Tgif2	TGFB-induced factor homeobox 2	0.9485	0.6069	0.8647
217707	Coq6	coenzyme Q6 homolog (yeast)	0.9485	0.3502	NA
106762	AW047481	expressed sequence AW047481	0.9485	0.5923	0.8584
73172	Dem1	defects in morphology 1 homolog (S. cerevisiae)	0.9485	0.3225	NA
64833	Acot10	acyl-CoA thioesterase 10	0.9485	0.7066	0.9059
21887	Tle3	transducin-like enhancer of split 3, homolog of Drosophila E(spl)	0.9485	0.3291	NA
100034361	Mfap1b	microfibrillar-associated protein 1B	0.9484	0.7938	0.9336
664883	Nova1	neuro-oncological ventral antigen 1	0.9484	0.7316	0.9153
432855	Zfhx2as	zinc finger homeobox 2, antisense	0.9484	0.5123	NA
387565	Cd300c	CD300C antigen	0.9484	0.8516	0.9537
75316	Taf1d	TATA box binding protein (Tbp)-associated factor, RNA polymerase I, D	0.9484	0.5404	0.8374
67010	Rbm7	RNA binding motif protein 7	0.9484	0.505	NA
66885	Acadsb	acyl-Coenzyme A dehydrogenase, short/branched chain	0.9484	0.4986	NA
23955	Nek4	NIMA (never in mitosis gene a)-related expressed kinase 4	0.9484	0.4623	NA
20382	Srsf2	serine/arginine-rich splicing factor 2	0.9484	0.5519	0.8432
211480	Kcnj14	potassium inwardly-rectifying channel, subfamily J, member 14	0.9483	0.6063	0.8645
67980	Gnpda2	glucosamine-6-phosphate deaminase 2	0.9483	0.5219	NA
66358	2310004I24Rik	RIKEN cDNA 2310004I24 gene	0.9483	0.5701	0.8492
100040276	Gm11595	predicted gene 11595	0.9482	0.7474	0.9199
545859	Vax2os2	Vax2 opposite strand transcript 2	0.9482	0.4215	NA
71982	Snx10	sorting nexin 10	0.9482	0.5669	0.8481
69188	Mll5	myeloid/lymphoid or mixed-lineage leukemia 5	0.9482	0.6347	0.8769
66299	2610019N06Rik	RIKEN cDNA 2610019N06 gene	0.9482	0.3778	NA
22371	Vwf	Von Willebrand factor homolog	0.9482	0.6673	0.8904
18554	Pcsk7	proprotein convertase subtilisin/kexin type 7	0.9482	0.5431	0.8389
620155	Gm6133	60S ribosomal protein L17 pseudogene	0.9481	0.615	0.8695
69029	1500032L24Rik	RIKEN cDNA 1500032L24 gene	0.9481	0.5124	NA
68708	Rabl2	RAB, member of RAS oncogene family-like 2	0.9481	0.2681	NA
67897	Rnmt	RNA (guanine-7-) methyltransferase	0.9481	0.5617	0.8465
67211	Armc10	armadillo repeat containing 10	0.9481	0.368	NA
14629	Gclc	glutamate-cysteine ligase, catalytic subunit	0.9481	0.4136	NA
100039133	Gm2058	predicted gene 2058	0.948	0.5213	NA
319615	Zfp944	zinc finger protein 944	0.948	0.6357	0.8771
229841	Cenpe	centromere protein E	0.948	0.5523	0.8433
211064	Alkbh1	alkB, alkylation repair homolog 1 (E. coli)	0.948	0.5891	0.8575
70681	Fam175a	family with sequence similarity 175, member A	0.948	0.4128	NA
70560	Wars2	tryptophanyl tRNA synthetase 2 (mitochondrial)	0.948	0.4873	NA
18587	Pde6b	phosphodiesterase 6B, cGMP, rod receptor, beta polypeptide	0.948	0.4436	NA
233812	BC030336	cDNA sequence BC030336	0.9479	0.3382	NA
67731	Fbxo32	F-box protein 32	0.9479	0.5414	0.8379
57444	Isg20	interferon-stimulated protein	0.9479	0.5984	0.8615

381760	Ssbp1	single-stranded DNA binding protein 1	0.9478	0.4504	NA
213236	Dnd1	dead end homolog 1 (zebrafish)	0.9478	0.5119	NA
70465	Wdr77	WD repeat domain 77	0.9478	0.3621	NA
69743	Casz1	castor homolog 1, zinc finger (Drosophila)	0.9478	0.6742	0.8924
14050	Eya3	eyes absent 3 homolog (Drosophila)	0.9478	0.2683	NA
12748	Clk2	CDC-like kinase 2	0.9478	0.3291	NA
212168	Zswim4	zinc finger, SWIM domain containing 4	0.9477	0.6439	0.8802
107250	Kazald1	Kazal-type serine peptidase inhibitor domain 1	0.9477	0.5326	0.8339
67075	Magt1	magnesium transporter 1	0.9477	0.4473	NA
66422	Dctpp1	dCTP pyrophosphatase 1	0.9477	0.4351	NA
19317	Qk	quaking	0.9477	0.701	0.9035
330941	AI593442	expressed sequence AI593442	0.9476	0.6763	0.8928
252903	Ap1s3	adaptor-related protein complex AP-1, sigma 3	0.9476	0.6778	0.8931
110876	Scn2a1	sodium channel, voltage-gated, type II, alpha 1	0.9476	0.6302	0.8748
19352	Rabggtb	RAB geranylgeranyl transferase, b subunit	0.9476	0.5959	0.8607
14950	H13	histocompatibility 13	0.9476	0.3825	NA
214763	E330016A19Rik	RIKEN cDNA E330016A19 gene	0.9475	0.7242	0.9126
211255	Kbtbd7	kelch repeat and BTB (POZ) domain containing 7	0.9475	0.4841	NA
107513	Ssr1	signal sequence receptor, alpha	0.9475	0.4019	NA
57443	Fbxo3	F-box protein 3	0.9475	0.6172	0.8698
22210	Ube2b	ubiquitin-conjugating enzyme E2B, RAD6 homology (S. cerevisiae)	0.9475	0.3291	NA
17933	Myt1l	myelin transcription factor 1-like	0.9475	0.2612	NA
14366	Fzd4	frizzled homolog 4 (Drosophila)	0.9475	0.3878	NA
239739	Lamp3	lysosomal-associated membrane protein 3	0.9474	0.1511	NA
100210	Gpn2	GPN-loop GTPase 2	0.9474	0.6147	0.8694
68232	1700120K04Rik	RIKEN cDNA 1700120K04 gene	0.9474	0.7848	0.9305
67148	Fam103a1	family with sequence similarity 103, member A1	0.9474	0.4829	NA
22755	Zfp93	zinc finger protein 93	0.9474	0.324	NA
19268	Ptprf	protein tyrosine phosphatase, receptor type, F	0.9474	0.4428	NA
751865	Sap25	sin3 associated polypeptide	0.9473	0.5883	0.8571
330216	Mblac1	metallo-beta-lactamase domain containing 1	0.9473	0.3628	NA
240726	Slco5a1	solute carrier organic anion transporter family, member 5A1	0.9473	0.7502	0.921
229571	Gm4858	predicted gene 4858	0.9473	0.8374	0.9479
219150	Hmbox1	homeobox containing 1	0.9473	0.7307	0.915
207304	Hectd1	HECT domain containing 1	0.9473	0.3636	NA
77110	Gpbp1l1	GC-rich promoter binding protein 1-like 1	0.9473	0.5682	0.8486
77040	Atg16l1	autophagy-related 16-like 1 (yeast)	0.9473	0.3141	NA
66439	2010012O05Rik	RIKEN cDNA 2010012O05 gene	0.9473	0.4156	NA
215113	Slc43a2	solute carrier family 43, member 2	0.9472	0.4025	NA
78455	Helz	helicase with zinc finger domain	0.9472	0.4237	NA
67384	Bag4	BCL2-associated athanogene 4	0.9472	0.3253	NA
53323	Ube2k	ubiquitin-conjugating enzyme E2K (UBC1 homolog, yeast)	0.9472	0.5284	0.8332
18636	Cfp	complement factor properdin	0.9472	0.4968	NA
432725	Gm5445	predicted gene 5445	0.9471	0.3232	NA
235323	Usp28	ubiquitin specific peptidase 28	0.9471	0.3768	NA
73660	Cabp4	calcium binding protein 4	0.9471	0.4688	NA
20614	Snap25	synaptosomal-associated protein 25	0.9471	0.3224	NA
380928	Lmo7	LIM domain only 7	0.947	0.5658	0.8478
319190	Hist2h2be	histone cluster 2, H2be	0.947	0.3475	NA

231999	Plekha8	pleckstrin homology domain containing, family A (phosphoinositide binding specific) member 8	0.947	0.4773	NA
67790	Rab39b	RAB39B, member RAS oncogene family	0.947	0.588	0.857
66892	Eif4e3	eukaryotic translation initiation factor 4E member 3	0.947	0.2374	NA
57837	Eral1	Era (G-protein)-like 1 (E. coli)	0.947	0.5524	0.8433
100037258	Dnajc3	DnaJ (Hsp40) homolog, subfamily C, member 3	0.9469	0.5369	0.8354
213452	Dstyk	dual serine/threonine and tyrosine protein kinase	0.9469	0.6147	0.8694
66500	Slc30a7	solute carrier family 30 (zinc transporter), member 7	0.9469	0.521	0.8286
22355	Vipr2	vasoactive intestinal peptide receptor 2	0.9469	0.748	0.9202
320727	Ipo8	importin 8	0.9468	0.4098	NA
235611	Plxnb1	plexin B1	0.9468	0.1875	NA
675812	Zfp605	zinc finger protein 605	0.9467	0.7543	0.9216
170779	Cd209d	CD209d antigen	0.9467	0.7905	0.9332
75778	Them4	thioesterase superfamily member 4	0.9467	0.3348	NA
71835	Lancl2	LanC (bacterial lantibiotic synthetase component C)-like 2	0.9467	0.5123	0.8262
68964	1500010J02Rik	RIKEN cDNA 1500010J02 gene	0.9467	0.334	NA
671641	Gm10063	predicted gene 10063	0.9466	0.464	NA
320456	B330016D10Rik	RIKEN cDNA B330016D10 gene	0.9466	0.7593	0.9229
242466	Zfp462	zinc finger protein 462	0.9466	0.644	0.8802
107197	AI462493	expressed sequence AI462493	0.9466	0.3678	NA
99480	Dnttip2	deoxynucleotidyltransferase, terminal, interacting protein 2	0.9466	0.2368	NA
52830	Pnrc2	proline-rich nuclear receptor coactivator 2	0.9466	0.3282	NA
18739	Pitpnm1	phosphatidylinositol transfer protein, membrane-associated 1	0.9466	0.4948	NA
72656	Ints8	integrator complex subunit 8	0.9465	0.4689	NA
18185	Nrl	neural retina leucine zipper gene	0.9465	0.6115	0.8673
140743	Rem2	rad and gem related GTP binding protein 2	0.9464	0.8503	0.9531
77853	Msl2	male-specific lethal 2 homolog (Drosophila)	0.9464	0.2244	NA
19664	Rbpj	recombination signal binding protein for immunoglobulin kappa J region	0.9464	0.5222	0.8293
269629	Lhfpl3	lipoma HMGIC fusion partner-like 3	0.9463	0.6467	0.8812
227801	Dennd1a	DENN/MADD domain containing 1A	0.9463	0.5742	0.8503
28146	Serp1	stress-associated endoplasmic reticulum protein 1	0.9463	0.4751	NA
226442	Zfp281	zinc finger protein 281	0.9462	0.3333	NA
208768	BC031781	cDNA sequence BC031781	0.9462	0.4765	NA
114863	Prosc	proline synthetase co-transcribed	0.9462	0.3304	NA
74737	Pcf11	cleavage and polyadenylation factor subunit homolog (S. cerevisiae)	0.9462	0.7189	0.9102
69017	Prrt2	proline-rich transmembrane protein 2	0.9462	0.4969	NA
20224	Sar1a	SAR1 gene homolog A (S. cerevisiae)	0.9462	0.6084	0.8657
19645	Rb1	retinoblastoma 1	0.9461	0.5988	0.8619
11538	Adnp	activity-dependent neuroprotective protein	0.9461	0.5257	0.832
66674	6330409N04Rik	RIKEN cDNA 6330409N04 gene	0.946	0.4672	NA
56430	Clip1	CAP-GLY domain containing linker protein 1	0.946	0.2796	NA
24050	Sep-03	septin 3	0.946	0.6818	0.8949
668936	Gm14217	predicted gene 14217	0.9459	0.5747	0.8504
434632	BC085271	cDNA sequence BC085271	0.9459	0.6211	0.8719
224656	Zfp523	zinc finger protein 523	0.9459	0.2656	NA
24047	Ccl19	chemokine (C-C motif) ligand 19	0.9459	0.7988	0.9361
100502680	LOC100502680	ubiquitin-conjugating enzyme E2 L3-like	0.9458	0.377	NA
100039043	Gm10731	predicted gene 10731	0.9458	0.4286	NA
217356	Tmc8	transmembrane channel-like gene family 8	0.9458	0.8467	0.9517
75906	Fam184a	family with sequence similarity 184, member A	0.9458	0.613	0.8682

72773	2810449G22Rik	RIKEN cDNA 2810449G22 gene	0.9458	0.7201	0.9106
68020	2810002N01Rik	RIKEN cDNA 2810002N01 gene	0.9458	0.5919	0.8584
56427	Tubd1	tubulin, delta 1	0.9458	0.699	0.903
26901	Deb1	differentially expressed in B16F10 1	0.9458	0.3909	NA
382913	Neil2	nei like 2 (E. coli)	0.9457	0.5412	0.8379
381678	Zcwpw1	zinc finger, CW type with PWWP domain 1	0.9457	0.6426	0.8794
231130	Tnip2	TNFAIP3 interacting protein 2	0.9457	0.1305	NA
20908	Stx3	syntaxin 3	0.9457	0.3842	NA
12565	Cdh9	cadherin 9	0.9457	0.4824	NA
100041585	Amd2	S-adenosylmethionine decarboxylase 2	0.9456	0.4158	NA
78373	Nudt17	nudix (nucleoside diphosphate linked moiety X)-type motif 17	0.9456	0.5429	0.8388
20104	Rps6	ribosomal protein S6	0.9456	0.2292	NA
16367	Irs1	insulin receptor substrate 1	0.9456	0.7486	0.9204
244650	Phlpp2	PH domain and leucine rich repeat protein phosphatase 2	0.9455	0.2165	NA
93746	Gprc5d	G protein-coupled receptor, family C, group 5, member D	0.9455	0.821	0.9433
56336	B4galt5	UDP-Gal:betaGlcNAc beta 1,4-galactosyltransferase, polypeptide 5	0.9455	0.7743	0.9266
54614	Prpf40b	PRP40 pre-mRNA processing factor 40 homolog B (yeast)	0.9455	0.4975	NA
320213	Senp5	SUMO/sentrin specific peptidase 5	0.9454	0.3755	NA
319586	Celf5	CUGBP, Elav-like family member 5	0.9454	0.4212	NA
230734	Yrdc	yrdC domain containing (E.coli)	0.9454	0.6018	0.8636
208146	Yeats2	YEATS domain containing 2	0.9454	0.2646	NA
72057	Phf10	PHD finger protein 10	0.9454	0.3835	NA
66694	Uqcrfs1	ubiquinol-cytochrome c reductase, Rieske iron-sulfur polypeptide 1	0.9454	0.4167	NA
57776	Ttyh1	tweety homolog 1 (Drosophila)	0.9454	0.4506	NA
381045	Ccdc58	coiled-coil domain containing 58	0.9453	0.5144	0.8272
271508	4933408B17Rik	RIKEN cDNA 4933408B17 gene	0.9453	0.4304	NA
232987	B9d2	B9 protein domain 2	0.9453	0.3665	NA
105525	AI480461	expressed sequence AI480461	0.9453	0.4735	NA
101351	A130022J15Rik	RIKEN cDNA A130022J15 gene	0.9453	0.4638	NA
71865	Fbxo30	F-box protein 30	0.9453	0.5071	0.8234
68177	Ebpl	emopamil binding protein-like	0.9453	0.3618	NA
66980	Zdhhc6	zinc finger, DHHC domain containing 6	0.9453	0.2327	NA
26409	Map3k7	mitogen-activated protein kinase kinase kinase 7	0.9453	0.5733	0.8501
22272	Uqcrq	ubiquinol-cytochrome c reductase, complex III subunit VII	0.9453	0.7786	0.9276
20304	Ccl5	chemokine (C-C motif) ligand 5	0.9453	0.7515	0.9213
626870	Gm11992	predicted gene 11992	0.9452	0.6269	0.8733
102141	Snx25	sorting nexin 25	0.9452	0.6478	0.8816
68691	1110028C15Rik	RIKEN cDNA 1110028C15 gene	0.9452	0.5325	0.8339
17188	Maz	MYC-associated zinc finger protein (purine-binding transcription factor)	0.9452	0.6611	0.8886
241688	6330439K17Rik	RIKEN cDNA 6330439K17 gene	0.9451	0.4408	NA
238871	Pde4d	phosphodiesterase 4D, cAMP specific	0.9451	0.5338	0.8343
69053	1810013L24Rik	RIKEN cDNA 1810013L24 gene	0.9451	0.6372	0.8776
63953	Dusp10	dual specificity phosphatase 10	0.9451	0.4952	NA
433586	Maml3	mastermind like 3 (Drosophila)	0.945	0.4883	NA
270106	Rpl13	ribosomal protein L13	0.945	0.6122	0.8678
244650	Phlpp2	PH domain and leucine rich repeat protein phosphatase 2	0.945	0.4697	NA
70363	Fam135b	family with sequence similarity 135, member B	0.945	0.7095	0.9069
19283	Ptprz1	protein tyrosine phosphatase, receptor type Z, polypeptide 1	0.945	0.4779	NA
234915	AK129341	cDNA sequence AK129341	0.9449	0.8919	0.9689

214601	Slc10a3	solute carrier family 10 (sodium/bile acid cotransporter family), member 3	0.9449	0.5325	0.8339
107652	Uap1	UDP-N-acetylglucosamine pyrophosphorylase 1	0.9449	0.2169	NA
72285	1810073O08Rik	RIKEN cDNA 1810073O08 gene	0.9449	0.7653	0.9251
67420	Far1	fatty acyl CoA reductase 1	0.9449	0.5605	0.8462
245841	Polr2h	polymerase (RNA) II (DNA directed) polypeptide H	0.9448	0.435	NA
232560	Caprin2	caprin family member 2	0.9448	0.72	0.9106
225608	Sh3tc2	SH3 domain and tetratricopeptide repeats 2	0.9448	0.6369	0.8776
70439	Taf15	TAF15 RNA polymerase II, TATA box binding protein (TBP)-associated factor	0.9448	0.4099	NA
52028	Bbs1	Bardet-Biedl syndrome 1 (human)	0.9448	0.5083	0.8242
17289	Mertk	c-mer proto-oncogene tyrosine kinase	0.9448	0.4613	NA
319944	Taf2	TAF2 RNA polymerase II, TATA box binding protein (TBP)-associated factor	0.9447	0.309	NA
226470	Zbtb41	zinc finger and BTB domain containing 41 homolog	0.9447	0.5971	0.8611
216551	1110067D22Rik	RIKEN cDNA 1110067D22 gene	0.9447	0.2921	NA
194268	9930104L06Rik	RIKEN cDNA 9930104L06 gene	0.9447	0.2132	NA
77613	Prss36	protease, serine, 36	0.9447	0.4404	NA
75788	Smurf1	SMAD specific E3 ubiquitin protein ligase 1	0.9447	0.4097	NA
71435	Arhgap21	Rho GTPase activating protein 21	0.9447	0.3919	NA
70556	Slc25a33	solute carrier family 25, member 33	0.9447	0.17	NA
67530	Uqcrb	ubiquinol-cytochrome c reductase binding protein	0.9447	0.2941	NA
66689	Klhl28	kelch-like 28 (Drosophila)	0.9447	0.3958	NA
18861	Pms2	postmeiotic segregation increased 2 (S. cerevisiae)	0.9447	0.574	0.8503
12454	Ccnk	cyclin K	0.9447	0.3885	NA
100042948	Gm15760	mitochondrial ribosomal protein S18B pseudogene	0.9446	0.8911	0.9687
668572	2610021A01Rik	RIKEN cDNA 2610021A01 gene	0.9446	0.5962	0.8609
399510	Map4k5	mitogen-activated protein kinase kinase kinase kinase 5	0.9446	0.4004	NA
216877	Dhx33	DEAH (Asp-Glu-Ala-His) box polypeptide 33	0.9446	0.2809	NA
52841	D2Bwg0886e	DNA segment, Chr 2, Brigham & Women's Genetics 0886 expressed	0.9446	0.8163	0.9417
18129	Notch2	Notch gene homolog 2 (Drosophila)	0.9446	0.5194	0.8282
15078	H3f3a	H3 histone, family 3A	0.9446	0.4982	0.8194
11733	Ank1	ankyrin 1, erythroid	0.9446	0.6798	0.8944
320162	Ccdc45	coiled-coil domain containing 45	0.9445	0.2572	NA
217335	Fbf1	Fas (TNFRSF6) binding factor 1	0.9445	0.4791	NA
117149	Tirap	toll-interleukin 1 receptor (TIR) domain-containing adaptor protein	0.9445	0.3	NA
77574	Fam115a	family with sequence similarity 115, member A	0.9445	0.5187	0.8282
68691	1110028C15Rik	RIKEN cDNA 1110028C15 gene	0.9445	0.3681	NA
50787	Hs6st3	heparan sulfate 6-O-sulfotransferase 3	0.9445	0.8118	0.9403
21885	Tle1	transducin-like enhancer of split 1, homolog of Drosophila E(spl)	0.9445	0.7448	0.9192
16528	Kcnk4	potassium channel, subfamily K, member 4	0.9445	0.4461	NA
381598	2610005L07Rik	cadherin 11 pseudogene	0.9444	0.3617	NA
319670	Eml5	echinoderm microtubule associated protein like 5	0.9444	0.4217	NA
218294	Cdc14b	CDC14 cell division cycle 14 homolog B (S. cerevisiae)	0.9444	0.1724	NA
74577	Glb1l	galactosidase, beta 1-like	0.9444	0.3532	NA
54673	Sh3glb1	SH3-domain GRB2-like B1 (endophilin)	0.9444	0.3057	NA
19317	Qk	quaking	0.9444	0.6777	0.8931
382030	Tmem188	transmembrane protein 188	0.9443	0.4575	NA
329986	BC080695	cDNA sequence BC080695	0.9443	0.6692	0.8904
244721	Zfp846	zinc finger protein 846	0.9443	0.3377	NA
208595	Gm9897	predicted gene 9897	0.9443	0.3743	NA
110816	Pwp2	PWP2 periodic tryptophan protein homolog (yeast)	0.9443	0.3346	NA

72194	Fbxl20	F-box and leucine-rich repeat protein 20	0.9443	0.2405	NA
67958	2610101N10Rik	RIKEN cDNA 2610101N10 gene	0.9443	0.3437	NA
66190	Acer3	alkaline ceramidase 3	0.9443	0.3442	NA
22276	Uros	uroporphyrinogen III synthase	0.9443	0.3519	NA
67006	Cisd2	CDGSH iron sulfur domain 2	0.9442	0.4154	NA
17937	Nab2	Ngfi-A binding protein 2	0.9442	0.5245	0.8306
12867	Cox7c	cytochrome c oxidase, subunit VIIc	0.9442	0.2268	NA
634650	Gbp11	guanylate binding protein 11	0.9441	0.6063	0.8645
672125	Gm14496	predicted gene 14496	0.944	0.624	0.8726
408067	Zfp874b	zinc finger protein 874b	0.944	0.7544	0.9216
320722	A330050F15Rik	RIKEN cDNA A330050F15 gene	0.944	0.514	0.8271
239318	Plcxd3	phosphatidylinositol-specific phospholipase C, X domain containing 3	0.944	0.5007	0.82
238871	Pde4d	phosphodiesterase 4D, cAMP specific	0.944	0.4678	NA
230316	Megf9	multiple EGF-like-domains 9	0.944	0.507	0.8234
74561	Nkx6-3	NK6 homeobox 3	0.944	0.4595	NA
50523	Lats2	large tumor suppressor 2	0.944	0.2015	NA
18521	Pcbp2	poly(rC) binding protein 2	0.944	0.3714	NA
15528	Hspe1	heat shock protein 1 (chaperonin 10)	0.944	0.8786	0.9628
15216	Hfe	hemochromatosis	0.944	0.6409	0.8788
100045367	LOC100045367	60S ribosomal protein L19-like	0.9439	0.1639	NA
319554	Idi1	isopentenyl-diphosphate delta isomerase	0.9439	0.2018	NA
223642	Zc3h3	zinc finger CCCH type containing 3	0.9439	0.5143	0.8272
74277	Chic2	cysteine-rich hydrophobic domain 2	0.9439	0.5535	0.844
72469	Plcd3	phospholipase C, delta 3	0.9439	0.2829	NA
67532	Mfap1a	microfibrillar-associated protein 1A	0.9439	0.2541	NA
15502	Dnaja1	DnaJ (Hsp40) homolog, subfamily A, member 1	0.9439	0.3133	NA
14696	Gnb4	guanine nucleotide binding protein (G protein), beta 4	0.9439	0.5164	0.8277
12180	Smyd1	SET and MYND domain containing 1	0.9439	0.7512	0.9213
70661	Sik3	SIK family kinase 3	0.9438	0.296	NA
66939	Aagab	alpha- and gamma-adaptin binding protein	0.9438	0.2947	NA
22751	Zfp90	zinc finger protein 90	0.9438	0.1975	NA
18187	Nrp2	neuropilin 2	0.9438	0.6505	0.8829
17886	Myh9	myosin, heavy polypeptide 9, non-muscle	0.9438	0.455	NA
70207	Taco1	translational activator of mitochondrially encoded cytochrome c oxidase I	0.9437	0.5701	0.8492
21645	Tcte1	t-complex-associated testis expressed 1	0.9437	0.7564	0.9223
17134	Mafg	v-maf musculoaponeurotic fibrosarcoma oncogene family, protein G (avian)	0.9437	0.2929	NA
12912	Creb1	cAMP responsive element binding protein 1	0.9437	0.3995	NA
217030	Synrg	synergin, gamma	0.9436	0.5004	0.8197
105833	Ccdc65	coiled-coil domain containing 65	0.9436	0.5666	0.8481
101118	Tmem168	transmembrane protein 168	0.9436	0.605	0.8645
75302	Asxl2	additional sex combs like 2 (Drosophila)	0.9436	0.3128	NA
69085	Zcchc9	zinc finger, CCHC domain containing 9	0.9436	0.2632	NA
20866	Stim1	stromal interaction molecule 1	0.9436	0.471	NA
12564	Cdh8	cadherin 8	0.9436	0.5372	0.8355
74778	Rrp7a	ribosomal RNA processing 7 homolog A (S. cerevisiae)	0.9435	0.3873	NA
73692	2410089E03Rik	RIKEN cDNA 2410089E03 gene	0.9435	0.5667	0.8481
239985	Arid1b	AT rich interactive domain 1B (SWI-like)	0.9434	0.377	NA
237459	Cdk17	cyclin-dependent kinase 17	0.9434	0.5601	0.846
12495	Entpd1	ectonucleoside triphosphate diphosphohydrolase 1	0.9434	0.6952	0.901

677044	Gm10653	ribsomal protein S2 pseudogene	0.9433	0.398	NA
241525	Ypel4	yippee-like 4 (Drosophila)	0.9433	0.4613	NA
213573	Efcab4a	EF-hand calcium binding domain 4A	0.9433	0.5104	0.8253
74264	1700045I19Rik	ring finger protein 138 pseudogene	0.9433	0.5698	0.8492
59013	Hnrnph1	heterogeneous nuclear ribonucleoprotein H1	0.9433	0.3824	NA
12532	Cdc25c	cell division cycle 25 homolog C (S. pombe)	0.9433	0.5686	0.8487
258418	Olfr469	olfactory receptor 469	0.9432	0.8386	0.9482
71921	2310058N22Rik	RIKEN cDNA 2310058N22 gene	0.9432	0.4764	NA
63959	Slc29a1	solute carrier family 29 (nucleoside transporters), member 1	0.9432	0.6069	0.8647
57312	Mrps31	mitochondrial ribosomal protein S31	0.9432	0.215	NA
13877	Erh	enhancer of rudimentary homolog (Drosophila)	0.9432	0.3186	NA
319277	A230046K03Rik	RIKEN cDNA A230046K03 gene	0.9431	0.4811	NA
269180	Inpp4a	inositol polyphosphate-4-phosphatase, type I	0.9431	0.5631	0.8465
116873	Stim2	stromal interaction molecule 2	0.9431	0.5641	0.8466
109284	C030046I01Rik	RIKEN cDNA C030046I01 gene	0.9431	0.4976	0.8193
76199	Med13l	mediator complex subunit 13-like	0.9431	0.6279	0.8739
73991	Atl1	atlastin GTPase 1	0.9431	0.3313	NA
69504	Zfp932	zinc finger protein 932	0.9431	0.2056	NA
68942	Chmp2b	chromatin modifying protein 2B	0.9431	0.4235	NA
66548	Adamtsl5	ADAMTS-like 5	0.9431	0.7532	0.9215
54204	Sep-01	septin 1	0.9431	0.6514	0.8833
16993	Lta4h	leukotriene A4 hydrolase	0.9431	0.3906	NA
245671	Klf8	Kruppel-like factor 8	0.943	0.5968	0.8611
70044	Tut1	terminal uridylyl transferase 1, U6 snRNA-specific	0.943	0.3222	NA
68832	1110057K04Rik	RIKEN cDNA 1110057K04 gene	0.943	0.5134	0.8268
67378	Bbs2	Bardet-Biedl syndrome 2 (human)	0.943	0.3336	NA
22210	Ube2b	ubiquitin-conjugating enzyme E2B, RAD6 homology (S. cerevisiae)	0.943	0.2461	NA
17717	ND2	NADH dehydrogenase subunit 2	0.943	0.4239	NA
13508	Dscam	Down syndrome cell adhesion molecule	0.943	0.3091	NA
11772	Ap2a2	adaptor protein complex AP-2, alpha 2 subunit	0.943	0.4487	NA
381101	BC048355	cDNA sequence BC048355	0.9429	0.4534	NA
219149	Xkr6	X Kell blood group precursor related family member 6 homolog	0.9429	0.5906	0.8579
170740	Zfp287	zinc finger protein 287	0.9429	0.6661	0.8904
108995	Tbc1d10c	TBC1 domain family, member 10c	0.9429	0.6219	0.8719
68031	Rnf146	ring finger protein 146	0.9429	0.766	0.9251
67111	Naaa	N-acylethanolamine acid amidase	0.9429	0.392	NA
54609	Ubqln2	ubiquilin 2	0.9429	0.2916	NA
20585	Hltf	helicase-like transcription factor	0.9429	0.4033	NA
319196	Ankrd5	ankyrin repeat domain 5	0.9428	0.6415	0.8789
227738	Lrsam1	leucine rich repeat and sterile alpha motif containing 1	0.9428	0.6598	0.8879
108800	Ston2	stonin 2	0.9428	0.6394	0.8783
54214	Golga4	golgi autoantigen, golgin subfamily a, 4	0.9428	0.3967	NA
11977	Atp7a	ATPase, Cu++ transporting, alpha polypeptide	0.9428	0.3894	NA
621976	Tmem170b	transmembrane protein 170B	0.9427	0.503	0.8215
233490	Crebzf	CREB/ATF bZIP transcription factor	0.9427	0.2884	NA
212508	Mtg1	mitochondrial GTPase 1 homolog (S. cerevisiae)	0.9427	0.289	NA
76425	2310003C23Rik	RIKEN cDNA 2310003C23 gene	0.9427	0.3445	NA
67392	4833420G17Rik	RIKEN cDNA 4833420G17 gene	0.9427	0.6992	0.9031
20401	Sh3bp1	SH3-domain binding protein 1	0.9427	0.635	0.877

12632	Cfl2	cofilin 2, muscle	0.9427	0.2379	NA
108745	2310051F07Rik	RIKEN cDNA 2310051F07 gene	0.9426	0.1998	NA
102545	Cmtm7	CKLF-like MARVEL transmembrane domain containing 7	0.9426	0.7769	0.9272
68092	Ncbp2	nuclear cap binding protein subunit 2	0.9426	0.6656	0.8904
66690	Tmem186	transmembrane protein 186	0.9426	0.2101	NA
53883	Celsr2	cadherin, EGF LAG seven-pass G-type receptor 2 (flamingo homolog, Drosophila)	0.9426	0.5904	0.8579
52480	D7Ertd715e	DNA segment, Chr 7, ERATO Doi 715, expressed	0.9426	0.6488	0.8818
12728	Clcn5	chloride channel 5	0.9426	0.5109	0.8255
230917	Tmem201	transmembrane protein 201	0.9425	0.6599	0.8879
224440	Setd4	SET domain containing 4	0.9425	0.2915	NA
105596	AU020094	expressed sequence AU020094	0.9425	0.8202	0.9431
77048	Ccdc41	coiled-coil domain containing 41	0.9425	0.2328	NA
21379	Tbrg4	transforming growth factor beta regulated gene 4	0.9425	0.3829	NA
16800	Arhgef2	rho/rac guanine nucleotide exchange factor (GEF) 2	0.9425	0.4439	NA
100038413	D130050E23Rik	RIKEN cDNA D130050E23 gene	0.9424	0.3071	NA
59015	Nup160	nucleoporin 160	0.9424	0.2752	NA
23871	Ets1	E26 avian leukemia oncogene 1, 5' domain	0.9424	0.5924	0.8584
22694	Zfp35	zinc finger protein 35	0.9424	0.5842	0.8546
433003	Gm5481	predicted gene 5481	0.9423	0.8353	0.9466
280408	Rilp	Rab interacting lysosomal protein	0.9423	0.2218	NA
69028	Mitd1	MIT, microtubule interacting and transport, domain containing 1	0.9423	0.4979	0.8193
234362	Zfp868	zinc finger protein 868	0.9422	0.5389	0.8366
225888	Suv420h1	suppressor of variegation 4-20 homolog 1 (Drosophila)	0.9422	0.5785	0.8519
73447	Wdr13	WD repeat domain 13	0.9422	0.1984	NA
69718	Ipmk	inositol polyphosphate multikinase	0.9422	0.28	NA
66263	1810014B01Rik	RIKEN cDNA 1810014B01 gene	0.9422	0.3599	NA
320560	Dennd5b	DENN/MADD domain containing 5B	0.9421	0.421	NA
243574	Kbtbd8	kelch repeat and BTB (POZ) domain containing 8	0.9421	0.5251	0.8312
216873	Spag7	sperm associated antigen 7	0.9421	0.2164	NA
74206	Sipa1l3	signal-induced proliferation-associated 1 like 3	0.9421	0.5166	0.8277
56194	Prpf40a	PRP40 pre-mRNA processing factor 40 homolog A (yeast)	0.9421	0.5071	0.8234
22144	Tuba3a	tubulin, alpha 3A	0.9421	0.6725	0.8922
20773	Sptlc2	serine palmitoyltransferase, long chain base subunit 2	0.9421	0.4354	NA
228356	1110051M20Rik	RIKEN cDNA 1110051M20 gene	0.942	0.4918	0.8156
228033	Atp5g3	ATP synthase, H+ transporting, mitochondrial F0 complex, subunit C3 (subunit 9)	0.942	0.5313	0.8339
140484	Pofut1	protein O-fucosyltransferase 1	0.942	0.3484	NA
79555	BC005537	cDNA sequence BC005537	0.942	0.3244	NA
69981	Tmem30a	transmembrane protein 30A	0.942	0.5894	0.8575
69918	2610020C07Rik	RIKEN cDNA 2610020C07 gene	0.942	0.4664	NA
56384	Letm1	leucine zipper-EF-hand containing transmembrane protein 1	0.942	0.6171	0.8698
26407	Map3k4	mitogen-activated protein kinase kinase kinase 4	0.942	0.2625	NA
13382	Dld	dihydrolipoamide dehydrogenase	0.942	0.5494	0.8414
619317	D930026N18Rik	RIKEN cDNA D930026N18 gene	0.9419	0.3343	NA
236285	Lancl3	LanC lantibiotic synthetase component C-like 3 (bacterial)	0.9419	0.5618	0.8465
98956	Nat10	N-acetyltransferase 10	0.9419	0.3498	NA
77300	Raph1	Ras association (RalGDS/AF-6) and pleckstrin homology domains 1	0.9419	0.3791	NA
73469	Rnf38	ring finger protein 38	0.9419	0.2118	NA
269536	Tex10	testis expressed gene 10	0.9418	0.1634	NA
258532	Olfr373	olfactory receptor 373	0.9418	0.6682	0.8904

67391	Fundc2	FUN14 domain containing 2	0.9418	0.3136	NA
66213	Med7	mediator complex subunit 7	0.9418	0.4454	NA
12366	Casp2	caspase 2	0.9418	0.2166	NA
791308	Gm9933	predicted gene 9933	0.9417	0.7223	0.9121
226105	Cyp2c70	cytochrome P450, family 2, subfamily c, polypeptide 70	0.9417	0.7102	0.9069
100504423	LOC100504423	hypothetical protein LOC100504423	0.9416	0.3233	NA
380669	Lin28b	lin-28 homolog B (C. elegans)	0.9416	0.5984	0.8615
242259	Slc44a5	solute carrier family 44, member 5	0.9416	0.4423	NA
229279	Hnrnpa3	heterogeneous nuclear ribonucleoprotein A3	0.9416	0.4237	NA
105377	Ankrd32	ankyrin repeat domain 32	0.9416	0.2527	NA
104349	Zfp119a	zinc finger protein 119a	0.9416	0.507	0.8234
68169	A930038C07Rik	RIKEN cDNA A930038C07 gene	0.9416	0.3693	NA
57376	Smarce1	SWI/SNF related, matrix associated, actin dependent regulator of chromatin, subfamily e, member 1	0.9416	0.5479	0.8406
333329	Cngb1	cyclic nucleotide gated channel beta 1	0.9415	0.3964	NA
226823	Kctd3	potassium channel tetramerisation domain containing 3	0.9415	0.5225	0.8294
105590	Zfp957	zinc finger protein 957	0.9415	0.5224	0.8293
20315	Cxcl12	chemokine (C-X-C motif) ligand 12	0.9415	0.5648	0.8471
17454	Mov10	Moloney leukemia virus 10	0.9415	0.6468	0.8812
14390	Gabpa	GA repeat binding protein, alpha	0.9415	0.5439	0.839
13483	Dpp6	dipeptidylpeptidase 6	0.9415	0.3961	NA
77996	D730039F16Rik	RIKEN cDNA D730039F16 gene	0.9414	0.3129	NA
64580	Ndst4	N-deacetylase/N-sulfotransferase (heparin glucosaminyl) 4	0.9414	0.4279	NA
243308	A430033K04Rik	RIKEN cDNA A430033K04 gene	0.9413	0.4372	NA
75029	Purg	purine-rich element binding protein G	0.9413	0.5223	0.8293
19656	Rbmxrt	RNA binding motif protein, X chromosome retrogene	0.9413	0.4084	NA
13714	Elk4	ELK4, member of ETS oncogene family	0.9412	0.5029	0.8214
13669	Eif3a	eukaryotic translation initiation factor 3, subunit A	0.9412	0.5003	0.8197
269113	Nup54	nucleoporin 54	0.9411	0.3443	NA
56784	Ralgapa1	Ral GTPase activating protein, alpha subunit 1	0.9411	0.2151	NA
16589	Uhmk1	U2AF homology motif (UHM) kinase 1	0.9411	0.6668	0.8904
629967	Gm11677	predicted gene 11677	0.941	0.5164	0.8277
624866	Lekr1	leucine, glutamate and lysine rich 1	0.941	0.2846	NA
245827	Fat2	FAT tumor suppressor homolog 2 (Drosophila)	0.941	0.5432	0.8389
228061	Agps	alkylglycerone phosphate synthase	0.941	0.2426	NA
114893	Dcun1d1	DCN1, defective in cullin neddylation 1, domain containing 1 (S. cerevisiae)	0.941	0.455	NA
114301	Palmd	palmdelphin	0.941	0.4761	0.8097
77533	C030034I22Rik	RIKEN cDNA C030034I22 gene	0.941	0.5397	0.8367
100233175	AK010878	cDNA sequence AK010878	0.9409	0.2648	NA
654824	Ankrd37	ankyrin repeat domain 37	0.9409	0.2433	NA
226751	Cdc42bpa	CDC42 binding protein kinase alpha	0.9409	0.6679	0.8904
105787	Prkaa1	protein kinase, AMP-activated, alpha 1 catalytic subunit	0.9409	0.2143	NA
72180	Zfp661	zinc finger protein 661	0.9409	0.2205	NA
66361	Zfand1	zinc finger, AN1-type domain 1	0.9409	0.3896	NA
13800	Enah	enabled homolog (Drosophila)	0.9409	0.557	0.8452
11640	Akap1	A kinase (PRKA) anchor protein 1	0.9409	0.4191	NA
246102	Rttn	rotatin	0.9408	0.5177	0.8278
114896	Afg3l1	AFG3(ATPase family gene 3)-like 1 (yeast)	0.9408	0.2658	NA
18120	Mrpl49	mitochondrial ribosomal protein L49	0.9408	0.2439	NA
76179	Usp31	ubiquitin specific peptidase 31	0.9407	0.1795	NA

66979	Pole4	polymerase (DNA-directed), epsilon 4 (p12 subunit)	0.9407	0.7695	0.9255
15528	Hspe1	heat shock protein 1 (chaperonin 10)	0.9407	0.241	NA
12753	Clock	circadian locomotor output cycles kaput	0.9407	0.4116	NA
12750	Clk4	CDC like kinase 4	0.9407	0.5732	0.8501
319783	A730056A06Rik	RIKEN cDNA A730056A06 gene	0.9406	0.6048	0.8644
70802	Pwwp2a	PWWP domain containing 2A	0.9406	0.3233	NA
67248	Rpl39	ribosomal protein L39	0.9406	0.4179	NA
56032	Nprl2	nitrogen permease regulator-like 2 (S. cerevisiae)	0.9406	0.2804	NA
20841	Zfp143	zinc finger protein 143	0.9406	0.3321	NA
12289	Cacna1d	calcium channel, voltage-dependent, L type, alpha 1D subunit	0.9406	0.4392	NA
100216343	LOC100216343	plasma membrane Ca2+ pump interacting protein	0.9405	0.7157	0.9087
208727	Hdac4	histone deacetylase 4	0.9405	0.3651	NA
16647	Kpna2	karyopherin (importin) alpha 2	0.9405	0.3905	NA
268448	Phf12	PHD finger protein 12	0.9404	0.5463	0.8395
234353	Psd3	pleckstrin and Sec7 domain containing 3	0.9404	0.5644	0.8468
67260	Lass4	LAG1 homolog, ceramide synthase 4	0.9404	0.4028	NA
27103	Eif2ak4	eukaryotic translation initiation factor 2 alpha kinase 4	0.9404	0.3478	NA
16798	Lats1	large tumor suppressor	0.9404	0.4292	NA
241489	Pde11a	phosphodiesterase 11A	0.9403	0.7126	0.9075
93759	Sirt1	sirtuin 1 (silent mating type information regulation 2, homolog) 1 (S. cerevisiae)	0.9403	0.2809	NA
75998	5033425G24Rik	RIKEN cDNA 5033425G24 gene	0.9403	0.7313	0.9153
22710	Zfp52	zinc finger protein 52	0.9403	0.5417	0.8379
16511	Kcnh2	potassium voltage-gated channel, subfamily H (eag-related), member 2	0.9403	0.6167	0.8698
12488	Cd2ap	CD2-associated protein	0.9403	0.3666	NA
11465	Actg1	actin, gamma, cytoplasmic 1	0.9403	0.4601	0.8011
104831	Ptpn23	protein tyrosine phosphatase, non-receptor type 23	0.9402	0.3558	NA
77018	Col25a1	collagen, type XXV, alpha 1	0.9402	0.8564	0.9554
74448	Arl13a	ADP-ribosylation factor-like 13A	0.9402	0.638	0.8776
67492	Anubl1	AN1, ubiquitin-like, homolog (Xenopus laevis)	0.9402	0.499	0.8197
12339	Capn7	calpain 7	0.9402	0.4143	NA
434729	Gm5635	predicted gene 5635	0.9401	0.4405	NA
76357	Trmt5	TRM5 tRNA methyltransferase 5 homolog (S. cerevisiae)	0.9401	0.339	NA
72899	Macrod2	MACRO domain containing 2	0.9401	0.5761	0.8506
71584	Gdpd2	glycerophosphodiester phosphodiesterase domain containing 2	0.9401	0.2256	NA
16069	Igj	immunoglobulin joining chain	0.9401	0.3006	NA
13592	Ebf2	early B-cell factor 2	0.9401	0.8581	0.9562
100042480	Nhsl2	NHS-like 2	0.94	0.6956	0.9012
100042014	Gm3625	predicted gene 3625	0.9399	0.6923	0.8993
330031	Gm5106	predicted gene 5106	0.9399	0.8801	0.9631
217366	Lrrc45	leucine rich repeat containing 45	0.9399	0.2584	NA
77519	Zfp266	zinc finger protein 266	0.9399	0.4682	0.8056
76971	2810007J24Rik	RIKEN cDNA 2810007J24 gene	0.9399	0.7392	0.9177
67211	Armc10	armadillo repeat containing 10	0.9399	0.2562	NA
19684	Rdx	radixin	0.9399	0.4363	NA
668096	Gm13698	predicted gene 13698	0.9398	0.3418	NA
80385	Tusc2	tumor suppressor candidate 2	0.9398	0.3855	NA
73124	Golim4	golgi integral membrane protein 4	0.9398	0.2641	NA
17710	COX3	cytochrome c oxidase subunit III	0.9398	0.532	0.8339
106627	AI646383	expressed sequence AI646383	0.9397	0.7277	0.9135

71941	Cars2	cysteinyl-tRNA synthetase 2 (mitochondrial)(putative)	0.9397	0.3684	NA
17449	Mdh1	malate dehydrogenase 1, NAD (soluble)	0.9397	0.4224	NA
12398	Cbfa2t3	core-binding factor, runt domain, alpha subunit 2, translocated to, 3 (human)	0.9397	0.7306	0.915
382019	Zfp882	zinc finger protein 882	0.9396	0.3945	NA
109113	Uhrf2	ubiquitin-like, containing PHD and RING finger domains 2	0.9396	0.6156	0.8697
67255	Zfp422	zinc finger protein 422	0.9396	0.4459	NA
67217	2810055F11Rik	RIKEN cDNA 2810055F11 gene	0.9396	0.397	NA
66568	Rwdd3	RWD domain containing 3	0.9396	0.6392	0.8782
16179	Irak1	interleukin-1 receptor-associated kinase 1	0.9396	0.2643	NA
224088	Atp13a3	ATPase type 13A3	0.9395	0.5477	0.8405
27364	Srr	serine racemase	0.9394	0.451	NA
77900	6720473M11Rik	RIKEN cDNA 6720473M11 gene	0.9393	0.733	0.9157
67785	Zmym4	zinc finger, MYM-type 4	0.9393	0.4035	NA
14407	Gabrg3	gamma-aminobutyric acid (GABA) A receptor, subunit gamma 3	0.9393	0.5725	0.8501
546711	LOC546711	40S ribosomal protein S25-like	0.9392	0.8311	0.9455
231327	Ppat	phosphoribosyl pyrophosphate amidotransferase	0.9392	0.6813	0.8949
217869	Eif5	eukaryotic translation initiation factor 5	0.9392	0.3753	NA
212326	Fam149a	family with sequence similarity 149, member A	0.9392	0.278	NA
77622	Apex2	apurinic/apyrimidinic endonuclease 2	0.9392	0.2184	NA
329015	Atg2a	ATG2 autophagy related 2 homolog A (S. cerevisiae)	0.9391	0.4722	0.8082
214952	Rhot2	ras homolog gene family, member T2	0.9391	0.2999	NA
18120	Mrpl49	mitochondrial ribosomal protein L49	0.9391	0.2122	NA
14933	Gyk	glycerol kinase	0.9391	0.4599	0.801
213541	Ythdf2	YTH domain family 2	0.939	0.2206	NA
170930	Sumo2	SMT3 suppressor of mif two 3 homolog 2 (yeast)	0.939	0.5732	0.8501
77929	Yipf6	Yip1 domain family, member 6	0.939	0.6963	0.9015
75894	Adal	adenosine deaminase-like	0.939	0.2329	NA
72469	Plcd3	phospholipase C, delta 3	0.939	0.1488	NA
17714	Grpel2	GrpE-like 2, mitochondrial	0.939	0.5132	0.8268
14563	Gdf5	growth differentiation factor 5	0.939	0.3726	NA
68153	Gtf2e2	general transcription factor II E, polypeptide 2 (beta subunit)	0.9389	0.318	NA
16004	Igf2r	insulin-like growth factor 2 receptor	0.9389	0.2218	NA
15499	Hsf1	heat shock factor 1	0.9389	0.3553	NA
12034	Phb2	prohibitin 2	0.9389	0.2197	NA
237500	Tmtc3	transmembrane and tetratricopeptide repeat containing 3	0.9388	0.4161	NA
234515	Inpp4b	inositol polyphosphate-4-phosphatase, type II	0.9388	0.5696	0.8491
16341	Eif3e	eukaryotic translation initiation factor 3, subunit E	0.9388	0.3772	NA
100038598	Gm10386	predicted gene 10386	0.9387	0.5234	0.8298
668339	Gm9112	predicted gene 9112	0.9387	0.6535	0.8848
234076	Tmco3	transmembrane and coiled-coil domains 3	0.9387	0.4548	0.7977
60364	Donson	downstream neighbor of SON	0.9387	0.3514	NA
230967	BC046331	cDNA sequence BC046331	0.9386	0.4552	0.7978
80913	Pum2	pumilio 2 (Drosophila)	0.9386	0.5454	0.839
78088	Ankrd56	ankyrin repeat domain 56	0.9386	0.5865	0.8559
74455	Nsun6	NOL1/NOP2/Sun domain family member 6	0.9386	0.7278	0.9135
14451	Gas1	growth arrest specific 1	0.9386	0.6388	0.8779
380718	Mks1	Meckel syndrome, type 1	0.9385	0.3832	NA
226844	Mfsd7b	major facilitator superfamily domain containing 7B	0.9385	0.3337	NA
76687	Spcs3	signal peptidase complex subunit 3 homolog (S. cerevisiae)	0.9385	0.7036	0.9046

74440	4933407C03Rik	RIKEN cDNA 4933407C03 gene	0.9385	0.4241	NA
68614	Letmd1	LETM1 domain containing 1	0.9385	0.2937	NA
66264	Ccdc28b	coiled coil domain containing 28B	0.9385	0.2384	NA
18029	Nfic	nuclear factor I/C	0.9385	0.4684	0.8056
14685	Gnat1	guanine nucleotide binding protein, alpha transducing 1	0.9385	0.5267	0.8325
384864	Gm1943	WD repeat domain 70 pseudogene	0.9384	0.2766	NA
240168	Rasgrp3	RAS, guanyl releasing protein 3	0.9384	0.6443	0.8802
20273	Scn8a	sodium channel, voltage-gated, type VIII, alpha	0.9384	0.4213	NA
320473	Heatr5b	HEAT repeat containing 5B	0.9383	0.6911	0.8989
244631	Pskh1	protein serine kinase H1	0.9383	0.5447	0.839
81004	Tbl1xr1	transducin (beta)-like 1X-linked receptor 1	0.9383	0.7114	0.907
19014	Med1	mediator complex subunit 1	0.9383	0.3282	NA
17714	Grpel2	GrpE-like 2, mitochondrial	0.9383	0.4776	0.81
100038585	F830223B06Rik	RIKEN cDNA F830223B06 gene	0.9382	0.6126	0.868
635253	Usp51	ubiquitin specific protease 51	0.9382	0.2947	NA
552890	LOC552890	hypothetical LOC552890	0.9382	0.4254	NA
213311	Fbxl21	F-box and leucine-rich repeat protein 21	0.9382	0.6882	0.8976
74276	Cldnd2	claudin domain containing 2	0.9382	0.7567	0.9225
69216	Ccdc23	coiled-coil domain containing 23	0.9382	0.579	0.8519
66641	Sike1	suppressor of IKBKE 1	0.9382	0.4297	NA
20646	Snrpn	small nuclear ribonucleoprotein N	0.9382	0.4756	0.8097
269061	Cpsf7	cleavage and polyadenylation specific factor 7	0.9381	0.2978	NA
231724	Rad9b	RAD9 homolog B (S. cerevisiae)	0.9381	0.3804	NA
210992	Lpcat1	lysophosphatidylcholine acyltransferase 1	0.9381	0.3369	NA
118451	Mrps2	mitochondrial ribosomal protein S2	0.9381	0.3673	NA
110893	Slc8a3	solute carrier family 8 (sodium/calcium exchanger), member 3	0.9381	0.4683	0.8056
99100	Cep152	centrosomal protein 152	0.9381	0.2486	NA
66109	Tspan13	tetraspanin 13	0.9381	0.2544	NA
56726	Sh3bgrl	SH3-binding domain glutamic acid-rich protein like	0.9381	0.3956	NA
23964	Odz2	odd Oz/ten-m homolog 2 (Drosophila)	0.9381	0.4336	NA
21804	Tgfb1i1	transforming growth factor beta 1 induced transcript 1	0.9381	0.638	0.8776
270672	Map3k15	mitogen-activated protein kinase kinase kinase 15	0.9379	0.4432	0.7931
210297	Lrch2	leucine-rich repeats and calponin homology (CH) domain containing 2	0.9379	0.3964	NA
114604	Prdm15	PR domain containing 15	0.9379	0.7362	0.9169
67071	Rps6ka6	ribosomal protein S6 kinase polypeptide 6	0.9379	0.7269	0.913
20937	Suv39h1	suppressor of variegation 3-9 homolog 1 (Drosophila)	0.9379	0.5679	0.8485
17125	Smad1	MAD homolog 1 (Drosophila)	0.9379	0.5356	0.835
12814	Col11a1	collagen, type XI, alpha 1	0.9379	0.7445	0.9192
233335	Synm	synemin, intermediate filament protein	0.9378	0.4357	NA
77115	6030451C04Rik	RIKEN cDNA 6030451C04 gene	0.9378	0.6715	0.8917
76080	Ttpal	tocopherol (alpha) transfer protein-like	0.9378	0.6974	0.9021
72413	Kcnmb2	potassium large conductance calcium-activated channel, subfamily M, beta member 2	0.9378	0.5294	0.8332
59038	Pxmp4	peroxisomal membrane protein 4	0.9378	0.7846	0.9304
13840	Epha6	Eph receptor A6	0.9377	0.6586	0.8869
12370	Casp8	caspase 8	0.9377	0.7665	0.9251
434128	Pnmal2	PNMA-like 2	0.9376	0.7707	0.9257
319251	9630001P10Rik	RIKEN cDNA 9630001P10 gene	0.9376	0.2197	NA
269704	Zfp664	zinc finger protein 664	0.9376	0.3044	NA
109676	Ank2	ankyrin 2, brain	0.9376	0.3717	NA

107182	Btaf1	BTAF1 RNA polymerase II, B-TFIID transcription factor-associated, (Mot1 homolog, S. cerevisiae)	0.9376	0.2974	NA
18786	Plaa	phospholipase A2, activating protein	0.9376	0.4471	0.7938
11990	Atrn	attractin	0.9376	0.4665	0.8049
432768	Gm5451	predicted gene 5451	0.9375	0.4695	0.8064
140630	Ube4a	ubiquitination factor E4A, UFD2 homolog (S. cerevisiae)	0.9375	0.2636	NA
69287	Odf3	outer dense fiber of sperm tails 3	0.9375	0.7087	0.9066
67894	Fam45a	family with sequence similarity 45, member A	0.9375	0.3085	NA
66980	Zdhhc6	zinc finger, DHHC domain containing 6	0.9375	0.3632	NA
52440	Tax1bp1	Tax1 (human T-cell leukemia virus type I) binding protein 1	0.9375	0.3866	NA
14105	Srsf10	serine/arginine-rich splicing factor 10	0.9375	0.1675	NA
13434	Trdmt1	tRNA aspartic acid methyltransferase 1	0.9375	0.1853	NA
331188	Zfp781	zinc finger protein 781	0.9374	0.5748	0.8504
70885	Ints10	integrator complex subunit 10	0.9374	0.3788	NA
27528	D0H4S114	DNA segment, human D4S114	0.9374	0.5446	0.839
19157	Cyth1	cytohesin 1	0.9374	0.6668	0.8904
14365	Fzd3	frizzled homolog 3 (Drosophila)	0.9374	0.4051	NA
105445	Dock9	dedicator of cytokinesis 9	0.9373	0.2908	NA
71787	Trnau1ap	tRNA selenocysteine 1 associated protein 1	0.9373	0.4418	0.7922
70604	Dnajb14	DnaJ (Hsp40) homolog, subfamily B, member 14	0.9373	0.3889	NA
66910	Tmem107	transmembrane protein 107	0.9373	0.3427	NA
19058	Ppp3r1	protein phosphatase 3, regulatory subunit B, alpha isoform (calcineurin B, type I)	0.9373	0.2769	NA
215085	Slc35f1	solute carrier family 35, member F1	0.9372	0.2651	NA
142682	Zcchc14	zinc finger, CCHC domain containing 14	0.9372	0.5415	0.8379
77739	Adamtsl1	ADAMTS-like 1	0.9372	0.555	0.8444
76233	Dnttip1	deoxynucleotidyltransferase, terminal, interacting protein 1	0.9372	0.2855	NA
76167	Snrnp35	small nuclear ribonucleoprotein 35 (U11/U12)	0.9372	0.4468	0.7938
74020	Cpne4	copine IV	0.9372	0.7329	0.9157
72252	1700022A21Rik	glycerol-3-phosphate dehydrogenase 1-like pseudogene	0.9372	0.9154	0.9753
56878	Rbms1	RNA binding motif, single stranded interacting protein 1	0.9372	0.3997	NA
54473	Tollip	toll interacting protein	0.9372	0.5294	0.8332
54326	Elovl2	elongation of very long chain fatty acids (FEN1/Elo2, SUR4/Elo3, yeast)-like 2	0.9372	0.4649	0.8044
20975	Synj2	synaptojanin 2	0.9372	0.6213	0.8719
19116	Prlr	prolactin receptor	0.9372	0.6239	0.8726
15893	Ica1	islet cell autoantigen 1	0.9372	0.3795	NA
14979	H2-Ke6	H2-K region expressed gene 6	0.9372	0.3869	NA
13424	Dync1h1	dynein cytoplasmic 1 heavy chain 1	0.9372	0.239	NA
69694	Tatdn1	TatD DNase domain containing 1	0.9371	0.6144	0.8691
12876	Cpe	carboxypeptidase E	0.9371	0.3133	NA
225998	Rorb	RAR-related orphan receptor beta	0.937	0.4886	0.8139
93878	Pcdhb7	protocadherin beta 7	0.937	0.343	NA
78787	Usp54	ubiquitin specific peptidase 54	0.937	0.409	NA
68234	2400009B08Rik	RIKEN cDNA 2400009B08 gene	0.937	0.7481	0.9202
15490	Hsd17b7	hydroxysteroid (17-beta) dehydrogenase 7	0.937	0.4573	0.799
13026	Pcyt1a	phosphate cytidylyltransferase 1, choline, alpha isoform	0.937	0.6479	0.8816
226352	Epb4.1l5	erythrocyte protein band 4.1-like 5	0.9369	0.3089	NA
110948	Hlcs	holocarboxylase synthetase (biotin- [propriony-Coenzyme A-carboxylase (ATP-hydrolysing)] ligase)	0.9369	0.1742	NA
52589	Ncald	neurocalcin delta	0.9369	0.6377	0.8776
15481	Hspa8	heat shock protein 8	0.9369	0.4486	0.7942
14593	Ggps1	geranylgeranyl diphosphate synthase 1	0.9369	0.3021	NA

14583	Gfpt1	glutamine fructose-6-phosphate transaminase 1	0.9369	0.2305	NA
110959	Nudt19	nudix (nucleoside diphosphate linked moiety X)-type motif 19	0.9368	0.625	0.8728
107986	Ddb2	damage specific DNA binding protein 2	0.9368	0.3201	NA
102442	Dennd4a	DENN/MADD domain containing 4A	0.9368	0.3019	NA
73331	1700034P13Rik	RIKEN cDNA 1700034P13 gene	0.9368	0.7021	0.9039
56398	1500003O03Rik	RIKEN cDNA 1500003O03 gene	0.9368	0.2831	NA
19934	Rpl22	ribosomal protein L22	0.9368	0.2616	NA
268903	Nrip1	nuclear receptor interacting protein 1	0.9367	0.182	NA
106504	Stk38	serine/threonine kinase 38	0.9367	0.6294	0.8745
104015	Synj1	synaptojanin 1	0.9367	0.3372	NA
75712	Tmem14a	transmembrane protein 14A	0.9367	0.3648	NA
72826	Fam76b	family with sequence similarity 76, member B	0.9367	0.2321	NA
66971	Cdk5rap1	CDK5 regulatory subunit associated protein 1	0.9367	0.5717	0.8497
17977	Ncoa1	nuclear receptor coactivator 1	0.9367	0.3575	NA
208618	Etl4	enhancer trap locus 4	0.9366	0.5919	0.8584
69944	2810021J22Rik	RIKEN cDNA 2810021J22 gene	0.9366	0.4144	NA
67224	Med29	mediator complex subunit 29	0.9366	0.2019	NA
26441	Psma4	proteasome (prosome, macropain) subunit, alpha type 4	0.9366	0.4957	0.8181
100036525	LOC100036525	hypothetical LOC100036525	0.9365	0.581	0.8527
231070	Insig1	insulin induced gene 1	0.9365	0.1657	NA
70054	Ccdc89	coiled-coil domain containing 89	0.9365	0.2719	NA
19281	Ptprt	protein tyrosine phosphatase, receptor type, T	0.9365	0.5421	0.8379
19273	Ptpru	protein tyrosine phosphatase, receptor type, U	0.9365	0.4493	0.7943
76742	Snx27	sorting nexin family member 27	0.9364	0.3464	NA
71916	Dus4l	dihydrouridine synthase 4-like (S. cerevisiae)	0.9364	0.3863	NA
69663	Ddx51	DEAD (Asp-Glu-Ala-Asp) box polypeptide 51	0.9364	0.1187	NA
67636	Lyrm5	LYR motif containing 5	0.9364	0.3603	NA
13383	Dlg1	discs, large homolog 1 (Drosophila)	0.9364	0.6216	0.8719
98741	Kcnb2	potassium voltage gated channel, Shab-related subfamily, member 2	0.9363	0.5086	0.8242
94220	Cnnm4	cyclin M4	0.9363	0.2347	NA
72982	Tmem138	transmembrane protein 138	0.9363	0.6585	0.8869
70790	Ubr5	ubiquitin protein ligase E3 component n-recognin 5	0.9363	0.3003	NA
353211	Prune2	prune homolog 2 (Drosophila)	0.9362	0.7746	0.9266
239188	Enox1	ecto-NOX disulfide-thiol exchanger 1	0.9362	0.896	0.9699
77106	Tmem181a	transmembrane protein 181A	0.9362	0.2243	NA
75424	Zfp820	zinc finger protein 820	0.9362	0.6048	0.8644
100042840	Gm16501	predicted gene 16501	0.9361	0.6252	0.8728
269702	Mphosph9	M-phase phosphoprotein 9	0.9361	0.77	0.9257
100986	Akap9	A kinase (PRKA) anchor protein (yotiao) 9	0.9361	0.2684	NA
93837	Dach2	dachshund 2 (Drosophila)	0.9361	0.355	NA
74610	Abcb8	ATP-binding cassette, sub-family B (MDR/TAP), member 8	0.9361	0.2812	NA
56440	Snx1	sorting nexin 1	0.9361	0.2739	NA
23966	Odz4	odd Oz/ten-m homolog 4 (Drosophila)	0.9361	0.2446	NA
277973	Slc9a5	solute carrier family 9 (sodium/hydrogen exchanger), member 5	0.936	0.3828	NA
223693	Tmem184b	transmembrane protein 184b	0.936	0.4248	NA
26939	Polr3e	polymerase (RNA) III (DNA directed) polypeptide E	0.936	0.3252	NA
17127	Smad3	MAD homolog 3 (Drosophila)	0.936	0.3905	NA
12643	Chad	chondroadherin	0.936	0.8445	0.9504
12190	Brca2	breast cancer 2	0.936	0.2574	NA

545611	Gm13298	predicted gene 13298	0.9359	0.4742	0.809
258392	Olfr190	olfactory receptor 190	0.9359	0.5037	0.8221
230235	6430704M03Rik	RIKEN cDNA 6430704M03 gene	0.9359	0.5062	0.823
80334	Kcnip4	Kv channel interacting protein 4	0.9359	0.5741	0.8503
547253	Parp14	poly (ADP-ribose) polymerase family, member 14	0.9358	0.2283	NA
320940	Atp11c	ATPase, class VI, type 11C	0.9358	0.4061	NA
226419	Dyrk3	dual-specificity tyrosine-(Y)-phosphorylation regulated kinase 3	0.9358	0.3413	NA
75387	Sirt4	sirtuin 4 (silent mating type information regulation 2 homolog) 4 (S. cerevisiae)	0.9358	0.3385	NA
22763	Zfr	zinc finger RNA binding protein	0.9358	0.7173	0.9096
21745	Tep1	telomerase associated protein 1	0.9358	0.4096	NA
18647	Cdk14	cyclin-dependent kinase 14	0.9358	0.263	NA
268741	Tox4	TOX high mobility group box family member 4	0.9357	0.629	0.8745
104015	Synj1	synaptojanin 1	0.9357	0.361	NA
93672	Il24	interleukin 24	0.9357	0.4158	NA
76646	Wdr38	WD repeat domain 38	0.9357	0.4798	0.8101
71682	Wdr27	WD repeat domain 27	0.9357	0.5723	0.85
69556	Bod1	biorientation of chromosomes in cell division 1	0.9357	0.4073	NA
30843	Fbxl12	F-box and leucine-rich repeat protein 12	0.9357	0.1571	NA
20844	Stam	signal transducing adaptor molecule (SH3 domain and ITAM motif) 1	0.9357	0.5265	0.8323
258581	Olfr1030	olfactory receptor 1030	0.9356	0.5565	0.8448
75580	Zbtb4	zinc finger and BTB domain containing 4	0.9356	0.6419	0.8791
21872	Tjp1	tight junction protein 1	0.9356	0.3955	NA
235493	BC031353	cDNA sequence BC031353	0.9355	0.1171	NA
69770	1600002K03Rik	RIKEN cDNA 1600002K03 gene	0.9355	0.3551	NA
244682	Cntn5	contactin 5	0.9354	0.2981	NA
210711	Mcmbp	MCM (minichromosome maintenance deficient) binding protein	0.9354	0.1183	NA
106618	Wdr90	WD repeat domain 90	0.9354	0.1308	NA
69181	Dyrk2	dual-specificity tyrosine-(Y)-phosphorylation regulated kinase 2	0.9354	0.3935	NA
26408	Map3k5	mitogen-activated protein kinase kinase kinase 5	0.9354	0.2552	NA
11773	Ap2m1	adaptor protein complex AP-2, mu1	0.9354	0.2583	NA
665251	Gm7555	predicted gene 7555	0.9353	0.3283	NA
209497	Tmem164	transmembrane protein 164	0.9353	0.6331	0.8758
52323	Klhl7	kelch-like 7 (Drosophila)	0.9353	0.2767	NA
633057	Gm7102	predicted gene 7102	0.9352	0.7337	0.9159
329628	Fat4	FAT tumor suppressor homolog 4 (Drosophila)	0.9352	0.2941	NA
320487	Heatr5a	HEAT repeat containing 5A	0.9352	0.2595	NA
105782	Scrib	scribbled homolog (Drosophila)	0.9352	0.1597	NA
97130	C77080	expressed sequence C77080	0.9352	0.1503	NA
67109	Zfp787	zinc finger protein 787	0.9352	0.1726	NA
545611	Gm13298	predicted gene 13298	0.9351	0.4006	NA
237716	Gpr75	G protein-coupled receptor 75	0.9351	0.6485	0.8817
235132	Zbtb44	zinc finger and BTB domain containing 44	0.9351	0.3574	NA
218103	Slc17a2	solute carrier family 17 (sodium phosphate), member 2	0.9351	0.7907	0.9333
75276	Ppp1r1c	protein phosphatase 1, regulatory (inhibitor) subunit 1C	0.9351	0.572	0.8499
74998	Rab11fip2	RAB11 family interacting protein 2 (class I)	0.9351	0.4135	NA
66593	Diablo	diablo homolog (Drosophila)	0.935	0.4598	0.801
18671	Abcb1a	ATP-binding cassette, sub-family B (MDR/TAP), member 1A	0.935	0.2978	NA
16646	Kpna1	karyopherin (importin) alpha 1	0.935	0.3663	NA
381062	2210404J11Rik	RIKEN cDNA 2210404J11 gene	0.9349	0.5563	0.8448

319195	Rpl17	ribosomal protein L17	0.9349	0.7869	0.9314
77053	Sun1	Sad1 and UNC84 domain containing 1	0.9349	0.5213	0.8286
74142	Lonp1	lon peptidase 1, mitochondrial	0.9349	0.1375	NA
320191	Hook3	hook homolog 3 (Drosophila)	0.9348	0.34	NA
319186	Hist1h2bm	histone cluster 1, H2bm	0.9348	0.2624	NA
233726	Ipo7	importin 7	0.9348	0.7	0.9033
109186	6720427I07Rik	RIKEN cDNA 6720427I07 gene	0.9348	0.3299	NA
97783	C76336	expressed sequence C76336	0.9348	0.5963	0.861
73174	Tbkbp1	TBK1 binding protein 1	0.9348	0.3305	NA
70461	Crtc3	CREB regulated transcription coactivator 3	0.9348	0.2688	NA
16522	Kcnj6	potassium inwardly-rectifying channel, subfamily J, member 6	0.9348	0.4198	0.7795
214254	Nudt15	nudix (nucleoside diphosphate linked moiety X)-type motif 15	0.9347	0.3718	NA
71833	Dcaf7	DDB1 and CUL4 associated factor 7	0.9347	0.4222	0.7801
66583	Exosc1	exosome component 1	0.9347	0.5524	0.8433
11538	Adnp	activity-dependent neuroprotective protein	0.9347	0.223	NA
665251	Gm7555	predicted gene 7555	0.9346	0.2918	NA
224938	Pja2	praja 2, RING-H2 motif containing	0.9346	0.4858	0.8127
69487	2310003L22Rik	RIKEN cDNA 2310003L22 gene	0.9346	0.4185	0.7787
13929	Amz2	archaelysin family metallopeptidase 2	0.9346	0.2451	NA
433791	Gm13251	predicted gene 13251	0.9345	0.7076	0.9064
228139	P2rx3	purinergic receptor P2X, ligand-gated ion channel, 3	0.9345	0.2321	NA
212127	2810046L04Rik	RIKEN cDNA 2810046L04 gene	0.9345	0.3618	NA
192160	Casc3	cancer susceptibility candidate 3	0.9345	0.06692	NA
105440	Kctd9	potassium channel tetramerisation domain containing 9	0.9345	0.2639	NA
19229	Ptk2b	PTK2 protein tyrosine kinase 2 beta	0.9345	0.6772	0.893
100038441	Gm10419	predicted gene 10419	0.9344	0.3777	NA
319211	Nol4	nucleolar protein 4	0.9344	0.676	0.8928
71764	C2cd2l	C2 calcium-dependent domain containing 2-like	0.9344	0.3304	NA
67134	Nop56	NOP56 ribonucleoprotein homolog (yeast)	0.9344	0.2591	NA
433966	5730422E09Rik	RIKEN cDNA 5730422E09 gene	0.9343	0.271	NA
381062	2210404J11Rik	RIKEN cDNA 2210404J11 gene	0.9343	0.2065	NA
320595	Phf8	PHD finger protein 8	0.9343	0.6309	0.8751
67952	Tomm20	translocase of outer mitochondrial membrane 20 homolog (yeast)	0.9343	0.5861	0.8556
18764	Pkd2	polycystic kidney disease 2	0.9343	0.2676	NA
15115	Hars	histidyl-tRNA synthetase	0.9343	0.5042	0.8221
320452	P4ha3	procollagen-proline, 2-oxoglutarate 4-dioxygenase (proline 4-hydroxylase), alpha polypeptide III	0.9342	0.6132	0.8683
231855	C330006K01Rik	RIKEN cDNA C330006K01 gene	0.9342	0.3836	NA
77827	Krba1	KRAB-A domain containing 1	0.9342	0.2529	NA
68278	Ddx39	DEAD (Asp-Glu-Ala-Asp) box polypeptide 39	0.9342	0.09889	NA
67102	D16Ertd472e	DNA segment, Chr 16, ERATO Doi 472, expressed	0.9342	0.1399	NA
67096	Mmachc	methylmalonic aciduria cblC type, with homocystinuria	0.9342	0.3096	NA
56453	Mbtps1	membrane-bound transcription factor peptidase, site 1	0.9342	0.1941	NA
381823	Apold1	apolipoprotein L domain containing 1	0.9341	0.4499	0.7943
20492	Slbp	stem-loop binding protein	0.9341	0.6	0.8632
231386	Ythdc1	YTH domain containing 1	0.934	0.2908	NA
76299	Erp44	endoplasmic reticulum protein 44	0.934	0.2516	NA
68339	Ccdc88c	coiled-coil domain containing 88C	0.934	0.5326	0.8339
57321	Terf2ip	telomeric repeat binding factor 2, interacting protein	0.934	0.6424	0.8793
14479	Usp15	ubiquitin specific peptidase 15	0.934	0.2606	NA

271697	Cdk15	cyclin-dependent kinase 15	0.9339	0.5188	0.8282
239985	Arid1b	AT rich interactive domain 1B (SWI-like)	0.9339	0.3462	NA
98402	Sh3bp4	SH3-domain binding protein 4	0.9339	0.7117	0.9073
69352	Necab1	N-terminal EF-hand calcium binding protein 1	0.9339	0.6062	0.8645
13610	S1pr3	sphingosine-1-phosphate receptor 3	0.9339	0.5451	0.839
381346	Gm13194	predicted gene 13194	0.9338	0.6638	0.8896
109135	Plekha5	pleckstrin homology domain containing, family A member 5	0.9338	0.3794	NA
74106	Dcaf6	DDB1 and CUL4 associated factor 6	0.9338	0.2656	NA
69928	Apitd1	apoptosis-inducing, TAF9-like domain 1	0.9338	0.4564	0.7987
67292	Pigc	phosphatidylinositol glycan anchor biosynthesis, class C	0.9338	0.5072	0.8234
54644	Otud5	OTU domain containing 5	0.9338	0.2426	NA
20649	Sntb1	syntrophin, basic 1	0.9338	0.4505	0.7945
19654	Rbm6	RNA binding motif protein 6	0.9338	0.3211	NA
12585	Cdr2	cerebellar degeneration-related 2	0.9338	0.3334	NA
382423	Atxn7l3b	ataxin 7-like 3B	0.9337	0.3536	NA
329165	Abi2	abl-interactor 2	0.9337	0.3479	NA
244713	Zfp317	zinc finger protein 317	0.9337	0.2678	NA
211586	Tfdp2	transcription factor Dp 2	0.9337	0.2329	NA
75729	4933432B09Rik	RIKEN cDNA 4933432B09 gene	0.9337	0.5378	0.8359
13171	Dbt	dihydrolipoamide branched chain transacylase E2	0.9337	0.5506	0.8424
545389	Cep170	centrosomal protein 170	0.9336	0.2609	NA
74238	Mterfd3	MTERF domain containing 3	0.9336	0.2827	NA
74154	Unkl	unkempt-like (Drosophila)	0.9336	0.5589	0.8459
11813	Apoc2	apolipoprotein C-II	0.9336	0.7332	0.9158
100049569	A730015C16Rik	RIKEN cDNA A730015C16 gene	0.9335	0.6667	0.8904
380705	Tmem102	transmembrane protein 102	0.9335	0.6602	0.888
232087	Mat2a	methionine adenosyltransferase II, alpha	0.9335	0.12	NA
76877	Rab36	RAB36, member RAS oncogene family	0.9335	0.6354	0.8771
72167	Thumpd2	THUMP domain containing 2	0.9335	0.218	NA
53885	Nphp1	nephronophthisis 1 (juvenile) homolog (human)	0.9335	0.6409	0.8788
30841	Kdm2b	lysine (K)-specific demethylase 2B	0.9335	0.3773	NA
18575	Pde1c	phosphodiesterase 1C	0.9335	0.3592	NA
14339	Aktip	thymoma viral proto-oncogene 1 interacting protein	0.9335	0.5382	0.8362
633640	Gm7120	predicted gene 7120	0.9334	0.42	0.7795
381038	Parl	presenilin associated, rhomboid-like	0.9334	0.4243	0.7819
231507	Plac8	placenta-specific 8	0.9334	0.7006	0.9033
212712	Satb2	special AT-rich sequence binding protein 2	0.9334	0.6041	0.8644
108058	Camk2d	calcium/calmodulin-dependent protein kinase II, delta	0.9334	0.5136	0.8268
70385	Ccdc99	coiled-coil domain containing 99	0.9334	0.655	0.8854
14433	Gapdh	glyceraldehyde-3-phosphate dehydrogenase	0.9334	0.1471	NA
546336	Prrg1	proline rich Gla (G-carboxyglutamic acid) 1	0.9333	0.5311	0.8339
277939	C2cd3	C2 calcium-dependent domain containing 3	0.9333	0.6794	0.8944
269261	Rpl12	ribosomal protein L12	0.9333	0.2807	NA
236727	Slc9a7	solute carrier family 9 (sodium/hydrogen exchanger), member 7	0.9333	0.4321	0.7859
235674	Acaa1b	acetyl-Coenzyme A acyltransferase 1B	0.9333	0.3209	NA
212531	Sh3bgrl2	SH3 domain binding glutamic acid-rich protein like 2	0.9333	0.3952	NA
98401	AI594674	expressed sequence AI594674	0.9333	0.4307	0.7846
72258	Kcnk10	potassium channel, subfamily K, member 10	0.9333	0.6295	0.8745
67252	Cap2	CAP, adenylate cyclase-associated protein, 2 (yeast)	0.9333	0.3457	NA

16599	Klf3		Kruppel-like factor 3 (basic)	0.9333	0.1863	NA
328110	Prpf39		PRP39 pre-mRNA processing factor 39 homolog (yeast)	0.9332	0.1592	NA
321022	Cdv3		carnitine deficiency-associated gene expressed in ventricle 3	0.9332	0.5858	0.8555
232533	Stk38l		serine/threonine kinase 38 like	0.9332	0.1626	NA
70047	Trnt1		tRNA nucleotidyl transferase, CCA-adding, 1	0.9332	0.5979	0.8613
19647	Rbbp6		retinoblastoma binding protein 6	0.9332	0.2031	NA
15357	Hmgcr		3-hydroxy-3-methylglutaryl-Coenzyme A reductase	0.9332	0.4081	0.773
674810	LOC674810		60S ribosomal protein L3-like	0.9331	0.3304	NA
319876	Cobll1		Cobl-like 1	0.9331	0.666	0.8904
237246	BC022960		cDNA sequence BC022960	0.9331	0.371	NA
232086	Tmem150a		transmembrane protein 150A	0.9331	0.2479	NA
69232	Qrich1		glutamine-rich 1	0.9331	0.4033	0.7708
66875	1200016B10Rik		RIKEN cDNA 1200016B10 gene	0.9331	0.236	NA
57912	Cdc42se1		CDC42 small effector 1	0.9331	0.6249	0.8728
21827	Thbs3		thrombospondin 3	0.9331	0.3825	NA
546143	Gm5918		predicted gene 5918	0.933	0.2775	NA
233833	Tnrc6a		trinucleotide repeat containing 6a	0.933	0.2425	NA
22034	Traf6		TNF receptor-associated factor 6	0.933	0.6063	0.8645
217588	Mbip		MAP3K12 binding inhibitory protein 1	0.9329	0.368	NA
93893	Pcdhb22		protocadherin beta 22	0.9329	0.2077	NA
68267	Slc25a22		solute carrier family 25 (mitochondrial carrier, glutamate), member 22	0.9329	0.8351	0.9466
258507	Olfr96		olfactory receptor 96	0.9328	0.8388	0.9482
68134	Upf3b		UPF3 regulator of nonsense transcripts homolog B (yeast)	0.9328	0.2837	NA
67712	Slc25a37		solute carrier family 25, member 37	0.9328	0.6108	0.8671
11637	Ak2		adenylate kinase 2	0.9328	0.3326	NA
217666	L2hgdh		L-2-hydroxyglutarate dehydrogenase	0.9327	0.7261	0.9126
170772	Glcci1		glucocorticoid induced transcript 1	0.9327	0.2491	NA
22210	Ube2b		ubiquitin-conjugating enzyme E2B, RAD6 homology (S. cerevisiae)	0.9327	0.3605	NA
13196	Asap1		ArfGAP with SH# domain, ankyrin repeat and PH domain1	0.9327	0.2699	NA
12234	Btrc		beta-transducin repeat containing protein	0.9327	0.3033	NA
629591	Gm6987		predicted pseudogene 6987	0.9326	0.8502	0.9531
329584	Gm14462		predicted gene 14462	0.9326	0.4009	0.7703
77956	A930026B05Rik		RIKEN cDNA A930026B05 gene	0.9326	0.4011	0.7703
66559	Metap1d		methionyl aminopeptidase type 1D (mitochondrial)	0.9326	0.1829	NA
18674	Slc25a3		solute carrier family 25 (mitochondrial carrier, phosphate carrier), member 3	0.9326	0.2645	NA
13560	E4f1		E4F transcription factor 1	0.9326	0.1489	NA
100040724	Mirg		miRNA containing gene	0.9325	0.3271	NA
237178	Ppef1		protein phosphatase with EF hand calcium-binding domain 1	0.9325	0.7416	0.918
57377	Mogs		mannosyl-oligosaccharide glucosidase	0.9325	0.1957	NA
28109	D10Wsu102e		DNA segment, Chr 10, Wayne State University 102, expressed	0.9325	0.3668	NA
12361	Cask		calcium/calmodulin-dependent serine protein kinase (MAGUK family)	0.9325	0.579	0.8519
11740	Slc25a5		solute carrier family 25 (mitochondrial carrier, adenine nucleotide translocator), member 5	0.9325	0.289	NA
595136	Ndufs5		NADH dehydrogenase (ubiquinone) Fe-S protein 5	0.9324	0.239	NA
77626	Smpd4		sphingomyelin phosphodiesterase 4	0.9324	0.1465	NA
17434	Mocs2		molybdenum cofactor synthesis 2	0.9324	0.2822	NA
268297	Scml4		sex comb on midleg-like 4 (Drosophila)	0.9323	0.6572	0.8863
71545	9030625G05Rik		RIKEN cDNA 9030625G05 gene	0.9323	0.5917	0.8584
67952	Tomm20		translocase of outer mitochondrial membrane 20 homolog (yeast)	0.9323	0.3999	0.7702
57438		Mar-07	membrane-associated ring finger (C3HC4) 7	0.9323	0.2514	NA

373864	Col27a1	collagen, type XXVII, alpha 1	0.9322	0.4099	0.7739
245684	Cnksr2	connector enhancer of kinase suppressor of Ras 2	0.9322	0.2543	NA
171273	Vmn1r217	vomeronasal 1 receptor 217	0.9322	0.865	0.9584
108655	Foxp1	forkhead box P1	0.9322	0.5694	0.8491
104776	Aldh6a1	aldehyde dehydrogenase family 6, subfamily A1	0.9322	0.5359	0.835
75616	2810008M24Rik	RIKEN cDNA 2810008M24 gene	0.9322	0.5995	0.8626
56484	Foxo3	forkhead box O3	0.9322	0.569	0.8488
433931	Pigg	phosphatidylinositol glycan anchor biosynthesis, class G	0.9321	0.5597	0.8459
233532	Rsf1	remodeling and spacing factor 1	0.9321	0.34	NA
70369	Bag5	BCL2-associated athanogene 5	0.9321	0.2999	NA
67640	4930465K10Rik	RIKEN cDNA 4930465K10 gene	0.9321	0.3415	NA
23833	Cd52	CD52 antigen	0.9321	0.7377	0.9172
22042	Tfrc	transferrin receptor	0.9321	0.2881	NA
11949	Atp5c1	ATP synthase, H+ transporting, mitochondrial F1 complex, gamma polypeptide 1	0.9321	0.7917	0.9333
328113	A230055C15	hypothetical protein A230055C15	0.932	0.3332	NA
244141	Nars2	asparaginyl-tRNA synthetase 2 (mitochondrial)(putative)	0.932	0.4714	0.8077
240595	Kcnv2	potassium channel, subfamily V, member 2	0.932	0.3518	NA
240038	Gm4944	predicted gene 4944	0.932	0.2596	NA
235132	Zbtb44	zinc finger and BTB domain containing 44	0.932	0.328	NA
234875	Ttc13	tetratricopeptide repeat domain 13	0.932	0.235	NA
14426	Galnt4	UDP-N-acetyl-alpha-D-galactosamine:polypeptide N-acetylgalactosaminyltransferase 4	0.932	0.1489	NA
320650	9530056K15Rik	RIKEN cDNA 9530056K15 gene	0.9319	0.2492	NA
100978	Nfxl1	nuclear transcription factor, X-box binding-like 1	0.9319	0.3318	NA
13207	Ddx5	DEAD (Asp-Glu-Ala-Asp) box polypeptide 5	0.9319	0.2312	NA
225358	Fam13b	family with sequence similarity 13, member B	0.9318	0.5319	0.8339
218734	3830406C13Rik	RIKEN cDNA 3830406C13 gene	0.9318	0.5208	0.8286
16880	Lifr	leukemia inhibitory factor receptor	0.9318	0.224	NA
258055	Olfr524	olfactory receptor 524	0.9317	0.7901	0.933
234371	Tmem161a	transmembrane protein 161A	0.9317	0.3926	0.7651
140781	Myh7	myosin, heavy polypeptide 7, cardiac muscle, beta	0.9317	0.6264	0.873
66433	Chchd7	coiled-coil-helix-coiled-coil-helix domain containing 7	0.9317	0.1627	NA
54122	Uevld	UEV and lactate/malate dehyrogenase domains	0.9317	0.5653	0.8476
15382	Hnrnpa1	heterogeneous nuclear ribonucleoprotein A1	0.9317	0.5775	0.8514
13221	Defa-rs12	defensin, alpha, related sequence 12	0.9317	0.775	0.9266
227746	Rabepk	Rab9 effector protein with kelch motifs	0.9316	0.2957	NA
224111	Ubxn7	UBX domain protein 7	0.9316	0.535	0.835
83431	Ndel1	nuclear distribution gene E-like homolog 1 (A. nidulans)	0.9316	0.4814	0.8108
76007	Zmym2	zinc finger, MYM-type 2	0.9316	0.269	NA
16801	Arhgef1	Rho guanine nucleotide exchange factor (GEF) 1	0.9316	0.5453	0.839
319189	Hist2h2bb	histone cluster 2, H2bb	0.9315	0.6755	0.8928
118454	Gjc2	gap junction protein, gamma 2	0.9315	0.06651	NA
100678	Psph	phosphoserine phosphatase	0.9315	0.2209	NA
69674	Mif4gd	MIF4G domain containing	0.9315	0.3151	NA
56380	Arid3b	AT rich interactive domain 3B (BRIGHT-like)	0.9315	0.5197	0.8284
16348	Invs	inversin	0.9315	0.4464	0.7938
212531	Sh3bgrl2	SH3 domain binding glutamic acid-rich protein like 2	0.9314	0.6744	0.8924
74035	Nol9	nucleolar protein 9	0.9314	0.723	0.9121
66204	Acyp1	acylphosphatase 1, erythrocyte (common) type	0.9314	0.8765	0.962
11545	Parp1	poly (ADP-ribose) polymerase family, member 1	0.9314	0.2542	NA

330660	Btbd16	BTB (POZ) domain containing 16	0.9313	0.4519	0.7951
240041	Zfp945	zinc finger protein 945	0.9313	0.2776	NA
224129	Adcy5	adenylate cyclase 5	0.9313	0.4625	0.8026
104625	Cnot6	CCR4-NOT transcription complex, subunit 6	0.9313	0.233	NA
78784	Celf3	VCUGBP, Elav-like family member 3	0.9313	0.4398	0.7908
74577	Glb1l	galactosidase, beta 1-like	0.9313	0.4118	0.7747
54343	Atf7ip	activating transcription factor 7 interacting protein	0.9313	0.314	NA
22612	Yes1	Yamaguchi sarcoma viral (v-yes) oncogene homolog 1	0.9313	0.5861	0.8556
20595	Smn1	survival motor neuron 1	0.9313	0.6514	0.8833
330908	Opcml	opioid binding protein/cell adhesion molecule-like	0.9311	0.4269	0.7825
229697	Cym	chymosin	0.9311	0.5802	0.8527
210711	Mcmbp	MCM (minichromosome maintenance deficient) binding protein	0.9311	0.1261	NA
72139	2610044O15Rik	RIKEN cDNA 2610044O15 gene	0.9311	0.665	0.8903
57138	Slc12a5	solute carrier family 12, member 5	0.9311	0.438	0.7894
20908	Stx3	syntaxin 3	0.9311	0.8083	0.9394
13688	Eif4ebp2	eukaryotic translation initiation factor 4E binding protein 2	0.9311	0.6203	0.8718
13367	Diap1	diaphanous homolog 1 (Drosophila)	0.9311	0.5102	0.8251
244810	AW551984	expressed sequence AW551984	0.931	0.3933	0.7655
240354	Malt1	mucosa associated lymphoid tissue lymphoma translocation gene 1	0.931	0.4061	0.7721
69851	2010007E15Rik	RIKEN cDNA 2010007E15 gene	0.931	0.6798	0.8944
67622	Mxra7	matrix-remodelling associated 7	0.931	0.6474	0.8814
24060	Slc35a1	solute carrier family 35 (CMP-sialic acid transporter), member 1	0.931	0.4117	0.7747
17129	Smad5	MAD homolog 5 (Drosophila)	0.931	0.383	NA
243937	Zfp536	zinc finger protein 536	0.9309	0.2039	NA
104859	Tecpr2	tectonin beta-propeller repeat containing 2	0.9309	0.2822	NA
59050	Nsa2	NSA2 ribosome biogenesis homolog (S. cerevisiae)	0.9309	0.2067	NA
14870	Gstp1	glutathione S-transferase, pi 1	0.9309	0.2486	NA
668319	Gm9104	predicted gene 9104	0.9308	0.156	NA
239731	Rimbp3	RIMS binding protein 3	0.9308	0.2226	NA
74741	5730419I09Rik	RIKEN cDNA 5730419I09 gene	0.9308	0.5886	0.8572
16876	Lhx9	LIM homeobox protein 9	0.9308	0.4381	0.7894
15975	Ifnar1	interferon (alpha and beta) receptor 1	0.9308	0.5636	0.8465
13496	Arid3a	AT rich interactive domain 3A (BRIGHT-like)	0.9308	0.4414	0.7921
545156	Kalrn	kalirin, RhoGEF kinase	0.9307	0.3644	NA
268729	Gm626	predicted gene 626	0.9307	0.1977	NA
112415	C030039L03Rik	RIKEN cDNA C030039L03 gene	0.9307	0.2697	NA
73349	1700042G15Rik	RIKEN cDNA 1700042G15 gene	0.9307	0.8351	0.9466
52666	Arhgef25	Rho guanine nucleotide exchange factor (GEF) 25	0.9307	0.1821	NA
399572	E430010N07Rik	RIKEN cDNA E430010N07 gene	0.9306	0.6386	0.8779
381677	Vgf	VGF nerve growth factor inducible	0.9306	0.2494	NA
224585	Zfp160	zinc finger protein 160	0.9306	0.5728	0.8501
67883	Uxs1	UDP-glucuronate decarboxylase 1	0.9306	0.4119	0.7747
100039707	Gm2382	predicted gene 2382	0.9305	0.2494	NA
277360	Prex1	phosphatidylinositol-3,4,5-trisphosphate-dependent Rac exchange factor 1	0.9305	0.3097	NA
75869	Arl5b	ADP-ribosylation factor-like 5B	0.9305	0.2837	NA
12862	Cox6a2	cytochrome c oxidase, subunit VI a, polypeptide 2	0.9305	0.6472	0.8812
225432	Rbm27	RNA binding motif protein 27	0.9304	0.492	0.8157
223254	Farp1	FERM, RhoGEF (Arhgef) and pleckstrin domain protein 1 (chondrocyte-derived)	0.9303	0.2592	NA
76899	Golga1	golgi autoantigen, golgin subfamily a, 1	0.9303	0.3399	NA

74230	1700016K19Rik	RIKEN cDNA 1700016K19 gene	0.9303	0.4203	0.7797
74190	1200009I06Rik	RIKEN cDNA 1200009I06 gene	0.9303	0.5518	0.8432
67118	Bfar	bifunctional apoptosis regulator	0.9303	0.3017	NA
26409	Map3k7	mitogen-activated protein kinase kinase kinase 7	0.9303	0.3872	0.7611
18582	Pde6d	phosphodiesterase 6D, cGMP-specific, rod, delta	0.9303	0.3327	NA
13000	Csnk2a2	casein kinase 2, alpha prime polypeptide	0.9303	0.2174	NA
226144	Erlin1	ER lipid raft associated 1	0.9302	0.1939	NA
75538	Fam71e1	family with sequence similarity 71, member E1	0.9302	0.3582	NA
68176	6230427J02Rik	RIKEN cDNA 6230427J02 gene	0.9302	0.6943	0.9006
15463	Agfg1	ArfGAP with FG repeats 1	0.9301	0.2256	NA
434632	BC085271	cDNA sequence BC085271	0.93	0.299	NA
235461	Fam63b	family with sequence similarity 63, member B	0.93	0.2723	NA
69606	Mtfmt	mitochondrial methionyl-tRNA formyltransferase	0.93	0.2185	NA
68268	Zdhhc21	zinc finger, DHHC domain containing 21	0.93	0.1467	NA
13998	Fgd6	FYVE, RhoGEF and PH domain containing 6	0.93	0.3967	0.7675
330301	Zfp786	zinc finger protein 786	0.9299	0.3766	NA
235567	Dnajc13	DnaJ (Hsp40) homolog, subfamily C, member 13	0.9299	0.1789	NA
105268	AU016916	expressed sequence AU016916	0.9299	0.1213	NA
73132	Slc25a16	solute carrier family 25 (mitochondrial carrier, Graves disease autoantigen), member 16	0.9299	0.3526	NA
20229	Sat1	spermidine/spermine N1-acetyl transferase 1	0.9299	0.5709	0.8492
233040	Fbxo27	F-box protein 27	0.9298	0.2195	NA
246694	Hps5	Hermansky-Pudlak syndrome 5 homolog (human)	0.9297	0.191	NA
14697	Gnb5	guanine nucleotide binding protein (G protein), beta 5	0.9297	0.5298	0.8332
100036524	C430017C20Rik	RIKEN cDNA C430017C20 gene	0.9296	0.5743	0.8503
380780	Serpina11	serine (or cysteine) peptidase inhibitor, clade A (alpha-1 antiproteinase, antitrypsin), member 11	0.9296	0.6985	0.9029
99003	Qser1	glutamine and serine rich 1	0.9296	0.2086	NA
71452	Ankrd40	ankyrin repeat domain 40	0.9296	0.5632	0.8465
16882	Lig3	ligase III, DNA, ATP-dependent	0.9296	0.3034	NA
14593	Ggps1	geranylgeranyl diphosphate synthase 1	0.9296	0.2599	NA
433387	Rpl21-ps1	ribosomal protein 21, pseudogene 1	0.9295	0.5647	0.8471
232333	Slc6a1	solute carrier family 6 (neurotransmitter transporter, GABA), member 1	0.9295	0.3003	NA
217379	Ubxn2a	UBX domain protein 2A	0.9295	0.3975	0.7683
77782	Polq	polymerase (DNA directed), theta	0.9295	0.1322	NA
74243	2210009G21Rik	RIKEN cDNA 2210009G21 gene	0.9295	0.3875	0.7611
20620	Plk2	polo-like kinase 2 (Drosophila)	0.9295	0.4321	0.7859
17190	Mbd1	methyl-CpG binding domain protein 1	0.9295	0.08595	NA
14915	Guca2a	guanylate cyclase activator 2a (guanylin)	0.9295	0.6221	0.8719
11911	Atf4	activating transcription factor 4	0.9295	0.4857	0.8127
233328	Lrrk1	leucine-rich repeat kinase 1	0.9294	0.4793	0.8101
228980	Taf4a	TAF4A RNA polymerase II, TATA box binding protein (TBP)-associated factor	0.9294	0.1898	NA
227731	Slc25a25	solute carrier family 25 (mitochondrial carrier, phosphate carrier), member 25	0.9294	0.3346	NA
110197	Dgkg	diacylglycerol kinase, gamma	0.9294	0.2824	NA
70620	Ube2v2	ubiquitin-conjugating enzyme E2 variant 2	0.9294	0.515	0.8275
65969	Cubn	cubilin (intrinsic factor-cobalamin receptor)	0.9294	0.7343	0.9162
29809	Rabgap1l	RAB GTPase activating protein 1-like	0.9294	0.1289	NA
21833	Thra	thyroid hormone receptor alpha	0.9294	0.198	NA
100503726	LOC100503726	hypothetical protein LOC100503726	0.9293	0.6348	0.8769
243911	Kirrel2	kin of IRRE like 2 (Drosophila)	0.9293	0.403	0.7708
74455	Nsun6	NOL1/NOP2/Sun domain family member 6	0.9293	0.3579	NA

12552	Cdh11	cadherin 11	0.9293	0.3479	NA
75180	4930538K18Rik	RIKEN cDNA 4930538K18 gene	0.9292	0.7106	0.9069
67427	Rps20	ribosomal protein S20	0.9292	0.3621	NA
67171	Dram2	VDNA-damage regulated autophagy modulator 2	0.9292	0.415	0.7764
53378	Sdcbp	syndecan binding protein	0.9292	0.412	0.7747
100038734	Gm10845	predicted gene 10845	0.9291	0.4576	0.7991
68497	1110018G07Rik	RIKEN cDNA 1110018G07 gene	0.9291	0.07919	NA
57321	Terf2ip	telomeric repeat binding factor 2, interacting protein	0.9291	0.2637	NA
170833	Hook2	hook homolog 2 (Drosophila)	0.929	0.2304	NA
54380	Smarcal1	SWI/SNF related matrix associated, actin dependent regulator of chromatin, subfamily a-like 1	0.929	0.2556	NA
53881	Slc5a3	solute carrier family 5 (inositol transporters), member 3	0.929	0.5546	0.8444
14924	Magi1	membrane associated guanylate kinase, WW and PDZ domain containing 1	0.929	0.1269	NA
78887	Sfi1	Sfi1 homolog, spindle assembly associated (yeast)	0.9289	0.8266	0.9448
55948	Sfn	stratifin	0.9289	0.711	0.907
237943	Gpatch8	G patch domain containing 8	0.9288	0.2097	NA
214580	Pstk	phosphoseryl-tRNA kinase	0.9288	0.2343	NA
109624	Cald1	caldesmon 1	0.9288	0.5356	0.835
74326	Hnrnpr	heterogeneous nuclear ribonucleoprotein R	0.9288	0.2219	NA
70356	St13	suppression of tumorigenicity 13	0.9288	0.1362	NA
67143	Ikzf5	IKAROS family zinc finger 5	0.9288	0.15	NA
56376	Pdlim5	PDZ and LIM domain 5	0.9288	0.5778	0.8514
23942	Mta2	metastasis-associated gene family, member 2	0.9288	0.4369	0.7888
11735	Ank3	ankyrin 3, epithelial	0.9288	0.3869	0.761
76484	Kndc1	kinase non-catalytic C-lobe domain (KIND) containing 1	0.9287	0.6258	0.8728
74112	Usp16	ubiquitin specific peptidase 16	0.9287	0.2329	NA
68792	Srpx2	sushi-repeat-containing protein, X-linked 2	0.9287	0.4151	0.7765
16536	Kcnq2	potassium voltage-gated channel, subfamily Q, member 2	0.9287	0.3968	0.7675
14772	Grk4	G protein-coupled receptor kinase 4	0.9287	0.1759	NA
12868	Cox8a	cytochrome c oxidase, subunit VIIIa	0.9287	0.3657	NA
320632	Snrnp200	small nuclear ribonucleoprotein 200 (U5)	0.9286	0.1297	NA
224648	Uhrf1bp1	UHRF1 (ICBP90) binding protein 1	0.9286	0.289	NA
72886	Ccdc94	coiled-coil domain containing 94	0.9286	0.3743	0.7548
64297	Gprc5b	G protein-coupled receptor, family C, group 5, member B	0.9286	0.5224	0.8293
19289	Igdcc3	immunoglobulin superfamily, DCC subclass, member 3	0.9286	0.3319	NA
217951	Tmem196	transmembrane protein 196	0.9285	0.4569	0.7987
209478	Tbc1d12	TBC1D12: TBC1 domain family, member 12	0.9285	0.6248	0.8728
381760	Ssbp1	single-stranded DNA binding protein 1	0.9284	0.191	NA
83603	Elovl4	elongation of very long chain fatty acids (FEN1/Elo2, SUR4/Elo3, yeast)-like 4	0.9284	0.07993	NA
76073	Pcgf5	polycomb group ring finger 5	0.9284	0.3726	0.7537
19646	Rbbp4	retinoblastoma binding protein 4	0.9284	0.654	0.8851
19248	Ptpn12	protein tyrosine phosphatase, non-receptor type 12	0.9284	0.2151	NA
272027	Tstd2	thiosulfate sulfurtransferase (rhodanese)-like domain containing 2	0.9283	0.4471	0.7938
216227	Slc17a8	solute carrier family 17 (sodium-dependent inorganic phosphate cotransporter), member 8	0.9283	0.5251	0.8312
210126	Lpp	LIM domain containing preferred translocation partner in lipoma	0.9283	0.7379	0.9173
99334	Zscan29	zinc finger SCAN domains 29	0.9283	0.3845	0.7606
71701	Pnpt1	polyribonucleotide nucleotidyltransferase 1	0.9283	0.08166	NA
52696	Zwint	ZW10 interactor	0.9283	0.2053	NA
14455	Gas5	growth arrest specific 5	0.9283	0.8654	0.9584
625098	Slc38a6	solute carrier family 38, member 6	0.9282	0.5043	0.8221

231912	Katnal1	katanin p60 subunit A-like 1	0.9282	0.4819	0.8108
212391	Lcor	ligand dependent nuclear receptor corepressor	0.9282	0.489	0.8142
56086	Set	SET nuclear oncogene	0.9282	0.2814	NA
18545	Pcp2	Purkinje cell protein 2 (L7)	0.9282	0.1872	NA
18294	Ogg1	8-oxoguanine DNA-glycosylase 1	0.9282	0.3445	NA
407824	BC020402	cDNA sequence BC020402	0.9281	0.4009	0.7703
69368	Wdfy1	WD repeat and FYVE domain containing 1	0.9281	0.4295	0.7843
626123	9130004C02Rik	RIKEN cDNA 9130004C02 gene	0.928	0.5696	0.8491
382030	Tmem188	transmembrane protein 188	0.928	0.2773	NA
381286	Serpinb3c	serine (or cysteine) peptidase inhibitor, clade B, member 3C	0.928	0.6673	0.8904
237436	Gas2l3	growth arrest-specific 2 like 3	0.928	0.3206	NA
207965	Gm71	predicted gene 71	0.928	0.1814	NA
100604	Lrrc8c	leucine rich repeat containing 8 family, member C	0.928	0.5129	0.8267
19090	Prkdc	protein kinase, DNA activated, catalytic polypeptide	0.928	0.2162	NA
319160	Hist1h4k	histone cluster 1, H4k	0.9279	0.6142	0.8691
231128	Fam193a	family with sequence similarity 193, member A	0.9279	0.259	NA
227682	Trub2	TruB pseudouridine (psi) synthase homolog 2 (E. coli)	0.9279	0.274	NA
217194	Klhl11	kelch-like 11 (Drosophila)	0.9279	0.7423	0.9184
78785	Clip4	CAP-GLY domain containing linker protein family, member 4	0.9279	0.5192	0.8282
74477	4933427D14Rik	RIKEN cDNA 4933427D14 gene	0.9279	0.3961	0.7671
71514	Sfpq	splicing factor proline/glutamine rich (polypyrimidine tract binding protein associated)	0.9279	0.2054	NA
68364	0610030E20Rik	RIKEN cDNA 0610030E20 gene	0.9279	0.2821	NA
21907	Nr2e1	nuclear receptor subfamily 2, group E, member 1	0.9279	0.1277	NA
15387	Hnrnpk	heterogeneous nuclear ribonucleoprotein K	0.9279	0.3395	NA
13370	Dio1	deiodinase, iodothyronine, type I	0.9279	0.641	0.8788
382099	Gm5161	predicted pseudogene 5161	0.9278	0.5746	0.8504
217718	Nek9	NIMA (never in mitosis gene a)-related expressed kinase 9	0.9278	0.07455	NA
74349	Fam160a2	family with sequence similarity 160, member A2	0.9278	0.3041	NA
68112	Sdccag3	serologically defined colon cancer antigen 3	0.9278	0.4624	0.8026
67921	Ube2f	ubiquitin-conjugating enzyme E2F (putative)	0.9278	0.3622	NA
52846	D1Bwg0212e	DNA segment, Chr 1, Brigham & Women's Genetics 0212 expressed	0.9278	0.3039	NA
72199	Mms19	MMS19 (MET18 S. cerevisiae)	0.9277	0.1733	NA
59024	Med12	mediator of RNA polymerase II transcription, subunit 12 homolog (yeast)	0.9277	0.3572	NA
320064	D130017N08Rik	RIKEN cDNA D130017N08 gene	0.9276	0.5851	0.855
238247	Arid4a	AT rich interactive domain 4A (RBP1-like)	0.9276	0.3812	0.7596
78283	Mtap7d2	MAP7 domain containing 2	0.9276	0.2835	NA
77979	B230104C14Rik	RIKEN cDNA B230104C14 gene	0.9276	0.6219	0.8719
71793	Ints12	integrator complex subunit 12	0.9276	0.2906	NA
68263	Pdhb	pyruvate dehydrogenase (lipoamide) beta	0.9276	0.2483	NA
67433	Ccdc127	coiled-coil domain containing 127	0.9276	0.3327	NA
54561	Nap1l3	nucleosome assembly protein 1-like 3	0.9276	0.2632	NA
19663	Rbpms	RNA binding protein gene with multiple splicing	0.9276	0.3914	0.7643
12291	Cacna1g	calcium channel, voltage-dependent, T type, alpha 1G subunit	0.9276	0.3859	0.761
319195	Rpl17	ribosomal protein L17	0.9275	0.5847	0.8549
241568	Lrrc4c	leucine rich repeat containing 4C	0.9275	0.2339	NA
237362	Npffr1	neuropeptide FF receptor 1	0.9275	0.7484	0.9204
235184	BC024479	cDNA sequence BC024479	0.9275	0.356	NA
208659	Fam20a	family with sequence similarity 20, member A	0.9275	0.5238	0.83
66226	Trappc2	trafficking protein particle complex 2	0.9275	0.4037	0.771

19704	Upf1	UPF1 regulator of nonsense transcripts homolog (yeast)	0.9275	0.5748	0.8504
19221	Ptgfrn	prostaglandin F2 receptor negative regulator	0.9275	0.1702	NA
13400	Dmpk	dystrophia myotonica-protein kinase	0.9275	0.7833	0.9299
11829	Aqp4	aquaporin 4	0.9275	0.3559	NA
232533	Stk38l	serine/threonine kinase 38 like	0.9274	0.2074	NA
78926	Gas2l1	growth arrest-specific 2 like 1	0.9274	0.771	0.9258
76251	0610007P08Rik	RIKEN cDNA 0610007P08 gene	0.9274	0.1938	NA
21687	Tek	endothelial-specific receptor tyrosine kinase	0.9274	0.4024	0.7707
330998	Ankrd34c	ankyrin repeat domain 34C	0.9273	0.4579	0.7994
75782	Lca5	Leber congenital amaurosis 5 (human)	0.9273	0.6232	0.8724
66165	Bccip	BRCA2 and CDKN1A interacting protein	0.9273	0.235	NA
407828	BC023969	cDNA sequence BC023969	0.9272	0.504	0.8221
330814	Lphn1	latrophilin 1	0.9272	0.451	0.7948
219189	1300010F03Rik	RIKEN cDNA 1300010F03 gene	0.9272	0.2774	NA
78921	9130019O22Rik	RIKEN cDNA 9130019O22 gene	0.9272	0.6479	0.8816
75422	Mettl5	methyltransferase like 5	0.9272	0.4137	0.7754
24128	Xrn2	5'-3' exoribonuclease 2	0.9272	0.1484	NA
258593	Olfr700	olfactory receptor 700	0.9271	0.655	0.8854
666173	Vps13b	vacuolar protein sorting 13B (yeast)	0.927	0.4972	0.8193
383436	Gm5244	ribosomal protein, large, P1 pseudogene	0.927	0.3024	NA
73945	Otud4	OTU domain containing 4	0.927	0.3191	NA
22185	U2af2	U2 small nuclear ribonucleoprotein auxiliary factor (U2AF) 2	0.927	0.5279	0.8332
20744	Strbp	spermatid perinuclear RNA binding protein	0.927	0.1929	NA
13684	Eif4e	eukaryotic translation initiation factor 4E	0.927	0.3472	NA
270627	Taf1	TAF1 RNA polymerase II, TATA box binding protein (TBP)-associated factor	0.9269	0.7925	0.9333
665466	Gm7644	predicted gene 7644	0.9268	0.7684	0.9255
269614	Pank4	pantothenate kinase 4	0.9268	0.258	NA
71833	Dcaf7	DDB1 and CUL4 associated factor 7	0.9268	0.4748	0.8094
66291	1810030N24Rik	RIKEN cDNA 1810030N24 gene	0.9268	0.3522	NA
27058	Srp9	signal recognition particle 9	0.9268	0.5205	0.8286
75747	Sesn3	sestrin 3	0.9267	0.4346	0.7879
67769	Gpatch2	G patch domain containing 2	0.9267	0.6414	0.8788
66432	Slc7a6os	solute carrier family 7, member 6 opposite strand	0.9267	0.2666	NA
21752	Tert	telomerase reverse transcriptase	0.9267	0.805	0.9381
83814	Nedd4l	neural precursor cell expressed, developmentally down-regulated gene 4-like	0.9266	0.2007	NA
78815	5031420N21Rik	RIKEN cDNA 5031420N21 gene	0.9266	0.5511	0.8428
22157	Tulp1	tubby like protein 1	0.9266	0.2759	NA
23885	Gmcl1	germ cell-less homolog 1 (Drosophila)	0.9265	0.2025	NA
320860	B130021B11Rik	RIKEN cDNA B130021B11 gene	0.9264	0.2466	NA
109880	Braf	Braf transforming gene	0.9264	0.1835	NA
104080	Nxph4	neurexophilin 4	0.9264	0.6365	0.8776
66384	Srp19	signal recognition particle 19	0.9264	0.5165	0.8277
14272	Fnta	farnesyltransferase, CAAX box, alpha	0.9264	0.2983	NA
56332	Amotl2	angiomotin-like 2	0.9263	0.07689	NA
19159	Cyth3	cytohesin 3	0.9263	0.4214	0.7798
12876	Cpe	carboxypeptidase E	0.9263	0.4477	0.7938
319955	Ercc6	excision repair cross-complementing rodent repair deficiency, complementation group 6	0.9262	0.1117	NA
245847	Amdhd2	amidohydrolase domain containing 2	0.9262	0.461	0.8018
213011	Zfp583	zinc finger protein 583	0.9262	0.2864	NA

546837	Cyp2j7-ps	cytochrome P450, family 2, subfamily j, polypeptide 7, pseudogene	0.9261	0.6304	0.8749
74365	Lonrf3	LON peptidase N-terminal domain and ring finger 3	0.9261	0.4308	0.7846
74076	4933406C10Rik	RIKEN cDNA 4933406C10 gene	0.9261	0.3365	NA
71780	Isyna1	myo-inositol 1-phosphate synthase A1	0.9261	0.4929	0.8159
67623	Tm7sf3	transmembrane 7 superfamily member 3	0.9261	0.4459	0.7938
19133	Prph2	peripherin 2	0.9261	0.2719	NA
11909	Atf2	activating transcription factor 2	0.9261	0.5355	0.835
435791	Gm13271	predicted gene 13271	0.926	0.6128	0.8681
387314	Tmtc1	transmembrane and tetratricopeptide repeat containing 1	0.926	0.4737	0.8088
278679	Apol7b	apolipoprotein L 7b	0.926	0.2616	NA
78902	4833447P13Rik	RIKEN cDNA 4833447P13 gene	0.926	0.5317	0.8339
77781	Epm2aip1	EPM2A (laforin) interacting protein 1	0.926	0.4543	0.7973
74132	Rnf6	ring finger protein (C3H2C3 type) 6	0.926	0.1668	NA
73668	Ttc21b	tetratricopeptide repeat domain 21B	0.926	0.2005	NA
70237	Bhlhb9	basic helix-loop-helix domain containing, class B9	0.926	0.4541	0.7973
68817	Ddi2	DNA-damage inducible protein 2	0.926	0.2909	NA
67540	4931428A05Rik	RIKEN cDNA 4931428A05 gene	0.926	0.6049	0.8644
110521	Hivep1	human immunodeficiency virus type I enhancer binding protein 1	0.9259	0.5287	0.8332
396184	Flrt1	fibronectin leucine rich transmembrane protein 1	0.9258	0.3604	0.7467
76022	Gon4l	gon-4-like (C.elegans)	0.9258	0.4075	0.7726
13006	Smc3	structural maintenace of chromosomes 3	0.9258	0.381	0.7594
100042947	Gm14816	predicted gene 14816	0.9257	0.7818	0.929
73728	Psd	pleckstrin and Sec7 domain containing	0.9257	0.4255	0.7823
70802	Pwwp2a	PWWP domain containing 2A	0.9257	0.4796	0.8101
791273	B630006N21Rik	RIKEN cDNA B630006N21 gene	0.9256	0.5437	0.839
237411	Zfp938	zinc finger protein 938	0.9256	0.397	0.7677
69940	Exoc1	exocyst complex component 1	0.9256	0.09913	NA
14011	Etv6	ets variant gene 6 (TEL oncogene)	0.9256	0.3018	NA
320644	C130030K03Rik	RIKEN cDNA C130030K03 gene	0.9255	0.5117	0.826
110876	Scn2a1	sodium channel, voltage-gated, type II, alpha 1	0.9255	0.3187	NA
76654	Upp2	uridine phosphorylase 2	0.9255	0.4877	0.8135
69612	2310037I24Rik	RIKEN cDNA 2310037I24 gene	0.9255	0.1778	NA
14732	Gpam	glycerol-3-phosphate acyltransferase, mitochondrial	0.9255	0.2785	NA
238799	Tnpo1	transportin 1	0.9254	0.3319	NA
235574	Atp2c1	ATPase, Ca++-sequestering	0.9254	0.1929	NA
68693	Hnrnpul2	heterogeneous nuclear ribonucleoprotein U-like 2	0.9254	0.1836	NA
67418	Ppil4	peptidylprolyl isomerase (cyclophilin)-like 4	0.9254	0.2024	NA
56220	Zfp386	zinc finger protein 386 (Kruppel-like)	0.9254	0.4159	0.7768
21406	Tcf12	transcription factor 12	0.9254	0.2861	NA
20473	Six3	sine oculis-related homeobox 3 homolog (Drosophila)	0.9254	0.6353	0.877
241950	Bbs12	Bardet-Biedl syndrome 12 (human)	0.9253	0.09603	NA
237868	Sarm1	sterile alpha and HEAT/Armadillo motif containing 1	0.9253	0.5455	0.839
170643	Kirrel	kin of IRRE like (Drosophila)	0.9253	0.1041	NA
108699	Chn1	chimerin (chimaerin) 1	0.9253	0.2748	NA
94089	Trim7	tripartite motif-containing 7	0.9253	0.7241	0.9126
83762	Otof	otoferlin	0.9253	0.6273	0.8736
17761	Mtap7	microtubule-associated protein 7	0.9253	0.1795	NA
17134	Mafg	v-maf musculoaponeurotic fibrosarcoma oncogene family, protein G (avian)	0.9253	0.1106	NA
75732	Iqcd	IQ motif containing D	0.9252	0.2322	NA

71900	Tmem106b	transmembrane protein 106B	0.9252	0.5264	0.8323
66272	Cox16	COX16 cytochrome c oxidase assembly homolog (S. cerevisiae)	0.9252	0.1231	NA
22680	Zfp207	zinc finger protein 207	0.9252	0.3924	0.765
245446	Slitrk4	SLIT and NTRK-like family, member 4	0.9251	0.3624	0.7483
74183	2310042D19Rik	RIKEN cDNA 2310042D19 gene	0.9251	0.5	0.8197
56348	Hsd17b12	hydroxysteroid (17-beta) dehydrogenase 12	0.9251	0.2709	NA
15493	Hsd3b2	hydroxy-delta-5-steroid dehydrogenase, 3 beta- and steroid delta-isomerase 2	0.9251	0.3821	0.7596
629059	Fam124a	family with sequence similarity 124, member A	0.925	0.3361	NA
434674	Gm5631	predicted gene 5631	0.925	0.4739	0.8088
329777	Pigk	phosphatidylinositol glycan anchor biosynthesis, class K	0.925	0.4294	0.7843
243302	Gm4963	predicted gene 4963	0.925	0.3605	0.7467
170936	Zfp369	zinc finger protein 369	0.925	0.4896	0.8145
71096	Sntg1	syntrophin, gamma 1	0.925	0.278	NA
67302	Zc3h13	zinc finger CCCH type containing 13	0.925	0.3052	NA
17996	Neb	nebulin	0.925	0.5561	0.8448
14276	Folr2	folate receptor 2 (fetal)	0.925	0.6961	0.9014
319965	Cc2d1b	coiled-coil and C2 domain containing 1B	0.9249	0.3001	NA
272589	Tbcel	tubulin folding cofactor E-like	0.9249	0.4835	0.8121
245880	Wasf3	WAS protein family, member 3	0.9249	0.4898	0.8145
217378	Dnajc27	DnaJ (Hsp40) homolog, subfamily C, member 27	0.9249	0.2681	NA
70661	Sik3	SIK family kinase 3	0.9249	0.15	NA
56380	Arid3b	AT rich interactive domain 3B (BRIGHT-like)	0.9249	0.3034	NA
280621	BC089491	cDNA sequence BC089491	0.9248	0.4861	0.8127
68277	2310057M21Rik	RIKEN cDNA 2310057M21 gene	0.9248	0.2746	NA
56382	Rab9	RAB9, member RAS oncogene family	0.9248	0.2292	NA
50774	Krtap5-1	keratin associated protein 5-1	0.9248	0.5949	0.86
23962	Oasl2	2'-5' oligoadenylate synthetase-like 2	0.9248	0.5681	0.8486
19165	Psen2	presenilin 2	0.9248	0.2224	NA
677044	Gm10653	ribsomal protein S2 pseudogene	0.9247	0.3613	0.7476
320713	Mysm1	myb-like, SWIRM and MPN domains 1	0.9247	0.2179	NA
260301	Otos	otospiralin	0.9247	0.4549	0.7977
228136	Zdhhc5	zinc finger, DHHC domain containing 5	0.9247	0.1917	NA
65973	Asph	aspartate-beta-hydroxylase	0.9247	0.2083	NA
57751	Rnf25	ring finger protein 25	0.9247	0.4664	0.8049
30940	Usp25	ubiquitin specific peptidase 25	0.9247	0.3799	0.7592
21821	Ift88	intraflagellar transport 88 homolog (Chlamydomonas)	0.9247	0.2497	NA
13876	Erg	avian erythroblastosis virus E-26 (v-ets) oncogene related	0.9247	0.7714	0.9258
320825	Samd5	sterile alpha motif domain containing 5	0.9246	0.1189	NA
67203	Nde1	nuclear distribution gene E homolog 1 (A nidulans)	0.9246	0.5467	0.8397
19014	Med1	mediator complex subunit 1	0.9246	0.1577	NA
11491	Adam17	a disintegrin and metallopeptidase domain 17	0.9246	0.4683	0.8056
74493	Tnks2	tankyrase, TRF1-interacting ankyrin-related ADP-ribose polymerase 2	0.9245	0.1602	NA
56354	Dnajc7	DnaJ (Hsp40) homolog, subfamily C, member 7	0.9245	0.1943	NA
12466	Cct6a	chaperonin containing Tcp1, subunit 6a (zeta)	0.9245	0.5237	0.83
672098	Gm9548	predicted gene 9548	0.9244	0.3716	0.7535
638008	Gm7227	predicted gene 7227	0.9244	0.3445	0.7359
244891	Scaper	S phase cyclin A-associated protein in the ER	0.9244	0.4465	0.7938
109036	6230415J03Rik	RIKEN cDNA 6230415J03 gene	0.9244	0.7653	0.9251
72508	Rps6kb1	ribosomal protein S6 kinase, polypeptide 1	0.9244	0.6331	0.8758

66471	Anp32e	acidic (leucine-rich) nuclear phosphoprotein 32 family, member E	0.9244	0.7002	0.9033
66249	Pno1	partner of NOB1 homolog (S. cerevisiae)	0.9244	0.121	NA
66167	Ccdc72	coiled-coil domain containing 72	0.9244	0.3426	0.7344
22240	Dpysl3	dihydropyrimidinase-like 3	0.9244	0.5753	0.8504
12298	Cacnb4	calcium channel, voltage-dependent, beta 4 subunit	0.9244	0.4136	0.7754
22202	Ube1y1	ubiquitin-activating enzyme E1, Chr Y 1	0.9243	0.3792	0.7583
396184	Flrt1	fibronectin leucine rich transmembrane protein 1	0.9242	0.2582	NA
216961	Coro6	coronin 6	0.9242	0.5803	0.8527
71492	Bbs7	Bardet-Biedl syndrome 7 (human)	0.9242	0.1104	NA
22284	Usp9x	ubiquitin specific peptidase 9, X chromosome	0.9242	0.485	0.8126
231380	Uba6	ubiquitin-like modifier activating enzyme 6	0.9241	0.4289	0.784
83557	Lin28a	lin-28 homolog A (C. elegans)	0.9241	0.6379	0.8776
75170	4930529F24Rik	RIKEN cDNA 4930529F24 gene	0.9241	0.553	0.8438
665610	Gm7710	predicted gene 7710	0.924	0.2551	NA
67878	Tmem33	transmembrane protein 33	0.924	0.3514	0.7395
56173	Cldn14	claudin 14	0.924	0.6959	0.9013
16706	Ksr1	kinase suppressor of ras 1	0.924	0.1686	NA
108902	B3gnt1	UDP-GlcNAc:betaGal beta-1,3-N-acetylglucosaminyltransferase 1	0.9239	0.2445	NA
103967	Dnm3	dynamin 3	0.9239	0.3544	0.7422
74080	Nmnat3	nicotinamide nucleotide adenylyltransferase 3	0.9239	0.4554	0.798
66209	1110054O05Rik	RIKEN cDNA 1110054O05 gene	0.9239	0.132	NA
12469	Cct8	chaperonin containing Tcp1, subunit 8 (theta)	0.9238	0.4333	0.7872
668319	Gm9104	predicted gene 9104	0.9237	0.519	0.8282
494448	Cbx6	chromobox homolog 6	0.9237	0.2793	NA
21453	Tcof1	Treacher Collins Franceschetti syndrome 1, homolog	0.9237	0.4615	0.802
66661	Srp72	signal recognition particle 72	0.9236	0.4425	0.7928
56417	Adar	adenosine deaminase, RNA-specific	0.9236	0.1949	NA
55935	Fnbp4	formin binding protein 4	0.9236	0.2137	NA
20807	Srf	serum response factor	0.9236	0.264	NA
17527	Mpv17	MpV17 mitochondrial inner membrane protein	0.9236	0.2308	NA
237860	Ssh2	slingshot homolog 2 (Drosophila)	0.9235	0.5616	0.8465
231147	Sh3tc1	SH3 domain and tetratricopeptide repeats 1	0.9235	0.323	NA
192216	Tmem47	transmembrane protein 47	0.9235	0.615	0.8695
108043	Chrnb3	cholinergic receptor, nicotinic, beta polypeptide 3	0.9235	0.1787	NA
66404	2410001C21Rik	RIKEN cDNA 2410001C21 gene	0.9235	0.547	0.8398
20530	Slc31a2	solute carrier family 31, member 2	0.9235	0.2703	NA
12388	Ctnnd1	catenin (cadherin associated protein), delta 1	0.9235	0.3938	0.7656
100043580	Gm4532	predicted gene 4532	0.9234	0.4701	0.8068
216543	Cep68	centrosomal protein 68	0.9234	0.07311	NA
100993	AW549542	expressed sequence AW549542	0.9234	0.4089	0.7734
75610	2010109A12Rik	RIKEN cDNA 2010109A12 gene	0.9234	0.4254	0.7823
66204	Acyp1	acylphosphatase 1, erythrocyte (common) type	0.9234	0.7568	0.9225
18213	Ntrk3	neurotrophic tyrosine kinase, receptor, type 3	0.9234	0.3778	0.7569
233865	D430042O09Rik	RIKEN cDNA D430042O09 gene	0.9233	0.1739	NA
224907	Dus3l	dihydrouridine synthase 3-like (S. cerevisiae)	0.9233	0.2152	NA
217262	Abca9	ATP-binding cassette, sub-family A (ABC1), member 9	0.9233	0.3505	0.7392
75410	Wbp7	WW domain binding protein 7	0.9233	0.1085	NA
71369	Krtap16-10	keratin associated protein 16-10	0.9233	0.5192	0.8282
15234	Hgf	hepatocyte growth factor	0.9233	0.4855	0.8127

80910	Gpr84	G protein-coupled receptor 84	0.9232	0.4765	0.8099
78618	Acap2	ArfGAP with coiled-coil, ankyrin repeat and PH domains 2	0.9232	0.3188	NA
26419	Mapk8	mitogen-activated protein kinase 8	0.9232	0.4684	0.8056
12480	Cd1d2	CD1d2 antigen	0.9232	0.5406	0.8375
192786	Rapgef6	Rap guanine nucleotide exchange factor (GEF) 6	0.9231	0.5511	0.8428
68995	Mcts1	malignant T cell amplified sequence 1	0.9231	0.4373	0.7891
66960	Fam188a	family with sequence similarity 188, member A	0.9231	0.2399	NA
53382	Txnl1	thioredoxin-like 1	0.9231	0.3153	NA
52653	Nudcd2	NudC domain containing 2	0.9231	0.2678	NA
18189	Nrxn1	neurexin I	0.9231	0.1921	NA
319448	Fndc3a	fibronectin type III domain containing 3A	0.923	0.5044	0.8221
238384	Slc24a4	solute carrier family 24 (sodium/potassium/calcium exchanger), member 4	0.923	0.2886	NA
212528	Trmt1	TRM1 tRNA methyltransferase 1 homolog (S. cerevisiae)	0.923	0.1233	NA
77781	Epm2aip1	EPM2A (laforin) interacting protein 1	0.923	0.127	NA
58243	Nap1l5	nucleosome assembly protein 1-like 5	0.923	0.403	0.7708
17878	Myf6	myogenic factor 6	0.923	0.3585	0.7457
11994	Pcdh15	protocadherin 15	0.923	0.528	0.8332
320158	Zmat4	zinc finger, matrin type 4	0.9229	0.2333	NA
270106	Rpl13	ribosomal protein L13	0.9229	0.3722	0.7536
83383	Tcfap4	transcription factor AP4	0.9229	0.2462	NA
80749	Lrfn1	leucine rich repeat and fibronectin type III domain containing 1	0.9229	0.4086	0.7732
64899	Lpin3	lipin 3	0.9229	0.4463	0.7938
19771	Rlbp1	retinaldehyde binding protein 1	0.9229	0.2528	NA
668814	Gm9376	predicted gene 9376	0.9228	0.4611	0.8019
269254	Setx	senataxin	0.9228	0.2178	NA
235626	Setd2	SET domain containing 2	0.9228	0.2267	NA
218333	BC018507	cDNA sequence BC018507	0.9228	0.2464	NA
102916	AI451458	expressed sequence AI451458	0.9228	0.2436	NA
102058	Exoc8	exocyst complex component 8	0.9228	0.5113	0.826
18718	Pip4k2a	phosphatidylinositol-5-phosphate 4-kinase, type II, alpha	0.9228	0.2886	NA
11834	Aqr	aquarius	0.9228	0.2754	NA
211468	Kcnh8	potassium voltage-gated channel, subfamily H (eag-related), member 8	0.9227	0.2157	NA
66628	Thg1l	tRNA-histidine guanylyltransferase 1-like (S. cerevisiae)	0.9227	0.3617	0.7477
268564	Zbtb1	zinc finger and BTB domain containing 1	0.9226	0.09081	NA
214345	Lrrc1	leucine rich repeat containing 1	0.9226	0.4543	0.7973
212285	Arap2	ArfGAP with RhoGAP domain, ankyrin repeat and PH domain 2	0.9226	0.6371	0.8776
106248	Qtrtd1	queuine tRNA-ribosyltransferase domain containing 1	0.9226	0.258	NA
70799	Cep192	centrosomal protein 192	0.9226	0.1666	NA
69902	Mrto4	MRT4, mRNA turnover 4, homolog (S. cerevisiae)	0.9226	0.1971	NA
66681	Pgm1	phosphoglucomutase 1	0.9226	0.5093	0.8247
11937	Atp2a1	ATPase, Ca++ transporting, cardiac muscle, fast twitch 1	0.9226	0.4435	0.7931
245902	Ccdc15	coiled-coil domain containing 15	0.9225	0.4191	0.7791
229279	Hnrnpa3	heterogeneous nuclear ribonucleoprotein A3	0.9225	0.1621	NA
69149	Kbtbd3	kelch repeat and BTB (POZ) domain containing 3	0.9225	0.1594	NA
67420	Far1	fatty acyl CoA reductase 1	0.9225	0.3954	0.7669
116972	Fam57a	family with sequence similarity 57, member A	0.9224	0.4007	0.7703
68473	Mobkl1a	MOB1, Mps One Binder kinase activator-like 1A (yeast)	0.9224	0.6897	0.8984
269423	3110057O12Rik	RIKEN cDNA 3110057O12 gene	0.9223	0.2097	NA
246179	Fktn	fukutin	0.9223	0.4251	0.7822

242409	Tmem8b	transmembrane protein 8B	0.9223	0.3092	NA
78751	Zc3h6	zinc finger CCCH type containing 6	0.9223	0.3273	0.7239
22592	Ercc5	excision repair cross-complementing rodent repair deficiency, complementation group 5	0.9223	0.304	NA
21812	Tgfbr1	transforming growth factor, beta receptor I	0.9223	0.4776	0.81
236727	Slc9a7	solute carrier family 9 (sodium/hydrogen exchanger), member 7	0.9222	0.4445	0.7936
68550	1110002N22Rik	RIKEN cDNA 1110002N22 gene	0.9222	0.3763	0.7557
12561	Cdh4	cadherin 4	0.9222	0.1873	NA
227674	Ddx31	DEAD/H (Asp-Glu-Ala-Asp/His) box polypeptide 31	0.9221	0.2477	NA
77922	A030005L19Rik	RIKEN cDNA A030005L19 gene	0.9221	0.3816	0.7596
353187	Nr1d2	nuclear receptor subfamily 1, group D, member 2	0.922	0.3907	0.7637
216238	Eea1	early endosome antigen 1	0.922	0.1185	NA
103537	Mbtd1	mbt domain containing 1	0.922	0.5453	0.839
67516	Kctd4	potassium channel tetramerisation domain containing 4	0.922	0.5834	0.854
271564	Vps13a	vacuolar protein sorting 13A (yeast)	0.9219	0.3232	0.7205
74602	4833428L15Rik	RIKEN cDNA 4833428L15 gene	0.9219	0.3889	0.7617
26878	B3galt2	UDP-Gal:betaGlcNAc beta 1,3-galactosyltransferase, polypeptide 2	0.9219	0.3802	0.7594
18176	Nras	neuroblastoma ras oncogene	0.9219	0.256	NA
100037278	Fam129c	family with sequence similarity 129, member C	0.9218	0.146	NA
240087	Mdc1	mediator of DNA damage checkpoint 1	0.9218	0.2538	NA
217410	Trib2	tribbles homolog 2 (Drosophila)	0.9218	0.5181	0.828
101190	AI853106	expressed sequence AI853106	0.9218	0.2195	NA
70974	Pgm2l1	phosphoglucomutase 2-like 1	0.9218	0.5891	0.8575
27354	Nbn	nibrin	0.9218	0.575	0.8504
20239	Atxn2	ataxin 2	0.9218	0.3698	0.7521
244209	Cyp2r1	cytochrome P450, family 2, subfamily r, polypeptide 1	0.9217	0.8399	0.9485
69923	Agk	acylglycerol kinase	0.9217	0.1738	NA
546648	Klhdc7b	kelch domain containing 7B	0.9216	0.6439	0.8802
320571	Atp8b5	ATPase, class I, type 8B, member 5	0.9216	0.4333	0.7872
77371	Sec24a	Sec24 related gene family, member A (S. cerevisiae)	0.9216	0.4492	0.7943
73852	D3Ertd751e	DNA segment, Chr 3, ERATO Doi 751, expressed	0.9216	0.6067	0.8646
71099	Tssk4	testis-specific serine kinase 4	0.9216	0.7196	0.9106
242235	Lrit3	leucine-rich repeat, immunoglobulin-like and transmembrane domains 3	0.9215	0.6021	0.8638
83691	Crispld1	cysteine-rich secretory protein LCCL domain containing 1	0.9215	0.4992	0.8197
67472	Mtfr1	mitochondrial fission regulator 1	0.9215	0.1237	NA
93681	Zfp192	zinc finger protein 192	0.9214	0.285	NA
70683	Utp20	UTP20, small subunit (SSU) processome component, homolog (yeast)	0.9214	0.1496	NA
54418	Fmn2	formin 2	0.9214	0.5031	0.8215
14852	Gspt1	G1 to S phase transition 1	0.9214	0.2314	NA
270198	Pfkfb4	6-phosphofructo-2-kinase/fructose-2,6-biphosphatase 4	0.9213	0.2667	NA
71740	Pvrl4	poliovirus receptor-related 4	0.9213	0.6008	0.8636
54678	Zfp108	zinc finger protein 108	0.9213	0.2943	NA
100040654	Gm2885	predicted gene 2885	0.9212	0.4716	0.8077
76566	Fam101b	family with sequence similarity 101, member B	0.9212	0.6905	0.8988
54371	Chst2	carbohydrate sulfotransferase 2	0.9212	0.562	0.8465
17756	Mtap2	microtubule-associated protein 2	0.9212	0.3311	0.7265
100042782	Gm14458	predicted gene 14458	0.9211	0.2668	NA
69798	1810044D09Rik	RIKEN cDNA 1810044D09 gene	0.9211	0.19	NA
381062	2210404J11Rik	RIKEN cDNA 2210404J11 gene	0.921	0.156	NA
211499	Tmem87a	transmembrane protein 87A	0.921	0.6342	0.8765

12070	Ngfrap1	nerve growth factor receptor (TNFRSF16) associated protein 1	0.921	0.1684	NA
240665	Ccnj	cyclin J	0.9209	0.3219	0.7202
231858	Radil	Ras association and DIL domains	0.9209	0.4458	0.7938
229227	4932438A13Rik	RIKEN cDNA 4932438A13 gene	0.9209	0.3152	0.7162
53608	Map3k6	mitogen-activated protein kinase kinase kinase 6	0.9209	0.6719	0.8919
20671	Sox17	SRY-box containing gene 17	0.9209	0.1684	NA
319269	A130040M12Rik	RIKEN cDNA A130040M12 gene	0.9208	0.447	0.7938
216613	Ccdc85a	coiled-coil domain containing 85A	0.9208	0.348	0.7385
380629	Heca	headcase homolog (Drosophila)	0.9207	0.235	NA
242408	4930412F15Rik	RIKEN cDNA 4930412F15 gene	0.9207	0.3118	NA
67727	Stx17	syntaxin 17	0.9207	0.4733	0.8088
66101	Ppih	peptidyl prolyl isomerase H	0.9207	0.3854	0.7607
414872	Zyg11b	zyg-ll homolog B (C. elegans)	0.9206	0.1314	NA
71254	Naif1	nuclear apoptosis inducing factor 1	0.9206	0.8265	0.9448
22658	Pcgf2	polycomb group ring finger 2	0.9206	0.3822	0.7596
207932	Urb1	URB1 ribosome biogenesis 1 homolog (S. cerevisiae)	0.9205	0.2799	NA
76857	Spopl	speckle-type POZ protein-like	0.9205	0.3623	0.7482
72017	Cyb5r1	cytochrome b5 reductase 1	0.9205	0.2768	NA
224829	Trerf1	transcriptional regulating factor 1	0.9204	0.6287	0.8745
57442	Kcne3	potassium voltage-gated channel, Isk-related subfamily, gene 3	0.9204	0.7352	0.9167
21429	Ubtf	upstream binding transcription factor, RNA polymerase I	0.9204	0.3598	0.7465
218490	Btf3	basic transcription factor 3	0.9203	0.3893	0.7624
80985	Trim44	tripartite motif-containing 44	0.9203	0.08576	NA
12340	Capza1	capping protein (actin filament) muscle Z-line, alpha 1	0.9203	0.2208	NA
242109	Zfp697	zinc finger protein 697	0.9202	0.4459	0.7938
235320	Zbtb16	zinc finger and BTB domain containing 16	0.9202	0.3311	0.7265
81898	Sf3b1	splicing factor 3b, subunit 1	0.9202	0.209	NA
72662	Dis3	DIS3 mitotic control homolog (S. cerevisiae)	0.9202	0.1823	NA
67429	Nudcd1	NudC domain containing 1	0.9202	0.2535	NA
103203	AI413759	expressed sequence AI413759	0.9201	0.3818	0.7596
54403	Slc4a4	solute carrier family 4 (anion exchanger), member 4	0.9201	0.3375	0.7312
675812	Zfp605	zinc finger protein 605	0.92	0.5168	0.8277
270210	Zfp651	zinc finger protein 651	0.92	0.3456	0.7361
56508	Rapgef4	Rap guanine nucleotide exchange factor (GEF) 4	0.92	0.5337	0.8343
56030	Tmem131	transmembrane protein 131	0.92	0.2039	NA
15387	Hnrnpk	heterogeneous nuclear ribonucleoprotein K	0.92	0.2968	NA
227120	Plcl1	phospholipase C-like 1	0.9199	0.5414	0.8379
224647	D17Wsu92e	DNA segment, Chr 17, Wayne State University 92, expressed	0.9199	0.5516	0.8431
103135	Pan2	PAN2 polyA specific ribonuclease subunit homolog (S. cerevisiae)	0.9199	0.1196	NA
59057	Zfp191	zinc finger protein 191	0.9199	0.3468	0.737
22026	Nr2c2	nuclear receptor subfamily 2, group C, member 2	0.9199	0.2131	NA
234663	Dync1li2	dynein, cytoplasmic 1 light intermediate chain 2	0.9198	0.1444	NA
109880	Braf	Braf transforming gene	0.9198	0.5699	0.8492
80750	N4bp1	NEDD4 binding protein 1	0.9198	0.4519	0.7951
80707	Wwox	WW domain-containing oxidoreductase	0.9198	0.2511	NA
68734	Smek1	SMEK homolog 1, suppressor of mek1 (Dictyostelium)	0.9198	0.2149	NA
68493	Ndufaf4	NADH dehydrogenase (ubiquinone) 1 alpha subcomplex, assembly factor 4	0.9198	0.1874	NA
66753	Erlec1	endoplasmic reticulum lectin 1	0.9198	0.2697	NA
57874	Ptplad1	protein tyrosine phosphatase-like A domain containing 1	0.9198	0.4745	0.809

12217	Bsn	bassoon	0.9198	0.3845	0.7606
217342	Ube2o	ubiquitin-conjugating enzyme E2O	0.9197	0.2041	NA
213011	Zfp583	zinc finger protein 583	0.9197	0.2225	NA
56758	Mbnl1	muscleblind-like 1 (Drosophila)	0.9197	0.2636	NA
30946	Abt1	activator of basal transcription 1	0.9197	0.6374	0.8776
320919	A230107N01Rik	RIKEN cDNA A230107N01 gene	0.9196	0.4812	0.8106
108062	Cstf2	cleavage stimulation factor, 3' pre-RNA subunit 2	0.9196	0.1074	NA
11308	Abi1	abl-interactor 1	0.9196	0.403	0.7708
269682	Golga3	golgi autoantigen, golgin subfamily a, 3	0.9195	0.321	0.7197
243548	Prickle2	prickle homolog 2 (Drosophila)	0.9195	0.3677	0.7515
227210	Ccnyl1	cyclin Y-like 1	0.9195	0.05554	NA
77890	6720407P12Rik	RIKEN cDNA 6720407P12 gene	0.9195	0.308	0.7112
22640	Zfp1	zinc finger protein 1	0.9195	0.3068	0.7108
13436	Dnmt3b	DNA methyltransferase 3B	0.9195	0.409	0.7735
244867	Arhgap20	Rho GTPase activating protein 20	0.9194	0.4264	0.7823
67270	Mrpl42	mitochondrial ribosomal protein L42	0.9194	0.1139	NA
50496	E2f6	E2F transcription factor 6	0.9194	0.07956	NA
15228	Foxg1	forkhead box G1	0.9194	0.3187	0.7182
12750	Clk4	CDC like kinase 4	0.9194	0.377	0.7563
666529	Gm8149	predicted gene 8149	0.9193	0.523	0.8295
321007	Serac1	serine active site containing 1	0.9193	0.2412	NA
319758	Rfx7	regulatory factor X, 7	0.9193	0.1331	NA
218756	Slc4a7	solute carrier family 4, sodium bicarbonate cotransporter, member 7	0.9193	0.195	NA
66140	Fam33a	family with sequence similarity 33, member A	0.9193	0.7198	0.9106
18720	Pip5k1a	phosphatidylinositol-4-phosphate 5-kinase, type 1 alpha	0.9193	0.4497	0.7943
12928	Crk	v-crk sarcoma virus CT10 oncogene homolog (avian)	0.9193	0.29	NA
101179	6430519N07Rik	RIKEN cDNA 6430519N07 gene	0.9192	0.7423	0.9184
70315	Hdac8	histone deacetylase 8	0.9192	0.5356	0.835
15214	Hey2	hairy/enhancer-of-split related with YRPW motif 2	0.9192	0.41	0.7739
13682	Eif4a2	eukaryotic translation initiation factor 4A2	0.9192	0.5628	0.8465
218850	D14Abb1e	DNA segment, Chr 14, Abbott 1 expressed	0.9191	0.2891	NA
211712	Pcdh9	protocadherin 9	0.9191	0.4681	0.8056
78547	E130304I02Rik	RIKEN cDNA E130304I02 gene	0.9191	0.3912	0.7643
54712	Plxnc1	plexin C1	0.9191	0.6205	0.8718
243906	Zfp14	zinc finger protein 14	0.919	0.1657	NA
83486	Rbm5	RNA binding motif protein 5	0.919	0.1268	NA
77215	Krtap5-3	keratin associated protein 5-3	0.919	0.5326	0.8339
18004	Nek1	NIMA (never in mitosis gene a)-related expressed kinase 1	0.919	0.3568	0.7443
667933	Gm8884	predicted gene 8884	0.9189	0.2897	NA
108946	Zzz3	zinc finger, ZZ domain containing 3	0.9189	0.1449	NA
71585	9130403I23Rik	RIKEN cDNA 9130403I23 gene	0.9189	0.4521	0.7951
68955	Srrm4	serine/arginine repetitive matrix 4	0.9189	0.1717	NA
66659	Acp6	acid phosphatase 6, lysophosphatidic	0.9189	0.07803	NA
266692	Cpne1	copine I	0.9188	0.4984	0.8196
217944	Rapgef5	Rap guanine nucleotide exchange factor (GEF) 5	0.9188	0.2711	NA
67035	Dnajb4	DnaJ (Hsp40) homolog, subfamily B, member 4	0.9188	0.426	0.7823
16597	Klf12	Kruppel-like factor 12	0.9188	0.3426	0.7344
329906	C030014L02	hypothetical protein C030014L02	0.9187	0.06981	NA
236511	Eif2c1	eukaryotic translation initiation factor 2C, 1	0.9187	0.3961	0.7671

320790	Chd7	chromodomain helicase DNA binding protein 7	0.9186	0.2477	NA
654788	4732440D04Rik	RIKEN cDNA 4732440D04 gene	0.9185	0.2586	NA
436493	H2-Gs10	MHC class I like protein GS10	0.9185	0.3802	0.7593
230598	Nrd1	nardilysin, N-arginine dibasic convertase, NRD convertase 1	0.9185	0.3352	0.73
218693	Paip1	polyadenylate binding protein-interacting protein 1	0.9185	0.2022	NA
71535	9030411M13Rik	RIKEN cDNA 9030411M13 gene	0.9185	0.4385	0.7896
18566	Pdcd1	programmed cell death 1	0.9185	0.7359	0.9169
217708	Lin52	lin-52 homolog (C. elegans)	0.9184	0.2748	NA
55993	Msh4	mutS homolog 4 (E. coli)	0.9184	0.2946	NA
18798	Plcb4	phospholipase C, beta 4	0.9184	0.1756	NA
18755	Prkch	protein kinase C, eta	0.9184	0.4621	0.8023
18176	Nras	neuroblastoma ras oncogene	0.9184	0.07682	NA
208647	Creb3l2	cAMP responsive element binding protein 3-like 2	0.9183	0.5108	0.8254
66276	1810009A15Rik	RIKEN cDNA 1810009A15 gene	0.9183	0.2727	NA
29807	Tpk1	thiamine pyrophosphokinase	0.9183	0.3877	0.7613
27279	Tnfrsf12a	tumor necrosis factor receptor superfamily, member 12a	0.9183	0.2533	NA
21689	Tekt1	tektin 1	0.9183	0.7071	0.9062
232334	Vgll4	vestigial like 4 (Drosophila)	0.9182	0.2875	NA
207304	Hectd1	HECT domain containing 1	0.9182	0.2291	NA
110304	Glra3	glycine receptor, alpha 3 subunit	0.9182	0.5951	0.8603
78523	Mrpl9	mitochondrial ribosomal protein L9	0.9182	0.2328	NA
52635	Esyt2	extended synaptotagmin-like protein 2	0.9182	0.1305	NA
16337	Insr	insulin receptor	0.9182	0.3895	0.7624
231151	Tada2b	transcriptional adaptor 2B	0.9181	0.5921	0.8584
26410	Map3k8	mitogen-activated protein kinase kinase kinase 8	0.9181	0.8331	0.9458
20855	Stc1	stanniocalcin 1	0.9181	0.7416	0.918
170772	Glcci1	glucocorticoid induced transcript 1	0.918	0.2375	NA
76972	2810008D09Rik	RIKEN cDNA 2810008D09 gene	0.918	0.4082	0.7731
66583	Exosc1	exosome component 1	0.918	0.3169	0.7174
66320	Tmem208	transmembrane protein 208	0.918	0.1984	NA
269180	Inpp4a	inositol polyphosphate-4-phosphatase, type I	0.9179	0.2106	NA
213541	Ythdf2	YTH domain family 2	0.9179	0.4963	0.8187
29863	Pde7b	phosphodiesterase 7B	0.9179	0.4737	0.8088
436049	Gm5741	predicted gene 5741	0.9178	0.08984	NA
66083	Setd6	SET domain containing 6	0.9178	0.09335	NA
76088	Dock8	dedicator of cytokinesis 8	0.9177	0.4732	0.8088
20817	Srpk2	serine/arginine-rich protein specific kinase 2	0.9177	0.2739	NA
100040599	Gm15319	predicted gene 15319	0.9176	0.5822	0.8533
214137	Arhgap29	Rho GTPase activating protein 29	0.9176	0.2805	NA
69181	Dyrk2	dual-specificity tyrosine-(Y)-phosphorylation regulated kinase 2	0.9176	0.3069	0.7108
54004	Diap2	diaphanous homolog 2 (Drosophila)	0.9176	0.3654	0.7496
18191	Nrxn3	neurexin III	0.9176	0.374	0.7545
100038385	F830115B05Rik	RIKEN cDNA F830115B05 gene	0.9175	0.5385	0.8364
442821	9330175M20Rik	RIKEN cDNA 9330175M20 gene	0.9175	0.2339	NA
320846	A530058N18Rik	RIKEN cDNA A530058N18 gene	0.9175	0.3579	0.7451
20983	Syt4	synaptotagmin IV	0.9175	0.381	0.7594
20088	Rps24	ribosomal protein S24	0.9175	0.3832	0.7599
11603	Agrn	agrin	0.9175	0.2183	NA
330409	Cecr2	cat eye syndrome chromosome region, candidate 2 homolog (human)	0.9174	0.6387	0.8779

328329	Mast4	microtubule associated serine/threonine kinase family member 4	0.9174	0.4253	0.7823
229898	Gbp5	guanylate binding protein 5	0.9174	0.3585	0.7457
207781	C2cd2	C2 calcium-dependent domain containing 2	0.9174	0.1306	NA
99138	Stard7	START domain containing 7	0.9174	0.1988	NA
27404	Abca8b	ATP-binding cassette, sub-family A (ABC1), member 8b	0.9174	0.07436	NA
18541	Pcnt	pericentrin (kendrin)	0.9174	0.1847	NA
12359	Cat	catalase	0.9174	0.3968	0.7675
402724	A330106F07Rik	RIKEN cDNA A330106F07 gene	0.9173	0.3123	0.7141
271377	Zbtb11	zinc finger and BTB domain containing 11	0.9173	0.2169	NA
229279	Hnrnpa3	heterogeneous nuclear ribonucleoprotein A3	0.9173	0.1732	NA
228787	Xkr7	X Kell blood group precursor related family member 7 homolog	0.9173	0.1676	NA
67382	Brd3	bromodomain containing 3	0.9173	0.2361	NA
56324	Stam2	signal transducing adaptor molecule (SH3 domain and ITAM motif) 2	0.9173	0.3273	0.7239
50779	Rgs6	regulator of G-protein signaling 6	0.9173	0.2001	NA
329324	Syt14	synaptotagmin XIV	0.9172	0.5848	0.8549
72465	Zfp131	zinc finger protein 131	0.9172	0.2997	0.7055
68185	Chchd8	coiled-coil-helix-coiled-coil-helix domain containing 8	0.9172	0.1779	NA
218236	Fam120a	family with sequence similarity 120, member A	0.9171	0.2441	NA
217995	Heatr1	HEAT repeat containing 1	0.9171	0.07073	NA
54673	Sh3glb1	SH3-domain GRB2-like B1 (endophilin)	0.9171	0.27	NA
100040711	Gm2921	predicted gene 2921	0.917	0.3242	0.7213
81702	Ankrd17	ankyrin repeat domain 17	0.917	0.2437	NA
78658	Ncapd3	non-SMC condensin II complex, subunit D3	0.917	0.1143	NA
68636	Fahd1	fumarylacetoacetate hydrolase domain containing 1	0.917	0.4733	0.8088
75210	Prr3	proline-rich polypeptide 3	0.9169	0.2386	NA
20779	Src	Rous sarcoma oncogene	0.9169	0.3792	0.7583
12572	Cdk7	cyclin-dependent kinase 7	0.9169	0.2547	NA
229503	BC023814	cDNA sequence BC023814	0.9168	0.09562	NA
114585	D17H6S53E	DNA segment, Chr 17, human D6S53E	0.9168	0.1949	NA
67771	Arpc5	actin related protein 2/3 complex, subunit 5	0.9168	0.3855	0.7607
17955	Nap1l4	nucleosome assembly protein 1-like 4	0.9168	0.6842	0.8956
14179	Fgf8	fibroblast growth factor 8	0.9168	0.05693	NA
330902	4930565O14	hypothetical protein 4930565O14	0.9167	0.3047	0.7093
213993	A630007B06Rik	RIKEN cDNA A630007B06 gene	0.9167	0.4715	0.8077
81910	Rrbp1	ribosome binding protein 1	0.9167	0.6628	0.8895
66075	Chchd3	coiled-coil-helix-coiled-coil-helix domain containing 3	0.9167	0.1555	NA
17294	Mest	mesoderm specific transcript	0.9167	0.1714	NA
330188	Ccdc63	coiled-coil domain containing 63	0.9166	0.4107	0.7742
217558	G2e3	G2/M-phase specific E3 ubiquitin ligase	0.9166	0.2382	NA
210027	Slc35f3	solute carrier family 35, member F3	0.9166	0.5098	0.825
68556	Uckl1	uridine-cytidine kinase 1-like 1	0.9166	0.4508	0.7946
27999	Fam3c	family with sequence similarity 3, member C	0.9166	0.1357	NA
22385	Baz1b	bromodomain adjacent to zinc finger domain, 1B	0.9166	0.1184	NA
19820	Rlim	ring finger protein, LIM domain interacting	0.9166	0.4369	0.7888
104570	Smek2	SMEK homolog 2, suppressor of mek1 (Dictyostelium)	0.9165	0.1546	NA
80743	Vps16	vacuolar protein sorting 16 (yeast)	0.9165	0.09988	NA
70549	Tln2	talin 2	0.9165	0.2605	NA
69612	2310037I24Rik	RIKEN cDNA 2310037I24 gene	0.9165	0.2192	NA
19013	Ppara	peroxisome proliferator activated receptor alpha	0.9165	0.4924	0.8157

100040288	Gm2692	predicted gene 2692	0.9164	0.6045	0.8644
218975	Mapk1ip1l	mitogen-activated protein kinase 1 interacting protein 1-like	0.9164	0.7028	0.9042
72565	Uaca	uveal autoantigen with coiled-coil domains and ankyrin repeats	0.9164	0.3701	0.7524
54473	Tollip	toll interacting protein	0.9164	0.2183	NA
66442	Spc25	SPC25, NDC80 kinetochore complex component, homolog (S. cerevisiae)	0.9163	0.3688	0.7521
100038635	Gm10621	predicted gene 10621	0.9162	0.4215	0.7798
320817	Atad2b	ATPase family, AAA domain containing 2B	0.9162	0.3389	0.732
100505283	LOC100505283	60S ribosomal protein L21-like	0.9161	0.1702	NA
238799	Tnpo1	transportin 1	0.9161	0.518	0.828
235442	Rab8b	RAB8B, member RAS oncogene family	0.9161	0.4267	0.7825
230863	Sh2d5	SH2 domain containing 5	0.9161	0.4156	0.7768
217340	Rnf157	ring finger protein 157	0.9161	0.547	0.8398
97230	C78228	expressed sequence C78228	0.9161	0.6733	0.8924
77469	C030034E14Rik	RIKEN cDNA C030034E14 gene	0.9161	0.3561	0.7443
68268	Zdhhc21	zinc finger, DHHC domain containing 21	0.9161	0.2718	NA
14660	Gls	glutaminase	0.9161	0.1327	NA
13618	Ednrb	endothelin receptor type B	0.9161	0.485	0.8126
238662	Gm4934	predicted gene 4934	0.916	0.6705	0.8912
237758	Zfp454	zinc finger protein 454	0.916	0.2435	NA
72927	Hepacam	hepatocyte cell adhesion molecule	0.916	0.5621	0.8465
72215	1700001P01Rik	RIKEN cDNA 1700001P01 gene	0.916	0.6992	0.9031
66559	Metap1d	methionyl aminopeptidase type 1D (mitochondrial)	0.916	0.04745	NA
16785	Rpsa	ribosomal protein SA	0.916	0.3313	0.7265
242050	Igsf10	immunoglobulin superfamily, member 10	0.9159	0.4597	0.801
210710	Gab3	growth factor receptor bound protein 2-associated protein 3	0.9159	0.1943	NA
71640	Zfp949	zinc finger protein 949	0.9159	0.4971	0.8193
19735	Rgs2	regulator of G-protein signaling 2	0.9159	0.06499	NA
18573	Pde1a	phosphodiesterase 1A, calmodulin-dependent	0.9159	0.3304	0.726
238161	Akap6	A kinase (PRKA) anchor protein 6	0.9158	0.2857	0.6982
80892	Zfhx4	zinc finger homeodomain 4	0.9158	0.2867	0.6982
68732	Lrrc16a	leucine rich repeat containing 16A	0.9158	0.5274	0.8331
12564	Cdh8	cadherin 8	0.9158	0.6629	0.8895
100040929	Gm10362	predicted gene 10362	0.9157	0.8689	0.9596
330010	Ttll10	tubulin tyrosine ligase-like family, member 10	0.9157	0.5955	0.8605
269589	Sytl1	synaptotagmin-like 1	0.9157	0.5267	0.8325
211673	Arfgef1	ADP-ribosylation factor guanine nucleotide-exchange factor 1(brefeldin A-inhibited)	0.9157	0.1171	NA
109637	Upk1a	uroplakin 1A	0.9157	0.3529	0.7408
73545	1700094D03Rik	RIKEN cDNA 1700094D03 gene	0.9157	0.123	NA
14167	Fgf12	fibroblast growth factor 12	0.9157	0.4198	0.7795
13177	Dci	dodecenoyl-Coenzyme A delta isomerase (3,2 trans-enoyl-Coenyme A isomerase)	0.9157	0.1621	NA
260299	Cadm4	cell adhesion molecule 4	0.9156	0.2964	0.7051
234728	Ftsjd1	FtsJ methyltransferase domain containing 1	0.9156	0.1064	NA
22355	Vipr2	vasoactive intestinal peptide receptor 2	0.9156	0.2021	NA
432628	Mfsd2b	major facilitator superfamily domain containing 2B	0.9155	0.2599	NA
195018	Zzef1	zinc finger, ZZ-type with EF hand domain 1	0.9155	0.2195	NA
170833	Hook2	hook homolog 2 (Drosophila)	0.9155	0.1483	NA
66283	Gkn1	gastrokine 1	0.9155	0.2458	NA
51797	Ctps	cytidine 5'-triphosphate synthase	0.9155	0.1581	NA
12617	Cenpc1	centromere protein C1	0.9155	0.2066	NA

75452	Ascc2	activating signal cointegrator 1 complex subunit 2	0.9154	0.342	0.7338
72091	Snhg7	small nucleolar RNA host gene (non-protein coding) 7	0.9154	0.1637	NA
665306	3930402G23Rik	RIKEN cDNA 3930402G23 gene	0.9153	0.7669	0.9253
268903	Nrip1	nuclear receptor interacting protein 1	0.9153	0.3055	0.7101
99543	Olfml3	olfactomedin-like 3	0.9153	0.3724	0.7536
17711	CYTB	cytochrome b	0.9153	0.3037	0.709
241322	Zbtb6	zinc finger and BTB domain containing 6	0.9152	0.1656	NA
75965	Zdhhc20	zinc finger, DHHC domain containing 20	0.9152	0.2385	NA
73953	4930421J07Rik	RIKEN cDNA 4930421J07 gene	0.9152	0.4001	0.7702
73713	Rbm20	RNA binding motif protein 20	0.9152	0.6294	0.8745
67074	Mon2	MON2 homolog (yeast)	0.9152	0.3715	0.7535
57264	Retn	resistin	0.9152	0.7226	0.9121
24014	Rnasel	ribonuclease L (2', 5'-oligoisoadenylate synthetase-dependent)	0.9152	0.5202	0.8286
433458	Gm13880	predicted gene 13880	0.9151	0.868	0.9595
107932	Chd4	chromodomain helicase DNA binding protein 4	0.9151	0.077	NA
76668	Mdh1b	malate dehydrogenase 1B, NAD (soluble)	0.9151	0.3387	0.732
73998	Herc3	hect domain and RLD 3	0.9151	0.1661	NA
56791	Ube2l6	ubiquitin-conjugating enzyme E2L 6	0.9151	0.2393	NA
20346	Sema3a	sema domain, immunoglobulin domain (Ig), short basic domain, secreted, (semaphorin) 3A	0.9151	0.4293	0.7843
11650	Alppl2	alkaline phosphatase, placental-like 2	0.9151	0.8495	0.953
108829	Jmjd1c	jumonji domain containing 1C	0.915	0.1205	NA
68092	Ncbp2	nuclear cap binding protein subunit 2	0.915	0.5668	0.8481
68364	0610030E20Rik	RIKEN cDNA 0610030E20 gene	0.9149	0.194	NA
12236	Bub1b	budding uninhibited by benzimidazoles 1 homolog, beta (S. cerevisiae)	0.9149	0.3609	0.7471
329872	Frem1	Fras1 related extracellular matrix protein 1	0.9148	0.2208	NA
269615	Plch2	phospholipase C, eta 2	0.9148	0.6204	0.8718
240442	Adnp2	ADNP homeobox 2	0.9148	0.3406	0.7328
225055	Fbxo11	F-box protein 11	0.9148	0.3391	0.7321
215493	A3galt2	alpha 1,3-galactosyltransferase 2 (isoglobotriaosylceramide synthase)	0.9148	0.325	0.7219
108943	Rg9mtd2	RNA (guanine-9-) methyltransferase domain containing 2	0.9148	0.508	0.8241
101994	Zfp828	zinc finger protein 828	0.9148	0.2256	NA
100047702	LOC100047702	battenin-like	0.9147	0.5767	0.8509
545156	Kalrn	kalirin, RhoGEF kinase	0.9147	0.09985	NA
66196	Myo19	myosin XIX	0.9147	0.1638	NA
22165	Txk	TXK tyrosine kinase	0.9147	0.4994	0.8197
20530	Slc31a2	solute carrier family 31, member 2	0.9147	0.2649	NA
12175	Bnip2	BCL2/adenovirus E1B interacting protein 2	0.9147	0.4538	0.797
223254	Farp1	FERM, RhoGEF (Arhgef) and pleckstrin domain protein 1 (chondrocyte-derived)	0.9146	0.5524	0.8433
207839	Galnt6	UDP-N-acetyl-alpha-D-galactosamine:polypeptide N-acetylgalactosaminyltransferase 6	0.9146	0.7038	0.9047
76295	Atp11b	ATPase, class VI, type 11B	0.9146	0.4653	0.8047
73898	4930429E23Rik	RIKEN cDNA 4930429E23 gene	0.9146	0.2463	NA
54562	Lrrc6	leucine rich repeat containing 6 (testis)	0.9146	0.3488	0.7388
20320	Nptn	neuroplastin	0.9146	0.3206	0.7196
100041412	Gm11938	predicted gene 11938	0.9145	0.6873	0.897
269198	Nbeal1	neurobeachin like 1	0.9145	0.1481	NA
79263	Trim39	tripartite motif-containing 39	0.9145	0.4071	0.7726
12663	Chml	choroideremia-like	0.9145	0.5639	0.8465
109181	Trip11	thyroid hormone receptor interactor 11	0.9144	0.06303	NA
70857	4921509J17Rik	RIKEN cDNA 4921509J17 gene	0.9144	0.2731	0.6904

58188	Vstm2b	V-set and transmembrane domain containing 2B	0.9144	0.8266	0.9448
381062	2210404J11Rik	RIKEN cDNA 2210404J11 gene	0.9143	0.2311	NA
278304	Zfp385c	zinc finger protein 385C	0.9143	0.1328	NA
212163	8030462N17Rik	RIKEN cDNA 8030462N17 gene	0.9143	0.3029	0.7088
53325	Banp	BTG3 associated nuclear protein	0.9142	0.1032	NA
52705	Krr1	KRR1, small subunit (SSU) processome component, homolog (yeast)	0.9142	0.113	NA
15486	Hsd17b2	hydroxysteroid (17-beta) dehydrogenase 2	0.9142	0.5956	0.8605
15446	Hpgd	hydroxyprostaglandin dehydrogenase 15 (NAD)	0.9142	0.1852	NA
13819	Epas1	endothelial PAS domain protein 1	0.9142	0.2174	NA
76815	Calcoco2	calcium binding and coiled-coil domain 2	0.9141	0.5675	0.8485
73683	Atg16l2	autophagy related 16 like 2 (S. cerevisiae)	0.9141	0.05186	NA
69077	Psmd11	proteasome (prosome, macropain) 26S subunit, non-ATPase, 11	0.9141	0.2074	NA
231600	Chfr	checkpoint with forkhead and ring finger domains	0.914	0.1371	NA
210293	Dock10	dedicator of cytokinesis 10	0.914	0.5031	0.8215
68722	1110029L17Rik	RIKEN cDNA 1110029L17 gene	0.914	0.2279	NA
226751	Cdc42bpa	CDC42 binding protein kinase alpha	0.9139	0.4906	0.8152
78016	Ccdc150	coiled-coil domain containing 150	0.9139	0.1688	NA
56086	Set	SET nuclear oncogene	0.9139	0.3939	0.7656
27418	Mkln1	muskelin 1, intracellular mediator containing kelch motifs	0.9139	0.3242	0.7212
18015	Nf1	neurofibromatosis 1	0.9139	0.5465	0.8396
13363	Dhh	desert hedgehog	0.9139	0.1849	NA
11431	Acp1	acid phosphatase 1, soluble	0.9139	0.2283	NA
382051	Pdp2	pyruvate dehyrogenase phosphatase catalytic subunit 2	0.9138	0.7086	0.9066
75605	Kdm5b	lysine (K)-specific demethylase 5B	0.9138	0.2321	NA
53325	Banp	BTG3 associated nuclear protein	0.9138	0.2293	NA
52575	Rg9mtd1	RNA (guanine-9-) methyltransferase domain containing 1	0.9138	0.3044	0.7091
140577	Ankrd6	ankyrin repeat domain 6	0.9137	0.4923	0.8157
108062	Cstf2	cleavage stimulation factor, 3' pre-RNA subunit 2	0.9137	0.342	0.7338
622474	Smok3b	sperm motility kinase 3B	0.9136	0.2839	0.6974
78757	Rictor	RPTOR independent companion of MTOR, complex 2	0.9136	0.1347	NA
56381	Spen	SPEN homolog, transcriptional regulator (Drosophila)	0.9136	0.07892	NA
12572	Cdk7	cyclin-dependent kinase 7	0.9136	0.4072	0.7726
382492	Gm12034	predicted gene 12034	0.9135	0.1233	NA
57264	Retn	resistin	0.9135	0.7521	0.9213
12449	Ccnf	cyclin F	0.9135	0.4352	0.7883
100043034	Gm13138	predicted gene 13138	0.9134	0.312	0.7139
353211	Prune2	prune homolog 2 (Drosophila)	0.9134	0.3306	0.726
330812	Rnf150	ring finger protein 150	0.9134	0.4617	0.8021
72190	2510009E07Rik	RIKEN cDNA 2510009E07 gene	0.9134	0.1355	NA
67472	Mtfr1	mitochondrial fission regulator 1	0.9134	0.1422	NA
269472	LOC269472	hypothetical LOC269472	0.9133	0.2398	NA
170823	Glmn	glomulin, FKBP associated protein	0.9133	0.1601	NA
78553	E130118H10Rik	RIKEN cDNA E130118H10 gene	0.9133	0.3255	0.7222
76238	Grhpr	glyoxylate reductase/hydroxypyruvate reductase	0.9133	0.3155	0.7165
18708	Pik3r1	phosphatidylinositol 3-kinase, regulatory subunit, polypeptide 1 (p85 alpha)	0.9133	0.4617	0.8021
100043231	Gm4303	predicted gene 4303	0.9132	0.8402	0.9485
73692	2410089E03Rik	RIKEN cDNA 2410089E03 gene	0.9132	0.2666	0.6862
67015	Ccdc91	coiled-coil domain containing 91	0.9132	0.5482	0.8406
628822	Gm6919	predicted gene 6919	0.9131	0.6008	0.8636

321006	Vprbp	Vpr (HIV-1) binding protein	0.9131	0.4003	0.7703
231583	Slc26a1	solute carrier family 26 (sulfate transporter), member 1	0.9131	0.4759	0.8097
432770	Rslcan18	regulator of sex-limitation candidate 18	0.913	0.3396	0.7325
230376	Haus6	HAUS augmin-like complex, subunit 6	0.913	0.2803	0.6954
76081	5830458C19Rik	RIKEN cDNA 5830458C19 gene	0.913	0.4674	0.8056
69617	Pitrm1	pitrilysin metallepetidase 1	0.913	0.2149	NA
18798	Plcb4	phospholipase C, beta 4	0.913	0.2282	NA
279067	Gm13777	predicted gene 13777	0.9129	0.3321	0.7269
238037	BC068281	cDNA sequence BC068281	0.9129	0.3783	0.7573
53623	Gria3	glutamate receptor, ionotropic, AMPA3 (alpha 3)	0.9129	0.1845	NA
27371	Sh2d2a	SH2 domain protein 2A	0.9129	0.6074	0.8648
258527	Olfr1368	olfactory receptor 1368	0.9128	0.512	0.8262
241943	Ccdc144b	coiled-coil domain containing 144B	0.9128	0.2689	0.6878
77570	3930401B19Rik	RIKEN cDNA 3930401B19 gene	0.9128	0.3931	0.7652
71950	Nanog	Nanog homeobox	0.9128	0.4792	0.8101
23894	Gtf2h2	general transcription factor II H, polypeptide 2	0.9128	0.3482	0.7385
17207	Mcf2l	mcf.2 transforming sequence-like	0.9128	0.3758	0.7556
101100	Ttll3	tubulin tyrosine ligase-like family, member 3	0.9127	0.2062	NA
22719	Zfp61	zinc finger protein 61	0.9127	0.4454	0.7938
320538	Ubn2	ubinuclein 2	0.9126	0.07838	NA
52705	Krr1	KRR1, small subunit (SSU) processome component, homolog (yeast)	0.9126	0.04904	NA
17910	Myo15	myosin XV	0.9126	0.3466	0.7369
622629	Gm10318	predicted gene 10318	0.9125	0.6549	0.8854
268527	Greb1	gene regulated by estrogen in breast cancer protein	0.9125	0.3948	0.7664
67121	Mastl	microtubule associated serine/threonine kinase-like	0.9125	0.4681	0.8056
271711	Tmem169	transmembrane protein 169	0.9124	0.63	0.8747
227334	Usp40	ubiquitin specific peptidase 40	0.9124	0.05854	NA
66385	Ppp1r7	protein phosphatase 1, regulatory (inhibitor) subunit 7	0.9124	0.4387	0.7899
51810	Hnrnpu	heterogeneous nuclear ribonucleoprotein U	0.9124	0.1489	NA
240255	Ythdc2	YTH domain containing 2	0.9123	0.176	NA
219257	Pcdh20	protocadherin 20	0.9123	0.4614	0.802
27226	Pla2g7	phospholipase A2, group VII (platelet-activating factor acetylhydrolase, plasma)	0.9123	0.4159	0.7768
13831	Epc1	enhancer of polycomb homolog 1 (Drosophila)	0.9123	0.4001	0.7702
229700	Rbm15	RNA binding motif protein 15	0.9122	0.2526	NA
72775	Fance	Fanconi anemia, complementation group E	0.9122	0.1196	NA
16988	Lst1	leukocyte specific transcript 1	0.9122	0.7107	0.9069
107358	Tm9sf3	transmembrane 9 superfamily member 3	0.9121	0.5112	0.8258
140810	Ttbk2	tau tubulin kinase 2	0.912	0.08969	NA
74319	1110005A03Rik	RIKEN cDNA 1110005A03 gene	0.912	0.2637	0.6843
66978	Luc7l	Luc7 homolog (S. cerevisiae)-like	0.912	0.223	NA
19290	Pura	purine rich element binding protein A	0.912	0.2136	NA
106052	Fbxo4	F-box protein 4	0.9119	0.1689	NA
11637	Ak2	adenylate kinase 2	0.9119	0.1518	NA
214230	Pak6	p21 protein (Cdc42/Rac)-activated kinase 6	0.9118	0.5203	0.8286
76626	Msi2	Musashi homolog 2 (Drosophila)	0.9118	0.09873	NA
19881	Rom1	rod outer segment membrane protein 1	0.9118	0.133	NA
16975	Lrp8	low density lipoprotein receptor-related protein 8, apolipoprotein e receptor	0.9118	0.07484	NA
15572	Elavl4	ELAV (embryonic lethal, abnormal vision, Drosophila)-like 4 (Hu antigen D)	0.9118	0.3378	0.7314
16876	Lhx9	LIM homeobox protein 9	0.9117	0.471	0.8076

207806	Gm608		predicted gene 608	0.9116	0.3365	0.7305
554160	BB283564		expressed sequence BB283564	0.9115	0.1612	NA
216154	Med16		mediator complex subunit 16	0.9115	0.1599	NA
21841	Tia1		cytotoxic granule-associated RNA binding protein 1	0.9115	0.3851	0.7607
75698	Fam35a		family with sequence similarity 35, member A	0.9114	0.3912	0.7643
74218	1700016H13Rik		RIKEN cDNA 1700016H13 gene	0.9114	0.7133	0.9076
70769	Nolc1		nucleolar and coiled-body phosphoprotein 1	0.9114	0.03181	NA
13436	Dnmt3b		DNA methyltransferase 3B	0.9114	0.231	NA
381066	Zfp948		zinc finger protein 948	0.9113	0.1938	NA
245537	Nlgn3		neuroligin 3	0.9113	0.4027	0.7708
112414	4930540M05Rik		RIKEN cDNA 4930540M05 gene	0.9113	0.5584	0.8459
12380	Cast		calpastatin	0.9113	0.06472	NA
100216455	Gm14124		predicted gene 14124	0.9112	0.7791	0.9278
232798	Leng8		leukocyte receptor cluster (LRC) member 8	0.9112	0.2548	0.6792
56717	Mtor		mechanistic target of rapamycin (serine/threonine kinase)	0.9112	0.1469	NA
330503	Gm5113		predicted gene 5113	0.9111	0.1486	NA
224703		Mar-02	membrane-associated ring finger (C3HC4) 2	0.9111	0.5579	0.8456
17763	Mtcp1		mature T-cell proliferation 1	0.9111	0.1946	NA
228913	Zfp217		zinc finger protein 217	0.911	0.4863	0.8127
67867	Lrrc28		leucine rich repeat containing 28	0.911	0.5513	0.843
26400	Map2k7		mitogen-activated protein kinase kinase 7	0.911	0.1886	NA
14806	Grik2		glutamate receptor, ionotropic, kainate 2 (beta 2)	0.911	0.08438	NA
380850	Gm5141		predicted gene 5141	0.9109	0.2675	0.6867
242773	Slc45a1		solute carrier family 45, member 1	0.9109	0.562	0.8465
75710	Rbm12		RNA binding motif protein 12	0.9109	0.1679	NA
12167	Bmpr1b		bone morphogenetic protein receptor, type 1B	0.9109	0.3579	0.7451
669149	Vmn2r88		vomeronasal 2, receptor 88	0.9108	0.2858	0.6982
107656	Krt9		keratin 9	0.9108	0.6226	0.872
78787	Usp54		ubiquitin specific peptidase 54	0.9108	0.4314	0.7852
72599	Pdia5		protein disulfide isomerase associated 5	0.9108	0.03344	NA
11492	Adam19		a disintegrin and metallopeptidase domain 19 (meltrin beta)	0.9108	0.2708	0.689
226747	Ahctf1		AT hook containing transcription factor 1	0.9107	0.135	NA
100177	Zmym6		zinc finger, MYM-type 6	0.9107	0.04756	NA
77371	Sec24a		Sec24 related gene family, member A (S. cerevisiae)	0.9107	0.1343	NA
632687		Mar-10	membrane-associated ring finger (C3HC4) 10	0.9106	0.7088	0.9066
214498	Cdc73		cell division cycle 73, Paf1/RNA polymerase II complex component, homolog (S. cerevisiae)	0.9106	0.3143	0.7155
16785	Rpsa		ribosomal protein SA	0.9106	0.2851	0.6979
73848	5430406J06Rik		RIKEN cDNA 5430406J06 gene	0.9105	0.0921	NA
100038443	9330195I24Rik		RIKEN cDNA 9330195I24 gene	0.9104	0.09103	NA
240058	Cpne5		copine V	0.9104	0.5191	0.8282
93891	Pcdhb20		protocadherin beta 20	0.9104	0.05219	NA
27027	Tspan32		tetraspanin 32	0.9104	0.561	0.8465
23792	Adam23		a disintegrin and metallopeptidase domain 23	0.9104	0.2519	0.6768
654424	A330050B17Rik		RIKEN cDNA A330050B17 gene	0.9103	0.4115	0.7747
238130	Dock4		dedicator of cytokinesis 4	0.9103	0.3592	0.7461
103405	4932439E07Rik		RIKEN cDNA 4932439E07 gene	0.9103	0.282	0.6967
22437	Xirp1		xin actin-binding repeat containing 1	0.9103	0.4655	0.8047
16561	Kif1b		kinesin family member 1B	0.9103	0.1022	NA
269784	Cntn4		contactin 4	0.9102	0.4156	0.7768

99183	AU018505	expressed sequence AU018505	0.9102	0.2028	NA
74843	Zmynd17	zinc finger, MYND domain containing 17	0.9102	0.3824	0.7596
57908	Zfp318	zinc finger protein 318	0.9102	0.1328	NA
18983	Cnot7	CCR4-NOT transcription complex, subunit 7	0.9102	0.2944	0.7042
269610	Chd5	chromodomain helicase DNA binding protein 5	0.9101	0.1364	NA
213819	Casd1	CAS1 domain containing 1	0.9101	0.7918	0.9333
71947	2310067B10Rik	RIKEN cDNA 2310067B10 gene	0.91	0.479	0.81
65945	Clstn1	calsyntenin 1	0.91	0.4745	0.809
17858	Mx2	myxovirus (influenza virus) resistance 2	0.91	0.3836	0.7599
100042970	Kifc1	kinesin family member C1	0.9099	0.2955	0.7046
329253	6530439I21	hypothetical protein 6530439I21	0.9099	0.3597	0.7465
238803	Zfp366	zinc finger protein 366	0.9099	0.6997	0.9032
226026	Smc5	structural maintenance of chromosomes 5	0.9099	0.4518	0.7951
68098	Rchy1	ring finger and CHY zinc finger domain containing 1	0.9099	0.2191	NA
66674	6330409N04Rik	RIKEN cDNA 6330409N04 gene	0.9099	0.4275	0.7831
66302	Fam82b	family with sequence similarity 82, member B	0.9099	0.1798	NA
17133	Maff	v-maf musculoaponeurotic fibrosarcoma oncogene family, protein F (avian)	0.9099	0.4099	0.7739
13531	Dub1	deubiquitinating enzyme 1	0.9099	0.5977	0.8611
231382	Tmprss11d	transmembrane protease, serine 11d	0.9098	0.3957	0.7671
22036	Traip	TRAF-interacting protein	0.9098	0.4895	0.8145
664609	Rhox4a	reproductive homeobox 4A	0.9097	0.6264	0.873
76983	Scfd1	Sec1 family domain containing 1	0.9097	0.1927	NA
18039	Nefl	neurofilament, light polypeptide	0.9097	0.03138	NA
245450	Slitrk2	SLIT and NTRK-like family, member 2	0.9096	0.2866	0.6982
100504975	LOC100504975	hypothetical protein LOC100504975	0.9095	0.2211	NA
432838	Gm5460	predicted gene 5460	0.9095	0.0578	NA
320365	Fry	furry homolog (Drosophila)	0.9095	0.07356	NA
223922	Atf7	activating transcription factor 7	0.9095	0.3682	0.7518
70680	3021401N23Rik	RIKEN cDNA 3021401N23 gene	0.9095	0.2467	0.6738
67897	Rnmt	RNA (guanine-7-) methyltransferase	0.9095	0.4736	0.8088
16792	Laptm5	lysosomal-associated protein transmembrane 5	0.9095	0.215	NA
207214	Larp4	La ribonucleoprotein domain family, member 4	0.9094	0.06384	NA
106200	Txndc11	thioredoxin domain containing 11	0.9094	0.3329	0.728
338467	Morc3	microrchidia 3	0.9093	0.2581	0.6815
217473	Ankmy2	ankyrin repeat and MYND domain containing 2	0.9093	0.5972	0.8611
216760	Mfap3	microfibrillar-associated protein 3	0.9093	0.1409	NA
83815	Cenpq	centromere protein Q	0.9093	0.3502	0.739
75754	9030607L02Rik	RIKEN cDNA 9030607L02 gene	0.9093	0.5085	0.8242
270328	Gsdmc3	gasdermin C3	0.9092	0.4916	0.8156
232449	Dera	2-deoxyribose-5-phosphate aldolase homolog (C. elegans)	0.9092	0.1494	NA
666445	Gm13035	predicted gene 13035	0.9091	0.251	0.6767
327860	Gm11961	predicted gene 11961	0.9091	0.3111	0.7135
320949	D830039M14Rik	RIKEN cDNA D830039M14 gene	0.9091	0.5545	0.8443
319513	Fam113a	family with sequence similarity 113, member A	0.9091	0.3317	0.7267
103554	Psme4	proteasome (prosome, macropain) activator subunit 4	0.9091	0.4516	0.7951
74030	Rin2	Ras and Rab interactor 2	0.9091	0.3634	0.7483
20908	Stx3	syntaxin 3	0.9091	0.292	0.7023
636808	Cntnap5a	contactin associated protein-like 5A	0.909	0.3428	0.7346
243937	Zfp536	zinc finger protein 536	0.909	0.2068	NA

77798	A930009A15Rik	RIKEN cDNA A930009A15 gene	0.909	0.3704	0.7525
66451	2610528J11Rik	RIKEN cDNA 2610528J11 gene	0.909	0.6505	0.8829
17222	Anapc1	anaphase promoting complex subunit 1	0.909	0.1129	NA
216227	Slc17a8	solute carrier family 17 (sodium-dependent inorganic phosphate cotransporter), member 8	0.9089	0.3902	0.7632
18584	Pde8a	phosphodiesterase 8A	0.9089	0.3613	0.7476
381290	Atp2b4	ATPase, Ca++ transporting, plasma membrane 4	0.9088	0.5871	0.8563
226519	Lamc1	laminin, gamma 1	0.9088	0.4415	0.7921
101148	B630005N14Rik	RIKEN cDNA B630005N14 gene	0.9088	0.3888	0.7616
319765	Igf2bp2	insulin-like growth factor 2 mRNA binding protein 2	0.9087	0.4786	0.81
242253	Wdr63	WD repeat domain 63	0.9087	0.2383	0.667
214922	Slc39a2	solute carrier family 39 (zinc transporter), member 2	0.9087	0.6763	0.8928
78177	Ninl	ninein-like	0.9087	0.4913	0.8156
18613	Pecam1	platelet/endothelial cell adhesion molecule 1	0.9087	0.3693	0.7521
384724	Cyp2t4	cytochrome P450, family 2, subfamily t, polypeptide 4	0.9085	0.4927	0.8158
320495	Ipcef1	interaction protein for cytohesin exchange factors 1	0.9085	0.3897	0.7626
225027	Srsf7	serine/arginine-rich splicing factor 7	0.9085	0.07217	NA
29819	Stau2	staufen (RNA binding protein) homolog 2 (Drosophila)	0.9085	0.2548	0.6792
14254	Flt1	FMS-like tyrosine kinase 1	0.9085	0.2066	NA
19210	Ptdss1	phosphatidylserine synthase 1	0.9084	0.1414	NA
217232	Cdc27	cell division cycle 27 homolog (S. cerevisiae)	0.9083	0.3521	0.7401
12417	Cbx3	chromobox homolog 3 (Drosophila HP1 gamma)	0.9083	0.1827	NA
264064	Cdk8	cyclin-dependent kinase 8	0.9082	0.5749	0.8504
224691	Zfp472	zinc finger protein 472	0.9082	0.04302	NA
72180	Zfp661	zinc finger protein 661	0.9082	0.1064	NA
67064	Chmp1b	chromatin modifying protein 1B	0.9082	0.228	NA
17355	Aff1	AF4/FMR2 family, member 1	0.9082	0.6054	0.8645
16531	Kcnma1	potassium large conductance calcium-activated channel, subfamily M, alpha member 1	0.9082	0.4053	0.7717
622116	Gm6287	predicted gene 6287	0.9081	0.3456	0.7361
242418	Dcaf10	DDB1 and CUL4 associated factor 10	0.9081	0.34	0.7326
93760	Arid1a	AT rich interactive domain 1A (SWI-like)	0.9081	0.162	NA
51938	Ccdc39	coiled-coil domain containing 39	0.9081	0.298	0.7051
14660	Gls	glutaminase	0.9081	0.336	0.7304
100043823	Gm4673	predicted gene 4673	0.908	0.3109	0.7135
94091	Trim11	tripartite motif-containing 11	0.908	0.1749	NA
75951	4930578M01Rik	RIKEN cDNA 4930578M01 gene	0.908	0.3605	0.7467
21408	Zfp354a	zinc finger protein 354A	0.908	0.2184	NA
15165	Hcn1	hyperpolarization-activated, cyclic nucleotide-gated K+ 1	0.908	0.7731	0.9263
14538	Gcnt2	glucosaminyl (N-acetyl) transferase 2, I-branching enzyme	0.908	0.1898	NA
11569	Aebp2	AE binding protein 2	0.908	0.1415	NA
319817	Rc3h2	ring finger and CCCH-type zinc finger domains 2	0.9079	0.258	0.6815
70924	4921511C10Rik	RIKEN cDNA 4921511C10 gene	0.9079	0.7197	0.9106
50764	Fbxo15	F-box protein 15	0.9079	0.3884	0.7616
23938	Map2k5	mitogen-activated protein kinase kinase 5	0.9079	0.09953	NA
320858	L3mbtl4	l(3)mbt-like 4 (Drosophila)	0.9078	0.3183	0.7182
80289	Lysmd3	LysM, putative peptidoglycan-binding, domain containing 3	0.9078	0.08883	NA
56351	Ptges3	prostaglandin E synthase 3 (cytosolic)	0.9078	0.1428	NA
19270	Ptprg	protein tyrosine phosphatase, receptor type, G	0.9078	0.4956	0.818
116848	Baz2a	bromodomain adjacent to zinc finger domain, 2A	0.9077	0.06875	NA
71703	Armcx3	armadillo repeat containing, X-linked 3	0.9077	0.2049	NA

69367	Glrx2	glutaredoxin 2 (thioltransferase)	0.9077	0.8723	0.9608
68915	Vars2	valyl-tRNA synthetase 2, mitochondrial (putative)	0.9077	0.3315	0.7265
66624	Spcs2	signal peptidase complex subunit 2 homolog (S. cerevisiae)	0.9077	0.1124	NA
30877	Gnl3	guanine nucleotide binding protein-like 3 (nucleolar)	0.9077	0.1589	NA
26432	Plod2	procollagen lysine, 2-oxoglutarate 5-dioxygenase 2	0.9077	0.4072	0.7726
637900	Gm12618	predicted gene 12618	0.9076	0.4841	0.8123
260315	Nav3	neuron navigator 3	0.9076	0.4804	0.8101
67561	Wdr48	WD repeat domain 48	0.9076	0.2903	0.7006
54216	Pcdh7	protocadherin 7	0.9076	0.3938	0.7656
268902	Robo2	roundabout homolog 2 (Drosophila)	0.9075	0.2575	0.6815
211499	Tmem87a	transmembrane protein 87A	0.9075	0.287	0.6983
56367	Scoc	short coiled-coil protein	0.9075	0.5357	0.835
264064	Cdk8	cyclin-dependent kinase 8	0.9074	0.3171	0.7176
67134	Nop56	NOP56 ribonucleoprotein homolog (yeast)	0.9074	0.2965	0.7051
17920	Myo6	myosin VI	0.9074	0.3355	0.7302
11730	Ang3	angiogenin, ribonuclease A family, member 3	0.9074	0.6885	0.8977
100043580	Gm4532	predicted gene 4532	0.9073	0.7162	0.909
68675	Fam172a	family with sequence similarity 172, member A	0.9073	0.5707	0.8492
19878	Rock2	Rho-associated coiled-coil containing protein kinase 2	0.9073	0.2533	0.6778
15002	H2-Ob	histocompatibility 2, O region beta locus	0.9073	0.8275	0.9449
667510	Gm8675	predicted gene 8675	0.9072	0.2224	NA
268490	Lsm12	LSM12 homolog (S. cerevisiae)	0.9072	0.1565	NA
214111	Slc24a1	solute carrier family 24 (sodium/potassium/calcium exchanger), member 1	0.9072	0.2169	NA
210925	Ints9	integrator complex subunit 9	0.9072	0.3805	0.7594
108155	Ogt	O-linked N-acetylglucosamine (GlcNAc) transferase (UDP-N-acetylglucosamine:polypeptide-N-acetylglucosaminyl transferase)	0.9072	0.1547	NA
56736	Rnf14	ring finger protein 14	0.9072	0.4263	0.7823
56459	Sae1	SUMO1 activating enzyme subunit 1	0.9072	0.288	0.6991
22589	Atrx	alpha thalassemia/mental retardation syndrome X-linked homolog (human)	0.9072	0.2882	0.6993
15481	Hspa8	heat shock protein 8	0.9072	0.369	0.7521
320528	Vps13c	vacuolar protein sorting 13C (yeast)	0.9071	0.3437	0.7356
69116	Ubr4	ubiquitin protein ligase E3 component n-recognin 4	0.9071	0.1607	NA
20534	Slc4a1ap	solute carrier family 4 (anion exchanger), member 1, adaptor protein	0.9071	0.6286	0.8745
17299	Mettl1	methyltransferase like 1	0.9071	0.5172	0.8277
100036569	Gm16525	predicted gene, 16525	0.907	0.09072	NA
665155	Srp54b	signal recognition particle 54B	0.907	0.6604	0.888
232533	Stk38l	serine/threonine kinase 38 like	0.9069	0.353	0.7409
70422	Ints2	integrator complex subunit 2	0.9069	0.2984	0.7052
26877	B3galt1	UDP-Gal:betaGlcNAc beta 1,3-galactosyltransferase, polypeptide 1	0.9069	0.1462	NA
12340	Capza1	capping protein (actin filament) muscle Z-line, alpha 1	0.9069	0.446	0.7938
635702	Naaladl2	N-acetylated alpha-linked acidic dipeptidase-like 2	0.9068	0.4737	0.8088
224836	Usp49	ubiquitin specific peptidase 49	0.9068	0.1802	NA
20747	Spop	speckle-type POZ protein	0.9068	0.4605	0.8014
19134	Prpf4b	PRP4 pre-mRNA processing factor 4 homolog B (yeast)	0.9068	0.31	0.713
14816	Grm1	glutamate receptor, metabotropic 1	0.9068	0.5003	0.8197
68564	Nufip2	nuclear fragile X mental retardation protein interacting protein 2	0.9067	0.0491	NA
75782	Lca5	Leber congenital amaurosis 5 (human)	0.9066	0.1473	NA
56403	Syncrip	synaptotagmin binding, cytoplasmic RNA interacting protein	0.9066	0.1465	NA
52468	Ctdsp2	CTD (carboxy-terminal domain, RNA polymerase II, polypeptide A) small phosphatase 2	0.9066	0.7684	0.9255
259032	Olfr1134	olfactory receptor 1134	0.9065	0.7491	0.9207

233765	Plekha7	pleckstrin homology domain containing, family A member 7	0.9065	0.04774	NA
208228	Mobkl2a	MOB1, Mps One Binder kinase activator-like 2A (yeast)	0.9065	0.4529	0.7962
117589	Asb7	ankyrin repeat and SOCS box-containing 7	0.9065	0.1263	NA
26450	Rbbp9	retinoblastoma binding protein 9	0.9065	0.3644	0.7491
552909	LOC552909	hypothetical LOC552909	0.9064	0.5769	0.851
226026	Smc5	structural maintenance of chromosomes 5	0.9064	0.3229	0.7204
114666	Krtap5-5	keratin associated protein 5-5	0.9064	0.6206	0.8718
73158	Larp1	La ribonucleoprotein domain family, member 1	0.9064	0.1252	NA
67727	Stx17	syntaxin 17	0.9064	0.4306	0.7846
19416	Rasd1	RAS, dexamethasone-induced 1	0.9064	0.2549	0.6793
12825	Col3a1	collagen, type III, alpha 1	0.9064	0.4265	0.7823
667350	Gm8587	predicted pseudogene 8587	0.9063	0.5392	0.8366
213393	8430408G22Rik	RIKEN cDNA 8430408G22 gene	0.9063	0.387	0.761
74320	Wdr33	WD repeat domain 33	0.9063	0.1858	NA
70591	5730455P16Rik	RIKEN cDNA 5730455P16 gene	0.9063	0.3552	0.7434
67163	Ccdc47	coiled-coil domain containing 47	0.9063	0.2369	0.6659
238161	Akap6	A kinase (PRKA) anchor protein 6	0.9061	0.5054	0.8229
77733	Rnf170	ring finger protein 170	0.9061	0.09214	NA
11814	Apoc3	apolipoprotein C-III	0.9061	0.701	0.9035
66505	Zmynd11	zinc finger, MYND domain containing 11	0.906	0.2815	0.6967
17957	Napb	N-ethylmaleimide sensitive fusion protein attachment protein beta	0.906	0.1746	NA
215335	Slc36a1	solute carrier family 36 (proton/amino acid symporter), member 1	0.9059	0.5974	0.8611
72925	Mar-01	membrane-associated ring finger (C3HC4) 1	0.9059	0.2719	0.6891
69657	2310047D07Rik	RIKEN cDNA 2310047D07 gene	0.9059	0.2103	NA
68718	Rnf166	ring finger protein 166	0.9059	0.055	NA
67230	Zfp329	zinc finger protein 329	0.9059	0.2971	0.7051
68371	Pbld1	phenazine biosynthesis-like protein domain containing 1	0.9058	0.2413	0.6692
12545	Cdc7	cell division cycle 7 (S. cerevisiae)	0.9058	0.3865	0.761
66614	Gpatch4	G patch domain containing 4	0.9057	0.1723	NA
24135	Zfp68	zinc finger protein 68	0.9057	0.1422	NA
21787	Tfg	Trk-fused gene	0.9057	0.1688	NA
108797	Mex3b	mex3 homolog B (C. elegans)	0.9056	0.1262	NA
27367	Rpl3	ribosomal protein L3	0.9056	0.1784	NA
319181	Hist1h2bg	histone cluster 1, H2bg	0.9055	0.3406	0.7328
226551	AI848100	expressed sequence AI848100	0.9055	0.2074	NA
667214	9930111J21Rik1	RIKEN cDNA 9930111J21 gene 1	0.9054	0.3704	0.7525
433182	Gm5506	predicted gene 5506	0.9053	0.4834	0.8121
227449	Zcchc2	zinc finger, CCHC domain containing 2	0.9053	0.3491	0.7388
72504	Taf4b	TAF4B RNA polymerase II, TATA box binding protein (TBP)-associated factor	0.9052	0.7265	0.9129
11541	Adora2b	adenosine A2b receptor	0.9052	0.4987	0.8197
75079	Zbtb49	zinc finger and BTB domain containing 49	0.9051	0.5105	0.8253
72461	Prcp	prolylcarboxypeptidase (angiotensinase C)	0.9051	0.2679	0.6871
215243	Traf3ip3	TRAF3 interacting protein 3	0.905	0.1639	NA
207227	Stxbp5l	syntaxin binding protein 5-like	0.905	0.223	0.654
110886	Gabra5	gamma-aminobutyric acid (GABA) A receptor, subunit alpha 5	0.905	0.4717	0.8077
68162	A930003A15Rik	RIKEN cDNA A930003A15 gene	0.905	0.09168	NA
18023	Nfe2l1	nuclear factor, erythroid derived 2,-like 1	0.905	0.5	0.8197
12611	Cebpg	CCAAT/enhancer binding protein (C/EBP), gamma	0.905	0.3225	0.7202
12412	Cbx1	chromobox homolog 1 (Drosophila HP1 beta)	0.905	0.4072	0.7726

320267	Fubp3	far upstream element (FUSE) binding protein 3	0.9049	0.4935	0.8163
18080	Nin	ninein	0.9049	0.1726	NA
104836	Cbll1	Casitas B-lineage lymphoma-like 1	0.9048	0.1625	NA
70956	Tex19.2	testis expressed gene 19.2	0.9048	0.5746	0.8504
19069	Nup88	nucleoporin 88	0.9048	0.1901	NA
216543	Cep68	centrosomal protein 68	0.9047	0.2339	0.6631
72438	2510016G02Rik	RIKEN cDNA 2510016G02 gene	0.9047	0.192	NA
11684	Alox12	arachidonate 12-lipoxygenase	0.9047	0.775	0.9266
227331	Gigyf2	GRB10 interacting GYF protein 2	0.9046	0.1756	NA
64085	Clstn2	calsyntenin 2	0.9046	0.1167	NA
330004	Gm833	predicted gene 833	0.9045	0.5791	0.8519
243764	Chrm2	cholinergic receptor, muscarinic 2, cardiac	0.9045	0.2983	0.7052
382406	Poc1b	POC1 centriolar protein homolog B (Chlamydomonas)	0.9044	0.1277	NA
235086	Igsf9b	immunoglobulin superfamily, member 9B	0.9044	0.3832	0.7599
53623	Gria3	glutamate receptor, ionotropic, AMPA3 (alpha 3)	0.9044	0.2826	0.6969
14423	Galnt1	UDP-N-acetyl-alpha-D-galactosamine:polypeptide N-acetylgalactosaminyltransferase 1	0.9044	0.4354	0.7883
192285	Phf21a	PHD finger protein 21A	0.9043	0.3473	0.7377
107885	Mthfs	5, 10-methenyltetrahydrofolate synthetase	0.9043	0.1739	NA
100336	Ppp1r8	protein phosphatase 1, regulatory (inhibitor) subunit 8	0.9043	0.4084	0.7731
74467	Pus10	pseudouridylate synthase 10	0.9043	0.2598	0.6815
76775	Slc10a7	solute carrier family 10 (sodium/bile acid cotransporter family), member 7	0.9042	0.6257	0.8728
67797	Snrnp48	small nuclear ribonucleoprotein 48 (U11/U12)	0.9042	0.2658	0.6856
67160	Eef1g	eukaryotic translation elongation factor 1 gamma	0.9042	0.4685	0.8056
54652	Cacna1f	calcium channel, voltage-dependent, alpha 1F subunit	0.9042	0.3628	0.7483
100040724	Mirg	miRNA containing gene	0.9041	0.1921	NA
229791	D3Bwg0562e	DNA segment, Chr 3, Brigham & Women's Genetics 0562 expressed	0.9041	0.5291	0.8332
217666	L2hgdh	L-2-hydroxyglutarate dehydrogenase	0.9041	0.3957	0.7671
102371	Gcom1	GRINL1A complex locus	0.9041	0.4816	0.8108
22026	Nr2c2	nuclear receptor subfamily 2, group C, member 2	0.9041	0.1944	NA
18628	Per3	period homolog 3 (Drosophila)	0.9041	0.103	NA
268970	Arhgap28	Rho GTPase activating protein 28	0.904	0.6315	0.8751
213109	Phf3	PHD finger protein 3	0.904	0.1141	NA
209011	Sirt7	sirtuin 7 (silent mating type information regulation 2, homolog) 7 (S. cerevisiae)	0.904	0.223	0.654
76286	1110006E14Rik	RIKEN cDNA 1110006E14 gene	0.904	0.2977	0.7051
18080	Nin	ninein	0.904	0.1776	NA
239336	Rxfp3	relaxin family peptide receptor 3	0.9039	0.2436	0.6714
213006	Mfsd4	major facilitator superfamily domain containing 4	0.9039	0.09272	NA
104401	Pcnxl3	pecanex-like 3 (Drosophila)	0.9039	0.2772	0.6925
52857	Gramd1a	GRAM domain containing 1A	0.9039	0.1854	NA
27223	Trp53bp1	transformation related protein 53 binding protein 1	0.9039	0.04431	NA
100502708	LOC100502708	hypothetical LOC100502708	0.9038	0.4169	0.7774
619331	Zfp551	zinc fingr protein 551	0.9038	0.4144	0.776
331461	Il1rapl1	interleukin 1 receptor accessory protein-like 1	0.9037	0.7597	0.9229
101351	A130022J15Rik	RIKEN cDNA A130022J15 gene	0.9037	0.03935	NA
72723	Zfp74	zinc finger protein 74	0.9037	0.3234	0.7206
67664	Rnf125	ring finger protein 125	0.9037	0.4047	0.7716
28006	D6Wsu116e	DNA segment, Chr 6, Wayne State University 116, expressed	0.9037	0.4659	0.8049
18213	Ntrk3	neurotrophic tyrosine kinase, receptor, type 3	0.9037	0.2837	0.6974
66950	Tmem206	transmembrane protein 206	0.9036	0.2757	0.6925

100504508	LOC100504508	protein SET-like	0.9035	0.1283	NA	
624866	Lekr1	leucine, glutamate and lysine rich 1	0.9035	0.2544		0.6792
101563	AI426330	expressed sequence AI426330	0.9035	0.1014	NA	
20871	Aurkc	aurora kinase C	0.9035	0.6353		0.877
12006	Axin2	axin2	0.9035	0.3885		0.7616
74025	Nphp3	nephronophthisis 3 (adolescent)	0.9034	0.2266		0.6554
71474	Ppp6r2	protein phosphatase 6, regulatory subunit 2	0.9034	0.153	NA	
66356	2310008H09Rik	RIKEN cDNA 2310008H09 gene	0.9034	0.1987	NA	
11909	Atf2	activating transcription factor 2	0.9034	0.2376		0.6663
170676	Peg10	paternally expressed 10	0.9033	0.7248		0.9126
105428	Fam149b	family with sequence similarity 149, member B	0.9033	0.244		0.6719
78330	Ndufv3	NADH dehydrogenase (ubiquinone) flavoprotein 3	0.9033	0.305		0.7097
70747	Tspan2	tetraspanin 2	0.9033	0.4581		0.7997
14674	Gna13	guanine nucleotide binding protein, alpha 13	0.9033	0.4009		0.7703
404311	Olfr209	olfactory receptor 209	0.9032	0.6674		0.8904
192193	Edem1	ER degradation enhancer, mannosidase alpha-like 1	0.9031	0.1287	NA	
53318	Pdlim3	PDZ and LIM domain 3	0.9031	0.4808		0.8103
19428	Rasl2-9-ps	RAS-like, family 2, locus 9, pseudogene	0.9031	0.8208		0.9433
74081	Cep350	centrosomal protein 350	0.903	0.576		0.8506
67568	Mrfap1	Morf4 family associated protein 1	0.903	0.2431		0.6708
17524	Mpp1	membrane protein, palmitoylated	0.903	0.2729		0.6902
14816	Grm1	glutamate receptor, metabotropic 1	0.903	0.2605		0.6815
381022	Mll2	myeloid/lymphoid or mixed-lineage leukemia 2	0.9029	0.3633		0.7483
223455	Mar-06	membrane-associated ring finger (C3HC4) 6	0.9029	0.2263		0.6549
78286	Nav2	neuron navigator 2	0.9029	0.4778		0.81
24067	Srp54a	signal recognition particle 54A	0.9029	0.5147		0.8273
19737	Rgs5	regulator of G-protein signaling 5	0.9029	0.3976		0.7684
19126	Prom1	prominin 1	0.9029	0.1854	NA	
791360	Gm9853	predicted gene 9853	0.9028	0.2603		0.6815
230257	Rod1	ROD1 regulator of differentiation 1 (S. pombe)	0.9028	0.4307		0.7846
170737	Znrf1	zinc and ring finger 1	0.9028	0.324		0.7211
78245	Acbd7	acyl-Coenzyme A binding domain containing 7	0.9028	0.05648	NA	
66308	2810021B07Rik	RIKEN cDNA 2810021B07 gene	0.9028	0.08476	NA	
17700	Mstn	myostatin	0.9028	0.4284		0.7838
230648	4732418C07Rik	RIKEN cDNA 4732418C07 gene	0.9027	0.1411	NA	
72128	2610008E11Rik	RIKEN cDNA 2610008E11 gene	0.9027	0.106	NA	
331487	Uprt	uracil phosphoribosyltransferase (FUR1) homolog (S. cerevisiae)	0.9026	0.3263		0.7227
219022	Ttc5	tetratricopeptide repeat domain 5	0.9026	0.1498	NA	
107815	Scml2	sex comb on midleg-like 2 (Drosophila)	0.9026	0.2147		0.6467
72588	2700012I20Rik	RIKEN cDNA 2700012I20 gene	0.9026	0.1798	NA	
54403	Slc4a4	solute carrier family 4 (anion exchanger), member 4	0.9026	0.2654		0.685
241769	Kcnk15	potassium channel, subfamily K, member 15	0.9025	0.5303		0.8333
16010	Igfbp4	insulin-like growth factor binding protein 4	0.9025	0.2335		0.6623
12808	Cobl	cordon-bleu	0.9025	0.4664		0.8049
12325	Camk2g	calcium/calmodulin-dependent protein kinase II gamma	0.9025	0.1011	NA	
20403	Itsn2	intersectin 2	0.9024	0.05444	NA	
170643	Kirrel	kin of IRRE like (Drosophila)	0.9023	0.4464		0.7938
67333	Stk35	serine/threonine kinase 35	0.9023	0.1761	NA	
19652	Rbm3	RNA binding motif protein 3	0.9023	0.1997		0.6343

320438	Alg6	asparagine-linked glycosylation 6 homolog (yeast, alpha-1,3,-glucosyltransferase)	0.9022	0.4507	0.7946
71599	Senp8	SUMO/sentrin specific peptidase 8	0.9022	0.5028	0.8214
20019	Polr1a	polymerase (RNA) I polypeptide A	0.9022	0.2553	0.6798
19127	Prop1	paired like homeodomain factor 1	0.9022	0.6028	0.8641
54383	Phc2	polyhomeotic-like 2 (Drosophila)	0.9021	0.1131	NA
26900	Ddx3y	DEAD (Asp-Glu-Ala-Asp) box polypeptide 3, Y-linked	0.9021	0.2198	0.6513
14405	Gabrg1	gamma-aminobutyric acid (GABA) A receptor, subunit gamma 1	0.9021	0.5347	0.8349
640382	LOC640382	hypothetical protein LOC640382	0.902	0.373	0.7538
432460	Gm5423	predicted gene 5423	0.902	0.2702	0.6884
269966	Nup98	nucleoporin 98	0.902	0.08861	NA
107970	Hist1h1t	histone cluster 1, H1t	0.902	0.2995	0.7054
80892	Zfhx4	zinc finger homeodomain 4	0.902	0.2377	0.6663
66832	Rsph3a	radial spoke 3A homolog (Chlamydomonas)	0.902	0.3553	0.7435
20340	Glg1	golgi apparatus protein 1	0.902	0.2864	0.6982
18572	Pdcd11	programmed cell death 11	0.902	0.1129	NA
11641	Akap2	A kinase (PRKA) anchor protein 2	0.902	0.1955	NA
93876	Pcdhb5	protocadherin beta 5	0.9019	0.2666	0.6862
26405	Map3k2	mitogen-activated protein kinase kinase kinase 2	0.9019	0.6058	0.8645
67911	Zfp169	zinc finger protein 169	0.9018	0.369	0.7521
67542	Cog6	component of oligomeric golgi complex 6	0.9018	0.3351	0.73
67531	5730408K05Rik	RIKEN cDNA 5730408K05 gene	0.9018	0.4909	0.8155
59050	Nsa2	NSA2 ribosome biogenesis homolog (S. cerevisiae)	0.9018	0.2876	0.6988
50769	Atp8a2	ATPase, aminophospholipid transporter-like, class I, type 8A, member 2	0.9018	0.6224	0.872
20442	St3gal1	ST3 beta-galactoside alpha-2,3-sialyltransferase 1	0.9018	0.1818	NA
211712	Pcdh9	protocadherin 9	0.9017	0.3989	0.7697
19386	Ranbp2	RAN binding protein 2	0.9017	0.1608	NA
18646	Prf1	perforin 1 (pore forming protein)	0.9016	0.5176	0.8278
237360	Adamts14	a disintegrin-like and metallopeptidase (reprolysin type) with thrombospondin type 1 motif, 14	0.9015	0.4826	0.8112
232875	Zscan18	zinc finger and SCAN domain containing 18	0.9015	0.04145	NA
101214	Tra2a	transformer 2 alpha homolog (Drosophila)	0.9015	0.2371	0.6659
78341	2900016J10Rik	RIKEN cDNA 2900016J10 gene	0.9015	0.05248	NA
68545	Ecscr	endothelial cell-specific chemotaxis regulator	0.9015	0.2066	0.6393
67254	2900011O08Rik	RIKEN cDNA 2900011O08 gene	0.9015	0.1221	NA
66950	Tmem206	transmembrane protein 206	0.9015	0.09342	NA
381668	Fbrsl1	fibrosin-like 1	0.9014	0.369	0.7521
241113	Prkag3	protein kinase, AMP-activated, gamma 3 non-catatlytic subunit	0.9014	0.526	0.832
74081	Cep350	centrosomal protein 350	0.9014	0.02295	NA
70963	4931402H11Rik	RIKEN cDNA 4931402H11 gene	0.9014	0.7507	0.9211
64934	Pes1	pescadillo homolog 1, containing BRCT domain (zebrafish)	0.9014	0.4138	0.7756
14256	Flt3l	FMS-like tyrosine kinase 3 ligand	0.9014	0.2249	0.654
320150	Zdhhc17	zinc finger, DHHC domain containing 17	0.9013	0.1249	NA
24047	Ccl19	chemokine (C-C motif) ligand 19	0.9013	0.5328	0.834
19266	Ptprd	protein tyrosine phosphatase, receptor type, D	0.9013	0.09898	NA
434729	Gm5635	predicted gene 5635	0.9012	0.5318	0.8339
218973	Wdhd1	WD repeat and HMG-box DNA binding protein 1	0.9012	0.1966	0.6315
208908	Ccdc62	coiled-coil domain containing 62	0.9012	0.1605	NA
20678	Sox5	SRY-box containing gene 5	0.9012	0.421	0.7798
12175	Bnip2	BCL2/adenovirus E1B interacting protein 2	0.9012	0.3967	0.7675
331021	9630017O17	hypothetical protein 9630017O17	0.9011	0.1486	NA

219131	Phf11	PHD finger protein 11	0.9011	0.5767	0.8509
77018	Col25a1	collagen, type XXV, alpha 1	0.9011	0.3735	0.754
246730	Oas1a	2'-5' oligoadenylate synthetase 1A	0.901	0.6472	0.8812
12337	Capn5	calpain 5	0.901	0.79	0.933
237943	Gpatch8	G patch domain containing 8	0.9009	0.02979	NA
22764	Zfx	zinc finger protein X-linked	0.9009	0.5816	0.853
15387	Hnrnpk	heterogeneous nuclear ribonucleoprotein K	0.9009	0.1818	NA
321006	Vprbp	Vpr (HIV-1) binding protein	0.9008	0.2217	0.6531
320384	B230334L07Rik	RIKEN cDNA B230334L07 gene	0.9008	0.302	0.708
319430	Gpr77	G protein-coupled receptor 77	0.9008	0.35	0.7389
243043	Kctd8	potassium channel tetramerisation domain containing 8	0.9008	0.2409	0.6687
192289	Tmlhe	trimethyllysine hydroxylase, epsilon	0.9008	0.2962	0.7051
69870	Polr3gl	polymerase (RNA) III (DNA directed) polypeptide G like	0.9008	0.1703	NA
64930	Tsc1	tuberous sclerosis 1	0.9008	0.1743	NA
100039982	Gm2533	predicted gene 2533	0.9007	0.05727	NA
623174	Gm6404	predicted gene 6404	0.9007	0.4406	0.7913
381067	Zfp229	zinc finger protein	0.9007	0.407	0.7726
328949	Mcc	mutated in colorectal cancers	0.9007	0.2408	0.6687
232288	Frmd4b	FERM domain containing 4B	0.9007	0.1053	NA
227731	Slc25a25	solute carrier family 25 (mitochondrial carrier, phosphate carrier), member 25	0.9007	0.309	0.7125
213956	Fam83f	family with sequence similarity 83, member F	0.9007	0.5539	0.8442
77371	Sec24a	Sec24 related gene family, member A (S. cerevisiae)	0.9007	0.5638	0.8465
68058	Chd1l	chromodomain helicase DNA binding protein 1-like	0.9007	0.1923	0.6283
56809	Gmeb1	glucocorticoid modulatory element binding protein 1	0.9007	0.2452	0.6728
21974	Top2b	topoisomerase (DNA) II beta	0.9007	0.07388	NA
231130	Tnip2	TNFAIP3 interacting protein 2	0.9006	0.1405	NA
233532	Rsf1	remodeling and spacing factor 1	0.9003	0.1784	NA
78785	Clip4	CAP-GLY domain containing linker protein family, member 4	0.9003	0.4038	0.7711
70999	Naa40	N(alpha)-acetyltransferase 40, NatD catalytic subunit, homolog (S. cerevisiae)	0.9003	0.2568	0.6809
56217	Mpp5	membrane protein, palmitoylated 5 (MAGUK p55 subfamily member 5)	0.9003	0.3433	0.7353
51813	Ccnc	cyclin C	0.9003	0.4303	0.7844
116837	Rims1	regulating synaptic membrane exocytosis 1	0.9002	0.5669	0.8481
19131	Prh1	proline rich protein HaeIII subfamily 1	0.9002	0.5753	0.8504
627096	LOC627096	hypothetical LOC627096	0.9001	0.6379	0.8776
224694	Zfp81	zinc finger protein 81	0.9001	0.425	0.7821
97541	Qars	glutaminyl-tRNA synthetase	0.9001	0.1839	NA
56200	Ddx21	DEAD (Asp-Glu-Ala-Asp) box polypeptide 21	0.9001	0.04803	NA
210711	Mcmbp	MCM (minichromosome maintenance deficient) binding protein	0.9	0.02928	NA
66075	Chchd3	coiled-coil-helix-coiled-coil-helix domain containing 3	0.9	0.1692	NA
51796	Srrm1	serine/arginine repetitive matrix 1	0.9	0.09098	NA
22003	Tpm1	tropomyosin 1, alpha	0.9	0.5753	0.8504
13813	Eomes	eomesodermin homolog (Xenopus laevis)	0.9	0.2648	0.6848
382161	Gm5165	predicted gene 5165	0.8999	0.3593	0.7461
218518	Marveld2	MARVEL (membrane-associating) domain containing 2	0.8999	0.06237	NA
216860	Neurl4	neuralized homolog 4 (Drosophila)	0.8999	0.2773	0.6925
213895	Bms1	BMS1 homolog, ribosome assembly protein (yeast)	0.8999	0.3536	0.7414
71200	Dydc2	DPY30 domain containing 2	0.8999	0.457	0.7987
69188	Mll5	myeloid/lymphoid or mixed-lineage leukemia 5	0.8999	0.1497	NA
12111	Bgn	biglycan	0.8999	0.8075	0.939

633947	Gm6225	predicted gene 6225	0.8998	0.3841	0.7603
75572	Acyp2	acylphosphatase 2, muscle type	0.8998	0.7899	0.933
71932	Ephx3	epoxide hydrolase 3	0.8998	0.4474	0.7938
12816	Col12a1	collagen, type XII, alpha 1	0.8998	0.1853	0.6223
100039826	Gm2444	predicted gene 2444	0.8997	0.3136	0.7152
108943	Rg9mtd2	RNA (guanine-9-) methyltransferase domain containing 2	0.8997	0.164	NA
107568	Wwp1	WW domain containing E3 ubiquitin protein ligase 1	0.8997	0.2643	0.6845
53963	D630030B22Rik	RIKEN cDNA D630030B22 gene	0.8997	0.2182	0.6502
14924	Magi1	membrane associated guanylate kinase, WW and PDZ domain containing 1	0.8997	0.2001	0.6345
224585	Zfp160	zinc finger protein 160	0.8996	0.1584	NA
68842	Tulp4	tubby like protein 4	0.8996	0.1622	NA
270669	Mbtps2	membrane-bound transcription factor peptidase, site 2	0.8995	0.4259	0.7823
238276	Akap5	A kinase (PRKA) anchor protein 5	0.8995	0.113	NA
214968	Sema6d	sema domain, transmembrane domain (TM), and cytoplasmic domain, (semaphorin) 6D	0.8995	0.5454	0.839
78833	Gins3	GINS complex subunit 3 (Psf3 homolog)	0.8994	0.4218	0.7799
635253	Usp51	ubiquitin specific protease 51	0.8993	0.08858	NA
625276	Gm6569	predicted gene 6569	0.8993	0.6409	0.8788
399619	A930027P06Rik	RIKEN cDNA A930027P06 gene	0.8993	0.3425	0.7343
229279	Hnrnpa3	heterogeneous nuclear ribonucleoprotein A3	0.8993	0.08065	NA
211378	6720489N17Rik	RIKEN cDNA 6720489N17 gene	0.8993	0.3018	0.708
76867	Rhbdd1	rhomboid domain containing 1	0.8993	0.5218	0.829
69399	1700025G04Rik	RIKEN cDNA 1700025G04 gene	0.8993	0.1137	NA
66948	Acad8	acyl-Coenzyme A dehydrogenase family, member 8	0.8993	0.2521	0.6771
56258	Hnrnph2	heterogeneous nuclear ribonucleoprotein H2	0.8993	0.2297	0.6596
226856	Lpgat1	lysophosphatidylglycerol acyltransferase 1	0.8992	0.3394	0.7322
216805	Flcn	folliculin	0.8992	0.03275	NA
207214	Larp4	La ribonucleoprotein domain family, member 4	0.8992	0.1286	NA
68018	Col4a3bp	collagen, type IV, alpha 3 (Goodpasture antigen) binding protein	0.8992	0.3863	0.761
16973	Lrp5	low density lipoprotein receptor-related protein 5	0.8992	0.3376	0.7312
12034	Phb2	prohibitin 2	0.8992	0.1288	NA
628110	LOC628110	similar to gonadotropin inducible ovarian transcription factor 1	0.8991	0.189	0.6266
269700	Gm15800	predicted gene 15800	0.8991	0.1048	NA
236790	Ddx26b	DEAD/H (Asp-Glu-Ala-Asp/His) box polypeptide 26B	0.8991	0.0984	NA
100041488	Gm11937	predicted gene 11937	0.899	0.3107	0.7135
231464	Cnot6l	CCR4-NOT transcription complex, subunit 6-like	0.899	0.2932	0.7028
78425	9530053H05Rik	RIKEN cDNA 9530053H05 gene	0.899	0.1719	NA
67474	Snap29	synaptosomal-associated protein 29	0.899	0.1828	0.6215
272322	Arntl2	aryl hydrocarbon receptor nuclear translocator-like 2	0.8989	0.555	0.8444
238057	Gdf7	growth differentiation factor 7	0.8989	0.6786	0.8938
110460	Acat2	acetyl-Coenzyme A acetyltransferase 2	0.8989	0.1558	NA
14282	Fosb	FBJ osteosarcoma oncogene B	0.8989	0.4789	0.81
75695	Rilpl1	Rab interacting lysosomal protein-like 1	0.8988	0.2227	0.654
59090	Midn	midnolin	0.8988	0.08104	NA
20822	Trove2	TROVE domain family, member 2	0.8988	0.06663	NA
14056	Ezh2	enhancer of zeste homolog 2 (Drosophila)	0.8988	0.1064	NA
259002	Olfr173	olfactory receptor 173	0.8987	0.5467	0.8397
211007	Trim41	tripartite motif-containing 41	0.8987	0.1142	NA
94118	Kifc5c-ps	kinesin family member C5C, pseudogene	0.8987	0.7721	0.9259
80890	Trim2	tripartite motif-containing 2	0.8987	0.3406	0.7328

75717	Cul5	cullin 5	0.8987	0.1314	NA
23947	Mid2	midline 2	0.8987	0.3439	0.7356
21750	Terf2	telomeric repeat binding factor 2	0.8987	0.3354	0.7302
380921	Dgkh	diacylglycerol kinase, eta	0.8986	0.1222	NA
331188	Zfp781	zinc finger protein 781	0.8986	0.3837	0.7599
224170	Dzip3	DAZ interacting protein 3, zinc finger	0.8986	0.2178	0.6499
19270	Ptprg	protein tyrosine phosphatase, receptor type, G	0.8986	0.3095	0.7128
637776	Gm7221	predicted gene 7221	0.8985	0.537	0.8354
239133	Dleu7	deleted in lymphocytic leukemia, 7	0.8985	0.2171	0.6491
216739	Acsl6	acyl-CoA synthetase long-chain family member 6	0.8985	0.4458	0.7938
58231	Stk4	serine/threonine kinase 4	0.8985	0.2858	0.6982
16765	Stmn1	stathmin 1	0.8985	0.2907	0.701
791365	6430601O08Rik	RIKEN cDNA 6430601O08 gene	0.8984	0.3868	0.761
215194	Kri1	KRI1 homolog (S. cerevisiae)	0.8984	0.1825	0.621
71492	Bbs7	Bardet-Biedl syndrome 7 (human)	0.8984	0.08879	NA
69239	2610034M16Rik	RIKEN cDNA 2610034M16 gene	0.8984	0.2449	0.6726
22253	Unc5c	unc-5 homolog C (C. elegans)	0.8984	0.2818	0.6967
20658	Son	Son DNA binding protein	0.8984	0.1201	NA
100037262	Gm12359	predicted gene 12359	0.8983	0.325	0.7219
239667	Dip2b	DIP2 disco-interacting protein 2 homolog B (Drosophila)	0.8983	0.3204	0.7194
78697	Pus7	pseudouridylate synthase 7 homolog (S. cerevisiae)	0.8983	0.2472	0.6743
76051	Ganc	glucosidase, alpha; neutral C	0.8983	0.03013	NA
15481	Hspa8	heat shock protein 8	0.8983	0.1226	NA
12613	Cel	carboxyl ester lipase	0.8983	0.3208	0.7196
239652	Zfp641	zinc finger protein 641	0.8982	0.2195	0.6513
75645	1700011F14Rik	RIKEN cDNA 1700011F14 gene	0.8982	0.3719	0.7535
56456	Actl6a	actin-like 6A	0.8982	0.3082	0.7112
271005	Klhdc1	kelch domain containing 1	0.8981	0.1154	NA
211468	Kcnh8	potassium voltage-gated channel, subfamily H (eag-related), member 8	0.8981	0.198	0.6329
81003	Trim23	tripartite motif-containing 23	0.8981	0.307	0.7108
53621	Cnot4	CCR4-NOT transcription complex, subunit 4	0.8981	0.1485	NA
71623	Krtap5-2	keratin associated protein 5-2	0.8979	0.3632	0.7483
15936	Ier2	immediate early response 2	0.8979	0.2192	0.6512
208936	Adamts18	a disintegrin-like and metallopeptidase (reprolysin type) with thrombospondin type 1 motif, 18	0.8978	0.651	0.8831
66202	1110059G10Rik	RIKEN cDNA 1110059G10 gene	0.8978	0.2097	0.6412
29810	Bag3	BCL2-associated athanogene 3	0.8978	0.1696	NA
668643	EG668643	predicted gene, EG668643	0.8977	0.6702	0.8912
14058	F10	coagulation factor X	0.8977	0.1121	NA
192287	Slc25a36	solute carrier family 25, member 36	0.8976	0.2002	0.6345
74025	Nphp3	nephronophthisis 3 (adolescent)	0.8976	0.3254	0.7222
67685	Dyx1c1	dyslexia susceptibility 1 candidate 1 homolog (human)	0.8976	0.04203	NA
16410	Itgav	integrin alpha V	0.8976	0.2768	0.6925
229055	Zbtb10	zinc finger and BTB domain containing 10	0.8975	0.2572	0.681
67942	Atp5g2	ATP synthase, H+ transporting, mitochondrial F0 complex, subunit C2 (subunit 9)	0.8975	0.1489	NA
67014	Mina	myc induced nuclear antigen	0.8975	0.03896	NA
94192	C1galt1	core 1 synthase, glycoprotein-N-acetylgalactosamine 3-beta-galactosyltransferase, 1	0.8974	0.5011	0.8202
67772	Chd8	chromodomain helicase DNA binding protein 8	0.8974	0.1778	0.6168
67300	Cltc	clathrin, heavy polypeptide (Hc)	0.8974	0.278	0.693
55989	Nop58	NOP58 ribonucleoprotein homolog (yeast)	0.8974	0.09146	NA

16764	Aff3	AF4/FMR2 family, member 3	0.8974	0.3062	0.7108
380728	Kcnh4	potassium voltage-gated channel, subfamily H (eag-related), member 4	0.8973	0.5343	0.8348
239510	Phf20l1	PHD finger protein 20-like 1	0.8973	0.5239	0.83
213696	Duoxa1	dual oxidase maturation factor 1	0.8972	0.7759	0.9266
102075	Plekhg4	pleckstrin homology domain containing, family G (with RhoGef domain) member 4	0.8972	0.07652	NA
11777	Ap3s1	adaptor-related protein complex 3, sigma 1 subunit	0.8972	0.2713	0.6891
668272	Gm9079	transmembrane emp24 domain trafficking protein 2 pseudogene	0.8971	0.1878	0.626
69815	Krtcap3	keratinocyte associated protein 3	0.8971	0.2041	0.6368
67876	Coq10b	coenzyme Q10 homolog B (S. cerevisiae)	0.8971	0.0898	NA
67187	Zmynd19	zinc finger, MYND domain containing 19	0.8971	0.246	0.6733
18040	Nefm	neurofilament, medium polypeptide	0.8971	0.118	NA
12877	Cpeb1	cytoplasmic polyadenylation element binding protein 1	0.8971	0.1667	NA
83814	Nedd4l	neural precursor cell expressed, developmentally down-regulated gene 4-like	0.897	0.4851	0.8126
67222	Srfbp1	serum response factor binding protein 1	0.897	0.1511	NA
109181	Trip11	thyroid hormone receptor interactor 11	0.8969	0.1308	NA
68488	1110002J07Rik	RIKEN cDNA 1110002J07 gene	0.8969	0.4728	0.8087
67136	Kbtbd4	kelch repeat and BTB (POZ) domain containing 4	0.8969	0.1087	NA
233871	Atxn2l	ataxin 2-like	0.8967	0.2603	0.6815
71970	Scand3	SCAN domain containing 3	0.8967	0.2156	0.6479
68348	Serpina1f	serine (or cysteine) peptidase inhibitor, clade A, member 1F	0.8967	0.6219	0.8719
58178	Sorcs1	VPS10 domain receptor protein SORCS 1	0.8967	0.187	0.6249
11658	Alcam	activated leukocyte cell adhesion molecule	0.8967	0.2859	0.6982
100042092	Gm3662	predicted gene 3662	0.8966	0.1165	NA
217337	Srp68	signal recognition particle 68	0.8966	0.2424	0.6704
71371	Arid5b	AT rich interactive domain 5B (MRF1-like)	0.8966	0.2028	0.6356
68146	Arl13b	ADP-ribosylation factor-like 13B	0.8966	0.4005	0.7703
380878	AF067063	cDNA sequence AF067063	0.8965	0.4193	0.7791
228602	4930402H24Rik	RIKEN cDNA 4930402H24 gene	0.8965	0.2424	0.6704
22070	Tpt1	tumor protein, translationally-controlled 1	0.8965	0.1818	0.6206
69513	1700030C10Rik	RIKEN cDNA 1700030C10 gene	0.8964	0.3541	0.7418
625360	BC147527	cDNA sequence BC147527	0.8963	0.7406	0.9179
15531	Ndst1	N-deacetylase/N-sulfotransferase (heparan glucosaminyl) 1	0.8963	0.1773	0.6167
328370	Rft1	RFT1 homolog (S. cerevisiae)	0.8962	0.1298	NA
214572	Prmt7	protein arginine N-methyltransferase 7	0.8962	0.2946	0.7044
104570	Smek2	SMEK homolog 2, suppressor of mek1 (Dictyostelium)	0.8962	0.3846	0.7606
52679	E2f7	E2F transcription factor 7	0.8962	0.5976	0.8611
13052	Cxadr	coxsackie virus and adenovirus receptor	0.8962	0.2624	0.6835
327885	C030046G05	hypothetical protein C030046G05	0.8961	0.211	0.6428
243308	A430033K04Rik	RIKEN cDNA A430033K04 gene	0.8961	0.2827	0.6969
241041	Gm4956	predicted gene 4956	0.8961	0.5593	0.8459
106205	Zc3h7a	zinc finger CCCH type containing 7 A	0.8961	0.1138	NA
52009	Hn1l	hematological and neurological expressed 1-like	0.8961	0.08767	NA
16600	Klf4	Kruppel-like factor 4 (gut)	0.8961	0.2532	0.6778
232539	Klhdc5	kelch domain containing 5	0.896	0.05978	NA
14284	Fosl2	fos-like antigen 2	0.896	0.221	0.6525
13846	Ephb4	Eph receptor B4	0.896	0.3295	0.7257
442812	B930049P21Rik	RIKEN cDNA B930049P21 gene	0.8959	0.21	0.6414
71951	Gpc2	glypican 2 (cerebroglycan)	0.8959	0.2125	0.6451
14287	Fpgs	folylpolyglutamyl synthetase	0.8959	0.2523	0.6775

329877	Dennd4c	DENN/MADD domain containing 4C	0.8958	0.1444	NA
30934	Tor1b	torsin family 1, member B	0.8958	0.09921	NA
16666	Krt16	keratin 16	0.8958	0.5234	0.8298
380912	Zfp395	zinc finger protein 395	0.8957	0.2874	0.6986
54137	Acrbp	proacrosin binding protein	0.8957	0.1026	NA
238505	Mtr	5-methyltetrahydrofolate-homocysteine methyltransferase	0.8956	0.1421	NA
78878	B230206F22Rik	RIKEN cDNA B230206F22 gene	0.8956	0.4842	0.8123
75423	Arl5a	ADP-ribosylation factor-like 5A	0.8956	0.2427	0.6705
73722	Lce1a2	late cornified envelope 1A2	0.8956	0.5622	0.8465
15519	Hsp90aa1	heat shock protein 90, alpha (cytosolic), class A member 1	0.8956	0.1041	NA
217558	G2e3	G2/M-phase specific E3 ubiquitin ligase	0.8955	0.4117	0.7747
195434	Utp14b	UTP14, U3 small nucleolar ribonucleoprotein, homolog B (yeast)	0.8955	0.6313	0.8751
66481	Rps21	ribosomal protein S21	0.8955	0.6772	0.893
66860	Tanc1	tetratricopeptide repeat, ankyrin repeat and coiled-coil containing 1	0.8954	0.302	0.708
319556	A330041J22Rik	RIKEN cDNA A330041J22 gene	0.8953	0.3569	0.7443
66609	Cryzl1	crystallin, zeta (quinone reductase)-like 1	0.8953	0.2156	0.6479
15163	Hcls1	hematopoietic cell specific Lyn substrate 1	0.8953	0.1108	NA
18803	Plcg1	phospholipase C, gamma 1	0.8952	0.2541	0.6788
67488	Calcoco1	calcium binding and coiled coil domain 1	0.8951	0.2671	0.6865
14457	Gas7	growth arrest specific 7	0.8951	0.3116	0.7137
12212	Chic1	cysteine-rich hydrophobic domain 1	0.8951	0.3563	0.7443
224139	Golgb1	golgi autoantigen, golgin subfamily b, macrogolgin 1	0.895	0.08095	NA
110417	Pigh	phosphatidylinositol glycan anchor biosynthesis, class H	0.895	0.109	NA
109624	Cald1	caldesmon 1	0.895	0.2586	0.6815
72102	Dusp11	dual specificity phosphatase 11 (RNA/RNP complex 1-interacting)	0.895	0.1697	0.611
68750	Rreb1	ras responsive element binding protein 1	0.895	0.1357	NA
83457	Fthl17	ferritin, heavy polypeptide-like 17	0.8949	0.2342	0.6633
21453	Tcof1	Treacher Collins Franceschetti syndrome 1, homolog	0.8949	0.1414	NA
100041241	Gm3227	predicted gene 3227	0.8948	0.5331	0.8341
270362	LOC270362	60S acidic ribosomal protein P1-like	0.8948	0.1822	0.6206
56316	Ggcx	gamma-glutamyl carboxylase	0.8948	0.1457	NA
21841	Tia1	cytotoxic granule-associated RNA binding protein 1	0.8948	0.08338	NA
17716	ND1	NADH dehydrogenase subunit 1	0.8948	0.1905	0.6275
230872	Crocc	ciliary rootlet coiled-coil, rootletin	0.8947	0.194	0.6299
15042	H2-T24	histocompatibility 2, T region locus 24	0.8947	0.8016	0.9373
13405	Dmd	dystrophin, muscular dystrophy	0.8947	0.03076	NA
225288	Fhod3	formin homology 2 domain containing 3	0.8946	0.1044	NA
56218	Patz1	POZ (BTB) and AT hook containing zinc finger 1	0.8946	0.3477	0.7383
93725	Ear10	eosinophil-associated, ribonuclease A family, member 10	0.8945	0.5395	0.8366
70661	Sik3	SIK family kinase 3	0.8944	0.1619	0.607
69562	Cdk13	cyclin-dependent kinase 13	0.8944	0.2065	0.6392
67429	Nudcd1	NudC domain containing 1	0.8944	0.5675	0.8485
83409	Robld3	roadblock domain containing 3	0.8943	0.2384	0.667
52626	Cdkn2aipnl	CDKN2A interacting protein N-terminal like	0.8943	0.3733	0.7538
50912	Exosc10	exosome component 10	0.8943	0.2604	0.6815
22779	Ikzf2	IKAROS family zinc finger 2	0.8943	0.2553	0.6798
22346	Vhl	von Hippel-Lindau tumor suppressor	0.8943	0.1289	NA
17755	Mtap1b	microtubule-associated protein 1B	0.8943	0.2365	0.6659
243725	Ppp1r9a	protein phosphatase 1, regulatory (inhibitor) subunit 9A	0.8942	0.5335	0.8343

140481	Man2a2	mannosidase 2, alpha 2	0.8942	0.2393	0.6679
67956	Setd8	SET domain containing (lysine methyltransferase) 8	0.8942	0.1856	0.6227
53959	AA914427	EST AA914427	0.8942	0.2011	0.635
70579	Zc3h11a	zinc finger CCCH type containing 11A	0.8941	0.1181	NA
66904	Pccb	propionyl Coenzyme A carboxylase, beta polypeptide	0.8941	0.06343	NA
66307	Isoc1	isochorismatase domain containing 1	0.8941	0.1457	NA
29863	Pde7b	phosphodiesterase 7B	0.8941	0.5229	0.8294
20020	Polr2a	polymerase (RNA) II (DNA directed) polypeptide A	0.8941	0.3766	0.7559
676974	LOC676974	glucose-6-phosphate isomerase-like	0.894	0.1474	NA
269378	Ahcy	S-adenosylhomocysteine hydrolase	0.894	0.353	0.7409
78785	Clip4	CAP-GLY domain containing linker protein family, member 4	0.894	0.1268	NA
69237	Gtpbp4	GTP binding protein 4	0.894	0.1383	NA
271887	BC066135	cDNA sequence BC066135	0.8939	0.5854	0.8551
18183	Nrg3	neuregulin 3	0.8939	0.5027	0.8214
14083	Ptk2	PTK2 protein tyrosine kinase 2	0.8939	0.4439	0.7931
240476	Zfp407	zinc finger protein 407	0.8938	0.5352	0.835
104570	Smek2	SMEK homolog 2, suppressor of mek1 (Dictyostelium)	0.8938	0.1166	NA
27973	Vkorc1	vitamin K epoxide reductase complex, subunit 1	0.8938	0.5289	0.8332
240614	Ranbp6	RAN binding protein 6	0.8937	0.2137	0.6465
238693	Zfp58	zinc finger protein 58	0.8937	0.2817	0.6967
70285	Rpf1	ribosome production factor 1 homolog (S. cerevisiae)	0.8937	0.2637	0.6843
13822	Epb4.1l2	erythrocyte protein band 4.1-like 2	0.8937	0.3337	0.7289
636187	LOC636187	60S ribosomal protein L21-like	0.8936	0.1734	0.6147
13518	Dst	dystonin	0.8936	0.6111	0.8672
209707	Lcorl	ligand dependent nuclear receptor corepressor-like	0.8935	0.2695	0.6878
108909	Aida	axin interactor, dorsalization associated	0.8934	0.0701	NA
17135	Mafk	v-maf musculoaponeurotic fibrosarcoma oncogene family, protein K (avian)	0.8934	0.06269	NA
170707	Usp48	ubiquitin specific peptidase 48	0.8933	0.2995	0.7054
103762	AI503301	expressed sequence AI503301	0.8933	0.1504	NA
71065	4933407O12Rik	RIKEN cDNA 4933407O12 gene	0.8933	0.3927	0.7651
17763	Mtcp1	mature T-cell proliferation 1	0.8933	0.2845	0.6977
13848	Ephb6	Eph receptor B6	0.8933	0.5748	0.8504
216190	Appl2	adaptor protein, phosphotyrosine interaction, PH domain and leucine zipper containing 2	0.8932	0.1009	NA
215690	Nav1	neuron navigator 1	0.8932	0.3186	0.7182
170459	Stard4	StAR-related lipid transfer (START) domain containing 4	0.8932	0.06826	NA
76510	Trappc9	trafficking protein particle complex 9	0.8932	0.494	0.8168
234023	Arglu1	arginine and glutamate rich 1	0.8931	0.1351	NA
75914	Exoc6b	exocyst complex component 6B	0.8931	0.4766	0.8099
12418	Cbx4	chromobox homolog 4 (Drosophila Pc class)	0.8931	0.184	0.6217
233033	Samd4b	sterile alpha motif domain containing 4B	0.893	0.2157	0.6479
229504	Isg20l2	interferon stimulated exonuclease gene 20-like 2	0.893	0.2025	0.6356
93871	Brwd1	bromodomain and WD repeat domain containing 1	0.893	0.04551	NA
76295	Atp11b	ATPase, class VI, type 11B	0.893	0.5194	0.8282
75678	Ippk	inositol 1,3,4,5,6-pentakisphosphate 2-kinase	0.8929	0.1206	NA
27176	Rpl7a	ribosomal protein L7A	0.8929	0.335	0.73
16568	Kif3a	kinesin family member 3A	0.8929	0.1585	0.603
619307	9430078G10Rik	RIKEN cDNA 9430078G10 gene	0.8928	0.2425	0.6705
236293	D630002G06Rik	RIKEN cDNA D630002G06 gene	0.8928	0.144	NA
66679	Rae1	RAE1 RNA export 1 homolog (S. pombe)	0.8928	0.1543	0.5975

22327	Vbp1	von Hippel-Lindau binding protein 1	0.8928	0.3454	0.736
15547	Trmt2a	TRM2 tRNA methyltransferase 2 homolog A (S. cerevisiae)	0.8928	0.03742	NA
237758	Zfp454	zinc finger protein 454	0.8927	0.4872	0.8133
226499	BC003331	cDNA sequence BC003331	0.8927	0.2381	0.6668
68789	Trmt61b	tRNA methyltransferase 61 homolog B (S. cerevisiae)	0.8926	0.3196	0.7189
53958	AU035318	EST AU035318	0.8926	0.2021	0.6356
544752	Tug1	taurine upregulated gene 1	0.8925	0.4867	0.8129
76897	Ralyl	RALY RNA binding protein-like	0.8924	0.4736	0.8088
66105	Ube2d3	ubiquitin-conjugating enzyme E2D 3 (UBC4/5 homolog, yeast)	0.8924	0.1444	NA
11546	Parp2	poly (ADP-ribose) polymerase family, member 2	0.8924	0.1992	0.6343
246179	Fktn	fukutin	0.8923	0.3138	0.7152
497097	Xkr4	X Kell blood group precursor related family member 4	0.8922	0.3489	0.7388
232966	Zfp114	zinc finger protein 114	0.8922	0.06888	NA
230753	Thrap3	thyroid hormone receptor associated protein 3	0.8922	0.6678	0.8904
108857	Ankhd1	ankyrin repeat and KH domain containing 1	0.8922	0.08243	NA
72554	Utp14a	UTP14, U3 small nucleolar ribonucleoprotein, homolog A (yeast)	0.8922	0.4459	0.7938
72117	Naa50	N(alpha)-acetyltransferase 50, NatE catalytic subunit	0.8922	0.4731	0.8088
71750	R3hdm2	R3H domain containing 2	0.8922	0.2966	0.7051
20318	Sdf4	stromal cell derived factor 4	0.8922	0.179	0.6181
16410	Itgav	integrin alpha V	0.8922	0.1265	NA
330267	Thsd7a	thrombospondin, type I, domain containing 7A	0.8921	0.1563	0.5994
107166	AI415730	expressed sequence AI415730	0.8921	0.04902	NA
66662	5730577I03Rik	zinc finger protein pseudogene	0.8921	0.3096	0.7129
74996	Usp47	ubiquitin specific peptidase 47	0.892	0.449	0.7943
29875	Iqgap1	IQ motif containing GTPase activating protein 1	0.892	0.1711	0.6118
239857	Cadm2	cell adhesion molecule 2	0.8919	0.1924	0.6283
227634	Camsap1	calmodulin regulated spectrin-associated protein 1	0.8919	0.06109	NA
75763	Dcaf17	DDB1 and CUL4 associated factor 17	0.8919	0.1755	0.6156
72562	Pcbd2	pterin 4 alpha carbinolamine dehydratase/dimerization cofactor of hepatocyte nuclear factor 1 alpha (TCF1) 2	0.8919	0.2584	0.6815
619309	A630081J09Rik	RIKEN cDNA A630081J09 gene	0.8918	0.2376	0.6663
270163	Myo9a	myosin IXa	0.8918	0.1252	NA
233276	Tubgcp5	tubulin, gamma complex associated protein 5	0.8918	0.3288	0.7253
216345	Zfc3h1	zinc finger, C3H1-type containing	0.8918	0.07773	NA
106068	Slc45a4	solute carrier family 45, member 4	0.8918	0.2203	0.6518
83925	Trps1	trichorhinophalangeal syndrome I (human)	0.8918	0.2289	0.6581
67139	Mis12	MIS12 homolog (yeast)	0.8918	0.3368	0.7305
59026	Huwe1	HECT, UBA and WWE domain containing 1	0.8918	0.1683	0.6095
12064	Bdnf	brain derived neurotrophic factor	0.8918	0.2393	0.6679
382090	4922501C03Rik	RIKEN cDNA 4922501C03 gene	0.8917	0.1231	NA
71675	0610010F05Rik	RIKEN cDNA 0610010F05 gene	0.8917	0.4757	0.8097
67620	Lrp2bp	Lrp2 binding protein	0.8917	0.4807	0.8103
67549	Gpr89	G protein-coupled receptor 89	0.8917	0.3137	0.7152
238021	Fscn2	fascin homolog 2, actin-bundling protein, retinal (Strongylocentrotus purpuratus)	0.8916	0.04267	NA
100503185	LOC100503185	BTB/POZ domain-containing protein 8-like	0.8915	0.3692	0.7521
382793	Mtx3	metaxin 3	0.8915	0.09014	NA
229542	Gatad2b	GATA zinc finger domain containing 2B	0.8915	0.2464	0.6734
108013	Celf4	CUGBP, Elav-like family member 4	0.8915	0.3007	0.7066
100040462	Mndal	myeloid nuclear differentiation antigen like	0.8914	0.1796	0.6187
269053	Gpr152	G protein-coupled receptor 152	0.8914	0.1105	NA

76781	Mettl4	methyltransferase like 4	0.8914	0.1382	NA
319638	Nt5dc1	5'-nucleotidase domain containing 1	0.8913	0.3069	0.7108
71163	Zfp626	zinc finger protein 626	0.8913	0.1536	0.5975
22172	Tyms-ps	thymidylate synthase, pseudogene	0.8913	0.1059	NA
319314	A930001C03Rik	RIKEN cDNA A930001C03 gene	0.8912	0.7128	0.9075
231326	Aasdh	aminoadipate-semialdehyde dehydrogenase	0.8912	0.1978	0.6329
214552	Cep164	centrosomal protein 164	0.8912	0.2757	0.6925
74302	Mtmr3	myotubularin related protein 3	0.8912	0.2212	0.6525
78889	Wsb1	WD repeat and SOCS box-containing 1	0.8911	0.1988	0.6336
22720	Zfp62	zinc finger protein 62	0.8911	0.3051	0.7098
11370	Acadvl	acyl-Coenzyme A dehydrogenase, very long chain	0.8911	0.1002	NA
320352	Lrrc31	leucine rich repeat containing 31	0.891	0.03932	NA
245886	Ankrd27	ankyrin repeat domain 27 (VPS9 domain)	0.891	0.5833	0.8538
242584	Wdr78	WD repeat domain 78	0.891	0.1526	0.5963
241296	Lrrc8a	leucine rich repeat containing 8A	0.891	0.4627	0.8028
76484	Kndc1	kinase non-catalytic C-lobe domain (KIND) containing 1	0.891	0.4043	0.7713
381305	Rc3h1	RING CCCH (C3H) domains 1	0.8908	0.3357	0.7302
72145	Wdfy3	WD repeat and FYVE domain containing 3	0.8908	0.2572	0.681
68235	2410066E13Rik	RIKEN cDNA 2410066E13 gene	0.8908	0.1347	NA
66616	Snx9	sorting nexin 9	0.8908	0.1685	0.6096
56036	Ccnl2	cyclin L2	0.8908	0.05852	NA
15202	Hemt1	hematopoietic cell transcript 1	0.8908	0.7385	0.9174
11790	Speg	SPEG complex locus	0.8908	0.2334	0.6623
622459	Gm12216	predicted gene 12216	0.8907	0.245	0.6727
319580	9630055L06Rik	RIKEN cDNA 9630055L06 gene	0.8907	0.1052	NA
68817	Ddi2	DNA-damage inducible protein 2	0.8907	0.416	0.7768
18072	Nhlh2	nescient helix loop helix 2	0.8907	0.1158	NA
435616	Gm5699	predicted gene 5699	0.8906	0.4684	0.8056
242851	Gnat3	guanine nucleotide binding protein, alpha transducing 3	0.8906	0.7026	0.9042
97820	4833439L19Rik	RIKEN cDNA 4833439L19 gene	0.8906	0.3263	0.7227
58800	Trpm7	transient receptor potential cation channel, subfamily M, member 7	0.8906	0.1345	NA
100042761	Gm10697	predicted gene 10697	0.8905	0.6194	0.8709
432628	Mfsd2b	major facilitator superfamily domain containing 2B	0.8905	0.1711	0.6118
27049	Etv3	ets variant gene 3	0.8905	0.09082	NA
544963	Iqgap2	IQ motif containing GTPase activating protein 2	0.8904	0.6114	0.8673
109575	Tbx10	T-box 10	0.8904	0.5982	0.8614
67579	Cpeb4	cytoplasmic polyadenylation element binding protein 4	0.8904	0.3363	0.7305
56009	Refbp2	RNA and export factor binding protein 2	0.8904	0.3179	0.718
319518	Pdpr	pyruvate dehydrogenase phosphatase regulatory subunit	0.8903	0.06736	NA
78244	Dnajc21	DnaJ (Hsp40) homolog, subfamily C, member 21	0.8903	0.1426	0.5877
58226	Cacna1h	calcium channel, voltage-dependent, T type, alpha 1H subunit	0.8903	0.04147	NA
18105	Nqo2	NAD(P)H dehydrogenase, quinone 2	0.8903	0.2555	0.6798
100039442	Gm2238	predicted gene 2238	0.8902	0.2613	0.6824
94109	Csmd1	CUB and Sushi multiple domains 1	0.8902	0.3695	0.7521
17975	Ncl	nucleolin	0.8902	0.3082	0.7112
233335	Synm	synemin, intermediate filament protein	0.8901	0.3633	0.7483
140917	Dclre1b	DNA cross-link repair 1B, PSO2 homolog (S. cerevisiae)	0.8901	0.3107	0.7135
69752	Zfp511	zinc finger protein 511	0.8901	0.1452	0.5899
21461	Tcp10b	t-complex protein 10b	0.8901	0.4276	0.7831

13175	Dclk1	doublecortin-like kinase 1	0.8901	0.08859	NA
67283	Slc25a19	solute carrier family 25 (mitochondrial thiamine pyrophosphate carrier), member 19	0.89	0.1613	0.607
17184	Matr3	matrin 3	0.89	0.2108	0.6426
12421	Rb1cc1	RB1-inducible coiled-coil 1	0.89	0.3163	0.7171
72139	2610044O15Rik	RIKEN cDNA 2610044O15 gene	0.8899	0.1284	NA
66923	Pbrm1	polybromo 1	0.8899	0.4696	0.8064
23881	G3bp2	GTPase activating protein (SH3 domain) binding protein 2	0.8899	0.1405	0.584
100040529	Gm2824	predicted gene 2824	0.8898	0.1393	0.5834
239364	Tspyl5	testis-specific protein, Y-encoded-like 5	0.8898	0.1998	0.6343
78433	A930006K02Rik	RIKEN cDNA A930006K02 gene	0.8898	0.3823	0.7596
66799	Ube2w	ubiquitin-conjugating enzyme E2W (putative)	0.8898	0.2439	0.6718
100503353	Gm14440	predicted gene 14440	0.8897	0.4739	0.8088
225888	Suv420h1	suppressor of variegation 4-20 homolog 1 (Drosophila)	0.8897	0.2974	0.7051
195208	Dcdc2a	doublecortin domain containing 2a	0.8897	0.1139	NA
107823	Whsc1	Wolf-Hirschhorn syndrome candidate 1 (human)	0.8897	0.2707	0.6889
69707	Iqcg	IQ motif containing G	0.8897	0.1636	0.6074
66854	Trim35	tripartite motif-containing 35	0.8897	0.706	0.9058
16439	Itpr2	inositol 1,4,5-triphosphate receptor 2	0.8897	0.06878	NA
100043489	1300002E11Rik	RIKEN cDNA 1300002E11 gene	0.8896	0.103	NA
544971	Bdp1	B double prime 1, subunit of RNA polymerase III transcription initiation factor IIIB	0.8896	0.09463	NA
70599	Ssfa2	sperm specific antigen 2	0.8896	0.281	0.6965
15569	Elavl2	ELAV (embryonic lethal, abnormal vision, Drosophila)-like 2 (Hu antigen B)	0.8896	0.2244	0.654
241447	Lass6	LAG1 homolog, ceramide synthase 6	0.8895	0.2996	0.7055
449000	Zfp960	zinc finger protein 960	0.8894	0.216	0.6479
319565	Syne2	synaptic nuclear envelope 2	0.8894	0.06209	NA
80905	Polh	polymerase (DNA directed), eta (RAD 30 related)	0.8894	0.2043	0.637
72140	Ccdc123	coiled-coil domain containing 123	0.8894	0.3108	0.7135
98758	Hnrnpf	heterogeneous nuclear ribonucleoprotein F	0.8893	0.5801	0.8527
77604	C430048L16Rik	RIKEN cDNA C430048L16 gene	0.8893	0.07083	NA
70361	Lman1	lectin, mannose-binding, 1	0.8893	0.4672	0.8054
22631	Ywhaz	tyrosine 3-monooxygenase/tryptophan 5-monooxygenase activation protein, zeta polypeptide	0.8893	0.4994	0.8197
19258	Ptpn4	protein tyrosine phosphatase, non-receptor type 4	0.8893	0.4264	0.7823
624784	Gm9855	thymine DNA glycosylase pseudogene	0.8892	0.5543	0.8443
381650	Thap6	THAP domain containing 6	0.8892	0.1023	NA
244885	Sh2d7	SH2 domain containing 7	0.8892	0.2746	0.6924
104082	Wdr7	WD repeat domain 7	0.8892	0.4238	0.7817
83397	Akap12	A kinase (PRKA) anchor protein (gravin) 12	0.8892	0.6381	0.8777
74552	Nipal3	NIPA-like domain containing 3	0.8892	0.2952	0.7046
69389	1700014N06Rik	RIKEN cDNA 1700014N06 gene	0.8892	0.7311	0.9153
68142	Ino80	INO80 homolog (S. cerevisiae)	0.8892	0.09177	NA
20349	Sema3e	sema domain, immunoglobulin domain (Ig), short basic domain, secreted, (semaphorin) 3E	0.8892	0.2803	0.6954
320508	Cachd1	cache domain containing 1	0.8891	0.103	NA
268706	Slc38a9	solute carrier family 38, member 9	0.8891	0.2675	0.6867
114564	Csprs	component of Sp100-rs	0.8891	0.5488	0.841
73229	3110052M02Rik	RIKEN cDNA 3110052M02 gene	0.8891	0.3379	0.7314
22630	Ywhaq	tyrosine 3-monooxygenase/tryptophan 5-monooxygenase activation protein, theta polypeptide	0.8891	0.3142	0.7155
239833	Lmln	leishmanolysin-like (metallopeptidase M8 family)	0.889	0.1542	0.5975
234967	Slc36a4	solute carrier family 36 (proton/amino acid symporter), member 4	0.889	0.275	0.6925
227644	Snapc4	small nuclear RNA activating complex, polypeptide 4	0.889	0.1007	NA

16000	Igf1	insulin-like growth factor 1	0.889	0.295	0.7045
328087	D930001B02	hypothetical protein D930001B02	0.8889	0.1738	0.6148
258750	Olfr551	olfactory receptor 551	0.8889	0.4667	0.8049
223828	Pphln1	periphilin 1	0.8889	0.283	0.697
16797	Lat	linker for activation of T cells	0.8889	0.3262	0.7227
14674	Gna13	guanine nucleotide binding protein, alpha 13	0.8889	0.2586	0.6815
432508	Cpsf6	cleavage and polyadenylation specific factor 6	0.8888	0.02578	NA
368203	Gm5136	predicted gene 5136	0.8888	0.6403	0.8786
214133	Tet2	tet oncogene family member 2	0.8888	0.1401	0.5834
67163	Ccdc47	coiled-coil domain containing 47	0.8888	0.5272	0.8329
271209	Rp1l1	retinitis pigmentosa 1 homolog (human)-like 1	0.8887	0.148	0.5911
217480	Dgkb	diacylglycerol kinase, beta	0.8887	0.1446	0.5892
93746	Gprc5d	G protein-coupled receptor, family C, group 5, member D	0.8887	0.2287	0.658
19714	Rev3l	REV3-like, catalytic subunit of DNA polymerase zeta RAD54 like (S. cerevisiae)	0.8887	0.109	NA
241175	Cntnap5b	contactin associated protein-like 5B	0.8886	0.2467	0.6738
74154	Unkl	unkempt-like (Drosophila)	0.8886	0.6258	0.8728
71840	Tekt4	tektin 4	0.8886	0.4216	0.7798
70750	Kdsr	3-ketodihydrosphingosine reductase	0.8886	0.187	0.6249
70160	Vps36	vacuolar protein sorting 36 (yeast)	0.8886	0.1792	0.6186
52463	Tet1	tet oncogene 1	0.8886	0.1532	0.5969
20425	Shmt1	serine hydroxymethyltransferase 1 (soluble)	0.8886	0.2758	0.6925
211484	Tsga10	testis specific 10	0.8885	0.1268	NA
100043272	5430417L22Rik	RIKEN cDNA 5430417L22 gene	0.8884	0.2863	0.6982
368202	Prss48	protease, serine, 48	0.8884	0.1304	0.5747
329831	Fam166b	family with sequence similarity 166, member B	0.8884	0.1175	NA
233895	Prr14	proline rich 14	0.8884	0.04075	NA
21871	Atp6v0a2	ATPase, H+ transporting, lysosomal V0 subunit A2	0.8884	0.1788	0.6181
100042453	Gm3852	predicted gene 3852	0.8883	0.2854	0.6982
245583	Tgif2lx1	TGFB-induced factor homeobox 2-like, X-linked 1	0.8883	0.516	0.8277
74023	Rd3	retinal degeneration 3	0.8883	0.2162	0.6479
71458	Bcor	BCL6 interacting corepressor	0.8883	0.2001	0.6345
236904	Klhl15	kelch-like 15 (Drosophila)	0.8882	0.5851	0.855
216971	BC017647	cDNA sequence BC017647	0.8882	0.359	0.7459
76890	Memo1	mediator of cell motility 1	0.8882	0.08593	NA
56808	Cacna2d2	calcium channel, voltage-dependent, alpha 2/delta subunit 2	0.8882	0.7113	0.907
21366	Slc6a6	solute carrier family 6 (neurotransmitter transporter, taurine), member 6	0.8882	0.09465	NA
269198	Nbeal1	neurobeachin like 1	0.8881	0.6037	0.8644
217705	Fam161b	family with sequence similarity 161, member B	0.8881	0.05574	NA
56708	Clcf1	cardiotrophin-like cytokine factor 1	0.8881	0.2164	0.6479
100303732	Gm14431	predicted gene 14431	0.888	0.3201	0.7191
19249	Ptpn13	protein tyrosine phosphatase, non-receptor type 13	0.888	0.1335	0.5774
320429	Trank1	tetratricopeptide repeat and ankyrin repeat containing 1	0.8879	0.5652	0.8474
71331	5430411C19Rik	RIKEN cDNA 5430411C19 gene	0.8879	0.08764	NA
626596	Rgs22	regulator of G-protein signalling 22	0.8878	0.2198	0.6513
338369	Tmem220	transmembrane protein 220	0.8878	0.3657	0.7499
116939	Pnpla3	patatin-like phospholipase domain containing 3	0.8878	0.1803	0.6197
78871	B230117O15Rik	RIKEN cDNA B230117O15 gene	0.8878	0.2854	0.6982
72108	Ddhd2	DDHD domain containing 2	0.8878	0.4742	0.809
20778	Scarb1	scavenger receptor class B, member 1	0.8878	0.1479	0.5911

268663	Cdhr2	cadherin-related family member 2	0.8877	0.4102	0.7739
78294	Rps27a	ribosomal protein S27A	0.8877	0.4337	0.7874
21423	Tcf3	transcription factor 3	0.8877	0.08291	NA
433102	Sfta2	surfactant associated 2	0.8876	0.3666	0.7504
235132	Zbtb44	zinc finger and BTB domain containing 44	0.8876	0.3177	0.7179
66253	Aig1	androgen-induced 1	0.8876	0.2844	0.6977
17913	Myo1c	myosin IC	0.8876	0.2229	0.654
230119	Zbtb5	zinc finger and BTB domain containing 5	0.8875	0.1916	0.6278
105522	Ankrd28	ankyrin repeat domain 28	0.8875	0.2124	0.6449
66254	Dimt1	DIM1 dimethyladenosine transferase 1-like (S. cerevisiae)	0.8875	0.06107	NA
58212	Srrm3	serine/arginine repetitive matrix 3	0.8875	0.2933	0.7028
19130	Prox1	prospero-related homeobox 1	0.8875	0.1272	0.5715
217030	Synrg	synergin, gamma	0.8874	0.2708	0.689
74352	Zfp84	zinc finger protein 84	0.8874	0.5171	0.8277
21416	Tcf7l2	transcription factor 7-like 2, T-cell specific, HMG-box	0.8874	0.6046	0.8644
22666	Zfp161	zinc finger protein 161	0.8873	0.2718	0.6891
381059	Gm1604b	predicted gene 1604b	0.8872	0.4272	0.7827
54632	Ftsj1	FtsJ homolog 1 (E. coli)	0.8872	0.2354	0.6654
77193	A930030B08Rik	RIKEN cDNA A930030B08 gene	0.8871	0.3501	0.739
67610	Rspry1	ring finger and SPRY domain containing 1	0.8871	0.08682	NA
56404	Trip4	thyroid hormone receptor interactor 4	0.8871	0.06635	NA
16971	Lrp1	low density lipoprotein receptor-related protein 1	0.8871	0.2225	0.654
76862	4833405L11Rik	RIKEN cDNA 4833405L11 gene	0.887	0.4572	0.799
17721	ND5	NADH dehydrogenase subunit 5	0.887	0.031	NA
14105	Srsf10	serine/arginine-rich splicing factor 10	0.887	0.1066	NA
286940	Flnb	filamin, beta	0.8869	0.1871	0.6249
259144	Olfr456	olfactory receptor 456	0.8869	0.6586	0.8869
226412	R3hdm1	R3H domain 1 (binds single-stranded nucleic acids)	0.8869	0.09145	NA
17931	Ppp1r12a	protein phosphatase 1, regulatory (inhibitor) subunit 12A	0.8869	0.3285	0.7253
99003	Qser1	glutamine and serine rich 1	0.8868	0.1254	0.5703
56637	Gsk3b	glycogen synthase kinase 3 beta	0.8868	0.1531	0.5966
20863	Stfa3	stefin A3	0.8868	0.6446	0.8802
12824	Col2a1	collagen, type II, alpha 1	0.8868	0.133	0.5772
319163	Hist1h2aa	histone cluster 1, H2aa	0.8867	0.5472	0.84
208111	C330019L16Rik	RIKEN cDNA C330019L16 gene	0.8867	0.3525	0.7406
11855	Arhgap5	Rho GTPase activating protein 5	0.8867	0.2478	0.6749
667118	Zbed6	zinc finger, BED domain containing 6	0.8866	0.3996	0.7699
268729	Gm626	predicted gene 626	0.8866	0.3537	0.7414
268721	2310021P13Rik	RIKEN cDNA 2310021P13 gene	0.8866	0.04751	NA
78921	9130019O22Rik	RIKEN cDNA 9130019O22 gene	0.8866	0.4806	0.8101
76174	6330526H18Rik	RIKEN cDNA 6330526H18 gene	0.8866	0.1861	0.6234
71175	Nipbl	Nipped-B homolog (Drosophila)	0.8866	0.3383	0.7317
328243	E230012P03	hypothetical protein E230012P03	0.8865	0.3279	0.7248
234915	AK129341	cDNA sequence AK129341	0.8865	0.04755	NA
68708	Rabl2	RAB, member of RAS oncogene family-like 2	0.8865	0.1663	0.6083
319164	Hist1h2ac	histone cluster 1, H2ac	0.8864	0.2688	0.6876
235661	Dync1li1	dynein cytoplasmic 1 light intermediate chain 1	0.8864	0.2103	0.6419
235542	Ppp2r3a	protein phosphatase 2, regulatory subunit B'', alpha	0.8864	0.4979	0.8193
75826	Senp2	SUMO/sentrin specific peptidase 2	0.8864	0.03212	NA

233490	Crebzf	CREB/ATF bZIP transcription factor	0.8863	0.1669	0.6083
93877	Pcdhb6	protocadherin beta 6	0.8863	0.03537	NA
76421	1700028K03Rik	RIKEN cDNA 1700028K03 gene	0.8863	0.1081	NA
74435	Lrriq3	leucine-rich repeats and IQ motif containing 3	0.8863	0.665	0.8903
73873	Fam161a	family with sequence similarity 161, member A	0.8863	0.1889	0.6266
20383	Srsf3	serine/arginine-rich splicing factor 3	0.8863	0.3884	0.7616
14800	Gria2	glutamate receptor, ionotropic, AMPA2 (alpha 2)	0.8863	0.271	0.689
66625	Sfrs18	serine/arginine-rich splicing factor 18	0.8862	0.1121	NA
57776	Ttyh1	tweety homolog 1 (Drosophila)	0.8862	0.01981	NA
54614	Prpf40b	PRP40 pre-mRNA processing factor 40 homolog B (yeast)	0.8862	0.2018	0.6356
16974	Lrp6	low density lipoprotein receptor-related protein 6	0.8862	0.271	0.6891
622665	Ccdc17	coiled-coil domain containing 17	0.8861	0.02362	NA
108071	Grm5	glutamate receptor, metabotropic 5	0.8861	0.2757	0.6925
68147	Gar1	GAR1 ribonucleoprotein homolog (yeast)	0.8861	0.02158	NA
68024	Hist1h2bc	histone cluster 1, H2bc	0.8861	0.1377	0.5815
72836	Pot1b	protection of telomeres 1B	0.886	0.4392	0.7906
70717	6330406I15Rik	RIKEN cDNA 6330406I15 gene	0.886	0.2202	0.6518
69260	Ing2	inhibitor of growth family, member 2	0.886	0.2122	0.6448
57259	Tob2	transducer of ERBB2, 2	0.886	0.43	0.7843
54367	Zfp326	zinc finger protein 326	0.886	0.1499	0.5938
103841	Cuedc1	CUE domain containing 1	0.8859	0.2724	0.6896
74201	Cep97	centrosomal protein 97	0.8859	0.2533	0.6778
67685	Dyx1c1	dyslexia susceptibility 1 candidate 1 homolog (human)	0.8859	0.01299	NA
20541	Slc8a1	solute carrier family 8 (sodium/calcium exchanger), member 1	0.8859	0.6549	0.8854
12351	Car4	carbonic anhydrase 4	0.8859	0.6917	0.8991
211948	Pde12	phosphodiesterase 12	0.8858	0.3417	0.7337
54137	Acrbp	proacrosin binding protein	0.8858	0.1756	0.6156
232811	Suv420h2	suppressor of variegation 4-20 homolog 2 (Drosophila)	0.8857	0.09183	NA
75124	Nxnl2	nucleoredoxin-like 2	0.8857	0.1216	0.5651
68952	Fam57b	family with sequence similarity 57, member B	0.8856	0.1248	0.5703
14583	Gfpt1	glutamine fructose-6-phosphate transaminase 1	0.8856	0.4511	0.7948
408058	BC048507	cDNA sequence BC048507	0.8855	0.7185	0.9102
71834	Zbtb43	zinc finger and BTB domain containing 43	0.8855	0.04291	NA
100042464	2610203C20Rik	RIKEN cDNA 2610203C20 gene	0.8854	0.1447	0.5893
269209	Stk36	serine/threonine kinase 36 (fused homolog, Drosophila)	0.8854	0.3071	0.7108
229357	Gpr149	G protein-coupled receptor 149	0.8854	0.2587	0.6815
100710	Pds5b	PDS5, regulator of cohesion maintenance, homolog B (S. cerevisiae)	0.8853	0.1998	0.6344
76897	Ralyl	RALY RNA binding protein-like	0.8853	0.4113	0.7747
72137	Wdsub1	WD repeat, SAM and U-box domain containing 1	0.8853	0.1323	0.5767
23984	Pde10a	phosphodiesterase 10A	0.8853	0.3496	0.7388
100038662	D130046C19Rik	RIKEN cDNA D130046C19 gene	0.8852	0.3483	0.7385
384783	Irs2	insulin receptor substrate 2	0.8852	0.1473	0.591
73453	1700067K01Rik	RIKEN cDNA 1700067K01 gene	0.8852	0.6351	0.877
18191	Nrxn3	neurexin III	0.8852	0.4106	0.7742
240614	Ranbp6	RAN binding protein 6	0.8851	0.2716	0.6891
225348	Wdr36	WD repeat domain 36	0.8851	0.2305	0.6601
210004	B3gntl1	UDP-GlcNAc:betaGal beta-1,3-N-acetylglucosaminyltransferase-like 1	0.8851	0.08133	NA
73183	5430402O13Rik	RIKEN cDNA 5430402O13 gene	0.8851	0.03672	NA
18578	Pde4b	phosphodiesterase 4B, cAMP specific	0.8851	0.4236	0.7816

12211	Birc6	baculoviral IAP repeat-containing 6	0.8851	0.09782	NA
67533	Ppfibp1	PTPRF interacting protein, binding protein 1 (liprin beta 1)	0.885	0.0252	NA
26428	Orc4	origin recognition complex, subunit 4	0.885	0.2197	0.6513
15528	Hspe1	heat shock protein 1 (chaperonin 10)	0.885	0.02699	NA
66101	Ppih	peptidyl prolyl isomerase H	0.8849	0.765	0.9251
320983	B130019D13Rik	RIKEN cDNA B130019D13 gene	0.8848	0.08367	NA
69161	Manbal	mannosidase, beta A, lysosomal-like	0.8848	0.1124	NA
14800	Gria2	glutamate receptor, ionotropic, AMPA2 (alpha 2)	0.8848	0.2445	0.6725
97455	C78859	expressed sequence C78859	0.8847	0.133	0.5772
620401	Gm16442	predicted gene 16442	0.8846	0.3269	0.7235
243274	Tmem132d	transmembrane protein 132D	0.8846	0.07856	NA
67526	Atg12	autophagy-related 12 (yeast)	0.8846	0.2275	0.6566
17933	Myt1l	myelin transcription factor 1-like	0.8846	0.3781	0.7573
14199	Fhl1	four and a half LIM domains 1	0.8846	0.09312	NA
13131	Dab1	disabled homolog 1 (Drosophila)	0.8846	0.4399	0.7908
224694	Zfp81	zinc finger protein 81	0.8845	0.5051	0.8226
97654	C81189	expressed sequence C81189	0.8845	0.2547	0.6792
170707	Usp48	ubiquitin specific peptidase 48	0.8844	0.07629	NA
101739	Psip1	PC4 and SFRS1 interacting protein 1	0.8844	0.4065	0.7726
11304	Abca4	ATP-binding cassette, sub-family A (ABC1), member 4	0.8844	0.172	0.6126
677113	LOC677113	40S ribosomal protein S24-like	0.8843	0.7564	0.9223
240641	Kif20b	kinesin family member 20B	0.8843	0.08124	NA
235106	Ntm	neurotrimin	0.8843	0.1588	0.6033
226418	Yod1	YOD1 OTU deubiquitinating enzyme 1 homologue (S. cerevisiae)	0.8843	0.08014	NA
106131	AA589532	expressed sequence AA589532	0.8843	0.2773	0.6925
24110	Usp18	ubiquitin specific peptidase 18	0.8843	0.4254	0.7823
22359	Vldlr	very low density lipoprotein receptor	0.8843	0.02649	NA
17706	ATP8	ATP synthase F0 subunit 8	0.8843	0.02831	NA
791388	AY512938	cDNA sequence AY512938	0.8842	0.2053	0.6382
72899	Macrod2	MACRO domain containing 2	0.8842	0.1254	0.5703
13618	Ednrb	endothelin receptor type B	0.8842	0.1317	0.5758
17128	Smad4	MAD homolog 4 (Drosophila)	0.8841	0.1658	0.6077
225004	BC027072	cDNA sequence BC027072	0.884	0.08233	NA
101706	Numa1	nuclear mitotic apparatus protein 1	0.884	0.09967	NA
68515	Myadml2	myeloid-associated differentiation marker-like 2	0.884	0.581	0.8527
67991	Nacc2	nucleus accumbens associated 2, BEN and BTB (POZ) domain containing	0.884	0.7992	0.9361
66139	Tmem8c	transmembrane protein 8C	0.884	0.364	0.7487
12983	Csf2rb	colony stimulating factor 2 receptor, beta, low-affinity (granulocyte-macrophage)	0.884	0.59	0.8578
12659	Ovgp1	oviductal glycoprotein 1	0.884	0.01023	NA
100041677	Gm13157	predicted gene 13157	0.8839	0.6097	0.8665
328417	Parp4	poly (ADP-ribose) polymerase family, member 4	0.8838	0.04236	NA
216965	Taok1	TAO kinase 1	0.8838	0.0248	NA
103012	6720401G13Rik	RIKEN cDNA 6720401G13 gene	0.8838	0.07365	NA
69743	Casz1	castor homolog 1, zinc finger (Drosophila)	0.8838	0.1121	0.5549
68703	Rere	arginine glutamic acid dipeptide (RE) repeats	0.8838	0.3138	0.7152
552906	LOC552906	hypothetical LOC552906	0.8837	0.1771	0.6165
99426	AW742560	expressed sequence AW742560	0.8837	0.1512	0.5943
67456	Ergic2	ERGIC and golgi 2	0.8837	0.3179	0.718
22648	Zfp11	zinc finger protein 11	0.8837	0.3599	0.7465

22021	Tpst1	protein-tyrosine sulfotransferase 1	0.8837	0.8565	0.9554
321003	Xpnpep3	X-prolyl aminopeptidase (aminopeptidase P) 3, putative	0.8836	0.4572	0.799
268890	Lsamp	limbic system-associated membrane protein	0.8836	0.2012	0.635
231207	Cpeb2	cytoplasmic polyadenylation element binding protein 2	0.8835	0.3678	0.7517
230259	E130308A19Rik	RIKEN cDNA E130308A19 gene	0.8835	0.3496	0.7388
225998	Rorb	RAR-related orphan receptor beta	0.8835	0.1033	NA
170823	Glmn	glomulin, FKBP associated protein	0.8835	0.1693	0.61
64291	Osbpl1a	oxysterol binding protein-like 1A	0.8835	0.228	0.6569
18763	Pkd1	polycystic kidney disease 1 homolog	0.8835	0.1812	0.6204
545459	Gm10766	predicted gene 10766	0.8833	0.1939	0.6299
52398	Sep-11	septin 11	0.8833	0.3385	0.7319
76707	Clasp1	CLIP associating protein 1	0.8832	0.07532	NA
320707	Atp2b3	ATPase, Ca++ transporting, plasma membrane 3	0.8831	0.2524	0.6775
110789	Gpr98	G protein-coupled receptor 98	0.8831	0.02392	NA
107368	Pdzd8	PDZ domain containing 8	0.8831	0.4028	0.7708
75458	Cklf	chemokine-like factor	0.8831	0.1868	0.6247
67300	Cltc	clathrin, heavy polypeptide (Hc)	0.8831	0.06093	NA
224598	Zfp758	zinc finger protein 758	0.883	0.4619	0.8022
104444	Rexo2	REX2, RNA exonuclease 2 homolog (S. cerevisiae)	0.883	0.1901	0.6274
70430	Tbce	tubulin-specific chaperone E	0.883	0.1596	0.6042
15571	Elavl3	ELAV (embryonic lethal, abnormal vision, Drosophila)-like 3 (Hu antigen C)	0.883	0.07898	NA
110058	Syt17	synaptotagmin XVII	0.8829	0.4286	0.7838
26366	Ceacam10	carcinoembryonic antigen-related cell adhesion molecule 10	0.8829	0.4743	0.809
74213	Rbm26	RNA binding motif protein 26	0.8828	0.2745	0.6924
53609	Clasrp	CLK4-associating serine/arginine rich protein	0.8827	0.1028	NA
12859	Cox5b	cytochrome c oxidase, subunit Vb	0.8827	0.1333	0.5773
101187	Parp11	poly (ADP-ribose) polymerase family, member 11	0.8826	0.2072	0.6398
73998	Herc3	hect domain and RLD 3	0.8826	0.1347	0.5779
66926	Trmt6	tRNA methyltransferase 6 homolog (S. cerevisiae)	0.8826	0.3145	0.7158
245386	Fam70a	family with sequence similarity 70, member A	0.8825	0.232	0.6618
269400	Rtel1	regulator of telomere elongation helicase 1	0.8824	0.09754	NA
214384	Myocd	myocardin	0.8824	0.4027	0.7708
77521	Mtus2	microtubule associated tumor suppressor candidate 2	0.8824	0.05583	NA
14113	Fbl	fibrillarin	0.8824	0.1119	0.5549
320595	Phf8	PHD finger protein 8	0.8823	0.1436	0.5879
67574	Alg13	asparagine-linked glycosylation 13 homolog (S. cerevisiae)	0.8823	0.107	0.5492
327958	Pitpnm3	PITPNM family member 3	0.8822	0.08411	NA
215008	Vezt	vezatin, adherens junctions transmembrane protein	0.8821	0.03792	NA
67006	Cisd2	CDGSH iron sulfur domain 2	0.8821	0.1033	NA
23919	Insl5	insulin-like 5	0.8821	0.2308	0.6601
268752	Wdfy2	WD repeat and FYVE domain containing 2	0.882	0.4247	0.7819
231805	Pilra	paired immunoglobin-like type 2 receptor alpha	0.882	0.5543	0.8443
213753	Zfp598	zinc finger protein 598	0.882	0.4281	0.7837
14812	Grin2b	glutamate receptor, ionotropic, NMDA2B (epsilon 2)	0.882	0.4718	0.8079
257973	Olfr207	olfactory receptor 207	0.8819	0.7182	0.91
68828	Sync	syncoilin	0.8818	0.4209	0.7798
23856	Dido1	death inducer-obliterator 1	0.8818	0.2104	0.642
13681	Eif4a1	eukaryotic translation initiation factor 4A1	0.8818	0.1356	0.5793
330361	AW146020	expressed sequence AW146020	0.8817	0.1158	0.558

20740	Spna2	spectrin alpha 2	0.8817	0.6167	0.8698
22589	Atrx	alpha thalassemia/mental retardation syndrome X-linked homolog (human)	0.8816	0.3055	0.7101
53379	Hnrnpa2b1	heterogeneous nuclear ribonucleoprotein A2/B1	0.8815	0.1523	0.5958
72338	Wdr89	WD repeat domain 89	0.8814	0.06815	NA
18087	Nktr	natural killer tumor recognition sequence	0.8814	0.01405	NA
17864	Mybl1	myeloblastosis oncogene-like 1	0.8814	0.2319	0.6618
194388	Tet3	tet oncogene family member 3	0.8813	0.5564	0.8448
69131	Cdk12	cyclin-dependent kinase 12	0.8813	0.175	0.6153
18004	Nek1	NIMA (never in mitosis gene a)-related expressed kinase 1	0.8813	0.1093	0.5529
14007	Celf2	CUGBP, Elav-like family member 2	0.8812	0.182	0.6206
74307	1700092M07Rik	RIKEN cDNA 1700092M07 gene	0.8811	0.1908	0.6276
93892	Pcdhb21	protocadherin beta 21	0.881	0.08336	NA
70359	Gtpbp3	GTP binding protein 3	0.881	0.3014	0.7077
19276	Ptprn2	protein tyrosine phosphatase, receptor type, N polypeptide 2	0.8809	0.185	0.6223
213054	Gabpb2	GA repeat binding protein, beta 2	0.8808	0.3632	0.7483
170742	Sertad3	SERTA domain containing 3	0.8808	0.482	0.8108
72672	Zfp518a	zinc finger protein 518A	0.8807	0.03185	NA
69930	Zfp715	zinc finger protein 715	0.8807	0.1116	0.5549
67842	2610027L16Rik	RIKEN cDNA 2610027L16 gene	0.8807	0.2774	0.6925
100043100	Zfp534	zinc finger protein 534	0.8806	0.6137	0.8688
239319	Card6	caspase recruitment domain family, member 6	0.8806	0.176	0.6156
100038734	Gm10845	predicted gene 10845	0.8805	0.3966	0.7675
18140	Uhrf1	ubiquitin-like, containing PHD and RING finger domains, 1	0.8805	0.5654	0.8476
71339	5430400D12Rik	RIKEN cDNA 5430400D12 gene	0.8804	0.06565	NA
20514	Slc1a5	solute carrier family 1 (neutral amino acid transporter), member 5	0.8804	0.2399	0.6685
319933	E230024E03Rik	RIKEN cDNA E230024E03 gene	0.8803	0.5506	0.8424
78672	9530057J20Rik	RIKEN cDNA 9530057J20 gene	0.8803	0.1819	0.6206
71512	9030419F21Rik	RIKEN cDNA 9030419F21 gene	0.8803	0.05966	NA
69668	Ccdc115	coiled-coil domain containing 115	0.8803	0.07681	NA
71643	4930422G04Rik	RIKEN cDNA 4930422G04 gene	0.8802	0.4643	0.8038
50796	Dmrt1	doublesex and mab-3 related transcription factor 1	0.8802	0.1287	0.5733
17245	Mdm1	transformed mouse 3T3 cell double minute 1	0.8802	0.266	0.6856
338366	Mia3	melanoma inhibitory activity 3	0.8801	0.4795	0.8101
235047	Zfp809	zinc finger protein 809	0.8801	0.1517	0.5948
223989	4921513D23Rik	RIKEN cDNA 4921513D23 gene	0.8801	0.4853	0.8127
208606	Rsrc2	arginine/serine-rich coiled-coil 2	0.8801	0.1146	0.5569
54169	Myst4	MYST histone acetyltransferase monocytic leukemia 4	0.8801	0.2489	0.6767
434008	Gm5567	predicted gene 5567	0.88	0.1872	0.6253
381290	Atp2b4	ATPase, Ca++ transporting, plasma membrane 4	0.88	0.3016	0.7079
320720	Fastkd1	FAST kinase domains 1	0.88	0.0603	NA
210544	Wdr67	WD repeat domain 67	0.8799	0.5681	0.8486
71064	4933406B15Rik	RIKEN cDNA 4933406B15 gene	0.8799	0.8406	0.9486
229776	Cdc14a	CDC14 cell division cycle 14 homolog A (S. cerevisiae)	0.8798	0.438	0.7894
215436	Slc35e3	solute carrier family 35, member E3	0.8798	0.2356	0.6654
24052	Sgcd	sarcoglycan, delta (dystrophin-associated glycoprotein)	0.8798	0.5375	0.8358
18987	Pou2f2	POU domain, class 2, transcription factor 2	0.8798	0.3061	0.7106
64291	Osbpl1a	oxysterol binding protein-like 1A	0.8797	0.2646	0.6845
20704	Serpina1e	serine (or cysteine) peptidase inhibitor, clade A, member 1E	0.8797	0.5666	0.8481
105377	Ankrd32	ankyrin repeat domain 32	0.8796	0.1676	0.6094

70480	5730407M17Rik	RIKEN cDNA 5730407M17 gene	0.8796	0.076	NA
68162	A930003A15Rik	RIKEN cDNA A930003A15 gene	0.8796	0.125	0.5703
26987	Eif4e2	eukaryotic translation initiation factor 4E member 2	0.8796	0.2848	0.6977
19951	Rpl32	ribosomal protein L32	0.8796	0.09939	0.5367
19134	Prpf4b	PRP4 pre-mRNA processing factor 4 homolog B (yeast)	0.8796	0.4244	0.7819
56356	Gltp	glycolipid transfer protein	0.8793	0.5093	0.8247
319885	Zcchc7	zinc finger, CCHC domain containing 7	0.8792	0.06388	NA
17925	Myo9b	myosin IXb	0.8792	0.02504	NA
76987	Hdhd2	haloacid dehalogenase-like hydrolase domain containing 2	0.8791	0.4316	0.7855
626150	Gm12271	predicted gene 12271	0.879	0.2748	0.6925
107477	Guca1b	guanylate cyclase activator 1B	0.879	0.3057	0.7104
66433	Chchd7	coiled-coil-helix-coiled-coil-helix domain containing 7	0.879	0.1131	0.5556
17311	Kitl	kit ligand	0.879	0.3367	0.7305
100040233	Gm10334	predicted gene 10334	0.8789	0.5733	0.8501
244871	Zc3h12c	zinc finger CCCH type containing 12C	0.8789	0.1842	0.6217
241656	Pak7	p21 protein (Cdc42/Rac)-activated kinase 7	0.8789	0.2502	0.6767
224826	Ubr2	ubiquitin protein ligase E3 component n-recognin 2	0.8789	0.3454	0.736
217265	Abca5	ATP-binding cassette, sub-family A (ABC1), member 5	0.8789	0.401	0.7703
78619	Zfp449	zinc finger protein 449	0.8789	0.1244	0.5694
545238	G430049J08Rik	RIKEN cDNA G430049J08 gene	0.8788	0.1242	0.5693
70546	Zdhhc2	zinc finger, DHHC domain containing 2	0.8788	0.0218	NA
16206	Lrig1	leucine-rich repeats and immunoglobulin-like domains 1	0.8788	0.2497	0.6767
12807	Hps3	Hermansky-Pudlak syndrome 3 homolog (human)	0.8788	0.01387	NA
69443	1700027J07Rik	RIKEN cDNA 1700027J07 gene	0.8787	0.6163	0.8698
14218	Sh3pxd2a	SH3 and PX domains 2A	0.8787	0.1319	0.5758
67429	Nudcd1	NudC domain containing 1	0.8786	0.8042	0.9381
545700	Vmn2r-ps14	vomeronasal 2, receptor, pseudogene 14	0.8785	0.4191	0.7791
78428	Wibg	within bgcn homolog (Drosophila)	0.8785	0.4947	0.8173
58182	Prokr1	prokineticin receptor 1	0.8785	0.4804	0.8101
56191	Tro	trophinin	0.8785	0.3808	0.7594
100042218	Gm3728	predicted gene 3728	0.8784	0.2763	0.6925
93885	Pcdhb14	protocadherin beta 14	0.8784	0.04517	NA
74200	2810403A07Rik	RIKEN cDNA 2810403A07 gene	0.8783	0.06412	NA
56220	Zfp386	zinc finger protein 386 (Kruppel-like)	0.8783	0.39	0.7629
70144	Lrch3	leucine-rich repeats and calponin homology (CH) domain containing 3	0.8781	0.1904	0.6275
227195	Ino80d	INO80 complex subunit D	0.878	0.1227	0.5667
110651	Rps6ka3	ribosomal protein S6 kinase polypeptide 3	0.878	0.4861	0.8127
73094	Sgip1	SH3-domain GRB2-like (endophilin) interacting protein 1	0.878	0.1737	0.6148
54611	Pde3a	phosphodiesterase 3A, cGMP inhibited	0.878	0.4832	0.8119
17248	Mdm4	transformed mouse 3T3 cell double minute 4	0.878	0.2588	0.6815
57294	Rps27	ribosomal protein S27	0.8779	0.1224	0.5658
17281	Fyco1	FYVE and coiled-coil domain containing 1	0.8779	0.0716	NA
16563	Kif2a	kinesin family member 2A	0.8779	0.09062	0.5229
16409	Itgam	integrin alpha M	0.8779	0.6166	0.8698
432971	A130088B03Rik	RIKEN cDNA A130088B03 gene	0.8778	0.1988	0.6336
108143	Taf9	TAF9 RNA polymerase II, TATA box binding protein (TBP)-associated factor	0.8778	0.09462	0.5318
100038641	Gm10452	predicted gene 10452	0.8777	0.2681	0.6871
246179	Fktn	fukutin	0.8777	0.2238	0.654
75029	Purg	purine-rich element binding protein G	0.8777	0.02687	NA

66310	Dpy30	dpy-30 homolog (C. elegans)	0.8777	0.08934	0.5223
22330	Vcl	vinculin	0.8777	0.1657	0.6077
17472	Gbp4	guanylate binding protein 4	0.8777	0.2913	0.7016
11947	Atp5b	ATP synthase, H+ transporting mitochondrial F1 complex, beta subunit	0.8777	0.02166	NA
321000	4933421E11Rik	RIKEN cDNA 4933421E11 gene	0.8776	0.09042	0.5229
16975	Lrp8	low density lipoprotein receptor-related protein 8, apolipoprotein e receptor	0.8776	0.3772	0.7563
11304	Abca4	ATP-binding cassette, sub-family A (ABC1), member 4	0.8776	0.1342	0.5779
240120	Zfp119b	zinc finger protein 119b	0.8775	0.512	0.8262
544696	D630037F22Rik	RIKEN cDNA D630037F22 gene	0.8773	0.2602	0.6815
434204	Whamm	WAS protein homolog associated with actin, golgi membranes and microtubules	0.8773	0.111	0.5549
52647	D15Ertd509e	DNA segment, Chr 15, ERATO Doi 509, expressed	0.8773	0.1932	0.6294
22715	Zfp57	zinc finger protein 57	0.8773	0.5346	0.8349
791393	A230065N10Rik	RIKEN cDNA A230065N10 gene	0.8772	0.3212	0.7199
19091	Prkg1	protein kinase, cGMP-dependent, type I	0.8772	0.1652	0.6077
77578	Bcl9	B-cell CLL/lymphoma 9	0.8771	0.1071	0.5492
26939	Polr3e	polymerase (RNA) III (DNA directed) polypeptide E	0.8771	0.2627	0.6838
57330	Gigyf1	GRB10 interacting GYF protein 1	0.877	0.182	0.6206
12313	Calm1	calmodulin 1	0.877	0.1021	0.5409
268729	Gm626	predicted gene 626	0.8769	0.3009	0.7068
56788	Scube2	signal peptide, CUB domain, EGF-like 2	0.8769	0.1473	0.591
12028	Bax	BCL2-associated X protein	0.8769	0.2822	0.6967
102731	4833445I07Rik	RIKEN cDNA 4833445I07 gene	0.8768	0.07708	NA
56066	Cxcl11	chemokine (C-X-C motif) ligand 11	0.8768	0.1296	0.5745
52410	D8Ertd769e	DNA segment, Chr 8, ERATO Doi 769, expressed	0.8768	0.08569	0.517
231637	Ssh1	slingshot homolog 1 (Drosophila)	0.8767	0.2255	0.654
229900	Gbp6	guanylate binding protein 6	0.8767	0.3041	0.709
74569	Ttc17	tetratricopeptide repeat domain 17	0.8766	0.1154	0.5578
208158	Map6d1	MAP6 domain containing 1	0.8765	0.5712	0.8494
76123	Gpsm2	G-protein signalling modulator 2 (AGS3-like, C. elegans)	0.8765	0.01174	NA
67011	Mettl6	methyltransferase like 6	0.8765	0.03794	NA
20147	Rs1	retinoschisis (X-linked, juvenile) 1 (human)	0.8765	0.03581	NA
16502	Kcnc1	potassium voltage gated channel, Shaw-related subfamily, member 1	0.8765	0.1248	0.5703
14172	Fgf18	fibroblast growth factor 18	0.8765	0.2226	0.654
383243	Olfr128	olfactory receptor 128	0.8764	0.7237	0.9125
74682	Wdr35	WD repeat domain 35	0.8764	0.03008	NA
20269	Scn3a	sodium channel, voltage-gated, type III, alpha	0.8764	0.1825	0.6209
14071	F9	coagulation factor IX	0.8764	0.5568	0.845
100504388	LOC100504388	histone acetyltransferase KAT2B-like	0.8763	0.6027	0.864
14087	Fanca	Fanconi anemia, complementation group A	0.8763	0.5057	0.823
242747	Zfp933	zinc finger protein 933	0.8762	0.5735	0.8501
11501	Adam8	a disintegrin and metallopeptidase domain 8	0.8762	0.127	0.5713
328099	Prps1l3	phosphoribosyl pyrophosphate synthetase 1-like 3	0.8761	0.02612	NA
240057	Syngap1	synaptic Ras GTPase activating protein 1 homolog (rat)	0.8761	0.1468	0.591
225363	Etf1	eukaryotic translation termination factor 1	0.8761	0.2599	0.6815
72020	Zfp654	zinc finger protein 654	0.8761	0.2832	0.6972
11554	Adrb1	adrenergic receptor, beta 1	0.8761	0.2082	0.6401
229782	Slc35a3	solute carrier family 35 (UDP-N-acetylglucosamine (UDP-GlcNAc) transporter), member 3	0.876	0.1546	0.5975
382019	Zfp882	zinc finger protein 882	0.8759	0.3435	0.7354
211006	Sepsecs	Sep (O-phosphoserine) tRNA:Sec (selenocysteine) tRNA synthase	0.8759	0.2588	0.6815

100910	Chpf2	chondroitin polymerizing factor 2	0.8759	0.155	0.5975
70465	Wdr77	WD repeat domain 77	0.8759	0.1943	0.6299
14776	Gpx2	glutathione peroxidase 2	0.8759	0.2327	0.6622
12942	Pcdha11	protocadherin alpha 11	0.8759	0.4502	0.7945
320022	Ccdc79	coiled-coil domain containing 79	0.8758	0.1266	0.5708
71567	Mcm9	minichromosome maintenance complex component 9	0.8758	0.1538	0.5975
67437	Ssr3	signal sequence receptor, gamma	0.8758	0.398	0.7684
19877	Rock1	Rho-associated coiled-coil containing protein kinase 1	0.8758	0.1893	0.627
320191	Hook3	hook homolog 3 (Drosophila)	0.8757	0.1042	0.5444
93699	Pcdhgb1	protocadherin gamma subfamily B, 1	0.8757	0.2641	0.6845
78558	Htra3	HtrA serine peptidase 3	0.8757	0.4601	0.8011
66371	Chmp4c	chromatin modifying protein 4C	0.8757	0.6161	0.8698
50766	Crim1	cysteine rich transmembrane BMP regulator 1 (chordin like)	0.8757	0.5934	0.8589
23871	Ets1	E26 avian leukemia oncogene 1, 5' domain	0.8757	0.1596	0.6042
100038491	A730068I03Rik	RIKEN cDNA A730068I03 gene	0.8755	0.3041	0.709
665186	Gm7534	predicted gene 7534	0.8755	0.4509	0.7946
17167	Marco	macrophage receptor with collagenous structure	0.8755	0.416	0.7768
100041121	Gm10144	predicted pseudogene 10144	0.8754	0.5291	0.8332
269870	Zfp446	zinc finger protein 446	0.8754	0.08626	0.5171
232035	Fam190a	family with sequence similarity 190, member A	0.8754	0.1115	0.5549
94040	Clmn	calmin	0.8754	0.01938	NA
27411	Slc14a2	solute carrier family 14 (urea transporter), member 2	0.8754	0.7075	0.9063
22141	Tub	tubby candidate gene	0.8754	0.007276	NA
17134	Mafg	v-maf musculoaponeurotic fibrosarcoma oncogene family, protein G (avian)	0.8754	0.1652	0.6077
330502	Zfp82	zinc finger protein 82	0.8753	0.5193	0.8282
78703	C330013J21Rik	RIKEN cDNA C330013J21 gene	0.8753	0.4096	0.7739
76020	5830409B07Rik	RIKEN cDNA 5830409B07 gene	0.8753	0.4787	0.81
72012	1600020E01Rik	RIKEN cDNA 1600020E01 gene	0.8753	0.02301	NA
54524	Syt6	synaptotagmin VI	0.8753	0.109	0.5529
11909	Atf2	activating transcription factor 2	0.8753	0.1628	0.6074
237754	Btnl9	butyrophilin-like 9	0.8752	0.5612	0.8465
66185	1110037F02Rik	RIKEN cDNA 1110037F02 gene	0.8752	0.28	0.6952
11428	Aco1	aconitase 1	0.8752	0.418	0.7785
209334	Gen1	Gen homolog 1, endonuclease (Drosophila)	0.8751	0.3758	0.7556
73674	Wdr75	WD repeat domain 75	0.8751	0.08576	0.517
69312	Lrrc67	leucine rich repeat containing 67	0.875	0.2798	0.6951
11861	Arl4a	ADP-ribosylation factor-like 4A	0.875	0.3295	0.7257
17082	Il1rl1	interleukin 1 receptor-like 1	0.8749	0.5289	0.8332
77316	C030011L09Rik	RIKEN cDNA C030011L09 gene	0.8748	0.2719	0.6891
19155	Npepps	aminopeptidase puromycin sensitive	0.8748	0.07262	NA
320204	4833442J19Rik	RIKEN cDNA 4833442J19 gene	0.8747	0.4054	0.7717
76286	1110006E14Rik	RIKEN cDNA 1110006E14 gene	0.8747	0.0763	NA
75953	Samd7	sterile alpha motif domain containing 7	0.8747	0.07712	NA
22171	Tyms	thymidylate synthase	0.8747	0.1852	0.6223
330149	Hfm1	HFM1, ATP-dependent DNA helicase homolog (S. cerevisiae)	0.8746	0.08046	0.5098
50772	Mapk6	mitogen-activated protein kinase 6	0.8746	0.2756	0.6925
235439	Herc1	hect (homologous to the E6-AP (UBE3A) carboxyl terminus) domain and RCC1 (CHC1)-like domain (RLD) 1	0.8745	0.03608	NA
74486	Osbpl10	oxysterol binding protein-like 10	0.8745	0.07322	NA
71069	Stox2	storkhead box 2	0.8745	0.08455	0.5154

56220	Zfp386	zinc finger protein 386 (Kruppel-like)	0.8745	0.1776	0.6167
216810	Tom1l2	target of myb1-like 2 (chicken)	0.8744	0.2312	0.6607
280668	Adam1a	a disintegrin and metallopeptidase domain 1a	0.8743	0.06959	NA
100045736	LOC100045736	hypothetical protein LOC100045736	0.8742	0.3837	0.7599
77799	Sla2	Src-like-adaptor 2	0.8742	0.08413	0.5154
237761	Ankrd43	ankyrin repeat domain 43	0.8741	0.1139	0.5569
234373	Sugp2	SURP and G patch domain containing 2	0.8741	0.02729	NA
233058	Zfp420	zinc finger protein 420	0.8741	0.1586	0.6033
229363	Gmps	guanine monophosphate synthetase	0.874	0.1853	0.6223
72125	Fam123a	family with sequence similarity 123, member A	0.874	0.09736	0.5352
383348	Kctd16	potassium channel tetramerisation domain containing 16	0.8739	0.1973	0.6324
230751	Oscp1	organic solute carrier partner 1	0.8739	0.1173	0.5603
224703	Mar-02	membrane-associated ring finger (C3HC4) 2	0.8739	0.4548	0.7977
110695	Aldh7a1	aldehyde dehydrogenase family 7, member A1	0.8739	0.3303	0.726
22722	Zfp64	zinc finger protein 64	0.8739	0.08034	0.5098
229658	Vangl1	vang-like 1 (van gogh, Drosophila)	0.8738	0.4075	0.7726
70510	Rnf167	ring finger protein 167	0.8738	0.06619	NA
544782	Gm12620	poly(A) binding protein, cytoplasmic 4 pseudogene	0.8737	0.138	0.5816
330267	Thsd7a	thrombospondin, type I, domain containing 7A	0.8737	0.3835	0.7599
70827	Trak2	trafficking protein, kinesin binding 2	0.8737	0.282	0.6967
19131	Prh1	proline rich protein HaeIII subfamily 1	0.8737	0.3368	0.7305
71782	Ankle2	ankyrin repeat and LEM domain containing 2	0.8736	0.4393	0.7906
791308	Gm9933	predicted gene 9933	0.8735	0.2376	0.6663
77290	9430078K24Rik	RIKEN cDNA 9430078K24 gene	0.8735	0.2531	0.6778
71128	4933417C20Rik	RIKEN cDNA 4933417C20 gene	0.8735	0.1755	0.6156
19726	Rfx3	regulatory factor X, 3 (influences HLA class II expression)	0.8735	0.2644	0.6845
628709	Gm10324	predicted gene 10324	0.8734	0.6828	0.8956
243753	2010107G12Rik	RIKEN cDNA 2010107G12 gene	0.8734	0.1012	0.5395
235067	Cypt4	cysteine-rich perinuclear theca 4	0.8734	0.4289	0.784
230594	Zcchc11	zinc finger, CCHC domain containing 11	0.8734	0.2327	0.6622
71907	Serpina9	serine (or cysteine) peptidase inhibitor, clade A (alpha-1 antiproteinase, antitrypsin), member 9	0.8734	0.4265	0.7823
12192	Zfp36l1	zinc finger protein 36, C3H type-like 1	0.8734	0.0337	NA
100041194	Ahnak2	AHNAK nucleoprotein 2	0.8733	0.3224	0.7202
381572	9430007A20Rik	RIKEN cDNA 9430007A20 gene	0.8733	0.4225	0.7802
78943	Ern1	endoplasmic reticulum (ER) to nucleus signalling 1	0.8733	0.1644	0.6076
72556	Zfp566	zinc finger protein 566	0.8733	0.1385	0.5825
22634	Plagl1	pleiomorphic adenoma gene-like 1	0.8733	0.1868	0.6247
16661	Krt10	keratin 10	0.8733	0.3655	0.7496
13367	Diap1	diaphanous homolog 1 (Drosophila)	0.8733	0.2652	0.6849
74322	Cxxc1	CXXC finger 1 (PHD domain)	0.8732	0.2224	0.654
108058	Camk2d	calcium/calmodulin-dependent protein kinase II, delta	0.8731	0.2119	0.6444
328734	Pisd-ps2	phosphatidylserine decarboxylase, pseudogene 2	0.873	0.2915	0.7018
242585	Slc35d1	solute carrier family 35 (UDP-glucuronic acid/UDP-N-acetylgalactosamine dual transporter), member D1	0.873	0.08739	0.5187
241324	Crb2	crumbs homolog 2 (Drosophila)	0.8729	0.02217	NA
211329	Ncoa7	nuclear receptor coactivator 7	0.8729	0.5099	0.8251
67120	Ttc14	tetratricopeptide repeat domain 14	0.8729	0.1575	0.6016
22762	Zfpm2	zinc finger protein, multitype 2	0.8729	0.06745	NA
246179	Fktn	fukutin	0.8728	0.2859	0.6982
244484	Wdr17	WD repeat domain 17	0.8728	0.06962	NA

19231	Ptma	prothymosin alpha	0.8728	0.3405	0.7328
18949	Pnn	pinin	0.8728	0.1008	0.5394
228410	Cstf3	cleavage stimulation factor, 3' pre-RNA, subunit 3	0.8727	0.105	0.5453
100039133	Gm2058	predicted gene 2058	0.8726	0.1869	0.6248
380924	Olfm4	olfactomedin 4	0.8726	0.1739	0.6148
380732	Gm885	predicted gene 885	0.8726	0.2393	0.6679
78672	9530057J20Rik	RIKEN cDNA 9530057J20 gene	0.8726	0.3251	0.7219
70470	Rprd1b	regulation of nuclear pre-mRNA domain containing 1B	0.8726	0.155	0.5975
11736	Ankfy1	ankyrin repeat and FYVE domain containing 1	0.8726	0.4366	0.7888
66923	Pbrm1	polybromo 1	0.8725	0.1371	0.5807
23984	Pde10a	phosphodiesterase 10A	0.8725	0.3887	0.7616
19712	Rest	RE1-silencing transcription factor	0.8725	0.3039	0.709
15159	Hccs	holocytochrome c synthetase	0.8725	0.02729	NA
280645	B3gat2	beta-1,3-glucuronyltransferase 2 (glucuronosyltransferase S)	0.8724	0.1342	0.5779
71807	Tars2	threonyl-tRNA synthetase 2, mitochondrial (putative)	0.8724	0.07843	0.5045
22057	Tob1	transducer of ErbB-2.1	0.8724	0.3566	0.7443
621080	AI429214	expressed sequence AI429214	0.8723	0.1312	0.5751
99512	Wdr47	WD repeat domain 47	0.8723	0.009148	NA
66313	Smurf2	SMAD specific E3 ubiquitin protein ligase 2	0.8722	0.3266	0.7232
26465	Zfp146	zinc finger protein 146	0.8722	0.5192	0.8282
208292	Zfp871	zinc finger protein 871	0.8721	0.06568	NA
78244	Dnajc21	DnaJ (Hsp40) homolog, subfamily C, member 21	0.8721	0.2059	0.6386
27362	Dnajb9	DnaJ (Hsp40) homolog, subfamily B, member 9	0.8721	0.1638	0.6074
14180	Fgf9	fibroblast growth factor 9	0.8721	0.1331	0.5772
791282	Gm10030	predicted gene 10030	0.872	0.1153	0.5578
234724	Tat	tyrosine aminotransferase	0.872	0.1249	0.5703
224250	Cldnd1	claudin domain containing 1	0.872	0.1401	0.5834
80707	Wwox	WW domain-containing oxidoreductase	0.872	0.167	0.6083
20619	Snap23	synaptosomal-associated protein 23	0.872	0.3422	0.7339
15354	Hmgb3	high mobility group box 3	0.872	0.1934	0.6295
11717	Ampd3	adenosine monophosphate deaminase 3	0.8719	0.233	0.6622
100041677	Gm13157	predicted gene 13157	0.8718	0.2773	0.6925
83453	Chrdl1	chordin-like 1	0.8718	0.2409	0.6687
78266	Zfp687	zinc finger protein 687	0.8718	0.04315	NA
53415	Htatip2	HIV-1 tat interactive protein 2, homolog (human)	0.8718	0.08446	0.5154
27381	Tcl1b2	T-cell leukemia/lymphoma 1B, 2	0.8718	0.7993	0.9361
22658	Pcgf2	polycomb group ring finger 2	0.8718	0.07309	0.4952
16000	Igf1	insulin-like growth factor 1	0.8718	0.09943	0.5367
13079	Cyp21a1	cytochrome P450, family 21, subfamily a, polypeptide 1	0.8718	0.5748	0.8504
12326	Camk4	calcium/calmodulin-dependent protein kinase IV	0.8718	0.3415	0.7337
258464	Olfr1384	olfactory receptor 1384	0.8717	0.07121	0.4932
192119	Dicer1	Dicer1, Dcr-1 homolog (Drosophila)	0.8717	0.05457	NA
67306	Fam164a	family with sequence similarity 164, member A	0.8717	0.1027	0.5424
100043305	Gm4349	SET domain, bifurcated 1 pseudogene	0.8716	0.3201	0.7191
208266	Dot1l	DOT1-like, histone H3 methyltransferase (S. cerevisiae)	0.8716	0.01338	NA
68617	1110012J17Rik	RIKEN cDNA 1110012J17 gene	0.8716	0.0135	NA
66369	Dus2l	dihydrouridine synthase 2-like (SMM1, S. cerevisiae)	0.8716	0.1502	0.5938
27371	Sh2d2a	SH2 domain protein 2A	0.8716	0.2691	0.6878
380614	Intu	inturned planar cell polarity effector homolog (Drosophila)	0.8715	0.2518	0.6767

27373	Csnk1e	casein kinase 1, epsilon	0.8715	0.385	0.7607
66614	Gpatch4	G patch domain containing 4	0.8714	0.1434	0.5879
16569	Kif3b	kinesin family member 3B	0.8714	0.2316	0.6612
15205	Hes1	hairy and enhancer of split 1 (Drosophila)	0.8714	0.3895	0.7624
14312	Brd2	bromodomain containing 2	0.8714	0.314	0.7153
628596	Gm6900	predicted gene 6900	0.8713	0.07365	0.4961
21848	Trim24	tripartite motif-containing 24	0.8713	0.2075	0.6398
15331	Hmgn2	high mobility group nucleosomal binding domain 2	0.8713	0.2368	0.6659
77361	9430085M18Rik	RIKEN cDNA 9430085M18 gene	0.8712	0.3587	0.7457
330812	Rnf150	ring finger protein 150	0.8711	0.08953	0.5223
229780	Ccdc76	coiled-coil domain containing 76	0.8711	0.1667	0.6083
106251	D230034L24Rik	RIKEN cDNA D230034L24 gene	0.8711	0.195	0.6304
80859	Nfkbiz	nuclear factor of kappa light polypeptide gene enhancer in B-cells inhibitor, zeta	0.8711	0.07297	0.4952
791333	Gm10050	predicted gene 10050	0.871	0.321	0.7197
121021	Cspg4	chondroitin sulfate proteoglycan 4	0.871	0.3947	0.7663
67161	Sclt1	sodium channel and clathrin linker 1	0.871	0.3198	0.719
237823	Pfas	phosphoribosylformylglycinamidine synthase (FGAR amidotransferase)	0.8709	0.08578	0.517
243385	Gprin3	GPRIN family member 3	0.8708	0.2028	0.6356
241656	Pak7	p21 protein (Cdc42/Rac)-activated kinase 7	0.8708	0.1901	0.6274
171286	Slc12a8	solute carrier family 12 (potassium/chloride transporters), member 8	0.8708	0.0558	NA
29876	Clic4	chloride intracellular channel 4 (mitochondrial)	0.8708	0.1653	0.6077
15251	Hif1a	hypoxia inducible factor 1, alpha subunit	0.8708	0.1836	0.6217
69309	Slc16a13	solute carrier family 16 (monocarboxylic acid transporters), member 13	0.8707	0.1393	0.5834
16565	Kif21b	kinesin family member 21B	0.8707	0.02248	NA
239528	Eif2c2	eukaryotic translation initiation factor 2C, 2	0.8706	0.1043	0.5444
212281	A530054K11Rik	RIKEN cDNA A530054K11 gene	0.8706	0.4399	0.7908
68564	Nufip2	nuclear fragile X mental retardation protein interacting protein 2	0.8706	0.4202	0.7797
27205	Podxl	podocalyxin-like	0.8706	0.07524	0.4992
14236	Foxn2	forkhead box N2	0.8706	0.164	0.6075
545471	Zfp345	zinc finger protein 345	0.8705	0.7775	0.9272
338523	Jhdm1d	jumonji C domain-containing histone demethylase 1 homolog D (S. cerevisiae)	0.8705	0.1106	0.5546
74121	Acoxl	acyl-Coenzyme A oxidase-like	0.8705	0.07248	0.4941
67163	Ccdc47	coiled-coil domain containing 47	0.8705	0.4045	0.7715
24086	Tlk2	tousled-like kinase 2 (Arabidopsis)	0.8705	0.1746	0.6148
100210	Gpn2	GPN-loop GTPase 2	0.8704	0.3379	0.7314
78438	A930028N01Rik	RIKEN cDNA A930028N01 gene	0.8703	0.1422	0.5871
74718	Snx16	sorting nexin 16	0.8703	0.3076	0.7112
18845	Plxna2	plexin A2	0.8703	0.02806	NA
15194	Htt	huntingtin	0.8703	0.1615	0.607
232157	Mobkl1b	MOB1, Mps One Binder kinase activator-like 1B (yeast)	0.8702	0.1702	0.6115
192197	Bcas3	breast carcinoma amplified sequence 3	0.8702	0.2298	0.6596
72685	Dnajc6	DnaJ (Hsp40) homolog, subfamily C, member 6	0.8702	0.2508	0.6767
12142	Prdm1	PR domain containing 1, with ZNF domain	0.8702	0.05177	NA
99470	Magi3	membrane associated guanylate kinase, WW and PDZ domain containing 3	0.87	0.1553	0.5984
100043468	Zfp955b	zinc finger protein 955B	0.8699	0.4007	0.7703
269424	Phf17	PHD finger protein 17	0.8699	0.1288	0.5733
242687	Wasf2	WAS protein family, member 2	0.8699	0.1622	0.607
20874	Slk	STE20-like kinase (yeast)	0.8698	0.1435	0.5879
11548	Adra1b	adrenergic receptor, alpha 1b	0.8698	0.3783	0.7573

100047183	LOC100047183	protein AHNAK2-like	0.8697	0.3593	0.7461
320538	Ubn2	ubinuclein 2	0.8697	0.04727	NA
110350	Dync2h1	dynein cytoplasmic 2 heavy chain 1	0.8697	0.07307	0.4952
237500	Tmtc3	transmembrane and tetratricopeptide repeat containing 3	0.8695	0.5978	0.8612
212943	Fam46a	family with sequence similarity 46, member A	0.8695	0.3665	0.7504
72795	Ttc19	tetratricopeptide repeat domain 19	0.8695	0.2866	0.6982
319240	9330159N05Rik	RIKEN cDNA 9330159N05 gene	0.8694	0.1037	0.5441
16504	Kcnc3	potassium voltage gated channel, Shaw-related subfamily, member 3	0.8694	0.5944	0.8597
403175	Tigd4	tigger transposable element derived 4	0.8693	0.146	0.591
72039	Mccc1	methylcrotonoyl-Coenzyme A carboxylase 1 (alpha)	0.8693	0.1414	0.5863
140780	Bmp2k	BMP2 inducible kinase	0.8692	0.05965	NA
68652	Tab2	TGF-beta activated kinase 1/MAP3K7 binding protein 2	0.8692	0.2215	0.6526
67897	Rnmt	RNA (guanine-7-) methyltransferase	0.8692	0.1932	0.6294
26920	Cep110	centrosomal protein 110	0.8692	0.3731	0.7538
233080	Ffar3	free fatty acid receptor 3	0.8691	0.5791	0.8519
107528	Magee1	melanoma antigen, family E, 1	0.8691	0.2568	0.6809
16009	Igfbp3	insulin-like growth factor binding protein 3	0.8691	0.2903	0.7006
71710	Lrrcc1	leucine rich repeat and coiled-coil domain containing 1	0.869	0.04866	NA
24069	Sufu	suppressor of fused homolog (Drosophila)	0.869	0.477	0.8099
18114	Rrp1	ribosomal RNA processing 1 homolog (S. cerevisiae)	0.869	0.1061	0.5468
14869	Gstp2	glutathione S-transferase, pi 2	0.869	0.03754	NA
230235	6430704M03Rik	RIKEN cDNA 6430704M03 gene	0.8689	0.233	0.6622
218503	Fcho2	FCH domain only 2	0.8689	0.1955	0.6304
100978	Nfxl1	nuclear transcription factor, X-box binding-like 1	0.8689	0.4152	0.7765
73207	3110068a07rik	RIKEN cDNA 3110068A07 gene	0.8689	0.3626	0.7483
19017	Ppargc1a	peroxisome proliferative activated receptor, gamma, coactivator 1 alpha	0.8689	0.03818	NA
16424	Itih1	inter-alpha trypsin inhibitor, heavy chain 1	0.8689	0.7615	0.9238
73852	D3Ertd751e	DNA segment, Chr 3, ERATO Doi 751, expressed	0.8688	0.1259	0.5703
21834	Thrb	thyroid hormone receptor beta	0.8688	0.1526	0.5963
100302432	Gm14435	predicted gene 14435	0.8687	0.228	0.6569
114666	Krtap5-5	keratin associated protein 5-5	0.8687	0.2515	0.6767
237761	Ankrd43	ankyrin repeat domain 43	0.8686	0.1731	0.6145
214150	Eif2c3	eukaryotic translation initiation factor 2C, 3	0.8686	0.3808	0.7594
170788	Crb1	crumbs homolog 1 (Drosophila)	0.8686	0.05827	NA
26920	Cep110	centrosomal protein 110	0.8686	0.04438	NA
667103	Gm13570	predicted gene 13570	0.8685	0.05095	NA
19739	Rgs9	regulator of G-protein signaling 9	0.8685	0.4681	0.8056
12995	Csnk2a1	casein kinase 2, alpha 1 polypeptide	0.8685	0.2071	0.6398
338349	Cntln	centlein, centrosomal protein	0.8684	0.2783	0.6934
319521	A730056I06Rik	RIKEN cDNA A730056I06 gene	0.8683	0.1408	0.5849
633385	Gm7111	predicted gene 7111	0.8682	0.09337	0.5293
272027	Tstd2	thiosulfate sulfurtransferase (rhodanese)-like domain containing 2	0.8682	0.09256	0.5271
100038587	Gm10278	predicted gene 10278	0.8681	0.06623	0.4837
258652	Olfr1131	olfactory receptor 1131	0.868	0.6072	0.8648
258436	Olfr458	olfactory receptor 458	0.868	0.477	0.8099
194735	4930430D24Rik	RIKEN cDNA 4930430D24 gene	0.868	0.6652	0.8903
73095	Slc25a42	solute carrier family 25, member 42	0.868	0.1019	0.5404
100039888	Gm11223	stathmin 1 pseudogene	0.8679	0.2386	0.6671
98661	AW061096	expressed sequence AW061096	0.8679	0.08678	0.5179

22691	Zscan2	zinc finger and SCAN domain containing 2	0.8679	0.3025	0.7087
56790	Fam48a	family with sequence similarity 48, member A	0.8678	0.1683	0.6095
11839	Areg	amphiregulin	0.8678	0.3458	0.7361
66208	Nenf	neuron derived neurotrophic factor	0.8677	0.08837	0.5202
100126228	Gm9856	predicted gene 9856	0.8676	0.01503	NA
67008	1600012F09Rik	RIKEN cDNA 1600012F09 gene	0.8676	0.1524	0.596
53374	Chst3	carbohydrate (chondroitin 6/keratan) sulfotransferase 3	0.8676	0.06606	0.483
407823	Baz2b	bromodomain adjacent to zinc finger domain, 2B	0.8675	0.03672	NA
231452	Sdad1	SDA1 domain containing 1	0.8675	0.1209	0.5643
106906	AI450241	expressed sequence AI450241	0.8675	0.1805	0.6198
67604	Get4	golgi to ER traffic protein 4 homolog (S. cerevisiae)	0.8675	0.0134	NA
12006	Axin2	axin2	0.8675	0.7572	0.9226
245174	Zfp937	zinc finger protein 937	0.8674	0.06857	0.4867
211347	Pank3	pantothenate kinase 3	0.8674	0.2031	0.6356
170707	Usp48	ubiquitin specific peptidase 48	0.8674	0.04578	NA
70693	Gpr125	G protein-coupled receptor 125	0.8674	0.5155	0.8277
230789	Fam76a	family with sequence similarity 76, member A	0.8673	0.266	0.6856
109959	Amy2a5	amylase 2a5	0.8673	0.2067	0.6393
27015	Polk	polymerase (DNA directed), kappa	0.8673	0.317	0.7176
26938	St6galnac5	ST6 (alpha-N-acetyl-neuraminyl-2,3-beta-galactosyl-1,3)-N-acetylgalactosaminide alpha-2,6-sialyltransferase 5	0.8673	0.06513	0.4816
18823	Plp1	proteolipid protein (myelin) 1	0.8673	0.07606	0.5008
12695	Inadl	InaD-like (Drosophila)	0.8673	0.2579	0.6815
232087	Mat2a	methionine adenosyltransferase II, alpha	0.8672	0.3652	0.7495
214552	Cep164	centrosomal protein 164	0.8672	0.1851	0.6223
72289	Malat1	metastasis associated lung adenocarcinoma transcript 1 (non-coding RNA)	0.8672	0.1732	0.6145
66410	Mterfd1	MTERF domain containing 1	0.8672	0.1795	0.6187
26369	Cetn1	centrin 1	0.8672	0.6219	0.8719
20747	Spop	speckle-type POZ protein	0.8672	0.2036	0.6362
224997	Dlgap1	discs, large (Drosophila) homolog-associated protein 1	0.8671	0.3273	0.7239
209645	Bend7	BEN domain containing 7	0.8671	0.09023	0.5229
83380	Prp2	proline rich protein 2	0.8671	0.2747	0.6925
73687	2410087M07Rik	RIKEN cDNA 2410087M07 gene	0.8671	0.4112	0.7746
66616	Snx9	sorting nexin 9	0.8671	0.1037	0.5441
18600	Padi2	peptidyl arginine deiminase, type II	0.8671	0.415	0.7764
338364	Trim65	tripartite motif-containing 65	0.867	0.3614	0.7476
17831	Muc2	mucin 2	0.867	0.6376	0.8776
677168	Gm9706	predicted gene 9706	0.8669	0.4758	0.8097
73178	Wasl	Wiskott-Aldrich syndrome-like (human)	0.8669	0.1099	0.5535
21414	Tcf7	transcription factor 7, T-cell specific	0.8669	0.1541	0.5975
16337	Insr	insulin receptor	0.8668	0.1716	0.6126
12607	Cebpz	CCAAT/enhancer binding protein zeta	0.8668	0.3031	0.7089
100041574	9030025P20Rik	RIKEN cDNA 9030025P20 gene	0.8667	0.03629	NA
16656	Hivep3	human immunodeficiency virus type I enhancer binding protein 3	0.8667	0.4786	0.81
328789	Lhfpl5	lipoma HMGIC fusion partner-like 5	0.8666	0.3186	0.7182
97954	C77847	expressed sequence C77847	0.8666	0.5389	0.8366
69761	1600015I10Rik	RIKEN cDNA 1600015I10 gene	0.8666	0.1273	0.5716
68070	Pdzd2	PDZ domain containing 2	0.8666	0.4549	0.7977
15379	Onecut1	one cut domain, family member 1	0.8666	0.3792	0.7583
77805	Esco1	establishment of cohesion 1 homolog 1 (S. cerevisiae)	0.8665	0.1929	0.6291

74481	Batf2	basic leucine zipper transcription factor, ATF-like 2	0.8665	0.4154	0.7767
20358	Sema6a	sema domain, transmembrane domain (TM), and cytoplasmic domain, (semaphorin) 6A	0.8665	0.1955	0.6304
242253	Wdr63	WD repeat domain 63	0.8664	0.1318	0.5758
75957	Mir17hg	MIR17 host gene 1 (non-protein coding)	0.8664	0.1044	0.5445
328156	C230037L09	hypothetical protein C230037L09	0.8663	0.08352	0.5144
69504	Zfp932	zinc finger protein 932	0.8663	0.1666	0.6083
17441	Mog	myelin oligodendrocyte glycoprotein	0.8663	0.6513	0.8833
436440	Gpr31c	G protein-coupled receptor 31, D17Leh66c region	0.8662	0.5323	0.8339
320011	Uggt1	UDP-glucose glycoprotein glucosyltransferase 1	0.8662	0.14	0.5834
66329	Susd3	sushi domain containing 3	0.8662	0.0904	0.5229
414098	C230096K16Rik	RIKEN cDNA C230096K16 gene	0.8661	0.1261	0.5703
331392	Gm5124	predicted pseudogene 5124	0.8661	0.3082	0.7112
320110	B230369F24Rik	RIKEN cDNA B230369F24 gene	0.8661	0.1585	0.603
12890	Cplx2	complexin 2	0.8661	0.295	0.7045
353130	Prss33	protease, serine, 33	0.866	0.09361	0.5296
103432	6430706H07Rik	RIKEN cDNA 6430706H07 gene	0.866	0.1115	0.5549
102243	AU024581	expressed sequence AU024581	0.866	0.1104	0.5539
66899	Fip1l1	FIP1 like 1 (S. cerevisiae)	0.866	0.3345	0.7298
227399	Ppip5k2	diphosphoinositol pentakisphosphate kinase 2	0.8659	0.137	0.5807
79555	BC005537	cDNA sequence BC005537	0.8659	0.1516	0.5948
319162	Hist3h2a	histone cluster 3, H2a	0.8657	0.04909	NA
56077	Dgke	diacylglycerol kinase, epsilon	0.8657	0.03832	NA
51796	Srrm1	serine/arginine repetitive matrix 1	0.8657	0.04638	NA
432442	Akap7	A kinase (PRKA) anchor protein 7	0.8655	0.04634	NA
71801	Plekhf2	pleckstrin homology domain containing, family F (with FYVE domain) member 2	0.8655	0.04321	NA
14349	Fv1	Friend virus susceptibility 1	0.8655	0.5422	0.838
78521	B230219D22Rik	RIKEN cDNA B230219D22 gene	0.8654	0.1238	0.5685
78323	2310046O06Rik	RIKEN cDNA 2310046O06 gene	0.8654	0.09518	0.5332
15490	Hsd17b7	hydroxysteroid (17-beta) dehydrogenase 7	0.8653	0.1476	0.591
319342	C230034O21Rik	RIKEN cDNA C230034O21 gene	0.8652	0.05136	0.4519
67230	Zfp329	zinc finger protein 329	0.8652	0.03916	NA
99334	Zscan29	zinc finger SCAN domains 29	0.8651	0.1679	0.6095
69131	Cdk12	cyclin-dependent kinase 12	0.8651	0.06825	0.486
73242	Atat1	alpha tubulin acetyltransferase 1	0.865	0.1112	0.5549
242466	Zfp462	zinc finger protein 462	0.8649	0.1502	0.5938
57342	Parva	parvin, alpha	0.8649	0.04552	NA
100039697	Gm2375	predicted gene 2375	0.8646	0.2932	0.7028
407788	BC051142	cDNA sequence BC051142	0.8646	0.1689	0.6096
320772	Mdga2	MAM domain containing glycosylphosphatidylinositol anchor 2	0.8646	0.1188	0.5622
71777	Ing3	inhibitor of growth family, member 3	0.8646	0.1576	0.6017
56722	Litaf	LPS-induced TN factor	0.8646	0.5663	0.8481
20445	St6galnac1	ST6 (alpha-N-acetyl-neuraminyl-2,3-beta-galactosyl-1,3)-N-acetylgalactosaminide alpha-2,6-sialyltransferase 1	0.8646	0.5356	0.835
14595	B4galt1	UDP-Gal:betaGlcNAc beta 1,4- galactosyltransferase, polypeptide 1	0.8646	0.09022	0.5229
70333	Cd3eap	CD3E antigen, epsilon polypeptide associated protein	0.8645	0.07084	0.4923
16408	Itgal	integrin alpha L	0.8645	0.324	0.7211
100042862	Gm4076	predicted gene 4076	0.8644	0.03501	NA
227648	Sec16a	SEC16 homolog A (S. cerevisiae)	0.8644	0.1112	0.5549
11431	Acp1	acid phosphatase 1, soluble	0.8644	0.2575	0.6815
228564	Frmd5	FERM domain containing 5	0.8643	0.07483	0.4981

78282	5330439K02Rik		RIKEN cDNA 5330439K02 gene	0.8642	0.1474	0.591
402752	C130068B02Rik		RIKEN cDNA C130068B02 gene	0.8641	0.06215	0.4771
382793	Mtx3		metaxin 3	0.8641	0.08049	0.5098
20616	Snap91		synaptosomal-associated protein 91	0.8641	0.08192	0.5131
16180	Il1rap		interleukin 1 receptor accessory protein	0.8641	0.11	0.5535
619937	Gm6115		predicted gene 6115	0.864	0.2853	0.6982
208595	Gm9897		predicted gene 9897	0.864	0.2065	0.6392
234214	Sorbs2		sorbin and SH3 domain containing 2	0.8639	0.2955	0.7046
110962	Mbd6		methyl-CpG binding domain protein 6	0.8639	0.07514	0.4989
76937	2810429I04Rik		RIKEN cDNA 2810429I04 gene	0.8639	0.3787	0.7578
100041694	Gm10451		predicted gene 10451	0.8638	0.08215	0.5139
72672	Zfp518a		zinc finger protein 518A	0.8638	0.2756	0.6925
16329	Inpp1		inositol polyphosphate-1-phosphatase	0.8638	0.1739	0.6148
17064	Cd93		CD93 antigen	0.8637	0.1431	0.5879
227357	Espnl		espin-like	0.8635	0.1148	0.5569
20585	Hltf		helicase-like transcription factor	0.8635	0.2407	0.6687
11426	Macf1		microtubule-actin crosslinking factor 1	0.8635	0.009985	NA
235472	Prtg		protogenin homolog (Gallus gallus)	0.8634	0.0892	0.522
78785	Clip4		CAP-GLY domain containing linker protein family, member 4	0.8634	0.4659	0.8049
58226	Cacna1h		calcium channel, voltage-dependent, T type, alpha 1H subunit	0.8634	0.2249	0.654
269623	C030048B08Rik		RIKEN cDNA C030048B08 gene	0.8632	0.2297	0.6596
56334	Tmed2		transmembrane emp24 domain trafficking protein 2	0.8632	0.2027	0.6356
666173	Vps13b		vacuolar protein sorting 13B (yeast)	0.8631	0.1013	0.5395
77875	Cyp4f41-ps		cytochrome P450, family 4, subfamily f, polypeptide 41 pseudogene	0.8631	0.08096	0.5105
21856	Timm44		translocase of inner mitochondrial membrane 44	0.8631	0.226	0.6545
54611	Pde3a		phosphodiesterase 3A, cGMP inhibited	0.863	0.1788	0.6181
18286	Odf2		outer dense fiber of sperm tails 2	0.863	0.1689	0.6096
77097	Tanc2		tetratricopeptide repeat, ankyrin repeat and coiled-coil containing 2	0.8629	0.3957	0.7671
68272	Rbm28		RNA binding motif protein 28	0.8629	0.1183	0.5609
237611	Stac3		SH3 and cysteine rich domain 3	0.8628	0.184	0.6217
171166	Mcoln3		mucolipin 3	0.8628	0.6305	0.8749
103817	AI662501		expressed sequence AI662501	0.8627	0.1079	0.551
13418	Dnajc1		DnaJ (Hsp40) homolog, subfamily C, member 1	0.8627	0.01897	NA
76007	Zmym2		zinc finger, MYM-type 2	0.8626	0.3074	0.711
72371	2210408I21Rik		RIKEN cDNA 2210408I21 gene	0.8626	0.1648	0.6077
20028	Pdc		phosducin	0.8626	0.1043	0.5444
18293	Ogdh		oxoglutarate dehydrogenase (lipoamide)	0.8626	0.07246	0.4941
16000	Igf1		insulin-like growth factor 1	0.8626	0.06817	0.4858
18000		Sep-02	septin 2	0.8625	0.2081	0.6401
228602	4930402H24Rik		RIKEN cDNA 4930402H24 gene	0.8624	0.3041	0.709
216991	Adap2		ArfGAP with dual PH domains 2	0.8624	0.1822	0.6206
103080		Sep-10	septin 10	0.8624	0.2549	0.6792
17355	Aff1		AF4/FMR2 family, member 1	0.8624	0.02912	NA
11907	Ate1		arginyltransferase 1	0.8622	0.2517	0.6767
53621	Cnot4		CCR4-NOT transcription complex, subunit 4	0.8621	0.4678	0.8056
229445	Ctso		cathepsin O	0.862	0.1844	0.6217
56637	Gsk3b		glycogen synthase kinase 3 beta	0.862	0.1256	0.5703
54526	Syt10		synaptotagmin X	0.862	0.6848	0.8959
20660	Sorl1		sortilin-related receptor, LDLR class A repeats-containing	0.862	0.1	0.5384

432999	A930007A09Rik	RIKEN cDNA A930007A09 gene	0.8619	0.1543	0.5975
67846	Tmem39a	transmembrane protein 39a	0.8619	0.06015	0.4728
67459	Nvl	nuclear VCP-like	0.8619	0.2822	0.6967
67058	2810428J06Rik	RIKEN cDNA 2810428J06 gene	0.8619	0.2083	0.6401
78887	Sfi1	Sfi1 homolog, spindle assembly associated (yeast)	0.8618	0.4253	0.7823
74842	4833419G08Rik	RIKEN cDNA 4833419G08 gene	0.8618	0.3073	0.711
71392	5430401F13Rik	RIKEN cDNA 5430401F13 gene	0.8618	0.2301	0.6599
208263	Tor1aip1	torsin A interacting protein 1	0.8617	0.1114	0.5549
67623	Tm7sf3	transmembrane 7 superfamily member 3	0.8617	0.001976	NA
22746	Zfp85-rs1	zinc finger protein 85, related sequence 1	0.8617	0.4347	0.788
17720	ND4L	NADH dehydrogenase subunit 4L	0.8617	0.004901	NA
235106	Ntm	neurotrimin	0.8616	0.1807	0.6198
71514	Sfpq	splicing factor proline/glutamine rich (polypyrimidine tract binding protein associated)	0.8616	0.2862	0.6982
21423	Tcf3	transcription factor 3	0.8616	0.05271	0.4554
100043364	Gm4392	predicted gene 4392	0.8615	0.254	0.6788
329506	Ctdspl2	CTD (carboxy-terminal domain, RNA polymerase II, polypeptide A) small phosphatase like 2	0.8615	0.1842	0.6217
76130	Las1l	LAS1-like (S. cerevisiae)	0.8615	0.01182	NA
17274	Rab8a	RAB8A, member RAS oncogene family	0.8615	0.3446	0.7359
16476	Jun	Jun oncogene	0.8615	0.02825	NA
229600	BC028528	cDNA sequence BC028528	0.8614	0.1456	0.5906
433961	Gm5565	predicted gene 5565	0.8613	0.1743	0.6148
328451	Gm5088	poly(A)-binding protein, cytoplasmic pseudogene	0.8613	0.4653	0.8047
18779	Pla2r1	phospholipase A2 receptor 1	0.8613	0.1839	0.6217
100101807	1700047I17Rik2	RIKEN cDNA 1700047I17 gene 2	0.8612	0.0655	0.4816
229488	Fam160a1	family with sequence similarity 160, member A1	0.8612	0.0581	0.4703
70808	4632415L05Rik	RRS1 ribosome biogenesis regulator homolog pseudogene	0.8612	0.1992	0.6343
243961	Shank1	SH3/ankyrin domain gene 1	0.8611	0.189	0.6266
12788	Cnga1	cyclic nucleotide gated channel alpha 1	0.861	0.1175	0.5606
237979	Sdk2	sidekick homolog 2 (chicken)	0.8609	0.4225	0.7802
83997	Slmap	sarcolemma associated protein	0.8609	0.2299	0.6596
75266	Tomm20l	translocase of outer mitochondrial membrane 20 homolog (yeast)-like	0.8609	0.08001	0.5096
192164	Pcdha12	protocadherin alpha 12	0.8608	0.09916	0.5367
77963	Hook1	hook homolog 1 (Drosophila)	0.8608	0.2214	0.6525
14811	Grin2a	glutamate receptor, ionotropic, NMDA2A (epsilon 1)	0.8607	0.3894	0.7624
224432	Scaf4	SR-related CTD-associated factor 4	0.8606	0.04063	0.4258
11920	Atm	ataxia telangiectasia mutated homolog (human)	0.8606	0.2801	0.6952
230235	6430704M03Rik	RIKEN cDNA 6430704M03 gene	0.8605	0.07692	0.5014
77200	5430403G16Rik	RIKEN cDNA 5430403G16 gene	0.8604	0.2953	0.7046
12283	Cab39	calcium binding protein 39	0.8604	0.1775	0.6167
233060	Zfp382	zinc finger protein 382	0.8603	0.07144	0.4934
69025	1500032P08Rik	RIKEN cDNA 1500032P08 gene	0.8603	0.1384	0.5825
12340	Capza1	capping protein (actin filament) muscle Z-line, alpha 1	0.8603	0.5292	0.8332
669389	Gm9456	predicted gene 9456	0.8602	0.2211	0.6525
106794	Dhx57	DEAH (Asp-Glu-Ala-Asp/His) box polypeptide 57	0.8602	0.01692	NA
77929	Yipf6	Yip1 domain family, member 6	0.8602	0.1595	0.6042
19716	Bex1	brain expressed gene 1	0.8602	0.218	0.6502
12890	Cplx2	complexin 2	0.8602	0.2741	0.6919
330695	Ctxn1	cortexin 1	0.8601	0.5914	0.8582
244745	Dpy19l1	dpy-19-like 1 (C. elegans)	0.8601	0.02298	NA

74559	Elovl7	ELOVL family member 7, elongation of long chain fatty acids (yeast)	0.8601	0.1202	0.5641
67728	Dph2	DPH2 homolog (S. cerevisiae)	0.8601	0.01125	NA
233274	Siglech	sialic acid binding Ig-like lectin H	0.86	0.06	0.4728
14913	Guca1a	guanylate cyclase activator 1a (retina)	0.8599	0.1016	0.5396
108946	Zzz3	zinc finger, ZZ domain containing 3	0.8598	0.06325	0.478
22634	Plagl1	pleiomorphic adenoma gene-like 1	0.8598	0.2305	0.6601
110651	Rps6ka3	ribosomal protein S6 kinase polypeptide 3	0.8597	0.1304	0.5747
58521	Eid1	EP300 interacting inhibitor of differentiation 1	0.8597	0.01594	NA
18145	Npc1	Niemann Pick type C1	0.8597	0.01889	NA
24127	Xrn1	5'-3' exoribonuclease 1	0.8596	0.1472	0.591
12367	Casp3	caspase 3	0.8596	0.1529	0.5966
243612	D630042P16Rik	RIKEN cDNA D630042P16 gene	0.8595	0.342	0.7338
235439	Herc1	hect (homologous to the E6-AP (UBE3A) carboxyl terminus) domain and RCC1 (CHC1)-like domain (RLD) 1	0.8595	0.3224	0.7202
56095	Ftsj3	FtsJ homolog 3 (E. coli)	0.8595	0.1881	0.6261
70715	6330405D24Rik	RIKEN cDNA 6330405D24 gene	0.8594	0.2402	0.6685
67972	Atp2b1	ATPase, Ca++ transporting, plasma membrane 1	0.8594	0.1549	0.5975
54378	Cacng6	calcium channel, voltage-dependent, gamma subunit 6	0.8594	0.5439	0.839
229363	Gmps	guanine monophosphate synthetase	0.8593	0.349	0.7388
74749	5830405M20Rik	RIKEN cDNA 5830405M20 gene	0.8593	0.3654	0.7496
64436	Inpp5e	inositol polyphosphate-5-phosphatase E	0.8593	0.1859	0.6232
13347	Dffa	DNA fragmentation factor, alpha subunit	0.8593	0.297	0.7051
11735	Ank3	ankyrin 3, epithelial	0.8593	0.003462	NA
70335	Reep6	receptor accessory protein 6	0.8592	0.3105	0.7135
67382	Brd3	bromodomain containing 3	0.8592	0.2121	0.6448
11722	Amy1	amylase 1, salivary	0.8592	0.04096	0.4265
100273	Osbpl9	oxysterol binding protein-like 9	0.8591	0.1961	0.6308
231863	Fbxl18	F-box and leucine-rich repeat protein 18	0.859	0.1973	0.6324
210853	Zfp947	zinc finger protein 947	0.859	0.4162	0.7769
17763	Mtcp1	mature T-cell proliferation 1	0.859	0.05412	0.4596
14172	Fgf18	fibroblast growth factor 18	0.859	0.1251	0.5703
235504	Slc17a5	solute carrier family 17 (anion/sugar transporter), member 5	0.8589	0.1402	0.5834
108989	Tpr	translocated promoter region	0.8589	0.4406	0.7913
56380	Arid3b	AT rich interactive domain 3B (BRIGHT-like)	0.8589	0.5931	0.8587
76683	1500002F19Rik	RIKEN cDNA 1500002F19 gene	0.8588	0.4494	0.7943
69350	1700003G18Rik	RIKEN cDNA 1700003G18 gene	0.8588	0.05948	0.4728
242557	Atg4c	autophagy-related 4C (yeast)	0.8587	0.2122	0.6448
21929	Tnfaip3	tumor necrosis factor, alpha-induced protein 3	0.8587	0.06079	0.4741
98682	Mfsd6	major facilitator superfamily domain containing 6	0.8586	0.1646	0.6076
16449	Jag1	jagged 1	0.8586	0.1086	0.5521
74355	Smchd1	SMC hinge domain containing 1	0.8585	0.05082	0.4519
69719	Cad	carbamoyl-phosphate synthetase 2, aspartate transcarbamylase, and dihydroorotase	0.8585	0.2362	0.6657
15364	Hmga2	high mobility group AT-hook 2	0.8585	0.0554	0.4626
99132	AI956758	expressed sequence AI956758	0.8584	0.3185	0.7182
73906	4833417C18Rik	RIKEN cDNA 4833417C18 gene	0.8584	0.6692	0.8904
69291	1700001L05Rik	RIKEN cDNA 1700001L05 gene	0.8584	0.05256	0.4549
22762	Zfpm2	zinc finger protein, multitype 2	0.8584	0.0373	0.4163
238271	Kcnh5	potassium voltage-gated channel, subfamily H (eag-related), member 5	0.8583	0.1436	0.588
72508	Rps6kb1	ribosomal protein S6 kinase, polypeptide 1	0.8583	0.009849	NA
26556	Homer1	homer homolog 1 (Drosophila)	0.8583	0.2027	0.6356

226856	Lpgat1	lysophosphatidylglycerol acyltransferase 1	0.8582	0.1083	0.552
208263	Tor1aip1	torsin A interacting protein 1	0.8582	0.5711	0.8493
30932	Zfp330	zinc finger protein 330	0.8582	0.3135	0.7152
12660	Chka	choline kinase alpha	0.8582	0.06803	0.4858
211660	Cspp1	centrosome and spindle pole associated protein 1	0.8581	0.02254	NA
71147	Oxsm	3-oxoacyl-ACP synthase, mitochondrial	0.8581	0.1887	0.6263
74254	Gpn1	GPN-loop GTPase 1	0.858	0.1604	0.6055
72179	Fbxl2	F-box and leucine-rich repeat protein 2	0.858	0.1124	0.5549
30940	Usp25	ubiquitin specific peptidase 25	0.858	0.0448	0.4353
233805	Dcun1d3	DCN1, defective in cullin neddylation 1, domain containing 3 (S. cerevisiae)	0.8579	0.4191	0.7791
23888	Gpc6	glypican 6	0.8579	0.2143	0.6467
22635	Zan	zonadhesin	0.8579	0.1335	0.5774
20674	Sox2	SRY-box containing gene 2	0.8579	0.09697	0.5346
69546	Mapk1ip1	mitogen-activated protein kinase 1 interacting protein 1	0.8578	0.03282	NA
53886	Cdkl2	cyclin-dependent kinase-like 2 (CDC2-related kinase)	0.8578	0.1674	0.6091
51886	Fubp1	far upstream element (FUSE) binding protein 1	0.8578	0.06595	0.483
338371	A730011L01Rik	RIKEN cDNA A730011L01 gene	0.8577	0.0957	0.5341
70729	Nos1ap	nitric oxide synthase 1 (neuronal) adaptor protein	0.8577	0.2842	0.6977
17123	Madcam1	mucosal vascular addressin cell adhesion molecule 1	0.8576	0.4664	0.8049
214899	Kdm5a	lysine (K)-specific demethylase 5A	0.8575	0.2229	0.654
17364	Trpm1	transient receptor potential cation channel, subfamily M, member 1	0.8575	0.1129	0.5554
381319	Batf3	basic leucine zipper transcription factor, ATF-like 3	0.8574	0.477	0.8099
18195	Nsf	N-ethylmaleimide sensitive fusion protein	0.8574	0.1587	0.6033
13821	Epb4.1l1	erythrocyte protein band 4.1-like 1	0.8574	0.208	0.6401
27395	Mrpl15	mitochondrial ribosomal protein L15	0.8573	0.05378	0.4594
100042539	Gm3893	predicted gene 3893	0.8572	0.1916	0.6278
18711	Pikfyve	phosphoinositide kinase, FYVE finger containing	0.8571	0.4334	0.7872
381832	Prpmp5	proline-rich protein MP5	0.857	0.433	0.7868
260423	Hist1h3f	histone cluster 1, H3f	0.857	0.5488	0.841
100040322	3830408C21Rik	RIKEN cDNA 3830408C21 gene	0.8569	0.0598	0.4728
93728	Pabpc5	poly(A) binding protein, cytoplasmic 5	0.8568	0.6212	0.8719
69277	3300002I08Rik	RIKEN cDNA 3300002I08 gene	0.8568	0.07814	0.5035
18508	Pax6	paired box gene 6	0.8568	0.1597	0.6042
675812	Zfp605	zinc finger protein 605	0.8567	0.1156	0.558
67225	Rnpc3	RNA-binding region (RNP1, RRM) containing 3	0.8567	0.2814	0.6967
74182	Gpcpd1	glycerophosphocholine phosphodiesterase GDE1 homolog (S. cerevisiae)	0.8566	0.2191	0.6512
240665	Ccnj	cyclin J	0.8565	0.1212	0.5649
53604	Zpbp	zona pellucida binding protein	0.8565	0.09597	0.5344
320854	9030203C11Rik	RIKEN cDNA 9030203C11 gene	0.8563	0.1612	0.607
319266	A130010J15Rik	RIKEN cDNA A130010J15 gene	0.8563	0.05667	0.4638
70432	Rufy2	RUN and FYVE domain-containing 2	0.8563	0.04	0.424
241494	Zfp385b	zinc finger protein 385B	0.8562	0.2989	0.7052
72930	Ppp2r2b	protein phosphatase 2 (formerly 2A), regulatory subunit B (PR 52), beta isoform	0.8561	0.08371	0.5144
13347	Dffa	DNA fragmentation factor, alpha subunit	0.8561	0.2619	0.6831
11512	Adcy6	adenylate cyclase 6	0.8561	0.0409	0.4264
77733	Rnf170	ring finger protein 170	0.856	0.0248	NA
69993	Chn2	chimerin (chimaerin) 2	0.856	0.01604	NA
545700	Vmn2r-ps14	vomeronasal 2, receptor, pseudogene 14	0.8558	0.1425	0.5872
53817	Bat1a	HLA-B-associated transcript 1A	0.8558	0.1956	0.6304

667141	Gm8479	thiopurine S-methyltransferase pseudogene	0.8557	0.2003	0.6345
100042173	Gm11968	Rps15a pseudogene	0.8555	0.06071	0.4741
320204	4833442J19Rik	RIKEN cDNA 4833442J19 gene	0.8555	0.2088	0.6401
67996	Srsf6	serine/arginine-rich splicing factor 6	0.8555	0.02882	NA
22042	Tfrc	transferrin receptor	0.8555	0.2494	0.6767
17122	Mxd4	Max dimerization protein 4	0.8555	0.02298	NA
234814	Mthfsd	methenyltetrahydrofolate synthetase domain containing	0.8554	0.0248	NA
78420	9530048J24Rik	RIKEN cDNA 9530048J24 gene	0.8554	0.1428	0.5879
68121	Cep70	centrosomal protein 70	0.8554	0.06238	0.4771
20017	Polr1b	polymerase (RNA) I polypeptide B	0.8554	0.1202	0.5641
12675	Chuk	conserved helix-loop-helix ubiquitous kinase	0.8552	0.02585	NA
320772	Mdga2	MAM domain containing glycosylphosphatidylinositol anchor 2	0.8551	0.05438	0.4601
51886	Fubp1	far upstream element (FUSE) binding protein 1	0.8551	0.137	0.5807
22764	Zfx	zinc finger protein X-linked	0.8551	0.1899	0.6273
93887	Pcdhb16	protocadherin beta 16	0.855	0.05136	0.4519
74648	S100pbp	S100P binding protein	0.855	0.2898	0.7004
320633	Zbtb26	zinc finger and BTB domain containing 26	0.8549	0.06774	0.4855
17761	Mtap7	microtubule-associated protein 7	0.8549	0.1851	0.6223
13518	Dst	dystonin	0.8549	0.006652	NA
434174	Hmg1l1	high-mobility group (nonhistone chromosomal) protein 1-like 1	0.8548	0.4021	0.7705
235283	Gramd1b	GRAM domain containing 1B	0.8548	0.2141	0.6467
231051	Mll3	myeloid/lymphoid or mixed-lineage leukemia 3	0.8548	0.02094	NA
30056	Timm9	translocase of inner mitochondrial membrane 9 homolog (yeast)	0.8547	0.09675	0.5345
20615	Snapin	SNAP-associated protein	0.8547	0.09818	0.5365
14546	Gdap10	ganglioside-induced differentiation-associated-protein 10	0.8547	0.01069	NA
16571	Kif4	kinesin family member 4	0.8546	0.03817	0.4193
235048	Zfp599	zinc finger protein 599	0.8545	0.424	0.7818
237542	Osbpl8	oxysterol binding protein-like 8	0.8544	0.05108	0.4519
17909	Myo10	myosin X	0.8544	0.06864	0.4867
218865	Chdh	choline dehydrogenase	0.8543	0.2044	0.637
52713	Ccdc59	coiled-coil domain containing 59	0.8543	0.158	0.6023
17245	Mdm1	transformed mouse 3T3 cell double minute 1	0.8543	0.2084	0.6401
628709	Gm10324	predicted gene 10324	0.8542	0.3924	0.765
57895	Ccdc126	coiled-coil domain containing 126	0.8542	0.2616	0.6831
360220	Speer4d	spermatogenesis associated glutamate (E)-rich protein 4d	0.854	0.5643	0.8467
228355	Madd	MAP-kinase activating death domain	0.854	0.5479	0.8406
57261	Brd4	bromodomain containing 4	0.8539	0.01989	NA
12168	Bmpr2	bone morphogenic protein receptor, type II (serine/threonine kinase)	0.8539	0.1794	0.6187
100043682	Gm10584	predicted gene 10584	0.8538	0.6171	0.8698
228880	Zmynd8	zinc finger, MYND-type containing 8	0.8538	0.06393	0.4802
77808	A930015G24Rik	RIKEN cDNA A930015G24 gene	0.8538	0.1094	0.5529
74564	9.13E+15	hypothetical 9130022E09	0.8538	0.06841	0.4864
71375	Foxn3	forkhead box N3	0.8538	0.04191	0.4285
18795	Plcb1	phospholipase C, beta 1	0.8538	0.2056	0.6386
210035	Tmem194	transmembrane protein 194	0.8537	0.04594	0.4381
171207	Arhgap4	Rho GTPase activating protein 4	0.8537	0.1026	0.5424
67973	Mphosph10	M-phase phosphoprotein 10 (U3 small nucleolar ribonucleoprotein)	0.8537	0.2943	0.7041
329506	Ctdspl2	CTD (carboxy-terminal domain, RNA polymerase II, polypeptide A) small phosphatase like 2	0.8536	0.0413	0.4282
226896	Tcfap2d	transcription factor AP-2, delta	0.8536	0.2007	0.6345

26932	Ppp2r5e	protein phosphatase 2, regulatory subunit B (B56), epsilon isoform	0.8536	0.2666	0.6862
68501	Nsmce2	non-SMC element 2 homolog (MMS21, S. cerevisiae)	0.8535	0.6064	0.8645
93697	Narg2	NMDA receptor-regulated gene 2	0.8534	0.05678	0.4643
382010	BC088983	cDNA sequence BC088983	0.8533	0.2977	0.7051
227699	Nup188	nucleoporin 188	0.8533	0.5155	0.8277
170651	Krtap16-1	keratin associated protein 16-1	0.8533	0.3377	0.7313
56224	Tspan5	tetraspanin 5	0.8533	0.1503	0.5938
19054	Ppp2r3d	protein phosphatase 2 (formerly 2A), regulatory subunit B'', delta	0.8533	0.009801	NA
18813	Pa2g4	proliferation-associated 2G4	0.8533	0.1484	0.5918
18626	Per1	period homolog 1 (Drosophila)	0.8533	0.1888	0.6266
16564	Kif21a	kinesin family member 21A	0.8533	0.03917	0.4218
22063	Trpc1	transient receptor potential cation channel, subfamily C, member 1	0.8532	0.05127	0.4519
170753	Zfp704	zinc finger protein 704	0.8531	0.3392	0.7321
52882	Rgs7bp	regulator of G-protein signalling 7 binding protein	0.8531	0.1821	0.6206
66799	Ube2w	ubiquitin-conjugating enzyme E2W (putative)	0.853	0.336	0.7303
26971	Pla2g2f	phospholipase A2, group IIF	0.853	0.66	0.8879
26413	Mapk1	mitogen-activated protein kinase 1	0.853	0.09422	0.5311
628951	Gm6936	predicted gene 6936	0.8529	0.2308	0.6601
217558	G2e3	G2/M-phase specific E3 ubiquitin ligase	0.8529	0.282	0.6967
215789	Phactr2	phosphatase and actin regulator 2	0.8529	0.31	0.713
622935	Krtap20-2	keratin associated protein 20-2	0.8528	0.3003	0.706
243906	Zfp14	zinc finger protein 14	0.8527	0.054	0.4596
21372	Tbl1x	transducin (beta)-like 1 X-linked	0.8526	0.03816	0.4193
20393	Sgk1	serum/glucocorticoid regulated kinase 1	0.8525	0.06527	0.4816
70227	Zfp619	zinc finger protein 619	0.8524	0.2948	0.7044
56640	Klk4	kallikrein related-peptidase 4 (prostase, enamel matrix, prostate)	0.8524	0.6118	0.8673
21353	Tank	TRAF family member-associated Nf-kappa B activator	0.8524	0.4222	0.7801
71820	Wdr34	WD repeat domain 34	0.8522	0.06474	0.4816
20563	Slit2	slit homolog 2 (Drosophila)	0.8522	0.007948	NA
319148	Hist1h3c	histone cluster 1, H3c	0.8521	0.566	0.8478
14806	Grik2	glutamate receptor, ionotropic, kainate 2 (beta 2)	0.8521	0.06885	0.487
231801	Agfg2	ArfGAP with FG repeats 2	0.852	0.1237	0.5682
66308	2810021B07Rik	RIKEN cDNA 2810021B07 gene	0.852	0.02624	0.3791
93882	Pcdhb11	protocadherin beta 11	0.8519	0.2356	0.6654
69384	Tmem89	transmembrane protein 89	0.8519	0.3291	0.7254
20747	Spop	speckle-type POZ protein	0.8519	0.08213	0.5139
20515	Slc20a1	solute carrier family 20, member 1	0.8519	0.263	0.6838
233335	Synm	synemin, intermediate filament protein	0.8518	0.03015	0.3947
110809	Srsf1	serine/arginine-rich splicing factor 1	0.8518	0.1057	0.5468
320226	4930473A06Rik	RIKEN cDNA 4930473A06 gene	0.8516	0.2814	0.6967
258294	Olfr1115	olfactory receptor 1115	0.8515	0.4126	0.7747
225027	Srsf7	serine/arginine-rich splicing factor 7	0.8515	0.09421	0.5311
80877	Lrba	LPS-responsive beige-like anchor	0.8515	0.05132	0.4519
77018	Col25a1	collagen, type XXV, alpha 1	0.8515	0.4126	0.7747
76763	Mospd2	motile sperm domain containing 2	0.8515	0.08514	0.5167
74200	2810403A07Rik	RIKEN cDNA 2810403A07 gene	0.8515	0.04162	0.4282
26428	Orc4	origin recognition complex, subunit 4	0.8515	0.0873	0.5187
21949	Tnfsf8	tumor necrosis factor (ligand) superfamily, member 8	0.8515	0.5187	0.8282
100042727	Gm3993	predicted gene 3993	0.8513	0.4352	0.7883

75220	4930535I16Rik	RIKEN cDNA 4930535I16 gene	0.8513	0.03591	0.4131
52231	Ankzf1	ankyrin repeat and zinc finger domain containing 1	0.8513	0.04184	0.4285
50933	Uchl3	ubiquitin carboxyl-terminal esterase L3 (ubiquitin thiolesterase)	0.8513	0.773	0.9263
544848	Gm5784	predicted gene 5784	0.8512	0.2156	0.6479
434179	Gm5595	predicted gene 5595	0.8512	0.6242	0.8728
320816	Ankrd16	ankyrin repeat domain 16	0.8512	0.135	0.5784
106344	Rfc4	replication factor C (activator 1) 4	0.8512	0.1589	0.6035
74558	Gvin1	GTPase, very large interferon inducible 1	0.8511	0.1517	0.5948
74478	Snx29	sorting nexin 29	0.8511	0.3191	0.7184
72098	Tmem68	transmembrane protein 68	0.8511	0.2299	0.6596
67522	1700097N02Rik	RIKEN cDNA 1700097N02 gene	0.8511	0.1223	0.5656
53612	Vti1b	vesicle transport through interaction with t-SNAREs 1B homolog	0.8511	0.06783	0.4855
17709	COX2	cytochrome c oxidase subunit II	0.851	0.08043	0.5098
15975	Ifnar1	interferon (alpha and beta) receptor 1	0.851	0.08312	0.5144
382073	Ccdc84	coiled-coil domain containing 84	0.8509	0.06206	0.4771
26875	Pclo	piccolo (presynaptic cytomatrix protein)	0.8509	0.03814	0.4193
17354	Mllt10	myeloid/lymphoid or mixed-lineage leukemia (trithorax homolog, Drosophila); translocated to, 10	0.8509	0.1219	0.5652
330260	Pon2	paraoxonase 2	0.8508	0.2592	0.6815
239510	Phf20l1	PHD finger protein 20-like 1	0.8508	0.06657	0.4847
52392	D1Ertd622e	DNA segment, Chr 1, ERATO Doi 622, expressed	0.8508	0.004426	NA
21778	Tex9	testis expressed gene 9	0.8508	0.1386	0.5825
54137	Acrbp	proacrosin binding protein	0.8506	0.02379	0.3766
20504	Slc17a1	solute carrier family 17 (sodium phosphate), member 1	0.8506	0.04244	0.4301
408068	Zfp738	zinc finger protein 738	0.8504	0.1574	0.6016
320506	Lmbrd2	LMBR1 domain containing 2	0.8504	0.2993	0.7053
66923	Pbrm1	polybromo 1	0.8504	0.363	0.7483
407795	BC030870	cDNA sequence BC030870	0.8503	0.3349	0.73
71274	4933433G15Rik	RIKEN cDNA 4933433G15 gene	0.8503	0.5165	0.8277
68795	Ubr3	ubiquitin protein ligase E3 component n-recognin 3	0.8502	0.03032	0.3954
18704	Pik3c2a	phosphatidylinositol 3-kinase, C2 domain containing, alpha polypeptide	0.8501	0.1469	0.591
238328	Vash1	vasohibin 1	0.85	0.3565	0.7443
399558	Flrt2	fibronectin leucine rich transmembrane protein 2	0.8498	0.1657	0.6077
23873	Faim	Fas apoptotic inhibitory molecule	0.8498	0.145	0.5896
320608	D930023I05Rik	RIKEN cDNA D930023I05 gene	0.8497	0.4697	0.8064
71601	Ceacam20	carcinoembryonic antigen-related cell adhesion molecule 20	0.8497	0.1106	0.5546
24013	Grk1	G protein-coupled receptor kinase 1	0.8497	0.2771	0.6925
16477	Junb	Jun-B oncogene	0.8497	0.4137	0.7754
66435	Uggt2	UDP-glucose glycoprotein glucosyltransferase 2	0.8496	0.1053	0.5459
20474	Six4	sine oculis-related homeobox 4 homolog (Drosophila)	0.8496	0.0641	0.4809
76763	Mospd2	motile sperm domain containing 2	0.8495	0.08503	0.5166
69294	Cst13	cystatin 13	0.8495	0.5334	0.8343
75985	Rab30	RAB30, member RAS oncogene family	0.8494	0.07861	0.5052
56404	Trip4	thyroid hormone receptor interactor 4	0.8494	0.2401	0.6685
16337	Insr	insulin receptor	0.8492	0.06947	0.4875
14260	Fmn1	formin 1	0.8492	0.2036	0.6362
97122	Hist2h4	histone cluster 2, H4	0.8491	0.1337	0.5776
328309	Gm9776	predicted gene 9776	0.849	0.7085	0.9066
106064	AW549877	expressed sequence AW549877	0.849	0.06077	0.4741
75605	Kdm5b	lysine (K)-specific demethylase 5B	0.849	0.04527	0.4353

13831	Epc1	enhancer of polycomb homolog 1 (Drosophila)	0.849	0.1042	0.5444
78211	4930558N11Rik	RIKEN cDNA 4930558N11 gene	0.8489	0.4793	0.8101
109050	6530418L21Rik	RIKEN cDNA 6530418L21 gene	0.8488	0.247	0.674
67370	Zfp606	zinc finger protein 606	0.8488	0.06915	0.4872
22643	Zfp101	zinc finger protein 101	0.8488	0.0224	0.3702
243846	Ccdc9	coiled-coil domain containing 9	0.8487	0.3376	0.7312
23959	Nt5e	5' nucleotidase, ecto	0.8487	0.0244	0.3782
402751	C430019N01Rik	RIKEN cDNA C430019N01 gene	0.8486	0.3556	0.7439
244713	Zfp317	zinc finger protein 317	0.8486	0.3151	0.7161
14173	Fgf2	fibroblast growth factor 2	0.8486	0.3034	0.709
320560	Dennd5b	DENN/MADD domain containing 5B	0.8484	0.3455	0.736
17960	Nat1	N-acetyl transferase 1	0.8484	0.1972	0.6324
319991	Kif6	kinesin family member 6	0.8483	0.3551	0.7434
234725	Zfp612	zinc finger protein 612	0.8483	0.009953	NA
67410	4930449I24Rik	RIKEN cDNA 4930449I24 gene	0.8483	0.3823	0.7596
11789	Apc	adenomatosis polyposis coli	0.8483	0.2896	0.7004
100504944	LOC100504944	hypothetical protein LOC100504944	0.8482	0.5574	0.8454
100041420	Gm3325	predicted gene 3325	0.8482	0.4699	0.8065
382236	Brwd3	bromodomain and WD repeat domain containing 3	0.8482	0.08321	0.5144
381695	N4bp2l2	NEDD4 binding protein 2-like 2	0.8482	0.1699	0.6112
270152	Amica1	adhesion molecule, interacts with CXADR antigen 1	0.8482	0.2439	0.6718
215999	Ccdc109a	coiled-coil domain containing 109A	0.8482	0.2886	0.6996
68070	Pdzd2	PDZ domain containing 2	0.8482	0.3028	0.7087
259086	Olfr609	olfactory receptor 609	0.8481	0.3393	0.7321
14455	Gas5	growth arrest specific 5	0.8481	0.1839	0.6217
12544	Cdc45	cell division cycle 45 homolog (S. cerevisiae)	0.8481	0.1784	0.618
12399	Runx3	runt related transcription factor 3	0.8481	0.3931	0.7652
319922	Vwc2	von Willebrand factor C domain containing 2	0.848	0.03491	0.4096
69391	1700018A14Rik	RIKEN cDNA 1700018A14 gene	0.848	0.2697	0.6878
100042057	Gm3643	predicted gene 3643	0.8479	0.1329	0.5772
11735	Ank3	ankyrin 3, epithelial	0.8479	0.2762	0.6925
329178	Unc80	unc-80 homolog (C. elegans)	0.8478	0.03799	0.4192
234362	Zfp868	zinc finger protein 868	0.8478	0.1432	0.5879
100041734	4930522L14Rik	RIKEN cDNA 4930522L14 gene	0.8477	0.2094	0.641
76933	Ifi27l2a	interferon, alpha-inducible protein 27 like 2A	0.8477	0.4568	0.7987
233529	Kctd14	potassium channel tetramerisation domain containing 14	0.8476	0.07351	0.4957
19212	Pter	phosphotriesterase related	0.8476	0.0366	0.4161
100233208	Gm10778	predicted gene 10778	0.8474	0.5328	0.834
70138	2210417A02Rik	RIKEN cDNA 2210417A02 gene	0.8474	0.1063	0.547
52906	Ahi1	Abelson helper integration site 1	0.8474	0.07504	0.4989
17116	Mab21l1	mab-21-like 1 (C. elegans)	0.8473	0.05379	0.4594
227960	Gca	grancalcin	0.8472	0.04473	0.4353
70425	Csnk1g3	casein kinase 1, gamma 3	0.8472	0.223	0.654
57753	Noc3l	nucleolar complex associated 3 homolog (S. cerevisiae)	0.8472	0.03123	0.3964
237560	Lrrc10	leucine rich repeat containing 10	0.8471	0.3115	0.7137
100042498	2210403K04Rik	RIKEN cDNA 2210403K04 gene	0.8469	0.2045	0.637
99334	Zscan29	zinc finger SCAN domains 29	0.8469	0.08467	0.5156
56490	Zbtb20	zinc finger and BTB domain containing 20	0.8469	0.05052	0.4517
18108	Nmt2	N-myristoyltransferase 2	0.8469	0.1049	0.5453

433416	Gm13547	predicted gene 13547	0.8468	0.3449	0.736
228602	4930402H24Rik	RIKEN cDNA 4930402H24 gene	0.8468	0.09643	0.5345
69546	Mapk1ip1	mitogen-activated protein kinase 1 interacting protein 1	0.8468	0.1915	0.6278
68642	Tmem216	transmembrane protein 216	0.8468	0.7472	0.9199
73449	1700066B19Rik	RIKEN cDNA 1700066B19 gene	0.8467	0.04393	0.4336
20716	Serpina3n	serine (or cysteine) peptidase inhibitor, clade A, member 3N	0.8467	0.241	0.6687
74053	Grip1	glutamate receptor interacting protein 1	0.8464	0.02888	0.3903
319387	Lphn3	latrophilin 3	0.8463	0.1852	0.6223
75985	Rab30	RAB30, member RAS oncogene family	0.8463	0.1158	0.558
18569	Pdcd4	programmed cell death 4	0.8463	0.1689	0.6096
100038746	LOC100038746	hypothetical LOC100038746	0.8462	0.00646	NA
74290	1700102J08Rik	RIKEN cDNA 1700102J08 gene	0.8461	0.1523	0.5958
109263	Rlf	rearranged L-myc fusion sequence	0.846	0.1113	0.5549
57784	Bin3	bridging integrator 3	0.846	0.09218	0.5263
50934	Slc7a8	solute carrier family 7 (cationic amino acid transporter, y+ system), member 8	0.846	0.2103	0.6419
382118	Zfp167	zinc finger protein 167	0.8459	0.209	0.6406
78797	Ndor1	NADPH dependent diflavin oxidoreductase 1	0.8459	0.1718	0.6126
75507	Pou5f2	POU domain class 5, transcription factor 2	0.8459	0.09362	0.5296
16616	Klk1b21	kallikrein 1-related peptidase b21	0.8459	0.4664	0.8049
225638	Alpk2	alpha-kinase 2	0.8458	0.03817	0.4193
75956	Srrm2	serine/arginine repetitive matrix 2	0.8458	0.3497	0.7388
107723	Slc12a6	solute carrier family 12, member 6	0.8457	0.2242	0.654
18028	Nfib	nuclear factor I/B	0.8457	0.2141	0.6467
15289	Hmgb1	high mobility group box 1	0.8457	0.2671	0.6865
14815	Nr3c1	nuclear receptor subfamily 3, group C, member 1	0.8457	0.203	0.6356
14884	Gtf2h1	general transcription factor II H, polypeptide 1	0.8456	0.01283	NA
24074	Taf7	TAF7 RNA polymerase II, TATA box binding protein (TBP)-associated factor	0.8455	0.2099	0.6413
102747	Lrrc49	leucine rich repeat containing 49	0.8454	0.09981	0.5377
14815	Nr3c1	nuclear receptor subfamily 3, group C, member 1	0.8454	0.05183	0.4525
110253	Triobp	TRIO and F-actin binding protein	0.8453	0.196	0.6307
70408	Polr3f	polymerase (RNA) III (DNA directed) polypeptide F	0.8453	0.08583	0.517
238722	Zfp72	zinc finger protein 72	0.8452	0.481	0.8104
67972	Atp2b1	ATPase, Ca++ transporting, plasma membrane 1	0.8452	0.2603	0.6815
214855	Arid5a	AT rich interactive domain 5A (MRF1-like)	0.8451	0.03728	0.4163
56298	Atl2	atlastin GTPase 2	0.8451	0.1763	0.6156
19360	Rad50	RAD50 homolog (S. cerevisiae)	0.8451	0.1272	0.5715
319177	Hist1h2ba	histone cluster 1, H2ba	0.845	0.03109	0.3964
230895	Vps13d	vacuolar protein sorting 13 D (yeast)	0.845	0.09668	0.5345
626058	E330020D12Rik	Riken cDNA E330020D12 gene	0.8449	0.6038	0.8644
67607	Zfp788	zinc finger protein 788	0.8448	0.7322	0.9157
11499	Adam5	a disintegrin and metallopeptidase domain 5	0.8448	0.1198	0.5633
242443	Grin3a	glutamate receptor ionotropic, NMDA3A	0.8447	0.1549	0.5975
207227	Stxbp5l	syntaxin binding protein 5-like	0.8447	0.1707	0.6115
52728	D17Zt10e	DNA segment, Chr 17, Zdenek Trachtulec 10 expressed	0.8447	0.184	0.6217
26895	Cops7b	COP9 (constitutive photomorphogenic) homolog, subunit 7b (Arabidopsis thaliana)	0.8447	0.1056	0.5468
269198	Nbeal1	neurobeachin like 1	0.8444	0.08821	0.5202
257871	Olfr1372-ps1	olfactory receptor 1372, pseudogene 1	0.8444	0.09872	0.5367
77087	Ankrd11	ankyrin repeat domain 11	0.8444	0.1103	0.5535
75555	1700020D05Rik	RIKEN cDNA 1700020D05 gene	0.8444	0.01838	0.351

72999	Insig2	insulin induced gene 2	0.8444	0.1877	0.626
59069	Tpm3	tropomyosin 3, gamma	0.8444	0.3238	0.721
546160	Fbxw25	F-box and WD-40 domain protein 25	0.8443	0.542	0.8379
114249	Npnt	nephronectin	0.8443	0.04398	0.4336
545700	Vmn2r-ps14	vomeronasal 2, receptor, pseudogene 14	0.8442	0.1734	0.6147
320076	C630001G18Rik	RIKEN cDNA C630001G18 gene	0.8442	0.2408	0.6687
69017	Prrt2	proline-rich transmembrane protein 2	0.8442	0.05564	0.4631
57748	Jmy	junction-mediating and regulatory protein	0.8442	0.1669	0.6083
19893	Rpgr	retinitis pigmentosa GTPase regulator	0.8442	0.03929	0.4222
320209	Ddx11	DEAD/H (Asp-Glu-Ala-Asp/His) box polypeptide 11 (CHL1-like helicase homolog, S. cerevisiae)	0.8441	0.04622	0.4389
19206	Ptch1	patched homolog 1	0.8441	0.7725	0.9261
229709	Ahcyl1	S-adenosylhomocysteine hydrolase-like 1	0.844	0.1833	0.6217
210135	Zfp180	zinc finger protein 180	0.844	0.05596	0.4631
74111	Rbm19	RNA binding motif protein 19	0.844	0.1973	0.6324
71781	Slc16a14	solute carrier family 16 (monocarboxylic acid transporters), member 14	0.844	0.008586	NA
77656	C430045I18Rik	RIKEN cDNA C430045I18 gene	0.8439	0.01388	NA
21915	Dtymk	deoxythymidylate kinase	0.8439	0.05055	0.4517
234135	Whsc1l1	Wolf-Hirschhorn syndrome candidate 1-like 1 (human)	0.8438	0.394	0.7657
70408	Polr3f	polymerase (RNA) III (DNA directed) polypeptide F	0.8437	0.3732	0.7538
666043	Gm7904	predicted gene 7904	0.8436	0.3007	0.7066
230721	Pabpc4	poly(A) binding protein, cytoplasmic 4	0.8436	0.04513	0.4353
80912	Pum1	pumilio 1 (Drosophila)	0.8435	0.05941	0.4728
407818	BC055308	cDNA sequence BC055308	0.8434	0.05391	0.4595
433966	5730422E09Rik	RIKEN cDNA 5730422E09 gene	0.8433	0.3283	0.7251
238799	Tnpo1	transportin 1	0.8433	0.4062	0.7721
75320	Etnk1	ethanolamine kinase 1	0.8432	0.283	0.697
70560	Wars2	tryptophanyl tRNA synthetase 2 (mitochondrial)	0.8431	0.1769	0.6164
19726	Rfx3	regulatory factor X, 3 (influences HLA class II expression)	0.8431	0.1885	0.6263
100191037	Krtap10-4	keratin associated protein 10-4	0.843	0.5626	0.8465
21413	Tcf4	transcription factor 4	0.8429	0.02232	0.3702
18315	Olfr18	olfactory receptor 18	0.8429	0.414	0.7757
626854	Gm6712	predicted gene 6712	0.8428	0.2333	0.6622
432870	Gm5464	predicted gene 5464	0.8428	0.2309	0.6601
74159	Acbd5	acyl-Coenzyme A binding domain containing 5	0.8427	0.3956	0.7671
329003	Zfp516	zinc finger protein 516	0.8426	0.2899	0.7004
52348	Vps37a	vacuolar protein sorting 37A (yeast)	0.8426	0.04464	0.4353
434350	9430091E24Rik	RIKEN cDNA 9430091E24 gene	0.8425	0.251	0.6767
353170	Txlng	taxilin gamma	0.8425	0.1681	0.6095
319986	A130042O14Rik	RIKEN cDNA A130042O14 gene	0.8425	0.2342	0.6633
74256	Cyld	cylindromatosis (turban tumor syndrome)	0.8425	0.3761	0.7556
12511	Cd6	CD6 antigen	0.8425	0.3951	0.7666
71519	Cyp2u1	cytochrome P450, family 2, subfamily u, polypeptide 1	0.8424	0.2886	0.6996
381318	Nsl1	NSL1, MIND kinetochore complex component, homolog (S. cerevisiae)	0.8423	0.03195	0.3992
240869	Zbtb37	zinc finger and BTB domain containing 37	0.8422	0.2407	0.6687
70020	Ino80b	INO80 complex subunit B	0.8422	0.08122	0.5107
24135	Zfp68	zinc finger protein 68	0.8422	0.2866	0.6982
269423	3110057O12Rik	RIKEN cDNA 3110057O12 gene	0.8421	0.1511	0.5942
18729	Pira6	paired-Ig-like receptor A6	0.842	0.483	0.8118
231125	Zfyve28	zinc finger, FYVE domain containing 28	0.8419	0.5043	0.8221

100504173	LOC100504173	hypothetical protein LOC100504173	0.8418	0.2541	0.6788
215751	BC013529	cDNA sequence BC013529	0.8418	0.2383	0.667
208366	Rpp40	ribonuclease P 40 subunit (human)	0.8418	0.0469	0.4432
330267	Thsd7a	thrombospondin, type I, domain containing 7A	0.8417	0.07271	0.495
77271	9430024F10Rik	RIKEN cDNA 9430024F10 gene	0.8417	0.07652	0.5014
12545	Cdc7	cell division cycle 7 (S. cerevisiae)	0.8417	0.006308	NA
240283	Dmxl1	Dmx-like 1	0.8416	0.2093	0.6409
235527	Plscr4	phospholipid scramblase 4	0.8415	0.4561	0.7986
212528	Trmt1	TRM1 tRNA methyltransferase 1 homolog (S. cerevisiae)	0.8415	0.1042	0.5444
83922	Tsga14	testis specific gene A14	0.8415	0.1943	0.6299
223672	Apol9a	apolipoprotein L 9a	0.8413	0.537	0.8354
114873	Dscaml1	Down syndrome cell adhesion molecule-like 1	0.8413	0.0656	0.4818
99503	AA517023	expressed sequence AA517023	0.8413	0.2759	0.6925
77117	Zfp934	zinc finger protein 934	0.8413	0.2446	0.6725
21985	Tpd52	tumor protein D52	0.8413	0.1116	0.5549
100043911	Ppp4r1l-ps	protein phosphatase 4, regulatory subunit 1-like, pseudogene	0.8412	0.03083	0.3964
232341	Wnk1	WNK lysine deficient protein kinase 1	0.8412	0.152	0.5954
230379	Acer2	alkaline ceramidase 2	0.8412	0.1415	0.5863
211612	Ptchd1	patched domain containing 1	0.8412	0.1649	0.6077
74554	9130002K18Rik	RIKEN cDNA 9130002K18 gene	0.8412	0.284	0.6975
329316	A930038G18	hypothetical protein A930038G18	0.841	0.2118	0.6444
69944	2810021J22Rik	RIKEN cDNA 2810021J22 gene	0.8409	0.1047	0.5452
17248	Mdm4	transformed mouse 3T3 cell double minute 4	0.8409	0.1919	0.6281
99470	Magi3	membrane associated guanylate kinase, WW and PDZ domain containing 3	0.8408	0.1591	0.6036
329581	Birc7	baculoviral IAP repeat-containing 7 (livin)	0.8406	0.4884	0.8138
72505	2610319H10Rik	RIKEN cDNA 2610319H10 gene	0.8405	0.02669	0.3813
20265	Scn1a	sodium channel, voltage-gated, type I, alpha	0.8405	0.05301	0.4565
56495	Asna1	arsA arsenite transporter, ATP-binding, homolog 1 (bacterial)	0.8404	0.07412	0.4962
65020	Zfp110	zinc finger protein 110	0.8403	0.05407	0.4596
19281	Ptprt	protein tyrosine phosphatase, receptor type, T	0.8402	0.2375	0.6663
229694	AI504432	expressed sequence AI504432	0.8401	0.1755	0.6156
233789	Smg1	SMG1 homolog, phosphatidylinositol 3-kinase-related kinase (C. elegans)	0.84	0.03285	0.4035
22685	Zfp239	zinc finger protein 239	0.84	0.07666	0.5014
382492	Gm12034	predicted gene 12034	0.8399	0.1409	0.5853
225644	Cplx4	complexin 4	0.8398	0.06537	0.4816
19652	Rbm3	RNA binding motif protein 3	0.8397	0.2013	0.635
78586	Srbd1	S1 RNA binding domain 1	0.8396	0.07614	0.5009
100683	Trrap	transformation/transcription domain-associated protein	0.8395	0.09077	0.5229
381290	Atp2b4	ATPase, Ca++ transporting, plasma membrane 4	0.8394	0.424	0.7818
12323	Camk2b	calcium/calmodulin-dependent protein kinase II, beta	0.8394	0.1255	0.5703
235300	Tmem136	transmembrane protein 136	0.8392	0.01434	0.3347
384382	A430108E01Rik	RIKEN cDNA A430108E01 gene	0.8391	0.2505	0.6767
234479	Gm4890	predicted gene 4890	0.839	0.2709	0.689
319734	Cacna2d4	calcium channel, voltage-dependent, alpha 2/delta subunit 4	0.8389	0.01423	0.3347
15242	Hhex	hematopoietically expressed homeobox	0.8389	0.08696	0.5184
107094	Rrp12	ribosomal RNA processing 12 homolog (S. cerevisiae)	0.8388	0.398	0.7685
76022	Gon4l	gon-4-like (C.elegans)	0.8388	0.112	0.5549
100043225	Gm16378	predicted gene 16378	0.8386	0.5171	0.8277
269389	Tox2	TOX high mobility group box family member 2	0.8386	0.06044	0.4739

207911	Mchr1	melanin-concentrating hormone receptor 1	0.8386	0.4682	0.8056
93871	Brwd1	bromodomain and WD repeat domain containing 1	0.8386	0.02036	0.3609
108154	Adamts6	a disintegrin-like and metallopeptidase (reprolysin type) with thrombospondin type 1 motif, 6	0.8385	0.3495	0.7388
628709	Gm10324	predicted gene 10324	0.8381	0.6167	0.8698
26416	Mapk14	mitogen-activated protein kinase 14	0.8381	0.2266	0.6554
18798	Plcb4	phospholipase C, beta 4	0.8381	0.1341	0.5779
433171	Gm10549	predicted gene 10549	0.838	0.06159	0.477
385454	Gm5396	predicted pseudogene 5396	0.8379	0.2205	0.6522
240869	Zbtb37	zinc finger and BTB domain containing 37	0.8379	0.146	0.591
18021	Nfatc3	nuclear factor of activated T-cells, cytoplasmic, calcineurin-dependent 3	0.8379	0.2269	0.6559
226866	Gm106	predicted gene 106	0.8378	0.1367	0.5807
53861	Zranb2	zinc finger, RAN-binding domain containing 2	0.8378	0.04173	0.4282
22367	Vrk1	vaccinia related kinase 1	0.8377	0.2108	0.6426
14593	Ggps1	geranylgeranyl diphosphate synthase 1	0.8377	0.04679	0.4424
74471	4933440N22Rik	RIKEN cDNA 4933440N22 gene	0.8376	0.02375	0.3764
69576	2310010M20Rik	RIKEN cDNA 2310010M20 gene	0.8376	0.2146	0.6467
217578	Baz1a	bromodomain adjacent to zinc finger domain 1A	0.8375	0.05498	0.4614
209683	Ttc28	tetratricopeptide repeat domain 28	0.8375	0.1159	0.558
218314	Zfp595	zinc finger protein 595	0.8374	0.4466	0.7938
75964	Trappc8	trafficking protein particle complex 8	0.8374	0.2687	0.6876
21418	Tcfap2a	transcription factor AP-2, alpha	0.8374	0.00467	NA
14660	Gls	glutaminase	0.8373	0.04909	0.4469
229776	Cdc14a	CDC14 cell division cycle 14 homolog A (S. cerevisiae)	0.8372	0.00391	NA
228005	Ppig	peptidyl-prolyl isomerase G (cyclophilin G)	0.8372	0.02442	0.3782
105689	Mycbp2	MYC binding protein 2	0.8372	0.04286	0.4315
12890	Cplx2	complexin 2	0.8372	0.2033	0.6361
100043902	Six3os1	Six3 opposite strand transcript 1	0.8371	0.1835	0.6217
319757	Smo	smoothened homolog (Drosophila)	0.8371	0.3637	0.7483
70948	Wdr20b	WD repeat domain 20b	0.8371	0.5037	0.8221
105203	BC016423	cDNA sequence BC016423	0.837	0.3066	0.7108
74568	Mlkl	mixed lineage kinase domain-like	0.837	0.4803	0.8101
16792	Laptm5	lysosomal-associated protein transmembrane 5	0.837	0.05839	0.4703
52463	Tet1	tet oncogene 1	0.8369	0.009174	0.3092
100039203	Gm2099	predicted gene 2099	0.8368	0.1467	0.591
330938	Dixdc1	DIX domain containing 1	0.8368	0.3209	0.7197
238317	C130039O16Rik	RIKEN cDNA C130039O16 gene	0.8368	0.334	0.7291
212285	Arap2	ArfGAP with RhoGAP domain, ankyrin repeat and PH domain 2	0.8368	0.1094	0.5529
77908	9230113P08Rik	RIKEN cDNA 9230113P08 gene	0.8368	0.4844	0.8123
67726	Fam114a2	family with sequence similarity 114, member A2	0.8368	0.1876	0.6259
240892	Dusp27	dual specificity phosphatase 27 (putative)	0.8367	0.08968	0.5224
23856	Dido1	death inducer-obliterator 1	0.8366	0.2877	0.6988
71385	5430434G16Rik	RIKEN cDNA 5430434G16 gene	0.8365	0.03933	0.4222
70568	Cpne3	copine III	0.8365	0.126	0.5703
14652	Glp1r	glucagon-like peptide 1 receptor	0.8365	0.5323	0.8339
214897	Csnk1g1	casein kinase 1, gamma 1	0.8363	0.07603	0.5008
67200	Ccdc77	coiled-coil domain containing 77	0.8363	0.1251	0.5703
381522	E230008N13Rik	RIKEN cDNA E230008N13 gene	0.8362	0.006126	NA
67154	Mtdh	metadherin	0.8362	0.03115	0.3964
320277	Spef2	sperm flagellar 2	0.8361	0.1657	0.6077

631323	Gm12250	predicted gene 12250	0.836	0.04768	0.4442
214253	Etnk2	ethanolamine kinase 2	0.836	0.2756	0.6925
29865	Cabp5	calcium binding protein 5	0.836	0.153	0.5966
434171	Gm5591	predicted gene 5591	0.8359	0.3283	0.7251
69129	Pex11c	peroxisomal biogenesis factor 11 gamma	0.8359	0.299	0.7052
16495	Kcna7	potassium voltage-gated channel, shaker-related subfamily, member 7	0.8358	0.2994	0.7054
13712	Elk1	ELK1, member of ETS oncogene family	0.8358	0.09148	0.5241
72269	Cda	cytidine deaminase	0.8357	0.5161	0.8277
320560	Dennd5b	DENN/MADD domain containing 5B	0.8355	0.2285	0.6577
638580	Gm7244	predicted gene 7244	0.8354	0.04494	0.4353
240505	Cdc42bpg	CDC42 binding protein kinase gamma (DMPK-like)	0.8354	0.2211	0.6525
71774	Shroom1	shroom family member 1	0.8354	0.3807	0.7594
319285	A430061O12Rik	RIKEN cDNA A430061O12 gene	0.8353	0.1093	0.5529
212281	A530054K11Rik	RIKEN cDNA A530054K11 gene	0.8353	0.237	0.6659
209239	Gan	giant axonal neuropathy	0.8353	0.1493	0.5938
13211	Dhx9	DEAH (Asp-Glu-Ala-His) box polypeptide 9	0.8352	0.2402	0.6685
22776	Zim1	zinc finger, imprinted 1	0.8351	0.06527	0.4816
72397	Rbm12b	RNA binding motif protein 12B	0.835	0.08478	0.5161
19244	Ptp4a2	protein tyrosine phosphatase 4a2	0.835	0.07116	0.4932
215632	Psd4	pleckstrin and Sec7 domain containing 4	0.8349	0.2242	0.654
218850	D14Abb1e	DNA segment, Chr 14, Abbott 1 expressed	0.8347	0.02165	0.367
215821	D10Bwg1379e	DNA segment, Chr 10, Brigham & Women's Genetics 1379 expressed	0.8347	0.3299	0.726
232174	Cyp26b1	cytochrome P450, family 26, subfamily b, polypeptide 1	0.8346	0.04825	0.4448
114863	Prosc	proline synthetase co-transcribed	0.8346	0.06465	0.4816
494519	Abpd	androgen binding protein delta	0.8343	0.5575	0.8454
385605	Rundc2a	RUN domain containing 2A	0.8343	0.469	0.8059
67263	Zswim6	zinc finger, SWIM domain containing 6	0.8343	0.005962	0.2803
79202	Tnfrsf22	tumor necrosis factor receptor superfamily, member 22	0.8342	0.7307	0.915
66244	Sdccag1	serologically defined colon cancer antigen 1	0.8342	0.02617	0.3791
18600	Padi2	peptidyl arginine deiminase, type II	0.8342	0.3057	0.7104
106369	Ypel1	yippee-like 1 (Drosophila)	0.8341	0.3206	0.7196
23958	Nr2e3	nuclear receptor subfamily 2, group E, member 3	0.8341	0.0105	0.3117
18572	Pdcd11	programmed cell death 11	0.8341	0.2459	0.6733
12902	Cr2	complement receptor 2	0.8341	0.1353	0.579
11552	Adra2b	adrenergic receptor, alpha 2b	0.8341	0.4032	0.7708
239857	Cadm2	cell adhesion molecule 2	0.834	0.03868	0.4213
54650	Sfmbt1	Scm-like with four mbt domains 1	0.834	0.08359	0.5144
12175	Bnip2	BCL2/adenovirus E1B interacting protein 2	0.834	0.5285	0.8332
272538	Tmco7	transmembrane and coiled-coil domains 7	0.8339	0.1711	0.6118
231646	Myo1h	myosin 1H	0.8339	0.2387	0.6671
210162	Zkscan2	zinc finger with KRAB and SCAN domains 2	0.8338	0.007448	0.2934
114675	4932431P20Rik	RIKEN cDNA 4932431P20 gene	0.8337	0.2514	0.6767
77207	8030425K09Rik	RIKEN cDNA 8030425K09 gene	0.8337	0.01467	0.3369
382793	Mtx3	metaxin 3	0.8336	0.256	0.6801
75173	4930544O15Rik	RIKEN cDNA 4930544O15 gene	0.8336	0.4268	0.7825
17329	Cxcl9	chemokine (C-X-C motif) ligand 9	0.8336	0.395	0.7666
14186	Fgfr4	fibroblast growth factor receptor 4	0.8336	0.05871	0.4712
319673	9330159M07Rik	RIKEN cDNA 9330159M07 gene	0.8335	0.02278	0.3702
72723	Zfp74	zinc finger protein 74	0.8335	0.04063	0.4258

245007	Zbtb38	zinc finger and BTB domain containing 38	0.8334	0.1789	0.6181
100515	Zfp518b	zinc finger protein 518B	0.8333	0.007227	0.2929
195576	Gm13229	predicted gene 13229	0.833	0.1743	0.6148
19212	Pter	phosphotriesterase related	0.8329	0.0254	0.3786
15361	Hmga1	high mobility group AT-hook 1	0.8329	0.009643	0.3112
12043	Bcl2	B-cell leukemia/lymphoma 2	0.8329	0.0281	0.3875
330177	Taok3	TAO kinase 3	0.8328	0.08784	0.5191
93761	Smarca1	SWI/SNF related, matrix associated, actin dependent regulator of chromatin, subfamily a, member 1	0.8327	0.09023	0.5229
235469	Zfp280d	zinc finger protein 280D	0.8326	0.001139	NA
66471	Anp32e	acidic (leucine-rich) nuclear phosphoprotein 32 family, member E	0.8326	0.1234	0.568
404337	Olfr1383	olfactory receptor 1383	0.8324	0.007987	0.3003
320150	Zdhhc17	zinc finger, DHHC domain containing 17	0.8324	0.1744	0.6148
21346	Tagln2	transgelin 2	0.8322	0.2073	0.6398
270120	Fat3	FAT tumor suppressor homolog 3 (Drosophila)	0.8321	0.1444	0.589
22290	Uty	ubiquitously transcribed tetratricopeptide repeat gene, Y chromosome	0.832	0.2719	0.6891
20384	Srsf5	serine/arginine-rich splicing factor 5	0.832	0.0307	0.3964
668923	Zfp442	zinc finger protein 442	0.8319	0.3651	0.7495
57246	Tbx20	T-box 20	0.8319	0.05841	0.4703
330323	Fam188b	family with sequence similarity 188, member B	0.8318	0.1905	0.6275
241694	Ralgapa2	Ral GTPase activating protein, alpha subunit 2 (catalytic)	0.8318	0.1281	0.5716
100113398	Adat3	adenosine deaminase, tRNA-specific 3, TAD2 homolog (S. cerevisiae)	0.8317	0.03002	0.3947
77068	5930436O19Rik	RIKEN cDNA 5930436O19 gene	0.8316	0.01612	0.3411
76508	2210015D19Rik	RIKEN cDNA 2210015D19 gene	0.8316	0.02534	0.3786
73884	Zdbf2	zinc finger, DBF-type containing 2	0.8316	0.2491	0.6767
22221	Ubp1	upstream binding protein 1	0.8316	0.05833	0.4703
13371	Dio2	deiodinase, iodothyronine, type II	0.8316	0.3206	0.7196
229473	D930015E06Rik	RIKEN cDNA D930015E06 gene	0.8315	0.05518	0.4616
70461	Crtc3	CREB regulated transcription coactivator 3	0.8315	0.01005	0.3112
235380	Dmxl2	Dmx-like 2	0.8313	0.07623	0.5011
23963	Odz1	odd Oz/ten-m homolog 1 (Drosophila)	0.8313	0.06768	0.4855
223604	Kcnk9	potassium channel, subfamily K, member 9	0.8311	0.3645	0.7492
215693	Zmat1	zinc finger, matrin type 1	0.8311	0.02474	0.3786
11931	Atp1b1	ATPase, Na+/K+ transporting, beta 1 polypeptide	0.8311	0.02004	0.3604
432572	Specc1	sperm antigen with calponin homology and coiled-coil domains 1	0.831	0.5482	0.8406
75881	4930579K19Rik	RIKEN cDNA 4930579K19 gene	0.831	0.1763	0.6156
338363	6030446N20Rik	RIKEN cDNA 6030446N20 gene	0.8309	0.1061	0.5468
75894	Adal	adenosine deaminase-like	0.8309	0.1263	0.5703
71844	Nupl1	nucleoporin like 1	0.8309	0.1758	0.6156
54427	Dnmt3l	DNA (cytosine-5-)-methyltransferase 3-like	0.8308	0.3195	0.7189
399616	A130078K24Rik	RIKEN cDNA A130078K24 gene	0.8307	0.2847	0.6977
258673	Olfr1428	olfactory receptor 1428	0.8307	0.2831	0.697
320541	Slc35e2	solute carrier family 35, member E2	0.8306	0.3287	0.7253
71726	Smug1	single-strand selective monofunctional uracil DNA glycosylase	0.8306	0.001446	NA
320615	Dopey1	dopey family member 1	0.8305	0.03074	0.3964
244431	Sgcz	sarcoglycan zeta	0.8305	0.3696	0.7521
224617	Tbc1d24	TBC1 domain family, member 24	0.8305	0.01133	0.3181
100662	D930016D06Rik	RIKEN cDNA D930016D06 gene	0.8305	0.01648	0.3416
74221	1700011F03Rik	RIKEN cDNA 1700011F03 gene	0.8304	0.26	0.6815
71301	4930593A02Rik	RIKEN cDNA 4930593A02 gene	0.8303	0.3315	0.7265

94352	Loxl2	lysyl oxidase-like 2	0.8302	0.2493	0.6767
76719	1700081L11Rik	RIKEN cDNA 1700081L11 gene	0.8302	0.5433	0.839
235542	Ppp2r3a	protein phosphatase 2, regulatory subunit B'', alpha	0.8301	0.09889	0.5367
235344	Sik2	salt inducible kinase 2	0.8301	0.5569	0.8452
110094	Phka2	phosphorylase kinase alpha 2	0.8301	0.2618	0.6831
54447	Asah2	N-acylsphingosine amidohydrolase 2	0.8299	0.1143	0.5569
109333	Pkn2	protein kinase N2	0.8298	0.1151	0.5578
399607	D230019N24Rik	RIKEN cDNA D230019N24 gene	0.8297	0.1287	0.5733
230379	Acer2	alkaline ceramidase 2	0.8297	0.08643	0.5176
58203	Zbp1	Z-DNA binding protein 1	0.8297	0.06857	0.4867
225888	Suv420h1	suppressor of variegation 4-20 homolog 1 (Drosophila)	0.8296	0.03298	0.4038
381760	Ssbp1	single-stranded DNA binding protein 1	0.8295	0.1464	0.591
50887	Hmgn5	high-mobility group nucleosome binding domain 5	0.8295	0.2486	0.6763
269593	Luzp1	leucine zipper protein 1	0.8294	0.1493	0.5938
12747	Clk1	CDC-like kinase 1	0.8294	0.08952	0.5223
11694	Alx3	aristaless-like homeobox 3	0.8294	0.39	0.7629
545156	Kalrn	kalirin, RhoGEF kinase	0.8293	0.2496	0.6767
319636	Fsd1l	fibronectin type III and SPRY domain containing 1-like	0.8293	0.1445	0.5892
78655	Eif3j	eukaryotic translation initiation factor 3, subunit J	0.8293	0.4017	0.7703
258484	Olfr1410	olfactory receptor 1410	0.8292	0.3477	0.7383
76279	Cyp2d26	cytochrome P450, family 2, subfamily d, polypeptide 26	0.8292	0.01209	0.3236
101401	Adamts9	a disintegrin-like and metallopeptidase (reprolysin type) with thrombospondin type 1 motif, 9	0.8291	0.4517	0.7951
17263	Meg3	maternally expressed 3	0.8291	0.02573	0.3786
21844	Tiam1	T-cell lymphoma invasion and metastasis 1	0.829	0.03154	0.3977
71368	5430431A17Rik	RIKEN cDNA 5430431A17 gene	0.8289	0.3438	0.7356
111173	Erc1	ELKS/RAB6-interacting/CAST family member 1	0.8288	0.0648	0.4816
665596	Hist1h2bq	histone cluster 1, H2bq	0.8287	0.2457	0.6733
114661	Prss28	protease, serine, 28	0.8286	0.2771	0.6925
22589	Atrx	alpha thalassemia/mental retardation syndrome X-linked homolog (human)	0.8286	0.1974	0.6325
664949	Gm7420	predicted gene 7420	0.8285	0.03345	0.4066
552912	LOC552912	hypothetical LOC552912	0.8284	0.07067	0.4921
328329	Mast4	microtubule associated serine/threonine kinase family member 4	0.8283	0.1402	0.5834
66932	Rexo1	REX1, RNA exonuclease 1 homolog (S. cerevisiae)	0.8283	0.03382	0.4086
14804	Grid2	glutamate receptor, ionotropic, delta 2	0.8283	0.3142	0.7155
14182	Fgfr1	fibroblast growth factor receptor 1	0.8283	0.3631	0.7483
102423	Hinfp	histone H4 transcription factor	0.8282	0.1561	0.5991
11605	Gla	galactosidase, alpha	0.8282	0.2125	0.6451
234413	Zfp961	zinc finger protein 961	0.828	0.1328	0.5771
30939	Pttg1	pituitary tumor-transforming gene 1	0.828	0.07462	0.4974
442834	D830031N03Rik	RIKEN cDNA D830031N03 gene	0.8279	0.2921	0.7023
431706	Zfp457	zinc finger protein 457	0.8279	0.3054	0.7101
217935	Wdr60	WD repeat domain 60	0.8279	0.004743	0.2643
19344	Rab5b	RAB5B, member RAS oncogene family	0.8279	0.1608	0.6063
227867	Epc2	enhancer of polycomb homolog 2 (Drosophila)	0.8276	0.03456	0.4091
83492	Gsdmc	gasdermin C	0.8276	0.2631	0.6838
56526	Sep-06	septin 6	0.8276	0.1086	0.5521
402769	LOC402769	hypothetical LOC402769	0.8275	0.2014	0.635
80744	Cwc22	CWC22 spliceosome-associated protein homolog (S. cerevisiae)	0.8275	0.228	0.6569
110616	Atxn3	ataxin 3	0.8273	0.1123	0.5549

320086	E430022K19Rik	RIKEN cDNA E430022K19 gene	0.8272	0.06064	0.4741
17260	Mef2c	myocyte enhancer factor 2C	0.8272	0.08715	0.5184
100038882	Isg15	ISG15 ubiquitin-like modifier	0.8271	0.2776	0.6925
664608	Rhox4g	reproductive homeobox 4G	0.8271	0.2818	0.6967
246256	Fcgr4	Fc receptor, IgG, low affinity IV	0.8271	0.519	0.8282
76499	Clasp2	CLIP associating protein 2	0.8271	0.07683	0.5014
72567	Bclaf1	BCL2-associated transcription factor 1	0.8271	0.05863	0.4711
320333	D830030K20Rik	RIKEN cDNA D830030K20 gene	0.827	0.01685	0.3424
232314	Ppp4r2	protein phosphatase 4, regulatory subunit 2	0.827	0.1222	0.5656
100316870	LOC100316870	DDB1 and CUL4 associated factor 5 pseudogene	0.8269	0.1557	0.599
319592	D030020J04Rik	RIKEN cDNA D030020J04 gene	0.8269	0.2743	0.692
12390	Cav2	caveolin 2	0.8269	0.4438	0.7931
209012	Ulk4	unc-51-like kinase 4 (C. elegans)	0.8268	0.1278	0.5716
52184	Odf2l	outer dense fiber of sperm tails 2-like	0.8268	0.2295	0.6594
619937	Gm6115	predicted gene 6115	0.8267	0.2966	0.7051
77604	C430048L16Rik	RIKEN cDNA C430048L16 gene	0.8267	0.09913	0.5367
212632	Iffo2	intermediate filament family orphan 2	0.8264	0.07868	0.5052
70320	2610008G14Rik	RIKEN cDNA 2610008G14 gene	0.8263	0.00323	0.227
54711	Plagl2	pleiomorphic adenoma gene-like 2	0.8263	0.1121	0.5549
252876	Gin1	gypsy retrotransposon integrase 1	0.8262	0.1143	0.5569
432964	K230010J24Rik	RIKEN cDNA K230010J24 gene	0.8261	0.2257	0.654
442802	C330011M18Rik	RIKEN cDNA C330011M18 gene	0.826	0.162	0.607
210126	Lpp	LIM domain containing preferred translocation partner in lipoma	0.826	0.0637	0.4794
338351	Akap17b	A kinase (PRKA) anchor protein 17B	0.8259	0.3087	0.712
229504	Isg20l2	interferon stimulated exonuclease gene 20-like 2	0.8259	0.2082	0.6401
16526	Kcnk2	potassium channel, subfamily K, member 2	0.8258	0.26	0.6815
14563	Gdf5	growth differentiation factor 5	0.8258	0.3723	0.7536
68484	Krtap16-8	keratin associated protein 16-8	0.8257	0.2178	0.6499
245867	Pcmtd2	protein-L-isoaspartate (D-aspartate) O-methyltransferase domain containing 2	0.8256	0.02841	0.3894
381314	Iars2	isoleucine-tRNA synthetase 2, mitochondrial	0.8255	0.2226	0.654
81840	Sorcs2	sortilin-related VPS10 domain containing receptor 2	0.8255	0.2363	0.6657
100045878	LOC100045878	t-cell receptor alpha chain V region RL-5-like	0.8254	0.4949	0.8175
100041487	Gm3366	predicted gene 3366	0.8253	0.1434	0.5879
100039796	Tgtp2	T-cell specific GTPase 2	0.8253	0.1623	0.607
71177	4933424B01Rik	RIKEN cDNA 4933424B01 gene	0.8252	0.009177	0.3092
67684	Luc7l3	LUC7-like 3 (S. cerevisiae)	0.8252	0.1706	0.6115
59050	Nsa2	NSA2 ribosome biogenesis homolog (S. cerevisiae)	0.8252	0.02508	0.3786
16517	Kcnj16	potassium inwardly-rectifying channel, subfamily J, member 16	0.8252	0.3183	0.7182
15368	Hmox1	heme oxygenase (decycling) 1	0.8252	0.06344	0.478
100019	Mdn1	midasin homolog (yeast)	0.825	0.07138	0.4933
75801	4930447C04Rik	RIKEN cDNA 4930447C04 gene	0.825	0.03536	0.4113
54711	Plagl2	pleiomorphic adenoma gene-like 2	0.825	0.06644	0.4842
404330	Olfr1198	olfactory receptor 1198	0.8248	0.5595	0.8459
208898	Unc13c	unc-13 homolog C (C. elegans)	0.8248	0.1773	0.6167
77744	6720463M24Rik	RIKEN cDNA 6720463M24 gene	0.8248	0.06003	0.4728
234358	Zfp930	zinc finger protein 930	0.8245	0.4162	0.7769
14396	Gabra3	gamma-aminobutyric acid (GABA) A receptor, subunit alpha 3	0.8245	0.2925	0.7024
319934	Sbf2	SET binding factor 2	0.8244	0.1891	0.6266
76214	6430710M23Rik	RIKEN cDNA 6430710M23 gene	0.8244	0.5081	0.8241

50793	Orc3	origin recognition complex, subunit 3	0.8244	0.09729	0.5352
234023	Arglu1	arginine and glutamate rich 1	0.8243	0.07998	0.5096
75560	Ep400	E1A binding protein p400	0.8243	0.02268	0.3702
17684	Cited2	Cbp/p300-interacting transactivator, with Glu/Asp-rich carboxy-terminal domain, 2	0.8243	0.02639	0.38
320015	A130082J08Rik	RIKEN cDNA A130082J08 gene	0.8242	0.2013	0.635
320110	B230369F24Rik	RIKEN cDNA B230369F24 gene	0.824	0.04423	0.4339
75818	4930478L05Rik	RIKEN cDNA 4930478L05 gene	0.824	0.08034	0.5098
622446	Gm6323	predicted gene 6323	0.8239	0.5489	0.841
15951	Ifi204	interferon activated gene 204	0.8237	0.2444	0.6724
380711	Rap1gap2	RAP1 GTPase activating protein 2	0.8236	0.2771	0.6925
319885	Zcchc7	zinc finger, CCHC domain containing 7	0.8235	0.03953	0.4222
238988	Erc2	ELKS/RAB6-interacting/CAST family member 2	0.8235	0.01428	0.3347
67302	Zc3h13	zinc finger CCCH type containing 13	0.8235	0.03713	0.4163
71434	5530400B01Rik	RIKEN cDNA 5530400B01 gene	0.8234	0.5205	0.8286
50770	Atp11a	ATPase, class VI, type 11A	0.8232	0.3996	0.7699
399579	6720462K09Rik	RIKEN cDNA 6720462K09 gene	0.823	0.01722	0.3452
378435	Mafa	v-maf musculoaponeurotic fibrosarcoma oncogene family, protein A (avian)	0.823	0.1011	0.5395
225872	Npas4	neuronal PAS domain protein 4	0.823	0.1131	0.5556
57432	Zc3h8	zinc finger CCCH type containing 8	0.823	0.1008	0.5394
13599	Ecel1	endothelin converting enzyme-like 1	0.823	0.2726	0.6897
54446	Nfat5	nuclear factor of activated T-cells 5	0.8228	0.3588	0.7457
433520	Gm14403	predicted gene 14403	0.8227	0.07905	0.5062
22226	Ucn	urocortin	0.8227	0.4631	0.8031
668620	Zfp936	zinc finger protein 936	0.8226	0.4569	0.7987
74004	Jakmip3	janus kinase and microtubule interacting protein 3	0.8226	0.2185	0.6506
627648	Klhl3	kelch-like 3 (Drosophila)	0.8223	0.09113	0.5232
66816	Thap2	THAP domain containing, apoptosis associated protein 2	0.8223	0.08764	0.5191
230257	Rod1	ROD1 regulator of differentiation 1 (S. pombe)	0.8222	0.2022	0.6356
21951	Tnks	tankyrase, TRF1-interacting ankyrin-related ADP-ribose polymerase	0.8222	0.07049	0.4917
227525	Dclre1c	DNA cross-link repair 1C, PSO2 homolog (S. cerevisiae)	0.8221	0.08982	0.5229
67781	Ilf2	interleukin enhancer binding factor 2	0.822	0.06218	0.4771
12499	Entpd5	ectonucleoside triphosphate diphosphohydrolase 5	0.822	0.007366	0.2933
241589	D430041D05Rik	RIKEN cDNA D430041D05 gene	0.8219	0.1953	0.6304
436230	BC065397	cDNA sequence BC065397	0.8216	0.04826	0.4448
319269	A130040M12Rik	RIKEN cDNA A130040M12 gene	0.8216	0.02881	0.3903
386400	LOC386400	carbonyl reductase [NADPH] 1-like	0.8215	0.5896	0.8575
56347	Eif3c	eukaryotic translation initiation factor 3, subunit C	0.8214	0.05067	0.4519
67289	3110021A11Rik	RIKEN cDNA 3110021A11 gene	0.8213	0.1161	0.558
11480	Acvr2a	activin receptor IIA	0.8211	0.118	0.5609
14265	Fmr1	fragile X mental retardation syndrome 1 homolog	0.821	0.2652	0.6849
75795	4930455D15Rik	RIKEN cDNA 4930455D15 gene	0.8209	0.644	0.8802
67263	Zswim6	zinc finger, SWIM domain containing 6	0.8209	0.05572	0.4631
23965	Odz3	odd Oz/ten-m homolog 3 (Drosophila)	0.8209	0.0154	0.3403
227058	Dnahc7b	dynein, axonemal, heavy chain 7B	0.8208	0.1983	0.6331
108797	Mex3b	mex3 homolog B (C. elegans)	0.8208	0.1506	0.5938
76964	2610028H24Rik	RIKEN cDNA 2610028H24 gene	0.8208	0.04074	0.4259
22064	Trpc2	transient receptor potential cation channel, subfamily C, member 2	0.8208	0.48	0.8101
83679	Pde4dip	phosphodiesterase 4D interacting protein (myomegalin)	0.8206	0.4448	0.7938
100101046	D230018H15Rik	RIKEN cDNA D230018H15 gene	0.8205	0.102	0.5406

381668	Fbrsl1	fibrosin-like 1	0.8205	0.02159	0.367
270627	Taf1	TAF1 RNA polymerase II, TATA box binding protein (TBP)-associated factor	0.8205	0.4486	0.7942
75840	4930558N01Rik	RIKEN cDNA 4930558N01 gene	0.8205	0.01267	0.3287
260315	Nav3	neuron navigator 3	0.8204	0.2532	0.6778
257971	Olfr1382	olfactory receptor 1382	0.8204	0.05305	0.4565
70354	Secisbp2l	SECIS binding protein 2-like	0.8204	0.345	0.736
26556	Homer1	homer homolog 1 (Drosophila)	0.8203	0.1088	0.5524
665005	Gm7444	ring finger protein 19A pseudogene	0.8202	0.04748	0.4442
74465	4933421H12Rik	RIKEN cDNA 4933421H12 gene	0.8202	0.3234	0.7206
67471	Gpatch1	G patch domain containing 1	0.8202	0.04502	0.4353
72611	Zfp655	zinc finger protein 655	0.8201	0.005153	0.2707
241794	Kcng1	potassium voltage-gated channel, subfamily G, member 1	0.82	0.332	0.7269
230757	5730409E04Rik	RIKEN cDNA 5730409E04Rik gene	0.8199	0.07229	0.4941
624918	Gm6537	predicted gene 6537	0.8198	0.1006	0.5394
210544	Wdr67	WD repeat domain 67	0.8198	0.382	0.7596
67946	Spata6	spermatogenesis associated 6	0.8198	0.002859	0.2228
20823	Ssb	Sjogren syndrome antigen B	0.8198	0.1597	0.6042
100046290	LOC100046290	60S ribosomal protein L21-like	0.8197	0.1101	0.5535
320362	9530085L11Rik	RIKEN cDNA 9530085L11 gene	0.8197	0.1885	0.6263
320022	Ccdc79	coiled-coil domain containing 79	0.8196	0.05219	0.4535
224763	Gm4831	predicted gene 4831	0.8196	0.3197	0.7189
73873	Fam161a	family with sequence similarity 161, member A	0.8195	0.1699	0.6112
100042149	Gm3696	predicted gene 3696	0.8194	0.2824	0.6969
667284	Gm8556	predicted gene 8556	0.8194	0.1601	0.605
97547	C81489	expressed sequence C81489	0.8194	0.1252	0.5703
20971	Sdc4	syndecan 4	0.8194	0.1938	0.6299
320840	Negr1	neuronal growth regulator 1	0.8193	0.04344	0.4329
234413	Zfp961	zinc finger protein 961	0.8193	0.005705	0.2775
208967	Thnsl1	threonine synthase-like 1 (bacterial)	0.8193	0.001009	0.1535
69743	Casz1	castor homolog 1, zinc finger (Drosophila)	0.8192	0.1169	0.5592
27223	Trp53bp1	transformation related protein 53 binding protein 1	0.8191	0.01809	0.3484
19244	Ptp4a2	protein tyrosine phosphatase 4a2	0.8191	0.04333	0.4324
381356	5930434B04Rik	RIKEN cDNA 5930434B04 gene	0.8189	0.008959	0.3086
227058	Dnahc7b	dynein, axonemal, heavy chain 7B	0.8189	0.03032	0.3954
117198	Ivns1abp	influenza virus NS1A binding protein	0.8189	0.2167	0.6482
18191	Nrxn3	neurexin III	0.8189	0.0738	0.4961
12212	Chic1	cysteine-rich hydrophobic domain 1	0.8188	0.2018	0.6356
15109	Hal	histidine ammonia lyase	0.8187	0.1473	0.591
212516	BC060267	cDNA sequence BC060267	0.8185	0.01204	0.3236
232941	Ppm1n	protein phosphatase, Mg2+/Mn2+ dependent, 1N (putative)	0.8184	0.004799	0.2647
67246	2810474O19Rik	RIKEN cDNA 2810474O19 gene	0.8184	0.1146	0.5569
53379	Hnrnpa2b1	heterogeneous nuclear ribonucleoprotein A2/B1	0.8184	0.3836	0.7599
384763	Zfp667	zinc finger protein 667	0.8183	0.2502	0.6767
75479	1700012D14Rik	RIKEN cDNA 1700012D14 gene	0.8183	0.01729	0.3452
56745	C1qtnf1	C1q and tumor necrosis factor related protein 1	0.8183	0.1726	0.6136
50771	Atp9b	ATPase, class II, type 9B	0.8182	0.1087	0.5521
16150	Ikbkb	inhibitor of kappaB kinase beta	0.8182	0.03602	0.4134
233529	Kctd14	potassium channel tetramerisation domain containing 14	0.8181	0.2523	0.6775
74471	4933440N22Rik	RIKEN cDNA 4933440N22 gene	0.8181	0.1604	0.6055

26875	Pclo	piccolo (presynaptic cytomatrix protein)	0.8181	0.03699	0.4161
16500	Kcnb1	potassium voltage gated channel, Shab-related subfamily, member 1	0.8181	0.05095	0.4519
665891	Krtap4-1	keratin associated protein 4-1	0.818	0.6033	0.8643
271457	Rab5a	RAB5A, member RAS oncogene family	0.818	0.1309	0.5751
100040320	Gm2710	predicted gene 2710	0.8179	0.1347	0.5779
234395	Ushbp1	Usher syndrome 1C binding protein 1	0.8179	0.36	0.7465
77246	9330177L23Rik	RIKEN cDNA 9330177L23 gene	0.8178	0.06307	0.478
347740	2900097C17Rik	RIKEN cDNA 2900097C17 gene	0.8177	0.02221	0.3702
72949	Ccnt2	cyclin T2	0.8177	0.1279	0.5716
20493	Slc10a1	solute carrier family 10 (sodium/bile acid cotransporter family), member 1	0.8176	0.116	0.558
15258	Hipk2	homeodomain interacting protein kinase 2	0.8174	0.03462	0.4091
14248	Flii	flightless I homolog (Drosophila)	0.8174	0.2554	0.6798
77938	Fam53b	family with sequence similarity 53, member B	0.8173	0.02968	0.3932
545156	Kalrn	kalirin, RhoGEF kinase	0.8171	0.1901	0.6274
107589	Mylk	myosin, light polypeptide kinase	0.8171	0.02751	0.3864
106585	Ankrd12	ankyrin repeat domain 12	0.8171	0.25	0.6767
435366	Gm5665	predicted gene 5665	0.817	0.3494	0.7388
56808	Cacna2d2	calcium channel, voltage-dependent, alpha 2/delta subunit 2	0.817	0.004901	0.2652
50754	Fbxw7	F-box and WD-40 domain protein 7	0.817	0.06178	0.4771
71330	Rcbtb1	regulator of chromosome condensation (RCC1) and BTB (POZ) domain containing protein 1	0.8169	0.1319	0.576
71063	Zfp597	zinc finger protein 597	0.8168	0.06767	0.4855
21960	Tnr	tenascin R	0.8168	0.03142	0.3966
18789	Papola	poly (A) polymerase alpha	0.8167	0.04814	0.4446
11647	Alpl	alkaline phosphatase, liver/bone/kidney	0.8166	0.03464	0.4091
328572	Ep300	E1A binding protein p300	0.8164	0.03572	0.4123
108686	Ccdc88a	coiled coil domain containing 88A	0.8164	0.1191	0.5626
70985	4931406E20Rik	RIKEN cDNA 4931406E20 gene	0.8164	0.04537	0.436
100041351	Gm3285	predicted gene 3285	0.8163	0.2027	0.6356
75114	4930516B21Rik	RIKEN cDNA 4930516B21 gene	0.8162	0.07707	0.5018
30878	Apln	apelin	0.816	0.131	0.5751
20333	Sec22b	SEC22 vesicle trafficking protein homolog B (S. cerevisiae)	0.816	0.01305	0.3305
64009	Syne1	synaptic nuclear envelope 1	0.8159	0.07772	0.5026
245860	Atg9a	autophagy-related 9A (yeast)	0.8158	0.004657	0.2642
100505360	LOC100505360	putative transposase element L1Md-A101/L1Md-A102/L1Md-A2-like	0.8157	0.3894	0.7624
85030	Tnfrsf25	tumor necrosis factor receptor superfamily, member 25	0.8157	0.2653	0.685
50759	Fbxo16	F-box protein 16	0.8157	0.08565	0.517
21676	Tead1	TEA domain family member 1	0.8157	0.2846	0.6977
328191	B230303A05Rik	RIKEN cDNA B230303A05 gene	0.8156	0.214	0.6467
320496	C230076A16Rik	RIKEN cDNA C230076A16 gene	0.8155	0.04548	0.4363
19699	Reln	reelin	0.8155	0.02802	0.3875
100039042	Gm2016	predicted gene 2016	0.8154	0.1979	0.6329
26556	Homer1	homer homolog 1 (Drosophila)	0.8154	0.163	0.6074
18027	Nfia	nuclear factor I/A	0.8154	0.1146	0.5569
74855	4930417G10Rik	RIKEN cDNA 4930417G10 gene	0.8153	0.01408	0.3347
19270	Ptprg	protein tyrosine phosphatase, receptor type, G	0.8152	0.09486	0.5323
18583	Pde7a	phosphodiesterase 7A	0.815	0.1633	0.6074
236511	Eif2c1	eukaryotic translation initiation factor 2C, 1	0.8149	0.1959	0.6307
19242	Ptn	pleiotrophin	0.8149	0.1592	0.604
258493	Olfr319	olfactory receptor 319	0.8148	0.6447	0.8803

93709	Pcdhga1	protocadherin gamma subfamily A, 1	0.8148	0.04116	0.4275
241915	Phc3	polyhomeotic-like 3 (Drosophila)	0.8147	0.1787	0.6181
216987	Utp6	UTP6, small subunit (SSU) processome component, homolog (yeast)	0.8147	0.1644	0.6076
73862	4930415F15Rik	RIKEN cDNA 4930415F15 gene	0.8147	0.0582	0.4703
20650	Sntb2	syntrophin, basic 2	0.8146	0.05627	0.4631
382421	Gm5176	predicted gene 5176	0.8145	0.2007	0.6345
194227	Gm13023	predicted gene 13023	0.8144	0.3935	0.7656
110829	Lims1	LIM and senescent cell antigen-like domains 1	0.8143	0.07188	0.494
83922	Tsga14	testis specific gene A14	0.8141	0.09471	0.5319
22682	Zfand5	zinc finger, AN1-type domain 5	0.8141	0.03647	0.4156
19883	Rora	RAR-related orphan receptor alpha	0.8141	0.05978	0.4728
225467	Pggt1b	protein geranylgeranyltransferase type I, beta subunit	0.814	0.08375	0.5144
22717	Zfp59	zinc finger protein 59	0.814	0.1422	0.5871
100504289	LOC100504289	hypothetical LOC100504289	0.8139	0.3039	0.709
20191	Ryr2	ryanodine receptor 2, cardiac	0.8137	0.005581	0.2775
78323	2310046O06Rik	RIKEN cDNA 2310046O06 gene	0.8136	0.04694	0.4433
75373	4930597O21Rik	RIKEN cDNA 4930597O21 gene	0.8134	0.5347	0.8349
66521	Rwdd1	RWD domain containing 1	0.8134	0.09418	0.5311
382118	Zfp167	zinc finger protein 167	0.8133	0.1219	0.5652
27392	Pign	phosphatidylinositol glycan anchor biosynthesis, class N	0.8133	0.2955	0.7046
73473	Iws1	IWS1 homolog (S. cerevisiae)	0.8132	0.2473	0.6744
319391	D230014I24Rik	RIKEN cDNA D230014I24 gene	0.8131	0.03647	0.4156
26382	Fgd2	FYVE, RhoGEF and PH domain containing 2	0.8131	0.463	0.8031
11622	Ahr	aryl-hydrocarbon receptor	0.8131	0.1521	0.5954
228880	Zmynd8	zinc finger, MYND-type containing 8	0.813	0.01728	0.3452
70745	6330418B08Rik	RIKEN cDNA 6330418B08 gene	0.8129	0.07277	0.4952
53859	Map3k14	mitogen-activated protein kinase kinase kinase 14	0.8129	0.1875	0.6259
26927	Foxl2	forkhead box L2	0.8129	0.128	0.5716
238247	Arid4a	AT rich interactive domain 4A (RBP1-like)	0.8127	0.2784	0.6934
71752	Gtf3c2	general transcription factor IIIC, polypeptide 2, beta	0.8127	0.167	0.6083
56531	Ylpm1	YLP motif containing 1	0.8127	0.2773	0.6925
26905	Eif2s3x	eukaryotic translation initiation factor 2, subunit 3, structural gene X-linked	0.8127	0.2076	0.6398
52478	D6Ertd131e	DNA segment, Chr 6, ERATO Doi 131, expressed	0.8126	0.02082	0.3609
76210	6430597G12Rik	RIKEN cDNA 6430597G12 gene	0.8125	0.003702	0.2432
13207	Ddx5	DEAD (Asp-Glu-Ala-Asp) box polypeptide 5	0.8124	0.2836	0.6974
75763	Dcaf17	DDB1 and CUL4 associated factor 17	0.8123	0.06411	0.4809
14783	Grb10	growth factor receptor bound protein 10	0.812	0.3327	0.7278
12648	Chd1	chromodomain helicase DNA binding protein 1	0.812	0.2255	0.654
622446	Gm6323	predicted gene 6323	0.8119	0.2059	0.6386
71148	Mier1	mesoderm induction early response 1 homolog (Xenopus laevis	0.8119	0.1031	0.5431
20400	Sh2d1a	SH2 domain protein 1A	0.8119	0.04848	0.445
226562	Prrc2c	proline-rich coiled-coil 2C	0.8118	0.05245	0.4547
94222	Olig3	oligodendrocyte transcription factor 3	0.8118	0.1574	0.6016
56223	Fscn3	fascin homolog 3, actin-bundling protein, testicular (Strongylocentrotus purpuratus)	0.8117	0.1459	0.591
52186	D8Ertd158e	DNA segment, Chr 8, ERATO Doi 158, expressed	0.8117	0.01743	0.3469
14265	Fmr1	fragile X mental retardation syndrome 1 homolog	0.8114	0.2363	0.6657
93890	Pcdhb19	protocadherin beta 19	0.8113	0.2597	0.6815
72154	Zfp157	zinc finger protein 157	0.8113	0.1331	0.5772
50907	Preb	prolactin regulatory element binding	0.8113	0.1099	0.5535

14681	Gnao1	guanine nucleotide binding protein, alpha O	0.8113	0.1299	0.5747
229694	AI504432	expressed sequence AI504432	0.8111	0.1435	0.5879
77018	Col25a1	collagen, type XXV, alpha 1	0.8111	0.07089	0.4923
237073	Rbm41	RNA binding motif protein 41	0.811	0.1981	0.6329
231214	Cc2d2a	coiled-coil and C2 domain containing 2A	0.811	0.01014	0.3115
100042679	Gm16386	predicted gene 16386	0.8109	0.3393	0.7321
71766	Raver1	ribonucleoprotein, PTB-binding 1	0.8109	0.2664	0.6862
54710	Hs3st3b1	heparan sulfate (glucosamine) 3-O-sulfotransferase 3B1	0.8109	0.1656	0.6077
14184	Fgfr3	fibroblast growth factor receptor 3	0.8109	0.3254	0.7222
399612	9630010G10Rik	RIKEN cDNA 9630010G10 gene	0.8107	0.03512	0.4105
227618	Lrrc26	leucine rich repeat containing 26	0.8107	0.584	0.8544
320237	Ncrna00086	non-protein coding RNA 86	0.8106	0.0127	0.3291
108737	Oxsr1	oxidative-stress responsive 1	0.8106	0.01225	0.3236
232790	Oscar	osteoclast associated receptor	0.8105	0.1193	0.5628
68744	Zfp740	zinc finger protein 740	0.8105	0.1368	0.5807
17708	COX1	cytochrome c oxidase subunit I	0.8105	0.03881	0.4213
667635	Trav7n-5	T cell receptor alpha variable 7N-5	0.8104	0.1764	0.6156
93873	Pcdhb2	protocadherin beta 2	0.8104	0.02616	0.3791
78781	Zc3hav1	zinc finger CCCH type, antiviral 1	0.8104	0.1471	0.591
100038882	Isg15	ISG15 ubiquitin-like modifier	0.8103	0.1179	0.5609
15572	Elavl4	ELAV (embryonic lethal, abnormal vision, Drosophila)-like 4 (Hu antigen D)	0.8103	0.04711	0.4435
20742	Spnb2	spectrin beta 2	0.8102	0.06194	0.4771
78774	4930529M08Rik	RIKEN cDNA 4930529M08 gene	0.8101	0.2325	0.6622
67199	Pfdn1	prefoldin 1	0.8101	0.03911	0.4217
619329	F420015M19Rik	RIKEN cDNA F420015M19 gene	0.8099	0.06507	0.4816
26367	Ceacam2	carcinoembryonic antigen-related cell adhesion molecule 2	0.8098	0.1085	0.5521
67200	Ccdc77	coiled-coil domain containing 77	0.8097	0.07149	0.4934
100042951	Gm4123	predicted gene 4123	0.8094	0.0452	0.4353
329478	C130078N14	hypothetical protein C130078N14	0.8094	0.05976	0.4728
17691	Sik1	salt inducible kinase 1	0.8094	0.2923	0.7024
233987	Zfp958	zinc finger protein 958	0.8093	0.09259	0.5271
106557	Ldhal6b	lactate dehydrogenase A-like 6B	0.8093	0.2909	0.7013
76220	6530402F18Rik	RIKEN cDNA 6530402F18 gene	0.8093	0.04903	0.4469
56711	Plag1	pleiomorphic adenoma gene 1	0.8092	0.1817	0.6206
231125	Zfyve28	zinc finger, FYVE domain containing 28	0.8091	0.01042	0.3117
26422	Nbea	neurobeachin	0.8091	0.07143	0.4934
235048	Zfp599	zinc finger protein 599	0.8089	0.1657	0.6077
19893	Rpgr	retinitis pigmentosa GTPase regulator	0.8089	0.04518	0.4353
217600	Rpl26-ps2	ribosomal protein L26, pseudogene 2	0.8086	0.579	0.8519
327959	Xaf1	XIAP associated factor 1	0.8085	0.07207	0.4941
73608	Marveld3	MARVEL (membrane-associating) domain containing 3	0.8085	0.02002	0.3604
68263	Pdhb	pyruvate dehydrogenase (lipoamide) beta	0.8085	0.04786	0.4443
12874	Cpd	carboxypeptidase D	0.8085	0.006679	0.288
12054	Bcl7b	B-cell CLL/lymphoma 7B	0.8085	0.1126	0.5549
270035	Letm2	leucine zipper-EF-hand containing transmembrane protein 2	0.8084	0.1558	0.599
243905	Zfp568	zinc finger protein 568	0.8083	0.06236	0.4771
213988	Tnrc6b	trinucleotide repeat containing 6b	0.8083	0.02938	0.3911
70012	Ccdc21	coiled-coil domain containing 21	0.8083	0.0777	0.5026
552902	LOC552902	hypothetical LOC552902	0.8082	0.01792	0.3475

64658	Mrps25	mitochondrial ribosomal protein S25	0.8081	0.01948	0.3565
381546	Ccdc24	coiled-coil domain containing 24	0.808	0.0372	0.4163
18784	Pla2g5	phospholipase A2, group V	0.808	0.03236	0.4017
100038492	F630048H11Rik	RIKEN cDNA F630048H11 gene	0.8077	0.2635	0.6842
432769	Zfp708	zinc finger protein 708	0.8077	0.2897	0.7004
269623	C030048B08Rik	RIKEN cDNA C030048B08 gene	0.8077	0.1302	0.5747
217558	G2e3	G2/M-phase specific E3 ubiquitin ligase	0.8077	0.2196	0.6513
53975	Ddx20	DEAD (Asp-Glu-Ala-Asp) box polypeptide 20	0.8077	0.228	0.6569
320497	A630065K11Rik	RIKEN cDNA A630065K11 gene	0.8076	0.05142	0.4519
16542	Kdr	kinase insert domain protein receptor	0.8076	0.003009	0.2228
654494	Gm7337	DAZ interacting protein 1 pseudogene	0.8075	0.1633	0.6074
16693	Krtap11-1	keratin associated protein 11-1	0.8075	0.3503	0.739
72767	2810427A07Rik	RIKEN cDNA 2810427A07 gene	0.8074	0.3725	0.7537
14356	Fxc1	fractured callus expressed transcript 1	0.8074	0.4488	0.7943
22196	Ube2i	ubiquitin-conjugating enzyme E2I	0.8073	0.04045	0.4257
26556	Homer1	homer homolog 1 (Drosophila)	0.8072	0.2275	0.6566
664783	Dux	double homeobox	0.8071	0.1329	0.5772
381760	Ssbp1	single-stranded DNA binding protein 1	0.807	0.06886	0.487
666464	Gm8120	predicted gene 8120	0.8069	0.101	0.5394
269704	Zfp664	zinc finger protein 664	0.8069	0.03948	0.4222
13609	S1pr1	sphingosine-1-phosphate receptor 1	0.8069	0.09553	0.5334
73242	Atat1	alpha tubulin acetyltransferase 1	0.8068	0.00999	0.3112
56706	Ccnl1	cyclin L1	0.8068	0.01352	0.3343
71536	8430437O03Rik	RIKEN cDNA 8430437O03 gene	0.8066	0.03247	0.4017
100047183	LOC100047183	protein AHNAK2-like	0.8065	0.04403	0.4336
72137	Wdsub1	WD repeat, SAM and U-box domain containing 1	0.8065	0.1345	0.5779
100038486	Gm10742	predicted gene 10742	0.8064	0.0485	0.445
271377	Zbtb11	zinc finger and BTB domain containing 11	0.8064	0.09533	0.5333
102219	AU018794	expressed sequence AU018794	0.8064	0.01255	0.327
16536	Kcnq2	potassium voltage-gated channel, subfamily Q, member 2	0.8064	0.04881	0.4469
13209	Ddx6	DEAD (Asp-Glu-Ala-Asp) box polypeptide 6	0.8064	0.05849	0.4706
100502641	LOC100502641	60S acidic ribosomal protein P1-like	0.8062	0.08046	0.5098
80292	Zxdc	ZXD family zinc finger C	0.8061	0.008349	0.3026
77789	A930007D18Rik	RIKEN cDNA A930007D18 gene	0.8059	0.05057	0.4517
20823	Ssb	Sjogren syndrome antigen B	0.8059	0.07293	0.4952
29807	Tpk1	thiamine pyrophosphokinase	0.8058	0.2761	0.6925
78283	Mtap7d2	MAP7 domain containing 2	0.8056	0.06506	0.4816
402771	C130090I23Rik	RIKEN cDNA C130090I23 gene	0.8055	0.01752	0.3469
192195	Ash1l	ash1 (absent, small, or homeotic)-like (Drosophila)	0.8055	0.01549	0.3403
107815	Scml2	sex comb on midleg-like 2 (Drosophila)	0.8055	0.0772	0.5024
18640	Pfkfb2	6-phosphofructo-2-kinase/fructose-2,6-biphosphatase 2	0.8055	0.006661	0.288
408067	Zfp874b	zinc finger protein 874b	0.8054	0.4014	0.7703
74407	Ttc25	tetratricopeptide repeat domain 25	0.8054	0.08116	0.5107
71713	Cdc40	cell division cycle 40 homolog (yeast)	0.8054	0.2192	0.6512
237940	Aoc2	amine oxidase, copper containing 2 (retina-specific)	0.8053	0.16	0.6045
227210	Ccnyl1	cyclin Y-like 1	0.8052	0.05781	0.469
66779	4933432I09Rik	RIKEN cDNA 4933432I09 gene	0.8052	0.3698	0.7521
13860	Eps8	epidermal growth factor receptor pathway substrate 8	0.8052	0.1668	0.6083
69445	1700023D09Rik	RIKEN cDNA 1700023D09 gene	0.8051	0.01192	0.3235

20744	Strbp	spermatid perinuclear RNA binding protein	0.8051	0.03116	0.3964
18213	Ntrk3	neurotrophic tyrosine kinase, receptor, type 3	0.8051	0.01195	0.3235
17931	Ppp1r12a	protein phosphatase 1, regulatory (inhibitor) subunit 12A	0.8051	0.01125	0.3181
242377	Pm20d2	peptidase M20 domain containing 2	0.805	0.05503	0.4614
619719	6430590A07Rik	RIKEN cDNA 6430590A07 gene	0.8049	0.04015	0.4248
69700	Col22a1	collagen, type XXII, alpha 1	0.8049	0.1189	0.5622
19242	Ptn	pleiotrophin	0.8049	0.09646	0.5345
381650	Thap6	THAP domain containing 6	0.8048	0.0129	0.3304
399623	5930433N17Rik	RIKEN cDNA 5930433N17 gene	0.8047	0.02945	0.3917
386463	Cdsn	corneodesmosin	0.8047	0.1632	0.6074
330788	Zfp866	zinc finger protein 866	0.8047	0.04086	0.4263
70981	4931423N10Rik	RIKEN cDNA 4931423N10 gene	0.8046	0.2236	0.654
16886	Limk2	LIM motif-containing protein kinase 2	0.8044	0.1967	0.6316
74852	4930402D18Rik	RIKEN cDNA 4930402D18 gene	0.8043	0.1291	0.5741
66344	Lce3b	late cornified envelope 3B	0.8043	0.4207	0.7798
338370	Nalcn	sodium leak channel, non-selective	0.8042	0.0241	0.3771
73192	Xpot	exportin, tRNA (nuclear export receptor for tRNAs)	0.8042	0.3466	0.7369
24046	Scn11a	sodium channel, voltage-gated, type XI, alpha	0.8042	0.1098	0.5535
23857	Dmtf1	cyclin D binding myb-like transcription factor 1	0.804	0.1194	0.5628
99003	Qser1	glutamine and serine rich 1	0.8039	0.02899	0.3903
320216	D230040J21Rik	RIKEN cDNA D230040J21 gene	0.8037	0.01982	0.3601
100502632	LOC100502632	zinc finger protein 709-like	0.8036	0.2672	0.6865
75972	5033423O07Rik	RIKEN cDNA 5033423O07 gene	0.8036	0.02806	0.3875
18768	Pkib	protein kinase inhibitor beta, cAMP dependent, testis specific	0.8034	0.5185	0.8282
217030	Synrg	synergin, gamma	0.8033	0.02048	0.3609
68968	Cdan1	congenital dyserythropoietic anemia, type I (human)	0.8033	0.02416	0.3771
320082	Fbxw21	F-box and WD-40 domain protein 21	0.8031	0.2065	0.6392
22325	Vav2	vav 2 oncogene	0.803	0.1235	0.568
242425	Gabbr2	gamma-aminobutyric acid (GABA) B receptor, 2	0.8029	0.1504	0.5938
72267	Lrrc8e	leucine rich repeat containing 8 family, member E	0.8029	0.61	0.8667
240752	Pik3c2b	phosphoinositide-3-kinase, class 2, beta polypeptide	0.8028	0.1828	0.6215
22688	Zfp26	zinc finger protein 26	0.8027	0.03182	0.3992
100040962	Gm3065	predicted gene 3065	0.8026	0.1015	0.5395
544678	2010015L04Rik	RIKEN cDNA 2010015L04 gene	0.8026	0.03886	0.4213
52850	Sgsm1	small G protein signaling modulator 1	0.8026	0.1398	0.5834
627214	Fam196a	family with sequence similarity 196, member A	0.8025	0.01121	0.3181
110257	Hba-a2	hemoglobin alpha, adult chain 2	0.8023	0.4492	0.7943
13002	Dnajc5	DnaJ (Hsp40) homolog, subfamily C, member 5	0.8023	0.05835	0.4703
78934	4930581F22Rik	RIKEN cDNA 4930581F22 gene	0.8022	0.3225	0.7202
17295	Met	met proto-oncogene	0.8021	0.03896	0.4215
233230	Mrgprb4	MAS-related GPR, member B4	0.802	0.6948	0.9009
17101	Lyst	lysosomal trafficking regulator	0.8019	0.1554	0.5984
667214	9930111J21Rik1	RIKEN cDNA 9930111J21 gene 1	0.8018	0.1543	0.5975
22608	Ybx1	Y box protein 1	0.8018	0.6934	0.9001
12326	Camk4	calcium/calmodulin-dependent protein kinase IV	0.8017	0.03436	0.4091
623169	Gm6402	ribosomal protein S17 pseudogene	0.8014	0.08566	0.517
104027	Synpo	synaptopodin	0.8013	0.1666	0.6083
93713	Pcdhga5	protocadherin gamma subfamily A, 5	0.8013	0.03801	0.4192
68943	Pink1	PTEN induced putative kinase 1	0.8012	0.1546	0.5975

637053	Vmn2r4	vomeronasal 2, receptor 4	0.8011	0.4796	0.8101
319236	9230105E10Rik	RIKEN cDNA 9230105E10 gene	0.8009	0.4961	0.8186
12014	Bach2	BTB and CNC homology 2	0.8009	0.5032	0.8215
68268	Zdhhc21	zinc finger, DHHC domain containing 21	0.8008	0.02663	0.3809
21968	Tom1	target of myb1 homolog (chicken)	0.8008	0.1266	0.5708
402733	A930009L07Rik	RIKEN cDNA A930009L07 gene	0.8004	0.0317	0.3985
66296	Haus2	HAUS augmin-like complex, subunit 2	0.8004	0.121	0.5647
241633	Atp8b4	ATPase, class I, type 8B, member 4	0.8002	0.6809	0.8949
238330	6430527G18Rik	RIKEN cDNA 6430527G18 gene	0.8002	0.06012	0.4728
26930	Ppnr	per-pentamer repeat gene	0.8002	0.07234	0.4941
100042554	Gm3902	predicted gene 3902	0.8	0.02821	0.3883
69706	Ppil5	peptidylprolyl isomerase (cyclophilin) like 5	0.7998	0.07816	0.5035
171261	Vmn1r87	vomeronasal 1 receptor 87	0.7996	0.5598	0.8459
74041	4632434I11Rik	RIKEN cDNA 4632434I11 gene	0.7996	0.03821	0.4194
432812	BC052688	cDNA sequence BC052688	0.7995	0.08447	0.5154
238317	C130039O16Rik	RIKEN cDNA C130039O16 gene	0.7994	0.1223	0.5656
100093715	Gm10404	predicted gene 10404	0.7993	0.2067	0.6393
245174	Zfp937	zinc finger protein 937	0.7993	0.1913	0.6278
100043767	Gm4633	predicted gene 4633	0.7992	0.6012	0.8636
208111	C330019L16Rik	RIKEN cDNA C330019L16 gene	0.7991	0.3399	0.7326
108155	Ogt	O-linked N-acetylglucosamine (GlcNAc) transferase (UDP-N-acetylglucosamine:polypeptide-N-acetylglucosaminyl transferase)	0.799	0.1753	0.6156
320698	C530042K13Rik	RIKEN cDNA C530042K13 gene	0.7989	0.1236	0.568
226957	Gm4850	THO complex 4 pseudogene	0.7989	0.5958	0.8606
207777	Bzrap1	benzodiazepine receptor associated protein 1	0.7988	0.01174	0.3214
14300	Frg1	FSHD region gene 1	0.7985	0.1155	0.5579
16648	Kpna3	karyopherin (importin) alpha 3	0.7984	0.03729	0.4163
77057	Ston1	stonin 1	0.7982	0.4618	0.8022
12914	Crebbp	CREB binding protein	0.7982	0.07243	0.4941
12561	Cdh4	cadherin 4	0.7982	0.1806	0.6198
114602	Zmynd10	zinc finger, MYND domain containing 10	0.7981	0.1128	0.5552
209692	Dhtkd1	dehydrogenase E1 and transketolase domain containing 1	0.798	0.07589	0.5007
94282	Sfxn5	sideroflexin 5	0.7979	0.06014	0.4728
69489	2310007J06Rik	RIKEN cDNA 2310007J06 gene	0.7979	0.04295	0.4315
320658	D430040L24Rik	RIKEN cDNA D430040L24 gene	0.7978	0.05142	0.4519
74613	4833412C15Rik	RIKEN cDNA 4833412C15 gene	0.7976	0.02184	0.369
216797	Prss38	protease, serine, 38	0.7975	0.002655	0.2224
72090	Entpd8	ectonucleoside triphosphate diphosphohydrolase 8	0.7975	0.479	0.81
11624	Ahrr	aryl-hydrocarbon receptor repressor	0.7975	0.2086	0.6401
74303	1700109K24Rik	RIKEN cDNA 1700109K24 gene	0.7972	0.01605	0.3408
100040999	Gm3086	RuvB-like protein 1 pseudogene	0.7971	0.006188	0.2833
224840	Treml4	triggering receptor expressed on myeloid cells-like 4	0.7971	0.03405	0.4091
29809	Rabgap1l	RAB GTPase activating protein 1-like	0.7971	0.007947	0.3002
404583	AL118220	expressed sequence AL118220	0.7968	0.0396	0.4222
347740	2900097C17Rik	RIKEN cDNA 2900097C17 gene	0.7967	0.2551	0.6796
18193	Nsd1	nuclear receptor-binding SET-domain protein 1	0.7967	0.1309	0.5751
70729	Nos1ap	nitric oxide synthase 1 (neuronal) adaptor protein	0.7965	0.09203	0.5263
11775	Ap3b2	adaptor-related protein complex 3, beta 2 subunit	0.7965	0.02189	0.3691
66880	Rsrc1	arginine/serine-rich coiled-coil 1	0.7963	0.07423	0.4962
170740	Zfp287	zinc finger protein 287	0.7962	0.1452	0.5897

65973	Asph	aspartate-beta-hydroxylase	0.7962	0.1927	0.6286
14166	Fgf11	fibroblast growth factor 11	0.796	0.09956	0.537
217698	Acot5	acyl-CoA thioesterase 5	0.7959	0.3944	0.7659
15381	Hnrnpc	heterogeneous nuclear ribonucleoprotein C	0.7958	0.1313	0.5751
381598	2610005L07Rik	cadherin 11 pseudogene	0.7952	0.1925	0.6283
70701	Nipal1	NIPA-like domain containing 1	0.7951	0.01296	0.3304
100503209	LOC100503209	hypothetical LOC100503209	0.795	0.01421	0.3347
381112	Arhgef33	Rho guanine nucleotide exchange factor (GEF) 33	0.795	0.05397	0.4596
94279	Sfxn2	sideroflexin 2	0.795	0.02474	0.3786
67102	D16Ertd472e	DNA segment, Chr 16, ERATO Doi 472, expressed	0.795	0.04394	0.4336
14792	Lpcat3	lysophosphatidylcholine acyltransferase 3	0.795	0.02552	0.3786
320870	E330013P08Rik	RIKEN cDNA E330013P08 gene	0.7949	0.02567	0.3786
219249	Tdrd3	tudor domain containing 3	0.7948	0.131	0.5751
240869	Zbtb37	zinc finger and BTB domain containing 37	0.7947	0.02938	0.3911
233490	Crebzf	CREB/ATF bZIP transcription factor	0.7947	0.09363	0.5296
78523	Mrpl9	mitochondrial ribosomal protein L9	0.7946	0.07464	0.4974
26908	Eif2s3y	eukaryotic translation initiation factor 2, subunit 3, structural gene Y-linked	0.7946	0.1958	0.6307
100042584	Gm16523	mitochondrial ribosomal protein L40 pseudogene	0.7945	0.05766	0.469
546024	Crxos1	Crx opposite strand transcript 1	0.7945	0.06812	0.4858
170936	Zfp369	zinc finger protein 369	0.7945	0.6008	0.8636
69671	Tmem52	transmembrane protein 52	0.7945	0.2981	0.7051
18616	Peg3	paternally expressed 3	0.7945	0.2014	0.635
18150	Npm3	nucleoplasmin 3	0.7945	0.4441	0.7931
434174	Hmg1l1	high-mobility group (nonhistone chromosomal) protein 1-like 1	0.7944	0.1735	0.6147
224826	Ubr2	ubiquitin protein ligase E3 component n-recognin 2	0.7944	0.6282	0.8743
22780	Ikzf3	IKAROS family zinc finger 3	0.7944	0.2697	0.6878
433759	Hdac1	histone deacetylase 1	0.7943	0.2026	0.6356
319565	Syne2	synaptic nuclear envelope 2	0.7941	0.2045	0.637
258772	Olfr821	olfactory receptor 821	0.7941	0.1902	0.6275
218756	Slc4a7	solute carrier family 4, sodium bicarbonate cotransporter, member 7	0.7941	0.08675	0.5179
232409	Clec2e	C-type lectin domain family 2, member e	0.794	0.1507	0.594
74100	Arpp21	cyclic AMP-regulated phosphoprotein, 21	0.7939	0.0986	0.5367
217258	Abca8a	ATP-binding cassette, sub-family A (ABC1), member 8a	0.7937	0.1072	0.5493
547168	Rhox7	reproductive homeobox 7	0.7935	0.3497	0.7388
328110	Prpf39	PRP39 pre-mRNA processing factor 39 homolog (yeast)	0.7935	0.1175	0.5606
103554	Psme4	proteasome (prosome, macropain) activator subunit 4	0.7934	0.08891	0.5211
56706	Ccnl1	cyclin L1	0.7934	0.2253	0.654
269774	Aak1	AP2 associated kinase 1	0.7933	0.003183	0.227
227058	Dnahc7b	dynein, axonemal, heavy chain 7B	0.7933	0.2205	0.6522
100040529	Gm2824	predicted gene 2824	0.7932	0.3904	0.7633
100502966	LOC100502966	zinc finger protein 717-like	0.7931	0.1458	0.591
627927	Gm6812	predicted gene 6812	0.7931	0.2788	0.6937
17263	Meg3	maternally expressed 3	0.7931	0.01413	0.3347
241134	9430031J16Rik	RIKEN cDNA 9430031J16 gene	0.793	0.0403	0.4253
13002	Dnajc5	DnaJ (Hsp40) homolog, subfamily C, member 5	0.7928	0.146	0.591
338349	Cntln	centlein, centrosomal protein	0.7926	0.1347	0.5779
68142	Ino80	INO80 homolog (S. cerevisiae)	0.7925	0.06364	0.4792
228598	Ebf4	early B-cell factor 4	0.7923	0.3214	0.7199
99094	AI849538	expressed sequence AI849538	0.7923	0.01893	0.353

70308	2610005M20Rik	RIKEN cDNA 2610005M20 gene	0.792	0.2609	0.682
22592	Ercc5	excision repair cross-complementing rodent repair deficiency, complementation group 5	0.7918	0.2326	0.6622
20911	Stxbp2	syntaxin binding protein 2	0.7917	0.006931	0.2918
100043316	Gm4354	predicted gene 4354	0.7915	0.2415	0.6694
104348	Zfp120	zinc finger protein 120	0.7914	0.1097	0.5535
208650	Cblb	Casitas B-lineage lymphoma b	0.7913	0.1841	0.6217
114677	4930590A17Rik	RIKEN cDNA 4930590A17 gene	0.7913	0.1305	0.5747
22114	Tssk1	testis-specific serine kinase 1	0.7913	0.05387	0.4594
93687	Csnk1a1	casein kinase 1, alpha 1	0.7912	0.1706	0.6115
21416	Tcf7l2	transcription factor 7-like 2, T-cell specific, HMG-box	0.7911	0.02938	0.3911
192786	Rapgef6	Rap guanine nucleotide exchange factor (GEF) 6	0.7909	0.008735	0.3086
320387	D930030O05Rik	RIKEN cDNA D930030O05 gene	0.7908	0.03012	0.3947
70571	Tcerg1l	transcription elongation regulator 1-like	0.7907	0.1561	0.5991
402749	B430316J06Rik	RIKEN cDNA B430316J06 gene	0.7906	0.2332	0.6622
218772	Rarb	retinoic acid receptor, beta	0.7906	0.0113	0.3181
402762	A630039O03Rik	RIKEN cDNA A630039O03 gene	0.7905	0.05486	0.4614
239570	Ttc38	tetratricopeptide repeat domain 38	0.7904	0.02282	0.3702
18798	Plcb4	phospholipase C, beta 4	0.7904	0.05614	0.4631
84092	Usp8	ubiquitin specific peptidase 8	0.7903	0.2903	0.7006
15366	Hmmr	hyaluronan mediated motility receptor (RHAMM)	0.7902	0.01048	0.3117
258525	Olfr1170	olfactory receptor 1170	0.7901	0.3961	0.7671
70497	Arhgap17	Rho GTPase activating protein 17	0.7901	0.1193	0.5628
12163	Bmp8a	bone morphogenetic protein 8a	0.7901	0.3879	0.7614
74675	Ptchd3	patched domain containing 3	0.7899	0.007235	0.2929
16561	Kif1b	kinesin family member 1B	0.7898	0.02499	0.3786
433759	Hdac1	histone deacetylase 1	0.7896	0.2924	0.7024
272636	Esyt3	extended synaptotagmin-like protein 3	0.7896	0.7981	0.936
72739	Zkscan3	zinc finger with KRAB and SCAN domains 3	0.7895	0.001066	0.1606
408192	Gm9839	predicted gene 9839	0.7894	0.3392	0.7321
545047	Gm5800	predicted gene 5800	0.7893	0.1994	0.6343
320150	Zdhhc17	zinc finger, DHHC domain containing 17	0.7892	0.1536	0.5975
67320	Iqcf4	IQ motif containing F4	0.7892	0.3452	0.736
75299	4930547M16Rik	RIKEN cDNA 4930547M16 gene	0.789	0.01795	0.3475
71795	Pitpnc1	phosphatidylinositol transfer protein, cytoplasmic 1	0.789	0.02477	0.3786
98740	BB116930	expressed sequence BB116930	0.7889	0.008615	0.3071
21958	Tnp1	transition protein 1	0.7889	0.08773	0.5191
100041585	Amd2	S-adenosylmethionine decarboxylase 2	0.7887	0.0709	0.4923
664987	LOC664987	novel KRAB box and zinc finger, C2H2 type domain containing protein	0.7886	0.1489	0.593
240672	Dusp5	dual specificity phosphatase 5	0.7886	0.1324	0.5768
104248	Cabin1	calcineurin binding protein 1	0.7886	0.02594	0.3786
73692	2410089E03Rik	RIKEN cDNA 2410089E03 gene	0.7886	0.02346	0.3748
72194	Fbxl20	F-box and leucine-rich repeat protein 20	0.7886	0.028	0.3875
67102	D16Ertd472e	DNA segment, Chr 16, ERATO Doi 472, expressed	0.7886	0.1509	0.5942
11624	Ahrr	aryl-hydrocarbon receptor repressor	0.7886	0.005254	0.2718
666183	Gm7968	predicted gene 7968	0.7884	0.02653	0.3809
630836	2010315B03Rik	RIKEN cDNA 2010315B03 gene	0.7884	0.009439	0.3112
271970	Arsj	arylsulfatase J	0.7883	0.08883	0.521
73852	D3Ertd751e	DNA segment, Chr 3, ERATO Doi 751, expressed	0.7883	0.01872	0.3527
14268	Fn1	fibronectin 1	0.7883	0.01444	0.3347

114301	Palmd	palmdelphin	0.7882	0.01672	0.3423
100040305	Gm2701	predicted gene 2701	0.7881	0.0718	0.494
54366	Ctnnal1	catenin (cadherin associated protein), alpha-like 1	0.7881	0.3208	0.7196
13107	Cyp2f2	cytochrome P450, family 2, subfamily f, polypeptide 2	0.7881	0.5734	0.8501
78124	4930458L03Rik	RIKEN cDNA 4930458L03 gene	0.7879	0.1711	0.6118
70897	Fam71d	family with sequence similarity 71, member D	0.7879	0.1415	0.5863
329908	Usp24	ubiquitin specific peptidase 24	0.7878	0.003383	0.2308
320929	4732460I02Rik	RIKEN cDNA 4732460I02 gene	0.7878	0.06166	0.477
72519	Tmem55a	transmembrane protein 55A	0.7878	0.07759	0.5026
27981	D4Wsu53e	DNA segment, Chr 4, Wayne State University 53, expressed	0.7877	0.05673	0.464
670833	Gm11213	predicted gene 11213	0.7876	0.5831	0.8538
76042	5830427D02Rik	RIKEN cDNA 5830427D02 gene	0.7875	0.01488	0.3375
227525	Dclre1c	DNA cross-link repair 1C, PSO2 homolog (S. cerevisiae)	0.7874	0.01869	0.3526
74180	Muc5b	mucin 5, subtype B, tracheobronchial	0.7874	0.4262	0.7823
640979	LOC640979	ig heavy chain V region B1-8/186-2-like	0.7872	0.01027	0.3117
22631	Ywhaz	tyrosine 3-monooxygenase/tryptophan 5-monooxygenase activation protein, zeta polypeptide	0.7872	0.2528	0.6778
403345	BC037438	cDNA sequence BC037438	0.7869	0.2112	0.6431
15251	Hif1a	hypoxia inducible factor 1, alpha subunit	0.7869	0.055	0.4614
76173	6330531I01Rik	RIKEN cDNA 6330531I01 gene	0.7868	0.2254	0.654
67040	Ddx17	DEAD (Asp-Glu-Ala-Asp) box polypeptide 17	0.7868	0.2083	0.6401
229096	Ythdf3	YTH domain family 3	0.7867	0.1015	0.5395
19418	Rasgrf2	RAS protein-specific guanine nucleotide-releasing factor 2	0.7866	0.1376	0.5815
229227	4932438A13Rik	RIKEN cDNA 4932438A13 gene	0.7865	0.044	0.4336
77800	4933406J10Rik	RIKEN cDNA 4933406J10 gene	0.7864	0.2667	0.6862
16764	Aff3	AF4/FMR2 family, member 3	0.7864	0.1688	0.6096
414069	BC024978	cDNA sequence BC024978	0.7863	0.07692	0.5014
15273	Hivep2	human immunodeficiency virus type I enhancer binding protein 2	0.7863	0.02609	0.3791
244495	Gm4975	predicted gene 4975	0.7862	0.08589	0.517
98662	AW061147	expressed sequence AW061147	0.7862	0.4286	0.7838
100504146	LOC100504146	zinc finger protein 709-like	0.7859	0.2848	0.6977
22061	Trp63	transformation related protein 63	0.7859	0.06677	0.485
245650	Gucy2f	guanylate cyclase 2f	0.7858	0.03116	0.3964
24084	Tekt2	tektin 2	0.7858	0.03169	0.3985
353282	Sfmbt2	Scm-like with four mbt domains 2	0.7856	0.006647	0.288
16504	Kcnc3	potassium voltage gated channel, Shaw-related subfamily, member 3	0.7856	0.02215	0.3702
16180	Il1rap	interleukin 1 receptor accessory protein	0.7855	0.575	0.8504
215789	Phactr2	phosphatase and actin regulator 2	0.7853	0.108	0.551
76500	Ip6k2	inositol hexaphosphate kinase 2	0.7853	0.184	0.6217
72344	Usp36	ubiquitin specific peptidase 36	0.7852	0.002449	0.2179
170826	Ppargc1b	peroxisome proliferative activated receptor, gamma, coactivator 1 beta	0.7851	0.219	0.6511
244059	Chd2	chromodomain helicase DNA binding protein 2	0.785	0.05265	0.4554
74473	4933433H22Rik	RIKEN cDNA 4933433H22 gene	0.7849	0.286	0.6982
26431	Git2	G protein-coupled receptor kinase-interactor 2	0.7849	0.06342	0.478
223827	Gxylt1	glucoside xylosyltransferase 1	0.7848	0.1897	0.6273
78876	B230214N19Rik	RIKEN cDNA B230214N19 gene	0.7846	0.08555	0.517
68750	Rreb1	ras responsive element binding protein 1	0.7846	0.03325	0.4053
100503311	1700027A23Rik	RIKEN cDNA 1700027A23 gene	0.7845	0.02261	0.3702
333789	N4bp2	NEDD4 binding protein 2	0.7845	0.03092	0.3964
78656	Brd8	bromodomain containing 8	0.7845	0.01094	0.3171

56036	Ccnl2	cyclin L2	0.7843	0.02271	0.3702
271981	Tbck	TBC1 domain containing kinase	0.7842	0.01896	0.3531
108857	Ankhd1	ankyrin repeat and KH domain containing 1	0.7842	0.01682	0.3424
24071	Synj2bp	synaptojanin 2 binding protein	0.7839	0.004212	0.2553
638247	9530082P21Rik	RIKEN cDNA 9530082P21 gene	0.7838	0.01881	0.353
67133	Gp2	glycoprotein 2 (zymogen granule membrane)	0.7838	0.7092	0.9069
66079	Tmem42	transmembrane protein 42	0.7838	0.001596	0.1903
320082	Fbxw21	F-box and WD-40 domain protein 21	0.7837	0.2673	0.6867
76096	5830468K08Rik	RIKEN cDNA 5830468K08 gene	0.7837	0.0606	0.4741
19799	Rn4.5s	4.5S RNA	0.7837	0.008917	0.3086
624696	Gm6522	predicted gene 6522	0.7836	0.2959	0.705
216613	Ccdc85a	coiled-coil domain containing 85A	0.7836	0.02514	0.3786
330401	Tmcc1	transmembrane and coiled coil domains 1	0.7833	0.03068	0.3964
98314	D2hgdh	D-2-hydroxyglutarate dehydrogenase	0.7833	0.03522	0.4108
70432	Rufy2	RUN and FYVE domain-containing 2	0.7833	0.006086	0.2833
24066	Spry4	sprouty homolog 4 (Drosophila)	0.7832	0.6571	0.8863
626848	Etohi1	ethanol induced 1	0.7831	0.1895	0.6272
118449	Synpo2	synaptopodin 2	0.7829	0.07796	0.5029
70828	4633401B06Rik	RIKEN cDNA 4633401B06 gene	0.7829	0.02951	0.392
622675	Zfp827	zinc finger protein 827	0.7828	0.07694	0.5014
229571	Gm4858	predicted gene 4858	0.7828	0.1809	0.6199
433294	A530098C11Rik	RIKEN cDNA A530098C11 gene	0.7827	0.05949	0.4728
52036	Ppp6r3	protein phosphatase 6, regulatory subunit 3	0.7826	0.2004	0.6345
15289	Hmgb1	high mobility group box 1	0.7824	0.0798	0.5091
381310	6330403A02Rik	RIKEN cDNA 6330403A02 gene	0.7822	0.5348	0.8349
233064	Wdr62	WD repeat domain 62	0.7822	0.107	0.5492
107338	Gbf1	golgi-specific brefeldin A-resistance factor 1	0.7821	0.06449	0.4816
56068	Ammecr1	Alport syndrome, mental retardation, midface hypoplasia and elliptocytosis chromosomal region gene 1 homolog (human)	0.7821	0.0562	0.4631
239420	Csmd3	CUB and Sushi multiple domains 3	0.782	0.2241	0.654
269587	Epb4.1	erythrocyte protein band 4.1	0.7818	0.3649	0.7493
227525	Dclre1c	DNA cross-link repair 1C, PSO2 homolog (S. cerevisiae)	0.7816	0.002652	0.2224
27028	Ermap	erythroblast membrane-associated protein	0.7815	0.02573	0.3786
192196	Luc7l2	LUC7-like 2 (S. cerevisiae)	0.7812	0.1547	0.5975
24000	Ptpn21	protein tyrosine phosphatase, non-receptor type 21	0.7812	0.01779	0.3475
12856	Cox17	cytochrome c oxidase, subunit XVII assembly protein homolog (yeast)	0.7812	0.1132	0.5557
319742	Mpzl3	myelin protein zero-like 3	0.781	0.2128	0.6453
627782	Gm6787	proteasome (prosome, macropain) subunit, beta type 7 pseudogene	0.7809	0.3448	0.736
541456	LOC541456	hypothetical LOC541456	0.7809	0.029	0.3903
216516	Ccdc157	coiled-coil domain containing 157	0.7809	0.0008529	0.148
106585	Ankrd12	ankyrin repeat domain 12	0.7808	0.08845	0.5202
76183	Celf6	CUGBP, Elav-like family member 6	0.7808	0.03408	0.4091
108115	Slco4a1	solute carrier organic anion transporter family, member 4a1	0.7807	0.002799	0.2228
229227	4932438A13Rik	RIKEN cDNA 4932438A13 gene	0.7806	0.05486	0.4614
105442	B130052P14Rik	RIKEN cDNA B130052P14 gene	0.7806	0.002726	0.2224
69207	Srsf11	serine/arginine-rich splicing factor 11	0.7806	0.07655	0.5014
26875	Pclo	piccolo (presynaptic cytomatrix protein)	0.7805	0.04044	0.4257
19017	Ppargc1a	peroxisome proliferative activated receptor, gamma, coactivator 1 alpha	0.7805	0.005111	0.2706
231214	Cc2d2a	coiled-coil and C2 domain containing 2A	0.7801	0.0311	0.3964
22157	Tulp1	tubby like protein 1	0.78	0.0388	0.4213

622175	E430024I08Rik	RIKEN cDNA E430024I08 gene	0.7798	0.1606	0.6058
14367	Fzd5	frizzled homolog 5 (Drosophila)	0.7798	0.03804	0.4193
100505237	LOC100505237	calcium-binding and coiled-coil domain-containing protein 2-like	0.7797	0.2722	0.6895
329178	Unc80	unc-80 homolog (C. elegans)	0.7797	0.01569	0.3403
319537	B230334C09Rik	RIKEN cDNA B230334C09 gene	0.7795	0.3943	0.7659
213084	Cdkl3	cyclin-dependent kinase-like 3	0.7795	0.02703	0.3847
433809	Rnf207	ring finger protein 207	0.7794	0.02266	0.3702
73709	2410125D13Rik	RIKEN cDNA 2410125D13 gene	0.7794	0.03308	0.404
52108	D9Ertd115e	DNA segment, Chr 9, ERATO Doi 115, expressed	0.7793	0.1062	0.5468
320478	Sox2ot	SOX2 overlapping transcript (non-protein coding)	0.7792	0.001596	0.1903
100041995	Gm3613	predicted gene 3613	0.7791	0.02034	0.3609
15201	Hells	helicase, lymphoid specific	0.7791	0.1747	0.6149
100503794	LOC100503794	hypothetical LOC100503794	0.779	0.1178	0.5609
76740	Efr3a	EFR3 homolog A (S. cerevisiae)	0.779	0.176	0.6156
195333	Gsc2	goosecoid homebox 2	0.7789	0.05257	0.4549
11920	Atm	ataxia telangiectasia mutated homolog (human)	0.7789	0.1098	0.5535
331046	Tgm4	transglutaminase 4 (prostate)	0.7788	0.003638	0.2427
26385	Grk6	G protein-coupled receptor kinase 6	0.7788	0.1154	0.5578
70832	4921504A21Rik	RIKEN cDNA 4921504A21 gene	0.7786	0.03158	0.398
18802	Plcd4	phospholipase C, delta 4	0.7786	0.2947	0.7044
319780	D030002E05Rik	RIKEN cDNA D030002E05 gene	0.7782	0.01995	0.3604
72772	Rint1	RAD50 interactor 1	0.7782	0.1906	0.6276
18607	Pdpk1	3-phosphoinositide dependent protein kinase 1	0.7782	0.01804	0.3482
319332	B930096F20Rik	RIKEN cDNA B930096F20 gene	0.7781	0.01727	0.3452
224656	Zfp523	zinc finger protein 523	0.7781	0.02505	0.3786
170788	Crb1	crumbs homolog 1 (Drosophila)	0.7781	0.01284	0.3301
13405	Dmd	dystrophin, muscular dystrophy	0.7781	0.06603	0.483
11994	Pcdh15	protocadherin 15	0.7781	0.01854	0.3517
20928	Abcc9	ATP-binding cassette, sub-family C (CFTR/MRP), member 9	0.7779	0.02142	0.3653
17294	Mest	mesoderm specific transcript	0.7779	0.05193	0.4525
100042165	BC005561	cDNA sequence BC005561	0.7776	0.003782	0.2433
622935	Krtap20-2	keratin associated protein 20-2	0.7776	0.07236	0.4941
381022	Mll2	myeloid/lymphoid or mixed-lineage leukemia 2	0.7775	0.02685	0.3832
72293	Nkd2	naked cuticle 2 homolog (Drosophila)	0.7775	0.159	0.6035
319520	Dusp4	dual specificity phosphatase 4	0.7774	0.06342	0.478
106622	AI605517	expressed sequence AI605517	0.7772	0.02404	0.377
16974	Lrp6	low density lipoprotein receptor-related protein 6	0.7772	0.01753	0.3469
258954	Olfr522	olfactory receptor 522	0.7771	0.1844	0.6217
231769	Sfswap	splicing factor, suppressor of white-apricot homolog (Drosophila)	0.7771	0.006519	0.288
15331	Hmgn2	high mobility group nucleosomal binding domain 2	0.7771	0.05862	0.4711
67848	Ddx55	DEAD (Asp-Glu-Ala-Asp) box polypeptide 55	0.777	0.08436	0.5154
94352	Loxl2	lysyl oxidase-like 2	0.7769	0.3385	0.7319
104103	Airn	antisense Igf2r RNA	0.7768	0.005749	0.2775
12530	Cdc25a	cell division cycle 25 homolog A (S. pombe)	0.7768	0.02551	0.3786
75019	Rnase10	ribonuclease, RNase A family, 10 (non-active)	0.7766	0.1585	0.603
70656	5730526G10Rik	RIKEN cDNA 5730526G10 gene	0.7766	0.00649	0.288
100502767	LOC100502767	hypothetical LOC100502767	0.7765	0.008877	0.3086
547150	6820431F20Rik	cadherin 11 pseudogene	0.7765	0.07355	0.4957
76233	Dnttip1	deoxynucleotidyltransferase, terminal, interacting protein 1	0.7764	0.0229	0.3708

100043484	Gm4470	predicted gene 4470	0.7763	0.137	0.5807
100048452	LOC100048452	hypothetical protein LOC100048452	0.7761	0.1341	0.5779
73386	1700048M11Rik	RIKEN cDNA 1700048M11 gene	0.7761	0.03962	0.4222
207921	A830093I24Rik	RIKEN cDNA A830093I24 gene	0.776	0.01402	0.3347
51922	D2Ertd612e	DNA segment, Chr 2, ERATO Doi 612, expressed	0.776	0.02023	0.3609
12647	Chat	choline acetyltransferase	0.7756	0.005848	0.278
399629	9530029O12Rik	RIKEN cDNA 9530029O12 gene	0.7755	0.005431	0.2775
21665	Tdg	thymine DNA glycosylase	0.7752	0.2085	0.6401
17686	Msh3	mutS homolog 3 (E. coli)	0.7751	0.2776	0.6925
100504534	LOC100504534	hypothetical LOC100504534	0.775	0.002054	0.2121
277939	C2cd3	C2 calcium-dependent domain containing 3	0.7748	0.04243	0.4301
21784	Tff1	trefoil factor 1	0.7747	0.5635	0.8465
73002	2900076A13Rik	RIKEN cDNA 2900076A13 gene	0.7746	0.01905	0.3531
226251	Ablim1	actin-binding LIM protein 1	0.7745	0.05416	0.4596
76491	Abhd14b	abhydrolase domain containing 14b	0.7744	0.03221	0.4005
234797	6430548M08Rik	RIKEN cDNA 6430548M08 gene	0.7743	0.2714	0.6891
240261	Ccdc112	coiled-coil domain containing 112	0.7742	0.1924	0.6283
670496	Gm11564	predicted gene 11564	0.7741	0.06088	0.4741
71169	Nbas	neuroblastoma amplified sequence	0.7741	0.1033	0.5433
68020	2810002N01Rik	RIKEN cDNA 2810002N01 gene	0.774	0.01219	0.3236
243872	Rpl7a-ps8	ribosomal protein L7A, pseudogene 8	0.7739	0.0009742	0.1524
353208	Zfp931	zinc finger protein 931	0.7737	0.0981	0.5365
75504	1700013F07Rik	RIKEN cDNA 1700013F07 gene	0.7737	0.1535	0.5975
30049	Scd3	stearoyl-coenzyme A desaturase 3	0.7737	0.02558	0.3786
26554	Cul3	cullin 3	0.7736	0.1064	0.5472
18383	Tnfrsf11b	tumor necrosis factor receptor superfamily, member 11b (osteoprotegerin)	0.7736	0.04376	0.4336
71409	Fmnl2	formin-like 2	0.7735	0.05473	0.4614
100038651	D130062J21Rik	RIKEN cDNA D130062J21 gene	0.7733	0.03574	0.4123
20979	Syt1	synaptotagmin I	0.7733	0.01443	0.3347
15132	Hbb-bh1	hemoglobin Z, beta-like embryonic chain	0.7733	0.377	0.7563
50873	Park2	Parkinson disease (autosomal recessive, juvenile) 2, parkin	0.7732	0.008676	0.3079
329251	Ppp1r12b	protein phosphatase 1, regulatory (inhibitor) subunit 12B	0.7731	0.1746	0.6148
100504857	LOC100504857	hypothetical LOC100504857	0.773	0.009033	0.3086
100038620	Gm10631	predicted gene 10631	0.773	0.2396	0.6681
12992	Csn1s2b	casein alpha s2-like B	0.773	0.2473	0.6743
69318	1700007K09Rik	RIKEN cDNA 1700007K09 gene	0.7728	0.5178	0.828
216725	Adamts2	a disintegrin-like and metallopeptidase (reprolysin type) with thrombospondin type 1 motif, 2	0.7727	0.6565	0.8859
19252	Dusp1	dual specificity phosphatase 1	0.7727	0.05787	0.469
16858	Lgals7	lectin, galactose binding, soluble 7	0.7726	0.2609	0.682
246103	Atxn7	ataxin 7	0.7725	0.01145	0.3202
70432	Rufy2	RUN and FYVE domain-containing 2	0.7724	0.005087	0.2706
665211	Gm14326	predicted gene 14326	0.7723	0.1812	0.6205
228998	Arfgap1	ADP-ribosylation factor GTPase activating protein 1	0.7721	0.01526	0.3403
66315	Senp7	SUMO1/sentrin specific peptidase 7	0.772	0.05915	0.4727
16598	Klf2	Kruppel-like factor 2 (lung)	0.772	0.04149	0.4282
546049	C330021F23Rik	RIKEN cDNA C330021F23 gene	0.7719	0.06894	0.487
231279	Guf1	GUF1 GTPase homolog (S. cerevisiae)	0.7719	0.06126	0.4758
14387	Gaa	glucosidase, alpha, acid	0.7719	0.0686	0.4867
620382	Gm6146	predicted gene 6146	0.7718	0.03	0.3947

75845	4930567K12Rik	RIKEN cDNA 4930567K12 gene	0.7717	0.298	0.7051
75302	Asxl2	additional sex combs like 2 (Drosophila)	0.7716	0.07355	0.4957
13405	Dmd	dystrophin, muscular dystrophy	0.7716	0.1647	0.6076
100043332	Gm4368	predicted gene 4368	0.7715	0.3111	0.7135
414113	D230040A04Rik	RIKEN cDNA D230040A04 gene	0.7712	0.08461	0.5154
17101	Lyst	lysosomal trafficking regulator	0.7712	0.1699	0.6112
211770	Trib1	tribbles homolog 1 (Drosophila)	0.7711	0.01049	0.3117
19263	Ptprb	protein tyrosine phosphatase, receptor type, B	0.771	0.004638	0.2642
19255	Ptpn2	protein tyrosine phosphatase, non-receptor type 2	0.771	0.07786	0.5028
231103	Gckr	glucokinase regulatory protein	0.7709	0.01856	0.3517
207806	Gm608	predicted gene 608	0.7708	0.1321	0.5763
100505153	LOC100505153	heterogeneous nuclear ribonucleoprotein A3-like	0.7707	0.1995	0.6343
404549	Ifna14	interferon, alpha 14	0.7707	0.4085	0.7731
628100	Fbxo39	F-box protein 39	0.7704	0.3535	0.7414
622480	Spocd1	SPOC domain containing 1	0.7704	0.173	0.6143
75202	Ncrna00085	non-protein coding RNA 85	0.7704	0.1055	0.5466
12953	Cry2	cryptochrome 2 (photolyase-like)	0.7704	0.008366	0.3026
630579	Zfp808	zinc finger protein 80	0.7702	0.1241	0.5692
100040872	Gm3014	predicted gene 3014	0.77	0.2145	0.6467
20871	Aurkc	aurora kinase C	0.77	0.02734	0.386
100036572	LOC100036572	hypothetical LOC100036572	0.7696	0.04241	0.4301
625237	Gm6566	predicted gene 6566	0.7695	0.1802	0.6195
75547	Akap13	A kinase (PRKA) anchor protein 13	0.7695	0.008212	0.3023
19668	Rbpjl	recombination signal binding protein for immunoglobulin kappa J region-like	0.7695	0.17	0.6113
30910	C230037E05Rik	RIKEN cDNA C230037E05 gene	0.7694	0.0009819	0.1524
56705	Ranbp9	RAN binding protein 9	0.7691	0.472	0.8081
18802	Plcd4	phospholipase C, delta 4	0.7691	0.08314	0.5144
622675	Zfp827	zinc finger protein 827	0.769	0.5293	0.8332
94279	Sfxn2	sideroflexin 2	0.7689	0.01982	0.3601
18159	Nppc	natriuretic peptide type C	0.7689	0.05014	0.4512
110862	Kcnq3	potassium voltage-gated channel, subfamily Q, member 3	0.7687	0.2582	0.6815
15331	Hmgn2	high mobility group nucleosomal binding domain 2	0.7684	0.06607	0.483
67956	Setd8	SET domain containing (lysine methyltransferase) 8	0.768	0.009301	0.3099
71186	4933417D19Rik	RIKEN cDNA 4933417D19 gene	0.7679	0.1907	0.6276
65970	Lima1	LIM domain and actin binding 1	0.7677	0.02691	0.3837
70791	Hars2	histidyl-tRNA synthetase 2, mitochondrial (putative)	0.7675	0.00208	0.2121
208583	Nek11	NIMA (never in mitosis gene a)-related expressed kinase 11	0.7674	0.08961	0.5224
72344	Usp36	ubiquitin specific peptidase 36	0.7674	0.02296	0.3708
214444	Cdk5rap2	CDK5 regulatory subunit associated protein 2	0.7673	0.1636	0.6074
104759	Pld4	phospholipase D family, member 4	0.7673	0.233	0.6622
72462	Rrp1b	ribosomal RNA processing 1 homolog B (S. cerevisiae)	0.7673	0.01519	0.3403
100504004	LOC100504004	putative transposase element L1Md-A101/L1Md-A102/L1Md-A2-like	0.7672	0.07882	0.5055
24001	Tiam2	T-cell lymphoma invasion and metastasis 2	0.7672	0.1745	0.6148
76613	1700069B07Rik	RIKEN cDNA 1700069B07 gene	0.7669	0.07573	0.5
17263	Meg3	maternally expressed 3	0.7665	0.06431	0.4816
12402	Cbl	Casitas B-lineage lymphoma	0.7663	0.01819	0.3486
77987	Ascc3	activating signal cointegrator 1 complex subunit 3	0.7662	0.08119	0.5107
170624	Dep1	diabetic embryopathy 1	0.7661	0.2028	0.6356
399617	A130019P10Rik	RIKEN cDNA A130019P10 gene	0.766	0.02917	0.3911

71101	4933407H18Rik	RIKEN cDNA 4933407H18 gene	0.766	0.2375	0.6663
13605	Ect2	ect2 oncogene	0.7656	0.01069	0.3144
73130	Tmed5	transmembrane emp24 protein transport domain containing 5	0.7655	0.01024	0.3117
12969	Crygf	crystallin, gamma F	0.7655	0.2383	0.667
74160	1300014J16Rik	RIKEN cDNA 1300014J16 gene	0.7654	0.1214	0.5651
78695	C030005K06Rik	RIKEN cDNA C030005K06 gene	0.7653	0.1876	0.6259
11640	Akap1	A kinase (PRKA) anchor protein 1	0.7652	0.02094	0.3618
18770	Pklr	pyruvate kinase liver and red blood cell	0.7651	0.2517	0.6767
668382	Pabpc1l2b-ps	poly(A) binding protein, cytoplasmic 1-like 2B, pseudogene	0.765	0.2971	0.7051
327760	4930565A17	hypothetical protein 4930565A17	0.765	0.2675	0.6867
71754	Cyp2d40	cytochrome P450, family 2, subfamily d, polypeptide 40	0.7649	0.3443	0.7358
333433	Gpd1l	glycerol-3-phosphate dehydrogenase 1-like	0.7648	0.05161	0.4524
239857	Cadm2	cell adhesion molecule 2	0.7645	0.05005	0.451
231042	Nupl2	nucleoporin like 2	0.7643	0.1852	0.6223
208084	Pif1	PIF1 5'-to-3' DNA helicase homolog (S. cerevisiae)	0.7643	0.09314	0.529
12660	Chka	choline kinase alpha	0.7642	0.01539	0.3403
13113	Cyp3a13	cytochrome P450, family 3, subfamily a, polypeptide 13	0.7641	0.0517	0.4525
19888	Rp1	retinitis pigmentosa 1 (human)	0.764	0.03101	0.3964
319317	Snhg11	small nucleolar RNA host gene 11	0.7639	0.03458	0.4091
22791	Dnajc2	DnaJ (Hsp40) homolog, subfamily C, member 2	0.7639	0.1882	0.6261
381065	Gm5145	small nuclear ribonucleoprotein polypeptide A pseudogene	0.7638	0.1428	0.5879
228094	Cerkl	ceramide kinase-like	0.7638	0.1506	0.5938
219105	Zmym5	zinc finger, MYM-type 5	0.7637	0.01436	0.3347
70425	Csnk1g3	casein kinase 1, gamma 3	0.7637	0.04965	0.4491
228357	Lrp4	low density lipoprotein receptor-related protein 4	0.7636	0.0003491	0.1365
193385	Fam65b	family with sequence similarity 65, member B	0.7636	0.00276	0.2224
54426	Hgfac	hepatocyte growth factor activator	0.7636	0.2435	0.6714
319984	Jph4	junctophilin 4	0.7635	0.01906	0.3531
242519	Ifna12	interferon alpha 12	0.7634	0.2337	0.6626
19893	Rpgr	retinitis pigmentosa GTPase regulator	0.7633	0.03022	0.3952
676882	Gm9693	predicted gene 9693	0.7631	0.1326	0.5771
100041870	Gm3556	predicted gene 3556	0.7628	0.00118	0.1652
100126203	6820426E19Rik	RIKEN cDNA 6820426E19 gene	0.7627	0.1879	0.626
109593	Lmo3	LIM domain only 3	0.7627	0.02648	0.3806
227292	Ctdsp1	CTD (carboxy-terminal domain, RNA polymerase II, polypeptide A) small phosphatase 1	0.7625	0.04412	0.4338
67367	Gcfc1	GC-rich sequence DNA-binding factor 1	0.7621	0.06551	0.4816
319385	D130061D10Rik	RIKEN cDNA D130061D10 gene	0.762	0.01123	0.3181
211429	Pla2g4b	phospholipase A2, group IVB (cytosolic)	0.762	0.04093	0.4265
71726	Smug1	single-strand selective monofunctional uracil DNA glycosylase	0.7619	0.09967	0.5372
654803	B130055M24Rik	RIKEN cDNA B130055M24 gene	0.7618	0.01083	0.3156
258955	Olfr531	olfactory receptor 531	0.7617	0.3548	0.7428
18703	Pigr	polymeric immunoglobulin receptor	0.7617	0.02835	0.3889
66797	Cntnap2	contactin associated protein-like 2	0.7612	0.3438	0.7356
66231	Thoc7	THO complex 7 homolog (Drosophila)	0.7612	0.1705	0.6115
668030	OTTMUSG00000016609	predicted gene, OTTMUSG00000016609	0.7611	0.07672	0.5014
240174	Thada	thyroid adenoma associated	0.7611	0.1483	0.5918
193217	BC018473	cDNA sequence BC018473	0.7611	0.2503	0.6767
330554	Fan1	FANCD2/FANCI-associated nuclease 1	0.7608	0.1014	0.5395
224405	Cyyr1	cysteine and tyrosine-rich protein 1	0.7608	0.1423	0.5872

75767	Rab11fip1	RAB11 family interacting protein 1 (class I)	0.7608	0.3384	0.7317
66259	Camk2n1	calcium/calmodulin-dependent protein kinase II inhibitor 1	0.7608	0.1143	0.5569
100303732	Gm14431	predicted gene 14431	0.7606	0.2279	0.6569
319269	A130040M12Rik	RIKEN cDNA A130040M12 gene	0.7605	0.01666	0.3419
17345	Mki67	antigen identified by monoclonal antibody Ki 67	0.7604	0.3818	0.7596
100303732	Gm14431	predicted gene 14431	0.7603	0.2864	0.6982
17755	Mtap1b	microtubule-associated protein 1B	0.7603	0.2246	0.654
15397	Hoxa11as	HOXA11 antisense RNA (non-protein coding)	0.7602	0.1263	0.5703
230861	Eif4g3	eukaryotic translation initiation factor 4 gamma, 3	0.7595	0.04441	0.4349
76873	4930447F24Rik	RIKEN cDNA 4930447F24 gene	0.7595	0.4421	0.7926
19263	Ptprb	protein tyrosine phosphatase, receptor type, B	0.7595	0.03683	0.4161
170822	Usp33	ubiquitin specific peptidase 33	0.7592	0.00295	0.2228
258383	Olfr1347	olfactory receptor 1347	0.7591	0.2209	0.6525
231470	Fras1	Fraser syndrome 1 homolog (human)	0.759	0.03367	0.408
22135	Tgoln2	trans-golgi network protein 2	0.7585	0.151	0.5942
72777	2810455B08Rik	RIKEN cDNA 2810455B08 gene	0.7584	0.0345	0.4091
218203	Mylip	myosin regulatory light chain interacting protein	0.758	0.07154	0.4934
72554	Utp14a	UTP14, U3 small nucleolar ribonucleoprotein, homolog A (yeast)	0.7579	0.04596	0.4381
56215	Acin1	apoptotic chromatin condensation inducer 1	0.7579	0.02267	0.3702
74175	Crct1	cysteine-rich C-terminal 1	0.7578	0.2681	0.6871
382066	Prdm10	PR domain containing 10	0.7577	0.1358	0.5794
100040852	Gm3002	alpha-takusan pseudogene	0.7576	0.1576	0.6017
667127	Gm15847	predicted gene 15847	0.7575	0.2492	0.6767
210172	Zfp526	zinc finger protein 526	0.7574	0.1988	0.6336
67120	Ttc14	tetratricopeptide repeat domain 14	0.7573	0.01965	0.3587
100465	Mobkl2c	MOB1, Mps One Binder kinase activator-like 2C (yeast)	0.7572	0.02412	0.3771
109294	Prex2	phosphatidylinositol-3,4,5-trisphosphate-dependent Rac exchange factor 2	0.7571	0.2506	0.6767
270627	Taf1	TAF1 RNA polymerase II, TATA box binding protein (TBP)-associated factor	0.757	0.008314	0.3025
93742	Pard3	par-3 (partitioning defective 3) homolog (C. elegans)	0.757	0.1835	0.6217
19283	Ptprz1	protein tyrosine phosphatase, receptor type Z, polypeptide 1	0.7569	0.06941	0.4874
544971	Bdp1	B double prime 1, subunit of RNA polymerase III transcription initiation factor IIIB	0.7568	0.06669	0.4848
70918	Nsun7	NOL1/NOP2/Sun domain family, member 7	0.7568	0.03747	0.417
100044236	Copg2as2	coatomer protein complex, subunit gamma 2, antisense 2	0.7564	0.2713	0.6891
50772	Mapk6	mitogen-activated protein kinase 6	0.7564	0.04152	0.4282
76421	1700028K03Rik	RIKEN cDNA 1700028K03 gene	0.7563	0.2183	0.6504
72459	Htatsf1	HIV TAT specific factor 1	0.7562	0.1478	0.5911
545798	Tmem233	transmembrane protein 233	0.756	0.4759	0.8097
258693	Olfr1443	olfactory receptor 1443	0.756	0.09068	0.5229
17951	Naip5	NLR family, apoptosis inhibitory protein 5	0.756	0.0281	0.3875
330490	Nlrp9c	NLR family, pyrin domain containing 9C	0.7557	0.004879	0.2652
320817	Atad2b	ATPase family, AAA domain containing 2B	0.7557	0.0754	0.4997
211134	Lzts1	leucine zipper, putative tumor suppressor 1	0.7555	0.2967	0.7051
229279	Hnrnpa3	heterogeneous nuclear ribonucleoprotein A3	0.7554	0.2409	0.6687
234733	Ddx19b	DEAD (Asp-Glu-Ala-Asp) box polypeptide 19b	0.7551	0.2874	0.6986
27416	Abcc5	ATP-binding cassette, sub-family C (CFTR/MRP), member 5	0.755	0.08867	0.5207
20742	Spnb2	spectrin beta 2	0.755	0.08342	0.5144
320129	Adrbk2	adrenergic receptor kinase, beta 2	0.7549	0.01531	0.3403
670912	Gm9510	predicted gene 9510	0.7548	0.1086	0.5521
320654	E330024J20Rik	RIKEN cDNA E330024J20 gene	0.7548	0.128	0.5716

667277	C1rb	complement component 1, r subcomponent B	0.7547	0.1339	0.5777
665211	Gm14326	predicted gene 14326	0.7545	0.1952	0.6304
109181	Trip11	thyroid hormone receptor interactor 11	0.7543	0.06266	0.4771
268281	Shprh	SNF2 histone linker PHD RING helicase	0.7542	0.2594	0.6815
17125	Smad1	MAD homolog 1 (Drosophila)	0.7542	0.1498	0.5938
100929	Tyw1	tRNA-yW synthesizing protein 1 homolog (S. cerevisiae)	0.7539	0.0005172	0.1365
319626	9530059O14Rik	RIKEN cDNA 9530059O14 gene	0.7538	0.03826	0.4197
51998	D17Ertd191e	DNA segment, Chr 17, ERATO Doi 191, expressed	0.7535	0.004527	0.2618
75884	4930572J10Rik	RIKEN cDNA 4930572J10 gene	0.7534	0.01173	0.3214
67133	Gp2	glycoprotein 2 (zymogen granule membrane)	0.7534	0.6743	0.8924
214162	Mll1	myeloid/lymphoid or mixed-lineage leukemia 1	0.7531	0.1808	0.6199
75299	4930547M16Rik	RIKEN cDNA 4930547M16 gene	0.7531	0.2845	0.6977
51795	Srpx	sushi-repeat-containing protein	0.753	0.004231	0.2553
399635	D230044B12Rik	RIKEN cDNA D230044B12 gene	0.7525	0.001119	0.1616
100043387	100043387	predicted gene, 100043387	0.7524	0.2369	0.6659
51929	D1Ertd704e	DNA segment, Chr 1, ERATO Doi 704, expressed	0.7523	0.1514	0.5947
93969	Klra22	killer cell lectin-like receptor subfamily A, member 22	0.7522	0.4365	0.7888
241627	Wdr76	WD repeat domain 76	0.7521	0.07455	0.4974
226781	Slc30a10	solute carrier family 30, member 10	0.752	0.1165	0.5581
402775	D130095D21Rik	RIKEN cDNA D130095D21 gene	0.7519	0.003922	0.2463
69116	Ubr4	ubiquitin protein ligase E3 component n-recognin 4	0.7518	0.001568	0.1903
209018	Vps8	vacuolar protein sorting 8 homolog (S. cerevisiae)	0.7517	0.005822	0.2775
72349	Dusp3	dual specificity phosphatase 3 (vaccinia virus phosphatase VH1-related)	0.7517	0.1305	0.5747
552875	AU015680	expressed sequence AU015680	0.7516	0.02646	0.3806
380714	Rph3al	rabphilin 3A-like (without C2 domains)	0.7513	0.3264	0.7228
212190	Ubxn10	UBX domain protein 10	0.7513	0.1637	0.6074
67420	Far1	fatty acyl CoA reductase 1	0.7512	0.06672	0.4848
109658	Txlna	taxilin alpha	0.7511	0.3027	0.7087
76918	3110001N23Rik	RIKEN cDNA 3110001N23 gene	0.7511	0.06275	0.4771
259058	Olfr646	olfactory receptor 646	0.7509	0.5915	0.8582
237886	Slfn9	schlafen 9	0.7509	0.2898	0.7004
22283	Ush2a	Usher syndrome 2A (autosomal recessive, mild) homolog (human)	0.7509	0.01305	0.3305
70557	5730416O20Rik	RIKEN cDNA 5730416O20 gene	0.7506	0.0171	0.3443
219181	Akap11	A kinase (PRKA) anchor protein 11	0.7505	0.1178	0.5609
666257	Gm8008	predicted gene 8008	0.7501	0.06917	0.4872
13656	Egr4	early growth response 4	0.7499	0.2127	0.6453
21784	Tff1	trefoil factor 1	0.7498	0.4996	0.8197
17756	Mtap2	microtubule-associated protein 2	0.7498	0.09319	0.5291
232339	Ankrd26	ankyrin repeat domain 26	0.7497	0.02009	0.3604
68655	Fndc1	fibronectin type III domain containing 1	0.7496	0.02774	0.3875
66921	Prpf38b	PRP38 pre-mRNA processing factor 38 (yeast) domain containing B	0.7496	0.2092	0.6409
216810	Tom1l2	target of myb1-like 2 (chicken)	0.7492	0.1258	0.5703
69256	Zfp397	zinc finger protein 397	0.749	0.01209	0.3236
56551	Txn2	thioredoxin 2	0.749	0.02161	0.367
77779	A930007I19Rik	RIKEN cDNA A930007I19 gene	0.7486	0.08509	0.5166
70090	2310005E17Rik	RIKEN cDNA 2310005E17 gene	0.7483	0.408	0.773
11941	Atp2b2	ATPase, Ca++ transporting, plasma membrane 2	0.7483	0.01248	0.3265
100041576	Gm3414	predicted gene 3414	0.7482	0.03599	0.4133
68168	A930009E08Rik	RIKEN cDNA A930009E08 gene	0.7475	0.01024	0.3117

17475	Mpdz	multiple PDZ domain protein	0.7475	0.0362	0.4143
231004	Samd11	sterile alpha motif domain containing 11	0.7474	0.00517	0.2707
14810	Grin1	glutamate receptor, ionotropic, NMDA1 (zeta 1)	0.7472	0.2682	0.6871
28022	D5Wsu152e	DNA segment, Chr 5, Wayne State University 152, expressed	0.747	0.3367	0.7305
665211	Gm14326	predicted gene 14326	0.7469	0.1709	0.6118
246048	Chodl	chondrolectin	0.7468	0.3721	0.7535
11637	Ak2	adenylate kinase 2	0.7468	0.03544	0.4117
214552	Cep164	centrosomal protein 164	0.7467	0.005801	0.2775
75605	Kdm5b	lysine (K)-specific demethylase 5B	0.7467	0.001257	0.1686
170707	Usp48	ubiquitin specific peptidase 48	0.7464	0.101	0.5394
319675	5830418K08Rik	RIKEN cDNA 5830418K08 gene	0.7463	0.01356	0.3343
20448	St6galnac4	ST6 (alpha-N-acetyl-neuraminyl-2,3-beta-galactosyl-1,3)-N-acetylgalactosaminide alpha-2,6-sialyltransferase 4	0.7462	0.0391	0.4217
77574	Fam115a	family with sequence similarity 115, member A	0.7459	0.0678	0.4855
28129	D11Wsu173e	DNA segment, Chr 11, Wayne State University 173, expressed	0.7458	0.02612	0.3791
18195	Nsf	N-ethylmaleimide sensitive fusion protein	0.7456	0.003573	0.2407
24132	Zfp53	zinc finger protein 53	0.7455	0.03516	0.4105
71398	5430427O19Rik	RIKEN cDNA 5430427O19 gene	0.7452	0.0584	0.4703
100041377	Gm3300	predicted gene 3300	0.7451	0.2388	0.6671
77391	9530003O04Rik	RIKEN cDNA 9530003O04 gene	0.7451	0.06666	0.4848
73712	Dmkn	dermokine	0.7451	0.1947	0.6304
434341	Nlrc5	NLR family, CARD domain containing 5	0.7449	0.333	0.728
17229	Tpsb2	tryptase beta 2	0.7449	0.02465	0.3786
108989	Tpr	translocated promoter region	0.7448	0.2765	0.6925
75984	5031415H12Rik	RIKEN cDNA 5031415H12 gene	0.7448	0.01389	0.3347
56215	Acin1	apoptotic chromatin condensation inducer 1	0.7447	0.2256	0.654
66356	2310008H09Rik	RIKEN cDNA 2310008H09 gene	0.7443	0.05338	0.4572
77951	Cyp20a1	cytochrome P450, family 20, subfamily A, polypeptide 1	0.7442	0.04783	0.4443
55983	Pdzrn3	PDZ domain containing RING finger 3	0.744	0.06798	0.4858
329954	Catsper4	cation channel, sperm associated 4	0.7439	0.0653	0.4816
15370	Nr4a1	nuclear receptor subfamily 4, group A, member 1	0.7437	0.1648	0.6077
19736	Rgs4	regulator of G-protein signaling 4	0.7436	0.0008506	0.148
320460	Vwc2l	von Willebrand factor C domain-containing protein 2-like	0.7435	0.0578	0.469
20401	Sh3bp1	SH3-domain binding protein 1	0.7435	0.03556	0.4117
264895	Acsf2	acyl-CoA synthetase family member 2	0.743	0.06712	0.4855
67441	Isoc2b	isochorismatase domain containing 2b	0.7429	0.1008	0.5394
66840	Wdr45l	Wdr45 like	0.7429	0.05992	0.4728
56376	Pdlim5	PDZ and LIM domain 5	0.7428	0.04069	0.4258
12823	Col19a1	collagen, type XIX, alpha 1	0.7428	0.007728	0.2969
328087	D930001B02	hypothetical protein D930001B02	0.7426	0.002144	0.2121
327942	Pigl	phosphatidylinositol glycan anchor biosynthesis, class L	0.7423	0.2527	0.6778
67939	Prorsd1	prolyl-tRNA synthetase domain containing 1	0.7423	0.004509	0.2618
235682	Zfp445	zinc finger protein 445	0.7422	0.03639	0.4154
70592	5730480H06Rik	RIKEN cDNA 5730480H06 gene	0.742	0.01029	0.3117
56790	Fam48a	family with sequence similarity 48, member A	0.742	0.1599	0.6042
12523	Cd84	CD84 antigen	0.742	0.01534	0.3403
230735	Epha10	Eph receptor A10	0.7418	0.02434	0.3777
107476	Acaca	acetyl-Coenzyme A carboxylase alpha	0.7416	0.09915	0.5367
93717	Pcdhga9	protocadherin gamma subfamily A, 9	0.7416	0.09133	0.5237
100039631	Gm2347	predicted gene 2347	0.7414	0.09072	0.5229

98685	1190005F20Rik	RIKEN cDNA 1190005F20 gene	0.7414	0.1014	0.5395
21406	Tcf12	transcription factor 12	0.7414	0.03969	0.4222
654439	D430004P15Rik	Riken cDNA D430004P15 gene	0.7413	0.01157	0.3208
258943	Olfr353	olfactory receptor 353	0.7413	0.5577	0.8454
232836	Galp	galanin-like peptide	0.7412	0.1211	0.5649
97868	C78704	expressed sequence C78704	0.7412	0.1274	0.5716
77938	Fam53b	family with sequence similarity 53, member B	0.741	0.005984	0.2803
258290	Olfr1143	olfactory receptor 1143	0.7409	0.2108	0.6426
238386	Btbd7	BTB (POZ) domain containing 7	0.7408	0.07217	0.4941
237943	Gpatch8	G patch domain containing 8	0.7408	0.001849	0.2019
319636	Fsd1l	fibronectin type III and SPRY domain containing 1-like	0.7407	0.01037	0.3117
237782	Smcr8	Smith-Magenis syndrome chromosome region, candidate 8 homolog (human)	0.7407	0.193	0.6291
74354	Lrguk	leucine-rich repeats and guanylate kinase domain containing	0.7407	0.01818	0.3486
207375	Fam120c	family with sequence similarity 120, member C	0.7405	0.06748	0.4855
14106	Foxh1	forkhead box H1	0.7405	0.2646	0.6845
53620	Vamp5	vesicle-associated membrane protein 5	0.7404	0.04497	0.4353
100040086	Gm2590	predicted gene 2590	0.7403	0.01401	0.3347
56715	Rabgef1	RAB guanine nucleotide exchange factor (GEF) 1	0.7403	0.2313	0.6608
269774	Aak1	AP2 associated kinase 1	0.7401	0.1336	0.5774
19250	Ptpn14	protein tyrosine phosphatase, non-receptor type 14	0.7401	0.01123	0.3181
625175	1700028E10Rik	RIKEN cDNA 1700028E10 gene	0.74	0.0319	0.3992
216274	Cep290	centrosomal protein 290	0.74	0.04518	0.4353
71389	Chd6	chromodomain helicase DNA binding protein 6	0.7399	0.08611	0.517
380912	Zfp395	zinc finger protein 395	0.7398	0.002768	0.2224
381022	Mll2	myeloid/lymphoid or mixed-lineage leukemia 2	0.7396	0.0749	0.4983
15962	Ifna1	interferon alpha 1	0.7393	0.03071	0.3964
73617	1700122H20Rik	RIKEN cDNA 1700122H20 gene	0.739	0.6201	0.8716
11908	Atf1	activating transcription factor 1	0.739	0.02705	0.3847
68721	1110032A03Rik	RIKEN cDNA 1110032A03 gene	0.7388	0.2705	0.6886
14673	Gna12	guanine nucleotide binding protein, alpha 12	0.7386	0.2604	0.6815
230249	AI314180	expressed sequence AI314180	0.7385	0.02492	0.3786
229937	Znhit6	zinc finger, HIT type 6	0.7385	0.09707	0.5347
320253	Mar-03	membrane-associated ring finger (C3HC4) 3	0.7383	0.1618	0.607
102991	AU022751	expressed sequence AU022751	0.7382	0.1008	0.5394
666190	Gm7972	predicted gene 7972	0.738	0.09593	0.5344
102623	AI414108	expressed sequence AI414108	0.7375	0.1788	0.6181
19652	Rbm3	RNA binding motif protein 3	0.7373	0.1384	0.5825
100043381	Gm14308	predicted gene 14308	0.737	0.2062	0.6388
22283	Ush2a	Usher syndrome 2A (autosomal recessive, mild) homolog (human)	0.7369	0.04802	0.4443
100504146	LOC100504146	zinc finger protein 709-like	0.7368	0.4269	0.7825
75725	Phf14	PHD finger protein 14	0.7367	0.01984	0.3601
73103	3110009E18Rik	RIKEN cDNA 3110009E18 gene	0.7366	0.03197	0.3992
15974	Ifnab	interferon alpha B	0.7366	0.4432	0.7931
100043089	Gm4221	predicted gene 4221	0.7364	0.05174	0.4525
76055	Mgea5	meningioma expressed antigen 5 (hyaluronidase)	0.7364	0.1164	0.5581
11994	Pcdh15	protocadherin 15	0.7364	0.1374	0.581
76251	0610007P08Rik	RIKEN cDNA 0610007P08 gene	0.7363	0.2578	0.6815
234549	Heatr3	HEAT repeat containing 3	0.7362	0.03881	0.4213
71521	Pds5a	PDS5, regulator of cohesion maintenance, homolog A (S. cerevisiae)	0.7361	0.3715	0.7535

26558	Homer3	homer homolog 3 (Drosophila)	0.7361	0.02181	0.3688
66457	2810002D19Rik	RIKEN cDNA 2810002D19 gene	0.7358	0.01254	0.327
665211	Gm14326	predicted gene 14326	0.7357	0.1752	0.6156
236915	Arhgef9	CDC42 guanine nucleotide exchange factor (GEF) 9	0.7356	0.1437	0.5881
403171	Banf2	barrier to autointegration factor 2	0.7355	0.2804	0.6954
110385	Pde4c	phosphodiesterase 4C, cAMP specific	0.7354	0.2153	0.6479
66195	Lce1g	late cornified envelope 1G	0.7354	0.583	0.8538
668880	Stard9	START domain containing 9	0.7352	0.002112	0.2121
329002	Zfp236	zinc finger protein 236	0.7351	0.04233	0.4301
68397	0610039H22Rik	RIKEN cDNA 0610039H22 gene	0.7344	0.007926	0.3002
109314	Prr9	proline rich 9	0.7342	0.06981	0.4885
674419	Rpl7a-ps5	ribosomal protein L7A, pseudogene 5	0.7341	0.3256	0.7222
76093	5830469G19Rik	RIKEN cDNA 5830469G19 gene	0.7338	0.001794	0.1999
69593	2310026I22Rik	RIKEN cDNA 2310026I22 gene	0.7337	0.04608	0.4382
207592	Tbc1d16	TBC1 domain family, member 16	0.7336	0.07292	0.4952
235582	Glyctk	glycerate kinase	0.7335	0.04598	0.4381
70831	Krtap31-1	keratin associated protein 31-1	0.7333	0.04989	0.4509
18087	Nktr	natural killer tumor recognition sequence	0.7333	0.03217	0.4003
621699	Gm6252	predicted gene 6252	0.7331	0.2916	0.7018
218343	Ttc37	tetratricopeptide repeat domain 37	0.7331	0.04152	0.4282
224014	Fgd4	FYVE, RhoGEF and PH domain containing 4	0.7329	0.1326	0.5771
52682	D11Ertd726e	DNA segment, Chr 11, ERATO Doi 726, expressed	0.7328	0.0007268	0.1472
20471	Six1	sine oculis-related homeobox 1 homolog (Drosophila)	0.7328	0.0009052	0.1513
170643	Kirrel	kin of IRRE like (Drosophila)	0.7327	0.007717	0.2969
232371	C1rl	complement component 1, r subcomponent-like	0.7326	0.297	0.7051
105260	D930043N17Rik	RIKEN cDNA D930043N17 gene	0.7326	0.007002	0.2924
100503757	LOC100503757	putative transposase element L1Md-A101/L1Md-A102/L1Md-A2-like	0.7325	0.04207	0.4285
628239	LOC628239	zinc finger protein 14-like	0.7325	0.1507	0.5938
269784	Cntn4	contactin 4	0.7323	0.02569	0.3786
227707	BC005624	cDNA sequence BC005624	0.7323	0.0247	0.3786
27278	Clnk	cytokine-dependent hematopoietic cell linker	0.7323	0.1781	0.6174
232889	Pla2g4c	phospholipase A2, group IVC (cytosolic, calcium-independent)	0.7322	0.1596	0.6042
12968	Cryge	crystallin, gamma E	0.7322	0.1412	0.5861
70885	Ints10	integrator complex subunit 10	0.7321	0.03073	0.3964
108816	4933409K07Rik	RIKEN cDNA 4933409K07 gene	0.7319	0.03563	0.412
258728	Olfr482	olfactory receptor 482	0.7318	0.1506	0.5938
328643	Vwa5b2	von Willebrand factor A domain containing 5B2	0.7317	0.2155	0.6479
78878	B230206F22Rik	RIKEN cDNA B230206F22 gene	0.7317	0.1303	0.5747
207596	Thsd4	thrombospondin, type I, domain containing 4	0.7315	0.04752	0.4442
75787	4930471M09Rik	RIKEN cDNA 4930471M09 gene	0.7315	0.005245	0.2718
319236	9230105E10Rik	RIKEN cDNA 9230105E10 gene	0.731	0.09946	0.5367
12519	Cd80	CD80 antigen	0.7306	0.02582	0.3786
328417	Parp4	poly (ADP-ribose) polymerase family, member 4	0.7305	0.00325	0.227
270163	Myo9a	myosin IXa	0.7305	0.2117	0.6443
548102	LOC548102	hypothetical LOC548102	0.7303	0.02358	0.375
99650	4933434E20Rik	RIKEN cDNA 4933434E20 gene	0.7302	0.03502	0.4097
435791	Gm13271	predicted gene 13271	0.7301	0.01401	0.3347
67375	Qprt	quinolinate phosphoribosyltransferase	0.73	0.5702	0.8492
100042166	Gm3704	predicted gene 3704	0.7297	0.1921	0.6281

12986	Csf3r		colony stimulating factor 3 receptor (granulocyte)	0.7297	0.02039	0.3609
12823	Col19a1		collagen, type XIX, alpha 1	0.7296	0.002283	0.2163
72925		Mar-01	membrane-associated ring finger (C3HC4) 1	0.7293	0.06479	0.4816
109359	Fam175b		family with sequence similarity 175, member B	0.7291	0.03844	0.4205
228859	Fitm2		fat storage-inducing transmembrane protein 2	0.7289	0.01376	0.3347
94090	Trim9		tripartite motif-containing 9	0.7287	0.01294	0.3304
210104	Zfp658		zinc finger protein 658	0.7285	0.1148	0.5569
98514	AU015232		expressed sequence AU015232	0.7284	0.01407	0.3347
16210	Impact		imprinted and ancient	0.7282	0.24	0.6685
100042588	Gm3916		predicted gene 3916	0.7281	0.003496	0.2375
67225	Rnpc3		RNA-binding region (RNP1, RRM) containing 3	0.728	0.1045	0.5445
50720	Sacs		sacsin	0.7278	0.01354	0.3343
100040412	LOC100040412		lysine-specific demethylase 5D-like	0.7277	0.1806	0.6198
80877	Lrba		LPS-responsive beige-like anchor	0.7277	0.01494	0.3382
109200	A430102J17Rik		RIKEN cDNA A430102J17 gene	0.7276	0.01984	0.3601
66756	4933411K20Rik		RIKEN cDNA 4933411K20 gene	0.7276	0.02468	0.3786
431706	Zfp457		zinc finger protein 457	0.7274	0.03692	0.4161
319457	C130045F17Rik		RIKEN cDNA C130045F17 gene	0.7274	0.03446	0.4091
320719	6030443J06Rik		RIKEN cDNA 6030443J06 gene	0.7272	0.1667	0.6083
328516	D430034N21		hypothetical protein D430034N21	0.7271	0.04902	0.4469
224139	Golgb1		golgi autoantigen, golgin subfamily b, macrogolgin 1	0.727	0.1169	0.5592
67768	N6amt1		N-6 adenine-specific DNA methyltransferase 1 (putative)	0.727	0.007022	0.2925
319276	A230038B01Rik		RIKEN cDNA A230038B01 gene	0.7268	0.1707	0.6116
68152	Fam133b		family with sequence similarity 133, member B	0.7268	0.08877	0.5209
279610	E530001F21Rik		RIKEN cDNA E530001F21 gene	0.7265	0.01597	0.3408
14768	Lancl1		LanC (bacterial lantibiotic synthetase component C)-like 1	0.7265	0.3721	0.7535
544678	2010015L04Rik		RIKEN cDNA 2010015L04 gene	0.7264	0.009511	0.3112
18992	Pou3f2		POU domain, class 3, transcription factor 2	0.7258	0.05906	0.4726
68564	Nufip2		nuclear fragile X mental retardation protein interacting protein 2	0.7256	0.02623	0.3791
399101	Snhg3		small nucleolar RNA host gene (non-protein coding) 3	0.7251	0.09344	0.5294
212124	E030019B06Rik		RIKEN cDNA E030019B06 gene	0.7251	0.004877	0.2652
665001	Gm14391		predicted gene 14391	0.725	0.1444	0.589
381591	L1td1		LINE-1 type transposase domain containing 1	0.725	0.008154	0.3021
242126	Slc22a15		solute carrier family 22 (organic anion/cation transporter), member 15	0.725	0.3	0.706
320141	9330156P08Rik		RIKEN cDNA 9330156P08 gene	0.7249	0.005756	0.2775
70717	6330406I15Rik		RIKEN cDNA 6330406I15 gene	0.7249	0.03754	0.417
13112	Cyp3a11		cytochrome P450, family 3, subfamily a, polypeptide 11	0.7249	0.1686	0.6096
73225	Fam118a		family with sequence similarity 118, member A	0.7247	0.1268	0.5709
20265	Scn1a		sodium channel, voltage-gated, type I, alpha	0.7245	0.099	0.5367
11994	Pcdh15		protocadherin 15	0.7245	0.02067	0.3609
74469	Taf7l		TAF7-like RNA polymerase II, TATA box binding protein (TBP)-associated factor	0.7243	0.05622	0.4631
78609	8030411F24Rik		RIKEN cDNA 8030411F24 gene	0.7242	0.1215	0.5651
67658	4930563F15Rik		RIKEN cDNA 4930563F15 gene	0.7241	0.122	0.5652
67225	Rnpc3		RNA-binding region (RNP1, RRM) containing 3	0.7239	0.05881	0.4712
50774	Krtap5-1		keratin associated protein 5-1	0.7234	0.2212	0.6525
53961	9530053J19Rik		RIKEN cDNA 9530053J19 gene	0.7233	0.01507	0.3388
75050	Kif27		kinesin family member 27	0.723	0.3412	0.7334
73020	2900073C17Rik		RIKEN cDNA 2900073C17 gene	0.7229	0.02476	0.3786
414084	Tnip3		TNFAIP3 interacting protein 3	0.7227	0.2324	0.6622

353208	Zfp931	zinc finger protein 931	0.7227	0.1342	0.5779
15109	Hal	histidine ammonia lyase	0.722	0.3171	0.7176
320032	C130093G08Rik	RIKEN cDNA C130093G08 gene	0.7219	0.0005659	0.1378
75693	3010001F23Rik	RIKEN cDNA 3010001F23 gene	0.7219	0.03255	0.4018
399614	C130009A20Rik	RIKEN cDNA C130009A20 gene	0.7215	0.0001559	0.09489
66329	Susd3	sushi domain containing 3	0.7215	0.6363	0.8774
18813	Pa2g4	proliferation-associated 2G4	0.7212	0.01761	0.3469
75739	Mpp7	membrane protein, palmitoylated 7 (MAGUK p55 subfamily member 7)	0.7205	0.01608	0.3408
73712	Dmkn	dermokine	0.7205	0.09097	0.5229
434232	Iqck	IQ motif containing K	0.7204	0.03068	0.3964
621852	Rhox3f	reproductive homeobox 3F	0.72	0.1422	0.5871
19266	Ptprd	protein tyrosine phosphatase, receptor type, D	0.72	0.1617	0.607
320202	Lefty2	left-right determination factor 2	0.7194	0.01669	0.3422
243842	Gltscr1	glioma tumor suppressor candidate region gene 1	0.7192	0.2737	0.6913
100503757	LOC100503757	putative transposase element L1Md-A101/L1Md-A102/L1Md-A2-like	0.7191	0.135	0.5784
106258	AI790442	expressed sequence AI790442	0.7191	0.3096	0.7129
69207	Srsf11	serine/arginine-rich splicing factor 11	0.7191	0.04397	0.4336
320327	A430106G13Rik	RIKEN cDNA A430106G13 gene	0.7189	0.002628	0.2224
434768	Rhox8	reproductive homeobox 8	0.7187	0.01218	0.3236
78202	4930557K07Rik	RIKEN cDNA 4930557K07 gene	0.7184	0.1739	0.6148
320692	9430037G07Rik	RIKEN cDNA 9430037G07 gene	0.718	0.0128	0.33
72267	Lrrc8e	leucine rich repeat containing 8 family, member E	0.7179	0.06018	0.4728
245596	Hdx	highly divergent homeobox	0.7178	0.07606	0.5008
75547	Akap13	A kinase (PRKA) anchor protein 13	0.7178	0.03937	0.4222
399604	C530014P21Rik	RIKEN cDNA C530014P21 gene	0.7175	0.01227	0.3236
319707	C430002N11Rik	RIKEN cDNA C430002N11 gene	0.7175	0.4803	0.8101
233987	Zfp958	zinc finger protein 958	0.7175	0.04566	0.4375
76798	2410137F16Rik	RIKEN cDNA 2410137F16 gene	0.7172	0.01648	0.3416
74051	Steap2	six transmembrane epithelial antigen of prostate 2	0.7172	0.04256	0.4301
102366	BB211804	expressed sequence BB211804	0.717	0.03491	0.4096
666747	Trim43b	tripartite motif-containing 43B	0.7169	0.2845	0.6977
171530	Ucn2	urocortin 2	0.7169	0.2336	0.6626
30959	Ddx25	DEAD (Asp-Glu-Ala-Asp) box polypeptide 25	0.7168	0.1182	0.5609
68026	2810417H13Rik	RIKEN cDNA 2810417H13 gene	0.7167	0.02563	0.3786
99458	BB166591	expressed sequence BB166591	0.7164	0.007579	0.294
77241	9430013L14Rik	RIKEN cDNA 9430013L14 gene	0.7162	0.02392	0.377
552873	LOC552873	hypothetical LOC552873	0.7161	0.01993	0.3604
78713	D530017H19Rik	RIKEN cDNA D530017H19 gene	0.7161	0.002539	0.2224
76223	Agbl3	ATP/GTP binding protein-like 3	0.716	0.01063	0.3138
229279	Hnrnpa3	heterogeneous nuclear ribonucleoprotein A3	0.7153	0.1934	0.6295
69479	1700029J07Rik	RIKEN cDNA 1700029J07 gene	0.7151	0.01571	0.3403
503851	B930025B16Rik	RIKEN cDNA B930025B16 gene	0.715	0.0003587	0.1365
52902	D10Bwg1070e	DNA segment, Chr 10, Brigham & Women's Genetics 1070 expressed	0.715	0.04248	0.4301
319254	9630039A02Rik	RIKEN cDNA 9630039A02 gene	0.7146	0.1455	0.5906
242602	BC055111	cDNA sequence BC055111	0.7143	0.382	0.7596
72719	2810040C05Rik	RIKEN cDNA 2810040C05 gene	0.7143	0.004618	0.2642
320262	A830073O21Rik	RIKEN cDNA A830073O21 gene	0.7142	0.2181	0.6502
226151	Fam178a	family with sequence similarity 178, member A	0.7142	0.07903	0.5062
194401	Mical3	microtubule associated monoxygenase, calponin and LIM domain containing 3	0.7142	0.0835	0.5144

70537	5730419F03Rik	RIKEN cDNA 5730419F03 gene	0.7137	0.3036	0.709
12969	Crygf	crystallin, gamma F	0.7134	0.2603	0.6815
30046	Zfp292	zinc finger protein 292	0.7133	0.002547	0.2224
18781	Pla2g2c	phospholipase A2, group IIC	0.7133	0.06319	0.478
622473	Ripply1	ripply1 homolog (zebrafish)	0.7132	0.1583	0.6029
63830	Kcnq1ot1	KCNQ1 overlapping transcript 1	0.7132	0.001366	0.1801
235533	Gk5	glycerol kinase 5 (putative)	0.7127	0.03774	0.417
70508	Bbx	bobby sox homolog (Drosophila)	0.7125	0.1318	0.5758
68867	Rnf122	ring finger protein 122	0.712	0.02312	0.3727
101100	Ttll3	tubulin tyrosine ligase-like family, member 3	0.7118	0.04315	0.4317
106967	4732423E21Rik	RIKEN cDNA 4732423E21 gene	0.7115	0.0111	0.3177
320238	A830054O07Rik	RIKEN cDNA A830054O07 gene	0.7114	0.03395	0.4091
216799	Nlrp3	NLR family, pyrin domain containing 3	0.7111	0.1925	0.6283
18106	Cd244	CD244 natural killer cell receptor 2B4	0.7109	0.3132	0.7152
215690	Nav1	neuron navigator 1	0.7107	0.03552	0.4117
237412	Gm4924	predicted gene 4924	0.7105	0.02406	0.377
140481	Man2a2	mannosidase 2, alpha 2	0.7101	0.05996	0.4728
244061	Gm4971	predicted gene 4971	0.7095	0.01436	0.3347
106459	BB163080	expressed sequence BB163080	0.7094	0.08534	0.517
320977	A330023F24Rik	RIKEN cDNA A330023F24 gene	0.7093	0.0007052	0.145
100434	Slc44a1	solute carrier family 44, member 1	0.7093	0.002904	0.2228
76265	Tsen54	tRNA splicing endonuclease 54 homolog (S. cerevisiae)	0.7093	0.02636	0.38
320943	B230213L16Rik	RIKEN cDNA B230213L16 gene	0.7092	0.01512	0.3394
60440	Iigp1	interferon inducible GTPase 1	0.7091	0.222	0.6535
71508	Zfp935	zinc finger protein 935	0.7085	0.04874	0.4464
26875	Pclo	piccolo (presynaptic cytomatrix protein)	0.7085	0.005182	0.2707
233919	Gpr26	G protein-coupled receptor 26	0.7079	0.01607	0.3408
212073	4831426I19Rik	RIKEN cDNA 4831426I19 gene	0.7075	0.000444	0.1365
13521	Slc26a2	solute carrier family 26 (sulfate transporter), member 2	0.7075	0.0009779	0.1524
14531	Gcm1	glial cells missing homolog 1 (Drosophila)	0.707	0.03532	0.411
27493	A230006K03Rik	RIKEN cDNA A230006K03 gene	0.7067	0.008097	0.302
234135	Whsc1l1	Wolf-Hirschhorn syndrome candidate 1-like 1 (human)	0.7065	0.15	0.5938
70753	6330415B21Rik	RIKEN cDNA 6330415B21 gene	0.7065	0.1028	0.5424
67295	Rab3c	RAB3C, member RAS oncogene family	0.7064	0.1146	0.5569
667666	Zfp600	zinc finger protein 600	0.7059	0.1916	0.6278
71059	Hexim2	hexamthylene bis-acetamide inducible 2	0.7059	0.01806	0.3482
76415	Fam187b	family with sequence similarity 187, member B	0.7056	0.1557	0.599
320586	A630089N07Rik	RIKEN cDNA A630089N07 gene	0.7054	0.0009942	0.1528
320615	Dopey1	dopey family member 1	0.7052	0.1482	0.5917
72987	2900057C01Rik	RIKEN cDNA 2900057C01 gene	0.7052	0.02595	0.3786
553096	LOC553096	hypothetical LOC553096	0.7051	0.0001813	0.1025
225659	Cep76	centrosomal protein 76	0.705	0.02012	0.3604
671132	Gm9519	predicted gene 9519	0.7049	0.2862	0.6982
17847	Usp34	ubiquitin specific peptidase 34	0.7046	0.04598	0.4381
18214	Ddr2	discoidin domain receptor family, member 2	0.7045	0.07402	0.4961
12050	Bcl2l2	BCL2-like 2	0.7038	0.5598	0.8459
102234	AU022229	expressed sequence AU022229	0.7036	0.005138	0.2707
639396	Gm7265	predicted gene 7265	0.7035	0.004883	0.2652
52145	D9Ertd720e	DNA segment, Chr 9, ERATO Doi 720, expressed	0.7032	0.1135	0.5564

12419	Cbx5	chromobox homolog 5 (Drosophila HP1a)	0.7031	0.07007	0.4899
664858	BB094273	expressed sequence BB094273	0.7029	0.1576	0.6017
72459	Htatsf1	HIV TAT specific factor 1	0.7023	0.0492	0.4469
72153	2610020P09Rik	RIKEN cDNA 2610020P09 gene	0.7023	0.07738	0.5026
76784	Mtif2	mitochondrial translational initiation factor 2	0.7019	0.04789	0.4443
237880	1700071K01Rik	RIKEN cDNA 1700071K01 gene	0.7018	0.04603	0.4381
73346	1700039I01Rik	RIKEN cDNA 1700039I01 gene	0.7018	0.1473	0.591
68189	5330431K02Rik	RIKEN cDNA 5330431K02 gene	0.7018	0.001407	0.1836
71449	Mettl13	methyltransferase like 13	0.7017	0.00753	0.294
16353	Ipw	imprinted gene in the Prader-Willi syndrome region	0.7013	0.03775	0.417
66203	Lce1m	late cornified envelope 1M	0.7012	0.2232	0.654
333193	BC053749	cDNA sequence BC053749	0.7011	0.01655	0.3416
52704	D11Ertd717e	DNA segment, Chr 11, ERATO Doi 717, expressed	0.7008	0.0007349	0.1472
268481	Krt222	keratin 222	0.7007	0.01181	0.3223
60532	Wtap	Wilms' tumour 1-associating protein	0.7006	0.007427	0.2934
12804	Cntfr	ciliary neurotrophic factor receptor	0.7002	0.01764	0.3469
77255	9430014F16Rik	RIKEN cDNA 9430014F16 gene	0.6999	0.08102	0.5105
100040346	Gm2724	predicted gene 2724	0.6998	0.1154	0.5578
628779	Hs3st4	heparan sulfate (glucosamine) 3-O-sulfotransferase 4	0.6998	0.06313	0.478
654493	8030494B02Rik	Riken cDNA 8030494B02 gene	0.6993	0.001576	0.1903
100434	Slc44a1	solute carrier family 44, member 1	0.6991	0.009021	0.3086
242474	D730040F13Rik	RIKEN cDNA D730040F13 gene	0.699	0.01783	0.3475
393082	Mettl7a2	methyltransferase like 7A2	0.6987	0.1496	0.5938
14281	Fos	FBJ osteosarcoma oncogene	0.6987	0.08207	0.5139
14255	Flt3	FMS-like tyrosine kinase 3	0.6982	0.006176	0.2833
72580	Zufsp	zinc finger with UFM1-specific peptidase domain	0.6981	0.03481	0.4094
238037	BC068281	cDNA sequence BC068281	0.6976	0.004339	0.2601
668357	Dmrtc1c2	DMRT-like family C1c2	0.6973	0.1387	0.5827
50875	Tmod3	tropomodulin 3	0.697	0.02759	0.3868
319377	D130012P04Rik	RIKEN cDNA D130012P04 gene	0.6968	0.06892	0.487
331046	Tgm4	transglutaminase 4 (prostate)	0.6964	0.06743	0.4855
77799	Sla2	Src-like-adaptor 2	0.6964	0.01222	0.3236
18191	Nrxn3	neurexin III	0.6956	0.00507	0.2706
15122	Hba-a1	hemoglobin alpha, adult chain 1	0.6955	0.2448	0.6726
629581	Gm6985	predicted pseudogene 6985	0.6954	0.3667	0.7504
94246	Arid4b	AT rich interactive domain 4B (RBP1-like)	0.6953	0.04718	0.4435
19276	Ptprn2	protein tyrosine phosphatase, receptor type, N polypeptide 2	0.6951	0.07403	0.4961
381405	Zfp663	zinc finger protein 663	0.6949	0.0176	0.3469
68655	Fndc1	fibronectin type III domain containing 1	0.6949	0.05055	0.4517
100043123	Gm11710	predicted gene 11710	0.6948	0.2188	0.6509
435791	Gm13271	predicted gene 13271	0.6947	0.0113	0.3181
320360	Ric3	resistance to inhibitors of cholinesterase 3 homolog (C. elegans)	0.6947	0.03409	0.4091
77945	Rpgrip1	retinitis pigmentosa GTPase regulator interacting protein 1	0.6946	0.003259	0.227
98541	AU019278	expressed sequence AU019278	0.6943	0.005503	0.2775
12802	Cnr2	cannabinoid receptor 2 (macrophage)	0.6942	0.2796	0.6951
331401	Thoc2	THO complex 2	0.694	0.08357	0.5144
74969	4930466K18Rik	RIKEN cDNA 4930466K18 gene	0.6938	0.1747	0.6149
330602	Gm5115	predicted gene 5115	0.6934	0.002358	0.2166
320731	A530030E21Rik	RIKEN cDNA A530030E21 gene	0.693	0.01364	0.3347

70290	2310079F09Rik	RIKEN cDNA 2310079F09 gene	0.6929	0.07305	0.4952
319888	5330437I02Rik	RIKEN cDNA 5330437I02 gene	0.6928	0.04378	0.4336
97149	C78893	expressed sequence C78893	0.6928	0.04915	0.4469
231760	Rimbp2	RIMS binding protein 2	0.6927	0.02342	0.3748
93871	Brwd1	bromodomain and WD repeat domain containing 1	0.6921	0.09647	0.5345
77188	A430105D02Rik	RIKEN cDNA A430105D02 gene	0.692	0.01658	0.3416
432442	Akap7	A kinase (PRKA) anchor protein 7	0.6918	0.06626	0.4837
56150	Mad2l1	MAD2 mitotic arrest deficient-like 1 (yeast)	0.6916	0.1414	0.5863
232566	Amn1	antagonist of mitotic exit network 1 homolog (S. cerevisiae)	0.6909	0.005604	0.2775
76964	2610028H24Rik	RIKEN cDNA 2610028H24 gene	0.6908	0.07609	0.5008
116811	Zim3	zinc finger, imprinted 3	0.6902	0.4056	0.7717
319742	Mpzl3	myelin protein zero-like 3	0.6899	0.4265	0.7823
81497	Btnl5	butyrophilin-like 5	0.6899	0.01683	0.3424
76265	Tsen54	tRNA splicing endonuclease 54 homolog (S. cerevisiae)	0.6898	0.08587	0.517
100504289	LOC100504289	hypothetical LOC100504289	0.6896	0.01412	0.3347
320495	Ipcef1	interaction protein for cytohesin exchange factors 1	0.6892	0.1065	0.5472
328580	Tubgcp6	tubulin, gamma complex associated protein 6	0.6881	0.09164	0.5244
77492	8030456M14Rik	RIKEN cDNA 8030456M14 gene	0.6881	0.07352	0.4957
77945	Rpgrip1	retinitis pigmentosa GTPase regulator interacting protein 1	0.6876	0.001527	0.1903
52139	D5Ertd560e	DNA segment, Chr 5, ERATO Doi 560, expressed	0.6871	0.01335	0.3338
102220	E330037G11Rik	RIKEN cDNA E330037G11 gene	0.686	0.01787	0.3475
13983	Esr2	estrogen receptor 2 (beta)	0.6858	0.02139	0.3652
13612	Edil3	EGF-like repeats and discoidin I-like domains 3	0.6854	0.07769	0.5026
223773	Zbed4	zinc finger, BED domain containing 4	0.6853	0.2001	0.6345
244059	Chd2	chromodomain helicase DNA binding protein 2	0.6843	0.04035	0.4255
226025	Trpm3	transient receptor potential cation channel, subfamily M, member 3	0.6838	0.09739	0.5352
80901	Cxcr6	chemokine (C-X-C motif) receptor 6	0.6837	0.08754	0.5189
224023	Klhl22	kelch-like 22 (Drosophila)	0.6834	0.1206	0.5643
71823	3300002A11Rik	RIKEN cDNA 3300002A11 gene	0.6834	0.002192	0.2121
20589	Ighmbp2	immunoglobulin mu binding protein 2	0.6833	0.01537	0.3403
56809	Gmeb1	glucocorticoid modulatory element binding protein 1	0.6831	0.0005485	0.1365
319660	Tmem195	transmembrane protein 195	0.6829	0.1127	0.5552
20619	Snap23	synaptosomal-associated protein 23	0.6828	0.01288	0.3303
77614	C030044M21Rik	RIKEN cDNA C030044M21 gene	0.6824	0.02262	0.3702
545238	G430049J08Rik	RIKEN cDNA G430049J08 gene	0.6822	0.024	0.377
20833	Ssrp1	structure specific recognition protein 1	0.6821	0.405	0.7716
13560	E4f1	E4F transcription factor 1	0.6821	0.05076	0.4519
664788	Gm7339	predicted gene 7339	0.6819	0.08893	0.5211
231855	C330006K01Rik	RIKEN cDNA C330006K01 gene	0.6819	0.02474	0.3786
28135	Cep63	centrosomal protein 63	0.6818	0.04328	0.4322
245855	BC026513	cDNA sequence BC026513	0.6815	0.003362	0.2303
65246	Xpo7	exportin 7	0.6814	0.04659	0.441
434825	LOC434825	hypothetical LOC434825	0.6812	0.01456	0.3365
100045736	LOC100045736	hypothetical protein LOC100045736	0.681	0.3213	0.7199
100043229	Gm10046	predicted gene 10046	0.6804	0.1622	0.607
210274	Shank2	SH3/ankyrin domain gene 2	0.6801	0.267	0.6865
11656	Alas2	aminolevulinic acid synthase 2, erythroid	0.6797	0.2895	0.7004
67926	Spert	spermatid associated	0.6795	0.1815	0.6206
100043232	3110099E03Rik	RIKEN cDNA 3110099E03 gene	0.679	0.1134	0.5562

100504699	LOC100504699	hypothetical LOC100504699	0.6786	0.01751	0.3469
320595	Phf8	PHD finger protein 8	0.6785	0.01757	0.3469
320383	B230317F23Rik	RIKEN cDNA B230317F23 gene	0.6785	0.07959	0.5084
654469	LOC654469	hypothetical LOC654469	0.6783	0.1101	0.5535
18616	Peg3	paternally expressed 3	0.6781	0.007137	0.2929
70458	2610318N02Rik	RIKEN cDNA 2610318N02 gene	0.6775	0.3776	0.7567
67118	Bfar	bifunctional apoptosis regulator	0.6772	0.03696	0.4161
664788	Gm7339	predicted gene 7339	0.6769	0.09143	0.5241
72392	Tmem175	transmembrane protein 175	0.6767	0.02554	0.3786
382090	4922501C03Rik	RIKEN cDNA 4922501C03 gene	0.6762	0.1211	0.5649
19276	Ptprn2	protein tyrosine phosphatase, receptor type, N polypeptide 2	0.6761	0.05868	0.4712
665211	Gm14326	predicted gene 14326	0.6754	0.05327	0.4568
100505360	LOC100505360	putative transposase element L1Md-A101/L1Md-A102/L1Md-A2-like	0.6751	0.04451	0.4349
328606	4731420N21	hypothetical protein 4731420N21	0.675	0.002224	0.2121
73874	4930419G24Rik	RIKEN cDNA 4930419G24 gene	0.6747	0.01578	0.3403
328399	A930018M24Rik	RIKEN cDNA A930018M24 gene	0.6744	0.00327	0.227
71930	2310043M15Rik	RIKEN cDNA 2310043M15 gene	0.6738	0.4662	0.8049
71163	Zfp626	zinc finger protein 626	0.6736	0.008126	0.302
109700	Itga1	integrin alpha 1	0.6733	0.05285	0.4558
56522	Papolb	poly (A) polymerase beta (testis specific)	0.6731	0.1467	0.591
17122	Mxd4	Max dimerization protein 4	0.6726	0.01246	0.3264
625175	1700028E10Rik	RIKEN cDNA 1700028E10 gene	0.6724	0.02557	0.3786
99946	6720422M22Rik	RIKEN cDNA 6720422M22 gene	0.6721	0.0022	0.2121
668825	Gm13931	predicted gene 13931	0.6719	0.2333	0.6622
245622	Fam199x	family with sequence similarity 199, X-linked	0.6718	0.1693	0.61
77090	Ocel1	occludin/ELL domain containing 1	0.6717	0.276	0.6925
223922	Atf7	activating transcription factor 7	0.6714	0.01819	0.3486
14219	Ctgf	connective tissue growth factor	0.6714	0.1278	0.5716
75739	Mpp7	membrane protein, palmitoylated 7 (MAGUK p55 subfamily member 7)	0.671	0.002312	0.2165
16874	Lhx6	LIM homeobox protein 6	0.6704	0.006586	0.288
320927	6720474J12Rik	RIKEN cDNA 6720474J12 gene	0.6703	0.01109	0.3177
109332	Cdcp1	CUB domain containing protein 1	0.6702	0.3742	0.7547
319386	D130062J10Rik	RIKEN cDNA D130062J10 gene	0.67	0.0349	0.4096
100041951	Gm3587	predicted gene 3587	0.6699	0.09273	0.5274
319424	E030004H24Rik	RIKEN cDNA E030004H24 gene	0.6699	0.003048	0.2228
21337	Tacr2	tachykinin receptor 2	0.6699	0.01421	0.3347
76464	Casc5	cancer susceptibility candidate 5	0.6698	0.08147	0.5121
27962	D9Wsu90e	DNA segment, Chr 9, Wayne State University 90, expressed	0.6698	0.0955	0.5334
13384	Mpp3	membrane protein, palmitoylated 3 (MAGUK p55 subfamily member 3)	0.6697	0.1239	0.5685
100381258	D930001M23Rik	Riken cDNA D930001M23 gene	0.6696	0.01384	0.3347
71511	9030601B04Rik	RIKEN cDNA 9030601B04 gene	0.6694	0.005986	0.2803
666023	Gm7894	predicted gene 7894	0.6692	0.0773	0.5026
327781	A130033P14	hypothetical protein A130033P14	0.6689	0.04393	0.4336
320319	E330018D03Rik	RIKEN cDNA E330018D03 gene	0.6688	0.002833	0.2228
80901	Cxcr6	chemokine (C-X-C motif) receptor 6	0.6686	0.008788	0.3086
77951	Cyp20a1	cytochrome P450, family 20, subfamily A, polypeptide 1	0.6686	0.01726	0.3452
268890	Lsamp	limbic system-associated membrane protein	0.6684	0.04022	0.425
12776	Ccr8	chemokine (C-C motif) receptor 8	0.6684	0.2162	0.6479
74491	5430420F09Rik	RIKEN cDNA 5430420F09 gene	0.668	0.0005261	0.1365

68672	1110035E04Rik	RIKEN cDNA 1110035E04 gene	0.6679	0.013	0.3305
14131	Fcgr3	Fc receptor, IgG, low affinity III	0.6679	0.1304	0.5747
56752	Aldh9a1	aldehyde dehydrogenase 9, subfamily A1	0.6676	0.3847	0.7606
100038410	Gm10536	predicted gene 10536	0.6675	0.3121	0.7139
622935	Krtap20-2	keratin associated protein 20-2	0.6672	0.06106	0.4744
100505360	LOC100505360	putative transposase element L1Md-A101/L1Md-A102/L1Md-A2-like	0.6671	0.02863	0.39
668253	Dleu2	deleted in lymphocytic leukemia, 2	0.6666	0.01194	0.3235
353211	Prune2	prune homolog 2 (Drosophila)	0.6665	0.05336	0.4572
217305	Cd300ld	CD300 molecule-like family member d	0.6663	0.1504	0.5938
22661	Zfp148	zinc finger protein 148	0.6656	0.02111	0.3629
73722	Lce1a2	late cornified envelope 1A2	0.6653	0.09942	0.5367
319454	A430071A18Rik	RIKEN cDNA A430071A18 gene	0.6642	0.003075	0.2228
67946	Spata6	spermatogenesis associated 6	0.6635	0.05914	0.4727
277854	Depdc5	DEP domain containing 5	0.6634	0.01473	0.3369
22239	Ugt8a	UDP galactosyltransferase 8A	0.6624	0.2069	0.6395
78103	8430431K14Rik	RIKEN cDNA 8430431K14 gene	0.6616	0.01764	0.3469
547431	Btnl2	butyrophilin-like 2	0.6615	0.05655	0.4638
103079	AA408251	expressed sequence AA408251	0.6614	0.07874	0.5052
99980	AA408865	expressed sequence AA408865	0.6606	0.05031	0.4516
333050	Ksr2	kinase suppressor of ras 2	0.6603	0.007118	0.2929
100040990	Gm3081	predicted gene 3081	0.6599	0.02421	0.3771
13423	Dnase2a	deoxyribonuclease II alpha	0.6594	0.1344	0.5779
100041184	Gm3187	predicted gene 3187	0.6592	0.04917	0.4469
100310809	Gm10509	zinc finger protein 51 pseudogene	0.6584	0.003727	0.2432
319506	7530428D23Rik	RIKEN cDNA 7530428D23 gene	0.6584	0.756	0.9223
319271	A130072N09Rik	RIKEN cDNA A130072N09 gene	0.6584	0.09815	0.5365
320171	D130019J16Rik	RIKEN cDNA D130019J16 gene	0.6583	0.01792	0.3475
29820	Tnfrsf19	tumor necrosis factor receptor superfamily, member 19	0.6577	0.1617	0.607
72386	2610035D17Rik	RIKEN cDNA 2610035D17 gene	0.6572	0.1708	0.6118
20592	Kdm5d	lysine (K)-specific demethylase 5D	0.6571	0.1475	0.591
67507	1700019N19Rik	RIKEN cDNA 1700019N19 gene	0.6562	0.4438	0.7931
15932	Idua	iduronidase, alpha-L-	0.6559	0.02364	0.3753
78439	A930037H05Rik	RIKEN cDNA A930037H05 gene	0.6558	0.01587	0.3406
219150	Hmbox1	homeobox containing 1	0.6557	0.1312	0.5751
434166	Gm5589	predicted gene 5589	0.6555	0.194	0.6299
83813	Tnk1	tyrosine kinase, non-receptor, 1	0.6555	0.1715	0.6124
76671	5330406M23Rik	RIKEN cDNA 5330406M23 gene	0.6553	0.007165	0.2929
20505	Slc34a1	solute carrier family 34 (sodium phosphate), member 1	0.6553	0.1199	0.5637
17364	Trpm1	transient receptor potential cation channel, subfamily M, member 1	0.6547	0.02802	0.3875
18390	Oprm1	opioid receptor, mu 1	0.6546	0.01887	0.353
327780	D430050E20Rik	RIKEN cDNA D430050E20 gene	0.6544	0.01864	0.3521
320057	A630057N01Rik	RIKEN cDNA A630057N01 gene	0.6544	0.002122	0.2121
246738	Dnajc28	DnaJ (Hsp40) homolog, subfamily C, member 28	0.6541	0.02537	0.3786
101100	Ttll3	tubulin tyrosine ligase-like family, member 3	0.6531	0.0008552	0.148
654438	A630031M23Rik	Riken cDNA A630031M23 gene	0.653	0.03436	0.4091
66399	Tsfm	Ts translation elongation factor, mitochondrial	0.6528	0.1099	0.5535
13176	Dcc	deleted in colorectal carcinoma	0.6526	0.02209	0.3702
15565	Htr6	5-hydroxytryptamine (serotonin) receptor 6	0.652	0.3405	0.7328
208982	Hmgcll1	3-hydroxymethyl-3-methylglutaryl-Coenzyme A lyase-like 1	0.6515	0.003832	0.2455

72343	2600002B07Rik	RIKEN cDNA 2600002B07 gene	0.651	0.04793	0.4443
99229	AU041474	expressed sequence AU041474	0.6504	0.05509	0.4616
110789	Gpr98	G protein-coupled receptor 98	0.6501	0.02664	0.3809
77333	C030007I01Rik	RIKEN cDNA C030007I01 gene	0.6498	0.01112	0.3178
19211	Pten	phosphatase and tensin homolog	0.649	0.05187	0.4525
68750	Rreb1	ras responsive element binding protein 1	0.6487	0.009622	0.3112
16409	Itgam	integrin alpha M	0.6478	0.02856	0.3896
231004	Samd11	sterile alpha motif domain containing 11	0.6477	0.0103	0.3117
78279	5330421C15Rik	RIKEN cDNA 5330421C15 gene	0.6477	0.03355	0.4072
67295	Rab3c	RAB3C, member RAS oncogene family	0.6476	0.1234	0.568
73796	4930402F11Rik	RIKEN cDNA 4930402F11 gene	0.6475	0.4658	0.8049
399602	9930024M15Rik	RIKEN cDNA 9930024M15 gene	0.6471	0.0002563	0.1196
235504	Slc17a5	solute carrier family 17 (anion/sugar transporter), member 5	0.6468	0.02501	0.3786
64654	Fgf23	fibroblast growth factor 23	0.6466	0.02887	0.3903
101028	BB070754	expressed sequence BB070754	0.6461	0.05125	0.4519
319864	D230035N22Rik	RIKEN cDNA D230035N22 gene	0.6457	0.2618	0.6831
56066	Cxcl11	chemokine (C-X-C motif) ligand 11	0.6457	0.1324	0.5768
654822	D330041H03Rik	RIKEN cDNA D330041H03 gene	0.6455	0.03136	0.3964
319366	C920008N22Rik	RIKEN cDNA C920008N22 gene	0.6453	0.0004863	0.1365
56397	Morf4l2	mortality factor 4 like 2	0.6445	0.08575	0.517
414326	D930050J11	hypothetical D930050J11	0.6441	0.002557	0.2224
234421	Cib3	calcium and integrin binding family member 3	0.6423	0.2903	0.7006
328456	6720482D04	hypothetical protein 6720482D04	0.6407	0.008797	0.3086
78233	6330522J23Rik	RIKEN cDNA 6330522J23 gene	0.6406	0.0008861	0.1508
234912	9230110C19Rik	RIKEN cDNA 9230110C19 gene	0.6401	0.1434	0.5879
319319	B230214O09Rik	RIKEN cDNA B230214O09 gene	0.6397	0.119	0.5626
12829	Col4a4	collagen, type IV, alpha 4	0.6394	0.09286	0.5277
19120	Prm3	protamine 3	0.6391	0.4194	0.7793
20753	Sprr1a	small proline-rich protein 1A	0.6373	0.006265	0.2842
216971	BC017647	cDNA sequence BC017647	0.6365	0.0627	0.4771
15129	Hbb-b1	hemoglobin, beta adult major chain	0.6362	0.1101	0.5535
239839	Ccdc14	coiled-coil domain containing 14	0.6357	0.04477	0.4353
11994	Pcdh15	protocadherin 15	0.6357	0.1135	0.5564
320583	C530030K21Rik	RIKEN cDNA C530030K21 gene	0.6355	0.02383	0.3768
99171	AU015584	expressed sequence AU015584	0.635	0.1287	0.5733
319893	A230057D06Rik	RIKEN cDNA A230057D06 gene	0.6345	0.03035	0.3954
23827	Bpnt1	bisphosphate 3'-nucleotidase 1	0.6345	0.07202	0.4941
14864	Gstm3	glutathione S-transferase, mu 3	0.6337	0.5377	0.8359
76359	2610028D06Rik	RIKEN cDNA 2610028D06 gene	0.6336	0.0002329	0.1196
100039855	Gm2461	predicted gene 2461	0.6331	0.009955	0.3112
671564	Rnf212	ring finger protein 212	0.633	0.1631	0.6074
622408	Gm6320	predicted gene 6320	0.6323	0.02042	0.3609
69131	Cdk12	cyclin-dependent kinase 12	0.6321	0.06224	0.4771
625530	Dub3	deubiquitinating enzyme 3	0.6318	0.6777	0.8931
228714	Csrp2bp	cysteine and glycine-rich protein 2 binding protein	0.6315	0.112	0.5549
237886	Slfn9	schlafen 9	0.6314	0.1194	0.5628
100041864	Gm3552	predicted gene 3552	0.6312	0.04719	0.4435
64095	Gpr35	G protein-coupled receptor 35	0.6312	0.3302	0.726
19672	Rcn1	reticulocalbin 1	0.631	0.4216	0.7798

100041098	Gm15431	predicted gene 15431	0.6305	0.001985	0.2121
381994	E030018B13Rik	RIKEN cDNA E030018B13 gene	0.6298	0.07687	0.5014
552901	LOC552901	hypothetical LOC552901	0.6293	0.001649	0.1903
108058	Camk2d	calcium/calmodulin-dependent protein kinase II, delta	0.629	0.1605	0.6057
320358	C430003N24Rik	RIKEN cDNA C430003N24 gene	0.6289	4.61E-05	0.04861
104103	Airn	antisense Igf2r RNA	0.6289	0.003097	0.2228
18115	Nnt	nicotinamide nucleotide transhydrogenase	0.6285	0.09336	0.5293
76561	Snx7	sorting nexin 7	0.6283	0.1759	0.6156
17833	Muc5ac	mucin 5, subtypes A and C, tracheobronchial/gastric	0.6283	0.3412	0.7334
101458	AA536887	expressed sequence AA536887	0.6281	0.006623	0.288
319862	E230008O15Rik	RIKEN cDNA E230008O15 gene	0.6277	0.0193	0.3548
544763	Hbq1b	hemoglobin, theta 1B	0.6276	0.06885	0.487
103067	AA522020	expressed sequence AA522020	0.6274	0.03033	0.3954
170731	Mfn2	mitofusin 2	0.6273	0.007355	0.2933
67647	4930523C07Rik	RIKEN cDNA 4930523C07 gene	0.6272	0.0867	0.5178
320693	C130021H21Rik	RIKEN cDNA C130021H21 gene	0.627	0.0321	0.3998
76665	1700123M08Rik	RIKEN cDNA 1700123M08 gene	0.627	0.01761	0.3469
51885	Tubgcp4	tubulin, gamma complex associated protein 4	0.6256	0.07369	0.4961
52473	D11Ertd80e	DNA segment, Chr 11, ERATO Doi 80, expressed	0.6254	0.002807	0.2228
319347	C230094B09Rik	RIKEN cDNA C230094B09 gene	0.6246	0.002216	0.2121
77528	C030014A21Rik	RIKEN cDNA C030014A21 gene	0.6245	0.0008067	0.148
15586	Hyal1	hyaluronoglucosaminidase 1	0.624	0.004211	0.2553
53417	Hif3a	hypoxia inducible factor 3, alpha subunit	0.6234	0.03241	0.4017
109905	Rap1a	RAS-related protein-1a	0.6223	0.5735	0.8501
100009609	Vmn2r65	vomeronasal 2, receptor 65	0.6216	0.1569	0.6011
77881	6030458E02Rik	RIKEN cDNA 6030458E02 gene	0.6216	0.02513	0.3786
67812	Ubxn4	UBX domain protein 4	0.6207	0.4341	0.7875
81879	Tcfcp2l1	transcription factor CP2-like 1	0.62	0.2704	0.6885
97715	C79015	expressed sequence C79015	0.6188	0.02858	0.3896
232685	AB041803	cDNA sequence AB041803	0.6187	0.007575	0.294
235854	Mrgpra4	MAS-related GPR, member A4	0.6181	0.004523	0.2618
328232	Gfod1	glucose-fructose oxidoreductase domain containing 1	0.6178	0.4147	0.776
210710	Gab3	growth factor receptor bound protein 2-associated protein 3	0.6175	0.02427	0.3771
320933	D230017M19Rik	RIKEN cDNA D230017M19 gene	0.6171	0.09634	0.5345
67014	Mina	myc induced nuclear antigen	0.6144	0.01634	0.3414
320715	C530043K16Rik	RIKEN cDNA C530043K16 gene	0.6141	0.03729	0.4163
50720	Sacs	sacsin	0.6141	0.1456	0.5906
16878	Lif	leukemia inhibitory factor	0.614	0.1957	0.6306
14201	Fhl3	four and a half LIM domains 3	0.6137	0.005899	0.2795
68070	Pdzd2	PDZ domain containing 2	0.613	0.01198	0.3235
666385	Gm8075	predicted gene 8075	0.6125	0.1631	0.6074
320878	Mical2	microtubule associated monoxygenase, calponin and LIM domain containing 2	0.6123	0.005389	0.2769
320135	BC049715	cDNA sequence BC049715	0.6121	0.004393	0.2611
77022	2700099C18Rik	NDC80 homolog, kinetochore complex component pseudogene	0.6117	0.09092	0.5229
70893	Glb1l3	galactosidase, beta 1 like 3	0.6111	0.0312	0.3964
13653	Egr1	early growth response 1	0.6088	0.1632	0.6074
553089	LOC553089	hypothetical LOC553089	0.6082	0.0008032	0.148
52680	D13Ertd787e	DNA segment, Chr 13, ERATO Doi 787, expressed	0.6079	0.0006259	0.145
20460	Stil	Scl/Tal1 interrupting locus	0.6077	0.1986	0.6336

269593	Luzp1	leucine zipper protein 1	0.6072	0.06941	0.4874
320798	4831440D22Rik	RIKEN cDNA 4831440D22 gene	0.6071	0.05732	0.4675
73757	4833421G17Rik	RIKEN cDNA 4833421G17 gene	0.6049	0.004909	0.2652
320365	Fry	furry homolog (Drosophila)	0.6043	0.2253	0.654
77599	5830420C07Rik	RIKEN cDNA 5830420C07 gene	0.6035	0.0005366	0.1365
242594	1700024P16Rik	RIKEN cDNA 1700024P16 gene	0.6021	0.2397	0.6681
223770	Brd1	bromodomain containing 1	0.6007	0.003111	0.2228
20611	Ssty1	spermiogenesis specific transcript on the Y 1	0.6001	0.2514	0.6767
235497	Leo1	Leo1, Paf1/RNA polymerase II complex component, homolog (S. cerevisiae)	0.5987	0.02463	0.3786
100503605	Beta-s	hemoglobin subunit beta-1-like	0.5985	0.1327	0.5771
264134	Ttc26	tetratricopeptide repeat domain 26	0.5981	0.01476	0.3369
11811	Apobec2	apolipoprotein B mRNA editing enzyme, catalytic polypeptide 2	0.598	0.001655	0.1903
80509	Med8	mediator of RNA polymerase II transcription, subunit 8 homolog (yeast)	0.5968	0.1139	0.5569
238123	Cog5	component of oligomeric golgi complex 5	0.5966	0.003884	0.2456
319791	9430011C21Rik	RIKEN cDNA 9430011C21 gene	0.5956	0.0009179	0.1513
227058	Dnahc7b	dynein, axonemal, heavy chain 7B	0.5954	0.09054	0.5229
68157	6720475J19Rik	RIKEN cDNA 6720475J19 gene	0.595	0.01601	0.3408
72400	Pinx1	PIN2/TERF1 interacting, telomerase inhibitor 1	0.5944	0.04577	0.4381
69236	2610034E01Rik	RIKEN cDNA 2610034E01 gene	0.5941	0.02099	0.3619
319603	A730009E18Rik	RIKEN cDNA A730009E18 gene	0.5933	0.0005382	0.1365
74100	Arpp21	cyclic AMP-regulated phosphoprotein, 21	0.593	0.001619	0.1903
12829	Col4a4	collagen, type IV, alpha 4	0.5929	0.1441	0.5889
214133	Tet2	tet oncogene family member 2	0.5925	0.000524	0.1365
14531	Gcm1	glial cells missing homolog 1 (Drosophila)	0.5925	0.02363	0.3753
667712	Gm8777	predicted gene 8777	0.5921	0.3954	0.7669
228139	P2rx3	purinergic receptor P2X, ligand-gated ion channel, 3	0.5916	0.2541	0.6788
13011	Cst7	cystatin F (leukocystatin)	0.5912	0.1372	0.5807
21412	Tcf21	transcription factor 21	0.591	0.6161	0.8698
76056	5830443J22Rik	RIKEN cDNA 5830443J22 gene	0.5908	0.002202	0.2121
207921	A830093I24Rik	RIKEN cDNA A830093I24 gene	0.5905	0.04508	0.4353
74571	Kcnk16	potassium channel, subfamily K, member 16	0.5901	0.1773	0.6167
638517	LOC638517	cyclic AMP-responsive element-binding protein 3-like protein 2-like	0.5892	0.005643	0.2775
213948	Atg9b	ATG9 autophagy related 9 homolog B (S. cerevisiae)	0.5881	0.2075	0.6398
73162	Otud3	OTU domain containing 3	0.5871	0.1422	0.5871
320135	BC049715	cDNA sequence BC049715	0.5858	0.003256	0.227
327984	E130101M22	hypothetical protein E130101M22	0.5856	0.0004883	0.1365
239036	4930596D02Rik	RIKEN cDNA 4930596D02 gene	0.5847	0.5101	0.8251
100502931	LOC100502931	hypothetical protein LOC100502931	0.5836	0.3505	0.7392
14051	Eya4	eyes absent 4 homolog (Drosophila)	0.5833	0.3017	0.708
227800	Rabgap1	RAB GTPase activating protein 1	0.5821	0.1979	0.6329
100038605	E030047D23Rik	RIKEN cDNA E030047D23 gene	0.582	0.01152	0.3208
100041290	D4Ertd617e	DNA segment, Chr 4, ERATO Doi 617, expressed	0.5814	0.111	0.5549
319800	C730048C13Rik	RIKEN cDNA C730048C13 gene	0.5805	0.008129	0.302
71960	Myh14	myosin, heavy polypeptide 14	0.5801	0.04288	0.4315
232685	AB041803	cDNA sequence AB041803	0.5778	0.003027	0.2228
54651	Usp27x	ubiquitin specific peptidase 27, X chromosome	0.5764	0.09786	0.5361
56419	Diap3	diaphanous homolog 3 (Drosophila)	0.5758	0.01004	0.3112
20503	Slc16a7	solute carrier family 16 (monocarboxylic acid transporters), member 7	0.5755	0.09827	0.5365
100039681	Zcwpw2	zinc finger, CW type with PWWP domain 2	0.5745	0.001127	0.1616

76968	2310046K23Rik	RIKEN cDNA 2310046K23 gene	0.5736	0.01401	0.3347
19893	Rpgr	retinitis pigmentosa GTPase regulator	0.5733	0.02933	0.3911
63830	Kcnq1ot1	KCNQ1 overlapping transcript 1	0.5726	0.002754	0.2224
78088	Ankrd56	ankyrin repeat domain 56	0.5725	0.1512	0.5943
333467	B020031M17Rik	RIKEN cDNA B020031M17 gene	0.5719	0.02564	0.3786
383032	Gm5215	predicted gene 5215	0.5718	0.1343	0.5779
16199	Il9r	interleukin 9 receptor	0.5704	0.08367	0.5144
114672	1700007E05Rik	RIKEN cDNA 1700007E05 gene	0.5703	0.01108	0.3177
67052	Ndc80	NDC80 homolog, kinetochore complex component (S. cerevisiae)	0.5702	0.01558	0.3403
74376	Myo18b	myosin XVIIIb	0.57	0.07689	0.5014
100019	Mdn1	midasin homolog (yeast)	0.5698	0.04182	0.4285
14531	Gcm1	glial cells missing homolog 1 (Drosophila)	0.5683	0.0831	0.5144
100503910	LOC100503910	hypothetical LOC100503910	0.5681	0.1697	0.611
12363	Casp4	caspase 4, apoptosis-related cysteine peptidase	0.5677	0.02396	0.377
320669	A230092J17Rik	RIKEN cDNA A230092J17 gene	0.5657	0.008168	0.3021
69311	1700008K24Rik	RIKEN cDNA 1700008K24 gene	0.5648	0.07551	0.4997
72358	2210411A11Rik	RIKEN cDNA 2210411A11 gene	0.5646	1.69E-05	0.03314
20608	Sstr4	somatostatin receptor 4	0.5621	0.4365	0.7888
26888	Clec4a2	C-type lectin domain family 4, member a2	0.5615	0.03423	0.4091
545861	Gm5878	predicted gene 5878	0.5612	0.1298	0.5747
319814	E130106K03Rik	RIKEN cDNA E130106K03 gene	0.5608	0.001518	0.1903
70449	2610209C05Rik	RIKEN cDNA 2610209C05 gene	0.5602	0.07063	0.4921
266620	Defb36	defensin beta 36	0.5592	0.3251	0.7219
98884	AI225934	expressed sequence AI225934	0.5587	0.002963	0.2228
208606	Rsrc2	arginine/serine-rich coiled-coil 2	0.5578	0.04739	0.4442
228839	Tgif2	TGFB-induced factor homeobox 2	0.5573	0.07137	0.4933
546896	Olfr455	olfactory receptor 455	0.557	0.001415	0.1836
12638	Cftr	cystic fibrosis transmembrane conductance regulator homolog	0.5559	0.05623	0.4631
100038680	Gm1971	predicted gene 1971	0.5557	0.000849	0.148
77767	Ermn	ermin, ERM-like protein	0.5557	0.02829	0.3886
18128	Notch1	Notch gene homolog 1 (Drosophila)	0.5535	0.03132	0.3964
75763	Dcaf17	DDB1 and CUL4 associated factor 17	0.5527	0.02556	0.3786
20147	Rs1	retinoschisis (X-linked, juvenile) 1 (human)	0.5527	0.05073	0.4519
382209	Rhox3a	reproductive homeobox 3A	0.552	0.1941	0.6299
100043188	Gm10696	predicted gene 10696	0.5511	0.02468	0.3786
100303645	LOC100303645	hypothetical LOC100303645	0.5508	0.06151	0.477
20284	Scrg1	scrapie responsive gene 1	0.5504	0.2077	0.64
239157	Pnma2	paraneoplastic antigen MA2	0.5502	0.04056	0.4257
15964	Ifna11	interferon alpha 11	0.5497	0.1467	0.591
29865	Cabp5	calcium binding protein 5	0.5493	0.08159	0.5122
100215	Gm11266	predicted gene 11266	0.5492	0.2757	0.6925
73719	Lce1c	late cornified envelope 1C	0.5479	0.04337	0.4326
231452	Sdad1	SDA1 domain containing 1	0.5464	0.03104	0.3964
320440	9530091C08Rik	RIKEN cDNA 9530091C08 gene	0.5458	0.136	0.5794
67504	Rnf151	ring finger protein 151	0.5438	0.1319	0.5758
97874	B430203I24Rik	RIKEN cDNA B430203I24 gene	0.5428	0.004593	0.2642
237029	4932411N23Rik	RIKEN cDNA 4932411N23 gene	0.5419	0.2457	0.6733
545861	Gm5878	predicted gene 5878	0.5413	0.1303	0.5747
258198	Olfr224	olfactory receptor 224	0.5403	0.1061	0.5468

433490	Defb45	defensin beta 45	0.5391	0.1775	0.6167
64661	Krtdap	keratinocyte differentiation associated protein	0.5376	0.1127	0.5552
241638	Prosapip1	ProSAPiP1 protein	0.5364	0.08024	0.5098
632971	Rergl	RERG/RAS-like	0.5337	0.05375	0.4594
15204	Herc2	hect (homologous to the E6-AP (UBE3A) carboxyl terminus) domain and RCC1 (CHC1)-like domain (RLD) 2	0.5324	0.1047	0.5452
320664	Cass4	Cas scaffolding protein family member 4	0.5321	0.09279	0.5276
22363	Vpreb2	pre-B lymphocyte gene 2	0.5316	0.1804	0.6198
11951	Atp5g1	ATP synthase, H+ transporting, mitochondrial F0 complex, subunit c1 (subunit 9)	0.5316	0.3344	0.7298
235281	Scn3b	sodium channel, voltage-gated, type III, beta	0.5312	0.07206	0.4941
266459	Gm5039	eukaryotic translation initiation factor 1A pseudogene	0.5299	0.4542	0.7973
433632	Gm5544	predicted gene 5544	0.5296	0.1433	0.5879
70973	4931431B13Rik	RIKEN cDNA 4931431B13 gene	0.5276	0.1612	0.607
100505352	LOC100505352	hypothetical LOC100505352	0.5271	0.002377	0.2166
627924	Gm6811	predicted gene 6811	0.5263	0.1257	0.5703
68416	Sycn	syncollin	0.5245	0.06764	0.4855
51873	D2Ertd127e	DNA segment, Chr 2, ERATO Doi 127, expressed	0.5239	0.01046	0.3117
271849	Shc4	SHC (Src homology 2 domain containing) family, member 4	0.5229	0.09108	0.5231
434223	Gm1966	predicted gene 1966	0.5214	0.318	0.718
434168	Gm5590	predicted gene 5590	0.5207	0.01661	0.3418
100043034	Gm13138	predicted gene 13138	0.52	0.2667	0.6862
74057	4933405D12Rik	RIKEN cDNA 4933405D12 gene	0.519	0.1441	0.589
74727	4930517G19Rik	RIKEN cDNA 4930517G19 gene	0.5189	0.1132	0.5557
624483	Gm6507	predicted gene 6507	0.5188	0.09015	0.5229
381157	Greb1l	growth regulation by estrogen in breast cancer-like	0.5183	2.61E-05	0.03314
104099	Itga9	integrin alpha 9	0.5182	2.30E-05	0.03314
76815	Calcoco2	calcium binding and coiled-coil domain 2	0.5182	0.04151	0.4282
380669	Lin28b	lin-28 homolog B (C. elegans)	0.5173	0.1908	0.6276
621852	Rhox3f	reproductive homeobox 3F	0.5159	0.2303	0.6601
69363	Spaca4	sperm acrosome associated 4	0.5159	0.1358	0.5794
667284	Gm8556	predicted gene 8556	0.5115	0.2504	0.6767
245403	Dcaf12l2	DDB1 and CUL4 associated factor 12-like 2	0.5112	0.1068	0.5485
624582	A730098P11Rik	RIKEN cDNA A730098P11 gene	0.5109	0.01099	0.3175
15186	Hdc	histidine decarboxylase	0.5102	0.0007009	0.145
546524	Gm5953	predicted gene 5953	0.5091	0.0834	0.5144
67703	Kirrel3	kin of IRRE like 3 (Drosophila)	0.5088	0.149	0.5934
109700	Itga1	integrin alpha 1	0.5075	0.1318	0.5758
18782	Pla2g2d	phospholipase A2, group IID	0.5072	0.06515	0.4816
74648	S100pbp	S100P binding protein	0.5058	0.002198	0.2121
74978	Lrriq1	leucine-rich repeats and IQ motif containing 1	0.5053	0.3492	0.7388
319691	D430022A14Rik	RIKEN cDNA D430022A14 gene	0.5051	0.03162	0.3981
239102	Zfhx2	zinc finger homeobox 2	0.5045	0.1006	0.5394
12819	Col15a1	collagen, type XV, alpha 1	0.5045	0.05975	0.4728
76196	6330575P09Rik	RIKEN cDNA 6330575P09 gene	0.504	0.035	0.4097
11834	Aqr	aquarius	0.5016	0.3159	0.7167
100043600	Gm4544	predicted gene 4544	0.5013	0.3447	0.7359
234594	Cnot1	CCR4-NOT transcription complex, subunit 1	0.4958	0.2981	0.7051
100042899	Gm4098	predicted gene 4098	0.495	0.0233	0.374
76469	Cmya5	cardiomyopathy associated 5	0.4948	0.02721	0.3854
100039432	Gm2233	predicted gene 2233	0.4915	0.01726	0.3452

14246	Flg	filaggrin	0.4883	0.08279	0.5144
434674	Gm5631	predicted gene 5631	0.4854	0.0002167	0.1183
68720	Lce1b	late cornified envelope 1B	0.4827	0.04776	0.4442
13009	Csrp3	cysteine and glycine-rich protein 3	0.4796	0.0002566	0.1196
16638	Klra7	killer cell lectin-like receptor, subfamily A, member 7	0.4788	0.07797	0.5029
12828	Col4a3	collagen, type IV, alpha 3	0.4758	0.001586	0.1903
52882	Rgs7bp	regulator of G-protein signalling 7 binding protein	0.4741	0.02236	0.3702
258964	Olfr541	olfactory receptor 541	0.4697	0.1547	0.5975
414120	E330018M18Rik	RIKEN cDNA E330018M18 gene	0.4693	0.07264	0.4947
545475	Defb28	defensin beta 28	0.464	0.177	0.6164
381974	Mrgprg	MAS-related GPR, member G	0.4639	0.09288	0.5277
16627	Klra1	killer cell lectin-like receptor, subfamily A, member 1	0.4629	0.1119	0.5549
66395	Ahnak	AHNAK nucleoprotein (desmoyokin)	0.4603	0.09264	0.5273
635756	Gm7166	predicted gene 7166	0.46	0.1276	0.5716
627871	Gm6802	predicted gene 6802	0.4582	0.0588	0.4712
380698	Obscn	obscurin, cytoskeletal calmodulin and titin-interacting RhoGEF	0.4582	0.1121	0.5549
18012	Neurod1	neurogenic differentiation 1	0.4577	0.1182	0.5609
21684	Tectb	tectorin beta	0.4543	0.2493	0.6767
233561	A430054B03	hypothetical protein A430054B03	0.4541	0.001766	0.1997
78790	4930414F18Rik	RIKEN cDNA 4930414F18 gene	0.4536	0.04017	0.4248
21667	Tdgf1	teratocarcinoma-derived growth factor 1	0.4531	0.1042	0.5444
245128	AU018091	expressed sequence AU018091	0.4526	0.1208	0.5643
12355	Nr1i3	nuclear receptor subfamily 1, group I, member 3	0.4526	0.03555	0.4117
384817	4930448N21Rik	RIKEN cDNA 4930448N21 gene	0.4456	0.1678	0.6095
100041781	Gm3510	predicted gene 3510	0.4437	0.2946	0.7044
67718	Lce1h	late cornified envelope 1H	0.4436	0.02924	0.3911
78391	2610524J22Rik	RIKEN cDNA 2610524J22 gene	0.4379	0.09692	0.5346
12096	Bglap	bone gamma carboxyglutamate protein	0.4361	0.2142	0.6467
20445	St6galnac1	ST6 (alpha-N-acetyl-neuraminyl-2,3-beta-galactosyl-1,3)-N-acetylgalactosaminide alpha-2,6-sialyltransferase 1	0.4355	0.05066	0.4519
69134	Fam25c	family with sequence similarity 25, member C	0.4348	0.01564	0.3403
100702	Mpa2l	macrophage activation 2 like	0.4273	0.08536	0.517
16612	Klk1	kallikrein 1	0.4244	0.1092	0.5529
70992	4931407E12Rik	RIKEN cDNA 4931407E12 gene	0.4237	0.02928	0.3911
67127	Lce1a1	late cornified envelope 1A1	0.4214	0.04253	0.4301
27204	Syn3	synapsin III	0.4212	0.08311	0.5144
319615	Zfp944	zinc finger protein 944	0.4152	0.3172	0.7176
73719	Lce1c	late cornified envelope 1C	0.4147	0.06711	0.4855
620253	Dcpp3	demilune cell and parotid protein 3	0.4137	0.3852	0.7607
381680	BC055004	cDNA sequence BC055004	0.4122	0.303	0.7088
620419	Zfp963	zinc finger protein 963	0.4076	0.198	0.6329
68616	Gdpd3	glycerophosphodiester phosphodiesterase domain containing 3	0.4067	0.1745	0.6148
230996	9430015G10Rik	RIKEN cDNA 9430015G10 gene	0.4051	0.3103	0.7134
245572	Tbx22	T-box 22	0.4033	0.06766	0.4855
80796	Calm4	calmodulin 4	0.4015	0.02597	0.3786
15130	Hbb-b2	hemoglobin, beta adult minor chain	0.4015	0.02539	0.3786
76585	Lce1i	late cornified envelope 1I	0.4013	0.02268	0.3702
404318	Olfr681	olfactory receptor 681	0.3986	0.06812	0.4858
246746	Cd300lf	CD300 antigen like family member F	0.3977	0.0489	0.4469
74050	4921525O09Rik	RIKEN cDNA 4921525O09 gene	0.3973	0.06212	0.4771

68694	Lce1e	late cornified envelope 1E	0.3944	0.02093	0.3618
58181	Il20	interleukin 20	0.3944	0.06477	0.4816
12829	Col4a4	collagen, type IV, alpha 4	0.3911	0.1057	0.5468
71831	1700007B14Rik	RIKEN cDNA 1700007B14 gene	0.3874	0.1028	0.5424
12314	Calm2	calmodulin 2	0.3868	0.3139	0.7153
258379	Olfr1284	olfactory receptor 1284	0.3849	0.1332	0.5772
21940	Cd27	CD27 antigen	0.3799	0.01841	0.3512
243659	Styk1	serine/threonine/tyrosine kinase 1	0.3788	0.2006	0.6345
67828	Lce1f	late cornified envelope 1F	0.3764	0.0143	0.3347
16061	Igh-VJ558	immunoglobulin heavy chain (J558 family)	0.3739	0.01275	0.3294
13654	Egr2	early growth response 2	0.3694	0.06869	0.4869
100504089	LOC100504089	calcium-binding protein p22-like	0.3662	0.2235	0.654
258097	Olfr1500	olfactory receptor 1500	0.3645	0.09798	0.5363
56092	Cts7	cathepsin 7	0.3602	0.05438	0.4601
71206	Katnal2	katanin p60 subunit A-like 2	0.3594	0.3081	0.7112
16061	Igh-VJ558	immunoglobulin heavy chain (J558 family)	0.3574	0.007337	0.2933
258744	Olfr875	olfactory receptor 875	0.3447	0.09924	0.5367
16867	Lhcgr	luteinizing hormone/choriogonadotropin receptor	0.3407	0.1029	0.5424
237890	Slfn14-ps	schlafen family member 14, pseudogene	0.3395	0.1693	0.61
69611	Lce1d	late cornified envelope 1D	0.3379	0.02876	0.3903
666021	2300003K06Rik	RIKEN cDNA 2300003K06 gene	0.3268	0.1078	0.5509
93689	Lmod1	leiomodin 1 (smooth muscle)	0.324	0.03443	0.4091
73252	1600022D10Rik	RIKEN cDNA 1600022D10 gene	0.3218	0.06589	0.4828
207854	Fmr1nb	fragile X mental retardation 1 neighbor	0.313	0.06387	0.4801
224079	Atp13a4	ATPase type 13A4	0.3083	0.1649	0.6077
98736	1700034H15Rik	RIKEN cDNA 1700034H15 gene	0.3021	0.1861	0.6234
22696	Zfp37	zinc finger protein 37	0.3013	0.3113	0.7135
68789	Trmt61b	tRNA methyltransferase 61 homolog B (S. cerevisiae)	0.2905	0.1156	0.558
224552	Vmn2r98	vomeronasal 2, receptor 98	0.2668	0.1685	0.6096
100043706	Gm4598	predicted gene 4598	0.2667	0.02922	0.3911
77394	9530025H10Rik	RIKEN cDNA 9530025H10 gene	0.2614	0.09673	0.5345
17427	Mns1	meiosis-specific nuclear structural protein 1	0.2543	0.01575	0.3403
84095	Pi4k2a	phosphatidylinositol 4-kinase type 2 alpha	0.2366	0.3137	0.7152
668501	Zfp507	zinc finger protein 507	0.2358	0.2897	0.7004
11808	Apoa4	apolipoprotein A-IV	0.1899	0.0973	0.5352
271221	5031414D18Rik	RIKEN cDNA 5031414D18 gene	0.1882	0.09902	0.5367
353371	Oxct2b	3-oxoacid CoA transferase 2B	0.1833	0.01235	0.3248
12836	Col7a1	collagen, type VII, alpha 1	0.1743	0.2868	0.6982
77936	BC065403	cDNA sequence BC065403	0.06927	0.001686	0.1919
