# Supplementary material for: Hif1a inactivation rescues photoreceptor degeneration induced by a chronic hypoxia-like stress
Source: Cell Death Differ. 2018 Apr 17;25(12):2071–85. doi: 10.1038/s41418-018-0094-7 (PMC6261999; doi:10.1038/s41418-018-0094-7)
Supplement: Supplementary file 4 — Supplemental File S3 [file 41418_2018_94_MOESM4_ESM.pdf]

Suppl. Table S3: Comparison: Average expression in $rod^{\Delta Vhl}$ divided by average expression in $rod^{\Delta Vhl;Hif1a}$

Entrez Gene ID [Agilent]	Gene Symbol [Agilent]	Description [Agilent]	ratio	pValue	fdr
71957	Cpsf3l	cleavage and polyadenylation specific factor 3-like	228.467	5.20E-09	1.00E-05
619297	C430049E01Rik	RIKEN cDNA C430049E01 gene	143.4103	5.39E-09	1.00E-05
223780	Adm2	adrenomedullin 2	70.77141	1.19E-06	0.0003443
13615	Edn2	endothelin 2	65.74622	3.12E-08	3.22E-05
76487	Ppp1r3g	protein phosphatase 1, regulatory (inhibitor) subunit 3G	59.03188	5.15E-06	0.0008176
213742	Xist	inactive X specific transcripts	35.23608	0.0301	0.1358
240913	Adamts4	a disintegrin-like and metallopeptidase (reprolysin type) with thrombospondin type 1 motif, 4	24.64268	0.0002889	0.008816
236220	LOC236220	hypothetical protein LOC236220	24.29543	1.34E-05	0.001452
11535	Adm	adrenomedullin	23.51834	6.34E-08	5.12E-05
16763	Lad1	ladinin	23.27747	3.38E-05	0.002625
67573	Loxl4	lysyl oxidase-like 4	22.3314	0.000251	0.008007
381359	Prdm12	PR domain containing 12	19.516	1.37E-05	0.001477
12051	Bcl3	B-cell leukemia/lymphoma 3	18.63238	4.52E-06	0.0007845
233187	Lim2	lens intrinsic membrane protein 2	16.98658	0.02373	0.1174
17339	Mip	major intrinsic protein of eye lens fiber	16.26016	0.02493	0.1209
21818	Tgm3	transglutaminase 3, E polypeptide	15.57632	0.0004757	0.01156
75290	4930557B15Rik	RIKEN cDNA 4930557B15 gene	14.29593	0.01069	0.07291
20856	Stc2	stanniocalcin 2	13.74948	5.69E-07	0.0002072
319506	7530428D23Rik	RIKEN cDNA 7530428D23 gene	13.50986	0.05511	0.1923
276829	Smtnl2	smoothelin-like 2	13.23802	2.79E-07	0.0001328
14663	Glycam1	glycosylation dependent cell adhesion molecule 1	12.98027	0.001268	0.02067
67573	Loxl4	lysyl oxidase-like 4	12.57545	1.99E-05	0.001868
15006	H2-Q1	histocompatibility 2, Q region locus 1	12.21001	9.49E-06	0.001179
68380	0610042G04Rik	RIKEN cDNA 0610042G04 gene	11.84834	3.10E-08	3.22E-05
14173	Fgf2	fibroblast growth factor 2	11.62656	7.27E-06	0.001022
192199	Rspo1	R-spondin homolog (Xenopus laevis)	11.33787	0.02042	0.1072
213742	Xist	inactive X specific transcripts	11.23974	0.02446	0.1195
12954	Cryaa	crystallin, alpha A	10.93016	0.01636	0.09371
100039660	Ect2l	epithelial cell transforming sequence 2 oncogene-like	10.84246	3.46E-05	0.002655
77998	Grifin	galectin-related inter-fiber protein	10.68262	0.01751	0.0978
15019	H2-Q8	histocompatibility 2, Q region locus 8	10.52078	2.15E-09	5.70E-06
238692	Zfp874a	zinc finger protein 874a	10.36807	4.21E-06	0.0007668
54382	Tcstv1	2-cell-stage, variable group, member 1	10.3114	0.0002324	0.007731
214301	Crygn	crystallin, gamma N	10.28595	0.0119	0.07773
12268	C4b	complement component 4B (Childo blood group)	10.17294	1.60E-05	0.001654
18073	Nid1	nidogen 1	10.14816	0.01537	0.0908
12962	Crybb3	crystallin, beta B3	10.12146	0.01193	0.07773
19013	Ppara	peroxisome proliferator activated receptor alpha	9.90099	4.40E-06	0.0007777
12609	Cebpd	CCAAT/enhancer binding protein (C/EBP), delta	9.469697	2.12E-06	0.000498
100043899	R3hdml	R3H domain containing-like	9.380863	0.001679	0.02483
434280	Gm5607	predicted gene 5607	9.337068	0.03643	0.1511
79554	Gltpd1	glycolipid transfer protein domain containing 1	9.107468	0.0001185	0.005227
94224	Srd5a2	steroid 5 alpha-reductase 2	9.107468	0.01744	0.09758

30923	Foxe3	forkhead box E3	9.066183	0.00489	0.04601
68662	Scgb3a1	secretoglobin, family 3A, member 1	9.049774	8.28E-05	0.004246
19153	Prx	periaxin	8.673027	0.004698	0.04511
14611	Gja3	gap junction protein, alpha 3	8.598452	0.01758	0.09797
74492	Kbtbd13	kelch repeat and BTB (POZ) domain containing 13	8.53971	0.002117	0.02798
13646	Klk1b22	kallikrein 1-related peptidase b22	8.503401	5.57E-05	0.00347
69291	1700001L05Rik	RIKEN cDNA 1700001L05 gene	8.230453	9.24E-07	0.000286
18542	Pcolce	procollagen C-endopeptidase enhancer protein	8.210181	0.0002328	0.007731
210321	BC048679	cDNA sequence BC048679	8.190008	0.02034	0.107
330004	Gm833	predicted gene 833	7.974482	1.19E-05	0.001363
12964	Cryga	crystallin, gamma A	7.727975	0.004877	0.04601
14184	Fgfr3	fibroblast growth factor receptor 3	7.558579	0.02041	0.1072
22422	Wnt7b	wingless-related MMTV integration site 7B	7.479432	0.02015	0.1066
15945	Cxcl10	chemokine (C-X-C motif) ligand 10	7.309942	0.0002254	0.007645
11807	Apoa2	apolipoprotein A-II	7.27802	0.02412	0.1186
70989	4931429I11Rik	RIKEN cDNA 4931429I11 gene	7.189073	5.82E-06	0.0008998
667034	Pnp2	purine-nucleoside phosphorylase 2	7.132668	4.67E-06	0.0007898
12182	Bst1	bone marrow stromal cell antigen 1	7.097232	8.52E-05	0.004277
56312	Nupr1	nuclear protein 1	7.017544	0.00638	0.05376
68616	Gdpd3	glycerophosphodiester phosphodiesterase domain containing 3	6.993007	0.01109	0.0746
14616	Gja8	gap junction protein, alpha 8	6.944444	0.02413	0.1186
12958	Cryba2	crystallin, beta A2	6.920415	0.03484	0.148
83961	Nrg4	neuregulin 4	6.85401	5.94E-05	0.003567
12310	Calca	calcitonin/calcitonin-related polypeptide, alpha	6.849315	2.76E-06	0.0005898
18050	Klk1b3	kallikrein 1-related peptidase b3	6.816633	7.95E-05	0.004179
620419	Zfp963	zinc finger protein 963	6.811989	0.01338	0.08342
12961	Crybb2	crystallin, beta B2	6.802721	0.03603	0.15
12960	Crybb1	crystallin, beta B1	6.788866	0.03826	0.1558
19013	Ppara	peroxisome proliferator activated receptor alpha	6.788866	0.0002929	0.008884
21956	Tnnt2	troponin T2, cardiac	6.784261	1.76E-07	0.000102
12960	Crybb1	crystallin, beta B1	6.779661	0.03436	0.1466
20296	Ccl2	chemokine (C-C motif) ligand 2	6.775068	0.0008039	0.01581
100041143	Gm3161	predicted gene 3161	6.644518	0.01489	0.08906
16453	Jak3	Janus kinase 3	6.618134	0.0001511	0.005969
12955	Cryab	crystallin, alpha B	6.56168	0.02461	0.1199
117167	Steap4	STEAP family member 4	6.447453	0.0006735	0.01424
12957	Cryba1	crystallin, beta A1	6.410256	0.02683	0.1267
266744	Lgsn	lengsin, lens protein with glutamine synthetase domain	6.402049	0.002452	0.03068
22300	Vmn2r123	vomeronasal 2, receptor 123	6.361323	8.91E-06	0.001137
100043229	Gm10046	predicted gene 10046	6.35324	5.48E-05	0.00347
235435	Lctl	lactase-like	6.297229	0.04553	0.1719
12965	Crygb	crystallin, gamma B	6.207325	0.006894	0.05627
112407	Egln3	EGL nine homolog 3 (C. elegans)	6.199628	1.08E-05	0.001282
18073	Nid1	nidogen 1	6.157635	0.01132	0.07536
12968	Cryge	crystallin, gamma E	6.153846	4.65E-07	0.0001961
116812	Zfp264	zinc finger protein 264	5.970149	0.0009015	0.01693
12630	Cfi	complement component factor i	5.913661	2.02E-05	0.00188
14863	Gstm2	glutathione S-transferase, mu 2	5.861665	0.008731	0.0644
14580	Gfap	glial fibrillary acidic protein	5.827506	5.14E-06	0.0008176

12774	Ccr5	chemokine (C-C motif) receptor 5	5.824112	4.43E-05	0.003078
232966	Zfp114	zinc finger protein 114	5.793743	2.22E-07	0.0001113
12961	Crybb2	crystallin, beta B2	5.77034	0.03877	0.1567
12955	Cryab	crystallin, alpha B	5.767013	0.01801	0.09928
94346	Tmem40	transmembrane protein 40	5.678592	0.03882	0.1567
94346	Tmem40	transmembrane protein 40	5.675369	0.04057	0.161
228796	Bpil3	bactericidal/permeability-increasing protein-like 3	5.675369	8.06E-05	0.004179
320893	6430562O15Rik	RIKEN cDNA 6430562O15 gene	5.656109	1.82E-06	0.0004567
58866	Treh	trehalase (brush-border membrane glycoprotein)	5.624297	3.00E-07	0.000136
14580	Gfap	glial fibrillary acidic protein	5.605381	2.27E-06	0.0005138
319189	Hist2h2bb	histone cluster 2, H2bb	5.524862	3.59E-05	0.002689
69291	1700001L05Rik	RIKEN cDNA 1700001L05 gene	5.494505	1.75E-06	0.0004549
12966	Crygc	crystallin, gamma C	5.414185	0.0123	0.07892
18742	Pitx3	paired-like homeodomain transcription factor 3	5.376344	0.02867	0.1319
69354	Slc38a4	solute carrier family 38, member 4	5.356186	0.01185	0.07745
69368	Wdfy1	WD repeat and FYVE domain containing 1	5.356186	7.77E-08	5.77E-05
620382	Gm6146	predicted gene 6146	5.333333	4.85E-06	0.0007936
667977	Gm8909	predicted gene 8909	5.327651	4.67E-07	0.0001961
100038632	F830016D02Rik	RIKEN cDNA F830016D02 gene	5.299417	0.01548	0.09117
12959	Cryba4	crystallin, beta A4	5.29661	0.06253	0.2086
100043600	Gm4544	predicted gene 4544	5.265929	0.04353	0.1676
14283	Fosl1	fos-like antigen 1	5.263158	8.74E-06	0.001132
103988	Gck	glucokinase	5.263158	4.87E-06	0.0007936
112407	Egln3	EGL nine homolog 3 (C. elegans)	5.243838	8.94E-06	0.001137
12966	Crygc	crystallin, gamma C	5.181347	0.02333	0.1165
12182	Bst1	bone marrow stromal cell antigen 1	5.076142	3.98E-05	0.002867
14580	Gfap	glial fibrillary acidic protein	5.053057	0.0001761	0.006593
20564	Slit3	slit homolog 3 (Drosophila)	5.053057	0.01198	0.07787
67410	4930449I24Rik	RIKEN cDNA 4930449I24 gene	5.053057	3.95E-06	0.0007327
12967	Crygd	crystallin, gamma D	5.027652	0.006782	0.05571
12832	Col5a2	collagen, type V, alpha 2	4.950495	1.77E-06	0.0004549
67712	Slc25a37	solute carrier family 25, member 37	4.92126	3.65E-06	0.0006967
75600	Calml4	calmodulin-like 4	4.911591	8.67E-06	0.001132
545798	Tmem233	transmembrane protein 233	4.859086	2.87E-06	0.0006064
232345	A2m	alpha-2-macroglobulin	4.786979	0.007723	0.06019
214105	Sox30	SRY-box containing gene 30	4.741584	6.62E-06	0.0009527
14462	Gata3	GATA binding protein 3	4.686036	0.0116	0.07642
108105	B3gnt5	UDP-GlcNAc:betaGal beta-1,3-N-acetylglucosaminyltransferase 5	4.681648	0.0394	0.1581
72267	Lrrc8e	leucine rich repeat containing 8 family, member E	4.621072	0.00197	0.027
75998	5033425G24Rik	RIKEN cDNA 5033425G24 gene	4.608295	6.49E-07	0.0002272
12959	Cryba4	crystallin, beta A4	4.587156	0.05952	0.2025
18073	Nid1	nidogen 1	4.578755	0.01	0.07008
108105	B3gnt5	UDP-GlcNAc:betaGal beta-1,3-N-acetylglucosaminyltransferase 5	4.537205	0.04444	0.1695
654812	Angptl7	angiopoietin-like 7	4.528986	0.02838	0.131
258692	Olfr1442	olfactory receptor 1442	4.50045	0.04862	0.1784
12075	Bfsp1	beaded filament structural protein 1, in lens-CP94	4.468275	0.04593	0.173
17345	Mki67	antigen identified by monoclonal antibody Ki 67	4.458315	0.01947	0.1045
15160	Serpind1	serine (or cysteine) peptidase inhibitor, clade D, member 1	4.43459	0.02428	0.119
21857	Timp1	tissue inhibitor of metalloproteinase 1	4.432624	6.65E-05	0.00373

12394	Runx1	runt related transcription factor 1	4.413063	0.001825	0.02573
100040022	LOC100040022	mCG1037230	4.403347	0.01718	0.09687
80885	Niacr1	niacin receptor 1	4.378284	0.00197	0.027
12266	C3	complement component 3	4.374453	0.0001392	0.005719
56727	Miox	myo-inositol oxygenase	4.329004	1.23E-05	0.001389
83961	Nrg4	neuregulin 4	4.329004	0.0003838	0.01027
67951	Tubb6	tubulin, beta 6	4.32339	0.01334	0.08332
12836	Col7a1	collagen, type VII, alpha 1	4.304778	0.3634	0.5986
329581	Birc7	baculoviral IAP repeat-containing 7 (livin)	4.301075	0.0007944	0.01572
12642	Ch25h	cholesterol 25-hydroxylase	4.286327	0.03592	0.1499
84095	Pi4k2a	phosphatidylinositol 4-kinase type 2 alpha	4.286327	0.305	0.5413
12484	Cd24a	CD24a antigen	4.275331	0.03869	0.1566
12966	Crygc	crystallin, gamma C	4.275331	0.02792	0.13
69706	Ppil5	peptidylprolyl isomerase (cyclophilin) like 5	4.271679	0.0004173	0.0108
57814	Kcne4	potassium voltage-gated channel, Isk-related subfamily, gene 4	4.244482	0.00145	0.02249
320323	A930038B10Rik	RIKEN cDNA A930038B10 gene	4.222973	1.07E-05	0.001268
12969	Crygf	crystallin, gamma F	4.205214	1.93E-05	0.001868
14526	Gcg	glucagon	4.201681	0.0257	0.1233
242620	Dmrta2	doublesex and mab-3 related transcription factor like family A2	4.194631	0.0451	0.1712
20377	Sfrp1	secreted frizzled-related protein 1	4.145937	0.02551	0.1227
224008	2310008H04Rik	RIKEN cDNA 2310008H04 gene	4.13394	6.43E-06	0.0009462
66812	Ppcdc	phosphopantothenoylcysteine decarboxylase	4.130525	0.002599	0.0316
14863	Gstm2	glutathione S-transferase, mu 2	4.127115	0.0177	0.09835
353211	Prune2	prune homolog 2 (Drosophila)	4.122012	1.98E-05	0.001868
668501	Zfp507	zinc finger protein 507	4.106776	0.2952	0.5311
74071	Ifltd1	intermediate filament tail domain containing 1	4.098361	0.09813	0.2764
100040018	Gm9835	predicted pseudogene 9835	4.083299	0.001013	0.01786
71004	4931440P22Rik	RIKEN cDNA 4931440P22 gene	4.056795	1.97E-05	0.001868
77090	Ocel1	occludin/ELL domain containing 1	4.043672	0.002091	0.02786
329977	Fhad1	forkhead-associated (FHA) phosphopeptide binding domain 1	4.00641	0.003849	0.03981
77619	Prelid2	PRELI domain containing 2	3.990423	2.39E-05	0.002088
80879	Slc16a3	solute carrier family 16 (monocarboxylic acid transporters), member 3	3.980892	7.99E-05	0.004179
64706	Scube1	signal peptide, CUB domain, EGF-like 1	3.976143	6.66E-05	0.00373
271424	Ip6k3	inositol hexaphosphate kinase 3	3.91696	3.52E-06	0.0006967
67410	4930449I24Rik	RIKEN cDNA 4930449I24 gene	3.900156	2.23E-05	0.002012
20730	Spink3	serine peptidase inhibitor, Kazal type 3	3.892565	0.0002246	0.007645
17380	Mme	membrane metallo endopeptidase	3.868472	0.002803	0.03286
26910	Figla	folliculogenesis specific basic helix-loop-helix	3.865481	0.09845	0.277
66643	Lix1	limb expression 1 homolog (chicken)	3.844675	0.01116	0.07479
100041089	Gm3134	predicted gene 3134	3.821169	0.0399	0.1592
80879	Slc16a3	solute carrier family 16 (monocarboxylic acid transporters), member 3	3.800836	8.54E-05	0.004277
71176	Fbxo24	F-box protein 24	3.776435	0.0003649	0.01009
22421	Wnt7a	wingless-related MMTV integration site 7A	3.775009	0.04094	0.1617
100689	Spon2	spondin 2, extracellular matrix protein	3.76506	0.0009111	0.01702
12550	Cdh1	cadherin 1	3.707824	0.0121	0.07827
76718	Catsperg2	cation channel, sperm-associated, gamma 2	3.703704	4.56E-05	0.003125
192136	5033411D12Rik	RIKEN cDNA 5033411D12 gene	3.68053	0.0002135	0.007384
15360	Hmgcs2	3-hydroxy-3-methylglutaryl-Coenzyme A synthase 2	3.677823	0.008097	0.0616
12970	Crygs	crystallin, gamma S	3.676471	0.04011	0.1596

20198	S100a4	S100 calcium binding protein A4	3.657644	0.04813	0.1775
100041358	Gm3289	predicted gene 3289	3.650968	0.006265	0.05321
12372	Casq1	calsequestrin 1	3.646973	0.0484	0.178
231832	Tmem184a	transmembrane protein 184a	3.645643	0.0001542	0.006064
621304	Gm6209	predicted gene 6209	3.615329	0.01542	0.0909
432494	Gm5427	predicted gene 5427	3.593245	0.002966	0.03402
104027	Synpo	synaptopodin	3.577818	0.0007943	0.01572
20198	S100a4	S100 calcium binding protein A4	3.55366	0.0486	0.1784
11670	Aldh3a1	aldehyde dehydrogenase family 3, subfamily A1	3.551136	0.022	0.1124
434341	Nlrc5	NLR family, CARD domain containing 5	3.549876	0.001719	0.0251
12829	Col4a4	collagen, type IV, alpha 4	3.548616	0.04785	0.177
71950	Nanog	Nanog homeobox	3.543586	0.006708	0.05537
72850	2900024J01Rik	RIKEN cDNA 2900024J01 gene	3.510004	6.22E-05	0.00362
14863	Gstm2	glutathione S-transferase, mu 2	3.489184	0.02083	0.1087
66101	Ppih	peptidyl prolyl isomerase H	3.484321	0.002465	0.0308
12258	Serping1	serine (or cysteine) peptidase inhibitor, clade G, member 1	3.475843	0.0002258	0.007645
100041143	Gm3161	predicted gene 3161	3.472222	0.1207	0.3148
109901	Cela1	chymotrypsin-like elastase family, member 1	3.434066	0.006728	0.05544
12702	Socs3	suppressor of cytokine signaling 3	3.414135	3.22E-05	0.002549
280635	Emilin3	elastin microfibril interfacer 3	3.318951	0.01565	0.0918
72361	Ces2g	carboxylesterase 2G	3.294893	0.03922	0.1577
241624	Exd1	exonuclease 3'-5' domain containing 1	3.255208	1.63E-05	0.001673
12258	Serping1	serine (or cysteine) peptidase inhibitor, clade G, member 1	3.241491	0.0001126	0.005054
74121	Acoxl	acyl-Coenzyme A oxidase-like	3.236246	2.56E-05	0.002171
74121	Acoxl	acyl-Coenzyme A oxidase-like	3.236246	3.97E-05	0.002867
112405	Egln1	EGL nine homolog 1 (C. elegans)	3.235199	4.81E-05	0.00322
69253	Hspb2	heat shock protein 2	3.231018	0.02179	0.1119
72088	Ush1c	Usher syndrome 1C homolog (human)	3.226847	3.34E-05	0.00261
73040	2900052N01Rik	RIKEN cDNA 2900052N01 gene	3.221649	2.13E-05	0.001972
22608	Ybx1	Y box protein 1	3.217503	0.00969	0.06856
102242	AU024180	expressed sequence AU024180	3.216468	0.01099	0.0742
791282	Gm10030	predicted gene 10030	3.215434	2.95E-07	0.000136
14183	Fgfr2	fibroblast growth factor receptor 2	3.208213	0.006606	0.05495
12954	Cryaa	crystallin, alpha A	3.203075	0.0206	0.1079
15007	H2-Q10	histocompatibility 2, Q region locus 10	3.198976	0.0001643	0.006304
18414	Osmr	oncostatin M receptor	3.180662	7.73E-05	0.004113
83457	Fthl17	ferritin, heavy polypeptide-like 17	3.171583	1.60E-05	0.001654
68355	2010204K13Rik	RIKEN cDNA 2010204K13 gene	3.151592	0.000118	0.005215
14962	Cfb	complement factor B	3.12989	0.02894	0.1327
69368	Wdfy1	WD repeat and FYVE domain containing 1	3.115265	2.01E-06	0.0004854
100039432	Gm2233	predicted gene 2233	3.115265	0.0001574	0.006103
327766	Tmem26	transmembrane protein 26	3.103662	0.001047	0.01821
12266	C3	complement component 3	3.083565	9.52E-05	0.004487
246177	Myo1g	myosin IG	3.082614	1.94E-05	0.001868
17873	Gadd45b	growth arrest and DNA-damage-inducible 45 beta	3.064664	1.68E-05	0.001696
24088	Tlr2	toll-like receptor 2	3.053435	0.001971	0.027
99543	Olfml3	olfactomedin-like 3	3.041363	0.003252	0.03607
23882	Gadd45g	growth arrest and DNA-damage-inducible 45 gamma	2.994909	6.47E-06	0.0009462
170706	Tmem37	transmembrane protein 37	2.993116	0.003775	0.03935

233561	A430054B03	hypothetical protein A430054B03	2.972652	0.0004043	0.0106
76905	Lrg1	leucine-rich alpha-2-glycoprotein 1	2.96824	0.00208	0.02786
72088	Ush1c	Usher syndrome 1C homolog (human)	2.96472	0.01487	0.08899
74645	Fam46c	family with sequence similarity 46, member C	2.96472	0.02271	0.1148
11812	Apoc1	apolipoprotein C-I	2.961208	7.01E-07	0.0002325
18295	Ogn	osteoglycin	2.961208	0.1118	0.3
11604	Agrp	agouti related protein	2.955083	0.0005646	0.01285
104099	Itga9	integrin alpha 9	2.95421	7.01E-07	0.0002325
20284	Scrg1	scrapie responsive gene 1	2.948113	0.03169	0.1399
105355	Slc17a3	solute carrier family 17 (sodium phosphate), member 3	2.941176	0.0001644	0.006304
270685	Mthfd1l	methylenetetrahydrofolate dehydrogenase (NADP+ dependent) 1-like	2.933412	8.27E-06	0.001114
666422	Gm8096	3-phosphoglycerate dehydrogenase pseudogene	2.9274	0.009253	0.06661
14862	Gstm1	glutathione S-transferase, mu 1	2.926544	0.002607	0.03161
75773	Adad2	adenosine deaminase domain containing 2	2.925688	0.004814	0.04562
100042782	Gm14458	predicted gene 14458	2.912904	2.51E-05	0.002162
768271	BE949265	cDNA sequence BE949265	2.896871	2.19E-05	0.002004
20293	Ccl12	chemokine (C-C motif) ligand 12	2.890173	0.009216	0.06646
236539	Phgdh	3-phosphoglycerate dehydrogenase	2.890173	0.007648	0.05989
63959	Slc29a1	solute carrier family 29 (nucleoside transporters), member 1	2.886003	8.74E-06	0.001132
14102	Fas	Fas (TNF receptor superfamily member 6)	2.883506	3.50E-05	0.002666
329502	Pla2g4e	phospholipase A2, group IVE	2.881014	0.001137	0.01911
12124	Bik	BCL2-interacting killer	2.875216	0.002359	0.03007
434729	Gm5635	predicted gene 5635	2.872738	3.55E-05	0.002677
71862	Gpr160	G protein-coupled receptor 160	2.866151	0.05517	0.1923
270685	Mthfd1l	methylenetetrahydrofolate dehydrogenase (NADP+ dependent) 1-like	2.864509	1.02E-05	0.001228
241490	Rbm45	RNA binding motif protein 45	2.855511	1.02E-05	0.001228
434729	Gm5635	predicted gene 5635	2.852253	0.0002815	0.008625
15360	Hmgcs2	3-hydroxy-3-methylglutaryl-Coenzyme A synthase 2	2.849003	0.008091	0.0616
22696	Zfp37	zinc finger protein 37	2.843332	0.3693	0.6042
75860	4930588N13Rik	RIKEN cDNA 4930588N13 gene	2.839296	0.00277	0.03265
625360	BC147527	cDNA sequence BC147527	2.839296	0.003414	0.03708
226527	BC026585	cDNA sequence BC026585	2.829655	0.0001444	0.005842
110253	Triobp	TRIO and F-actin binding protein	2.828854	5.96E-05	0.003567
102265	AV026068	expressed sequence AV026068	2.811358	0.09312	0.2679
56012	Pgam2	phosphoglycerate mutase 2	2.807412	0.001982	0.02709
74199	Vit	vitrin	2.807412	0.03962	0.1586
11670	Aldh3a1	aldehyde dehydrogenase family 3, subfamily A1	2.800336	0.02129	0.1103
102436	Lars2	leucyl-tRNA synthetase, mitochondrial	2.798769	5.56E-08	4.69E-05
236539	Phgdh	3-phosphoglycerate dehydrogenase	2.798769	0.01106	0.07445
14972	H2-K1	histocompatibility 2, K1, K region	2.794857	5.67E-06	0.0008851
17831	Muc2	mucin 2	2.790957	0.004573	0.04461
72778	Dnajc22	DnaJ (Hsp40) homolog, subfamily C, member 22	2.776235	0.009086	0.06599
245450	Slitrk2	SLIT and NTRK-like family, member 2	2.772387	1.27E-06	0.0003513
319555	Nwd1	NACHT and WD repeat domain containing 1	2.771619	0.07498	0.234
67410	4930449I24Rik	RIKEN cDNA 4930449I24 gene	2.760906	3.70E-06	0.0006967
21950	Tnfsf9	tumor necrosis factor (ligand) superfamily, member 9	2.760144	9.95E-06	0.001224
210876	Vmn2r111	vomeronasal 2, receptor 111	2.748008	0.01396	0.08557
56847	Aldh1a3	aldehyde dehydrogenase family 1, subfamily A3	2.746498	0.0106	0.07262
209195	Clic6	chloride intracellular channel 6	2.746498	0.0007025	0.01459

17133	Maff	v-maf musculoaponeurotic fibrosarcoma oncogene family, protein F (avian)	2.745744	8.78E-06	0.001132
218630	Ccno	cyclin O	2.743484	0.0002011	0.007214
67315	Ceacam12	carcinoembryonic antigen-related cell adhesion molecule 12	2.742732	0.1445	0.3497
666048	Gm12824	predicted gene 12824	2.738226	0.007412	0.05875
70258	1500035N22Rik	RIKEN cDNA 1500035N22 gene	2.727769	0.002592	0.03154
18933	Prrx1	paired related homeobox 1	2.726281	0.0737	0.2317
18400	Slc22a18	solute carrier family 22 (organic cation transporter), member 18	2.724796	0.03035	0.1366
27371	Sh2d2a	SH2 domain protein 2A	2.718869	8.67E-05	0.004321
69706	Ppil5	peptidylprolyl isomerase (cyclophilin) like 5	2.715178	3.46E-05	0.002655
15403	Hoxa6	homeobox A6	2.709293	0.0001568	0.006103
20391	Sgca	sarcoglycan, alpha (dystrophin-associated glycoprotein)	2.708559	0.01207	0.07821
12614	Celsr1	cadherin, EGF LAG seven-pass G-type receptor 1 (flamingo homolog, Drosophila)	2.707093	0.03736	0.1532
626175	Gm6654	predicted pseudogene 6654	2.703433	0.001691	0.02489
84004	Mcam	melanoma cell adhesion molecule	2.695418	0.001652	0.0246
71846	Syce2	synaptonemal complex central element protein 2	2.693966	5.17E-05	0.003366
17380	Mme	membrane metallo endopeptidase	2.683843	0.1026	0.2841
21785	Tff2	trefoil factor 2 (spasmolytic protein 1)	2.678811	0.001821	0.02572
631145	4932442L08Rik	RIKEN cDNA 4932442L08 gene	2.672368	0.002056	0.0277
408063	BC062258	cDNA sequence BC062258	2.655337	5.86E-07	0.0002091
12654	Chi3l1	chitinase 3-like 1	2.646203	0.0001568	0.006103
67551	4933409L14Rik	RIKEN cDNA 4933409L14 gene	2.639916	0.1854	0.405
20848	Stat3	signal transducer and activator of transcription 3	2.618487	0.0001474	0.005884
73424	1700064H15Rik	RIKEN cDNA 1700064H15 gene	2.617801	0.0009837	0.01768
20971	Sdc4	syndecan 4	2.61233	0.0002136	0.007384
94352	Loxl2	lysyl oxidase-like 2	2.611648	0.004124	0.04164
66898	Baiap2l1	BAI1-associated protein 2-like 1	2.602134	0.0002334	0.007739
218215	Rnf144b	ring finger protein 144B	2.590674	0.0001071	0.004886
379043	Raet1e	retinoic acid early transcript 1E	2.588662	0.1628	0.375
72386	2610035D17Rik	RIKEN cDNA 2610035D17 gene	2.585315	0.001133	0.01908
71206	Katnal2	katanin p60 subunit A-like 2	2.579979	0.3387	0.5745
18115	Nnt	nicotinamide nucleotide transhydrogenase	2.579314	0.01358	0.08418
71162	4933421I07Rik	RIKEN cDNA 4933421I07 gene	2.576656	1.97E-05	0.001868
14864	Gstm3	glutathione S-transferase, mu 3	2.572016	0.002216	0.02883
17909	Myo10	myosin X	2.565418	0.0004026	0.01059
78523	Mrpl9	mitochondrial ribosomal protein L9	2.56476	3.64E-05	0.002706
12444	Ccnd2	cyclin D2	2.561475	0.009865	0.06942
380878	AF067063	cDNA sequence AF067063	2.561475	3.12E-05	0.002493
240913	Adamts4	a disintegrin-like and metallopeptidase (reprolysin type) with thrombospondin type 1 motif, 4	2.554931	0.002098	0.02786
266620	Defb36	defensin beta 36	2.551671	0.03537	0.1485
71930	2310043M15Rik	RIKEN cDNA 2310043M15 gene	2.55102	0.0423	0.1646
73649	Cybrd1	cytochrome b reductase 1	2.546473	0.005649	0.05027
26436	Psg16	pregnancy specific glycoprotein 16	2.53614	0.0001133	0.005071
633640	Gm7120	predicted gene 7120	2.53357	1.74E-05	0.00175
66141	Ifitm3	interferon induced transmembrane protein 3	2.531646	5.71E-05	0.003526
20200	S100a6	S100 calcium binding protein A6 (calcyclin)	2.529724	0.009503	0.06781
236285	Lancl3	LanC lantibiotic synthetase component C-like 3 (bacterial)	2.524615	0.0456	0.1721
70415	2610018G03Rik	RIKEN cDNA 2610018G03 gene	2.517623	0.004409	0.04342
15486	Hsd17b2	hydroxysteroid (17-beta) dehydrogenase 2	2.51699	0.0007078	0.01462
224079	Atp13a4	ATPase type 13A4	2.51004	0.0003546	0.009944

20390	Sftpd	surfactant associated protein D	2.50941	0.1418	0.3462
18566	Pdcd1	programmed cell death 1	2.508151	0.02282	0.1151
207839	Galnt6	UDP-N-acetyl-alpha-D-galactosamine:polypeptide N-acetylgalactosaminyltransferase 6	2.508151	0.002619	0.03169
15512	Hspa2	heat shock protein 2	2.501251	4.35E-05	0.003073
414120	E330018M18Rik	RIKEN cDNA E330018M18 gene	2.5	0.03876	0.1567
18214	Ddr2	discoidin domain receptor family, member 2	2.49501	8.71E-05	0.004321
272636	Esyt3	extended synaptotagmin-like protein 3	2.491901	0.06023	0.2038
234130	Dkk4	dickkopf homolog 4 (Xenopus laevis)	2.49066	0.09825	0.2765
20848	Stat3	signal transducer and activator of transcription 3	2.4888	2.33E-05	0.002062
105355	Slc17a3	solute carrier family 17 (sodium phosphate), member 3	2.485707	0.004432	0.04355
100043272	5430417L22Rik	RIKEN cDNA 5430417L22 gene	2.485089	7.71E-05	0.004113
74249	Lrrc2	leucine rich repeat containing 2	2.479544	0.0001454	0.005848
71583	9130008F23Rik	RIKEN cDNA 9130008F23 gene	2.470966	5.82E-05	0.003555
58214	Cst10	cystatin 10 (chondrocytes)	2.44858	0.1265	0.3238
319207	Pgbd1	piggyBac transposable element derived 1	2.439619	0.001475	0.0227
19128	Pros1	protein S (alpha)	2.431907	0.0009794	0.01765
69382	1700024P04Rik	RIKEN cDNA 1700024P04 gene	2.427184	0.001547	0.02348
19725	Rfx2	regulatory factor X, 2 (influences HLA class II expression)	2.42483	0.0002426	0.007862
21938	Tnfrsf1b	tumor necrosis factor receptor superfamily, member 1b	2.42483	0.05118	0.1838
214425	Cilp	cartilage intermediate layer protein, nucleotide pyrophosphohydrolase	2.422481	0.02588	0.124
100504089	LOC100504089	calcium-binding protein p22-like	2.421308	0.2748	0.5102
226041	Pgm5	phosphoglucomutase 5	2.41838	0.01328	0.08312
14107	Fat1	FAT tumor suppressor homolog 1 (Drosophila)	2.416626	0.01615	0.09307
16453	Jak3	Janus kinase 3	2.414293	8.34E-06	0.001114
14184	Fgfr3	fibroblast growth factor receptor 3	2.412545	0.002094	0.02786
236285	Lancl3	LanC lantibiotic synthetase component C-like 3 (bacterial)	2.407318	4.07E-05	0.002919
258165	Olfr965	olfactory receptor 965	2.407318	0.06516	0.2139
50909	C1ra	complement component 1, r subcomponent A	2.402114	0.001041	0.01814
19013	Ppara	peroxisome proliferator activated receptor alpha	2.40096	8.90E-05	0.004353
14865	Gstm4	glutathione S-transferase, mu 4	2.397507	0.0008019	0.01579
633640	Gm7120	predicted gene 7120	2.382087	0.000157	0.006103
50909	C1ra	complement component 1, r subcomponent A	2.381519	2.79E-05	0.002312
14362	Fzd1	frizzled homolog 1 (Drosophila)	2.376991	1.30E-05	0.001431
12738	Cldn2	claudin 2	2.374733	0.2326	0.4639
21930	Tnfaip6	tumor necrosis factor alpha induced protein 6	2.372479	0.0006377	0.01372
23833	Cd52	CD52 antigen	2.372479	0.0002303	0.007731
100041734	4930522L14Rik	RIKEN cDNA 4930522L14 gene	2.36016	0.0002561	0.008115
329316	A930038G18	hypothetical protein A930038G18	2.352388	0.0003207	0.009405
15002	H2-Ob	histocompatibility 2, O region beta locus	2.350176	0.03426	0.1464
14963	H2-Bl	histocompatibility 2, blastocyst	2.349624	0.0002452	0.007904
12483	Cd22	CD22 antigen	2.347969	0.05217	0.1856
245884	Fam71f2	family with sequence similarity 71, member F2	2.344666	9.40E-05	0.004481
57746	Piwil2	piwi-like homolog 2 (Drosophila)	2.342469	0.005337	0.04865
17084	Ly86	lymphocyte antigen 86	2.339729	7.64E-05	0.004089
263803	Pkn3	protein kinase N3	2.336449	0.04682	0.175
12505	Cd44	CD44 antigen	2.329373	0.01038	0.07177
70186	Fam162a	family with sequence similarity 162, member A	2.325041	4.30E-06	0.0007747
11910	Atf3	activating transcription factor 3	2.32396	2.21E-05	0.002004
66338	Cdrt4	CMT1A duplicated region transcript 4	2.317497	0.08915	0.261

242481	Palm2	paralemmin 2	2.317497	0.03722	0.1529
545648	Gm13272	predicted gene 13272	2.317497	0.00183	0.02575
20716	Serpina3n	serine (or cysteine) peptidase inhibitor, clade A, member 3N	2.31696	0.0009113	0.01702
56306	Fam60a	family with sequence similarity 60, member A	2.316423	0.0001786	0.006621
230996	9430015G10Rik	RIKEN cDNA 9430015G10 gene	2.314815	0.341	0.5768
16403	Itga6	integrin alpha 6	2.313744	0.02833	0.131
21937	Tnfrsf1a	tumor necrosis factor receptor superfamily, member 1a	2.313744	2.52E-06	0.0005559
252830	Obox6	oocyte specific homeobox 6	2.312139	7.84E-06	0.001087
231805	Pilra	paired immunoglobin-like type 2 receptor alpha	2.310536	0.02743	0.1287
14961	H2-Ab1	histocompatibility 2, class II antigen A, beta 1	2.30521	0.003023	0.03442
20852	Stat6	signal transducer and activator of transcription 6	2.303086	0.03325	0.144
56868	Psg23	pregnancy-specific glycoprotein 23	2.303086	0.0001705	0.006482
14407	Gabrg3	gamma-aminobutyric acid (GABA) A receptor, subunit gamma 3	2.298322	0.0002413	0.007862
245855	BC026513	cDNA sequence BC026513	2.291476	3.28E-06	0.0006613
12829	Col4a4	collagen, type IV, alpha 4	2.290426	0.04306	0.1664
218215	Rnf144b	ring finger protein 144B	2.289902	0.0001392	0.005719
14961	H2-Ab1	histocompatibility 2, class II antigen A, beta 1	2.289377	0.002686	0.03213
15130	Hbb-b2	hemoglobin, beta adult minor chain	2.286237	0.01859	0.1013
14972	H2-K1	histocompatibility 2, K1, K region	2.285714	1.33E-05	0.001447
75853	4930592I03Rik	RIKEN cDNA 4930592I03 gene	2.284148	0.007706	0.06013
230099	Car9	carbonic anhydrase 9	2.281542	0.05923	0.2016
13654	Egr2	early growth response 2	2.278943	0.04271	0.1656
18012	Neurod1	neurogenic differentiation 1	2.272727	0.09787	0.276
20664	Sox1	SRY-box containing gene 1	2.270148	0.05013	0.1818
668880	Stard9	START domain containing 9	2.268603	3.01E-05	0.002424
67606	Fibin	fin bud initiation factor homolog (zebrafish)	2.263468	1.67E-05	0.001694
26415	Mapk13	mitogen-activated protein kinase 13	2.262955	0.0005266	0.01231
667742	Fam38b	family with sequence similarity 38, member B	2.262443	0.03251	0.142
11876	Artn	artemin	2.260398	0.0007927	0.01572
94352	Loxl2	lysyl oxidase-like 2	2.257846	0.001512	0.02315
244864	Layn	layilin	2.257336	0.007466	0.05901
74341	G630025P09Rik	RIKEN cDNA G630025P09 gene	2.252252	6.71E-05	0.00373
12314	Calm2	calmodulin 2	2.250225	0.3814	0.615
100038504	Gm10369	predicted gene 10369	2.248201	0.05074	0.1828
101477	AA960618	expressed sequence AA960618	2.242655	0.0992	0.2779
12829	Col4a4	collagen, type IV, alpha 4	2.241147	0.01062	0.07262
68813	Dock5	dedicator of cytokinesis 5	2.241147	0.04211	0.1642
17970	Ncf2	neutrophil cytosolic factor 2	2.240645	0.001902	0.02652
21817	Tgm2	transglutaminase 2, C polypeptide	2.238639	0.0001384	0.005711
14936	Gys1	glycogen synthase 1, muscle	2.238138	0.0001617	0.006242
54139	Irf6	interferon regulatory factor 6	2.235636	0.0001172	0.005193
14166	Fgf11	fibroblast growth factor 11	2.234637	0.003091	0.03492
545056	Gm5801	ubiquitin-conjugating enzyme E2, J2 homolog pseudogene	2.232143	0.003993	0.04078
19672	Rcn1	reticulocalbin 1	2.225189	0.1755	0.3923
13685	Eif4ebp1	eukaryotic translation initiation factor 4E binding protein 1	2.223705	6.71E-05	0.00373
53318	Pdlim3	PDZ and LIM domain 3	2.221235	0.001106	0.01881
242608	Podn	podocan	2.220742	0.03526	0.1484
30955	Pik3cg	phosphoinositide-3-kinase, catalytic, gamma polypeptide	2.219756	0.04293	0.1662
15007	H2-Q10	histocompatibility 2, Q region locus 10	2.217787	0.000631	0.01363

14114	Fbln1	fibulin 1	2.215821	0.2851	0.5204
16477	Junb	Jun-B oncogene	2.213369	0.0005865	0.01321
278795	Lrrc10b	leucine rich repeat containing 10B	2.212389	0.06284	0.2093
50905	Il17rb	interleukin 17 receptor B	2.207993	0.002301	0.02961
58521	Eid1	EP300 interacting inhibitor of differentiation 1	2.204586	2.37E-05	0.002087
72415	Sgol1	shugoshin-like 1 (S. pombe)	2.201673	0.0002892	0.008816
21884	Fabp9	fatty acid binding protein 9, testis	2.197802	0.0002043	0.00726
16161	Il12rb1	interleukin 12 receptor, beta 1	2.196354	0.008391	0.06283
666317	Prl2c1	Prolactin family 2, subfamily c, member 1	2.194908	8.02E-06	0.001102
76238	Grhpr	glyoxylate reductase/hydroxypyruvate reductase	2.192982	7.36E-05	0.003971
22041	Trf	transferrin	2.192502	0.01266	0.08063
71738	Mamdc2	MAM domain containing 2	2.190101	6.62E-05	0.00373
17476	Mpeg1	macrophage expressed gene 1	2.189142	0.02705	0.1272
94352	Loxl2	lysyl oxidase-like 2	2.188663	7.18E-05	0.003894
78004	Prr15	proline rich 15	2.187227	0.000552	0.01265
208936	Adamts18	a disintegrin-like and metallopeptidase (reprolysin type) with thrombospondin type 1 motif, 18	2.185792	0.01724	0.09706
27218	Slamf1	signaling lymphocytic activation molecule family member 1	2.185315	0.2061	0.4324
14862	Gstm1	glutathione S-transferase, mu 1	2.178649	0.00336	0.0368
319269	A130040M12Rik	RIKEN cDNA A130040M12 gene	2.172024	0.0008472	0.01638
212108	Rln3	relaxin 3	2.171553	0.00304	0.03449
12505	Cd44	CD44 antigen	2.169668	0.001373	0.02174
17345	Mki67	antigen identified by monoclonal antibody Ki 67	2.169197	0.01255	0.0802
243755	Slc13a4	solute carrier family 13 (sodium/sulfate symporters), member 4	2.168727	0.1465	0.3524
235472	Prtg	protogenin homolog (Gallus gallus)	2.164502	0.00193	0.02675
635756	Gm7166	predicted gene 7166	2.164034	0.1451	0.3505
12353	Car6	carbonic anhydrase 6	2.163098	0.06897	0.2221
24117	Wif1	Wnt inhibitory factor 1	2.16263	0.1665	0.3808
219033	Ang4	angiogenin, ribonuclease A family, member 4	2.158429	0.0007351	0.01495
12345	Capzb	capping protein (actin filament) muscle Z-line, beta	2.155637	0.01601	0.09269
12870	Cp	ceruloplasmin	2.155637	0.0003552	0.009945
208439	Klhl29	kelch-like 29 (Drosophila)	2.154244	4.61E-06	0.0007898
18755	Prkch	protein kinase C, eta	2.153316	0.002556	0.03136
625098	Slc38a6	solute carrier family 38, member 6	2.152853	0.000143	0.005828
621852	Rhox3f	reproductive homeobox 3F	2.151926	0.1815	0.4002
12803	Cntf	ciliary neurotrophic factor	2.151	0.0005018	0.01194
319236	9230105E10Rik	RIKEN cDNA 9230105E10 gene	2.150075	0.001	0.01779
108832	5430405G05Rik	RIKEN cDNA 5430405G05 gene	2.149151	0.000177	0.0066
12363	Casp4	caspase 4, apoptosis-related cysteine peptidase	2.148228	0.04125	0.1624
15530	Hspg2	perlecan (heparan sulfate proteoglycan 2)	2.148228	0.0849	0.2526
320685	Dctd	dCMP deaminase	2.140869	0.01549	0.09117
100038552	4732463B04Rik	RIKEN cDNA 4732463B04 gene	2.139953	0.01511	0.08978
76747	Dapl1	death associated protein-like 1	2.138123	0.0836	0.25
320159	Fam179a	family with sequence similarity 179, member A	2.138123	0.0006823	0.01439
414801	Itprip	inositol 1,4,5-triphosphate receptor interacting protein	2.126302	0.001667	0.02472
622384	Fabp5l2	fatty acid binding protein 5-like 2	2.122692	0.0696	0.2232
20608	Sstr4	somatostatin receptor 4	2.122241	0.3166	0.5533
52502	Carhsp1	calcium regulated heat stable protein 1	2.120891	0.01471	0.08842
330004	Gm833	predicted gene 833	2.116402	0.0002943	0.008884
57275	Lenep	lens epithelial protein	2.109705	0.02425	0.1189

320383	B230317F23Rik	RIKEN cDNA B230317F23 gene	2.109705	0.004631	0.04474
50934	Slc7a8	solute carrier family 7 (cationic amino acid transporter, y+ system), member 8	2.107482	0.0003276	0.009549
11639	Ak4	adenylate kinase 4	2.106594	0.001471	0.02267
21937	Tnfrsf1a	tumor necrosis factor receptor superfamily, member 1a	2.105706	8.51E-05	0.004277
18452	P4ha2	procollagen-proline, 2-oxoglutarate 4-dioxygenase (proline 4-hydroxylase), alpha II polypeptide	2.103934	2.25E-05	0.002016
67712	Slc25a37	solute carrier family 25, member 37	2.103492	2.60E-05	0.002182
14936	Gys1	glycogen synthase 1, muscle	2.102607	9.12E-05	0.004411
20613	Snai1	snail homolog 1 (Drosophila)	2.102607	0.1033	0.2851
21817	Tgm2	transglutaminase 2, C polypeptide	2.098636	9.01E-05	0.004391
18011	Neurl1a	neuralized homolog 1A (Drosophila)	2.097755	6.81E-05	0.003738
76722	Ckmt2	creatine kinase, mitochondrial 2	2.096876	0.06663	0.2172
18440	P2rx6	purinergic receptor P2X, ligand-gated ion channel, 6	2.095557	0.03525	0.1484
56185	Hao2	hydroxyacid oxidase 2	2.095557	5.97E-05	0.003567
70904	4921515G04Rik	RIKEN cDNA 4921515G04 gene	2.095557	0.06554	0.2147
15586	Hyal1	hyaluronoglucosaminidase 1	2.094241	4.12E-06	0.0007578
18712	Pim1	proviral integration site 1	2.094241	0.0003604	0.01002
20204	Prrx2	paired related homeobox 2	2.091613	0.0009527	0.0174
56857	Slc37a2	solute carrier family 37 (glycerol-3-phosphate transporter), member 2	2.088555	0.00107	0.01845
320135	BC049715	cDNA sequence BC049715	2.087247	0.000134	0.0056
16592	Fabp5	fatty acid binding protein 5, epidermal	2.086811	0.08248	0.2477
78771	Mctp1	multiple C2 domains, transmembrane 1	2.084202	0.008678	0.06419
227753	Gsn	gelsolin	2.083333	0.0373	0.153
225659	Cep76	centrosomal protein 76	2.0803	9.37E-05	0.004481
77570	3930401B19Rik	RIKEN cDNA 3930401B19 gene	2.079434	0.001742	0.02527
231125	Zfyve28	zinc finger, FYVE domain containing 28	2.079434	1.20E-05	0.001365
432855	Zfhx2as	zinc finger homeobox 2, antisense	2.079434	5.58E-05	0.00347
66892	Eif4e3	eukaryotic translation initiation factor 4E member 3	2.072109	8.61E-06	0.001132
14468	Gbp1	guanylate binding protein 1	2.070822	0.0005881	0.01323
14735	Gpc4	glypican 4	2.069965	0.08074	0.2444
320528	Vps13c	vacuolar protein sorting 13C (yeast)	2.06441	0.004885	0.04601
73914	Irak3	interleukin-1 receptor-associated kinase 3	2.063132	0.002713	0.03222
26968	Islr	immunoglobulin superfamily containing leucine-rich repeat	2.061856	0.05964	0.2026
50874	Tmod4	tropomodulin 4	2.061431	0.003657	0.03867
666385	Gm8075	predicted gene 8075	2.05846	0.02145	0.1108
170484	Nphs2	nephrosis 2 homolog, podocin (human)	2.058037	0.04116	0.1622
228026	Pdk1	pyruvate dehydrogenase kinase, isoenzyme 1	2.056767	5.56E-05	0.00347
317677	Gm5077	predicted gene 5077	2.056767	0.0005035	0.01195
230145	Galnt12	UDP-N-acetyl-alpha-D-galactosamine:polypeptide N-acetylgalactosaminyltransferase 12	2.055498	0.081	0.2448
381868	Gm1082	predicted gene 1082	2.054654	0.00824	0.06224
12971	Crym	crystallin, mu	2.053388	4.49E-05	0.003086
22041	Trf	transferrin	2.053388	0.009641	0.06834
100041143	Gm3161	predicted gene 3161	2.053388	0.02361	0.1171
15964	Ifna11	interferon alpha 11	2.051282	0.1536	0.3633
16693	Krtap11-1	keratin associated protein 11-1	2.04918	0.007946	0.0611
219134	Shisa2	shisa homolog 2 (Xenopus laevis)	2.047502	0.0004345	0.01101
545652	Gm13275	predicted gene 13275	2.047083	0.002165	0.02829
226421	5430435G22Rik	RIKEN cDNA 5430435G22 gene	2.044572	0.0002363	0.00779
226041	Pgm5	phosphoglucomutase 5	2.044154	0.08881	0.2605
14469	Gbp2	guanylate binding protein 2	2.043318	0.0002434	0.007862

18011	Neurl1a	neuralized homolog 1A (Drosophila)	2.043318	8.31E-06	0.001114
66058	Tmem176a	transmembrane protein 176A	2.0404	3.52E-05	0.002666
14609	Gja1	gap junction protein, alpha 1	2.039984	0.1503	0.3581
21858	Timp2	tissue inhibitor of metalloproteinase 2	2.039984	0.001093	0.01866
16165	Il13ra2	interleukin 13 receptor, alpha 2	2.037075	0.005274	0.0483
54526	Syt10	synaptotagmin X	2.03376	0.1366	0.3383
108832	5430405G05Rik	RIKEN cDNA 5430405G05 gene	2.03252	8.85E-05	0.004348
50908	C1s	complement component 1, s subcomponent	2.032107	0.0001743	0.006578
58521	Eid1	EP300 interacting inhibitor of differentiation 1	2.030045	4.12E-05	0.00294
67269	Agtpbp1	ATP/GTP binding protein 1	2.029633	0.0004349	0.01101
11622	Ahr	aryl-hydrocarbon receptor	2.028398	0.001489	0.02289
74589	Kbtbd12	kelch repeat and BTB (POZ) domain containing 12	2.027986	0.008971	0.06552
226781	Slc30a10	solute carrier family 30, member 10	2.023882	0.02818	0.1307
12223	Btc	betacellulin, epidermal growth factor family member	2.023063	0.005492	0.04947
110958	D6Mm5e	DNA segment, Chr 6, Miriam Meisler 5, expressed	2.021836	0.03262	0.1423
21788	Tfpi	tissue factor pathway inhibitor	2.021427	0.001015	0.01786
625237	Gm6566	predicted gene 6566	2.017349	0.0004276	0.01095
12355	Nr1i3	nuclear receptor subfamily 1, group I, member 3	2.016942	0.08967	0.2617
67946	Spata6	spermatogenesis associated 6	2.015316	0.01142	0.07571
12826	Col4a1	collagen, type IV, alpha 1	2.012477	0.004244	0.04243
20533	Slc4a1	solute carrier family 4 (anion exchanger), member 1	2.012477	0.1264	0.3236
94226	S1pr5	sphingosine-1-phosphate receptor 5	2.010859	0.0004754	0.01156
11639	Ak4	adenylate kinase 4	2.006421	5.10E-05	0.003347
50766	Crim1	cysteine rich transmembrane BMP regulator 1 (chordin like)	1.996805	0.03369	0.1451
320135	BC049715	cDNA sequence BC049715	1.994416	0.0001971	0.007119
14695	Gnb3	guanine nucleotide binding protein (G protein), beta 3	1.988467	0.0002184	0.007482
78558	Htra3	HtrA serine peptidase 3	1.986887	0.0009491	0.01738
67304	3110070M22Rik	RIKEN cDNA 3110070M22 gene	1.986097	3.83E-05	0.002808
26570	Slc7a11	solute carrier family 7 (cationic amino acid transporter, y+ system), member 11	1.985703	0.16	0.3717
12575	Cdkn1a	cyclin-dependent kinase inhibitor 1A (P21)	1.98334	0.0005134	0.01212
67504	Rnf151	ring finger protein 151	1.98334	0.09362	0.2688
258689	Olfr1466	olfactory receptor 1466	1.982554	0.08047	0.244
11951	Atp5g1	ATP synthase, H+ transporting, mitochondrial F0 complex, subunit c1 (subunit 9)	1.979414	0.2948	0.5311
73542	Tssk5	testis-specific serine kinase 5	1.977457	0.005475	0.04936
76408	Abcc3	ATP-binding cassette, sub-family C (CFTR/MRP), member 3	1.977457	0.0007347	0.01495
16792	Laptm5	lysosomal-associated protein transmembrane 5	1.977066	0.0003662	0.0101
69634	Clybl	citrate lyase beta like	1.976675	0.0002323	0.007731
66599	Rdm1	RAD52 motif 1	1.973165	0.0001143	0.00509
110959	Nudt19	nudix (nucleoside diphosphate linked moiety X)-type motif 19	1.971998	0.0003094	0.009116
78353	2500002B13Rik	RIKEN cDNA 2500002B13 gene	1.97122	0.0008596	0.01655
77890	6720407P12Rik	RIKEN cDNA 6720407P12 gene	1.967342	0.0005168	0.01218
18726	Lilra6	leukocyte immunoglobulin-like receptor, subfamily A (with TM domain), member 6	1.966182	0.01559	0.0916
70560	Wars2	tryptophanyl tRNA synthetase 2 (mitochondrial)	1.964637	1.12E-05	0.0013
67434	Ankrd33b	ankyrin repeat domain 33B	1.963479	0.01002	0.07014
69820	1810059H22Rik	RIKEN cDNA 1810059H22 gene	1.960016	0.0002691	0.008396
98733	Obsl1	obscurin-like 1	1.958097	0.007098	0.05707
18817	Plk1	polo-like kinase 1 (Drosophila)	1.957713	3.04E-06	0.0006265
71145	Scara5	scavenger receptor class A, member 5 (putative)	1.955417	0.04201	0.164
16423	Cd47	CD47 antigen (Rh-related antigen, integrin-associated signal transducer)	1.952362	0.0001823	0.006688

15162	Hck	hemopoietic cell kinase	1.950078	0.0003045	0.009046
18140	Uhrf1	ubiquitin-like, containing PHD and RING finger domains, 1	1.950078	0.009728	0.06872
67896	Ccdc80	coiled-coil domain containing 80	1.949698	0.02632	0.1252
97458	C80012	expressed sequence C80012	1.949318	0.000353	0.009914
17919	Myo5b	myosin VB	1.948938	0.0006083	0.01349
208439	Klhl29	kelch-like 29 (Drosophila)	1.948938	0.0001305	0.005517
12803	Cntf	ciliary neurotrophic factor	1.945904	0.0006592	0.01403
100049162	BC001981	cDNA sequence BC001981	1.945525	0.2452	0.479
320528	Vps13c	vacuolar protein sorting 13C (yeast)	1.944012	0.0008279	0.01609
100041781	Gm3510	predicted gene 3510	1.943635	0.3802	0.6136
227545	5430407P10Rik	RIKEN cDNA 5430407P10 gene	1.943257	0.0002134	0.007384
237360	Adamts14	a disintegrin-like and metallopeptidase (reprolysin type) with thrombospondin type 1 motif, 14	1.942125	9.03E-06	0.00114
394435	Ugt1a6b	UDP glucuronosyltransferase 1 family, polypeptide A6B	1.942125	0.0003621	0.01005
14726	Pdpn	podoplanin	1.934236	0.0004521	0.01118
211896	Depdc7	DEP domain containing 7	1.932741	0.0253	0.1221
12549	Arhgap31	Rho GTPase activating protein 31	1.931248	4.63E-05	0.003151
668147	Gm13284	predicted gene 13284	1.931248	0.001134	0.01908
12338	Capn6	calpain 6	1.929385	0.02394	0.118
67269	Agtpbp1	ATP/GTP binding protein 1	1.928268	0.0003327	0.009606
56295	Higd1a	HIG1 domain family, member 1A	1.927525	5.45E-05	0.00347
329727	Dennd2c	DENN/MADD domain containing 2C	1.927154	0.002983	0.03413
207819	4930539E08Rik	RIKEN cDNA 4930539E08 gene	1.925298	0.1078	0.2936
15016	H2-Q5	histocompatibility 2, Q region locus 5	1.922338	0.0006333	0.01366
78790	4930414F18Rik	RIKEN cDNA 4930414F18 gene	1.921599	0.141	0.3449
229228	Nudt6	nudix (nucleoside diphosphate linked moiety X)-type motif 6	1.920861	0.0001303	0.005517
231103	Gckr	glucokinase regulatory protein	1.920123	0.001129	0.01906
76486	Ly6k	lymphocyte antigen 6 complex, locus K	1.918281	0.00202	0.02731
76293	Mfap4	microfibrillar-associated protein 4	1.917546	0.3662	0.6018
14864	Gstm3	glutathione S-transferase, mu 3	1.913509	0.2922	0.5281
232560	Caprin2	caprin family member 2	1.913509	0.005689	0.05041
243914	Lgi4	leucine-rich repeat LGI family, member 4	1.912046	0.0001575	0.006103
246084	Defb35	defensin beta 35	1.909855	0.4487	0.6744
12969	Crygf	crystallin, gamma F	1.90949	0.00791	0.06104
14114	Fbln1	fibulin 1	1.90949	0.274	0.5096
11830	Aqp5	aquaporin 5	1.909126	0.04504	0.1711
26432	Plod2	procollagen lysine, 2-oxoglutarate 5-dioxygenase 2	1.908761	0.006362	0.05366
319269	A130040M12Rik	RIKEN cDNA A130040M12 gene	1.908397	0.002729	0.03237
245269	E130304F04Rik	RIKEN cDNA E130304F04 gene	1.908033	3.56E-06	0.0006967
320664	Cass4	Cas scaffolding protein family member 4	1.907305	0.2058	0.432
74580	Pyroxd2	pyridine nucleotide-disulphide oxidoreductase domain 2	1.906214	0.006769	0.0557
67269	Agtpbp1	ATP/GTP binding protein 1	1.905488	0.0002431	0.007862
14468	Gbp1	guanylate binding protein 1	1.904399	0.001342	0.02145
12862	Cox6a2	cytochrome c oxidase, subunit VI a, polypeptide 2	1.903674	5.37E-05	0.003458
16952	Anxa1	annexin A1	1.90295	0.02768	0.1295
20135	Rrm2	ribonucleotide reductase M2	1.899335	0.01658	0.09464
14587	Gfra3	glial cell line derived neurotrophic factor family receptor alpha 3	1.897173	0.006603	0.05494
23827	Bpnt1	bisphosphate 3'-nucleotidase 1	1.896813	0.02145	0.1108
229534	Pbxip1	pre-B-cell leukemia transcription factor interacting protein 1	1.896813	0.0003002	0.008976
547431	Btnl2	butyrophilin-like 2	1.896454	0.02946	0.1341

100042265	Gm9790	predicted gene 9790	1.895375	6.65E-05	0.00373
11534	Adk	adenosine kinase	1.895016	0.0003758	0.01017
240444	Kcng2	potassium voltage-gated channel, subfamily G, member 2	1.894657	0.01529	0.09052
14275	Folr1	folate receptor 1 (adult)	1.893939	0.00395	0.04055
57435	Plin4	perilipin 4	1.893939	0.01882	0.1022
170768	Pfkfb3	6-phosphofructo-2-kinase/fructose-2,6-biphosphatase 3	1.893222	0.007546	0.05939
20969	Sdc1	syndecan 1	1.892864	0.09911	0.2779
80290	Gpr146	G protein-coupled receptor 146	1.892864	0.0002615	0.008258
252903	Ap1s3	adaptor-related protein complex AP-1, sigma 3	1.891074	8.91E-05	0.004353
68794	Flnc	filamin C, gamma	1.890002	0.000797	0.01572
18824	Plp2	proteolipid protein 2	1.889645	0.006207	0.05304
231125	Zfyve28	zinc finger, FYVE domain containing 28	1.886437	9.06E-05	0.004392
100040235	LOC100040235	y-linked testis-specific protein 1-like	1.88537	0.5205	0.7287
18824	Plp2	proteolipid protein 2	1.882885	0.0003061	0.009062
23962	Oasl2	2'-5' oligoadenylate synthetase-like 2	1.880053	0.001622	0.02427
100042484	4732419C18Rik	RIKEN cDNA 4732419C18 gene	1.878287	0.01369	0.08462
72772	Rint1	RAD50 interactor 1	1.875821	0.004975	0.04658
22352	Vim	vimentin	1.875469	0.004939	0.04629
21788	Tfpi	tissue factor pathway inhibitor	1.873712	8.06E-05	0.004179
66066	Gng11	guanine nucleotide binding protein (G protein), gamma 11	1.873361	0.0006452	0.01383
225187	Ankrd29	ankyrin repeat domain 29	1.870208	0.0006265	0.01363
17969	Ncf1	neutrophil cytosolic factor 1	1.869508	0.1185	0.3111
12827	Col4a2	collagen, type IV, alpha 2	1.869159	0.01151	0.07605
20675	Sox3	SRY-box containing gene 3	1.86881	0.001462	0.02261
11830	Aqp5	aquaporin 5	1.865324	0.06916	0.2224
58198	Sall1	sal-like 1 (Drosophila)	1.863238	0.0556	0.1929
16007	Cyr61	cysteine rich protein 61	1.862891	1.51E-05	0.001591
229534	Pbxip1	pre-B-cell leukemia transcription factor interacting protein 1	1.862891	8.34E-05	0.004264
63873	Trpv4	transient receptor potential cation channel, subfamily V, member 4	1.861504	0.008588	0.06377
211151	Churc1	churchill domain containing 1	1.860465	1.88E-05	0.001847
212124	E030019B06Rik	RIKEN cDNA E030019B06 gene	1.859427	0.000684	0.0144
16997	Ltbp2	latent transforming growth factor beta binding protein 2	1.858736	0.3942	0.6252
30963	Ptpla	protein tyrosine phosphatase-like (proline instead of catalytic arginine), member a	1.854943	9.20E-05	0.004417
80876	Ifitm2	interferon induced transmembrane protein 2	1.853568	0.004563	0.04454
108052	Slc14a1	solute carrier family 14 (urea transporter), member 1	1.853568	0.0005508	0.01264
13836	Epha2	Eph receptor A2	1.852881	0.002286	0.02951
14867	Gstm6	glutathione S-transferase, mu 6	1.851852	1.94E-05	0.001868
69376	Zpbp2	zona pellucida binding protein 2	1.851852	0.002484	0.03083
22626	Slc23a3	solute carrier family 23 (nucleobase transporters), member 3	1.849112	0.00212	0.02798
229534	Pbxip1	pre-B-cell leukemia transcription factor interacting protein 1	1.848771	6.71E-05	0.00373
15040	H2-T23	histocompatibility 2, T region locus 23	1.848087	0.002128	0.028
69550	Bst2	bone marrow stromal cell antigen 2	1.847063	4.84E-05	0.00322
52502	Carhsp1	calcium regulated heat stable protein 1	1.84604	0.01664	0.09487
320608	D930023I05Rik	RIKEN cDNA D930023I05 gene	1.84604	0.03987	0.1592
13175	Dclk1	doublecortin-like kinase 1	1.845359	0.0002254	0.007645
71914	Antxr2	anthrax toxin receptor 2	1.843658	0.0006303	0.01363
100529082	Gm11127	predicted gene 11127	1.843318	0.003975	0.04069
74589	Kbtbd12	kelch repeat and BTB (POZ) domain containing 12	1.842639	0.005584	0.04996
624483	Gm6507	predicted gene 6507	1.842639	0.1458	0.3513

17123	Madcam1	mucosal vascular addressin cell adhesion molecule 1	1.840604	0.002876	0.03352
68151	Wls	wntless homolog (Drosophila)	1.839588	0.04804	0.1775
26410	Map3k8	mitogen-activated protein kinase kinase kinase 8	1.83925	0.0004638	0.01139
67392	4833420G17Rik	RIKEN cDNA 4833420G17 gene	1.838235	2.28E-05	0.002033
626058	E330020D12Rik	Riken cDNA E330020D12 gene	1.837897	0.1334	0.3339
18018	Nfatc1	nuclear factor of activated T-cells, cytoplasmic, calcineurin-dependent 1	1.835873	0.002471	0.03081
69573	2310016C08Rik	RIKEN cDNA 2310016C08 gene	1.834189	6.11E-05	0.003591
16423	Cd47	CD47 antigen (Rh-related antigen, integrin-associated signal transducer)	1.832845	0.001376	0.02176
434402	Gm5617	predicted gene 5617	1.832509	8.48E-05	0.004277
13835	Epha1	Eph receptor A1	1.831502	0.002077	0.02786
171279	Vmn1r216	vomeronasal 1 receptor 216	1.830496	0.1375	0.3395
100040377	LOC100040377	hypothetical protein LOC100040377	1.830161	0.0002398	0.007862
110058	Syt17	synaptotagmin XVII	1.829157	0.003765	0.03935
13846	Ephb4	Eph receptor B4	1.828822	0.004588	0.04469
53381	Prdx4	peroxiredoxin 4	1.828822	0.0001191	0.005236
226041	Pgm5	phosphoglucomutase 5	1.828154	0.1228	0.3185
14555	Gpd1	glycerol-3-phosphate dehydrogenase 1 (soluble)	1.827819	0.07005	0.224
67392	4833420G17Rik	RIKEN cDNA 4833420G17 gene	1.827151	0.0004822	0.01163
381232	5830416P10Rik	RIKEN cDNA 5830416P10 gene	1.827151	0.02824	0.1308
56722	Litaf	LPS-induced TN factor	1.823819	0.02311	0.1159
107817	Jmjd6	jumonji domain containing 6	1.823487	4.98E-05	0.003291
69573	2310016C08Rik	RIKEN cDNA 2310016C08 gene	1.822822	3.97E-05	0.002867
69479	1700029J07Rik	RIKEN cDNA 1700029J07 gene	1.822157	0.001455	0.02253
67217	2810055F11Rik	RIKEN cDNA 2810055F11 gene	1.82083	3.49E-05	0.002666
16773	Lama2	laminin, alpha 2	1.819505	0.05062	0.1828
12306	Anxa2	annexin A2	1.819174	0.0004314	0.01101
378460	Pram1	PML-RAR alpha-regulated adaptor molecule 1	1.818843	8.51E-05	0.004277
67099	Fam119a	family with sequence similarity 119, member A	1.818512	0.0004498	0.01115
227545	5430407P10Rik	RIKEN cDNA 5430407P10 gene	1.818512	0.001011	0.01786
227631	Sohlh1	spermatogenesis and oogenesis specific basic helix-loop-helix 1	1.817191	0.08702	0.2571
14635	Galk1	galactokinase 1	1.81653	0.0003345	0.009635
72141	Adpgk	ADP-dependent glucokinase	1.811922	0.01167	0.07669
14264	Fmod	fibromodulin	1.810938	0.182	0.4009
18431	Oca2	oculocutaneous albinism II	1.810938	0.0884	0.2597
319251	9630001P10Rik	RIKEN cDNA 9630001P10 gene	1.8093	0.0003887	0.01038
12830	Col4a5	collagen, type IV, alpha 5	1.808318	0.02024	0.1069
57370	B4galt3	UDP-Gal:betaGlcNAc beta 1,4-galactosyltransferase, polypeptide 3	1.808318	0.03035	0.1366
107993	Bfsp2	beaded filament structural protein 2, phakinin	1.806685	0.4337	0.6607
16669	Krt19	keratin 19	1.80538	0.02336	0.1166
11745	Anxa3	annexin A3	1.805054	0.1255	0.3224
407790	Ndufa4l2	NADH dehydrogenase (ubiquinone) 1 alpha subcomplex, 4-like 2	1.803427	0.003313	0.03649
17919	Myo5b	myosin VB	1.801802	0.02232	0.1134
58998	Pvrl3	poliovirus receptor-related 3	1.801153	0.002076	0.02786
547349	LOC547349	similar to MHC class I antigen precursor	1.800504	0.001739	0.02525
24001	Tiam2	T-cell lymphoma invasion and metastasis 2	1.80018	0.01805	0.09939
230674	Kdm4a	lysine (K)-specific demethylase 4A	1.80018	0.0005972	0.01338
73671	Sult6b1	sulfotransferase family, cytosolic, 6B, member 1	1.799856	0.0006307	0.01363
20692	Sparc	secreted acidic cysteine rich glycoprotein	1.799208	0.001138	0.01912
231805	Pilra	paired immunoglobin-like type 2 receptor alpha	1.798885	0.02019	0.1068

17916	Myo1f	myosin IF	1.795655	0.02457	0.1198
245050	Fam198a	family with sequence similarity 198, member A	1.795332	0.0003728	0.01011
320933	D230017M19Rik	RIKEN cDNA D230017M19 gene	1.795332	0.124	0.3204
16206	Lrig1	leucine-rich repeats and immunoglobulin-like domains 1	1.794044	6.83E-05	0.003738
19207	Ptch2	patched homolog 2	1.792757	0.1478	0.3544
58181	Il20	interleukin 20	1.791152	0.2302	0.4614
84035	Kremen1	kringle containing transmembrane protein 1	1.790831	0.008568	0.0637
20491	Sla	src-like adaptor	1.79051	0.002933	0.03379
100042149	Gm3696	predicted gene 3696	1.789549	0.06199	0.2073
100041184	Gm3187	predicted gene 3187	1.789229	0.016	0.09268
14130	Fcgr2b	Fc receptor, IgG, low affinity IIb	1.786991	0.01043	0.07196
223650	Eppk1	epiplakin 1	1.786991	0.01294	0.08175
20692	Sparc	secreted acidic cysteine rich glycoprotein	1.786671	0.03714	0.1527
78286	Nav2	neuron navigator 2	1.786033	0.1908	0.413
12763	Cmah	cytidine monophospho-N-acetylneuraminic acid hydroxylase	1.785077	0.2436	0.477
67052	Ndc80	NDC80 homolog, kinetochore complex component (S. cerevisiae)	1.785077	0.0741	0.2322
110095	Pygl	liver glycogen phosphorylase	1.785077	0.02353	0.1169
12053	Bcl6	B-cell leukemia/lymphoma 6	1.784758	2.33E-05	0.002062
17181	Matn2	matrilin 2	1.784758	0.09174	0.2655
100169874	Gm11110	predicted gene 11110	1.784121	0.0005217	0.01224
16773	Lama2	laminin, alpha 2	1.783803	0.03446	0.1468
229228	Nudt6	nudix (nucleoside diphosphate linked moiety X)-type motif 6	1.781261	0.001413	0.02219
497106	Rnase12	ribonuclease, RNase A family, 12 (non-active)	1.780944	0.006318	0.05346
266459	Gm5039	eukaryotic translation initiation factor 1A pseudogene	1.779676	0.3957	0.6262
75627	Snapc1	small nuclear RNA activating complex, polypeptide 1	1.778726	1.96E-05	0.001868
72215	1700001P01Rik	RIKEN cDNA 1700001P01 gene	1.77841	0.005867	0.05145
234594	Cnot1	CCR4-NOT transcription complex, subunit 1	1.778094	0.3836	0.6162
327959	Xaf1	XIAP associated factor 1	1.777462	0.0002157	0.007417
13051	Cx3cr1	chemokine (C-X3-C) receptor 1	1.775568	0.001922	0.0267
16792	Laptm5	lysosomal-associated protein transmembrane 5	1.774308	0.000929	0.01717
17476	Mpeg1	macrophage expressed gene 1	1.774308	0.0001706	0.006482
116847	Prelp	proline arginine-rich end leucine-rich repeat	1.773679	0.1398	0.3432
210029	Metrnl	meteorin, glial cell differentiation regulator-like	1.773679	0.06985	0.2237
13175	Dclk1	doublecortin-like kinase 1	1.772107	0.001369	0.02171
66756	4933411K20Rik	RIKEN cDNA 4933411K20 gene	1.771793	0.005377	0.04883
330108	4732457N14	hypothetical protein 4732457N14	1.771479	0.1504	0.3582
94279	Sfxn2	sideroflexin 2	1.769912	0.0001087	0.00492
67306	Fam164a	family with sequence similarity 164, member A	1.768972	9.95E-05	0.004628
56722	Litaf	LPS-induced TN factor	1.768347	0.001016	0.01786
12983	Csf2rb	colony stimulating factor 2 receptor, beta, low-affinity (granulocyte-macrophage)	1.767721	0.04173	0.1635
13610	S1pr3	sphingosine-1-phosphate receptor 3	1.762736	0.0003413	0.009703
13731	Emp2	epithelial membrane protein 2	1.762425	0.04815	0.1775
381489	Rxfp1	relaxin/insulin-like family peptide receptor 1	1.762115	0.01721	0.09696
320295	C920006O11Rik	RIKEN cDNA C920006O11 gene	1.760253	0.002531	0.03114
633640	Gm7120	predicted gene 7120	1.757778	0.001007	0.01786
72136	Chst14	carbohydrate (N-acetylgalactosamine 4-0) sulfotransferase 14	1.756852	0.005074	0.04712
18858	Pmp22	peripheral myelin protein 22	1.756543	0.002668	0.032
110606	Fntb	farnesyltransferase, CAAX box, beta	1.755002	0.02411	0.1186
207474	Kctd12b	potassium channel tetramerisation domain containing 12b	1.755002	0.0001453	0.005848

217154	Stac2	SH3 and cysteine rich domain 2	1.755002	3.43E-05	0.002655
72565	Uaca	uveal autoantigen with coiled-coil domains and ankyrin repeats	1.754694	0.0004661	0.01141
223646	Naprt1	nicotinate phosphoribosyltransferase domain containing 1	1.753463	0.000648	0.01387
60322	Chst7	carbohydrate (N-acetylglucosamino) sulfotransferase 7	1.752234	0.07211	0.2283
71368	5430431A17Rik	RIKEN cDNA 5430431A17 gene	1.75162	0.09155	0.2652
78713	D530017H19Rik	RIKEN cDNA D530017H19 gene	1.75162	0.001628	0.02433
104759	Pld4	phospholipase D family, member 4	1.751007	0.003869	0.03992
67991	Nacc2	nucleus accumbens associated 2, BEN and BTB (POZ) domain containing	1.750088	0.2897	0.5258
12828	Col4a3	collagen, type IV, alpha 3	1.749781	0.008895	0.06523
17750	Mt2	metallothionein 2	1.748863	0.0009123	0.01702
116811	Zim3	zinc finger, imprinted 3	1.748252	0.1628	0.375
71994	Cnn3	calponin 3, acidic	1.747946	0.0003946	0.01045
22324	Vav1	vav 1 oncogene	1.747335	0.007284	0.05811
30794	Pdlim4	PDZ and LIM domain 4	1.74703	0.0009856	0.01769
268935	Scube3	signal peptide, CUB domain, EGF-like 3	1.74581	0.02072	0.1083
17888	Myh6	myosin, heavy polypeptide 6, cardiac muscle, alpha	1.745201	0.005991	0.05214
619289	Rfx8	regulatory factor X 8	1.744592	0.05302	0.1873
17132	Maf	avian musculoaponeurotic fibrosarcoma (v-maf) AS42 oncogene homolog	1.744287	0.04619	0.1735
408065	Zfp456	zinc finger protein 456	1.743071	0.002543	0.03125
381974	Mrgprg	MAS-related GPR, member G	1.74216	0.2756	0.5111
231125	Zfyve28	zinc finger, FYVE domain containing 28	1.741553	0.0001138	0.00508
15013	H2-Q2	histocompatibility 2, Q region locus 2	1.740947	0.003255	0.03607
242481	Palm2	paralemmin 2	1.740644	0.1062	0.2906
12527	Cd9	CD9 antigen	1.739736	2.45E-05	0.002132
20341	Selenbp1	selenium binding protein 1	1.73913	0.000372	0.01011
60531	Npvf	neuropeptide VF precursor	1.738526	0.4369	0.6632
22339	Vegfa	vascular endothelial growth factor A	1.737318	0.009535	0.06794
16323	Inhba	inhibin beta-A	1.737016	0.007914	0.06104
69634	Clybl	citrate lyase beta like	1.734906	0.000391	0.0104
70843	Krt28	keratin 28	1.734906	0.001058	0.01831
17289	Mertk	c-mer proto-oncogene tyrosine kinase	1.734605	2.88E-05	0.002364
320004	A930002H24Rik	RIKEN cDNA A930002H24 gene	1.734305	0.007885	0.06097
69382	1700024P04Rik	RIKEN cDNA 1700024P04 gene	1.733403	0.03495	0.1482
15024	H2-T10	histocompatibility 2, T region locus 10	1.732802	0.002607	0.03161
22774	Zic4	zinc finger protein of the cerebellum 4	1.732802	0.2203	0.4501
628900	Serpina3i	serine (or cysteine) peptidase inhibitor, clade A, member 3I	1.731602	0.1315	0.3317
225187	Ankrd29	ankyrin repeat domain 29	1.729805	0.001072	0.01846
73649	Cybrd1	cytochrome b reductase 1	1.72831	0.0137	0.08462
630499	H2-K2	histocompatibility 2, K region locus 2	1.728011	0.001965	0.027
21859	Timp3	tissue inhibitor of metalloproteinase 3	1.726221	0.007027	0.05677
630499	H2-K2	histocompatibility 2, K region locus 2	1.726221	0.002622	0.03171
22419	Wnt5b	wingless-related MMTV integration site 5B	1.725626	0.03964	0.1586
68157	6720475J19Rik	RIKEN cDNA 6720475J19 gene	1.725626	0.03601	0.15
329002	Zfp236	zinc finger protein 236	1.724733	0.0008922	0.01687
68797	Pdgfrl	platelet-derived growth factor receptor-like	1.724435	0.0002698	0.008405
435791	Gm13271	predicted gene 13271	1.724435	0.002627	0.03175
68487	Tmem140	transmembrane protein 140	1.724138	0.005426	0.04902
12176	Bnip3	BCL2/adenovirus E1B interacting protein 3	1.722356	0.001218	0.02012
216725	Adamts2	a disintegrin-like and metallopeptidase (reprolysin type) with thrombospondin type 1 motif, 2	1.720578	0.2846	0.5201

84035	Kremen1	kringle containing transmembrane protein 1	1.719395	0.0001068	0.004885
18034	Nfkb2	nuclear factor of kappa light polypeptide gene enhancer in B-cells 2, p49/p100	1.718508	0.006703	0.05537
71760	Agxt2l1	alanine-glyoxylate aminotransferase 2-like 1	1.718508	0.01449	0.08758
69305	Dcps	decapping enzyme, scavenger	1.718213	1.31E-05	0.001441
26888	Clec4a2	C-type lectin domain family 4, member a2	1.717623	0.2329	0.464
20852	Stat6	signal transducer and activator of transcription 6	1.717328	9.04E-05	0.004392
103743	Tmem98	transmembrane protein 98	1.714972	0.0003314	0.009606
14633	Gli2	GLI-Kruppel family member GLI2	1.714384	0.04285	0.166
100382	AW011738	expressed sequence AW011738	1.710864	0.03169	0.1399
110835	Chrna5	cholinergic receptor, nicotinic, alpha polypeptide 5	1.710279	0.006046	0.05238
56392	Shoc2	soc-2 (suppressor of clear) homolog (C. elegans)	1.709694	0.006327	0.05346
613117	4930571B16Rik	RIKEN cDNA 4930571B16 gene	1.708817	0.02775	0.1296
71726	Smug1	single-strand selective monofunctional uracil DNA glycosylase	1.708234	0.04878	0.1787
52231	Ankzf1	ankyrin repeat and zinc finger domain containing 1	1.707942	0.0002955	0.008884
76166	6330545A04Rik	RIKEN cDNA 6330545A04 gene	1.707359	0.0253	0.1221
67454	Ikbip	IKBKB interacting protein	1.706485	0.0001439	0.005842
666794	Rbm24	RNA binding motif protein 24	1.705611	0.06709	0.2183
14728	Lilrb4	leukocyte immunoglobulin-like receptor, subfamily B, member 4	1.702417	0.3246	0.5612
380930	9330188P03Rik	RIKEN cDNA 9330188P03 gene	1.702417	0.001097	0.01868
65973	Asph	aspartate-beta-hydroxylase	1.70068	0.01673	0.09513
67622	Mxra7	matrix-remodelling associated 7	1.70068	0.02289	0.1152
403175	Tigd4	tigger transposable element derived 4	1.699524	0.01783	0.09876
11727	Ang	angiogenin, ribonuclease, RNase A family, 5	1.699235	0.0006304	0.01363
16854	Lgals3	lectin, galactose binding, soluble 3	1.698081	0.03546	0.1487
67860	S100a16	S100 calcium binding protein A16	1.697505	0.0003419	0.009707
12983	Csf2rb	colony stimulating factor 2 receptor, beta, low-affinity (granulocyte-macrophage)	1.696929	0.1208	0.3149
108097	Prkab2	protein kinase, AMP-activated, beta 2 non-catalytic subunit	1.696641	0.001069	0.01845
319888	5330437I02Rik	RIKEN cDNA 5330437I02 gene	1.695203	0.07474	0.2334
76088	Dock8	dedicator of cytokinesis 8	1.694915	0.00355	0.03803
434782	Gm5637	predicted pseudogene 5637	1.694628	0.00163	0.02435
99543	Olfml3	olfactomedin-like 3	1.694054	0.02132	0.1104
11542	Adora3	adenosine A3 receptor	1.693767	0.03322	0.144
56277	Tmem45a	transmembrane protein 45a	1.693767	0.08894	0.2606
100042092	Gm3662	predicted gene 3662	1.69348	0.0008291	0.01609
13636	Efna1	ephrin A1	1.693193	0.0002935	0.008884
67784	Plxnd1	plexin D1	1.692047	0.001749	0.0253
630836	2010315B03Rik	RIKEN cDNA 2010315B03 gene	1.690903	0.003779	0.03935
216974	Proca1	protein interacting with cyclin A1	1.690331	0.001245	0.02045
19221	Ptgfrn	prostaglandin F2 receptor negative regulator	1.68976	2.21E-05	0.002004
72807	Zfp429	zinc finger protein 429	1.68976	0.001151	0.01929
14275	Folr1	folate receptor 1 (adult)	1.689475	0.003084	0.03489
22117	Tst	thiosulfate sulfurtransferase, mitochondrial	1.689189	0.001858	0.02606
242125	BC037703	cDNA sequence BC037703	1.689189	0.0512	0.1838
12262	C1qc	complement component 1, q subcomponent, C chain	1.68691	0.002289	0.02953
69668	Ccdc115	coiled-coil domain containing 115	1.686625	3.02E-05	0.002424
319615	Zfp944	zinc finger protein 944	1.685488	0.5393	0.7436
74465	4933421H12Rik	RIKEN cDNA 4933421H12 gene	1.684636	0.1023	0.2834
11624	Ahrr	aryl-hydrocarbon receptor repressor	1.684352	0.0005791	0.01306
19041	Ppl	periplakin	1.684352	0.1188	0.3115

16008	Igfbp2	insulin-like growth factor binding protein 2	1.682369	0.0003276	0.009549
223650	Eppk1	epiplakin 1	1.680108	0.01174	0.07694
67532	Mfap1a	microfibrillar-associated protein 1A	1.679543	2.55E-05	0.002171
67608	Narf	nuclear prelamin A recognition factor	1.678416	0.0003325	0.009606
13846	Ephb4	Eph receptor B4	1.67729	0.01334	0.08332
93694	Clec2d	C-type lectin domain family 2, member d	1.67729	0.003874	0.03996
100503257	LOC100503257	hypothetical protein LOC100503257	1.677008	0.23	0.4612
77945	Rpgrip1	retinitis pigmentosa GTPase regulator interacting protein 1	1.676727	0.002017	0.02731
52348	Vps37a	vacuolar protein sorting 37A (yeast)	1.675603	0.000125	0.00537
217353	Tmc6	transmembrane channel-like gene family 6	1.675322	0.01945	0.1044
11622	Ahr	aryl-hydrocarbon receptor	1.675042	0.005379	0.04883
240667	Sec31b	Sec31 homolog B (S. cerevisiae)	1.675042	0.0005268	0.01231
27368	Tbl2	transducin (beta)-like 2	1.67392	9.47E-05	0.004481
622282	Gm6306	predicted gene 6306	1.67336	0.0001275	0.005439
80837	Rhoj	ras homolog gene family, member J	1.6728	0.05139	0.1841
20525	Slc2a1	solute carrier family 2 (facilitated glucose transporter), member 1	1.67252	1.67E-05	0.001694
229445	Ctso	cathepsin O	1.669728	0.0006679	0.01414
100038740	Gm10325	predicted gene 10325	1.669449	0.0006909	0.01446
171281	Acot3	acyl-CoA thioesterase 3	1.668892	0.07098	0.2259
246746	Cd300lf	CD300 antigen like family member F	1.666944	0.2845	0.52
270160	Rab39	RAB39, member RAS oncogene family	1.665279	0.0008985	0.0169
17295	Met	met proto-oncogene	1.664724	0.003719	0.039
77333	C030007I01Rik	RIKEN cDNA C030007I01 gene	1.664447	0.0943	0.2698
15013	H2-Q2	histocompatibility 2, Q region locus 2	1.663894	0.01061	0.07262
666048	Gm12824	predicted gene 12824	1.663617	0.08514	0.253
245572	Tbx22	T-box 22	1.663063	0.3369	0.5726
434674	Gm5631	predicted gene 5631	1.66251	0.0003023	0.008994
66775	Ptplad2	protein tyrosine phosphatase-like A domain containing 2	1.662234	0.01844	0.1008
319537	B230334C09Rik	RIKEN cDNA B230334C09 gene	1.66113	0.1502	0.3581
13506	Dsc2	desmocollin 2	1.660578	0.004472	0.04388
100038410	Gm10536	predicted gene 10536	1.660302	0.2593	0.4945
19039	Lgals3bp	lectin, galactoside-binding, soluble, 3 binding protein	1.659751	0.0004274	0.01095
666257	Gm8008	predicted gene 8008	1.659476	0.0134	0.08343
67776	Vwa5a	von Willebrand factor A domain containing 5A	1.6592	0.000446	0.0111
67454	Ikbip	IKBKB interacting protein	1.658925	8.43E-05	0.004277
30046	Zfp292	zinc finger protein 292	1.65755	0.0004379	0.01101
384009	Glipr2	GLI pathogenesis-related 2	1.65755	0.00178	0.02544
80285	Parp9	poly (ADP-ribose) polymerase family, member 9	1.657275	0.0001992	0.007179
16949	Loxl1	lysyl oxidase-like 1	1.657001	0.004374	0.04317
20130	Rras	Harvey rat sarcoma oncogene, subgroup R	1.657001	0.0003291	0.009577
17702	Msx2	homeobox, msh-like 2	1.656726	0.06737	0.2189
18826	Lcp1	lymphocyte cytosolic protein 1	1.655903	0.0003648	0.01009
27368	Tbl2	transducin (beta)-like 2	1.654533	0.0001472	0.005884
68588	Cthrc1	collagen triple helix repeat containing 1	1.653986	0.008331	0.06257
12412	Cbx1	chromobox homolog 1 (Drosophila HP1 beta)	1.652346	0.004632	0.04474
83453	Chrdl1	chordin-like 1	1.652073	0.003398	0.03698
16008	Igfbp2	insulin-like growth factor binding protein 2	1.651528	0.0003819	0.01025
69376	Zpbp2	zona pellucida binding protein 2	1.651255	0.125	0.3217
100044874	LOC100044874	h-2 class I histocompatibility antigen, K-W28 alpha chain-like	1.651255	0.03264	0.1423

74249	Lrrc2	leucine rich repeat containing 2	1.650165	0.004341	0.04296
77037	Mrap	melanocortin 2 receptor accessory protein	1.649893	0.01736	0.09735
12260	C1qb	complement component 1, q subcomponent, beta polypeptide	1.649349	0.005409	0.04894
11624	Ahrr	aryl-hydrocarbon receptor repressor	1.649077	0.02498	0.121
233328	Lrrk1	leucine-rich repeat kinase 1	1.648533	0.002136	0.02807
11920	Atm	ataxia telangiectasia mutated homolog (human)	1.647718	0.01251	0.08
104816	Aspg	asparaginase homolog (S. cerevisiae)	1.647175	0.08276	0.2482
65969	Cubn	cubilin (intrinsic factor-cobalamin receptor)	1.646904	0.02942	0.134
11834	Aqr	aquarius	1.646362	0.4571	0.6805
66214	1190002H23Rik	RIKEN cDNA 1190002H23 gene	1.646091	7.78E-06	0.001085
74365	Lonrf3	LON peptidase N-terminal domain and ring finger 3	1.643655	0.00305	0.03457
103080	Sep-10	septin 10	1.642845	0.01402	0.08575
50496	E2f6	E2F transcription factor 6	1.641228	0.002462	0.03079
22117	Tst	thiosulfate sulfurtransferase, mitochondrial	1.64042	0.005306	0.04847
433490	Defb45	defensin beta 45	1.640151	0.124	0.3204
68190	5330426P16Rik	RIKEN cDNA 5330426P16 gene	1.639613	0.02106	0.1094
14182	Fgfr1	fibroblast growth factor receptor 1	1.639344	0.006082	0.05249
70370	Fbln7	fibulin 7	1.638538	0.02467	0.1201
14726	Pdpn	podoplanin	1.637733	0.001862	0.02607
235504	Slc17a5	solute carrier family 17 (anion/sugar transporter), member 5	1.637197	0.1245	0.3212
12176	Bnip3	BCL2/adenovirus E1B interacting protein 3	1.636126	0.0009534	0.0174
20611	Ssty1	spermiogenesis specific transcript on the Y 1	1.636126	0.3846	0.617
14012	Mpzl2	myelin protein zero-like 2	1.635858	0.01311	0.08256
22339	Vegfa	vascular endothelial growth factor A	1.635858	0.000823	0.01603
216019	Hkdc1	hexokinase domain containing 1	1.635323	0.004665	0.045
240725	Sulf1	sulfatase 1	1.635323	0.002522	0.03112
67795	Rnls	renalase, FAD-dependent amine oxidase	1.634521	0.0007724	0.01545
20871	Aurkc	aurora kinase C	1.63372	0.001156	0.01934
50765	Trfr2	transferrin receptor 2	1.633453	0.00172	0.0251
66715	4921515J06Rik	RIKEN cDNA 4921515J06 gene	1.631588	0.01271	0.08084
68753	Mybphl	myosin binding protein H-like	1.631588	0.01215	0.07841
320736	E130203B14Rik	RIKEN cDNA E130203B14 gene	1.630789	0.04806	0.1775
114584	Clic1	chloride intracellular channel 1	1.630523	5.52E-05	0.00347
73690	Glipr1	GLI pathogenesis-related 1 (glioma)	1.630258	0.02803	0.1303
16952	Anxa1	annexin A1	1.629726	0.07985	0.243
13713	Elk3	ELK3, member of ETS oncogene family	1.629461	0.008685	0.06421
624582	A730098P11Rik	RIKEN cDNA A730098P11 gene	1.628664	0.02855	0.1316
72669	2810032G03Rik	RIKEN cDNA 2810032G03 gene	1.627604	0.007909	0.06104
16909	Lmo2	LIM domain only 2	1.627075	0.0005223	0.01224
632971	Rergl	RERG/RAS-like	1.627075	0.1775	0.3948
387345	Tas2r113	taste receptor, type 2, member 113	1.626545	0.1412	0.3453
16391	Irf9	interferon regulatory factor 9	1.626016	7.20E-05	0.003894
97122	Hist2h4	histone cluster 2, H4	1.625752	0.02789	0.1298
228839	Tgif2	TGFB-induced factor homeobox 2	1.625752	0.01484	0.08886
105785	Kdelr3	KDEL (Lys-Asp-Glu-Leu) endoplasmic reticulum protein retention receptor 3	1.625488	0.01757	0.09797
320910	Itgb8	integrin beta 8	1.625223	0.003613	0.03842
11853	Rhoc	ras homolog gene family, member C	1.624431	0.0001078	0.004893
19285	Ptrf	polymerase I and transcript release factor	1.62206	0.002267	0.02932
17313	Mgp	matrix Gla protein	1.621797	8.47E-05	0.004277

226250	Afap1l2	actin filament associated protein 1-like 2	1.621008	0.01365	0.08444
106762	AW047481	expressed sequence AW047481	1.620483	0.002933	0.03379
66905	Plin3	perilipin 3	1.62022	0.001094	0.01867
18080	Nin	ninein	1.619958	5.99E-05	0.003567
66079	Tmem42	transmembrane protein 42	1.619695	6.04E-05	0.00358
76459	Car12	carbonic anyhydrase 12	1.619433	0.07295	0.2301
238252	Gpr135	G protein-coupled receptor 135	1.618909	0.000683	0.01439
18828	Plscr2	phospholipid scramblase 2	1.618385	0.0247	0.1202
13032	Ctsc	cathepsin C	1.617861	6.09E-05	0.003587
15900	Irf8	interferon regulatory factor 8	1.617861	0.0004377	0.01101
16819	Lcn2	lipocalin 2	1.617861	0.02025	0.1069
27381	Tcl1b2	T-cell leukemia/lymphoma 1B, 2	1.617599	0.4541	0.6779
57741	Noc2l	nucleolar complex associated 2 homolog (S. cerevisiae)	1.617599	0.0001867	0.006796
58861	Cysltr1	cysteinyl leukotriene receptor 1	1.617338	0.108	0.294
170768	Pfkfb3	6-phosphofructo-2-kinase/fructose-2,6-biphosphatase 3	1.617076	7.46E-05	0.004005
234155	Mboat4	membrane bound O-acyltransferase domain containing 4	1.616292	0.1755	0.3922
81910	Rrbp1	ribosome binding protein 1	1.614466	0.05207	0.1855
67099	Fam119a	family with sequence similarity 119, member A	1.612643	0.0002948	0.008884
13731	Emp2	epithelial membrane protein 2	1.612123	0.07409	0.2322
17117	Amacr	alpha-methylacyl-CoA racemase	1.612123	0.0003717	0.01011
70240	Ufsp1	UFM1-specific peptidase 1	1.611863	0.0007365	0.01496
239368	BC030476	cDNA sequence BC030476	1.611604	0.00135	0.02151
12176	Bnip3	BCL2/adenovirus E1B interacting protein 3	1.611344	0.0008603	0.01655
17000	Ltbr	lymphotoxin B receptor	1.608493	0.0006173	0.01361
17919	Myo5b	myosin VB	1.606942	0.001127	0.01906
13009	Csrp3	cysteine and glycine-rich protein 3	1.606684	0.03266	0.1424
99929	Tiparp	TCDD-inducible poly(ADP-ribose) polymerase	1.606684	0.000611	0.0135
14675	Gna14	guanine nucleotide binding protein, alpha 14	1.606426	0.05295	0.1873
64899	Lpin3	lipin 3	1.606426	0.000513	0.01212
208263	Tor1aip1	torsin A interacting protein 1	1.606426	0.02287	0.1152
26401	Map3k1	mitogen-activated protein kinase kinase kinase 1	1.606168	0.003652	0.03863
117109	Pop5	processing of precursor 5, ribonuclease P/MRP family (S. cerevisiae)	1.606168	0.03336	0.1442
70652	Tmem144	transmembrane protein 144	1.60591	0.0003212	0.009405
68603	Pmvk	phosphomevalonate kinase	1.605652	0.0002421	0.007862
228094	Cerkl	ceramide kinase-like	1.605394	0.03833	0.1559
17229	Tpsb2	tryptase beta 2	1.605136	0.06341	0.2103
234395	Ushbp1	Usher syndrome 1C binding protein 1	1.604621	0.05424	0.1903
545030	Wdfy4	WD repeat and FYVE domain containing 4	1.604621	0.0009801	0.01765
18858	Pmp22	peripheral myelin protein 22	1.604364	0.0005381	0.01247
54353	Skap2	src family associated phosphoprotein 2	1.603592	0.00061	0.0135
12259	C1qa	complement component 1, q subcomponent, alpha polypeptide	1.603335	0.000954	0.0174
338369	Tmem220	transmembrane protein 220	1.603335	0.0165	0.09433
77945	Rpgrip1	retinitis pigmentosa GTPase regulator interacting protein 1	1.602821	0.001581	0.02378
228778	6820408C15Rik	RIKEN cDNA 6820408C15 gene	1.602821	0.01074	0.07309
547253	Parp14	poly (ADP-ribose) polymerase family, member 14	1.602051	0.0007783	0.01551
71742	Ulk3	unc-51-like kinase 3 (C. elegans)	1.601537	0.0109	0.07377
239530	Gpr20	G protein-coupled receptor 20	1.600768	0.03773	0.1542
224224	Impg2	interphotoreceptor matrix proteoglycan 2	1.600512	0.001819	0.02572
235461	Fam63b	family with sequence similarity 63, member B	1.599744	0.0004833	0.01164

67621	Bend5	BEN domain containing 5	1.598721	0.0002049	0.00726
338367	Myo1d	myosin ID	1.597699	0.0004206	0.01085
16576	Kif7	kinesin family member 7	1.596679	0.003006	0.03428
71726	Smug1	single-strand selective monofunctional uracil DNA glycosylase	1.596679	2.91E-05	0.002377
11607	Agtr1a	angiotensin II receptor, type 1a	1.595914	0.06038	0.2041
16949	Loxl1	lysyl oxidase-like 1	1.594388	0.009682	0.06853
63873	Trpv4	transient receptor potential cation channel, subfamily V, member 4	1.59388	0.01101	0.07421
100041487	Gm3366	predicted gene 3366	1.593625	0.04512	0.1712
14693	Gnb2	guanine nucleotide binding protein (G protein), beta 2	1.59185	0.00861	0.06388
69640	Fam83g	family with sequence similarity 83, member G	1.59185	0.0269	0.1268
327959	Xaf1	XIAP associated factor 1	1.591596	0.008916	0.06529
100042092	Gm3662	predicted gene 3662	1.591596	0.001287	0.02083
19091	Prkg1	protein kinase, cGMP-dependent, type I	1.590584	0.004618	0.04473
81015	Vmn1r56	vomeronasal 1 receptor 56	1.589825	0.2126	0.4403
232334	Vgll4	vestigial like 4 (Drosophila)	1.589572	0.0004618	0.01136
13011	Cst7	cystatin F (leukocystatin)	1.587302	0.3085	0.5448
76568	Ift46	intraflagellar transport 46 homolog (Chlamydomonas)	1.587302	7.20E-06	0.001021
12036	Bcat2	branched chain aminotransferase 2, mitochondrial	1.586798	0.002881	0.03352
68328	Rab13	RAB13, member RAS oncogene family	1.586798	0.0002547	0.008098
15482	Hspa1l	heat shock protein 1-like	1.586546	0.02206	0.1125
214424	Parp16	poly (ADP-ribose) polymerase family, member 16	1.585791	0.01745	0.09762
29810	Bag3	BCL2-associated athanogene 3	1.58554	0.0003764	0.01017
13167	Dbi	diazepam binding inhibitor	1.585289	0.0009198	0.01704
94242	Tinagl1	tubulointerstitial nephritis antigen-like 1	1.585289	0.05511	0.1923
210027	Slc35f3	solute carrier family 35, member F3	1.585037	0.01666	0.09491
17178	Fxyd3	FXYD domain-containing ion transport regulator 3	1.584786	0.0008183	0.01602
69513	1700030C10Rik	RIKEN cDNA 1700030C10 gene	1.584535	0.0008597	0.01655
12606	Cebpa	CCAAT/enhancer binding protein (C/EBP), alpha	1.584284	0.01887	0.1023
14537	Gcnt1	glucosaminyl (N-acetyl) transferase 1, core 2	1.583782	0.03047	0.1368
100040294	Gm2694	predicted gene 2694	1.583782	0.001019	0.01788
15368	Hmox1	heme oxygenase (decycling) 1	1.58303	0.1121	0.3006
230145	Galnt12	UDP-N-acetyl-alpha-D-galactosamine:polypeptide N-acetylgalactosaminyltransferase 12	1.58303	0.1187	0.3114
21877	Tk1	thymidine kinase 1	1.582779	0.136	0.3379
70788	Klhl30	kelch-like 30 (Drosophila)	1.582779	0.001963	0.027
54325	Elovl1	elongation of very long chain fatty acids (FEN1/Elo2, SUR4/Elo3, yeast)-like 1	1.581778	0.04787	0.177
71943	Tom1l1	target of myb1-like 1 (chicken)	1.581778	0.02985	0.1351
627924	Gm6811	predicted gene 6811	1.581778	0.2951	0.5311
69675	Pxdn	peroxidasin homolog (Drosophila)	1.581528	0.03255	0.1421
104444	Rexo2	REX2, RNA exonuclease 2 homolog (S. cerevisiae)	1.581528	0.0007824	0.01553
98396	Slc41a1	solute carrier family 41, member 1	1.581278	0.001049	0.01822
229841	Cenpe	centromere protein E	1.580528	0.002095	0.02786
381994	E030018B13Rik	RIKEN cDNA E030018B13 gene	1.579779	0.1184	0.3111
70355	Gprc5c	G protein-coupled receptor, family C, group 5, member C	1.57928	0.01375	0.08484
22673	Zfp185	zinc finger protein 185	1.578781	0.07996	0.2433
70358	Steap1	six transmembrane epithelial antigen of the prostate 1	1.578781	0.03402	0.1459
258505	Olfr97	olfactory receptor 97	1.578034	0.08882	0.2605
26401	Map3k1	mitogen-activated protein kinase kinase kinase 1	1.577785	0.004513	0.04416
245572	Tbx22	T-box 22	1.577536	0.3597	0.5947
56524	Mpp6	membrane protein, palmitoylated 6 (MAGUK p55 subfamily member 6)	1.577287	0.002007	0.02726

14919	Gucy2e	guanylate cyclase 2e	1.57679	0.0002013	0.007214
15242	Hhex	hematopoietically expressed homeobox	1.57679	0.00667	0.05528
107747	Aldh1l1	aldehyde dehydrogenase 1 family, member L1	1.57679	0.0004772	0.01158
14531	Gcm1	glial cells missing homolog 1 (Drosophila)	1.576541	0.2012	0.4272
14867	Gstm6	glutathione S-transferase, mu 6	1.575299	0.003587	0.03823
319651	Usp37	ubiquitin specific peptidase 37	1.574803	0.0006593	0.01403
75777	Ttc23l	tetratricopeptide repeat domain 23-like	1.574555	0.2004	0.4258
77022	2700099C18Rik	NDC80 homolog, kinetochore complex component pseudogene	1.574555	0.2451	0.4788
97064	Wwtr1	WW domain containing transcription regulator 1	1.574307	0.0002996	0.00897
233571	P2ry6	pyrimidinergic receptor P2Y, G-protein coupled, 6	1.574307	0.0202	0.1068
171286	Slc12a8	solute carrier family 12 (potassium/chloride transporters), member 8	1.574059	0.01809	0.09956
22146	Tuba1c	tubulin, alpha 1C	1.572574	0.002389	0.03025
664862	Gpr137b-ps	G protein-coupled receptor 137B, pseudogene	1.57208	0.004577	0.04461
319276	A230038B01Rik	RIKEN cDNA A230038B01 gene	1.571833	0.1124	0.3011
15205	Hes1	hairy and enhancer of split 1 (Drosophila)	1.571586	0.02535	0.1222
21337	Tacr2	tachykinin receptor 2	1.571092	0.1219	0.3169
94175	Hrg	histidine-rich glycoprotein	1.571092	0.03329	0.1442
619329	F420015M19Rik	RIKEN cDNA F420015M19 gene	1.569859	0.00808	0.0616
666464	Gm8120	predicted gene 8120	1.568874	0.01335	0.08332
26886	Cenph	centromere protein H	1.568135	0.1527	0.3621
252967	Ropn1l	ropporin 1-like	1.56789	0.01324	0.08299
19124	Procr	protein C receptor, endothelial	1.567644	0.00434	0.04296
64297	Gprc5b	G protein-coupled receptor, family C, group 5, member B	1.567644	0.02907	0.1331
320004	A930002H24Rik	RIKEN cDNA A930002H24 gene	1.567644	0.002439	0.03058
11593	Aga	aspartylglucosaminidase	1.567398	0.00182	0.02572
107373	Fam111a	family with sequence similarity 111, member A	1.567398	0.1531	0.3626
22146	Tuba1c	tubulin, alpha 1C	1.566661	0.002979	0.0341
83701	Srrt	serrate RNA effector molecule homolog (Arabidopsis)	1.566661	0.0003021	0.008994
171504	Apob48r	apolipoprotein B48 receptor	1.566416	0.001684	0.02485
18733	Lilrb3	leukocyte immunoglobulin-like receptor, subfamily B (with TM and ITIM domains), member 3	1.566171	0.2202	0.45
208922	Cpeb3	cytoplasmic polyadenylation element binding protein 3	1.564945	0.001086	0.01856
13112	Cyp3a11	cytochrome P450, family 3, subfamily a, polypeptide 11	1.564456	0.1085	0.2946
65963	Tmem176b	transmembrane protein 176B	1.563722	0.001779	0.02544
239122	Setdb2	SET domain, bifurcated 2	1.563722	0.0008301	0.01609
74319	1110005A03Rik	RIKEN cDNA 1110005A03 gene	1.563477	0.001274	0.02073
21898	Tlr4	toll-like receptor 4	1.562988	0.01751	0.0978
12580	Cdkn2c	cyclin-dependent kinase inhibitor 2C (p18, inhibits CDK4)	1.561768	0.0001066	0.004885
269423	3110057O12Rik	RIKEN cDNA 3110057O12 gene	1.561768	0.01574	0.09219
545047	Gm5800	predicted gene 5800	1.561768	0.0771	0.2379
72057	Phf10	PHD finger protein 10	1.56128	0.0002641	0.008323
74856	4930418C01Rik	RIKEN cDNA 4930418C01 gene	1.560549	0.5723	0.7664
16774	Lama3	laminin, alpha 3	1.559819	0.03879	0.1567
107272	Psat1	phosphoserine aminotransferase 1	1.559089	0.00116	0.01937
319757	Smo	smoothened homolog (Drosophila)	1.558603	0.1081	0.2942
331046	Tgm4	transglutaminase 4 (prostate)	1.558118	0.02515	0.1216
16194	Il6ra	interleukin 6 receptor, alpha	1.557875	0.00277	0.03265
16874	Lhx6	LIM homeobox protein 6	1.557875	0.1354	0.337
67216	Mboat2	membrane bound O-acyltransferase domain containing 2	1.557875	0.001535	0.02342
68603	Pmvk	phosphomevalonate kinase	1.557875	4.40E-05	0.003078

319765	Igf2bp2	insulin-like growth factor 2 mRNA binding protein 2	1.557875	0.009274	0.06668
12633	Cflar	CASP8 and FADD-like apoptosis regulator	1.557632	0.0003695	0.01011
77558	9330179D12Rik	RIKEN cDNA 9330179D12 gene	1.557147	0.07096	0.2259
320085	B830012L14Rik	RIKEN cDNA B830012L14 gene	1.556905	0.08092	0.2447
109624	Cald1	caldesmon 1	1.55642	0.01801	0.09928
72947	Agxt2l2	alanine-glyoxylate aminotransferase 2-like 2	1.555694	0.002127	0.028
140497	AF251705	cDNA sequence AF251705	1.555694	0.0009894	0.0177
235582	Glyctk	glycerate kinase	1.554968	0.01208	0.07822
100041230	Hist1h4m	histone cluster 1, H4m	1.554968	0.3609	0.5958
72982	Tmem138	transmembrane protein 138	1.554002	0.0005401	0.01249
170750	Xpnpep1	X-prolyl aminopeptidase (aminopeptidase P) 1, soluble	1.554002	5.10E-05	0.003347
654822	D330041H03Rik	RIKEN cDNA D330041H03 gene	1.554002	0.01092	0.07384
14768	Lancl1	LanC (bacterial lantibiotic synthetase component C)-like 1	1.553277	0.2402	0.4729
20193	S100a1	S100 calcium binding protein A1	1.552795	0.0001212	0.005257
791308	Gm9933	predicted gene 9933	1.552795	0.003697	0.03891
56213	Htra1	HtrA serine peptidase 1	1.552554	9.97E-05	0.004628
15214	Hey2	hairy/enhancer-of-split related with YRPW motif 2	1.55159	0.02108	0.1094
319153	Hist1h3i	histone cluster 1, H3i	1.551109	0.0169	0.09578
545700	Vmn2r-ps14	vomeronasal 2, receptor, pseudogene 14	1.550628	0.0553	0.1925
12257	Tspo	translocator protein	1.550388	0.0003817	0.01025
30935	Tor3a	torsin family 3, member A	1.549907	9.45E-05	0.004481
67451	Pkp2	plakophilin 2	1.549907	0.0001473	0.005884
20024	Sub1	SUB1 homolog (S. cerevisiae)	1.549667	0.0004352	0.01101
266632	Irak4	interleukin-1 receptor-associated kinase 4	1.548947	0.03721	0.1529
16476	Jun	Jun oncogene	1.548707	0.0008888	0.01684
404330	Olfr1198	olfactory receptor 1198	1.548467	0.09974	0.2789
639774	Skint8	selection and upkeep of intraepithelial T cells 8	1.548227	0.496	0.7111
224805	Aars2	alanyl-tRNA synthetase 2, mitochondrial (putative)	1.547509	0.01014	0.07071
72040	Cdhr5	cadherin-related family member 5	1.546551	0.008283	0.06238
207965	Gm71	predicted gene 71	1.546073	2.61E-05	0.002182
100042198	Gm3716	predicted gene 3716	1.545595	0.0009196	0.01704
22601	Yap1	yes-associated protein 1	1.54464	0.0004501	0.01115
19120	Prm3	protamine 3	1.544163	0.4849	0.7033
74482	Ifitm7	interferon induced transmembrane protein 7	1.544163	0.006111	0.0526
19255	Ptpn2	protein tyrosine phosphatase, non-receptor type 2	1.54321	0.002098	0.02786
195531	Gm13152	predicted gene 13152	1.542258	0.06145	0.2061
18008	Nes	nestin	1.54202	0.002867	0.03348
12153	Bmp1	bone morphogenetic protein 1	1.54107	0.00462	0.04473
18783	Pla2g4a	phospholipase A2, group IVA (cytosolic, calcium-dependent)	1.54107	0.0008958	0.01687
319791	9430011C21Rik	RIKEN cDNA 9430011C21 gene	1.540357	0.002471	0.03081
12977	Csf1	colony stimulating factor 1 (macrophage)	1.538935	0.001536	0.02342
14066	F3	coagulation factor III	1.538935	0.0132	0.08286
242700	Il28ra	interleukin 28 receptor alpha	1.538935	0.0408	0.1614
74174	Gtsf1	gametocyte specific factor 1	1.538698	0.187	0.4076
14187	Akr1b8	aldo-keto reductase family 1, member B8	1.538225	0.0001933	0.007022
69073	1810019J16Rik	RIKEN cDNA 1810019J16 gene	1.537752	0.01608	0.09296
13040	Ctss	cathepsin S	1.537043	0.005159	0.04763
17349	Mlf1	myeloid leukemia factor 1	1.536807	0.0005136	0.01212
109218	Tmem139	transmembrane protein 139	1.53657	0.05144	0.1842

213326	Scyl2	SCY1-like 2 (S. cerevisiae)	1.53657	0.0003729	0.01011
18654	Pgf	placental growth factor	1.535862	0.06075	0.2048
19118	Prm1	protamine 1	1.535862	0.4525	0.677
20657	Sod3	superoxide dismutase 3, extracellular	1.535627	0.1113	0.2993
100041951	Gm3587	predicted gene 3587	1.535627	0.1064	0.291
12177	Bnip3l	BCL2/adenovirus E1B interacting protein 3-like	1.535391	0.0006249	0.01363
78388	Mvp	major vault protein	1.535391	0.006252	0.05321
208795	Tmem63a	transmembrane protein 63a	1.535155	0.005005	0.04674
27392	Pign	phosphatidylinositol glycan anchor biosynthesis, class N	1.534684	0.03607	0.1501
20660	Sorl1	sortilin-related receptor, LDLR class A repeats-containing	1.534448	0.001011	0.01786
232966	Zfp114	zinc finger protein 114	1.533507	6.21E-05	0.00362
52570	Ccdc69	coiled-coil domain containing 69	1.533272	0.004294	0.04274
230971	Megf6	multiple EGF-like-domains 6	1.533037	0.0442	0.1691
19329	Rab17	RAB17, member RAS oncogene family	1.532802	0.1827	0.4016
21815	Tgif1	TGFB-induced factor homeobox 1	1.531863	0.003587	0.03823
18080	Nin	ninein	1.531394	6.70E-05	0.00373
55983	Pdzrn3	PDZ domain containing RING finger 3	1.53069	0.1215	0.3162
20868	Stk10	serine/threonine kinase 10	1.530222	0.001097	0.01868
65973	Asph	aspartate-beta-hydroxylase	1.529988	0.002292	0.02954
16627	Klra1	killer cell lectin-like receptor, subfamily A, member 1	1.529754	0.4066	0.636
170753	Zfp704	zinc finger protein 704	1.52952	0.02796	0.1301
78878	B230206F22Rik	RIKEN cDNA B230206F22 gene	1.529286	0.08078	0.2444
11761	Aox1	aldehyde oxidase 1	1.528818	0.0004465	0.0111
246747	Adig	adipogenin	1.528117	0.001959	0.02699
269120	Optc	opticin	1.527884	0.5601	0.7587
16439	Itpr2	inositol 1,4,5-triphosphate receptor 2	1.52765	0.0001748	0.006581
108114	Slc22a7	solute carrier family 22 (organic anion transporter), member 7	1.526718	0.03767	0.1541
57342	Parva	parvin, alpha	1.526485	0.0002142	0.007384
14701	Gng12	guanine nucleotide binding protein (G protein), gamma 12	1.525553	0.0006044	0.01344
66889	Rnf128	ring finger protein 128	1.525553	0.01049	0.07219
11853	Rhoc	ras homolog gene family, member C	1.525088	0.0008657	0.01661
100041098	Gm15431	predicted gene 15431	1.525088	0.008154	0.06182
20862	Stfa2	stefin A2	1.523926	0.0486	0.1784
69675	Pxdn	peroxidasin homolog (Drosophila)	1.523926	0.006079	0.05249
382018	Unc13a	unc-13 homolog A (C. elegans)	1.523926	0.01733	0.09727
333467	B020031M17Rik	RIKEN cDNA B020031M17 gene	1.522302	0.1474	0.354
17972	Ncf4	neutrophil cytosolic factor 4	1.52207	0.01359	0.08427
75472	1700009P17Rik	RIKEN cDNA 1700009P17 gene	1.521607	0.0131	0.08253
21346	Tagln2	transgelin 2	1.521375	0.001564	0.02361
654824	Ankrd37	ankyrin repeat domain 37	1.521375	0.0001127	0.005054
78896	1500015O10Rik	RIKEN cDNA 1500015O10 gene	1.521144	0.4023	0.6322
12508	Cd53	CD53 antigen	1.520681	0.03566	0.1492
56016	Hebp2	heme binding protein 2	1.520681	0.02063	0.108
434168	Gm5590	predicted gene 5590	1.519988	0.2009	0.4267
68178	Cgnl1	cingulin-like 1	1.519526	0.01983	0.1056
70693	Gpr125	G protein-coupled receptor 125	1.518372	0.04719	0.1758
666704	Samd1	sterile alpha motif domain containing 1	1.518372	0.0006448	0.01383
56524	Mpp6	membrane protein, palmitoylated 6 (MAGUK p55 subfamily member 6)	1.517681	0.001426	0.02229
13052	Cxadr	coxsackie virus and adenovirus receptor	1.51722	0.0001193	0.005236

14734	Gpc3	glypican 3	1.51699	0.0003436	0.009724
211612	Ptchd1	patched domain containing 1	1.51699	0.004608	0.04472
64297	Gprc5b	G protein-coupled receptor, family C, group 5, member B	1.51607	0.001212	0.02004
380912	Zfp395	zinc finger protein 395	1.51607	0.001389	0.02189
14991	H2-M3	histocompatibility 2, M region locus 3	1.514463	0.003723	0.03903
16206	Lrig1	leucine-rich repeats and immunoglobulin-like domains 1	1.514463	3.72E-05	0.002743
433182	Gm5506	predicted gene 5506	1.51263	0.009028	0.06578
66491	Polr2l	polymerase (RNA) II (DNA directed) polypeptide L	1.512173	0.01023	0.07112
70458	2610318N02Rik	RIKEN cDNA 2610318N02 gene	1.512173	0.3678	0.6028
14282	Fosb	FBJ osteosarcoma oncogene B	1.511487	0.00368	0.03885
622408	Gm6320	predicted gene 6320	1.511487	0.1863	0.4067
73748	Gadl1	glutamate decarboxylase-like 1	1.509434	0.3143	0.5508
268481	Krt222	keratin 222	1.509434	0.1651	0.3787
320204	4833442J19Rik	RIKEN cDNA 4833442J19 gene	1.508978	0.003	0.03425
19039	Lgals3bp	lectin, galactoside-binding, soluble, 3 binding protein	1.508296	0.000337	0.009646
109624	Cald1	caldesmon 1	1.507613	0.03012	0.1359
435333	LOC435333	similar to monoclonal antibody heavy chain	1.507613	0.007268	0.05807
12608	Cebpb	CCAAT/enhancer binding protein (C/EBP), beta	1.506478	0.0007634	0.01534
791273	B630006N21Rik	RIKEN cDNA B630006N21 gene	1.505571	0.03895	0.1571
240725	Sulf1	sulfatase 1	1.505344	0.00381	0.0395
238317	C130039O16Rik	RIKEN cDNA C130039O16 gene	1.504438	0.04613	0.1734
71145	Scara5	scavenger receptor class A, member 5 (putative)	1.503759	0.007924	0.06104
104027	Synpo	synaptopodin	1.503533	0.01848	0.1009
434246	Trim72	tripartite motif-containing 72	1.503533	0.08263	0.2479
384059	Tlr12	toll-like receptor 12	1.502855	0.02839	0.131
17240	Mdfi	MyoD family inhibitor	1.502404	0.04148	0.163
328035	Fads6	fatty acid desaturase domain family, member 6	1.501953	0.3825	0.6157
234362	Zfp868	zinc finger protein 868	1.501727	0.002576	0.0315
74376	Myo18b	myosin XVIIIb	1.501276	0.3288	0.5652
66857	Plbd1	phospholipase B domain containing 1	1.500825	0.1356	0.3372
106878	2010002N04Rik	RIKEN cDNA 2010002N04 gene	1.500825	0.004805	0.04556
13648	Klk1b9	kallikrein 1-related peptidase b9	1.5006	0.4679	0.6897
16878	Lif	leukemia inhibitory factor	1.5006	0.25	0.4841
78376	Ng23	Ng23 protein	1.500375	0.1912	0.4132
18805	Pld1	phospholipase D1	1.50015	0.03009	0.1358
381925	Ppapdc1a	phosphatidic acid phosphatase type 2 domain containing 1A	1.4997	0.2111	0.4385
235631	Prss50	protease, serine, 50	1.499475	0.3923	0.6239
15507	Hspb1	heat shock protein 1	1.49925	0.005733	0.05063
66902	Mtap	methylthioadenosine phosphorylase	1.49925	0.03148	0.1393
12633	Cflar	CASP8 and FADD-like apoptosis regulator	1.499026	0.002788	0.03278
69660	Tmbim1	transmembrane BAX inhibitor motif containing 1	1.497903	0.0001647	0.006304
75472	1700009P17Rik	RIKEN cDNA 1700009P17 gene	1.497903	0.006907	0.05627
21416	Tcf7l2	transcription factor 7-like 2, T-cell specific, HMG-box	1.497679	0.01101	0.07421
329384	Ptrh1	peptidyl-tRNA hydrolase 1 homolog (S. cerevisiae)	1.49723	0.003866	0.03991
14955	H19	H19 fetal liver mRNA	1.497006	0.001453	0.02251
213208	Il20rb	interleukin 20 receptor beta	1.496558	0.07562	0.2354
67009	Ttc23	tetratricopeptide repeat domain 23	1.496334	0.001548	0.02348
20538	Slc6a2	solute carrier family 6 (neurotransmitter transporter, noradrenalin), member 2	1.495886	0.2166	0.4455
100040880	Gm3020	predicted gene 3020	1.495439	0.2064	0.4326

100041241	Gm3227	predicted gene 3227	1.494768	0.07831	0.2399
13363	Dhh	desert hedgehog	1.493429	9.87E-05	0.004628
52535	Mett11d1	methyltransferase 11 domain containing 1	1.493429	0.001301	0.02099
17150	Mfap2	microfibrillar-associated protein 2	1.493206	0.01313	0.0826
20193	S100a1	S100 calcium binding protein A1	1.492983	6.82E-05	0.003738
259300	Ehd2	EH-domain containing 2	1.492983	0.001949	0.02695
66350	Pla2g12a	phospholipase A2, group XIIA	1.492315	0.001914	0.02665
330695	Ctxn1	cortexin 1	1.491424	0.03375	0.1452
17314	Mgmt	O-6-methylguanine-DNA methyltransferase	1.491202	0.1128	0.3017
431706	Zfp457	zinc finger protein 457	1.490757	0.03241	0.1417
76566	Fam101b	family with sequence similarity 101, member B	1.490535	0.08571	0.2541
268739	Arhgef40	Rho guanine nucleotide exchange factor (GEF) 40	1.490535	0.006106	0.0526
71918	Zcchc24	zinc finger, CCHC domain containing 24	1.490091	0.000173	0.006555
80859	Nfkbiz	nuclear factor of kappa light polypeptide gene enhancer in B-cells inhibitor, zeta	1.490091	0.002104	0.02789
15018	H2-Q7	histocompatibility 2, Q region locus 7	1.489203	0.003548	0.03803
333452	Gm5132	predicted gene 5132	1.489203	0.08169	0.2462
12638	Cftr	cystic fibrosis transmembrane conductance regulator homolog	1.48876	0.173	0.3891
13649	Egfr	epidermal growth factor receptor	1.488317	0.3961	0.6266
72925	Mar-01	membrane-associated ring finger (C3HC4) 1	1.488317	0.1529	0.3625
116914	Slc19a2	solute carrier family 19 (thiamine transporter), member 2	1.488095	0.002124	0.028
110557	H2-Q6	histocompatibility 2, Q region locus 6	1.487652	0.004798	0.04552
100039042	Gm2016	predicted gene 2016	1.487431	0.01127	0.07522
107581	Col16a1	collagen, type XVI, alpha 1	1.486768	0.002378	0.03016
107581	Col16a1	collagen, type XVI, alpha 1	1.486768	0.00814	0.06176
16624	Klk1b8	kallikrein 1-related peptidase b8	1.486105	0.05393	0.1896
17698	Msn	moesin	1.485884	0.002406	0.03037
21949	Tnfsf8	tumor necrosis factor (ligand) superfamily, member 8	1.485663	0.1389	0.3418
78323	2310046O06Rik	RIKEN cDNA 2310046O06 gene	1.485663	0.003227	0.03596
13167	Dbi	diazepam binding inhibitor	1.485443	0.0006352	0.01368
102366	BB211804	expressed sequence BB211804	1.485443	0.01772	0.09839
72462	Rrp1b	ribosomal RNA processing 1 homolog B (S. cerevisiae)	1.485222	0.006119	0.05261
100043335	Gm4371	eukaryotic translation initiation factor 3, subunit I pseudogene	1.485001	0.1194	0.3127
77870	E130116L18Rik	RIKEN cDNA E130116L18 gene	1.484781	0.537	0.7416
233274	Siglech	sialic acid binding Ig-like lectin H	1.484561	0.009017	0.06573
12521	Cd82	CD82 antigen	1.48412	0.007018	0.05676
226432	Ipo9	importin 9	1.483239	9.44E-05	0.004481
331046	Tgm4	transglutaminase 4 (prostate)	1.48236	0.001377	0.02176
110606	Fntb	farnesyltransferase, CAAX box, beta	1.481701	0.0267	0.1264
228839	Tgif2	TGFB-induced factor homeobox 2	1.481481	0.2443	0.4778
68153	Gtf2e2	general transcription factor II E, polypeptide 2 (beta subunit)	1.481262	0.0006596	0.01403
53622	Krt85	keratin 85	1.481043	0.02605	0.1244
64654	Fgf23	fibroblast growth factor 23	1.480823	0.2096	0.4366
14314	Fstl1	follistatin-like 1	1.480604	0.00601	0.05219
104080	Nxph4	neurexophilin 4	1.479071	0.03603	0.15
380712	Tlcd2	TLC domain containing 2	1.478852	7.39E-05	0.003978
56615	Mgst1	microsomal glutathione S-transferase 1	1.478634	0.06303	0.2095
20619	Snap23	synaptosomal-associated protein 23	1.478415	0.1642	0.3775
14230	Fkbp10	FK506 binding protein 10	1.478197	0.005399	0.04892
78829	Tsc22d4	TSC22 domain family, member 4	1.47776	0.09925	0.2779

233744	Spon1	spondin 1, (f-spondin) extracellular matrix protein	1.477541	0.006828	0.05599
22035	Tnfsf10	tumor necrosis factor (ligand) superfamily, member 10	1.477323	0.1912	0.4132
16199	Il9r	interleukin 9 receptor	1.477105	0.155	0.3648
103067	AA522020	expressed sequence AA522020	1.477105	0.2279	0.4587
238037	BC068281	cDNA sequence BC068281	1.477105	0.006431	0.0541
434280	Gm5607	predicted gene 5607	1.476887	0.2825	0.5179
18129	Notch2	Notch gene homolog 2 (Drosophila)	1.475797	0.001266	0.02067
12521	Cd82	CD82 antigen	1.475144	0.001984	0.02709
56615	Mgst1	microsomal glutathione S-transferase 1	1.475144	0.04822	0.1777
217344	Rhbdf2	rhomboid 5 homolog 2 (Drosophila)	1.475144	0.001884	0.02634
380705	Tmem102	transmembrane protein 102	1.475144	0.03907	0.1573
621080	AI429214	expressed sequence AI429214	1.475144	0.00357	0.03815
11504	Adamts1	a disintegrin-like and metallopeptidase (reprolysin type) with thrombospondin type 1 motif, 1	1.474709	0.01911	0.1031
207175	Cetn4	centrin 4	1.474491	0.003243	0.03607
338417	Scgb1c1	secretoglobin, family 1C, member 1	1.474491	0.4791	0.6986
21401	Tcea3	transcription elongation factor A (SII), 3	1.474057	0.06189	0.2072
22271	Upp1	uridine phosphorylase 1	1.473188	0.08009	0.2434
60344	Fign	fidgetin	1.473188	0.0489	0.179
56078	Car5b	carbonic anhydrase 5b, mitochondrial	1.472971	0.004357	0.0431
71660	Rarres2	retinoic acid receptor responder (tazarotene induced) 2	1.472971	0.09908	0.2779
97908	Hist1h3g	histone cluster 1, H3g	1.472971	0.03496	0.1482
328424	Kcnrg	potassium channel regulator	1.472971	0.1308	0.3306
64058	Perp	PERP, TP53 apoptosis effector	1.472754	0.1666	0.381
14598	Ggt1	gamma-glutamyltransferase 1	1.472537	0.1051	0.2885
640979	LOC640979	ig heavy chain V region B1-8/186-2-like	1.472537	0.0007261	0.01488
72947	Agxt2l2	alanine-glyoxylate aminotransferase 2-like 2	1.47232	0.002946	0.03387
78323	2310046O06Rik	RIKEN cDNA 2310046O06 gene	1.47232	0.001212	0.02004
100503215	LOC100503215	hypothetical protein LOC100503215	1.471887	0.04911	0.1795
113845	Vmn1r48	vomeronasal 1 receptor 48	1.47167	0.03507	0.1484
23880	Fyb	FYN binding protein	1.471237	0.2987	0.5348
545260	Arsi	arylsulfatase i	1.470588	0.3388	0.5745
12177	Bnip3l	BCL2/adenovirus E1B interacting protein 3-like	1.470372	0.0001431	0.005828
67092	Gatm	glycine amidinotransferase (L-arginine:glycine amidinotransferase)	1.469724	0.001985	0.02709
231125	Zfyve28	zinc finger, FYVE domain containing 28	1.469508	0.1015	0.2823
100043484	Gm4470	predicted gene 4470	1.469292	0.1471	0.3535
14412	Slc6a13	solute carrier family 6 (neurotransmitter transporter, GABA), member 13	1.46886	0.289	0.525
74627	4930413E15Rik	RIKEN cDNA 4930413E15 gene	1.46886	0.2881	0.5239
56050	Cyp39a1	cytochrome P450, family 39, subfamily a, polypeptide 1	1.468644	0.01778	0.09857
669389	Gm9456	predicted gene 9456	1.468644	0.02451	0.1197
272551	Gins2	GINS complex subunit 2 (Psf2 homolog)	1.468429	0.002097	0.02786
69386	Hist1h4h	histone cluster 1, H4h	1.468213	0.006904	0.05627
67454	Ikbip	IKBKB interacting protein	1.467782	0.0008959	0.01687
94242	Tinagl1	tubulointerstitial nephritis antigen-like 1	1.467567	0.03199	0.1407
76096	5830468K08Rik	RIKEN cDNA 5830468K08 gene	1.467136	0.006983	0.05654
60527	Fads3	fatty acid desaturase 3	1.466921	0.001782	0.02545
22329	Vcam1	vascular cell adhesion molecule 1	1.466706	0.03424	0.1464
12362	Casp1	caspase 1	1.466276	0.09088	0.264
67507	1700019N19Rik	RIKEN cDNA 1700019N19 gene	1.464987	0.4868	0.7048
74410	Ttll11	tubulin tyrosine ligase-like family, member 11	1.463915	0.01078	0.07326

100038941	Vmn2r121	vomeronasal 2, receptor 121	1.463915	0.06159	0.2064
12661	Chl1	cell adhesion molecule with homology to L1CAM	1.4637	0.00488	0.04601
171506	H1foo	H1 histone family, member O, oocyte-specific	1.463486	0.01663	0.09482
20445	St6galnac1	ST6 (alpha-N-acetyl-neuraminyl-2,3-beta-galactosyl-1,3)-N-acetylgalactosaminide alpha-2,6-sialyltransferase 1	1.463272	0.3748	0.6089
56226	Espn	espin	1.461561	0.03158	0.1397
234839	Fam38a	family with sequence similarity 38, member A	1.461561	0.006949	0.05649
448987	Fbxl7	F-box and leucine-rich repeat protein 7	1.461347	0.07928	0.2418
638517	LOC638517	cyclic AMP-responsive element-binding protein 3-like protein 2-like	1.461347	0.07424	0.2326
231583	Slc26a1	solute carrier family 26 (sulfate transporter), member 1	1.46092	0.008946	0.06544
252967	Ropn1l	ropporin 1-like	1.46092	0.04166	0.1634
216616	Efemp1	epidermal growth factor-containing fibulin-like extracellular matrix protein 1	1.460707	0.006314	0.05346
171286	Slc12a8	solute carrier family 12 (potassium/chloride transporters), member 8	1.460494	0.0009869	0.01769
18213	Ntrk3	neurotrophic tyrosine kinase, receptor, type 3	1.460067	0.0006562	0.014
14131	Fcgr3	Fc receptor, IgG, low affinity III	1.459854	0.01941	0.1043
77634	Snapc3	small nuclear RNA activating complex, polypeptide 3	1.459854	0.00176	0.02539
320825	Samd5	sterile alpha motif domain containing 5	1.459641	0.0005279	0.01231
13631	Eef2k	eukaryotic elongation factor-2 kinase	1.459215	0.01493	0.08913
17150	Mfap2	microfibrillar-associated protein 2	1.459215	0.0146	0.08797
65970	Lima1	LIM domain and actin binding 1	1.459215	0.002258	0.02923
75605	Kdm5b	lysine (K)-specific demethylase 5B	1.458789	0.00224	0.02906
268741	Tox4	TOX high mobility group box family member 4	1.458789	0.06619	0.2163
100043074	Gm4211	predicted gene 4211	1.458789	0.002713	0.03222
69430	1700048O20Rik	RIKEN cDNA 1700048O20 gene	1.458576	0.1049	0.2882
213056	Fam126b	family with sequence similarity 126, member B	1.458576	0.001004	0.01783
231452	Sdad1	SDA1 domain containing 1	1.458151	0.2013	0.4273
381045	Ccdc58	coiled-coil domain containing 58	1.457938	0.003611	0.03841
78439	A930037H05Rik	RIKEN cDNA A930037H05 gene	1.457726	0.05818	0.1991
171095	Il17rc	interleukin 17 receptor C	1.457513	0.07163	0.2272
403346	A730062M13Rik	RIKEN cDNA A730062M13 gene	1.457513	0.3207	0.5572
78781	Zc3hav1	zinc finger CCCH type, antiviral 1	1.457089	0.0596	0.2026
27015	Polk	polymerase (DNA directed), kappa	1.456664	0.02645	0.1256
12521	Cd82	CD82 antigen	1.45624	0.01702	0.09625
384198	Fam47e	family with sequence similarity 47, member E	1.45624	0.3928	0.6241
20197	S100a3	S100 calcium binding protein A3	1.456028	0.0003387	0.009658
235281	Scn3b	sodium channel, voltage-gated, type III, beta	1.456028	0.317	0.5536
72433	Rab38	RAB38, member of RAS oncogene family	1.455816	0.07324	0.2306
93871	Brwd1	bromodomain and WD repeat domain containing 1	1.455816	0.05424	0.1903
246079	Defb9	defensin beta 9	1.455604	0.4941	0.71
18217	Ntsr2	neurotensin receptor 2	1.455392	0.03936	0.1581
320662	Casc1	cancer susceptibility candidate 1	1.455392	0.003806	0.0395
12047	Bcl2a1d	B-cell leukemia/lymphoma 2 related protein A1d	1.45518	0.0338	0.1453
239405	Rspo2	R-spondin 2 homolog (Xenopus laevis)	1.45518	0.06764	0.2194
106258	AI790442	expressed sequence AI790442	1.454757	0.3398	0.5755
209200	Dtx3l	deltex 3-like (Drosophila)	1.454757	0.009214	0.06646
268564	Zbtb1	zinc finger and BTB domain containing 1	1.454757	0.00288	0.03352
78403	2900041M22Rik	RIKEN cDNA 2900041M22 gene	1.454334	0.01112	0.07467
213211	Rnf26	ring finger protein 26	1.453911	0.09171	0.2655
628900	Serpina3i	serine (or cysteine) peptidase inhibitor, clade A, member 3I	1.453911	0.01384	0.08511
56226	Espn	espin	1.4537	0.05619	0.1942

68119	Cmtm3	CKLF-like MARVEL transmembrane domain containing 3	1.4537	0.00274	0.03242
21632	Tcrg-V1	T-cell receptor gamma, variable 1	1.453488	0.1396	0.343
99503	AA517023	expressed sequence AA517023	1.453066	0.009219	0.06646
68867	Rnf122	ring finger protein 122	1.452855	0.02018	0.1067
54366	Ctnnal1	catenin (cadherin associated protein), alpha-like 1	1.452222	0.2125	0.4403
70745	6330418B08Rik	RIKEN cDNA 6330418B08 gene	1.452222	0.07152	0.227
83554	Fstl3	follistatin-like 3	1.4518	0.001804	0.0256
240752	Pik3c2b	phosphoinositide-3-kinase, class 2, beta polypeptide	1.451379	0.03971	0.1587
56485	Slc2a5	solute carrier family 2 (facilitated glucose transporter), member 5	1.451168	0.1571	0.3673
68239	Krt42	keratin 42	1.451168	0.02214	0.1128
52118	Pvr	poliovirus receptor	1.450958	0.008544	0.0636
219151	Scara3	scavenger receptor class A, member 3	1.450537	0.00427	0.04262
76281	Tax1bp3	Tax1 (human T-cell leukemia virus type I) binding protein 3	1.450326	0.00291	0.0337
18619	Penk	preproenkephalin	1.449906	0.2981	0.5341
216343	Tph2	tryptophan hydroxylase 2	1.449906	0.1886	0.4097
76469	Cmya5	cardiomyopathy associated 5	1.449485	0.2294	0.4606
70355	Gprc5c	G protein-coupled receptor, family C, group 5, member C	1.449275	0.05042	0.1824
15251	Hif1a	hypoxia inducible factor 1, alpha subunit	1.449065	0.007931	0.06104
70673	Prdm16	PR domain containing 16	1.448646	0.0308	0.1374
19255	Ptpn2	protein tyrosine phosphatase, non-receptor type 2	1.447387	0.00595	0.05195
14067	F5	coagulation factor V	1.447178	0.4282	0.6552
12177	Bnip3l	BCL2/adenovirus E1B interacting protein 3-like	1.445922	0.001348	0.0215
50873	Park2	Parkinson disease (autosomal recessive, juvenile) 2, parkin	1.445922	0.0004883	0.01171
18983	Cnot7	CCR4-NOT transcription complex, subunit 7	1.445504	0.002346	0.03
16852	Lgals1	lectin, galactose binding, soluble 1	1.44446	0.09073	0.2638
70508	Bbx	bobby sox homolog (Drosophila)	1.444252	0.1535	0.3632
15228	Foxg1	forkhead box G1	1.444043	0.002156	0.02823
78878	B230206F22Rik	RIKEN cDNA B230206F22 gene	1.443835	0.05976	0.2028
16409	Itgam	integrin alpha M	1.443001	0.2181	0.4472
83493	Sacm1l	SAC1 (suppressor of actin mutations 1, homolog)-like (S. cerevisiae)	1.443001	0.00599	0.05214
436022	6030429G01Rik	RIKEN cDNA 6030429G01 gene	1.443001	0.08973	0.2619
20347	Sema3b	sema domain, immunoglobulin domain (Ig), short basic domain, secreted, (semaphorin) 3B	1.442793	0.006049	0.05238
66929	Asf1b	ASF1 anti-silencing function 1 homolog B (S. cerevisiae)	1.442793	0.001744	0.02528
70537	5730419F03Rik	RIKEN cDNA 5730419F03 gene	1.442377	0.3147	0.551
19224	Ptgs1	prostaglandin-endoperoxide synthase 1	1.441961	0.04853	0.1783
77956	A930026B05Rik	RIKEN cDNA A930026B05 gene	1.441753	0.009954	0.06981
269423	3110057O12Rik	RIKEN cDNA 3110057O12 gene	1.441753	0.01585	0.09248
12919	Crhbp	corticotropin releasing hormone binding protein	1.441338	0.2709	0.5061
216974	Proca1	protein interacting with cyclin A1	1.441338	0.04685	0.175
71918	Zcchc24	zinc finger, CCHC domain containing 24	1.44113	0.0001275	0.005439
66729	Ankrd61	ankyrin repeat domain 61	1.440507	0.006865	0.05612
12215	Bsg	basigin	1.439885	0.007507	0.05922
622640	Gm6337	predicted gene 6337	1.439885	0.2787	0.5143
104444	Rexo2	REX2, RNA exonuclease 2 homolog (S. cerevisiae)	1.43947	0.001259	0.02061
109593	Lmo3	LIM domain only 3	1.43947	0.01134	0.07546
218121	Mboat1	membrane bound O-acyltransferase domain containing 1	1.439263	0.1585	0.3695
100342	Fam46b	family with sequence similarity 46, member B	1.438228	0.06131	0.2059
238393	Serpina3f	serine (or cysteine) peptidase inhibitor, clade A, member 3F	1.438021	0.002384	0.03021
77041	Arsk	arylsulfatase K	1.437401	0.002089	0.02786

75805	Nln	neurolysin (metallopeptidase M3 family)	1.437195	6.82E-05	0.003738
13003	Vcan	versican	1.436988	0.4281	0.6551
20229	Sat1	spermidine/spermine N1-acetyl transferase 1	1.436782	0.009361	0.06712
81910	Rrbp1	ribosome binding protein 1	1.436782	0.001274	0.02073
70294	Rnf126	ring finger protein 126	1.436575	0.00671	0.05537
330260	Pon2	paraoxonase 2	1.435956	0.002829	0.03309
100038846	Gm1973	predicted gene 1973	1.43575	0.3325	0.5686
18128	Notch1	Notch gene homolog 1 (Drosophila)	1.435338	0.2467	0.4807
12946	Cr1l	complement component (3b/4b) receptor 1-like	1.434926	0.007072	0.05694
628705	Gm6907	predicted gene 6907	1.434514	0.0398	0.159
100037278	Fam129c	family with sequence similarity 129, member C	1.434514	0.0005908	0.01328
71839	Osgin1	oxidative stress induced growth inhibitor 1	1.434309	0.03416	0.1462
218865	Chdh	choline dehydrogenase	1.434309	0.02618	0.1248
403347	F830014O18Rik	RIKEN cDNA F830014O18 gene	1.434309	0.06341	0.2103
11854	Rhod	ras homolog gene family, member D	1.434103	2.66E-05	0.002212
67512	Agpat2	1-acylglycerol-3-phosphate O-acyltransferase 2 (lysophosphatidic acid acyltransferase, beta)	1.433692	0.002696	0.03218
320405	Cadps2	Ca2+-dependent activator protein for secretion 2	1.433692	0.006312	0.05346
18797	Plcb3	phospholipase C, beta 3	1.433075	0.002988	0.03416
215456	Gpat2	glycerol-3-phosphate acyltransferase 2, mitochondrial	1.432254	0.003781	0.03935
66395	Ahnak	AHNAK nucleoprotein (desmoyokin)	1.432049	0.07614	0.2365
70572	Ipo5	importin 5	1.431844	0.002088	0.02786
270192	Rab6b	RAB6B, member RAS oncogene family	1.431844	0.0001669	0.006375
12931	Crlf1	cytokine receptor-like factor 1	1.431434	0.00644	0.05413
12978	Csf1r	colony stimulating factor 1 receptor	1.431434	0.00954	0.06794
12759	Clu	clusterin	1.431229	0.005244	0.04813
210135	Zfp180	zinc finger protein 180	1.431229	0.002188	0.02851
99929	Tiparp	TCDD-inducible poly(ADP-ribose) polymerase	1.430615	0.004206	0.04219
21677	Tead2	TEA domain family member 2	1.430411	0.0005443	0.01255
380912	Zfp395	zinc finger protein 395	1.430411	0.006644	0.05522
380912	Zfp395	zinc finger protein 395	1.430411	0.0312	0.1384
20305	Ccl6	chemokine (C-C motif) ligand 6	1.430001	0.1025	0.2837
12229	Btk	Bruton agammaglobulinemia tyrosine kinase	1.42898	0.001082	0.01851
17130	Smad6	MAD homolog 6 (Drosophila)	1.428367	0.01588	0.09256
68339	Ccdc88c	coiled-coil domain containing 88C	1.428367	0.05064	0.1828
77800	4933406J10Rik	RIKEN cDNA 4933406J10 gene	1.428163	0.1111	0.2991
11813	Apoc2	apolipoprotein C-II	1.427756	0.2908	0.5269
17025	Alad	aminolevulinate, delta-, dehydratase	1.427552	0.003984	0.04075
57765	Tbx21	T-box 21	1.427348	0.0004329	0.01101
215627	Zbtb8b	zinc finger and BTB domain containing 8b	1.427348	0.02105	0.1094
50530	Mfap5	microfibrillar associated protein 5	1.427144	0.1147	0.3051
74091	Npl	N-acetylneuraminate pyruvate lyase	1.427144	0.01863	0.1015
21677	Tead2	TEA domain family member 2	1.426941	0.003458	0.0374
66922	Rras2	related RAS viral (r-ras) oncogene homolog 2	1.426737	0.0111	0.07461
74747	Ddit4	DNA-damage-inducible transcript 4	1.42633	0.0005699	0.01292
12499	Entpd5	ectonucleoside triphosphate diphosphohydrolase 5	1.425923	0.00248	0.03083
78829	Tsc22d4	TSC22 domain family, member 4	1.42572	0.003325	0.03658
27392	Pign	phosphatidylinositol glycan anchor biosynthesis, class N	1.425517	0.246	0.48
16776	Lama5	laminin, alpha 5	1.425314	0.0168	0.09544
212974	Athl1	ATH1, acid trehalase-like 1 (yeast)	1.425314	0.01631	0.09362

100306953	4122401K19Rik	RIKEN cDNA 4122401K19 gene	1.424704	0.009184	0.0664
11829	Aqp4	aquaporin 4	1.424096	0.0004384	0.01101
70449	2610209C05Rik	RIKEN cDNA 2610209C05 gene	1.424096	0.2279	0.4588
100039027	Gm2011	predicted gene 2011	1.42369	0.03272	0.1426
233335	Synm	synemin, intermediate filament protein	1.423488	0.006686	0.0553
56795	Arl10	ADP-ribosylation factor-like 10	1.423082	0.1066	0.2912
67464	Entpd4	ectonucleoside triphosphate diphosphohydrolase 4	1.423082	0.003578	0.03817
57916	Tnfrsf13b	tumor necrosis factor receptor superfamily, member 13b	1.422071	0.03709	0.1526
74004	Jakmip3	janus kinase and microtubule interacting protein 3	1.421868	0.05369	0.189
12177	Bnip3l	BCL2/adenovirus E1B interacting protein 3-like	1.42106	0.00192	0.0267
19132	Prph	peripherin	1.42106	0.1046	0.2876
13631	Eef2k	eukaryotic elongation factor-2 kinase	1.420858	0.006316	0.05346
13713	Elk3	ELK3, member of ETS oncogene family	1.420253	0.009616	0.06824
15275	Hk1	hexokinase 1	1.420253	0.04755	0.1765
20498	Slc12a4	solute carrier family 12, member 4	1.420051	0.00397	0.04069
70572	Ipo5	importin 5	1.420051	0.0006933	0.01448
665225	Gm7544	predicted gene 7544	1.420051	0.2607	0.4959
12512	Cd63	CD63 antigen	1.419849	0.006522	0.05458
13400	Dmpk	dystrophia myotonica-protein kinase	1.419648	0.1981	0.4232
19255	Ptpn2	protein tyrosine phosphatase, non-receptor type 2	1.419446	0.001432	0.02232
108655	Foxp1	forkhead box P1	1.419245	0.03252	0.142
67784	Plxnd1	plexin D1	1.418842	0.009834	0.06929
12799	Cnp	2',3'-cyclic nucleotide 3' phosphodiesterase	1.418641	0.02394	0.118
56215	Acin1	apoptotic chromatin condensation inducer 1	1.41844	0.01004	0.07018
68612	Ube2c	ubiquitin-conjugating enzyme E2C	1.41844	0.08436	0.2514
27204	Syn3	synapsin III	1.418037	0.4707	0.6922
18576	Pde3b	phosphodiesterase 3B, cGMP-inhibited	1.417033	0.004229	0.04235
233328	Lrrk1	leucine-rich repeat kinase 1	1.417033	0.04508	0.1712
170721	Papln	papilin, proteoglycan-like sulfated glycoprotein	1.416431	0.04416	0.169
14867	Gstm6	glutathione S-transferase, mu 6	1.415829	0.01686	0.09566
18390	Oprm1	opioid receptor, mu 1	1.415228	0.2659	0.5015
21667	Tdgf1	teratocarcinoma-derived growth factor 1	1.415228	0.4394	0.6654
328353	D030051J21Rik	RIKEN cDNA D030051J21 gene	1.415228	0.05116	0.1838
229900	Gbp6	guanylate binding protein 6	1.415028	0.002701	0.03218
54219	Cd320	CD320 antigen	1.414827	0.004754	0.04537
71994	Cnn3	calponin 3, acidic	1.414827	0.001025	0.01793
15288	Hmbs	hydroxymethylbilane synthase	1.414627	0.0005767	0.01303
20439	Siah2	seven in absentia 2	1.414627	0.0002085	0.007333
100126228	Gm9856	predicted gene 9856	1.414627	0.001243	0.02044
74969	4930466K18Rik	RIKEN cDNA 4930466K18 gene	1.414427	0.3071	0.5434
72465	Zfp131	zinc finger protein 131	1.414227	0.008431	0.06306
246728	Oas2	2'-5' oligoadenylate synthetase 2	1.413827	0.0599	0.2031
72318	Cyth4	cytohesin 4	1.413428	0.02241	0.1138
69080	Gmppa	GDP-mannose pyrophosphorylase A	1.413028	0.005517	0.0496
433966	5730422E09Rik	RIKEN cDNA 5730422E09 gene	1.413028	0.01261	0.08035
171095	Il17rc	interleukin 17 receptor C	1.412828	0.01727	0.09712
242109	Zfp697	zinc finger protein 697	1.41223	0.01431	0.08686
70333	Cd3eap	CD3E antigen, epsilon polypeptide associated protein	1.41203	0.001667	0.02472
20409	Ostf1	osteoclast stimulating factor 1	1.411831	0.004677	0.04506

50722	Dkkl1	dickkopf-like 1	1.411433	0.005961	0.05198
100669	9930105H17Rik	RIKEN cDNA 9930105H17 gene	1.411433	0.007891	0.06099
15932	Idua	iduronidase, alpha-L-	1.411233	0.2352	0.4673
214944	Mobkl2b	MOB1, Mps One Binder kinase activator-like 2B (yeast)	1.411034	0.00202	0.02731
17960	Nat1	N-acetyl transferase 1	1.410835	0.001524	0.02329
67473	Slc47a1	solute carrier family 47, member 1	1.410835	0.02422	0.1188
73916	Ift57	intraflagellar transport 57 homolog (Chlamydomonas)	1.410636	0.002186	0.02851
252864	Dusp15	dual specificity phosphatase-like 15	1.410238	0.03585	0.1497
79554	Gltpd1	glycolipid transfer protein domain containing 1	1.409841	0.003193	0.03567
66494	Prelid1	PRELI domain containing 1	1.409642	0.0002084	0.007333
77614	C030044M21Rik	RIKEN cDNA C030044M21 gene	1.409245	0.0402	0.1599
240638	Slc16a12	solute carrier family 16 (monocarboxylic acid transporters), member 12	1.409245	0.4724	0.6934
76718	Catsperg2	cation channel, sperm-associated, gamma 2	1.408649	0.2178	0.447
74100	Arpp21	cyclic AMP-regulated phosphoprotein, 21	1.408252	0.003704	0.03894
140703	Emid1	EMI domain containing 1	1.408054	0.07232	0.2287
13206	Ddx4	DEAD (Asp-Glu-Ala-Asp) box polypeptide 4	1.407856	0.09296	0.2677
70274	Ly6g6e	lymphocyte antigen 6 complex, locus G6E	1.407856	0.05505	0.1922
13713	Elk3	ELK3, member of ETS oncogene family	1.40746	0.006561	0.05472
77271	9430024F10Rik	RIKEN cDNA 9430024F10 gene	1.40746	0.006856	0.05609
11650	Alppl2	alkaline phosphatase, placental-like 2	1.406866	0.3874	0.6195
230766	Fam167b	family with sequence similarity 167, member B	1.406668	0.1344	0.3353
252973	Grhl2	grainyhead-like 2 (Drosophila)	1.406272	0.07267	0.2294
239096	Cdh24	cadherin-like 24	1.405679	0.003173	0.03551
12406	Serpinh1	serine (or cysteine) peptidase inhibitor, clade H, member 1	1.405481	0.03095	0.1378
320655	Pgap3	post-GPI attachment to proteins 3	1.405481	0.001797	0.02553
12984	Csf2rb2	colony stimulating factor 2 receptor, beta 2, low-affinity (granulocyte-macrophage)	1.405284	0.009505	0.06781
14683	Gnas	GNAS (guanine nucleotide binding protein, alpha stimulating) complex locus	1.405086	0.4043	0.634
229214	Qrfpr	pyroglutamylated RFamide peptide receptor	1.404692	0.002757	0.03254
319899	Dock6	dedicator of cytokinesis 6	1.404692	0.0002501	0.007993
52639	Wipi1	WD repeat domain, phosphoinositide interacting 1	1.404494	0.00456	0.04453
72400	Pinx1	PIN2/TERF1 interacting, telomerase inhibitor 1	1.404297	0.2182	0.4474
19054	Ppp2r3d	protein phosphatase 2 (formerly 2A), regulatory subunit B'', delta	1.403903	0.01849	0.1009
56215	Acin1	apoptotic chromatin condensation inducer 1	1.403706	0.2169	0.4456
78244	Dnajc21	DnaJ (Hsp40) homolog, subfamily C, member 21	1.403706	0.008274	0.06237
52588	Tspan14	tetraspanin 14	1.403509	0.001316	0.02117
109019	Obfc2a	oligonucleotide/oligosaccharide-binding fold containing 2A	1.403509	0.03045	0.1368
74471	4933440N22Rik	RIKEN cDNA 4933440N22 gene	1.403312	0.0097	0.0686
100465	Mobkl2c	MOB1, Mps One Binder kinase activator-like 2C (yeast)	1.403312	0.001478	0.02273
213311	Fbxl21	F-box and leucine-rich repeat protein 21	1.403312	0.003275	0.03624
76742	Snx27	sorting nexin family member 27	1.402721	0.004196	0.04215
11867	Arpc1b	actin related protein 2/3 complex, subunit 1B	1.402328	0.07515	0.2344
12978	Csf1r	colony stimulating factor 1 receptor	1.402328	0.005338	0.04865
14854	Gss	glutathione synthetase	1.401738	0.1048	0.2879
14451	Gas1	growth arrest specific 1	1.401345	0.01662	0.09481
56188	Fxyd1	FXYD domain-containing ion transport regulator 1	1.401149	0.002322	0.02979
76629	Wbscr28	Williams-Beuren syndrome chromosome region 28 (human)	1.400953	0.2181	0.4472
65963	Tmem176b	transmembrane protein 176B	1.400756	0.004418	0.04344
14707	Gng5	guanine nucleotide binding protein (G protein), gamma 5	1.40056	0.001166	0.01944
56228	Ube2j1	ubiquitin-conjugating enzyme E2, J1	1.40056	0.0002675	0.008376

102448	Xylb	xylulokinase homolog (H. influenzae)	1.40056	0.00147	0.02267
225608	Sh3tc2	SH3 domain and tetratricopeptide repeats 2	1.40056	0.02781	0.1297
230824	Grhl3	grainyhead-like 3 (Drosophila)	1.400364	0.01691	0.09581
74568	Mlkl	mixed lineage kinase domain-like	1.399776	0.1611	0.373
17768	Mthfd2	methylenetetrahydrofolate dehydrogenase (NAD+ dependent), methenyltetrahydrofolate cyclohydrolase	1.39958	0.005878	0.05151
67448	Plxdc2	plexin domain containing 2	1.39958	0.005898	0.05163
79201	Tnfrsf23	tumor necrosis factor receptor superfamily, member 23	1.39958	0.2019	0.428
16770	Lalba	lactalbumin, alpha	1.399384	0.022	0.1124
72267	Lrrc8e	leucine rich repeat containing 8 family, member E	1.398993	0.03093	0.1378
319158	Hist1h4i	histone cluster 1, H4i	1.398993	0.01616	0.09307
13732	Emp3	epithelial membrane protein 3	1.397624	0.06747	0.219
71729	Rgs12	regulator of G-protein signaling 12	1.397624	0.05667	0.1955
14119	Fbn2	fibrillin 2	1.397429	0.1569	0.3671
238333	Samd15	sterile alpha motif domain containing 15	1.397038	0.1758	0.3928
271849	Shc4	SHC (Src homology 2 domain containing) family, member 4	1.397038	0.3893	0.6212
13848	Ephb6	Eph receptor B6	1.396843	0.05278	0.1869
217353	Tmc6	transmembrane channel-like gene family 6	1.396843	0.06124	0.2058
100702	Mpa2l	macrophage activation 2 like	1.396648	0.4813	0.7
107503	Atf5	activating transcription factor 5	1.396648	0.0006304	0.01363
171382	Trpm8	transient receptor potential cation channel, subfamily M, member 8	1.396258	0.2575	0.4926
67860	S100a16	S100 calcium binding protein A16	1.396063	0.003574	0.03815
223696	Tomm22	translocase of outer mitochondrial membrane 22 homolog (yeast)	1.396063	0.01095	0.07401
319236	9230105E10Rik	RIKEN cDNA 9230105E10 gene	1.396063	0.3512	0.5867
67075	Magt1	magnesium transporter 1	1.395868	0.02887	0.1324
75840	4930558N01Rik	RIKEN cDNA 4930558N01 gene	1.395868	0.007786	0.06048
239157	Pnma2	paraneoplastic antigen MA2	1.395673	0.239	0.4716
64177	Trpv6	transient receptor potential cation channel, subfamily V, member 6	1.395479	0.1621	0.3743
78771	Mctp1	multiple C2 domains, transmembrane 1	1.395284	0.2069	0.433
11829	Aqp4	aquaporin 4	1.395089	0.002293	0.02954
66101	Ppih	peptidyl prolyl isomerase H	1.395089	0.02013	0.1066
72459	Htatsf1	HIV TAT specific factor 1	1.395089	0.06116	0.2056
241989	Pabpc4l	poly(A) binding protein, cytoplasmic 4-like	1.395089	0.01786	0.09884
26934	Racgap1	Rac GTPase-activating protein 1	1.3947	0.007294	0.05811
107272	Psat1	phosphoserine aminotransferase 1	1.3947	0.0004467	0.0111
14127	Fcer1g	Fc receptor, IgE, high affinity I, gamma polypeptide	1.394506	0.01212	0.07833
16427	Itih4	inter alpha-trypsin inhibitor, heavy chain 4	1.394506	0.05399	0.1898
69987	1700026L06Rik	RIKEN cDNA 1700026L06 gene	1.394506	0.2048	0.4311
18992	Pou3f2	POU domain, class 3, transcription factor 2	1.394311	0.04378	0.1681
68303	Fam114a1	family with sequence similarity 114, member A1	1.393922	0.004602	0.04471
75114	4930516B21Rik	RIKEN cDNA 4930516B21 gene	1.393922	0.144	0.3489
432825	Gm5458	predicted gene 5458	1.393728	0.3208	0.5573
53404	Atoh7	atonal homolog 7 (Drosophila)	1.39334	0.07691	0.2375
53608	Map3k6	mitogen-activated protein kinase kinase kinase 6	1.39334	0.03738	0.1532
83679	Pde4dip	phosphodiesterase 4D interacting protein (myomegalin)	1.393146	0.001029	0.01795
58182	Prokr1	prokineticin receptor 1	1.392952	0.3712	0.6062
18103	Nme2	non-metastatic cells 2, protein (NM23B) expressed in	1.392758	0.001302	0.02099
231440	Parm1	prostate androgen-regulated mucin-like protein 1	1.392758	0.00892	0.06529
22774	Zic4	zinc finger protein of the cerebellum 4	1.392564	0.5259	0.7331
83454	Nxf2	nuclear RNA export factor 2	1.392564	0.04846	0.1781

93897	Fzd10	frizzled homolog 10 (Drosophila)	1.39237	0.01002	0.07015
229571	Gm4858	predicted gene 4858	1.392176	0.1006	0.2806
13039	Ctsl	cathepsin L	1.391982	0.006233	0.05313
192656	Ripk2	receptor (TNFRSF)-interacting serine-threonine kinase 2	1.391982	0.01111	0.07461
229214	Qrfpr	pyroglutamylated RFamide peptide receptor	1.391982	0.002618	0.03169
12159	Bmp4	bone morphogenetic protein 4	1.391401	0.3815	0.615
13866	Erbb2	v-erb-b2 erythroblastic leukemia viral oncogene homolog 2, neuro/glioblastoma derived oncogene homolog (avian)	1.391401	0.003688	0.03888
207175	Cetn4	centrin 4	1.391014	0.009076	0.06595
18030	Nfil3	nuclear factor, interleukin 3, regulated	1.390821	0.0001814	0.00667
64934	Pes1	pescadillo homolog 1, containing BRCT domain (zebrafish)	1.390821	0.02911	0.1332
382083	Snx22	sorting nexin 22	1.390627	0.0275	0.1289
11928	Atp1a1	ATPase, Na+/K+ transporting, alpha 1 polypeptide	1.390241	0.001287	0.02083
14794	Spsb2	splA/ryanodine receptor domain and SOCS box containing 2	1.389854	0.01078	0.07326
74055	Plce1	phospholipase C, epsilon 1	1.389854	0.002169	0.02832
66395	Ahnak	AHNAK nucleoprotein (desmoyokin)	1.389661	0.3715	0.6064
14369	Fzd7	frizzled homolog 7 (Drosophila)	1.388889	0.004495	0.04408
51798	Ech1	enoyl coenzyme A hydratase 1, peroxisomal	1.388889	0.0006951	0.0145
15937	Ier3	immediate early response 3	1.388696	0.002138	0.02807
319565	Syne2	synaptic nuclear envelope 2	1.38831	0.002378	0.03016
19242	Ptn	pleiotrophin	1.388118	0.04399	0.1686
22325	Vav2	vav 2 oncogene	1.387155	0.004705	0.04511
67075	Magt1	magnesium transporter 1	1.387155	0.02895	0.1327
17480	Mpl	myeloproliferative leukemia virus oncogene	1.386963	0.007951	0.0611
12368	Casp6	caspase 6	1.38677	0.0002415	0.007862
75984	5031415H12Rik	RIKEN cDNA 5031415H12 gene	1.38677	0.01212	0.07833
69571	2310034O05Rik	RIKEN cDNA 2310034O05 gene	1.386578	0.1107	0.2984
16543	Mdfic	MyoD family inhibitor domain containing	1.386194	0.1406	0.3443
66961	Neat1	nuclear paraspeckle assembly transcript 1 (non-protein coding)	1.386194	0.1031	0.2848
71226	4933433G19Rik	RIKEN cDNA 4933433G19 gene	1.386001	0.02805	0.1304
66494	Prelid1	PRELI domain containing 1	1.385617	0.004724	0.04516
192212	Prom2	prominin 2	1.385617	0.6617	0.8258
228550	Itpka	inositol 1,4,5-trisphosphate 3-kinase A	1.385617	0.01698	0.09609
26408	Map3k5	mitogen-activated protein kinase kinase kinase 5	1.385425	0.002822	0.03304
71893	Noxo1	NADPH oxidase organizer 1	1.385425	0.01365	0.08444
20356	Sema5a	sema domain, seven thrombospondin repeats (type 1 and type 1-like), transmembrane domain (TM) and short cytoplasmic domain, (semaphorin) 5A	1.385233	0.001329	0.02132
76571	Styxl1	serine/threonine/tyrosine interacting-like 1	1.385233	0.00347	0.03747
545475	Defb28	defensin beta 28	1.385042	0.5977	0.7834
13036	Ctsh	cathepsin H	1.38485	0.01024	0.07112
27756	Lsm2	LSM2 homolog, U6 small nuclear RNA associated (S. cerevisiae)	1.38485	0.1358	0.3374
209200	Dtx3l	deltex 3-like (Drosophila)	1.38485	0.01331	0.08321
16504	Kcnc3	potassium voltage gated channel, Shaw-related subfamily, member 3	1.384466	0.03512	0.1484
54324	Arhgef5	Rho guanine nucleotide exchange factor (GEF) 5	1.384466	0.01973	0.1051
228608	Smox	spermine oxidase	1.384466	0.005529	0.04963
231162	Cytl1	cytokine-like 1	1.384466	0.03377	0.1452
674419	Rpl7a-ps5	ribosomal protein L7A, pseudogene 5	1.384466	0.2983	0.5343
66181	Nop10	NOP10 ribonucleoprotein homolog (yeast)	1.3837	0.00178	0.02544
70355	Gprc5c	G protein-coupled receptor, family C, group 5, member C	1.3837	0.07436	0.2328
12495	Entpd1	ectonucleoside triphosphate diphosphohydrolase 1	1.383509	0.05441	0.1907
75495	Morn5	MORN repeat containing 5	1.383317	0.01274	0.08085

75695	Rilpl1	Rab interacting lysosomal protein-like 1	1.383126	0.003256	0.03607
22763	Zfr	zinc finger RNA binding protein	1.382935	0.062	0.2073
76441	Daam2	dishevelled associated activator of morphogenesis 2	1.382743	0.03066	0.1371
98267	Stk17b	serine/threonine kinase 17b (apoptosis-inducing)	1.38217	0.01498	0.0893
260296	Trim61	tripartite motif-containing 61	1.38217	0.2777	0.5134
100038577	Gm10790	predicted gene 10790	1.38217	0.1238	0.3203
12822	Col18a1	collagen, type XVIII, alpha 1	1.381979	0.3521	0.5874
14457	Gas7	growth arrest specific 7	1.381979	0.008339	0.06257
80901	Cxcr6	chemokine (C-X-C motif) receptor 6	1.381597	0.1218	0.3168
231986	Jazf1	JAZF zinc finger 1	1.381406	0.02092	0.109
214359	Tmem51	transmembrane protein 51	1.381025	0.005775	0.0509
75580	Zbtb4	zinc finger and BTB domain containing 4	1.380834	0.001724	0.02511
22371	Vwf	Von Willebrand factor homolog	1.380262	0.0151	0.08974
18103	Nme2	non-metastatic cells 2, protein (NM23B) expressed in	1.37931	0.001967	0.027
223604	Kcnk9	potassium channel, subfamily K, member 9	1.37931	0.1329	0.3337
64009	Syne1	synaptic nuclear envelope 1	1.37855	0.003392	0.03695
233115	Dpy19l3	dpy-19-like 3 (C. elegans)	1.37855	0.001028	0.01795
382118	Zfp167	zinc finger protein 167	1.37836	0.05137	0.1841
110253	Triobp	TRIO and F-actin binding protein	1.37798	0.1051	0.2885
11832	Aqp7	aquaporin 7	1.3776	0.1262	0.3235
17294	Mest	mesoderm specific transcript	1.37741	0.01525	0.09032
66855	Tcf25	transcription factor 25 (basic helix-loop-helix)	1.37741	0.02098	0.1091
12494	Cd38	CD38 antigen	1.377031	0.06894	0.222
108978	4930555G01Rik	RIKEN cDNA 4930555G01 gene	1.376842	0.2672	0.5027
13649	Egfr	epidermal growth factor receptor	1.376462	0.009063	0.06591
75736	Bcl2l12	BCL2-like 12 (proline rich)	1.376273	0.001042	0.01814
246738	Dnajc28	DnaJ (Hsp40) homolog, subfamily C, member 28	1.376084	0.07073	0.2254
432442	Akap7	A kinase (PRKA) anchor protein 7	1.376084	0.1571	0.3674
20474	Six4	sine oculis-related homeobox 4 homolog (Drosophila)	1.375705	0.0002749	0.008505
11491	Adam17	a disintegrin and metallopeptidase domain 17	1.375327	0.01945	0.1044
360220	Speer4d	spermatogenesis associated glutamate (E)-rich protein 4d	1.375327	0.05744	0.1971
17841	Mup2	major urinary protein 2	1.375138	0.1156	0.3064
78244	Dnajc21	DnaJ (Hsp40) homolog, subfamily C, member 21	1.374948	0.03051	0.1369
54357	Epb4.1l4b	erythrocyte protein band 4.1-like 4b	1.374193	0.2393	0.4718
109267	Srcrb4d	scavenger receptor cysteine rich domain containing, group B (4 domains)	1.373815	0.02637	0.1254
414084	Tnip3	TNFAIP3 interacting protein 3	1.373815	0.3957	0.6262
71059	Hexim2	hexamthylene bis-acetamide inducible 2	1.373626	0.22	0.4499
17916	Myo1f	myosin IF	1.372872	0.01812	0.09967
207683	Igsf11	immunoglobulin superfamily, member 11	1.372872	0.006596	0.05491
17259	Mef2b	myocyte enhancer factor 2B	1.372495	0.04911	0.1795
15368	Hmox1	heme oxygenase (decycling) 1	1.37193	0.03446	0.1468
67724	Pop1	processing of precursor 1, ribonuclease P/MRP family, (S. cerevisiae)	1.37193	0.007659	0.0599
70355	Gprc5c	G protein-coupled receptor, family C, group 5, member C	1.37193	0.09071	0.2638
20619	Snap23	synaptosomal-associated protein 23	1.371366	0.003837	0.03973
170799	Rtkn2	rhotekin 2	1.371366	0.01129	0.07532
18176	Nras	neuroblastoma ras oncogene	1.371178	0.002204	0.02869
72358	2210411A11Rik	RIKEN cDNA 2210411A11 gene	1.37099	0.0005311	0.01237
239102	Zfhx2	zinc finger homeobox 2	1.37099	0.4528	0.6772
20871	Aurkc	aurora kinase C	1.370802	0.03474	0.1478

330010	Ttll10	tubulin tyrosine ligase-like family, member 10	1.370802	0.01988	0.1057
63872	Zfp296	zinc finger protein 296	1.370614	0.05872	0.2005
72240	1600014C23Rik	RIKEN cDNA 1600014C23 gene	1.370238	0.01545	0.09107
13168	Dbil5	diazepam binding inhibitor-like 5	1.369863	0.005713	0.05051
69974	2810405F15Rik	RIKEN cDNA 2810405F15 gene	1.369113	0.01889	0.1024
57748	Jmy	junction-mediating and regulatory protein	1.368925	0.04443	0.1695
74185	Gbe1	glucan (1,4-alpha-), branching enzyme 1	1.368925	0.001436	0.02235
72287	Plekhf1	pleckstrin homology domain containing, family F (with FYVE domain) member 1	1.368176	0.03277	0.1428
12476	Cd151	CD151 antigen	1.367989	0.0006098	0.0135
22329	Vcam1	vascular cell adhesion molecule 1	1.367989	0.01223	0.07871
195046	Nlrp1a	NLR family, pyrin domain containing 1A	1.367802	0.002029	0.02739
19317	Qk	quaking	1.367615	0.04563	0.1721
23828	Bves	blood vessel epicardial substance	1.367054	0.001985	0.02709
70001	1700028B04Rik	RIKEN cDNA 1700028B04 gene	1.366867	0.004883	0.04601
268595	D430019H16Rik	RIKEN cDNA D430019H16 gene	1.366867	0.01323	0.08297
260296	Trim61	tripartite motif-containing 61	1.366494	0.1343	0.3353
12661	Chl1	cell adhesion molecule with homology to L1CAM	1.366307	0.03111	0.1382
14999	H2-DMb1	histocompatibility 2, class II, locus Mb1	1.366307	0.116	0.3073
20852	Stat6	signal transducer and activator of transcription 6	1.366307	0.01731	0.09722
22333	Vdac1	voltage-dependent anion channel 1	1.36612	0.008082	0.0616
78586	Srbd1	S1 RNA binding domain 1	1.36612	0.01736	0.09735
224405	Cyyr1	cysteine and tyrosine-rich protein 1	1.365934	0.1452	0.3505
442802	C330011M18Rik	RIKEN cDNA C330011M18 gene	1.365934	0.0426	0.1652
103220	BC030307	cDNA sequence BC030307	1.365747	0.03924	0.1577
109594	Lmo1	LIM domain only 1	1.365747	0.0003347	0.009635
100041121	Gm10144	predicted pseudogene 10144	1.365561	0.0923	0.2662
12841	Col9a3	collagen, type IX, alpha 3	1.365374	0.03113	0.1382
66707	Nkapl	NFKB activating protein-like	1.365374	0.01829	0.1003
381286	Serpinb3c	serine (or cysteine) peptidase inhibitor, clade B, member 3C	1.365374	0.1336	0.334
67623	Tm7sf3	transmembrane 7 superfamily member 3	1.365001	0.001407	0.02215
72284	Oraov1	oral cancer overexpressed 1	1.365001	0.1331	0.3338
320698	C530042K13Rik	RIKEN cDNA C530042K13 gene	1.365001	0.09131	0.2648
71355	Col24a1	collagen, type XXIV, alpha 1	1.364815	0.03283	0.1428
15203	Heph	hephaestin	1.364443	0.01838	0.1006
74564	9.13E+15	hypothetical 9130022E09	1.364443	0.007036	0.05679
218518	Marveld2	MARVEL (membrane-associating) domain containing 2	1.364256	0.009463	0.0676
12822	Col18a1	collagen, type XVIII, alpha 1	1.36407	0.2353	0.4674
231147	Sh3tc1	SH3 domain and tetratricopeptide repeats 1	1.36407	0.01233	0.0791
402751	C430019N01Rik	RIKEN cDNA C430019N01 gene	1.36407	0.09652	0.2737
76448	2310014H01Rik	RIKEN cDNA 2310014H01 gene	1.363698	0.2212	0.4511
27279	Tnfrsf12a	tumor necrosis factor receptor superfamily, member 12a	1.363327	0.003506	0.03774
66494	Prelid1	PRELI domain containing 1	1.363327	0.001154	0.01931
72512	Tmem173	transmembrane protein 173	1.363327	0.02975	0.1349
15442	Hpse	heparanase	1.363141	0.008542	0.0636
19011	Endou	endonuclease, polyU-specific	1.362955	0.07828	0.2399
74491	5430420F09Rik	RIKEN cDNA 5430420F09 gene	1.362955	0.03181	0.1401
16362	Irf1	interferon regulatory factor 1	1.362769	0.0009794	0.01765
76281	Tax1bp3	Tax1 (human T-cell leukemia virus type I) binding protein 3	1.362583	0.006116	0.05261
229320	Clrn1	clarin 1	1.362583	0.002579	0.0315

214855	Arid5a	AT rich interactive domain 5A (MRF1-like)	1.362398	0.006896	0.05627
30940	Usp25	ubiquitin specific peptidase 25	1.362212	0.003425	0.03714
245638	Tbc1d8b	TBC1 domain family, member 8B	1.361841	0.03333	0.1442
11933	Atp1b3	ATPase, Na+/K+ transporting, beta 3 polypeptide	1.360544	0.008702	0.06429
100009609	Vmn2r65	vomeronasal 2, receptor 65	1.360544	0.5226	0.7305
241950	Bbs12	Bardet-Biedl syndrome 12 (human)	1.360359	0.0006118	0.01351
242939	Cpz	carboxypeptidase Z	1.360174	0.2686	0.5038
78829	Tsc22d4	TSC22 domain family, member 4	1.359989	0.03836	0.156
12628	Cfh	complement component factor h	1.359804	0.0231	0.1158
14066	F3	coagulation factor III	1.359804	0.001237	0.02037
14969	H2-Eb1	histocompatibility 2, class II antigen E beta	1.359434	0.2067	0.4328
100038847	Gm10406	predicted gene 10406	1.359434	0.3512	0.5867
18802	Plcd4	phospholipase C, delta 4	1.35925	0.1125	0.3012
11481	Acvr2b	activin receptor IIB	1.35888	0.005059	0.04703
67621	Bend5	BEN domain containing 5	1.35888	0.01584	0.09248
73016	Kremen2	kringle containing transmembrane protein 2	1.35888	0.01281	0.08122
93761	Smarca1	SWI/SNF related, matrix associated, actin dependent regulator of chromatin, subfamily a, member 1	1.358511	0.01781	0.09869
64099	Parvg	parvin, gamma	1.358327	0.1784	0.3961
20147	Rs1	retinoschisis (X-linked, juvenile) 1 (human)	1.358142	0.3012	0.537
14807	Grik3	glutamate receptor, ionotropic, kainate 3	1.357958	0.03347	0.1445
12560	Cdh3	cadherin 3	1.357773	0.2811	0.5162
16617	Klk1b24	kallikrein 1-related peptidase b24	1.357773	0.1075	0.293
15277	Hk2	hexokinase 2	1.357589	0.0005421	0.01252
381405	Zfp663	zinc finger protein 663	1.35722	0.266	0.5015
399604	C530014P21Rik	RIKEN cDNA C530014P21 gene	1.35722	0.005216	0.04794
404318	Olfr681	olfactory receptor 681	1.35722	0.232	0.4633
71586	Ifih1	interferon induced with helicase C domain 1	1.357036	0.01317	0.08275
69590	Gpx8	glutathione peroxidase 8 (putative)	1.356852	0.00199	0.02711
12367	Casp3	caspase 3	1.356484	0.009017	0.06573
22177	Tyrobp	TYRO protein tyrosine kinase binding protein	1.355565	0.01443	0.08735
67320	Iqcf4	IQ motif containing F4	1.355381	0.4516	0.6764
258493	Olfr319	olfactory receptor 319	1.355381	0.4336	0.6607
13590	Lefty1	left right determination factor 1	1.355197	0.1715	0.3872
216188	Aldh1l2	aldehyde dehydrogenase 1 family, member L2	1.355014	0.01204	0.07813
192166	Sardh	sarcosine dehydrogenase	1.35483	0.01962	0.1049
226422	Rab7l1	RAB7, member RAS oncogene family-like 1	1.35483	0.004074	0.04135
218693	Paip1	polyadenylate binding protein-interacting protein 1	1.354646	0.00804	0.06145
241769	Kcnk15	potassium channel, subfamily K, member 15	1.354646	0.09525	0.2715
21991	Tpi1	triosephosphate isomerase 1	1.354463	0.02345	0.1168
243362	Stard13	StAR-related lipid transfer (START) domain containing 13	1.354096	0.002934	0.03379
13008	Csrp2	cysteine and glycine-rich protein 2	1.35373	0.02915	0.1333
105501	Abhd4	abhydrolase domain containing 4	1.35373	0.0006817	0.01439
56068	Ammecr1	Alport syndrome, mental retardation, midface hypoplasia and elliptocytosis chromosomal region gene 1 homolog (human)	1.353546	0.04758	0.1765
66395	Ahnak	AHNAK nucleoprotein (desmoyokin)	1.353546	0.5248	0.7321
72723	Zfp74	zinc finger protein 74	1.352997	0.003094	0.03492
22359	Vldlr	very low density lipoprotein receptor	1.352814	0.01906	0.103
228785	Mylk2	myosin, light polypeptide kinase 2, skeletal muscle	1.352631	0.3459	0.5812
83997	Slmap	sarcolemma associated protein	1.352448	0.02908	0.1331
207683	Igsf11	immunoglobulin superfamily, member 11	1.352448	0.06697	0.218

17686	Msh3	mutS homolog 3 (E. coli)	1.352082	0.003932	0.0404
20527	Slc2a3	solute carrier family 2 (facilitated glucose transporter), member 3	1.351899	0.01275	0.08087
55932	Gbp3	guanylate binding protein 3	1.351899	0.01874	0.1019
11898	Ass1	argininosuccinate synthetase 1	1.351717	0.02818	0.1307
13665	Eif2s1	eukaryotic translation initiation factor 2, subunit 1 alpha	1.351717	0.04994	0.1815
432870	Gm5464	predicted gene 5464	1.350804	0.1395	0.3429
20324	Sdpr	serum deprivation response	1.350621	0.1298	0.329
626575	Gm6687	predicted gene 6687	1.350621	0.09493	0.2709
213311	Fbxl21	F-box and leucine-rich repeat protein 21	1.350257	0.1299	0.3292
18080	Nin	ninein	1.350074	0.00947	0.06762
20196	S100a13	S100 calcium binding protein A13	1.350074	0.002101	0.02786
67726	Fam114a2	family with sequence similarity 114, member A2	1.349528	0.03708	0.1526
233335	Synm	synemin, intermediate filament protein	1.349528	0.015	0.08937
11746	Anxa4	annexin A4	1.349346	0.005879	0.05151
246256	Fcgr4	Fc receptor, IgG, low affinity IV	1.349346	0.3405	0.5762
71956	Rnf135	ring finger protein 135	1.348982	0.0131	0.08253
100039855	Gm2461	predicted gene 2461	1.348982	0.129	0.3276
66949	Trim59	tripartite motif-containing 59	1.348618	0.01056	0.07245
66952	2310030G06Rik	RIKEN cDNA 2310030G06 gene	1.348618	0.07842	0.24
26927	Foxl2	forkhead box L2	1.348436	0.0139	0.08531
68234	2400009B08Rik	RIKEN cDNA 2400009B08 gene	1.348436	0.1117	0.2999
207607	Ccdc40	coiled-coil domain containing 40	1.348436	0.02726	0.128
71544	Arhgap42	Rho GTPase activating protein 42	1.348254	0.003235	0.03603
70784	Rasl12	RAS-like, family 12	1.347891	0.05844	0.1998
380912	Zfp395	zinc finger protein 395	1.347709	0.02579	0.1236
386463	Cdsn	corneodesmosin	1.347709	0.08385	0.2504
94090	Trim9	tripartite motif-containing 9	1.347527	0.002784	0.03275
320731	A530030E21Rik	RIKEN cDNA A530030E21 gene	1.347527	0.2804	0.5158
640530	Gm7298	predicted gene 7298	1.347527	0.3683	0.6032
14735	Gpc4	glypican 4	1.347346	0.02195	0.1123
17110	Lyz1	lysozyme 1	1.347346	0.07774	0.239
20649	Sntb1	syntrophin, basic 1	1.347346	0.1046	0.2876
67512	Agpat2	1-acylglycerol-3-phosphate O-acyltransferase 2 (lysophosphatidic acid acyltransferase, beta)	1.347346	0.01567	0.09181
270152	Amica1	adhesion molecule, interacts with CXADR antigen 1	1.347346	0.1141	0.304
101437	Dhx32	DEAH (Asp-Glu-Ala-His) box polypeptide 32	1.347164	0.03419	0.1462
629557	Gm6981	glyceraldehyde-3-phosphate dehydrogenase pseudogene	1.347164	0.04813	0.1775
100047183	LOC100047183	protein AHNAK2-like	1.346983	0.02455	0.1198
19329	Rab17	RAB17, member RAS oncogene family	1.34662	0.1577	0.3683
232533	Stk38l	serine/threonine kinase 38 like	1.346439	0.003728	0.03906
74051	Steap2	six transmembrane epithelial antigen of prostate 2	1.346257	0.1259	0.3231
68968	Cdan1	congenital dyserythropoietic anemia, type I (human)	1.346076	0.008973	0.06552
70511	Fam86	family with sequence similarity 86	1.345895	0.001161	0.01937
12483	Cd22	CD22 antigen	1.345714	0.1298	0.329
16889	Lipa	lysosomal acid lipase A	1.345533	0.00768	0.06001
234362	Zfp868	zinc finger protein 868	1.345533	0.0229	0.1152
626870	Gm11992	predicted gene 11992	1.345533	0.08397	0.2507
277360	Prex1	phosphatidylinositol-3,4,5-trisphosphate-dependent Rac exchange factor 1	1.345352	0.0009142	0.01702
320022	Ccdc79	coiled-coil domain containing 79	1.345352	0.02465	0.12
216850	Kdm6b	KDM1 lysine (K)-specific demethylase 6B	1.345171	0.01532	0.09061

17756	Mtap2	microtubule-associated protein 2	1.34499	0.08645	0.2557
77057	Ston1	stonin 1	1.34499	0.2789	0.5144
67926	Spert	spermatid associated	1.344809	0.387	0.6191
70005	1700029I01Rik	RIKEN cDNA 1700029I01 gene	1.344809	0.08229	0.2474
108897	Aif1l	allograft inflammatory factor 1-like	1.344628	0.001676	0.02482
66170	Chchd5	coiled-coil-helix-coiled-coil-helix domain containing 5	1.344267	0.00339	0.03695
258830	Olfr103	olfactory receptor 103	1.344086	0.3399	0.5757
11815	Apod	apolipoprotein D	1.343905	0.003811	0.0395
57265	Fzd2	frizzled homolog 2 (Drosophila)	1.342823	0.07658	0.2369
15170	Ptpn6	protein tyrosine phosphatase, non-receptor type 6	1.342642	0.1438	0.3486
66913	Kdelr2	KDEL (Lys-Asp-Glu-Leu) endoplasmic reticulum protein retention receptor 2	1.342642	0.003922	0.04035
109857	Cbr3	carbonyl reductase 3	1.342642	0.002738	0.03242
26377	Dapp1	dual adaptor for phosphotyrosine and 3-phosphoinositides 1	1.342462	0.03891	0.157
59289	Ccbp2	chemokine binding protein 2	1.342462	0.01775	0.0984
19245	Ptp4a3	protein tyrosine phosphatase 4a3	1.342102	0.03514	0.1484
100042173	Gm11968	Rps15a pseudogene	1.341922	0.01192	0.07773
241638	Prosapip1	ProSAPiP1 protein	1.340662	0.4361	0.6627
100039681	Zcwpw2	zinc finger, CW type with PWWP domain 2	1.340483	0.0317	0.1399
78233	6330522J23Rik	RIKEN cDNA 6330522J23 gene	1.340303	0.03784	0.1546
98365	Slamf9	SLAM family member 9	1.340303	0.01339	0.08343
242747	Zfp933	zinc finger protein 933	1.339944	0.08634	0.2556
17319	Mif	macrophage migration inhibitory factor	1.339764	0.01115	0.07475
13848	Ephb6	Eph receptor B6	1.339585	0.03504	0.1484
20591	Kdm5c	lysine (K)-specific demethylase 5C	1.339585	0.1387	0.3416
319508	Syt15	synaptotagmin XV	1.339585	0.01345	0.08358
24064	Spry2	sprouty homolog 2 (Drosophila)	1.339405	0.1622	0.3745
243362	Stard13	StAR-related lipid transfer (START) domain containing 13	1.339226	0.04873	0.1786
12192	Zfp36l1	zinc finger protein 36, C3H type-like 1	1.338867	0.006151	0.05272
381175	Ccdc68	coiled-coil domain containing 68	1.338867	0.3303	0.5668
385605	Rundc2a	RUN domain containing 2A	1.338867	0.2448	0.4784
16162	Il12rb2	interleukin 12 receptor, beta 2	1.338509	0.1454	0.3508
319160	Hist1h4k	histone cluster 1, H4k	1.338509	0.1047	0.2877
319164	Hist1h2ac	histone cluster 1, H2ac	1.33833	0.08284	0.2483
382035	Pabpn1l	poly(A)binding protein nuclear 1-like	1.338151	0.006093	0.05254
235320	Zbtb16	zinc finger and BTB domain containing 16	1.337793	0.05011	0.1817
242705	E2f2	E2F transcription factor 2	1.337793	0.01227	0.07887
100041489	Gm3367	predicted gene 3367	1.337614	0.02236	0.1136
21407	Tcf15	transcription factor 15	1.337435	0.01015	0.07076
16188	Il3ra	interleukin 3 receptor, alpha chain	1.337256	0.1503	0.3582
20708	Serpinb6b	serine (or cysteine) peptidase inhibitor, clade B, member 6b	1.337256	0.1122	0.3007
21922	Clec3b	C-type lectin domain family 3, member b	1.336362	0.2126	0.4403
14300	Frg1	FSHD region gene 1	1.33547	0.02476	0.1204
52139	D5Ertd560e	DNA segment, Chr 5, ERATO Doi 560, expressed	1.33547	0.02178	0.1119
12419	Cbx5	chromobox homolog 5 (Drosophila HP1a)	1.335113	0.165	0.3787
319504	Nrcam	neuron-glia-CAM-related cell adhesion molecule	1.335113	0.007862	0.0609
16542	Kdr	kinase insert domain protein receptor	1.334935	0.002429	0.03056
217830	9030617O03Rik	RIKEN cDNA 9030617O03 gene	1.334757	0.02975	0.1349
20979	Syt1	synaptotagmin I	1.334223	0.03591	0.1499
71521	Pds5a	PDS5, regulator of cohesion maintenance, homolog A (S. cerevisiae)	1.334045	0.4168	0.645

76742	Snx27	sorting nexin family member 27	1.334045	0.0005688	0.01291
320495	Ipcef1	interaction protein for cytohesin exchange factors 1	1.333867	0.2051	0.4314
26450	Rbbp9	retinoblastoma binding protein 9	1.333689	0.00778	0.06048
50779	Rgs6	regulator of G-protein signaling 6	1.333333	0.00118	0.01962
75767	Rab11fip1	RAB11 family interacting protein 1 (class I)	1.333333	0.1596	0.3709
66129	1110018J18Rik	RIKEN cDNA 1110018J18 gene	1.3328	0.009061	0.06591
78458	1700064N11Rik	RIKEN cDNA 1700064N11 gene	1.332623	0.2208	0.4507
224170	Dzip3	DAZ interacting protein 3, zinc finger	1.332268	0.01461	0.08799
11641	Akap2	A kinase (PRKA) anchor protein 2	1.33209	0.02475	0.1204
17754	Mtap1a	microtubule-associated protein 1 A	1.33209	0.01273	0.08085
327942	Pigl	phosphatidylinositol glycan anchor biosynthesis, class L	1.331735	0.2641	0.5
170788	Crb1	crumbs homolog 1 (Drosophila)	1.331558	0.02821	0.1308
23849	Klf6	Kruppel-like factor 6	1.331381	0.01539	0.09088
73608	Marveld3	MARVEL (membrane-associating) domain containing 3	1.331381	0.004321	0.0429
16410	Itgav	integrin alpha V	1.331203	0.01768	0.09835
94089	Trim7	tripartite motif-containing 7	1.331203	0.1045	0.2875
78550	E130119H09Rik	RIKEN cDNA E130119H09 gene	1.330672	0.01506	0.0896
68396	Nat8	N-acetyltransferase 8 (GCN5-related, putative)	1.330495	0.1177	0.3099
17294	Mest	mesoderm specific transcript	1.330141	0.0002478	0.007959
18442	P2ry2	purinergic receptor P2Y, G-protein coupled 2	1.329787	0.1652	0.3789
14182	Fgfr1	fibroblast growth factor receptor 1	1.329257	0.07516	0.2344
170742	Sertad3	SERTA domain containing 3	1.329257	0.2213	0.4513
67946	Spata6	spermatogenesis associated 6	1.32908	0.03431	0.1465
11922	Neurod6	neurogenic differentiation 6	1.328727	0.5687	0.7635
67141	Fbxo5	F-box protein 5	1.328551	0.009016	0.06573
381319	Batf3	basic leucine zipper transcription factor, ATF-like 3	1.328551	0.2502	0.4841
56173	Cldn14	claudin 14	1.328374	0.351	0.5866
235497	Leo1	Leo1, Paf1/RNA polymerase II complex component, homolog (S. cerevisiae)	1.328374	0.2564	0.4914
12663	Chml	choroideremia-like	1.328198	0.1142	0.3042
545007	ENSMUSG00000068790	predicted gene, ENSMUSG00000068790	1.328198	0.4125	0.6412
83560	Tex14	testis expressed gene 14	1.328021	0.03152	0.1394
319188	Hist1h2bp	histone cluster 1, H2bp	1.327845	0.106	0.2903
11730	Ang3	angiogenin, ribonuclease A family, member 3	1.327492	0.3069	0.5433
107522	Ece2	endothelin converting enzyme 2	1.327492	0.02142	0.1107
77056	Tmco4	transmembrane and coiled-coil domains 4	1.327316	0.01226	0.07887
19215	Ptgds	prostaglandin D2 synthase (brain)	1.326612	0.3174	0.5539
24082	Gm16516	predicted gene, Gm16516	1.326612	0.01191	0.07773
268490	Lsm12	LSM12 homolog (S. cerevisiae)	1.326612	0.01152	0.0761
238317	C130039O16Rik	RIKEN cDNA C130039O16 gene	1.32626	0.04157	0.1632
382209	Rhox3a	reproductive homeobox 3A	1.32626	0.5573	0.7566
12476	Cd151	CD151 antigen	1.326084	0.001771	0.02542
30791	Slc39a1	solute carrier family 39 (zinc transporter), member 1	1.326084	0.002583	0.0315
76763	Mospd2	motile sperm domain containing 2	1.326084	0.006241	0.05318
70808	4632415L05Rik	RRS1 ribosome biogenesis regulator homolog pseudogene	1.325381	0.01195	0.07775
74843	Zmynd17	zinc finger, MYND domain containing 17	1.325381	0.04101	0.1619
319177	Hist1h2ba	histone cluster 1, H2ba	1.325205	0.003393	0.03695
68545	Ecscr	endothelial cell-specific chemotaxis regulator	1.32503	0.02891	0.1325
233905	Zfp646	zinc finger protein 646	1.324854	0.2624	0.4978
214133	Tet2	tet oncogene family member 2	1.324679	0.02766	0.1294

22682	Zfand5	zinc finger, AN1-type domain 5	1.324503	0.062	0.2073
66835	Snord123	small nucleolar RNA, C/D box 123	1.324503	0.01929	0.1038
27205	Podxl	podocalyxin-like	1.324153	0.002371	0.03015
654455	Defb48-ps	defensin beta 48 pseudogene	1.324153	0.182	0.4009
18791	Plat	plasminogen activator, tissue	1.323977	0.01061	0.07262
338368	Fam109b	family with sequence similarity 109, member B	1.323977	0.09238	0.2663
545007	ENSMUSG00000068790	predicted gene, ENSMUSG00000068790	1.323802	0.3044	0.5406
11668	Aldh1a1	aldehyde dehydrogenase family 1, subfamily A1	1.323627	0.04758	0.1765
16195	Il6st	interleukin 6 signal transducer	1.323452	0.001371	0.02171
22289	Kdm6a	4lysine (K)-specific demethylase 6A	1.323452	0.01124	0.07511
53859	Map3k14	mitogen-activated protein kinase kinase kinase 14	1.323452	0.1169	0.3087
229658	Vangl1	vang-like 1 (van gogh, Drosophila)	1.323452	0.11	0.2973
71754	Cyp2d40	cytochrome P450, family 2, subfamily d, polypeptide 40	1.323276	0.321	0.5574
218100	Zfp322a	zinc finger protein 322A	1.322926	0.01775	0.0984
667284	Gm8556	predicted gene 8556	1.322751	0.6799	0.8362
14388	Gab1	growth factor receptor bound protein 2-associated protein 1	1.322227	0.01798	0.09919
94279	Sfxn2	sideroflexin 2	1.322052	0.002592	0.03154
442812	B930049P21Rik	RIKEN cDNA B930049P21 gene	1.322052	0.01507	0.08962
100503757	LOC100503757	putative transposase element L1Md-A101/L1Md-A102/L1Md-A2-like	1.322052	0.07202	0.2282
106869	Tnfaip8	tumor necrosis factor, alpha-induced protein 8	1.321877	0.002395	0.03026
319660	Tmem195	transmembrane protein 195	1.321877	0.3934	0.6244
12305	Ddr1	discoidin domain receptor family, member 1	1.321702	0.001993	0.02713
320701	Fam19a4	family with sequence similarity 19, member A4	1.321702	0.04511	0.1712
102614	Rpp25	ribonuclease P 25 subunit (human)	1.321528	0.03179	0.1401
70320	2610008G14Rik	RIKEN cDNA 2610008G14 gene	1.321353	0.001924	0.02672
15481	Hspa8	heat shock protein 8	1.321178	0.0122	0.07862
52639	Wipi1	WD repeat domain, phosphoinositide interacting 1	1.321178	0.005836	0.0513
140577	Ankrd6	ankyrin repeat domain 6	1.321178	0.07832	0.2399
380712	Tlcd2	TLC domain containing 2	1.320829	0.04769	0.1766
19736	Rgs4	regulator of G-protein signaling 4	1.320655	0.0009176	0.01704
107607	Nod1	nucleotide-binding oligomerization domain containing 1	1.320655	0.04356	0.1676
77767	Ermn	ermin, ERM-like protein	1.320481	0.2953	0.5311
224530	Acat3	acetyl-Coenzyme A acetyltransferase 3	1.320481	0.009109	0.06608
12452	Ccng2	cyclin G2	1.320132	0.03992	0.1592
320798	4831440D22Rik	RIKEN cDNA 4831440D22 gene	1.319958	0.319	0.5554
216445	Arhgap9	Rho GTPase activating protein 9	1.319784	0.02356	0.117
13641	Efnb1	ephrin B1	1.319609	0.004872	0.04601
14368	Fzd6	frizzled homolog 6 (Drosophila)	1.319435	0.2308	0.4621
100043823	Gm4673	predicted gene 4673	1.319087	0.01612	0.09302
19249	Ptpn13	protein tyrosine phosphatase, non-receptor type 13	1.318913	0.009427	0.06739
19276	Ptprn2	protein tyrosine phosphatase, receptor type, N polypeptide 2	1.318739	0.03946	0.1582
76665	1700123M08Rik	RIKEN cDNA 1700123M08 gene	1.318739	0.1142	0.3043
227612	A830007P12Rik	RIKEN cDNA A830007P12 gene	1.318739	0.08393	0.2506
100216343	LOC100216343	plasma membrane Ca2+ pump interacting protein	1.318739	0.1658	0.3797
226422	Rab7l1	RAB7, member RAS oncogene family-like 1	1.318565	0.01592	0.09258
14083	Ptk2	PTK2 protein tyrosine kinase 2	1.318392	0.1727	0.3887
212670	Catsper2	cation channel, sperm associated 2	1.318044	0.02655	0.126
76432	2310001H17Rik	RIKEN cDNA 2310001H17 gene	1.317697	0.2673	0.5029
114301	Palmd	palmdelphin	1.317697	0.007269	0.05807

329003	Zfp516	zinc finger protein 516	1.317697	0.07735	0.2384
56349	Net1	neuroepithelial cell transforming gene 1	1.317523	0.01484	0.08886
72789	Veph1	ventricular zone expressed PH domain homolog 1 (zebrafish)	1.317523	0.3007	0.5366
22363	Vpreb2	pre-B lymphocyte gene 2	1.317349	0.5978	0.7835
93742	Pard3	par-3 (partitioning defective 3) homolog (C. elegans)	1.317349	0.2372	0.4694
17536	Meis2	Meis homeobox 2	1.317176	0.01711	0.09661
218772	Rarb	retinoic acid receptor, beta	1.317176	0.005694	0.05041
234582	Ccdc102a	coiled-coil domain containing 102A	1.317176	0.009263	0.06666
56289	Rassf1	Ras association (RalGDS/AF-6) domain family member 1	1.317003	0.002565	0.03145
66253	Aig1	androgen-induced 1	1.316829	0.03301	0.1434
18242	Oat	ornithine aminotransferase	1.316656	0.00455	0.04446
100039258	Gm10290	glyceraldehyde-3-phosphate dehydrogenase pseudogene	1.316656	0.01707	0.09645
79362	Bhlhe41	basic helix-loop-helix family, member e41	1.316309	0.02006	0.1064
20963	Sykb	spleen tyrosine kinase	1.316136	0.1811	0.3998
76056	5830443J22Rik	RIKEN cDNA 5830443J22 gene	1.316136	0.1338	0.3344
208618	Etl4	enhancer trap locus 4	1.315789	0.1278	0.3257
20591	Kdm5c	lysine (K)-specific demethylase 5C	1.315616	0.05352	0.1885
328087	D930001B02	hypothetical protein D930001B02	1.315616	0.04601	0.1731
57028	Pdxp	pyridoxal (pyridoxine, vitamin B6) phosphatase	1.31527	0.005326	0.04862
319158	Hist1h4i	histone cluster 1, H4i	1.315097	0.0007798	0.01552
100037283	Rnaset2a	ribonuclease T2A	1.315097	0.004922	0.04622
232201	Arhgap25	Rho GTPase activating protein 25	1.314924	0.02124	0.1101
69922	Vrk2	vaccinia related kinase 2	1.314752	0.08212	0.247
319986	A130042O14Rik	RIKEN cDNA A130042O14 gene	1.314752	0.05559	0.1929
24110	Usp18	ubiquitin specific peptidase 18	1.314233	0.04105	0.1621
67073	Pi4k2b	phosphatidylinositol 4-kinase type 2 beta	1.314233	0.0167	0.09504
14011	Etv6	ets variant gene 6 (TEL oncogene)	1.31406	0.01287	0.08153
68348	Serpina1f	serine (or cysteine) peptidase inhibitor, clade A, member 1F	1.313888	0.3544	0.5891
320183	Msrb3	methionine sulfoxide reductase B3	1.313888	0.007072	0.05694
78892	Crispld2	cysteine-rich secretory protein LCCL domain containing 2	1.313715	0.004669	0.04501
229279	Hnrnpa3	heterogeneous nuclear ribonucleoprotein A3	1.313715	0.303	0.539
14711	Gnmt	glycine N-methyltransferase	1.313543	0.01386	0.08514
19712	Rest	RE1-silencing transcription factor	1.313543	0.01012	0.07058
20818	Srprb	signal recognition particle receptor, B subunit	1.313543	0.08443	0.2516
71795	Pitpnc1	phosphatidylinositol transfer protein, cytoplasmic 1	1.313198	0.08103	0.2448
238037	BC068281	cDNA sequence BC068281	1.313025	0.05965	0.2026
240427	Setbp1	SET binding protein 1	1.31268	0.0792	0.2417
321022	Cdv3	carnitine deficiency-associated gene expressed in ventricle 3	1.31268	0.1202	0.3139
69068	1810011O10Rik	RIKEN cDNA 1810011O10 gene	1.312508	0.04939	0.1803
330323	Fam188b	family with sequence similarity 188, member B	1.312508	0.23	0.4612
14674	Gna13	guanine nucleotide binding protein, alpha 13	1.311992	0.03076	0.1374
19354	Rac2	RAS-related C3 botulinum substrate 2	1.311475	0.03865	0.1565
72719	2810040C05Rik	RIKEN cDNA 2810040C05 gene	1.311303	0.01839	0.1006
212516	BC060267	cDNA sequence BC060267	1.311132	0.1944	0.4181
20908	Stx3	syntaxin 3	1.31096	0.007444	0.05895
118449	Synpo2	synaptopodin 2	1.310788	0.1012	0.2817
18193	Nsd1	nuclear receptor-binding SET-domain protein 1	1.310444	0.1599	0.3715
27027	Tspan32	tetraspanin 32	1.310273	0.1007	0.2806
78921	9130019O22Rik	RIKEN cDNA 9130019O22 gene	1.310273	0.01686	0.09566

93688	Klhl1	kelch-like 1 (Drosophila)	1.310101	0.001234	0.02035
243373	AI854703	expressed sequence AI854703	1.310101	0.03899	0.1571
71755	Dhdh	dihydrodiol dehydrogenase (dimeric)	1.309929	0.02993	0.1353
76281	Tax1bp3	Tax1 (human T-cell leukemia virus type I) binding protein 3	1.309929	0.002476	0.03081
233335	Synm	synemin, intermediate filament protein	1.309929	0.03101	0.1379
330004	Gm833	predicted gene 833	1.309929	0.1224	0.3179
432825	Gm5458	predicted gene 5458	1.309929	0.3652	0.6009
77634	Snapc3	small nuclear RNA activating complex, polypeptide 3	1.309758	0.01108	0.07453
320237	Ncrna00086	non-protein coding RNA 86	1.309758	0.04954	0.1806
399558	Flrt2	fibronectin leucine rich transmembrane protein 2	1.309758	0.05224	0.1856
432812	BC052688	cDNA sequence BC052688	1.309586	0.2557	0.4906
74153	Uba7	ubiquitin-like modifier activating enzyme 7	1.309415	0.002415	0.03043
56863	Cldn9	claudin 9	1.309243	0.07742	0.2386
106585	Ankrd12	ankyrin repeat domain 12	1.309243	0.1076	0.2932
259300	Ehd2	EH-domain containing 2	1.308729	0.08679	0.2566
13639	Efna4	ephrin A4	1.308558	0.02198	0.1123
320398	Lrig3	leucine-rich repeats and immunoglobulin-like domains 3	1.308558	0.007073	0.05694
14176	Fgf5	fibroblast growth factor 5	1.308387	0.02775	0.1296
59050	Nsa2	NSA2 ribosome biogenesis homolog (S. cerevisiae)	1.308387	0.001772	0.02542
21818	Tgm3	transglutaminase 3, E polypeptide	1.308216	0.3916	0.623
16779	Lamb2	laminin, beta 2	1.308044	0.01413	0.08619
192657	Ell2	elongation factor RNA polymerase II 2	1.308044	0.03606	0.1501
11475	Acta2	actin, alpha 2, smooth muscle, aorta	1.307702	0.4914	0.7079
66356	2310008H09Rik	RIKEN cDNA 2310008H09 gene	1.307702	0.1089	0.2953
402749	B430316J06Rik	RIKEN cDNA B430316J06 gene	1.307702	0.09572	0.2723
66795	Atg10	autophagy-related 10 (yeast)	1.307531	0.003365	0.03683
15289	Hmgb1	high mobility group box 1	1.307019	0.08169	0.2462
65972	Ifi30	interferon gamma inducible protein 30	1.307019	0.01426	0.08667
22661	Zfp148	zinc finger protein 148	1.306848	0.09808	0.2763
71585	9130403I23Rik	RIKEN cDNA 9130403I23 gene	1.306506	0.01432	0.08686
68861	1190002N15Rik	RIKEN cDNA 1190002N15 gene	1.306336	0.04577	0.1725
621852	Rhox3f	reproductive homeobox 3F	1.306336	0.2313	0.4629
56448	Cyp2d22	cytochrome P450, family 2, subfamily d, polypeptide 22	1.306165	0.07323	0.2306
21766	Tex261	testis expressed gene 261	1.305483	0.002438	0.03058
230809	Pdik1l	PDLIM1 interacting kinase 1 like	1.305313	0.04918	0.1797
18789	Papola	poly (A) polymerase alpha	1.305142	0.07448	0.2329
320405	Cadps2	Ca2+-dependent activator protein for secretion 2	1.305142	0.009934	0.06972
11877	Arvcf	armadillo repeat gene deleted in velo-cardio-facial syndrome	1.304461	0.1793	0.3972
22771	Zic1	zinc finger protein of the cerebellum 1	1.304121	0.2498	0.484
78177	Ninl	ninein-like	1.303951	0.1102	0.2975
320332	Hist4h4	histone cluster 4, H4	1.303951	0.1707	0.3861
404311	Olfr209	olfactory receptor 209	1.303781	0.3465	0.582
666752	Gm8273	predicted gene 8273	1.303441	0.009314	0.06691
19317	Qk	quaking	1.303271	0.007332	0.05829
227800	Rabgap1	RAB GTPase activating protein 1	1.303271	0.5641	0.7613
16410	Itgav	integrin alpha V	1.302932	0.06548	0.2146
104445	Cdc42ep1	CDC42 effector protein (Rho GTPase binding) 1	1.302932	0.02103	0.1093
100045736	LOC100045736	hypothetical protein LOC100045736	1.302932	0.5076	0.7193
238799	Tnpo1	transportin 1	1.302762	0.2094	0.4364

208924	A730045E13Rik	RIKEN cDNA A730045E13 gene	1.302592	0.2425	0.4755
244141	Nars2	asparaginyl-tRNA synthetase 2 (mitochondrial)(putative)	1.302592	0.006529	0.05458
14132	Fcgrt	Fc receptor, IgG, alpha chain transporter	1.302423	0.0263	0.1252
171580	Mical1	microtubule associated monoxygenase, calponin and LIM domain containing 1	1.302423	0.01766	0.09827
101437	Dhx32	DEAH (Asp-Glu-Ala-His) box polypeptide 32	1.302083	0.0737	0.2317
245128	AU018091	expressed sequence AU018091	1.302083	0.6033	0.7871
16974	Lrp6	low density lipoprotein receptor-related protein 6	1.301914	0.018	0.09928
14919	Gucy2e	guanylate cyclase 2e	1.301406	0.01176	0.07703
16651	Sspn	sarcospan	1.301406	0.02134	0.1104
99470	Magi3	membrane associated guanylate kinase, WW and PDZ domain containing 3	1.301406	0.02405	0.1184
19416	Rasd1	RAS, dexamethasone-induced 1	1.301236	0.0436	0.1676
333193	BC053749	cDNA sequence BC053749	1.301236	0.2922	0.5281
27973	Vkorc1	vitamin K epoxide reductase complex, subunit 1	1.301067	0.1431	0.3479
17686	Msh3	mutS homolog 3 (E. coli)	1.300898	0.3458	0.5812
77115	6030451C04Rik	RIKEN cDNA 6030451C04 gene	1.300898	0.1607	0.3725
328156	C230037L09	hypothetical protein C230037L09	1.300728	0.0156	0.09162
27276	Plekhb1	pleckstrin homology domain containing, family B (evectins) member 1	1.300559	0.04751	0.1765
102141	Snx25	sorting nexin 25	1.300052	0.1419	0.3462
269132	Glt25d2	glycosyltransferase 25 domain containing 2	1.300052	0.002337	0.0299
66075	Chchd3	coiled-coil-helix-coiled-coil-helix domain containing 3	1.299883	0.002605	0.03161
17122	Mxd4	Max dimerization protein 4	1.299714	0.09978	0.279
19155	Npepps	aminopeptidase puromycin sensitive	1.299714	0.007085	0.05701
21858	Timp2	tissue inhibitor of metalloproteinase 2	1.299714	0.006777	0.0557
70020	Ino80b	INO80 complex subunit B	1.299714	0.06264	0.2089
338320	Mia2	melanoma inhibitory activity 2	1.299714	0.05356	0.1886
381716	1700015F17Rik	RIKEN cDNA 1700015F17 gene	1.299714	0.04443	0.1695
105268	AU016916	expressed sequence AU016916	1.299545	0.02574	0.1235
237886	Slfn9	schlafen 9	1.299376	0.3066	0.5432
11569	Aebp2	AE binding protein 2	1.299039	0.03041	0.1368
22003	Tpm1	tropomyosin 1, alpha	1.299039	0.2332	0.4644
27386	Npas3	neuronal PAS domain protein 3	1.299039	0.001884	0.02634
18115	Nnt	nicotinamide nucleotide transhydrogenase	1.29887	0.002973	0.03406
22342	Lin7b	lin-7 homolog B (C. elegans)	1.29887	0.03681	0.152
277360	Prex1	phosphatidylinositol-3,4,5-trisphosphate-dependent Rac exchange factor 1	1.29887	0.00304	0.03449
54381	Pgcp	plasma glutamate carboxypeptidase	1.298533	0.02605	0.1244
319156	Hist1h4d	histone cluster 1, H4d	1.298533	0.1453	0.3507
21961	Tns1	tensin 1	1.298364	0.01933	0.104
26559	Hunk	hormonally upregulated Neu-associated kinase	1.298364	0.02947	0.1341
217837	Itpk1	inositol 1,3,4-triphosphate 5/6 kinase	1.298364	0.07201	0.2282
74057	4933405D12Rik	RIKEN cDNA 4933405D12 gene	1.298196	0.5287	0.7354
268935	Scube3	signal peptide, CUB domain, EGF-like 3	1.298027	0.08859	0.2599
19024	Ppfibp2	PTPRF interacting protein, binding protein 2 (liprin beta 2)	1.297859	0.02322	0.1161
19049	Ppp1r1b	protein phosphatase 1, regulatory (inhibitor) subunit 1B	1.297859	0.05462	0.1912
72536	Tagap	T-cell activation Rho GTPase-activating protein	1.297859	0.005039	0.04691
57342	Parva	parvin, alpha	1.29769	0.04368	0.1679
71742	Ulk3	unc-51-like kinase 3 (C. elegans)	1.297522	0.007863	0.0609
74744	5830408C22Rik	RIKEN cDNA 5830408C22 gene	1.297522	0.1435	0.3485
117149	Tirap	toll-interleukin 1 receptor (TIR) domain-containing adaptor protein	1.297353	0.002508	0.03102
271005	Klhdc1	kelch domain containing 1	1.297353	0.009945	0.06978

328417	Parp4	poly (ADP-ribose) polymerase family, member 4	1.297185	0.01634	0.09371
671564	Rnf212	ring finger protein 212	1.296849	0.1549	0.3647
14652	Glp1r	glucagon-like peptide 1 receptor	1.29668	0.4468	0.6722
16180	Il1rap	interleukin 1 receptor accessory protein	1.29668	0.5423	0.7461
22354	Vipr1	vasoactive intestinal peptide receptor 1	1.29668	0.04217	0.1643
13733	Emr1	EGF-like module containing, mucin-like, hormone receptor-like sequence 1	1.296512	0.289	0.525
19731	Rgl1	ral guanine nucleotide dissociation stimulator,-like 1	1.296512	0.003717	0.039
665891	Krtap4-1	keratin associated protein 4-1	1.296512	0.3472	0.5826
18158	Nppb	natriuretic peptide type B	1.296344	0.0476	0.1765
20401	Sh3bp1	SH3-domain binding protein 1	1.296344	0.1098	0.2969
70435	Inf2	inverted formin, FH2 and WH2 domain containing	1.296344	0.01087	0.07365
268291	Rnf217	ring finger protein 217	1.296176	0.02665	0.1262
12577	Cdkn1c	cyclin-dependent kinase inhibitor 1C (P57)	1.296008	0.08165	0.2462
66991	2410004A20Rik	RIKEN cDNA 2410004A20 gene	1.296008	0.02597	0.1242
100039707	Gm2382	predicted gene 2382	1.29584	0.003373	0.0369
231872	Aimp2	aminoacyl tRNA synthetase complex-interacting multifunctional protein 2	1.295672	0.0007063	0.01462
270035	Letm2	leucine zipper-EF-hand containing transmembrane protein 2	1.295672	0.1396	0.343
320790	Chd7	chromodomain helicase DNA binding protein 7	1.295672	0.01077	0.07326
69178	Snx5	sorting nexin 5	1.295505	0.07364	0.2315
216161	Sbno2	strawberry notch homolog 2 (Drosophila)	1.295337	0.05593	0.1937
19700	Rem1	rad and gem related GTP binding protein 1	1.295169	0.2898	0.526
109263	Rlf	rearranged L-myc fusion sequence	1.295169	0.04997	0.1816
12519	Cd80	CD80 antigen	1.294834	0.09651	0.2737
71004	4931440P22Rik	RIKEN cDNA 4931440P22 gene	1.294834	0.03674	0.1519
13019	Ctf1	cardiotrophin 1	1.294666	0.01598	0.09265
319159	Hist1h4j	histone cluster 1, H4j	1.294666	0.1008	0.2809
13649	Egfr	epidermal growth factor receptor	1.294498	0.08689	0.2568
66659	Acp6	acid phosphatase 6, lysophosphatidic	1.294498	0.001015	0.01786
330790	Hapln4	hyaluronan and proteoglycan link protein 4	1.294498	0.1239	0.3204
624696	Gm6522	predicted gene 6522	1.294498	0.3256	0.5622
381678	Zcwpw1	zinc finger, CW type with PWWP domain 1	1.294331	0.06603	0.216
15163	Hcls1	hematopoietic cell specific Lyn substrate 1	1.294163	0.002797	0.03286
16412	Itgb1	integrin beta 1 (fibronectin receptor beta)	1.293828	0.1523	0.3613
71186	4933417D19Rik	RIKEN cDNA 4933417D19 gene	1.293828	0.06467	0.2129
16322	Inha	inhibin alpha	1.293661	0.01326	0.08305
109700	Itga1	integrin alpha 1	1.293661	0.5961	0.7823
638833	Gm7251	glyceraldehyde-3-phosphate dehydrogenase pseudogene	1.293661	0.07317	0.2305
319603	A730009E18Rik	RIKEN cDNA A730009E18 gene	1.293494	0.1361	0.3379
195333	Gsc2	goosecoid homebox 2	1.293326	0.04395	0.1686
229898	Gbp5	guanylate binding protein 5	1.293326	0.06154	0.2063
241113	Prkag3	protein kinase, AMP-activated, gamma 3 non-catatlytic subunit	1.293159	0.1561	0.3659
100039697	Gm2375	predicted gene 2375	1.293159	0.1086	0.2948
17354	Mllt10	myeloid/lymphoid or mixed-lineage leukemia (trithorax homolog, Drosophila); translocated to, 10	1.292992	0.112	0.3005
73824	Snhg6	small nucleolar RNA host gene (non-protein coding) 6	1.292825	0.02642	0.1255
81497	Btnl5	butyrophilin-like 5	1.292658	0.05215	0.1856
234733	Ddx19b	DEAD (Asp-Glu-Ala-Asp) box polypeptide 19b	1.292658	0.3206	0.5572
74025	Nphp3	nephronophthisis 3 (adolescent)	1.292324	0.06521	0.214
74142	Lonp1	lon peptidase 1, mitochondrial	1.292324	0.001185	0.01967
212442	Lactb2	lactamase, beta 2	1.292324	0.0177	0.09835

14356	Fxc1	fractured callus expressed transcript 1	1.292157	0.3628	0.5979
382253	Cdkl5	cyclin-dependent kinase-like 5	1.292157	0.02845	0.1312
52463	Tet1	tet oncogene 1	1.29199	0.04291	0.1661
21748	Terc	telomerase RNA component	1.291823	0.1343	0.3353
78892	Crispld2	cysteine-rich secretory protein LCCL domain containing 2	1.291656	0.05547	0.1927
210710	Gab3	growth factor receptor bound protein 2-associated protein 3	1.291656	0.2281	0.459
15064	Mr1	major histocompatibility complex, class I-related	1.291489	0.01401	0.08574
19317	Qk	quaking	1.291322	0.1201	0.3139
73453	1700067K01Rik	RIKEN cDNA 1700067K01 gene	1.291322	0.2726	0.5081
100505122	LOC100505122	hypothetical LOC100505122	1.291322	0.008654	0.06405
195208	Dcdc2a	doublecortin domain containing 2a	1.291156	0.005609	0.05007
16409	Itgam	integrin alpha M	1.290822	0.2574	0.4926
232449	Dera	2-deoxyribose-5-phosphate aldolase homolog (C. elegans)	1.290822	0.01818	0.0998
236573	Gbp9	guanylate-binding protein 9	1.290656	0.02413	0.1186
544678	2010015L04Rik	RIKEN cDNA 2010015L04 gene	1.290489	0.01942	0.1043
545013	Gm5797	predicted gene 5797	1.290489	0.3878	0.6197
65973	Asph	aspartate-beta-hydroxylase	1.290323	0.001692	0.02489
69761	1600015I10Rik	RIKEN cDNA 1600015I10 gene	1.290323	0.05432	0.1905
51873	D2Ertd127e	DNA segment, Chr 2, ERATO Doi 127, expressed	1.290156	0.227	0.4584
18515	Pbx2	pre B-cell leukemia transcription factor 2	1.289823	0.002101	0.02786
50772	Mapk6	mitogen-activated protein kinase 6	1.289823	0.1458	0.3514
78088	Ankrd56	ankyrin repeat domain 56	1.289823	0.566	0.762
244723	Olfm2	olfactomedin 2	1.289823	0.1939	0.4172
665211	Gm14326	predicted gene 14326	1.289324	0.2489	0.4832
53378	Sdcbp	syndecan binding protein	1.289158	0.05536	0.1925
20363	Sepp1	selenoprotein P, plasma, 1	1.288992	0.01748	0.09774
99470	Magi3	membrane associated guanylate kinase, WW and PDZ domain containing 3	1.288992	0.01423	0.08658
67739	Slc48a1	solute carrier family 48 (heme transporter), member 1	1.28866	0.003805	0.0395
667141	Gm8479	thiopurine S-methyltransferase pseudogene	1.28866	0.09065	0.2637
216792	A230051G13Rik	RIKEN cDNA A230051G13 gene	1.288494	0.01989	0.1057
545238	G430049J08Rik	RIKEN cDNA G430049J08 gene	1.288328	0.5134	0.7237
66170	Chchd5	coiled-coil-helix-coiled-coil-helix domain containing 5	1.288162	0.007411	0.05875
100198	H6pd	hexose-6-phosphate dehydrogenase (glucose 1-dehydrogenase)	1.288162	0.1682	0.3832
22354	Vipr1	vasoactive intestinal peptide receptor 1	1.287996	0.0139	0.08531
12054	Bcl7b	B-cell CLL/lymphoma 7B	1.28783	0.07681	0.2374
28200	Dhrs4	dehydrogenase/reductase (SDR family) member 4	1.28783	0.01465	0.08814
52013	D19Ertd386e	DNA segment, Chr 19, ERATO Doi 386, expressed	1.28783	0.0184	0.1007
68703	Rere	arginine glutamic acid dipeptide (RE) repeats	1.28783	0.08721	0.2575
12831	Col5a1	collagen, type V, alpha 1	1.287664	0.04881	0.1787
103142	Rdh9	retinol dehydrogenase 9	1.287664	0.02882	0.1323
18028	Nfib	nuclear factor I/B	1.287498	0.1551	0.3648
73687	2410087M07Rik	RIKEN cDNA 2410087M07 gene	1.287498	0.2902	0.5264
101148	B630005N14Rik	RIKEN cDNA B630005N14 gene	1.286836	0.04903	0.1794
100503757	LOC100503757	putative transposase element L1Md-A101/L1Md-A102/L1Md-A2-like	1.286008	0.2346	0.4664
21406	Tcf12	transcription factor 12	1.285843	0.09032	0.2631
93880	Pcdhb9	protocadherin beta 9	1.285843	0.07634	0.2367
18938	Ppp1r14b	protein phosphatase 1, regulatory (inhibitor) subunit 14B	1.285512	0.01796	0.09916
216864	Mgl2	macrophage galactose N-acetyl-galactosamine specific lectin 2	1.285347	0.001563	0.02361
619331	Zfp551	zinc fingr protein 551	1.285347	0.1732	0.3894

69870	Polr3gl	polymerase (RNA) III (DNA directed) polypeptide G like	1.285182	0.008633	0.06401
226866	Gm106	predicted gene 106	1.285182	0.06	0.2032
26559	Hunk	hormonally upregulated Neu-associated kinase	1.284852	0.01396	0.08557
54366	Ctnnal1	catenin (cadherin associated protein), alpha-like 1	1.284852	0.02776	0.1296
70354	Secisbp2l	SECIS binding protein 2-like	1.284687	0.108	0.294
70361	Lman1	lectin, mannose-binding, 1	1.284687	0.09974	0.2789
76763	Mospd2	motile sperm domain containing 2	1.284687	0.01065	0.07272
320923	Mtap7d3	MAP7 domain containing 3	1.284687	0.07933	0.2419
84094	Plvap	plasmalemma vesicle associated protein	1.284522	0.0062	0.05304
231287	Atp10d	ATPase, class V, type 10D	1.284522	0.2842	0.5199
245945	Rbm47	RNA binding motif protein 47	1.284522	0.2649	0.5008
545366	BC026782	cDNA sequence BC026782	1.284522	0.1006	0.2806
12705	Cited1	Cbp/p300-interacting transactivator with Glu/Asp-rich carboxy-terminal domain 1	1.284357	0.004604	0.04471
66395	Ahnak	AHNAK nucleoprotein (desmoyokin)	1.284357	0.383	0.6161
93840	Vangl2	vang-like 2 (van gogh, Drosophila)	1.284027	0.05168	0.1848
58207	Slc43a3	solute carrier family 43, member 3	1.283862	0.1273	0.325
246081	Defb11	defensin beta 11	1.283862	0.2269	0.4584
574428	Zmynd15	zinc finger, MYND-type containing 15	1.283862	0.01575	0.09219
16596	Klf1	Kruppel-like factor 1 (erythroid)	1.283697	0.1151	0.3058
18301	Fxyd5	FXYD domain-containing ion transport regulator 5	1.283532	0.09906	0.2779
16764	Aff3	AF4/FMR2 family, member 3	1.283368	0.2606	0.4959
110611	Hdlbp	high density lipoprotein (HDL) binding protein	1.283368	0.02197	0.1123
100040608	Fancf	Fanconi anemia, complementation group F	1.283203	0.03303	0.1434
110596	Rgnef	Rho-guanine nucleotide exchange factor	1.283038	0.02001	0.1063
170756	Slc24a6	solute carrier family 24 (sodium/potassium/calcium exchanger), member 6	1.283038	0.225	0.4561
26934	Racgap1	Rac GTPase-activating protein 1	1.282709	0.08058	0.2441
67704	1810037I17Rik	RIKEN cDNA 1810037I17 gene	1.282709	0.01381	0.08504
100040671	Gm2897	predicted gene 2897	1.282709	0.3503	0.5861
67554	Slc25a30	solute carrier family 25, member 30	1.28238	0.007705	0.06013
244059	Chd2	chromodomain helicase DNA binding protein 2	1.28238	0.1947	0.4186
69384	Tmem89	transmembrane protein 89	1.282216	0.02527	0.1221
72690	Grrp1	glycine/arginine rich protein 1	1.282216	0.01169	0.0768
244864	Layn	layilin	1.282216	0.04225	0.1645
252903	Ap1s3	adaptor-related protein complex AP-1, sigma 3	1.281887	0.1037	0.2859
70045	2610528A11Rik	RIKEN cDNA 2610528A11 gene	1.281723	0.1521	0.3612
66251	Arfgap3	ADP-ribosylation factor GTPase activating protein 3	1.281394	0.006006	0.05219
69772	Bdh2	3-hydroxybutyrate dehydrogenase, type 2	1.281394	0.02334	0.1165
11898	Ass1	argininosuccinate synthetase 1	1.280738	0.05381	0.1893
71207	Nudt4	nudix (nucleoside diphosphate linked moiety X)-type motif 4	1.280738	0.001712	0.02505
75016	4930480K23Rik	RIKEN cDNA 4930480K23 gene	1.280738	0.1098	0.2969
78816	Gmip	Gem-interacting protein	1.28041	0.03544	0.1487
100049569	A730015C16Rik	RIKEN cDNA A730015C16 gene	1.28041	0.2465	0.4806
59050	Nsa2	NSA2 ribosome biogenesis homolog (S. cerevisiae)	1.280246	0.01324	0.08299
231452	Sdad1	SDA1 domain containing 1	1.280246	0.03056	0.137
75064	Zcchc13	zinc finger, CCHC domain containing 13	1.279918	0.04688	0.1751
320022	Ccdc79	coiled-coil domain containing 79	1.279754	0.05401	0.1898
17755	Mtap1b	microtubule-associated protein 1B	1.279591	0.2618	0.4969
56347	Eif3c	eukaryotic translation initiation factor 3, subunit C	1.279591	0.05042	0.1824
19348	Kif20a	kinesin family member 20A	1.279427	0.004646	0.04486

105450	Mmrn2	multimerin 2	1.279427	0.04704	0.1755
383243	Olfr128	olfactory receptor 128	1.279427	0.6169	0.7969
14725	Lrp2	low density lipoprotein receptor-related protein 2	1.279263	0.01436	0.08708
319625	Galm	galactose mutarotase	1.279263	0.1022	0.2833
100043682	Gm10584	predicted gene 10584	1.2791	0.4803	0.6993
16562	Kif1c	kinesin family member 1C	1.278936	0.005582	0.04996
71670	Acy3	aspartoacylase (aminoacylase) 3	1.278936	0.01899	0.1027
30049	Scd3	stearoyl-coenzyme A desaturase 3	1.278609	0.04356	0.1676
218734	3830406C13Rik	RIKEN cDNA 3830406C13 gene	1.278609	0.1055	0.2892
20509	Slc19a1	solute carrier family 19 (sodium/hydrogen exchanger), member 1	1.278445	0.0367	0.1517
70810	Krt25	keratin 25	1.278445	0.05607	0.194
100702	Mpa2l	macrophage activation 2 like	1.278445	0.1261	0.3233
74318	Hopx	HOP homeobox	1.277955	0.01166	0.07664
18648	Pgam1	phosphoglycerate mutase 1	1.277792	0.03828	0.1558
218214	Kdm1b	lysine (K)-specific demethylase 1B	1.277466	0.01382	0.08508
14810	Grin1	glutamate receptor, ionotropic, NMDA1 (zeta 1)	1.277302	0.1685	0.3835
16855	Lgals4	lectin, galactose binding, soluble 4	1.277302	0.1592	0.3704
18451	P4ha1	procollagen-proline, 2-oxoglutarate 4-dioxygenase (proline 4-hydroxylase), alpha 1 polypeptide	1.277302	0.02468	0.1201
29865	Cabp5	calcium binding protein 5	1.277302	0.4838	0.7021
14109	Fau	Finkel-Biskis-Reilly murine sarcoma virus (FBR-MuSV) ubiquitously expressed (fox derived)	1.277139	0.02081	0.1087
21750	Terf2	telomeric repeat binding factor 2	1.27665	0.05729	0.1968
73230	Bmper	BMP-binding endothelial regulator	1.27665	0.1278	0.3258
667277	C1rb	complement component 1, r subcomponent B	1.27665	0.01037	0.07173
12927	Bcar1	breast cancer anti-estrogen resistance 1	1.276487	0.000521	0.01224
15381	Hnrnpc	heterogeneous nuclear ribonucleoprotein C	1.276487	0.08982	0.262
20018	Polr1d	polymerase (RNA) I polypeptide D	1.276487	0.007111	0.05713
269774	Aak1	AP2 associated kinase 1	1.276324	0.002673	0.03204
667572	Gm8709	glyceraldehyde-3-phosphate dehydrogenase pseudogene	1.276324	0.1286	0.327
16367	Irs1	insulin receptor substrate 1	1.276161	0.1456	0.351
66968	Plin5	perilipin 5	1.276161	0.1792	0.3972
12010	B2m	beta-2 microglobulin	1.275998	0.02871	0.132
18230	Nxn	nucleoredoxin	1.275998	0.002378	0.03016
93742	Pard3	par-3 (partitioning defective 3) homolog (C. elegans)	1.275998	0.01954	0.1047
74485	4933430H15Rik	RIKEN cDNA 4933430H15 gene	1.275836	0.3117	0.5482
434325	Tmem221	transmembrane protein 221	1.275673	0.5022	0.7152
270627	Taf1	TAF1 RNA polymerase II, TATA box binding protein (TBP)-associated factor	1.27551	0.5188	0.728
319160	Hist1h4k	histone cluster 1, H4k	1.27551	0.1617	0.3737
26930	Ppnr	per-pentamer repeat gene	1.275348	0.0263	0.1252
217430	Pqlc3	PQ loop repeat containing	1.275348	0.1955	0.4192
73182	Pear1	platelet endothelial aggregation receptor 1	1.275185	0.1317	0.3319
20345	Selplg	selectin, platelet (p-selectin) ligand	1.27486	0.08065	0.2442
240672	Dusp5	dual specificity phosphatase 5	1.27486	0.1406	0.3443
545861	Gm5878	predicted gene 5878	1.27486	0.5061	0.7183
270627	Taf1	TAF1 RNA polymerase II, TATA box binding protein (TBP)-associated factor	1.274697	0.05404	0.1898
28114	Nsun2	NOL1/NOP2/Sun domain family member 2	1.274535	0.00168	0.02484
58859	Efemp2	epidermal growth factor-containing fibulin-like extracellular matrix protein 2	1.274535	0.01254	0.08011
74559	Elovl7	ELOVL family member 7, elongation of long chain fatty acids (yeast)	1.274535	0.06856	0.2211
399619	A930027P06Rik	RIKEN cDNA A930027P06 gene	1.274535	0.2469	0.481
232933	Ccdc61	coiled-coil domain containing 61	1.273723	0.026	0.1242

26921	Map4k4	mitogen-activated protein kinase kinase kinase kinase 4	1.273561	0.1054	0.289
100040259	Gm16379	predicted pseudogene 16379	1.273561	0.01055	0.07244
17250	Abcc1	ATP-binding cassette, sub-family C (CFTR/MRP), member 1	1.273399	0.0006672	0.01414
66578	2610039C10Rik	RIKEN cDNA 2610039C10 gene	1.273399	0.05912	0.2014
78103	8430431K14Rik	RIKEN cDNA 8430431K14 gene	1.273399	0.1545	0.3644
230757	5730409E04Rik	RIKEN cDNA 5730409E04Rik gene	1.273399	0.04089	0.1616
26369	Cetn1	centrin 1	1.273237	0.3506	0.5864
319757	Smo	smoothened homolog (Drosophila)	1.273237	0.01651	0.09433
230073	Ddx58	DEAD (Asp-Glu-Ala-Asp) box polypeptide 58	1.27275	0.04829	0.1778
219132	D14Ertd668e	DNA segment, Chr 14, ERATO Doi 668, expressed	1.272588	0.06968	0.2234
84682	Cox4i2	cytochrome c oxidase subunit IV isoform 2	1.272427	0.0338	0.1453
240479	Fam69c	family with sequence similarity 69, member C	1.272427	0.1726	0.3885
213948	Atg9b	ATG9 autophagy related 9 homolog B (S. cerevisiae)	1.272265	0.5619	0.7602
620986	Gm6195	predicted pseudogene 6195	1.272103	0.002799	0.03286
16913	Psmb8	proteasome (prosome, macropain) subunit, beta type 8 (large multifunctional peptidase 7)	1.271941	0.063	0.2095
76566	Fam101b	family with sequence similarity 101, member B	1.271941	0.07688	0.2375
320496	C230076A16Rik	RIKEN cDNA C230076A16 gene	1.271779	0.04398	0.1686
14531	Gcm1	glial cells missing homolog 1 (Drosophila)	1.271617	0.2609	0.4961
329152	Hecw2	HECT, C2 and WW domain containing E3 ubiquitin protein ligase 2	1.271456	0.3024	0.5385
545611	Gm13298	predicted gene 13298	1.271456	0.4522	0.677
217069	Trim25	tripartite motif-containing 25	1.271294	0.02659	0.1261
102589	AI835735	expressed sequence AI835735	1.270971	0.621	0.7994
20345	Selplg	selectin, platelet (p-selectin) ligand	1.27081	0.05547	0.1927
100039316	Gm9843	predicted gene 9843	1.27081	0.01589	0.09256
16978	Lrrfip1	leucine rich repeat (in FLII) interacting protein 1	1.270325	0.0553	0.1925
105278	Cdk20	cyclin-dependent kinase 20	1.270325	0.009522	0.06791
108960	Irak2	interleukin-1 receptor-associated kinase 2	1.270164	0.0369	0.1521
12263	C2	complement component 2 (within H-2S)	1.270003	0.04707	0.1755
72459	Htatsf1	HIV TAT specific factor 1	1.269841	0.2068	0.4329
76815	Calcoco2	calcium binding and coiled-coil domain 2	1.26968	0.4432	0.669
269823	Pon3	paraoxonase 3	1.26968	0.2478	0.4821
58998	Pvrl3	poliovirus receptor-related 3	1.269519	0.07804	0.2394
93687	Csnk1a1	casein kinase 1, alpha 1	1.269519	0.2172	0.4462
332397	Nanos1	nanos homolog 1 (Drosophila)	1.269519	0.004459	0.04378
59010	Sqrdl	sulfide quinone reductase-like (yeast)	1.269036	0.2274	0.4586
76123	Gpsm2	G-protein signalling modulator 2 (AGS3-like, C. elegans)	1.269036	0.00042	0.01084
13650	Rhbdf1	rhomboid family 1 (Drosophila)	1.268875	0.04286	0.166
14919	Gucy2e	guanylate cyclase 2e	1.268875	0.005765	0.05085
258383	Olfr1347	olfactory receptor 1347	1.268875	0.2532	0.4878
17215	Mcm3	minichromosome maintenance deficient 3 (S. cerevisiae)	1.268714	0.05513	0.1923
102644	Oaf	OAF homolog (Drosophila)	1.268714	0.1274	0.325
244650	Phlpp2	PH domain and leucine rich repeat protein phosphatase 2	1.268714	0.05793	0.1984
434174	Hmg1l1	high-mobility group (nonhistone chromosomal) protein 1-like 1	1.268714	0.1797	0.3979
75605	Kdm5b	lysine (K)-specific demethylase 5B	1.268553	0.01579	0.09229
14370	Fzd8	frizzled homolog 8 (Drosophila)	1.268392	0.02881	0.1323
28135	Cep63	centrosomal protein 63	1.268392	0.01293	0.0817
545156	Kalrn	kalirin, RhoGEF kinase	1.268231	0.1499	0.3577
18552	Pcsk5	proprotein convertase subtilisin/kexin type 5	1.26807	0.03495	0.1482
19823	Rnf7	ring finger protein 7	1.26807	0.01092	0.07384

100019	Mdn1	midasin homolog (yeast)	1.26807	0.3471	0.5825
77018	Col25a1	collagen, type XXV, alpha 1	1.267909	0.2367	0.4689
230837	Asap3	ArfGAP with SH3 domain, ankyrin repeat and PH domain 3	1.267427	0.1184	0.3111
231130	Tnip2	TNFAIP3 interacting protein 2	1.267427	0.001424	0.02229
22350	Ezr	ezrin	1.267267	0.2174	0.4464
100040999	Gm3086	RuvB-like protein 1 pseudogene	1.267267	0.01129	0.07532
77951	Cyp20a1	cytochrome P450, family 20, subfamily A, polypeptide 1	1.266945	0.2423	0.4753
75423	Arl5a	ADP-ribosylation factor-like 5A	1.266785	0.03993	0.1592
67122	Nrarp	Notch-regulated ankyrin repeat protein	1.266464	0.07982	0.243
195576	Gm13229	predicted gene 13229	1.266464	0.1072	0.2925
667572	Gm8709	glyceraldehyde-3-phosphate dehydrogenase pseudogene	1.266464	0.07864	0.2405
140703	Emid1	EMI domain containing 1	1.266304	0.09359	0.2688
626150	Gm12271	predicted gene 12271	1.265983	0.04016	0.1598
67622	Mxra7	matrix-remodelling associated 7	1.265663	0.02184	0.112
70550	5730416F02Rik	RIKEN cDNA 5730416F02 gene	1.265663	0.2267	0.4582
67397	Erp29	endoplasmic reticulum protein 29	1.265502	0.00488	0.04601
12097	Bglap2	bone gamma-carboxyglutamate protein 2	1.265342	0.1219	0.3169
64929	Scel	sciellin	1.265022	0.3278	0.5642
76223	Agbl3	ATP/GTP binding protein-like 3	1.265022	0.1169	0.3088
70571	Tcerg1l	transcription elongation regulator 1-like	1.264702	0.09791	0.276
16331	Inpp5d	inositol polyphosphate-5-phosphatase D	1.264542	0.03162	0.1398
98660	Atp1a2	ATPase, Na+/K+ transporting, alpha 2 polypeptide	1.264542	0.2384	0.4708
433294	A530098C11Rik	RIKEN cDNA A530098C11 gene	1.264542	0.2795	0.515
621699	Gm6252	predicted gene 6252	1.264542	0.3181	0.5545
319186	Hist1h2bm	histone cluster 1, H2bm	1.264063	0.005582	0.04996
227289	Gpbar1	G protein-coupled bile acid receptor 1	1.263584	0.0344	0.1466
320943	B230213L16Rik	RIKEN cDNA B230213L16 gene	1.263584	0.03915	0.1576
66129	1110018J18Rik	RIKEN cDNA 1110018J18 gene	1.263264	0.007282	0.05811
70290	2310079F09Rik	RIKEN cDNA 2310079F09 gene	1.263264	0.2184	0.4477
215690	Nav1	neuron navigator 1	1.262945	0.01639	0.09385
381572	9430007A20Rik	RIKEN cDNA 9430007A20 gene	1.262626	0.2089	0.4358
67238	2810453I06Rik	RIKEN cDNA 2810453I06 gene	1.262467	0.01759	0.09797
20465	Sim2	single-minded homolog 2 (Drosophila)	1.262307	0.2603	0.4957
223254	Farp1	FERM, RhoGEF (Arhgef) and pleckstrin domain protein 1 (chondrocyte-derived)	1.262307	0.009112	0.06608
26459	Slc27a5	solute carrier family 27 (fatty acid transporter), member 5	1.262148	0.0554	0.1926
242474	D730040F13Rik	RIKEN cDNA D730040F13 gene	1.262148	0.08506	0.2528
100040320	Gm2710	predicted gene 2710	1.262148	0.09499	0.271
20442	St3gal1	ST3 beta-galactoside alpha-2,3-sialyltransferase 1	1.261989	0.03104	0.1379
213389	Prdm9	PR domain containing 9	1.261989	0.03534	0.1484
18048	Klk1b4	kallikrein 1-related pepidase b4	1.26167	0.2973	0.5334
12977	Csf1	colony stimulating factor 1 (macrophage)	1.261511	0.2293	0.4604
140499	Ube2j2	ubiquitin-conjugating enzyme E2, J2 homolog (yeast)	1.261511	0.01116	0.07479
17294	Mest	mesoderm specific transcript	1.261352	0.007783	0.06048
15208	Hes5	hairy and enhancer of split 5 (Drosophila)	1.260875	0.01589	0.09256
68576	Hbxip	hepatitis B virus x interacting protein	1.260716	0.005616	0.05009
66940	Shisa5	shisa homolog 5 (Xenopus laevis)	1.260557	0.01393	0.08544
22042	Tfrc	transferrin receptor	1.260398	0.1434	0.3482
26941	Slc9a3r1	solute carrier family 9 (sodium/hydrogen exchanger), member 3 regulator 1	1.260398	0.01838	0.1006
19276	Ptprn2	protein tyrosine phosphatase, receptor type, N polypeptide 2	1.260239	0.342	0.5775

97122	Hist2h4	histone cluster 2, H4	1.260239	0.1964	0.4206
100040382	Gm2745	predicted gene 2745	1.260239	0.1572	0.3675
57913	Lrdd	leucine-rich and death domain containing	1.259922	0.1356	0.3372
56293	Amac1	acyl-malonyl condensing enzyme 1	1.259763	0.2496	0.4837
232748	Fam115c	family with sequence similarity 115, member C	1.259763	0.1087	0.295
12530	Cdc25a	cell division cycle 25 homolog A (S. pombe)	1.259604	0.07563	0.2354
13681	Eif4a1	eukaryotic translation initiation factor 4A1	1.259604	0.02755	0.1291
18230	Nxn	nucleoredoxin	1.259604	0.02986	0.1351
72611	Zfp655	zinc finger protein 655	1.259604	0.01336	0.08337
210126	Lpp	LIM domain containing preferred translocation partner in lipoma	1.259604	0.1039	0.2865
353170	Txlng	taxilin gamma	1.259604	0.07306	0.2303
67526	Atg12	autophagy-related 12 (yeast)	1.259446	0.0776	0.2389
270669	Mbtps2	membrane-bound transcription factor peptidase, site 2	1.259446	0.09685	0.2743
15107	Hadh	hydroxyacyl-Coenzyme A dehydrogenase	1.259287	0.02547	0.1226
20357	Sema5b	sema domain, seven thrombospondin repeats (type 1 and type 1-like), transmembrane domain (TM) and short cytoplasmic domain, (semaphorin) 5B	1.259287	0.07188	0.2279
56752	Aldh9a1	aldehyde dehydrogenase 9, subfamily A1	1.259287	0.606	0.7891
73608	Marveld3	MARVEL (membrane-associating) domain containing 3	1.259287	0.02384	0.1177
100038635	Gm10621	predicted gene 10621	1.259287	0.0473	0.176
111173	Erc1	ELKS/RAB6-interacting/CAST family member 1	1.259129	0.1044	0.2873
319469	A230056J06Rik	RIKEN cDNA A230056J06 gene	1.259129	0.09994	0.2792
116701	Fgfrl1	fibroblast growth factor receptor-like 1	1.25897	0.06521	0.214
12365	Casp14	caspase 14	1.258812	0.3097	0.5462
100039220	Gm12751	predicted gene 12751	1.258812	0.008056	0.0615
72042	Cotl1	coactosin-like 1 (Dictyostelium)	1.258653	0.02348	0.1169
99586	Dpyd	dihydropyrimidine dehydrogenase	1.258653	0.01565	0.0918
18669	Abcb1b	ATP-binding cassette, sub-family B (MDR/TAP), member 1B	1.258495	0.01303	0.08218
381280	Hjurp	Holliday junction recognition protein	1.258336	0.4439	0.6695
319236	9230105E10Rik	RIKEN cDNA 9230105E10 gene	1.258178	0.3097	0.5462
12153	Bmp1	bone morphogenetic protein 1	1.257862	0.0449	0.1708
20315	Cxcl12	chemokine (C-X-C motif) ligand 12	1.257862	0.1512	0.3595
64685	Nmi	N-myc (and STAT) interactor	1.257862	0.04774	0.1767
93892	Pcdhb21	protocadherin beta 21	1.257862	0.01073	0.07306
26905	Eif2s3x	eukaryotic translation initiation factor 2, subunit 3, structural gene X-linked	1.257703	0.0114	0.07568
67410	4930449I24Rik	RIKEN cDNA 4930449I24 gene	1.257703	0.1541	0.3637
100036569	Gm16525	predicted gene, 16525	1.257703	0.1327	0.3333
216795	Wnt9a	wingless-type MMTV integration site 9A	1.257545	0.01288	0.08153
16413	Itgb1bp1	integrin beta 1 binding protein 1	1.257387	0.0601	0.2034
20401	Sh3bp1	SH3-domain binding protein 1	1.257387	0.1117	0.2999
66775	Ptplad2	protein tyrosine phosphatase-like A domain containing 2	1.257387	0.1623	0.3746
72446	Prr5l	proline rich 5 like	1.257229	0.05071	0.1828
11637	Ak2	adenylate kinase 2	1.257071	0.1387	0.3416
11758	Prdx6	peroxiredoxin 6	1.257071	0.007335	0.05829
13531	Dub1	deubiquitinating enzyme 1	1.256913	0.2457	0.4796
16543	Mdfic	MyoD family inhibitor domain containing	1.256913	0.2316	0.4631
109754	Cyb5r3	cytochrome b5 reductase 3	1.256913	0.004282	0.04269
140481	Man2a2	mannosidase 2, alpha 2	1.256755	0.2173	0.4463
385454	Gm5396	predicted pseudogene 5396	1.256755	0.1315	0.3317
11423	Ache	acetylcholinesterase	1.256597	0.08376	0.2503
14792	Lpcat3	lysophosphatidylcholine acyltransferase 3	1.256597	0.07731	0.2384

78785	Clip4	CAP-GLY domain containing linker protein family, member 4	1.256597	0.7884	0.8995
226025	Trpm3	transient receptor potential cation channel, subfamily M, member 3	1.256597	0.06481	0.2132
140859	Nek8	NIMA (never in mitosis gene a)-related expressed kinase 8	1.256439	0.003254	0.03607
20700	Serpina1a	serine (or cysteine) peptidase inhibitor, clade A, member 1A	1.256281	0.1449	0.3503
75033	Mei4	meiosis-specific, MEI4 homolog (S. cerevisiae)	1.256281	0.4022	0.6321
78420	9530048J24Rik	RIKEN cDNA 9530048J24 gene	1.256281	0.2322	0.4635
83453	Chrdl1	chordin-like 1	1.256124	0.009595	0.0682
212073	4831426I19Rik	RIKEN cDNA 4831426I19 gene	1.256124	0.008654	0.06405
69287	Odf3	outer dense fiber of sperm tails 3	1.255966	0.1714	0.3871
224705	Vps52	vacuolar protein sorting 52 (yeast)	1.255966	0.225	0.4561
12490	Cd34	CD34 antigen	1.25565	0.02396	0.118
63830	Kcnq1ot1	KCNQ1 overlapping transcript 1	1.25565	0.008916	0.06529
19250	Ptpn14	protein tyrosine phosphatase, non-receptor type 14	1.255493	0.01578	0.09228
210544	Wdr67	WD repeat domain 67	1.255493	0.3931	0.6243
319996	Casc4	cancer susceptibility candidate 4	1.255493	0.008294	0.06244
18596	Pdgfrb	platelet derived growth factor receptor, beta polypeptide	1.255178	0.0446	0.1699
246228	Vwa1	von Willebrand factor A domain containing 1	1.255178	0.1482	0.355
55942	Sertad1	SERTA domain containing 1	1.25502	0.008767	0.06456
75299	4930547M16Rik	RIKEN cDNA 4930547M16 gene	1.254705	0.02033	0.107
319269	A130040M12Rik	RIKEN cDNA A130040M12 gene	1.254705	0.2099	0.4369
13215	Defb2	defensin beta 2	1.254548	0.6923	0.8435
72388	Ripk4	receptor-interacting serine-threonine kinase 4	1.25439	0.05025	0.182
234577	Cpne2	copine II	1.25439	0.06561	0.2149
231668	Vsig10	V-set and immunoglobulin domain containing 10	1.254233	0.09412	0.2696
56735	Krt71	keratin 71	1.254076	0.1782	0.3958
110075	Bmp3	bone morphogenetic protein 3	1.254076	0.02373	0.1174
225579	Slc27a6	solute carrier family 27 (fatty acid transporter), member 6	1.254076	0.1143	0.3044
667284	Gm8556	predicted gene 8556	1.254076	0.1228	0.3185
67956	Setd8	SET domain containing (lysine methyltransferase) 8	1.253761	0.1406	0.3443
68337	Crip2	cysteine rich protein 2	1.253604	0.001276	0.02073
211770	Trib1	tribbles homolog 1 (Drosophila)	1.253604	0.04944	0.1803
100038847	Gm10406	predicted gene 10406	1.253604	0.3826	0.6157
54391	Rfk	riboflavin kinase	1.253447	0.001655	0.0246
75712	Tmem14a	transmembrane protein 14A	1.253447	0.1749	0.3916
67389	Fam132a	family with sequence similarity 132, member A	1.25329	0.005199	0.04785
622675	Zfp827	zinc finger protein 827	1.25329	0.118	0.3104
19712	Rest	RE1-silencing transcription factor	1.253133	0.1509	0.359
20833	Ssrp1	structure specific recognition protein 1	1.253133	0.5146	0.7247
19332	Rab20	RAB20, member RAS oncogene family	1.252976	0.07969	0.2428
67724	Pop1	processing of precursor 1, ribonuclease P/MRP family, (S. cerevisiae)	1.252819	0.01006	0.07023
18938	Ppp1r14b	protein phosphatase 1, regulatory (inhibitor) subunit 14B	1.252662	0.01926	0.1037
68385	Tlcd1	TLC domain containing 1	1.252662	0.04158	0.1632
109294	Prex2	phosphatidylinositol-3,4,5-trisphosphate-dependent Rac exchange factor 2	1.252662	0.236	0.4684
319513	Fam113a	family with sequence similarity 113, member A	1.252662	0.014	0.08574
108737	Oxsr1	oxidative-stress responsive 1	1.252505	0.01384	0.08511
338523	Jhdm1d	jumonji C domain-containing histone demethylase 1 homolog D (S. cerevisiae)	1.252505	0.0532	0.1877
19823	Rnf7	ring finger protein 7	1.252348	0.008872	0.06515
59027	Nampt	nicotinamide phosphoribosyltransferase	1.252191	0.02831	0.131
78309	Cul9	cullin 9	1.252191	0.04595	0.173

14198	Fhit	fragile histidine triad gene	1.252035	0.2861	0.5215
71994	Cnn3	calponin 3, acidic	1.252035	0.00746	0.059
77074	4930426I24Rik	RIKEN cDNA 4930426I24 gene	1.252035	0.3341	0.5697
12566	Cdk2	cyclin-dependent kinase 2	1.251878	0.03055	0.137
108655	Foxp1	forkhead box P1	1.251878	0.307	0.5433
77053	Sun1	Sad1 and UNC84 domain containing 1	1.251721	0.1094	0.296
433594	Gm5537	phosphoglycerate kinase 1-like	1.251721	0.03025	0.1363
74048	4632428N05Rik	RIKEN cDNA 4632428N05 gene	1.251564	0.04895	0.1791
60611	Foxj2	forkhead box J2	1.251251	0.06369	0.2108
72701	Zfp618	zinc fingerprotein 618	1.251251	0.2372	0.4694
77533	C030034I22Rik	RIKEN cDNA C030034I22 gene	1.251251	0.01073	0.07306
100042588	Gm3916	predicted gene 3916	1.251251	0.07942	0.2421
14254	Flt1	FMS-like tyrosine kinase 1	1.251095	0.05219	0.1856
11979	Atp7b	ATPase, Cu++ transporting, beta polypeptide	1.250938	0.1144	0.3044
12388	Ctnnd1	catenin (cadherin associated protein), delta 1	1.250938	0.06397	0.2113
77533	C030034I22Rik	RIKEN cDNA C030034I22 gene	1.250938	0.02595	0.1242
15204	Herc2	hect (homologous to the E6-AP (UBE3A) carboxyl terminus) domain and RCC1 (CHC1)-like domain (RLD) 2	1.250782	0.5752	0.7688
17064	Cd93	CD93 antigen	1.250782	0.1157	0.3065
30049	Scd3	stearoyl-coenzyme A desaturase 3	1.250782	0.05044	0.1824
236900	Pdk3	pyruvate dehydrogenase kinase, isoenzyme 3	1.250782	0.04444	0.1695
11652	Akt2	thymoma viral proto-oncogene 2	1.250625	0.007779	0.06048
217578	Baz1a	bromodomain adjacent to zinc finger domain 1A	1.250625	0.05974	0.2028
75050	Kif27	kinesin family member 27	1.250313	0.4806	0.6994
70885	Ints10	integrator complex subunit 10	1.250156	0.01735	0.09735
75019	Rnase10	ribonuclease, RNase A family, 10 (non-active)	1.25	0.2877	0.5234
22634	Plagl1	pleiomorphic adenoma gene-like 1	1.249844	0.05556	0.1929
114654	Ly6g6d	lymphocyte antigen 6 complex, locus G6D	1.249844	0.0321	0.1408
14910	Gt(ROSA)26Sor	gene trap ROSA 26, Philippe Soriano	1.249688	0.03058	0.137
436440	Gpr31c	G protein-coupled receptor 31, D17Leh66c region	1.249688	0.3856	0.6177
20704	Serpina1e	serine (or cysteine) peptidase inhibitor, clade A, member 1E	1.249531	0.1412	0.3452
78412	3110062M04Rik	RIKEN cDNA 3110062M04 gene	1.249375	0.01632	0.09362
12480	Cd1d2	CD1d2 antigen	1.249219	0.1248	0.3215
227737	Fam129b	family with sequence similarity 129, member B	1.249219	0.01084	0.07355
381059	Gm1604b	predicted gene 1604b	1.249219	0.1438	0.3487
381101	BC048355	cDNA sequence BC048355	1.249063	0.03061	0.137
100042834	Gm4055	predicted gene 4055	1.249063	0.04489	0.1708
11994	Pcdh15	protocadherin 15	1.248751	0.2797	0.5152
19155	Npepps	aminopeptidase puromycin sensitive	1.248751	0.01625	0.0934
66628	Thg1l	tRNA-histidine guanylyltransferase 1-like (S. cerevisiae)	1.248751	0.07356	0.2314
76055	Mgea5	meningioma expressed antigen 5 (hyaluronidase)	1.248751	0.2975	0.5334
104174	Gldc	glycine decarboxylase	1.248751	0.06515	0.2139
14433	Gapdh	glyceraldehyde-3-phosphate dehydrogenase	1.248284	0.1279	0.3258
68240	Rpa3	replication protein A3	1.248284	0.005575	0.04995
77391	9530003O04Rik	RIKEN cDNA 9530003O04 gene	1.248284	0.2208	0.4507
22635	Zan	zonadhesin	1.248128	0.09446	0.2701
100009600	Zglp1	zinc finger, GATA-like protein 1	1.248128	0.05297	0.1873
13609	S1pr1	sphingosine-1-phosphate receptor 1	1.247972	0.2053	0.4316
56228	Ube2j1	ubiquitin-conjugating enzyme E2, J1	1.247972	0.01012	0.07058
110862	Kcnq3	potassium voltage-gated channel, subfamily Q, member 3	1.247972	0.2094	0.4364

100038443	9330195I24Rik	RIKEN cDNA 9330195I24 gene	1.247972	0.008218	0.06214
67464	Entpd4	ectonucleoside triphosphate diphosphohydrolase 4	1.247816	0.003041	0.03449
71877	Efhc1	EF-hand domain (C-terminal) containing 1	1.247816	0.07649	0.2367
21955	Tnnt1	troponin T1, skeletal, slow	1.247661	0.003776	0.03935
319239	Npsr1	neuropeptide S receptor 1	1.247661	0.2213	0.4513
78977	Popdc3	popeye domain containing 3	1.247505	0.0129	0.08162
18032	Nfix	nuclear factor I/X	1.247349	0.04859	0.1784
240479	Fam69c	family with sequence similarity 69, member C	1.247349	0.142	0.3463
258954	Olfr522	olfactory receptor 522	1.247349	0.2568	0.4918
50529	Mrps7	mitchondrial ribosomal protein S7	1.247194	0.004036	0.0411
108655	Foxp1	forkhead box P1	1.247194	0.05226	0.1856
77609	Ccdc151	coiled-coil domain containing 151	1.247038	0.009848	0.06936
240283	Dmxl1	Dmx-like 1	1.246883	0.05572	0.1932
74737	Pcf11	cleavage and polyadenylation factor subunit homolog (S. cerevisiae)	1.246727	0.1473	0.3538
99480	Dnttip2	deoxynucleotidyltransferase, terminal, interacting protein 2	1.246727	0.01194	0.07773
13664	Eif1a	eukaryotic translation initiation factor 1A	1.246572	0.009614	0.06824
17701	Msx1	homeobox, msh-like 1	1.246417	0.1756	0.3924
21356	Tapbp	TAP binding protein	1.246417	0.02354	0.117
21429	Ubtf	upstream binding transcription factor, RNA polymerase I	1.246417	0.06445	0.2125
78902	4833447P13Rik	RIKEN cDNA 4833447P13 gene	1.246417	0.1436	0.3485
381544	Gm1661	predicted gene 1661	1.246417	0.3813	0.6149
100038712	Gm10516	predicted gene 10516	1.246417	0.04199	0.1639
107885	Mthfs	5, 10-methenyltetrahydrofolate synthetase	1.246261	0.05174	0.1849
108797	Mex3b	mex3 homolog B (C. elegans)	1.246261	0.187	0.4076
225888	Suv420h1	suppressor of variegation 4-20 homolog 1 (Drosophila)	1.246106	0.1228	0.3185
326618	Tpm4	tropomyosin 4	1.246106	0.005303	0.04847
791308	Gm9933	predicted gene 9933	1.246106	0.1627	0.375
19659	Rbp1	retinol binding protein 1, cellular	1.245795	0.03768	0.1541
20917	Suclg2	succinate-Coenzyme A ligase, GDP-forming, beta subunit	1.245795	0.002234	0.02902
52184	Odf2l	outer dense fiber of sperm tails 2-like	1.245795	0.2549	0.4896
53871	Pkd2l2	polycystic kidney disease 2-like 2	1.245795	0.2301	0.4612
109280	9330176C04Rik	solute carrier family 22 (organic cation transporter), member 13 gene:pseudogene	1.245795	0.01309	0.08249
14673	Gna12	guanine nucleotide binding protein, alpha 12	1.24564	0.2807	0.5161
14087	Fanca	Fanconi anemia, complementation group A	1.245485	0.2848	0.5202
17387	Mmp14	matrix metallopeptidase 14 (membrane-inserted)	1.245485	0.01052	0.07234
15481	Hspa8	heat shock protein 8	1.24533	0.05048	0.1825
72454	Ccdc71	coiled-coil domain containing 71	1.24533	0.01197	0.07782
23985	Slc26a4	solute carrier family 26, member 4	1.245175	0.7527	0.8804
102209	Snapc2	small nuclear RNA activating complex, polypeptide 2	1.245175	0.01966	0.1049
232539	Klhdc5	kelch domain containing 5	1.24502	0.002679	0.03209
14804	Grid2	glutamate receptor, ionotropic, delta 2	1.244865	0.2797	0.5152
434204	Whamm	WAS protein homolog associated with actin, golgi membranes and microtubules	1.24471	0.03519	0.1484
63828	Fn3k	fructosamine 3 kinase	1.244555	0.003496	0.03767
225341	Lims2	LIM and senescent cell antigen like domains 2	1.244555	0.3228	0.5592
18798	Plcb4	phospholipase C, beta 4	1.2444	0.1317	0.3319
77053	Sun1	Sad1 and UNC84 domain containing 1	1.2444	0.03192	0.1404
320460	Vwc2l	von Willebrand factor C domain-containing protein 2-like	1.2444	0.1075	0.293
230119	Zbtb5	zinc finger and BTB domain containing 5	1.244245	0.04001	0.1594
11596	Ager	advanced glycosylation end product-specific receptor	1.244091	0.0738	0.2318

14260	Fmn1	formin 1	1.243936	0.2019	0.428
15962	Ifna1	interferon alpha 1	1.243936	0.08493	0.2526
17858	Mx2	myxovirus (influenza virus) resistance 2	1.243936	0.05088	0.183
209334	Gen1	Gen homolog 1, endonuclease (Drosophila)	1.243626	0.09758	0.2755
16855	Lgals4	lectin, galactose binding, soluble 4	1.243472	0.1814	0.4001
16353	Ipw	imprinted gene in the Prader-Willi syndrome region	1.243317	0.3852	0.6175
63986	Gmfg	glia maturation factor, gamma	1.243317	0.3183	0.5548
235406	Snx33	sorting nexin 33	1.243317	0.01415	0.08627
23802	Amfr	autocrine motility factor receptor	1.243163	0.02816	0.1307
67341	Ascl4	achaete-scute complex homolog 4 (Drosophila)	1.243163	0.133	0.3338
108682	Gpt2	glutamic pyruvate transaminase (alanine aminotransferase) 2	1.242854	0.003491	0.03763
16978	Lrrfip1	leucine rich repeat (in FLII) interacting protein 1	1.242699	0.0146	0.08796
627872	Dnahc7a	dynein, axonemal, heavy chain 7A	1.242699	0.0203	0.107
102103	Mtus1	mitochondrial tumor suppressor 1	1.242545	0.02897	0.1327
257635	Sdsl	serine dehydratase-like	1.242545	0.008458	0.06313
69207	Srsf11	serine/arginine-rich splicing factor 11	1.24239	0.2027	0.429
14815	Nr3c1	nuclear receptor subfamily 3, group C, member 1	1.242082	0.06801	0.2202
56484	Foxo3	forkhead box O3	1.242082	0.201	0.4268
20583	Snai2	snail homolog 2 (Drosophila)	1.241927	0.2368	0.469
23986	Peci	peroxisomal delta3, delta2-enoyl-Coenzyme A isomerase	1.241927	0.1606	0.3723
27055	Fkbp9	FK506 binding protein 9	1.241927	0.01773	0.09839
66307	Isoc1	isochorismatase domain containing 1	1.241927	0.042	0.1639
71844	Nupl1	nucleoporin like 1	1.241927	0.1498	0.3577
12167	Bmpr1b	bone morphogenetic protein receptor, type 1B	1.241773	0.05493	0.1919
68488	1110002J07Rik	RIKEN cDNA 1110002J07 gene	1.241465	0.1754	0.3921
81489	Dnajb1	DnaJ (Hsp40) homolog, subfamily B, member 1	1.241465	0.002144	0.02813
114249	Npnt	nephronectin	1.241465	0.0384	0.156
74032	Sdr42e1	short chain dehydrogenase/reductase family 42E, member 1	1.241311	0.02036	0.107
258290	Olfr1143	olfactory receptor 1143	1.241311	0.5607	0.7594
320387	D930030O05Rik	RIKEN cDNA D930030O05 gene	1.241311	0.07958	0.2425
12479	Cd1d1	CD1d1 antigen	1.241157	0.0103	0.07142
15186	Hdc	histidine decarboxylase	1.241157	0.2793	0.5149
268977	Ltbp1	latent transforming growth factor beta binding protein 1	1.241157	0.3704	0.6052
269704	Zfp664	zinc finger protein 664	1.241157	0.04423	0.1691
66251	Arfgap3	ADP-ribosylation factor GTPase activating protein 3	1.241003	0.07765	0.239
106869	Tnfaip8	tumor necrosis factor, alpha-induced protein 8	1.241003	0.04493	0.1708
66180	Leprel4	leprecan-like 4	1.240849	0.02474	0.1203
216456	Gls2	glutaminase 2 (liver, mitochondrial)	1.240849	0.001317	0.02117
15561	Htr3a	5-hydroxytryptamine (serotonin) receptor 3A	1.240695	0.06818	0.2206
65973	Asph	aspartate-beta-hydroxylase	1.240695	0.02129	0.1103
227737	Fam129b	family with sequence similarity 129, member B	1.240695	0.008087	0.0616
214239	A430105I19Rik	RIKEN cDNA A430105I19 gene	1.240541	0.05876	0.2006
219150	Hmbox1	homeobox containing 1	1.240541	0.4824	0.7011
74756	5830408B19Rik	RIKEN cDNA 5830408B19 gene	1.240387	0.09563	0.2722
434171	Gm5591	predicted gene 5591	1.240387	0.4047	0.6343
13078	Cyp1b1	cytochrome P450, family 1, subfamily b, polypeptide 1	1.240233	0.1304	0.33
12335	Capn3	calpain 3	1.240079	0.09958	0.2785
18755	Prkch	protein kinase C, eta	1.240079	0.176	0.3929
432999	A930007A09Rik	RIKEN cDNA A930007A09 gene	1.240079	0.06939	0.2228

102502	Pls1	plastin 1 (I-isoform)	1.239618	0.3015	0.5374
106628	Trip10	thyroid hormone receptor interactor 10	1.239618	0.005515	0.0496
100040736	9130206I24Rik	RIKEN cDNA 9130206I24 gene	1.239618	0.02803	0.1303
18624	Pepd	peptidase D	1.239465	0.002716	0.03224
109108	Slc30a9	solute carrier family 30 (zinc transporter), member 9	1.239465	0.08909	0.261
20357	Sema5b	sema domain, seven thrombospondin repeats (type 1 and type 1-like), transmembrane domain (TM) and short cytoplasmic domain, (semaphorin) 5B	1.239311	0.04644	0.1742
109333	Pkn2	protein kinase N2	1.239311	0.102	0.2829
19210	Ptdss1	phosphatidylserine synthase 1	1.239157	0.01098	0.07419
56745	C1qtnf1	C1q and tumor necrosis factor related protein 1	1.239004	0.0855	0.2537
19152	Prtn3	proteinase 3	1.23885	0.2374	0.4696
74682	Wdr35	WD repeat domain 35	1.23885	0.008657	0.06405
243084	Tmprss11e	transmembrane protease, serine 11e	1.23885	0.5581	0.757
17390	Mmp2	matrix metallopeptidase 2	1.238543	0.2197	0.4495
68861	1190002N15Rik	RIKEN cDNA 1190002N15 gene	1.238543	0.0172	0.09696
382421	Gm5176	predicted gene 5176	1.238543	0.1662	0.3803
15896	Icam2	intercellular adhesion molecule 2	1.23839	0.05006	0.1817
210027	Slc35f3	solute carrier family 35, member F3	1.238237	0.3379	0.5735
22359	Vldlr	very low density lipoprotein receptor	1.238083	0.006266	0.05321
109552	Sri	sorcin	1.238083	0.01404	0.08578
228545	Vps18	vacuolar protein sorting 18 (yeast)	1.237624	0.07449	0.2329
70021	Nt5dc2	5'-nucleotidase domain containing 2	1.237317	0.1991	0.4244
74761	Mxra8	matrix-remodelling associated 8	1.237317	0.2011	0.427
68024	Hist1h2bc	histone cluster 1, H2bc	1.237164	0.02786	0.1298
268527	Greb1	gene regulated by estrogen in breast cancer protein	1.237164	0.1145	0.3047
20340	Glg1	golgi apparatus protein 1	1.237011	0.03533	0.1484
211612	Ptchd1	patched domain containing 1	1.237011	0.03403	0.1459
11548	Adra1b	adrenergic receptor, alpha 1b	1.236858	0.2577	0.4928
58998	Pvrl3	poliovirus receptor-related 3	1.236858	0.01661	0.09477
193286	BC049762	cDNA sequence BC049762	1.236858	0.1609	0.3726
381062	2210404J11Rik	RIKEN cDNA 2210404J11 gene	1.236858	0.07883	0.2409
50764	Fbxo15	F-box protein 15	1.236552	0.02882	0.1323
545989	Gm5901	predicted gene 5901	1.236552	0.227	0.4584
17920	Myo6	myosin VI	1.2364	0.02458	0.1199
84092	Usp8	ubiquitin specific peptidase 8	1.236247	0.4411	0.6669
226957	Gm4850	THO complex 4 pseudogene	1.236247	0.5506	0.7511
107476	Acaca	acetyl-Coenzyme A carboxylase alpha	1.236094	0.5112	0.7219
16977	Lrrc23	leucine rich repeat containing 23	1.235788	0.07691	0.2375
52668	Ifi27l1	interferon, alpha-inducible protein 27 like 1	1.235636	0.01717	0.09684
227682	Trub2	TruB pseudouridine (psi) synthase homolog 2 (E. coli)	1.235636	0.005078	0.04714
67289	3110021A11Rik	RIKEN cDNA 3110021A11 gene	1.235483	0.02972	0.1348
72999	Insig2	insulin induced gene 2	1.235483	0.1112	0.2993
21346	Tagln2	transgelin 2	1.23533	0.1828	0.4016
14367	Fzd5	frizzled homolog 5 (Drosophila)	1.235178	0.02671	0.1264
56808	Cacna2d2	calcium channel, voltage-dependent, alpha 2/delta subunit 2	1.235178	0.001464	0.02264
58222	Rab37	RAB37, member of RAS oncogene family	1.234873	0.06413	0.2117
622446	Gm6323	predicted gene 6323	1.234873	0.2293	0.4604
21646	Tcte2	t-complex-associated testis expressed 2	1.23472	0.1476	0.3542
52187	Rragd	Ras-related GTP binding D	1.23472	0.2527	0.4872
219181	Akap11	A kinase (PRKA) anchor protein 11	1.234416	0.268	0.5035

23881	G3bp2	GTPase activating protein (SH3 domain) binding protein 2	1.234263	0.02333	0.1165
219022	Ttc5	tetratricopeptide repeat domain 5	1.234263	0.01441	0.0873
67246	2810474O19Rik	RIKEN cDNA 2810474O19 gene	1.234111	0.08947	0.2613
319259	9930021D14Rik	RIKEN cDNA 9930021D14 gene	1.234111	0.1354	0.3369
353172	Gars	glycyl-tRNA synthetase	1.234111	0.04371	0.1679
60411	Cenpk	centromere protein K	1.233959	0.06475	0.213
68070	Pdzd2	PDZ domain containing 2	1.233959	0.2544	0.4892
207958	Alg11	asparagine-linked glycosylation 11 homolog (yeast, alpha-1,2-mannosyltransferase)	1.233959	0.1272	0.3249
326623	Tnfsf15	tumor necrosis factor (ligand) superfamily, member 15	1.233806	0.2211	0.4511
66365	Ccdc90b	coiled-coil domain containing 90B	1.233654	0.03084	0.1375
72123	2010109K11Rik	RIKEN cDNA 2010109K11 gene	1.233502	0.01123	0.07509
17454	Mov10	Moloney leukemia virus 10	1.23335	0.05779	0.198
235256	Olfr149	olfactory receptor 149	1.23335	0.5634	0.761
16912	Psmb9	proteasome (prosome, macropain) subunit, beta type 9 (large multifunctional peptidase 2)	1.233198	0.152	0.3609
69034	4930579G22Rik	RIKEN cDNA 4930579G22 gene	1.233198	0.007942	0.0611
171171	Ntng2	netrin G2	1.232894	0.17	0.3852
212516	BC060267	cDNA sequence BC060267	1.232894	0.009921	0.06966
319162	Hist3h2a	histone cluster 3, H2a	1.232894	0.003241	0.03607
71956	Rnf135	ring finger protein 135	1.232742	0.04358	0.1676
18028	Nfib	nuclear factor I/B	1.232286	0.0173	0.09722
50779	Rgs6	regulator of G-protein signaling 6	1.232286	0.01361	0.08428
69332	Lelp1	late cornified envelope-like proline-rich 1	1.231831	0.3041	0.5404
70546	Zdhhc2	zinc finger, DHHC domain containing 2	1.231831	0.006667	0.05528
319601	Zfp653	zinc finger protein 653	1.231831	0.004148	0.04183
330260	Pon2	paraoxonase 2	1.231679	0.2018	0.428
668339	Gm9112	predicted gene 9112	1.231679	0.184	0.403
74245	Ctbs	chitobiase, di-N-acetyl-	1.231527	0.03151	0.1394
93717	Pcdhga9	protocadherin gamma subfamily A, 9	1.231375	0.3364	0.572
14067	F5	coagulation factor V	1.231224	0.5705	0.7645
106344	Rfc4	replication factor C (activator 1) 4	1.231224	0.1069	0.2918
54188	Cpsf4	cleavage and polyadenylation specific factor 4	1.231072	0.05272	0.1868
101497	Plekhg2	pleckstrin homology domain containing, family G (with RhoGef domain) member 2	1.231072	0.02379	0.1175
140792	Colec12	collectin sub-family member 12	1.231072	0.2589	0.4942
67437	Ssr3	signal sequence receptor, gamma	1.230921	0.2743	0.5099
192976	BC046404	cDNA sequence BC046404	1.230921	0.041	0.1619
227682	Trub2	TruB pseudouridine (psi) synthase homolog 2 (E. coli)	1.230921	0.04142	0.1628
80749	Lrfn1	leucine rich repeat and fibronectin type III domain containing 1	1.230769	0.3606	0.5954
98685	1190005F20Rik	RIKEN cDNA 1190005F20 gene	1.230769	0.2448	0.4784
109108	Slc30a9	solute carrier family 30 (zinc transporter), member 9	1.230769	0.03632	0.1509
319508	Syt15	synaptotagmin XV	1.230769	0.02286	0.1152
672125	Gm14496	predicted gene 14496	1.230769	0.3211	0.5575
21391	Tbxas1	thromboxane A synthase 1, platelet	1.230618	0.07107	0.2261
12445	Ccnd3	cyclin D3	1.230466	0.0006996	0.01456
13829	Epb4.9	erythrocyte protein band 4.9	1.230466	0.09317	0.268
66521	Rwdd1	RWD domain containing 1	1.230466	0.1064	0.291
67683	2610029G23Rik	RIKEN cDNA 2610029G23 gene	1.230466	0.02533	0.1222
192976	BC046404	cDNA sequence BC046404	1.230466	0.01606	0.09289
19073	Srgn	serglycin	1.230315	0.1584	0.3695
26362	Axl	AXL receptor tyrosine kinase	1.230315	0.02939	0.134

399510	Map4k5	mitogen-activated protein kinase kinase kinase kinase 5	1.230164	0.03096	0.1379
545861	Gm5878	predicted gene 5878	1.230164	0.5454	0.7478
68458	Ppp1r14a	protein phosphatase 1, regulatory (inhibitor) subunit 14A	1.230012	0.07409	0.2322
74155	Errfi1	ERBB receptor feedback inhibitor 1	1.230012	0.003914	0.04032
57246	Tbx20	T-box 20	1.22971	0.1397	0.343
107970	Hist1h1t	histone cluster 1, H1t	1.22971	0.09789	0.276
319342	C230034O21Rik	RIKEN cDNA C230034O21 gene	1.22971	0.01435	0.08704
19823	Rnf7	ring finger protein 7	1.229407	0.003385	0.03695
12499	Entpd5	ectonucleoside triphosphate diphosphohydrolase 5	1.229105	0.05185	0.1851
15493	Hsd3b2	hydroxy-delta-5-steroid dehydrogenase, 3 beta- and steroid delta-isomerase 2	1.229105	0.2748	0.5102
11541	Adora2b	adenosine A2b receptor	1.228954	0.2483	0.4824
100041019	Gm3099	predicted gene 3099	1.228954	0.4431	0.669
18176	Nras	neuroblastoma ras oncogene	1.228803	0.01917	0.1033
67513	2610002J02Rik	RIKEN cDNA 2610002J02 gene	1.228803	0.05187	0.1851
11686	Alox12b	arachidonate 12-lipoxygenase, 12R type	1.228652	0.1761	0.3931
68828	Sync	syncoilin	1.228652	0.244	0.4775
104479	Ccdc117	coiled-coil domain containing 117	1.228652	0.06804	0.2202
669149	Vmn2r88	vomeronasal 2, receptor 88	1.228652	0.127	0.3245
23880	Fyb	FYN binding protein	1.228501	0.1817	0.4005
20352	Sema4b	sema domain, immunoglobulin domain (Ig), transmembrane domain (TM) and short cytoplasmic domain, (semaphorin) 4B	1.22835	0.02085	0.1088
71207	Nudt4	nudix (nucleoside diphosphate linked moiety X)-type motif 4	1.228199	0.008264	0.06231
54402	Stk19	serine/threonine kinase 19	1.228049	0.004689	0.04508
67457	Frmd8	FERM domain containing 8	1.228049	0.02489	0.1208
71398	5430427O19Rik	RIKEN cDNA 5430427O19 gene	1.228049	0.1545	0.3644
78294	Rps27a	ribosomal protein S27A	1.228049	0.274	0.5096
21682	Tec	tec protein tyrosine kinase	1.227747	0.03737	0.1532
16412	Itgb1	integrin beta 1 (fibronectin receptor beta)	1.227596	0.009213	0.06646
70829	Ccdc93	coiled-coil domain containing 93	1.227596	0.01721	0.09696
497210	Tpt1p	tumor protein, translationally-controlled 1 pseudogene	1.227596	0.001857	0.02606
622935	Krtap20-2	keratin associated protein 20-2	1.227596	0.2028	0.429
116891	Derl2	Der1-like domain family, member 2	1.227446	0.02156	0.1111
17826	Mtvr2	mammary tumor virus receptor 2	1.227295	0.01124	0.07509
103537	Mbtd1	mbt domain containing 1	1.227295	0.2303	0.4615
98711	Rdh10	retinol dehydrogenase 10 (all-trans)	1.227144	0.01819	0.09984
100678	Psph	phosphoserine phosphatase	1.227144	0.004932	0.04628
223752	Gramd4	GRAM domain containing 4	1.227144	0.002476	0.03081
170624	Dep1	diabetic embryopathy 1	1.226994	0.2608	0.496
76815	Calcoco2	calcium binding and coiled-coil domain 2	1.226693	0.09213	0.266
14284	Fosl2	fos-like antigen 2	1.226242	0.04298	0.1662
66102	Cxcl16	chemokine (C-X-C motif) ligand 16	1.226091	0.07108	0.2261
67513	2610002J02Rik	RIKEN cDNA 2610002J02 gene	1.225941	0.02864	0.1318
70225	Ppil3	peptidylprolyl isomerase (cyclophilin)-like 3	1.225941	0.02013	0.1066
75224	4930528J11Rik	RIKEN cDNA 4930528J11 gene	1.225941	0.04859	0.1784
67685	Dyx1c1	dyslexia susceptibility 1 candidate 1 homolog (human)	1.225791	0.02448	0.1196
73713	Rbm20	RNA binding motif protein 20	1.225791	0.1969	0.4215
20425	Shmt1	serine hydroxymethyltransferase 1 (soluble)	1.22519	0.3852	0.6175
382769	Gm5196	predicted gene 5196	1.22519	0.02663	0.1262
544717	1190007I07Rik	RIKEN cDNA 1190007I07 gene	1.22519	0.2226	0.4527
544707	Gm5779	predicted gene 5779	1.22504	0.04074	0.1613

28193	Reep3		receptor accessory protein 3	1.22489	0.008922	0.06529
72658	2700097O09Rik		RIKEN cDNA 2700097O09 gene	1.22489	0.004741	0.0453
434768	Rhox8		reproductive homeobox 8	1.22489	0.1112	0.2993
17129	Smad5		MAD homolog 5 (Drosophila)	1.22474	0.07304	0.2303
22232	Slc35a2		solute carrier family 35 (UDP-galactose transporter), member A2	1.22474	0.06706	0.2182
74025	Nphp3		nephronophthisis 3 (adolescent)	1.22474	0.05029	0.1821
77134	Hnrnpa0		heterogeneous nuclear ribonucleoprotein A0	1.22474	0.09692	0.2743
435791	Gm13271		predicted gene 13271	1.22474	0.2143	0.4426
15289	Hmgb1		high mobility group box 1	1.22459	0.2054	0.4317
15273	Hivep2		human immunodeficiency virus type I enhancer binding protein 2	1.22444	0.05219	0.1856
58231	Stk4		serine/threonine kinase 4	1.22444	0.04339	0.1672
14629	Gclc		glutamate-cysteine ligase, catalytic subunit	1.22429	0.04217	0.1643
319991	Kif6		kinesin family member 6	1.22429	0.1181	0.3106
14119	Fbn2		fibrillin 2	1.22414	0.3969	0.6273
217830	9030617O03Rik		RIKEN cDNA 9030617O03 gene	1.22414	0.05792	0.1984
11499	Adam5		a disintegrin and metallopeptidase domain 5	1.223691	0.0802	0.2435
12572	Cdk7		cyclin-dependent kinase 7	1.223691	0.1382	0.3407
19012	Ppap2a		phosphatidic acid phosphatase type 2A	1.223691	0.1443	0.3494
242519	Ifna12		interferon alpha 12	1.223691	0.3665	0.6019
19662	Rbp4		retinol binding protein 4, plasma	1.223541	0.01588	0.09256
12125	Bcl2l11		BCL2-like 11 (apoptosis facilitator)	1.223391	0.1084	0.2945
59042	Cope		coatomer protein complex, subunit epsilon	1.223391	0.04116	0.1622
105352	Dusp22		dual specificity phosphatase 22	1.223391	0.01402	0.08575
75985	Rab30		RAB30, member RAS oncogene family	1.223242	0.09902	0.2779
80752	Fam20c		family with sequence similarity 20, member C	1.223242	0.1531	0.3626
78425	9530053H05Rik		RIKEN cDNA 9530053H05 gene	1.223092	0.09228	0.2662
111173	Erc1		ELKS/RAB6-interacting/CAST family member 1	1.223092	0.05199	0.1854
77951	Cyp20a1		cytochrome P450, family 20, subfamily A, polypeptide 1	1.222942	0.285	0.5203
109232	Sccpdh		saccharopine dehydrogenase (putative)	1.222942	0.01381	0.08504
319675	5830418K08Rik		RIKEN cDNA 5830418K08 gene	1.222942	0.1006	0.2805
54204		Sep-01	septin 1	1.222793	0.04295	0.1662
104383	Rcor2		REST corepressor 2	1.222793	0.04815	0.1775
242785	Klhl21		kelch-like 21 (Drosophila)	1.222793	0.06329	0.21
15275	Hk1		hexokinase 1	1.222643	0.06988	0.2237
69745	Pold4		polymerase (DNA-directed), delta 4	1.222494	0.0268	0.1267
215493	A3galt2		alpha 1,3-galactosyltransferase 2 (isoglobotriaosylceramide synthase)	1.222494	0.08983	0.262
12156	Bmp2		bone morphogenetic protein 2	1.222344	0.5564	0.7557
320595	Phf8		PHD finger protein 8	1.222344	0.3363	0.572
54396	Irgm2		immunity-related GTPase family M member 2	1.222195	0.105	0.2883
101351	A130022J15Rik		RIKEN cDNA A130022J15 gene	1.222195	0.007881	0.06096
54418	Fmn2		formin 2	1.222046	0.1337	0.3343
242687	Wasf2		WAS protein family, member 2	1.222046	0.03391	0.1456
319163	Hist1h2aa		histone cluster 1, H2aa	1.222046	0.1433	0.3482
19331	Rab19		RAB19, member RAS oncogene family	1.221896	0.02211	0.1127
66840	Wdr45l		Wdr45 like	1.221896	0.1628	0.375
18000		Sep-02	septin 2	1.221747	0.1522	0.3613
56440	Snx1		sorting nexin 1	1.221747	0.02434	0.1192
67916	Ppap2b		phosphatidic acid phosphatase type 2B	1.221747	0.0598	0.2029
382073	Ccdc84		coiled-coil domain containing 84	1.221747	0.05312	0.1875

68142	Ino80	INO80 homolog (S. cerevisiae)	1.221598	0.07637	0.2367
18822	Plod1	procollagen-lysine, 2-oxoglutarate 5-dioxygenase 1	1.221449	0.1656	0.3795
217258	Abca8a	ATP-binding cassette, sub-family A (ABC1), member 8a	1.221449	0.1618	0.374
227867	Epc2	enhancer of polycomb homolog 2 (Drosophila)	1.221449	0.06743	0.219
382066	Prdm10	PR domain containing 10	1.221299	0.2945	0.5306
22689	Zfp27	zinc finger protein 27	1.22115	0.02984	0.1351
83922	Tsga14	testis specific gene A14	1.22115	0.1661	0.3803
13838	Epha4	Eph receptor A4	1.221001	0.0568	0.1958
20723	Serpinb9	serine (or cysteine) peptidase inhibitor, clade B, member 9	1.221001	0.08157	0.246
81877	Tnxb	tenascin XB	1.220852	0.06541	0.2145
215789	Phactr2	phosphatase and actin regulator 2	1.220852	0.2079	0.4342
18822	Plod1	procollagen-lysine, 2-oxoglutarate 5-dioxygenase 1	1.220703	0.0829	0.2484
23801	Aloxe3	arachidonate lipoxygenase 3	1.220703	0.01665	0.09487
69216	Ccdc23	coiled-coil domain containing 23	1.220703	0.1914	0.4135
170472	Recql5	RecQ protein-like 5	1.220703	0.1763	0.3933
259058	Olfr646	olfactory receptor 646	1.220703	0.7108	0.8547
102141	Snx25	sorting nexin 25	1.220256	0.01472	0.08843
213211	Rnf26	ring finger protein 26	1.220256	0.00501	0.04676
209837	Slc38a5	solute carrier family 38, member 5	1.220107	0.1696	0.3848
100042342	Gm10375	predicted gene 10375	1.220107	0.4536	0.6776
19276	Ptprn2	protein tyrosine phosphatase, receptor type, N polypeptide 2	1.219959	0.394	0.625
21460	Tcp10a	t-complex protein 10a	1.219959	0.2567	0.4917
271849	Shc4	SHC (Src homology 2 domain containing) family, member 4	1.21981	0.2174	0.4464
109359	Fam175b	family with sequence similarity 175, member B	1.219066	0.2116	0.4391
16598	Klf2	Kruppel-like factor 2 (lung)	1.218918	0.175	0.3917
22772	Zic2	zinc finger protein of the cerebellum 2	1.218918	0.3157	0.5524
68152	Fam133b	family with sequence similarity 133, member B	1.218918	0.3261	0.5626
237504	Rassf9	Ras association (RalGDS/AF-6) domain family (N-terminal) member 9	1.218918	0.3024	0.5385
70419	2810408A11Rik	RIKEN cDNA 2810408A11 gene	1.218769	0.01577	0.09222
243219	2900026A02Rik	RIKEN cDNA 2900026A02 gene	1.218769	0.3317	0.5678
13033	Ctsd	cathepsin D	1.218324	0.1078	0.2937
66315	Senp7	SUMO1/sentrin specific peptidase 7	1.218175	0.1836	0.4026
328451	Gm5088	poly(A)-binding protein, cytoplasmic pseudogene	1.218027	0.2786	0.514
11827	Aqp2	aquaporin 2	1.217878	0.2249	0.456
22791	Dnajc2	DnaJ (Hsp40) homolog, subfamily C, member 2	1.217878	0.3356	0.5713
11520	Plin2	perilipin 2	1.21773	0.02675	0.1265
67121	Mastl	microtubule associated serine/threonine kinase-like	1.21773	0.2233	0.4537
12417	Cbx3	chromobox homolog 3 (Drosophila HP1 gamma)	1.217582	0.0722	0.2284
12587	Mia1	melanoma inhibitory activity 1	1.217285	0.2159	0.4446
66624	Spcs2	signal peptidase complex subunit 2 homolog (S. cerevisiae)	1.217285	0.02362	0.1171
170790	Mlc1	megalencephalic leukoencephalopathy with subcortical cysts 1 homolog (human)	1.217285	0.001508	NA
217935	Wdr60	WD repeat domain 60	1.217285	0.01191	0.07773
21925	Tnnc2	troponin C2, fast	1.217137	0.09347	0.2686
57776	Ttyh1	tweety homolog 1 (Drosophila)	1.216841	0.003107	0.03497
67701	Wfdc2	WAP four-disulfide core domain 2	1.216841	0.1452	0.3505
67885	1500011K16Rik	RIKEN cDNA 1500011K16 gene	1.216841	0.01698	0.09609
18552	Pcsk5	proprotein convertase subtilisin/kexin type 5	1.216693	0.06007	0.2034
27280	Phlda3	pleckstrin homology-like domain, family A, member 3	1.216693	0.03358	0.1449
106389	Eaf2	ELL associated factor 2	1.216693	0.06136	0.206

72640	Mex3a	mex3 homolog A (C. elegans)	1.216397	0.07577	0.2357
67689	Aldh3b1	aldehyde dehydrogenase 3 family, member B1	1.216249	0.09176	0.2655
78781	Zc3hav1	zinc finger CCCH type, antiviral 1	1.215953	0.06773	0.2196
11603	Agrn	agrin	1.215805	0.02729	0.1281
77707	9130604C24Rik	RIKEN cDNA 9130604C24 gene	1.215658	0.1111	0.2991
109929	Zbtb25	zinc finger and BTB domain containing 25	1.215658	0.03839	0.156
21907	Nr2e1	nuclear receptor subfamily 2, group E, member 1	1.215362	0.002642	0.03185
67486	Polr3g	polymerase (RNA) III (DNA directed) polypeptide G	1.215362	0.0375	0.1535
319638	Nt5dc1	5'-nucleotidase domain containing 1	1.215362	0.1277	0.3256
638008	Gm7227	predicted gene 7227	1.215362	0.0388	0.1567
12837	Col8a1	collagen, type VIII, alpha 1	1.215214	0.4623	0.6849
100039631	Gm2347	predicted gene 2347	1.214919	0.2476	0.482
19737	Rgs5	regulator of G-protein signaling 5	1.214772	0.1197	0.3132
231093	Agbl5	ATP/GTP binding protein-like 5	1.214624	0.02364	0.1172
628596	Gm6900	predicted gene 6900	1.214477	0.02218	0.1129
18191	Nrxn3	neurexin III	1.214329	0.05315	0.1876
231042	Nupl2	nucleoporin like 2	1.214329	0.2587	0.494
19893	Rpgr	retinitis pigmentosa GTPase regulator	1.214182	0.3813	0.615
14263	Fmo5	flavin containing monooxygenase 5	1.214034	0.2046	0.4309
236915	Arhgef9	CDC42 guanine nucleotide exchange factor (GEF) 9	1.213887	0.3739	0.6082
78689	Naa35	N(alpha)-acetyltransferase 35, NatC auxiliary subunit	1.213592	0.01872	0.1018
101612	Grwd1	glutamate-rich WD repeat containing 1	1.213592	0.2604	0.4959
50794	Klf13	Kruppel-like factor 13	1.213445	0.2606	0.4959
236293	D630002G06Rik	RIKEN cDNA D630002G06 gene	1.213445	0.1081	0.294
12111	Bgn	biglycan	1.213298	0.629	0.8043
93960	Nkd1	naked cuticle 1 homolog (Drosophila)	1.213298	0.02868	0.1319
228775	Trib3	tribbles homolog 3 (Drosophila)	1.213298	0.05043	0.1824
234371	Tmem161a	transmembrane protein 161A	1.213298	0.01965	0.1049
320669	A230092J17Rik	RIKEN cDNA A230092J17 gene	1.213298	0.078	0.2394
624784	Gm9855	thymine DNA glycosylase pseudogene	1.213151	0.35	0.5859
63913	Fam129a	family with sequence similarity 129, member A	1.213003	0.4623	0.6849
75547	Akap13	A kinase (PRKA) anchor protein 13	1.213003	0.03332	0.1442
624367	Gm6498	glyceraldehyde-3-phosphate dehydrogenase pseudogene	1.213003	0.09567	0.2722
18704	Pik3c2a	phosphatidylinositol 3-kinase, C2 domain containing, alpha polypeptide	1.212856	0.1626	0.3749
71729	Rgs12	regulator of G-protein signaling 12	1.212709	0.08571	0.2541
83962	Btbd1	BTB (POZ) domain containing 1	1.212709	0.02603	0.1243
235587	Parp3	poly (ADP-ribose) polymerase family, member 3	1.212709	0.2062	0.4324
14433	Gapdh	glyceraldehyde-3-phosphate dehydrogenase	1.212415	0.1464	0.3522
13138	Dag1	dystroglycan 1	1.212121	0.08758	0.2582
66356	2310008H09Rik	RIKEN cDNA 2310008H09 gene	1.212121	0.02936	0.1339
72285	1810073O08Rik	RIKEN cDNA 1810073O08 gene	1.212121	0.3125	0.5491
72927	Hepacam	hepatocyte cell adhesion molecule	1.212121	0.1122	0.3006
17961	Nat2	N-acetyltransferase 2 (arylamine N-acetyltransferase)	1.211974	0.03611	0.1501
18111	Nnat	neuronatin	1.211974	0.1964	0.4206
252838	Tox	thymocyte selection-associated high mobility group box	1.211827	0.04685	0.175
320244	Ttll5	tubulin tyrosine ligase-like family, member 5	1.211827	0.1106	0.2982
56419	Diap3	diaphanous homolog 3 (Drosophila)	1.211681	0.2944	0.5305
68428	Steap3	STEAP family member 3	1.211681	0.04994	0.1815
22630	Ywhaq	tyrosine 3-monooxygenase/tryptophan 5-monooxygenase activation protein, theta polypeptide	1.211534	0.09465	0.2705

320817	Atad2b	ATPase family, AAA domain containing 2B	1.211534	0.1547	0.3646
20186	Nr1h4	nuclear receptor subfamily 1, group H, member 4	1.211387	0.2287	0.4597
212442	Lactb2	lactamase, beta 2	1.211387	0.09396	0.2695
22755	Zfp93	zinc finger protein 93	1.21124	0.007127	0.0572
22771	Zic1	zinc finger protein of the cerebellum 1	1.21124	0.1334	0.3339
70729	Nos1ap	nitric oxide synthase 1 (neuronal) adaptor protein	1.21124	0.1302	0.3297
215160	Rhbdd2	rhomboid domain containing 2	1.21124	0.009584	0.06817
13002	Dnajc5	DnaJ (Hsp40) homolog, subfamily C, member 5	1.211094	0.03666	0.1517
16784	Lamp2	lysosomal-associated membrane protein 2	1.211094	0.1031	0.2848
228139	P2rx3	purinergic receptor P2X, ligand-gated ion channel, 3	1.211094	0.7014	0.8494
231130	Tnip2	TNFAIP3 interacting protein 2	1.211094	0.008107	0.06164
19766	Ripk1	receptor (TNFRSF)-interacting serine-threonine kinase 1	1.210947	0.03713	0.1527
71823	3300002A11Rik	RIKEN cDNA 3300002A11 gene	1.2108	0.08769	0.2583
72523	2700005E23Rik	RIKEN cDNA 2700005E23 gene	1.210507	0.3329	0.5689
114585	D17H6S53E	DNA segment, Chr 17, human D6S53E	1.210507	0.08938	0.2612
381113	Cdkl4	cyclin-dependent kinase-like 4	1.210507	0.1861	0.4063
21856	Timm44	translocase of inner mitochondrial membrane 44	1.210361	0.01817	0.0998
19242	Ptn	pleiotrophin	1.210214	0.1532	0.3627
72338	Wdr89	WD repeat domain 89	1.210214	0.04641	0.1742
71617	9130011E15Rik	RIKEN cDNA 9130011E15 gene	1.210068	0.4018	0.632
70138	2210417A02Rik	RIKEN cDNA 2210417A02 gene	1.209921	0.3929	0.6241
72293	Nkd2	naked cuticle 2 homolog (Drosophila)	1.209775	0.1008	0.2809
218506	Mrps27	mitochondrial ribosomal protein S27	1.209775	0.1623	0.3746
20677	Sox4	SRY-box containing gene 4	1.209629	0.02995	0.1354
56356	Gltp	glycolipid transfer protein	1.209629	0.2652	0.501
75859	4930568D16Rik	RIKEN cDNA 4930568D16 gene	1.209629	0.7002	0.8486
171469	Gpr37l1	G protein-coupled receptor 37-like 1	1.209629	0.2336	0.4649
77087	Ankrd11	ankyrin repeat domain 11	1.209482	0.04842	0.1781
13025	Ctla2b	cytotoxic T lymphocyte-associated protein 2 beta	1.209336	0.05172	0.1849
19024	Ppfibp2	PTPRF interacting protein, binding protein 2 (liprin beta 2)	1.209336	0.1499	0.3577
11816	Apoe	apolipoprotein E	1.20919	0.005955	0.05198
238330	6430527G18Rik	RIKEN cDNA 6430527G18 gene	1.208605	0.1419	0.3462
14247	Fli1	Friend leukemia integration 1	1.208459	0.1195	0.3129
76964	2610028H24Rik	RIKEN cDNA 2610028H24 gene	1.208313	0.1524	0.3616
216516	Ccdc157	coiled-coil domain containing 157	1.208167	0.02568	0.1233
380997	Cyp2d12	cytochrome P450, family 2, subfamily d, polypeptide 12	1.208021	0.1785	0.3963
21665	Tdg	thymine DNA glycosylase	1.207584	0.2447	0.4784
13418	Dnajc1	DnaJ (Hsp40) homolog, subfamily C, member 1	1.207438	0.02661	0.1261
14852	Gspt1	G1 to S phase transition 1	1.207438	0.01212	0.07833
232533	Stk38l	serine/threonine kinase 38 like	1.207438	0.03517	0.1484
15229	Foxd1	forkhead box D1	1.207292	0.3637	0.5989
69216	Ccdc23	coiled-coil domain containing 23	1.207292	0.01693	0.09591
12633	Cflar	CASP8 and FADD-like apoptosis regulator	1.207146	0.06745	0.219
72750	Fam117b	family with sequence similarity 117, member B	1.207001	0.04477	0.1705
114675	4932431P20Rik	RIKEN cDNA 4932431P20 gene	1.207001	0.2299	0.4612
58178	Sorcs1	VPS10 domain receptor protein SORCS 1	1.206855	0.06411	0.2117
18027	Nfia	nuclear factor I/A	1.206564	0.2086	0.4353
67330	1700047M11Rik	RIKEN cDNA 1700047M11 gene	1.206564	0.02372	0.1174
381549	Zfp69	zinc finger protein 69	1.206418	0.2804	0.5157

20908	Stx3	syntaxin 3	1.206273	0.4513	0.6761
114230	Aipl1	aryl hydrocarbon receptor-interacting protein-like 1	1.206273	0.0703	0.2246
114601	Ehbp1l1	EH domain binding protein 1-like 1	1.206273	0.05081	0.1829
403178	Plcxd1	phosphatidylinositol-specific phospholipase C, X domain containing 1	1.206273	0.04115	0.1622
20779	Src	Rous sarcoma oncogene	1.206127	0.1743	0.3908
67204	Eif2s2	eukaryotic translation initiation factor 2, subunit 2 (beta)	1.205982	0.01657	0.09464
68632	Myct1	myc target 1	1.205982	0.3347	0.5705
83436	Plekha2	pleckstrin homology domain-containing, family A (phosphoinositide binding specific) member 2	1.205982	0.01576	0.09222
269955	Rccd1	RCC1 domain containing 1	1.205982	0.03645	0.1512
103841	Cuedc1	CUE domain containing 1	1.205836	0.1533	0.3629
239408	Tmem74	transmembrane protein 74	1.205836	0.0819	0.2466
12007	Azgp1	alpha-2-glycoprotein 1, zinc	1.205691	0.3625	0.5976
329470	Accs	1-aminocyclopropane-1-carboxylate synthase homolog (Arabidopsis)(non-functional)	1.205546	0.1325	0.3331
112422	2610305D13Rik	RIKEN cDNA 2610305D13 gene	1.2054	0.1759	0.3929
170459	Stard4	StAR-related lipid transfer (START) domain containing 4	1.2054	0.03814	0.1554
100037282	Rsph3b	radial spoke 3B homolog (Chlamydomonas)	1.2054	0.002673	NA
245038	Dclk3	doublecortin-like kinase 3	1.20511	0.07133	0.2266
574428	Zmynd15	zinc finger, MYND-type containing 15	1.20511	0.168	0.383
52463	Tet1	tet oncogene 1	1.204964	0.04438	0.1695
232430	Crebl2	cAMP responsive element binding protein-like 2	1.204964	0.04823	0.1777
223922	Atf7	activating transcription factor 7	1.204819	0.1818	0.4007
16562	Kif1c	kinesin family member 1C	1.204384	0.2577	0.4928
54485	Dll4	delta-like 4 (Drosophila)	1.204384	0.5918	0.7799
11606	Agt	angiotensinogen (serpin peptidase inhibitor, clade A, member 8)	1.204239	0.1444	0.3495
21678	Tead3	TEA domain family member 3	1.204239	0.1494	0.357
74039	Nfam1	Nfat activating molecule with ITAM motif 1	1.204239	0.3128	0.5493
257913	Olfr141	olfactory receptor 141	1.204239	0.234	0.4655
12426	Cckbr	cholecystokinin B receptor	1.204094	0.1148	0.3052
16190	Il4ra	interleukin 4 receptor, alpha	1.204094	0.1171	0.309
67041	Oxct1	3-oxoacid CoA transferase 1	1.204094	0.004378	NA
13051	Cx3cr1	chemokine (C-X3-C) receptor 1	1.203949	0.2365	0.4688
75698	Fam35a	family with sequence similarity 35, member A	1.203949	0.0342	0.1462
242050	Igsf10	immunoglobulin superfamily, member 10	1.203949	0.1663	0.3805
21645	Tcte1	t-complex-associated testis expressed 1	1.203804	0.3925	0.624
66953	Cdca7	cell division cycle associated 7	1.203804	0.156	0.3658
231912	Katnal1	katanin p60 subunit A-like 1	1.203804	0.1329	0.3336
18476	Pafah1b3	platelet-activating factor acetylhydrolase, isoform 1b, subunit 3	1.203659	0.06361	0.2107
84094	Plvap	plasmalemma vesicle associated protein	1.203659	0.05819	0.1991
210126	Lpp	LIM domain containing preferred translocation partner in lipoma	1.203659	0.4167	0.645
237221	Gemin8	gem (nuclear organelle) associated protein 8	1.203659	0.00573	NA
270163	Myo9a	myosin IXa	1.203659	0.4567	0.6801
546143	Gm5918	predicted gene 5918	1.203659	0.01765	0.09827
74577	Glb1l	galactosidase, beta 1-like	1.203514	0.03528	0.1484
67282	Ccdc53	coiled-coil domain containing 53	1.203369	0.01054	0.07239
74218	1700016H13Rik	RIKEN cDNA 1700016H13 gene	1.203369	0.4827	0.7013
11652	Akt2	thymoma viral proto-oncogene 2	1.203225	0.1577	0.3684
235533	Gk5	glycerol kinase 5 (putative)	1.203225	0.2322	0.4635
22720	Zfp62	zinc finger protein 62	1.20308	0.1469	0.353
54371	Chst2	carbohydrate sulfotransferase 2	1.202935	0.2311	0.4625

78284	Creb3l4	cAMP responsive element binding protein 3-like 4	1.20279	0.07314	0.2304
83436	Plekha2	pleckstrin homology domain-containing, family A (phosphoinositide binding specific) member 2	1.20279	0.1507	0.3587
66253	Aig1	androgen-induced 1	1.202646	0.1137	0.3033
17684	Cited2	Cbp/p300-interacting transactivator, with Glu/Asp-rich carboxy-terminal domain, 2	1.202501	0.102	0.2829
11491	Adam17	a disintegrin and metallopeptidase domain 17	1.202357	0.01758	0.09797
56348	Hsd17b12	hydroxysteroid (17-beta) dehydrogenase 12	1.202357	0.0696	0.2232
215819	Nhsl1	NHS-like 1	1.202357	0.2933	0.5294
244417	Gm501	predicted gene 501	1.202357	0.1442	0.3492
386612	Thoc6	THO complex 6 homolog (Drosophila)	1.202357	0.02289	0.1152
20932	Surf4	surfeit gene 4	1.202212	0.02829	0.131
666190	Gm7972	predicted gene 7972	1.202212	0.3678	0.6028
52440	Tax1bp1	Tax1 (human T-cell leukemia virus type I) binding protein 1	1.202068	0.03533	0.1484
22070	Tpt1	tumor protein, translationally-controlled 1	1.201923	0.04792	0.1771
239528	Eif2c2	eukaryotic translation initiation factor 2C, 2	1.201923	0.3362	0.572
14871	Gstt1	glutathione S-transferase, theta 1	1.201779	0.04825	0.1777
19212	Pter	phosphotriesterase related	1.201779	0.02652	0.1258
170728	Rtn4ip1	reticulon 4 interacting protein 1	1.201779	0.07773	0.239
22418	Wnt5a	wingless-related MMTV integration site 5A	1.201634	0.05528	0.1925
16510	Kcnh1	potassium voltage-gated channel, subfamily H (eag-related), member 1	1.20149	0.3766	0.6106
66077	Aurkaip1	aurora kinase A interacting protein 1	1.20149	0.04058	0.161
69942	Rnf113a1	ring finger protein 113A1	1.20149	0.006131	NA
78521	B230219D22Rik	RIKEN cDNA B230219D22 gene	1.201346	0.08458	0.2519
100201	Tmem64	transmembrane protein 64	1.201346	0.003036	NA
57028	Pdxp	pyridoxal (pyridoxine, vitamin B6) phosphatase	1.201201	0.06204	0.2074
72137	Wdsub1	WD repeat, SAM and U-box domain containing 1	1.201201	0.1716	0.3873
16456	F11r	F11 receptor	1.200913	0.04184	0.1638
108151	Sema3d	sema domain, immunoglobulin domain (Ig), short basic domain, secreted, (semaphorin) 3D	1.200913	0.2515	0.4858
269338	Vps39	vacuolar protein sorting 39 (yeast)	1.200913	0.1232	0.3192
26416	Mapk14	mitogen-activated protein kinase 14	1.200768	0.26	0.4953
226252	Fam160b1	family with sequence similarity 160, member B1	1.200768	0.05458	0.1911
19301	Pxmp2	peroxisomal membrane protein 2	1.20048	0.1548	0.3647
69666	Psmg4	proteasome (prosome, macropain) assembly chaperone 4	1.20048	0.08022	0.2436
67664	Rnf125	ring finger protein 125	1.200336	0.09957	0.2785
237412	Gm4924	predicted gene 4924	1.200336	0.1991	0.4244
259277	Klk8	kallikrein related-peptidase 8	1.200336	0.4358	0.6626
66471	Anp32e	acidic (leucine-rich) nuclear phosphoprotein 32 family, member E	1.200048	0.1305	0.3302
74048	4632428N05Rik	RIKEN cDNA 4632428N05 gene	1.199904	0.09293	0.2677
20503	Slc16a7	solute carrier family 16 (monocarboxylic acid transporters), member 7	1.19976	0.6449	0.8146
15212	Hexb	hexosaminidase B	1.199616	0.01615	0.09307
70961	4921532D01Rik	RIKEN cDNA 4921532D01 gene	1.199616	0.2652	0.501
71409	Fmnl2	formin-like 2	1.199616	0.3928	0.6241
260423	Hist1h3f	histone cluster 1, H3f	1.199616	0.513	0.7233
30058	Timm8a1	translocase of inner mitochondrial membrane 8 homolog a1 (yeast)	1.199472	0.1692	0.3843
66365	Ccdc90b	coiled-coil domain containing 90B	1.199328	0.02256	0.1143
382019	Zfp882	zinc finger protein 882	1.199328	0.1617	0.3737
12155	Bmp15	bone morphogenetic protein 15	1.199185	0.158	0.3689
22165	Txk	TXK tyrosine kinase	1.199185	0.3666	0.6019
70973	4931431B13Rik	RIKEN cDNA 4931431B13 gene	1.199185	0.7203	0.8602
67685	Dyx1c1	dyslexia susceptibility 1 candidate 1 homolog (human)	1.198897	0.01087	0.07363

633947	Gm6225	predicted gene 6225	1.198897	0.162	0.3743
53608	Map3k6	mitogen-activated protein kinase kinase kinase 6	1.198753	0.3648	0.6003
71138	Tmem217	transmembrane protein 217	1.198753	0.2343	0.4659
552902	LOC552902	hypothetical LOC552902	1.198753	0.03656	0.1514
54199	Ccrl2	chemokine (C-C motif) receptor-like 2	1.19861	0.2735	0.5094
18641	Pfkl	phosphofructokinase, liver, B-type	1.198466	0.1799	0.398
20720	Serpine2	serine (or cysteine) peptidase inhibitor, clade E, member 2	1.198466	0.4032	0.6332
75547	Akap13	A kinase (PRKA) anchor protein 13	1.198466	0.1645	0.378
80901	Cxcr6	chemokine (C-X-C motif) receptor 6	1.198466	0.2365	0.4688
68185	Chchd8	coiled-coil-helix-coiled-coil-helix domain containing 8	1.198322	0.02858	0.1316
382019	Zfp882	zinc finger protein 882	1.198322	0.01005	0.07023
12346	Car1	carbonic anhydrase 1	1.198179	0.2748	0.5102
67880	Dcxr	dicarbonyl L-xylulose reductase	1.198179	0.1989	0.4242
18845	Plxna2	plexin A2	1.198035	0.009053	0.06591
11536	Gpr182	G protein-coupled receptor 182	1.197892	0.0805	0.244
21452	Tcn2	transcobalamin 2	1.197748	0.02045	0.1074
66682	Trappc5	trafficking protein particle complex 5	1.197748	0.05146	0.1843
71275	4933437F05Rik	RIKEN cDNA 4933437F05 gene	1.197748	0.09596	0.2727
108058	Camk2d	calcium/calmodulin-dependent protein kinase II, delta	1.197748	0.5448	0.7474
18088	Nkx2-2	NK2 transcription factor related, locus 2 (Drosophila)	1.197605	0.2353	0.4674
70686	Dusp16	dual specificity phosphatase 16	1.197605	0.249	0.4832
12520	Cd81	CD81 antigen	1.197461	0.006388	NA
13527	Dtna	dystrobrevin alpha	1.197461	0.02615	0.1247
15366	Hmmr	hyaluronan mediated motility receptor (RHAMM)	1.197461	0.307	0.5433
73020	2900073C17Rik	RIKEN cDNA 2900073C17 gene	1.197318	0.182	0.4009
11783	Apaf1	apoptotic peptidase activating factor 1	1.197175	0.1142	0.3042
14674	Gna13	guanine nucleotide binding protein, alpha 13	1.197175	0.04581	0.1727
74649	Cpa5	carboxypeptidase A5	1.197175	0.159	0.3701
83675	Bicc1	bicaudal C homolog 1 (Drosophila)	1.197175	0.1101	0.2974
12808	Cobl	cordon-bleu	1.197031	0.2517	0.4861
26558	Homer3	homer homolog 3 (Drosophila)	1.197031	0.1976	0.4225
237073	Rbm41	RNA binding motif protein 41	1.197031	0.2172	0.4462
383032	Gm5215	predicted gene 5215	1.196888	0.6237	0.801
66578	2610039C10Rik	RIKEN cDNA 2610039C10 gene	1.196745	0.04848	0.1782
217864	Rcor1	REST corepressor 1	1.196745	0.1585	0.3696
12995	Csnk2a1	casein kinase 2, alpha 1 polypeptide	1.196602	0.2629	0.4984
15201	Hells	helicase, lymphoid specific	1.196602	0.3271	0.5636
15936	Ier2	immediate early response 2	1.196602	0.138	0.3405
170721	Papln	papilin, proteoglycan-like sulfated glycoprotein	1.196602	0.1156	0.3064
269999	Orai3	ORAI calcium release-activated calcium modulator 3	1.196602	0.2705	0.5056
26377	Dapp1	dual adaptor for phosphotyrosine and 3-phosphoinositides 1	1.196458	0.2491	0.4832
78465	1700084C01Rik	RIKEN cDNA 1700084C01 gene	1.196458	0.3081	0.5443
16440	Itpr3	inositol 1,4,5-triphosphate receptor 3	1.196315	0.01615	0.09307
13169	Dbnl	drebrin-like	1.196172	0.03559	0.149
14678	Gnai2	guanine nucleotide binding protein (G protein), alpha inhibiting 2	1.196029	0.03406	0.1459
70828	4633401B06Rik	RIKEN cDNA 4633401B06 gene	1.196029	0.2024	0.4286
14113	Fbl	fibrillarin	1.195886	0.02405	0.1184
17136	Mag	myelin-associated glycoprotein	1.195886	0.1763	0.3933
70579	Zc3h11a	zinc finger CCCH type containing 11A	1.195743	0.06518	0.2139

170770	Bbc3	BCL2 binding component 3	1.195743	0.04037	0.1604
545005	LOC545005	hypothetical protein LOC545005	1.195743	0.5079	0.7193
207375	Fam120c	family with sequence similarity 120, member C	1.1956	0.2533	0.488
70356	St13	suppression of tumorigenicity 13	1.195457	0.003979	NA
387131	Ssxb9	synovial sarcoma, X member B, breakpoint 9	1.195457	0.2857	0.521
68957	Paqr6	progestin and adipoQ receptor family member VI	1.195314	0.07868	0.2406
72123	2010109K11Rik	RIKEN cDNA 2010109K11 gene	1.195314	0.06314	0.2098
258772	Olfr821	olfactory receptor 821	1.195314	0.2678	0.5034
386612	Thoc6	THO complex 6 homolog (Drosophila)	1.195314	0.02037	0.1071
20449	St8sia1	ST8 alpha-N-acetyl-neuraminide alpha-2,8-sialyltransferase 1	1.195172	0.2452	0.479
17988	Ndrg1	N-myc downstream regulated gene 1	1.195029	0.004452	NA
69008	Cab39l	calcium binding protein 39-like	1.195029	0.06877	0.2216
22135	Tgoln2	trans-golgi network protein 2	1.194886	0.4121	0.6408
72388	Ripk4	receptor-interacting serine-threonine kinase 4	1.194886	0.1365	0.3383
73668	Ttc21b	tetratricopeptide repeat domain 21B	1.194886	0.06482	0.2132
11565	Adssl1	adenylosuccinate synthetase like 1	1.194458	0.05248	0.1862
30053	Reg3d	regenerating islet-derived 3 delta	1.194458	0.5779	0.7704
241627	Wdr76	WD repeat domain 76	1.194458	0.1842	0.4033
11443	Chrnb1	cholinergic receptor, nicotinic, beta polypeptide 1 (muscle)	1.194315	0.063	0.2095
21808	Tgfb2	transforming growth factor, beta 2	1.194315	0.05958	0.2026
74143	Opa1	optic atrophy 1 homolog (human)	1.194315	0.01606	0.09289
100043736	Gm10762	predicted gene 10762	1.194315	0.2954	0.5311
399616	A130078K24Rik	RIKEN cDNA A130078K24 gene	1.194172	0.2874	0.5229
66910	Tmem107	transmembrane protein 107	1.19403	0.0306	0.137
74307	1700092M07Rik	RIKEN cDNA 1700092M07 gene	1.19403	0.1027	0.2842
98363	Efhd1	EF hand domain containing 1	1.19403	0.1016	0.2823
67674	Trmt112	tRNA methyltransferase 11-2 homolog (S. cerevisiae)	1.193887	0.05601	0.1939
231633	Tmem119	transmembrane protein 119	1.193887	0.1569	0.3671
13661	Ehf	ets homologous factor	1.193602	0.3615	0.5963
26385	Grk6	G protein-coupled receptor kinase 6	1.193602	0.2612	0.4963
100037283	Rnaset2a	ribonuclease T2A	1.193602	0.02689	0.1268
16797	Lat	linker for activation of T cells	1.193317	0.171	0.3865
69724	Rnaseh2a	ribonuclease H2, large subunit	1.193317	0.09755	0.2755
664968	2210411K11Rik	RIKEN cDNA 2210411K11 gene	1.193317	0.182	0.4009
21808	Tgfb2	transforming growth factor, beta 2	1.193175	0.02315	0.116
269389	Tox2	TOX high mobility group box family member 2	1.193033	0.111	0.2989
15944	Irgm1	immunity-related GTPase family M member 1	1.19289	0.03046	0.1368
22773	Zic3	zinc finger protein of the cerebellum 3	1.19289	0.286	0.5215
27354	Nbn	nibrin	1.19289	0.1743	0.3908
67118	Bfar	bifunctional apoptosis regulator	1.19289	0.328	0.5645
319195	Rpl17	ribosomal protein L17	1.19289	0.1616	0.3737
16601	Klf9	Kruppel-like factor 9	1.192748	0.02978	0.1349
320538	Ubn2	ubinuclein 2	1.192748	0.0644	0.2124
18176	Nras	neuroblastoma ras oncogene	1.192606	0.03058	0.137
75212	Rnf121	ring finger protein 121	1.192606	0.01792	0.09903
12739	Cldn3	claudin 3	1.192464	0.3584	0.5934
245403	Dcaf12l2	DDB1 and CUL4 associated factor 12-like 2	1.192464	0.6416	0.8127
26895	Cops7b	COP9 (constitutive photomorphogenic) homolog, subunit 7b (Arabidopsis thaliana)	1.192321	0.1127	0.3015
71962	Gatsl3	GATS protein-like 3	1.192321	0.2654	0.5012

214897	Csnk1g1	casein kinase 1, gamma 1	1.192321	0.05202	0.1855
207521	Dtx4	deltex 4 homolog (Drosophila)	1.191895	0.005967	NA
212933	Pm20d1	peptidase M20 domain containing 1	1.191753	0.1089	0.2952
17128	Smad4	MAD homolog 4 (Drosophila)	1.191611	0.04717	0.1758
74158	Josd1	Josephin domain containing 1	1.191611	0.01016	NA
110960	Tars	threonyl-tRNA synthetase	1.191611	0.009691	NA
58187	Cldn10	claudin 10	1.191327	0.3633	0.5985
78560	Gpr124	G protein-coupled receptor 124	1.191327	0.02595	0.1242
277468	Slc39a12	solute carrier family 39 (zinc transporter), member 12	1.191327	0.281	0.5162
69307	Pxt1	peroxisomal, testis specific 1	1.191185	0.1263	0.3236
230259	E130308A19Rik	RIKEN cDNA E130308A19 gene	1.191185	0.171	0.3865
223922	Atf7	activating transcription factor 7	1.191043	0.3117	0.5482
225182	Rbbp8	retinoblastoma binding protein 8	1.191043	0.08788	0.2586
228019	Mettl8	methyltransferase like 8	1.191043	0.03405	0.1459
214424	Parp16	poly (ADP-ribose) polymerase family, member 16	1.190902	0.1025	0.2837
56190	Rbm38	RNA binding motif protein 38	1.19076	0.4222	0.6501
226359	C1ql2	complement component 1, q subcomponent-like 2	1.19076	0.2226	0.4528
16367	Irs1	insulin receptor substrate 1	1.190618	0.04075	0.1613
70160	Vps36	vacuolar protein sorting 36 (yeast)	1.190618	0.08792	0.2586
12496	Entpd2	ectonucleoside triphosphate diphosphohydrolase 2	1.190476	0.1334	0.3339
74243	2210009G21Rik	RIKEN cDNA 2210009G21 gene	1.190476	0.1721	0.3878
229473	D930015E06Rik	RIKEN cDNA D930015E06 gene	1.190476	0.117	0.3089
434174	Hmg1l1	high-mobility group (nonhistone chromosomal) protein 1-like 1	1.190476	0.329	0.5656
56791	Ube2l6	ubiquitin-conjugating enzyme E2L 6	1.190334	0.01691	0.09581
229279	Hnrnpa3	heterogeneous nuclear ribonucleoprotein A3	1.190334	0.4811	0.6999
381314	Iars2	isoleucine-tRNA synthetase 2, mitochondrial	1.190334	0.3646	0.6
545007	ENSMUSG00000068790	predicted gene, ENSMUSG00000068790	1.190193	0.5866	0.7763
24004	Rai2	retinoic acid induced 2	1.18991	0.04221	0.1644
225644	Cplx4	complexin 4	1.18991	0.04038	0.1604
22631	Ywhaz	tyrosine 3-monooxygenase/tryptophan 5-monooxygenase activation protein, zeta polypeptide	1.189768	0.3875	0.6195
29865	Cabp5	calcium binding protein 5	1.189768	0.1024	0.2836
70370	Fbln7	fibulin 7	1.189768	0.2248	0.4559
213389	Prdm9	PR domain containing 9	1.189768	0.04598	0.1731
268709	Fam107a	family with sequence similarity 107, member A	1.189768	0.4104	0.6395
100043902	Six3os1	Six3 opposite strand transcript 1	1.189768	0.6258	0.8024
16784	Lamp2	lysosomal-associated membrane protein 2	1.189626	0.03683	0.152
277744	Gm694	predicted gene 694	1.189626	0.1835	0.4026
380732	Gm885	predicted gene 885	1.189626	0.373	0.6072
12228	Btg3	B-cell translocation gene 3	1.189485	0.07623	0.2365
13110	Cyp2j6	cytochrome P450, family 2, subfamily j, polypeptide 6	1.189485	0.02941	0.134
232533	Stk38l	serine/threonine kinase 38 like	1.189485	0.2749	0.5103
19271	Ptprj	protein tyrosine phosphatase, receptor type, J	1.189202	0.514	0.7243
269951	Idh2	isocitrate dehydrogenase 2 (NADP+), mitochondrial	1.189202	0.03516	0.1484
75299	4930547M16Rik	RIKEN cDNA 4930547M16 gene	1.189061	0.09123	0.2647
75732	Iqcd	IQ motif containing D	1.189061	0.03318	0.1439
235461	Fam63b	family with sequence similarity 63, member B	1.189061	0.2256	0.4566
70425	Csnk1g3	casein kinase 1, gamma 3	1.188919	0.2566	0.4916
11629	Aif1	allograft inflammatory factor 1	1.188778	0.1523	0.3613
16974	Lrp6	low density lipoprotein receptor-related protein 6	1.188637	0.09401	0.2695

22229	Ucp3	uncoupling protein 3 (mitochondrial, proton carrier)	1.188637	0.1986	0.4238
28010	Miip	migration and invasion inhibitory protein	1.188637	0.2579	0.4929
100042659	Gm3952	predicted gene 3952	1.188637	0.6245	0.8016
12390	Cav2	caveolin 2	1.188354	0.3145	0.5508
76938	Rbm17	RNA binding motif protein 17	1.188354	0.03341	0.1444
100502982	LOC100502982	hypothetical LOC100502982	1.188354	0.02558	0.1229
12331	Cap1	CAP, adenylate cyclase-associated protein 1 (yeast)	1.188213	0.06788	0.2199
320923	Mtap7d3	MAP7 domain containing 3	1.188213	0.2546	0.4894
353282	Sfmbt2	Scm-like with four mbt domains 2	1.188213	0.08002	0.2433
13436	Dnmt3b	DNA methyltransferase 3B	1.187931	0.08782	0.2586
101118	Tmem168	transmembrane protein 168	1.187931	0.3174	0.5539
215493	A3galt2	alpha 1,3-galactosyltransferase 2 (isoglobotriaosylceramide synthase)	1.187931	0.135	0.3363
19353	Rac1	RAS-related C3 botulinum substrate 1	1.18779	0.009867	NA
52713	Ccdc59	coiled-coil domain containing 59	1.187648	0.1413	0.3455
14613	Gja5	gap junction protein, alpha 5	1.187507	0.1601	0.3717
20446	St6galnac2	ST6 (alpha-N-acetyl-neuraminyl-2,3-beta-galactosyl-1,3)-N-acetylgalactosaminide alpha-2,6-sialyltransferase 2	1.187507	0.09785	0.2759
170947	Myoz3	myozenin 3	1.187507	0.4816	0.7003
791360	Gm9853	predicted gene 9853	1.187507	0.09664	0.274
20681	Sox8	SRY-box containing gene 8	1.187366	0.07895	0.2412
216551	1110067D22Rik	RIKEN cDNA 1110067D22 gene	1.187366	0.02931	0.1338
15245	Hhip	Hedgehog-interacting protein	1.187225	0.1621	0.3743
16774	Lama3	laminin, alpha 3	1.187225	0.0998	0.279
66047	Mrpl54	mitochondrial ribosomal protein L54	1.187225	0.04673	0.1748
12506	Cd48	CD48 antigen	1.187085	0.0762	0.2365
14387	Gaa	glucosidase, alpha, acid	1.187085	0.1927	0.4153
11676	Aldoc	aldolase C, fructose-bisphosphate	1.186944	0.1678	0.3828
105559	Mbnl2	muscleblind-like 2	1.186944	0.1572	0.3675
319765	Igf2bp2	insulin-like growth factor 2 mRNA binding protein 2	1.186803	0.03944	0.1582
26395	Map2k1	mitogen-activated protein kinase kinase 1	1.186662	0.04606	0.1732
70110	Ifi35	interferon-induced protein 35	1.186662	0.07922	0.2417
227683	Coq4	coenzyme Q4 homolog (yeast)	1.18638	0.0357	0.1493
14455	Gas5	growth arrest specific 5	1.18624	0.1364	0.3382
70361	Lman1	lectin, mannose-binding, 1	1.18624	0.02141	0.1107
72137	Wdsub1	WD repeat, SAM and U-box domain containing 1	1.18624	0.02666	0.1262
27419	Naglu	alpha-N-acetylglucosaminidase (Sanfilippo disease IIIB)	1.185958	0.0366	0.1515
97484	Cog8	component of oligomeric golgi complex 8	1.185958	0.1869	0.4075
106740	LOC106740	hypothetical LOC106740	1.185958	0.06295	0.2095
235854	Mrgpra4	MAS-related GPR, member A4	1.185958	0.2437	0.4772
16337	Insr	insulin receptor	1.185818	0.077	0.2377
66838	0610009L18Rik	RIKEN cDNA 0610009L18 gene	1.185818	0.146	0.3515
66939	Aagab	alpha- and gamma-adaptin binding protein	1.185818	0.01811	0.09964
109889	Mzf1	myeloid zinc finger 1	1.185818	0.1326	0.3332
223770	Brd1	bromodomain containing 1	1.185818	0.3455	0.581
72773	2810449G22Rik	RIKEN cDNA 2810449G22 gene	1.185677	0.2903	0.5264
213539	Bag2	BCL2-associated athanogene 2	1.185677	0.02839	0.131
270627	Taf1	TAF1 RNA polymerase II, TATA box binding protein (TBP)-associated factor	1.185677	0.5451	0.7477
338368	Fam109b	family with sequence similarity 109, member B	1.185677	0.1017	0.2824
69953	2810025M15Rik	RIKEN cDNA 2810025M15 gene	1.185536	0.02685	0.1267
100039258	Gm10290	glyceraldehyde-3-phosphate dehydrogenase pseudogene	1.185536	0.2658	0.5014

68214	Gsto2	glutathione S-transferase omega 2	1.185396	0.1521	0.3612
56217	Mpp5	membrane protein, palmitoylated 5 (MAGUK p55 subfamily member 5)	1.185255	0.1722	0.388
106052	Fbxo4	F-box protein 4	1.185255	0.03831	0.1559
240660	Tmem20	transmembrane protein 20	1.185255	0.04072	0.1613
11568	Aebp1	AE binding protein 1	1.185115	0.171	0.3865
17127	Smad3	MAD homolog 3 (Drosophila)	1.185115	0.2079	0.4342
18595	Pdgfra	platelet derived growth factor receptor, alpha polypeptide	1.185115	0.1572	0.3675
18685	Phtf1	putative homeodomain transcription factor 1	1.185115	0.006107	NA
20512	Slc1a3	solute carrier family 1 (glial high affinity glutamate transporter), member 3	1.185115	0.04697	0.1753
74753	5830415F09Rik	RIKEN cDNA 5830415F09 gene	1.185115	0.3561	0.5907
78928	Pigt	phosphatidylinositol glycan anchor biosynthesis, class T	1.185115	0.3175	0.5539
20615	Snapin	SNAP-associated protein	1.184975	0.09167	0.2654
66313	Smurf2	SMAD specific E3 ubiquitin protein ligase 2	1.184975	0.2654	0.5011
636808	Cntnap5a	contactin associated protein-like 5A	1.184694	0.1331	0.3339
100039528	1110002E22Rik	RIKEN cDNA 1110002E22 gene	1.184694	0.2424	0.4755
56526	Sep-06	septin 6	1.184553	0.2127	0.4405
67163	Ccdc47	coiled-coil domain containing 47	1.184553	0.2943	0.5305
76311	1110019D14Rik	RIKEN cDNA 1110019D14 gene	1.184553	0.1146	0.3049
69547	Nkpd1	NTPase, KAP family P-loop domain containing 1	1.184413	0.1296	0.3286
83433	Trem2	triggering receptor expressed on myeloid cells 2	1.184413	0.1286	0.327
229600	BC028528	cDNA sequence BC028528	1.184273	0.2168	0.4456
320076	C630001G18Rik	RIKEN cDNA C630001G18 gene	1.184273	0.1111	0.2991
100041677	Gm13157	predicted gene 13157	1.184273	0.06908	0.2223
14950	H13	histocompatibility 13	1.184133	0.04717	0.1758
68191	5330430P22Rik	RIKEN cDNA 5330430P22 gene	1.184133	0.2422	0.4753
320110	B230369F24Rik	RIKEN cDNA B230369F24 gene	1.184133	0.1842	0.4033
13865	Nr2f1	nuclear receptor subfamily 2, group F, member 1	1.183992	0.02113	0.1097
66873	Tril	TLR4 interactor with leucine-rich repeats	1.183992	0.3167	0.5534
381760	Ssbp1	single-stranded DNA binding protein 1	1.183992	0.2086	0.4353
77857	9430065F17Rik	RIKEN cDNA 9430065F17 gene	1.183852	0.3105	0.5468
14600	Ghr	growth hormone receptor	1.183712	0.02454	0.1198
16648	Kpna3	karyopherin (importin) alpha 3	1.183712	0.1718	0.3875
18028	Nfib	nuclear factor I/B	1.183712	0.09775	0.2759
18571	Pdcd6ip	programmed cell death 6 interacting protein	1.183712	0.04487	0.1707
72554	Utp14a	UTP14, U3 small nucleolar ribonucleoprotein, homolog A (yeast)	1.183572	0.09584	0.2725
78405	Ntf5	neurotrophin 5	1.183572	0.3026	0.5387
320586	A630089N07Rik	RIKEN cDNA A630089N07 gene	1.183572	0.07648	0.2367
15223	Foxj1	forkhead box J1	1.183432	0.02692	0.1269
433182	Gm5506	predicted gene 5506	1.183432	0.01556	NA
69551	2310022B05Rik	RIKEN cDNA 2310022B05 gene	1.183292	0.04863	0.1784
223752	Gramd4	GRAM domain containing 4	1.183292	0.01264	NA
667433	Gm8630	predicted gene 8630	1.183292	0.334	0.5697
100504534	LOC100504534	hypothetical LOC100504534	1.183292	0.0196	0.1049
20682	Sox9	SRY-box containing gene 9	1.183152	0.2143	0.4427
71069	Stox2	storkhead box 2	1.183152	0.0321	0.1408
107995	Cdc20	cell division cycle 20 homolog (S. cerevisiae)	1.183152	0.1109	0.2986
239857	Cadm2	cell adhesion molecule 2	1.183152	0.1423	0.3467
627096	LOC627096	hypothetical LOC627096	1.183152	0.4523	0.677
57764	Ntn4	netrin 4	1.183012	0.2277	0.4587

59020	Pdzk1	PDZ domain containing 1	1.182872	0.06041	0.2041
74030	Rin2	Ras and Rab interactor 2	1.182872	0.195	0.4188
231382	Tmprss11d	transmembrane protease, serine 11d	1.182872	0.2684	0.5038
68750	Rreb1	ras responsive element binding protein 1	1.182592	0.1905	0.4126
94220	Cnnm4	cyclin M4	1.182592	0.01367	NA
237782	Smcr8	Smith-Magenis syndrome chromosome region, candidate 8 homolog (human)	1.182592	0.5011	0.7146
628779	Hs3st4	heparan sulfate (glucosamine) 3-O-sulfotransferase 4	1.182592	0.4089	0.6382
100503637	LOC100503637	envelope glycoprotein-like	1.182592	0.05435	0.1905
71820	Wdr34	WD repeat domain 34	1.182452	0.02384	0.1177
218311	Zfp455	zinc finger protein 455	1.182452	0.1333	0.3339
100502931	LOC100502931	hypothetical protein LOC100502931	1.182452	0.7707	0.8898
24063	Spry1	sprouty homolog 1 (Drosophila)	1.182173	0.02595	0.1242
66191	Ier3ip1	immediate early response 3 interacting protein 1	1.182173	0.0692	0.2224
109181	Trip11	thyroid hormone receptor interactor 11	1.182173	0.271	0.5063
11837	Rplp0	ribosomal protein, large, P0	1.182033	0.03973	0.1588
22063	Trpc1	transient receptor potential cation channel, subfamily C, member 1	1.182033	0.1355	0.337
212285	Arap2	ArfGAP with RhoGAP domain, ankyrin repeat and PH domain 2	1.182033	0.131	0.3308
14453	Gas2	growth arrest specific 2	1.181893	0.0596	0.2026
17095	Lyl1	lymphoblastomic leukemia 1	1.181893	0.2275	0.4586
67255	Zfp422	zinc finger protein 422	1.181893	0.3364	0.572
103079	AA408251	expressed sequence AA408251	1.181893	0.5113	0.722
19352	Rabggtb	RAB geranylgeranyl transferase, b subunit	1.181754	0.1436	0.3485
57738	Slc15a2	solute carrier family 15 (H+/peptide transporter), member 2	1.181754	0.05066	0.1828
233987	Zfp958	zinc finger protein 958	1.181754	0.2397	0.4722
14827	Pdia3	protein disulfide isomerase associated 3	1.181474	0.2856	0.521
18861	Pms2	postmeiotic segregation increased 2 (S. cerevisiae)	1.181474	0.01397	NA
70753	6330415B21Rik	RIKEN cDNA 6330415B21 gene	1.181474	0.5259	0.7331
70885	Ints10	integrator complex subunit 10	1.181474	0.2693	0.5047
219022	Ttc5	tetratricopeptide repeat domain 5	1.181474	0.04335	0.1672
14467	Gbas	glioblastoma amplified sequence	1.181335	0.03505	0.1484
11758	Prdx6	peroxiredoxin 6	1.181195	0.1402	0.3438
219249	Tdrd3	tudor domain containing 3	1.181195	0.2677	0.5032
13560	E4f1	E4F transcription factor 1	1.181056	0.3397	0.5754
20493	Slc10a1	solute carrier family 10 (sodium/bile acid cotransporter family), member 1	1.181056	0.1419	0.3462
67013	Oma1	OMA1 homolog, zinc metallopeptidase (S. cerevisiae)	1.181056	0.05189	0.1851
72567	Bclaf1	BCL2-associated transcription factor 1	1.181056	0.1168	0.3087
17060	Blnk	B-cell linker	1.180916	0.3104	0.5468
50927	Nasp	nuclear autoantigenic sperm protein (histone-binding)	1.180916	0.4303	0.6572
54644	Otud5	OTU domain containing 5	1.180916	0.02293	0.1153
98845	Eps8l2	EPS8-like 2	1.180916	0.4362	0.6628
64295	Tmub1	transmembrane and ubiquitin-like domain containing 1	1.180777	0.01059	NA
13176	Dcc	deleted in colorectal carcinoma	1.180638	0.3088	0.545
77569	Limch1	LIM and calponin homology domains 1	1.180359	0.1065	0.2911
231151	Tada2b	transcriptional adaptor 2B	1.180359	0.3043	0.5405
404331	Olfr1252	olfactory receptor 1252	1.18022	0.3939	0.6248
14118	Fbn1	fibrillin 1	1.18008	0.5404	0.7446
68328	Rab13	RAB13, member RAS oncogene family	1.18008	0.07219	0.2284
12867	Cox7c	cytochrome c oxidase, subunit VIIc	1.179802	0.3011	0.537
104725	1110002B05Rik	RIKEN cDNA 1110002B05 gene	1.179663	0.03104	0.1379

67460	Decr1	2,4-dienoyl CoA reductase 1, mitochondrial	1.179523	0.08017	0.2435
67884	1810043G02Rik	RIKEN cDNA 1810043G02 gene	1.179523	0.04632	0.174
229285	Spg20	spastic paraplegia 20, spartin (Troyer syndrome) homolog (human)	1.179523	0.1637	0.3767
67154	Mtdh	metadherin	1.179384	0.09216	0.266
73712	Dmkn	dermokine	1.179384	0.6091	0.7914
11652	Akt2	thymoma viral proto-oncogene 2	1.179245	0.006049	NA
76042	5830427D02Rik	RIKEN cDNA 5830427D02 gene	1.179106	0.106	0.2902
266690	Cyb5r4	cytochrome b5 reductase 4	1.178828	0.03231	0.1414
320926	C730029A08Rik	RIKEN cDNA C730029A08 gene	1.178828	0.1249	0.3217
100039203	Gm2099	predicted gene 2099	1.178828	0.166	0.3801
21816	Tgm1	transglutaminase 1, K polypeptide	1.178689	0.5231	0.7309
54325	Elovl1	elongation of very long chain fatty acids (FEN1/Elo2, SUR4/Elo3, yeast)-like 1	1.178689	0.1176	0.3097
67706	Tmem179b	transmembrane protein 179B	1.178689	0.08405	0.2508
320712	Abi3bp	ABI gene family, member 3 (NESH) binding protein	1.17855	0.4216	0.6495
12475	Cd14	CD14 antigen	1.178412	0.07198	0.2282
18802	Plcd4	phospholipase C, delta 4	1.178412	0.08922	0.2611
56516	Rbms2	RNA binding motif, single stranded interacting protein 2	1.178412	0.02701	0.1272
99980	AA408865	expressed sequence AA408865	1.178412	0.509	0.72
117198	Ivns1abp	influenza virus NS1A binding protein	1.178412	0.4639	0.6865
228608	Smox	spermine oxidase	1.178412	0.06737	0.2189
14634	Gli3	GLI-Kruppel family member GLI3	1.178273	0.1093	0.296
78330	Ndufv3	NADH dehydrogenase (ubiquinone) flavoprotein 3	1.178273	0.08359	0.25
101490	Inpp5f	inositol polyphosphate-5-phosphatase F	1.178273	0.415	0.6435
244745	Dpy19l1	dpy-19-like 1 (C. elegans)	1.178273	0.03378	0.1453
19729	Slc50a1	solute carrier family 50 (sugar transporter), member 1	1.177995	0.1102	0.2975
77630	Prdm8	PR domain containing 8	1.177995	0.01077	NA
50720	Sacs	sacsin	1.177856	0.5596	0.7582
68718	Rnf166	ring finger protein 166	1.177718	0.02413	NA
114606	Tle6	transducin-like enhancer of split 6, homolog of Drosophila E(spl)	1.177718	0.01805	NA
381668	Fbrsl1	fibrosin-like 1	1.177718	0.06615	0.2163
16337	Insr	insulin receptor	1.177579	0.2698	0.5049
627927	Gm6812	predicted gene 6812	1.177579	0.5357	0.7409
67338	Rffl	ring finger and FYVE like domain containing protein	1.17744	0.1968	0.4213
217030	Synrg	synergin, gamma	1.17744	0.1878	0.4087
436493	H2-Gs10	MHC class I like protein GS10	1.17744	0.4075	0.6368
26885	Casp8ap2	caspase 8 associated protein 2	1.177302	0.06389	0.2112
98514	AU015232	expressed sequence AU015232	1.177302	0.2186	0.4478
14674	Gna13	guanine nucleotide binding protein, alpha 13	1.177163	0.3569	0.5916
74080	Nmnat3	nicotinamide nucleotide adenylyltransferase 3	1.177163	0.2288	0.4598
545700	Vmn2r-ps14	vomeronasal 2, receptor, pseudogene 14	1.177163	0.1827	0.4016
14701	Gng12	guanine nucleotide binding protein (G protein), gamma 12	1.177024	0.01292	NA
70984	4931406C07Rik	RIKEN cDNA 4931406C07 gene	1.177024	0.03433	0.1465
70044	Tut1	terminal uridylyl transferase 1, U6 snRNA-specific	1.176747	0.0161	NA
100042545	Gm3896	predicted gene 3896	1.176747	0.4104	0.6395
12684	Cideb	cell death-inducing DNA fragmentation factor, alpha subunit-like effector B	1.176609	0.2387	0.4711
378954	3000002C10Rik	glyceraldehyde-3-phosphate dehydrogenase pseudogene	1.176609	0.2362	0.4687
57896	Krcc1	lysine-rich coiled-coil 1	1.176332	0.1317	0.3319
76499	Clasp2	CLIP associating protein 2	1.176332	0.09347	0.2686
22439	Xk	Kell blood group precursor (McLeod phenotype) homolog	1.176194	0.2284	0.4594

68743	Anln	anillin, actin binding protein	1.176194	0.1505	0.3584
20928	Abcc9	ATP-binding cassette, sub-family C (CFTR/MRP), member 9	1.176056	0.1812	0.3999
73712	Dmkn	dermokine	1.176056	0.605	0.7884
101706	Numa1	nuclear mitotic apparatus protein 1	1.176056	0.06455	0.2127
170779	Cd209d	CD209d antigen	1.176056	0.4316	0.6589
216964	Trp53i13	transformation related protein 53 inducible protein 13	1.176056	0.04066	0.1613
14579	Gem	GTP binding protein (gene overexpressed in skeletal muscle)	1.175917	0.1375	0.3395
67429	Nudcd1	NudC domain containing 1	1.175917	0.3141	0.5507
70530	Lrfn2	leucine rich repeat and fibronectin type III domain containing 2	1.175917	0.2543	0.4892
76740	Efr3a	EFR3 homolog A (S. cerevisiae)	1.175917	0.3823	0.6156
102234	AU022229	expressed sequence AU022229	1.175917	0.1296	0.3287
100037260	9430060I03Rik	RIKEN cDNA 9430060I03 gene	1.175779	0.04653	0.1744
27276	Plekhb1	pleckstrin homology domain containing, family B (evectins) member 1	1.175641	0.02834	0.131
71545	9030625G05Rik	RIKEN cDNA 9030625G05 gene	1.175641	0.2371	0.4693
64658	Mrps25	mitochondrial ribosomal protein S25	1.175503	0.06843	0.2209
71147	Oxsm	3-oxoacyl-ACP synthase, mitochondrial	1.175364	0.1671	0.3819
107476	Acaca	acetyl-Coenzyme A carboxylase alpha	1.175364	0.3938	0.6248
381306	BC055324	cDNA sequence BC055324	1.175364	0.05753	0.1974
171261	Vmn1r87	vomeronasal 1 receptor 87	1.175226	0.6961	0.8459
56421	Pfkp	phosphofructokinase, platelet	1.175088	0.05041	0.1824
320343	Lypd6	LY6/PLAUR domain containing 6	1.175088	0.0142	NA
66399	Tsfm	Ts translation elongation factor, mitochondrial	1.17495	0.5612	0.7597
74315	Rnf145	ring finger protein 145	1.17495	0.02593	NA
11941	Atp2b2	ATPase, Ca++ transporting, plasma membrane 2	1.174812	0.1226	0.3184
218454	Lhfpl2	lipoma HMGIC fusion partner-like 2	1.174812	0.04168	0.1634
74486	Osbpl10	oxysterol binding protein-like 10	1.174674	0.05222	0.1856
321006	Vprbp	Vpr (HIV-1) binding protein	1.174674	0.05578	0.1934
58867	Syngr4	synaptogyrin 4	1.174536	0.2162	0.445
15191	Hdgf	hepatoma-derived growth factor	1.174398	0.05058	0.1827
66404	2410001C21Rik	RIKEN cDNA 2410001C21 gene	1.17426	0.189	0.4103
67178	Zmat5	zinc finger, matrin type 5	1.17426	0.07079	0.2255
72014	Btbd17	BTB (POZ) domain containing 17	1.17426	0.2148	0.4435
77805	Esco1	establishment of cohesion 1 homolog 1 (S. cerevisiae)	1.174122	0.2448	0.4784
75964	Trappc8	trafficking protein particle complex 8	1.173985	0.4372	0.6636
319865	E130114P18Rik	RIKEN cDNA E130114P18 gene	1.173985	0.07833	0.2399
17346	Mknk1	MAP kinase-interacting serine/threonine kinase 1	1.173847	0.1697	0.3849
68922	Dnaic1	dynein, axonemal, intermediate chain 1	1.173847	0.09663	0.274
71750	R3hdm2	R3H domain containing 2	1.173847	0.2017	0.428
213673	9530068E07Rik	RIKEN cDNA 9530068E07 gene	1.173847	0.005574	NA
277333	Gm5069	glyceraldehyde-3-phosphate dehydrogenase pseudogene	1.173847	0.04085	0.1615
268291	Rnf217	ring finger protein 217	1.173709	0.009357	NA
99132	AI956758	expressed sequence AI956758	1.173571	0.334	0.5697
552901	LOC552901	hypothetical LOC552901	1.173571	0.08353	0.2499
17527	Mpv17	MpV17 mitochondrial inner membrane protein	1.173433	0.04057	0.161
19878	Rock2	Rho-associated coiled-coil containing protein kinase 2	1.173433	0.1243	0.321
74190	1200009I06Rik	RIKEN cDNA 1200009I06 gene	1.173433	0.3404	0.5762
626359	Wdr93	WD repeat domain 93	1.173433	0.1547	0.3646
56351	Ptges3	prostaglandin E synthase 3 (cytosolic)	1.173296	0.07915	0.2416
66109	Tspan13	tetraspanin 13	1.173158	0.01792	NA

74570	Zkscan1	zinc finger with KRAB and SCAN domains 1	1.173158	0.1724	0.3882
104080	Nxph4	neurexophilin 4	1.173158	0.2743	0.5099
319366	C920008N22Rik	RIKEN cDNA C920008N22 gene	1.173158	0.1015	0.2822
15213	Hey1	hairy/enhancer-of-split related with YRPW motif 1	1.173021	0.0102	NA
93709	Pcdhga1	protocadherin gamma subfamily A, 1	1.173021	0.08152	0.2459
239833	Lmln	leishmanolysin-like (metallopeptidase M8 family)	1.173021	0.05492	0.1919
27028	Ermap	erythroblast membrane-associated protein	1.172883	0.2307	0.4621
52685	Cd300lg	CD300 antigen like family member G	1.172883	0.3723	0.6068
59044	Rnf130	ring finger protein 130	1.172883	0.03264	0.1423
64436	Inpp5e	inositol polyphosphate-5-phosphatase E	1.172883	0.3218	0.5582
74347	4632415K11Rik	RIKEN cDNA 4632415K11 gene	1.172883	0.1245	0.3212
93893	Pcdhb22	protocadherin beta 22	1.172883	0.03415	0.1462
328370	Rft1	RFT1 homolog (S. cerevisiae)	1.172883	0.01531	NA
18081	Ninj1	ninjurin 1	1.172745	0.04226	0.1645
241589	D430041D05Rik	RIKEN cDNA D430041D05 gene	1.172745	0.1986	0.4238
19205	Ptbp1	polypyrimidine tract binding protein 1	1.17247	0.1639	0.377
77188	A430105D02Rik	RIKEN cDNA A430105D02 gene	1.172333	0.2555	0.4903
208213	Tmem132c	transmembrane protein 132C	1.172333	0.09746	0.2754
214804	Syde2	synapse defective 1, Rho GTPase, homolog 2 (C. elegans)	1.172058	0.08478	0.2523
217700	Acot6	acyl-CoA thioesterase 6	1.172058	0.1211	0.3156
235533	Gk5	glycerol kinase 5 (putative)	1.172058	0.3932	0.6243
544872	Gm5786	predicted pseudogene 5786	1.172058	0.1755	0.3922
56088	Psmg1	proteasome (prosome, macropain) assembly chaperone 1	1.171921	0.009053	NA
328243	E230012P03	hypothetical protein E230012P03	1.171921	0.2394	0.472
104086	Cyp27a1	cytochrome P450, family 27, subfamily a, polypeptide 1	1.171783	0.2404	0.4731
11747	Anxa5	annexin A5	1.171646	0.00978	NA
76483	Lmf1	lipase maturation factor 1	1.171646	0.04908	0.1795
21827	Thbs3	thrombospondin 3	1.171509	0.08577	0.2542
22634	Plagl1	pleiomorphic adenoma gene-like 1	1.171509	0.2205	0.4504
66568	Rwdd3	RWD domain containing 3	1.171372	0.1451	0.3505
73692	2410089E03Rik	RIKEN cDNA 2410089E03 gene	1.171372	0.1758	0.3928
73847	Fam110a	family with sequence similarity 110, member A	1.171372	0.06339	0.2103
233765	Plekha7	pleckstrin homology domain containing, family A member 7	1.171372	0.04405	0.1687
271887	BC066135	cDNA sequence BC066135	1.171372	0.3641	0.5994
15903	Id3	inhibitor of DNA binding 3	1.171234	0.04824	0.1777
67623	Tm7sf3	transmembrane 7 superfamily member 3	1.171234	0.1647	0.3781
69047	Atp2c2	ATPase, Ca++ transporting, type 2C, member 2	1.171097	0.2844	0.52
70394	Kptn	kaptin	1.171097	0.1186	0.3113
74842	4833419G08Rik	RIKEN cDNA 4833419G08 gene	1.17096	0.2039	0.4302
230917	Tmem201	transmembrane protein 201	1.17096	0.249	0.4832
23836	Cdh20	cadherin 20	1.170823	0.04569	0.1723
69743	Casz1	castor homolog 1, zinc finger (Drosophila)	1.170823	0.1247	0.3214
23871	Ets1	E26 avian leukemia oncogene 1, 5' domain	1.170686	0.2278	0.4587
83815	Cenpq	centromere protein Q	1.170686	0.1778	0.3952
232314	Ppp4r2	protein phosphatase 4, regulatory subunit 2	1.170686	0.1478	0.3544
78294	Rps27a	ribosomal protein S27A	1.170412	0.07811	0.2396
12517	Cd72	CD72 antigen	1.170275	0.08323	0.2493
71921	2310058N22Rik	RIKEN cDNA 2310058N22 gene	1.170275	0.1557	0.3654
75953	Samd7	sterile alpha motif domain containing 7	1.170275	0.07903	0.2413

19668	Rbpjl	recombination signal binding protein for immunoglobulin kappa J region-like	1.170138	0.3868	0.6189
22781	Ikzf4	IKAROS family zinc finger 4	1.170001	0.1228	0.3185
107585	Dio3	deiodinase, iodothyronine type III	1.170001	0.6568	0.8223
381065	Gm5145	small nuclear ribonucleoprotein polypeptide A pseudogene	1.170001	0.1881	0.409
17319	Mif	macrophage migration inhibitory factor	1.169864	0.03865	0.1565
13983	Esr2	estrogen receptor 2 (beta)	1.169727	0.4075	0.6368
14815	Nr3c1	nuclear receptor subfamily 3, group C, member 1	1.169727	0.3468	0.5822
18212	Ntrk2	neurotrophic tyrosine kinase, receptor, type 2	1.169727	0.09726	0.275
66948	Acad8	acyl-Coenzyme A dehydrogenase family, member 8	1.169727	0.06738	0.2189
68038	Chid1	chitinase domain containing 1	1.169727	0.2695	0.5047
72591	2700022O18Rik	RIKEN cDNA 2700022O18 gene	1.169727	0.2455	0.4793
13423	Dnase2a	deoxyribonuclease II alpha	1.169591	0.4565	0.6801
15365	Hmga2-ps1	high mobility group AT-hook 2, pseudogene 1	1.169591	0.1298	0.329
16904	Gzmm	granzyme M (lymphocyte met-ase 1)	1.169591	0.08935	0.2612
18120	Mrpl49	mitochondrial ribosomal protein L49	1.169591	0.0223	NA
381455	6430573P05Rik	RIKEN cDNA 6430573P05 gene	1.169591	0.03285	NA
68942	Chmp2b	chromatin modifying protein 2B	1.169454	0.04553	0.1719
69563	2310015B20Rik	RIKEN cDNA 2310015B20 gene	1.169454	0.03908	0.1573
71445	5530601H04Rik	RIKEN cDNA 5530601H04 gene	1.169454	0.1839	0.403
257871	Olfr1372-ps1	olfactory receptor 1372, pseudogene 1	1.169454	0.1313	0.3312
11695	Alx4	aristaless-like homeobox 4	1.169317	0.4881	0.7056
110052	Dek	DEK oncogene (DNA binding)	1.169317	0.01888	NA
110637	Grik4	glutamate receptor, ionotropic, kainate 4	1.169317	0.06839	0.2209
76100	5830454E08Rik	RIKEN cDNA 5830454E08 gene	1.16918	0.04765	0.1765
98884	AI225934	expressed sequence AI225934	1.16918	0.3391	0.5747
675985	Rps6-ps2	ribosomal protein S6, pseudogene 2	1.16918	0.08695	0.2569
15452	Hprt	hypoxanthine guanine phosphoribosyl transferase	1.168907	0.04777	0.1768
99426	AW742560	expressed sequence AW742560	1.168907	0.1415	0.3457
19418	Rasgrf2	RAS protein-specific guanine nucleotide-releasing factor 2	1.16877	0.3887	0.6204
67878	Tmem33	transmembrane protein 33	1.16877	0.07549	0.2352
227099	Pms1	postmeiotic segregation increased 1 (S. cerevisiae)	1.16877	0.0705	0.2249
20116	Rps8	ribosomal protein S8	1.168361	0.0915	0.2652
69953	2810025M15Rik	RIKEN cDNA 2810025M15 gene	1.168361	0.09179	0.2655
72349	Dusp3	dual specificity phosphatase 3 (vaccinia virus phosphatase VH1-related)	1.168224	0.4637	0.6863
320244	Ttll5	tubulin tyrosine ligase-like family, member 5	1.168224	0.3252	0.5618
14538	Gcnt2	glucosaminyl (N-acetyl) transferase 2, I-branching enzyme	1.168088	0.08262	0.2479
16880	Lifr	leukemia inhibitory factor receptor	1.168088	0.09697	0.2744
60510	Syt9	synaptotagmin IX	1.167951	0.03048	NA
70040	2610037D02Rik	RIKEN cDNA 2610037D02 gene	1.167951	0.6926	0.8437
399558	Flrt2	fibronectin leucine rich transmembrane protein 2	1.167951	0.01974	NA
14201	Fhl3	four and a half LIM domains 3	1.167815	0.3263	0.5628
244061	Gm4971	predicted gene 4971	1.167815	0.2162	0.445
503692	Aym1	activator of yeast meiotic promoters 1	1.167815	0.2114	0.4389
66357	Ostc	oligosaccharyltransferase complex subunit	1.167679	0.04904	0.1794
67991	Nacc2	nucleus accumbens associated 2, BEN and BTB (POZ) domain containing	1.167679	0.04763	0.1765
16898	Rps2	ribosomal protein S2	1.167542	0.0461	0.1733
230979	Tnfrsf14	tumor necrosis factor receptor superfamily, member 14 (herpesvirus entry mediator)	1.16727	0.6127	0.794
12175	Bnip2	BCL2/adenovirus E1B interacting protein 2	1.166997	0.6646	0.8279
17434	Mocs2	molybdenum cofactor synthesis 2	1.166997	0.08848	0.2598

269784	Cntn4	contactin 4	1.166861	0.2466	0.4807
14390	Gabpa	GA repeat binding protein, alpha	1.166725	0.1707	0.3861
268482	Krt12	keratin 12	1.166725	0.7447	0.8759
623174	Gm6404	predicted gene 6404	1.166725	0.2471	0.4812
12579	Cdkn2b	cyclin-dependent kinase inhibitor 2B (p15, inhibits CDK4)	1.166453	0.2263	0.4578
108857	Ankhd1	ankyrin repeat and KH domain containing 1	1.166453	0.1208	0.3149
252875	Mios	missing oocyte, meiosis regulator, homolog (Drosophila)	1.166453	0.1016	0.2823
18019	Nfatc2	nuclear factor of activated T-cells, cytoplasmic, calcineurin-dependent 2	1.166317	0.3665	0.6019
20265	Scn1a	sodium channel, voltage-gated, type I, alpha	1.166317	0.4148	0.6434
56066	Cxcl11	chemokine (C-X-C motif) ligand 11	1.166317	0.5164	0.7262
100047183	LOC100047183	protein AHNAK2-like	1.166317	0.05294	0.1873
16917	Lmx1b	LIM homeobox transcription factor 1 beta	1.166181	0.4006	0.6309
170756	Slc24a6	solute carrier family 24 (sodium/potassium/calcium exchanger), member 6	1.166181	0.2589	0.4942
321022	Cdv3	carnitine deficiency-associated gene expressed in ventricle 3	1.166181	0.1746	0.3912
545700	Vmn2r-ps14	vomeronasal 2, receptor, pseudogene 14	1.166181	0.2994	0.5351
56358	Copz2	coatomer protein complex, subunit zeta 2	1.166045	0.0936	0.2688
66105	Ube2d3	ubiquitin-conjugating enzyme E2D 3 (UBC4/5 homolog, yeast)	1.166045	0.06698	0.218
108655	Foxp1	forkhead box P1	1.166045	0.1556	0.3654
320701	Fam19a4	family with sequence similarity 19, member A4	1.166045	0.1792	0.3972
17886	Myh9	myosin, heavy polypeptide 9, non-muscle	1.165909	0.1606	0.3724
109700	Itga1	integrin alpha 1	1.165909	0.4161	0.6447
233490	Crebzf	CREB/ATF bZIP transcription factor	1.165909	0.08948	0.2613
16535	Kcnq1	potassium voltage-gated channel, subfamily Q, member 1	1.165773	0.5269	0.7339
57432	Zc3h8	zinc finger CCCH type containing 8	1.165637	0.1752	0.3919
66756	4933411K20Rik	RIKEN cDNA 4933411K20 gene	1.165637	0.03253	NA
80292	Zxdc	ZXD family zinc finger C	1.165637	0.1699	0.3851
628919	Gm6934	predicted gene 6934	1.165637	0.3844	0.6168
18114	Rrp1	ribosomal RNA processing 1 homolog (S. cerevisiae)	1.165501	0.1118	0.3
18798	Plcb4	phospholipase C, beta 4	1.165501	0.2605	0.4959
18087	Nktr	natural killer tumor recognition sequence	1.165365	0.2949	0.5311
668936	Gm14217	predicted gene 14217	1.165365	0.1869	0.4075
100042539	Gm3893	predicted gene 3893	1.165365	0.03402	NA
17215	Mcm3	minichromosome maintenance deficient 3 (S. cerevisiae)	1.16523	0.01498	NA
52589	Ncald	neurocalcin delta	1.16523	0.3046	0.541
353504	Dio3os	deiodinase, iodothyronine type III, opposite strand	1.165094	0.4799	0.6989
100273	Osbpl9	oxysterol binding protein-like 9	1.164958	0.2831	0.5187
19703	Renbp	renin binding protein	1.164822	0.1889	0.4103
108037	Shmt2	serine hydroxymethyltransferase 2 (mitochondrial)	1.164822	0.09111	0.2644
68352	Aspdh	aspartate dehydrogenase domain containing	1.164687	0.173	0.3891
216274	Cep290	centrosomal protein 290	1.164687	0.3231	0.5595
11982	Atp10a	ATPase, class V, type 10A	1.164551	0.1335	0.3339
14081	Acsl1	acyl-CoA synthetase long-chain family member 1	1.164551	0.1793	0.3972
16668	Krt18	keratin 18	1.164551	0.03295	NA
76267	Fads1	fatty acid desaturase 1	1.164415	0.03807	NA
385138	BC061237	cDNA sequence BC061237	1.164415	0.5485	0.7498
59052	Mettl9	methyltransferase like 9	1.16428	0.05466	0.1912
622459	Gm12216	predicted gene 12216	1.16428	0.139	0.3418
15199	Hebp1	heme binding protein 1	1.164144	0.05427	0.1903
67647	4930523C07Rik	RIKEN cDNA 4930523C07 gene	1.164144	0.5977	0.7834

114666	Krtap5-5	keratin associated protein 5-5	1.163873	0.05659	0.1953
243937	Zfp536	zinc finger protein 536	1.163873	0.09144	0.265
382051	Pdp2	pyruvate dehyrogenase phosphatase catalytic subunit 2	1.163873	0.3372	0.5727
71720	Osbpl3	oxysterol binding protein-like 3	1.163738	0.1183	0.3109
77018	Col25a1	collagen, type XXV, alpha 1	1.163738	0.4711	0.6924
19729	Slc50a1	solute carrier family 50 (sugar transporter), member 1	1.163603	0.1686	0.3835
229694	AI504432	expressed sequence AI504432	1.163603	0.2825	0.5179
242960	Fbxl5	F-box and leucine-rich repeat protein 5	1.163603	0.09686	0.2743
13714	Elk4	ELK4, member of ETS oncogene family	1.163332	0.2774	0.5131
71339	5430400D12Rik	RIKEN cDNA 5430400D12 gene	1.163332	0.07344	0.2311
71929	Tmem123	transmembrane protein 123	1.163332	0.008868	NA
78283	Mtap7d2	MAP7 domain containing 2	1.163332	0.1985	0.4237
22409	Wnt10a	wingless related MMTV integration site 10a	1.163061	0.3557	0.5905
54519	Apbb1ip	amyloid beta (A4) precursor protein-binding, family B, member 1 interacting protein	1.163061	0.3262	0.5628
73379	Dcbld2	discoidin, CUB and LCCL domain containing 2	1.163061	0.2789	0.5143
75452	Ascc2	activating signal cointegrator 1 complex subunit 2	1.163061	0.1304	0.33
83922	Tsga14	testis specific gene A14	1.163061	0.3165	0.5532
67313	5730559C18Rik	RIKEN cDNA 5730559C18 gene	1.162926	0.6361	0.8088
13026	Pcyt1a	phosphate cytidylyltransferase 1, choline, alpha isoform	1.162791	0.3219	0.5583
13448	Dok1	docking protein 1	1.162791	0.05352	0.1885
18607	Pdpk1	3-phosphoinositide dependent protein kinase 1	1.162791	0.1502	0.3581
53333	Tomm40	translocase of outer mitochondrial membrane 40 homolog (yeast)	1.162791	0.2285	0.4594
21376	Tbrg1	transforming growth factor beta regulated gene 1	1.162656	0.09336	0.2684
211922	Fam116a	family with sequence similarity 116, member A	1.162656	0.06238	0.2082
217342	Ube2o	ubiquitin-conjugating enzyme E2O	1.162656	0.05225	0.1856
13162	Slc6a3	solute carrier family 6 (neurotransmitter transporter, dopamine), member 3	1.16252	0.5803	0.7724
67474	Snap29	synaptosomal-associated protein 29	1.16252	0.05731	0.1968
18221	Nudc	nuclear distribution gene C homolog (Aspergillus)	1.162385	0.03607	NA
73137	Prrc1	proline-rich coiled-coil 1	1.162385	0.05224	0.1856
385138	BC061237	cDNA sequence BC061237	1.162385	0.3555	0.5903
70791	Hars2	histidyl-tRNA synthetase 2, mitochondrial (putative)	1.162115	0.0663	0.2165
64659	Mrps14	mitochondrial ribosomal protein S14	1.16198	0.01378	NA
54125	Polm	polymerase (DNA directed), mu	1.161845	0.09559	0.2722
22154	Tubb5	tubulin, beta 5	1.16171	0.1683	0.3833
76784	Mtif2	mitochondrial translational initiation factor 2	1.16171	0.383	0.6161
622935	Krtap20-2	keratin associated protein 20-2	1.16171	0.5842	0.7745
69577	Fastkd3	FAST kinase domains 3	1.161575	0.01002	NA
237886	Slfn9	schlafen 9	1.161575	0.4689	0.6909
55938	Apom	apolipoprotein M	1.16144	0.2156	0.4442
229541	Dennd4b	DENN/MADD domain containing 4B	1.16144	0.06256	0.2086
20516	Slc20a2	solute carrier family 20, member 2	1.161305	0.1718	0.3876
208647	Creb3l2	cAMP responsive element binding protein 3-like 2	1.161305	0.2186	0.4478
328099	Prps1l3	phosphoribosyl pyrophosphate synthetase 1-like 3	1.161305	0.06064	0.2047
19142	Prss12	protease, serine, 12 neurotrypsin (motopsin)	1.16117	0.4155	0.6441
80877	Lrba	LPS-responsive beige-like anchor	1.16117	0.1589	0.3701
234094	Arhgef10	Rho guanine nucleotide exchange factor (GEF) 10	1.16117	0.1955	0.4192
67459	Nvl	nuclear VCP-like	1.161036	0.2684	0.5038
246103	Atxn7	ataxin 7	1.161036	0.1955	0.4193
18569	Pdcd4	programmed cell death 4	1.160901	0.3807	0.6143

75805	Nln	neurolysin (metallopeptidase M3 family)	1.160901	0.3972	0.6277
407816	BC023202	cDNA sequence BC023202	1.160901	0.1197	0.3132
13002	Dnajc5	DnaJ (Hsp40) homolog, subfamily C, member 5	1.160766	0.1646	0.378
14088	Fancc	Fanconi anemia, complementation group C	1.160766	0.1995	0.425
22186	Uba52	ubiquitin A-52 residue ribosomal protein fusion product 1	1.160766	0.05896	0.201
70422	Ints2	integrator complex subunit 2	1.160766	0.3434	0.5789
16513	Kcnj10	potassium inwardly-rectifying channel, subfamily J, member 10	1.160631	0.1077	0.2934
16785	Rpsa	ribosomal protein SA	1.160631	0.1346	0.3357
19698	Relb	avian reticuloendotheliosis viral (v-rel) oncogene related B	1.160631	0.1172	0.309
170736	Parvb	parvin, beta	1.160497	0.1004	0.2802
19249	Ptpn13	protein tyrosine phosphatase, non-receptor type 13	1.160362	0.02402	NA
22032	Traf4	TNF receptor associated factor 4	1.160362	0.1614	0.3734
97998	Deptor	DEP domain containing MTOR-interacting protein	1.160362	0.2526	0.4872
213233	Tapbpl	TAP binding protein-like	1.160362	0.1087	0.295
66899	Fip1l1	FIP1 like 1 (S. cerevisiae)	1.160227	0.3559	0.5906
68564	Nufip2	nuclear fragile X mental retardation protein interacting protein 2	1.160227	0.3675	0.6026
83813	Tnk1	tyrosine kinase, non-receptor, 1	1.160227	0.281	0.5162
11908	Atf1	activating transcription factor 1	1.160093	0.2459	0.4797
22158	Tulp3	tubby-like protein 3	1.160093	0.04929	NA
71607	Snx20	sorting nexin 20	1.160093	0.1449	0.3503
218975	Mapk1ip1l	mitogen-activated protein kinase 1 interacting protein 1-like	1.160093	0.5893	0.7784
21454	Tcp1	t-complex protein 1	1.159958	0.07664	0.237
66679	Rae1	RAE1 RNA export 1 homolog (S. pombe)	1.159958	0.0684	0.2209
72083	Mzt2	mitotic spindle organizing protein 2	1.159958	0.03794	NA
116891	Derl2	Der1-like domain family, member 2	1.159958	0.0356	NA
235469	Zfp280d	zinc finger protein 280D	1.159958	0.0562	0.1942
433961	Gm5565	predicted gene 5565	1.159958	0.3018	0.5377
381933	6430531B16Rik	RIKEN cDNA 6430531B16 gene	1.159824	0.1455	0.3509
30945	Rnf19a	ring finger protein 19A	1.159555	0.01321	NA
331623	Bend3	BEN domain containing 3	1.159555	0.148	0.3547
13205	Ddx3x	DEAD/H (Asp-Glu-Ala-Asp/His) box polypeptide 3, X-linked	1.15942	0.174	0.3904
13831	Epc1	enhancer of polycomb homolog 1 (Drosophila)	1.15942	0.1768	0.3938
16847	Lepr	leptin receptor	1.15942	0.5534	0.7534
11431	Acp1	acid phosphatase 1, soluble	1.159152	0.2237	0.4543
14433	Gapdh	glyceraldehyde-3-phosphate dehydrogenase	1.158883	0.1547	0.3646
233335	Synm	synemin, intermediate filament protein	1.158883	0.06919	0.2224
16840	Lect1	leukocyte cell derived chemotaxin 1	1.158749	0.06049	0.2043
72588	2700012I20Rik	RIKEN cDNA 2700012I20 gene	1.158749	0.1317	0.3319
21350	Tal2	T-cell acute lymphocytic leukemia 2	1.158614	0.5821	0.7731
70604	Dnajb14	DnaJ (Hsp40) homolog, subfamily B, member 14	1.15848	0.06374	0.2109
70612	5730494N06Rik	RIKEN cDNA 5730494N06 gene	1.15848	0.01289	NA
71834	Zbtb43	zinc finger and BTB domain containing 43	1.15848	0.1036	0.2858
14158	Fert2	fer (fms/fps related) protein kinase, testis specific 2	1.158346	0.03649	NA
21917	Tmpo	thymopoietin	1.158346	0.1254	0.3224
547150	6820431F20Rik	cadherin 11 pseudogene	1.158346	0.2788	0.5143
15587	Hyal2	hyaluronoglucosaminidase 2	1.158212	0.1776	0.3949
67199	Pfdn1	prefoldin 1	1.158212	0.1294	0.3284
208898	Unc13c	unc-13 homolog C (C. elegans)	1.158212	0.1172	0.309
20444	St3gal2	ST3 beta-galactoside alpha-2,3-sialyltransferase 2	1.158078	0.1604	0.3722

214444	Cdk5rap2	CDK5 regulatory subunit associated protein 2	1.158078	0.3929	0.6241
100502854	LOC100502854	hypothetical LOC100502854	1.158078	0.06943	0.2229
74254	Gpn1	GPN-loop GTPase 1	1.157809	0.1191	0.3121
74318	Hopx	HOP homeobox	1.157809	0.05622	0.1942
219105	Zmym5	zinc finger, MYM-type 5	1.157809	0.2051	0.4314
77578	Bcl9	B-cell CLL/lymphoma 9	1.157675	0.1472	0.3536
230857	Ece1	endothelin converting enzyme 1	1.157675	0.354	0.5888
338350	9330129D05Rik	RIKEN cDNA 9330129D05 gene	1.157675	0.2198	0.4497
433586	Maml3	mastermind like 3 (Drosophila)	1.157675	0.2663	0.5017
19823	Rnf7	ring finger protein 7	1.157541	0.04039	NA
60532	Wtap	Wilms' tumour 1-associating protein	1.157541	0.0216	NA
68431	Fbxl15	F-box and leucine-rich repeat protein 15	1.157541	0.08547	0.2537
105722	Ano6	anoctamin 6	1.157541	0.1299	0.3292
192195	Ash1l	ash1 (absent, small, or homeotic)-like (Drosophila)	1.157541	0.09986	0.279
19652	Rbm3	RNA binding motif protein 3	1.157407	0.4597	0.683
70873	4921517L17Rik	RIKEN cDNA 4921517L17 gene	1.157407	0.1405	0.3442
619937	Gm6115	predicted gene 6115	1.157407	0.2858	0.5211
67397	Erp29	endoplasmic reticulum protein 29	1.157273	0.0105	NA
66932	Rexo1	REX1, RNA exonuclease 1 homolog (S. cerevisiae)	1.15714	0.1256	0.3225
546164	Gm5921	predicted gene 5921	1.15714	0.06603	0.216
70510	Rnf167	ring finger protein 167	1.157006	0.1678	0.3828
259144	Olfr456	olfactory receptor 456	1.157006	0.5868	0.7763
20974	Syngr3	synaptogyrin 3	1.156872	0.2657	0.5014
24135	Zfp68	zinc finger protein 68	1.156872	0.05995	0.2031
30932	Zfp330	zinc finger protein 330	1.156872	0.3328	0.5688
64658	Mrps25	mitochondrial ribosomal protein S25	1.156872	0.02655	NA
80385	Tusc2	tumor suppressor candidate 2	1.156872	0.07787	0.2392
554327	2610042L04Rik	RIKEN cDNA 2610042L04 gene	1.156872	0.6591	0.8238
78695	C030005K06Rik	RIKEN cDNA C030005K06 gene	1.156604	0.4149	0.6435
17087	Ly96	lymphocyte antigen 96	1.15647	0.129	0.3277
69034	4930579G22Rik	RIKEN cDNA 4930579G22 gene	1.15647	0.05086	NA
17878	Myf6	myogenic factor 6	1.156203	0.2464	0.4804
21390	Tbxa2r	thromboxane A2 receptor	1.156203	0.09515	0.2712
21827	Thbs3	thrombospondin 3	1.156203	0.1398	0.3432
100515	Zfp518b	zinc finger protein 518B	1.156069	0.2545	0.4892
100503185	LOC100503185	BTB/POZ domain-containing protein 8-like	1.156069	0.2648	0.5008
52276	Cdca8	cell division cycle associated 8	1.155936	0.2953	0.5311
71772	Plbd2	phospholipase B domain containing 2	1.155936	0.1266	0.324
238799	Tnpo1	transportin 1	1.155936	0.09561	0.2722
319862	E230008O15Rik	RIKEN cDNA E230008O15 gene	1.155936	0.388	0.6199
12992	Csn1s2b	casein alpha s2-like B	1.155669	0.7013	0.8494
93878	Pcdhb7	protocadherin beta 7	1.155669	0.03433	NA
319169	Hist1h2ak	histone cluster 1, H2ak	1.155535	0.04601	NA
101502	Hsd3b7	hydroxy-delta-5-steroid dehydrogenase, 3 beta- and steroid delta-isomerase 7	1.155402	0.04533	NA
110596	Rgnef	Rho-guanine nucleotide exchange factor	1.155402	0.1937	0.417
212281	A530054K11Rik	RIKEN cDNA A530054K11 gene	1.155402	0.2889	0.525
12479	Cd1d1	CD1d1 antigen	1.155268	0.03136	NA
17188	Maz	MYC-associated zinc finger protein (purine-binding transcription factor)	1.154868	0.319	0.5554
68957	Paqr6	progestin and adipoQ receptor family member VI	1.154734	0.3933	0.6244

110954	Rpl10	ribosomal protein 10	1.154734	0.01917	NA
224024	Scarf2	scavenger receptor class F, member 2	1.154734	0.1694	0.3845
17974	Nck2	non-catalytic region of tyrosine kinase adaptor protein 2	1.154601	0.1421	0.3463
27404	Abca8b	ATP-binding cassette, sub-family A (ABC1), member 8b	1.154601	0.0218	NA
14546	Gdap10	ganglioside-induced differentiation-associated-protein 10	1.154335	0.1535	0.3631
56275	Rbm14	RNA binding motif protein 14	1.154335	0.391	0.6228
77756	A230101C19Rik	RIKEN cDNA A230101C19 gene	1.154335	0.4196	0.6475
217558	G2e3	G2/M-phase specific E3 ubiquitin ligase	1.154335	0.3739	0.6082
234796	Klhl36	kelch-like 36 (Drosophila)	1.154335	0.07225	0.2285
666173	Vps13b	vacuolar protein sorting 13B (yeast)	1.154335	0.1289	0.3276
21752	Tert	telomerase reverse transcriptase	1.154201	0.6066	0.7895
330812	Rnf150	ring finger protein 150	1.154201	0.1334	0.3339
13024	Ctla2a	cytotoxic T lymphocyte-associated protein 2 alpha	1.154068	0.1058	0.2899
70757	Ptplb	protein tyrosine phosphatase-like (proline instead of catalytic arginine), member b	1.154068	0.05626	NA
241226	Itga8	integrin alpha 8	1.154068	0.1706	0.3861
677044	Gm10653	ribsomal protein S2 pseudogene	1.153935	0.06463	0.2128
13079	Cyp21a1	cytochrome P450, family 21, subfamily a, polypeptide 1	1.153669	0.5702	0.7642
20349	Sema3e	sema domain, immunoglobulin domain (Ig), short basic domain, secreted, (semaphorin) 3E	1.153669	0.1654	0.3792
56706	Ccnl1	cyclin L1	1.153669	0.09245	0.2665
620155	Gm6133	60S ribosomal protein L17 pseudogene	1.153669	0.1358	0.3374
52392	D1Ertd622e	DNA segment, Chr 1, ERATO Doi 622, expressed	1.153536	0.005201	NA
69752	Zfp511	zinc finger protein 511	1.153536	0.2219	0.4523
73183	5430402O13Rik	RIKEN cDNA 5430402O13 gene	1.153536	0.02407	NA
17191	Mbd2	methyl-CpG binding domain protein 2	1.153403	0.07815	0.2397
382010	BC088983	cDNA sequence BC088983	1.15327	0.3655	0.6011
619937	Gm6115	predicted gene 6115	1.15327	0.4737	0.6945
70425	Csnk1g3	casein kinase 1, gamma 3	1.153137	0.3096	0.5462
218772	Rarb	retinoic acid receptor, beta	1.153137	0.04922	NA
546336	Prrg1	proline rich Gla (G-carboxyglutamic acid) 1	1.153137	0.1852	0.4048
664858	BB094273	expressed sequence BB094273	1.153137	0.4844	0.7029
21917	Tmpo	thymopoietin	1.152871	0.05086	NA
12493	Cd37	CD37 antigen	1.152738	0.6186	0.7978
21915	Dtymk	deoxythymidylate kinase	1.152738	0.07769	0.239
208777	Sned1	sushi, nidogen and EGF-like domains 1	1.152738	0.6136	0.7948
238021	Fscn2	fascin homolog 2, actin-bundling protein, retinal (Strongylocentrotus purpuratus)	1.152738	0.03386	NA
329977	Fhad1	forkhead-associated (FHA) phosphopeptide binding domain 1	1.152738	0.3433	0.5787
382097	Gm1123	predicted gene 1123	1.152738	0.3296	0.5662
69993	Chn2	chimerin (chimaerin) 2	1.152605	0.09532	0.2716
210711	Mcmbp	MCM (minichromosome maintenance deficient) binding protein	1.152472	0.02067	NA
14451	Gas1	growth arrest specific 1	1.152339	0.09629	0.2733
14865	Gstm4	glutathione S-transferase, mu 4	1.152339	0.1906	0.4128
66176	Nat9	N-acetyltransferase 9 (GCN5-related, putative)	1.152339	0.07757	0.2389
319673	9330159M07Rik	RIKEN cDNA 9330159M07 gene	1.152339	0.09276	0.2673
384179	Gm5292	predicted gene 5292	1.152339	0.1609	0.3726
17951	Naip5	NLR family, apoptosis inhibitory protein 5	1.152206	0.2472	0.4813
70369	Bag5	BCL2-associated athanogene 5	1.152206	0.05537	NA
232889	Pla2g4c	phospholipase A2, group IVC (cytosolic, calcium-independent)	1.152206	0.611	0.7928
75690	Vsig10l	ZV-set and immunoglobulin domain containing 10 like	1.152074	0.2174	0.4464
17772	Mtm1	X-linked myotubular myopathy gene 1	1.151941	0.2942	0.5304

20364	Sepw1	selenoprotein W, muscle 1	1.151941	0.1432	0.3481
170459	Stard4	StAR-related lipid transfer (START) domain containing 4	1.151941	0.2003	0.4258
218952	Fermt2	fermitin family homolog 2 (Drosophila)	1.151941	0.134	0.3346
27267	Cars	cysteinyl-tRNA synthetase	1.151808	0.1558	0.3656
54123	Irf7	interferon regulatory factor 7	1.151808	0.4168	0.645
235504	Slc17a5	solute carrier family 17 (anion/sugar transporter), member 5	1.151808	0.1568	0.367
52428	Rhpn2	rhophilin, Rho GTPase binding protein 2	1.151676	0.06153	NA
108015	Chrnb4	cholinergic receptor, nicotinic, beta polypeptide 4	1.151676	0.1857	0.4056
108989	Tpr	translocated promoter region	1.151676	0.5785	0.7708
235302	D630033O11Rik	RIKEN cDNA D630033O11 gene	1.151676	0.1244	0.321
56637	Gsk3b	glycogen synthase kinase 3 beta	1.151543	0.1433	0.3482
234878	BC021891	cDNA sequence BC021891	1.151543	0.5038	0.7165
236069	Gm13238	predicted gene 13238	1.151543	0.1989	0.4241
434825	LOC434825	hypothetical LOC434825	1.151543	0.1902	0.4121
18802	Plcd4	phospholipase C, delta 4	1.15141	0.1194	0.3127
380924	Olfm4	olfactomedin 4	1.15141	0.3052	0.5417
21824	Thbd	thrombomodulin	1.151278	0.2031	0.4293
13177	Dci	dodecenoyl-Coenzyme A delta isomerase (3,2 trans-enoyl-Coenyme A isomerase)	1.151145	0.04245	NA
16828	Ldha	lactate dehydrogenase A	1.151145	0.1475	0.3541
28077	Med10	mediator of RNA polymerase II transcription, subunit 10 homolog (NUT2, S. cerevisiae)	1.151145	0.09506	0.2711
214812	Zfp609	zinc finger protein 609	1.151013	0.4246	0.6522
244421	Lonrf1	LON peptidase N-terminal domain and ring finger 1	1.151013	0.1157	0.3065
13002	Dnajc5	DnaJ (Hsp40) homolog, subfamily C, member 5	1.15088	0.5495	0.7506
16522	Kcnj6	potassium inwardly-rectifying channel, subfamily J, member 6	1.15088	0.1819	0.4007
19266	Ptprd	protein tyrosine phosphatase, receptor type, D	1.15088	0.5372	0.7417
69537	Dnase1l1	deoxyribonuclease 1-like 1	1.15088	0.2191	0.4485
58222	Rab37	RAB37, member of RAS oncogene family	1.150748	0.1567	0.3668
67912	1600012H06Rik	RIKEN cDNA 1600012H06 gene	1.150748	0.09847	0.277
75995	5033417F24Rik	RIKEN cDNA 5033417F24 gene	1.150748	0.2366	0.4689
226551	AI848100	expressed sequence AI848100	1.150748	0.1406	0.3443
240476	Zfp407	zinc finger protein 407	1.150748	0.387	0.6191
27078	B9d1	B9 protein domain 1	1.150616	0.005372	NA
76633	1700112E06Rik	RIKEN cDNA 1700112E06 gene	1.150616	0.1098	0.2969
66616	Snx9	sorting nexin 9	1.150483	0.2096	0.4366
381810	Lpar5	lysophosphatidic acid receptor 5	1.150483	0.4183	0.6463
20588	Smarcc1	SWI/SNF related, matrix associated, actin dependent regulator of chromatin, subfamily c, member 1	1.150219	0.08902	0.2608
69444	Lyzl6	lysozyme-like 6	1.150219	0.4162	0.6448
246710	Rhobtb2	Rho-related BTB domain containing 2	1.150219	0.05976	NA
100040852	Gm3002	alpha-takusan pseudogene	1.150219	0.5472	0.7488
71729	Rgs12	regulator of G-protein signaling 12	1.150086	0.421	0.6488
27965	Spg21	spastic paraplegia 21 homolog (human)	1.149954	0.1164	0.3078
73910	Arhgap18	Rho GTPase activating protein 18	1.149954	0.03952	NA
216527	Ccm2	cerebral cavernous malformation 2 homolog (human)	1.149954	0.01968	NA
229694	AI504432	expressed sequence AI504432	1.149954	0.3318	0.5679
404337	Olfr1383	olfactory receptor 1383	1.149954	0.2485	0.4827
12390	Cav2	caveolin 2	1.149822	0.3099	0.5464
14071	F9	coagulation factor IX	1.149822	0.5618	0.7602
21894	Tln1	talin 1	1.14969	0.05743	NA
74023	Rd3	retinal degeneration 3	1.14969	0.2069	0.433

77613	Prss36	protease, serine, 36	1.14969	0.09957	0.2785
100978	Nfxl1	nuclear transcription factor, X-box binding-like 1	1.14969	0.4968	0.7118
100504173	LOC100504173	hypothetical protein LOC100504173	1.14969	0.3961	0.6266
13163	Daxx	Fas death domain-associated protein	1.149557	0.1043	0.2871
240058	Cpne5	copine V	1.149557	0.3727	0.6069
12043	Bcl2	B-cell leukemia/lymphoma 2	1.149425	0.2528	0.4874
56398	1500003O03Rik	RIKEN cDNA 1500003O03 gene	1.149425	0.1236	0.32
64654	Fgf23	fibroblast growth factor 23	1.149425	0.3099	0.5464
69219	Ddah1	dimethylarginine dimethylaminohydrolase 1	1.149425	0.1476	0.3542
320878	Mical2	microtubule associated monoxygenase, calponin and LIM domain containing 2	1.149425	0.2055	0.4317
414085	9330151L19Rik	RIKEN cDNA 9330151L19 gene	1.149425	0.0301	NA
73242	Atat1	alpha tubulin acetyltransferase 1	1.149293	0.03289	NA
227449	Zcchc2	zinc finger, CCHC domain containing 2	1.149293	0.2171	0.4461
230899	Nppa	natriuretic peptide type A	1.149293	0.7598	0.8836
242585	Slc35d1	solute carrier family 35 (UDP-glucuronic acid/UDP-N-acetylgalactosamine dual transporter), member D1	1.149293	0.1205	0.3143
237459	Cdk17	cyclin-dependent kinase 17	1.149161	0.1848	0.4042
319173	Hist1h2af	histone cluster 1, H2af	1.149161	0.06814	NA
12660	Chka	choline kinase alpha	1.149029	0.2755	0.511
54630	Prickle3	prickle homolog 3 (Drosophila)	1.149029	0.08199	0.2468
73192	Xpot	exportin, tRNA (nuclear export receptor for tRNAs)	1.149029	0.4117	0.6407
52478	D6Ertd131e	DNA segment, Chr 6, ERATO Doi 131, expressed	1.148897	0.1139	0.3037
26400	Map2k7	mitogen-activated protein kinase kinase 7	1.148765	0.179	0.3971
60440	Iigp1	interferon inducible GTPase 1	1.148765	0.5543	0.7539
110595	Timp4	tissue inhibitor of metalloproteinase 4	1.148765	0.2492	0.4834
14758	Gpm6b	glycoprotein m6b	1.148633	0.0964	0.2736
319887	E030030I06Rik	RIKEN cDNA E030030I06 gene	1.148633	0.05551	NA
330490	Nlrp9c	NLR family, pyrin domain containing 9C	1.148633	0.1986	0.4238
11431	Acp1	acid phosphatase 1, soluble	1.148501	0.1223	0.3177
14670	Gnl1	guanine nucleotide binding protein-like 1	1.148501	0.01059	NA
70568	Cpne3	copine III	1.148501	0.3391	0.5747
67885	1500011K16Rik	RIKEN cDNA 1500011K16 gene	1.148369	0.02388	NA
72343	2600002B07Rik	RIKEN cDNA 2600002B07 gene	1.148369	0.4513	0.6761
117599	Helb	helicase (DNA) B	1.148369	0.07792	0.2393
16599	Klf3	Kruppel-like factor 3 (basic)	1.148237	0.08269	0.248
21353	Tank	TRAF family member-associated Nf-kappa B activator	1.148237	0.5381	0.7425
100038385	F830115B05Rik	RIKEN cDNA F830115B05 gene	1.148237	0.4346	0.6616
229488	Fam160a1	family with sequence similarity 160, member A1	1.148106	0.2647	0.5006
12348	Car11	carbonic anhydrase 11	1.147974	0.08102	0.2448
69707	Iqcg	IQ motif containing G	1.147974	0.1626	0.3748
83383	Tcfap4	transcription factor AP4	1.147974	0.03081	NA
68394	Ccdc163	coiled-coil domain containing 163	1.147842	0.2153	0.4439
71584	Gdpd2	glycerophosphodiester phosphodiesterase domain containing 2	1.147842	0.1019	0.2826
71274	4933433G15Rik	RIKEN cDNA 4933433G15 gene	1.14771	0.6751	0.8334
83962	Btbd1	BTB (POZ) domain containing 1	1.14771	0.3077	0.544
100604	Lrrc8c	leucine rich repeat containing 8 family, member C	1.14771	0.2249	0.4559
101148	B630005N14Rik	RIKEN cDNA B630005N14 gene	1.14771	0.2152	0.4438
331401	Thoc2	THO complex 2	1.14771	0.4934	0.7095
20823	Ssb	Sjogren syndrome antigen B	1.147579	0.2705	0.5056
83679	Pde4dip	phosphodiesterase 4D interacting protein (myomegalin)	1.147447	0.6509	0.8186

12144	Blm	Bloom syndrome, RecQ helicase-like	1.147315	0.09692	0.2743
29876	Clic4	chloride intracellular channel 4 (mitochondrial)	1.147315	0.143	0.3479
320919	A230107N01Rik	RIKEN cDNA A230107N01 gene	1.147315	0.2526	0.4872
56534	Hspb3	heat shock protein 3	1.147184	0.2988	0.5348
545198	LOC545198	hypothetical LOC545198	1.147184	0.1204	0.3143
666043	Gm7904	predicted gene 7904	1.147052	0.3842	0.6166
54725	Cadm1	cell adhesion molecule 1	1.146921	0.1057	0.2895
209039	Tenc1	tensin like C1 domain-containing phosphatase	1.146921	0.304	0.5403
58238	Fam181b	family with sequence similarity 181, member B	1.146789	0.1492	0.3568
384482	Gm5316	predicted gene 5316	1.146789	0.1084	0.2945
72012	1600020E01Rik	RIKEN cDNA 1600020E01 gene	1.146657	0.07224	NA
207592	Tbc1d16	TBC1 domain family, member 16	1.146657	0.3776	0.6115
226849	Ppp2r5a	protein phosphatase 2, regulatory subunit B (B56), alpha isoform	1.146657	0.0573	NA
269608	Plekhg5	pleckstrin homology domain containing, family G (with RhoGef domain) member 5	1.146657	0.3183	0.5547
20476	Six6	sine oculis-related homeobox 6 homolog (Drosophila)	1.146526	0.1251	0.322
21354	Tap1	transporter 1, ATP-binding cassette, sub-family B (MDR/TAP)	1.146526	0.4129	0.6417
56838	Ccl28	chemokine (C-C motif) ligand 28	1.146395	0.421	0.6488
59050	Nsa2	NSA2 ribosome biogenesis homolog (S. cerevisiae)	1.146395	0.2512	0.4855
103554	Psme4	proteasome (prosome, macropain) activator subunit 4	1.146395	0.3079	0.5442
232566	Amn1	antagonist of mitotic exit network 1 homolog (S. cerevisiae)	1.146395	0.3237	0.5603
414098	C230096K16Rik	RIKEN cDNA C230096K16 gene	1.146395	0.1933	0.4165
214579	Aldh5a1	aldhehyde dehydrogenase family 5, subfamily A1	1.146263	0.09206	0.2659
56223	Fscn3	fascin homolog 3, actin-bundling protein, testicular (Strongylocentrotus purpuratus)	1.146132	0.3327	0.5688
56708	Clcf1	cardiotrophin-like cytokine factor 1	1.146132	0.2261	0.4575
74140	Tm9sf1	transmembrane 9 superfamily member 1	1.145869	0.2276	0.4586
226499	BC003331	cDNA sequence BC003331	1.145869	0.2308	0.4621
319314	A930001C03Rik	RIKEN cDNA A930001C03 gene	1.145869	0.6051	0.7884
329416	Nostrin	nitric oxide synthase trafficker	1.145869	0.1881	0.4091
17319	Mif	macrophage migration inhibitory factor	1.145738	0.3326	0.5687
20132	Rrh	retinal pigment epithelium derived rhodopsin homolog	1.145738	0.2409	0.4736
21968	Tom1	target of myb1 homolog (chicken)	1.145738	0.3176	0.5539
13589	Mapre1	microtubule-associated protein, RP/EB family, member 1	1.145607	0.05637	NA
17172	Ascl1	achaete-scute complex homolog 1 (Drosophila)	1.145607	0.1863	0.4065
105377	Ankrd32	ankyrin repeat domain 32	1.145607	0.1253	0.3222
319167	Hist1h2ag	histone cluster 1, H2ag	1.145607	0.05423	NA
24084	Tekt2	tektin 2	1.145475	0.1592	0.3704
17364	Trpm1	transient receptor potential cation channel, subfamily M, member 1	1.145344	0.4236	0.6513
68481	Mpzl1	myelin protein zero-like 1	1.145344	0.04841	NA
20102	Rps4x	ribosomal protein S4, X-linked	1.145082	0.1405	0.3442
73049	2900054C01Rik	RIKEN cDNA 2900054C01 gene	1.145082	0.1308	0.3306
224705	Vps52	vacuolar protein sorting 52 (yeast)	1.145082	0.06341	NA
332397	Nanos1	nanos homolog 1 (Drosophila)	1.145082	0.1727	0.3887
67429	Nudcd1	NudC domain containing 1	1.144951	0.1318	0.332
74519	Cyp2j9	cytochrome P450, family 2, subfamily j, polypeptide 9	1.144951	0.04053	NA
69928	Apitd1	apoptosis-inducing, TAF9-like domain 1	1.14482	0.1547	0.3646
101187	Parp11	poly (ADP-ribose) polymerase family, member 11	1.144689	0.2662	0.5016
15191	Hdgf	hepatoma-derived growth factor	1.144558	0.2075	0.434
18700	Piga	phosphatidylinositol glycan anchor biosynthesis, class A	1.144558	0.06142	NA
74191	P2ry13	purinergic receptor P2Y, G-protein coupled 13	1.144558	0.4024	0.6323

81840	Sorcs2	sortilin-related VPS10 domain containing receptor 2	1.144558	0.4721	0.6932
13709	Elf1	E74-like factor 1	1.144296	0.1399	0.3432
16855	Lgals4	lectin, galactose binding, soluble 4	1.144165	0.361	0.5958
19883	Rora	RAR-related orphan receptor alpha	1.144165	0.1664	0.3806
27176	Rpl7a	ribosomal protein L7A	1.144165	0.1688	0.3839
216799	Nlrp3	NLR family, pyrin domain containing 3	1.144034	0.6381	0.81
67956	Setd8	SET domain containing (lysine methyltransferase) 8	1.143903	0.05575	NA
216965	Taok1	TAO kinase 1	1.143903	0.04593	NA
231050	Galnt11	UDP-N-acetyl-alpha-D-galactosamine:polypeptide N-acetylgalactosaminyltransferase 11	1.143903	0.1988	0.4241
104248	Cabin1	calcineurin binding protein 1	1.143772	0.3793	0.613
623172	Gm6403	predicted gene 6403	1.143772	0.4658	0.6876
102626	Mapkapk3	mitogen-activated protein kinase-activated protein kinase 3	1.143641	0.2377	0.4699
11799	Birc5	baculoviral IAP repeat-containing 5	1.143511	0.3513	0.5867
59030	Mkks	McKusick-Kaufman syndrome protein	1.143511	0.0553	NA
74062	Speer8-ps1	spermatogenesis associated glutamate (E)-rich protein 8, pseudogene 1	1.143511	0.3489	0.5848
74277	Chic2	cysteine-rich hydrophobic domain 2	1.143511	0.2632	0.4989
15461	Hras1	Harvey rat sarcoma virus oncogene 1	1.14338	0.344	0.5794
17191	Mbd2	methyl-CpG binding domain protein 2	1.14338	0.01245	NA
320244	Ttll5	tubulin tyrosine ligase-like family, member 5	1.14338	0.07518	NA
545459	Gm10766	predicted gene 10766	1.14338	0.1824	0.4013
20502	Slc16a2	solute carrier family 16 (monocarboxylic acid transporters), member 2	1.143249	0.05302	NA
76646	Wdr38	WD repeat domain 38	1.143249	0.4163	0.6449
211484	Tsga10	testis specific 10	1.143249	0.1541	0.3637
100046950	LOC100046950	hypothetical LOC100046950	1.143249	0.5016	0.7148
13207	Ddx5	DEAD (Asp-Glu-Ala-Asp) box polypeptide 5	1.143118	0.5246	0.732
57756	Fhl5	four and a half LIM domains 5	1.142857	0.4789	0.6985
319480	Itga11	integrin alpha 11	1.142857	0.3062	0.5428
22255	Uncx	UNC homeobox	1.142727	0.249	0.4832
408192	Gm9839	predicted gene 9839	1.142727	0.4776	0.6976
665211	Gm14326	predicted gene 14326	1.142727	0.4767	0.6966
12325	Camk2g	calcium/calmodulin-dependent protein kinase II gamma	1.142596	0.06921	NA
17167	Marco	macrophage receptor with collagenous structure	1.142596	0.4997	0.7143
27411	Slc14a2	solute carrier family 14 (urea transporter), member 2	1.142596	0.7394	0.8727
193742	Abhd16a	abhydrolase domain containing 16A	1.142596	0.136	0.3377
15464	Hrc	histidine rich calcium binding protein	1.142465	0.06861	NA
14433	Gapdh	glyceraldehyde-3-phosphate dehydrogenase	1.142335	0.3113	0.5477
66662	5730577I03Rik	zinc finger protein pseudogene	1.142335	0.3279	0.5643
74365	Lonrf3	LON peptidase N-terminal domain and ring finger 3	1.142335	0.07097	NA
93686	Rbfox2	RNA binding protein, fox-1 homolog (C. elegans) 2	1.142335	0.1637	0.3767
225020	Fez2	fasciculation and elongation protein zeta 2 (zygin II)	1.142335	0.03279	NA
319934	Sbf2	SET binding factor 2	1.142335	0.458	0.6814
27528	D0H4S114	DNA segment, human D4S114	1.142204	0.2447	0.4784
68672	1110035E04Rik	RIKEN cDNA 1110035E04 gene	1.142204	0.2834	0.5192
235086	Igsf9b	immunoglobulin superfamily, member 9B	1.142204	0.5264	0.7336
330938	Dixdc1	DIX domain containing 1	1.142204	0.4391	0.665
50774	Krtap5-1	keratin associated protein 5-1	1.142074	0.6023	0.7866
67382	Brd3	bromodomain containing 3	1.142074	0.3602	0.5951
71853	Pdia6	protein disulfide isomerase associated 6	1.142074	0.03669	NA
102093	Phkb	phosphorylase kinase beta	1.142074	0.3335	0.5693

234757	BC024137	cDNA sequence BC024137	1.141944	0.4255	0.6529
100034361	Mfap1b	microfibrillar-associated protein 1B	1.141944	0.5084	0.7198
13057	Cyba	cytochrome b-245, alpha polypeptide	1.141813	0.06162	NA
14276	Folr2	folate receptor 2 (fetal)	1.141813	0.4432	0.669
15458	Hpx	hemopexin	1.141813	0.2954	0.5312
15968	Ifna5	interferon alpha 5	1.141813	0.566	0.762
19698	Relb	avian reticuloendotheliosis viral (v-rel) oncogene related B	1.141813	0.1701	0.3853
71720	Osbpl3	oxysterol binding protein-like 3	1.141813	0.137	0.3387
321006	Vprbp	Vpr (HIV-1) binding protein	1.141813	0.2632	0.4989
100038433	9130213A22Rik	RIKEN cDNA 9130213A22 gene	1.141813	0.4618	0.6847
66510	Rnf181	ring finger protein 181	1.141683	0.1396	0.3429
433586	Maml3	mastermind like 3 (Drosophila)	1.141683	0.2715	0.507
108012	Ap1s2	adaptor-related protein complex 1, sigma 2 subunit	1.141553	0.307	0.5433
93722	Pcdhga10	protocadherin gamma subfamily A, 10	1.141422	0.2254	0.4565
242022	Frem2	Fras1 related extracellular matrix protein 2	1.141422	0.05051	NA
93887	Pcdhb16	protocadherin beta 16	1.141292	0.08432	NA
271047	Serpina3b	serine (or cysteine) peptidase inhibitor, clade A, member 3B	1.141292	0.4407	0.6666
433171	Gm10549	predicted gene 10549	1.141292	0.3161	0.5529
791088	C630016N16Rik	RIKEN cDNA C630016N16 gene	1.141292	0.1327	0.3333
12977	Csf1	colony stimulating factor 1 (macrophage)	1.141162	0.61	0.792
16898	Rps2	ribosomal protein S2	1.141162	0.2235	0.454
103220	BC030307	cDNA sequence BC030307	1.141162	0.2595	0.4946
100503311	1700027A23Rik	RIKEN cDNA 1700027A23 gene	1.141162	0.2413	0.4741
53378	Sdcbp	syndecan binding protein	1.141031	0.1276	0.3255
68691	1110028C15Rik	RIKEN cDNA 1110028C15 gene	1.141031	0.1243	0.321
68836	Mrpl52	mitochondrial ribosomal protein L52	1.141031	0.1457	0.3511
114602	Zmynd10	zinc finger, MYND domain containing 10	1.141031	0.3752	0.6092
18439	P2rx7	purinergic receptor P2X, ligand-gated ion channel, 7	1.140901	0.7074	0.8532
20163	Rsu1	Ras suppressor protein 1	1.140901	0.04621	NA
70717	6330406I15Rik	RIKEN cDNA 6330406I15 gene	1.140901	0.2417	0.4747
319370	Fam100b	family with sequence similarity 100, member B	1.140901	0.04696	NA
384864	Gm1943	WD repeat domain 70 pseudogene	1.140901	0.0949	0.2709
67399	Pdlim7	PDZ and LIM domain 7	1.140771	0.1195	0.3129
77994	2810055G20Rik	RIKEN cDNA 2810055G20 gene	1.140771	0.2621	0.4973
105083	Pelo	pelota homolog (Drosophila)	1.140771	0.02899	NA
109264	Me3	malic enzyme 3, NADP(+)-dependent, mitochondrial	1.140771	0.08973	NA
11974	Atp6v0e	ATPase, H+ transporting, lysosomal V0 subunit E	1.140641	0.03081	NA
14431	Gamt	guanidinoacetate methyltransferase	1.140641	0.3325	0.5686
22631	Ywhaz	tyrosine 3-monooxygenase/tryptophan 5-monooxygenase activation protein, zeta polypeptide	1.140641	0.4825	0.7012
23794	Adamts5	a disintegrin-like and metallopeptidase (reprolysin type) with thrombospondin type 1 motif, 5 (aggrecanase-2)	1.140641	0.2177	0.4468
53975	Ddx20	DEAD (Asp-Glu-Ala-Asp) box polypeptide 20	1.140641	0.4902	0.7072
66609	Cryzl1	crystallin, zeta (quinone reductase)-like 1	1.140641	0.09802	0.2761
76850	Eif2c4	eukaryotic translation initiation factor 2C, 4	1.140641	0.1216	0.3165
93883	Pcdhb12	protocadherin beta 12	1.140641	0.08293	NA
209478	Tbc1d12	TBC1D12: TBC1 domain family, member 12	1.140641	0.4249	0.6525
56484	Foxo3	forkhead box O3	1.140511	0.1454	0.3508
103817	AI662501	expressed sequence AI662501	1.140511	0.1243	0.321
20335	Sec61g	SEC61, gamma subunit	1.140381	0.1432	0.3481
71458	Bcor	BCL6 interacting corepressor	1.140381	0.1607	0.3725

76987	Hdhd2		haloacid dehalogenase-like hydrolase domain containing 2	1.140381	0.403	0.633
432971	A130088B03Rik		RIKEN cDNA A130088B03 gene	1.140381	0.2975	0.5334
545363	Gm5833		predicted gene 5833	1.140381	0.4548	0.6784
22320	Vamp8		vesicle-associated membrane protein 8	1.140251	0.07716	NA
22722	Zfp64		zinc finger protein 64	1.140121	0.06396	NA
73172	Dem1		defects in morphology 1 homolog (S. cerevisiae)	1.140121	0.08502	NA
232157	Mobkl1b		MOB1, Mps One Binder kinase activator-like 1B (yeast)	1.140121	0.1814	0.4001
27278	Clnk		cytokine-dependent hematopoietic cell linker	1.139991	0.4978	0.7127
67771	Arpc5		actin related protein 2/3 complex, subunit 5	1.139991	0.2064	0.4326
17151	Ccndbp1		cyclin D-type binding-protein 1	1.139861	0.1101	0.2974
17709	COX2		cytochrome c oxidase subunit II	1.139861	0.1856	0.4054
226548	Aph1a		anterior pharynx defective 1a homolog (C. elegans)	1.139861	0.1021	0.283
432770	Rslcan18		regulator of sex-limitation candidate 18	1.139861	0.1902	0.4121
100303732	Gm14431		predicted gene 14431	1.139861	0.1864	0.4067
58178	Sorcs1		VPS10 domain receptor protein SORCS 1	1.139601	0.1847	0.4041
67248	Rpl39		ribosomal protein L39	1.139601	0.1299	0.3292
76947	2310030N02Rik		RIKEN cDNA 2310030N02 gene	1.139601	0.03484	NA
223527	Eny2		enhancer of yellow 2 homolog (Drosophila)	1.139601	0.0626	NA
16495	Kcna7		potassium voltage-gated channel, shaker-related subfamily, member 7	1.139471	0.4228	0.6507
67005	Polr3k		polymerase (RNA) III (DNA directed) polypeptide K	1.139471	0.1267	0.3241
654469	LOC654469		hypothetical LOC654469	1.139471	0.4407	0.6666
78655	Eif3j		eukaryotic translation initiation factor 3, subunit J	1.139341	0.4939	0.7098
100040322	3830408C21Rik		RIKEN cDNA 3830408C21 gene	1.139341	0.1087	0.295
57438		Mar-07	membrane-associated ring finger (C3HC4) 7	1.139212	0.1019	0.2827
71949	Lass5		LAG1 homolog, ceramide synthase 5	1.139212	0.04082	NA
101095	Zfp282		zinc finger protein 282	1.139212	0.1845	0.4037
15078	H3f3a		H3 histone, family 3A	1.139082	0.1761	0.3931
56224	Tspan5		tetraspanin 5	1.139082	0.1676	0.3827
66379	2310016M24Rik		RIKEN cDNA 2310016M24 gene	1.139082	0.07408	NA
50918	Myadm		myeloid-associated differentiation marker	1.138952	0.09508	NA
67247	Mosc2		MOCO sulphurase C-terminal domain containing 2	1.138952	0.03738	NA
211006	Sepsecs		Sep (O-phosphoserine) tRNA:Sec (selenocysteine) tRNA synthase	1.138952	0.266	0.5015
66880	Rsrc1		arginine/serine-rich coiled-coil 1	1.138822	0.2454	0.4792
381823	Apold1		apolipoprotein L domain containing 1	1.138822	0.1863	0.4065
67760	Slc38a2		solute carrier family 38, member 2	1.138693	0.1132	0.3025
69123	1810022C23Rik		RIKEN cDNA 1810022C23 gene	1.138693	0.05612	NA
211577	Mrgprf		MAS-related GPR, member F	1.138693	0.4984	0.713
12916	Crem		cAMP responsive element modulator	1.138563	0.1621	0.3743
59047	Pnkp		polynucleotide kinase 3'- phosphatase	1.138563	0.03449	NA
209361	Taf3		TAF3 RNA polymerase II, TATA box binding protein (TBP)-associated factor	1.138563	0.03517	NA
210925	Ints9		integrator complex subunit 9	1.138563	0.3002	0.536
22632	Yy1		YY1 transcription factor	1.138434	0.04427	NA
666445	Gm13035		predicted gene 13035	1.138434	0.132	0.3324
102545	Cmtm7		CKLF-like MARVEL transmembrane domain containing 7	1.138304	0.2823	0.5176
14950	H13		histocompatibility 13	1.138174	0.1635	0.3763
18753	Prkcd		protein kinase C, delta	1.138174	0.09226	NA
66230	Mrps28		mitochondrial ribosomal protein S28	1.138174	0.08934	NA
224912	Crb3		crumbs homolog 3 (Drosophila)	1.138045	0.3911	0.6229
14869	Gstp2		glutathione S-transferase, pi 2	1.137915	0.08568	NA

57394	Tmem27	transmembrane protein 27	1.137915	0.09762	NA
73998	Herc3	hect domain and RLD 3	1.137915	0.1557	0.3655
77207	8030425K09Rik	RIKEN cDNA 8030425K09 gene	1.137915	0.1326	0.3332
100039781	Hrct1	histidine rich carboxyl terminus 1	1.137915	0.2852	0.5205
12340	Capza1	capping protein (actin filament) muscle Z-line, alpha 1	1.137786	0.06707	NA
14579	Gem	GTP binding protein (gene overexpressed in skeletal muscle)	1.137786	0.1789	0.397
17984	Ndn	necdin	1.137786	0.1768	0.3938
68736	1110034B05Rik	RIKEN cDNA 1110034B05 gene	1.137786	0.08935	NA
75958	5033403F01Rik	RIKEN cDNA 5033403F01 gene	1.137786	0.2232	0.4537
12540	Cdc42	cell division cycle 42 homolog (S. cerevisiae)	1.137656	0.0459	NA
20619	Snap23	synaptosomal-associated protein 23	1.137527	0.4189	0.6468
67997	Ddx59	DEAD (Asp-Glu-Ala-Asp) box polypeptide 59	1.137527	0.1426	0.3472
243961	Shank1	SH3/ankyrin domain gene 1	1.137527	0.2921	0.5281
382492	Gm12034	predicted gene 12034	1.137527	0.2538	0.4885
58172	Sertad2	SERTA domain containing 2	1.137398	0.2613	0.4964
69129	Pex11c	peroxisomal biogenesis factor 11 gamma	1.137398	0.2426	0.4755
74090	Paqr5	progestin and adipoQ receptor family member V	1.137398	0.2688	0.504
77422	C330018D20Rik	RIKEN cDNA C330018D20 gene	1.137398	0.1422	0.3466
277333	Gm5069	glyceraldehyde-3-phosphate dehydrogenase pseudogene	1.137398	0.3838	0.6163
432964	K230010J24Rik	RIKEN cDNA K230010J24 gene	1.137398	0.1516	0.3602
72519	Tmem55a	transmembrane protein 55A	1.137268	0.2522	0.4867
13003	Vcan	versican	1.137139	0.3439	0.5794
93746	Gprc5d	G protein-coupled receptor, family C, group 5, member D	1.137139	0.3756	0.6094
56488	Nxt1	NTF2-related export protein 1	1.13701	0.02654	NA
72333	Palld	palladin, cytoskeletal associated protein	1.13688	0.2	0.4255
72828	Ubash3b	ubiquitin associated and SH3 domain containing, B	1.13688	0.06413	NA
225363	Etf1	eukaryotic translation termination factor 1	1.136751	0.2952	0.5311
665306	3930402G23Rik	RIKEN cDNA 3930402G23 gene	1.136751	0.6843	0.8389
100505153	LOC100505153	heterogeneous nuclear ribonucleoprotein A3-like	1.136751	0.5219	0.7296
27418	Mkln1	muskelin 1, intracellular mediator containing kelch motifs	1.136493	0.2331	0.4643
70686	Dusp16	dual specificity phosphatase 16	1.136493	0.04429	NA
100502767	LOC100502767	hypothetical LOC100502767	1.136493	0.1884	0.4094
14793	Cdca3	cell division cycle associated 3	1.136364	0.2327	0.4639
17274	Rab8a	RAB8A, member RAS oncogene family	1.136364	0.4296	0.6566
18746	Pkm2	pyruvate kinase, muscle	1.136364	0.2336	0.4649
18798	Plcb4	phospholipase C, beta 4	1.136364	0.1011	NA
21807	Tsc22d1	TSC22 domain family, member 1	1.136364	0.01806	NA
75302	Asxl2	additional sex combs like 2 (Drosophila)	1.136364	0.384	0.6165
107971	Frs3	fibroblast growth factor receptor substrate 3	1.136364	0.7215	0.8605
629557	Gm6981	glyceraldehyde-3-phosphate dehydrogenase pseudogene	1.136364	0.2203	0.4501
68021	Bphl	biphenyl hydrolase-like (serine hydrolase, breast epithelial mucin-associated antigen)	1.136235	0.1364	0.3382
213956	Fam83f	family with sequence similarity 83, member F	1.136105	0.5065	0.7183
320693	C130021H21Rik	RIKEN cDNA C130021H21 gene	1.135976	0.646	0.8153
13200	Ddost	dolichyl-di-phosphooligosaccharide-protein glycotransferase	1.135847	0.1273	0.325
100504975	LOC100504975	hypothetical protein LOC100504975	1.135847	0.2338	0.4652
22722	Zfp64	zinc finger protein 64	1.135718	0.2869	0.5224
224014	Fgd4	FYVE, RhoGEF and PH domain containing 4	1.135718	0.4114	0.6405
11482	Acvrl1	activin A receptor, type II-like 1	1.135589	0.05861	NA
68026	2810417H13Rik	RIKEN cDNA 2810417H13 gene	1.135589	0.4103	0.6394

72205	Eml2	echinoderm microtubule associated protein like 2	1.135589	0.0618	NA
67252	Cap2	CAP, adenylate cyclase-associated protein, 2 (yeast)	1.13546	0.1263	0.3235
232685	AB041803	cDNA sequence AB041803	1.13546	0.3529	0.5879
242570	Raver2	ribonucleoprotein, PTB-binding 2	1.13546	0.07887	NA
12953	Cry2	cryptochrome 2 (photolyase-like)	1.135332	0.2736	0.5094
20402	Zfp106	zinc finger protein 106	1.135332	0.08529	NA
66460	Sys1	SYS1 Golgi-localized integral membrane protein homolog (S. cerevisiae)	1.135332	0.0821	NA
544736	Glipr1l3	GLI pathogenesis-related 1 like 3	1.135332	0.3987	0.6292
56637	Gsk3b	glycogen synthase kinase 3 beta	1.135203	0.2531	0.4876
654438	A630031M23Rik	Riken cDNA A630031M23 gene	1.135203	0.3288	0.5652
12843	Col1a2	collagen, type I, alpha 2	1.135074	0.6315	0.8059
78586	Srbd1	S1 RNA binding domain 1	1.135074	0.1132	0.3025
217337	Srp68	signal recognition particle 68	1.135074	0.1189	0.3118
242466	Zfp462	zinc finger protein 462	1.135074	0.2267	0.4582
67861	Akr1b10	aldo-keto reductase family 1, member B10 (aldose reductase)	1.134945	0.05999	NA
93713	Pcdhga5	protocadherin gamma subfamily A, 5	1.134945	0.1456	0.3511
225888	Suv420h1	suppressor of variegation 4-20 homolog 1 (Drosophila)	1.134945	0.3908	0.6227
330902	4930565O14	hypothetical protein 4930565O14	1.134816	0.1389	0.3418
26407	Map3k4	mitogen-activated protein kinase kinase kinase 4	1.134687	0.05047	NA
229363	Gmps	guanine monophosphate synthetase	1.134687	0.5195	0.7282
104776	Aldh6a1	aldehyde dehydrogenase family 6, subfamily A1	1.134559	0.2958	0.5315
234797	6430548M08Rik	RIKEN cDNA 6430548M08 gene	1.134559	0.5945	0.7816
100502680	LOC100502680	ubiquitin-conjugating enzyme E2 L3-like	1.134559	0.1603	0.3721
14593	Ggps1	geranylgeranyl diphosphate synthase 1	1.13443	0.07736	NA
18191	Nrxn3	neurexin III	1.13443	0.2051	0.4314
93691	Klf7	Kruppel-like factor 7 (ubiquitous)	1.13443	0.1208	0.3149
11465	Actg1	actin, gamma, cytoplasmic 1	1.134301	0.14	0.3434
57377	Mogs	mannosyl-oligosaccharide glucosidase	1.134301	0.02431	NA
109264	Me3	malic enzyme 3, NADP(+)-dependent, mitochondrial	1.134301	0.06439	NA
407824	BC020402	cDNA sequence BC020402	1.134301	0.3316	0.5678
18231	Nxph1	neurexophilin 1	1.134173	0.3208	0.5573
67169	Nradd	neurotrophin receptor associated death domain	1.134173	0.2091	0.4359
74026	Msl1	male-specific lethal 1 homolog (Drosophila)	1.134173	0.1738	0.3902
76892	Rnft1	ring finger protein, transmembrane 1	1.134173	0.1949	0.4187
320226	4930473A06Rik	RIKEN cDNA 4930473A06 gene	1.134173	0.5628	0.7609
14782	Gsr	glutathione reductase	1.134044	0.1043	NA
56520	Nme4	non-metastatic cells 4, protein expressed in	1.134044	0.09148	NA
234309	Cbr4	carbonyl reductase 4	1.134044	0.1848	0.4043
16498	Kcnab2	potassium voltage-gated channel, shaker-related subfamily, beta member 2	1.133915	0.5432	0.7465
107056	AW742475	expressed sequence AW742475	1.133915	0.427	0.6541
110880	Scn4a	sodium channel, voltage-gated, type IV, alpha	1.133915	0.3312	0.5675
320411	A730089K16Rik	RIKEN cDNA A730089K16 gene	1.133915	0.5949	0.7818
67317	1700022I11Rik	RIKEN cDNA 1700022I11 gene	1.133787	0.1612	0.373
270328	Gsdmc3	gasdermin C3	1.133787	0.4999	0.7144
56335	Mettl3	methyltransferase like 3	1.133658	0.04693	NA
65107	Lrp10	low-density lipoprotein receptor-related protein 10	1.133658	0.09399	NA
622446	Gm6323	predicted gene 6323	1.13353	0.6164	0.7965
16876	Lhx9	LIM homeobox protein 9	1.133401	0.3876	0.6197
66483	Rpl36al	ribosomal protein L36A-like	1.133401	0.03943	NA

545551	BC021767	cingulin-like	1.133401	0.3407	0.5764
636901	LOC636901	40S ribosomal protein SA-like	1.133401	0.03078	NA
140781	Myh7	myosin, heavy polypeptide 7, cardiac muscle, beta	1.133273	0.3259	0.5626
15132	Hbb-bh1	hemoglobin Z, beta-like embryonic chain	1.133144	0.4093	0.6386
71919	Rpap3	RNA polymerase II associated protein 3	1.133144	0.04999	NA
269954	Ttll13	tubulin tyrosine ligase-like family, member 13	1.133144	0.236	0.4685
272589	Tbcel	tubulin folding cofactor E-like	1.133144	0.2444	0.478
70750	Kdsr	3-ketodihydrosphingosine reductase	1.133016	0.133	0.3338
227613	Tubb2c	tubulin, beta 2C	1.133016	0.04177	NA
319513	Fam113a	family with sequence similarity 113, member A	1.133016	0.3091	0.5455
12359	Cat	catalase	1.132888	0.3974	0.6278
14395	Gabra2	gamma-aminobutyric acid (GABA) A receptor, subunit alpha 2	1.132888	0.3824	0.6157
24055	Sh3bp2	SH3-domain binding protein 2	1.132888	0.1622	0.3744
230721	Pabpc4	poly(A) binding protein, cytoplasmic 4	1.132759	0.2315	0.463
320707	Atp2b3	ATPase, Ca++ transporting, plasma membrane 3	1.132759	0.1973	0.4219
66396	Ccdc82	coiled-coil domain containing 82	1.132631	0.03563	NA
78372	Snrnp25	small nuclear ribonucleoprotein 25 (U11/U12)	1.132631	0.05931	NA
16418	Eif6	eukaryotic translation initiation factor 6	1.132503	0.3142	0.5508
19360	Rad50	RAD50 homolog (S. cerevisiae)	1.132503	0.1895	0.4113
69010	Anapc13	anaphase promoting complex subunit 13	1.132375	0.1225	0.318
75560	Ep400	E1A binding protein p400	1.132375	0.09467	NA
100561	Slc15a4	solute carrier family 15, member 4	1.132375	0.01499	NA
70560	Wars2	tryptophanyl tRNA synthetase 2 (mitochondrial)	1.132246	0.3592	0.5944
332579	Card9	caspase recruitment domain family, member 9	1.132246	0.2987	0.5348
22685	Zfp239	zinc finger protein 239	1.132118	0.3271	0.5636
28135	Cep63	centrosomal protein 63	1.132118	0.5543	0.7539
30926	Glrx3	glutaredoxin 3	1.132118	0.1707	0.3861
107358	Tm9sf3	transmembrane 9 superfamily member 3	1.132118	0.3952	0.6258
545136	Fam186b	family with sequence similarity 186, member B	1.132118	0.471	0.6923
68795	Ubr3	ubiquitin protein ligase E3 component n-recognin 3	1.13199	0.2933	0.5294
209683	Ttc28	tetratricopeptide repeat domain 28	1.13199	0.4795	0.6989
12725	Clcn3	chloride channel 3	1.131862	0.2231	0.4536
19290	Pura	purine rich element binding protein A	1.131862	0.2276	0.4586
23894	Gtf2h2	general transcription factor II H, polypeptide 2	1.131862	0.2284	0.4594
69171	1810031K17Rik	RIKEN cDNA 1810031K17 gene	1.131862	0.03912	NA
244650	Phlpp2	PH domain and leucine rich repeat protein phosphatase 2	1.131862	0.04094	NA
67897	Rnmt	RNA (guanine-7-) methyltransferase	1.131734	0.2368	0.469
217558	G2e3	G2/M-phase specific E3 ubiquitin ligase	1.131734	0.4569	0.6804
230459	Cyp2j13	cytochrome P450, family 2, subfamily j, polypeptide 13	1.131734	0.2546	0.4893
238276	Akap5	A kinase (PRKA) anchor protein 5	1.131734	0.1459	0.3515
26378	Decr2	2-4-dienoyl-Coenzyme A reductase 2, peroxisomal	1.131606	0.1925	0.415
67456	Ergic2	ERGIC and golgi 2	1.131606	0.199	0.4242
67898	Pef1	penta-EF hand domain containing 1	1.131478	0.09579	NA
102623	AI414108	expressed sequence AI414108	1.131478	0.489	0.7061
268470	Ube2z	ubiquitin-conjugating enzyme E2Z (putative)	1.131478	0.03848	NA
13209	Ddx6	DEAD (Asp-Glu-Ala-Asp) box polypeptide 6	1.13135	0.2738	0.5094
19727	Rfxank	regulatory factor X-associated ankyrin-containing protein	1.13135	0.2594	0.4945
57376	Smarce1	SWI/SNF related, matrix associated, actin dependent regulator of chromatin, subfamily e, member 1	1.13135	0.1739	0.3904
67941	Rps27l	ribosomal protein S27-like	1.13135	0.05351	NA

70839	P2ry12	purinergic receptor P2Y, G-protein coupled 12	1.13135	0.2739	0.5095
239555	Smcr7l	Smith-Magenis syndrome chromosome region, candidate 7-like (human)	1.13135	0.04812	NA
327860	Gm11961	predicted gene 11961	1.13135	0.3174	0.5539
54380	Smarcal1	SWI/SNF related matrix associated, actin dependent regulator of chromatin, subfamily a-like 1	1.131222	0.09858	NA
68278	Ddx39	DEAD (Asp-Glu-Ala-Asp) box polypeptide 39	1.131222	0.02187	NA
97874	B430203I24Rik	RIKEN cDNA B430203I24 gene	1.131222	0.3755	0.6094
107375	Slc25a45	solute carrier family 25, member 45	1.131222	0.3872	0.6193
319170	Hist1h2an	histone cluster 1, H2an	1.131222	0.0694	NA
15194	Htt	huntingtin	1.131094	0.2094	0.4364
22762	Zfpm2	zinc finger protein, multitype 2	1.131094	0.02861	NA
100191037	Krtap10-4	keratin associated protein 10-4	1.131094	0.661	0.8252
56338	Txnip	thioredoxin interacting protein	1.130966	0.1896	0.4113
56812	Dnajb2	DnaJ (Hsp40) homolog, subfamily B, member 2	1.130966	0.1588	0.3701
194401	Mical3	microtubule associated monoxygenase, calponin and LIM domain containing 3	1.130966	0.4757	0.6962
100504388	LOC100504388	histone acetyltransferase KAT2B-like	1.130966	0.5624	0.7606
16578	Kif9	kinesin family member 9	1.13071	0.2236	0.4541
66231	Thoc7	THO complex 7 homolog (Drosophila)	1.13071	0.1719	0.3877
68915	Vars2	valyl-tRNA synthetase 2, mitochondrial (putative)	1.13071	0.08313	NA
77569	Limch1	LIM and calponin homology domains 1	1.13071	0.05918	NA
68066	Slc25a39	solute carrier family 25, member 39	1.130582	0.2168	0.4456
216991	Adap2	ArfGAP with dual PH domains 2	1.130582	0.426	0.6534
17067	Ly6c1	lymphocyte antigen 6 complex, locus C1	1.130454	0.1226	NA
19663	Rbpms	RNA binding protein gene with multiple splicing	1.130454	0.08628	NA
320508	Cachd1	cache domain containing 1	1.130327	0.04925	NA
68020	2810002N01Rik	RIKEN cDNA 2810002N01 gene	1.130199	0.1242	0.3209
72691	Calhm2	calcium homeostasis modulator 2	1.130199	0.3429	0.5782
272359	Irf2bp1	interferon regulatory factor 2 binding protein 1	1.130071	0.1968	0.4213
14696	Gnb4	guanine nucleotide binding protein (G protein), beta 4	1.129944	0.3084	0.5447
66371	Chmp4c	chromatin modifying protein 4C	1.129944	0.5938	0.7811
20509	Slc19a1	solute carrier family 19 (sodium/hydrogen exchanger), member 1	1.129816	0.07725	NA
54132	Pdlim1	PDZ and LIM domain 1 (elfin)	1.129816	0.5086	0.7198
106459	BB163080	expressed sequence BB163080	1.129816	0.5129	0.7232
230514	Leprot	leptin receptor overlapping transcript	1.129816	0.02531	NA
270362	LOC270362	60S acidic ribosomal protein P1-like	1.129816	0.1853	0.4048
100038491	A730068I03Rik	RIKEN cDNA A730068I03 gene	1.129816	0.6043	0.7879
16001	Igf1r	insulin-like growth factor I receptor	1.129688	0.2303	0.4615
67337	Cstf1	cleavage stimulation factor, 3' pre-RNA, subunit 1	1.129688	0.156	0.3659
67501	Ccdc50	coiled-coil domain containing 50	1.129688	0.2687	0.504
59002	Wdr8	WD repeat domain 8	1.129561	0.1426	0.3472
209683	Ttc28	tetratricopeptide repeat domain 28	1.129561	0.1616	0.3737
319800	C730048C13Rik	RIKEN cDNA C730048C13 gene	1.129433	0.4557	0.6793
328580	Tubgcp6	tubulin, gamma complex associated protein 6	1.129433	0.5423	0.7461
100047044	LOC100047044	hypothetical protein LOC100047044	1.129433	0.5696	0.7638
14423	Galnt1	UDP-N-acetyl-alpha-D-galactosamine:polypeptide N-acetylgalactosaminyltransferase 1	1.129305	0.4137	0.6425
20674	Sox2	SRY-box containing gene 2	1.129305	0.2125	0.4403
171463	Il17rd	interleukin 17 receptor D	1.129305	0.2405	0.4731
22004	Tpm2	tropomyosin 2, beta	1.129178	0.1325	0.3332
64009	Syne1	synaptic nuclear envelope 1	1.129178	0.2103	0.4374
211389	Suox	sulfite oxidase	1.12905	0.1125	NA

17966	Nbr1	neighbor of Brca1 gene 1	1.128923	0.1628	0.375
18111	Nnat	neuronatin	1.128923	0.4922	0.7086
67819	Derl1	Der1-like domain family, member 1	1.128923	0.03521	NA
71567	Mcm9	minichromosome maintenance complex component 9	1.128923	0.2345	0.4661
73710	Tubb2b	tubulin, beta 2B	1.128923	0.04816	NA
230459	Cyp2j13	cytochrome P450, family 2, subfamily j, polypeptide 13	1.128923	0.4137	0.6425
320204	4833442J19Rik	RIKEN cDNA 4833442J19 gene	1.128923	0.3335	0.5693
100503238	LOC100503238	hypothetical LOC100503238	1.128923	0.1885	0.4097
18538	Pcna	proliferating cell nuclear antigen	1.128796	0.05974	NA
243872	Rpl7a-ps8	ribosomal protein L7A, pseudogene 8	1.128796	0.3145	0.5508
71891	Cdadc1	cytidine and dCMP deaminase domain containing 1	1.128668	0.1147	NA
209497	Tmem164	transmembrane protein 164	1.128668	0.07877	NA
229517	Slc25a44	solute carrier family 25, member 44	1.128668	0.3371	0.5727
228913	Zfp217	zinc finger protein 217	1.128541	0.3712	0.6062
23890	Gpr34	G protein-coupled receptor 34	1.128413	0.2889	0.5249
233046	Rasgrp4	RAS guanyl releasing protein 4	1.128413	0.3632	0.5983
667250	Gm12657	predicted gene 12657	1.128413	0.07599	NA
53817	Bat1a	HLA-B-associated transcript 1A	1.128286	0.2524	0.487
72562	Pcbd2	pterin 4 alpha carbinolamine dehydratase/dimerization cofactor of hepatocyte nuclear factor 1 alpha (TCF1) 2	1.128286	0.2476	0.4819
17775	Laptm4a	lysosomal-associated protein transmembrane 4A	1.128159	0.2809	0.5162
20019	Polr1a	polymerase (RNA) I polypeptide A	1.128159	0.195	0.4188
54445	Unc93b1	unc-93 homolog B1 (C. elegans)	1.128159	0.1369	0.3386
118454	Gjc2	gap junction protein, gamma 2	1.128159	0.1171	NA
67213	Cmtm6	CKLF-like MARVEL transmembrane domain containing 6	1.128032	0.1089	NA
233812	BC030336	cDNA sequence BC030336	1.128032	0.1365	0.3383
13002	Dnajc5	DnaJ (Hsp40) homolog, subfamily C, member 5	1.127904	0.01736	NA
676894	Gm9694	predicted gene 9694	1.127904	0.4175	0.6457
66101	Ppih	peptidyl prolyl isomerase H	1.12765	0.2434	0.4767
78250	Iqch	IQ motif containing H	1.12765	0.5958	0.7822
225912	Cybasc3	cytochrome b, ascorbate dependent 3	1.12765	0.1356	0.3372
328788	Gm749	predicted gene 749	1.12765	0.5552	0.7547
17254	Slc3a2	solute carrier family 3 (activators of dibasic and neutral amino acid transport), member 2	1.127523	0.09982	NA
245638	Tbc1d8b	TBC1 domain family, member 8B	1.127523	0.3275	0.5639
328265	A530001N23Rik	RIKEN cDNA A530001N23 gene	1.127523	0.5272	0.7341
15505	Hsph1	heat shock 105kDa/110kDa protein 1	1.127396	0.09303	NA
226169	Pprc1	peroxisome proliferative activated receptor, gamma, coactivator-related 1	1.127396	0.1508	0.3588
14433	Gapdh	glyceraldehyde-3-phosphate dehydrogenase	1.127269	0.1673	0.3821
22319	Vamp3	vesicle-associated membrane protein 3	1.127269	0.07189	NA
64010	Sav1	salvador homolog 1 (Drosophila)	1.127269	0.05685	NA
171210	Acot2	acyl-CoA thioesterase 2	1.127269	0.4183	0.6463
229791	D3Bwg0562e	DNA segment, Chr 3, Brigham & Women's Genetics 0562 expressed	1.127269	0.1322	NA
16419	Itgb5	integrin beta 5	1.127142	0.1702	0.3854
22196	Ube2i	ubiquitin-conjugating enzyme E2I	1.127142	0.3201	0.5566
232941	Ppm1n	protein phosphatase, Mg2+/Mn2+ dependent, 1N (putative)	1.127142	0.141	0.3449
633387	LOC633387	nucleophosmin-like	1.127015	0.06336	NA
12334	Capn2	calpain 2	1.126888	0.04612	NA
20516	Slc20a2	solute carrier family 20, member 2	1.126888	0.1831	0.402
321022	Cdv3	carnitine deficiency-associated gene expressed in ventricle 3	1.126888	0.04944	NA
56336	B4galt5	UDP-Gal:betaGlcNAc beta 1,4-galactosyltransferase, polypeptide 5	1.126761	0.04534	NA

246782	Atpaf2	ATP synthase mitochondrial F1 complex assembly factor 2	1.126761	0.1387	0.3416
74154	Unkl	unkempt-like (Drosophila)	1.126634	0.1837	0.4027
215472	Gm4792	predicted gene 4792	1.126634	0.4959	0.7111
320799	Zhx3	zinc fingers and homeoboxes 3	1.126634	0.02868	NA
18193	Nsd1	nuclear receptor-binding SET-domain protein 1	1.126507	0.06578	NA
18810	Plec	plectin	1.126507	0.2209	0.4507
20224	Sar1a	SAR1 gene homolog A (S. cerevisiae)	1.126507	0.2949	0.5311
210035	Tmem194	transmembrane protein 194	1.12638	0.2515	0.4858
214444	Cdk5rap2	CDK5 regulatory subunit associated protein 2	1.12638	0.3518	0.5871
56456	Actl6a	actin-like 6A	1.126253	0.322	0.5584
667350	Gm8587	predicted pseudogene 8587	1.126253	0.4045	0.6342
101700	Trim68	tripartite motif-containing 68	1.126126	0.1173	NA
333433	Gpd1l	glycerol-3-phosphate dehydrogenase 1-like	1.126126	0.3732	0.6074
66606	Lrrc57	leucine rich repeat containing 57	1.125999	0.148	0.3547
100039181	LOC100039181	uncharacterized protein C4orf3 homolog	1.125999	0.07419	NA
20643	Snrpe	small nuclear ribonucleoprotein E	1.125873	0.1334	NA
110893	Slc8a3	solute carrier family 8 (sodium/calcium exchanger), member 3	1.125873	0.1311	NA
70432	Rufy2	RUN and FYVE domain-containing 2	1.125746	0.302	0.5378
76113	Lpo	lactoperoxidase	1.125746	0.5439	0.7471
20732	Spint1	serine protease inhibitor, Kunitz type 1	1.125619	0.371	0.6059
22658	Pcgf2	polycomb group ring finger 2	1.125619	0.1586	0.3697
68416	Sycn	syncollin	1.125619	0.5758	0.769
69538	Antxr1	anthrax toxin receptor 1	1.125492	0.09563	NA
328092	6530401N04Rik	RIKEN cDNA 6530401N04 gene	1.125492	0.1348	NA
17217	Mcm4	minichromosome maintenance deficient 4 homolog (S. cerevisiae)	1.125366	0.0378	NA
56336	B4galt5	UDP-Gal:betaGlcNAc beta 1,4-galactosyltransferase, polypeptide 5	1.125366	0.5779	0.7704
76138	Ccdc138	coiled-coil domain containing 138	1.125366	0.132	NA
80707	Wwox	WW domain-containing oxidoreductase	1.125366	0.27	0.505
104318	Csnk1d	casein kinase 1, delta	1.125366	0.2014	0.4275
234023	Arglu1	arginine and glutamate rich 1	1.125366	0.2225	0.4527
242819	Rundc3b	RUN domain containing 3B	1.125366	0.2199	0.4497
100042539	Gm3893	predicted gene 3893	1.125366	0.4049	0.6345
20287	Sct	secretin	1.125239	0.4058	0.6354
22362	Vpreb1	pre-B lymphocyte gene 1	1.125239	0.3839	0.6164
228911	Tshz2	teashirt zinc finger family member 2	1.125239	0.06796	NA
19317	Qk	quaking	1.125113	0.2054	0.4317
27973	Vkorc1	vitamin K epoxide reductase complex, subunit 1	1.125113	0.05513	NA
71951	Gpc2	glypican 2 (cerebroglycan)	1.125113	0.1689	0.384
74012	Rap2b	RAP2B, member of RAS oncogene family	1.125113	0.3408	0.5765
170654	Krtap16-4	keratin associated protein 16-4	1.125113	0.4814	0.7
11911	Atf4	activating transcription factor 4	1.124859	0.248	0.4822
98740	BB116930	expressed sequence BB116930	1.124859	0.1108	NA
140579	Elmo2	engulfment and cell motility 2, ced-12 homolog (C. elegans)	1.124859	0.1123	NA
319168	Hist1h2ah	histone cluster 1, H2ah	1.124859	0.07151	NA
320595	Phf8	PHD finger protein 8	1.124859	0.4208	0.6487
28193	Reep3	receptor accessory protein 3	1.124733	0.1702	0.3854
56531	Ylpm1	YLP motif containing 1	1.124733	0.4311	0.6581
74721	4930519P11Rik	RIKEN cDNA 4930519P11 gene	1.124733	0.4934	0.7095
216613	Ccdc85a	coiled-coil domain containing 85A	1.124733	0.1487	0.356

12995	Csnk2a1	casein kinase 2, alpha 1 polypeptide	1.124606	0.3496	0.5854
67268	2900073G15Rik	RIKEN cDNA 2900073G15 gene	1.124606	0.05939	NA
117589	Asb7	ankyrin repeat and SOCS box-containing 7	1.124606	0.06795	NA
213499	Fbxo42	F-box protein 42	1.124606	0.1675	0.3825
18451	P4ha1	procollagen-proline, 2-oxoglutarate 4-dioxygenase (proline 4-hydroxylase), alpha 1 polypeptide	1.12448	0.04416	NA
338364	Trim65	tripartite motif-containing 65	1.12448	0.4148	0.6434
14229	Fkbp5	FK506 binding protein 5	1.124353	0.1917	0.4139
67939	Prorsd1	prolyl-tRNA synthetase domain containing 1	1.124353	0.2547	0.4895
268420	Alkbh5	alkB, alkylation repair homolog 5 (E. coli)	1.124353	0.3665	0.6019
545123	Cyp2d11	cytochrome P450, family 2, subfamily d, polypeptide 11	1.124353	0.2735	0.5094
217340	Rnf157	ring finger protein 157	1.124227	0.4694	0.6914
69131	Cdk12	cyclin-dependent kinase 12	1.124101	0.06945	NA
232371	C1rl	complement component 1, r subcomponent-like	1.124101	0.6362	0.8088
14451	Gas1	growth arrest specific 1	1.123974	0.04807	NA
74670	Zfp943	zinc finger prtoein 943	1.123974	0.1532	0.3627
277939	C2cd3	C2 calcium-dependent domain containing 3	1.123974	0.377	0.611
20088	Rps24	ribosomal protein S24	1.123596	0.2314	0.4629
21372	Tbl1x	transducin (beta)-like 1 X-linked	1.123596	0.1769	0.3939
56349	Net1	neuroepithelial cell transforming gene 1	1.123596	0.1661	0.3802
229357	Gpr149	G protein-coupled receptor 149	1.123596	0.2222	0.4526
11907	Ate1	arginyltransferase 1	1.123469	0.3764	0.6103
72748	Hdhd3	haloacid dehalogenase-like hydrolase domain containing 3	1.123469	0.2059	0.432
29807	Tpk1	thiamine pyrophosphokinase	1.123343	0.4303	0.6572
53374	Chst3	carbohydrate (chondroitin 6/keratan) sulfotransferase 3	1.123343	0.1542	0.3639
75693	3010001F23Rik	RIKEN cDNA 3010001F23 gene	1.123343	0.4827	0.7013
544963	Iqgap2	IQ motif containing GTPase activating protein 2	1.123343	0.5839	0.7745
100040288	Gm2692	predicted gene 2692	1.123343	0.4839	0.7022
18707	Pik3cd	phosphatidylinositol 3-kinase catalytic delta polypeptide	1.123217	0.3659	0.6015
67332	Snrpd3	small nuclear ribonucleoprotein D3	1.123217	0.09433	NA
330502	Zfp82	zinc finger protein 82	1.123217	0.395	0.6256
19782	Rmrp	RNA component of mitochondrial RNAase P	1.123091	0.649	0.8173
67778	Zfp639	zinc finger protein 639	1.123091	0.0598	NA
72572	Spats2	spermatogenesis associated, serine-rich 2	1.123091	0.2097	0.4367
73822	F630110N24Rik	RIKEN cDNA F630110N24 gene	1.123091	0.7048	0.8516
353504	Dio3os	deiodinase, iodothyronine type III, opposite strand	1.123091	0.5929	0.7805
207728	Pde2a	phosphodiesterase 2A, cGMP-stimulated	1.122965	0.1296	NA
18813	Pa2g4	proliferation-associated 2G4	1.122839	0.2482	0.4824
67200	Ccdc77	coiled-coil domain containing 77	1.122839	0.3453	0.5809
791365	6430601O08Rik	RIKEN cDNA 6430601O08 gene	1.122839	0.327	0.5636
67733	Itgb3bp	integrin beta 3 binding protein (beta3-endonexin)	1.122712	0.1899	0.4118
393082	Mettl7a2	methyltransferase like 7A2	1.122712	0.6262	0.8027
17955	Nap1l4	nucleosome assembly protein 1-like 4	1.122586	0.1071	NA
66165	Bccip	BRCA2 and CDKN1A interacting protein	1.122586	0.04434	NA
70419	2810408A11Rik	RIKEN cDNA 2810408A11 gene	1.122586	0.4954	0.7111
230734	Yrdc	yrdC domain containing (E.coli)	1.122586	0.2794	0.515
18631	Pex11a	peroxisomal biogenesis factor 11 alpha	1.12246	0.1549	0.3647
22195	Ube2l3	ubiquitin-conjugating enzyme E2L 3	1.12246	0.3236	0.5602
67628	Anp32b	acidic (leucine-rich) nuclear phosphoprotein 32 family, member B	1.12246	0.05867	NA
13850	Ephx2	epoxide hydrolase 2, cytoplasmic	1.122209	0.184	0.403

66231	Thoc7	THO complex 7 homolog (Drosophila)	1.122209	0.3852	0.6175
78655	Eif3j	eukaryotic translation initiation factor 3, subunit J	1.122209	0.07649	NA
12611	Cebpg	CCAAT/enhancer binding protein (C/EBP), gamma	1.122083	0.2029	0.4292
15975	Ifnar1	interferon (alpha and beta) receptor 1	1.122083	0.4301	0.6571
17863	Myb	myeloblastosis oncogene	1.122083	0.1375	NA
56494	Gosr2	golgi SNAP receptor complex member 2	1.122083	0.01315	NA
99458	BB166591	expressed sequence BB166591	1.122083	0.3664	0.6019
12289	Cacna1d	calcium channel, voltage-dependent, L type, alpha 1D subunit	1.121957	0.04008	NA
14387	Gaa	glucosidase, alpha, acid	1.121957	0.4784	0.6982
66346	1700029P11Rik	RIKEN cDNA 1700029P11 gene	1.121957	0.5014	0.7146
243274	Tmem132d	transmembrane protein 132D	1.121957	0.229	0.46
245007	Zbtb38	zinc finger and BTB domain containing 38	1.121957	0.4187	0.6467
225164	Mib1	mindbomb homolog 1 (Drosophila)	1.121831	0.2001	0.4256
328516	D430034N21	hypothetical protein D430034N21	1.121831	0.5578	0.7568
228913	Zfp217	zinc finger protein 217	1.121705	0.2379	0.4702
238123	Cog5	component of oligomeric golgi complex 5	1.121705	0.4376	0.6641
258728	Olfr482	olfactory receptor 482	1.121705	0.6658	0.8286
57815	Spata5	spermatogenesis associated 5	1.121579	0.1747	0.3914
68691	1110028C15Rik	RIKEN cDNA 1110028C15 gene	1.121579	0.3225	0.5589
80744	Cwc22	CWC22 spliceosome-associated protein homolog (S. cerevisiae)	1.121579	0.4723	0.6933
14385	Slc37a4	solute carrier family 37 (glucose-6-phosphate transporter), member 4	1.121453	0.05658	NA
75570	Nhej1	nonhomologous end-joining factor 1	1.121202	0.158	0.3689
77798	A930009A15Rik	RIKEN cDNA A930009A15 gene	1.121202	0.2662	0.5016
80509	Med8	mediator of RNA polymerase II transcription, subunit 8 homolog (yeast)	1.121202	0.07739	NA
84652	Fam126a	family with sequence similarity 126, member A	1.121202	0.5312	0.7372
14751	Gpi1	glucose phosphate isomerase 1	1.121076	0.2399	0.4724
17931	Ppp1r12a	protein phosphatase 1, regulatory (inhibitor) subunit 12A	1.121076	0.2456	0.4794
52186	D8Ertd158e	DNA segment, Chr 8, ERATO Doi 158, expressed	1.121076	0.159	0.3703
73486	1700084J12Rik	RIKEN cDNA 1700084J12 gene	1.121076	0.4916	0.7079
78926	Gas2l1	growth arrest-specific 2 like 1	1.121076	0.576	0.769
328035	Fads6	fatty acid desaturase domain family, member 6	1.121076	0.102	NA
434858	Gm5643	heterogeneous nuclear ribonucleoprotein A1 pseudogene	1.121076	0.1508	NA
66167	Ccdc72	coiled-coil domain containing 72	1.120951	0.1174	NA
68272	Rbm28	RNA binding motif protein 28	1.120951	0.3722	0.6068
76826	Nubpl	nucleotide binding protein-like	1.120951	0.1229	NA
229488	Fam160a1	family with sequence similarity 160, member A1	1.120951	0.2101	0.437
14679	Gnai3	guanine nucleotide binding protein (G protein), alpha inhibiting 3	1.120699	0.1041	NA
19730	Ralgds	ral guanine nucleotide dissociation stimulator	1.120699	0.1322	NA
50793	Orc3	origin recognition complex, subunit 3	1.120699	0.3272	0.5636
76670	Ttc18	tetratricopeptide repeat domain 18	1.120699	0.1225	NA
78134	Lpar4	lysophosphatidic acid receptor 4	1.120699	0.5802	0.7724
108101	Fermt3	fermitin family homolog 3 (Drosophila)	1.120699	0.4497	0.6747
227298	Fam134a	family with sequence similarity 134, member A	1.120699	0.183	0.402
236904	Klhl15	kelch-like 15 (Drosophila)	1.120699	0.3196	0.5562
67509	1810063B07Rik	RIKEN cDNA 1810063B07 gene	1.120574	0.2284	0.4594
68982	1500015A07Rik	RIKEN cDNA 1500015A07 gene	1.120574	0.2687	0.5039
212127	2810046L04Rik	RIKEN cDNA 2810046L04 gene	1.120574	0.7622	0.885
71795	Pitpnc1	phosphatidylinositol transfer protein, cytoplasmic 1	1.120448	0.1836	0.4026
105348	Golm1	golgi membrane protein 1	1.120448	0.1778	0.3952

71163	Zfp626	zinc finger protein 626	1.120323	0.05974	NA
76936	Hnrnpm	heterogeneous nuclear ribonucleoprotein M	1.120323	0.08352	NA
15944	Irgm1	immunity-related GTPase family M member 1	1.120197	0.374	0.6082
72085	Osgepl1	O-sialoglycoprotein endopeptidase-like 1	1.120197	0.07706	NA
20742	Spnb2	spectrin beta 2	1.120072	0.545	0.7477
67200	Ccdc77	coiled-coil domain containing 77	1.120072	0.3429	0.5782
69802	Cox11	COX11 homolog, cytochrome c oxidase assembly protein (yeast)	1.120072	0.1146	NA
408070	B930036G03Rik	RIKEN cDNA B930036G03 gene	1.120072	0.05376	NA
12912	Creb1	cAMP responsive element binding protein 1	1.119946	0.1516	NA
21848	Trim24	tripartite motif-containing 24	1.119946	0.2897	0.5258
68617	1110012J17Rik	RIKEN cDNA 1110012J17 gene	1.119946	0.1072	NA
231713	Naa25	N(alpha)-acetyltransferase 25, NatB auxiliary subunit	1.119946	0.1547	NA
233912	Armc5	armadillo repeat containing 5	1.119946	0.1295	NA
67693	2310003F16Rik	RIKEN cDNA 2310003F16 gene	1.119821	0.09619	NA
72194	Fbxl20	F-box and leucine-rich repeat protein 20	1.119821	0.4235	0.6513
22003	Tpm1	tropomyosin 1, alpha	1.119695	0.2046	0.4309
408068	Zfp738	zinc finger protein 738	1.119695	0.3255	0.5621
12035	Bcat1	branched chain aminotransferase 1, cytosolic	1.11957	0.1747	0.3913
17222	Anapc1	anaphase promoting complex subunit 1	1.11957	0.1217	NA
20091	Rps3a	ribosomal protein S3A	1.11957	0.4542	0.6779
71101	4933407H18Rik	RIKEN cDNA 4933407H18 gene	1.11957	0.6277	0.8037
70510	Rnf167	ring finger protein 167	1.119445	0.1271	NA
258693	Olfr1443	olfactory receptor 1443	1.119445	0.4248	0.6524
319176	Hist2h2ac	histone cluster 2, H2ac	1.119445	0.09124	NA
66435	Uggt2	UDP-glucose glycoprotein glucosyltransferase 2	1.119319	0.1951	0.4188
12805	Cntn1	contactin 1	1.119194	0.08757	NA
66832	Rsph3a	radial spoke 3A homolog (Chlamydomonas)	1.119069	0.2973	0.5334
76687	Spcs3	signal peptidase complex subunit 3 homolog (S. cerevisiae)	1.119069	0.5139	0.7242
230279	6330416G13Rik	RIKEN cDNA 6330416G13 gene	1.119069	0.3997	0.63
545366	BC026782	cDNA sequence BC026782	1.119069	0.4172	0.6455
19344	Rab5b	RAB5B, member RAS oncogene family	1.118944	0.4889	0.7061
68087	Dcakd	dephospho-CoA kinase domain containing	1.118819	0.2953	0.5311
27395	Mrpl15	mitochondrial ribosomal protein L15	1.118693	0.188	0.4088
245880	Wasf3	WAS protein family, member 3	1.118693	0.3843	0.6167
320020	6330415G19Rik	RIKEN cDNA 6330415G19 gene	1.118693	0.7299	0.8664
19229	Ptk2b	PTK2 protein tyrosine kinase 2 beta	1.118568	0.5581	0.757
385109	Gm1499	predicted gene 1499	1.118568	0.3337	0.5695
19165	Psen2	presenilin 2	1.118443	0.1079	NA
26419	Mapk8	mitogen-activated protein kinase 8	1.118443	0.3442	0.5797
64291	Osbpl1a	oxysterol binding protein-like 1A	1.118443	0.3534	0.5882
243937	Zfp536	zinc finger protein 536	1.118443	0.07619	NA
545013	Gm5797	predicted gene 5797	1.118443	0.6979	0.8471
18028	Nfib	nuclear factor I/B	1.118318	0.2502	0.4841
67078	Pgp	phosphoglycolate phosphatase	1.118318	0.1548	NA
68995	Mcts1	malignant T cell amplified sequence 1	1.118318	0.3246	0.5612
74159	Acbd5	acyl-Coenzyme A binding domain containing 5	1.118318	0.537	0.7416
68185	Chchd8	coiled-coil-helix-coiled-coil-helix domain containing 8	1.118193	0.0752	NA
70408	Polr3f	polymerase (RNA) III (DNA directed) polypeptide F	1.118193	0.6213	0.7994
224823	Rrp36	ribosomal RNA processing 36 homolog (S. cerevisiae)	1.118193	0.1691	0.3843

12445	Ccnd3	cyclin D3	1.118068	0.09512	NA	
13875	Erf	Ets2 repressor factor	1.118068	0.08531	NA	
106585	Ankrd12	ankyrin repeat domain 12	1.118068	0.3988		0.6292
328606	4731420N21	hypothetical protein 4731420N21	1.118068	0.1301	NA	
108686	Ccdc88a	coiled coil domain containing 88A	1.117943	0.3946		0.6254
226351	Tmem185b	transmembrane protein 185B	1.117943	0.1807		0.3994
240832	Tor1aip2	torsin A interacting protein 2	1.117943	0.0492	NA	
16475	Jub	ajuba	1.117818	0.2578		0.4928
20741	Spnb1	spectrin beta 1	1.117818	0.1793		0.3972
218314	Zfp595	zinc finger protein 595	1.117818	0.2772		0.5129
223435	Trio	triple functional domain (PTPRF interacting)	1.117693	0.6573		0.8223
20587	Smarcb1	SWI/SNF related, matrix associated, actin dependent regulator of chromatin, subfamily b, member 1	1.117568	0.1336	NA	
68114	Mum1	melanoma associated antigen (mutated) 1	1.117568	0.28		0.5154
632883	LOC632883	protein transport protein Sec61 subunit gamma-like	1.117568	0.2426		0.4755
28071	Twistnb	TWIST neighbor	1.117443	0.2987		0.5348
56351	Ptges3	prostaglandin E synthase 3 (cytosolic)	1.117443	0.203		0.4293
66431	1810049H13Rik	RIKEN cDNA 1810049H13 gene	1.117443	0.2032		0.4294
73067	Tmem192	transmembrane protein 192	1.117443	0.1373	NA	
329828	AI464131	expressed sequence AI464131	1.117443	0.4408		0.6667
380855	Rsl1	regulator of sex limited protein 1	1.117318	0.1057	NA	
622175	E430024I08Rik	RIKEN cDNA E430024I08 gene	1.117318	0.4755		0.6961
12175	Bnip2	BCL2/adenovirus E1B interacting protein 2	1.117194	0.3653		0.601
21413	Tcf4	transcription factor 4	1.117194	0.0554	NA	
67469	Abhd5	abhydrolase domain containing 5	1.117194	0.3545		0.5892
15516	Hsp90ab1	heat shock protein 90 alpha (cytosolic), class B member 1	1.117069	0.2816		0.5168
22121	Rpl13a	ribosomal protein L13A	1.117069	0.0869	NA	
71803	Slc25a18	solute carrier family 25 (mitochondrial carrier), member 18	1.117069	0.5306		0.7368
19075	Prim1	DNA primase, p49 subunit	1.116944	0.09054	NA	
71268	Lrrfip2	leucine rich repeat (in FLII) interacting protein 2	1.116944	0.4874		0.7053
328971	Spink10	serine peptidase inhibitor, Kazal type 10	1.116944	0.4223		0.6501
667250	Gm12657	predicted gene 12657	1.116944	0.2088		0.4355
14739	S1pr2	sphingosine-1-phosphate receptor 2	1.116819	0.381		0.6147
72093	2010320M18Rik	RIKEN cDNA 2010320M18 gene	1.116819	0.3906		0.6226
233806	Tmem159	transmembrane protein 159	1.116819	0.2501		0.4841
241303	Fam78a	family with sequence similarity 78, member A	1.116819	0.2382		0.4706
27273	Pdk4	pyruvate dehydrogenase kinase, isoenzyme 4	1.116695	0.4524		0.677
64138	Ctsz	cathepsin Z	1.116695	0.1982		0.4233
67993	Nudt12	nudix (nucleoside diphosphate linked moiety X)-type motif 12	1.116695	0.372		0.6068
71966	Nkiras2	NFKB inhibitor interacting Ras-like protein 2	1.116695	0.3106		0.5469
234378	Klhl26	kelch-like 26 (Drosophila)	1.116695	0.1355	NA	
13607	Eda	ectodysplasin-A	1.11657	0.6502		0.8182
66971	Cdk5rap1	CDK5 regulatory subunit associated protein 1	1.11657	0.1352	NA	
215615	Rnpep	arginyl aminopeptidase (aminopeptidase B)	1.11657	0.2003		0.4258
238328	Vash1	vasohibin 1	1.11657	0.5833		0.7739
270624	Spin4	spindlin family, member 4	1.11657	0.07337	NA	
12729	Clns1a	chloride channel, nucleotide-sensitive, 1A	1.116445	0.2392		0.4718
22661	Zfp148	zinc finger protein 148	1.116445	0.237		0.4693
68440	Dusp23	dual specificity phosphatase 23	1.116445	0.4387		0.6648
211488	Ado	2-aminoethanethiol (cysteamine) dioxygenase	1.116445	0.295		0.5311

654796	9530036O11Rik	RIKEN cDNA 9530036O11Rik	1.116445	0.2194	0.449
17865	Mybl2	myeloblastosis oncogene-like 2	1.116321	0.3011	0.537
18293	Ogdh	oxoglutarate dehydrogenase (lipoamide)	1.116321	0.2152	0.4438
30060	Mfi2	antigen p97 (melanoma associated) identified by monoclonal antibodies 133.2 and 96.5	1.116321	0.5551	0.7546
66175	Mustn1	musculoskeletal, embryonic nuclear protein 1	1.116321	0.6293	0.8043
387314	Tmtc1	transmembrane and tetratricopeptide repeat containing 1	1.116321	0.17	NA
11465	Actg1	actin, gamma, cytoplasmic 1	1.116196	0.1707	NA
12607	Cebpz	CCAAT/enhancer binding protein zeta	1.116196	0.3595	0.5946
12867	Cox7c	cytochrome c oxidase, subunit VIIc	1.116196	0.06546	NA
13841	Epha7	Eph receptor A7	1.116071	0.2098	0.4368
17063	Muc13	mucin 13, epithelial transmembrane	1.116071	0.3029	0.539
19170	Psmb1	proteasome (prosome, macropain) subunit, beta type 1	1.116071	0.1347	NA
244059	Chd2	chromodomain helicase DNA binding protein 2	1.116071	0.265	0.5008
15976	Ifnar2	interferon (alpha and beta) receptor 2	1.115947	0.1857	0.4056
73363	1700056E22Rik	RIKEN cDNA 1700056E22 gene	1.115947	0.08002	NA
109658	Txlna	taxilin alpha	1.115947	0.7077	0.8534
242377	Pm20d2	peptidase M20 domain containing 2	1.115947	0.3041	0.5404
98488	Gtf3c3	general transcription factor IIIC, polypeptide 3	1.115822	0.06816	NA
237387	Lrrc3	leucine rich repeat containing 3	1.115822	0.2873	0.5228
74435	Lrriq3	leucine-rich repeats and IQ motif containing 3	1.115698	0.7017	0.8495
228061	Agps	alkylglycerone phosphate synthase	1.115698	0.08312	NA
67117	Dynlt3	dynein light chain Tctex-type 3	1.115573	0.2544	0.4892
94192	C1galt1	core 1 synthase, glycoprotein-N-acetylgalactosamine 3-beta-galactosyltransferase, 1	1.115573	0.4942	0.71
258294	Olfr1115	olfactory receptor 1115	1.115573	0.6478	0.8166
433375	Creg1	cellular repressor of E1A-stimulated genes 1	1.115573	0.1644	NA
20729	Spin1	spindlin 1	1.115449	0.1434	NA
30928	Zfp238	zinc finger protein 238	1.115449	0.1236	NA
67844	Rab32	RAB32, member RAS oncogene family	1.115449	0.1203	NA
74614	4833422F24Rik	RIKEN cDNA 4833422F24 gene	1.115449	0.5934	0.7808
73795	4930405D01Rik	RIKEN cDNA 4930405D01 gene	1.115325	0.6726	0.8323
229707	Fam40a	family with sequence similarity 40, member A	1.115325	0.06339	NA
270210	Zfp651	zinc finger protein 651	1.115325	0.2308	0.4621
436049	Gm5741	predicted gene 5741	1.115325	0.1329	NA
627110	Tubb2a-ps2	tubulin, beta 2a, pseudogene 2	1.115325	0.1458	NA
100039826	Gm2444	predicted gene 2444	1.115325	0.3416	0.5772
13436	Dnmt3b	DNA methyltransferase 3B	1.1152	0.2457	0.4796
19014	Med1	mediator complex subunit 1	1.115076	0.03897	NA
223254	Farp1	FERM, RhoGEF (Arhgef) and pleckstrin domain protein 1 (chondrocyte-derived)	1.115076	0.6028	0.7868
20905	Sts	steroid sulfatase	1.114951	0.4436	0.6694
100336	Ppp1r8	protein phosphatase 1, regulatory (inhibitor) subunit 8	1.114951	0.384	0.6165
100900	Hscb	HscB iron-sulfur cluster co-chaperone homolog (E. coli)	1.114951	0.1663	NA
231863	Fbxl18	F-box and leucine-rich repeat protein 18	1.114951	0.3204	0.5571
18969	Pola2	polymerase (DNA directed), alpha 2	1.114827	0.5394	0.7437
69241	Polr2d	polymerase (RNA) II (DNA directed) polypeptide D	1.114827	0.1286	NA
100040990	Gm3081	predicted gene 3081	1.114827	0.4944	0.7101
53605	Nap1l1	nucleosome assembly protein 1-like 1	1.114703	0.05251	NA
217674	Gphb5	glycoprotein hormone beta 5	1.114703	0.4416	0.6675
241289	Gm347	predicted gene 347	1.114703	0.06178	NA
69066	1810010H24Rik	RIKEN cDNA 1810010H24 gene	1.114579	0.2018	0.428

73363	1700056E22Rik	RIKEN cDNA 1700056E22 gene	1.114579	0.2753	0.5107
12613	Cel	carboxyl ester lipase	1.114454	0.2918	0.528
18145	Npc1	Niemann Pick type C1	1.114454	0.09386	NA
50868	Keap1	kelch-like ECH-associated protein 1	1.114454	0.2168	0.4456
64339	Fndc4	fibronectin type III domain containing 4	1.114454	0.05527	NA
67781	Ilf2	interleukin enhancer binding factor 2	1.114454	0.3099	0.5464
396184	Flrt1	fibronectin leucine rich transmembrane protein 1	1.114454	0.1376	NA
434204	Whamm	WAS protein homolog associated with actin, golgi membranes and microtubules	1.114454	0.6184	0.7976
20702	Serpina1c	serine (or cysteine) peptidase inhibitor, clade A, member 1C	1.11433	0.504	0.7166
27356	Insl6	insulin-like 6	1.11433	0.4797	0.6989
11837	Rplp0	ribosomal protein, large, P0	1.114206	0.1029	NA
14700	Gng10	guanine nucleotide binding protein (G protein), gamma 10	1.114206	0.08932	NA
19201	Pstpip2	proline-serine-threonine phosphatase-interacting protein 2	1.114206	0.3585	0.5934
69009	Thap7	THAP domain containing 7	1.114082	0.4857	0.7041
16800	Arhgef2	rho/rac guanine nucleotide exchange factor (GEF) 2	1.113958	0.1655	NA
57810	Cdon	cell adhesion molecule-related/down-regulated by oncogenes	1.113958	0.4859	0.7043
67008	1600012F09Rik	RIKEN cDNA 1600012F09 gene	1.113958	0.2047	0.431
71177	4933424B01Rik	RIKEN cDNA 4933424B01 gene	1.113958	0.1033	NA
109082	Fbxw17	F-box and WD-40 domain protein 17	1.113834	0.2792	0.5148
13445	Cdk2ap1	CDK2 (cyclin-dependent kinase 2)-associated protein 1	1.11371	0.05316	NA
73296	Rhobtb3	Rho-related BTB domain containing 3	1.11371	0.2107	0.438
78802	Ttc30a1	tetratricopeptide repeat domain 30A1	1.11371	0.08301	NA
93891	Pcdhb20	protocadherin beta 20	1.11371	0.1268	NA
214685	Chadl	chondroadherin-like	1.11371	0.197	0.4215
20318	Sdf4	stromal cell derived factor 4	1.113586	0.2423	0.4753
380711	Rap1gap2	RAP1 GTPase activating protein 2	1.113586	0.5174	0.7269
11461	Actb	actin, beta	1.113462	0.2842	0.52
64540	Tspan4	tetraspanin 4	1.113462	0.1506	NA
23876	Fbln5	fibulin 5	1.113338	0.6537	0.8207
56739	Rec8	REC8 homolog (yeast)	1.113338	0.4957	0.7111
319939	Tns3	tensin 3	1.113338	0.166	NA
654795	Sdr39u1	short chain dehydrogenase/reductase family 39U, member 1	1.113338	0.3261	0.5626
101351	A130022J15Rik	RIKEN cDNA A130022J15 gene	1.113214	0.203	0.4292
333473	Zfp36l3	zinc finger protein 36, C3H type-like 3	1.113214	0.5362	0.7413
14709	Gng8	guanine nucleotide binding protein (G protein), gamma 8	1.11309	0.5029	0.7158
75420	Secisbp2	SECIS binding protein 2	1.11309	0.2411	0.4738
12332	Capg	capping protein (actin filament), gelsolin-like	1.112966	0.2904	0.5265
16785	Rpsa	ribosomal protein SA	1.112966	0.1875	0.4081
56771	Med20	mediator complex subunit 20	1.112966	0.2122	0.44
69358	Lrrc51	leucine rich repeat containing 51	1.112966	0.1918	0.414
81500	Sil1	endoplasmic reticulum chaperone SIL1 homolog (S. cerevisiae)	1.112966	0.107	NA
103432	6430706H07Rik	RIKEN cDNA 6430706H07 gene	1.112966	0.346	0.5814
209225	Zfp710	zinc finger protein 710	1.112966	0.1028	NA
217869	Eif5	eukaryotic translation initiation factor 5	1.112966	0.2636	0.4994
233276	Tubgcp5	tubulin, gamma complex associated protein 5	1.112966	0.4703	0.692
13436	Dnmt3b	DNA methyltransferase 3B	1.112842	0.3067	0.5432
396184	Flrt1	fibronectin leucine rich transmembrane protein 1	1.112842	0.1556	NA
67732	Iah1	isoamyl acetate-hydrolyzing esterase 1 homolog (S. cerevisiae)	1.112718	0.2084	0.4351
71163	Zfp626	zinc finger protein 626	1.112718	0.3285	0.565

77744	6720463M24Rik	RIKEN cDNA 6720463M24 gene	1.112718	0.0907	NA
69274	Ctdspl	CTD (carboxy-terminal domain, RNA polymerase II, polypeptide A) small phosphatase-like	1.112595	0.3069	0.5433
75695	Rilpl1	Rab interacting lysosomal protein-like 1	1.112595	0.1002	NA
77125	Il33	interleukin 33	1.112595	0.2321	0.4635
108837	Ibtk	inhibitor of Bruton agammaglobulinemia tyrosine kinase	1.112595	0.2371	0.4693
75552	Paqr9	progestin and adipoQ receptor family member IX	1.112471	0.3406	0.5764
229725	Clcc1	chloride channel CLIC-like 1	1.112347	0.08252	NA
54216	Pcdh7	protocadherin 7	1.112223	0.3991	0.6295
67096	Mmachc	methylmalonic aciduria cblC type, with homocystinuria	1.112223	0.1281	NA
553096	LOC553096	hypothetical LOC553096	1.112223	0.1019	NA
11855	Arhgap5	Rho GTPase activating protein 5	1.1121	0.3755	0.6094
13808	Eno3	enolase 3, beta muscle	1.1121	0.3027	0.5388
110789	Gpr98	G protein-coupled receptor 98	1.1121	0.558	0.757
66220	Zdhhc12	zinc finger, DHHC domain containing 12	1.111976	0.1113	NA
77908	9230113P08Rik	RIKEN cDNA 9230113P08 gene	1.111976	0.7493	0.8785
320358	C430003N24Rik	RIKEN cDNA C430003N24 gene	1.111976	0.2096	0.4366
13595	Ebp	phenylalkylamine Ca2+ antagonist (emopamil) binding protein	1.111852	0.2537	0.4885
17722	ND6	NADH dehydrogenase subunit 6	1.111852	0.5817	0.7728
76407	Sun5	Sad1 and UNC84 domain containing 5	1.111852	0.4222	0.6501
546164	Gm5921	predicted gene 5921	1.111852	0.326	0.5626
100503302	LOC100503302	40S ribosomal protein S19-like	1.111852	0.2281	0.459
70605	Zdhhc24	zinc finger, DHHC domain containing 24	1.111729	0.4279	0.655
193796	Kdm4b	lysine (K)-specific demethylase 4B	1.111729	0.334	0.5697
215693	Zmat1	zinc finger, matrin type 1	1.111729	0.2185	0.4478
385380	Tex28	testis expressed 28	1.111729	0.3925	0.624
17190	Mbd1	methyl-CpG binding domain protein 1	1.111605	0.05473	NA
17261	Mef2d	myocyte enhancer factor 2D	1.111605	0.3165	0.5532
68106	Nt5c3l	5'-nucleotidase, cytosolic III-like	1.111605	0.1296	NA
233335	Synm	synemin, intermediate filament protein	1.111605	0.1171	NA
171207	Arhgap4	Rho GTPase activating protein 4	1.111482	0.1177	NA
229543	Ints3	integrator complex subunit 3	1.111482	0.3263	0.5628
67091	Trappc6a	trafficking protein particle complex 6A	1.111358	0.07697	NA
69976	Galk2	galactokinase 2	1.111358	0.2278	0.4587
223722	Mcat	malonyl CoA:ACP acyltransferase (mitochondrial)	1.111358	0.2798	0.5152
12896	Cpt2	carnitine palmitoyltransferase 2	1.111235	0.2161	0.4448
14748	Gpr3	G-protein coupled receptor 3	1.111235	0.5414	0.7455
21414	Tcf7	transcription factor 7, T-cell specific	1.111235	0.3016	0.5375
16155	Il10rb	interleukin 10 receptor, beta	1.111111	0.07265	NA
56404	Trip4	thyroid hormone receptor interactor 4	1.111111	0.4864	0.7045
69263	Rfc3	replication factor C (activator 1) 3	1.111111	0.3155	0.5522
217306	Cd300e	CD300e antigen	1.111111	0.5461	0.7482
380629	Heca	headcase homolog (Drosophila)	1.111111	0.2405	0.4732
19212	Pter	phosphotriesterase related	1.110864	0.1527	NA
65103	Arl6ip6	ADP-ribosylation factor-like 6 interacting protein 6	1.110864	0.2873	0.5228
66263	1810014B01Rik	RIKEN cDNA 1810014B01 gene	1.110864	0.1044	NA
69752	Zfp511	zinc finger protein 511	1.110864	0.1392	NA
234373	Sugp2	SURP and G patch domain containing 2	1.110864	0.1745	NA
320319	E330018D03Rik	RIKEN cDNA E330018D03 gene	1.110864	0.4787	0.6984
433416	Gm13547	predicted gene 13547	1.110864	0.6289	0.8043

677044	Gm10653	ribsomal protein S2 pseudogene	1.110864	0.1102	NA
12317	Calr	calreticulin	1.110741	0.1326	NA
51793	Ddah2	dimethylarginine dimethylaminohydrolase 2	1.110741	0.1297	NA
30959	Ddx25	DEAD (Asp-Glu-Ala-Asp) box polypeptide 25	1.110618	0.62	0.7991
52838	Dnlz	DNL-type zinc finger	1.110618	0.2308	0.4621
74132	Rnf6	ring finger protein (C3H2C3 type) 6	1.110618	0.05474	NA
319181	Hist1h2bg	histone cluster 1, H2bg	1.110618	0.4173	0.6456
68137	Kdelr1	KDEL (Lys-Asp-Glu-Leu) endoplasmic reticulum protein retention receptor 1	1.110494	0.1198	NA
330188	Ccdc63	coiled-coil domain containing 63	1.110494	0.4225	0.6503
18186	Nrp1	neuropilin 1	1.110371	0.1899	NA
19730	Ralgds	ral guanine nucleotide dissociation stimulator	1.110371	0.5116	0.7223
100855	Tbc1d14	TBC1 domain family, member 14	1.110371	0.02923	NA
101543	Wtip	WT1-interacting protein	1.110371	0.05632	NA
210622	Pamr1	peptidase domain containing associated with muscle regeneration 1	1.110371	0.1791	NA
54352	Irx5	Iroquois related homeobox 5 (Drosophila)	1.110248	0.4819	0.7006
76251	0610007P08Rik	RIKEN cDNA 0610007P08 gene	1.110248	0.1296	NA
226856	Lpgat1	lysophosphatidylglycerol acyltransferase 1	1.110248	0.1186	NA
12042	Bcl10	B-cell leukemia/lymphoma 10	1.110124	0.2501	0.4841
106759	Ticam1	toll-like receptor adaptor molecule 1	1.110124	0.4808	0.6996
16478	Jund	Jun proto-oncogene related gene d	1.110001	0.2066	0.4327
21665	Tdg	thymine DNA glycosylase	1.110001	0.2361	0.4685
55927	Hes6	hairy and enhancer of split 6 (Drosophila)	1.110001	0.07229	NA
66641	Sike1	suppressor of IKBKE 1	1.110001	0.1343	NA
78921	9130019O22Rik	RIKEN cDNA 9130019O22 gene	1.110001	0.5285	0.7353
320119	Rps6kc1	ribosomal protein S6 kinase polypeptide 1	1.110001	0.3761	0.6099
15939	Ier5	immediate early response 5	1.109878	0.3307	0.5673
21453	Tcof1	Treacher Collins Franceschetti syndrome 1, homolog	1.109878	0.2465	0.4806
21788	Tfpi	tissue factor pathway inhibitor	1.109878	0.6475	0.8163
26901	Deb1	differentially expressed in B16F10 1	1.109878	0.1199	NA
240028	Lnpep	leucyl/cystinyl aminopeptidase	1.109755	0.1661	NA
15109	Hal	histidine ammonia lyase	1.109508	0.7374	0.8714
19283	Ptprz1	protein tyrosine phosphatase, receptor type Z, polypeptide 1	1.109508	0.2043	0.4306
217666	L2hgdh	L-2-hydroxyglutarate dehydrogenase	1.109508	0.3106	0.5469
66092	Ghitm	growth hormone inducible transmembrane protein	1.109385	0.1109	NA
69008	Cab39l	calcium binding protein 39-like	1.109385	0.3104	0.5468
71228	Dlg5	discs, large homolog 5 (Drosophila)	1.109385	0.03925	NA
107815	Scml2	sex comb on midleg-like 2 (Drosophila)	1.109385	0.2143	0.4426
236794	Slc9a6	solute carrier family 9 (sodium/hydrogen exchanger), member 6	1.109385	0.1643	NA
432572	Specc1	sperm antigen with calponin homology and coiled-coil domains 1	1.109385	0.7612	0.8843
19340	Rab3d	RAB3D, member RAS oncogene family	1.109262	0.3927	0.6241
12034	Phb2	prohibitin 2	1.109139	0.1861	NA
320343	Lypd6	LY6/PLAUR domain containing 6	1.109139	0.2844	0.52
320615	Dopey1	dopey family member 1	1.109139	0.6554	0.8212
16408	Itgal	integrin alpha L	1.109016	0.5893	0.7784
20678	Sox5	SRY-box containing gene 5	1.109016	0.3928	0.6241
665189	Gm7536	predicted gene 7536	1.109016	0.2194	0.449
11682	Alk	anaplastic lymphoma kinase	1.108893	0.09781	NA
217333	Trim47	tripartite motif-containing 47	1.108893	0.3008	0.5367
217480	Dgkb	diacylglycerol kinase, beta	1.108893	0.2179	0.447

434758	Rhox3h	reproductive homeobox 3H	1.108893	0.5347	0.7401
232440	H2afj	H2A histone family, member J	1.10877	0.1481	NA
233893	Zfp764	zinc finger protein 764	1.10877	0.09872	NA
100042757	Gm4013	predicted gene 4013	1.10877	0.3509	0.5866
16172	Il17ra	interleukin 17 receptor A	1.108647	0.5418	0.7457
23950	Dnajb6	DnaJ (Hsp40) homolog, subfamily B, member 6	1.108647	0.1135	NA
30956	Aass	aminoadipate-semialdehyde synthase	1.108525	0.1777	NA
228911	Tshz2	teashirt zinc finger family member 2	1.108525	0.3755	0.6094
16430	Stt3a	STT3, subunit of the oligosaccharyltransferase complex, homolog A (S. cerevisiae)	1.108402	0.3718	0.6067
16890	Lipe	lipase, hormone sensitive	1.108402	0.3081	0.5443
18674	Slc25a3	solute carrier family 25 (mitochondrial carrier, phosphate carrier), member 3	1.108402	0.1087	NA
59038	Pxmp4	peroxisomal membrane protein 4	1.108402	0.2854	0.5208
68263	Pdhb	pyruvate dehydrogenase (lipoamide) beta	1.108402	0.1835	NA
104110	Adcy4	adenylate cyclase 4	1.108402	0.4269	0.6541
552912	LOC552912	hypothetical LOC552912	1.108402	0.213	0.4408
670912	Gm9510	predicted gene 9510	1.108402	0.5951	0.782
17125	Smad1	MAD homolog 1 (Drosophila)	1.108279	0.5384	0.7428
72938	Hspb11	heat shock protein family B (small), member 11	1.108279	0.1035	NA
77106	Tmem181a	transmembrane protein 181A	1.108279	0.06366	NA
217410	Trib2	tribbles homolog 2 (Drosophila)	1.108279	0.4858	0.7043
50782	Rgs11	regulator of G-protein signaling 11	1.108156	0.2144	0.4427
56706	Ccnl1	cyclin L1	1.108156	0.5793	0.7714
241053	Rpl12-ps1	ribosomal protein L12, pseudogene 1	1.108156	0.204	NA
14783	Grb10	growth factor receptor bound protein 10	1.108033	0.6185	0.7978
57321	Terf2ip	telomeric repeat binding factor 2, interacting protein	1.108033	0.532	0.7378
232089	Elmod3	ELMO/CED-12 domain containing 3	1.108033	0.1968	NA
671535	Parp10	poly (ADP-ribose) polymerase family, member 10	1.108033	0.1278	NA
19230	Twf1	twinfilin, actin-binding protein, homolog 1 (Drosophila)	1.10791	0.03994	NA
402733	A930009L07Rik	RIKEN cDNA A930009L07 gene	1.10791	0.3594	0.5946
433102	Sfta2	surfactant associated 2	1.10791	0.406	0.6356
13543	Dvl2	dishevelled 2, dsh homolog (Drosophila)	1.107788	0.1312	NA
74569	Ttc17	tetratricopeptide repeat domain 17	1.107788	0.2221	0.4526
259086	Olfr609	olfactory receptor 609	1.107788	0.63	0.8048
66848	Fuca2	fucosidase, alpha-L- 2, plasma	1.107665	0.2224	0.4527
677044	Gm10653	ribsomal protein S2 pseudogene	1.107665	0.2415	0.4745
71685	Galnt14	UDP-N-acetyl-alpha-D-galactosamine:polypeptide N-acetylgalactosaminyltransferase 14	1.10742	0.3578	0.5926
353169	Slc2a12	solute carrier family 2 (facilitated glucose transporter), member 12	1.10742	0.07314	NA
26556	Homer1	homer homolog 1 (Drosophila)	1.107297	0.4801	0.6991
30926	Glrx3	glutaredoxin 3	1.107297	0.1493	NA
70470	Rprd1b	regulation of nuclear pre-mRNA domain containing 1B	1.107297	0.2785	0.514
76983	Scfd1	Sec1 family domain containing 1	1.107297	0.08226	NA
97761	Sgsm2	small G protein signaling modulator 2	1.107297	0.119	NA
57258	Xpo4	exportin 4	1.107174	0.1044	NA
73068	Fut11	fucosyltransferase 11	1.107174	0.345	0.5804
75284	Bcdin3d	BCDIN3 domain containing	1.107174	0.2459	0.4797
108014	Srsf9	serine/arginine-rich splicing factor 9	1.107174	0.151	NA
12283	Cab39	calcium binding protein 39	1.107052	0.3314	0.5677
20610	Sumo3	SMT3 suppressor of mif two 3 homolog 3 (yeast)	1.107052	0.2308	0.4621
53885	Nphp1	nephronophthisis 1 (juvenile) homolog (human)	1.107052	0.6227	0.8003

668382	Pabpc1l2b-ps	poly(A) binding protein, cytoplasmic 1-like 2B, pseudogene	1.107052	0.6893	0.8415
16418	Eif6	eukaryotic translation initiation factor 6	1.106929	0.3722	0.6068
27176	Rpl7a	ribosomal protein L7A	1.106929	0.5845	0.7749
665610	Gm7710	predicted gene 7710	1.106929	0.2274	0.4586
100504876	LOC100504876	60S ribosomal protein L7a-like	1.106929	0.04684	NA
22253	Unc5c	unc-5 homolog C (C. elegans)	1.106807	0.2741	0.5097
99382	Abtb2	ankyrin repeat and BTB (POZ) domain containing 2	1.106807	0.2042	NA
76108	Rap2a	RAS related protein 2a	1.106684	0.03713	NA
77987	Ascc3	activating signal cointegrator 1 complex subunit 3	1.106684	0.4514	0.6761
620695	Gm13889	predicted gene 13889	1.106684	0.03933	NA
12554	Cdh13	cadherin 13	1.106562	0.3028	0.5388
17772	Mtm1	X-linked myotubular myopathy gene 1	1.106562	0.1622	NA
26927	Foxl2	forkhead box L2	1.106562	0.3555	0.5903
71777	Ing3	inhibitor of growth family, member 3	1.106562	0.2591	0.4943
72711	2810037O22Rik	RIKEN cDNA 2810037O22 gene	1.106562	0.2841	0.5199
625540	Gm6598	predicted gene 6598	1.106562	0.3703	0.6052
16542	Kdr	kinase insert domain protein receptor	1.106439	0.4008	0.6309
70375	Ica1l	islet cell autoantigen 1-like	1.106439	0.2713	0.5068
71599	Senp8	SUMO/sentrin specific peptidase 8	1.106439	0.4967	0.7116
268739	Arhgef40	Rho guanine nucleotide exchange factor (GEF) 40	1.106439	0.1961	NA
12840	Col9a2	collagen, type IX, alpha 2	1.106317	0.3796	0.6132
55934	rp9	retinitis pigmentosa 9 (human)	1.106317	0.1636	NA
68312	Gstm7	glutathione S-transferase, mu 7	1.106317	0.5229	0.7307
68397	0610039H22Rik	RIKEN cDNA 0610039H22 gene	1.106317	0.3544	0.5891
74182	Gpcpd1	glycerophosphocholine phosphodiesterase GDE1 homolog (S. cerevisiae)	1.106317	0.5238	0.7314
77255	9430014F16Rik	RIKEN cDNA 9430014F16 gene	1.106317	0.581	0.7726
17113	M6pr	mannose-6-phosphate receptor, cation dependent	1.106195	0.03711	NA
235416	Lman1l	lectin, mannose-binding 1 like	1.106195	0.2945	0.5306
16956	Lpl	lipoprotein lipase	1.106072	0.2321	0.4635
16973	Lrp5	low density lipoprotein receptor-related protein 5	1.106072	0.3727	0.6069
19943	Rpl28	ribosomal protein L28	1.106072	0.06899	NA
20661	Sort1	sortilin 1	1.106072	0.5929	0.7805
237353	Sh3rf3	SH3 domain containing ring finger 3	1.106072	0.1538	NA
108909	Aida	axin interactor, dorsalization associated	1.105828	0.2548	0.4896
110842	Etfa	electron transferring flavoprotein, alpha polypeptide	1.105828	0.2802	0.5156
320267	Fubp3	far upstream element (FUSE) binding protein 3	1.105828	0.4277	0.6548
16480	Jup	junction plakoglobin	1.105705	0.7568	0.8824
52670	Cpsf4l	cleavage and polyadenylation specific factor 4-like	1.105705	0.6487	0.8172
69638	Enho	energy homeostasis associated	1.105705	0.07826	NA
66548	Adamtsl5	ADAMTS-like 5	1.105583	0.6312	0.8058
70296	Tbc1d13	TBC1 domain family, member 13	1.105583	0.3815	0.615
103710	Slc35e4	solute carrier family 35, member E4	1.105583	0.2994	0.5351
14081	Acsl1	acyl-CoA synthetase long-chain family member 1	1.105461	0.2002	NA
16156	Il11	interleukin 11	1.105461	0.3215	0.558
56707	Zfp111	zinc finger protein 111	1.105461	0.3317	0.5678
98733	Obsl1	obscurin-like 1	1.105461	0.2098	NA
17155	Man1a	mannosidase 1, alpha	1.105339	0.5418	0.7457
19047	Ppp1cc	protein phosphatase 1, catalytic subunit, gamma isoform	1.105339	0.2992	0.5348
70503	Ddo	D-aspartate oxidase	1.105339	0.2041	NA

83771	Tas1r3	taste receptor, type 1, member 3	1.105339	0.4975	0.7125
223453	Dap	death-associated protein	1.105339	0.2527	0.4872
231855	C330006K01Rik	RIKEN cDNA C330006K01 gene	1.105339	0.5197	0.7282
11550	Adra1d	adrenergic receptor, alpha 1d	1.105217	0.2807	0.5161
12169	Bmx	BMX non-receptor tyrosine kinase	1.105217	0.4064	0.6359
57342	Parva	parvin, alpha	1.105217	0.1124	NA
72008	Zfyve19	zinc finger, FYVE domain containing 19	1.105217	0.1019	NA
76937	2810429I04Rik	RIKEN cDNA 2810429I04 gene	1.105217	0.5494	0.7506
77113	Klhl2	kelch-like 2, Mayven (Drosophila)	1.105217	0.1191	NA
216543	Cep68	centrosomal protein 68	1.105217	0.3483	0.5839
237320	Aldh8a1	aldehyde dehydrogenase 8 family, member A1	1.105217	0.6046	0.7881
12523	Cd84	CD84 antigen	1.105094	0.3737	0.6081
67925	1700066D14Rik	RIKEN cDNA 1700066D14 gene	1.105094	0.7789	0.8935
223978	Cpped1	calcineurin-like phosphoesterase domain containing 1	1.105094	0.2385	0.4708
380836	Mrs2	MRS2 magnesium homeostasis factor homolog (S. cerevisiae)	1.105094	0.1978	NA
100039478	Gm11810	predicted gene 11810	1.105094	0.3078	0.544
27058	Srp9	signal recognition particle 9	1.104972	0.3281	0.5645
28146	Serp1	stress-associated endoplasmic reticulum protein 1	1.104972	0.2618	0.4969
65116	Prrg2	proline-rich Gla (G-carboxyglutamic acid) polypeptide 2	1.104972	0.2798	0.5153
77462	Tmem116	transmembrane protein 116	1.104972	0.2751	0.5105
97654	C81189	expressed sequence C81189	1.104972	0.4728	0.6936
233887	Zfp553	zinc finger protein 553	1.104972	0.07963	NA
327954	Dnahc2	dynein, axonemal, heavy chain 2	1.104972	0.496	0.7111
381356	5930434B04Rik	RIKEN cDNA 5930434B04 gene	1.104972	0.1469	NA
20846	Stat1	signal transducer and activator of transcription 1	1.10485	0.3812	0.6149
70237	Bhlhb9	basic helix-loop-helix domain containing, class B9	1.10485	0.3527	0.5877
328779	Hs3st6	heparan sulfate (glucosamine) 3-O-sulfotransferase 6	1.10485	0.3416	0.5772
638532	Gm7241	predicted pseudogene 7241	1.10485	0.1221	NA
19889	Rp2h	retinitis pigmentosa 2 homolog (human)	1.104728	0.3013	0.5371
30934	Tor1b	torsin family 1, member B	1.104728	0.3748	0.6089
30935	Tor3a	torsin family 3, member A	1.104728	0.391	0.6228
67972	Atp2b1	ATPase, Ca++ transporting, plasma membrane 1	1.104728	0.4607	0.6837
107650	Pi4kb	phosphatidylinositol 4-kinase, catalytic, beta polypeptide	1.104728	0.2117	NA
20383	Srsf3	serine/arginine-rich splicing factor 3	1.104606	0.4192	0.6472
75465	Dynlrb2	dynein light chain roadblock-type 2	1.104606	0.3814	0.615
101604	E430018J23Rik	RIKEN cDNA E430018J23 gene	1.104606	0.1161	NA
213391	Rassf4	Ras association (RalGDS/AF-6) domain family member 4	1.104606	0.2573	0.4925
13849	Ephx1	epoxide hydrolase 1, microsomal	1.104484	0.2136	NA
72836	Pot1b	protection of telomeres 1B	1.104484	0.486	0.7043
628012	Gm16441	predicted pseudogene 16441	1.104484	0.6867	0.8402
68758	Abhd11	abhydrolase domain containing 11	1.104362	0.2527	0.4872
77775	A430103D13Rik	RIKEN cDNA A430103D13 gene	1.104362	0.2658	0.5015
16985	Lsp1	lymphocyte specific 1	1.10424	0.1529	NA
74749	5830405M20Rik	RIKEN cDNA 5830405M20 gene	1.10424	0.5632	0.7609
232341	Wnk1	WNK lysine deficient protein kinase 1	1.10424	0.3923	0.6238
16210	Impact	imprinted and ancient	1.104118	0.6496	0.8177
72147	Zbtb46	zinc finger and BTB domain containing 46	1.104118	0.3315	0.5677
266692	Cpne1	copine I	1.104118	0.5918	0.7799
78151	4930404H24Rik	RIKEN cDNA 4930404H24 gene	1.103996	0.6066	0.7895

212508	Mtg1	mitochondrial GTPase 1 homolog (S. cerevisiae)	1.103996	0.1047	NA
237943	Gpatch8	G patch domain containing 8	1.103996	0.1558	NA
100502683	LOC100502683	40S ribosomal protein S29-like	1.103996	0.2078	NA
11987	Slc7a1	solute carrier family 7 (cationic amino acid transporter, y+ system), member 1	1.103875	0.2746	0.5101
66240	Kcne1l	potassium voltage-gated channel, Isk-related family, member 1-like, pseudogene	1.103875	0.2848	0.5202
67264	Ndufb8	NADH dehydrogenase (ubiquinone) 1 beta subcomplex 8	1.103875	0.1378	NA
68612	Ube2c	ubiquitin-conjugating enzyme E2C	1.103875	0.672	0.832
270106	Rpl13	ribosomal protein L13	1.103875	0.2661	0.5016
12695	Inadl	InaD-like (Drosophila)	1.103753	0.5688	0.7635
22388	Wdr1	WD repeat domain 1	1.103753	0.1282	NA
100210	Gpn2	GPN-loop GTPase 2	1.103753	0.1342	NA
107242	AI837181	expressed sequence AI837181	1.103753	0.4006	0.6309
11856	Arhgap6	Rho GTPase activating protein 6	1.103631	0.3773	0.6113
14874	Gstz1	glutathione transferase zeta 1 (maleylacetoacetate isomerase)	1.103631	0.2049	NA
15975	Ifnar1	interferon (alpha and beta) receptor 1	1.103631	0.2483	0.4824
18247	Oaz2-ps	ornithine decarboxylase antizyme 2, pseudogene	1.103631	0.1997	NA
56417	Adar	adenosine deaminase, RNA-specific	1.103631	0.1072	NA
70963	4931402H11Rik	RIKEN cDNA 4931402H11 gene	1.103631	0.659	0.8238
73738	Haus7	HAUS augmin-like complex, subunit 7	1.103631	0.1303	NA
228139	P2rx3	purinergic receptor P2X, ligand-gated ion channel, 3	1.103631	0.1982	NA
100088	Rcc1	regulator of chromosome condensation 1	1.103509	0.1043	NA
223693	Tmem184b	transmembrane protein 184b	1.103509	0.2228	NA
26459	Slc27a5	solute carrier family 27 (fatty acid transporter), member 5	1.103387	0.1335	NA
27756	Lsm2	LSM2 homolog, U6 small nuclear RNA associated (S. cerevisiae)	1.103387	0.1469	NA
67212	Mrpl55	mitochondrial ribosomal protein L55	1.103387	0.2554	0.4902
73845	Ankrd42	ankyrin repeat domain 42	1.103387	0.3602	0.5951
108150	Galnt7	UDP-N-acetyl-alpha-D-galactosamine: polypeptide N-acetylgalactosaminyltransferase 7	1.103387	0.2123	NA
245532	Awat2	acyl-CoA wax alcohol acyltransferase 2	1.103387	0.5463	0.7482
100040086	Gm2590	predicted gene 2590	1.103387	0.2857	0.5211
20088	Rps24	ribosomal protein S24	1.103266	0.3272	0.5636
57815	Spata5	spermatogenesis associated 5	1.103266	0.1937	NA
214572	Prmt7	protein arginine N-methyltransferase 7	1.103266	0.322	0.5585
19989	Rpl7	ribosomal protein L7	1.103144	0.2785	0.514
22590	Xpa	xeroderma pigmentosum, complementation group A	1.103144	0.1645	NA
267019	Rps15a	ribosomal protein S15A	1.103144	0.09319	NA
70448	2610204G22Rik	RIKEN cDNA 2610204G22 gene	1.103022	0.1406	NA
73449	1700066B19Rik	RIKEN cDNA 1700066B19 gene	1.103022	0.3785	0.6121
214162	Mll1	myeloid/lymphoid or mixed-lineage leukemia 1	1.103022	0.5646	0.7616
217370	BC017643	cDNA sequence BC017643	1.103022	0.05566	NA
268420	Alkbh5	alkB, alkylation repair homolog 5 (E. coli)	1.102901	0.1632	NA
100038580	6820445E23Rik	RIKEN cDNA 6820445E23 gene	1.102901	0.4319	0.6591
18453	P4hb	prolyl 4-hydroxylase, beta polypeptide	1.102779	0.1229	NA
65971	Tbata	thymus, brain and testes associated	1.102779	0.6914	0.8428
67246	2810474O19Rik	RIKEN cDNA 2810474O19 gene	1.102779	0.296	0.5317
106967	4732423E21Rik	RIKEN cDNA 4732423E21 gene	1.102779	0.3657	0.6013
218490	Btf3	basic transcription factor 3	1.102779	0.2978	0.5337
14531	Gcm1	glial cells missing homolog 1 (Drosophila)	1.102657	0.5181	0.7273
52680	D13Ertd787e	DNA segment, Chr 13, ERATO Doi 787, expressed	1.102657	0.2419	0.4748
21453	Tcof1	Treacher Collins Franceschetti syndrome 1, homolog	1.102536	0.1688	NA

83602	Gtf2a1	general transcription factor II A, 1	1.102536	0.426	0.6534
214987	Chtf8	CTF8, chromosome transmission fidelity factor 8 homolog (S. cerevisiae)	1.102536	0.1238	NA
215690	Nav1	neuron navigator 1	1.102536	0.4165	0.645
544971	Bdp1	B double prime 1, subunit of RNA polymerase III transcription initiation factor IIIB	1.102536	0.4561	0.6795
16828	Ldha	lactate dehydrogenase A	1.102414	0.2681	0.5035
244418	D8Ertd82e	DNA segment, Chr 8, ERATO Doi 82, expressed	1.102414	0.2672	0.5027
320039	A030010E16Rik	RIKEN cDNA A030010E16 gene	1.102414	0.2875	0.523
70544	5730437N04Rik	RIKEN cDNA 5730437N04 gene	1.102293	0.1146	NA
101358	Fbxl14	F-box and leucine-rich repeat protein 14	1.102293	0.3117	0.5482
552873	LOC552873	hypothetical LOC552873	1.102293	0.4064	0.6359
69363	Spaca4	sperm acrosome associated 4	1.102171	0.7444	0.8758
99377	Sall4	sal-like 4 (Drosophila)	1.102171	0.4525	0.677
100101807	1700047I17Rik2	RIKEN cDNA 1700047I17 gene 2	1.102171	0.1379	NA
56711	Plag1	pleiomorphic adenoma gene 1	1.10205	0.5648	0.7617
210711	Mcmbp	MCM (minichromosome maintenance deficient) binding protein	1.10205	0.1729	NA
319148	Hist1h3c	histone cluster 1, H3c	1.10205	0.6813	0.8369
19125	Prodh	proline dehydrogenase	1.101928	0.4528	0.6772
22323	Vasp	vasodilator-stimulated phosphoprotein	1.101928	0.06372	NA
66313	Smurf2	SMAD specific E3 ubiquitin protein ligase 2	1.101928	0.2479	0.4821
673094	Cd99	CD99 antigen	1.101928	0.3096	0.5462
17955	Nap1l4	nucleosome assembly protein 1-like 4	1.101564	0.6634	0.8269
26921	Map4k4	mitogen-activated protein kinase kinase kinase kinase 4	1.101564	0.1505	NA
76022	Gon4l	gon-4-like (C.elegans)	1.101564	0.2985	0.5345
227707	BC005624	cDNA sequence BC005624	1.101564	0.4272	0.6543
231841	AA881470	EST AA881470	1.101564	0.4813	0.7
100042464	2610203C20Rik	RIKEN cDNA 2610203C20 gene	1.101564	0.5427	0.7463
17872	Ppp1r15a	protein phosphatase 1, regulatory (inhibitor) subunit 15A	1.101443	0.2031	NA
76646	Wdr38	WD repeat domain 38	1.101443	0.276	0.5115
217030	Synrg	synergin, gamma	1.101443	0.3217	0.5582
99311	Commd7	COMM domain containing 7	1.101322	0.2037	NA
12804	Cntfr	ciliary neurotrophic factor receptor	1.101079	0.5382	0.7426
224794	Enpp4	ectonucleotide pyrophosphatase/phosphodiesterase 4	1.101079	0.1555	NA
12398	Cbfa2t3	core-binding factor, runt domain, alpha subunit 2, translocated to, 3 (human)	1.100837	0.6773	0.8348
18207	Nthl1	nth (endonuclease III)-like 1 (E.coli)	1.100837	0.2173	NA
22781	Ikzf4	IKAROS family zinc finger 4	1.100837	0.2886	0.5248
29815	Bcar3	breast cancer anti-estrogen resistance 3	1.100837	0.1553	NA
75320	Etnk1	ethanolamine kinase 1	1.100837	0.5823	0.7732
225745	Haus1	HAUS augmin-like complex, subunit 1	1.100837	0.2005	NA
192216	Tmem47	transmembrane protein 47	1.100715	0.5453	0.7478
434674	Gm5631	predicted gene 5631	1.100715	0.4236	0.6513
14431	Gamt	guanidinoacetate methyltransferase	1.100473	0.4452	0.6708
66111	Tmed3	transmembrane emp24 domain containing 3	1.100473	0.244	NA
217721	Mfsd7c	major facilitator superfamily domain containing 7C	1.100473	0.5291	0.7356
20511	Slc1a2	solute carrier family 1 (glial high affinity glutamate transporter), member 2	1.100352	0.5684	0.7634
21416	Tcf7l2	transcription factor 7-like 2, T-cell specific, HMG-box	1.100352	0.367	0.6022
93886	Pcdhb15	protocadherin beta 15	1.100352	0.08808	NA
212862	Chpt1	choline phosphotransferase 1	1.100352	0.3193	0.5558
229782	Slc35a3	solute carrier family 35 (UDP-N-acetylglucosamine (UDP-GlcNAc) transporter), member 3	1.100352	0.1991	NA
100041488	Gm11937	predicted gene 11937	1.100352	0.333	0.5689

17001	Ltc4s	leukotriene C4 synthase	1.100231	0.3514	0.5868
18759	Prkci	protein kinase C, iota	1.100231	0.04223	NA
21894	Tln1	talin 1	1.100231	0.4819	0.7006
54473	Tollip	toll interacting protein	1.100231	0.3347	0.5705
68463	Mrpl14	mitochondrial ribosomal protein L14	1.100231	0.221	NA
14560	Gdf10	growth differentiation factor 10	1.10011	0.4416	0.6675
70951	Spata1	spermatogenesis associated 1	1.10011	0.3991	0.6295
104303	Arl1	ADP-ribosylation factor-like 1	1.10011	0.3543	0.589
208266	Dot1l	DOT1-like, histone H3 methyltransferase (S. cerevisiae)	1.10011	0.197	NA
224090	Tmem44	transmembrane protein 44	1.10011	0.2683	0.5036
72554	Utp14a	UTP14, U3 small nucleolar ribonucleoprotein, homolog A (yeast)	1.099989	0.4386	0.6647
76826	Nubpl	nucleotide binding protein-like	1.099989	0.282	0.5174
214601	Slc10a3	solute carrier family 10 (sodium/bile acid cotransporter family), member 3	1.099868	0.2828	0.5182
16006	Igfbp1	insulin-like growth factor binding protein 1	1.099747	0.5006	0.7145
22691	Zscan2	zinc finger and SCAN domain containing 2	1.099747	0.567	0.7625
29861	Dpf1	D4, zinc and double PHD fingers family 1	1.099626	0.2645	0.5006
66132	1110008L16Rik	RIKEN cDNA 1110008L16 gene	1.099626	0.5033	0.716
76174	6330526H18Rik	RIKEN cDNA 6330526H18 gene	1.099626	0.3512	0.5867
98402	Sh3bp4	SH3-domain binding protein 4	1.099626	0.4531	0.6774
621818	Gm9785	BMI1-like	1.099626	0.5378	0.7424
12928	Crk	v-crk sarcoma virus CT10 oncogene homolog (avian)	1.099505	0.3019	0.5377
17252	Rdh11	retinol dehydrogenase 11	1.099505	0.06413	NA
74349	Fam160a2	family with sequence similarity 160, member A2	1.099505	0.1342	NA
76846	Rps9	ribosomal protein S9	1.099505	0.1137	NA
105976	AU022793	expressed sequence AU022793	1.099505	0.8171	0.9163
19188	Psme2	proteasome (prosome, macropain) 28 subunit, beta	1.099384	0.1101	NA
212670	Catsper2	cation channel, sperm associated 2	1.099384	0.2781	0.5137
75751	Ipo4	importin 4	1.099263	0.2466	NA
170707	Usp48	ubiquitin specific peptidase 48	1.099263	0.5707	0.7647
100125931	A130049A11Rik	RIKEN cDNA A130049A11 gene	1.099263	0.4269	0.6541
18028	Nfib	nuclear factor I/B	1.099143	0.1869	NA
20104	Rps6	ribosomal protein S6	1.099143	0.2095	NA
66614	Gpatch4	G patch domain containing 4	1.099143	0.1752	NA
93877	Pcdhb6	protocadherin beta 6	1.099143	0.09836	NA
107045	Lars	leucyl-tRNA synthetase	1.099143	0.1655	NA
622935	Krtap20-2	keratin associated protein 20-2	1.099143	0.5784	0.7707
100038969	Gm14958	predicted gene 14958	1.099143	0.3577	0.5926
14828	Hspa5	heat shock protein 5	1.099022	0.1169	NA
52705	Krr1	KRR1, small subunit (SSU) processome component, homolog (yeast)	1.098901	0.06523	NA
218756	Slc4a7	solute carrier family 4, sodium bicarbonate cotransporter, member 7	1.098901	0.4471	0.6725
237880	1700071K01Rik	RIKEN cDNA 1700071K01 gene	1.098901	0.6394	0.8111
319780	D030002E05Rik	RIKEN cDNA D030002E05 gene	1.098901	0.3551	0.5898
14433	Gapdh	glyceraldehyde-3-phosphate dehydrogenase	1.09878	0.4328	0.66
20901	Strap	serine/threonine kinase receptor associated protein	1.09878	0.2492	NA
80898	Erap1	endoplasmic reticulum aminopeptidase 1	1.09878	0.2966	0.5325
104457	0610010K14Rik	RIKEN cDNA 0610010K14 gene	1.09878	0.1787	NA
223775	Pim3	proviral integration site 3	1.09878	0.465	0.687
226251	Ablim1	actin-binding LIM protein 1	1.09878	0.489	0.7061
269261	Rpl12	ribosomal protein L12	1.09878	0.1306	NA

384864	Gm1943	WD repeat domain 70 pseudogene	1.09878	0.2362	NA
433050	Rpl26-ps4	ribosomal protein L26, pseudogene 4	1.09878	0.1453	NA
14055	Ezh1	enhancer of zeste homolog 1 (Drosophila)	1.09866	0.06145	NA
15108	Hsd17b10	hydroxysteroid (17-beta) dehydrogenase 10	1.09866	0.3302	0.5668
78889	Wsb1	WD repeat and SOCS box-containing 1	1.09866	0.3086	0.5449
208366	Rpp40	ribonuclease P 40 subunit (human)	1.09866	0.491	0.7078
225266	Klhl14	kelch-like 14 (Drosophila)	1.09866	0.5697	0.7639
243846	Ccdc9	coiled-coil domain containing 9	1.09866	0.5341	0.7395
21991	Tpi1	triosephosphate isomerase 1	1.098539	0.2135	NA
71782	Ankle2	ankyrin repeat and LEM domain containing 2	1.098539	0.611	0.7928
208967	Thnsl1	threonine synthase-like 1 (bacterial)	1.098539	0.2147	NA
320150	Zdhhc17	zinc finger, DHHC domain containing 17	1.098539	0.5653	0.7618
18247	Oaz2-ps	ornithine decarboxylase antizyme 2, pseudogene	1.098418	0.1459	NA
20448	St6galnac4	ST6 (alpha-N-acetyl-neuraminyl-2,3-beta-galactosyl-1,3)-N-acetylgalactosaminide alpha-2,6-sialyltransferase 4	1.098418	0.5006	0.7145
23938	Map2k5	mitogen-activated protein kinase kinase 5	1.098418	0.176	NA
627871	Gm6802	predicted gene 6802	1.098418	0.843	0.9294
67160	Eef1g	eukaryotic translation elongation factor 1 gamma	1.098298	0.4215	0.6494
67282	Ccdc53	coiled-coil domain containing 53	1.098298	0.1623	NA
21985	Tpd52	tumor protein D52	1.098177	0.4411	0.6669
67064	Chmp1b	chromatin modifying protein 1B	1.098177	0.2587	0.494
67528	Nudt7	nudix (nucleoside diphosphate linked moiety X)-type motif 7	1.098177	0.1894	NA
70757	Ptplb	protein tyrosine phosphatase-like (proline instead of catalytic arginine), member b	1.098177	0.1718	NA
73167	Arhgap8	Rho GTPase activating protein 8	1.098177	0.1474	NA
268756	Gulo	gulonolactone (L-) oxidase	1.098177	0.2654	0.5011
16564	Kif21a	kinesin family member 21A	1.098056	0.1965	NA
110213	Tmbim6	transmembrane BAX inhibitor motif containing 6	1.098056	0.1756	NA
665992	Krtap4-8	keratin associated protein 4-8	1.098056	0.4631	0.6858
11852	Rhob	ras homolog gene family, member B	1.097936	0.1705	NA
107022	Gramd3	GRAM domain containing 3	1.097936	0.3018	0.5377
214048	Larp1b	La ribonucleoprotein domain family, member 1B	1.097936	0.1891	NA
217262	Abca9	ATP-binding cassette, sub-family A (ABC1), member 9	1.097936	0.2212	NA
382492	Gm12034	predicted gene 12034	1.097936	0.1366	NA
66536	Nipsnap3b	nipsnap homolog 3B (C. elegans)	1.097815	0.2077	NA
68092	Ncbp2	nuclear cap binding protein subunit 2	1.097815	0.4594	0.6826
218850	D14Abb1e	DNA segment, Chr 14, Abbott 1 expressed	1.097815	0.2629	0.4984
16348	Invs	inversin	1.097695	0.3677	0.6028
20598	Smpd2	sphingomyelin phosphodiesterase 2, neutral	1.097695	0.3517	0.5871
51800	Bok	BCL2-related ovarian killer protein	1.097574	0.2677	0.5032
54326	Elovl2	elongation of very long chain fatty acids (FEN1/Elo2, SUR4/Elo3, yeast)-like 2	1.097574	0.3715	0.6064
11637	Ak2	adenylate kinase 2	1.097454	0.239	NA
26413	Mapk1	mitogen-activated protein kinase 1	1.097454	0.3726	0.6069
272589	Tbcel	tubulin folding cofactor E-like	1.097454	0.08287	NA
434203	Slc28a1	solute carrier family 28 (sodium-coupled nucleoside transporter), member 1	1.097454	0.6017	0.7863
12389	Cav1	caveolin 1, caveolae protein	1.097333	0.3	0.5357
16796	Lasp1	LIM and SH3 protein 1	1.097333	0.5505	0.7511
76183	Celf6	CUGBP, Elav-like family member 6	1.097333	0.3509	0.5866
80708	Pacsin3	protein kinase C and casein kinase substrate in neurons 3	1.097333	0.205	NA
216505	Pik3ip1	phosphoinositide-3-kinase interacting protein 1	1.097333	0.2437	NA
246179	Fktn	fukutin	1.097333	0.5092	0.7202

14760	Gpr19	G protein-coupled receptor 19	1.097213	0.1953	NA	
26357	Abcg2	ATP-binding cassette, sub-family G (WHITE), member 2	1.097213	0.1164	NA	
70461	Crtc3	CREB regulated transcription coactivator 3	1.097213	0.3663		0.6019
76233	Dnttip1	deoxynucleotidyltransferase, terminal, interacting protein 1	1.097213	0.4534		0.6774
213084	Cdkl3	cyclin-dependent kinase-like 3	1.097213	0.3034		0.5395
227154	Stradb	STE20-related kinase adaptor beta	1.097213	0.167	NA	
22640	Zfp1	zinc finger protein 1	1.097093	0.2895		0.5256
70693	Gpr125	G protein-coupled receptor 125	1.097093	0.2447	NA	
338371	A730011L01Rik	RIKEN cDNA A730011L01 gene	1.097093	0.2525	NA	
13445	Cdk2ap1	CDK2 (cyclin-dependent kinase 2)-associated protein 1	1.096972	0.2568	NA	
19328	Rab12	RAB12, member RAS oncogene family	1.096972	0.1895	NA	
21767	Tex264	testis expressed gene 264	1.096972	0.1841	NA	
233016	Blvrb	biliverdin reductase B (flavin reductase (NADPH))	1.096972	0.3316		0.5678
622116	Gm6287	predicted gene 6287	1.096972	0.4017		0.6319
17134	Mafg	v-maf musculoaponeurotic fibrosarcoma oncogene family, protein G (avian)	1.096852	0.3177		0.5541
50912	Exosc10	exosome component 10	1.096852	0.3156		0.5524
72931	2900010J23Rik	RIKEN cDNA 2900010J23 gene	1.096852	0.09064	NA	
77574	Fam115a	family with sequence similarity 115, member A	1.096852	0.6148		0.7954
12567	Cdk4	cyclin-dependent kinase 4	1.096732	0.1465	NA	
17532	Mras	muscle and microspikes RAS	1.096732	0.4082		0.6375
30940	Usp25	ubiquitin specific peptidase 25	1.096611	0.2899		0.5261
66523	2810004N23Rik	RIKEN cDNA 2810004N23 gene	1.096611	0.06712	NA	
67902	Sumf2	sulfatase modifying factor 2	1.096611	0.4466		0.672
228005	Ppig	peptidyl-prolyl isomerase G (cyclophilin G)	1.096611	0.2489	NA	
269954	Ttll13	tubulin tyrosine ligase-like family, member 13	1.096611	0.7467		0.877
13839	Epha5	Eph receptor A5	1.096491	0.204	NA	
20615	Snapin	SNAP-associated protein	1.096491	0.1901	NA	
66614	Gpatch4	G patch domain containing 4	1.096491	0.2546	NA	
75985	Rab30	RAB30, member RAS oncogene family	1.096491	0.4038		0.6337
320595	Phf8	PHD finger protein 8	1.096491	0.0637	NA	
12908	Crat	carnitine acetyltransferase	1.096371	0.2229	NA	
271786	Galnt13	UDP-N-acetyl-alpha-D-galactosamine:polypeptide N-acetylgalactosaminyltransferase 13	1.096371	0.2653	NA	
108071	Grm5	glutamate receptor, metabotropic 5	1.096251	0.3143		0.5508
223864	Rapgef3	Rap guanine nucleotide exchange factor (GEF) 3	1.096251	0.3851		0.6175
224742	Abcf1	ATP-binding cassette, sub-family F (GCN20), member 1	1.096251	0.2975		0.5334
16443	Itsn1	intersectin 1 (SH3 domain protein 1A)	1.096131	0.1504	NA	
23965	Odz3	odd Oz/ten-m homolog 3 (Drosophila)	1.096131	0.3673		0.6024
75202	Ncrna00085	non-protein coding RNA 85	1.096131	0.3066		0.5432
107652	Uap1	UDP-N-acetylglucosamine pyrophosphorylase 1	1.096131	0.1091	NA	
71365	Pdss2	prenyl (solanesyl) diphosphate synthase, subunit 2	1.096011	0.3247		0.5612
106581	Itfg3	integrin alpha FG-GAP repeat containing 3	1.096011	0.2538	NA	
215194	Kri1	KRI1 homolog (S. cerevisiae)	1.096011	0.2959		0.5316
15547	Trmt2a	TRM2 tRNA methyltransferase 2 homolog A (S. cerevisiae)	1.09589	0.1185	NA	
545861	Gm5878	predicted gene 5878	1.09589	0.2464	NA	
14158	Fert2	fer (fms/fps related) protein kinase, testis specific 2	1.09577	0.1684	NA	
14789	Leprel2	leprecan-like 2	1.09577	0.1414	NA	
30058	Timm8a1	translocase of inner mitochondrial membrane 8 homolog a1 (yeast)	1.09577	0.106	NA	
246154	Vasn	vasorin	1.09577	0.2861		0.5215
328456	6720482D04	hypothetical protein 6720482D04	1.09577	0.4849		0.7033

23980	Pebp1	phosphatidylethanolamine binding protein 1	1.09565	0.4864	0.7045
26556	Homer1	homer homolog 1 (Drosophila)	1.09565	0.4607	0.6837
68533	Mphosph6	M phase phosphoprotein 6	1.09565	0.1944	NA
73225	Fam118a	family with sequence similarity 118, member A	1.09565	0.5537	0.7536
77739	Adamtsl1	ADAMTS-like 1	1.09565	0.4437	0.6694
224250	Cldnd1	claudin domain containing 1	1.09565	0.2443	NA
234135	Whsc1l1	Wolf-Hirschhorn syndrome candidate 1-like 1 (human)	1.09565	0.6675	0.8294
268996	Ss18	synovial sarcoma translocation, Chromosome 18	1.09565	0.05235	NA
329584	Gm14462	predicted gene 14462	1.09565	0.3836	0.6162
12925	Crip1	cysteine-rich protein 1 (intestinal)	1.09553	0.3133	0.5495
13478	Dpagt1	dolichyl-phosphate (UDP-N-acetylglucosamine) acetylglucosaminephosphotransferase 1 (GlcNAc-1-P transferase)	1.09553	0.246	NA
56369	Apip	APAF1 interacting protein	1.09553	0.2149	NA
78438	A930028N01Rik	RIKEN cDNA A930028N01 gene	1.09553	0.3706	0.6054
625342	Gm13315	lactate dehydrogenase A pseudogene	1.09553	0.459	0.6824
21689	Tekt1	tektin 1	1.09541	0.688	0.8408
68763	1110038B12Rik	RIKEN cDNA 1110038B12 gene	1.09541	0.2239	NA
75763	Dcaf17	DDB1 and CUL4 associated factor 17	1.09541	0.3818	0.6152
103677	Smg6	Smg-6 homolog, nonsense mediated mRNA decay factor (C. elegans)	1.09541	0.2428	NA
19241	Tmsb4x	thymosin, beta 4, X chromosome	1.09529	0.143	NA
20021	Polr2c	polymerase (RNA) II (DNA directed) polypeptide C	1.09529	0.1024	NA
234549	Heatr3	HEAT repeat containing 3	1.09529	0.4869	0.7049
791393	A230065N10Rik	RIKEN cDNA A230065N10 gene	1.09529	0.5284	0.7353
100041870	Gm3556	predicted gene 3556	1.09529	0.1307	NA
14660	Gls	glutaminase	1.09517	0.3239	0.5603
68845	Pih1d1	PIH1 domain containing 1	1.09517	0.2201	NA
109305	Orai1	ORAI calcium release-activated calcium modulator 1	1.09517	0.4208	0.6487
226562	Prrc2c	proline-rich coiled-coil 2C	1.09517	0.2963	0.5322
242291	Impad1	inositol monophosphatase domain containing 1	1.09517	0.2127	NA
100040220	Gm9892	predicted gene 9892	1.09517	0.1368	NA
12564	Cdh8	cadherin 8	1.09505	0.1866	NA
20751	Spr	sepiapterin reductase	1.09505	0.08748	NA
69757	Leng1	leukocyte receptor cluster (LRC) member 1	1.09505	0.1927	NA
242484	D630039A03Rik	RIKEN cDNA D630039A03 gene	1.09493	0.7574	0.8827
434008	Gm5567	predicted gene 5567	1.09493	0.3774	0.6113
100038651	D130062J21Rik	RIKEN cDNA D130062J21 gene	1.09493	0.4678	0.6897
12399	Runx3	runt related transcription factor 3	1.094811	0.6711	0.8317
12725	Clcn3	chloride channel 3	1.094811	0.1929	NA
228859	Fitm2	fat storage-inducing transmembrane protein 2	1.094811	0.4797	0.6989
320541	Slc35e2	solute carrier family 35, member E2	1.094811	0.5757	0.769
381066	Zfp948	zinc finger protein 948	1.094691	0.198	NA
433966	5730422E09Rik	RIKEN cDNA 5730422E09 gene	1.094691	0.4406	0.6666
104103	Airn	antisense Igf2r RNA	1.094571	0.4262	0.6535
546071	Mast3	microtubule associated serine/threonine kinase 3	1.094571	0.4798	0.6989
11669	Aldh2	aldehyde dehydrogenase 2, mitochondrial	1.094451	0.1753	NA
19951	Rpl32	ribosomal protein L32	1.094451	0.1598	NA
20823	Ssb	Sjogren syndrome antigen B	1.094451	0.5534	0.7534
23955	Nek4	NIMA (never in mitosis gene a)-related expressed kinase 4	1.094451	0.2531	NA
57294	Rps27	ribosomal protein S27	1.094451	0.4494	0.6746
105837	Mtbp	Mdm2, transformed 3T3 cell double minute p53 binding protein	1.094451	0.2352	NA

14563	Gdf5	growth differentiation factor 5	1.094331	0.7911		0.9009
17686	Msh3	mutS homolog 3 (E. coli)	1.094331	0.1448	NA	
20833	Ssrp1	structure specific recognition protein 1	1.094331	0.1382	NA	
66073	Txndc12	thioredoxin domain containing 12 (endoplasmic reticulum)	1.094331	0.1229	NA	
71743	Coasy	Coenzyme A synthase	1.094331	0.1366	NA	
100040276	Gm11595	predicted gene 11595	1.094331	0.6324		0.8065
26356	Ing1	inhibitor of growth family, member 1	1.094212	0.1384	NA	
55949	Eef1b2	eukaryotic translation elongation factor 1 beta 2	1.094212	0.1761	NA	
66235	Eif1ax	eukaryotic translation initiation factor 1A, X-linked	1.094212	0.1133	NA	
67433	Ccdc127	coiled-coil domain containing 127	1.094212	0.248	NA	
80743	Vps16	vacuolar protein sorting 16 (yeast)	1.094212	0.1113	NA	
244682	Cntn5	contactin 5	1.094212	0.378		0.6117
78329	2310010J17Rik	RIKEN cDNA 2310010J17 gene	1.094092	0.2149	NA	
225207	Zfp521	zinc finger protein 521	1.094092	0.3363		0.572
12972	Cryz	crystallin, zeta	1.093972	0.1231	NA	
245622	Fam199x	family with sequence similarity 199, X-linked	1.093972	0.7488		0.8784
319468	Ppm1h	protein phosphatase 1H (PP2C domain containing)	1.093972	0.176	NA	
14106	Foxh1	forkhead box H1	1.093853	0.5255		0.7326
217011	Nle1	notchless homolog 1 (Drosophila)	1.093853	0.1564	NA	
18655	Pgk1	phosphoglycerate kinase 1	1.093733	0.3515		0.5869
22276	Uros	uroporphyrinogen III synthase	1.093733	0.7125		0.8554
72739	Zkscan3	zinc finger with KRAB and SCAN domains 3	1.093733	0.3466		0.5821
216971	BC017647	cDNA sequence BC017647	1.093733	0.6966		0.8463
227195	Ino80d	INO80 complex subunit D	1.093733	0.2911		0.5271
56809	Gmeb1	glucocorticoid modulatory element binding protein 1	1.093613	0.331		0.5674
69692	Hddc2	HD domain containing 2	1.093613	0.1138	NA	
210992	Lpcat1	lysophosphatidylcholine acyltransferase 1	1.093613	0.5041		0.7166
13666	Eif2ak3	eukaryotic translation initiation factor 2 alpha kinase 3	1.093494	0.2149	NA	
68048	Aen	apoptosis enhancing nuclease	1.093494	0.1286	NA	
71787	Trnau1ap	tRNA selenocysteine 1 associated protein 1	1.093494	0.2687	NA	
74302	Mtmr3	myotubularin related protein 3	1.093494	0.3439		0.5794
93896	Glp2r	glucagon-like peptide 2 receptor	1.093494	0.706		0.8524
231503	Tmem150c	transmembrane protein 150C	1.093494	0.3747		0.6088
732482	Gm9731	Fam58a pseudogene	1.093494	0.1133	NA	
70717	6330406I15Rik	RIKEN cDNA 6330406I15 gene	1.093374	0.4402		0.6662
110417	Pigh	phosphatidylinositol glycan anchor biosynthesis, class H	1.093374	0.2129	NA	
233552	Gdpd5	glycerophosphodiester phosphodiesterase domain containing 5	1.093374	0.2944		0.5306
70661	Sik3	SIK family kinase 3	1.093255	0.1565	NA	
245424	Gpr101	G protein-coupled receptor 101	1.093255	0.2979		0.5337
16470	Ush1g	Usher syndrome 1G homolog (human)	1.093135	0.3692		0.6041
70772	Ggnbp1	gametogenetin binding protein 1	1.093135	0.4491		0.6745
192678	Rassf3	Ras association (RalGDS/AF-6) domain family member 3	1.093135	0.4355		0.6623
67203	Nde1	nuclear distribution gene E homolog 1 (A nidulans)	1.093016	0.3275		0.564
233064	Wdr62	WD repeat domain 62	1.093016	0.3564		0.5912
347722	Agap1	ArfGAP with GTPase domain, ankyrin repeat and PH domain 1	1.093016	0.3456		0.5812
66193	1110049F12Rik	RIKEN cDNA 1110049F12 gene	1.092896	0.2138	NA	
68701	Dysfip1	dysferlin interacting protein 1	1.092896	0.5886		0.7778
72823	Pard3b	par-3 partitioning defective 3 homolog B (C. elegans)	1.092896	0.6481		0.8167
75692	Nr2c2ap	nuclear receptor 2C2-associated protein	1.092896	0.3877		0.6197

69269	Scnm1	sodium channel modifier 1	1.092777	0.3427	0.5782
269623	C030048B08Rik	RIKEN cDNA C030048B08 gene	1.092777	0.4386	0.6647
12028	Bax	BCL2-associated X protein	1.092657	0.5213	0.7292
67160	Eef1g	eukaryotic translation elongation factor 1 gamma	1.092657	0.5633	0.7609
69671	Tmem52	transmembrane protein 52	1.092657	0.6219	0.7997
57810	Cdon	cell adhesion molecule-related/down-regulated by oncogenes	1.092538	0.1959	NA
18768	Pkib	protein kinase inhibitor beta, cAMP dependent, testis specific	1.092419	0.7533	0.8809
83396	Glis2	GLIS family zinc finger 2	1.092419	0.2385	NA
100705	Acacb	acetyl-Coenzyme A carboxylase beta	1.092419	0.5293	0.7357
67561	Wdr48	WD repeat domain 48	1.092299	0.3322	0.5682
230257	Rod1	ROD1 regulator of differentiation 1 (S. pombe)	1.092299	0.4968	0.7118
338350	9330129D05Rik	RIKEN cDNA 9330129D05 gene	1.092299	0.3383	0.5742
11758	Prdx6	peroxiredoxin 6	1.09218	0.1823	NA
19106	Eif2ak2	eukaryotic translation initiation factor 2-alpha kinase 2	1.09218	0.4712	0.6924
20104	Rps6	ribosomal protein S6	1.09218	0.1268	NA
66069	Snupn	snurportin 1	1.09218	0.1216	NA
97213	C77137	expressed sequence C77137	1.09218	0.5384	0.7428
626391	Zfp951	zinc finger protein 951	1.09218	0.7118	0.8553
665095	Cyp2j8-ps	cytochrome P450, family 2, subfamily j, polypeptide 8, pseudogene	1.09218	0.1408	NA
14186	Fgfr4	fibroblast growth factor receptor 4	1.091941	0.4323	0.6594
72828	Ubash3b	ubiquitin associated and SH3 domain containing, B	1.091941	0.1649	NA
99334	Zscan29	zinc finger SCAN domains 29	1.091941	0.331	0.5674
223773	Zbed4	zinc finger, BED domain containing 4	1.091941	0.7491	0.8784
268741	Tox4	TOX high mobility group box family member 4	1.091941	0.3259	0.5626
67884	1810043G02Rik	RIKEN cDNA 1810043G02 gene	1.091822	0.2744	NA
68957	Paqr6	progestin and adipoQ receptor family member VI	1.091822	0.4895	0.7066
214922	Slc39a2	solute carrier family 39 (zinc transporter), member 2	1.091822	0.7465	0.877
100038941	Vmn2r121	vomeronasal 2, receptor 121	1.091822	0.4559	0.6794
23871	Ets1	E26 avian leukemia oncogene 1, 5' domain	1.091703	0.584	0.7745
72438	2510016G02Rik	RIKEN cDNA 2510016G02 gene	1.091703	0.3605	0.5954
75686	Nudt16	nudix (nucleoside diphosphate linked moiety X)-type motif 16	1.091703	0.2196	NA
106200	Txndc11	thioredoxin domain containing 11	1.091703	0.3666	0.6019
211007	Trim41	tripartite motif-containing 41	1.091703	0.3205	0.5571
100043232	3110099E03Rik	RIKEN cDNA 3110099E03 gene	1.091703	0.6415	0.8127
69288	Rhobtb1	Rho-related BTB domain containing 1	1.091584	0.5089	0.72
70661	Sik3	SIK family kinase 3	1.091584	0.1576	NA
97683	C76332	expressed sequence C76332	1.091584	0.7522	0.8803
230810	Slc30a2	solute carrier family 30 (zinc transporter), member 2	1.091584	0.2974	0.5334
277010	Marveld1	MARVEL (membrane-associating) domain containing 1	1.091584	0.2469	NA
330401	Tmcc1	transmembrane and coiled coil domains 1	1.091584	0.2936	0.5295
12845	Comp	cartilage oligomeric matrix protein	1.091465	0.5971	0.7832
595136	Ndufs5	NADH dehydrogenase (ubiquinone) Fe-S protein 5	1.091465	0.1769	NA
100045736	LOC100045736	hypothetical protein LOC100045736	1.091465	0.599	0.7845
15374	Hn1	hematological and neurological expressed sequence 1	1.091346	0.4161	0.6447
21676	Tead1	TEA domain family member 1	1.091346	0.3303	0.5668
23950	Dnajb6	DnaJ (Hsp40) homolog, subfamily B, member 6	1.091346	0.3549	0.5896
74230	1700016K19Rik	RIKEN cDNA 1700016K19 gene	1.091346	0.5453	0.7478
14396	Gabra3	gamma-aminobutyric acid (GABA) A receptor, subunit alpha 3	1.091227	0.6062	0.7891
69923	Agk	acylglycerol kinase	1.091227	0.2184	NA

70985	4931406E20Rik	RIKEN cDNA 4931406E20 gene	1.091227	0.4045	0.6342
83704	Slc12a9	solute carrier family 12 (potassium/chloride transporters), member 9	1.091227	0.2745	NA
233280	Nipa1	non imprinted in Prader-Willi/Angelman syndrome 1 homolog (human)	1.091227	0.2153	NA
237336	Tbpl1	TATA box binding protein-like 1	1.091227	0.1703	NA
667847	Gm8842	predicted gene 8842	1.091227	0.3502	0.5861
12905	Cradd	CASP2 and RIPK1 domain containing adaptor with death domain	1.091107	0.1185	NA
27176	Rpl7a	ribosomal protein L7A	1.091107	0.1139	NA
66789	Alg14	asparagine-linked glycosylation 14 homolog (yeast)	1.091107	0.1367	NA
69944	2810021J22Rik	RIKEN cDNA 2810021J22 gene	1.091107	0.4253	0.6527
71063	Zfp597	zinc finger protein 597	1.091107	0.4913	0.7079
75304	4930563E22Rik	RIKEN cDNA 4930563E22 gene	1.091107	0.2708	NA
236749	Gm4907	predicted gene 4907	1.091107	0.2175	NA
243912	Hspb6	heat shock protein, alpha-crystallin-related, B6	1.091107	0.285	NA
76142	Ppp1r14c	protein phosphatase 1, regulatory (inhibitor) subunit 14c	1.090988	0.6787	0.8357
17925	Myo9b	myosin IXb	1.090869	0.08808	NA
73674	Wdr75	WD repeat domain 75	1.090869	0.2617	NA
105522	Ankrd28	ankyrin repeat domain 28	1.090869	0.3902	0.6223
211134	Lzts1	leucine zipper, putative tumor suppressor 1	1.090869	0.7535	0.8811
21871	Atp6v0a2	ATPase, H+ transporting, lysosomal V0 subunit A2	1.09075	0.259	NA
22026	Nr2c2	nuclear receptor subfamily 2, group C, member 2	1.09075	0.343	0.5783
232089	Elmod3	ELMO/CED-12 domain containing 3	1.09075	0.2447	NA
239759	Liph	lipase, member H	1.09075	0.564	0.7613
12035	Bcat1	branched chain aminotransferase 1, cytosolic	1.090631	0.3689	0.6038
21917	Tmpo	thymopoietin	1.090631	0.2468	NA
67889	Rbm18	RNA binding motif protein 18	1.090631	0.1247	NA
108960	Irak2	interleukin-1 receptor-associated kinase 2	1.090631	0.2179	NA
245860	Atg9a	autophagy-related 9A (yeast)	1.090631	0.444	0.6696
668253	Dleu2	deleted in lymphocytic leukemia, 2	1.090631	0.1501	NA
14595	B4galt1	UDP-Gal:betaGlcNAc beta 1,4- galactosyltransferase, polypeptide 1	1.090513	0.3508	0.5866
77219	Ptgr2	prostaglandin reductase 2	1.090513	0.1582	NA
106952	Arap3	ArfGAP with RhoGAP domain, ankyrin repeat and PH domain 3	1.090513	0.5085	0.7198
224904	2410015M20Rik	RIKEN cDNA 2410015M20 gene	1.090513	0.1084	NA
14884	Gtf2h1	general transcription factor II H, polypeptide 1	1.090394	0.2253	NA
20042	Rps12	ribosomal protein S12	1.090394	0.1908	NA
71853	Pdia6	protein disulfide isomerase associated 6	1.090394	0.1547	NA
94044	Bcl2l13	BCL2-like 13 (apoptosis facilitator)	1.090394	0.3417	0.5772
225358	Fam13b	family with sequence similarity 13, member B	1.090394	0.4706	0.6922
56726	Sh3bgrl	SH3-binding domain glutamic acid-rich protein like	1.090275	0.4106	0.6396
66684	Tceal8	transcription elongation factor A (SII)-like 8	1.090275	0.3083	0.5446
70314	Rabep2	rabaptin, RAB GTPase binding effector protein 2	1.090156	0.2106	NA
72140	Ccdc123	coiled-coil domain containing 123	1.090156	0.5337	0.7392
74243	2210009G21Rik	RIKEN cDNA 2210009G21 gene	1.090156	0.3387	0.5745
258852	Olfr1341	olfactory receptor 1341	1.090156	0.7486	0.8783
75530	Lyrm7	LYR motif containing 7	1.090037	0.5193	0.7282
213649	Arhgef19	Rho guanine nucleotide exchange factor (GEF) 19	1.090037	0.4429	0.6688
93737	Pard6g	par-6 partitioning defective 6 homolog gamma (C. elegans)	1.089918	0.2421	NA
69657	2310047D07Rik	RIKEN cDNA 2310047D07 gene	1.089799	0.2743	NA
70804	Pgrmc2	progesterone receptor membrane component 2	1.089799	0.1154	NA
232089	Elmod3	ELMO/CED-12 domain containing 3	1.089799	0.3125	0.5491

12572	Cdk7	cyclin-dependent kinase 7	1.089681	0.2977	NA	
16768	Lag3	lymphocyte-activation gene 3	1.089681	0.4849		0.7033
20729	Spin1	spindlin 1	1.089681	0.09668	NA	
67861	Akr1b10	aldo-keto reductase family 1, member B10 (aldose reductase)	1.089681	0.1425	NA	
83962	Btbd1	BTB (POZ) domain containing 1	1.089681	0.08824	NA	
22027	Hsp90b1	heat shock protein 90, beta (Grp94), member 1	1.089562	0.1551	NA	
70508	Bbx	bobby sox homolog (Drosophila)	1.089562	0.1605	NA	
72153	2610020P09Rik	RIKEN cDNA 2610020P09 gene	1.089562	0.6726		0.8323
50850	Spast	spastin	1.089443	0.1432	NA	
101646	B830008H07Rik	RIKEN cDNA B830008H07 gene	1.089443	0.2506	NA	
321003	Xpnpep3	X-prolyl aminopeptidase (aminopeptidase P) 3, putative	1.089443	0.5802		0.7724
13038	Ctsk	cathepsin K	1.089325	0.267	NA	
15078	H3f3a	H3 histone, family 3A	1.089325	0.1847	NA	
20818	Srprb	signal recognition particle receptor, B subunit	1.089325	0.4871		0.705
20850	Stat5a	signal transducer and activator of transcription 5A	1.089325	0.289	NA	
270166	Clpx	caseinolytic peptidase X (E.coli)	1.089325	0.2319	NA	
320554	Tcp11l1	t-complex 11 like 1	1.089325	0.4444		0.67
22064	Trpc2	transient receptor potential cation channel, subfamily C, member 2	1.089206	0.7285		0.8655
380601	Fastkd5	FAST kinase domains 5	1.089206	0.1318	NA	
12741	Cldn5	claudin 5	1.089087	0.4933		0.7095
20467	Sin3b	transcriptional regulator, SIN3B (yeast)	1.089087	0.1233	NA	
56380	Arid3b	AT rich interactive domain 3B (BRIGHT-like)	1.089087	0.4903		0.7073
635470	Gm14407	predicted gene 14407	1.089087	0.1445	NA	
66510	Rnf181	ring finger protein 181	1.088969	0.1967	NA	
114301	Palmd	palmdelphin	1.088969	0.3407		0.5764
18643	Pfn1	profilin 1	1.08885	0.3801		0.6135
72987	2900057C01Rik	RIKEN cDNA 2900057C01 gene	1.08885	0.5001		0.7144
107250	Kazald1	Kazal-type serine peptidase inhibitor domain 1	1.08885	0.3572		0.5919
319149	Hist1h3d	histone cluster 1, H3d	1.08885	0.2557	NA	
19942	Rpl27	ribosomal protein L27	1.088732	0.1473	NA	
100986	Akap9	A kinase (PRKA) anchor protein (yotiao) 9	1.088732	0.2538	NA	
246179	Fktn	fukutin	1.088732	0.4495		0.6746
329178	Unc80	unc-80 homolog (C. elegans)	1.088732	0.2037	NA	
378937	Lrrc24	leucine rich repeat containing 24	1.088732	0.3686		0.6034
68870	Ak8	adenylate kinase 8	1.088495	0.3599		0.5948
70544	5730437N04Rik	RIKEN cDNA 5730437N04 gene	1.088495	0.08395	NA	
71330	Rcbtb1	regulator of chromosome condensation (RCC1) and BTB (POZ) domain containing protein 1	1.088495	0.5006		0.7145
80912	Pum1	pumilio 1 (Drosophila)	1.088495	0.3504		0.5862
93742	Pard3	par-3 (partitioning defective 3) homolog (C. elegans)	1.088495	0.4208		0.6487
378702	Serf2	small EDRK-rich factor 2	1.088495	0.2055	NA	
22755	Zfp93	zinc finger protein 93	1.088376	0.2847	NA	
112406	Egln2	EGL nine homolog 2 (C. elegans)	1.088376	0.2236	NA	
627908	LOC627908	hypothetical LOC627908	1.088376	0.1716	NA	
17913	Myo1c	myosin IC	1.088258	0.3257		0.5625
24071	Synj2bp	synaptojanin 2 binding protein	1.088258	0.5621		0.7604
68581	Tmed10	transmembrane emp24-like trafficking protein 10 (yeast)	1.088258	0.2532	NA	
26364	Cd97	CD97 antigen	1.088139	0.2339	NA	
66864	Clec14a	C-type lectin domain family 14, member a	1.088139	0.6709		0.8316
56218	Patz1	POZ (BTB) and AT hook containing zinc finger 1	1.088021	0.4193		0.6473

66369	Dus2l	dihydrouridine synthase 2-like (SMM1, S. cerevisiae)	1.088021	0.3664	0.6019
14007	Celf2	CUGBP, Elav-like family member 2	1.087903	0.2904	NA
27386	Npas3	neuronal PAS domain protein 3	1.087903	0.2761	NA
67603	Dusp6	dual specificity phosphatase 6	1.087903	0.1963	NA
77209	8030453O22Rik	RIKEN cDNA 8030453O22 gene	1.087903	0.6431	0.8136
104303	Arl1	ADP-ribosylation factor-like 1	1.087903	0.3159	0.5526
218343	Ttc37	tetratricopeptide repeat domain 37	1.087903	0.592	0.7801
18195	Nsf	N-ethylmaleimide sensitive fusion protein	1.087784	0.2818	NA
20588	Smarcc1	SWI/SNF related, matrix associated, actin dependent regulator of chromatin, subfamily c, member 1	1.087784	0.5581	0.757
54188	Cpsf4	cleavage and polyadenylation specific factor 4	1.087784	0.2605	NA
73680	Zbtb8a	zinc finger and BTB domain containing 8a	1.087784	0.4929	0.7092
216197	Ckap4	cytoskeleton-associated protein 4	1.087784	0.7496	0.8785
224703	Mar-02	membrane-associated ring finger (C3HC4) 2	1.087784	0.6731	0.8325
20265	Scn1a	sodium channel, voltage-gated, type I, alpha	1.087666	0.1747	NA
21379	Tbrg4	transforming growth factor beta regulated gene 4	1.087666	0.2899	NA
22433	Xbp1	X-box binding protein 1	1.087666	0.2065	NA
68514	Efha1	EF hand domain family A1	1.087666	0.16	NA
75884	4930572J10Rik	RIKEN cDNA 4930572J10 gene	1.087666	0.5403	0.7446
69399	1700025G04Rik	RIKEN cDNA 1700025G04 gene	1.087548	0.3536	0.5885
76574	Mfsd2a	major facilitator superfamily domain containing 2A	1.087548	0.5095	0.7205
239796	1600021P15Rik	RIKEN cDNA 1600021P15 gene	1.087548	0.6435	0.8137
16975	Lrp8	low density lipoprotein receptor-related protein 8, apolipoprotein e receptor	1.087429	0.5694	0.7638
67610	Rspry1	ring finger and SPRY domain containing 1	1.087429	0.2002	NA
319370	Fam100b	family with sequence similarity 100, member B	1.087429	0.1967	NA
74111	Rbm19	RNA binding motif protein 19	1.087311	0.5681	0.7634
218914	Wapal	wings apart-like homolog (Drosophila)	1.087311	0.3068	NA
382406	Poc1b	POC1 centriolar protein homolog B (Chlamydomonas)	1.087311	0.2735	NA
15446	Hpgd	hydroxyprostaglandin dehydrogenase 15 (NAD)	1.087193	0.5619	0.7602
56376	Pdlim5	PDZ and LIM domain 5	1.087193	0.61	0.792
67432	Hoga1	4-hydroxy-2-oxoglutarate aldolase 1	1.087193	0.4805	0.6993
67738	Ppid	peptidylprolyl isomerase D (cyclophilin D)	1.087193	0.1673	NA
269593	Luzp1	leucine zipper protein 1	1.087193	0.4812	0.7
380614	Intu	inturned planar cell polarity effector homolog (Drosophila)	1.087193	0.4605	0.6837
71774	Shroom1	shroom family member 1	1.087075	0.5632	0.7609
268395	Mpg	N-methylpurine-DNA glycosylase	1.087075	0.391	0.6228
16782	Lamc2	laminin, gamma 2	1.086957	0.3421	0.5775
18795	Plcb1	phospholipase C, beta 1	1.086957	0.4744	0.695
67707	Mrpl24	mitochondrial ribosomal protein L24	1.086957	0.105	NA
67726	Fam114a2	family with sequence similarity 114, member A2	1.086957	0.1333	NA
319934	Sbf2	SET binding factor 2	1.086957	0.4612	0.6841
100039982	Gm2533	predicted gene 2533	1.086957	0.2701	NA
258662	Olfr738	olfactory receptor 738	1.086838	0.7293	0.8661
319386	D130062J10Rik	RIKEN cDNA D130062J10 gene	1.086838	0.5904	0.7791
12123	Hrk	harakiri, BCL2 interacting protein (contains only BH3 domain)	1.08672	0.6684	0.8296
19982	Rpl36a	ribosomal protein L36A	1.08672	0.2227	NA
20911	Stxbp2	syntaxin binding protein 2	1.08672	0.2741	NA
94178	Mcoln1	mucolipin 1	1.086602	0.1852	NA
20719	Serpinb6a	serine (or cysteine) peptidase inhibitor, clade B, member 6a	1.086484	0.452	0.6768
230582	Cyb5rl	cytochrome b5 reductase-like	1.086484	0.5701	0.7641

639606	LOC639606	40S ribosomal protein S2-like	1.086484	0.3544	0.5891
100038587	Gm10278	predicted gene 10278	1.086484	0.1612	NA
14433	Gapdh	glyceraldehyde-3-phosphate dehydrogenase	1.086366	0.3018	NA
97086	Nhedc2	Na+/H+ exchanger domain containing 2	1.086366	0.4332	0.6604
68750	Rreb1	ras responsive element binding protein 1	1.086248	0.3738	0.6081
212898	Dse	dermatan sulfate epimerase	1.086248	0.3721	0.6068
14297	Fxn	frataxin	1.08613	0.3089	NA
19877	Rock1	Rho-associated coiled-coil containing protein kinase 1	1.08613	0.5159	0.7259
213573	Efcab4a	EF-hand calcium binding domain 4A	1.08613	0.4861	0.7044
319192	Hist2h2aa2	histone cluster 2, H2aa2	1.08613	0.1781	NA
56453	Mbtps1	membrane-bound transcription factor peptidase, site 1	1.086012	0.09304	NA
56458	Foxo1	forkhead box O1	1.086012	0.4202	0.6482
57170	Dolpp1	dolichyl pyrophosphate phosphatase 1	1.086012	0.1884	NA
66191	Ier3ip1	immediate early response 3 interacting protein 1	1.086012	0.2716	NA
81897	Tlr9	toll-like receptor 9	1.086012	0.2771	NA
108767	Pnrc1	proline-rich nuclear receptor coactivator 1	1.086012	0.3889	0.6206
268281	Shprh	SNF2 histone linker PHD RING helicase	1.086012	0.7351	0.8702
664608	Rhox4g	reproductive homeobox 4G	1.086012	0.6815	0.837
50771	Atp9b	ATPase, class II, type 9B	1.085894	0.578	0.7704
67889	Rbm18	RNA binding motif protein 18	1.085894	0.5455	0.7478
75580	Zbtb4	zinc finger and BTB domain containing 4	1.085894	0.5459	0.7481
11632	Aip	aryl-hydrocarbon receptor-interacting protein	1.085776	0.4742	0.6948
109332	Cdcp1	CUB domain containing protein 1	1.085776	0.8196	0.9178
271377	Zbtb11	zinc finger and BTB domain containing 11	1.085776	0.3844	0.6167
19946	Rpl30	ribosomal protein L30	1.085658	0.2596	NA
22612	Yes1	Yamaguchi sarcoma viral (v-yes) oncogene homolog 1	1.085658	0.5239	0.7315
27176	Rpl7a	ribosomal protein L7A	1.085658	0.1317	NA
67263	Zswim6	zinc finger, SWIM domain containing 6	1.085658	0.2153	NA
67865	Rgs10	regulator of G-protein signalling 10	1.085658	0.2148	NA
73490	Mipol1	mirror-image polydactyly gene 1 homolog (human)	1.085658	0.2985	NA
218490	Btf3	basic transcription factor 3	1.085658	0.09736	NA
245583	Tgif2lx1	TGFB-induced factor homeobox 2-like, X-linked 1	1.085658	0.6096	0.7917
100043225	Gm16378	predicted gene 16378	1.085658	0.7115	0.8551
16971	Lrp1	low density lipoprotein receptor-related protein 1	1.085541	0.2317	NA
69053	1810013L24Rik	RIKEN cDNA 1810013L24 gene	1.085541	0.5301	0.7364
71409	Fmnl2	formin-like 2	1.085541	0.6179	0.7973
72727	B3gat3	beta-1,3-glucuronyltransferase 3 (glucuronosyltransferase I)	1.085541	0.2975	NA
12466	Cct6a	chaperonin containing Tcp1, subunit 6a (zeta)	1.085423	0.498	0.7127
74053	Grip1	glutamate receptor interacting protein 1	1.085423	0.359	0.5941
75881	4930579K19Rik	RIKEN cDNA 4930579K19 gene	1.085423	0.4935	0.7095
208084	Pif1	PIF1 5'-to-3' DNA helicase homolog (S. cerevisiae)	1.085423	0.6807	0.8364
233107	Kctd15	potassium channel tetramerisation domain containing 15	1.085423	0.209	NA
236576	Spry3	sprouty homolog 3 (Drosophila)	1.085423	0.3046	NA
320720	Fastkd1	FAST kinase domains 1	1.085423	0.1254	NA
100040591	Kcnj13	potassium inwardly-rectifying channel, subfamily J, member 13	1.085423	0.7965	0.9043
17957	Napb	N-ethylmaleimide sensitive fusion protein attachment protein beta	1.085305	0.3934	0.6244
20362	Sep-08	septin 8	1.085305	0.6171	0.797
22070	Tpt1	tumor protein, translationally-controlled 1	1.085305	0.2299	NA
71385	5430434G16Rik	RIKEN cDNA 5430434G16 gene	1.085305	0.3129	NA

73191	Fezf1	Fez family zinc finger 1	1.085305	0.7601	0.8837
213054	Gabpb2	GA repeat binding protein, beta 2	1.085305	0.5902	0.779
230903	Fbxo44	F-box protein 44	1.085305	0.5688	0.7635
320492	A830018L16Rik	RIKEN cDNA A830018L16 gene	1.085305	0.5898	0.7786
64817	Svep1	sushi, von Willebrand factor type A, EGF and pentraxin domain containing 1	1.085187	0.5426	0.7463
67605	Akt1s1	AKT1 substrate 1 (proline-rich)	1.085187	0.3782	0.6118
75747	Sesn3	sestrin 3	1.085187	0.4639	0.6865
79044	Mrps34	mitochondrial ribosomal protein S34	1.085187	0.1112	NA
103819	AI663975	expressed sequence AI663975	1.085187	0.2349	NA
319195	Rpl17	ribosomal protein L17	1.085187	0.4205	0.6484
11920	Atm	ataxia telangiectasia mutated homolog (human)	1.085069	0.5511	0.7513
12859	Cox5b	cytochrome c oxidase, subunit Vb	1.084952	0.3081	NA
13388	Dll1	delta-like 1 (Drosophila)	1.084952	0.3367	0.5723
20445	St6galnac1	ST6 (alpha-N-acetyl-neuraminyl-2,3-beta-galactosyl-1,3)-N-acetylgalactosaminide alpha-2,6-sialyltransferase 1	1.084952	0.6799	0.8362
93790	Nipa2	non imprinted in Prader-Willi/Angelman syndrome 2 homolog (human)	1.084952	0.1264	NA
16646	Kpna1	karyopherin (importin) alpha 1	1.084834	0.2519	NA
22004	Tpm2	tropomyosin 2, beta	1.084834	0.1522	NA
16400	Itga3	integrin alpha 3	1.084716	0.5511	0.7513
68235	2410066E13Rik	RIKEN cDNA 2410066E13 gene	1.084716	0.3907	0.6227
69192	Dhx16	DEAH (Asp-Glu-Ala-His) box polypeptide 16	1.084716	0.2883	NA
231842	Amz1	archaelysin family metallopeptidase 1	1.084716	0.5386	0.7429
548102	LOC548102	hypothetical LOC548102	1.084716	0.4064	0.6359
17869	Myc	myelocytomatosis oncogene	1.084599	0.4366	0.6631
67030	Fancl	Fanconi anemia, complementation group L	1.084599	0.3672	0.6024
67236	Cinp	cyclin-dependent kinase 2 interacting protein	1.084599	0.4566	0.6801
229782	Slc35a3	solute carrier family 35 (UDP-N-acetylglucosamine (UDP-GlcNAc) transporter), member 3	1.084599	0.4143	0.643
11877	Arvcf	armadillo repeat gene deleted in velo-cardio-facial syndrome	1.084481	0.4734	0.6942
67471	Gpatch1	G patch domain containing 1	1.084481	0.3465	0.582
74268	Aven	apoptosis, caspase activation inhibitor	1.084481	0.2651	NA
233332	Adamts17	a disintegrin-like and metallopeptidase (reprolysin type) with thrombospondin type 1 motif, 17	1.084481	0.4636	0.6863
246278	Cd207	CD207 antigen	1.084481	0.532	0.7378
17299	Mettl1	methyltransferase like 1	1.084363	0.4383	0.6645
17342	Mitf	microphthalmia-associated transcription factor	1.084363	0.3035	NA
22061	Trp63	transformation related protein 63	1.084363	0.66	0.8245
71790	Anxa9	annexin A9	1.084363	0.7458	0.8766
12986	Csf3r	colony stimulating factor 3 receptor (granulocyte)	1.084246	0.599	0.7845
66616	Snx9	sorting nexin 9	1.084246	0.3973	0.6277
69008	Cab39l	calcium binding protein 39-like	1.084246	0.159	NA
74473	4933433H22Rik	RIKEN cDNA 4933433H22 gene	1.084246	0.7122	0.8553
208777	Sned1	sushi, nidogen and EGF-like domains 1	1.084246	0.8241	0.9204
623483	LOC623483	60S ribosomal protein L22-like	1.084246	0.2417	NA
64833	Acot10	acyl-CoA thioesterase 10	1.084128	0.5416	0.7457
69260	Ing2	inhibitor of growth family, member 2	1.084128	0.3902	0.6223
70737	Cgn	cingulin	1.084128	0.4149	0.6435
106582	Nrm	nurim (nuclear envelope membrane protein)	1.084128	0.4173	0.6456
210544	Wdr67	WD repeat domain 67	1.084128	0.7371	0.8712
14221	Fjx1	four jointed box 1 (Drosophila)	1.084011	0.3016	NA
14593	Ggps1	geranylgeranyl diphosphate synthase 1	1.084011	0.4065	0.6359
16697	LOC16697	keratin associated protein LOC16697	1.084011	0.5556	0.7549

217356	Tmc8	transmembrane channel-like gene family 8	1.084011	0.7926	0.9016
20090	Rps29	ribosomal protein S29	1.083893	0.2411	NA
20747	Spop	speckle-type POZ protein	1.083893	0.4832	0.7014
110379	Sec13	SEC13 homolog (S. cerevisiae)	1.083893	0.3445	0.5799
18803	Plcg1	phospholipase C, gamma 1	1.083776	0.3946	0.6254
22070	Tpt1	tumor protein, translationally-controlled 1	1.083776	0.4381	0.6644
52040	Ppp1r10	protein phosphatase 1, regulatory subunit 10	1.083776	0.512	0.7225
227210	Ccnyl1	cyclin Y-like 1	1.083776	0.5338	0.7392
546049	C330021F23Rik	RIKEN cDNA C330021F23 gene	1.083776	0.5825	0.7734
100039895	Gm2479	predicted gene 2479	1.083776	0.6792	0.8358
14366	Fzd4	frizzled homolog 4 (Drosophila)	1.083658	0.3342	NA
13885	Esd	esterase D/formylglutathione hydrolase	1.083541	0.1921	NA
16562	Kif1c	kinesin family member 1C	1.083541	0.374	0.6082
70097	Sash1	SAM and SH3 domain containing 1	1.083541	0.3225	NA
74096	Hvcn1	hydrogen voltage-gated channel 1	1.083541	0.5303	0.7366
78751	Zc3h6	zinc finger CCCH type containing 6	1.083541	0.3743	0.6084
230917	Tmem201	transmembrane protein 201	1.083541	0.4696	0.6916
269037	Gm672	predicted gene 672	1.083541	0.4303	0.6572
330216	Mblac1	metallo-beta-lactamase domain containing 1	1.083541	0.2243	NA
11423	Ache	acetylcholinesterase	1.083424	0.5555	0.7548
100041576	Gm3414	predicted gene 3414	1.083424	0.4798	0.6989
13560	E4f1	E4F transcription factor 1	1.083306	0.2614	NA
27224	Tceb3	transcription elongation factor B (SIII), polypeptide 3	1.083306	0.2938	NA
56724	Cript	cysteine-rich PDZ-binding protein	1.083306	0.358	0.593
329777	Pigk	phosphatidylinositol glycan anchor biosynthesis, class K	1.083306	0.3709	0.6059
100041231	Gm3219	B-cell CLL/lymphoma 7C pseudogene	1.083306	0.5894	0.7784
20378	Frzb	frizzled-related protein	1.083189	0.1776	NA
66694	Uqcrfs1	ubiquinol-cytochrome c reductase, Rieske iron-sulfur polypeptide 1	1.083189	0.1822	NA
67501	Ccdc50	coiled-coil domain containing 50	1.083189	0.669	0.83
68039	Nmb	neuromedin B	1.083189	0.3968	0.6273
12442	Ccnb2	cyclin B2	1.083072	0.1991	NA
13626	Eed	embryonic ectoderm development	1.083072	0.2117	NA
19231	Ptma	prothymosin alpha	1.083072	0.5776	0.7702
235459	Gtf2a2	general transcription factor II A, 2	1.083072	0.3427	0.5782
105377	Ankrd32	ankyrin repeat domain 32	1.082954	0.355	0.5897
232370	Clstn3	calsyntenin 3	1.082954	0.2553	NA
237979	Sdk2	sidekick homolog 2 (chicken)	1.082954	0.6332	0.8071
403345	BC037438	cDNA sequence BC037438	1.082837	0.6881	0.8408
408022	Ccdc111	coiled-coil domain containing 111	1.082837	0.4351	0.662
14732	Gpam	glycerol-3-phosphate acyltransferase, mitochondrial	1.08272	0.4788	0.6985
76425	2310003C23Rik	RIKEN cDNA 2310003C23 gene	1.08272	0.2666	NA
78266	Zfp687	zinc finger protein 687	1.08272	0.1691	NA
73711	Fam125a	family with sequence similarity 125, member A	1.082485	0.3375	NA
224648	Uhrf1bp1	UHRF1 (ICBP90) binding protein 1	1.082485	0.1788	NA
633385	Gm7111	predicted gene 7111	1.082485	0.3492	0.5851
21423	Tcf3	transcription factor 3	1.082368	0.2374	NA
56175	Bace2	beta-site APP-cleaving enzyme 2	1.082368	0.7441	0.8756
57357	Srd5a3	steroid 5 alpha-reductase 3	1.082368	0.4289	0.656
79566	Sh3bp5l	SH3 binding domain protein 5 like	1.082368	0.09577	NA

83409	Robld3	roadblock domain containing 3	1.082368	0.5103	0.7212
242687	Wasf2	WAS protein family, member 2	1.082368	0.4322	0.6594
245847	Amdhd2	amidohydrolase domain containing 2	1.082368	0.4061	0.6356
381229	Ccdc147	coiled-coil domain containing 147	1.082368	0.4935	0.7095
433931	Pigg	phosphatidylinositol glycan anchor biosynthesis, class G	1.082368	0.5189	0.728
14158	Fert2	fer (fms/fps related) protein kinase, testis specific 2	1.082251	0.5544	0.754
66832	Rsph3a	radial spoke 3A homolog (Chlamydomonas)	1.082251	0.3758	0.6095
68911	Pygo2	pygopus 2	1.082251	0.1206	NA
237221	Gemin8	gem (nuclear organelle) associated protein 8	1.082251	0.3968	0.6273
674810	LOC674810	60S ribosomal protein L3-like	1.082251	0.5012	0.7146
11553	Adra2c	adrenergic receptor, alpha 2c	1.082134	0.5173	0.7268
17134	Mafg	v-maf musculoaponeurotic fibrosarcoma oncogene family, protein G (avian)	1.082134	0.3673	0.6024
83964	Jam3	junction adhesion molecule 3	1.082134	0.1876	NA
433966	5730422E09Rik	RIKEN cDNA 5730422E09 gene	1.082134	0.638	0.8099
11489	Adam12	a disintegrin and metallopeptidase domain 12 (meltrin alpha)	1.082017	0.3748	0.6089
14255	Flt3	FMS-like tyrosine kinase 3	1.082017	0.4513	0.6761
229096	Ythdf3	YTH domain family 3	1.082017	0.6881	0.8408
11981	Atp9a	ATPase, class II, type 9A	1.0819	0.4863	0.7045
68750	Rreb1	ras responsive element binding protein 1	1.0819	0.3142	NA
72248	1700014B07Rik	RIKEN cDNA 1700014B07 gene	1.0819	0.4897	0.7067
75593	2410003K15Rik	RIKEN cDNA 2410003K15 gene	1.0819	0.248	NA
223732	Ldoc1l	leucine zipper, down-regulated in cancer 1-like	1.0819	0.3444	NA
244913	Gm4978	ribosomal protein L7A pseudogene	1.0819	0.1921	NA
26441	Psma4	proteasome (prosome, macropain) subunit, alpha type 4	1.081783	0.3402	NA
71128	4933417C20Rik	RIKEN cDNA 4933417C20 gene	1.081783	0.5341	0.7396
321022	Cdv3	carnitine deficiency-associated gene expressed in ventricle 3	1.081783	0.2102	NA
66585	Snrnp40	small nuclear ribonucleoprotein 40 (U5)	1.081666	0.3198	NA
74287	Kcmf1	potassium channel modulatory factor 1	1.081666	0.2489	NA
12404	Cbln1	cerebellin 1 precursor protein	1.081549	0.3913	0.623
106648	Cyp4f15	cytochrome P450, family 4, subfamily f, polypeptide 15	1.081549	0.8462	0.9311
230718	Nt5c1a	5'-nucleotidase, cytosolic IA	1.081549	0.7985	0.9053
665669	Gm7742	predicted gene 7742	1.081549	0.4036	0.6335
56404	Trip4	thyroid hormone receptor interactor 4	1.081432	0.2236	NA
67211	Armc10	armadillo repeat containing 10	1.081432	0.1259	NA
230861	Eif4g3	eukaryotic translation initiation factor 4 gamma, 3	1.081432	0.6081	0.7905
73738	Haus7	HAUS augmin-like complex, subunit 7	1.081315	0.3013	NA
109095	Rbm15b	RNA binding motif protein 15B	1.081315	0.505	0.7174
382793	Mtx3	metaxin 3	1.081315	0.5792	0.7714
12703	Socs1	suppressor of cytokine signaling 1	1.081198	0.4112	0.6403
16528	Kcnk4	potassium channel, subfamily K, member 4	1.081198	0.3797	0.6133
57874	Ptplad1	protein tyrosine phosphatase-like A domain containing 1	1.081198	0.5108	0.7216
67102	D16Ertd472e	DNA segment, Chr 16, ERATO Doi 472, expressed	1.081198	0.3756	0.6094
97541	Qars	glutaminyl-tRNA synthetase	1.081198	0.1801	NA
330662	Dock1	dedicator of cytokinesis 1	1.081198	0.3119	NA
20866	Stim1	stromal interaction molecule 1	1.081081	0.3073	NA
67628	Anp32b	acidic (leucine-rich) nuclear phosphoprotein 32 family, member B	1.081081	0.1222	NA
73847	Fam110a	family with sequence similarity 110, member A	1.081081	0.3184	NA
12038	Bche	butyrylcholinesterase	1.080964	0.3451	NA
94184	Pdxdc1	pyridoxal-dependent decarboxylase domain containing 1	1.080964	0.2408	NA

54678	Zfp108	zinc finger protein 108	1.080847	0.3347	NA
66240	Kcne1l	potassium voltage-gated channel, Isk-related family, member 1-like, pseudogene	1.080847	0.4775	0.6974
106504	Stk38	serine/threonine kinase 38	1.080847	0.518	0.7273
239691	AU021092	expressed sequence AU021092	1.080847	0.4921	0.7084
279067	Gm13777	predicted gene 13777	1.080847	0.464	0.6866
11690	Alox5ap	arachidonate 5-lipoxygenase activating protein	1.080731	0.2788	NA
20649	Sntb1	syntrophin, basic 1	1.080731	0.4552	0.6787
53881	Slc5a3	solute carrier family 5 (inositol transporters), member 3	1.080731	0.6104	0.7924
67883	Uxs1	UDP-glucuronate decarboxylase 1	1.080731	0.6341	0.8075
73692	2410089E03Rik	RIKEN cDNA 2410089E03 gene	1.080731	0.536	0.7411
223978	Cpped1	calcineurin-like phosphoesterase domain containing 1	1.080731	0.2401	NA
229644	Trim45	tripartite motif-containing 45	1.080731	0.2427	NA
15382	Hnrnpa1	heterogeneous nuclear ribonucleoprotein A1	1.080614	0.3057	NA
22779	Ikzf2	IKAROS family zinc finger 2	1.080614	0.3219	NA
56398	1500003O03Rik	RIKEN cDNA 1500003O03 gene	1.080614	0.177	NA
71743	Coasy	Coenzyme A synthase	1.080614	0.1436	NA
74355	Smchd1	SMC hinge domain containing 1	1.080614	0.2728	NA
14359	Fxr1	fragile X mental retardation gene 1, autosomal homolog	1.080497	0.4648	0.687
76964	2610028H24Rik	RIKEN cDNA 2610028H24 gene	1.080497	0.721	0.8603
246316	Lgi2	leucine-rich repeat LGI family, member 2	1.080497	0.4966	0.7116
627798	Gm6790	tumor protein, translationally-controlled 1 pseudogene	1.080497	0.5229	0.7307
12890	Cplx2	complexin 2	1.08038	0.4902	0.7072
66420	Polr2e	polymerase (RNA) II (DNA directed) polypeptide E	1.08038	0.3044	NA
210530	Leprel1	leprecan-like 1	1.08038	0.4982	0.7128
16976	Lrpap1	low density lipoprotein receptor-related protein associated protein 1	1.080264	0.1286	NA
22245	Uck1	uridine-cytidine kinase 1	1.080264	0.2485	NA
24051	Sgcb	sarcoglycan, beta (dystrophin-associated glycoprotein)	1.080264	0.2749	NA
74692	4930442P07Rik	RIKEN cDNA 4930442P07 gene	1.080264	0.6716	0.8318
77644	C330007P06Rik	RIKEN cDNA C330007P06 gene	1.080264	0.2222	NA
235442	Rab8b	RAB8B, member RAS oncogene family	1.080264	0.4979	0.7127
319618	Dcp1b	DCP1 decapping enzyme homolog b (S. cerevisiae)	1.080264	0.124	NA
24068	Sra1	steroid receptor RNA activator 1	1.080147	0.2651	NA
56334	Tmed2	transmembrane emp24 domain trafficking protein 2	1.080147	0.3861	0.6183
121022	Mrps6	mitochondrial ribosomal protein S6	1.080147	0.2145	NA
67288	Srek1ip1	splicing regulatory glutamine/lysine-rich protein 1interacting protein 1	1.08003	0.3783	0.612
70729	Nos1ap	nitric oxide synthase 1 (neuronal) adaptor protein	1.08003	0.5213	0.7292
233529	Kctd14	potassium channel tetramerisation domain containing 14	1.08003	0.4336	0.6607
56380	Arid3b	AT rich interactive domain 3B (BRIGHT-like)	1.079914	0.432	0.6592
229007	Zgpat	zinc finger, CCCH-type with G patch domain	1.079914	0.465	0.687
622474	Smok3b	sperm motility kinase 3B	1.079914	0.3021	NA
100047702	LOC100047702	battenin-like	1.079914	0.6283	0.8039
18212	Ntrk2	neurotrophic tyrosine kinase, receptor, type 2	1.079797	0.4944	0.7101
19726	Rfx3	regulatory factor X, 3 (influences HLA class II expression)	1.079797	0.5308	0.7369
51813	Ccnc	cyclin C	1.079797	0.5682	0.7634
76995	1700095A13Rik	RIKEN cDNA 1700095A13 gene	1.079797	0.654	0.8208
320279	C630007K24Rik	RIKEN cDNA C630007K24 gene	1.079797	0.3699	0.6048
69556	Bod1	biorientation of chromosomes in cell division 1	1.07968	0.3109	NA
231326	Aasdh	aminoadipate-semialdehyde dehydrogenase	1.07968	0.3665	0.6019
22185	U2af2	U2 small nuclear ribonucleoprotein auxiliary factor (U2AF) 2	1.079564	0.3555	NA

83602	Gtf2a1	general transcription factor II A, 1	1.079564	0.4185	0.6466
103236	Csnk1g2	casein kinase 1, gamma 2	1.079564	0.4746	0.6951
227154	Stradb	STE20-related kinase adaptor beta	1.079447	0.3253	NA
20729	Spin1	spindlin 1	1.079331	0.4532	0.6774
57320	Park7	Parkinson disease (autosomal recessive, early onset) 7	1.079331	0.2434	NA
66860	Tanc1	tetratricopeptide repeat, ankyrin repeat and coiled-coil containing 1	1.079331	0.512	0.7225
77591	Ddx10	DEAD (Asp-Glu-Ala-Asp) box polypeptide 10	1.079331	0.3997	0.63
57912	Cdc42se1	CDC42 small effector 1	1.079214	0.2437	NA
245857	Ssh3	slingshot homolog 3 (Drosophila)	1.079098	0.3079	NA
381379	Med19	mediator of RNA polymerase II transcription, subunit 19 homolog (yeast)	1.079098	0.3393	NA
17305	Mfng	MFNG O-fucosylpeptide 3-beta-N-acetylglucosaminyltransferase	1.078981	0.3371	NA
67727	Stx17	syntaxin 17	1.078981	0.459	0.6824
68472	Tmem126b	transmembrane protein 126B	1.078981	0.28	NA
68636	Fahd1	fumarylacetoacetate hydrolase domain containing 1	1.078981	0.5323	0.738
72931	2900010J23Rik	RIKEN cDNA 2900010J23 gene	1.078981	0.1546	NA
227334	Usp40	ubiquitin specific peptidase 40	1.078981	0.1943	NA
12354	Car7	carbonic anhydrase 7	1.078865	0.7077	0.8534
19273	Ptpru	protein tyrosine phosphatase, receptor type, U	1.078865	0.4936	0.7097
19726	Rfx3	regulatory factor X, 3 (influences HLA class II expression)	1.078865	0.4535	0.6774
56695	Pnkd	paroxysmal nonkinesiogenic dyskinesia	1.078865	0.2	NA
217335	Fbf1	Fas (TNFRSF6) binding factor 1	1.078865	0.5141	0.7244
100302432	Gm14435	predicted gene 14435	1.078865	0.5512	0.7515
50523	Lats2	large tumor suppressor 2	1.078749	0.6003	0.7851
55934	rp9	retinitis pigmentosa 9 (human)	1.078749	0.1963	NA
67222	Srfbp1	serum response factor binding protein 1	1.078749	0.08411	NA
69312	Lrrc67	leucine rich repeat containing 67	1.078749	0.3596	NA
98417	Cnih4	cornichon homolog 4 (Drosophila)	1.078749	0.2937	NA
268656	Sptlc1	serine palmitoyltransferase, long chain base subunit 1	1.078749	0.3261	NA
320352	Lrrc31	leucine rich repeat containing 31	1.078749	0.2445	NA
54673	Sh3glb1	SH3-domain GRB2-like B1 (endophilin)	1.078632	0.2889	NA
71511	9030601B04Rik	RIKEN cDNA 9030601B04 gene	1.078632	0.5075	0.7192
74469	Taf7l	TAF7-like RNA polymerase II, TATA box binding protein (TBP)-associated factor	1.078632	0.6494	0.8177
213019	Pdlim2	PDZ and LIM domain 2	1.078632	0.4635	0.6863
227738	Lrsam1	leucine rich repeat and sterile alpha motif containing 1	1.078632	0.6587	0.8235
70445	Cd248	CD248 antigen, endosialin	1.078516	0.6802	0.8363
18053	Ngfr	nerve growth factor receptor (TNFR superfamily, member 16)	1.0784	0.442	0.6678
71990	Ddx54	DEAD (Asp-Glu-Ala-Asp) box polypeptide 54	1.0784	0.3836	0.6162
98303	D630023F18Rik	RIKEN cDNA D630023F18 gene	1.0784	0.2426	NA
241770	Rims4	regulating synaptic membrane exocytosis 4	1.0784	0.713	0.8557
13131	Dab1	disabled homolog 1 (Drosophila)	1.078283	0.6601	0.8246
22240	Dpysl3	dihydropyrimidinase-like 3	1.078283	0.3349	NA
53861	Zranb2	zinc finger, RAN-binding domain containing 2	1.078283	0.452	0.6768
67026	Thap4	THAP domain containing 4	1.078283	0.445	0.6706
114896	Afg3l1	AFG3(ATPase family gene 3)-like 1 (yeast)	1.078283	0.3685	NA
269623	C030048B08Rik	RIKEN cDNA C030048B08 gene	1.078283	0.5753	0.7688
270106	Rpl13	ribosomal protein L13	1.078283	0.5621	0.7604
382099	Gm5161	predicted pseudogene 5161	1.078283	0.5467	0.7484
16549	Khsrp	KH-type splicing regulatory protein	1.078167	0.4542	0.6779
23807	Arih2	ariadne homolog 2 (Drosophila)	1.078167	0.1096	NA

71910	Ppapdc1b	phosphatidic acid phosphatase type 2 domain containing 1B	1.078167	0.3798	0.6134
78755	Fam122b	family with sequence similarity 122, member B	1.078167	0.5982	0.7837
232987	B9d2	B9 protein domain 2	1.078167	0.2396	NA
235330	Ttc12	tetratricopeptide repeat domain 12	1.078167	0.3235	NA
381306	BC055324	cDNA sequence BC055324	1.078167	0.3727	0.6069
14548	Mrps33	mitochondrial ribosomal protein S33	1.078051	0.2163	NA
68048	Aen	apoptosis enhancing nuclease	1.078051	0.1573	NA
68327	0610007P22Rik	RIKEN cDNA 0610007P22 gene	1.078051	0.2131	NA
19268	Ptprf	protein tyrosine phosphatase, receptor type, F	1.077935	0.3935	0.6245
230648	4732418C07Rik	RIKEN cDNA 4732418C07 gene	1.077935	0.3297	NA
631906	Gm7075	predicted gene 7075	1.077935	0.5457	0.748
53872	Caprin1	cell cycle associated protein 1	1.077818	0.2129	NA
58802	Kcnmb4	potassium large conductance calcium-activated channel, subfamily M, beta member 4	1.077818	0.5429	0.7464
67106	Zbtb8os	zinc finger and BTB domain containing 8 opposite strand	1.077818	0.3567	NA
68039	Nmb	neuromedin B	1.077818	0.403	0.633
212632	Iffo2	intermediate filament family orphan 2	1.077818	0.3992	0.6295
320869	4732415M23Rik	RIKEN cDNA 4732415M23 gene	1.077818	0.4835	0.7017
328699	Gabrr3	gamma-aminobutyric acid (GABA) receptor, rho 3	1.077818	0.5396	0.7439
58865	Tdh	L-threonine dehydrogenase	1.077702	0.7421	0.8743
107528	Magee1	melanoma antigen, family E, 1	1.077702	0.5942	0.7814
246293	Klhl8	kelch-like 8 (Drosophila)	1.077702	0.113	NA
14714	Gnrh1	gonadotropin releasing hormone 1	1.077586	0.517	0.7265
53310	Dlg3	discs, large homolog 3 (Drosophila)	1.077586	0.1972	NA
64656	Mrps23	mitochondrial ribosomal protein S23	1.077586	0.3592	NA
71990	Ddx54	DEAD (Asp-Glu-Ala-Asp) box polypeptide 54	1.077586	0.3732	NA
78887	Sfi1	Sfi1 homolog, spindle assembly associated (yeast)	1.077586	0.77	0.8895
232333	Slc6a1	solute carrier family 6 (neurotransmitter transporter, GABA), member 1	1.077586	0.4233	0.651
237611	Stac3	SH3 and cysteine rich domain 3	1.077586	0.5006	0.7145
11431	Acp1	acid phosphatase 1, soluble	1.07747	0.3415	NA
12167	Bmpr1b	bone morphogenetic protein receptor, type 1B	1.07747	0.3622	NA
19043	Ppm1b	protein phosphatase 1B, magnesium dependent, beta isoform	1.07747	0.2922	NA
67869	Paip2	polyadenylate-binding protein-interacting protein 2	1.07747	0.3746	0.6088
68436	Rpl34	ribosomal protein L34	1.07747	0.4041	0.6339
106369	Ypel1	yippee-like 1 (Drosophila)	1.07747	0.4042	0.634
668144	Gm9000	predicted gene 9000	1.07747	0.2905	NA
12370	Casp8	caspase 8	1.077354	0.4889	0.7061
67345	Herc4	hect domain and RLD 4	1.077354	0.2848	NA
52065	Mfhas1	malignant fibrous histiocytoma amplified sequence 1	1.077238	0.2842	NA
66868	Mfsd1	major facilitator superfamily domain containing 1	1.077238	0.4166	0.645
72886	Ccdc94	coiled-coil domain containing 94	1.077238	0.3675	NA
76737	Creld2	cysteine-rich with EGF-like domains 2	1.077122	0.2155	NA
432555	Gm5431	predicted gene 5431	1.077122	0.4142	0.643
22003	Tpm1	tropomyosin 1, alpha	1.077006	0.2703	NA
24069	Sufu	suppressor of fused homolog (Drosophila)	1.077006	0.5757	0.769
58227	Fam184b	family with sequence similarity 184, member B	1.077006	0.5205	0.7287
70533	Btf3l4	basic transcription factor 3-like 4	1.077006	0.2035	NA
106251	D230034L24Rik	RIKEN cDNA D230034L24 gene	1.077006	0.2974	NA
432754	LOC432754	similar to glyceraldehyde-3-phosphate dehydrogenase	1.077006	0.5159	0.7259
17113	M6pr	mannose-6-phosphate receptor, cation dependent	1.07689	0.1381	NA

18226	Nup62	nucleoporin 62	1.07689	0.2414	NA
21968	Tom1	target of myb1 homolog (chicken)	1.07689	0.1675	NA
26428	Orc4	origin recognition complex, subunit 4	1.07689	0.4461	0.6718
69131	Cdk12	cyclin-dependent kinase 12	1.07689	0.7263	0.8641
76894	Mett5d1	methyltransferase 5 domain containing 1	1.07689	0.3929	0.6241
104570	Smek2	SMEK homolog 2, suppressor of mek1 (Dictyostelium)	1.07689	0.4344	0.6613
214189	Scgn	secretagogin, EF-hand calcium binding protein	1.07689	0.6032	0.7871
12540	Cdc42	cell division cycle 42 homolog (S. cerevisiae)	1.076774	0.2349	NA
20250	Scd2	stearoyl-Coenzyme A desaturase 2	1.076774	0.1878	NA
51944	D2Ertd750e	DNA segment, Chr 2, ERATO Doi 750, expressed	1.076774	0.6062	0.7891
73419	1700052N19Rik	RIKEN cDNA 1700052N19 gene	1.076774	0.2166	NA
12467	Cct6b	chaperonin containing Tcp1, subunit 6b (zeta)	1.076658	0.5207	0.7289
56361	Pus1	pseudouridine synthase 1	1.076658	0.3977	0.6281
102414	Clk3	CDC-like kinase 3	1.076658	0.31	NA
319586	Celf5	CUGBP, Elav-like family member 5	1.076658	0.5077	0.7193
546663	Gm5963	predicted pseudogene 5963	1.076658	0.4333	0.6604
100039795	Ildr2	immunoglobulin-like domain containing receptor 2	1.076658	0.4554	0.6791
12982	Csf2ra	colony stimulating factor 2 receptor, alpha, low-affinity (granulocyte-macrophage)	1.076542	0.3281	NA
18436	P2rx1	purinergic receptor P2X, ligand-gated ion channel, 1	1.076542	0.8143	0.9145
20346	Sema3a	sema domain, immunoglobulin domain (Ig), short basic domain, secreted, (semaphorin) 3A	1.076542	0.5732	0.767
20911	Stxbp2	syntaxin binding protein 2	1.076542	0.5662	0.762
56809	Gmeb1	glucocorticoid modulatory element binding protein 1	1.076542	0.2736	NA
66942	Ddx18	DEAD (Asp-Glu-Ala-Asp) box polypeptide 18	1.076542	0.323	NA
328633	4930515I15	hypothetical protein 4930515I15	1.076542	0.5918	0.7799
14566	Gdf9	growth differentiation factor 9	1.076426	0.4989	0.7135
67006	Cisd2	CDGSH iron sulfur domain 2	1.076426	0.3027	NA
67059	Ola1	Obg-like ATPase 1	1.076426	0.4701	0.692
235574	Atp2c1	ATPase, Ca++-sequestering	1.076426	0.2338	NA
266690	Cyb5r4	cytochrome b5 reductase 4	1.076426	0.4249	0.6524
57315	Wdr46	WD repeat domain 46	1.07631	0.2193	NA
67097	Rps10	ribosomal protein S10	1.076195	0.3286	NA
104570	Smek2	SMEK homolog 2, suppressor of mek1 (Dictyostelium)	1.076195	0.2726	NA
231999	Plekha8	pleckstrin homology domain containing, family A (phosphoinositide binding specific) member 8	1.076195	0.3119	NA
242894	Actr3b	ARP3 actin-related protein 3 homolog B (yeast)	1.076195	0.3034	NA
277854	Depdc5	DEP domain containing 5	1.076195	0.6027	0.7868
384783	Irs2	insulin receptor substrate 2	1.076195	0.4089	0.6382
621312	Gm6211	predicted gene 6211	1.076195	0.1448	NA
52150	Kcnk6	potassium inwardly-rectifying channel, subfamily K, member 6	1.076079	0.4951	0.7108
67789	Dalrd3	DALR anticodon binding domain containing 3	1.076079	0.6033	0.7871
19364	Rad51l3	RAD51-like 3 (S. cerevisiae)	1.075963	0.3253	NA
19707	Reps1	RalBP1 associated Eps domain containing protein	1.075963	0.2829	NA
619547	Rpl34-ps1	ribosomal protein L34, pseudogene 1	1.075963	0.2345	NA
12890	Cplx2	complexin 2	1.075847	0.6318	0.806
21681	Thoc4	THO complex 4	1.075847	0.2151	NA
68991	Ssu72	Ssu72 RNA polymerase II CTD phosphatase homolog (yeast)	1.075847	0.3285	NA
105734	Tigd5	tigger transposable element derived 5	1.075847	0.6161	0.7965
385380	Tex28	testis expressed 28	1.075847	0.7329	0.8684
67898	Pef1	penta-EF hand domain containing 1	1.075731	0.1933	NA
217030	Synrg	synergin, gamma	1.075731	0.4763	0.6965

217057	Ptrh2	peptidyl-tRNA hydrolase 2	1.075731	0.247	NA
14457	Gas7	growth arrest specific 7	1.075616	0.727	0.8645
71766	Raver1	ribonucleoprotein, PTB-binding 1	1.075616	0.6693	0.8303
52882	Rgs7bp	regulator of G-protein signalling 7 binding protein	1.0755	0.8451	0.9307
66487	2010107H07Rik	RIKEN cDNA 2010107H07 gene	1.075384	0.2626	NA
68507	Ppfia4	protein tyrosine phosphatase, receptor type, f polypeptide (PTPRF), interacting protein (liprin), alpha 4	1.075384	0.4911	0.7078
101602	AI467606	expressed sequence AI467606	1.075384	0.5467	0.7484
108735	Sft2d2	SFT2 domain containing 2	1.075384	0.122	NA
12914	Crebbp	CREB binding protein	1.075269	0.6448	0.8146
67203	Nde1	nuclear distribution gene E homolog 1 (A nidulans)	1.075269	0.6025	0.7867
74244	Atg7	autophagy-related 7 (yeast)	1.075269	0.5917	0.7799
102060	Gadd45gip1	growth arrest and DNA-damage-inducible, gamma interacting protein 1	1.075269	0.4649	0.687
19888	Rp1	retinitis pigmentosa 1 (human)	1.075153	0.4818	0.7004
22762	Zfpm2	zinc finger protein, multitype 2	1.075153	0.2286	NA
76613	1700069B07Rik	RIKEN cDNA 1700069B07 gene	1.075153	0.6513	0.8188
214899	Kdm5a	lysine (K)-specific demethylase 5A	1.075153	0.4844	0.7029
665596	Hist1h2bq	histone cluster 1, H2bq	1.075153	0.544	0.7471
29869	Ulk2	Unc-51 like kinase 2 (C. elegans)	1.075038	0.2274	NA
56431	Dstn	destrin	1.075038	0.4104	0.6395
66847	Hint3	histidine triad nucleotide binding protein 3	1.075038	0.3958	0.6264
228033	Atp5g3	ATP synthase, H+ transporting, mitochondrial F0 complex, subunit C3 (subunit 9)	1.075038	0.4978	0.7127
28081	D11Wsu99e	DNA segment, Chr 11, Wayne State University 99, expressed	1.074922	0.3674	NA
170829	Tram2	translocating chain-associating membrane protein 2	1.074922	0.5637	0.7611
320150	Zdhhc17	zinc finger, DHHC domain containing 17	1.074922	0.6788	0.8357
13000	Csnk2a2	casein kinase 2, alpha prime polypeptide	1.074807	0.3673	NA
68484	Krtap16-8	keratin associated protein 16-8	1.074807	0.6664	0.8289
74145	F13a1	coagulation factor XIII, A1 subunit	1.074807	0.8043	0.9083
107435	Hat1	histone aminotransferase 1	1.074807	0.5	0.7144
15531	Ndst1	N-deacetylase/N-sulfotransferase (heparan glucosaminyl) 1	1.074691	0.4716	0.6927
217198	Plekhh3	pleckstrin homology domain containing, family H (with MyTH4 domain) member 3	1.074691	0.1651	NA
353156	Egfl7	EGF-like domain 7	1.074691	0.21	NA
57320	Park7	Parkinson disease (autosomal recessive, early onset) 7	1.074576	0.2004	NA
68550	1110002N22Rik	RIKEN cDNA 1110002N22 gene	1.074576	0.3712	NA
107702	Rnh1	ribonuclease/angiogenin inhibitor 1	1.074576	0.3163	NA
269682	Golga3	golgi autoantigen, golgin subfamily a, 3	1.074576	0.2479	NA
68695	Hddc3	HD domain containing 3	1.07446	0.1507	NA
434446	Ccdc13	coiled-coil domain containing 13	1.07446	0.5445	0.7473
14151	Fech	ferrochelatase	1.074345	0.1219	NA
93840	Vangl2	vang-like 2 (van gogh, Drosophila)	1.074345	0.6094	0.7917
192662	Arhgdia	Rho GDP dissociation inhibitor (GDI) alpha	1.074345	0.3609	NA
100415785	Gm11559	predicted gene 11559	1.074345	0.4084	0.6377
58172	Sertad2	SERTA domain containing 2	1.074229	0.2296	NA
382030	Tmem188	transmembrane protein 188	1.074229	0.3162	NA
11459	Acta1	actin, alpha 1, skeletal muscle	1.074114	0.5053	0.7176
80750	N4bp1	NEDD4 binding protein 1	1.074114	0.4894	0.7065
192185	Nadk	NAD kinase	1.074114	0.3161	NA
328309	Gm9776	predicted gene 9776	1.074114	0.5595	0.7582
330660	Btbd16	BTB (POZ) domain containing 16	1.074114	0.3214	NA
378702	Serf2	small EDRK-rich factor 2	1.074114	0.1371	NA

624086	A230045G11Rik	RIKEN cDNA A230045G11 gene	1.074114	0.143	NA
20091	Rps3a	ribosomal protein S3A	1.073998	0.1582	NA
27362	Dnajb9	DnaJ (Hsp40) homolog, subfamily B, member 9	1.073998	0.3929	NA
70769	Nolc1	nucleolar and coiled-body phosphoprotein 1	1.073998	0.2191	NA
338349	Cntln	centlein, centrosomal protein	1.073998	0.5942	0.7814
546100	Gm5914	predicted gene 5914	1.073998	0.3482	NA
100502966	LOC100502966	zinc finger protein 717-like	1.073998	0.6246	0.8016
18383	Tnfrsf11b	tumor necrosis factor receptor superfamily, member 11b (osteoprotegerin)	1.073883	0.5913	0.7798
69188	Mll5	myeloid/lymphoid or mixed-lineage leukemia 5	1.073883	0.3787	NA
100678	Psph	phosphoserine phosphatase	1.073883	0.4297	0.6567
22631	Ywhaz	tyrosine 3-monooxygenase/tryptophan 5-monooxygenase activation protein, zeta polypeptide	1.073768	0.4677	0.6897
381059	Gm1604b	predicted gene 1604b	1.073768	0.5462	0.7482
12326	Camk4	calcium/calmodulin-dependent protein kinase IV	1.073653	0.5733	0.767
20641	Snrpd1	small nuclear ribonucleoprotein D1	1.073653	0.2079	NA
67006	Cisd2	CDGSH iron sulfur domain 2	1.073653	0.2576	NA
67867	Lrrc28	leucine rich repeat containing 28	1.073653	0.2896	NA
72584	Cul4b	cullin 4B	1.073653	0.4622	0.6849
214133	Tet2	tet oncogene family member 2	1.073653	0.3353	NA
11837	Rplp0	ribosomal protein, large, P0	1.073537	0.5776	0.7702
15288	Hmbs	hydroxymethylbilane synthase	1.073537	0.329	NA
20585	Hltf	helicase-like transcription factor	1.073537	0.6048	0.7883
30934	Tor1b	torsin family 1, member B	1.073537	0.3602	NA
57441	Gmnn	geminin	1.073537	0.2671	NA
67139	Mis12	MIS12 homolog (yeast)	1.073537	0.5208	0.7289
73844	Ankrd45	ankyrin repeat domain 45	1.073537	0.707	0.853
624866	Lekr1	leucine, glutamate and lysine rich 1	1.073537	0.4318	0.6591
11937	Atp2a1	ATPase, Ca++ transporting, cardiac muscle, fast twitch 1	1.073422	0.4323	0.6594
67542	Cog6	component of oligomeric golgi complex 6	1.073422	0.4785	0.6982
235442	Rab8b	RAB8B, member RAS oncogene family	1.073422	0.3271	NA
237073	Rbm41	RNA binding motif protein 41	1.073422	0.5466	0.7484
654795	Sdr39u1	short chain dehydrogenase/reductase family 39U, member 1	1.073422	0.1521	NA
14020	Evi5	ecotropic viral integration site 5	1.073307	0.2437	NA
14198	Fhit	fragile histidine triad gene	1.073307	0.6051	0.7884
18205	Ntf3	neurotrophin 3	1.073307	0.8058	0.9092
68371	Pbld1	phenazine biosynthesis-like protein domain containing 1	1.073307	0.4528	0.6772
214137	Arhgap29	Rho GTPase activating protein 29	1.073307	0.3116	NA
218503	Fcho2	FCH domain only 2	1.073307	0.5368	0.7416
67974	Ccny	cyclin Y	1.073192	0.6842	0.8388
74026	Msl1	male-specific lethal 1 homolog (Drosophila)	1.073192	0.2069	NA
19317	Qk	quaking	1.073077	0.4918	0.708
99696	Ankrd50	ankyrin repeat domain 50	1.073077	0.6603	0.8246
140499	Ube2j2	ubiquitin-conjugating enzyme E2, J2 homolog (yeast)	1.073077	0.1864	NA
242083	Ppm1l	protein phosphatase 1 (formerly 2C)-like	1.073077	0.2892	NA
20280	Scp2	sterol carrier protein 2, liver	1.072961	0.424	0.6517
67367	Gcfc1	GC-rich sequence DNA-binding factor 1	1.072961	0.6151	0.7956
72050	Kdelc1	KDEL (Lys-Asp-Glu-Leu) containing 1	1.072846	0.8157	0.9154
245000	Atr	ataxia telangiectasia and Rad3 related	1.072846	0.3281	NA
13714	Elk4	ELK4, member of ETS oncogene family	1.072731	0.6039	0.7876
68634	Tm2d3	TM2 domain containing 3	1.072731	0.2636	NA

170833	Hook2	hook homolog 2 (Drosophila)	1.072731	0.3306	NA
12315	Calm3	calmodulin 3	1.072616	0.2095	NA
24059	Slco2a1	solute carrier organic anion transporter family, member 2a1	1.072616	0.5317	0.7378
73251	Setd7	SET domain containing (lysine methyltransferase) 7	1.072616	0.3534	NA
74255	Smu1	smu-1 suppressor of mec-8 and unc-52 homolog (C. elegans)	1.072616	0.3441	NA
109113	Uhrf2	ubiquitin-like, containing PHD and RING finger domains 2	1.072616	0.5662	0.762
19108	Prkx	protein kinase, X-linked	1.072501	0.3666	NA
20416	Shc1	src homology 2 domain-containing transforming protein C1	1.072501	0.4973	0.7123
27384	Akr1c13	aldo-keto reductase family 1, member C13	1.072501	0.5298	0.7361
78934	4930581F22Rik	RIKEN cDNA 4930581F22 gene	1.072501	0.5767	0.7696
17210	Mcl1	myeloid cell leukemia sequence 1	1.072386	0.1978	NA
17826	Mtvr2	mammary tumor virus receptor 2	1.072386	0.3716	NA
66923	Pbrm1	polybromo 1	1.072386	0.6734	0.8326
69582	Plekhm2	pleckstrin homology domain containing, family M (with RUN domain) member 2	1.072386	0.2018	NA
15473	Hrsp12	heat-responsive protein 12	1.072271	0.4383	0.6645
64435	Fcamr	Fc receptor, IgA, IgM, high affinity	1.072271	0.8948	0.9549
79196	Osbpl5	oxysterol binding protein-like 5	1.072271	0.164	NA
98732	Rab3gap2	RAB3 GTPase activating protein subunit 2	1.072271	0.383	NA
20088	Rps24	ribosomal protein S24	1.072156	0.5688	0.7635
66323	1700001K19Rik	RIKEN cDNA 1700001K19 gene	1.072156	0.5498	0.7507
73103	3110009E18Rik	RIKEN cDNA 3110009E18 gene	1.072156	0.445	0.6706
100710	Pds5b	PDS5, regulator of cohesion maintenance, homolog B (S. cerevisiae)	1.072156	0.4032	NA
236920	Stard8	START domain containing 8	1.072156	0.4115	0.6406
21411	Tcf20	transcription factor 20	1.072041	0.507	0.7187
54615	Npff	neuropeptide FF-amide peptide precursor	1.072041	0.4228	0.6507
83984	Tssk6	testis-specific serine kinase 6	1.072041	0.676	0.8337
223455	Mar-06	membrane-associated ring finger (C3HC4) 6	1.072041	0.3962	NA
14872	Gstt2	glutathione S-transferase, theta 2	1.071926	0.3594	NA
19946	Rpl30	ribosomal protein L30	1.071926	0.4229	0.6507
53380	Psmd10	proteasome (prosome, macropain) 26S subunit, non-ATPase, 10	1.071926	0.3473	NA
69639	Exosc8	exosome component 8	1.071926	0.265	NA
234135	Whsc1l1	Wolf-Hirschhorn syndrome candidate 1-like 1 (human)	1.071926	0.7278	0.8652
381038	Parl	presenilin associated, rhomboid-like	1.071811	0.4219	0.6498
11637	Ak2	adenylate kinase 2	1.071582	0.3685	NA
16828	Ldha	lactate dehydrogenase A	1.071582	0.4998	0.7143
70093	Ube2q1	ubiquitin-conjugating enzyme E2Q (putative) 1	1.071582	0.1353	NA
66921	Prpf38b	PRP38 pre-mRNA processing factor 38 (yeast) domain containing B	1.071467	0.7108	0.8547
67023	Use1	unconventional SNARE in the ER 1 homolog (S. cerevisiae)	1.071467	0.3354	NA
77219	Ptgr2	prostaglandin reductase 2	1.071467	0.3011	NA
80748	BC004004	cDNA sequence BC004004	1.071467	0.2632	NA
381290	Atp2b4	ATPase, Ca++ transporting, plasma membrane 4	1.071467	0.5945	0.7816
13384	Mpp3	membrane protein, palmitoylated 3 (MAGUK p55 subfamily member 3)	1.071352	0.7616	0.8845
14194	Fh1	fumarate hydratase 1	1.071352	0.1431	NA
18763	Pkd1	polycystic kidney disease 1 homolog	1.071352	0.3812	NA
74440	4933407C03Rik	RIKEN cDNA 4933407C03 gene	1.071352	0.3692	NA
217708	Lin52	lin-52 homolog (C. elegans)	1.071352	0.5687	0.7635
380753	Atxn7l1	ataxin 7-like 1	1.071352	0.6931	0.8439
18764	Pkd2	polycystic kidney disease 2	1.071237	0.3313	NA
20249	Scd1	stearoyl-Coenzyme A desaturase 1	1.071237	0.5563	0.7556

30910	C230037E05Rik	RIKEN cDNA C230037E05 gene	1.071237	0.2847	NA
67971	Tppp3	tubulin polymerization-promoting protein family member 3	1.071237	0.3416	NA
72293	Nkd2	naked cuticle 2 homolog (Drosophila)	1.071237	0.653	0.8204
74097	Pop7	processing of precursor 7, ribonuclease P family, (S. cerevisiae)	1.071237	0.246	NA
77034	2510039O18Rik	RIKEN cDNA 2510039O18 gene	1.071237	0.1642	NA
93684	Sep-15	selenoprotein	1.071237	0.1282	NA
230379	Acer2	alkaline ceramidase 2	1.071237	0.4704	0.692
238799	Tnpo1	transportin 1	1.071237	0.6362	0.8088
13661	Ehf	ets homologous factor	1.071123	0.5735	0.7672
20638	Snrpb	small nuclear ribonucleoprotein B	1.071123	0.5079	0.7193
54342	Gnpnat1	glucosamine-phosphate N-acetyltransferase 1	1.071123	0.4107	NA
230761	Zfp362	zinc finger protein 362	1.071123	0.4793	0.6987
231997	Fkbp14	FK506 binding protein 14	1.071123	0.2947	NA
22428	Dctn6	dynactin 6	1.071008	0.1537	NA
76299	Erp44	endoplasmic reticulum protein 44	1.071008	0.3646	NA
77492	8030456M14Rik	RIKEN cDNA 8030456M14 gene	1.071008	0.4718	0.6928
240892	Dusp27	dual specificity phosphatase 27 (putative)	1.071008	0.5969	0.783
243612	D630042P16Rik	RIKEN cDNA D630042P16 gene	1.071008	0.6637	0.8272
14588	Gfra4	glial cell line derived neurotrophic factor family receptor alpha 4	1.070893	0.6901	0.8418
16145	Igtp	interferon gamma induced GTPase	1.070893	0.4927	0.7091
67035	Dnajb4	DnaJ (Hsp40) homolog, subfamily B, member 4	1.070893	0.5526	0.7526
209011	Sirt7	sirtuin 7 (silent mating type information regulation 2, homolog) 7 (S. cerevisiae)	1.070893	0.4239	0.6516
319266	A130010J15Rik	RIKEN cDNA A130010J15 gene	1.070893	0.4379	0.6643
347740	2900097C17Rik	RIKEN cDNA 2900097C17 gene	1.070893	0.7094	0.8542
21813	Tgfbr2	transforming growth factor, beta receptor II	1.070778	0.5191	0.7281
66979	Pole4	polymerase (DNA-directed), epsilon 4 (p12 subunit)	1.070778	0.6884	0.8409
73757	4833421G17Rik	RIKEN cDNA 4833421G17 gene	1.070778	0.5916	0.7799
629159	1700008J07Rik	RIKEN cDNA 1700008J07 gene	1.070778	0.4957	0.7111
16190	Il4ra	interleukin 4 receptor, alpha	1.070664	0.604	0.7876
22026	Nr2c2	nuclear receptor subfamily 2, group C, member 2	1.070664	0.5161	0.726
26897	Acot1	acyl-CoA thioesterase 1	1.070664	0.5305	0.7368
66320	Tmem208	transmembrane protein 208	1.070664	0.2544	NA
74268	Aven	apoptosis, caspase activation inhibitor	1.070664	0.3664	NA
240411	Loxhd1	lipoxygenase homology domains 1	1.070664	0.761	0.8842
431706	Zfp457	zinc finger protein 457	1.070664	0.6948	0.845
52357	Wwc2	WW, C2 and coiled-coil domain containing 2	1.070549	0.4097	NA
76355	Tgds	TDP-glucose 4,6-dehydratase	1.070549	0.5503	0.7511
103583	Fbxw11	F-box and WD-40 domain protein 11	1.070549	0.1701	NA
106200	Txndc11	thioredoxin domain containing 11	1.070549	0.1616	NA
192231	Hexim1	hexamethylene bis-acetamide inducible 1	1.070549	0.2763	NA
230936	Phf13	PHD finger protein 13	1.070549	0.1961	NA
100042959	Gm4130	predicted gene 4130	1.070549	0.5102	0.7212
18105	Nqo2	NAD(P)H dehydrogenase, quinone 2	1.070435	0.4752	0.6958
21855	Timm17b	translocase of inner mitochondrial membrane 17b	1.070435	0.2173	NA
53417	Hif3a	hypoxia inducible factor 3, alpha subunit	1.070435	0.6705	0.8313
68268	Zdhhc21	zinc finger, DHHC domain containing 21	1.070435	0.4546	0.6782
72180	Zfp661	zinc finger protein 661	1.070435	0.2574	NA
110893	Slc8a3	solute carrier family 8 (sodium/calcium exchanger), member 3	1.070435	0.5932	0.7808
209378	Itih5	inter-alpha (globulin) inhibitor H5	1.070435	0.5592	0.7579

12552	Cdh11	cadherin 11	1.07032	0.4342	0.6612
68969	Eif1b	eukaryotic translation initiation factor 1B	1.07032	0.2974	NA
217294	BC006965	cDNA sequence BC006965	1.07032	0.5553	0.7548
100503659	Dos	downstream of Stk11	1.07032	0.491	0.7078
14225	Fkbp1a	FK506 binding protein 1a	1.070205	0.5736	0.7672
71709	Syde1	synapse defective 1, Rho GTPase, homolog 1 (C. elegans)	1.070205	0.4831	0.7014
75452	Ascc2	activating signal cointegrator 1 complex subunit 2	1.070205	0.3778	NA
546519	Gm12581	predicted gene 12581	1.070205	0.5327	0.7384
16796	Lasp1	LIM and SH3 protein 1	1.070091	0.2099	NA
17688	Msh6	mutS homolog 6 (E. coli)	1.070091	0.1756	NA
58809	Rnase4	ribonuclease, RNase A family 4	1.070091	0.5694	0.7638
66541	Immp1l	IMP1 inner mitochondrial membrane peptidase-like (S. cerevisiae)	1.070091	0.2972	NA
231642	Alkbh2	alkB, alkylation repair homolog 2 (E. coli)	1.070091	0.2828	NA
320348	C130051F05Rik	RIKEN cDNA C130051F05 gene	1.070091	0.6834	0.8384
414069	BC024978	cDNA sequence BC024978	1.070091	0.6266	0.803
13555	E2f1	E2F transcription factor 1	1.069976	0.5506	0.7511
14569	Gdi2	guanosine diphosphate (GDP) dissociation inhibitor 2	1.069976	0.2091	NA
20817	Srpk2	serine/arginine-rich protein specific kinase 2	1.069976	0.3473	NA
75717	Cul5	cullin 5	1.069976	0.2622	NA
76916	4930455C21Rik	RIKEN cDNA 4930455C21 gene	1.069976	0.4828	0.7013
210992	Lpcat1	lysophosphatidylcholine acyltransferase 1	1.069976	0.427	0.6541
17122	Mxd4	Max dimerization protein 4	1.069862	0.3285	NA
18618	Pemt	phosphatidylethanolamine N-methyltransferase	1.069862	0.6648	0.8279
21833	Thra	thyroid hormone receptor alpha	1.069862	0.4169	NA
57914	Crlf2	cytokine receptor-like factor 2	1.069862	0.1728	NA
114893	Dcun1d1	DCN1, defective in cullin neddylation 1, domain containing 1 (S. cerevisiae)	1.069862	0.4257	0.6532
268301	Ankrd57	ankyrin repeat domain 57	1.069862	0.2078	NA
22330	Vcl	vinculin	1.069748	0.5727	0.7666
67568	Mrfap1	Morf4 family associated protein 1	1.069748	0.4623	0.6849
71508	Zfp935	zinc finger protein 935	1.069748	0.6622	0.8261
72129	Pex13	peroxisomal biogenesis factor 13	1.069748	0.1601	NA
74487	5430405H02Rik	RIKEN cDNA 5430405H02 gene	1.069748	0.3864	NA
231912	Katnal1	katanin p60 subunit A-like 1	1.069748	0.3103	NA
319169	Hist1h2ak	histone cluster 1, H2ak	1.069748	0.4198	NA
319618	Dcp1b	DCP1 decapping enzyme homolog b (S. cerevisiae)	1.069748	0.164	NA
666183	Gm7968	predicted gene 7968	1.069748	0.5278	0.7346
20085	Rps19	ribosomal protein S19	1.069633	0.2885	NA
23857	Dmtf1	cyclin D binding myb-like transcription factor 1	1.069633	0.705	0.8517
56486	Gabarap	gamma-aminobutyric acid receptor associated protein	1.069633	0.2134	NA
58520	0610007P14Rik	RIKEN cDNA 0610007P14 gene	1.069633	0.1917	NA
66162	Bola2	bolA-like 2 (E. coli)	1.069633	0.2756	NA
269997	Zfp747	zinc finger protein 747	1.069633	0.1546	NA
100504871	LOC100504871	ATP synthase lipid-binding protein, mitochondrial-like	1.069633	0.5194	0.7282
57279	Slc25a20	solute carrier family 25 (mitochondrial carnitine/acylcarnitine translocase), member 20	1.069519	0.508	0.7194
66923	Pbrm1	polybromo 1	1.069519	0.6554	0.8212
72117	Naa50	N(alpha)-acetyltransferase 50, NatE catalytic subunit	1.069519	0.6739	0.833
56709	Dnajb12	DnaJ (Hsp40) homolog, subfamily B, member 12	1.069404	0.2202	NA
228071	Sestd1	SEC14 and spectrin domains 1	1.069404	0.429	0.656
320253	Mar-03	membrane-associated ring finger (C3HC4) 3	1.069404	0.7083	0.8537

67477	Abhd15	abhydrolase domain containing 15	1.06929	0.6877	0.8407
75646	Rai14	retinoic acid induced 14	1.06929	0.3798	NA
13877	Erh	enhancer of rudimentary homolog (Drosophila)	1.069176	0.2671	NA
244667	Disc1	disrupted in schizophrenia 1	1.069176	0.3021	NA
13427	Dync1i2	dynein cytoplasmic 1 intermediate chain 2	1.069061	0.2934	NA
74075	Syce1	synaptonemal complex central element protein 1	1.069061	0.7148	0.857
21833	Thra	thyroid hormone receptor alpha	1.068947	0.4064	NA
56228	Ube2j1	ubiquitin-conjugating enzyme E2, J1	1.068947	0.6147	0.7953
67963	Npc2	Niemann Pick type C2	1.068947	0.3112	NA
73873	Fam161a	family with sequence similarity 161, member A	1.068947	0.4931	0.7093
93728	Pabpc5	poly(A) binding protein, cytoplasmic 5	1.068947	0.51	0.721
209086	Samd9l	sterile alpha motif domain containing 9-like	1.068947	0.4702	0.692
230972	Arhgef16	Rho guanine nucleotide exchange factor (GEF) 16	1.068947	0.5319	0.7378
11641	Akap2	A kinase (PRKA) anchor protein 2	1.068833	0.61	0.792
20185	Ncor1	nuclear receptor co-repressor 1	1.068833	0.4621	0.6849
23966	Odz4	odd Oz/ten-m homolog 4 (Drosophila)	1.068833	0.225	NA
71710	Lrrcc1	leucine rich repeat and coiled-coil domain containing 1	1.068833	0.4279	NA
72500	Ier5l	immediate early response 5-like	1.068833	0.1801	NA
20475	Six5	sine oculis-related homeobox 5 homolog (Drosophila)	1.068719	0.5429	0.7464
23872	Ets2	E26 avian leukemia oncogene 2, 3' domain	1.068719	0.2582	NA
69259	Kctd5	potassium channel tetramerisation domain containing 5	1.068719	0.5289	0.7356
66500	Slc30a7	solute carrier family 30 (zinc transporter), member 7	1.068604	0.4623	0.6849
72018	Fundc1	FUN14 domain containing 1	1.068604	0.2071	NA
208595	Gm9897	predicted gene 9897	1.068604	0.5204	0.7287
67045	Riok2	RIO kinase 2 (yeast)	1.06849	0.2121	NA
71389	Chd6	chromodomain helicase DNA binding protein 6	1.06849	0.7228	0.8615
74241	Chpf	chondroitin polymerizing factor	1.06849	0.3344	NA
78381	2310047N11Rik	RIKEN cDNA 2310047N11 gene	1.06849	0.7101	0.8543
56397	Morf4l2	mortality factor 4 like 2	1.068376	0.7903	0.9007
225876	Kdm2a	lysine (K)-specific demethylase 2A	1.068376	0.6249	0.8017
229906	Gtf2b	general transcription factor IIB	1.068376	0.3062	NA
269774	Aak1	AP2 associated kinase 1	1.068376	0.7622	0.885
12558	Cdh2	cadherin 2	1.068262	0.6	0.7851
226499	BC003331	cDNA sequence BC003331	1.068262	0.3007	NA
14979	H2-Ke6	H2-K region expressed gene 6	1.068148	0.3387	NA
26462	Txnrd2	thioredoxin reductase 2	1.068148	0.3001	NA
242418	Dcaf10	DDB1 and CUL4 associated factor 10	1.068148	0.5007	0.7145
20603	Sms	spermine synthase	1.068034	0.2481	NA
192285	Phf21a	PHD finger protein 21A	1.068034	0.5758	0.769
319454	A430071A18Rik	RIKEN cDNA A430071A18 gene	1.068034	0.4764	0.6965
503610	Zdhhc18	zinc finger, DHHC domain containing 18	1.068034	0.3802	NA
11421	Ace	angiotensin I converting enzyme (peptidyl-dipeptidase A) 1	1.06792	0.7605	0.884
19731	Rgl1	ral guanine nucleotide dissociation stimulator,-like 1	1.06792	0.3024	NA
20511	Slc1a2	solute carrier family 1 (glial high affinity glutamate transporter), member 2	1.06792	0.563	0.7609
66988	Lap3	leucine aminopeptidase 3	1.06792	0.245	NA
67463	Poc5	POC5 centriolar protein homolog (Chlamydomonas)	1.06792	0.1356	NA
106064	AW549877	expressed sequence AW549877	1.06792	0.6397	0.8112
108116	Slco3a1	solute carrier organic anion transporter family, member 3a1	1.067806	0.2363	NA
245174	Zfp937	zinc finger protein 937	1.067806	0.3843	NA

269424	Phf17	PHD finger protein 17	1.067806	0.4572	0.6806
380780	Serpina11	serine (or cysteine) peptidase inhibitor, clade A (alpha-1 antiproteinase, antitrypsin), member 11	1.067806	0.6658	0.8286
20937	Suv39h1	suppressor of variegation 3-9 homolog 1 (Drosophila)	1.067692	0.5358	0.7409
208718	Dis3l2	DIS3 mitotic control homolog (S. cerevisiae)-like 2	1.067692	0.2488	NA
240261	Ccdc112	coiled-coil domain containing 112	1.067692	0.7388	0.8722
14581	Gfi1	growth factor independent 1	1.067578	0.6177	0.7971
19035	Ppib	peptidylprolyl isomerase B	1.067578	0.247	NA
19899	Rpl18	ribosomal protein L18	1.067578	0.2024	NA
56378	Arpc3	actin related protein 2/3 complex, subunit 3	1.067578	0.3081	NA
66799	Ube2w	ubiquitin-conjugating enzyme E2W (putative)	1.067578	0.4882	0.7056
102243	AU024581	expressed sequence AU024581	1.067578	0.4608	0.6838
433375	Creg1	cellular repressor of E1A-stimulated genes 1	1.067578	0.4022	NA
15381	Hnrnpc	heterogeneous nuclear ribonucleoprotein C	1.067464	0.2864	NA
18753	Prkcd	protein kinase C, delta	1.067464	0.1641	NA
66488	Fam136a	family with sequence similarity 136, member A	1.067464	0.2522	NA
69099	1810009N02Rik	RIKEN cDNA 1810009N02 gene	1.067464	0.4426	0.6686
70747	Tspan2	tetraspanin 2	1.06735	0.5772	0.77
52398	Sep-11	septin 11	1.067236	0.6275	0.8036
64136	Sdf2l1	stromal cell-derived factor 2-like 1	1.067236	0.5273	0.7342
66356	2310008H09Rik	RIKEN cDNA 2310008H09 gene	1.067236	0.244	NA
74325	Cltb	clathrin, light polypeptide (Lcb)	1.067236	0.4482	0.6738
210529	Mettl14	methyltransferase like 14	1.067236	0.4141	NA
218203	Mylip	myosin regulatory light chain interacting protein	1.067236	0.6233	0.8007
100042982	Gm4146	predicted gene 4146	1.067236	0.5667	0.7625
15569	Elavl2	ELAV (embryonic lethal, abnormal vision, Drosophila)-like 2 (Hu antigen B)	1.067122	0.4911	0.7078
230751	Oscp1	organic solute carrier partner 1	1.067122	0.3387	NA
76974	1190003J15Rik	RIKEN cDNA 1190003J15 gene	1.067008	0.7263	0.8641
81701	Egfl8	EGF-like domain 8	1.067008	0.2218	NA
225743	Rnf165	ring finger protein 165	1.067008	0.508	0.7194
22240	Dpysl3	dihydropyrimidinase-like 3	1.066894	0.5682	0.7634
56150	Mad2l1	MAD2 mitotic arrest deficient-like 1 (yeast)	1.066894	0.7988	0.9054
233919	Gpr26	G protein-coupled receptor 26	1.066894	0.678	0.8351
69710	Arap1	ArfGAP with RhoGAP domain, ankyrin repeat and PH domain 1	1.06678	0.4653	0.6872
71148	Mier1	mesoderm induction early response 1 homolog (Xenopus laevis	1.06678	0.6541	0.8209
71446	Wrb	tryptophan rich basic protein	1.06678	0.6188	0.7979
104570	Smek2	SMEK homolog 2, suppressor of mek1 (Dictyostelium)	1.06678	0.5956	0.7822
108154	Adamts6	a disintegrin-like and metallopeptidase (reprolysin type) with thrombospondin type 1 motif, 6	1.06678	0.7206	0.8602
632764	5730471H19Rik	RIKEN cDNA 5730471H19 gene	1.06678	0.5032	0.716
67204	Eif2s2	eukaryotic translation initiation factor 2, subunit 2 (beta)	1.066667	0.3369	NA
108013	Celf4	CUGBP, Elav-like family member 4	1.066667	0.6264	0.8028
194908	Pld6	phospholipase D family, member 6	1.066667	0.5672	0.7627
233186	Siglec5	sialic acid binding Ig-like lectin 5	1.066667	0.6364	0.8089
26568	Slc27a3	solute carrier family 27 (fatty acid transporter), member 3	1.066553	0.3155	NA
72895	Setd5	SET domain containing 5	1.066553	0.3148	NA
75210	Prr3	proline-rich polypeptide 3	1.066553	0.4389	NA
110954	Rpl10	ribosomal protein 10	1.066553	0.322	NA
208211	Alg1	asparagine-linked glycosylation 1 homolog (yeast, beta-1,4-mannosyltransferase)	1.066553	0.3266	NA
19303	Pxn	paxillin	1.066439	0.5046	0.7171
66170	Chchd5	coiled-coil-helix-coiled-coil-helix domain containing 5	1.066439	0.2501	NA

66260	Tmem54	transmembrane protein 54	1.066439	0.796	0.904
77870	E130116L18Rik	RIKEN cDNA E130116L18 gene	1.066439	0.6889	0.8413
94185	Tnfrsf21	tumor necrosis factor receptor superfamily, member 21	1.066439	0.566	0.762
54135	Lsr	lipolysis stimulated lipoprotein receptor	1.066325	0.4362	NA
77095	D330022H12Rik	RIKEN cDNA D330022H12 gene	1.066325	0.532	0.7378
79555	BC005537	cDNA sequence BC005537	1.066325	0.5851	0.7753
226026	Smc5	structural maintenance of chromosomes 5	1.066325	0.4272	NA
269472	LOC269472	hypothetical LOC269472	1.066325	0.644	0.814
12238	Commd3	COMM domain containing 3	1.066212	0.16	NA
54650	Sfmbt1	Scm-like with four mbt domains 1	1.066212	0.4827	0.7013
66139	Tmem8c	transmembrane protein 8C	1.066212	0.6774	0.8348
67863	Slc25a11	solute carrier family 25 (mitochondrial carrier oxoglutarate carrier), member 11	1.066212	0.2387	NA
320560	Dennd5b	DENN/MADD domain containing 5B	1.066212	0.6666	0.8289
14455	Gas5	growth arrest specific 5	1.066098	0.8569	0.9367
18221	Nudc	nuclear distribution gene C homolog (Aspergillus)	1.066098	0.3605	NA
19697	Rela	v-rel reticuloendotheliosis viral oncogene homolog A (avian)	1.066098	0.3554	NA
20132	Rrh	retinal pigment epithelium derived rhodopsin homolog	1.066098	0.2788	NA
66308	2810021B07Rik	RIKEN cDNA 2810021B07 gene	1.066098	0.2961	NA
69511	Klk12	kallikrein related-peptidase 12	1.066098	0.7655	0.8868
72113	Adck1	aarF domain containing kinase 1	1.066098	0.1832	NA
110948	Hlcs	holocarboxylase synthetase (biotin- [propriony-Coenzyme A-carboxylase (ATP-hydrolysing)] ligase)	1.066098	0.3835	NA
100216474	Ttll2	tubulin tyrosine ligase-like family, member 2	1.066098	0.7154	0.8575
78251	Zfp712	zinc finger protein 712	1.065984	0.3249	NA
243764	Chrm2	cholinergic receptor, muscarinic 2, cardiac	1.065984	0.4945	0.7102
73750	Whrn	whirlin	1.065871	0.6183	0.7976
16886	Limk2	LIM motif-containing protein kinase 2	1.065757	0.6589	0.8238
53604	Zpbp	zona pellucida binding protein	1.065757	0.5028	0.7158
110198	Akr7a5	aldo-keto reductase family 7, member A5 (aflatoxin aldehyde reductase)	1.065757	0.4757	0.6962
68147	Gar1	GAR1 ribonucleoprotein homolog (yeast)	1.065644	0.1875	NA
68188	Sympk	symplekin	1.06553	0.5028	0.7158
72124	Seh1l	SEH1-like (S. cerevisiae	1.06553	0.388	NA
382864	Colq	collagen-like tail subunit (single strand of homotrimer) of asymmetric acetylcholinesterase	1.06553	0.4994	0.714
546648	Klhdc7b	kelch domain containing 7B	1.06553	0.6951	0.8451
66491	Polr2l	polymerase (RNA) II (DNA directed) polypeptide L	1.065417	0.4255	NA
223527	Eny2	enhancer of yellow 2 homolog (Drosophila)	1.065417	0.2091	NA
14137	Fdft1	farnesyl diphosphate farnesyl transferase 1	1.065303	0.4531	NA
66294	Fam3a	family with sequence similarity 3, member A	1.065303	0.3815	NA
67753	4930579C15Rik	RIKEN cDNA 4930579C15 gene	1.065303	0.806	0.9093
72392	Tmem175	transmembrane protein 175	1.065303	0.6975	0.8468
229003	BC006779	cDNA sequence BC006779	1.065303	0.5004	0.7145
545389	Cep170	centrosomal protein 170	1.065303	0.4596	0.6829
11416	Slc33a1	solute carrier family 33 (acetyl-CoA transporter), member 1	1.06519	0.3938	NA
50724	Sap30l	SAP30-like	1.06519	0.2187	NA
52377	Rcn3	reticulocalbin 3, EF-hand calcium binding domain	1.06519	0.5873	0.7767
53620	Vamp5	vesicle-associated membrane protein 5	1.06519	0.6605	0.8247
69674	Mif4gd	MIF4G domain containing	1.06519	0.3453	NA
402771	C130090I23Rik	RIKEN cDNA C130090I23 gene	1.06519	0.5025	0.7155
14933	Gyk	glycerol kinase	1.065076	0.4388	NA
26436	Psg16	pregnancy specific glycoprotein 16	1.065076	0.3738	NA

27681	Snf8	SNF8, ESCRT-II complex subunit, homolog (S. cerevisiae)	1.065076	0.4092	NA
70829	Ccdc93	coiled-coil domain containing 93	1.065076	0.6453	0.8148
73469	Rnf38	ring finger protein 38	1.065076	0.218	NA
80890	Trim2	tripartite motif-containing 2	1.065076	0.5903	0.7791
230917	Tmem201	transmembrane protein 201	1.065076	0.6108	0.7927
71301	4930593A02Rik	RIKEN cDNA 4930593A02 gene	1.064963	0.7443	0.8758
72112	Ppp1r14d	protein phosphatase 1, regulatory (inhibitor) subunit 14D	1.064963	0.7028	0.8502
234683	Elmo3	engulfment and cell motility 3, ced-12 homolog (C. elegans)	1.064963	0.5885	0.7778
320333	D830030K20Rik	RIKEN cDNA D830030K20 gene	1.064963	0.7869	0.8984
100502708	LOC100502708	hypothetical LOC100502708	1.064963	0.6371	0.8093
73075	Ppil6	peptidylprolyl isomerase (cyclophilin)-like 6	1.064849	0.4422	NA
320674	4921534A09Rik	RIKEN cDNA 4921534A09 gene	1.064849	0.7184	0.8593
18015	Nf1	neurofibromatosis 1	1.064736	0.6245	0.8016
50927	Nasp	nuclear autoantigenic sperm protein (histone-binding)	1.064736	0.3402	NA
67136	Kbtbd4	kelch repeat and BTB (POZ) domain containing 4	1.064736	0.3094	NA
97149	C78893	expressed sequence C78893	1.064736	0.7185	0.8594
110197	Dgkg	diacylglycerol kinase, gamma	1.064736	0.519	0.7281
18399	Slc22a6	solute carrier family 22 (organic anion transporter), member 6	1.064623	0.7452	0.8762
67533	Ppfibp1	PTPRF interacting protein, binding protein 1 (liprin beta 1)	1.064623	0.253	NA
69034	4930579G22Rik	RIKEN cDNA 4930579G22 gene	1.064623	0.5779	0.7704
69146	Gsdmd	gasdermin D	1.064623	0.5612	0.7597
239985	Arid1b	AT rich interactive domain 1B (SWI-like)	1.064623	0.3927	NA
69076	Triap1	TP53 regulated inhibitor of apoptosis 1	1.064509	0.2945	NA
225131	Wac	WW domain containing adaptor with coiled-coil	1.064509	0.2468	NA
12034	Phb2	prohibitin 2	1.064396	0.237	NA
71817	Tmem50a	transmembrane protein 50A	1.064396	0.3446	NA
71883	Coq2	coenzyme Q2 homolog, prenyltransferase (yeast)	1.064396	0.208	NA
224090	Tmem44	transmembrane protein 44	1.064396	0.7578	0.8829
233545	2210018M11Rik	RIKEN cDNA 2210018M11 gene	1.064396	0.3489	NA
12070	Ngfrap1	nerve growth factor receptor (TNFRSF16) associated protein 1	1.064283	0.2178	NA
13205	Ddx3x	DEAD/H (Asp-Glu-Ala-Asp/His) box polypeptide 3, X-linked	1.064283	0.3015	NA
13844	Ephb2	Eph receptor B2	1.064283	0.6683	0.8296
52009	Hn1l	hematological and neurological expressed 1-like	1.064283	0.4578	NA
56760	Clec1b	C-type lectin domain family 1, member b	1.064283	0.7604	0.8839
320502	Lmod3	leiomodin 3 (fetal)	1.064283	0.5632	0.7609
330474	Zc3h4	zinc finger CCCH-type containing 4	1.064283	0.47	0.692
100046302	LOC100046302	protein disulfide-isomerase A6-like	1.064283	0.5204	0.7287
15481	Hspa8	heat shock protein 8	1.064169	0.4905	0.7075
19244	Ptp4a2	protein tyrosine phosphatase 4a2	1.064169	0.5242	0.7317
58186	Rad18	RAD18 homolog (S. cerevisiae)	1.064169	0.4097	NA
64934	Pes1	pescadillo homolog 1, containing BRCT domain (zebrafish)	1.064169	0.1986	NA
66861	Dnajc10	DnaJ (Hsp40) homolog, subfamily C, member 10	1.064169	0.2524	NA
77371	Sec24a	Sec24 related gene family, member A (S. cerevisiae)	1.064169	0.668	0.8296
213402	Armc2	armadillo repeat containing 2	1.064169	0.3311	NA
270097	Vat1l	vesicle amine transport protein 1 homolog-like (T. californica)	1.064169	0.3527	NA
12704	Cit	citron	1.064056	0.6462	0.8155
77361	9430085M18Rik	RIKEN cDNA 9430085M18 gene	1.064056	0.6458	0.8151
399572	E430010N07Rik	RIKEN cDNA E430010N07 gene	1.064056	0.6106	0.7925
13712	Elk1	ELK1, member of ETS oncogene family	1.063943	0.5658	0.762

14168	Fgf13	fibroblast growth factor 13	1.063943	0.6842	0.8388
74383	Ubap2l	ubiquitin associated protein 2-like	1.063943	0.3594	NA
78211	4930558N11Rik	RIKEN cDNA 4930558N11 gene	1.063943	0.7309	0.8669
108654	4933403F05Rik	RIKEN cDNA 4933403F05 gene	1.063943	0.3511	NA
242687	Wasf2	WAS protein family, member 2	1.063943	0.3506	NA
665434	Gm7634	predicted gene 7634	1.063943	0.5355	0.7408
21843	Tial1	Tia1 cytotoxic granule-associated RNA binding protein-like 1	1.06383	0.479	0.6985
66642	Ctnnbl1	catenin, beta like 1	1.06383	0.1461	NA
108017	Fxyd4	FXYD domain-containing ion transport regulator 4	1.063717	0.7302	0.8664
108900	Fam72a	family with sequence similarity 72, member A	1.063717	0.3997	NA
574437	Xlr3b	X-linked lymphocyte-regulated 3B	1.063717	0.8543	0.9352
21928	Tnfaip2	tumor necrosis factor, alpha-induced protein 2	1.063603	0.7775	0.8928
68554	1110001A16Rik	RIKEN cDNA 1110001A16 gene	1.063603	0.2699	NA
227120	Plcl1	phospholipase C-like 1	1.063603	0.5649	0.7617
243371	Lrrc61	leucine rich repeat containing 61	1.063603	0.3194	NA
328855	E330032C10Rik	RIKEN cDNA E330032C10 gene	1.063603	0.4225	NA
19364	Rad51l3	RAD51-like 3 (S. cerevisiae)	1.06349	0.47	0.692
66506	Psmg3	proteasome (prosome, macropain) assembly chaperone 3	1.06349	0.4258	NA
330059	A430017K17	hypothetical protein A430017K17	1.06349	0.4382	NA
56354	Dnajc7	DnaJ (Hsp40) homolog, subfamily C, member 7	1.063377	0.38	NA
67168	Lpar6	lysophosphatidic acid receptor 6	1.063377	0.5754	0.7689
67333	Stk35	serine/threonine kinase 35	1.063377	0.5169	0.7265
208449	Sgms1	sphingomyelin synthase 1	1.063377	0.4294	NA
59048	C1galt1c1	C1GALT1-specific chaperone 1	1.063264	0.3099	NA
66202	1110059G10Rik	RIKEN cDNA 1110059G10 gene	1.063264	0.3904	NA
68176	6230427J02Rik	RIKEN cDNA 6230427J02 gene	1.063264	0.7762	0.8923
68859	1190007F08Rik	RIKEN cDNA 1190007F08 gene	1.063264	0.589	0.7782
70591	5730455P16Rik	RIKEN cDNA 5730455P16 gene	1.063264	0.4694	NA
80880	Kank3	KN motif and ankyrin repeat domains 3	1.063264	0.5863	0.7761
231571	Rpap2	RNA polymerase II associated protein 2	1.063264	0.3329	NA
100217450	Snora47	small nucleolar RNA, H/ACA box 47	1.063264	0.7614	0.8844
12015	Bad	BCL2-associated agonist of cell death	1.063151	0.3807	NA
16777	Lamb1	laminin B1	1.063151	0.3756	NA
17906	Myl2	myosin, light polypeptide 2, regulatory, cardiac, slow	1.063151	0.6374	0.8096
83492	Gsdmc	gasdermin C	1.063151	0.7224	0.8612
624713	Gm6525	ribosomal protein L36a pseudogene	1.063151	0.24	NA
19716	Bex1	brain expressed gene 1	1.063038	0.5881	0.7775
71966	Nkiras2	NFKB inhibitor interacting Ras-like protein 2	1.063038	0.336	NA
330189	Tmem120b	transmembrane protein 120B	1.063038	0.4549	NA
620592	Tmem28	transmembrane protein 28	1.063038	0.4299	NA
15974	Ifnab	interferon alpha B	1.062925	0.8251	0.9207
67516	Kctd4	potassium channel tetramerisation domain containing 4	1.062925	0.6	0.7851
229227	4932438A13Rik	RIKEN cDNA 4932438A13 gene	1.062925	0.7026	0.8502
243931	Tshz3	teashirt zinc finger family member 3	1.062925	0.4259	NA
16578	Kif9	kinesin family member 9	1.062812	0.5498	0.7507
54201	Zfp316	zinc finger protein 316	1.062812	0.5056	0.7179
109129	Mmadhc	methylmalonic aciduria (cobalamin deficiency) cblD type, with homocystinuria	1.062812	0.2393	NA
380701	Slc47a2	solute carrier family 47, member 2	1.062812	0.5987	0.7842
432508	Cpsf6	cleavage and polyadenylation specific factor 6	1.062812	0.332	NA

100040872	Gm3014	predicted gene 3014	1.062812	0.747	0.8772
67103	Ptgr1	prostaglandin reductase 1	1.062699	0.5285	0.7353
78792	4930432F04Rik	RIKEN cDNA 4930432F04 gene	1.062699	0.3482	NA
446101	Xrra1	X-ray radiation resistance associated 1	1.062699	0.6876	0.8407
22785	Slc30a4	solute carrier family 30 (zinc transporter), member 4	1.062586	0.2517	NA
77044	Arid2	AT rich interactive domain 2 (ARID, RFX-like)	1.062586	0.6468	0.8158
110385	Pde4c	phosphodiesterase 4C, cAMP specific	1.062586	0.7489	0.8784
241520	Fam171b	family with sequence similarity 171, member B	1.062586	0.3523	NA
18674	Slc25a3	solute carrier family 25 (mitochondrial carrier, phosphate carrier), member 3	1.062473	0.4943	0.7101
66700	Vps24	vacuolar protein sorting 24 (yeast)	1.062473	0.2963	NA
73747	1110034G24Rik	RIKEN cDNA 1110034G24 gene	1.062473	0.2482	NA
140570	Plxnb2	plexin B2	1.062473	0.5065	0.7183
227525	Dclre1c	DNA cross-link repair 1C, PSO2 homolog (S. cerevisiae)	1.062473	0.4338	NA
100036572	LOC100036572	hypothetical LOC100036572	1.062473	0.588	0.7775
100043580	Gm4532	predicted gene 4532	1.062473	0.5896	0.7786
22680	Zfp207	zinc finger protein 207	1.062361	0.5242	0.7317
77087	Ankrd11	ankyrin repeat domain 11	1.062361	0.1994	NA
233863	Gtf3c1	general transcription factor III C 1	1.062361	0.4204	NA
319285	A430061O12Rik	RIKEN cDNA A430061O12 gene	1.062361	0.5021	0.7152
16568	Kif3a	kinesin family member 3A	1.062248	0.3021	NA
53421	Sec61a1	Sec61 alpha 1 subunit (S. cerevisiae)	1.062248	0.2643	NA
67897	Rnmt	RNA (guanine-7-) methyltransferase	1.062248	0.6001	0.7851
68721	1110032A03Rik	RIKEN cDNA 1110032A03 gene	1.062248	0.8517	0.9337
75597	Ndufaf2	NADH dehydrogenase (ubiquinone) 1 alpha subcomplex, assembly factor 2	1.062248	0.4575	NA
79362	Bhlhe41	basic helix-loop-helix family, member e41	1.062248	0.7598	0.8836
215015	Fam20b	family with sequence similarity 20, member B	1.062248	0.4956	0.7111
16150	Ikbkb	inhibitor of kappaB kinase beta	1.062135	0.6002	0.7851
16706	Ksr1	kinase suppressor of ras 1	1.062135	0.6637	0.8272
52521	Zfp622	zinc finger protein 622	1.062135	0.2302	NA
52585	Dhrs1	dehydrogenase/reductase (SDR family) member 1	1.062135	0.4477	NA
56376	Pdlim5	PDZ and LIM domain 5	1.062135	0.6472	0.8162
68558	Ankra2	ankyrin repeat, family A (RFXANK-like), 2	1.062135	0.4609	NA
70887	Dmrtc1a	DMRT-like family C1a	1.062135	0.634	0.8075
73067	Tmem192	transmembrane protein 192	1.062022	0.2371	NA
73830	Eif3k	eukaryotic translation initiation factor 3, subunit K	1.062022	0.3296	NA
74302	Mtmr3	myotubularin related protein 3	1.062022	0.3282	NA
231672	Fbxw8	F-box and WD-40 domain protein 8	1.062022	0.4939	0.7098
22351	Vill	villin-like	1.061909	0.7561	0.8822
76927	1700021C14Rik	RIKEN cDNA 1700021C14 gene	1.061909	0.4012	NA
667510	Gm8675	predicted gene 8675	1.061909	0.591	0.7796
73852	D3Ertd751e	DNA segment, Chr 3, ERATO Doi 751, expressed	1.061797	0.5979	0.7835
110094	Phka2	phosphorylase kinase alpha 2	1.061797	0.7369	0.8711
230789	Fam76a	family with sequence similarity 76, member A	1.061797	0.6076	0.7902
22367	Vrk1	vaccinia related kinase 1	1.061684	0.6725	0.8323
72306	Zfp777	zinc finger protein 777	1.061684	0.3898	NA
18578	Pde4b	phosphodiesterase 4B, cAMP specific	1.061571	0.61	0.792
22217	Usp12	ubiquitin specific peptidase 12	1.061571	0.3466	NA
54709	Eif3i	eukaryotic translation initiation factor 3, subunit I	1.061571	0.4932	0.7095
241296	Lrrc8a	leucine rich repeat containing 8A	1.061571	0.6507	0.8185

670211	Gm12508	predicted gene 12508	1.061571	0.3717	NA
12400	Cbfb	core binding factor beta	1.061458	0.3885	NA
21953	Tnni2	troponin I, skeletal, fast 2	1.061458	0.7935	0.9021
50754	Fbxw7	F-box and WD-40 domain protein 7	1.061458	0.5465	0.7484
66358	2310004I24Rik	RIKEN cDNA 2310004I24 gene	1.061458	0.5234	0.7311
192187	Stab1	stabilin 1	1.061458	0.627	0.8031
12323	Camk2b	calcium/calmodulin-dependent protein kinase II, beta	1.061346	0.605	0.7884
20515	Slc20a1	solute carrier family 20, member 1	1.061346	0.759	0.8831
69309	Slc16a13	solute carrier family 16 (monocarboxylic acid transporters), member 13	1.061346	0.576	0.769
56788	Scube2	signal peptide, CUB domain, EGF-like 2	1.061233	0.4965	0.7116
59125	Nek7	NIMA (never in mitosis gene a)-related expressed kinase 7	1.061233	0.3785	NA
67211	Armc10	armadillo repeat containing 10	1.061233	0.2955	NA
71924	Tube1	epsilon-tubulin 1	1.061233	0.2069	NA
233490	Crebzf	CREB/ATF bZIP transcription factor	1.061233	0.4083	NA
320795	Pkn1	protein kinase N1	1.061233	0.388	NA
623169	Gm6402	ribosomal protein S17 pseudogene	1.061233	0.641	0.8124
14661	Glud1	glutamate dehydrogenase 1	1.061121	0.3511	NA
66293	1810032O08Rik	RIKEN cDNA 1810032O08 gene	1.061121	0.2721	NA
66923	Pbrm1	polybromo 1	1.061121	0.4392	NA
103583	Fbxw11	F-box and WD-40 domain protein 11	1.061121	0.268	NA
675812	Zfp605	zinc finger protein 605	1.061121	0.3667	NA
69597	Afg3l2	AFG3(ATPase family gene 3)-like 2 (yeast)	1.061008	0.3166	NA
70557	5730416O20Rik	RIKEN cDNA 5730416O20 gene	1.061008	0.5922	0.7801
224763	Gm4831	predicted gene 4831	1.061008	0.6999	0.8485
234723	Txnl4b	thioredoxin-like 4B	1.061008	0.4691	NA
270906	Prr11	proline rich 11	1.061008	0.2025	NA
546024	Crxos1	Crx opposite strand transcript 1	1.061008	0.4879	0.7056
238690	Zfp458	zinc finger protein 458	1.060895	0.4232	NA
16563	Kif2a	kinesin family member 2A	1.060783	0.371	NA
67148	Fam103a1	family with sequence similarity 103, member A1	1.060783	0.4168	NA
93672	Il24	interleukin 24	1.060783	0.3986	NA
272027	Tstd2	thiosulfate sulfurtransferase (rhodanese)-like domain containing 2	1.060783	0.4785	NA
19263	Ptprb	protein tyrosine phosphatase, receptor type, B	1.06067	0.561	0.7597
73103	3110009E18Rik	RIKEN cDNA 3110009E18 gene	1.06067	0.5085	0.7198
56086	Set	SET nuclear oncogene	1.060558	0.5806	0.7725
69020	Zfp707	zinc finger protein 707	1.060558	0.2779	NA
69257	Elf2	E74-like factor 2	1.060558	0.3676	NA
69480	Ttc9	tetratricopeptide repeat domain 9	1.060558	0.2659	NA
665268	1600029O15Rik	ribosomal protein L17 pseudogene	1.060558	0.3266	NA
21847	Klf10	Kruppel-like factor 10	1.060445	0.3484	NA
56496	Tspan6	tetraspanin 6	1.060445	0.3066	NA
75079	Zbtb49	zinc finger and BTB domain containing 49	1.060445	0.593	0.7806
272350	Gm5065	predicted gene 5065	1.060445	0.7516	0.8799
21423	Tcf3	transcription factor 3	1.060333	0.3317	NA
22210	Ube2b	ubiquitin-conjugating enzyme E2B, RAD6 homology (S. cerevisiae)	1.060333	0.4023	NA
64652	Nisch	nischarin	1.060333	0.3702	NA
67420	Far1	fatty acyl CoA reductase 1	1.060333	0.7049	0.8517
98314	D2hgdh	D-2-hydroxyglutarate dehydrogenase	1.060333	0.652	0.8194
268822	Adck5	aarF domain containing kinase 5	1.060333	0.3711	NA

22030	Traf2	TNF receptor-associated factor 2	1.060221	0.3936	NA
64291	Osbpl1a	oxysterol binding protein-like 1A	1.060221	0.6061	0.7891
71609	Tradd	TNFRSF1A-associated via death domain	1.060221	0.467	NA
75291	Zbtb3	zinc finger and BTB domain containing 3	1.060221	0.4176	NA
12798	Cnn2	calponin 2	1.060108	0.6265	0.8029
16009	Igfbp3	insulin-like growth factor binding protein 3	1.060108	0.7101	0.8543
20218	Khdrbs1	KH domain containing, RNA binding, signal transduction associated 1	1.060108	0.4461	NA
66313	Smurf2	SMAD specific E3 ubiquitin protein ligase 2	1.060108	0.5047	0.7171
70430	Tbce	tubulin-specific chaperone E	1.060108	0.543	0.7464
232339	Ankrd26	ankyrin repeat domain 26	1.060108	0.5009	0.7146
16818	Lck	lymphocyte protein tyrosine kinase	1.059996	0.4844	NA
16638	Klra7	killer cell lectin-like receptor, subfamily A, member 7	1.059883	0.897	0.9557
72091	Snhg7	small nucleolar RNA host gene (non-protein coding) 7	1.059883	0.4591	NA
19131	Prh1	proline rich protein HaeIII subfamily 1	1.059771	0.7137	0.8564
56368	Cyb561d2	cytochrome b-561 domain containing 2	1.059771	0.2672	NA
69306	Efcab9	EF-hand calcium binding domain 9	1.059771	0.7897	0.9005
216156	Wdr18	WD repeat domain 18	1.059771	0.587	0.7765
227937	Pkp4	plakophilin 4	1.059771	0.3661	NA
268880	AI480653	expressed sequence AI480653	1.059771	0.5064	0.7183
24109	Ubl3	ubiquitin-like 3	1.059659	0.4303	NA
77782	Polq	polymerase (DNA directed), theta	1.059659	0.3315	NA
78611	Btbd19	BTB (POZ) domain containing 19	1.059659	0.5185	0.7278
192196	Luc7l2	LUC7-like 2 (S. cerevisiae)	1.059547	0.666	0.8286
330361	AW146020	expressed sequence AW146020	1.059547	0.3666	NA
330788	Zfp866	zinc finger protein 866	1.059547	0.6618	0.8258
13449	Dok2	docking protein 2	1.059434	0.5386	0.7429
19340	Rab3d	RAB3D, member RAS oncogene family	1.059434	0.5627	0.7607
69757	Leng1	leukocyte receptor cluster (LRC) member 1	1.059434	0.4089	NA
211499	Tmem87a	transmembrane protein 87A	1.059434	0.7466	0.877
269774	Aak1	AP2 associated kinase 1	1.059434	0.7845	0.8967
269870	Zfp446	zinc finger protein 446	1.059434	0.5113	0.722
100042453	Gm3852	predicted gene 3852	1.059434	0.6355	0.8084
13849	Ephx1	epoxide hydrolase 1, microsomal	1.059322	0.6001	0.7851
20563	Slit2	slit homolog 2 (Drosophila)	1.059322	0.414	NA
56332	Amotl2	angiomotin-like 2	1.059322	0.2598	NA
58217	Trem1	triggering receptor expressed on myeloid cells 1	1.059322	0.5027	0.7157
59069	Tpm3	tropomyosin 3, gamma	1.059322	0.244	NA
68106	Nt5c3l	5'-nucleotidase, cytosolic III-like	1.059322	0.3242	NA
224022	Slc7a4	solute carrier family 7 (cationic amino acid transporter, y+ system), member 4	1.059322	0.4798	NA
235661	Dync1li1	dynein cytoplasmic 1 light intermediate chain 1	1.059322	0.5582	0.757
623174	Gm6404	predicted gene 6404	1.059322	0.5416	0.7457
13660	Ehd1	EH-domain containing 1	1.05921	0.2026	NA
14269	Fnbp1	formin binding protein 1	1.05921	0.5552	0.7547
21415	Tcf7l1	transcription factor 7-like 1 (T-cell specific, HMG box)	1.05921	0.598	0.7835
22194	Ube2e1	ubiquitin-conjugating enzyme E2E 1, UBC4/5 homolog (yeast)	1.05921	0.4135	NA
56790	Fam48a	family with sequence similarity 48, member A	1.05921	0.8029	0.9075
108699	Chn1	chimerin (chimaerin) 1	1.05921	0.4042	NA
227746	Rabepk	Rab9 effector protein with kelch motifs	1.05921	0.5157	0.7257
234309	Cbr4	carbonyl reductase 4	1.05921	0.4162	NA

242406	Rgp1	RGP1 retrograde golgi transport homolog (S. cerevisiae)	1.05921	0.789	0.8999
319915	A830049F12Rik	RIKEN cDNA A830049F12 gene	1.05921	0.6002	0.7851
15361	Hmga1	high mobility group AT-hook 1	1.059098	0.6151	0.7956
18023	Nfe2l1	nuclear factor, erythroid derived 2,-like 1	1.059098	0.6858	0.8398
18508	Pax6	paired box gene 6	1.059098	0.6564	0.8221
67378	Bbs2	Bardet-Biedl syndrome 2 (human)	1.059098	0.4	NA
71856	Wfdc3	WAP four-disulfide core domain 3	1.059098	0.8022	0.9069
74852	4930402D18Rik	RIKEN cDNA 4930402D18 gene	1.059098	0.6375	0.8097
208092	Chmp6	chromatin modifying protein 6	1.059098	0.5973	0.7833
100039024	LOC100039024	arf-GAP with SH3 domain, ANK repeat and PH domain-containing protein 1-like	1.059098	0.5475	0.7489
19045	Ppp1ca	protein phosphatase 1, catalytic subunit, alpha isoform	1.058985	0.3609	NA
20586	Smarca4	SWI/SNF related, matrix associated, actin dependent regulator of chromatin, subfamily a, member 4	1.058985	0.4436	NA
110826	Etfb	electron transferring flavoprotein, beta polypeptide	1.058985	0.4147	NA
65247	Asb1	ankyrin repeat and SOCS box-containing 1	1.058873	0.6313	0.8059
231123	Haus3	HAUS augmin-like complex, subunit 3	1.058873	0.3123	NA
234076	Tmco3	transmembrane and coiled-coil domains 3	1.058873	0.3833	NA
26384	Gnpda1	glucosamine-6-phosphate deaminase 1	1.058761	0.6081	0.7905
56031	Ppie	peptidylprolyl isomerase E (cyclophilin E)	1.058761	0.5837	0.7743
75029	Purg	purine-rich element binding protein G	1.058761	0.5153	0.7255
75763	Dcaf17	DDB1 and CUL4 associated factor 17	1.058761	0.7314	0.8674
224129	Adcy5	adenylate cyclase 5	1.058761	0.4993	NA
320360	Ric3	resistance to inhibitors of cholinesterase 3 homolog (C. elegans)	1.058761	0.7833	0.8961
17132	Maf	avian musculoaponeurotic fibrosarcoma (v-maf) AS42 oncogene homolog	1.058649	0.725	0.8633
69737	Ttl	tubulin tyrosine ligase	1.058649	0.316	NA
93697	Narg2	NMDA receptor-regulated gene 2	1.058649	0.4057	NA
17281	Fyco1	FYVE and coiled-coil domain containing 1	1.058537	0.5005	NA
22184	Zrsr2	zinc finger (CCCH type), RNA binding motif and serine/arginine rich 2	1.058537	0.4558	NA
66169	Tomm7	translocase of outer mitochondrial membrane 7 homolog (yeast)	1.058537	0.3996	NA
70325	Pigw	phosphatidylinositol glycan anchor biosynthesis, class W	1.058537	0.7565	0.8822
70350	Basp1	brain abundant, membrane attached signal protein 1	1.058537	0.5677	0.7632
241694	Ralgapa2	Ral GTPase activating protein, alpha subunit 2 (catalytic)	1.058537	0.6112	0.793
11773	Ap2m1	adaptor protein complex AP-2, mu1	1.058425	0.5441	0.7471
23980	Pebp1	phosphatidylethanolamine binding protein 1	1.058425	0.3409	NA
27981	D4Wsu53e	DNA segment, Chr 4, Wayne State University 53, expressed	1.058425	0.5911	0.7796
207393	Elfn2	leucine rich repeat and fibronectin type III, extracellular 2	1.058425	0.5696	0.7638
209692	Dhtkd1	dehydrogenase E1 and transketolase domain containing 1	1.058425	0.7067	0.8528
226610	Fam78b	family with sequence similarity 78, member B	1.058425	0.4877	NA
53817	Bat1a	HLA-B-associated transcript 1A	1.058313	0.3141	NA
56327	Arl2	ADP-ribosylation factor-like 2	1.058313	0.4865	NA
56522	Papolb	poly (A) polymerase beta (testis specific)	1.058313	0.7936	0.9021
71683	Gypc	glycophorin C	1.058313	0.7709	0.8898
208618	Etl4	enhancer trap locus 4	1.058313	0.6203	0.7991
209462	Hace1	HECT domain and ankyrin repeat containing, E3 ubiquitin protein ligase 1	1.058313	0.5259	0.7331
216820	Dhrs7b	dehydrogenase/reductase (SDR family) member 7B	1.058313	0.3343	NA
414326	D930050J11	hypothetical D930050J11	1.058313	0.6237	0.801
22704	Zfp46	zinc finger protein 46	1.058201	0.2686	NA
68098	Rchy1	ring finger and CHY zinc finger domain containing 1	1.058201	0.399	NA
30954	Siva1	SIVA1, apoptosis-inducing factor	1.058089	0.4578	NA
68708	Rabl2	RAB, member of RAS oncogene family-like 2	1.058089	0.4053	NA

69596	2310035K24Rik	RIKEN cDNA 2310035K24 gene	1.058089	0.4148	NA
667338	Gm8580	ribosomal protein L29 pseudogene	1.058089	0.4586	NA
12808	Cobl	cordon-bleu	1.057977	0.5337	0.7392
19337	Rab33a	RAB33A, member of RAS oncogene family	1.057977	0.4074	NA
24047	Ccl19	chemokine (C-C motif) ligand 19	1.057977	0.7724	0.8906
98221	Eif3m	eukaryotic translation initiation factor 3, subunit M	1.057977	0.3522	NA
277978	Exoc3l	exocyst complex component 3-like	1.057977	0.6862	0.8399
19047	Ppp1cc	protein phosphatase 1, catalytic subunit, gamma isoform	1.057865	0.5868	0.7763
56275	Rbm14	RNA binding motif protein 14	1.057865	0.2879	NA
12402	Cbl	Casitas B-lineage lymphoma	1.057753	0.5542	0.7539
22003	Tpm1	tropomyosin 1, alpha	1.057753	0.5537	0.7536
76867	Rhbdd1	rhomboid domain containing 1	1.057753	0.683	0.838
224585	Zfp160	zinc finger protein 160	1.057753	0.7514	0.8798
245688	Rbbp7	retinoblastoma binding protein 7	1.057753	0.4922	NA
68465	Adipor2	adiponectin receptor 2	1.057641	0.3569	NA
100037258	Dnajc3	DnaJ (Hsp40) homolog, subfamily C, member 3	1.057641	0.4927	NA
66548	Adamtsl5	ADAMTS-like 5	1.05753	0.6629	0.8266
76602	1700040D17Rik	RIKEN cDNA 1700040D17 gene	1.05753	0.271	NA
434460	Gm5623	predicted gene 5623	1.05753	0.4358	NA
20111	Rps6ka1	ribosomal protein S6 kinase polypeptide 1	1.057418	0.6015	0.7862
64143	Ralb	v-ral simian leukemia viral oncogene homolog B (ras related)	1.057418	0.5933	0.7808
68023	Pdf	peptide deformylase (mitochondrial)	1.057418	0.3377	NA
76457	Ccdc134	coiled-coil domain containing 134	1.057418	0.417	NA
239570	Ttc38	tetratricopeptide repeat domain 38	1.057418	0.7426	0.8746
380732	Gm885	predicted gene 885	1.057418	0.4422	NA
59050	Nsa2	NSA2 ribosome biogenesis homolog (S. cerevisiae)	1.057306	0.3268	NA
65945	Clstn1	calsyntenin 1	1.057306	0.7238	0.8624
54409	Ramp2	receptor (calcitonin) activity modifying protein 2	1.057194	0.3896	NA
68527	Ucma	upper zone of growth plate and cartilage matrix associated	1.057194	0.4882	NA
210766	Brcc3	BRCA1/BRCA2-containing complex, subunit 3	1.057194	0.5288	0.7354
19719	Rfng	RFNG O-fucosylpeptide 3-beta-N-acetylglucosaminyltransferase	1.057082	0.7124	0.8553
58212	Srrm3	serine/arginine repetitive matrix 3	1.057082	0.5774	0.7702
17245	Mdm1	transformed mouse 3T3 cell double minute 1	1.056971	0.5216	0.7295
23831	Car14	carbonic anhydrase 14	1.056971	0.4435	NA
67869	Paip2	polyadenylate-binding protein-interacting protein 2	1.056971	0.4044	NA
68092	Ncbp2	nuclear cap binding protein subunit 2	1.056971	0.6715	0.8318
69593	2310026I22Rik	RIKEN cDNA 2310026I22 gene	1.056971	0.764	0.8862
77622	Apex2	apurinic/apyrimidinic endonuclease 2	1.056971	0.5892	0.7784
260299	Cadm4	cell adhesion molecule 4	1.056971	0.4585	NA
16402	Itga5	integrin alpha 5 (fibronectin receptor alpha)	1.056859	0.6843	0.8389
19084	Prkar1a	protein kinase, cAMP dependent regulatory, type I, alpha	1.056859	0.3211	NA
57373	D930014E17Rik	RIKEN cDNA D930014E17 gene	1.056859	0.4117	NA
67236	Cinp	cyclin-dependent kinase 2 interacting protein	1.056859	0.5508	0.7511
72344	Usp36	ubiquitin specific peptidase 36	1.056859	0.5984	0.7838
14248	Flii	flightless I homolog (Drosophila)	1.056747	0.7792	0.8935
69743	Casz1	castor homolog 1, zinc finger (Drosophila)	1.056747	0.6026	0.7867
75220	4930535I16Rik	RIKEN cDNA 4930535I16 gene	1.056747	0.5265	0.7337
75292	Prkd3	protein kinase D3	1.056747	0.491	NA
331026	Gmppb	GDP-mannose pyrophosphorylase B	1.056747	0.2807	NA

12366	Casp2	caspase 2	1.056636	0.2577	NA
20813	Srp14	signal recognition particle 14	1.056636	0.2658	NA
72999	Insig2	insulin induced gene 2	1.056636	0.3299	NA
331392	Gm5124	predicted pseudogene 5124	1.056636	0.6904	0.8421
13367	Diap1	diaphanous homolog 1 (Drosophila)	1.056524	0.5697	0.7639
24061	Smc1a	structural maintenance of chromosomes 1A	1.056524	0.4733	NA
106794	Dhx57	DEAH (Asp-Glu-Ala-Asp/His) box polypeptide 57	1.056524	0.4359	NA
216152	BC005764	cDNA sequence BC005764	1.056524	0.4749	NA
236690	Nyx	nyctalopin	1.056524	0.4093	NA
382118	Zfp167	zinc finger protein 167	1.056524	0.7813	0.895
20170	Hps6	Hermansky-Pudlak syndrome 6	1.056412	0.4316	NA
72614	Pih1d2	PIH1 domain containing 2	1.056412	0.486	NA
105440	Kctd9	potassium channel tetramerisation domain containing 9	1.056412	0.5024	NA
231769	Sfswap	splicing factor, suppressor of white-apricot homolog (Drosophila)	1.056412	0.6732	0.8325
268417	Zkscan17	zinc finger with KRAB and SCAN domains 17	1.056412	0.275	NA
14687	Gnaz	guanine nucleotide binding protein, alpha z subunit	1.056301	0.6801	0.8363
67923	Tceb1	transcription elongation factor B (SIII), polypeptide 1	1.056301	0.3836	NA
76816	Sdccag8	serologically defined colon cancer antigen 8	1.056301	0.7821	0.8953
102442	Dennd4a	DENN/MADD domain containing 4A	1.056301	0.4023	NA
216613	Ccdc85a	coiled-coil domain containing 85A	1.056301	0.4914	NA
338362	Ust	uronyl-2-sulfotransferase	1.056301	0.5955	0.7821
18550	Furin	furin (paired basic amino acid cleaving enzyme)	1.056189	0.5012	NA
19247	Ptpn11	protein tyrosine phosphatase, non-receptor type 11	1.056189	0.274	NA
52626	Cdkn2aipnl	CDKN2A interacting protein N-terminal like	1.056189	0.5655	0.762
57915	Tbc1d1	TBC1 domain family, member 1	1.056189	0.4688	NA
67487	Dhx40	DEAH (Asp-Glu-Ala-His) box polypeptide 40	1.056189	0.4528	NA
433667	Ankrd13c	ankyrin repeat domain 13c	1.056189	0.3686	NA
26992	Brd7	bromodomain containing 7	1.056078	0.4303	NA
57312	Mrps31	mitochondrial ribosomal protein S31	1.056078	0.2849	NA
241794	Kcng1	potassium voltage-gated channel, subfamily G, member 1	1.056078	0.7733	0.891
545718	Gm13141	predicted gene 13141	1.056078	0.4349	NA
14356	Fxc1	fractured callus expressed transcript 1	1.055966	0.3153	NA
56356	Gltp	glycolipid transfer protein	1.055966	0.6141	0.795
59079	Erbb2ip	Erbb2 interacting protein	1.055966	0.4625	NA
67942	Atp5g2	ATP synthase, H+ transporting, mitochondrial F0 complex, subunit C2 (subunit 9)	1.055966	0.4405	NA
67955	Sugt1	SGT1, suppressor of G2 allele of SKP1 (S. cerevisiae)	1.055966	0.2939	NA
671641	Gm10063	predicted gene 10063	1.055966	0.1892	NA
12469	Cct8	chaperonin containing Tcp1, subunit 8 (theta)	1.055855	0.5185	NA
20878	Aurka	aurora kinase A	1.055855	0.6415	0.8127
26370	Cetn2	centrin 2	1.055855	0.2871	NA
26931	Ppp2r5c	protein phosphatase 2, regulatory subunit B (B56), gamma isoform	1.055855	0.3756	NA
76668	Mdh1b	malate dehydrogenase 1B, NAD (soluble)	1.055855	0.6329	0.8068
13397	Dlx6as	distal-less homeobox 6, antisense	1.055743	0.7681	0.8883
19989	Rpl7	ribosomal protein L7	1.055743	0.2225	NA
26419	Mapk8	mitogen-activated protein kinase 8	1.055743	0.6016	0.7863
54383	Phc2	polyhomeotic-like 2 (Drosophila)	1.055743	0.4248	NA
56066	Cxcl11	chemokine (C-X-C motif) ligand 11	1.055743	0.5247	0.7321
76500	Ip6k2	inositol hexaphosphate kinase 2	1.055743	0.7972	0.9048
319564	C230012O17Rik	RIKEN cDNA C230012O17 gene	1.055743	0.7693	0.8892

338366	Mia3	melanoma inhibitory activity 3	1.055743	0.7845	0.8967
14755	Pigq	phosphatidylinositol glycan anchor biosynthesis, class Q	1.055632	0.3263	NA
20102	Rps4x	ribosomal protein S4, X-linked	1.055632	0.3117	NA
22230	Ufd1l	ubiquitin fusion degradation 1 like	1.055632	0.4708	NA
67842	2610027L16Rik	RIKEN cDNA 2610027L16 gene	1.055632	0.6006	0.7854
68667	Trpm4	transient receptor potential cation channel, subfamily M, member 4	1.055632	0.4885	NA
74205	Acsl3	acyl-CoA synthetase long-chain family member 3	1.055632	0.6694	0.8303
16443	Itsn1	intersectin 1 (SH3 domain protein 1A)	1.05552	0.5159	NA
20595	Smn1	survival motor neuron 1	1.05552	0.692	0.8433
69072	Ebna1bp2	EBNA1 binding protein 2	1.05552	0.5906	0.7792
216829	Mmgt2	membrane magnesium transporter 2	1.05552	0.4174	NA
382423	Atxn7l3b	ataxin 7-like 3B	1.05552	0.519	NA
11736	Ankfy1	ankyrin repeat and FYVE domain containing 1	1.055409	0.7389	0.8723
19847	Rnu1b6	U1b6 small nuclear RNA	1.055409	0.7397	0.8728
22210	Ube2b	ubiquitin-conjugating enzyme E2B, RAD6 homology (S. cerevisiae)	1.055409	0.4656	NA
24132	Zfp53	zinc finger protein 53	1.055409	0.6335	0.8073
66320	Tmem208	transmembrane protein 208	1.055409	0.299	NA
67890	Ufm1	ubiquitin-fold modifier 1	1.055409	0.4494	NA
69568	Vkorc1l1	vitamin K epoxide reductase complex, subunit 1-like 1	1.055409	0.4554	NA
70568	Cpne3	copine III	1.055409	0.5019	NA
193740	Hspa1a	heat shock protein 1A	1.055409	0.4709	NA
320541	Slc35e2	solute carrier family 35, member E2	1.055409	0.5085	NA
665618	Gm7715	predicted gene 7715	1.055409	0.6442	0.8142
56036	Ccnl2	cyclin L2	1.055298	0.5042	NA
28077	Med10	mediator of RNA polymerase II transcription, subunit 10 homolog (NUT2, S. cerevisiae)	1.055186	0.4295	NA
68050	Akirin1	akirin 1	1.055186	0.4288	NA
227801	Dennd1a	DENN/MADD domain containing 1A	1.055186	0.5224	NA
100502641	LOC100502641	60S acidic ribosomal protein P1-like	1.055186	0.6854	0.8397
17441	Mog	myelin oligodendrocyte glycoprotein	1.055075	0.8747	0.9452
19188	Psme2	proteasome (prosome, macropain) 28 subunit, beta	1.055075	0.3169	NA
19654	Rbm6	RNA binding motif protein 6	1.055075	0.3662	NA
21761	Morf4l1	mortality factor 4 like 1	1.055075	0.4702	NA
66593	Diablo	diablo homolog (Drosophila)	1.055075	0.6019	0.7863
71679	Atp5h	ATP synthase, H+ transporting, mitochondrial F0 complex, subunit d	1.055075	0.5175	NA
209212	Osgin2	oxidative stress induced growth inhibitor family member 2	1.055075	0.2933	NA
21687	Tek	endothelial-specific receptor tyrosine kinase	1.054964	0.6792	0.8358
24060	Slc35a1	solute carrier family 35 (CMP-sialic acid transporter), member 1	1.054964	0.3831	NA
15356	Hmgcl	3-hydroxy-3-methylglutaryl-Coenzyme A lyase	1.054852	0.1883	NA
15572	Elavl4	ELAV (embryonic lethal, abnormal vision, Drosophila)-like 4 (Hu antigen D)	1.054852	0.6095	0.7917
16179	Irak1	interleukin-1 receptor-associated kinase 1	1.054852	0.4986	NA
18701	Pigf	phosphatidylinositol glycan anchor biosynthesis, class F	1.054852	0.4582	NA
53600	Timm23	translocase of inner mitochondrial membrane 23 homolog (yeast)	1.054852	0.3174	NA
69071	Tmem97	transmembrane protein 97	1.054852	0.4245	NA
80914	Uck2	uridine-cytidine kinase 2	1.054852	0.2187	NA
243302	Gm4963	predicted gene 4963	1.054852	0.532	0.7378
271305	Phf21b	PHD finger protein 21B	1.054852	0.3839	NA
19230	Twf1	twinfilin, actin-binding protein, homolog 1 (Drosophila)	1.054741	0.3646	NA
64659	Mrps14	mitochondrial ribosomal protein S14	1.054741	0.4244	NA
93885	Pcdhb14	protocadherin beta 14	1.054741	0.3525	NA

216739	Acsl6	acyl-CoA synthetase long-chain family member 6	1.054741	0.6743	0.8331
20280	Scp2	sterol carrier protein 2, liver	1.05463	0.2215	NA
75786	Ckap5	cytoskeleton associated protein 5	1.05463	0.2592	NA
26425	Nubp1	nucleotide binding protein 1	1.054519	0.1937	NA
54004	Diap2	diaphanous homolog 2 (Drosophila)	1.054519	0.5312	NA
57329	Otor	otoraplin	1.054519	0.4734	NA
71375	Foxn3	forkhead box N3	1.054519	0.5036	NA
217473	Ankmy2	ankyrin repeat and MYND domain containing 2	1.054519	0.7572	0.8826
230793	Ahdc1	AT hook, DNA binding motif, containing 1	1.054519	0.5537	0.7536
664894	Gm13215	predicted gene 13215	1.054519	0.6077	0.7902
20112	Rps6ka2	ribosomal protein S6 kinase, polypeptide 2	1.054407	0.5651	0.7618
23961	Oas1b	2'-5' oligoadenylate synthetase 1B	1.054407	0.4776	NA
546546	Serpina3h	serine (or cysteine) peptidase inhibitor, clade A, member 3H	1.054407	0.8658	0.9416
26894	Cops7a	COP9 (constitutive photomorphogenic) homolog, subunit 7a (Arabidopsis thaliana)	1.054296	0.6281	0.8039
66753	Erlec1	endoplasmic reticulum lectin 1	1.054296	0.489	NA
72692	Hnrpll	heterogeneous nuclear ribonucleoprotein L-like	1.054296	0.461	NA
320362	9530085L11Rik	RIKEN cDNA 9530085L11 gene	1.054296	0.7272	0.8647
100043272	5430417L22Rik	RIKEN cDNA 5430417L22 gene	1.054296	0.6875	0.8407
17207	Mcf2l	mcf.2 transforming sequence-like	1.054185	0.5972	0.7832
20511	Slc1a2	solute carrier family 1 (glial high affinity glutamate transporter), member 2	1.054185	0.5191	NA
69581	Rhou	ras homolog gene family, member U	1.054185	0.5127	NA
224671	Btbd9	BTB (POZ) domain containing 9	1.054185	0.3117	NA
13929	Amz2	archaelysin family metallopeptidase 2	1.054074	0.3557	NA
52856	Gtpbp5	GTP binding protein 5	1.054074	0.4593	NA
54380	Smarcal1	SWI/SNF related matrix associated, actin dependent regulator of chromatin, subfamily a-like 1	1.054074	0.4014	NA
100038641	Gm10452	predicted gene 10452	1.054074	0.6893	0.8415
99167	Ssx2ip	synovial sarcoma, X breakpoint 2 interacting protein	1.053963	0.5586	0.7573
224640	Lemd2	LEM domain containing 2	1.053963	0.3649	NA
14977	Slc39a7	solute carrier family 39 (zinc transporter), member 7	1.053852	0.5433	0.7466
19943	Rpl28	ribosomal protein L28	1.053852	0.5111	NA
67229	Prpf18	PRP18 pre-mRNA processing factor 18 homolog (yeast)	1.053852	0.2702	NA
76721	1700085B13Rik	RIKEN cDNA 1700085B13 gene	1.053852	0.7426	0.8746
212153	2610015P09Rik	RIKEN cDNA 2610015P09 gene	1.053852	0.4341	NA
12802	Cnr2	cannabinoid receptor 2 (macrophage)	1.053741	0.8411	0.9287
19799	Rn4.5s	4.5S RNA	1.053741	0.6248	0.8016
235047	Zfp809	zinc finger protein 809	1.053741	0.566	0.762
30954	Siva1	SIVA1, apoptosis-inducing factor	1.05363	0.4344	NA
67270	Mrpl42	mitochondrial ribosomal protein L42	1.05363	0.3861	NA
27207	Rps11	ribosomal protein S11	1.053519	0.2562	NA
52466	Slc46a1	solute carrier family 46, member 1	1.053519	0.5753	0.7688
117606	Boc	biregional cell adhesion molecule-related/down-regulated by oncogenes (Cdon) binding protein	1.053519	0.5384	NA
232969	Zfp428	zinc finger protein 428	1.053519	0.4174	NA
12916	Crem	cAMP responsive element modulator	1.053408	0.4719	NA
19411	Rarg	retinoic acid receptor, gamma	1.053408	0.5481	0.7496
74256	Cyld	cylindromatosis (turban tumor syndrome)	1.053408	0.8041	0.9083
101739	Psip1	PC4 and SFRS1 interacting protein 1	1.053408	0.7749	0.8918
217149	Cisd3	CDGSH iron sulfur domain 3	1.053408	0.4144	NA
243043	Kctd8	potassium channel tetramerisation domain containing 8	1.053408	0.4985	NA
269470	Wdr3	WD repeat domain 3	1.053408	0.4378	NA

14086	Fscn1	fascin homolog 1, actin bundling protein (Strongylocentrotus purpuratus)	1.053297	0.6303	0.8051
69236	2610034E01Rik	RIKEN cDNA 2610034E01 gene	1.053297	0.8205	0.9183
77862	Thyn1	thymocyte nuclear protein 1	1.053297	0.4407	NA
102162	Taf5l	TAF5-like RNA polymerase II, p300/CBP-associated factor (PCAF)-associated factor	1.053297	0.2337	NA
319880	Tmcc3	transmembrane and coiled coil domains 3	1.053297	0.6261	0.8026
626858	Gm6713	predicted gene 6713	1.053297	0.8272	0.9218
15387	Hnrnpk	heterogeneous nuclear ribonucleoprotein K	1.053186	0.5577	0.7568
208228	Mobkl2a	MOB1, Mps One Binder kinase activator-like 2A (yeast)	1.053186	0.657	0.8223
218973	Wdhd1	WD repeat and HMG-box DNA binding protein 1	1.053186	0.6748	0.8334
239827	Pigz	phosphatidylinositol glycan anchor biosynthesis, class Z	1.053186	0.5442	0.7472
243872	Rpl7a-ps8	ribosomal protein L7A, pseudogene 8	1.053186	0.2594	NA
16414	Itgb2	integrin beta 2	1.053075	0.697	0.8466
16506	Kcnd1	potassium voltage-gated channel, Shal-related family, member 1	1.053075	0.6336	0.8073
20844	Stam	signal transducing adaptor molecule (SH3 domain and ITAM motif) 1	1.053075	0.6537	0.8207
269941	Chsy1	chondroitin sulfate synthase 1	1.053075	0.3011	NA
16975	Lrp8	low density lipoprotein receptor-related protein 8, apolipoprotein e receptor	1.052964	0.4245	NA
19877	Rock1	Rho-associated coiled-coil containing protein kinase 1	1.052964	0.6871	0.8405
67245	Peli1	pellino 1	1.052964	0.3292	NA
102570	Slc22a13	solute carrier family 22 (organic cation transporter), member 13	1.052964	0.5683	0.7634
232685	AB041803	cDNA sequence AB041803	1.052964	0.6773	0.8348
100042899	Gm4098	predicted gene 4098	1.052964	0.8253	0.9208
13688	Eif4ebp2	eukaryotic translation initiation factor 4E binding protein 2	1.052853	0.6894	0.8415
66588	Cmpk1	cytidine monophosphate (UMP-CMP) kinase 1	1.052853	0.5167	NA
77808	A930015G24Rik	RIKEN cDNA A930015G24 gene	1.052853	0.5346	NA
244238	Mrgpre	MAS-related GPR, member E	1.052853	0.5544	0.7539
11538	Adnp	activity-dependent neuroprotective protein	1.052742	0.6217	0.7996
13682	Eif4a2	eukaryotic translation initiation factor 4A2	1.052742	0.7317	0.8677
54152	Dnalc4	dynein, axonemal, light chain 4	1.052742	0.4168	NA
107771	Bmyc	brain expressed myelocytomatosis oncogene	1.052742	0.5011	NA
211770	Trib1	tribbles homolog 1 (Drosophila)	1.052742	0.2442	NA
213541	Ythdf2	YTH domain family 2	1.052742	0.6874	0.8407
13819	Epas1	endothelial PAS domain protein 1	1.052632	0.6095	0.7917
69071	Tmem97	transmembrane protein 97	1.052632	0.5551	0.7546
69981	Tmem30a	transmembrane protein 30A	1.052632	0.6294	0.8043
93735	Wnt16	wingless-related MMTV integration site 16	1.052632	0.7705	0.8897
192786	Rapgef6	Rap guanine nucleotide exchange factor (GEF) 6	1.052632	0.5925	0.7802
223978	Cpped1	calcineurin-like phosphoesterase domain containing 1	1.052632	0.4011	NA
230235	6430704M03Rik	RIKEN cDNA 6430704M03 gene	1.052632	0.4705	NA
12321	Calu	calumenin	1.052521	0.5688	0.7635
57783	Tnip1	TNFAIP3 interacting protein 1	1.052521	0.4217	NA
70480	5730407M17Rik	RIKEN cDNA 5730407M17 gene	1.052521	0.4754	NA
71213	Cage1	cancer antigen 1	1.052521	0.6231	0.8005
71514	Sfpq	splicing factor proline/glutamine rich (polypyrimidine tract binding protein associated)	1.052521	0.3852	NA
224109	Lrrc33	leucine rich repeat containing 33	1.052521	0.5563	0.7556
319195	Rpl17	ribosomal protein L17	1.052521	0.7754	0.892
434632	BC085271	cDNA sequence BC085271	1.052521	0.6518	0.8193
544782	Gm12620	poly(A) binding protein, cytoplasmic 4 pseudogene	1.052521	0.5397	NA
20362	Sep-08	septin 8	1.05241	0.3752	NA
27425	Atp5l	ATP synthase, H+ transporting, mitochondrial F0 complex, subunit g	1.05241	0.7805	0.8946

53859	Map3k14	mitogen-activated protein kinase kinase kinase 14	1.05241	0.4445	NA
70397	Tmem70	transmembrane protein 70	1.05241	0.3071	NA
75758	9130401M01Rik	RIKEN cDNA 9130401M01 gene	1.05241	0.5828	0.7736
245841	Polr2h	polymerase (RNA) II (DNA directed) polypeptide H	1.05241	0.4613	NA
625603	Gm6607	40S ribosomal protein S20 pseudogene	1.05241	0.5812	0.7726
381598	2610005L07Rik	cadherin 11 pseudogene	1.052299	0.2669	NA
20747	Spop	speckle-type POZ protein	1.052189	0.6532	0.8205
75422	Mettl5	methyltransferase like 5	1.052189	0.5135	NA
319728	E030042N06Rik	RIKEN cDNA E030042N06 gene	1.052189	0.4509	NA
402752	C130068B02Rik	RIKEN cDNA C130068B02 gene	1.052189	0.4685	NA
12333	Capn1	calpain 1	1.051967	0.6775	0.8348
18038	Nfkbil1	nuclear factor of kappa light polypeptide gene enhancer in B-cells inhibitor-like 1	1.051967	0.4293	NA
67106	Zbtb8os	zinc finger and BTB domain containing 8 opposite strand	1.051967	0.5755	0.7689
94282	Sfxn5	sideroflexin 5	1.051967	0.7598	0.8836
105670	Rcbtb2	regulator of chromosome condensation (RCC1) and BTB (POZ) domain containing protein 2	1.051967	0.4694	NA
210104	Zfp658	zinc finger protein 658	1.051967	0.7504	0.879
213522	Plekhg6	pleckstrin homology domain containing, family G (with RhoGef domain) member 6	1.051967	0.6129	0.7941
218952	Fermt2	fermitin family homolog 2 (Drosophila)	1.051967	0.5173	NA
668272	Gm9079	transmembrane emp24 domain trafficking protein 2 pseudogene	1.051967	0.5154	NA
11826	Aqp1	aquaporin 1	1.051857	0.5956	0.7822
66412	Arrdc4	arrestin domain containing 4	1.051857	0.4972	NA
66985	Rassf7	Ras association (RalGDS/AF-6) domain family (N-terminal) member 7	1.051857	0.4268	NA
68277	2310057M21Rik	RIKEN cDNA 2310057M21 gene	1.051857	0.3888	NA
74996	Usp47	ubiquitin specific peptidase 47	1.051857	0.7375	0.8714
77881	6030458E02Rik	RIKEN cDNA 6030458E02 gene	1.051857	0.7923	0.9016
381110	Fam82a1	family with sequence similarity 82, member A1	1.051857	0.5319	NA
11844	Arf5	ADP-ribosylation factor 5	1.051746	0.4113	NA
67528	Nudt7	nudix (nucleoside diphosphate linked moiety X)-type motif 7	1.051746	0.4214	NA
68735	Mrps18c	mitochondrial ribosomal protein S18C	1.051746	0.4659	NA
68755	Cgrrf1	cell growth regulator with ring finger domain 1	1.051746	0.5344	NA
229588	Gm128	predicted gene 128	1.051746	0.4495	NA
100505237	LOC100505237	calcium-binding and coiled-coil domain-containing protein 2-like	1.051746	0.8389	0.9273
57267	Apba3	amyloid beta (A4) precursor protein-binding, family A, member 3	1.051635	0.6834	0.8384
67752	4930579J09Rik	RIKEN cDNA 4930579J09 gene	1.051525	0.5455	NA
99512	Wdr47	WD repeat domain 47	1.051525	0.3343	NA
106344	Rfc4	replication factor C (activator 1) 4	1.051525	0.5046	NA
403187	Opa3	optic atrophy 3 (human)	1.051525	0.7219	0.8609
434179	Gm5595	predicted gene 5595	1.051525	0.4026	NA
436188	Gm5751	predicted gene 5751	1.051525	0.7658	0.8869
19263	Ptprb	protein tyrosine phosphatase, receptor type, B	1.051414	0.3997	NA
22169	Cmpk2	cytidine monophosphate (UMP-CMP) kinase 2, mitochondrial	1.051414	0.5135	NA
75475	Oplah	5-oxoprolinase (ATP-hydrolysing)	1.051414	0.6062	0.7891
242466	Zfp462	zinc finger protein 462	1.051414	0.5878	0.7773
268663	Cdhr2	cadherin-related family member 2	1.051414	0.7263	0.8641
100038554	Gm15348	predicted gene 15348	1.051414	0.3308	NA
19057	Ppp3cc	protein phosphatase 3, catalytic subunit, gamma isoform	1.051304	0.3551	NA
140740	Sec63	SEC63-like (S. cerevisiae)	1.051304	0.5506	NA
24074	Taf7	TAF7 RNA polymerase II, TATA box binding protein (TBP)-associated factor	1.051193	0.6524	0.8198
56401	Lepre1	leprecan 1	1.051193	0.7003	0.8486

68058	Chd1l	chromodomain helicase DNA binding protein 1-like	1.051193	0.4473	NA
20443	St3gal4	ST3 beta-galactoside alpha-2,3-sialyltransferase 4	1.051083	0.5491	NA
20444	St3gal2	ST3 beta-galactoside alpha-2,3-sialyltransferase 2	1.051083	0.3703	NA
21781	Tfdp1	transcription factor Dp 1	1.051083	0.5518	NA
77889	Lbh	limb-bud and heart	1.050972	0.4578	NA
11931	Atp1b1	ATPase, Na+/K+ transporting, beta 1 polypeptide	1.050862	0.5421	NA
67579	Cpeb4	cytoplasmic polyadenylation element binding protein 4	1.050862	0.746	0.8767
68183	Bcas2	breast carcinoma amplified sequence 2	1.050862	0.4018	NA
70054	Ccdc89	coiled-coil domain containing 89	1.050862	0.4468	NA
109115	Supt3h	suppressor of Ty 3 homolog (S. cerevisiae)	1.050862	0.5541	NA
170644	Ubn1	ubinuclein 1	1.050862	0.5803	0.7724
240084	Cchcr1	coiled-coil alpha-helical rod protein 1	1.050862	0.5993	0.7846
22754	Zfp92	zinc finger protein 92	1.050751	0.5993	0.7846
69168	Bola1	bolA-like 1 (E. coli)	1.050751	0.4559	NA
69902	Mrto4	MRT4, mRNA turnover 4, homolog (S. cerevisiae)	1.050751	0.4402	NA
76261	0610040J01Rik	RIKEN cDNA 0610040J01 gene	1.050751	0.6513	0.8188
229615	Pias3	protein inhibitor of activated STAT 3	1.050751	0.7197	0.8599
17129	Smad5	MAD homolog 5 (Drosophila)	1.050641	0.3188	NA
56087	Dnahc10	dynein, axonemal, heavy chain 10	1.050641	0.7764	0.8924
65105	Arl6ip4	ADP-ribosylation factor-like 6 interacting protein 4	1.050641	0.2894	NA
68169	A930038C07Rik	RIKEN cDNA A930038C07 gene	1.050641	0.5574	NA
217995	Heatr1	HEAT repeat containing 1	1.050641	0.3637	NA
11848	Rhoa	ras homolog gene family, member A	1.050531	0.4116	NA
13043	Cttn	cortactin	1.050531	0.3513	NA
13400	Dmpk	dystrophia myotonica-protein kinase	1.050531	0.6163	0.7965
19012	Ppap2a	phosphatidic acid phosphatase type 2A	1.050531	0.3988	NA
19989	Rpl7	ribosomal protein L7	1.050531	0.3491	NA
24086	Tlk2	tousled-like kinase 2 (Arabidopsis)	1.050531	0.6139	0.795
58805	Mlxipl	MLX interacting protein-like	1.050531	0.7649	0.8866
216971	BC017647	cDNA sequence BC017647	1.050531	0.6727	0.8323
270669	Mbtps2	membrane-bound transcription factor peptidase, site 2	1.050531	0.5793	0.7715
329934	Foxo6	forkhead box O6	1.050531	0.8438	0.9299
18750	Prkca	protein kinase C, alpha	1.05042	0.3932	NA
72522	Atxn7l2	ataxin 7-like 2	1.05042	0.7282	0.8653
19325	Rab10	RAB10, member RAS oncogene family	1.05031	0.5593	NA
52700	Txndc17	thioredoxin domain containing 17	1.05031	0.4771	NA
70144	Lrch3	leucine-rich repeats and calponin homology (CH) domain containing 3	1.05031	0.5975	0.7834
230895	Vps13d	vacuolar protein sorting 13 D (yeast)	1.05031	0.6009	0.7858
330267	Thsd7a	thrombospondin, type I, domain containing 7A	1.05031	0.5529	NA
100040711	Gm2921	predicted gene 2921	1.05031	0.723	0.8616
55950	Bri3	brain protein I3	1.0502	0.4179	NA
67582	Slc25a26	solute carrier family 25 (mitochondrial carrier, phosphate carrier), member 26	1.0502	0.6256	0.8023
68652	Tab2	TGF-beta activated kinase 1/MAP3K7 binding protein 2	1.0502	0.711	0.8547
235682	Zfp445	zinc finger protein 445	1.0502	0.722	0.8609
22185	U2af2	U2 small nuclear ribonucleoprotein auxiliary factor (U2AF) 2	1.050089	0.7225	0.8612
68634	Tm2d3	TM2 domain containing 3	1.050089	0.3561	NA
56088	Psmg1	proteasome (prosome, macropain) assembly chaperone 1	1.049979	0.4387	NA
20301	Ccl27a	chemokine (C-C motif) ligand 27A	1.049869	0.4609	NA
52647	D15Ertd509e	DNA segment, Chr 15, ERATO Doi 509, expressed	1.049869	0.6348	0.8078

66056	Zfp524	zinc finger protein 524	1.049869	0.5026	NA
67025	Rpl11	ribosomal protein L11	1.049869	0.5862	0.7761
105513	Chmp7	CHMP family, member 7	1.049869	0.6298	0.8047
109305	Orai1	ORAI calcium release-activated calcium modulator 1	1.049869	0.6711	0.8317
353287	Clec18a	C-type lectin domain family 18, member A	1.049869	0.8009	0.9064
68339	Ccdc88c	coiled-coil domain containing 88C	1.049759	0.5293	NA
73174	Tbkbp1	TBK1 binding protein 1	1.049759	0.4538	NA
75642	1700020C07Rik	RIKEN cDNA 1700020C07 gene	1.049759	0.8899	0.9522
76820	Fam49a	family with sequence similarity 49, member A	1.049759	0.5995	0.7847
240641	Kif20b	kinesin family member 20B	1.049759	0.5137	NA
638247	9530082P21Rik	RIKEN cDNA 9530082P21 gene	1.049759	0.5081	NA
75782	Lca5	Leber congenital amaurosis 5 (human)	1.049648	0.7654	0.8868
94218	Cnnm3	cyclin M3	1.049648	0.5283	NA
380928	Lmo7	LIM domain only 7	1.049648	0.659	0.8238
381760	Ssbp1	single-stranded DNA binding protein 1	1.049648	0.646	0.8153
15979	Ifngr1	interferon gamma receptor 1	1.049538	0.366	NA
18572	Pdcd11	programmed cell death 11	1.049538	0.7473	0.8773
57261	Brd4	bromodomain containing 4	1.049538	0.4829	NA
67690	Prss37	protease, serine, 37	1.049538	0.6364	0.8089
74525	8430419L09Rik	RIKEN cDNA 8430419L09 gene	1.049538	0.8521	0.9339
208595	Gm9897	predicted gene 9897	1.049538	0.4861	NA
264064	Cdk8	cyclin-dependent kinase 8	1.049538	0.5146	NA
328274	Zfp459	zinc finger protein 459	1.049538	0.6144	0.795
56486	Gabarap	gamma-aminobutyric acid receptor associated protein	1.049428	0.4069	NA
67581	Tbc1d23	TBC1 domain family, member 23	1.049428	0.3174	NA
228019	Mettl8	methyltransferase like 8	1.049428	0.5226	NA
330602	Gm5115	predicted gene 5115	1.049428	0.6294	0.8043
18007	Neo1	neogenin	1.049318	0.4644	NA
72098	Tmem68	transmembrane protein 68	1.049318	0.7375	0.8714
103203	AI413759	expressed sequence AI413759	1.049318	0.5235	NA
104263	Kdm3a	lysine (K)-specific demethylase 3A	1.049318	0.4182	NA
106627	AI646383	expressed sequence AI646383	1.049318	0.7608	0.8842
240058	Cpne5	copine V	1.049318	0.5813	0.7727
408058	BC048507	cDNA sequence BC048507	1.049318	0.8578	0.9368
17134	Mafg	v-maf musculoaponeurotic fibrosarcoma oncogene family, protein G (avian)	1.049208	0.6338	0.8074
21343	Taf6	TAF6 RNA polymerase II, TATA box binding protein (TBP)-associated factor	1.049208	0.3006	NA
75062	Sf3a3	splicing factor 3a, subunit 3	1.049208	0.3829	NA
106757	Tmem146	transmembrane protein 146	1.049208	0.7199	0.8599
669393	Gm9457	predicted gene 9457	1.049208	0.3829	NA
18260	Ocln	occludin	1.049098	0.8173	0.9164
19262	Ptpra	protein tyrosine phosphatase, receptor type, A	1.049098	0.3307	NA
20733	Spint2	serine protease inhibitor, Kunitz type 2	1.049098	0.5066	NA
76453	Prss23	protease, serine, 23	1.049098	0.5774	0.7702
217864	Rcor1	REST corepressor 1	1.049098	0.3861	NA
56430	Clip1	CAP-GLY domain containing linker protein 1	1.048988	0.3543	NA
75608	Chmp4b	chromatin modifying protein 4B	1.048988	0.3751	NA
237400	Mex3d	mex3 homolog D (C. elegans)	1.048988	0.4216	NA
380686	Cnrip1	cannabinoid receptor interacting protein 1	1.048988	0.4565	NA
58231	Stk4	serine/threonine kinase 4	1.048878	0.6855	0.8397

80891	Fcrls	Fc receptor-like S, scavenger receptor	1.048878	0.5361	NA
108664	Atp6v1h	ATPase, H+ transporting, lysosomal V1 subunit H	1.048878	0.5276	NA
209773	Dennd2a	DENN/MADD domain containing 2A	1.048878	0.3239	NA
226548	Aph1a	anterior pharynx defective 1a homolog (C. elegans)	1.048878	0.6643	0.8277
236643	Sytl5	synaptotagmin-like 5	1.048878	0.6808	0.8365
12616	Cenpb	centromere protein B	1.048768	0.6417	0.8128
16678	Krt1	keratin 1	1.048768	0.7836	0.8962
68059	Tm9sf2	transmembrane 9 superfamily member 2	1.048768	0.5921	0.7801
80906	Kcnip2	Kv channel-interacting protein 2	1.048768	0.8114	0.9127
223267	A2ld1	AIG2-like domain 1	1.048768	0.4713	NA
224111	Ubxn7	UBX domain protein 7	1.048768	0.6163	0.7965
228880	Zmynd8	zinc finger, MYND-type containing 8	1.048768	0.6552	0.8212
319565	Syne2	synaptic nuclear envelope 2	1.048768	0.7907	0.9009
434632	BC085271	cDNA sequence BC085271	1.048768	0.4332	NA
12488	Cd2ap	CD2-associated protein	1.048658	0.5181	NA
53883	Celsr2	cadherin, EGF LAG seven-pass G-type receptor 2 (flamingo homolog, Drosophila)	1.048658	0.4407	NA
66536	Nipsnap3b	nipsnap homolog 3B (C. elegans)	1.048658	0.716	0.8578
104184	Blmh	bleomycin hydrolase	1.048658	0.3854	NA
269109	Dpp10	dipeptidylpeptidase 10	1.048658	0.5978	0.7835
29819	Stau2	staufen (RNA binding protein) homolog 2 (Drosophila)	1.048548	0.4355	NA
75784	1700007G11Rik	RIKEN cDNA 1700007G11 gene	1.048548	0.5826	0.7735
108755	Lyrm2	LYR motif containing 2	1.048548	0.5394	NA
110304	Glra3	glycine receptor, alpha 3 subunit	1.048548	0.8095	0.9116
13929	Amz2	archaelysin family metallopeptidase 2	1.048438	0.4116	NA
70478	Mipep	mitochondrial intermediate peptidase	1.048438	0.4995	NA
18100	Mrpl40	mitochondrial ribosomal protein L40	1.048328	0.4049	NA
71983	Tmco6	transmembrane and coiled-coil domains 6	1.048328	0.6172	0.797
75995	5033417F24Rik	RIKEN cDNA 5033417F24 gene	1.048328	0.7046	0.8514
333654	Ppp1r13l	protein phosphatase 1, regulatory (inhibitor) subunit 13 like	1.048328	0.4823	NA
24071	Synj2bp	synaptojanin 2 binding protein	1.048218	0.5326	NA
28084	Vps25	vacuolar protein sorting 25 (yeast)	1.048218	0.6466	0.8156
56690	Mlycd	malonyl-CoA decarboxylase	1.048218	0.3517	NA
66885	Acadsb	acyl-Coenzyme A dehydrogenase, short/branched chain	1.048218	0.4789	NA
69072	Ebna1bp2	EBNA1 binding protein 2	1.048218	0.426	NA
18174	Slc11a2	solute carrier family 11 (proton-coupled divalent metal ion transporters), member 2	1.048108	0.5901	0.7788
22757	Zkscan5	zinc finger with KRAB and SCAN domains 5	1.048108	0.5539	NA
195209	Gm22	predicted gene 22	1.048108	0.7578	0.8829
269261	Rpl12	ribosomal protein L12	1.048108	0.371	NA
18518	Igbp1	immunoglobulin (CD79A) binding protein 1	1.047998	0.4907	NA
94221	Gopc	golgi associated PDZ and coiled-coil motif containing	1.047998	0.5293	NA
100043534	Gm11686	predicted gene 11686	1.047998	0.4715	NA
104069	Sncb	synuclein, beta	1.047889	0.693	0.8439
319876	Cobll1	Cobl-like 1	1.047889	0.7489	0.8784
77697	Mmab	methylmalonic aciduria (cobalamin deficiency) type B homolog (human)	1.047779	0.6124	0.7937
228812	Pigu	phosphatidylinositol glycan anchor biosynthesis, class U	1.047779	0.5214	NA
238123	Cog5	component of oligomeric golgi complex 5	1.047779	0.7349	0.87
28084	Vps25	vacuolar protein sorting 25 (yeast)	1.047669	0.6679	0.8296
78827	5830426C09Rik	RIKEN cDNA 5830426C09 gene	1.047669	0.7015	0.8494
264895	Acsf2	acyl-CoA synthetase family member 2	1.047669	0.7192	0.8597

652925	4930420K17Rik	RIKEN cDNA 4930420K17 gene	1.047669	0.5076	NA
670496	Gm11564	predicted gene 11564	1.047669	0.687	0.8404
100041724	Gm15217	predicted gene 15217	1.047669	0.6779	0.8351
20005	Rpl9	ribosomal protein L9	1.047559	0.4662	NA
70153	2210016F16Rik	RIKEN cDNA 2210016F16 gene	1.047559	0.4459	NA
109660	Ctrl	chymotrypsin-like	1.047559	0.6991	0.8478
216395	Tmem5	transmembrane protein 5	1.047559	0.6161	0.7965
216760	Mfap3	microfibrillar-associated protein 3	1.047559	0.4058	NA
229504	Isg20l2	interferon stimulated exonuclease gene 20-like 2	1.047559	0.6338	0.8074
13853	Epm2a	epilepsy, progressive myoclonic epilepsy, type 2 gene alpha	1.047449	0.7765	0.8924
66525	Timm50	translocase of inner mitochondrial membrane 50 homolog (yeast)	1.047449	0.6429	0.8135
66950	Tmem206	transmembrane protein 206	1.047449	0.442	NA
67381	Med4	mediator of RNA polymerase II transcription, subunit 4 homolog (yeast)	1.047449	0.5558	NA
107829	Thoc5	THO complex 5	1.047449	0.6422	0.8132
381062	2210404J11Rik	RIKEN cDNA 2210404J11 gene	1.047449	0.4896	NA
72611	Zfp655	zinc finger protein 655	1.04734	0.3863	NA
74104	Abcb6	ATP-binding cassette, sub-family B (MDR/TAP), member 6	1.04734	0.543	NA
114666	Krtap5-5	keratin associated protein 5-5	1.04734	0.4606	NA
212285	Arap2	ArfGAP with RhoGAP domain, ankyrin repeat and PH domain 2	1.04734	0.8089	0.9112
268996	Ss18	synovial sarcoma translocation, Chromosome 18	1.04734	0.3816	NA
21958	Tnp1	transition protein 1	1.04723	0.7984	0.9053
70681	Fam175a	family with sequence similarity 175, member A	1.04723	0.5977	0.7834
225207	Zfp521	zinc finger protein 521	1.04723	0.5347	NA
22194	Ube2e1	ubiquitin-conjugating enzyme E2E 1, UBC4/5 homolog (yeast)	1.04712	0.5635	NA
66374	2310011J03Rik	RIKEN cDNA 2310011J03 gene	1.04712	0.4965	NA
67254	2900011O08Rik	RIKEN cDNA 2900011O08 gene	1.04712	0.5473	NA
69577	Fastkd3	FAST kinase domains 3	1.04712	0.5386	NA
103140	Gstt3	glutathione S-transferase, theta 3	1.04712	0.5022	NA
107686	Snrpd2	small nuclear ribonucleoprotein D2	1.04712	0.4884	NA
13806	Eno1	enolase 1, alpha non-neuron	1.047011	0.5798	NA
14088	Fancc	Fanconi anemia, complementation group C	1.047011	0.5814	NA
66589	Ube2v1	ubiquitin-conjugating enzyme E2 variant 1	1.047011	0.7586	0.8831
192287	Slc25a36	solute carrier family 25, member 36	1.047011	0.7187	0.8594
229709	Ahcyl1	S-adenosylhomocysteine hydrolase-like 1	1.047011	0.7447	0.8759
26556	Homer1	homer homolog 1 (Drosophila)	1.046901	0.7894	0.9003
58202	Cobra1	cofactor of BRCA1	1.046901	0.3729	NA
59290	Gpa33	glycoprotein A33 (transmembrane)	1.046901	0.8103	0.9121
67087	Ctnnbip1	catenin beta interacting protein 1	1.046901	0.4545	NA
266690	Cyb5r4	cytochrome b5 reductase 4	1.046901	0.71	0.8543
320965	4831440E17Rik	RIKEN cDNA 4831440E17 gene	1.046901	0.6925	0.8437
12142	Prdm1	PR domain containing 1, with ZNF domain	1.046792	0.424	NA
30932	Zfp330	zinc finger protein 330	1.046792	0.5927	0.7804
67452	Pnpla8	patatin-like phospholipase domain containing 8	1.046792	0.4048	NA
226178	D19Wsu162e	DNA segment, Chr 19, Wayne State University 162, expressed	1.046792	0.5062	NA
380993	Zfat	zinc finger and AT hook domain containing	1.046792	0.7014	0.8494
19216	Ptger1	prostaglandin E receptor 1 (subtype EP1)	1.046682	0.7803	0.8944
72007	Fndc3b	fibronectin type III domain containing 3B	1.046682	0.4197	NA
72440	5930416I19Rik	RIKEN cDNA 5930416I19 gene	1.046682	0.6339	0.8075
76857	Spopl	speckle-type POZ protein-like	1.046682	0.6088	0.7912

171257	Vmn1r195	vomeronasal 1 receptor 195	1.046682	0.8732	0.9449
623131	Prr19	proline rich 19	1.046682	0.766	0.8871
22172	Tyms-ps	thymidylate synthase, pseudogene	1.046572	0.5655	NA
628100	Fbxo39	F-box protein 39	1.046572	0.8155	0.9153
14084	Faf1	Fas-associated factor 1	1.046463	0.5951	NA
67302	Zc3h13	zinc finger CCCH type containing 13	1.046463	0.6724	0.8323
75725	Phf14	PHD finger protein 14	1.046463	0.559	NA
226151	Fam178a	family with sequence similarity 178, member A	1.046463	0.7934	0.9021
13367	Diap1	diaphanous homolog 1 (Drosophila)	1.046353	0.7721	0.8906
15258	Hipk2	homeodomain interacting protein kinase 2	1.046353	0.4144	NA
18987	Pou2f2	POU domain, class 2, transcription factor 2	1.046353	0.7032	0.8504
22346	Vhl	von Hippel-Lindau tumor suppressor	1.046353	0.4948	NA
72180	Zfp661	zinc finger protein 661	1.046353	0.365	NA
229279	Hnrnpa3	heterogeneous nuclear ribonucleoprotein A3	1.046353	0.516	NA
21974	Top2b	topoisomerase (DNA) II beta	1.046244	0.5011	NA
26926	Aifm1	apoptosis-inducing factor, mitochondrion-associated 1	1.046244	0.3252	NA
73668	Ttc21b	tetratricopeptide repeat domain 21B	1.046244	0.3461	NA
76483	Lmf1	lipase maturation factor 1	1.046244	0.7457	0.8766
215445	Rab11fip3	RAB11 family interacting protein 3 (class II)	1.046244	0.5729	NA
270120	Fat3	FAT tumor suppressor homolog 3 (Drosophila)	1.046244	0.6506	0.8184
384309	Trim56	tripartite motif-containing 56	1.046244	0.73	0.8664
435791	Gm13271	predicted gene 13271	1.046244	0.6795	0.8359
17246	Mdm2	transformed mouse 3T3 cell double minute 2	1.046135	0.2554	NA
27057	Ncoa4	nuclear receptor coactivator 4	1.046135	0.3742	NA
28295	D10Jhu81e	DNA segment, Chr 10, Johns Hopkins University 81 expressed	1.046135	0.4651	NA
52036	Ppp6r3	protein phosphatase 6, regulatory subunit 3	1.046135	0.8302	0.9234
53886	Cdkl2	cyclin-dependent kinase-like 2 (CDC2-related kinase)	1.046135	0.6285	0.8039
108943	Rg9mtd2	RNA (guanine-9-) methyltransferase domain containing 2	1.046135	0.7103	0.8544
210297	Lrch2	leucine-rich repeats and calponin homology (CH) domain containing 2	1.046135	0.5351	NA
212772	2700007P21Rik	RIKEN cDNA 2700007P21 gene	1.046135	0.5229	NA
217695	Zfyve1	zinc finger, FYVE domain containing 1	1.046135	0.5303	NA
331487	Uprt	uracil phosphoribosyltransferase (FUR1) homolog (S. cerevisiae)	1.046135	0.7671	0.8876
11671	Aldh3a2	aldehyde dehydrogenase family 3, subfamily A2	1.046025	0.5472	NA
19656	Rbmxrt	RNA binding motif protein, X chromosome retrogene	1.046025	0.4462	NA
67997	Ddx59	DEAD (Asp-Glu-Ala-Asp) box polypeptide 59	1.046025	0.5089	NA
192662	Arhgdia	Rho GDP dissociation inhibitor (GDI) alpha	1.046025	0.5876	NA
320633	Zbtb26	zinc finger and BTB domain containing 26	1.046025	0.7556	0.8821
13665	Eif2s1	eukaryotic translation initiation factor 2, subunit 1 alpha	1.045916	0.4079	NA
73161	3110035C09Rik	RIKEN cDNA 3110035C09 gene	1.045916	0.8104	0.9122
73261	1700037C18Rik	RIKEN cDNA 1700037C18 gene	1.045916	0.5967	NA
192169	Ufsp2	UFM1-specific peptidase 2	1.045916	0.4807	NA
27643	Ubl4	ubiquitin-like 4	1.045806	0.4696	NA
114666	Krtap5-5	keratin associated protein 5-5	1.045806	0.8379	0.927
637916	LOC637916	midline-1-like	1.045806	0.7597	0.8836
11994	Pcdh15	protocadherin 15	1.045697	0.8667	0.9421
18861	Pms2	postmeiotic segregation increased 2 (S. cerevisiae)	1.045697	0.6776	0.8348
27041	G3bp1	Ras-GTPase-activating protein SH3-domain binding protein 1	1.045697	0.4126	NA
71911	Bdh1	3-hydroxybutyrate dehydrogenase, type 1	1.045697	0.501	NA
219189	1300010F03Rik	RIKEN cDNA 1300010F03 gene	1.045697	0.5649	NA

241447	Lass6	LAG1 homolog, ceramide synthase 6	1.045697	0.7214	0.8605
396184	Flrt1	fibronectin leucine rich transmembrane protein 1	1.045697	0.7379	0.8715
20603	Sms	spermine synthase	1.045588	0.6328	0.8068
52855	Lair1	leukocyte-associated Ig-like receptor 1	1.045588	0.5722	NA
60321	Wbp11	WW domain binding protein 11	1.045588	0.4524	NA
74296	1700093J21Rik	RIKEN cDNA 1700093J21 gene	1.045588	0.4831	NA
241621	Gm13981	predicted gene 13981	1.045588	0.3831	NA
14763	Gpr37	G protein-coupled receptor 37	1.045478	0.5267	NA
74043	Pex26	peroxisomal biogenesis factor 26	1.045478	0.3103	NA
75826	Senp2	SUMO/sentrin specific peptidase 2	1.045478	0.4846	NA
225339	Ammecr1l	AMME chromosomal region gene 1-like	1.045478	0.2802	NA
234356	Csgalnact1	chondroitin sulfate N-acetylgalactosaminyltransferase 1	1.045478	0.5738	NA
15354	Hmgb3	high mobility group box 3	1.04526	0.5959	NA
27393	Mrpl39	mitochondrial ribosomal protein L39	1.04526	0.4937	NA
67872	Nsmce4a	non-SMC element 4 homolog A (S. cerevisiae)	1.04526	0.3486	NA
68592	Syf2	SYF2 homolog, RNA splicing factor (S. cerevisiae)	1.04526	0.6676	0.8294
68675	Fam172a	family with sequence similarity 172, member A	1.04526	0.7834	0.8961
75847	Ispd	isoprenoid synthase domain containing	1.04526	0.6684	0.8296
78943	Ern1	endoplasmic reticulum (ER) to nucleus signalling 1	1.04526	0.7096	0.8542
12616	Cenpb	centromere protein B	1.045151	0.6647	0.8279
20318	Sdf4	stromal cell derived factor 4	1.045151	0.5743	NA
21780	Tfam	transcription factor A, mitochondrial	1.045151	0.6105	0.7924
52563	Cdc23	CDC23 (cell division cycle 23, yeast, homolog)	1.045151	0.6164	0.7965
55960	Ebag9	estrogen receptor-binding fragment-associated gene 9	1.045151	0.5537	NA
65099	Irak1bp1	interleukin-1 receptor-associated kinase 1 binding protein 1	1.045151	0.3347	NA
68736	1110034B05Rik	RIKEN cDNA 1110034B05 gene	1.045151	0.6599	0.8245
77065	Ints7	integrator complex subunit 7	1.045151	0.6231	0.8005
239217	Kctd12	potassium channel tetramerisation domain containing 12	1.045151	0.4499	NA
74326	Hnrnpr	heterogeneous nuclear ribonucleoprotein R	1.045041	0.502	NA
74393	4933403G14Rik	RIKEN cDNA 4933403G14 gene	1.045041	0.4698	NA
319814	E130106K03Rik	RIKEN cDNA E130106K03 gene	1.045041	0.7956	0.9036
329421	Myo3b	myosin IIIB	1.045041	0.6885	0.841
402735	B230114P17Rik	RIKEN cDNA B230114P17 gene	1.045041	0.6928	0.8438
12421	Rb1cc1	RB1-inducible coiled-coil 1	1.044932	0.7166	0.8583
242653	Cldn19	claudin 19	1.044932	0.8581	0.9369
319832	Tmem229a	transmembrane protein 229A	1.044932	0.6552	0.8212
67011	Mettl6	methyltransferase like 6	1.044823	0.4451	NA
74481	Batf2	basic leucine zipper transcription factor, ATF-like 2	1.044823	0.8304	0.9234
235041	Kank2	KN motif and ankyrin repeat domains 2	1.044823	0.4867	NA
235542	Ppp2r3a	protein phosphatase 2, regulatory subunit B'', alpha	1.044823	0.6256	0.8023
330171	Kctd10	potassium channel tetramerisation domain containing 10	1.044823	0.7325	0.8681
18120	Mrpl49	mitochondrial ribosomal protein L49	1.044714	0.4704	NA
68441	Rraga	Ras-related GTP binding A	1.044714	0.4415	NA
70349	Copb1	coatomer protein complex, subunit beta 1	1.044714	0.5	NA
73710	Tubb2b	tubulin, beta 2B	1.044714	0.8419	0.9289
74316	Isca2	iron-sulfur cluster assembly 2 homolog (S. cerevisiae)	1.044714	0.4454	NA
23794	Adamts5	a disintegrin-like and metallopeptidase (reprolysin type) with thrombospondin type 1 motif, 5 (aggrecanase-2)	1.044605	0.6972	0.8467
67773	Myst1	MYST histone acetyltransferase 1	1.044605	0.3318	NA
70026	Tspo2	translocator protein 2	1.044605	0.8116	0.9128

216860	Neurl4	neuralized homolog 4 (Drosophila)	1.044605	0.6519	0.8193
229707	Fam40a	family with sequence similarity 40, member A	1.044605	0.6965	0.8463
627626	3110082D06Rik	RIKEN cDNA 3110082D06 gene	1.044605	0.8528	0.9342
17164	Mapkapk2	MAP kinase-activated protein kinase 2	1.044496	0.6394	0.8111
19848	Rnu2	U2 small nuclear RNA	1.044496	0.8751	0.9453
72096	Mettl10	methyltransferase like 10	1.044496	0.4328	NA
75613	Med25	mediator of RNA polymerase II transcription, subunit 25 homolog (yeast)	1.044496	0.7299	0.8664
215114	Hip1	huntingtin interacting protein 1	1.044496	0.6629	0.8266
240038	Gm4944	predicted gene 4944	1.044496	0.6354	0.8084
66844	Ormdl2	ORM1-like 2 (S. cerevisiae)	1.044386	0.478	NA
71890	Mad2l2	MAD2 mitotic arrest deficient-like 2 (yeast)	1.044386	0.4588	NA
215201	Trmt2b	TRM2 tRNA methyltransferase 2 homolog B (S. cerevisiae)	1.044386	0.5555	NA
226856	Lpgat1	lysophosphatidylglycerol acyltransferase 1	1.044386	0.6471	0.8161
270106	Rpl13	ribosomal protein L13	1.044386	0.5773	NA
100039864	Snhg12	small nucleolar RNA host gene 12	1.044386	0.5391	NA
15451	Hpn	hepsin	1.044277	0.578	NA
56314	Zfp113	zinc finger protein 113	1.044277	0.5969	NA
75565	Ccdc101	coiled-coil domain containing 101	1.044277	0.6134	0.7947
170930	Sumo2	SMT3 suppressor of mif two 3 homolog 2 (yeast)	1.044277	0.4861	NA
328789	Lhfpl5	lipoma HMGIC fusion partner-like 5	1.044277	0.8318	0.9245
628596	Gm6900	predicted gene 6900	1.044277	0.6325	0.8065
18458	Pabpc1	poly(A) binding protein, cytoplasmic 1	1.044168	0.5325	NA
52846	D1Bwg0212e	DNA segment, Chr 1, Brigham & Women's Genetics 0212 expressed	1.044168	0.4227	NA
67305	Gpx7	glutathione peroxidase 7	1.044168	0.7624	0.8851
93757	Immp2l	IMP2 inner mitochondrial membrane peptidase-like (S. cerevisiae)	1.044168	0.6573	0.8223
18029	Nfic	nuclear factor I/C	1.044059	0.7023	0.85
20973	Syngr2	synaptogyrin 2	1.044059	0.573	NA
66074	Tmem167	transmembrane protein 167	1.044059	0.5324	NA
67669	l7Rn6	lethal, Chr 7, Rinchik 6	1.044059	0.5939	NA
68497	1110018G07Rik	RIKEN cDNA 1110018G07 gene	1.044059	0.3744	NA
81535	Sgpp1	sphingosine-1-phosphate phosphatase 1	1.044059	0.4273	NA
269254	Setx	senataxin	1.044059	0.5375	NA
270151	Nlrx1	NLR family member X1	1.044059	0.7095	0.8542
329872	Frem1	Fras1 related extracellular matrix protein 1	1.044059	0.6078	NA
66213	Med7	mediator complex subunit 7	1.04395	0.5719	NA
226751	Cdc42bpa	CDC42 binding protein kinase alpha	1.04395	0.7724	0.8906
80905	Polh	polymerase (DNA directed), eta (RAD 30 related)	1.043841	0.6787	0.8357
108673	Ccdc86	coiled-coil domain containing 86	1.043841	0.5257	NA
238393	Serpina3f	serine (or cysteine) peptidase inhibitor, clade A, member 3F	1.043841	0.8094	0.9116
14239	Foxs1	forkhead box S1	1.043732	0.6149	NA
16351	Ipp	IAP promoted placental gene	1.043732	0.3958	NA
22646	Zfp105	zinc finger protein 105	1.043732	0.4157	NA
73666	Thoc3	THO complex 3	1.043732	0.6215	0.7996
80985	Trim44	tripartite motif-containing 44	1.043732	0.5489	NA
85029	Rpph1	ribonuclease P RNA component H1	1.043732	0.8406	0.9283
243725	Ppp1r9a	protein phosphatase 1, regulatory (inhibitor) subunit 9A	1.043732	0.7904	0.9007
20664	Sox1	SRY-box containing gene 1	1.043623	0.8836	0.9495
51929	D1Ertd704e	DNA segment, Chr 1, ERATO Doi 704, expressed	1.043623	0.8128	0.9136
233870	Tufm	Tu translation elongation factor, mitochondrial	1.043623	0.5157	NA

74666	4930432K21Rik	RIKEN cDNA 4930432K21 gene	1.043515	0.6285	0.8039
229279	Hnrnpa3	heterogeneous nuclear ribonucleoprotein A3	1.043515	0.6348	0.8078
11465	Actg1	actin, gamma, cytoplasmic 1	1.043406	0.6368	0.8091
13404	Dmc1	DMC1 dosage suppressor of mck1 homolog, meiosis-specific homologous recombination (yeast)	1.043406	0.8522	0.9339
22022	Tpst2	protein-tyrosine sulfotransferase 2	1.043406	0.4734	NA
232337	Zfp637	zinc finger protein 637	1.043406	0.4518	NA
554160	BB283564	expressed sequence BB283564	1.043406	0.62	0.7991
12804	Cntfr	ciliary neurotrophic factor receptor	1.043297	0.6567	0.8223
12824	Col2a1	collagen, type II, alpha 1	1.043297	0.6169	NA
16011	Igfbp5	insulin-like growth factor binding protein 5	1.043297	0.574	NA
20088	Rps24	ribosomal protein S24	1.043297	0.644	0.814
21961	Tns1	tensin 1	1.043297	0.5889	NA
66840	Wdr45l	Wdr45 like	1.043297	0.6036	NA
69089	Oxa1l	oxidase assembly 1-like	1.043297	0.4487	NA
78887	Sfi1	Sfi1 homolog, spindle assembly associated (yeast)	1.043297	0.8783	0.9465
26430	Parg	poly (ADP-ribose) glycohydrolase	1.043188	0.3731	NA
69723	Rpain	RPA interacting protein	1.043188	0.3294	NA
632687	Mar-10	membrane-associated ring finger (C3HC4) 10	1.043188	0.8972	0.9557
15220	Foxq1	forkhead box Q1	1.043079	0.7238	0.8624
66254	Dimt1	DIM1 dimethyladenosine transferase 1-like (S. cerevisiae)	1.043079	0.5345	NA
67109	Zfp787	zinc finger protein 787	1.043079	0.4607	NA
225058	Gm4832	predicted gene 4832	1.043079	0.602	NA
56176	Pigp	phosphatidylinositol glycan anchor biosynthesis, class P	1.04297	0.4712	NA
268396	Sh3pxd2b	SH3 and PX domains 2B	1.04297	0.6786	0.8357
12567	Cdk4	cyclin-dependent kinase 4	1.042862	0.564	NA
19671	Rce1	RCE1 homolog, prenyl protein peptidase (S. cerevisiae)	1.042862	0.6326	0.8067
22420	Wnt6	wingless-related MMTV integration site 6	1.042862	0.8137	0.9141
56086	Set	SET nuclear oncogene	1.042862	0.4249	NA
72454	Ccdc71	coiled-coil domain containing 71	1.042862	0.455	NA
105014	Rdh14	retinol dehydrogenase 14 (all-trans and 9-cis)	1.042862	0.5376	NA
268469	Zfp652	zinc finger protein 652	1.042862	0.4092	NA
50723	Icosl	icos ligand	1.042753	0.7081	0.8534
72171	Shq1	SHQ1 homolog (S. cerevisiae)	1.042753	0.5626	NA
619307	9430078G10Rik	RIKEN cDNA 9430078G10 gene	1.042753	0.7242	0.8626
100042554	Gm3902	predicted gene 3902	1.042753	0.6558	0.8216
16795	Large	like-glycosyltransferase	1.042644	0.5519	NA
21923	Tnc	tenascin C	1.042644	0.8207	0.9184
22764	Zfx	zinc finger protein X-linked	1.042644	0.8111	0.9124
57776	Ttyh1	tweety homolog 1 (Drosophila)	1.042644	0.5451	NA
77779	A930007I19Rik	RIKEN cDNA A930007I19 gene	1.042644	0.7845	0.8967
94091	Trim11	tripartite motif-containing 11	1.042644	0.4123	NA
103737	Pex12	peroxisomal biogenesis factor 12	1.042644	0.4061	NA
237943	Gpatch8	G patch domain containing 8	1.042644	0.5342	NA
242408	4930412F15Rik	RIKEN cDNA 4930412F15 gene	1.042644	0.7189	0.8597
11430	Acox1	acyl-Coenzyme A oxidase 1, palmitoyl	1.042535	0.4236	NA
56044	Rala	v-ral simian leukemia viral oncogene homolog A (ras related)	1.042535	0.3767	NA
66874	1200014J11Rik	RIKEN cDNA 1200014J11 gene	1.042535	0.6683	0.8296
67830	Rer1	RER1 retention in endoplasmic reticulum 1 homolog (S. cerevisiae)	1.042535	0.3959	NA
667214	9930111J21Rik1	RIKEN cDNA 9930111J21 gene 1	1.042535	0.7578	0.8829

22393	Wfs1	Wolfram syndrome 1 homolog (human)	1.042427	0.5601	NA
68975	Med27	mediator complex subunit 27	1.042427	0.4422	NA
81630	Zbtb22	zinc finger and BTB domain containing 22	1.042427	0.5164	NA
16157	Il11ra1	interleukin 11 receptor, alpha chain 1	1.042318	0.4895	NA
54152	Dnalc4	dynein, axonemal, light chain 4	1.042318	0.4806	NA
56043	Akr1e1	aldo-keto reductase family 1, member E1	1.042209	0.4942	NA
67150	Rnf141	ring finger protein 141	1.042209	0.3317	NA
102580	Alg9	asparagine-linked glycosylation 9 homolog (yeast, alpha 1,2 mannosyltransferase)	1.042209	0.6103	NA
108143	Taf9	TAF9 RNA polymerase II, TATA box binding protein (TBP)-associated factor	1.042209	0.5338	NA
16848	Lfng	LFNG O-fucosylpeptide 3-beta-N-acetylglucosaminyltransferase	1.042101	0.8059	0.9092
73178	Wasl	Wiskott-Aldrich syndrome-like (human)	1.042101	0.6941	0.8445
208967	Thnsl1	threonine synthase-like 1 (bacterial)	1.042101	0.3689	NA
230890	Gm436	predicted gene 436	1.042101	0.7641	0.8862
12912	Creb1	cAMP responsive element binding protein 1	1.041992	0.7138	0.8564
22282	Usf2	upstream transcription factor 2	1.041992	0.5625	NA
53319	Nxf1	nuclear RNA export factor 1 homolog (S. cerevisiae)	1.041992	0.5025	NA
67838	Dnajb11	DnaJ (Hsp40) homolog, subfamily B, member 11	1.041992	0.3647	NA
12667	Chrd	chordin	1.041884	0.7747	0.8916
17118	Marcks	myristoylated alanine rich protein kinase C substrate	1.041884	0.5766	NA
52666	Arhgef25	Rho guanine nucleotide exchange factor (GEF) 25	1.041884	0.4306	NA
240476	Zfp407	zinc finger protein 407	1.041884	0.523	NA
268729	Gm626	predicted gene 626	1.041884	0.7758	0.8922
14667	Gm2a	GM2 ganglioside activator protein	1.041775	0.5435	NA
21672	Prdx2	peroxiredoxin 2	1.041775	0.4804	NA
27226	Pla2g7	phospholipase A2, group VII (platelet-activating factor acetylhydrolase, plasma)	1.041775	0.7024	0.85
66249	Pno1	partner of NOB1 homolog (S. cerevisiae)	1.041775	0.4141	NA
67846	Tmem39a	transmembrane protein 39a	1.041775	0.5532	NA
73467	1700066M21Rik	RIKEN cDNA 1700066M21 gene	1.041775	0.6075	NA
224088	Atp13a3	ATPase type 13A3	1.041775	0.7871	0.8985
234724	Tat	tyrosine aminotransferase	1.041775	0.645	0.8146
328699	Gabrr3	gamma-aminobutyric acid (GABA) receptor, rho 3	1.041775	0.6513	0.8188
26374	Rfwd2	ring finger and WD repeat domain 2	1.041667	0.4211	NA
69786	Tprkb	Tp53rk binding protein	1.041667	0.4316	NA
108115	Slco4a1	solute carrier organic anion transporter family, member 4a1	1.041667	0.3472	NA
223665	C030006K11Rik	RIKEN cDNA C030006K11 gene	1.041667	0.7002	0.8486
66845	Mrpl33	mitochondrial ribosomal protein L33	1.041558	0.5284	NA
66890	Lman2	lectin, mannose-binding 2	1.041558	0.3624	NA
78406	2900041H08Rik	RIKEN cDNA 2900041H08 gene	1.041558	0.7524	0.8803
67911	Zfp169	zinc finger protein 169	1.04145	0.7945	0.9027
209630	Frmd4a	FERM domain containing 4A	1.04145	0.724	0.8625
211948	Pde12	phosphodiesterase 12	1.04145	0.6544	0.821
212986	Scfd2	Sec1 family domain containing 2	1.04145	0.589	NA
436332	Gm5766	ribosomal protein L7a pseudogene	1.04145	0.3535	NA
18131	Notch3	Notch gene homolog 3 (Drosophila)	1.041341	0.6851	0.8394
69161	Manbal	mannosidase, beta A, lysosomal-like	1.041341	0.5748	NA
70552	Lrrc56	leucine rich repeat containing 56	1.041341	0.6794	0.8359
75665	Ccdc64	coiled-coil domain containing 64	1.041341	0.7722	0.8906
17711	CYTB	cytochrome b	1.041233	0.6791	0.8358
19283	Ptprz1	protein tyrosine phosphatase, receptor type Z, polypeptide 1	1.041233	0.5485	NA

20055	Rps16	ribosomal protein S16	1.041233	0.551	NA
230584	Yipf1	Yip1 domain family, member 1	1.041233	0.553	NA
269587	Epb4.1	erythrocyte protein band 4.1	1.041233	0.8862	0.9506
16515	Kcnj12	potassium inwardly-rectifying channel, subfamily J, member 12	1.041124	0.6819	0.8374
71740	Pvrl4	poliovirus receptor-related 4	1.041124	0.6993	0.848
74375	Gcc1	golgi coiled coil 1	1.041124	0.7216	0.8607
208650	Cblb	Casitas B-lineage lymphoma b	1.041124	0.8392	0.9275
15312	Hmgn1	high mobility group nucleosomal binding domain 1	1.041016	0.7171	0.8585
101869	Unc45a	unc-45 homolog A (C. elegans)	1.041016	0.546	NA
103806	Maml1	mastermind like 1 (Drosophila)	1.041016	0.4633	NA
18181	Nrf1	nuclear respiratory factor 1	1.040908	0.6878	0.8407
244958	Mrap2	melanocortin 2 receptor accessory protein 2	1.040908	0.669	0.83
67050	Nkap	NFKB activating protein	1.040799	0.6197	NA
230582	Cyb5rl	cytochrome b5 reductase-like	1.040799	0.6912	0.8427
258055	Olfr524	olfactory receptor 524	1.040799	0.8878	0.9513
329360	Gm757	predicted gene 757	1.040799	0.8781	0.9465
637053	Vmn2r4	vomeronasal 2, receptor 4	1.040799	0.9273	0.9704
654439	D430004P15Rik	Riken cDNA D430004P15 gene	1.040799	0.7704	0.8896
13642	Efnb2	ephrin B2	1.040691	0.6208	NA
14590	Ggh	gamma-glutamyl hydrolase	1.040691	0.443	NA
16504	Kcnc3	potassium voltage gated channel, Shaw-related subfamily, member 3	1.040691	0.8471	0.9317
67026	Thap4	THAP domain containing 4	1.040691	0.4383	NA
217869	Eif5	eukaryotic translation initiation factor 5	1.040691	0.5877	NA
226861	Hhat	hedgehog acyltransferase	1.040691	0.7056	0.8521
235459	Gtf2a2	general transcription factor II A, 2	1.040691	0.4744	NA
19377	Rai1	retinoic acid induced 1	1.040583	0.533	NA
20382	Srsf2	serine/arginine-rich splicing factor 2	1.040583	0.6977	0.8469
67210	Gatad1	GATA zinc finger domain containing 1	1.040583	0.4448	NA
109272	Mybpc1	myosin binding protein C, slow-type	1.040583	0.833	0.9247
20588	Smarcc1	SWI/SNF related, matrix associated, actin dependent regulator of chromatin, subfamily c, member 1	1.040474	0.791	0.9009
20874	Slk	STE20-like kinase (yeast)	1.040474	0.6906	0.8422
66420	Polr2e	polymerase (RNA) II (DNA directed) polypeptide E	1.040474	0.4679	NA
73728	Psd	pleckstrin and Sec7 domain containing	1.040474	0.686	0.8399
74106	Dcaf6	DDB1 and CUL4 associated factor 6	1.040474	0.5852	NA
75712	Tmem14a	transmembrane protein 14A	1.040474	0.49	NA
76971	2810007J24Rik	RIKEN cDNA 2810007J24 gene	1.040474	0.7775	0.8928
20333	Sec22b	SEC22 vesicle trafficking protein homolog B (S. cerevisiae)	1.040366	0.3266	NA
20480	Clpb	ClpB caseinolytic peptidase B homolog (E. coli)	1.040366	0.7668	0.8874
28075	Pppde2	PPPDE peptidase domain containing 2	1.040366	0.6857	0.8398
192173	Fam195b	family with sequence similarity 195, member B	1.040366	0.5351	NA
209361	Taf3	TAF3 RNA polymerase II, TATA box binding protein (TBP)-associated factor	1.040366	0.708	0.8534
17865	Mybl2	myeloblastosis oncogene-like 2	1.040258	0.576	NA
22129	Ttc3	tetratricopeptide repeat domain 3	1.040258	0.7689	0.889
76871	4930422N03Rik	RIKEN cDNA 4930422N03 gene	1.040258	0.6866	0.8402
110796	Tshz1	teashirt zinc finger family member 1	1.040258	0.6229	NA
227120	Plcl1	phospholipase C-like 1	1.040258	0.7625	0.8852
230908	Tardbp	TAR DNA binding protein	1.040258	0.4057	NA
381760	Ssbp1	single-stranded DNA binding protein 1	1.040258	0.5546	NA
100503637	LOC100503637	envelope glycoprotein-like	1.040258	0.5847	NA

22210	Ube2b	ubiquitin-conjugating enzyme E2B, RAD6 homology (S. cerevisiae)	1.04015	0.5281	NA
26936	Mprip	myosin phosphatase Rho interacting protein	1.04015	0.6211	NA
56009	Refbp2	RNA and export factor binding protein 2	1.04015	0.6994	0.848
67698	Fam174a	family with sequence similarity 174, member A	1.04015	0.5036	NA
70423	Tspan15	tetraspanin 15	1.04015	0.5316	NA
74504	Fam53a	family with sequence similarity 53, member A	1.04015	0.5039	NA
75604	Tm4sf5	transmembrane 4 superfamily member 5	1.04015	0.7496	0.8785
76866	Morn1	MORN repeat containing 1	1.04015	0.7403	0.8731
99683	Sec24b	Sec24 related gene family, member B (S. cerevisiae)	1.04015	0.4425	NA
224807	Tmem63b	transmembrane protein 63b	1.04015	0.7833	0.8961
546837	Cyp2j7-ps	cytochrome P450, family 2, subfamily j, polypeptide 7, pseudogene	1.04015	0.7838	0.8964
73830	Eif3k	eukaryotic translation initiation factor 3, subunit K	1.040042	0.5342	NA
79566	Sh3bp5l	SH3 binding domain protein 5 like	1.040042	0.4296	NA
102866	Pls3	plastin 3 (T-isoform)	1.040042	0.5249	NA
108899	2700081O15Rik	RIKEN cDNA 2700081O15 gene	1.040042	0.7299	0.8664
235323	Usp28	ubiquitin specific peptidase 28	1.040042	0.5869	NA
245841	Polr2h	polymerase (RNA) II (DNA directed) polypeptide H	1.040042	0.633	NA
22153	Tubb4	tubulin, beta 4	1.039933	0.7301	0.8664
22234	Ugcg	UDP-glucose ceramide glucosyltransferase	1.039933	0.5149	NA
407243	Tmem189	transmembrane protein 189	1.039933	0.8386	0.9273
26554	Cul3	cullin 3	1.039825	0.7773	0.8928
73212	3110082I17Rik	RIKEN cDNA 3110082I17 gene	1.039825	0.5668	NA
213541	Ythdf2	YTH domain family 2	1.039825	0.458	NA
634650	Gbp11	guanylate binding protein 11	1.039825	0.8494	0.9329
11964	Atp6v1a	ATPase, H+ transporting, lysosomal V1 subunit A	1.039717	0.5856	NA
12517	Cd72	CD72 antigen	1.039717	0.6928	0.8438
52696	Zwint	ZW10 interactor	1.039717	0.5167	NA
67458	Ergic1	endoplasmic reticulum-golgi intermediate compartment (ERGIC) 1	1.039717	0.6433	NA
69544	Wdr5b	WD repeat domain 5B	1.039717	0.5398	NA
214763	E330016A19Rik	RIKEN cDNA E330016A19 gene	1.039717	0.8053	0.9089
238161	Akap6	A kinase (PRKA) anchor protein 6	1.039717	0.743	0.8748
14172	Fgf18	fibroblast growth factor 18	1.039609	0.6113	NA
21750	Terf2	telomeric repeat binding factor 2	1.039609	0.6789	0.8357
67725	Nudt13	nudix (nucleoside diphosphate linked moiety X)-type motif 13	1.039609	0.5738	NA
71640	Zfp949	zinc finger protein 949	1.039609	0.7609	0.8842
101604	E430018J23Rik	RIKEN cDNA E430018J23 gene	1.039609	0.5183	NA
329506	Ctdspl2	CTD (carboxy-terminal domain, RNA polymerase II, polypeptide A) small phosphatase like 2	1.039609	0.7712	0.8901
382030	Tmem188	transmembrane protein 188	1.039609	0.5809	NA
20054	Rps15	ribosomal protein S15	1.039501	0.5443	NA
20663	Sos2	son of sevenless homolog 2 (Drosophila)	1.039501	0.4611	NA
21408	Zfp354a	zinc finger protein 354A	1.039501	0.5205	NA
21888	Tle4	transducin-like enhancer of split 4, homolog of Drosophila E(spl)	1.039501	0.6714	0.8318
54411	Atp6ap1	ATPase, H+ transporting, lysosomal accessory protein 1	1.039501	0.7417	0.874
57394	Tmem27	transmembrane protein 27	1.039501	0.6381	NA
67763	Prpsap1	phosphoribosyl pyrophosphate synthetase-associated protein 1	1.039501	0.4897	NA
72047	Ddx42	DEAD (Asp-Glu-Ala-Asp) box polypeptide 42	1.039501	0.4043	NA
77799	Sla2	Src-like-adaptor 2	1.039501	0.8246	0.9205
99334	Zscan29	zinc finger SCAN domains 29	1.039501	0.6646	0.8279
233826	Palb2	partner and localizer of BRCA2	1.039501	0.5549	NA

407788	BC051142	cDNA sequence BC051142	1.039501	0.7843	0.8967
13361	Dhfr	dihydrofolate reductase	1.039393	0.6068	NA
26572	Cops3	COP9 (constitutive photomorphogenic) homolog, subunit 3 (Arabidopsis thaliana)	1.039393	0.5495	NA
73102	Slc22a23	solute carrier family 22, member 23	1.039393	0.4911	NA
74343	Crtc2	CREB regulated transcription coactivator 2	1.039393	0.5193	NA
107305	Vps37c	vacuolar protein sorting 37C (yeast)	1.039393	0.7131	0.8559
171166	Mcoln3	mucolipin 3	1.039393	0.903	0.9588
194388	Tet3	tet oncogene family member 3	1.039393	0.8492	0.9328
224897	Dpp9	dipeptidylpeptidase 9	1.039393	0.7667	0.8874
330788	Zfp866	zinc finger protein 866	1.039393	0.6581	0.8229
20187	Ryk	receptor-like tyrosine kinase	1.039285	0.606	NA
22666	Zfp161	zinc finger protein 161	1.039285	0.6409	NA
67903	Gipc1	GIPC PDZ domain containing family, member 1	1.039285	0.7086	0.8537
68427	Slc39a13	solute carrier family 39 (metal ion transporter), member 13	1.039285	0.6393	NA
71702	Cdc5l	cell division cycle 5-like (S. pombe)	1.039285	0.3827	NA
107686	Snrpd2	small nuclear ribonucleoprotein D2	1.039285	0.4936	NA
22427	Wrn	Werner syndrome homolog (human)	1.039177	0.7326	0.8682
66626	5730403B10Rik	RIKEN cDNA 5730403B10 gene	1.039177	0.7563	0.8822
268527	Greb1	gene regulated by estrogen in breast cancer protein	1.039177	0.6397	NA
436230	BC065397	cDNA sequence BC065397	1.039177	0.688	0.8408
100233175	AK010878	cDNA sequence AK010878	1.039177	0.5228	NA
226654	Tstd1	thiosulfate sulfurtransferase (rhodanese)-like domain containing 1	1.039069	0.6245	NA
353237	Pcdhac2	protocadherin alpha subfamily C, 2	1.039069	0.6568	0.8223
100503209	LOC100503209	hypothetical LOC100503209	1.039069	0.6861	0.8399
19017	Ppargc1a	peroxisome proliferative activated receptor, gamma, coactivator 1 alpha	1.038961	0.64	NA
20893	Bhlhe40	basic helix-loop-helix family, member e40	1.038961	0.5493	NA
67711	Nsmce1	non-SMC element 1 homolog (S. cerevisiae)	1.038961	0.5463	NA
72043	Sulf2	sulfatase 2	1.038961	0.5708	NA
230235	6430704M03Rik	RIKEN cDNA 6430704M03 gene	1.038961	0.6181	NA
56233	Hdac7	histone deacetylase 7	1.038853	0.6805	0.8364
223754	Tbc1d22a	TBC1 domain family, member 22a	1.038853	0.4104	NA
19266	Ptprd	protein tyrosine phosphatase, receptor type, D	1.038745	0.5991	NA
22377	Wbp1	WW domain binding protein 1	1.038745	0.5678	NA
22589	Atrx	alpha thalassemia/mental retardation syndrome X-linked homolog (human)	1.038745	0.759	0.8831
66256	Ssr2	signal sequence receptor, beta	1.038745	0.7264	0.8641
71833	Dcaf7	DDB1 and CUL4 associated factor 7	1.038745	0.7498	0.8787
217356	Tmc8	transmembrane channel-like gene family 8	1.038745	0.8101	0.912
319748	Zfp865	zinc finger protein 865	1.038745	0.7651	0.8866
329506	Ctdspl2	CTD (carboxy-terminal domain, RNA polymerase II, polypeptide A) small phosphatase like 2	1.038745	0.7074	0.8532
21887	Tle3	transducin-like enhancer of split 3, homolog of Drosophila E(spl)	1.038637	0.3844	NA
27979	Eif3b	eukaryotic translation initiation factor 3, subunit B	1.038637	0.449	NA
30838	Fbxw4	F-box and WD-40 domain protein 4	1.038637	0.5476	NA
97848	Serpinb6c	serine (or cysteine) peptidase inhibitor, clade B, member 6c	1.038637	0.5844	NA
19387	Rangap1	RAN GTPase activating protein 1	1.038529	0.6275	NA
66333	Aqp11	aquaporin 11	1.038529	0.5582	NA
70396	Asnsd1	asparagine synthetase domain containing 1	1.038529	0.4638	NA
80749	Lrfn1	leucine rich repeat and fibronectin type III domain containing 1	1.038529	0.7072	0.853
109314	Prr9	proline rich 9	1.038529	0.8029	0.9075
214779	Zfp879	zinc figer protein 879	1.038529	0.5438	NA

245386	Fam70a	family with sequence similarity 70, member A	1.038529	0.6515	NA
18045	Nfyb	nuclear transcription factor-Y beta	1.038422	0.6569	NA
23947	Mid2	midline 2	1.038422	0.7517	0.8799
67972	Atp2b1	ATPase, Ca++ transporting, plasma membrane 1	1.038422	0.7019	0.8496
170718	Idh3b	isocitrate dehydrogenase 3 (NAD+) beta	1.038422	0.6559	NA
214254	Nudt15	nudix (nucleoside diphosphate linked moiety X)-type motif 15	1.038422	0.8067	0.9098
100039359	Gm10399	predicted gene 10399	1.038422	0.6447	NA
13637	Efna2	ephrin A2	1.038314	0.5016	NA
26556	Homer1	homer homolog 1 (Drosophila)	1.038314	0.7378	0.8715
66101	Ppih	peptidyl prolyl isomerase H	1.038314	0.6811	0.8368
66495	Ndufb3	NADH dehydrogenase (ubiquinone) 1 beta subcomplex 3	1.038314	0.4971	NA
70315	Hdac8	histone deacetylase 8	1.038314	0.5795	NA
228012	Tlk1	tousled-like kinase 1	1.038314	0.5552	NA
26458	Slc27a2	solute carrier family 27 (fatty acid transporter), member 2	1.038206	0.7368	0.8711
27416	Abcc5	ATP-binding cassette, sub-family C (CFTR/MRP), member 5	1.038206	0.8453	0.9307
11993	Aup1	ancient ubiquitous protein 1	1.03799	0.7053	0.8518
16179	Irak1	interleukin-1 receptor-associated kinase 1	1.03799	0.5654	NA
21787	Tfg	Trk-fused gene	1.03799	0.5607	NA
53332	Mtmr1	myotubularin related protein 1	1.03799	0.4163	NA
54683	Prdx5	peroxiredoxin 5	1.03799	0.4304	NA
228852	Ppp1r16b	protein phosphatase 1, regulatory (inhibitor) subunit 16B	1.03799	0.7608	0.8842
22241	Ulk1	Unc-51 like kinase 1 (C. elegans)	1.037883	0.5315	NA
24051	Sgcb	sarcoglycan, beta (dystrophin-associated glycoprotein)	1.037883	0.5285	NA
100715	Papd4	PAP associated domain containing 4	1.037883	0.6182	NA
29805	Znhit2-ps	zinc finger, HIT domain containing 2, pseudogene	1.037775	0.5407	NA
69890	Zfp219	zinc finger protein 219	1.037775	0.777	0.8927
102545	Cmtm7	CKLF-like MARVEL transmembrane domain containing 7	1.037775	0.8294	0.923
114677	4930590A17Rik	RIKEN cDNA 4930590A17 gene	1.037775	0.7995	0.9057
100503670	Rpl5	ribosomal protein L5	1.037775	0.67	0.8308
14231	Fkbp7	FK506 binding protein 7	1.037667	0.4428	NA
21351	Taldo1	transaldolase 1	1.037667	0.4227	NA
67922	Fam32a	family with sequence similarity 32, member A	1.037667	0.6105	NA
100503359	LOC100503359	hypothetical LOC100503359	1.037667	0.9479	0.9794
14924	Magi1	membrane associated guanylate kinase, WW and PDZ domain containing 1	1.03756	0.6651	NA
73719	Lce1c	late cornified envelope 1C	1.03756	0.8729	0.9449
108735	Sft2d2	SFT2 domain containing 2	1.03756	0.5363	NA
100041694	Gm10451	predicted gene 10451	1.03756	0.7825	0.8955
67287	Parp6	poly (ADP-ribose) polymerase family, member 6	1.037452	0.6207	NA
69311	1700008K24Rik	RIKEN cDNA 1700008K24 gene	1.037452	0.9085	0.9616
110279	Bcr	breakpoint cluster region	1.037452	0.6728	0.8323
19015	Ppard	peroxisome proliferator activator receptor delta	1.037344	0.7218	0.8608
75796	Cdyl2	chromodomain protein, Y chromosome-like 2	1.037344	0.7924	0.9016
216877	Dhx33	DEAH (Asp-Glu-Ala-His) box polypeptide 33	1.037237	0.6275	NA
269233	Fam171a1	family with sequence similarity 171, member A1	1.037237	0.6801	0.8363
67387	Unc50	unc-50 homolog (C. elegans)	1.037129	0.528	NA
69638	Enho	energy homeostasis associated	1.037129	0.5409	NA
76179	Usp31	ubiquitin specific peptidase 31	1.037129	0.4698	NA
268934	Grm4	glutamate receptor, metabotropic 4	1.037129	0.7648	0.8866
382913	Neil2	nei like 2 (E. coli)	1.037129	0.6431	NA

13821	Epb4.1l1	erythrocyte protein band 4.1-like 1	1.037022	0.7561	0.8822
26422	Nbea	neurobeachin	1.037022	0.6824	0.8378
66549	Aggf1	angiogenic factor with G patch and FHA domains 1	1.037022	0.4752	NA
69740	Dph5	DPH5 homolog (S. cerevisiae)	1.037022	0.4704	NA
72117	Naa50	N(alpha)-acetyltransferase 50, NatE catalytic subunit	1.037022	0.6297	NA
102866	Pls3	plastin 3 (T-isoform)	1.037022	0.476	NA
19336	Rab24	RAB24, member RAS oncogene family	1.036914	0.435	NA
55950	Bri3	brain protein I3	1.036914	0.4625	NA
56412	2610024G14Rik	RIKEN cDNA 2610024G14 gene	1.036914	0.5771	NA
56490	Zbtb20	zinc finger and BTB domain containing 20	1.036914	0.5787	NA
68212	Tmbim4	transmembrane BAX inhibitor motif containing 4	1.036914	0.5559	NA
103784	Wdr92	WD repeat domain 92	1.036914	0.6618	NA
228769	Psmf1	proteasome (prosome, macropain) inhibitor subunit 1	1.036914	0.7713	0.8902
12168	Bmpr2	bone morphogenic protein receptor, type II (serine/threonine kinase)	1.036807	0.7668	0.8874
16369	Irs3	insulin receptor substrate 3	1.036807	0.7717	0.8904
19718	Rfc2	replication factor C (activator 1) 2	1.036807	0.6655	NA
22213	Ube2g2	ubiquitin-conjugating enzyme E2G 2	1.036807	0.5862	NA
216443	Mars	methionine-tRNA synthetase	1.036807	0.5675	NA
233490	Crebzf	CREB/ATF bZIP transcription factor	1.036807	0.7368	0.8711
243819	Ppp6r1	protein phosphatase 6, regulatory subunit 1	1.036807	0.7895	0.9003
330502	Zfp82	zinc finger protein 82	1.036807	0.5174	NA
12387	Ctnnb1	catenin (cadherin associated protein), beta 1	1.036699	0.5505	NA
20166	Rtkn	rhotekin	1.036699	0.577	NA
50523	Lats2	large tumor suppressor 2	1.036699	0.615	NA
67980	Gnpda2	glucosamine-6-phosphate deaminase 2	1.036699	0.6055	NA
69957	Cdc16	CDC16 cell division cycle 16 homolog (S. cerevisiae)	1.036699	0.4346	NA
71902	Cand1	cullin associated and neddylation disassociated 1	1.036699	0.3988	NA
216853	Wrap53	WD repeat containing, antisense to TP53	1.036699	0.5101	NA
230594	Zcchc11	zinc finger, CCHC domain containing 11	1.036699	0.7512	0.8797
232156	Slc4a5	solute carrier family 4, sodium bicarbonate cotransporter, member 5	1.036699	0.8321	0.9245
381827	1700073E17Rik	ribosomal protein L7 pseudogene	1.036699	0.4976	NA
619883	Gm6109	predicted gene 6109	1.036699	0.4799	NA
18213	Ntrk3	neurotrophic tyrosine kinase, receptor, type 3	1.036592	0.7194	0.8597
74213	Rbm26	RNA binding motif protein 26	1.036592	0.7397	0.8728
235380	Dmxl2	Dmx-like 2	1.036592	0.7186	0.8594
19286	Pts	6-pyruvoyl-tetrahydropterin synthase	1.036484	0.7319	0.8678
20505	Slc34a1	solute carrier family 34 (sodium phosphate), member 1	1.036484	0.9303	0.9715
67553	Gstcd	glutathione S-transferase, C-terminal domain containing	1.036484	0.6887	0.8411
109880	Braf	Braf transforming gene	1.036484	0.8077	0.9104
226118	AI606181	expressed sequence AI606181	1.036484	0.6494	NA
238330	6430527G18Rik	RIKEN cDNA 6430527G18 gene	1.036484	0.5833	NA
13491	Drd4	dopamine receptor D4	1.036377	0.5618	NA
66360	Bbip1	BBSome interacting protein 1	1.036377	0.674	NA
66500	Slc30a7	solute carrier family 30 (zinc transporter), member 7	1.036377	0.6471	NA
71591	Zfp251	zinc finger protein 251	1.036377	0.5175	NA
80861	Dhx58	DEXH (Asp-Glu-X-His) box polypeptide 58	1.036377	0.6716	NA
101985	AA960436	expressed sequence AA960436	1.036377	0.7719	0.8906
269604	Gpr157	G protein-coupled receptor 157	1.036377	0.8757	0.9457
19173	Psmb5	proteasome (prosome, macropain) subunit, beta type 5	1.036269	0.579	NA

20496	Slc12a2	solute carrier family 12, member 2	1.036269	0.6754	NA
192176	Flna	filamin, alpha	1.036269	0.5907	NA
211187	Lrtm2	leucine-rich repeats and transmembrane domains 2	1.036269	0.7346	0.8697
215789	Phactr2	phosphatase and actin regulator 2	1.036269	0.7876	0.8989
207818	Smagp	small cell adhesion glycoprotein	1.036162	0.7549	0.8816
268395	Mpg	N-methylpurine-DNA glycosylase	1.036162	0.5139	NA
20605	Sstr1	somatostatin receptor 1	1.036055	0.7437	0.8754
21676	Tead1	TEA domain family member 1	1.036055	0.8234	0.92
66487	2010107H07Rik	RIKEN cDNA 2010107H07 gene	1.036055	0.562	NA
80892	Zfhx4	zinc finger homeodomain 4	1.036055	0.6341	NA
432767	Gm5450	predicted gene 5450	1.036055	0.5947	NA
76210	6430597G12Rik	RIKEN cDNA 6430597G12 gene	1.035947	0.4825	NA
114886	Cygb	cytoglobin	1.035947	0.6196	NA
100042583	Gm10268	predicted gene 10268	1.035947	0.569	NA
67996	Srsf6	serine/arginine-rich splicing factor 6	1.03584	0.7086	0.8537
108946	Zzz3	zinc finger, ZZ domain containing 3	1.03584	0.5122	NA
118452	Baalc	brain and acute leukemia, cytoplasmic	1.03584	0.7548	0.8816
100505360	LOC100505360	putative transposase element L1Md-A101/L1Md-A102/L1Md-A2-like	1.03584	0.8545	0.9352
13627	Eef1a1	eukaryotic translation elongation factor 1 alpha 1	1.035733	0.7087	0.8537
74451	Pgs1	phosphatidylglycerophosphate synthase 1	1.035733	0.5795	NA
100929	Tyw1	tRNA-yW synthesizing protein 1 homolog (S. cerevisiae)	1.035733	0.6694	NA
13684	Eif4e	eukaryotic translation initiation factor 4E	1.035626	0.6492	NA
18717	Pip5k1c	phosphatidylinositol-4-phosphate 5-kinase, type 1 gamma	1.035626	0.7984	0.9053
67331	Atp8b3	ATPase, class I, type 8B, member 3	1.035626	0.679	NA
108099	Prkag2	protein kinase, AMP-activated, gamma 2 non-catalytic subunit	1.035626	0.6573	NA
54198	Snx3	sorting nexin 3	1.035518	0.6707	NA
80751	Rnf34	ring finger protein 34	1.035518	0.5318	NA
238328	Vash1	vasohibin 1	1.035518	0.7031	0.8504
667635	Trav7n-5	T cell receptor alpha variable 7N-5	1.035518	0.8244	0.9204
19346	Rab6	RAB6, member RAS oncogene family	1.035411	0.536	NA
66725	Lrrk2	leucine-rich repeat kinase 2	1.035411	0.6115	NA
71623	Krtap5-2	keratin associated protein 5-2	1.035411	0.7086	0.8537
76824	Fam54b	family with sequence similarity 54, member B	1.035411	0.6559	NA
111175	Pecr	peroxisomal trans-2-enoyl-CoA reductase	1.035411	0.7515	0.8799
320916	Wscd2	WSC domain containing 2	1.035411	0.701	0.8492
13496	Arid3a	AT rich interactive domain 3A (BRIGHT-like)	1.035304	0.7452	0.8762
16870	Lhx2	LIM homeobox protein 2	1.035304	0.5276	NA
18082	Nipsnap1	4-nitrophenylphosphatase domain and non-neuronal SNAP25-like protein homolog 1 (C. elegans)	1.035304	0.5633	NA
52504	Cenpo	centromere protein O	1.035304	0.697	0.8466
71538	Fbxo9	f-box protein 9	1.035304	0.5569	NA
117197	Cno	cappuccino	1.035304	0.5863	NA
223666	Arhgap39	Rho GTPase activating protein 39	1.035304	0.5894	NA
100039968	Gm12942	predicted gene 12942	1.035304	0.5344	NA
18141	Nup50	nucleoporin 50	1.035197	0.5534	NA
103266	AI597468	expressed sequence AI597468	1.035197	0.6115	NA
214253	Etnk2	ethanolamine kinase 2	1.035197	0.8602	0.9381
217666	L2hgdh	L-2-hydroxyglutarate dehydrogenase	1.035197	0.6174	NA
259279	Tubgcp3	tubulin, gamma complex associated protein 3	1.035197	0.3734	NA
338348	Ttc16	tetratricopeptide repeat domain 16	1.035197	0.5785	NA

13877	Erh	enhancer of rudimentary homolog (Drosophila)	1.03509	0.4801	NA
20358	Sema6a	sema domain, transmembrane domain (TM), and cytoplasmic domain, (semaphorin) 6A	1.03509	0.644	NA
67225	Rnpc3	RNA-binding region (RNP1, RRM) containing 3	1.03509	0.8579	0.9368
70598	Filip1	filamin A interacting protein 1	1.03509	0.7096	0.8542
225876	Kdm2a	lysine (K)-specific demethylase 2A	1.03509	0.7627	0.8853
241275	Noxa1	NADPH oxidase activator 1	1.03509	0.7127	0.8555
246179	Fktn	fukutin	1.03509	0.8058	0.9092
330817	Dhps	deoxyhypusine synthase	1.03509	0.6699	NA
11958	Atp5k	ATP synthase, H+ transporting, mitochondrial F1F0 complex, subunit e	1.034982	0.5931	NA
27275	Nufip1	nuclear fragile X mental retardation protein interacting protein 1	1.034982	0.6698	NA
67684	Luc7l3	LUC7-like 3 (S. cerevisiae)	1.034982	0.8093	0.9115
77574	Fam115a	family with sequence similarity 115, member A	1.034982	0.6661	NA
215449	Rap1b	RAS related protein 1b	1.034982	0.4177	NA
666938	Bend4	BEN domain containing 4	1.034982	0.7068	0.8528
12443	Ccnd1	cyclin D1	1.034875	0.8141	0.9144
17184	Matr3	matrin 3	1.034875	0.7677	0.8879
67187	Zmynd19	zinc finger, MYND domain containing 19	1.034875	0.7278	0.8652
80752	Fam20c	family with sequence similarity 20, member C	1.034875	0.761	0.8842
228368	Slc35c1	solute carrier family 35, member C1	1.034875	0.5856	NA
234725	Zfp612	zinc finger protein 612	1.034875	0.6421	NA
100041154	Gm3167	predicted gene 3167	1.034875	0.4003	NA
100505098	LOC100505098	60S ribosomal protein L19-like	1.034875	0.5475	NA
11534	Adk	adenosine kinase	1.034768	0.8233	0.92
232811	Suv420h2	suppressor of variegation 4-20 homolog 2 (Drosophila)	1.034768	0.6547	NA
246735	AY074887	cDNA sequence AY074887	1.034768	0.8304	0.9234
100040529	Gm2824	predicted gene 2824	1.034768	0.873	0.9449
18194	Nsdhl	NAD(P) dependent steroid dehydrogenase-like	1.034661	0.7052	0.8518
75782	Lca5	Leber congenital amaurosis 5 (human)	1.034661	0.6301	NA
109575	Tbx10	T-box 10	1.034661	0.8906	0.9525
116748	Lsm10	U7 snRNP-specific Sm-like protein LSM10	1.034661	0.6204	NA
78372	Snrnp25	small nuclear ribonucleoprotein 25 (U11/U12)	1.034554	0.6273	NA
11477	Acvr1	activin A receptor, type 1	1.034447	0.6344	NA
269704	Zfp664	zinc finger protein 664	1.034447	0.5353	NA
330914	Arhgap32	Rho GTPase activating protein 32	1.034447	0.7212	0.8603
100048452	LOC100048452	hypothetical protein LOC100048452	1.034447	0.8498	0.933
13885	Esd	esterase D/formylglutathione hydrolase	1.03434	0.579	NA
67604	Get4	golgi to ER traffic protein 4 homolog (S. cerevisiae)	1.03434	0.5901	NA
74238	Mterfd3	MTERF domain containing 3	1.03434	0.5786	NA
107605	Rdh1	retinol dehydrogenase 1 (all trans)	1.03434	0.8226	0.9196
380732	Gm885	predicted gene 885	1.03434	0.8041	0.9083
494448	Cbx6	chromobox homolog 6	1.03434	0.5778	NA
11774	Ap3b1	adaptor-related protein complex 3, beta 1 subunit	1.034233	0.52	NA
66637	Tsen15	tRNA splicing endonuclease 15 homolog (S. cerevisiae)	1.034233	0.5838	NA
66824	Pycard	PYD and CARD domain containing	1.034233	0.718	0.859
67371	Gtf3c6	general transcription factor IIIC, polypeptide 6, alpha	1.034233	0.5941	NA
106639	Vmac	vimentin-type intermediate filament associated coiled-coil protein	1.034233	0.6314	NA
116871	Mta3	metastasis associated 3	1.034233	0.7432	0.8749
100040591	Kcnj13	potassium inwardly-rectifying channel, subfamily J, member 13	1.034233	0.9109	0.9628
12212	Chic1	cysteine-rich hydrophobic domain 1	1.034126	0.7589	0.8831

224055	Rtp2	receptor transporter protein 2	1.034126	0.9232	0.9688
245688	Rbbp7	retinoblastoma binding protein 7	1.034126	0.4877	NA
17152	Mak	male germ cell-associated kinase	1.034019	0.7042	0.8511
20365	Serf1	small EDRK-rich factor 1	1.034019	0.6572	NA
268860	Abat	4-aminobutyrate aminotransferase	1.034019	0.5277	NA
100045367	LOC100045367	60S ribosomal protein L19-like	1.034019	0.5075	NA
67177	Cdt1	chromatin licensing and DNA replication factor 1	1.033912	0.6965	NA
70617	5730508B09Rik	RIKEN cDNA 5730508B09 gene	1.033912	0.659	NA
72301	1810041L15Rik	RIKEN cDNA 1810041L15 gene	1.033912	0.7657	0.8869
106143	Cggbp1	CGG triplet repeat binding protein 1	1.033912	0.709	0.8541
216805	Flcn	folliculin	1.033912	0.486	NA
242037	Gm410	predicted gene 410	1.033912	0.8752	0.9454
16619	Klk1b27	kallikrein 1-related peptidase b27	1.033805	0.8169	0.9162
29811	Ndrg2	N-myc downstream regulated gene 2	1.033805	0.5416	NA
52250	Reep1	receptor accessory protein 1	1.033805	0.4941	NA
66836	Tmem223	transmembrane protein 223	1.033805	0.5363	NA
68801	Elovl5	ELOVL family member 5, elongation of long chain fatty acids (yeast)	1.033805	0.5383	NA
207667	Skor1	SKI family transcriptional corepressor 1	1.033805	0.7785	0.8934
213491	D4Ertd22e	DNA segment, Chr 4, ERATO Doi 22, expressed	1.033805	0.7666	0.8874
433813	Pusl1	pseudouridylate synthase-like 1	1.033805	0.6002	NA
12581	Cdkn2d	cyclin-dependent kinase inhibitor 2D (p19, inhibits CDK4)	1.033699	0.5591	NA
13046	Celf1	CUGBP, Elav-like family member 1	1.033699	0.774	0.8913
20422	Shfm1	split hand/foot malformation (ectrodactyly) type 1	1.033699	0.5508	NA
212163	8030462N17Rik	RIKEN cDNA 8030462N17 gene	1.033699	0.7886	0.8997
270156	AU019823	expressed sequence AU019823	1.033699	0.6679	NA
280408	Rilp	Rab interacting lysosomal protein	1.033699	0.4655	NA
16880	Lifr	leukemia inhibitory factor receptor	1.033592	0.6875	NA
59001	Pole3	polymerase (DNA directed), epsilon 3 (p17 subunit)	1.033592	0.7527	0.8804
74388	Dpp8	dipeptidylpeptidase 8	1.033592	0.6989	NA
74934	Armc4	armadillo repeat containing 4	1.033592	0.8741	0.9452
224647	D17Wsu92e	DNA segment, Chr 17, Wayne State University 92, expressed	1.033592	0.8087	0.9111
232536	Mrps35	mitochondrial ribosomal protein S35	1.033592	0.6321	NA
14651	Hagh	hydroxyacyl glutathione hydrolase	1.033485	0.6556	NA
67089	Psmc6	proteasome (prosome, macropain) 26S subunit, ATPase, 6	1.033485	0.5372	NA
67111	Naaa	N-acylethanolamine acid amidase	1.033485	0.684	NA
93719	Ear6	eosinophil-associated, ribonuclease A family, member 6	1.033485	0.8635	0.9401
15384	Hnrnpab	heterogeneous nuclear ribonucleoprotein A/B	1.033378	0.5725	NA
19921	Rpl19	ribosomal protein L19	1.033378	0.6324	NA
66940	Shisa5	shisa homolog 5 (Xenopus laevis)	1.033378	0.8042	0.9083
71512	9030419F21Rik	RIKEN cDNA 9030419F21 gene	1.033378	0.6569	NA
74160	1300014J16Rik	RIKEN cDNA 1300014J16 gene	1.033378	0.8382	0.9271
243872	Rpl7a-ps8	ribosomal protein L7A, pseudogene 8	1.033378	0.6537	NA
433273	Gm5523	glyceraldehyde-3-phosphate dehydrogenase pseudogene	1.033378	0.6567	NA
67832	Brix1	BRX1, biogenesis of ribosomes, homolog (S. cerevisiae)	1.033271	0.5581	NA
68028	Rpl22l1	ribosomal protein L22 like 1	1.033271	0.6283	NA
74895	4930455F23Rik	RIKEN cDNA 4930455F23 gene	1.033271	0.4534	NA
231571	Rpap2	RNA polymerase II associated protein 2	1.033271	0.6487	NA
100040766	Heatr7b1	HEAT repeat containing 7B1	1.033271	0.9134	0.9639
26894	Cops7a	COP9 (constitutive photomorphogenic) homolog, subunit 7a (Arabidopsis thaliana)	1.033165	0.5681	NA

57376	Smarce1	SWI/SNF related, matrix associated, actin dependent regulator of chromatin, subfamily e, member 1	1.033165	0.4859	NA
71306	Mfap3l	microfibrillar-associated protein 3-like	1.033165	0.589	NA
227399	Ppip5k2	diphosphoinositol pentakisphosphate kinase 2	1.033165	0.7097	0.8542
233274	Siglech	sialic acid binding Ig-like lectin H	1.033165	0.8019	0.9069
67067	Romo1	reactive oxygen species modulator 1	1.033058	0.6229	NA
69773	1810026J23Rik	RIKEN cDNA 1810026J23 gene	1.033058	0.737	0.8712
72699	Lime1	Lck interacting transmembrane adaptor 1	1.033058	0.6957	NA
241489	Pde11a	phosphodiesterase 11A	1.033058	0.7934	0.9021
11947	Atp5b	ATP synthase, H+ transporting mitochondrial F1 complex, beta subunit	1.032951	0.5857	NA
13819	Epas1	endothelial PAS domain protein 1	1.032951	0.5807	NA
14055	Ezh1	enhancer of zeste homolog 1 (Drosophila)	1.032951	0.6227	NA
18570	Pdcd6	programmed cell death 6	1.032951	0.4631	NA
26417	Mapk3	mitogen-activated protein kinase 3	1.032951	0.7729	0.891
66077	Aurkaip1	aurora kinase A interacting protein 1	1.032951	0.5963	NA
67205	Utp11l	UTP11-like, U3 small nucleolar ribonucleoprotein, (yeast)	1.032951	0.5571	NA
72542	Pgam5	phosphoglycerate mutase family member 5	1.032844	0.5625	NA
330267	Thsd7a	thrombospondin, type I, domain containing 7A	1.032844	0.6149	NA
14411	Slc6a12	solute carrier family 6 (neurotransmitter transporter, betaine/GABA), member 12	1.032738	0.7885	0.8997
20042	Rps12	ribosomal protein S12	1.032738	0.7607	0.8842
67118	Bfar	bifunctional apoptosis regulator	1.032738	0.616	NA
69627	Fam89a	family with sequence similarity 89, member A	1.032738	0.7192	0.8597
74610	Abcb8	ATP-binding cassette, sub-family B (MDR/TAP), member 8	1.032738	0.5578	NA
217698	Acot5	acyl-CoA thioesterase 5	1.032738	0.8954	0.9552
14356	Fxc1	fractured callus expressed transcript 1	1.032631	0.645	NA
19881	Rom1	rod outer segment membrane protein 1	1.032631	0.6029	NA
20704	Serpina1e	serine (or cysteine) peptidase inhibitor, clade A, member 1E	1.032631	0.8753	0.9454
21968	Tom1	target of myb1 homolog (chicken)	1.032631	0.5774	NA
54397	Ppt2	palmitoyl-protein thioesterase 2	1.032631	0.7874	0.8987
63954	Rbp7	retinol binding protein 7, cellular	1.032631	0.6568	NA
68145	Etaa1	Ewing's tumor-associated antigen 1	1.032631	0.7121	0.8553
70532	5730433K22Rik	RIKEN cDNA 5730433K22 gene	1.032631	0.6826	NA
108946	Zzz3	zinc finger, ZZ domain containing 3	1.032631	0.575	NA
109136	Mmaa	methylmalonic aciduria (cobalamin deficiency) type A	1.032631	0.6556	NA
227525	Dclre1c	DNA cross-link repair 1C, PSO2 homolog (S. cerevisiae)	1.032631	0.6702	NA
381668	Fbrsl1	fibrosin-like 1	1.032631	0.7477	0.8775
629499	4922505G16Rik	RIKEN cDNA 4922505G16 gene	1.032631	0.7562	0.8822
11350	Abl1	c-abl oncogene 1, non-receptor tyrosine kinase	1.032525	0.6329	NA
71793	Ints12	integrator complex subunit 12	1.032525	0.6108	NA
75129	4930524J08Rik	RIKEN cDNA 4930524J08 gene	1.032525	0.6002	NA
234374	Ddx49	DEAD (Asp-Glu-Ala-Asp) box polypeptide 49	1.032525	0.542	NA
17975	Ncl	nucleolin	1.032418	0.6616	NA
19982	Rpl36a	ribosomal protein L36A	1.032418	0.51	NA
74013	Rftn2	raftlin family member 2	1.032418	0.6418	NA
17329	Cxcl9	chemokine (C-X-C motif) ligand 9	1.032311	0.8263	0.9211
30878	Apln	apelin	1.032311	0.842	0.9289
434179	Gm5595	predicted gene 5595	1.032311	0.6712	NA
12915	Atf6b	activating transcription factor 6 beta	1.032205	0.7935	0.9021
16772	Lama1	laminin, alpha 1	1.032205	0.6967	NA
22157	Tulp1	tubby like protein 1	1.032205	0.7129	0.8557

24128	Xrn2	5'-3' exoribonuclease 2	1.032205	0.5136	NA
69870	Polr3gl	polymerase (RNA) III (DNA directed) polypeptide G like	1.032205	0.6204	NA
320384	B230334L07Rik	RIKEN cDNA B230334L07 gene	1.032205	0.7174	0.8587
74201	Cep97	centrosomal protein 97	1.032098	0.793	0.9019
232035	Fam190a	family with sequence similarity 190, member A	1.032098	0.6848	NA
328417	Parp4	poly (ADP-ribose) polymerase family, member 4	1.032098	0.57	NA
23950	Dnajb6	DnaJ (Hsp40) homolog, subfamily B, member 6	1.031992	0.8377	0.927
263876	Spata2	spermatogenesis associated 2	1.031992	0.7138	NA
503851	B930025B16Rik	RIKEN cDNA B930025B16 gene	1.031992	0.6611	NA
669149	Vmn2r88	vomeronasal 2, receptor 88	1.031992	0.801	0.9064
15258	Hipk2	homeodomain interacting protein kinase 2	1.031885	0.774	0.8913
18986	Pou2f1	POU domain, class 2, transcription factor 1	1.031885	0.8385	0.9272
66072	Sdhaf2	succinate dehydrogenase complex assembly factor 2	1.031885	0.6625	NA
66552	2010106G01Rik	RIKEN cDNA 2010106G01 gene	1.031885	0.6609	NA
107734	Mrpl30	mitochondrial ribosomal protein L30	1.031885	0.5889	NA
320238	A830054O07Rik	RIKEN cDNA A830054O07 gene	1.031885	0.8245	0.9204
100503304	LOC100503304	STAT3-interacting protein as a repressor-like	1.031885	0.6658	NA
16993	Lta4h	leukotriene A4 hydrolase	1.031779	0.6505	NA
67526	Atg12	autophagy-related 12 (yeast)	1.031779	0.5477	NA
76952	Nt5c2	5'-nucleotidase, cytosolic II	1.031779	0.6589	NA
14923	Guk1	guanylate kinase 1	1.031672	0.5433	NA
18590	Pdgfa	platelet derived growth factor, alpha	1.031672	0.6816	NA
54403	Slc4a4	solute carrier family 4 (anion exchanger), member 4	1.031672	0.7662	0.8872
67785	Zmym4	zinc finger, MYM-type 4	1.031672	0.6497	NA
71517	9030624J02Rik	RIKEN cDNA 9030624J02 gene	1.031672	0.6994	NA
170788	Crb1	crumbs homolog 1 (Drosophila)	1.031672	0.7275	0.8649
227656	Rexo4	REX4, RNA exonuclease 4 homolog (S. cerevisiae)	1.031672	0.5744	NA
432479	4930404N11Rik	RIKEN cDNA 4930404N11 gene	1.031672	0.7104	NA
627049	Zfp800	zinc finger protein 800	1.031672	0.7564	0.8822
50789	Fbxl3	F-box and leucine-rich repeat protein 3	1.031566	0.7003	NA
212090	Tmem60	transmembrane protein 60	1.031566	0.5747	NA
353287	Clec18a	C-type lectin domain family 18, member A	1.031566	0.8538	0.9348
14375	Xrcc6	X-ray repair complementing defective repair in Chinese hamster cells 6	1.03146	0.6557	NA
72569	Bbs5	Bardet-Biedl syndrome 5 (human)	1.03146	0.7268	0.8644
242406	Rgp1	RGP1 retrograde golgi transport homolog (S. cerevisiae)	1.03146	0.7929	0.9018
243905	Zfp568	zinc finger protein 568	1.03146	0.7361	0.8707
11881	Arsb	arylsulfatase B	1.031353	0.8412	0.9287
14056	Ezh2	enhancer of zeste homolog 2 (Drosophila)	1.031353	0.6489	NA
58233	Dnaja4	DnaJ (Hsp40) homolog, subfamily A, member 4	1.031353	0.5298	NA
170930	Sumo2	SMT3 suppressor of mif two 3 homolog 2 (yeast)	1.031353	0.7939	0.9023
232286	Tmf1	TATA element modulatory factor 1	1.031353	0.7472	0.8773
271564	Vps13a	vacuolar protein sorting 13A (yeast)	1.031353	0.539	NA
640382	LOC640382	hypothetical protein LOC640382	1.031353	0.8525	0.934
100504508	LOC100504508	protein SET-like	1.031353	0.6548	NA
11541	Adora2b	adenosine A2b receptor	1.031247	0.7062	NA
56278	Gkap1	G kinase anchoring protein 1	1.031247	0.6015	NA
60315	Myg1	melanocyte proliferating gene 1	1.031247	0.7197	NA
66966	Trit1	tRNA isopentenyltransferase 1	1.031247	0.7291	0.866
68250	Fam96a	family with sequence similarity 96, member A	1.031247	0.6609	NA

71331	5430411C19Rik	RIKEN cDNA 5430411C19 gene	1.031247	0.6385	NA
76899	Golga1	golgi autoantigen, golgin subfamily a, 1	1.031247	0.5539	NA
76942	Lypd5	Ly6/Plaur domain containing 5	1.031247	0.7651	0.8866
107476	Acaca	acetyl-Coenzyme A carboxylase alpha	1.031247	0.5805	NA
20054	Rps15	ribosomal protein S15	1.03114	0.7119	NA
20183	Rxrg	retinoid X receptor gamma	1.03114	0.7708	0.8898
26901	Deb1	differentially expressed in B16F10 1	1.03114	0.595	NA
13653	Egr1	early growth response 1	1.031034	0.9154	0.965
20442	St3gal1	ST3 beta-galactoside alpha-2,3-sialyltransferase 1	1.031034	0.6297	NA
54673	Sh3glb1	SH3-domain GRB2-like B1 (endophilin)	1.031034	0.7563	0.8822
207704	Gtpbp10	GTP-binding protein 10 (putative)	1.031034	0.5754	NA
226646	Ndufs2	NADH dehydrogenase (ubiquinone) Fe-S protein 2	1.031034	0.7075	NA
381598	2610005L07Rik	cadherin 11 pseudogene	1.031034	0.8325	0.9246
654801	Zfp784	zinc finger protein 784	1.031034	0.5089	NA
77809	Lrrc42	leucine rich repeat containing 42	1.030928	0.6371	NA
12648	Chd1	chromodomain helicase DNA binding protein 1	1.030822	0.8626	0.9395
93670	Tac4	tachykinin 4	1.030715	0.728	0.8652
21812	Tgfbr1	transforming growth factor, beta receptor I	1.030609	0.8303	0.9234
52874	D19Bwg1357e	DNA segment, Chr 19, Brigham & Women's Genetics 1357 expressed	1.030609	0.7242	NA
68011	Snrpg	small nuclear ribonucleoprotein polypeptide G	1.030609	0.6078	NA
70351	Ppp4r1	protein phosphatase 4, regulatory subunit 1	1.030609	0.5881	NA
104215	Rhoq	ras homolog gene family, member Q	1.030609	0.6817	NA
105193	Nhlrc1	NHL repeat containing 1	1.030609	0.7787	0.8935
106947	Slc39a3	solute carrier family 39 (zinc transporter), member 3	1.030609	0.5824	NA
225027	Srsf7	serine/arginine-rich splicing factor 7	1.030609	0.6672	NA
16149	Cd74	CD74 antigen (invariant polypeptide of major histocompatibility complex, class II antigen-associated)	1.030503	0.872	0.9446
71769	Bbs10	Bardet-Biedl syndrome 10 (human)	1.030503	0.6073	NA
230125	Mcart1	mitochondrial carrier triple repeat 1	1.030503	0.6709	NA
100039442	Gm2238	predicted gene 2238	1.030503	0.7774	0.8928
20747	Spop	speckle-type POZ protein	1.030397	0.7248	NA
53323	Ube2k	ubiquitin-conjugating enzyme E2K (UBC1 homolog, yeast)	1.030397	0.6952	NA
73158	Larp1	La ribonucleoprotein domain family, member 1	1.030397	0.6489	NA
108155	Ogt	O-linked N-acetylglucosamine (GlcNAc) transferase (UDP-N-acetylglucosamine:polypeptide-N-acetylglucosaminyl transferase)	1.030397	0.878	0.9465
211329	Ncoa7	nuclear receptor coactivator 7	1.030397	0.7801	0.8943
12226	Btg1	B-cell translocation gene 1, anti-proliferative	1.030291	0.5653	NA
57138	Slc12a5	solute carrier family 12, member 5	1.030291	0.7044	NA
67295	Rab3c	RAB3C, member RAS oncogene family	1.030291	0.8986	0.9565
67532	Mfap1a	microfibrillar-associated protein 1A	1.030291	0.5548	NA
218734	3830406C13Rik	RIKEN cDNA 3830406C13 gene	1.030291	0.6855	NA
230075	Ndufb6	NADH dehydrogenase (ubiquinone) 1 beta subcomplex, 6	1.030291	0.5963	NA
236266	Alms1	Alstrom syndrome 1 homolog (human)	1.030291	0.833	0.9247
75731	5133401N09Rik	RIKEN cDNA 5133401N09 gene	1.030184	0.698	NA
77644	C330007P06Rik	RIKEN cDNA C330007P06 gene	1.030184	0.8497	0.9329
77697	Mmab	methylmalonic aciduria (cobalamin deficiency) type B homolog (human)	1.030184	0.7466	0.877
278679	Apol7b	apolipoprotein L 7b	1.030184	0.7059	NA
381695	N4bp2l2	NEDD4 binding protein 2-like 2	1.030184	0.7771	0.8928
629967	Gm11677	predicted gene 11677	1.030184	0.7215	NA
676882	Gm9693	predicted gene 9693	1.030184	0.8749	0.9452
18590	Pdgfa	platelet derived growth factor, alpha	1.030078	0.6518	NA

54125	Polm	polymerase (DNA directed), mu	1.030078	0.7888	0.8998
56458	Foxo1	forkhead box O1	1.030078	0.5277	NA
57321	Terf2ip	telomeric repeat binding factor 2, interacting protein	1.030078	0.6485	NA
66848	Fuca2	fucosidase, alpha-L- 2, plasma	1.030078	0.7717	0.8904
98221	Eif3m	eukaryotic translation initiation factor 3, subunit M	1.030078	0.6018	NA
278507	Wfikkn2	WAP, follistatin/kazal, immunoglobulin, kunitz and netrin domain containing 2	1.030078	0.7494	0.8785
67636	Lyrm5	LYR motif containing 5	1.029972	0.7138	NA
70726	Angptl6	angiopoietin-like 6	1.029972	0.6639	NA
72103	Aplf	aprataxin and PNKP like factor	1.029972	0.6262	NA
72672	Zfp518a	zinc finger protein 518A	1.029972	0.8657	0.9416
225416	Gm4838	predicted gene 4838	1.029972	0.6077	NA
231296	Lrrc66	leucine rich repeat containing 66	1.029972	0.6996	NA
235132	Zbtb44	zinc finger and BTB domain containing 44	1.029972	0.692	NA
15387	Hnrnpk	heterogeneous nuclear ribonucleoprotein K	1.029866	0.7192	NA
18715	Pim2	proviral integration site 2	1.029866	0.7546	0.8815
20855	Stc1	stanniocalcin 1	1.029866	0.8815	0.9481
26441	Psma4	proteasome (prosome, macropain) subunit, alpha type 4	1.029866	0.7232	NA
56191	Tro	trophinin	1.029866	0.8709	0.9441
70012	Ccdc21	coiled-coil domain containing 21	1.029866	0.8197	0.9178
77766	Elp4	elongation protein 4 homolog (S. cerevisiae)	1.029866	0.6933	NA
100041995	Gm3613	predicted gene 3613	1.029866	0.7821	0.8953
12825	Col3a1	collagen, type III, alpha 1	1.02976	0.8511	0.9335
14156	Fen1	flap structure specific endonuclease 1	1.02976	0.5855	NA
17524	Mpp1	membrane protein, palmitoylated	1.02976	0.6816	NA
18412	Sqstm1	sequestosome 1	1.02976	0.8503	0.9332
21769	Zfand3	zinc finger, AN1-type domain 3	1.02976	0.5289	NA
230904	Fbxo2	F-box protein 2	1.02976	0.6893	NA
11750	Anxa7	annexin A7	1.029654	0.7166	NA
18108	Nmt2	N-myristoyltransferase 2	1.029654	0.7835	0.8961
23983	Pcbp1	poly(rC) binding protein 1	1.029654	0.6394	NA
27103	Eif2ak4	eukaryotic translation initiation factor 2 alpha kinase 4	1.029654	0.7023	NA
53614	Reck	reversion-inducing-cysteine-rich protein with kazal motifs	1.029548	0.742	0.8743
69743	Casz1	castor homolog 1, zinc finger (Drosophila)	1.029548	0.8026	0.9073
70396	Asnsd1	asparagine synthetase domain containing 1	1.029548	0.5701	NA
105428	Fam149b	family with sequence similarity 149, member B	1.029548	0.8384	0.9272
224273	Crybg3	beta-gamma crystallin domain containing 3	1.029548	0.7126	NA
18119	Nodal	nodal	1.029442	0.9059	0.9601
67080	1700019D03Rik	RIKEN cDNA 1700019D03 gene	1.029442	0.651	NA
232853	Zfp954	zinc finger protein 954	1.029442	0.7545	0.8815
239364	Tspyl5	testis-specific protein, Y-encoded-like 5	1.029442	0.7981	0.9053
381062	2210404J11Rik	RIKEN cDNA 2210404J11 gene	1.029442	0.6583	NA
66381	Rnf113a2	ring finger protein 113A2	1.029336	0.519	NA
76484	Kndc1	kinase non-catalytic C-lobe domain (KIND) containing 1	1.029336	0.8524	0.934
211712	Pcdh9	protocadherin 9	1.029336	0.8187	0.9174
232430	Crebl2	cAMP responsive element binding protein-like 2	1.029336	0.8132	0.9139
319604	Fam168a	family with sequence similarity 168, member A	1.029336	0.5658	NA
11936	Fxyd2	FXYD domain-containing ion transport regulator 2	1.02923	0.8262	0.9211
13605	Ect2	ect2 oncogene	1.02923	0.6921	NA
19205	Ptbp1	polypyrimidine tract binding protein 1	1.02923	0.5681	NA

20743	Spnb3	spectrin beta 3	1.02923	0.7385	0.872
26390	Mapkbp1	mitogen-activated protein kinase binding protein 1	1.02923	0.7545	0.8815
192191	Med9	mediator of RNA polymerase II transcription, subunit 9 homolog (yeast)	1.02923	0.5915	NA
232790	Oscar	osteoclast associated receptor	1.02923	0.8031	0.9076
272465	Fam70b	family with sequence similarity 70, member B	1.02923	0.5477	NA
368202	Prss48	protease, serine, 48	1.02923	0.75	0.8788
50772	Mapk6	mitogen-activated protein kinase 6	1.029124	0.7995	0.9057
67134	Nop56	NOP56 ribonucleoprotein homolog (yeast)	1.029124	0.7232	NA
381832	Prpmp5	proline-rich protein MP5	1.029124	0.8732	0.9449
14733	Gpc1	glypican 1	1.029018	0.646	NA
16706	Ksr1	kinase suppressor of ras 1	1.029018	0.6998	NA
20641	Snrpd1	small nuclear ribonucleoprotein D1	1.029018	0.6753	NA
28084	Vps25	vacuolar protein sorting 25 (yeast)	1.029018	0.7996	0.9057
54422	Barhl1	BarH-like 1 (Drosophila)	1.029018	0.8728	0.9449
67115	Rpl14	ribosomal protein L14	1.029018	0.7301	NA
76421	1700028K03Rik	RIKEN cDNA 1700028K03 gene	1.029018	0.8873	0.9512
110954	Rpl10	ribosomal protein 10	1.029018	0.7783	0.8934
223691	Eif3l	eukaryotic translation initiation factor 3, subunit L	1.029018	0.6255	NA
628596	Gm6900	predicted gene 6900	1.029018	0.5467	NA
102115	Dohh	deoxyhypusine hydroxylase/monooxygenase	1.028912	0.6216	NA
230696	AU022252	expressed sequence AU022252	1.028912	0.6401	NA
269198	Nbeal1	neurobeachin like 1	1.028912	0.7848	0.8969
320506	Lmbrd2	LMBR1 domain containing 2	1.028912	0.8673	0.9424
13869	Erbb4	v-erb-a erythroblastic leukemia viral oncogene homolog 4 (avian)	1.028807	0.752	0.8802
18024	Nfe2l2	nuclear factor, erythroid derived 2, like 2	1.028807	0.6423	NA
20585	Hltf	helicase-like transcription factor	1.028807	0.5589	NA
101471	Phrf1	PHD and ring finger domains 1	1.028807	0.6894	NA
333050	Ksr2	kinase suppressor of ras 2	1.028807	0.8116	0.9128
14548	Mrps33	mitochondrial ribosomal protein S33	1.028701	0.821	0.9186
18477	Prdx1	peroxiredoxin 1	1.028701	0.6068	NA
19663	Rbpms	RNA binding protein gene with multiple splicing	1.028701	0.6026	NA
53325	Banp	BTG3 associated nuclear protein	1.028701	0.671	NA
67530	Uqcrb	ubiquinol-cytochrome c reductase binding protein	1.028701	0.5496	NA
242584	Wdr78	WD repeat domain 78	1.028701	0.6888	NA
100504289	LOC100504289	hypothetical LOC100504289	1.028701	0.8343	0.9251
97547	C81489	expressed sequence C81489	1.028595	0.8663	0.9418
107734	Mrpl30	mitochondrial ribosomal protein L30	1.028595	0.6244	NA
225326	Pik3c3	phosphoinositide-3-kinase, class 3	1.028595	0.7122	NA
229504	Isg20l2	interferon stimulated exonuclease gene 20-like 2	1.028595	0.8229	0.9198
14824	Grn	granulin	1.028489	0.7342	NA
15382	Hnrnpa1	heterogeneous nuclear ribonucleoprotein A1	1.028489	0.7983	0.9053
29873	Cspg5	chondroitin sulfate proteoglycan 5	1.028489	0.6397	NA
56363	Tmeff2	transmembrane protein with EGF-like and two follistatin-like domains 2	1.028489	0.7597	0.8836
67444	Ilkap	integrin-linked kinase-associated serine/threonine phosphatase 2C	1.028489	0.5953	NA
67728	Dph2	DPH2 homolog (S. cerevisiae)	1.028489	0.6514	NA
71713	Cdc40	cell division cycle 40 homolog (yeast)	1.028489	0.8685	0.9428
72140	Ccdc123	coiled-coil domain containing 123	1.028489	0.7437	NA
76411	1700019E19Rik	RIKEN cDNA 1700019E19 gene	1.028489	0.597	NA
216613	Ccdc85a	coiled-coil domain containing 85A	1.028489	0.7073	NA

233826	Palb2	partner and localizer of BRCA2	1.028489	0.7798	0.894
353282	Sfmbt2	Scm-like with four mbt domains 2	1.028489	0.851	0.9334
434350	9430091E24Rik	RIKEN cDNA 9430091E24 gene	1.028489	0.8589	0.9373
66590	Farsa	phenylalanyl-tRNA synthetase, alpha subunit	1.028383	0.709	NA
69546	Mapk1ip1	mitogen-activated protein kinase 1 interacting protein 1	1.028383	0.6924	NA
224105	Pak2	p21 protein (Cdc42/Rac)-activated kinase 2	1.028383	0.6385	NA
237436	Gas2l3	growth arrest-specific 2 like 3	1.028383	0.7379	NA
239273	Abcc4	ATP-binding cassette, sub-family C (CFTR/MRP), member 4	1.028383	0.7808	0.8948
338370	Nalcn	sodium leak channel, non-selective	1.028383	0.7122	NA
12785	Cnbp	cellular nucleic acid binding protein	1.028278	0.7137	NA
50773	Nt5c	5',3'-nucleotidase, cytosolic	1.028278	0.6143	NA
73242	Atat1	alpha tubulin acetyltransferase 1	1.028278	0.7731	0.891
208177	Phldb2	pleckstrin homology-like domain, family B, member 2	1.028278	0.8379	0.927
229937	Znhit6	zinc finger, HIT type 6	1.028278	0.5793	NA
11911	Atf4	activating transcription factor 4	1.028172	0.6232	NA
13430	Dnm2	dynamin 2	1.028172	0.7964	0.9043
67511	Tmed9	transmembrane emp24 protein transport domain containing 9	1.028172	0.747	NA
223745	Gm4825	predicted pseudogene 4825	1.028172	0.5108	NA
623483	LOC623483	60S ribosomal protein L22-like	1.028172	0.7435	NA
100043911	Ppp4r1l-ps	protein phosphatase 4, regulatory subunit 1-like, pseudogene	1.028172	0.7735	0.8911
14218	Sh3pxd2a	SH3 and PX domains 2A	1.028066	0.8164	0.916
15950	Ifi203	interferon activated gene 203	1.028066	0.8919	0.9536
56749	Dhodh	dihydroorotate dehydrogenase	1.028066	0.6406	NA
67469	Abhd5	abhydrolase domain containing 5	1.028066	0.6414	NA
69576	2310010M20Rik	RIKEN cDNA 2310010M20 gene	1.028066	0.8612	0.9387
74196	Ttc27	tetratricopeptide repeat domain 27	1.028066	0.7681	0.8883
83815	Cenpq	centromere protein Q	1.028066	0.6558	NA
93761	Smarca1	SWI/SNF related, matrix associated, actin dependent regulator of chromatin, subfamily a, member 1	1.028066	0.6373	NA
235626	Setd2	SET domain containing 2	1.028066	0.6425	NA
237436	Gas2l3	growth arrest-specific 2 like 3	1.028066	0.5418	NA
17101	Lyst	lysosomal trafficking regulator	1.027961	0.829	0.9227
22375	Wars	tryptophanyl-tRNA synthetase	1.027961	0.8442	0.9303
54722	Dfna5	deafness, autosomal dominant 5 (human)	1.027961	0.6696	NA
74648	S100pbp	S100P binding protein	1.027961	0.8608	0.9385
84652	Fam126a	family with sequence similarity 126, member A	1.027961	0.6971	NA
98496	Pid1	phosphotyrosine interaction domain containing 1	1.027961	0.7085	NA
218885	Oxnad1	oxidoreductase NAD-binding domain containing 1	1.027961	0.6371	NA
15510	Hspd1	heat shock protein 1 (chaperonin)	1.027855	0.5674	NA
28106	D17Wsu104e	DNA segment, Chr 17, Wayne State University 104, expressed	1.027855	0.7122	NA
67163	Ccdc47	coiled-coil domain containing 47	1.027855	0.7816	0.8951
108071	Grm5	glutamate receptor, metabotropic 5	1.027855	0.8611	0.9386
67399	Pdlim7	PDZ and LIM domain 7	1.027749	0.7774	0.8928
67938	Myl12b	myosin, light chain 12B, regulatory	1.027749	0.6619	NA
74840	Manf	mesencephalic astrocyte-derived neurotrophic factor	1.027749	0.7227	NA
83997	Slmap	sarcolemma associated protein	1.027749	0.689	NA
18035	Nfkbia	nuclear factor of kappa light polypeptide gene enhancer in B-cells inhibitor, alpha	1.027644	0.5206	NA
69392	1700024P12Rik	RIKEN cDNA 1700024P12 gene	1.027644	0.8356	0.926
232164	Paip2b	poly(A) binding protein interacting protein 2B	1.027644	0.642	NA
268729	Gm626	predicted gene 626	1.027644	0.5497	NA

20826	Nhp2l1	NHP2 non-histone chromosome protein 2-like 1 (S. cerevisiae)	1.027538	0.5384	NA
234912	9230110C19Rik	RIKEN cDNA 9230110C19 gene	1.027538	0.9249	0.9694
11890	Asgr2	asialoglycoprotein receptor 2	1.027432	0.8803	0.9478
12033	Bcap29	B-cell receptor-associated protein 29	1.027432	0.7758	0.8922
19116	Prlr	prolactin receptor	1.027432	0.8238	0.9202
28036	Larp7	La ribonucleoprotein domain family, member 7	1.027432	0.6456	NA
53600	Timm23	translocase of inner mitochondrial membrane 23 homolog (yeast)	1.027432	0.7072	NA
56703	Pigo	phosphatidylinositol glycan anchor biosynthesis, class O	1.027432	0.6465	NA
56736	Rnf14	ring finger protein 14	1.027432	0.8337	0.9248
100756	Usp30	ubiquitin specific peptidase 30	1.027432	0.601	NA
170833	Hook2	hook homolog 2 (Drosophila)	1.027432	0.6763	NA
226162	Dpcd	deleted in primary ciliary dyskinesia	1.027432	0.6033	NA
381310	6330403A02Rik	RIKEN cDNA 6330403A02 gene	1.027432	0.9545	0.9821
67025	Rpl11	ribosomal protein L11	1.027327	0.7094	NA
68294	Mfsd10	major facilitator superfamily domain containing 10	1.027327	0.7164	NA
211429	Pla2g4b	phospholipase A2, group IVB (cytosolic)	1.027327	0.761	0.8842
267019	Rps15a	ribosomal protein S15A	1.027327	0.5766	NA
14775	Gpx1	glutathione peroxidase 1	1.027221	0.779	0.8935
231889	Bud31	BUD31 homolog (yeast)	1.027221	0.502	NA
16330	Inpp5b	inositol polyphosphate-5-phosphatase B	1.027116	0.6998	NA
78797	Ndor1	NADPH dependent diflavin oxidoreductase 1	1.027116	0.8211	0.9186
100434	Slc44a1	solute carrier family 44, member 1	1.027116	0.8321	0.9245
319277	A230046K03Rik	RIKEN cDNA A230046K03 gene	1.027116	0.7217	NA
338351	Akap17b	A kinase (PRKA) anchor protein 17B	1.027116	0.8819	0.9483
14339	Aktip	thymoma viral proto-oncogene 1 interacting protein	1.02701	0.8052	0.9089
17449	Mdh1	malate dehydrogenase 1, NAD (soluble)	1.02701	0.7058	NA
19211	Pten	phosphatase and tensin homolog	1.02701	0.8997	0.957
21355	Tap2	transporter 2, ATP-binding cassette, sub-family B (MDR/TAP)	1.02701	0.6937	NA
238023	Hexdc	hexosaminidase (glycosyl hydrolase family 20, catalytic domain) containing	1.02701	0.7769	0.8927
270110	Irf2bp2	interferon regulatory factor 2 binding protein 2	1.02701	0.7985	0.9053
23897	Hax1	HCLS1 associated X-1	1.026905	0.7904	0.9007
24083	Gm16515	predicted gene, Gm16515	1.026905	0.6555	NA
72590	Ppme1	protein phosphatase methylesterase 1	1.026905	0.7835	0.8961
76809	Bri3bp	Bri3 binding protein	1.026905	0.6256	NA
18844	Plxna1	plexin A1	1.026799	0.8695	0.9435
19946	Rpl30	ribosomal protein L30	1.026799	0.5597	NA
66427	Cyb5b	cytochrome b5 type B	1.026799	0.6309	NA
70281	2310068J16Rik	RIKEN cDNA 2310068J16 gene	1.026799	0.7649	0.8866
237940	Aoc2	amine oxidase, copper containing 2 (retina-specific)	1.026799	0.8373	0.927
432768	Gm5451	predicted gene 5451	1.026799	0.8169	0.9162
15957	Ifit1	interferon-induced protein with tetratricopeptide repeats 1	1.026694	0.8775	0.9464
19699	Reln	reelin	1.026694	0.7693	0.8892
67171	Dram2	VDNA-damage regulated autophagy modulator 2	1.026694	0.791	0.9009
67357	1700092C02Rik	RIKEN cDNA 1700092C02 gene	1.026694	0.8847	0.95
67857	Ppp6c	protein phosphatase 6, catalytic subunit	1.026694	0.7819	0.8953
68135	Eif3h	eukaryotic translation initiation factor 3, subunit H	1.026694	0.7178	NA
104401	Pcnxl3	pecanex-like 3 (Drosophila)	1.026694	0.6368	NA
226757	Wdr26	WD repeat domain 26	1.026694	0.6661	NA
233895	Prr14	proline rich 14	1.026694	0.6293	NA

12511	Cd6	CD6 antigen	1.026589	0.8373	0.927
66880	Rsrc1	arginine/serine-rich coiled-coil 1	1.026589	0.7513	NA
67248	Rpl39	ribosomal protein L39	1.026589	0.7173	NA
70369	Bag5	BCL2-associated athanogene 5	1.026589	0.6625	NA
72341	2610002I17Rik	RIKEN cDNA 2610002I17	1.026589	0.7106	NA
76820	Fam49a	family with sequence similarity 49, member A	1.026589	0.6557	NA
93881	Pcdhb10	protocadherin beta 10	1.026589	0.6728	NA
229731	Slc25a24	solute carrier family 25 (mitochondrial carrier, phosphate carrier), member 24	1.026589	0.7305	NA
231413	Grsf1	G-rich RNA sequence binding factor 1	1.026589	0.6922	NA
402724	A330106F07Rik	RIKEN cDNA A330106F07 gene	1.026589	0.8286	0.9225
12764	Cmas	cytidine monophospho-N-acetylneuraminic acid synthetase	1.026483	0.7641	0.8862
21982	Tmem165	transmembrane protein 165	1.026483	0.6655	NA
27207	Rps11	ribosomal protein S11	1.026483	0.6499	NA
67826	Snap47	synaptosomal-associated protein, 47	1.026483	0.755	NA
77631	4930554H23Rik	RIKEN cDNA 4930554H23 gene	1.026483	0.7286	NA
107951	Cdk9	cyclin-dependent kinase 9 (CDC2-related kinase)	1.026483	0.6622	NA
117109	Pop5	processing of precursor 5, ribonuclease P/MRP family (S. cerevisiae)	1.026483	0.6408	NA
26965	Cul1	cullin 1	1.026378	0.7758	0.8922
72505	2610319H10Rik	RIKEN cDNA 2610319H10 gene	1.026378	0.7739	0.8913
76561	Snx7	sorting nexin 7	1.026378	0.8096	0.9116
668096	Gm13698	predicted gene 13698	1.026378	0.7172	NA
12322	Camk2a	calcium/calmodulin-dependent protein kinase II alpha	1.026273	0.7817	0.8951
21855	Timm17b	translocase of inner mitochondrial membrane 17b	1.026273	0.5703	NA
64436	Inpp5e	inositol polyphosphate-5-phosphatase E	1.026273	0.7733	0.891
67287	Parp6	poly (ADP-ribose) polymerase family, member 6	1.026273	0.7121	NA
74477	4933427D14Rik	RIKEN cDNA 4933427D14 gene	1.026273	0.7512	NA
75580	Zbtb4	zinc finger and BTB domain containing 4	1.026273	0.5818	NA
118449	Synpo2	synaptopodin 2	1.026273	0.8055	0.9091
239027	Arhgap22	Rho GTPase activating protein 22	1.026273	0.8222	0.9194
240087	Mdc1	mediator of DNA damage checkpoint 1	1.026273	0.6541	NA
381175	Ccdc68	coiled-coil domain containing 68	1.026273	0.8889	0.9519
14312	Brd2	bromodomain containing 2	1.026167	0.8097	0.9116
15081	H3f3b	H3 histone, family 3B	1.026167	0.7518	NA
69694	Tatdn1	TatD DNase domain containing 1	1.026167	0.7744	0.8916
74164	Nfx1	nuclear transcription factor, X-box binding 1	1.026167	0.6062	NA
76425	2310003C23Rik	RIKEN cDNA 2310003C23 gene	1.026167	0.6049	NA
208908	Ccdc62	coiled-coil domain containing 62	1.026167	0.7339	NA
225348	Wdr36	WD repeat domain 36	1.026167	0.7584	NA
22139	Ttr	transthyretin	1.026062	0.9219	0.9682
74392	Specc1l	sperm antigen with calponin homology and coiled-coil domains 1-like	1.026062	0.8206	0.9184
75212	Rnf121	ring finger protein 121	1.026062	0.7384	NA
107071	Wdr74	WD repeat domain 74	1.026062	0.791	0.9009
218210	Nup153	nucleoporin 153	1.026062	0.6993	NA
677073	Gm16477	ribosomal protein L7a pseudogene	1.026062	0.5648	NA
69487	2310003L22Rik	RIKEN cDNA 2310003L22 gene	1.025957	0.6826	NA
69841	2010002M09Rik	RIKEN cDNA 2010002M09 gene	1.025957	0.7263	NA
70439	Taf15	TAF15 RNA polymerase II, TATA box binding protein (TBP)-associated factor	1.025957	0.6612	NA
72230	Zfp558	zinc finger protein 558	1.025957	0.9225	0.9683
73247	1600027N09Rik	RIKEN cDNA 1600027N09 gene	1.025957	0.7266	NA

78339	Ttyh3	tweety homolog 3 (Drosophila)	1.025957	0.6494	NA
80707	Wwox	WW domain-containing oxidoreductase	1.025957	0.7263	NA
269800	Zfp384	zinc finger protein 384	1.025957	0.7737	0.8912
327780	D430050E20Rik	RIKEN cDNA D430050E20 gene	1.025957	0.8721	0.9446
327885	C030046G05	hypothetical protein C030046G05	1.025957	0.7757	0.8922
78774	4930529M08Rik	RIKEN cDNA 4930529M08 gene	1.025851	0.9094	0.9622
102182	Prmt10	protein arginine methyltransferase 10 (putative)	1.025851	0.6417	NA
12417	Cbx3	chromobox homolog 3 (Drosophila HP1 gamma)	1.025746	0.6154	NA
67673	Tceb2	transcription elongation factor B (SIII), polypeptide 2	1.025746	0.6342	NA
74167	Nudt9	nudix (nucleoside diphosphate linked moiety X)-type motif 9	1.025746	0.7005	NA
19183	Psmc3ip	proteasome (prosome, macropain) 26S subunit, ATPase 3, interacting protein	1.025641	0.7318	NA
57423	Atp5j2	ATP synthase, H+ transporting, mitochondrial F0 complex, subunit F2	1.025641	0.6217	NA
67490	1810074P20Rik	RIKEN cDNA 1810074P20 gene	1.025641	0.6643	NA
74711	Ttll9	tubulin tyrosine ligase-like family, member 9	1.025641	0.8593	0.9375
654788	4732440D04Rik	RIKEN cDNA 4732440D04 gene	1.025641	0.7581	NA
19941	Rpl26	ribosomal protein L26	1.025536	0.6999	NA
75422	Mettl5	methyltransferase like 5	1.025536	0.7017	NA
116940	Tgs1	trimethylguanosine synthase homolog (S. cerevisiae)	1.025536	0.7858	0.8977
57751	Rnf25	ring finger protein 25	1.025431	0.7824	0.8955
70417	Megf10	multiple EGF-like-domains 10	1.025431	0.6685	NA
110886	Gabra5	gamma-aminobutyric acid (GABA) A receptor, subunit alpha 5	1.025431	0.8016	0.9069
18432	Mybbp1a	MYB binding protein (P160) 1a	1.025326	0.6907	NA
20729	Spin1	spindlin 1	1.025326	0.8335	0.9248
69109	Fam58b	family with sequence similarity 58, member B	1.025326	0.545	NA
69754	Fbxo7	F-box protein 7	1.025326	0.6738	NA
20637	Snrnp70	small nuclear ribonucleoprotein 70 (U1)	1.02522	0.8675	0.9425
68051	Nutf2	nuclear transport factor 2	1.02522	0.7154	NA
68059	Tm9sf2	transmembrane 9 superfamily member 2	1.02522	0.7026	NA
68738	Acss1	acyl-CoA synthetase short-chain family member 1	1.02522	0.893	0.9541
78473	Skap1	src family associated phosphoprotein 1	1.02522	0.8801	0.9478
235415	Cplx3	complexin 3	1.02522	0.8636	0.9402
100043902	Six3os1	Six3 opposite strand transcript 1	1.02522	0.772	NA
66309	Tmem128	transmembrane protein 128	1.025115	0.703	NA
66827	Ttc1	tetratricopeptide repeat domain 1	1.025115	0.6051	NA
226412	R3hdm1	R3H domain 1 (binds single-stranded nucleic acids)	1.025115	0.7355	NA
320858	L3mbtl4	l(3)mbt-like 4 (Drosophila)	1.025115	0.7268	NA
66720	Klhl10	kelch-like 10 (Drosophila)	1.02501	0.8592	0.9375
72190	2510009E07Rik	RIKEN cDNA 2510009E07 gene	1.02501	0.758	NA
74569	Ttc17	tetratricopeptide repeat domain 17	1.02501	0.8783	0.9465
435376	Atp6ap1l	ATPase, H+ transporting, lysosomal accessory protein 1-like	1.02501	0.7923	0.9016
17703	Msx3	homeobox, msh-like 3	1.024905	0.8097	0.9116
67223	Rrp15	ribosomal RNA processing 15 homolog (S. cerevisiae)	1.024905	0.7649	NA
74407	Ttc25	tetratricopeptide repeat domain 25	1.024905	0.8356	0.926
12385	Ctnna1	catenin (cadherin associated protein), alpha 1	1.0248	0.5638	NA
16450	Jag2	jagged 2	1.0248	0.7876	0.8989
17761	Mtap7	microtubule-associated protein 7	1.0248	0.8497	0.9329
26417	Mapk3	mitogen-activated protein kinase 3	1.0248	0.7438	NA
27050	Rps3	ribosomal protein S3	1.0248	0.7553	NA
104009	Qsox1	quiescin Q6 sulfhydryl oxidase 1	1.0248	0.7564	NA

109731	Maob	monoamine oxidase B	1.0248	0.8401	0.928
219140	Spata13	spermatogenesis associated 13	1.0248	0.7952	0.9034
17242	Mdk	midkine	1.024695	0.779	0.8935
72075	Ogfr	opioid growth factor receptor	1.024695	0.7368	NA
72108	Ddhd2	DDHD domain containing 2	1.024695	0.7314	NA
237761	Ankrd43	ankyrin repeat domain 43	1.024695	0.7998	0.9059
238384	Slc24a4	solute carrier family 24 (sodium/potassium/calcium exchanger), member 4	1.024695	0.8387	0.9273
258028	Olfr901	olfactory receptor 901	1.024695	0.9624	0.9846
20652	Soat1	sterol O-acyltransferase 1	1.02459	0.8576	0.9368
53959	AA914427	EST AA914427	1.02459	0.808	0.9107
66358	2310004I24Rik	RIKEN cDNA 2310004I24 gene	1.02459	0.6551	NA
67588	Rnf41	ring finger protein 41	1.02459	0.7458	NA
74613	4833412C15Rik	RIKEN cDNA 4833412C15 gene	1.02459	0.8276	0.9222
628110	LOC628110	similar to gonadotropin inducible ovarian transcription factor 1	1.02459	0.8028	0.9075
18106	Cd244	CD244 natural killer cell receptor 2B4	1.024485	0.9291	0.9711
20815	Srpk1	serine/arginine-rich protein specific kinase 1	1.024485	0.6552	NA
20447	St6galnac3	ST6 (alpha-N-acetyl-neuraminyl-2,3-beta-galactosyl-1,3)-N-acetylgalactosaminide alpha-2,6-sialyltransferase 3	1.02438	0.8984	0.9565
26451	Rpl27a	ribosomal protein L27A	1.02438	0.5772	NA
65020	Zfp110	zinc finger protein 110	1.02438	0.8206	0.9184
66590	Farsa	phenylalanyl-tRNA synthetase, alpha subunit	1.02438	0.6039	NA
105446	Gmpr2	guanosine monophosphate reductase 2	1.02438	0.7433	NA
231070	Insig1	insulin induced gene 1	1.02438	0.7744	NA
100041953	Gm10094	predicted gene 10094	1.02438	0.6137	NA
23894	Gtf2h2	general transcription factor II H, polypeptide 2	1.024275	0.7721	NA
54426	Hgfac	hepatocyte growth factor activator	1.024275	0.9159	0.965
58800	Trpm7	transient receptor potential cation channel, subfamily M, member 7	1.024275	0.732	NA
241062	Pgap1	post-GPI attachment to proteins 1	1.024275	0.7758	NA
624866	Lekr1	leucine, glutamate and lysine rich 1	1.024275	0.8128	0.9136
625175	1700028E10Rik	RIKEN cDNA 1700028E10 gene	1.024275	0.8676	0.9425
52014	Nus1	nuclear undecaprenyl pyrophosphate synthase 1 homolog (S. cerevisiae)	1.02417	0.769	NA
69077	Psmd11	proteasome (prosome, macropain) 26S subunit, non-ATPase, 11	1.02417	0.7339	NA
72083	Mzt2	mitotic spindle organizing protein 2	1.02417	0.7773	NA
93762	Smarca5	SWI/SNF related, matrix associated, actin dependent regulator of chromatin, subfamily a, member 5	1.02417	0.6057	NA
228366	Gyltl1b	glycosyltransferase-like 1B	1.02417	0.9181	0.9663
12978	Csf1r	colony stimulating factor 1 receptor	1.024066	0.845	0.9307
20529	Slc31a1	solute carrier family 31, member 1	1.024066	0.6771	NA
22619	Siae	sialic acid acetylesterase	1.024066	0.8044	0.9084
228491	Zfp770	zinc finger protein 770	1.024066	0.682	NA
320862	A730054J21Rik	RIKEN cDNA A730054J21 gene	1.024066	0.9119	0.9635
11811	Apobec2	apolipoprotein B mRNA editing enzyme, catalytic polypeptide 2	1.023961	0.8794	0.9472
104836	Cbll1	Casitas B-lineage lymphoma-like 1	1.023961	0.7971	0.9047
228993	Slc17a9	solute carrier family 17, member 9	1.023961	0.7532	NA
654493	8030494B02Rik	Riken cDNA 8030494B02 gene	1.023961	0.8652	0.9412
13178	Dck	deoxycytidine kinase	1.023856	0.8816	0.9481
19271	Ptprj	protein tyrosine phosphatase, receptor type, J	1.023856	0.8719	0.9446
26875	Pclo	piccolo (presynaptic cytomatrix protein)	1.023856	0.7388	NA
57752	Tacc2	transforming, acidic coiled-coil containing protein 2	1.023856	0.6249	NA
66052	Sdhc	succinate dehydrogenase complex, subunit C, integral membrane protein	1.023856	0.7123	NA
74290	1700102J08Rik	RIKEN cDNA 1700102J08 gene	1.023856	0.8473	0.9317

235439	Herc1	hect (homologous to the E6-AP (UBE3A) carboxyl terminus) domain and RCC1 (CHC1)-like domain (RLD) 1	1.023856	0.6615	NA
19070	Mobkl3	MOB1, Mps One Binder kinase activator-like 3 (yeast)	1.023751	0.789	0.8999
58911	Sumf1	sulfatase modifying factor 1	1.023646	0.7149	NA
70478	Mipep	mitochondrial intermediate peptidase	1.023646	0.7695	NA
71116	Stx18	syntaxin 18	1.023646	0.7069	NA
243833	Zfp128	zinc finger protein 128	1.023646	0.7092	NA
14227	Fkbp2	FK506 binding protein 2	1.023541	0.639	NA
18607	Pdpk1	3-phosphoinositide dependent protein kinase 1	1.023541	0.7457	NA
30938	Fgd3	FYVE, RhoGEF and PH domain containing 3	1.023541	0.8136	0.9141
57330	Gigyf1	GRB10 interacting GYF protein 1	1.023541	0.8226	0.9196
70497	Arhgap17	Rho GTPase activating protein 17	1.023541	0.8913	0.9531
71769	Bbs10	Bardet-Biedl syndrome 10 (human)	1.023541	0.7366	NA
218793	Ube2e2	ubiquitin-conjugating enzyme E2E 2 (UBC4/5 homolog, yeast)	1.023541	0.7267	NA
67542	Cog6	component of oligomeric golgi complex 6	1.023437	0.718	NA
70003	1700028I16Rik	RIKEN cDNA 1700028I16 gene	1.023437	0.8784	0.9466
71520	Grap	GRB2-related adaptor protein	1.023437	0.882	0.9483
628847	Gm6921	predicted pseudogene 6921	1.023437	0.7922	0.9016
13353	Dgcr6	DiGeorge syndrome critical region gene 6	1.023332	0.7601	NA
16201	Ilf3	interleukin enhancer binding factor 3	1.023332	0.6684	NA
68523	Fam96b	family with sequence similarity 96, member B	1.023332	0.6368	NA
72479	Hsdl2	hydroxysteroid dehydrogenase like 2	1.023332	0.6544	NA
100929	Tyw1	tRNA-yW synthesizing protein 1 homolog (S. cerevisiae)	1.023332	0.7928	0.9017
12558	Cdh2	cadherin 2	1.023227	0.8769	0.9462
56376	Pdlim5	PDZ and LIM domain 5	1.023227	0.6892	NA
217265	Abca5	ATP-binding cassette, sub-family A (ABC1), member 5	1.023227	0.8809	0.9478
66448	Mrpl20	mitochondrial ribosomal protein L20	1.023123	0.715	NA
69672	Txndc15	thioredoxin domain containing 15	1.023123	0.6234	NA
241118	Accn4	amiloride-sensitive cation channel 4, pituitary	1.023123	0.7519	NA
381305	Rc3h1	RING CCCH (C3H) domains 1	1.023123	0.7249	NA
11694	Alx3	aristaless-like homeobox 3	1.023018	0.9279	0.9705
22408	Wnt1	wingless-related MMTV integration site 1	1.023018	0.8707	0.9441
75801	4930447C04Rik	RIKEN cDNA 4930447C04 gene	1.023018	0.7871	NA
192173	Fam195b	family with sequence similarity 195, member B	1.023018	0.811	0.9124
215387	Ncaph	non-SMC condensin I complex, subunit H	1.023018	0.7782	NA
380714	Rph3al	rabphilin 3A-like (without C2 domains)	1.023018	0.9462	0.9787
432582	E130309D14Rik	RIKEN cDNA E130309D14 gene	1.023018	0.8555	0.9356
100040563	Dynlt1c	dynein light chain Tctex-type 1C	1.023018	0.766	NA
29864	Rnf11	ring finger protein 11	1.022913	0.7888	NA
217707	Coq6	coenzyme Q6 homolog (yeast)	1.022913	0.6798	NA
50791	Magi2	membrane associated guanylate kinase, WW and PDZ domain containing 2	1.022809	0.7442	NA
56351	Ptges3	prostaglandin E synthase 3 (cytosolic)	1.022809	0.6432	NA
74646	Spsb1	splA/ryanodine receptor domain and SOCS box containing 1	1.022809	0.8873	0.9512
75044	4930506A18Rik	RIKEN cDNA 4930506A18 gene	1.022809	0.8848	0.95
110208	Pgd	phosphogluconate dehydrogenase	1.022809	0.6611	NA
245867	Pcmtd2	protein-L-isoaspartate (D-aspartate) O-methyltransferase domain containing 2	1.022809	0.8056	0.9091
319880	Tmcc3	transmembrane and coiled coil domains 3	1.022809	0.7468	NA
100042371	Gm3807	predicted gene 3807	1.022809	0.8809	0.9478
22228	Ucp2	uncoupling protein 2 (mitochondrial, proton carrier)	1.022704	0.8623	0.9393
66398	Commd5	COMM domain containing 5	1.022704	0.6141	NA

66830	Nacc1	nucleus accumbens associated 1, BEN and BTB (POZ) domain containing	1.022704	0.7777	NA
68642	Tmem216	transmembrane protein 216	1.022704	0.6201	NA
241118	Accn4	amiloride-sensitive cation channel 4, pituitary	1.022704	0.7683	NA
407785	Ndufs6	NADH dehydrogenase (ubiquinone) Fe-S protein 6	1.022704	0.8278	0.9222
17192	Mbd3	methyl-CpG binding domain protein 3	1.022599	0.7074	NA
56321	Aatf	apoptosis antagonizing transcription factor	1.022599	0.799	0.9055
59057	Zfp191	zinc finger protein 191	1.022599	0.8016	0.9069
210973	Kbtbd2	kelch repeat and BTB (POZ) domain containing 2	1.022599	0.6843	NA
23986	Peci	peroxisomal delta3, delta2-enoyl-Coenzyme A isomerase	1.022495	0.7511	NA
66647	Ndnl2	necdin-like 2	1.022495	0.7342	NA
654472	Gm12070	glyceraldehyde-3-phosphate dehydrogenase pseudogene	1.022495	0.8307	0.9235
13819	Epas1	endothelial PAS domain protein 1	1.02239	0.6814	NA
27367	Rpl3	ribosomal protein L3	1.02239	0.7986	0.9053
67939	Prorsd1	prolyl-tRNA synthetase domain containing 1	1.02239	0.8009	0.9064
68050	Akirin1	akirin 1	1.02239	0.7371	NA
68349	Ndufs3	NADH dehydrogenase (ubiquinone) Fe-S protein 3	1.02239	0.6254	NA
100215	Gm11266	predicted gene 11266	1.02239	0.9741	0.9895
226519	Lamc1	laminin, gamma 1	1.02239	0.7998	0.9059
319215	4932413F04Rik	RIKEN cDNA 4932413F04 gene	1.02239	0.9017	0.958
100040632	AA684185	expressed sequence AA684185	1.02239	0.7833	NA
14055	Ezh1	enhancer of zeste homolog 1 (Drosophila)	1.022286	0.897	0.9557
18146	Npdc1	neural proliferation, differentiation and control gene 1	1.022286	0.6334	NA
67883	Uxs1	UDP-glucuronate decarboxylase 1	1.022286	0.7857	NA
75430	3200002M19Rik	RIKEN cDNA 3200002M19 gene	1.022286	0.7584	NA
80281	Cttnbp2nl	CTTNBP2 N-terminal like	1.022286	0.6704	NA
108043	Chrnb3	cholinergic receptor, nicotinic, beta polypeptide 3	1.022286	0.76	NA
72278	Ccpg1	cell cycle progression 1	1.022181	0.7973	NA
319195	Rpl17	ribosomal protein L17	1.022181	0.7171	NA
320860	B130021B11Rik	RIKEN cDNA B130021B11 gene	1.022181	0.8129	0.9136
382139	Gm1715	predicted gene 1715	1.022181	0.9054	0.9598
384179	Gm5292	predicted gene 5292	1.022181	0.6226	NA
19664	Rbpj	recombination signal binding protein for immunoglobulin kappa J region	1.022077	0.7818	NA
72901	2900011F02Rik	RIKEN cDNA 2900011F02 gene	1.022077	0.8338	0.9249
213988	Tnrc6b	trinucleotide repeat containing 6b	1.022077	0.7345	NA
269941	Chsy1	chondroitin sulfate synthase 1	1.022077	0.6592	NA
100038734	Gm10845	predicted gene 10845	1.022077	0.8492	0.9328
231279	Guf1	GUF1 GTPase homolog (S. cerevisiae)	1.021972	0.8705	0.9441
170759	Atp13a1	ATPase type 13A1	1.021868	0.7844	NA
237758	Zfp454	zinc finger protein 454	1.021868	0.8503	0.9332
11552	Adra2b	adrenergic receptor, alpha 2b	1.021764	0.9116	0.9633
11785	Apbb1	amyloid beta (A4) precursor protein-binding, family B, member 1	1.021764	0.8926	0.9539
69318	1700007K09Rik	RIKEN cDNA 1700007K09 gene	1.021764	0.8043	0.9083
74136	Sec14l1	SEC14-like 1 (S. cerevisiae)	1.021764	0.8629	0.9397
223337	Ugt3a2	UDP glycosyltransferases 3 family, polypeptide A2	1.021764	0.9429	0.9768
547008	Gm6010	predicted gene 6010	1.021764	0.7595	NA
56459	Sae1	SUMO1 activating enzyme subunit 1	1.021659	0.8526	0.934
67703	Kirrel3	kin of IRRE like 3 (Drosophila)	1.021659	0.8421	0.929
76954	St5	suppression of tumorigenicity 5	1.021659	0.7736	NA
214345	Lrrc1	leucine rich repeat containing 1	1.021659	0.835	0.9256

231128	Fam193a	family with sequence similarity 193, member A	1.021659	0.6263	NA
237433	Gm4925	predicted gene 4925	1.021659	0.8813	0.9481
27416	Abcc5	ATP-binding cassette, sub-family C (CFTR/MRP), member 5	1.021555	0.7271	NA
74116	Pi16	peptidase inhibitor 16	1.021555	0.8491	0.9328
211253	Mtrf1	mitochondrial translational release factor 1	1.021555	0.774	NA
268857	Nlrc3	NLR family, CARD domain containing 3	1.021555	0.8841	0.9497
16452	Jak2	Janus kinase 2	1.02145	0.7942	NA
67109	Zfp787	zinc finger protein 787	1.02145	0.8621	0.9392
229644	Trim45	tripartite motif-containing 45	1.02145	0.8342	0.9251
230801	Pigv	phosphatidylinositol glycan anchor biosynthesis, class V	1.02145	0.7363	NA
17986	Ndp	Norrie disease (pseudoglioma) (human)	1.021346	0.7796	NA
66432	Slc7a6os	solute carrier family 7, member 6 opposite strand	1.021346	0.7083	NA
69391	1700018A14Rik	RIKEN cDNA 1700018A14 gene	1.021346	0.8715	0.9443
140489	Bhlhe23	basic helix-loop-helix family, member e23	1.021346	0.8768	0.9462
229055	Zbtb10	zinc finger and BTB domain containing 10	1.021346	0.8058	NA
229279	Hnrnpa3	heterogeneous nuclear ribonucleoprotein A3	1.021346	0.7288	NA
20646	Snrpn	small nuclear ribonucleoprotein N	1.021242	0.809	0.9113
11603	Agrn	agrin	1.021138	0.9205	0.9676
16010	Igfbp4	insulin-like growth factor binding protein 4	1.021138	0.8329	0.9247
78757	Rictor	RPTOR independent companion of MTOR, complex 2	1.021138	0.7199	NA
105782	Scrib	scribbled homolog (Drosophila)	1.021138	0.6737	NA
666676	Gm8230	predicted gene 8230	1.021138	0.8343	0.9251
16558	Kif16b	kinesin family member 16B	1.021033	0.8003	NA
19893	Rpgr	retinitis pigmentosa GTPase regulator	1.021033	0.8367	0.9268
21872	Tjp1	tight junction protein 1	1.021033	0.8261	0.9211
22183	Zrsr1	zinc finger (CCCH type), RNA binding motif and serine/arginine rich 1	1.021033	0.8082	NA
68350	Mul1	mitochondrial ubiquitin ligase activator of NFKB 1	1.021033	0.7559	NA
72899	Macrod2	MACRO domain containing 2	1.021033	0.7806	NA
229791	D3Bwg0562e	DNA segment, Chr 3, Brigham & Women's Genetics 0562 expressed	1.021033	0.8338	0.9248
406217	Bex4	brain expressed gene 4	1.021033	0.74	NA
629557	Gm6981	glyceraldehyde-3-phosphate dehydrogenase pseudogene	1.021033	0.8179	0.9169
12048	Bcl2l1	BCL2-like 1	1.020929	0.8253	0.9208
12864	Cox6c	cytochrome c oxidase, subunit VIc	1.020929	0.7227	NA
74741	5730419I09Rik	RIKEN cDNA 5730419I09 gene	1.020929	0.8183	0.9173
214669	L3mbtl2	l(3)mbt-like 2 (Drosophila)	1.020929	0.8516	0.9337
57773	Wdr4	WD repeat domain 4	1.020825	0.6288	NA
69654	Dctn2	dynactin 2	1.020825	0.7878	NA
104348	Zfp120	zinc finger protein 120	1.020825	0.8778	0.9464
239336	Rxfp3	relaxin family peptide receptor 3	1.020825	0.8621	0.9392
100039133	Gm2058	predicted gene 2058	1.020825	0.8289	0.9226
19063	Ppt1	palmitoyl-protein thioesterase 1	1.020721	0.7229	NA
19347	Dennd5a	DENN/MADD domain containing 5A	1.020721	0.7614	NA
67267	2900010M23Rik	RIKEN cDNA 2900010M23 gene	1.020721	0.7696	NA
83925	Trps1	trichorhinophalangeal syndrome I (human)	1.020721	0.8177	0.9168
22764	Zfx	zinc finger protein X-linked	1.020616	0.8601	0.938
57776	Ttyh1	tweety homolog 1 (Drosophila)	1.020616	0.7489	NA
67131	Acbd4	acyl-Coenzyme A binding domain containing 4	1.020616	0.7601	NA
109342	Slc5a10	solute carrier family 5 (sodium/glucose cotransporter), member 10	1.020616	0.9411	0.9762
226409	Zranb3	zinc finger, RAN-binding domain containing 3	1.020616	0.8284	0.9224

20393	Sgk1	serum/glucocorticoid regulated kinase 1	1.020512	0.7682	NA
69736	Nup37	nucleoporin 37	1.020512	0.772	NA
69790	Med30	mediator complex subunit 30	1.020512	0.6873	NA
142682	Zcchc14	zinc finger, CCHC domain containing 14	1.020512	0.8716	0.9444
241303	Fam78a	family with sequence similarity 78, member A	1.020512	0.9009	0.9577
474156	Zbtb9	zinc finger and BTB domain containing 9	1.020512	0.8726	0.9448
17532	Mras	muscle and microspikes RAS	1.020408	0.8395	0.9277
19982	Rpl36a	ribosomal protein L36A	1.020408	0.7476	NA
20473	Six3	sine oculis-related homeobox 3 homolog (Drosophila)	1.020408	0.8899	0.9522
27360	Add3	adducin 3 (gamma)	1.020408	0.6964	NA
66656	Eef1d	eukaryotic translation elongation factor 1 delta (guanine nucleotide exchange protein)	1.020408	0.6064	NA
68401	G6pc3	glucose 6 phosphatase, catalytic, 3	1.020408	0.8073	NA
68734	Smek1	SMEK homolog 1, suppressor of mek1 (Dictyostelium)	1.020408	0.7652	NA
70823	Hmgxb4	HMG box domain containing 4	1.020408	0.6866	NA
94067	Mrpl43	mitochondrial ribosomal protein L43	1.020408	0.6631	NA
98193	Dcaf8	DDB1 and CUL4 associated factor 8	1.020408	0.6999	NA
320806	Gfm2	G elongation factor, mitochondrial 2	1.020408	0.6761	NA
12934	Dpysl2	dihydropyrimidinase-like 2	1.020304	0.6469	NA
14180	Fgf9	fibroblast growth factor 9	1.020304	0.8576	0.9368
14998	H2-DMa	histocompatibility 2, class II, locus DMa	1.020304	0.8787	0.9467
26914	H2afy	H2A histone family, member Y	1.020304	0.8521	0.9339
67131	Acbd4	acyl-Coenzyme A binding domain containing 4	1.020304	0.7634	NA
71974	Prmt3	protein arginine N-methyltransferase 3	1.020304	0.8898	0.9522
79565	Wbscr27	Williams Beuren syndrome chromosome region 27 (human)	1.020304	0.8228	0.9198
668940	Myh7b	myosin, heavy chain 7B, cardiac muscle, beta	1.020304	0.8893	0.9521
18571	Pdcd6ip	programmed cell death 6 interacting protein	1.0202	0.7872	NA
54375	Azin1	antizyme inhibitor 1	1.0202	0.8836	0.9495
64085	Clstn2	calsyntenin 2	1.0202	0.8013	NA
66511	2500003M10Rik	RIKEN cDNA 2500003M10 gene	1.0202	0.7769	NA
69171	1810031K17Rik	RIKEN cDNA 1810031K17 gene	1.0202	0.7134	NA
12895	Cpt1b	carnitine palmitoyltransferase 1b, muscle	1.020096	0.8425	0.9292
18481	Pak3	p21 protein (Cdc42/Rac)-activated kinase 3	1.020096	0.7645	NA
56297	Arl6	ADP-ribosylation factor-like 6	1.020096	0.7881	NA
230709	Zmpste24	zinc metallopeptidase, STE24 homolog (S. cerevisiae)	1.020096	0.6684	NA
232187	Smyd5	SET and MYND domain containing 5	1.020096	0.8724	0.9447
246154	Vasn	vasorin	1.020096	0.7725	NA
18976	Pomc	pro-opiomelanocortin-alpha	1.019992	0.7718	NA
19058	Ppp3r1	protein phosphatase 3, regulatory subunit B, alpha isoform (calcineurin B, type I)	1.019992	0.8074	NA
23863	Dand5	DAN domain family, member 5	1.019992	0.7457	NA
53610	Nono	non-POU-domain-containing, octamer binding protein	1.019992	0.7756	NA
54378	Cacng6	calcium channel, voltage-dependent, gamma subunit 6	1.019992	0.9467	0.9789
56347	Eif3c	eukaryotic translation initiation factor 3, subunit C	1.019992	0.7731	NA
66201	Vta1	Vps20-associated 1 homolog (S. cerevisiae)	1.019992	0.8143	NA
68283	9530077C05Rik	RIKEN cDNA 9530077C05 gene	1.019992	0.7426	NA
77469	C030034E14Rik	RIKEN cDNA C030034E14 gene	1.019992	0.913	0.9638
110012	Gm16517	predicted gene, Gm16517	1.019992	0.8259	0.9211
212679	Mars2	methionine-tRNA synthetase 2 (mitochondrial)	1.019992	0.8163	NA
213236	Dnd1	dead end homolog 1 (zebrafish)	1.019992	0.8458	0.931
225004	BC027072	cDNA sequence BC027072	1.019992	0.7032	NA

20336	Exoc4	exocyst complex component 4	1.019888	0.8598	0.9379
66156	Anapc11	anaphase promoting complex subunit 11	1.019888	0.6751	NA
66212	Sec61b	Sec61 beta subunit	1.019888	0.7774	NA
71169	Nbas	neuroblastoma amplified sequence	1.019888	0.8834	0.9494
72096	Mettl10	methyltransferase like 10	1.019888	0.7263	NA
73124	Golim4	golgi integral membrane protein 4	1.019888	0.7462	NA
74102	Slc35a5	solute carrier family 35, member A5	1.019888	0.8846	0.9499
77246	9330177L23Rik	RIKEN cDNA 9330177L23 gene	1.019888	0.833	0.9247
107358	Tm9sf3	transmembrane 9 superfamily member 3	1.019888	0.8402	0.9281
238271	Kcnh5	potassium voltage-gated channel, subfamily H (eag-related), member 5	1.019888	0.8136	NA
243780	E330009J07Rik	RIKEN cDNA E330009J07 gene	1.019888	0.7307	NA
268445	Ankrd13b	ankyrin repeat domain 13b	1.019888	0.7939	NA
56297	Arl6	ADP-ribosylation factor-like 6	1.019784	0.8508	0.9334
66997	Psmd12	proteasome (prosome, macropain) 26S subunit, non-ATPase, 12	1.019784	0.6221	NA
102747	Lrrc49	leucine rich repeat containing 49	1.019784	0.8546	0.9352
229279	Hnrnpa3	heterogeneous nuclear ribonucleoprotein A3	1.019784	0.7906	NA
234214	Sorbs2	sorbin and SH3 domain containing 2	1.019784	0.8724	0.9447
319448	Fndc3a	fibronectin type III domain containing 3A	1.019784	0.8579	0.9368
100039252	Gm12693	predicted gene 12693	1.019784	0.709	NA
19291	Purb	purine rich element binding protein B	1.01968	0.8483	0.9323
20501	Slc16a1	solute carrier family 16 (monocarboxylic acid transporters), member 1	1.01968	0.8971	0.9557
23844	Clca3	chloride channel calcium activated 3	1.01968	0.8772	0.9464
76222	Magef1	melanoma antigen family F, 1	1.01968	0.6562	NA
114663	Impa2	inositol (myo)-1(or 4)-monophosphatase 2	1.01968	0.8735	0.945
100042947	Gm14816	predicted gene 14816	1.01968	0.9355	0.9732
30960	Vapa	vesicle-associated membrane protein, associated protein A	1.019576	0.7074	NA
68758	Abhd11	abhydrolase domain containing 11	1.019576	0.8234	0.92
18720	Pip5k1a	phosphatidylinositol-4-phosphate 5-kinase, type 1 alpha	1.019472	0.7953	NA
66568	Rwdd3	RWD domain containing 3	1.019472	0.8605	0.9382
73827	1110012D08Rik	RIKEN cDNA 1110012D08 gene	1.019472	0.7965	NA
75302	Asxl2	additional sex combs like 2 (Drosophila)	1.019472	0.8059	NA
246179	Fktn	fukutin	1.019472	0.8485	0.9325
60595	Actn4	actinin alpha 4	1.019368	0.7771	NA
66244	Sdccag1	serologically defined colon cancer antigen 1	1.019368	0.7771	NA
67891	Rpl4	ribosomal protein L4	1.019368	0.8381	0.927
100210	Gpn2	GPN-loop GTPase 2	1.019368	0.8287	0.9226
101592	Eftud1	elongation factor Tu GTP binding domain containing 1	1.019368	0.7556	NA
625276	Gm6569	predicted gene 6569	1.019368	0.9589	0.9836
633057	Gm7102	predicted gene 7102	1.019368	0.9088	0.9618
69029	1500032L24Rik	RIKEN cDNA 1500032L24 gene	1.019264	0.7996	NA
74778	Rrp7a	ribosomal RNA processing 7 homolog A (S. cerevisiae)	1.019264	0.7593	NA
109077	Ints5	integrator complex subunit 5	1.019264	0.8774	0.9464
110074	Dut	deoxyuridine triphosphatase	1.019264	0.8245	NA
246196	Zfp277	zinc finger protein 277	1.019264	0.7237	NA
100039133	Gm2058	predicted gene 2058	1.019264	0.8895	0.9521
15465	Hrh1	histamine receptor H1	1.01916	0.8927	0.9539
16950	Loxl3	lysyl oxidase-like 3	1.01916	0.8233	NA
18187	Nrp2	neuropilin 2	1.01916	0.7292	NA
192157	Socs7	suppressor of cytokine signaling 7	1.01916	0.7288	NA

217951	Tmem196	transmembrane protein 196	1.01916	0.8445	0.9305
12695	Inadl	InaD-like (Drosophila)	1.019056	0.8216	NA
54427	Dnmt3l	DNA (cytosine-5-)-methyltransferase 3-like	1.019056	0.9055	0.9598
71131	Zfp689	zinc finger protein 689	1.019056	0.8903	0.9524
14082	Fadd	Fas (TNFRSF6)-associated via death domain	1.018953	0.8504	0.9332
67669	l7Rn6	lethal, Chr 7, Rinchik 6	1.018953	0.6808	NA
68312	Gstm7	glutathione S-transferase, mu 7	1.018953	0.8999	0.957
75758	9130401M01Rik	RIKEN cDNA 9130401M01 gene	1.018953	0.6957	NA
78903	Wrnip1	Werner helicase interacting protein 1	1.018953	0.6814	NA
108098	Med21	mediator complex subunit 21	1.018953	0.7452	NA
234734	Aars	alanyl-tRNA synthetase	1.018953	0.8028	NA
245676	Gm4997	predicted gene 4997	1.018953	0.6853	NA
319195	Rpl17	ribosomal protein L17	1.018953	0.7319	NA
330657	Prss53	protease, serine, 53	1.018953	0.8739	0.9451
100041377	Gm3300	predicted gene 3300	1.018953	0.933	0.9725
13007	Csrp1	cysteine and glycine-rich protein 1	1.018849	0.7087	NA
67503	1700001G17Rik	RIKEN cDNA 1700001G17 gene	1.018849	0.7809	NA
68174	4930534H18Rik	RIKEN cDNA 4930534H18 gene	1.018849	0.9307	0.9715
230577	Pars2	prolyl-tRNA synthetase (mitochondrial)(putative)	1.018849	0.8889	0.9519
53627	Porcn	porcupine homolog (Drosophila)	1.018745	0.7559	NA
65102	Nif3l1	Ngg1 interacting factor 3-like 1 (S. pombe)	1.018745	0.7253	NA
66933	1700025L06Rik	RIKEN cDNA 1700025L06 gene	1.018745	0.9349	0.973
72667	Zfp444	zinc finger protein 444	1.018745	0.8004	NA
78832	2700078E11Rik	RIKEN cDNA 2700078E11 gene	1.018745	0.741	NA
269997	Zfp747	zinc finger protein 747	1.018745	0.7337	NA
319195	Rpl17	ribosomal protein L17	1.018745	0.7108	NA
70680	3021401N23Rik	RIKEN cDNA 3021401N23 gene	1.018641	0.7809	NA
75914	Exoc6b	exocyst complex component 6B	1.018641	0.9165	0.9652
97487	Cmtm4	CKLF-like MARVEL transmembrane domain containing 4	1.018641	0.7804	NA
232491	Pyroxd1	pyridine nucleotide-disulphide oxidoreductase domain 1	1.018641	0.7433	NA
433809	Rnf207	ring finger protein 207	1.018641	0.8257	NA
12352	Car5a	carbonic anhydrase 5a, mitochondrial	1.018537	0.9384	0.9746
66266	Eapp	E2F-associated phosphoprotein	1.018537	0.7827	NA
12261	C1qbp	complement component 1, q subcomponent binding protein	1.018434	0.7013	NA
14083	Ptk2	PTK2 protein tyrosine kinase 2	1.018434	0.7847	NA
70873	4921517L17Rik	RIKEN cDNA 4921517L17 gene	1.018434	0.8664	0.9418
212514	Spice1	spindle and centriole associated protein 1	1.018434	0.7891	NA
233058	Zfp420	zinc finger protein 420	1.018434	0.8906	0.9525
12492	Scarb2	scavenger receptor class B, member 2	1.01833	0.7706	NA
14687	Gnaz	guanine nucleotide binding protein, alpha z subunit	1.01833	0.8434	0.9298
50793	Orc3	origin recognition complex, subunit 3	1.01833	0.709	NA
54405	Ndufa1	NADH dehydrogenase (ubiquinone) 1 alpha subcomplex, 1	1.01833	0.7043	NA
69116	Ubr4	ubiquitin protein ligase E3 component n-recognin 4	1.01833	0.8075	NA
108160	Fam50a	family with sequence similarity 50, member A	1.01833	0.6578	NA
226751	Cdc42bpa	CDC42 binding protein kinase alpha	1.01833	0.7524	NA
231997	Fkbp14	FK506 binding protein 14	1.01833	0.8578	0.9368
234577	Cpne2	copine II	1.01833	0.8202	NA
271457	Rab5a	RAB5A, member RAS oncogene family	1.01833	0.8903	0.9524
67826	Snap47	synaptosomal-associated protein, 47	1.018226	0.8727	0.9448

217331	Unk	unkempt homolog (Drosophila)	1.018226	0.7734	NA
100042165	BC005561	cDNA sequence BC005561	1.018226	0.8495	0.9329
63856	Taf8	TAF8 RNA polymerase II, TATA box binding protein (TBP)-associated factorq	1.018123	0.6763	NA
70291	2510049J12Rik	RIKEN cDNA 2510049J12 gene	1.018123	0.7918	NA
70790	Ubr5	ubiquitin protein ligase E3 component n-recognin 5	1.018123	0.7839	NA
72726	Tbcc	tubulin-specific chaperone C	1.018123	0.8062	NA
99730	Taf13	TAF13 RNA polymerase II, TATA box binding protein (TBP)-associated factor	1.018123	0.8842	0.9497
106906	AI450241	expressed sequence AI450241	1.018123	0.8105	NA
11532	Adh5	alcohol dehydrogenase 5 (class III), chi polypeptide	1.018019	0.8397	0.9278
16000	Igf1	insulin-like growth factor 1	1.018019	0.9132	0.9639
18220	Nucb1	nucleobindin 1	1.018019	0.843	0.9294
77599	5830420C07Rik	RIKEN cDNA 5830420C07 gene	1.018019	0.8248	NA
93960	Nkd1	naked cuticle 1 homolog (Drosophila)	1.018019	0.912	0.9635
232974	Gm4881	predicted gene 4881	1.018019	0.9146	0.9645
12453	Ccni	cyclin I	1.017915	0.7319	NA
22330	Vcl	vinculin	1.017915	0.7941	NA
76220	6530402F18Rik	RIKEN cDNA 6530402F18 gene	1.017915	0.852	0.9339
213211	Rnf26	ring finger protein 26	1.017915	0.9047	0.9597
234967	Slc36a4	solute carrier family 36 (proton/amino acid symporter), member 4	1.017915	0.8713	0.9443
433926	Lrrc8b	leucine rich repeat containing 8 family, member B	1.017915	0.9068	0.9607
67736	Ccdc130	coiled-coil domain containing 130	1.017812	0.8687	0.943
67797	Snrnp48	small nuclear ribonucleoprotein 48 (U11/U12)	1.017812	0.8079	NA
102462	Imp3	IMP3, U3 small nucleolar ribonucleoprotein, homolog (yeast)	1.017812	0.7174	NA
217615	Ctage5	CTAGE family, member 5	1.017812	0.7755	NA
231600	Chfr	checkpoint with forkhead and ring finger domains	1.017812	0.7831	NA
68730	Dus1l	dihydrouridine synthase 1-like (S. cerevisiae)	1.017708	0.7685	NA
69961	2810432D09Rik	RIKEN cDNA 2810432D09 gene	1.017708	0.7254	NA
234814	Mthfsd	methenyltetrahydrofolate synthetase domain containing	1.017708	0.777	NA
67604	Get4	golgi to ER traffic protein 4 homolog (S. cerevisiae)	1.017605	0.708	NA
74167	Nudt9	nudix (nucleoside diphosphate linked moiety X)-type motif 9	1.017605	0.7381	NA
270802	BC048403	cDNA sequence BC048403	1.017605	0.8501	0.9332
20740	Spna2	spectrin alpha 2	1.017501	0.9524	0.9814
67680	Sdhb	succinate dehydrogenase complex, subunit B, iron sulfur (Ip)	1.017501	0.7638	NA
69221	2410006H16Rik	RIKEN cDNA 2410006H16 gene	1.017501	0.8009	NA
71065	4933407O12Rik	RIKEN cDNA 4933407O12 gene	1.017501	0.9195	0.9672
234479	Gm4890	predicted gene 4890	1.017501	0.9162	0.9652
23792	Adam23	a disintegrin and metallopeptidase domain 23	1.017397	0.8771	0.9463
66592	Stoml2	stomatin (Epb7.2)-like 2	1.017397	0.7573	NA
72324	Plxdc1	plexin domain containing 1	1.017397	0.807	NA
270096	Mon1b	MON1 homolog b (yeast)	1.017397	0.7766	NA
100041294	Gm3258	predicted gene 3258	1.017397	0.7884	NA
20918	Eif1	eukaryotic translation initiation factor 1	1.017294	0.8893	0.9521
69191	Pdia2	protein disulfide isomerase associated 2	1.017294	0.9265	0.97
71960	Myh14	myosin, heavy polypeptide 14	1.017294	0.8086	NA
74778	Rrp7a	ribosomal RNA processing 7 homolog A (S. cerevisiae)	1.017294	0.8855	0.9504
15260	Hira	histone cell cycle regulation defective homolog A (S. cerevisiae)	1.017191	0.8167	NA
50798	Gne	glucosamine	1.017191	0.8328	NA
66055	0610009D07Rik	RIKEN cDNA 0610009D07 gene	1.017191	0.8078	NA
66352	Blzf1	basic leucine zipper nuclear factor 1	1.017191	0.7267	NA

67158	Sft2d3	SFT2 domain containing 3	1.017191	0.8714	0.9443
69537	Dnase1l1	deoxyribonuclease 1-like 1	1.017191	0.8807	0.9478
76416	Znrd1as	ZNRD1 antisense RNA	1.017191	0.7563	NA
78651	Lsm6	LSM6 homolog, U6 small nuclear RNA associated (S. cerevisiae)	1.017191	0.7361	NA
26932	Ppp2r5e	protein phosphatase 2, regulatory subunit B (B56), epsilon isoform	1.017087	0.9092	0.9621
56209	Gde1	glycerophosphodiester phosphodiesterase 1	1.017087	0.8228	NA
69731	Gemin7	gem (nuclear organelle) associated protein 7	1.017087	0.7952	NA
212989	Best2	bestrophin 2	1.017087	0.9814	0.9921
231148	Ablim2	actin-binding LIM protein 2	1.017087	0.8987	0.9565
382090	4922501C03Rik	RIKEN cDNA 4922501C03 gene	1.017087	0.9429	0.9768
382236	Brwd3	bromodomain and WD repeat domain containing 3	1.017087	0.8759	0.9458
17937	Nab2	Ngfi-A binding protein 2	1.016984	0.9051	0.9597
19289	Igdcc3	immunoglobulin superfamily, DCC subclass, member 3	1.016984	0.8294	NA
56421	Pfkp	phosphofructokinase, platelet	1.016984	0.8501	0.9332
66681	Pgm1	phosphoglucomutase 1	1.016984	0.8573	0.9368
230257	Rod1	ROD1 regulator of differentiation 1 (S. pombe)	1.016984	0.902	0.9581
19739	Rgs9	regulator of G-protein signaling 9	1.01688	0.8509	0.9334
20174	Ruvbl2	RuvB-like protein 2	1.01688	0.8144	NA
67781	Ilf2	interleukin enhancer binding factor 2	1.01688	0.7828	NA
110789	Gpr98	G protein-coupled receptor 98	1.01688	0.7001	NA
17977	Ncoa1	nuclear receptor coactivator 1	1.016777	0.8278	NA
53895	Clpp	caseinolytic peptidase, ATP-dependent, proteolytic subunit homolog (E. coli)	1.016777	0.792	NA
67306	Fam164a	family with sequence similarity 164, member A	1.016777	0.7877	NA
69445	1700023D09Rik	RIKEN cDNA 1700023D09 gene	1.016777	0.8596	0.9377
108989	Tpr	translocated promoter region	1.016777	0.9265	0.97
224912	Crb3	crumbs homolog 3 (Drosophila)	1.016777	0.9128	0.9638
225280	Ino80c	INO80 complex subunit C	1.016777	0.8627	0.9396
226823	Kctd3	potassium channel tetramerisation domain containing 3	1.016777	0.7033	NA
407803	BC051226	cDNA sequence BC051226	1.016777	0.8648	0.9409
68394	Ccdc163	coiled-coil domain containing 163	1.016673	0.8076	NA
229658	Vangl1	vang-like 1 (van gogh, Drosophila)	1.016673	0.7947	NA
16998	Ltbp3	latent transforming growth factor beta binding protein 3	1.01657	0.8438	NA
58887	Repin1	replication initiator 1	1.01657	0.7376	NA
66830	Nacc1	nucleus accumbens associated 1, BEN and BTB (POZ) domain containing	1.01657	0.9255	0.9696
69833	Polr2f	polymerase (RNA) II (DNA directed) polypeptide F	1.01657	0.8075	NA
72544	Exosc6	exosome component 6	1.01657	0.813	NA
80880	Kank3	KN motif and ankyrin repeat domains 3	1.01657	0.8197	NA
53332	Mtmr1	myotubularin related protein 1	1.016467	0.8815	0.9481
56382	Rab9	RAB9, member RAS oncogene family	1.016467	0.8261	NA
75079	Zbtb49	zinc finger and BTB domain containing 49	1.016467	0.9083	0.9615
75430	3200002M19Rik	RIKEN cDNA 3200002M19 gene	1.016467	0.7993	NA
75563	Dnali1	dynein, axonemal, light intermediate polypeptide 1	1.016467	0.9001	0.9571
56044	Rala	v-ral simian leukemia viral oncogene homolog A (ras related)	1.016363	0.8691	0.9432
66627	Ogfod2	2-oxoglutarate and iron-dependent oxygenase domain containing 2	1.016363	0.7171	NA
68539	Tmem109	transmembrane protein 109	1.016363	0.7898	NA
69274	Ctdspl	CTD (carboxy-terminal domain, RNA polymerase II, polypeptide A) small phosphatase-like	1.016363	0.841	NA
76510	Trappc9	trafficking protein particle complex 9	1.016363	0.8525	0.934
229776	Cdc14a	CDC14 cell division cycle 14 homolog A (S. cerevisiae)	1.016363	0.9139	0.9642
319319	B230214O09Rik	RIKEN cDNA B230214O09 gene	1.016363	0.9532	0.9815

629557	Gm6981	glyceraldehyde-3-phosphate dehydrogenase pseudogene	1.016363	0.8618	0.9391
15490	Hsd17b7	hydroxysteroid (17-beta) dehydrogenase 7	1.01626	0.8583	0.937
19988	Rpl6	ribosomal protein L6	1.01626	0.8176	NA
57745	Zfp112	zinc finger protein 112	1.01626	0.9533	0.9815
68653	Samm50	sorting and assembly machinery component 50 homolog (S. cerevisiae)	1.01626	0.8371	NA
71137	Rfx4	regulatory factor X, 4 (influences HLA class II expression)	1.01626	0.9104	0.9625
74144	Robo4	roundabout homolog 4 (Drosophila)	1.01626	0.8644	0.9407
93681	Zfp192	zinc finger protein 192	1.01626	0.8788	0.9467
114604	Prdm15	PR domain containing 15	1.01626	0.9303	0.9715
407819	BC031181	cDNA sequence BC031181	1.01626	0.7714	NA
16528	Kcnk4	potassium channel, subfamily K, member 4	1.016157	0.8451	NA
52468	Ctdsp2	CTD (carboxy-terminal domain, RNA polymerase II, polypeptide A) small phosphatase 2	1.016157	0.8387	NA
56358	Copz2	coatomer protein complex, subunit zeta 2	1.016157	0.9102	0.9625
72569	Bbs5	Bardet-Biedl syndrome 5 (human)	1.016157	0.7856	NA
224703	Mar-02	membrane-associated ring finger (C3HC4) 2	1.016157	0.8452	NA
54451	Cpsf3	cleavage and polyadenylation specificity factor 3	1.016054	0.7551	NA
66192	Lage3	L antigen family, member 3	1.016054	0.8438	NA
66899	Fip1l1	FIP1 like 1 (S. cerevisiae)	1.016054	0.8398	NA
140780	Bmp2k	BMP2 inducible kinase	1.016054	0.8429	NA
12777	Ccr10	chemokine (C-C motif) receptor 10	1.01595	0.883	0.949
17347	Mknk2	MAP kinase-interacting serine/threonine kinase 2	1.01595	0.8114	NA
24058	Sigirr	single immunoglobulin and toll-interleukin 1 receptor (TIR) domain	1.01595	0.9047	0.9597
30947	Adat1	adenosine deaminase, tRNA-specific 1	1.01595	0.8749	0.9452
54137	Acrbp	proacrosin binding protein	1.01595	0.8783	0.9465
66498	Dda1	DET1 and DDB1 associated 1	1.01595	0.8438	NA
66587	Fastk	Fas-activated serine/threonine kinase	1.01595	0.8328	NA
67248	Rpl39	ribosomal protein L39	1.01595	0.9059	0.9601
69524	Esam	endothelial cell-specific adhesion molecule	1.01595	0.8776	0.9464
227674	Ddx31	DEAD/H (Asp-Glu-Ala-Asp/His) box polypeptide 31	1.01595	0.8228	NA
15488	Hsd17b4	hydroxysteroid (17-beta) dehydrogenase 4	1.015847	0.7565	NA
53312	Nub1	negative regulator of ubiquitin-like proteins 1	1.015847	0.8455	NA
72198	Skiv2l2	superkiller viralicidic activity 2-like 2 (S. cerevisiae)	1.015847	0.7147	NA
102423	Hinfp	histone H4 transcription factor	1.015847	0.9053	0.9597
210544	Wdr67	WD repeat domain 67	1.015847	0.8148	NA
217305	Cd300ld	CD300 molecule-like family member d	1.015847	0.952	0.9811
22644	Rnf103	ring finger protein 103	1.015744	0.9361	0.9734
23939	Mapk7	mitogen-activated protein kinase 7	1.015744	0.8397	NA
66980	Zdhhc6	zinc finger, DHHC domain containing 6	1.015744	0.8414	NA
504193	Npcd	neuronal pentraxin chromo domain	1.015744	0.8704	0.9441
19989	Rpl7	ribosomal protein L7	1.015641	0.8867	0.9508
60613	Kcnq4	potassium voltage-gated channel, subfamily Q, member 4	1.015641	0.8375	NA
75616	2810008M24Rik	RIKEN cDNA 2810008M24 gene	1.015641	0.7454	NA
93888	Pcdhb17	protocadherin beta 17	1.015641	0.805	NA
208884	Zdhhc9	zinc finger, DHHC domain containing 9	1.015641	0.8305	NA
66273	1810020D17Rik	RIKEN cDNA 1810020D17 gene	1.015538	0.8488	NA
69256	Zfp397	zinc finger protein 397	1.015538	0.8983	0.9565
664808	Gm7349	predicted gene 7349	1.015538	0.8678	0.9426
668224	Gm9054	predicted gene 9054	1.015538	0.9254	0.9696
72580	Zufsp	zinc finger with UFM1-specific peptidase domain	1.015435	0.9233	0.9688

99712	Cept1	choline/ethanolaminephosphotransferase 1	1.015435	0.8036	NA
100017	Ldlrap1	low density lipoprotein receptor adaptor protein 1	1.015435	0.871	0.9441
108652	Slc35b3	solute carrier family 35, member B3	1.015435	0.8189	NA
329910	Acot11	acyl-CoA thioesterase 11	1.015435	0.7671	NA
16511	Kcnh2	potassium voltage-gated channel, subfamily H (eag-related), member 2	1.015332	0.9032	0.9588
19206	Ptch1	patched homolog 1	1.015332	0.8645	0.9407
436332	Gm5766	ribosomal protein L7a pseudogene	1.015332	0.7145	NA
13017	Ctbp2	C-terminal binding protein 2	1.015228	0.7422	NA
76233	Dnttip1	deoxynucleotidyltransferase, terminal, interacting protein 1	1.015228	0.8061	NA
105442	B130052P14Rik	RIKEN cDNA B130052P14 gene	1.015228	0.8991	0.9568
402775	D130095D21Rik	RIKEN cDNA D130095D21 gene	1.015228	0.7801	NA
58193	Extl2	exostoses (multiple)-like 2	1.015125	0.7704	NA
59090	Midn	midnolin	1.015125	0.8154	NA
68015	Trap1	TNF receptor-associated protein 1	1.015125	0.8236	NA
109791	Clps	colipase, pancreatic	1.015125	0.9518	0.981
230848	Zbtb40	zinc finger and BTB domain containing 40	1.015125	0.8213	NA
50527	Ero1l	ERO1-like (S. cerevisiae)	1.015022	0.8876	0.9513
56428	Mtch2	mitochondrial carrier homolog 2 (C. elegans)	1.015022	0.8134	NA
71904	Paqr7	progestin and adipoQ receptor family member VII	1.015022	0.814	NA
77605	H2afv	H2A histone family, member V	1.015022	0.776	NA
217692	Sipa1l1	signal-induced proliferation-associated 1 like 1	1.015022	0.76	NA
319481	Wdr59	WD repeat domain 59	1.015022	0.8124	NA
20826	Nhp2l1	NHP2 non-histone chromosome protein 2-like 1 (S. cerevisiae)	1.014919	0.7936	NA
52850	Sgsm1	small G protein signaling modulator 1	1.014919	0.9042	0.9595
67848	Ddx55	DEAD (Asp-Glu-Ala-Asp) box polypeptide 55	1.014919	0.9221	0.9683
268288	Samd3	sterile alpha motif domain containing 3	1.014919	0.9209	0.9679
347740	2900097C17Rik	RIKEN cDNA 2900097C17 gene	1.014919	0.9079	0.9614
20679	Sox6	SRY-box containing gene 6	1.014816	0.9022	0.9583
21380	Tbx1	T-box 1	1.014816	0.9203	0.9675
52276	Cdca8	cell division cycle associated 8	1.014816	0.8856	0.9504
77048	Ccdc41	coiled-coil domain containing 41	1.014816	0.7541	NA
105428	Fam149b	family with sequence similarity 149, member B	1.014816	0.8516	NA
207304	Hectd1	HECT domain containing 1	1.014816	0.8672	0.9424
229473	D930015E06Rik	RIKEN cDNA D930015E06 gene	1.014816	0.8352	NA
19704	Upf1	UPF1 regulator of nonsense transcripts homolog (yeast)	1.014713	0.8502	NA
23825	Banf1	barrier to autointegration factor 1	1.014713	0.8636	NA
50878	Stag3	stromal antigen 3	1.014713	0.8424	NA
50996	Pdcd7	programmed cell death 7	1.014713	0.7988	NA
67729	Mansc1	MANSC domain containing 1	1.014713	0.9268	0.9701
71175	Nipbl	Nipped-B homolog (Drosophila)	1.014713	0.8786	0.9467
192775	Kcnh6	potassium voltage-gated channel, subfamily H (eag-related), member 6	1.014713	0.9135	0.964
209357	Gtf2h3	general transcription factor IIH, polypeptide 3	1.014713	0.8216	NA
234076	Tmco3	transmembrane and coiled-coil domains 3	1.014713	0.9153	0.9649
382034	Gse1	genetic suppressor element 1	1.014713	0.9145	0.9645
12902	Cr2	complement receptor 2	1.01461	0.9277	0.9705
17420	Mnat1	menage a trois 1	1.01461	0.8178	NA
20866	Stim1	stromal interaction molecule 1	1.01461	0.8675	0.9425
56369	Apip	APAF1 interacting protein	1.01461	0.8291	NA
69944	2810021J22Rik	RIKEN cDNA 2810021J22 gene	1.01461	0.8906	0.9525

71685	Galnt14	UDP-N-acetyl-alpha-D-galactosamine:polypeptide N-acetylgalactosaminyltransferase 14	1.01461	0.827	NA
78303	Hist3h2ba	histone cluster 3, H2ba	1.01461	0.7931	NA
107815	Scml2	sex comb on midleg-like 2 (Drosophila)	1.01461	0.9039	0.9592
109754	Cyb5r3	cytochrome b5 reductase 3	1.01461	0.8954	0.9552
20650	Sntb2	syntrophin, basic 2	1.014507	0.8589	NA
21780	Tfam	transcription factor A, mitochondrial	1.014507	0.8309	NA
72355	2210021J22Rik	RIKEN cDNA 2210021J22 gene	1.014507	0.8214	NA
208638	Slc25a38	solute carrier family 25, member 38	1.014507	0.7791	NA
629557	Gm6981	glyceraldehyde-3-phosphate dehydrogenase pseudogene	1.014507	0.9181	0.9663
12677	Vsx2	visual system homeobox 2	1.014405	0.763	NA
18003	Nedd9	neural precursor cell expressed, developmentally down-regulated gene 9	1.014405	0.8466	NA
233833	Tnrc6a	trinucleotide repeat containing 6a	1.014405	0.8092	NA
245405	Gm4987	predicted gene 4987	1.014405	0.8458	NA
12340	Capza1	capping protein (actin filament) muscle Z-line, alpha 1	1.014302	0.8604	NA
20892	Stra13	stimulated by retinoic acid 13	1.014302	0.8615	NA
26451	Rpl27a	ribosomal protein L27A	1.014302	0.8067	NA
66439	2010012O05Rik	RIKEN cDNA 2010012O05 gene	1.014302	0.8746	0.9452
67710	Polr2g	polymerase (RNA) II (DNA directed) polypeptide G	1.014302	0.751	NA
72777	2810455B08Rik	RIKEN cDNA 2810455B08 gene	1.014302	0.8974	0.9558
242316	Gdf6	growth differentiation factor 6	1.014302	0.9279	0.9705
245595	Zfp711	zinc finger protein 711	1.014302	0.843	NA
268958	Capn11	calpain 11	1.014302	0.9126	0.9638
16785	Rpsa	ribosomal protein SA	1.014199	0.881	0.9479
66164	Nip7	nuclear import 7 homolog (S. cerevisiae)	1.014199	0.742	NA
74478	Snx29	sorting nexin 29	1.014199	0.8558	NA
75036	4930488B01Rik	RIKEN cDNA 4930488B01 gene	1.014199	0.7895	NA
75812	Tasp1	taspase, threonine aspartase 1	1.014199	0.8329	NA
268729	Gm626	predicted gene 626	1.014199	0.92	0.9674
100041585	Amd2	S-adenosylmethionine decarboxylase 2	1.014199	0.8897	0.9521
27388	Ptdss2	phosphatidylserine synthase 2	1.014096	0.9117	0.9634
68837	Foxk2	forkhead box K2	1.014096	0.8479	NA
100043404	Gm4416	predicted gene 4416	1.014096	0.8194	NA
12790	Cnga3	cyclic nucleotide gated channel alpha 3	1.013993	0.8877	0.9513
17967	Ncam1	neural cell adhesion molecule 1	1.013993	0.9007	0.9577
20775	Sqle	squalene epoxidase	1.013993	0.8671	NA
24070	Mpdu1	mannose-P-dolichol utilization defect 1	1.013993	0.9019	0.9581
69798	1810044D09Rik	RIKEN cDNA 1810044D09 gene	1.013993	0.8696	NA
69920	Polr2i	polymerase (RNA) II (DNA directed) polypeptide I	1.013993	0.8422	NA
381346	Gm13194	predicted gene 13194	1.013993	0.9086	0.9617
67695	Ost4	oligosaccharyltransferase 4 homolog (S. cerevisiae)	1.01389	0.8869	0.951
78102	8430426J06Rik	RIKEN cDNA 8430426J06 gene	1.01389	0.9379	0.9743
106046	AW413774	expressed sequence AW413774	1.01389	0.9309	0.9715
215748	Cnksr3	Cnksr family member 3	1.01389	0.802	NA
241568	Lrrc4c	leucine rich repeat containing 4C	1.01389	0.82	NA
20021	Polr2c	polymerase (RNA) II (DNA directed) polypeptide C	1.013788	0.9164	0.9652
22142	Tuba1a	tubulin, alpha 1A	1.013788	0.9136	0.9641
108829	Jmjd1c	jumonji domain containing 1C	1.013788	0.8325	NA
209018	Vps8	vacuolar protein sorting 8 homolog (S. cerevisiae)	1.013788	0.8236	NA
14479	Usp15	ubiquitin specific peptidase 15	1.013685	0.8327	NA

14852	Gspt1	G1 to S phase transition 1	1.013685	0.8332	NA
52838	Dnlz	DNL-type zinc finger	1.013685	0.8405	NA
66172	Med11	mediator of RNA polymerase II transcription, subunit 11 homolog (S. cerevisiae)	1.013685	0.8701	NA
11819	Nr2f2	nuclear receptor subfamily 2, group F, member 2	1.013582	0.8776	0.9464
14390	Gabpa	GA repeat binding protein, alpha	1.013582	0.8686	NA
16842	Lef1	lymphoid enhancer binding factor 1	1.013582	0.9158	0.965
74466	4933427G17Rik	RIKEN cDNA 4933427G17 gene	1.013582	0.9243	0.9692
216169	Fam108a	family with sequence similarity 108, member A	1.013582	0.883	0.949
235043	Tmem205	transmembrane protein 205	1.013582	0.8928	0.9539
235441	Usp3	ubiquitin specific peptidase 3	1.013582	0.7846	NA
14252	Flot2	flotillin 2	1.013479	0.8282	NA
16561	Kif1b	kinesin family member 1B	1.013479	0.8321	NA
24000	Ptpn21	protein tyrosine phosphatase, non-receptor type 21	1.013479	0.9052	0.9597
66399	Tsfm	Ts translation elongation factor, mitochondrial	1.013479	0.8959	0.9553
69702	Ndufaf1	NADH dehydrogenase (ubiquinone) 1 alpha subcomplex, assembly factor 1	1.013479	0.804	NA
70382	Kctd2	potassium channel tetramerisation domain containing 2	1.013479	0.8783	0.9465
71348	5430439M09Rik	RIKEN cDNA 5430439M09 gene	1.013479	0.8434	NA
72614	Pih1d2	PIH1 domain containing 2	1.013479	0.8286	NA
109168	Atl3	atlastin GTPase 3	1.013479	0.8947	0.9549
17714	Grpel2	GrpE-like 2, mitochondrial	1.013377	0.8653	NA
57370	B4galt3	UDP-Gal:betaGlcNAc beta 1,4-galactosyltransferase, polypeptide 3	1.013377	0.8509	NA
214572	Prmt7	protein arginine N-methyltransferase 7	1.013377	0.8201	NA
243510	Ccdc142	coiled-coil domain containing 142	1.013377	0.8483	NA
100043404	Gm4416	predicted gene 4416	1.013377	0.8687	NA
19826	Rnps1	ribonucleic acid binding protein S1	1.013274	0.8986	0.9565
73736	Fcf1	FCF1 small subunit (SSU) processome component homolog (S. cerevisiae)	1.013274	0.8506	NA
209966	Pgbd5	piggyBac transposable element derived 5	1.013274	0.7533	NA
100126203	6820426E19Rik	RIKEN cDNA 6820426E19 gene	1.013274	0.9555	0.9824
68709	Cilp2	cartilage intermediate layer protein 2	1.013171	0.888	0.9513
414116	D630024D03Rik	RIKEN cDNA D630024D03 gene	1.013171	0.9224	0.9683
56480	Tbk1	TANK-binding kinase 1	1.013069	0.8562	NA
57264	Retn	resistin	1.013069	0.9497	0.9801
103213	Traf3ip2	TRAF3 interacting protein 2	1.013069	0.8978	0.956
223989	4921513D23Rik	RIKEN cDNA 4921513D23 gene	1.013069	0.932	0.972
319817	Rc3h2	ring finger and CCCH-type zinc finger domains 2	1.013069	0.8571	NA
16882	Lig3	ligase III, DNA, ATP-dependent	1.012966	0.8514	NA
17165	Mapkapk5	MAP kinase-activated protein kinase 5	1.012966	0.7998	NA
18646	Prf1	perforin 1 (pore forming protein)	1.012966	0.9245	0.9692
19244	Ptp4a2	protein tyrosine phosphatase 4a2	1.012966	0.8713	NA
19684	Rdx	radixin	1.012966	0.8794	NA
24013	Grk1	G protein-coupled receptor kinase 1	1.012966	0.9326	0.9724
67057	Yaf2	YY1 associated factor 2	1.012966	0.7966	NA
76375	Det1	de-etiolated homolog 1 (Arabidopsis)	1.012966	0.8998	0.957
99349	Dnajc24	DnaJ (Hsp40) homolog, subfamily C, member 24	1.012966	0.8678	NA
101488	Slco2b1	solute carrier organic anion transporter family, member 2b1	1.012966	0.9267	0.9701
107197	AI462493	expressed sequence AI462493	1.012966	0.8787	NA
107686	Snrpd2	small nuclear ribonucleoprotein D2	1.012966	0.8716	NA
233210	Prr12	proline rich 12	1.012966	0.9024	0.9584
26433	Plod3	procollagen-lysine, 2-oxoglutarate 5-dioxygenase 3	1.012863	0.8853	0.9503

27967	Cherp	calcium homeostasis endoplasmic reticulum protein	1.012863	0.8104	NA
73261	1700037C18Rik	RIKEN cDNA 1700037C18 gene	1.012863	0.8889	0.9519
107476	Acaca	acetyl-Coenzyme A carboxylase alpha	1.012863	0.8143	NA
108159	Ubxn8	UBX domain protein 8	1.012863	0.8931	0.9541
320854	9030203C11Rik	RIKEN cDNA 9030203C11 gene	1.012863	0.8918	0.9535
381560	Xkr8	X Kell blood group precursor related family member 8 homolog	1.012863	0.8922	0.9536
19038	Ppic	peptidylprolyl isomerase C	1.012761	0.9128	0.9638
67168	Lpar6	lysophosphatidic acid receptor 6	1.012761	0.9157	0.965
67733	Itgb3bp	integrin beta 3 binding protein (beta3-endonexin)	1.012761	0.8686	NA
107271	Yars	tyrosyl-tRNA synthetase	1.012761	0.8987	0.9565
19359	Rad23b	RAD23b homolog (S. cerevisiae)	1.012658	0.8469	NA
53896	Slc7a10	solute carrier family 7 (cationic amino acid transporter, y+ system), member 10	1.012658	0.9754	0.9901
319719	4732471D19Rik	RIKEN cDNA 4732471D19 gene	1.012658	0.8166	NA
17931	Ppp1r12a	protein phosphatase 1, regulatory (inhibitor) subunit 12A	1.012556	0.9191	0.9669
20851	Stat5b	signal transducer and activator of transcription 5B	1.012556	0.8744	NA
69903	Rasip1	Ras interacting protein 1	1.012556	0.901	0.9577
72125	Fam123a	family with sequence similarity 123, member A	1.012556	0.8602	NA
72925	Mar-01	membrane-associated ring finger (C3HC4) 1	1.012556	0.8865	0.9507
619331	Zfp551	zinc fingr protein 551	1.012556	0.8784	NA
19701	Ren1	renin 1 structural	1.012453	0.9289	0.971
66234	Sc4mol	sterol-C4-methyl oxidase-like	1.012453	0.854	NA
72826	Fam76b	family with sequence similarity 76, member B	1.012453	0.8723	NA
94064	Mrpl27	mitochondrial ribosomal protein L27	1.012453	0.8697	NA
216154	Med16	mediator complex subunit 16	1.012453	0.8945	0.9548
239570	Ttc38	tetratricopeptide repeat domain 38	1.012453	0.8994	0.957
432572	Specc1	sperm antigen with calponin homology and coiled-coil domains 1	1.012453	0.8501	NA
552877	LOC552877	hypothetical LOC552877	1.012453	0.8425	NA
16981	Lrrn3	leucine rich repeat protein 3, neuronal	1.012351	0.8212	NA
17762	Mapt	microtubule-associated protein tau	1.012351	0.81	NA
216987	Utp6	UTP6, small subunit (SSU) processome component, homolog (yeast)	1.012351	0.919	0.9669
231464	Cnot6l	CCR4-NOT transcription complex, subunit 6-like	1.012351	0.7961	NA
235542	Ppp2r3a	protein phosphatase 2, regulatory subunit B'', alpha	1.012351	0.9335	0.9726
624918	Gm6537	predicted gene 6537	1.012351	0.9093	0.9622
21405	Hnf1a	HNF1 homeobox A	1.012248	0.9105	0.9626
22335	Vdac3	voltage-dependent anion channel 3	1.012248	0.8316	NA
56551	Txn2	thioredoxin 2	1.012248	0.9198	0.9673
70533	Btf3l4	basic transcription factor 3-like 4	1.012248	0.8134	NA
75616	2810008M24Rik	RIKEN cDNA 2810008M24 gene	1.012248	0.9147	0.9645
226747	Ahctf1	AT hook containing transcription factor 1	1.012248	0.822	NA
56495	Asna1	arsA arsenite transporter, ATP-binding, homolog 1 (bacterial)	1.012146	0.8826	NA
67236	Cinp	cyclin-dependent kinase 2 interacting protein	1.012146	0.9515	0.981
26384	Gnpda1	glucosamine-6-phosphate deaminase 1	1.012043	0.8573	NA
67263	Zswim6	zinc finger, SWIM domain containing 6	1.012043	0.9051	0.9597
69918	2610020C07Rik	RIKEN cDNA 2610020C07 gene	1.012043	0.8552	NA
207214	Larp4	La ribonucleoprotein domain family, member 4	1.012043	0.8094	NA
225888	Suv420h1	suppressor of variegation 4-20 homolog 1 (Drosophila)	1.012043	0.8951	0.9551
230596	Prpf38a	PRP38 pre-mRNA processing factor 38 (yeast) domain containing A	1.012043	0.8068	NA
233890	Zfp768	zinc finger protein 768	1.012043	0.838	NA
18249	Obp1a	odorant binding protein Ia	1.011941	0.9527	0.9814

66205	Cd302	CD302 antigen	1.011941	0.9213	0.968
66482	Exoc2	exocyst complex component 2	1.011941	0.8665	NA
72124	Seh1l	SEH1-like (S. cerevisiae	1.011941	0.9065	0.9604
72482	Acbd6	acyl-Coenzyme A binding domain containing 6	1.011941	0.8109	NA
74237	Tubgcp2	tubulin, gamma complex associated protein 2	1.011941	0.8819	NA
219170	AU021034	expressed sequence AU021034	1.011941	0.9721	0.9888
378435	Mafa	v-maf musculoaponeurotic fibrosarcoma oncogene family, protein A (avian)	1.011941	0.9156	0.965
12812	Coil	coilin	1.011839	0.8579	NA
69742	Tm2d2	TM2 domain containing 2	1.011839	0.8398	NA
70432	Rufy2	RUN and FYVE domain-containing 2	1.011839	0.829	NA
244144	Usp35	ubiquitin specific peptidase 35	1.011839	0.9204	0.9675
320538	Ubn2	ubinuclein 2	1.011839	0.8071	NA
219150	Hmbox1	homeobox containing 1	1.011736	0.93	0.9715
278304	Zfp385c	zinc finger protein 385C	1.011736	0.8503	NA
667766	Gm8801	protein phosphatase 1, regulatory subunit 10 pseudogene	1.011736	0.896	0.9553
100044339	LOC100044339	zinc finger protein 764-like	1.011736	0.9083	0.9615
19134	Prpf4b	PRP4 pre-mRNA processing factor 4 homolog B (yeast)	1.011634	0.9428	0.9768
22343	Lin7c	lin-7 homolog C (C. elegans)	1.011634	0.9069	0.9607
56086	Set	SET nuclear oncogene	1.011634	0.9163	0.9652
56461	Kcnip3	Kv channel interacting protein 3, calsenilin	1.011634	0.8806	NA
56715	Rabgef1	RAB guanine nucleotide exchange factor (GEF) 1	1.011634	0.9607	0.9842
75871	Zfp821	zinc finger protein 821	1.011634	0.8616	NA
76854	Gpr30	G protein-coupled receptor 30	1.011634	0.8822	NA
209497	Tmem164	transmembrane protein 164	1.011634	0.9392	0.9751
211480	Kcnj14	potassium inwardly-rectifying channel, subfamily J, member 14	1.011634	0.9263	0.97
227634	Camsap1	calmodulin regulated spectrin-associated protein 1	1.011634	0.7996	NA
257633	Acsf3	acyl-CoA synthetase family member 3	1.011634	0.8862	NA
19042	Ppm1a	protein phosphatase 1A, magnesium dependent, alpha isoform	1.011531	0.8292	NA
20610	Sumo3	SMT3 suppressor of mif two 3 homolog 3 (yeast)	1.011531	0.9306	0.9715
21425	Tcfeb	transcription factor EB	1.011531	0.9322	0.9721
21934	Tnfrsf11a	tumor necrosis factor receptor superfamily, member 11a	1.011531	0.8447	NA
22038	Plscr1	phospholipid scramblase 1	1.011531	0.9067	0.9606
66060	0610010O12Rik	RIKEN cDNA 0610010O12 gene	1.011531	0.8976	0.9559
66480	Rpl15	ribosomal protein L15	1.011531	0.7998	NA
75930	4930567H12Rik	RIKEN cDNA 4930567H12 gene	1.011531	0.972	0.9887
494504	Apcdd1	adenomatosis polyposis coli down-regulated 1	1.011531	0.9043	0.9595
17979	Ncoa3	nuclear receptor coactivator 3	1.011429	0.9252	0.9696
26875	Pclo	piccolo (presynaptic cytomatrix protein)	1.011429	0.9225	0.9683
56517	Slc22a21	solute carrier family 22 (organic cation transporter), member 21	1.011429	0.8973	0.9558
66406	Sac3d1	SAC3 domain containing 1	1.011429	0.8156	NA
74347	4632415K11Rik	RIKEN cDNA 4632415K11 gene	1.011429	0.8724	NA
96935	Susd4	sushi domain containing 4	1.011429	0.871	NA
100169	Phactr4	phosphatase and actin regulator 4	1.011429	0.8227	NA
237943	Gpatch8	G patch domain containing 8	1.011429	0.8403	NA
20438	Siah1b	seven in absentia 1B	1.011327	0.8606	NA
22099	Tsn	translin	1.011327	0.8398	NA
22151	Tubb2a	tubulin, beta 2A	1.011327	0.8886	NA
56794	Hacl1	2-hydroxyacyl-CoA lyase 1	1.011327	0.8203	NA
72992	2900076A07Rik	RIKEN cDNA 2900076A07 gene	1.011327	0.8995	0.957

320497	A630065K11Rik	RIKEN cDNA A630065K11 gene	1.011327	0.9144	0.9645
12567	Cdk4	cyclin-dependent kinase 4	1.011225	0.8925	NA
20286	Zc3h7b	zinc finger CCCH type containing 7B	1.011225	0.8409	NA
52662	D18Ertd653e	DNA segment, Chr 18, ERATO Doi 653, expressed	1.011225	0.9078	0.9614
57440	Ehd3	EH-domain containing 3	1.011225	0.8703	NA
68344	Tmem174	transmembrane protein 174	1.011225	0.9495	0.9801
69546	Mapk1ip1	mitogen-activated protein kinase 1 interacting protein 1	1.011225	0.8927	NA
71365	Pdss2	prenyl (solanesyl) diphosphate synthase, subunit 2	1.011225	0.8139	NA
102774	Bbs4	Bardet-Biedl syndrome 4 (human)	1.011225	0.848	NA
28036	Larp7	La ribonucleoprotein domain family, member 7	1.011122	0.8518	NA
52468	Ctdsp2	CTD (carboxy-terminal domain, RNA polymerase II, polypeptide A) small phosphatase 2	1.011122	0.9707	0.988
66868	Mfsd1	major facilitator superfamily domain containing 1	1.011122	0.9156	0.965
76813	Armc6	armadillo repeat containing 6	1.011122	0.914	0.9643
85030	Tnfrsf25	tumor necrosis factor receptor superfamily, member 25	1.011122	0.9556	0.9824
269952	D330012F22Rik	RIKEN cDNA D330012F22 gene	1.011122	0.9247	0.9693
665055	Gm7467	predicted gene 7467	1.011122	0.8875	NA
791333	Gm10050	predicted gene 10050	1.011122	0.9434	0.977
19353	Rac1	RAS-related C3 botulinum substrate 1	1.01102	0.8556	NA
29816	Hip1r	huntingtin interacting protein 1 related	1.01102	0.9098	0.9624
52432	Ppp2r2d	protein phosphatase 2, regulatory subunit B, delta isoform	1.01102	0.8446	NA
66915	Myeov2	myeloma overexpressed 2	1.01102	0.9072	0.9608
71701	Pnpt1	polyribonucleotide nucleotidyltransferase 1	1.01102	0.8242	NA
13207	Ddx5	DEAD (Asp-Glu-Ala-Asp) box polypeptide 5	1.010918	0.8226	NA
26409	Map3k7	mitogen-activated protein kinase kinase kinase 7	1.010918	0.9	0.9571
214968	Sema6d	sema domain, transmembrane domain (TM), and cytoplasmic domain, (semaphorin) 6D	1.010918	0.9496	0.9801
239739	Lamp3	lysosomal-associated membrane protein 3	1.010918	0.8469	NA
319195	Rpl17	ribosomal protein L17	1.010918	0.8471	NA
329178	Unc80	unc-80 homolog (C. elegans)	1.010918	0.8909	NA
329324	Syt14	synaptotagmin XIV	1.010918	0.9113	0.9632
435784	Gm12471	predicted gene 12471	1.010918	0.9198	0.9673
13205	Ddx3x	DEAD/H (Asp-Glu-Ala-Asp/His) box polypeptide 3, X-linked	1.010816	0.8516	NA
19663	Rbpms	RNA binding protein gene with multiple splicing	1.010816	0.9194	0.9672
67674	Trmt112	tRNA methyltransferase 11-2 homolog (S. cerevisiae)	1.010816	0.86	NA
75610	2010109A12Rik	RIKEN cDNA 2010109A12 gene	1.010816	0.9521	0.9811
76775	Slc10a7	solute carrier family 10 (sodium/bile acid cotransporter family), member 7	1.010816	0.9621	0.9846
319518	Pdpr	pyruvate dehydrogenase phosphatase regulatory subunit	1.010816	0.8513	NA
18786	Plaa	phospholipase A2, activating protein	1.010714	0.9016	NA
20482	Skil	SKI-like	1.010714	0.9498	0.9801
66834	Acot13	acyl-CoA thioesterase 13	1.010714	0.8428	NA
67862	2310033P09Rik	RIKEN cDNA 2310033P09 gene	1.010714	0.8824	NA
72201	Otud6b	OTU domain containing 6B	1.010714	0.8752	NA
93687	Csnk1a1	casein kinase 1, alpha 1	1.010714	0.8715	NA
226861	Hhat	hedgehog acyltransferase	1.010714	0.9731	0.9892
237758	Zfp454	zinc finger protein 454	1.010714	0.8706	NA
15574	Hus1	Hus1 homolog (S. pombe)	1.010611	0.8584	NA
22402	Wisp1	WNT1 inducible signaling pathway protein 1	1.010611	0.9354	0.9731
70420	2610034B18Rik	RIKEN cDNA 2610034B18 gene	1.010611	0.9282	0.9705
497097	Xkr4	X Kell blood group precursor related family member 4	1.010611	0.9224	0.9683
53622	Krt85	keratin 85	1.010509	0.9442	0.9775

66299	2610019N06Rik	RIKEN cDNA 2610019N06 gene	1.010509	0.9244	0.9692
74450	Pank2	pantothenate kinase 2	1.010509	0.8173	NA
270106	Rpl13	ribosomal protein L13	1.010509	0.8554	NA
13877	Erh	enhancer of rudimentary homolog (Drosophila)	1.010407	0.9221	0.9683
26889	Cln8	ceroid-lipofuscinosis, neuronal 8	1.010407	0.8627	NA
212427	A730008H23Rik	RIKEN cDNA A730008H23 gene	1.010407	0.8802	NA
432763	Prr7	proline rich 7 (synaptic)	1.010407	0.8946	NA
20220	Sap18	Sin3-associated polypeptide 18	1.010305	0.8661	NA
27207	Rps11	ribosomal protein S11	1.010305	0.8373	NA
56046	Uqcc	ubiquinol-cytochrome c reductase complex chaperone, CBP3 homolog (yeast)	1.010305	0.8503	NA
66229	Rpl7l1	ribosomal protein L7-like 1	1.010305	0.9147	0.9645
98386	Lbr	lamin B receptor	1.010305	0.9008	NA
12928	Crk	v-crk sarcoma virus CT10 oncogene homolog (avian)	1.010203	0.9424	0.9767
19043	Ppm1b	protein phosphatase 1B, magnesium dependent, beta isoform	1.010203	0.8763	NA
67101	2310039H08Rik	RIKEN cDNA 2310039H08 gene	1.010203	0.857	NA
67230	Zfp329	zinc finger protein 329	1.010203	0.8727	NA
69641	Wdr20a	WD repeat domain 20A	1.010203	0.9046	NA
329954	Catsper4	cation channel, sperm associated 4	1.010203	0.91	0.9624
13211	Dhx9	DEAH (Asp-Glu-Ala-His) box polypeptide 9	1.010101	0.9598	0.9838
16500	Kcnb1	potassium voltage gated channel, Shab-related subfamily, member 1	1.010101	0.9001	NA
16539	Kcns2	K+ voltage-gated channel, subfamily S, 2	1.010101	0.9343	0.9728
66481	Rps21	ribosomal protein S21	1.010101	0.9629	0.9847
71678	Brox	BRO1 domain and CAAX motif containing	1.010101	0.9474	0.9792
192970	Dhrs11	dehydrogenase/reductase (SDR family) member 11	1.010101	0.9582	0.9835
215008	Vezt	vezatin, adherens junctions transmembrane protein	1.010101	0.8454	NA
319468	Ppm1h	protein phosphatase 1H (PP2C domain containing)	1.010101	0.8742	NA
11958	Atp5k	ATP synthase, H+ transporting, mitochondrial F1F0 complex, subunit e	1.009999	0.8624	NA
76303	Osbp	oxysterol binding protein	1.009999	0.9278	0.9705
231889	Bud31	BUD31 homolog (yeast)	1.009999	0.9	NA
233033	Samd4b	sterile alpha motif domain containing 4B	1.009999	0.8987	NA
271209	Rp1l1	retinitis pigmentosa 1 homolog (human)-like 1	1.009999	0.8987	NA
328801	Zfp414	zinc finger protein 414	1.009999	0.8973	NA
54401	Ywhab	tyrosine 3-monooxygenase/tryptophan 5-monooxygenase activation protein, beta polypeptide	1.009897	0.9139	0.9642
68219	Nudt21	nudix (nucleoside diphosphate linked moiety X)-type motif 21	1.009897	0.8694	NA
69731	Gemin7	gem (nuclear organelle) associated protein 7	1.009897	0.8563	NA
226778	Mark1	MAP/microtubule affinity-regulating kinase 1	1.009897	0.8871	NA
226830	Smyd2	SET and MYND domain containing 2	1.009897	0.8664	NA
19047	Ppp1cc	protein phosphatase 1, catalytic subunit, gamma isoform	1.009795	0.8866	NA
69228	Zfp746	zinc finger protein 746	1.009795	0.9063	NA
74127	Krt80	keratin 80	1.009795	0.9431	0.9768
234730	Fuk	fucokinase	1.009795	0.866	NA
268970	Arhgap28	Rho GTPase activating protein 28	1.009795	0.8798	NA
628951	Gm6936	predicted gene 6936	1.009795	0.9415	0.9765
665533	Gm13004	60S ribosomal protein L31 pseudogene	1.009795	0.8381	NA
56470	Rgs19	regulator of G-protein signaling 19	1.009693	0.876	NA
71985	Acad10	acyl-Coenzyme A dehydrogenase family, member 10	1.009693	0.9114	0.9632
74648	S100pbp	S100P binding protein	1.009693	0.9245	0.9692
170823	Glmn	glomulin, FKBP associated protein	1.009693	0.9271	0.9704
192193	Edem1	ER degradation enhancer, mannosidase alpha-like 1	1.009693	0.8792	NA

225583	A730017C20Rik	RIKEN cDNA A730017C20 gene	1.009693	0.9478	0.9793
14183	Fgfr2	fibroblast growth factor receptor 2	1.009591	0.9336	0.9726
24017	Rnf13	ring finger protein 13	1.009591	0.8547	NA
72795	Ttc19	tetratricopeptide repeat domain 19	1.009591	0.9318	0.972
268749	Rnf31	ring finger protein 31	1.009591	0.8811	NA
432879	Gm5465	predicted gene 5465	1.009591	0.9375	0.9742
433791	Gm13251	predicted gene 13251	1.009591	0.9533	0.9815
18389	Oprl1	opioid receptor-like 1	1.009489	0.9684	0.9867
109186	6720427I07Rik	RIKEN cDNA 6720427I07 gene	1.009489	0.9081	NA
381650	Thap6	THAP domain containing 6	1.009489	0.9537	0.9817
15239	Hgs	HGF-regulated tyrosine kinase substrate	1.009387	0.8749	NA
56032	Nprl2	nitrogen permease regulator-like 2 (S. cerevisiae)	1.009387	0.8784	NA
66622	Ubr7	ubiquitin protein ligase E3 component n-recognin 7 (putative)	1.009387	0.8558	NA
67058	2810428J06Rik	RIKEN cDNA 2810428J06 gene	1.009387	0.9417	0.9765
71836	1700012A16Rik	RIKEN cDNA 1700012A16 gene	1.009387	0.9693	0.9872
106407	Osta	organic solute transporter alpha	1.009387	0.9393	0.9751
114889	Vsx1	visual system homeobox 1 homolog (zebrafish)	1.009387	0.8834	NA
226823	Kctd3	potassium channel tetramerisation domain containing 3	1.009387	0.9181	0.9663
22259	Nr1h3	nuclear receptor subfamily 1, group H, member 3	1.009285	0.9045	NA
27398	Mrpl2	mitochondrial ribosomal protein L2	1.009285	0.9014	NA
71295	4933431K14Rik	RIKEN cDNA 4933431K14 gene	1.009285	0.8901	NA
71900	Tmem106b	transmembrane protein 106B	1.009285	0.933	0.9725
216565	Ehbp1	EH domain binding protein 1	1.009285	0.9501	0.9803
12757	Clta	clathrin, light polypeptide (Lca)	1.009184	0.8892	NA
15239	Hgs	HGF-regulated tyrosine kinase substrate	1.009184	0.8796	NA
19944	Rpl29	ribosomal protein L29	1.009184	0.8873	NA
22134	Tgoln1	trans-golgi network protein	1.009184	0.8841	NA
66231	Thoc7	THO complex 7 homolog (Drosophila)	1.009184	0.8668	NA
66394	Nosip	nitric oxide synthase interacting protein	1.009184	0.8852	NA
104721	Ddx1	DEAD (Asp-Glu-Ala-Asp) box polypeptide 1	1.009184	0.8573	NA
209462	Hace1	HECT domain and ankyrin repeat containing, E3 ubiquitin protein ligase 1	1.009184	0.8789	NA
545725	Mterf	mitochondrial transcription termination factor	1.009184	0.8903	NA
12890	Cplx2	complexin 2	1.009082	0.9201	0.9674
19126	Prom1	prominin 1	1.009082	0.9165	NA
20182	Rxrb	retinoid X receptor beta	1.009082	0.9006	NA
21762	Psmd2	proteasome (prosome, macropain) 26S subunit, non-ATPase, 2	1.009082	0.8821	NA
67427	Rps20	ribosomal protein S20	1.009082	0.9078	NA
246229	Bivm	basic, immunoglobulin-like variable motif containing	1.009082	0.9194	0.9672
12015	Bad	BCL2-associated agonist of cell death	1.00898	0.8892	NA
12796	Camp	cathelicidin antimicrobial peptide	1.00898	0.9305	0.9715
18222	Numb	numb gene homolog (Drosophila)	1.00898	0.9238	0.9689
18718	Pip4k2a	phosphatidylinositol-5-phosphate 4-kinase, type II, alpha	1.00898	0.8932	NA
20112	Rps6ka2	ribosomal protein S6 kinase, polypeptide 2	1.00898	0.9291	0.9711
23938	Map2k5	mitogen-activated protein kinase kinase 5	1.00898	0.9105	NA
50917	Galns	galactosamine (N-acetyl)-6-sulfate sulfatase	1.00898	0.9356	0.9732
56470	Rgs19	regulator of G-protein signaling 19	1.00898	0.9202	0.9674
76167	Snrnp35	small nuclear ribonucleoprotein 35 (U11/U12)	1.00898	0.9146	NA
76263	Gstk1	glutathione S-transferase kappa 1	1.00898	0.8932	NA
269513	Nkain3	Na+/K+ transporting ATPase interacting 3	1.00898	0.8877	NA

100038746	LOC100038746	hypothetical LOC100038746	1.00898	0.877	NA
11764	Ap1b1	adaptor protein complex AP-1, beta 1 subunit	1.008878	0.852	NA
19384	Ran	RAN, member RAS oncogene family	1.008878	0.8741	NA
68591	Mocos	molybdenum cofactor sulfurase	1.008878	0.9031	NA
69389	1700014N06Rik	RIKEN cDNA 1700014N06 gene	1.008878	0.9775	0.9908
71562	Afmid	arylformamidase	1.008878	0.9319	0.972
72075	Ogfr	opioid growth factor receptor	1.008878	0.9104	NA
234728	Ftsjd1	FtsJ methyltransferase domain containing 1	1.008878	0.8614	NA
14062	F2r	coagulation factor II (thrombin) receptor	1.008776	0.9229	0.9686
53610	Nono	non-POU-domain-containing, octamer binding protein	1.008776	0.8742	NA
67489	Ap4b1	adaptor-related protein complex AP-4, beta 1	1.008776	0.9004	NA
72392	Tmem175	transmembrane protein 175	1.008776	0.8998	NA
16512	Kcnh3	potassium voltage-gated channel, subfamily H (eag-related), member 3	1.008675	0.9699	0.9876
24100	Tpra1	transmembrane protein, adipocyte asscociated 1	1.008675	0.9447	0.9776
26446	Psmb3	proteasome (prosome, macropain) subunit, beta type 3	1.008675	0.9005	NA
56375	B4galt4	UDP-Gal:betaGlcNAc beta 1,4-galactosyltransferase, polypeptide 4	1.008675	0.9086	NA
60406	Sap30	sin3 associated polypeptide	1.008675	0.9251	0.9695
106672	AI413582	expressed sequence AI413582	1.008675	0.8633	NA
18294	Ogg1	8-oxoguanine DNA-glycosylase 1	1.008573	0.9085	NA
68364	0610030E20Rik	RIKEN cDNA 0610030E20 gene	1.008573	0.8981	NA
109637	Upk1a	uroplakin 1A	1.008573	0.9121	NA
212880	Ddx46	DEAD (Asp-Glu-Ala-Asp) box polypeptide 46	1.008573	0.8988	NA
16531	Kcnma1	potassium large conductance calcium-activated channel, subfamily M, alpha member 1	1.008471	0.8667	NA
20750	Spp1	secreted phosphoprotein 1	1.008471	0.9179	NA
67105	1700034H14Rik	RIKEN cDNA 1700034H14 gene	1.008471	0.8664	NA
67484	Eepd1	endonuclease/exonuclease/phosphatase family domain containing 1	1.008471	0.8426	NA
213827	Arcn1	archain 1	1.008471	0.9484	0.9796
238205	Lrfn5	leucine rich repeat and fibronectin type III domain containing 5	1.008471	0.9404	0.9757
11692	Gfer	growth factor, erv1 (S. cerevisiae)-like (augmenter of liver regeneration)	1.008369	0.869	NA
13006	Smc3	structural maintenace of chromosomes 3	1.008369	0.9316	0.9719
15171	Hcrt	hypocretin	1.008369	0.9127	NA
53333	Tomm40	translocase of outer mitochondrial membrane 40 homolog (yeast)	1.008369	0.9185	NA
114641	Rpl31	ribosomal protein L31	1.008369	0.8534	NA
231134	Dok7	docking protein 7	1.008369	0.9439	0.9773
246102	Rttn	rotatin	1.008369	0.933	0.9725
11844	Arf5	ADP-ribosylation factor 5	1.008268	0.8782	NA
22294	Uxt	ubiquitously expressed transcript	1.008268	0.9266	0.97
22666	Zfp161	zinc finger protein 161	1.008268	0.9147	NA
76161	6330527O06Rik	RIKEN cDNA 6330527O06 gene	1.008268	0.9138	NA
239559	A4galt	alpha 1,4-galactosyltransferase	1.008268	0.9711	0.9882
240613	9930021J03Rik	RIKEN cDNA 9930021J03 gene	1.008268	0.8907	NA
244579	Tox3	TOX high mobility group box family member 3	1.008268	0.9222	NA
21366	Slc6a6	solute carrier family 6 (neurotransmitter transporter, taurine), member 6	1.008166	0.919	NA
26363	Btd	biotinidase	1.008166	0.9211	NA
54712	Plxnc1	plexin C1	1.008166	0.9646	0.9854
94064	Mrpl27	mitochondrial ribosomal protein L27	1.008166	0.9023	NA
11981	Atp9a	ATPase, class II, type 9A	1.008065	0.9516	0.981
66894	Wwp2	WW domain containing E3 ubiquitin protein ligase 2	1.008065	0.9103	NA
104082	Wdr7	WD repeat domain 7	1.008065	0.9665	0.9858

106489	Sft2d1	SFT2 domain containing 1	1.008065	0.9281	0.9705
11554	Adrb1	adrenergic receptor, beta 1	1.007963	0.9347	0.973
66844	Ormdl2	ORM1-like 2 (S. cerevisiae)	1.007963	0.9022	NA
67840	Mrp63	mitochondrial ribosomal protein 63	1.007963	0.8813	NA
107766	Haao	3-hydroxyanthranilate 3,4-dioxygenase	1.007963	0.9562	0.9824
117198	Ivns1abp	influenza virus NS1A binding protein	1.007963	0.94	0.9755
215999	Ccdc109a	coiled-coil domain containing 109A	1.007963	0.9585	0.9836
227298	Fam134a	family with sequence similarity 134, member A	1.007963	0.9202	NA
235086	Igsf9b	immunoglobulin superfamily, member 9B	1.007963	0.9512	0.9809
26939	Polr3e	polymerase (RNA) III (DNA directed) polypeptide E	1.007861	0.9334	0.9726
69747	Zswim7	zinc finger, SWIM-type containing 7	1.007861	0.8821	NA
215085	Slc35f1	solute carrier family 35, member F1	1.00776	0.9555	0.9824
232946	Bloc1s3	biogenesis of lysosome-related organelles complex-1, subunit 3	1.00776	0.9418	0.9765
69277	3300002I08Rik	RIKEN cDNA 3300002I08 gene	1.007658	0.9263	NA
77241	9430013L14Rik	RIKEN cDNA 9430013L14 gene	1.007658	0.9494	0.9801
109815	H47	histocompatibility 47	1.007658	0.8685	NA
226751	Cdc42bpa	CDC42 binding protein kinase alpha	1.007658	0.93	0.9715
320706	9830001H06Rik	RIKEN cDNA 9830001H06 gene	1.007658	0.9149	NA
14043	Ext2	exostoses (multiple) 2	1.007557	0.9101	NA
66101	Ppih	peptidyl prolyl isomerase H	1.007557	0.8875	NA
66433	Chchd7	coiled-coil-helix-coiled-coil-helix domain containing 7	1.007557	0.9348	0.973
69878	Snrpf	small nuclear ribonucleoprotein polypeptide F	1.007557	0.9261	NA
240869	Zbtb37	zinc finger and BTB domain containing 37	1.007557	0.9592	0.9836
269019	Stk32a	serine/threonine kinase 32A	1.007557	0.929	NA
22648	Zfp11	zinc finger protein 11	1.007455	0.9513	0.9809
51869	Rif1	Rap1 interacting factor 1 homolog (yeast)	1.007455	0.9078	NA
56486	Gabarap	gamma-aminobutyric acid receptor associated protein	1.007455	0.9229	NA
65246	Xpo7	exportin 7	1.007455	0.8961	NA
70178	Fam108c	family with sequence similarity 108, member C	1.007455	0.9061	NA
227331	Gigyf2	GRB10 interacting GYF protein 2	1.007455	0.9253	NA
227580	C1ql3	C1q-like 3	1.007455	0.9171	NA
52705	Krr1	KRR1, small subunit (SSU) processome component, homolog (yeast)	1.007354	0.8992	NA
56407	Trpc4ap	transient receptor potential cation channel, subfamily C, member 4 associated protein	1.007354	0.9014	NA
68135	Eif3h	eukaryotic translation initiation factor 3, subunit H	1.007354	0.9212	NA
121021	Cspg4	chondroitin sulfate proteoglycan 4	1.007354	0.9621	0.9846
211347	Pank3	pantothenate kinase 3	1.007354	0.9527	0.9814
243308	A430033K04Rik	RIKEN cDNA A430033K04 gene	1.007354	0.9486	0.9798
319155	Hist1h4c	histone cluster 1, H4c	1.007354	0.955	0.9824
100038474	K230015D01Rik	RIKEN cDNA K230015D01 gene	1.007354	0.9553	0.9824
19981	Rpl37a	ribosomal protein L37a	1.007252	0.9411	0.9762
52463	Tet1	tet oncogene 1	1.007252	0.9556	0.9824
56386	B4galt6	UDP-Gal:betaGlcNAc beta 1,4-galactosyltransferase, polypeptide 6	1.007252	0.9452	0.978
74322	Cxxc1	CXXC finger 1 (PHD domain)	1.007252	0.934	0.9727
74498	Gorasp1	golgi reassembly stacking protein 1	1.007252	0.9161	NA
382867	Zfp488	zinc finger protein 488	1.007252	0.9379	0.9743
12511	Cd6	CD6 antigen	1.007151	0.9766	0.9905
14265	Fmr1	fragile X mental retardation syndrome 1 homolog	1.007151	0.9684	0.9867
17463	Psmd7	proteasome (prosome, macropain) 26S subunit, non-ATPase, 7	1.007151	0.8859	NA
70047	Trnt1	tRNA nucleotidyl transferase, CCA-adding, 1	1.007151	0.9525	0.9814

103172	Chchd10	coiled-coil-helix-coiled-coil-helix domain containing 10	1.007151	0.9067	NA
208715	Hmgcs1	3-hydroxy-3-methylglutaryl-Coenzyme A synthase 1	1.007151	0.9185	NA
224143	Poglut1	protein O-glucosyltransferase 1	1.007151	0.9166	NA
16880	Lifr	leukemia inhibitory factor receptor	1.007049	0.891	NA
30957	Mapk8ip3	mitogen-activated protein kinase 8 interacting protein 3	1.007049	0.9426	0.9767
53869	Rab11a	RAB11a, member RAS oncogene family	1.007049	0.9239	NA
66117	1110001J03Rik	RIKEN cDNA 1110001J03 gene	1.007049	0.9075	NA
68079	Pdcd2l	programmed cell death 2-like	1.007049	0.8938	NA
105445	Dock9	dedicator of cytokinesis 9	1.007049	0.932	NA
109246	Tspan9	tetraspanin 9	1.007049	0.9652	0.9856
226525	Rasal2	RAS protein activator like 2	1.007049	0.9591	0.9836
230101	Gba2	glucosidase beta 2	1.007049	0.8595	NA
19656	Rbmxrt	RNA binding motif protein, X chromosome retrogene	1.006948	0.9096	NA
26431	Git2	G protein-coupled receptor kinase-interactor 2	1.006948	0.9696	0.9874
54195	Gucy1b3	guanylate cyclase 1, soluble, beta 3	1.006948	0.9056	NA
56187	Rabggta	Rab geranylgeranyl transferase, a subunit	1.006948	0.9109	NA
70676	Gulp1	GULP, engulfment adaptor PTB domain containing 1	1.006948	0.9466	0.9788
78783	Brpf1	bromodomain and PHD finger containing, 1	1.006948	0.8926	NA
229542	Gatad2b	GATA zinc finger domain containing 2B	1.006948	0.9495	0.9801
11784	Apba2	amyloid beta (A4) precursor protein-binding, family A, member 2	1.006847	0.9195	NA
11865	Arntl	aryl hydrocarbon receptor nuclear translocator-like	1.006847	0.9258	NA
18572	Pdcd11	programmed cell death 11	1.006847	0.9124	NA
65019	Rpl23	ribosomal protein L23	1.006847	0.9064	NA
69125	Cnot8	CCR4-NOT transcription complex, subunit 8	1.006847	0.8802	NA
76491	Abhd14b	abhydrolase domain containing 14b	1.006847	0.9443	0.9776
77975	Tmem50b	transmembrane protein 50B	1.006847	0.8921	NA
100877	AV074028	expressed sequence AV074028	1.006847	0.9506	0.9806
218314	Zfp595	zinc finger protein 595	1.006847	0.9711	0.9882
218734	3830406C13Rik	RIKEN cDNA 3830406C13 gene	1.006847	0.9276	NA
242521	Klhl9	kelch-like 9 (Drosophila)	1.006847	0.9201	NA
545474	Scrt2	scratch homolog 2, zinc finger protein (Drosophila)	1.006847	0.9494	0.9801
11409	Acads	acyl-Coenzyme A dehydrogenase, short chain	1.006745	0.956	0.9824
11488	Adam11	a disintegrin and metallopeptidase domain 11	1.006745	0.9446	0.9776
12848	Cops2	COP9 (constitutive photomorphogenic) homolog, subunit 2 (Arabidopsis thaliana)	1.006745	0.919	NA
22218	Sumo1	SMT3 suppressor of mif two 3 homolog 1 (yeast)	1.006745	0.9062	NA
66580	Esf1	ESF1, nucleolar pre-rRNA processing protein, homolog (S. cerevisiae)	1.006745	0.9045	NA
67763	Prpsap1	phosphoribosyl pyrophosphate synthetase-associated protein 1	1.006745	0.9082	NA
74777	Sepn1	selenoprotein N, 1	1.006745	0.9589	0.9836
78016	Ccdc150	coiled-coil domain containing 150	1.006745	0.9277	NA
110326	Tas1r1	taste receptor, type 1, member 1	1.006745	0.9437	0.9772
218138	Gmds	GDP-mannose 4, 6-dehydratase	1.006745	0.927	NA
240444	Kcng2	potassium voltage-gated channel, subfamily G, member 2	1.006745	0.9652	0.9856
545238	G430049J08Rik	RIKEN cDNA G430049J08 gene	1.006745	0.9637	0.9849
14115	Fbln2	fibulin 2	1.006644	0.9431	0.9768
18567	Pdcd2	programmed cell death 2	1.006644	0.8787	NA
22719	Zfp61	zinc finger protein 61	1.006644	0.9639	0.9849
54710	Hs3st3b1	heparan sulfate (glucosamine) 3-O-sulfotransferase 3B1	1.006644	0.9546	0.9822
66938	1700029G01Rik	RIKEN cDNA 1700029G01 gene	1.006644	0.9039	NA
67437	Ssr3	signal sequence receptor, gamma	1.006644	0.8862	NA

78808	Stxbp5	syntaxin binding protein 5 (tomosyn)	1.006644	0.9532	0.9815
231386	Ythdc1	YTH domain containing 1	1.006644	0.9315	NA
231807	BC037034	cDNA sequence BC037034	1.006644	0.9567	0.9824
243272	Sbno1	sno, strawberry notch homolog 1 (Drosophila)	1.006644	0.9163	NA
12017	Bag1	BCL2-associated athanogene 1	1.006543	0.9334	NA
13356	Dgcr2	DiGeorge syndrome critical region gene 2	1.006543	0.9	NA
20336	Exoc4	exocyst complex component 4	1.006543	0.9352	NA
71968	Wdr73	WD repeat domain 73	1.006543	0.9119	NA
74455	Nsun6	NOL1/NOP2/Sun domain family member 6	1.006543	0.9306	NA
231760	Rimbp2	RIMS binding protein 2	1.006543	0.968	0.9866
17828	Muted	muted	1.006441	0.9303	NA
20609	Sstr5	somatostatin receptor 5	1.006441	0.9659	0.9858
67897	Rnmt	RNA (guanine-7-) methyltransferase	1.006441	0.936	NA
70024	Mcm10	minichromosome maintenance deficient 10 (S. cerevisiae)	1.006441	0.9435	0.9771
72992	2900076A07Rik	RIKEN cDNA 2900076A07 gene	1.006441	0.9779	0.9909
76932	Arfip2	ADP-ribosylation factor interacting protein 2	1.006441	0.9232	NA
78908	Igsf3	immunoglobulin superfamily, member 3	1.006441	0.9556	0.9824
100910	Chpf2	chondroitin polymerizing factor 2	1.006441	0.9388	NA
241128	Fam124b	family with sequence similarity 124, member B	1.006441	0.9557	0.9824
13542	Dvl1	dishevelled, dsh homolog 1 (Drosophila)	1.00634	0.9064	NA
23966	Odz4	odd Oz/ten-m homolog 4 (Drosophila)	1.00634	0.9588	0.9836
72649	Tmem209	transmembrane protein 209	1.00634	0.8982	NA
544696	D630037F22Rik	RIKEN cDNA D630037F22 gene	1.00634	0.9561	0.9824
12313	Calm1	calmodulin 1	1.006239	0.9596	0.9838
27493	A230006K03Rik	RIKEN cDNA A230006K03 gene	1.006239	0.9801	0.9917
66126	Elof1	elongation factor 1 homolog (ELF1, S. cerevisiae)	1.006239	0.9084	NA
77559	Agl	amylo-1,6-glucosidase, 4-alpha-glucanotransferase	1.006239	0.9248	NA
140887	Lnx2	ligand of numb-protein X 2	1.006239	0.8919	NA
224902	Safb2	scaffold attachment factor B2	1.006239	0.9179	NA
330171	Kctd10	potassium channel tetramerisation domain containing 10	1.006239	0.9195	NA
19283	Ptprz1	protein tyrosine phosphatase, receptor type Z, polypeptide 1	1.006137	0.9693	0.9872
19934	Rpl22	ribosomal protein L22	1.006137	0.9308	NA
24075	Taf10	TAF10 RNA polymerase II, TATA box binding protein (TBP)-associated factor	1.006137	0.9249	NA
74355	Smchd1	SMC hinge domain containing 1	1.006137	0.9494	0.9801
329659	E130311K13Rik	RIKEN cDNA E130311K13 gene	1.006137	0.9582	0.9835
442821	9330175M20Rik	RIKEN cDNA 9330175M20 gene	1.006137	0.9408	NA
21336	Tacr1	tachykinin receptor 1	1.006036	0.958	0.9834
94067	Mrpl43	mitochondrial ribosomal protein L43	1.006036	0.8959	NA
227733	Pip5kl1	phosphatidylinositol-4-phosphate 5-kinase-like 1	1.006036	0.9707	0.988
243963	Zfp473	zinc finger protein 473	1.006036	0.937	NA
12544	Cdc45	cell division cycle 45 homolog (S. cerevisiae)	1.005935	0.9508	0.9808
19941	Rpl26	ribosomal protein L26	1.005935	0.9109	NA
66871	Cpne8	copine VIII	1.005935	0.8966	NA
67738	Ppid	peptidylprolyl isomerase D (cyclophilin D)	1.005935	0.924	NA
103468	Nup107	nucleoporin 107	1.005935	0.9186	NA
108853	Mtrf1l	mitochondrial translational release factor 1-like	1.005935	0.9332	NA
237759	Col23a1	collagen, type XXIII, alpha 1	1.005935	0.9094	NA
240396	Mex3c	mex3 homolog C (C. elegans)	1.005935	0.9315	NA
17763	Mtcp1	mature T-cell proliferation 1	1.005834	0.9348	NA

67398	Srpr	signal recognition particle receptor ('docking protein')	1.005834	0.9361	NA
72310	Nkg7	natural killer cell group 7 sequence	1.005834	0.963	0.9848
74202	Fblim1	filamin binding LIM protein 1	1.005834	0.9716	0.9885
76900	Ssbp4	single stranded DNA binding protein 4	1.005834	0.9496	0.9801
78697	Pus7	pseudouridylate synthase 7 homolog (S. cerevisiae)	1.005834	0.9509	0.9808
381022	Mll2	myeloid/lymphoid or mixed-lineage leukemia 2	1.005834	0.9665	0.9858
15463	Agfg1	ArfGAP with FG repeats 1	1.005733	0.9274	NA
66997	Psmd12	proteasome (prosome, macropain) 26S subunit, non-ATPase, 12	1.005733	0.9539	0.9818
71989	Rpusd4	RNA pseudouridylate synthase domain containing 4	1.005733	0.9104	NA
94232	Ubqln4	ubiquilin 4	1.005733	0.9474	0.9792
216558	Ugp2	UDP-glucose pyrophosphorylase 2	1.005733	0.9019	NA
11739	Slc25a4	solute carrier family 25 (mitochondrial carrier, adenine nucleotide translocator), member 4	1.005632	0.9086	NA
14870	Gstp1	glutathione S-transferase, pi 1	1.005632	0.9322	NA
28295	D10Jhu81e	DNA segment, Chr 10, Johns Hopkins University 81 expressed	1.005632	0.9034	NA
100972	Rab28	RAB28, member RAS oncogene family	1.005632	0.9382	NA
229279	Hnrnpa3	heterogeneous nuclear ribonucleoprotein A3	1.005632	0.938	NA
276770	Eif5a	eukaryotic translation initiation factor 5A	1.005632	0.9391	NA
13426	Dync1i1	dynein cytoplasmic 1 intermediate chain 1	1.00553	0.9232	NA
23984	Pde10a	phosphodiesterase 10A	1.00553	0.9712	0.9882
52206	Anapc4	anaphase promoting complex subunit 4	1.00553	0.8987	NA
67921	Ube2f	ubiquitin-conjugating enzyme E2F (putative)	1.00553	0.9434	NA
77733	Rnf170	ring finger protein 170	1.00553	0.9277	NA
238662	Gm4934	predicted gene 4934	1.00553	0.979	0.9911
66779	4933432I09Rik	RIKEN cDNA 4933432I09 gene	1.005429	0.9846	0.9934
71838	Phf7	PHD finger protein 7	1.005429	0.9548	0.9823
76007	Zmym2	zinc finger, MYM-type 2	1.005429	0.9352	NA
216028	Lrrtm3	leucine rich repeat transmembrane neuronal 3	1.005429	0.9589	0.9836
216705	Clint1	clathrin interactor 1	1.005429	0.9464	NA
228136	Zdhhc5	zinc finger, DHHC domain containing 5	1.005429	0.9333	NA
330863	Trim67	tripartite motif-containing 67	1.005429	0.9794	0.9912
16785	Rpsa	ribosomal protein SA	1.005328	0.9611	0.9846
66676	Tmed7	transmembrane emp24 protein transport domain containing 7	1.005328	0.9615	0.9846
81018	Rnf114	ring finger protein 114	1.005328	0.9662	0.9858
223732	Ldoc1l	leucine zipper, down-regulated in cancer 1-like	1.005328	0.9355	NA
228961	Npepl1	aminopeptidase-like 1	1.005328	0.9513	0.981
12361	Cask	calcium/calmodulin-dependent serine protein kinase (MAGUK family)	1.005227	0.9692	0.9872
27367	Rpl3	ribosomal protein L3	1.005227	0.9544	0.9821
50788	Fbxl8	F-box and leucine-rich repeat protein 8	1.005227	0.9533	0.9815
67658	4930563F15Rik	RIKEN cDNA 4930563F15 gene	1.005227	0.9704	0.9879
69065	Chac1	ChaC, cation transport regulator-like 1 (E. coli)	1.005227	0.9772	0.9907
108800	Ston2	stonin 2	1.005227	0.9646	0.9854
224481	Tfb1m	transcription factor B1, mitochondrial	1.005227	0.952	NA
230767	Iqcc	IQ motif containing C	1.005227	0.954	0.9818
319463	C230057M02Rik	RIKEN cDNA C230057M02 gene	1.005227	0.9601	0.9838
27054	Sec23b	SEC23B (S. cerevisiae)	1.005126	0.9254	NA
243369	Sspo	SCO-spondin	1.005126	0.9688	0.9871
20810	Srm	spermidine synthase	1.005025	0.9593	0.9836
57267	Apba3	amyloid beta (A4) precursor protein-binding, family A, member 3	1.005025	0.9644	0.9854
66190	Acer3	alkaline ceramidase 3	1.005025	0.9317	NA

66242	Mrps16	mitochondrial ribosomal protein S16	1.005025	0.9158	NA
66387	Nudt8	nudix (nucleoside diphosphate linked moiety X)-type motif 8	1.005025	0.9253	NA
110809	Srsf1	serine/arginine-rich splicing factor 1	1.005025	0.9592	0.9836
225283	Rprd1a	regulation of nuclear pre-mRNA domain containing 1A	1.005025	0.9005	NA
11428	Aco1	aconitase 1	1.004924	0.9481	NA
18004	Nek1	NIMA (never in mitosis gene a)-related expressed kinase 1	1.004924	0.9591	0.9836
19428	Rasl2-9-ps	RAS-like, family 2, locus 9, pseudogene	1.004924	0.9537	NA
19935	Mrpl23	mitochondrial ribosomal protein L23	1.004924	0.9478	NA
53605	Nap1l1	nucleosome assembly protein 1-like 1	1.004924	0.9358	NA
75403	1010001B22Rik	RIKEN cDNA 1010001B22 gene	1.004924	0.9793	0.9912
319885	Zcchc7	zinc finger, CCHC domain containing 7	1.004924	0.9431	NA
381933	6430531B16Rik	RIKEN cDNA 6430531B16 gene	1.004924	0.9625	0.9846
432940	Fam105b	family with sequence similarity 105, member B	1.004924	0.9413	NA
73121	Fam101a	family with sequence similarity 101, member A	1.004823	0.969	0.9871
74302	Mtmr3	myotubularin related protein 3	1.004823	0.9601	0.9838
75729	4933432B09Rik	RIKEN cDNA 4933432B09 gene	1.004823	0.9682	0.9867
17973	Nck1	non-catalytic region of tyrosine kinase adaptor protein 1	1.004722	0.9358	NA
50781	Dkk3	dickkopf homolog 3 (Xenopus laevis)	1.004722	0.9621	0.9846
53951	Ccdc75	coiled-coil domain containing 75	1.004722	0.9307	NA
66320	Tmem208	transmembrane protein 208	1.004722	0.9271	NA
75355	4930553P18Rik	RIKEN cDNA 4930553P18 gene	1.004722	0.9654	0.9856
106042	Prickle1	prickle homolog 1 (Drosophila)	1.004722	0.9392	NA
107869	Cth	cystathionase (cystathionine gamma-lyase)	1.004722	0.9078	NA
210711	Mcmbp	MCM (minichromosome maintenance deficient) binding protein	1.004722	0.9401	NA
224617	Tbc1d24	TBC1 domain family, member 24	1.004722	0.9595	0.9837
231326	Aasdh	aminoadipate-semialdehyde dehydrogenase	1.004722	0.9415	NA
383815	Rps24-ps2	ribosomal protein S24, pseudogene 2	1.004722	0.9507	NA
11769	Ap1s1	adaptor protein complex AP-1, sigma 1	1.004621	0.9428	NA
51797	Ctps	cytidine 5'-triphosphate synthase	1.004621	0.9426	NA
59043	Wsb2	WD repeat and SOCS box-containing 2	1.004621	0.9453	NA
71182	4933417G07Rik	RIKEN cDNA 4933417G07 gene	1.004621	0.9648	0.9855
73689	Bloc1s2	biogenesis of lysosome-related organelles complex-1, subunit 2	1.004621	0.9211	NA
237553	Trhde	TRH-degrading enzyme	1.004621	0.9327	NA
18679	Phka1	phosphorylase kinase alpha 1	1.00452	0.9619	0.9846
28028	Mrpl50	mitochondrial ribosomal protein L50	1.00452	0.915	NA
67768	N6amt1	N-6 adenine-specific DNA methyltransferase 1 (putative)	1.00452	0.9565	NA
71970	Scand3	SCAN domain containing 3	1.00452	0.9669	0.986
93836	Rnf111	ring finger 111	1.00452	0.9474	NA
105855	Nckap1l	NCK associated protein 1 like	1.00452	0.9628	0.9847
269881	Map3k10	mitogen-activated protein kinase kinase kinase 10	1.00452	0.976	0.9903
320634	Ocrl	oculocerebrorenal syndrome of Lowe	1.00452	0.9567	NA
27998	Exosc5	exosome component 5	1.004419	0.9543	NA
66793	Efcab1	EF hand calcium binding domain 1	1.004419	0.9828	0.9926
66928	3110001D03Rik	RIKEN cDNA 3110001D03 gene	1.004419	0.9307	NA
269105	Gm5048	predicted gene 5048	1.004419	0.9459	NA
381356	5930434B04Rik	RIKEN cDNA 5930434B04 gene	1.004419	0.9279	NA
100038538	Gm10767	predicted gene 10767	1.004419	0.945	NA
14272	Fnta	farnesyltransferase, CAAX box, alpha	1.004319	0.9233	NA
17190	Mbd1	methyl-CpG binding domain protein 1	1.004319	0.9807	0.992

60411	Cenpk	centromere protein K	1.004319	0.9775	0.9908
67031	Upf3a	UPF3 regulator of nonsense transcripts homolog A (yeast)	1.004319	0.9516	NA
207704	Gtpbp10	GTP-binding protein 10 (putative)	1.004319	0.9392	NA
208111	C330019L16Rik	RIKEN cDNA C330019L16 gene	1.004319	0.9736	0.9894
53945	Slc40a1	solute carrier family 40 (iron-regulated transporter), member 1	1.004218	0.9656	0.9857
66492	Zmat2	zinc finger, matrin type 2	1.004218	0.9371	NA
17101	Lyst	lysosomal trafficking regulator	1.004117	0.98	0.9917
18000	Sep-02	septin 2	1.004117	0.943	NA
20520	Slc22a5	solute carrier family 22 (organic cation transporter), member 5	1.004117	0.9421	NA
66222	Serpinb1a	serine (or cysteine) peptidase inhibitor, clade B, member 1a	1.004117	0.9809	0.992
66421	2410004B18Rik	RIKEN cDNA 2410004B18 gene	1.004117	0.9571	NA
102693	Phldb1	pleckstrin homology-like domain, family B, member 1	1.004117	0.9609	NA
107823	Whsc1	Wolf-Hirschhorn syndrome candidate 1 (human)	1.004117	0.9473	NA
233812	BC030336	cDNA sequence BC030336	1.004117	0.959	NA
329831	Fam166b	family with sequence similarity 166, member B	1.004117	0.9736	0.9894
18798	Plcb4	phospholipase C, beta 4	1.004016	0.973	0.9891
19122	Prnp	prion protein	1.004016	0.9559	NA
22195	Ube2l3	ubiquitin-conjugating enzyme E2L 3	1.004016	0.9333	NA
99229	AU041474	expressed sequence AU041474	1.004016	0.9686	0.9869
654472	Gm12070	glyceraldehyde-3-phosphate dehydrogenase pseudogene	1.004016	0.9711	0.9882
21834	Thrb	thyroid hormone receptor beta	1.003915	0.9705	0.988
67115	Rpl14	ribosomal protein L14	1.003915	0.9486	NA
83814	Nedd4l	neural precursor cell expressed, developmentally down-regulated gene 4-like	1.003915	0.9507	NA
214952	Rhot2	ras homolog gene family, member T2	1.003915	0.9725	0.9889
100049077	LOC100049077	hypothetical protein LOC100049077	1.003915	0.964	0.985
17165	Mapkapk5	MAP kinase-activated protein kinase 5	1.003814	0.9285	NA
72003	Synpr	synaptoporin	1.003814	0.9727	0.9889
109778	Blvra	biliverdin reductase A	1.003814	0.9556	NA
171486	Cd99l2	CD99 antigen-like 2	1.003814	0.9605	NA
258507	Olfr96	olfactory receptor 96	1.003814	0.9924	0.9967
320940	Atp11c	ATPase, class VI, type 11C	1.003814	0.968	0.9866
11843	Arf4	ADP-ribosylation factor 4	1.003714	0.9575	NA
12462	Cct3	chaperonin containing Tcp1, subunit 3 (gamma)	1.003714	0.9537	NA
12465	Cct5	chaperonin containing Tcp1, subunit 5 (epsilon)	1.003714	0.9327	NA
20544	Slc9a1	solute carrier family 9 (sodium/hydrogen exchanger), member 1	1.003714	0.9864	0.9943
24018	Rngtt	RNA guanylyltransferase and 5'-phosphatase	1.003714	0.9399	NA
67042	Ift27	intraflagellar transport 27 homolog (Chlamydomonas)	1.003714	0.9612	NA
67048	Vma21	VMA21 vacuolar H+-ATPase homolog (S. cerevisiae)	1.003714	0.9557	NA
73192	Xpot	exportin, tRNA (nuclear export receptor for tRNAs)	1.003714	0.9476	NA
104859	Tecpr2	tectonin beta-propeller repeat containing 2	1.003714	0.959	NA
381833	Prb1	proline-rich protein BstNI subfamily 1	1.003714	0.9674	0.9864
26434	Prnd	prion protein dublet	1.003613	0.978	0.9909
73293	Ccdc103	coiled-coil domain containing 103	1.003613	0.9751	0.9901
101401	Adamts9	a disintegrin-like and metallopeptidase (reprolysin type) with thrombospondin type 1 motif, 9	1.003613	0.9726	0.9889
109154	Mlec	malectin	1.003613	0.9536	NA
56693	Crtap	cartilage associated protein	1.003512	0.9546	NA
67019	Actr6	ARP6 actin-related protein 6 homolog (yeast)	1.003512	0.9538	NA
69535	2310004N24Rik	RIKEN cDNA 2310004N24 gene	1.003512	0.9628	NA
76594	Dnajc18	DnaJ (Hsp40) homolog, subfamily C, member 18	1.003512	0.9418	NA

78246	Phf23	PHD finger protein 23	1.003512	0.9641	NA
105428	Fam149b	family with sequence similarity 149, member B	1.003512	0.9576	NA
20773	Sptlc2	serine palmitoyltransferase, long chain base subunit 2	1.003412	0.9627	NA
52906	Ahi1	Abelson helper integration site 1	1.003412	0.9675	NA
12461	Cct2	chaperonin containing Tcp1, subunit 2 (beta)	1.003311	0.9497	NA
13143	Dapk2	death-associated protein kinase 2	1.003311	0.9778	0.9909
56305	Pitpnb	phosphatidylinositol transfer protein, beta	1.003311	0.9771	0.9907
93746	Gprc5d	G protein-coupled receptor, family C, group 5, member D	1.003311	0.9816	0.9922
217119	Xylt2	xylosyltransferase II	1.003311	0.9804	0.9918
268859	Rbfox1	RNA binding protein, fox-1 homolog (C. elegans) 1	1.003311	0.9651	NA
319521	A730056I06Rik	RIKEN cDNA A730056I06 gene	1.003311	0.9745	0.9898
17283	Men1	multiple endocrine neoplasia 1	1.00321	0.9593	NA
21349	Tal1	T-cell acute lymphocytic leukemia 1	1.00321	0.9827	0.9926
66272	Cox16	COX16 cytochrome c oxidase assembly homolog (S. cerevisiae)	1.00321	0.958	NA
110842	Etfa	electron transferring flavoprotein, alpha polypeptide	1.00321	0.9512	NA
14027	Evpl	envoplakin	1.00311	0.9779	0.9909
20195	S100a11	S100 calcium binding protein A11 (calgizzarin)	1.00311	0.9809	0.992
67420	Far1	fatty acyl CoA reductase 1	1.00311	0.987	0.9944
69683	2310044H10Rik	RIKEN cDNA 2310044H10 gene	1.00311	0.9823	0.9924
98193	Dcaf8	DDB1 and CUL4 associated factor 8	1.00311	0.9644	NA
194388	Tet3	tet oncogene family member 3	1.00311	0.9884	0.9949
13822	Epb4.1l2	erythrocyte protein band 4.1-like 2	1.003009	0.9845	0.9934
19291	Purb	purine rich element binding protein B	1.003009	0.962	NA
50927	Nasp	nuclear autoantigenic sperm protein (histone-binding)	1.003009	0.9676	NA
56463	Snd1	staphylococcal nuclease and tudor domain containing 1	1.003009	0.968	NA
66272	Cox16	COX16 cytochrome c oxidase assembly homolog (S. cerevisiae)	1.003009	0.9627	NA
66882	Bzw1	basic leucine zipper and W2 domains 1	1.003009	0.9764	0.9905
68001	1110004E09Rik	RIKEN cDNA 1110004E09 gene	1.003009	0.9538	NA
71715	Dhx35	DEAH (Asp-Glu-Ala-His) box polypeptide 35	1.003009	0.968	NA
71833	Dcaf7	DDB1 and CUL4 associated factor 7	1.003009	0.9796	0.9913
72103	Aplf	aprataxin and PNKP like factor	1.003009	0.9649	NA
625123	Gm6557	predicted gene 6557	1.003009	0.9759	0.9903
100043060	Gm4200	predicted gene 4200	1.003009	0.9886	0.9949
11848	Rhoa	ras homolog gene family, member A	1.002908	0.9552	NA
56299	Fkbpl	FK506 binding protein-like	1.002908	0.962	NA
68281	4930430F08Rik	RIKEN cDNA 4930430F08 gene	1.002908	0.9689	NA
74577	Glb1l	galactosidase, beta 1-like	1.002908	0.9702	NA
268933	Wdr24	WD repeat domain 24	1.002908	0.9773	0.9908
20980	Syt2	synaptotagmin II	1.002808	0.9766	0.9905
22036	Traip	TRAF-interacting protein	1.002808	0.985	0.9936
54720	Rcan1	regulator of calcineurin 1	1.002808	0.9735	NA
68572	Ict1	immature colon carcinoma transcript 1	1.002808	0.9678	NA
72555	Shisa9	shisa homolog 9 (Xenopus laevis)	1.002808	0.9726	NA
103963	Rpn1	ribophorin I	1.002808	0.9592	NA
16650	Kpna6	karyopherin (importin) alpha 6	1.002707	0.9454	NA
19280	Ptprs	protein tyrosine phosphatase, receptor type, S	1.002707	0.9635	NA
57784	Bin3	bridging integrator 3	1.002707	0.9797	0.9914
66384	Srp19	signal recognition particle 19	1.002707	0.9774	0.9908
69217	Plekha4	pleckstrin homology domain containing, family A (phosphoinositide binding specific) member 4	1.002707	0.9727	NA

216821	Tmem11	transmembrane protein 11	1.002707	0.952	NA	
234371	Tmem161a	transmembrane protein 161A	1.002707	0.9686	NA	
14950	H13	histocompatibility 13	1.002607	0.9599	NA	
74471	4933440N22Rik	RIKEN cDNA 4933440N22 gene	1.002607	0.9816		0.9922
109241	Mbd5	methyl-CpG binding domain protein 5	1.002607	0.9767		0.9905
224691	Zfp472	zinc finger protein 472	1.002607	0.9702	NA	
235416	Lman1l	lectin, mannose-binding 1 like	1.002607	0.976		0.9903
15159	Hccs	holocytochrome c synthetase	1.002506	0.9823		0.9924
81845	Gpank1	G patch domain and ankyrin repeats 1	1.002506	0.963	NA	
544696	D630037F22Rik	RIKEN cDNA D630037F22 gene	1.002506	0.9747	NA	
17904	Myl6	myosin, light polypeptide 6, alkali, smooth muscle and non-muscle	1.002406	0.9691	NA	
66416	Ndufa7	NADH dehydrogenase (ubiquinone) 1 alpha subcomplex, 7 (B14.5a)	1.002406	0.969	NA	
97130	C77080	expressed sequence C77080	1.002406	0.9631	NA	
106821	AI314976	expressed sequence AI314976	1.002406	0.9625	NA	
218294	Cdc14b	CDC14 cell division cycle 14 homolog B (S. cerevisiae)	1.002406	0.9687	NA	
100040654	Gm2885	predicted gene 2885	1.002406	0.9868		0.9944
13435	Dnmt3a	DNA methyltransferase 3A	1.002305	0.9685	NA	
22142	Tuba1a	tubulin, alpha 1A	1.002305	0.9808		0.992
70005	1700029I01Rik	RIKEN cDNA 1700029I01 gene	1.002305	0.9813		0.9921
18951	Sep-05	septin 5	1.002205	0.9873		0.9945
66209	1110054O05Rik	RIKEN cDNA 1110054O05 gene	1.002205	0.9729	NA	
70047	Trnt1	tRNA nucleotidyl transferase, CCA-adding, 1	1.002205	0.9791	NA	
72787	Tmem48	transmembrane protein 48	1.002205	0.9778	NA	
235040	Atg4d	autophagy-related 4D (yeast)	1.002205	0.9728	NA	
268752	Wdfy2	WD repeat and FYVE domain containing 2	1.002205	0.9897		0.9955
100038620	Gm10631	predicted gene 10631	1.002205	0.9905		0.9957
17434	Mocs2	molybdenum cofactor synthesis 2	1.002104	0.9674	NA	
27357	Gyg	glycogenin	1.002104	0.9685	NA	
66067	Gtpbp8	GTP-binding protein 8 (putative)	1.002104	0.9774	NA	
74493	Tnks2	tankyrase, TRF1-interacting ankyrin-related ADP-ribose polymerase 2	1.002104	0.9752	NA	
76781	Mettl4	methyltransferase like 4	1.002104	0.9801	NA	
80987	Nckipsd	NCK interacting protein with SH3 domain	1.002104	0.9866		0.9943
108653	Rimklb	ribosomal modification protein rimK-like family member B	1.002104	0.9873		0.9945
330222	Sdk1	sidekick homolog 1 (chicken)	1.002104	0.9811		0.992
17293	Mesp2	mesoderm posterior 2	1.002004	0.9844		0.9934
18703	Pigr	polymeric immunoglobulin receptor	1.002004	0.9899		0.9955
19243	Ptp4a1	protein tyrosine phosphatase 4a1	1.002004	0.9829		0.9926
71131	Zfp689	zinc finger protein 689	1.002004	0.9806	NA	
381832	Prpmp5	proline-rich protein MP5	1.002004	0.9871		0.9945
27357	Gyg	glycogenin	1.001904	0.97	NA	
71722	Cic	capicua homolog (Drosophila)	1.001904	0.9753	NA	
76960	Bcas1	breast carcinoma amplified sequence 1	1.001904	0.984		0.9932
226151	Fam178a	family with sequence similarity 178, member A	1.001904	0.9762	NA	
192285	Phf21a	PHD finger protein 21A	1.001803	0.9743	NA	
227743	Mapkap1	mitogen-activated protein kinase associated protein 1	1.001703	0.9775	NA	
233489	Picalm	phosphatidylinositol binding clathrin assembly protein	1.001703	0.9852		0.9937
243510	Ccdc142	coiled-coil domain containing 142	1.001703	0.9926		0.9968
100044951	LOC100044951	hypothetical LOC100044951	1.001703	0.9767	NA	
17762	Mapt	microtubule-associated protein tau	1.001603	0.9819	NA	

30953	Schip1	schwannomin interacting protein 1	1.001603	0.9877	0.9946
75729	4933432B09Rik	RIKEN cDNA 4933432B09 gene	1.001603	0.9875	0.9946
78651	Lsm6	LSM6 homolog, U6 small nuclear RNA associated (S. cerevisiae)	1.001603	0.9754	NA
258353	Olfr521	olfactory receptor 521	1.001603	0.9865	0.9943
13528	Dtnb	dystrobrevin, beta	1.001502	0.986	0.9941
66768	Pacrgl	PARK2 co-regulated-like	1.001502	0.9761	NA
110157	Raf1	v-raf-leukemia viral oncogene 1	1.001502	0.9762	NA
268451	Rab11fip4	RAB11 family interacting protein 4 (class II)	1.001502	0.9853	NA
16416	Itgb3	integrin beta 3	1.001402	0.9943	0.9974
52665	Echdc1	enoyl Coenzyme A hydratase domain containing 1	1.001402	0.9885	0.9949
66410	Mterfd1	MTERF domain containing 1	1.001402	0.9883	0.9949
66682	Trappc5	trafficking protein particle complex 5	1.001402	0.9808	NA
67433	Ccdc127	coiled-coil domain containing 127	1.001402	0.9888	0.9949
78618	Acap2	ArfGAP with coiled-coil, ankyrin repeat and PH domains 2	1.001402	0.9859	NA
101568	Vrk3	vaccinia related kinase 3	1.001402	0.9781	NA
230587	Glis1	GLIS family zinc finger 1	1.001402	0.9874	0.9946
246782	Atpaf2	ATP synthase mitochondrial F1 complex assembly factor 2	1.001402	0.9796	NA
329739	Fam102b	family with sequence similarity 102, member B	1.001402	0.9893	0.9954
12464	Cct4	chaperonin containing Tcp1, subunit 4 (delta)	1.001302	0.984	NA
13992	Khdrbs3	KH domain containing, RNA binding, signal transduction associated 3	1.001302	0.985	NA
52123	Agpat5	1-acylglycerol-3-phosphate O-acyltransferase 5 (lysophosphatidic acid acyltransferase, epsilon)	1.001302	0.9819	NA
58175	Rgs20	regulator of G-protein signaling 20	1.001302	0.9936	0.9971
66152	Uqcr10	ubiquinol-cytochrome c reductase, complex III subunit X	1.001302	0.983	NA
68581	Tmed10	transmembrane emp24-like trafficking protein 10 (yeast)	1.001302	0.9838	NA
69574	Cmbl	carboxymethylenebutenolidase-like (Pseudomonas)	1.001302	0.9873	NA
234730	Fuk	fucokinase	1.001302	0.9801	NA
66083	Setd6	SET domain containing 6	1.001201	0.9834	NA
68198	Ndufb2	NADH dehydrogenase (ubiquinone) 1 beta subcomplex, 2	1.001201	0.983	NA
70807	Arrdc2	arrestin domain containing 2	1.001201	0.993	0.997
108086	Rnf216	ring finger protein 216	1.001201	0.9869	NA
228136	Zdhhc5	zinc finger, DHHC domain containing 5	1.001201	0.9911	0.996
238505	Mtr	5-methyltetrahydrofolate-homocysteine methyltransferase	1.001201	0.9891	0.9952
384061	Fndc5	fibronectin type III domain containing 5	1.001201	0.9867	NA
666173	Vps13b	vacuolar protein sorting 13B (yeast)	1.001201	0.9909	0.996
67163	Ccdc47	coiled-coil domain containing 47	1.001101	0.9943	0.9974
75869	Arl5b	ADP-ribosylation factor-like 5B	1.001101	0.988	NA
244219	Zfp668	zinc finger protein 668	1.001101	0.9901	0.9956
276952	Rasl10b	RAS-like, family 10, member B	1.001101	0.9936	0.9971
547267	Gm6030	predicted gene 6030	1.001101	0.9849	NA
100042049	LOC100042049	ribosomal protein L22 like 1 pseudogene	1.001101	0.9872	NA
16549	Khsrp	KH-type splicing regulatory protein	1.001001	0.9938	0.9972
30841	Kdm2b	lysine (K)-specific demethylase 2B	1.001001	0.9907	NA
71787	Trnau1ap	tRNA selenocysteine 1 associated protein 1	1.001001	0.9821	NA
333315	Frem3	Fras1 related extracellular matrix protein 3	1.001001	0.9908	NA
19159	Cyth3	cytohesin 3	1.000901	0.9937	0.9972
19171	Psmb10	proteasome (prosome, macropain) subunit, beta type 10	1.000901	0.9856	NA
54484	Mkrn1	makorin, ring finger protein, 1	1.000901	0.9863	NA
56433	Vps29	vacuolar protein sorting 29 (S. pombe)	1.000901	0.9845	NA
80981	Arl4d	ADP-ribosylation factor-like 4D	1.000901	0.9909	NA

99183	AU018505	expressed sequence AU018505	1.000901	0.9881	NA
104457	0610010K14Rik	RIKEN cDNA 0610010K14 gene	1.000901	0.9852	NA
106618	Wdr90	WD repeat domain 90	1.000901	0.9851	NA
227059	Slc39a10	solute carrier family 39 (zinc transporter), member 10	1.000901	0.9936	0.9971
269180	Inpp4a	inositol polyphosphate-4-phosphatase, type I	1.000901	0.9893	NA
77781	Epm2aip1	EPM2A (laforin) interacting protein 1	1.000801	0.9877	NA
108062	Cstf2	cleavage stimulation factor, 3' pre-RNA subunit 2	1.000801	0.9887	NA
230936	Phf13	PHD finger protein 13	1.000801	0.9945	0.9974
70997	Spef1	sperm flagellar 1	1.0007	0.9913	NA
104884	Tdp1	tyrosyl-DNA phosphodiesterase 1	1.0007	0.9904	NA
192285	Phf21a	PHD finger protein 21A	1.0007	0.9925	NA
100504423	LOC100504423	hypothetical protein LOC100504423	1.0007	0.9924	NA
11539	Adora1	adenosine A1 receptor	1.0006	0.9899	NA
15289	Hmgb1	high mobility group box 1	1.0006	0.9937	NA
58802	Kcnmb4	potassium large conductance calcium-activated channel, subfamily M, beta member 4	1.0006	0.9931	NA
67945	Rpl41	ribosomal protein L41	1.0006	0.9937	NA
76967	2700049A03Rik	RIKEN cDNA 2700049A03 gene	1.0006	0.9919	NA
319887	E030030I06Rik	RIKEN cDNA E030030I06 gene	1.0006	0.997	0.9985
381038	Parl	presenilin associated, rhomboid-like	1.0006	0.991	NA
66290	Atp6v1g1	ATPase, H+ transporting, lysosomal V1 subunit G1	1.0005	0.9919	NA
194655	Klf11	Kruppel-like factor 11	1.0005	0.998	0.999
668158	Ccdc85c	coiled-coil domain containing 85C	1.0005	0.9978	0.999
12014	Bach2	BTB and CNC homology 2	1.0004	0.999	0.9994
19347	Dennd5a	DENN/MADD domain containing 5A	1.0004	0.9935	NA
101489	Ric8	resistance to inhibitors of cholinesterase 8 homolog (C. elegans)	1.0004	0.9953	NA
211798	Mfsd9	major facilitator superfamily domain containing 9	1.0004	0.9971	0.9985
230459	Cyp2j13	cytochrome P450, family 2, subfamily j, polypeptide 13	1.0004	0.9946	NA
668137	Gm8994	predicted gene 8994	1.0004	0.9958	NA
14172	Fgf18	fibroblast growth factor 18	1.0003	0.997	NA
20317	Serpinf1	serine (or cysteine) peptidase inhibitor, clade F, member 1	1.0003	0.9988	0.9993
27207	Rps11	ribosomal protein S11	1.0003	0.9965	NA
51886	Fubp1	far upstream element (FUSE) binding protein 1	1.0003	0.9965	NA
74754	Dhcr24	24-dehydrocholesterol reductase	1.0003	0.9966	NA
13194	Ddb1	damage specific DNA binding protein 1	1.0002	0.9974	NA
54411	Atp6ap1	ATPase, H+ transporting, lysosomal accessory protein 1	1.0002	0.9986	0.9993
66258	Mrps17	mitochondrial ribosomal protein S17	1.0002	0.9965	NA
67279	Med31	mediator of RNA polymerase II transcription, subunit 31 homolog (yeast)	1.0002	0.9965	NA
70552	Lrrc56	leucine rich repeat containing 56	1.0002	0.9979	0.999
71733	Susd2	sushi domain containing 2	1.0002	0.9983	0.9992
77528	C030014A21Rik	RIKEN cDNA C030014A21 gene	1.0002	0.9985	0.9993
108143	Taf9	TAF9 RNA polymerase II, TATA box binding protein (TBP)-associated factor	1.0002	0.9983	NA
232341	Wnk1	WNK lysine deficient protein kinase 1	1.0002	0.9972	NA
239157	Pnma2	paraneoplastic antigen MA2	1.0002	0.9974	NA
83397	Akap12	A kinase (PRKA) anchor protein (gravin) 12	1.0001	0.9998	0.9999
329693	Fcrl5	Fc receptor-like 5	1.0001	0.9994	0.9998
13929	Amz2	archaelysin family metallopeptidase 2	1	0.9982	NA
15975	Ifnar1	interferon (alpha and beta) receptor 1	1	0.9951	NA
16536	Kcnq2	potassium voltage-gated channel, subfamily Q, member 2	1	0.9989	0.9994
20316	Sdf2	stromal cell derived factor 2	1	0.9951	NA

21406	Tcf12	transcription factor 12	1	0.9967	0.9983
21761	Morf4l1	mortality factor 4 like 1	1	0.996	NA
22764	Zfx	zinc finger protein X-linked	1	0.992	NA
26443	Psma6	proteasome (prosome, macropain) subunit, alpha type 6	1	0.9966	NA
30937	Lmcd1	LIM and cysteine-rich domains 1	1	0.9991	0.9995
57276	Vsig2	V-set and immunoglobulin domain containing 2	1	0.9997	0.9999
67549	Gpr89	G protein-coupled receptor 89	1	0.9959	NA
69126	1810022K09Rik	RIKEN cDNA 1810022K09 gene	1	0.9991	NA
69318	1700007K09Rik	RIKEN cDNA 1700007K09 gene	1	1	1
69860	Eif1ad	eukaryotic translation initiation factor 1A domain containing	1	0.9978	NA
69940	Exoc1	exocyst complex component 1	1	0.9936	NA
74901	Kbtbd11	kelch repeat and BTB (POZ) domain containing 11	1	0.9993	NA
76508	2210015D19Rik	RIKEN cDNA 2210015D19 gene	1	0.9984	NA
77044	Arid2	AT rich interactive domain 2 (ARID, RFX-like)	1	0.9976	NA
105352	Dusp22	dual specificity phosphatase 22	1	0.997	NA
107477	Guca1b	guanylate cyclase activator 1B	1	0.9998	0.9999
110784	Nr3c2	nuclear receptor subfamily 3, group C, member 2	1	0.9987	0.9993
140499	Ube2j2	ubiquitin-conjugating enzyme E2, J2 homolog (yeast)	1	0.9997	NA
218811	Sec24c	Sec24 related gene family, member C (S. cerevisiae)	1	0.9994	NA
229363	Gmps	guanine monophosphate synthetase	1	0.997	NA
237759	Col23a1	collagen, type XXIII, alpha 1	1	0.9967	NA
319197	Gpr4	G protein-coupled receptor 4	1	0.9983	0.9992
12660	Chka	choline kinase alpha	0.999001	0.99	NA
12793	Cnih	cornichon homolog (Drosophila)	0.999001	0.9895	NA
13123	Cyp7b1	cytochrome P450, family 7, subfamily b, polypeptide 1	0.999001	0.9922	NA
14050	Eya3	eyes absent 3 homolog (Drosophila)	0.999001	0.9777	NA
14200	Fhl2	four and a half LIM domains 2	0.999001	0.9911	0.996
16202	Ilk	integrin linked kinase	0.999001	0.9902	0.9956
16597	Klf12	Kruppel-like factor 12	0.999001	0.9946	0.9974
17756	Mtap2	microtubule-associated protein 2	0.999001	0.9917	NA
18769	Pkig	protein kinase inhibitor, gamma	0.999001	0.9929	0.9969
18970	Polb	polymerase (DNA directed), beta	0.999001	0.9848	NA
19934	Rpl22	ribosomal protein L22	0.999001	0.9801	NA
20534	Slc4a1ap	solute carrier family 4 (anion exchanger), member 1, adaptor protein	0.999001	0.9932	0.997
22592	Ercc5	excision repair cross-complementing rodent repair deficiency, complementation group 5	0.999001	0.9963	0.9981
22668	Sf1	splicing factor 1	0.999001	0.9772	NA
23999	Twf2	twinfilin, actin-binding protein, homolog 2 (Drosophila)	0.999001	0.9919	0.9964
28030	Gfm1	G elongation factor, mitochondrial 1	0.999001	0.9749	NA
30957	Mapk8ip3	mitogen-activated protein kinase 8 interacting protein 3	0.999001	0.9944	0.9974
52430	Echdc2	enoyl Coenzyme A hydratase domain containing 2	0.999001	0.9925	NA
52683	Ncaph2	non-SMC condensin II complex, subunit H2	0.999001	0.995	0.9976
53881	Slc5a3	solute carrier family 5 (inositol transporters), member 3	0.999001	0.9881	NA
54139	Irf6	interferon regulatory factor 6	0.999001	0.9934	0.9971
54204	Sep-01	septin 1	0.999001	0.995	0.9976
54342	Gnpnat1	glucosamine-phosphate N-acetyltransferase 1	0.999001	0.9955	0.9978
56452	Orc6	origin recognition complex, subunit 6	0.999001	0.9813	NA
56503	Ankrd49	ankyrin repeat domain 49	0.999001	0.9865	NA
64931	Folr4	folate receptor 4 (delta)	0.999001	0.9925	0.9967
66142	Cox7b	cytochrome c oxidase subunit VIIb	0.999001	0.9851	NA

66515	Cul7	cullin 7	0.999001	0.9887	NA
66881	Pcyox1	prenylcysteine oxidase 1	0.999001	0.9798	NA
67211	Armc10	armadillo repeat containing 10	0.999001	0.9929	NA
67295	Rab3c	RAB3C, member RAS oncogene family	0.999001	0.9952	0.9977
67702	Rnf149	ring finger protein 149	0.999001	0.9958	0.998
68036	Zfp706	zinc finger protein 706	0.999001	0.9821	NA
68250	Fam96a	family with sequence similarity 96, member A	0.999001	0.9897	NA
69719	Cad	carbamoyl-phosphate synthetase 2, aspartate transcarbamylase, and dihydroorotase	0.999001	0.9945	0.9974
70207	Taco1	translational activator of mitochondrially encoded cytochrome c oxidase I	0.999001	0.9933	0.9971
71807	Tars2	threonyl-tRNA synthetase 2, mitochondrial (putative)	0.999001	0.9943	NA
72323	Asb6	ankyrin repeat and SOCS box-containing 6	0.999001	0.9905	0.9957
72667	Zfp444	zinc finger protein 444	0.999001	0.9919	0.9964
72893	2900040C04Rik	RIKEN cDNA 2900040C04 gene	0.999001	0.9948	0.9975
73658	Spns1	spinster homolog 1 (Drosophila)	0.999001	0.9878	NA
74164	Nfx1	nuclear transcription factor, X-box binding 1	0.999001	0.9871	NA
77038	Arfgap2	ADP-ribosylation factor GTPase activating protein 2	0.999001	0.9897	0.9955
78248	Armcx1	armadillo repeat containing, X-linked 1	0.999001	0.9926	NA
83945	Dnaja3	DnaJ (Hsp40) homolog, subfamily A, member 3	0.999001	0.9875	NA
97820	4833439L19Rik	RIKEN cDNA 4833439L19 gene	0.999001	0.9964	0.9981
100609	Nsun5	NOL1/NOP2/Sun domain family, member 5	0.999001	0.9921	NA
108058	Camk2d	calcium/calmodulin-dependent protein kinase II, delta	0.999001	0.9946	0.9974
140810	Ttbk2	tau tubulin kinase 2	0.999001	0.984	NA
170930	Sumo2	SMT3 suppressor of mif two 3 homolog 2 (yeast)	0.999001	0.9925	0.9967
208618	Etl4	enhancer trap locus 4	0.999001	0.9939	0.9972
217207	Dhx8	DEAH (Asp-Glu-Ala-His) box polypeptide 8	0.999001	0.982	NA
217232	Cdc27	cell division cycle 27 homolog (S. cerevisiae)	0.999001	0.9965	0.9981
219024	Tmem55b	transmembrane protein 55b	0.999001	0.9948	0.9975
224023	Klhl22	kelch-like 22 (Drosophila)	0.999001	0.9963	0.9981
229877	Rap1gds1	RAP1, GTP-GDP dissociation stimulator 1	0.999001	0.9856	NA
230959	Ajap1	adherens junction associated protein 1	0.999001	0.9873	NA
241915	Phc3	polyhomeotic-like 3 (Drosophila)	0.999001	0.9874	NA
319211	Nol4	nucleolar protein 4	0.999001	0.9963	0.9981
320162	Ccdc45	coiled-coil domain containing 45	0.999001	0.98	NA
320929	4732460I02Rik	RIKEN cDNA 4732460I02 gene	0.999001	0.9954	0.9978
320946	A930035D04Rik	RIKEN cDNA A930035D04 gene	0.999001	0.9894	0.9954
338523	Jhdm1d	jumonji C domain-containing histone demethylase 1 homolog D (S. cerevisiae)	0.999001	0.9959	0.9981
407823	Baz2b	bromodomain adjacent to zinc finger domain, 2B	0.999001	0.9934	NA
100042150	Nrg2	neuregulin 2	0.999001	0.9917	NA
11364	Acadm	acyl-Coenzyme A dehydrogenase, medium chain	0.998004	0.9782	NA
11674	Aldoa	aldolase A, fructose-bisphosphate	0.998004	0.972	NA
11881	Arsb	arylsulfatase B	0.998004	0.9805	NA
12068	Bet1	blocked early in transport 1 homolog (S. cerevisiae)	0.998004	0.9831	NA
13382	Dld	dihydrolipoamide dehydrogenase	0.998004	0.9855	0.9938
18521	Pcbp2	poly(rC) binding protein 2	0.998004	0.9803	NA
18711	Pikfyve	phosphoinositide kinase, FYVE finger containing	0.998004	0.9858	0.994
18949	Pnn	pinin	0.998004	0.9801	NA
21345	Tagln	transgelin	0.998004	0.9846	0.9934
21976	Top3b	topoisomerase (DNA) III beta	0.998004	0.9802	NA
26374	Rfwd2	ring finger and WD repeat domain 2	0.998004	0.9571	NA

50753	Fbxo8	F-box protein 8	0.998004	0.9833	0.9928
63955	Cables1	CDK5 and Abl enzyme substrate 1	0.998004	0.9828	0.9926
66212	Sec61b	Sec61 beta subunit	0.998004	0.9739	NA
66665	5730528L13Rik	RIKEN cDNA 5730528L13 gene	0.998004	0.9665	NA
66942	Ddx18	DEAD (Asp-Glu-Ala-Asp) box polypeptide 18	0.998004	0.9745	NA
66990	Tmem134	transmembrane protein 134	0.998004	0.9727	NA
67015	Ccdc91	coiled-coil domain containing 91	0.998004	0.9875	0.9946
67511	Tmed9	transmembrane emp24 protein transport domain containing 9	0.998004	0.9704	NA
68146	Arl13b	ADP-ribosylation factor-like 13B	0.998004	0.9872	0.9945
68818	Zfand2b	zinc finger, AN1 type domain 2B	0.998004	0.9731	NA
69202	Ptms	parathymosin	0.998004	0.9834	0.9928
70960	4921531P14Rik	RIKEN cDNA 4921531P14 gene	0.998004	0.9919	0.9964
72203	2610507I01Rik	RIKEN cDNA 2610507I01 gene	0.998004	0.9788	0.9911
72252	1700022A21Rik	glycerol-3-phosphate dehydrogenase 1-like pseudogene	0.998004	0.9961	0.9981
72254	1700030K09Rik	RIKEN cDNA 1700030K09 gene	0.998004	0.9705	NA
72568	Lin9	lin-9 homolog (C. elegans)	0.998004	0.9732	NA
74585	Sppl3	signal peptide peptidase 3	0.998004	0.9798	NA
75458	Cklf	chemokine-like factor	0.998004	0.9773	NA
75725	Phf14	PHD finger protein 14	0.998004	0.9665	NA
75906	Fam184a	family with sequence similarity 184, member A	0.998004	0.9843	0.9933
76429	Lhpp	phospholysine phosphohistidine inorganic pyrophosphate phosphatase	0.998004	0.9826	NA
76522	Naa38	N(alpha)-acetyltransferase 38, NatC auxiliary subunit	0.998004	0.9736	NA
76965	Slitrk1	SLIT and NTRK-like family, member 1	0.998004	0.9854	0.9938
77579	Myh10	myosin, heavy polypeptide 10, non-muscle	0.998004	0.981	NA
80886	Senp3	SUMO/sentrin specific peptidase 3	0.998004	0.9763	NA
81909	Zfpl1	zinc finger like protein 1	0.998004	0.9791	0.9911
101206	Tada3	transcriptional adaptor 3	0.998004	0.9882	0.9949
209018	Vps8	vacuolar protein sorting 8 homolog (S. cerevisiae)	0.998004	0.9869	0.9944
224432	Scaf4	SR-related CTD-associated factor 4	0.998004	0.979	NA
228361	Ambra1	autophagy/beclin 1 regulator 1	0.998004	0.9704	NA
230379	Acer2	alkaline ceramidase 2	0.998004	0.9789	0.9911
230903	Fbxo44	F-box protein 44	0.998004	0.9882	0.9949
237898	Usp32	ubiquitin specific peptidase 32	0.998004	0.9642	NA
241915	Phc3	polyhomeotic-like 3 (Drosophila)	0.998004	0.9887	0.9949
268780	Egflam	EGF-like, fibronectin type III and laminin G domains	0.998004	0.9842	0.9933
269224	Pask	PAS domain containing serine/threonine kinase	0.998004	0.9785	NA
270076	Gcdh	glutaryl-Coenzyme A dehydrogenase	0.998004	0.9802	NA
433375	Creg1	cellular repressor of E1A-stimulated genes 1	0.998004	0.986	0.9941
434377	Zfp560	zinc finger protein 560	0.998004	0.972	NA
435684	Shf	Src homology 2 domain containing F	0.998004	0.9886	0.9949
626596	Rgs22	regulator of G-protein signalling 22	0.998004	0.9772	NA
100040851	Gm9767	predicted gene 9767	0.998004	0.9744	NA
12163	Bmp8a	bone morphogenetic protein 8a	0.997009	0.9899	0.9955
14345	Fut4	fucosyltransferase 4	0.997009	0.9865	0.9943
14731	Gpaa1	GPI anchor attachment protein 1	0.997009	0.9825	0.9926
16337	Insr	insulin receptor	0.997009	0.9794	0.9912
17083	Tmed1	transmembrane emp24 domain containing 1	0.997009	0.9604	NA
17355	Aff1	AF4/FMR2 family, member 1	0.997009	0.9604	NA
18670	Abcb4	ATP-binding cassette, sub-family B (MDR/TAP), member 4	0.997009	0.9642	NA

19942	Rpl27	ribosomal protein L27	0.997009	0.9755	0.9901
20103	Rps5	ribosomal protein S5	0.997009	0.9755	0.9901
22169	Cmpk2	cytidine monophosphate (UMP-CMP) kinase 2, mitochondrial	0.997009	0.9664	NA
23873	Faim	Fas apoptotic inhibitory molecule	0.997009	0.9697	0.9875
30840	Fbxl6	F-box and leucine-rich repeat protein 6	0.997009	0.9804	0.9918
53382	Txnl1	thioredoxin-like 1	0.997009	0.9567	NA
53424	Tsnax	translin-associated factor X	0.997009	0.9588	NA
53605	Nap1l1	nucleosome assembly protein 1-like 1	0.997009	0.9533	NA
56030	Tmem131	transmembrane protein 131	0.997009	0.9661	NA
56095	Ftsj3	FtsJ homolog 3 (E. coli)	0.997009	0.9811	0.992
56330	Pdcd5	programmed cell death 5	0.997009	0.9454	NA
56505	Ruvbl1	RuvB-like protein 1	0.997009	0.9487	NA
57316	C1d	C1D nuclear receptor co-repressor	0.997009	0.9823	0.9924
57905	Isy1	ISY1 splicing factor homolog (S. cerevisiae)	0.997009	0.955	NA
59008	Anapc5	anaphase-promoting complex subunit 5	0.997009	0.9548	NA
66294	Fam3a	family with sequence similarity 3, member A	0.997009	0.9416	NA
66617	Mettl11a	methyltransferase like 11A	0.997009	0.9726	NA
67980	Gnpda2	glucosamine-6-phosphate deaminase 2	0.997009	0.9549	NA
68501	Nsmce2	non-SMC element 2 homolog (MMS21, S. cerevisiae)	0.997009	0.9579	NA
68770	Phtf2	putative homeodomain transcription factor 2	0.997009	0.9692	NA
69162	Sec31a	Sec31 homolog A (S. cerevisiae)	0.997009	0.9716	NA
69188	Mll5	myeloid/lymphoid or mixed-lineage leukemia 5	0.997009	0.9738	0.9895
69748	Aldh16a1	aldehyde dehydrogenase 16 family, member A1	0.997009	0.9551	NA
70315	Hdac8	histone deacetylase 8	0.997009	0.9784	0.9911
71711	Mus81	MUS81 endonuclease homolog (yeast)	0.997009	0.9787	0.9911
72003	Synpr	synaptoporin	0.997009	0.9632	NA
72446	Prr5l	proline rich 5 like	0.997009	0.9837	0.993
73598	1700001O22Rik	RIKEN cDNA 1700001O22 gene	0.997009	0.9764	0.9905
74008	Arsg	arylsulfatase G	0.997009	0.9606	NA
74479	Snx11	sorting nexin 11	0.997009	0.9817	0.9922
75202	Ncrna00085	non-protein coding RNA 85	0.997009	0.9895	0.9954
75565	Ccdc101	coiled-coil domain containing 101	0.997009	0.9647	NA
78305	1500032F14Rik	RIKEN cDNA 1500032F14 gene	0.997009	0.9808	0.992
78428	Wibg	within bgcn homolog (Drosophila)	0.997009	0.9843	0.9933
100434	Slc44a1	solute carrier family 44, member 1	0.997009	0.9854	0.9938
104776	Aldh6a1	aldehyde dehydrogenase family 6, subfamily A1	0.997009	0.9519	NA
107372	C030016D13Rik	RIKEN cDNA C030016D13 gene	0.997009	0.9739	NA
107951	Cdk9	cyclin-dependent kinase 9 (CDC2-related kinase)	0.997009	0.9844	0.9933
108737	Oxsr1	oxidative-stress responsive 1	0.997009	0.9812	0.992
109658	Txlna	taxilin alpha	0.997009	0.9685	NA
110651	Rps6ka3	ribosomal protein S6 kinase polypeptide 3	0.997009	0.9731	NA
213350	Pddc1	Parkinson disease 7 domain containing 1	0.997009	0.9768	0.9905
217366	Lrrc45	leucine rich repeat containing 45	0.997009	0.9714	NA
228355	Madd	MAP-kinase activating death domain	0.997009	0.9927	0.9968
231051	Mll3	myeloid/lymphoid or mixed-lineage leukemia 3	0.997009	0.9622	NA
231861	Tnrc18	trinucleotide repeat containing 18	0.997009	0.9831	0.9927
234865	Nup133	nucleoporin 133	0.997009	0.9537	NA
237300	Gm4922	predicted gene 4922	0.997009	0.9831	0.9927
258851	Olfr1339	olfactory receptor 1339	0.997009	0.9903	0.9957

260298	Fev	FEV (ETS oncogene family)	0.997009	0.9811	0.992
268749	Rnf31	ring finger protein 31	0.997009	0.9687	NA
269378	Ahcy	S-adenosylhomocysteine hydrolase	0.997009	0.9706	0.988
320683	Zfp629	zinc finger protein 629	0.997009	0.9501	NA
320722	A330050F15Rik	RIKEN cDNA A330050F15 gene	0.997009	0.9676	NA
433313	Rpl17-ps1	ribosomal protein L17, pseudogene 1	0.997009	0.9535	NA
434215	Lrrc32	leucine rich repeat containing 32	0.997009	0.967	NA
100046290	LOC100046290	60S ribosomal protein L21-like	0.997009	0.9789	0.9911
12180	Smyd1	SET and MYND domain containing 1	0.996016	0.9847	0.9934
12564	Cdh8	cadherin 8	0.996016	0.9862	0.9942
14661	Glud1	glutamate dehydrogenase 1	0.996016	0.9385	NA
15893	Ica1	islet cell autoantigen 1	0.996016	0.9411	NA
16988	Lst1	leukocyte specific transcript 1	0.996016	0.9852	0.9937
18100	Mrpl40	mitochondrial ribosomal protein L40	0.996016	0.9422	NA
20091	Rps3a	ribosomal protein S3A	0.996016	0.918	NA
20354	Sema4d	sema domain, immunoglobulin domain (Ig), transmembrane domain (TM) and short cytoplasmic domain, (semaphorin) 4D	0.996016	0.9635	0.9849
22694	Zfp35	zinc finger protein 35	0.996016	0.9728	0.9889
23970	Pacsin2	protein kinase C and casein kinase substrate in neurons 2	0.996016	0.9384	NA
53331	Stx7	syntaxin 7	0.996016	0.9452	NA
53607	Snrpa	small nuclear ribonucleoprotein polypeptide A	0.996016	0.961	NA
56040	Rplp1	ribosomal protein, large, P1	0.996016	0.9439	NA
56434	Tspan3	tetraspanin 3	0.996016	0.9308	NA
64385	Cyp4f14	cytochrome P450, family 4, subfamily f, polypeptide 14	0.996016	0.9722	0.9888
66333	Aqp11	aquaporin 11	0.996016	0.9484	NA
66414	Ndufa12	NADH dehydrogenase (ubiquinone) 1 alpha subcomplex, 12	0.996016	0.9244	NA
66455	Cnpy4	canopy 4 homolog (zebrafish)	0.996016	0.955	NA
66689	Klhl28	kelch-like 28 (Drosophila)	0.996016	0.951	NA
67014	Mina	myc induced nuclear antigen	0.996016	0.9427	NA
67150	Rnf141	ring finger protein 141	0.996016	0.9645	0.9854
67300	Cltc	clathrin, heavy polypeptide (Hc)	0.996016	0.9654	0.9856
68526	Gpr155	G protein-coupled receptor 155	0.996016	0.9559	NA
71393	Kctd6	potassium channel tetramerisation domain containing 6	0.996016	0.946	NA
71785	Pdgfd	platelet-derived growth factor, D polypeptide	0.996016	0.96	NA
71835	Lancl2	LanC (bacterial lantibiotic synthetase component C)-like 2	0.996016	0.9653	0.9856
72023	Cyb561d1	cytochrome b-561 domain containing 1	0.996016	0.9467	NA
72171	Shq1	SHQ1 homolog (S. cerevisiae)	0.996016	0.9793	0.9912
72181	Nsun4	NOL1/NOP2/Sun domain family, member 4	0.996016	0.9671	0.9862
72309	Tmem158	transmembrane protein 158	0.996016	0.9243	NA
74112	Usp16	ubiquitin specific peptidase 16	0.996016	0.9533	NA
74206	Sipa1l3	signal-induced proliferation-associated 1 like 3	0.996016	0.9532	NA
103284	Zc3h10	zinc finger CCCH type containing 10	0.996016	0.9496	NA
106068	Slc45a4	solute carrier family 45, member 4	0.996016	0.9626	0.9846
106512	Gpsm3	G-protein signalling modulator 3 (AGS3-like, C. elegans)	0.996016	0.9442	NA
108062	Cstf2	cleavage stimulation factor, 3' pre-RNA subunit 2	0.996016	0.9739	0.9895
110651	Rps6ka3	ribosomal protein S6 kinase polypeptide 3	0.996016	0.9854	0.9938
114863	Prosc	proline synthetase co-transcribed	0.996016	0.9429	NA
140559	Igsf8	immunoglobulin superfamily, member 8	0.996016	0.9723	0.9889
192652	Wdr81	WD repeat domain 81	0.996016	0.9433	NA
215708	Fam73a	family with sequence similarity 73, member A	0.996016	0.928	NA

216767	Mrpl22	mitochondrial ribosomal protein L22	0.996016	0.9349	NA
217031	Tada2a	transcriptional adaptor 2A	0.996016	0.9293	NA
231717	Fam109a	family with sequence similarity 109, member A	0.996016	0.9223	NA
239420	Csmd3	CUB and Sushi multiple domains 3	0.996016	0.9837	0.9931
242126	Slc22a15	solute carrier family 22 (organic anion/cation transporter), member 15	0.996016	0.9431	NA
243983	Zdhhc13	zinc finger, DHHC domain containing 13	0.996016	0.9494	NA
244646	Pkd1l3	polycystic kidney disease 1 like 3	0.996016	0.9897	0.9955
246277	Csad	cysteine sulfinic acid decarboxylase	0.996016	0.9607	NA
258527	Olfr1368	olfactory receptor 1368	0.996016	0.9734	0.9893
319240	9330159N05Rik	RIKEN cDNA 9330159N05 gene	0.996016	0.9725	0.9889
380768	Gm1568	predicted gene 1568	0.996016	0.9506	NA
382117	D9Ertd402e	DNA segment, Chr 9, ERATO Doi 402, expressed	0.996016	0.9499	NA
435684	Shf	Src homology 2 domain containing F	0.996016	0.9625	0.9846
100503794	LOC100503794	hypothetical LOC100503794	0.996016	0.9776	0.9909
100505360	LOC100505360	putative transposase element L1Md-A101/L1Md-A102/L1Md-A2-like	0.996016	0.9803	0.9918
12361	Cask	calcium/calmodulin-dependent serine protein kinase (MAGUK family)	0.995025	0.9447	NA
12589	Ift81	intraflagellar transport 81 homolog (Chlamydomonas)	0.995025	0.9655	0.9857
13383	Dlg1	discs, large homolog 1 (Drosophila)	0.995025	0.9753	0.9901
13521	Slc26a2	solute carrier family 26 (sulfate transporter), member 2	0.995025	0.9566	0.9824
14913	Guca1a	guanylate cyclase activator 1a (retina)	0.995025	0.9576	0.9832
15446	Hpgd	hydroxyprostaglandin dehydrogenase 15 (NAD)	0.995025	0.9618	0.9846
17534	Mrc2	mannose receptor, C type 2	0.995025	0.9739	0.9895
19053	Ppp2cb	protein phosphatase 2 (formerly 2A), catalytic subunit, beta isoform	0.995025	0.909	NA
19330	Rab18	RAB18, member RAS oncogene family	0.995025	0.9425	NA
19878	Rock2	Rho-associated coiled-coil containing protein kinase 2	0.995025	0.9171	NA
19988	Rpl6	ribosomal protein L6	0.995025	0.9247	NA
20655	Sod1	superoxide dismutase 1, soluble	0.995025	0.9485	NA
20773	Sptlc2	serine palmitoyltransferase, long chain base subunit 2	0.995025	0.9512	NA
21983	Tpbg	trophoblast glycoprotein	0.995025	0.943	NA
22688	Zfp26	zinc finger protein 26	0.995025	0.9698	0.9875
23963	Odz1	odd Oz/ten-m homolog 1 (Drosophila)	0.995025	0.953	NA
26427	Creb3l1	cAMP responsive element binding protein 3-like 1	0.995025	0.9425	NA
27370	Rps26	ribosomal protein S26	0.995025	0.934	NA
51796	Srrm1	serine/arginine repetitive matrix 1	0.995025	0.9289	NA
54126	Arhgef7	Rho guanine nucleotide exchange factor (GEF7)	0.995025	0.9224	NA
56430	Clip1	CAP-GLY domain containing linker protein 1	0.995025	0.9183	NA
57813	Tk2	thymidine kinase 2, mitochondrial	0.995025	0.9464	NA
66070	Cwc15	CWC15 homolog (S. cerevisiae)	0.995025	0.918	NA
66302	Fam82b	family with sequence similarity 82, member B	0.995025	0.9404	NA
66556	Drap1	Dr1 associated protein 1 (negative cofactor 2 alpha)	0.995025	0.9155	NA
66766	4933425O20Rik	RIKEN cDNA 4933425O20 gene	0.995025	0.9667	0.9859
67120	Ttc14	tetratricopeptide repeat domain 14	0.995025	0.9661	0.9858
67228	Wdr85	WD repeat domain 85	0.995025	0.9394	NA
67630	Samd8	sterile alpha motif domain containing 8	0.995025	0.9679	0.9866
68520	Zfyve21	zinc finger, FYVE domain containing 21	0.995025	0.9484	NA
69724	Rnaseh2a	ribonuclease H2, large subunit	0.995025	0.945	NA
70683	Utp20	UTP20, small subunit (SSU) processome component, homolog (yeast)	0.995025	0.9528	0.9814
72068	Cnot2	CCR4-NOT transcription complex, subunit 2	0.995025	0.9365	NA
72587	Pan3	PAN3 polyA specific ribonuclease subunit homolog (S. cerevisiae)	0.995025	0.9175	NA

73826	Poldip3	polymerase (DNA-directed), delta interacting protein 3	0.995025	0.9355	NA
74100	Arpp21	cyclic AMP-regulated phosphoprotein, 21	0.995025	0.9633	0.9848
76573	1700027D21Rik	RIKEN cDNA 1700027D21 gene	0.995025	0.9621	0.9846
76740	Efr3a	EFR3 homolog A (S. cerevisiae)	0.995025	0.941	NA
77799	Sla2	Src-like-adaptor 2	0.995025	0.9647	0.9854
77996	D730039F16Rik	RIKEN cDNA D730039F16 gene	0.995025	0.9727	0.9889
78304	Lsmd1	LSM domain containing 1	0.995025	0.8928	NA
83762	Otof	otoferlin	0.995025	0.9715	0.9885
94353	Hmgn3	high mobility group nucleosomal binding domain 3	0.995025	0.9413	NA
97550	C130081A10Rik	RIKEN cDNA C130081A10 gene	0.995025	0.9534	NA
102124	E130303B06Rik	RIKEN cDNA E130303B06 gene	0.995025	0.9347	NA
110821	Pcca	propionyl-Coenzyme A carboxylase, alpha polypeptide	0.995025	0.9522	NA
192197	Bcas3	breast carcinoma amplified sequence 3	0.995025	0.964	0.985
207596	Thsd4	thrombospondin, type I, domain containing 4	0.995025	0.9792	0.9912
210035	Tmem194	transmembrane protein 194	0.995025	0.9417	NA
211673	Arfgef1	ADP-ribosylation factor guanine nucleotide-exchange factor 1(brefeldin A-inhibited)	0.995025	0.9218	NA
213011	Zfp583	zinc finger protein 583	0.995025	0.9468	NA
216818	Gm4802	predicted gene 4802	0.995025	0.9317	NA
223664	Lrrc14	leucine rich repeat containing 14	0.995025	0.9742	0.9895
223773	Zbed4	zinc finger, BED domain containing 4	0.995025	0.9307	NA
224045	Eif2b5	eukaryotic translation initiation factor 2B, subunit 5 epsilon	0.995025	0.9603	0.984
224897	Dpp9	dipeptidylpeptidase 9	0.995025	0.9633	0.9849
240518	Peli3	pellino 3	0.995025	0.9701	0.9877
243300	6430598A04Rik	RIKEN cDNA 6430598A04 gene	0.995025	0.9452	NA
243308	A430033K04Rik	RIKEN cDNA A430033K04 gene	0.995025	0.9763	0.9905
268709	Fam107a	family with sequence similarity 107, member A	0.995025	0.9788	0.9911
272396	Tarsl2	threonyl-tRNA synthetase-like 2	0.995025	0.9393	NA
319457	C130045F17Rik	RIKEN cDNA C130045F17 gene	0.995025	0.9755	0.9901
320237	Ncrna00086	non-protein coding RNA 86	0.995025	0.9622	0.9846
320632	Snrnp200	small nuclear ribonucleoprotein 200 (U5)	0.995025	0.9306	NA
330998	Ankrd34c	ankyrin repeat domain 34C	0.995025	0.9466	NA
333789	N4bp2	NEDD4 binding protein 2	0.995025	0.9553	0.9824
381126	Fam59a	family with sequence similarity 59, member A	0.995025	0.9431	NA
434232	Iqck	IQ motif containing K	0.995025	0.9819	0.9924
625281	Gm6570	predicted gene 6570	0.995025	0.9575	0.9832
100038683	Gm10775	predicted gene 10775	0.995025	0.9224	NA
100043597	Srcap	Snf2-related CREBBP activator protein	0.995025	0.965	0.9856
15371	Hmx1	H6 homeobox 1	0.994036	0.9402	NA
19027	Sypl	synaptophysin-like protein	0.994036	0.9136	NA
19141	Lgmn	legumain	0.994036	0.9327	NA
19344	Rab5b	RAB5B, member RAS oncogene family	0.994036	0.9665	0.9858
19653	Rbm4	RNA binding motif protein 4	0.994036	0.9081	NA
20068	Rps17	ribosomal protein S17	0.994036	0.9051	NA
20351	Sema4a	sema domain, immunoglobulin domain (Ig), transmembrane domain (TM) and short cytoplasmic domain, (semaphorin) 4A	0.994036	0.911	NA
24010	Ik	IK cytokine	0.994036	0.9004	NA
52145	D9Ertd720e	DNA segment, Chr 9, ERATO Doi 720, expressed	0.994036	0.9784	0.9911
56692	Mapksp1	MAPK scaffold protein 1	0.994036	0.8939	NA
56874	Rnf32	ring finger protein 32	0.994036	0.9193	NA
65105	Arl6ip4	ADP-ribosylation factor-like 6 interacting protein 4	0.994036	0.8933	NA

66070	Cwc15	CWC15 homolog (S. cerevisiae)	0.994036	0.9311	NA
66131	Tipin	timeless interacting protein	0.994036	0.9357	NA
66282	1810029B16Rik	RIKEN cDNA 1810029B16 gene	0.994036	0.917	NA
66594	Uqcr11	ubiquinol-cytochrome c reductase, complex III subunit XI	0.994036	0.9164	NA
66733	Kcng4	potassium voltage-gated channel, subfamily G, member 4	0.994036	0.9558	0.9824
66863	Lztr1	leucine-zipper-like transcriptional regulator, 1	0.994036	0.9344	NA
68189	5330431K02Rik	RIKEN cDNA 5330431K02 gene	0.994036	0.9462	NA
68276	Toe1	target of EGR1, member 1 (nuclear)	0.994036	0.9564	0.9824
68614	Letmd1	LETM1 domain containing 1	0.994036	0.9224	NA
68915	Vars2	valyl-tRNA synthetase 2, mitochondrial (putative)	0.994036	0.942	0.9765
68964	1500010J02Rik	RIKEN cDNA 1500010J02 gene	0.994036	0.9217	NA
69358	Lrrc51	leucine rich repeat containing 51	0.994036	0.9406	NA
70120	Yars2	tyrosyl-tRNA synthetase 2 (mitochondrial)	0.994036	0.9109	NA
71276	Ccdc57	coiled-coil domain containing 57	0.994036	0.9418	0.9765
71767	Tysnd1	trypsin domain containing 1	0.994036	0.9285	NA
73130	Tmed5	transmembrane emp24 protein transport domain containing 5	0.994036	0.9636	0.9849
74114	Crot	carnitine O-octanoyltransferase	0.994036	0.9203	NA
75754	9030607L02Rik	RIKEN cDNA 9030607L02 gene	0.994036	0.9765	0.9905
76642	1700113A16Rik	RIKEN cDNA 1700113A16 gene	0.994036	0.9433	NA
78934	4930581F22Rik	RIKEN cDNA 4930581F22 gene	0.994036	0.9781	0.991
80981	Arl4d	ADP-ribosylation factor-like 4D	0.994036	0.9429	0.9768
93890	Pcdhb19	protocadherin beta 19	0.994036	0.9755	0.9901
101471	Phrf1	PHD and ring finger domains 1	0.994036	0.9303	NA
101685	Spty2d1	SPT2, Suppressor of Ty, domain containing 1 (S. cerevisiae)	0.994036	0.9528	0.9814
102323	Dcun1d2	DCN1, defective in cullin neddylation 1, domain containing 2 (S. cerevisiae)	0.994036	0.9112	NA
103012	6720401G13Rik	RIKEN cDNA 6720401G13 gene	0.994036	0.9632	0.9848
104570	Smek2	SMEK homolog 2, suppressor of mek1 (Dictyostelium)	0.994036	0.9078	NA
105203	BC016423	cDNA sequence BC016423	0.994036	0.974	0.9895
107182	Btaf1	BTAF1 RNA polymerase II, B-TFIID transcription factor-associated, (Mot1 homolog, S. cerevisiae)	0.994036	0.9191	NA
108689	Obfc1	oligonucleotide/oligosaccharide-binding fold containing 1	0.994036	0.9281	NA
109042	Prkcdbp	protein kinase C, delta binding protein	0.994036	0.9302	NA
109672	Cyb5	cytochrome b-5	0.994036	0.9147	NA
114664	Hsd17b11	hydroxysteroid (17-beta) dehydrogenase 11	0.994036	0.9655	0.9856
170460	Stard5	StAR-related lipid transfer (START) domain containing 5	0.994036	0.9167	NA
208263	Tor1aip1	torsin A interacting protein 1	0.994036	0.949	0.9801
209086	Samd9l	sterile alpha motif domain containing 9-like	0.994036	0.9509	0.9808
215335	Slc36a1	solute carrier family 36 (proton/amino acid symporter), member 1	0.994036	0.9706	0.988
215494	C85492	expressed sequence C85492	0.994036	0.9336	NA
226356	Gm101	predicted gene 101	0.994036	0.9778	0.9909
228019	Mettl8	methyltransferase like 8	0.994036	0.9386	NA
230737	Gnl2	guanine nucleotide binding protein-like 2 (nucleolar)	0.994036	0.8973	NA
233865	D430042O09Rik	RIKEN cDNA D430042O09 gene	0.994036	0.9102	NA
234135	Whsc1l1	Wolf-Hirschhorn syndrome candidate 1-like 1 (human)	0.994036	0.9166	NA
239250	Slitrk6	SLIT and NTRK-like family, member 6	0.994036	0.9259	NA
244810	AW551984	expressed sequence AW551984	0.994036	0.9502	0.9804
245880	Wasf3	WAS protein family, member 3	0.994036	0.9639	0.9849
258593	Olfr700	olfactory receptor 700	0.994036	0.9768	0.9905
320560	Dennd5b	DENN/MADD domain containing 5B	0.994036	0.9722	0.9888
329251	Ppp1r12b	protein phosphatase 1, regulatory (inhibitor) subunit 12B	0.994036	0.9744	0.9897

353170	Txlng	taxilin gamma	0.994036	0.9463	0.9787
380967	Tmem106c	transmembrane protein 106C	0.994036	0.9626	0.9846
381201	Gm962	predicted gene 962	0.994036	0.9385	NA
382423	Atxn7l3b	ataxin 7-like 3B	0.994036	0.9049	NA
434179	Gm5595	predicted gene 5595	0.994036	0.9276	NA
622665	Ccdc17	coiled-coil domain containing 17	0.994036	0.9089	NA
11818	Apoh	apolipoprotein H	0.993049	0.962	0.9846
12190	Brca2	breast cancer 2	0.993049	0.9201	NA
12454	Ccnk	cyclin K	0.993049	0.9122	NA
14085	Fah	fumarylacetoacetate hydrolase	0.993049	0.9139	NA
14784	Grb2	growth factor receptor bound protein 2	0.993049	0.9516	0.981
15275	Hk1	hexokinase 1	0.993049	0.8951	NA
16588	Kin	antigenic determinant of rec-A protein	0.993049	0.9051	NA
17116	Mab21l1	mab-21-like 1 (C. elegans)	0.993049	0.9447	0.9776
17308	Mgat1	mannoside acetylglucosaminyltransferase 1	0.993049	0.9089	NA
18232	Nxph2	neurexophilin 2	0.993049	0.9566	0.9824
18573	Pde1a	phosphodiesterase 1A, calmodulin-dependent	0.993049	0.9536	0.9817
19210	Ptdss1	phosphatidylserine synthase 1	0.993049	0.9577	0.9832
19211	Pten	phosphatase and tensin homolog	0.993049	0.9235	NA
19674	Rcvrn	recoverin	0.993049	0.9409	0.9761
19739	Rgs9	regulator of G-protein signaling 9	0.993049	0.9419	0.9765
20055	Rps16	ribosomal protein S16	0.993049	0.9309	NA
20238	Atxn1	ataxin 1	0.993049	0.9466	0.9788
22202	Ube1y1	ubiquitin-activating enzyme E1, Chr Y 1	0.993049	0.923	NA
23789	Coro1b	coronin, actin binding protein 1B	0.993049	0.9475	0.9792
23971	Papss1	3'-phosphoadenosine 5'-phosphosulfate synthase 1	0.993049	0.9115	NA
30050	Fbxw2	F-box and WD-40 domain protein 2	0.993049	0.9462	0.9787
52331	Stbd1	starch binding domain 1	0.993049	0.9235	NA
56193	Plek	pleckstrin	0.993049	0.9383	0.9746
56384	Letm1	leucine zipper-EF-hand containing transmembrane protein 1	0.993049	0.9001	NA
56420	Ppp4c	protein phosphatase 4, catalytic subunit	0.993049	0.9425	0.9767
56550	Ube2d2	ubiquitin-conjugating enzyme E2D 2	0.993049	0.9225	NA
57895	Ccdc126	coiled-coil domain containing 126	0.993049	0.9567	0.9824
57912	Cdc42se1	CDC42 small effector 1	0.993049	0.9444	0.9776
58244	Stx6	syntaxin 6	0.993049	0.8979	NA
64339	Fndc4	fibronectin type III domain containing 4	0.993049	0.9199	NA
66314	Tpd52l2	tumor protein D52-like 2	0.993049	0.9372	0.9741
66433	Chchd7	coiled-coil-helix-coiled-coil-helix domain containing 7	0.993049	0.8803	NA
66511	2500003M10Rik	RIKEN cDNA 2500003M10 gene	0.993049	0.9096	NA
66611	Ribc1	RIB43A domain with coiled-coils 1	0.993049	0.9131	NA
67074	Mon2	MON2 homolog (yeast)	0.993049	0.9493	0.9801
67143	Ikzf5	IKAROS family zinc finger 5	0.993049	0.8546	NA
68036	Zfp706	zinc finger protein 706	0.993049	0.9095	NA
68133	Gcsh	glycine cleavage system protein H (aminomethyl carrier)	0.993049	0.9136	NA
68365	Rab14	RAB14, member RAS oncogene family	0.993049	0.8942	NA
68936	Fam165b	family with sequence similarity 165, member B	0.993049	0.965	0.9856
69408	Dnajc17	DnaJ (Hsp40) homolog, subfamily C, member 17	0.993049	0.9595	0.9837
71436	Flrt3	fibronectin leucine rich transmembrane protein 3	0.993049	0.8994	NA
71900	Tmem106b	transmembrane protein 106B	0.993049	0.9561	0.9824

72008	Zfyve19	zinc finger, FYVE domain containing 19	0.993049	0.8899	NA
72615	Anks3	ankyrin repeat and sterile alpha motif domain containing 3	0.993049	0.8979	NA
75705	Eif4b	eukaryotic translation initiation factor 4B	0.993049	0.9295	NA
75841	Rnf139	ring finger protein 139	0.993049	0.9175	NA
77733	Rnf170	ring finger protein 170	0.993049	0.9348	0.973
80860	Ghdc	GH3 domain containing	0.993049	0.9516	0.981
97159	A430005L14Rik	RIKEN cDNA A430005L14 gene	0.993049	0.8791	NA
98417	Cnih4	cornichon homolog 4 (Drosophila)	0.993049	0.919	NA
102632	Acad11	acyl-Coenzyme A dehydrogenase family, member 11	0.993049	0.8939	NA
103978	Gpc5	glypican 5	0.993049	0.9584	0.9836
108030	Lin7a	lin-7 homolog A (C. elegans)	0.993049	0.8818	NA
113865	Vmn1r25	vomeronasal 1 receptor 25	0.993049	0.9575	0.9832
171262	Vmn1r70	vomeronasal 1 receptor 70	0.993049	0.9606	0.9842
209018	Vps8	vacuolar protein sorting 8 homolog (S. cerevisiae)	0.993049	0.9313	NA
211556	Ap1ar	adaptor-related protein complex 1 associated regulatory protein	0.993049	0.9694	0.9873
212281	A530054K11Rik	RIKEN cDNA A530054K11 gene	0.993049	0.9638	0.9849
217732	2310044G17Rik	RIKEN cDNA 2310044G17 gene	0.993049	0.8853	NA
218989	6720456H20Rik	RIKEN cDNA 6720456H20 gene	0.993049	0.8907	NA
223753	Cerk	ceramide kinase	0.993049	0.9013	NA
227522	Rpp38	ribonuclease P/MRP 38 subunit (human)	0.993049	0.8891	NA
227867	Epc2	enhancer of polycomb homolog 2 (Drosophila)	0.993049	0.9166	NA
230249	AI314180	expressed sequence AI314180	0.993049	0.9605	0.9841
230279	6330416G13Rik	RIKEN cDNA 6330416G13 gene	0.993049	0.9466	0.9788
233073	U2af1l4	U2 small nuclear RNA auxiliary factor 1-like 4	0.993049	0.9211	NA
241489	Pde11a	phosphodiesterase 11A	0.993049	0.9674	0.9864
330173	2610524H06Rik	RIKEN cDNA 2610524H06 gene	0.993049	0.9215	NA
347740	2900097C17Rik	RIKEN cDNA 2900097C17 gene	0.993049	0.9754	0.9901
381760	Ssbp1	single-stranded DNA binding protein 1	0.993049	0.8903	NA
621967	Gm6272	predicted pseudogene 6272	0.993049	0.9738	0.9895
100041194	Ahnak2	AHNAK nucleoprotein 2	0.993049	0.9662	0.9858
11778	Ap3s2	adaptor-related protein complex 3, sigma 2 subunit	0.992063	0.8941	NA
13508	Dscam	Down syndrome cell adhesion molecule	0.992063	0.8903	NA
14312	Brd2	bromodomain containing 2	0.992063	0.8751	NA
14964	H2-D1	histocompatibility 2, D region locus 1	0.992063	0.9361	0.9734
15364	Hmga2	high mobility group AT-hook 2	0.992063	0.9181	NA
16432	Itm2b	integral membrane protein 2B	0.992063	0.8974	NA
16578	Kif9	kinesin family member 9	0.992063	0.9553	0.9824
16842	Lef1	lymphoid enhancer binding factor 1	0.992063	0.9542	0.9819
17436	Me1	malic enzyme 1, NADP(+)-dependent, cytosolic	0.992063	0.944	0.9774
18213	Ntrk3	neurotrophic tyrosine kinase, receptor, type 3	0.992063	0.9203	NA
19130	Prox1	prospero-related homeobox 1	0.992063	0.9315	0.9719
19164	Psen1	presenilin 1	0.992063	0.9252	NA
19702	Ren2	renin 2 tandem duplication of Ren1	0.992063	0.9637	0.9849
22276	Uros	uroporphyrinogen III synthase	0.992063	0.871	NA
22294	Uxt	ubiquitously expressed transcript	0.992063	0.9375	0.9742
22596	Xrcc5	X-ray repair complementing defective repair in Chinese hamster cells 5	0.992063	0.8317	NA
22724	Zbtb7b	zinc finger and BTB domain containing 7B	0.992063	0.8799	NA
30938	Fgd3	FYVE, RhoGEF and PH domain containing 3	0.992063	0.9626	0.9846
52466	Slc46a1	solute carrier family 46, member 1	0.992063	0.934	0.9727

54151	Cyhr1	cysteine and histidine rich 1	0.992063	0.8486	NA
56418	Ykt6	YKT6 homolog (S. Cerevisiae)	0.992063	0.9306	0.9715
56422	Hbs1l	Hbs1-like (S. cerevisiae)	0.992063	0.8861	NA
56550	Ube2d2	ubiquitin-conjugating enzyme E2D 2	0.992063	0.847	NA
58869	Pex5l	peroxisomal biogenesis factor 5-like	0.992063	0.9171	NA
63985	Gmfb	glia maturation factor, beta	0.992063	0.8816	NA
66291	1810030N24Rik	RIKEN cDNA 1810030N24 gene	0.992063	0.904	NA
66594	Uqcr11	ubiquinol-cytochrome c reductase, complex III subunit XI	0.992063	0.8711	NA
66795	Atg10	autophagy-related 10 (yeast)	0.992063	0.936	0.9734
67179	Ccdc25	coiled-coil domain containing 25	0.992063	0.9296	0.9713
67333	Stk35	serine/threonine kinase 35	0.992063	0.9335	0.9726
68047	Mpnd	MPN domain containing	0.992063	0.926	NA
68564	Nufip2	nuclear fragile X mental retardation protein interacting protein 2	0.992063	0.9095	NA
68611	Mrpl28	mitochondrial ribosomal protein L28	0.992063	0.921	NA
69104	Mar-05	membrane-associated ring finger (C3HC4) 5	0.992063	0.9265	0.97
69104	Mar-05	membrane-associated ring finger (C3HC4) 5	0.992063	0.8666	NA
69234	Zfp688	zinc finger protein 688	0.992063	0.9114	NA
70699	Nup205	nucleoporin 205	0.992063	0.8628	NA
71169	Nbas	neuroblastoma amplified sequence	0.992063	0.9257	NA
74154	Unkl	unkempt-like (Drosophila)	0.992063	0.9683	0.9867
74386	Rmi1	RMI1, RecQ mediated genome instability 1, homolog (S. cerevisiae)	0.992063	0.8506	NA
74675	Ptchd3	patched domain containing 3	0.992063	0.922	NA
75778	Them4	thioesterase superfamily member 4	0.992063	0.8783	NA
76366	Mtif3	mitochondrial translational initiation factor 3	0.992063	0.9344	0.9729
77359	9430063H18Rik	RIKEN cDNA 9430063H18 gene	0.992063	0.9232	NA
101100	Ttll3	tubulin tyrosine ligase-like family, member 3	0.992063	0.9477	0.9792
104923	Adi1	acireductone dioxygenase 1	0.992063	0.9075	NA
108934	BC024659	cDNA sequence BC024659	0.992063	0.8812	NA
109674	Ampd2	adenosine monophosphate deaminase 2	0.992063	0.8749	NA
213573	Efcab4a	EF-hand calcium binding domain 4A	0.992063	0.936	0.9734
215751	BC013529	cDNA sequence BC013529	0.992063	0.9448	0.9777
217379	Ubxn2a	UBX domain protein 2A	0.992063	0.9232	NA
219103	Cenpj	centromere protein J	0.992063	0.9023	NA
226896	Tcfap2d	transcription factor AP-2, delta	0.992063	0.9206	NA
233103	4931406P16Rik	RIKEN cDNA 4931406P16 gene	0.992063	0.8499	NA
235611	Plxnb1	plexin B1	0.992063	0.9146	NA
240263	Fem1c	fem-1 homolog c (C.elegans)	0.992063	0.8987	NA
245405	Gm4987	predicted gene 4987	0.992063	0.8981	NA
257921	Olfr1229	olfactory receptor 1229	0.992063	0.9517	0.981
259105	Olfr549	olfactory receptor 549	0.992063	0.9828	0.9926
319455	Pld5	phospholipase D family, member 5	0.992063	0.9613	0.9846
320360	Ric3	resistance to inhibitors of cholinesterase 3 homolog (C. elegans)	0.992063	0.9625	0.9846
353310	Zfp703	zinc finger protein 703	0.992063	0.9001	NA
386655	Eid2	EP300 interacting inhibitor of differentiation 2	0.992063	0.9179	NA
545611	Gm13298	predicted gene 13298	0.992063	0.909	NA
12398	Cbfa2t3	core-binding factor, runt domain, alpha subunit 2, translocated to, 3 (human)	0.99108	0.9275	0.9705
12518	Cd79a	CD79A antigen (immunoglobulin-associated alpha)	0.99108	0.9318	0.972
13033	Ctsd	cathepsin D	0.99108	0.8982	NA
14208	Ppm1g	protein phosphatase 1G (formerly 2C), magnesium-dependent, gamma isoform	0.99108	0.9026	NA

15115	Hars	histidyl-tRNA synthetase	0.99108	0.9162	0.9651
15247	Hiat1	hippocampus abundant gene transcript 1	0.99108	0.8647	NA
15312	Hmgn1	high mobility group nucleosomal binding domain 1	0.99108	0.9281	0.9705
16438	Itpr1	inositol 1,4,5-triphosphate receptor 1	0.99108	0.9068	NA
16871	Lhx3	LIM homeobox protein 3	0.99108	0.8915	NA
18213	Ntrk3	neurotrophic tyrosine kinase, receptor, type 3	0.99108	0.9031	NA
18717	Pip5k1c	phosphatidylinositol-4-phosphate 5-kinase, type 1 gamma	0.99108	0.9384	0.9746
19172	Psmb4	proteasome (prosome, macropain) subunit, beta type 4	0.99108	0.8912	NA
19401	Rara	retinoic acid receptor, alpha	0.99108	0.9144	NA
20024	Sub1	SUB1 homolog (S. cerevisiae)	0.99108	0.8923	NA
21353	Tank	TRAF family member-associated Nf-kappa B activator	0.99108	0.921	0.9679
22213	Ube2g2	ubiquitin-conjugating enzyme E2G 2	0.99108	0.9402	0.9757
28129	D11Wsu173e	DNA segment, Chr 11, Wayne State University 173, expressed	0.99108	0.9424	0.9767
30805	Slc22a4	solute carrier family 22 (organic cation transporter), member 4	0.99108	0.9297	0.9714
54446	Nfat5	nuclear factor of activated T-cells 5	0.99108	0.9676	0.9864
54635	Pdgfc	platelet-derived growth factor, C polypeptide	0.99108	0.9327	0.9724
56707	Zfp111	zinc finger protein 111	0.99108	0.9005	NA
66185	1110037F02Rik	RIKEN cDNA 1110037F02 gene	0.99108	0.942	0.9765
67067	Romo1	reactive oxygen species modulator 1	0.99108	0.8559	NA
67231	Tbc1d20	TBC1 domain family, member 20	0.99108	0.9325	0.9724
67308	Mrpl46	mitochondrial ribosomal protein L46	0.99108	0.9013	NA
67326	1700037H04Rik	RIKEN cDNA 1700037H04 gene	0.99108	0.8923	NA
67790	Rab39b	RAB39B, member RAS oncogene family	0.99108	0.9301	0.9715
67921	Ube2f	ubiquitin-conjugating enzyme E2F (putative)	0.99108	0.8741	NA
68499	Mrpl53	mitochondrial ribosomal protein L53	0.99108	0.8578	NA
68607	Serhl	serine hydrolase-like	0.99108	0.8998	NA
68918	1190005I06Rik	RIKEN cDNA 1190005I06 gene	0.99108	0.8962	NA
69085	Zcchc9	zinc finger, CCHC domain containing 9	0.99108	0.8894	NA
71213	Cage1	cancer antigen 1	0.99108	0.9183	0.9664
73062	Ppp1r16a	protein phosphatase 1, regulatory (inhibitor) subunit 16A	0.99108	0.9425	0.9767
73442	Hspa12a	heat shock protein 12A	0.99108	0.9535	0.9816
73754	Thap1	THAP domain containing, apoptosis associated protein 1	0.99108	0.9007	NA
76959	Chmp5	chromatin modifying protein 5	0.99108	0.8844	NA
89867	Sec16b	SEC16 homolog B (S. cerevisiae)	0.99108	0.9389	0.9749
93690	Gpr45	G protein-coupled receptor 45	0.99108	0.971	0.9882
98432	Phlpp1	PH domain and leucine rich repeat protein phosphatase 1	0.99108	0.8817	NA
108943	Rg9mtd2	RNA (guanine-9-) methyltransferase domain containing 2	0.99108	0.8672	NA
116848	Baz2a	bromodomain adjacent to zinc finger domain, 2A	0.99108	0.8793	NA
140721	Caskin2	CASK-interacting protein 2	0.99108	0.8927	NA
207667	Skor1	SKI family transcriptional corepressor 1	0.99108	0.9246	0.9693
211550	Tifa	TRAF-interacting protein with forkhead-associated domain	0.99108	0.8887	NA
212528	Trmt1	TRM1 tRNA methyltransferase 1 homolog (S. cerevisiae)	0.99108	0.9164	0.9652
234159	Gm4889	predicted gene 4889	0.99108	0.8807	NA
238130	Dock4	dedicator of cytokinesis 4	0.99108	0.9589	0.9836
319939	Tns3	tensin 3	0.99108	0.914	NA
330267	Thsd7a	thrombospondin, type I, domain containing 7A	0.99108	0.956	0.9824
621542	Gm6238	predicted pseudogene 6238	0.99108	0.8165	NA
621580	Gm13308	predicted gene 13308	0.99108	0.9073	NA
625054	Gm6548	eukaryotic translation elongation factor 1 alpha 1 pseudogene	0.99108	0.9137	NA

667214	9930111J21Rik1	RIKEN cDNA 9930111J21 gene 1	0.99108	0.9258	0.9698
671641	Gm10063	predicted gene 10063	0.99108	0.9188	0.9668
675749	Gm10693	predicted pseudogene 10693	0.99108	0.9738	0.9895
100505330	LOC100505330	NHP2-like protein 1-like	0.99108	0.8791	NA
12616	Cenpb	centromere protein B	0.990099	0.8766	NA
12951	Crx	cone-rod homeobox containing gene	0.990099	0.9215	0.968
16728	L1cam	L1 cell adhesion molecule	0.990099	0.9431	0.9768
16970	Lrmp	lymphoid-restricted membrane protein	0.990099	0.9229	0.9686
18148	Npm1	nucleophosmin 1	0.990099	0.8474	NA
21420	Tcfap2c	transcription factor AP-2, gamma	0.990099	0.8471	NA
21844	Tiam1	T-cell lymphoma invasion and metastasis 1	0.990099	0.8953	NA
22687	Zfp259	zinc finger protein 259	0.990099	0.8592	NA
22758	Zscan12	zinc finger and SCAN domain containing 12	0.990099	0.8449	NA
23888	Gpc6	glypican 6	0.990099	0.9451	0.978
23961	Oas1b	2'-5' oligoadenylate synthetase 1B	0.990099	0.839	NA
24045	Scamp3	secretory carrier membrane protein 3	0.990099	0.8495	NA
26569	Slc27a4	solute carrier family 27 (fatty acid transporter), member 4	0.990099	0.8497	NA
28126	Nop16	NOP16 nucleolar protein homolog (yeast)	0.990099	0.9198	0.9673
30841	Kdm2b	lysine (K)-specific demethylase 2B	0.990099	0.8455	NA
54608	Abhd2	abhydrolase domain containing 2	0.990099	0.9424	0.9767
56473	Fads2	fatty acid desaturase 2	0.990099	0.9042	NA
60530	Fignl1	fidgetin-like 1	0.990099	0.9131	0.9638
66213	Med7	mediator complex subunit 7	0.990099	0.8701	NA
66860	Tanc1	tetratricopeptide repeat, ankyrin repeat and coiled-coil containing 1	0.990099	0.9463	0.9787
67025	Rpl11	ribosomal protein L11	0.990099	0.9068	NA
68162	A930003A15Rik	RIKEN cDNA A930003A15 gene	0.990099	0.9178	0.9662
68954	1500012K07Rik	RIKEN cDNA 1500012K07 gene	0.990099	0.9132	0.9639
69627	Fam89a	family with sequence similarity 89, member A	0.990099	0.9162	0.9651
70090	2310005E17Rik	RIKEN cDNA 2310005E17 gene	0.990099	0.975	0.9901
71371	Arid5b	AT rich interactive domain 5B (MRF1-like)	0.990099	0.8505	NA
71481	Alpk1	alpha-kinase 1	0.990099	0.9121	0.9635
71787	Trnau1ap	tRNA selenocysteine 1 associated protein 1	0.990099	0.9127	0.9638
72194	Fbxl20	F-box and leucine-rich repeat protein 20	0.990099	0.8429	NA
72205	Eml2	echinoderm microtubule associated protein like 2	0.990099	0.8557	NA
72722	Fam98a	family with sequence similarity 98, member A	0.990099	0.821	NA
74107	Cep55	centrosomal protein 55	0.990099	0.8996	NA
74356	4931428F04Rik	RIKEN cDNA 4931428F04 gene	0.990099	0.8433	NA
74407	Ttc25	tetratricopeptide repeat domain 25	0.990099	0.9144	0.9645
76332	Cog2	component of oligomeric golgi complex 2	0.990099	0.9116	0.9633
76547	Tmem101	transmembrane protein 101	0.990099	0.8779	NA
76779	Cluap1	clusterin associated protein 1	0.990099	0.8353	NA
76799	2510006D16Rik	RIKEN cDNA 2510006D16 gene	0.990099	0.8479	NA
77644	C330007P06Rik	RIKEN cDNA C330007P06 gene	0.990099	0.8994	NA
79263	Trim39	tripartite motif-containing 39	0.990099	0.9289	0.971
83924	Gpr137b	G protein-coupled receptor 137B	0.990099	0.8844	NA
98662	AW061147	expressed sequence AW061147	0.990099	0.9733	0.9893
108013	Celf4	CUGBP, Elav-like family member 4	0.990099	0.9253	0.9696
108954	Ppp1r15b	protein phosphatase 1, regulatory (inhibitor) subunit 15b	0.990099	0.91	0.9624
109113	Uhrf2	ubiquitin-like, containing PHD and RING finger domains 2	0.990099	0.8441	NA

114600	Gm4736	predicted gene 4736	0.990099	0.9555	0.9824
192185	Nadk	NAD kinase	0.990099	0.9238	0.9689
214498	Cdc73	cell division cycle 73, Paf1/RNA polymerase II complex component, homolog (S. cerevisiae)	0.990099	0.9262	0.97
214505	Gnptg	N-acetylglucosamine-1-phosphotransferase, gamma subunit	0.990099	0.8392	NA
214779	Zfp879	zinc figer protein 879	0.990099	0.9061	0.9602
217340	Rnf157	ring finger protein 157	0.990099	0.9006	NA
223989	4921513D23Rik	RIKEN cDNA 4921513D23 gene	0.990099	0.9388	0.9749
227095	Hibch	3-hydroxyisobutyryl-Coenzyme A hydrolase	0.990099	0.8526	NA
232337	Zfp637	zinc finger protein 637	0.990099	0.8844	NA
233733	Galntl4	UDP-N-acetyl-alpha-D-galactosamine:polypeptide N-acetylgalactosaminyltransferase-like 4	0.990099	0.9147	0.9645
237877	Atad5	ATPase family, AAA domain containing 5	0.990099	0.8644	NA
268816	Gm628	predicted gene 628	0.990099	0.9564	0.9824
269152	Kif26b	kinesin family member 26B	0.990099	0.9484	0.9796
320141	9330156P08Rik	RIKEN cDNA 9330156P08 gene	0.990099	0.9148	0.9645
380664	Lemd3	LEM domain containing 3	0.990099	0.8906	NA
433520	Gm14403	predicted gene 14403	0.990099	0.9172	0.9657
544752	Tug1	taurine upregulated gene 1	0.990099	0.9616	0.9846
544971	Bdp1	B double prime 1, subunit of RNA polymerase III transcription initiation factor IIIB	0.990099	0.8993	NA
621407	Gm9970	predicted gene 9970	0.990099	0.9261	0.97
622645	Tmem200c	transmembrane protein 200C	0.990099	0.9456	0.9783
625347	Gm6578	mitochondrial ribosomal protein L32 pseudogene	0.990099	0.8033	NA
100040305	Gm2701	predicted gene 2701	0.990099	0.9374	0.9742
11686	Alox12b	arachidonate 12-lipoxygenase, 12R type	0.98912	0.9476	0.9792
11924	Neurog2	neurogenin 2	0.98912	0.8594	NA
13617	Ednra	endothelin receptor type A	0.98912	0.8848	NA
13823	Epb4.1l3	erythrocyte protein band 4.1-like 3	0.98912	0.8742	NA
14718	Got1	glutamate oxaloacetate transaminase 1, soluble	0.98912	0.856	NA
15267	Hist2h2aa1	histone cluster 2, H2aa1	0.98912	0.8323	NA
15525	Hspa4	heat shock protein 4	0.98912	0.7837	NA
16069	Igj	immunoglobulin joining chain	0.98912	0.9159	0.965
18021	Nfatc3	nuclear factor of activated T-cells, cytoplasmic, calcineurin-dependent 3	0.98912	0.9563	0.9824
18577	Pde4a	phosphodiesterase 4A, cAMP specific	0.98912	0.9302	0.9715
20014	Rpn2	ribophorin II	0.98912	0.8854	NA
21888	Tle4	transducin-like enhancer of split 4, homolog of Drosophila E(spl)	0.98912	0.8964	NA
26426	Nubp2	nucleotide binding protein 2	0.98912	0.897	0.9557
26961	Rpl8	ribosomal protein L8	0.98912	0.8489	NA
27407	Abcf2	ATP-binding cassette, sub-family F (GCN20), member 2	0.98912	0.9108	0.9628
52575	Rg9mtd1	RNA (guanine-9-) methyltransferase domain containing 1	0.98912	0.865	NA
53380	Psmd10	proteasome (prosome, macropain) 26S subunit, non-ATPase, 10	0.98912	0.9012	0.9578
56531	Ylpm1	YLP motif containing 1	0.98912	0.8936	NA
66422	Dctpp1	dCTP pyrophosphatase 1	0.98912	0.8763	NA
67201	Glod4	glyoxalase domain containing 4	0.98912	0.8042	NA
67676	Rpp21	ribonuclease P 21 subunit (human)	0.98912	0.8086	NA
68073	Fam173b	family with sequence similarity 173, member B	0.98912	0.8786	NA
68347	0610011F06Rik	RIKEN cDNA 0610011F06 gene	0.98912	0.8901	NA
68796	Tmem214	transmembrane protein 214	0.98912	0.8885	NA
68846	Rnf208	ring finger protein 208	0.98912	0.9046	0.9596
73005	2900072G11Rik	RIKEN cDNA 2900072G11 gene	0.98912	0.9638	0.9849
73830	Eif3k	eukaryotic translation initiation factor 3, subunit K	0.98912	0.8437	NA

74096	Hvcn1	hydrogen voltage-gated channel 1	0.98912	0.931	0.9715
74150	Slc35f5	solute carrier family 35, member F5	0.98912	0.8476	NA
74325	Cltb	clathrin, light polypeptide (Lcb)	0.98912	0.7803	NA
78373	Nudt17	nudix (nucleoside diphosphate linked moiety X)-type motif 17	0.98912	0.936	0.9734
78891	Scyl1	SCY1-like 1 (S. cerevisiae)	0.98912	0.8297	NA
83767	Wasf1	WASP family 1	0.98912	0.8526	NA
83921	Tmem2	transmembrane protein 2	0.98912	0.897	NA
98238	Lrrc59	leucine rich repeat containing 59	0.98912	0.9353	0.9731
105148	Iars	isoleucine-tRNA synthetase	0.98912	0.8175	NA
216440	Os9	amplified in osteosarcoma	0.98912	0.8674	NA
217201	Rundc1	RUN domain containing 1	0.98912	0.8434	NA
239719	Mkl2	MKL/myocardin-like 2	0.98912	0.835	NA
241846	Lsm14b	LSM14 homolog B (SCD6, S. cerevisiae)	0.98912	0.8841	NA
245866	Ift52	intraflagellar transport 52 homolog (Chlamydomonas)	0.98912	0.7969	NA
278279	Tmtc2	transmembrane and tetratricopeptide repeat containing 2	0.98912	0.8569	NA
384281	Gatc	glutamyl-tRNA(Gln) amidotransferase, subunit C homolog (bacterial)	0.98912	0.8787	NA
442803	A830005F24Rik	RIKEN cDNA A830005F24 gene	0.98912	0.8937	NA
574437	Xlr3b	X-linked lymphocyte-regulated 3B	0.98912	0.9786	0.9911
11821	Aprt	adenine phosphoribosyl transferase	0.988142	0.8374	NA
11841	Arf2	ADP-ribosylation factor 2	0.988142	0.8495	NA
13434	Trdmt1	tRNA aspartic acid methyltransferase 1	0.988142	0.8269	NA
13871	Ercc2	excision repair cross-complementing rodent repair deficiency, complementation group 2	0.988142	0.8195	NA
15982	Ifrd1	interferon-related developmental regulator 1	0.988142	0.8727	NA
16151	Ikbkg	inhibitor of kappaB kinase gamma	0.988142	0.8407	NA
16536	Kcnq2	potassium voltage-gated channel, subfamily Q, member 2	0.988142	0.9214	0.968
16569	Kif3b	kinesin family member 3B	0.988142	0.908	0.9614
16681	Krt2	keratin 2	0.988142	0.9533	0.9815
17330	Minpp1	multiple inositol polyphosphate histidine phosphatase 1	0.988142	0.8571	NA
17762	Mapt	microtubule-associated protein tau	0.988142	0.8188	NA
18736	Pou1f1	POU domain, class 1, transcription factor 1	0.988142	0.943	0.9768
19087	Prkar2a	protein kinase, cAMP dependent regulatory, type II alpha	0.988142	0.9308	0.9715
19206	Ptch1	patched homolog 1	0.988142	0.9811	0.992
20595	Smn1	survival motor neuron 1	0.988142	0.8378	NA
20729	Spin1	spindlin 1	0.988142	0.8171	NA
22385	Baz1b	bromodomain adjacent to zinc finger domain, 1B	0.988142	0.8339	NA
22717	Zfp59	zinc finger protein 59	0.988142	0.924	0.969
22782	Slc30a1	solute carrier family 30 (zinc transporter), member 1	0.988142	0.8576	NA
23808	Ash2l	ash2 (absent, small, or homeotic)-like (Drosophila)	0.988142	0.8292	NA
24018	Rngtt	RNA guanylyltransferase and 5'-phosphatase	0.988142	0.9277	0.9705
26442	Psma5	proteasome (prosome, macropain) subunit, alpha type 5	0.988142	0.7883	NA
30957	Mapk8ip3	mitogen-activated protein kinase 8 interacting protein 3	0.988142	0.8008	NA
50708	Hist1h1c	histone cluster 1, H1c	0.988142	0.8206	NA
50785	Hs6st1	heparan sulfate 6-O-sulfotransferase 1	0.988142	0.9316	0.9719
54613	St3gal6	ST3 beta-galactoside alpha-2,3-sialyltransferase 6	0.988142	0.9254	0.9696
64340	Dhx38	DEAH (Asp-Glu-Ala-His) box polypeptide 38	0.988142	0.8507	NA
66314	Tpd52l2	tumor protein D52-like 2	0.988142	0.8935	0.9543
66771	4933439F18Rik	RIKEN cDNA 4933439F18 gene	0.988142	0.7607	NA
66897	Naa16	N(alpha)-acetyltransferase 16, NatA auxiliary subunit	0.988142	0.8914	NA
66960	Fam188a	family with sequence similarity 188, member A	0.988142	0.8595	NA

67673	Tceb2	transcription elongation factor B (SIII), polypeptide 2	0.988142	0.7843	NA
68011	Snrpg	small nuclear ribonucleoprotein polypeptide G	0.988142	0.81	NA
68375	Ndufa8	NADH dehydrogenase (ubiquinone) 1 alpha subcomplex, 8	0.988142	0.8874	0.9512
68436	Rpl34	ribosomal protein L34	0.988142	0.8967	0.9557
69372	Mocs3	molybdenum cofactor synthesis 3	0.988142	0.852	NA
69788	1600023N17Rik	RIKEN cDNA 1600023N17 gene	0.988142	0.8704	NA
69792	Med6	mediator of RNA polymerase II transcription, subunit 6 homolog (yeast)	0.988142	0.8092	NA
70088	Meaf6	MYST/Esa1-associated factor 6	0.988142	0.8694	NA
71448	Tmem80	transmembrane protein 80	0.988142	0.8444	NA
72106	Jmjd8	jumonji domain containing 8	0.988142	0.9231	0.9687
72469	Plcd3	phospholipase C, delta 3	0.988142	0.8447	NA
76497	Ppp1r11	protein phosphatase 1, regulatory (inhibitor) subunit 11	0.988142	0.8789	NA
77312	C030010L15Rik	RIKEN cDNA C030010L15 gene	0.988142	0.9305	0.9715
77629	Sphkap	SPHK1 interactor, AKAP domain containing	0.988142	0.87	NA
78425	9530053H05Rik	RIKEN cDNA 9530053H05 gene	0.988142	0.8961	0.9553
93873	Pcdhb2	protocadherin beta 2	0.988142	0.8781	NA
94062	Mrpl3	mitochondrial ribosomal protein L3	0.988142	0.8166	NA
94181	Nans	N-acetylneuraminic acid synthase (sialic acid synthase)	0.988142	0.857	NA
105171	Arrdc3	arrestin domain containing 3	0.988142	0.9103	0.9625
108857	Ankhd1	ankyrin repeat and KH domain containing 1	0.988142	0.7945	NA
109275	Actr5	ARP5 actin-related protein 5 homolog (yeast)	0.988142	0.8977	0.956
192651	Zfp286	zinc finger protein 286	0.988142	0.8435	NA
208820	Gm11818	predicted gene 11818	0.988142	0.8732	NA
215476	C330019G07Rik	RIKEN cDNA C330019G07 gene	0.988142	0.95	0.9802
217365	Nploc4	nuclear protein localization 4 homolog (S. cerevisiae)	0.988142	0.9027	0.9585
224129	Adcy5	adenylate cyclase 5	0.988142	0.8889	0.9519
224454	Zdhhc14	zinc finger, DHHC domain containing 14	0.988142	0.8732	NA
226098	Hectd2	HECT domain containing 2	0.988142	0.8584	NA
226153	Peo1	progressive external ophthalmoplegia 1 (human)	0.988142	0.8497	NA
230908	Tardbp	TAR DNA binding protein	0.988142	0.9122	0.9635
233189	Ctu1	cytosolic thiouridylase subunit 1 homolog (S. pombe)	0.988142	0.9034	0.9589
237211	Fancb	Fanconi anemia, complementation group B	0.988142	0.8704	NA
278240	Spin2	spindlin family, member 2	0.988142	0.8897	NA
319493	A430078G23Rik	RIKEN cDNA A430078G23 gene	0.988142	0.9015	0.9578
381694	B3galtl	beta 1,3-galactosyltransferase-like	0.988142	0.9025	0.9584
403187	Opa3	optic atrophy 3 (human)	0.988142	0.8692	NA
545253	Gm5820	predicted gene 5820	0.988142	0.9674	0.9864
627788	Gm6788	predicted gene 6788	0.988142	0.935	0.973
11502	Adam9	a disintegrin and metallopeptidase domain 9 (meltrin gamma)	0.987167	0.8143	NA
12095	Bglap-rs1	bone gamma-carboxyglutamate protein, related sequence 1	0.987167	0.8948	0.9549
13057	Cyba	cytochrome b-245, alpha polypeptide	0.987167	0.812	NA
14376	Ganab	alpha glucosidase 2 alpha neutral subunit	0.987167	0.789	NA
15211	Hexa	hexosaminidase A	0.987167	0.8037	NA
15387	Hnrnpk	heterogeneous nuclear ribonucleoprotein K	0.987167	0.8632	NA
18218	Dusp8	dual specificity phosphatase 8	0.987167	0.9118	0.9634
19414	Rasa3	RAS p21 protein activator 3	0.987167	0.7968	NA
19726	Rfx3	regulatory factor X, 3 (influences HLA class II expression)	0.987167	0.892	0.9536
20289	Scx	scleraxis	0.987167	0.8628	NA
21745	Tep1	telomerase associated protein 1	0.987167	0.8587	NA

21804	Tgfb1i1	transforming growth factor beta 1 induced transcript 1	0.987167	0.8818	NA
21927	Tnfaip1	tumor necrosis factor, alpha-induced protein 1 (endothelial)	0.987167	0.7982	NA
22629	Ywhah	tyrosine 3-monooxygenase/tryptophan 5-monooxygenase activation protein, eta polypeptide	0.987167	0.8354	NA
24069	Sufu	suppressor of fused homolog (Drosophila)	0.987167	0.9518	0.981
26409	Map3k7	mitogen-activated protein kinase kinase kinase 7	0.987167	0.906	0.9602
26451	Rpl27a	ribosomal protein L27A	0.987167	0.8503	NA
27058	Srp9	signal recognition particle 9	0.987167	0.91	0.9624
56280	Mrpl37	mitochondrial ribosomal protein L37	0.987167	0.8676	NA
56529	Sec11a	SEC11 homolog A (S. cerevisiae)	0.987167	0.8622	NA
58250	Chst11	carbohydrate sulfotransferase 11	0.987167	0.8815	0.9481
59007	Ngly1	N-glycanase 1	0.987167	0.9132	0.9639
66075	Chchd3	coiled-coil-helix-coiled-coil-helix domain containing 3	0.987167	0.8637	NA
66343	Tmem177	transmembrane protein 177	0.987167	0.895	0.955
66706	Ndufaf3	NADH dehydrogenase (ubiquinone) 1 alpha subcomplex, assembly factor 3	0.987167	0.8852	0.9503
68841	1110054M08Rik	RIKEN cDNA 1110054M08 gene	0.987167	0.887	0.951
71840	Tekt4	tektin 4	0.987167	0.9296	0.9713
74374	Clec16a	C-type lectin domain family 16, member A	0.987167	0.8316	NA
76438	Rftn1	raftlin lipid raft linker 1	0.987167	0.8479	NA
76477	Pcolce2	procollagen C-endopeptidase enhancer 2	0.987167	0.9065	0.9604
76510	Trappc9	trafficking protein particle complex 9	0.987167	0.9461	0.9787
76918	3110001N23Rik	RIKEN cDNA 3110001N23 gene	0.987167	0.9379	0.9743
98758	Hnrnpf	heterogeneous nuclear ribonucleoprotein F	0.987167	0.9532	0.9815
104112	Acly	ATP citrate lyase	0.987167	0.8428	NA
104303	Arl1	ADP-ribosylation factor-like 1	0.987167	0.7488	NA
104625	Cnot6	CCR4-NOT transcription complex, subunit 6	0.987167	0.8369	NA
107733	Mrpl41	mitochondrial ribosomal protein L41	0.987167	0.7856	NA
109785	Pgm3	phosphoglucomutase 3	0.987167	0.7866	NA
193385	Fam65b	family with sequence similarity 65, member B	0.987167	0.9098	0.9624
211961	Asxl3	additional sex combs like 3 (Drosophila)	0.987167	0.8515	NA
231646	Myo1h	myosin 1H	0.987167	0.9332	0.9726
231874	Ccz1	CCZ1 vacuolar protein trafficking and biogenesis associated homolog (S. cerevisiae)	0.987167	0.8112	NA
235028	Zfp426	zinc finger protein 426	0.987167	0.8292	NA
239510	Phf20l1	PHD finger protein 20-like 1	0.987167	0.9483	0.9796
246221	Mpst	mercaptopyruvate sulfurtransferase	0.987167	0.9222	0.9683
319377	D130012P04Rik	RIKEN cDNA D130012P04 gene	0.987167	0.9464	0.9787
320011	Uggt1	UDP-glucose glycoprotein glucosyltransferase 1	0.987167	0.7966	NA
320158	Zmat4	zinc finger, matrin type 4	0.987167	0.7992	NA
433864	Nom1	nucleolar protein with MIF4G domain 1	0.987167	0.8544	NA
494519	Abpd	androgen binding protein delta	0.987167	0.9624	0.9846
545487	Gm14439	predicted gene 14439	0.987167	0.7899	NA
11845	Arf6	ADP-ribosylation factor 6	0.986193	0.8433	NA
13489	Drd2	dopamine receptor D2	0.986193	0.9208	0.9678
14489	Mtpn	myotrophin	0.986193	0.8628	NA
15361	Hmga1	high mobility group AT-hook 1	0.986193	0.7507	NA
17160	Man2b2	mannosidase 2, alpha B2	0.986193	0.8986	0.9565
18575	Pde1c	phosphodiesterase 1C	0.986193	0.9281	0.9705
18582	Pde6d	phosphodiesterase 6D, cGMP-specific, rod, delta	0.986193	0.8551	NA
19089	Prkcsh	protein kinase C substrate 80K-H	0.986193	0.7884	NA
19243	Ptp4a1	protein tyrosine phosphatase 4a1	0.986193	0.8378	NA

19687	Rfc1	replication factor C (activator 1) 1	0.986193	0.7582	NA
19735	Rgs2	regulator of G-protein signaling 2	0.986193	0.7852	NA
20403	Itsn2	intersectin 2	0.986193	0.7774	NA
20524	Slc25a17	solute carrier family 25 (mitochondrial carrier, peroxisomal membrane protein), member 17	0.986193	0.8178	NA
20630	Snrpc	U1 small nuclear ribonucleoprotein C	0.986193	0.8129	NA
22031	Traf3	TNF receptor-associated factor 3	0.986193	0.7581	NA
23922	Jtb	jumping translocation breakpoint	0.986193	0.7932	NA
26398	Map2k4	mitogen-activated protein kinase kinase 4	0.986193	0.8659	NA
50498	Ebi3	Epstein-Barr virus induced gene 3	0.986193	0.8654	NA
51801	Ramp1	receptor (calcitonin) activity modifying protein 1	0.986193	0.8494	NA
52064	Coq5	coenzyme Q5 homolog, methyltransferase (yeast)	0.986193	0.8823	0.9485
54713	Fezf2	Fez family zinc finger 2	0.986193	0.8725	0.9448
58226	Cacna1h	calcium channel, voltage-dependent, T type, alpha 1H subunit	0.986193	0.8203	NA
59004	Pias4	protein inhibitor of activated STAT 4	0.986193	0.917	0.9655
59029	Psmd14	proteasome (prosome, macropain) 26S subunit, non-ATPase, 14	0.986193	0.9128	0.9638
66266	Eapp	E2F-associated phosphoprotein	0.986193	0.7455	NA
66859	Slc16a9	solute carrier family 16 (monocarboxylic acid transporters), member 9	0.986193	0.9309	0.9715
69038	1810006K21Rik	RIKEN cDNA 1810006K21 gene	0.986193	0.7721	NA
71960	Myh14	myosin, heavy polypeptide 14	0.986193	0.9211	0.968
78653	Bola3	bolA-like 3 (E. coli)	0.986193	0.8269	NA
100213	Rusc2	RUN and SH3 domain containing 2	0.986193	0.8233	NA
106633	Ift140	intraflagellar transport 140 homolog (Chlamydomonas)	0.986193	0.91	0.9624
108012	Ap1s2	adaptor-related protein complex 1, sigma 2 subunit	0.986193	0.8659	NA
108067	Eif2b3	eukaryotic translation initiation factor 2B, subunit 3	0.986193	0.8427	NA
109036	6230415J03Rik	RIKEN cDNA 6230415J03 gene	0.986193	0.942	0.9765
109181	Trip11	thyroid hormone receptor interactor 11	0.986193	0.8619	NA
116837	Rims1	regulating synaptic membrane exocytosis 1	0.986193	0.9379	0.9743
140629	Ubox5	U box domain containing 5	0.986193	0.8179	NA
170731	Mfn2	mitofusin 2	0.986193	0.9399	0.9755
192196	Luc7l2	LUC7-like 2 (S. cerevisiae)	0.986193	0.8534	NA
193116	Slu7	SLU7 splicing factor homolog (S. cerevisiae)	0.986193	0.8188	NA
218333	BC018507	cDNA sequence BC018507	0.986193	0.8458	NA
223701	Mkl1	MKL (megakaryoblastic leukemia)/myocardin-like 1	0.986193	0.823	NA
226751	Cdc42bpa	CDC42 binding protein kinase alpha	0.986193	0.9185	0.9666
228356	1110051M20Rik	RIKEN cDNA 1110051M20 gene	0.986193	0.9045	0.9596
230259	E130308A19Rik	RIKEN cDNA E130308A19 gene	0.986193	0.84	NA
231470	Fras1	Fraser syndrome 1 homolog (human)	0.986193	0.9296	0.9713
233204	Tbc1d17	TBC1 domain family, member 17	0.986193	0.8695	NA
234664	Nae1	NEDD8 activating enzyme E1 subunit 1	0.986193	0.8697	0.9436
240817	5830403L16Rik	RIKEN cDNA 5830403L16 gene	0.986193	0.9145	0.9645
243385	Gprin3	GPRIN family member 3	0.986193	0.8441	NA
246198	Mllt6	myeloid/lymphoid or mixed-lineage leukemia (trithorax homolog, Drosophila); translocated to, 6	0.986193	0.8288	NA
320615	Dopey1	dopey family member 1	0.986193	0.8479	NA
328133	Slc39a9	solute carrier family 39 (zinc transporter), member 9	0.986193	0.8429	NA
448850	Znhit3	zinc finger, HIT type 3	0.986193	0.8786	0.9467
544817	Arhgap27	Rho GTPase activating protein 27	0.986193	0.9554	0.9824
723988	A330062J17Rik	RIKEN cDNA A330062J17 gene	0.986193	0.8698	NA
100039317	Gm2155	predicted gene 2155	0.986193	0.9338	0.9727
11658	Alcam	activated leukocyte cell adhesion molecule	0.985222	0.8992	0.9568

12796	Camp	cathelicidin antimicrobial peptide	0.985222	0.85	NA
13813	Eomes	eomesodermin homolog (Xenopus laevis)	0.985222	0.8158	NA
14816	Grm1	glutamate receptor, metabotropic 1	0.985222	0.8932	0.9541
16177	Il1r1	interleukin 1 receptor, type I	0.985222	0.8378	NA
19719	Rfng	RFNG O-fucosylpeptide 3-beta-N-acetylglucosaminyltransferase	0.985222	0.8173	NA
21803	Tgfb1	transforming growth factor, beta 1	0.985222	0.9181	0.9663
27364	Srr	serine racemase	0.985222	0.8922	0.9536
27366	Txnl4a	thioredoxin-like 4A	0.985222	0.7893	NA
28075	Pppde2	PPPDE peptidase domain containing 2	0.985222	0.8576	NA
30931	Tor1a	torsin family 1, member A (torsin A)	0.985222	0.8007	NA
53330	Vamp4	vesicle-associated membrane protein 4	0.985222	0.8562	NA
56258	Hnrnph2	heterogeneous nuclear ribonucleoprotein H2	0.985222	0.8687	0.943
56438	Rbx1	ring-box 1	0.985222	0.8023	NA
57294	Rps27	ribosomal protein S27	0.985222	0.7998	NA
66174	Nudt14	nudix (nucleoside diphosphate linked moiety X)-type motif 14	0.985222	0.848	NA
66409	Rsl1d1	ribosomal L1 domain containing 1	0.985222	0.7206	NA
66462	2810428I15Rik	RIKEN cDNA 2810428I15 gene	0.985222	0.8372	NA
66887	Lonp2	lon peptidase 2, peroxisomal	0.985222	0.8064	NA
67186	Rplp2	ribosomal protein, large P2	0.985222	0.8753	0.9454
68094	Smarcc2	SWI/SNF related, matrix associated, actin dependent regulator of chromatin, subfamily c, member 2	0.985222	0.8417	NA
68215	Fam98b	family with sequence similarity 98, member B	0.985222	0.87	0.9438
68939	Rasl11b	RAS-like, family 11, member B	0.985222	0.8896	0.9521
69150	Snx4	sorting nexin 4	0.985222	0.8668	0.9421
70999	Naa40	N(alpha)-acetyltransferase 40, NatD catalytic subunit, homolog (S. cerevisiae)	0.985222	0.868	0.9427
74044	Ttf2	transcription termination factor, RNA polymerase II	0.985222	0.8574	NA
74778	Rrp7a	ribosomal RNA processing 7 homolog A (S. cerevisiae)	0.985222	0.7073	NA
74841	Usp38	ubiquitin specific peptidase 38	0.985222	0.7665	NA
74883	4930461C15Rik	RIKEN cDNA 4930461C15 gene	0.985222	0.9053	0.9597
75544	1700016K05Rik	RIKEN cDNA 1700016K05 gene	0.985222	0.8994	0.957
75732	Iqcd	IQ motif containing D	0.985222	0.7965	NA
76282	Gpt	glutamic pyruvic transaminase, soluble	0.985222	0.7899	NA
76429	Lhpp	phospholysine phosphohistidine inorganic pyrophosphate phosphatase	0.985222	0.9343	0.9728
83557	Lin28a	lin-28 homolog A (C. elegans)	0.985222	0.9014	0.9578
94246	Arid4b	AT rich interactive domain 4B (RBP1-like)	0.985222	0.929	0.971
99982	Kdm1a	lysine (K)-specific demethylase 1A	0.985222	0.8083	NA
103517	BB187676	expressed sequence BB187676	0.985222	0.8739	0.9451
103694	Tmed4	transmembrane emp24 protein transport domain containing 4	0.985222	0.8059	NA
104910	Slc25a47	solute carrier family 25, member 47	0.985222	0.9035	0.9589
118451	Mrps2	mitochondrial ribosomal protein S2	0.985222	0.884	0.9497
170772	Glcci1	glucocorticoid induced transcript 1	0.985222	0.9127	0.9638
210719	Mkx	mohawk homeobox	0.985222	0.8326	NA
212528	Trmt1	TRM1 tRNA methyltransferase 1 homolog (S. cerevisiae)	0.985222	0.7549	NA
215512	Fam117a	family with sequence similarity 117, memberA	0.985222	0.8118	NA
226470	Zbtb41	zinc finger and BTB domain containing 41 homolog	0.985222	0.8937	0.9544
228139	P2rx3	purinergic receptor P2X, ligand-gated ion channel, 3	0.985222	0.8083	NA
229593	Golph3l	golgi phosphoprotein 3-like	0.985222	0.7897	NA
230162	Zfp189	zinc finger protein 189	0.985222	0.775	NA
328108	Fam179b	family with sequence similarity 179, member B	0.985222	0.8989	0.9566
330814	Lphn1	latrophilin 1	0.985222	0.7406	NA

474156	Zbtb9	zinc finger and BTB domain containing 9	0.985222	0.834	NA
666955	Gm8378	predicted gene 8378	0.985222	0.8846	0.9499
667572	Gm8709	glyceraldehyde-3-phosphate dehydrogenase pseudogene	0.985222	0.9003	0.9573
676974	LOC676974	glucose-6-phosphate isomerase-like	0.985222	0.9121	0.9635
100042970	Kifc1	kinesin family member C1	0.985222	0.8655	0.9415
11769	Ap1s1	adaptor protein complex AP-1, sigma 1	0.984252	0.81	NA
11994	Pcdh15	protocadherin 15	0.984252	0.8968	0.9557
12457	Ccrn4l	CCR4 carbon catabolite repression 4-like (S. cerevisiae)	0.984252	0.7605	NA
13841	Epha7	Eph receptor A7	0.984252	0.8254	NA
18036	Nfkbib	nuclear factor of kappa light polypeptide gene enhancer in B-cells inhibitor, beta	0.984252	0.9094	0.9622
18036	Nfkbib	nuclear factor of kappa light polypeptide gene enhancer in B-cells inhibitor, beta	0.984252	0.8252	NA
18241	Gpr143	G protein-coupled receptor 143	0.984252	0.9657	0.9857
18706	Pik3ca	phosphatidylinositol 3-kinase, catalytic, alpha polypeptide	0.984252	0.8777	0.9464
19025	Ctsa	cathepsin A	0.984252	0.7098	NA
20544	Slc9a1	solute carrier family 9 (sodium/hydrogen exchanger), member 1	0.984252	0.7744	NA
20947	Swap70	SWA-70 protein	0.984252	0.8132	NA
20972	Syngr1	synaptogyrin 1	0.984252	0.8258	NA
22095	Tshr	thyroid stimulating hormone receptor	0.984252	0.9048	0.9597
27414	Sergef	secretion regulating guanine nucleotide exchange factor	0.984252	0.8491	NA
27632	Rdbp	RD RNA-binding protein	0.984252	0.833	NA
28006	D6Wsu116e	DNA segment, Chr 6, Wayne State University 116, expressed	0.984252	0.9049	0.9597
30877	Gnl3	guanine nucleotide binding protein-like 3 (nucleolar)	0.984252	0.7698	NA
50884	Nckap1	NCK-associated protein 1	0.984252	0.8329	NA
57354	Cramp1l	Crm, cramped-like (Drosophila)	0.984252	0.7746	NA
58226	Cacna1h	calcium channel, voltage-dependent, T type, alpha 1H subunit	0.984252	0.822	NA
58230	Rnf8	ring finger protein 8	0.984252	0.7404	NA
59026	Huwe1	HECT, UBA and WWE domain containing 1	0.984252	0.82	NA
66815	Ccdc109b	coiled-coil domain containing 109B	0.984252	0.8246	NA
67008	1600012F09Rik	RIKEN cDNA 1600012F09 gene	0.984252	0.7893	NA
67390	Rnmtl1	RNA methyltransferase like 1	0.984252	0.7906	NA
67841	Atg3	autophagy-related 3 (yeast)	0.984252	0.8455	NA
67998	Fam134c	family with sequence similarity 134, member C	0.984252	0.857	0.9367
68618	1110012L19Rik	RIKEN cDNA 1110012L19 gene	0.984252	0.8195	NA
68916	Cdkal1	CDK5 regulatory subunit associated protein 1-like 1	0.984252	0.7678	NA
71991	Ercc8	excision repaiross-complementing rodent repair deficiency, complementation group 8	0.984252	0.8197	NA
74919	4930471M23Rik	RIKEN cDNA 4930471M23 gene	0.984252	0.81	NA
75602	1810062O18Rik	RIKEN cDNA 1810062O18 gene	0.984252	0.8283	NA
76800	Usp42	ubiquitin specific peptidase 42	0.984252	0.8394	NA
76889	Adck4	aarF domain containing kinase 4	0.984252	0.8209	NA
76890	Memo1	mediator of cell motility 1	0.984252	0.7781	NA
78308	Gpr108	G protein-coupled receptor 108	0.984252	0.696	NA
78830	Slc25a12	solute carrier family 25 (mitochondrial carrier, Aralar), member 12	0.984252	0.7849	NA
94043	Tm2d1	TM2 domain containing 1	0.984252	0.7931	NA
101476	Plekha1	pleckstrin homology domain containing, family A (phosphoinositide binding specific) member 1	0.984252	0.8947	0.9549
102220	E330037G11Rik	RIKEN cDNA E330037G11 gene	0.984252	0.9376	0.9742
102278	Cpne7	copine VII	0.984252	0.7719	NA
106557	Ldhal6b	lactate dehydrogenase A-like 6B	0.984252	0.9335	0.9726
107733	Mrpl41	mitochondrial ribosomal protein L41	0.984252	0.7343	NA
110816	Pwp2	PWP2 periodic tryptophan protein homolog (yeast)	0.984252	0.7995	NA

116870	Mta1	metastasis associated 1	0.984252	0.8509	0.9334
209318	Gps1	G protein pathway suppressor 1	0.984252	0.7654	NA
212307	Mapre2	microtubule-associated protein, RP/EB family, member 2	0.984252	0.7098	NA
223827	Gxylt1	glucoside xylosyltransferase 1	0.984252	0.9263	0.97
226122	Ubtd1	ubiquitin domain containing 1	0.984252	0.8961	0.9553
229675	Rsbn1	rosbin, round spermatid basic protein 1	0.984252	0.8516	NA
231474	Paqr3	progestin and adipoQ receptor family member III	0.984252	0.8838	0.9496
243374	Gimap8	GTPase, IMAP family member 8	0.984252	0.908	0.9614
263764	Creg2	cellular repressor of E1A-stimulated genes 2	0.984252	0.8363	NA
271377	Zbtb11	zinc finger and BTB domain containing 11	0.984252	0.8082	NA
280645	B3gat2	beta-1,3-glucuronyltransferase 2 (glucuronosyltransferase S)	0.984252	0.7981	NA
328234	Rnf182	ring finger protein 182	0.984252	0.837	NA
100039043	Gm10731	predicted gene 10731	0.984252	0.8594	0.9375
11518	Add1	adducin 1 (alpha)	0.983284	0.7106	NA
11545	Parp1	poly (ADP-ribose) polymerase family, member 1	0.983284	0.8048	NA
12212	Chic1	cysteine-rich hydrophobic domain 1	0.983284	0.8465	0.9313
12748	Clk2	CDC-like kinase 2	0.983284	0.7343	NA
13612	Edil3	EGF-like repeats and discoidin I-like domains 3	0.983284	0.9233	0.9688
14058	F10	coagulation factor X	0.983284	0.8503	0.9332
16705	Krtap9-1	keratin associated protein 9-1	0.983284	0.8973	0.9558
16881	Lig1	ligase I, DNA, ATP-dependent	0.983284	0.7071	NA
17749	Polr2k	polymerase (RNA) II (DNA directed) polypeptide K	0.983284	0.783	NA
19650	Rbl1	retinoblastoma-like 1 (p107)	0.983284	0.8022	NA
20301	Ccl27a	chemokine (C-C motif) ligand 27A	0.983284	0.7147	NA
22590	Xpa	xeroderma pigmentosum, complementation group A	0.983284	0.7835	NA
27222	Atp1a4	ATPase, Na+/K+ transporting, alpha 4 polypeptide	0.983284	0.9217	0.9681
50995	Uba2	ubiquitin-like modifier activating enzyme 2	0.983284	0.7269	NA
51796	Srrm1	serine/arginine repetitive matrix 1	0.983284	0.8459	0.931
54399	Bet1l	blocked early in transport 1 homolog (S. cerevisiae)-like	0.983284	0.7951	NA
63953	Dusp10	dual specificity phosphatase 10	0.983284	0.8279	NA
64292	Ptges	prostaglandin E synthase	0.983284	0.8901	0.9523
66148	Dnajc15	DnaJ (Hsp40) homolog, subfamily C, member 15	0.983284	0.747	NA
66308	2810021B07Rik	RIKEN cDNA 2810021B07 gene	0.983284	0.8163	NA
66454	Nmnat1	nicotinamide nucleotide adenylyltransferase 1	0.983284	0.7996	NA
66691	Gapvd1	GTPase activating protein and VPS9 domains 1	0.983284	0.7168	NA
66855	Tcf25	transcription factor 25 (basic helix-loop-helix)	0.983284	0.8363	NA
67703	Kirrel3	kin of IRRE like 3 (Drosophila)	0.983284	0.8775	0.9464
68095	Ociad1	OCIA domain containing 1	0.983284	0.7533	NA
68196	Hsbp1	heat shock factor binding protein 1	0.983284	0.7755	NA
69104	Mar-05	membrane-associated ring finger (C3HC4) 5	0.983284	0.8762	0.946
71667	0610007L01Rik	RIKEN cDNA 0610007L01 gene	0.983284	0.7472	NA
71982	Snx10	sorting nexin 10	0.983284	0.8543	0.9352
74019	Traf3ip1	TRAF3 interacting protein 1	0.983284	0.9282	0.9705
74320	Wdr33	WD repeat domain 33	0.983284	0.854	0.935
74533	Gzf1	GDNF-inducible zinc finger protein 1	0.983284	0.7489	NA
76467	Msrb2	methionine sulfoxide reductase B2	0.983284	0.7864	NA
76626	Msi2	Musashi homolog 2 (Drosophila)	0.983284	0.8472	0.9317
80902	Zfp202	zinc finger protein 202	0.983284	0.8175	NA
99349	Dnajc24	DnaJ (Hsp40) homolog, subfamily C, member 24	0.983284	0.8002	NA

100705	Acacb	acetyl-Coenzyme A carboxylase beta	0.983284	0.8715	0.9443
101966	D8Ertd738e	DNA segment, Chr 8, ERATO Doi 738, expressed	0.983284	0.7548	NA
101994	Zfp828	zinc finger protein 828	0.983284	0.7992	NA
104130	Ndufb11	NADH dehydrogenase (ubiquinone) 1 beta subcomplex, 11	0.983284	0.8588	0.9373
107823	Whsc1	Wolf-Hirschhorn syndrome candidate 1 (human)	0.983284	0.8803	0.9478
108096	Slco1a5	solute carrier organic anion transporter family, member 1a5	0.983284	0.928	0.9705
108148	Galnt2	UDP-N-acetyl-alpha-D-galactosamine:polypeptide N-acetylgalactosaminyltransferase 2	0.983284	0.7623	NA
109801	Glo1	glyoxalase 1	0.983284	0.8366	NA
114641	Rpl31	ribosomal protein L31	0.983284	0.7335	NA
209239	Gan	giant axonal neuropathy	0.983284	0.8955	0.9552
211666	Mgst2	microsomal glutathione S-transferase 2	0.983284	0.8956	0.9552
215693	Zmat1	zinc finger, matrin type 1	0.983284	0.7475	NA
216543	Cep68	centrosomal protein 68	0.983284	0.7522	NA
216560	AV249152	expressed sequence AV249152	0.983284	0.8257	NA
216858	Kctd11	potassium channel tetramerisation domain containing 11	0.983284	0.845	0.9307
227102	Ormdl1	ORM1-like 1 (S. cerevisiae)	0.983284	0.7098	NA
229681	St7l	suppression of tumorigenicity 7-like	0.983284	0.7734	NA
235028	Zfp426	zinc finger protein 426	0.983284	0.8462	NA
235431	Coro2b	coronin, actin binding protein, 2B	0.983284	0.7331	NA
235582	Glyctk	glycerate kinase	0.983284	0.8214	NA
242425	Gabbr2	gamma-aminobutyric acid (GABA) B receptor, 2	0.983284	0.919	0.9669
268390	Ahsa2	AHA1, activator of heat shock protein ATPase homolog 2 (yeast)	0.983284	0.7951	NA
269113	Nup54	nucleoporin 54	0.983284	0.7976	NA
319586	Celf5	CUGBP, Elav-like family member 5	0.983284	0.8086	NA
319933	E230024E03Rik	RIKEN cDNA E230024E03 gene	0.983284	0.9352	0.9731
330409	Cecr2	cat eye syndrome chromosome region, candidate 2 homolog (human)	0.983284	0.87	0.9438
433771	2310028O11Rik	RIKEN cDNA 2310028O11 gene	0.983284	0.7355	NA
621998	Gm6277	predicted gene 6277	0.983284	0.9158	0.965
626391	Zfp951	zinc finger protein 951	0.983284	0.8943	0.9548
100041879	Gm3561	predicted gene 3561	0.983284	0.8848	0.95
12568	Cdk5	cyclin-dependent kinase 5	0.982318	0.7206	NA
16004	Igf2r	insulin-like growth factor 2 receptor	0.982318	0.7452	NA
16859	Lgals9	lectin, galactose binding, soluble 9	0.982318	0.9039	0.9592
17847	Usp34	ubiquitin specific peptidase 34	0.982318	0.9108	0.9628
18117	Cox4nb	COX4 neighbor	0.982318	0.7143	NA
18201	Nsmaf	neutral sphingomyelinase (N-SMase) activation associated factor	0.982318	0.7297	NA
18472	Pafah1b1	platelet-activating factor acetylhydrolase, isoform 1b, subunit 1	0.982318	0.7705	NA
18645	Pfn2	profilin 2	0.982318	0.7843	NA
19126	Prom1	prominin 1	0.982318	0.8519	0.9338
19647	Rbbp6	retinoblastoma binding protein 6	0.982318	0.8221	NA
19941	Rpl26	ribosomal protein L26	0.982318	0.7502	NA
20055	Rps16	ribosomal protein S16	0.982318	0.8377	0.927
21427	Vps72	vacuolar protein sorting 72 (yeast)	0.982318	0.8934	0.9543
27426	Nagpa	N-acetylglucosamine-1-phosphodiester alpha-N-acetylglucosaminidase	0.982318	0.886	0.9506
50907	Preb	prolactin regulatory element binding	0.982318	0.8879	0.9513
51810	Hnrnpu	heterogeneous nuclear ribonucleoprotein U	0.982318	0.8286	NA
54484	Mkrn1	makorin, ring finger protein, 1	0.982318	0.8333	0.9247
57750	Wdr12	WD repeat domain 12	0.982318	0.7603	NA
59021	Rab2a	RAB2A, member RAS oncogene family	0.982318	0.8059	NA

66330	1700020L24Rik	RIKEN cDNA 1700020L24 gene	0.982318	0.8721	0.9446
67067	Romo1	reactive oxygen species modulator 1	0.982318	0.8046	NA
67678	Lsm3	LSM3 homolog, U6 small nuclear RNA associated (S. cerevisiae)	0.982318	0.8061	NA
68944	Tmco1	transmembrane and coiled-coil domains 1	0.982318	0.7075	NA
69962	2810422O20Rik	RIKEN cDNA 2810422O20 gene	0.982318	0.8361	NA
72503	2610507B11Rik	RIKEN cDNA 2610507B11 gene	0.982318	0.712	NA
73836	Slc35b2	solute carrier family 35, member B2	0.982318	0.7585	NA
75292	Prkd3	protein kinase D3	0.982318	0.7238	NA
75665	Ccdc64	coiled-coil domain containing 64	0.982318	0.7521	NA
75951	4930578M01Rik	RIKEN cDNA 4930578M01 gene	0.982318	0.864	0.9403
77827	Krba1	KRAB-A domain containing 1	0.982318	0.7279	NA
77929	Yipf6	Yip1 domain family, member 6	0.982318	0.8896	0.9521
78885	Coro7	coronin 7	0.982318	0.7998	NA
109050	6530418L21Rik	RIKEN cDNA 6530418L21 gene	0.982318	0.8925	0.9538
110391	Qdpr	quinoid dihydropteridine reductase	0.982318	0.8044	NA
114615	Elac1	elaC homolog 1 (E. coli)	0.982318	0.7709	NA
114863	Prosc	proline synthetase co-transcribed	0.982318	0.874	0.9452
114896	Afg3l1	AFG3(ATPase family gene 3)-like 1 (yeast)	0.982318	0.7352	NA
215476	C330019G07Rik	RIKEN cDNA C330019G07 gene	0.982318	0.8323	0.9245
229714	Gpr61	G protein-coupled receptor 61	0.982318	0.7263	NA
237222	Ofd1	oral-facial-digital syndrome 1 gene homolog (human)	0.982318	0.7845	NA
243529	H1fx	H1 histone family, member X	0.982318	0.8942	0.9548
245828	Trappc1	trafficking protein particle complex 1	0.982318	0.8483	0.9323
269614	Pank4	pantothenate kinase 4	0.982318	0.7759	NA
279618	Gm715	predicted pseudogene 715	0.982318	0.7642	NA
321000	4933421E11Rik	RIKEN cDNA 4933421E11 gene	0.982318	0.7777	NA
100041351	Gm3285	predicted gene 3285	0.982318	0.9198	0.9673
100043364	Gm4392	predicted gene 4392	0.982318	0.8638	0.9402
100043902	Six3os1	Six3 opposite strand transcript 1	0.982318	0.9033	0.9588
100504004	LOC100504004	putative transposase element L1Md-A101/L1Md-A102/L1Md-A2-like	0.982318	0.913	0.9638
12995	Csnk2a1	casein kinase 2, alpha 1 polypeptide	0.981354	0.6488	NA
14009	Etv1	ets variant gene 1	0.981354	0.8703	0.944
16443	Itsn1	intersectin 1 (SH3 domain protein 1A)	0.981354	0.8621	0.9392
16561	Kif1b	kinesin family member 1B	0.981354	0.8475	0.9318
16616	Klk1b21	kallikrein 1-related peptidase b21	0.981354	0.9223	0.9683
18004	Nek1	NIMA (never in mitosis gene a)-related expressed kinase 1	0.981354	0.8448	0.9306
18032	Nfix	nuclear factor I/X	0.981354	0.8842	0.9497
18074	Nid2	nidogen 2	0.981354	0.9403	0.9757
18132	Notch4	Notch gene homolog 4 (Drosophila)	0.981354	0.7717	NA
18618	Pemt	phosphatidylethanolamine N-methyltransferase	0.981354	0.8857	0.9505
20847	Stat2	signal transducer and activator of transcription 2	0.981354	0.7143	NA
20933	Med22	mediator complex subunit 22	0.981354	0.8521	0.9339
21990	Tph1	tryptophan hydroxylase 1	0.981354	0.8685	0.9428
22715	Zfp57	zinc finger protein 57	0.981354	0.8705	0.9441
22746	Zfp85-rs1	zinc finger protein 85, related sequence 1	0.981354	0.9035	0.9589
23950	Dnajb6	DnaJ (Hsp40) homolog, subfamily B, member 6	0.981354	0.8649	0.941
24013	Grk1	G protein-coupled receptor kinase 1	0.981354	0.7817	NA
54201	Zfp316	zinc finger protein 316	0.981354	0.9065	0.9604
54614	Prpf40b	PRP40 pre-mRNA processing factor 40 homolog B (yeast)	0.981354	0.8503	0.9332

55989	Nop58	NOP58 ribonucleoprotein homolog (yeast)	0.981354	0.766	NA
56314	Zfp113	zinc finger protein 113	0.981354	0.8417	0.9289
56395	Tmem115	transmembrane protein 115	0.981354	0.866	0.9418
66413	Psmd6	proteasome (prosome, macropain) 26S subunit, non-ATPase, 6	0.981354	0.7566	NA
66525	Timm50	translocase of inner mitochondrial membrane 50 homolog (yeast)	0.981354	0.8496	0.9329
67125	Tspan31	tetraspanin 31	0.981354	0.8298	0.9233
67866	Wfdc1	WAP four-disulfide core domain 1	0.981354	0.9097	0.9624
68644	Abhd14a	abhydrolase domain containing 14A	0.981354	0.8768	0.9462
70312	2510012J08Rik	RIKEN cDNA 2510012J08 gene	0.981354	0.699	NA
70465	Wdr77	WD repeat domain 77	0.981354	0.8054	NA
72371	2210408I21Rik	RIKEN cDNA 2210408I21 gene	0.981354	0.7998	NA
72416	Lrpprc	leucine-rich PPR-motif containing	0.981354	0.6984	NA
73825	Klraq1	KLRAQ motif containing 1	0.981354	0.8305	0.9234
74148	1300001I01Rik	RIKEN cDNA 1300001I01 gene	0.981354	0.7293	NA
74630	4930432N10Rik	RIKEN cDNA 4930432N10 gene	0.981354	0.8567	0.9365
78926	Gas2l1	growth arrest-specific 2 like 1	0.981354	0.7709	NA
81018	Rnf114	ring finger protein 114	0.981354	0.7114	NA
83397	Akap12	A kinase (PRKA) anchor protein (gravin) 12	0.981354	0.759	NA
107932	Chd4	chromodomain helicase DNA binding protein 4	0.981354	0.7077	NA
216119	Ybey	ybeY metallopeptidase	0.981354	0.8129	NA
225896	Ubxn1	UBX domain protein 1	0.981354	0.7259	NA
233532	Rsf1	remodeling and spacing factor 1	0.981354	0.7804	NA
235559	Topbp1	topoisomerase (DNA) II binding protein 1	0.981354	0.7872	NA
236848	BC023829	cDNA sequence BC023829	0.981354	0.7479	NA
238988	Erc2	ELKS/RAB6-interacting/CAST family member 2	0.981354	0.8505	0.9332
242557	Atg4c	autophagy-related 4C (yeast)	0.981354	0.7709	NA
243373	AI854703	expressed sequence AI854703	0.981354	0.8449	0.9307
269116	Nfasc	neurofascin	0.981354	0.8419	0.9289
277333	Gm5069	glyceraldehyde-3-phosphate dehydrogenase pseudogene	0.981354	0.8091	NA
319554	Idi1	isopentenyl-diphosphate delta isomerase	0.981354	0.7663	NA
320495	Ipcef1	interaction protein for cytohesin exchange factors 1	0.981354	0.8507	0.9333
380608	Tagap1	T-cell activation GTPase activating protein 1	0.981354	0.8554	0.9356
381314	Iars2	isoleucine-tRNA synthetase 2, mitochondrial	0.981354	0.7986	NA
385658	Fam55c	family with sequence similarity 55, member C	0.981354	0.7457	NA
435285	Krtap4-16	keratin associated protein 4-16	0.981354	0.9161	0.9651
666676	Gm8230	predicted gene 8230	0.981354	0.813	NA
100038637	Gm10134	predicted gene 10134	0.981354	0.8816	0.9481
100045878	LOC100045878	t-cell receptor alpha chain V region RL-5-like	0.981354	0.9496	0.9801
100504289	LOC100504289	hypothetical LOC100504289	0.981354	0.935	0.973
12006	Axin2	axin2	0.980392	0.8829	0.949
12291	Cacna1g	calcium channel, voltage-dependent, T type, alpha 1G subunit	0.980392	0.7285	NA
12552	Cdh11	cadherin 11	0.980392	0.8374	0.927
13097	Cyp2c38	cytochrome P450, family 2, subfamily c, polypeptide 38	0.980392	0.9217	0.9681
14681	Gnao1	guanine nucleotide binding protein, alpha O	0.980392	0.9032	0.9588
15926	Idh1	isocitrate dehydrogenase 1 (NADP+), soluble	0.980392	0.8487	0.9326
17128	Smad4	MAD homolog 4 (Drosophila)	0.980392	0.685	NA
17385	Mmp11	matrix metallopeptidase 11	0.980392	0.8235	0.92
17385	Mmp11	matrix metallopeptidase 11	0.980392	0.7936	NA
18181	Nrf1	nuclear respiratory factor 1	0.980392	0.7875	NA

19944	Rpl29	ribosomal protein L29	0.980392	0.7985	NA
20239	Atxn2	ataxin 2	0.980392	0.8218	0.9191
21411	Tcf20	transcription factor 20	0.980392	0.773	NA
23892	Grem1	gremlin 1	0.980392	0.86	0.938
23994	Dazap2	DAZ associated protein 2	0.980392	0.728	NA
26961	Rpl8	ribosomal protein L8	0.980392	0.7412	NA
27261	Dok3	docking protein 3	0.980392	0.8635	0.9401
27397	Mrpl17	mitochondrial ribosomal protein L17	0.980392	0.7731	NA
54141	Spag5	sperm associated antigen 5	0.980392	0.8745	0.9452
56353	Rybp	RING1 and YY1 binding protein	0.980392	0.7157	NA
56360	Acot9	acyl-CoA thioesterase 9	0.980392	0.8098	NA
60532	Wtap	Wilms' tumour 1-associating protein	0.980392	0.8869	0.951
64144	Mllt1	myeloid/lymphoid or mixed-lineage leukemia (trithorax homolog, Drosophila); translocated to, 1	0.980392	0.8218	0.9191
66193	1110049F12Rik	RIKEN cDNA 1110049F12 gene	0.980392	0.6719	NA
66309	Tmem128	transmembrane protein 128	0.980392	0.9012	0.9578
66999	Med28	mediator of RNA polymerase II transcription, subunit 28 homolog (yeast)	0.980392	0.6146	NA
67135	2310021H06Rik	RIKEN cDNA 2310021H06 gene	0.980392	0.9316	0.9719
67500	Ccar1	cell division cycle and apoptosis regulator 1	0.980392	0.7542	NA
67792	Rgs8	regulator of G-protein signaling 8	0.980392	0.8363	0.9265
67839	Gpsm1	G-protein signalling modulator 1 (AGS3-like, C. elegans)	0.980392	0.9201	0.9674
68955	Srrm4	serine/arginine repetitive matrix 4	0.980392	0.773	NA
69028	Mitd1	MIT, microtubule interacting and transport, domain containing 1	0.980392	0.788	NA
69051	Pycr2	pyrroline-5-carboxylate reductase family, member 2	0.980392	0.7472	NA
69723	Rpain	RPA interacting protein	0.980392	0.7764	NA
70231	Gorasp2	golgi reassembly stacking protein 2	0.980392	0.7936	NA
75614	2610019E17Rik	RIKEN cDNA 2610019E17 gene	0.980392	0.8708	0.9441
76612	Lrrc27	leucine rich repeat containing 27	0.980392	0.8059	NA
85308	Fam158a	family with sequence similarity 158, member A	0.980392	0.7825	NA
98238	Lrrc59	leucine rich repeat containing 59	0.980392	0.8267	0.9215
100163	Pafah2	platelet-activating factor acetylhydrolase 2	0.980392	0.7527	NA
109065	1110034A24Rik	RIKEN cDNA 1110034A24 gene	0.980392	0.8233	0.92
111175	Pecr	peroxisomal trans-2-enoyl-CoA reductase	0.980392	0.7882	NA
140630	Ube4a	ubiquitination factor E4A, UFD2 homolog (S. cerevisiae)	0.980392	0.8107	NA
170625	Snx18	sorting nexin 18	0.980392	0.659	NA
211064	Alkbh1	alkB, alkylation repair homolog 1 (E. coli)	0.980392	0.838	0.927
215814	Ccdc28a	coiled-coil domain containing 28A	0.980392	0.7528	NA
218820	Zfp503	zinc finger protein 503	0.980392	0.8215	0.9189
226414	Dars	aspartyl-tRNA synthetase	0.980392	0.8713	0.9443
227960	Gca	grancalcin	0.980392	0.8031	NA
235493	BC031353	cDNA sequence BC031353	0.980392	0.6771	NA
246779	Il27	interleukin 27	0.980392	0.9001	0.9572
320478	Sox2ot	SOX2 overlapping transcript (non-protein coding)	0.980392	0.7962	NA
320949	D830039M14Rik	RIKEN cDNA D830039M14 gene	0.980392	0.8107	NA
620592	Tmem28	transmembrane protein 28	0.980392	0.8045	NA
100038734	Gm10845	predicted gene 10845	0.980392	0.7266	NA
100043489	1300002E11Rik	RIKEN cDNA 1300002E11 gene	0.980392	0.7899	NA
11556	Adrb3	adrenergic receptor, beta 3	0.979432	0.8838	0.9496
12021	Bard1	BRCA1 associated RING domain 1	0.979432	0.816	0.9156
12942	Pcdha11	protocadherin alpha 11	0.979432	0.8966	0.9557

13185	Dscr3	Down syndrome critical region gene 3	0.979432	0.7017	NA
14533	Bloc1s1	biogenesis of lysosome-related organelles complex-1, subunit 1	0.979432	0.7445	NA
14924	Magi1	membrane associated guanylate kinase, WW and PDZ domain containing 1	0.979432	0.7285	NA
17684	Cited2	Cbp/p300-interacting transactivator, with Glu/Asp-rich carboxy-terminal domain, 2	0.979432	0.8935	0.9543
18195	Nsf	N-ethylmaleimide sensitive fusion protein	0.979432	0.779	NA
19981	Rpl37a	ribosomal protein L37a	0.979432	0.8392	0.9275
20742	Spnb2	spectrin beta 2	0.979432	0.8679	0.9426
22259	Nr1h3	nuclear receptor subfamily 1, group H, member 3	0.979432	0.7695	NA
23989	Med24	mediator complex subunit 24	0.979432	0.8681	0.9427
24071	Synj2bp	synaptojanin 2 binding protein	0.979432	0.6905	NA
26936	Mprip	myosin phosphatase Rho interacting protein	0.979432	0.7956	NA
28018	Ubfd1	ubiquitin family domain containing 1	0.979432	0.672	NA
30949	Lcmt1	leucine carboxyl methyltransferase 1	0.979432	0.6731	NA
56378	Arpc3	actin related protein 2/3 complex, subunit 3	0.979432	0.6743	NA
57808	Rpl35a	ribosomal protein L35A	0.979432	0.5848	NA
66048	Tmem93	transmembrane protein 93	0.979432	0.7191	NA
66087	Tmem111	transmembrane protein 111	0.979432	0.8273	0.9219
66118	Sarnp	SAP domain containing ribonucleoprotein	0.979432	0.7415	NA
66155	Ufc1	ubiquitin-fold modifier conjugating enzyme 1	0.979432	0.7002	NA
66179	1110031I02Rik	RIKEN cDNA 1110031I02 gene	0.979432	0.7109	NA
66446	Exosc7	exosome component 7	0.979432	0.7633	NA
66449	Pam16	presequence translocase-asssociated motor 16 homolog (S. cerevisiae)	0.979432	0.7182	NA
66461	Ptpmt1	protein tyrosine phosphatase, mitochondrial 1	0.979432	0.6823	NA
66914	Vps28	vacuolar protein sorting 28 (yeast)	0.979432	0.7125	NA
66935	Cir1	corepressor interacting with RBPJ, 1	0.979432	0.8294	0.923
66973	Mrps18b	mitochondrial ribosomal protein S18B	0.979432	0.8196	0.9178
67273	Ndufa10	NADH dehydrogenase (ubiquinone) 1 alpha subcomplex 10	0.979432	0.7836	NA
67285	Cwc27	CWC27 spliceosome-associated protein homolog (S. cerevisiae)	0.979432	0.7685	NA
67382	Brd3	bromodomain containing 3	0.979432	0.7422	NA
68112	Sdccag3	serologically defined colon cancer antigen 3	0.979432	0.878	0.9465
68170	B230118H07Rik	RIKEN cDNA B230118H07 gene	0.979432	0.7066	NA
68842	Tulp4	tubby like protein 4	0.979432	0.7746	NA
69443	1700027J07Rik	RIKEN cDNA 1700027J07 gene	0.979432	0.9308	0.9715
69540	Klk10	kallikrein related-peptidase 10	0.979432	0.8741	0.9452
69684	Aarsd1	alanyl-tRNA synthetase domain containing 1	0.979432	0.7837	NA
70101	Cyp4f16	cytochrome P450, family 4, subfamily f, polypeptide 16	0.979432	0.8721	0.9446
70359	Gtpbp3	GTP binding protein 3	0.979432	0.8807	0.9478
71665	Fuca1	fucosidase, alpha-L- 1, tissue	0.979432	0.805	NA
71807	Tars2	threonyl-tRNA synthetase 2, mitochondrial (putative)	0.979432	0.6575	NA
71843	R3hcc1	R3H domain and coiled-coil containing 1	0.979432	0.7332	NA
72068	Cnot2	CCR4-NOT transcription complex, subunit 2	0.979432	0.651	NA
72930	Ppp2r2b	protein phosphatase 2 (formerly 2A), regulatory subunit B (PR 52), beta isoform	0.979432	0.7539	NA
73736	Fcf1	FCF1 small subunit (SSU) processome component homolog (S. cerevisiae)	0.979432	0.7324	NA
74434	Sohlh2	spermatogenesis and oogenesis specific basic helix-loop-helix 2	0.979432	0.8658	0.9416
75482	Hspb9	heat shock protein, alpha-crystallin-related, B9	0.979432	0.8538	0.9348
77371	Sec24a	Sec24 related gene family, member A (S. cerevisiae)	0.979432	0.9063	0.9604
102093	Phkb	phosphorylase kinase beta	0.979432	0.7852	NA
212163	8030462N17Rik	RIKEN cDNA 8030462N17 gene	0.979432	0.8744	0.9452
213109	Phf3	PHD finger protein 3	0.979432	0.7647	NA

214469	Fam168b	family with sequence similarity 168, member B	0.979432	0.6593	NA
231855	C330006K01Rik	RIKEN cDNA C330006K01 gene	0.979432	0.7731	NA
232798	Leng8	leukocyte receptor cluster (LRC) member 8	0.979432	0.8858	0.9505
233802	Thumpd1	THUMP domain containing 1	0.979432	0.691	NA
242681	Rab42-ps	RAB42, member RAS oncogene family, pseudogene	0.979432	0.75	NA
243963	Zfp473	zinc finger protein 473	0.979432	0.7892	NA
317750	Slc24a5	solute carrier family 24, member 5	0.979432	0.8693	0.9434
399629	9530029O12Rik	RIKEN cDNA 9530029O12 gene	0.979432	0.7733	NA
640374	Gm7293	glyceraldehyde-3-phosphate dehydrogenase pseudogene	0.979432	0.8379	0.927
665434	Gm7634	predicted gene 7634	0.979432	0.6537	NA
100046692	LOC100046692	40S ribosomal protein S10-like	0.979432	0.8965	0.9556
13726	Emd	emerin	0.978474	0.8566	0.9365
14682	Gnaq	guanine nucleotide binding protein, alpha q polypeptide	0.978474	0.6981	NA
14886	Gtf2i	general transcription factor II I	0.978474	0.6793	NA
15526	Hspa9	heat shock protein 9	0.978474	0.6932	NA
15566	Htr7	5-hydroxytryptamine (serotonin) receptor 7	0.978474	0.842	0.9289
16553	Kif13a	kinesin family member 13A	0.978474	0.6031	NA
16825	Ldb1	LIM domain binding 1	0.978474	0.7288	NA
18139	Zfml	zinc finger, matrin-like	0.978474	0.6777	NA
18263	Odc1	ornithine decarboxylase, structural 1	0.978474	0.7807	NA
18549	Pcsk2	proprotein convertase subtilisin/kexin type 2	0.978474	0.8153	0.9151
21428	Mlx	MAX-like protein X	0.978474	0.7057	NA
22209	Ube2a	ubiquitin-conjugating enzyme E2A, RAD6 homolog (S. cerevisiae)	0.978474	0.6714	NA
22404	Wiz	widely-interspaced zinc finger motifs	0.978474	0.6784	NA
23879	Fxr2	fragile X mental retardation, autosomal homolog 2	0.978474	0.7177	NA
26408	Map3k5	mitogen-activated protein kinase kinase kinase 5	0.978474	0.8939	0.9546
27369	Dguok	deoxyguanosine kinase	0.978474	0.6682	NA
50776	Polg2	polymerase (DNA directed), gamma 2, accessory subunit	0.978474	0.6619	NA
50876	Tmod2	tropomodulin 2	0.978474	0.7414	NA
56438	Rbx1	ring-box 1	0.978474	0.6767	NA
58875	Hibadh	3-hydroxyisobutyrate dehydrogenase	0.978474	0.7589	NA
59040	Rhot1	ras homolog gene family, member T1	0.978474	0.705	NA
60455	Tmem8	transmembrane protein 8 (five membrane-spanning domains)	0.978474	0.6636	NA
66279	Tmem218	transmembrane protein 218	0.978474	0.7048	NA
66314	Tpd52l2	tumor protein D52-like 2	0.978474	0.6431	NA
66925	Sdhd	succinate dehydrogenase complex, subunit D, integral membrane protein	0.978474	0.6786	NA
66980	Zdhhc6	zinc finger, DHHC domain containing 6	0.978474	0.6643	NA
67281	Rpl37	ribosomal protein L37	0.978474	0.7677	NA
67978	Tctn2	tectonic family member 2	0.978474	0.6867	NA
68268	Zdhhc21	zinc finger, DHHC domain containing 21	0.978474	0.6663	NA
68342	Ndufb10	NADH dehydrogenase (ubiquinone) 1 beta subcomplex, 10	0.978474	0.7208	NA
68493	Ndufaf4	NADH dehydrogenase (ubiquinone) 1 alpha subcomplex, assembly factor 4	0.978474	0.7267	NA
70998	Phf6	PHD finger protein 6	0.978474	0.7163	NA
71941	Cars2	cysteinyl-tRNA synthetase 2 (mitochondrial)(putative)	0.978474	0.7547	NA
72805	Zfp839	zinc finger protein 839	0.978474	0.8762	0.946
73431	1700052K11Rik	RIKEN cDNA 1700052K11 gene	0.978474	0.7538	NA
75430	3200002M19Rik	RIKEN cDNA 3200002M19 gene	0.978474	0.8485	0.9325
76688	Arfrp1	ADP-ribosylation factor related protein 1	0.978474	0.6562	NA
76857	Spopl	speckle-type POZ protein-like	0.978474	0.7895	NA

77300	Raph1	Ras association (RalGDS/AF-6) and pleckstrin homology domains 1	0.978474	0.7635	NA
78785	Clip4	CAP-GLY domain containing linker protein family, member 4	0.978474	0.7219	NA
78938	Fbxo34	F-box protein 34	0.978474	0.8574	0.9368
80294	Pofut2	protein O-fucosyltransferase 2	0.978474	0.9098	0.9624
81004	Tbl1xr1	transducin (beta)-like 1X-linked receptor 1	0.978474	0.8961	0.9553
97287	Mtmr14	myotubularin related protein 14	0.978474	0.7321	NA
99237	Tm9sf4	transmembrane 9 superfamily protein member 4	0.978474	0.6969	NA
100061	Lrrc19	leucine rich repeat containing 19	0.978474	0.8969	0.9557
100978	Nfxl1	nuclear transcription factor, X-box binding-like 1	0.978474	0.8061	0.9093
101240	Wdr91	WD repeat domain 91	0.978474	0.779	NA
140858	Wdr5	WD repeat domain 5	0.978474	0.621	NA
193452	Zfp184	zinc finger protein 184 (Kruppel-like)	0.978474	0.7809	NA
193670	Rnf185	ring finger protein 185	0.978474	0.5795	NA
210148	Slc30a6	solute carrier family 30 (zinc transporter), member 6	0.978474	0.6127	NA
215201	Trmt2b	TRM2 tRNA methyltransferase 2 homolog B (S. cerevisiae)	0.978474	0.8184	0.9173
223593	E430025E21Rik	RIKEN cDNA E430025E21 gene	0.978474	0.7078	NA
224440	Setd4	SET domain containing 4	0.978474	0.7342	NA
229715	Amigo1	adhesion molecule with Ig like domain 1	0.978474	0.7724	NA
233187	Lim2	lens intrinsic membrane protein 2	0.978474	0.9051	0.9597
235132	Zbtb44	zinc finger and BTB domain containing 44	0.978474	0.8452	0.9307
242702	Myom3	myomesin family, member 3	0.978474	0.8208	0.9184
268469	Zfp652	zinc finger protein 652	0.978474	0.7964	NA
269023	Zfp608	zinc finger protein 608	0.978474	0.8135	0.9141
269401	Znf512b	zinc finger protein 512B	0.978474	0.6718	NA
277203	Tm4sf19	transmembrane 4 L six family member 19	0.978474	0.9272	0.9704
319991	Kif6	kinesin family member 6	0.978474	0.888	0.9513
381287	A530032D15Rik	RIKEN cDNA A530032D15Rik gene	0.978474	0.9022	0.9583
622459	Gm12216	predicted gene 12216	0.978474	0.8814	0.9481
666731	Trim43c	tripartite motif-containing 43C	0.978474	0.6717	NA
100038347	Fam174b	family with sequence similarity 174, member B	0.978474	0.7513	NA
100040631	Dynlt1e	dynein light chain Tctex-type 1E	0.978474	0.6285	NA
11632	Aip	aryl-hydrocarbon receptor-interacting protein	0.977517	0.8625	0.9395
11858	Rnd2	Rho family GTPase 2	0.977517	0.669	NA
11941	Atp2b2	ATPase, Ca++ transporting, plasma membrane 2	0.977517	0.6707	NA
12539	Cdc37	cell division cycle 37 homolog (S. cerevisiae)	0.977517	0.7934	0.9021
12709	Ckb	creatine kinase, brain	0.977517	0.7552	NA
14432	Gap43	growth associated protein 43	0.977517	0.7807	NA
14630	Gclm	glutamate-cysteine ligase, modifier subunit	0.977517	0.841	0.9287
14790	Grcc10	gene rich cluster, C10 gene	0.977517	0.7012	NA
15185	Hdac6	histone deacetylase 6	0.977517	0.7542	NA
15958	Ifit2	interferon-induced protein with tetratricopeptide repeats 2	0.977517	0.6353	NA
16392	Isl1	ISL1 transcription factor, LIM/homeodomain	0.977517	0.7895	NA
18671	Abcb1a	ATP-binding cassette, sub-family B (MDR/TAP), member 1A	0.977517	0.758	NA
18747	Prkaca	protein kinase, cAMP dependent, catalytic, alpha	0.977517	0.8738	0.9451
22030	Traf2	TNF receptor-associated factor 2	0.977517	0.7192	NA
22187	Ubb	ubiquitin B	0.977517	0.7926	0.9016
26893	Cops6	COP9 (constitutive photomorphogenic) homolog, subunit 6 (Arabidopsis thaliana)	0.977517	0.7632	NA
26903	Dysf	dysferlin	0.977517	0.8368	0.9269
52552	Parp8	poly (ADP-ribose) polymerase family, member 8	0.977517	0.7697	NA

56298	Atl2	atlastin GTPase 2	0.977517	0.8336	0.9248
56382	Rab9	RAB9, member RAS oncogene family	0.977517	0.6964	NA
57784	Bin3	bridging integrator 3	0.977517	0.7657	NA
57869	Adck2	aarF domain containing kinase 2	0.977517	0.7708	NA
66505	Zmynd11	zinc finger, MYND domain containing 11	0.977517	0.8115	0.9127
66916	Ndufb7	NADH dehydrogenase (ubiquinone) 1 beta subcomplex, 7	0.977517	0.7782	NA
66999	Med28	mediator of RNA polymerase II transcription, subunit 28 homolog (yeast)	0.977517	0.74	NA
67242	Gemin6	gem (nuclear organelle) associated protein 6	0.977517	0.8555	0.9356
67602	Necap1	NECAP endocytosis associated 1	0.977517	0.7299	NA
67769	Gpatch2	G patch domain containing 2	0.977517	0.8765	0.9462
67942	Atp5g2	ATP synthase, H+ transporting, mitochondrial F0 complex, subunit C2 (subunit 9)	0.977517	0.595	NA
68170	B230118H07Rik	RIKEN cDNA B230118H07 gene	0.977517	0.7457	NA
68262	Agpat4	1-acylglycerol-3-phosphate O-acyltransferase 4 (lysophosphatidic acid acyltransferase, delta)	0.977517	0.6805	NA
68493	Ndufaf4	NADH dehydrogenase (ubiquinone) 1 alpha subcomplex, assembly factor 4	0.977517	0.726	NA
68563	Dpm3	dolichyl-phosphate mannosyltransferase polypeptide 3	0.977517	0.6185	NA
68988	Prpf31	PRP31 pre-mRNA processing factor 31 homolog (yeast)	0.977517	0.7382	NA
69136	Tusc1	tumor suppressor candidate 1	0.977517	0.607	NA
71946	Endod1	endonuclease domain containing 1	0.977517	0.7414	NA
72320	2510003E04Rik	RIKEN cDNA 2510003E04 gene	0.977517	0.8579	0.9368
72514	Fgfbp3	fibroblast growth factor binding protein 3	0.977517	0.7946	NA
73683	Atg16l2	autophagy related 16 like 2 (S. cerevisiae)	0.977517	0.7751	NA
75787	4930471M09Rik	RIKEN cDNA 4930471M09 gene	0.977517	0.842	0.9289
76302	Pcnp	PEST proteolytic signal containing nuclear protein	0.977517	0.6754	NA
77578	Bcl9	B-cell CLL/lymphoma 9	0.977517	0.8013	0.9066
78232	Trappc6b	trafficking protein particle complex 6B	0.977517	0.7827	NA
78321	Ankrd23	ankyrin repeat domain 23	0.977517	0.8021	0.9069
98314	D2hgdh	D-2-hydroxyglutarate dehydrogenase	0.977517	0.735	NA
98682	Mfsd6	major facilitator superfamily domain containing 6	0.977517	0.6624	NA
98758	Hnrnpf	heterogeneous nuclear ribonucleoprotein F	0.977517	0.7092	NA
106522	Pkdcc	protein kinase domain containing, cytoplasmic	0.977517	0.812	0.9129
107732	Mrpl10	mitochondrial ribosomal protein L10	0.977517	0.7865	NA
212503	Paox	polyamine oxidase (exo-N4-amino)	0.977517	0.8236	0.92
226594	Rcsd1	RCSD domain containing 1	0.977517	0.8528	0.9342
228869	Ncoa5	nuclear receptor coactivator 5	0.977517	0.7158	NA
234699	Edc4	enhancer of mRNA decapping 4	0.977517	0.6711	NA
237422	Ric8b	resistance to inhibitors of cholinesterase 8 homolog B (C. elegans)	0.977517	0.6844	NA
269378	Ahcy	S-adenosylhomocysteine hydrolase	0.977517	0.8214	0.9188
320500	Tmem215	transmembrane protein 215	0.977517	0.8115	0.9127
382090	4922501C03Rik	RIKEN cDNA 4922501C03 gene	0.977517	0.7405	NA
383766	Gm1332	predicted gene 1332	0.977517	0.8343	0.9251
384382	A430108E01Rik	RIKEN cDNA A430108E01 gene	0.977517	0.8981	0.9563
386400	LOC386400	carbonyl reductase [NADPH] 1-like	0.977517	0.9564	0.9824
620631	Ttc30a2	tetratricopeptide repeat domain 30A2	0.977517	0.765	NA
100041294	Gm3258	predicted gene 3258	0.977517	0.7226	NA
12181	Bop1	block of proliferation 1	0.976563	0.8223	0.9194
12626	Cetn3	centrin 3	0.976563	0.7971	0.9047
14359	Fxr1	fragile X mental retardation gene 1, autosomal homolog	0.976563	0.6443	NA
15504	Dnajb3	DnaJ (Hsp40) homolog, subfamily B, member 3	0.976563	0.835	0.9256
18673	Phb	prohibitin	0.976563	0.8422	0.929

19243	Ptp4a1	protein tyrosine phosphatase 4a1	0.976563	0.8381	0.927
20226	Sars	seryl-aminoacyl-tRNA synthetase	0.976563	0.8286	0.9225
21849	Trim28	tripartite motif-containing 28	0.976563	0.8167	0.9162
22327	Vbp1	von Hippel-Lindau binding protein 1	0.976563	0.8537	0.9348
26416	Mapk14	mitogen-activated protein kinase 14	0.976563	0.7026	NA
27375	Tjp3	tight junction protein 3	0.976563	0.8332	0.9247
28028	Mrpl50	mitochondrial ribosomal protein L50	0.976563	0.7712	NA
50907	Preb	prolactin regulatory element binding	0.976563	0.8419	0.9289
53609	Clasrp	CLK4-associating serine/arginine rich protein	0.976563	0.7617	NA
53817	Bat1a	HLA-B-associated transcript 1A	0.976563	0.8529	0.9343
53893	Nudt5	nudix (nucleoside diphosphate linked moiety X)-type motif 5	0.976563	0.7455	NA
54214	Golga4	golgi autoantigen, golgin subfamily a, 4	0.976563	0.7999	0.9059
56397	Morf4l2	mortality factor 4 like 2	0.976563	0.6091	NA
56406	Ncoa6	nuclear receptor coactivator 6	0.976563	0.7597	NA
56460	Pkp3	plakophilin 3	0.976563	0.8881	0.9513
57783	Tnip1	TNFAIP3 interacting protein 1	0.976563	0.6765	NA
58523	Elp2	elongation protein 2 homolog (S. cerevisiae)	0.976563	0.5802	NA
66185	1110037F02Rik	RIKEN cDNA 1110037F02 gene	0.976563	0.846	0.9311
66367	2310022A10Rik	RIKEN cDNA 2310022A10 gene	0.976563	0.6798	NA
66473	Ctrb1	chymotrypsinogen B1	0.976563	0.8737	0.945
66589	Ube2v1	ubiquitin-conjugating enzyme E2 variant 1	0.976563	0.8329	0.9247
67119	Ccdc159	coiled-coil domain containing 159	0.976563	0.7736	NA
67370	Zfp606	zinc finger protein 606	0.976563	0.7123	NA
67556	Pigm	phosphatidylinositol glycan anchor biosynthesis, class M	0.976563	0.7273	NA
67808	Tprgl	transformation related protein 63 regulated like	0.976563	0.5632	NA
68017	Ftsj2	FtsJ homolog 2 (E. coli)	0.976563	0.7823	NA
68112	Sdccag3	serologically defined colon cancer antigen 3	0.976563	0.7861	NA
68118	9430023L20Rik	RIKEN cDNA 9430023L20 gene	0.976563	0.7427	NA
68201	Ccdc34	coiled-coil domain containing 34	0.976563	0.7465	NA
69225	Carkd	carbohydrate kinase domain containing	0.976563	0.5733	NA
69804	Tmem147	transmembrane protein 147	0.976563	0.6064	NA
69975	2810405F17Rik	RIKEN cDNA 2810405F17 gene	0.976563	0.7269	NA
70827	Trak2	trafficking protein, kinesin binding 2	0.976563	0.827	0.9218
71536	8430437O03Rik	RIKEN cDNA 8430437O03 gene	0.976563	0.7983	0.9053
71911	Bdh1	3-hydroxybutyrate dehydrogenase, type 1	0.976563	0.572	NA
71916	Dus4l	dihydrouridine synthase 4-like (S. cerevisiae)	0.976563	0.7243	NA
72050	Kdelc1	KDEL (Lys-Asp-Glu-Leu) containing 1	0.976563	0.7569	NA
73828	Dcaf4	DDB1 and CUL4 associated factor 4	0.976563	0.7689	NA
74453	Ccdc11	coiled-coil domain containing 11	0.976563	0.706	NA
76014	Zc3h18	zinc finger CCCH-type containing 18	0.976563	0.8434	0.9298
76573	1700027D21Rik	RIKEN cDNA 1700027D21 gene	0.976563	0.7861	0.8978
76773	Wdyhv1	WDYHV motif containing 1	0.976563	0.6146	NA
77462	Tmem116	transmembrane protein 116	0.976563	0.8033	0.9078
77853	Msl2	male-specific lethal 2 homolog (Drosophila)	0.976563	0.7381	NA
78925	Srd5a1	steroid 5 alpha-reductase 1	0.976563	0.6788	NA
93760	Arid1a	AT rich interactive domain 1A (SWI-like)	0.976563	0.7415	NA
98386	Lbr	lamin B receptor	0.976563	0.6738	NA
106205	Zc3h7a	zinc finger CCCH type containing 7 A	0.976563	0.7529	NA
208643	Eif4g1	eukaryotic translation initiation factor 4, gamma 1	0.976563	0.7785	NA

228730	Plk1s1	polo-like kinase 1 substrate 1	0.976563	0.6211	NA
230700	Foxj3	forkhead box J3	0.976563	0.6529	NA
237542	Osbpl8	oxysterol binding protein-like 8	0.976563	0.7528	NA
240853	Gm4953	ATP synthase, H+ transporting, mitochondrial F0 complex, subunit d pseudogene	0.976563	0.7726	NA
243302	Gm4963	predicted gene 4963	0.976563	0.7148	NA
258818	Olfr629	olfactory receptor 629	0.976563	0.8331	0.9247
268934	Grm4	glutamate receptor, metabotropic 4	0.976563	0.9584	0.9836
269180	Inpp4a	inositol polyphosphate-4-phosphatase, type I	0.976563	0.7387	NA
269702	Mphosph9	M-phase phosphoprotein 9	0.976563	0.9164	0.9652
276852	D11Wsu47e	DNA segment, Chr 11, Wayne State University 47, expressed	0.976563	0.6774	NA
320191	Hook3	hook homolog 3 (Drosophila)	0.976563	0.7415	NA
327749	Gm5079	predicted gene 5079	0.976563	0.8776	0.9464
380702	Shisa6	shisa homolog 6 (Xenopus laevis)	0.976563	0.7257	NA
672284	Nkx1-1	NK1 transcription factor related, locus 1 (Drosophila)	0.976563	0.8389	0.9273
100038402	BC025933	cDNA sequence BC025933	0.976563	0.9244	0.9692
11927	Atox1	ATX1 (antioxidant protein 1) homolog 1 (yeast)	0.97561	0.7014	NA
11966	Atp6v1b2	ATPase, H+ transporting, lysosomal V1 subunit B2	0.97561	0.7551	NA
13110	Cyp2j6	cytochrome P450, family 2, subfamily j, polypeptide 6	0.97561	0.8681	0.9427
13684	Eif4e	eukaryotic translation initiation factor 4E	0.97561	0.6557	NA
16865	Eif2d	eukaryotic translation initiation factor 2D	0.97561	0.7311	NA
17067	Ly6c1	lymphocyte antigen 6 complex, locus C1	0.97561	0.7118	NA
18441	P2ry1	purinergic receptor P2Y, G-protein coupled 1	0.97561	0.7525	NA
19186	Psme1	proteasome (prosome, macropain) 28 subunit, alpha	0.97561	0.7661	NA
20677	Sox4	SRY-box containing gene 4	0.97561	0.7743	0.8916
21682	Tec	tec protein tyrosine kinase	0.97561	0.7335	NA
22658	Pcgf2	polycomb group ring finger 2	0.97561	0.819	0.9174
22781	Ikzf4	IKAROS family zinc finger 4	0.97561	0.941	0.9762
23829	C1ql1	complement component 1, q subcomponent-like 1	0.97561	0.8959	0.9553
24012	Rgs7	regulator of G protein signaling 7	0.97561	0.7405	NA
52477	Angel2	angel homolog 2 (Drosophila)	0.97561	0.591	NA
52837	Tmx4	thioredoxin-related transmembrane protein 4	0.97561	0.7742	NA
54122	Uevld	UEV and lactate/malate dehyrogenase domains	0.97561	0.8	0.9059
55991	Panx1	pannexin 1	0.97561	0.6818	NA
56469	Pias1	protein inhibitor of activated STAT 1	0.97561	0.7045	NA
56637	Gsk3b	glycogen synthase kinase 3 beta	0.97561	0.6539	NA
59047	Pnkp	polynucleotide kinase 3'- phosphatase	0.97561	0.6875	NA
66406	Sac3d1	SAC3 domain containing 1	0.97561	0.5863	NA
66624	Spcs2	signal peptidase complex subunit 2 homolog (S. cerevisiae)	0.97561	0.8534	0.9345
66701	Spryd4	SPRY domain containing 4	0.97561	0.7159	NA
66945	Sdha	succinate dehydrogenase complex, subunit A, flavoprotein (Fp)	0.97561	0.6609	NA
67115	Rpl14	ribosomal protein L14	0.97561	0.7649	NA
67418	Ppil4	peptidylprolyl isomerase (cyclophilin)-like 4	0.97561	0.6238	NA
67772	Chd8	chromodomain helicase DNA binding protein 8	0.97561	0.7687	NA
68168	A930009E08Rik	RIKEN cDNA A930009E08 gene	0.97561	0.7242	NA
68194	Ndufb4	NADH dehydrogenase (ubiquinone) 1 beta subcomplex 4	0.97561	0.6411	NA
68832	1110057K04Rik	RIKEN cDNA 1110057K04 gene	0.97561	0.7725	NA
69894	2010107G23Rik	RIKEN cDNA 2010107G23 gene	0.97561	0.5924	NA
70681	Fam175a	family with sequence similarity 175, member A	0.97561	0.6966	NA
71752	Gtf3c2	general transcription factor IIIC, polypeptide 2, beta	0.97561	0.7649	NA

72195	Supt7l	suppressor of Ty 7 (S. cerevisiae)-like	0.97561	0.7487	NA
72265	Tram1	translocating chain-associating membrane protein 1	0.97561	0.8071	0.91
73316	Calr3	calreticulin 3	0.97561	0.7805	0.8946
74137	Nuak2	NUAK family, SNF1-like kinase, 2	0.97561	0.8244	0.9204
74610	Abcb8	ATP-binding cassette, sub-family B (MDR/TAP), member 8	0.97561	0.8312	0.9239
74781	Wipi2	WD repeat domain, phosphoinositide interacting 2	0.97561	0.6875	NA
78890	2310079F23Rik	RIKEN cDNA 2310079F23 gene	0.97561	0.7595	NA
79560	Ublcp1	ubiquitin-like domain containing CTD phosphatase 1	0.97561	0.6208	NA
83796	Smarcd2	SWI/SNF related, matrix associated, actin dependent regulator of chromatin, subfamily d, member 2	0.97561	0.7668	NA
98053	Gtf2f1	general transcription factor IIF, polypeptide 1	0.97561	0.6779	NA
108645	Mat2b	methionine adenosyltransferase II, beta	0.97561	0.6672	NA
194309	Vps37d	vacuolar protein sorting 37D (yeast)	0.97561	0.5768	NA
215051	Bud13	BUD13 homolog (yeast)	0.97561	0.8043	0.9083
230967	BC046331	cDNA sequence BC046331	0.97561	0.7527	NA
231798	Lrch4	leucine-rich repeats and calponin homology (CH) domain containing 4	0.97561	0.8791	0.947
240215	Slc4a9	solute carrier family 4, sodium bicarbonate cotransporter, member 9	0.97561	0.6726	NA
319984	Jph4	junctophilin 4	0.97561	0.8755	0.9455
330031	Gm5106	predicted gene 5106	0.97561	0.9406	0.976
381510	Dpy19l4	dpy-19-like 4 (C. elegans)	0.97561	0.7651	NA
384185	Arl9	ADP-ribosylation factor-like 9	0.97561	0.8638	0.9402
433416	Gm13547	predicted gene 13547	0.97561	0.903	0.9588
436062	Fam92b	family with sequence similarity 92, member B	0.97561	0.8196	0.9178
621580	Gm13308	predicted gene 13308	0.97561	0.7277	NA
100044236	Copg2as2	coatomer protein complex, subunit gamma 2, antisense 2	0.97561	0.9081	0.9614
11298	Aanat	arylalkylamine N-acetyltransferase	0.974659	0.8375	0.927
11605	Gla	galactosidase, alpha	0.974659	0.8629	0.9397
12953	Cry2	cryptochrome 2 (photolyase-like)	0.974659	0.5641	NA
13166	Dbh	dopamine beta hydroxylase	0.974659	0.8871	0.9511
14281	Fos	FBJ osteosarcoma oncogene	0.974659	0.8734	0.9449
15465	Hrh1	histamine receptor H1	0.974659	0.8733	0.9449
17237	Mgrn1	mahogunin, ring finger 1	0.974659	0.6973	NA
18012	Neurod1	neurogenic differentiation 1	0.974659	0.7479	NA
18263	Odc1	ornithine decarboxylase, structural 1	0.974659	0.8007	0.9064
18606	Enpp2	ectonucleotide pyrophosphatase/phosphodiesterase 2	0.974659	0.6889	NA
19988	Rpl6	ribosomal protein L6	0.974659	0.8041	0.9083
20133	Rrm1	ribonucleotide reductase M1	0.974659	0.6959	NA
20620	Plk2	polo-like kinase 2 (Drosophila)	0.974659	0.7702	0.8895
20872	Stk16	serine/threonine kinase 16	0.974659	0.8035	0.9079
21968	Tom1	target of myb1 homolog (chicken)	0.974659	0.8853	0.9503
22694	Zfp35	zinc finger protein 35	0.974659	0.6558	NA
24070	Mpdu1	mannose-P-dolichol utilization defect 1	0.974659	0.5904	NA
26429	Orc5	origin recognition complex, subunit 5	0.974659	0.8421	0.929
53614	Reck	reversion-inducing-cysteine-rich protein with kazal motifs	0.974659	0.7563	NA
54170	Rragc	Ras-related GTP binding C	0.974659	0.7567	NA
55988	Snx12	sorting nexin 12	0.974659	0.7702	0.8895
55990	Fmo2	flavin containing monooxygenase 2	0.974659	0.835	0.9256
57913	Lrdd	leucine-rich and death domain containing	0.974659	0.7169	NA
58220	Pard6b	par-6 (partitioning defective 6) homolog beta (C. elegans)	0.974659	0.8147	0.9148
59008	Anapc5	anaphase-promoting complex subunit 5	0.974659	0.775	0.8918

64050	Yeats4	YEATS domain containing 4	0.974659	0.7814	0.8951
66192	Lage3	L antigen family, member 3	0.974659	0.6736	NA
66200	Commd6	COMM domain containing 6	0.974659	0.6899	NA
66641	Sike1	suppressor of IKBKE 1	0.974659	0.7626	NA
66866	Nhlrc2	NHL repeat containing 2	0.974659	0.5585	NA
66973	Mrps18b	mitochondrial ribosomal protein S18B	0.974659	0.7172	NA
67474	Snap29	synaptosomal-associated protein 29	0.974659	0.6661	NA
68041	Mid1ip1	Mid1 interacting protein 1 (gastrulation specific G12-like (zebrafish))	0.974659	0.6555	NA
68180	Hyi	hydroxypyruvate isomerase homolog (E. coli)	0.974659	0.7653	NA
68193	Rpl24	ribosomal protein L24	0.974659	0.6638	NA
68544	2310036O22Rik	RIKEN cDNA 2310036O22 gene	0.974659	0.724	NA
68796	Tmem214	transmembrane protein 214	0.974659	0.7054	NA
69009	Thap7	THAP domain containing 7	0.974659	0.741	NA
69017	Prrt2	proline-rich transmembrane protein 2	0.974659	0.8213	0.9188
70300	Fuz	fuzzy homolog (Drosophila)	0.974659	0.6464	NA
71952	2410016O06Rik	RIKEN cDNA 2410016O06 gene	0.974659	0.6799	NA
72183	Snx6	sorting nexin 6	0.974659	0.7577	NA
72289	Malat1	metastasis associated lung adenocarcinoma transcript 1 (non-coding RNA)	0.974659	0.8303	0.9234
72754	Arhgef10l	Rho guanine nucleotide exchange factor (GEF) 10-like	0.974659	0.6491	NA
73516	1700086O06Rik	RIKEN cDNA 1700086O06 gene	0.974659	0.7012	NA
74229	Paqr8	progestin and adipoQ receptor family member VIII	0.974659	0.7493	NA
75705	Eif4b	eukaryotic translation initiation factor 4B	0.974659	0.8517	0.9337
76671	5330406M23Rik	RIKEN cDNA 5330406M23 gene	0.974659	0.8108	0.9123
76798	2410137F16Rik	RIKEN cDNA 2410137F16 gene	0.974659	0.8244	0.9204
101214	Tra2a	transformer 2 alpha homolog (Drosophila)	0.974659	0.7466	NA
102058	Exoc8	exocyst complex component 8	0.974659	0.8228	0.9198
109229	Fam118b	family with sequence similarity 118, member B	0.974659	0.584	NA
109676	Ank2	ankyrin 2, brain	0.974659	0.6976	NA
110265	Msra	methionine sulfoxide reductase A	0.974659	0.6158	NA
170762	Nup155	nucleoporin 155	0.974659	0.6225	NA
209446	Tcfe3	transcription factor E3	0.974659	0.7017	NA
219189	1300010F03Rik	RIKEN cDNA 1300010F03 gene	0.974659	0.7862	0.8979
223648	2410075B13Rik	RIKEN cDNA 2410075B13 gene	0.974659	0.7049	NA
225215	Rsl24d1	ribosomal L24 domain containing 1	0.974659	0.7554	NA
227800	Rabgap1	RAB GTPase activating protein 1	0.974659	0.8862	0.9506
228602	4930402H24Rik	RIKEN cDNA 4930402H24 gene	0.974659	0.8107	0.9123
229487	Pet112l	PET112-like (yeast)	0.974659	0.7724	0.8906
229571	Gm4858	predicted gene 4858	0.974659	0.8961	0.9553
230088	B230312A22Rik	RIKEN cDNA B230312A22 gene	0.974659	0.7769	0.8927
230234	BC026590	cDNA sequence BC026590	0.974659	0.6375	NA
232174	Cyp26b1	cytochrome P450, family 26, subfamily b, polypeptide 1	0.974659	0.7527	NA
239319	Card6	caspase recruitment domain family, member 6	0.974659	0.8696	0.9436
241035	Pkhd1	polycystic kidney and hepatic disease 1	0.974659	0.838	0.927
380959	Alg10b	asparagine-linked glycosylation 10 homolog B (yeast, alpha-1,2-glucosyltransferase)	0.974659	0.7459	NA
381410	Zfp408	zinc finger protein 408	0.974659	0.8001	0.906
668459	Gm9182	predicted gene 9182	0.974659	0.6427	NA
12421	Rb1cc1	RB1-inducible coiled-coil 1	0.97371	0.6793	NA
12561	Cdh4	cadherin 4	0.97371	0.7163	NA
13559	E2f5	E2F transcription factor 5	0.97371	0.7655	0.8868

14155	Fem1b	feminization 1 homolog b (C. elegans)	0.97371	0.6451	NA
14265	Fmr1	fragile X mental retardation syndrome 1 homolog	0.97371	0.8895	0.9521
17344	Pias2	protein inhibitor of activated STAT 2	0.97371	0.6147	NA
18802	Plcd4	phospholipase C, delta 4	0.97371	0.8695	0.9435
18986	Pou2f1	POU domain, class 2, transcription factor 1	0.97371	0.7048	NA
19166	Psma2	proteasome (prosome, macropain) subunit, alpha type 2	0.97371	0.6028	NA
19294	Pvrl2	poliovirus receptor-related 2	0.97371	0.7194	NA
19349	Rab7	RAB7, member RAS oncogene family	0.97371	0.7101	NA
19355	Rad1	RAD1 homolog (S. pombe)	0.97371	0.6512	NA
20090	Rps29	ribosomal protein S29	0.97371	0.7494	NA
20168	Rtn3	reticulon 3	0.97371	0.8444	0.9304
20867	Stip1	stress-induced phosphoprotein 1	0.97371	0.7732	0.891
21853	Timeless	timeless homolog (Drosophila)	0.97371	0.6753	NA
22324	Vav1	vav 1 oncogene	0.97371	0.9539	0.9818
23954	Nek3	NIMA (never in mitosis gene a)-related expressed kinase 3	0.97371	0.7472	NA
26938	St6galnac5	ST6 (alpha-N-acetyl-neuraminyl-2,3-beta-galactosyl-1,3)-N-acetylgalactosaminide alpha-2,6-sialyltransferase 5	0.97371	0.6099	NA
27050	Rps3	ribosomal protein S3	0.97371	0.727	NA
51788	H2afz	H2A histone family, member Z	0.97371	0.6191	NA
52690	Setd3	SET domain containing 3	0.97371	0.5884	NA
54137	Acrbp	proacrosin binding protein	0.97371	0.7915	0.9012
54197	Rnf5	ring finger protein 5	0.97371	0.6863	NA
54637	Praf2	PRA1 domain family 2	0.97371	0.7265	NA
56506	Cib2	calcium and integrin binding family member 2	0.97371	0.5994	NA
56844	Tssc4	tumor-suppressing subchromosomal transferable fragment 4	0.97371	0.6671	NA
57837	Eral1	Era (G-protein)-like 1 (E. coli)	0.97371	0.7387	NA
58233	Dnaja4	DnaJ (Hsp40) homolog, subfamily A, member 4	0.97371	0.8765	0.9462
66270	Fam134b	family with sequence similarity 134, member B	0.97371	0.7414	NA
66390	Slmo2	slowmo homolog 2 (Drosophila)	0.97371	0.6875	NA
66853	Pnpla2	patatin-like phospholipase domain containing 2	0.97371	0.7911	0.9009
67291	Ccdc137	coiled-coil domain containing 137	0.97371	0.8076	0.9104
68051	Nutf2	nuclear transport factor 2	0.97371	0.5414	NA
68121	Cep70	centrosomal protein 70	0.97371	0.7411	NA
68975	Med27	mediator complex subunit 27	0.97371	0.8202	0.9181
69234	Zfp688	zinc finger protein 688	0.97371	0.6436	NA
69806	Slc39a11	solute carrier family 39 (metal ion transporter), member 11	0.97371	0.6905	NA
71624	4833411C07Rik	RIKEN cDNA 4833411C07 gene	0.97371	0.7558	NA
71932	Ephx3	epoxide hydrolase 3	0.97371	0.8426	0.9293
72145	Wdfy3	WD repeat and FYVE domain containing 3	0.97371	0.7981	0.9053
72167	Thumpd2	THUMP domain containing 2	0.97371	0.6968	NA
73390	Msl3l2	male-specific lethal 3-like 2 (Drosophila)	0.97371	0.7378	NA
74098	0610037L13Rik	RIKEN cDNA 0610037L13 gene	0.97371	0.679	NA
74335	Xrcc3	X-ray repair complementing defective repair in Chinese hamster cells 3	0.97371	0.7361	NA
74467	Pus10	pseudouridylate synthase 10	0.97371	0.7639	0.8862
75991	Slain2	SLAIN motif family, member 2	0.97371	0.6115	NA
76020	5830409B07Rik	RIKEN cDNA 5830409B07 gene	0.97371	0.8615	0.9388
76895	Bicd2	bicaudal D homolog 2 (Drosophila)	0.97371	0.5369	NA
79043	Spsb3	splA/ryanodine receptor domain and SOCS box containing 3	0.97371	0.6209	NA
98366	Smap1	stromal membrane-associated protein 1	0.97371	0.6303	NA
102334	Ankrd10	ankyrin repeat domain 10	0.97371	0.63	NA

103733	Tubg1	tubulin, gamma 1	0.97371	0.7406	NA
104836	Cbll1	Casitas B-lineage lymphoma-like 1	0.97371	0.8006	0.9064
109181	Trip11	thyroid hormone receptor interactor 11	0.97371	0.6012	NA
209318	Gps1	G protein pathway suppressor 1	0.97371	0.6298	NA
211255	Kbtbd7	kelch repeat and BTB (POZ) domain containing 7	0.97371	0.7923	0.9016
227723	Prrc2b	proline-rich coiled-coil 2B	0.97371	0.5864	NA
229007	Zgpat	zinc finger, CCCH-type with G patch domain	0.97371	0.6751	NA
232337	Zfp637	zinc finger protein 637	0.97371	0.6645	NA
233805	Dcun1d3	DCN1, defective in cullin neddylation 1, domain containing 3 (S. cerevisiae)	0.97371	0.8747	0.9452
234700	Nrn1l	neuritin 1-like	0.97371	0.6206	NA
234797	6430548M08Rik	RIKEN cDNA 6430548M08 gene	0.97371	0.7512	NA
234964	Ccdc67	coiled-coil domain containing 67	0.97371	0.8533	0.9345
236511	Eif2c1	eukaryotic translation initiation factor 2C, 1	0.97371	0.8567	0.9365
269831	Tspan12	tetraspanin 12	0.97371	0.7526	NA
320472	Ppm1e	protein phosphatase 1E (PP2C domain containing)	0.97371	0.5854	NA
381280	Hjurp	Holliday junction recognition protein	0.97371	0.7766	0.8925
433273	Gm5523	glyceraldehyde-3-phosphate dehydrogenase pseudogene	0.97371	0.7507	NA
664883	Nova1	neuro-oncological ventral antigen 1	0.97371	0.8437	0.9299
668592	Gm9258	predicted gene 9258	0.97371	0.8013	0.9066
677368	LOC677368	hypothetical protein LOC677368	0.97371	0.8455	0.9309
11973	Atp6v1e1	ATPase, H+ transporting, lysosomal V1 subunit E1	0.972763	0.6775	NA
12418	Cbx4	chromobox homolog 4 (Drosophila Pc class)	0.972763	0.7812	0.8949
13030	Ctsb	cathepsin B	0.972763	0.6509	NA
13096	Cyp2c37	cytochrome P450, family 2. subfamily c, polypeptide 37	0.972763	0.8428	0.9294
13543	Dvl2	dishevelled 2, dsh homolog (Drosophila)	0.972763	0.8852	0.9503
14129	Fcgr1	Fc receptor, IgG, high affinity I	0.972763	0.8661	0.9418
15505	Hsph1	heat shock 105kDa/110kDa protein 1	0.972763	0.6408	NA
16911	Lmo4	LIM domain only 4	0.972763	0.5457	NA
17448	Mdh2	malate dehydrogenase 2, NAD (mitochondrial)	0.972763	0.7985	0.9053
17713	Grpel1	GrpE-like 1, mitochondrial	0.972763	0.5416	NA
18126	Nos2	nitric oxide synthase 2, inducible	0.972763	0.7562	0.8822
20194	S100a10	S100 calcium binding protein A10 (calpactin)	0.972763	0.699	NA
22083	Ctr9	Ctr9, Paf1/RNA polymerase II complex component, homolog (S. cerevisiae)	0.972763	0.7536	0.8811
22253	Unc5c	unc-5 homolog C (C. elegans)	0.972763	0.7442	NA
22717	Zfp59	zinc finger protein 59	0.972763	0.8188	0.9174
26611	Rcn2	reticulocalbin 2	0.972763	0.7785	0.8934
30947	Adat1	adenosine deaminase, tRNA-specific 1	0.972763	0.8277	0.9222
52708	Zfp410	zinc finger protein 410	0.972763	0.7366	NA
54721	Tyk2	tyrosine kinase 2	0.972763	0.8051	0.9089
56085	Ubqln1	ubiquilin 1	0.972763	0.5831	NA
56199	Abcb10	ATP-binding cassette, sub-family B (MDR/TAP), member 10	0.972763	0.7006	NA
56445	Dnaja2	DnaJ (Hsp40) homolog, subfamily A, member 2	0.972763	0.6923	NA
59025	Usp14	ubiquitin specific peptidase 14	0.972763	0.6196	NA
66091	Ndufa3	NADH dehydrogenase (ubiquinone) 1 alpha subcomplex, 3	0.972763	0.6531	NA
66121	Chchd1	coiled-coil-helix-coiled-coil-helix domain containing 1	0.972763	0.701	NA
66128	Mrps36	mitochondrial ribosomal protein S36	0.972763	0.5304	NA
66821	Bcs1l	BCS1-like (yeast)	0.972763	0.7201	NA
66841	Etfdh	electron transferring flavoprotein, dehydrogenase	0.972763	0.6074	NA
67393	Cxxc5	CXXC finger 5	0.972763	0.5895	NA

67495	Tmem167b	transmembrane protein 167B	0.972763	0.6968	NA	
68273	Pomgnt1	protein O-linked mannose beta1,2-N-acetylglucosaminyltransferase	0.972763	0.7057	NA	
68708	Rabl2	RAB, member of RAS oncogene family-like 2	0.972763	0.5741	NA	
68816	Ppil1	peptidylprolyl isomerase (cyclophilin)-like 1	0.972763	0.6674	NA	
71133	4933422A05Rik	RIKEN cDNA 4933422A05 gene	0.972763	0.8928		0.9539
74352	Zfp84	zinc finger protein 84	0.972763	0.678	NA	
74513	Neto2	neuropilin (NRP) and tolloid (TLL)-like 2	0.972763	0.7572		0.8826
76551	Ccdc6	coiled-coil domain containing 6	0.972763	0.719	NA	
99650	4933434E20Rik	RIKEN cDNA 4933434E20 gene	0.972763	0.5609	NA	
101835	AW146154	expressed sequence AW146154	0.972763	0.6378	NA	
103844	Inca1	inhibitor of CDK, cyclin A1 interacting protein 1	0.972763	0.802		0.9069
105298	Epdr1	ependymin related protein 1 (zebrafish)	0.972763	0.6365	NA	
217219	Fam171a2	family with sequence similarity 171, member A2	0.972763	0.7626		0.8853
223770	Brd1	bromodomain containing 1	0.972763	0.7584		0.883
224247	E330017A01Rik	RIKEN cDNA E330017A01 gene	0.972763	0.8944		0.9548
224703	Mar-02	membrane-associated ring finger (C3HC4) 2	0.972763	0.8734		0.9449
227700	Sh3glb2	SH3-domain GRB2-like endophilin B2	0.972763	0.6403	NA	
233789	Smg1	SMG1 homolog, phosphatidylinositol 3-kinase-related kinase (C. elegans)	0.972763	0.6779	NA	
240121	Fsd1	fibronectin type 3 and SPRY domain-containing protein	0.972763	0.7506		0.8792
241311	Zbtb34	zinc finger and BTB domain containing 34	0.972763	0.8108		0.9123
269061	Cpsf7	cleavage and polyadenylation specific factor 7	0.972763	0.713	NA	
320583	C530030K21Rik	RIKEN cDNA C530030K21 gene	0.972763	0.891		0.9529
382062	AB124611	cDNA sequence AB124611	0.972763	0.7561		0.8822
433759	Hdac1	histone deacetylase 1	0.972763	0.5607	NA	
434179	Gm5595	predicted gene 5595	0.972763	0.6874	NA	
445007	Nup85	nucleoporin 85	0.972763	0.5564	NA	
628324	S100a2	S100 calcium binding protein A2	0.972763	0.8961		0.9553
11834	Aqr	aquarius	0.971817	0.741	NA	
11886	Asah1	N-acylsphingosine amidohydrolase 1	0.971817	0.8036		0.9079
11949	Atp5c1	ATP synthase, H+ transporting, mitochondrial F1 complex, gamma polypeptide 1	0.971817	0.8785		0.9466
12237	Bub3	budding uninhibited by benzimidazoles 3 homolog (S. cerevisiae)	0.971817	0.6281	NA	
13660	Ehd1	EH-domain containing 1	0.971817	0.6884	NA	
13804	Endog	endonuclease G	0.971817	0.6769	NA	
13972	Gnb1l	guanine nucleotide binding protein (G protein), beta polypeptide 1-like	0.971817	0.6627	NA	
14248	Flii	flightless I homolog (Drosophila)	0.971817	0.7409	NA	
18029	Nfic	nuclear factor I/C	0.971817	0.6654	NA	
18033	Nfkb1	nuclear factor of kappa light polypeptide gene enhancer in B-cells 1, p105	0.971817	0.8367		0.9268
18826	Lcp1	lymphocyte cytosolic protein 1	0.971817	0.8261		0.9211
19009	Pou6f1	POU domain, class 6, transcription factor 1	0.971817	0.6982	NA	
19090	Prkdc	protein kinase, DNA activated, catalytic polypeptide	0.971817	0.6333	NA	
20203	S100b	S100 protein, beta polypeptide, neural	0.971817	0.6059	NA	
20480	Clpb	ClpB caseinolytic peptidase B homolog (E. coli)	0.971817	0.8169		0.9162
52551	Sgta	small glutamine-rich tetratricopeptide repeat (TPR)-containing, alpha	0.971817	0.6177	NA	
64209	Herpud1	homocysteine-inducible, endoplasmic reticulum stress-inducible, ubiquitin-like domain member 1	0.971817	0.6379	NA	
65960	Twsg1	twisted gastrulation homolog 1 (Drosophila)	0.971817	0.782		0.8953
66308	2810021B07Rik	RIKEN cDNA 2810021B07 gene	0.971817	0.6362	NA	
66884	Appbp2	amyloid beta precursor protein (cytoplasmic tail) binding protein 2	0.971817	0.611	NA	
67288	Srek1ip1	splicing regulatory glutamine/lysine-rich protein 1interacting protein 1	0.971817	0.6454	NA	
67422	Dhdds	dehydrodolichyl diphosphate synthase	0.971817	0.7023	NA	

67552	H2afy3	H2A histone family, member Y3	0.971817	0.5903	NA
68089	Arpc4	actin related protein 2/3 complex, subunit 4	0.971817	0.7771	0.8928
68567	Cgref1	cell growth regulator with EF hand domain 1	0.971817	0.7208	NA
68810	Nexn	nexilin	0.971817	0.7882	0.8994
68979	Nol11	nucleolar protein 11	0.971817	0.5874	NA
69106	Stoml1	stomatin-like 1	0.971817	0.6464	NA
69349	1700008O03Rik	RIKEN cDNA 1700008O03 gene	0.971817	0.6299	NA
70252	2010000I03Rik	RIKEN cDNA 2010000I03 gene	0.971817	0.8455	0.9309
71524	8430432A02Rik	RIKEN cDNA 8430432A02 gene	0.971817	0.6674	NA
71778	Klhl5	kelch-like 5 (Drosophila)	0.971817	0.6297	NA
71997	1500002O20Rik	RIKEN cDNA 1500002O20 gene	0.971817	0.8471	0.9317
73174	Tbkbp1	TBK1 binding protein 1	0.971817	0.8778	0.9464
73823	4930401B11Rik	RIKEN cDNA 4930401B11 gene	0.971817	0.6258	NA
74178	Stk40	serine/threonine kinase 40	0.971817	0.6183	NA
74479	Snx11	sorting nexin 11	0.971817	0.7231	NA
74729	Setmar	SET domain and mariner transposase fusion gene	0.971817	0.7783	0.8934
76306	1110021L09Rik	RIKEN cDNA 1110021L09 gene	0.971817	0.5899	NA
78895	Pus7l	pseudouridylate synthase 7 homolog (S. cerevisiae)-like	0.971817	0.696	NA
81907	Tmem108	transmembrane protein 108	0.971817	0.855	0.9353
98541	AU019278	expressed sequence AU019278	0.971817	0.736	NA
98932	Myl9	myosin, light polypeptide 9, regulatory	0.971817	0.5844	NA
103537	Mbtd1	mbt domain containing 1	0.971817	0.6038	NA
110948	Hlcs	holocarboxylase synthetase (biotin- [propriony-Coenzyme A-carboxylase (ATP-hydrolysing)] ligase)	0.971817	0.7136	NA
116972	Fam57a	family with sequence similarity 57, member A	0.971817	0.8477	0.9319
140481	Man2a2	mannosidase 2, alpha 2	0.971817	0.7856	0.8976
140482	Zfp358	zinc finger protein 358	0.971817	0.7073	NA
209318	Gps1	G protein pathway suppressor 1	0.971817	0.5754	NA
209354	Eif2b1	eukaryotic translation initiation factor 2B, subunit 1 (alpha)	0.971817	0.6282	NA
213498	Arhgef11	Rho guanine nucleotide exchange factor (GEF) 11	0.971817	0.709	NA
214345	Lrrc1	leucine rich repeat containing 1	0.971817	0.6269	NA
214627	Papd5	PAP associated domain containing 5	0.971817	0.7135	NA
216439	Agap2	ArfGAP with GTPase domain, ankyrin repeat and PH domain 2	0.971817	0.517	NA
217980	Larp4b	La ribonucleoprotein domain family, member 4B	0.971817	0.6857	NA
225288	Fhod3	formin homology 2 domain containing 3	0.971817	0.657	NA
229603	Otud7b	OTU domain containing 7B	0.971817	0.7425	0.8746
229776	Cdc14a	CDC14 cell division cycle 14 homolog A (S. cerevisiae)	0.971817	0.6046	NA
231659	Gcn1l1	GCN1 general control of amino-acid synthesis 1-like 1 (yeast)	0.971817	0.663	NA
232821	Ccdc106	coiled-coil domain containing 106	0.971817	0.6826	NA
234366	Gatad2a	GATA zinc finger domain containing 2A	0.971817	0.7777	0.893
237775	Zfp867	zinc finger protein 867	0.971817	0.783	0.896
237781	Smcr7	Smith-Magenis syndrome chromosome region, candidate 7 homolog (human)	0.971817	0.5553	NA
269514	Fbxl4	F-box and leucine-rich repeat protein 4	0.971817	0.6181	NA
319475	Zfp672	zinc finger protein 672	0.971817	0.6182	NA
319885	Zcchc7	zinc finger, CCHC domain containing 7	0.971817	0.7642	0.8862
320150	Zdhhc17	zinc finger, DHHC domain containing 17	0.971817	0.6715	NA
353258	Ltv1	LTV1 homolog (S. cerevisiae)	0.971817	0.5364	NA
666113	Gm7935	predicted pseudogene 7935	0.971817	0.8855	0.9504
11949	Atp5c1	ATP synthase, H+ transporting, mitochondrial F1 complex, gamma polypeptide 1	0.970874	0.6792	NA
13134	Dach1	dachshund 1 (Drosophila)	0.970874	0.7653	0.8867

14357	Dtx1	deltex 1 homolog (Drosophila)	0.970874	0.7279	NA
14797	Aes	amino-terminal enhancer of split	0.970874	0.7221	NA
18245	Oaz1	ornithine decarboxylase antizyme 1	0.970874	0.7184	NA
18483	Palm	paralemmin	0.970874	0.7891	0.9
19179	Psmc1	protease (prosome, macropain) 26S subunit, ATPase 1	0.970874	0.7363	0.8708
20684	Sp100	nuclear antigen Sp100	0.970874	0.8587	0.9373
21821	Ift88	intraflagellar transport 88 homolog (Chlamydomonas)	0.970874	0.607	NA
22057	Tob1	transducer of ErbB-2.1	0.970874	0.828	0.9222
22143	Tuba1b	tubulin, alpha 1B	0.970874	0.7476	0.8775
22592	Ercc5	excision repair cross-complementing rodent repair deficiency, complementation group 5	0.970874	0.7085	NA
22687	Zfp259	zinc finger protein 259	0.970874	0.5572	NA
23837	Cfdp1	craniofacial development protein 1	0.970874	0.64	NA
24045	Scamp3	secretory carrier membrane protein 3	0.970874	0.5054	NA
50877	Neu3	neuraminidase 3	0.970874	0.7003	NA
52682	D11Ertd726e	DNA segment, Chr 11, ERATO Doi 726, expressed	0.970874	0.8798	0.9475
53325	Banp	BTG3 associated nuclear protein	0.970874	0.7936	0.9021
54216	Pcdh7	protocadherin 7	0.970874	0.7773	0.8928
66048	Tmem93	transmembrane protein 93	0.970874	0.5222	NA
66201	Vta1	Vps20-associated 1 homolog (S. cerevisiae)	0.970874	0.8952	0.9551
66361	Zfand1	zinc finger, AN1-type domain 1	0.970874	0.6805	NA
66691	Gapvd1	GTPase activating protein and VPS9 domains 1	0.970874	0.5278	NA
66816	Thap2	THAP domain containing, apoptosis associated protein 2	0.970874	0.7863	0.8979
67226	Tmem19	transmembrane protein 19	0.970874	0.6226	NA
67731	Fbxo32	F-box protein 32	0.970874	0.7397	0.8728
67892	1810063B05Rik	RIKEN cDNA 1810063B05 gene	0.970874	0.5955	NA
68230	1700102H20Rik	RIKEN cDNA 1700102H20 gene	0.970874	0.7743	0.8916
68981	Snrpa1	small nuclear ribonucleoprotein polypeptide A'	0.970874	0.5759	NA
69807	Trim32	tripartite motif-containing 32	0.970874	0.6132	NA
70238	Rnf168	ring finger protein 168	0.970874	0.6215	NA
71673	Rnf215	ring finger protein 215	0.970874	0.5386	NA
72039	Mccc1	methylcrotonoyl-Coenzyme A carboxylase 1 (alpha)	0.970874	0.7785	0.8934
72567	Bclaf1	BCL2-associated transcription factor 1	0.970874	0.6861	NA
72803	2810454L23Rik	RIKEN cDNA 2810454L23 gene	0.970874	0.6973	NA
74235	1700020G03Rik	RIKEN cDNA 1700020G03 gene	0.970874	0.824	0.9204
74600	Mrpl47	mitochondrial ribosomal protein L47	0.970874	0.7196	NA
76916	4930455C21Rik	RIKEN cDNA 4930455C21 gene	0.970874	0.7035	NA
77134	Hnrnpa0	heterogeneous nuclear ribonucleoprotein A0	0.970874	0.5788	NA
77462	Tmem116	transmembrane protein 116	0.970874	0.7703	0.8895
77604	C430048L16Rik	RIKEN cDNA C430048L16 gene	0.970874	0.6114	NA
104015	Synj1	synaptojanin 1	0.970874	0.6898	NA
110006	Gusb	glucuronidase, beta	0.970874	0.6592	NA
217716	Mlh3	mutL homolog 3 (E coli)	0.970874	0.5802	NA
224111	Ubxn7	UBX domain protein 7	0.970874	0.8326	0.9246
225849	Ppp2r5b	protein phosphatase 2, regulatory subunit B (B56), beta isoform	0.970874	0.4522	NA
227292	Ctdsp1	CTD (carboxy-terminal domain, RNA polymerase II, polypeptide A) small phosphatase 1	0.970874	0.8324	0.9245
232944	Mark4	MAP/microtubule affinity-regulating kinase 4	0.970874	0.8318	0.9245
234875	Ttc13	tetratricopeptide repeat domain 13	0.970874	0.6108	NA
272158	Poln	DNA polymerase N	0.970874	0.9014	0.9578
319317	Snhg11	small nucleolar RNA host gene 11	0.970874	0.8456	0.9309

320014	B930025P03Rik	RIKEN cDNA B930025P03 gene	0.970874	0.754	0.8812
545600	Gm12372	predicted gene 12372	0.970874	0.5495	NA
100039086	Gm15032	predicted gene 15032	0.970874	0.8991	0.9568
100040016	Rhox2e	reproductive homeobox 2E	0.970874	0.8675	0.9425
100043305	Gm4349	SET domain, bifurcated 1 pseudogene	0.970874	0.8358	0.9261
11518	Add1	adducin 1 (alpha)	0.969932	0.7366	0.871
11564	Adsl	adenylosuccinate lyase	0.969932	0.7662	0.8872
12593	Cdyl	chromodomain protein, Y chromosome-like	0.969932	0.5125	NA
13480	Dpm1	dolichol-phosphate (beta-D) mannosyltransferase 1	0.969932	0.6369	NA
13722	Aimp1	aminoacyl tRNA synthetase complex-interacting multifunctional protein 1	0.969932	0.5252	NA
14976	H2-Ke2	H2-K region expressed gene 2	0.969932	0.6751	NA
16332	Inppl1	inositol polyphosphate phosphatase-like 1	0.969932	0.5881	NA
18189	Nrxn1	neurexin I	0.969932	0.7098	NA
18424	Otx2	orthodenticle homolog 2 (Drosophila)	0.969932	0.7151	NA
19108	Prkx	protein kinase, X-linked	0.969932	0.7077	NA
20851	Stat5b	signal transducer and activator of transcription 5B	0.969932	0.7125	NA
22042	Tfrc	transferrin receptor	0.969932	0.7964	0.9043
23802	Amfr	autocrine motility factor receptor	0.969932	0.5597	NA
26382	Fgd2	FYVE, RhoGEF and PH domain containing 2	0.969932	0.8316	0.9244
27402	Pdhx	pyruvate dehydrogenase complex, component X	0.969932	0.5323	NA
51938	Ccdc39	coiled-coil domain containing 39	0.969932	0.7573	0.8826
52036	Ppp6r3	protein phosphatase 6, regulatory subunit 3	0.969932	0.6646	NA
56409	Nudt3	nudix (nucleotide diphosphate linked moiety X)-type motif 3	0.969932	0.5231	NA
56473	Fads2	fatty acid desaturase 2	0.969932	0.7508	0.8793
56525	Zfp235	zinc finger protein 235	0.969932	0.6288	NA
56788	Scube2	signal peptide, CUB domain, EGF-like 2	0.969932	0.7811	0.8949
64655	Mrps22	mitochondrial ribosomal protein S22	0.969932	0.5757	NA
64704	Htra2	HtrA serine peptidase 2	0.969932	0.6404	NA
66212	Sec61b	Sec61 beta subunit	0.969932	0.7694	0.8892
66273	1810020D17Rik	RIKEN cDNA 1810020D17 gene	0.969932	0.6662	NA
66492	Zmat2	zinc finger, matrin type 2	0.969932	0.6769	NA
66538	Rps19bp1	ribosomal protein S19 binding protein 1	0.969932	0.6563	NA
67899	Cmc1	COX assembly mitochondrial protein homolog (S. cerevisiae)	0.969932	0.543	NA
69126	1810022K09Rik	RIKEN cDNA 1810022K09 gene	0.969932	0.7013	NA
69195	Tmem121	transmembrane protein 121	0.969932	0.6772	NA
69250	2610029K11Rik	RIKEN cDNA 2610029K11 gene	0.969932	0.6658	NA
71777	Ing3	inhibitor of growth family, member 3	0.969932	0.6219	NA
75624	Metap1	methionyl aminopeptidase 1	0.969932	0.7244	NA
78321	Ankrd23	ankyrin repeat domain 23	0.969932	0.6122	NA
80883	Ntng1	netrin G1	0.969932	0.6909	NA
83429	Ctns	cystinosis, nephropathic	0.969932	0.5356	NA
93871	Brwd1	bromodomain and WD repeat domain containing 1	0.969932	0.6941	NA
98403	Zfp451	zinc finger protein 451	0.969932	0.6241	NA
101943	Sf3b3	splicing factor 3b, subunit 3	0.969932	0.6534	NA
103724	Tbc1d10a	TBC1 domain family, member 10a	0.969932	0.603	NA
106131	AA589532	expressed sequence AA589532	0.969932	0.7982	0.9053
208718	Dis3l2	DIS3 mitotic control homolog (S. cerevisiae)-like 2	0.969932	0.7281	0.8652
210998	D15Ertd621e	DNA segment, Chr 15, ERATO Doi 621, expressed	0.969932	0.6166	NA
211468	Kcnh8	potassium voltage-gated channel, subfamily H (eag-related), member 8	0.969932	0.8745	0.9452

211660	Cspp1	centrosome and spindle pole associated protein 1	0.969932	0.6076	NA
214137	Arhgap29	Rho GTPase activating protein 29	0.969932	0.7666	0.8874
216846	Cntrob	centrobin, centrosomal BRCA2 interacting protein	0.969932	0.636	NA
217038	Mrm1	mitochondrial rRNA methyltransferase 1 homolog (S. cerevisiae)	0.969932	0.6776	NA
217216	BC030867	cDNA sequence BC030867	0.969932	0.7976	0.905
218850	D14Abb1e	DNA segment, Chr 14, Abbott 1 expressed	0.969932	0.681	NA
223723	Ttll12	tubulin tyrosine ligase-like family, member 12	0.969932	0.6314	NA
225887	Ndufs8	NADH dehydrogenase (ubiquinone) Fe-S protein 8	0.969932	0.6638	NA
244199	Ovch2	ovochymase 2	0.969932	0.8859	0.9505
244871	Zc3h12c	zinc finger CCCH type containing 12C	0.969932	0.7325	0.8682
381280	Hjurp	Holliday junction recognition protein	0.969932	0.7027	NA
432530	Adcy1	adenylate cyclase 1	0.969932	0.6617	NA
627648	Klhl3	kelch-like 3 (Drosophila)	0.969932	0.7634	0.8858
11677	Akr1b3	aldo-keto reductase family 1, member B3 (aldose reductase)	0.968992	0.7955	0.9035
13074	Cyp17a1	cytochrome P450, family 17, subfamily a, polypeptide 1	0.968992	0.8806	0.9478
13389	Dll3	delta-like 3 (Drosophila)	0.968992	0.6769	NA
14159	Fes	feline sarcoma oncogene	0.968992	0.759	0.8831
14160	Lgr5	leucine rich repeat containing G protein coupled receptor 5	0.968992	0.6094	NA
14360	Fyn	Fyn proto-oncogene	0.968992	0.623	NA
16502	Kcnc1	potassium voltage gated channel, Shaw-related subfamily, member 1	0.968992	0.7251	0.8633
16648	Kpna3	karyopherin (importin) alpha 3	0.968992	0.8105	0.9122
16814	Lbx1	ladybird homeobox homolog 1 (Drosophila)	0.968992	0.8728	0.9449
17311	Kitl	kit ligand	0.968992	0.8144	0.9146
17692	Msl3	male-specific lethal 3 homolog (Drosophila)	0.968992	0.56	NA
18212	Ntrk2	neurotrophic tyrosine kinase, receptor, type 2	0.968992	0.6468	NA
19344	Rab5b	RAB5B, member RAS oncogene family	0.968992	0.6548	NA
19386	Ranbp2	RAN binding protein 2	0.968992	0.6621	NA
20472	Six2	sine oculis-related homeobox 2 homolog (Drosophila)	0.968992	0.8282	0.9223
21386	Tbx3	T-box 3	0.968992	0.6372	NA
21814	Tgfbr3	transforming growth factor, beta receptor III	0.968992	0.7066	NA
22248	Unc119	unc-119 homolog (C. elegans)	0.968992	0.79	0.9007
26992	Brd7	bromodomain containing 7	0.968992	0.4658	NA
28185	Tomm70a	translocase of outer mitochondrial membrane 70 homolog A (yeast)	0.968992	0.7739	0.8913
30925	Slamf6	SLAM family member 6	0.968992	0.8796	0.9473
50907	Preb	prolactin regulatory element binding	0.968992	0.5998	NA
56692	Mapksp1	MAPK scaffold protein 1	0.968992	0.8068	0.9099
65098	Zfand6	zinc finger, AN1-type domain 6	0.968992	0.7525	0.8803
65116	Prrg2	proline-rich Gla (G-carboxyglutamic acid) polypeptide 2	0.968992	0.5866	NA
66257	Nicn1	nicolin 1	0.968992	0.7051	NA
66480	Rpl15	ribosomal protein L15	0.968992	0.7337	0.869
66520	2610001J05Rik	RIKEN cDNA 2610001J05 gene	0.968992	0.5923	NA
66799	Ube2w	ubiquitin-conjugating enzyme E2W (putative)	0.968992	0.8278	0.9222
67187	Zmynd19	zinc finger, MYND domain containing 19	0.968992	0.5504	NA
68193	Rpl24	ribosomal protein L24	0.968992	0.7183	0.8592
68515	Myadml2	myeloid-associated differentiation marker-like 2	0.968992	0.7108	NA
68598	Dnajc8	DnaJ (Hsp40) homolog, subfamily C, member 8	0.968992	0.6914	NA
68767	ORF19	open reading frame 19	0.968992	0.6865	NA
69397	1700019A02Rik	RIKEN cDNA 1700019A02 gene	0.968992	0.8324	0.9245
69821	Mterfd2	MTERF domain containing 2	0.968992	0.61	NA

70235	Poc1a	POC1 centriolar protein homolog A (Chlamydomonas)	0.968992	0.6306	NA
72117	Naa50	N(alpha)-acetyltransferase 50, NatE catalytic subunit	0.968992	0.7309	0.8669
72322	Xpo5	exportin 5	0.968992	0.627	NA
73072	BC068157	cDNA sequence BC068157	0.968992	0.7685	0.8886
73545	1700094D03Rik	RIKEN cDNA 1700094D03 gene	0.968992	0.6016	NA
76246	Rtf1	Rtf1, Paf1/RNA polymerase II complex component, homolog (S. cerevisiae)	0.968992	0.5225	NA
76614	Immt	inner membrane protein, mitochondrial	0.968992	0.58	NA
77006	Ddrgk1	DDRGK domain containing 1	0.968992	0.747	0.8772
93876	Pcdhb5	protocadherin beta 5	0.968992	0.6888	NA
107976	Bre	brain and reproductive organ-expressed protein	0.968992	0.6656	NA
108072	Grm6	glutamate receptor, metabotropic 6	0.968992	0.558	NA
108911	Rcc2	regulator of chromosome condensation 2	0.968992	0.537	NA
209018	Vps8	vacuolar protein sorting 8 homolog (S. cerevisiae)	0.968992	0.7934	0.9021
224902	Safb2	scaffold attachment factor B2	0.968992	0.7387	0.8722
226976	4632411B12Rik	RIKEN cDNA 4632411B12 gene	0.968992	0.5284	NA
227449	Zcchc2	zinc finger, CCHC domain containing 2	0.968992	0.6737	NA
227738	Lrsam1	leucine rich repeat and sterile alpha motif containing 1	0.968992	0.6561	NA
227835	Gtdc1	glycosyltransferase-like domain containing 1	0.968992	0.5292	NA
229599	Gm129	predicted gene 129	0.968992	0.7791	0.8935
229700	Rbm15	RNA binding motif protein 15	0.968992	0.6603	NA
232785	Zfp783	zinc finger protein 783	0.968992	0.6737	NA
234663	Dync1li2	dynein, cytoplasmic 1 light intermediate chain 2	0.968992	0.5702	NA
240057	Syngap1	synaptic Ras GTPase activating protein 1 homolog (rat)	0.968992	0.8157	0.9154
242553	Kank4	KN motif and ankyrin repeat domains 4	0.968992	0.8571	0.9367
244431	Sgcz	sarcoglycan zeta	0.968992	0.8748	0.9452
277089	Gm5068	predicted gene 5068	0.968992	0.8049	0.9086
286942	Kif19a	kinesin family member 19A	0.968992	0.8108	0.9123
320171	D130019J16Rik	RIKEN cDNA D130019J16 gene	0.968992	0.8299	0.9233
320816	Ankrd16	ankyrin repeat domain 16	0.968992	0.6645	NA
328530	D030063E12	hypothetical protein D030063E12	0.968992	0.7198	0.8599
360216	Zranb1	zinc finger, RAN-binding domain containing 1	0.968992	0.5777	NA
382793	Mtx3	metaxin 3	0.968992	0.6151	NA
399599	Ccdc87	coiled-coil domain containing 87	0.968992	0.7755	0.892
432713	Gm5441	predicted gene 5441	0.968992	0.7697	0.8895
494448	Cbx6	chromobox homolog 6	0.968992	0.7822	0.8954
641340	Nrbf2	nuclear receptor binding factor 2	0.968992	0.5789	NA
11441	Chrna7	cholinergic receptor, nicotinic, alpha polypeptide 7	0.968054	0.7514	0.8798
11610	Agtrap	angiotensin II, type I receptor-associated protein	0.968054	0.7577	0.8829
11814	Apoc3	apolipoprotein C-III	0.968054	0.8731	0.9449
12545	Cdc7	cell division cycle 7 (S. cerevisiae)	0.968054	0.7494	0.8785
12651	Chkb	choline kinase beta	0.968054	0.5721	NA
13712	Elk1	ELK1, member of ETS oncogene family	0.968054	0.7779	0.8931
14534	Kat2a	K(lysine) acetyltransferase 2A	0.968054	0.7703	0.8895
14802	Gria4	glutamate receptor, ionotropic, AMPA4 (alpha 4)	0.968054	0.6676	NA
16502	Kcnc1	potassium voltage gated channel, Shaw-related subfamily, member 1	0.968054	0.7437	0.8754
16569	Kif3b	kinesin family member 3B	0.968054	0.6639	NA
17256	Mea1	male enhanced antigen 1	0.968054	0.6243	NA
17864	Mybl1	myeloblastosis oncogene-like 1	0.968054	0.8493	0.9329
17979	Ncoa3	nuclear receptor coactivator 3	0.968054	0.6316	NA

18583	Pde7a	phosphodiesterase 7A	0.968054	0.797	0.9047
20318	Sdf4	stromal cell derived factor 4	0.968054	0.7212	0.8603
21939	Cd40	CD40 antigen	0.968054	0.8084	0.9108
22123	Psmd3	proteasome (prosome, macropain) 26S subunit, non-ATPase, 3	0.968054	0.6057	NA
22608	Ybx1	Y box protein 1	0.968054	0.544	NA
24113	Vax2	ventral anterior homeobox containing gene 2	0.968054	0.684	NA
27373	Csnk1e	casein kinase 1, epsilon	0.968054	0.7407	0.8733
54611	Pde3a	phosphodiesterase 3A, cGMP inhibited	0.968054	0.8626	0.9395
56743	Lat2	linker for activation of T cells family, member 2	0.968054	0.7797	0.894
66310	Dpy30	dpy-30 homolog (C. elegans)	0.968054	0.656	NA
66962	2310047B19Rik	RIKEN cDNA 2310047B19 gene	0.968054	0.6326	NA
67674	Trmt112	tRNA methyltransferase 11-2 homolog (S. cerevisiae)	0.968054	0.5452	NA
67676	Rpp21	ribonuclease P 21 subunit (human)	0.968054	0.6184	NA
67771	Arpc5	actin related protein 2/3 complex, subunit 5	0.968054	0.5751	NA
68051	Nutf2	nuclear transport factor 2	0.968054	0.4372	NA
68366	Tmem129	transmembrane protein 129	0.968054	0.6311	NA
68646	1110020G09Rik	RIKEN cDNA 1110020G09 gene	0.968054	0.4832	NA
68927	Ptcd2	pentatricopeptide repeat domain 2	0.968054	0.514	NA
69113	Alkbh3	alkB, alkylation repair homolog 3 (E. coli)	0.968054	0.4518	NA
69902	Mrto4	MRT4, mRNA turnover 4, homolog (S. cerevisiae)	0.968054	0.5707	NA
70620	Ube2v2	ubiquitin-conjugating enzyme E2 variant 2	0.968054	0.5421	NA
71238	Acn9	ACN9 homolog (S. cerevisiae)	0.968054	0.5841	NA
71941	Cars2	cysteinyl-tRNA synthetase 2 (mitochondrial)(putative)	0.968054	0.6904	NA
71954	Suds3	suppressor of defective silencing 3 homolog (S. cerevisiae)	0.968054	0.7734	0.8911
72522	Atxn7l2	ataxin 7-like 2	0.968054	0.7993	0.9057
74123	Foxp4	forkhead box P4	0.968054	0.6553	NA
75909	Tmem49	transmembrane protein 49	0.968054	0.4568	NA
76577	Faf2	Fas associated factor family member 2	0.968054	0.6295	NA
78893	Cnot10	CCR4-NOT transcription complex, subunit 10	0.968054	0.6371	NA
81702	Ankrd17	ankyrin repeat domain 17	0.968054	0.6744	NA
81896	Ift122	intraflagellar transport 122 homolog (Chlamydomonas)	0.968054	0.6543	NA
98952	Fam102a	family with sequence similarity 102, member A	0.968054	0.575	NA
99650	4933434E20Rik	RIKEN cDNA 4933434E20 gene	0.968054	0.8341	0.9251
100226	Stx12	syntaxin 12	0.968054	0.5408	NA
102294	Cyp4v3	cytochrome P450, family 4, subfamily v, polypeptide 3	0.968054	0.5754	NA
107392	Brms1	breast cancer metastasis-suppressor 1	0.968054	0.7231	0.8618
114713	Rasa2	RAS p21 protein activator 2	0.968054	0.6272	NA
114875	Plcz1	phospholipase C, zeta 1	0.968054	0.7913	0.901
192159	Prpf8	pre-mRNA processing factor 8	0.968054	0.593	NA
207952	Klhl25	kelch-like 25 (Drosophila)	0.968054	0.6568	NA
209268	Igsf1	immunoglobulin superfamily, member 1	0.968054	0.8137	0.9141
235036	Ppan	peter pan homolog (Drosophila)	0.968054	0.6188	NA
237339	L3mbtl3	l(3)mbt-like 3 (Drosophila)	0.968054	0.582	NA
239606	Slc2a13	solute carrier family 2 (facilitated glucose transporter), member 13	0.968054	0.5997	NA
239985	Arid1b	AT rich interactive domain 1B (SWI-like)	0.968054	0.7191	0.8597
244059	Chd2	chromodomain helicase DNA binding protein 2	0.968054	0.6295	NA
258652	Olfr1131	olfactory receptor 1131	0.968054	0.9046	0.9596
269400	Rtel1	regulator of telomere elongation helicase 1	0.968054	0.583	NA
380787	A230065H16Rik	RIKEN cDNA A230065H16 gene	0.968054	0.8007	0.9064

381356	5930434B04Rik	RIKEN cDNA 5930434B04 gene	0.968054	0.7586	0.8831
381629	0610007C21Rik	RIKEN cDNA 0610007C21 gene	0.968054	0.6467	NA
383436	Gm5244	ribosomal protein, large, P1 pseudogene	0.968054	0.6657	NA
553089	LOC553089	hypothetical LOC553089	0.968054	0.7734	0.8911
100041686	Gm15427	predicted pseudogene 15427	0.968054	0.7004	NA
100043468	Zfp955b	zinc finger protein 955B	0.968054	0.8584	0.937
11433	Acp5	acid phosphatase 5, tartrate resistant	0.967118	0.8321	0.9245
11496	Adam22	a disintegrin and metallopeptidase domain 22	0.967118	0.5361	NA
12166	Bmpr1a	bone morphogenetic protein receptor, type 1A	0.967118	0.5887	NA
12684	Cideb	cell death-inducing DNA fragmentation factor, alpha subunit-like effector B	0.967118	0.775	0.8918
13855	Epn2	epsin 2	0.967118	0.7401	0.8729
14705	Bscl2	Bernardinelli-Seip congenital lipodystrophy 2 homolog (human)	0.967118	0.7673	0.8878
14841	Gsg2	germ cell-specific gene 2	0.967118	0.838	0.927
16330	Inpp5b	inositol polyphosphate-5-phosphatase B	0.967118	0.6006	NA
16872	Lhx4	LIM homeobox protein 4	0.967118	0.7472	0.8773
16995	Ltb4r1	leukotriene B4 receptor 1	0.967118	0.7401	0.8729
17156	Man1a2	mannosidase, alpha, class 1A, member 2	0.967118	0.6275	NA
17933	Myt1l	myelin transcription factor 1-like	0.967118	0.5935	NA
18647	Cdk14	cyclin-dependent kinase 14	0.967118	0.5944	NA
20320	Nptn	neuroplastin	0.967118	0.7078	0.8534
21924	Tnnc1	troponin C, cardiac/slow skeletal	0.967118	0.6886	NA
22062	Trp73	transformation related protein 73	0.967118	0.886	0.9506
22196	Ube2i	ubiquitin-conjugating enzyme E2I	0.967118	0.5341	NA
22652	Mkrn3	makorin, ring finger protein, 3	0.967118	0.5739	NA
23984	Pde10a	phosphodiesterase 10A	0.967118	0.8371	0.927
26356	Ing1	inhibitor of growth family, member 1	0.967118	0.6785	NA
26939	Polr3e	polymerase (RNA) III (DNA directed) polypeptide E	0.967118	0.5113	NA
29807	Tpk1	thiamine pyrophosphokinase	0.967118	0.5416	NA
53598	Dctn3	dynactin 3	0.967118	0.4107	NA
54160	Copg2	coatomer protein complex, subunit gamma 2	0.967118	0.5338	NA
54189	Rabep1	rabaptin, RAB GTPase binding effector protein 1	0.967118	0.6241	NA
56207	Uchl5	ubiquitin carboxyl-terminal esterase L5	0.967118	0.563	NA
56274	Stk3	serine/threonine kinase 3 (Ste20, yeast homolog)	0.967118	0.8724	0.9447
56307	Metap2	methionine aminopeptidase 2	0.967118	0.5804	NA
57230	Sap30bp	SAP30 binding protein	0.967118	0.7851	0.8972
64898	Lpin2	lipin 2	0.967118	0.7102	0.8543
66108	Ndufa9	NADH dehydrogenase (ubiquinone) 1 alpha subcomplex, 9	0.967118	0.5296	NA
66176	Nat9	N-acetyltransferase 9 (GCN5-related, putative)	0.967118	0.7074	0.8532
66225	Llph	LLP homolog, long-term synaptic facilitation (Aplysia)	0.967118	0.6101	NA
67151	Psmd9	proteasome (prosome, macropain) 26S subunit, non-ATPase, 9	0.967118	0.5659	NA
67967	Pold3	polymerase (DNA-directed), delta 3, accessory subunit	0.967118	0.4798	NA
68137	Kdelr1	KDEL (Lys-Asp-Glu-Leu) endoplasmic reticulum protein retention receptor 1	0.967118	0.6552	NA
68537	Mrpl13	mitochondrial ribosomal protein L13	0.967118	0.495	NA
68585	Rtn4	reticulon 4	0.967118	0.7697	0.8895
68980	Wdr53	WD repeat domain 53	0.967118	0.4485	NA
69227	2810407C02Rik	RIKEN cDNA 2810407C02 gene	0.967118	0.6658	NA
69612	2310037I24Rik	RIKEN cDNA 2310037I24 gene	0.967118	0.6354	NA
70579	Zc3h11a	zinc finger CCCH type containing 11A	0.967118	0.6599	NA
71367	Chst9	carbohydrate (N-acetylgalactosamine 4-0) sulfotransferase 9	0.967118	0.7955	0.9035

71891	Cdadc1	cytidine and dCMP deaminase domain containing 1	0.967118	0.4384	NA
72630	Hspa12b	heat shock protein 12B	0.967118	0.6653	NA
74020	Cpne4	copine IV	0.967118	0.8453	0.9307
74617	Scpep1	serine carboxypeptidase 1	0.967118	0.6245	NA
74777	Sepn1	selenoprotein N, 1	0.967118	0.7218	0.8608
77040	Atg16l1	autophagy-related 16-like 1 (yeast)	0.967118	0.5899	NA
78914	Nadsyn1	NAD synthetase 1	0.967118	0.6207	NA
103405	4932439E07Rik	RIKEN cDNA 4932439E07 gene	0.967118	0.7072	0.853
106894	Hmgxb3	HMG box domain containing 3	0.967118	0.5532	NA
108707	1810008A18Rik	RIKEN cDNA 1810008A18 gene	0.967118	0.5854	NA
117005	Olfr74	olfactory receptor 74	0.967118	0.8985	0.9565
192120	Bspry	B-box and SPRY domain containing	0.967118	0.8328	0.9247
207806	Gm608	predicted gene 608	0.967118	0.8583	0.937
224656	Zfp523	zinc finger protein 523	0.967118	0.5291	NA
224836	Usp49	ubiquitin specific peptidase 49	0.967118	0.6823	NA
226252	Fam160b1	family with sequence similarity 160, member B1	0.967118	0.5093	NA
227695	D2Wsu81e	DNA segment, Chr 2, Wayne State University 81, expressed	0.967118	0.6475	NA
232947	Lrrc68	leucine rich repeat containing 68	0.967118	0.696	NA
233744	Spon1	spondin 1, (f-spondin) extracellular matrix protein	0.967118	0.6356	NA
234825	Klhdc4	kelch domain containing 4	0.967118	0.5901	NA
237500	Tmtc3	transmembrane and tetratricopeptide repeat containing 3	0.967118	0.8468	0.9316
238405	Adam6b	a disintegrin and metallopeptidase domain 6B	0.967118	0.9354	0.9731
268902	Robo2	roundabout homolog 2 (Drosophila)	0.967118	0.7256	0.8636
270163	Myo9a	myosin IXa	0.967118	0.6093	NA
381067	Zfp229	zinc finger protein	0.967118	0.7724	0.8906
435392	Gm5670	predicted gene 5670	0.967118	0.8414	0.9288
546052	Gm5908	predicted gene 5908	0.967118	0.5139	NA
619605	Zcchc17	zinc finger, CCHC domain containing 17	0.967118	0.4995	NA
100038999	Gm13552	predicted gene 13552	0.967118	0.6442	NA
100504876	LOC100504876	60S ribosomal protein L7a-like	0.967118	0.6098	NA
11861	Arl4a	ADP-ribosylation factor-like 4A	0.966184	0.77	0.8895
12757	Clta	clathrin, light polypeptide (Lca)	0.966184	0.4214	NA
12846	Comt	catechol-O-methyltransferase	0.966184	0.6652	NA
13548	Dyrk1a	dual-specificity tyrosine-(Y)-phosphorylation regulated kinase 1a	0.966184	0.6967	0.8463
13669	Eif3a	eukaryotic translation initiation factor 3, subunit A	0.966184	0.725	0.8633
14042	Ext1	exostoses (multiple) 1	0.966184	0.5991	NA
14376	Ganab	alpha glucosidase 2 alpha neutral subunit	0.966184	0.4384	NA
14588	Gfra4	glial cell line derived neurotrophic factor family receptor alpha 4	0.966184	0.7694	0.8892
16578	Kif9	kinesin family member 9	0.966184	0.8138	0.9141
16704	Krtap8-2	keratin associated protein 8-2	0.966184	0.7839	0.8964
18854	Pml	promyelocytic leukemia	0.966184	0.4938	NA
19088	Prkar2b	protein kinase, cAMP dependent regulatory, type II beta	0.966184	0.5394	NA
20840	Stac	src homology three (SH3) and cysteine rich domain	0.966184	0.8153	0.9151
27371	Sh2d2a	SH2 domain protein 2A	0.966184	0.688	NA
51786	Cpsf2	cleavage and polyadenylation specific factor 2	0.966184	0.5843	NA
52712	Zkscan6	zinc finger with KRAB and SCAN domains 6	0.966184	0.5279	NA
54127	Rps28	ribosomal protein S28	0.966184	0.741	0.8736
56350	Arl3	ADP-ribosylation factor-like 3	0.966184	0.6716	NA
66248	Alg5	asparagine-linked glycosylation 5 homolog (yeast, dolichyl-phosphate beta-glucosyltransferase)	0.966184	0.6088	NA

66433	Chchd7	coiled-coil-helix-coiled-coil-helix domain containing 7	0.966184	0.6504	NA
66665	5730528L13Rik	RIKEN cDNA 5730528L13 gene	0.966184	0.6697	NA
68606	Ppm1f	protein phosphatase 1F (PP2C domain containing)	0.966184	0.5203	NA
68776	Taf11	TAF11 RNA polymerase II, TATA box binding protein (TBP)-associated factor	0.966184	0.5399	NA
68875	Tmcc2	transmembrane and coiled-coil domains 2	0.966184	0.6225	NA
68910	Zfp467	zinc finger protein 467	0.966184	0.4845	NA
68972	Tatdn3	TatD DNase domain containing 3	0.966184	0.5618	NA
69534	Avpi1	arginine vasopressin-induced 1	0.966184	0.629	NA
69662	2310061I04Rik	RIKEN cDNA 2310061I04 gene	0.966184	0.6863	NA
69663	Ddx51	DEAD (Asp-Glu-Ala-Asp) box polypeptide 51	0.966184	0.5434	NA
70310	Plscr3	phospholipid scramblase 3	0.966184	0.7194	0.8597
70802	Pwwp2a	PWWP domain containing 2A	0.966184	0.6725	NA
71198	Otud1	OTU domain containing 1	0.966184	0.6595	NA
71766	Raver1	ribonucleoprotein, PTB-binding 1	0.966184	0.8571	0.9367
72284	Oraov1	oral cancer overexpressed 1	0.966184	0.5568	NA
73130	Tmed5	transmembrane emp24 protein transport domain containing 5	0.966184	0.7179	0.859
73293	Ccdc103	coiled-coil domain containing 103	0.966184	0.8282	0.9223
76233	Dnttip1	deoxynucleotidyltransferase, terminal, interacting protein 1	0.966184	0.4209	NA
77701	Lcn12	lipocalin 12	0.966184	0.7724	0.8906
77805	Esco1	establishment of cohesion 1 homolog 1 (S. cerevisiae)	0.966184	0.5564	NA
98404	AI597479	expressed sequence AI597479	0.966184	0.5167	NA
101612	Grwd1	glutamate-rich WD repeat containing 1	0.966184	0.6739	NA
109075	Exosc4	exosome component 4	0.966184	0.6481	NA
109135	Plekha5	pleckstrin homology domain containing, family A member 5	0.966184	0.6503	NA
109161	Ube2q2	ubiquitin-conjugating enzyme E2Q (putative) 2	0.966184	0.6701	NA
116871	Mta3	metastasis associated 3	0.966184	0.596	NA
170458	Gpha2	glycoprotein hormone alpha 2	0.966184	0.8727	0.9448
194735	4930430D24Rik	RIKEN cDNA 4930430D24 gene	0.966184	0.9012	0.9578
212168	Zswim4	zinc finger, SWIM domain containing 4	0.966184	0.8071	0.91
214663	Slc25a29	solute carrier family 25 (mitochondrial carrier, palmitoylcarnitine transporter), member 29	0.966184	0.6967	0.8464
218756	Slc4a7	solute carrier family 4, sodium bicarbonate cotransporter, member 7	0.966184	0.6421	NA
227210	Ccnyl1	cyclin Y-like 1	0.966184	0.7848	0.8969
228790	Asxl1	additional sex combs like 1 (Drosophila)	0.966184	0.5158	NA
230316	Megf9	multiple EGF-like-domains 9	0.966184	0.7628	0.8853
235300	Tmem136	transmembrane protein 136	0.966184	0.5015	NA
236082	Dhrsx	dehydrogenase/reductase (SDR family) X chromosome	0.966184	0.7121	0.8553
239408	Tmem74	transmembrane protein 74	0.966184	0.7196	0.8599
240185	9430020K01Rik	RIKEN cDNA 9430020K01 gene	0.966184	0.7079	0.8534
244484	Wdr17	WD repeat domain 17	0.966184	0.7051	0.8517
268420	Alkbh5	alkB, alkylation repair homolog 5 (E. coli)	0.966184	0.4841	NA
319740	Zfyve27	zinc finger, FYVE domain containing 27	0.966184	0.4841	NA
320234	Ccdc66	coiled-coil domain containing 66	0.966184	0.6482	NA
328066	C920021A13	hypothetical protein C920021A13	0.966184	0.8662	0.9418
494448	Cbx6	chromobox homolog 6	0.966184	0.6034	NA
100169878	Gm10941	predicted gene 10941	0.966184	0.7342	0.8694
12340	Capza1	capping protein (actin filament) muscle Z-line, alpha 1	0.965251	0.8762	0.946
13382	Dld	dihydrolipoamide dehydrogenase	0.965251	0.5084	NA
14238	Foxf2	forkhead box F2	0.965251	0.8323	0.9245
14885	Gtf2h4	general transcription factor II H, polypeptide 4	0.965251	0.5465	NA

16341	Eif3e	eukaryotic translation initiation factor 3, subunit E	0.965251	0.6684	NA
16570	Kif3c	kinesin family member 3C	0.965251	0.4674	NA
16857	Lgals6	lectin, galactose binding, soluble 6	0.965251	0.5755	NA
17763	Mtcp1	mature T-cell proliferation 1	0.965251	0.5441	NA
17763	Mtcp1	mature T-cell proliferation 1	0.965251	0.6502	NA
18575	Pde1c	phosphodiesterase 1C	0.965251	0.6172	NA
18975	Polg	polymerase (DNA directed), gamma	0.965251	0.5696	NA
19133	Prph2	peripherin 2	0.965251	0.6131	NA
22428	Dctn6	dynactin 6	0.965251	0.4757	NA
22724	Zbtb7b	zinc finger and BTB domain containing 7B	0.965251	0.8438	0.9299
24050	Sep-03	septin 3	0.965251	0.7117	0.8553
26875	Pclo	piccolo (presynaptic cytomatrix protein)	0.965251	0.653	NA
50529	Mrps7	mitchondrial ribosomal protein S7	0.965251	0.6124	NA
50817	Solh	small optic lobes homolog (Drosophila)	0.965251	0.8457	0.9309
50849	Rnf10	ring finger protein 10	0.965251	0.6778	NA
50873	Park2	Parkinson disease (autosomal recessive, juvenile) 2, parkin	0.965251	0.7951	0.9033
54217	Rpl36	ribosomal protein L36	0.965251	0.6502	NA
56403	Syncrip	synaptotagmin binding, cytoplasmic RNA interacting protein	0.965251	0.5691	NA
57230	Sap30bp	SAP30 binding protein	0.965251	0.702	0.8498
57434	Xrcc2	X-ray repair complementing defective repair in Chinese hamster cells 2	0.965251	0.7633	0.8857
57916	Tnfrsf13b	tumor necrosis factor receptor superfamily, member 13b	0.965251	0.8252	0.9207
66078	Tsen34	tRNA splicing endonuclease 34 homolog (S. cerevisiae)	0.965251	0.5783	NA
66336	Cenpp	centromere protein P	0.965251	0.7204	0.8602
66489	Rpl35	ribosomal protein L35	0.965251	0.6642	NA
66497	2610528E23Rik	RIKEN cDNA 2610528E23 gene	0.965251	0.62	NA
66609	Cryzl1	crystallin, zeta (quinone reductase)-like 1	0.965251	0.5556	NA
66931	1700010I14Rik	RIKEN cDNA 1700010I14 gene	0.965251	0.7635	0.8858
67673	Tceb2	transcription elongation factor B (SIII), polypeptide 2	0.965251	0.4588	NA
67701	Wfdc2	WAP four-disulfide core domain 2	0.965251	0.7527	0.8804
67811	Poldip2	polymerase (DNA-directed), delta interacting protein 2	0.965251	0.7224	0.8612
68046	2700062C07Rik	RIKEN cDNA 2700062C07 gene	0.965251	0.4588	NA
69116	Ubr4	ubiquitin protein ligase E3 component n-recognin 4	0.965251	0.5448	NA
69190	Dym	dymeclin	0.965251	0.6747	NA
69470	Tmem127	transmembrane protein 127	0.965251	0.5615	NA
69665	2310043J07Rik	RIKEN cDNA 2310043J07 gene	0.965251	0.8802	0.9478
71950	Nanog	Nanog homeobox	0.965251	0.776	0.8922
72354	Ttc4	tetratricopeptide repeat domain 4	0.965251	0.5984	NA
74493	Tnks2	tankyrase, TRF1-interacting ankyrin-related ADP-ribose polymerase 2	0.965251	0.5878	NA
74764	Klc4	kinesin light chain 4	0.965251	0.7121	0.8553
75124	Nxnl2	nucleoredoxin-like 2	0.965251	0.5314	NA
75221	Dpp3	dipeptidylpeptidase 3	0.965251	0.6765	NA
75734	Mff	mitochondrial fission factor	0.965251	0.4858	NA
76093	5830469G19Rik	RIKEN cDNA 5830469G19 gene	0.965251	0.6495	NA
76563	Qrsl1	glutaminyl-tRNA synthase (glutamine-hydrolyzing)-like 1	0.965251	0.5158	NA
80748	BC004004	cDNA sequence BC004004	0.965251	0.4851	NA
101563	AI426330	expressed sequence AI426330	0.965251	0.5013	NA
106021	Topors	topoisomerase I binding, arginine/serine-rich	0.965251	0.6144	NA
108100	Baiap2	brain-specific angiogenesis inhibitor 1-associated protein 2	0.965251	0.7364	0.8708
218613	Mier3	mesoderm induction early response 1, family member 3	0.965251	0.6838	0.8386

227290	Aamp	angio-associated migratory protein	0.965251	0.4889	NA
230648	4732418C07Rik	RIKEN cDNA 4732418C07 gene	0.965251	0.5351	NA
234967	Slc36a4	solute carrier family 36 (proton/amino acid symporter), member 4	0.965251	0.7324	0.868
237782	Smcr8	Smith-Magenis syndrome chromosome region, candidate 8 homolog (human)	0.965251	0.5253	NA
240614	Ranbp6	RAN binding protein 6	0.965251	0.7523	0.8803
264064	Cdk8	cyclin-dependent kinase 8	0.965251	0.8593	0.9375
268354	Fam19a2	family with sequence similarity 19, member A2	0.965251	0.5882	NA
268566	Gphn	gephyrin	0.965251	0.4851	NA
269702	Mphosph9	M-phase phosphoprotein 9	0.965251	0.719	0.8597
331021	9630017O17	hypothetical protein 9630017O17	0.965251	0.6011	NA
399607	D230019N24Rik	RIKEN cDNA D230019N24 gene	0.965251	0.7581	0.8829
11352	Abl2	v-abl Abelson murine leukemia viral oncogene homolog 2 (arg, Abelson-related gene)	0.96432	0.3929	NA
11516	Adcyap1	adenylate cyclase activating polypeptide 1	0.96432	0.5733	NA
11842	Arf3	ADP-ribosylation factor 3	0.96432	0.4001	NA
12868	Cox8a	cytochrome c oxidase, subunit VIIIa	0.96432	0.7059	0.8524
13196	Asap1	ArfGAP with SH# domain, ankyrin repeat and PH domain1	0.96432	0.4682	NA
13680	Ddx19a	DEAD (Asp-Glu-Ala-Asp) box polypeptide 19a	0.96432	0.6547	NA
15490	Hsd17b7	hydroxysteroid (17-beta) dehydrogenase 7	0.96432	0.6388	NA
15519	Hsp90aa1	heat shock protein 90, alpha (cytosolic), class A member 1	0.96432	0.5715	NA
17305	Mfng	MFNG O-fucosylpeptide 3-beta-N-acetylglucosaminyltransferase	0.96432	0.6335	NA
17967	Ncam1	neural cell adhesion molecule 1	0.96432	0.5219	NA
18148	Npm1	nucleophosmin 1	0.96432	0.6175	NA
18438	P2rx4	purinergic receptor P2X, ligand-gated ion channel 4	0.96432	0.5781	NA
18968	Pola1	polymerase (DNA directed), alpha 1	0.96432	0.6359	NA
19046	Ppp1cb	protein phosphatase 1, catalytic subunit, beta isoform	0.96432	0.6459	NA
19691	Recql	RecQ protein-like	0.96432	0.5188	NA
26931	Ppp2r5c	protein phosphatase 2, regulatory subunit B (B56), gamma isoform	0.96432	0.6145	NA
27225	Ddx24	DEAD (Asp-Glu-Ala-Asp) box polypeptide 24	0.96432	0.561	NA
54403	Slc4a4	solute carrier family 4 (anion exchanger), member 4	0.96432	0.6982	0.8473
54473	Tollip	toll interacting protein	0.96432	0.4989	NA
56692	Mapksp1	MAPK scaffold protein 1	0.96432	0.4614	NA
56716	Mlst8	MTOR associated protein, LST8 homolog (S. cerevisiae)	0.96432	0.6594	NA
58867	Syngr4	synaptogyrin 4	0.96432	0.8345	0.9252
59045	Stard3	START domain containing 3	0.96432	0.4565	NA
64296	Abhd8	abhydrolase domain containing 8	0.96432	0.6202	NA
66226	Trappc2	trafficking protein particle complex 2	0.96432	0.7338	0.8691
66278	1810013D10Rik	RIKEN cDNA 1810013D10 gene	0.96432	0.632	NA
66440	Cdc26	cell division cycle 26	0.96432	0.6167	NA
66943	Pqlc1	PQ loop repeat containing 1	0.96432	0.5821	NA
66975	2410002O22Rik	RIKEN cDNA 2410002O22 gene	0.96432	0.5798	NA
67290	3110040N11Rik	RIKEN cDNA 3110040N11 gene	0.96432	0.566	NA
68020	2810002N01Rik	RIKEN cDNA 2810002N01 gene	0.96432	0.6268	NA
68031	Rnf146	ring finger protein 146	0.96432	0.8301	0.9234
68133	Gcsh	glycine cleavage system protein H (aminomethyl carrier)	0.96432	0.4919	NA
68559	Pdrg1	p53 and DNA damage regulated 1	0.96432	0.4825	NA
69765	1500004F05Rik	RIKEN cDNA 1500004F05 gene	0.96432	0.7582	0.8829
70239	Gtf3c5	general transcription factor IIIC, polypeptide 5	0.96432	0.5661	NA
70533	Btf3l4	basic transcription factor 3-like 4	0.96432	0.5545	NA
72831	Dhx30	DEAH (Asp-Glu-Ala-His) box polypeptide 30	0.96432	0.5673	NA

74239	Iqce	IQ motif containing E	0.96432	0.7369	0.8711
74255	Smu1	smu-1 suppressor of mec-8 and unc-52 homolog (C. elegans)	0.96432	0.5285	NA
75416	Nop14	NOP14 nucleolar protein homolog (yeast)	0.96432	0.7452	0.8762
76479	Smndc1	survival motor neuron domain containing 1	0.96432	0.8249	0.9206
76707	Clasp1	CLIP associating protein 1	0.96432	0.4852	NA
84113	Ptov1	prostate tumor over expressed gene 1	0.96432	0.6829	0.838
94090	Trim9	tripartite motif-containing 9	0.96432	0.7582	0.8829
94186	Strn3	striatin, calmodulin binding protein 3	0.96432	0.6235	NA
94213	Ddx50	DEAD (Asp-Glu-Ala-Asp) box polypeptide 50	0.96432	0.5724	NA
99650	4933434E20Rik	RIKEN cDNA 4933434E20 gene	0.96432	0.7003	0.8486
100206	Adprhl2	ADP-ribosylhydrolase like 2	0.96432	0.6533	NA
101023	Zfp513	zinc finger protein 513	0.96432	0.421	NA
103836	Zfp692	zinc finger protein 692	0.96432	0.712	0.8553
107770	Tm6sf2	transmembrane 6 superfamily member 2	0.96432	0.7259	0.8639
107939	Pom121	nuclear pore membrane protein 121	0.96432	0.531	NA
108800	Ston2	stonin 2	0.96432	0.743	0.8748
171207	Arhgap4	Rho GTPase activating protein 4	0.96432	0.7029	0.8502
212167	Pion	pigeon homolog (Drosophila)	0.96432	0.6034	NA
217140	Scrn2	secernin 2	0.96432	0.6194	NA
218699	Pxk	PX domain containing serine/threonine kinase	0.96432	0.6637	NA
224829	Trerf1	transcriptional regulating factor 1	0.96432	0.7199	0.8599
225995	D030056L22Rik	RIKEN cDNA D030056L22 gene	0.96432	0.4513	NA
226971	Plekhb2	pleckstrin homology domain containing, family B (evectins) member 2	0.96432	0.7669	0.8875
231014	9330182L06Rik	RIKEN cDNA 9330182L06 gene	0.96432	0.5698	NA
231214	Cc2d2a	coiled-coil and C2 domain containing 2A	0.96432	0.7157	0.8577
233902	Fbxl19	F-box and leucine-rich repeat protein 19	0.96432	0.5798	NA
241627	Wdr76	WD repeat domain 76	0.96432	0.8766	0.9462
244373	Erlin2	ER lipid raft associated 2	0.96432	0.5828	NA
244885	Sh2d7	SH2 domain containing 7	0.96432	0.6646	NA
319391	D230014I24Rik	RIKEN cDNA D230014I24 gene	0.96432	0.7901	0.9007
319691	D430022A14Rik	RIKEN cDNA D430022A14 gene	0.96432	0.8743	0.9452
330721	Nek5	NIMA (never in mitosis gene a)-related expressed kinase 5	0.96432	0.9341	0.9728
442834	D830031N03Rik	RIKEN cDNA D830031N03 gene	0.96432	0.8371	0.927
541456	LOC541456	hypothetical LOC541456	0.96432	0.7053	0.8518
630579	Zfp808	zinc finger protein 80	0.96432	0.8535	0.9346
100303732	Gm14431	predicted gene 14431	0.96432	0.8719	0.9446
100504663	Atg14	VATG14 autophagy related 14 homolog (S. cerevisiae)	0.96432	0.4562	NA
11302	Aatk	apoptosis-associated tyrosine kinase	0.963391	0.5197	NA
11440	Chrna6	cholinergic receptor, nicotinic, alpha polypeptide 6	0.963391	0.5606	NA
11936	Fxyd2	FXYD domain-containing ion transport regulator 2	0.963391	0.7293	0.8661
12380	Cast	calpastatin	0.963391	0.6354	NA
12847	Copa	coatomer protein complex subunit alpha	0.963391	0.562	NA
13405	Dmd	dystrophin, muscular dystrophy	0.963391	0.8258	0.9211
14137	Fdft1	farnesyl diphosphate farnesyl transferase 1	0.963391	0.6466	NA
14588	Gfra4	glial cell line derived neurotrophic factor family receptor alpha 4	0.963391	0.7174	0.8587
15469	Prmt1	protein arginine N-methyltransferase 1	0.963391	0.6449	NA
15904	Id4	inhibitor of DNA binding 4	0.963391	0.6751	0.8334
16404	Itga7	integrin alpha 7	0.963391	0.5743	NA
17957	Napb	N-ethylmaleimide sensitive fusion protein attachment protein beta	0.963391	0.6408	NA

18087	Nktr	natural killer tumor recognition sequence	0.963391	0.5224	NA
18181	Nrf1	nuclear respiratory factor 1	0.963391	0.5397	NA
19896	Rpl10a	ribosomal protein L10A	0.963391	0.5837	NA
20930	Surf1	surfeit gene 1	0.963391	0.613	NA
20975	Synj2	synaptojanin 2	0.963391	0.8144	0.9146
21665	Tdg	thymine DNA glycosylase	0.963391	0.775	0.8918
22321	Vars	valyl-tRNA synthetase	0.963391	0.8268	0.9216
23936	Lynx1	Ly6/neurotoxin 1	0.963391	0.6781	0.8352
24047	Ccl19	chemokine (C-C motif) ligand 19	0.963391	0.8546	0.9352
28081	D11Wsu99e	DNA segment, Chr 11, Wayne State University 99, expressed	0.963391	0.6312	NA
30055	Timm13	translocase of inner mitochondrial membrane 13 homolog (yeast)	0.963391	0.539	NA
50794	Klf13	Kruppel-like factor 13	0.963391	0.7222	0.8611
58186	Rad18	RAD18 homolog (S. cerevisiae)	0.963391	0.6587	NA
66046	Ndufb5	NADH dehydrogenase (ubiquinone) 1 beta subcomplex, 5	0.963391	0.5071	NA
66078	Tsen34	tRNA splicing endonuclease 34 homolog (S. cerevisiae)	0.963391	0.5898	NA
66585	Snrnp40	small nuclear ribonucleoprotein 40 (U5)	0.963391	0.5511	NA
66596	Gtf3a	general transcription factor III A	0.963391	0.5431	NA
67003	Uqcrc2	ubiquinol cytochrome c reductase core protein 2	0.963391	0.3936	NA
67489	Ap4b1	adaptor-related protein complex AP-4, beta 1	0.963391	0.5799	NA
67671	Rpl38	ribosomal protein L38	0.963391	0.7067	0.8528
67738	Ppid	peptidylprolyl isomerase D (cyclophilin D)	0.963391	0.7575	0.8827
67755	Ddx47	DEAD (Asp-Glu-Ala-Asp) box polypeptide 47	0.963391	0.5831	NA
68722	1110029L17Rik	RIKEN cDNA 1110029L17 gene	0.963391	0.5969	NA
70715	6330405D24Rik	RIKEN cDNA 6330405D24 gene	0.963391	0.8296	0.9231
71146	Golga7b	golgi autoantigen, golgin subfamily a, 7B	0.963391	0.6898	0.8418
71679	Atp5h	ATP synthase, H+ transporting, mitochondrial F0 complex, subunit d	0.963391	0.5253	NA
71746	Rgl3	ral guanine nucleotide dissociation stimulator-like 3	0.963391	0.6746	0.8332
73694	2410091C18Rik	RIKEN cDNA 2410091C18 gene	0.963391	0.4786	NA
74192	Arpc5l	actin related protein 2/3 complex, subunit 5-like	0.963391	0.5154	NA
74204	Xpo6	exportin 6	0.963391	0.6942	0.8445
74303	1700109K24Rik	RIKEN cDNA 1700109K24 gene	0.963391	0.7635	0.8858
74552	Nipal3	NIPA-like domain containing 3	0.963391	0.5163	NA
74629	4930426L09Rik	RIKEN cDNA 4930426L09 gene	0.963391	0.7909	0.9009
74718	Snx16	sorting nexin 16	0.963391	0.3742	NA
75516	Ttc32	tetratricopeptide repeat domain 32	0.963391	0.6705	0.8313
78317	Ccdc88b	coiled-coil domain containing 88B	0.963391	0.6526	NA
79464	Lias	lipoic acid synthetase	0.963391	0.4537	NA
80284	BC003266	cDNA sequence BC003266	0.963391	0.5468	NA
81601	Kat5	K(lysine) acetyltransferase 5	0.963391	0.4897	NA
97230	C78228	expressed sequence C78228	0.963391	0.8411	0.9287
104156	Etv5	ets variant gene 5	0.963391	0.6883	0.8409
106529	Tecr	trans-2,3-enoyl-CoA reductase	0.963391	0.7398	0.8728
108888	Atad3a	ATPase family, AAA domain containing 3A	0.963391	0.4962	NA
114255	Dok4	docking protein 4	0.963391	0.5765	NA
223433	Fam105a	family with sequence similarity 105, member A	0.963391	0.791	0.9009
228960	Stx16	syntaxin 16	0.963391	0.4214	NA
234852	Chmp1a	chromatin modifying protein 1A	0.963391	0.3469	NA
235047	Zfp809	zinc finger protein 809	0.963391	0.7033	0.8504
235459	Gtf2a2	general transcription factor II A, 2	0.963391	0.5683	NA

245174	Zfp937	zinc finger protein 937	0.963391	0.8017	0.9069
245880	Wasf3	WAS protein family, member 3	0.963391	0.6842	0.8388
269633	Wdr86	WD repeat domain 86	0.963391	0.8891	0.952
278097	Armcx6	armadillo repeat containing, X-linked 6	0.963391	0.8333	0.9247
320328	B130024M06Rik	RIKEN cDNA B130024M06 gene	0.963391	0.7287	0.8656
383770	Gm11449	predicted gene 11449	0.963391	0.74	0.8729
384724	Cyp2t4	cytochrome P450, family 2, subfamily t, polypeptide 4	0.963391	0.8192	0.9175
399617	A130019P10Rik	RIKEN cDNA A130019P10 gene	0.963391	0.7252	0.8634
622208	Gm6297	predicted gene 6297	0.963391	0.6727	0.8323
639396	Gm7265	predicted gene 7265	0.963391	0.7037	0.8507
100043580	Gm4532	predicted gene 4532	0.963391	0.853	0.9343
12493	Cd37	CD37 antigen	0.962464	0.5783	NA
12890	Cplx2	complexin 2	0.962464	0.7517	0.8799
13544	Dvl3	dishevelled 3, dsh homolog (Drosophila)	0.962464	0.847	0.9317
13998	Fgd6	FYVE, RhoGEF and PH domain containing 6	0.962464	0.5952	NA
14211	Smc2	structural maintenance of chromosomes 2	0.962464	0.5847	NA
15353	Hmg20b	high mobility group 20 B	0.962464	0.4784	NA
18175	Nrap	nebulin-related anchoring protein	0.962464	0.73	0.8664
18347	Olfr48	olfactory receptor 48	0.962464	0.9213	0.968
19177	Psmb7	proteasome (prosome, macropain) subunit, beta type 7	0.962464	0.6597	0.8244
19401	Rara	retinoic acid receptor, alpha	0.962464	0.5811	NA
20532	Slc3a1	solute carrier family 3, member 1	0.962464	0.6447	NA
22644	Rnf103	ring finger protein 103	0.962464	0.5412	NA
24099	Tnfsf13b	tumor necrosis factor (ligand) superfamily, member 13b	0.962464	0.7417	0.874
26405	Map3k2	mitogen-activated protein kinase kinase kinase 2	0.962464	0.6758	0.8335
54357	Epb4.1l4b	erythrocyte protein band 4.1-like 4b	0.962464	0.6908	0.8424
55993	Msh4	mutS homolog 4 (E. coli)	0.962464	0.7732	0.891
56371	Fzr1	fizzy/cell division cycle 20 related 1 (Drosophila)	0.962464	0.6767	0.8343
56529	Sec11a	SEC11 homolog A (S. cerevisiae)	0.962464	0.6398	NA
57437	Golga7	golgi autoantigen, golgin subfamily a, 7	0.962464	0.4649	NA
64945	Cldn12	claudin 12	0.962464	0.4956	NA
67102	D16Ertd472e	DNA segment, Chr 16, ERATO Doi 472, expressed	0.962464	0.8243	0.9204
67434	Ankrd33b	ankyrin repeat domain 33B	0.962464	0.4755	NA
67574	Alg13	asparagine-linked glycosylation 13 homolog (S. cerevisiae)	0.962464	0.561	NA
67843	Slc35a4	solute carrier family 35, member A4	0.962464	0.6804	0.8364
68079	Pdcd2l	programmed cell death 2-like	0.962464	0.4063	NA
68198	Ndufb2	NADH dehydrogenase (ubiquinone) 1 beta subcomplex, 2	0.962464	0.6753	0.8334
68597	1110021J02Rik	RIKEN cDNA 1110021J02 gene	0.962464	0.7096	0.8542
68971	1500001M20Rik	RIKEN cDNA 1500001M20 gene	0.962464	0.5496	NA
68981	Snrpa1	small nuclear ribonucleoprotein polypeptide A'	0.962464	0.5416	NA
69399	1700025G04Rik	RIKEN cDNA 1700025G04 gene	0.962464	0.631	NA
69612	2310037I24Rik	RIKEN cDNA 2310037I24 gene	0.962464	0.5502	NA
69674	Mif4gd	MIF4G domain containing	0.962464	0.5974	NA
70155	Ogfrl1	opioid growth factor receptor-like 1	0.962464	0.6028	NA
71330	Rcbtb1	regulator of chromosome condensation (RCC1) and BTB (POZ) domain containing protein 1	0.962464	0.5265	NA
72320	2510003E04Rik	RIKEN cDNA 2510003E04 gene	0.962464	0.4867	NA
72865	Cxx1c	CAAX box 1 homolog C (human)	0.962464	0.711	0.8547
73634	1700125H20Rik	RIKEN cDNA 1700125H20 gene	0.962464	0.7596	0.8836
75770	Brsk2	BR serine/threonine kinase 2	0.962464	0.7353	0.8703

76524	Cln6	ceroid-lipofuscinosis, neuronal 6	0.962464	0.7027	0.8502
76974	1190003J15Rik	RIKEN cDNA 1190003J15 gene	0.962464	0.7937	0.9022
77717	6030408B16Rik	RIKEN cDNA 6030408B16 gene	0.962464	0.7483	0.8781
77929	Yipf6	Yip1 domain family, member 6	0.962464	0.6343	NA
80890	Trim2	tripartite motif-containing 2	0.962464	0.4038	NA
93841	Uchl4	ubiquitin carboxyl-terminal esterase L4	0.962464	0.6061	NA
106795	Tcf19	transcription factor 19	0.962464	0.6608	0.825
107029	Me2	malic enzyme 2, NAD(+)-dependent, mitochondrial	0.962464	0.5213	NA
170638	Hpcal4	hippocalcin-like 4	0.962464	0.72	0.86
192786	Rapgef6	Rap guanine nucleotide exchange factor (GEF) 6	0.962464	0.5356	NA
217715	Eif2b2	eukaryotic translation initiation factor 2B, subunit 2 beta	0.962464	0.4569	NA
218232	Ptpdc1	protein tyrosine phosphatase domain containing 1	0.962464	0.5749	NA
223918	Spryd3	SPRY domain containing 3	0.962464	0.4959	NA
227697	Dolk	dolichol kinase	0.962464	0.449	NA
230376	Haus6	HAUS augmin-like complex, subunit 6	0.962464	0.6513	NA
231866	Zfp12	zinc finger protein 12	0.962464	0.8261	0.9211
232236	C130022K22Rik	RIKEN cDNA C130022K22 gene	0.962464	0.4971	NA
269999	Orai3	ORAI calcium release-activated calcium modulator 3	0.962464	0.607	NA
320262	A830073O21Rik	RIKEN cDNA A830073O21 gene	0.962464	0.8583	0.937
320299	Iqcb1	IQ calmodulin-binding motif containing 1	0.962464	0.6842	0.8388
320560	Dennd5b	DENN/MADD domain containing 5B	0.962464	0.6752	0.8334
329628	Fat4	FAT tumor suppressor homolog 4 (Drosophila)	0.962464	0.5632	NA
338363	6030446N20Rik	RIKEN cDNA 6030446N20 gene	0.962464	0.5422	NA
667338	Gm8580	ribosomal protein L29 pseudogene	0.962464	0.7305	0.8668
100042014	Gm3625	predicted gene 3625	0.962464	0.8245	0.9204
11431	Acp1	acid phosphatase 1, soluble	0.961538	0.6495	0.8177
11480	Acvr2a	activin receptor IIA	0.961538	0.7964	0.9043
13000	Csnk2a2	casein kinase 2, alpha prime polypeptide	0.961538	0.5771	NA
14812	Grin2b	glutamate receptor, ionotropic, NMDA2B (epsilon 2)	0.961538	0.7996	0.9057
15441	Hp1bp3	heterochromatin protein 1, binding protein 3	0.961538	0.4543	NA
15571	Elavl3	ELAV (embryonic lethal, abnormal vision, Drosophila)-like 3 (Hu antigen C)	0.961538	0.4976	NA
20624	Eftud2	elongation factor Tu GTP binding domain containing 2	0.961538	0.5271	NA
22194	Ube2e1	ubiquitin-conjugating enzyme E2E 1, UBC4/5 homolog (yeast)	0.961538	0.6397	NA
22608	Ybx1	Y box protein 1	0.961538	0.5964	NA
24051	Sgcb	sarcoglycan, beta (dystrophin-associated glycoprotein)	0.961538	0.5701	NA
24075	Taf10	TAF10 RNA polymerase II, TATA box binding protein (TBP)-associated factor	0.961538	0.674	0.833
27056	Irf5	interferon regulatory factor 5	0.961538	0.7476	0.8775
27374	Prmt5	protein arginine N-methyltransferase 5	0.961538	0.5879	NA
52696	Zwint	ZW10 interactor	0.961538	0.6464	NA
60534	Fancg	Fanconi anemia, complementation group G	0.961538	0.7123	0.8553
64384	Sirt3	sirtuin 3 (silent mating type information regulation 2, homolog) 3 (S. cerevisiae)	0.961538	0.4066	NA
66052	Sdhc	succinate dehydrogenase complex, subunit C, integral membrane protein	0.961538	0.6162	NA
66125	Sf3b5	splicing factor 3b, subunit 5	0.961538	0.4649	NA
66152	Uqcr10	ubiquinol-cytochrome c reductase, complex III subunit X	0.961538	0.4128	NA
66441	Magohb	mago-nashi homolog B (Drosophila)	0.961538	0.5649	NA
67157	2610301B20Rik	RIKEN cDNA 2610301B20 gene	0.961538	0.769	0.8891
67338	Rffl	ring finger and FYVE like domain containing protein	0.961538	0.7284	0.8654
68229	AI846148	expressed sequence AI846148	0.961538	0.6199	NA
68291	Mto1	mitochondrial translation optimization 1 homolog (S. cerevisiae)	0.961538	0.469	NA

68316	Apoo	apolipoprotein O	0.961538	0.4375	NA
68479	Phf5a	PHD finger protein 5A	0.961538	0.5525	NA
69617	Pitrm1	pitrilysin metallepetidase 1	0.961538	0.5993	NA
69632	Arhgef12	Rho guanine nucleotide exchange factor (GEF) 12	0.961538	0.5543	NA
69938	Scrn1	secernin 1	0.961538	0.3722	NA
70233	Cd2bp2	CD2 antigen (cytoplasmic tail) binding protein 2	0.961538	0.4817	NA
71889	Epn3	epsin 3	0.961538	0.7404	0.8731
72795	Ttc19	tetratricopeptide repeat domain 19	0.961538	0.4602	NA
73845	Ankrd42	ankyrin repeat domain 42	0.961538	0.6573	0.8223
76582	Ipo11	importin 11	0.961538	0.419	NA
78100	8430410K20Rik	RIKEN cDNA 8430410K20 gene	0.961538	0.5555	NA
80291	Rilpl2	Rab interacting lysosomal protein-like 2	0.961538	0.5409	NA
81003	Trim23	tripartite motif-containing 23	0.961538	0.7471	0.8772
83925	Trps1	trichorhinophalangeal syndrome I (human)	0.961538	0.6548	0.8212
97827	Exd2	exonuclease 3'-5' domain containing 2	0.961538	0.5691	NA
104318	Csnk1d	casein kinase 1, delta	0.961538	0.3383	NA
107035	Fbxo38	F-box protein 38	0.961538	0.3445	NA
112406	Egln2	EGL nine homolog 2 (C. elegans)	0.961538	0.4624	NA
210162	Zkscan2	zinc finger with KRAB and SCAN domains 2	0.961538	0.7038	0.8508
214763	E330016A19Rik	RIKEN cDNA E330016A19 gene	0.961538	0.8299	0.9233
216131	Trappc10	trafficking protein particle complex 10	0.961538	0.7275	0.8649
216459	Myl6b	myosin, light polypeptide 6B	0.961538	0.6986	0.8476
217708	Lin52	lin-52 homolog (C. elegans)	0.961538	0.5389	NA
217874	BC048943	cDNA sequence BC048943	0.961538	0.5474	NA
233987	Zfp958	zinc finger protein 958	0.961538	0.7675	0.8879
234624	A330008L17Rik	RIKEN cDNA A330008L17 gene	0.961538	0.6689	0.83
235283	Gramd1b	GRAM domain containing 1B	0.961538	0.7561	0.8822
239134	Gucy1b2	guanylate cyclase 1, soluble, beta 2	0.961538	0.908	0.9614
242584	Wdr78	WD repeat domain 78	0.961538	0.7502	0.8789
244666	Gm505	predicted gene 505	0.961538	0.6916	0.843
269639	Zfp512	zinc finger protein 512	0.961538	0.5897	NA
381236	Lipo1	lipase, member O1	0.961538	0.6628	0.8266
432768	Gm5451	predicted gene 5451	0.961538	0.6312	NA
640636	Gm7303	predicted gene 7303	0.961538	0.7619	0.8847
100042480	Nhsl2	NHS-like 2	0.961538	0.8331	0.9247
100043316	Gm4354	predicted gene 4354	0.961538	0.8009	0.9064
100044171	LOC100044171	hypothetical LOC100044171	0.961538	0.6827	0.838
11810	Apobec1	apolipoprotein B mRNA editing enzyme, catalytic polypeptide 1	0.960615	0.8378	0.927
11908	Atf1	activating transcription factor 1	0.960615	0.6788	0.8357
11908	Atf1	activating transcription factor 1	0.960615	0.4939	NA
12297	Cacnb3	calcium channel, voltage-dependent, beta 3 subunit	0.960615	0.7254	0.8636
14017	Evi2a	ecotropic viral integration site 2a	0.960615	0.7645	0.8864
15166	Hcn2	hyperpolarization-activated, cyclic nucleotide-gated K+ 2	0.960615	0.7173	0.8586
16485	Kcna1	potassium voltage-gated channel, shaker-related subfamily, member 1	0.960615	0.5974	NA
16815	Lbx2	ladybird homeobox homolog 2 (Drosophila)	0.960615	0.71	0.8543
18806	Pld2	phospholipase D2	0.960615	0.4716	NA
19044	Ppox	protoporphyrinogen oxidase	0.960615	0.7498	0.8787
19173	Psmb5	proteasome (prosome, macropain) subunit, beta type 5	0.960615	0.736	0.8707
19227	Pthlh	parathyroid hormone-like peptide	0.960615	0.8093	0.9115

21754	Tesk1	testis specific protein kinase 1	0.960615	0.4417	NA
22068	Trpc6	transient receptor potential cation channel, subfamily C, member 6	0.960615	0.7013	0.8494
23856	Dido1	death inducer-obliterator 1	0.960615	0.8333	0.9247
26414	Mapk10	mitogen-activated protein kinase 10	0.960615	0.615	NA
26440	Psma1	proteasome (prosome, macropain) subunit, alpha type 1	0.960615	0.471	NA
26893	Cops6	COP9 (constitutive photomorphogenic) homolog, subunit 6 (Arabidopsis thaliana)	0.960615	0.5765	NA
51812	Mcrs1	microspherule protein 1	0.960615	0.6022	NA
52704	D11Ertd717e	DNA segment, Chr 11, ERATO Doi 717, expressed	0.960615	0.6957	0.8456
53416	Stk39	serine/threonine kinase 39, STE20/SPS1 homolog (yeast)	0.960615	0.5122	NA
56747	Sez6l	seizure related 6 homolog like	0.960615	0.619	NA
57436	Gabarapl1	gamma-aminobutyric acid (GABA) A receptor-associated protein-like 1	0.960615	0.6122	NA
64657	Mrps10	mitochondrial ribosomal protein S10	0.960615	0.6314	NA
64704	Htra2	HtrA serine peptidase 2	0.960615	0.5181	NA
65113	Ndfip1	Nedd4 family interacting protein 1	0.960615	0.5995	NA
66053	Ppil2	peptidylprolyl isomerase (cyclophilin)-like 2	0.960615	0.61	NA
66151	Prr13	proline rich 13	0.960615	0.4184	NA
66164	Nip7	nuclear import 7 homolog (S. cerevisiae)	0.960615	0.6898	0.8418
66496	Ppdpf	pancreatic progenitor cell differentiation and proliferation factor homolog (zebrafish)RIKEN cDNA 2700038C09 gene	0.960615	0.5694	NA
66932	Rexo1	REX1, RNA exonuclease 1 homolog (S. cerevisiae)	0.960615	0.8148	0.9149
66989	Kctd20	potassium channel tetramerisation domain containing 20	0.960615	0.4862	NA
67225	Rnpc3	RNA-binding region (RNP1, RRM) containing 3	0.960615	0.7343	0.8694
67442	Retsat	retinol saturase (all trans retinol 13,14 reductase)	0.960615	0.666	0.8286
69572	Mfsd3	major facilitator superfamily domain containing 3	0.960615	0.5338	NA
69690	2310057B04Rik	RIKEN cDNA 2310057B04 gene	0.960615	0.6077	NA
69718	Ipmk	inositol polyphosphate multikinase	0.960615	0.4217	NA
72290	Lsm11	U7 snRNP-specific Sm-like protein LSM11	0.960615	0.5757	NA
73284	Ddit4l	DNA-damage-inducible transcript 4-like	0.960615	0.6265	NA
74427	Eaf1	ELL associated factor 1	0.960615	0.6022	NA
76709	Arpc2	actin related protein 2/3 complex, subunit 2	0.960615	0.5655	NA
78279	5330421C15Rik	RIKEN cDNA 5330421C15 gene	0.960615	0.828	0.9222
78668	E130112N10Rik	RIKEN cDNA E130112N10 gene	0.960615	0.7491	0.8784
83486	Rbm5	RNA binding motif protein 5	0.960615	0.4827	NA
98258	Txndc9	thioredoxin domain containing 9	0.960615	0.543	NA
100662	D930016D06Rik	RIKEN cDNA D930016D06 gene	0.960615	0.6088	NA
104771	Jkamp	JNK1/MAPK8-associated membrane protein	0.960615	0.3575	NA
105638	Dph3	DPH3 homolog (KTI11, S. cerevisiae)	0.960615	0.6878	0.8407
108841	Rdh13	retinol dehydrogenase 13 (all-trans and 9-cis)	0.960615	0.5924	NA
109359	Fam175b	family with sequence similarity 175, member B	0.960615	0.5448	NA
114715	Spred1	sprouty protein with EVH-1 domain 1, related sequence	0.960615	0.5478	NA
171212	Galnt10	UDP-N-acetyl-alpha-D-galactosamine:polypeptide N-acetylgalactosaminyltransferase 10	0.960615	0.6253	NA
192292	Nrbp1	nuclear receptor binding protein 1	0.960615	0.5375	NA
207227	Stxbp5l	syntaxin binding protein 5-like	0.960615	0.7506	0.8792
208768	BC031781	cDNA sequence BC031781	0.960615	0.6163	NA
209224	Enox2	ecto-NOX disulfide-thiol exchanger 2	0.960615	0.6173	NA
211446	Exoc3	exocyst complex component 3	0.960615	0.4717	NA
216881	Wscd1	WSC domain containing 1	0.960615	0.8036	0.9079
217379	Ubxn2a	UBX domain protein 2A	0.960615	0.6423	0.8133
224903	Safb	scaffold attachment factor B	0.960615	0.5545	NA
226251	Ablim1	actin-binding LIM protein 1	0.960615	0.5753	NA

227731	Slc25a25	solute carrier family 25 (mitochondrial carrier, phosphate carrier), member 25	0.960615	0.7112	0.8549
232970	Phldb3	pleckstrin homology-like domain, family B, member 3	0.960615	0.6828	0.838
234023	Arglu1	arginine and glutamate rich 1	0.960615	0.6005	NA
234159	Gm4889	predicted gene 4889	0.960615	0.4615	NA
237716	Gpr75	G protein-coupled receptor 75	0.960615	0.8245	0.9204
277939	C2cd3	C2 calcium-dependent domain containing 3	0.960615	0.8185	0.9173
320333	D830030K20Rik	RIKEN cDNA D830030K20 gene	0.960615	0.7919	0.9014
11569	Aebp2	AE binding protein 2	0.959693	0.54	NA
12055	Bcl7c	B-cell CLL/lymphoma 7C	0.959693	0.6296	NA
12647	Chat	choline acetyltransferase	0.959693	0.6801	0.8363
13383	Dlg1	discs, large homolog 1 (Drosophila)	0.959693	0.7722	0.8906
14219	Ctgf	connective tissue growth factor	0.959693	0.8363	0.9265
17827	Mtx1	metaxin 1	0.959693	0.5479	NA
17937	Nab2	Ngfi-A binding protein 2	0.959693	0.6573	0.8223
18099	Nlk	nemo like kinase	0.959693	0.3712	NA
18751	Prkcb	protein kinase C, beta	0.959693	0.5534	NA
19185	Psmd4	proteasome (prosome, macropain) 26S subunit, non-ATPase, 4	0.959693	0.5468	NA
19376	Rab34	RAB34, member of RAS oncogene family	0.959693	0.4586	NA
20383	Srsf3	serine/arginine-rich splicing factor 3	0.959693	0.6121	NA
22038	Plscr1	phospholipid scramblase 1	0.959693	0.6367	0.8091
22591	Xpc	xeroderma pigmentosum, complementation group C	0.959693	0.381	NA
23959	Nt5e	5' nucleotidase, ecto	0.959693	0.5286	NA
23964	Odz2	odd Oz/ten-m homolog 2 (Drosophila)	0.959693	0.5199	NA
30059	Timm10	translocase of inner mitochondrial membrane 10 homolog (yeast)	0.959693	0.5052	NA
30933	Tor2a	torsin family 2, member A	0.959693	0.4307	NA
52830	Pnrc2	proline-rich nuclear receptor coactivator 2	0.959693	0.4015	NA
54525	Syt7	synaptotagmin VII	0.959693	0.8377	0.927
54723	Tfip11	tuftelin interacting protein 11	0.959693	0.6394	0.8111
55992	Trim3	tripartite motif-containing 3	0.959693	0.7466	0.877
56322	Timm22	translocase of inner mitochondrial membrane 22 homolog (yeast)	0.959693	0.3637	NA
56787	Ascl3	achaete-scute complex homolog 3 (Drosophila)	0.959693	0.7033	0.8504
59093	Pcbp3	poly(rC) binding protein 3	0.959693	0.541	NA
65962	Slc9a3r2	solute carrier family 9 (sodium/hydrogen exchanger), member 3 regulator 2	0.959693	0.7263	0.8641
66593	Diablo	diablo homolog (Drosophila)	0.959693	0.4787	NA
66971	Cdk5rap1	CDK5 regulatory subunit associated protein 1	0.959693	0.7629	0.8854
67225	Rnpc3	RNA-binding region (RNP1, RRM) containing 3	0.959693	0.8359	0.9262
67530	Uqcrb	ubiquinol-cytochrome c reductase binding protein	0.959693	0.4329	NA
68292	Stt3b	STT3, subunit of the oligosaccharyltransferase complex, homolog B (S. cerevisiae)	0.959693	0.428	NA
68401	G6pc3	glucose 6 phosphatase, catalytic, 3	0.959693	0.5924	NA
69554	Klhdc2	kelch domain containing 2	0.959693	0.6551	0.8212
70230	3300002P13Rik	RIKEN cDNA 3300002P13 gene	0.959693	0.7537	0.8812
70779	Prdm5	PR domain containing 5	0.959693	0.4746	NA
71986	Ddx28	DEAD (Asp-Glu-Ala-Asp) box polypeptide 28	0.959693	0.6975	0.8468
72612	2700029M09Rik	RIKEN cDNA 2700029M09 gene	0.959693	0.5616	NA
74256	Cyld	cylindromatosis (turban tumor syndrome)	0.959693	0.5457	NA
74600	Mrpl47	mitochondrial ribosomal protein L47	0.959693	0.7691	0.8891
75744	Svip	small VCP/p97-interacting protein	0.959693	0.4122	NA
76688	Arfrp1	ADP-ribosylation factor related protein 1	0.959693	0.5696	NA
78541	Asb8	ankyrin repeat and SOCS box-containing 8	0.959693	0.574	NA

78651	Lsm6	LSM6 homolog, U6 small nuclear RNA associated (S. cerevisiae)	0.959693	0.3398	NA
97820	4833439L19Rik	RIKEN cDNA 4833439L19 gene	0.959693	0.4136	NA
100087	Kti12	KTI12 homolog, chromatin associated (S. cerevisiae)	0.959693	0.527	NA
107227	Macrod1	MACRO domain containing 1	0.959693	0.6414	0.8126
112403	Dom3z	DOM-3 homolog Z (C. elegans)	0.959693	0.6148	NA
192170	Eif4a3	eukaryotic translation initiation factor 4A3	0.959693	0.7212	0.8603
213006	Mfsd4	major facilitator superfamily domain containing 4	0.959693	0.4865	NA
217692	Sipa1l1	signal-induced proliferation-associated 1 like 1	0.959693	0.4971	NA
228829	Phf20	PHD finger protein 20	0.959693	0.3794	NA
228994	Ythdf1	YTH domain family 1	0.959693	0.45	NA
229503	BC023814	cDNA sequence BC023814	0.959693	0.6129	NA
231637	Ssh1	slingshot homolog 1 (Drosophila)	0.959693	0.6897	0.8417
231861	Tnrc18	trinucleotide repeat containing 18	0.959693	0.5381	NA
236537	Zfp352	zinc finger protein 352	0.959693	0.5143	NA
320808	Dcaf5	DDB1 and CUL4 associated factor 5	0.959693	0.8321	0.9245
381305	Rc3h1	RING CCCH (C3H) domains 1	0.959693	0.5149	NA
404634	H2afy2	H2A histone family, member Y2	0.959693	0.4337	NA
433287	Gm15455	predicted gene 15455	0.959693	0.5683	NA
677113	LOC677113	40S ribosomal protein S24-like	0.959693	0.8997	0.957
100042720	Gm3988	predicted gene 3988	0.959693	0.3711	NA
11479	Acvr1b	activin A receptor, type 1B	0.958773	0.8243	0.9204
13537	Dusp2	dual specificity phosphatase 2	0.958773	0.7578	0.8829
14260	Fmn1	formin 1	0.958773	0.8148	0.9149
14894	Gtl3	gene trap locus 3	0.958773	0.4291	NA
16660	Krt31	keratin 31	0.958773	0.7792	0.8935
17169	Mark3	MAP/microtubule affinity-regulating kinase 3	0.958773	0.3546	NA
18029	Nfic	nuclear factor I/C	0.958773	0.6503	0.8183
18194	Nsdhl	NAD(P) dependent steroid dehydrogenase-like	0.958773	0.6682	0.8296
18744	Pja1	praja1, RING-H2 motif containing	0.958773	0.3726	NA
18769	Pkig	protein kinase inhibitor, gamma	0.958773	0.6764	0.834
19735	Rgs2	regulator of G-protein signaling 2	0.958773	0.552	NA
19822	Rnf4	ring finger protein 4	0.958773	0.4983	NA
20616	Snap91	synaptosomal-associated protein 91	0.958773	0.5774	NA
21975	Top3a	topoisomerase (DNA) III alpha	0.958773	0.631	0.8057
22040	Trex1	three prime repair exonuclease 1	0.958773	0.5404	NA
23945	Mgll	monoglyceride lipase	0.958773	0.6749	0.8334
24127	Xrn1	5'-3' exoribonuclease 1	0.958773	0.5839	NA
28042	Ept1	ethanolaminephosphotransferase 1 (CDP-ethanolamine-specific)	0.958773	0.3668	NA
50912	Exosc10	exosome component 10	0.958773	0.5037	NA
52882	Rgs7bp	regulator of G-protein signalling 7 binding protein	0.958773	0.7359	0.8707
54367	Zfp326	zinc finger protein 326	0.958773	0.6141	NA
56310	Gps2	G protein pathway suppressor 2	0.958773	0.6056	NA
66397	Sar1b	SAR1 gene homolog B (S. cerevisiae)	0.958773	0.6103	NA
66725	Lrrk2	leucine-rich repeat kinase 2	0.958773	0.7334	0.8688
67493	Mett10d	methyltransferase 10 domain containing	0.958773	0.5724	NA
69697	2310057J16Rik	RIKEN cDNA 2310057J16 gene	0.958773	0.7061	0.8525
71099	Tssk4	testis-specific serine kinase 4	0.958773	0.7225	0.8612
72141	Adpgk	ADP-dependent glucokinase	0.958773	0.5309	NA
72148	2610019F03Rik	RIKEN cDNA 2610019F03 gene	0.958773	0.5945	NA

72155	Cenpn	centromere protein N	0.958773	0.6546	0.8212
73447	Wdr13	WD repeat domain 13	0.958773	0.5257	NA
74412	Gle1	GLE1 RNA export mediator (yeast)	0.958773	0.605	NA
76829	Dok5	docking protein 5	0.958773	0.7724	0.8906
98314	D2hgdh	D-2-hydroxyglutarate dehydrogenase	0.958773	0.7122	0.8553
102857	Slc6a8	solute carrier family 6 (neurotransmitter transporter, creatine), member 8	0.958773	0.4051	NA
107435	Hat1	histone aminotransferase 1	0.958773	0.6986	0.8476
107701	Sf3b4	splicing factor 3b, subunit 4	0.958773	0.7135	0.8562
170460	Stard5	StAR-related lipid transfer (START) domain containing 5	0.958773	0.7839	0.8964
213575	Dync2li1	dynein cytoplasmic 2 light intermediate chain 1	0.958773	0.6722	0.8322
215243	Traf3ip3	TRAF3 interacting protein 3	0.958773	0.591	NA
216805	Flcn	folliculin	0.958773	0.4314	NA
224132	Dirc2	disrupted in renal carcinoma 2 (human)	0.958773	0.6678	0.8296
224139	Golgb1	golgi autoantigen, golgin subfamily b, macrogolgin 1	0.958773	0.4829	NA
226144	Erlin1	ER lipid raft associated 1	0.958773	0.5171	NA
235330	Ttc12	tetratricopeptide repeat domain 12	0.958773	0.7292	0.8661
238266	Syt16	synaptotagmin XVI	0.958773	0.5106	NA
245670	Rragb	Ras-related GTP binding B	0.958773	0.6973	0.8467
268721	2310021P13Rik	RIKEN cDNA 2310021P13 gene	0.958773	0.6318	0.806
269113	Nup54	nucleoporin 54	0.958773	0.3877	NA
269881	Map3k10	mitogen-activated protein kinase kinase kinase 10	0.958773	0.6787	0.8357
319734	Cacna2d4	calcium channel, voltage-dependent, alpha 2/delta subunit 4	0.958773	0.572	NA
327956	Vmo1	vitelline membrane outer layer 1 homolog (chicken)	0.958773	0.655	0.8212
330217	Gal3st4	galactose-3-O-sulfotransferase 4	0.958773	0.8266	0.9214
381823	Apold1	apolipoprotein L domain containing 1	0.958773	0.5584	NA
384569	Nova2	neuro-oncological ventral antigen 2	0.958773	0.7571	0.8825
414072	BC031361	cDNA sequence BC031361	0.958773	0.4379	NA
435616	Gm5699	predicted gene 5699	0.958773	0.7068	0.8528
791318	Gm10125	predicted gene 10125	0.958773	0.6827	0.838
12345	Capzb	capping protein (actin filament) muscle Z-line, beta	0.957854	0.5978	NA
12450	Ccng1	cyclin G1	0.957854	0.4574	NA
13800	Enah	enabled homolog (Drosophila)	0.957854	0.5449	NA
14694	Gnb2l1	guanine nucleotide binding protein (G protein), beta polypeptide 2 like 1	0.957854	0.7024	0.85
14706	Gng4	guanine nucleotide binding protein (G protein), gamma 4	0.957854	0.6671	0.8292
14773	Grk5	G protein-coupled receptor kinase 5	0.957854	0.4432	NA
15361	Hmga1	high mobility group AT-hook 1	0.957854	0.6936	0.8442
16661	Krt10	keratin 10	0.957854	0.784	0.8964
17203	Mc5r	melanocortin 5 receptor	0.957854	0.8642	0.9405
18189	Nrxn1	neurexin I	0.957854	0.6089	NA
20530	Slc31a2	solute carrier family 31, member 2	0.957854	0.6684	0.8296
21685	Tef	thyrotroph embryonic factor	0.957854	0.5562	NA
21929	Tnfaip3	tumor necrosis factor, alpha-induced protein 3	0.957854	0.528	NA
22019	Tpp2	tripeptidyl peptidase II	0.957854	0.6929	0.8439
22193	Ube2e3	ubiquitin-conjugating enzyme E2E 3, UBC4/5 homolog (yeast)	0.957854	0.4855	NA
22196	Ube2i	ubiquitin-conjugating enzyme E2I	0.957854	0.4329	NA
28022	D5Wsu152e	DNA segment, Chr 5, Wayne State University 152, expressed	0.957854	0.8561	0.9361
54624	Paf1	Paf1, RNA polymerase II associated factor, homolog (S. cerevisiae)	0.957854	0.4158	NA
56041	Uso1	USO1 homolog, vesicle docking protein (yeast)	0.957854	0.3882	NA
65106	Arl6ip5	ADP-ribosylation factor-like 6 interacting protein 5	0.957854	0.4618	NA

66116	Cml1	camello-like 1	0.957854	0.5477	NA
66591	Mad2l1bp	MAD2L1 binding protein	0.957854	0.366	NA
67017	2010011I20Rik	RIKEN cDNA 2010011I20 gene	0.957854	0.3512	NA
67224	Med29	mediator complex subunit 29	0.957854	0.465	NA
67292	Pigc	phosphatidylinositol glycan anchor biosynthesis, class C	0.957854	0.6823	0.8378
67568	Mrfap1	Morf4 family associated protein 1	0.957854	0.5401	NA
67609	4930453N24Rik	RIKEN cDNA 4930453N24 gene	0.957854	0.5054	NA
67836	Wdr83	WD repeat domain containing 83	0.957854	0.6696	0.8305
67952	Tomm20	translocase of outer mitochondrial membrane 20 homolog (yeast)	0.957854	0.5576	NA
68316	Apoo	apolipoprotein O	0.957854	0.5577	NA
68379	Ciz1	CDKN1A interacting zinc finger protein 1	0.957854	0.4833	NA
68628	Fbxw9	F-box and WD-40 domain protein 9	0.957854	0.6335	0.8073
69159	Rhebl1	Ras homolog enriched in brain like 1	0.957854	0.3819	NA
69276	Sec62	SEC62 homolog (S. cerevisiae)	0.957854	0.3875	NA
69431	1700022N22Rik	RIKEN cDNA 1700022N22 gene	0.957854	0.6898	0.8418
69726	Smyd3	SET and MYND domain containing 3	0.957854	0.5603	NA
69871	2010007H12Rik	RIKEN cDNA 2010007H12 gene	0.957854	0.6491	0.8174
69882	2010321M09Rik	RIKEN cDNA 2010321M09 gene	0.957854	0.4935	NA
70316	Ndufab1	NADH dehydrogenase (ubiquinone) 1, alpha/beta subcomplex, 1	0.957854	0.6026	NA
72026	Trmu	tRNA 5-methylaminomethyl-2-thiouridylate methyltransferase	0.957854	0.4695	NA
72399	Brap	BRCA1 associated protein	0.957854	0.3468	NA
72736	Tmx1	thioredoxin-related transmembrane protein 1	0.957854	0.5833	NA
80517	Herpud2	HERPUD family member 2	0.957854	0.362	NA
104896	AI852580	expressed sequence AI852580	0.957854	0.6802	0.8363
108903	Tbcd	tubulin-specific chaperone d	0.957854	0.5858	NA
118451	Mrps2	mitochondrial ribosomal protein S2	0.957854	0.3791	NA
170755	Sgk3	serum/glucocorticoid regulated kinase 3	0.957854	0.6512	0.8188
193813	Mcfd2	multiple coagulation factor deficiency 2	0.957854	0.4182	NA
216558	Ugp2	UDP-glucose pyrophosphorylase 2	0.957854	0.4281	NA
216618	Ccdc104	coiled-coil domain containing 104	0.957854	0.4636	NA
229211	Acad9	acyl-Coenzyme A dehydrogenase family, member 9	0.957854	0.3958	NA
229227	4932438A13Rik	RIKEN cDNA 4932438A13 gene	0.957854	0.6833	0.8384
231571	Rpap2	RNA polymerase II associated protein 2	0.957854	0.5249	NA
233802	Thumpd1	THUMP domain containing 1	0.957854	0.3541	NA
236904	Klhl15	kelch-like 15 (Drosophila)	0.957854	0.8579	0.9368
238247	Arid4a	AT rich interactive domain 4A (RBP1-like)	0.957854	0.7778	0.893
269514	Fbxl4	F-box and leucine-rich repeat protein 4	0.957854	0.4907	NA
384763	Zfp667	zinc finger protein 667	0.957854	0.7171	0.8585
386649	Nsfl1c	NSFL1 (p97) cofactor (p47)	0.957854	0.3972	NA
435965	Lrp3	low density lipoprotein receptor-related protein 3	0.957854	0.8046	0.9084
654470	Tctn1	tectonic family member 1	0.957854	0.6894	0.8415
668030	OTTMUSG00000016609	predicted gene, OTTMUSG00000016609	0.957854	0.7881	0.8994
100042332	2810410L24Rik	RIKEN cDNA 2810410L24 gene	0.957854	0.3985	NA
100502698	1700021K19Rik	RIKEN cDNA 1700021K19 gene	0.957854	0.4843	NA
12160	Bmp5	bone morphogenetic protein 5	0.956938	0.5036	NA
12226	Btg1	B-cell translocation gene 1, anti-proliferative	0.956938	0.6165	0.7965
12282	Hyou1	hypoxia up-regulated 1	0.956938	0.6876	0.8407
12537	Cdk11b	cyclin-dependent kinase 11B	0.956938	0.4127	NA
12892	Cpox	coproporphyrinogen oxidase	0.956938	0.4952	NA

13548	Dyrk1a	dual-specificity tyrosine-(Y)-phosphorylation regulated kinase 1a	0.956938	0.663	0.8266
13714	Elk4	ELK4, member of ETS oncogene family	0.956938	0.6001	NA
17169	Mark3	MAP/microtubule affinity-regulating kinase 3	0.956938	0.4116	NA
17532	Mras	muscle and microspikes RAS	0.956938	0.7384	0.8719
18555	Cdk16	cyclin-dependent kinase 16	0.956938	0.7439	0.8756
19157	Cyth1	cytohesin 1	0.956938	0.4938	NA
19775	Xpr1	xenotropic and polytropic retrovirus receptor 1	0.956938	0.7109	0.8547
20469	Sipa1	signal-induced proliferation associated gene 1	0.956938	0.343	NA
20683	Sp1	trans-acting transcription factor 1	0.956938	0.3927	NA
21853	Timeless	timeless homolog (Drosophila)	0.956938	0.6905	0.8421
22003	Tpm1	tropomyosin 1, alpha	0.956938	0.4751	NA
22247	Umps	uridine monophosphate synthetase	0.956938	0.4911	NA
22709	Zfp51	zinc finger protein 51	0.956938	0.4287	NA
28015	Grinl1a	glutamate receptor, ionotropic, N-methyl D-aspartate-like 1A	0.956938	0.3599	NA
53599	Cd164	CD164 antigen	0.956938	0.4007	NA
54169	Myst4	MYST histone acetyltransferase monocytic leukemia 4	0.956938	0.7234	0.8619
56294	Ptpn9	protein tyrosine phosphatase, non-receptor type 9	0.956938	0.6722	0.8322
56386	B4galt6	UDP-Gal:betaGlcNAc beta 1,4-galactosyltransferase, polypeptide 6	0.956938	0.5485	NA
57754	Cend1	cell cycle exit and neuronal differentiation 1	0.956938	0.361	NA
57912	Cdc42se1	CDC42 small effector 1	0.956938	0.7206	0.8602
65961	Utp3	UTP3, small subunit (SSU) processome component, homolog (S. cerevisiae)	0.956938	0.4213	NA
66177	Ubl5	ubiquitin-like 5	0.956938	0.4416	NA
67936	Wdr55	WD repeat domain 55	0.956938	0.4709	NA
69786	Tprkb	Tp53rk binding protein	0.956938	0.456	NA
69956	Ptcd3	pentatricopeptide repeat domain 3	0.956938	0.425	NA
70575	Gfod2	glucose-fructose oxidoreductase domain containing 2	0.956938	0.502	NA
71745	Cul2	cullin 2	0.956938	0.4958	NA
73490	Mipol1	mirror-image polydactyly gene 1 homolog (human)	0.956938	0.8201	0.918
73886	4930431P19Rik	RIKEN cDNA 4930431P19 gene	0.956938	0.543	NA
75216	4930534B04Rik	RIKEN cDNA 4930534B04 gene	0.956938	0.6376	0.8097
76260	Ttc8	tetratricopeptide repeat domain 8	0.956938	0.5903	NA
76897	Ralyl	RALY RNA binding protein-like	0.956938	0.674	0.833
77068	5930436O19Rik	RIKEN cDNA 5930436O19 gene	0.956938	0.5424	NA
77480	Kidins220	kinase D-interacting substrate 220	0.956938	0.7321	0.8678
77590	Chst15	carbohydrate (N-acetylgalactosamine 4-sulfate 6-O) sulfotransferase 15	0.956938	0.6056	NA
77987	Ascc3	activating signal cointegrator 1 complex subunit 3	0.956938	0.4208	NA
94245	Dtnbp1	dystrobrevin binding protein 1	0.956938	0.5629	NA
100177	Zmym6	zinc finger, MYM-type 6	0.956938	0.3637	NA
105193	Nhlrc1	NHL repeat containing 1	0.956938	0.7112	0.8549
107094	Rrp12	ribosomal RNA processing 12 homolog (S. cerevisiae)	0.956938	0.8119	0.9129
223186	Gm4822	predicted pseudogene 4822	0.956938	0.6857	0.8398
223739	5031439G07Rik	RIKEN cDNA 5031439G07 gene	0.956938	0.6851	0.8394
224023	Klhl22	kelch-like 22 (Drosophila)	0.956938	0.4285	NA
224598	Zfp758	zinc finger protein 758	0.956938	0.807	0.91
233410	Zfp592	zinc finger protein 592	0.956938	0.4338	NA
234959	Med17	mediator complex subunit 17	0.956938	0.5603	NA
240332	Slc6a7	solute carrier family 6 (neurotransmitter transporter, L-proline), member 7	0.956938	0.4836	NA
277343	Wfdc8	WAP four-disulfide core domain 8	0.956938	0.8248	0.9206
328825	Gm5093	predicted gene 5093	0.956938	0.8322	0.9245

329934	Foxo6	forkhead box O6	0.956938	0.6904	0.8421
332993	Gm5129	predicted gene 5129	0.956938	0.7294	0.8661
407818	BC055308	cDNA sequence BC055308	0.956938	0.5832	NA
433938	Mn1	meningioma 1	0.956938	0.4836	NA
627214	Fam196a	family with sequence similarity 196, member A	0.956938	0.5916	NA
654409	4932416H05Rik	RIKEN cDNA 4932416H05 gene	0.956938	0.6179	0.7973
11477	Acvr1	activin A receptor, type 1	0.956023	0.5827	NA
11839	Areg	amphiregulin	0.956023	0.7256	0.8636
13424	Dync1h1	dynein cytoplasmic 1 heavy chain 1	0.956023	0.5004	NA
13667	Eif2b4	eukaryotic translation initiation factor 2B, subunit 4 delta	0.956023	0.5346	NA
14235	Foxm1	forkhead box M1	0.956023	0.79	0.9007
19175	Psmb6	proteasome (prosome, macropain) subunit, beta type 6	0.956023	0.4054	NA
19181	Psmc2	proteasome (prosome, macropain) 26S subunit, ATPase 2	0.956023	0.4364	NA
19664	Rbpj	recombination signal binding protein for immunoglobulin kappa J region	0.956023	0.7382	0.8718
19682	Rdh5	retinol dehydrogenase 5	0.956023	0.8322	0.9245
22221	Ubp1	upstream binding protein 1	0.956023	0.7353	0.8703
22258	Usp4	ubiquitin specific peptidase 4 (proto-oncogene)	0.956023	0.4433	NA
27060	Tcirg1	T-cell, immune regulator 1, ATPase, H+ transporting, lysosomal V0 protein A3	0.956023	0.6306	0.8054
27355	X99384	cDNA sequence X99384	0.956023	0.4798	NA
27364	Srr	serine racemase	0.956023	0.4839	NA
50768	Dlc1	deleted in liver cancer 1	0.956023	0.6327	0.8067
51897	Atg13	ATG13 autophagy related 13 homolog (S. cerevisiae)	0.956023	0.4504	NA
56208	Becn1	beclin 1, autophagy related	0.956023	0.522	NA
59016	Thap11	THAP domain containing 11	0.956023	0.4259	NA
60596	Gucy1a3	guanylate cyclase 1, soluble, alpha 3	0.956023	0.3799	NA
66597	Trim13	tripartite motif-containing 13	0.956023	0.5716	NA
66711	Sbds	Shwachman-Bodian-Diamond syndrome homolog (human)	0.956023	0.4263	NA
66878	Riok3	RIO kinase 3 (yeast)	0.956023	0.3474	NA
67874	Rprm	reprimo, TP53 dependent G2 arrest mediator candidate	0.956023	0.5648	NA
67949	Mki67ip	Mki67 (FHA domain) interacting nucleolar phosphoprotein	0.956023	0.3635	NA
68815	Btbd10	BTB (POZ) domain containing 10	0.956023	0.359	NA
69048	Slc30a5	solute carrier family 30 (zinc transporter), member 5	0.956023	0.4113	NA
69962	2810422O20Rik	RIKEN cDNA 2810422O20 gene	0.956023	0.6056	0.7887
70425	Csnk1g3	casein kinase 1, gamma 3	0.956023	0.5083	NA
70656	5730526G10Rik	RIKEN cDNA 5730526G10 gene	0.956023	0.6739	0.833
70853	Vwa3b	von Willebrand factor A domain containing 3B	0.956023	0.7853	0.8973
71832	Csl	citrate synthase like	0.956023	0.4554	NA
72017	Cyb5r1	cytochrome b5 reductase 1	0.956023	0.5886	NA
72774	Neil1	nei endonuclease VIII-like 1 (E. coli)	0.956023	0.6392	0.811
74123	Foxp4	forkhead box P4	0.956023	0.6018	0.7863
74915	Atp6v1e2	ATPase, H+ transporting, lysosomal V1 subunit E2	0.956023	0.8398	0.9278
75507	Pou5f2	POU domain class 5, transcription factor 2	0.956023	0.6575	0.8224
75723	Amotl1	angiomotin-like 1	0.956023	0.6412	0.8126
77316	C030011L09Rik	RIKEN cDNA C030011L09 gene	0.956023	0.7591	0.8831
77605	H2afv	H2A histone family, member V	0.956023	0.5391	NA
78610	Uvrag	UV radiation resistance associated gene	0.956023	0.425	NA
80884	Maged2	melanoma antigen, family D, 2	0.956023	0.5203	NA
98766	Ubac1	ubiquitin associated domain containing 1	0.956023	0.3865	NA
103850	Nt5m	5',3'-nucleotidase, mitochondrial	0.956023	0.3833	NA

104871	Spata7	spermatogenesis associated 7	0.956023	0.5169	NA
108156	Mthfd1	methylenetetrahydrofolate dehydrogenase (NADP+ dependent), methenyltetrahydrofolate cyclohydrolase, formyltetrahydrofolate synthase	0.956023	0.5011	NA
108679	Cops8	COP9 (constitutive photomorphogenic) homolog, subunit 8 (Arabidopsis thaliana)	0.956023	0.6792	0.8358
109205	Sobp	sine oculis-binding protein homolog (Drosophila)	0.956023	0.4026	NA
109346	Ankrd39	ankyrin repeat domain 39	0.956023	0.5017	NA
170753	Zfp704	zinc finger protein 704	0.956023	0.6364	0.8089
209707	Lcorl	ligand dependent nuclear receptor corepressor-like	0.956023	0.7171	0.8585
217218	Atxn7l3	ataxin 7-like 3	0.956023	0.7701	0.8895
217684	4933426M11Rik	RIKEN cDNA 4933426M11 gene	0.956023	0.334	NA
227619	Man1b1	mannosidase, alpha, class 1B, member 1	0.956023	0.5131	NA
234776	Atmin	ATM interactor	0.956023	0.3813	NA
244152	Tsku	tsukushin	0.956023	0.7017	0.8495
246694	Hps5	Hermansky-Pudlak syndrome 5 homolog (human)	0.956023	0.3976	NA
269473	Lrig2	leucine-rich repeats and immunoglobulin-like domains 2	0.956023	0.7259	0.8638
271508	4933408B17Rik	RIKEN cDNA 4933408B17 gene	0.956023	0.5356	NA
279610	E530001F21Rik	RIKEN cDNA E530001F21 gene	0.956023	0.7255	0.8636
286940	Flnb	filamin, beta	0.956023	0.5366	NA
320679	Samd12	sterile alpha motif domain containing 12	0.956023	0.4328	NA
381259	Als2cr4	amyotrophic lateral sclerosis 2 (juvenile) chromosome region, candidate 4	0.956023	0.7533	0.8809
384525	Gm5321	predicted gene 5321	0.956023	0.2673	NA
545611	Gm13298	predicted gene 13298	0.956023	0.6987	0.8476
100040632	AA684185	expressed sequence AA684185	0.956023	0.6563	0.822
11487	Adam10	a disintegrin and metallopeptidase domain 10	0.95511	0.4918	NA
12017	Bag1	BCL2-associated athanogene 1	0.95511	0.4638	NA
12398	Cbfa2t3	core-binding factor, runt domain, alpha subunit 2, translocated to, 3 (human)	0.95511	0.7974	0.9049
12632	Cfl2	cofilin 2, muscle	0.95511	0.4837	NA
12709	Ckb	creatine kinase, brain	0.95511	0.4587	NA
12789	Cnga2	cyclic nucleotide gated channel alpha 2	0.95511	0.832	0.9245
12866	Cox7a2	cytochrome c oxidase, subunit VIIa 2	0.95511	0.5331	NA
14182	Fgfr1	fibroblast growth factor receptor 1	0.95511	0.828	0.9222
14784	Grb2	growth factor receptor bound protein 2	0.95511	0.4488	NA
14791	Emg1	EMG1 nucleolar protein homolog (S. cerevisiae)	0.95511	0.4771	NA
16600	Klf4	Kruppel-like factor 4 (gut)	0.95511	0.5252	NA
17761	Mtap7	microtubule-associated protein 7	0.95511	0.4816	NA
18519	Kat2b	K(lysine) acetyltransferase 2B	0.95511	0.7165	0.8583
19182	Psmc3	proteasome (prosome, macropain) 26S subunit, ATPase 3	0.95511	0.466	NA
20787	Srebf1	sterol regulatory element binding transcription factor 1	0.95511	0.667	0.8292
21960	Tnr	tenascin R	0.95511	0.4848	NA
23918	Impdh2	inosine 5'-phosphate dehydrogenase 2	0.95511	0.3228	NA
27008	Micall1	microtubule associated monoxygenase, calponin and LIM domain containing -like 1	0.95511	0.3898	NA
56316	Ggcx	gamma-glutamyl carboxylase	0.95511	0.5891	NA
56551	Txn2	thioredoxin 2	0.95511	0.3426	NA
56786	Tmem9b	TMEM9 domain family, member B	0.95511	0.4538	NA
56878	Rbms1	RNA binding motif, single stranded interacting protein 1	0.95511	0.5654	NA
59009	Sh3rf1	SH3 domain containing ring finger 1	0.95511	0.5558	NA
66084	Rmnd1	required for meiotic nuclear division 1 homolog (S. cerevisiae)	0.95511	0.5008	NA
66311	Cenpw	centromere protein W	0.95511	0.6293	0.8043
66680	3230401D17Rik	RIKEN cDNA 3230401D17 gene	0.95511	0.3644	NA
66882	Bzw1	basic leucine zipper and W2 domains 1	0.95511	0.3514	NA

67145	Tomm34	translocase of outer mitochondrial membrane 34	0.95511	0.4278	NA
67905	Ppm1m	protein phosphatase 1M	0.95511	0.5934	0.7808
68298	Ncapd2	non-SMC condensin I complex, subunit D2	0.95511	0.4467	NA
68936	Fam165b	family with sequence similarity 165, member B	0.95511	0.487	NA
69020	Zfp707	zinc finger protein 707	0.95511	0.5172	NA
69718	Ipmk	inositol polyphosphate multikinase	0.95511	0.6568	0.8223
70123	2210013O21Rik	RIKEN cDNA 2210013O21 gene	0.95511	0.3416	NA
71927	Itfg1	integrin alpha FG-GAP repeat containing 1	0.95511	0.3443	NA
76932	Arfip2	ADP-ribosylation factor interacting protein 2	0.95511	0.5233	NA
97244	C80140	expressed sequence C80140	0.95511	0.6813	0.8369
98682	Mfsd6	major facilitator superfamily domain containing 6	0.95511	0.5504	NA
98741	Kcnb2	potassium voltage gated channel, Shab-related subfamily, member 2	0.95511	0.7155	0.8576
219249	Tdrd3	tudor domain containing 3	0.95511	0.5288	NA
235132	Zbtb44	zinc finger and BTB domain containing 44	0.95511	0.6658	0.8286
240055	Neurl1b	neuralized homolog 1b (Drosophila)	0.95511	0.7079	0.8534
243302	Gm4963	predicted gene 4963	0.95511	0.3003	NA
244144	Usp35	ubiquitin specific peptidase 35	0.95511	0.5487	NA
244216	Zfp771	zinc finger protein 771	0.95511	0.4868	NA
244631	Pskh1	protein serine kinase H1	0.95511	0.6405	0.8119
252870	Usp7	ubiquitin specific peptidase 7	0.95511	0.452	NA
319748	Zfp865	zinc finger protein 865	0.95511	0.7343	0.8694
432768	Gm5451	predicted gene 5451	0.95511	0.6447	0.8146
433702	Ncbp1	nuclear cap binding protein subunit 1	0.95511	0.5712	NA
654472	Gm12070	glyceraldehyde-3-phosphate dehydrogenase pseudogene	0.95511	0.6485	0.817
100039199	Gm2095	predicted gene 2095	0.95511	0.6434	0.8136
100042862	Gm4076	predicted gene 4076	0.95511	0.6451	0.8147
100505291	LOC100505291	60S ribosomal protein L36-like	0.95511	0.6422	0.8132
11501	Adam8	a disintegrin and metallopeptidase domain 8	0.954198	0.507	NA
12005	Axin1	axin 1	0.954198	0.6563	0.822
12023	Barx2	BarH-like homeobox 2	0.954198	0.7084	0.8537
12295	Cacnb1	calcium channel, voltage-dependent, beta 1 subunit	0.954198	0.6791	0.8358
12877	Cpeb1	cytoplasmic polyadenylation element binding protein 1	0.954198	0.6324	0.8065
14367	Fzd5	frizzled homolog 5 (Drosophila)	0.954198	0.8576	0.9368
14377	G6pc	glucose-6-phosphatase, catalytic	0.954198	0.8386	0.9272
14778	Gpx3	glutathione peroxidase 3	0.954198	0.8875	0.9512
15278	Tfb2m	transcription factor B2, mitochondrial	0.954198	0.4142	NA
15289	Hmgb1	high mobility group box 1	0.954198	0.5866	0.7763
16765	Stmn1	stathmin 1	0.954198	0.4801	NA
16969	Zbtb7a	zinc finger and BTB domain containing 7a	0.954198	0.6892	0.8415
17300	Foxc1	forkhead box C1	0.954198	0.6883	0.8409
18618	Pemt	phosphatidylethanolamine N-methyltransferase	0.954198	0.6439	0.814
18777	Lypla1	lysophospholipase 1	0.954198	0.5062	NA
19271	Ptprj	protein tyrosine phosphatase, receptor type, J	0.954198	0.8462	0.9311
27041	G3bp1	Ras-GTPase-activating protein SH3-domain binding protein 1	0.954198	0.6016	0.7862
28080	Atp5o	ATP synthase, H+ transporting, mitochondrial F1 complex, O subunit	0.954198	0.48	NA
51885	Tubgcp4	tubulin, gamma complex associated protein 4	0.954198	0.4839	NA
53375	Mtx2	metaxin 2	0.954198	0.6117	0.7933
54128	Pmm2	phosphomannomutase 2	0.954198	0.7413	0.8738
54611	Pde3a	phosphodiesterase 3A, cGMP inhibited	0.954198	0.5947	0.7817

54632	Ftsj1	FtsJ homolog 1 (E. coli)	0.954198	0.369	NA
56444	Actr10	ARP10 actin-related protein 10 homolog (S. cerevisiae)	0.954198	0.2728	NA
56612	Pfdn5	prefoldin 5	0.954198	0.39	NA
64424	Polr1e	polymerase (RNA) I polypeptide E	0.954198	0.5066	NA
64436	Inpp5e	inositol polyphosphate-5-phosphatase E	0.954198	0.4921	NA
65019	Rpl23	ribosomal protein L23	0.954198	0.3239	NA
66355	Gmpr	guanosine monophosphate reductase	0.954198	0.6748	0.8333
67283	Slc25a19	solute carrier family 25 (mitochondrial thiamine pyrophosphate carrier), member 19	0.954198	0.5653	NA
67382	Brd3	bromodomain containing 3	0.954198	0.455	NA
67665	Dctn4	dynactin 4	0.954198	0.2905	NA
68140	Tigd2	tigger transposable element derived 2	0.954198	0.3503	NA
68910	Zfp467	zinc finger protein 467	0.954198	0.5757	NA
69697	2310057J16Rik	RIKEN cDNA 2310057J16 gene	0.954198	0.5525	NA
70573	Tbccd1	TBCC domain containing 1	0.954198	0.449	NA
71776	Tha1	threonine aldolase 1	0.954198	0.6414	0.8126
72881	Zdhhc4	zinc finger, DHHC domain containing 4	0.954198	0.6933	0.844
73024	2900064A13Rik	RIKEN cDNA 2900064A13 gene	0.954198	0.3725	NA
73139	Cenpv	centromere protein V	0.954198	0.4839	NA
76972	2810008D09Rik	RIKEN cDNA 2810008D09 gene	0.954198	0.5688	NA
77766	Elp4	elongation protein 4 homolog (S. cerevisiae)	0.954198	0.5599	NA
94282	Sfxn5	sideroflexin 5	0.954198	0.6939	0.8444
98999	Znfx1	zinc finger, NFX1-type containing 1	0.954198	0.4792	NA
108912	Cdca2	cell division cycle associated 2	0.954198	0.7178	0.859
109145	Gins4	GINS complex subunit 4 (Sld5 homolog)	0.954198	0.5536	NA
140741	Gpr6	G protein-coupled receptor 6	0.954198	0.7375	0.8714
195018	Zzef1	zinc finger, ZZ-type with EF hand domain 1	0.954198	0.4535	NA
208628	Kntc1	kinetochore associated 1	0.954198	0.8164	0.916
211499	Tmem87a	transmembrane protein 87A	0.954198	0.7461	0.8767
211586	Tfdp2	transcription factor Dp 2	0.954198	0.6361	0.8088
213550	Dis3l	DIS3 mitotic control homolog (S. cerevisiae)-like	0.954198	0.4343	NA
233871	Atxn2l	ataxin 2-like	0.954198	0.5673	NA
269003	Sap130	Sin3A associated protein	0.954198	0.5449	NA
319385	D130061D10Rik	RIKEN cDNA D130061D10 gene	0.954198	0.6958	0.8456
320772	Mdga2	MAM domain containing glycosylphosphatidylinositol anchor 2	0.954198	0.6621	0.826
328110	Prpf39	PRP39 pre-mRNA processing factor 39 homolog (yeast)	0.954198	0.3153	NA
328572	Ep300	E1A binding protein p300	0.954198	0.665	0.828
338355	Fkbp15	FK506 binding protein 15	0.954198	0.6039	0.7876
399635	D230044B12Rik	RIKEN cDNA D230044B12 gene	0.954198	0.5553	NA
12905	Cradd	CASP2 and RIPK1 domain containing adaptor with death domain	0.953289	0.3305	NA
13495	Drg2	developmentally regulated GTP binding protein 2	0.953289	0.6136	0.7948
13865	Nr2f1	nuclear receptor subfamily 2, group F, member 1	0.953289	0.5719	NA
14128	Fcer2a	Fc receptor, IgE, low affinity II, alpha polypeptide	0.953289	0.8018	0.9069
14853	Gspt2	G1 to S phase transition 2	0.953289	0.3827	NA
14958	H1f0	H1 histone family, member 0	0.953289	0.5998	0.785
15259	Hipk3	homeodomain interacting protein kinase 3	0.953289	0.5104	NA
16527	Kcnk3	potassium channel, subfamily K, member 3	0.953289	0.7142	0.8566
16798	Lats1	large tumor suppressor	0.953289	0.6195	0.7986
17690	Msi1	Musashi homolog 1(Drosophila)	0.953289	0.7396	0.8728
26371	Ciao1	cytosolic iron-sulfur protein assembly 1 homolog (S. cerevisiae)	0.953289	0.5449	NA

26448	Stk30	serine/threonine kinase 30	0.953289	0.7297	0.8663
26922	Mecr	mitochondrial trans-2-enoyl-CoA reductase	0.953289	0.5372	NA
29864	Rnf11	ring finger protein 11	0.953289	0.4396	NA
53334	Gosr1	golgi SNAP receptor complex member 1	0.953289	0.5406	NA
53413	Exoc7	exocyst complex component 7	0.953289	0.4991	NA
56249	Actr8	ARP8 actin-related protein 8 homolog (S. cerevisiae)	0.953289	0.419	NA
56320	Dbn1	drebrin 1	0.953289	0.5241	NA
57294	Rps27	ribosomal protein S27	0.953289	0.5936	0.781
57808	Rpl35a	ribosomal protein L35A	0.953289	0.4454	NA
66361	Zfand1	zinc finger, AN1-type domain 1	0.953289	0.6241	0.8013
66377	Ndufc1	NADH dehydrogenase (ubiquinone) 1, subcomplex unknown, 1	0.953289	0.4286	NA
66674	6330409N04Rik	RIKEN cDNA 6330409N04 gene	0.953289	0.6294	0.8043
67804	Snx2	sorting nexin 2	0.953289	0.3761	NA
68519	Eml1	echinoderm microtubule associated protein like 1	0.953289	0.2624	NA
68525	Evc2	Ellis van Creveld syndrome 2 homolog (human)	0.953289	0.7713	0.8902
69161	Manbal	mannosidase, beta A, lysosomal-like	0.953289	0.598	0.7835
69195	Tmem121	transmembrane protein 121	0.953289	0.5435	NA
69587	Pcgf3	polycomb group ring finger 3	0.953289	0.7918	0.9013
70227	Zfp619	zinc finger protein 619	0.953289	0.6996	0.8482
70451	Dhrs13	dehydrogenase/reductase (SDR family) member 13	0.953289	0.3726	NA
70564	5730469M10Rik	RIKEN cDNA 5730469M10 gene	0.953289	0.5011	NA
70797	Ankib1	ankyrin repeat and IBR domain containing 1	0.953289	0.4411	NA
71452	Ankrd40	ankyrin repeat domain 40	0.953289	0.5677	NA
71947	2310067B10Rik	RIKEN cDNA 2310067B10 gene	0.953289	0.799	0.9055
72344	Usp36	ubiquitin specific peptidase 36	0.953289	0.5429	NA
72543	Fam125b	family with sequence similarity 125, member B	0.953289	0.5316	NA
72873	2900006K08Rik	RIKEN cDNA 2900006K08 gene	0.953289	0.7436	0.8753
73721	1110017D15Rik	RIKEN cDNA 1110017D15 gene	0.953289	0.7861	0.8978
74117	Actr3	ARP3 actin-related protein 3 homolog (yeast)	0.953289	0.3874	NA
74164	Nfx1	nuclear transcription factor, X-box binding 1	0.953289	0.6429	0.8135
75173	4930544O15Rik	RIKEN cDNA 4930544O15 gene	0.953289	0.7139	0.8564
75769	4833424O15Rik	RIKEN cDNA 4833424O15 gene	0.953289	0.5798	0.772
75894	Adal	adenosine deaminase-like	0.953289	0.3738	NA
76022	Gon4l	gon-4-like (C.elegans)	0.953289	0.6637	0.8272
77407	Rab35	RAB35, member RAS oncogene family	0.953289	0.4009	NA
79264	Krit1	KRIT1, ankyrin repeat containing	0.953289	0.3002	NA
80733	Car15	carbonic anhydrase 15	0.953289	0.5463	NA
80795	Selk	selenoprotein K	0.953289	0.4461	NA
83380	Prp2	proline rich protein 2	0.953289	0.6865	0.8402
97884	B3galnt2	UDP-GalNAc:betaGlcNAc beta 1,3-galactosaminyltransferase, polypeptide 2	0.953289	0.3753	NA
104111	Adcy3	adenylate cyclase 3	0.953289	0.6651	0.828
104248	Cabin1	calcineurin binding protein 1	0.953289	0.5308	NA
105559	Mbnl2	muscleblind-like 2	0.953289	0.6054	0.7886
106326	Osbpl11	oxysterol binding protein-like 11	0.953289	0.3225	NA
107328	Trpt1	tRNA phosphotransferase 1	0.953289	0.4347	NA
107951	Cdk9	cyclin-dependent kinase 9 (CDC2-related kinase)	0.953289	0.55	NA
108909	Aida	axin interactor, dorsalization associated	0.953289	0.3875	NA
109284	C030046I01Rik	RIKEN cDNA C030046I01 gene	0.953289	0.5258	NA
170707	Usp48	ubiquitin specific peptidase 48	0.953289	0.6226	0.8003

212127	2810046L04Rik	RIKEN cDNA 2810046L04 gene	0.953289	0.5121	NA
217449	Ttc15	tetratricopeptide repeat domain 15	0.953289	0.396	NA
225049	Ttc7	tetratricopeptide repeat domain 7	0.953289	0.6247	0.8016
228788	BC020535	cDNA sequence BC020535	0.953289	0.6879	0.8407
231430	Cox18	COX18 cytochrome c oxidase assembly homolog (S. cerevisiae)	0.953289	0.6114	0.7931
235379	Gldn	gliomedin	0.953289	0.6074	0.79
243906	Zfp14	zinc finger protein 14	0.953289	0.5224	NA
245902	Ccdc15	coiled-coil domain containing 15	0.953289	0.6613	0.8254
268449	Rpl23a	ribosomal protein L23A	0.953289	0.3554	NA
270066	Slc35e1	solute carrier family 35, member E1	0.953289	0.7087	0.8537
321008	6330408A02Rik	RIKEN cDNA 6330408A02 gene	0.953289	0.5089	NA
625929	Gm6636	predicted gene 6636	0.953289	0.6601	0.8246
626055	Gm15645	predicted gene 15645	0.953289	0.5816	0.7728
637004	Vmn2r3	vomeronasal 2, receptor 3	0.953289	0.8357	0.926
672214	Gm10136	predicted pseudogene 10136	0.953289	0.3776	NA
11994	Pcdh15	protocadherin 15	0.952381	0.6291	0.8043
12449	Ccnf	cyclin F	0.952381	0.6733	0.8326
12631	Cfl1	cofilin 1, non-muscle	0.952381	0.6663	0.8288
12995	Csnk2a1	casein kinase 2, alpha 1 polypeptide	0.952381	0.4101	NA
13638	Efna3	ephrin A3	0.952381	0.6999	0.8485
14272	Fnta	farnesyltransferase, CAAX box, alpha	0.952381	0.5676	0.7631
14751	Gpi1	glucose phosphate isomerase 1	0.952381	0.5613	NA
17125	Smad1	MAD homolog 1 (Drosophila)	0.952381	0.8334	0.9247
17713	Grpel1	GrpE-like 1, mitochondrial	0.952381	0.3399	NA
18096	Nkx6-1	NK6 homeobox 1	0.952381	0.6484	0.817
19072	Prep	prolyl endopeptidase	0.952381	0.4802	NA
19182	Psmc3	proteasome (prosome, macropain) 26S subunit, ATPase 3	0.952381	0.6211	0.7994
19684	Rdx	radixin	0.952381	0.5592	NA
20688	Sp4	trans-acting transcription factor 4	0.952381	0.5911	0.7796
21881	Tkt	transketolase	0.952381	0.6003	0.7851
22376	Was	Wiskott-Aldrich syndrome homolog (human)	0.952381	0.8188	0.9174
22385	Baz1b	bromodomain adjacent to zinc finger domain, 1B	0.952381	0.4423	NA
23945	Mgll	monoglyceride lipase	0.952381	0.56	NA
57317	Srsf4	serine/arginine-rich splicing factor 4	0.952381	0.3617	NA
59050	Nsa2	NSA2 ribosome biogenesis homolog (S. cerevisiae)	0.952381	0.756	0.8822
66194	Pycrl	pyrroline-5-carboxylate reductase-like	0.952381	0.6605	0.8247
66258	Mrps17	mitochondrial ribosomal protein S17	0.952381	0.2799	NA
66396	Ccdc82	coiled-coil domain containing 82	0.952381	0.322	NA
66632	Atpbd4	ATP binding domain 4	0.952381	0.4431	NA
66773	Gm17019	predicted gene	0.952381	0.9012	0.9578
67048	Vma21	VMA21 vacuolar H+-ATPase homolog (S. cerevisiae)	0.952381	0.7159	0.8578
67049	Pus3	pseudouridine synthase 3	0.952381	0.3473	NA
67130	Ndufa6	NADH dehydrogenase (ubiquinone) 1 alpha subcomplex, 6 (B14)	0.952381	0.3453	NA
67249	Tbc1d19	TBC1 domain family, member 19	0.952381	0.2884	NA
67870	Enoph1	enolase-phosphatase 1	0.952381	0.4037	NA
68283	9530077C05Rik	RIKEN cDNA 9530077C05 gene	0.952381	0.6086	0.791
68379	Ciz1	CDKN1A interacting zinc finger protein 1	0.952381	0.4221	NA
68475	Ssna1	Sjogren's syndrome nuclear autoantigen 1	0.952381	0.5017	NA
68507	Ppfia4	protein tyrosine phosphatase, receptor type, f polypeptide (PTPRF), interacting protein (liprin), alpha 4	0.952381	0.3625	NA

69318	1700007K09Rik	RIKEN cDNA 1700007K09 gene	0.952381	0.6726	0.8323
69601	Dab2ip	disabled homolog 2 (Drosophila) interacting protein	0.952381	0.4891	NA
70123	2210013O21Rik	RIKEN cDNA 2210013O21 gene	0.952381	0.3648	NA
72469	Plcd3	phospholipase C, delta 3	0.952381	0.4675	NA
73166	Tm7sf2	transmembrane 7 superfamily member 2	0.952381	0.5662	NA
73341	Arhgef6	Rac/Cdc42 guanine nucleotide exchange factor (GEF) 6	0.952381	0.5134	NA
74998	Rab11fip2	RAB11 family interacting protein 2 (class I)	0.952381	0.5187	NA
76007	Zmym2	zinc finger, MYM-type 2	0.952381	0.7786	0.8935
76025	Cant1	calcium activated nucleotidase 1	0.952381	0.642	0.8132
77254	Yif1b	Yip1 interacting factor homolog B (S. cerevisiae)	0.952381	0.5957	0.7822
78619	Zfp449	zinc finger protein 449	0.952381	0.5005	NA
78787	Usp54	ubiquitin specific peptidase 54	0.952381	0.586	0.7761
94066	Mrpl36	mitochondrial ribosomal protein L36	0.952381	0.3703	NA
110147	Ehmt2	euchromatic histone lysine N-methyltransferase 2	0.952381	0.3667	NA
110391	Qdpr	quinoid dihydropteridine reductase	0.952381	0.5803	0.7724
170643	Kirrel	kin of IRRE like (Drosophila)	0.952381	0.3316	NA
216169	Fam108a	family with sequence similarity 108, member A	0.952381	0.6864	0.8402
223649	Nrbp2	nuclear receptor binding protein 2	0.952381	0.4431	NA
224796	Clic5	chloride intracellular channel 5	0.952381	0.6698	0.8307
225010	Lclat1	lysocardiolipin acyltransferase 1	0.952381	0.4033	NA
226026	Smc5	structural maintenance of chromosomes 5	0.952381	0.6761	0.8337
231670	Fbxo21	F-box protein 21	0.952381	0.5071	NA
232910	Ap2s1	adaptor-related protein complex 2, sigma 1 subunit	0.952381	0.5124	NA
240067	Zfp952	zinc finger protein 952	0.952381	0.6402	0.8116
242253	Wdr63	WD repeat domain 63	0.952381	0.655	0.8212
320713	Mysm1	myb-like, SWIRM and MPN domains 1	0.952381	0.547	NA
380718	Mks1	Meckel syndrome, type 1	0.952381	0.4065	NA
380918	Siah3	seven in absentia homolog 3 (Drosophila)	0.952381	0.6276	0.8037
433003	Gm5481	predicted gene 5481	0.952381	0.8201	0.918
625054	Gm6548	eukaryotic translation elongation factor 1 alpha 1 pseudogene	0.952381	0.5726	0.7665
626231	Gm6658	predicted gene 6658	0.952381	0.83	0.9233
627214	Fam196a	family with sequence similarity 196, member A	0.952381	0.3109	NA
11538	Adnp	activity-dependent neuroprotective protein	0.951475	0.4498	NA
13602	Sparcl1	SPARC-like 1	0.951475	0.6115	0.7932
13709	Elf1	E74-like factor 1	0.951475	0.4324	NA
14911	Thumpd3	THUMP domain containing 3	0.951475	0.4361	NA
16563	Kif2a	kinesin family member 2A	0.951475	0.7097	0.8542
18415	Hspa4l	heat shock protein 4 like	0.951475	0.2714	NA
18775	Prl3d1	prolactin family 3, subfamily d, member 1	0.951475	0.8218	0.9191
19156	Psap	prosaposin	0.951475	0.5555	NA
20523	Slc25a14	solute carrier family 25 (mitochondrial carrier, brain), member 14	0.951475	0.3576	NA
22222	Ubr1	ubiquitin protein ligase E3 component n-recognin 1	0.951475	0.658	0.8229
26940	Ecsit	ECSIT homolog (Drosophila)	0.951475	0.2632	NA
30843	Fbxl12	F-box and leucine-rich repeat protein 12	0.951475	0.337	NA
52468	Ctdsp2	CTD (carboxy-terminal domain, RNA polymerase II, polypeptide A) small phosphatase 2	0.951475	0.5917	0.7799
55936	Ctps2	cytidine 5'-triphosphate synthase 2	0.951475	0.4338	NA
56438	Rbx1	ring-box 1	0.951475	0.3147	NA
56469	Pias1	protein inhibitor of activated STAT 1	0.951475	0.6355	0.8084
56705	Ranbp9	RAN binding protein 9	0.951475	0.4353	NA

60530	Fignl1	fidgetin-like 1	0.951475	0.6315	0.8059
66046	Ndufb5	NADH dehydrogenase (ubiquinone) 1 beta subcomplex, 5	0.951475	0.4659	NA
66098	Chchd6	coiled-coil-helix-coiled-coil-helix domain containing 6	0.951475	0.4971	NA
66128	Mrps36	mitochondrial ribosomal protein S36	0.951475	0.2866	NA
66407	Mrps15	mitochondrial ribosomal protein S15	0.951475	0.275	NA
66890	Lman2	lectin, mannose-binding 2	0.951475	0.4365	NA
68598	Dnajc8	DnaJ (Hsp40) homolog, subfamily C, member 8	0.951475	0.6878	0.8407
69064	1810014F10Rik	RIKEN cDNA 1810014F10 gene	0.951475	0.4475	NA
69352	Necab1	N-terminal EF-hand calcium binding protein 1	0.951475	0.7399	0.8729
69655	Cd164l2	CD164 sialomucin-like 2	0.951475	0.765	0.8866
69665	2310043J07Rik	RIKEN cDNA 2310043J07 gene	0.951475	0.8326	0.9246
71678	Brox	BRO1 domain and CAAX motif containing	0.951475	0.6642	0.8276
71779	Mar-08	membrane-associated ring finger (C3HC4) 8	0.951475	0.3952	NA
72199	Mms19	MMS19 (MET18 S. cerevisiae)	0.951475	0.4965	NA
72775	Fance	Fanconi anemia, complementation group E	0.951475	0.4122	NA
74414	Polr3c	polymerase (RNA) III (DNA directed) polypeptide C	0.951475	0.4978	NA
74720	Tmem114	transmembrane protein 114	0.951475	0.627	0.8031
74776	Ppa2	pyrophosphatase (inorganic) 2	0.951475	0.3203	NA
75425	Tti1	Tel2 interacting protein 1 homolog (S. pombe)	0.951475	0.346	NA
76089	Rapgef2	Rap guanine nucleotide exchange factor (GEF) 2	0.951475	0.3254	NA
76167	Snrnp35	small nuclear ribonucleoprotein 35 (U11/U12)	0.951475	0.3956	NA
76987	Hdhd2	haloacid dehalogenase-like hydrolase domain containing 2	0.951475	0.7948	0.903
78286	Nav2	neuron navigator 2	0.951475	0.7548	0.8816
101540	Prkd2	protein kinase D2	0.951475	0.6068	0.7896
103149	Upb1	ureidopropionase, beta	0.951475	0.69	0.8418
103554	Psme4	proteasome (prosome, macropain) activator subunit 4	0.951475	0.7746	0.8916
106952	Arap3	ArfGAP with RhoGAP domain, ankyrin repeat and PH domain 3	0.951475	0.4506	NA
170737	Znrf1	zinc and ring finger 1	0.951475	0.5702	0.7642
225289	AW554918	expressed sequence AW554918	0.951475	0.4237	NA
225348	Wdr36	WD repeat domain 36	0.951475	0.499	NA
231724	Rad9b	RAD9 homolog B (S. cerevisiae)	0.951475	0.7445	0.8758
269198	Nbeal1	neurobeachin like 1	0.951475	0.5415	NA
271457	Rab5a	RAB5A, member RAS oncogene family	0.951475	0.5828	0.7736
271981	Tbck	TBC1 domain containing kinase	0.951475	0.5651	0.7618
272027	Tstd2	thiosulfate sulfurtransferase (rhodanese)-like domain containing 2	0.951475	0.6813	0.8369
276905	Armc7	armadillo repeat containing 7	0.951475	0.6465	0.8155
320534	Tmem104	transmembrane protein 104	0.951475	0.649	0.8173
329877	Dennd4c	DENN/MADD domain containing 4C	0.951475	0.6342	0.8075
330814	Lphn1	latrophilin 1	0.951475	0.6533	0.8205
11479	Acvr1b	activin A receptor, type 1B	0.95057	0.4647	NA
11789	Apc	adenomatosis polyposis coli	0.95057	0.7415	0.8739
12411	Cbs	cystathionine beta-synthase	0.95057	0.648	0.8166
12929	Crkl	v-crk sarcoma virus CT10 oncogene homolog (avian)-like	0.95057	0.4807	NA
13385	Dlg4	discs, large homolog 4 (Drosophila)	0.95057	0.5363	NA
13680	Ddx19a	DEAD (Asp-Glu-Ala-Asp) box polypeptide 19a	0.95057	0.6508	0.8186
14797	Aes	amino-terminal enhancer of split	0.95057	0.5376	NA
15185	Hdac6	histone deacetylase 6	0.95057	0.4262	NA
19739	Rgs9	regulator of G-protein signaling 9	0.95057	0.8369	0.9269
20022	Polr2j	polymerase (RNA) II (DNA directed) polypeptide J	0.95057	0.2487	NA

20479	Vps4b	vacuolar protein sorting 4b (yeast)	0.95057	0.4514	NA
20788	Srebf2	sterol regulatory element binding factor 2	0.95057	0.5073	NA
22272	Uqcrq	ubiquinol-cytochrome c reductase, complex III subunit VII	0.95057	0.5318	NA
26399	Map2k6	mitogen-activated protein kinase kinase 6	0.95057	0.5118	NA
53901	Rcan2	regulator of calcineurin 2	0.95057	0.3179	NA
55944	Eif3d	eukaryotic translation initiation factor 3, subunit D	0.95057	0.4422	NA
56200	Ddx21	DEAD (Asp-Glu-Ala-Asp) box polypeptide 21	0.95057	0.4757	NA
66687	Tbc1d15	TBC1 domain family, member 15	0.95057	0.6926	0.8437
66870	Serbp1	serpine1 mRNA binding protein 1	0.95057	0.5631	0.7609
68115	9430016H08Rik	RIKEN cDNA 9430016H08 gene	0.95057	0.3483	NA
68977	Haghl	hydroxyacylglutathione hydrolase-like	0.95057	0.3935	NA
69232	Qrich1	glutamine-rich 1	0.95057	0.484	NA
70427	Mier2	mesoderm induction early response 1, family member 2	0.95057	0.701	0.8492
71653	4930506M07Rik	RIKEN cDNA 4930506M07 gene	0.95057	0.541	NA
71865	Fbxo30	F-box protein 30	0.95057	0.4513	NA
74360	Cep57	centrosomal protein 57	0.95057	0.3494	NA
75387	Sirt4	sirtuin 4 (silent mating type information regulation 2 homolog) 4 (S. cerevisiae)	0.95057	0.4586	NA
75689	Higd1b	HIG1 domain family, member 1B	0.95057	0.4924	NA
76850	Eif2c4	eukaryotic translation initiation factor 2C, 4	0.95057	0.5521	0.7522
83431	Ndel1	nuclear distribution gene E-like homolog 1 (A. nidulans)	0.95057	0.3353	NA
94093	Trim33	tripartite motif-containing 33	0.95057	0.4903	NA
94190	Ophn1	oligophrenin 1	0.95057	0.4028	NA
101095	Zfp282	zinc finger protein 282	0.95057	0.6753	0.8334
102566	Ano10	anoctamin 10	0.95057	0.605	0.7884
110532	Adarb1	adenosine deaminase, RNA-specific, B1	0.95057	0.2194	NA
170719	Oxr1	oxidation resistance 1	0.95057	0.5719	0.766
192786	Rapgef6	Rap guanine nucleotide exchange factor (GEF) 6	0.95057	0.7307	0.8669
211401	Mtss1	metastasis suppressor 1	0.95057	0.4937	NA
214254	Nudt15	nudix (nucleoside diphosphate linked moiety X)-type motif 15	0.95057	0.4601	NA
214897	Csnk1g1	casein kinase 1, gamma 1	0.95057	0.2966	NA
226352	Epb4.1l5	erythrocyte protein band 4.1-like 5	0.95057	0.6175	0.7971
230075	Ndufb6	NADH dehydrogenase (ubiquinone) 1 beta subcomplex, 6	0.95057	0.3402	NA
230767	Iqcc	IQ motif containing C	0.95057	0.6485	0.817
240869	Zbtb37	zinc finger and BTB domain containing 37	0.95057	0.6031	0.787
244895	C230081A13Rik	RIKEN cDNA C230081A13 gene	0.95057	0.3365	NA
244962	Snx14	sorting nexin 14	0.95057	0.3993	NA
252966	Cables2	CDK5 and Abl enzyme substrate 2	0.95057	0.4122	NA
264134	Ttc26	tetratricopeptide repeat domain 26	0.95057	0.582	0.773
270066	Slc35e1	solute carrier family 35, member E1	0.95057	0.4011	NA
319944	Taf2	TAF2 RNA polymerase II, TATA box binding protein (TBP)-associated factor	0.95057	0.4989	NA
320184	Lrrc58	leucine rich repeat containing 58	0.95057	0.5579	0.7569
666372	Gm8066	predicted gene 8066	0.95057	0.5828	0.7736
11977	Atp7a	ATPase, Cu++ transporting, alpha polypeptide	0.949668	0.4775	NA
12894	Cpt1a	carnitine palmitoyltransferase 1a, liver	0.949668	0.6009	0.7858
13643	Efnb3	ephrin B3	0.949668	0.53	NA
13800	Enah	enabled homolog (Drosophila)	0.949668	0.6436	0.8137
14583	Gfpt1	glutamine fructose-6-phosphate transaminase 1	0.949668	0.7286	0.8655
14593	Ggps1	geranylgeranyl diphosphate synthase 1	0.949668	0.4106	NA
15077	Hist2h3c1	histone cluster 2, H3c1	0.949668	0.563	0.7609

15502	Dnaja1	DnaJ (Hsp40) homolog, subfamily A, member 1	0.949668	0.3483	NA
17149	Magoh	mago-nashi homolog, proliferation-associated (Drosophila)	0.949668	0.3777	NA
17763	Mtcp1	mature T-cell proliferation 1	0.949668	0.6931	0.8439
18729	Pira6	paired-Ig-like receptor A6	0.949668	0.8379	0.927
19179	Psmc1	protease (prosome, macropain) 26S subunit, ATPase 1	0.949668	0.3439	NA
19360	Rad50	RAD50 homolog (S. cerevisiae)	0.949668	0.3438	NA
20104	Rps6	ribosomal protein S6	0.949668	0.4585	NA
20273	Scn8a	sodium channel, voltage-gated, type VIII, alpha	0.949668	0.6231	0.8005
20397	Sgpl1	sphingosine phosphate lyase 1	0.949668	0.2992	NA
20646	Snrpn	small nuclear ribonucleoprotein N	0.949668	0.5348	NA
21841	Tia1	cytotoxic granule-associated RNA binding protein 1	0.949668	0.4966	NA
28199	Dcaf11	DDB1 and CUL4 associated factor 11	0.949668	0.3122	NA
52174	Tmem222	transmembrane protein 222	0.949668	0.3081	NA
52717	Anapc16	anaphase promoting complex subunit 16	0.949668	0.2004	NA
52840	Dbndd2	dysbindin (dystrobrevin binding protein 1) domain containing 2	0.949668	0.4423	NA
54151	Cyhr1	cysteine and histidine rich 1	0.949668	0.5111	NA
54371	Chst2	carbohydrate sulfotransferase 2	0.949668	0.7829	0.896
54636	Wdr45	WD repeat domain 45	0.949668	0.3372	NA
66246	Osgep	O-sialoglycoprotein endopeptidase	0.949668	0.2767	NA
66314	Tpd52l2	tumor protein D52-like 2	0.949668	0.5503	0.7511
66391	2310061J03Rik	RIKEN cDNA 2310061J03 gene	0.949668	0.5032	NA
66508	2400001E08Rik	RIKEN cDNA 2400001E08 gene	0.949668	0.4758	NA
67952	Tomm20	translocase of outer mitochondrial membrane 20 homolog (yeast)	0.949668	0.6311	0.8058
70713	Gpr137c	G protein-coupled receptor 137C	0.949668	0.7354	0.8703
71147	Oxsm	3-oxoacyl-ACP synthase, mitochondrial	0.949668	0.5732	0.767
71532	9030418K01Rik	RIKEN cDNA 9030418K01 gene	0.949668	0.5592	0.7579
71804	Fam54a	family with sequence similarity 54, member A	0.949668	0.7168	0.8584
73635	1700113I22Rik	RIKEN cDNA 1700113I22 gene	0.949668	0.5653	0.7618
73991	Atl1	atlastin GTPase 1	0.949668	0.5228	NA
74198	Dtx2	deltex 2 homolog (Drosophila)	0.949668	0.3683	NA
74763	Nat15	N-acetyltransferase 15 (GCN5-related, putative)	0.949668	0.3518	NA
75497	Fabp12	fatty acid binding protein 12	0.949668	0.3533	NA
75553	Zc3h14	zinc finger CCCH type containing 14	0.949668	0.3633	NA
76719	1700081L11Rik	RIKEN cDNA 1700081L11 gene	0.949668	0.6437	0.8139
77789	A930007D18Rik	RIKEN cDNA A930007D18 gene	0.949668	0.6176	0.7971
78287	Zfyve20	zinc finger, FYVE domain containing 20	0.949668	0.2817	NA
85305	Kars	lysyl-tRNA synthetase	0.949668	0.5208	NA
93759	Sirt1	sirtuin 1 (silent mating type information regulation 2, homolog) 1 (S. cerevisiae)	0.949668	0.4027	NA
103968	Plin1	perilipin 1	0.949668	0.7793	0.8936
109674	Ampd2	adenosine monophosphate deaminase 2	0.949668	0.6116	0.7932
114774	Pawr	PRKC, apoptosis, WT1, regulator	0.949668	0.6399	0.8113
210789	Tbc1d4	TBC1 domain family, member 4	0.949668	0.5004	NA
212503	Paox	polyamine oxidase (exo-N4-amino)	0.949668	0.5105	NA
218975	Mapk1ip1l	mitogen-activated protein kinase 1 interacting protein 1-like	0.949668	0.4826	NA
228715	Gm561	predicted gene 561	0.949668	0.2754	NA
230126	Shb	src homology 2 domain-containing transforming protein B	0.949668	0.6264	0.8028
231207	Cpeb2	cytoplasmic polyadenylation element binding protein 2	0.949668	0.7122	0.8553
233812	BC030336	cDNA sequence BC030336	0.949668	0.5353	NA
233899	Gm166	predicted gene 166	0.949668	0.6291	0.8043

235315	Rnf214	ring finger protein 214	0.949668	0.3997	NA
237313	Il20ra	interleukin 20 receptor, alpha	0.949668	0.5476	0.749
237775	Zfp867	zinc finger protein 867	0.949668	0.4681	NA
238377	Gpr68	G protein-coupled receptor 68	0.949668	0.6165	0.7965
239510	Phf20l1	PHD finger protein 20-like 1	0.949668	0.4851	NA
666938	Bend4	BEN domain containing 4	0.949668	0.6425	0.8134
100042786	Gm16381	predicted gene 16381	0.949668	0.306	NA
12842	Col1a1	collagen, type I, alpha 1	0.948767	0.6937	0.8442
16697	LOC16697	keratin associated protein LOC16697	0.948767	0.412	NA
17364	Trpm1	transient receptor potential cation channel, subfamily M, member 1	0.948767	0.6217	0.7996
17904	Myl6	myosin, light polypeptide 6, alkali, smooth muscle and non-muscle	0.948767	0.5494	0.7506
18231	Nxph1	neurexophilin 1	0.948767	0.6474	0.8162
18292	Sebox	SEBOX homeobox	0.948767	0.4248	NA
18584	Pde8a	phosphodiesterase 8A	0.948767	0.529	NA
19211	Pten	phosphatase and tensin homolog	0.948767	0.4849	NA
19270	Ptprg	protein tyrosine phosphatase, receptor type, G	0.948767	0.7046	0.8514
19988	Rpl6	ribosomal protein L6	0.948767	0.6973	0.8467
21771	Cirh1a	cirrhosis, autosomal recessive 1A (human)	0.948767	0.3587	NA
22143	Tuba1b	tubulin, alpha 1B	0.948767	0.636	0.8087
29875	Iqgap1	IQ motif containing GTPase activating protein 1	0.948767	0.6357	0.8084
52335	Atxn1l	ataxin 1-like	0.948767	0.3053	NA
56036	Ccnl2	cyclin L2	0.948767	0.4212	NA
56451	Suclg1	succinate-CoA ligase, GDP-forming, alpha subunit	0.948767	0.4306	NA
56530	Cnpy2	canopy 2 homolog (zebrafish)	0.948767	0.2556	NA
66368	Rtcd1	RNA terminal phosphate cyclase domain 1	0.948767	0.537	0.7416
66700	Vps24	vacuolar protein sorting 24 (yeast)	0.948767	0.6236	0.801
67471	Gpatch1	G patch domain containing 1	0.948767	0.4242	NA
68021	Bphl	biphenyl hydrolase-like (serine hydrolase, breast epithelial mucin-associated antigen)	0.948767	0.2825	NA
68097	Dynll2	dynein light chain LC8-type 2	0.948767	0.41	NA
68193	Rpl24	ribosomal protein L24	0.948767	0.6096	0.7917
69908	Rab3b	RAB3B, member RAS oncogene family	0.948767	0.4625	NA
71928	2310047K21Rik	RIKEN cDNA 2310047K21 gene	0.948767	0.4719	NA
74931	4930481A15Rik	RIKEN cDNA 4930481A15 gene	0.948767	0.5683	0.7634
75316	Taf1d	TATA box binding protein (Tbp)-associated factor, RNA polymerase I, D	0.948767	0.5345	NA
76251	0610007P08Rik	RIKEN cDNA 0610007P08 gene	0.948767	0.7085	0.8537
76897	Ralyl	RALY RNA binding protein-like	0.948767	0.59	0.7788
78365	1500016L03Rik	RIKEN cDNA 1500016L03 gene	0.948767	0.6815	0.837
78798	Eml4	echinoderm microtubule associated protein like 4	0.948767	0.3895	NA
94223	Dgcr8	DiGeorge syndrome critical region gene 8	0.948767	0.4617	NA
99152	Anapc2	anaphase promoting complex subunit 2	0.948767	0.5198	NA
99696	Ankrd50	ankyrin repeat domain 50	0.948767	0.3959	NA
102787	AW552889	expressed sequence AW552889	0.948767	0.83	0.9233
105638	Dph3	DPH3 homolog (KTI11, S. cerevisiae)	0.948767	0.3797	NA
106338	Nsun3	NOL1/NOP2/Sun domain family member 3	0.948767	0.8423	0.9291
110749	Chaf1b	chromatin assembly factor 1, subunit B (p60)	0.948767	0.6728	0.8323
212862	Chpt1	choline phosphotransferase 1	0.948767	0.7133	0.8559
213350	Pddc1	Parkinson disease 7 domain containing 1	0.948767	0.6738	0.833
218975	Mapk1ip1l	mitogen-activated protein kinase 1 interacting protein 1-like	0.948767	0.5701	0.7641
232157	Mobkl1b	MOB1, Mps One Binder kinase activator-like 1B (yeast)	0.948767	0.7453	0.8762

237178	Ppef1	protein phosphatase with EF hand calcium-binding domain 1	0.948767	0.7816	0.8951
241694	Ralgapa2	Ral GTPase activating protein, alpha subunit 2 (catalytic)	0.948767	0.4706	NA
242409	Tmem8b	transmembrane protein 8B	0.948767	0.7556	0.8821
243548	Prickle2	prickle homolog 2 (Drosophila)	0.948767	0.5322	NA
245578	Pcdh11x	protocadherin 11 X-linked	0.948767	0.6794	0.8359
268980	Strn	striatin, calmodulin binding protein	0.948767	0.5436	0.7468
319636	Fsd1l	fibronectin type III and SPRY domain containing 1-like	0.948767	0.6339	0.8075
320110	B230369F24Rik	RIKEN cDNA B230369F24 gene	0.948767	0.6032	0.7871
320216	D230040J21Rik	RIKEN cDNA D230040J21 gene	0.948767	0.6017	0.7863
330369	Fbxo41	F-box protein 41	0.948767	0.4915	NA
432939	Gm5468	predicted gene 5468	0.948767	0.6385	0.8103
100504872	LOC100504872	60S ribosomal protein L32-like	0.948767	0.4004	NA
11544	Adprh	ADP-ribosylarginine hydrolase	0.947867	0.4977	NA
11958	Atp5k	ATP synthase, H+ transporting, mitochondrial F1F0 complex, subunit e	0.947867	0.3313	NA
12879	Cys1	cystin 1	0.947867	0.574	0.7675
14105	Srsf10	serine/arginine-rich splicing factor 10	0.947867	0.5607	0.7594
14756	Gpld1	glycosylphosphatidylinositol specific phospholipase D1	0.947867	0.6865	0.8402
16319	Incenp	inner centromere protein	0.947867	0.3122	NA
16401	Itga4	integrin alpha 4	0.947867	0.3874	NA
19891	Rpa2	replication protein A2	0.947867	0.4414	NA
20768	Sephs2	selenophosphate synthetase 2	0.947867	0.6051	0.7884
21340	Taf1b	TATA box binding protein (Tbp)-associated factor, RNA polymerase I, B	0.947867	0.636	0.8087
21652	Phf1	PHD finger protein 1	0.947867	0.583	0.7737
22163	Tnfrsf4	tumor necrosis factor receptor superfamily, member 4	0.947867	0.5279	NA
22412	Wnt9b	wingless-type MMTV integration site 9B	0.947867	0.8084	0.9108
22628	Ywhag	tyrosine 3-monooxygenase/tryptophan 5-monooxygenase activation protein, gamma polypeptide	0.947867	0.319	NA
51922	D2Ertd612e	DNA segment, Chr 2, ERATO Doi 612, expressed	0.947867	0.5725	0.7665
52653	Nudcd2	NudC domain containing 2	0.947867	0.4693	NA
56445	Dnaja2	DnaJ (Hsp40) homolog, subfamily A, member 2	0.947867	0.441	NA
56790	Fam48a	family with sequence similarity 48, member A	0.947867	0.4029	NA
57357	Srd5a3	steroid 5 alpha-reductase 3	0.947867	0.4892	NA
60425	Doc2g	double C2, gamma	0.947867	0.5253	NA
66059	Krtcap2	keratinocyte associated protein 2	0.947867	0.257	NA
66615	Atg4b	autophagy-related 4B (yeast)	0.947867	0.4775	NA
67895	Ppa1	pyrophosphatase (inorganic) 1	0.947867	0.3801	NA
67973	Mphosph10	M-phase phosphoprotein 10 (U3 small nucleolar ribonucleoprotein)	0.947867	0.409	NA
67979	Atad1	ATPase family, AAA domain containing 1	0.947867	0.4167	NA
68232	1700120K04Rik	RIKEN cDNA 1700120K04 gene	0.947867	0.6119	0.7935
68259	Ift80	intraflagellar transport 80 homolog (Chlamydomonas)	0.947867	0.2916	NA
68846	Rnf208	ring finger protein 208	0.947867	0.6118	0.7933
72061	2010111I01Rik	RIKEN cDNA 2010111I01 gene	0.947867	0.3977	NA
72568	Lin9	lin-9 homolog (C. elegans)	0.947867	0.6661	0.8286
73112	3110003A17Rik	RIKEN cDNA 3110003A17 gene	0.947867	0.4626	NA
78285	5330426L24Rik	RIKEN cDNA 5330426L24 gene	0.947867	0.567	0.7625
97287	Mtmr14	myotubularin related protein 14	0.947867	0.5424	0.7461
98415	Nucks1	nuclear casein kinase and cyclin-dependent kinase substrate 1	0.947867	0.5781	0.7704
104307	Rnu12	RNA U12, small nuclear	0.947867	0.5052	NA
104681	Slc16a6	solute carrier family 16 (monocarboxylic acid transporters), member 6	0.947867	0.2852	NA
105504	Exoc5	exocyst complex component 5	0.947867	0.3681	NA

106504	Stk38	serine/threonine kinase 38	0.947867	0.4341	NA	
210376	Mtmr9	myotubularin related protein 9	0.947867	0.4048	NA	
210710	Gab3	growth factor receptor bound protein 2-associated protein 3	0.947867	0.5649		0.7617
211739	Vstm2a	V-set and transmembrane domain containing 2A	0.947867	0.3231	NA	
213819	Casd1	CAS1 domain containing 1	0.947867	0.4552	NA	
216198	Tcp11l2	t-complex 11 (mouse) like 2	0.947867	0.4665	NA	
216227	Slc17a8	solute carrier family 17 (sodium-dependent inorganic phosphate cotransporter), member 8	0.947867	0.5752		0.7688
219149	Xkr6	X Kell blood group precursor related family member 6 homolog	0.947867	0.5025	NA	
223774	Alg12	asparagine-linked glycosylation 12 homolog (yeast, alpha-1,6-mannosyltransferase)	0.947867	0.6757		0.8335
223828	Pphln1	periphilin 1	0.947867	0.4491	NA	
227674	Ddx31	DEAD/H (Asp-Glu-Ala-Asp/His) box polypeptide 31	0.947867	0.3208	NA	
232853	Zfp954	zinc finger protein 954	0.947867	0.4815	NA	
235623	Scap	SREBF chaperone	0.947867	0.2883	NA	
237107	Gnl3l	guanine nucleotide binding protein-like 3 (nucleolar)-like	0.947867	0.6951		0.8451
238803	Zfp366	zinc finger protein 366	0.947867	0.7902		0.9007
241593	Pin1-ps1	peptidylprolyl cis/trans isomerase, NIMA-interacting 1, pseudogene 1	0.947867	0.3023	NA	
242662	Rims3	regulating synaptic membrane exocytosis 3	0.947867	0.6217		0.7996
242735	Lrrc38	leucine rich repeat containing 38	0.947867	0.412	NA	
266781	Snx17	sorting nexin 17	0.947867	0.3927	NA	
276919	Gemin4	gem (nuclear organelle) associated protein 4	0.947867	0.6285		0.8039
320429	Trank1	tetratricopeptide repeat and ankyrin repeat containing 1	0.947867	0.8378		0.927
330817	Dhps	deoxyhypusine synthase	0.947867	0.3562	NA	
338365	Slc41a2	solute carrier family 41, member 2	0.947867	0.2817	NA	
378430	Nanos2	nanos homolog 2 (Drosophila)	0.947867	0.7266		0.8643
382089	Ripply2	ripply2 homolog (zebrafish)	0.947867	0.4658	NA	
545156	Kalrn	kalirin, RhoGEF kinase	0.947867	0.4157	NA	
619719	6430590A07Rik	RIKEN cDNA 6430590A07 gene	0.947867	0.7169		0.8585
667682	Gm8759	predicted gene 8759	0.947867	0.3688	NA	
674321	LOC674321	glycine cleavage system H protein, mitochondrial-like	0.947867	0.4315	NA	
100137727	9130230N09Rik	RIKEN cDNA 9130230N09 gene	0.947867	0.4916	NA	
12396	Cbfa2t2	core-binding factor, runt domain, alpha subunit 2, translocated to, 2 (human)	0.94697	0.2087	NA	
12507	Cd5	CD5 antigen	0.94697	0.6172		0.797
13191	Dctn1	dynactin 1	0.94697	0.3715	NA	
14148	Fdx1	ferredoxin 1	0.94697	0.2493	NA	
14782	Gsr	glutathione reductase	0.94697	0.4743	NA	
15460	Hr	hairless	0.94697	0.5946		0.7816
15468	Prmt2	protein arginine N-methyltransferase 2	0.94697	0.3512	NA	
18109	Mycn	v-myc myelocytomatosis viral related oncogene, neuroblastoma derived (avian)	0.94697	0.4723	NA	
19018	Scand1	SCAN domain-containing 1	0.94697	0.4381	NA	
19207	Ptch2	patched homolog 2	0.94697	0.7919		0.9014
21915	Dtymk	deoxythymidylate kinase	0.94697	0.4903	NA	
22209	Ube2a	ubiquitin-conjugating enzyme E2A, RAD6 homolog (S. cerevisiae)	0.94697	0.2177	NA	
22758	Zscan12	zinc finger and SCAN domain containing 12	0.94697	0.4056	NA	
23808	Ash2l	ash2 (absent, small, or homeotic)-like (Drosophila)	0.94697	0.2896	NA	
26987	Eif4e2	eukaryotic translation initiation factor 4E member 2	0.94697	0.6856		0.8398
50887	Hmgn5	high-mobility group nucleosome binding domain 5	0.94697	0.7452		0.8762
54138	Atxn10	ataxin 10	0.94697	0.4151	NA	
56278	Gkap1	G kinase anchoring protein 1	0.94697	0.2412	NA	
56471	Stmn4	stathmin-like 4	0.94697	0.4922	NA	

65247	Asb1	ankyrin repeat and SOCS box-containing 1	0.94697	0.2977	NA
67028	2610002M06Rik	RIKEN cDNA 2610002M06 gene	0.94697	0.4513	NA
67540	4931428A05Rik	RIKEN cDNA 4931428A05 gene	0.94697	0.6674	0.8293
67665	Dctn4	dynactin 4	0.94697	0.2167	NA
67933	Hcfc2	host cell factor C2	0.94697	0.5723	0.7664
67988	Tmx3	thioredoxin-related transmembrane protein 3	0.94697	0.594	0.7814
69434	Snhg10	small nucleolar RNA host gene (non-protein coding) 10	0.94697	0.4617	NA
69478	2300009A05Rik	RIKEN cDNA 2300009A05 gene	0.94697	0.3368	NA
72185	Dbndd1	dysbindin (dystrobrevin binding protein 1) domain containing 1	0.94697	0.4741	NA
72425	2410042D21Rik	RIKEN cDNA 2410042D21 gene	0.94697	0.4615	NA
72949	Ccnt2	cyclin T2	0.94697	0.6194	0.7985
75458	Cklf	chemokine-like factor	0.94697	0.3473	NA
76893	Lass2	LAG1 homolog, ceramide synthase 2	0.94697	0.4242	NA
83485	Ngrn	neugrin, neurite outgrowth associated	0.94697	0.2881	NA
94245	Dtnbp1	dystrobrevin binding protein 1	0.94697	0.3263	NA
98258	Txndc9	thioredoxin domain containing 9	0.94697	0.5292	0.7357
114672	1700007E05Rik	RIKEN cDNA 1700007E05 gene	0.94697	0.7377	0.8715
192651	Zfp286	zinc finger protein 286	0.94697	0.4436	NA
208869	Dock3	dedicator of cyto-kinesis 3	0.94697	0.5973	0.7833
214931	Fbxl16	F-box and leucine-rich repeat protein 16	0.94697	0.3418	NA
223701	Mkl1	MKL (megakaryoblastic leukemia)/myocardin-like 1	0.94697	0.5658	0.762
224656	Zfp523	zinc finger protein 523	0.94697	0.6945	0.8447
226757	Wdr26	WD repeat domain 26	0.94697	0.3461	NA
230085	N28178	expressed sequence N28178	0.94697	0.5157	NA
239099	Homez	homeodomain leucine zipper-encoding gene	0.94697	0.4279	NA
252907	Vmn1r192	vomeronasal 1 receptor 192	0.94697	0.6839	0.8386
269700	Gm15800	predicted gene 15800	0.94697	0.3306	NA
319387	Lphn3	latrophilin 3	0.94697	0.6743	0.8331
381337	Fam178b	family with sequence similarity 178, member B	0.94697	0.6815	0.837
625730	Gm6616	predicted gene 6616	0.94697	0.5427	0.7463
100042332	2810410L24Rik	RIKEN cDNA 2810410L24 gene	0.94697	0.5109	NA
100042498	2210403K04Rik	RIKEN cDNA 2210403K04 gene	0.94697	0.6542	0.8209
100042958	Gm4129	predicted gene 4129	0.94697	0.5347	0.7401
11957	Atp5j	ATP synthase, H+ transporting, mitochondrial F0 complex, subunit F	0.946074	0.2362	NA
14562	Gdf3	growth differentiation factor 3	0.946074	0.7017	0.8495
14806	Grik2	glutamate receptor, ionotropic, kainate 2 (beta 2)	0.946074	0.6077	0.7902
16764	Aff3	AF4/FMR2 family, member 3	0.946074	0.7372	0.8712
17527	Mpv17	MpV17 mitochondrial inner membrane protein	0.946074	0.4571	NA
20224	Sar1a	SAR1 gene homolog A (S. cerevisiae)	0.946074	0.3108	NA
20273	Scn8a	sodium channel, voltage-gated, type VIII, alpha	0.946074	0.7186	0.8594
22196	Ube2i	ubiquitin-conjugating enzyme E2I	0.946074	0.5288	0.7354
22627	Ywhae	tyrosine 3-monooxygenase/tryptophan 5-monooxygenase activation protein, epsilon polypeptide	0.946074	0.4389	NA
52829	D4Bwg0951e	DNA segment, Chr 4, Brigham & Women's Genetics 0951 expressed	0.946074	0.3869	NA
52892	Sco1	SCO cytochrome oxidase deficient homolog 1 (yeast)	0.946074	0.5481	0.7496
56372	1110004F10Rik	RIKEN cDNA 1110004F10 gene	0.946074	0.5099	NA
56378	Arpc3	actin related protein 2/3 complex, subunit 3	0.946074	0.2257	NA
59046	Arpp19	cAMP-regulated phosphoprotein 19	0.946074	0.7149	0.857
66530	Ubxn6	UBX domain protein 6	0.946074	0.4545	NA
66934	Dsn1	DSN1, MIND kinetochore complex component, homolog (S. cerevisiae)	0.946074	0.5654	0.7619

67445	C1qtnf4	C1q and tumor necrosis factor related protein 4	0.946074	0.5813	0.7727
67469	Abhd5	abhydrolase domain containing 5	0.946074	0.4064	NA
68152	Fam133b	family with sequence similarity 133, member B	0.946074	0.3591	NA
68364	0610030E20Rik	RIKEN cDNA 0610030E20 gene	0.946074	0.466	NA
71392	5430401F13Rik	RIKEN cDNA 5430401F13 gene	0.946074	0.7295	0.8662
71805	Nup93	nucleoporin 93	0.946074	0.2945	NA
71835	Lancl2	LanC (bacterial lantibiotic synthetase component C)-like 2	0.946074	0.2606	NA
72108	Ddhd2	DDHD domain containing 2	0.946074	0.7516	0.8799
72421	Ttc30b	tetratricopeptide repeat domain 30B	0.946074	0.5237	0.7314
72843	Prdm4	PR domain containing 4	0.946074	0.3551	NA
73095	Slc25a42	solute carrier family 25, member 42	0.946074	0.4773	NA
73166	Tm7sf2	transmembrane 7 superfamily member 2	0.946074	0.5807	0.7725
73533	1700080G18Rik	RIKEN cDNA 1700080G18 gene	0.946074	0.4652	NA
74340	Ahcyl2	S-adenosylhomocysteine hydrolase-like 2	0.946074	0.2367	NA
74438	Clvs1	clavesin 1	0.946074	0.5653	0.7618
76251	0610007P08Rik	RIKEN cDNA 0610007P08 gene	0.946074	0.6494	0.8177
83768	Dpp7	dipeptidylpeptidase 7	0.946074	0.4231	NA
99045	Mrps26	mitochondrial ribosomal protein S26	0.946074	0.3744	NA
99167	Ssx2ip	synovial sarcoma, X breakpoint 2 interacting protein	0.946074	0.1746	NA
101883	Tmem149	transmembrane protein 149	0.946074	0.541	0.7451
102339	Cog4	component of oligomeric golgi complex 4	0.946074	0.3266	NA
105833	Ccdc65	coiled-coil domain containing 65	0.946074	0.4266	NA
209456	Trp53bp2	transformation related protein 53 binding protein 2	0.946074	0.7359	0.8707
210293	Dock10	dedicator of cytokinesis 10	0.946074	0.6543	0.8209
217125	Samd14	sterile alpha motif domain containing 14	0.946074	0.4643	NA
223921	Aaas	achalasia, adrenocortical insufficiency, alacrimia	0.946074	0.5304	0.7368
225326	Pik3c3	phosphoinositide-3-kinase, class 3	0.946074	0.6127	0.794
227058	Dnahc7b	dynein, axonemal, heavy chain 7B	0.946074	0.781	0.8949
228859	Fitm2	fat storage-inducing transmembrane protein 2	0.946074	0.3133	NA
230752	Fam176b	family with sequence similarity 176, member B	0.946074	0.3111	NA
232314	Ppp4r2	protein phosphatase 4, regulatory subunit 2	0.946074	0.4644	NA
234740	Tmem231	transmembrane protein 231	0.946074	0.6767	0.8343
239336	Rxfp3	relaxin family peptide receptor 3	0.946074	0.6619	0.8258
239556	Cacna1i	calcium channel, voltage-dependent, alpha 1I subunit	0.946074	0.6619	0.8259
280621	BC089491	cDNA sequence BC089491	0.946074	0.4955	NA
319965	Cc2d1b	coiled-coil and C2 domain containing 1B	0.946074	0.4722	NA
320534	Tmem104	transmembrane protein 104	0.946074	0.4229	NA
380753	Atxn7l1	ataxin 7-like 1	0.946074	0.4541	NA
384719	Gm5341	predicted pseudogene 5341	0.946074	0.7027	0.8502
622301	Rhox2h	reproductive homeobox 2H	0.946074	0.3151	NA
11429	Aco2	aconitase 2, mitochondrial	0.94518	0.4394	NA
12652	Chga	chromogranin A	0.94518	0.4544	NA
15939	Ier5	immediate early response 5	0.94518	0.6476	0.8165
16548	Khk	ketohexokinase	0.94518	0.4653	NA
18574	Pde1b	phosphodiesterase 1B, Ca2+-calmodulin dependent	0.94518	0.4737	NA
20084	Rps18	ribosomal protein S18	0.94518	0.2835	NA
21854	Timm17a	translocase of inner mitochondrial membrane 17a	0.94518	0.2568	NA
22225	Usp5	ubiquitin specific peptidase 5 (isopeptidase T)	0.94518	0.3535	NA
23881	G3bp2	GTPase activating protein (SH3 domain) binding protein 2	0.94518	0.3619	NA

51875	Tmem141	transmembrane protein 141	0.94518	0.4507	NA
56220	Zfp386	zinc finger protein 386 (Kruppel-like)	0.94518	0.4907	NA
56338	Txnip	thioredoxin interacting protein	0.94518	0.384	NA
56382	Rab9	RAB9, member RAS oncogene family	0.94518	0.3096	NA
57138	Slc12a5	solute carrier family 12, member 5	0.94518	0.532	0.7378
57294	Rps27	ribosomal protein S27	0.94518	0.4095	NA
66302	Fam82b	family with sequence similarity 82, member B	0.94518	0.4203	NA
66493	Mrpl51	mitochondrial ribosomal protein L51	0.94518	0.1899	NA
66513	Tab1	TGF-beta activated kinase 1/MAP3K7 binding protein 1	0.94518	0.4602	NA
67943	Mesdc2	mesoderm development candidate 2	0.94518	0.3495	NA
68203	Diras2	DIRAS family, GTP-binding RAS-like 2	0.94518	0.4087	NA
68949	1500012F01Rik	RIKEN cDNA 1500012F01 gene	0.94518	0.2672	NA
69101	Ydjc	YdjC homolog (bacterial)	0.94518	0.4371	NA
69834	Rab43	RAB43, member RAS oncogene family	0.94518	0.3143	NA
71073	4933421O10Rik	RIKEN cDNA 4933421O10 gene	0.94518	0.6165	0.7965
72139	2610044O15Rik	RIKEN cDNA 2610044O15 gene	0.94518	0.4473	NA
73386	1700048M11Rik	RIKEN cDNA 1700048M11 gene	0.94518	0.6941	0.8445
74111	Rbm19	RNA binding motif protein 19	0.94518	0.4154	NA
74919	4930471M23Rik	RIKEN cDNA 4930471M23 gene	0.94518	0.5811	0.7726
75339	Mphosph8	M-phase phosphoprotein 8	0.94518	0.2172	NA
77116	Mtmr2	myotubularin related protein 2	0.94518	0.4537	NA
78294	Rps27a	ribosomal protein S27A	0.94518	0.568	0.7633
80795	Selk	selenoprotein K	0.94518	0.3202	NA
97954	C77847	expressed sequence C77847	0.94518	0.7639	0.8862
100336	Ppp1r8	protein phosphatase 1, regulatory (inhibitor) subunit 8	0.94518	0.5742	0.7677
100382	AW011738	expressed sequence AW011738	0.94518	0.5082	NA
106389	Eaf2	ELL associated factor 2	0.94518	0.565	0.7618
118454	Gjc2	gap junction protein, gamma 2	0.94518	0.4181	NA
194388	Tet3	tet oncogene family member 3	0.94518	0.8177	0.9168
211712	Pcdh9	protocadherin 9	0.94518	0.6023	0.7866
216150	Cdc34	cell division cycle 34 homolog (S. cerevisiae)	0.94518	0.2964	NA
216810	Tom1l2	target of myb1-like 2 (chicken)	0.94518	0.7811	0.8949
219158	2610301G19Rik	RIKEN cDNA 2610301G19 gene	0.94518	0.3798	NA
225888	Suv420h1	suppressor of variegation 4-20 homolog 1 (Drosophila)	0.94518	0.398	NA
228714	Csrp2bp	cysteine and glycine-rich protein 2 binding protein	0.94518	0.6835	0.8384
229780	Ccdc76	coiled-coil domain containing 76	0.94518	0.5693	0.7638
239337	Adamts12	a disintegrin-like and metallopeptidase (reprolysin type) with thrombospondin type 1 motif, 12	0.94518	0.7465	0.877
244713	Zfp317	zinc finger protein 317	0.94518	0.7777	0.893
244867	Arhgap20	Rho GTPase activating protein 20	0.94518	0.5858	0.776
266614	Ly6g5b	lymphocyte antigen 6 complex, locus G5B	0.94518	0.4725	NA
320983	B130019D13Rik	RIKEN cDNA B130019D13 gene	0.94518	0.6277	0.8037
338366	Mia3	melanoma inhibitory activity 3	0.94518	0.3636	NA
382038	Urb2	URB2 ribosome biogenesis 2 homolog (S. cerevisiae)	0.94518	0.5393	0.7436
382221	Gm1141	predicted gene 1141	0.94518	0.7926	0.9016
382562	Pfn4	profilin family, member 4	0.94518	0.4153	NA
433904	Ociad2	OCIA domain containing 2	0.94518	0.507	NA
436230	BC065397	cDNA sequence BC065397	0.94518	0.2805	NA
631470	Gm7065	predicted gene 7065	0.94518	0.3679	NA
664987	LOC664987	novel KRAB box and zinc finger, C2H2 type domain containing protein	0.94518	0.7539	0.8812

100038371	Zfp389	zinc finger protein 389	0.94518	0.5037	NA
100043468	Zfp955b	zinc finger protein 955B	0.94518	0.5528	0.7528
11861	Arl4a	ADP-ribosylation factor-like 4A	0.944287	0.4433	NA
12095	Bglap-rs1	bone gamma-carboxyglutamate protein, related sequence 1	0.944287	0.6745	0.8332
13144	Dapk3	death-associated protein kinase 3	0.944287	0.7037	0.8507
13663	Ei24	etoposide induced 2.4 mRNA	0.944287	0.5169	0.7265
14470	Rabac1	Rab acceptor 1 (prenylated)	0.944287	0.4737	NA
15461	Hras1	Harvey rat sarcoma virus oncogene 1	0.944287	0.4268	NA
16532	Kcnu1	potassium channel, subfamily U, member 1	0.944287	0.5446	0.7474
16835	Ldlr	low density lipoprotein receptor	0.944287	0.4236	NA
17938	Naca	nascent polypeptide-associated complex alpha polypeptide	0.944287	0.281	NA
18784	Pla2g5	phospholipase A2, group V	0.944287	0.6015	0.7862
27223	Trp53bp1	transformation related protein 53 binding protein 1	0.944287	0.5691	0.7638
29849	Olfr159	olfactory receptor 159	0.944287	0.5735	0.7672
50755	Fbxo18	F-box protein 18	0.944287	0.2753	NA
56700	0610031J06Rik	RIKEN cDNA 0610031J06 gene	0.944287	0.5959	0.7822
59038	Pxmp4	peroxisomal membrane protein 4	0.944287	0.7686	0.8886
66197	Cks2	CDC28 protein kinase regulatory subunit 2	0.944287	0.5297	0.7361
66317	Wdr61	WD repeat domain 61	0.944287	0.2359	NA
66405	Mcts2	malignant T cell amplified sequence 2	0.944287	0.2395	NA
66467	Gtf2h5	general transcription factor IIH, polypeptide 5	0.944287	0.4886	NA
67054	Paics	phosphoribosylaminoimidazole carboxylase, phosphoribosylaminoribosylaminoimidazole, succinocarboxamide synthetase	0.944287	0.3311	NA
67369	Qpctl	glutaminyl-peptide cyclotransferase-like	0.944287	0.5967	0.7828
67399	Pdlim7	PDZ and LIM domain 7	0.944287	0.6551	0.8212
68202	Ndufa5	NADH dehydrogenase (ubiquinone) 1 alpha subcomplex, 5	0.944287	0.3367	NA
68552	1110003E01Rik	RIKEN cDNA 1110003E01 gene	0.944287	0.2505	NA
70428	Polr3b	polymerase (RNA) III (DNA directed) polypeptide B	0.944287	0.2048	NA
70592	5730480H06Rik	RIKEN cDNA 5730480H06 gene	0.944287	0.4923	NA
71745	Cul2	cullin 2	0.944287	0.1865	NA
72184	Klhl35	kelch-like 35 (Drosophila)	0.944287	0.5936	0.781
72685	Dnajc6	DnaJ (Hsp40) homolog, subfamily C, member 6	0.944287	0.7192	0.8597
74455	Nsun6	NOL1/NOP2/Sun domain family member 6	0.944287	0.6072	0.79
75669	Pik3r4	phosphatidylinositol 3 kinase, regulatory subunit, polypeptide 4, p150	0.944287	0.3904	NA
76178	6330578E17Rik	RIKEN cDNA 6330578E17 gene	0.944287	0.5196	0.7282
76178	6330578E17Rik	RIKEN cDNA 6330578E17 gene	0.944287	0.4858	NA
76295	Atp11b	ATPase, class VI, type 11B	0.944287	0.7194	0.8597
83435	Plekha3	pleckstrin homology domain-containing, family A (phosphoinositide binding specific) member 3	0.944287	0.2416	NA
94219	Cnnm2	cyclin M2	0.944287	0.3236	NA
101100	Ttll3	tubulin tyrosine ligase-like family, member 3	0.944287	0.6385	0.8103
108671	Dnajc9	DnaJ (Hsp40) homolog, subfamily C, member 9	0.944287	0.2768	NA
114896	Afg3l1	AFG3(ATPase family gene 3)-like 1 (yeast)	0.944287	0.2588	NA
211945	Plekhh1	pleckstrin homology domain containing, family H (with MyTH4 domain) member 1	0.944287	0.4702	NA
214952	Rhot2	ras homolog gene family, member T2	0.944287	0.3544	NA
227624	B230208H17Rik	RIKEN cDNA B230208H17 gene	0.944287	0.5202	0.7287
227644	Snapc4	small nuclear RNA activating complex, polypeptide 4	0.944287	0.448	NA
230868	Igsf21	immunoglobin superfamily, member 21	0.944287	0.3798	NA
232906	Grlf1	glucocorticoid receptor DNA binding factor 1	0.944287	0.3348	NA
233020	Hipk4	homeodomain interacting protein kinase 4	0.944287	0.6756	0.8335
233315	Mtmr10	myotubularin related protein 10	0.944287	0.3618	NA

239528	Eif2c2	eukaryotic translation initiation factor 2C, 2	0.944287	0.5614	0.7598
240186	Zfp438	zinc finger protein 438	0.944287	0.6107	0.7926
242409	Tmem8b	transmembrane protein 8B	0.944287	0.5669	0.7625
268783	Mtmr12	myotubularin related protein 12	0.944287	0.3888	NA
319901	Dsel	dermatan sulfate epimerase-like	0.944287	0.5019	NA
319922	Vwc2	von Willebrand factor C domain containing 2	0.944287	0.5456	0.7479
329152	Hecw2	HECT, C2 and WW domain containing E3 ubiquitin protein ligase 2	0.944287	0.3812	NA
622534	Gm13611	predicted gene 13611	0.944287	0.5208	0.7289
100040599	Gm15319	predicted gene 15319	0.944287	0.6532	0.8205
100042784	Prdm11	PR domain containing 11	0.944287	0.5916	0.7799
11886	Asah1	N-acylsphingosine amidohydrolase 1	0.943396	0.5367	0.7416
12032	Bcan	brevican	0.943396	0.4514	NA
12867	Cox7c	cytochrome c oxidase, subunit VIIc	0.943396	0.6957	0.8456
16525	Kcnk1	potassium channel, subfamily K, member 1	0.943396	0.4543	NA
18541	Pcnt	pericentrin (kendrin)	0.943396	0.239	NA
19119	Prm2	protamine 2	0.943396	0.8279	0.9222
19133	Prph2	peripherin 2	0.943396	0.4147	NA
19139	Prps1	phosphoribosyl pyrophosphate synthetase 1	0.943396	0.24	NA
19167	Psma3	proteasome (prosome, macropain) subunit, alpha type 3	0.943396	0.2557	NA
21807	Tsc22d1	TSC22 domain family, member 1	0.943396	0.3979	NA
22411	Wnt11	wingless-related MMTV integration site 11	0.943396	0.6263	0.8028
26394	Lypla2	lysophospholipase 2	0.943396	0.3638	NA
26428	Orc4	origin recognition complex, subunit 4	0.943396	0.6449	0.8146
26912	Gcat	glycine C-acetyltransferase (2-amino-3-ketobutyrate-coenzyme A ligase)	0.943396	0.5232	0.7309
28088	D10Wsu52e	DNA segment, Chr 10, Wayne State University 52, expressed	0.943396	0.2516	NA
50721	Sirt6	sirtuin 6 (silent mating type information regulation 2, homolog) 6 (S. cerevisiae)	0.943396	0.6388	0.8106
51813	Ccnc	cyclin C	0.943396	0.3652	NA
57319	Smpdl3a	sphingomyelin phosphodiesterase, acid-like 3A	0.943396	0.6297	0.8046
66199	Commd4	COMM domain containing 4	0.943396	0.3145	NA
66306	Fam53c	family with sequence similarity 53, member C	0.943396	0.297	NA
66818	9130011J15Rik	RIKEN cDNA 9130011J15 gene	0.943396	0.5006	0.7145
68083	Pak1ip1	PAK1 interacting protein 1	0.943396	0.3345	NA
68323	Nudt22	nudix (nucleoside diphosphate linked moiety X)-type motif 22	0.943396	0.4211	NA
69955	Fars2	phenylalanine-tRNA synthetase 2 (mitochondrial)	0.943396	0.2342	NA
70380	Mospd1	motile sperm domain containing 1	0.943396	0.3795	NA
72238	Tbc1d5	TBC1 domain family, member 5	0.943396	0.4058	NA
74102	Slc35a5	solute carrier family 35, member A5	0.943396	0.504	0.7166
74122	Tmem43	transmembrane protein 43	0.943396	0.4768	NA
75234	Rnf19b	ring finger protein 19B	0.943396	0.5826	0.7735
75740	Egfem1	EGF-like and EMI domain containing 1	0.943396	0.6122	0.7937
76179	Usp31	ubiquitin specific peptidase 31	0.943396	0.1733	NA
78658	Ncapd3	non-SMC condensin II complex, subunit D3	0.943396	0.2689	NA
98828	Cdc123	cell division cycle 123 homolog (S. cerevisiae)	0.943396	0.1918	NA
102122	Fam192a	family with sequence similarity 192, member A	0.943396	0.288	NA
110829	Lims1	LIM and senescent cell antigen-like domains 1	0.943396	0.5649	0.7617
218038	Amph	amphiphysin	0.943396	0.2012	NA
227715	Exosc2	exosome component 2	0.943396	0.4327	NA
228543	Rhov	ras homolog gene family, member V	0.943396	0.6285	0.8039
228564	Frmd5	FERM domain containing 5	0.943396	0.5215	0.7293

233040	Fbxo27	F-box protein 27	0.943396	0.2992	NA
238317	C130039O16Rik	RIKEN cDNA C130039O16 gene	0.943396	0.5941	0.7814
241727	Snph	syntaphilin	0.943396	0.4745	NA
245305	B230307C23Rik	RIKEN cDNA B230307C23 gene	0.943396	0.496	0.7111
381582	Gm5151	predicted gene 5151	0.943396	0.7319	0.8678
381591	L1td1	LINE-1 type transposase domain containing 1	0.943396	0.6832	0.8382
449521	Zfp213	zinc finger protein 213	0.943396	0.3967	NA
626903	Gm6718	predicted gene 6718	0.943396	0.6232	0.8006
100038991	Gm2000	predicted gene 2000	0.943396	0.584	0.7745
100041114	Gm3146	predicted gene 3146	0.943396	0.6141	0.795
100042584	Gm16523	mitochondrial ribosomal protein L40 pseudogene	0.943396	0.6087	0.7911
11426	Macf1	microtubule-actin crosslinking factor 1	0.942507	0.2332	NA
12175	Bnip2	BCL2/adenovirus E1B interacting protein 2	0.942507	0.6304	0.8051
13041	Ctsw	cathepsin W	0.942507	0.7615	0.8845
13640	Efna5	ephrin A5	0.942507	0.2554	NA
13642	Efnb2	ephrin B2	0.942507	0.6753	0.8334
13723	Emb	embigin	0.942507	0.2058	NA
15980	Ifngr2	interferon gamma receptor 2	0.942507	0.5483	0.7497
16876	Lhx9	LIM homeobox protein 9	0.942507	0.4254	NA
16911	Lmo4	LIM domain only 4	0.942507	0.6211	0.7994
17685	Msh2	mutS homolog 2 (E. coli)	0.942507	0.2839	NA
19131	Prh1	proline rich protein HaeIII subfamily 1	0.942507	0.734	0.8692
19324	Rab1	RAB1, member RAS oncogene family	0.942507	0.2859	NA
20301	Ccl27a	chemokine (C-C motif) ligand 27A	0.942507	0.1959	NA
20955	Vamp7	vesicle-associated membrane protein 7	0.942507	0.3903	NA
23924	Katna1	katanin p60 (ATPase-containing) subunit A1	0.942507	0.3527	NA
26875	Pclo	piccolo (presynaptic cytomatrix protein)	0.942507	0.5949	0.7818
52686	Mettl2	methyltransferase like 2	0.942507	0.6572	0.8223
53612	Vti1b	vesicle transport through interaction with t-SNAREs 1B homolog	0.942507	0.4806	NA
56292	Naa10	N(alpha)-acetyltransferase 10, NatA catalytic subunitNalpha acetyltransferase 10	0.942507	0.486	0.7043
56317	Anapc7	anaphase promoting complex subunit 7	0.942507	0.6661	0.8286
56374	Tmem59	transmembrane protein 59	0.942507	0.4433	NA
66074	Tmem167	transmembrane protein 167	0.942507	0.4147	NA
66152	Uqcr10	ubiquinol-cytochrome c reductase, complex III subunit X	0.942507	0.2994	NA
67300	Cltc	clathrin, heavy polypeptide (Hc)	0.942507	0.3439	NA
67510	Fam18b	family with sequence similarity 18, member B	0.942507	0.5021	0.7152
67529	Fgfr1op2	FGFR1 oncogene partner 2	0.942507	0.4582	NA
68519	Eml1	echinoderm microtubule associated protein like 1	0.942507	0.6462	0.8155
68675	Fam172a	family with sequence similarity 172, member A	0.942507	0.2951	NA
69094	Tmem160	transmembrane protein 160	0.942507	0.3353	NA
69270	Gins1	GINS complex subunit 1 (Psf1 homolog)	0.942507	0.4768	NA
69944	2810021J22Rik	RIKEN cDNA 2810021J22 gene	0.942507	0.3279	NA
74134	Cyp2s1	cytochrome P450, family 2, subfamily s, polypeptide 1	0.942507	0.5909	0.7794
74352	Zfp84	zinc finger protein 84	0.942507	0.6451	0.8147
75678	Ippk	inositol 1,3,4,5,6-pentakisphosphate 2-kinase	0.942507	0.4436	NA
76688	Arfrp1	ADP-ribosylation factor related protein 1	0.942507	0.3083	NA
76976	2900062L11Rik	RIKEN cDNA 2900062L11 gene	0.942507	0.2975	NA
77891	Ube2s	ubiquitin-conjugating enzyme E2S	0.942507	0.2908	NA
77965	A930033C23Rik	RIKEN cDNA A930033C23 gene	0.942507	0.4493	NA

77980	Sbf1	SET binding factor 1	0.942507	0.3335	NA
80292	Zxdc	ZXD family zinc finger C	0.942507	0.2574	NA
97998	Deptor	DEP domain containing MTOR-interacting protein	0.942507	0.6913	0.8428
100494	Zfand2a	zinc finger, AN1-type domain 2A	0.942507	0.1981	NA
103768	Tubg2	tubulin, gamma 2	0.942507	0.4008	NA
107568	Wwp1	WW domain containing E3 ubiquitin protein ligase 1	0.942507	0.6072	0.79
192232	Hps4	Hermansky-Pudlak syndrome 4 homolog (human)	0.942507	0.3553	NA
192289	Tmlhe	trimethyllysine hydroxylase, epsilon	0.942507	0.5343	0.7397
214901	Chtf18	CTF18, chromosome transmission fidelity factor 18 homolog (S. cerevisiae)	0.942507	0.4878	0.7055
215951	Lace1	lactation elevated 1	0.942507	0.5923	0.7802
217143	Gpr179	G protein-coupled receptor 179	0.942507	0.3616	NA
219181	Akap11	A kinase (PRKA) anchor protein 11	0.942507	0.3152	NA
224650	Anks1	ankyrin repeat and SAM domain containing 1	0.942507	0.3524	NA
230393	BC057079	cDNA sequence BC057079	0.942507	0.3922	NA
231148	Ablim2	actin-binding LIM protein 2	0.942507	0.4284	NA
234593	Ndrg4	N-myc downstream regulated gene 4	0.942507	0.5082	0.7197
237823	Pfas	phosphoribosylformylglycinamidine synthase (FGAR amidotransferase)	0.942507	0.5627	0.7608
246691	Prok1	prokineticin 1	0.942507	0.3194	NA
277396	Klhl23	kelch-like 23 (Drosophila)	0.942507	0.496	0.7111
320202	Lefty2	left-right determination factor 2	0.942507	0.7035	0.8506
380629	Heca	headcase homolog (Drosophila)	0.942507	0.3287	NA
381644	Cep135	centrosomal protein 135	0.942507	0.4005	NA
606735	A330069E16Rik	RIKEN cDNA A330069E16 gene	0.942507	0.4748	NA
791358	Gm10065	predicted gene 10065	0.942507	0.3994	NA
100381258	D930001M23Rik	Riken cDNA D930001M23 gene	0.942507	0.6688	0.83
11790	Speg	SPEG complex locus	0.94162	0.4573	NA
11858	Rnd2	Rho family GTPase 2	0.94162	0.3459	NA
11990	Atrn	attractin	0.94162	0.4908	0.7077
12895	Cpt1b	carnitine palmitoyltransferase 1b, muscle	0.94162	0.5539	0.7537
13000	Csnk2a2	casein kinase 2, alpha prime polypeptide	0.94162	0.3942	NA
15289	Hmgb1	high mobility group box 1	0.94162	0.2851	NA
16000	Igf1	insulin-like growth factor 1	0.94162	0.3405	NA
17126	Smad2	MAD homolog 2 (Drosophila)	0.94162	0.4137	NA
17153	Mal	myelin and lymphocyte protein, T-cell differentiation protein	0.94162	0.756	0.8822
18046	Nfyc	nuclear transcription factor-Y gamma	0.94162	0.4058	NA
18174	Slc11a2	solute carrier family 11 (proton-coupled divalent metal ion transporters), member 2	0.94162	0.4423	NA
19017	Ppargc1a	peroxisome proliferative activated receptor, gamma, coactivator 1 alpha	0.94162	0.5013	0.7146
19143	St14	suppression of tumorigenicity 14 (colon carcinoma)	0.94162	0.449	NA
20392	Sgce	sarcoglycan, epsilon	0.94162	0.448	NA
20869	Stk11	serine/threonine kinase 11	0.94162	0.4663	NA
21371	Tbca	tubulin cofactor A	0.94162	0.715	0.8571
21763	Tex2	testis expressed gene 2	0.94162	0.2013	NA
22654	Zfp13	zinc finger protein 13	0.94162	0.371	NA
24044	Scamp2	secretory carrier membrane protein 2	0.94162	0.3443	NA
51812	Mcrs1	microspherule protein 1	0.94162	0.5455	0.7478
52064	Coq5	coenzyme Q5 homolog, methyltransferase (yeast)	0.94162	0.5447	0.7474
53621	Cnot4	CCR4-NOT transcription complex, subunit 4	0.94162	0.4615	NA
56455	Dynll1	dynein light chain LC8-type 1	0.94162	0.4768	0.6966
56542	Ick	intestinal cell kinase	0.94162	0.5496	0.7507

56811	Dkk2	dickkopf homolog 2 (Xenopus laevis)	0.94162	0.7177	0.8589
57753	Noc3l	nucleolar complex associated 3 homolog (S. cerevisiae)	0.94162	0.3711	NA
66104	Tceal6	transcription elongation factor A (SII)-like 6	0.94162	0.2836	NA
66451	2610528J11Rik	RIKEN cDNA 2610528J11 gene	0.94162	0.776	0.8922
66464	Taf12	TAF12 RNA polymerase II, TATA box binding protein (TBP)-associated factor	0.94162	0.4286	NA
66559	Metap1d	methionyl aminopeptidase type 1D (mitochondrial)	0.94162	0.4481	NA
67203	Nde1	nuclear distribution gene E homolog 1 (A nidulans)	0.94162	0.2406	NA
67522	1700097N02Rik	RIKEN cDNA 1700097N02 gene	0.94162	0.6142	0.795
67738	Ppid	peptidylprolyl isomerase D (cyclophilin D)	0.94162	0.4439	NA
67851	1700021F05Rik	RIKEN cDNA 1700021F05 gene	0.94162	0.2755	NA
68423	Ankrd13d	ankyrin repeat domain 13 family, member D	0.94162	0.3635	NA
68879	Prpf6	PRP6 pre-mRNA splicing factor 6 homolog (yeast)	0.94162	0.3221	NA
69527	Mrps9	mitochondrial ribosomal protein S9	0.94162	0.367	NA
71461	Ptk7	PTK7 protein tyrosine kinase 7	0.94162	0.6073	0.79
71883	Coq2	coenzyme Q2 homolog, prenyltransferase (yeast)	0.94162	0.3437	NA
73822	F630110N24Rik	RIKEN cDNA F630110N24 gene	0.94162	0.4386	NA
74025	Nphp3	nephronophthisis 3 (adolescent)	0.94162	0.843	0.9294
74781	Wipi2	WD repeat domain, phosphoinositide interacting 2	0.94162	0.4357	NA
76214	6430710M23Rik	RIKEN cDNA 6430710M23 gene	0.94162	0.8461	0.9311
77290	9430078K24Rik	RIKEN cDNA 9430078K24 gene	0.94162	0.4829	0.7013
77922	A030005L19Rik	RIKEN cDNA A030005L19 gene	0.94162	0.4957	0.7111
83885	Slc25a2	solute carrier family 25 (mitochondrial carrier, ornithine transporter) member 2	0.94162	0.7996	0.9057
84505	Setdb1	SET domain, bifurcated 1	0.94162	0.2197	NA
99412	Golga2	golgi autoantigen, golgin subfamily a, 2	0.94162	0.1734	NA
102680	Slc6a20a	solute carrier family 6 (neurotransmitter transporter), member 20A	0.94162	0.7503	0.879
114643	Oas1c	2'-5' oligoadenylate synthetase 1C	0.94162	0.5861	0.7761
223697	Sun2	Sad1 and UNC84 domain containing 2	0.94162	0.4026	NA
234683	Elmo3	engulfment and cell motility 3, ced-12 homolog (C. elegans)	0.94162	0.5118	0.7223
241296	Lrrc8a	leucine rich repeat containing 8A	0.94162	0.2736	NA
243616	Slc6a11	solute carrier family 6 (neurotransmitter transporter, GABA), member 11	0.94162	0.4364	NA
276846	Pigs	phosphatidylinositol glycan anchor biosynthesis, class S	0.94162	0.2632	NA
278507	Wfikkn2	WAP, follistatin/kazal, immunoglobulin, kunitz and netrin domain containing 2	0.94162	0.6397	0.8112
319636	Fsd1l	fibronectin type III and SPRY domain containing 1-like	0.94162	0.6202	0.7991
330485	Tmem145	transmembrane protein 145	0.94162	0.4161	NA
381921	Taok2	TAO kinase 2	0.94162	0.7266	0.8643
383712	Gm13637	predicted gene 13637	0.94162	0.4115	NA
552909	LOC552909	hypothetical LOC552909	0.94162	0.7276	0.865
668489	Gm9199	glycine cleavage system protein H (aminomethyl carrier) pseudogene	0.94162	0.6878	0.8407
11776	Ap3d1	adaptor-related protein complex 3, delta 1 subunit	0.940734	0.1718	NA
11843	Arf4	ADP-ribosylation factor 4	0.940734	0.3433	NA
12468	Cct7	chaperonin containing Tcp1, subunit 7 (eta)	0.940734	0.4031	NA
12922	Crhr2	corticotropin releasing hormone receptor 2	0.940734	0.5819	0.7729
16649	Kpna4	karyopherin (importin) alpha 4	0.940734	0.3126	NA
17145	Mageb1	melanoma antigen, family B, 1	0.940734	0.7744	0.8916
17263	Meg3	maternally expressed 3	0.940734	0.4579	NA
18607	Pdpk1	3-phosphoinositide dependent protein kinase 1	0.940734	0.541	0.7451
18762	Prkcz	protein kinase C, zeta	0.940734	0.458	NA
19732	Rgl2	ral guanine nucleotide dissociation stimulator-like 2	0.940734	0.4358	NA
19775	Xpr1	xenotropic and polytropic retrovirus receptor 1	0.940734	0.2101	NA

20603	Sms	spermine synthase	0.940734	0.1428	NA
20843	Stag2	stromal antigen 2	0.940734	0.4011	NA
21781	Tfdp1	transcription factor Dp 1	0.940734	0.4763	0.6965
22184	Zrsr2	zinc finger (CCCH type), RNA binding motif and serine/arginine rich 2	0.940734	0.3564	NA
26919	Zfp346	zinc finger protein 346	0.940734	0.4508	NA
55994	Smad9	MAD homolog 9 (Drosophila)	0.940734	0.5471	0.7487
66094	Lsm7	LSM7 homolog, U6 small nuclear RNA associated (S. cerevisiae)	0.940734	0.159	NA
66541	Immp1l	IMP1 inner mitochondrial membrane peptidase-like (S. cerevisiae)	0.940734	0.5763	0.7693
66854	Trim35	tripartite motif-containing 35	0.940734	0.2032	NA
67803	Limd2	LIM domain containing 2	0.940734	0.6676	0.8294
68255	Tmem86b	transmembrane protein 86B	0.940734	0.578	0.7704
69912	Nup43	nucleoporin 43	0.940734	0.3344	NA
71159	4933416I08Rik	RIKEN cDNA 4933416I08 gene	0.940734	0.685	0.8394
93696	Chrac1	chromatin accessibility complex 1	0.940734	0.2235	NA
93765	Ube2n	ubiquitin-conjugating enzyme E2N	0.940734	0.1694	NA
99375	Cul4a	cullin 4A	0.940734	0.4222	NA
101314	6720456B07Rik	RIKEN cDNA 6720456B07 gene	0.940734	0.359	NA
102423	Hinfp	histone H4 transcription factor	0.940734	0.4163	NA
107513	Ssr1	signal sequence receptor, alpha	0.940734	0.4214	NA
108802	Calr4	calreticulin 4	0.940734	0.537	0.7416
112414	4930540M05Rik	RIKEN cDNA 4930540M05 gene	0.940734	0.79	0.9007
170760	Acbd3	acyl-Coenzyme A binding domain containing 3	0.940734	0.4489	NA
210293	Dock10	dedicator of cytokinesis 10	0.940734	0.4144	NA
216136	Ilvbl	ilvB (bacterial acetolactate synthase)-like	0.940734	0.3929	NA
216148	Shc2	SHC (Src homology 2 domain containing) transforming protein 2	0.940734	0.4949	0.7106
216742	Fnip1	folliculin interacting protein 1	0.940734	0.2782	NA
217700	Acot6	acyl-CoA thioesterase 6	0.940734	0.489	0.7061
224613	Flywch1	FLYWCH-type zinc finger 1	0.940734	0.5816	0.7728
229504	Isg20l2	interferon stimulated exonuclease gene 20-like 2	0.940734	0.4079	NA
230751	Oscp1	organic solute carrier partner 1	0.940734	0.2091	NA
232854	Zfp418	zinc finger protein 418	0.940734	0.5547	0.7542
233067	Lrfn3	leucine rich repeat and fibronectin type III domain containing 3	0.940734	0.4723	NA
234344	Naf1	nuclear assembly factor 1 homolog (S. cerevisiae)	0.940734	0.3571	NA
234463	Tmem184c	transmembrane protein 184C	0.940734	0.3626	NA
237403	Lingo3	leucine rich repeat and Ig domain containing 3	0.940734	0.5367	0.7416
237560	Lrrc10	leucine rich repeat containing 10	0.940734	0.7012	0.8493
245537	Nlgn3	neuroligin 3	0.940734	0.5422	0.746
245671	Klf8	Kruppel-like factor 8	0.940734	0.5266	0.7337
319636	Fsd1l	fibronectin type III and SPRY domain containing 1-like	0.940734	0.5543	0.7539
319638	Nt5dc1	5'-nucleotidase domain containing 1	0.940734	0.4959	0.7111
382252	A830080D01Rik	RIKEN cDNA A830080D01 gene	0.940734	0.2464	NA
384281	Gatc	glutamyl-tRNA(Gln) amidotransferase, subunit C homolog (bacterial)	0.940734	0.588	0.7775
100101457	A830035O19Rik	RIKEN cDNA A830035O19 gene	0.940734	0.509	0.72
12798	Cnn2	calponin 2	0.93985	0.4891	0.7062
13855	Epn2	epsin 2	0.93985	0.2786	NA
14595	B4galt1	UDP-Gal:betaGlcNAc beta 1,4- galactosyltransferase, polypeptide 1	0.93985	0.2218	NA
14904	Gtpbp1	GTP binding protein 1	0.93985	0.5363	0.7413
15925	Ide	insulin degrading enzyme	0.93985	0.2455	NA
16653	Kras	v-Ki-ras2 Kirsten rat sarcoma viral oncogene homolog	0.93985	0.4554	NA

17428	Mnt	max binding protein	0.93985	0.3441	NA
18514	Pbx1	pre B-cell leukemia transcription factor 1	0.93985	0.5808	0.7725
20384	Srsf5	serine/arginine-rich splicing factor 5	0.93985	0.45	NA
20411	Sorbs1	sorbin and SH3 domain containing 1	0.93985	0.5852	0.7755
20448	St6galnac4	ST6 (alpha-N-acetyl-neuraminyl-2,3-beta-galactosyl-1,3)-N-acetylgalactosaminide alpha-2,6-sialyltransferase 4	0.93985	0.5242	0.7317
20666	Sox11	SRY-box containing gene 11	0.93985	0.472	0.6931
21951	Tnks	tankyrase, TRF1-interacting ankyrin-related ADP-ribose polymerase	0.93985	0.5685	0.7635
22088	Tsg101	tumor susceptibility gene 101	0.93985	0.3761	NA
22630	Ywhaq	tyrosine 3-monooxygenase/tryptophan 5-monooxygenase activation protein, theta polypeptide	0.93985	0.2645	NA
26444	Psma7	proteasome (prosome, macropain) subunit, alpha type 7	0.93985	0.4105	NA
26757	Dpysl4	dihydropyrimidinase-like 4	0.93985	0.4311	NA
30057	Timm8b	translocase of inner mitochondrial membrane 8 homolog b (yeast)	0.93985	0.2965	NA
50754	Fbxw7	F-box and WD-40 domain protein 7	0.93985	0.3194	NA
50880	Scly	selenocysteine lyase	0.93985	0.4682	0.6901
51789	Tnk2	tyrosine kinase, non-receptor, 2	0.93985	0.1937	NA
52504	Cenpo	centromere protein O	0.93985	0.39	NA
53883	Celsr2	cadherin, EGF LAG seven-pass G-type receptor 2 (flamingo homolog, Drosophila)	0.93985	0.7992	0.9056
54351	Rai12	retinoic acid induced 12	0.93985	0.5404	0.7446
55987	Cpxm2	carboxypeptidase X 2 (M14 family)	0.93985	0.7859	0.8978
56375	B4galt4	UDP-Gal:betaGlcNAc beta 1,4-galactosyltransferase, polypeptide 4	0.93985	0.5011	0.7146
56736	Rnf14	ring finger protein 14	0.93985	0.1466	NA
56878	Rbms1	RNA binding motif, single stranded interacting protein 1	0.93985	0.4278	NA
59003	Maea	macrophage erythroblast attacher	0.93985	0.3105	NA
59009	Sh3rf1	SH3 domain containing ring finger 1	0.93985	0.3388	NA
66054	Cndp2	CNDP dipeptidase 2 (metallopeptidase M20 family)	0.93985	0.5533	0.7534
66958	Tmx2	thioredoxin-related transmembrane protein 2	0.93985	0.4956	0.7111
67713	Dnajc19	DnaJ (Hsp40) homolog, subfamily C, member 19	0.93985	0.416	NA
69539	Trnp1	TMF1-regulated nuclear protein 1	0.93985	0.5847	0.775
69605	Lnp	limb and neural patterns	0.93985	0.667	0.8292
70461	Crtc3	CREB regulated transcription coactivator 3	0.93985	0.3693	NA
70870	4921516I12Rik	RIKEN cDNA 4921516I12 gene	0.93985	0.374	NA
71514	Sfpq	splicing factor proline/glutamine rich (polypyrimidine tract binding protein associated)	0.93985	0.6369	0.8091
72144	Slc37a3	solute carrier family 37 (glycerol-3-phosphate transporter), member 3	0.93985	0.2358	NA
72344	Usp36	ubiquitin specific peptidase 36	0.93985	0.5254	0.7326
72831	Dhx30	DEAH (Asp-Glu-Ala-His) box polypeptide 30	0.93985	0.4566	NA
73261	1700037C18Rik	RIKEN cDNA 1700037C18 gene	0.93985	0.6439	0.814
74035	Nol9	nucleolar protein 9	0.93985	0.7587	0.8831
74254	Gpn1	GPN-loop GTPase 1	0.93985	0.2384	NA
75051	4930578N16Rik	RIKEN cDNA 4930578N16 gene	0.93985	0.4321	NA
77264	Zfp142	zinc finger protein 142	0.93985	0.5695	0.7638
96957	Tmem62	transmembrane protein 62	0.93985	0.6413	0.8126
170791	Rbm39	RNA binding motif protein 39	0.93985	0.4403	NA
170823	Glmn	glomulin, FKBP associated protein	0.93985	0.3528	NA
171508	Creld1	cysteine-rich with EGF-like domains 1	0.93985	0.466	NA
192950	Nacad	NAC alpha domain containing	0.93985	0.4478	NA
216134	Pdxk	pyridoxal (pyridoxine, vitamin B6) kinase	0.93985	0.5278	0.7346
216157	ORF61	open reading frame 61	0.93985	0.438	NA
218397	Rasa1	RAS p21 protein activator 1	0.93985	0.537	0.7416
229524	Msto1	misato homolog 1 (Drosophila)	0.93985	0.4035	NA

232210	8430410A17Rik	RIKEN cDNA 8430410A17 gene	0.93985	0.3461	NA
232944	Mark4	MAP/microtubule affinity-regulating kinase 4	0.93985	0.4632	0.6859
237400	Mex3d	mex3 homolog D (C. elegans)	0.93985	0.3416	NA
257926	Olfr544	olfactory receptor 544	0.93985	0.667	0.8292
260305	Nphp4	nephronophthisis 4 (juvenile) homolog (human)	0.93985	0.4255	NA
384214	Ephx4	epoxide hydrolase 4	0.93985	0.2793	NA
404583	AL118220	expressed sequence AL118220	0.93985	0.5003	0.7144
626578	Gbp10	guanylate-binding protein 10	0.93985	0.6784	0.8355
634012	LOC634012	ubiquitin-conjugating enzyme E2 Q2-like	0.93985	0.3838	NA
668272	Gm9079	transmembrane emp24 domain trafficking protein 2 pseudogene	0.93985	0.2992	NA
100036525	LOC100036525	hypothetical LOC100036525	0.93985	0.5363	0.7413
100504262	LOC100504262	hypothetical LOC100504262	0.93985	0.4974	0.7123
100505352	LOC100505352	hypothetical LOC100505352	0.93985	0.566	0.762
11735	Ank3	ankyrin 3, epithelial	0.938967	0.6643	0.8277
13860	Eps8	epidermal growth factor receptor pathway substrate 8	0.938967	0.6033	0.7871
14068	F7	coagulation factor VII	0.938967	0.6776	0.8348
14539	Opn1mw	opsin 1 (cone pigments), medium-wave-sensitive (color blindness, deutan)	0.938967	0.2446	NA
17993	Ndufs4	NADH dehydrogenase (ubiquinone) Fe-S protein 4	0.938967	0.1635	NA
18041	Nfs1	nitrogen fixation gene 1 (S. cerevisiae)	0.938967	0.3253	NA
18150	Npm3	nucleoplasmin 3	0.938967	0.8513	0.9336
18163	Ctnnd2	catenin (cadherin associated protein), delta 2	0.938967	0.5101	0.7211
20540	Slc7a7	solute carrier family 7 (cationic amino acid transporter, y+ system), member 7	0.938967	0.5288	0.7354
20621	Snn	stannin	0.938967	0.2581	NA
20832	Ssr4	signal sequence receptor, delta	0.938967	0.173	NA
22066	Trpc4	transient receptor potential cation channel, subfamily C, member 4	0.938967	0.3109	NA
26754	Cops5	COP9 (constitutive photomorphogenic) homolog, subunit 5 (Arabidopsis thaliana)	0.938967	0.1602	NA
27494	Amot	angiomotin	0.938967	0.3494	NA
55992	Trim3	tripartite motif-containing 3	0.938967	0.5371	0.7417
65971	Tbata	thymus, brain and testes associated	0.938967	0.6345	0.8076
66511	2500003M10Rik	RIKEN cDNA 2500003M10 gene	0.938967	0.276	NA
67016	Tbc1d2b	TBC1 domain family, member 2B	0.938967	0.5297	0.7361
67241	Smc6	structural maintenance of chromosomes 6	0.938967	0.3417	NA
68184	Denr	density-regulated protein	0.938967	0.2777	NA
68449	Tbc1d10b	TBC1 domain family, member 10b	0.938967	0.2147	NA
68795	Ubr3	ubiquitin protein ligase E3 component n-recognin 3	0.938967	0.4322	NA
68889	Ubac2	ubiquitin associated domain containing 2	0.938967	0.4361	NA
69890	Zfp219	zinc finger protein 219	0.938967	0.7145	0.8567
69934	Rg9mtd3	RNA (guanine-9-) methyltransferase domain containing 3	0.938967	0.289	NA
72486	Rnf219	ring finger protein 219	0.938967	0.6322	0.8064
72508	Rps6kb1	ribosomal protein S6 kinase, polypeptide 1	0.938967	0.355	NA
75605	Kdm5b	lysine (K)-specific demethylase 5B	0.938967	0.469	0.6909
76187	Adhfe1	alcohol dehydrogenase, iron containing, 1	0.938967	0.5165	0.7263
78581	Utp23	UTP23, small subunit (SSU) processome component, homolog (yeast)	0.938967	0.3004	NA
78920	Dlst	dihydrolipoamide S-succinyltransferase (E2 component of 2-oxo-glutarate complex)	0.938967	0.3787	NA
83382	Siglece	sialic acid binding Ig-like lectin E	0.938967	0.731	0.8669
103135	Pan2	PAN2 polyA specific ribonuclease subunit homolog (S. cerevisiae)	0.938967	0.3396	NA
107976	Bre	brain and reproductive organ-expressed protein	0.938967	0.1993	NA
108653	Rimklb	ribosomal modification protein rimK-like family member B	0.938967	0.3806	NA
110350	Dync2h1	dynein cytoplasmic 2 heavy chain 1	0.938967	0.4157	NA

110809	Srsf1	serine/arginine-rich splicing factor 1	0.938967	0.3011	NA
111975	Igf2as	insulin-like growth factor 2, antisense	0.938967	0.5059	0.7181
114570	Crip3	cysteine-rich protein 3	0.938967	0.6267	0.803
214580	Pstk	phosphoseryl-tRNA kinase	0.938967	0.2962	NA
214917	Fam173a	family with sequence similarity 173, member A	0.938967	0.2824	NA
226591	Tiprl	TIP41, TOR signalling pathway regulator-like (S. cerevisiae)	0.938967	0.4222	NA
230597	Zfyve9	zinc finger, FYVE domain containing 9	0.938967	0.2686	NA
269593	Luzp1	leucine zipper protein 1	0.938967	0.2789	NA
269966	Nup98	nucleoporin 98	0.938967	0.1693	NA
320376	Bcorl1	BCL6 co-repressor-like 1	0.938967	0.3662	NA
381884	Slc6a16	solute carrier family 6, member 16	0.938967	0.5754	0.7689
433940	BC057022	cDNA sequence BC057022	0.938967	0.4226	NA
546711	LOC546711	40S ribosomal protein S25-like	0.938967	0.7752	0.8918
12339	Capn7	calpain 7	0.938086	0.4558	0.6794
12747	Clk1	CDC-like kinase 1	0.938086	0.5213	0.7292
14236	Foxn2	forkhead box N2	0.938086	0.5805	0.7725
14287	Fpgs	folylpolyglutamyl synthetase	0.938086	0.3816	NA
15165	Hcn1	hyperpolarization-activated, cyclic nucleotide-gated K+ 1	0.938086	0.8257	0.9211
15568	Elavl1	ELAV (embryonic lethal, abnormal vision, Drosophila)-like 1 (Hu antigen R)	0.938086	0.177	NA
17151	Ccndbp1	cyclin D-type binding-protein 1	0.938086	0.2969	NA
18795	Plcb1	phospholipase C, beta 1	0.938086	0.5741	0.7676
19736	Rgs4	regulator of G-protein signaling 4	0.938086	0.4781	0.6979
19821	Rnf2	ring finger protein 2	0.938086	0.2346	NA
20334	Sec23a	SEC23A (S. cerevisiae)	0.938086	0.4388	NA
22375	Wars	tryptophanyl-tRNA synthetase	0.938086	0.2341	NA
22751	Zfp90	zinc finger protein 90	0.938086	0.2694	NA
23792	Adam23	a disintegrin and metallopeptidase domain 23	0.938086	0.2973	NA
23897	Hax1	HCLS1 associated X-1	0.938086	0.3285	NA
26920	Cep110	centrosomal protein 110	0.938086	0.635	0.8079
27784	Commd8	COMM domain containing 8	0.938086	0.5145	0.7247
50774	Krtap5-1	keratin associated protein 5-1	0.938086	0.5964	0.7825
53621	Cnot4	CCR4-NOT transcription complex, subunit 4	0.938086	0.6598	0.8244
53890	Sart3	squamous cell carcinoma antigen recognized by T-cells 3	0.938086	0.2353	NA
53961	9530053J19Rik	RIKEN cDNA 9530053J19 gene	0.938086	0.5864	0.7762
56174	Nagk	N-acetylglucosamine kinase	0.938086	0.5033	0.716
56384	Letm1	leucine zipper-EF-hand containing transmembrane protein 1	0.938086	0.6408	0.8121
56527	Mast1	microtubule associated serine/threonine kinase 1	0.938086	0.6295	0.8044
59050	Nsa2	NSA2 ribosome biogenesis homolog (S. cerevisiae)	0.938086	0.3704	NA
59054	Mrps30	mitochondrial ribosomal protein S30	0.938086	0.1573	NA
66401	Nudt2	nudix (nucleoside diphosphate linked moiety X)-type motif 2	0.938086	0.2779	NA
66592	Stoml2	stomatin (Epb7.2)-like 2	0.938086	0.181	NA
66821	Bcs1l	BCS1-like (yeast)	0.938086	0.4485	NA
67534	Ttll4	tubulin tyrosine ligase-like family, member 4	0.938086	0.5176	0.727
67615	Ube2r2	ubiquitin-conjugating enzyme E2R 2	0.938086	0.2887	NA
67878	Tmem33	transmembrane protein 33	0.938086	0.5573	0.7566
69019	Spcs1	signal peptidase complex subunit 1 homolog (S. cerevisiae)	0.938086	0.1391	NA
70078	Nol7	nucleolar protein 7	0.938086	0.4534	0.6774
70601	Ecd	ecdysoneless homolog (Drosophila)	0.938086	0.3504	NA
71373	Prr16	proline rich 16	0.938086	0.6567	0.8223

72699	Lime1	Lck interacting transmembrane adaptor 1	0.938086	0.3894	NA
73945	Otud4	OTU domain containing 4	0.938086	0.4482	NA
74384	4932433N03Rik	RIKEN cDNA 4932433N03 gene	0.938086	0.3382	NA
74637	Shpk	sedoheptulokinase	0.938086	0.3578	NA
75624	Metap1	methionyl aminopeptidase 1	0.938086	0.2293	NA
77215	Krtap5-3	keratin associated protein 5-3	0.938086	0.664	0.8275
78317	Ccdc88b	coiled-coil domain containing 88B	0.938086	0.3323	NA
101206	Tada3	transcriptional adaptor 3	0.938086	0.4149	NA
101513	2700078K21Rik	RIKEN cDNA 2700078K21 gene	0.938086	0.382	NA
102247	Agpat6	1-acylglycerol-3-phosphate O-acyltransferase 6 (lysophosphatidic acid acyltransferase, zeta)	0.938086	0.3419	NA
103466	Nt5dc3	5'-nucleotidase domain containing 3	0.938086	0.3603	NA
106572	Rab31	RAB31, member RAS oncogene family	0.938086	0.5806	0.7725
192652	Wdr81	WD repeat domain 81	0.938086	0.2569	NA
214133	Tet2	tet oncogene family member 2	0.938086	0.3046	NA
214290	Zcchc6	zinc finger, CCHC domain containing 6	0.938086	0.2467	NA
217232	Cdc27	cell division cycle 27 homolog (S. cerevisiae)	0.938086	0.6128	0.7941
218461	Pde8b	phosphodiesterase 8B	0.938086	0.2722	NA
225215	Rsl24d1	ribosomal L24 domain containing 1	0.938086	0.5634	0.761
228410	Cstf3	cleavage stimulation factor, 3' pre-RNA, subunit 3	0.938086	0.3879	NA
320301	E530011L22Rik	RIKEN cDNA E530011L22 gene	0.938086	0.2746	NA
320977	A330023F24Rik	RIKEN cDNA A330023F24 gene	0.938086	0.5779	0.7704
327781	A130033P14	hypothetical protein A130033P14	0.938086	0.7468	0.877
338359	Supv3l1	suppressor of var1, 3-like 1 (S. cerevisiae)	0.938086	0.2795	NA
378462	Morn2	MORN repeat containing 2	0.938086	0.6079	0.7904
594844	Tceal3	transcription elongation factor A (SII)-like 3	0.938086	0.2112	NA
791379	C030009H01Rik	RIKEN cDNA C030009H01 gene	0.938086	0.6466	0.8156
100039815	Gm2436	predicted gene 2436	0.938086	0.2975	NA
12308	Calb2	calbindin 2	0.937207	0.443	0.6689
12570	Cdk5r2	cyclin-dependent kinase 5, regulatory subunit 2 (p39)	0.937207	0.422	NA
13222	Defa-rs2	defensin, alpha, related sequence 2	0.937207	0.5051	0.7174
13859	Eps15l1	epidermal growth factor receptor pathway substrate 15-like 1	0.937207	0.6304	0.8051
14105	Srsf10	serine/arginine-rich splicing factor 10	0.937207	0.3612	NA
14148	Fdx1	ferredoxin 1	0.937207	0.1854	NA
14401	Gabrb2	gamma-aminobutyric acid (GABA) A receptor, subunit beta 2	0.937207	0.3505	NA
14407	Gabrg3	gamma-aminobutyric acid (GABA) A receptor, subunit gamma 3	0.937207	0.4729	0.6937
17999	Nedd4	neural precursor cell expressed, developmentally down-regulated 4	0.937207	0.3305	NA
18040	Nefm	neurofilament, medium polypeptide	0.937207	0.3379	NA
18749	Prkacb	protein kinase, cAMP dependent, catalytic, beta	0.937207	0.2692	NA
19356	Rad17	RAD17 homolog (S. pombe)	0.937207	0.368	NA
20312	Cx3cl1	chemokine (C-X3-C motif) ligand 1	0.937207	0.4301	NA
20742	Spnb2	spectrin beta 2	0.937207	0.2471	NA
21848	Trim24	tripartite motif-containing 24	0.937207	0.3773	NA
21917	Tmpo	thymopoietin	0.937207	0.5959	0.7822
22718	Zfp60	zinc finger protein 60	0.937207	0.576	0.769
23991	Cib1	calcium and integrin binding 1 (calmyrin)	0.937207	0.2403	NA
24017	Rnf13	ring finger protein 13	0.937207	0.2211	NA
26891	Cops4	COP9 (constitutive photomorphogenic) homolog, subunit 4 (Arabidopsis thaliana)	0.937207	0.2663	NA
52004	Cdk2ap2	CDK2-associated protein 2	0.937207	0.5007	0.7145
54712	Plxnc1	plexin C1	0.937207	0.1652	NA

56426	Pdcd10	programmed cell death 10	0.937207	0.3411	NA
56734	Tulp2	tubby-like protein 2	0.937207	0.5991	0.7846
66082	Abhd6	abhydrolase domain containing 6	0.937207	0.2488	NA
66972	Slc25a23	solute carrier family 25 (mitochondrial carrier; phosphate carrier), member 23	0.937207	0.736	0.8707
67230	Zfp329	zinc finger protein 329	0.937207	0.5643	0.7615
67544	Fam120b	family with sequence similarity 120, member B	0.937207	0.3828	NA
67871	Mrrf	mitochondrial ribosome recycling factor	0.937207	0.4829	0.7013
67877	Naa20	N(alpha)-acetyltransferase 20, NatB catalytic subunit	0.937207	0.1978	NA
67994	Mrps11	mitochondrial ribosomal protein S11	0.937207	0.3556	NA
68052	Rps13	ribosomal protein S13	0.937207	0.4503	0.6752
68075	1520402A15Rik	RIKEN cDNA 1520402A15 gene	0.937207	0.5336	0.7392
68938	Aspscr1	alveolar soft part sarcoma chromosome region, candidate 1 (human)	0.937207	0.6586	0.8235
68948	1500011H22Rik	RIKEN cDNA 1500011H22 gene	0.937207	0.1541	NA
69562	Cdk13	cyclin-dependent kinase 13	0.937207	0.4003	NA
70004	1700028J19Rik	RIKEN cDNA 1700028J19 gene	0.937207	0.6065	0.7894
71474	Ppp6r2	protein phosphatase 6, regulatory subunit 2	0.937207	0.5563	0.7556
71567	Mcm9	minichromosome maintenance complex component 9	0.937207	0.4034	NA
71991	Ercc8	excision repaiross-complementing rodent repair deficiency, complementation group 8	0.937207	0.6154	0.7958
72844	Kctd17	potassium channel tetramerisation domain containing 17	0.937207	0.368	NA
72972	Gcap14	granule cell antiserum positive 14	0.937207	0.2224	NA
72981	Prkrir	protein-kinase, interferon-inducible double stranded RNA dependent inhibitor, repressor of (P58 repressor)	0.937207	0.3553	NA
76375	Det1	de-etiolated homolog 1 (Arabidopsis)	0.937207	0.3725	NA
77031	Slc9a8	solute carrier family 9 (sodium/hydrogen exchanger), member 8	0.937207	0.3226	NA
78541	Asb8	ankyrin repeat and SOCS box-containing 8	0.937207	0.2358	NA
80913	Pum2	pumilio 2 (Drosophila)	0.937207	0.6337	0.8074
93765	Ube2n	ubiquitin-conjugating enzyme E2N	0.937207	0.5737	0.7672
102791	Tcta	T-cell leukemia translocation altered gene	0.937207	0.612	0.7935
104112	Acly	ATP citrate lyase	0.937207	0.4994	0.714
170755	Sgk3	serum/glucocorticoid regulated kinase 3	0.937207	0.1606	NA
213895	Bms1	BMS1 homolog, ribosome assembly protein (yeast)	0.937207	0.4915	0.7079
215085	Slc35f1	solute carrier family 35, member F1	0.937207	0.345	NA
216134	Pdxk	pyridoxal (pyridoxine, vitamin B6) kinase	0.937207	0.5476	0.749
217866	Cdc42bpb	CDC42 binding protein kinase beta	0.937207	0.6219	0.7997
224826	Ubr2	ubiquitin protein ligase E3 component n-recognin 2	0.937207	0.699	0.8477
228359	Arhgap1	Rho GTPase activating protein 1	0.937207	0.6252	0.8019
241556	Tspan18	tetraspanin 18	0.937207	0.5533	0.7534
245631	Mum1l1	melanoma associated antigen (mutated) 1-like 1	0.937207	0.4201	NA
260297	Prrt1	proline-rich transmembrane protein 1	0.937207	0.5434	0.7466
269181	Mgat4a	mannoside acetylglucosaminyltransferase 4, isoenzyme A	0.937207	0.4443	NA
269870	Zfp446	zinc finger protein 446	0.937207	0.4435	NA
380752	Tssc1	tumor suppressing subtransferable candidate 1	0.937207	0.5646	0.7616
406218	Panx2	pannexin 2	0.937207	0.6377	0.8097
432628	Mfsd2b	major facilitator superfamily domain containing 2B	0.937207	0.3353	NA
433424	Gm13476	predicted gene 13476	0.937207	0.6003	0.7852
435518	Pdxk-ps	pyridoxal (pyridoxine, vitamin B6) kinase, pseudogene	0.937207	0.7679	0.8882
622629	Gm10318	predicted gene 10318	0.937207	0.7055	0.852
664949	Gm7420	predicted gene 7420	0.937207	0.4173	NA
100043133	9130023H24Rik	RIKEN cDNA 9130023H24 gene	0.937207	0.5276	0.7345
11305	Abca2	ATP-binding cassette, sub-family A (ABC1), member 2	0.93633	0.5432	0.7465

11550	Adra1d	adrenergic receptor, alpha 1d	0.93633	0.6165	0.7965
12876	Cpe	carboxypeptidase E	0.93633	0.5065	0.7183
13047	Cux1	cut-like homeobox 1	0.93633	0.6847	0.8392
13116	Cyp46a1	cytochrome P450, family 46, subfamily a, polypeptide 1	0.93633	0.6612	0.8254
13131	Dab1	disabled homolog 1 (Drosophila)	0.93633	0.534	0.7394
14194	Fh1	fumarate hydratase 1	0.93633	0.1985	NA
14533	Bloc1s1	biogenesis of lysosome-related organelles complex-1, subunit 1	0.93633	0.2255	NA
14547	Gdap2	ganglioside-induced differentiation-associated-protein 2	0.93633	0.6202	0.7991
16536	Kcnq2	potassium voltage-gated channel, subfamily Q, member 2	0.93633	0.2305	NA
16764	Aff3	AF4/FMR2 family, member 3	0.93633	0.4371	0.6635
18429	Oxt	oxytocin	0.93633	0.2998	NA
18975	Polg	polymerase (DNA directed), gamma	0.93633	0.1791	NA
19252	Dusp1	dual specificity phosphatase 1	0.93633	0.7157	0.8577
19271	Ptprj	protein tyrosine phosphatase, receptor type, J	0.93633	0.2941	NA
20588	Smarcc1	SWI/SNF related, matrix associated, actin dependent regulator of chromatin, subfamily c, member 1	0.93633	0.7008	0.8492
22682	Zfand5	zinc finger, AN1-type domain 5	0.93633	0.3937	NA
26396	Map2k2	mitogen-activated protein kinase kinase 2	0.93633	0.3858	NA
26466	Zfp260	zinc finger protein 260	0.93633	0.191	NA
27050	Rps3	ribosomal protein S3	0.93633	0.2757	NA
27062	Cadps	Ca2+-dependent secretion activator	0.93633	0.406	NA
52633	Nit2	nitrilase family, member 2	0.93633	0.4014	NA
56298	Atl2	atlastin GTPase 2	0.93633	0.2637	NA
59053	Brp16	brain protein 16	0.93633	0.5128	0.7232
63958	Ube4b	ubiquitination factor E4B, UFD2 homolog (S. cerevisiae)	0.93633	0.3184	NA
66082	Abhd6	abhydrolase domain containing 6	0.93633	0.5709	0.7647
66315	Senp7	SUMO1/sentrin specific peptidase 7	0.93633	0.5154	0.7256
67044	Higd2a	HIG1 domain family, member 2A	0.93633	0.2872	NA
67198	Spats2l	spermatogenesis associated, serine-rich 2-like	0.93633	0.5243	0.7317
69163	Mrpl44	mitochondrial ribosomal protein L44	0.93633	0.2601	NA
70675	Vcpip1	valosin containing protein (p97)/p47 complex interacting protein 1	0.93633	0.4275	NA
71151	Eri2	exoribonuclease 2	0.93633	0.7553	0.882
71999	Fbxo22	F-box protein 22	0.93633	0.1253	NA
72515	Wdr43	WD repeat domain 43	0.93633	0.2874	NA
74157	Ftsjd2	FtsJ methyltransferase domain containing 2	0.93633	0.2067	NA
75266	Tomm20l	translocase of outer mitochondrial membrane 20 homolog (yeast)-like	0.93633	0.4908	0.7077
76950	2900001G08Rik	RIKEN cDNA 2900001G08 gene	0.93633	0.6535	0.8206
78670	Plekhj1	pleckstrin homology domain containing, family J member 1	0.93633	0.5897	0.7786
80888	Hspb8	heat shock protein 8	0.93633	0.337	NA
80911	Acox3	acyl-Coenzyme A oxidase 3, pristanoyl	0.93633	0.5396	0.7439
94184	Pdxdc1	pyridoxal-dependent decarboxylase domain containing 1	0.93633	0.3624	NA
94230	Cpsf1	cleavage and polyadenylation specific factor 1	0.93633	0.515	0.7251
101476	Plekha1	pleckstrin homology domain containing, family A (phosphoinositide binding specific) member 1	0.93633	0.3603	NA
101685	Spty2d1	SPT2, Suppressor of Ty, domain containing 1 (S. cerevisiae)	0.93633	0.1388	NA
102060	Gadd45gip1	growth arrest and DNA-damage-inducible, gamma interacting protein 1	0.93633	0.462	0.6848
102657	Cd276	CD276 antigen	0.93633	0.5961	0.7823
104346	Gas8	growth arrest specific 8	0.93633	0.2938	NA
106393	Srl	sarcalumenin	0.93633	0.682	0.8374
107975	Pacs1	phosphofurin acidic cluster sorting protein 1	0.93633	0.6209	0.7994
108077	Skiv2l	superkiller viralicidic activity 2-like (S. cerevisiae)	0.93633	0.3521	NA

108660	Rnf187	ring finger protein 187	0.93633	0.2113	NA
170789	Acot8	acyl-CoA thioesterase 8	0.93633	0.3528	NA
171567	Nme7	non-metastatic cells 7, protein expressed in (nucleoside-diphosphate kinase)	0.93633	0.5455	0.7478
208188	Ghsr	growth hormone secretagogue receptor	0.93633	0.431	NA
214951	Rhbdl1	rhomboid, veinlet-like 1 (Drosophila)	0.93633	0.5585	0.7573
225845	Pla2g16	phospholipase A2, group XVI	0.93633	0.5925	0.7802
227634	Camsap1	calmodulin regulated spectrin-associated protein 1	0.93633	0.5711	0.765
238076	Kcns3	potassium voltage-gated channel, delayed-rectifier, subfamily S, member 3	0.93633	0.5831	0.7738
239760	Gm4943	predicted pseudogene 4943	0.93633	0.3222	NA
269585	Zscan20	zinc finger and SCAN domains 20	0.93633	0.4764	0.6965
277743	Fam131c	family with sequence similarity 131, member C	0.93633	0.8197	0.9178
329731	Fam19a3	family with sequence similarity 19, member A3	0.93633	0.3963	NA
383341	Gm5239	ubiquitin A-52 residue ribosomal protein fusion product 1 pseudogene	0.93633	0.421	NA
384701	Dub2a	deubiquitinating enzyme 2a	0.93633	0.8098	0.9117
442829	Ccin	calicin	0.93633	0.4551	0.6787
100038760	LOC100038760	hypothetical LOC100038760	0.93633	0.4047	NA
12408	Cbr1	carbonyl reductase 1	0.935454	0.2857	NA
12724	Clcn2	chloride channel 2	0.935454	0.2499	NA
12728	Clcn5	chloride channel 5	0.935454	0.4702	0.692
12913	Creb3	cAMP responsive element binding protein 3	0.935454	0.1182	NA
14466	Gba	glucosidase, beta, acid	0.935454	0.5055	0.7178
16661	Krt10	keratin 10	0.935454	0.1961	NA
17933	Myt1l	myelin transcription factor 1-like	0.935454	0.6045	0.7881
18148	Npm1	nucleophosmin 1	0.935454	0.3334	NA
18634	Pex7	peroxisomal biogenesis factor 7	0.935454	0.3173	NA
19296	Pvt1	plasmacytoma variant translocation 1	0.935454	0.4848	0.7033
19342	Rab4b	RAB4B, member RAS oncogene family	0.935454	0.6114	0.7931
19383	Raly	hnRNP-associated with lethal yellow	0.935454	0.3549	NA
19826	Rnps1	ribonucleic acid binding protein S1	0.935454	0.511	0.7218
21416	Tcf7l2	transcription factor 7-like 2, T-cell specific, HMG-box	0.935454	0.7589	0.8831
22348	Slc32a1	solute carrier family 32 (GABA vesicular transporter), member 1	0.935454	0.6208	0.7994
52592	Brms1l	breast cancer metastasis-suppressor 1-like	0.935454	0.2103	NA
55951	Brp44l	brain protein 44-like	0.935454	0.2818	NA
56710	Dbc1	deleted in bladder cancer 1 (human)	0.935454	0.4363	0.6629
56812	Dnajb2	DnaJ (Hsp40) homolog, subfamily B, member 2	0.935454	0.2295	NA
66329	Susd3	sushi domain containing 3	0.935454	0.2668	NA
66496	Ppdpf	pancreatic progenitor cell differentiation and proliferation factor homolog (zebrafish)RIKEN cDNA 2700038C09 gene	0.935454	0.2947	NA
66854	Trim35	tripartite motif-containing 35	0.935454	0.8624	0.9394
66874	1200014J11Rik	RIKEN cDNA 1200014J11 gene	0.935454	0.6946	0.8448
66912	Bzw2	basic leucine zipper and W2 domains 2	0.935454	0.2823	NA
67263	Zswim6	zinc finger, SWIM domain containing 6	0.935454	0.7373	0.8713
68149	Otub2	OTU domain, ubiquitin aldehyde binding 2	0.935454	0.2852	NA
68194	Ndufb4	NADH dehydrogenase (ubiquinone) 1 beta subcomplex 4	0.935454	0.207	NA
68961	Phkg2	phosphorylase kinase, gamma 2 (testis)	0.935454	0.4615	0.6845
70767	Prpf3	PRP3 pre-mRNA processing factor 3 homolog (yeast)	0.935454	0.2475	NA
72157	Pgm2	phosphoglucomutase 2	0.935454	0.4679	0.6897
73373	Phospho2	phosphatase, orphan 2	0.935454	0.3682	NA
73389	Hbp1	high mobility group box transcription factor 1	0.935454	0.29	NA
76863	Dcun1d5	DCN1, defective in cullin neddylation 1, domain containing 5 (S. cerevisiae)	0.935454	0.2108	NA

78825	Pppde1	PPPDE peptidase domain containing 1	0.935454	0.5701	0.7641
81904	Cacng7	calcium channel, voltage-dependent, gamma subunit 7	0.935454	0.5435	0.7467
98956	Nat10	N-acetyltransferase 10	0.935454	0.1849	NA
101861	Ints4	integrator complex subunit 4	0.935454	0.5854	0.7756
105833	Ccdc65	coiled-coil domain containing 65	0.935454	0.4915	0.7079
107823	Whsc1	Wolf-Hirschhorn syndrome candidate 1 (human)	0.935454	0.4715	0.6926
170439	Elovl6	ELOVL family member 6, elongation of long chain fatty acids (yeast)	0.935454	0.2074	NA
170749	Mtmr4	myotubularin related protein 4	0.935454	0.302	NA
208146	Yeats2	YEATS domain containing 2	0.935454	0.2579	NA
212531	Sh3bgrl2	SH3 domain binding glutamic acid-rich protein like 2	0.935454	0.6035	0.7872
212627	Prpsap2	phosphoribosyl pyrophosphate synthetase-associated protein 2	0.935454	0.3052	NA
213484	Nudt18	nudix (nucleoside diphosphate linked moiety X)-type motif 18	0.935454	0.7911	0.9009
214063	Dnajc16	DnaJ (Hsp40) homolog, subfamily C, member 16	0.935454	0.3208	NA
218294	Cdc14b	CDC14 cell division cycle 14 homolog B (S. cerevisiae)	0.935454	0.36	NA
224997	Dlgap1	discs, large (Drosophila) homolog-associated protein 1	0.935454	0.4532	0.6774
228787	Xkr7	X Kell blood group precursor related family member 7 homolog	0.935454	0.3999	NA
229722	5330417C22Rik	RIKEN cDNA 5330417C22 gene	0.935454	0.547	0.7487
230484	Usp1	ubiquitin specific peptidase 1	0.935454	0.3639	NA
237761	Ankrd43	ankyrin repeat domain 43	0.935454	0.4349	0.6619
277010	Marveld1	MARVEL (membrane-associating) domain containing 1	0.935454	0.3197	NA
320178	4921529L05Rik	RIKEN cDNA 4921529L05 gene	0.935454	0.7121	0.8553
332937	Tcfap2e	transcription factor AP-2, epsilon	0.935454	0.4928	0.7092
381792	2310040G24Rik	RIKEN cDNA 2310040G24 gene	0.935454	0.5212	0.7292
382090	4922501C03Rik	RIKEN cDNA 4922501C03 gene	0.935454	0.5267	0.7337
414095	B130034C11Rik	RIKEN cDNA B130034C11 gene	0.935454	0.4862	0.7045
545428	Ccdc141	coiled-coil domain containing 141	0.935454	0.7144	0.8567
626683	Gm11362	predicted gene 11362	0.935454	0.6604	0.8247
627049	Zfp800	zinc finger protein 800	0.935454	0.4477	0.6732
100042218	Gm3728	predicted gene 3728	0.935454	0.5044	0.7169
11750	Anxa7	annexin A7	0.934579	0.4138	NA
12390	Cav2	caveolin 2	0.934579	0.6172	0.797
13048	Cux2	cut-like homeobox 2	0.934579	0.4034	NA
13202	Ddt	D-dopachrome tautomerase	0.934579	0.2147	NA
17716	ND1	NADH dehydrogenase subunit 1	0.934579	0.4767	0.6966
17773	Mtnr1a	melatonin receptor 1A	0.934579	0.6304	0.8051
17876	Myef2	myelin basic protein expression factor 2, repressor	0.934579	0.3442	NA
18104	Nqo1	NAD(P)H dehydrogenase, quinone 1	0.934579	0.5157	0.7257
20115	Rps7	ribosomal protein S7	0.934579	0.3468	NA
20983	Syt4	synaptotagmin IV	0.934579	0.428	0.6551
22402	Wisp1	WNT1 inducible signaling pathway protein 1	0.934579	0.3494	NA
22693	Zfp30	zinc finger protein 30	0.934579	0.3476	NA
26896	Med14	mediator complex subunit 14	0.934579	0.3558	NA
52637	Cisd1	CDGSH iron sulfur domain 1	0.934579	0.1443	NA
56200	Ddx21	DEAD (Asp-Glu-Ala-Asp) box polypeptide 21	0.934579	0.2545	NA
57295	Icmt	isoprenylcysteine carboxyl methyltransferase	0.934579	0.4533	0.6774
57837	Eral1	Era (G-protein)-like 1 (E. coli)	0.934579	0.457	0.6804
64933	Ap3m2	adaptor-related protein complex 3, mu 2 subunit	0.934579	0.2685	NA
66268	Pigyl	phosphatidylinositol glycan anchor biosynthesis, class Y-like	0.934579	0.2119	NA
66271	Tmem126a	transmembrane protein 126A	0.934579	0.401	NA

66400	Alkbh7	alkB, alkylation repair homolog 7 (E. coli)	0.934579	0.2915	NA
67106	Zbtb8os	zinc finger and BTB domain containing 8 opposite strand	0.934579	0.4695	0.6916
67680	Sdhb	succinate dehydrogenase complex, subunit B, iron sulfur (Ip)	0.934579	0.1851	NA
68077	Gltscr2	glioma tumor suppressor candidate region gene 2	0.934579	0.4646	0.687
70807	Arrdc2	arrestin domain containing 2	0.934579	0.5841	0.7745
71131	Zfp689	zinc finger protein 689	0.934579	0.4462	0.6718
71890	Mad2l2	MAD2 mitotic arrest deficient-like 2 (yeast)	0.934579	0.585	0.7753
72325	1300018I17Rik	RIKEN cDNA 1300018I17 gene	0.934579	0.1813	NA
73078	Pmpcb	peptidase (mitochondrial processing) beta	0.934579	0.2612	NA
77110	Gpbp1l1	GC-rich promoter binding protein 1-like 1	0.934579	0.4043	NA
78341	2900016J10Rik	RIKEN cDNA 2900016J10 gene	0.934579	0.2164	NA
79560	Ublcp1	ubiquitin-like domain containing CTD phosphatase 1	0.934579	0.2327	NA
94186	Strn3	striatin, calmodulin binding protein 3	0.934579	0.4746	0.6951
99311	Commd7	COMM domain containing 7	0.934579	0.3302	NA
105239	Rnf44	ring finger protein 44	0.934579	0.2078	NA
108067	Eif2b3	eukaryotic translation initiation factor 2B, subunit 3	0.934579	0.4792	0.6986
110809	Srsf1	serine/arginine-rich splicing factor 1	0.934579	0.3375	NA
110920	Hspa13	heat shock protein 70 family, member 13	0.934579	0.6094	0.7917
193838	Eme2	essential meiotic endonuclease 1 homolog 2 (S. pombe)	0.934579	0.3432	NA
211586	Tfdp2	transcription factor Dp 2	0.934579	0.2672	NA
212276	Zfp748	zinc finger protein 748	0.934579	0.4233	0.651
212531	Sh3bgrl2	SH3 domain binding glutamic acid-rich protein like 2	0.934579	0.5726	0.7666
213990	Agap3	ArfGAP with GTPase domain, ankyrin repeat and PH domain 3	0.934579	0.2251	NA
216154	Med16	mediator complex subunit 16	0.934579	0.378	NA
217517	Stxbp6	syntaxin binding protein 6 (amisyn)	0.934579	0.2681	NA
225742	St8sia5	ST8 alpha-N-acetyl-neuraminide alpha-2,8-sialyltransferase 5	0.934579	0.3521	NA
236732	Rbm10	RNA binding motif protein 10	0.934579	0.1907	NA
269529	Fbxo10	F-box protein 10	0.934579	0.3382	NA
280287	Kiss1	KiSS-1 metastasis-suppressor	0.934579	0.6985	0.8476
328162	Trmt61a	tRNA methyltransferase 61 homolog A (S. cerevisiae)	0.934579	0.4532	0.6774
381802	Tsen2	tRNA splicing endonuclease 2 homolog (S. cerevisiae)	0.934579	0.1732	NA
434282	Gm5608	predicted gene 5608	0.934579	0.6176	0.7971
606496	Gsk3a	glycogen synthase kinase 3 alpha	0.934579	0.3998	NA
666185	Gm7969	predicted gene 7969	0.934579	0.2625	NA
100040353	2810416G20Rik	RIKEN cDNA 2810416G20 gene	0.934579	0.5447	0.7474
100043431	Gm4430	predicted gene 4430	0.934579	0.637	0.8092
100504965	LOC100504965	hypothetical LOC100504965	0.934579	0.5192	0.7282
12336	Capns1	calpain, small subunit 1	0.933707	0.5016	0.7148
12576	Cdkn1b	cyclin-dependent kinase inhibitor 1B	0.933707	0.2897	NA
13830	Stom	stomatin	0.933707	0.6632	0.8268
13885	Esd	esterase D/formylglutathione hydrolase	0.933707	0.4886	0.706
14079	Fabp2	fatty acid binding protein 2, intestinal	0.933707	0.7165	0.8583
14408	Gabrr1	gamma-aminobutyric acid (GABA) C receptor, subunit rho 1	0.933707	0.3114	NA
15116	Has1	hyaluronan synthase1	0.933707	0.4611	0.6841
17912	Myo1b	myosin IB	0.933707	0.4154	NA
20844	Stam	signal transducing adaptor molecule (SH3 domain and ITAM motif) 1	0.933707	0.4519	0.6767
21399	Tcea1	transcription elongation factor A (SII) 1	0.933707	0.4758	0.6962
21408	Zfp354a	zinc finger protein 354A	0.933707	0.41	NA
22698	Zfp39	zinc finger protein 39	0.933707	0.4391	0.6651

26413	Mapk1	mitogen-activated protein kinase 1	0.933707	0.2439	NA
29812	Ndrg3	N-myc downstream regulated gene 3	0.933707	0.2115	NA
52530	Nhp2	NHP2 ribonucleoprotein homolog (yeast)	0.933707	0.4165	0.645
59126	Nek6	NIMA (never in mitosis gene a)-related expressed kinase 6	0.933707	0.1915	NA
60364	Donson	downstream neighbor of SON	0.933707	0.4104	NA
64660	Mrps24	mitochondrial ribosomal protein S24	0.933707	0.2677	NA
67043	Syap1	synapse associated protein 1	0.933707	0.2363	NA
67914	Coq9	coenzyme Q9 homolog (yeast)	0.933707	0.4166	0.645
67922	Fam32a	family with sequence similarity 32, member A	0.933707	0.4488	0.6744
68226	Efcab2	EF-hand calcium binding domain 2	0.933707	0.4407	0.6666
68792	Srpx2	sushi-repeat-containing protein, X-linked 2	0.933707	0.4367	0.6631
69721	Nkiras1	NFKB inhibitor interacting Ras-like protein 1	0.933707	0.4209	0.6488
71435	Arhgap21	Rho GTPase activating protein 21	0.933707	0.3864	NA
71529	9030409G11Rik	RIKEN cDNA 9030409G11 gene	0.933707	0.507	0.7187
71723	Dhx34	DEAH (Asp-Glu-Ala-His) box polypeptide 34	0.933707	0.3435	NA
73218	3110056O03Rik	RIKEN cDNA 3110056O03 gene	0.933707	0.2093	NA
74020	Cpne4	copine IV	0.933707	0.3218	NA
101113	Snx21	sorting nexin family member 21	0.933707	0.338	NA
110078	Pygb	brain glycogen phosphorylase	0.933707	0.5962	0.7824
212986	Scfd2	Sec1 family domain containing 2	0.933707	0.5294	0.7358
217718	Nek9	NIMA (never in mitosis gene a)-related expressed kinase 9	0.933707	0.1365	NA
227738	Lrsam1	leucine rich repeat and sterile alpha motif containing 1	0.933707	0.4017	NA
230025	Prdm13	PR domain containing 13	0.933707	0.4044	NA
230959	Ajap1	adherens junction associated protein 1	0.933707	0.3721	NA
233011	Itpkc	inositol 1,4,5-trisphosphate 3-kinase C	0.933707	0.4016	NA
233870	Tufm	Tu translation elongation factor, mitochondrial	0.933707	0.4652	0.687
244668	Sipa1l2	signal-induced proliferation-associated 1 like 2	0.933707	0.1088	NA
245877	Mtap7d1	microtubule-associated protein 7 domain containing 1	0.933707	0.2389	NA
319758	Rfx7	regulatory factor X, 7	0.933707	0.5515	0.7517
380773	1810035L17Rik	RIKEN cDNA 1810035L17 gene	0.933707	0.4559	0.6794
407786	Taf9b	TAF9B RNA polymerase II, TATA box binding protein (TBP)-associated factor	0.933707	0.2859	NA
432768	Gm5451	predicted gene 5451	0.933707	0.6381	0.81
433091	Pnpla1	patatin-like phospholipase domain containing 1	0.933707	0.5168	0.7265
433486	Gm14151	predicted gene 14151	0.933707	0.223	NA
13618	Ednrb	endothelin receptor type B	0.932836	0.5861	0.7761
13996	Etohd2	ethanol decreased 2	0.932836	0.6453	0.8148
14179	Fgf8	fibroblast growth factor 8	0.932836	0.2503	NA
14433	Gapdh	glyceraldehyde-3-phosphate dehydrogenase	0.932836	0.408	NA
14708	Gng7	guanine nucleotide binding protein (G protein), gamma 7	0.932836	0.6426	0.8134
17187	Max	Max protein	0.932836	0.1272	NA
19229	Ptk2b	PTK2 protein tyrosine kinase 2 beta	0.932836	0.4789	0.6985
20842	Stag1	stromal antigen 1	0.932836	0.3409	NA
22051	Trip6	thyroid hormone receptor interactor 6	0.932836	0.4017	NA
50789	Fbxl3	F-box and leucine-rich repeat protein 3	0.932836	0.3243	NA
53611	Vti1a	vesicle transport through interaction with t-SNAREs homolog 1A (yeast)	0.932836	0.171	NA
53975	Ddx20	DEAD (Asp-Glu-Ala-Asp) box polypeptide 20	0.932836	0.1483	NA
55947	Dclre1a	DNA cross-link repair 1A, PSO2 homolog (S. cerevisiae)	0.932836	0.2142	NA
56698	Phax	phosphorylated adaptor for RNA export	0.932836	0.6151	0.7956
56745	C1qtnf1	C1q and tumor necrosis factor related protein 1	0.932836	0.5884	0.7777

57437	Golga7	golgi autoantigen, golgin subfamily a, 7	0.932836	0.2137	NA
66184	Rps4y2	ribosomal protein S4, Y-linked 2	0.932836	0.276	NA
66875	1200016B10Rik	RIKEN cDNA 1200016B10 gene	0.932836	0.282	NA
67126	Atp5e	ATP synthase, H+ transporting, mitochondrial F1 complex, epsilon subunit	0.932836	0.2964	NA
67160	Eef1g	eukaryotic translation elongation factor 1 gamma	0.932836	0.3275	NA
67181	Ctdnep1	CTD nuclear envelope phosphatase 1	0.932836	0.327	NA
68114	Mum1	melanoma associated antigen (mutated) 1	0.932836	0.2672	NA
68275	Rpa1	replication protein A1	0.932836	0.2152	NA
69920	Polr2i	polymerase (RNA) II (DNA directed) polypeptide I	0.932836	0.2486	NA
71093	Atoh8	atonal homolog 8 (Drosophila)	0.932836	0.6226	0.8003
71750	R3hdm2	R3H domain containing 2	0.932836	0.2634	NA
72175	Mfsd8	major facilitator superfamily domain containing 8	0.932836	0.3134	NA
73683	Atg16l2	autophagy related 16 like 2 (S. cerevisiae)	0.932836	0.3798	NA
74252	Armc1	armadillo repeat containing 1	0.932836	0.155	NA
75764	Slx1b	SLX1 structure-specific endonuclease subunit homolog B (S. cerevisiae)	0.932836	0.4979	0.7127
76041	Ccdc125	coiled-coil domain containing 125	0.932836	0.3846	NA
77697	Mmab	methylmalonic aciduria (cobalamin deficiency) type B homolog (human)	0.932836	0.4433	0.669
78255	Ralgps2	Ral GEF with PH domain and SH3 binding motif 2	0.932836	0.2641	NA
93889	Pcdhb18	protocadherin beta 18	0.932836	0.4796	0.6989
99334	Zscan29	zinc finger SCAN domains 29	0.932836	0.4729	0.6937
103836	Zfp692	zinc finger protein 692	0.932836	0.438	0.6643
105525	AI480461	expressed sequence AI480461	0.932836	0.5254	0.7326
112418	1700102P08Rik	RIKEN cDNA 1700102P08 gene	0.932836	0.5193	0.7282
210146	Irgq	immunity-related GTPase family, Q	0.932836	0.1556	NA
211286	Cln5	ceroid-lipofuscinosis, neuronal 5	0.932836	0.15	NA
213827	Arcn1	archain 1	0.932836	0.2387	NA
218194	Phactr1	phosphatase and actin regulator 1	0.932836	0.1046	NA
224727	Bat3	HLA-B-associated transcript 3	0.932836	0.4756	0.6962
225049	Ttc7	tetratricopeptide repeat domain 7	0.932836	0.5909	0.7794
230673	Ipo13	importin 13	0.932836	0.5788	0.7711
231672	Fbxw8	F-box and WD-40 domain protein 8	0.932836	0.5963	0.7824
236899	Pcyt1b	phosphate cytidylyltransferase 1, choline, beta isoform	0.932836	0.3299	NA
242915	Fam59b	family with sequence similarity 59, member B	0.932836	0.5013	0.7146
268449	Rpl23a	ribosomal protein L23A	0.932836	0.2057	NA
269587	Epb4.1	erythrocyte protein band 4.1	0.932836	0.6403	0.8117
320292	Rasgef1b	RasGEF domain family, member 1B	0.932836	0.285	NA
320878	Mical2	microtubule associated monoxygenase, calponin and LIM domain containing 2	0.932836	0.3338	NA
622320	Kctd21	potassium channel tetramerisation domain containing 21	0.932836	0.4368	0.6632
667127	Gm15847	predicted gene 15847	0.932836	0.6201	0.7991
14391	Gabpb1	GA repeat binding protein, beta 1	0.931966	0.5193	0.7282
14420	Galc	galactosylceramidase	0.931966	0.4341	0.6612
14470	Rabac1	Rab acceptor 1 (prenylated)	0.931966	0.267	NA
17257	Mecp2	methyl CpG binding protein 2	0.931966	0.1446	NA
18972	Pold2	polymerase (DNA directed), delta 2, regulatory subunit	0.931966	0.587	0.7765
19299	Abcd3	ATP-binding cassette, sub-family D (ALD), member 3	0.931966	0.1799	NA
20541	Slc8a1	solute carrier family 8 (sodium/calcium exchanger), member 1	0.931966	0.8272	0.9218
20656	Sod2	superoxide dismutase 2, mitochondrial	0.931966	0.1385	NA
21333	Tac1	tachykinin 1	0.931966	0.2743	NA
21402	Skp1a	S-phase kinase-associated protein 1A	0.931966	0.2443	NA

22260	Nr1h2	nuclear receptor subfamily 1, group H, member 2	0.931966	0.304	NA
22278	Usf1	upstream transcription factor 1	0.931966	0.3999	NA
23797	Akt3	thymoma viral proto-oncogene 3	0.931966	0.2018	NA
26913	Gprin1	G protein-regulated inducer of neurite outgrowth 1	0.931966	0.483	0.7014
27660	1700088E04Rik	RIKEN cDNA 1700088E04 gene	0.931966	0.2266	NA
29877	Hdgfrp3	hepatoma-derived growth factor, related protein 3	0.931966	0.3431	NA
56317	Anapc7	anaphase promoting complex subunit 7	0.931966	0.325	NA
66042	Sostdc1	sclerostin domain containing 1	0.931966	0.6771	0.8346
66124	Josd2	Josephin domain containing 2	0.931966	0.5954	0.7821
66400	Alkbh7	alkB, alkylation repair homolog 7 (E. coli)	0.931966	0.2274	NA
66423	2410022L05Rik	RIKEN cDNA 2410022L05 gene	0.931966	0.2391	NA
66867	Hmg20a	high mobility group 20A	0.931966	0.2368	NA
67440	Mtpap	mitochondrial poly(A) polymerase	0.931966	0.1358	NA
68953	Chmp2a	chromatin modifying protein 2A	0.931966	0.2596	NA
69934	Rg9mtd3	RNA (guanine-9-) methyltransferase domain containing 3	0.931966	0.1564	NA
70620	Ube2v2	ubiquitin-conjugating enzyme E2 variant 2	0.931966	0.3567	NA
70847	4733401D01Rik	RIKEN cDNA 4733401D01 gene	0.931966	0.7787	0.8935
72900	Ndufv2	NADH dehydrogenase (ubiquinone) flavoprotein 2	0.931966	0.2007	NA
75398	Mrpl32	mitochondrial ribosomal protein L32	0.931966	0.2296	NA
76943	Psapl1	prosaposin-like 1	0.931966	0.4286	0.6557
78394	Ddx52	DEAD (Asp-Glu-Ala-Asp) box polypeptide 52	0.931966	0.3217	NA
78929	Polr3h	polymerase (RNA) III (DNA directed) polypeptide H	0.931966	0.3903	NA
80913	Pum2	pumilio 2 (Drosophila)	0.931966	0.5514	0.7517
97031	Tprn	taperin	0.931966	0.4651	0.687
102991	AU022751	expressed sequence AU022751	0.931966	0.761	0.8842
103425	Ncln	nicalin homolog (zebrafish)	0.931966	0.4345	0.6614
104416	Bap1	Brca1 associated protein 1	0.931966	0.3391	NA
104522	AU040972	expressed sequence AU040972	0.931966	0.5996	0.7848
108927	Lhfp	lipoma HMGIC fusion partner	0.931966	0.3933	NA
170638	Hpcal4	hippocalcin-like 4	0.931966	0.5461	0.7482
210009	Mtrr	5-methyltetrahydrofolate-homocysteine methyltransferase reductase	0.931966	0.2759	NA
216767	Mrpl22	mitochondrial ribosomal protein L22	0.931966	0.3693	NA
226154	Lzts2	leucine zipper, putative tumor suppressor 2	0.931966	0.3249	NA
227699	Nup188	nucleoporin 188	0.931966	0.3518	NA
228836	Dlgap4	discs, large homolog-associated protein 4 (Drosophila)	0.931966	0.3188	NA
231887	Pdap1	PDGFA associated protein 1	0.931966	0.1252	NA
231999	Plekha8	pleckstrin homology domain containing, family A (phosphoinositide binding specific) member 8	0.931966	0.3347	NA
232679	Zc3hc1	zinc finger, C3HC type 1	0.931966	0.4534	0.6774
237730	Fbll1	fibrillarin-like 1	0.931966	0.5022	0.7152
239167	D930020E02Rik	RIKEN cDNA D930020E02 gene	0.931966	0.7358	0.8706
320634	Ocrl	oculocerebrorenal syndrome of Lowe	0.931966	0.1513	NA
330409	Cecr2	cat eye syndrome chromosome region, candidate 2 homolog (human)	0.931966	0.7208	0.8603
330941	AI593442	expressed sequence AI593442	0.931966	0.6575	0.8224
381338	Lonrf2	LON peptidase N-terminal domain and ring finger 2	0.931966	0.4961	0.7111
386750	Slitrk3	SLIT and NTRK-like family, member 3	0.931966	0.2877	NA
668166	Zxdb	zinc finger, X-linked, duplicated B	0.931966	0.3281	NA
100503330	LOC100503330	hypothetical LOC100503330	0.931966	0.4229	0.6507
100504699	LOC100504699	hypothetical LOC100504699	0.931966	0.5924	0.7802
100505024	LOC100505024	homeobox protein TGIF2-like	0.931966	0.7128	0.8556

12153	Bmp1	bone morphogenetic protein 1	0.931099	0.6918	0.8432
12386	Ctnna2	catenin (cadherin associated protein), alpha 2	0.931099	0.2451	NA
13016	Ctbp1	C-terminal binding protein 1	0.931099	0.2349	NA
13063	Cycs	cytochrome c, somatic	0.931099	0.2212	NA
14645	Glul	glutamate-ammonia ligase (glutamine synthetase)	0.931099	0.2306	NA
14761	Gpr27	G protein-coupled receptor 27	0.931099	0.7542	0.8813
16593	Klc1	kinesin light chain 1	0.931099	0.4412	0.667
16978	Lrrfip1	leucine rich repeat (in FLII) interacting protein 1	0.931099	0.2388	NA
16998	Ltbp3	latent transforming growth factor beta binding protein 3	0.931099	0.4417	0.6675
17714	Grpel2	GrpE-like 2, mitochondrial	0.931099	0.4429	0.6689
19714	Rev3l	REV3-like, catalytic subunit of DNA polymerase zeta RAD54 like (S. cerevisiae)	0.931099	0.3804	NA
20810	Srm	spermidine synthase	0.931099	0.5065	0.7183
21885	Tle1	transducin-like enhancer of split 1, homolog of Drosophila E(spl)	0.931099	0.7029	0.8502
22697	Zscan21	zinc finger and SCAN domain containing 21	0.931099	0.1739	NA
22793	Zyx	zyxin	0.931099	0.2966	NA
23797	Akt3	thymoma viral proto-oncogene 3	0.931099	0.6464	0.8155
26397	Map2k3	mitogen-activated protein kinase kinase 3	0.931099	0.3778	NA
27045	Nit1	nitrilase 1	0.931099	0.1542	NA
30941	Usp21	ubiquitin specific peptidase 21	0.931099	0.4489	0.6745
50780	Rgs3	regulator of G-protein signaling 3	0.931099	0.3149	NA
53356	Eif3g	eukaryotic translation initiation factor 3, subunit G	0.931099	0.1958	NA
54170	Rragc	Ras-related GTP binding C	0.931099	0.3724	NA
56177	Olfm1	olfactomedin 1	0.931099	0.2454	NA
56296	Dmrtb1	DMRT-like family B with proline-rich C-terminal, 1	0.931099	0.5708	0.7647
56335	Mettl3	methyltransferase like 3	0.931099	0.5493	0.7506
66404	2410001C21Rik	RIKEN cDNA 2410001C21 gene	0.931099	0.3345	NA
66653	Brf2	BRF2, subunit of RNA polymerase III transcription initiation factor, BRF1-like	0.931099	0.4388	0.6648
66921	Prpf38b	PRP38 pre-mRNA processing factor 38 (yeast) domain containing B	0.931099	0.394	NA
67749	4930583H14Rik	RIKEN cDNA 4930583H14 gene	0.931099	0.3657	NA
69064	1810014F10Rik	RIKEN cDNA 1810014F10 gene	0.931099	0.1799	NA
69556	Bod1	biorientation of chromosomes in cell division 1	0.931099	0.1677	NA
69886	2610002J23Rik	RIKEN cDNA 2610002J23 gene	0.931099	0.3908	NA
70036	2700023E23Rik	RIKEN cDNA 2700023E23 gene	0.931099	0.2623	NA
70257	2010107E04Rik	RIKEN cDNA 2010107E04 gene	0.931099	0.3548	NA
70363	Fam135b	family with sequence similarity 135, member B	0.931099	0.5357	0.7409
71146	Golga7b	golgi autoantigen, golgin subfamily a, 7B	0.931099	0.7499	0.8788
72128	2610008E11Rik	RIKEN cDNA 2610008E11 gene	0.931099	0.26	NA
72672	Zfp518a	zinc finger protein 518A	0.931099	0.4944	0.7101
75540	Fpgt	fucose-1-phosphate guanylyltransferase	0.931099	0.4167	0.645
78653	Bola3	bolA-like 3 (E. coli)	0.931099	0.1984	NA
78785	Clip4	CAP-GLY domain containing linker protein family, member 4	0.931099	0.5405	0.7447
80283	Abtb1	ankyrin repeat and BTB (POZ) domain containing 1	0.931099	0.1642	NA
80889	Mesdc1	mesoderm development candidate 1	0.931099	0.2966	NA
94254	Wbscr16	Williams-Beuren syndrome chromosome region 16 homolog (human)	0.931099	0.3585	NA
98376	Gorab	golgin, RAB6-interacting	0.931099	0.2323	NA
104027	Synpo	synaptopodin	0.931099	0.4715	0.6927
105847	Lmf2	lipase maturation factor 2	0.931099	0.5525	0.7526
106025	Sharpin	SHANK-associated RH domain interacting protein	0.931099	0.2011	NA
108902	B3gnt1	UDP-GlcNAc:betaGal beta-1,3-N-acetylglucosaminyltransferase 1	0.931099	0.1831	NA

170574	Sp7	Sp7 transcription factor 7	0.931099	0.7753	0.8919
170759	Atp13a1	ATPase type 13A1	0.931099	0.4855	0.704
170930	Sumo2	SMT3 suppressor of mif two 3 homolog 2 (yeast)	0.931099	0.3844	NA
207227	Stxbp5l	syntaxin binding protein 5-like	0.931099	0.3887	NA
208092	Chmp6	chromatin modifying protein 6	0.931099	0.3275	NA
211922	Fam116a	family with sequence similarity 116, member A	0.931099	0.2385	NA
213056	Fam126b	family with sequence similarity 126, member B	0.931099	0.263	NA
214968	Sema6d	sema domain, transmembrane domain (TM), and cytoplasmic domain, (semaphorin) 6D	0.931099	0.7383	0.8719
216438	Mar-09	membrane-associated ring finger (C3HC4) 9	0.931099	0.4009	0.631
217779	Lysmd1	LysM, putative peptidoglycan-binding, domain containing 1	0.931099	0.2173	NA
223648	2410075B13Rik	RIKEN cDNA 2410075B13 gene	0.931099	0.2592	NA
224697	Adamts10	a disintegrin-like and metallopeptidase (reprolysin type) with thrombospondin type 1 motif, 10	0.931099	0.5124	0.7229
229905	Ccbl2	cysteine conjugate-beta lyase 2	0.931099	0.4437	0.6694
245828	Trappc1	trafficking protein particle complex 1	0.931099	0.1687	NA
329068	A330044H09	hypothetical protein A330044H09	0.931099	0.2498	NA
329165	Abi2	abl-interactor 2	0.931099	0.3905	NA
382793	Mtx3	metaxin 3	0.931099	0.6867	0.8402
402737	A130014A01Rik	RIKEN cDNA A130014A01 gene	0.931099	0.35	NA
433693	Akirin2	akirin 2	0.931099	0.3008	NA
100503353	Gm14440	predicted gene 14440	0.931099	0.6597	0.8244
11640	Akap1	A kinase (PRKA) anchor protein 1	0.930233	0.4912	0.7078
11733	Ank1	ankyrin 1, erythroid	0.930233	0.307	NA
13486	Dr1	down-regulator of transcription 1	0.930233	0.5194	0.7282
14395	Gabra2	gamma-aminobutyric acid (GABA) A receptor, subunit alpha 2	0.930233	0.5993	0.7846
14840	Gsg1	germ cell-specific gene 1	0.930233	0.2697	NA
16434	Itpa	inosine triphosphatase (nucleoside triphosphate pyrophosphatase)	0.930233	0.2037	NA
19243	Ptp4a1	protein tyrosine phosphatase 4a1	0.930233	0.2978	NA
19744	Rheb	Ras homolog enriched in brain	0.930233	0.6558	0.8216
20639	Snrpb2	U2 small nuclear ribonucleoprotein B	0.930233	0.3213	NA
22195	Ube2l3	ubiquitin-conjugating enzyme E2L 3	0.930233	0.1834	NA
27226	Pla2g7	phospholipase A2, group VII (platelet-activating factor acetylhydrolase, plasma)	0.930233	0.3714	NA
30056	Timm9	translocase of inner mitochondrial membrane 9 homolog (yeast)	0.930233	0.3025	NA
53867	Col5a3	collagen, type V, alpha 3	0.930233	0.6171	0.797
54351	Rai12	retinoic acid induced 12	0.930233	0.187	NA
56389	Stx5a	syntaxin 5A	0.930233	0.2101	NA
56494	Gosr2	golgi SNAP receptor complex member 2	0.930233	0.3638	NA
64424	Polr1e	polymerase (RNA) I polypeptide E	0.930233	0.4316	0.6589
66144	Atp6v1f	ATPase, H+ transporting, lysosomal V1 subunit F	0.930233	0.1461	NA
66354	Snw1	SNW domain containing 1	0.930233	0.2063	NA
66498	Dda1	DET1 and DDB1 associated 1	0.930233	0.2825	NA
67116	Cuedc2	CUE domain containing 2	0.930233	0.2944	NA
67952	Tomm20	translocase of outer mitochondrial membrane 20 homolog (yeast)	0.930233	0.331	NA
68235	2410066E13Rik	RIKEN cDNA 2410066E13 gene	0.930233	0.6684	0.8296
69090	Ascc1	activating signal cointegrator 1 complex subunit 1	0.930233	0.5786	0.7708
70383	Cox10	COX10 homolog, cytochrome c oxidase assembly protein, heme A: farnesyltransferase (yeast)	0.930233	0.6856	0.8398
72665	2810039B14Rik	RIKEN cDNA 2810039B14 gene	0.930233	0.3733	NA
72902	Spock3	sparc/osteonectin, cwcv and kazal-like domains proteoglycan 3	0.930233	0.8064	0.9095
74366	4932422M17Rik	RIKEN cDNA 4932422M17 gene	0.930233	0.3236	NA
74743	5830403F22Rik	RIKEN cDNA 5830403F22 gene	0.930233	0.7331	0.8685

75146	Tmem180	transmembrane protein 180	0.930233	0.3431	NA
76000	5033430I15Rik	RIKEN cDNA 5033430I15 gene	0.930233	0.2343	NA
77254	Yif1b	Yip1 interacting factor homolog B (S. cerevisiae)	0.930233	0.2911	NA
101190	AI853106	expressed sequence AI853106	0.930233	0.5399	0.7442
105203	BC016423	cDNA sequence BC016423	0.930233	0.4612	0.6841
170758	Rac3	RAS-related C3 botulinum substrate 3	0.930233	0.4174	0.6456
217039	Ggnbp2	gametogenetin binding protein 2	0.930233	0.369	NA
224997	Dlgap1	discs, large (Drosophila) homolog-associated protein 1	0.930233	0.5555	0.7548
229584	Pogz	pogo transposable element with ZNF domain	0.930233	0.2032	NA
229663	Csde1	cold shock domain containing E1, RNA binding	0.930233	0.2226	NA
230796	Wdtc1	WD and tetratricopeptide repeats 1	0.930233	0.3165	NA
232816	Zfp628	zinc finger protein 628	0.930233	0.4502	0.6752
235441	Usp3	ubiquitin specific peptidase 3	0.930233	0.4797	0.6989
319332	B930096F20Rik	RIKEN cDNA B930096F20 gene	0.930233	0.4003	0.6308
381375	Dfnb59	deafness, autosomal recessive 59 (human)	0.930233	0.4678	0.6897
627035	Gm6729	predicted gene 6729	0.930233	0.6245	0.8016
100504758	LOC100504758	hypothetical LOC100504758	0.930233	0.5413	0.7455
12350	Car3	carbonic anhydrase 3	0.929368	0.703	0.8503
15529	Sdc2	syndecan 2	0.929368	0.1357	NA
16523	Kcnj8	potassium inwardly-rectifying channel, subfamily J, member 8	0.929368	0.516	0.7259
19385	Ranbp1	RAN binding protein 1	0.929368	0.3268	NA
19716	Bex1	brain expressed gene 1	0.929368	0.2426	NA
20513	Slc1a6	solute carrier family 1 (high affinity aspartate/glutamate transporter), member 6	0.929368	0.3234	NA
20931	Surf2	surfeit gene 2	0.929368	0.2096	NA
21753	Tes	testis derived transcript	0.929368	0.1377	NA
56194	Prpf40a	PRP40 pre-mRNA processing factor 40 homolog A (yeast)	0.929368	0.5391	0.7435
56196	Tdp2	tyrosyl-DNA phosphodiesterase 2	0.929368	0.22	NA
56224	Tspan5	tetraspanin 5	0.929368	0.4223	0.6502
57264	Retn	resistin	0.929368	0.6606	0.8248
60507	Qtrt1	queuine tRNA-ribosyltransferase 1	0.929368	0.4529	0.6772
66163	Mrpl4	mitochondrial ribosomal protein L4	0.929368	0.1516	NA
67326	1700037H04Rik	RIKEN cDNA 1700037H04 gene	0.929368	0.6216	0.7996
67381	Med4	mediator of RNA polymerase II transcription, subunit 4 homolog (yeast)	0.929368	0.318	NA
67563	Narfl	nuclear prelamin A recognition factor-like	0.929368	0.4065	0.6359
68117	Apool	apolipoprotein O-like	0.929368	0.2981	NA
69161	Manbal	mannosidase, beta A, lysosomal-like	0.929368	0.4533	0.6774
69237	Gtpbp4	GTP binding protein 4	0.929368	0.3626	NA
70266	Ccbl1	cysteine conjugate-beta lyase 1	0.929368	0.1579	NA
71382	Pex1	peroxisomal biogenesis factor 1	0.929368	0.2856	NA
71703	Armcx3	armadillo repeat containing, X-linked 3	0.929368	0.3053	NA
71752	Gtf3c2	general transcription factor IIIC, polypeptide 2, beta	0.929368	0.4728	0.6936
72039	Mccc1	methylcrotonoyl-Coenzyme A carboxylase 1 (alpha)	0.929368	0.3487	NA
72055	Slc38a10	solute carrier family 38, member 10	0.929368	0.3134	NA
72258	Kcnk10	potassium channel, subfamily K, member 10	0.929368	0.6205	0.7991
74018	Als2	amyotrophic lateral sclerosis 2 (juvenile) homolog (human)	0.929368	0.5083	0.7197
74570	Zkscan1	zinc finger with KRAB and SCAN domains 1	0.929368	0.3947	0.6254
75234	Rnf19b	ring finger protein 19B	0.929368	0.3016	NA
76484	Kndc1	kinase non-catalytic C-lobe domain (KIND) containing 1	0.929368	0.6268	0.803
76808	Rpl18a	ribosomal protein L18A	0.929368	0.2478	NA

79555	BC005537	cDNA sequence BC005537	0.929368	0.3707	NA
80280	Cdk5rap3	CDK5 regulatory subunit associated protein 3	0.929368	0.3478	NA
100910	Chpf2	chondroitin polymerizing factor 2	0.929368	0.4996	0.7142
101867	Rrp8	ribosomal RNA processing 8, methyltransferase, homolog (yeast)	0.929368	0.46	0.6833
106073	Mfsd5	major facilitator superfamily domain containing 5	0.929368	0.3231	NA
107513	Ssr1	signal sequence receptor, alpha	0.929368	0.2196	NA
107732	Mrpl10	mitochondrial ribosomal protein L10	0.929368	0.3797	NA
217737	Ahsa1	AHA1, activator of heat shock protein ATPase homolog 1 (yeast)	0.929368	0.303	NA
225791	Zadh2	zinc binding alcohol dehydrogenase, domain containing 2	0.929368	0.1904	NA
227615	Tmem203	transmembrane protein 203	0.929368	0.18	NA
232196	C87436	expressed sequence C87436	0.929368	0.2069	NA
236511	Eif2c1	eukaryotic translation initiation factor 2C, 1	0.929368	0.5198	0.7283
240066	Zfp870	zinc finger protein 870	0.929368	0.52	0.7284
242669	Adc	arginine decarboxylase	0.929368	0.4646	0.687
245446	Slitrk4	SLIT and NTRK-like family, member 4	0.929368	0.3873	0.6194
272538	Tmco7	transmembrane and coiled-coil domains 7	0.929368	0.6344	0.8076
319615	Zfp944	zinc finger protein 944	0.929368	0.3977	0.6281
320024	Nceh1	arylacetamide deacetylase-like 1	0.929368	0.1485	NA
327826	Frs2	fibroblast growth factor receptor substrate 2	0.929368	0.2521	NA
353190	Edc3	enhancer of mRNA decapping 3 homolog (S. cerevisiae)	0.929368	0.4444	0.67
381350	BC061194	cDNA sequence BC061194	0.929368	0.7119	0.8553
414077	BC056474	cDNA sequence BC056474	0.929368	0.4547	0.6783
100503605	Beta-s	hemoglobin subunit beta-1-like	0.929368	0.7969	0.9047
11304	Abca4	ATP-binding cassette, sub-family A (ABC1), member 4	0.928505	0.2899	NA
11444	Chrnb2	cholinergic receptor, nicotinic, beta polypeptide 2 (neuronal)	0.928505	0.305	NA
13190	Dct	dopachrome tautomerase	0.928505	0.8071	0.91
14680	Gnal	guanine nucleotide binding protein, alpha stimulating, olfactory type	0.928505	0.2179	NA
16002	Igf2	insulin-like growth factor 2	0.928505	0.5803	0.7724
16494	Kcna6	potassium voltage-gated channel, shaker-related, subfamily, member 6	0.928505	0.6186	0.7978
17268	Meis1	Meis homeobox 1	0.928505	0.2359	NA
17274	Rab8a	RAB8A, member RAS oncogene family	0.928505	0.4706	0.6922
17710	COX3	cytochrome c oxidase subunit III	0.928505	0.3768	NA
17720	ND4L	NADH dehydrogenase subunit 4L	0.928505	0.5897	0.7786
18585	Pde9a	phosphodiesterase 9A	0.928505	0.3467	NA
20218	Khdrbs1	KH domain containing, RNA binding, signal transduction associated 1	0.928505	0.2581	NA
20370	Sez6	seizure related gene 6	0.928505	0.6464	0.8155
21780	Tfam	transcription factor A, mitochondrial	0.928505	0.3681	NA
22317	Vamp1	vesicle-associated membrane protein 1	0.928505	0.4409	0.6668
22379	Fmnl3	formin-like 3	0.928505	0.5148	0.725
26423	Nr5a1	nuclear receptor subfamily 5, group A, member 1	0.928505	0.7571	0.8825
26896	Med14	mediator complex subunit 14	0.928505	0.2314	NA
27274	Zfp354b	zinc finger protein 354B	0.928505	0.1736	NA
28000	Prpf19	PRP19/PSO4 pre-mRNA processing factor 19 homolog (S. cerevisiae)	0.928505	0.3049	NA
50994	Mtag2	metastasis associated gene 2	0.928505	0.3864	0.6186
52683	Ncaph2	non-SMC condensin II complex, subunit H2	0.928505	0.2521	NA
54633	Pqbp1	polyglutamine binding protein 1	0.928505	0.3915	0.623
56443	Arpc1a	actin related protein 2/3 complex, subunit 1A	0.928505	0.2655	NA
56535	Pex3	peroxisomal biogenesis factor 3	0.928505	0.2758	NA
57330	Gigyf1	GRB10 interacting GYF protein 1	0.928505	0.3679	NA

66092	Ghitm	growth hormone inducible transmembrane protein	0.928505	0.3372	NA
66218	Ndufb9	NADH dehydrogenase (ubiquinone) 1 beta subcomplex, 9	0.928505	0.1259	NA
66911	Nudt16l1	nudix (nucleoside diphosphate linked moiety X)-type motif 16-like 1	0.928505	0.4605	0.6837
67112	Fgf22	fibroblast growth factor 22	0.928505	0.5002	0.7144
67238	2810453I06Rik	RIKEN cDNA 2810453I06 gene	0.928505	0.1313	NA
67824	Nmral1	NmrA-like family domain containing 1	0.928505	0.3203	NA
68027	Tmem178	transmembrane protein 178	0.928505	0.1775	NA
68045	2700060E02Rik	RIKEN cDNA 2700060E02 gene	0.928505	0.127	NA
68512	Tomm5	translocase of outer mitochondrial membrane 5 homolog (yeast)	0.928505	0.1361	NA
69528	1700030J22Rik	RIKEN cDNA 1700030J22 gene	0.928505	0.4806	0.6994
69743	Casz1	castor homolog 1, zinc finger (Drosophila)	0.928505	0.2931	NA
70536	Qpct	glutaminyl-peptide cyclotransferase (glutaminyl cyclase)	0.928505	0.2773	NA
71041	Pcgf6	polycomb group ring finger 6	0.928505	0.3047	NA
72273	2210404O07Rik	RIKEN cDNA 2210404O07 gene	0.928505	0.3572	NA
73205	3110043O21Rik	RIKEN cDNA 3110043O21 gene	0.928505	0.181	NA
73274	Gpbp1	GC-rich promoter binding protein 1	0.928505	0.2397	NA
73873	Fam161a	family with sequence similarity 161, member A	0.928505	0.624	0.8012
74868	Tmem65	transmembrane protein 65	0.928505	0.2629	NA
75406	Ndufs7	NADH dehydrogenase (ubiquinone) Fe-S protein 7	0.928505	0.2392	NA
77264	Zfp142	zinc finger protein 142	0.928505	0.2002	NA
77781	Epm2aip1	EPM2A (laforin) interacting protein 1	0.928505	0.5013	0.7146
93840	Vangl2	vang-like 2 (van gogh, Drosophila)	0.928505	0.5977	0.7834
100121	Tdrd7	tudor domain containing 7	0.928505	0.1646	NA
103534	Mgat4b	mannoside acetylglucosaminyltransferase 4, isoenzyme B	0.928505	0.3609	NA
107368	Pdzd8	PDZ domain containing 8	0.928505	0.2794	NA
109331	Rnf20	ring finger protein 20	0.928505	0.2111	NA
215999	Ccdc109a	coiled-coil domain containing 109A	0.928505	0.5143	0.7245
217194	Klhl11	kelch-like 11 (Drosophila)	0.928505	0.7699	0.8895
217371	Rab40b	Rab40b, member RAS oncogene family	0.928505	0.2836	NA
217558	G2e3	G2/M-phase specific E3 ubiquitin ligase	0.928505	0.5665	0.7622
224938	Pja2	praja 2, RING-H2 motif containing	0.928505	0.3362	NA
225055	Fbxo11	F-box protein 11	0.928505	0.4046	0.6342
231380	Uba6	ubiquitin-like modifier activating enzyme 6	0.928505	0.5254	0.7326
231821	Adap1	ArfGAP with dual PH domains 1	0.928505	0.3366	NA
234915	AK129341	cDNA sequence AK129341	0.928505	0.315	NA
241274	Pnpla7	patatin-like phospholipase domain containing 7	0.928505	0.4955	0.7111
244891	Scaper	S phase cyclin A-associated protein in the ER	0.928505	0.4671	0.6892
269536	Tex10	testis expressed gene 10	0.928505	0.1492	NA
276852	D11Wsu47e	DNA segment, Chr 11, Wayne State University 47, expressed	0.928505	0.2141	NA
620709	Lcn6	lipocalin 6	0.928505	0.7308	0.8669
668661	2410002F23Rik	RIKEN cDNA 2410002F23 gene	0.928505	0.261	NA
100040298	Gm15501	predicted pseudogene 15501	0.928505	0.435	0.6619
100044832	LOC100044832	mucosal pentraxin-like	0.928505	0.69	0.8418
11304	Abca4	ATP-binding cassette, sub-family A (ABC1), member 4	0.927644	0.2869	NA
12330	Canx	calnexin	0.927644	0.3789	0.6126
13841	Epha7	Eph receptor A7	0.927644	0.2467	NA
14660	Gls	glutaminase	0.927644	0.2506	NA
16907	Lmnb2	lamin B2	0.927644	0.5168	0.7265
17766	Nudt1	nudix (nucleoside diphosphate linked moiety X)-type motif 1	0.927644	0.336	NA

18191	Nrxn3	neurexin III	0.927644	0.4567	0.6801
19647	Rbbp6	retinoblastoma binding protein 6	0.927644	0.2309	NA
20970	Sdc3	syndecan 3	0.927644	0.2118	NA
27361	Sepx1	selenoprotein X 1	0.927644	0.181	NA
50497	Hspa14	heat shock protein 14	0.927644	0.1526	NA
52421	D4Ertd681e	DNA segment, Chr 4, ERATO Doi 681, expressed	0.927644	0.6935	0.8442
54524	Syt6	synaptotagmin VI	0.927644	0.3801	0.6135
54636	Wdr45	WD repeat domain 45	0.927644	0.1103	NA
56075	Pdss1	prenyl (solanesyl) diphosphate synthase, subunit 1	0.927644	0.2351	NA
56748	Nfu1	NFU1 iron-sulfur cluster scaffold homolog (S. cerevisiae)	0.927644	0.14	NA
57782	Rbak	RB-associated KRAB repressor	0.927644	0.2467	NA
59032	Ppp2r3c	protein phosphatase 2, regulatory subunit B'', gamma	0.927644	0.08456	NA
66225	Llph	LLP homolog, long-term synaptic facilitation (Aplysia)	0.927644	0.4591	0.6825
66456	2810001G20Rik	RIKEN cDNA 2810001G20 gene	0.927644	0.3724	0.6068
68597	1110021J02Rik	RIKEN cDNA 1110021J02 gene	0.927644	0.1096	NA
69187	Erp27	endoplasmic reticulum protein 27	0.927644	0.5312	0.7372
70611	Fbxo33	F-box protein 33	0.927644	0.3722	0.6068
71452	Ankrd40	ankyrin repeat domain 40	0.927644	0.2683	NA
71923	2310047M10Rik	RIKEN cDNA 2310047M10 gene	0.927644	0.4108	0.6399
74244	Atg7	autophagy-related 7 (yeast)	0.927644	0.2601	NA
74385	Mudeng	MU-2/AP1M2 domain containing, death-inducing	0.927644	0.2389	NA
75373	4930597O21Rik	RIKEN cDNA 4930597O21 gene	0.927644	0.849	0.9328
75734	Mff	mitochondrial fission factor	0.927644	0.2407	NA
76539	D19Ertd737e	DNA segment, Chr 19, ERATO Doi 737, expressed	0.927644	0.2132	NA
76820	Fam49a	family with sequence similarity 49, member A	0.927644	0.1974	NA
77116	Mtmr2	myotubularin related protein 2	0.927644	0.1379	NA
77446	Heg1	HEG homolog 1 (zebrafish)	0.927644	0.7212	0.8603
78070	Cpt1c	carnitine palmitoyltransferase 1c	0.927644	0.4831	0.7014
78703	C330013J21Rik	RIKEN cDNA C330013J21 gene	0.927644	0.4444	0.67
83673	Snhg1	small nucleolar RNA host gene (non-protein coding) 1	0.927644	0.4074	0.6367
101831	C230052I12Rik	RIKEN cDNA C230052I12 gene	0.927644	0.6317	0.806
109136	Mmaa	methylmalonic aciduria (cobalamin deficiency) type A	0.927644	0.4462	0.6718
114661	Prss28	protease, serine, 28	0.927644	0.5816	0.7728
170772	Glcci1	glucocorticoid induced transcript 1	0.927644	0.1714	NA
208158	Map6d1	MAP6 domain containing 1	0.927644	0.7204	0.8602
210044	Adcy2	adenylate cyclase 2	0.927644	0.311	NA
212123	Dcaf15	DDB1 and CUL4 associated factor 15	0.927644	0.5547	0.7542
213993	A630007B06Rik	RIKEN cDNA A630007B06 gene	0.927644	0.5894	0.7784
217893	Pacs2	phosphofurin acidic cluster sorting protein 2	0.927644	0.3963	0.6267
227197	Ndufs1	NADH dehydrogenase (ubiquinone) Fe-S protein 1	0.927644	0.2061	NA
227210	Ccnyl1	cyclin Y-like 1	0.927644	0.1066	NA
229725	Clcc1	chloride channel CLIC-like 1	0.927644	0.6754	0.8334
231042	Nupl2	nucleoporin like 2	0.927644	0.2122	NA
232409	Clec2e	C-type lectin domain family 2, member e	0.927644	0.6543	0.8209
232791	Cnot3	CCR4-NOT transcription complex, subunit 3	0.927644	0.6025	0.7867
244579	Tox3	TOX high mobility group box family member 3	0.927644	0.3706	0.6054
268445	Ankrd13b	ankyrin repeat domain 13b	0.927644	0.7546	0.8815
270672	Map3k15	mitogen-activated protein kinase kinase kinase 15	0.927644	0.5124	0.7229
277939	C2cd3	C2 calcium-dependent domain containing 3	0.927644	0.4291	0.6562

279572	Tlr13	toll-like receptor 13	0.927644	0.5812	0.7726
319675	5830418K08Rik	RIKEN cDNA 5830418K08 gene	0.927644	0.3653	NA
329559	Zfp335	zinc finger protein 335	0.927644	0.3342	NA
627004	Gm12770	predicted gene 12770	0.927644	0.5483	0.7497
100046282	LOC100046282	selenoprotein K pseudogene	0.927644	0.1526	NA
100113398	Adat3	adenosine deaminase, tRNA-specific 3, TAD2 homolog (S. cerevisiae)	0.927644	0.362	NA
12724	Clcn2	chloride channel 2	0.926784	0.2649	NA
12870	Cp	ceruloplasmin	0.926784	0.3028	NA
13043	Cttn	cortactin	0.926784	0.6023	0.7866
13226	Defa-rs7	defensin, alpha, related sequence 7	0.926784	0.2914	NA
13518	Dst	dystonin	0.926784	0.09317	NA
13726	Emd	emerin	0.926784	0.2518	NA
15200	Hbegf	heparin-binding EGF-like growth factor	0.926784	0.14	NA
16000	Igf1	insulin-like growth factor 1	0.926784	0.2235	NA
16370	Irs4	insulin receptor substrate 4	0.926784	0.6758	0.8335
16666	Krt16	keratin 16	0.926784	0.6572	0.8223
16783	Lamp1	lysosomal-associated membrane protein 1	0.926784	0.2695	NA
16801	Arhgef1	Rho guanine nucleotide exchange factor (GEF) 1	0.926784	0.564	0.7613
17470	Cd200	CD200 antigen	0.926784	0.2567	NA
20454	St3gal5	ST3 beta-galactoside alpha-2,3-sialyltransferase 5	0.926784	0.06428	NA
20463	Cox7a2l	cytochrome c oxidase subunit VIIa polypeptide 2-like	0.926784	0.1689	NA
22017	Tpmt	thiopurine methyltransferase	0.926784	0.2995	NA
22116	Tsks	testis-specific serine kinase substrate	0.926784	0.308	NA
23965	Odz3	odd Oz/ten-m homolog 3 (Drosophila)	0.926784	0.5903	0.7791
28109	D10Wsu102e	DNA segment, Chr 10, Wayne State University 102, expressed	0.926784	0.456	0.6794
53379	Hnrnpa2b1	heterogeneous nuclear ribonucleoprotein A2/B1	0.926784	0.4526	0.6771
66043	Atp5d	ATP synthase, H+ transporting, mitochondrial F1 complex, delta subunit	0.926784	0.3866	0.6189
66264	Ccdc28b	coiled coil domain containing 28B	0.926784	0.1261	NA
66855	Tcf25	transcription factor 25 (basic helix-loop-helix)	0.926784	0.07174	NA
66950	Tmem206	transmembrane protein 206	0.926784	0.4006	0.6309
66975	2410002O22Rik	RIKEN cDNA 2410002O22 gene	0.926784	0.1815	NA
67383	2410127L17Rik	RIKEN cDNA 2410127L17 gene	0.926784	0.1274	NA
68177	Ebpl	emopamil binding protein-like	0.926784	0.2027	NA
68742	Tmem219	transmembrane protein 219	0.926784	0.2623	NA
68744	Zfp740	zinc finger protein 740	0.926784	0.6572	0.8223
68817	Ddi2	DNA-damage inducible protein 2	0.926784	0.1568	NA
70951	Spata1	spermatogenesis associated 1	0.926784	0.8419	0.9289
74108	Parn	poly(A)-specific ribonuclease (deadenylation nuclease)	0.926784	0.1802	NA
97613	C85181	expressed sequence C85181	0.926784	0.3294	NA
97848	Serpinb6c	serine (or cysteine) peptidase inhibitor, clade B, member 6c	0.926784	0.5135	0.7238
102595	Plekho2	pleckstrin homology domain containing, family O member 2	0.926784	0.1339	NA
102607	Snx19	sorting nexin 19	0.926784	0.2941	NA
110750	Cse1l	chromosome segregation 1-like (S. cerevisiae)	0.926784	0.5679	0.7633
207777	Bzrap1	benzodiazepine receptor associated protein 1	0.926784	0.3362	NA
208748	Prrg3	proline rich Gla (G-carboxyglutamic acid) 3 (transmembrane)	0.926784	0.4276	0.6547
214552	Cep164	centrosomal protein 164	0.926784	0.4516	0.6764
225743	Rnf165	ring finger protein 165	0.926784	0.3794	0.6131
231834	Snx8	sorting nexin 8	0.926784	0.2103	NA
239706	BC024814	cDNA sequence BC024814	0.926784	0.459	0.6824

270049	Galntl6	UDP-N-acetyl-alpha-D-galactosamine:polypeptide N-acetylgalactosaminyltransferase-like 6	0.926784	0.4678	0.6897
319580	9630055L06Rik	RIKEN cDNA 9630055L06 gene	0.926784	0.5948	0.7817
320011	Uggt1	UDP-glucose glycoprotein glucosyltransferase 1	0.926784	0.4119	0.6408
320234	Ccdc66	coiled-coil domain containing 66	0.926784	0.23	NA
327984	E130101M22	hypothetical protein E130101M22	0.926784	0.4763	0.6965
330485	Tmem145	transmembrane protein 145	0.926784	0.2348	NA
381085	Tbc1d22b	TBC1 domain family, member 22B	0.926784	0.5311	0.7372
404549	Ifna14	interferon, alpha 14	0.926784	0.8186	0.9174
628779	Hs3st4	heparan sulfate (glucosamine) 3-O-sulfotransferase 4	0.926784	0.2365	NA
100503774	LOC100503774	hypothetical LOC100503774	0.926784	0.1675	NA
11464	Actc1	actin, alpha, cardiac muscle 1	0.925926	0.3301	NA
11757	Prdx3	peroxiredoxin 3	0.925926	0.2927	NA
12292	Cacna1s	calcium channel, voltage-dependent, L type, alpha 1S subunit	0.925926	0.3212	NA
12850	Coq7	demethyl-Q 7	0.925926	0.5693	0.7638
13496	Arid3a	AT rich interactive domain 3A (BRIGHT-like)	0.925926	0.4124	0.6411
13682	Eif4a2	eukaryotic translation initiation factor 4A2	0.925926	0.1608	NA
14000	Drosha	drosha, ribonuclease type III	0.925926	0.08957	NA
14164	Fgf1	fibroblast growth factor 1	0.925926	0.5324	0.7381
15270	H2afx	H2A histone family, member X	0.925926	0.4199	0.6478
16980	Lrrn2	leucine rich repeat protein 2, neuronal	0.925926	0.3262	NA
18002	Nedd8	neural precursor cell expressed, developmentally down-regulated gene 8	0.925926	0.2492	NA
21985	Tpd52	tumor protein D52	0.925926	0.06963	NA
22017	Tpmt	thiopurine methyltransferase	0.925926	0.1905	NA
22171	Tyms	thymidylate synthase	0.925926	0.509	0.72
26414	Mapk10	mitogen-activated protein kinase 10	0.925926	0.3568	NA
27281	Hrasls	HRAS-like suppressor	0.925926	0.1972	NA
52635	Esyt2	extended synaptotagmin-like protein 2	0.925926	0.2717	NA
53317	Plrg1	pleiotropic regulator 1, PRL1 homolog (Arabidopsis)	0.925926	0.1486	NA
56191	Tro	trophinin	0.925926	0.2107	NA
56212	Rhog	ras homolog gene family, member G	0.925926	0.4034	0.6333
56374	Tmem59	transmembrane protein 59	0.925926	0.342	NA
56422	Hbs1l	Hbs1-like (S. cerevisiae)	0.925926	0.3815	0.615
66050	0610009B22Rik	RIKEN cDNA 0610009B22 gene	0.925926	0.2113	NA
66233	Dmap1	DNA methyltransferase 1-associated protein 1	0.925926	0.1986	NA
66245	Hspbp1	HSPA (heat shock 70kDa) binding protein, cytoplasmic cochaperone 1	0.925926	0.4938	0.7098
66457	2810002D19Rik	RIKEN cDNA 2810002D19 gene	0.925926	0.5769	0.7697
67134	Nop56	NOP56 ribonucleoprotein homolog (yeast)	0.925926	0.2212	NA
67515	Ttc33	tetratricopeptide repeat domain 33	0.925926	0.1838	NA
67675	Cuta	cutA divalent cation tolerance homolog (E. coli)	0.925926	0.5238	0.7314
69917	Obfc2b	oligonucleotide/oligosaccharide-binding fold containing 2B	0.925926	0.4826	0.7012
70088	Meaf6	MYST/Esa1-associated factor 6	0.925926	0.09517	NA
70574	Cpm	carboxypeptidase M	0.925926	0.5389	0.7433
72635	Lins	lines homolog (Drosophila)	0.925926	0.2761	NA
73833	Fam98c	family with sequence similarity 98, member C	0.925926	0.3044	NA
74343	Crtc2	CREB regulated transcription coactivator 2	0.925926	0.5743	0.7678
75099	Lysmd4	LysM, putative peptidoglycan-binding, domain containing 4	0.925926	0.3104	NA
76246	Rtf1	Rtf1, Paf1/RNA polymerase II complex component, homolog (S. cerevisiae)	0.925926	0.361	0.5958
77521	Mtus2	microtubule associated tumor suppressor candidate 2	0.925926	0.5064	0.7183
94112	Med15	mediator complex subunit 15	0.925926	0.1721	NA

195040	Tmem199	transmembrane protein 199	0.925926	0.1758	NA
207304	Hectd1	HECT domain containing 1	0.925926	0.2979	NA
213452	Dstyk	dual serine/threonine and tyrosine protein kinase	0.925926	0.4752	0.6958
214459	Fnbp1l	formin binding protein 1-like	0.925926	0.4121	0.6408
218335	Clptm1l	CLPTM1-like	0.925926	0.2994	NA
218441	Zfyve16	zinc finger, FYVE domain containing 16	0.925926	0.2248	NA
227720	Nup214	nucleoporin 214	0.925926	0.6368	0.8091
230598	Nrd1	nardilysin, N-arginine dibasic convertase, NRD convertase 1	0.925926	0.4709	0.6922
231931	Gimap6	GTPase, IMAP family member 6	0.925926	0.4627	0.6853
232784	Zfp212	Zinc finger protein 212	0.925926	0.1825	NA
235344	Sik2	salt inducible kinase 2	0.925926	0.3235	NA
241547	Harbi1	harbinger transposase derived 1	0.925926	0.2239	NA
245522	Zc4h2	zinc finger, C4H2 domain containing	0.925926	0.4037	0.6336
319352	C530028O21Rik	RIKEN cDNA C530028O21 gene	0.925926	0.417	0.6452
320078	Olfml2b	olfactomedin-like 2B	0.925926	0.4645	0.687
320302	Glt28d2	glycosyltransferase 28 domain containing 2	0.925926	0.4041	0.6339
434394	Gm5614	predicted gene 5614	0.925926	0.3402	NA
545859	Vax2os2	Vax2 opposite strand transcript 2	0.925926	0.2816	NA
627967	Gm6816	predicted gene 6816	0.925926	0.4034	0.6333
11857	Arhgdib	Rho, GDP dissociation inhibitor (GDI) beta	0.925069	0.5645	0.7616
12292	Cacna1s	calcium channel, voltage-dependent, L type, alpha 1S subunit	0.925069	0.516	0.7259
12460	Ccs	copper chaperone for superoxide dismutase	0.925069	0.1865	NA
12532	Cdc25c	cell division cycle 25 homolog C (S. pombe)	0.925069	0.4651	0.687
12750	Clk4	CDC like kinase 4	0.925069	0.4506	0.6754
13972	Gnb1l	guanine nucleotide binding protein (G protein), beta polypeptide 1-like	0.925069	0.3916	0.623
14719	Got2	glutamate oxaloacetate transaminase 2, mitochondrial	0.925069	0.1672	NA
14866	Gstm5	glutathione S-transferase, mu 5	0.925069	0.3798	0.6134
17155	Man1a	mannosidase 1, alpha	0.925069	0.5574	0.7566
18557	Cdk18	cyclin-dependent kinase 18	0.925069	0.197	NA
20191	Ryr2	ryanodine receptor 2, cardiac	0.925069	0.3726	0.6069
20467	Sin3b	transcriptional regulator, SIN3B (yeast)	0.925069	0.479	0.6985
21769	Zfand3	zinc finger, AN1-type domain 3	0.925069	0.3464	NA
22187	Ubb	ubiquitin B	0.925069	0.3947	0.6254
26356	Ing1	inhibitor of growth family, member 1	0.925069	0.4503	0.6752
27083	Xlr4b	X-linked lymphocyte-regulated 4B	0.925069	0.8611	0.9386
27373	Csnk1e	casein kinase 1, epsilon	0.925069	0.6144	0.795
27373	Csnk1e	casein kinase 1, epsilon	0.925069	0.2478	NA
54218	B3galt4	UDP-Gal:betaGlcNAc beta 1,3-galactosyltransferase, polypeptide 4	0.925069	0.4728	0.6936
56330	Pdcd5	programmed cell death 5	0.925069	0.2113	NA
56367	Scoc	short coiled-coil protein	0.925069	0.6173	0.797
66105	Ube2d3	ubiquitin-conjugating enzyme E2D 3 (UBC4/5 homolog, yeast)	0.925069	0.3305	NA
67219	Med18	mediator of RNA polymerase II transcription, subunit 18 homolog (yeast)	0.925069	0.1321	NA
68133	Gcsh	glycine cleavage system protein H (aminomethyl carrier)	0.925069	0.4299	0.6569
71434	5530400B01Rik	RIKEN cDNA 5530400B01 gene	0.925069	0.7851	0.8972
71449	Mettl13	methyltransferase like 13	0.925069	0.4286	0.6557
72133	Trub1	TruB pseudouridine (psi) synthase homolog 1 (E. coli)	0.925069	0.5644	0.7615
75573	2310007L24Rik	RIKEN cDNA 2310007L24 gene	0.925069	0.5726	0.7665
75957	Mir17hg	MIR17 host gene 1 (non-protein coding)	0.925069	0.3482	NA
80877	Lrba	LPS-responsive beige-like anchor	0.925069	0.1647	NA

80892	Zfhx4	zinc finger homeodomain 4	0.925069	0.3703	0.6052
98985	Clp1	CLP1, cleavage and polyadenylation factor I subunit, homolog (S. cerevisiae)	0.925069	0.4006	0.6309
107986	Ddb2	damage specific DNA binding protein 2	0.925069	0.2958	NA
192156	Mvd	mevalonate (diphospho) decarboxylase	0.925069	0.5234	0.7311
208624	Alg3	asparagine-linked glycosylation 3 homolog (yeast, alpha-1,3-mannosyltransferase)	0.925069	0.6136	0.7948
209416	Gpkow	G patch domain and KOW motifs	0.925069	0.2701	NA
216166	Plk5	polo-like kinase 5 (Drosophila)	0.925069	0.3169	NA
224742	Abcf1	ATP-binding cassette, sub-family F (GCN20), member 1	0.925069	0.2034	NA
229697	Cym	chymosin	0.925069	0.5381	0.7425
230753	Thrap3	thyroid hormone receptor associated protein 3	0.925069	0.7767	0.8926
246048	Chodl	chondrolectin	0.925069	0.5047	0.7171
246696	Slc25a28	solute carrier family 25, member 28	0.925069	0.2425	NA
246703	Apoa1bp	apolipoprotein A-I binding protein	0.925069	0.3264	NA
319347	C230094B09Rik	RIKEN cDNA C230094B09 gene	0.925069	0.4651	0.687
320717	Pptc7	PTC7 protein phosphatase homolog (S. cerevisiae)	0.925069	0.2045	NA
330050	Fam185a	family with sequence similarity 185, member A	0.925069	0.5259	0.7331
654494	Gm7337	DAZ interacting protein 1 pseudogene	0.925069	0.5755	0.7689
11820	App	amyloid beta (A4) precursor protein	0.924214	0.328	NA
11889	Asgr1	asialoglycoprotein receptor 1	0.924214	0.5029	0.7158
11975	Atp6v0a1	ATPase, H+ transporting, lysosomal V0 subunit A1	0.924214	0.2822	NA
14799	Gria1	glutamate receptor, ionotropic, AMPA1 (alpha 1)	0.924214	0.2966	NA
16492	Kcna4	potassium voltage-gated channel, shaker-related subfamily, member 4	0.924214	0.6754	0.8334
19088	Prkar2b	protein kinase, cAMP dependent regulatory, type II beta	0.924214	0.4265	0.6537
19305	Pex5	peroxisomal biogenesis factor 5	0.924214	0.3326	NA
19338	Rab33b	RAB33B, member of RAS oncogene family	0.924214	0.265	NA
23972	Papss2	3'-phosphoadenosine 5'-phosphosulfate synthase 2	0.924214	0.4541	0.6779
24015	Abce1	ATP-binding cassette, sub-family E (OABP), member 1	0.924214	0.1879	NA
26374	Rfwd2	ring finger and WD repeat domain 2	0.924214	0.3156	NA
50797	Copb2	coatomer protein complex, subunit beta 2 (beta prime)	0.924214	0.2702	NA
52864	Slx4	SLX4 structure-specific endonuclease subunit homolog (S. cerevisiae)	0.924214	0.1352	NA
56513	Pard6a	par-6 (partitioning defective 6,) homolog alpha (C. elegans)	0.924214	0.2718	NA
65019	Rpl23	ribosomal protein L23	0.924214	0.4773	0.6972
66713	Actr2	ARP2 actin-related protein 2 homolog (yeast)	0.924214	0.2239	NA
66881	Pcyox1	prenylcysteine oxidase 1	0.924214	0.2831	NA
67384	Bag4	BCL2-associated athanogene 4	0.924214	0.2003	NA
67467	1200011I18Rik	RIKEN cDNA 1200011I18 gene	0.924214	0.1033	NA
68521	Fam189b	family with sequence similarity 189, member B	0.924214	0.2673	NA
68833	Pdcl3	phosducin-like 3	0.924214	0.542	0.7459
69219	Ddah1	dimethylarginine dimethylaminohydrolase 1	0.924214	0.2567	NA
69310	Pacrg	PARK2 co-regulated	0.924214	0.24	NA
71913	Tmem79	transmembrane protein 79	0.924214	0.5246	0.732
71963	Cdca4	cell division cycle associated 4	0.924214	0.3831	0.6161
72053	Tmub2	transmembrane and ubiquitin-like domain containing 2	0.924214	0.1879	NA
73173	Pcdh18	protocadherin 18	0.924214	0.2233	NA
74051	Steap2	six transmembrane epithelial antigen of prostate 2	0.924214	0.2158	NA
74370	Rptor	regulatory associated protein of MTOR, complex 1	0.924214	0.1334	NA
74901	Kbtbd11	kelch repeat and BTB (POZ) domain containing 11	0.924214	0.5039	0.7165
75454	Phpt1	phosphohistidine phosphatase 1	0.924214	0.2268	NA
76501	Commd9	COMM domain containing 9	0.924214	0.3247	NA

78283	Mtap7d2	MAP7 domain containing 2	0.924214	0.4046	0.6342
78670	Plekhj1	pleckstrin homology domain containing, family J member 1	0.924214	0.5443	0.7472
97863	C78339	expressed sequence C78339	0.924214	0.5163	0.7261
98170	Tmem132a	transmembrane protein 132A	0.924214	0.1107	NA
107767	Scamp1	secretory carrier membrane protein 1	0.924214	0.1775	NA
108863	4833420G11Rik	RIKEN cDNA 4833420G11 gene	0.924214	0.2872	NA
110876	Scn2a1	sodium channel, voltage-gated, type II, alpha 1	0.924214	0.4559	0.6794
170736	Parvb	parvin, beta	0.924214	0.687	0.8404
216363	Rab3ip	RAB3A interacting protein	0.924214	0.11	NA
224807	Tmem63b	transmembrane protein 63b	0.924214	0.247	NA
225467	Pggt1b	protein geranylgeranyltransferase type I, beta subunit	0.924214	0.4194	0.6474
226539	Dars2	aspartyl-tRNA synthetase 2 (mitochondrial)	0.924214	0.513	0.7233
230500	Efcab7	EF-hand calcium binding domain 7	0.924214	0.7327	0.8682
231659	Gcn1l1	GCN1 general control of amino-acid synthesis 1-like 1 (yeast)	0.924214	0.3551	0.5898
234797	6430548M08Rik	RIKEN cDNA 6430548M08 gene	0.924214	0.3834	0.6162
245695	Tceanc	transcription elongation factor A (SII) N-terminal and central domain containing	0.924214	0.6846	0.8392
268480	Rapgefl1	Rap guanine nucleotide exchange factor (GEF)-like 1	0.924214	0.3597	0.5947
268706	Slc38a9	solute carrier family 38, member 9	0.924214	0.1109	NA
268859	Rbfox1	RNA binding protein, fox-1 homolog (C. elegans) 1	0.924214	0.1854	NA
321008	6330408A02Rik	RIKEN cDNA 6330408A02 gene	0.924214	0.2387	NA
368203	Gm5136	predicted gene 5136	0.924214	0.6825	0.8378
387524	Znrf2	zinc and ring finger 2	0.924214	0.1879	NA
12424	Cck	cholecystokinin	0.923361	0.1703	NA
13244	Degs1	degenerative spermatocyte homolog 1 (Drosophila)	0.923361	0.1958	NA
13426	Dync1i1	dynein cytoplasmic 1 intermediate chain 1	0.923361	0.4887	0.7061
13528	Dtnb	dystrobrevin, beta	0.923361	0.246	NA
14164	Fgf1	fibroblast growth factor 1	0.923361	0.3006	NA
14268	Fn1	fibronectin 1	0.923361	0.3974	0.6278
14570	Arhgdig	Rho GDP dissociation inhibitor (GDI) gamma	0.923361	0.3898	0.6219
18105	Nqo2	NAD(P)H dehydrogenase, quinone 2	0.923361	0.1926	NA
18472	Pafah1b1	platelet-activating factor acetylhydrolase, isoform 1b, subunit 1	0.923361	0.2065	NA
20272	Scn7a	sodium channel, voltage-gated, type VII, alpha	0.923361	0.4981	0.7127
21689	Tekt1	tektin 1	0.923361	0.3863	0.6185
21841	Tia1	cytotoxic granule-associated RNA binding protein 1	0.923361	0.4507	0.6756
22334	Vdac2	voltage-dependent anion channel 2	0.923361	0.1041	NA
22355	Vipr2	vasoactive intestinal peptide receptor 2	0.923361	0.5864	0.7761
22678	Zfp2	zinc finger protein 2	0.923361	0.3909	0.6227
23854	Def8	differentially expressed in FDCP 8	0.923361	0.1749	NA
27395	Mrpl15	mitochondrial ribosomal protein L15	0.923361	0.2607	NA
56543	Kcnd3	potassium voltage-gated channel, Shal-related family, member 3	0.923361	0.4207	0.6486
56748	Nfu1	NFU1 iron-sulfur cluster scaffold homolog (S. cerevisiae)	0.923361	0.1419	NA
66154	Tmem14c	transmembrane protein 14C	0.923361	0.2134	NA
66241	Tmem9	transmembrane protein 9	0.923361	0.4201	0.6479
68525	Evc2	Ellis van Creveld syndrome 2 homolog (human)	0.923361	0.2962	NA
69149	Kbtbd3	kelch repeat and BTB (POZ) domain containing 3	0.923361	0.2746	NA
70832	4921504A21Rik	RIKEN cDNA 4921504A21 gene	0.923361	0.4637	0.6863
71707	Ubiad1	UbiA prenyltransferase domain containing 1	0.923361	0.09622	NA
72508	Rps6kb1	ribosomal protein S6 kinase, polypeptide 1	0.923361	0.5633	0.7609
72549	Reep4	receptor accessory protein 4	0.923361	0.6213	0.7994

73132	Slc25a16	solute carrier family 25 (mitochondrial carrier, Graves disease autoantigen), member 16	0.923361	0.3541	0.5888
73722	Lce1a2	late cornified envelope 1A2	0.923361	0.7367	0.8711
75504	1700013F07Rik	RIKEN cDNA 1700013F07 gene	0.923361	0.5868	0.7763
75625	Mageh1	melanoma antigen, family H, 1	0.923361	0.3198	NA
76308	Rab1b	RAB1B, member RAS oncogene family	0.923361	0.3192	NA
76980	Ube2ql1	ubiquitin-conjugating enzyme E2Q family-like 1	0.923361	0.5434	0.7466
77900	6720473M11Rik	RIKEN cDNA 6720473M11 gene	0.923361	0.6859	0.8398
78124	4930458L03Rik	RIKEN cDNA 4930458L03 gene	0.923361	0.5885	0.7778
80289	Lysmd3	LysM, putative peptidoglycan-binding, domain containing 3	0.923361	0.32	NA
99003	Qser1	glutamine and serine rich 1	0.923361	0.3962	0.6267
107823	Whsc1	Wolf-Hirschhorn syndrome candidate 1 (human)	0.923361	0.4327	0.6599
107869	Cth	cystathionase (cystathionine gamma-lyase)	0.923361	0.339	NA
114641	Rpl31	ribosomal protein L31	0.923361	0.2074	NA
171273	Vmn1r217	vomeronasal 1 receptor 217	0.923361	0.6312	0.8058
216963	Git1	G protein-coupled receptor kinase-interactor 1	0.923361	0.3959	0.6264
228880	Zmynd8	zinc finger, MYND-type containing 8	0.923361	0.4054	0.6351
229521	Syt11	synaptotagmin XI	0.923361	0.45	0.6751
230103	Npr2	natriuretic peptide receptor 2	0.923361	0.2444	NA
238386	Btbd7	BTB (POZ) domain containing 7	0.923361	0.4365	0.663
269999	Orai3	ORAI calcium release-activated calcium modulator 3	0.923361	0.5778	0.7704
338337	Cog3	component of oligomeric golgi complex 3	0.923361	0.2032	NA
621976	Tmem170b	transmembrane protein 170B	0.923361	0.3811	0.6148
100039138	Gm2061	predicted gene 2061	0.923361	0.5423	0.7461
100043685	Gm4588	predicted gene 4588	0.923361	0.2854	NA
100043885	Gm4710	predicted gene 4710	0.923361	0.6714	0.8318
11972	Atp6v0d1	ATPase, H+ transporting, lysosomal V0 subunit D1	0.922509	0.3629	0.598
12069	Bex2	brain expressed X-linked 2	0.922509	0.1958	NA
12561	Cdh4	cadherin 4	0.922509	0.3773	0.6113
12859	Cox5b	cytochrome c oxidase, subunit Vb	0.922509	0.1628	NA
13010	Cst3	cystatin C	0.922509	0.4119	0.6408
13557	E2f3	E2F transcription factor 3	0.922509	0.2722	NA
13859	Eps15l1	epidermal growth factor receptor pathway substrate 15-like 1	0.922509	0.1431	NA
14660	Gls	glutaminase	0.922509	0.5724	0.7665
14732	Gpam	glycerol-3-phosphate acyltransferase, mitochondrial	0.922509	0.347	0.5825
14803	Grid1	glutamate receptor, ionotropic, delta 1	0.922509	0.6789	0.8357
14816	Grm1	glutamate receptor, metabotropic 1	0.922509	0.4961	0.7111
16865	Eif2d	eukaryotic translation initiation factor 2D	0.922509	0.2281	NA
17192	Mbd3	methyl-CpG binding domain protein 3	0.922509	0.2644	NA
17248	Mdm4	transformed mouse 3T3 cell double minute 4	0.922509	0.5356	0.7408
17283	Men1	multiple endocrine neoplasia 1	0.922509	0.2887	NA
18163	Ctnnd2	catenin (cadherin associated protein), delta 2	0.922509	0.4189	0.6468
18738	Pitpna	phosphatidylinositol transfer protein, alpha	0.922509	0.06096	NA
19079	Prkab1	protein kinase, AMP-activated, beta 1 non-catalytic subunit	0.922509	0.6344	0.8076
19265	Ptprcap	protein tyrosine phosphatase, receptor type, C polypeptide-associated protein	0.922509	0.4138	0.6425
19820	Rlim	ring finger protein, LIM domain interacting	0.922509	0.3896	0.6216
20753	Sprr1a	small proline-rich protein 1A	0.922509	0.7662	0.8872
21371	Tbca	tubulin cofactor A	0.922509	0.1081	NA
22284	Usp9x	ubiquitin specific peptidase 9, X chromosome	0.922509	0.5506	0.7511
22780	Ikzf3	IKAROS family zinc finger 3	0.922509	0.6473	0.8162

29808	Mga		MAX gene associated	0.922509	0.25	NA
30839	Fbxw5		F-box and WD-40 domain protein 5	0.922509	0.3143	NA
50505	Ercc4		excision repair cross-complementing rodent repair deficiency, complementation group 4	0.922509	0.1919	NA
50760	Fbxo17		F-box protein 17	0.922509	0.4506	0.6754
50935	St6galnac6		ST6 (alpha-N-acetyl-neuraminyl-2,3-beta-galactosyl-1,3)-N-acetylgalactosaminide alpha-2,6-sialyltransferase 6	0.922509	0.285	NA
54131	Irf3		interferon regulatory factor 3	0.922509	0.2546	NA
54418	Fmn2		formin 2	0.922509	0.3028	NA
54473	Tollip		toll interacting protein	0.922509	0.1584	NA
57438		Mar-07	membrane-associated ring finger (C3HC4) 7	0.922509	0.4027	0.6326
60365	Rbm8a		RNA binding motif protein 8a	0.922509	0.3151	NA
66390	Slmo2		slowmo homolog 2 (Drosophila)	0.922509	0.3322	0.5682
67030	Fancl		Fanconi anemia, complementation group L	0.922509	0.3023	NA
67207	Lsm1		LSM1 homolog, U6 small nuclear RNA associated (S. cerevisiae)	0.922509	0.3296	NA
67890	Ufm1		ubiquitin-fold modifier 1	0.922509	0.2814	NA
68032	Tmem85		transmembrane protein 85	0.922509	0.1137	NA
68099	Fam92a		family with sequence similarity 92, member A	0.922509	0.3219	NA
68192	Leprotl1		leptin receptor overlapping transcript-like 1	0.922509	0.1692	NA
68310	Zmym1		zinc finger, MYM domain containing 1	0.922509	0.1851	NA
69710	Arap1		ArfGAP with RhoGAP domain, ankyrin repeat and PH domain 1	0.922509	0.5487	0.7501
69930	Zfp715		zinc finger protein 715	0.922509	0.2738	NA
72611	Zfp655		zinc finger protein 655	0.922509	0.2775	NA
74849	4930412F12Rik		RIKEN cDNA 4930412F12 gene	0.922509	0.3299	NA
77305	Wdr82		WD repeat domain containing 82	0.922509	0.1426	NA
77519	Zfp266		zinc finger protein 266	0.922509	0.2951	NA
93679	Trim8		tripartite motif-containing 8	0.922509	0.1986	NA
102032	AI316807		expressed sequence AI316807	0.922509	0.2358	NA
110651	Rps6ka3		ribosomal protein S6 kinase polypeptide 3	0.922509	0.5172	0.7267
207932	Urb1		URB1 ribosome biogenesis 1 homolog (S. cerevisiae)	0.922509	0.3133	NA
217708	Lin52		lin-52 homolog (C. elegans)	0.922509	0.2402	NA
225058	Gm4832		predicted gene 4832	0.922509	0.6455	0.8149
231991	Creb5		cAMP responsive element binding protein 5	0.922509	0.5421	0.7459
233865	D430042O09Rik		RIKEN cDNA D430042O09 gene	0.922509	0.4048	0.6344
240442	Adnp2		ADNP homeobox 2	0.922509	0.3677	0.6027
243867	Fbxo46		F-box protein 46	0.922509	0.2144	NA
331004	Slc9a9		solute carrier family 9 (sodium/hydrogen exchanger), member 9	0.922509	0.4887	0.7061
381290	Atp2b4		ATPase, Ca++ transporting, plasma membrane 4	0.922509	0.6379	0.8099
383348	Kctd16		potassium channel tetramerisation domain containing 16	0.922509	0.5089	0.72
666945	Gm10638		predicted gene 10638	0.922509	0.3334	NA
100126824	Sco2		SCO cytochrome oxidase deficient homolog 2 (yeast)	0.922509	0.378	0.6117
12349	Car2		carbonic anhydrase 2	0.921659	0.3388	0.5745
12386	Ctnna2		catenin (cadherin associated protein), alpha 2	0.921659	0.1922	NA
12632	Cfl2		cofilin 2, muscle	0.921659	0.1924	NA
17965	Nbl1		neuroblastoma, suppression of tumorigenicity 1	0.921659	0.3701	0.605
22166	Txn1		thioredoxin 1	0.921659	0.2357	NA
26413	Mapk1		mitogen-activated protein kinase 1	0.921659	0.2468	NA
30951	Cbx8		chromobox homolog 8 (Drosophila Pc class)	0.921659	0.6735	0.8328
52137	D7Ertd595e		DNA segment, Chr 7, ERATO Doi 595, expressed	0.921659	0.3944	0.6252
53328	Pgrmc1		progesterone receptor membrane component 1	0.921659	0.2292	NA
53761	Prrc2a		proline-rich coiled-coil 2A	0.921659	0.496	0.7111

56264	Cpxm1	carboxypeptidase X 1 (M14 family)	0.921659	0.4372	0.6636
56351	Ptges3	prostaglandin E synthase 3 (cytosolic)	0.921659	0.3502	0.5861
56705	Ranbp9	RAN binding protein 9	0.921659	0.2072	NA
66612	Ormdl3	ORM1-like 3 (S. cerevisiae)	0.921659	0.1893	NA
66911	Nudt16l1	nudix (nucleoside diphosphate linked moiety X)-type motif 16-like 1	0.921659	0.1784	NA
66965	Ctu2	cytosolic thiouridylase subunit 2 homolog (S. pombe)	0.921659	0.1092	NA
67170	2610306M01Rik	RIKEN cDNA 2610306M01 gene	0.921659	0.4333	0.6604
67375	Qprt	quinolinate phosphoribosyltransferase	0.921659	0.8593	0.9375
67468	Mmd	monocyte to macrophage differentiation-associated	0.921659	0.2339	NA
68480	1110007C09Rik	RIKEN cDNA 1110007C09 gene	0.921659	0.2373	NA
68731	Rbfa	ribosome binding factor A	0.921659	0.4499	0.675
68857	Dtwd2	DTW domain containing 2	0.921659	0.4054	0.6351
71361	Aifm2	apoptosis-inducing factor, mitochondrion-associated 2	0.921659	0.3867	0.6189
72119	Tpx2	TPX2, microtubule-associated protein homolog (Xenopus laevis)	0.921659	0.4494	0.6746
72170	Chchd4	coiled-coil-helix-coiled-coil-helix domain containing 4	0.921659	0.2838	NA
73723	Sh3bgrl3	SH3 domain binding glutamic acid-rich protein-like 3	0.921659	0.4014	0.6316
78433	A930006K02Rik	RIKEN cDNA A930006K02 gene	0.921659	0.5058	0.7181
94315	Prcc	papillary renal cell carcinoma (translocation-associated)	0.921659	0.3642	0.5996
104831	Ptpn23	protein tyrosine phosphatase, non-receptor type 23	0.921659	0.2244	NA
105260	D930043N17Rik	RIKEN cDNA D930043N17 gene	0.921659	0.4419	0.6677
108888	Atad3a	ATPase family, AAA domain containing 3A	0.921659	0.5073	0.719
109778	Blvra	biliverdin reductase A	0.921659	0.2643	NA
140743	Rem2	rad and gem related GTP binding protein 2	0.921659	0.7421	0.8743
207165	Bptf	bromodomain PHD finger transcription factor	0.921659	0.1769	NA
210356	Nckap5	NCK-associated protein 5	0.921659	0.1842	NA
212483	Fam193b	family with sequence similarity 193, member B	0.921659	0.2673	NA
215210	Tmem120a	transmembrane protein 120A	0.921659	0.1324	NA
216238	Eea1	early endosome antigen 1	0.921659	0.2537	NA
217732	2310044G17Rik	RIKEN cDNA 2310044G17 gene	0.921659	0.5326	0.7383
219135	Mtmr6	myotubularin related protein 6	0.921659	0.1498	NA
223435	Trio	triple functional domain (PTPRF interacting)	0.921659	0.1929	NA
226180	Ina	internexin neuronal intermediate filament protein, alpha	0.921659	0.449	0.6745
227054	Rpl23a-ps1	ribosomal protein 23A, pseudogene 1	0.921659	0.1552	NA
228366	Gyltl1b	glycosyltransferase-like 1B	0.921659	0.4908	0.7077
232566	Amn1	antagonist of mitotic exit network 1 homolog (S. cerevisiae)	0.921659	0.258	NA
232989	Hnrnpul1	heterogeneous nuclear ribonucleoprotein U-like 1	0.921659	0.4917	0.708
233900	Rnf40	ring finger protein 40	0.921659	0.2841	NA
235339	Dlat	dihydrolipoamide S-acetyltransferase (E2 component of pyruvate dehydrogenase complex)	0.921659	0.1485	NA
243376	Doxl2	diamine oxidase-like protein 2	0.921659	0.669	0.83
244058	Rgma	RGM domain family, member A	0.921659	0.5095	0.7205
252870	Usp7	ubiquitin specific peptidase 7	0.921659	0.2318	NA
252876	Gin1	gypsy retrotransposon integrase 1	0.921659	0.5922	0.7801
269209	Stk36	serine/threonine kinase 36 (fused homolog, Drosophila)	0.921659	0.3969	0.6273
277468	Slc39a12	solute carrier family 39 (zinc transporter), member 12	0.921659	0.5482	0.7496
330319	Wipf3	WAS/WASL interacting protein family, member 3	0.921659	0.2515	NA
11994	Pcdh15	protocadherin 15	0.92081	0.4015	0.6317
13132	Dab2	disabled homolog 2 (Drosophila)	0.92081	0.6723	0.8322
13389	Dll3	delta-like 3 (Drosophila)	0.92081	0.4043	0.634
13593	Ebf3	early B-cell factor 3	0.92081	0.09829	NA

13627	Eef1a1	eukaryotic translation elongation factor 1 alpha 1	0.92081	0.4432	0.669
14683	Gnas	GNAS (guanine nucleotide binding protein, alpha stimulating) complex locus	0.92081	0.2121	NA
14697	Gnb5	guanine nucleotide binding protein (G protein), beta 5	0.92081	0.499	0.7135
16923	Sh2b3	SH2B adaptor protein 3	0.92081	0.423	0.6508
16987	Lss	lanosterol synthase	0.92081	0.4147	0.6433
17158	Man2a1	mannosidase 2, alpha 1	0.92081	0.2282	NA
17159	Man2b1	mannosidase 2, alpha B1	0.92081	0.2563	NA
17259	Mef2b	myocyte enhancer factor 2B	0.92081	0.6946	0.8448
17261	Mef2d	myocyte enhancer factor 2D	0.92081	0.5355	0.7408
17721	ND5	NADH dehydrogenase subunit 5	0.92081	0.445	0.6706
17777	Mttp	microsomal triglyceride transfer protein	0.92081	0.1533	NA
21400	Tcea2	transcription elongation factor A (SII), 2	0.92081	0.5	0.7144
21664	Phlda1	pleckstrin homology-like domain, family A, member 1	0.92081	0.3694	0.6043
21750	Terf2	telomeric repeat binding factor 2	0.92081	0.319	NA
30795	Fkbp3	FK506 binding protein 3	0.92081	0.09322	NA
66663	Uba5	ubiquitin-like modifier activating enzyme 5	0.92081	0.1128	NA
68626	Elac2	elaC homolog 2 (E. coli)	0.92081	0.3023	NA
70335	Reep6	receptor accessory protein 6	0.92081	0.6342	0.8075
70834	Spag9	sperm associated antigen 9	0.92081	0.2105	NA
70957	4921530L18Rik	RIKEN cDNA 4921530L18 gene	0.92081	0.645	0.8146
72056	1810055G02Rik	RIKEN cDNA 1810055G02 gene	0.92081	0.2771	NA
74334	Ranbp10	RAN binding protein 10	0.92081	0.2017	NA
74775	Lmbr1l	limb region 1 like	0.92081	0.3412	0.5768
76687	Spcs3	signal peptidase complex subunit 3 homolog (S. cerevisiae)	0.92081	0.1735	NA
76787	Ppfia3	protein tyrosine phosphatase, receptor type, f polypeptide (PTPRF), interacting protein (liprin), alpha 3	0.92081	0.3478	0.5832
77031	Slc9a8	solute carrier family 9 (sodium/hydrogen exchanger), member 8	0.92081	0.7249	0.8633
77583	Notum	notum pectinacetylesterase homolog (Drosophila)	0.92081	0.6283	0.8039
99890	Prmt6	protein arginine N-methyltransferase 6	0.92081	0.3316	0.5678
103967	Dnm3	dynamin 3	0.92081	0.2756	NA
107338	Gbf1	golgi-specific brefeldin A-resistance factor 1	0.92081	0.6036	0.7873
109785	Pgm3	phosphoglucomutase 3	0.92081	0.3332	0.5691
110749	Chaf1b	chromatin assembly factor 1, subunit B (p60)	0.92081	0.4132	0.642
140489	Bhlhe23	basic helix-loop-helix family, member e23	0.92081	0.1267	NA
213084	Cdkl3	cyclin-dependent kinase-like 3	0.92081	0.2361	NA
213582	Mtap9	microtubule-associated protein 9	0.92081	0.2546	NA
216869	Arrb2	arrestin, beta 2	0.92081	0.265	NA
227094	Tmem194b	transmembrane protein 194B	0.92081	0.4876	0.7055
234515	Inpp4b	inositol polyphosphate-4-phosphatase, type II	0.92081	0.4288	0.6558
235072	Sep-07	septin 7	0.92081	0.2444	NA
237052	Tceal1	transcription elongation factor A (SII)-like 1	0.92081	0.4076	0.6369
238693	Zfp58	zinc finger protein 58	0.92081	0.499	0.7135
239393	Lrp12	low density lipoprotein-related protein 12	0.92081	0.208	NA
240505	Cdc42bpg	CDC42 binding protein kinase gamma (DMPK-like)	0.92081	0.5577	0.7568
269397	Ss18l1	synovial sarcoma translocation gene on chromosome 18-like 1	0.92081	0.206	NA
330812	Rnf150	ring finger protein 150	0.92081	0.5766	0.7695
381801	Tatdn2	TatD DNase domain containing 2	0.92081	0.4728	0.6936
382051	Pdp2	pyruvate dehyrogenase phosphatase catalytic subunit 2	0.92081	0.3435	0.5789
385354	Frmd7	FERM domain containing 7	0.92081	0.5862	0.7761
434128	Pnmal2	PNMA-like 2	0.92081	0.7132	0.8559

11781	Ap4m1	adaptor-related protein complex AP-4, mu 1	0.919963	0.3467	0.5821
11836	Araf	v-raf murine sarcoma 3611 viral oncogene homolog	0.919963	0.4133	0.642
12049	Bcl2l10	Bcl2-like 10	0.919963	0.3919	0.6234
12660	Chka	choline kinase alpha	0.919963	0.2688	NA
13063	Cycs	cytochrome c, somatic	0.919963	0.1437	NA
13356	Dgcr2	DiGeorge syndrome critical region gene 2	0.919963	0.5473	0.7488
13805	Eng	endoglin	0.919963	0.3125	NA
19651	Rbl2	retinoblastoma-like 2	0.919963	0.2212	NA
19896	Rpl10a	ribosomal protein L10A	0.919963	0.2432	NA
20405	Sh3gl1	SH3-domain GRB2-like 1	0.919963	0.4276	0.6547
22141	Tub	tubby candidate gene	0.919963	0.5857	0.776
22630	Ywhaq	tyrosine 3-monooxygenase/tryptophan 5-monooxygenase activation protein, theta polypeptide	0.919963	0.2926	NA
24105	Rbck1	RanBP-type and C3HC4-type zinc finger containing 1	0.919963	0.3524	0.5875
50493	Txnrd1	thioredoxin reductase 1	0.919963	0.2235	NA
52902	D10Bwg1070e	DNA segment, Chr 10, Brigham & Women's Genetics 1070 expressed	0.919963	0.573	0.7669
56282	Mrpl12	mitochondrial ribosomal protein L12	0.919963	0.1189	NA
56839	Lgi1	leucine-rich repeat LGI family, member 1	0.919963	0.2993	NA
66537	Pomp	proteasome maturation protein	0.919963	0.286	NA
66631	Hiatl1	hippocampus abundant transcript-like 1	0.919963	0.3842	0.6166
67112	Fgf22	fibroblast growth factor 22	0.919963	0.5041	0.7166
67958	2610101N10Rik	RIKEN cDNA 2610101N10 gene	0.919963	0.2561	NA
68877	Maf1	MAF1 homolog (S. cerevisiae)	0.919963	0.3137	NA
69227	2810407C02Rik	RIKEN cDNA 2810407C02 gene	0.919963	0.373	0.6072
69714	Tfpt	TCF3 (E2A) fusion partner	0.919963	0.5814	0.7728
70057	2210008F06Rik	RIKEN cDNA 2210008F06 gene	0.919963	0.451	0.6759
70408	Polr3f	polymerase (RNA) III (DNA directed) polypeptide F	0.919963	0.3568	0.5916
74112	Usp16	ubiquitin specific peptidase 16	0.919963	0.144	NA
74125	Armc8	armadillo repeat containing 8	0.919963	0.3443	0.5797
74600	Mrpl47	mitochondrial ribosomal protein L47	0.919963	0.1965	NA
76804	Kdm4c	lysine (K)-specific demethylase 4C	0.919963	0.2005	NA
77971	A930037O16Rik	RIKEN cDNA A930037O16 gene	0.919963	0.1361	NA
79264	Krit1	KRIT1, ankyrin repeat containing	0.919963	0.3122	NA
81703	Jdp2	Jun dimerization protein 2	0.919963	0.414	0.6427
100019	Mdn1	midasin homolog (yeast)	0.919963	0.3109	NA
110173	Manba	mannosidase, beta A, lysosomal	0.919963	0.2472	NA
170711	Otud7a	OTU domain containing 7A	0.919963	0.4204	0.6483
207615	Wdr37	WD repeat domain 37	0.919963	0.1652	NA
213027	Evi5l	ecotropic viral integration site 5 like	0.919963	0.1494	NA
224020	Pi4ka	phosphatidylinositol 4-kinase, catalytic, alpha polypeptide	0.919963	0.1305	NA
232156	Slc4a5	solute carrier family 4, sodium bicarbonate cotransporter, member 5	0.919963	0.4359	0.6626
233904	Setd1a	SET domain containing 1A	0.919963	0.2023	NA
268319	BC025920	zinc finger protein pseudogene	0.919963	0.2853	NA
100093715	Gm10404	predicted gene 10404	0.919963	0.5727	0.7666
12861	Cox6a1	cytochrome c oxidase, subunit VI a, polypeptide 1	0.919118	0.2616	NA
18479	Pak1	p21 protein (Cdc42/Rac)-activated kinase 1	0.919118	0.285	NA
19338	Rab33b	RAB33B, member of RAS oncogene family	0.919118	0.3837	0.6163
20020	Polr2a	polymerase (RNA) II (DNA directed) polypeptide A	0.919118	0.3429	0.5782
20116	Rps8	ribosomal protein S8	0.919118	0.4319	0.6591
24136	Zeb2	zinc finger E-box binding homeobox 2	0.919118	0.2847	NA

28064	Yipf3	Yip1 domain family, member 3	0.919118	0.3454	0.5809
54003	Nell2	NEL-like 2 (chicken)	0.919118	0.6171	0.797
54218	B3galt4	UDP-Gal:betaGlcNAc beta 1,3-galactosyltransferase, polypeptide 4	0.919118	0.472	0.6931
55943	Stx8	syntaxin 8	0.919118	0.2411	NA
56325	Abcb9	ATP-binding cassette, sub-family B (MDR/TAP), member 9	0.919118	0.4189	0.6468
56543	Kcnd3	potassium voltage-gated channel, Shal-related family, member 3	0.919118	0.3195	0.556
60441	Mrpl38	mitochondrial ribosomal protein L38	0.919118	0.3725	0.6068
66085	Eif3f	eukaryotic translation initiation factor 3, subunit F	0.919118	0.169	NA
66359	Fam36a	family with sequence similarity 36, member A	0.919118	0.1823	NA
66385	Ppp1r7	protein phosphatase 1, regulatory (inhibitor) subunit 7	0.919118	0.1727	NA
66531	2310061C15Rik	RIKEN cDNA 2310061C15 gene	0.919118	0.2393	NA
68564	Nufip2	nuclear fragile X mental retardation protein interacting protein 2	0.919118	0.6567	0.8223
70223	Nars	asparaginyl-tRNA synthetase	0.919118	0.3386	0.5745
72701	Zfp618	zinc fingerprotein 618	0.919118	0.2361	NA
74005	6330412A17Rik	RIKEN cDNA 6330412A17 gene	0.919118	0.422	0.6499
74246	Gale	galactose-4-epimerase, UDP	0.919118	0.2422	NA
75538	Fam71e1	family with sequence similarity 71, member E1	0.919118	0.259	NA
93969	Klra22	killer cell lectin-like receptor subfamily A, member 22	0.919118	0.8468	0.9316
94282	Sfxn5	sideroflexin 5	0.919118	0.1958	NA
103012	6720401G13Rik	RIKEN cDNA 6720401G13 gene	0.919118	0.278	NA
105833	Ccdc65	coiled-coil domain containing 65	0.919118	0.622	0.7997
214547	She	src homology 2 domain-containing transforming protein E	0.919118	0.3796	0.6132
217166	Nr1d1	nuclear receptor subfamily 1, group D, member 1	0.919118	0.501	0.7146
223690	Ankrd54	ankyrin repeat domain 54	0.919118	0.3982	0.6287
224129	Adcy5	adenylate cyclase 5	0.919118	0.1252	NA
227638	Qsox2	quiescin Q6 sulfhydryl oxidase 2	0.919118	0.3144	0.5508
228545	Vps18	vacuolar protein sorting 18 (yeast)	0.919118	0.1807	NA
232493	Gys2	glycogen synthase 2	0.919118	0.6632	0.8268
233056	Zfp790	zinc finger protein 790	0.919118	0.5932	0.7808
235033	Rdh8	retinol dehydrogenase 8	0.919118	0.2806	NA
240025	Dact2	dapper homolog 2, antagonist of beta-catenin (xenopus)	0.919118	0.6288	0.8042
264064	Cdk8	cyclin-dependent kinase 8	0.919118	0.2638	NA
268319	BC025920	zinc finger protein pseudogene	0.919118	0.3341	0.5697
319845	Bbs9	Bardet-Biedl syndrome 9 (human)	0.919118	0.07878	NA
320907	B430105G09Rik	RIKEN cDNA B430105G09 gene	0.919118	0.2984	NA
329918	Skint9	selection and upkeep of intraepithelial T cells 9	0.919118	0.7652	0.8867
399603	Fam84b	family with sequence similarity 84, member B	0.919118	0.4704	0.692
434438	Ccdc36	coiled-coil domain containing 36	0.919118	0.6939	0.8444
544710	Krtap10-10	keratin associated protein 10-10	0.919118	0.6653	0.8282
100038598	Gm10386	predicted gene 10386	0.919118	0.4347	0.6616
12326	Camk4	calcium/calmodulin-dependent protein kinase IV	0.918274	0.46	0.6833
12874	Cpd	carboxypeptidase D	0.918274	0.303	NA
14563	Gdf5	growth differentiation factor 5	0.918274	0.1609	NA
15183	Hdac3	histone deacetylase 3	0.918274	0.2427	NA
16531	Kcnma1	potassium large conductance calcium-activated channel, subfamily M, alpha member 1	0.918274	0.4071	0.6365
17977	Ncoa1	nuclear receptor coactivator 1	0.918274	0.07248	NA
18021	Nfatc3	nuclear factor of activated T-cells, cytoplasmic, calcineurin-dependent 3	0.918274	0.2775	NA
18114	Rrp1	ribosomal RNA processing 1 homolog (S. cerevisiae)	0.918274	0.1306	NA
20300	Ccl25	chemokine (C-C motif) ligand 25	0.918274	0.3074	0.5437

20492	Slbp	stem-loop binding protein	0.918274	0.5489	0.7502
20617	Snca	synuclein, alpha	0.918274	0.2699	NA
20698	Sphk1	sphingosine kinase 1	0.918274	0.4119	0.6408
22036	Traip	TRAF-interacting protein	0.918274	0.5179	0.7273
26411	Map4k1	mitogen-activated protein kinase kinase kinase kinase 1	0.918274	0.3683	0.6032
51788	H2afz	H2A histone family, member Z	0.918274	0.1099	NA
58237	Nkain4	Na+/K+ transporting ATPase interacting 4	0.918274	0.2358	NA
59287	Ncstn	nicastrin	0.918274	0.4879	0.7056
67767	Jagn1	jagunal homolog 1 (Drosophila)	0.918274	0.1357	NA
68316	Apoo	apolipoprotein O	0.918274	0.1986	NA
68767	ORF19	open reading frame 19	0.918274	0.1788	NA
68929	Mospd3	motile sperm domain containing 3	0.918274	0.2906	NA
70239	Gtf3c5	general transcription factor IIIC, polypeptide 5	0.918274	0.1708	NA
71957	Cpsf3l	cleavage and polyadenylation specific factor 3-like	0.918274	0.2249	NA
73713	Rbm20	RNA binding motif protein 20	0.918274	0.6919	0.8433
73750	Whrn	whirlin	0.918274	0.2999	NA
76137	Ccdc90a	coiled-coil domain containing 90A	0.918274	0.4495	0.6746
107065	Lrrtm2	leucine rich repeat transmembrane neuronal 2	0.918274	0.2094	NA
118445	Klf16	Kruppel-like factor 16	0.918274	0.3171	0.5537
219257	Pcdh20	protocadherin 20	0.918274	0.5321	0.7378
223776	1300018J18Rik	RIKEN cDNA 1300018J18 gene	0.918274	0.2003	NA
228421	Kif18a	kinesin family member 18A	0.918274	0.4823	0.701
228788	BC020535	cDNA sequence BC020535	0.918274	0.4041	0.6339
228966	Ppp1r3d	protein phosphatase 1, regulatory subunit 3D	0.918274	0.2807	NA
231803	Mepce	methylphosphate capping enzyme	0.918274	0.2793	NA
233073	U2af1l4	U2 small nuclear RNA auxiliary factor 1-like 4	0.918274	0.4021	0.6321
237500	Tmtc3	transmembrane and tetratricopeptide repeat containing 3	0.918274	0.173	NA
240023	Pnldc1	poly(A)-specific ribonuclease (PARN)-like domain containing 1	0.918274	0.1393	NA
279653	Pcdh19	protocadherin 19	0.918274	0.2678	NA
320333	D830030K20Rik	RIKEN cDNA D830030K20 gene	0.918274	0.3836	0.6162
338354	Zfp780b	zinc finger protein 780B	0.918274	0.4614	0.6845
433458	Gm13880	predicted gene 13880	0.918274	0.8429	0.9294
545156	Kalrn	kalirin, RhoGEF kinase	0.918274	0.2599	NA
635702	Naaladl2	N-acetylated alpha-linked acidic dipeptidase-like 2	0.918274	0.6055	0.7887
791344	E330037I15Rik	RIKEN cDNA E330037I15 gene	0.918274	0.7905	0.9007
11909	Atf2	activating transcription factor 2	0.917431	0.4007	0.6309
11991	Hnrnpd	heterogeneous nuclear ribonucleoprotein D	0.917431	0.227	NA
13047	Cux1	cut-like homeobox 1	0.917431	0.1565	NA
13136	Cd55	CD55 antigen	0.917431	0.5357	0.7409
14269	Fnbp1	formin binding protein 1	0.917431	0.5104	0.7213
14365	Fzd3	frizzled homolog 3 (Drosophila)	0.917431	0.2992	NA
14628	Ostm1	osteopetrosis associated transmembrane protein 1	0.917431	0.2154	NA
15234	Hgf	hepatocyte growth factor	0.917431	0.4767	0.6966
16151	Ikbkg	inhibitor of kappaB kinase gamma	0.917431	0.2979	NA
17991	Ndufa2	NADH dehydrogenase (ubiquinone) 1 alpha subcomplex, 2	0.917431	0.2072	NA
18693	Pick1	protein interacting with C kinase 1	0.917431	0.3306	0.5671
18751	Prkcb	protein kinase C, beta	0.917431	0.1811	NA
18787	Serpine1	serine (or cysteine) peptidase inhibitor, clade E, member 1	0.917431	0.871	0.9441
19866	Rnu7	U7 small nuclear RNA	0.917431	0.1639	NA

20729	Spin1	spindlin 1	0.917431	0.152	NA
27096	Trappc3	trafficking protein particle complex 3	0.917431	0.1451	NA
56205	Ensa	endosulfine alpha	0.917431	0.166	NA
56462	Mtch1	mitochondrial carrier homolog 1 (C. elegans)	0.917431	0.2977	NA
56541	Habp4	hyaluronic acid binding protein 4	0.917431	0.2923	NA
66839	0610009O20Rik	RIKEN cDNA 0610009O20 gene	0.917431	0.2737	NA
67088	Cand2	cullin-associated and neddylation-dissociated 2 (putative)	0.917431	0.2644	NA
67295	Rab3c	RAB3C, member RAS oncogene family	0.917431	0.1787	NA
67370	Zfp606	zinc finger protein 606	0.917431	0.2997	NA
67976	Trabd	TraB domain containing	0.917431	0.3175	0.5539
69536	Hemk1	HemK methyltransferase family member 1	0.917431	0.4583	0.6816
70456	Brp44	brain protein 44	0.917431	0.08603	NA
72149	Strada	STE20-related kinase adaptor alpha	0.917431	0.1215	NA
73381	Cmtm2a	CKLF-like MARVEL transmembrane domain containing 2A	0.917431	0.3226	0.559
74018	Als2	amyotrophic lateral sclerosis 2 (juvenile) homolog (human)	0.917431	0.4403	0.6663
75273	Pelp1	proline, glutamic acid and leucine rich protein 1	0.917431	0.2813	NA
75458	Cklf	chemokine-like factor	0.917431	0.2736	NA
76044	Ncapg2	non-SMC condensin II complex, subunit G2	0.917431	0.4046	0.6342
76217	Jakmip2	janus kinase and microtubule interacting protein 2	0.917431	0.2961	NA
77220	Tmem200a	transmembrane protein 200A	0.917431	0.2974	NA
100608	Noc4l	nucleolar complex associated 4 homolog (S. cerevisiae)	0.917431	0.2743	NA
192734	AI646023	expressed sequence AI646023	0.917431	0.2412	NA
211401	Mtss1	metastasis suppressor 1	0.917431	0.1539	NA
213773	Tbl3	transducin (beta)-like 3	0.917431	0.41	0.6392
213980	Fbxw10	F-box and WD-40 domain protein 10	0.917431	0.3916	0.623
216860	Neurl4	neuralized homolog 4 (Drosophila)	0.917431	0.2921	NA
218271	B4galt7	xylosylprotein beta1,4-galactosyltransferase, polypeptide 7 (galactosyltransferase I)	0.917431	0.2672	NA
231830	Micall2	MICAL-like 2	0.917431	0.5822	0.7731
232087	Mat2a	methionine adenosyltransferase II, alpha	0.917431	0.53	0.7363
381605	Tbc1d2	TBC1 domain family, member 2	0.917431	0.4008	0.631
381605	Tbc1d2	TBC1 domain family, member 2	0.917431	0.4638	0.6864
670833	Gm11213	predicted gene 11213	0.917431	0.8174	0.9165
100038485	Gm10398	predicted gene 10398	0.917431	0.8577	0.9368
11308	Abi1	abl-interactor 1	0.91659	0.4384	0.6646
11512	Adcy6	adenylate cyclase 6	0.91659	0.3292	0.5657
12419	Cbx5	chromobox homolog 5 (Drosophila HP1a)	0.91659	0.212	NA
13190	Dct	dopachrome tautomerase	0.91659	0.7751	0.8918
13358	Slc25a1	solute carrier family 25 (mitochondrial carrier, citrate transporter), member 1	0.91659	0.4045	0.6342
14724	Gp1bb	glycoprotein Ib, beta polypeptide	0.91659	0.4136	0.6423
16161	Il12rb1	interleukin 12 receptor, beta 1	0.91659	0.7138	0.8564
18948	Pnmt	phenylethanolamine-N-methyltransferase	0.91659	0.4653	0.6872
19248	Ptpn12	protein tyrosine phosphatase, non-receptor type 12	0.91659	0.2411	NA
19646	Rbbp4	retinoblastoma binding protein 4	0.91659	0.2892	NA
20410	Sorbs3	sorbin and SH3 domain containing 3	0.91659	0.5804	0.7725
20807	Srf	serum response factor	0.91659	0.1184	NA
28071	Twistnb	TWIST neighbor	0.91659	0.269	NA
52469	Ccdc56	coiled-coil domain containing 56	0.91659	0.1308	NA
56214	Scamp4	secretory carrier membrane protein 4	0.91659	0.167	NA
64656	Mrps23	mitochondrial ribosomal protein S23	0.91659	0.1633	NA

66274	1810012P15Rik	RIKEN cDNA 1810012P15 gene	0.91659	0.2106	NA
66705	Dnase1l2	deoxyribonuclease 1-like 2	0.91659	0.4916	0.7079
67188	2700046G09Rik	RIKEN cDNA 2700046G09 gene	0.91659	0.1345	NA
67661	Ift172	intraflagellar transport 172 homolog (Chlamydomonas)	0.91659	0.2571	NA
67737	Ttc39d	tetratricopeptide repeat domain 39D	0.91659	0.6713	0.8318
68035	Rbm42	RNA binding motif protein 42	0.91659	0.1733	NA
68067	3010026O09Rik	RIKEN cDNA 3010026O09 gene	0.91659	0.2062	NA
68292	Stt3b	STT3, subunit of the oligosaccharyltransferase complex, homolog B (S. cerevisiae)	0.91659	0.2684	NA
68705	Gtf2f2	general transcription factor IIF, polypeptide 2	0.91659	0.1199	NA
68966	Ngdn	neuroguidin, EIF4E binding protein	0.91659	0.212	NA
70561	Txndc16	thioredoxin domain containing 16	0.91659	0.1433	NA
71765	Klhdc3	kelch domain containing 3	0.91659	0.3125	0.5491
73239	3110054G05Rik	RIKEN cDNA 3110054G05 gene	0.91659	0.6745	0.8332
74770	Hhatl	hedgehog acyltransferase-like	0.91659	0.5667	0.7625
76580	Mib2	mindbomb homolog 2 (Drosophila)	0.91659	0.1688	NA
78913	Ltn1	listerin E3 ubiquitin protein ligase 1	0.91659	0.3676	0.6026
100210	Gpn2	GPN-loop GTPase 2	0.91659	0.3503	0.5861
106564	Ppcs	phosphopantothenoylcysteine synthetase	0.91659	0.2524	NA
106931	Kctd1	potassium channel tetramerisation domain containing 1	0.91659	0.3388	0.5745
192197	Bcas3	breast carcinoma amplified sequence 3	0.91659	0.4033	0.6333
208727	Hdac4	histone deacetylase 4	0.91659	0.3186	0.5551
217119	Xylt2	xylosyltransferase II	0.91659	0.3411	0.5768
225432	Rbm27	RNA binding motif protein 27	0.91659	0.355	0.5897
236844	Gm4910	predicted pseudogene 4910	0.91659	0.09391	NA
269784	Cntn4	contactin 4	0.91659	0.3204	0.5571
319748	Zfp865	zinc finger protein 865	0.91659	0.4036	0.6335
320742	A230072C01Rik	RIKEN cDNA A230072C01 gene	0.91659	0.4402	0.6663
320927	6720474J12Rik	RIKEN cDNA 6720474J12 gene	0.91659	0.5249	0.7322
321007	Serac1	serine active site containing 1	0.91659	0.3669	0.6022
381353	Gm996	predicted gene 996	0.91659	0.6902	0.8419
414075	BC049265	cDNA sequence BC049265	0.91659	0.6114	0.7931
545156	Kalrn	kalirin, RhoGEF kinase	0.91659	0.5516	0.7518
791286	Gm9886	predicted gene 9886	0.91659	0.6453	0.8148
11566	Adss	adenylosuccinate synthetase, non muscle	0.915751	0.2137	NA
11807	Apoa2	apolipoprotein A-II	0.915751	0.3493	0.5852
11950	Atp5f1	ATP synthase, H+ transporting, mitochondrial F0 complex, subunit B1	0.915751	0.0792	NA
12289	Cacna1d	calcium channel, voltage-dependent, L type, alpha 1D subunit	0.915751	0.2637	NA
12675	Chuk	conserved helix-loop-helix ubiquitous kinase	0.915751	0.2085	NA
14405	Gabrg1	gamma-aminobutyric acid (GABA) A receptor, subunit gamma 1	0.915751	0.5118	0.7223
17207	Mcf2l	mcf.2 transforming sequence-like	0.915751	0.1082	NA
17874	Myd88	myeloid differentiation primary response gene 88	0.915751	0.1844	NA
17967	Ncam1	neural cell adhesion molecule 1	0.915751	0.1557	NA
18484	Pam	peptidylglycine alpha-amidating monooxygenase	0.915751	0.149	NA
20005	Rpl9	ribosomal protein L9	0.915751	0.1928	NA
20269	Scn3a	sodium channel, voltage-gated, type III, alpha	0.915751	0.4257	0.6532
20539	Slc7a5	solute carrier family 7 (cationic amino acid transporter, y+ system), member 5	0.915751	0.1226	NA
20807	Srf	serum response factor	0.915751	0.2559	NA
20975	Synj2	synaptojanin 2	0.915751	0.6041	0.7876
22700	Zfp40	zinc finger protein 40	0.915751	0.1819	NA

26885	Casp8ap2	caspase 8 associated protein 2	0.915751	0.2291	NA	
27366	Txnl4a	thioredoxin-like 4A	0.915751	0.07489	NA	
52132	Ccdc97	coiled-coil domain containing 97	0.915751	0.1247	NA	
54137	Acrbp	proacrosin binding protein	0.915751	0.3007		0.5366
54364	Rpp30	ribonuclease P/MRP 30 subunit (human)	0.915751	0.3114		0.5479
54646	Ppp1r3f	protein phosphatase 1, regulatory (inhibitor) subunit 3F	0.915751	0.1865	NA	
55978	Ift20	intraflagellar transport 20 homolog (Chlamydomonas)	0.915751	0.3353		0.5711
58240	Hs1bp3	HCLS1 binding protein 3	0.915751	0.3282		0.5646
66163	Mrpl4	mitochondrial ribosomal protein L4	0.915751	0.3479		0.5834
67144	Lrrc40	leucine rich repeat containing 40	0.915751	0.3272		0.5636
68164	A430106A12Rik	RIKEN cDNA A430106A12 gene	0.915751	0.2722	NA	
69151	Lzic	leucine zipper and CTNNBIP1 domain containing	0.915751	0.3622		0.5971
70248	Dazap1	DAZ associated protein 1	0.915751	0.1342	NA	
72054	Cyp4f18	cytochrome P450, family 4, subfamily f, polypeptide 18	0.915751	0.5131		0.7234
72776	Sass6	spindle assembly 6 homolog (C. elegans)	0.915751	0.3106		0.5469
72948	Tppp	tubulin polymerization promoting protein	0.915751	0.1892	NA	
76179	Usp31	ubiquitin specific peptidase 31	0.915751	0.4468		0.6722
77018	Col25a1	collagen, type XXV, alpha 1	0.915751	0.4476		0.6731
78523	Mrpl9	mitochondrial ribosomal protein L9	0.915751	0.2752	NA	
80909	Gatsl2	GATS protein-like 2	0.915751	0.175	NA	
98660	Atp1a2	ATPase, Na+/K+ transporting, alpha 2 polypeptide	0.915751	0.7101		0.8543
140483	Hnmt	histamine N-methyltransferase	0.915751	0.4896		0.7067
224647	D17Wsu92e	DNA segment, Chr 17, Wayne State University 92, expressed	0.915751	0.08714	NA	
224671	Btbd9	BTB (POZ) domain containing 9	0.915751	0.118	NA	
224694	Zfp81	zinc finger protein 81	0.915751	0.5941		0.7814
229363	Gmps	guanine monophosphate synthetase	0.915751	0.2903		0.5264
230073	Ddx58	DEAD (Asp-Glu-Ala-Asp) box polypeptide 58	0.915751	0.6709		0.8316
241943	Ccdc144b	coiled-coil domain containing 144B	0.915751	0.3075		0.5437
270198	Pfkfb4	6-phosphofructo-2-kinase/fructose-2,6-biphosphatase 4	0.915751	0.2621	NA	
282619	Sbsn	suprabasin	0.915751	0.4242		0.6519
319801	9630033F20Rik	RIKEN cDNA 9630033F20 gene	0.915751	0.1066	NA	
381990	Zbtb2	zinc finger and BTB domain containing 2	0.915751	0.1225	NA	
382007	Adam26b	a disintegrin and metallopeptidase domain 26B	0.915751	0.5366		0.7416
626848	Etohi1	ethanol induced 1	0.915751	0.612		0.7935
666185	Gm7969	predicted gene 7969	0.915751	0.2213	NA	
13557	E2f3	E2F transcription factor 3	0.914913	0.529		0.7356
15254	Hint1	histidine triad nucleotide binding protein 1	0.914913	0.5002		0.7144
15369	Hmox2	heme oxygenase (decycling) 2	0.914913	0.2899		0.5261
18521	Pcbp2	poly(rC) binding protein 2	0.914913	0.1004	NA	
19744	Rheb	Ras homolog enriched in brain	0.914913	0.6681		0.8296
19858	Rnu3b1	U3B small nuclear RNA 1	0.914913	0.697		0.8466
20504	Slc17a1	solute carrier family 17 (sodium phosphate), member 1	0.914913	0.2448	NA	
20842	Stag1	stromal antigen 1	0.914913	0.06404	NA	
20916	Sucla2	succinate-Coenzyme A ligase, ADP-forming, beta subunit	0.914913	0.1614	NA	
21933	Tnfrsf10b	tumor necrosis factor receptor superfamily, member 10b	0.914913	0.5944		0.7816
24014	Rnasel	ribonuclease L (2', 5'-oligoisoadenylate synthetase-dependent)	0.914913	0.4878		0.7055
53623	Gria3	glutamate receptor, ionotropic, AMPA3 (alpha 3)	0.914913	0.2482	NA	
54638	Ccdc22	coiled-coil domain containing 22	0.914913	0.1449	NA	
58246	Slc35b4	solute carrier family 35, member B4	0.914913	0.334		0.5697

66404	2410001C21Rik	RIKEN cDNA 2410001C21 gene	0.914913	0.4881	0.7056
66496	Ppdpf	pancreatic progenitor cell differentiation and proliferation factor homolog (zebrafish)RIKEN cDNA 2700038C09 gene	0.914913	0.1677	NA
68565	Mrps18a	mitochondrial ribosomal protein S18A	0.914913	0.1401	NA
69893	2010305A19Rik	RIKEN cDNA 2010305A19 gene	0.914913	0.3372	0.5727
69932	2810004I08Rik	RIKEN cDNA 2810004I08 gene	0.914913	0.1588	NA
71519	Cyp2u1	cytochrome P450, family 2, subfamily u, polypeptide 1	0.914913	0.554	0.7539
73368	Col20a1	collagen, type XX, alpha 1	0.914913	0.4605	0.6837
74563	Rasgef1c	RasGEF domain family, member 1C	0.914913	0.4245	0.6522
74570	Zkscan1	zinc finger with KRAB and SCAN domains 1	0.914913	0.1819	NA
75424	Zfp820	zinc finger protein 820	0.914913	0.5694	0.7638
75659	Wdr54	WD repeat domain 54	0.914913	0.6328	0.8068
76915	Mnd1	meiotic nuclear divisions 1 homolog (S. cerevisiae)	0.914913	0.427	0.6541
76983	Scfd1	Sec1 family domain containing 1	0.914913	0.1804	NA
79221	Hdac9	histone deacetylase 9	0.914913	0.2598	NA
81000	Rad54l2	RAD54 like 2 (S. cerevisiae)	0.914913	0.2816	NA
97212	Hadha	hydroxyacyl-Coenzyme A dehydrogenase/3-ketoacyl-Coenzyme A thiolase/enoyl-Coenzyme A hydratase (trifunctional protein), alpha subunit	0.914913	0.4435	0.6693
100710	Pds5b	PDS5, regulator of cohesion maintenance, homolog B (S. cerevisiae)	0.914913	0.3069	0.5433
101359	Prrt4	proline-rich transmembrane protein 4	0.914913	0.4511	0.6761
108657	Rnpepl1	arginyl aminopeptidase (aminopeptidase B)-like 1	0.914913	0.2722	NA
109093	Rars2	arginyl-tRNA synthetase 2, mitochondrial	0.914913	0.1088	NA
109202	A930024E05Rik	RIKEN cDNA A930024E05 gene	0.914913	0.5125	0.723
170771	Khdrbs2	KH domain containing, RNA binding, signal transduction associated 2	0.914913	0.3737	0.608
170772	Glcci1	glucocorticoid induced transcript 1	0.914913	0.215	NA
192232	Hps4	Hermansky-Pudlak syndrome 4 homolog (human)	0.914913	0.1288	NA
207214	Larp4	La ribonucleoprotein domain family, member 4	0.914913	0.4354	0.6623
212398	Frat2	frequently rearranged in advanced T-cell lymphomas 2	0.914913	0.3926	0.624
228005	Ppig	peptidyl-prolyl isomerase G (cyclophilin G)	0.914913	0.1129	NA
229672	Bcl2l15	BCLl2-like 15	0.914913	0.3595	0.5946
239857	Cadm2	cell adhesion molecule 2	0.914913	0.4543	0.6779
244495	Gm4975	predicted gene 4975	0.914913	0.6428	0.8135
268294	Zbtb24	zinc finger and BTB domain containing 24	0.914913	0.08692	NA
320452	P4ha3	procollagen-proline, 2-oxoglutarate 4-dioxygenase (proline 4-hydroxylase), alpha polypeptide III	0.914913	0.4229	0.6507
320473	Heatr5b	HEAT repeat containing 5B	0.914913	0.5921	0.7801
329154	Ankrd44	ankyrin repeat domain 44	0.914913	0.2184	NA
338355	Fkbp15	FK506 binding protein 15	0.914913	0.6037	0.7873
414073	BC040756	cDNA sequence BC040756	0.914913	0.2499	NA
432467	Hnrnph3	heterogeneous nuclear ribonucleoprotein H3	0.914913	0.2131	NA
664609	Rhox4a	reproductive homeobox 4A	0.914913	0.7002	0.8486
675812	Zfp605	zinc finger protein 605	0.914913	0.6258	0.8024
14455	Gas5	growth arrest specific 5	0.914077	0.2839	0.5197
15182	Hdac2	histone deacetylase 2	0.914077	0.5055	0.7178
15257	Hipk1	homeodomain interacting protein kinase 1	0.914077	0.5177	0.7272
16834	Cog1	component of oligomeric golgi complex 1	0.914077	0.2925	0.5284
17828	Muted	muted	0.914077	0.09076	NA
17997	Nedd1	neural precursor cell expressed, developmentally down-regulated gene 1	0.914077	0.1425	NA
19085	Prkar1b	protein kinase, cAMP dependent regulatory, type I beta	0.914077	0.4358	0.6626
20499	Slc12a7	solute carrier family 12, member 7	0.914077	0.3354	0.5713
22224	Usp10	ubiquitin specific peptidase 10	0.914077	0.2045	NA
22288	Utrn	utrophin	0.914077	0.6602	0.8246

22637	Zap70	zeta-chain (TCR) associated protein kinase	0.914077	0.1425	NA
22779	Ikzf2	IKAROS family zinc finger 2	0.914077	0.2405	NA
23856	Dido1	death inducer-obliterator 1	0.914077	0.1701	NA
50784	Ppap2c	phosphatidic acid phosphatase type 2C	0.914077	0.1201	NA
52892	Sco1	SCO cytochrome oxidase deficient homolog 1 (yeast)	0.914077	0.4187	0.6467
54387	Mcm3ap	minichromosome maintenance deficient 3 (S. cerevisiae) associated protein	0.914077	0.3082	0.5444
57247	Zfp276	zinc finger protein (C2H2 type) 276	0.914077	0.2617	NA
66236	1500011B03Rik	RIKEN cDNA 1500011B03 gene	0.914077	0.09795	NA
67759	5033414D02Rik	RIKEN cDNA 5033414D02 gene	0.914077	0.2062	NA
69727	Usp46	ubiquitin specific peptidase 46	0.914077	0.1989	NA
70099	Smc4	structural maintenance of chromosomes 4	0.914077	0.1685	NA
71684	Rbm43	RNA binding motif protein 43	0.914077	0.1818	NA
73288	Ccdc132	coiled-coil domain containing 132	0.914077	0.2414	NA
74411	Ppapdc2	phosphatidic acid phosphatase type 2 domain containing 2	0.914077	0.1423	NA
75479	1700012D14Rik	RIKEN cDNA 1700012D14 gene	0.914077	0.2368	NA
76608	Hectd3	HECT domain containing 3	0.914077	0.3206	0.5572
77683	Ehmt1	euchromatic histone methyltransferase 1	0.914077	0.1597	NA
80886	Senp3	SUMO/sentrin specific peptidase 3	0.914077	0.08135	NA
103199	Fig4	FIG4 homolog (S. cerevisiae)	0.914077	0.33	0.5665
108068	Grm2	glutamate receptor, metabotropic 2	0.914077	0.4935	0.7095
110751	Adam33	a disintegrin and metallopeptidase domain 33	0.914077	0.6498	0.8179
110920	Hspa13	heat shock protein 70 family, member 13	0.914077	0.1664	NA
193043	Zfp3	zinc finger protein 3	0.914077	0.2145	NA
194227	Gm13023	predicted gene 13023	0.914077	0.7068	0.8528
224907	Dus3l	dihydrouridine synthase 3-like (S. cerevisiae)	0.914077	0.17	NA
229937	Znhit6	zinc finger, HIT type 6	0.914077	0.6402	0.8116
233977	Ppfia1	protein tyrosine phosphatase, receptor type, f polypeptide (PTPRF), interacting protein (liprin), alpha 1	0.914077	0.07429	NA
234396	Ankle1	ankyrin repeat and LEM domain containing 1	0.914077	0.4727	0.6936
269643	Ppp2r2c	protein phosphatase 2 (formerly 2A), regulatory subunit B (PR 52), gamma isoform	0.914077	0.1104	NA
271127	Adamts16	a disintegrin-like and metallopeptidase (reprolysin type) with thrombospondin type 1 motif, 16	0.914077	0.4929	0.7092
271711	Tmem169	transmembrane protein 169	0.914077	0.5407	0.7448
381582	Gm5151	predicted gene 5151	0.914077	0.2093	NA
100233208	Gm10778	predicted gene 10778	0.914077	0.1759	NA
100503659	Dos	downstream of Stk11	0.914077	0.3061	0.5427
11465	Actg1	actin, gamma, cytoplasmic 1	0.913242	0.4309	0.658
11797	Birc2	baculoviral IAP repeat-containing 2	0.913242	0.2103	NA
11964	Atp6v1a	ATPase, H+ transporting, lysosomal V1 subunit A	0.913242	0.287	0.5225
12847	Copa	coatomer protein complex subunit alpha	0.913242	0.4436	0.6694
14199	Fhl1	four and a half LIM domains 1	0.913242	0.1888	NA
14260	Fmn1	formin 1	0.913242	0.5306	0.7368
14800	Gria2	glutamate receptor, ionotropic, AMPA2 (alpha 2)	0.913242	0.3785	0.6121
15929	Idh3g	isocitrate dehydrogenase 3 (NAD+), gamma	0.913242	0.1654	NA
16170	Il16	interleukin 16	0.913242	0.4966	0.7116
18185	Nrl	neural retina leucine zipper gene	0.913242	0.4951	0.7108
18537	Pcmt1	protein-L-isoaspartate (D-aspartate) O-methyltransferase 1	0.913242	0.1913	NA
19193	Pipox	pipecolic acid oxidase	0.913242	0.3915	0.623
20084	Rps18	ribosomal protein S18	0.913242	0.1497	NA
22025	Nr2c1	nuclear receptor subfamily 2, group C, member 1	0.913242	0.1701	NA
27878	Tada1	transcriptional adaptor 1	0.913242	0.09436	NA

28075	Pppde2	PPPDE peptidase domain containing 2	0.913242	0.2953	0.5311
53317	Plrg1	pleiotropic regulator 1, PRL1 homolog (Arabidopsis)	0.913242	0.1542	NA
56702	Hist1h1b	histone cluster 1, H1b	0.913242	0.2042	NA
66408	Aptx	aprataxin	0.913242	0.1293	NA
66559	Metap1d	methionyl aminopeptidase type 1D (mitochondrial)	0.913242	0.4076	0.6369
67063	2810432L12Rik	RIKEN cDNA 2810432L12 gene	0.913242	0.1619	NA
67727	Stx17	syntaxin 17	0.913242	0.4942	0.71
68229	AI846148	expressed sequence AI846148	0.913242	0.3285	0.565
68295	0610011L14Rik	RIKEN cDNA 0610011L14 gene	0.913242	0.1971	NA
68477	Rmnd5a	required for meiotic nuclear division 5 homolog A (S. cerevisiae)	0.913242	0.486	0.7043
68926	Ubap2	ubiquitin-associated protein 2	0.913242	0.1314	NA
69025	1500032P08Rik	RIKEN cDNA 1500032P08 gene	0.913242	0.4641	0.6866
72599	Pdia5	protein disulfide isomerase associated 5	0.913242	0.09762	NA
72978	Cnih3	cornichon homolog 3 (Drosophila)	0.913242	0.3183	0.5547
73942	Fam151b	family with sequence similarity 151, member B	0.913242	0.2818	0.5171
76824	Fam54b	family with sequence similarity 54, member B	0.913242	0.096	NA
78656	Brd8	bromodomain containing 8	0.913242	0.2764	0.512
78938	Fbxo34	F-box protein 34	0.913242	0.1345	NA
93843	Pnck	pregnancy upregulated non-ubiquitously expressed CaM kinase	0.913242	0.2414	NA
105732	Fam83h	family with sequence similarity 83, member H	0.913242	0.3499	0.5858
106014	Fam19a5	family with sequence similarity 19, member A5	0.913242	0.1206	NA
108687	Edem2	ER degradation enhancer, mannosidase alpha-like 2	0.913242	0.5379	0.7424
110196	Fdps	farnesyl diphosphate synthetase	0.913242	0.1225	NA
116905	Dph1	DPH1 homolog (S. cerevisiae)	0.913242	0.5095	0.7205
117171	1110038F14Rik	RIKEN cDNA 1110038F14 gene	0.913242	0.1667	NA
170935	Grid2ip	glutamate receptor, ionotropic, delta 2 (Grid2) interacting protein 1	0.913242	0.4607	0.6837
207781	C2cd2	C2 calcium-dependent domain containing 2	0.913242	0.1664	NA
210172	Zfp526	zinc finger protein 526	0.913242	0.6498	0.8179
214585	Spg11	spastic paraplegia 11	0.913242	0.1538	NA
224938	Pja2	praja 2, RING-H2 motif containing	0.913242	0.4069	0.6363
228413	Prrg4	proline rich Gla (G-carboxyglutamic acid) 4 (transmembrane)	0.913242	0.8006	0.9064
229589	Prune	prune homolog (Drosophila)	0.913242	0.3686	0.6035
232227	Iqsec1	IQ motif and Sec7 domain 1	0.913242	0.4264	0.6537
240832	Tor1aip2	torsin A interacting protein 2	0.913242	0.2905	0.5266
244059	Chd2	chromodomain helicase DNA binding protein 2	0.913242	0.6732	0.8325
245666	Iqsec2	IQ motif and Sec7 domain 2	0.913242	0.247	NA
260301	Otos	otospiralin	0.913242	0.6532	0.8205
269233	Fam171a1	family with sequence similarity 171, member A1	0.913242	0.2253	NA
269252	Gtf3c4	general transcription factor IIIC, polypeptide 4	0.913242	0.3938	0.6248
320436	C030009J22Rik	RIKEN cDNA C030009J22 gene	0.913242	0.1635	NA
497255	LOC497255	hypothetical LOC497255	0.913242	0.407	0.6364
544696	D630037F22Rik	RIKEN cDNA D630037F22 gene	0.913242	0.2088	NA
11416	Slc33a1	solute carrier family 33 (acetyl-CoA transporter), member 1	0.912409	0.2395	NA
12055	Bcl7c	B-cell CLL/lymphoma 7C	0.912409	0.1659	NA
12848	Cops2	COP9 (constitutive photomorphogenic) homolog, subunit 2 (Arabidopsis thaliana)	0.912409	0.2386	NA
13171	Dbt	dihydrolipoamide branched chain transacylase E2	0.912409	0.3658	0.6014
14371	Fzd9	frizzled homolog 9 (Drosophila)	0.912409	0.4069	0.6363
14869	Gstp2	glutathione S-transferase, pi 2	0.912409	0.2659	NA
16179	Irak1	interleukin-1 receptor-associated kinase 1	0.912409	0.1779	NA

16834	Cog1	component of oligomeric golgi complex 1	0.912409	0.0956	NA
17119	Mxd1	MAX dimerization protein 1	0.912409	0.1067	NA
18617	Rhox5	reproductive homeobox 5	0.912409	0.3497	0.5855
21385	Tbx2	T-box 2	0.912409	0.1552	NA
26406	Map3k3	mitogen-activated protein kinase kinase kinase 3	0.912409	0.4427	0.6687
53323	Ube2k	ubiquitin-conjugating enzyme E2K (UBC1 homolog, yeast)	0.912409	0.04826	NA
54604	Pcnx	pecanex homolog (Drosophila)	0.912409	0.1689	NA
58243	Nap1l5	nucleosome assembly protein 1-like 5	0.912409	0.2994	0.5351
60364	Donson	downstream neighbor of SON	0.912409	0.07516	NA
64176	Sv2b	synaptic vesicle glycoprotein 2 b	0.912409	0.1899	NA
66343	Tmem177	transmembrane protein 177	0.912409	0.3653	0.601
66469	2810405K02Rik	RIKEN cDNA 2810405K02 gene	0.912409	0.258	NA
66583	Exosc1	exosome component 1	0.912409	0.3223	0.5587
66660	Sltm	SAFB-like, transcription modulator	0.912409	0.1311	NA
68047	Mpnd	MPN domain containing	0.912409	0.2622	NA
68090	Yif1a	Yip1 interacting factor homolog A (S. cerevisiae)	0.912409	0.3568	0.5916
68295	0610011L14Rik	RIKEN cDNA 0610011L14 gene	0.912409	0.3296	0.5662
68598	Dnajc8	DnaJ (Hsp40) homolog, subfamily C, member 8	0.912409	0.2719	0.5073
72080	2010317E24Rik	RIKEN cDNA 2010317E24 gene	0.912409	0.6345	0.8076
74015	Fcho1	FCH domain only 1	0.912409	0.6061	0.7891
75641	1700029I15Rik	RIKEN cDNA 1700029I15 gene	0.912409	0.3065	0.5431
104885	Tmem179	transmembrane protein 179	0.912409	0.1812	NA
109689	Arrb1	arrestin, beta 1	0.912409	0.1772	NA
214791	Sertad4	SERTA domain containing 4	0.912409	0.2918	0.528
234515	Inpp4b	inositol polyphosphate-4-phosphatase, type II	0.912409	0.2279	NA
235567	Dnajc13	DnaJ (Hsp40) homolog, subfamily C, member 13	0.912409	0.2219	NA
236598	LOC236598	28S ribosomal RNA	0.912409	0.5249	0.7322
239038	Lrit2	leucine-rich repeat, immunoglobulin-like and transmembrane domains 2	0.912409	0.1695	NA
243813	Leng9	leukocyte receptor cluster (LRC) member 9	0.912409	0.1701	NA
380728	Kcnh4	potassium voltage-gated channel, subfamily H (eag-related), member 4	0.912409	0.6741	0.833
628239	LOC628239	zinc finger protein 14-like	0.912409	0.7067	0.8528
11992	Auh	AU RNA binding protein/enoyl-coenzyme A hydratase	0.911577	0.1279	NA
12236	Bub1b	budding uninhibited by benzimidazoles 1 homolog, beta (S. cerevisiae)	0.911577	0.5473	0.7488
14167	Fgf12	fibroblast growth factor 12	0.911577	0.361	0.5958
15257	Hipk1	homeodomain interacting protein kinase 1	0.911577	0.5016	0.7148
16832	Ldhb	lactate dehydrogenase B	0.911577	0.073	NA
17125	Smad1	MAD homolog 1 (Drosophila)	0.911577	0.4211	0.6488
17388	Mmp15	matrix metallopeptidase 15	0.911577	0.4099	0.6391
18604	Pdk2	pyruvate dehydrogenase kinase, isoenzyme 2	0.911577	0.4797	0.6989
18771	Pknox1	Pbx/knotted 1 homeobox	0.911577	0.3835	0.6162
19134	Prpf4b	PRP4 pre-mRNA processing factor 4 homolog B (yeast)	0.911577	0.3603	0.5951
20539	Slc7a5	solute carrier family 7 (cationic amino acid transporter, y+ system), member 5	0.911577	0.5348	0.7401
22746	Zfp85-rs1	zinc finger protein 85, related sequence 1	0.911577	0.261	NA
26413	Mapk1	mitogen-activated protein kinase 1	0.911577	0.05683	NA
28113	Tinf2	Terf1 (TRF1)-interacting nuclear factor 2	0.911577	0.1445	NA
50706	Postn	periostin, osteoblast specific factor	0.911577	0.38	0.6135
54354	Rassf5	Ras association (RalGDS/AF-6) domain family member 5	0.911577	0.1475	NA
56177	Olfm1	olfactomedin 1	0.911577	0.2699	0.505
57423	Atp5j2	ATP synthase, H+ transporting, mitochondrial F0 complex, subunit F2	0.911577	0.08952	NA

66292	Mrps21	mitochondrial ribosomal protein S21	0.911577	0.1229	NA
66310	Dpy30	dpy-30 homolog (C. elegans)	0.911577	0.1762	NA
67197	Zcrb1	zinc finger CCHC-type and RNA binding motif 1	0.911577	0.5471	0.7487
67222	Srfbp1	serum response factor binding protein 1	0.911577	0.4039	0.6338
67694	Ift74	intraflagellar transport 74 homolog (Chlamydomonas)	0.911577	0.1159	NA
69726	Smyd3	SET and MYND domain containing 3	0.911577	0.1486	NA
70472	Atad2	ATPase family, AAA domain containing 2	0.911577	0.6653	0.8282
71452	Ankrd40	ankyrin repeat domain 40	0.911577	0.4444	0.67
71706	Slc46a3	solute carrier family 46, member 3	0.911577	0.1772	NA
72278	Ccpg1	cell cycle progression 1	0.911577	0.1327	NA
75104	Mmd2	monocyte to macrophage differentiation-associated 2	0.911577	0.3824	0.6156
75420	Secisbp2	SECIS binding protein 2	0.911577	0.2983	0.5343
75739	Mpp7	membrane protein, palmitoylated 7 (MAGUK p55 subfamily member 7)	0.911577	0.2208	NA
75746	Morc4	microrchidia 4	0.911577	0.1841	NA
76073	Pcgf5	polycomb group ring finger 5	0.911577	0.2252	NA
77629	Sphkap	SPHK1 interactor, AKAP domain containing	0.911577	0.6392	0.811
78935	Saal1	serum amyloid A-like 1	0.911577	0.3055	0.5419
104600	AW125324	expressed sequence AW125324	0.911577	0.3143	0.5508
107701	Sf3b4	splicing factor 3b, subunit 4	0.911577	0.2027	NA
108058	Camk2d	calcium/calmodulin-dependent protein kinase II, delta	0.911577	0.3849	0.6173
108745	2310051F07Rik	RIKEN cDNA 2310051F07 gene	0.911577	0.1182	NA
192119	Dicer1	Dicer1, Dcr-1 homolog (Drosophila)	0.911577	0.1592	NA
213012	Abhd10	abhydrolase domain containing 10	0.911577	0.4344	0.6613
223881	Rnd1	Rho family GTPase 1	0.911577	0.174	NA
224829	Trerf1	transcriptional regulating factor 1	0.911577	0.5039	0.7165
234396	Ankle1	ankyrin repeat and LEM domain containing 1	0.911577	0.4647	0.687
244608	Ccdc113	coiled-coil domain containing 113	0.911577	0.2503	NA
245020	Tmem22	transmembrane protein 22	0.911577	0.2189	NA
319211	Nol4	nucleolar protein 4	0.911577	0.6257	0.8023
329015	Atg2a	ATG2 autophagy related 2 homolog A (S. cerevisiae)	0.911577	0.3034	0.5395
381626	Rbm33	RNA binding motif protein 33	0.911577	0.176	NA
544752	Tug1	taurine upregulated gene 1	0.911577	0.2515	NA
100040599	Gm15319	predicted gene 15319	0.911577	0.3428	0.5782
100042693	Gm3970	predicted gene 3970	0.911577	0.4736	0.6944
100044509	Gm14378	predicted gene 14378	0.911577	0.4959	0.7111
11305	Abca2	ATP-binding cassette, sub-family A (ABC1), member 2	0.910747	0.2926	0.5284
11419	Accn2	amiloride-sensitive cation channel 2, neuronal	0.910747	0.3363	0.572
11666	Abcd1	ATP-binding cassette, sub-family D (ALD), member 1	0.910747	0.6526	0.82
11782	Ap4s1	adaptor-related protein complex AP-4, sigma 1	0.910747	0.2416	NA
11840	Arf1	ADP-ribosylation factor 1	0.910747	0.2353	NA
11950	Atp5f1	ATP synthase, H+ transporting, mitochondrial F0 complex, subunit B1	0.910747	0.1293	NA
12857	Cox4i1	cytochrome c oxidase subunit IV isoform 1	0.910747	0.3402	0.576
13056	Cyb561	cytochrome b-561	0.910747	0.3235	0.5601
16508	Kcnd2	potassium voltage-gated channel, Shal-related family, member 2	0.910747	0.1776	NA
17925	Myo9b	myosin IXb	0.910747	0.2082	NA
18039	Nefl	neurofilament, light polypeptide	0.910747	0.1272	NA
18190	Nrxn2	neurexin II	0.910747	0.1251	NA
18293	Ogdh	oxoglutarate dehydrogenase (lipoamide)	0.910747	0.3403	0.5762
18807	Pld3	phospholipase D family, member 3	0.910747	0.2475	NA

18971	Pold1	polymerase (DNA directed), delta 1, catalytic subunit	0.910747	0.2175	NA
19181	Psmc2	proteasome (prosome, macropain) 26S subunit, ATPase 2	0.910747	0.06121	NA
19369	Raet1b	retinoic acid early transcript beta	0.910747	0.5677	0.7632
20482	Skil	SKI-like	0.910747	0.1777	NA
20615	Snapin	SNAP-associated protein	0.910747	0.1249	NA
21834	Thrb	thyroid hormone receptor beta	0.910747	0.372	0.6068
21843	Tial1	Tia1 cytotoxic granule-associated RNA binding protein-like 1	0.910747	0.1053	NA
22384	Eif4h	eukaryotic translation initiation factor 4H	0.910747	0.1288	NA
23856	Dido1	death inducer-obliterator 1	0.910747	0.2587	0.494
23918	Impdh2	inosine 5'-phosphate dehydrogenase 2	0.910747	0.1305	NA
28040	D6Wsu163e	DNA segment, Chr 6, Wayne State University 163, expressed	0.910747	0.09347	NA
53320	Folh1	folate hydrolase	0.910747	0.1692	NA
53624	Cldn7	claudin 7	0.910747	0.2618	0.4969
54711	Plagl2	pleiomorphic adenoma gene-like 2	0.910747	0.5557	0.755
56273	Pex14	peroxisomal biogenesis factor 14	0.910747	0.5046	0.7171
58799	Crbn	cereblon	0.910747	0.2148	NA
59022	Edf1	endothelial differentiation-related factor 1	0.910747	0.2155	NA
64242	Ngb	neuroglobin	0.910747	0.2215	NA
66340	Psenen	presenilin enhancer 2 homolog (C. elegans)	0.910747	0.2977	0.5337
67028	2610002M06Rik	RIKEN cDNA 2610002M06 gene	0.910747	0.3249	0.5615
67856	Echdc3	enoyl Coenzyme A hydratase domain containing 3	0.910747	0.3295	0.5662
68162	A930003A15Rik	RIKEN cDNA A930003A15 gene	0.910747	0.1349	NA
68165	Fdx1l	ferredoxin 1-like	0.910747	0.1496	NA
71472	Usp19	ubiquitin specific peptidase 19	0.910747	0.551	0.7513
72151	Rfc5	replication factor C (activator 1) 5	0.910747	0.2027	NA
74100	Arpp21	cyclic AMP-regulated phosphoprotein, 21	0.910747	0.5964	0.7825
74120	Zfp263	zinc finger protein 263	0.910747	0.1318	NA
74617	Scpep1	serine carboxypeptidase 1	0.910747	0.1328	NA
76611	1700071A11Rik	RIKEN cDNA 1700071A11 gene	0.910747	0.3773	0.6113
80883	Ntng1	netrin G1	0.910747	0.1291	NA
93871	Brwd1	bromodomain and WD repeat domain containing 1	0.910747	0.1575	NA
97114	Hist2h3c2-ps	histone cluster 2, H3c2, pseudogene	0.910747	0.3992	0.6295
97961	Nol12	nucleolar protein 12	0.910747	0.331	0.5674
103573	Xpo1	exportin 1, CRM1 homolog (yeast)	0.910747	0.1816	NA
216049	Zfp365	zinc finger protein 365	0.910747	0.1664	NA
219105	Zmym5	zinc finger, MYM-type 5	0.910747	0.2005	NA
225027	Srsf7	serine/arginine-rich splicing factor 7	0.910747	0.3487	0.5845
227693	Zer1	zer-1 homolog (C. elegans)	0.910747	0.196	NA
232878	Zscan22	zinc finger and SCAN domain containing 22	0.910747	0.2868	0.5222
233532	Rsf1	remodeling and spacing factor 1	0.910747	0.2068	NA
233902	Fbxl19	F-box and leucine-rich repeat protein 19	0.910747	0.1541	NA
242506	Frmd3	FERM domain containing 3	0.910747	0.2784	0.514
319211	Nol4	nucleolar protein 4	0.910747	0.1905	NA
320632	Snrnp200	small nuclear ribonucleoprotein 200 (U5)	0.910747	0.03213	NA
381085	Tbc1d22b	TBC1 domain family, member 22B	0.910747	0.3522	0.5874
384619	Ccdc155	coiled-coil domain containing 155	0.910747	0.2567	NA
406218	Panx2	pannexin 2	0.910747	0.5383	0.7428
545156	Kalrn	kalirin, RhoGEF kinase	0.910747	0.3578	0.5926
668661	2410002F23Rik	RIKEN cDNA 2410002F23 gene	0.910747	0.2122	NA

675812	Zfp605	zinc finger protein 605	0.910747	0.507	0.7187
11488	Adam11	a disintegrin and metallopeptidase domain 11	0.909918	0.2662	0.5016
13001	Csnk2b	casein kinase 2, beta polypeptide	0.909918	0.07724	NA
14373	G0s2	G0/G1 switch gene 2	0.909918	0.2944	0.5306
14380	G6pd2	glucose-6-phosphate dehydrogenase 2	0.909918	0.5465	0.7484
14583	Gfpt1	glutamine fructose-6-phosphate transaminase 1	0.909918	0.187	NA
14686	Gnat2	guanine nucleotide binding protein, alpha transducing 2	0.909918	0.2167	NA
16468	Jarid2	jumonji, AT rich interactive domain 2	0.909918	0.1036	NA
17127	Smad3	MAD homolog 3 (Drosophila)	0.909918	0.1054	NA
17342	Mitf	microphthalmia-associated transcription factor	0.909918	0.1579	NA
17855	Mvk	mevalonate kinase	0.909918	0.1252	NA
18016	Nf2	neurofibromatosis 2	0.909918	0.3465	0.582
18044	Nfya	nuclear transcription factor-Y alpha	0.909918	0.2688	0.504
18514	Pbx1	pre B-cell leukemia transcription factor 1	0.909918	0.1984	NA
19246	Ptpn1	protein tyrosine phosphatase, non-receptor type 1	0.909918	0.1171	NA
19367	Rad9	RAD9 homolog (S. pombe)	0.909918	0.1489	NA
19720	Trim27	tripartite motif-containing 27	0.909918	0.2568	0.4918
20320	Nptn	neuroplastin	0.909918	0.1033	NA
20534	Slc4a1ap	solute carrier family 4 (anion exchanger), member 1, adaptor protein	0.909918	0.1603	NA
20602	Ncor2	nuclear receptor co-repressor 2	0.909918	0.4078	0.637
20909	Stx4a	syntaxin 4A (placental)	0.909918	0.09809	NA
20909	Stx4a	syntaxin 4A (placental)	0.909918	0.103	NA
21374	Tbp	TATA box binding protein	0.909918	0.3071	0.5434
21770	Ppp2r5d	protein phosphatase 2, regulatory subunit B (B56), delta isoform	0.909918	0.5708	0.7647
21892	Tll1	tolloid-like	0.909918	0.4363	0.6629
21912	Tspan7	tetraspanin 7	0.909918	0.265	0.5008
22282	Usf2	upstream transcription factor 2	0.909918	0.2918	0.528
23988	Pin1	protein (peptidyl-prolyl cis/trans isomerase) NIMA-interacting 1	0.909918	0.1677	NA
54376	Cacng3	calcium channel, voltage-dependent, gamma subunit 3	0.909918	0.5625	0.7607
56457	Clptm1	cleft lip and palate associated transmembrane protein 1	0.909918	0.335	0.5709
56697	Akap10	A kinase (PRKA) anchor protein 10	0.909918	0.2127	NA
57080	Gtf2ird1	general transcription factor II I repeat domain-containing 1	0.909918	0.09372	NA
58180	Hic2	hypermethylated in cancer 2	0.909918	0.4711	0.6924
64707	Suv39h2	suppressor of variegation 3-9 homolog 2 (Drosophila)	0.909918	0.3999	0.6302
66340	Psenen	presenilin enhancer 2 homolog (C. elegans)	0.909918	0.2816	0.5168
66822	Fbxo25	F-box protein 25	0.909918	0.06801	NA
66967	Edem3	ER degradation enhancer, mannosidase alpha-like 3	0.909918	0.1359	NA
67143	Ikzf5	IKAROS family zinc finger 5	0.909918	0.1492	NA
67206	2810013P06Rik	RIKEN cDNA 2810013P06 gene	0.909918	0.2509	NA
67619	Nob1	NIN1/RPN12 binding protein 1 homolog (S. cerevisiae)	0.909918	0.3777	0.6116
67775	Rtp4	receptor transporter protein 4	0.909918	0.5005	0.7145
67819	Derl1	Der1-like domain family, member 1	0.909918	0.03606	NA
67966	Zcchc10	zinc finger, CCHC domain containing 10	0.909918	0.1114	NA
69519	Rwdd2a	RWD domain containing 2A	0.909918	0.3108	0.547
69713	Pin4	protein (peptidyl-prolyl cis/trans isomerase) NIMA-interacting, 4 (parvulin)	0.909918	0.1811	NA
73754	Thap1	THAP domain containing, apoptosis associated protein 1	0.909918	0.1143	NA
77656	C430045I18Rik	RIKEN cDNA C430045I18 gene	0.909918	0.1176	NA
78688	Nol3	nucleolar protein 3 (apoptosis repressor with CARD domain)	0.909918	0.3363	0.572
78912	Sp2	Sp2 transcription factor	0.909918	0.2743	0.5099

93742	Pard3	par-3 (partitioning defective 3) homolog (C. elegans)	0.909918	0.1205	NA
99887	Tmem56	transmembrane protein 56	0.909918	0.2514	NA
102193	Zdhhc7	zinc finger, DHHC domain containing 7	0.909918	0.2683	0.5036
109079	Sephs1	selenophosphate synthetase 1	0.909918	0.1574	NA
192164	Pcdha12	protocadherin alpha 12	0.909918	0.1745	NA
210853	Zfp947	zinc finger protein 947	0.909918	0.5872	0.7766
212880	Ddx46	DEAD (Asp-Glu-Ala-Asp) box polypeptide 46	0.909918	0.05201	NA
218544	Sgtb	small glutamine-rich tetratricopeptide repeat (TPR)-containing, beta	0.909918	0.3806	0.6141
234686	Fhod1	formin homology 2 domain containing 1	0.909918	0.3317	0.5678
240068	Zfp563	zinc finger protein 563	0.909918	0.09494	NA
258750	Olfr551	olfactory receptor 551	0.909918	0.5772	0.77
319229	Sctr	secretin receptor	0.909918	0.3671	0.6024
319424	E030004H24Rik	RIKEN cDNA E030004H24 gene	0.909918	0.4211	0.6488
319583	Lig4	ligase IV, DNA, ATP-dependent	0.909918	0.192	NA
319984	Jph4	junctophilin 4	0.909918	0.2278	NA
320311	Rnf152	ring finger protein 152	0.909918	0.2176	NA
320650	9530056K15Rik	RIKEN cDNA 9530056K15 gene	0.909918	0.2243	NA
100041677	Gm13157	predicted gene 13157	0.909918	0.4602	0.6835
100043381	Gm14308	predicted gene 14308	0.909918	0.6775	0.8348
100043732	Gm4613	60S ribosomal protein L37a pseudogene	0.909918	0.3276	0.564
12562	Cdh5	cadherin 5	0.909091	0.2846	0.5201
17207	Mcf2l	mcf.2 transforming sequence-like	0.909091	0.3931	0.6243
19157	Cyth1	cytohesin 1	0.909091	0.5608	0.7595
19893	Rpgr	retinitis pigmentosa GTPase regulator	0.909091	0.2537	0.4885
22195	Ube2l3	ubiquitin-conjugating enzyme E2L 3	0.909091	0.1779	NA
56454	Aldh18a1	aldehyde dehydrogenase 18 family, member A1	0.909091	0.402	0.6321
59057	Zfp191	zinc finger protein 191	0.909091	0.1394	NA
66158	Cxx1a	CAAX box 1 homolog A (human)	0.909091	0.4804	0.6993
67845	Rnf115	ring finger protein 115	0.909091	0.418	0.6462
68404	Nrn1	neuritin 1	0.909091	0.1611	NA
69724	Rnaseh2a	ribonuclease H2, large subunit	0.909091	0.1626	NA
70083	Metrn	meteorin, glial cell differentiation regulator	0.909091	0.3215	0.558
70382	Kctd2	potassium channel tetramerisation domain containing 2	0.909091	0.1041	NA
70527	Stambp	STAM binding protein	0.909091	0.1088	NA
71699	Slc41a3	solute carrier family 41, member 3	0.909091	0.4404	0.6664
73766	4833420D23Rik	RIKEN cDNA 4833420D23 gene	0.909091	0.2012	NA
75782	Lca5	Leber congenital amaurosis 5 (human)	0.909091	0.369	0.6039
75838	4930560O18Rik	RIKEN cDNA 4930560O18 gene	0.909091	0.5509	0.7512
80281	Cttnbp2nl	CTTNBP2 N-terminal like	0.909091	0.1421	NA
94089	Trim7	tripartite motif-containing 7	0.909091	0.6218	0.7997
100532	Rell1	RELT-like 1	0.909091	0.1157	NA
104732	4930427A07Rik	RIKEN cDNA 4930427A07 gene	0.909091	0.3726	0.6069
107515	Lgr4	leucine-rich repeat-containing G protein-coupled receptor 4	0.909091	0.2782	0.5138
210853	Zfp947	zinc finger protein 947	0.909091	0.1131	NA
212706	N4bp3	NEDD4 binding protein 3	0.909091	0.131	NA
216739	Acsl6	acyl-CoA synthetase long-chain family member 6	0.909091	0.04618	NA
217364	Engase	endo-beta-N-acetylglucosaminidase	0.909091	0.2523	0.4868
217666	L2hgdh	L-2-hydroxyglutarate dehydrogenase	0.909091	0.5451	0.7477
223499	Dcaf13	DDB1 and CUL4 associated factor 13	0.909091	0.3297	0.5663

224893	Zfp959	zinc finger protein 959	0.909091	0.1494	NA
230376	Haus6	HAUS augmin-like complex, subunit 6	0.909091	0.6551	0.8212
231506	Lin54	lin-54 homolog (C. elegans)	0.909091	0.09206	NA
232232	Hdac11	histone deacetylase 11	0.909091	0.3885	0.6203
235184	BC024479	cDNA sequence BC024479	0.909091	0.2419	NA
245684	Cnksr2	connector enhancer of kinase suppressor of Ras 2	0.909091	0.2764	0.512
327762	Dna2	DNA replication helicase 2 homolog (yeast)	0.909091	0.2368	NA
380928	Lmo7	LIM domain only 7	0.909091	0.4881	0.7056
414077	BC056474	cDNA sequence BC056474	0.909091	0.313	0.5493
433752	AA415398	expressed sequence AA415398	0.909091	0.1774	NA
100039043	Gm10731	predicted gene 10731	0.909091	0.2565	0.4916
11636	Ak1	adenylate kinase 1	0.908265	0.1147	NA
12050	Bcl2l2	BCL2-like 2	0.908265	0.1478	NA
12057	Opn1sw	opsin 1 (cone pigments), short-wave-sensitive (color blindness, tritan)	0.908265	0.2224	NA
12291	Cacna1g	calcium channel, voltage-dependent, T type, alpha 1G subunit	0.908265	0.266	0.5015
12807	Hps3	Hermansky-Pudlak syndrome 3 homolog (human)	0.908265	0.104	NA
13480	Dpm1	dolichol-phosphate (beta-D) mannosyltransferase 1	0.908265	0.1991	NA
13483	Dpp6	dipeptidylpeptidase 6	0.908265	0.2821	0.5174
17220	Mcm7	minichromosome maintenance deficient 7 (S. cerevisiae)	0.908265	0.105	NA
17896	Myl4	myosin, light polypeptide 4	0.908265	0.3624	0.5974
18263	Odc1	ornithine decarboxylase, structural 1	0.908265	0.503	0.7158
18991	Pou3f1	POU domain, class 3, transcription factor 1	0.908265	0.1618	NA
20208	Saa1	serum amyloid A 1	0.908265	0.4907	0.7077
20841	Zfp143	zinc finger protein 143	0.908265	0.2598	0.495
22232	Slc35a2	solute carrier family 35 (UDP-galactose transporter), member A2	0.908265	0.1292	NA
22318	Vamp2	vesicle-associated membrane protein 2	0.908265	0.1798	NA
53860	Sep-09	septin 9	0.908265	0.1918	NA
53872	Caprin1	cell cycle associated protein 1	0.908265	0.4131	0.642
55980	Impa1	inositol (myo)-1(or 4)-monophosphatase 1	0.908265	0.1808	NA
56323	Dnajb5	DnaJ (Hsp40) homolog, subfamily B, member 5	0.908265	0.1744	NA
57908	Zfp318	zinc finger protein 318	0.908265	0.1732	NA
59035	Carm1	coactivator-associated arginine methyltransferase 1	0.908265	0.2774	0.5131
68304	Kdelc2	KDEL (Lys-Asp-Glu-Leu) containing 2	0.908265	0.5955	0.7821
69085	Zcchc9	zinc finger, CCHC domain containing 9	0.908265	0.4375	0.6639
69155	1810030O07Rik	RIKEN cDNA 1810030O07 gene	0.908265	0.1504	NA
69482	Nup35	nucleoporin 35	0.908265	0.1409	NA
72981	Prkrir	protein-kinase, interferon-inducible double stranded RNA dependent inhibitor, repressor of (P58 repressor)	0.908265	0.3419	0.5774
74340	Ahcyl2	S-adenosylhomocysteine hydrolase-like 2	0.908265	0.4708	0.6922
75170	4930529F24Rik	RIKEN cDNA 4930529F24 gene	0.908265	0.482	0.7006
76279	Cyp2d26	cytochrome P450, family 2, subfamily d, polypeptide 26	0.908265	0.5336	0.7392
76650	Srxn1	sulfiredoxin 1 homolog (S. cerevisiae)	0.908265	0.2325	NA
93790	Nipa2	non imprinted in Prader-Willi/Angelman syndrome 2 homolog (human)	0.908265	0.09444	NA
99890	Prmt6	protein arginine N-methyltransferase 6	0.908265	0.1291	NA
106059	A430088P11Rik	RIKEN cDNA A430088P11 gene	0.908265	0.474	0.6947
140484	Pofut1	protein O-fucosyltransferase 1	0.908265	0.1387	NA
170740	Zfp287	zinc finger protein 287	0.908265	0.5806	0.7725
192174	Rwdd4a	RWD domain containing 4A	0.908265	0.5086	0.7198
214058	Megf11	multiple EGF-like-domains 11	0.908265	0.5669	0.7625
225724	Mapk4	mitogen-activated protein kinase 4	0.908265	0.3581	0.5931

226641	Atf6	activating transcription factor 6	0.908265	0.2757	0.5112
230752	Fam176b	family with sequence similarity 176, member B	0.908265	0.2738	0.5094
236451	Gm4902	predicted gene 4902	0.908265	0.5786	0.7708
239037	Lrit1	leucine-rich repeat, immunoglobulin-like and transmembrane domains 1	0.908265	0.1029	NA
240174	Thada	thyroid adenoma associated	0.908265	0.5468	0.7486
243725	Ppp1r9a	protein phosphatase 1, regulatory (inhibitor) subunit 9A	0.908265	0.07007	NA
244958	Mrap2	melanocortin 2 receptor accessory protein 2	0.908265	0.2495	0.4836
272031	E130309F12Rik	RIKEN cDNA E130309F12 gene	0.908265	0.2166	NA
320870	E330013P08Rik	RIKEN cDNA E330013P08 gene	0.908265	0.3239	0.5603
338467	Morc3	microrchidia 3	0.908265	0.2505	0.4845
622629	Gm10318	predicted gene 10318	0.908265	0.5316	0.7376
100505017	LOC100505017	putative Pol polyprotein-like	0.908265	0.568	0.7633
11717	Ampd3	adenosine monophosphate deaminase 3	0.907441	0.5472	0.7487
12328	Caml	calcium modulating ligand	0.907441	0.1222	NA
12753	Clock	circadian locomotor output cycles kaput	0.907441	0.193	NA
16976	Lrpap1	low density lipoprotein receptor-related protein associated protein 1	0.907441	0.06396	NA
17975	Ncl	nucleolin	0.907441	0.4357	0.6626
19027	Sypl	synaptophysin-like protein	0.907441	0.1309	NA
19296	Pvt1	plasmacytoma variant translocation 1	0.907441	0.3907	0.6227
19791	Rn18s	18S ribosomal RNA	0.907441	0.2566	0.4916
20255	Scg3	secretogranin III	0.907441	0.1924	NA
20492	Slbp	stem-loop binding protein	0.907441	0.5007	0.7145
22401	Zmat3	zinc finger matrin type 3	0.907441	0.2681	0.5035
50492	Thop1	thimet oligopeptidase 1	0.907441	0.4091	0.6384
51789	Tnk2	tyrosine kinase, non-receptor, 2	0.907441	0.386	0.6182
52897	Rbfox3	RNA binding protein, fox-1 homolog (C. elegans) 3	0.907441	0.5228	0.7307
59043	Wsb2	WD repeat and SOCS box-containing 2	0.907441	0.1535	NA
66589	Ube2v1	ubiquitin-conjugating enzyme E2 variant 1	0.907441	0.06245	NA
66797	Cntnap2	contactin associated protein-like 2	0.907441	0.3444	0.5799
67123	Ubap1	ubiquitin-associated protein 1	0.907441	0.1774	NA
67488	Calcoco1	calcium binding and coiled coil domain 1	0.907441	0.4795	0.6988
68917	Hint2	histidine triad nucleotide binding protein 2	0.907441	0.07865	NA
69906	Slc25a32	solute carrier family 25, member 32	0.907441	0.2723	0.5079
70454	Cenpl	centromere protein L	0.907441	0.3899	0.622
71592	Pogk	pogo transposable element with KRAB domain	0.907441	0.268	0.5035
72046	Urgcp	upregulator of cell proliferation	0.907441	0.3869	0.6191
72852	Mblac2	metallo-beta-lactamase domain containing 2	0.907441	0.3049	0.5413
72962	Tymp	thymidine phosphorylase	0.907441	0.2746	0.5101
74081	Cep350	centrosomal protein 350	0.907441	0.5636	0.7611
74696	4930519A11Rik	RIKEN cDNA 4930519A11 gene	0.907441	0.6533	0.8205
74766	Yipf2	Yip1 domain family, member 2	0.907441	0.1877	NA
75409	Slitrk5	SLIT and NTRK-like family, member 5	0.907441	0.2444	0.478
75698	Fam35a	family with sequence similarity 35, member A	0.907441	0.2003	NA
76485	Glt8d1	glycosyltransferase 8 domain containing 1	0.907441	0.0837	NA
77329	C030010C08Rik	RIKEN cDNA C030010C08 gene	0.907441	0.2872	0.5226
78454	9530077C14Rik	RIKEN cDNA 9530077C14 gene	0.907441	0.1399	NA
80732	Mynn	myoneurin	0.907441	0.05086	NA
83703	Dbr1	debranching enzyme homolog 1 (S. cerevisiae)	0.907441	0.3297	0.5663
105596	AU020094	expressed sequence AU020094	0.907441	0.4348	0.6617

106264	0610012G03Rik	RIKEN cDNA 0610012G03 gene	0.907441	0.4581	0.6814
108705	Pttg1ip	pituitary tumor-transforming 1 interacting protein	0.907441	0.09124	NA
170707	Usp48	ubiquitin specific peptidase 48	0.907441	0.2104	NA
191578	Helq	helicase, POLQ-like	0.907441	0.1792	NA
208501	1810043H04Rik	RIKEN cDNA 1810043H04 gene	0.907441	0.6514	0.8188
217364	Engase	endo-beta-N-acetylglucosaminidase	0.907441	0.1055	NA
223255	Stk24	serine/threonine kinase 24 (STE20 homolog, yeast)	0.907441	0.1409	NA
224824	Pex6	peroxisomal biogenesis factor 6	0.907441	0.2358	0.4682
229709	Ahcyl1	S-adenosylhomocysteine hydrolase-like 1	0.907441	0.2306	NA
232236	C130022K22Rik	RIKEN cDNA C130022K22 gene	0.907441	0.06062	NA
235106	Ntm	neurotrimin	0.907441	0.3883	0.6202
268451	Rab11fip4	RAB11 family interacting protein 4 (class II)	0.907441	0.5218	0.7296
277463	Gpr107	G protein-coupled receptor 107	0.907441	0.233	NA
328121	Abhd12b	abhydrolase domain containing 12B	0.907441	0.584	0.7745
330301	Zfp786	zinc finger protein 786	0.907441	0.3627	0.5978
407795	BC030870	cDNA sequence BC030870	0.907441	0.5127	0.7232
100505195	LOC100505195	hypothetical LOC100505195	0.907441	0.6176	0.7971
11737	Anp32a	acidic (leucine-rich) nuclear phosphoprotein 32 family, member A	0.906618	0.08434	NA
11838	Arc	activity regulated cytoskeletal-associated protein	0.906618	0.311	0.5473
12231	Btn1a1	butyrophilin, subfamily 1, member A1	0.906618	0.4981	0.7127
12669	Chrm1	cholinergic receptor, muscarinic 1, CNS	0.906618	0.2086	NA
14396	Gabra3	gamma-aminobutyric acid (GABA) A receptor, subunit alpha 3	0.906618	0.08011	NA
14699	Gngt1	guanine nucleotide binding protein (G protein), gamma transducing activity polypeptide 1	0.906618	0.3425	0.5781
16372	Irx2	Iroquois related homeobox 2 (Drosophila)	0.906618	0.3221	0.5585
17210	Mcl1	myeloid cell leukemia sequence 1	0.906618	0.1456	NA
17918	Myo5a	myosin VA	0.906618	0.09732	NA
18710	Pik3r3	phosphatidylinositol 3 kinase, regulatory subunit, polypeptide 3 (p55)	0.906618	0.2183	NA
18974	Pole2	polymerase (DNA directed), epsilon 2 (p59 subunit)	0.906618	0.5013	0.7146
19819	Rnaseh1	ribonuclease H1	0.906618	0.1031	NA
22668	Sf1	splicing factor 1	0.906618	0.3356	0.5713
22710	Zfp52	zinc finger protein 52	0.906618	0.4251	0.6525
23934	Ly6h	lymphocyte antigen 6 complex, locus H	0.906618	0.131	NA
26394	Lypla2	lysophospholipase 2	0.906618	0.1504	NA
27387	Sh2d3c	SH2 domain containing 3C	0.906618	0.2535	0.4883
27966	Rrp9	RRP9, small subunit (SSU) processome component, homolog (yeast)	0.906618	0.3537	0.5885
28169	Agpat3	1-acylglycerol-3-phosphate O-acyltransferase 3	0.906618	0.1246	NA
52589	Ncald	neurocalcin delta	0.906618	0.2062	NA
54633	Pqbp1	polyglutamine binding protein 1	0.906618	0.2915	0.5277
66618	Snrnp27	small nuclear ribonucleoprotein 27 (U4/U6.U5)	0.906618	0.104	NA
67040	Ddx17	DEAD (Asp-Glu-Ala-Asp) box polypeptide 17	0.906618	0.1528	NA
67553	Gstcd	glutathione S-transferase, C-terminal domain containing	0.906618	0.2324	0.4638
68721	1110032A03Rik	RIKEN cDNA 1110032A03 gene	0.906618	0.07046	NA
68992	Zfp580	zinc finger protein 580	0.906618	0.3619	0.5969
69207	Srsf11	serine/arginine-rich splicing factor 11	0.906618	0.5019	0.715
69456	Commd10	COMM domain containing 10	0.906618	0.2074	NA
70625	Med26	mediator complex subunit 26	0.906618	0.08915	NA
71900	Tmem106b	transmembrane protein 106B	0.906618	0.1425	NA
72607	Usp13	ubiquitin specific peptidase 13 (isopeptidase T-3)	0.906618	0.1925	NA
72747	Ttc39c	tetratricopeptide repeat domain 39C	0.906618	0.1112	NA

74257	Tspan17	tetraspanin 17	0.906618	0.09875	NA
76130	Las1l	LAS1-like (S. cerevisiae)	0.906618	0.1828	NA
76551	Ccdc6	coiled-coil domain containing 6	0.906618	0.5277	0.7346
77721	Mrps5	mitochondrial ribosomal protein S5	0.906618	0.2744	0.51
102731	4833445I07Rik	RIKEN cDNA 4833445I07 gene	0.906618	0.1884	NA
105689	Mycbp2	MYC binding protein 2	0.906618	0.2454	0.4792
110460	Acat2	acetyl-Coenzyme A acetyltransferase 2	0.906618	0.1903	NA
207352	Sec23ip	Sec23 interacting protein	0.906618	0.1701	NA
208583	Nek11	NIMA (never in mitosis gene a)-related expressed kinase 11	0.906618	0.626	0.8026
225724	Mapk4	mitogen-activated protein kinase 4	0.906618	0.1483	NA
228355	Madd	MAP-kinase activating death domain	0.906618	0.3526	0.5876
231225	Tapt1	transmembrane anterior posterior transformation 1	0.906618	0.1683	NA
239554	Foxred2	FAD-dependent oxidoreductase domain containing 2	0.906618	0.1781	NA
239839	Ccdc14	coiled-coil domain containing 14	0.906618	0.3295	0.5662
240063	Zfp811	zinc finger protein 811	0.906618	0.1392	NA
241066	Carf	calcium response factor	0.906618	0.3943	0.6252
269587	Epb4.1	erythrocyte protein band 4.1	0.906618	0.1311	NA
269589	Sytl1	synaptotagmin-like 1	0.906618	0.3318	0.5678
327900	Ubtd2	ubiquitin domain containing 2	0.906618	0.1977	NA
330324	6430584L05	hypothetical protein 6430584L05	0.906618	0.5336	0.7392
353130	Prss33	protease, serine, 33	0.906618	0.5223	0.7301
382427	Best3	bestrophin 3	0.906618	0.5062	0.7183
665001	Gm14391	predicted gene 14391	0.906618	0.6803	0.8364
100038850	A130082M07Rik	RIKEN cDNA A130082M07 gene	0.906618	0.4914	0.7079
100040297	Gm2695	predicted gene 2695	0.906618	0.291	0.5271
14570	Arhgdig	Rho GDP dissociation inhibitor (GDI) gamma	0.905797	0.3938	0.6248
15193	Hdgfrp2	hepatoma-derived growth factor, related protein 2	0.905797	0.1076	NA
19357	Rad21	RAD21 homolog (S. pombe)	0.905797	0.1808	NA
20466	Sin3a	transcriptional regulator, SIN3A (yeast)	0.905797	0.2959	0.5316
20939	Sva	seminal vesicle antigen	0.905797	0.5077	0.7193
22365	Vps45	vacuolar protein sorting 45 (yeast)	0.905797	0.2797	0.5152
29812	Ndrg3	N-myc downstream regulated gene 3	0.905797	0.2774	0.5131
50780	Rgs3	regulator of G-protein signaling 3	0.905797	0.3396	0.5754
56248	Ak3	adenylate kinase 3	0.905797	0.1689	NA
56310	Gps2	G protein pathway suppressor 2	0.905797	0.368	0.603
56334	Tmed2	transmembrane emp24 domain trafficking protein 2	0.905797	0.1205	NA
56717	Mtor	mechanistic target of rapamycin (serine/threonine kinase)	0.905797	0.1934	NA
57355	BC051019	cDNA sequence BC051019	0.905797	0.5761	0.7691
65967	Eefsec	eukaryotic elongation factor, selenocysteine-tRNA-specific	0.905797	0.3523	0.5874
66153	Fbxo36	F-box protein 36	0.905797	0.1942	NA
66203	Lce1m	late cornified envelope 1M	0.905797	0.7461	0.8767
66223	Mrpl35	mitochondrial ribosomal protein L35	0.905797	0.1872	NA
66826	Taz	tafazzin	0.905797	0.1453	NA
67151	Psmd9	proteasome (prosome, macropain) 26S subunit, non-ATPase, 9	0.905797	0.1186	NA
67260	Lass4	LAG1 homolog, ceramide synthase 4	0.905797	0.1962	NA
69724	Rnaseh2a	ribonuclease H2, large subunit	0.905797	0.2669	0.5024
70638	Fam189a1	family with sequence similarity 189, member A1	0.905797	0.2827	0.518
70954	4922502B01Rik	RIKEN cDNA 4922502B01 gene	0.905797	0.3509	0.5866
72345	Fam123b	family with sequence similarity 123, member B	0.905797	0.1872	NA

76793	Snip1	Smad nuclear interacting protein 1	0.905797	0.22	NA
93717	Pcdhga9	protocadherin gamma subfamily A, 9	0.905797	0.2405	0.4731
101533	Klk9	kallikrein related-peptidase 9	0.905797	0.4877	0.7055
104401	Pcnxl3	pecanex-like 3 (Drosophila)	0.905797	0.4143	0.643
105787	Prkaa1	protein kinase, AMP-activated, alpha 1 catalytic subunit	0.905797	0.1535	NA
110323	Cox6b1	cytochrome c oxidase, subunit VIb polypeptide 1	0.905797	0.08292	NA
170472	Recql5	RecQ protein-like 5	0.905797	0.5128	0.7232
211468	Kcnh8	potassium voltage-gated channel, subfamily H (eag-related), member 8	0.905797	0.2123	NA
212980	Slc45a3	solute carrier family 45, member 3	0.905797	0.4464	0.6719
216766	Gemin5	gem (nuclear organelle) associated protein 5	0.905797	0.2845	0.52
229699	Slc16a4	solute carrier family 16 (monocarboxylic acid transporters), member 4	0.905797	0.4052	0.6349
243339	Tmem130	transmembrane protein 130	0.905797	0.08839	NA
258384	Olfr1350	olfactory receptor 1350	0.905797	0.5818	0.7729
269994	Gsg1l	GSG1-like	0.905797	0.2257	NA
276952	Rasl10b	RAS-like, family 10, member B	0.905797	0.3048	0.5412
347740	2900097C17Rik	RIKEN cDNA 2900097C17 gene	0.905797	0.1314	NA
666945	Gm10638	predicted gene 10638	0.905797	0.3453	0.5809
667370	I830012O16Rik	RIKEN cDNA I830012O16 gene	0.905797	0.2885	0.5245
100042761	Gm10697	predicted gene 10697	0.905797	0.5358	0.7409
13014	Cstb	cystatin B	0.904977	0.06119	NA
13619	Phc1	polyhomeotic-like 1 (Drosophila)	0.904977	0.2541	0.489
13799	En2	engrailed 2	0.904977	0.2152	NA
14654	Glra1	glycine receptor, alpha 1 subunit	0.904977	0.3849	0.6173
15469	Prmt1	protein arginine N-methyltransferase 1	0.904977	0.1095	NA
15500	Hsf2	heat shock factor 2	0.904977	0.1479	NA
16443	Itsn1	intersectin 1 (SH3 domain protein 1A)	0.904977	0.2147	NA
19099	Mapk8ip1	mitogen-activated protein kinase 8 interacting protein 1	0.904977	0.2607	0.4959
19345	Rab5c	RAB5C, member RAS oncogene family	0.904977	0.1705	NA
19377	Rai1	retinoic acid induced 1	0.904977	0.2258	0.4569
21770	Ppp2r5d	protein phosphatase 2, regulatory subunit B (B56), delta isoform	0.904977	0.212	NA
22272	Uqcrq	ubiquinol-cytochrome c reductase, complex III subunit VII	0.904977	0.5522	0.7523
24131	Ldb3	LIM domain binding 3	0.904977	0.4649	0.687
26943	Serinc3	serine incorporator 3	0.904977	0.1847	NA
52023	Pibf1	progesterone immunomodulatory binding factor 1	0.904977	0.4385	0.6647
52538	Acaa2	acetyl-Coenzyme A acyltransferase 2 (mitochondrial 3-oxoacyl-Coenzyme A thiolase)	0.904977	0.1992	NA
56175	Bace2	beta-site APP-cleaving enzyme 2	0.904977	0.7941	0.9024
56220	Zfp386	zinc finger protein 386 (Kruppel-like)	0.904977	0.3336	0.5693
56398	1500003O03Rik	RIKEN cDNA 1500003O03 gene	0.904977	0.2748	0.5102
56453	Mbtps1	membrane-bound transcription factor peptidase, site 1	0.904977	0.3106	0.5469
66136	Znrd1	zinc ribbon domain containing, 1	0.904977	0.07316	NA
67188	2700046G09Rik	RIKEN cDNA 2700046G09 gene	0.904977	0.2902	0.5264
67623	Tm7sf3	transmembrane 7 superfamily member 3	0.904977	0.4901	0.7072
67742	Samsn1	SAM domain, SH3 domain and nuclear localization signals, 1	0.904977	0.07824	NA
67803	Limd2	LIM domain containing 2	0.904977	0.4113	0.6403
68531	1110020A21Rik	RIKEN cDNA 1110020A21 gene	0.904977	0.1165	NA
69549	2310009B15Rik	RIKEN cDNA 2310009B15 gene	0.904977	0.1123	NA
72183	Snx6	sorting nexin 6	0.904977	0.1784	NA
73316	Calr3	calreticulin 3	0.904977	0.313	0.5493
73447	Wdr13	WD repeat domain 13	0.904977	0.2928	0.5287

74718	Snx16	sorting nexin 16	0.904977	0.2	NA
76788	Klhdc10	kelch domain containing 10	0.904977	0.07679	NA
77127	A930001A20Rik	RIKEN cDNA A930001A20 gene	0.904977	0.08789	NA
216156	Wdr18	WD repeat domain 18	0.904977	0.2952	0.5311
217410	Trib2	tribbles homolog 2 (Drosophila)	0.904977	0.2497	0.4838
217827	BC002230	cDNA sequence BC002230	0.904977	0.3673	0.6024
226255	Atrnl1	attractin like 1	0.904977	0.3836	0.6162
226977	Actr1b	ARP1 actin-related protein 1 homolog B, centractin beta (yeast)	0.904977	0.3127	0.5493
229524	Msto1	misato homolog 1 (Drosophila)	0.904977	0.1689	NA
229584	Pogz	pogo transposable element with ZNF domain	0.904977	0.1181	NA
230735	Epha10	Eph receptor A10	0.904977	0.4585	0.6817
234353	Psd3	pleckstrin and Sec7 domain containing 3	0.904977	0.1169	NA
234729	Vac14	Vac14 homolog (S. cerevisiae)	0.904977	0.4806	0.6994
239570	Ttc38	tetratricopeptide repeat domain 38	0.904977	0.7095	0.8542
263876	Spata2	spermatogenesis associated 2	0.904977	0.2191	NA
319876	Cobll1	Cobl-like 1	0.904977	0.496	0.7111
320060	B230308N11Rik	RIKEN cDNA B230308N11 gene	0.904977	0.2353	0.4674
328019	4933400C05Rik	RIKEN cDNA 4933400C05 gene	0.904977	0.5117	0.7223
432442	Akap7	A kinase (PRKA) anchor protein 7	0.904977	0.1859	NA
631323	Gm12250	predicted gene 12250	0.904977	0.2123	NA
11883	Arsa	arylsulfatase A	0.904159	0.07706	NA
11938	Atp2a2	ATPase, Ca++ transporting, cardiac muscle, slow twitch 2	0.904159	0.2951	0.5311
12728	Clcn5	chloride channel 5	0.904159	0.3948	0.6254
13839	Epha5	Eph receptor A5	0.904159	0.5064	0.7183
14534	Kat2a	K(lysine) acetyltransferase 2A	0.904159	0.5817	0.7728
14712	Gnpat	glyceronephosphate O-acyltransferase	0.904159	0.076	NA
16418	Eif6	eukaryotic translation initiation factor 6	0.904159	0.46	0.6833
16594	Klc2	kinesin light chain 2	0.904159	0.06359	NA
16882	Lig3	ligase III, DNA, ATP-dependent	0.904159	0.2704	0.5056
17423	Ndst2	N-deacetylase/N-sulfotransferase (heparan glucosaminyl) 2	0.904159	0.1066	NA
18950	Pnp	purine-nucleoside phosphorylase	0.904159	0.09282	NA
19046	Ppp1cb	protein phosphatase 1, catalytic subunit, beta isoform	0.904159	0.2611	0.4963
19325	Rab10	RAB10, member RAS oncogene family	0.904159	0.1633	NA
29876	Clic4	chloride intracellular channel 4 (mitochondrial)	0.904159	0.4672	0.6892
50721	Sirt6	sirtuin 6 (silent mating type information regulation 2, homolog) 6 (S. cerevisiae)	0.904159	0.317	0.5536
54153	Rasa4	RAS p21 protein activator 4	0.904159	0.2077	NA
56878	Rbms1	RNA binding motif, single stranded interacting protein 1	0.904159	0.3174	0.5539
59005	Trappc2l	trafficking protein particle complex 2-like	0.904159	0.1562	NA
66674	6330409N04Rik	RIKEN cDNA 6330409N04 gene	0.904159	0.2549	0.4896
67383	2410127L17Rik	RIKEN cDNA 2410127L17 gene	0.904159	0.04305	NA
68732	Lrrc16a	leucine rich repeat containing 16A	0.904159	0.3631	0.5983
68865	Arv1	ARV1 homolog (yeast)	0.904159	0.1465	NA
69178	Snx5	sorting nexin 5	0.904159	0.1022	NA
69482	Nup35	nucleoporin 35	0.904159	0.09211	NA
70497	Arhgap17	Rho GTPase activating protein 17	0.904159	0.05085	NA
71340	Riok1	RIO kinase 1 (yeast)	0.904159	0.1758	NA
71982	Snx10	sorting nexin 10	0.904159	0.222	0.4524
73062	Ppp1r16a	protein phosphatase 1, regulatory (inhibitor) subunit 16A	0.904159	0.188	NA
75608	Chmp4b	chromatin modifying protein 4B	0.904159	0.05628	NA

75691	Anks6	ankyrin repeat and sterile alpha motif domain containing 6	0.904159	0.4578	0.6812
77976	Nuak1	NUAK family, SNF1-like kinase, 1	0.904159	0.3415	0.5772
78408	Fam131a	family with sequence similarity 131, member A	0.904159	0.07303	NA
78777	2410002I01Rik	RIKEN cDNA 2410002I01 gene	0.904159	0.1772	NA
80287	Apobec3	apolipoprotein B mRNA editing enzyme, catalytic polypeptide 3	0.904159	0.4906	0.7075
83410	Cstf2t	cleavage stimulation factor, 3' pre-RNA subunit 2, tau	0.904159	0.05262	NA
94187	Zfp423	zinc finger protein 423	0.904159	0.08269	NA
99100	Cep152	centrosomal protein 152	0.904159	0.3049	0.5413
104001	Rtn1	reticulon 1	0.904159	0.2085	NA
114893	Dcun1d1	DCN1, defective in cullin neddylation 1, domain containing 1 (S. cerevisiae)	0.904159	0.1325	NA
170789	Acot8	acyl-CoA thioesterase 8	0.904159	0.119	NA
194237	Rimkla	ribosomal modification protein rimK-like family member A	0.904159	0.1594	NA
195733	Grhl1	grainyhead-like 1 (Drosophila)	0.904159	0.1468	NA
208518	Cep78	centrosomal protein 78	0.904159	0.227	0.4584
213760	Prepl	prolyl endopeptidase-like	0.904159	0.09978	NA
215436	Slc35e3	solute carrier family 35, member E3	0.904159	0.3239	0.5603
218820	Zfp503	zinc finger protein 503	0.904159	0.4522	0.677
226517	Smg7	Smg-7 homolog, nonsense mediated mRNA decay factor (C. elegans)	0.904159	0.1847	NA
231086	Hadhb	hydroxyacyl-Coenzyme A dehydrogenase/3-ketoacyl-Coenzyme A thiolase/enoyl-Coenzyme A hydratase (trifunctional protein), beta subunit	0.904159	0.05779	NA
269295	Rtn4rl2	reticulon 4 receptor-like 2	0.904159	0.261	0.4961
272790	Magee2	melanoma antigen, family E, 2	0.904159	0.3318	0.5678
320103	A730080H06Rik	RIKEN cDNA A730080H06 gene	0.904159	0.5069	0.7186
328108	Fam179b	family with sequence similarity 179, member B	0.904159	0.1446	NA
381903	Alg8	asparagine-linked glycosylation 8 homolog (yeast, alpha-1,3-glucosyltransferase)	0.904159	0.1089	NA
665186	Gm7534	predicted gene 7534	0.904159	0.4361	0.6627
672098	Gm9548	predicted gene 9548	0.904159	0.314	0.5505
100038486	Gm10742	predicted gene 10742	0.904159	0.39	0.6221
100502723	Tsga8	testis specific gene A8	0.904159	0.3669	0.6022
11793	Atg5	autophagy-related 5 (yeast)	0.903342	0.08873	NA
16979	Lrrn1	leucine rich repeat protein 1, neuronal	0.903342	0.2037	NA
17691	Sik1	salt inducible kinase 1	0.903342	0.5066	0.7184
18033	Nfkb1	nuclear factor of kappa light polypeptide gene enhancer in B-cells 1, p105	0.903342	0.6649	0.828
18854	Pml	promyelocytic leukemia	0.903342	0.2358	0.4682
19052	Ppp2ca	protein phosphatase 2 (formerly 2A), catalytic subunit, alpha isoform	0.903342	0.05007	NA
20535	Slc4a2	solute carrier family 4 (anion exchanger), member 2	0.903342	0.4438	0.6694
22690	Zfp28	zinc finger protein 28	0.903342	0.4352	0.662
23934	Ly6h	lymphocyte antigen 6 complex, locus H	0.903342	0.2008	NA
56216	Stx1b	syntaxin 1B	0.903342	0.2848	0.5202
56284	Mrpl19	mitochondrial ribosomal protein L19	0.903342	0.2749	0.5103
66054	Cndp2	CNDP dipeptidase 2 (metallopeptidase M20 family)	0.903342	0.3474	0.5827
66204	Acyp1	acylphosphatase 1, erythrocyte (common) type	0.903342	0.8043	0.9083
66537	Pomp	proteasome maturation protein	0.903342	0.05721	NA
66730	4921531C22Rik	RIKEN cDNA 4921531C22 gene	0.903342	0.2435	0.477
66849	Ppp1r2	protein phosphatase 1, regulatory (inhibitor) subunit 2	0.903342	0.16	NA
68421	Lmbrd1	LMBR1 domain containing 1	0.903342	0.1622	NA
69185	Dtwd1	DTW domain containing 1	0.903342	0.3312	0.5675
72475	Ssbp3	single-stranded DNA binding protein 3	0.903342	0.03129	NA
74383	Ubap2l	ubiquitin associated protein 2-like	0.903342	0.203	NA
75608	Chmp4b	chromatin modifying protein 4B	0.903342	0.1316	NA

75623	1700029F09Rik	RIKEN cDNA 1700029F09 gene	0.903342	0.2717	0.5071
76192	Abhd12	abhydrolase domain containing 12	0.903342	0.3758	0.6095
76219	6530401D17Rik	RIKEN cDNA 6530401D17 gene	0.903342	0.1503	NA
77626	Smpd4	sphingomyelin phosphodiesterase 4	0.903342	0.1284	NA
78245	Acbd7	acyl-Coenzyme A binding domain containing 7	0.903342	0.1547	NA
78785	Clip4	CAP-GLY domain containing linker protein family, member 4	0.903342	0.2829	0.5183
79456	Recql4	RecQ protein-like 4	0.903342	0.1619	NA
81601	Kat5	K(lysine) acetyltransferase 5	0.903342	0.1089	NA
103220	BC030307	cDNA sequence BC030307	0.903342	0.42	0.6478
114873	Dscaml1	Down syndrome cell adhesion molecule-like 1	0.903342	0.2856	0.521
170757	Eltd1	EGF, latrophilin seven transmembrane domain containing 1	0.903342	0.498	0.7127
209815	Tbc1d25	TBC1 domain family, member 25	0.903342	0.09917	NA
217353	Tmc6	transmembrane channel-like gene family 6	0.903342	0.5447	0.7474
217463	Snx13	sorting nexin 13	0.903342	0.04772	NA
231380	Uba6	ubiquitin-like modifier activating enzyme 6	0.903342	0.3245	0.5612
233876	Hirip3	HIRA interacting protein 3	0.903342	0.07873	NA
234814	Mthfsd	methenyltetrahydrofolate synthetase domain containing	0.903342	0.158	NA
243025	Tmem156	transmembrane protein 156	0.903342	0.2252	0.4562
246133	Kcne2	potassium voltage-gated channel, Isk-related subfamily, gene 2	0.903342	0.07623	NA
269060	Dagla	diacylglycerol lipase, alpha	0.903342	0.4494	0.6746
278097	Armcx6	armadillo repeat containing, X-linked 6	0.903342	0.2197	0.4495
319807	3110047P20Rik	RIKEN cDNA 3110047P20 gene	0.903342	0.4546	0.6783
320772	Mdga2	MAM domain containing glycosylphosphatidylinositol anchor 2	0.903342	0.2817	0.517
353208	Zfp931	zinc finger protein 931	0.903342	0.6655	0.8283
414066	BC037032	cDNA Sequence BC037032	0.903342	0.4703	0.692
11909	Atf2	activating transcription factor 2	0.902527	0.3862	0.6185
12296	Cacnb2	calcium channel, voltage-dependent, beta 2 subunit	0.902527	0.375	0.6091
12839	Col9a1	collagen, type IX, alpha 1	0.902527	0.2223	0.4526
13831	Epc1	enhancer of polycomb homolog 1 (Drosophila)	0.902527	0.07766	NA
14313	Fst	follistatin	0.902527	0.2852	0.5205
14402	Gabrb3	gamma-aminobutyric acid (GABA) A receptor, subunit beta 3	0.902527	0.08038	NA
15499	Hsf1	heat shock factor 1	0.902527	0.2157	0.4443
17769	Mthfr	5,10-methylenetetrahydrofolate reductase	0.902527	0.03459	NA
18127	Nos3	nitric oxide synthase 3, endothelial cell	0.902527	0.1974	NA
19082	Prkag1	protein kinase, AMP-activated, gamma 1 non-catalytic subunit	0.902527	0.0913	NA
20227	Sart1	squamous cell carcinoma antigen recognized by T-cells 1	0.902527	0.2273	0.4586
20658	Son	Son DNA binding protein	0.902527	0.0946	NA
20853	Stau1	staufen (RNA binding protein) homolog 1 (Drosophila)	0.902527	0.3458	0.5812
21835	Thrsp	thyroid hormone responsive SPOT14 homolog (Rattus)	0.902527	0.1513	NA
22273	Uqcrc1	ubiquinol-cytochrome c reductase core protein 1	0.902527	0.3598	0.5948
24046	Scn11a	sodium channel, voltage-gated, type XI, alpha	0.902527	0.4279	0.655
27416	Abcc5	ATP-binding cassette, sub-family C (CFTR/MRP), member 5	0.902527	0.0631	NA
28035	Usp39	ubiquitin specific peptidase 39	0.902527	0.319	0.5554
52389	Gpr123	G protein-coupled receptor 123	0.902527	0.3609	0.5957
54616	Extl3	exostoses (multiple)-like 3	0.902527	0.03181	NA
56469	Pias1	protein inhibitor of activated STAT 1	0.902527	0.2024	NA
67053	Rpp14	ribonuclease P 14 subunit (human)	0.902527	0.1897	NA
67788	6330577E15Rik	RIKEN cDNA 6330577E15 gene	0.902527	0.1229	NA
69082	Zc3h15	zinc finger CCCH-type containing 15	0.902527	0.185	NA

69309	Slc16a13	solute carrier family 16 (monocarboxylic acid transporters), member 13	0.902527	0.4606	0.6837
70785	Dennd1c	DENN/MADD domain containing 1C	0.902527	0.401	0.6311
72349	Dusp3	dual specificity phosphatase 3 (vaccinia virus phosphatase VH1-related)	0.902527	0.04598	NA
72429	Dnajc25	DnaJ (Hsp40) homolog, subfamily C, member 25	0.902527	0.2551	0.4898
74479	Snx11	sorting nexin 11	0.902527	0.07104	NA
74910	4930480E11Rik	RIKEN cDNA 4930480E11 gene	0.902527	0.5736	0.7672
76245	6530415H11Rik	RIKEN cDNA 6530415H11 gene	0.902527	0.1328	NA
101197	Zfp956	zinc finger protein 956	0.902527	0.09858	NA
108907	Nusap1	nucleolar and spindle associated protein 1	0.902527	0.4425	0.6685
109711	Actn1	actinin, alpha 1	0.902527	0.4363	0.6629
114249	Npnt	nephronectin	0.902527	0.6213	0.7994
192986	Cyb5d2	cytochrome b5 domain containing 2	0.902527	0.1396	NA
212377	Mms22l	MMS22-like, DNA repair protein	0.902527	0.4219	0.6498
216292	BC067068	cDNA sequence BC067068	0.902527	0.09789	NA
216549	Aftph	aftiphilin	0.902527	0.1698	NA
232077	Foxi3	forkhead box I3	0.902527	0.7926	0.9016
239029	Antxrl	anthrax toxin receptor-like	0.902527	0.5845	0.7749
239114	Il17d	interleukin 17D	0.902527	0.3944	0.6252
242557	Atg4c	autophagy-related 4C (yeast)	0.902527	0.5617	0.76
270110	Irf2bp2	interferon regulatory factor 2 binding protein 2	0.902527	0.2919	0.528
319659	A730091E23Rik	RIKEN cDNA A730091E23 gene	0.902527	0.364	0.5994
329908	Usp24	ubiquitin specific peptidase 24	0.902527	0.2112	0.4386
100043697	Gm9959	predicted gene 9959	0.902527	0.3446	0.5799
100043699	Gm4593	predicted gene 4593	0.902527	0.501	0.7146
11540	Adora2a	adenosine A2a receptor	0.901713	0.2477	0.482
11909	Atf2	activating transcription factor 2	0.901713	0.4265	0.6537
12304	Pdia4	protein disulfide isomerase associated 4	0.901713	0.339	0.5747
12788	Cnga1	cyclic nucleotide gated channel alpha 1	0.901713	0.3835	0.6162
13198	Ddit3	DNA-damage inducible transcript 3	0.901713	0.1184	NA
15572	Elavl4	ELAV (embryonic lethal, abnormal vision, Drosophila)-like 4 (Hu antigen D)	0.901713	0.3573	0.5922
18799	Plcd1	phospholipase C, delta 1	0.901713	0.07909	NA
19344	Rab5b	RAB5B, member RAS oncogene family	0.901713	0.3213	0.5577
20116	Rps8	ribosomal protein S8	0.901713	0.4467	0.6722
20589	Ighmbp2	immunoglobulin mu binding protein 2	0.901713	0.2134	0.4417
20778	Scarb1	scavenger receptor class B, member 1	0.901713	0.3133	0.5495
21809	Tgfb3	transforming growth factor, beta 3	0.901713	0.1537	NA
21945	Dedd	death effector domain-containing	0.901713	0.2077	0.4342
50769	Atp8a2	ATPase, aminophospholipid transporter-like, class I, type 8A, member 2	0.901713	0.6178	0.7972
53872	Caprin1	cell cycle associated protein 1	0.901713	0.2078	0.4342
56613	Rps6ka4	ribosomal protein S6 kinase, polypeptide 4	0.901713	0.1935	NA
57278	Bcam	basal cell adhesion molecule	0.901713	0.4283	0.6554
66690	Tmem186	transmembrane protein 186	0.901713	0.03763	NA
67149	Nkain1	Na+/K+ transporting ATPase interacting 1	0.901713	0.3693	0.6042
67774	Loh12cr1	loss of heterozygosity, 12, chromosomal region 1 homolog (human)	0.901713	0.3028	0.5388
68202	Ndufa5	NADH dehydrogenase (ubiquinone) 1 alpha subcomplex, 5	0.901713	0.1081	NA
70118	Srrd	SRR1 domain containing	0.901713	0.15	NA
70441	2610100L16Rik	RIKEN cDNA 2610100L16 gene	0.901713	0.1254	NA
72650	2810006K23Rik	RIKEN cDNA 2810006K23 gene	0.901713	0.1576	NA
74252	Armc1	armadillo repeat containing 1	0.901713	0.0651	NA

74376	Myo18b	myosin XVIIIb	0.901713	0.5768	0.7696
74558	Gvin1	GTPase, very large interferon inducible 1	0.901713	0.2647	0.5006
75599	Pcdh1	protocadherin 1	0.901713	0.317	0.5536
76294	Asb5	ankyrin repeat and SOCs box-containing 5	0.901713	0.4577	0.6812
76952	Nt5c2	5'-nucleotidase, cytosolic II	0.901713	0.1358	NA
77059	4931408D14Rik	RIKEN cDNA 4931408D14 gene	0.901713	0.4902	0.7072
80910	Gpr84	G protein-coupled receptor 84	0.901713	0.3775	0.6114
83946	Phip	pleckstrin homology domain interacting protein	0.901713	0.09117	NA
99526	Usp53	ubiquitin specific peptidase 53	0.901713	0.2976	0.5335
100993	AW549542	expressed sequence AW549542	0.901713	0.2426	0.4755
107173	Gpr137	G protein-coupled receptor 137	0.901713	0.3947	0.6254
110355	Adrbk1	adrenergic receptor kinase, beta 1	0.901713	0.2606	0.4959
194952	Jmjd4	jumonji domain containing 4	0.901713	0.2728	0.5084
210004	B3gntl1	UDP-GlcNAc:betaGal beta-1,3-N-acetylglucosaminyltransferase-like 1	0.901713	0.383	0.6161
223881	Rnd1	Rho family GTPase 1	0.901713	0.4397	0.6658
224023	Klhl22	kelch-like 22 (Drosophila)	0.901713	0.1384	NA
227058	Dnahc7b	dynein, axonemal, heavy chain 7B	0.901713	0.54	0.7443
234740	Tmem231	transmembrane protein 231	0.901713	0.2166	0.4455
237754	Btnl9	butyrophilin-like 9	0.901713	0.7721	0.8906
237847	Rtn4rl1	reticulon 4 receptor-like 1	0.901713	0.3835	0.6162
239667	Dip2b	DIP2 disco-interacting protein 2 homolog B (Drosophila)	0.901713	0.2896	0.5258
240168	Rasgrp3	RAS, guanyl releasing protein 3	0.901713	0.5335	0.7392
258272	Olfr1402	olfactory receptor 1402	0.901713	0.656	0.8218
268890	Lsamp	limbic system-associated membrane protein	0.901713	0.06083	NA
268932	Caskin1	CASK interacting protein 1	0.901713	0.1793	NA
269523	Vcp	valosin containing protein	0.901713	0.05019	NA
319945	Flad1	RFad1, flavin adenine dinucleotide synthetase, homolog (yeast)	0.901713	0.2856	0.521
353208	Zfp931	zinc finger protein 931	0.901713	0.5458	0.748
353342	Peg13	paternally expressed 13	0.901713	0.06778	NA
497652	Acd	adrenocortical dysplasia	0.901713	0.05498	NA
552875	AU015680	expressed sequence AU015680	0.901713	0.3933	0.6244
664805	Skor2	SKI family transcriptional corepressor 2	0.901713	0.44	0.6661
11685	Alox12e	arachidonate lipoxygenase, epidermal	0.900901	0.5333	0.739
11722	Amy1	amylase 1, salivary	0.900901	0.1312	NA
14167	Fgf12	fibroblast growth factor 12	0.900901	0.1578	NA
16210	Impact	imprinted and ancient	0.900901	0.1276	NA
17769	Mthfr	5,10-methylenetetrahydrofolate reductase	0.900901	0.3615	0.5963
20254	Scg2	secretogranin II	0.900901	0.05727	NA
20418	Shc3	src homology 2 domain-containing transforming protein C3	0.900901	0.2893	0.5255
20872	Stk16	serine/threonine kinase 16	0.900901	0.03198	NA
22630	Ywhaq	tyrosine 3-monooxygenase/tryptophan 5-monooxygenase activation protein, theta polypeptide	0.900901	0.06473	NA
22637	Zap70	zeta-chain (TCR) associated protein kinase	0.900901	0.1856	NA
26404	Map3k12	mitogen-activated protein kinase kinase kinase 12	0.900901	0.1965	NA
29813	Zfp385a	zinc finger protein 385A	0.900901	0.5632	0.7609
30937	Lmcd1	LIM and cysteine-rich domains 1	0.900901	0.4021	0.6321
52841	D2Bwg0886e	DNA segment, Chr 2, Brigham & Women's Genetics 0886 expressed	0.900901	0.6878	0.8407
53963	D630030B22Rik	RIKEN cDNA D630030B22 gene	0.900901	0.2393	0.4718
66283	Gkn1	gastrokine 1	0.900901	0.2381	0.4705
66917	Chordc1	cysteine and histidine-rich domain (CHORD)-containing, zinc-binding protein 1	0.900901	0.1139	NA

67364	1700121C10Rik	RIKEN cDNA 1700121C10 gene	0.900901	0.3348	0.5706
67792	Rgs8	regulator of G-protein signaling 8	0.900901	0.05394	NA
68260	Trmt12	tRNA methyltranferase 12 homolog (S. cerevisiae)	0.900901	0.2748	0.5102
71151	Eri2	exoribonuclease 2	0.900901	0.2062	0.4325
71837	1700003E16Rik	RIKEN cDNA 1700003E16 gene	0.900901	0.3908	0.6227
72301	1810041L15Rik	RIKEN cDNA 1810041L15 gene	0.900901	0.06739	NA
72357	2210016L21Rik	RIKEN cDNA 2210016L21 gene	0.900901	0.08882	NA
72511	2610316D01Rik	RIKEN cDNA 2610316D01 gene	0.900901	0.2392	0.4718
73953	4930421J07Rik	RIKEN cDNA 4930421J07 gene	0.900901	0.3717	0.6066
77958	A930023M06Rik	RIKEN cDNA A930023M06 gene	0.900901	0.261	0.4961
77963	Hook1	hook homolog 1 (Drosophila)	0.900901	0.409	0.6383
83431	Ndel1	nuclear distribution gene E-like homolog 1 (A. nidulans)	0.900901	0.2761	0.5115
93683	Glce	glucuronyl C5-epimerase	0.900901	0.1218	NA
99003	Qser1	glutamine and serine rich 1	0.900901	0.1652	NA
99371	Arfgef2	ADP-ribosylation factor guanine nucleotide-exchange factor 2 (brefeldin A-inhibited)	0.900901	0.1704	NA
110109	Nop2	NOP2 nucleolar protein homolog (yeast)	0.900901	0.1981	NA
110521	Hivep1	human immunodeficiency virus type I enhancer binding protein 1	0.900901	0.1755	NA
110695	Aldh7a1	aldehyde dehydrogenase family 7, member A1	0.900901	0.5179	0.7273
170936	Zfp369	zinc finger protein 369	0.900901	0.133	NA
214742	Rcor3	REST corepressor 3	0.900901	0.2233	0.4537
217082	Hlf	hepatic leukemia factor	0.900901	0.1997	NA
226442	Zfp281	zinc finger protein 281	0.900901	0.1041	NA
228602	4930402H24Rik	RIKEN cDNA 4930402H24 gene	0.900901	0.5327	0.7384
230119	Zbtb5	zinc finger and BTB domain containing 5	0.900901	0.1476	NA
231915	Uspl1	ubiquitin specific peptidase like 1	0.900901	0.06377	NA
233075	Gm4883	predicted gene 4883	0.900901	0.2015	0.4276
234463	Tmem184c	transmembrane protein 184C	0.900901	0.14	NA
245446	Slitrk4	SLIT and NTRK-like family, member 4	0.900901	0.04006	NA
269437	Plch1	phospholipase C, eta 1	0.900901	0.1213	NA
319172	Hist1h2ab	histone cluster 1, H2ab	0.900901	0.5169	0.7265
319262	Fchsd1	FCH and double SH3 domains 1	0.900901	0.136	NA
380664	Lemd3	LEM domain containing 3	0.900901	0.2508	0.4849
100042784	Prdm11	PR domain containing 11	0.900901	0.6666	0.8289
11740	Slc25a5	solute carrier family 25 (mitochondrial carrier, adenine nucleotide translocator), member 5	0.90009	0.2513	0.4856
13430	Dnm2	dynamin 2	0.90009	0.1771	NA
13628	Eef1a2	eukaryotic translation elongation factor 1 alpha 2	0.90009	0.4184	0.6464
13845	Ephb3	Eph receptor B3	0.90009	0.4464	0.6719
14030	Ewsr1	Ewing sarcoma breakpoint region 1	0.90009	0.2502	0.4841
14409	Gabrr2	gamma-aminobutyric acid (GABA) C receptor, subunit rho 2	0.90009	0.3492	0.5851
14605	Tsc22d3	TSC22 domain family, member 3	0.90009	0.1323	NA
18148	Npm1	nucleophosmin 1	0.90009	0.2246	0.4556
18761	Prkcq	protein kinase C, theta	0.90009	0.2044	0.4308
19052	Ppp2ca	protein phosphatase 2 (formerly 2A), catalytic subunit, alpha isoform	0.90009	0.1658	NA
19309	Pygm	muscle glycogen phosphorylase	0.90009	0.3299	0.5664
19346	Rab6	RAB6, member RAS oncogene family	0.90009	0.1601	NA
19661	Rbp3	retinol binding protein 3, interstitial	0.90009	0.228	0.4588
21389	Tbx6	T-box 6	0.90009	0.4006	0.6309
22042	Tfrc	transferrin receptor	0.90009	0.4652	0.687
22337	Vdr	vitamin D receptor	0.90009	0.6533	0.8205

22417	Wnt4	wingless-related MMTV integration site 4	0.90009	0.3445	0.5799
52615	Suz12	suppressor of zeste 12 homolog (Drosophila)	0.90009	0.09828	NA
66147	Necap2	NECAP endocytosis associated 2	0.90009	0.2203	0.4501
66964	Golt1b	golgi transport 1 homolog B (S. cerevisiae)	0.90009	0.1493	NA
67596	5830405N20Rik	RIKEN cDNA 5830405N20 gene	0.90009	0.475	0.6957
69162	Sec31a	Sec31 homolog A (S. cerevisiae)	0.90009	0.0923	NA
69379	C8g	complement component 8, gamma polypeptide	0.90009	0.3012	0.537
70247	Psmd1	proteasome (prosome, macropain) 26S subunit, non-ATPase, 1	0.90009	0.09272	NA
70426	Tekt5	tektin 5	0.90009	0.5329	0.7386
72899	Macrod2	MACRO domain containing 2	0.90009	0.07732	NA
74430	4930452B06Rik	RIKEN cDNA 4930452B06 gene	0.90009	0.3389	0.5745
75173	4930544O15Rik	RIKEN cDNA 4930544O15 gene	0.90009	0.146	NA
76383	1700012L04Rik	RIKEN cDNA 1700012L04 gene	0.90009	0.535	0.7403
77252	9430038I01Rik	RIKEN cDNA 9430038I01 gene	0.90009	0.3683	0.6032
78455	Helz	helicase with zinc finger domain	0.90009	0.1906	NA
93739	Gabarapl2	gamma-aminobutyric acid (GABA) A receptor-associated protein-like 2	0.90009	0.2065	0.4326
101197	Zfp956	zinc finger protein 956	0.90009	0.06995	NA
195727	Nhs	Nance-Horan syndrome (human)	0.90009	0.3042	0.5404
211986	Tmem18	transmembrane protein 18	0.90009	0.02959	NA
214384	Myocd	myocardin	0.90009	0.6493	0.8177
214895	Lman2l	lectin, mannose-binding 2-like	0.90009	0.06253	NA
215705	Arrdc1	arrestin domain containing 1	0.90009	0.2104	0.4376
224619	Traf7	TNF receptor-associated factor 7	0.90009	0.05506	NA
228807	Zfp341	zinc finger protein 341	0.90009	0.2143	0.4426
230866	C230096C10Rik	RIKEN cDNA C230096C10 gene	0.90009	0.275	0.5104
230917	Tmem201	transmembrane protein 201	0.90009	0.1952	0.4189
243274	Tmem132d	transmembrane protein 132D	0.90009	0.1842	NA
245474	Dkc1	dyskeratosis congenita 1, dyskerin homolog (human)	0.90009	0.2657	0.5014
268706	Slc38a9	solute carrier family 38, member 9	0.90009	0.2648	0.5006
320717	Pptc7	PTC7 protein phosphatase homolog (S. cerevisiae)	0.90009	0.3778	0.6116
380969	Nckap5l	NCK-associated protein 5-like	0.90009	0.4489	0.6745
382014	Ano8	anoctamin 8	0.90009	0.2738	0.5094
432582	E130309D14Rik	RIKEN cDNA E130309D14 gene	0.90009	0.1999	0.4255
667103	Gm13570	predicted gene 13570	0.90009	0.4862	0.7045
668173	Pex10	peroxisomal biogenesis factor 10	0.90009	0.2316	0.4631
100033459	Pydc3	pyrin domain containing 3	0.90009	0.2939	0.5301
100042480	Nhsl2	NHS-like 2	0.90009	0.1375	NA
100047133	LOC100047133	hypothetical LOC100047133	0.90009	0.2578	0.4928
11655	Alas1	aminolevulinic acid synthase 1	0.899281	0.1133	NA
14724	Gp1bb	glycoprotein Ib, beta polypeptide	0.899281	0.3497	0.5856
14870	Gstp1	glutathione S-transferase, pi 1	0.899281	0.2439	0.4774
17357	Marcksl1	MARCKS-like 1	0.899281	0.09866	NA
18472	Pafah1b1	platelet-activating factor acetylhydrolase, isoform 1b, subunit 1	0.899281	0.1436	NA
19363	Rad51l1	RAD51-like 1 (S. cerevisiae)	0.899281	0.4487	0.6744
19773	Rln1	relaxin 1	0.899281	0.5165	0.7263
20440	St6gal1	beta galactoside alpha 2,6 sialyltransferase 1	0.899281	0.1336	NA
20441	St3gal3	ST3 beta-galactoside alpha-2,3-sialyltransferase 3	0.899281	0.2156	0.4442
22110	Tspyl1	testis-specific protein, Y-encoded-like 1	0.899281	0.04848	NA
22344	Vezf1	vascular endothelial zinc finger 1	0.899281	0.07236	NA

22355	Vipr2	vasoactive intestinal peptide receptor 2	0.899281	0.05848	NA
26405	Map3k2	mitogen-activated protein kinase kinase kinase 2	0.899281	0.5515	0.7517
29867	Cabp1	calcium binding protein 1	0.899281	0.1899	NA
30045	Dnajc12	DnaJ (Hsp40) homolog, subfamily C, member 12	0.899281	0.2179	0.447
54343	Atf7ip	activating transcription factor 7 interacting protein	0.899281	0.1498	NA
54614	Prpf40b	PRP40 pre-mRNA processing factor 40 homolog B (yeast)	0.899281	0.1172	NA
58188	Vstm2b	V-set and transmembrane domain containing 2B	0.899281	0.321	0.5574
58235	Pvrl1	poliovirus receptor-related 1	0.899281	0.09592	NA
58239	Dexi	dexamethasone-induced transcript	0.899281	0.0948	NA
66714	4921524J17Rik	RIKEN cDNA 4921524J17 gene	0.899281	0.2274	0.4586
68477	Rmnd5a	required for meiotic nuclear division 5 homolog A (S. cerevisiae)	0.899281	0.06586	NA
69072	Ebna1bp2	EBNA1 binding protein 2	0.899281	0.2499	0.484
69080	Gmppa	GDP-mannose pyrophosphorylase A	0.899281	0.2815	0.5168
69536	Hemk1	HemK methyltransferase family member 1	0.899281	0.3145	0.5509
70571	Tcerg1l	transcription elongation regulator 1-like	0.899281	0.2569	0.492
71912	Jsrp1	junctional sarcoplasmic reticulum protein 1	0.899281	0.14	NA
72607	Usp13	ubiquitin specific peptidase 13 (isopeptidase T-3)	0.899281	0.0655	NA
73094	Sgip1	SH3-domain GRB2-like (endophilin) interacting protein 1	0.899281	0.3225	0.5589
73181	Nfatc4	nuclear factor of activated T-cells, cytoplasmic, calcineurin-dependent 4	0.899281	0.3428	0.5782
74375	Gcc1	golgi coiled coil 1	0.899281	0.1464	NA
74718	Snx16	sorting nexin 16	0.899281	0.5077	0.7193
77038	Arfgap2	ADP-ribosylation factor GTPase activating protein 2	0.899281	0.07344	NA
77582	Mboat7	membrane bound O-acyltransferase domain containing 7	0.899281	0.08476	NA
103236	Csnk1g2	casein kinase 1, gamma 2	0.899281	0.08471	NA
108760	Galntl1	UDP-N-acetyl-alpha-D-galactosamine:polypeptide N-acetylgalactosaminyltransferase-like 1	0.899281	0.15	NA
212139	Cc2d1a	coiled-coil and C2 domain containing 1A	0.899281	0.2622	0.4975
215351	Senp6	SUMO/sentrin specific peptidase 6	0.899281	0.09394	NA
215653	Rassf2	Ras association (RalGDS/AF-6) domain family member 2	0.899281	0.3955	0.6261
218194	Phactr1	phosphatase and actin regulator 1	0.899281	0.03472	NA
219181	Akap11	A kinase (PRKA) anchor protein 11	0.899281	0.08944	NA
230872	Crocc	ciliary rootlet coiled-coil, rootletin	0.899281	0.1768	NA
231014	9330182L06Rik	RIKEN cDNA 9330182L06 gene	0.899281	0.1008	NA
319945	Flad1	RFad1, flavin adenine dinucleotide synthetase, homolog (yeast)	0.899281	0.2593	0.4945
320714	D030016E14Rik	RIKEN cDNA D030016E14 gene	0.899281	0.09443	NA
414872	Zyg11b	zyg-ll homolog B (C. elegans)	0.899281	0.4014	0.6316
545938	Zfp607	zinc finger proten 607	0.899281	0.08248	NA
12091	Glb1	galactosidase, beta 1	0.898473	0.2862	0.5216
12653	Chgb	chromogranin B	0.898473	0.07724	NA
13350	Dgat1	diacylglycerol O-acyltransferase 1	0.898473	0.1523	NA
13629	Eef2	eukaryotic translation elongation factor 2	0.898473	0.1614	NA
16396	Itch	itchy, E3 ubiquitin protein ligase	0.898473	0.1373	NA
18613	Pecam1	platelet/endothelial cell adhesion molecule 1	0.898473	0.302	0.5379
18637	Pfdn2	prefoldin 2	0.898473	0.2633	0.499
18673	Phb	prohibitin	0.898473	0.1873	NA
20028	Pdc	phosducin	0.898473	0.327	0.5636
21388	Tbx5	T-box 5	0.898473	0.3269	0.5635
24056	Sh3bp5	SH3-domain binding protein 5 (BTK-associated)	0.898473	0.2676	0.5031
26951	Zw10	ZW10 homolog (Drosophila), centromere/kinetochore protein	0.898473	0.08544	NA
27425	Atp5l	ATP synthase, H+ transporting, mitochondrial F0 complex, subunit g	0.898473	0.1039	NA

27886	Dgcr14	DiGeorge syndrome critical region gene 14	0.898473	0.194	0.4173
28019	Ing4	inhibitor of growth family, member 4	0.898473	0.05798	NA
50524	Sall2	sal-like 2 (Drosophila)	0.898473	0.04564	NA
57431	Dnajc4	DnaJ (Hsp40) homolog, subfamily C, member 4	0.898473	0.1251	NA
57908	Zfp318	zinc finger protein 318	0.898473	0.1264	NA
59013	Hnrnph1	heterogeneous nuclear ribonucleoprotein H1	0.898473	0.2382	0.4706
67224	Med29	mediator complex subunit 29	0.898473	0.2028	0.4291
67547	Slc39a8	solute carrier family 39 (metal ion transporter), member 8	0.898473	0.3654	0.601
67871	Mrrf	mitochondrial ribosome recycling factor	0.898473	0.2743	0.5099
68505	1110014N23Rik	RIKEN cDNA 1110014N23 gene	0.898473	0.3785	0.6121
68524	Wipf2	WAS/WASL interacting protein family, member 2	0.898473	0.07775	NA
69179	Tmem110	transmembrane protein 110	0.898473	0.214	0.4424
70604	Dnajb14	DnaJ (Hsp40) homolog, subfamily B, member 14	0.898473	0.1338	NA
71086	4933412E12Rik	RIKEN cDNA 4933412E12 gene	0.898473	0.3049	0.5413
71667	0610007L01Rik	RIKEN cDNA 0610007L01 gene	0.898473	0.1469	NA
71885	2310003H01Rik	RIKEN cDNA 2310003H01 gene	0.898473	0.5035	0.7162
72179	Fbxl2	F-box and leucine-rich repeat protein 2	0.898473	0.2624	0.4978
72946	Lrrc47	leucine rich repeat containing 47	0.898473	0.04109	NA
99011	Pomt1	protein-O-mannosyltransferase 1	0.898473	0.3655	0.6011
100683	Trrap	transformation/transcription domain-associated protein	0.898473	0.2992	0.5348
104130	Ndufb11	NADH dehydrogenase (ubiquinone) 1 beta subcomplex, 11	0.898473	0.269	0.5043
106583	Scaf8	SR-related CTD-associated factor 8	0.898473	0.08534	NA
107569	Nt5c3	5'-nucleotidase, cytosolic III	0.898473	0.06859	NA
195522	Zfp691	zinc finger protein 691	0.898473	0.3593	0.5946
210106	Papd7	PAP associated domain containing 7	0.898473	0.1264	NA
217944	Rapgef5	Rap guanine nucleotide exchange factor (GEF) 5	0.898473	0.2277	0.4586
228980	Taf4a	TAF4A RNA polymerase II, TATA box binding protein (TBP)-associated factor	0.898473	0.0946	NA
233726	Ipo7	importin 7	0.898473	0.5658	0.762
233824	Cog7	component of oligomeric golgi complex 7	0.898473	0.2837	0.5194
240756	Klhl12	kelch-like 12 (Drosophila)	0.898473	0.07852	NA
381334	Gal3st2	galactose-3-O-sulfotransferase 2	0.898473	0.3754	0.6094
381820	2700089E24Rik	RIKEN cDNA 2700089E24 gene	0.898473	0.08233	NA
100504857	LOC100504857	hypothetical LOC100504857	0.898473	0.2376	0.4698
11767	Ap1m1	adaptor-related protein complex AP-1, mu subunit 1	0.897666	0.2018	0.428
11841	Arf2	ADP-ribosylation factor 2	0.897666	0.1266	NA
12778	Cxcr7	chemokine (C-X-C motif) receptor 7	0.897666	0.2954	0.5312
14456	Gas6	growth arrest specific 6	0.897666	0.1712	NA
17444	Grap2	GRB2-related adaptor protein 2	0.897666	0.1284	NA
17846	Commd1	COMM domain containing 1	0.897666	0.08643	NA
18389	Oprl1	opioid receptor-like 1	0.897666	0.1914	0.4135
20382	Srsf2	serine/arginine-rich splicing factor 2	0.897666	0.2252	0.4562
20671	Sox17	SRY-box containing gene 17	0.897666	0.1585	NA
20912	Stxbp3a	syntaxin binding protein 3A	0.897666	0.08036	NA
21413	Tcf4	transcription factor 4	0.897666	0.3714	0.6063
54006	Deaf1	deformed epidermal autoregulatory factor 1 (Drosophila)	0.897666	0.2092	0.4362
55979	Agpat1	1-acylglycerol-3-phosphate O-acyltransferase 1 (lysophosphatidic acid acyltransferase, alpha)	0.897666	0.0632	NA
66993	Smarcd3	SWI/SNF related, matrix associated, actin dependent regulator of chromatin, subfamily d, member 3	0.897666	0.1586	NA
68515	Myadml2	myeloid-associated differentiation marker-like 2	0.897666	0.1822	NA
69091	Vps26b	vacuolar protein sorting 26 homolog B (yeast)	0.897666	0.1622	NA

70925	Cdkn2aip	CDKN2A interacting protein	0.897666	0.1226	NA	
71279	Slc29a3	solute carrier family 29 (nucleoside transporters), member 3	0.897666	0.5018		0.715
71529	9030409G11Rik	RIKEN cDNA 9030409G11 gene	0.897666	0.09695	NA	
78267	Klhdc8b	kelch domain containing 8B	0.897666	0.08702	NA	
109006	Ciapin1	cytokine induced apoptosis inhibitor 1	0.897666	0.2099		0.4369
207742	Rnf43	ring finger protein 43	0.897666	0.4388		0.6648
216190	Appl2	adaptor protein, phosphotyrosine interaction, PH domain and leucine zipper containing 2	0.897666	0.1501	NA	
218877	Sema3g	sema domain, immunoglobulin domain (Ig), short basic domain, secreted, (semaphorin) 3G	0.897666	0.1782	NA	
223918	Spryd3	SPRY domain containing 3	0.897666	0.4014		0.6316
230157	Tmeff1	transmembrane protein with EGF-like and two follistatin-like domains 1	0.897666	0.04999	NA	
233875	Ino80e	INO80 complex subunit E	0.897666	0.3129		0.5493
237253	Lrp11	low density lipoprotein receptor-related protein 11	0.897666	0.08973	NA	
245596	Hdx	highly divergent homeobox	0.897666	0.2701		0.5052
320129	Adrbk2	adrenergic receptor kinase, beta 2	0.897666	0.06324	NA	
320840	Negr1	neuronal growth regulator 1	0.897666	0.1314	NA	
381352	Mamdc4	MAM domain containing 4	0.897666	0.2418		0.4747
381738	Gm1060	predicted gene 1060	0.897666	0.3532		0.5879
665211	Gm14326	predicted gene 14326	0.897666	0.5161		0.726
12212	Chic1	cysteine-rich hydrophobic domain 1	0.896861	0.3876		0.6196
12816	Col12a1	collagen, type XII, alpha 1	0.896861	0.2549		0.4896
13106	Cyp2e1	cytochrome P450, family 2, subfamily e, polypeptide 1	0.896861	0.7486		0.8783
14561	Gdf11	growth differentiation factor 11	0.896861	0.3781		0.6118
14706	Gng4	guanine nucleotide binding protein (G protein), gamma 4	0.896861	0.4865		0.7045
14724	Gp1bb	glycoprotein Ib, beta polypeptide	0.896861	0.354		0.5888
16154	Il10ra	interleukin 10 receptor, alpha	0.896861	0.2664		0.5018
17309	Mgat3	mannoside acetylglucosaminyltransferase 3	0.896861	0.1706	NA	
17758	Mtap4	microtubule-associated protein 4	0.896861	0.0618	NA	
19062	Inpp5k	inositol polyphosphate 5-phosphatase K	0.896861	0.2189		0.4483
22029	Traf1	TNF receptor-associated factor 1	0.896861	0.4805		0.6993
23900	Hcst	hematopoietic cell signal transducer	0.896861	0.4665		0.6885
27053	Asns	asparagine synthetase	0.896861	0.2951		0.5311
52120	Hgsnat	heparan-alpha-glucosaminide N-acetyltransferase	0.896861	0.06456	NA	
52398	Sep-11	septin 11	0.896861	0.05567	NA	
53321	Cntnap1	contactin associated protein-like 1	0.896861	0.1851		0.4046
53323	Ube2k	ubiquitin-conjugating enzyme E2K (UBC1 homolog, yeast)	0.896861	0.08087	NA	
56459	Sae1	SUMO1 activating enzyme subunit 1	0.896861	0.331		0.5674
57895	Ccdc126	coiled-coil domain containing 126	0.896861	0.2127		0.4405
66071	Ethe1	ethylmalonic encephalopathy 1	0.896861	0.1695	NA	
66471	Anp32e	acidic (leucine-rich) nuclear phosphoprotein 32 family, member E	0.896861	0.5582		0.757
66625	Sfrs18	serine/arginine-rich splicing factor 18	0.896861	0.202		0.4281
67157	2610301B20Rik	RIKEN cDNA 2610301B20 gene	0.896861	0.06945	NA	
67468	Mmd	monocyte to macrophage differentiation-associated	0.896861	0.06453	NA	
68205	Urm1	ubiquitin related modifier 1 homolog (S. cerevisiae)	0.896861	0.09613	NA	
68346	Sirt5	sirtuin 5 (silent mating type information regulation 2 homolog) 5 (S. cerevisiae)	0.896861	0.1096	NA	
71816	Rnf180	ring finger protein 180	0.896861	0.3249		0.5615
73473	Iws1	IWS1 homolog (S. cerevisiae)	0.896861	0.6204		0.7991
74257	Tspan17	tetraspanin 17	0.896861	0.1899		0.4118
74585	Sppl3	signal peptide peptidase 3	0.896861	0.14	NA	
76482	3110002H16Rik	RIKEN cDNA 3110002H16 gene	0.896861	0.2597		0.495

78906	9130017N09Rik	RIKEN cDNA 9130017N09 gene	0.896861	0.5115	0.7221
80904	Dtx3	deltex 3 homolog (Drosophila)	0.896861	0.3696	0.6046
94214	Spock2	sparc/osteonectin, cwcv and kazal-like domains proteoglycan 2	0.896861	0.139	NA
104015	Synj1	synaptojanin 1	0.896861	0.1276	NA
107371	Exoc6	exocyst complex component 6	0.896861	0.2866	0.522
107513	Ssr1	signal sequence receptor, alpha	0.896861	0.2023	0.4284
192160	Casc3	cancer susceptibility candidate 3	0.896861	0.02225	NA
209011	Sirt7	sirtuin 7 (silent mating type information regulation 2, homolog) 7 (S. cerevisiae)	0.896861	0.1205	NA
210172	Zfp526	zinc finger protein 526	0.896861	0.4259	0.6533
210582	Coq10a	coenzyme Q10 homolog A (yeast)	0.896861	0.07573	NA
212448	9330159F19Rik	RIKEN cDNA 9330159F19 gene	0.896861	0.08326	NA
215707	Ccdc92	coiled-coil domain containing 92	0.896861	0.1202	NA
219094	Khnyn	KH and NYN domain containing	0.896861	0.1903	0.4122
224092	Lsg1	large subunit GTPase 1 homolog (S. cerevisiae)	0.896861	0.1109	NA
228850	Ralgapb	Ral GTPase activating protein, beta subunit (non-catalytic)	0.896861	0.09119	NA
228998	Arfgap1	ADP-ribosylation factor GTPase activating protein 1	0.896861	0.2225	0.4527
229227	4932438A13Rik	RIKEN cDNA 4932438A13 gene	0.896861	0.2785	0.514
230753	Thrap3	thyroid hormone receptor associated protein 3	0.896861	0.6219	0.7997
231464	Cnot6l	CCR4-NOT transcription complex, subunit 6-like	0.896861	0.4534	0.6774
233651	Dchs1	dachsous 1 (Drosophila)	0.896861	0.3128	0.5493
235106	Ntm	neurotrimin	0.896861	0.216	0.4448
239556	Cacna1i	calcium channel, voltage-dependent, alpha 1I subunit	0.896861	0.3855	0.6176
241520	Fam171b	family with sequence similarity 171, member B	0.896861	0.2667	0.5021
241656	Pak7	p21 protein (Cdc42/Rac)-activated kinase 7	0.896861	0.3879	0.6198
245877	Mtap7d1	microtubule-associated protein 7 domain containing 1	0.896861	0.05505	NA
268373	Ppia	peptidylprolyl isomerase A	0.896861	0.1534	NA
320435	Rinl	Ras and Rab interactor-like	0.896861	0.5003	0.7144
100039128	LOC100039128	nuclear pore complex protein Nup93-like	0.896861	0.454	0.6778
100041585	Amd2	S-adenosylmethionine decarboxylase 2	0.896861	0.05453	NA
13498	Atn1	atrophin 1	0.896057	0.3135	0.5498
13876	Erg	avian erythroblastosis virus E-26 (v-ets) oncogene related	0.896057	0.6171	0.797
14225	Fkbp1a	FK506 binding protein 1a	0.896057	0.1795	0.3975
14547	Gdap2	ganglioside-induced differentiation-associated-protein 2	0.896057	0.1237	NA
14751	Gpi1	glucose phosphate isomerase 1	0.896057	0.1295	NA
16581	Kifc2	kinesin family member C2	0.896057	0.1326	NA
18803	Plcg1	phospholipase C, gamma 1	0.896057	0.2425	0.4755
18998	Pou4f3	POU domain, class 4, transcription factor 3	0.896057	0.3991	0.6295
20183	Rxrg	retinoid X receptor gamma	0.896057	0.3401	0.5759
20379	Sfrp4	secreted frizzled-related protein 4	0.896057	0.1495	NA
27410	Abca3	ATP-binding cassette, sub-family A (ABC1), member 3	0.896057	0.03696	NA
52397	Zfp644	zinc finger protein 644	0.896057	0.07741	NA
57908	Zfp318	zinc finger protein 318	0.896057	0.1602	NA
59028	Rcl1	RNA terminal phosphate cyclase-like 1	0.896057	0.1875	0.4082
66983	Zfp830	zinc finger protein 830	0.896057	0.04654	NA
67062	Mcart6	mitochondrial carrier triple repeat 6	0.896057	0.4232	0.651
70082	Lysmd2	LysM, putative peptidoglycan-binding, domain containing 2	0.896057	0.1358	NA
70233	Cd2bp2	CD2 antigen (cytoplasmic tail) binding protein 2	0.896057	0.1205	NA
70432	Rufy2	RUN and FYVE domain-containing 2	0.896057	0.1799	0.3981
70434	2610201A13Rik	RIKEN cDNA 2610201A13 gene	0.896057	0.1207	NA

70967	4931408A02Rik	RIKEN cDNA 4931408A02 gene	0.896057	0.07657	NA
71728	Stk11ip	serine/threonine kinase 11 interacting protein	0.896057	0.2941	0.5303
71860	Wdr16	WD repeat domain 16	0.896057	0.3863	0.6185
78108	4930414L22Rik	RIKEN cDNA 4930414L22 gene	0.896057	0.3704	0.6052
79233	Zfp319	zinc finger protein 319	0.896057	0.1919	0.4141
94187	Zfp423	zinc finger protein 423	0.896057	0.1952	0.4189
102058	Exoc8	exocyst complex component 8	0.896057	0.1172	NA
105892	9030619P08Rik	RIKEN cDNA 9030619P08 gene	0.896057	0.3747	0.6089
107723	Slc12a6	solute carrier family 12, member 6	0.896057	0.539	0.7434
108653	Rimklb	ribosomal modification protein rimK-like family member B	0.896057	0.1155	NA
211556	Ap1ar	adaptor-related protein complex 1 associated regulatory protein	0.896057	0.2318	0.4633
212111	Inpp5a	inositol polyphosphate-5-phosphatase A	0.896057	0.1098	NA
228801	U46068	cDNA sequence U46068	0.896057	0.5626	0.7607
233271	Luzp2	leucine zipper protein 2	0.896057	0.2293	0.4604
242425	Gabbr2	gamma-aminobutyric acid (GABA) B receptor, 2	0.896057	0.3522	0.5874
244209	Cyp2r1	cytochrome P450, family 2, subfamily r, polypeptide 1	0.896057	0.705	0.8517
260315	Nav3	neuron navigator 3	0.896057	0.4465	0.672
268970	Arhgap28	Rho GTPase activating protein 28	0.896057	0.5692	0.7638
319476	Lrtm1	leucine-rich repeats and transmembrane domains 1	0.896057	0.1459	NA
11350	Abl1	c-abl oncogene 1, non-receptor tyrosine kinase	0.895255	0.3667	0.6021
11765	Ap1g1	adaptor protein complex AP-1, gamma 1 subunit	0.895255	0.03453	NA
11907	Ate1	arginyltransferase 1	0.895255	0.07428	NA
12183	Bpgm	2,3-bisphosphoglycerate mutase	0.895255	0.1057	NA
12234	Btrc	beta-transducin repeat containing protein	0.895255	0.1367	NA
12752	Cln3	ceroid lipofuscinosis, neuronal 3, juvenile (Batten, Spielmeyer-Vogt disease)	0.895255	0.3559	0.5906
13199	Ddn	dendrin	0.895255	0.515	0.7251
13481	Dpm2	dolichol-phosphate (beta-D) mannosyltransferase 2	0.895255	0.09229	NA
14450	Gart	phosphoribosylglycinamide formyltransferase	0.895255	0.1628	NA
16521	Kcnj5	potassium inwardly-rectifying channel, subfamily J, member 5	0.895255	0.04275	NA
17764	Mtf1	metal response element binding transcription factor 1	0.895255	0.4089	0.6382
18491	Pappa	pregnancy-associated plasma protein A	0.895255	0.2102	0.4372
19014	Med1	mediator complex subunit 1	0.895255	0.1016	NA
20821	Trim21	tripartite motif-containing 21	0.895255	0.2479	0.4821
21422	Tcfcp2	transcription factor CP2	0.895255	0.02396	NA
22193	Ube2e3	ubiquitin-conjugating enzyme E2E 3, UBC4/5 homolog (yeast)	0.895255	0.04691	NA
22776	Zim1	zinc finger, imprinted 1	0.895255	0.2697	0.5048
23997	Psmd13	proteasome (prosome, macropain) 26S subunit, non-ATPase, 13	0.895255	0.06151	NA
50772	Mapk6	mitogen-activated protein kinase 6	0.895255	0.1761	0.3931
50916	Irx4	Iroquois related homeobox 4 (Drosophila)	0.895255	0.1798	0.398
52815	Ldhd	lactate dehydrogenase D	0.895255	0.3068	0.5433
57295	Icmt	isoprenylcysteine carboxyl methyltransferase	0.895255	0.3201	0.5566
64213	St7	suppression of tumorigenicity 7	0.895255	0.04695	NA
68626	Elac2	elaC homolog 2 (E. coli)	0.895255	0.1212	NA
68972	Tatdn3	TatD DNase domain containing 3	0.895255	0.02825	NA
69770	1600002K03Rik	RIKEN cDNA 1600002K03 gene	0.895255	0.2686	0.5038
70661	Sik3	SIK family kinase 3	0.895255	0.3175	0.5539
72102	Dusp11	dual specificity phosphatase 11 (RNA/RNP complex 1-interacting)	0.895255	0.1758	0.3928
73158	Larp1	La ribonucleoprotein domain family, member 1	0.895255	0.382	0.6153
74200	2810403A07Rik	RIKEN cDNA 2810403A07 gene	0.895255	0.163	NA

76454	Fbxo31	F-box protein 31	0.895255	0.1594	NA
107999	Gtpbp6	GTP binding protein 6 (putative)	0.895255	0.2482	0.4824
116972	Fam57a	family with sequence similarity 57, member A	0.895255	0.2587	0.494
193736	Zbtb12	zinc finger and BTB domain containing 12	0.895255	0.03885	NA
208080	Gm514	predicted gene 514	0.895255	0.05131	NA
211548	Nomo1	nodal modulator 1	0.895255	0.1381	NA
214764	2700050L05Rik	RIKEN cDNA 2700050L05 gene	0.895255	0.1834	0.4025
216873	Spag7	sperm associated antigen 7	0.895255	0.1952	0.4189
227696	Phyhd1	phytanoyl-CoA dioxygenase domain containing 1	0.895255	0.03076	NA
228432	Ano3	anoctamin 3	0.895255	0.2865	0.5218
230775	Bai2	brain-specific angiogenesis inhibitor 2	0.895255	0.2138	0.4422
232807	Ppp1r12c	protein phosphatase 1, regulatory (inhibitor) subunit 12C	0.895255	0.1794	0.3974
232976	Zfp574	zinc finger protein 574	0.895255	0.3549	0.5897
234404	Nxnl1	nucleoredoxin-like 1	0.895255	0.3526	0.5876
234854	Cdk10	cyclin-dependent kinase 10	0.895255	0.1037	NA
242864	Napepld	N-acyl phosphatidylethanolamine phospholipase D	0.895255	0.1005	NA
243771	Parp12	poly (ADP-ribose) polymerase family, member 12	0.895255	0.353	0.5879
271564	Vps13a	vacuolar protein sorting 13A (yeast)	0.895255	0.1794	0.3974
13175	Dclk1	doublecortin-like kinase 1	0.894454	0.169	0.3842
13490	Drd3	dopamine receptor D3	0.894454	0.6619	0.8258
14177	Fgf6	fibroblast growth factor 6	0.894454	0.557	0.7562
18771	Pknox1	Pbx/knotted 1 homeobox	0.894454	0.0184	NA
19116	Prlr	prolactin receptor	0.894454	0.6091	0.7914
26396	Map2k2	mitogen-activated protein kinase kinase 2	0.894454	0.06593	NA
27081	Zfp275	zinc finger protein 275	0.894454	0.1077	NA
27215	Azi2	5-azacytidine induced gene 2	0.894454	0.03954	NA
53965	9430099O15Rik	RIKEN cDNA 9430099O15 gene	0.894454	0.4287	0.6558
54217	Rpl36	ribosomal protein L36	0.894454	0.2382	0.4706
56471	Stmn4	stathmin-like 4	0.894454	0.1767	0.3938
56876	Nelf	nasal embryonic LHRH factor	0.894454	0.1198	NA
65254	Dpysl5	dihydropyrimidinase-like 5	0.894454	0.528	0.7349
70266	Ccbl1	cysteine conjugate-beta lyase 1	0.894454	0.2106	0.4379
70620	Ube2v2	ubiquitin-conjugating enzyme E2 variant 2	0.894454	0.4231	0.651
71474	Ppp6r2	protein phosphatase 6, regulatory subunit 2	0.894454	0.1499	NA
71682	Wdr27	WD repeat domain 27	0.894454	0.4367	0.6632
71910	Ppapdc1b	phosphatidic acid phosphatase type 2 domain containing 1B	0.894454	0.04542	NA
74004	Jakmip3	janus kinase and microtubule interacting protein 3	0.894454	0.5508	0.7511
74320	Wdr33	WD repeat domain 33	0.894454	0.1507	NA
74412	Gle1	GLE1 RNA export mediator (yeast)	0.894454	0.1957	0.4196
94332	Cadm3	cell adhesion molecule 3	0.894454	0.3163	0.553
97640	C78653	expressed sequence C78653	0.894454	0.5643	0.7615
107260	Otub1	OTU domain, ubiquitin aldehyde binding 1	0.894454	0.1678	0.3828
107371	Exoc6	exocyst complex component 6	0.894454	0.1039	NA
110639	Prps2	phosphoribosyl pyrophosphate synthetase 2	0.894454	0.479	0.6985
140740	Sec63	SEC63-like (S. cerevisiae)	0.894454	0.1146	NA
170930	Sumo2	SMT3 suppressor of mif two 3 homolog 2 (yeast)	0.894454	0.1761	0.3931
214505	Gnptg	N-acetylglucosamine-1-phosphotransferase, gamma subunit	0.894454	0.04134	NA
216578	Papolg	poly(A) polymerase gamma	0.894454	0.06716	NA
226025	Trpm3	transient receptor potential cation channel, subfamily M, member 3	0.894454	0.3816	0.615

228662	Btbd3	BTB (POZ) domain containing 3	0.894454	0.426	0.6534
230157	Tmeff1	transmembrane protein with EGF-like and two follistatin-like domains 1	0.894454	0.1845	0.4037
230584	Yipf1	Yip1 domain family, member 1	0.894454	0.3314	0.5677
230603	Ttc39a	tetratricopeptide repeat domain 39A	0.894454	0.3061	0.5427
230779	Serinc2	serine incorporator 2	0.894454	0.5526	0.7526
234353	Psd3	pleckstrin and Sec7 domain containing 3	0.894454	0.3926	0.624
234736	Rfwd3	ring finger and WD repeat domain 3	0.894454	0.05554	NA
243923	Rgs9bp	regulator of G-protein signalling 9 binding protein	0.894454	0.3379	0.5735
259302	Srgap3	SLIT-ROBO Rho GTPase activating protein 3	0.894454	0.04965	NA
268281	Shprh	SNF2 histone linker PHD RING helicase	0.894454	0.2795	0.515
320106	Slc38a11	solute carrier family 38, member 11	0.894454	0.5439	0.7471
328505	Skint7	selection and upkeep of intraepithelial T cells 7	0.894454	0.282	0.5174
330959	Snapc5	small nuclear RNA activating complex, polypeptide 5	0.894454	0.203	0.4292
381822	1190002F15Rik	RIKEN cDNA 1190002F15 gene	0.894454	0.3309	0.5674
399602	9930024M15Rik	RIKEN cDNA 9930024M15 gene	0.894454	0.2373	0.4694
668572	2610021A01Rik	RIKEN cDNA 2610021A01 gene	0.894454	0.2771	0.5126
12018	Bak1	BCL2-antagonist/killer 1	0.893655	0.2065	0.4326
12314	Calm2	calmodulin 2	0.893655	0.1683	0.3833
12896	Cpt2	carnitine palmitoyltransferase 2	0.893655	0.5668	0.7625
13872	Ercc3	excision repair cross-complementing rodent repair deficiency, complementation group 3	0.893655	0.2364	0.4688
14612	Gja4	gap junction protein, alpha 4	0.893655	0.3684	0.6033
15257	Hipk1	homeodomain interacting protein kinase 1	0.893655	0.4168	0.645
16866	Lhb	luteinizing hormone beta	0.893655	0.7006	0.849
17356	Mllt4	myeloid/lymphoid or mixed-lineage leukemia (trithorax homolog, Drosophila); translocated to, 4	0.893655	0.3602	0.5951
18514	Pbx1	pre B-cell leukemia transcription factor 1	0.893655	0.06903	NA
18563	Pcx	pyruvate carboxylase	0.893655	0.2634	0.4991
18583	Pde7a	phosphodiesterase 7A	0.893655	0.08674	NA
20463	Cox7a2l	cytochrome c oxidase subunit VIIa polypeptide 2-like	0.893655	0.1121	NA
23805	Apc2	adenomatosis polyposis coli 2	0.893655	0.2492	0.4833
27015	Polk	polymerase (DNA directed), kappa	0.893655	0.2313	0.4629
51792	Ppp2r1a	protein phosphatase 2 (formerly 2A), regulatory subunit A (PR 65), alpha isoform	0.893655	0.4777	0.6976
52512	D14Ertd670e	DNA segment, Chr 14, ERATO Doi 670, expressed	0.893655	0.4641	0.6866
54609	Ubqln2	ubiquilin 2	0.893655	0.2661	0.5016
64294	Itm2c	integral membrane protein 2C	0.893655	0.04522	NA
67027	Mkrn2	makorin, ring finger protein, 2	0.893655	0.1566	NA
69186	1810027O10Rik	RIKEN cDNA 1810027O10 gene	0.893655	0.09592	NA
71200	Dydc2	DPY30 domain containing 2	0.893655	0.4246	0.6522
71240	Osbpl7	oxysterol binding protein-like 7	0.893655	0.4053	0.6351
71701	Pnpt1	polyribonucleotide nucleotidyltransferase 1	0.893655	0.02345	NA
72397	Rbm12b	RNA binding motif protein 12B	0.893655	0.2384	0.4707
74006	Dnm1l	dynamin 1-like	0.893655	0.1559	NA
75617	Rps25	ribosomal protein S25	0.893655	0.04365	NA
75710	Rbm12	RNA binding motif protein 12	0.893655	0.1078	NA
77110	Gpbp1l1	GC-rich promoter binding protein 1-like 1	0.893655	0.1276	NA
80911	Acox3	acyl-Coenzyme A oxidase 3, pristanoyl	0.893655	0.048	NA
81904	Cacng7	calcium channel, voltage-dependent, gamma subunit 7	0.893655	0.04576	NA
93874	Pcdhb3	protocadherin beta 3	0.893655	0.1665	0.3808
100910	Chpf2	chondroitin polymerizing factor 2	0.893655	0.2063	0.4325
104349	Zfp119a	zinc finger protein 119a	0.893655	0.2305	0.4618

106369	Ypel1	yippee-like 1 (Drosophila)	0.893655	0.5699	0.764
108958	Fam73b	family with sequence similarity 73, member B	0.893655	0.03925	NA
110172	Slc35b1	solute carrier family 35, member B1	0.893655	0.08367	NA
211949	Spsb4	splA/ryanodine receptor domain and SOCS box containing 4	0.893655	0.1144	NA
226089	C030046E11Rik	RIKEN cDNA C030046E11 gene	0.893655	0.1628	NA
235627	Nbeal2	neurobeachin-like 2	0.893655	0.3323	0.5684
245650	Gucy2f	guanylate cyclase 2f	0.893655	0.3504	0.5862
268510	Mgat5b	mannoside acetylglucosaminyltransferase 5, isoenzyme B	0.893655	0.3572	0.5919
629732	Rps8-ps1	ribosomal protein S8, pseudogene 1	0.893655	0.1009	NA
11370	Acadvl	acyl-Coenzyme A dehydrogenase, very long chain	0.892857	0.1205	NA
11548	Adra1b	adrenergic receptor, alpha 1b	0.892857	0.4568	0.6803
12576	Cdkn1b	cyclin-dependent kinase inhibitor 1B	0.892857	0.3751	0.6091
13638	Efna3	ephrin A3	0.892857	0.3735	0.6078
13716	Ell	elongation factor RNA polymerase II	0.892857	0.05145	NA
16572	Kif5a	kinesin family member 5A	0.892857	0.06204	NA
19069	Nup88	nucleoporin 88	0.892857	0.1453	NA
20740	Spna2	spectrin alpha 2	0.892857	0.06348	NA
21673	Dntt	deoxynucleotidyltransferase, terminal	0.892857	0.526	0.7332
23806	Arih1	ariadne ubiquitin-conjugating enzyme E2 binding protein homolog 1 (Drosophila)	0.892857	0.04215	NA
23853	Def6	differentially expressed in FDCP 6	0.892857	0.3281	0.5646
23885	Gmcl1	germ cell-less homolog 1 (Drosophila)	0.892857	0.08003	NA
26757	Dpysl4	dihydropyrimidinase-like 4	0.892857	0.1299	NA
28113	Tinf2	Terf1 (TRF1)-interacting nuclear factor 2	0.892857	0.04076	NA
52163	Camk1	calcium/calmodulin-dependent protein kinase I	0.892857	0.4456	0.6711
56491	Vapb	vesicle-associated membrane protein, associated protein B and C	0.892857	0.2271	0.4584
56491	Vapb	vesicle-associated membrane protein, associated protein B and C	0.892857	0.03969	NA
58810	Akr1a4	aldo-keto reductase family 1, member A4 (aldehyde reductase)	0.892857	0.1391	NA
59022	Edf1	endothelial differentiation-related factor 1	0.892857	0.05151	NA
66156	Anapc11	anaphase promoting complex subunit 11	0.892857	0.3711	0.606
66356	2310008H09Rik	RIKEN cDNA 2310008H09 gene	0.892857	0.5758	0.769
66449	Pam16	presequence translocase-asssociated motor 16 homolog (S. cerevisiae)	0.892857	0.1192	NA
66935	Cir1	corepressor interacting with RBPJ, 1	0.892857	0.4883	0.7056
67582	Slc25a26	solute carrier family 25 (mitochondrial carrier, phosphate carrier), member 26	0.892857	0.1715	0.3872
68043	N6amt2	N-6 adenine-specific DNA methyltransferase 2 (putative)	0.892857	0.1126	NA
69716	Trip13	thyroid hormone receptor interactor 13	0.892857	0.2682	0.5036
69875	Ndufa11	NADH dehydrogenase (ubiquinone) 1 alpha subcomplex 11	0.892857	0.08928	NA
72605	Car10	carbonic anhydrase 10	0.892857	0.0667	NA
72729	Cdc42se2	CDC42 small effector 2	0.892857	0.05491	NA
73130	Tmed5	transmembrane emp24 protein transport domain containing 5	0.892857	0.2922	0.5281
75579	2310034G01Rik	RIKEN cDNA 2310034G01 gene	0.892857	0.2651	0.5009
75965	Zdhhc20	zinc finger, DHHC domain containing 20	0.892857	0.1794	0.3974
76332	Cog2	component of oligomeric golgi complex 2	0.892857	0.1454	NA
102566	Ano10	anoctamin 10	0.892857	0.3083	0.5446
103511	Fam26e	family with sequence similarity 26, member E	0.892857	0.4067	0.636
110695	Aldh7a1	aldehyde dehydrogenase family 7, member A1	0.892857	0.1062	NA
140765	Tmprss3	transmembrane protease, serine 3	0.892857	0.5864	0.7761
208666	Diras1	DIRAS family, GTP-binding RAS-like 1	0.892857	0.2249	0.456
209630	Frmd4a	FERM domain containing 4A	0.892857	0.09332	NA
223669	Zfp7	zinc finger protein 7	0.892857	0.07871	NA

224624	Rab40c	Rab40c, member RAS oncogene family	0.892857	0.1317	NA
226090	Ermp1	endoplasmic reticulum metallopeptidase 1	0.892857	0.1674	0.3824
235584	Dusp7	dual specificity phosphatase 7	0.892857	0.1395	NA
243743	Plxna4	plexin A4	0.892857	0.1278	NA
244418	D8Ertd82e	DNA segment, Chr 8, ERATO Doi 82, expressed	0.892857	0.3853	0.6175
320404	Itpkb	inositol 1,4,5-trisphosphate 3-kinase B	0.892857	0.1746	0.3913
387343	Tas2r109	taste receptor, type 2, member 109	0.892857	0.5894	0.7784
399614	C130009A20Rik	RIKEN cDNA C130009A20 gene	0.892857	0.07773	NA
13990	Smarcad1	SWI/SNF-related, matrix-associated actin-dependent regulator of chromatin, subfamily a, containing DEAD/H box 1	0.892061	0.1557	NA
14772	Grk4	G protein-coupled receptor kinase 4	0.892061	0.03382	NA
17312	Clec10a	C-type lectin domain family 10, member A	0.892061	0.382	0.6153
17765	Mtf2	metal response element binding transcription factor 2	0.892061	0.04325	NA
18045	Nfyb	nuclear transcription factor-Y beta	0.892061	0.1982	0.4233
20399	Sh2b1	SH2B adaptor protein 1	0.892061	0.07986	NA
20713	Serpini1	serine (or cysteine) peptidase inhibitor, clade I, member 1	0.892061	0.2332	0.4644
20912	Stxbp3a	syntaxin binding protein 3A	0.892061	0.5928	0.7805
26403	Map3k11	mitogen-activated protein kinase kinase kinase 11	0.892061	0.4437	0.6694
51886	Fubp1	far upstream element (FUSE) binding protein 1	0.892061	0.3836	0.6162
56807	Scamp5	secretory carrier membrane protein 5	0.892061	0.415	0.6435
57816	Tesc	tescalcin	0.892061	0.1523	NA
59092	Pcbp4	poly(rC) binding protein 4	0.892061	0.214	0.4424
64930	Tsc1	tuberous sclerosis 1	0.892061	0.1901	0.412
65103	Arl6ip6	ADP-ribosylation factor-like 6 interacting protein 6	0.892061	0.4356	0.6625
66112	Mosc1	MOCO sulphurase C-terminal domain containing 1	0.892061	0.4246	0.6522
66138	Wbscr22	Williams Beuren syndrome chromosome region 22	0.892061	0.2221	0.4524
66362	Exosc3	exosome component 3	0.892061	0.1116	NA
67655	Ctdp1	CTD (carboxy-terminal domain, RNA polymerase II, polypeptide A) phosphatase, subunit 1	0.892061	0.1513	NA
67930	1700101I11Rik	RIKEN cDNA 1700101I11 gene	0.892061	0.267	0.5026
68045	2700060E02Rik	RIKEN cDNA 2700060E02 gene	0.892061	0.1455	NA
68051	Nutf2	nuclear transport factor 2	0.892061	0.3388	0.5745
68241	Fam195a	family with sequence similarity 195, member A	0.892061	0.1317	NA
68251	5430437P03Rik	RIKEN cDNA 5430437P03 gene	0.892061	0.02981	NA
68473	Mobkl1a	MOB1, Mps One Binder kinase activator-like 1A (yeast)	0.892061	0.6379	0.8099
68490	Zfp579	zinc finger protein 579	0.892061	0.07754	NA
70727	Rasgef1a	RasGEF domain family, member 1A	0.892061	0.0971	NA
70930	Nol8	nucleolar protein 8	0.892061	0.1971	0.4217
71116	Stx18	syntaxin 18	0.892061	0.1223	NA
71240	Osbpl7	oxysterol binding protein-like 7	0.892061	0.05535	NA
72993	Appl1	adaptor protein, phosphotyrosine interaction, PH domain and leucine zipper containing 1	0.892061	0.1276	NA
73884	Zdbf2	zinc finger, DBF-type containing 2	0.892061	0.4246	0.6522
74270	Usp20	ubiquitin specific peptidase 20	0.892061	0.09362	NA
74552	Nipal3	NIPA-like domain containing 3	0.892061	0.3937	0.6248
77428	9430083A17Rik	RIKEN cDNA 9430083A17 gene	0.892061	0.1018	NA
78547	E130304I02Rik	RIKEN cDNA E130304I02 gene	0.892061	0.4647	0.687
80877	Lrba	LPS-responsive beige-like anchor	0.892061	0.1784	0.3961
83691	Crispld1	cysteine-rich secretory protein LCCL domain containing 1	0.892061	0.3309	0.5674
103098	Slc6a15	solute carrier family 6 (neurotransmitter transporter), member 15	0.892061	0.2694	0.5047
109151	Chd9	chromodomain helicase DNA binding protein 9	0.892061	0.04278	NA
216001	Cbara1	calcium binding atopy-related autoantigen 1	0.892061	0.1681	0.3831

218639	Arl15	ADP-ribosylation factor-like 15	0.892061	0.4573	0.6807
223753	Cerk	ceramide kinase	0.892061	0.04678	NA
225870	Rin1	Ras and Rab interactor 1	0.892061	0.7039	0.8508
228852	Ppp1r16b	protein phosphatase 1, regulatory (inhibitor) subunit 16B	0.892061	0.552	0.7522
235627	Nbeal2	neurobeachin-like 2	0.892061	0.2127	0.4404
237082	Nxt2	nuclear transport factor 2-like export factor 2	0.892061	0.1402	NA
246085	Defb10	defensin beta 10	0.892061	0.2286	0.4596
276919	Gemin4	gem (nuclear organelle) associated protein 4	0.892061	0.3968	0.6273
319740	Zfyve27	zinc finger, FYVE domain containing 27	0.892061	0.1434	NA
320309	1520401A03Rik	RIKEN cDNA 1520401A03 gene	0.892061	0.693	0.8439
330780	E130110O22Rik	RIKEN cDNA E130110O22 gene	0.892061	0.1068	NA
380921	Dgkh	diacylglycerol kinase, eta	0.892061	0.07531	NA
12293	Cacna2d1	calcium channel, voltage-dependent, alpha2/delta subunit 1	0.891266	0.1012	NA
13196	Asap1	ArfGAP with SH# domain, ankyrin repeat and PH domain1	0.891266	0.167	0.3816
16519	Kcnj3	potassium inwardly-rectifying channel, subfamily J, member 3	0.891266	0.4618	0.6847
18591	Pdgfb	platelet derived growth factor, B polypeptide	0.891266	0.2969	0.5329
18636	Cfp	complement factor properdin	0.891266	0.2154	0.444
19882	Mst1r	macrophage stimulating 1 receptor (c-met-related tyrosine kinase)	0.891266	0.1361	NA
22381	Wbp5	WW domain binding protein 5	0.891266	0.08521	NA
22670	Trim26	tripartite motif-containing 26	0.891266	0.02896	NA
54561	Nap1l3	nucleosome assembly protein 1-like 3	0.891266	0.1151	NA
56150	Mad2l1	MAD2 mitotic arrest deficient-like 1 (yeast)	0.891266	0.412	0.6408
56464	Ctsf	cathepsin F	0.891266	0.2023	0.4284
59008	Anapc5	anaphase-promoting complex subunit 5	0.891266	0.2473	0.4816
66626	5730403B10Rik	RIKEN cDNA 5730403B10 gene	0.891266	0.09686	NA
66966	Trit1	tRNA isopentenyltransferase 1	0.891266	0.1023	NA
67255	Zfp422	zinc finger protein 422	0.891266	0.1973	0.4219
69181	Dyrk2	dual-specificity tyrosine-(Y)-phosphorylation regulated kinase 2	0.891266	0.1784	0.3961
93747	Echs1	enoyl Coenzyme A hydratase, short chain, 1, mitochondrial	0.891266	0.1167	NA
105727	Slc38a1	solute carrier family 38, member 1	0.891266	0.1418	NA
140904	Caln1	calneuron 1	0.891266	0.1176	NA
217127	Myst2	MYST histone acetyltransferase 2	0.891266	0.1078	NA
217682	3830431G21Rik	RIKEN cDNA 3830431G21 gene	0.891266	0.158	0.3689
218442	Serinc5	serine incorporator 5	0.891266	0.06775	NA
226025	Trpm3	transient receptor potential cation channel, subfamily M, member 3	0.891266	0.6624	0.8262
252972	Tpcn1	two pore channel 1	0.891266	0.1691	0.3843
320806	Gfm2	G elongation factor, mitochondrial 2	0.891266	0.1925	0.415
381418	Ctxn2	cortexin 2	0.891266	0.3129	0.5493
619941	Gm13770	predicted gene 13770	0.891266	0.3697	0.6046
665211	Gm14326	predicted gene 14326	0.891266	0.6018	0.7863
100047292	LOC100047292	pleckstrin homology domain-containing family A member 7-like	0.891266	0.1194	NA
100504944	LOC100504944	hypothetical protein LOC100504944	0.891266	0.7486	0.8783
12340	Capza1	capping protein (actin filament) muscle Z-line, alpha 1	0.890472	0.4059	0.6354
12695	Inadl	InaD-like (Drosophila)	0.890472	0.1951	0.4188
14123	Fbrs	fibrosin	0.890472	0.3331	0.569
16709	Ktn1	kinectin 1	0.890472	0.1246	NA
16801	Arhgef1	Rho guanine nucleotide exchange factor (GEF) 1	0.890472	0.1845	0.4037
17179	Matk	megakaryocyte-associated tyrosine kinase	0.890472	0.2679	0.5035
17968	Ncam2	neural cell adhesion molecule 2	0.890472	0.32	0.5566

17977	Ncoa1	nuclear receptor coactivator 1	0.890472	0.07065	NA
18760	Prkd1	protein kinase D1	0.890472	0.0665	NA
19272	Ptprk	protein tyrosine phosphatase, receptor type, K	0.890472	0.2151	0.4438
22083	Ctr9	Ctr9, Paf1/RNA polymerase II complex component, homolog (S. cerevisiae)	0.890472	0.1469	NA
56772	Mllt11	myeloid/lymphoid or mixed-lineage leukemia (trithorax homolog, Drosophila); translocated to, 11	0.890472	0.03527	NA
57277	Slurp1	secreted Ly6/Plaur domain containing 1	0.890472	0.7924	0.9016
58208	Bcl11b	B-cell leukemia/lymphoma 11B	0.890472	0.3979	0.6282
58994	Smpd3	sphingomyelin phosphodiesterase 3, neutral	0.890472	0.1262	NA
66276	1810009A15Rik	RIKEN cDNA 1810009A15 gene	0.890472	0.1042	NA
66629	Golph3	golgi phosphoprotein 3	0.890472	0.2279	0.4588
68034	Fam122a	family with sequence similarity 122, member A	0.890472	0.3206	0.5572
68770	Phtf2	putative homeodomain transcription factor 2	0.890472	0.596	0.7823
70821	4921507P07Rik	RIKEN cDNA 4921507P07 gene	0.890472	0.5399	0.7442
72236	Tsnaxip1	translin-associated factor X (Tsnax) interacting protein 1	0.890472	0.4854	0.7038
72552	Hsdl1	hydroxysteroid dehydrogenase like 1	0.890472	0.3094	0.5459
72749	Tonsl	tonsoku-like, DNA repair protein	0.890472	0.1996	0.425
74147	Ehhadh	enoyl-Coenzyme A, hydratase/3-hydroxyacyl Coenzyme A dehydrogenase	0.890472	0.2207	0.4506
77097	Tanc2	tetratricopeptide repeat, ankyrin repeat and coiled-coil containing 2	0.890472	0.5832	0.7739
78784	Celf3	VCUGBP, Elav-like family member 3	0.890472	0.198	0.423
81879	Tcfcp2l1	transcription factor CP2-like 1	0.890472	0.668	0.8296
102442	Dennd4a	DENN/MADD domain containing 4A	0.890472	0.5306	0.7368
216345	Zfc3h1	zinc finger, C3H1-type containing	0.890472	0.181	0.3996
217328	Myo15b	myosin XVB	0.890472	0.5637	0.7611
230866	C230096C10Rik	RIKEN cDNA C230096C10 gene	0.890472	0.2	0.4255
237988	Cdr2l	cerebellar degeneration-related protein 2-like	0.890472	0.09002	NA
239318	Plcxd3	phosphatidylinositol-specific phospholipase C, X domain containing 3	0.890472	0.2573	0.4925
243547	Grip2	glutamate receptor interacting protein 2	0.890472	0.3607	0.5955
280411	Lix1l	Lix1-like	0.890472	0.02704	NA
320504	5930403N24Rik	RIKEN cDNA 5930403N24 gene	0.890472	0.0558	NA
630146	Cd101	CD101 antigen	0.890472	0.7198	0.8599
100043474	Gm4461	predicted gene 4461	0.890472	0.6141	0.795
11490	Adam15	a disintegrin and metallopeptidase domain 15 (metargidin)	0.88968	0.07244	NA
12531	Cdc25b	cell division cycle 25 homolog B (S. pombe)	0.88968	0.04291	NA
13207	Ddx5	DEAD (Asp-Glu-Ala-Asp) box polypeptide 5	0.88968	0.2208	0.4507
14057	Sfxn1	sideroflexin 1	0.88968	0.2432	0.4764
14600	Ghr	growth hormone receptor	0.88968	0.1925	0.415
14950	H13	histocompatibility 13	0.88968	0.04254	NA
16647	Kpna2	karyopherin (importin) alpha 2	0.88968	0.105	NA
17156	Man1a2	mannosidase, alpha, class 1A, member 2	0.88968	0.2197	0.4495
18632	Pex11b	peroxisomal biogenesis factor 11 beta	0.88968	0.06685	NA
19418	Rasgrf2	RAS protein-specific guanine nucleotide-releasing factor 2	0.88968	0.05145	NA
20926	Supt6h	suppressor of Ty 6 homolog (S. cerevisiae)	0.88968	0.09878	NA
21828	Thbs4	thrombospondin 4	0.88968	0.6049	0.7884
22034	Traf6	TNF receptor-associated factor 6	0.88968	0.3469	0.5823
26419	Mapk8	mitogen-activated protein kinase 8	0.88968	0.06843	NA
27681	Snf8	SNF8, ESCRT-II complex subunit, homolog (S. cerevisiae)	0.88968	0.052	NA
50701	Elane	elastase, neutrophil expressed	0.88968	0.5768	0.7696
56380	Arid3b	AT rich interactive domain 3B (BRIGHT-like)	0.88968	0.3247	0.5612
66236	1500011B03Rik	RIKEN cDNA 1500011B03 gene	0.88968	0.2401	0.4728

66268	Pigyl	phosphatidylinositol glycan anchor biosynthesis, class Y-like	0.88968	0.2803	0.5157
66736	Ttc35	tetratricopeptide repeat domain 35	0.88968	0.0618	NA
66817	Tmem170	transmembrane protein 170	0.88968	0.1434	NA
67070	Lsm14a	LSM14 homolog A (SCD6, S. cerevisiae)	0.88968	0.4064	0.6359
68050	Akirin1	akirin 1	0.88968	0.08168	NA
68126	Fahd2a	fumarylacetoacetate hydrolase domain containing 2A	0.88968	0.0554	NA
71492	Bbs7	Bardet-Biedl syndrome 7 (human)	0.88968	0.08223	NA
71718	Telo2	TEL2, telomere maintenance 2, homolog (S. cerevisiae)	0.88968	0.1318	NA
72162	Dhx36	DEAH (Asp-Glu-Ala-His) box polypeptide 36	0.88968	0.04401	NA
72480	Tspyl4	TSPY-like 4	0.88968	0.1036	NA
73739	Cby1	chibby homolog 1 (Drosophila)	0.88968	0.2652	0.501
74200	2810403A07Rik	RIKEN cDNA 2810403A07 gene	0.88968	0.1398	NA
74571	Kcnk16	potassium channel, subfamily K, member 16	0.88968	0.4253	0.6527
76051	Ganc	glucosidase, alpha; neutral C	0.88968	0.07801	NA
83672	Sytl3	synaptotagmin-like 3	0.88968	0.4875	0.7054
101488	Slco2b1	solute carrier organic anion transporter family, member 2b1	0.88968	0.3885	0.6203
103711	Pnpo	pyridoxine 5'-phosphate oxidase	0.88968	0.05408	NA
106622	AI605517	expressed sequence AI605517	0.88968	0.2045	0.4308
108911	Rcc2	regulator of chromosome condensation 2	0.88968	0.1795	0.3974
110962	Mbd6	methyl-CpG binding domain protein 6	0.88968	0.2412	0.4741
212114	Nhlrc3	NHL repeat containing 3	0.88968	0.3583	0.5933
216134	Pdxk	pyridoxal (pyridoxine, vitamin B6) kinase	0.88968	0.05177	NA
217124	Ppp1r9b	protein phosphatase 1, regulatory subunit 9B	0.88968	0.1551	0.3648
233080	Ffar3	free fatty acid receptor 3	0.88968	0.6682	0.8296
235674	Acaa1b	acetyl-Coenzyme A acyltransferase 1B	0.88968	0.06444	NA
241656	Pak7	p21 protein (Cdc42/Rac)-activated kinase 7	0.88968	0.3277	0.5641
246317	Neto1	neuropilin (NRP) and tolloid (TLL)-like 1	0.88968	0.2932	0.5292
320571	Atp8b5	ATPase, class I, type 8B, member 5	0.88968	0.2138	0.4422
380850	Gm5141	predicted gene 5141	0.88968	0.2029	0.4292
380977	A330009N23Rik	RIKEN cDNA A330009N23 gene	0.88968	0.2836	0.5194
16000	Igf1	insulin-like growth factor 1	0.888889	0.1826	0.4015
17933	Myt1l	myelin transcription factor 1-like	0.888889	0.08003	NA
19076	Prim2	DNA primase, p58 subunit	0.888889	0.5024	0.7154
19210	Ptdss1	phosphatidylserine synthase 1	0.888889	0.2691	0.5043
22121	Rpl13a	ribosomal protein L13A	0.888889	0.26	0.4953
23936	Lynx1	Ly6/neurotoxin 1	0.888889	0.4879	0.7056
66143	Eef1e1	eukaryotic translation elongation factor 1 epsilon 1	0.888889	0.2333	0.4644
66645	Pspc1	paraspeckle protein 1	0.888889	0.1298	NA
67458	Ergic1	endoplasmic reticulum-golgi intermediate compartment (ERGIC) 1	0.888889	0.1716	0.3874
67681	Mrpl18	mitochondrial ribosomal protein L18	0.888889	0.1328	NA
68970	Dcaf12	DDB1 and CUL4 associated factor 12	0.888889	0.09163	NA
70495	Atp6ap2	ATPase, H+ transporting, lysosomal accessory protein 2	0.888889	0.1151	NA
71863	1700019O17Rik	RIKEN cDNA 1700019O17 gene	0.888889	0.4358	0.6626
72656	Ints8	integrator complex subunit 8	0.888889	0.1612	0.373
76073	Pcgf5	polycomb group ring finger 5	0.888889	0.2203	0.4501
76612	Lrrc27	leucine rich repeat containing 27	0.888889	0.293	0.5291
76968	2310046K23Rik	RIKEN cDNA 2310046K23 gene	0.888889	0.51	0.721
77093	A330094K24Rik	RIKEN cDNA A330094K24 gene	0.888889	0.2443	0.4778
104662	Tsr1	TSR1, 20S rRNA accumulation, homolog (yeast)	0.888889	0.1045	NA

105827	Amigo2	adhesion molecule with Ig like domain 2	0.888889	0.0835	NA
107656	Krt9	keratin 9	0.888889	0.6746	0.8332
208846	Daam1	dishevelled associated activator of morphogenesis 1	0.888889	0.2575	0.4926
212569	Zfp273	zinc finger protein 273	0.888889	0.05893	NA
234779	Plcg2	phospholipase C, gamma 2	0.888889	0.173	0.3891
245404	Dcaf12l1	DDB1 and CUL4 associated factor 12-like 1	0.888889	0.1682	0.3831
259302	Srgap3	SLIT-ROBO Rho GTPase activating protein 3	0.888889	0.2063	0.4325
320265	Fam19a1	family with sequence similarity 19, member A1	0.888889	0.2123	0.4401
330401	Tmcc1	transmembrane and coiled coil domains 1	0.888889	0.03302	NA
407802	BC028789	cDNA sequence BC028789	0.888889	0.7215	0.8605
675578	Gm9648	predicted gene 9648	0.888889	0.2289	0.4599
13858	Eps15	epidermal growth factor receptor pathway substrate 15	0.888099	0.3196	0.5562
14228	Fkbp4	FK506 binding protein 4	0.888099	0.1008	NA
14886	Gtf2i	general transcription factor II I	0.888099	0.4323	0.6594
18996	Pou4f1	POU domain, class 4, transcription factor 1	0.888099	0.2653	0.501
23945	Mgll	monoglyceride lipase	0.888099	0.1118	NA
26987	Eif4e2	eukaryotic translation initiation factor 4E member 2	0.888099	0.1133	NA
27204	Syn3	synapsin III	0.888099	0.4717	0.6928
54698	Crtam	cytotoxic and regulatory T cell molecule	0.888099	0.2722	0.5077
66286	Sec11c	SEC11 homolog C (S. cerevisiae)	0.888099	0.1803	0.3986
67263	Zswim6	zinc finger, SWIM domain containing 6	0.888099	0.3599	0.5948
67667	Alkbh8	alkB, alkylation repair homolog 8 (E. coli)	0.888099	0.1587	0.3699
67747	Ribc2	RIB43A domain with coiled-coils 2	0.888099	0.2407	0.4734
72515	Wdr43	WD repeat domain 43	0.888099	0.0832	NA
74203	Eif4enif1	eukaryotic translation initiation factor 4E nuclear import factor 1	0.888099	0.08705	NA
75705	Eif4b	eukaryotic translation initiation factor 4B	0.888099	0.1587	0.3699
76787	Ppfia3	protein tyrosine phosphatase, receptor type, f polypeptide (PTPRF), interacting protein (liprin), alpha 3	0.888099	0.3265	0.563
76898	B3gat1	beta-1,3-glucuronyltransferase 1 (glucuronosyltransferase P)	0.888099	0.2659	0.5015
76917	Flywch2	FLYWCH family member 2	0.888099	0.07373	NA
101142	Itfg2	integrin alpha FG-GAP repeat containing 2	0.888099	0.09093	NA
170835	Inpp5j	inositol polyphosphate 5-phosphatase J	0.888099	0.06046	NA
207495	Baiap2l2	BAI1-associated protein 2-like 2	0.888099	0.4769	0.6966
212531	Sh3bgrl2	SH3 domain binding glutamic acid-rich protein like 2	0.888099	0.1829	0.4017
224598	Zfp758	zinc finger protein 758	0.888099	0.2227	0.4529
226139	Cox15	COX15 homolog, cytochrome c oxidase assembly protein (yeast)	0.888099	0.3962	0.6267
230777	Hcrtr1	hypocretin (orexin) receptor 1	0.888099	0.2272	0.4586
231699	Oas1e	2'-5' oligoadenylate synthetase 1E	0.888099	0.1774	0.3946
238323	Rps6kl1	ribosomal protein S6 kinase-like 1	0.888099	0.07028	NA
239122	Setdb2	SET domain, bifurcated 2	0.888099	0.6094	0.7917
252876	Gin1	gypsy retrotransposon integrase 1	0.888099	0.5464	0.7484
258319	Olfr187	olfactory receptor 187	0.888099	0.5881	0.7775
320277	Spef2	sperm flagellar 2	0.888099	0.4099	0.6391
380684	Nefh	neurofilament, heavy polypeptide	0.888099	0.2858	0.5212
100042202	Gm3718	predicted gene 3718	0.888099	0.4565	0.6801
13857	Epor	erythropoietin receptor	0.887311	0.2285	0.4595
14584	Gfpt2	glutamine fructose-6-phosphate transaminase 2	0.887311	0.5437	0.7469
14788	Gpr162	G protein-coupled receptor 162	0.887311	0.2502	0.4841
16956	Lpl	lipoprotein lipase	0.887311	0.1836	0.4026
26448	Stk30	serine/threonine kinase 30	0.887311	0.034	NA

52245	Commd2	COMM domain containing 2	0.887311	0.3205	0.5571
54393	Gabbr1	gamma-aminobutyric acid (GABA) B receptor, 1	0.887311	0.2925	0.5284
54563	Nup210	nucleoporin 210	0.887311	0.04173	NA
60363	Cldn15	claudin 15	0.887311	0.09948	NA
67180	Yipf5	Yip1 domain family, member 5	0.887311	0.101	NA
67286	Rabl5	RAB, member of RAS oncogene family-like 5	0.887311	0.2112	0.4386
69860	Eif1ad	eukaryotic translation initiation factor 1A domain containing	0.887311	0.08804	NA
70377	Derl3	Der1-like domain family, member 3	0.887311	0.2922	0.5281
71728	Stk11ip	serine/threonine kinase 11 interacting protein	0.887311	0.2271	0.4585
72139	2610044O15Rik	RIKEN cDNA 2610044O15 gene	0.887311	0.5206	0.7288
73331	1700034P13Rik	RIKEN cDNA 1700034P13 gene	0.887311	0.4351	0.6619
74148	1300001I01Rik	RIKEN cDNA 1300001I01 gene	0.887311	0.2373	0.4695
77604	C430048L16Rik	RIKEN cDNA C430048L16 gene	0.887311	0.2591	0.4943
97423	R74862	expressed sequence R74862	0.887311	0.05476	NA
101739	Psip1	PC4 and SFRS1 interacting protein 1	0.887311	0.2067	0.4328
106338	Nsun3	NOL1/NOP2/Sun domain family member 3	0.887311	0.2462	0.4802
109900	Asl	argininosuccinate lyase	0.887311	0.2398	0.4724
211652	Wwc1	WW, C2 and coiled-coil domain containing 1	0.887311	0.3175	0.5539
225638	Alpk2	alpha-kinase 2	0.887311	0.07512	NA
226139	Cox15	COX15 homolog, cytochrome c oxidase assembly protein (yeast)	0.887311	0.121	NA
229776	Cdc14a	CDC14 cell division cycle 14 homolog A (S. cerevisiae)	0.887311	0.0957	NA
233879	Asphd1	aspartate beta-hydroxylase domain containing 1	0.887311	0.1166	NA
234388	Ccdc124	coiled-coil domain containing 124	0.887311	0.1534	0.363
320360	Ric3	resistance to inhibitors of cholinesterase 3 homolog (C. elegans)	0.887311	0.1938	0.4171
399623	5930433N17Rik	RIKEN cDNA 5930433N17 gene	0.887311	0.2047	0.431
404580	AL117821	expressed sequence AL117821	0.887311	0.1869	0.4075
635504	Gm7160	predicted gene 7160	0.887311	0.1002	NA
12151	Bmi1	Bmi1 polycomb ring finger oncogene	0.886525	0.0741	NA
12695	Inadl	InaD-like (Drosophila)	0.886525	0.06218	NA
13864	Nr2f6	nuclear receptor subfamily 2, group F, member 6	0.886525	0.1998	0.4253
16499	Kcnab3	potassium voltage-gated channel, shaker-related subfamily, beta member 3	0.886525	0.02404	NA
17120	Mad1l1	mitotic arrest deficient 1-like 1	0.886525	0.245	0.4788
17391	Mmp24	matrix metallopeptidase 24	0.886525	0.2842	0.5199
18197	Nsg2	neuron specific gene family member 2	0.886525	0.0187	NA
26949	Vat1	vesicle amine transport protein 1 homolog (T californica)	0.886525	0.4656	0.6874
50753	Fbxo8	F-box protein 8	0.886525	0.07102	NA
52679	E2f7	E2F transcription factor 7	0.886525	0.3595	0.5946
53858	Rwdd2b	RWD domain containing 2B	0.886525	0.1689	0.384
68501	Nsmce2	non-SMC element 2 homolog (MMS21, S. cerevisiae)	0.886525	0.606	0.7891
71096	Sntg1	syntrophin, gamma 1	0.886525	0.05184	NA
74016	Phf19	PHD finger protein 19	0.886525	0.2851	0.5204
74772	Atp13a2	ATPase type 13A2	0.886525	0.1278	NA
76173	6330531I01Rik	RIKEN cDNA 6330531I01 gene	0.886525	0.5088	0.72
104130	Ndufb11	NADH dehydrogenase (ubiquinone) 1 beta subcomplex, 11	0.886525	0.1972	0.4218
105590	Zfp957	zinc finger protein 957	0.886525	0.2043	0.4306
110829	Lims1	LIM and senescent cell antigen-like domains 1	0.886525	0.2085	0.4352
114142	Foxp2	forkhead box P2	0.886525	0.2785	0.514
114229	Kiss1r	KISS1 receptor	0.886525	0.2487	0.483
211446	Exoc3	exocyst complex component 3	0.886525	0.3574	0.5923

216825	Usp22	ubiquitin specific peptidase 22	0.886525	0.3558	0.5906
224585	Zfp160	zinc finger protein 160	0.886525	0.221	0.4509
227699	Nup188	nucleoporin 188	0.886525	0.2821	0.5174
229615	Pias3	protein inhibitor of activated STAT 3	0.886525	0.3184	0.5549
233532	Rsf1	remodeling and spacing factor 1	0.886525	0.1882	0.4091
237711	Eml6	echinoderm microtubule associated protein like 6	0.886525	0.07054	NA
238871	Pde4d	phosphodiesterase 4D, cAMP specific	0.886525	0.2534	0.488
239667	Dip2b	DIP2 disco-interacting protein 2 homolog B (Drosophila)	0.886525	0.08497	NA
244585	Rpgrip1l	Rpgrip1-like	0.886525	0.1194	NA
319322	Sf3b2	splicing factor 3b, subunit 2	0.886525	0.09359	NA
320961	A630026N12Rik	RIKEN cDNA A630026N12 gene	0.886525	0.1572	0.3675
654424	A330050B17Rik	RIKEN cDNA A330050B17 gene	0.886525	0.3426	0.5781
100042786	Gm16381	predicted gene 16381	0.886525	0.5972	0.7833
100043089	Gm4221	predicted gene 4221	0.886525	0.376	0.6099
100503168	LOC100503168	hypothetical LOC100503168	0.886525	0.5098	0.7208
11630	Aim1	absent in melanoma 1	0.88574	0.2808	0.5162
16573	Kif5b	kinesin family member 5B	0.88574	0.4502	0.6752
16579	Kifap3	kinesin-associated protein 3	0.88574	0.0173	NA
18577	Pde4a	phosphodiesterase 4A, cAMP specific	0.88574	0.1191	NA
19703	Renbp	renin binding protein	0.88574	0.2906	0.5266
20167	Rtn2	reticulon 2 (Z-band associated protein)	0.88574	0.06372	NA
20346	Sema3a	sema domain, immunoglobulin domain (Ig), short basic domain, secreted, (semaphorin) 3A	0.88574	0.1999	0.4255
24067	Srp54a	signal recognition particle 54A	0.88574	0.5078	0.7193
27425	Atp5l	ATP synthase, H+ transporting, mitochondrial F0 complex, subunit g	0.88574	0.1002	NA
29869	Ulk2	Unc-51 like kinase 2 (C. elegans)	0.88574	0.2041	0.4305
55948	Sfn	stratifin	0.88574	0.3669	0.6022
56626	Poll	polymerase (DNA directed), lambda	0.88574	0.1751	0.3919
66661	Srp72	signal recognition particle 72	0.88574	0.1495	0.3573
67426	Adck3	aarF domain containing kinase 3	0.88574	0.2301	0.4612
67453	Slc25a46	solute carrier family 25, member 46	0.88574	0.3224	0.5588
67900	Mtfp1	mitochondrial fission process 1	0.88574	0.07309	NA
68097	Dynll2	dynein light chain LC8-type 2	0.88574	0.09195	NA
69713	Pin4	protein (peptidyl-prolyl cis/trans isomerase) NIMA-interacting, 4 (parvulin)	0.88574	0.05455	NA
70285	Rpf1	ribosome production factor 1 homolog (S. cerevisiae)	0.88574	0.1425	0.347
70640	Dcp2	DCP2 decapping enzyme homolog (S. cerevisiae)	0.88574	0.07672	NA
71751	Map3k13	mitogen-activated protein kinase kinase kinase 13	0.88574	0.2364	0.4688
74094	Tjap1	tight junction associated protein 1	0.88574	0.0995	NA
75497	Fabp12	fatty acid binding protein 12	0.88574	0.04905	NA
75914	Exoc6b	exocyst complex component 6B	0.88574	0.04753	NA
109054	Pfdn4	prefoldin 4	0.88574	0.2485	0.4827
114714	Rad51c	RAD51 homolog c (S. cerevisiae)	0.88574	0.4472	0.6726
140500	Acap3	ArfGAP with coiled-coil, ankyrin repeat and PH domains 3	0.88574	0.2233	0.4537
170731	Mfn2	mitofusin 2	0.88574	0.06403	NA
171209	Accn3	amiloride-sensitive cation channel 3	0.88574	0.2973	0.5334
215113	Slc43a2	solute carrier family 43, member 2	0.88574	0.1745	0.3912
217026	Heatr6	HEAT repeat containing 6	0.88574	0.0902	NA
224093	Fam43a	family with sequence similarity 43, member A	0.88574	0.202	0.4281
226016	Fam108b	family with sequence similarity 108, member B	0.88574	0.07647	NA
226043	Cbwd1	COBW domain containing 1	0.88574	0.03358	NA

230657	Tmem69	transmembrane protein 69	0.88574	0.3956	0.6262
230815	Man1c1	mannosidase, alpha, class 1C, member 1	0.88574	0.1464	0.3522
244721	Zfp846	zinc finger protein 846	0.88574	0.07594	NA
320163	4930525G20Rik	RIKEN cDNA 4930525G20 gene	0.88574	0.1626	0.3748
330192	Vps37b	vacuolar protein sorting 37B (yeast)	0.88574	0.1205	NA
627626	3110082D06Rik	RIKEN cDNA 3110082D06 gene	0.88574	0.2038	0.4302
11990	Atrn	attractin	0.884956	0.05551	NA
13368	Dffb	DNA fragmentation factor, beta subunit	0.884956	0.0799	NA
13393	Dlx3	distal-less homeobox 3	0.884956	0.3615	0.5963
13488	Drd1a	dopamine receptor D1A	0.884956	0.1743	0.3908
13601	Ecm1	extracellular matrix protein 1	0.884956	0.4304	0.6572
16905	Lmna	lamin A	0.884956	0.2084	0.4351
17425	Foxk1	forkhead box K1	0.884956	0.02312	NA
19158	Cyth2	cytohesin 2	0.884956	0.1093	NA
21429	Ubtf	upstream binding transcription factor, RNA polymerase I	0.884956	0.1983	0.4234
54393	Gabbr1	gamma-aminobutyric acid (GABA) B receptor, 1	0.884956	0.1042	NA
57444	Isg20	interferon-stimulated protein	0.884956	0.1268	0.3241
64113	Moap1	modulator of apoptosis 1	0.884956	0.1657	0.3796
66262	Ing5	inhibitor of growth family, member 5	0.884956	0.08834	NA
66743	Rnf220	ring finger protein 220	0.884956	0.02767	NA
66882	Bzw1	basic leucine zipper and W2 domains 1	0.884956	0.1289	0.3276
66998	Psmd5	proteasome (prosome, macropain) 26S subunit, non-ATPase, 5	0.884956	0.1012	NA
67569	Mgat4c	mannosyl (alpha-1,3-)-glycoprotein beta-1,4-N-acetylglucosaminyltransferase, isozyme C (putative)	0.884956	0.2883	0.5242
71492	Bbs7	Bardet-Biedl syndrome 7 (human)	0.884956	0.02362	NA
73723	Sh3bgrl3	SH3 domain binding glutamic acid-rich protein-like 3	0.884956	0.1549	0.3647
76577	Faf2	Fas associated factor family member 2	0.884956	0.0772	NA
76969	Chst1	carbohydrate (keratan sulfate Gal-6) sulfotransferase 1	0.884956	0.02521	NA
77032	2610029I01Rik	RIKEN cDNA 2610029I01 gene	0.884956	0.1378	0.3401
100764	1110008J03Rik	RIKEN cDNA 1110008J03 gene	0.884956	0.2301	0.4612
103742	1810046J19Rik	RIKEN cDNA 1810046J19 gene	0.884956	0.1454	0.3508
104799	Vipar	VPS33B interacting protein, apical-basolateral polarity regulator	0.884956	0.08375	NA
109200	A430102J17Rik	RIKEN cDNA A430102J17 gene	0.884956	0.367	0.6022
218629	Dhx29	DEAH (Asp-Glu-Ala-His) box polypeptide 29	0.884956	0.02044	NA
223435	Trio	triple functional domain (PTPRF interacting)	0.884956	0.05839	NA
224694	Zfp81	zinc finger protein 81	0.884956	0.2667	0.5021
230649	Atpaf1	ATP synthase mitochondrial F1 complex assembly factor 1	0.884956	0.3773	0.6113
230676	Szt2	seizure threshold 2	0.884956	0.1231	NA
230753	Thrap3	thyroid hormone receptor associated protein 3	0.884956	0.03884	NA
231841	AA881470	EST AA881470	0.884956	0.2926	0.5284
233877	Kctd13	potassium channel tetramerisation domain containing 13	0.884956	0.3778	0.6116
235180	Fez1	fasciculation and elongation protein zeta 1 (zygin I)	0.884956	0.2056	0.4319
241322	Zbtb6	zinc finger and BTB domain containing 6	0.884956	0.1247	NA
269682	Golga3	golgi autoantigen, golgin subfamily a, 3	0.884956	0.2074	0.4337
330812	Rnf150	ring finger protein 150	0.884956	0.3567	0.5916
333050	Ksr2	kinase suppressor of ras 2	0.884956	0.273	0.5086
333315	Frem3	Fras1 related extracellular matrix protein 3	0.884956	0.1331	0.3338
382823	D13Mgi7	DNA Segment, Chr 13, Mouse Genome Informatics 7	0.884956	0.3912	0.6229
13591	Ebf1	early B-cell factor 1	0.884173	0.1178	NA
13644	Efs	embryonal Fyn-associated substrate	0.884173	0.2362	0.4687

14683	Gnas	GNAS (guanine nucleotide binding protein, alpha stimulating) complex locus	0.884173	0.05784	NA
17978	Ncoa2	nuclear receptor coactivator 2	0.884173	0.1244	0.3211
19365	Rad52	RAD52 homolog (S. cerevisiae)	0.884173	0.1033	NA
20090	Rps29	ribosomal protein S29	0.884173	0.08509	NA
20181	Rxra	retinoid X receptor alpha	0.884173	0.4736	0.6944
20317	Serpinf1	serine (or cysteine) peptidase inhibitor, clade F, member 1	0.884173	0.3038	0.5401
22235	Ugdh	UDP-glucose dehydrogenase	0.884173	0.1026	NA
22378	Wbp2	WW domain binding protein 2	0.884173	0.1681	0.3831
24131	Ldb3	LIM domain binding 3	0.884173	0.6549	0.8212
26562	Ncdn	neurochondrin	0.884173	0.4181	0.6462
27984	Efhd2	EF hand domain containing 2	0.884173	0.3037	0.5401
53970	Rfx5	regulatory factor X, 5 (influences HLA class II expression)	0.884173	0.1849	0.4043
54130	Actr1a	ARP1 actin-related protein 1 homolog A, centractin alpha (yeast)	0.884173	0.1984	0.4237
56390	Sssca1	Sjogren's syndrome/scleroderma autoantigen 1 homolog (human)	0.884173	0.1714	0.3871
56508	Rapgef4	Rap guanine nucleotide exchange factor (GEF) 4	0.884173	0.5012	0.7146
56808	Cacna2d2	calcium channel, voltage-dependent, alpha 2/delta subunit 2	0.884173	0.701	0.8492
59040	Rhot1	ras homolog gene family, member T1	0.884173	0.1569	0.3671
67379	Dedd2	death effector domain-containing DNA binding protein 2	0.884173	0.1063	NA
67803	Limd2	LIM domain containing 2	0.884173	0.1016	NA
68263	Pdhb	pyruvate dehydrogenase (lipoamide) beta	0.884173	0.2627	0.4982
68510	Ints1	integrator complex subunit 1	0.884173	0.1577	0.3684
69226	Snx24	sorting nexing 24	0.884173	0.4537	0.6776
69823	Fyttd1	forty-two-three domain containing 1	0.884173	0.2953	0.5311
71601	Ceacam20	carcinoembryonic antigen-related cell adhesion molecule 20	0.884173	0.1208	NA
73229	3110052M02Rik	RIKEN cDNA 3110052M02 gene	0.884173	0.3349	0.5706
73660	Cabp4	calcium binding protein 4	0.884173	0.1277	0.3257
74351	Ddx23	DEAD (Asp-Glu-Ala-Asp) box polypeptide 23	0.884173	0.374	0.6082
74534	8430428J23Rik	RIKEN cDNA 8430428J23 gene	0.884173	0.2746	0.5101
108797	Mex3b	mex3 homolog B (C. elegans)	0.884173	0.1128	NA
217558	G2e3	G2/M-phase specific E3 ubiquitin ligase	0.884173	0.4096	0.6389
218236	Fam120a	family with sequence similarity 120, member A	0.884173	0.2205	0.4504
226971	Plekhb2	pleckstrin homology domain containing, family B (evectins) member 2	0.884173	0.3458	0.5812
230235	6430704M03Rik	RIKEN cDNA 6430704M03 gene	0.884173	0.1512	0.3595
239546	Zfp647	zinc finger protein 647	0.884173	0.03506	NA
258464	Olfr1384	olfactory receptor 1384	0.884173	0.3975	0.6279
320213	Senp5	SUMO/sentrin specific peptidase 5	0.884173	0.07411	NA
320727	Ipo8	importin 8	0.884173	0.1552	0.3649
328580	Tubgcp6	tubulin, gamma complex associated protein 6	0.884173	0.2584	0.4937
381693	Wdr95	WD40 repeat domain 95	0.884173	0.2902	0.5264
613262	BC029722	cDNA sequence BC029722	0.884173	0.06757	NA
12013	Bach1	BTB and CNC homology 1	0.883392	0.191	0.413
12048	Bcl2l1	BCL2-like 1	0.883392	0.5198	0.7283
13494	Drg1	developmentally regulated GTP binding protein 1	0.883392	0.06729	NA
14000	Drosha	drosha, ribonuclease type III	0.883392	0.188	0.4088
14403	Gabrd	gamma-aminobutyric acid (GABA) A receptor, subunit delta	0.883392	0.1317	0.3319
15161	Hcfc1	host cell factor C1	0.883392	0.07311	NA
15166	Hcn2	hyperpolarization-activated, cyclic nucleotide-gated K+ 2	0.883392	0.3631	0.5983
17939	Naga	N-acetyl galactosaminidase, alpha	0.883392	0.3008	0.5367
18488	Cntn3	contactin 3	0.883392	0.1772	0.3944

19231	Ptma	prothymosin alpha	0.883392	0.1077	NA
22222	Ubr1	ubiquitin protein ligase E3 component n-recognin 1	0.883392	0.2377	0.4699
23989	Med24	mediator complex subunit 24	0.883392	0.1451	0.3505
27425	Atp5l	ATP synthase, H+ transporting, mitochondrial F0 complex, subunit g	0.883392	0.1156	NA
52443	Mrpl48	mitochondrial ribosomal protein L48	0.883392	0.1002	NA
58248	1700123O20Rik	RIKEN cDNA 1700123O20 gene	0.883392	0.05728	NA
66970	Ssbp2	single-stranded DNA binding protein 2	0.883392	0.05216	NA
69876	Thap3	THAP domain containing, apoptosis associated protein 3	0.883392	0.1119	NA
69886	2610002J23Rik	RIKEN cDNA 2610002J23 gene	0.883392	0.021	NA
71691	Pnmal1	PNMA-like 1	0.883392	0.06701	NA
71834	Zbtb43	zinc finger and BTB domain containing 43	0.883392	0.01287	NA
72244	1600014C10Rik	RIKEN cDNA 1600014C10 gene	0.883392	0.2685	0.5038
75288	Slc35f4	solute carrier family 35, member F4	0.883392	0.08423	NA
76795	Tbc1d9b	TBC1 domain family, member 9B	0.883392	0.1335	0.3339
77767	Ermn	ermin, ERM-like protein	0.883392	0.4583	0.6816
81907	Tmem108	transmembrane protein 108	0.883392	0.1603	0.3721
99889	Arfip1	ADP-ribosylation factor interacting protein 1	0.883392	0.4802	0.6991
114143	Atp6v0b	ATPase, H+ transporting, lysosomal V0 subunit B	0.883392	0.1608	0.3725
170767	Rfxap	regulatory factor X-associated protein	0.883392	0.2097	0.4366
209131	Snx30	sorting nexin family member 30	0.883392	0.2038	0.4302
212999	Tnpo2	transportin 2 (importin 3, karyopherin beta 2b)	0.883392	0.307	0.5433
230861	Eif4g3	eukaryotic translation initiation factor 4 gamma, 3	0.883392	0.07597	NA
235907	Zfp71-rs1	zinc finger protein 71, related sequence	0.883392	0.167	0.3817
242667	Dlgap3	discs, large (Drosophila) homolog-associated protein 3	0.883392	0.4785	0.6982
243923	Rgs9bp	regulator of G-protein signalling 9 binding protein	0.883392	0.2743	0.5099
243961	Shank1	SH3/ankyrin domain gene 1	0.883392	0.1712	0.387
14084	Faf1	Fas-associated factor 1	0.882613	0.2519	0.4863
14256	Flt3l	FMS-like tyrosine kinase 3 ligand	0.882613	0.2556	0.4904
15331	Hmgn2	high mobility group nucleosomal binding domain 2	0.882613	0.2272	0.4586
15444	Hpca	hippocalcin	0.882613	0.396	0.6265
16560	Kif1a	kinesin family member 1A	0.882613	0.4813	0.7
16716	Ky	kyphoscoliosis peptidase	0.882613	0.3056	0.5421
18263	Odc1	ornithine decarboxylase, structural 1	0.882613	0.1494	0.3571
18611	Pea15a	phosphoprotein enriched in astrocytes 15A	0.882613	0.255	0.4897
18997	Pou4f2	POU domain, class 4, transcription factor 2	0.882613	0.07518	NA
19192	Psme3	proteaseome (prosome, macropain) 28 subunit, 3	0.882613	0.5061	0.7183
20530	Slc31a2	solute carrier family 31, member 2	0.882613	0.2265	0.458
23877	Fiz1	Flt3 interacting zinc finger protein 1	0.882613	0.3143	0.5508
27425	Atp5l	ATP synthase, H+ transporting, mitochondrial F0 complex, subunit g	0.882613	0.01796	NA
56433	Vps29	vacuolar protein sorting 29 (S. pombe)	0.882613	0.1559	0.3658
66277	Klf15	Kruppel-like factor 15	0.882613	0.5603	0.7589
66566	Ntpcr	nucleoside-triphosphatase, cancer-related	0.882613	0.08705	NA
66978	Luc7l	Luc7 homolog (S. cerevisiae)-like	0.882613	0.07294	NA
67845	Rnf115	ring finger protein 115	0.882613	0.09281	NA
67959	Puf60	poly-U binding splicing factor 60	0.882613	0.1285	0.327
68033	Cox19	COX19 cytochrome c oxidase assembly homolog (S. cerevisiae)	0.882613	0.07748	NA
68275	Rpa1	replication protein A1	0.882613	0.09436	NA
68473	Mobkl1a	MOB1, Mps One Binder kinase activator-like 1A (yeast)	0.882613	0.3374	0.573
69993	Chn2	chimerin (chimaerin) 2	0.882613	0.2145	0.443

70556	Slc25a33	solute carrier family 25, member 33	0.882613	0.01986	NA
71865	Fbxo30	F-box protein 30	0.882613	0.06981	NA
72020	Zfp654	zinc finger protein 654	0.882613	0.4072	0.6366
74369	Mei1	meiosis defective 1	0.882613	0.09084	NA
78786	4833439F03Rik	RIKEN cDNA 4833439F03 gene	0.882613	0.1523	0.3613
103844	Inca1	inhibitor of CDK, cyclin A1 interacting protein 1	0.882613	0.2438	0.4773
107526	Gimap4	GTPase, IMAP family member 4	0.882613	0.5148	0.725
108116	Slco3a1	solute carrier organic anion transporter family, member 3a1	0.882613	0.154	0.3636
108723	Card11	caspase recruitment domain family, member 11	0.882613	0.3396	0.5753
142688	Asb13	ankyrin repeat and SOCS box-containing 13	0.882613	0.05827	NA
210982	BC032203	cDNA sequence BC032203	0.882613	0.0205	NA
212326	Fam149a	family with sequence similarity 149, member A	0.882613	0.07576	NA
232227	Iqsec1	IQ motif and Sec7 domain 1	0.882613	0.1249	0.3217
241324	Crb2	crumbs homolog 2 (Drosophila)	0.882613	0.07228	NA
243382	Ppm1k	protein phosphatase 1K (PP2C domain containing)	0.882613	0.1162	0.3075
269855	A430110N23Rik	RIKEN cDNA A430110N23 gene	0.882613	0.2225	0.4527
319801	9630033F20Rik	RIKEN cDNA 9630033F20 gene	0.882613	0.04664	NA
319955	Ercc6	excision repair cross-complementing rodent repair deficiency, complementation group 6	0.882613	0.1004	NA
320429	Trank1	tetratricopeptide repeat and ankyrin repeat containing 1	0.882613	0.3996	0.63
320982	Arl4c	ADP-ribosylation factor-like 4C	0.882613	0.3681	0.6031
381546	Ccdc24	coiled-coil domain containing 24	0.882613	0.3952	0.6258
12663	Chml	choroideremia-like	0.881834	0.08982	NA
12752	Cln3	ceroid lipofuscinosis, neuronal 3, juvenile (Batten, Spielmeyer-Vogt disease)	0.881834	0.2208	0.4507
13131	Dab1	disabled homolog 1 (Drosophila)	0.881834	0.1262	0.3235
14539	Opn1mw	opsin 1 (cone pigments), medium-wave-sensitive (color blindness, deutan)	0.881834	0.1599	0.3715
18508	Pax6	paired box gene 6	0.881834	0.317	0.5536
19288	Ptx3	pentraxin related gene	0.881834	0.2122	0.44
50759	Fbxo16	F-box protein 16	0.881834	0.2189	0.4483
52850	Sgsm1	small G protein signaling modulator 1	0.881834	0.05998	NA
54194	Akap8l	A kinase (PRKA) anchor protein 8-like	0.881834	0.1666	0.3809
56542	Ick	intestinal cell kinase	0.881834	0.1261	0.3234
65114	Vps35	vacuolar protein sorting 35	0.881834	0.0858	NA
66849	Ppp1r2	protein phosphatase 1, regulatory (inhibitor) subunit 2	0.881834	0.05936	NA
67120	Ttc14	tetratricopeptide repeat domain 14	0.881834	0.3905	0.6226
67453	Slc25a46	solute carrier family 25, member 46	0.881834	0.04294	NA
68209	Rnaseh2c	ribonuclease H2, subunit C	0.881834	0.1468	0.353
74616	Scrn3	secernin 3	0.881834	0.1964	0.4206
75687	Fam65a	family with sequence similarity 65, member A	0.881834	0.02491	NA
76366	Mtif3	mitochondrial translational initiation factor 3	0.881834	0.2443	0.4778
76987	Hdhd2	haloacid dehalogenase-like hydrolase domain containing 2	0.881834	0.6988	0.8476
81840	Sorcs2	sortilin-related VPS10 domain containing receptor 2	0.881834	0.1115	0.2995
83603	Elovl4	elongation of very long chain fatty acids (FEN1/Elo2, SUR4/Elo3, yeast)-like 4	0.881834	0.3596	0.5947
94218	Cnnm3	cyclin M3	0.881834	0.1548	0.3647
108083	Pip4k2b	phosphatidylinositol-5-phosphate 4-kinase, type II, beta	0.881834	0.0518	NA
108995	Tbc1d10c	TBC1 domain family, member 10c	0.881834	0.1231	0.319
116891	Derl2	Der1-like domain family, member 2	0.881834	0.03557	NA
117160	Ttyh2	tweety homolog 2 (Drosophila)	0.881834	0.4702	0.692
210094	Iglon5	IgLON family member 5	0.881834	0.1082	NA
216151	Polrmt	polymerase (RNA) mitochondrial (DNA directed)	0.881834	0.07161	NA

223669	Zfp7	zinc finger protein 7	0.881834	0.1467	0.3528
226123	Morn4	MORN repeat containing 4	0.881834	0.1555	0.3652
228858	Gdap1l1	ganglioside-induced differentiation-associated protein 1-like 1	0.881834	0.02934	NA
232087	Mat2a	methionine adenosyltransferase II, alpha	0.881834	0.02025	NA
234069	Pcid2	PCI domain containing 2	0.881834	0.3531	0.5879
242607	Slc1a7	solute carrier family 1 (glutamate transporter), member 7	0.881834	0.4024	0.6324
245537	Nlgn3	neuroligin 3	0.881834	0.2036	0.4299
320404	Itpkb	inositol 1,4,5-trisphosphate 3-kinase B	0.881834	0.4713	0.6925
380684	Nefh	neurofilament, heavy polypeptide	0.881834	0.06133	NA
380785	Begain	brain-enriched guanylate kinase-associated	0.881834	0.128	0.326
433004	B830017H08Rik	RIKEN cDNA B830017H08 gene	0.881834	0.2998	0.5355
494468	Armcx5	armadillo repeat containing, X-linked 5	0.881834	0.02909	NA
629147	Ctxn3	cortexin 3	0.881834	0.3073	0.5435
100041799	Gm3515	predicted gene 3515	0.881834	0.6859	0.8398
100042916	Gm4107	predicted gene 4107	0.881834	0.4247	0.6522
14256	Flt3l	FMS-like tyrosine kinase 3 ligand	0.881057	0.2332	0.4644
17196	Mbp	myelin basic protein	0.881057	0.04864	NA
17364	Trpm1	transient receptor potential cation channel, subfamily M, member 1	0.881057	0.5612	0.7597
17706	ATP8	ATP synthase F0 subunit 8	0.881057	0.3699	0.6048
21418	Tcfap2a	transcription factor AP-2, alpha	0.881057	0.1183	0.3109
58242	Nudt11	nudix (nucleoside diphosphate linked moiety X)-type motif 11	0.881057	0.1196	0.3131
66089	Rmnd5b	required for meiotic nuclear division 5 homolog B (S. cerevisiae)	0.881057	0.3239	0.5603
67068	Dynlrb1	dynein light chain roadblock-type 1	0.881057	0.08124	NA
67072	Cdc37l1	cell division cycle 37 homolog (S. cerevisiae)-like 1	0.881057	0.04943	NA
69786	Tprkb	Tp53rk binding protein	0.881057	0.2045	0.4308
70381	Tecpr1	tectonin beta-propeller repeat containing 1	0.881057	0.104	NA
71592	Pogk	pogo transposable element with KRAB domain	0.881057	0.3846	0.617
74653	4930444A02Rik	RIKEN cDNA 4930444A02 gene	0.881057	0.1725	0.3884
77219	Ptgr2	prostaglandin reductase 2	0.881057	0.09968	NA
77938	Fam53b	family with sequence similarity 53, member B	0.881057	0.2805	0.5159
83558	Tex11	testis expressed gene 11	0.881057	0.2366	0.4689
110380	Shroom2	shroom family member 2	0.881057	0.07795	NA
170826	Ppargc1b	peroxisome proliferative activated receptor, gamma, coactivator 1 beta	0.881057	0.4791	0.6985
207854	Fmr1nb	fragile X mental retardation 1 neighbor	0.881057	0.8371	0.927
210105	Zfp719	zinc finger protein 719	0.881057	0.0263	NA
212198	Wdr25	WD repeat domain 25	0.881057	0.1897	0.4115
212390	Klhl32	kelch-like 32 (Drosophila)	0.881057	0.3163	0.553
242050	Igsf10	immunoglobulin superfamily, member 10	0.881057	0.4846	0.7031
243634	Ano2	anoctamin 2	0.881057	0.06768	NA
272465	Fam70b	family with sequence similarity 70, member B	0.881057	0.212	0.4398
319783	A730056A06Rik	RIKEN cDNA A730056A06 gene	0.881057	0.2292	0.4604
320015	A130082J08Rik	RIKEN cDNA A130082J08 gene	0.881057	0.4871	0.705
320473	Heatr5b	HEAT repeat containing 5B	0.881057	0.09291	NA
320615	Dopey1	dopey family member 1	0.881057	0.1657	0.3797
328734	Pisd-ps2	phosphatidylserine decarboxylase, pseudogene 2	0.881057	0.4297	0.6567
100041352	Gm9880	predicted gene 9880	0.881057	0.7103	0.8544
11492	Adam19	a disintegrin and metallopeptidase domain 19 (meltrin beta)	0.880282	0.04092	NA
12631	Cfl1	cofilin 1, non-muscle	0.880282	0.03231	NA
13831	Epc1	enhancer of polycomb homolog 1 (Drosophila)	0.880282	0.2026	0.4288

16647	Kpna2	karyopherin (importin) alpha 2	0.880282	0.05414	NA
18191	Nrxn3	neurexin III	0.880282	0.1999	0.4255
18477	Prdx1	peroxiredoxin 1	0.880282	0.1289	0.3276
18599	Padi1	peptidyl arginine deiminase, type I	0.880282	0.3503	0.5862
19655	Rbmx	RNA binding motif protein, X chromosome	0.880282	0.08665	NA
20510	Slc1a1	solute carrier family 1 (neuronal/epithelial high affinity glutamate transporter, system Xag), member 1	0.880282	0.3305	0.5671
20643	Snrpe	small nuclear ribonucleoprotein E	0.880282	0.0351	NA
20907	Stx1a	syntaxin 1A (brain)	0.880282	0.3337	0.5695
22286	Utf1	undifferentiated embryonic cell transcription factor 1	0.880282	0.3515	0.5869
50762	Fbxo6	F-box protein 6	0.880282	0.06114	NA
52846	D1Bwg0212e	DNA segment, Chr 1, Brigham & Women's Genetics 0212 expressed	0.880282	0.09184	NA
53618	Fut8	fucosyltransferase 8	0.880282	0.07624	NA
58991	Ghrl	ghrelin	0.880282	0.03607	NA
67530	Uqcrb	ubiquinol-cytochrome c reductase binding protein	0.880282	0.04437	NA
67785	Zmym4	zinc finger, MYM-type 4	0.880282	0.07012	NA
70028	Dopey2	dopey family member 2	0.880282	0.2136	0.4419
70297	Gcc2	GRIP and coiled-coil domain containing 2	0.880282	0.05134	NA
73862	4930415F15Rik	RIKEN cDNA 4930415F15 gene	0.880282	0.05651	NA
77980	Sbf1	SET binding factor 1	0.880282	0.3878	0.6197
78658	Ncapd3	non-SMC condensin II complex, subunit D3	0.880282	0.1502	0.3581
98445	AI844869	expressed sequence AI844869	0.880282	0.02572	NA
224630	Bnip1	BCL2/adenovirus E1B interacting protein 1	0.880282	0.44	0.6661
231832	Tmem184a	transmembrane protein 184a	0.880282	0.6879	0.8407
237362	Npffr1	neuropeptide FF receptor 1	0.880282	0.6656	0.8284
241112	Gm216	predicted gene 216	0.880282	0.4453	0.6709
100044193	LOC100044193	hypothetical protein LOC100044193	0.880282	0.1941	0.4177
11306	Abcb7	ATP-binding cassette, sub-family B (MDR/TAP), member 7	0.879507	0.2001	0.4256
11431	Acp1	acid phosphatase 1, soluble	0.879507	0.5524	0.7524
11655	Alas1	aminolevulinic acid synthase 1	0.879507	0.171	0.3865
18013	Neurod2	neurogenic differentiation 2	0.879507	0.05584	NA
18045	Nfyb	nuclear transcription factor-Y beta	0.879507	0.06785	NA
19270	Ptprg	protein tyrosine phosphatase, receptor type, G	0.879507	0.4779	0.6978
23830	Capn10	calpain 10	0.879507	0.127	0.3245
23958	Nr2e3	nuclear receptor subfamily 2, group E, member 3	0.879507	0.1244	0.321
50755	Fbxo18	F-box protein 18	0.879507	0.04053	NA
57444	Isg20	interferon-stimulated protein	0.879507	0.2013	0.4273
57750	Wdr12	WD repeat domain 12	0.879507	0.03534	NA
67695	Ost4	oligosaccharyltransferase 4 homolog (S. cerevisiae)	0.879507	0.132	0.3324
72121	Dennd2d	DENN/MADD domain containing 2D	0.879507	0.2546	0.4894
72181	Nsun4	NOL1/NOP2/Sun domain family, member 4	0.879507	0.4021	0.6321
74159	Acbd5	acyl-Coenzyme A binding domain containing 5	0.879507	0.1028	0.2843
77832	Tchp	trichoplein, keratin filament binding	0.879507	0.03122	NA
100317	AU040320	expressed sequence AU040320	0.879507	0.03604	NA
109959	Amy2a5	amylase 2a5	0.879507	0.3005	0.5365
209645	Bend7	BEN domain containing 7	0.879507	0.07989	NA
228777	Nrsn2	neurensin 2	0.879507	0.215	0.4437
229474	Fhdc1	FH2 domain containing 1	0.879507	0.3054	0.5419
231580	Gak	cyclin G associated kinase	0.879507	0.02892	NA
235633	Als2cl	ALS2 C-terminal like	0.879507	0.3683	0.6032

239364	Tspyl5	testis-specific protein, Y-encoded-like 5	0.879507	0.1883	0.4092
269994	Gsg1l	GSG1-like	0.879507	0.4979	0.7127
319653	Slc25a40	solute carrier family 25, member 40	0.879507	0.08227	NA
320080	Zbtb39	zinc finger and BTB domain containing 39	0.879507	0.1347	0.3357
327655	Ppip5k1	diphosphoinositol pentakisphosphate kinase 1	0.879507	0.3931	0.6243
330177	Taok3	TAO kinase 3	0.879507	0.123	0.319
666060	Frmpd1	FERM and PDZ domain containing 1	0.879507	0.1878	0.4087
100047123	LOC100047123	hypothetical protein LOC100047123	0.879507	0.3228	0.5592
100502705	LOC100502705	hypothetical LOC100502705	0.879507	0.09947	NA
11989	Slc7a3	solute carrier family 7 (cationic amino acid transporter, y+ system), member 3	0.878735	0.1415	0.3457
13219	Defa-rs10	defensin, alpha, related sequence 10	0.878735	0.09468	NA
14077	Fabp3	fatty acid binding protein 3, muscle and heart	0.878735	0.0339	NA
16413	Itgb1bp1	integrin beta 1 binding protein 1	0.878735	0.1695	0.3847
17718	ND3	NADH dehydrogenase subunit 3	0.878735	0.2152	0.4439
17954	Nap1l2	nucleosome assembly protein 1-like 2	0.878735	0.05432	NA
18587	Pde6b	phosphodiesterase 6B, cGMP, rod receptor, beta polypeptide	0.878735	0.1681	0.3831
19400	Rapsn	receptor-associated protein of the synapse	0.878735	0.4685	0.6904
20044	Rps14	ribosomal protein S14	0.878735	0.1687	0.3839
21417	Zeb1	zinc finger E-box binding homeobox 1	0.878735	0.08879	NA
22065	Trpc3	transient receptor potential cation channel, subfamily C, member 3	0.878735	0.05472	NA
50759	Fbxo16	F-box protein 16	0.878735	0.3756	0.6094
52710	Gpr172b	G protein-coupled receptor 172B	0.878735	0.214	0.4424
52850	Sgsm1	small G protein signaling modulator 1	0.878735	0.4577	0.6812
57259	Tob2	transducer of ERBB2, 2	0.878735	0.2988	0.5348
66291	1810030N24Rik	RIKEN cDNA 1810030N24 gene	0.878735	0.025	NA
66597	Trim13	tripartite motif-containing 13	0.878735	0.1042	0.2871
67150	Rnf141	ring finger protein 141	0.878735	0.04806	NA
67184	Ndufa13	NADH dehydrogenase (ubiquinone) 1 alpha subcomplex, 13	0.878735	0.3263	0.5628
67557	Larp6	La ribonucleoprotein domain family, member 6	0.878735	0.0451	NA
68693	Hnrnpul2	heterogeneous nuclear ribonucleoprotein U-like 2	0.878735	0.04305	NA
68737	Angel1	angel homolog 1 (Drosophila)	0.878735	0.08796	NA
71323	Rassf8	Ras association (RalGDS/AF-6) domain family (N-terminal) member 8	0.878735	0.1504	0.3583
73174	Tbkbp1	TBK1 binding protein 1	0.878735	0.2371	0.4693
75547	Akap13	A kinase (PRKA) anchor protein 13	0.878735	0.06334	NA
97351	E230006M18Rik	RIKEN cDNA E230006M18 gene	0.878735	0.1469	0.353
99939	BB085087	expressed sequence BB085087	0.878735	0.2855	0.5209
103136	Pwp1	PWP1 homolog (S. cerevisiae)	0.878735	0.06678	NA
103573	Xpo1	exportin 1, CRM1 homolog (yeast)	0.878735	0.0481	NA
108121	U2af1	U2 small nuclear ribonucleoprotein auxiliary factor (U2AF) 1	0.878735	0.02033	NA
114641	Rpl31	ribosomal protein L31	0.878735	0.154	0.3636
208606	Rsrc2	arginine/serine-rich coiled-coil 2	0.878735	0.1723	0.388
215193	AA408296	expressed sequence AA408296	0.878735	0.03086	NA
216892	Spns2	spinster homolog 2 (Drosophila)	0.878735	0.07158	NA
217705	Fam161b	family with sequence similarity 161, member B	0.878735	0.06906	NA
241525	Ypel4	yippee-like 4 (Drosophila)	0.878735	0.1051	0.2885
272031	E130309F12Rik	RIKEN cDNA E130309F12 gene	0.878735	0.2785	0.514
327814	Ppfia2	protein tyrosine phosphatase, receptor type, f polypeptide (PTPRF), interacting protein (liprin), alpha 2	0.878735	0.02591	NA
331461	Il1rapl1	interleukin 1 receptor accessory protein-like 1	0.878735	0.1261	0.3233
433022	Plcxd2	phosphatidylinositol-specific phospholipase C, X domain containing 2	0.878735	0.09308	NA

11946	Atp5a1	ATP synthase, H+ transporting, mitochondrial F1 complex, alpha subunit 1	0.877963	0.05908	NA
12050	Bcl2l2	BCL2-like 2	0.877963	0.8391	0.9274
13807	Eno2	enolase 2, gamma neuronal	0.877963	0.04631	NA
14007	Celf2	CUGBP, Elav-like family member 2	0.877963	0.2779	0.5135
14528	Gch1	GTP cyclohydrolase 1	0.877963	0.2506	0.4847
17193	Mbd4	methyl-CpG binding domain protein 4	0.877963	0.04902	NA
17880	Myh11	myosin, heavy polypeptide 11, smooth muscle	0.877963	0.445	0.6706
19769	Rit1	Ras-like without CAAX 1	0.877963	0.04051	NA
20394	Scg5	secretogranin V	0.877963	0.03586	NA
22129	Ttc3	tetratricopeptide repeat domain 3	0.877963	0.2419	0.4748
22275	Urod	uroporphyrinogen decarboxylase	0.877963	0.02566	NA
27061	Bcap31	B-cell receptor-associated protein 31	0.877963	0.0313	NA
30050	Fbxw2	F-box and WD-40 domain protein 2	0.877963	0.0567	NA
67059	Ola1	Obg-like ATPase 1	0.877963	0.04962	NA
67155	Smarca2	SWI/SNF related, matrix associated, actin dependent regulator of chromatin, subfamily a, member 2	0.877963	0.04571	NA
67416	Armcx2	armadillo repeat containing, X-linked 2	0.877963	0.04089	NA
67420	Far1	fatty acyl CoA reductase 1	0.877963	0.2326	0.4639
67618	Aasdhppt	aminoadipate-semialdehyde dehydrogenase-phosphopantetheinyl transferase	0.877963	0.1003	0.2798
69462	2300005B03Rik	RIKEN cDNA 2300005B03 gene	0.877963	0.4664	0.6885
70235	Poc1a	POC1 centriolar protein homolog A (Chlamydomonas)	0.877963	0.2273	0.4586
71810	Ranbp3	RAN binding protein 3	0.877963	0.04358	NA
74513	Neto2	neuropilin (NRP) and tolloid (TLL)-like 2	0.877963	0.2083	0.4349
78921	9130019O22Rik	RIKEN cDNA 9130019O22 gene	0.877963	0.4021	0.6321
104458	Rars	arginyl-tRNA synthetase	0.877963	0.3214	0.5578
105988	Espl1	extra spindle poles-like 1 (S. cerevisiae)	0.877963	0.3558	0.5906
109910	Zfp91	zinc finger protein 91	0.877963	0.1409	0.3447
114715	Spred1	sprouty protein with EVH-1 domain 1, related sequence	0.877963	0.181	0.3996
210982	BC032203	cDNA sequence BC032203	0.877963	0.3239	0.5603
227648	Sec16a	SEC16 homolog A (S. cerevisiae)	0.877963	0.03325	NA
231003	Klhl17	kelch-like 17 (Drosophila)	0.877963	0.1101	0.2974
231327	Ppat	phosphoribosyl pyrophosphate amidotransferase	0.877963	0.3277	0.5642
231915	Uspl1	ubiquitin specific peptidase like 1	0.877963	0.02339	NA
234413	Zfp961	zinc finger protein 961	0.877963	0.2737	0.5094
238328	Vash1	vasohibin 1	0.877963	0.02658	NA
330836	Slc7a6	solute carrier family 7 (cationic amino acid transporter, y+ system), member 6	0.877963	0.1045	0.2874
381112	Arhgef33	Rho guanine nucleotide exchange factor (GEF) 33	0.877963	0.3375	0.573
100134990	LOC100134990	selenoprotein K pseudogene	0.877963	0.139	0.3418
12300	Cacng2	calcium channel, voltage-dependent, gamma subunit 2	0.877193	0.291	0.5271
12371	Casp9	caspase 9	0.877193	0.05718	NA
12373	Casq2	calsequestrin 2	0.877193	0.133	0.3338
12631	Cfl1	cofilin 1, non-muscle	0.877193	0.1154	0.3062
12974	Cs	citrate synthase	0.877193	0.04612	NA
14169	Fgf14	fibroblast growth factor 14	0.877193	0.3731	0.6072
15184	Hdac5	histone deacetylase 5	0.877193	0.07401	NA
17301	Foxd2	forkhead box D2	0.877193	0.2037	0.43
19056	Ppp3cb	protein phosphatase 3, catalytic subunit, beta isoform	0.877193	0.07397	NA
19240	Tmsb10	thymosin, beta 10	0.877193	0.08684	NA
19358	Rad23a	RAD23a homolog (S. cerevisiae)	0.877193	0.3014	0.5372
21812	Tgfbr1	transforming growth factor, beta receptor I	0.877193	0.06667	NA

22643	Zfp101	zinc finger protein 101	0.877193	0.03339	NA
26896	Med14	mediator complex subunit 14	0.877193	0.02446	NA
26914	H2afy	H2A histone family, member Y	0.877193	0.2732	0.5088
27883	D16H22S680E	DNA segment, Chr 16, human D22S680E, expressed	0.877193	0.06238	NA
28105	Trim36	tripartite motif-containing 36	0.877193	0.05143	NA
53357	Pla2g6	phospholipase A2, group VI	0.877193	0.2935	0.5294
56470	Rgs19	regulator of G-protein signaling 19	0.877193	0.1024	0.2836
56699	Cdc42ep4	CDC42 effector protein (Rho GTPase binding) 4	0.877193	0.06314	NA
56741	Igdcc4	immunoglobulin superfamily, DCC subclass, member 4	0.877193	0.0911	NA
69549	2310009B15Rik	RIKEN cDNA 2310009B15 gene	0.877193	0.1814	0.4001
70052	Prpf4	PRP4 pre-mRNA processing factor 4 homolog (yeast)	0.877193	0.1441	0.349
71566	9030425E11Rik	RIKEN cDNA 9030425E11 gene	0.877193	0.1721	0.3878
72084	Pigx	phosphatidylinositol glycan anchor biosynthesis, class X	0.877193	0.04945	NA
72545	2700008G24Rik	RIKEN cDNA 2700008G24 gene	0.877193	0.05486	NA
73420	1700054N08Rik	RIKEN cDNA 1700054N08 gene	0.877193	0.09267	NA
83603	Elovl4	elongation of very long chain fatty acids (FEN1/Elo2, SUR4/Elo3, yeast)-like 4	0.877193	0.1416	0.3459
94061	Mrpl1	mitochondrial ribosomal protein L1	0.877193	0.05843	NA
108079	Prkaa2	protein kinase, AMP-activated, alpha 2 catalytic subunit	0.877193	0.1023	0.2835
217588	Mbip	MAP3K12 binding inhibitory protein 1	0.877193	0.09825	0.2765
224523	4732491K20Rik	RIKEN cDNA 4732491K20 gene	0.877193	0.1798	0.398
230654	Lrrc41	leucine rich repeat containing 41	0.877193	0.284	0.5198
231329	Polr2b	polymerase (RNA) II (DNA directed) polypeptide B	0.877193	0.08525	NA
231549	Lrrc8d	leucine rich repeat containing 8D	0.877193	0.1977	0.4225
231630	Ficd	FIC domain containing	0.877193	0.1307	0.3305
232836	Galp	galanin-like peptide	0.877193	0.5507	0.7511
233064	Wdr62	WD repeat domain 62	0.877193	0.4606	0.6837
239647	Fam113b	family with sequence similarity 113, member B	0.877193	0.08565	NA
320844	Amigo3	adhesion molecule with Ig like domain 3	0.877193	0.3096	0.5461
381404	Pabpc1l	poly(A) binding protein, cytoplasmic 1-like	0.877193	0.3427	0.5782
382245	Tmem29	transmembrane protein 29	0.877193	0.09905	0.2779
435145	AI848285	expressed sequence AI848285	0.877193	0.3116	0.5481
545055	Cma2	chymase 2, mast cell	0.877193	0.6433	0.8136
622404	Ccdc107	coiled-coil domain containing 107	0.877193	0.07657	NA
668917	LOC668917	hypothetical LOC668917	0.877193	0.5652	0.7618
11432	Acp2	acid phosphatase 2, lysosomal	0.876424	0.04451	NA
11640	Akap1	A kinase (PRKA) anchor protein 1	0.876424	0.09441	0.2701
11651	Akt1	thymoma viral proto-oncogene 1	0.876424	0.1736	0.3899
11792	Apex1	apurinic/apyrimidinic endonuclease 1	0.876424	0.1093	0.296
13405	Dmd	dystrophin, muscular dystrophy	0.876424	0.2698	0.5049
13476	Reep5	receptor accessory protein 5	0.876424	0.1551	0.3648
13859	Eps15l1	epidermal growth factor receptor pathway substrate 15-like 1	0.876424	0.05671	NA
14319	Fth1	ferritin heavy chain 1	0.876424	0.04539	NA
14545	Gdap1	ganglioside-induced differentiation-associated-protein 1	0.876424	0.1153	0.3061
17112	Tm4sf1	transmembrane 4 superfamily member 1	0.876424	0.1205	0.3143
17304	Mfge8	milk fat globule-EGF factor 8 protein	0.876424	0.1743	0.3908
18578	Pde4b	phosphodiesterase 4B, cAMP specific	0.876424	0.2514	0.4856
20912	Stxbp3a	syntaxin binding protein 3A	0.876424	0.04723	NA
21885	Tle1	transducin-like enhancer of split 1, homolog of Drosophila E(spl)	0.876424	0.09677	0.2741
26931	Ppp2r5c	protein phosphatase 2, regulatory subunit B (B56), gamma isoform	0.876424	0.531	0.7371

27999	Fam3c	family with sequence similarity 3, member C	0.876424	0.02264	NA
56424	Stub1	STIP1 homology and U-Box containing protein 1	0.876424	0.1183	0.3109
56447	Copz1	coatomer protein complex, subunit zeta 1	0.876424	0.07163	NA
58799	Crbn	cereblon	0.876424	0.3144	0.5508
66114	Dnajc30	DnaJ (Hsp40) homolog, subfamily C, member 30	0.876424	0.02236	NA
68040	Zfp593	zinc finger protein 593	0.876424	0.06752	NA
68671	Pcyt2	phosphate cytidylyltransferase 2, ethanolamine	0.876424	0.02416	NA
72654	Ccdc12	coiled-coil domain containing 12	0.876424	0.03189	NA
73432	1700061N14Rik	RIKEN cDNA 1700061N14 gene	0.876424	0.2159	0.4446
74126	Syvn1	synovial apoptosis inhibitor 1, synoviolin	0.876424	0.03544	NA
74769	Pik3cb	phosphatidylinositol 3-kinase, catalytic, beta polypeptide	0.876424	0.1072	0.2926
78202	4930557K07Rik	RIKEN cDNA 4930557K07 gene	0.876424	0.6551	0.8212
79059	Nme3	non-metastatic cells 3, protein expressed in	0.876424	0.1009	0.2809
106957	Slc39a6	solute carrier family 39 (metal ion transporter), member 6	0.876424	0.02737	NA
117146	Ube3b	ubiquitin protein ligase E3B	0.876424	0.05493	NA
216831	Arhgap44	Rho GTPase activating protein 44	0.876424	0.03944	NA
216987	Utp6	UTP6, small subunit (SSU) processome component, homolog (yeast)	0.876424	0.2051	0.4314
232333	Slc6a1	solute carrier family 6 (neurotransmitter transporter, GABA), member 1	0.876424	0.0495	NA
269878	Megf8	multiple EGF-like-domains 8	0.876424	0.07478	NA
338363	6030446N20Rik	RIKEN cDNA 6030446N20 gene	0.876424	0.28	0.5154
432516	Myo1a	myosin IA	0.876424	0.1502	0.3581
613254	AA465934	expressed sequence AA465934	0.876424	0.5462	0.7482
668212	Efr3b	EFR3 homolog B (S. cerevisiae)	0.876424	0.01787	NA
12723	Clcn1	chloride channel 1	0.875657	0.227	0.4584
13135	Dad1	defender against cell death 1	0.875657	0.09166	0.2654
13360	Dhcr7	7-dehydrocholesterol reductase	0.875657	0.303	0.539
16590	Kit	kit oncogene	0.875657	0.1676	0.3826
16969	Zbtb7a	zinc finger and BTB domain containing 7a	0.875657	0.01269	NA
18628	Per3	period homolog 3 (Drosophila)	0.875657	0.1632	0.3758
22025	Nr2c1	nuclear receptor subfamily 2, group C, member 1	0.875657	0.174	0.3905
26556	Homer1	homer homolog 1 (Drosophila)	0.875657	0.03139	NA
28017	D7Wsu130e	DNA segment, Chr 7, Wayne State University 130, expressed	0.875657	0.3265	0.563
66183	1110032A04Rik	RIKEN cDNA 1110032A04 gene	0.875657	0.4465	0.672
68952	Fam57b	family with sequence similarity 57, member B	0.875657	0.145	0.3505
74476	4933439C10Rik	RIKEN cDNA 4933439C10 gene	0.875657	0.2106	0.4379
76628	1700112J16Rik	RIKEN cDNA 1700112J16 gene	0.875657	0.08006	NA
77045	Bcl7a	B-cell CLL/lymphoma 7A	0.875657	0.02707	NA
97485	C88045	expressed sequence C88045	0.875657	0.2046	0.4309
98766	Ubac1	ubiquitin associated domain containing 1	0.875657	0.1257	0.3227
170752	Bco2	beta-carotene oxygenase 2	0.875657	0.04659	NA
170787	Hdac10	histone deacetylase 10	0.875657	0.2005	0.426
207565	Camkk2	calcium/calmodulin-dependent protein kinase kinase 2, beta	0.875657	0.0448	NA
231510	Agpat9	1-acylglycerol-3-phosphate O-acyltransferase 9	0.875657	0.1005	0.2803
233058	Zfp420	zinc finger protein 420	0.875657	0.2331	0.4643
381626	Rbm33	RNA binding motif protein 33	0.875657	0.1064	0.291
382137	Fdxacb1	ferredoxin-fold anticodon binding domain containing 1	0.875657	0.08701	NA
432842	LOC432842	hypothetical LOC432842	0.875657	0.2144	0.4427
11737	Anp32a	acidic (leucine-rich) nuclear phosphoprotein 32 family, member A	0.874891	0.1553	0.3651
11983	Atpif1	ATPase inhibitory factor 1	0.874891	0.04857	NA

12211	Birc6	baculoviral IAP repeat-containing 6	0.874891	0.08571	NA
12569	Cdk5r1	cyclin-dependent kinase 5, regulatory subunit 1 (p35)	0.874891	0.03228	NA
14026	Evl	Ena-vasodilator stimulated phosphoprotein	0.874891	0.02035	NA
16319	Incenp	inner centromere protein	0.874891	0.05328	NA
20256	Clec11a	C-type lectin domain family 11, member a	0.874891	0.2041	0.4304
21763	Tex2	testis expressed gene 2	0.874891	0.278	0.5137
21969	Top1	topoisomerase (DNA) I	0.874891	0.1923	0.4148
22064	Trpc2	transient receptor potential cation channel, subfamily C, member 2	0.874891	0.0487	NA
22690	Zfp28	zinc finger protein 28	0.874891	0.09626	0.2733
26385	Grk6	G protein-coupled receptor kinase 6	0.874891	0.2262	0.4575
28071	Twistnb	TWIST neighbor	0.874891	0.21	0.437
30944	Zfp354c	zinc finger protein 354C	0.874891	0.3456	0.5812
52513	Ddx56	DEAD (Asp-Glu-Ala-Asp) box polypeptide 56	0.874891	0.2268	0.4582
56017	Slc2a8	solute carrier family 2, (facilitated glucose transporter), member 8	0.874891	0.02126	NA
56070	Tcerg1	transcription elongation regulator 1 (CA150)	0.874891	0.1831	0.402
64453	Zfp280b	zinc finger protein 280B	0.874891	0.01641	NA
66419	Mrpl11	mitochondrial ribosomal protein L11	0.874891	0.04274	NA
66704	Rbm4b	RNA binding motif protein 4B	0.874891	0.04434	NA
66877	Crnkl1	Crn, crooked neck-like 1 (Drosophila)	0.874891	0.09884	0.2777
67881	Mdp1	magnesium-dependent phosphatase 1	0.874891	0.1191	0.312
68044	Chac2	ChaC, cation transport regulator homolog 2 (E. coli)	0.874891	0.05788	NA
68682	Slc44a2	solute carrier family 44, member 2	0.874891	0.04166	NA
72935	Ddx41	DEAD (Asp-Glu-Ala-Asp) box polypeptide 41	0.874891	0.2639	0.4999
73046	Glrx5	glutaredoxin 5 homolog (S. cerevisiae)	0.874891	0.03585	NA
74187	Katnb1	katanin p80 (WD40-containing) subunit B 1	0.874891	0.08184	NA
74354	Lrguk	leucine-rich repeats and guanylate kinase domain containing	0.874891	0.3913	0.623
77771	Csrnp3	cysteine-serine-rich nuclear protein 3	0.874891	0.06804	NA
94246	Arid4b	AT rich interactive domain 4B (RBP1-like)	0.874891	0.3461	0.5814
99296	Hrh3	histamine receptor H3	0.874891	0.2325	0.4638
100737	Dcun1d4	DCN1, defective in cullin neddylation 1, domain containing 4 (S. cerevisiae)	0.874891	0.1461	0.3517
108123	Napg	N-ethylmaleimide sensitive fusion protein attachment protein gamma	0.874891	0.1018	0.2826
108937	Rnf169	ring finger protein 169	0.874891	0.01205	NA
207921	A830093I24Rik	RIKEN cDNA A830093I24 gene	0.874891	0.4957	0.7111
216148	Shc2	SHC (Src homology 2 domain containing) transforming protein 2	0.874891	0.1363	0.3382
227731	Slc25a25	solute carrier family 25 (mitochondrial carrier, phosphate carrier), member 25	0.874891	0.127	0.3245
230935	Dnajc11	DnaJ (Hsp40) homolog, subfamily C, member 11	0.874891	0.2728	0.5084
233056	Zfp790	zinc finger protein 790	0.874891	0.1264	0.3236
235169	Foxred1	FAD-dependent oxidoreductase domain containing 1	0.874891	0.04252	NA
242773	Slc45a1	solute carrier family 45, member 1	0.874891	0.1449	0.3504
245190	Gm4980	predicted gene 4980	0.874891	0.4731	0.6939
321006	Vprbp	Vpr (HIV-1) binding protein	0.874891	0.356	0.5907
353170	Txlng	taxilin gamma	0.874891	0.03111	NA
399603	Fam84b	family with sequence similarity 84, member B	0.874891	0.3542	0.5889
665211	Gm14326	predicted gene 14326	0.874891	0.56	0.7586
100039286	Gm2138	predicted gene 2138	0.874891	0.1114	0.2993
100041585	Amd2	S-adenosylmethionine decarboxylase 2	0.874891	0.01832	NA
12301	Cacybp	calcyclin binding protein	0.874126	0.08373	NA
12823	Col19a1	collagen, type XIX, alpha 1	0.874126	0.2077	0.4342
13830	Stom	stomatin	0.874126	0.1288	0.3274

14104	Fasn	fatty acid synthase	0.874126	0.08642	0.2557
14464	Gata5	GATA binding protein 5	0.874126	0.3128	0.5493
14814	Grin2d	glutamate receptor, ionotropic, NMDA2D (epsilon 4)	0.874126	0.5254	0.7326
15901	Id1	inhibitor of DNA binding 1	0.874126	0.3087	0.5449
18211	Ntrk1	neurotrophic tyrosine kinase, receptor, type 1	0.874126	0.4151	0.6436
18516	Pbx3	pre B-cell leukemia transcription factor 3	0.874126	0.02988	NA
18709	Pik3r2	phosphatidylinositol 3-kinase, regulatory subunit, polypeptide 2 (p85 beta)	0.874126	0.3055	0.5419
21838	Thy1	thymus cell antigen 1, theta	0.874126	0.07497	NA
22186	Uba52	ubiquitin A-52 residue ribosomal protein fusion product 1	0.874126	0.1428	0.3475
22648	Zfp11	zinc finger protein 11	0.874126	0.1767	0.3938
50926	Hnrpdl	heterogeneous nuclear ribonucleoprotein D-like	0.874126	0.113	0.3022
52410	D8Ertd769e	DNA segment, Chr 8, ERATO Doi 769, expressed	0.874126	0.1793	0.3972
57743	Sec61a2	Sec61, alpha subunit 2 (S. cerevisiae)	0.874126	0.1813	0.4
66586	Crls1	cardiolipin synthase 1	0.874126	0.1825	0.4015
66776	Pisd-ps3	phosphatidylserine decarboxylase, pseudogene 3	0.874126	0.2242	0.4548
70008	Ace2	angiotensin I converting enzyme (peptidyl-dipeptidase A) 2	0.874126	0.5643	0.7615
74252	Armc1	armadillo repeat containing 1	0.874126	0.02041	NA
104885	Tmem179	transmembrane protein 179	0.874126	0.08586	0.2544
109652	Acy1	aminoacylase 1	0.874126	0.2319	0.4633
117589	Asb7	ankyrin repeat and SOCS box-containing 7	0.874126	0.377	0.611
194590	Reps2	RALBP1 associated Eps domain containing protein 2	0.874126	0.06135	NA
209003	Rbmx2	RNA binding motif protein, X-linked 2	0.874126	0.2489	0.4832
216810	Tom1l2	target of myb1-like 2 (chicken)	0.874126	0.1163	0.3077
230784	Sesn2	sestrin 2	0.874126	0.2496	0.4836
240174	Thada	thyroid adenoma associated	0.874126	0.3762	0.61
240613	9930021J03Rik	RIKEN cDNA 9930021J03 gene	0.874126	0.1396	0.3429
269695	Rnft2	ring finger protein, transmembrane 2	0.874126	0.04556	NA
319430	Gpr77	G protein-coupled receptor 77	0.874126	0.3355	0.5713
326622	Upf2	UPF2 regulator of nonsense transcripts homolog (yeast)	0.874126	0.09085	0.2639
434437	Amt	aminomethyltransferase	0.874126	0.2714	0.5068
100040724	Mirg	miRNA containing gene	0.874126	0.1215	0.3163
11891	Rab27a	RAB27A, member RAS oncogene family	0.873362	0.3968	0.6273
13645	Egf	epidermal growth factor	0.873362	0.1397	0.343
14776	Gpx2	glutathione peroxidase 2	0.873362	0.2545	0.4893
16648	Kpna3	karyopherin (importin) alpha 3	0.873362	0.04757	NA
18286	Odf2	outer dense fiber of sperm tails 2	0.873362	0.1943	0.4179
18597	Pdha1	pyruvate dehydrogenase E1 alpha 1	0.873362	0.122	0.317
19258	Ptpn4	protein tyrosine phosphatase, non-receptor type 4	0.873362	0.2903	0.5264
19275	Ptprn	protein tyrosine phosphatase, receptor type, N	0.873362	0.3916	0.623
54160	Copg2	coatomer protein complex, subunit gamma 2	0.873362	0.05179	NA
68453	Gpihbp1	GPI-anchored HDL-binding protein 1	0.873362	0.2809	0.5162
70134	2210011C24Rik	RIKEN cDNA 2210011C24 gene	0.873362	0.4884	0.7058
73571	1700096K18Rik	RIKEN cDNA 1700096K18 gene	0.873362	0.3312	0.5675
74251	Ankrd9	ankyrin repeat domain 9	0.873362	0.1529	0.3624
80748	BC004004	cDNA sequence BC004004	0.873362	0.1693	0.3843
97112	Nmd3	NMD3 homolog (S. cerevisiae)	0.873362	0.2087	0.4354
140484	Pofut1	protein O-fucosyltransferase 1	0.873362	0.3	0.5357
170461	Stard6	StAR-related lipid transfer (START) domain containing 6	0.873362	0.09343	0.2686
229055	Zbtb10	zinc finger and BTB domain containing 10	0.873362	0.1453	0.3507

235604	Camkv	CaM kinase-like vesicle-associated	0.873362	0.04615	NA
268297	Scml4	sex comb on midleg-like 4 (Drosophila)	0.873362	0.4583	0.6816
269788	Lhfpl4	lipoma HMGIC fusion partner-like protein 4	0.873362	0.2971	0.5332
277854	Depdc5	DEP domain containing 5	0.873362	0.5842	0.7746
353502	Hcfc1r1	host cell factor C1 regulator 1 (XPO1-dependent)	0.873362	0.1001	0.2795
381022	Mll2	myeloid/lymphoid or mixed-lineage leukemia 2	0.873362	0.157	0.3672
625249	Gpx4	glutathione peroxidase 4	0.873362	0.2516	0.4859
11790	Speg	SPEG complex locus	0.8726	0.02595	NA
11863	Arnt	aryl hydrocarbon receptor nuclear translocator	0.8726	0.03519	NA
16211	Kpnb1	karyopherin (importin) beta 1	0.8726	0.0834	0.2497
16467	Atcay	ataxia, cerebellar, Cayman type homolog (human)	0.8726	0.1692	0.3843
18514	Pbx1	pre B-cell leukemia transcription factor 1	0.8726	0.1572	0.3675
18530	Pcdh8	protocadherin 8	0.8726	0.1525	0.3616
20597	Smpd1	sphingomyelin phosphodiesterase 1, acid lysosomal	0.8726	0.1208	0.3149
26456	Sema4g	sema domain, immunoglobulin domain (Ig), transmembrane domain (TM) and short cytoplasmic domain, (semaphorin) 4G	0.8726	0.2458	0.4796
30853	Mlf2	myeloid leukemia factor 2	0.8726	0.08003	0.2433
50787	Hs6st3	heparan sulfate 6-O-sulfotransferase 3	0.8726	0.4173	0.6456
50790	Acsl4	acyl-CoA synthetase long-chain family member 4	0.8726	0.05833	NA
58244	Stx6	syntaxin 6	0.8726	0.04533	NA
66168	Grina	glutamate receptor, ionotropic, N-methyl D-aspartate-associated protein 1 (glutamate binding)	0.8726	0.2991	0.5348
66383	Iscu	IscU iron-sulfur cluster scaffold homolog (E. coli)	0.8726	0.02697	NA
66385	Ppp1r7	protein phosphatase 1, regulatory (inhibitor) subunit 7	0.8726	0.2163	0.4452
66865	Pmpca	peptidase (mitochondrial processing) alpha	0.8726	0.06371	NA
68971	1500001M20Rik	RIKEN cDNA 1500001M20 gene	0.8726	0.4723	0.6933
70615	Ankrd24	ankyrin repeat domain 24	0.8726	0.04437	NA
75137	Rprd2	regulation of nuclear pre-mRNA domain containing 2	0.8726	0.2538	0.4885
83922	Tsga14	testis specific gene A14	0.8726	0.01896	NA
114674	Gtf2ird2	GTF2I repeat domain containing 2	0.8726	0.08428	0.2513
209586	Nudcd3	NudC domain containing 3	0.8726	0.06635	NA
217733	Tmem63c	transmembrane protein 63c	0.8726	0.1653	0.379
225998	Rorb	RAR-related orphan receptor beta	0.8726	0.05893	NA
241528	Lrrc55	leucine rich repeat containing 55	0.8726	0.2773	0.513
241919	Slc7a14	solute carrier family 7 (cationic amino acid transporter, y+ system), member 14	0.8726	0.08986	0.262
245607	Gprasp2	G protein-coupled receptor associated sorting protein 2	0.8726	0.03618	NA
319909	Ism1	isthmin 1 homolog (zebrafish)	0.8726	0.1683	0.3833
320720	Fastkd1	FAST kinase domains 1	0.8726	0.1358	0.3375
360213	Trim46	tripartite motif-containing 46	0.8726	0.1237	0.3201
399548	Scn4b	sodium channel, type IV, beta	0.8726	0.0922	0.2661
432769	Zfp708	zinc finger protein 708	0.8726	0.5336	0.7392
544922	Zkscan4	zinc finger with KRAB and SCAN domains 4	0.8726	0.07637	NA
624219	Gm6484	predicted gene 6484	0.8726	0.4242	0.6519
100038734	Gm10845	predicted gene 10845	0.8726	0.2213	0.4513
12395	Runx1t1	runt-related transcription factor 1; translocated to, 1 (cyclin D-related)	0.87184	0.06843	NA
12416	Cbx2	chromobox homolog 2 (Drosophila Pc class)	0.87184	0.3754	0.6094
12617	Cenpc1	centromere protein C1	0.87184	0.08785	0.2586
13717	Eln	elastin	0.87184	0.4999	0.7144
18072	Nhlh2	nescient helix loop helix 2	0.87184	0.1232	0.3192
18633	Pex16	peroxisomal biogenesis factor 16	0.87184	0.1469	0.353
19158	Cyth2	cytohesin 2	0.87184	0.25	0.4841

20658	Son	Son DNA binding protein	0.87184	0.05392	NA
21418	Tcfap2a	transcription factor AP-2, alpha	0.87184	0.02171	NA
26386	Hsf4	heat shock transcription factor 4	0.87184	0.4782	0.6979
55988	Snx12	sorting nexin 12	0.87184	0.02428	NA
66061	Tctex1d2	Tctex1 domain containing 2	0.87184	0.09082	0.2639
66335	Atp6v1c1	ATPase, H+ transporting, lysosomal V1 subunit C1	0.87184	0.06124	NA
67666	Hapln3	hyaluronan and proteoglycan link protein 3	0.87184	0.08725	0.2575
69635	Dapk1	death associated protein kinase 1	0.87184	0.1333	0.3339
69792	Med6	mediator of RNA polymerase II transcription, subunit 6 homolog (yeast)	0.87184	0.09794	0.276
71973	Rbpms2	RNA binding protein with multiple splicing 2	0.87184	0.04619	NA
72313	Fryl	furry homolog-like (Drosophila)	0.87184	0.2004	0.4259
76654	Upp2	uridine phosphorylase 2	0.87184	0.1531	0.3627
77521	Mtus2	microtubule associated tumor suppressor candidate 2	0.87184	0.1041	0.2869
84095	Pi4k2a	phosphatidylinositol 4-kinase type 2 alpha	0.87184	0.07877	0.2407
170731	Mfn2	mitofusin 2	0.87184	0.02021	NA
212518	Sprn	shadow of prion protein	0.87184	0.1692	0.3843
215090	Maneal	mannosidase, endo-alpha-like	0.87184	0.03921	NA
231327	Ppat	phosphoribosyl pyrophosphate amidotransferase	0.87184	0.07993	0.2433
234086	Erich1	glutamate-rich 1	0.87184	0.1163	0.3077
269774	Aak1	AP2 associated kinase 1	0.87184	0.0141	NA
320737	4732416N19Rik	RIKEN cDNA 4732416N19 gene	0.87184	0.1589	0.3701
331401	Thoc2	THO complex 2	0.87184	0.3981	0.6286
629378	Dact3	dapper homolog 3, antagonist of beta-catenin (xenopus)	0.87184	0.05024	NA
667452	Dcdc2b	doublecortin domain containing 2b	0.87184	0.5378	0.7424
12296	Cacnb2	calcium channel, voltage-dependent, beta 2 subunit	0.87108	0.2128	0.4406
12307	Calb1	calbindin 1	0.87108	0.1369	0.3386
13204	Dhx15	DEAH (Asp-Glu-Ala-His) box polypeptide 15	0.87108	0.2174	0.4464
13728	Mark2	MAP/microtubule affinity-regulating kinase 2	0.87108	0.3863	0.6185
14618	Gjb1	gap junction protein, beta 1	0.87108	0.4801	0.6991
14860	Gsta4	glutathione S-transferase, alpha 4	0.87108	0.2251	0.4562
17524	Mpp1	membrane protein, palmitoylated	0.87108	0.02987	NA
17717	ND2	NADH dehydrogenase subunit 2	0.87108	0.2809	0.5162
17995	Ndufv1	NADH dehydrogenase (ubiquinone) flavoprotein 1	0.87108	0.1415	0.3457
18185	Nrl	neural retina leucine zipper gene	0.87108	0.08654	0.256
18545	Pcp2	Purkinje cell protein 2 (L7)	0.87108	0.1315	0.3316
27362	Dnajb9	DnaJ (Hsp40) homolog, subfamily B, member 9	0.87108	0.04289	NA
55982	Paxip1	PAX interacting (with transcription-activation domain) protein 1	0.87108	0.06157	NA
56442	Serinc1	serine incorporator 1	0.87108	0.06425	NA
66384	Srp19	signal recognition particle 19	0.87108	0.2428	0.4757
68832	1110057K04Rik	RIKEN cDNA 1110057K04 gene	0.87108	0.1015	0.2821
69709	2410017P09Rik	RIKEN cDNA 2410017P09 gene	0.87108	0.2618	0.4969
70719	Hmha1	histocompatibility (minor) HA-1	0.87108	0.06782	NA
71392	5430401F13Rik	RIKEN cDNA 5430401F13 gene	0.87108	0.3144	0.5508
73225	Fam118a	family with sequence similarity 118, member A	0.87108	0.1071	0.2924
74443	P4htm	prolyl 4-hydroxylase, transmembrane (endoplasmic reticulum)	0.87108	0.1802	0.3984
74694	Tbc1d30	TBC1 domain family, member 30	0.87108	0.1531	0.3627
76686	Clip3	CAP-GLY domain containing linker protein 3	0.87108	0.04493	NA
81896	Ift122	intraflagellar transport 122 homolog (Chlamydomonas)	0.87108	0.04287	NA
110854	Ppp2r4	protein phosphatase 2A, regulatory subunit B (PR 53)	0.87108	0.2738	0.5094

170748	BC017612	cDNA sequence BC017612	0.87108	0.057	NA
228998	Arfgap1	ADP-ribosylation factor GTPase activating protein 1	0.87108	0.2674	0.503
232975	Atp1a3	ATPase, Na+/K+ transporting, alpha 3 polypeptide	0.87108	0.3195	0.556
235134	Nfrkb	nuclear factor related to kappa B binding protein	0.87108	0.05464	NA
241075	Plekhm3	pleckstrin homology domain containing, family M, member 3	0.87108	0.01837	NA
245537	Nlgn3	neuroligin 3	0.87108	0.281	0.5162
268706	Slc38a9	solute carrier family 38, member 9	0.87108	0.0304	NA
320191	Hook3	hook homolog 3 (Drosophila)	0.87108	0.1357	0.3374
320376	Bcorl1	BCL6 co-repressor-like 1	0.87108	0.07795	0.2393
381352	Mamdc4	MAM domain containing 4	0.87108	0.189	0.4103
381813	Prmt8	protein arginine N-methyltransferase 8	0.87108	0.0826	0.2479
399612	9630010G10Rik	RIKEN cDNA 9630010G10 gene	0.87108	0.3275	0.564
11864	Arnt2	aryl hydrocarbon receptor nuclear translocator 2	0.870322	0.4981	0.7127
11909	Atf2	activating transcription factor 2	0.870322	0.03155	NA
12569	Cdk5r1	cyclin-dependent kinase 5, regulatory subunit 1 (p35)	0.870322	0.02199	NA
14149	Fdxr	ferredoxin reductase	0.870322	0.0314	NA
14154	Fem1a	feminization 1 homolog a (C. elegans)	0.870322	0.05702	NA
14615	Gjc1	gap junction protein, gamma 1	0.870322	0.06997	NA
15558	Htr2a	5-hydroxytryptamine (serotonin) receptor 2A	0.870322	0.1267	0.3241
17218	Mcm5	minichromosome maintenance deficient 5, cell division cycle 46 (S. cerevisiae)	0.870322	0.2109	0.4383
18046	Nfyc	nuclear transcription factor-Y gamma	0.870322	0.1073	0.2928
18604	Pdk2	pyruvate dehydrogenase kinase, isoenzyme 2	0.870322	0.08375	0.2503
19384	Ran	RAN, member RAS oncogene family	0.870322	0.01467	NA
20924	Supt5h	suppressor of Ty 5 homolog (S. cerevisiae)	0.870322	0.04709	NA
22770	Zhx1	zinc fingers and homeoboxes 1	0.870322	0.05551	NA
26379	Esrra	estrogen related receptor, alpha	0.870322	0.06026	NA
54375	Azin1	antizyme inhibitor 1	0.870322	0.0251	NA
58996	Arhgap23	Rho GTPase activating protein 23	0.870322	0.07847	0.24
67010	Rbm7	RNA binding motif protein 7	0.870322	0.1282	0.3264
68852	Lrrn4cl	LRRN4 C-terminal like	0.870322	0.0581	NA
70974	Pgm2l1	phosphoglucomutase 2-like 1	0.870322	0.06372	NA
71296	4933436C20Rik	RIKEN cDNA 4933436C20 gene	0.870322	0.1909	0.413
71599	Senp8	SUMO/sentrin specific peptidase 8	0.870322	0.186	0.406
71710	Lrrcc1	leucine rich repeat and coiled-coil domain containing 1	0.870322	0.09706	0.2746
72662	Dis3	DIS3 mitotic control homolog (S. cerevisiae)	0.870322	0.05215	NA
74521	Ppp4r4	protein phosphatase 4, regulatory subunit 4	0.870322	0.09196	0.2658
75607	Wnk2	WNK lysine deficient protein kinase 2	0.870322	0.04231	NA
93834	Peli2	pellino 2	0.870322	0.02614	NA
211535	Ccdc114	coiled-coil domain containing 114	0.870322	0.3131	0.5494
224694	Zfp81	zinc finger protein 81	0.870322	0.3331	0.569
225028	Map4k3	mitogen-activated protein kinase kinase kinase kinase 3	0.870322	0.01695	NA
231214	Cc2d2a	coiled-coil and C2 domain containing 2A	0.870322	0.0666	NA
235956	Zfp825	zinc finger protein 825	0.870322	0.025	NA
245944	Vps54	vacuolar protein sorting 54 (yeast)	0.870322	0.07846	0.24
382620	Tmed8	transmembrane emp24 domain containing 8	0.870322	0.09144	0.265
622512	Gm6328	predicted gene 6328	0.870322	0.3676	0.6026
12293	Cacna2d1	calcium channel, voltage-dependent, alpha2/delta subunit 1	0.869565	0.05793	NA
12326	Camk4	calcium/calmodulin-dependent protein kinase IV	0.869565	0.07448	0.2329
12351	Car4	carbonic anhydrase 4	0.869565	0.3522	0.5874

16495	Kcna7	potassium voltage-gated channel, shaker-related subfamily, member 7	0.869565	0.4615	0.6845
18010	Neu1	neuraminidase 1	0.869565	0.0153	NA
18707	Pik3cd	phosphatidylinositol 3-kinase catalytic delta polypeptide	0.869565	0.2341	0.4655
19647	Rbbp6	retinoblastoma binding protein 6	0.869565	0.1314	0.3316
20975	Synj2	synaptojanin 2	0.869565	0.2746	0.5101
22067	Trpc5	transient receptor potential cation channel, subfamily C, member 5	0.869565	0.3355	0.5713
22141	Tub	tubby candidate gene	0.869565	0.007193	NA
22178	Tyrp1	tyrosinase-related protein 1	0.869565	0.6767	0.8343
23969	Pacsin1	protein kinase C and casein kinase substrate in neurons 1	0.869565	0.2164	0.4452
57314	Th1l	TH1-like homolog (Drosophila)	0.869565	0.2354	0.4675
59015	Nup160	nucleoporin 160	0.869565	0.04455	NA
66293	1810032O08Rik	RIKEN cDNA 1810032O08 gene	0.869565	0.07022	0.2244
66388	Cutc	cutC copper transporter homolog (E.coli)	0.869565	0.2374	0.4696
67239	Rpf2	ribosome production factor 2 homolog (S. cerevisiae)	0.869565	0.0635	NA
68839	Ankrd46	ankyrin repeat domain 46	0.869565	0.1937	0.417
69137	2200002K05Rik	RIKEN cDNA 2200002K05 gene	0.869565	0.04121	NA
69539	Trnp1	TMF1-regulated nuclear protein 1	0.869565	0.1258	0.3229
72308	Brf1	BRF1 homolog, subunit of RNA polymerase III transcription initiation factor IIIB (S. cerevisiae)	0.869565	0.05775	NA
74197	Gtf2e1	general transcription factor II E, polypeptide 1 (alpha subunit)	0.869565	0.03587	NA
75785	Klhl24	kelch-like 24 (Drosophila)	0.869565	0.234	0.4655
83674	Cnnm1	cyclin M1	0.869565	0.05783	NA
93686	Rbfox2	RNA binding protein, fox-1 homolog (C. elegans) 2	0.869565	0.1207	0.3149
103210	D630004K10Rik	RIKEN cDNA D630004K10 gene	0.869565	0.1787	0.3966
104718	Ttc7b	tetratricopeptide repeat domain 7B	0.869565	0.01429	NA
107934	Celsr3	cadherin, EGF LAG seven-pass G-type receptor 3 (flamingo homolog, Drosophila)	0.869565	0.04691	NA
140742	Sesn1	sestrin 1	0.869565	0.02288	NA
227334	Usp40	ubiquitin specific peptidase 40	0.869565	0.1236	0.3199
230233	Ikbkap	inhibitor of kappa light polypeptide enhancer in B-cells, kinase complex-associated protein	0.869565	0.01424	NA
232875	Zscan18	zinc finger and SCAN domain containing 18	0.869565	0.03237	NA
319477	6030419C18Rik	RIKEN cDNA 6030419C18 gene	0.869565	0.3842	0.6167
100042150	Nrg2	neuregulin 2	0.869565	0.09117	0.2645
11735	Ank3	ankyrin 3, epithelial	0.86881	0.007694	NA
13018	Ctcf	CCCTC-binding factor	0.86881	0.05773	NA
14270	Srgap2	SLIT-ROBO Rho GTPase activating protein 2	0.86881	0.2961	0.5318
15081	H3f3b	H3 histone, family 3B	0.86881	0.1161	0.3075
15114	Hap1	huntingtin-associated protein 1	0.86881	0.2562	0.4913
17535	Mre11a	meiotic recombination 11 homolog A (S. cerevisiae)	0.86881	0.06058	NA
21885	Tle1	transducin-like enhancer of split 1, homolog of Drosophila E(spl)	0.86881	0.1658	0.3797
21987	Tpd52l1	tumor protein D52-like 1	0.86881	0.251	0.4851
22066	Trpc4	transient receptor potential cation channel, subfamily C, member 4	0.86881	0.05383	NA
22173	Tyr	tyrosinase	0.86881	0.5307	0.7369
22368	Trpv2	transient receptor potential cation channel, subfamily V, member 2	0.86881	0.1414	0.3456
22634	Plagl1	pleiomorphic adenoma gene-like 1	0.86881	0.1964	0.4206
50795	Sh3bgr	SH3-binding domain glutamic acid-rich protein	0.86881	0.06829	0.2207
52323	Klhl7	kelch-like 7 (Drosophila)	0.86881	0.06239	NA
56370	Tagln3	transgelin 3	0.86881	0.04301	NA
58249	Fibp	fibroblast growth factor (acidic) intracellular binding protein	0.86881	0.1211	0.3154
66125	Sf3b5	splicing factor 3b, subunit 5	0.86881	0.01185	NA
66801	Prkrip1	Prkr interacting protein 1 (IL11 inducible)	0.86881	0.1382	0.3407

67414	Mfn1	mitofusin 1	0.86881	0.03549	NA
67618	Aasdhppt	aminoadipate-semialdehyde dehydrogenase-phosphopantetheinyl transferase	0.86881	0.09878	0.2776
68332	Sdhaf1	succinate dehydrogenase complex assembly factor 1	0.86881	0.0196	NA
68644	Abhd14a	abhydrolase domain containing 14A	0.86881	0.1043	0.2871
68897	Disp1	dispatched homolog 1 (Drosophila)	0.86881	0.1388	0.3418
69035	Zdhhc3	zinc finger, DHHC domain containing 3	0.86881	0.08884	0.2605
72084	Pigx	phosphatidylinositol glycan anchor biosynthesis, class X	0.86881	0.02773	NA
72930	Ppp2r2b	protein phosphatase 2 (formerly 2A), regulatory subunit B (PR 52), beta isoform	0.86881	0.1388	0.3417
75410	Wbp7	WW domain binding protein 7	0.86881	0.02048	NA
78755	Fam122b	family with sequence similarity 122, member B	0.86881	0.06205	NA
79263	Trim39	tripartite motif-containing 39	0.86881	0.02974	NA
81004	Tbl1xr1	transducin (beta)-like 1X-linked receptor 1	0.86881	0.05566	NA
106840	Unc119b	unc-119 homolog B (C. elegans)	0.86881	0.3313	0.5675
208228	Mobkl2a	MOB1, Mps One Binder kinase activator-like 2A (yeast)	0.86881	0.2767	0.5123
209707	Lcorl	ligand dependent nuclear receptor corepressor-like	0.86881	0.2439	0.4774
210573	Tmem151b	transmembrane protein 151B	0.86881	0.01495	NA
226352	Epb4.1l5	erythrocyte protein band 4.1-like 5	0.86881	0.2922	0.5281
246103	Atxn7	ataxin 7	0.86881	0.3551	0.5898
257917	Olfr314	olfactory receptor 314	0.86881	0.7537	0.8812
552906	LOC552906	hypothetical LOC552906	0.86881	0.175	0.3917
12700	Cish	cytokine inducible SH2-containing protein	0.868056	0.1273	0.325
16531	Kcnma1	potassium large conductance calcium-activated channel, subfamily M, alpha member 1	0.868056	0.1076	0.2932
16647	Kpna2	karyopherin (importin) alpha 2	0.868056	0.07077	0.2255
16775	Lama4	laminin, alpha 4	0.868056	0.3874	0.6195
17764	Mtf1	metal response element binding transcription factor 1	0.868056	0.02682	NA
18245	Oaz1	ornithine decarboxylase antizyme 1	0.868056	0.2199	0.4497
19335	Rab23	RAB23, member RAS oncogene family	0.868056	0.2733	0.5091
19395	Rasgrp2	RAS, guanyl releasing protein 2	0.868056	0.07392	0.232
20450	St8sia2	ST8 alpha-N-acetyl-neuraminide alpha-2,8-sialyltransferase 2	0.868056	0.2606	0.4959
20732	Spint1	serine protease inhibitor, Kunitz type 1	0.868056	0.4493	0.6746
20778	Scarb1	scavenger receptor class B, member 1	0.868056	0.1395	0.3429
29861	Dpf1	D4, zinc and double PHD fingers family 1	0.868056	0.08243	0.2477
50758	Fbxl17	F-box and leucine-rich repeat protein 17	0.868056	0.0854	0.2535
52708	Zfp410	zinc finger protein 410	0.868056	0.01256	NA
56697	Akap10	A kinase (PRKA) anchor protein 10	0.868056	0.227	0.4584
63859	Impg1	interphotoreceptor matrix proteoglycan 1	0.868056	0.3043	0.5405
66437	Fis1	fission 1 (mitochondrial outer membrane) homolog (yeast)	0.868056	0.02053	NA
66797	Cntnap2	contactin associated protein-like 2	0.868056	0.05931	NA
67455	Klhl13	kelch-like 13 (Drosophila)	0.868056	0.1344	0.3353
67824	Nmral1	NmrA-like family domain containing 1	0.868056	0.06491	0.2134
67878	Tmem33	transmembrane protein 33	0.868056	0.04909	NA
70650	Zcchc8	zinc finger, CCHC domain containing 8	0.868056	0.03308	NA
71027	Tmem30c	transmembrane protein 30C	0.868056	0.1604	0.3721
72023	Cyb561d1	cytochrome b-561 domain containing 1	0.868056	0.1743	0.3908
72865	Cxx1c	CAAX box 1 homolog C (human)	0.868056	0.2201	0.45
72961	Slc17a7	solute carrier family 17 (sodium-dependent inorganic phosphate cotransporter), member 7	0.868056	0.4386	0.6647
74286	Tbc1d21	TBC1 domain family, member 21	0.868056	0.4742	0.6948
74635	4930425O10Rik	RIKEN cDNA 4930425O10 gene	0.868056	0.3369	0.5726
94187	Zfp423	zinc finger protein 423	0.868056	0.1886	0.4097

102920	Cenpi	centromere protein I	0.868056	0.4863	0.7045
105835	Sgsm3	small G protein signaling modulator 3	0.868056	0.2041	0.4304
192654	Pla2g15	phospholipase A2, group XV	0.868056	0.1081	0.2941
210108	D130043K22Rik	RIKEN cDNA D130043K22 gene	0.868056	0.2601	0.4954
212647	Aldh4a1	aldehyde dehydrogenase 4 family, member A1	0.868056	0.04484	NA
217473	Ankmy2	ankyrin repeat and MYND domain containing 2	0.868056	0.2052	0.4314
224014	Fgd4	FYVE, RhoGEF and PH domain containing 4	0.868056	0.04878	NA
226105	Cyp2c70	cytochrome P450, family 2, subfamily c, polypeptide 70	0.868056	0.509	0.72
228357	Lrp4	low density lipoprotein receptor-related protein 4	0.868056	0.008015	NA
235527	Plscr4	phospholipid scramblase 4	0.868056	0.4852	0.7036
241694	Ralgapa2	Ral GTPase activating protein, alpha subunit 2 (catalytic)	0.868056	0.2052	0.4314
319864	D230035N22Rik	RIKEN cDNA D230035N22 gene	0.868056	0.7092	0.8542
320351	D230037D09Rik	RIKEN cDNA D230037D09 gene	0.868056	0.07579	0.2357
11749	Anxa6	annexin A6	0.867303	0.1742	0.3907
15081	H3f3b	H3 histone, family 3B	0.867303	0.08844	0.2598
16524	Kcnj9	potassium inwardly-rectifying channel, subfamily J, member 9	0.867303	0.04244	NA
18173	Slc11a1	solute carrier family 11 (proton-coupled divalent metal ion transporters), member 1	0.867303	0.3222	0.5585
21335	Tacc3	transforming, acidic coiled-coil containing protein 3	0.867303	0.09156	0.2652
23937	Mab21l2	mab-21-like 2 (C. elegans)	0.867303	0.1821	0.4009
29863	Pde7b	phosphodiesterase 7B	0.867303	0.3146	0.5509
30059	Timm10	translocase of inner mitochondrial membrane 10 homolog (yeast)	0.867303	0.0355	NA
50776	Polg2	polymerase (DNA directed), gamma 2, accessory subunit	0.867303	0.2724	0.5079
67057	Yaf2	YY1 associated factor 2	0.867303	0.01993	NA
68666	Svop	SV2 related protein	0.867303	0.09569	0.2723
68760	Synpo2l	synaptopodin 2-like	0.867303	0.3838	0.6164
76205	Stard3nl	STARD3 N-terminal like	0.867303	0.06015	NA
76380	Ccdc46	coiled-coil domain containing 46	0.867303	0.1468	0.353
78785	Clip4	CAP-GLY domain containing linker protein family, member 4	0.867303	0.2095	0.4364
140492	Kcnn2	potassium intermediate/small conductance calcium-activated channel, subfamily N, member 2	0.867303	0.03127	NA
217265	Abca5	ATP-binding cassette, sub-family A (ABC1), member 5	0.867303	0.06836	0.2209
233208	Scaf1	SR-related CTD-associated factor 1	0.867303	0.2051	0.4314
242506	Frmd3	FERM domain containing 3	0.867303	0.05353	NA
260409	Cdc42ep3	CDC42 effector protein (Rho GTPase binding) 3	0.867303	0.006832	NA
320100	Relt	RELT tumor necrosis factor receptor	0.867303	0.2391	0.4717
100039684	5031434O11Rik	RIKEN cDNA 5031434O11 gene	0.867303	0.1868	0.4075
11514	Adcy8	adenylate cyclase 8	0.866551	0.2421	0.4751
12298	Cacnb4	calcium channel, voltage-dependent, beta 4 subunit	0.866551	0.1327	0.3333
14391	Gabpb1	GA repeat binding protein, beta 1	0.866551	0.1694	0.3845
14461	Gata2	GATA binding protein 2	0.866551	0.2666	0.5021
15369	Hmox2	heme oxygenase (decycling) 2	0.866551	0.06276	0.2092
16364	Irf4	interferon regulatory factor 4	0.866551	0.4832	0.7014
17690	Msi1	Musashi homolog 1(Drosophila)	0.866551	0.2293	0.4605
18647	Cdk14	cyclin-dependent kinase 14	0.866551	0.2979	0.5337
20671	Sox17	SRY-box containing gene 17	0.866551	0.2119	0.4396
20672	Sox18	SRY-box containing gene 18	0.866551	0.314	0.5505
21823	Th	tyrosine hydroxylase	0.866551	0.09009	0.2626
54447	Asah2	N-acylsphingosine amidohydrolase 2	0.866551	0.3085	0.5448
58208	Bcl11b	B-cell leukemia/lymphoma 11B	0.866551	0.5068	0.7186
66086	0610037P05Rik	RIKEN cDNA 0610037P05 gene	0.866551	0.01017	NA

68053	Ubxn2b	UBX domain protein 2B	0.866551	0.03667	NA
68263	Pdhb	pyruvate dehydrogenase (lipoamide) beta	0.866551	0.1007	0.2806
68631	Cryl1	crystallin, lambda 1	0.866551	0.2238	0.4543
69349	1700008O03Rik	RIKEN cDNA 1700008O03 gene	0.866551	0.2325	0.4638
71907	Serpina9	serine (or cysteine) peptidase inhibitor, clade A (alpha-1 antiproteinase, antitrypsin), member 9	0.866551	0.5432	0.7465
73834	Atp6v1d	ATPase, H+ transporting, lysosomal V1 subunit D	0.866551	0.02463	NA
74267	Iqcf1	IQ motif containing F1	0.866551	0.1281	0.3263
83797	Smarcd1	SWI/SNF related, matrix associated, actin dependent regulator of chromatin, subfamily d, member 1	0.866551	0.3214	0.5579
102954	Nudt10	nudix (nucleoside diphosphate linked moiety X)-type motif 10	0.866551	0.02688	NA
207182	Ggt7	gamma-glutamyltransferase 7	0.866551	0.1376	0.3395
208258	Ankrd33	ankyrin repeat domain 33	0.866551	0.2795	0.515
214158	Trim38	tripartite motif-containing 38	0.866551	0.06677	0.2174
216177	AU041133	expressed sequence AU041133	0.866551	0.08787	0.2586
231876	Lmtk2	lemur tyrosine kinase 2	0.866551	0.5735	0.7672
236792	Mmgt1	membrane magnesium transporter 1	0.866551	0.1255	0.3225
243168	Hsd17b13	hydroxysteroid (17-beta) dehydrogenase 13	0.866551	0.2931	0.5292
269615	Plch2	phospholipase C, eta 2	0.866551	0.02026	NA
330941	AI593442	expressed sequence AI593442	0.866551	0.04191	NA
434156	Eid2b	EP300 interacting inhibitor of differentiation 2B	0.866551	0.06728	0.2188
100040346	Gm2724	predicted gene 2724	0.866551	0.5585	0.7573
100045292	LOC100045292	hypothetical protein LOC100045292	0.866551	0.05818	NA
12858	Cox5a	cytochrome c oxidase, subunit Va	0.865801	0.009649	NA
14806	Grik2	glutamate receptor, ionotropic, kainate 2 (beta 2)	0.865801	0.1201	0.3139
19240	Tmsb10	thymosin, beta 10	0.865801	0.07757	0.2389
20383	Srsf3	serine/arginine-rich splicing factor 3	0.865801	0.04675	NA
21981	Ppp1r13b	protein phosphatase 1, regulatory (inhibitor) subunit 13B	0.865801	0.06736	0.2189
26420	Mapk9	mitogen-activated protein kinase 9	0.865801	0.2569	0.492
27277	Golga5	golgi autoantigen, golgin subfamily a, 5	0.865801	0.009399	NA
54126	Arhgef7	Rho guanine nucleotide exchange factor (GEF7)	0.865801	0.02568	NA
54612	Sfrp5	secreted frizzled-related sequence protein 5	0.865801	0.2793	0.5149
54650	Sfmbt1	Scm-like with four mbt domains 1	0.865801	0.1517	0.3603
56279	Fam69b	family with sequence similarity 69, member B	0.865801	0.08496	0.2526
56364	Zmym3	zinc finger, MYM-type 3	0.865801	0.03739	NA
58988	Rps6kb2	ribosomal protein S6 kinase, polypeptide 2	0.865801	0.2578	0.4928
59058	Bhlhe22	basic helix-loop-helix family, member e22	0.865801	0.1595	0.3709
67391	Fundc2	FUN14 domain containing 2	0.865801	0.104	0.2865
69379	C8g	complement component 8, gamma polypeptide	0.865801	0.1189	0.3118
70503	Ddo	D-aspartate oxidase	0.865801	0.1177	0.3099
75974	Dock11	dedicator of cytokinesis 11	0.865801	0.07387	0.232
98682	Mfsd6	major facilitator superfamily domain containing 6	0.865801	0.1952	0.4189
105351	AW209491	expressed sequence AW209491	0.865801	0.02342	NA
109979	Art3	ADP-ribosyltransferase 3	0.865801	0.1182	0.3107
192198	Lrrc4	leucine rich repeat containing 4	0.865801	0.02016	NA
214597	Sidt2	SID1 transmembrane family, member 2	0.865801	0.04719	NA
217648	Gm527	predicted gene 527	0.865801	0.05587	NA
218194	Phactr1	phosphatase and actin regulator 1	0.865801	0.01261	NA
218460	Wdr41	WD repeat domain 41	0.865801	0.03121	NA
219189	1300010F03Rik	RIKEN cDNA 1300010F03 gene	0.865801	0.1708	0.3865
228998	Arfgap1	ADP-ribosylation factor GTPase activating protein 1	0.865801	0.169	0.3842

230603	Ttc39a	tetratricopeptide repeat domain 39A	0.865801	0.04595	NA
240665	Ccnj	cyclin J	0.865801	0.1566	0.3667
270893	Tmem132e	transmembrane protein 132E	0.865801	0.1925	0.415
280668	Adam1a	a disintegrin and metallopeptidase domain 1a	0.865801	0.04532	NA
381067	Zfp229	zinc finger protein	0.865801	0.1131	0.3023
11307	Abcg1	ATP-binding cassette, sub-family G (WHITE), member 1	0.865052	0.006203	NA
13170	Dbp	D site albumin promoter binding protein	0.865052	0.2379	0.4702
14232	Fkbp8	FK506 binding protein 8	0.865052	0.06563	0.2149
14702	Gng2	guanine nucleotide binding protein (G protein), gamma 2	0.865052	0.08123	0.2452
14738	Gpr12	G-protein coupled receptor 12	0.865052	0.04276	NA
16201	Ilf3	interleukin enhancer binding factor 3	0.865052	0.1108	0.2985
16371	Irx1	Iroquois related homeobox 1 (Drosophila)	0.865052	0.2233	0.4537
16451	Jak1	Janus kinase 1	0.865052	0.05398	NA
17318	Mid1	midline 1	0.865052	0.2501	0.4841
17850	Mut	methylmalonyl-Coenzyme A mutase	0.865052	0.008144	NA
18183	Nrg3	neuregulin 3	0.865052	0.3697	0.6046
19014	Med1	mediator complex subunit 1	0.865052	0.2151	0.4438
19708	Dpf2	D4, zinc and double PHD fingers family 2	0.865052	0.03734	NA
20528	Slc2a4	solute carrier family 2 (facilitated glucose transporter), member 4	0.865052	0.1437	0.3486
23859	Dlg2	discs, large homolog 2 (Drosophila)	0.865052	0.1619	0.3741
52202	Rbm34	RNA binding motif protein 34	0.865052	0.09027	0.263
53892	Ppm1d	protein phosphatase 1D magnesium-dependent, delta isoform	0.865052	0.02081	NA
66194	Pycrl	pyrroline-5-carboxylate reductase-like	0.865052	0.1292	0.328
66686	Dcbld1	discoidin, CUB and LCCL domain containing 1	0.865052	0.06274	0.2092
67298	Gprasp1	G protein-coupled receptor associated sorting protein 1	0.865052	0.01664	NA
67770	5830433M19Rik	RIKEN cDNA 5830433M19 gene	0.865052	0.1	0.2792
67893	Tmem86a	transmembrane protein 86A	0.865052	0.1016	0.2824
74011	Slc25a27	solute carrier family 25, member 27	0.865052	0.02767	NA
78330	Ndufv3	NADH dehydrogenase (ubiquinone) flavoprotein 3	0.865052	0.239	0.4717
99138	Stard7	START domain containing 7	0.865052	0.04846	NA
109880	Braf	Braf transforming gene	0.865052	0.04862	NA
110902	Chrna2	cholinergic receptor, nicotinic, alpha polypeptide 2 (neuronal)	0.865052	0.2022	0.4284
116873	Stim2	stromal interaction molecule 2	0.865052	0.1884	0.4095
170787	Hdac10	histone deacetylase 10	0.865052	0.115	0.3055
227707	BC005624	cDNA sequence BC005624	0.865052	0.0415	NA
228564	Frmd5	FERM domain containing 5	0.865052	0.01897	NA
242748	Ptchd2	patched domain containing 2	0.865052	0.2542	0.4891
245007	Zbtb38	zinc finger and BTB domain containing 38	0.865052	0.2186	0.4478
260315	Nav3	neuron navigator 3	0.865052	0.218	0.4472
331474	Rgag4	retrotransposon gag domain containing 4	0.865052	0.1428	0.3475
442801	Arhgef15	Rho guanine nucleotide exchange factor (GEF) 15	0.865052	0.447	0.6724
641376	Tomm40l	translocase of outer mitochondrial membrane 40 homolog-like (yeast)	0.865052	0.02501	NA
13589	Mapre1	microtubule-associated protein, RP/EB family, member 1	0.864304	0.07313	0.2304
13803	Enc1	ectodermal-neural cortex 1	0.864304	0.1225	0.3182
14245	Lpin1	lipin 1	0.864304	0.09084	0.2639
14405	Gabrg1	gamma-aminobutyric acid (GABA) A receptor, subunit gamma 1	0.864304	0.08146	0.2458
14786	Grb7	growth factor receptor bound protein 7	0.864304	0.2541	0.489
15384	Hnrnpab	heterogeneous nuclear ribonucleoprotein A/B	0.864304	0.05559	0.1929
18559	Pctp	phosphatidylcholine transfer protein	0.864304	0.4177	0.6459

18616	Peg3	paternally expressed 3	0.864304	0.277	0.5126
18762	Prkcz	protein kinase C, zeta	0.864304	0.1576	0.3682
20668	Sox13	SRY-box containing gene 13	0.864304	0.2488	0.483
22122	Tsta3	tissue specific transplantation antigen P35B	0.864304	0.1115	0.2995
22200	Uba3	ubiquitin-like modifier activating enzyme 3	0.864304	0.1679	0.3829
27221	Chaf1a	chromatin assembly factor 1, subunit A (p150)	0.864304	0.1564	0.3663
29859	Sult4a1	sulfotransferase family 4A, member 1	0.864304	0.2987	0.5348
56077	Dgke	diacylglycerol kinase, epsilon	0.864304	0.07997	0.2433
57344	As3mt	arsenic (+3 oxidation state) methyltransferase	0.864304	0.0112	NA
64602	Ireb2	iron responsive element binding protein 2	0.864304	0.1825	0.4014
67109	Zfp787	zinc finger protein 787	0.864304	0.05521	0.1924
67391	Fundc2	FUN14 domain containing 2	0.864304	0.02706	NA
70465	Wdr77	WD repeat domain 77	0.864304	0.06205	0.2074
94190	Ophn1	oligophrenin 1	0.864304	0.301	0.5369
101100	Ttll3	tubulin tyrosine ligase-like family, member 3	0.864304	0.07437	0.2328
214150	Eif2c3	eukaryotic translation initiation factor 2C, 3	0.864304	0.3614	0.5962
217325	Llgl2	lethal giant larvae homolog 2 (Drosophila)	0.864304	0.0426	NA
225362	Reep2	receptor accessory protein 2	0.864304	0.2056	0.4319
232023	Vopp1	vesicular, overexpressed in cancer, prosurvival protein 1	0.864304	0.164	0.3771
268345	Kcnc2	potassium voltage gated channel, Shaw-related subfamily, member 2	0.864304	0.08227	0.2473
319196	Ankrd5	ankyrin repeat domain 5	0.864304	0.332	0.5681
319615	Zfp944	zinc finger protein 944	0.864304	0.02415	NA
329554	Gm826	predicted gene 826	0.864304	0.2348	0.4665
407812	Zfp941	zinc finger protein 941	0.864304	0.06284	0.2093
14077	Fabp3	fatty acid binding protein 3, muscle and heart	0.863558	0.04012	NA
17131	Smad7	MAD homolog 7 (Drosophila)	0.863558	0.2926	0.5284
20841	Zfp143	zinc finger protein 143	0.863558	0.07751	0.2388
21778	Tex9	testis expressed gene 9	0.863558	0.1589	0.3701
23921	Sh2b2	SH2B adaptor protein 2	0.863558	0.1873	0.4079
52915	Zmiz2	zinc finger, MIZ-type containing 2	0.863558	0.08089	0.2447
57439	Tmem183a	transmembrane protein 183A	0.863558	0.01368	NA
58194	Sh3kbp1	SH3-domain kinase binding protein 1	0.863558	0.07546	0.2351
66540	Fam107b	family with sequence similarity 107, member B	0.863558	0.01447	NA
67434	Ankrd33b	ankyrin repeat domain 33B	0.863558	0.344	0.5794
68134	Upf3b	UPF3 regulator of nonsense transcripts homolog B (yeast)	0.863558	0.03929	NA
69017	Prrt2	proline-rich transmembrane protein 2	0.863558	0.08399	0.2507
69215	Sat2	spermidine/spermine N1-acetyl transferase 2	0.863558	0.08179	0.2463
70357	Kcnip1	Kv channel-interacting protein 1	0.863558	0.2966	0.5325
71955	2400003C14Rik	RIKEN cDNA 2400003C14 gene	0.863558	0.05805	0.1988
73722	Lce1a2	late cornified envelope 1A2	0.863558	0.4735	0.6944
74304	1700094M24Rik	RIKEN cDNA 1700094M24 gene	0.863558	0.6348	0.8078
110454	Ly6a	lymphocyte antigen 6 complex, locus A	0.863558	0.2479	0.4821
112419	2010002M12Rik	RIKEN cDNA 2010002M12 gene	0.863558	0.06039	0.2041
228140	Tnks1bp1	tankyrase 1 binding protein 1	0.863558	0.05961	0.2026
228602	4930402H24Rik	RIKEN cDNA 4930402H24 gene	0.863558	0.2059	0.4321
230796	Wdtc1	WD and tetratricopeptide repeats 1	0.863558	0.3723	0.6068
234684	Lrrc29	leucine rich repeat containing 29	0.863558	0.3259	0.5626
236915	Arhgef9	CDC42 guanine nucleotide exchange factor (GEF) 9	0.863558	0.02477	NA
239554	Foxred2	FAD-dependent oxidoreductase domain containing 2	0.863558	0.02796	NA

269788	Lhfpl4	lipoma HMGIC fusion partner-like protein 4	0.863558	0.02319	NA
320714	D030016E14Rik	RIKEN cDNA D030016E14 gene	0.863558	0.2957	0.5314
433941	Gm5561	ribosomal protein L29 pseudogene	0.863558	0.07596	0.2361
665113	Tnik	TRAF2 and NCK interacting kinase	0.863558	0.03132	NA
11771	Ap2a1	adaptor protein complex AP-2, alpha 1 subunit	0.862813	0.1505	0.3584
11833	Aqp8	aquaporin 8	0.862813	0.4265	0.6537
12868	Cox8a	cytochrome c oxidase, subunit VIIIa	0.862813	0.03957	NA
15902	Id2	inhibitor of DNA binding 2	0.862813	0.0778	0.2391
17175	Masp2	mannan-binding lectin serine peptidase 2	0.862813	0.2914	0.5276
19281	Ptprt	protein tyrosine phosphatase, receptor type, T	0.862813	0.2659	0.5015
20927	Abcc8	ATP-binding cassette, sub-family C (CFTR/MRP), member 8	0.862813	0.08262	0.2479
22594	Xrcc1	X-ray repair complementing defective repair in Chinese hamster cells 1	0.862813	0.04719	NA
23790	Coro1c	coronin, actin binding protein 1C	0.862813	0.03214	NA
26987	Eif4e2	eukaryotic translation initiation factor 4E member 2	0.862813	0.1048	0.2879
29871	Scmh1	sex comb on midleg homolog 1	0.862813	0.1343	0.3353
64451	Dip2a	DIP2 disco-interacting protein 2 homolog A (Drosophila)	0.862813	0.008728	NA
65103	Arl6ip6	ADP-ribosylation factor-like 6 interacting protein 6	0.862813	0.19	0.4118
66576	Uqcrh	ubiquinol-cytochrome c reductase hinge protein	0.862813	0.04928	NA
67296	Socs4	suppressor of cytokine signaling 4	0.862813	0.285	0.5203
67569	Mgat4c	mannosyl (alpha-1,3-)-glycoprotein beta-1,4-N-acetylglucosaminyltransferase, isozyme C (putative)	0.862813	0.2182	0.4474
71897	Lypd6b	LY6/PLAUR domain containing 6B	0.862813	0.2894	0.5256
71996	1600014K23Rik	RIKEN cDNA 1600014K23 gene	0.862813	0.3568	0.5916
72720	Zfp248	zinc finger protein 248	0.862813	0.2036	0.4299
73744	Man2c1	mannosidase, alpha, class 2C, member 1	0.862813	0.03818	NA
77480	Kidins220	kinase D-interacting substrate 220	0.862813	0.04991	NA
78514	Arhgap10	Rho GTPase activating protein 10	0.862813	0.3754	0.6094
106298	Rrn3	RRN3 RNA polymerase I transcription factor homolog (yeast)	0.862813	0.01926	NA
109648	Npy	neuropeptide Y	0.862813	0.18	0.3981
112415	C030039L03Rik	RIKEN cDNA C030039L03 gene	0.862813	0.09556	0.2722
225131	Wac	WW domain containing adaptor with coiled-coil	0.862813	0.1478	0.3544
244653	Hydin	hydrocephalus inducing	0.862813	0.5614	0.7598
268930	Pkmyt1	protein kinase, membrane associated tyrosine/threonine 1	0.862813	0.06144	0.2061
319755	6330566A10Rik	RIKEN cDNA 6330566A10 gene	0.862813	0.4593	0.6826
320604	A730037C10Rik	RIKEN cDNA A730037C10 gene	0.862813	0.3405	0.5762
330222	Sdk1	sidekick homolog 1 (chicken)	0.862813	0.1617	0.3737
665180	Clec2l	C-type lectin domain family, member L	0.862813	0.329	0.5655
100038441	Gm10419	predicted gene 10419	0.862813	0.0498	NA
12447	Ccne1	cyclin E1	0.862069	0.02739	NA
12752	Cln3	ceroid lipofuscinosis, neuronal 3, juvenile (Batten, Spielmeyer-Vogt disease)	0.862069	0.05579	0.1934
14381	G6pdx	glucose-6-phosphate dehydrogenase X-linked	0.862069	0.1108	0.2986
14562	Gdf3	growth differentiation factor 3	0.862069	0.1871	0.4076
15560	Htr2c	5-hydroxytryptamine (serotonin) receptor 2C	0.862069	0.5505	0.7511
16568	Kif3a	kinesin family member 3A	0.862069	0.03008	NA
18127	Nos3	nitric oxide synthase 3, endothelial cell	0.862069	0.02727	NA
22701	Zfp41	zinc finger protein 41	0.862069	0.1339	0.3346
26878	B3galt2	UDP-Gal:betaGlcNAc beta 1,3-galactosyltransferase, polypeptide 2	0.862069	0.2276	0.4586
52715	Ccdc43	coiled-coil domain containing 43	0.862069	0.1335	0.3339
54375	Azin1	antizyme inhibitor 1	0.862069	0.07431	0.2328
54380	Smarcal1	SWI/SNF related matrix associated, actin dependent regulator of chromatin, subfamily a-like 1	0.862069	0.01703	NA

57274	Slc16a8	solute carrier family 16 (monocarboxylic acid transporters), member 8	0.862069	0.4973	0.7123
66065	Hsd17b14	hydroxysteroid (17-beta) dehydrogenase 14	0.862069	0.3102	0.5467
66282	1810029B16Rik	RIKEN cDNA 1810029B16 gene	0.862069	0.1055	0.2891
66849	Ppp1r2	protein phosphatase 1, regulatory (inhibitor) subunit 2	0.862069	0.1295	0.3286
67062	Mcart6	mitochondrial carrier triple repeat 6	0.862069	0.1719	0.3876
67815	Sec14l2	SEC14-like 2 (S. cerevisiae)	0.862069	0.08517	0.253
68031	Rnf146	ring finger protein 146	0.862069	0.06297	0.2095
71675	0610010F05Rik	RIKEN cDNA 0610010F05 gene	0.862069	0.3421	0.5775
74376	Myo18b	myosin XVIIIb	0.862069	0.4649	0.687
75098	4930515G13Rik	RIKEN cDNA 4930515G13 gene	0.862069	0.5418	0.7457
78787	Usp54	ubiquitin specific peptidase 54	0.862069	0.3126	0.5492
100732	Mapre3	microtubule-associated protein, RP/EB family, member 3	0.862069	0.08436	0.2514
107769	Tm6sf1	transmembrane 6 superfamily member 1	0.862069	0.02205	NA
108147	Atic	5-aminoimidazole-4-carboxamide ribonucleotide formyltransferase/IMP cyclohydrolase	0.862069	0.1084	0.2946
171429	Slc26a6	solute carrier family 26, member 6	0.862069	0.18	0.3981
225115	Svil	supervillin	0.862069	0.1042	0.287
225372	Apbb3	amyloid beta (A4) precursor protein-binding, family B, member 3	0.862069	0.02314	NA
228788	BC020535	cDNA sequence BC020535	0.862069	0.01449	NA
272322	Arntl2	aryl hydrocarbon receptor nuclear translocator-like 2	0.862069	0.1682	0.3831
320707	Atp2b3	ATPase, Ca++ transporting, plasma membrane 3	0.862069	0.1389	0.3418
327951	Cyb5d1	cytochrome b5 domain containing 1	0.862069	0.1871	0.4077
382384	Odf3l2	outer dense fiber of sperm tails 3-like 2	0.862069	0.5508	0.7511
100040529	Gm2824	predicted gene 2824	0.862069	0.2152	0.4438
11732	Ank	progressive ankylosis	0.861326	0.05452	0.191
12161	Bmp6	bone morphogenetic protein 6	0.861326	0.6465	0.8156
12426	Cckbr	cholecystokinin B receptor	0.861326	0.07395	0.2321
12876	Cpe	carboxypeptidase E	0.861326	0.04596	NA
17766	Nudt1	nudix (nucleoside diphosphate linked moiety X)-type motif 1	0.861326	0.1283	0.3265
19267	Ptpre	protein tyrosine phosphatase, receptor type, E	0.861326	0.05057	0.1827
19428	Rasl2-9-ps	RAS-like, family 2, locus 9, pseudogene	0.861326	0.6886	0.841
20713	Serpini1	serine (or cysteine) peptidase inhibitor, clade I, member 1	0.861326	0.05132	0.1841
22784	Slc30a3	solute carrier family 30 (zinc transporter), member 3	0.861326	0.2173	0.4463
26380	Esrrb	estrogen related receptor, beta	0.861326	0.1672	0.382
26412	Map4k2	mitogen-activated protein kinase kinase kinase kinase 2	0.861326	0.1685	0.3835
59288	Dctn5	dynactin 5	0.861326	0.01831	NA
67040	Ddx17	DEAD (Asp-Glu-Ala-Asp) box polypeptide 17	0.861326	0.5535	0.7534
67095	Trak1	trafficking protein, kinesin binding 1	0.861326	0.2426	0.4755
67576	4930429B21Rik	RIKEN cDNA 4930429B21 gene	0.861326	0.1074	0.293
68040	Zfp593	zinc finger protein 593	0.861326	0.0504	0.1824
68276	Toe1	target of EGR1, member 1 (nuclear)	0.861326	0.0347	NA
70556	Slc25a33	solute carrier family 25, member 33	0.861326	0.2042	0.4305
71291	4933435E02Rik	RIKEN cDNA 4933435E02 gene	0.861326	0.3953	0.6258
75007	Fam63a	family with sequence similarity 63, member A	0.861326	0.02372	NA
76133	6230400D17Rik	RIKEN cDNA 6230400D17 gene	0.861326	0.138	0.3405
76982	3110035E14Rik	RIKEN cDNA 3110035E14 gene	0.861326	0.231	0.4624
77066	5930409G06Rik	RIKEN cDNA 5930409G06 gene	0.861326	0.23	0.4612
77300	Raph1	Ras association (RalGDS/AF-6) and pleckstrin homology domains 1	0.861326	0.02839	NA
93875	Pcdhb4	protocadherin beta 4	0.861326	0.1366	0.3383
94065	Mrpl34	mitochondrial ribosomal protein L34	0.861326	0.03384	NA

103406	Zfr2	zinc finger RNA binding protein 2	0.861326	0.1736	0.3899
230657	Tmem69	transmembrane protein 69	0.861326	0.2844	0.52
232934	Mypop	Myb-related transcription factor, partner of profilin	0.861326	0.1508	0.3588
236848	BC023829	cDNA sequence BC023829	0.861326	0.1105	0.2979
237831	Slc13a5	solute carrier family 13 (sodium-dependent citrate transporter), member 5	0.861326	0.1028	0.2843
239114	Il17d	interleukin 17D	0.861326	0.08995	0.2622
268936	Brpf3	bromodomain and PHD finger containing, 3	0.861326	0.06282	0.2093
319594	Hif1an	hypoxia-inducible factor 1, alpha subunit inhibitor	0.861326	0.092	0.2658
320129	Adrbk2	adrenergic receptor kinase, beta 2	0.861326	0.1749	0.3916
328087	D930001B02	hypothetical protein D930001B02	0.861326	0.1236	0.3199
333088	Kcp	kielin/chordin-like protein	0.861326	0.203	0.4292
433319	Gm5528	predicted gene 5528	0.861326	0.1084	0.2945
474160	BC033916	cDNA sequence BC033916	0.861326	0.1054	0.2891
100041481	Gm3364	predicted gene 3364	0.861326	0.5594	0.7581
13854	Epn1	epsin 1	0.860585	0.02011	NA
14251	Flot1	flotillin 1	0.860585	0.03985	NA
17173	Ascl2	achaete-scute complex homolog 2 (Drosophila)	0.860585	0.1405	0.3442
19246	Ptpn1	protein tyrosine phosphatase, non-receptor type 1	0.860585	0.08031	0.2437
20481	Ski	ski sarcoma viral oncogene homolog (avian)	0.860585	0.4448	0.6705
27425	Atp5l	ATP synthase, H+ transporting, mitochondrial F0 complex, subunit g	0.860585	0.01904	NA
55946	Ap3m1	adaptor-related protein complex 3, mu 1 subunit	0.860585	0.02421	NA
65246	Xpo7	exportin 7	0.860585	0.384	0.6165
66443	Tnfaip8l1	tumor necrosis factor, alpha-induced protein 8-like 1	0.860585	0.04739	0.1762
67388	1110008F13Rik	RIKEN cDNA 1110008F13 gene	0.860585	0.01272	NA
71217	4933431I19Rik	RIKEN cDNA 4933431I19 gene	0.860585	0.1507	0.3587
71242	Spata24	spermatogenesis associated 24	0.860585	0.3793	0.613
71336	Rbks	ribokinase	0.860585	0.02616	NA
71617	9130011E15Rik	RIKEN cDNA 9130011E15 gene	0.860585	0.368	0.603
72022	Slc35f2	solute carrier family 35, member F2	0.860585	0.1705	0.3859
72502	Cwf19l1	CWF19-like 1, cell cycle control (S. pombe)	0.860585	0.0249	NA
72729	Cdc42se2	CDC42 small effector 2	0.860585	0.07462	0.2332
73825	Klraq1	KLRAQ motif containing 1	0.860585	0.2069	0.433
73848	5430406J06Rik	RIKEN cDNA 5430406J06 gene	0.860585	0.1116	0.2997
78334	Cdk19	cyclin-dependent kinase 19	0.860585	0.02377	NA
80883	Ntng1	netrin G1	0.860585	0.2367	0.4689
213006	Mfsd4	major facilitator superfamily domain containing 4	0.860585	0.2949	0.5311
217847	Serpina10	serine (or cysteine) peptidase inhibitor, clade A (alpha-1 antiproteinase, antitrypsin), member 10	0.860585	0.394	0.6249
224840	Treml4	triggering receptor expressed on myeloid cells-like 4	0.860585	0.2266	0.4582
228807	Zfp341	zinc finger protein 341	0.860585	0.09919	0.2779
239618	Pdzrn4	PDZ domain containing RING finger 4	0.860585	0.05084	0.1829
243621	Iqsec3	IQ motif and Sec7 domain 3	0.860585	0.005589	NA
243842	Gltscr1	glioma tumor suppressor candidate region gene 1	0.860585	0.6142	0.795
258484	Olfr1410	olfactory receptor 1410	0.860585	0.3513	0.5867
269630	5031425E22Rik	RIKEN cDNA 5031425E22 gene	0.860585	0.07998	0.2433
320213	Senp5	SUMO/sentrin specific peptidase 5	0.860585	0.2475	0.4817
382985	Rrm2b	ribonucleotide reductase M2 B (TP53 inducible)	0.860585	0.09759	0.2755
553127	Cxx1b	CAAX box 1 homolog B (human)	0.860585	0.07247	0.229
100033459	Pydc3	pyrin domain containing 3	0.860585	0.1153	0.306
12193	Zfp36l2	zinc finger protein 36, C3H type-like 2	0.859845	0.09631	0.2734

12904	Crabp2	cellular retinoic acid binding protein II	0.859845	0.04069	NA
16565	Kif21b	kinesin family member 21B	0.859845	0.02097	NA
17364	Trpm1	transient receptor potential cation channel, subfamily M, member 1	0.859845	0.3175	0.5539
18711	Pikfyve	phosphoinositide kinase, FYVE finger containing	0.859845	0.09906	0.2779
19359	Rad23b	RAD23b homolog (S. cerevisiae)	0.859845	0.01824	NA
20257	Stmn2	stathmin-like 2	0.859845	0.03246	NA
22634	Plagl1	pleiomorphic adenoma gene-like 1	0.859845	0.1203	0.3141
30853	Mlf2	myeloid leukemia factor 2	0.859845	0.3068	0.5433
50926	Hnrpdl	heterogeneous nuclear ribonucleoprotein D-like	0.859845	0.1489	0.3563
52076	Tmem38b	transmembrane protein 38B	0.859845	0.324	0.5604
66049	Rogdi	rogdi homolog (Drosophila)	0.859845	0.1646	0.378
67201	Glod4	glyoxalase domain containing 4	0.859845	0.1017	0.2826
68089	Arpc4	actin related protein 2/3 complex, subunit 4	0.859845	0.2738	0.5094
71132	Cabyr	calcium-binding tyrosine-(Y)-phosphorylation regulated (fibrousheathin 2)	0.859845	0.1266	0.3239
76559	Atg2b	ATG2 autophagy related 2 homolog B (S. cerevisiae)	0.859845	0.02063	NA
83679	Pde4dip	phosphodiesterase 4D interacting protein (myomegalin)	0.859845	0.04453	0.1697
98910	Usp6nl	USP6 N-terminal like	0.859845	0.06842	0.2209
214084	Slc18a2	solute carrier family 18 (vesicular monoamine), member 2	0.859845	0.04085	NA
218543	Srsf12	serine/arginine-rich splicing factor 12	0.859845	0.2447	0.4784
227377	Farp2	FERM, RhoGEF and pleckstrin domain protein 2	0.859845	0.06751	0.2191
228866	Pcif1	PDX1 C-terminal inhibiting factor 1	0.859845	0.3416	0.5772
229517	Slc25a44	solute carrier family 25, member 44	0.859845	0.1312	0.3312
242667	Dlgap3	discs, large (Drosophila) homolog-associated protein 3	0.859845	0.1418	0.3462
330474	Zc3h4	zinc finger CCCH-type containing 4	0.859845	0.01435	NA
381654	C87414	expressed sequence C87414	0.859845	0.3854	0.6176
408062	Zfp873	zinc finger protein 873	0.859845	0.08301	0.2487
619317	D930026N18Rik	RIKEN cDNA D930026N18 gene	0.859845	0.05023	0.1819
667933	Gm8884	predicted gene 8884	0.859845	0.2711	0.5065
11496	Adam22	a disintegrin and metallopeptidase domain 22	0.859107	0.1091	0.2956
11836	Araf	v-raf murine sarcoma 3611 viral oncogene homolog	0.859107	0.05256	0.1863
14680	Gnal	guanine nucleotide binding protein, alpha stimulating, olfactory type	0.859107	0.1556	0.3654
16205	Gimap1	GTPase, IMAP family member 1	0.859107	0.4152	0.6436
19084	Prkar1a	protein kinase, cAMP dependent regulatory, type I, alpha	0.859107	0.0914	0.265
19679	Pitpnm2	phosphatidylinositol transfer protein, membrane-associated 2	0.859107	0.2086	0.4353
20788	Srebf2	sterol regulatory element binding factor 2	0.859107	0.4867	0.7047
22589	Atrx	alpha thalassemia/mental retardation syndrome X-linked homolog (human)	0.859107	0.2593	0.4945
30938	Fgd3	FYVE, RhoGEF and PH domain containing 3	0.859107	0.377	0.611
50932	Mink1	misshapen-like kinase 1 (zebrafish)	0.859107	0.1233	0.3194
52028	Bbs1	Bardet-Biedl syndrome 1 (human)	0.859107	0.01204	NA
53324	Nptx2	neuronal pentraxin 2	0.859107	0.3338	0.5696
66570	Cenpm	centromere protein M	0.859107	0.4427	0.6687
66634	Mcm8	minichromosome maintenance deficient 8 (S. cerevisiae)	0.859107	0.05675	0.1957
70458	2610318N02Rik	RIKEN cDNA 2610318N02 gene	0.859107	0.1269	0.3244
70524	5730414N17Rik	RIKEN cDNA 5730414N17 gene	0.859107	0.184	0.403
71017	4933407E14Rik	RIKEN cDNA 4933407E14 gene	0.859107	0.05714	0.1964
72693	Zcchc12	zinc finger, CCHC domain containing 12	0.859107	0.02228	NA
73242	Atat1	alpha tubulin acetyltransferase 1	0.859107	0.3472	0.5826
76877	Rab36	RAB36, member RAS oncogene family	0.859107	0.3645	0.5999
77963	Hook1	hook homolog 1 (Drosophila)	0.859107	0.4985	0.7132

100383	Bsdc1	BSD domain containing 1	0.859107	0.0592	0.2016
140546	Eri3	exoribonuclease 3	0.859107	0.2738	0.5094
195646	Hs3st2	heparan sulfate (glucosamine) 3-O-sulfotransferase 2	0.859107	0.3356	0.5713
224694	Zfp81	zinc finger protein 81	0.859107	0.04758	0.1765
230822	A330049M08Rik	RIKEN cDNA A330049M08 gene	0.859107	0.2168	0.4456
238247	Arid4a	AT rich interactive domain 4A (RBP1-like)	0.859107	0.1272	0.3249
268729	Gm626	predicted gene 626	0.859107	0.2715	0.507
381522	E230008N13Rik	RIKEN cDNA E230008N13 gene	0.859107	0.1476	0.3542
432486	Gnptab	N-acetylglucosamine-1-phosphate transferase, alpha and beta subunits	0.859107	0.03274	NA
668253	Dleu2	deleted in lymphocytic leukemia, 2	0.859107	0.4088	0.6382
100041799	Gm3515	predicted gene 3515	0.859107	0.1486	0.3559
11515	Adcy9	adenylate cyclase 9	0.858369	0.03095	NA
12009	Azi1	5-azacytidine induced gene 1	0.858369	0.1298	0.329
12750	Clk4	CDC like kinase 4	0.858369	0.1838	0.4028
13842	Epha8	Eph receptor A8	0.858369	0.09916	0.2779
15388	Hnrnpl	heterogeneous nuclear ribonucleoprotein L	0.858369	0.0623	0.208
17216	Mcm2	minichromosome maintenance deficient 2 mitotin (S. cerevisiae)	0.858369	0.07729	0.2384
19893	Rpgr	retinitis pigmentosa GTPase regulator	0.858369	0.1134	0.3028
20972	Syngr1	synaptogyrin 1	0.858369	0.2987	0.5348
22340	Vegfb	vascular endothelial growth factor B	0.858369	0.01706	NA
22682	Zfand5	zinc finger, AN1-type domain 5	0.858369	0.035	NA
56292	Naa10	N(alpha)-acetyltransferase 10, NatA catalytic subunitNalpha acetyltransferase 10	0.858369	0.1197	0.3132
56364	Zmym3	zinc finger, MYM-type 3	0.858369	0.06108	0.2055
56737	Alg2	asparagine-linked glycosylation 2 homolog (yeast, alpha-1,3-mannosyltransferase)	0.858369	0.124	0.3205
66366	Ergic3	ERGIC and golgi 3	0.858369	0.09407	0.2696
66427	Cyb5b	cytochrome b5 type B	0.858369	0.07718	0.2381
66460	Sys1	SYS1 Golgi-localized integral membrane protein homolog (S. cerevisiae)	0.858369	0.06115	0.2056
67059	Ola1	Obg-like ATPase 1	0.858369	0.0431	0.1665
72775	Fance	Fanconi anemia, complementation group E	0.858369	0.1274	0.325
72899	Macrod2	MACRO domain containing 2	0.858369	0.1344	0.3353
74342	Lrrtm1	leucine rich repeat transmembrane neuronal 1	0.858369	0.09335	0.2684
76484	Kndc1	kinase non-catalytic C-lobe domain (KIND) containing 1	0.858369	0.04061	NA
78912	Sp2	Sp2 transcription factor	0.858369	0.1829	0.4017
93734	Mpv17l	Mpv17 transgene, kidney disease mutant-like	0.858369	0.1583	0.3694
101185	Pot1a	protection of telomeres 1A	0.858369	0.4417	0.6675
108058	Camk2d	calcium/calmodulin-dependent protein kinase II, delta	0.858369	0.0327	NA
116939	Pnpla3	patatin-like phospholipase domain containing 3	0.858369	0.1054	0.2889
140723	Cacng5	calcium channel, voltage-dependent, gamma subunit 5	0.858369	0.1131	0.3023
170461	Stard6	StAR-related lipid transfer (START) domain containing 6	0.858369	0.1943	0.4179
211484	Tsga10	testis specific 10	0.858369	0.1636	0.3765
216705	Clint1	clathrin interactor 1	0.858369	0.0639	0.2112
233724	Tmem41b	transmembrane protein 41B	0.858369	0.01656	NA
259053	Olfr362	olfactory receptor 362	0.858369	0.5757	0.769
320327	A430106G13Rik	RIKEN cDNA A430106G13 gene	0.858369	0.07958	0.2425
380694	Ccnjl	cyclin J-like	0.858369	0.06791	0.2199
433297	Gm5526	predicted pseudogene 5526	0.858369	0.04839	0.178
666244	Tmsb15b1	thymosin beta 15b1	0.858369	0.03675	NA
668303	Kif26a	kinesin family member 26A	0.858369	0.1152	0.3058
11772	Ap2a2	adaptor protein complex AP-2, alpha 2 subunit	0.857633	0.06462	0.2128

13117	Cyp4a10	cytochrome P450, family 4, subfamily a, polypeptide 10	0.857633	0.4593	0.6826
14559	Gdf1	growth differentiation factor 1	0.857633	0.0248	NA
16324	Inhbb	inhibin beta-B	0.857633	0.146	0.3516
19366	Rad54l	RAD54 like (S. cerevisiae)	0.857633	0.2991	0.5348
53897	Gal3st1	galactose-3-O-sulfotransferase 1	0.857633	0.2802	0.5156
56784	Ralgapa1	Ral GTPase activating protein, alpha subunit 1	0.857633	0.015	NA
58180	Hic2	hypermethylated in cancer 2	0.857633	0.02299	NA
58200	Ppp1r1a	protein phosphatase 1, regulatory (inhibitor) subunit 1A	0.857633	0.2061	0.4324
66208	Nenf	neuron derived neurotrophic factor	0.857633	0.04759	0.1765
67102	D16Ertd472e	DNA segment, Chr 16, ERATO Doi 472, expressed	0.857633	0.02092	NA
67708	1810048J11Rik	RIKEN cDNA 1810048J11 gene	0.857633	0.03022	NA
68968	Cdan1	congenital dyserythropoietic anemia, type I (human)	0.857633	0.4071	0.6365
69487	2310003L22Rik	RIKEN cDNA 2310003L22 gene	0.857633	0.1508	0.3588
69601	Dab2ip	disabled homolog 2 (Drosophila) interacting protein	0.857633	0.3298	0.5664
69938	Scrn1	secernin 1	0.857633	0.05137	0.1841
72000	1600016N20Rik	RIKEN cDNA 1600016N20 gene	0.857633	0.3928	0.6241
77463	C030014O09Rik	RIKEN cDNA C030014O09 gene	0.857633	0.2823	0.5177
77832	Tchp	trichoplein, keratin filament binding	0.857633	0.04695	0.1753
78038	Mccc2	methylcrotonoyl-Coenzyme A carboxylase 2 (beta)	0.857633	0.04881	0.1787
78651	Lsm6	LSM6 homolog, U6 small nuclear RNA associated (S. cerevisiae)	0.857633	0.222	0.4523
207278	Fchsd2	FCH and double SH3 domains 2	0.857633	0.03493	NA
224019	Tmem191c	transmembrane protein 191C	0.857633	0.1353	0.3368
224860	Plcl2	phospholipase C-like 2	0.857633	0.08567	0.2541
228607	Mavs	mitochondrial antiviral signaling protein	0.857633	0.0598	0.2029
240843	Fam5b	family with sequence similarity 5, member B	0.857633	0.2045	0.4308
269424	Phf17	PHD finger protein 17	0.857633	0.00639	NA
277250	Kdm3b	KDM3B lysine (K)-specific demethylase 3B	0.857633	0.1222	0.3176
319586	Celf5	CUGBP, Elav-like family member 5	0.857633	0.2826	0.5179
320007	Sidt1	SID1 transmembrane family, member 1	0.857633	0.09912	0.2779
320469	9930014A18Rik	RIKEN cDNA 9930014A18 gene	0.857633	0.1713	0.387
328113	A230055C15	hypothetical protein A230055C15	0.857633	0.077	0.2377
330064	Slc5a6	solute carrier family 5 (sodium-dependent vitamin transporter), member 6	0.857633	0.009888	NA
381921	Taok2	TAO kinase 2	0.857633	0.2324	0.4638
408190	Wfdc13	WAP four-disulfide core domain 13	0.857633	0.6228	0.8003
11465	Actg1	actin, gamma, cytoplasmic 1	0.856898	0.2228	0.453
11787	Apbb2	amyloid beta (A4) precursor protein-binding, family B, member 2	0.856898	0.005376	NA
12794	Cnih2	cornichon homolog 2 (Drosophila)	0.856898	0.06458	0.2127
12854	Cort	cortistatin	0.856898	0.1935	0.4167
15168	Hcn3	hyperpolarization-activated, cyclic nucleotide-gated K+ 3	0.856898	0.224	0.4546
15502	Dnaja1	DnaJ (Hsp40) homolog, subfamily A, member 1	0.856898	0.2018	0.428
18227	Nr4a2	nuclear receptor subfamily 4, group A, member 2	0.856898	0.03277	NA
18784	Pla2g5	phospholipase A2, group V	0.856898	0.1007	0.2806
20661	Sort1	sortilin 1	0.856898	0.07282	0.2298
20965	Syn2	synapsin II	0.856898	0.01787	NA
21419	Tcfap2b	transcription factor AP-2 beta	0.856898	0.02393	NA
22348	Slc32a1	solute carrier family 32 (GABA vesicular transporter), member 1	0.856898	0.04297	0.1662
30948	Bin1	bridging integrator 1	0.856898	0.01697	NA
50787	Hs6st3	heparan sulfate 6-O-sulfotransferase 3	0.856898	0.0783	0.2399
52014	Nus1	nuclear undecaprenyl pyrophosphate synthase 1 homolog (S. cerevisiae)	0.856898	0.1847	0.4041

56805	Zbtb33	zinc finger and BTB domain containing 33	0.856898	0.02433	NA
66132	1110008L16Rik	RIKEN cDNA 1110008L16 gene	0.856898	0.1389	0.3418
66246	Osgep	O-sialoglycoprotein endopeptidase	0.856898	0.01419	NA
66403	Asf1a	ASF1 anti-silencing function 1 homolog A (S. cerevisiae)	0.856898	0.1203	0.3141
68161	A930005H10Rik	RIKEN cDNA A930005H10 gene	0.856898	0.01448	NA
68732	Lrrc16a	leucine rich repeat containing 16A	0.856898	0.2576	0.4927
73086	Rps6ka5	ribosomal protein S6 kinase, polypeptide 5	0.856898	0.1136	0.3032
74168	Zdhhc16	zinc finger, DHHC domain containing 16	0.856898	0.005537	NA
93871	Brwd1	bromodomain and WD repeat domain containing 1	0.856898	0.03199	NA
100090	Zbtb48	zinc finger and BTB domain containing 48	0.856898	0.2166	0.4455
102442	Dennd4a	DENN/MADD domain containing 4A	0.856898	0.01164	NA
104418	Dgkz	diacylglycerol kinase zeta	0.856898	0.1897	0.4114
105859	Csdc2	cold shock domain containing C2, RNA binding	0.856898	0.238	0.4704
170731	Mfn2	mitofusin 2	0.856898	0.01912	NA
170738	Kcnh7	potassium voltage-gated channel, subfamily H (eag-related), member 7	0.856898	0.0527	0.1867
216810	Tom1l2	target of myb1-like 2 (chicken)	0.856898	0.03151	NA
229672	Bcl2l15	BCLl2-like 15	0.856898	0.2974	0.5334
230753	Thrap3	thyroid hormone receptor associated protein 3	0.856898	0.05226	0.1856
231801	Agfg2	ArfGAP with FG repeats 2	0.856898	0.113	0.3022
232670	Tspan33	tetraspanin 33	0.856898	0.03276	NA
239188	Enox1	ecto-NOX disulfide-thiol exchanger 1	0.856898	0.7549	0.8816
242418	Dcaf10	DDB1 and CUL4 associated factor 10	0.856898	0.00971	NA
257902	Olfr704	olfactory receptor 704	0.856898	0.6315	0.8059
320208	Tmem91	transmembrane protein 91	0.856898	0.09192	0.2658
320817	Atad2b	ATPase family, AAA domain containing 2B	0.856898	0.06598	0.2159
664883	Nova1	neuro-oncological ventral antigen 1	0.856898	0.2226	0.4528
100043231	Gm4303	predicted gene 4303	0.856898	0.695	0.8451
12933	Crmp1	collapsin response mediator protein 1	0.856164	0.03918	0.1576
16924	Lnx1	ligand of numb-protein X 1	0.856164	0.2071	0.4332
18293	Ogdh	oxoglutarate dehydrogenase (lipoamide)	0.856164	0.1065	0.2911
19088	Prkar2b	protein kinase, cAMP dependent regulatory, type II beta	0.856164	0.04352	0.1676
19092	Prkg2	protein kinase, cGMP-dependent, type II	0.856164	0.06319	0.2099
19335	Rab23	RAB23, member RAS oncogene family	0.856164	0.07576	0.2357
20266	Scn1b	sodium channel, voltage-gated, type I, beta	0.856164	0.247	0.4812
21461	Tcp10b	t-complex protein 10b	0.856164	0.2724	0.5079
22355	Vipr2	vasoactive intestinal peptide receptor 2	0.856164	0.02019	NA
23996	Psmc4	proteasome (prosome, macropain) 26S subunit, ATPase, 4	0.856164	0.02278	NA
56150	Mad2l1	MAD2 mitotic arrest deficient-like 1 (yeast)	0.856164	0.04994	0.1815
56210	Rev1	REV1 homolog (S. cerevisiae)	0.856164	0.154	0.3636
56631	Trim17	tripartite motif-containing 17	0.856164	0.3385	0.5744
66096	0910001L09Rik	RIKEN cDNA 0910001L09 gene	0.856164	0.03126	NA
68729	Trim37	tripartite motif-containing 37	0.856164	0.00928	NA
73207	3110068a07rik	RIKEN cDNA 3110068A07 gene	0.856164	0.3062	0.5428
74309	Osbp2	oxysterol binding protein 2	0.856164	0.1783	0.396
74648	S100pbp	S100P binding protein	0.856164	0.2934	0.5294
104662	Tsr1	TSR1, 20S rRNA accumulation, homolog (yeast)	0.856164	0.08924	0.2611
108013	Celf4	CUGBP, Elav-like family member 4	0.856164	0.0407	0.1613
116837	Rims1	regulating synaptic membrane exocytosis 1	0.856164	0.1437	0.3486
213417	Klhdc8a	kelch domain containing 8A	0.856164	0.1645	0.3779

219132	D14Ertd668e	DNA segment, Chr 14, ERATO Doi 668, expressed	0.856164	0.3295	0.5662
223227	Sox21	SRY-box containing gene 21	0.856164	0.211	0.4385
227157	Mpp4	membrane protein, palmitoylated 4 (MAGUK p55 subfamily member 4)	0.856164	0.07873	0.2406
244329	Mcph1	microcephaly, primary autosomal recessive 1	0.856164	0.1382	0.3407
270162	Elmod1	ELMO domain containing 1	0.856164	0.00635	NA
320802	6330512M04Rik	RIKEN cDNA 6330512M04 gene	0.856164	0.3369	0.5726
13131	Dab1	disabled homolog 1 (Drosophila)	0.855432	0.06374	0.2109
13492	Drd5	dopamine receptor D5	0.855432	0.1325	0.3331
14425	Galnt3	UDP-N-acetyl-alpha-D-galactosamine:polypeptide N-acetylgalactosaminyltransferase 3	0.855432	0.1698	0.385
17751	Mt3	metallothionein 3	0.855432	0.1136	0.3032
20744	Strbp	spermatid perinuclear RNA binding protein	0.855432	0.09102	0.2642
20910	Stxbp1	syntaxin binding protein 1	0.855432	0.06126	0.2058
21338	Tacr3	tachykinin receptor 3	0.855432	0.1016	0.2823
22380	Wbp4	WW domain binding protein 4	0.855432	0.01596	NA
24047	Ccl19	chemokine (C-C motif) ligand 19	0.855432	0.3818	0.6151
66049	Rogdi	rogdi homolog (Drosophila)	0.855432	0.08914	0.261
66774	4933439G12Rik	RIKEN cDNA 4933439G12 gene	0.855432	0.3816	0.615
66994	1500031L02Rik	RIKEN cDNA 1500031L02 gene	0.855432	0.0201	NA
67048	Vma21	VMA21 vacuolar H+-ATPase homolog (S. cerevisiae)	0.855432	0.1472	0.3535
72657	2700094K13Rik	RIKEN cDNA 2700094K13 gene	0.855432	0.01184	NA
75645	1700011F14Rik	RIKEN cDNA 1700011F14 gene	0.855432	0.3887	0.6204
94332	Cadm3	cell adhesion molecule 3	0.855432	0.2136	0.4419
108807	4933415E08Rik	RIKEN cDNA 4933415E08 gene	0.855432	0.08564	0.254
218232	Ptpdc1	protein tyrosine phosphatase domain containing 1	0.855432	0.03728	0.153
223828	Pphln1	periphilin 1	0.855432	0.1612	0.373
227526	Cdnf	cerebral dopamine neurotrophic factor	0.855432	0.03575	0.1494
231946	D330028D13Rik	RIKEN cDNA D330028D13 gene	0.855432	0.2076	0.4341
233878	Sez6l2	seizure related 6 homolog like 2	0.855432	0.04506	0.1711
240869	Zbtb37	zinc finger and BTB domain containing 37	0.855432	0.4061	0.6357
320840	Negr1	neuronal growth regulator 1	0.855432	0.02121	NA
12151	Bmi1	Bmi1 polycomb ring finger oncogene	0.854701	0.03848	0.1562
14766	Gpr56	G protein-coupled receptor 56	0.854701	0.1333	0.3339
14816	Grm1	glutamate receptor, metabotropic 1	0.854701	0.1011	0.2813
16826	Ldb2	LIM domain binding 2	0.854701	0.07148	0.2269
17719	ND4	NADH dehydrogenase subunit 4	0.854701	0.0726	0.2293
18107	Nmt1	N-myristoyltransferase 1	0.854701	0.04585	0.1727
18302	Oit3	oncoprotein induced transcript 3	0.854701	0.1498	0.3577
18984	Por	P450 (cytochrome) oxidoreductase	0.854701	0.2399	0.4724
19016	Pparg	peroxisome proliferator activated receptor gamma	0.854701	0.2082	0.4349
20360	Sema6c	sema domain, transmembrane domain (TM), and cytoplasmic domain, (semaphorin) 6C	0.854701	0.2514	0.4857
20370	Sez6	seizure related gene 6	0.854701	0.2077	0.4342
21652	Phf1	PHD finger protein 1	0.854701	0.04943	0.1803
22157	Tulp1	tubby like protein 1	0.854701	0.1062	0.2906
50911	Exosc9	exosome component 9	0.854701	0.2996	0.5352
54393	Gabbr1	gamma-aminobutyric acid (GABA) B receptor, 1	0.854701	0.1267	0.324
56403	Syncrip	synaptotagmin binding, cytoplasmic RNA interacting protein	0.854701	0.06966	0.2234
56441	Nat6	N-acetyltransferase 6	0.854701	0.2222	0.4526
56738	Mocs1	molybdenum cofactor synthesis 1	0.854701	0.1943	0.4179
66576	Uqcrh	ubiquinol-cytochrome c reductase hinge protein	0.854701	0.01635	NA

66970	Ssbp2	single-stranded DNA binding protein 2	0.854701	0.03516	0.1484
70599	Ssfa2	sperm specific antigen 2	0.854701	0.1171	0.309
73112	3110003A17Rik	RIKEN cDNA 3110003A17 gene	0.854701	0.02745	NA
77041	Arsk	arylsulfatase K	0.854701	0.06183	0.207
106877	Afap1l1	actin filament associated protein 1-like 1	0.854701	0.04548	0.1718
218832	Polr3a	polymerase (RNA) III (DNA directed) polypeptide A	0.854701	0.07782	0.2391
232878	Zscan22	zinc finger and SCAN domain containing 22	0.854701	0.1476	0.3542
244672	Cwf19l2	CWF19-like 2, cell cycle control (S. pombe)	0.854701	0.1051	0.2884
269854	Nat14	N-acetyltransferase 14	0.854701	0.09851	0.277
272381	Lrrc4b	leucine rich repeat containing 4B	0.854701	0.08809	0.2589
320858	L3mbtl4	l(3)mbt-like 4 (Drosophila)	0.854701	0.03175	NA
332175	Zdhhc23	zinc finger, DHHC domain containing 23	0.854701	0.05138	0.1841
414113	D230040A04Rik	RIKEN cDNA D230040A04 gene	0.854701	0.2933	0.5294
100504473	LOC100504473	RNA-binding protein Musashi homolog 2-like	0.854701	0.1594	0.3708
12041	Bckdk	branched chain ketoacid dehydrogenase kinase	0.853971	0.1273	0.3249
14897	Trip12	thyroid hormone receptor interactor 12	0.853971	0.1029	0.2844
16924	Lnx1	ligand of numb-protein X 1	0.853971	0.01967	NA
17135	Mafk	v-maf musculoaponeurotic fibrosarcoma oncogene family, protein K (avian)	0.853971	0.04086	0.1615
18588	Pde6g	phosphodiesterase 6G, cGMP-specific, rod, gamma	0.853971	0.07602	0.2362
18795	Plcb1	phospholipase C, beta 1	0.853971	0.08244	0.2477
19341	Rab4a	RAB4A, member RAS oncogene family	0.853971	0.05528	0.1925
20269	Scn3a	sodium channel, voltage-gated, type III, alpha	0.853971	0.06298	0.2095
28018	Ubfd1	ubiquitin family domain containing 1	0.853971	0.03343	0.1444
54394	Crlf3	cytokine receptor-like factor 3	0.853971	0.02945	NA
54562	Lrrc6	leucine rich repeat containing 6 (testis)	0.853971	0.3197	0.5562
55951	Brp44l	brain protein 44-like	0.853971	0.0413	0.1626
66349	Atp5sl	ATP5S-like	0.853971	0.04254	0.1651
68080	Gpn3	GPN-loop GTPase 3	0.853971	0.03848	0.1562
72393	Faim2	Fas apoptotic inhibitory molecule 2	0.853971	0.007223	NA
74213	Rbm26	RNA binding motif protein 26	0.853971	0.04168	0.1634
77573	Vps33a	vacuolar protein sorting 33A (yeast)	0.853971	0.05122	0.1839
101923	BB212172	expressed sequence BB212172	0.853971	0.03067	NA
102098	Arhgef18	rho/rac guanine nucleotide exchange factor (GEF) 18	0.853971	0.008656	NA
103762	AI503301	expressed sequence AI503301	0.853971	0.06298	0.2095
106766	Stap2	signal transducing adaptor family member 2	0.853971	0.04576	0.1725
208650	Cblb	Casitas B-lineage lymphoma b	0.853971	0.01998	NA
213993	A630007B06Rik	RIKEN cDNA A630007B06 gene	0.853971	0.01741	NA
228889	Ddx27	DEAD (Asp-Glu-Ala-Asp) box polypeptide 27	0.853971	0.01557	NA
269799	Clec4a1	C-type lectin domain family 4, member a1	0.853971	0.5315	0.7375
319415	Hs3st5	heparan sulfate (glucosamine) 3-O-sulfotransferase 5	0.853971	0.05961	0.2026
319893	A230057D06Rik	RIKEN cDNA A230057D06 gene	0.853971	0.438	0.6643
432731	Zfp187	zinc finger protein 187	0.853971	0.00661	NA
432779	Lrrc14b	leucine rich repeat containing 14B	0.853971	0.1236	0.3199
100504346	Gm13304	predicted gene 13304	0.853971	0.4342	0.6612
11517	Adcyap1r1	adenylate cyclase activating polypeptide 1 receptor 1	0.853242	0.243	0.4761
11984	Atp6v0c	ATPase, H+ transporting, lysosomal V0 subunit C	0.853242	0.1306	0.3302
17708	COX1	cytochrome c oxidase subunit I	0.853242	0.2063	0.4325
18526	Pcdh10	protocadherin 10	0.853242	0.02548	NA
19184	Psmc5	protease (prosome, macropain) 26S subunit, ATPase 5	0.853242	0.07981	0.243

20586	Smarca4	SWI/SNF related, matrix associated, actin dependent regulator of chromatin, subfamily a, member 4	0.853242	0.01539	NA
26373	Clcn7	chloride channel 7	0.853242	0.02352	NA
51944	D2Ertd750e	DNA segment, Chr 2, ERATO Doi 750, expressed	0.853242	0.1619	0.3741
53902	Rcan3	regulator of calcineurin 3	0.853242	0.1602	0.3719
66832	Rsph3a	radial spoke 3A homolog (Chlamydomonas)	0.853242	0.2454	0.4792
67134	Nop56	NOP56 ribonucleoprotein homolog (yeast)	0.853242	0.04009	0.1596
67666	Hapln3	hyaluronan and proteoglycan link protein 3	0.853242	0.05993	0.2031
69350	1700003G18Rik	RIKEN cDNA 1700003G18 gene	0.853242	0.2315	0.4631
72461	Prcp	prolylcarboxypeptidase (angiotensinase C)	0.853242	0.09365	0.2689
72661	Serp2	stress-associated endoplasmic reticulum protein family member 2	0.853242	0.004	NA
72821	Scn2b	sodium channel, voltage-gated, type II, beta	0.853242	0.0224	NA
74081	Cep350	centrosomal protein 350	0.853242	0.005879	NA
74123	Foxp4	forkhead box P4	0.853242	0.4741	0.6947
74665	Lrrc48	leucine rich repeat containing 48	0.853242	0.226	0.4572
94280	Sfxn3	sideroflexin 3	0.853242	0.04927	0.1799
105844	Card10	caspase recruitment domain family, member 10	0.853242	0.05535	0.1925
110876	Scn2a1	sodium channel, voltage-gated, type II, alpha 1	0.853242	0.03245	0.1418
114565	Zfp295	zinc finger protein 295	0.853242	0.05192	0.1852
234353	Psd3	pleckstrin and Sec7 domain containing 3	0.853242	0.1513	0.3597
240916	Vsig8	V-set and immunoglobulin domain containing 8	0.853242	0.02781	NA
320268	B930095G15Rik	RIKEN cDNA B930095G15 gene	0.853242	0.02421	NA
11600	Angpt1	angiopoietin 1	0.852515	0.1175	0.3096
12659	Ovgp1	oviductal glycoprotein 1	0.852515	0.04248	0.165
12727	Clcn4-2	chloride channel 4-2	0.852515	0.01972	NA
13518	Dst	dystonin	0.852515	0.465	0.687
14567	Gdi1	guanosine diphosphate (GDP) dissociation inhibitor 1	0.852515	0.2363	0.4687
16180	Il1rap	interleukin 1 receptor accessory protein	0.852515	0.1676	0.3826
18005	Nek2	NIMA (never in mitosis gene a)-related expressed kinase 2	0.852515	0.3789	0.6126
20271	Scn5a	sodium channel, voltage-gated, type V, alpha	0.852515	0.2686	0.5038
22034	Traf6	TNF receptor-associated factor 6	0.852515	0.0817	0.2462
27801	Zdhhc8	zinc finger, DHHC domain containing 8	0.852515	0.1284	0.3268
67302	Zc3h13	zinc finger CCCH type containing 13	0.852515	0.0419	0.1638
70747	Tspan2	tetraspanin 2	0.852515	0.08736	0.2577
73043	2900064F13Rik	RIKEN cDNA 2900064F13 gene	0.852515	0.1076	0.2932
75296	Fgfr1op	Fgfr1 oncogene partner	0.852515	0.01012	NA
77018	Col25a1	collagen, type XXV, alpha 1	0.852515	0.3468	0.5823
78600	Pde6h	phosphodiesterase 6H, cGMP-specific, cone, gamma	0.852515	0.03093	0.1378
93760	Arid1a	AT rich interactive domain 1A (SWI-like)	0.852515	0.3746	0.6088
208198	Btbd2	BTB (POZ) domain containing 2	0.852515	0.1592	0.3704
218440	Ankrd34b	ankyrin repeat domain 34B	0.852515	0.3639	0.5992
226844	Mfsd7b	major facilitator superfamily domain containing 7B	0.852515	0.05953	0.2025
232078	Thnsl2	threonine synthase-like 2 (bacterial)	0.852515	0.07074	0.2254
235050	Zfp810	zinc finger protein 810	0.852515	0.03047	0.1368
237940	Aoc2	amine oxidase, copper containing 2 (retina-specific)	0.852515	0.03992	0.1592
244548	Elmod2	ELMO domain containing 2	0.852515	0.01418	NA
270151	Nlrx1	NLR family member X1	0.852515	0.3014	0.5372
272381	Lrrc4b	leucine rich repeat containing 4B	0.852515	0.1096	0.2965
11519	Add2	adducin 2 (beta)	0.851789	0.0752	0.2344
11787	Apbb2	amyloid beta (A4) precursor protein-binding, family B, member 2	0.851789	0.0722	0.2284

14186	Fgfr4	fibroblast growth factor receptor 4	0.851789	0.2813	0.5165
18642	Pfkm	phosphofructokinase, muscle	0.851789	0.1141	0.304
18813	Pa2g4	proliferation-associated 2G4	0.851789	0.1263	0.3235
27406	Abcf3	ATP-binding cassette, sub-family F (GCN20), member 3	0.851789	0.06982	0.2237
56018	Stard10	START domain containing 10	0.851789	0.08447	0.2517
57434	Xrcc2	X-ray repair complementing defective repair in Chinese hamster cells 2	0.851789	0.03859	0.1564
58909	Fam13a	family with sequence similarity 13, member A	0.851789	0.01291	NA
66664	Tmem41a	transmembrane protein 41a	0.851789	0.02744	NA
67307	Pbld2	phenazine biosynthesis-like protein domain containing 2	0.851789	0.1352	0.3366
69834	Rab43	RAB43, member RAS oncogene family	0.851789	0.03058	0.137
70031	Cmtm8	CKLF-like MARVEL transmembrane domain containing 8	0.851789	0.1295	0.3286
72307	2510002D24Rik	RIKEN cDNA 2510002D24 gene	0.851789	0.08978	0.2619
74194	Rnd3	Rho family GTPase 3	0.851789	0.03372	0.1452
78070	Cpt1c	carnitine palmitoyltransferase 1c	0.851789	0.06822	0.2206
117600	Srgap1	SLIT-ROBO Rho GTPase activating protein 1	0.851789	0.1369	0.3386
212390	Klhl32	kelch-like 32 (Drosophila)	0.851789	0.2812	0.5165
231946	D330028D13Rik	RIKEN cDNA D330028D13 gene	0.851789	0.07405	0.2322
232879	Zbtb45	zinc finger and BTB domain containing 45	0.851789	0.2716	0.5071
233724	Tmem41b	transmembrane protein 41B	0.851789	0.0658	0.2154
241732	Tspyl3	TSPY-like 3	0.851789	0.04766	0.1765
258532	Olfr373	olfactory receptor 373	0.851789	0.2268	0.4582
268903	Nrip1	nuclear receptor interacting protein 1	0.851789	0.0473	0.176
320556	C230085N15Rik	RIKEN cDNA C230085N15 gene	0.851789	0.1438	0.3486
328778	Rab26	RAB26, member RAS oncogene family	0.851789	0.1285	0.3269
329763	Gm5105	predicted gene 5105	0.851789	0.134	0.3347
677168	Gm9706	predicted gene 9706	0.851789	0.2666	0.5021
100043585	Gm4535	predicted gene 4535	0.851789	0.02967	0.1347
100046223	LOC100046223	40S ribosomal protein S15-like	0.851789	0.154	0.3636
100303645	LOC100303645	hypothetical LOC100303645	0.851789	0.3538	0.5885
13170	Dbp	D site albumin promoter binding protein	0.851064	0.09401	0.2695
14348	Fut9	fucosyltransferase 9	0.851064	0.05591	0.1936
14406	Gabrg2	gamma-aminobutyric acid (GABA) A receptor, subunit gamma 2	0.851064	0.01512	NA
22712	Zfp54	zinc finger protein 54	0.851064	0.04714	0.1758
50760	Fbxo17	F-box protein 17	0.851064	0.08613	0.2552
51960	Kctd18	potassium channel tetramerisation domain containing 18	0.851064	0.05118	0.1838
52897	Rbfox3	RNA binding protein, fox-1 homolog (C. elegans) 3	0.851064	0.2393	0.4718
56526	Sep-06	septin 6	0.851064	0.03098	0.1379
56533	Rgs17	regulator of G-protein signaling 17	0.851064	0.01835	NA
57295	Icmt	isoprenylcysteine carboxyl methyltransferase	0.851064	0.144	0.3489
64082	Popdc2	popeye domain containing 2	0.851064	0.1482	0.355
67046	Tbc1d7	TBC1 domain family, member 7	0.851064	0.1173	0.3092
70059	Degs2	degenerative spermatocyte homolog 2 (Drosophila), lipid desaturase	0.851064	0.1827	0.4016
71732	Vps11	vacuolar protein sorting 11 (yeast)	0.851064	0.06397	0.2113
71881	2310001A20Rik	RIKEN cDNA 2310001A20 gene	0.851064	0.1104	0.2979
75415	Arhgap12	Rho GTPase activating protein 12	0.851064	0.0309	0.1378
76901	Phf15	PHD finger protein 15	0.851064	0.01653	NA
83814	Nedd4l	neural precursor cell expressed, developmentally down-regulated gene 4-like	0.851064	0.1892	0.4107
116940	Tgs1	trimethylguanosine synthase homolog (S. cerevisiae)	0.851064	0.03841	0.156
170716	Cyp4f13	cytochrome P450, family 4, subfamily f, polypeptide 13	0.851064	0.05482	0.1916

227333	Dgkd	diacylglycerol kinase, delta	0.851064	0.03325	0.144
227446	2310035C23Rik	RIKEN cDNA 2310035C23 gene	0.851064	0.1013	0.2818
228858	Gdap1l1	ganglioside-induced differentiation-associated protein 1-like 1	0.851064	0.07844	0.24
240354	Malt1	mucosa associated lymphoid tissue lymphoma translocation gene 1	0.851064	0.09499	0.271
280645	B3gat2	beta-1,3-glucuronyltransferase 2 (glucuronosyltransferase S)	0.851064	0.05712	0.1964
319807	3110047P20Rik	RIKEN cDNA 3110047P20 gene	0.851064	0.03643	0.1511
546954	Gm6003	predicted gene 6003	0.851064	0.6571	0.8223
621381	Gm6222	predicted pseudogene 6222	0.851064	0.01976	NA
11434	Acr	acrosin prepropeptide	0.85034	0.1333	0.3339
14394	Gabra1	gamma-aminobutyric acid (GABA) A receptor, subunit alpha 1	0.85034	0.07004	0.224
14805	Grik1	glutamate receptor, ionotropic, kainate 1	0.85034	0.1901	0.412
15331	Hmgn2	high mobility group nucleosomal binding domain 2	0.85034	0.2668	0.5023
16574	Kif5c	kinesin family member 5C	0.85034	0.02188	NA
17760	Mtap6	microtubule-associated protein 6	0.85034	0.09958	0.2785
22230	Ufd1l	ubiquitin fusion degradation 1 like	0.85034	0.1241	0.3206
27223	Trp53bp1	transformation related protein 53 binding protein 1	0.85034	0.01437	NA
53623	Gria3	glutamate receptor, ionotropic, AMPA3 (alpha 3)	0.85034	0.03822	0.1557
54217	Rpl36	ribosomal protein L36	0.85034	0.1371	0.339
56045	Samhd1	SAM domain and HD domain, 1	0.85034	0.02314	NA
56632	Sphk2	sphingosine kinase 2	0.85034	0.06107	0.2055
67266	Fam69a	family with sequence similarity 69, member A	0.85034	0.04993	0.1815
70208	Med23	mediator complex subunit 23	0.85034	0.04121	0.1623
70209	Tmem143	transmembrane protein 143	0.85034	0.1607	0.3725
71702	Cdc5l	cell division cycle 5-like (S. pombe)	0.85034	0.1405	0.3442
71978	Ppp2r2a	protein phosphatase 2 (formerly 2A), regulatory subunit B (PR 52), alpha isoform	0.85034	0.03827	0.1558
74498	Gorasp1	golgi reassembly stacking protein 1	0.85034	0.09783	0.2759
94063	Mrpl16	mitochondrial ribosomal protein L16	0.85034	0.03878	0.1567
101458	AA536887	expressed sequence AA536887	0.85034	0.06161	0.2064
105246	Brd9	bromodomain containing 9	0.85034	0.04407	0.1688
107566	Arl2bp	ADP-ribosylation factor-like 2 binding protein	0.85034	0.01495	NA
114128	Laptm4b	lysosomal-associated protein transmembrane 4B	0.85034	0.06931	0.2227
208194	Exog	endo/exonuclease (5'-3'), endonuclease G-like	0.85034	0.1699	0.3851
211383	Fam123c	family with sequence similarity 123, member C	0.85034	0.2502	0.4841
216080	Ube2d1	ubiquitin-conjugating enzyme E2D 1, UBC4/5 homolog (yeast)	0.85034	0.1128	0.3019
216724	Rufy1	RUN and FYVE domain containing 1	0.85034	0.04949	0.1805
232989	Hnrnpul1	heterogeneous nuclear ribonucleoprotein U-like 1	0.85034	0.06285	0.2093
233410	Zfp592	zinc finger protein 592	0.85034	0.01316	NA
240119	St6gal2	beta galactoside alpha 2,6 sialyltransferase 2	0.85034	0.126	0.3231
242736	Pramef8	PRAME family member 8	0.85034	0.07622	0.2365
268448	Phf12	PHD finger protein 12	0.85034	0.02937	0.1339
319478	Cxxc4	CXXC finger 4	0.85034	0.08235	0.2475
319974	Auts2	autism susceptibility candidate 2	0.85034	0.3272	0.5636
330908	Opcml	opioid binding protein/cell adhesion molecule-like	0.85034	0.06714	0.2184
332359	Tigd3	tigger transposable element derived 3	0.85034	0.09852	0.277
338349	Cntln	centlein, centrosomal protein	0.85034	0.02227	NA
11677	Akr1b3	aldo-keto reductase family 1, member B3 (aldose reductase)	0.849618	0.04507	0.1711
12497	Entpd6	ectonucleoside triphosphate diphosphohydrolase 6	0.849618	0.2365	0.4688
12522	Cd83	CD83 antigen	0.849618	0.1356	0.3372
12563	Cdh6	cadherin 6	0.849618	0.1276	0.3255

14417	Gad2	glutamic acid decarboxylase 2	0.849618	0.01024	NA
14685	Gnat1	guanine nucleotide binding protein, alpha transducing 1	0.849618	0.0674	0.2189
19888	Rp1	retinitis pigmentosa 1 (human)	0.849618	0.03232	0.1414
20322	Sord	sorbitol dehydrogenase	0.849618	0.1054	0.2889
20463	Cox7a2l	cytochrome c oxidase subunit VIIa polypeptide 2-like	0.849618	0.01218	NA
23988	Pin1	protein (peptidyl-prolyl cis/trans isomerase) NIMA-interacting 1	0.849618	0.265	0.5008
53379	Hnrnpa2b1	heterogeneous nuclear ribonucleoprotein A2/B1	0.849618	0.4667	0.6887
53970	Rfx5	regulatory factor X, 5 (influences HLA class II expression)	0.849618	0.3124	0.5491
57775	Usp29	ubiquitin specific peptidase 29	0.849618	0.03118	0.1384
66297	2610017I09Rik	RIKEN cDNA 2610017I09 gene	0.849618	0.09281	0.2674
66959	Dusp26	dual specificity phosphatase 26 (putative)	0.849618	0.01093	NA
67414	Mfn1	mitofusin 1	0.849618	0.01904	NA
67487	Dhx40	DEAH (Asp-Glu-Ala-His) box polypeptide 40	0.849618	0.07073	0.2254
67671	Rpl38	ribosomal protein L38	0.849618	0.1612	0.373
69232	Qrich1	glutamine-rich 1	0.849618	0.07388	0.232
69297	Lrrc46	leucine rich repeat containing 46	0.849618	0.1452	0.3506
71643	4930422G04Rik	RIKEN cDNA 4930422G04 gene	0.849618	0.5181	0.7273
75612	Gns	glucosamine (N-acetyl)-6-sulfatase	0.849618	0.007179	NA
77480	Kidins220	kinase D-interacting substrate 220	0.849618	0.1137	0.3035
104806	Fancm	Fanconi anemia, complementation group M	0.849618	0.1051	0.2885
207806	Gm608	predicted gene 608	0.849618	0.3321	0.5682
213011	Zfp583	zinc finger protein 583	0.849618	0.1852	0.4047
227357	Espnl	espin-like	0.849618	0.1373	0.3393
227659	Slc2a6	solute carrier family 2 (facilitated glucose transporter), member 6	0.849618	0.3948	0.6254
320747	Lingo4	leucine rich repeat and Ig domain containing 4	0.849618	0.1527	0.3621
11546	Parp2	poly (ADP-ribose) polymerase family, member 2	0.848896	0.0636	0.2107
18187	Nrp2	neuropilin 2	0.848896	0.1133	0.3026
19652	Rbm3	RNA binding motif protein 3	0.848896	0.2406	0.4733
20181	Rxra	retinoid X receptor alpha	0.848896	0.1397	0.343
30052	Pcsk1n	proprotein convertase subtilisin/kexin type 1 inhibitor	0.848896	0.1153	0.3061
30930	Vps26a	vacuolar protein sorting 26 homolog A (yeast)	0.848896	0.07256	0.2292
66724	Tab3	TGF-beta activated kinase 1/MAP3K7 binding protein 3	0.848896	0.04538	0.1717
66861	Dnajc10	DnaJ (Hsp40) homolog, subfamily C, member 10	0.848896	0.2339	0.4654
67102	D16Ertd472e	DNA segment, Chr 16, ERATO Doi 472, expressed	0.848896	0.02313	NA
67422	Dhdds	dehydrodolichyl diphosphate synthase	0.848896	0.006207	NA
69656	Pir	pirin	0.848896	0.09983	0.279
70081	2210404O09Rik	RIKEN cDNA 2210404O09 gene	0.848896	0.1128	0.3017
72899	Macrod2	MACRO domain containing 2	0.848896	0.08007	0.2434
83679	Pde4dip	phosphodiesterase 4D interacting protein (myomegalin)	0.848896	0.02101	NA
98970	Fibcd1	fibrinogen C domain containing 1	0.848896	0.277	0.5126
107769	Tm6sf1	transmembrane 6 superfamily member 1	0.848896	0.1424	0.3468
194268	9930104L06Rik	RIKEN cDNA 9930104L06 gene	0.848896	0.07665	0.237
214812	Zfp609	zinc finger protein 609	0.848896	0.1581	0.369
215335	Slc36a1	solute carrier family 36 (proton/amino acid symporter), member 1	0.848896	0.2592	0.4945
215821	D10Bwg1379e	DNA segment, Chr 10, Brigham & Women's Genetics 1379 expressed	0.848896	0.436	0.6627
227541	Camk1d	calcium/calmodulin-dependent protein kinase ID	0.848896	0.2794	0.515
234072	Adprhl1	ADP-ribosylhydrolase like 1	0.848896	0.3537	0.5885
234695	Rltpr	RGD motif, leucine rich repeats, tropomodulin domain and proline-rich containing	0.848896	0.2263	0.4577
319642	Rab9b	RAB9B, member RAS oncogene family	0.848896	0.01654	NA

320827	C530008M17Rik	RIKEN cDNA C530008M17 gene	0.848896	0.03948	0.1582
329972	Spata21	spermatogenesis associated 21	0.848896	0.7723	0.8906
338372	Map3k9	mitogen-activated protein kinase kinase kinase 9	0.848896	0.2033	0.4296
100037262	Gm12359	predicted gene 12359	0.848896	0.1771	0.3943
11432	Acp2	acid phosphatase 2, lysosomal	0.848176	0.1781	0.3957
11773	Ap2m1	adaptor protein complex AP-2, mu1	0.848176	0.03511	0.1484
11804	Aplp2	amyloid beta (A4) precursor-like protein 2	0.848176	0.008685	NA
14676	Gna15	guanine nucleotide binding protein, alpha 15	0.848176	0.4274	0.6545
14802	Gria4	glutamate receptor, ionotropic, AMPA4 (alpha 4)	0.848176	0.1782	0.3958
16704	Krtap8-2	keratin associated protein 8-2	0.848176	0.276	0.5115
18391	Sigmar1	sigma non-opioid intracellular receptor 1	0.848176	0.09478	0.2707
19277	Ptpro	protein tyrosine phosphatase, receptor type, O	0.848176	0.01716	NA
20400	Sh2d1a	SH2 domain protein 1A	0.848176	0.04177	0.1636
24116	Whsc2	Wolf-Hirschhorn syndrome candidate 2 (human)	0.848176	0.01603	NA
54004	Diap2	diaphanous homolog 2 (Drosophila)	0.848176	0.154	0.3636
54196	Pabpn1	poly(A) binding protein, nuclear 1	0.848176	0.0114	NA
68002	1110058L19Rik	RIKEN cDNA 1110058L19 gene	0.848176	0.04838	0.178
69089	Oxa1l	oxidase assembly 1-like	0.848176	0.03331	0.1442
70387	Ttc9c	tetratricopeptide repeat domain 9C	0.848176	0.1088	0.2951
71887	Ppm1j	protein phosphatase 1J	0.848176	0.2367	0.4689
73368	Col20a1	collagen, type XX, alpha 1	0.848176	0.07837	0.24
83669	Wdr6	WD repeat domain 6	0.848176	0.1422	0.3465
97387	Strn4	striatin, calmodulin binding protein 4	0.848176	0.09671	0.2741
104079	Nxph3	neurexophilin 3	0.848176	0.08948	0.2613
109959	Amy2a5	amylase 2a5	0.848176	0.05407	0.1899
110094	Phka2	phosphorylase kinase alpha 2	0.848176	0.08677	0.2566
216233	Socs2	suppressor of cytokine signaling 2	0.848176	0.01218	NA
217779	Lysmd1	LysM, putative peptidoglycan-binding, domain containing 1	0.848176	0.0422	0.1644
217826	Kcnk13	potassium channel, subfamily K, member 13	0.848176	0.4037	0.6336
232975	Atp1a3	ATPase, Na+/K+ transporting, alpha 3 polypeptide	0.848176	0.3235	0.5601
268902	Robo2	roundabout homolog 2 (Drosophila)	0.848176	0.05696	0.1961
381979	Brsk1	BR serine/threonine kinase 1	0.848176	0.0152	NA
12721	Coro1a	coronin, actin binding protein 1A	0.847458	0.08276	0.2482
12921	Crhr1	corticotropin releasing hormone receptor 1	0.847458	0.1375	0.3395
14559	Gdf1	growth differentiation factor 1	0.847458	0.4379	0.6643
16443	Itsn1	intersectin 1 (SH3 domain protein 1A)	0.847458	0.4689	0.6908
19334	Rab22a	RAB22A, member RAS oncogene family	0.847458	0.09489	0.2709
19713	Ret	ret proto-oncogene	0.847458	0.03646	0.1512
20359	Sema6b	sema domain, transmembrane domain (TM), and cytoplasmic domain, (semaphorin) 6B	0.847458	0.1753	0.392
21892	Tll1	tolloid-like	0.847458	0.1657	0.3797
21944	Tnfsf12	tumor necrosis factor (ligand) superfamily, member 12	0.847458	0.1542	0.3639
23917	Impdh1	inosine 5'-phosphate dehydrogenase 1	0.847458	0.1122	0.3006
56856	Insm2	insulinoma-associated 2	0.847458	0.2043	0.4306
57258	Xpo4	exportin 4	0.847458	0.1686	0.3836
64379	Irx6	Iroquois related homeobox 6 (Drosophila)	0.847458	0.04971	0.1811
67077	1700019N12Rik	RIKEN cDNA 1700019N12 gene	0.847458	0.02535	0.1222
70549	Tln2	talin 2	0.847458	0.05302	0.1873
70757	Ptplb	protein tyrosine phosphatase-like (proline instead of catalytic arginine), member b	0.847458	0.03303	0.1434
72054	Cyp4f18	cytochrome P450, family 4, subfamily f, polypeptide 18	0.847458	0.3039	0.5401

73389	Hbp1	high mobility group box transcription factor 1	0.847458	0.1713	0.387
73998	Herc3	hect domain and RLD 3	0.847458	0.01723	NA
74143	Opa1	optic atrophy 1 homolog (human)	0.847458	0.004628	NA
75430	3200002M19Rik	RIKEN cDNA 3200002M19 gene	0.847458	0.06994	0.2238
78656	Brd8	bromodomain containing 8	0.847458	0.01047	NA
94253	Hecw1	HECT, C2 and WW domain containing E3 ubiquitin protein ligase 1	0.847458	0.2363	0.4687
101631	Pwwp2b	PWWP domain containing 2B	0.847458	0.07019	0.2244
103551	E130012A19Rik	RIKEN cDNA E130012A19 gene	0.847458	0.06792	0.2199
170677	Cdhr1	cadherin-related family member 1	0.847458	0.01277	NA
231801	Agfg2	ArfGAP with FG repeats 2	0.847458	0.1362	0.338
235330	Ttc12	tetratricopeptide repeat domain 12	0.847458	0.3513	0.5867
246317	Neto1	neuropilin (NRP) and tolloid (TLL)-like 1	0.847458	0.003293	NA
269019	Stk32a	serine/threonine kinase 32A	0.847458	0.08927	0.2611
270190	Ephb1	Eph receptor B1	0.847458	0.1386	0.3414
272009	Srsf13b	serine/arginine-rich splicing factor 13B	0.847458	0.02599	0.1242
319263	Pcmtd1	protein-L-isoaspartate (D-aspartate) O-methyltransferase domain containing 1	0.847458	0.2266	0.4582
319535	Zfp182	zinc finger protein 182	0.847458	0.08854	0.2599
319713	Ablim3	actin binding LIM protein family, member 3	0.847458	0.04507	0.1711
380916	Lrch1	leucine-rich repeats and calponin homology (CH) domain containing 1	0.847458	0.04087	0.1615
432611	Dnaic2	dynein, axonemal, intermediate chain 2	0.847458	0.2719	0.5074
547160	Gm14484	predicted gene 14484	0.847458	0.3067	0.5432
11899	Astn1	astrotactin 1	0.84674	0.009616	NA
12876	Cpe	carboxypeptidase E	0.84674	0.04416	0.169
17755	Mtap1b	microtubule-associated protein 1B	0.84674	0.1158	0.3066
18049	Ngf	nerve growth factor	0.84674	0.3718	0.6067
19300	Abcd4	ATP-binding cassette, sub-family D (ALD), member 4	0.84674	0.02296	0.1153
20147	Rs1	retinoschisis (X-linked, juvenile) 1 (human)	0.84674	0.02798	0.1302
20515	Slc20a1	solute carrier family 20, member 1	0.84674	0.01951	NA
21389	Tbx6	T-box 6	0.84674	0.1219	0.3169
23948	Mmp17	matrix metallopeptidase 17	0.84674	0.08792	0.2586
26564	Ror2	receptor tyrosine kinase-like orphan receptor 2	0.84674	0.3818	0.6152
54369	Nme6	non-metastatic cells 6, protein expressed in (nucleoside-diphosphate kinase)	0.84674	0.1909	0.413
56436	Adrm1	adhesion regulating molecule 1	0.84674	0.0705	0.2249
56515	Rnf138	ring finger protein 138	0.84674	0.1874	0.408
66869	Zfp869	zinc finger protein 869	0.84674	0.03361	0.1449
67834	Idh3a	isocitrate dehydrogenase 3 (NAD+) alpha	0.84674	0.0215	0.111
70796	Zdhhc1	zinc finger, DHHC domain containing 1	0.84674	0.231	0.4624
110033	Kif22	kinesin family member 22	0.84674	0.03046	0.1368
213603	Slc44a3	solute carrier family 44, member 3	0.84674	0.5507	0.7511
217882	AW555464	expressed sequence AW555464	0.84674	0.1087	0.295
223642	Zc3h3	zinc finger CCCH type containing 3	0.84674	0.05666	0.1955
230125	Mcart1	mitochondrial carrier triple repeat 1	0.84674	0.0267	0.1264
235344	Sik2	salt inducible kinase 2	0.84674	0.4877	0.7055
239099	Homez	homeodomain leucine zipper-encoding gene	0.84674	0.06415	0.2117
260297	Prrt1	proline-rich transmembrane protein 1	0.84674	0.01867	NA
272347	Zfp398	zinc finger protein 398	0.84674	0.009855	NA
12921	Crhr1	corticotropin releasing hormone receptor 1	0.846024	0.2965	0.5324
13982	Esr1	estrogen receptor 1 (alpha)	0.846024	0.1374	0.3393
16408	Itgal	integrin alpha L	0.846024	0.2362	0.4687

22215	Ube3a	ubiquitin protein ligase E3A	0.846024	0.2275	0.4586
22223	Uchl1	ubiquitin carboxy-terminal hydrolase L1	0.846024	0.1034	0.2853
22247	Umps	uridine monophosphate synthetase	0.846024	0.03417	0.1462
22264	Prap1	proline-rich acidic protein 1	0.846024	0.1146	0.3048
24086	Tlk2	tousled-like kinase 2 (Arabidopsis)	0.846024	0.08874	0.2603
57257	Vav3	vav 3 oncogene	0.846024	0.07656	0.2368
66603	Sip1	survival of motor neuron protein interacting protein 1	0.846024	0.1291	0.3278
67153	Rnaseh2b	ribonuclease H2, subunit B	0.846024	0.07683	0.2374
68108	9430008C03Rik	RIKEN cDNA 9430008C03 gene	0.846024	0.03957	0.1585
69046	Isca1	iron-sulfur cluster assembly 1 homolog (S. cerevisiae)	0.846024	0.1315	0.3317
69239	2610034M16Rik	RIKEN cDNA 2610034M16 gene	0.846024	0.01147	NA
72584	Cul4b	cullin 4B	0.846024	0.02046	0.1074
74482	Ifitm7	interferon induced transmembrane protein 7	0.846024	0.2501	0.4841
74530	9030612E09Rik	RIKEN cDNA 9030612E09 gene	0.846024	0.229	0.46
75552	Paqr9	progestin and adipoQ receptor family member IX	0.846024	0.05182	0.1851
78552	E130201H02Rik	Y box protein 1 pseudogene	0.846024	0.3962	0.6266
94040	Clmn	calmin	0.846024	0.009909	NA
114679	Selm	selenoprotein M	0.846024	0.01746	NA
217351	Tnrc6c	trinucleotide repeat containing 6C	0.846024	0.04725	0.1759
232286	Tmf1	TATA element modulatory factor 1	0.846024	0.08039	0.2439
244886	AI118078	expressed sequence AI118078	0.846024	0.06669	0.2173
320394	Cenpt	centromere protein T	0.846024	0.08854	0.2599
329165	Abi2	abl-interactor 2	0.846024	0.02053	0.1076
381633	Gm1673	predicted gene 1673	0.846024	0.02207	0.1125
621893	Hist2h2ab	histone cluster 2, H2ab	0.846024	0.439	0.665
623661	Lipt1	lipoyltransferase 1	0.846024	0.09236	0.2663
13429	Dnm1	dynamin 1	0.845309	0.2972	0.5334
14270	Srgap2	SLIT-ROBO Rho GTPase activating protein 2	0.845309	0.1022	0.2833
14586	Gfra2	glial cell line derived neurotrophic factor family receptor alpha 2	0.845309	0.01974	0.1052
16493	Kcna5	potassium voltage-gated channel, shaker-related subfamily, member 5	0.845309	0.01983	0.1056
17395	Mmp9	matrix metallopeptidase 9	0.845309	0.1996	0.425
51885	Tubgcp4	tubulin, gamma complex associated protein 4	0.845309	0.0172	NA
56048	Lgals8	lectin, galactose binding, soluble 8	0.845309	0.1255	0.3225
56428	Mtch2	mitochondrial carrier homolog 2 (C. elegans)	0.845309	0.1803	0.3986
66296	Haus2	HAUS augmin-like complex, subunit 2	0.845309	0.1611	0.373
66646	Rpe	ribulose-5-phosphate-3-epimerase	0.845309	0.1728	0.3888
67727	Stx17	syntaxin 17	0.845309	0.03208	0.1408
69714	Tfpt	TCF3 (E2A) fusion partner	0.845309	0.2572	0.4924
69743	Casz1	castor homolog 1, zinc finger (Drosophila)	0.845309	0.06349	0.2105
70802	Pwwp2a	PWWP domain containing 2A	0.845309	0.03524	0.1484
70891	Spdya	speedy homolog A (Xenopus laevis)	0.845309	0.03958	0.1585
70924	4921511C10Rik	RIKEN cDNA 4921511C10 gene	0.845309	0.5452	0.7478
75141	Rasd2	RASD family, member 2	0.845309	0.05255	0.1863
75276	Ppp1r1c	protein phosphatase 1, regulatory (inhibitor) subunit 1C	0.845309	0.1787	0.3966
100072	Camta1	calmodulin binding transcription activator 1	0.845309	0.006317	NA
100710	Pds5b	PDS5, regulator of cohesion maintenance, homolog B (S. cerevisiae)	0.845309	0.1032	0.285
229004	Gmeb2	glucocorticoid modulatory element binding protein 2	0.845309	0.04236	0.1647
230577	Pars2	prolyl-tRNA synthetase (mitochondrial)(putative)	0.845309	0.04667	0.1747
241576	Ldlrad3	low density lipoprotein receptor class A domain containing 3	0.845309	0.2418	0.4747

242443	Grin3a	glutamate receptor ionotropic, NMDA3A	0.845309	0.04137	0.1627
268448	Phf12	PHD finger protein 12	0.845309	0.1868	0.4075
328110	Prpf39	PRP39 pre-mRNA processing factor 39 homolog (yeast)	0.845309	0.2328	0.464
329993	Gm438	predicted gene 438	0.845309	0.2479	0.4821
434178	Zfp141	zinc finger protein 141	0.845309	0.3075	0.5437
12039	Bckdha	branched chain ketoacid dehydrogenase E1, alpha polypeptide	0.844595	0.07741	0.2386
12660	Chka	choline kinase alpha	0.844595	0.08331	0.2495
13139	Dgka	diacylglycerol kinase, alpha	0.844595	0.01642	NA
13429	Dnm1	dynamin 1	0.844595	0.1326	0.3332
13829	Epb4.9	erythrocyte protein band 4.9	0.844595	0.416	0.6447
14704	Gng3	guanine nucleotide binding protein (G protein), gamma 3	0.844595	0.09726	0.275
16580	Kifc5b	kinesin family member C5B	0.844595	0.2847	0.5202
18738	Pitpna	phosphatidylinositol transfer protein, alpha	0.844595	0.0135	NA
19691	Recql	RecQ protein-like	0.844595	0.09202	0.2658
22327	Vbp1	von Hippel-Lindau binding protein 1	0.844595	0.1474	0.354
53886	Cdkl2	cyclin-dependent kinase-like 2 (CDC2-related kinase)	0.844595	0.04399	0.1686
56839	Lgi1	leucine-rich repeat LGI family, member 1	0.844595	0.1583	0.3693
57785	Rangrf	RAN guanine nucleotide release factor	0.844595	0.06881	0.2217
59069	Tpm3	tropomyosin 3, gamma	0.844595	0.3101	0.5467
64450	Gpr85	G protein-coupled receptor 85	0.844595	0.01535	NA
66958	Tmx2	thioredoxin-related transmembrane protein 2	0.844595	0.07163	0.2272
71384	5430440L12Rik	RIKEN cDNA 5430440L12 gene	0.844595	0.2405	0.4732
71901	2310028H24Rik	RIKEN cDNA 2310028H24 gene	0.844595	0.02206	0.1125
72477	Tmem87b	transmembrane protein 87B	0.844595	0.07399	0.2321
108816	4933409K07Rik	RIKEN cDNA 4933409K07 gene	0.844595	0.3591	0.5943
208968	Zfp280c	zinc finger protein 280C	0.844595	0.1013	0.2818
235048	Zfp599	zinc finger protein 599	0.844595	0.01782	NA
239102	Zfhx2	zinc finger homeobox 2	0.844595	0.2664	0.5018
243961	Shank1	SH3/ankyrin domain gene 1	0.844595	0.1549	0.3647
330671	B4galnt4	beta-1,4-N-acetyl-galactosaminyl transferase 4	0.844595	0.1957	0.4196
381269	Mreg	melanoregulin	0.844595	0.07293	0.2301
414093	A830082N09Rik	RIKEN cDNA A830082N09 gene	0.844595	0.02705	0.1272
100043726	Gm4610	predicted gene 4610	0.844595	0.196	0.42
100101806	Srp54c	signal recognition particle 54C	0.844595	0.02467	0.1201
14400	Gabrb1	gamma-aminobutyric acid (GABA) A receptor, subunit beta 1	0.843882	0.09311	0.2679
15331	Hmgn2	high mobility group nucleosomal binding domain 2	0.843882	0.01348	NA
15525	Hspa4	heat shock protein 4	0.843882	0.01234	NA
18125	Nos1	nitric oxide synthase 1, neuronal	0.843882	0.14	0.3435
19054	Ppp2r3d	protein phosphatase 2 (formerly 2A), regulatory subunit B'', delta	0.843882	0.006123	NA
19201	Pstpip2	proline-serine-threonine phosphatase-interacting protein 2	0.843882	0.614	0.795
20286	Zc3h7b	zinc finger CCCH type containing 7B	0.843882	0.02277	0.115
21393	Tcap	titin-cap	0.843882	0.0583	0.1994
27784	Commd8	COMM domain containing 8	0.843882	0.03677	0.1519
27984	Efhd2	EF hand domain containing 2	0.843882	0.0488	0.1787
50915	Grb14	growth factor receptor bound protein 14	0.843882	0.1497	0.3576
53420	Syt5	synaptotagmin V	0.843882	0.04116	0.1622
56550	Ube2d2	ubiquitin-conjugating enzyme E2D 2	0.843882	0.05684	0.1959
60533	Cd274	CD274 antigen	0.843882	0.12	0.3137
66880	Rsrc1	arginine/serine-rich coiled-coil 1	0.843882	0.02035	0.107

67443	Map1lc3b	microtubule-associated protein 1 light chain 3 beta	0.843882	0.101	0.2811
67465	Sf3a1	splicing factor 3a, subunit 1	0.843882	0.006689	NA
70285	Rpf1	ribosome production factor 1 homolog (S. cerevisiae)	0.843882	0.1717	0.3874
72825	Mon1a	MON1 homolog A (yeast)	0.843882	0.0303	0.1365
97440	B3gnt9-ps	UDP-GlcNAc:betaGal beta-1,3-N-acetylglucosaminyltransferase 9, pseudogene	0.843882	0.1785	0.3963
100317	AU040320	expressed sequence AU040320	0.843882	0.2806	0.516
105675	Ppif	peptidylprolyl isomerase F (cyclophilin F)	0.843882	0.09841	0.2769
194231	Cnksr1	connector enhancer of kinase suppressor of Ras 1	0.843882	0.2317	0.4631
212391	Lcor	ligand dependent nuclear receptor corepressor	0.843882	0.1332	0.3339
225192	Hrh4	histamine receptor H4	0.843882	0.4454	0.671
231855	C330006K01Rik	RIKEN cDNA C330006K01 gene	0.843882	0.01113	NA
236266	Alms1	Alstrom syndrome 1 homolog (human)	0.843882	0.2562	0.4913
238722	Zfp72	zinc finger protein 72	0.843882	0.4197	0.6475
329540	8430427H17Rik	RIKEN cDNA 8430427H17 gene	0.843882	0.01707	0.09647
338521	Fa2h	fatty acid 2-hydroxylase	0.843882	0.3743	0.6084
100040962	Gm3065	predicted gene 3065	0.843882	0.2331	0.4643
14105	Srsf10	serine/arginine-rich splicing factor 10	0.84317	0.148	0.3547
14527	Gcgr	glucagon receptor	0.84317	0.3328	0.5689
19298	Pex19	peroxisomal biogenesis factor 19	0.84317	0.1204	0.3143
22589	Atrx	alpha thalassemia/mental retardation syndrome X-linked homolog (human)	0.84317	0.1342	0.3351
29859	Sult4a1	sulfotransferase family 4A, member 1	0.84317	0.02948	0.1341
50795	Sh3bgr	SH3-binding domain glutamic acid-rich protein	0.84317	0.0392	0.1577
56371	Fzr1	fizzy/cell division cycle 20 related 1 (Drosophila)	0.84317	0.1271	0.3246
64337	Gng13	guanine nucleotide binding protein (G protein), gamma 13	0.84317	0.07267	0.2294
65079	Rtn4r	reticulon 4 receptor	0.84317	0.2168	0.4456
67166	Arl8b	ADP-ribosylation factor-like 8B	0.84317	0.02484	0.1206
70831	Krtap31-1	keratin associated protein 31-1	0.84317	0.3108	0.547
71413	5430410E06Rik	RIKEN cDNA 5430410E06 gene	0.84317	0.5078	0.7193
73327	1700040I03Rik	RIKEN cDNA 1700040I03 gene	0.84317	0.06369	0.2108
74330	Dnajc14	DnaJ (Hsp40) homolog, subfamily C, member 14	0.84317	0.1366	0.3383
74386	Rmi1	RMI1, RecQ mediated genome instability 1, homolog (S. cerevisiae)	0.84317	0.0201	0.1065
94111	Mepe	matrix extracellular phosphoglycoprotein with ASARM motif (bone)	0.84317	0.3605	0.5954
104010	Cdh22	cadherin 22	0.84317	0.02899	0.1328
109161	Ube2q2	ubiquitin-conjugating enzyme E2Q (putative) 2	0.84317	0.07473	0.2334
218035	Vps41	vacuolar protein sorting 41 (yeast)	0.84317	0.0297	0.1348
230908	Tardbp	TAR DNA binding protein	0.84317	0.003587	NA
231871	Daglb	diacylglycerol lipase, beta	0.84317	0.07305	0.2303
232223	Txnrd3	thioredoxin reductase 3	0.84317	0.02387	0.1178
320207	Pik3r5	phosphoinositide-3-kinase, regulatory subunit 5, p101	0.84317	0.1554	0.3651
433882	Gm16223	predicted gene 16223	0.84317	0.621	0.7994
552890	LOC552890	hypothetical LOC552890	0.84317	0.04548	0.1718
629678	Gm6994	predicted gene 6994	0.84317	0.2709	0.5062
12728	Clcn5	chloride channel 5	0.84246	0.08728	0.2575
13004	Ncan	neurocan	0.84246	0.1466	0.3526
14347	Fut7	fucosyltransferase 7	0.84246	0.04796	0.1772
15331	Hmgn2	high mobility group nucleosomal binding domain 2	0.84246	0.1198	0.3134
15569	Elavl2	ELAV (embryonic lethal, abnormal vision, Drosophila)-like 2 (Hu antigen B)	0.84246	0.1697	0.3849
18640	Pfkfb2	6-phosphofructo-2-kinase/fructose-2,6-biphosphatase 2	0.84246	0.02278	0.115
22688	Zfp26	zinc finger protein 26	0.84246	0.1918	0.4139

27059	Sh3d19	SH3 domain protein D19	0.84246	0.2926	0.5284
50770	Atp11a	ATPase, class VI, type 11A	0.84246	0.103	0.2848
53870	Cntn6	contactin 6	0.84246	0.05839	0.1996
54645	Gripap1	GRIP1 associated protein 1	0.84246	0.01579	0.09228
54648	Ccdc120	coiled-coil domain containing 120	0.84246	0.1551	0.3648
67278	2900092E17Rik	RIKEN cDNA 2900092E17 gene	0.84246	0.01146	NA
67873	Mri1	methylthioribose-1-phosphate isomerase homolog (S. cerevisiae)	0.84246	0.03648	0.1512
69024	Snx15	sorting nexin 15	0.84246	0.07044	0.2249
74734	Rhoh	ras homolog gene family, member H	0.84246	0.1742	0.3908
75086	4930520P13Rik	RIKEN cDNA 4930520P13 gene	0.84246	0.1804	0.3986
80515	A030009H04Rik	RIKEN cDNA A030009H04 gene	0.84246	0.06647	0.2169
106707	Rpusd1	RNA pseudouridylate synthase domain containing 1	0.84246	0.1691	0.3843
192167	Nlgn1	neuroligin 1	0.84246	0.05083	0.1829
225875	Lrfn4	leucine rich repeat and fibronectin type III domain containing 4	0.84246	0.05871	0.2005
320105	D030036P13Rik	RIKEN cDNA D030036P13 gene	0.84246	0.09672	0.2741
381511	Pdp1	pyruvate dehyrogenase phosphatase catalytic subunit 1	0.84246	0.03873	0.1566
432725	Gm5445	predicted gene 5445	0.84246	0.02004	0.1064
434057	Nat8b	N-acetyltransferase 8B	0.84246	0.1423	0.3467
625638	Fam43b	family with sequence similarity 43, member B	0.84246	0.1563	0.3663
11980	Atp8a1	ATPase, aminophospholipid transporter (APLT), class I, type 8A, member 1	0.841751	0.4251	0.6526
12585	Cdr2	cerebellar degeneration-related 2	0.841751	0.1333	0.3339
14772	Grk4	G protein-coupled receptor kinase 4	0.841751	0.02332	0.1165
17355	Aff1	AF4/FMR2 family, member 1	0.841751	0.3495	0.5854
19765	Ralbp1	ralA binding protein 1	0.841751	0.008304	NA
20935	Surf6	surfeit gene 6	0.841751	0.03125	0.1385
27993	Imp4	IMP4, U3 small nucleolar ribonucleoprotein, homolog (yeast)	0.841751	0.09736	0.2752
56324	Stam2	signal transducing adaptor molecule (SH3 domain and ITAM motif) 2	0.841751	0.1171	0.309
57442	Kcne3	potassium voltage-gated channel, Isk-related subfamily, gene 3	0.841751	0.557	0.7562
67367	Gcfc1	GC-rich sequence DNA-binding factor 1	0.841751	0.03176	0.14
69276	Sec62	SEC62 homolog (S. cerevisiae)	0.841751	0.03285	0.1429
71472	Usp19	ubiquitin specific peptidase 19	0.841751	0.03123	0.1385
72759	Tmem135	transmembrane protein 135	0.841751	0.05505	0.1922
74525	8430419L09Rik	RIKEN cDNA 8430419L09 gene	0.841751	0.4121	0.6408
107324	AV313155	expressed sequence AV313155	0.841751	0.1703	0.3856
114642	Brdt	bromodomain, testis-specific	0.841751	0.1395	0.3429
170643	Kirrel	kin of IRRE like (Drosophila)	0.841751	0.3133	0.5495
209707	Lcorl	ligand dependent nuclear receptor corepressor-like	0.841751	0.06955	0.2231
213753	Zfp598	zinc finger protein 598	0.841751	0.1953	0.4189
223527	Eny2	enhancer of yellow 2 homolog (Drosophila)	0.841751	0.02241	0.1138
237831	Slc13a5	solute carrier family 13 (sodium-dependent citrate transporter), member 5	0.841751	0.06449	0.2126
240614	Ranbp6	RAN binding protein 6	0.841751	0.1422	0.3465
246257	Ovca2	candidate tumor suppressor in ovarian cancer 2	0.841751	0.04527	0.1715
277973	Slc9a5	solute carrier family 9 (sodium/hydrogen exchanger), member 5	0.841751	0.06321	0.2099
320799	Zhx3	zinc fingers and homeoboxes 3	0.841751	0.02878	0.1322
606497	Otx2os1	Otx2 opposite strand transcript 1	0.841751	0.1361	0.338
100233208	Gm10778	predicted gene 10778	0.841751	0.4336	0.6607
14385	Slc37a4	solute carrier family 37 (glucose-6-phosphate transporter), member 4	0.841043	0.02149	0.111
14787	Rhpn1	rhophilin, Rho GTPase binding protein 1	0.841043	0.1375	0.3395
16876	Lhx9	LIM homeobox protein 9	0.841043	0.1381	0.3405

17537	Meis3	Meis homeobox 3	0.841043	0.1609	0.3726
19302	Pxmp3	peroxisomal membrane protein 3	0.841043	0.02414	0.1186
20665	Sox10	SRY-box containing gene 10	0.841043	0.3894	0.6214
22152	Tubb3	tubulin, beta 3	0.841043	0.03291	0.1431
23821	Bace1	beta-site APP cleaving enzyme 1	0.841043	0.006411	NA
24057	Sh3yl1	Sh3 domain YSC-like 1	0.841043	0.009442	NA
55935	Fnbp4	formin binding protein 4	0.841043	0.0394	0.1581
56417	Adar	adenosine deaminase, RNA-specific	0.841043	0.03021	0.1361
56468	Socs5	suppressor of cytokine signaling 5	0.841043	0.01635	0.09371
67254	2900011O08Rik	RIKEN cDNA 2900011O08 gene	0.841043	0.01595	0.0926
67412	6330407J23Rik	RIKEN cDNA 6330407J23 gene	0.841043	0.008857	NA
70122	Mllt3	myeloid/lymphoid or mixed-lineage leukemia (trithorax homolog, Drosophila); translocated to, 3	0.841043	0.04297	0.1662
71436	Flrt3	fibronectin leucine rich transmembrane protein 3	0.841043	0.1187	0.3114
72650	2810006K23Rik	RIKEN cDNA 2810006K23 gene	0.841043	0.1093	0.2959
78286	Nav2	neuron navigator 2	0.841043	0.1829	0.4017
106248	Qtrtd1	queuine tRNA-ribosyltransferase domain containing 1	0.841043	0.05742	0.1971
215705	Arrdc1	arrestin domain containing 1	0.841043	0.1809	0.3995
226123	Morn4	MORN repeat containing 4	0.841043	0.01768	0.09835
228983	Osbpl2	oxysterol binding protein-like 2	0.841043	0.05475	0.1915
234542	Rtbdn	retbindin	0.841043	0.05887	0.2008
243833	Zfp128	zinc finger protein 128	0.841043	0.1162	0.3075
545192	Baiap3	BAI1-associated protein 3	0.841043	0.4284	0.6554
665563	Mthfd2l	methylenetetrahydrofolate dehydrogenase (NADP+ dependent) 2-like	0.841043	0.02716	0.1277
100043100	Zfp534	zinc finger protein 534	0.841043	0.4076	0.6369
12162	Bmp7	bone morphogenetic protein 7	0.840336	0.4129	0.6418
12909	Crcp	calcitonin gene-related peptide-receptor component protein	0.840336	0.04536	0.1717
14226	Fkbp1b	FK506 binding protein 1b	0.840336	0.1569	0.3671
18546	Pcp4	Purkinje cell protein 4	0.840336	0.01051	NA
20431	Pmel	premelanosome protein	0.840336	0.3868	0.6189
22084	Tsc2	tuberous sclerosis 2	0.840336	0.02599	0.1242
22685	Zfp239	zinc finger protein 239	0.840336	0.08369	0.2502
23919	Insl5	insulin-like 5	0.840336	0.05968	0.2027
53313	Atp2a3	ATPase, Ca++ transporting, ubiquitous	0.840336	0.1308	0.3306
53626	Insm1	insulinoma-associated 1	0.840336	0.05021	0.1819
54711	Plagl2	pleiomorphic adenoma gene-like 2	0.840336	0.1439	0.3489
56758	Mbnl1	muscleblind-like 1 (Drosophila)	0.840336	0.02436	0.1192
57080	Gtf2ird1	general transcription factor II I repeat domain-containing 1	0.840336	0.03955	0.1585
66112	Mosc1	MOCO sulphurase C-terminal domain containing 1	0.840336	0.1245	0.3211
67095	Trak1	trafficking protein, kinesin binding 1	0.840336	0.007242	NA
67672	0610040B10Rik	RIKEN cDNA 0610040B10 gene	0.840336	0.1612	0.373
69504	Zfp932	zinc finger protein 932	0.840336	0.1539	0.3636
70857	4921509J17Rik	RIKEN cDNA 4921509J17 gene	0.840336	0.01845	0.1008
72016	1600002H07Rik	RIKEN cDNA 1600002H07 gene	0.840336	0.02056	0.1077
72338	Wdr89	WD repeat domain 89	0.840336	0.1986	0.4238
73737	1110008P14Rik	RIKEN cDNA 1110008P14 gene	0.840336	0.07731	0.2384
74022	Glyr1	glyoxylate reductase 1 homolog (Arabidopsis)	0.840336	0.00919	NA
80976	Syt13	synaptotagmin XIII	0.840336	0.0113	NA
100223	9630041G16Rik	RIKEN cDNA 9630041G16 gene	0.840336	0.008868	NA
103537	Mbtd1	mbt domain containing 1	0.840336	0.01451	0.08763

105372	Utp15	UTP15, U3 small nucleolar ribonucleoprotein, homolog (yeast)	0.840336	0.1507	0.3587
216867	Slc16a11	solute carrier family 16 (monocarboxylic acid transporters), member 11	0.840336	0.0218	0.1119
229722	5330417C22Rik	RIKEN cDNA 5330417C22 gene	0.840336	0.07474	0.2334
234595	Slc38a7	solute carrier family 38, member 7	0.840336	0.1751	0.3919
236727	Slc9a7	solute carrier family 9 (sodium/hydrogen exchanger), member 7	0.840336	0.01371	0.08467
237411	Zfp938	zinc finger protein 938	0.840336	0.1605	0.3723
320365	Fry	furry homolog (Drosophila)	0.840336	0.01892	0.1025
381199	Tmem151a	transmembrane protein 151A	0.840336	0.2996	0.5352
433759	Hdac1	histone deacetylase 1	0.840336	0.2363	0.4687
11836	Araf	v-raf murine sarcoma 3611 viral oncogene homolog	0.839631	0.009926	NA
13176	Dcc	deleted in colorectal carcinoma	0.839631	0.1566	0.3667
13549	Dyrk1b	dual-specificity tyrosine-(Y)-phosphorylation regulated kinase 1b	0.839631	0.3831	0.6161
14169	Fgf14	fibroblast growth factor 14	0.839631	0.03867	0.1565
14459	Gast	gastrin	0.839631	0.4187	0.6467
20536	Slc4a3	solute carrier family 4 (anion exchanger), member 3	0.839631	0.1323	0.3329
22201	Uba1	ubiquitin-like modifier activating enzyme 1	0.839631	0.02444	0.1195
53893	Nudt5	nudix (nucleoside diphosphate linked moiety X)-type motif 5	0.839631	0.2034	0.4298
69900	Mfsd11	major facilitator superfamily domain containing 11	0.839631	0.07259	0.2293
70127	Dpf3	D4, zinc and double PHD fingers, family 3	0.839631	0.0306	0.137
71764	C2cd2l	C2 calcium-dependent domain containing 2-like	0.839631	0.01825	0.1001
73047	Camk2n2	calcium/calmodulin-dependent protein kinase II inhibitor 2	0.839631	0.02374	0.1174
75533	Nme5	non-metastatic cells 5, protein expressed in (nucleoside-diphosphate kinase)	0.839631	0.1873	0.4079
110332	4921523A10Rik	RIKEN cDNA 4921523A10 gene	0.839631	0.1873	0.4079
110834	Chrna3	cholinergic receptor, nicotinic, alpha polypeptide 3	0.839631	0.2326	0.4639
208691	Eif5a2	eukaryotic translation initiation factor 5A2	0.839631	0.08152	0.2459
218203	Mylip	myosin regulatory light chain interacting protein	0.839631	0.07563	0.2354
227622	BC029214	cDNA sequence BC029214	0.839631	0.03515	0.1484
229512	Smg5	Smg-5 homolog, nonsense mediated mRNA decay factor (C. elegans)	0.839631	0.02423	0.1188
230991	B930041F14Rik	RIKEN cDNA B930041F14 gene	0.839631	0.04536	0.1717
239857	Cadm2	cell adhesion molecule 2	0.839631	0.3055	0.542
259302	Srgap3	SLIT-ROBO Rho GTPase activating protein 3	0.839631	0.2563	0.4913
320846	A530058N18Rik	RIKEN cDNA A530058N18 gene	0.839631	0.04415	0.169
667055	Gm9992	predicted gene 9992	0.839631	0.2224	0.4527
100045653	LOC100045653	hypothetical LOC100045653	0.839631	0.3523	0.5874
12121	Bicd1	bicaudal D homolog 1 (Drosophila)	0.838926	0.1193	0.3124
14430	Galt	galactose-1-phosphate uridyl transferase	0.838926	0.08072	0.2444
18548	Pcsk1	proprotein convertase subtilisin/kexin type 1	0.838926	0.05516	0.1923
20295	Ccl17	chemokine (C-C motif) ligand 17	0.838926	0.3303	0.5668
21909	Tlx2	T-cell leukemia, homeobox 2	0.838926	0.2154	0.444
52808	Tspyl2	TSPY-like 2	0.838926	0.1682	0.3831
71059	Hexim2	hexamthylene bis-acetamide inducible 2	0.838926	0.02033	0.107
76916	4930455C21Rik	RIKEN cDNA 4930455C21 gene	0.838926	0.04516	0.1712
93699	Pcdhgb1	protocadherin gamma subfamily B, 1	0.838926	0.1813	0.4
94281	Sfxn4	sideroflexin 4	0.838926	0.05712	0.1964
99371	Arfgef2	ADP-ribosylation factor guanine nucleotide-exchange factor 2 (brefeldin A-inhibited)	0.838926	0.04203	0.164
99633	Lphn2	latrophilin 2	0.838926	0.2344	0.466
102075	Plekhg4	pleckstrin homology domain containing, family G (with RhoGef domain) member 4	0.838926	0.0241	0.1186
231868	E130309D02Rik	RIKEN cDNA E130309D02 gene	0.838926	0.08759	0.2582
234413	Zfp961	zinc finger protein 961	0.838926	0.12	0.3138

235606	Apeh	acylpeptide hydrolase	0.838926	0.008427	NA
241944	D3Ertd254e	DNA segment, Chr 3, ERATO Doi 254, expressed	0.838926	0.006983	NA
263406	Plekhg3	pleckstrin homology domain containing, family G (with RhoGef domain) member 3	0.838926	0.2114	0.4389
320082	Fbxw21	F-box and WD-40 domain protein 21	0.838926	0.3828	0.6161
360216	Zranb1	zinc finger, RAN-binding domain containing 1	0.838926	0.0237	0.1174
381310	6330403A02Rik	RIKEN cDNA 6330403A02 gene	0.838926	0.1103	0.2976
381411	Accsl	1-aminocyclopropane-1-carboxylate synthase homolog (Arabidopsis)(non-functional)-like	0.838926	0.02952	0.1342
13370	Dio1	deiodinase, iodothyronine, type I	0.838223	0.2156	0.4442
13690	Eif4g2	eukaryotic translation initiation factor 4, gamma 2	0.838223	0.06322	0.2099
14617	Gjd2	gap junction protein, delta 2	0.838223	0.09924	0.2779
17475	Mpdz	multiple PDZ domain protein	0.838223	0.1477	0.3544
18754	Prkce	protein kinase C, epsilon	0.838223	0.1001	0.2795
19281	Ptprt	protein tyrosine phosphatase, receptor type, T	0.838223	0.1405	0.3442
19341	Rab4a	RAB4A, member RAS oncogene family	0.838223	0.03281	0.1428
20744	Strbp	spermatid perinuclear RNA binding protein	0.838223	0.1265	0.3238
22724	Zbtb7b	zinc finger and BTB domain containing 7B	0.838223	0.06098	0.2053
52898	Rnasek	ribonuclease, RNase K	0.838223	0.1254	0.3224
55984	Camkk1	calcium/calmodulin-dependent protein kinase kinase 1, alpha	0.838223	0.007469	NA
66661	Srp72	signal recognition particle 72	0.838223	0.01133	NA
66849	Ppp1r2	protein phosphatase 1, regulatory (inhibitor) subunit 2	0.838223	0.1353	0.3368
72634	Tdrkh	tudor and KH domain containing protein	0.838223	0.06821	0.2206
73473	Iws1	IWS1 homolog (S. cerevisiae)	0.838223	0.1035	0.2855
107586	Ovol2	ovo-like 2 (Drosophila)	0.838223	0.1418	0.3462
192775	Kcnh6	potassium voltage-gated channel, subfamily H (eag-related), member 6	0.838223	0.1436	0.3485
207181	Rbms3	RNA binding motif, single stranded interacting protein	0.838223	0.09028	0.263
207920	Esrp1	epithelial splicing regulatory protein 1	0.838223	0.06653	0.2169
218877	Sema3g	sema domain, immunoglobulin domain (Ig), short basic domain, secreted, (semaphorin) 3G	0.838223	0.2475	0.4817
240058	Cpne5	copine V	0.838223	0.04202	0.164
242362	Manea	mannosidase, endo-alpha	0.838223	0.1182	0.3107
242506	Frmd3	FERM domain containing 3	0.838223	0.02661	0.1261
243272	Sbno1	sno, strawberry notch homolog 1 (Drosophila)	0.838223	0.05627	0.1944
319739	B230303O12Rik	RIKEN cDNA B230303O12 gene	0.838223	0.04184	0.1638
320127	Dgki	diacylglycerol kinase, iota	0.838223	0.1312	0.3312
320840	Negr1	neuronal growth regulator 1	0.838223	0.117	0.3089
381511	Pdp1	pyruvate dehyrogenase phosphatase catalytic subunit 1	0.838223	0.00321	NA
664862	Gpr137b-ps	G protein-coupled receptor 137B, pseudogene	0.838223	0.03548	0.1487
100038585	F830223B06Rik	RIKEN cDNA F830223B06 gene	0.838223	0.1762	0.3931
12903	Crabp1	cellular retinoic acid binding protein I	0.837521	0.1488	0.3561
15206	Hes2	hairy and enhancer of split 2 (Drosophila)	0.837521	0.3948	0.6254
15525	Hspa4	heat shock protein 4	0.837521	0.002578	NA
16663	Krt13	keratin 13	0.837521	0.4499	0.6749
19646	Rbbp4	retinoblastoma binding protein 4	0.837521	0.248	0.4822
22201	Uba1	ubiquitin-like modifier activating enzyme 1	0.837521	0.0328	0.1428
24050	Sep-03	septin 3	0.837521	0.261	0.4961
50769	Atp8a2	ATPase, aminophospholipid transporter-like, class I, type 8A, member 2	0.837521	0.02574	0.1235
56198	Heyl	hairy/enhancer-of-split related with YRPW motif-like	0.837521	0.3371	0.5727
68273	Pomgnt1	protein O-linked mannose beta1,2-N-acetylglucosaminyltransferase	0.837521	0.04987	0.1815
69256	Zfp397	zinc finger protein 397	0.837521	0.08775	0.2584
69504	Zfp932	zinc finger protein 932	0.837521	0.006655	NA

70047	Trnt1	tRNA nucleotidyl transferase, CCA-adding, 1	0.837521	0.02125	0.1101
75620	2810422J05Rik	RIKEN cDNA 2810422J05 gene	0.837521	0.02632	0.1252
75660	Lin37	lin-37 homolog (C. elegans)	0.837521	0.1224	0.318
75974	Dock11	dedicator of cytokinesis 11	0.837521	0.04291	0.1661
108121	U2af1	U2 small nuclear ribonucleoprotein auxiliary factor (U2AF) 1	0.837521	0.05187	0.1851
192174	Rwdd4a	RWD domain containing 4A	0.837521	0.3059	0.5424
216028	Lrrtm3	leucine rich repeat transmembrane neuronal 3	0.837521	0.007192	NA
217843	Unc79	unc-79 homolog (C. elegans)	0.837521	0.07095	0.2259
225631	Onecut2	one cut domain, family member 2	0.837521	0.03435	0.1466
233168	AI987944	expressed sequence AI987944	0.837521	0.09895	0.2779
235312	C1qtnf5	C1q and tumor necrosis factor related protein 5	0.837521	0.2597	0.495
239570	Ttc38	tetratricopeptide repeat domain 38	0.837521	0.04187	0.1638
360013	Myo18a	myosin XVIIIA	0.837521	0.07043	0.2249
545554	Ankrd34a	ankyrin repeat domain 34A	0.837521	0.1594	0.3707
623474	Rad54b	RAD54 homolog B (S. cerevisiae)	0.837521	0.03488	0.1481
14528	Gch1	GTP cyclohydrolase 1	0.83682	0.2328	0.4639
15568	Elavl1	ELAV (embryonic lethal, abnormal vision, Drosophila)-like 1 (Hu antigen R)	0.83682	0.1032	0.285
17184	Matr3	matrin 3	0.83682	0.1335	0.3339
18987	Pou2f2	POU domain, class 2, transcription factor 2	0.83682	0.1589	0.3701
29858	Pmm1	phosphomannomutase 1	0.83682	0.01594	0.0926
54161	Copg	coatomer protein complex, subunit gamma	0.83682	0.1774	0.3945
55946	Ap3m1	adaptor-related protein complex 3, mu 1 subunit	0.83682	0.04563	0.1721
67180	Yipf5	Yip1 domain family, member 5	0.83682	0.02049	0.1075
70546	Zdhhc2	zinc finger, DHHC domain containing 2	0.83682	0.02046	0.1074
71000	4931440J10Rik	RIKEN cDNA 4931440J10 gene	0.83682	0.6457	0.815
75647	1700025E21Rik	RIKEN cDNA 1700025E21 gene	0.83682	0.2153	0.4439
77128	A930001N09Rik	RIKEN cDNA A930001N09 gene	0.83682	0.2578	0.4928
108155	Ogt	O-linked N-acetylglucosamine (GlcNAc) transferase (UDP-N-acetylglucosamine:polypeptide-N-acetylglucosaminyl transferase)	0.83682	0.04198	0.1639
170952	Prima1	proline rich membrane anchor 1	0.83682	0.05824	0.1992
217887	BC022687	cDNA sequence BC022687	0.83682	0.05165	0.1847
226265	Eno4	enolase 4	0.83682	0.2247	0.4557
238161	Akap6	A kinase (PRKA) anchor protein 6	0.83682	0.02178	0.1119
277414	Trp53i11	transformation related protein 53 inducible protein 11	0.83682	0.03494	0.1482
319934	Sbf2	SET binding factor 2	0.83682	0.05513	0.1923
319953	Ttll1	tubulin tyrosine ligase-like 1	0.83682	0.2888	0.5249
381022	Mll2	myeloid/lymphoid or mixed-lineage leukemia 2	0.83682	0.2606	0.4959
381979	Brsk1	BR serine/threonine kinase 1	0.83682	0.01293	0.0817
382056	Crtc1	CREB regulated transcription coactivator 1	0.83682	0.01496	0.08923
407831	Tmem204	transmembrane protein 204	0.83682	0.1838	0.4028
17855	Mvk	mevalonate kinase	0.83612	0.07373	0.2317
19395	Rasgrp2	RAS, guanyl releasing protein 2	0.83612	0.02614	0.1247
52028	Bbs1	Bardet-Biedl syndrome 1 (human)	0.83612	0.07318	0.2305
66658	Ccdc51	coiled-coil domain containing 51	0.83612	0.005966	NA
67923	Tceb1	transcription elongation factor B (SIII), polypeptide 1	0.83612	0.03994	0.1592
69217	Plekha4	pleckstrin homology domain containing, family A (phosphoinositide binding specific) member 4	0.83612	0.03014	0.1359
71835	Lancl2	LanC (bacterial lantibiotic synthetase component C)-like 2	0.83612	0.09404	0.2695
75341	4930564C03Rik	RIKEN cDNA 4930564C03 gene	0.83612	0.3088	0.545
75387	Sirt4	sirtuin 4 (silent mating type information regulation 2 homolog) 4 (S. cerevisiae)	0.83612	0.03314	0.1437
81013	Vmn1r65	vomeronasal 1 receptor 65	0.83612	0.5267	0.7337

83924	Gpr137b	G protein-coupled receptor 137B	0.83612	0.1148	0.3052
107746	Rapgef1	Rap guanine nucleotide exchange factor (GEF) 1	0.83612	0.0353	0.1484
140483	Hnmt	histamine N-methyltransferase	0.83612	0.1524	0.3615
208990	Npb	neuropeptide B	0.83612	0.03716	0.1527
211323	Nrg1	neuregulin 1	0.83612	0.009712	0.06863
231605	Galnt9	UDP-N-acetyl-alpha-D-galactosamine:polypeptide N-acetylgalactosaminyltransferase 9	0.83612	0.2099	0.4369
234407	Glt25d1	glycosyltransferase 25 domain containing 1	0.83612	0.1217	0.3167
240120	Zfp119b	zinc finger protein 119b	0.83612	0.29	0.5262
240261	Ccdc112	coiled-coil domain containing 112	0.83612	0.317	0.5536
11775	Ap3b2	adaptor-related protein complex 3, beta 2 subunit	0.835422	0.1577	0.3683
11852	Rhob	ras homolog gene family, member B	0.835422	0.3302	0.5668
11938	Atp2a2	ATPase, Ca++ transporting, cardiac muscle, slow twitch 2	0.835422	0.006369	NA
14009	Etv1	ets variant gene 1	0.835422	0.2384	0.4708
15932	Idua	iduronidase, alpha-L-	0.835422	0.03566	0.1492
16490	Kcna2	potassium voltage-gated channel, shaker-related subfamily, member 2	0.835422	0.3129	0.5493
19256	Ptpn20	protein tyrosine phosphatase, non-receptor type 20	0.835422	0.1352	0.3367
19762	Rit2	Ras-like without CAAX 2	0.835422	0.0692	0.2224
22045	Trhr	thyrotropin releasing hormone receptor	0.835422	0.03189	0.1404
50786	Hs6st2	heparan sulfate 6-O-sulfotransferase 2	0.835422	0.04066	0.1613
50932	Mink1	misshapen-like kinase 1 (zebrafish)	0.835422	0.07775	0.239
57875	Angptl4	angiopoietin-like 4	0.835422	0.178	0.3955
67713	Dnajc19	DnaJ (Hsp40) homolog, subfamily C, member 19	0.835422	0.3518	0.5871
68904	Abhd13	abhydrolase domain containing 13	0.835422	0.1344	0.3353
69215	Sat2	spermidine/spermine N1-acetyl transferase 2	0.835422	0.01162	0.07651
70317	Arl16	ADP-ribosylation factor-like 16	0.835422	0.04444	0.1695
71735	Lrwd1	leucine-rich repeats and WD repeat domain containing 1	0.835422	0.0751	0.2342
75901	Dcp1a	DCP1 decapping enzyme homolog A (S. cerevisiae)	0.835422	0.01632	0.09362
84585	Rnf123	ring finger protein 123	0.835422	0.1366	0.3383
207565	Camkk2	calcium/calmodulin-dependent protein kinase kinase 2, beta	0.835422	0.008213	NA
213053	Slc39a14	solute carrier family 39 (zinc transporter), member 14	0.835422	0.09323	0.2681
217378	Dnajc27	DnaJ (Hsp40) homolog, subfamily C, member 27	0.835422	0.2224	0.4527
235439	Herc1	hect (homologous to the E6-AP (UBE3A) carboxyl terminus) domain and RCC1 (CHC1)-like domain (RLD) 1	0.835422	0.3365	0.572
244310	Dlgap2	discs, large (Drosophila) homolog-associated protein 2	0.835422	0.03221	0.1412
268481	Krt222	keratin 222	0.835422	0.06293	0.2095
268512	Slc26a11	solute carrier family 26, member 11	0.835422	0.0254	0.1223
319475	Zfp672	zinc finger protein 672	0.835422	0.0419	0.1638
328329	Mast4	microtubule associated serine/threonine kinase family member 4	0.835422	0.02353	0.1169
329908	Usp24	ubiquitin specific peptidase 24	0.835422	0.08302	0.2487
330177	Taok3	TAO kinase 3	0.835422	0.08751	0.258
11775	Ap3b2	adaptor-related protein complex 3, beta 2 subunit	0.834725	0.1469	0.353
14585	Gfra1	glial cell line derived neurotrophic factor family receptor alpha 1	0.834725	0.02034	0.107
14657	Glra4	glycine receptor, alpha 4 subunit	0.834725	0.008007	NA
15248	Hic1	hypermethylated in cancer 1	0.834725	0.1176	0.3096
18612	Etv4	ets variant gene 4 (E1A enhancer binding protein, E1AF)	0.834725	0.1696	0.3848
22668	Sf1	splicing factor 1	0.834725	0.01193	0.07773
26398	Map2k4	mitogen-activated protein kinase kinase 4	0.834725	0.08418	0.2511
26920	Cep110	centrosomal protein 110	0.834725	0.0234	0.1167
54369	Nme6	non-metastatic cells 6, protein expressed in (nucleoside-diphosphate kinase)	0.834725	0.08407	0.2508
59024	Med12	mediator of RNA polymerase II transcription, subunit 12 homolog (yeast)	0.834725	0.02458	0.1199

66146	Tmem57	transmembrane protein 57	0.834725	0.007764	NA
66412	Arrdc4	arrestin domain containing 4	0.834725	0.07111	0.2262
67466	Pdcl	phosducin-like	0.834725	0.1177	0.3099
70028	Dopey2	dopey family member 2	0.834725	0.01619	0.0932
80907	Lactb	lactamase, beta	0.834725	0.0138	0.08504
93736	Aff4	AF4/FMR2 family, member 4	0.834725	0.566	0.762
106565	Dlk2	delta-like 2 homolog (Drosophila)	0.834725	0.09744	0.2754
114640	Pth2	parathyroid hormone 2	0.834725	0.1793	0.3972
226982	Eif5b	eukaryotic translation initiation factor 5B	0.834725	0.03475	0.1478
241041	Gm4956	predicted gene 4956	0.834725	0.4303	0.6572
269630	5031425E22Rik	RIKEN cDNA 5031425E22 gene	0.834725	0.2652	0.501
319263	Pcmtd1	protein-L-isoaspartate (D-aspartate) O-methyltransferase domain containing 1	0.834725	0.08993	0.2622
319655	Podxl2	podocalyxin-like 2	0.834725	0.13	0.3293
100041864	Gm3552	predicted gene 3552	0.834725	0.4728	0.6936
12323	Camk2b	calcium/calmodulin-dependent protein kinase II, beta	0.834028	0.002279	NA
13618	Ednrb	endothelin receptor type B	0.834028	0.05141	0.1841
14672	Gna11	guanine nucleotide binding protein, alpha 11	0.834028	0.0355	0.1488
19434	Rax	retina and anterior neural fold homeobox	0.834028	0.1113	0.2993
20408	Sh3gl3	SH3-domain GRB2-like 3	0.834028	0.0307	0.1372
56045	Samhd1	SAM domain and HD domain, 1	0.834028	0.003058	NA
60345	Nrip2	nuclear receptor interacting protein 2	0.834028	0.1764	0.3935
66425	Pcp4l1	Purkinje cell protein 4-like 1	0.834028	0.009194	0.06642
67801	Pllp	plasma membrane proteolipid	0.834028	0.3421	0.5775
68778	1110038D17Rik	RIKEN cDNA 1110038D17 gene	0.834028	0.03091	0.1378
69824	Glod5	glyoxalase domain containing 5	0.834028	0.2784	0.514
72114	Zbed3	zinc finger, BED domain containing 3	0.834028	0.07933	0.2419
73178	Wasl	Wiskott-Aldrich syndrome-like (human)	0.834028	0.07574	0.2357
73202	3110067C02Rik	RIKEN cDNA 3110067C02 gene	0.834028	0.05584	0.1935
75541	1700019G17Rik	RIKEN cDNA 1700019G17 gene	0.834028	0.06165	0.2065
76653	Cby3	chibby homolog 3 (Drosophila)	0.834028	0.1434	0.3483
78739	9530062K07Rik	RIKEN cDNA 9530062K07 gene	0.834028	0.1084	0.2945
98258	Txndc9	thioredoxin domain containing 9	0.834028	0.1762	0.3932
99922	AW552393	expressed sequence AW552393	0.834028	0.371	0.6059
110876	Scn2a1	sodium channel, voltage-gated, type II, alpha 1	0.834028	0.0846	0.2519
116905	Dph1	DPH1 homolog (S. cerevisiae)	0.834028	0.03725	0.153
207181	Rbms3	RNA binding motif, single stranded interacting protein	0.834028	0.1825	0.4015
210673	Prrt3	proline-rich transmembrane protein 3	0.834028	0.1108	0.2986
212541	Rho	rhodopsin	0.834028	0.07549	0.2352
237858	Tusc5	tumor suppressor candidate 5	0.834028	0.08196	0.2468
269717	Orai2	ORAI calcium release-activated calcium modulator 2	0.834028	0.02028	0.107
320661	D5Ertd579e	DNA segment, Chr 5, ERATO Doi 579, expressed	0.834028	0.5588	0.7574
11932	Atp1b2	ATPase, Na+/K+ transporting, beta 2 polypeptide	0.833333	0.0941	0.2696
14065	F2rl3	coagulation factor II (thrombin) receptor-like 3	0.833333	0.2566	0.4916
19014	Med1	mediator complex subunit 1	0.833333	0.01146	0.07585
22360	Nrsn1	neurensin 1	0.833333	0.02396	0.118
26358	Aldh1a7	aldehyde dehydrogenase family 1, subfamily A7	0.833333	0.1699	0.3851
29866	Cabp2	calcium binding protein 2	0.833333	0.07821	0.2398
51799	Rundc3a	RUN domain containing 3A	0.833333	0.07212	0.2283
52857	Gramd1a	GRAM domain containing 1A	0.833333	0.03181	0.1401

53330	Vamp4	vesicle-associated membrane protein 4	0.833333	0.04322	0.1668
68910	Zfp467	zinc finger protein 467	0.833333	0.008792	0.06472
69499	Tsr2	TSR2, 20S rRNA accumulation, homolog (S. cerevisiae)	0.833333	0.007353	NA
72108	Ddhd2	DDHD domain containing 2	0.833333	0.01006	0.07024
72193	Scaf11	SR-related CTD-associated factor 11	0.833333	0.01905	0.1029
76183	Celf6	CUGBP, Elav-like family member 6	0.833333	0.07707	0.2379
77593	Usp45	ubiquitin specific petidase 45	0.833333	0.1024	0.2837
83925	Trps1	trichorhinophalangeal syndrome I (human)	0.833333	0.1161	0.3075
100129	Gpr153	G protein-coupled receptor 153	0.833333	0.0249	0.1208
216874	Camta2	calmodulin binding transcription activator 2	0.833333	0.008549	0.06362
223658	Heatr7a	HEAT repeat containing 7A	0.833333	0.3191	0.5556
12286	Cacna1a	calcium channel, voltage-dependent, P/Q type, alpha 1A subunit	0.832639	0.0207	0.1083
13121	Cyp51	cytochrome P450, family 51	0.832639	0.02026	0.1069
16168	Il15	interleukin 15	0.832639	0.2453	0.4791
16497	Kcnab1	potassium voltage-gated channel, shaker-related subfamily, beta member 1	0.832639	0.02218	0.1129
18209	Ntn3	netrin 3	0.832639	0.1364	0.3382
22059	Trp53	transformation related protein 53	0.832639	0.1148	0.3052
22775	Zik1	zinc finger protein interacting with K protein 1	0.832639	0.01787	0.09884
73699	Ppp2r1b	protein phosphatase 2 (formerly 2A), regulatory subunit A (PR 65), beta isoform	0.832639	0.2795	0.515
73728	Psd	pleckstrin and Sec7 domain containing	0.832639	0.08088	0.2447
73893	Tmem202	transmembrane protein 202	0.832639	0.05466	0.1912
78617	Cstad	CSA-conditional, T cell activation-dependent protein	0.832639	0.02181	0.1119
94275	Maged1	melanoma antigen, family D, 1	0.832639	0.01486	0.08896
104348	Zfp120	zinc finger protein 120	0.832639	0.03568	0.1492
110593	Prdm2	PR domain containing 2, with ZNF domain	0.832639	0.353	0.5879
208292	Zfp871	zinc finger protein 871	0.832639	0.03485	0.148
211378	6720489N17Rik	RIKEN cDNA 6720489N17 gene	0.832639	0.1116	0.2997
319304	A730081D07Rik	RIKEN cDNA A730081D07 gene	0.832639	0.06903	0.2222
320165	Tacc1	transforming, acidic coiled-coil containing protein 1	0.832639	0.0641	0.2117
791275	Gm9885	predicted gene 9885	0.832639	0.04659	0.1745
100502627	LOC100502627	hypothetical LOC100502627	0.832639	0.01681	0.09547
11777	Ap3s1	adaptor-related protein complex 3, sigma 1 subunit	0.831947	0.04944	0.1803
14677	Gnai1	guanine nucleotide binding protein (G protein), alpha inhibiting 1	0.831947	0.05264	0.1866
20589	Ighmbp2	immunoglobulin mu binding protein 2	0.831947	0.01117	0.07479
20666	Sox11	SRY-box containing gene 11	0.831947	0.05607	0.194
29867	Cabp1	calcium binding protein 1	0.831947	0.08106	0.2448
51799	Rundc3a	RUN domain containing 3A	0.831947	0.03646	0.1512
52626	Cdkn2aipnl	CDKN2A interacting protein N-terminal like	0.831947	0.009833	0.06929
56013	Srcin1	SRC kinase signaling inhibitor 1	0.831947	0.03102	0.1379
57276	Vsig2	V-set and immunoglobulin domain containing 2	0.831947	0.0655	0.2147
65962	Slc9a3r2	solute carrier family 9 (sodium/hydrogen exchanger), member 3 regulator 2	0.831947	0.1822	0.401
66662	5730577I03Rik	zinc finger protein pseudogene	0.831947	0.2789	0.5144
72865	Cxx1c	CAAX box 1 homolog C (human)	0.831947	0.04337	0.1672
73094	Sgip1	SH3-domain GRB2-like (endophilin) interacting protein 1	0.831947	0.2254	0.4565
105245	Txndc5	thioredoxin domain containing 5	0.831947	0.2134	0.4416
110446	Acat1	acetyl-Coenzyme A acetyltransferase 1	0.831947	0.01227	0.07887
226518	Nmnat2	nicotinamide nucleotide adenylyltransferase 2	0.831947	0.1869	0.4075
227622	BC029214	cDNA sequence BC029214	0.831947	0.08799	0.2587
237175	Gpr64	G protein-coupled receptor 64	0.831947	0.2164	0.4452

100038570	Gm11744	predicted gene 11744	0.831947	0.02483	0.1206
12794	Cnih2	cornichon homolog 2 (Drosophila)	0.831255	0.04944	0.1803
15531	Ndst1	N-deacetylase/N-sulfotransferase (heparan glucosaminyl) 1	0.831255	0.1551	0.3648
17537	Meis3	Meis homeobox 3	0.831255	0.1156	0.3064
19060	Ppp5c	protein phosphatase 5, catalytic subunit	0.831255	0.5833	0.7739
20350	Sema3f	sema domain, immunoglobulin domain (Ig), short basic domain, secreted, (semaphorin) 3F	0.831255	0.03264	0.1423
20404	Sh3gl2	SH3-domain GRB2-like 2	0.831255	0.3039	0.5401
56449	Csda	cold shock domain protein A	0.831255	0.08987	0.262
66241	Tmem9	transmembrane protein 9	0.831255	0.04842	0.1781
67620	Lrp2bp	Lrp2 binding protein	0.831255	0.03711	0.1526
70981	4931423N10Rik	RIKEN cDNA 4931423N10 gene	0.831255	0.2613	0.4964
73582	1700106N22Rik	RIKEN cDNA 1700106N22 gene	0.831255	0.02449	0.1197
74173	1700012B15Rik	RIKEN cDNA 1700012B15 gene	0.831255	0.05522	0.1924
75607	Wnk2	WNK lysine deficient protein kinase 2	0.831255	0.1263	0.3235
75795	4930455D15Rik	RIKEN cDNA 4930455D15 gene	0.831255	0.6499	0.818
77519	Zfp266	zinc finger protein 266	0.831255	0.03731	0.153
83965	Enpp5	ectonucleotide pyrophosphatase/phosphodiesterase 5	0.831255	0.008408	0.06292
170707	Usp48	ubiquitin specific peptidase 48	0.831255	0.006753	0.05559
225913	Dak	dihydroxyacetone kinase 2 homolog (yeast)	0.831255	0.07644	0.2367
238673	Zfp367	zinc finger protein 367	0.831255	0.02185	0.112
238693	Zfp58	zinc finger protein 58	0.831255	0.01333	0.08332
329795	Tmem67	transmembrane protein 67	0.831255	0.006042	0.05238
433256	Acsl5	acyl-CoA synthetase long-chain family member 5	0.831255	0.03288	0.143
100502641	LOC100502641	60S acidic ribosomal protein P1-like	0.831255	0.05221	0.1856
12315	Calm3	calmodulin 3	0.830565	0.1185	0.3111
14809	Grik5	glutamate receptor, ionotropic, kainate 5 (gamma 2)	0.830565	0.1516	0.3602
16563	Kif2a	kinesin family member 2A	0.830565	0.04238	0.1648
17524	Mpp1	membrane protein, palmitoylated	0.830565	0.03739	0.1532
18739	Pitpnm1	phosphatidylinositol transfer protein, membrane-associated 1	0.830565	0.06181	0.207
23992	Prkra	protein kinase, interferon inducible double stranded RNA dependent activator	0.830565	0.05331	0.188
56205	Ensa	endosulfine alpha	0.830565	0.02836	0.131
59049	Slc22a17	solute carrier family 22 (organic cation transporter), member 17	0.830565	0.09178	0.2655
64051	Sv2a	synaptic vesicle glycoprotein 2 a	0.830565	0.1188	0.3115
73692	2410089E03Rik	RIKEN cDNA 2410089E03 gene	0.830565	0.05387	0.1894
96979	Ptges2	prostaglandin E synthase 2	0.830565	0.08943	0.2613
107065	Lrrtm2	leucine rich repeat transmembrane neuronal 2	0.830565	0.06367	0.2108
117150	Pip4k2c	phosphatidylinositol-5-phosphate 4-kinase, type II, gamma	0.830565	0.05048	0.1825
195046	Nlrp1a	NLR family, pyrin domain containing 1A	0.830565	0.579	0.7713
208117	Aph1b	anterior pharynx defective 1b homolog (C. elegans)	0.830565	0.04734	0.176
208990	Npb	neuropeptide B	0.830565	0.0351	0.1484
245469	Pdzd4	PDZ domain containing 4	0.830565	0.07568	0.2355
327987	Med13	mediator complex subunit 13	0.830565	0.2555	0.4903
403185	4932443I19Rik	RIKEN cDNA 4932443I19 gene	0.830565	0.15	0.3578
504193	Npcd	neuronal pentraxin chromo domain	0.830565	0.0329	0.1431
619301	G630016D24Rik	RIKEN cDNA G630016D24 gene	0.830565	0.2433	0.4766
13384	Mpp3	membrane protein, palmitoylated 3 (MAGUK p55 subfamily member 3)	0.829876	0.03632	0.1509
13437	Dnpep	aspartyl aminopeptidase	0.829876	0.009216	0.06646
13733	Emr1	EGF-like module containing, mucin-like, hormone receptor-like sequence 1	0.829876	0.6712	0.8317
14265	Fmr1	fragile X mental retardation syndrome 1 homolog	0.829876	0.07203	0.2282

15289	Hmgb1	high mobility group box 1	0.829876	0.07137	0.2267
15931	Ids	iduronate 2-sulfatase	0.829876	0.02288	0.1152
16068	Il18bp	interleukin 18 binding protein	0.829876	0.02984	0.1351
16494	Kcna6	potassium voltage-gated channel, shaker-related, subfamily, member 6	0.829876	0.01539	0.09088
16518	Kcnj2	potassium inwardly-rectifying channel, subfamily J, member 2	0.829876	0.1608	0.3726
17201	Mc3r	melanocortin 3 receptor	0.829876	0.1112	0.2993
19023	Ppef2	protein phosphatase, EF hand calcium-binding domain 2	0.829876	0.02019	0.1068
26367	Ceacam2	carcinoembryonic antigen-related cell adhesion molecule 2	0.829876	0.2068	0.4329
27399	Ip6k1	inositol hexaphosphate kinase 1	0.829876	0.03678	0.1519
27965	Spg21	spastic paraplegia 21 homolog (human)	0.829876	0.1326	0.3332
30051	Spdef	SAM pointed domain containing ets transcription factor	0.829876	0.04273	0.1656
56715	Rabgef1	RAB guanine nucleotide exchange factor (GEF) 1	0.829876	0.02238	0.1137
64383	Sirt2	sirtuin 2 (silent mating type information regulation 2, homolog) 2 (S. cerevisiae)	0.829876	0.08377	0.2503
66849	Ppp1r2	protein phosphatase 1, regulatory (inhibitor) subunit 2	0.829876	0.3509	0.5866
69440	Fam116b	family with sequence similarity 116, member B	0.829876	0.07442	0.2328
72898	Asphd2	aspartate beta-hydroxylase domain containing 2	0.829876	0.01967	0.105
72960	Top1mt	DNA topoisomerase 1, mitochondrial	0.829876	0.05351	0.1885
74596	Cds1	CDP-diacylglycerol synthase 1	0.829876	0.1091	0.2956
84004	Mcam	melanoma cell adhesion molecule	0.829876	0.1816	0.4004
98710	Rabif	RAB interacting factor	0.829876	0.07401	0.2321
101809	Spred3	sprouty-related, EVH1 domain containing 3	0.829876	0.06803	0.2202
104681	Slc16a6	solute carrier family 16 (monocarboxylic acid transporters), member 6	0.829876	0.1627	0.375
208258	Ankrd33	ankyrin repeat domain 33	0.829876	0.00626	0.05321
211914	Asap2	ArfGAP with SH3 domain, ankyrin repeat and PH domain 2	0.829876	0.01194	0.07773
230661	Tesk2	testis-specific kinase 2	0.829876	0.006441	0.05413
230991	B930041F14Rik	RIKEN cDNA B930041F14 gene	0.829876	0.05191	0.1852
233908	Fus	fusion, derived from t(12;16) malignant liposarcoma (human)	0.829876	0.002682	NA
245886	Ankrd27	ankyrin repeat domain 27 (VPS9 domain)	0.829876	0.4059	0.6354
270109	Pcnxl2	pecanex-like 2 (Drosophila)	0.829876	0.05865	0.2003
319278	A230050P20Rik	RIKEN cDNA A230050P20 gene	0.829876	0.03983	0.159
547176	Zc3h12b	zinc finger CCCH-type containing 12B	0.829876	0.4678	0.6897
619441	BC096441	cDNA sequence BC096441	0.829876	0.01773	0.09839
100040724	Mirg	miRNA containing gene	0.829876	0.06651	0.2169
12583	Cdo1	cysteine dioxygenase 1, cytosolic	0.829187	0.02087	0.1089
15129	Hbb-b1	hemoglobin, beta adult major chain	0.829187	0.4546	0.6782
16506	Kcnd1	potassium voltage-gated channel, Shal-related family, member 1	0.829187	0.06784	0.2199
17318	Mid1	midline 1	0.829187	0.1584	0.3695
18203	Ntan1	N-terminal Asn amidase	0.829187	0.005036	0.04691
20680	Sox7	SRY-box containing gene 7	0.829187	0.2225	0.4527
21942	Tnfrsf9	tumor necrosis factor receptor superfamily, member 9	0.829187	0.2604	0.4958
22033	Traf5	TNF receptor-associated factor 5	0.829187	0.009889	0.06952
50875	Tmod3	tropomodulin 3	0.829187	0.179	0.397
52897	Rbfox3	RNA binding protein, fox-1 homolog (C. elegans) 3	0.829187	0.003929	NA
54377	Cacng4	calcium channel, voltage-dependent, gamma subunit 4	0.829187	0.03191	0.1404
56309	Mycbp	c-myc binding protein	0.829187	0.1736	0.3899
56373	Cpb2	carboxypeptidase B2 (plasma)	0.829187	0.0548	0.1916
66140	Fam33a	family with sequence similarity 33, member A	0.829187	0.4462	0.6718
67426	Adck3	aarF domain containing kinase 3	0.829187	0.06775	0.2197
69815	Krtcap3	keratinocyte associated protein 3	0.829187	0.05363	0.1888

70454	Cenpl	centromere protein L	0.829187	0.09896	0.2779
77836	Mlana	melan-A	0.829187	0.5428	0.7463
108100	Baiap2	brain-specific angiogenesis inhibitor 1-associated protein 2	0.829187	0.04149	0.163
171429	Slc26a6	solute carrier family 26, member 6	0.829187	0.006334	0.0535
218341	Rfesd	Rieske (Fe-S) domain containing	0.829187	0.04744	0.1763
225655	Slmo1	slowmo homolog 1 (Drosophila)	0.829187	0.008341	0.06257
226856	Lpgat1	lysophosphatidylglycerol acyltransferase 1	0.829187	0.03813	0.1554
234267	Gpm6a	glycoprotein m6a	0.829187	0.2238	0.4543
235534	Acpl2	acid phosphatase-like 2	0.829187	0.1371	0.339
269717	Orai2	ORAI calcium release-activated calcium modulator 2	0.829187	0.01101	0.07421
319455	Pld5	phospholipase D family, member 5	0.829187	0.2906	0.5266
333605	Frmpd4	FERM and PDZ domain containing 4	0.829187	0.04587	0.1728
667118	Zbed6	zinc finger, BED domain containing 6	0.829187	0.2821	0.5174
12894	Cpt1a	carnitine palmitoyltransferase 1a, liver	0.8285	0.004669	NA
20230	Satb1	special AT-rich sequence binding protein 1	0.8285	0.004911	0.04616
20744	Strbp	spermatid perinuclear RNA binding protein	0.8285	0.04343	0.1673
22092	Rsph1	radial spoke head 1 homolog (Chlamydomonas)	0.8285	0.37	0.605
27401	Skp2	S-phase kinase-associated protein 2 (p45)	0.8285	0.1951	0.4189
50770	Atp11a	ATPase, class VI, type 11A	0.8285	0.02279	0.115
50796	Dmrt1	doublesex and mab-3 related transcription factor 1	0.8285	0.06868	0.2214
58212	Srrm3	serine/arginine repetitive matrix 3	0.8285	0.06256	0.2086
66598	3110001I22Rik	RIKEN cDNA 3110001I22 gene	0.8285	0.081	0.2448
66648	5730494M16Rik	RIKEN cDNA 5730494M16 gene	0.8285	0.1208	0.3149
66700	Vps24	vacuolar protein sorting 24 (yeast)	0.8285	0.05541	0.1926
68018	Col4a3bp	collagen, type IV, alpha 3 (Goodpasture antigen) binding protein	0.8285	0.122	0.317
74464	Zswim5	zinc finger, SWIM domain containing 5	0.8285	0.1691	0.3843
76080	Ttpal	tocopherol (alpha) transfer protein-like	0.8285	0.2902	0.5264
77531	Anks1b	ankyrin repeat and sterile alpha motif domain containing 1B	0.8285	0.04075	0.1613
83766	Actl6b	actin-like 6B	0.8285	0.387	0.6191
117600	Srgap1	SLIT-ROBO Rho GTPase activating protein 1	0.8285	0.2268	0.4582
215114	Hip1	huntingtin interacting protein 1	0.8285	0.003506	NA
223870	Senp1	SUMO1/sentrin specific peptidase 1	0.8285	0.1602	0.3719
224674	Slc37a1	solute carrier family 37 (glycerol-3-phosphate transporter), member 1	0.8285	0.1065	0.2911
234384	Mpv17l2	MPV17 mitochondrial membrane protein-like 2	0.8285	0.07779	0.2391
234889	Gucy1a2	guanylate cyclase 1, soluble, alpha 2	0.8285	0.1849	0.4043
236604	Pisd-ps1	phosphatidylserine decarboxylase, pseudogene 1	0.8285	0.04456	0.1698
242669	Adc	arginine decarboxylase	0.8285	0.0577	0.1979
244551	Nanos3	nanos homolog 3 (Drosophila)	0.8285	0.04191	0.1638
435366	Gm5665	predicted gene 5665	0.8285	0.3284	0.5648
100048658	Ddx43	DEAD (Asp-Glu-Ala-Asp) box polypeptide 43	0.8285	0.1957	0.4196
11773	Ap2m1	adaptor protein complex AP-2, mu1	0.827815	0.04325	0.1669
17936	Nab1	Ngfi-A binding protein 1	0.827815	0.09924	0.2779
20276	Scnn1a	sodium channel, nonvoltage-gated 1 alpha	0.827815	0.155	0.3648
26557	Homer2	homer homolog 2 (Drosophila)	0.827815	0.0938	0.2692
26874	Abcd2	ATP-binding cassette, sub-family D (ALD), member 2	0.827815	0.02526	0.1221
52858	Cdipt	CDP-diacylglycerol--inositol 3-phosphatidyltransferase (phosphatidylinositol synthase)	0.827815	0.006111	0.0526
53857	Tuba8	tubulin, alpha 8	0.827815	0.03051	0.1369
57890	Il17re	interleukin 17 receptor E	0.827815	0.107	0.2921
66171	Pgls	6-phosphogluconolactonase	0.827815	0.02319	0.1161

66196	Myo19	myosin XIX	0.827815	0.01222	0.07871
66445	Cyc1	cytochrome c-1	0.827815	0.008168	0.06187
74149	Zfp946	zinc finger protein 946	0.827815	0.01886	0.1023
74180	Muc5b	mucin 5, subtype B, tracheobronchial	0.827815	0.2256	0.4566
76630	Stambpl1	STAM binding protein like 1	0.827815	0.01903	0.1029
80297	Spnb4	spectrin beta 4	0.827815	0.01622	0.09332
93882	Pcdhb11	protocadherin beta 11	0.827815	0.1482	0.3551
209446	Tcfe3	transcription factor E3	0.827815	0.1078	0.2937
229317	Eif2a	eukaryotic translation initiation factor 2A	0.827815	0.04055	0.161
238831	Ppwd1	peptidylprolyl isomerase domain and WD repeat containing 1	0.827815	0.0402	0.1599
242259	Slc44a5	solute carrier family 44, member 5	0.827815	0.06864	0.2214
242509	Bnc2	basonuclin 2	0.827815	0.02	0.1062
16421	Itgb7	integrin beta 7	0.82713	0.2644	0.5005
16650	Kpna6	karyopherin (importin) alpha 6	0.82713	0.06279	0.2093
20602	Ncor2	nuclear receptor co-repressor 2	0.82713	0.01959	0.1049
23893	Grem2	gremlin 2 homolog, cysteine knot superfamily (Xenopus laevis)	0.82713	0.04804	0.1775
66237	Atp6v1g2	ATPase, H+ transporting, lysosomal V1 subunit G2	0.82713	0.1443	0.3493
66799	Ube2w	ubiquitin-conjugating enzyme E2W (putative)	0.82713	0.15	0.3577
75605	Kdm5b	lysine (K)-specific demethylase 5B	0.82713	0.03004	0.1357
97868	C78704	expressed sequence C78704	0.82713	0.206	0.4323
105663	Thtpa	thiamine triphosphatase	0.82713	0.02376	0.1175
107368	Pdzd8	PDZ domain containing 8	0.82713	0.2186	0.4478
107477	Guca1b	guanylate cyclase activator 1B	0.82713	0.02153	0.1111
114716	Spred2	sprouty-related, EVH1 domain containing 2	0.82713	0.1272	0.3249
211488	Ado	2-aminoethanethiol (cysteamine) dioxygenase	0.82713	0.006978	0.05654
232813	Shisa7	shisa homolog 7 (Xenopus laevis)	0.82713	0.01371	0.08467
234413	Zfp961	zinc finger protein 961	0.82713	0.0123	0.07892
279766	Rhbdd3	rhomboid domain containing 3	0.82713	0.01201	0.07796
353170	Txlng	taxilin gamma	0.82713	0.06363	0.2107
574418	Serinc4	serine incorporator 4	0.82713	0.02768	0.1295
11744	Anxa11	annexin A11	0.826446	0.1276	0.3255
17175	Masp2	mannan-binding lectin serine peptidase 2	0.826446	0.1444	0.3495
18107	Nmt1	N-myristoyltransferase 1	0.826446	0.04515	0.1712
18762	Prkcz	protein kinase C, zeta	0.826446	0.006722	0.05541
20910	Stxbp1	syntaxin binding protein 1	0.826446	0.01043	0.07196
27403	Abca7	ATP-binding cassette, sub-family A (ABC1), member 7	0.826446	0.05508	0.1923
29819	Stau2	staufen (RNA binding protein) homolog 2 (Drosophila)	0.826446	0.03684	0.152
52389	Gpr123	G protein-coupled receptor 123	0.826446	0.2018	0.428
54607	Socs6	suppressor of cytokine signaling 6	0.826446	0.05184	0.1851
69641	Wdr20a	WD repeat domain 20A	0.826446	0.01636	0.09371
70897	Fam71d	family with sequence similarity 71, member D	0.826446	0.3993	0.6296
71354	Wdr31	WD repeat domain 31	0.826446	0.07761	0.2389
75444	1700011I03Rik	RIKEN cDNA 1700011I03 gene	0.826446	0.1768	0.3938
78752	Csgalnact2	chondroitin sulfate N-acetylgalactosaminyltransferase 2	0.826446	0.0562	0.1942
101179	6430519N07Rik	RIKEN cDNA 6430519N07 gene	0.826446	0.5175	0.7269
107831	Bai1	brain-specific angiogenesis inhibitor 1	0.826446	0.01535	0.09072
227399	Ppip5k2	diphosphoinositol pentakisphosphate kinase 2	0.826446	0.2849	0.5202
230775	Bai2	brain-specific angiogenesis inhibitor 2	0.826446	0.2553	0.4902
232146	Fam176a	family with sequence similarity 176, member A	0.826446	0.01424	0.08658

243910	Nfkbid	nuclear factor of kappa light polypeptide gene enhancer in B-cells inhibitor, delta	0.826446	0.1307	0.3304
245386	Fam70a	family with sequence similarity 70, member A	0.826446	0.01519	0.09001
245827	Fat2	FAT tumor suppressor homolog 2 (Drosophila)	0.826446	0.01028	0.0714
319179	Hist1h2be	histone cluster 1, H2be	0.826446	0.04828	0.1778
320057	A630057N01Rik	RIKEN cDNA A630057N01 gene	0.826446	0.2019	0.428
14708	Gng7	guanine nucleotide binding protein (G protein), gamma 7	0.825764	0.1246	0.3213
16519	Kcnj3	potassium inwardly-rectifying channel, subfamily J, member 3	0.825764	0.2267	0.4582
17330	Minpp1	multiple inositol polyphosphate histidine phosphatase 1	0.825764	0.05697	0.1961
17996	Neb	nebulin	0.825764	0.184	0.403
20016	Polr1c	polymerase (RNA) I polypeptide C	0.825764	0.01296	0.08184
20222	Sf3a2	splicing factor 3a, subunit 2	0.825764	0.2436	0.4771
20927	Abcc8	ATP-binding cassette, sub-family C (CFTR/MRP), member 8	0.825764	0.036	0.15
26950	Vsnl1	visinin-like 1	0.825764	0.0277	0.1295
52108	D9Ertd115e	DNA segment, Chr 9, ERATO Doi 115, expressed	0.825764	0.3251	0.5618
57340	Jph3	junctophilin 3	0.825764	0.05838	0.1996
66926	Trmt6	tRNA methyltransferase 6 homolog (S. cerevisiae)	0.825764	0.01384	0.08511
67071	Rps6ka6	ribosomal protein S6 kinase polypeptide 6	0.825764	0.3343	0.5699
68792	Srpx2	sushi-repeat-containing protein, X-linked 2	0.825764	0.2639	0.4999
69655	Cd164l2	CD164 sialomucin-like 2	0.825764	0.2112	0.4386
69851	2010007E15Rik	RIKEN cDNA 2010007E15 gene	0.825764	0.3405	0.5762
70373	1700020O03Rik	RIKEN cDNA 1700020O03 gene	0.825764	0.006473	0.05428
71703	Armcx3	armadillo repeat containing, X-linked 3	0.825764	0.1459	0.3514
71770	Ap2b1	adaptor-related protein complex 2, beta 1 subunit	0.825764	0.1173	0.3093
71911	Bdh1	3-hydroxybutyrate dehydrogenase, type 1	0.825764	0.2027	0.429
76897	Ralyl	RALY RNA binding protein-like	0.825764	0.02869	0.1319
98388	Chst10	carbohydrate sulfotransferase 10	0.825764	0.04909	0.1795
114641	Rpl31	ribosomal protein L31	0.825764	0.03042	0.1368
218103	Slc17a2	solute carrier family 17 (sodium phosphate), member 2	0.825764	0.4448	0.6706
226744	Cnst	consortin, connexin sorting protein	0.825764	0.02119	0.1099
242691	Gpatch3	G patch domain containing 3	0.825764	0.1393	0.3424
100041479	Gm3363	predicted gene 3363	0.825764	0.309	0.5453
100303732	Gm14431	predicted gene 14431	0.825764	0.4501	0.6751
13180	Pcbd1	pterin 4 alpha carbinolamine dehydratase/dimerization cofactor of hepatocyte nuclear factor 1 alpha (TCF1) 1	0.825083	0.01748	0.09774
17183	Matn4	matrilin 4	0.825083	0.09767	0.2757
17754	Mtap1a	microtubule-associated protein 1 A	0.825083	0.1492	0.3567
20430	Cyfip1	cytoplasmic FMR1 interacting protein 1	0.825083	0.09865	0.2773
22761	Zfpm1	zinc finger protein, multitype 1	0.825083	0.02597	0.1242
23943	Esyt1	extended synaptotagmin-like protein 1	0.825083	0.08643	0.2557
27380	Tcl1b4	T-cell leukemia/lymphoma 1B, 4	0.825083	0.3507	0.5864
28250	Slco1a4	solute carrier organic anion transporter family, member 1a4	0.825083	0.08587	0.2544
60597	Mapk8ip2	mitogen-activated protein kinase 8 interacting protein 2	0.825083	0.05707	0.1963
66930	Fank1	fibronectin type 3 and ankyrin repeat domains 1	0.825083	0.06373	0.2109
67864	Yipf4	Yip1 domain family, member 4	0.825083	0.02433	0.1192
68874	Klhdc9	kelch domain containing 9	0.825083	0.1489	0.3563
72168	Aifm3	apoptosis-inducing factor, mitochondrion-associated 3	0.825083	0.06209	0.2074
73721	1110017D15Rik	RIKEN cDNA 1110017D15 gene	0.825083	0.03044	0.1368
75089	Uhrf1bp1l	UHRF1 (ICBP90) binding protein 1-like	0.825083	0.002437	NA
75972	5033423O07Rik	RIKEN cDNA 5033423O07 gene	0.825083	0.08729	0.2575
76217	Jakmip2	janus kinase and microtubule interacting protein 2	0.825083	0.006667	0.05528

107321	Lpxn	leupaxin	0.825083	0.02071	0.1083
211480	Kcnj14	potassium inwardly-rectifying channel, subfamily J, member 14	0.825083	0.03427	0.1464
227058	Dnahc7b	dynein, axonemal, heavy chain 7B	0.825083	0.01349	0.08382
239652	Zfp641	zinc finger protein 641	0.825083	0.04724	0.1759
244654	Mtss1l	metastasis suppressor 1-like	0.825083	0.07591	0.236
320208	Tmem91	transmembrane protein 91	0.825083	0.07241	0.2289
328365	Zmiz1	zinc finger, MIZ-type containing 1	0.825083	0.1179	0.3103
353235	Pcdha8	protocadherin alpha 8	0.825083	0.01848	0.1009
19294	Pvrl2	poliovirus receptor-related 2	0.824402	0.09455	0.2703
22249	Unc13b	unc-13 homolog B (C. elegans)	0.824402	0.006506	0.05453
57296	Psmd8	proteasome (prosome, macropain) 26S subunit, non-ATPase, 8	0.824402	0.004934	0.04628
59014	Rrs1	RRS1 ribosome biogenesis regulator homolog (S. cerevisiae)	0.824402	0.008487	0.06328
67911	Zfp169	zinc finger protein 169	0.824402	0.05011	0.1817
68184	Denr	density-regulated protein	0.824402	0.09864	0.2773
69179	Tmem110	transmembrane protein 110	0.824402	0.08455	0.2519
72282	1810062G17Rik	RIKEN cDNA 1810062G17 gene	0.824402	0.3392	0.5747
72674	Adipor1	adiponectin receptor 1	0.824402	0.001333	NA
73761	4833415N18Rik	RIKEN cDNA 4833415N18 gene	0.824402	0.339	0.5747
74691	Tdrd9	tudor domain containing 9	0.824402	0.05925	0.2017
76199	Med13l	mediator complex subunit 13-like	0.824402	0.1586	0.3697
214489	BC003965	cDNA sequence BC003965	0.824402	0.01004	0.07018
225655	Slmo1	slowmo homolog 1 (Drosophila)	0.824402	0.0572	0.1965
230777	Hcrtr1	hypocretin (orexin) receptor 1	0.824402	0.02583	0.1238
239170	Fam160b2	family with sequence similarity 160, member B2	0.824402	0.3155	0.5522
242425	Gabbr2	gamma-aminobutyric acid (GABA) B receptor, 2	0.824402	0.1722	0.388
327958	Pitpnm3	PITPNM family member 3	0.824402	0.06839	0.2209
12217	Bsn	bassoon	0.823723	0.09299	0.2677
15357	Hmgcr	3-hydroxy-3-methylglutaryl-Coenzyme A reductase	0.823723	0.04018	0.1599
15531	Ndst1	N-deacetylase/N-sulfotransferase (heparan glucosaminyl) 1	0.823723	0.05311	0.1875
16679	Krt86	keratin 86	0.823723	0.5765	0.7695
18188	Nrtn	neurturin	0.823723	0.01808	0.09955
20185	Ncor1	nuclear receptor co-repressor 1	0.823723	0.1096	0.2965
60599	Trp53inp1	transformation related protein 53 inducible nuclear protein 1	0.823723	0.06553	0.2147
66514	Asrgl1	asparaginase like 1	0.823723	0.2356	0.4678
67039	Rbm25	RNA binding motif protein 25	0.823723	0.1216	0.3164
70911	Phyhipl	phytanoyl-CoA hydroxylase interacting protein-like	0.823723	0.1596	0.371
71132	Cabyr	calcium-binding tyrosine-(Y)-phosphorylation regulated (fibrousheathin 2)	0.823723	0.1558	0.3657
72017	Cyb5r1	cytochrome b5 reductase 1	0.823723	0.08575	0.2542
75561	1700016J18Rik	RIKEN cDNA 1700016J18 gene	0.823723	0.08028	0.2437
76081	5830458C19Rik	RIKEN cDNA 5830458C19 gene	0.823723	0.1147	0.305
117592	B3galt6	UDP-Gal:betaGal beta 1,3-galactosyltransferase, polypeptide 6	0.823723	0.1035	0.2856
208104	Mlxip	MLX interacting protein	0.823723	0.2002	0.4258
215446	Entpd3	ectonucleoside triphosphate diphosphohydrolase 3	0.823723	0.08176	0.2463
268759	9930012K11Rik	RIKEN cDNA 9930012K11 gene	0.823723	0.1996	0.425
319466	9330199G10Rik	RIKEN cDNA 9330199G10 gene	0.823723	0.4118	0.6408
329260	Dennd1b	DENN/MADD domain containing 1B	0.823723	0.03923	0.1577
620709	Lcn6	lipocalin 6	0.823723	0.2767	0.5123
14007	Celf2	CUGBP, Elav-like family member 2	0.823045	0.03704	0.1525
20420	Shd	src homology 2 domain-containing transforming protein D	0.823045	0.06148	0.2062

20977	Syp	synaptophysin	0.823045	0.03634	0.1509
26938	St6galnac5	ST6 (alpha-N-acetyl-neuraminyl-2,3-beta-galactosyl-1,3)-N-acetylgalactosaminide alpha-2,6-sialyltransferase 5	0.823045	0.1155	0.3063
57780	Fxyd7	FXYD domain-containing ion transport regulator 7	0.823045	0.1405	0.3442
65254	Dpysl5	dihydropyrimidinase-like 5	0.823045	0.3179	0.5543
66337	1700025K23Rik	RIKEN cDNA 1700025K23 gene	0.823045	0.004889	0.04601
66505	Zmynd11	zinc finger, MYND domain containing 11	0.823045	0.01681	0.09547
66874	1200014J11Rik	RIKEN cDNA 1200014J11 gene	0.823045	0.02766	0.1294
67538	Zswim3	zinc finger, SWIM domain containing 3	0.823045	0.07526	0.2346
67948	Fbxo28	F-box protein 28	0.823045	0.005172	0.0477
70310	Plscr3	phospholipid scramblase 3	0.823045	0.2778	0.5135
75533	Nme5	non-metastatic cells 5, protein expressed in (nucleoside-diphosphate kinase)	0.823045	0.01913	0.1032
107566	Arl2bp	ADP-ribosylation factor-like 2 binding protein	0.823045	0.0135	0.08384
207785	Csrnp2	cysteine-serine-rich nuclear protein 2	0.823045	0.03505	0.1484
224650	Anks1	ankyrin repeat and SAM domain containing 1	0.823045	0.04789	0.1771
232431	Gprc5a	G protein-coupled receptor, family C, group 5, member A	0.823045	0.1916	0.4137
277854	Depdc5	DEP domain containing 5	0.823045	0.1346	0.3356
319592	D030020J04Rik	RIKEN cDNA D030020J04 gene	0.823045	0.2371	0.4693
329986	BC080695	cDNA sequence BC080695	0.823045	0.1243	0.3209
434249	Gm5602	predicted gene 5602	0.823045	0.03722	0.1529
11647	Alpl	alkaline phosphatase, liver/bone/kidney	0.822368	0.1212	0.3156
14811	Grin2a	glutamate receptor, ionotropic, NMDA2A (epsilon 1)	0.822368	0.132	0.3323
14812	Grin2b	glutamate receptor, ionotropic, NMDA2B (epsilon 2)	0.822368	0.2235	0.4541
17263	Meg3	maternally expressed 3	0.822368	0.1912	0.4132
18720	Pip5k1a	phosphatidylinositol-4-phosphate 5-kinase, type 1 alpha	0.822368	0.01259	0.0803
18749	Prkacb	protein kinase, cAMP dependent, catalytic, beta	0.822368	0.03839	0.156
64378	Gpr88	G-protein coupled receptor 88	0.822368	0.0163	0.09362
71675	0610010F05Rik	RIKEN cDNA 0610010F05 gene	0.822368	0.01513	0.08982
72154	Zfp157	zinc finger protein 157	0.822368	0.1821	0.4009
72795	Ttc19	tetratricopeptide repeat domain 19	0.822368	0.01466	0.08819
227721	Ppapdc3	phosphatidic acid phosphatase type 2 domain containing 3	0.822368	0.02347	0.1169
233529	Kctd14	potassium channel tetramerisation domain containing 14	0.822368	0.3835	0.6162
268747	Lrrc16b	leucine rich repeat containing 16B	0.822368	0.09344	0.2686
268903	Nrip1	nuclear receptor interacting protein 1	0.822368	0.08121	0.2452
381318	Nsl1	NSL1, MIND kinetochore complex component, homolog (S. cerevisiae)	0.822368	0.02343	0.1168
434234	2610020H08Rik	RIKEN cDNA 2610020H08 gene	0.822368	0.04399	0.1686
545554	Ankrd34a	ankyrin repeat domain 34A	0.822368	0.01299	0.08197
628431	Hmgb1-rs17	high mobility group box 1, related sequence 17	0.822368	0.07761	0.2389
11789	Apc	adenomatosis polyposis coli	0.821693	0.1824	0.4014
12165	Gdf2	growth differentiation factor 2	0.821693	0.3105	0.5468
13047	Cux1	cut-like homeobox 1	0.821693	0.01274	0.08085
16491	Kcna3	potassium voltage-gated channel, shaker-related subfamily, member 3	0.821693	0.01208	0.07821
18393	Orc2	origin recognition complex, subunit 2	0.821693	0.02201	0.1124
18767	Pkia	protein kinase inhibitor, alpha	0.821693	0.1761	0.3931
66648	5730494M16Rik	RIKEN cDNA 5730494M16 gene	0.821693	0.03922	0.1577
66810	Rbm22	RNA binding motif protein 22	0.821693	0.03688	0.1521
67996	Srsf6	serine/arginine-rich splicing factor 6	0.821693	0.04077	0.1614
71448	Tmem80	transmembrane protein 80	0.821693	0.003652	0.03863
71519	Cyp2u1	cytochrome P450, family 2, subfamily u, polypeptide 1	0.821693	0.1053	0.2888
72097	2010300C02Rik	RIKEN cDNA 2010300C02 gene	0.821693	0.145	0.3504

72273	2210404O07Rik	RIKEN cDNA 2210404O07 gene	0.821693	0.04118	0.1622
73002	2900076A13Rik	RIKEN cDNA 2900076A13 gene	0.821693	0.05065	0.1828
77189	A430105J06Rik	RIKEN cDNA A430105J06 gene	0.821693	0.1256	0.3225
78795	Armc9	armadillo repeat containing 9	0.821693	0.005629	0.05017
117229	Stk33	serine/threonine kinase 33	0.821693	0.1	0.2793
225895	Taf6l	TAF6-like RNA polymerase II, p300/CBP-associated factor (PCAF)-associated factor	0.821693	0.1833	0.4023
228356	1110051M20Rik	RIKEN cDNA 1110051M20 gene	0.821693	0.02723	0.1279
239410	A930017M01Rik	Smg-5 homolog, nonsense mediated mRNA decay factor pseudogene	0.821693	0.009067	0.06591
258778	Olfr921	olfactory receptor 921	0.821693	0.3412	0.5769
319613	Sybu	syntabulin (syntaxin-interacting)	0.821693	0.003113	0.03501
320534	Tmem104	transmembrane protein 104	0.821693	0.299	0.5348
13048	Cux2	cut-like homeobox 2	0.821018	0.01388	0.0853
15289	Hmgb1	high mobility group box 1	0.821018	0.03176	0.14
19933	Rpl21	ribosomal protein L21	0.821018	0.009601	0.06822
20623	Snrk	SNF related kinase	0.821018	0.02767	0.1295
53602	Hpcal1	hippocalcin-like 1	0.821018	0.04073	0.1613
54524	Syt6	synaptotagmin VI	0.821018	0.01557	0.0915
54610	Tbc1d8	TBC1 domain family, member 8	0.821018	0.02584	0.1238
65111	Dap3	death associated protein 3	0.821018	0.07225	0.2285
69780	Smap2	stromal membrane-associated GTPase-activating protein 2	0.821018	0.03609	0.1501
70701	Nipal1	NIPA-like domain containing 1	0.821018	0.003572	0.03815
71745	Cul2	cullin 2	0.821018	0.1143	0.3044
73301	Ttc29	tetratricopeptide repeat domain 29	0.821018	0.4755	0.6961
74711	Ttll9	tubulin tyrosine ligase-like family, member 9	0.821018	0.08406	0.2508
76186	6330563C09Rik	RIKEN cDNA 6330563C09 gene	0.821018	0.05474	0.1915
99738	Kcnc4	potassium voltage gated channel, Shaw-related subfamily, member 4	0.821018	0.1602	0.3719
209683	Ttc28	tetratricopeptide repeat domain 28	0.821018	0.006721	0.05541
213464	Rbbp5	retinoblastoma binding protein 5	0.821018	0.06074	0.2048
216344	Rab21	RAB21, member RAS oncogene family	0.821018	0.03901	0.1571
235067	Cypt4	cysteine-rich perinuclear theca 4	0.821018	0.2763	0.5119
243853	Fkrp	fukutin related protein	0.821018	0.02914	0.1333
436583	Snora74a	small nucleolar RNA, H/ACA box 74A	0.821018	0.3486	0.5844
544678	2010015L04Rik	RIKEN cDNA 2010015L04 gene	0.821018	0.01738	0.09739
12164	Bmp8b	bone morphogenetic protein 8b	0.820345	0.2142	0.4426
13840	Epha6	Eph receptor A6	0.820345	0.1647	0.3782
14169	Fgf14	fibroblast growth factor 14	0.820345	0.09314	0.268
17258	Mef2a	myocyte enhancer factor 2A	0.820345	0.3817	0.615
20361	Sema7a	sema domain, immunoglobulin domain (Ig), and GPI membrane anchor, (semaphorin) 7A	0.820345	0.02436	0.1192
65115	Bean1	brain expressed, associated with Nedd4, 1	0.820345	0.0715	0.227
66788	5430414B12Rik	RIKEN cDNA 5430414B12 gene	0.820345	0.07747	0.2387
66999	Med28	mediator of RNA polymerase II transcription, subunit 28 homolog (yeast)	0.820345	0.007111	0.05713
67466	Pdcl	phosducin-like	0.820345	0.01939	0.1042
67531	5730408K05Rik	RIKEN cDNA 5730408K05 gene	0.820345	0.09916	0.2779
68082	Dusp19	dual specificity phosphatase 19	0.820345	0.07619	0.2365
108073	Grm7	glutamate receptor, metabotropic 7	0.820345	0.007931	0.06104
108138	Xrcc4	X-ray repair complementing defective repair in Chinese hamster cells 4	0.820345	0.01404	0.08578
208440	Dip2c	DIP2 disco-interacting protein 2 homolog C (Drosophila)	0.820345	0.02554	0.1228
214932	Cecr5	cat eye syndrome chromosome region, candidate 5 homolog (human)	0.820345	0.05092	0.1831
217310	C630004H02Rik	RIKEN cDNA C630004H02 gene	0.820345	0.03169	0.1399

223642	Zc3h3	zinc finger CCCH type containing 3	0.820345	0.02867	0.1319
229801	Tram1l1	translocation associated membrane protein 1-like 1	0.820345	0.01286	0.08147
230751	Oscp1	organic solute carrier partner 1	0.820345	0.05995	0.2031
244713	Zfp317	zinc finger protein 317	0.820345	0.02854	0.1316
320858	L3mbtl4	l(3)mbt-like 4 (Drosophila)	0.820345	0.1735	0.3897
100036521	Gm16039	predicted gene 16039	0.820345	0.2126	0.4404
12343	Capza2	capping protein (actin filament) muscle Z-line, alpha 2	0.819672	0.04008	0.1596
18190	Nrxn2	neurexin II	0.819672	0.08101	0.2448
19253	Ptpn18	protein tyrosine phosphatase, non-receptor type 18	0.819672	0.02949	0.1341
53951	Ccdc75	coiled-coil domain containing 75	0.819672	0.01052	0.07234
56405	Dusp14	dual specificity phosphatase 14	0.819672	0.03524	0.1484
69816	2010001M09Rik	RIKEN cDNA 2010001M09 gene	0.819672	0.04869	0.1786
73296	Rhobtb3	Rho-related BTB domain containing 3	0.819672	0.0323	0.1414
76527	Il34	interleukin 34	0.819672	0.02941	0.134
76781	Mettl4	methyltransferase like 4	0.819672	0.03979	0.1589
78885	Coro7	coronin 7	0.819672	0.01445	0.08743
109032	Sp110	Sp110 nuclear body protein	0.819672	0.172	0.3878
170763	Zfp87	zinc finger protein 87	0.819672	0.02359	0.1171
385343	Rhox1	reproductive homeobox 1	0.819672	0.4098	0.6391
668923	Zfp442	zinc finger protein 442	0.819672	0.2887	0.5248
100034363	Tmsb15b2	thymosin beta 15b2	0.819672	0.009121	0.06612
14073	Faah	fatty acid amide hydrolase	0.819001	0.07441	0.2328
20845	Star	steroidogenic acute regulatory protein	0.819001	0.1886	0.4097
22239	Ugt8a	UDP galactosyltransferase 8A	0.819001	0.1451	0.3505
50799	Slc25a13	solute carrier family 25 (mitochondrial carrier, adenine nucleotide translocator), member 13	0.819001	0.1715	0.3872
53625	B3gnt2	UDP-GlcNAc:betaGal beta-1,3-N-acetylglucosaminyltransferase 2	0.819001	0.03062	0.137
56089	Ramp3	receptor (calcitonin) activity modifying protein 3	0.819001	0.02551	0.1227
60525	Acss2	acyl-CoA synthetase short-chain family member 2	0.819001	0.007856	0.0609
60532	Wtap	Wilms' tumour 1-associating protein	0.819001	0.03098	0.1379
69837	Pcgf1	polycomb group ring finger 1	0.819001	0.04983	0.1814
71310	Tbc1d9	TBC1 domain family, member 9	0.819001	0.03969	0.1587
71564	Izumo4	IZUMO family member 4	0.819001	0.06617	0.2163
71902	Cand1	cullin associated and neddylation disassociated 1	0.819001	0.02558	0.1229
94045	P2rx5	purinergic receptor P2X, ligand-gated ion channel, 5	0.819001	0.06363	0.2107
99029	AI596198	expressed sequence AI596198	0.819001	0.007616	0.05976
99681	Tchh	trichohyalin	0.819001	0.3577	0.5926
114644	Slc13a3	solute carrier family 13 (sodium-dependent dicarboxylate transporter), member 3	0.819001	0.4133	0.642
170729	Scrt1	scratch homolog 1, zinc finger protein (Drosophila)	0.819001	0.02265	0.1146
208151	Tmem132b	transmembrane protein 132B	0.819001	0.3153	0.5519
225998	Rorb	RAR-related orphan receptor beta	0.819001	0.08733	0.2576
228714	Csrp2bp	cysteine and glycine-rich protein 2 binding protein	0.819001	0.007988	0.06128
234854	Cdk10	cyclin-dependent kinase 10	0.819001	0.02167	0.1115
243385	Gprin3	GPRIN family member 3	0.819001	0.1615	0.3735
330286	D630045J12Rik	RIKEN cDNA D630045J12 gene	0.819001	0.0461	0.1733
330863	Trim67	tripartite motif-containing 67	0.819001	0.01023	0.07112
381511	Pdp1	pyruvate dehyrogenase phosphatase catalytic subunit 1	0.819001	0.01962	0.1049
408067	Zfp874b	zinc finger protein 874b	0.819001	0.005998	0.05218
100039757	Gm10153	predicted gene 10153	0.819001	0.2837	0.5195
11803	Aplp1	amyloid beta (A4) precursor-like protein 1	0.818331	0.09478	0.2707

12405	Cbln2	cerebellin 2 precursor protein	0.818331	0.00801	0.06134
12988	Csk	c-src tyrosine kinase	0.818331	0.07021	0.2244
22652	Mkrn3	makorin, ring finger protein, 3	0.818331	0.01195	0.07775
58184	Rqcd1	rcd1 (required for cell differentiation) homolog 1 (S. pombe)	0.818331	0.006569	0.05475
67768	N6amt1	N-6 adenine-specific DNA methyltransferase 1 (putative)	0.818331	0.04525	0.1715
67920	Mak16	MAK16 homolog (S. cerevisiae)	0.818331	0.06637	0.2167
68014	Zwilch	Zwilch, kinetochore associated, homolog (Drosophila)	0.818331	0.03141	0.1391
78751	Zc3h6	zinc finger CCCH type containing 6	0.818331	0.1087	0.295
99326	Garnl3	GTPase activating RANGAP domain-like 3	0.818331	0.01792	0.09903
110524	Dgkq	diacylglycerol kinase, theta	0.818331	0.01484	0.08886
170822	Usp33	ubiquitin specific peptidase 33	0.818331	0.003692	0.03888
192188	Stab2	stabilin 2	0.818331	0.03352	0.1446
208884	Zdhhc9	zinc finger, DHHC domain containing 9	0.818331	0.2276	0.4586
209131	Snx30	sorting nexin family member 30	0.818331	0.02099	0.1092
238057	Gdf7	growth differentiation factor 7	0.818331	0.3415	0.5772
242384	Lingo2	leucine rich repeat and Ig domain containing 2	0.818331	0.03202	0.1408
319822	Smyd4	SET and MYND domain containing 4	0.818331	0.06617	0.2163
319974	Auts2	autism susceptibility candidate 2	0.818331	0.001607	0.02408
381199	Tmem151a	transmembrane protein 151A	0.818331	0.2249	0.4559
11735	Ank3	ankyrin 3, epithelial	0.817661	0.09053	0.2636
12313	Calm1	calmodulin 1	0.817661	0.06111	0.2056
12545	Cdc7	cell division cycle 7 (S. cerevisiae)	0.817661	0.2069	0.433
14061	F2	coagulation factor II	0.817661	0.3749	0.609
14299	Ncs1	neuronal calcium sensor 1	0.817661	0.01902	0.1028
19065	Ppyr1	pancreatic polypeptide receptor 1	0.817661	0.2697	0.5049
22750	Zfp9	zinc finger protein 9	0.817661	0.05699	0.1962
50780	Rgs3	regulator of G-protein signaling 3	0.817661	0.007672	0.05997
66790	Grtp1	GH regulated TBC protein 1	0.817661	0.01317	0.08275
67128	Ube2g1	ubiquitin-conjugating enzyme E2G 1 (UBC7 homolog, C. elegans)	0.817661	0.01939	0.1042
67746	4930577N17Rik	RIKEN cDNA 4930577N17 gene	0.817661	0.1564	0.3664
68420	Ankrd13a	ankyrin repeat domain 13a	0.817661	0.04212	0.1642
68910	Zfp467	zinc finger protein 467	0.817661	0.01605	0.09289
71263	Mro	maestro	0.817661	0.1798	0.398
72098	Tmem68	transmembrane protein 68	0.817661	0.04189	0.1638
75941	4930570B17Rik	RIKEN cDNA 4930570B17 gene	0.817661	0.06757	0.2192
78937	Avl9	AVL9 homolog (S. cerevisiase)	0.817661	0.02756	0.1291
104175	Sbk1	SH3-binding kinase 1	0.817661	0.06387	0.2112
223626	4930572J05Rik	RIKEN cDNA 4930572J05 gene	0.817661	0.2619	0.4971
224824	Pex6	peroxisomal biogenesis factor 6	0.817661	0.1378	0.3401
320127	Dgki	diacylglycerol kinase, iota	0.817661	0.03477	0.1478
320705	Bend6	BEN domain containing 6	0.817661	0.005827	0.05127
381983	Lmtk3	lemur tyrosine kinase 3	0.817661	0.03872	0.1566
629581	Gm6985	predicted pseudogene 6985	0.817661	0.5688	0.7635
20981	Syt3	synaptotagmin III	0.816993	0.2753	0.5108
66743	Rnf220	ring finger protein 220	0.816993	0.07342	0.2311
75578	Fggy	FGGY carbohydrate kinase domain containing	0.816993	0.1534	0.3631
76071	Jakmip1	janus kinase and microtubule interacting protein 1	0.816993	0.0292	0.1334
77036	1700109H08Rik	RIKEN cDNA 1700109H08 gene	0.816993	0.01727	0.09712
78672	9530057J20Rik	RIKEN cDNA 9530057J20 gene	0.816993	0.04885	0.1788

105439	Slain1	SLAIN motif family, member 1	0.816993	0.04645	0.1742
109050	6530418L21Rik	RIKEN cDNA 6530418L21 gene	0.816993	0.003394	0.03695
109270	Prr5	proline rich 5 (renal)	0.816993	0.2491	0.4832
216233	Socs2	suppressor of cytokine signaling 2	0.816993	0.03393	0.1456
216453	Rdh19	retinol dehydrogenase 19	0.816993	0.4196	0.6475
217369	Uts2r	urotensin 2 receptor	0.816993	0.2178	0.447
232288	Frmd4b	FERM domain containing 4B	0.816993	0.04979	0.1813
237759	Col23a1	collagen, type XXIII, alpha 1	0.816993	0.06625	0.2164
399566	Btbd6	BTB (POZ) domain containing 6	0.816993	0.04698	0.1753
100038604	3426406K10Rik	RIKEN cDNA 3426406K10 gene	0.816993	0.478	0.6978
12869	Cox8b	cytochrome c oxidase, subunit VIIIb	0.816327	0.521	0.7291
71781	Slc16a14	solute carrier family 16 (monocarboxylic acid transporters), member 14	0.816327	0.00755	0.05939
73130	Tmed5	transmembrane emp24 protein transport domain containing 5	0.816327	0.0385	0.1562
75710	Rbm12	RNA binding motif protein 12	0.816327	0.05096	0.1832
75956	Srrm2	serine/arginine repetitive matrix 2	0.816327	0.1433	0.3482
93966	Hemgn	hemogen	0.816327	0.1132	0.3025
99890	Prmt6	protein arginine N-methyltransferase 6	0.816327	0.02619	0.1248
241308	Ralgps1	Ral GEF with PH domain and SH3 binding motif 1	0.816327	0.2757	0.5112
320487	Heatr5a	HEAT repeat containing 5A	0.816327	0.005937	0.05187
435965	Lrp3	low density lipoprotein receptor-related protein 3	0.816327	0.06034	0.204
636187	LOC636187	60S ribosomal protein L21-like	0.816327	0.0259	0.124
100505360	LOC100505360	putative transposase element L1Md-A101/L1Md-A102/L1Md-A2-like	0.816327	0.3339	0.5697
15122	Hba-a1	hemoglobin alpha, adult chain 1	0.815661	0.4866	0.7047
15476	Hs3st1	heparan sulfate (glucosamine) 3-O-sulfotransferase 1	0.815661	0.04492	0.1708
16169	Il15ra	interleukin 15 receptor, alpha chain	0.815661	0.3102	0.5467
58988	Rps6kb2	ribosomal protein S6 kinase, polypeptide 2	0.815661	0.1365	0.3383
67894	Fam45a	family with sequence similarity 45, member A	0.815661	0.006937	0.05646
68512	Tomm5	translocase of outer mitochondrial membrane 5 homolog (yeast)	0.815661	0.006988	0.05655
69156	Comtd1	catechol-O-methyltransferase domain containing 1	0.815661	0.006898	0.05627
71704	Arhgef3	Rho guanine nucleotide exchange factor (GEF) 3	0.815661	0.01672	0.09513
71984	Sars2	seryl-aminoacyl-tRNA synthetase 2	0.815661	0.09816	0.2764
93842	Igsf9	immunoglobulin superfamily, member 9	0.815661	0.1781	0.3958
192192	Shkbp1	Sh3kbp1 binding protein 1	0.815661	0.05249	0.1862
207740	Fam100a	family with sequence similarity 100, member A	0.815661	0.06451	0.2126
224814	Abcc10	ATP-binding cassette, sub-family C (CFTR/MRP), member 10	0.815661	0.01477	0.08861
226841	Vash2	vasohibin 2	0.815661	0.09917	0.2779
233060	Zfp382	zinc finger protein 382	0.815661	0.09429	0.2698
238871	Pde4d	phosphodiesterase 4D, cAMP specific	0.815661	0.0432	0.1668
319807	3110047P20Rik	RIKEN cDNA 3110047P20 gene	0.815661	0.04247	0.165
381922	D830044I16Rik	RIKEN cDNA D830044I16 gene	0.815661	0.03056	0.137
432769	Zfp708	zinc finger protein 708	0.815661	0.09131	0.2648
668178	Gm10035	predicted gene 10035	0.815661	0.02036	0.107
100503357	LOC100503357	hypothetical LOC100503357	0.815661	0.3959	0.6264
12322	Camk2a	calcium/calmodulin-dependent protein kinase II alpha	0.814996	0.0893	0.2612
20017	Polr1b	polymerase (RNA) I polypeptide B	0.814996	0.02357	0.117
20677	Sox4	SRY-box containing gene 4	0.814996	0.1368	0.3385
21357	Tarbp2	TAR (HIV) RNA binding protein 2	0.814996	0.004318	0.0429
52040	Ppp1r10	protein phosphatase 1, regulatory subunit 10	0.814996	0.2064	0.4325
56055	Gtpbp2	GTP binding protein 2	0.814996	0.01891	0.1024

56381	Spen	SPEN homolog, transcriptional regulator (Drosophila)	0.814996	0.002932	0.03379
63958	Ube4b	ubiquitination factor E4B, UFD2 homolog (S. cerevisiae)	0.814996	0.01044	0.072
68146	Arl13b	ADP-ribosylation factor-like 13B	0.814996	0.00702	0.05676
69181	Dyrk2	dual-specificity tyrosine-(Y)-phosphorylation regulated kinase 2	0.814996	0.02777	0.1296
80707	Wwox	WW domain-containing oxidoreductase	0.814996	0.06589	0.2156
84653	Hes7	hairy and enhancer of split 7 (Drosophila)	0.814996	0.09212	0.266
93896	Glp2r	glucagon-like peptide 2 receptor	0.814996	0.05016	0.1818
102916	AI451458	expressed sequence AI451458	0.814996	0.03848	0.1562
104582	Rprml	reprimo-like	0.814996	0.1549	0.3647
113868	Acaa1a	acetyl-Coenzyme A acyltransferase 1A	0.814996	0.02313	0.1159
212398	Frat2	frequently rearranged in advanced T-cell lymphomas 2	0.814996	0.08795	0.2587
230770	Tmem39b	transmembrane protein 39b	0.814996	0.01032	0.07152
245174	Zfp937	zinc finger protein 937	0.814996	0.05151	0.1844
621239	Nhlrc4	NHL repeat containing 4	0.814996	0.04481	0.1706
626231	Gm6658	predicted gene 6658	0.814996	0.08469	0.2522
666105	Gm7932	predicted gene 7932	0.814996	0.2695	0.5047
100504362	Gm1987	predicted gene 1987	0.814996	0.2058	0.432
13063	Cycs	cytochrome c, somatic	0.814332	0.02522	0.1219
14165	Fgf10	fibroblast growth factor 10	0.814332	0.05165	0.1847
14658	Glrb	glycine receptor, beta subunit	0.814332	0.03551	0.1488
19771	Rlbp1	retinaldehyde binding protein 1	0.814332	0.05435	0.1905
53412	Ppp1r3c	protein phosphatase 1, regulatory (inhibitor) subunit 3C	0.814332	0.01677	0.09534
66970	Ssbp2	single-stranded DNA binding protein 2	0.814332	0.1241	0.3207
76338	Rab2b	RAB2B, member RAS oncogene family	0.814332	0.002337	0.0299
76373	Zfp773	zinc finger protein 773	0.814332	0.03199	0.1407
78894	Aacs	acetoacetyl-CoA synthetase	0.814332	0.01086	0.07361
211978	Zfyve26	zinc finger, FYVE domain containing 26	0.814332	0.03323	0.144
213006	Mfsd4	major facilitator superfamily domain containing 4	0.814332	0.004111	0.04157
226418	Yod1	YOD1 OTU deubiquitinating enzyme 1 homologue (S. cerevisiae)	0.814332	0.02094	0.1091
242860	Rsbn1l	round spermatid basic protein 1-like	0.814332	0.07442	0.2328
268882	Fbxo45	F-box protein 45	0.814332	0.06125	0.2058
320046	F730043M19Rik	RIKEN cDNA F730043M19 gene	0.814332	0.01258	0.0803
329679	Fnip2	folliculin interacting protein 2	0.814332	0.0344	0.1466
434778	Ccdc160	coiled-coil domain containing 160	0.814332	0.04425	0.1691
665251	Gm7555	predicted gene 7555	0.814332	0.008346	0.06258
12504	Cd4	CD4 antigen	0.81367	0.2521	0.4866
15444	Hpca	hippocalcin	0.81367	0.0376	0.1539
20404	Sh3gl2	SH3-domain GRB2-like 2	0.81367	0.2506	0.4846
68728	Trp53inp2	transformation related protein 53 inducible nuclear protein 2	0.81367	0.00785	0.0609
71436	Flrt3	fibronectin leucine rich transmembrane protein 3	0.81367	0.1044	0.2872
71640	Zfp949	zinc finger protein 949	0.81367	0.002306	0.02965
75209	Sv2c	synaptic vesicle glycoprotein 2c	0.81367	0.05204	0.1855
76965	Slitrk1	SLIT and NTRK-like family, member 1	0.81367	0.02972	0.1348
116939	Pnpla3	patatin-like phospholipase domain containing 3	0.81367	0.04964	0.1809
171429	Slc26a6	solute carrier family 26, member 6	0.81367	0.0207	0.1083
226777	C130074G19Rik	RIKEN cDNA C130074G19 gene	0.81367	0.2696	0.5048
235610	Atrip	ATR interacting protein	0.81367	0.08384	0.2504
263876	Spata2	spermatogenesis associated 2	0.81367	0.09058	0.2636
639281	Gm10382	predicted gene 10382	0.81367	0.2552	0.49

665270	Plb1	phospholipase B1	0.81367	0.1066	0.2913
14419	Gal	galanin	0.813008	0.04007	0.1596
18020	Nfatc2ip	nuclear factor of activated T-cells, cytoplasmic, calcineurin-dependent 2 interacting protein	0.813008	0.01575	0.09219
19876	Robo1	roundabout homolog 1 (Drosophila)	0.813008	0.05914	0.2014
29856	Smtn	smoothelin	0.813008	0.0115	0.07605
56195	Ptbp2	polypyrimidine tract binding protein 2	0.813008	0.08263	0.2479
56422	Hbs1l	Hbs1-like (S. cerevisiae)	0.813008	0.06503	0.2136
57785	Rangrf	RAN guanine nucleotide release factor	0.813008	0.2123	0.4401
67276	Eri1	exoribonuclease 1	0.813008	0.005757	0.0508
67311	Nanp	N-acetylneuraminic acid phosphatase	0.813008	0.09592	0.2726
69606	Mtfmt	mitochondrial methionyl-tRNA formyltransferase	0.813008	0.005682	0.05039
73451	Zfp763	zinc finger protein 763	0.813008	0.06536	0.2144
140557	Smc1b	structural maintenance of chromosomes 1B	0.813008	0.2863	0.5217
212996	Wbscr17	Williams-Beuren syndrome chromosome region 17 homolog (human)	0.813008	0.03773	0.1542
214058	Megf11	multiple EGF-like-domains 11	0.813008	0.06474	0.213
223601	Fam49b	family with sequence similarity 49, member B	0.813008	0.007189	0.0576
224019	Tmem191c	transmembrane protein 191C	0.813008	0.01043	0.07196
225861	Snx32	sorting nexin 32	0.813008	0.06144	0.2061
282663	Serpinb1b	serine (or cysteine) peptidase inhibitor, clade B, member 1b	0.813008	0.3362	0.572
14536	Nr6a1	nuclear receptor subfamily 6, group A, member 1	0.812348	0.2072	0.4333
17260	Mef2c	myocyte enhancer factor 2C	0.812348	0.02257	0.1143
18682	Phkg1	phosphorylase kinase gamma 1	0.812348	0.175	0.3917
26942	Spag1	sperm associated antigen 1	0.812348	0.09939	0.2782
27222	Atp1a4	ATPase, Na+/K+ transporting, alpha 4 polypeptide	0.812348	0.1198	0.3133
50929	Il22	interleukin 22	0.812348	0.2991	0.5348
52840	Dbndd2	dysbindin (dystrobrevin binding protein 1) domain containing 2	0.812348	0.02781	0.1297
57811	Rgr	retinal G protein coupled receptor	0.812348	0.3904	0.6224
58234	Shank3	SH3/ankyrin domain gene 3	0.812348	0.05227	0.1856
69642	2310046A06Rik	RIKEN cDNA 2310046A06 gene	0.812348	0.2202	0.45
71517	9030624J02Rik	RIKEN cDNA 9030624J02 gene	0.812348	0.1189	0.3117
72179	Fbxl2	F-box and leucine-rich repeat protein 2	0.812348	0.1232	0.3192
74105	Gga2	golgi associated, gamma adaptin ear containing, ARF binding protein 2	0.812348	0.0116	0.07642
74376	Myo18b	myosin XVIIIb	0.812348	0.2319	0.4633
75697	C2cd4b	C2 calcium-dependent domain containing 4B	0.812348	0.1732	0.3893
76792	2410131K14Rik	RIKEN cDNA 2410131K14 gene	0.812348	0.05414	0.1901
108071	Grm5	glutamate receptor, metabotropic 5	0.812348	0.06496	0.2135
212281	A530054K11Rik	RIKEN cDNA A530054K11 gene	0.812348	0.003681	0.03885
216874	Camta2	calmodulin binding transcription activator 2	0.812348	0.08047	0.244
320522	Bhlha9	basic helix-loop-helix family, member a9	0.812348	0.007783	0.06048
100502841	5430411K18Rik	RIKEN cDNA 5430411K18 gene	0.812348	0.005591	0.05
12283	Cab39	calcium binding protein 39	0.811688	0.004711	0.04511
12834	Col6a2	collagen, type VI, alpha 2	0.811688	0.2584	0.4937
14664	Slc6a9	solute carrier family 6 (neurotransmitter transporter, glycine), member 9	0.811688	0.02021	0.1068
17242	Mdk	midkine	0.811688	0.07964	0.2427
19085	Prkar1b	protein kinase, cAMP dependent regulatory, type I beta	0.811688	0.01949	0.1045
20215	Sag	retinal S-antigen	0.811688	0.006956	0.05649
72633	2810019C22Rik	RIKEN cDNA 2810019C22 gene	0.811688	0.1103	0.2977
94249	Slc24a3	solute carrier family 24 (sodium/potassium/calcium exchanger), member 3	0.811688	0.005249	0.04814
105727	Slc38a1	solute carrier family 38, member 1	0.811688	0.07628	0.2366

211329	Ncoa7	nuclear receptor coactivator 7	0.811688	0.3831	0.6161
216021	Stox1	storkhead box 1	0.811688	0.0667	0.2173
217071	Gm525	predicted gene 525	0.811688	0.05205	0.1855
227634	Camsap1	calmodulin regulated spectrin-associated protein 1	0.811688	0.01258	0.0803
236733	Usp11	ubiquitin specific peptidase 11	0.811688	0.1057	0.2896
381353	Gm996	predicted gene 996	0.811688	0.006063	0.05241
100041261	Gm3238	predicted gene 3238	0.811688	0.07309	0.2303
100505283	LOC100505283	60S ribosomal protein L21-like	0.811688	0.009267	0.06666
11777	Ap3s1	adaptor-related protein complex 3, sigma 1 subunit	0.81103	0.00664	0.05521
14571	Gpd2	glycerol phosphate dehydrogenase 2, mitochondrial	0.81103	0.02916	0.1333
14858	Gsta2	glutathione S-transferase, alpha 2 (Yc2)	0.81103	0.4963	0.7114
17391	Mmp24	matrix metallopeptidase 24	0.81103	0.09642	0.2736
20935	Surf6	surfeit gene 6	0.81103	0.03947	0.1582
22283	Ush2a	Usher syndrome 2A (autosomal recessive, mild) homolog (human)	0.81103	0.103	0.2848
27389	Dusp13	dual specificity phosphatase 13	0.81103	0.1695	0.3847
29858	Pmm1	phosphomannomutase 1	0.81103	0.1258	0.3229
51795	Srpx	sushi-repeat-containing protein	0.81103	0.03051	0.1369
56291	Styx	serine/threonine/tyrosine interaction protein	0.81103	0.0025	0.03096
69046	Isca1	iron-sulfur cluster assembly 1 homolog (S. cerevisiae)	0.81103	0.00897	0.06552
70025	Acot7	acyl-CoA thioesterase 7	0.81103	0.03337	0.1443
71801	Plekhf2	pleckstrin homology domain containing, family F (with FYVE domain) member 2	0.81103	0.008759	0.06456
72076	Mospd4	motile sperm domain containing 4	0.81103	0.2647	0.5006
73610	Zfp433	RIKEN cDNA 1700123A16 gene	0.81103	0.2001	0.4256
78797	Ndor1	NADPH dependent diflavin oxidoreductase 1	0.81103	0.04328	0.167
99094	AI849538	expressed sequence AI849538	0.81103	0.2364	0.4688
100535	Oas1d	2'-5' oligoadenylate synthetase 1D	0.81103	0.4266	0.6537
217069	Trim25	tripartite motif-containing 25	0.81103	0.09892	0.2779
226419	Dyrk3	dual-specificity tyrosine-(Y)-phosphorylation regulated kinase 3	0.81103	0.04056	0.161
229011	Samd10	sterile alpha motif domain containing 10	0.81103	0.3495	0.5853
232086	Tmem150a	transmembrane protein 150A	0.81103	0.03335	0.1442
240753	Plekha6	pleckstrin homology domain containing, family A member 6	0.81103	0.03044	0.1368
244958	Mrap2	melanocortin 2 receptor accessory protein 2	0.81103	0.06052	0.2044
276920	Ccdc42	coiled-coil domain containing 42	0.81103	0.2393	0.4718
320692	9430037G07Rik	RIKEN cDNA 9430037G07 gene	0.81103	0.08759	0.2582
320719	6030443J06Rik	RIKEN cDNA 6030443J06 gene	0.81103	0.295	0.5311
328329	Mast4	microtubule associated serine/threonine kinase family member 4	0.81103	0.1695	0.3847
382867	Zfp488	zinc finger protein 488	0.81103	0.0214	0.1107
399579	6720462K09Rik	RIKEN cDNA 6720462K09 gene	0.81103	0.02714	0.1276
654818	C030030A07Rik	RIKEN cDNA C030030A07 gene	0.81103	0.563	0.7609
675851	LOC675851	NADH dehydrogenase [ubiquinone] 1 subunit C2-like	0.81103	0.008335	0.06257
14068	F7	coagulation factor VII	0.810373	0.1409	0.3447
18772	Pkp1	plakophilin 1	0.810373	0.007294	0.05811
67639	4930474N09Rik	RIKEN cDNA 4930474N09 gene	0.810373	0.1102	0.2976
68055	Atp5s	ATP synthase, H+ transporting, mitochondrial F0 complex, subunit s	0.810373	0.02182	0.112
68920	1110065P20Rik	RIKEN cDNA 1110065P20 gene	0.810373	0.001114	0.01893
75770	Brsk2	BR serine/threonine kinase 2	0.810373	0.007638	0.05983
77675	5033406O09Rik	RIKEN cDNA 5033406O09 gene	0.810373	0.0232	0.1161
107522	Ece2	endothelin converting enzyme 2	0.810373	0.08377	0.2503
108089	Rnf144a	ring finger protein 144A	0.810373	0.005391	0.04891

210503	Zfp677	zinc finger protein 677	0.810373	0.01034	0.07162
212943	Fam46a	family with sequence similarity 46, member A	0.810373	0.1261	0.3234
213012	Abhd10	abhydrolase domain containing 10	0.810373	0.01081	0.07336
239731	Rimbp3	RIMS binding protein 3	0.810373	0.02008	0.1064
387314	Tmtc1	transmembrane and tetratricopeptide repeat containing 1	0.810373	0.01726	0.09712
11800	Api5	apoptosis inhibitor 5	0.809717	0.03043	0.1368
13349	Darc	Duffy blood group, chemokine receptor	0.809717	0.01172	0.0769
16173	Il18	interleukin 18	0.809717	0.02632	0.1252
59126	Nek6	NIMA (never in mitosis gene a)-related expressed kinase 6	0.809717	0.007778	0.06048
70458	2610318N02Rik	RIKEN cDNA 2610318N02 gene	0.809717	0.01379	0.085
75697	C2cd4b	C2 calcium-dependent domain containing 4B	0.809717	0.117	0.3089
77877	6030458C11Rik	RIKEN cDNA 6030458C11 gene	0.809717	0.008393	0.06283
80915	Dusp12	dual specificity phosphatase 12	0.809717	0.01376	0.08486
107448	Unc5a	unc-5 homolog A (C. elegans)	0.809717	0.02323	0.1162
170740	Zfp287	zinc finger protein 287	0.809717	0.00853	0.06354
213119	Itga10	integrin, alpha 10	0.809717	0.03765	0.154
223672	Apol9a	apolipoprotein L 9a	0.809717	0.2414	0.4744
329641	6030405A18Rik	RIKEN cDNA 6030405A18 gene	0.809717	0.01964	0.1049
619441	BC096441	cDNA sequence BC096441	0.809717	0.002696	0.03218
12337	Capn5	calpain 5	0.809061	0.3054	0.5419
52822	Rufy3	RUN and FYVE domain containing 3	0.809061	0.00101	0.01786
66202	1110059G10Rik	RIKEN cDNA 1110059G10 gene	0.809061	0.01177	0.07703
66776	Pisd-ps3	phosphatidylserine decarboxylase, pseudogene 3	0.809061	0.1623	0.3746
67252	Cap2	CAP, adenylate cyclase-associated protein, 2 (yeast)	0.809061	0.02704	0.1272
71367	Chst9	carbohydrate (N-acetylgalactosamine 4-0) sulfotransferase 9	0.809061	0.07651	0.2367
72649	Tmem209	transmembrane protein 209	0.809061	0.02986	0.1351
76257	Slc38a3	solute carrier family 38, member 3	0.809061	0.05808	0.1989
110119	Mpi	mannose phosphate isomerase	0.809061	0.01503	0.08953
235293	Sc5d	sterol-C5-desaturase (fungal ERG3, delta-5-desaturase) homolog (S. cerevisae)	0.809061	0.001507	0.02311
237615	Ankrd52	ankyrin repeat domain 52	0.809061	0.06232	0.208
260302	Gga3	golgi associated, gamma adaptin ear containing, ARF binding protein 3	0.809061	0.01154	0.07615
331532	Tceal5	transcription elongation factor A (SII)-like 5	0.809061	0.002014	0.02731
333050	Ksr2	kinase suppressor of ras 2	0.809061	0.5174	0.7269
13195	Ddc	dopa decarboxylase	0.808407	0.2274	0.4586
14472	Gbx2	gastrulation brain homeobox 2	0.808407	0.0674	0.2189
20964	Syn1	synapsin I	0.808407	0.01802	0.09932
21341	Taf1c	TATA box binding protein (Tbp)-associated factor, RNA polymerase I, C	0.808407	0.04314	0.1666
23912	Rhof	ras homolog gene family, member f	0.808407	0.05003	0.1817
66437	Fis1	fission 1 (mitochondrial outer membrane) homolog (yeast)	0.808407	0.01541	0.0909
67480	Cwc25	CWC25 spliceosome-associated protein homolog (S. cerevisiae)	0.808407	0.01391	0.08534
68299	Vps53	vacuolar protein sorting 53 (yeast)	0.808407	0.06625	0.2164
100763	Ube3c	ubiquitin protein ligase E3C	0.808407	0.004825	0.04568
110616	Atxn3	ataxin 3	0.808407	0.03385	0.1454
227736	1700019L03Rik	RIKEN cDNA 1700019L03 gene	0.808407	0.05292	0.1873
240255	Ythdc2	YTH domain containing 2	0.808407	0.04087	0.1615
320032	C130093G08Rik	RIKEN cDNA C130093G08 gene	0.808407	0.06209	0.2074
330908	Opcml	opioid binding protein/cell adhesion molecule-like	0.808407	0.3208	0.5573
11433	Acp5	acid phosphatase 5, tartrate resistant	0.807754	0.2677	0.5032
12662	Chm	choroidermia	0.807754	0.03364	0.145

16449	Jag1	jagged 1	0.807754	0.04413	0.169
20618	Sncg	synuclein, gamma	0.807754	0.02704	0.1272
21749	Terf1	telomeric repeat binding factor 1	0.807754	0.02237	0.1137
26420	Mapk9	mitogen-activated protein kinase 9	0.807754	0.002031	0.0274
30056	Timm9	translocase of inner mitochondrial membrane 9 homolog (yeast)	0.807754	0.04359	0.1676
66373	Lsm5	LSM5 homolog, U6 small nuclear RNA associated (S. cerevisiae)	0.807754	0.0501	0.1817
67037	Pmf1	polyamine-modulated factor 1	0.807754	0.1659	0.3799
67439	Xab2	XPA binding protein 2	0.807754	0.06313	0.2098
70603	Mutyh	mutY homolog (E. coli)	0.807754	0.01062	0.07262
76376	Slc24a2	solute carrier family 24 (sodium/potassium/calcium exchanger), member 2	0.807754	0.02498	0.121
239170	Fam160b2	family with sequence similarity 160, member B2	0.807754	0.1403	0.3439
319582	6430573F11Rik	RIKEN cDNA 6430573F11 gene	0.807754	0.005004	0.04674
328949	Mcc	mutated in colorectal cancers	0.807754	0.003692	0.03888
329152	Hecw2	HECT, C2 and WW domain containing E3 ubiquitin protein ligase 2	0.807754	0.1322	0.3327
381290	Atp2b4	ATPase, Ca++ transporting, plasma membrane 4	0.807754	0.1919	0.4141
636931	Trim71	tripartite motif-containing 71	0.807754	0.1567	0.3668
18616	Peg3	paternally expressed 3	0.807103	0.2371	0.4693
19260	Ptpn22	protein tyrosine phosphatase, non-receptor type 22 (lymphoid)	0.807103	0.0188	0.1021
22719	Zfp61	zinc finger protein 61	0.807103	0.0007038	0.0146
55963	Slc1a4	solute carrier family 1 (glutamate/neutral amino acid transporter), member 4	0.807103	0.3597	0.5947
66648	5730494M16Rik	RIKEN cDNA 5730494M16 gene	0.807103	0.1022	0.2833
70385	Ccdc99	coiled-coil domain containing 99	0.807103	0.2531	0.4878
70549	Tln2	talin 2	0.807103	0.04994	0.1815
210933	Bai3	brain-specific angiogenesis inhibitor 3	0.807103	0.008556	0.06364
241070	Gpr1	G protein-coupled receptor 1	0.807103	0.2613	0.4964
432731	Zfp187	zinc finger protein 187	0.807103	0.08541	0.2535
100101046	D230018H15Rik	RIKEN cDNA D230018H15 gene	0.807103	0.1292	0.328
12745	Clgn	calmegin	0.806452	0.00455	0.04446
12891	Cpne6	copine VI	0.806452	0.02008	0.1064
13082	Cyp26a1	cytochrome P450, family 26, subfamily a, polypeptide 1	0.806452	0.04196	0.1639
14025	Bcl11a	B-cell CLL/lymphoma 11A (zinc finger protein)	0.806452	0.0342	0.1462
22390	Wee1	WEE 1 homolog 1 (S. pombe)	0.806452	0.007327	0.05829
65112	Pmepa1	prostate transmembrane protein, androgen induced 1	0.806452	0.004335	0.04296
66477	Usmg5	upregulated during skeletal muscle growth 5	0.806452	0.003929	0.04039
66885	Acadsb	acyl-Coenzyme A dehydrogenase, short/branched chain	0.806452	0.1838	0.4028
75564	Rsph9	radial spoke head 9 homolog (Chlamydomonas)	0.806452	0.07797	0.2393
101565	6330503K22Rik	RIKEN cDNA 6330503K22 gene	0.806452	0.008094	0.0616
106039	Gga1	golgi associated, gamma adaptin ear containing, ARF binding protein 1	0.806452	0.07646	0.2367
170743	Tlr7	toll-like receptor 7	0.806452	0.5429	0.7464
215303	Camk1g	calcium/calmodulin-dependent protein kinase I gamma	0.806452	0.02942	0.134
230935	Dnajc11	DnaJ (Hsp40) homolog, subfamily C, member 11	0.806452	0.05586	0.1935
231238	Sel1l3	sel-1 suppressor of lin-12-like 3 (C. elegans)	0.806452	0.005543	0.04974
242505	Rasef	RAS and EF hand domain containing	0.806452	0.1749	0.3916
244556	Zfp791	zinc finger protein 791	0.806452	0.241	0.4737
271970	Arsj	arylsulfatase J	0.806452	0.2547	0.4895
380780	Serpina11	serine (or cysteine) peptidase inhibitor, clade A (alpha-1 antiproteinase, antitrypsin), member 11	0.806452	0.2415	0.4745
381438	Gm5148	predicted gene 5148	0.806452	0.004286	0.04269
100038570	Gm11744	predicted gene 11744	0.806452	0.02333	0.1165
12890	Cplx2	complexin 2	0.805802	0.01837	0.1006

19228	Pth1r	parathyroid hormone 1 receptor	0.805802	0.04368	0.1679
20262	Stmn3	stathmin-like 3	0.805802	0.01881	0.1022
54006	Deaf1	deformed epidermal autoregulatory factor 1 (Drosophila)	0.805802	0.04255	0.1651
66904	Pccb	propionyl Coenzyme A carboxylase, beta polypeptide	0.805802	0.02033	0.107
72145	Wdfy3	WD repeat and FYVE domain containing 3	0.805802	0.2749	0.5103
73919	Lyrm1	LYR motif containing 1	0.805802	0.01738	0.09738
75827	4930542N06Rik	RIKEN cDNA 4930542N06 gene	0.805802	0.2278	0.4587
104943	Fam110c	family with sequence similarity 110, member C	0.805802	0.1739	0.3903
242235	Lrit3	leucine-rich repeat, immunoglobulin-like and transmembrane domains 3	0.805802	0.2528	0.4874
385668	Lca5l	Leber congenital amaurosis 5-like	0.805802	0.1121	0.3006
12455	Ccnt1	cyclin T1	0.805153	0.2218	0.4521
14688	Gnb1	guanine nucleotide binding protein (G protein), beta 1	0.805153	0.02365	0.1172
14772	Grk4	G protein-coupled receptor kinase 4	0.805153	0.01664	0.09487
16885	Limk1	LIM-domain containing, protein kinase	0.805153	0.02751	0.1289
26409	Map3k7	mitogen-activated protein kinase kinase kinase 7	0.805153	0.06209	0.2074
56149	Grasp	GRP1 (general receptor for phosphoinositides 1)-associated scaffold protein	0.805153	0.06216	0.2076
58194	Sh3kbp1	SH3-domain kinase binding protein 1	0.805153	0.04614	0.1734
67475	Ero1lb	ERO1-like beta (S. cerevisiae)	0.805153	0.01709	0.09654
68725	1110032F04Rik	RIKEN cDNA 1110032F04 gene	0.805153	0.0638	0.211
73122	Tgfbrap1	transforming growth factor, beta receptor associated protein 1	0.805153	0.1768	0.3938
75986	Agmat	agmatine ureohydrolase (agmatinase)	0.805153	0.06989	0.2237
78754	Galntl2	UDP-N-acetyl-alpha-D-galactosamine:polypeptide N-acetylgalactosaminyltransferase-like 2	0.805153	0.09085	0.2639
78815	5031420N21Rik	RIKEN cDNA 5031420N21 gene	0.805153	0.1363	0.3381
207921	A830093I24Rik	RIKEN cDNA A830093I24 gene	0.805153	0.0514	0.1841
228536	Bahd1	bromo adjacent homology domain containing 1	0.805153	0.01055	0.07245
320595	Phf8	PHD finger protein 8	0.805153	0.08266	0.248
330427	9330102E08Rik	RIKEN cDNA 9330102E08 gene	0.805153	0.0268	0.1267
407812	Zfp941	zinc finger protein 941	0.805153	0.04734	0.176
433759	Hdac1	histone deacetylase 1	0.805153	0.09211	0.266
433771	2310028O11Rik	RIKEN cDNA 2310028O11 gene	0.805153	0.0006072	0.01348
632793	Vmn1r204	vomeronasal 1 receptor 204	0.805153	0.2685	0.5038
13497	Drp2	dystrophin related protein 2	0.804505	0.06439	0.2124
14089	Fap	fibroblast activation protein	0.804505	0.3856	0.6177
18777	Lypla1	lysophospholipase 1	0.804505	0.06673	0.2174
19362	Rad51ap1	RAD51 associated protein 1	0.804505	0.009172	0.06634
20604	Sst	somatostatin	0.804505	0.2022	0.4284
26372	Clcn6	chloride channel 6	0.804505	0.06701	0.2181
27412	Peg12	paternally expressed 12	0.804505	0.09574	0.2723
69035	Zdhhc3	zinc finger, DHHC domain containing 3	0.804505	0.05913	0.2014
75939	4930579G24Rik	RIKEN cDNA 4930579G24 gene	0.804505	0.01329	0.08316
100434	Slc44a1	solute carrier family 44, member 1	0.804505	0.07085	0.2256
107239	Carns1	carnosine synthase 1	0.804505	0.0179	0.09899
242083	Ppm1l	protein phosphatase 1 (formerly 2C)-like	0.804505	0.02501	0.1211
243897	Ggn	gametogenetin	0.804505	0.05708	0.1963
353025	Caps2	calcyphosphine 2	0.804505	0.2956	0.5314
621239	Nhlrc4	NHL repeat containing 4	0.804505	0.1551	0.3648
100048345	LOC100048345	hypothetical protein LOC100048345	0.804505	0.09981	0.279
14823	Grm8	glutamate receptor, metabotropic 8	0.803859	0.001825	0.02573
70335	Reep6	receptor accessory protein 6	0.803859	0.1063	0.2906

71972	Dnmbp	dynamin binding protein	0.803859	0.2392	0.4718
74076	4933406C10Rik	RIKEN cDNA 4933406C10 gene	0.803859	0.1827	0.4016
76505	1500009C09Rik	RIKEN cDNA 1500009C09 gene	0.803859	0.004771	0.04543
77938	Fam53b	family with sequence similarity 53, member B	0.803859	0.05127	0.184
78655	Eif3j	eukaryotic translation initiation factor 3, subunit J	0.803859	0.02815	0.1307
109242	Kif24	kinesin family member 24	0.803859	0.05151	0.1844
237868	Sarm1	sterile alpha and HEAT/Armadillo motif containing 1	0.803859	0.1993	0.4247
320865	Cdh18	cadherin 18	0.803859	0.02179	0.1119
402729	9330184L24Rik	RIKEN cDNA 9330184L24 gene	0.803859	0.2642	0.5002
432460	Gm5423	predicted gene 5423	0.803859	0.09908	0.2779
433485	Tmem90b	transmembrane protein 90B	0.803859	0.2717	0.5071
100504146	LOC100504146	zinc finger protein 709-like	0.803859	0.2959	0.5316
100504720	LOC100504720	hypothetical LOC100504720	0.803859	0.1286	0.327
13612	Edil3	EGF-like repeats and discoidin I-like domains 3	0.803213	0.02836	0.131
14397	Gabra4	gamma-aminobutyric acid (GABA) A receptor, subunit alpha 4	0.803213	0.03394	0.1457
16373	Irx3	Iroquois related homeobox 3 (Drosophila)	0.803213	0.01136	0.07556
20423	Shh	sonic hedgehog	0.803213	0.1083	0.2945
29809	Rabgap1l	RAB GTPase activating protein 1-like	0.803213	0.005374	0.04883
59041	Stk25	serine/threonine kinase 25 (yeast)	0.803213	0.002701	0.03218
67460	Decr1	2,4-dienoyl CoA reductase 1, mitochondrial	0.803213	0.0183	0.1003
70292	Afap1	actin filament associated protein 1	0.803213	0.0118	0.07717
71361	Aifm2	apoptosis-inducing factor, mitochondrion-associated 2	0.803213	0.04525	0.1715
71805	Nup93	nucleoporin 93	0.803213	0.0712	0.2263
73863	4930415O20Rik	RIKEN cDNA 4930415O20 gene	0.803213	0.1638	0.3767
74007	Btbd11	BTB (POZ) domain containing 11	0.803213	0.03143	0.1391
74020	Cpne4	copine IV	0.803213	0.003278	0.03625
74522	Morc2a	microrchidia 2A	0.803213	0.001121	0.019
75029	Purg	purine-rich element binding protein G	0.803213	0.1047	0.2877
76421	1700028K03Rik	RIKEN cDNA 1700028K03 gene	0.803213	0.07815	0.2397
78593	Nrip3	nuclear receptor interacting protein 3	0.803213	0.006805	0.05588
106064	AW549877	expressed sequence AW549877	0.803213	0.01897	0.1027
107377	AW492981	expressed sequence AW492981	0.803213	0.1954	0.4191
244416	Ppp1r3b	protein phosphatase 1, regulatory (inhibitor) subunit 3B	0.803213	0.2407	0.4735
260305	Nphp4	nephronophthisis 4 (juvenile) homolog (human)	0.803213	0.08201	0.2468
18551	Pcsk4	proprotein convertase subtilisin/kexin type 4	0.802568	0.02442	0.1194
24057	Sh3yl1	Sh3 domain YSC-like 1	0.802568	0.1942	0.4177
30044	Opn4	opsin 4 (melanopsin)	0.802568	0.0938	0.2692
56642	Ankrd2	ankyrin repeat domain 2 (stretch responsive muscle)	0.802568	0.01564	0.09179
70603	Mutyh	mutY homolog (E. coli)	0.802568	0.03395	0.1457
71233	Enkur	enkurin, TRPC channel interacting protein	0.802568	0.01523	0.09027
72535	Aldh1b1	aldehyde dehydrogenase 1 family, member B1	0.802568	0.1166	0.3082
75739	Mpp7	membrane protein, palmitoylated 7 (MAGUK p55 subfamily member 7)	0.802568	0.08066	0.2442
102293	AW111846	expressed sequence AW111846	0.802568	0.0082	0.06206
216233	Socs2	suppressor of cytokine signaling 2	0.802568	0.006663	0.05528
242851	Gnat3	guanine nucleotide binding protein, alpha transducing 3	0.802568	0.4679	0.6897
100039284	Gm2137	predicted gene 2137	0.802568	0.05141	0.1841
11513	Adcy7	adenylate cyclase 7	0.801925	0.02333	0.1165
12035	Bcat1	branched chain aminotransferase 1, cytosolic	0.801925	0.01127	0.07522
12928	Crk	v-crk sarcoma virus CT10 oncogene homolog (avian)	0.801925	0.5961	0.7823

22761	Zfpm1	zinc finger protein, multitype 1	0.801925	0.03609	0.1501
67149	Nkain1	Na+/K+ transporting ATPase interacting 1	0.801925	0.06246	0.2084
67832	Brix1	BRX1, biogenesis of ribosomes, homolog (S. cerevisiae)	0.801925	0.01157	0.07629
70584	Pak4	p21 protein (Cdc42/Rac)-activated kinase 4	0.801925	0.1736	0.3899
76415	Fam187b	family with sequence similarity 187, member B	0.801925	0.3991	0.6295
101122	Rpusd3	RNA pseudouridylate synthase domain containing 3	0.801925	0.02505	0.1212
103767	A530047J11Rik	RIKEN cDNA A530047J11 gene	0.801925	0.06032	0.204
110351	Rap1gap	Rap1 GTPase-activating protein	0.801925	0.01495	0.08918
215654	Cdh12	cadherin 12	0.801925	0.02798	0.1302
234678	D230025D16Rik	RIKEN cDNA D230025D16 gene	0.801925	0.01474	0.0885
241201	Cdh7	cadherin 7, type 2	0.801925	0.01033	0.07155
380840	Lyrm4	LYR motif containing 4	0.801925	0.002653	0.03192
380856	AA987161	expressed sequence AA987161	0.801925	0.04678	0.175
433804	Gm13154	predicted gene 13154	0.801925	0.3723	0.6068
545975	Lass3	LAG1 homolog, ceramide synthase 3	0.801925	0.06667	0.2173
546849	Gm13178	predicted gene 13178	0.801925	0.327	0.5636
629059	Fam124a	family with sequence similarity 124, member A	0.801925	0.02219	0.1129
12122	Bid	BH3 interacting domain death agonist	0.801282	0.1647	0.3782
12140	Fabp7	fatty acid binding protein 7, brain	0.801282	0.06959	0.2232
16560	Kif1a	kinesin family member 1A	0.801282	0.004838	0.04577
60367	Il1rapl2	interleukin 1 receptor accessory protein-like 2	0.801282	0.08802	0.2587
75740	Egfem1	EGF-like and EMI domain containing 1	0.801282	0.03834	0.1559
77328	C030009O12Rik	RIKEN cDNA C030009O12 gene	0.801282	0.0083	0.06246
77889	Lbh	limb-bud and heart	0.801282	0.03187	0.1403
212307	Mapre2	microtubule-associated protein, RP/EB family, member 2	0.801282	0.04255	0.1651
216810	Tom1l2	target of myb1-like 2 (chicken)	0.801282	0.1362	0.338
12902	Cr2	complement receptor 2	0.800641	0.4363	0.6629
13497	Drp2	dystrophin related protein 2	0.800641	0.02082	0.1087
14201	Fhl3	four and a half LIM domains 3	0.800641	0.2486	0.4828
17069	Ly6e	lymphocyte antigen 6 complex, locus E	0.800641	0.1263	0.3235
18377	Omg	oligodendrocyte myelin glycoprotein	0.800641	0.00338	0.03693
18554	Pcsk7	proprotein convertase subtilisin/kexin type 7	0.800641	0.01243	0.07958
18973	Pole	polymerase (DNA directed), epsilon	0.800641	0.03347	0.1445
20536	Slc4a3	solute carrier family 4 (anion exchanger), member 3	0.800641	0.02352	0.1169
20822	Trove2	TROVE domain family, member 2	0.800641	0.0161	0.09296
20981	Syt3	synaptotagmin III	0.800641	0.1882	0.4091
26383	Fto	fat mass and obesity associated	0.800641	0.04047	0.1607
64011	Nrgn	neurogranin	0.800641	0.1022	0.2833
68166	Spire1	spire homolog 1 (Drosophila)	0.800641	0.006454	0.0542
70827	Trak2	trafficking protein, kinesin binding 2	0.800641	0.02976	0.1349
70892	Ttll7	tubulin tyrosine ligase-like family, member 7	0.800641	0.1097	0.2966
71637	4930413F20Rik	RIKEN cDNA 4930413F20 gene	0.800641	0.2952	0.5311
73852	D3Ertd751e	DNA segment, Chr 3, ERATO Doi 751, expressed	0.800641	0.21	0.437
101772	Ano1	anoctamin 1, calcium activated chloride channel	0.800641	0.05713	0.1964
103268	Cep57l1	centrosomal protein 57-like 1	0.800641	0.08352	0.2499
170651	Krtap16-1	keratin associated protein 16-1	0.800641	0.1826	0.4015
216856	Nlgn2	neuroligin 2	0.800641	0.01018	0.07094
232288	Frmd4b	FERM domain containing 4B	0.800641	0.02824	0.1308
277432	Vstm2l	V-set and transmembrane domain containing 2-like	0.800641	0.01298	0.08194

321007	Serac1	serine active site containing 1	0.800641	0.007898	0.06102
432838	Gm5460	predicted gene 5460	0.800641	0.009633	0.06833
545172	Rpl21-ps5	ribosomal protein L21, pseudogene 5	0.800641	0.004185	0.04209
100041979	Gm3604	predicted gene 3604	0.800641	0.01453	0.08767
12696	Cirbp	cold inducible RNA binding protein	0.8	0.005407	0.04894
15560	Htr2c	5-hydroxytryptamine (serotonin) receptor 2C	0.8	0.02837	0.131
19734	Rgs16	regulator of G-protein signaling 16	0.8	0.0229	0.1152
22630	Ywhaq	tyrosine 3-monooxygenase/tryptophan 5-monooxygenase activation protein, theta polypeptide	0.8	0.01224	0.07879
22671	Rnf112	ring finger protein 112	0.8	0.05064	0.1828
27393	Mrpl39	mitochondrial ribosomal protein L39	0.8	0.03865	0.1565
54338	Slc23a2	solute carrier family 23 (nucleobase transporters), member 2	0.8	0.002921	0.03377
54447	Asah2	N-acylsphingosine amidohydrolase 2	0.8	0.188	0.4088
67048	Vma21	VMA21 vacuolar H+-ATPase homolog (S. cerevisiae)	0.8	0.04784	0.177
67574	Alg13	asparagine-linked glycosylation 13 homolog (S. cerevisiae)	0.8	0.01959	0.1049
71721	Fam13c	family with sequence similarity 13, member C	0.8	0.3549	0.5896
71766	Raver1	ribonucleoprotein, PTB-binding 1	0.8	0.2419	0.4748
74551	Pck2	phosphoenolpyruvate carboxykinase 2 (mitochondrial)	0.8	0.004383	0.04321
76832	Hyls1	hydrolethalus syndrome 1	0.8	0.007719	0.06019
76890	Memo1	mediator of cell motility 1	0.8	0.007057	0.05689
102153	C230098O21Rik	RIKEN cDNA C230098O21 gene	0.8	0.06445	0.2125
107508	Eprs	glutamyl-prolyl-tRNA synthetase	0.8	0.09562	0.2722
208144	Dhx37	DEAH (Asp-Glu-Ala-His) box polypeptide 37	0.8	0.1113	0.2993
216441	Slc26a10	solute carrier family 26, member 10	0.8	0.1949	0.4187
225929	Patl1	protein associated with topoisomerase II homolog 1 (yeast)	0.8	0.008975	0.06552
231712	Trafd1	TRAF type zinc finger domain containing 1	0.8	0.009588	0.06817
232943	Klc3	kinesin light chain 3	0.8	0.1055	0.2892
14337	Ftl2	ferritin light chain 2	0.799361	0.0359	0.1498
15951	Ifi204	interferon activated gene 204	0.799361	0.1918	0.4139
20319	Sfrp2	secreted frizzled-related protein 2	0.799361	0.1502	0.3581
53958	AU035318	EST AU035318	0.799361	0.01793	0.09904
70762	Dclk2	doublecortin-like kinase 2	0.799361	0.04423	0.1691
74189	Phactr3	phosphatase and actin regulator 3	0.799361	0.04261	0.1652
75396	Spp2	secreted phosphoprotein 2	0.799361	0.1218	0.3168
78177	Ninl	ninein-like	0.799361	0.08717	0.2574
270118	Maml2	mastermind like 2 (Drosophila)	0.799361	0.01202	0.07803
319953	Ttll1	tubulin tyrosine ligase-like 1	0.799361	0.07104	0.2261
668620	Zfp936	zinc finger protein 936	0.799361	0.3378	0.5735
14348	Fut9	fucosyltransferase 9	0.798722	0.04808	0.1775
17968	Ncam2	neural cell adhesion molecule 2	0.798722	0.1263	0.3236
65257	Asb3	ankyrin repeat and SOCS box-containing 3	0.798722	0.281	0.5162
66373	Lsm5	LSM5 homolog, U6 small nuclear RNA associated (S. cerevisiae)	0.798722	0.02637	0.1254
67590	Tctn3	tectonic family member 3	0.798722	0.002664	0.03197
68642	Tmem216	transmembrane protein 216	0.798722	0.6281	0.8039
69191	Pdia2	protein disulfide isomerase associated 2	0.798722	0.03642	0.1511
75956	Srrm2	serine/arginine repetitive matrix 2	0.798722	0.2628	0.4984
76505	1500009C09Rik	RIKEN cDNA 1500009C09 gene	0.798722	0.01005	0.07023
105559	Mbnl2	muscleblind-like 2	0.798722	0.1507	0.3587
193217	BC018473	cDNA sequence BC018473	0.798722	0.3847	0.617
218460	Wdr41	WD repeat domain 41	0.798722	0.01787	0.09884

224139	Golgb1	golgi autoantigen, golgin subfamily b, macrogolgin 1	0.798722	0.2969	0.5329
240595	Kcnv2	potassium channel, subfamily V, member 2	0.798722	0.009794	0.06908
246746	Cd300lf	CD300 antigen like family member F	0.798722	0.00887	0.06515
16493	Kcna5	potassium voltage-gated channel, shaker-related subfamily, member 5	0.798085	0.01551	0.09123
18216	Ntsr1	neurotensin receptor 1	0.798085	0.09012	0.2626
20190	Ryr1	ryanodine receptor 1, skeletal muscle	0.798085	0.2047	0.431
22156	Tuft1	tuftelin 1	0.798085	0.01037	0.07173
30924	Angptl3	angiopoietin-like 3	0.798085	0.06939	0.2228
64292	Ptges	prostaglandin E synthase	0.798085	0.1943	0.4179
76701	Ctrc	chymotrypsin C (caldecrin)	0.798085	0.2894	0.5256
76884	Cyfip2	cytoplasmic FMR1 interacting protein 2	0.798085	0.002662	0.03197
77371	Sec24a	Sec24 related gene family, member A (S. cerevisiae)	0.798085	0.04425	0.1691
77560	9330198I05Rik	RIKEN cDNA 9330198I05 gene	0.798085	0.1913	0.4134
78506	Efha2	EF-hand domain family, member A2	0.798085	0.2695	0.5047
225523	Cep120	centrosomal protein 120	0.798085	0.007741	0.0603
333329	Cngb1	cyclic nucleotide gated channel beta 1	0.798085	0.04993	0.1815
12751	Tpp1	tripeptidyl peptidase I	0.797448	0.0007959	0.01572
20926	Supt6h	suppressor of Ty 6 homolog (S. cerevisiae)	0.797448	0.292	0.5281
29856	Smtn	smoothelin	0.797448	0.03242	0.1417
64242	Ngb	neuroglobin	0.797448	0.09855	0.2771
66734	Map1lc3a	microtubule-associated protein 1 light chain 3 alpha	0.797448	0.01366	0.08445
68187	Fam135a	family with sequence similarity 135, member A	0.797448	0.01601	0.09269
68777	Tmem53	transmembrane protein 53	0.797448	0.03245	0.1418
69315	1700001L19Rik	RIKEN cDNA 1700001L19 gene	0.797448	0.01513	0.08982
73681	Trmt11	tRNA methyltransferase 11 homolog (S. cerevisiae)	0.797448	0.01742	0.09755
75735	Pank1	pantothenate kinase 1	0.797448	0.03225	0.1413
77015	Mpped2	metallophosphoesterase domain containing 2	0.797448	0.01858	0.1013
99045	Mrps26	mitochondrial ribosomal protein S26	0.797448	0.05853	0.2
218693	Paip1	polyadenylate binding protein-interacting protein 1	0.797448	0.1326	0.3332
319520	Dusp4	dual specificity phosphatase 4	0.797448	0.1032	0.285
16647	Kpna2	karyopherin (importin) alpha 2	0.796813	0.0209	0.1089
17119	Mxd1	MAX dimerization protein 1	0.796813	0.007462	0.059
17776	Mast2	microtubule associated serine/threonine kinase 2	0.796813	0.01676	0.09528
53619	Blcap	bladder cancer associated protein homolog (human)	0.796813	0.2021	0.4282
72368	2310045N01Rik	RIKEN cDNA 2310045N01 gene	0.796813	0.009921	0.06966
73168	3110027N22Rik	RIKEN cDNA 3110027N22 gene	0.796813	0.01549	0.09117
75424	Zfp820	zinc finger protein 820	0.796813	0.2694	0.5047
94229	Slc4a10	solute carrier family 4, sodium bicarbonate cotransporter-like, member 10	0.796813	0.268	0.5035
229011	Samd10	sterile alpha motif domain containing 10	0.796813	0.1451	0.3505
240665	Ccnj	cyclin J	0.796813	0.02354	0.117
320021	C430042M11Rik	RIKEN cDNA C430042M11 gene	0.796813	0.08066	0.2442
18162	Npr3	natriuretic peptide receptor 3	0.796178	0.02163	0.1114
18952	Sep-04	septin 4	0.796178	0.03306	0.1435
19293	Pvalb	parvalbumin	0.796178	0.002577	0.0315
19293	Pvalb	parvalbumin	0.796178	0.006555	0.05472
20667	Sox12	SRY-box containing gene 12	0.796178	0.05561	0.1929
22256	Ung	uracil DNA glycosylase	0.796178	0.06885	0.2218
54607	Socs6	suppressor of cytokine signaling 6	0.796178	0.04599	0.1731
59032	Ppp2r3c	protein phosphatase 2, regulatory subunit B'', gamma	0.796178	0.009359	0.06712

67235	Zkscan14	zinc finger with KRAB and SCAN domains 14	0.796178	0.02416	0.1186
77652	Zfp955a	zinc finger protein 955A	0.796178	0.001295	0.02091
78899	4932443L11Rik	RIKEN cDNA 4932443L11 gene	0.796178	0.009892	0.06952
242800	Ttc34	tetratricopeptide repeat domain 34	0.796178	0.1995	0.4249
269610	Chd5	chromodomain helicase DNA binding protein 5	0.796178	0.005178	0.04773
385668	Lca5l	Leber congenital amaurosis 5-like	0.796178	0.0619	0.2072
12994	Csn3	casein kappa	0.795545	0.1513	0.3596
14048	Eya1	eyes absent 1 homolog (Drosophila)	0.795545	0.1537	0.3634
14426	Galnt4	UDP-N-acetyl-alpha-D-galactosamine:polypeptide N-acetylgalactosaminyltransferase 4	0.795545	0.00417	0.04196
18191	Nrxn3	neurexin III	0.795545	0.06468	0.2129
20355	Sema4f	sema domain, immunoglobulin domain (Ig), TM domain, and short cytoplasmic domain	0.795545	0.1286	0.327
20437	Siah1a	seven in absentia 1A	0.795545	0.03033	0.1366
20698	Sphk1	sphingosine kinase 1	0.795545	0.1406	0.3443
53376	Usp2	ubiquitin specific peptidase 2	0.795545	0.02265	0.1146
56504	Srpk3	serine/arginine-rich protein specific kinase 3	0.795545	0.04868	0.1786
66717	Ccdc96	coiled-coil domain containing 96	0.795545	0.01957	0.1049
69131	Cdk12	cyclin-dependent kinase 12	0.795545	0.04243	0.1649
70948	Wdr20b	WD repeat domain 20b	0.795545	0.3385	0.5744
78754	Galntl2	UDP-N-acetyl-alpha-D-galactosamine:polypeptide N-acetylgalactosaminyltransferase-like 2	0.795545	0.004712	0.04511
80720	Pbx4	pre-B-cell leukemia homeobox 4	0.795545	0.05434	0.1905
105000	Dnalc1	dynein, axonemal, light chain 1	0.795545	0.4483	0.6739
213262	Fstl5	follistatin-like 5	0.795545	0.01959	0.1049
219072	Haus4	HAUS augmin-like complex, subunit 4	0.795545	0.0141	0.0861
245944	Vps54	vacuolar protein sorting 54 (yeast)	0.795545	0.004279	0.04269
270201	Klhl18	kelch-like 18 (Drosophila)	0.795545	0.01853	0.1011
338374	Il28b	interleukin 28B	0.795545	0.1372	0.339
668319	Gm9104	predicted gene 9104	0.795545	0.001574	0.02374
100040834	Gm2990	predicted gene 2990	0.795545	0.006137	0.05265
100503813	LOC100503813	hypothetical protein LOC100503813	0.795545	0.04673	0.1748
14058	F10	coagulation factor X	0.794913	0.5181	0.7273
18430	Oxtr	oxytocin receptor	0.794913	0.1589	0.3701
19652	Rbm3	RNA binding motif protein 3	0.794913	0.02535	0.1222
27059	Sh3d19	SH3 domain protein D19	0.794913	0.06949	0.2231
54611	Pde3a	phosphodiesterase 3A, cGMP inhibited	0.794913	0.2525	0.4872
67220	Plekho1	pleckstrin homology domain containing, family O member 1	0.794913	0.002452	0.03068
76252	Atp6v0e2	ATPase, H+ transporting, lysosomal V0 subunit E2	0.794913	0.005272	0.0483
77766	Elp4	elongation protein 4 homolog (S. cerevisiae)	0.794913	0.06789	0.2199
104360	Isl2	insulin related protein 2 (islet 2)	0.794913	0.03237	0.1416
105710	BB086117	expressed sequence BB086117	0.794913	0.006011	0.05219
107723	Slc12a6	solute carrier family 12, member 6	0.794913	0.006664	0.05528
207792	BC034090	cDNA sequence BC034090	0.794913	0.03137	0.139
216799	Nlrp3	NLR family, pyrin domain containing 3	0.794913	0.3904	0.6224
237782	Smcr8	Smith-Magenis syndrome chromosome region, candidate 8 homolog (human)	0.794913	0.02859	0.1317
269198	Nbeal1	neurobeachin like 1	0.794913	0.4025	0.6324
320320	B230343J05Rik	RIKEN cDNA B230343J05 gene	0.794913	0.008325	0.06257
432995	Gm5480	predicted gene 5480	0.794913	0.1608	0.3726
624855	Gm6531	predicted gene 6531	0.794913	0.05007	0.1817
12815	Col11a2	collagen, type XI, alpha 2	0.794281	0.1747	0.3914
13870	Ercc1	excision repair cross-complementing rodent repair deficiency, complementation group 1	0.794281	0.02918	0.1334

16621	Klkb1	kallikrein B, plasma 1	0.794281	0.002772	0.03265
19877	Rock1	Rho-associated coiled-coil containing protein kinase 1	0.794281	0.1211	0.3154
22283	Ush2a	Usher syndrome 2A (autosomal recessive, mild) homolog (human)	0.794281	0.05651	0.1951
23806	Arih1	ariadne ubiquitin-conjugating enzyme E2 binding protein homolog 1 (Drosophila)	0.794281	0.008025	0.06137
26914	H2afy	H2A histone family, member Y	0.794281	0.0892	0.261
52055	Rab11fip5	RAB11 family interacting protein 5 (class I)	0.794281	0.004593	0.04469
66078	Tsen34	tRNA splicing endonuclease 34 homolog (S. cerevisiae)	0.794281	0.001891	0.02642
67620	Lrp2bp	Lrp2 binding protein	0.794281	0.1488	0.3561
72046	Urgcp	upregulator of cell proliferation	0.794281	0.05067	0.1828
212439	AA986860	expressed sequence AA986860	0.794281	0.002518	0.0311
236727	Slc9a7	solute carrier family 9 (sodium/hydrogen exchanger), member 7	0.794281	0.0456	0.1721
320616	B130006D01Rik	RIKEN cDNA B130006D01 gene	0.794281	0.4183	0.6463
16520	Kcnj4	potassium inwardly-rectifying channel, subfamily J, member 4	0.793651	0.08957	0.2615
19261	Sirpa	signal-regulatory protein alpha	0.793651	0.002898	0.03363
19784	Rprl2	ribonuclease P RNA-like 2	0.793651	0.2663	0.5018
20273	Scn8a	sodium channel, voltage-gated, type VIII, alpha	0.793651	0.2506	0.4846
56744	Pf4	platelet factor 4	0.793651	0.3885	0.6203
70551	Tmtc4	transmembrane and tetratricopeptide repeat containing 4	0.793651	0.006211	0.05304
71354	Wdr31	WD repeat domain 31	0.793651	0.05274	0.1868
76132	6230409E13Rik	RIKEN cDNA 6230409E13 gene	0.793651	0.0145	0.08758
76295	Atp11b	ATPase, class VI, type 11B	0.793651	0.02639	0.1254
76898	B3gat1	beta-1,3-glucuronyltransferase 1 (glucuronosyltransferase P)	0.793651	0.0255	0.1227
78779	Spata2L	spermatogenesis associated 2-like	0.793651	0.06308	0.2096
100637	N4bp2l1	NEDD4 binding protein 2-like 1	0.793651	0.01478	0.08862
170483	Grin3b	glutamate receptor, ionotropic, NMDA3B	0.793651	0.006452	0.0542
228598	Ebf4	early B-cell factor 4	0.793651	0.04672	0.1748
238205	Lrfn5	leucine rich repeat and fibronectin type III domain containing 5	0.793651	0.2249	0.4559
320938	Tnpo3	transportin 3	0.793651	0.08101	0.2448
381107	Tmem232	transmembrane protein 232	0.793651	0.07845	0.24
665251	Gm7555	predicted gene 7555	0.793651	0.005445	0.04916
14654	Glra1	glycine receptor, alpha 1 subunit	0.793021	0.03255	0.1421
15259	Hipk3	homeodomain interacting protein kinase 3	0.793021	0.006676	0.0553
16443	Itsn1	intersectin 1 (SH3 domain protein 1A)	0.793021	0.1652	0.3789
16790	Anpep	alanyl (membrane) aminopeptidase	0.793021	0.192	0.4141
56089	Ramp3	receptor (calcitonin) activity modifying protein 3	0.793021	0.03002	0.1356
64707	Suv39h2	suppressor of variegation 3-9 homolog 2 (Drosophila)	0.793021	0.1607	0.3725
66787	4933433P14Rik	RIKEN cDNA 4933433P14 gene	0.793021	0.02005	0.1064
77524	C030017B01Rik	RIKEN cDNA C030017B01 gene	0.793021	0.03639	0.1511
78774	4930529M08Rik	RIKEN cDNA 4930529M08 gene	0.793021	0.1041	0.2868
93695	Gpnmb	glycoprotein (transmembrane) nmb	0.793021	0.412	0.6408
97165	Hmgb2	high mobility group box 2	0.793021	0.03892	0.157
210035	Tmem194	transmembrane protein 194	0.793021	0.05561	0.1929
217325	Llgl2	lethal giant larvae homolog 2 (Drosophila)	0.793021	0.02095	0.1091
234847	Spg7	spastic paraplegia 7 homolog (human)	0.793021	0.002661	0.03197
242253	Wdr63	WD repeat domain 63	0.793021	0.05882	0.2007
244550	Podnl1	podocan-like 1	0.793021	0.05702	0.1963
270757	Bpil2	bactericidal/permeability-increasing protein-like 2	0.793021	0.1561	0.3659
383103	Fam18a	family with sequence similarity 18, member A	0.793021	0.008366	0.0627
666539	Gm8154	predicted gene 8154	0.793021	0.3473	0.5827

11923	Neurod4	neurogenic differentiation 4	0.792393	0.1492	0.3567
20648	Snta1	syntrophin, acidic 1	0.792393	0.04919	0.1797
29863	Pde7b	phosphodiesterase 7B	0.792393	0.0296	0.1345
50767	Pnpla6	patatin-like phospholipase domain containing 6	0.792393	0.03516	0.1484
74440	4933407C03Rik	RIKEN cDNA 4933407C03 gene	0.792393	0.006101	0.05258
100637	N4bp2l1	NEDD4 binding protein 2-like 1	0.792393	0.008648	0.06405
107684	Coro2a	coronin, actin binding protein 2A	0.792393	0.01914	0.1032
212712	Satb2	special AT-rich sequence binding protein 2	0.792393	0.1592	0.3704
269053	Gpr152	G protein-coupled receptor 152	0.792393	0.006137	0.05265
11798	Xiap	X-linked inhibitor of apoptosis	0.791766	0.004113	0.04157
16469	Jrk	jerky	0.791766	0.04756	0.1765
17175	Masp2	mannan-binding lectin serine peptidase 2	0.791766	0.4738	0.6946
18475	Pafah1b2	platelet-activating factor acetylhydrolase, isoform 1b, subunit 2	0.791766	0.009894	0.06952
18708	Pik3r1	phosphatidylinositol 3-kinase, regulatory subunit, polypeptide 1 (p85 alpha)	0.791766	0.1119	0.3002
66532	Rep15	RAB15 effector protein	0.791766	0.04417	0.169
71745	Cul2	cullin 2	0.791766	0.01049	0.07219
140629	Ubox5	U box domain containing 5	0.791766	0.03349	0.1446
207686	A330021E22Rik	RIKEN cDNA A330021E22 gene	0.791766	0.0937	0.269
207952	Klhl25	kelch-like 25 (Drosophila)	0.791766	0.03795	0.1549
353237	Pcdhac2	protocadherin alpha subfamily C, 2	0.791766	0.01838	0.1006
382985	Rrm2b	ribonucleotide reductase M2 B (TP53 inducible)	0.791766	0.3789	0.6126
544752	Tug1	taurine upregulated gene 1	0.791766	0.01354	0.084
12310	Calca	calcitonin/calcitonin-related polypeptide, alpha	0.791139	0.009324	0.06696
12801	Cnr1	cannabinoid receptor 1 (brain)	0.791139	0.006688	0.0553
19724	Rfx1	regulatory factor X, 1 (influences HLA class II expression)	0.791139	0.007857	0.0609
19885	Rorc	RAR-related orphan receptor gamma	0.791139	0.01581	0.09235
20333	Sec22b	SEC22 vesicle trafficking protein homolog B (S. cerevisiae)	0.791139	0.01354	0.08399
29820	Tnfrsf19	tumor necrosis factor receptor superfamily, member 19	0.791139	0.01996	0.106
66090	Ypel3	yippee-like 3 (Drosophila)	0.791139	0.01432	0.08686
67037	Pmf1	polyamine-modulated factor 1	0.791139	0.1664	0.3807
70710	6330407I18Rik	RIKEN cDNA 6330407I18 gene	0.791139	0.01491	0.08913
72508	Rps6kb1	ribosomal protein S6 kinase, polypeptide 1	0.791139	0.002429	0.03056
74166	Tmem38a	transmembrane protein 38A	0.791139	0.00942	0.06737
74189	Phactr3	phosphatase and actin regulator 3	0.791139	0.1007	0.2806
76719	1700081L11Rik	RIKEN cDNA 1700081L11 gene	0.791139	0.3237	0.5602
78369	Icam4	intercellular adhesion molecule 4, Landsteiner-Wiener blood group	0.791139	0.082	0.2468
80334	Kcnip4	Kv channel interacting protein 4	0.791139	0.0465	0.1743
108112	Eif4ebp3	eukaryotic translation initiation factor 4E binding protein 3	0.791139	0.004688	0.04508
214111	Slc24a1	solute carrier family 24 (sodium/potassium/calcium exchanger), member 1	0.791139	0.02628	0.1252
231279	Guf1	GUF1 GTPase homolog (S. cerevisiae)	0.791139	0.02758	0.1291
100040322	3830408C21Rik	RIKEN cDNA 3830408C21 gene	0.791139	0.09504	0.2711
13113	Cyp3a13	cytochrome P450, family 3, subfamily a, polypeptide 13	0.790514	0.2093	0.4363
20662	Sos1	son of sevenless homolog 1 (Drosophila)	0.790514	0.09919	0.2779
21339	Taf1a	TATA box binding protein (Tbp)-associated factor, RNA polymerase I, A	0.790514	0.01086	0.07362
68925	Rpap1	RNA polymerase II associated protein 1	0.790514	0.0113	0.07532
71389	Chd6	chromodomain helicase DNA binding protein 6	0.790514	0.007605	0.0597
74103	Nebl	nebulette	0.790514	0.07638	0.2367
76376	Slc24a2	solute carrier family 24 (sodium/potassium/calcium exchanger), member 2	0.790514	0.003712	0.03898
76469	Cmya5	cardiomyopathy associated 5	0.790514	0.1685	0.3835

208677	Creb3l3	cAMP responsive element binding protein 3-like 3	0.790514	0.0133	0.08316
232943	Klc3	kinesin light chain 3	0.790514	0.03207	0.1408
234395	Ushbp1	Usher syndrome 1C binding protein 1	0.790514	0.00476	0.04539
12361	Cask	calcium/calmodulin-dependent serine protein kinase (MAGUK family)	0.789889	0.07388	0.232
12952	Cry1	cryptochrome 1 (photolyase-like)	0.789889	0.4016	0.6318
52430	Echdc2	enoyl Coenzyme A hydratase domain containing 2	0.789889	0.003498	0.03767
53602	Hpcal1	hippocalcin-like 1	0.789889	0.02685	0.1267
68724	Arl8a	ADP-ribosylation factor-like 8A	0.789889	0.004187	0.04209
76886	Fam81a	family with sequence similarity 81, member A	0.789889	0.05212	0.1856
107895	Mgat5	mannoside acetylglucosaminyltransferase 5	0.789889	0.04639	0.1741
110351	Rap1gap	Rap1 GTPase-activating protein	0.789889	0.05959	0.2026
217664	Mgat2	mannoside acetylglucosaminyltransferase 2	0.789889	0.03685	0.152
320700	A930033H14Rik	RIKEN cDNA A930033H14 gene	0.789889	0.04466	0.1701
751865	Sap25	sin3 associated polypeptide	0.789889	0.05139	0.1841
100038595	Gm16287	predicted gene 16287	0.789889	0.01417	0.08633
12874	Cpd	carboxypeptidase D	0.789266	0.09428	0.2698
14810	Grin1	glutamate receptor, ionotropic, NMDA1 (zeta 1)	0.789266	0.07841	0.24
67890	Ufm1	ubiquitin-fold modifier 1	0.789266	0.009311	0.06691
68566	Caly	calcyon neuron-specific vesicular protein	0.789266	0.02452	0.1197
71833	Dcaf7	DDB1 and CUL4 associated factor 7	0.789266	0.04376	0.168
72097	2010300C02Rik	RIKEN cDNA 2010300C02 gene	0.789266	0.1595	0.3709
99031	Osbpl6	oxysterol binding protein-like 6	0.789266	0.004371	0.04317
208285	Cyp4f17	cytochrome P450, family 4, subfamily f, polypeptide 17	0.789266	0.03668	0.1517
235130	Adamts15	a disintegrin-like and metallopeptidase (reprolysin type) with thrombospondin type 1 motif, 15	0.789266	0.001018	0.01788
238331	Zdhhc22	zinc finger, DHHC-type containing 22	0.789266	0.07224	0.2285
327655	Ppip5k1	diphosphoinositol pentakisphosphate kinase 1	0.789266	0.003537	0.03793
329274	Fam163a	family with sequence similarity 163, member A	0.789266	0.01073	0.07306
12918	Crh	corticotropin releasing hormone	0.788644	0.06143	0.2061
13347	Dffa	DNA fragmentation factor, alpha subunit	0.788644	0.09243	0.2665
14159	Fes	feline sarcoma oncogene	0.788644	0.01517	0.08993
56177	Olfm1	olfactomedin 1	0.788644	0.0009137	0.01702
65111	Dap3	death associated protein 3	0.788644	0.001011	0.01786
74211	1700017B05Rik	RIKEN cDNA 1700017B05 gene	0.788644	0.09298	0.2677
75572	Acyp2	acylphosphatase 2, muscle type	0.788644	0.4713	0.6925
100102	Pcsk9	proprotein convertase subtilisin/kexin type 9	0.788644	0.108	0.294
117229	Stk33	serine/threonine kinase 33	0.788644	0.01195	0.07775
229949	Ak5	adenylate kinase 5	0.788644	0.06856	0.2211
231570	A830010M20Rik	RIKEN cDNA A830010M20 gene	0.788644	0.3437	0.5791
237213	Glra2	glycine receptor, alpha 2 subunit	0.788644	0.02529	0.1221
270084	Lpcat2	lysophosphatidylcholine acyltransferase 2	0.788644	0.02496	0.121
381914	B230311B06Rik	RIKEN cDNA B230311B06 gene	0.788644	0.1898	0.4116
545527	Fam194a	family with sequence similarity 194, member A	0.788644	0.01923	0.1036
16536	Kcnq2	potassium voltage-gated channel, subfamily Q, member 2	0.788022	0.08629	0.2555
22156	Tuft1	tuftelin 1	0.788022	0.01541	0.0909
70103	Znhit1	zinc finger, HIT domain containing 1	0.788022	0.04362	0.1677
70974	Pgm2l1	phosphoglucomutase 2-like 1	0.788022	0.1328	0.3334
226413	Lct	lactase	0.788022	0.04724	0.1759
232816	Zfp628	zinc finger protein 628	0.788022	0.1815	0.4002
240261	Ccdc112	coiled-coil domain containing 112	0.788022	0.05755	0.1974

327992	Hsf5	heat shock transcription factor family member 5	0.788022	0.1894	0.4111
407243	Tmem189	transmembrane protein 189	0.788022	0.527	0.7339
629016	Zfp953	zinc finger protein 953	0.788022	0.07119	0.2263
665155	Srp54b	signal recognition particle 54B	0.788022	0.2872	0.5226
666529	Gm8149	predicted gene 8149	0.788022	0.09196	0.2658
100039861	Gm2464	predicted gene 2464	0.788022	0.07506	0.2342
100504389	LOC100504389	hypothetical LOC100504389	0.788022	0.01551	0.09123
12615	Cenpa	centromere protein A	0.787402	0.02782	0.1297
13433	Dnmt1	DNA methyltransferase (cytosine-5) 1	0.787402	0.01504	0.08955
22717	Zfp59	zinc finger protein 59	0.787402	0.0282	0.1307
76872	Ccdc116	coiled-coil domain containing 116	0.787402	0.2336	0.4649
76963	2310076G05Rik	RIKEN cDNA 2310076G05 gene	0.787402	0.05819	0.1991
77286	Nkrf	NF-kappaB repressing factor	0.787402	0.02023	0.1069
78796	Zcchc4	zinc finger, CCHC domain containing 4	0.787402	0.09043	0.2633
114642	Brdt	bromodomain, testis-specific	0.787402	0.01438	0.08713
244694	Kdm4d	lysine (K)-specific demethylase 4D	0.787402	0.1094	0.296
319574	9330133O14Rik	RIKEN cDNA 9330133O14 gene	0.787402	0.04973	0.1811
14057	Sfxn1	sideroflexin 1	0.786782	0.008158	0.06182
15893	Ica1	islet cell autoantigen 1	0.786782	0.02189	0.1121
16780	Lamb3	laminin, beta 3	0.786782	0.1227	0.3185
56695	Pnkd	paroxysmal nonkinesiogenic dyskinesia	0.786782	0.1682	0.3831
66329	Susd3	sushi domain containing 3	0.786782	0.7146	0.8568
66435	Uggt2	UDP-glucose glycoprotein glucosyltransferase 2	0.786782	0.0007099	0.01464
71535	9030411M13Rik	RIKEN cDNA 9030411M13 gene	0.786782	0.1808	0.3995
77683	Ehmt1	euchromatic histone methyltransferase 1	0.786782	0.192	0.4141
110175	Ggct	gamma-glutamyl cyclotransferase	0.786782	0.005422	0.04901
110891	Slc8a2	solute carrier family 8 (sodium/calcium exchanger), member 2	0.786782	0.08433	0.2514
113849	Vmn1r52	vomeronasal 1 receptor 52	0.786782	0.2591	0.4943
232798	Leng8	leukocyte receptor cluster (LRC) member 8	0.786782	0.1152	0.3058
243753	2010107G12Rik	RIKEN cDNA 2010107G12 gene	0.786782	0.0266	0.1261
245403	Dcaf12l2	DDB1 and CUL4 associated factor 12-like 2	0.786782	0.3267	0.5633
434130	Ccdc8	coiled-coil domain containing 8	0.786782	0.1915	0.4136
100233175	AK010878	cDNA sequence AK010878	0.786782	0.02348	0.1169
16589	Uhmk1	U2AF homology motif (UHM) kinase 1	0.786164	0.2035	0.4298
19051	Gsbs	G substrate	0.786164	0.1045	0.2875
60525	Acss2	acyl-CoA synthetase short-chain family member 2	0.786164	0.03528	0.1484
70799	Cep192	centrosomal protein 192	0.786164	0.01424	0.08658
72792	2810459M11Rik	RIKEN cDNA 2810459M11 gene	0.786164	0.08166	0.2462
100182	Akna	AT-hook transcription factor	0.786164	0.01978	0.1054
106763	Ttbk1	tau tubulin kinase 1	0.786164	0.05602	0.1939
226180	Ina	internexin neuronal intermediate filament protein, alpha	0.786164	0.01059	0.0726
245440	Gm4988	predicted gene 4988	0.786164	0.0004397	0.01102
245468	Pnma3	paraneoplastic antigen MA3	0.786164	0.00657	0.05475
329906	C030014L02	hypothetical protein C030014L02	0.786164	0.005399	0.04892
620401	Gm16442	predicted gene 16442	0.786164	0.1307	0.3305
628822	Gm6919	predicted gene 6919	0.786164	0.1103	0.2976
16334	Ins2	insulin II	0.785546	0.0573	0.1968
26558	Homer3	homer homolog 3 (Drosophila)	0.785546	0.08014	0.2435
51960	Kctd18	potassium channel tetramerisation domain containing 18	0.785546	0.05966	0.2026

66204	Acyp1	acylphosphatase 1, erythrocyte (common) type	0.785546	0.221	0.4509
93692	Glrx	glutaredoxin	0.785546	0.008438	0.06309
105670	Rcbtb2	regulator of chromosome condensation (RCC1) and BTB (POZ) domain containing protein 2	0.785546	0.004107	0.04155
109079	Sephs1	selenophosphate synthetase 1	0.785546	0.005683	0.05039
233529	Kctd14	potassium channel tetramerisation domain containing 14	0.785546	0.008732	0.0644
246707	Emilin2	elastin microfibril interfacer 2	0.785546	0.1435	0.3485
331524	Xkrx	X Kell blood group precursor related X linked	0.785546	0.03531	0.1484
100503619	LOC100503619	hypothetical LOC100503619	0.785546	0.08018	0.2435
14226	Fkbp1b	FK506 binding protein 1b	0.784929	0.01887	0.1023
20660	Sorl1	sortilin-related receptor, LDLR class A repeats-containing	0.784929	0.01392	0.08539
51902	Rnf24	ring finger protein 24	0.784929	0.0008691	0.01662
56622	Adam21	a disintegrin and metallopeptidase domain 21	0.784929	0.005453	0.04921
66206	1110059E24Rik	RIKEN cDNA 1110059E24 gene	0.784929	0.007543	0.05939
212531	Sh3bgrl2	SH3 domain binding glutamic acid-rich protein like 2	0.784929	0.002586	0.0315
214230	Pak6	p21 protein (Cdc42/Rac)-activated kinase 6	0.784929	0.2756	0.5111
226541	Klhl20	kelch-like 20 (Drosophila)	0.784929	0.001841	0.02587
234353	Psd3	pleckstrin and Sec7 domain containing 3	0.784929	0.01926	0.1037
270091	Lrrc36	leucine rich repeat containing 36	0.784929	0.01271	0.08084
320206	A730028G07Rik	RIKEN cDNA A730028G07 gene	0.784929	0.06096	0.2053
11303	Abca1	ATP-binding cassette, sub-family A (ABC1), member 1	0.784314	0.04757	0.1765
20500	Slc13a2	solute carrier family 13 (sodium-dependent dicarboxylate transporter), member 2	0.784314	0.2469	0.481
23874	Farsb	phenylalanyl-tRNA synthetase, beta subunit	0.784314	0.005207	0.04791
66926	Trmt6	tRNA methyltransferase 6 homolog (S. cerevisiae)	0.784314	0.3008	0.5367
76138	Ccdc138	coiled-coil domain containing 138	0.784314	0.1221	0.3173
94191	Adarb2	adenosine deaminase, RNA-specific, B2	0.784314	0.369	0.6039
104709	Pik3r6	phosphoinositide-3-kinase, regulatory subunit 6	0.784314	0.1517	0.3603
216622	4931440F15Rik	RIKEN cDNA 4931440F15 gene	0.784314	0.08319	0.2492
226970	Arhgef4	Rho guanine nucleotide exchange factor (GEF) 4	0.784314	0.008719	0.06438
231858	Radil	Ras association and DIL domains	0.784314	0.06207	0.2074
270163	Myo9a	myosin IXa	0.784314	0.02921	0.1334
13601	Ecm1	extracellular matrix protein 1	0.783699	0.02014	0.1066
18536	Pcm1	pericentriolar material 1	0.783699	0.004374	0.04317
20018	Polr1d	polymerase (RNA) I polypeptide D	0.783699	0.007912	0.06104
66526	2210012G02Rik	RIKEN cDNA 2210012G02 gene	0.783699	0.157	0.3673
67071	Rps6ka6	ribosomal protein S6 kinase polypeptide 6	0.783699	0.02101	0.1092
240034	Zfp760	zinc finger protein 760	0.783699	0.008846	0.06503
331461	Il1rapl1	interleukin 1 receptor accessory protein-like 1	0.783699	0.03835	0.1559
14768	Lancl1	LanC (bacterial lantibiotic synthetase component C)-like 1	0.783085	0.0002101	0.007372
20648	Snta1	syntrophin, acidic 1	0.783085	0.01835	0.1006
21825	Thbs1	thrombospondin 1	0.783085	0.1623	0.3746
66167	Ccdc72	coiled-coil domain containing 72	0.783085	0.00296	0.03397
67640	4930465K10Rik	RIKEN cDNA 4930465K10 gene	0.783085	0.037	0.1524
71096	Sntg1	syntrophin, gamma 1	0.783085	0.02186	0.112
73720	Cst6	cystatin E/M	0.783085	0.06553	0.2147
74838	Naa15	N(alpha)-acetyltransferase 15, NatA auxiliary subunit	0.783085	0.05325	0.1878
77875	Cyp4f41-ps	cytochrome P450, family 4, subfamily f, polypeptide 41 pseudogene	0.783085	0.2037	0.43
94109	Csmd1	CUB and Sushi multiple domains 1	0.783085	0.04194	0.1639
227094	Tmem194b	transmembrane protein 194B	0.783085	0.4221	0.65
232313	Gxylt2	glucoside xylosyltransferase 2	0.783085	0.2062	0.4324

320124	B930095M22Rik	RIKEN cDNA B930095M22 gene	0.783085	0.02778	0.1296
332934	Zmynd12	zinc finger, MYND domain containing 12	0.783085	0.03523	0.1484
407821	Znrf3	zinc and ring finger 3	0.783085	0.09491	0.2709
100505283	LOC100505283	60S ribosomal protein L21-like	0.783085	0.001691	0.02489
11994	Pcdh15	protocadherin 15	0.782473	0.001931	0.02675
12833	Col6a1	collagen, type VI, alpha 1	0.782473	0.08934	0.2612
18993	Pou3f3	POU domain, class 3, transcription factor 3	0.782473	0.1213	0.3159
53418	B4galt2	UDP-Gal:betaGlcNAc beta 1,4- galactosyltransferase, polypeptide 2	0.782473	0.01315	0.08273
56079	Astn2	astrotactin 2	0.782473	0.1463	0.352
68905	1110065H08Rik	RIKEN cDNA 1110065H08 gene	0.782473	0.06917	0.2224
83398	Ndst3	N-deacetylase/N-sulfotransferase (heparan glucosaminyl) 3	0.782473	0.002089	0.02786
208076	Pknox2	Pbx/knotted 1 homeobox 2	0.782473	0.02313	0.1159
211147	Mar-11	membrane-associated ring finger (C3HC4) 11	0.782473	0.06099	0.2053
233649	Cnga4	cyclic nucleotide gated channel alpha 4	0.782473	0.05018	0.1819
353155	Gjd3	gap junction protein, delta 3	0.782473	0.03134	0.1389
665934	Gm7854	predicted gene 7854	0.782473	0.01698	0.0961
13347	Dffa	DNA fragmentation factor, alpha subunit	0.781861	0.03505	0.1484
16906	Lmnb1	lamin B1	0.781861	0.06066	0.2047
56307	Metap2	methionine aminopeptidase 2	0.781861	0.006349	0.05358
67564	Tmem35	transmembrane protein 35	0.781861	0.05531	0.1925
69459	Ubl7	ubiquitin-like 7 (bone marrow stromal cell-derived)	0.781861	0.1625	0.3747
71901	2310028H24Rik	RIKEN cDNA 2310028H24 gene	0.781861	0.1808	0.3994
228875	Slc35c2	solute carrier family 35, member C2	0.781861	0.03282	0.1428
235584	Dusp7	dual specificity phosphatase 7	0.781861	0.02159	0.1112
380614	Intu	inturned planar cell polarity effector homolog (Drosophila)	0.781861	0.008003	0.06132
637900	Gm12618	predicted gene 12618	0.781861	0.04279	0.1658
100043387	100043387	predicted gene, 100043387	0.781861	0.2751	0.5105
13447	Doc2b	double C2, beta	0.78125	0.001145	0.01923
17929	Myom1	myomesin 1	0.78125	0.06653	0.2169
20308	Ccl9	chemokine (C-C motif) ligand 9	0.78125	0.05752	0.1974
23969	Pacsin1	protein kinase C and casein kinase substrate in neurons 1	0.78125	0.009546	0.06795
50783	Lsm4	LSM4 homolog, U6 small nuclear RNA associated (S. cerevisiae)	0.78125	0.0007354	0.01495
66848	Fuca2	fucosidase, alpha-L- 2, plasma	0.78125	0.0053	0.04847
67161	Sclt1	sodium channel and clathrin linker 1	0.78125	0.0507	0.1828
67417	Ears2	glutamyl-tRNA synthetase 2 (mitochondrial)(putative)	0.78125	0.008315	0.06255
68943	Pink1	PTEN induced putative kinase 1	0.78125	0.184	0.403
71828	Gtf2a1l	general transcription factor IIA, 1-like	0.78125	0.1214	0.3161
77940	A930004D18Rik	RIKEN cDNA A930004D18 gene	0.78125	0.1752	0.3919
96957	Tmem62	transmembrane protein 62	0.78125	0.002097	0.02786
103288	AI836737	expressed sequence AI836737	0.78125	0.1398	0.3432
233045	C130030J05	hypothetical protein C130030J05	0.78125	0.1644	0.3778
240690	St18	suppression of tumorigenicity 18	0.78125	0.0219	0.1121
319737	D130051D11Rik	RIKEN cDNA D130051D11 gene	0.78125	0.3217	0.5581
14269	Fnbp1	formin binding protein 1	0.78064	0.1737	0.3901
23886	Gdf15	growth differentiation factor 15	0.78064	0.0591	0.2014
24086	Tlk2	tousled-like kinase 2 (Arabidopsis)	0.78064	0.005522	0.0496
66204	Acyp1	acylphosphatase 1, erythrocyte (common) type	0.78064	0.4493	0.6746
68197	Ndufc2	NADH dehydrogenase (ubiquinone) 1, subcomplex unknown, 2	0.78064	0.006574	0.05476
71648	Optn	optineurin	0.78064	0.04087	0.1615

215798	Gpr126	G protein-coupled receptor 126	0.78064	0.05099	0.1833
223473	Nipal2	NIPA-like domain containing 2	0.78064	0.1709	0.3865
232664	Ccdc136	coiled-coil domain containing 136	0.78064	0.007245	0.05797
319587	4930539J05Rik	RIKEN cDNA 4930539J05 gene	0.78064	0.05005	0.1817
320951	Pisd	phosphatidylserine decarboxylase	0.78064	0.02543	0.1225
353187	Nr1d2	nuclear receptor subfamily 1, group D, member 2	0.78064	0.03365	0.145
353187	Nr1d2	nuclear receptor subfamily 1, group D, member 2	0.78064	0.08104	0.2448
668311	Gm9099	predicted gene 9099	0.78064	0.119	0.3118
13347	Dffa	DNA fragmentation factor, alpha subunit	0.780031	0.1163	0.3077
24136	Zeb2	zinc finger E-box binding homeobox 2	0.780031	0.1019	0.2828
53414	Bysl	bystin-like	0.780031	0.002439	0.03058
66226	Trappc2	trafficking protein particle complex 2	0.780031	0.5234	0.7311
66686	Dcbld1	discoidin, CUB and LCCL domain containing 1	0.780031	0.007995	0.06131
69847	Wnk4	WNK lysine deficient protein kinase 4	0.780031	0.002523	0.03112
73852	D3Ertd751e	DNA segment, Chr 3, ERATO Doi 751, expressed	0.780031	0.09074	0.2638
94047	Cecr6	cat eye syndrome chromosome region, candidate 6 homolog (human)	0.780031	0.1935	0.4168
227648	Sec16a	SEC16 homolog A (S. cerevisiae)	0.780031	0.03208	0.1408
619309	A630081J09Rik	RIKEN cDNA A630081J09 gene	0.780031	0.03896	0.1571
668319	Gm9104	predicted gene 9104	0.780031	0.07156	0.2271
66671	Ccnh	cyclin H	0.779423	0.01002	0.07015
69368	Wdfy1	WD repeat and FYVE domain containing 1	0.779423	0.03107	0.1381
76915	Mnd1	meiotic nuclear divisions 1 homolog (S. cerevisiae)	0.779423	0.06074	0.2048
212555	Pqlc2	PQ loop repeat containing 2	0.779423	0.00637	0.0537
229706	Slc6a17	solute carrier family 6 (neurotransmitter transporter), member 17	0.779423	0.02772	0.1295
238988	Erc2	ELKS/RAB6-interacting/CAST family member 2	0.779423	0.0253	0.1221
432442	Akap7	A kinase (PRKA) anchor protein 7	0.779423	0.01548	0.09117
432989	D930007P13Rik	Riken cDNA D930007P13 gene	0.779423	0.1148	0.3052
18793	Plaur	plasminogen activator, urokinase receptor	0.778816	0.4646	0.687
19326	Rab11b	RAB11B, member RAS oncogene family	0.778816	0.1084	0.2945
19711	Resp18	regulated endocrine-specific protein 18	0.778816	0.002775	0.03267
20148	Dhrs3	dehydrogenase/reductase (SDR family) member 3	0.778816	0.00107	0.01845
20338	Sel1l	sel-1 suppressor of lin-12-like (C. elegans)	0.778816	0.1157	0.3066
26381	Esrrg	estrogen-related receptor gamma	0.778816	0.01318	0.08275
140919	Slc17a6	solute carrier family 17 (sodium-dependent inorganic phosphate cotransporter), member 6	0.778816	0.02482	0.1206
233670	Olfr6	olfactory receptor 6	0.778816	0.6752	0.8334
270028	Fam155a	family with sequence similarity 155, member A	0.778816	0.008154	0.06182
271377	Zbtb11	zinc finger and BTB domain containing 11	0.778816	0.09058	0.2636
19270	Ptprg	protein tyrosine phosphatase, receptor type, G	0.77821	0.09428	0.2698
56873	Lmbr1	limb region 1	0.77821	0.1499	0.3577
64580	Ndst4	N-deacetylase/N-sulfotransferase (heparin glucosaminyl) 4	0.77821	0.05355	0.1886
67657	Rabl3	RAB, member of RAS oncogene family-like 3	0.77821	0.006899	0.05627
76080	Ttpal	tocopherol (alpha) transfer protein-like	0.77821	0.1964	0.4206
210789	Tbc1d4	TBC1 domain family, member 4	0.77821	0.02035	0.107
240726	Slco5a1	solute carrier organic anion transporter family, member 5A1	0.77821	0.2729	0.5085
319998	Tmem198	transmembrane protein 198	0.77821	0.01542	0.0909
12821	Col17a1	collagen, type XVII, alpha 1	0.777605	0.0134	0.08343
18457	Pldn	pallidin	0.777605	0.005682	0.05039
18823	Plp1	proteolipid protein (myelin) 1	0.777605	0.004511	0.04416
22370	Vtn	vitronectin	0.777605	0.009189	0.06641

99003	Qser1		glutamine and serine rich 1	0.777605	0.005667	0.05039
110593	Prdm2		PR domain containing 2, with ZNF domain	0.777605	0.007447	0.05895
117148	Necab2		N-terminal EF-hand calcium binding protein 2	0.777605	0.0175	0.0978
237246	BC022960		cDNA sequence BC022960	0.777605	0.008003	0.06132
666340	Gm8054		Rpl21 pseudogene	0.777605	0.001795	0.02551
11363	Acadl		acyl-Coenzyme A dehydrogenase, long-chain	0.777001	0.01244	0.0796
13117	Cyp4a10		cytochrome P450, family 4, subfamily a, polypeptide 10	0.777001	0.2321	0.4635
17248	Mdm4		transformed mouse 3T3 cell double minute 4	0.777001	0.172	0.3878
18952		Sep-04	septin 4	0.777001	0.003006	0.03428
52857	Gramd1a		GRAM domain containing 1A	0.777001	0.03429	0.1465
54607	Socs6		suppressor of cytokine signaling 6	0.777001	0.04501	0.1711
227157	Mpp4		membrane protein, palmitoylated 4 (MAGUK p55 subfamily member 4)	0.777001	0.004127	0.04164
233071	Arhgap33		Rho GTPase activating protein 33	0.777001	0.1335	0.334
240041	Zfp945		zinc finger protein 945	0.777001	0.02698	0.1271
328949	Mcc		mutated in colorectal cancers	0.777001	0.0226	0.1144
574519	Vax2os1		Vax2 opposite strand transcript 1	0.777001	0.04604	0.1732
20606	Sstr2		somatostatin receptor 2	0.776398	0.1449	0.3503
57908	Zfp318		zinc finger protein 318	0.776398	0.01148	0.076
72296	Rusc1		RUN and SH3 domain containing 1	0.776398	0.0226	0.1144
76303	Osbp		oxysterol binding protein	0.776398	0.01454	0.08767
77938	Fam53b		family with sequence similarity 53, member B	0.776398	0.001247	0.02045
223726	Mpped1		metallophosphoesterase domain containing 1	0.776398	0.04757	0.1765
234353	Psd3		pleckstrin and Sec7 domain containing 3	0.776398	0.01512	0.0898
268534	Sntg2		syntrophin, gamma 2	0.776398	0.02157	0.1112
270086	Ogfod1		2-oxoglutarate and iron-dependent oxygenase domain containing 1	0.776398	0.03681	0.152
12491	Cd36		CD36 antigen	0.775795	0.1105	0.2979
12671	Chrm3		cholinergic receptor, muscarinic 3, cardiac	0.775795	0.01142	0.07571
17910	Myo15		myosin XV	0.775795	0.01473	0.08846
66995	Zcchc18		zinc finger, CCHC domain containing 18	0.775795	0.0323	0.1414
67015	Ccdc91		coiled-coil domain containing 91	0.775795	0.03065	0.1371
76367	Trp53rk		transformation related protein 53 regulating kinase	0.775795	0.007478	0.05907
114332	Lyve1		lymphatic vessel endothelial hyaluronan receptor 1	0.775795	0.01888	0.1024
240038	Gm4944		predicted gene 4944	0.775795	0.01236	0.0792
319804	Glt1d1		glycosyltransferase 1 domain containing 1	0.775795	0.053	0.1873
552880	LOC552880		hypothetical LOC552880	0.775795	0.1476	0.3541
16000	Igf1		insulin-like growth factor 1	0.775194	0.008133	0.06173
18605	Enpp1		ectonucleotide pyrophosphatase/phosphodiesterase 1	0.775194	0.1909	0.413
21374	Tbp		TATA box binding protein	0.775194	0.0009182	0.01704
58175	Rgs20		regulator of G-protein signaling 20	0.775194	0.009683	0.06853
67374	Jam2		junction adhesion molecule 2	0.775194	0.0006316	0.01363
71876	Mlf1ip		myeloid leukemia factor 1 interacting protein	0.775194	0.02493	0.1209
76862	4833405L11Rik		RIKEN cDNA 4833405L11 gene	0.775194	0.1407	0.3443
104394	E2f4		E2F transcription factor 4	0.775194	0.4329	0.66
215890	Clvs2		clavesin 2	0.775194	0.0134	0.08343
230126	Shb		src homology 2 domain-containing transforming protein B	0.775194	0.1972	0.4219
231602	P2rx2		purinergic receptor P2X, ligand-gated ion channel, 2	0.775194	0.03651	0.1513
244721	Zfp846		zinc finger protein 846	0.775194	0.004411	0.04342
268747	Lrrc16b		leucine rich repeat containing 16B	0.775194	0.05776	0.198
269629	Lhfpl3		lipoma HMGIC fusion partner-like 3	0.775194	0.0321	0.1408

320609	Fam40b	family with sequence similarity 40, member B	0.775194	0.02289	0.1152
330503	Gm5113	predicted gene 5113	0.775194	0.003331	0.03661
11484	Aspa	aspartoacylase	0.774593	0.03473	0.1477
18781	Pla2g2c	phospholipase A2, group IIC	0.774593	0.2932	0.5292
56220	Zfp386	zinc finger protein 386 (Kruppel-like)	0.774593	0.07709	0.2379
75571	Spata9	spermatogenesis associated 9	0.774593	0.1256	0.3225
207212	Arhgef17	Rho guanine nucleotide exchange factor (GEF) 17	0.774593	0.0006041	0.01344
223827	Gxylt1	glucoside xylosyltransferase 1	0.774593	0.001718	0.0251
244349	Myst3	MYST histone acetyltransferase (monocytic leukemia) 3	0.774593	0.004593	0.04469
320644	C130030K03Rik	RIKEN cDNA C130030K03 gene	0.774593	0.0478	0.1769
331474	Rgag4	retrotransposon gag domain containing 4	0.774593	0.006972	0.05654
11306	Abcb7	ATP-binding cassette, sub-family B (MDR/TAP), member 7	0.773994	0.07994	0.2433
12404	Cbln1	cerebellin 1 precursor protein	0.773994	0.00506	0.04703
19025	Ctsa	cathepsin A	0.773994	0.01866	0.1016
19746	Rhd	Rh blood group, D antigen	0.773994	0.3222	0.5585
63993	Slc5a7	solute carrier family 5 (choline transporter), member 7	0.773994	0.01585	0.09248
104601	Mycbpap	MYCBP associated protein	0.773994	0.002493	0.03092
109904	Mcf2	mcf.2 transforming sequence	0.773994	0.0134	0.08343
211151	Churc1	churchill domain containing 1	0.773994	0.001937	0.02682
234857	Spire2	spire homolog 2 (Drosophila)	0.773994	0.07816	0.2397
319535	Zfp182	zinc finger protein 182	0.773994	0.07379	0.2318
320563	Islr2	immunoglobulin superfamily containing leucine-rich repeat 2	0.773994	0.001808	0.02562
21944	Tnfsf12	tumor necrosis factor (ligand) superfamily, member 12	0.773395	0.21	0.437
56427	Tubd1	tubulin, delta 1	0.773395	0.04127	0.1625
78282	5330439K02Rik	RIKEN cDNA 5330439K02 gene	0.773395	0.02957	0.1343
108699	Chn1	chimerin (chimaerin) 1	0.773395	0.005522	0.0496
207615	Wdr37	WD repeat domain 37	0.773395	0.01212	0.07833
245468	Pnma3	paraneoplastic antigen MA3	0.773395	0.03523	0.1484
269784	Cntn4	contactin 4	0.773395	0.05303	0.1873
627939	Gm6813	predicted gene 6813	0.773395	0.0115	0.07605
16917	Lmx1b	LIM homeobox transcription factor 1 beta	0.772798	0.01268	0.08069
20401	Sh3bp1	SH3-domain binding protein 1	0.772798	0.02197	0.1123
20451	St8sia3	ST8 alpha-N-acetyl-neuraminide alpha-2,8-sialyltransferase 3	0.772798	0.002992	0.03419
67492	Anubl1	AN1, ubiquitin-like, homolog (Xenopus laevis)	0.772798	0.08208	0.2469
70450	Unc13d	unc-13 homolog D (C. elegans)	0.772798	0.004025	0.04108
76357	Trmt5	TRM5 tRNA methyltransferase 5 homolog (S. cerevisiae)	0.772798	0.008958	0.0655
78933	Agbl4	ATP/GTP binding protein-like 4	0.772798	0.2489	0.4832
170676	Peg10	paternally expressed 10	0.772798	0.3371	0.5727
232217	4933427D06Rik	RIKEN cDNA 4933427D06 gene	0.772798	0.09516	0.2712
244911	C2cd4a	C2 calcium-dependent domain containing 4A	0.772798	0.08095	0.2448
269713	Clip2	CAP-GLY domain containing linker protein 2	0.772798	0.1166	0.3082
319477	6030419C18Rik	RIKEN cDNA 6030419C18 gene	0.772798	0.06024	0.2038
381650	Thap6	THAP domain containing 6	0.772798	0.0004333	0.01101
14688	Gnb1	guanine nucleotide binding protein (G protein), beta 1	0.772201	0.009747	0.06883
27390	Mmel1	membrane metallo-endopeptidase-like 1	0.772201	0.004685	0.04508
70113	Odf3b	outer dense fiber of sperm tails 3B	0.772201	0.02956	0.1343
71302	Arhgap26	Rho GTPase activating protein 26	0.772201	0.2585	0.4937
109934	Abr	active BCR-related gene	0.772201	0.04435	0.1694
207425	Wdr11	WD repeat domain 11	0.772201	0.07849	0.2401

209707	Lcorl	ligand dependent nuclear receptor corepressor-like	0.772201	0.03167	0.1399
218440	Ankrd34b	ankyrin repeat domain 34B	0.772201	0.1122	0.3006
240880	Scyl3	SCY1-like 3 (S. cerevisiae)	0.772201	0.003531	0.03789
373864	Col27a1	collagen, type XXVII, alpha 1	0.772201	0.01022	0.07112
383563	Gpr25	G protein-coupled receptor 25	0.772201	0.4989	0.7135
11418	Accn1	amiloride-sensitive cation channel 1, neuronal (degenerin)	0.771605	0.001348	0.0215
14407	Gabrg3	gamma-aminobutyric acid (GABA) A receptor, subunit gamma 3	0.771605	0.2647	0.5006
18286	Odf2	outer dense fiber of sperm tails 2	0.771605	0.01364	0.08441
21375	Tbr1	T-box brain gene 1	0.771605	0.01071	0.07301
69564	Itgb1bp3	integrin beta 1 binding protein 3	0.771605	0.08934	0.2612
71302	Arhgap26	Rho GTPase activating protein 26	0.771605	0.003779	0.03935
75873	4930568B11Rik	RIKEN cDNA 4930568B11 gene	0.771605	0.0381	0.1554
76157	Slc35d3	solute carrier family 35, member D3	0.771605	0.02847	0.1313
217734	Pomt2	protein-O-mannosyltransferase 2	0.771605	0.0029	0.03363
226182	Taf5	TAF5 RNA polymerase II, TATA box binding protein (TBP)-associated factor	0.771605	0.01221	0.07866
320368	A730063M14Rik	RIKEN cDNA A730063M14 gene	0.771605	0.05984	0.2029
12369	Casp7	caspase 7	0.77101	0.0006851	0.0144
13824	Epb4.1l4a	erythrocyte protein band 4.1-like 4a	0.77101	0.05372	0.189
16658	Mafb	v-maf musculoaponeurotic fibrosarcoma oncogene family, protein B (avian)	0.77101	0.006776	0.0557
18536	Pcm1	pericentriolar material 1	0.77101	0.01979	0.1054
19879	Slc22a8	solute carrier family 22 (organic anion transporter), member 8	0.77101	0.2468	0.4809
50490	Nox4	NADPH oxidase 4	0.77101	0.3188	0.5554
66673	Sorcs3	sortilin-related VPS10 domain containing receptor 3	0.77101	0.0009105	0.01702
67065	Polr3d	polymerase (RNA) III (DNA directed) polypeptide D	0.77101	0.01228	0.07892
69602	Otop3	otopetrin 3	0.77101	0.02567	0.1233
70713	Gpr137c	G protein-coupled receptor 137C	0.77101	0.1076	0.2932
80877	Lrba	LPS-responsive beige-like anchor	0.77101	0.01049	0.07219
140475	Bsnd	Bartter syndrome, infantile, with sensorineural deafness (Barttin)	0.77101	0.04546	0.1718
228003	Kbtbd10	kelch repeat and BTB (POZ) domain containing 10	0.77101	0.06096	0.2053
231002	Plekhn1	pleckstrin homology domain containing, family N member 1	0.77101	0.07013	0.2243
269642	Nat8l	N-acetyltransferase 8-like	0.77101	0.007233	0.05791
353047	Plekhm1	pleckstrin homology domain containing, family M (with RUN domain) member 1	0.77101	0.009991	0.07002
11790	Speg	SPEG complex locus	0.770416	0.03389	0.1456
18639	Pfkfb1	6-phosphofructo-2-kinase/fructose-2,6-biphosphatase 1	0.770416	0.007635	0.05983
27029	Sgsh	N-sulfoglucosamine sulfohydrolase (sulfamidase)	0.770416	0.1323	0.3328
66411	Tbcb	tubulin folding cofactor B	0.770416	0.01596	0.0926
76376	Slc24a2	solute carrier family 24 (sodium/potassium/calcium exchanger), member 2	0.770416	0.1934	0.4166
104252	Cdc42ep2	CDC42 effector protein (Rho GTPase binding) 2	0.770416	0.02416	0.1186
237860	Ssh2	slingshot homolog 2 (Drosophila)	0.770416	0.1174	0.3094
320709	Tmem117	transmembrane protein 117	0.770416	0.01401	0.08574
383196	Gm5222	predicted gene 5222	0.770416	0.2348	0.4666
11831	Aqp6	aquaporin 6	0.769823	0.005038	0.04691
12716	Ckmt1	creatine kinase, mitochondrial 1, ubiquitous	0.769823	0.002513	0.03106
13446	Doc2a	double C2, alpha	0.769823	0.007505	0.05922
17760	Mtap6	microtubule-associated protein 6	0.769823	0.003594	0.03829
66459	Pigy	phosphatidylinositol glycan anchor biosynthesis, class Y	0.769823	0.0102	0.07106
67036	Mrpl45	mitochondrial ribosomal protein L45	0.769823	0.001584	0.02378
71997	1500002O20Rik	RIKEN cDNA 1500002O20 gene	0.769823	0.003093	0.03492
73724	Mcee	methylmalonyl CoA epimerase	0.769823	0.03113	0.1382

75040	Efcab10	EF-hand calcium binding domain 10	0.769823	0.1731	0.3892
227659	Slc2a6	solute carrier family 2 (facilitated glucose transporter), member 6	0.769823	0.02435	0.1192
329504	Lcmt2	leucine carboxyl methyltransferase 2	0.769823	0.001294	0.02091
380714	Rph3al	rabphilin 3A-like (without C2 domains)	0.769823	0.07623	0.2365
12336	Capns1	calpain, small subunit 1	0.769231	0.01964	0.1049
26561	Mmp23	matrix metallopeptidase 23	0.769231	0.03741	0.1533
76498	Paqr4	progestin and adipoQ receptor family member IV	0.769231	0.00137	0.02171
103765	Tmem17	transmembrane protein 17	0.769231	0.004062	0.0413
232664	Ccdc136	coiled-coil domain containing 136	0.769231	0.08163	0.2461
245886	Ankrd27	ankyrin repeat domain 27 (VPS9 domain)	0.769231	0.008444	0.06311
402762	A630039O03Rik	RIKEN cDNA A630039O03 gene	0.769231	0.03311	0.1436
12307	Calb1	calbindin 1	0.76864	0.001342	0.02145
18227	Nr4a2	nuclear receptor subfamily 4, group A, member 2	0.76864	0.06075	0.2048
20479	Vps4b	vacuolar protein sorting 4b (yeast)	0.76864	0.02985	0.1351
51801	Ramp1	receptor (calcitonin) activity modifying protein 1	0.76864	0.2614	0.4965
74309	Osbp2	oxysterol binding protein 2	0.76864	0.01505	0.08958
77864	Ypel2	yippee-like 2 (Drosophila)	0.76864	0.0009666	0.01756
140580	Elmo1	engulfment and cell motility 1, ced-12 homolog (C. elegans)	0.76864	0.04195	0.1639
230082	Nol6	nucleolar protein family 6 (RNA-associated)	0.76864	0.007689	0.06005
235044	BC018242	cDNA sequence BC018242	0.76864	0.09781	0.2759
280287	Kiss1	KiSS-1 metastasis-suppressor	0.76864	0.07801	0.2394
100503726	LOC100503726	hypothetical protein LOC100503726	0.76864	0.07673	0.2372
12662	Chm	choroidermia	0.768049	0.003649	0.03863
17035	Lxn	latexin	0.768049	0.04693	0.1753
17263	Meg3	maternally expressed 3	0.768049	0.03791	0.1548
18526	Pcdh10	protocadherin 10	0.768049	0.005298	0.04847
78833	Gins3	GINS complex subunit 3 (Psf3 homolog)	0.768049	0.06633	0.2166
225160	Thoc1	THO complex 1	0.768049	0.0351	0.1484
230996	9430015G10Rik	RIKEN cDNA 9430015G10 gene	0.768049	0.03065	0.1371
241494	Zfp385b	zinc finger protein 385B	0.768049	0.06498	0.2135
268670	Zfp759	zinc finger protein 759	0.768049	0.003146	0.03525
330788	Zfp866	zinc finger protein 866	0.768049	0.001027	0.01795
353188	Adam32	a disintegrin and metallopeptidase domain 32	0.768049	0.02295	0.1153
12337	Capn5	calpain 5	0.76746	0.008586	0.06377
21849	Trim28	tripartite motif-containing 28	0.76746	0.08807	0.2588
26879	B3galnt1	UDP-GalNAc:betaGlcNAc beta 1,3-galactosaminyltransferase, polypeptide 1	0.76746	0.01048	0.07219
68817	Ddi2	DNA-damage inducible protein 2	0.76746	0.0297	0.1348
140494	Atp6v0a4	ATPase, H+ transporting, lysosomal V0 subunit A4	0.76746	0.1163	0.3077
212943	Fam46a	family with sequence similarity 46, member A	0.76746	0.002137	0.02807
217431	Nol10	nucleolar protein 10	0.76746	0.08529	0.2533
328789	Lhfpl5	lipoma HMGIC fusion partner-like 5	0.76746	0.02377	0.1175
331461	Il1rapl1	interleukin 1 receptor accessory protein-like 1	0.76746	0.4077	0.637
402728	Pax6os1	Pax6 opposite strand transcript 1	0.76746	0.02191	0.1121
13603	Opn3	opsin 3	0.766871	0.03726	0.153
16765	Stmn1	stathmin 1	0.766871	0.02295	0.1153
21334	Tac2	tachykinin 2	0.766871	0.003989	0.04076
63993	Slc5a7	solute carrier family 5 (choline transporter), member 7	0.766871	0.01527	0.09044
68267	Slc25a22	solute carrier family 25 (mitochondrial carrier, glutamate), member 22	0.766871	0.3885	0.6203
76832	Hyls1	hydrolethalus syndrome 1	0.766871	0.06141	0.2061

102595	Plekho2	pleckstrin homology domain containing, family O member 2	0.766871	0.03009	0.1358
231042	Nupl2	nucleoporin like 2	0.766871	0.01893	0.1025
239650	AI836003	expressed sequence AI836003	0.766871	0.0892	0.261
319190	Hist2h2be	histone cluster 2, H2be	0.766871	0.007023	0.05676
544678	2010015L04Rik	RIKEN cDNA 2010015L04 gene	0.766871	0.02054	0.1076
28240	Trpm2	transient receptor potential cation channel, subfamily M, member 2	0.766284	0.03648	0.1512
66101	Ppih	peptidyl prolyl isomerase H	0.766284	0.06411	0.2117
224344	Rbm11	RNA binding motif protein 11	0.766284	0.0918	0.2655
243328	Slc29a4	solute carrier family 29 (nucleoside transporters), member 4	0.766284	0.02161	0.1113
333329	Cngb1	cyclic nucleotide gated channel beta 1	0.766284	0.0007507	0.01515
333329	Cngb1	cyclic nucleotide gated channel beta 1	0.766284	0.002438	0.03058
433387	Rpl21-ps1	ribosomal protein 21, pseudogene 1	0.766284	0.05072	0.1828
627939	Gm6813	predicted gene 6813	0.766284	0.003146	0.03525
11766	Ap1g2	adaptor protein complex AP-1, gamma 2 subunit	0.765697	0.01454	0.08767
80982	9930013L23Rik	RIKEN cDNA 9930013L23 gene	0.765697	0.3611	0.5958
239420	Csmd3	CUB and Sushi multiple domains 3	0.765697	0.2323	0.4638
239985	Arid1b	AT rich interactive domain 1B (SWI-like)	0.765697	0.06647	0.2169
330070	Gm5107	predicted gene 5107	0.765697	0.1808	0.3994
620760	2900079G21Rik	RIKEN cDNA 2900079G21 gene	0.765697	0.2301	0.4612
100040462	Mndal	myeloid nuclear differentiation antigen like	0.765697	0.01879	0.1021
11863	Arnt	aryl hydrocarbon receptor nuclear translocator	0.765111	0.0163	0.09362
12311	Calcr	calcitonin receptor	0.765111	0.152	0.361
14573	Gdnf	glial cell line derived neurotrophic factor	0.765111	0.1296	0.3286
19733	Rgn	regucalcin	0.765111	0.04696	0.1753
26445	Psmb2	proteasome (prosome, macropain) subunit, beta type 2	0.765111	0.01734	0.09732
58243	Nap1l5	nucleosome assembly protein 1-like 5	0.765111	0.003519	0.03783
67958	2610101N10Rik	RIKEN cDNA 2610101N10 gene	0.765111	0.02184	0.112
75352	4930550L24Rik	RIKEN cDNA 4930550L24 gene	0.765111	0.1139	0.3037
208111	C330019L16Rik	RIKEN cDNA C330019L16 gene	0.765111	0.2202	0.45
240514	Ccdc85b	coiled-coil domain containing 85B	0.765111	0.1931	0.4161
13483	Dpp6	dipeptidylpeptidase 6	0.764526	0.007405	0.05875
21916	Tmod1	tropomodulin 1	0.764526	0.01556	0.09149
59025	Usp14	ubiquitin specific peptidase 14	0.764526	0.005672	0.05039
80838	Hist1h1a	histone cluster 1, H1a	0.764526	0.1654	0.3792
216011	Lrrc20	leucine rich repeat containing 20	0.764526	0.03417	0.1462
225898	Eml3	echinoderm microtubule associated protein like 3	0.764526	0.001793	0.02551
234358	Zfp930	zinc finger protein 930	0.764526	0.2281	0.459
503690	A930005G22Rik	RIKEN cDNA A930005G22 gene	0.764526	0.0006036	0.01344
54601	Foxo4	forkhead box O4	0.763942	0.253	0.4876
75180	4930538K18Rik	RIKEN cDNA 4930538K18 gene	0.763942	0.2238	0.4543
77996	D730039F16Rik	RIKEN cDNA D730039F16 gene	0.763942	0.001831	0.02575
234889	Gucy1a2	guanylate cyclase 1, soluble, alpha 2	0.763942	0.05417	0.1901
239618	Pdzrn4	PDZ domain containing RING finger 4	0.763942	0.02155	0.1111
100041677	Gm13157	predicted gene 13157	0.763942	0.2164	0.4452
100042480	Nhsl2	NHS-like 2	0.763942	0.1139	0.3037
11945	Atp4b	ATPase, H+/K+ exchanging, beta polypeptide	0.763359	0.4027	0.6326
16431	Itm2a	integral membrane protein 2A	0.763359	0.03079	0.1374
18196	Nsg1	neuron specific gene family member 1	0.763359	0.01841	0.1007
27220	Cartpt	CART prepropeptide	0.763359	0.003386	0.03695

69952	2810011L19Rik	RIKEN cDNA 2810011L19 gene	0.763359	0.05942	0.2022
99946	6720422M22Rik	RIKEN cDNA 6720422M22 gene	0.763359	0.02681	0.1267
233752	Insc	inscuteable homolog (Drosophila)	0.763359	0.03077	0.1374
234138	BC019943	cDNA sequence BC019943	0.763359	0.03524	0.1484
239552	Apol8	apolipoprotein L 8	0.763359	0.007095	0.05707
243911	Kirrel2	kin of IRRE like 2 (Drosophila)	0.763359	0.006667	0.05528
14664	Slc6a9	solute carrier family 6 (neurotransmitter transporter, glycine), member 9	0.762777	0.02879	0.1322
15251	Hif1a	hypoxia inducible factor 1, alpha subunit	0.762777	0.05316	0.1876
16363	Irf2	interferon regulatory factor 2	0.762777	0.03845	0.1561
56527	Mast1	microtubule associated serine/threonine kinase 1	0.762777	0.004915	0.04618
68014	Zwilch	Zwilch, kinetochore associated, homolog (Drosophila)	0.762777	0.005917	0.05177
69863	Ttc39b	tetratricopeptide repeat domain 39B	0.762777	0.04829	0.1778
72716	2810047C21Rik1	RIKEN cDNA 2810047C21 gene 1	0.762777	0.0141	0.08608
78553	E130118H10Rik	RIKEN cDNA E130118H10 gene	0.762777	0.02825	0.1308
317717	Sec22a	SEC22 vesicle trafficking protein homologe A (S. cerevisiae)	0.762777	0.006261	0.05321
16372	Irx2	Iroquois related homeobox 2 (Drosophila)	0.762195	0.01726	0.09712
20419	Shcbp1	Shc SH2-domain binding protein 1	0.762195	0.05381	0.1893
68556	Uckl1	uridine-cytidine kinase 1-like 1	0.762195	0.02402	0.1183
209601	4922501L14Rik	RIKEN cDNA 4922501L14 gene	0.762195	0.008623	0.06396
225997	Trpm6	transient receptor potential cation channel, subfamily M, member 6	0.762195	0.1627	0.375
246104	Rhbdl3	rhomboid, veinlet-like 3 (Drosophila)	0.762195	0.02342	0.1168
317755	Zar1	zygote arrest 1	0.762195	0.02177	0.1119
320111	Prr18	proline rich region 18	0.762195	0.03204	0.1408
14917	Gucy2c	guanylate cyclase 2c	0.761615	0.3671	0.6024
114564	Csprs	component of Sp100-rs	0.761615	0.1724	0.3882
140917	Dclre1b	DNA cross-link repair 1B, PSO2 homolog (S. cerevisiae)	0.761615	0.05259	0.1864
232440	H2afj	H2A histone family, member J	0.761615	0.0003406	0.009697
380669	Lin28b	lin-28 homolog B (C. elegans)	0.761615	0.04831	0.1778
14004	Chchd2	coiled-coil-helix-coiled-coil-helix domain containing 2	0.761035	0.03597	0.1499
64074	Smoc2	SPARC related modular calcium binding 2	0.761035	0.006972	0.05654
67809	Fam82a2	family with sequence similarity 82, member A2	0.761035	0.01273	0.08085
239017	Ogdhl	oxoglutarate dehydrogenase-like	0.761035	0.1018	0.2826
384261	Gm5296	predicted gene 5296	0.761035	0.002005	0.02724
408067	Zfp874b	zinc finger protein 874b	0.761035	0.1556	0.3654
654803	B130055M24Rik	RIKEN cDNA B130055M24 gene	0.761035	0.02075	0.1084
73813	Fam83e	family with sequence similarity 83, member E	0.760456	0.09077	0.2639
192196	Luc7l2	LUC7-like 2 (S. cerevisiae)	0.760456	0.01177	0.07703
240064	Zfp799	zinc finger protein 799	0.760456	0.03852	0.1562
241688	6330439K17Rik	RIKEN cDNA 6330439K17 gene	0.760456	0.006127	0.05263
320209	Ddx11	DEAD/H (Asp-Glu-Ala-Asp/His) box polypeptide 11 (CHL1-like helicase homolog, S. cerevisiae)	0.760456	0.07146	0.2269
13371	Dio2	deiodinase, iodothyronine, type II	0.759878	0.08913	0.261
17436	Me1	malic enzyme 1, NADP(+)-dependent, cytosolic	0.759878	0.01163	0.07651
18016	Nf2	neurofibromatosis 2	0.759878	0.08943	0.2613
50709	Hist1h1e	histone cluster 1, H1e	0.759878	0.1193	0.3125
54006	Deaf1	deformed epidermal autoregulatory factor 1 (Drosophila)	0.759878	0.02843	0.1312
55985	Cxcl13	chemokine (C-X-C motif) ligand 13	0.759878	0.04249	0.165
66011	Ranbp17	RAN binding protein 17	0.759878	0.1343	0.3353
233065	Alkbh6	alkB, alkylation repair homolog 6 (E. coli)	0.759878	0.003106	0.03497
235599	6430571L13Rik	RIKEN cDNA 6430571L13 gene	0.759878	0.006132	0.05265

269615	Plch2	phospholipase C, eta 2	0.759878	0.1716	0.3873
18203	Ntan1	N-terminal Asn amidase	0.759301	0.001357	0.02157
66797	Cntnap2	contactin associated protein-like 2	0.759301	0.006074	0.05248
69732	2410018L13Rik	RIKEN cDNA 2410018L13 gene	0.759301	0.01449	0.08758
74722	4930517J16Rik	RIKEN cDNA 4930517J16 gene	0.759301	0.0117	0.07682
75394	0610040F04Rik	RIKEN cDNA 0610040F04 gene	0.759301	0.01065	0.07272
77938	Fam53b	family with sequence similarity 53, member B	0.759301	0.0331	0.1436
218030	Pou6f2	POU domain, class 6, transcription factor 2	0.759301	0.0664	0.2167
228765	Sdcbp2	syndecan binding protein (syntenin) 2	0.759301	0.107	0.2921
70956	Tex19.2	testis expressed gene 19.2	0.758725	0.3653	0.601
102941	B630019K06Rik	RIKEN cDNA B630019K06 gene	0.758725	0.001771	0.02542
225115	Svil	supervillin	0.758725	0.001583	0.02378
320678	Iffo1	intermediate filament family orphan 1	0.758725	0.0004589	0.0113
13411	Dnahc11	dynein, axonemal, heavy chain 11	0.75815	0.0381	0.1554
15216	Hfe	hemochromatosis	0.75815	0.005126	0.0474
22145	Tuba4a	tubulin, alpha 4A	0.75815	0.04505	0.1711
54525	Syt7	synaptotagmin VII	0.75815	0.01414	0.08626
72275	2200002D01Rik	RIKEN cDNA 2200002D01 gene	0.75815	0.02924	0.1335
78929	Polr3h	polymerase (RNA) III (DNA directed) polypeptide H	0.75815	0.08057	0.2441
107166	AI415730	expressed sequence AI415730	0.75815	0.002581	0.0315
208982	Hmgcll1	3-hydroxymethyl-3-methylglutaryl-Coenzyme A lyase-like 1	0.75815	0.01129	0.07532
241589	D430041D05Rik	RIKEN cDNA D430041D05 gene	0.75815	0.01592	0.09258
319622	Itpripl2	inositol 1,4,5-triphosphate receptor interacting protein-like 2	0.75815	0.004712	0.04511
667370	I830012O16Rik	RIKEN cDNA I830012O16 gene	0.75815	0.2575	0.4926
11551	Adra2a	adrenergic receptor, alpha 2a	0.757576	0.001294	0.02091
13809	Enpep	glutamyl aminopeptidase	0.757576	0.05537	0.1925
20353	Sema4c	sema domain, immunoglobulin domain (Ig), transmembrane domain (TM) and short cytoplasmic domain, (semaphorin) 4C	0.757576	0.02548	0.1226
58804	Cdc42ep5	CDC42 effector protein (Rho GTPase binding) 5	0.757576	0.0703	0.2246
59056	Evc	Ellis van Creveld gene homolog (human)	0.757576	0.03774	0.1542
66259	Camk2n1	calcium/calmodulin-dependent protein kinase II inhibitor 1	0.757576	0.1561	0.3659
67374	Jam2	junction adhesion molecule 2	0.757576	0.0006878	0.01445
71091	Cdkl1	cyclin-dependent kinase-like 1 (CDC2-related kinase)	0.757576	0.01342	0.08348
78249	Gpr115	G protein-coupled receptor 115	0.757576	0.02688	0.1268
80334	Kcnip4	Kv channel interacting protein 4	0.757576	0.0004217	0.01086
233230	Mrgprb4	MAS-related GPR, member B4	0.757576	0.6478	0.8166
238831	Ppwd1	peptidylprolyl isomerase domain and WD repeat containing 1	0.757576	0.007593	0.05963
381217	Fam189a2	family with sequence similarity 189, member A2	0.757576	0.03103	0.1379
11736	Ankfy1	ankyrin repeat and FYVE domain containing 1	0.757002	0.01885	0.1023
16591	Kl	klotho	0.757002	0.001448	0.02247
17833	Muc5ac	mucin 5, subtypes A and C, tracheobronchial/gastric	0.757002	0.2371	0.4693
57911	Gsdma	gasdermin A	0.757002	0.008246	0.06224
68428	Steap3	STEAP family member 3	0.757002	0.01251	0.08
68808	1110046J04Rik	RIKEN cDNA 1110046J04 gene	0.757002	0.01616	0.09307
71369	Krtap16-10	keratin associated protein 16-10	0.757002	0.1252	0.3221
107449	Unc5b	unc-5 homolog B (C. elegans)	0.757002	0.03119	0.1384
212996	Wbscr17	Williams-Beuren syndrome chromosome region 17 homolog (human)	0.757002	0.04329	0.167
225392	Rell2	RELT-like 2	0.757002	0.005609	0.05007
232680	Cpa2	carboxypeptidase A2, pancreatic	0.757002	0.004041	0.04113
270086	Ogfod1	2-oxoglutarate and iron-dependent oxygenase domain containing 1	0.757002	0.05709	0.1963

19895	Rpia	ribose 5-phosphate isomerase A	0.75643	0.0003501	0.009863
22341	Vegfc	vascular endothelial growth factor C	0.75643	0.004705	0.04511
67654	4930558C23Rik	RIKEN cDNA 4930558C23 gene	0.75643	0.06985	0.2237
74359	4931414P19Rik	RIKEN cDNA 4931414P19 gene	0.75643	0.05121	0.1839
217303	Cd300a	CD300A antigen	0.75643	0.372	0.6068
331513	Gm9927	predicted gene 9927	0.75643	0.03458	0.1473
14163	Fgd1	FYVE, RhoGEF and PH domain containing 1	0.755858	0.03168	0.1399
71971	Zswim1	zinc finger, SWIM domain containing 1	0.755858	0.05076	0.1828
76406	1700019B03Rik	RIKEN cDNA 1700019B03 gene	0.755858	0.1613	0.3732
78088	Ankrd56	ankyrin repeat domain 56	0.755858	0.16	0.3716
193003	Pirt	phosphoinositide-interacting regulator of transient receptor potential channels	0.755858	0.04111	0.1622
211712	Pcdh9	protocadherin 9	0.755858	0.005107	0.04732
213819	Casd1	CAS1 domain containing 1	0.755858	0.3445	0.5799
338351	Akap17b	A kinase (PRKA) anchor protein 17B	0.755858	0.004219	0.04227
381409	Cdh26	cadherin-like 26	0.755858	0.1118	0.3
384071	Slc25a34	solute carrier family 25, member 34	0.755858	0.2332	0.4644
666244	Tmsb15b1	thymosin beta 15b1	0.755858	0.00403	0.04108
14859	Gsta3	glutathione S-transferase, alpha 3	0.755287	0.008023	0.06137
57248	Ly6i	lymphocyte antigen 6 complex, locus I	0.755287	0.2336	0.4649
71602	Myo1e	myosin IE	0.755287	0.03206	0.1408
71721	Fam13c	family with sequence similarity 13, member C	0.755287	0.008199	0.06206
74634	4930423D22Rik	RIKEN cDNA 4930423D22 gene	0.755287	0.1903	0.4122
76080	Ttpal	tocopherol (alpha) transfer protein-like	0.755287	0.0003957	0.01045
236790	Ddx26b	DEAD/H (Asp-Glu-Ala-Asp/His) box polypeptide 26B	0.755287	0.002473	0.03081
242297	Fam110b	family with sequence similarity 110, member B	0.755287	0.005724	0.05058
59024	Med12	mediator of RNA polymerase II transcription, subunit 12 homolog (yeast)	0.754717	0.02063	0.108
72413	Kcnmb2	potassium large conductance calcium-activated channel, subfamily M, beta member 2	0.754717	0.05409	0.1899
75555	1700020D05Rik	RIKEN cDNA 1700020D05 gene	0.754717	0.00547	0.04934
224813	Gm88	predicted gene 88	0.754717	0.004937	0.04629
319556	A330041J22Rik	RIKEN cDNA A330041J22 gene	0.754717	0.1961	0.4201
622434	Arhgef26	Rho guanine nucleotide exchange factor (GEF) 26	0.754717	0.001057	0.0183
12351	Car4	carbonic anhydrase 4	0.754148	0.3273	0.5636
15114	Hap1	huntingtin-associated protein 1	0.754148	0.002936	0.03379
23859	Dlg2	discs, large homolog 2 (Drosophila)	0.754148	0.0001791	0.006624
23908	Hs2st1	heparan sulfate 2-O-sulfotransferase 1	0.754148	0.1897	0.4114
54652	Cacna1f	calcium channel, voltage-dependent, alpha 1F subunit	0.754148	0.01114	0.07475
209584	Tyw3	tRNA-yW synthesizing protein 3 homolog (S. cerevisiae)	0.754148	0.01954	0.1047
330409	Cecr2	cat eye syndrome chromosome region, candidate 2 homolog (human)	0.754148	0.02926	0.1336
13837	Epha3	Eph receptor A3	0.75358	0.01194	0.07773
14421	B4galnt1	beta-1,4-N-acetyl-galactosaminyl transferase 1	0.75358	0.08081	0.2445
20562	Slit1	slit homolog 1 (Drosophila)	0.75358	0.006211	0.05304
56399	Akap8	A kinase (PRKA) anchor protein 8	0.75358	0.01804	0.09937
76357	Trmt5	TRM5 tRNA methyltransferase 5 homolog (S. cerevisiae)	0.75358	0.002392	0.03026
78309	Cul9	cullin 9	0.75358	0.01213	0.07836
80893	Tmprss5	transmembrane protease, serine 5 (spinesin)	0.75358	0.1239	0.3204
93961	B3galt5	UDP-Gal:betaGlcNAc beta 1,3-galactosyltransferase, polypeptide 5	0.75358	0.003024	0.03442
110542	Amhr2	anti-Mullerian hormone type 2 receptor	0.75358	0.04639	0.1741
117160	Ttyh2	tweety homolog 2 (Drosophila)	0.75358	0.03283	0.1428
227325	Dner	delta/notch-like EGF-related receptor	0.75358	0.01028	0.0714

641387	1700120B22Rik	RIKEN cDNA 1700120B22 gene	0.75358	0.2141	0.4425
18846	Plxna3	plexin A3	0.753012	0.01669	0.09502
77117	Zfp934	zinc finger protein 934	0.753012	0.04761	0.1765
100072	Camta1	calmodulin binding transcription activator 1	0.753012	0.0009138	0.01702
381072	Abca17	ATP-binding cassette, sub-family A (ABC1), member 17	0.753012	0.2835	0.5193
381835	Gm1078	predicted gene 1078	0.753012	0.01813	0.09971
627191	Tmem90a	transmembrane protein 90a	0.753012	0.03248	0.1419
640502	LOC640502	UDP-N-acetylhexosamine pyrophosphorylase-like	0.753012	0.003609	0.03841
100042948	Gm15760	mitochondrial ribosomal protein S18B pseudogene	0.753012	0.5293	0.7358
20452	St8sia4	ST8 alpha-N-acetyl-neuraminide alpha-2,8-sialyltransferase 4	0.752445	0.07339	0.231
30952	Cngb3	cyclic nucleotide gated channel beta 3	0.752445	0.01398	0.08567
331188	Zfp781	zinc finger protein 781	0.752445	0.143	0.3479
20028	Pdc	phosducin	0.75188	0.01636	0.09371
56229	Thsd1	thrombospondin, type I, domain 1	0.75188	0.004632	0.04474
74855	4930417G10Rik	RIKEN cDNA 4930417G10 gene	0.75188	0.03585	0.1497
12850	Coq7	demethyl-Q 7	0.751315	0.005108	0.04732
17540	Mrvi1	MRV integration site 1	0.751315	0.02189	0.1121
72386	2610035D17Rik	RIKEN cDNA 2610035D17 gene	0.751315	0.0257	0.1233
14938	Gzma	granzyme A	0.750751	0.4678	0.6897
26877	B3galt1	UDP-Gal:betaGlcNAc beta 1,3-galactosyltransferase, polypeptide 1	0.750751	0.002187	0.02851
75813	4930484H19Rik	RIKEN cDNA 4930484H19 gene	0.750751	0.06989	0.2237
242202	Pde5a	phosphodiesterase 5A, cGMP-specific	0.750751	0.1873	0.4079
328309	Gm9776	predicted gene 9776	0.750751	0.2974	0.5334
15552	Htr1d	5-hydroxytryptamine (serotonin) receptor 1D	0.750188	0.01514	0.08983
20508	Slc18a3	solute carrier family 18 (vesicular monoamine), member 3	0.750188	0.007454	0.05899
70918	Nsun7	NOL1/NOP2/Sun domain family, member 7	0.750188	0.04154	0.1631
330463	Zfp78	zinc finger protein 78	0.750188	0.0002357	0.007786
546160	Fbxw25	F-box and WD-40 domain protein 25	0.750188	0.4344	0.6613
66569	Gdpd1	glycerophosphodiester phosphodiesterase domain containing 1	0.749625	0.0153	0.09055
75429	Fam183b	family with sequence similarity 183, member B	0.749625	0.03498	0.1483
76156	Fam131b	family with sequence similarity 131, member B	0.749625	0.07405	0.2322
77531	Anks1b	ankyrin repeat and sterile alpha motif domain containing 1B	0.749625	0.04511	0.1712
97455	C78859	expressed sequence C78859	0.749625	0.003622	0.03849
105727	Slc38a1	solute carrier family 38, member 1	0.749625	0.1183	0.3109
116838	Rims2	regulating synaptic membrane exocytosis 2	0.749625	0.007869	0.06092
225600	Pde6a	phosphodiesterase 6A, cGMP-specific, rod, alpha	0.749625	0.007927	0.06104
231602	P2rx2	purinergic receptor P2X, ligand-gated ion channel, 2	0.749625	0.3233	0.5598
232035	Fam190a	family with sequence similarity 190, member A	0.749625	0.0173	0.09722
319893	A230057D06Rik	RIKEN cDNA A230057D06 gene	0.749625	0.06847	0.221
768252	Foxl2os	forkhead box L2 opposite strand transcript	0.749625	0.4295	0.6566
12006	Axin2	axin2	0.749064	0.5373	0.7418
15528	Hspe1	heat shock protein 1 (chaperonin 10)	0.749064	0.0002037	0.007259
19892	Rpe65	retinal pigment epithelium 65	0.749064	0.2704	0.5056
24135	Zfp68	zinc finger protein 68	0.749064	0.03017	0.1361
68999	Anapc10	anaphase promoting complex subunit 10	0.749064	0.002483	0.03083
72780	Rspo3	R-spondin 3 homolog (Xenopus laevis)	0.749064	0.2201	0.45
78749	Filip1l	filamin A interacting protein 1-like	0.749064	0.001749	0.0253
109979	Art3	ADP-ribosyltransferase 3	0.749064	0.07267	0.2294
383563	Gpr25	G protein-coupled receptor 25	0.749064	0.005504	0.04955

654805	F930015N05Rik	RIKEN cDNA F930015N05 gene	0.749064	0.1165	0.308
100101807	1700047I17Rik2	RIKEN cDNA 1700047I17 gene 2	0.749064	0.0281	0.1305
23937	Mab21l2	mab-21-like 2 (C. elegans)	0.748503	0.03575	0.1494
59308	Emcn	endomucin	0.748503	0.09916	0.2779
69449	1700027A15Rik	RIKEN cDNA 1700027A15 gene	0.748503	0.04385	0.1683
69794	1600027J07Rik	RIKEN cDNA 1600027J07 gene	0.748503	0.1141	0.304
227632	Kcnt1	potassium channel, subfamily T, member 1	0.748503	0.001909	0.02661
320095	6430550D23Rik	RIKEN cDNA 6430550D23 gene	0.748503	0.01828	0.1003
433956	Heatr2	HEAT repeat containing 2	0.748503	0.000435	0.01101
626123	9130004C02Rik	RIKEN cDNA 9130004C02 gene	0.748503	0.03845	0.1561
100039888	Gm11223	stathmin 1 pseudogene	0.748503	0.01815	0.09974
100041412	Gm11938	predicted gene 11938	0.748503	0.2589	0.4942
20745	Spock1	sparc/osteonectin, cwcv and kazal-like domains proteoglycan 1	0.747943	0.0177	0.09835
70769	Nolc1	nucleolar and coiled-body phosphoprotein 1	0.747943	0.01341	0.08343
71780	Isyna1	myo-inositol 1-phosphate synthase A1	0.747943	0.00924	0.06654
72371	2210408I21Rik	RIKEN cDNA 2210408I21 gene	0.747943	0.08364	0.2501
215001	Wfikkn1	WAP, FS, Ig, KU, and NTR-containing protein 1	0.747943	0.0328	0.1428
229487	Pet112l	PET112-like (yeast)	0.747943	0.02628	0.1252
319758	Rfx7	regulatory factor X, 7	0.747943	0.03579	0.1495
399101	Snhg3	small nucleolar RNA host gene (non-protein coding) 3	0.747943	0.09612	0.2729
18378	Omp	olfactory marker protein	0.747384	0.1085	0.2946
56846	Necab3	N-terminal EF-hand calcium binding protein 3	0.747384	0.02381	0.1176
72434	Lypd3	Ly6/Plaur domain containing 3	0.747384	0.4483	0.6739
72621	Pdzd11	PDZ domain containing 11	0.747384	0.01682	0.09548
74288	Spem1	sperm maturation 1	0.747384	0.003282	0.03627
83395	Sp6	trans-acting transcription factor 6	0.747384	0.2736	0.5094
214575	Tdrd5	tudor domain containing 5	0.747384	0.0191	0.1031
216848	Chd3	chromodomain helicase DNA binding protein 3	0.747384	0.0005955	0.01337
13592	Ebf2	early B-cell factor 2	0.746826	0.05973	0.2028
16559	Kif17	kinesin family member 17	0.746826	0.02463	0.12
16922	Phyh	phytanoyl-CoA hydroxylase	0.746826	0.0004255	0.01094
59033	Slc4a8	solute carrier family 4 (anion exchanger), member 8	0.746826	0.001369	0.02171
208634	Tspan10	tetraspanin 10	0.746826	0.3341	0.5697
229672	Bcl2l15	BCLl2-like 15	0.746826	0.1733	0.3896
239673	4732456N10Rik	RIKEN cDNA 4732456N10 gene	0.746826	0.3989	0.6294
319670	Eml5	echinoderm microtubule associated protein like 5	0.746826	0.009892	0.06952
319924	Apba1	amyloid beta (A4) precursor protein binding, family A, member 1	0.746826	0.002948	0.03387
330577	Fam154b	family with sequence similarity 154, member B	0.746826	0.0003708	0.01011
100043309	4732414G09Rik	RIKEN cDNA 4732414G09 gene	0.746826	0.02289	0.1152
12316	Aspm	asp (abnormal spindle)-like, microcephaly associated (Drosophila)	0.746269	0.02746	0.1288
13614	Edn1	endothelin 1	0.746269	0.01099	0.0742
16582	Kifc3	kinesin family member C3	0.746269	0.005648	0.05027
20185	Ncor1	nuclear receptor co-repressor 1	0.746269	0.001721	0.0251
27390	Mmel1	membrane metallo-endopeptidase-like 1	0.746269	0.006387	0.0538
74542	9030625N01Rik	RIKEN cDNA 9030625N01 gene	0.746269	0.03608	0.1501
77200	5430403G16Rik	RIKEN cDNA 5430403G16 gene	0.746269	0.0292	0.1334
227933	Ccdc148	coiled-coil domain containing 148	0.746269	0.08786	0.2586
279029	Gm711	predicted gene 711	0.746269	0.07045	0.2249
11754	Aoc3	amine oxidase, copper containing 3	0.745712	0.07049	0.2249

56705	Ranbp9	RAN binding protein 9	0.745712	0.5048	0.7172
76286	1110006E14Rik	RIKEN cDNA 1110006E14 gene	0.745712	0.009957	0.06981
241263	Gpr158	G protein-coupled receptor 158	0.745712	0.01257	0.08025
241656	Pak7	p21 protein (Cdc42/Rac)-activated kinase 7	0.745712	0.01972	0.1051
20199	S100a5	S100 calcium binding protein A5	0.745156	0.1208	0.3149
23850	Pappa2	pappalysin 2	0.745156	0.00212	0.02798
26381	Esrrg	estrogen-related receptor gamma	0.745156	0.001411	0.02219
65256	Asb2	ankyrin repeat and SOCS box-containing 2	0.745156	0.04252	0.1651
72041	Alkbh4	alkB, alkylation repair homolog 4 (E. coli)	0.745156	0.002163	0.02829
74665	Lrrc48	leucine rich repeat containing 48	0.745156	0.004611	0.04473
227620	Uap1l1	UDP-N-acteylglucosamine pyrophosphorylase 1-like 1	0.745156	0.01022	0.07112
100042342	Gm10375	predicted gene 10375	0.745156	0.3718	0.6067
18750	Prkca	protein kinase C, alpha	0.744602	0.003489	0.03763
29862	Ninj2	ninjurin 2	0.744602	0.001959	0.02699
74041	4632434I11Rik	RIKEN cDNA 4632434I11 gene	0.744602	0.03402	0.1459
94090	Trim9	tripartite motif-containing 9	0.744602	0.04655	0.1744
109323	C1qtnf7	C1q and tumor necrosis factor related protein 7	0.744602	0.1004	0.2802
228714	Csrp2bp	cysteine and glycine-rich protein 2 binding protein	0.744602	0.0005279	0.01231
12672	Chrm4	cholinergic receptor, muscarinic 4	0.744048	0.1771	0.3942
17196	Mbp	myelin basic protein	0.744048	0.02321	0.1161
18813	Pa2g4	proliferation-associated 2G4	0.744048	0.0231	0.1158
19094	Mapk11	mitogen-activated protein kinase 11	0.744048	0.01043	0.07196
21886	Tle2	transducin-like enhancer of split 2, homolog of Drosophila E(spl)	0.744048	0.007341	0.05832
56363	Tmeff2	transmembrane protein with EGF-like and two follistatin-like domains 2	0.744048	0.01765	0.09827
70387	Ttc9c	tetratricopeptide repeat domain 9C	0.744048	0.002468	0.03081
71751	Map3k13	mitogen-activated protein kinase kinase kinase 13	0.744048	0.1165	0.308
114874	Ddhd1	DDHD domain containing 1	0.744048	0.0038	0.03947
213989	Tmem82	transmembrane protein 82	0.744048	0.09055	0.2636
243574	Kbtbd8	kelch repeat and BTB (POZ) domain containing 8	0.744048	0.02645	0.1256
18102	Nme1	non-metastatic cells 1, protein (NM23A) expressed in	0.743494	0.0001857	0.006796
19259	Ptpn5	protein tyrosine phosphatase, non-receptor type 5	0.743494	0.002537	0.03119
13401	Dmwd	dystrophia myotonica-containing WD repeat motif	0.742942	0.006849	0.05607
15953	Ifi47	interferon gamma inducible protein 47	0.742942	0.08018	0.2435
17005	Ltk	leukocyte tyrosine kinase	0.742942	0.03526	0.1484
18071	Nhlh1	nescient helix loop helix 1	0.742942	0.04769	0.1766
18676	Phf2	PHD finger protein 2	0.742942	0.02217	0.1129
67472	Mtfr1	mitochondrial fission regulator 1	0.742942	0.00179	0.02551
67586	Ubxn11	UBX domain protein 11	0.742942	0.05521	0.1924
67705	1810058I24Rik	RIKEN cDNA 1810058I24 gene	0.742942	0.2387	0.4712
225994	BC016495	cDNA sequence BC016495	0.742942	0.08216	0.247
246746	Cd300lf	CD300 antigen like family member F	0.742942	0.004341	0.04296
12889	Cplx1	complexin 1	0.74239	0.008118	0.06166
18979	Pon1	paraoxonase 1	0.74239	0.314	0.5505
54216	Pcdh7	protocadherin 7	0.74239	0.07187	0.2279
69002	1500026H17Rik	RIKEN cDNA 1500026H17 gene	0.74239	0.01173	0.07691
104886	Rab15	RAB15, member RAS oncogene family	0.74239	0.1169	0.3088
258718	Olfr513	olfactory receptor 513	0.74239	0.3978	0.6282
19894	Rph3a	rabphilin 3A	0.74184	0.1443	0.3494
27361	Sepx1	selenoprotein X 1	0.74184	0.006337	0.0535

67472	Mtfr1	mitochondrial fission regulator 1	0.74184	0.00134	0.02144
74525	8430419L09Rik	RIKEN cDNA 8430419L09 gene	0.74184	0.001963	0.027
109349	Fam163b	family with sequence similarity 163, member B	0.74184	0.00192	0.0267
100503563	LOC100503563	60S ribosomal protein L21-like	0.74184	0.003923	0.04035
17168	Nprl3	nitrogen permease regulator-like 3 (S. cerevisiae)	0.74129	0.07617	0.2365
17748	Mt1	metallothionein 1	0.74129	0.009221	0.06646
18779	Pla2r1	phospholipase A2 receptor 1	0.74129	0.005254	0.04816
74337	Palm3	paralemmin 3	0.74129	0.003029	0.03442
77795	A930010G16Rik	RIKEN cDNA A930010G16 gene	0.74129	0.03214	0.141
140477	Dmbx1	diencephalon/mesencephalon homeobox 1	0.74129	0.03303	0.1434
381570	Oog2	oogenesin 2	0.74129	0.3211	0.5575
654810	D630032N06Rik	RIKEN cDNA D630032N06 gene	0.74129	0.2918	0.528
18166	Npy1r	neuropeptide Y receptor Y1	0.740741	0.005714	0.05051
50997	Mpp2	membrane protein, palmitoylated 2 (MAGUK p55 subfamily member 2)	0.740741	0.2396	0.4722
69926	Dnahc17	dynein, axonemal, heavy chain 17	0.740741	0.03786	0.1546
74183	2310042D19Rik	RIKEN cDNA 2310042D19 gene	0.740741	0.05857	0.2001
228942	Cbln4	cerebellin 4 precursor protein	0.740741	0.04876	0.1787
241846	Lsm14b	LSM14 homolog B (SCD6, S. cerevisiae)	0.740741	0.02782	0.1297
100043072	A230070E04Rik	RIKEN cDNA A230070E04 gene	0.740741	0.003971	0.04069
20733	Spint2	serine protease inhibitor, Kunitz type 2	0.740192	0.0003474	0.009816
54161	Copg	coatomer protein complex, subunit gamma	0.740192	0.007296	0.05811
74264	1700045I19Rik	ring finger protein 138 pseudogene	0.740192	0.04377	0.1681
74330	Dnajc14	DnaJ (Hsp40) homolog, subfamily C, member 14	0.740192	0.05239	0.186
230822	A330049M08Rik	RIKEN cDNA A330049M08 gene	0.740192	0.04443	0.1695
15467	Eif2ak1	eukaryotic translation initiation factor 2 alpha kinase 1	0.739645	0.07436	0.2328
23856	Dido1	death inducer-obliterator 1	0.739645	0.01421	0.08649
56177	Olfm1	olfactomedin 1	0.739645	0.001655	0.0246
69694	Tatdn1	TatD DNase domain containing 1	0.739645	0.1828	0.4016
72244	1600014C10Rik	RIKEN cDNA 1600014C10 gene	0.739645	0.007364	0.05845
94216	Col4a6	collagen, type IV, alpha 6	0.739645	0.1089	0.2952
231727	B3gnt4	UDP-GlcNAc:betaGal beta-1,3-N-acetylglucosaminyltransferase 4	0.739645	0.05779	0.198
12014	Bach2	BTB and CNC homology 2	0.739098	0.01061	0.07262
59012	Moxd1	monooxygenase, DBH-like 1	0.739098	0.005678	0.05039
60409	Trappc4	trafficking protein particle complex 4	0.739098	0.0004259	0.01094
66119	Tomm6	translocase of outer mitochondrial membrane 6 homolog (yeast)	0.739098	0.0003781	0.0102
74579	4833419E13Rik	RIKEN cDNA 4833419E13 gene	0.739098	0.4381	0.6644
74626	Tmem81	transmembrane protein 81	0.739098	0.04386	0.1683
19218	Ptger3	prostaglandin E receptor 3 (subtype EP3)	0.738552	0.114	0.304
22364	Vpreb3	pre-B lymphocyte gene 3	0.738552	0.02177	0.1119
66965	Ctu2	cytosolic thiouridylase subunit 2 homolog (S. pombe)	0.738552	0.01381	0.08504
67800	Dgat2	diacylglycerol O-acyltransferase 2	0.738552	0.0008069	0.01585
215378	Fam5c	family with sequence similarity 5, member C	0.738552	0.01091	0.07384
277414	Trp53i11	transformation related protein 53 inducible protein 11	0.738552	0.08474	0.2522
399591	Tmsb15l	thymosin beta 15b like	0.738552	0.003027	0.03442
100036524	C430017C20Rik	RIKEN cDNA C430017C20 gene	0.738552	0.06067	0.2047
12121	Bicd1	bicaudal D homolog 1 (Drosophila)	0.738007	0.02738	0.1285
16869	Lhx1	LIM homeobox protein 1	0.738007	0.0129	0.08162
27416	Abcc5	ATP-binding cassette, sub-family C (CFTR/MRP), member 5	0.738007	0.05502	0.1922
71517	9030624J02Rik	RIKEN cDNA 9030624J02 gene	0.738007	0.05708	0.1963

73898	4930429E23Rik	RIKEN cDNA 4930429E23 gene	0.738007	0.006124	0.05263
242642	Hpdl	4-hydroxyphenylpyruvate dioxygenase-like	0.738007	0.01197	0.07782
381073	Npw	neuropeptide W	0.738007	0.1305	0.3302
18578	Pde4b	phosphodiesterase 4B, cAMP specific	0.737463	0.005839	0.0513
67525	Trim62	tripartite motif-containing 62	0.737463	0.03973	0.1588
77929	Yipf6	Yip1 domain family, member 6	0.737463	0.003975	0.04069
224908	Prr22	proline rich 22	0.737463	0.02154	0.1111
328329	Mast4	microtubule associated serine/threonine kinase family member 4	0.737463	0.0161	0.09296
15370	Nr4a1	nuclear receptor subfamily 4, group A, member 1	0.73692	0.07847	0.24
58188	Vstm2b	V-set and transmembrane domain containing 2B	0.73692	0.4295	0.6566
67935	Ces5a	carboxylesterase 5A	0.73692	0.2778	0.5135
107522	Ece2	endothelin converting enzyme 2	0.73692	0.002062	0.02773
230863	Sh2d5	SH2 domain containing 5	0.73692	0.008126	0.0617
319613	Sybu	syntabulin (syntaxin-interacting)	0.73692	0.03906	0.1573
11554	Adrb1	adrenergic receptor, beta 1	0.736377	0.0006628	0.01406
17168	Nprl3	nitrogen permease regulator-like 3 (S. cerevisiae)	0.736377	0.06652	0.2169
67704	1810037I17Rik	RIKEN cDNA 1810037I17 gene	0.736377	0.05397	0.1897
68070	Pdzd2	PDZ domain containing 2	0.736377	0.1706	0.3861
69893	2010305A19Rik	RIKEN cDNA 2010305A19 gene	0.736377	0.007848	0.0609
108124	Napa	N-ethylmaleimide sensitive fusion protein attachment protein alpha	0.736377	0.001431	0.02232
433766	Trim63	tripartite motif-containing 63	0.736377	0.01145	0.07583
13118	Cyp4a12b	cytochrome P450, family 4, subfamily a, polypeptide 12B	0.735835	0.08894	0.2606
59031	Chst12	carbohydrate sulfotransferase 12	0.735835	0.06847	0.221
77531	Anks1b	ankyrin repeat and sterile alpha motif domain containing 1B	0.735835	0.0009991	0.01779
94118	Kifc5c-ps	kinesin family member C5C, pseudogene	0.735835	0.2562	0.4913
271697	Cdk15	cyclin-dependent kinase 15	0.735835	0.003142	0.03525
331045	A530083I20Rik	RIKEN cDNA A530083I20 gene	0.735835	0.000401	0.01056
414115	D330050I16Rik	RIKEN cDNA D330050I16 gene	0.735835	0.08288	0.2484
20724	Serpinb5	serine (or cysteine) peptidase inhibitor, clade B, member 5	0.735294	0.2328	0.4639
21948	Cd70	CD70 antigen	0.735294	0.1092	0.2958
57266	Cxcl14	chemokine (C-X-C motif) ligand 14	0.735294	0.0009792	0.01765
72135	Pygo1	pygopus 1	0.735294	0.1562	0.366
80286	Tusc3	tumor suppressor candidate 3	0.735294	0.03024	0.1363
217151	Arl5c	ADP-ribosylation factor-like 5C	0.735294	0.006533	0.05458
56742	Psrc1	proline/serine-rich coiled-coil 1	0.734754	0.0219	0.1121
385674	Zfp174	zinc finger protein 174	0.734754	0.006154	0.05272
403174	A930005I04Rik	RIKEN cDNA A930005I04 gene	0.734754	0.02212	0.1127
668763	Gm9340	predicted gene 9340	0.734754	0.05893	0.201
14069	F8	coagulation factor VIII	0.734214	0.1632	0.3758
66447	Mgst3	microsomal glutathione S-transferase 3	0.734214	0.001426	0.02229
69982	Spink2	serine peptidase inhibitor, Kazal type 2	0.734214	0.1256	0.3225
101122	Rpusd3	RNA pseudouridylate synthase domain containing 3	0.734214	0.003515	0.03781
170643	Kirrel	kin of IRRE like (Drosophila)	0.734214	0.01657	0.09464
381434	C230057A21Rik	RIKEN cDNA C230057A21 gene	0.734214	0.5143	0.7245
383295	Ypel5	yippee-like 5 (Drosophila)	0.734214	0.0009785	0.01765
666168	Cyp4a31	cytochrome P450, family 4, subfamily a, polypeptide 31	0.734214	0.388	0.6199
11906	Zfhx3	zinc finger homeobox 3	0.733676	0.008336	0.06257
13549	Dyrk1b	dual-specificity tyrosine-(Y)-phosphorylation regulated kinase 1b	0.733676	0.0401	0.1596
54384	Mtmr7	myotubularin related protein 7	0.733676	0.0002785	0.008559

83671	Sytl2	synaptotagmin-like 2	0.733676	0.001122	0.019
100038413	D130050E23Rik	RIKEN cDNA D130050E23 gene	0.733676	0.02961	0.1345
12224	Klf5	Kruppel-like factor 5	0.733138	0.05908	0.2014
12810	Coch	coagulation factor C homolog (Limulus polyphemus)	0.733138	0.0004927	0.01177
17687	Msh5	mutS homolog 5 (E. coli)	0.733138	0.02106	0.1094
19417	Rasgrf1	RAS protein-specific guanine nucleotide-releasing factor 1	0.733138	0.07055	0.225
30944	Zfp354c	zinc finger protein 354C	0.733138	0.001469	0.02267
56323	Dnajb5	DnaJ (Hsp40) homolog, subfamily B, member 5	0.733138	0.02787	0.1298
67917	Zcchc3	zinc finger, CCHC domain containing 3	0.733138	0.005516	0.0496
68468	Ly6g6c	lymphocyte antigen 6 complex, locus G6C	0.733138	0.1534	0.363
100038662	D130046C19Rik	RIKEN cDNA D130046C19 gene	0.733138	0.01153	0.0761
11556	Adrb3	adrenergic receptor, beta 3	0.732601	0.05223	0.1856
12814	Col11a1	collagen, type XI, alpha 1	0.732601	0.1496	0.3575
80915	Dusp12	dual specificity phosphatase 12	0.732601	0.0007446	0.01508
219228	Pcdh17	protocadherin 17	0.732601	0.009408	0.06731
243262	Oas1f	2'-5' oligoadenylate synthetase 1F	0.732064	0.01915	0.1032
100038875	Gm15450	predicted gene 15450	0.732064	0.009712	0.06863
17258	Mef2a	myocyte enhancer factor 2A	0.731529	0.04901	0.1793
20192	Ryr3	ryanodine receptor 3	0.731529	0.004032	0.04108
66995	Zcchc18	zinc finger, CCHC domain containing 18	0.731529	0.001792	0.02551
67337	Cstf1	cleavage stimulation factor, 3' pre-RNA, subunit 1	0.731529	0.01286	0.08147
67937	Tmem59l	transmembrane protein 59-like	0.731529	0.0004096	0.0107
84506	Hamp	hepcidin antimicrobial peptide	0.731529	0.02875	0.1321
102913	6330509M05Rik	RIKEN cDNA 6330509M05 gene	0.731529	0.00468	0.04506
233905	Zfp646	zinc finger protein 646	0.731529	0.0006934	0.01448
54124	Cks1b	CDC28 protein kinase 1b	0.730994	0.003108	0.03497
67968	Ooep	oocyte expressed protein homolog (dog)	0.730994	0.5753	0.7688
12823	Col19a1	collagen, type XIX, alpha 1	0.73046	0.2055	0.4317
16494	Kcna6	potassium voltage-gated channel, shaker-related, subfamily, member 6	0.73046	0.1365	0.3383
78672	9530057J20Rik	RIKEN cDNA 9530057J20 gene	0.73046	0.02207	0.1125
229096	Ythdf3	YTH domain family 3	0.73046	0.03463	0.1474
230163	Aldob	aldolase B, fructose-bisphosphate	0.73046	0.001436	0.02235
338349	Cntln	centlein, centrosomal protein	0.73046	0.04143	0.1629
16858	Lgals7	lectin, galactose binding, soluble 7	0.729927	0.2877	0.5234
19279	Ptprr	protein tyrosine phosphatase, receptor type, R	0.729927	0.0002665	0.008363
68243	A930018P22Rik	RIKEN cDNA A930018P22 gene	0.729927	0.01431	0.08686
74549	Mau2	MAU2 chromatid cohesion factor homolog (C. elegans)	0.729927	0.01066	0.07278
77552	Shisa4	shisa homolog 4 (Xenopus laevis)	0.729927	0.02866	0.1319
320271	Scai	suppressor of cancer cell invasion	0.729927	0.211	0.4384
20300	Ccl25	chemokine (C-C motif) ligand 25	0.729395	0.1561	0.3659
23920	Insrr	insulin receptor-related receptor	0.729395	0.08977	0.2619
77741	6720483E21Rik	RIKEN cDNA 6720483E21 gene	0.729395	0.04697	0.1753
16656	Hivep3	human immunodeficiency virus type I enhancer binding protein 3	0.728863	0.02491	0.1209
68142	Ino80	INO80 homolog (S. cerevisiae)	0.728863	0.0009599	0.01749
246086	Onecut3	one cut domain, family member 3	0.728863	0.000735	0.01495
13197	Gadd45a	growth arrest and DNA-damage-inducible 45 alpha	0.728332	0.000743	0.01508
14245	Lpin1	lipin 1	0.728332	0.008914	0.06529
22144	Tuba3a	tubulin, alpha 3A	0.728332	0.05352	0.1885
106565	Dlk2	delta-like 2 homolog (Drosophila)	0.728332	0.05289	0.1872

320438	Alg6	asparagine-linked glycosylation 6 homolog (yeast, alpha-1,3,-glucosyltransferase)	0.728332	0.02641	0.1255
665536	Gm7676	interferon induced transmembrane protein 1 pseudogene	0.728332	0.151	0.3591
100046456	LOC100046456	zinc finger protein 91-like	0.728332	0.3522	0.5874
12950	Hapln1	hyaluronan and proteoglycan link protein 1	0.727802	0.00807	0.06158
14325	Ftl1	ferritin light chain 1	0.727802	0.00615	0.05272
27376	Slc25a10	solute carrier family 25 (mitochondrial carrier, dicarboxylate transporter), member 10	0.727802	0.1552	0.3648
50887	Hmgn5	high-mobility group nucleosome binding domain 5	0.727802	0.002402	0.03034
67847	Sncaip	synuclein, alpha interacting protein (synphilin)	0.727802	0.0177	0.09835
69294	Cst13	cystatin 13	0.727802	0.5409	0.7451
170938	Zfp617	zinc finger protein 617	0.727802	0.0002846	0.008706
330149	Hfm1	HFM1, ATP-dependent DNA helicase homolog (S. cerevisiae)	0.727802	0.004062	0.0413
624860	Gm12253	predicted gene 12253	0.727802	0.01407	0.08598
100504146	LOC100504146	zinc finger protein 709-like	0.727802	0.3375	0.573
22756	Zfp94	zinc finger protein 94	0.727273	0.01959	0.1049
56527	Mast1	microtubule associated serine/threonine kinase 1	0.727273	0.01589	0.09256
110257	Hba-a2	hemoglobin alpha, adult chain 2	0.727273	0.2562	0.4913
330119	Adamts3	a disintegrin-like and metallopeptidase (reprolysin type) with thrombospondin type 1 motif, 3	0.727273	0.03072	0.1373
434624	LOC434624	ferritin light chain 1-like	0.727273	0.06299	0.2095
14296	Frat1	frequently rearranged in advanced T-cell lymphomas	0.726744	0.001502	0.02306
23942	Mta2	metastasis-associated gene family, member 2	0.726744	0.0003687	0.01011
26457	Slc27a1	solute carrier family 27 (fatty acid transporter), member 1	0.726744	0.01609	0.09296
56222	Cited4	Cbp/p300-interacting transactivator, with Glu/Asp-rich carboxy-terminal domain, 4	0.726744	0.02785	0.1298
66975	2410002O22Rik	RIKEN cDNA 2410002O22 gene	0.726744	0.006937	0.05646
73332	Ccdc30	coiled-coil domain containing 30	0.726744	0.004219	0.04227
231570	A830010M20Rik	RIKEN cDNA A830010M20 gene	0.726744	0.008027	0.06137
666182	Gm7967	predicted gene 7967	0.726744	0.0503	0.1821
18707	Pik3cd	phosphatidylinositol 3-kinase catalytic delta polypeptide	0.726216	0.2325	0.4638
69608	Sec24d	Sec24 related gene family, member D (S. cerevisiae)	0.726216	0.004099	0.04152
77085	3010027C24Rik	RIKEN cDNA 3010027C24 gene	0.726216	0.0709	0.2258
213119	Itga10	integrin, alpha 10	0.726216	0.001631	0.02435
229759	Olfm3	olfactomedin 3	0.726216	0.01141	0.07568
17341	Bhlha15	basic helix-loop-helix family, member a15	0.725689	0.04859	0.1784
20965	Syn2	synapsin II	0.725689	0.05608	0.194
223473	Nipal2	NIPA-like domain containing 2	0.725689	0.01123	0.07509
20913	Stxbp4	syntaxin binding protein 4	0.725163	0.02697	0.1271
22689	Zfp27	zinc finger protein 27	0.725163	0.005359	0.04877
71909	Haus5	HAUS augmin-like complex, subunit 5	0.725163	0.003431	0.03718
72160	Tmem163	transmembrane protein 163	0.725163	0.095	0.271
208117	Aph1b	anterior pharynx defective 1b homolog (C. elegans)	0.725163	0.003356	0.03678
258097	Olfr1500	olfactory receptor 1500	0.725163	0.5928	0.7805
328440	Npm2	nucleophosmin/nucleoplasmin 2	0.725163	0.02997	0.1355
12501	Cd3e	CD3 antigen, epsilon polypeptide	0.724638	0.2347	0.4665
14204	Il4i1	interleukin 4 induced 1	0.724638	0.09512	0.2712
70616	Sugp1	SURP and G patch domain containing 1	0.724638	0.001554	0.0235
223917	Krt79	keratin 79	0.724638	0.06416	0.2117
381270	Mar-04	membrane-associated ring finger (C3HC4) 4	0.724638	0.01591	0.09258
383619	Aim2	absent in melanoma 2	0.724638	0.2702	0.5053
665635	LOC665635	hypothetical protein LOC665635	0.724638	0.003334	0.03662
667230	Gm8526	predicted gene 8526	0.724638	0.003884	0.04004

14915	Guca2a	guanylate cyclase activator 2a (guanylin)	0.724113	0.3347	0.5705
211147	Mar-11	membrane-associated ring finger (C3HC4) 11	0.724113	0.0009296	0.01717
225872	Npas4	neuronal PAS domain protein 4	0.724113	0.06822	0.2206
78834	Zfp623	zinc finger protein 623	0.723589	0.01312	0.0826
103712	6330403K07Rik	RIKEN cDNA 6330403K07 gene	0.723589	0.0008803	0.01676
245049	Myrip	myosin VIIA and Rab interacting protein	0.723589	0.000732	0.01495
11474	Actn3	actinin alpha 3	0.723066	0.2494	0.4835
18223	Numbl	numb-like	0.723066	0.0001769	0.0066
29857	Mapk12	mitogen-activated protein kinase 12	0.723066	0.01068	0.07286
56401	Lepre1	leprecan 1	0.723066	0.03964	0.1586
74762	Mdga1	MAM domain containing glycosylphosphatidylinositol anchor 1	0.723066	0.001827	0.02574
233575	Pgap2	post-GPI attachment to proteins 2	0.723066	0.003188	0.03563
234366	Gatad2a	GATA zinc finger domain containing 2A	0.723066	0.0002128	0.007384
330635	3830612M24	hypothetical protein 3830612M24	0.723066	0.02886	0.1324
18164	Nptx1	neuronal pentraxin 1	0.722543	0.03077	0.1374
54120	Gipc2	GIPC PDZ domain containing family, member 2	0.722543	0.02313	0.1159
66978	Luc7l	Luc7 homolog (S. cerevisiae)-like	0.722543	0.0004911	0.01175
170740	Zfp287	zinc finger protein 287	0.722543	0.1382	0.3407
209550	Rad51ap2	RAD51 associated protein 2	0.722543	0.005923	0.05177
234788	Slc38a8	solute carrier family 38, member 8	0.722543	0.3164	0.5531
258611	Olfr297	olfactory receptor 297	0.722543	0.07115	0.2262
12359	Cat	catalase	0.722022	0.007511	0.05922
233274	Siglech	sialic acid binding Ig-like lectin H	0.722022	0.08284	0.2483
382843	Rpl21-ps4	ribosomal protein L21, pseudogene 4	0.722022	0.0003677	0.01011
17926	Myoc	myocilin	0.721501	0.06333	0.2101
66204	Acyp1	acylphosphatase 1, erythrocyte (common) type	0.721501	0.0312	0.1384
73032	Ttc9b	tetratricopeptide repeat domain 9B	0.721501	0.05072	0.1828
72745	Tmem161b	transmembrane protein 161B	0.720981	0.0179	0.09897
16012	Igfbp6	insulin-like growth factor binding protein 6	0.720461	0.009577	0.06815
54208	Arl6ip1	ADP-ribosylation factor-like 6 interacting protein 1	0.720461	0.002635	0.03179
74015	Fcho1	FCH domain only 1	0.720461	0.003127	0.03512
223732	Ldoc1l	leucine zipper, down-regulated in cancer 1-like	0.720461	0.3172	0.5537
17245	Mdm1	transformed mouse 3T3 cell double minute 1	0.719942	0.02255	0.1143
29809	Rabgap1l	RAB GTPase activating protein 1-like	0.719942	0.001334	0.02139
59036	Dact1	dapper homolog 1, antagonist of beta-catenin (xenopus)	0.719942	0.007985	0.06128
72507	Dzip1l	DAZ interacting protein 1-like	0.719942	0.0421	0.1642
12447	Ccne1	cyclin E1	0.719424	0.00535	0.04871
50525	Spag6	sperm associated antigen 6	0.719424	0.193	0.4159
72129	Pex13	peroxisomal biogenesis factor 13	0.719424	0.005243	0.04813
230674	Kdm4a	lysine (K)-specific demethylase 4A	0.719424	0.003422	0.03713
76478	Haus8	4HAUS augmin-like complex, subunit 8	0.718907	0.0002261	0.007645
208659	Fam20a	family with sequence similarity 20, member A	0.718907	0.01049	0.07219
331188	Zfp781	zinc finger protein 781	0.718907	0.02671	0.1264
18612	Etv4	ets variant gene 4 (E1A enhancer binding protein, E1AF)	0.718391	0.02367	0.1173
24074	Taf7	TAF7 RNA polymerase II, TATA box binding protein (TBP)-associated factor	0.718391	0.007172	0.05751
74195	Elp3	elongation protein 3 homolog (S. cerevisiae)	0.718391	0.0004099	0.0107
100434	Slc44a1	solute carrier family 44, member 1	0.718391	0.0161	0.09296
105653	Phyhip	phytanoyl-CoA hydroxylase interacting protein	0.718391	0.02681	0.1267
243906	Zfp14	zinc finger protein 14	0.718391	0.004708	0.04511

629591	Gm6987	predicted pseudogene 6987	0.718391	0.3133	0.5495
665466	Gm7644	predicted gene 7644	0.718391	0.2422	0.4753
791383	Gm9962	predicted gene 9962	0.718391	0.1649	0.3784
68268	Zdhhc21	zinc finger, DHHC domain containing 21	0.717875	0.08493	0.2526
74490	Mamstr	MEF2 activating motif and SAP domain containing transcriptional regulator	0.717875	0.03615	0.1503
329251	Ppp1r12b	protein phosphatase 1, regulatory (inhibitor) subunit 12B	0.717875	0.09561	0.2722
399568	BC052040	cDNA sequence BC052040	0.717875	0.08942	0.2613
11438	Chrna4	cholinergic receptor, nicotinic, alpha polypeptide 4	0.71736	0.0396	0.1585
19777	C80913	expressed sequence C80913	0.71736	0.001737	0.02525
27214	Dbf4	DBF4 homolog (S. cerevisiae)	0.71736	0.007856	0.0609
269336	Ccdc32	coiled-coil domain containing 32	0.71736	0.003347	0.03672
320654	E330024J20Rik	RIKEN cDNA E330024J20 gene	0.71736	0.1309	0.3307
434423	Dppa5a	developmental pluripotency associated 5A	0.71736	0.1121	0.3006
654818	C030030A07Rik	RIKEN cDNA C030030A07 gene	0.71736	0.3984	0.6289
100504017	LOC100504017	hypothetical LOC100504017	0.71736	0.1433	0.3482
19415	Rasal1	RAS protein activator like 1 (GAP1 like)	0.716846	0.04995	0.1815
53422	Ybx2	Y box protein 2	0.716846	0.003489	0.03763
67704	1810037I17Rik	RIKEN cDNA 1810037I17 gene	0.716846	0.04722	0.1759
69681	Cdk3-ps	cyclin-dependent kinase 3, pseudogene	0.716846	0.1923	0.4148
72834	2810468N07Rik	RIKEN cDNA 2810468N07 gene	0.716846	0.0009979	0.01779
105853	Mal2	mal, T-cell differentiation protein 2	0.716846	0.0007172	0.01473
100039210	Gm2102	predicted gene 2102	0.716846	0.03496	0.1482
100043380	Gm4402	predicted gene 4402	0.716846	0.2325	0.4638
100316870	LOC100316870	DDB1 and CUL4 associated factor 5 pseudogene	0.716846	0.04759	0.1765
17161	Maoa	monoamine oxidase A	0.716332	0.004235	0.04236
70893	Glb1l3	galactosidase, beta 1 like 3	0.716332	0.1734	0.3896
100073351	Yy2	Yy2 transcription factor	0.716332	0.0321	0.1408
17537	Meis3	Meis homeobox 3	0.71582	0.04543	0.1718
18071	Nhlh1	nescient helix loop helix 1	0.71582	0.1787	0.3966
26366	Ceacam10	carcinoembryonic antigen-related cell adhesion molecule 10	0.71582	0.1332	0.3339
545758	Gm5868	predicted gene 5868	0.71582	0.0007762	0.01551
237625	Pla2g3	phospholipase A2, group III	0.715308	0.007303	0.05814
331480	Gm5126	predicted pseudogene 5126	0.715308	0.006841	0.05605
13193	Dcx	doublecortin	0.714796	0.001956	0.02699
19055	Ppp3ca	protein phosphatase 3, catalytic subunit, alpha isoform	0.714796	0.0272	0.1278
67876	Coq10b	coenzyme Q10 homolog B (S. cerevisiae)	0.714796	0.02012	0.1066
211378	6720489N17Rik	RIKEN cDNA 6720489N17 gene	0.714796	0.05634	0.1946
225642	Grp	gastrin releasing peptide	0.714796	0.02459	0.1199
237038	Nox1	NADPH oxidase 1	0.714796	0.09712	0.2746
16329	Inpp1	inositol polyphosphate-1-phosphatase	0.714286	0.02857	0.1316
18169	Npy6r	neuropeptide Y receptor Y6	0.714286	0.0807	0.2443
54384	Mtmr7	myotubularin related protein 7	0.714286	0.006268	0.05321
68713	Ifitm1	interferon induced transmembrane protein 1	0.714286	0.1254	0.3224
73009	2900057B20Rik	RIKEN cDNA 2900057B20 gene	0.714286	0.6479	0.8166
73407	Tepp	testis, prostate and placenta expressed	0.714286	0.001276	0.02073
74762	Mdga1	MAM domain containing glycosylphosphatidylinositol anchor 1	0.714286	0.03955	0.1585
75426	Igfbpl1	insulin-like growth factor binding protein-like 1	0.714286	0.004207	0.04219
98401	AI594674	expressed sequence AI594674	0.714286	0.006816	0.05594
194126	Mtmr11	myotubularin related protein 11	0.714286	0.01162	0.07651

239845	Gpr156	G protein-coupled receptor 156	0.714286	0.001286	0.02083
332110	Mapk15	mitogen-activated protein kinase 15	0.714286	0.07733	0.2384
11847	Arg2	arginase type II	0.713776	0.0007014	0.01458
104886	Rab15	RAB15, member RAS oncogene family	0.713776	0.0005361	0.01246
230779	Serinc2	serine incorporator 2	0.713776	0.04458	0.1698
100042314	Gm10639	predicted gene 10639	0.713776	0.1692	0.3843
100045796	LOC100045796	cysteine desulfurase, mitochondrial-like	0.713776	0.00793	0.06104
15528	Hspe1	heat shock protein 1 (chaperonin 10)	0.713267	0.3161	0.5529
69699	2310079G19Rik	RIKEN cDNA 2310079G19 gene	0.713267	0.2702	0.5053
72090	Entpd8	ectonucleoside triphosphate diphosphohydrolase 8	0.713267	0.2163	0.4452
93681	Zfp192	zinc finger protein 192	0.713267	0.01706	0.09643
231290	Slc10a4	solute carrier family 10 (sodium/bile acid cotransporter family), member 4	0.713267	0.02398	0.1181
13134	Dach1	dachshund 1 (Drosophila)	0.712758	0.008458	0.06313
21810	Tgfbi	transforming growth factor, beta induced	0.712251	0.1368	0.3385
208990	Npb	neuropeptide B	0.712251	0.03375	0.1452
258866	Olfr1008	olfactory receptor 1008	0.712251	0.08912	0.261
268379	Abca13	ATP-binding cassette, sub-family A (ABC1), member 13	0.712251	0.01177	0.07705
15483	Hsd11b1	hydroxysteroid 11-beta dehydrogenase 1	0.711744	0.003732	0.03906
19645	Rb1	retinoblastoma 1	0.711744	0.03964	0.1586
72049	Tnfrsf13c	tumor necrosis factor receptor superfamily, member 13c	0.711744	0.04168	0.1634
104362	Meig1	meiosis expressed gene 1	0.711744	0.002128	0.028
170571	Cntnap4	contactin associated protein-like 4	0.711744	0.02392	0.118
64293	Stk32b	serine/threonine kinase 32B	0.711238	0.003252	0.03607
70646	Naa30	N(alpha)-acetyltransferase 30, NatC catalytic subunit	0.711238	0.0006545	0.01398
15042	H2-T24	histocompatibility 2, T region locus 24	0.710732	0.353	0.5879
22349	Vil1	villin 1	0.710732	6.67E-05	0.00373
71566	9030425E11Rik	RIKEN cDNA 9030425E11 gene	0.710732	0.04555	0.172
72219	Fam75d3	family with sequence similarity 75, member D3	0.710732	0.331	0.5674
14681	Gnao1	guanine nucleotide binding protein, alpha O	0.710227	0.005186	0.04778
16409	Itgam	integrin alpha M	0.710227	0.1771	0.3942
26562	Ncdn	neurochondrin	0.710227	0.002299	0.02959
58234	Shank3	SH3/ankyrin domain gene 3	0.710227	0.03332	0.1442
71693	Colec11	collectin sub-family member 11	0.710227	0.04529	0.1715
74597	4833418N02Rik	RIKEN cDNA 4833418N02 gene	0.710227	0.0005609	0.01278
102371	Gcom1	GRINL1A complex locus	0.710227	0.03666	0.1517
230098	E130306D19Rik	RIKEN cDNA E130306D19 gene	0.710227	0.01511	0.08978
433408	Gm13375	predicted gene 13375	0.710227	0.005813	0.05117
100040972	Tceal7	transcription elongation factor A (SII)-like 7	0.710227	0.0735	0.2312
11770	Fabp4	fatty acid binding protein 4, adipocyte	0.709723	0.005673	0.05039
214575	Tdrd5	tudor domain containing 5	0.709723	0.007328	0.05829
226255	Atrnl1	attractin like 1	0.709723	0.001537	0.02342
100042951	Gm4123	predicted gene 4123	0.709723	0.04744	0.1763
13340	Slc29a2	solute carrier family 29 (nucleoside transporters), member 2	0.70922	0.009371	0.06713
19417	Rasgrf1	RAS protein-specific guanine nucleotide-releasing factor 1	0.70922	0.003529	0.03789
213469	Lgi3	leucine-rich repeat LGI family, member 3	0.70922	0.002753	0.03254
671132	Gm9519	predicted gene 9519	0.70922	0.1401	0.3435
23928	Lamc3	laminin gamma 3	0.708717	0.01099	0.0742
26549	Itgb1bp2	integrin beta 1 binding protein 2	0.708717	0.05213	0.1856
104362	Meig1	meiosis expressed gene 1	0.708717	0.0009996	0.01779

320557	Fam169a	family with sequence similarity 169, member A	0.708717	0.008726	0.0644
100043772	Gm4636	predicted gene 4636	0.708717	0.01262	0.08038
100504462	LOC100504462	hypothetical LOC100504462	0.708717	0.1454	0.3508
11480	Acvr2a	activin receptor IIA	0.708215	0.01341	0.08343
434768	Rhox8	reproductive homeobox 8	0.708215	0.1018	0.2826
16918	Mycl1	v-myc myelocytomatosis viral oncogene homolog 1, lung carcinoma derived (avian)	0.707714	0.1137	0.3033
17897	Myl3	myosin, light polypeptide 3	0.707714	0.3065	0.5431
18739	Pitpnm1	phosphatidylinositol transfer protein, membrane-associated 1	0.707714	0.01583	0.09244
58992	F12	coagulation factor XII (Hageman factor)	0.707714	0.1798	0.398
116733	Vps4a	vacuolar protein sorting 4a (yeast)	0.707714	0.05689	0.196
381677	Vgf	VGF nerve growth factor inducible	0.707714	0.0007499	0.01515
434166	Gm5589	predicted gene 5589	0.707714	0.2657	0.5014
544888	Fam181a	family with sequence similarity 181, member A	0.707714	0.04406	0.1687
628709	Gm10324	predicted gene 10324	0.707714	0.2887	0.5248
12227	Btg2	B-cell translocation gene 2, anti-proliferative	0.707214	0.006305	0.05345
66447	Mgst3	microsomal glutathione S-transferase 3	0.707214	0.002578	0.0315
77794	Adamtsl2	ADAMTS-like 2	0.707214	0.03709	0.1526
233781	Xylt1	xylosyltransferase 1	0.707214	0.0422	0.1644
328399	A930018M24Rik	RIKEN cDNA A930018M24 gene	0.707214	0.011	0.0742
16428	Itk	IL2-inducible T-cell kinase	0.706714	0.01169	0.0768
20686	Spa17	sperm autoantigenic protein 17	0.706714	0.002634	0.03179
14049	Eya2	eyes absent 2 homolog (Drosophila)	0.706215	0.0037	0.03891
16939	Lor	loricrin	0.706215	0.0005122	0.01212
52679	E2f7	E2F transcription factor 7	0.706215	0.05809	0.1989
72029	Cnpy3	canopy 3 homolog (zebrafish)	0.706215	0.0004856	0.01168
78871	B230117O15Rik	RIKEN cDNA B230117O15 gene	0.705716	0.0113	0.07532
104923	Adi1	acireductone dioxygenase 1	0.705716	0.00177	0.02542
107589	Mylk	myosin, light polypeptide kinase	0.705716	0.0219	0.1121
228876	Zfp334	zinc finger protein 334	0.705716	0.0123	0.07892
545085	Wdr70	WD repeat domain 70	0.705716	0.004784	0.04548
100504710	LOC100504710	camello-like 3-like	0.705716	0.1501	0.358
18627	Per2	period homolog 2 (Drosophila)	0.705219	0.01362	0.08428
20541	Slc8a1	solute carrier family 8 (sodium/calcium exchanger), member 1	0.705219	0.01476	0.08857
69726	Smyd3	SET and MYND domain containing 3	0.705219	0.004087	0.04146
215418	Csrnp1	cysteine-serine-rich nuclear protein 1	0.705219	0.001793	0.02551
12428	Ccna2	cyclin A2	0.704722	0.0009764	0.01765
626082	LOC626082	hypothetical protein LOC626082	0.704722	0.004857	0.04593
66605	1700017N19Rik	RIKEN cDNA 1700017N19 gene	0.704225	0.0162	0.09321
73719	Lce1c	late cornified envelope 1C	0.704225	0.5269	0.7339
75209	Sv2c	synaptic vesicle glycoprotein 2c	0.704225	0.002371	0.03015
231858	Radil	Ras association and DIL domains	0.704225	0.006469	0.05427
70567	5730455O13Rik	RIKEN cDNA 5730455O13 gene	0.70373	0.0008782	0.01676
70626	5730522E02Rik	RIKEN cDNA 5730522E02 gene	0.70373	0.03236	0.1416
72027	Slc39a4	solute carrier family 39 (zinc transporter), member 4	0.70373	0.03897	0.1571
192897	Itgb4	integrin beta 4	0.70373	0.006849	0.05607
224997	Dlgap1	discs, large (Drosophila) homolog-associated protein 1	0.70373	0.03018	0.1361
237636	Npc1l1	NPC1-like 1	0.70373	0.004619	0.04473
242747	Zfp933	zinc finger protein 933	0.70373	0.125	0.3218
244049	Mctp2	multiple C2 domains, transmembrane 2	0.70373	0.06973	0.2235

353211	Prune2	prune homolog 2 (Drosophila)	0.70373	0.1441	0.3491
16691	Krt8	keratin 8	0.703235	0.09291	0.2676
19933	Rpl21	ribosomal protein L21	0.703235	0.009781	0.06901
59009	Sh3rf1	SH3 domain containing ring finger 1	0.703235	0.2383	0.4707
233575	Pgap2	post-GPI attachment to proteins 2	0.703235	0.001413	0.02219
21854	Timm17a	translocase of inner mitochondrial membrane 17a	0.702741	0.0003958	0.01045
27377	Yme1l1	YME1-like 1 (S. cerevisiae)	0.702741	0.01594	0.0926
52480	D7Ertd715e	DNA segment, Chr 7, ERATO Doi 715, expressed	0.702741	0.07971	0.2428
73709	2410125D13Rik	RIKEN cDNA 2410125D13 gene	0.702741	0.001076	0.01848
81879	Tcfcp2l1	transcription factor CP2-like 1	0.702741	0.3081	0.5443
320365	Fry	furry homolog (Drosophila)	0.702741	0.3692	0.6041
13417	Dnahc8	dynein, axonemal, heavy chain 8	0.702247	0.04871	0.1786
192161	Pcdha9	protocadherin alpha 9	0.702247	0.004072	0.04135
217217	Asb16	ankyrin repeat and SOCS box-containing 16	0.702247	0.005681	0.05039
668110	Syce1l	synaptonemal complex central element protein 1 like	0.702247	0.001079	0.01849
57778	Fmnl1	formin-like 1	0.701754	0.1174	0.3094
227541	Camk1d	calcium/calmodulin-dependent protein kinase ID	0.701754	0.0003662	0.0101
15528	Hspe1	heat shock protein 1 (chaperonin 10)	0.701262	4.61E-05	0.003145
66573	Dzip1	DAZ interacting protein 1	0.701262	0.04025	0.16
77974	Rdh12	retinol dehydrogenase 12	0.701262	0.007159	0.05744
17992	Ndufa4	NADH dehydrogenase (ubiquinone) 1 alpha subcomplex, 4	0.700771	0.001212	0.02004
109225	Ms4a7	membrane-spanning 4-domains, subfamily A, member 7	0.700771	0.09676	0.2741
232016	Ccdc129	coiled-coil domain containing 129	0.700771	0.002606	0.03161
72832	Crtac1	cartilage acidic protein 1	0.70028	0.004169	0.04196
93842	Igsf9	immunoglobulin superfamily, member 9	0.70028	0.002881	0.03352
212190	Ubxn10	UBX domain protein 10	0.70028	0.07535	0.2348
319317	Snhg11	small nucleolar RNA host gene 11	0.70028	0.006458	0.0542
17932	Myt1	myelin transcription factor 1	0.69979	0.1541	0.3637
213121	Ankrd35	ankyrin repeat domain 35	0.69979	0.007512	0.05922
216797	Prss38	protease, serine, 38	0.69979	0.0007619	0.01532
271944	C2cd4d	C2 calcium-dependent domain containing 4D	0.69979	0.01004	0.07018
100041639	Gm3448	predicted gene 3448	0.69979	0.0695	0.2231
20462	Tra2b	transformer 2 beta homolog (Drosophila)	0.699301	0.3941	0.6251
75668	Rasl10a	RAS-like, family 10, member A	0.699301	0.006225	0.05309
208836	Fanci	Fanconi anemia, complementation group I	0.699301	0.1522	0.3613
319742	Mpzl3	myelin protein zero-like 3	0.699301	0.09847	0.277
171167	Fut10	fucosyltransferase 10	0.698812	0.00024	0.007862
64380	Ms4a4c	membrane-spanning 4-domains, subfamily A, member 4C	0.698324	0.01862	0.1015
69211	2310081J21Rik	RIKEN cDNA 2310081J21 gene	0.698324	0.1356	0.3372
12797	Cnn1	calponin 1	0.697837	0.2762	0.5116
19419	Rasgrp1	RAS guanyl releasing protein 1	0.697837	0.0002268	0.007657
56695	Pnkd	paroxysmal nonkinesiogenic dyskinesia	0.697837	0.03594	0.1499
57425	U90926	cDNA sequence U90926	0.697837	0.115	0.3056
68799	Rgmb	RGM domain family, member B	0.697837	0.004234	0.04236
320658	D430040L24Rik	RIKEN cDNA D430040L24 gene	0.697837	0.008251	0.06224
545276	Gal3st3	galactose-3-O-sulfotransferase 3	0.697837	0.0636	0.2107
13405	Dmd	dystrophin, muscular dystrophy	0.69735	0.0001995	0.007179
72148	2610019F03Rik	RIKEN cDNA 2610019F03 gene	0.69735	0.00846	0.06313
18102	Nme1	non-metastatic cells 1, protein (NM23A) expressed in	0.696864	0.0001638	0.006304

93879	Pcdhb8	protocadherin beta 8	0.696864	0.03726	0.153
106407	Osta	organic solute transporter alpha	0.696864	0.03502	0.1484
12936	Pcdha4	protocadherin alpha 4	0.696379	0.006825	0.05599
72883	2900035I09Rik	RIKEN cDNA 2900035I09 gene	0.696379	0.01174	0.07693
81898	Sf3b1	splicing factor 3b, subunit 1	0.696379	0.006217	0.05304
240753	Plekha6	pleckstrin homology domain containing, family A member 6	0.696379	0.0008744	0.0167
58245	Gpr180	G protein-coupled receptor 180	0.695894	0.01115	0.07478
230594	Zcchc11	zinc finger, CCHC domain containing 11	0.695894	0.02031	0.107
17202	Mc4r	melanocortin 4 receptor	0.69541	0.00703	0.05677
17930	Myom2	myomesin 2	0.69541	0.003969	0.04069
20689	Sall3	sal-like 3 (Drosophila)	0.69541	0.009173	0.06634
70319	2600006K01Rik	RIKEN cDNA 2600006K01 gene	0.69541	0.06383	0.2111
71494	8430406P12Rik	RIKEN cDNA 8430406P12 gene	0.69541	0.004794	0.04552
15235	Mst1	macrophage stimulating 1 (hepatocyte growth factor-like)	0.694927	0.176	0.3929
53311	Mybph	myosin binding protein H	0.694927	0.372	0.6068
66438	Hamp2	hepcidin antimicrobial peptide 2	0.694927	0.07773	0.239
76654	Upp2	uridine phosphorylase 2	0.694927	0.1054	0.2889
236428	BC026762	cDNA sequence BC026762	0.694927	0.1681	0.3831
13813	Eomes	eomesodermin homolog (Xenopus laevis)	0.694444	0.0008294	0.01609
22715	Zfp57	zinc finger protein 57	0.694444	0.03745	0.1534
78600	Pde6h	phosphodiesterase 6H, cGMP-specific, cone, gamma	0.694444	8.55E-05	0.004277
319931	A330032B11Rik	RIKEN cDNA A330032B11 gene	0.694444	0.163	0.3754
69189	1810033B17Rik	RIKEN cDNA 1810033B17 gene	0.693963	0.034	0.1458
211232	Cpne9	copine family member IX	0.693963	0.005693	0.05041
230863	Sh2d5	SH2 domain containing 5	0.693963	0.001942	0.02686
382014	Ano8	anoctamin 8	0.693963	0.03089	0.1377
434232	Iqck	IQ motif containing K	0.693963	0.07899	0.2412
546134	Gramd2	GRAM domain containing 2	0.693963	0.0001554	0.006088
620949	Dph3b-ps	DPH3B, KTI11 homolog B (S. cerevisiae), pseudogene	0.693963	0.248	0.4822
18121	Nog	noggin	0.693481	0.1331	0.3338
56357	Ivd	isovaleryl coenzyme A dehydrogenase	0.693481	0.0001277	0.005439
74561	Nkx6-3	NK6 homeobox 3	0.693481	0.002272	0.02938
76022	Gon4l	gon-4-like (C.elegans)	0.693481	0.1896	0.4113
192663	Abcg4	ATP-binding cassette, sub-family G (WHITE), member 4	0.693481	0.001775	0.02544
14945	Gzmk	granzyme K	0.693001	0.1491	0.3566
21846	Tie1	tyrosine kinase with immunoglobulin-like and EGF-like domains 1	0.693001	0.04082	0.1615
76286	1110006E14Rik	RIKEN cDNA 1110006E14 gene	0.693001	0.01071	0.07301
210541	Gm581	predicted gene 581	0.693001	0.1127	0.3015
402767	A830052D11Rik	RIKEN cDNA A830052D11 gene	0.693001	0.03587	0.1498
100043450	AA387883	expressed sequence AA387883	0.693001	0.05202	0.1855
53422	Ybx2	Y box protein 2	0.692042	0.002891	0.03358
54632	Ftsj1	FtsJ homolog 1 (E. coli)	0.692042	0.01773	0.09839
110084	Dnahc1	dynein, axonemal, heavy chain 1	0.692042	0.0347	0.1476
279499	Kctd19	potassium channel tetramerisation domain containing 19	0.692042	0.03662	0.1516
319875	Tmprss11bnl	transmembrane protease, serine 11b N terminal like	0.692042	0.0804	0.2439
66375	Dhrs7	dehydrogenase/reductase (SDR family) member 7	0.691563	0.0007332	0.01495
192663	Abcg4	ATP-binding cassette, sub-family G (WHITE), member 4	0.691563	0.001682	0.02484
675440	Gm13430	predicted gene 13430	0.691563	0.2983	0.5344
68802	Mypn	myopalladin	0.691085	0.2786	0.514

212326	Fam149a	family with sequence similarity 149, member A	0.691085	9.94E-05	0.004628
271209	Rp1l1	retinitis pigmentosa 1 homolog (human)-like 1	0.691085	0.1673	0.3822
69367	Glrx2	glutaredoxin 2 (thioltransferase)	0.690608	0.1668	0.3812
83672	Sytl3	synaptotagmin-like 3	0.690608	0.03858	0.1564
74448	Arl13a	ADP-ribosylation factor-like 13A	0.690131	0.01151	0.07605
228410	Cstf3	cleavage stimulation factor, 3' pre-RNA, subunit 3	0.690131	0.00284	0.0332
11656	Alas2	aminolevulinic acid synthase 2, erythroid	0.689655	0.2723	0.5079
667414	Gm8618	predicted gene 8618	0.689655	0.2519	0.4864
14710	Gngt2	guanine nucleotide binding protein (G protein), gamma transducing activity polypeptide 2	0.68918	0.000984	0.01768
57443	Fbxo3	F-box protein 3	0.68918	0.01493	0.08913
435766	Tnni3k	TNNI3 interacting kinase	0.68918	0.2144	0.4428
21784	Tff1	trefoil factor 1	0.688705	0.18	0.3982
93728	Pabpc5	poly(A) binding protein, cytoplasmic 5	0.688705	0.003097	0.03493
240216	E230025N22Rik	Riken cDNA E230025N22 gene	0.688705	0.07248	0.229
320469	9930014A18Rik	RIKEN cDNA 9930014A18 gene	0.688705	0.01758	0.09797
67876	Coq10b	coenzyme Q10 homolog B (S. cerevisiae)	0.688231	0.0002768	0.008535
72351	Ptar1	protein prenyltransferase alpha subunit repeat containing 1	0.688231	0.002097	0.02786
224826	Ubr2	ubiquitin protein ligase E3 component n-recognin 2	0.688231	0.4877	0.7055
66526	2210012G02Rik	RIKEN cDNA 2210012G02 gene	0.687758	0.00211	0.02793
66169	Tomm7	translocase of outer mitochondrial membrane 7 homolog (yeast)	0.687285	0.006981	0.05654
73010	Gpr22	G protein-coupled receptor 22	0.687285	0.02144	0.1108
244859	Ankk1	ankyrin repeat and kinase domain containing 1	0.687285	0.07207	0.2283
628709	Gm10324	predicted gene 10324	0.687285	0.2138	0.4422
16988	Lst1	leukocyte specific transcript 1	0.686813	0.009343	0.06707
66039	D14Ertd449e	DNA segment, Chr 14, ERATO Doi 449, expressed	0.686813	0.003375	0.0369
67127	Lce1a1	late cornified envelope 1A1	0.686813	0.3201	0.5566
74760	Rab3il1	RAB3A interacting protein (rabin3)-like 1	0.686813	0.02682	0.1267
100038452	Gm13372	predicted gene 13372	0.686813	0.1547	0.3646
20646	Snrpn	small nuclear ribonucleoprotein N	0.686342	0.0005741	0.01299
22353	Vip	vasoactive intestinal polypeptide	0.686342	0.003561	0.03811
51966	D11Bhm181e	DNA segment, Chr 11, Boehm 181, expressed	0.685871	0.006555	0.05472
75788	Smurf1	SMAD specific E3 ubiquitin protein ligase 1	0.685871	0.002872	0.03349
80794	Cblc	Casitas B-lineage lymphoma c	0.685871	0.04759	0.1765
268807	Klhl38	kelch-like 38 (Drosophila)	0.685871	0.005747	0.05073
329509	1810024B03Rik	RIKEN cDNA 1810024B03 gene	0.685871	0.276	0.5115
215474	Sec22c	SEC22 vesicle trafficking protein homolog C (S. cerevisiae)	0.685401	0.005604	0.05006
57742	Abhd1	abhydrolase domain containing 1	0.684932	0.01737	0.09735
219131	Phf11	PHD finger protein 11	0.684932	0.09999	0.2792
328977	Zfp532	zinc finger protein 532	0.684932	0.001855	0.02604
26372	Clcn6	chloride channel 6	0.684463	0.02377	0.1175
100038759	LOC100038759	hypothetical LOC100038759	0.684463	0.05073	0.1828
73103	3110009E18Rik	RIKEN cDNA 3110009E18 gene	0.683995	0.06524	0.214
75718	Vwa5b1	von Willebrand factor A domain containing 5B1	0.683995	0.01732	0.09727
432478	Tmprss9	transmembrane protease, serine 9	0.683995	0.007651	0.05989
56176	Pigp	phosphatidylinositol glycan anchor biosynthesis, class P	0.683527	0.0007685	0.01542
382161	Gm5165	predicted gene 5165	0.683527	0.004414	0.04343
100041639	Gm3448	predicted gene 3448	0.683527	0.06985	0.2237
230779	Serinc2	serine incorporator 2	0.68306	0.01758	0.09797
232855	Zfp772	zinc finger protein 772	0.68306	0.003567	0.03815

432628	Mfsd2b	major facilitator superfamily domain containing 2B	0.68306	0.01561	0.09164
60599	Trp53inp1	transformation related protein 53 inducible nuclear protein 1	0.682594	0.0407	0.1613
259002	Olfr173	olfactory receptor 173	0.682594	0.06396	0.2113
622657	Gm6340	predicted pseudogene 6340	0.682594	0.0002184	0.007482
20725	Serpinb8	serine (or cysteine) peptidase inhibitor, clade B, member 8	0.682128	0.1201	0.3139
20897	Stra6	stimulated by retinoic acid gene 6	0.682128	0.0282	0.1307
66949	Trim59	tripartite motif-containing 59	0.682128	0.003464	0.03744
93884	Pcdhb13	protocadherin beta 13	0.682128	0.007952	0.0611
98256	Kmo	kynurenine 3-monooxygenase (kynurenine 3-hydroxylase)	0.682128	0.0004422	0.01105
16691	Krt8	keratin 8	0.681663	0.08617	0.2552
20425	Shmt1	serine hydroxymethyltransferase 1 (soluble)	0.681663	0.05688	0.1959
226791	Lyplal1	lysophospholipase-like 1	0.681663	0.005115	0.04734
16571	Kif4	kinesin family member 4	0.681199	0.07796	0.2393
64075	Smoc1	SPARC related modular calcium binding 1	0.681199	0.0145	0.08758
74405	Efhc2	EF-hand domain (C-terminal) containing 2	0.681199	0.0235	0.1169
432987	Gm5478	predicted pseudogene 5478	0.681199	0.009863	0.06942
26943	Serinc3	serine incorporator 3	0.680735	0.0004656	0.01141
224792	Gpr116	G protein-coupled receptor 116	0.680735	0.01623	0.09336
233405	Vps33b	vacuolar protein sorting 33B (yeast)	0.680735	0.009066	0.06591
14601	Ghrh	growth hormone releasing hormone	0.680272	0.01156	0.07622
66573	Dzip1	DAZ interacting protein 1	0.680272	0.004764	0.0454
72169	Trim29	tripartite motif-containing 29	0.680272	0.1462	0.3519
98878	Ehd4	EH-domain containing 4	0.680272	0.0001948	0.007059
229697	Cym	chymosin	0.680272	0.01351	0.08387
20514	Slc1a5	solute carrier family 1 (neutral amino acid transporter), member 5	0.67981	0.001131	0.01908
67344	Tctex1d1	Tctex1 domain containing 1	0.67981	0.04732	0.176
72269	Cda	cytidine deaminase	0.67981	0.2813	0.5166
78547	E130304I02Rik	RIKEN cDNA E130304I02 gene	0.67981	0.009236	0.06654
320091	Ano4	anoctamin 4	0.67981	0.01135	0.07552
320609	Fam40b	family with sequence similarity 40, member B	0.67981	0.0005466	0.01257
653016	Gm7325	predicted gene 7325	0.67981	0.02835	0.131
30806	Adamts8	a disintegrin-like and metallopeptidase (reprolysin type) with thrombospondin type 1 motif, 8	0.679348	0.008247	0.06224
77072	6720420G18Rik	RIKEN cDNA 6720420G18 gene	0.679348	0.2036	0.4299
78751	Zc3h6	zinc finger CCCH type containing 6	0.679348	0.02118	0.1099
100037396	D030047H15Rik	RIKEN cDNA D030047H15 gene	0.679348	0.1649	0.3784
15051	H2-T9	histocompatibility 2, T region locus 9	0.678887	0.09599	0.2727
76117	Arhgap15	Rho GTPase activating protein 15	0.678887	0.000886	0.01681
76265	Tsen54	tRNA splicing endonuclease 54 homolog (S. cerevisiae)	0.678887	0.003641	0.03858
114304	Slc28a3	solute carrier family 28 (sodium-coupled nucleoside transporter), member 3	0.678887	0.05983	0.2029
215280	Wipf1	WAS/WASL interacting protein family, member 1	0.678887	0.01171	0.07683
624710	Gm6524	katanin p60 (ATPase-containing) subunit A1 pseudogene	0.678887	0.003688	0.03888
14859	Gsta3	glutathione S-transferase, alpha 3	0.678426	0.005006	0.04674
20315	Cxcl12	chemokine (C-X-C motif) ligand 12	0.678426	0.008375	0.06275
244281	Myo16	myosin XVI	0.678426	8.19E-05	0.004218
26371	Ciao1	cytosolic iron-sulfur protein assembly 1 homolog (S. cerevisiae)	0.677966	0.002062	0.02773
66634	Mcm8	minichromosome maintenance deficient 8 (S. cerevisiae)	0.677966	0.001335	0.02139
69269	Scnm1	sodium channel modifier 1	0.677966	0.002585	0.0315
18600	Padi2	peptidyl arginine deiminase, type II	0.677507	0.0324	0.1417
233066	AI428936	expressed sequence AI428936	0.677507	0.003124	0.0351

574403	Fam196b	family with sequence similarity 196, member B	0.677507	7.98E-05	0.004179
791388	AY512938	cDNA sequence AY512938	0.677507	0.003459	0.0374
100038882	Isg15	ISG15 ubiquitin-like modifier	0.677507	0.0108	0.07334
15207	Hes3	hairy and enhancer of split 3 (Drosophila)	0.677048	0.007293	0.05811
414872	Zyg11b	zyg-ll homolog B (C. elegans)	0.677048	0.0003712	0.01011
209012	Ulk4	unc-51-like kinase 4 (C. elegans)	0.67659	0.1449	0.3504
403344	C230006B20	hypothetical LOC403344	0.67659	0.007557	0.05942
76561	Snx7	sorting nexin 7	0.675676	0.4912	0.7078
214084	Slc18a2	solute carrier family 18 (vesicular monoamine), member 2	0.675676	0.006058	0.05239
330355	Dnahc6	dynein, axonemal, heavy chain 6	0.675676	0.005085	0.04718
69784	1500009L16Rik	RIKEN cDNA 1500009L16 gene	0.675219	0.006409	0.05396
71059	Hexim2	hexamthylene bis-acetamide inducible 2	0.675219	0.01335	0.08332
72404	Wdr44	WD repeat domain 44	0.675219	0.01964	0.1049
18830	Pltp	phospholipid transfer protein	0.674764	0.01759	0.09797
72413	Kcnmb2	potassium large conductance calcium-activated channel, subfamily M, beta member 2	0.674764	0.002017	0.02731
68393	Mogat1	monoacylglycerol O-acyltransferase 1	0.674309	0.002227	0.02895
72383	Cnfn	cornifelin	0.674309	0.05554	0.1929
239133	Dleu7	deleted in lymphocytic leukemia, 7	0.674309	0.001553	0.0235
544848	Gm5784	predicted gene 5784	0.673854	0.02776	0.1296
100303744	Sprr2a2	small proline-rich protein 2A2	0.673854	0.0007985	0.01574
18667	Pgr	progesterone receptor	0.673401	0.0029	0.03363
67419	3632451O06Rik	RIKEN cDNA 3632451O06 gene	0.673401	0.03175	0.14
71998	Slc25a35	solute carrier family 25, member 35	0.673401	0.0008797	0.01676
246709	Rgs13	regulator of G-protein signaling 13	0.673401	0.0007105	0.01464
329941	Col8a2	collagen, type VIII, alpha 2	0.673401	0.1586	0.3697
14415	Gad1	glutamic acid decarboxylase 1	0.672948	0.00022	0.007521
237859	Ccdc55	coiled-coil domain containing 55	0.672948	0.004948	0.04635
258661	Olfr740	olfactory receptor 740	0.672948	0.114	0.3039
71972	Dnmbp	dynamin binding protein	0.672495	0.006274	0.05323
241230	St8sia6	ST8 alpha-N-acetyl-neuraminide alpha-2,8-sialyltransferase 6	0.672495	0.009111	0.06608
241494	Zfp385b	zinc finger protein 385B	0.672495	0.003781	0.03935
100042510	Gm3877	predicted gene 3877	0.672495	0.002713	0.03222
24030	Mrps12	mitochondrial ribosomal protein S12	0.672043	9.94E-05	0.004628
69489	2310007J06Rik	RIKEN cDNA 2310007J06 gene	0.672043	0.001593	0.02389
72899	Macrod2	MACRO domain containing 2	0.672043	0.001013	0.01786
75209	Sv2c	synaptic vesicle glycoprotein 2c	0.672043	0.1965	0.4207
192289	Tmlhe	trimethyllysine hydroxylase, epsilon	0.672043	0.02414	0.1186
17350	Mlh1	mutL homolog 1 (E. coli)	0.671592	0.003027	0.03442
102142	AI662245	expressed sequence AI662245	0.671592	0.04172	0.1635
54124	Cks1b	CDC28 protein kinase 1b	0.671141	0.0002406	0.007862
214106	4933430I17Rik	RIKEN cDNA 4933430I17 gene	0.671141	0.005958	0.05198
244853	Fam55d	family with sequence similarity 55, member D	0.671141	7.17E-05	0.003894
85031	Pla1a	phospholipase A1 member A	0.670691	0.06872	0.2215
14406	Gabrg2	gamma-aminobutyric acid (GABA) A receptor, subunit gamma 2	0.670241	0.0004947	0.0118
110911	Cds2	CDP-diacylglycerol synthase (phosphatidate cytidylyltransferase) 2	0.670241	0.002704	0.03218
16204	Fabp6	fatty acid binding protein 6, ileal (gastrotropin)	0.669792	0.1403	0.3439
52728	D17Zt10e	DNA segment, Chr 17, Zdenek Trachtulec 10 expressed	0.669792	0.08048	0.244
75763	Dcaf17	DDB1 and CUL4 associated factor 17	0.669792	0.001697	0.02494
212391	Lcor	ligand dependent nuclear receptor corepressor	0.669344	0.007422	0.05881

14069	F8	coagulation factor VIII	0.668896	0.001238	0.02037
21684	Tectb	tectorin beta	0.668896	0.5368	0.7416
320438	Alg6	asparagine-linked glycosylation 6 homolog (yeast, alpha-1,3,-glucosyltransferase)	0.668896	0.0004374	0.01101
328643	Vwa5b2	von Willebrand factor A domain containing 5B2	0.668896	0.005641	0.05026
15379	Onecut1	one cut domain, family member 1	0.668449	0.06077	0.2049
66610	Abi3	ABI gene family, member 3	0.668449	0.0527	0.1867
74215	Prss51	protease, serine, 51	0.667557	0.1782	0.3959
76421	1700028K03Rik	RIKEN cDNA 1700028K03 gene	0.667557	0.05157	0.1845
12032	Bcan	brevican	0.667111	0.001425	0.02229
53972	Ngef	neuronal guanine nucleotide exchange factor	0.667111	0.0004956	0.01181
239151	Gm600	predicted gene 600	0.667111	0.6352	0.8082
635253	Usp51	ubiquitin specific protease 51	0.667111	0.0004503	0.01115
14544	Gda	guanine deaminase	0.666667	0.005129	0.0474
16994	Ltb	lymphotoxin B	0.666667	0.003208	0.03582
22147	Tuba3b	tubulin, alpha 3B	0.666667	0.0227	0.1148
71003	Prss41	protease, serine, 41	0.666667	0.002087	0.02786
258248	Olfr576	olfactory receptor 576	0.666667	0.06067	0.2047
415115	Neurl2	neuralized-like 2 (Drosophila)	0.666667	0.002974	0.03406
100041420	Gm3325	predicted gene 3325	0.666667	0.06752	0.2191
12021	Bard1	BRCA1 associated RING domain 1	0.666223	0.1156	0.3064
52882	Rgs7bp	regulator of G-protein signalling 7 binding protein	0.666223	0.0007693	0.01542
54601	Foxo4	forkhead box O4	0.666223	0.08213	0.247
69327	1700007K13Rik	RIKEN cDNA 1700007K13 gene	0.666223	0.003629	0.03854
328424	Kcnrg	potassium channel regulator	0.666223	0.008765	0.06456
57811	Rgr	retinal G protein coupled receptor	0.665779	0.2209	0.4507
67973	Mphosph10	M-phase phosphoprotein 10 (U3 small nucleolar ribonucleoprotein)	0.665779	0.01281	0.08122
217149	Cisd3	CDGSH iron sulfur domain 3	0.665779	0.00936	0.06712
67272	Cmtm5	CKLF-like MARVEL transmembrane domain containing 5	0.665336	0.07435	0.2328
70103	Znhit1	zinc finger, HIT domain containing 1	0.665336	0.001264	0.02067
75894	Adal	adenosine deaminase-like	0.665336	0.08859	0.2599
170942	Erdr1	erythroid differentiation regulator 1	0.665336	0.08776	0.2584
233913	BC017158	cDNA sequence BC017158	0.665336	0.0001806	0.006665
239170	Fam160b2	family with sequence similarity 160, member B2	0.665336	0.004578	0.04461
383592	Gm1305	predicted gene 1305	0.664894	0.4741	0.6947
664787	LOC664787	similar to Sp110 nuclear body protein	0.664894	0.07288	0.2299
79565	Wbscr27	Williams Beuren syndrome chromosome region 27 (human)	0.664452	0.362	0.597
105243	Slc9a3	solute carrier family 9 (sodium/hydrogen exchanger), member 3	0.664452	0.0044	0.04335
93835	Amn	amnionless	0.664011	0.006012	0.05219
245578	Pcdh11x	protocadherin 11 X-linked	0.664011	0.03477	0.1478
17771	Mtl5	metallothionein-like 5, testis-specific (tesmin)	0.66357	0.0735	0.2312
69060	Pnlip	pancreatic lipase	0.66357	0.005365	0.0488
353499	Tmc4	transmembrane channel-like gene family 4	0.66357	0.0007123	0.01466
100502632	LOC100502632	zinc finger protein 709-like	0.66357	0.03552	0.1488
12565	Cdh9	cadherin 9	0.66313	0.0001204	0.005257
15898	Icam5	intercellular adhesion molecule 5, telencephalin	0.66313	0.0008821	0.01678
50753	Fbxo8	F-box protein 8	0.66313	0.01111	0.07461
109821	F11	coagulation factor XI	0.66313	0.006688	0.0553
18600	Padi2	peptidyl arginine deiminase, type II	0.662691	0.03747	0.1534
69134	Fam25c	family with sequence similarity 25, member C	0.662691	0.2769	0.5126

381157	Greb1l	growth regulation by estrogen in breast cancer-like	0.662691	0.002375	0.03016
320086	E430022K19Rik	RIKEN cDNA E430022K19 gene	0.662252	0.002393	0.03026
22192	Ube2m	ubiquitin-conjugating enzyme E2M (UBC12 homolog, yeast)	0.661376	0.001552	0.0235
27412	Peg12	paternally expressed 12	0.661376	0.04994	0.1815
81600	Chia	chitinase, acidic	0.661376	0.02835	0.131
21784	Tff1	trefoil factor 1	0.660939	0.1309	0.3307
69367	Glrx2	glutaredoxin 2 (thioltransferase)	0.660939	0.4542	0.6779
14813	Grin2c	glutamate receptor, ionotropic, NMDA2C (epsilon 3)	0.660502	0.003458	0.0374
77318	Ankrd55	ankyrin repeat domain 55	0.660502	0.002121	0.02798
213393	8430408G22Rik	RIKEN cDNA 8430408G22 gene	0.660066	0.07578	0.2357
16514	Kcnj11	potassium inwardly rectifying channel, subfamily J, member 11	0.659631	0.0004695	0.01147
108903	Tbcd	tubulin-specific chaperone d	0.659631	0.005032	0.04691
67272	Cmtm5	CKLF-like MARVEL transmembrane domain containing 5	0.659196	0.01944	0.1044
83924	Gpr137b	G protein-coupled receptor 137B	0.659196	0.0002516	0.008012
100042727	Gm3993	predicted gene 3993	0.659196	0.03705	0.1526
170720	Card14	caspase recruitment domain family, member 14	0.658762	0.09102	0.2642
271842	Rpusd2	RNA pseudouridylate synthase domain containing 2	0.658762	0.0747	0.2334
23854	Def8	differentially expressed in FDCP 8	0.65703	0.04115	0.1622
17436	Me1	malic enzyme 1, NADP(+)-dependent, cytosolic	0.656599	0.01106	0.07445
18549	Pcsk2	proprotein convertase subtilisin/kexin type 2	0.656599	0.001197	0.01985
225651	Mppe1	metallophosphoesterase 1	0.656599	0.017	0.09616
171543	Bmf	BCL2 modifying factor	0.656168	0.08539	0.2535
18037	Nfkbie	nuclear factor of kappa light polypeptide gene enhancer in B-cells inhibitor, epsilon	0.655308	0.002545	0.03125
29806	Limd1	LIM domains containing 1	0.655308	0.0001735	0.006559
75058	4930519H02Rik	RIKEN cDNA 4930519H02 gene	0.655308	0.07044	0.2249
27385	Magel2	melanoma antigen, family L, 2	0.654879	0.0002957	0.008884
98661	AW061096	expressed sequence AW061096	0.65445	0.001082	0.01851
266692	Cpne1	copine I	0.65445	0.0006539	0.01398
223915	Krt73	keratin 73	0.654022	0.02702	0.1272
320876	D930002L09Rik	RIKEN cDNA D930002L09 gene	0.654022	0.08801	0.2587
18704	Pik3c2a	phosphatidylinositol 3-kinase, C2 domain containing, alpha polypeptide	0.653595	0.02114	0.1097
69700	Col22a1	collagen, type XXII, alpha 1	0.653595	0.01482	0.08882
76022	Gon4l	gon-4-like (C.elegans)	0.653595	0.003087	0.03491
170735	Arr3	arrestin 3, retinal	0.653595	0.0002691	0.008396
242721	Klhdc7a	kelch domain containing 7A	0.653595	0.1651	0.3787
544848	Gm5784	predicted gene 5784	0.653595	0.004787	0.04548
100046290	LOC100046290	60S ribosomal protein L21-like	0.653595	0.003314	0.03649
14857	Gsta1	glutathione S-transferase, alpha 1 (Ya)	0.653168	0.1289	0.3276
114741	Supt16h	suppressor of Ty 16 homolog (S. cerevisiae)	0.653168	0.006047	0.05238
269356	Slc4a11	solute carrier family 4, sodium bicarbonate transporter-like, member 11	0.653168	0.02499	0.121
21946	Pglyrp1	peptidoglycan recognition protein 1	0.652742	0.009373	0.06713
20348	Sema3c	sema domain, immunoglobulin domain (Ig), short basic domain, secreted, (semaphorin) 3C	0.652316	0.04013	0.1597
99031	Osbpl6	oxysterol binding protein-like 6	0.652316	0.01534	0.09072
269109	Dpp10	dipeptidylpeptidase 10	0.652316	0.0008112	0.0159
14255	Flt3	FMS-like tyrosine kinase 3	0.65189	0.0001455	0.005848
27049	Etv3	ets variant gene 3	0.65189	0.001648	0.02456
71870	Ccdc19	coiled-coil domain containing 19	0.65189	0.0108	0.07335
72632	2700090O03Rik	RIKEN cDNA 2700090O03 gene	0.65189	0.0002139	0.007384
380654	4930485B16Rik	RIKEN cDNA 4930485B16 gene	0.65189	0.09382	0.2692

626854	Gm6712	predicted gene 6712	0.65189	0.00669	0.0553
381062	2210404J11Rik	RIKEN cDNA 2210404J11 gene	0.651466	0.004995	0.04672
210741	Kcnk12	potassium channel, subfamily K, member 12	0.651042	0.03532	0.1484
245865	Spag4	sperm associated antigen 4	0.650618	0.0017	0.02495
790956	LOC790956	5.8S ribosomal RNA	0.650195	0.3944	0.6252
12865	Cox7a1	cytochrome c oxidase, subunit VIIa 1	0.649773	0.001765	0.02542
76880	6430411K18Rik	RIKEN cDNA 6430411K18 gen	0.649773	0.01533	0.09067
319229	Sctr	secretin receptor	0.649773	0.1767	0.3938
18036	Nfkbib	nuclear factor of kappa light polypeptide gene enhancer in B-cells inhibitor, beta	0.649351	0.08473	0.2522
68127	B230217C12Rik	RIKEN cDNA B230217C12 gene	0.648929	0.0003585	0.00998
76072	Rnf183	ring finger protein 183	0.648929	0.01321	0.0829
216961	Coro6	coronin 6	0.648508	0.01494	0.08913
227525	Dclre1c	DNA cross-link repair 1C, PSO2 homolog (S. cerevisiae)	0.648508	0.001445	0.02247
12795	Plk3	polo-like kinase 3 (Drosophila)	0.648088	0.0008228	0.01603
233057	Zfp940	zinc finger protein 940	0.648088	0.001351	0.02151
328092	6530401N04Rik	RIKEN cDNA 6530401N04 gene	0.648088	0.0004863	0.01168
19649	Robo3	roundabout homolog 3 (Drosophila)	0.647668	0.01135	0.07552
434484	Sp140	Sp140 nuclear body protein	0.647668	0.01217	0.07852
18155	Pnoc	prepronociceptin	0.647249	0.01228	0.07892
27029	Sgsh	N-sulfoglucosamine sulfohydrolase (sulfamidase)	0.647249	0.06954	0.2231
80292	Zxdc	ZXD family zinc finger C	0.647249	0.1424	0.3467
627939	Gm6813	predicted gene 6813	0.647249	0.0001753	0.006589
664788	Gm7339	predicted gene 7339	0.647249	0.1769	0.3939
24067	Srp54a	signal recognition particle 54A	0.646412	0.001705	0.025
26943	Serinc3	serine incorporator 3	0.646412	3.24E-05	0.002549
74455	Nsun6	NOL1/NOP2/Sun domain family member 6	0.646412	0.06322	0.2099
238247	Arid4a	AT rich interactive domain 4A (RBP1-like)	0.646412	0.004203	0.04219
56760	Clec1b	C-type lectin domain family 1, member b	0.645995	0.2077	0.4342
75880	4930592A05Rik	RIKEN cDNA 4930592A05 gene	0.645995	0.05072	0.1828
11828	Aqp3	aquaporin 3	0.645578	0.2024	0.4285
382019	Zfp882	zinc finger protein 882	0.645578	6.57E-05	0.00373
76184	Abca6	ATP-binding cassette, sub-family A (ABC1), member 6	0.645161	0.0197	0.1051
170735	Arr3	arrestin 3, retinal	0.645161	0.0003968	0.01046
791373	B930078G14Rik	RIKEN cDNA B930078G14 gene	0.64433	0.2385	0.4708
12064	Bdnf	brain derived neurotrophic factor	0.643915	0.000434	0.01101
20607	Sstr3	somatostatin receptor 3	0.642674	0.006213	0.05304
231004	Samd11	sterile alpha motif domain containing 11	0.642674	0.0002667	0.008363
100038882	Isg15	ISG15 ubiquitin-like modifier	0.642674	0.01797	0.09919
240041	Zfp945	zinc finger protein 945	0.642261	0.03684	0.152
69454	Clic3	chloride intracellular channel 3	0.641849	0.01447	0.08752
333182	Cox6b2	cytochrome c oxidase subunit VIb polypeptide 2	0.641849	0.001544	0.02346
53415	Htatip2	HIV-1 tat interactive protein 2, homolog (human)	0.641437	0.05776	0.198
434179	Gm5595	predicted gene 5595	0.641437	0.156	0.3658
19222	Ptgir	prostaglandin I receptor (IP)	0.641026	0.005488	0.04946
244666	Gm505	predicted gene 505	0.641026	0.0747	0.2334
13086	Cyp2a4	cytochrome P450, family 2, subfamily a, polypeptide 4	0.640205	0.2186	0.4478
67196	Ube2t	ubiquitin-conjugating enzyme E2T (putative)	0.640205	0.2397	0.4722
77979	B230104C14Rik	RIKEN cDNA B230104C14 gene	0.640205	0.01215	0.07841
18108	Nmt2	N-myristoyltransferase 2	0.639386	0.006058	0.05239

433323	Sgpp2	sphingosine-1-phosphate phosphotase 2	0.638978	0.004745	0.04532
628709	Gm10324	predicted gene 10324	0.638978	0.01853	0.1011
234915	AK129341	cDNA sequence AK129341	0.63857	0.2675	0.5031
338352	Nell1	NEL-like 1 (chicken)	0.63857	0.0007501	0.01515
57390	Psors1c2	psoriasis susceptibility 1 candidate 2 (human)	0.638162	0.00825	0.06224
15397	Hoxa11as	HOXA11 antisense RNA (non-protein coding)	0.637755	0.04235	0.1647
67529	Fgfr1op2	FGFR1 oncogene partner 2	0.636537	0.01207	0.07821
70884	Ccdc81	coiled-coil domain containing 81	0.636537	0.0002991	0.00897
243308	A430033K04Rik	RIKEN cDNA A430033K04 gene	0.636537	0.002614	0.03167
100039672	Msmp	microseminoprotein, prostate associated	0.636537	0.4085	0.6378
102219	AU018794	expressed sequence AU018794	0.636132	0.005982	0.05212
30806	Adamts8	a disintegrin-like and metallopeptidase (reprolysin type) with thrombospondin type 1 motif, 8	0.635728	0.08685	0.2567
244757	Glb1l2	galactosidase, beta 1-like 2	0.635728	0.0003799	0.01024
22018	Tpo	thyroid peroxidase	0.635324	0.002582	0.0315
22174	Tyro3	TYRO3 protein tyrosine kinase 3	0.634921	0.0006305	0.01363
22190	Ubc	ubiquitin C	0.634921	0.002245	0.0291
100038415	F830034J09Rik	RIKEN cDNA F830034J09 gene	0.634921	0.1475	0.3541
19361	Rad51	RAD51 homolog (S. cerevisiae)	0.634115	0.0003812	0.01025
20776	Tmie	transmembrane inner ear	0.634115	0.003245	0.03607
30785	Cttnbp2	cortactin binding protein 2	0.634115	0.001579	0.02378
69698	2310046K01Rik	RIKEN cDNA 2310046K01 gene	0.634115	0.06542	0.2145
73884	Zdbf2	zinc finger, DBF-type containing 2	0.634115	0.1248	0.3216
664788	Gm7339	predicted gene 7339	0.634115	0.1491	0.3566
100038605	E030047D23Rik	RIKEN cDNA E030047D23 gene	0.634115	0.06222	0.2078
11752	Anxa8	annexin A8	0.633312	0.0417	0.1634
57740	Stk32c	serine/threonine kinase 32C	0.633312	0.08745	0.2579
20471	Six1	sine oculis-related homeobox 1 homolog (Drosophila)	0.632911	0.0002323	0.007731
69863	Ttc39b	tetratricopeptide repeat domain 39B	0.632911	0.06304	0.2096
110877	Slc18a1	solute carrier family 18 (vesicular monoamine), member 1	0.632911	0.1434	0.3482
239435	Aard	alanine and arginine rich domain containing protein	0.632911	0.001426	0.02229
15950	Ifi203	interferon activated gene 203	0.632111	0.1414	0.3456
68947	Chst8	carbohydrate (N-acetylgalactosamine 4-0) sulfotransferase 8	0.631712	0.0004571	0.01127
22144	Tuba3a	tubulin, alpha 3A	0.630517	0.003453	0.0374
68895	Rasl11a	RAS-like, family 11, member A	0.630517	0.0007815	0.01553
224116	Muc20	mucin 20	0.630517	0.0488	0.1787
381853	Gipr	gastric inhibitory polypeptide receptor	0.630517	0.002422	0.03051
66300	Prr24	proline rich 24	0.629723	8.28E-06	0.001114
105005	Fam84a	family with sequence similarity 84, member A	0.629723	0.001961	0.027
104103	Airn	antisense Igf2r RNA	0.629327	0.004597	0.0447
319610	9530083O12Rik	RIKEN cDNA 9530083O12 gene	0.629327	0.1115	0.2995
19339	Rab3a	RAB3A, member RAS oncogene family	0.628141	0.0001335	0.0056
71799	Ptcd1	pentatricopeptide repeat domain 1	0.628141	0.00138	0.02178
76640	1700113H08Rik	RIKEN cDNA 1700113H08 gene	0.628141	0.04962	0.1808
269344	Ell3	elongation factor RNA polymerase II-like 3	0.627746	0.002114	0.02797
17472	Gbp4	guanylate binding protein 4	0.627353	0.01137	0.07561
14121	Fbp1	fructose bisphosphatase 1	0.625391	0.001741	0.02527
258392	Olfr190	olfactory receptor 190	0.625	0.005838	0.0513
407828	BC023969	cDNA sequence BC023969	0.625	0.00621	0.05304
12286	Cacna1a	calcium channel, voltage-dependent, P/Q type, alpha 1A subunit	0.62422	0.001047	0.01821

76073	Pcgf5	polycomb group ring finger 5	0.62383	4.86E-05	0.00322
16502	Kcnc1	potassium voltage gated channel, Shaw-related subfamily, member 1	0.622278	0.0004357	0.01101
75619	Fastkd2	FAST kinase domains 2	0.622278	0.01361	0.08428
230576	Ttc22	tetratricopeptide repeat domain 22	0.622278	0.0235	0.1169
100039307	Gm2149	predicted gene 2149	0.621891	0.01743	0.09758
94217	Lrp1b	low density lipoprotein-related protein 1B (deleted in tumors)	0.621504	0.5441	0.7471
12409	Cbr2	carbonyl reductase 2	0.621118	0.005295	0.04847
18610	Pdyn	prodynorphin	0.621118	0.0007447	0.01508
623279	Dok6	docking protein 6	0.621118	0.02052	0.1076
100294583	Fam150b	family with sequence similarity 150, member B	0.621118	0.005327	0.04862
70835	Prss22	protease, serine, 22	0.620732	0.1363	0.3381
214240	Disp2	dispatched homolog 2 (Drosophila)	0.620732	6.15E-06	0.0009207
20259	Scin	scinderin	0.620347	0.01592	0.09258
70470	Rprd1b	regulation of nuclear pre-mRNA domain containing 1B	0.620347	0.0114	0.07568
76619	1700087I21Rik	RIKEN cDNA 1700087I21 gene	0.620347	0.003029	0.03442
100039948	Rhox2c	reproductive homeobox 2C	0.620347	0.04255	0.1651
74270	Usp20	ubiquitin specific peptidase 20	0.619579	0.0467	0.1748
258623	Olfr123	olfactory receptor 123	0.619579	0.1309	0.3307
320116	C030019I05Rik	RIKEN cDNA C030019I05 gene	0.619579	0.02748	0.1288
214552	Cep164	centrosomal protein 164	0.619195	0.0001068	0.004885
319998	Tmem198	transmembrane protein 198	0.618812	0.00035	0.009863
107350	AW112010	expressed sequence AW112010	0.618429	0.003822	0.03959
329504	Lcmt2	leucine carboxyl methyltransferase 2	0.618429	0.001725	0.02511
13860	Eps8	epidermal growth factor receptor pathway substrate 8	0.617284	5.77E-05	0.003535
17147	Mageb3	melanoma antigen, family B, 3	0.616903	0.2935	0.5294
14205	Figf	c-fos induced growth factor	0.616523	0.003416	0.03708
20687	Sp3	trans-acting transcription factor 3	0.616523	0.2078	0.4342
320775	B230319C09Rik	RIKEN cDNA B230319C09 gene	0.616143	0.002646	0.03188
629756	Wfdc10	WAP four-disulfide core domain 10	0.616143	0.001765	0.02542
12458	Ccr6	chemokine (C-C motif) receptor 6	0.615764	0.05537	0.1925
70405	Calml3	calmodulin-like 3	0.615764	0.02824	0.1308
99151	Cercam	cerebral endothelial cell adhesion molecule	0.615764	0.05246	0.1862
22174	Tyro3	TYRO3 protein tyrosine kinase 3	0.615385	0.09089	0.264
75578	Fggy	FGGY carbohydrate kinase domain containing	0.615385	0.03854	0.1563
14800	Gria2	glutamate receptor, ionotropic, AMPA2 (alpha 2)	0.614628	0.0008088	0.01587
20913	Stxbp4	syntaxin binding protein 4	0.614628	0.002529	0.03114
56219	Extl1	exostoses (multiple)-like 1	0.614628	0.01142	0.07571
58864	Tssk3	testis-specific serine kinase 3	0.614628	0.04682	0.175
329160	9130024F11Rik	RIKEN cDNA 9130024F11 gene	0.614628	0.002367	0.03015
212684	Pdzd7	PDZ domain containing 7	0.614251	0.0007699	0.01542
545428	Ccdc141	coiled-coil domain containing 141	0.614251	0.2252	0.4562
11689	Alox5	arachidonate 5-lipoxygenase	0.613874	0.005711	0.05051
15564	Htr5b	5-hydroxytryptamine (serotonin) receptor 5B	0.613874	0.1532	0.3628
54634	Magix	MAGI family member, X-linked	0.613874	0.09273	0.2672
635253	Usp51	ubiquitin specific protease 51	0.613874	8.05E-05	0.004179
15478	Hs3st3a1	heparan sulfate (glucosamine) 3-O-sulfotransferase 3A1	0.613497	0.003337	0.03664
434794	Xlr4a	X-linked lymphocyte-regulated 4A	0.613497	0.2647	0.5006
619842	Gm12633	zinc finger protein 352 pseudogene	0.613497	0.251	0.4851
66793	Efcab1	EF hand calcium binding domain 1	0.613121	0.04931	0.18

668809	Gm9372	predicted gene 9372	0.613121	0.1677	0.3827
72504	Taf4b	TAF4B RNA polymerase II, TATA box binding protein (TBP)-associated factor	0.61237	0.11	0.2973
231004	Samd11	sterile alpha motif domain containing 11	0.61237	0.006013	0.05219
69849	2010007H06Rik	RIKEN cDNA 2010007H06 gene	0.611995	0.003291	0.03631
75677	Cldn22	claudin 22	0.611995	0.00273	0.03237
16664	Krt14	keratin 14	0.611621	0.01468	0.08828
71263	Mro	maestro	0.611621	0.03442	0.1467
73961	4930447J18Rik	RIKEN cDNA 4930447J18 gene	0.611621	0.06004	0.2033
69329	1700003M02Rik	RIKEN cDNA 1700003M02 gene	0.611247	0.001986	0.02709
73430	1700049G17Rik	RIKEN cDNA 1700049G17 gene	0.610874	0.0002311	0.007731
100042057	Gm3643	predicted gene 3643	0.610874	0.001822	0.02572
21954	Tnni3	troponin I, cardiac 3	0.610501	0.006525	0.05458
241514	Zfp804a	zinc finger protein 804A	0.610128	0.3855	0.6176
16665	Krt15	keratin 15	0.609756	0.003987	0.04076
237806	Dnahc9	dynein, axonemal, heavy chain 9	0.609756	0.01205	0.07813
58807	Slco1c1	solute carrier organic anion transporter family, member 1c1	0.609385	0.003769	0.03935
629091	LOC629091	hypothetical protein LOC629091	0.608273	0.05083	0.1829
74221	1700011F03Rik	RIKEN cDNA 1700011F03 gene	0.607903	0.03944	0.1582
12412	Cbx1	chromobox homolog 1 (Drosophila HP1 beta)	0.607533	0.001248	0.02045
631105	Gm7056	predicted gene 7056	0.607533	0.4577	0.6812
107684	Coro2a	coronin, actin binding protein 2A	0.606796	0.1388	0.3418
14284	Fosl2	fos-like antigen 2	0.606061	0.1945	0.4182
93837	Dach2	dachshund 2 (Drosophila)	0.606061	0.0001216	0.005257
67887	Tmem66	transmembrane protein 66	0.605694	0.0003668	0.0101
74363	4931430N09Rik	RIKEN cDNA 4931430N09 gene	0.605327	0.001161	0.01937
80718	Rab27b	RAB27b, member RAS oncogene family	0.604961	0.0002811	0.008625
433215	BC048609	cDNA sequence BC048609	0.604961	0.005419	0.04901
13599	Ecel1	endothelin converting enzyme-like 1	0.603865	0.002354	0.03004
76789	Mzt1	mitotic spindle organizing protein 1	0.603865	0.016	0.09268
21463	Tcp11	t-complex protein 11	0.6035	0.0009332	0.01718
77035	Jmjd5	jumonji domain containing 5	0.6035	4.42E-05	0.003078
218763	Lrrc3b	leucine rich repeat containing 3B	0.6035	0.1131	0.3023
257932	Olfr332	olfactory receptor 332	0.6035	0.269	0.5043
319545	D430020J02Rik	RIKEN cDNA D430020J02 gene	0.6035	0.1975	0.4224
75578	Fggy	FGGY carbohydrate kinase domain containing	0.603136	1.79E-05	0.001788
224090	Tmem44	transmembrane protein 44	0.602773	0.01166	0.07664
15959	Ifit3	interferon-induced protein with tetratricopeptide repeats 3	0.60241	0.001354	0.02154
100043335	Gm4371	eukaryotic translation initiation factor 3, subunit I pseudogene	0.602047	0.001318	0.02117
268567	Tmem229b	transmembrane protein 229B	0.601685	0.001537	0.02342
329650	Med12l	mediator of RNA polymerase II transcription, subunit 12 homolog (yeast)-like	0.601323	0.06489	0.2134
353211	Prune2	prune homolog 2 (Drosophila)	0.600962	0.006207	0.05304
11857	Arhgdib	Rho, GDP dissociation inhibitor (GDI) beta	0.600601	0.001001	0.01779
19259	Ptpn5	protein tyrosine phosphatase, non-receptor type 5	0.600601	0.0009633	0.01752
101694	AI854517	expressed sequence AI854517	0.600601	0.0001339	0.0056
24135	Zfp68	zinc finger protein 68	0.60024	2.50E-05	0.002162
98878	Ehd4	EH-domain containing 4	0.60024	3.59E-05	0.002689
215384	Fcgbp	Fc fragment of IgG binding protein	0.60024	0.005061	0.04703
15983	Ifrd2	interferon-related developmental regulator 2	0.59988	0.005554	0.0498
75818	4930478L05Rik	RIKEN cDNA 4930478L05 gene	0.59952	0.003401	0.03699

242960	Fbxl5	F-box and leucine-rich repeat protein 5	0.59952	0.0005403	0.01249
328191	B230303A05Rik	RIKEN cDNA B230303A05 gene	0.599161	0.02981	0.1351
433466	Jmjd7	jumonji domain containing 7	0.599161	1.52E-05	0.001591
100041574	9030025P20Rik	RIKEN cDNA 9030025P20 gene	0.598444	0.0004792	0.01159
18168	Npy5r	neuropeptide Y receptor Y5	0.598086	0.005921	0.05177
68338	Golt1a	golgi transport 1 homolog A (S. cerevisiae)	0.597372	0.05463	0.1912
76872	Ccdc116	coiled-coil domain containing 116	0.597372	0.02287	0.1152
100036538	BC028777	cDNA sequence BC028777	0.597372	0.06229	0.208
11601	Angpt2	angiopoietin 2	0.596659	0.001793	0.02551
330554	Fan1	FANCD2/FANCI-associated nuclease 1	0.596659	0.0002032	0.007259
638580	Gm7244	predicted gene 7244	0.596303	0.0001759	0.006593
107351	Kank1	KN motif and ankyrin repeat domains 1	0.595948	0.0003433	0.009724
24053	Sgcg	sarcoglycan, gamma (dystrophin-associated glycoprotein)	0.595238	0.1264	0.3236
72350	Fam164c	family with sequence similarity 164, member C	0.595238	0.001777	0.02544
80796	Calm4	calmodulin 4	0.595238	0.2316	0.4631
210417	Thsd7b	thrombospondin, type I, domain containing 7B	0.594884	0.002149	0.02816
56523	Pmfbp1	polyamine modulated factor 1 binding protein 1	0.594177	0.003632	0.03855
75578	Fggy	FGGY carbohydrate kinase domain containing	0.594177	0.0002113	0.007384
434797	Gm5640	predicted gene 5640	0.594177	0.08561	0.254
269629	Lhfpl3	lipoma HMGIC fusion partner-like 3	0.593472	0.0001351	0.0056
320095	6430550D23Rik	RIKEN cDNA 6430550D23 gene	0.59312	0.007543	0.05939
100042840	Gm16501	predicted gene 16501	0.59312	0.006945	0.05649
16333	Ins1	insulin I	0.592768	0.01594	0.0926
66166	S100a14	S100 calcium binding protein A14	0.592768	0.01911	0.1031
18019	Nfatc2	nuclear factor of activated T-cells, cytoplasmic, calcineurin-dependent 2	0.591716	0.0006303	0.01363
237987	Otop2	otopetrin 2	0.591366	0.06031	0.204
19744	Rheb	Ras homolog enriched in brain	0.591017	0.2053	0.4316
240041	Zfp945	zinc finger protein 945	0.591017	0.004069	0.04134
73941	4930412L05Rik	RIKEN cDNA 4930412L05 gene	0.590667	0.0006894	0.01446
13179	Dcn	decorin	0.589971	0.02364	0.1172
93837	Dach2	dachshund 2 (Drosophila)	0.588928	0.0109	0.07377
547103	Gm10674	predicted gene 10674	0.588928	0.02489	0.1208
105332	AW011956	expressed sequence AW011956	0.588235	0.04346	0.1674
639653	Gm7276	predicted gene 7276	0.588235	4.84E-05	0.00322
108069	Grm3	glutamate receptor, metabotropic 3	0.587889	0.001539	0.02343
23956	Neu2	neuraminidase 2	0.587544	0.0005481	0.01259
66805	Tspan1	tetraspanin 1	0.587199	0.02934	0.1338
20715	Serpina3g	serine (or cysteine) peptidase inhibitor, clade A, member 3G	0.586854	0.003226	0.03596
67014	Mina	myc induced nuclear antigen	0.586854	0.003799	0.03947
74354	Lrguk	leucine-rich repeats and guanylate kinase domain containing	0.58651	0.01466	0.08819
100302730	5830417I10Rik	RIKEN cDNA 5830417I10 gene	0.58651	0.0009768	0.01765
68052	Rps13	ribosomal protein S13	0.586166	0.000383	0.01026
67182	Pdzk1ip1	PDZK1 interacting protein 1	0.585823	0.05536	0.1925
382571	Kcnf1	potassium voltage-gated channel, subfamily F, member 1	0.585823	0.01456	0.08775
258186	Olfr75-ps1	olfactory receptor 75, pseudogene 1	0.58548	0.0125	0.07994
232400	BC048546	cDNA sequence BC048546	0.584795	0.0007965	0.01572
252864	Dusp15	dual specificity phosphatase-like 15	0.584795	0.3222	0.5585
229905	Ccbl2	cysteine conjugate-beta lyase 2	0.584454	0.1153	0.306
20304	Ccl5	chemokine (C-C motif) ligand 5	0.583771	0.02624	0.1251

68789	Trmt61b	tRNA methyltransferase 61 homolog B (S. cerevisiae)	0.583771	9.88E-05	0.004628
664876	Gm7385	predicted gene 7385	0.583771	0.2493	0.4835
320873	Cdh10	cadherin 10	0.581395	8.75E-05	0.004331
192236	Hps1	Hermansky-Pudlak syndrome 1 homolog (human)	0.581058	0.0007222	0.01482
66039	D14Ertd449e	DNA segment, Chr 14, ERATO Doi 449, expressed	0.57971	0.003636	0.03856
16687	Krt6a	keratin 6A	0.579374	0.1279	0.3259
22021	Tpst1	protein-tyrosine sulfotransferase 1	0.579374	0.4133	0.642
109032	Sp110	Sp110 nuclear body protein	0.579374	0.000482	0.01163
240334	Pcyox1l	prenylcysteine oxidase 1 like	0.578704	0.000625	0.01363
70405	Calml3	calmodulin-like 3	0.578369	0.006324	0.05346
226407	Rab3gap1	RAB3 GTPase activating protein subunit 1	0.578369	0.001074	0.01846
381406	2810408M09Rik	RIKEN cDNA 2810408M09 gene	0.578369	0.004786	0.04548
108075	Ltbp4	latent transforming growth factor beta binding protein 4	0.578035	0.0004136	0.01075
14237	Foxd4	forkhead box D4	0.577701	0.05655	0.1952
267019	Rps15a	ribosomal protein S15A	0.577701	0.0008959	0.01687
216225	Slc5a8	solute carrier family 5 (iodide transporter), member 8	0.577367	0.07644	0.2367
77193	A930030B08Rik	RIKEN cDNA A930030B08 gene	0.576701	0.001618	0.02422
245555	C77370	expressed sequence C77370	0.576037	0.05165	0.1847
69282	1700001J03Rik	RIKEN cDNA 1700001J03 gene	0.575705	0.0002075	0.007323
26918	Ern2	endoplasmic reticulum (ER) to nucleus signalling 2	0.575043	0.1082	0.2943
53322	Nucb2	nucleobindin 2	0.574383	0.0009436	0.01733
67886	Camsap1l1	calmodulin regulated spectrin-associated protein 1-like 1	0.574383	0.003211	0.03582
381062	2210404J11Rik	RIKEN cDNA 2210404J11 gene	0.574053	0.0002069	0.007318
60345	Nrip2	nuclear receptor interacting protein 2	0.573066	0.0001707	0.006482
11752	Anxa8	annexin A8	0.572082	0.03601	0.15
16538	Kcns1	K+ voltage-gated channel, subfamily S, 1	0.571429	0.03871	0.1566
226438	Igfn1	immunoglobulin-like and fibronectin type III domain containing 1	0.571429	0.00151	0.02312
108816	4933409K07Rik	RIKEN cDNA 4933409K07 gene	0.571102	0.3006	0.5366
214552	Cep164	centrosomal protein 164	0.571102	8.16E-05	0.004217
100126243	A030001D20Rik	RIKEN cDNA A030001D20 gene	0.570451	5.75E-05	0.003535
76265	Tsen54	tRNA splicing endonuclease 54 homolog (S. cerevisiae)	0.569801	0.0129	0.08162
71338	Tprg	transformation related protein 63 regulated	0.569476	0.2609	0.4961
76933	Ifi27l2a	interferon, alpha-inducible protein 27 like 2A	0.568828	0.01112	0.07467
114873	Dscaml1	Down syndrome cell adhesion molecule-like 1	0.568505	0.02306	0.1158
14349	Fv1	Friend virus susceptibility 1	0.568182	0.0149	0.08912
381476	B930007M17Rik	RIKEN cDNA B930007M17 gene	0.568182	0.2401	0.4728
17199	Mc1r	melanocortin 1 receptor	0.567859	0.000123	0.005296
83453	Chrdl1	chordin-like 1	0.567215	0.0003568	0.009947
27413	Abcb11	ATP-binding cassette, sub-family B (MDR/TAP), member 11	0.566893	0.0009928	0.01774
76658	1700123K08Rik	RIKEN cDNA 1700123K08 gene	0.566893	0.0002496	0.007991
433215	BC048609	cDNA sequence BC048609	0.566251	0.009612	0.06824
17700	Mstn	myostatin	0.565291	0.0123	0.07893
243078	Tecrl	trans-2,3-enoyl-CoA reductase-like	0.564972	0.0002718	0.008451
13107	Cyp2f2	cytochrome P450, family 2, subfamily f, polypeptide 2	0.564334	0.0233	0.1165
66326	Dnajc5b	DnaJ (Hsp40) homolog, subfamily C, member 5 beta	0.563698	0.1879	0.4088
76560	Prss8	protease, serine, 8 (prostasin)	0.563698	0.02428	0.119
79235	Lrat	lecithin-retinol acyltransferase (phosphatidylcholine-retinol-O-acyltransferase)	0.56338	0.1813	0.4001
58807	Slco1c1	solute carrier organic anion transporter family, member 1c1	0.563063	0.0002937	0.008884
11997	Akr1b7	aldo-keto reductase family 1, member B7	0.56243	0.001754	0.02535

224671	Btbd9	BTB (POZ) domain containing 9	0.562114	0.0005745	0.01299
15235	Mst1	macrophage stimulating 1 (hepatocyte growth factor-like)	0.560852	0.05454	0.191
210274	Shank2	SH3/ankyrin domain gene 2	0.560538	0.2281	0.459
331529	Gm5128	predicted gene 5128	0.560224	0.4478	0.6733
65112	Pmepa1	prostate transmembrane protein, androgen induced 1	0.557724	4.85E-05	0.00322
382111	Susd5	sushi domain containing 5	0.557724	0.03364	0.145
68311	Lypd2	Ly6/Plaur domain containing 2	0.557103	0.1531	0.3626
18626	Per1	period homolog 1 (Drosophila)	0.556483	0.000475	0.01156
79202	Tnfrsf22	tumor necrosis factor receptor superfamily, member 22	0.556483	0.1493	0.3568
63828	Fn3k	fructosamine 3 kinase	0.556174	0.0006911	0.01446
67607	Zfp788	zinc finger protein 788	0.554631	0.1809	0.3995
386454	Rnf39	ring finger protein 39	0.554631	0.02504	0.1212
18510	Pax8	paired box gene 8	0.554017	0.007186	0.0576
93730	Lztfl1	leucine zipper transcription factor-like 1	0.554017	0.0001153	0.005121
20901	Strap	serine/threonine kinase receptor associated protein	0.553097	5.46E-05	0.00347
71334	5430416G10Rik	RIKEN cDNA 5430416G10 gene	0.553097	5.37E-05	0.003458
80288	Bcl9l	B-cell CLL/lymphoma 9-like	0.553097	0.001106	0.01881
320082	Fbxw21	F-box and WD-40 domain protein 21	0.552792	0.01645	0.09414
68720	Lce1b	late cornified envelope 1B	0.552181	0.1172	0.3091
270058	Mtap1s	microtubule-associated protein 1S	0.552181	0.0002435	0.007862
237010	Klhl4	kelch-like 4 (Drosophila)	0.551876	0.002331	0.02986
257632	Nod2	nucleotide-binding oligomerization domain containing 2	0.551876	0.06709	0.2183
97783	C76336	expressed sequence C76336	0.551572	6.36E-05	0.003678
20514	Slc1a5	solute carrier family 1 (neutral amino acid transporter), member 5	0.551268	7.98E-05	0.004179
26971	Pla2g2f	phospholipase A2, group IIF	0.551268	0.1339	0.3346
226245	9930023K05Rik	RIKEN cDNA 9930023K05 gene	0.551268	0.01667	0.09492
193385	Fam65b	family with sequence similarity 65, member B	0.550661	0.03937	0.1581
100041244	Gm3230	predicted gene 3230	0.550358	0.2489	0.4832
20861	Stfa1	stefin A1	0.550055	0.04355	0.1676
22215	Ube3a	ubiquitin protein ligase E3A	0.549753	0.0007788	0.01551
73673	2410076I21Rik	RIKEN cDNA 2410076I21 gene	0.549451	0.0002496	0.007991
64452	Slc5a4a	solute carrier family 5, member 4a	0.548847	0.2737	0.5094
20614	Snap25	synaptosomal-associated protein 25	0.548246	0.01104	0.07436
13482	Dpp4	dipeptidylpeptidase 4	0.547645	0.008046	0.06145
100039239	Gm2115	predicted gene 2115	0.547345	0.001266	0.02067
69036	Zg16	zymogen granule protein 16	0.545852	0.3781	0.6118
107581	Col16a1	collagen, type XVI, alpha 1	0.545852	0.1773	0.3945
100216455	Gm14124	predicted gene 14124	0.545256	0.06979	0.2237
71764	C2cd2l	C2 calcium-dependent domain containing 2-like	0.544959	5.95E-05	0.003567
216859	Acap1	ArfGAP with coiled-coil, ankyrin repeat and PH domains 1	0.544959	0.02419	0.1187
338349	Cntln	centlein, centrosomal protein	0.544959	0.007572	0.05949
100043034	Gm13138	predicted gene 13138	0.544959	3.15E-05	0.002512
546886	Ccdc42b	coiled-coil domain containing 42B	0.544662	0.2553	0.4902
74558	Gvin1	GTPase, very large interferon inducible 1	0.543774	0.0561	0.194
224833	AI661453	expressed sequence AI661453	0.543774	0.01962	0.1049
21462	Tcp10c	t-complex protein 10c	0.542888	0.007296	0.05811
319711	E230029C05Rik	RIKEN cDNA E230029C05 gene	0.542888	0.0001346	0.0056
19378	Aldh1a2	aldehyde dehydrogenase family 1, subfamily A2	0.542005	0.005041	0.04691
320064	D130017N08Rik	RIKEN cDNA D130017N08 gene	0.541126	0.002437	0.03058

224833	AI661453	expressed sequence AI661453	0.540249	0.07557	0.2354
22268	Upk1b	uroplakin 1B	0.539957	0.0444	0.1695
209012	Ulk4	unc-51-like kinase 4 (C. elegans)	0.539957	0.0003369	0.009646
12409	Cbr2	carbonyl reductase 2	0.539665	0.002499	0.03096
23827	Bpnt1	bisphosphate 3'-nucleotidase 1	0.539374	0.0004727	0.01153
269952	D330012F22Rik	RIKEN cDNA D330012F22 gene	0.538503	0.00307	0.03478
353211	Prune2	prune homolog 2 (Drosophila)	0.538503	0.01319	0.08279
633093	Gm7104	REX1, RNA exonuclease 1 homolog pseudogene	0.538503	0.1322	0.3326
67425	Eps8l1	EPS8-like 1	0.537924	0.03798	0.155
103775	Slc25a41	solute carrier family 25, member 41	0.537057	0.002503	0.03098
69976	Galk2	galactokinase 2	0.536193	0.002235	0.02902
242773	Slc45a1	solute carrier family 45, member 1	0.535619	0.000598	0.01338
216227	Slc17a8	solute carrier family 17 (sodium-dependent inorganic phosphate cotransporter), member 8	0.535332	0.002529	0.03114
76560	Prss8	protease, serine, 8 (prostasin)	0.533903	0.06149	0.2062
319622	Itpripl2	inositol 1,4,5-triphosphate receptor interacting protein-like 2	0.533618	0.0004796	0.01159
66522	Pgpep1	pyroglutamyl-peptidase I	0.532481	0.0003085	0.009106
244198	Olfml1	olfactomedin-like 1	0.531067	0.1348	0.3359
22436	Xdh	xanthine dehydrogenase	0.530786	0.002918	0.03375
14178	Fgf7	fibroblast growth factor 7	0.529661	9.19E-05	0.004417
24102	Trex2	three prime repair exonuclease 2	0.529661	0.05245	0.1862
26388	Ifi202b	interferon activated gene 202B	0.529381	0.1153	0.3061
258757	Olfr1013	olfactory receptor 1013	0.528262	0.2677	0.5032
15559	Htr2b	5-hydroxytryptamine (serotonin) receptor 2B	0.527983	0.0002107	0.007382
66044	Dtd1	D-tyrosyl-tRNA deacylase 1 homolog (S. cerevisiae)	0.527704	0.0008563	0.01654
320456	B330016D10Rik	RIKEN cDNA B330016D10 gene	0.527426	0.005335	0.04865
668643	EG668643	predicted gene, EG668643	0.527148	0.01718	0.09687
13730	Emp1	epithelial membrane protein 1	0.526039	0.02177	0.1119
20269	Scn3a	sodium channel, voltage-gated, type III, alpha	0.526039	0.02577	0.1235
56773	Chst5	carbohydrate (N-acetylglucosamine 6-O) sulfotransferase 5	0.526039	0.2056	0.4319
353188	Adam32	a disintegrin and metallopeptidase domain 32	0.525762	0.01023	0.07112
16688	Krt6b	keratin 6B	0.525486	0.09697	0.2744
217116	Spata20	spermatogenesis associated 20	0.52521	0.004767	0.04541
12288	Cacna1c	calcium channel, voltage-dependent, L type, alpha 1C subunit	0.524934	0.2589	0.4942
114671	4930444G20Rik	RIKEN cDNA 4930444G20 gene	0.524109	0.1635	0.3763
140795	P2ry14	purinergic receptor P2Y, G-protein coupled, 14	0.524109	0.002529	0.03114
94222	Olig3	oligodendrocyte transcription factor 3	0.523834	0.004096	0.04151
67859	2310002J15Rik	RIKEN cDNA 2310002J15 gene	0.523286	0.02175	0.1119
68655	Fndc1	fibronectin type III domain containing 1	0.523013	0.0009245	0.01711
238130	Dock4	dedicator of cytokinesis 4	0.522739	5.94E-05	0.003567
241134	9430031J16Rik	RIKEN cDNA 9430031J16 gene	0.522739	0.0004683	0.01145
12294	Cacna2d3	calcium channel, voltage-dependent, alpha2/delta subunit 3	0.520021	2.61E-05	0.002182
636931	Trim71	tripartite motif-containing 71	0.519481	0.008322	0.06257
110310	Krt7	keratin 7	0.519211	0.001685	0.02485
100039227	Gm10394	predicted gene 10394	0.519211	0.006268	0.05321
93730	Lztfl1	leucine zipper transcription factor-like 1	0.518135	2.05E-06	0.0004889
67020	Tmem88	transmembrane protein 88	0.517866	8.20E-05	0.004218
108803	4933402P03Rik	RIKEN cDNA 4933402P03 gene	0.517866	0.04227	0.1645
59036	Dact1	dapper homolog 1, antagonist of beta-catenin (xenopus)	0.517063	0.0009174	0.01704
12767	Cxcr4	chemokine (C-X-C motif) receptor 4	0.516529	0.03073	0.1373

76585	Lce1i	late cornified envelope 1I	0.516262	0.191	0.413
668110	Syce1l	synaptonemal complex central element protein 1 like	0.513875	0.0003145	0.009254
67085	1700024G13Rik	RIKEN cDNA 1700024G13 gene	0.513611	0.02392	0.118
68888	Gkn3	gastrokine 3	0.513611	0.002738	0.03242
320415	Gchfr	GTP cyclohydrolase I feedback regulator	0.513611	5.59E-05	0.00347
243834	Zfp324	zinc finger protein 324	0.513347	0.0001093	0.004925
272322	Arntl2	aryl hydrocarbon receptor nuclear translocator-like 2	0.513084	0.004308	0.04286
17444	Grap2	GRB2-related adaptor protein 2	0.510986	0.1728	0.3888
668605	Gm9265	predicted pseudogene 9265	0.510725	1.02E-05	0.001228
665389	Gm7616	predicted gene 7616	0.510465	0.03511	0.1484
668239	Gm12270	predicted gene 12270	0.509944	1.25E-06	0.0003502
11522	Adh1	alcohol dehydrogenase 1 (class I)	0.508647	0.01361	0.08428
100503910	LOC100503910	hypothetical LOC100503910	0.508647	0.05174	0.1849
20887	Sult1a1	sulfotransferase family 1A, phenol-preferring, member 1	0.506586	9.21E-05	0.004417
77481	C030048H21Rik	RIKEN cDNA C030048H21 gene	0.506329	0.001446	0.02247
68233	1700125D06Rik	RIKEN cDNA 1700125D06 gene	0.506073	0.05989	0.2031
195434	Utp14b	UTP14, U3 small nucleolar ribonucleoprotein, homolog B (yeast)	0.504541	0.02338	0.1166
140474	Muc4	mucin 4	0.503778	0.02263	0.1146
269593	Luzp1	leucine zipper protein 1	0.503778	0.1182	0.3107
26447	Poli	polymerase (DNA directed), iota	0.503018	0.006858	0.05609
72585	Lypd1	Ly6/Plaur domain containing 1	0.502008	0.0002049	0.00726
93837	Dach2	dachshund 2 (Drosophila)	0.502008	0.007248	0.05797
11486	Ada	adenosine deaminase	0.501505	0.004369	0.04317
108075	Ltbp4	latent transforming growth factor beta binding protein 4	0.50025	0.004714	0.04511
68655	Fndc1	fibronectin type III domain containing 1	0.5	3.93E-05	0.002859
622139	Gm6289	predicted gene 6289	0.4995	0.006956	0.05649
207686	A330021E22Rik	RIKEN cDNA A330021E22 gene	0.499002	0.0006219	0.01363
328092	6530401N04Rik	RIKEN cDNA 6530401N04 gene	0.497265	0.0002144	0.007384
20348	Sema3c	sema domain, immunoglobulin domain (Ig), short basic domain, secreted, (semaphorin) 3C	0.496771	0.05297	0.1873
268482	Krt12	keratin 12	0.496771	0.2556	0.4905
66161	Pop4	processing of precursor 4, ribonuclease P/MRP family, (S. cerevisiae)	0.495786	0.0008855	0.01681
381218	4430402I18Rik	RIKEN cDNA 4430402I18 gene	0.494805	0.09587	0.2725
329253	6530439I21	hypothetical protein 6530439I21	0.494315	0.004436	0.04357
13052	Cxadr	coxsackie virus and adenovirus receptor	0.493583	0.0003374	0.009646
100034684	BC100530	cDNA sequence BC100530	0.489956	0.01551	0.09123
16663	Krt13	keratin 13	0.489716	0.01646	0.09419
16816	Lcat	lecithin cholesterol acyltransferase	0.489476	0.4692	0.6912
233038	Nccrp1	non-specific cytotoxic cell receptor protein 1 homolog (zebrafish)	0.488998	0.01479	0.08867
237926	Rsad1	radical S-adenosyl methionine domain containing 1	0.488759	0.0007058	0.01462
117147	Acsm1	acyl-CoA synthetase medium-chain family member 1	0.488043	0.001999	0.02718
26465	Zfp146	zinc finger protein 146	0.487567	0.002855	0.03335
22301	Vmn2r89	vomeronasal 2, receptor 89	0.487329	0.01814	0.09973
73463	Als2cr11	amyotrophic lateral sclerosis 2 (juvenile) chromosome region, candidate 11 (human)	0.486855	0.001118	0.01897
56365	Clcnkb	chloride channel Kb	0.485201	0.0004429	0.01105
381413	Gpr176	G protein-coupled receptor 176	0.484496	1.83E-05	0.00182
114564	Csprs	component of Sp100-rs	0.483793	0.04196	0.1639
20887	Sult1a1	sulfotransferase family 1A, phenol-preferring, member 1	0.483559	6.20E-05	0.00362
330554	Fan1	FANCD2/FANCI-associated nuclease 1	0.483325	0.02435	0.1192
71898	Apol9b	apolipoprotein L 9b	0.481928	0.005898	0.05163

236193	Zfp709	zinc finger protein 709	0.480769	5.72E-05	0.003526
74463	Exoc3l2	exocyst complex component 3-like 2	0.480307	0.007567	0.05948
69368	Wdfy1	WD repeat and FYVE domain containing 1	0.480077	7.10E-06	0.001014
233406	Prc1	protein regulator of cytokinesis 1	0.479616	0.0001528	0.006022
18101	Nmbr	neuromedin B receptor	0.478927	0.0006207	0.01362
319271	A130072N09Rik	RIKEN cDNA A130072N09 gene	0.478698	0.0236	0.1171
623166	Prr23a	proline rich 23A	0.47824	0.1803	0.3986
70725	6330411D24Rik	RIKEN cDNA 6330411D24 gene	0.475059	0.01375	0.08486
214253	Etnk2	ethanolamine kinase 2	0.475059	0.00123	0.0203
71981	Tdrd12	tudor domain containing 12	0.474608	7.83E-05	0.004143
77596	Gpr110	G protein-coupled receptor 110	0.474608	0.03549	0.1488
71908	Cldn23	claudin 23	0.473934	0.0001864	0.006796
246730	Oas1a	2'-5' oligoadenylate synthetase 1A	0.473709	0.005217	0.04794
319830	1500004A13Rik	RIKEN cDNA 1500004A13 gene	0.47259	0.0009336	0.01718
229595	Adamtsl4	ADAMTS-like 4	0.471921	1.03E-05	0.001228
627367	Vmn2r97	vomeronasal 2, receptor 97	0.471476	0.1709	0.3865
68854	Asb11	ankyrin repeat and SOCS box-containing 11	0.471254	0.00166	0.02466
320226	4930473A06Rik	RIKEN cDNA 4930473A06 gene	0.471254	0.000265	0.008339
76426	1700029J11Rik	RIKEN cDNA 1700029J11 gene	0.470588	0.03704	0.1525
80718	Rab27b	RAB27b, member RAS oncogene family	0.469043	0.0001587	0.006139
217109	Utp18	UTP18, small subunit (SSU) processome component, homolog (yeast)	0.468604	0.1258	0.3228
26388	Ifi202b	interferon activated gene 202B	0.467508	0.09778	0.2759
66255	Hsbp1l1	heat shock factor binding protein 1-like 1	0.46729	0.000954	0.0174
16869	Lhx1	LIM homeobox protein 1	0.466418	0.0001091	0.004925
100039796	Tgtp2	T-cell specific GTPase 2	0.466418	0.00199	0.02711
16992	Lta	lymphotoxin A	0.465549	0.003288	0.03631
67828	Lce1f	late cornified envelope 1F	0.465549	0.1227	0.3185
234290	BC030500	cDNA sequence BC030500	0.465333	1.42E-05	0.001503
11684	Alox12	arachidonate 12-lipoxygenase	0.463392	0.1362	0.338
67855	Asprv1	aspartic peptidase, retroviral-like 1	0.461255	0.0317	0.1399
56546	Sec1	secretory blood group 1	0.460617	0.0003056	0.009062
72556	Zfp566	zinc finger protein 566	0.459982	8.85E-05	0.004348
73332	Ccdc30	coiled-coil domain containing 30	0.458926	0.002483	0.03083
72061	2010111I01Rik	RIKEN cDNA 2010111I01 gene	0.457247	0.2759	0.5114
75617	Rps25	ribosomal protein S25	0.457247	1.30E-05	0.001431
68694	Lce1e	late cornified envelope 1E	0.456413	0.06811	0.2204
54200	Sult2b1	sulfotransferase family, cytosolic, 2B, member 1	0.456204	0.0002236	0.007631
211623	Plac9	placenta specific 9	0.456204	1.87E-05	0.001846
70918	Nsun7	NOL1/NOP2/Sun domain family, member 7	0.455789	0.01239	0.07935
18167	Npy2r	neuropeptide Y receptor Y2	0.454752	0.3102	0.5467
207798	Gramd1c	GRAM domain containing 1C	0.453926	0.005371	0.04883
269378	Ahcy	S-adenosylhomocysteine hydrolase	0.451671	0.000439	0.01101
15492	Hsd3b1	hydroxy-delta-5-steroid dehydrogenase, 3 beta- and steroid delta-isomerase 1	0.450045	0.2483	0.4824
16328	Cep250	centrosomal protein 250	0.449438	0.0001545	0.006066
16688	Krt6b	keratin 6B	0.449438	0.04528	0.1715
106757	Tmem146	transmembrane protein 146	0.448632	0.02065	0.1081
67182	Pdzk1ip1	PDZK1 interacting protein 1	0.448029	0.002811	0.03293
66583	Exosc1	exosome component 1	0.447628	0.0004033	0.01059
18216	Ntsr1	neurotensin receptor 1	0.447427	0.378	0.6117

19763	Ring1	ring finger protein 1	0.445831	0.0004797	0.01159
232813	Shisa7	shisa homolog 7 (Xenopus laevis)	0.445434	0.002445	0.03063
54672	Gpr97	G protein-coupled receptor 97	0.440141	0.005402	0.04892
11496	Adam22	a disintegrin and metallopeptidase domain 22	0.439754	0.08623	0.2553
71874	2310007B03Rik	RIKEN cDNA 2310007B03 gene	0.438789	0.002902	0.03363
94180	Acsbg1	acyl-CoA synthetase bubblegum family member 1	0.437445	0.002094	0.02786
13656	Egr4	early growth response 4	0.437254	0.0007286	0.01491
320152	4930412C18Rik	RIKEN cDNA 4930412C18 gene	0.437254	0.0002422	0.007862
69611	Lce1d	late cornified envelope 1D	0.437063	0.2149	0.4435
100042166	Gm3704	predicted gene 3704	0.436872	0.01064	0.07272
547349	LOC547349	similar to MHC class I antigen precursor	0.43554	9.05E-07	0.0002847
17285	Meox1	mesenchyme homeobox 1	0.433839	0.003257	0.03607
74424	Tmc5	transmembrane channel-like gene family 5	0.433276	0.0004403	0.01102
75136	Rsph10b2	radial spoke head 10 homolog B (Chlamydomonas)	0.431406	0.002756	0.03254
13711	Elf5	E74-like factor 5	0.430108	0.001024	0.01793
110308	Krt5	keratin 5	0.429553	0.04138	0.1628
76464	Casc5	cancer susceptibility candidate 5	0.428082	0.1372	0.339
59095	Fxyd6	FXYD domain-containing ion transport regulator 6	0.426257	3.70E-05	0.002737
58203	Zbp1	Z-DNA binding protein 1	0.424809	6.34E-05	0.003678
100043609	Gm14207	predicted gene 14207	0.424809	0.2007	0.4264
58801	Pmaip1	phorbol-12-myristate-13-acetate-induced protein 1	0.422297	4.44E-05	0.003078
58185	Rsad2	radical S-adenosyl methionine domain containing 2	0.421053	0.007624	0.05979
66402	Sln	sarcolipin	0.420345	0.1598	0.3715
68231	H2afb1	H2A histone family, member B1	0.417885	0.0002557	0.008114
67722	4921517D21Rik	RIKEN cDNA 4921517D21 gene	0.417537	0.09412	0.2696
18553	Pcsk6	proprotein convertase subtilisin/kexin type 6	0.4158	8.07E-07	0.0002627
20863	Stfa3	stefin A3	0.415455	0.03425	0.1464
13852	Stx2	syntaxin 2	0.415282	6.63E-05	0.00373
24053	Sgcg	sarcoglycan, gamma (dystrophin-associated glycoprotein)	0.414594	0.01138	0.07561
545471	Zfp345	zinc finger protein 345	0.413052	0.04823	0.1777
66353	Riiad1	regulatory subunit of type II PKA R-subunit (RIIa) domain containing 1	0.412541	8.69E-05	0.004321
17287	Mep1a	meprin 1 alpha	0.412031	0.05248	0.1862
104271	Tex15	testis expressed gene 15	0.410172	0.002355	0.03004
231507	Plac8	placenta-specific 8	0.409836	0.006086	0.0525
74176	Tgm5	transglutaminase 5	0.407498	0.001424	0.02229
53868	Rab25	RAB25, member RAS oncogene family	0.407	0.01452	0.08763
77883	6030498E09Rik	RIKEN cDNA 6030498E09 gene	0.406669	0.004818	0.04563
329636	Gm5103	predicted gene 5103	0.405515	0.005236	0.0481
269033	4930503L19Rik	RIKEN cDNA 4930503L19 gene	0.404367	0.0008415	0.01629
12683	Cidea	cell death-inducing DNA fragmentation factor, alpha subunit-like effector A	0.40404	5.28E-05	0.00343
12490	Cd34	CD34 antigen	0.403877	0.0003909	0.0104
234366	Gatad2a	GATA zinc finger domain containing 2A	0.403063	4.46E-05	0.003079
101807	AV356131	expressed sequence AV356131	0.402901	4.79E-07	0.0001961
100038492	F630048H11Rik	RIKEN cDNA F630048H11 gene	0.402901	0.0002948	0.008884
330734	B930018H19	hypothetical protein B930018H19	0.401606	6.74E-05	0.003733
100038666	Gm12298	predicted gene 12298	0.400962	0.004704	0.04511
77883	6030498E09Rik	RIKEN cDNA 6030498E09 gene	0.399202	0.005618	0.05009
78685	B430105A11Rik	RIKEN cDNA B430105A11 gene	0.398883	1.55E-06	0.0004105
214704	Iqub	IQ motif and ubiquitin domain containing	0.396511	0.004778	0.04547

100038417	Spink13	serine peptidase inhibitor, Kazal type 13	0.39604	4.40E-05	0.003078
17427	Mns1	meiosis-specific nuclear structural protein 1	0.395101	0.06129	0.2059
69930	Zfp715	zinc finger protein 715	0.394789	0.01873	0.1018
26905	Eif2s3x	eukaryotic translation initiation factor 2, subunit 3, structural gene X-linked	0.393082	0.04515	0.1712
75761	Apol7a	apolipoprotein L 7a	0.390778	0.008702	0.06429
67374	Jam2	junction adhesion molecule 2	0.390016	0.004625	0.04474
71141	4933407L21Rik	RIKEN cDNA 4933407L21 gene	0.387898	0.0001862	0.006796
94179	Krt23	keratin 23	0.387747	0.000332	0.009606
12555	Cdh15	cadherin 15	0.386399	3.21E-06	0.0006551
546144	Wdr72	WD repeat domain 72	0.386399	0.0002915	0.00887
232983	Cxcl17	chemokine (C-X-C motif) ligand 17	0.384615	0.005852	0.05137
625353	Vmn2r35	vomeronasal 2, receptor 35	0.384615	0.006022	0.05224
69121	Chrdl2	chordin-like 2	0.380807	0.03438	0.1466
211135	D130040H23Rik	RIKEN cDNA D130040H23 gene	0.380084	0.0008866	0.01681
73906	4833417C18Rik	RIKEN cDNA 4833417C18 gene	0.379939	0.01187	0.07759
53324	Nptx2	neuronal pentraxin 2	0.378931	0.0001319	0.005564
76359	2610028D06Rik	RIKEN cDNA 2610028D06 gene	0.378788	0.0001077	0.004893
21873	Tjp2	tight junction protein 2	0.376932	5.51E-06	0.0008672
100041290	D4Ertd617e	DNA segment, Chr 4, ERATO Doi 617, expressed	0.374672	0.002702	0.03218
11846	Arg1	arginase, liver	0.371058	0.0005561	0.0127
239083	Ccnb1ip1	cyclin B1 interacting protein 1	0.369276	0.01106	0.07445
228770	Rspo4	R-spondin family, member 4	0.366703	0.0008659	0.01661
664994	Isoc2a	isochorismatase domain containing 2a	0.365898	0.0017	0.02495
12509	Cd59a	CD59a antigen	0.365364	9.19E-08	6.32E-05
228576	Mall	mal, T-cell differentiation protein-like	0.365364	0.002754	0.03254
12234	Btrc	beta-transducin repeat containing protein	0.364431	1.97E-06	0.0004808
14842	Gsx1	GS homeobox 1	0.356633	0.0009311	0.01718
80907	Lactb	lactamase, beta	0.356252	1.11E-05	0.001293
170765	Ripply3	ripply3 homolog (zebrafish)	0.355999	6.63E-05	0.00373
246787	Slc5a2	solute carrier family 5 (sodium/glucose cotransporter), member 2	0.355492	0.001125	0.01904
238024	Fn3krp	fructosamine 3 kinase related protein	0.355114	0.002886	0.03355
13214	Defb1	defensin beta 1	0.354233	0.0001422	0.005814
69055	1810007D17Rik	RIKEN cDNA 1810007D17 gene	0.354233	0.02088	0.1089
449000	Zfp960	zinc finger protein 960	0.353732	4.20E-05	0.002987
74175	Crct1	cysteine-rich C-terminal 1	0.352983	0.03977	0.1589
76229	Vmn2r29	vomeronasal 2, receptor 29	0.352858	0.004904	0.04612
114479	Slc5a5	solute carrier family 5 (sodium iodide symporter), member 5	0.352485	0.000512	0.01212
76509	1600029D21Rik	RIKEN cDNA 1600029D21 gene	0.349773	0.001582	0.02378
67441	Isoc2b	isochorismatase domain containing 2b	0.349162	0.0001444	0.005842
214531	Tmprss13	transmembrane protease, serine 13	0.348797	0.001285	0.02083
76509	1600029D21Rik	RIKEN cDNA 1600029D21 gene	0.348675	0.002683	0.03212
277345	Wfdc16	WAP four-disulfide core domain 16	0.347705	0.1005	0.2802
27219	Sgk2	serum/glucocorticoid regulated kinase 2	0.346141	0.002311	0.02969
14873	Gsto1	glutathione S-transferase omega 1	0.345901	1.38E-05	0.001478
109978	Art4	ADP-ribosyltransferase 4	0.345423	0.1013	0.2818
66042	Sostdc1	sclerostin domain containing 1	0.344471	3.23E-05	0.002549
234358	Zfp930	zinc finger protein 930	0.343879	5.91E-06	0.0009031
100174921	4930505N22Rik	RIKEN cDNA 4930505N22 gene	0.341413	0.09224	0.2662
100040049	1810010D01Rik	RIKEN cDNA 1810010D01 gene	0.340368	9.49E-05	0.004481

23795	Agr2	anterior gradient 2 (Xenopus laevis)	0.338639	0.0001291	0.005485
73333	Slc25a31	solute carrier family 25 (mitochondrial carrier; adenine nucleotide translocator), member 31	0.3367	0.08715	0.2574
67446	Dusp28	dual specificity phosphatase 28	0.336474	0.001709	0.02505
246229	Bivm	basic, immunoglobulin-like variable motif containing	0.333111	0.3232	0.5598
100043332	Gm4368	predicted gene 4368	0.332668	0.0001199	0.005252
100505078	LOC100505078	hypothetical protein LOC100505078	0.331016	0.03225	0.1413
71413	5430410E06Rik	RIKEN cDNA 5430410E06 gene	0.330142	0.06497	0.2135
72785	2810474C18Rik	RIKEN cDNA 2810474C18 gene	0.329381	0.003295	0.03632
14428	Galr2	galanin receptor 2	0.327439	0.01334	0.08332
68307	Lrriq4	leucine-rich repeats and IQ motif containing 4	0.326158	0.04644	0.1742
77803	Fam159b	family with sequence similarity 159, member B	0.325521	0.004503	0.04412
17002	Ltf	lactotransferrin	0.323206	0.0007144	0.01469
18261	Ocm	oncomodulin	0.323206	4.87E-06	0.0007936
110855	Pde6c	phosphodiesterase 6C, cGMP specific, cone, alpha prime	0.322893	5.41E-07	0.0002009
70227	Zfp619	zinc finger protein 619	0.321337	3.65E-05	0.002706
100043407	Gm4419	predicted gene 4419	0.315358	4.25E-05	0.003013
72373	Psca	prostate stem cell antigen	0.314169	0.003857	0.03987
16061	Igh-VJ558	immunoglobulin heavy chain (J558 family)	0.3125	0.02277	0.115
67133	Gp2	glycoprotein 2 (zymogen granule membrane)	0.311915	0.004159	0.0419
241490	Rbm45	RNA binding motif protein 45	0.311818	0.0002126	0.007384
12040	Bckdhb	branched chain ketoacid dehydrogenase E1, beta polypeptide	0.306748	0.2911	0.5271
55936	Ctps2	cytidine 5'-triphosphate synthase 2	0.306091	1.52E-06	0.0004105
71078	Adam30	a disintegrin and metallopeptidase domain 30	0.305064	0.07672	0.2372
110855	Pde6c	phosphodiesterase 6C, cGMP specific, cone, alpha prime	0.304971	4.92E-06	0.0007936
72461	Prcp	prolylcarboxypeptidase (angiotensinase C)	0.30303	4.47E-06	0.0007825
108124	Napa	N-ethylmaleimide sensitive fusion protein attachment protein alpha	0.302755	2.39E-06	0.0005336
244071	Agbl1	ATP/GTP binding protein-like 1	0.30175	0.05224	0.1856
77090	Ocel1	occludin/ELL domain containing 1	0.301659	5.18E-07	0.0002009
668814	Gm9376	predicted gene 9376	0.301205	2.19E-06	0.0005025
84112	Sucnr1	succinate receptor 1	0.300842	0.008283	0.06238
69117	Adh6a	alcohol dehydrogenase 6A (class V)	0.299312	0.03989	0.1592
667373	Gm14446	predicted gene 14446	0.295946	0.0004901	0.01174
17219	Mcm6	minichromosome maintenance deficient 6 (MIS5 homolog, S. pombe) (S. cerevisiae)	0.295159	1.89E-07	0.0001034
20202	S100a9	S100 calcium binding protein A9 (calgranulin B)	0.293083	0.09066	0.2637
74725	4930524B17Rik	RIKEN cDNA 4930524B17 gene	0.291036	0.05347	0.1885
327768	A330049N07Rik	RIKEN cDNA A330049N07 gene	0.285144	0.003919	0.04035
672682	Gm9573	predicted gene 9573	0.283046	0.001504	0.02308
84111	Gpr87	G protein-coupled receptor 87	0.282087	0.007039	0.0568
258354	Olfr168	olfactory receptor 168	0.280348	0.1367	0.3383
13710	Elf3	E74-like factor 3	0.278862	0.0002312	0.007731
66809	Krt20	keratin 20	0.278242	4.65E-05	0.003153
114662	Prss29	protease, serine, 29	0.277701	0.09136	0.2649
223332	Ranbp3l	RAN binding protein 3-like	0.275028	0.105	0.2883
319742	Mpzl3	myelin protein zero-like 3	0.271887	0.001022	0.01792
258408	Olfr463	olfactory receptor 463	0.27049	0.1068	0.2916
234199	Fgl1	fibrinogen-like protein 1	0.269324	0.0001271	0.005439
20908	Stx3	syntaxin 3	0.267237	8.85E-07	0.0002834
109820	Pgc	progastricsin (pepsinogen C)	0.267094	0.0001208	0.005257
66757	Adat2	adenosine deaminase, tRNA-specific 2, TAD2 homolog (S. cerevisiae)	0.266525	1.99E-05	0.001868

72723	Zfp74	zinc finger protein 74	0.265675	1.56E-05	0.001628
22702	Zfp42	zinc finger protein 42	0.26455	0.1012	0.2815
231655	Oasl1	2'-5' oligoadenylate synthetase-like 1	0.262398	0.000395	0.01045
50500	Ttpa	tocopherol (alpha) transfer protein	0.261986	0.09798	0.2761
408061	BC051408	cDNA sequence BC051408	0.261986	0.0553	0.1925
94094	Trim34a	tripartite motif-containing 34A	0.261575	0.004096	0.04151
328561	Apol10b	apolipoprotein L 10b	0.261575	0.0126	0.08032
27411	Slc14a2	solute carrier family 14 (urea transporter), member 2	0.259202	0.04153	0.1631
106610	AI504002	expressed sequence AI504002	0.256016	0.3012	0.537
433470	AA467197	expressed sequence AA467197	0.249439	0.0009487	0.01738
272643	Prss43	protease, serine, 43	0.243962	0.07398	0.2321
108812	Als2cr12	amyotrophic lateral sclerosis 2 (juvenile) chromosome region, candidate 12 (human)	0.242542	2.97E-05	0.002405
100042761	Gm10697	predicted gene 10697	0.242072	0.09357	0.2688
12319	Car8	carbonic anhydrase 8	0.236742	0.0002367	0.00779
22290	Uty	ubiquitously transcribed tetratricopeptide repeat gene, Y chromosome	0.236351	0.07591	0.236
77044	Arid2	AT rich interactive domain 2 (ARID, RFX-like)	0.231911	0.05096	0.1832
67133	Gp2	glycoprotein 2 (zymogen granule membrane)	0.231803	0.005198	0.04785
98763	AA407782	expressed sequence AA407782	0.230415	0.2845	0.5201
20592	Kdm5d	lysine (K)-specific demethylase 5D	0.228311	0.1232	0.3192
434540	Psg20	pregnancy-specific glycoprotein 20	0.226809	0.09825	0.2765
67483	1700028P14Rik	RIKEN cDNA 1700028P14 gene	0.224215	4.77E-06	0.0007936
52020	Umodl1	uromodulin-like 1	0.220022	0.0002343	0.007755
76568	Ift46	intraflagellar transport 46 homolog (Chlamydomonas)	0.219202	1.11E-05	0.001293
22290	Uty	ubiquitously transcribed tetratricopeptide repeat gene, Y chromosome	0.217108	0.01627	0.09352
382109	Fbxw26	F-box and WD-40 domain protein 26	0.215332	0.09533	0.2716
545548	Lce3a	late cornified envelope 3A	0.21322	0.002165	0.02829
76808	Rpl18a	ribosomal protein L18A	0.21254	0.008113	0.06165
67483	1700028P14Rik	RIKEN cDNA 1700028P14 gene	0.209688	3.67E-06	0.0006967
171167	Fut10	fucosyltransferase 10	0.20903	1.21E-06	0.0003443
232801	Lilra5	leukocyte immunoglobulin-like receptor, subfamily A (with TM domain), member 5	0.206143	0.0961	0.2729
234404	Nxnl1	nucleoredoxin-like 1	0.202061	1.04E-07	6.88E-05
73162	Otud3	OTU domain containing 3	0.201491	0.1462	0.3519
78609	8030411F24Rik	RIKEN cDNA 8030411F24 gene	0.20145	2.52E-05	0.002162
13067	Cyct	cytochrome c, testis	0.200924	0.0002585	0.008176
67588	Rnf41	ring finger protein 41	0.19857	2.60E-06	0.0005677
12319	Car8	carbonic anhydrase 8	0.198177	5.94E-06	0.0009031
72902	Spock3	sparc/osteonectin, cwcv and kazal-like domains proteoglycan 3	0.197824	0.01586	0.09253
231507	Plac8	placenta-specific 8	0.194212	0.0001051	0.004852
234138	BC019943	cDNA sequence BC019943	0.18997	1.72E-07	0.000102
107047	Psmg2	proteasome (prosome, macropain) assembly chaperone 2	0.185288	0.03946	0.1582
66442	Spc25	SPC25, NDC80 kinetochore complex component, homolog (S. cerevisiae)	0.183385	0.0001349	0.0056
234199	Fgl1	fibrinogen-like protein 1	0.177778	0.0005564	0.0127
75080	4930518C09Rik	RIKEN cDNA 4930518C09 gene	0.175593	0.01449	0.08758
18261	Ocm	oncomodulin	0.175101	1.41E-05	0.001503
74528	8430406I07Rik	RIKEN cDNA 8430406I07 gene	0.172117	0.00656	0.05472
16181	Il1rn	interleukin 1 receptor antagonist	0.169005	0.09192	0.2658
19223	Ptgis	prostaglandin I2 (prostacyclin) synthase	0.167196	2.71E-06	0.0005846
234138	BC019943	cDNA sequence BC019943	0.165017	4.28E-08	3.78E-05
100040412	LOC100040412	lysine-specific demethylase 5D-like	0.15674	0.09181	0.2655

71687	Tmem25	transmembrane protein 25	0.155958	7.66E-08	5.77E-05
229613	6330549D23Rik	RIKEN cDNA 6330549D23 gene	0.142005	4.86E-07	0.0001961
68668	Klk5	kallikrein related-peptidase 5	0.133958	0.0122	0.07864
209760	Tmc7	transmembrane channel-like gene family 7	0.1288	1.24E-05	0.001395
76681	Trim12a	tripartite motif-containing 12A	0.126968	0.0001357	0.005612
73061	3110007F17Rik	RIKEN cDNA 3110007F17 gene	0.124844	0.005393	0.04891
70979	4931417G12Rik	RIKEN cDNA 4931417G12 gene	0.122564	2.47E-08	3.05E-05
140709	Emid2	EMI domain containing 2	0.112499	9.73E-07	0.0002868
94216	Col4a6	collagen, type IV, alpha 6	0.112259	0.0003704	0.01011
269209	Stk36	serine/threonine kinase 36 (fused homolog, Drosophila)	0.103199	4.68E-06	0.0007898
100040410	Gm14957	predicted gene 14957	0.102428	1.31E-07	8.38E-05
72461	Prcp	prolylcarboxypeptidase (angiotensinase C)	0.100908	9.91E-09	1.53E-05
214523	Tmprss4	transmembrane protease, serine 4	0.083752	2.07E-08	2.75E-05
26450	Rbbp9	retinoblastoma binding protein 9	0.072993	0.06114	0.2056
17921	Myo7a	myosin VIIA	0.071174	3.64E-08	3.55E-05
74602	4833428L15Rik	RIKEN cDNA 4833428L15 gene	0.069204	2.38E-07	0.0001164
67705	1810058I24Rik	RIKEN cDNA 1810058I24 gene	0.057637	9.13E-06	0.001146
72154	Zfp157	zinc finger protein 157	0.052994	2.11E-07	0.0001113
73061	3110007F17Rik	RIKEN cDNA 3110007F17 gene	0.052165	3.63E-10	1.35E-06
109685	Hyal3	hyaluronoglucosaminidase 3	0.052002	3.57E-06	0.0006967
30939	Pttg1	pituitary tumor-transforming gene 1	0.051282	3.72E-06	0.0006967
26900	Ddx3y	DEAD (Asp-Glu-Ala-Asp) box polypeptide 3, Y-linked	0.049188	0.06557	0.2148
26908	Eif2s3y	eukaryotic translation initiation factor 2, subunit 3, structural gene Y-linked	0.039002	0.06617	0.2163
14537	Gcnt1	glucosaminyl (N-acetyl) transferase 1, core 2	0.034868	3.59E-11	2.22E-07
75764	Slx1b	SLX1 structure-specific endonuclease subunit homolog B (S. cerevisiae)	0.033979	4.22E-09	9.80E-06
17996	Neb	nebulin	0.031526	0.09391	0.2694
69301	1700008P20Rik	RIKEN cDNA 1700008P20 gene	0.029446	1.95E-06	0.0004808
54631	Nphs1	nephrosis 1 homolog, nephrin (human)	0.027556	4.80E-07	0.0001961
433100	AA388235	expressed sequence AA388235	0.02736	3.36E-11	2.22E-07
258198	Olfr224	olfactory receptor 224	0.027056	2.80E-05	0.002314
30939	Pttg1	pituitary tumor-transforming gene 1	0.026254	1.81E-10	8.40E-07
69301	1700008P20Rik	RIKEN cDNA 1700008P20 gene	0.02474	2.20E-07	0.0001113
80509	Med8	mediator of RNA polymerase II transcription, subunit 8 homolog (yeast)	0.024588	1.54E-06	0.0004105
76681	Trim12a	tripartite motif-containing 12A	0.014134	8.54E-09	1.44E-05
329478	C130078N14	hypothetical protein C130078N14	0.007267	1.52E-12	2.82E-08
